# Supplementary material for: Living with relatives offsets the harm caused by pathogens in natural populations
Source: eLife. 2021 Jul 26;10:e66649. doi: 10.7554/eLife.66649 (PMC8313236; doi:10.7554/eLife.66649)
Supplement: Supplementary file 1. — Tables S1-19 can be found in excel format at the open science framework DOI 10.17605/OSF.IO/Q3ANE. Table S1: The references screened for effect sizes of within-group relatedness on mortality and pathogen abundance and whether they were included in analyses. Table S2: Data used for analysis of the effect of within-group relatedness on mortality and pathogen abundance (r). Table S3: Data on whether species typically live with kin or non-kin. Table S4: Data used for analysis of the effect of within-group relatedness on variance in mortality and pathogen abundance (LnCVR). Table S5: The overall effect of within-group relatedness on mortality and pathogen abundance on Zr. Table S6: The effect of pathogen presence and whether mortality or pathogen abundance was measured on Zr. Table S7: The effect of pathogen manipulation on Zr. Table S8: The effect of relatedness manipulations and pathogen presence on Zr. Table S9: The effect of kin structure and pathogen presence measures on Zr. Table S10: The overall effect of within-group relatedness on variance (LnCVR) mortality and pathogen abundances. Table S11: The effect of pathogen manipulation on LnCVR. Table S12: The effect of pathogen manipulation and whether mortality or pathogen abundance was measured on LnCVR. Table S13: The effect of relatedness manipulations and pathogen presence on LnCVR. Table S14: The effect of kin structure and pathogen presence measures on Zr excluding potential effects of inbreeding. Table S15: Effect of parasite manipulations and fitness measures on LnCVR excluding potential effects of inbreeding. Table S16: Laboratory effects on Zr. Table S17: Laboratory effects on LnCVR. Table S18: The sensitivity of analyses on Zr to the statistical techniques used in primary studies. Table S19: Data on within-group relatedness on mortality in ostrich chicks. [file elife-66649-supp1.zip › SIinformation.html]

SIinformation.utf8


# **Supplementary Information:** Living with relatives offsets the harm caused by pathogens in natural populations

Hanna M. Bensch1, Emily O’Connor1 & Charlie K. Cornwallis1

1Department of Biology, Lund University, SE-223 62, Lund, Sweden Corresponding author: Charlie Cornwallis **Email**: charlie.cornwallis@biol.lu.se

# Supplementary Tables

## **Table S1:** The references screened for effect sizes of within-group relatedness on mortality and pathogen abundance and whether they were included in analyses.

|  | Search | Date\_screened | AU | Pub\_Year | Title | Abstract | Relevance\_score | Abstract\_of\_interest | Excluded\_on\_abstract\_extra\_info | Excluded\_on\_fulltext\_extra\_info | References\_checked | Included\_final\_analysis |
| --- | --- | --- | --- | --- | --- | --- | --- | --- | --- | --- | --- | --- |
| 1 | 2017 | 43004 | Weiser, EL; Grueber, CE; Kennedy, ES; Jamieson, IG | 2016 | Unexpected positive and negative effects of continuing inbreeding in one of the world’s most inbred wild animals | “Inbreeding depression, the reduced fitness of offspring of related individuals, is a central theme in evolutionary biology. Inbreeding effects are influenced by the genetic makeup of a population, which is driven by any history of genetic bottlenecks and genetic drift. The Chatham Island black robin represents a case of extreme inbreeding following two severe population bottlenecks. We tested whether inbreeding measured by a 20-year pedigree predicted variation in fitness among individuals, despite the high mean level of inbreeding and low genetic diversity in this species. We found that paternal and maternal inbreeding reduced fledgling survival and individual inbreeding reduced juvenile survival, indicating that inbreeding depression affects even this highly inbred population. Close inbreeding also reduced survival for fledglings with less-inbred mothers, but unexpectedly improved survival for fledglings with highly inbred mothers. This counterintuitive interaction could not be explained by various potentially confounding variables. We propose a genetic mechanism, whereby a highly inbred chick with a highly inbred parent inherits a”“proven”" genotype and thus experiences a fitness advantage, which could explain the interaction. The positive and negative effects we found emphasize that continuing inbreeding can have important effects on individual fitness, even in populations that are already highly inbred." | 1 | no | inbreeding ind | NA | no | no |
| 2 | 2017 | 43004 | Spielman, D; Brook, BW; Briscoe, DA; Frankham, R | 2004 | Does inbreeding and loss of genetic diversity decrease disease resistance? | Inbreeding and loss of genetic diversity are predicted to decrease the resistance of species to disease. However, this issue is controversial and there is limited rigorous scientific evidence available. To test whether inbreeding and loss of genetic diversity affect a host’s resistance to disease, Drosophila melanogaster populations with different levels of inbreeding and genetic diversity were exposed separately to ( a) thuringiensin, an insecticidal toxin produced by some strains of Bacillus thuringiensis, and (b) live Serratia marcescens bacteria. Inbreeding and loss of genetic diversity significantly reduced resistance of D. melanogaster to both the thuringiensin toxin and live Serratia marcescens. For both, the best fitting relationships between resistance and inbreeding were curvilinear. As expected, there was wide variation among replicate inbred populations in disease resistance. Lowered resistances to both the toxin and the pathogen in inbred populations were due to specific resistance alleles, rather than generalized inbreeding effects, as correlations between resistance and population fitness were low or negative. Wildlife managers should strive to minimise inbreeding and loss of genetic diversity within threatened populations and to minimise exposure of inbred populations to disease. | 2 | no | inbreeding | NA | no | no |
| 3 | 2017 | 43004 | Aguirre, JD; Marshall, DJ | 2012 | Genetic diversity increases population productivity in a sessile marine invertebrate | Reductions in genetic diversity can have widespread ecological consequences: populations with higher genetic diversity are more stable, productive and resistant to disturbance or disease than populations with lower genetic diversity. These ecological effects of genetic diversity differ from the more familiar evolutionary consequences of depleting genetic diversity, because ecological effects manifest within a single generation. If common, genetic diversity effects have the potential to change the way we view and manage populations, but our understanding of these effects is far from complete, and the role of genetic diversity in sexually reproducing animals remains unclear. Here, we examined the effects of genetic diversity in a sexually reproducing marine invertebrate in the field. We manipulated the genetic diversity of experimental populations and then measured individual survival, growth, and fecundity, as well as the size of offspring produced by individuals in high and low genetic diversity populations. Overall, we found greater genetic diversity increased performance across all metrics, and that complementarity effects drove the increased productivity of our high-diversity populations. Our results show that differences in genetic diversity among populations can have pervasive effects on population productivity within remarkably short periods of time. | 3 | yes | make mean out of populations? | NA | yes | yes |
| 4 | 2017 | 43004 | Keller, LF; Arcese, P | 1998 | No evidence for inbreeding avoidance in a natural population of song sparrows (Melospiza melodia) | We studied mate choice and inbreeding avoidance in a natural population of song sparrows (Melospiza melodia) on Mandarte Island, Canada. Inbreeding occurred regularly: 59% of all matings were between known relatives. We tested for inbreeding avoidance by comparing the observed levels of inbreeding to those expected if mate choice had been random with respect to relatedness. Independent of our assumptions about the availability of mates in the random mating model, we found that the expected and observed distributions of inbreeding coefficients were similar. as was the expected and observed frequency of close (f greater than or equal to 0.125) inbreeding. Furthermore, there was no difference in relatedness of observed pairs and those that would have resulted had birds mated instead with their nearest neighbors. The only evidence to suggest any inbreeding avoidance was a reduced rate of parent-offspring matings as compared to one random mating model but not the other. Hence, despite substantial inbreeding depression in this population, we found little evidence for inbreeding avoidance through mate choice. We present a simple model to suggest that variation in inbreeding avoidance behaviors in birds may arise from differences in survival rates: in species with low survival rates, the costs of forfeiting matings to avoid inbreeding may exceed the costs of inbreeding. | 4 | no | NA | NA | no | no |
| 5 | 2017 | 43004 | Brzeski, KE; Rabon, DR; Chamberlain, MJ; Waits, LP; Taylor, SS | 2014 | Inbreeding and inbreeding depression in endangered red wolves (Canis rufus) | In natural populations, the expression and severity of inbreeding depression can vary widely across taxa. Describing processes that influence the extent of inbreeding and inbreeding depression aid in our understanding of the evolutionary history of mating systems such as cooperative breeding and nonrandom mate selection. Such findings also help shape wildlife conservation theory because inbreeding depression reduces the viability of small populations. We evaluated the extent of inbreeding and inbreeding depression in a small, re-introduced population of red wolves (Canis rufus) in North Carolina. Since red wolves were first re-introduced in 1987, pedigree inbreeding coefficients (f) increased considerably and almost every wild born wolf was inbred (average f = 0.154 and max f = 0.383). The large inbreeding coefficients were due to both background relatedness associated with few founders and numerous close relative matings. Inbreeding depression was most evident for adult body size and generally absent for direct fitness measures such as reproductive success and survival; no lethal equivalents (LE = 0.00) were detected in juvenile survival. The lack of strong inbreeding depression in direct measures of fitness could be due to a founder effect or because there were no outbred individuals for comparison. Our results highlight the variable expression of inbreeding depression across traits and the need to measure a number of different traits when evaluating inbreeding depression in a wild population. | 5 | no | only one pop | NA | no | no |
| 6 | 2017 | 43004 | Margulis, SW | 1998 | Differential effects of inbreeding at juvenile and adult life-history stages in Peromyscus polionotus | Inbreeding depression traditionally has been measured at early life-history stages, such as neonatal or juvenile viability or deficits in growth and development. I investigated additional fitness components by evaluating impact of inbreeding on reproductive success of adult females in a captive colony of oldfield mice (Peromyscus polionotus subgriseus and P. p. rhoadsi). By first conducting analyses on only inbreeding of litters (i.e., relatedness of parents) and then adding maternal effects (mother’s inbreeding coefficient independent of parental relatedness, parity, and maternal inactivity), I illustrated effect of inbreeding on life-history characters that were manifested in adulthood. Young in inbred litters (f > 0.1) tended to be smaller than young in outbred Litters, but inbred litters did not exhibit any decrements in survival probability. Number of young raised to weaning from inbred and outbred Litters did not differ. Inbred females (inbreeding coefficient f > 0.1) reared significantly fewer young than did outbred females (f < 0.1) (independent of whether parents were related and therefore independent of whether young themselves were inbred). Differences between the two subspecies were found for a number of fitness traits. These differences are attributed to chance differences in genetic makeup of founder stocks (founder effect). Examining inbreeding effects only at a single life-history stage (i.e., during the juvenile period) and only on a single fitness trait (juvenile viability) may result in serious underestimation of the extent of inbreeding depression. | 6 | no | NA | NA | no | no |
| 7 | 2017 | 43004 | Borg, AA; Pedersen, SA; Jensen, H; Westerdahl, H | 2011 | Variation in MHC genotypes in two populations of house sparrow (Passer domesticus) with different population histories | Small populations are likely to have a low genetic ability for disease resistance due to loss of genetic variation through inbreeding and genetic drift. In vertebrates, the highest genetic diversity of the immune system is located at genes within the major histocompatibility complex (MHC). Interestingly, parasite-mediated selection is thought to potentially maintain variation at MHC loci even in populations that are monomorphic at other loci. Therefore, general loss of genetic variation in the genome may not necessarily be associated with low variation at MHC loci. We evaluated inter- and intrapopulation variation in MHC genotypes between an inbred (Aldra) and a relatively outbred population (Hestmannoy) of house sparrows (Passer domesticus) in a metapopulation at Helgeland, Norway. Genomic (gDNA) and transcribed (cDNA) alleles of functional MHC class I and IIB loci, along with neutral noncoding microsatellite markers, were analyzed to obtain relevant estimates of genetic variation. We found lower allelic richness in microsatellites in the inbred population, but high genetic variation in MHC class I and IIB loci in both populations. This suggests that also the inbred population could be under balancing selection to maintain genetic variation for pathogen resistance. | 7 | no | interesting study! - for discussion? | NA | no | no |
| 8 | 2017 | 43004 | Brekke, P; Bennett, PM; Wang, JL; Pettorelli, N; Ewen, JG | 2010 | Sensitive males: inbreeding depression in an endangered bird | Attempts to conserve threatened species by establishing new populations via reintroduction are controversial. Theory predicts that genetic bottlenecks result in increased mating between relatives and inbreeding depression. However, few studies of wild sourced reintroductions have carefully examined these genetic consequences. Our study assesses inbreeding and inbreeding depression in a free-living reintroduced population of an endangered New Zealand bird, the hihi (Notiomystis cincta). Using molecular sexing and marker-based inbreeding coefficients estimated from 19 autosomal microsatellite loci, we show that (i) inbreeding depresses offspring survival, (ii) male embryos are more inbred on average than female embryos, (iii) the effect of inbreeding depression is male-biased and (iv) this population has a substantial genetic load. Male susceptibility to inbreeding during embryo and nestling development may be due to size dimorphism, resulting in faster growth rates and more stressful development for male embryos and nestlings compared with females. This work highlights the effects of inbreeding at early life-history stages and the repercussions for the long-term population viability of threatened species. | 8 | no | NA | NA | no | no |
| 9 | 2017 | 43004 | Philip, VM; Sokoloff, G; Ackert-Bicknell, CL; Striz, M; Branstetter, L; Beckmann, MA; Spence, JS; Jackson, BL; Galloway, LD; Barker, P; Wymore, AM; Hunsicker, PR; Durtschi, DC; Shaw, GS; Shinpock, S; Manly, KF; Miller, DR; Donohue, KD; Culiat, CT; Churchill, GA; Lariviere, WR; Palmer, AA; O’Hara, BF; Voy, BH; Chesler, EJ | 2011 | Genetic analysis in the Collaborative Cross breeding population | Genetic reference populations in model organisms are critical resources for systems genetic analysis of disease related phenotypes. The breeding history of these inbred panels may influence detectable allelic and phenotypic diversity. The existing panel of common inbred strains reflects historical selection biases, and existing recombinant inbred panels have low allelic diversity. All such populations may be subject to consequences of inbreeding depression. The Collaborative Cross (CC) is a mouse reference population with high allelic diversity that is being constructed using a randomized breeding design that systematically outcrosses eight founder strains, followed by inbreeding to obtain new recombinant inbred strains. Five of the eight founders are common laboratory strains, and three are wild-derived. Since its inception, the partially inbred CC has been characterized for physiological, morphological, and behavioral traits. The construction of this population provided a unique opportunity to observe phenotypic variation as new allelic combinations arose through intercrossing and inbreeding to create new stable genetic combinations. Processes including inbreeding depression and its impact on allelic and phenotypic diversity were assessed. Phenotypic variation in the CC breeding population exceeds that of existing mouse genetic reference populations due to both high founder genetic diversity and novel epistatic combinations. However, some focal evidence of allele purging was detected including a suggestive QTL for litter size in a location of changing allele frequency. Despite these inescapable pressures, high diversity and precision for genetic mapping remain. These results demonstrate the potential of the CC population once completed and highlight implications for development of related populations. | 9 | no | NA | NA | no | no |
| 10 | 2017 | 43004 | Ross-Gillespie, A; O’Riain, MJ; Keller, LF | 2007 | Viral epizootic reveals inbreeding depression in a habitually inbreeding mammal | “Inbreeding is typically detrimental to fitness. However, some animal populations are reported to inbreed without incurring inbreeding depression, ostensibly due to past”“purging”" of deleterious alleles. Challenging this is the position that purging can, at best, only adapt a population to a particular environment | 10 | no | only one pop | NA | no | no |
| 11 | 2017 | 43004 | Townsend, SM; Jamieson, IG | 2013 | Molecular and pedigree measures of relatedness provide similar estimates of inbreeding depression in a bottlenecked population | Individual-based estimates of the degree of inbreeding or parental relatedness from pedigrees provide a critical starting point for studies of inbreeding depression, but in practice wild pedigrees are difficult to obtain. Because inbreeding increases the proportion of genomewide loci that are identical by descent, inbreeding variation within populations has the potential to generate observable correlations between heterozygosity measured using molecular markers and a variety of fitness related traits. Termed heterozygosity-fitness correlations (HFCs), these correlations have been observed in a wide variety of taxa. The difficulty of obtaining wild pedigree data, however, means that empirical investigations of how pedigree inbreeding influences HFCs are rare. Here, we assess evidence for inbreeding depression in three life-history traits (hatching and fledging success and juvenile survival) in an isolated population of Stewart Island robins using both pedigree- and molecular-derived measures of relatedness. We found results from the two measures were highly correlated and supported evidence for significant but weak inbreeding depression. However, standardized effect sizes for inbreeding depression based on the pedigree-based kin coefficients (k) were greater and had smaller standard errors than those based on molecular genetic measures of relatedness (RI), particularly for hatching and fledging success. Nevertheless, the results presented here support the use of molecular-based measures of relatedness in bottlenecked populations when information regarding inbreeding depression is desired but pedigree data on relatedness are unavailable. | 11 | no | only one pop | NA | no | no |
| 12 | 2017 | 43004 | Huchard, E; Schliehe-Diecks, S; Kappeler, PM; Kraus, C | 2017 | The inbreeding strategy of a solitary primate, Microcebus murinus | Inbreeding depression may be common in nature, reflecting either the failure of inbreeding avoidance strategies or inbreeding tolerance when avoidance is costly. The combined assessment of inbreeding risk, avoidance and depression is therefore fundamental to evaluate the inbreeding strategy of a population, that is how individuals respond to the risk of inbreeding. Here, we use the demographic and genetic monitoring of 10 generations of wild grey mouse lemurs (Microcebus murinus), small primates from Madagascar with overlapping generations, to examine their inbreeding strategy. Grey mouse lemurs have retained ancestral mammalian traits, including solitary lifestyle, polygynandry and male-biased dispersal, and may therefore offer a representative example of the inbreeding strategy of solitary mammals. The occurrence of close kin among candidate mates was frequent in young females (similar to 37%, most often the father) and uncommon in young males (similar to 6%) due to male-biased dispersal. However, close kin consistently represented a tiny fraction of candidate mates (<1%) across age and sex categories. Mating biases favouring partners with intermediate relatedness were detectable in yearling females and adult males, possibly partly caused by avoidance of daughter-father matings. Finally, inbreeding depression, assessed as the effect of heterozygosity on survival, was undetectable using a capture-mark-recapture study. Overall, these results indicate that sex-biased dispersal is a primary inbreeding avoidance mechanism at the population level, and mating biases represent an additional strategy that may mitigate residual inbreeding costs at the individual level. Combined, these mechanisms explain the rarity of inbreeding and the lack of detectable inbreeding depression in this large, genetically diverse population. | 12 | no | NA | NA | no | no |
| 13 | 2017 | 43004 | Reid, JM; Arcese, P; Bocedi, G; Duthie, AB; Wolak, ME; Keller, LF | 2015 | Resolving the conundrum of inbreeding depression but no inbreeding avoidance: Estimating sex-specific selection on inbreeding by song sparrows (Melospiza melodia) | “Inbreeding avoidance among interacting females and males is not always observed despite inbreeding depression in offspring fitness, creating an apparent”“inbreeding paradox.”" This paradox could be resolved if selection against inbreeding was in fact weak, despite inbreeding depression. However, the net magnitude and direction of selection on the degree to which females and males inbreed by pairing with relatives has not been explicitly estimated. We used long-term pedigree data to estimate phenotypic selection gradients on the degree of inbreeding that female and male song sparrows (Melospiza melodia) expressed by forming socially persistent breeding pairs with relatives. Fitness was measured as the total numbers of offspring and grand offspring contributed to the population, and as corresponding expected numbers of identical-by-descent allele copies, thereby accounting for variation in offspring survival, reproduction, and relatedness associated with variation in parental inbreeding. Estimated selection gradients on the degree to which individuals paired with relatives were weakly positive in females, but negative in males that formed at least one socially persistent pairing. However, males that paired had higher mean fitness than males that remained socially unpaired. These analyses suggest that net selection against inbreeding may be weak in both sexes despite strong inbreeding depression, thereby resolving the "“inbreeding paradox.”"" | 13 | no | NA | NA | no | no |
| 14 | 2017 | 43004 | Francisco, FD; Santiago, LR; Arias, MC | 2013 | Molecular genetic diversity in populations of the stingless bee Plebeia remota: A case study | Genetic diversity is a major component of the biological diversity of an ecosystem. The survival of a population may be seriously threatened if its genetic diversity values are low. In this work, we measured the genetic diversity of the stingless bee Plebeia remota based on molecular data obtained by analyzing 15 microsatellite loci and sequencing two mitochondrial genes. Population structure and genetic diversity differed depending on the molecular marker analyzed: microsatellites showed low population structure and moderate to high genetic diversity, while mitochondrial DNA (mtDNA) showed high population structure and low diversity in three populations. Queen philopatry and male dispersal behavior are discussed as the main reasons for these findings. | 14 | maybe | any fitness measure? | no fitness measure | no | no |
| 15 | 2017 | 43004 | Olson, LE; Blumstein, DT; Pollinger, JR; Wayne, RK | 2012 | No evidence of inbreeding avoidance despite demonstrated survival costs in a polygynous rodent | Individuals are generally predicted to avoid inbreeding because of detrimental fitness effects. However, several recent studies have shown that limited inbreeding is tolerated by some vertebrate species. Here, we examine the costs and benefits of inbreeding in a largely polygynous rodent, the yellow-bellied marmot (Marmota flaviventris). We use a pedigree constructed from 8 years of genetic data to determine the relatedness of all marmots in our study population and examine offspring survival, annual male reproductive success, relatedness between breeding pairs and the effects of group composition on likelihood of male reproduction to assess inbreeding in this species. We found decreased survival in inbred offspring, but equal net reproductive success among males that inbred and those that avoided it. Relatedness between breeding pairs was greater than that expected by chance, indicating that marmots do not appear to avoid breeding with relatives. Further, male marmots do not avoid inbreeding: males mate with equal frequency in groups composed of both related and unrelated females and in groups composed of only female relatives. Our results demonstrate that inbreeding can be tolerated in a polygynous species if the reproductive costs of inbreeding are low and individuals that mate indiscriminately do not suffer decreased reproductive success. | 15 | no | individual level | NA | no | no |
| 16 | 2017 | 43004 | Postma, E; Martini, L; Martini, P | 2010 | Inbred women in a small and isolated Swiss village have fewer children | Despite overwhelming evidence for a negative effect of inbreeding on fitness in plants and nonhuman animals, the exact nature of its effect in humans remains subject to debate. To obtain a better understanding of the effects of inbreeding on reproductive success in humans, we reconstructed the genealogies of the current inhabitants of a small and isolated Swiss village and used these to estimate the level of inbreeding of both members of all married couples, as well as their relatedness (i.e. the level of inbreeding of their offspring). Although there was no effect of parental relatedness on the number of children a couple had, we found that inbred mothers, but not inbred fathers, had significantly fewer children. Thus, although related couples did not have fewer children themselves, their inbred daughters did leave them with fewer grandchildren. Thereby, we provide evidence for the existence of inbreeding depression in human fertility, also in relatively outbred and egalitarian communities. | 16 | no | human | NA | no | no |
| 17 | 2017 | 43004 | Caballero, A; Bravo, I; Wang, J | 2017 | Inbreeding load and purging: implications for the short-term survival and the conservation management of small populations | Using computer simulations, we evaluate the effects of genetic purging of inbreeding load in small populations, assuming genetic models of deleterious mutations which account for the typical amount of load empirically observed. Our results show that genetic purging efficiently removes the inbreeding load of both lethal and non-lethal mutations, reducing the amount of inbreeding depression relative to that expected without selection. We find that the minimum effective population size to avoid severe inbreeding depression in the short term is of the order of N-e approximate to 70 for a wide range of species’ reproductive rates. We also carried out simulations of captive breeding populations where two contrasting management methods are performed, one avoiding inbreeding (equalisation of parental contributions (EC)) and the other forcing it (circular sib mating (CM)). We show that, for the inbreeding loads considered, CM leads to unacceptably high extinction risks and, as a result, to lower genetic diversity than EC. Thus we conclude that methods aimed at enhancing purging by intentional inbreeding should not be generally advised in captive breeding conservation programmes. | 17 | no | NA | NA | no | no |
| 18 | 2017 | 43004 | KELLER, LF; ARCESE, P; SMITH, JNM; HOCHACHKA, WM; STEARNS, SC | 1994 | SELECTION AGAINST INBRED SONG SPARROWS DURING A NATURAL-POPULATION BOTTLENECK | THE genetic and demographic consequences of population subdivision have received considerable attention from conservation biologists. In particular, losses of genetic variability and reduced viability and fecundity due to inbreeding (inbreeding depression) are of concern(1-3). Studies of domestic, laboratory(4,5) and zoo populations(2,6,7) have shown inbreeding depression in a variety of traits related to fitness. Consequently, inbreeding depression is widely accepted as a fact. Recently, however, the relative impact of inbreeding on the viability of natural populations has been questioned(8-10). Work on the cheetah (Acinonyx jubatus), for example, has emphasized the overwhelming importance of environmental factors on mortality in the wild(9,10). Here we report that song sparrows (Melospiza melodia) that survived a severe population bottleneck were a non-random subset of the pre-crash population with respect to inbreeding, and that natural selection favoured outbred individuals. Thus, inbreeding depression was expressed in the face of an environmental challenge. Such challenges are also likely to be faced by inbred populations of endangered species. We suggest that environmental and genetic effects on survival may interact and, as a consequence, that their effects on individuals and populations should not be considered independently. | 18 | no | interesting study! - for discussion? | NA | no | no |
| 19 | 2017 | 43004 | Townsend, AK; Clark, AB; McGowan, KJ; Miller, AD; Buckles, EL | 2010 | Condition, innate immunity and disease mortality of inbred crows | Cooperatively breeding American crows (Corvus brachyrhynchos) suffer a severe disease-mediated survival cost from inbreeding, but the proximate mechanisms linking inbreeding to disease are unknown. Here, we examine indices of nestling body condition and innate immunocompetence in relationship to inbreeding and disease mortality. Using an estimate of microsatellite heterozygosity that predicts inbreeding in this population, we show that inbred crows were in relatively poor condition as nestlings, and that body condition index measured in the first 2-33 days after hatching, in addition to inbreeding index, predicted disease probability in the first 34 months of life. Inbred nestlings also mounted a weaker response along one axis of innate immunity: the proportion of bacteria killed in a microbiocidal assay increased as heterozygosity index increased. Relatively poor body condition and low innate immunocompetence are two mechanisms that might predispose inbred crows to ultimate disease mortality. A better understanding of condition-mediated inbreeding depression can guide efforts to minimize disease costs of inbreeding in small populations. | 19 | no | individual level | NA | no | no |
| 20 | 2017 | 43004 | Nichols, HJ; Cant, MA; Hoffman, JI; Sanderson, JL | 2014 | Evidence for frequent incest in a cooperatively breeding mammal | As breeding between relatives often results in inbreeding depression, inbreeding avoidance is widespread in the animal kingdom. However, inbreeding avoidance may entail fitness costs. For example, dispersal away from relatives may reduce survival. How these conflicting selection pressures are resolved is challenging to investigate, but theoretical models predict that inbreeding should occur frequently in some systems. Despite this, few studies have found evidence of regular incest in mammals, even in social species where relatives are spatio-temporally clustered and opportunities for inbreeding frequently arise. We used genetic parentage assignments together with relatedness data to quantify inbreeding rates in a wild population of banded mongooses, a cooperatively breeding carnivore. We show that females regularly conceive to close relatives, including fathers and brothers. We suggest that the costs of inbreeding avoidance may sometimes outweigh the benefits, even in cooperatively breeding species where strong within-group incest avoidance is considered to be the norm. | 20 | no | NA | NA | no | no |
| 21 | 2017 | 43004 | Johnson, HE; Mills, LS; Wehausen, JD; Stephenson, TR; Luikart, G | 2011 | Translating Effects of Inbreeding Depression on Component Vital Rates to Overall Population Growth in Endangered Bighorn Sheep | Evidence of inbreeding depression is commonly detected from the fitness traits of animals, yet its effects on population growth rates of endangered species are rarely assessed. We examined whether inbreeding depression was affecting Sierra Nevada bighorn sheep (Ovis canadensis sierrae), a subspecies listed as endangered under the U.S. Endangered Species Act. Our objectives were to characterize genetic variation in this subspecies; test whether inbreeding depression affects bighorn sheep vital rates (adult survival and female fecundity); evaluate whether inbreeding depression may limit subspecies recovery; and examine the potential for genetic management to increase population growth rates. Genetic variation in 4 populations of Sierra Nevada bighorn sheep was among the lowest reported for any wild bighorn sheep population, and our results suggest that inbreeding depression has reduced adult female fecundity. Despite this population sizes and growth rates predicted from matrix-based projection models demonstrated that inbreeding depression would not substantially inhibit the recovery of Sierra Nevada bighorn sheep populations in the next approximately 8 bighorn sheep generations (48 years). Furthermore, simulations of genetic rescue within the subspecies did not suggest that such activities would appreciably increase population sizes or growth rates during the period we modeled (10 bighorn sheep generations, 60 years). Only simulations that augmented the Mono Basin population with genetic variation from other subspecies, which is not currently a management option, predicted significant increases in population size. Although we recommend that recovery activities should minimize future losses of genetic variation, genetic effects within these endangered populationseither negative (inbreeding depression) or positive (within subspecies genetic rescue)appear unlikely to dramatically compromise or stimulate short-term conservation efforts. The distinction between detecting the effects of inbreeding depression on a component vital rate (e.g., fecundity) and the effects of inbreeding depression on population growth underscores the importance of quantifying inbreeding costs relative to population dynamics to effectively manage endangered populations. | 21 | no | no good fitness measure (they use fecundity) and difference between groups? | NA | no | no |
| 22 | 2017 | 43004 | HARTT, L; HAEFNER, JW | 1995 | INBREEDING DEPRESSION EFFECTS ON EXTINCTION TIME IN A PREDATOR-PREY SYSTEM | Traditional methods of assessing population viability ignore both genetic-demographic interactions as well as community level dynamics. We address these deficiencies by presenting a model that investigates the effects of predation on a prey population experiencing inbreeding depression. Beginning with a simple Lotka-Volterra predator-prey system, we rewrite prey per capita mortality as a function of inbreeding. Inbreeding varies as a function of population size. Using computer simulation, we find that prey extinction times are inversely related to the level of inbreeding depression with and without predation. For all but very low levels of inbreeding depression, predation appreciably reduces persistence time. At moderate levels of inbreeding, predators go extinct before prey. When migration is introduced at low and moderate rates, persistence times only improve for those populations with low inbreeding depression measures. At a higher migration rate, persistence times are lengthened for low and moderately depressed prey populations. Increasing birth rates produce a visible, though noisy, trend towards increased times to extinction for low to moderate levels of inbreeding. | 22 | no | NA | NA | no | no |
| 23 | 2017 | 43004 | Marshall, TC; Spalton, JA | 2000 | Simultaneous inbreeding and outbreeding depression in reintroduced Arabian oryx | In most species the offspring of closely related parents have reduced fitness compared with the offspring of unrelated parents, a phenomenon known as inbreeding depression. However if parents are very distantly related, their offspring may also have reduced fitness. This pattern, outbreeding depression, has been most commonly observed in plants and only rarely in animals. Here we examine the consequences of inbreeding and outbreeding on juvenile survival of reintroduced Arabian oryx (Oryx leucoryx) in Oman, a population with a small number of founders drawn from a number of sources. Using microsatellite-based measures of inbreeding and outbreeding, there was no apparent relationship between inbreeding or outbreeding and survival when inbreeding and outbreeding were tested in separate statistical models. However when inbreeding and outbreeding were tested in the same statistical model, we found simultaneous inbreeding depression and outbreeding depression acting on juvenile survival. Outbreeding depression may be more common in vertebrates than previously supposed, and conservation strategies that seek to maximize the genetic diversity of managed populations may risk mixing lineages that are sufficiently differentiated to cause outbreeding depression among descendants. | 23 | no | NA | NA | no | no |
| 24 | 2017 | 43004 | Halverson, MA; Skelly, DK; Caccone, A | 2006 | Inbreeding linked to amphibian survival in the wild but not in the laboratory | We examined the effects of inbreeding on the performance of wood frog (Rana sylvatica) larvae in the field and in the laboratory. We used microsatellite analysis to establish the parentage and degree of inbreeding of the larvae. Two different estimators of inbreeding were used. The first was based on average multilocus heterozygosity, and the second was based on a molecular relatedness estimator. The estimators were highly correlated, and both showed a significant negative relationship between inbreeding and survival in the wild. However, there was no evidence that inbreeding influenced growth or development in the wild. Neither was there any evidence that inbreeding affected survival, growth, or development in the laboratory. These results suggest that, for wood frogs, inbreeding has a bigger effect on fitness in the wild than in captivity and that measurements of survival are more sensitive than measures of growth or development. | 24 | no | NA | NA | no | no |
| 25 | 2017 | 43004 | Overall, ADJ; Byrne, KA; Pilkington, JG; Pemberton, JM | 2005 | Heterozygosity, inbreeding and neonatal traits in Soay sheep on St Kilda | We investigated whether birth weight and neonatal survival, a period within which 24% of all mortalities occur, were correlated with levels of inbreeding in St Kilda Soay sheep, using pedigree inbreeding coefficients and four marker-based estimators of inbreeding. None of the inbreeding estimators, either of the offspring, or of their mothers, explained significant variation in a lamb’s birth weight or probability of surviving the neonatal period, suggesting low inbreeding depression for these traits. We evaluated the correlation between the marker-based measures of inbreeding and inbreeding coefficients obtained from the Soay pedigree, where paternal links were inferred using the same panel of microsatellite markers. Even when using a relatively complete portion of the pedigree, in which all individuals had known maternal and paternal grandparents, the correlation was found to be weak (r = -0.207, where mean f = 0.0168). These results add support to the recent prediction that when the mean and variance in inbreeding are low in a population, heterozygosity-fitness correlations can be very weak or even undetectable. The pursuit of more detailed pedigrees offers the best prospect for identifying inbreeding depression within this study population. | 25 | no | NA | NA | no | no |
| 26 | 2017 | 43004 | Heber, S; Varsani, A; Kuhn, S; Girg, A; Kempenaers, B; Briskie, J | 2013 | The genetic rescue of two bottlenecked South Island robin populations using translocations of inbred donors | Populations forced through bottlenecks typically lose genetic variation and exhibit inbreeding depression. ‘Genetic rescue’ techniques that introduce individuals from outbred populations can be highly effective in reversing the deleterious effects of inbreeding, but have limited application for the majority of endangered species, which survive only in a few bottlenecked populations. We tested the effectiveness of using highly inbred populations as donors to rescue two isolated and bottlenecked populations of the South Island robin (Petroica australis). Reciprocal translocations significantly increased heterozygosity and allelic diversity. Increased genetic diversity was accompanied by increased juvenile survival and recruitment, sperm quality, and immunocompetence of hybrid individuals (crosses between the two populations) compared with inbred control individuals (crosses within each population). Our results confirm that the implementation of ‘genetic rescue’ using bottlenecked populations as donors provides a way of preserving endangered species and restoring their viability when outbred donor populations no longer exist. | 26 | no | NA | NA | no | no |
| 27 | 2017 | 43004 | Ilmonen, P; Penn, DJ; Damjanovich, K; Clarke, J; Lamborn, D; Morrison, L; Ghotbi, L; Potts, WK | 2008 | Experimental infection magnifies inbreeding depression in house mice | It is often assumed that inbreeding reduces resistance to pathogens, yet there are few experimental tests of this idea in vertebrates, and no tests for the effects of moderate levels of inbreeding more commonly found in nature. We mated wild-derived mice with siblings or first cousins and compared the resistance of their offspring to Salmonella infection with outbred controls under laboratory and seminatural conditions. In the laboratory, full-sib inbreeding reduced resistance to Salmonella and survivorship, whereas first-cousin inbreeding had no detectable effects. In competitive population enclosures, we found that first-cousin inbreeding reduced male fitness by 57% in infected vs. only 34% in noninfected control populations. Our study provides experimental evidence that inbreeding reduces resistance and ability to survive pathogenic infection, and moreover, it shows that even moderate inbreeding can cause significant fitness declines under naturalistic conditions of social stress, and especially with exposure to infectious agents. | 27 | no | individual level | NA | no | no |
| 28 | 2017 | 43004 | Townsend, AK; Clark, AB; McGowan, KJ; Buckles, EL; Miller, AD; Lovette, IJ | 2009 | Disease-mediated inbreeding depression in a large, open population of cooperative crows | Disease-mediated inbreeding depression is a potential cost of living in groups with kin, but its general magnitude in wild populations is unclear. We examined the relationships between inbreeding, survival and disease for 312 offspring, produced by 35 parental pairs, in a large, open population of cooperatively breeding American crows (Corvus brachyrhynchos). Genetic analyses of parentage, parental relatedness coefficients and pedigree information suggested that 23 per cent of parental dyads were first- or second-order kin. Heterozygosity-heterozygosity correlations suggested that a microsatellite-based index of individual heterozygosity predicted individual genome-wide heterozygosity in this population. After excluding birds that died traumatically, survival probability was lower for relatively inbred birds during the 2-50 months after banding: the hazard rate for the most inbred birds was 170 per cent higher than that for the least inbred birds across the range of inbreeding index values. Birds that died with disease symptoms had higher inbreeding indices than birds with other fates. Our results suggest that avoidance of close inbreeding and the absence of inbreeding depression in large, open populations should not be assumed in taxa with kin-based social systems, and that microsatellite-based indices of individual heterozygosity can be an appropriate tool for examining the inbreeding depression in populations where incest and close inbreeding occur. | 28 | no | only one pop | NA | no | no |
| 29 | 2017 | 43004 | EGAN, VT; GRANT, WS | 1993 | BREEDING THE STRIPED PUFF ADDER BITIS-ARIETANS - INBREEDING AVOIDANCE | Inbreeding, the loss of genetic variability, is the most important genetic consideration in the propagation of small populations. The loss of vigour, reduced fecundity or decreased survival are often consequences of inbreeding and may lead to the extinction of a population or race. There is now a captive population of a rare, striped morph of the puff adder that originated from a single mating, and the avoidance of inbreeding is uppermost in devising a successful breeding strategy to propagate this commercially-valuable variant. We outline a breeding programme that produces true-breeding striped puff adders without increasing levels of inbreeding. | 29 | no | NA | NA | no | no |
| 30 | 2017 | 43004 | Woods, JH; Wang, T; Aitken, SN | 2002 | Effects of inbreeding on coastal Douglas-fir: Nursery performance | In advanced generation seed orchards, low levels of inbreeding may be inevitable as relatedness among individuals in breeding populations increases with each generation. Unlike selfing, low level inbreeding can produce relatively large number of viable seeds. Following previous study on the effects of inbreeding on coastal Douglas-fir (Pseudotsuga menziesii var. menziesii) filled seed production, the present study investigated inbreeding on nursery performance over various crosstypes, including outcrosses (inbreeding coefficient F=0), crosses between half-sibs (F=0.125), between full-sibs (F=0.25), between parents and offspring (F=0.25), and selfing (F=0.5). Significant differences were found among cross-types for germination, seedling mortality, seedling diameter and height, and nursery cull rate. Inbreeding also increased among-family genetic variability. Cumulative losses of seedlings at the nursery stage were 18, 33, 31, 36 and 43%, respectively for the above types of crosses. This result indicates that seeds with low levels of inbreeding may produce relatively large numbers of seedlings that meet nursery culling standards and could be used for reforestation, resulting in negative impacts on the genetic gain realized in field plantations. | 30 | no | individual level? | NA | no | no |
| 31 | 2017 | 43004 | Noren, K; Godoy, E; Dalen, L; Meijer, T; Angerbjorn, A | 2016 | Inbreeding depression in a critically endangered carnivore | Harmful effects arising from matings between relatives (inbreeding) is a long-standing observation that is well founded in theory. Empirical evidence for inbreeding depression in natural populations is however rare because of the challenges of assembling pedigrees supplemented with fitness traits. We examined the occurrence of inbreeding and subsequent inbreeding depression using a unique data set containing a genetically verified pedigree with individual fitness traits for a critically endangered arctic fox (Vulpes lagopus) population. The study covered nine years and was comprised of 33 litters with a total of 205 individuals. We recorded that the present population was founded by only five individuals. Over the study period, the population exhibited a tenfold increase in average inbreeding coefficient with a final level corresponding to half-sib matings. Inbreeding mainly occurred between cousins, but we also observed two cases of full-sib matings. The pedigree data demonstrated clear evidence of inbreeding depression on traditional fitness traits where inbred individuals displayed reduced survival and reproduction. Fitness traits were however differently affected by the fluctuating resource abundande. Inbred individuals born at low-quality years displayed reduced first-year survival, while inbred individuals born at high-quality years were less likely to reproduce. The documentation of inbreeding depression in fundamental fitness traits suggests that inbreeding depression can limit population recovery. Introducing new genetic material to promote a genetic rescue effect may thus be necessary for population long-term persistence. | 31 | no | only one pop | NA | no | no |
| 32 | 2017 | 43004 | Kanthaswamy, S; Smith, DG | 2002 | Assessment of genetic management at three specific-pathogen-free rhesus macaque (Macaca mulatta) colonies | Genetic management is required to maintain genetic diversity by minimizing inbreeding and genetic subdivision in colonies of animals bred for biomedical research. Polymorphic short tandem repeat (STR) loci are useful for genetic management because they facilitate parentage assignments, genetic characterization of individuals, and estimates of baseline population genetic parameters. Using highly informative STR loci, we estimated gene diversity and F-statistics to determine the level of genetic heterogeneity and genetic structure of three specific-pathogen-free (SPF) rhesus macaque (Macaca mulatta) colonies. Effective population sizes, variance in male reproductive success, and rate of decrease in genetic variability also were estimated for two of the three colonies. We documented the overall success of genetic management in maintaining genetic diversity in captive colonies. We report that even genetically managed SPF colonies, despite maintaining high and stable levels of gene diversity (over 0.75), are prone to genetic subdivision due to different management strategies, founder effects, genetic isolation, and drift. These processes are accelerated by the high variances in male reproductive success and low adult sex ratios that are typical of captive rhesus macaque breeding groups, both of which reduce the effective population sizes of these groups. | 32 | no | no good fitness measure | NA | no | no |
| 33 | 2017 | 43004 | Agnarsson, I; Aviles, L; Maddison, WP | 2013 | Loss of genetic variability in social spiders: genetic and phylogenetic consequences of population subdivision and inbreeding | 8 The consequences of population subdivision and inbreeding have been studied in many organisms, particularly in plants. However, most studies focus on the short-term consequences, such as inbreeding depression. To investigate the consequences of both population fragmentation and inbreeding for genetic variability in the longer term, we here make use of a natural inbreeding experiment in spiders, where sociality and accompanying population subdivision and inbreeding have evolved repeatedly. We use mitochondrial and nuclear data to infer phylogenetic relationships among 170 individuals of Anelosimus spiders representing 23 species. We then compare relative mitochondrial and nuclear genetic variability of the inbred social species and their outbred relatives. We focus on four independently derived social species and four subsocial species, including two outbredinbred sister species pairs. We find that social species have 50% reduced mitochondrial sequence divergence. As inbreeding is not expected to reduce genetic variability in the maternally inherited mitochondrial genome, this suggests the loss of variation due to strong population subdivision, founder effects, small effective population sizes (colonies as individuals) and lineage turnover. Social species have < 10% of the nuclear genetic variability of the outbred species, also suggesting the loss of genetic variability through founder effects and/or inbreeding. Inbred sociality hence may result in reduction in variability through various processes. Sociality in most Anelosimus species probably arose relatively recently (0.12 mya), with even the oldest social lineages having failed to diversify. This is consistent with the hypothesis that inbred spider sociality represents an evolutionary dead end. Heterosis underlies a species potential to respond to environmental change and/or disease. Inbreeding and loss of genetic variability may thus limit diversification in social Anelosimus lineages and similarly pose a threat to many wild populations subject to habitat fragmentation or reduced population sizes. | 33 | no | NA | NA | no | no |
| 34 | 2017 | 43004 | Ilmonen, P; Stundner, G; Thoss, M; Penn, DJ | 2009 | Females prefer the scent of outbred males: good-genes-as-heterozygosity? | Background: There is increasing interest to determine the relative importance of non-additive genetic benefits as opposed to additive ones for the evolution of mating preferences and maintenance of genetic variation in sexual ornaments. The ‘good-genes-as-heterozygosity’ hypothesis predicts that females should prefer to mate with more heterozygous males to gain more heterozygous (and less inbred) offspring. Heterozygosity increases males’ sexual ornamentation, mating success and reproduction success, yet few experiments have tested whether females are preferentially attracted to heterozygous males, and none have tested whether females’ own heterozygosity influences their preferences. Outbred females might have the luxury of being more choosey, but on the other hand, inbred females might have more to gain by mating with heterozygous males. We manipulated heterozygosity in wild-derived house mice (Mus musculus musculus) through inbreeding and tested whether the females are more attracted to the scent of outbred versus inbred males, and whether females’ own inbreeding status affects their preferences. We also tested whether infecting both inbred and outbred males with Salmonella would magnify females’ preferences for outbred males. Results: Females showed a significant preference for outbred males, and this preference was more pronounced among inbred females. We found no evidence that Salmonella infection increased the relative attractiveness of outbred versus inbred males; however, we found no evidence that inbreeding affected males’ disease resistance in this study. Conclusion: Our findings support the idea that females are more attracted to outbred males, and they suggest that such preferences may be stronger among inbred than outbred females, which is consistent with the ‘good-genes-as-heterozygosity’ hypothesis. It is unclear whether this odour preference reflects females’ actual mating preferences, though it suggests that future studies should consider females’ as well as males’ heterozygosity. Our study has implications for efforts to understand how mate choice can provide genetic benefits without eroding genetic diversity (lek paradox), and also conservation efforts to determine the fitness consequences of inbreeding and the maintenance of genetic diversity in small, inbred populations. | 34 | no | NA | NA | no | no |
| 35 | 2017 | 43004 | Townsend, SM; Jamieson, IG | 2013 | INBREEDING INFLUENCES WITHIN-BROOD HETEROZYGOSITY-FITNESS CORRELATIONS (HFCS) IN AN ISOLATED PASSERINE POPULATION | Molecular estimates of inbreeding may be made using genetic markers such as microsatellites, however the interpretation of resulting heterozygosity-fitness correlations (HFCs) with respect to inbreeding depression is not straightforward. We investigated the relationship between pedigree-determined inbreeding coefficients (f) and HFCs in a closely monitored, reintroduced population of Stewart Island robins (Petroica australis rakiura) on Ulva Island, New Zealand. Using a full sibling design, we focused on differences in juvenile survival associated specifically with individual sibling variation in standardized multilocus heterozygosity (SH) when expected f was identical. We found that within broods, siblings with higher SH at microsatellite loci experienced a higher probability of juvenile survival. This effect, however, was detected primarily within broods that experienced inbreeding or when inbreeding had occurred in their pedigree histories (i.e., at the parents’ level). Thus we show, for the first time in a wild population, that the strength of an HFC is partially dependent on the presence of inbreeding events in the recent pedigree history. Our results illustrate the importance of realized effects of inbreeding on genetic variation and fitness and the value of full-sibling designs for the study of HFCs in the context of small, inbred populations. | 35 | no | only one pop | NA | no | no |
| 36 | 2017 | 43004 | Cena, CJ; Morgan, GE; Malette, MD; Heath, DD | 2006 | Inbreeding, outbreeding and environmental effects on genetic diversity in 46 walleye (Sander vitreus) populations | Genetic diversity is recognized as an important population attribute for both conservation and evolutionary purposes; however, the functional relationships between the environment, genetic diversity, and fitness-related traits are poorly understood. We examined relationships between selected lake parameters and population genetic diversity measures in 46 walleye (Sander vitreus) populations across the province of Ontario, Canada, and then tested for relationships between six life history traits (in three categories: growth, reproductive investment, and mortality) that are closely related to fitness, and genetic diversity measures (heterozygosity, d(2), and Wright’s inbreeding coefficient). Positive relationships were observed between lake surface area, growing degree days, number of species, and hatchery supplementation versus genetic diversity. Walleye early growth rate was the only life history trait significantly correlated with population heterozygosity in both males and females. The relationship between F-IS and male early growth rate was negative and significant (P < 0.01) and marginally nonsignificant for females (P = 0.06), consistent with inbreeding depression effects. Only one significant relationship was observed for d(2): female early growth rate (P < 0.05). Stepwise regression models showed that surface area and heterozygosity had a significant effect on female early growth rate, while hatchery supplementation, surface area and heterozygosity had a significant effect on male early growth rate. The strong relationship between lake parameters, such as surface area, and hatchery supplementation, versus genetic diversity suggests inbreeding and outbreeding in some of the populations; however, the weak relationships between genetic diversity and life history traits indicate that inbreeding and outbreeding depression are not yet seriously impacting Ontario walleye populations. | 36 | yes | NA | inbreeding | no | no |
| 37 | 2017 | 43004 | Weyrauch, SL; Grubb, TC | 2006 | Effects of the interaction between genetic diversity and UV-B radiation on wood frog fitness | Genetic diversity may buffer amphibian populations against environmental vicissitudes. We hypothesized that wood frogs (Rana sylvatica) from populations with lower genetic diversity are more susceptible to ultraviolet-B (UV-B) radiation than those from populations with higher diversity. We used RAPD markers to obtain genetic diversity estimates for 12 wood frog populations. We reared larval wood frogs from these populations and exposed experimental groups of eggs and larvae to one of three treatments: unfiltered sunlight, sunlight filtered through a UV-B-blocking filter (Mylar), and sunlight filtered through a UV-B-transmitting filter (acetate). In groups exposed to UV-B, larval mortality and deformity rates increased significantly, but egg mortality did not We found a significant negative relationship between genetic diversity and egg mortality, larval mortality, and deformity rates. Furthermore, the interaction between UV-B treatment and genetic diversity significantly affected larval mortality. Populations with low genetic diversity experienced higher larval mortality rates when exposed to UV-B than did populations with high genetic diversity. This is the first time an interaction between genetic diversity and an environmental stressor has been documented in amphibians. Differences in genetic diversity among populations, coupled with environmental stressors, may help explain patterns of amphibian decline. | 37 | yes | effects by environmental effects as well! | NA | no | yes |
| 38 | 2017 | 43004 | Vilas, C; San Miguel, E; Amaro, R; Garcia, C | 2006 | Relative contribution of inbreeding depression and eroded adaptive diversify to extinction risk in small populations of shore Campion | To study the relative importance of inbreeding depression and the loss of adaptive diversity in determining the extinction risk of small populations, we carried out an experiment in which we crossed and self-fertilized founder plants from a single, large population of shore campion (Silene littorea Brot). We used the seeds these plants produced to colonize 18 new locations within the distribution area of the species. The reintroduced populations were of three kinds: inbred and genetically homogeneous, each made up of selfed seed from a single plant; inbred and mixed, made up of a mixture of selfed seeds from all founder plants; and outbred and mixed, made up of a mixture of seeds obtained in outcrosses between the founders. We compared the inbred homogeneous populations with the inbred mixed to measure the effect of genetic diversity among individuals and the inbred mixed with the outbred mixed to measure the effect of inbreeding. Reintroduction success was seriously limited by inbreeding, whereas it was not affected by genetic diversity. This observation and the nonsignificant interaction between family and reintroduction location for individual plant characters suggest that the fixation of overall deleterious genes causing inbreeding depression posed a snore serious threat to the short-term survival of the populations than the loss of genes involved in genotype and environment interactions. Thus, reintroduction success was related to adaptive diversity. Preventing such fixation might be the most important consideration in the genetic management and conservation of shore campion populations. | 38 | probably yes | “comparison bewteen”“inbred”" with "“inbred mixed”“?” | inbreeding | no | no |
| 39 | 2017 | 43004 | He, TH; Lamont, BB | 2010 | High microsatellite genetic diversity fails to predict greater population resistance to extreme drought | There is intense debate whether genetic diversity measured via neutral molecular markers can be used as a surrogate for fitness and as an indirect estimate of the amount of genetic variation for fitness-related traits in a population. Here, we measured microsatellite DNA genetic diversity (before the onset of drought) and mortality after prolonged drought in 15 populations of Banksia hookeriana in the species-rich southwestern Australian flora, to test the relationship between population genetic diversity and resistance to extreme climate fluctuations. Number of alleles per locus varied from 5.2 to 8.2 at eleven microsatellite loci among 30 individuals in each population. Mortality varied from 25 to 50% in individual populations after prolonged drought. Lower mortality was not observed in populations with higher genetic diversity, but in populations with lower genetic diversity. Thus, higher microsatellite genetic diversity fails to predict lower population mortality during extreme drought in B. hookeriana. Our results imply that it may be misleading to use studies of neutral genetic variation exclusively as the basis for inferring population and species capacity for resisting extreme climate events and for species conservation and management decisions. | 39 | yes | of interest | NA | yes | yes |
| 40 | 2017 | 43004 | Wang, T; Aitken, SN; Woods, JH; Polsson, K; Magnussen, S | 2004 | Effects of inbreeding on coastal Douglas fir growth and yield in operational plantations: a model-based approach | In advanced generation seed orchards, tradeoffs exist between genetic gain obtained by selecting the best related individuals for seed orchard populations, and potential losses due to subsequent inbreeding between these individuals. Although inbreeding depression for growth rate is strong in most forest tree species at the individual tree level, the effect of a small proportion of inbreds in seed lots on final stand yield may be less important. The effects of inbreeding on wood production of mature stands cannot be assessed empirically in the short term, thus such effects were simulated for coastal Douglas fir [Pseudotsuga menziesii var. menziesii (Mirb.) Franco] using an individual-tree growth and yield model TASS (Tree and Stand Simulator). The simulations were based on seed set, nursery culling rates, and 10-year-old field test performance for trees resulting from crosses between unrelated individuals and for inbred trees produced through mating between half-sibs, full-sibs, parents and offspring and self-pollination. Results indicate that inclusion of a small proportion of related clones in seed orchards will have relatively low impacts on stand yields due to low probability of related individuals mating, lower probability of producing acceptable seedlings from related matings than from unrelated matings, and a greater probability of competition-induced mortality for slower growing inbred individuals than for outcrossed trees. Thus, competition reduces the losses expected due to inbreeding depression at harvest, particularly on better sites with higher planting densities and longer rotations. Slightly higher breeding values for related clones than unrelated clones would offset or exceed the effects of inbreeding resulting from related matings. Concerns regarding the maintenance of genetic diversity are more likely to limit inclusion of related clones in orchards than inbreeding depression for final stand yield. | 40 | no | NA | NA | no | no |
| 41 | 2017 | 43004 | Charpentier, MJE; Williams, CV; Drea, CM | 2008 | Inbreeding depression in ring-tailed lemurs (Lemur catta): genetic diversity predicts parasitism, immunocompetence, and survivorship | The consequences of inbreeding have been well studied in a variety of taxa, revealing that inbreeding has major negative impacts in numerous species, both in captivity and in the wild; however, as trans-generational health data are difficult to obtain for long-lived, free-ranging species, similar analyses are generally lacking for nonhuman primates. Here, we examined the long-term effects of inbreeding on numerous health estimates in a captive colony of ring-tailed lemurs (Lemur catta), housed under semi-natural conditions. This vulnerable strepsirrhine primate is endemic to Madagascar, a threatened hotspot of biodiversity; consequently, this captive population represents an important surrogate. Despite significant attention to maintaining the genetic diversity of captive animals, breeding colonies invariably suffer from various degrees of inbreeding. We used neutral heterozygosity as an estimate of inbreeding and showed that our results reflect genome-wide inbreeding, rather than local genetic effects. In particular, we found that genetic diversity affects several fitness correlates, including the prevalence and burden of Cuterebra parasites and a third (N = 6) of the blood parameters analyzed, some of which reflect immunocompetence. As a final validation of inbreeding depression in this captive colony, we showed that, compared to outbred individuals, inbred lemurs were more likely to die earlier from diseases. Through these analyses, we highlight the importance of monitoring genetic variation in captive animals-a key objective for conservation geneticists-and provide insight into the potential negative consequences faced by small or isolated populations in the wild. | 41 | no | only one pop | NA | no | no |
| 42 | 2017 | 43004 | Whiteman, NK; Matson, KD; Bollmer, JL; Parker, PG | 2006 | Disease ecology in the Galapagos Hawk (Buteo galapagoensis): host genetic diversity, parasite load and natural antibodies | An increased susceptibility to disease is one hypothesis explaining how inbreeding hastens extinction in island endemics and threatened species. Experimental studies show that disease resistance declines as inbreeding increases, but data from in situ wildlife systems are scarce. Genetic diversity increases with island size across the entire range of an extremely inbred Galapagos endemic bird, providing the context for a natural experiment examining the effects of inbreeding on disease susceptibility. Extremely inbred populations of Galapagos hawks had higher parasite abundances than relatively outbred populations. We found a significant island effect on constitutively produced natural antibody (NAb) levels and inbred populations generally harboured lower average and less variable NAb levels than relatively outbred populations. Furthermore, NAb levels explained abundance of amblyceran lice, which encounter the host immune system. This is the first study linking inbreeding, innate immunity and parasite load in an endemic, in situ wildlife population and provides a clear framework for assessment of disease risk in a Galapagos endemic. | 42 | yes | from thesis | no good gd measure | no | no |
| 43 | 2017 | 43004 | Aguirre, JD; Marshall, DJ | 2012 | Does genetic diversity reduce sibling competition? | An enduring hypothesis for the proximal benefits of sex is that recombination increases the genetic variation among offspring and that this genetic variation increases offspring performance. A corollary of this hypothesis is that mothers that mate multiply increase genetic variation within a clutch and gain benefits due to genetic diversity alone. Many studies have demonstrated that multiple mating can increase offspring performance, but most attribute this increase to sexual selection and the role of genetic diversity has received less attention. Here, we used a breeding design to generate populations of full-siblings, half-siblings, and unrelated individuals of the solitary ascidian Ciona intestinalis. Importantly, we preclude the potentially confounding influences of maternal effects and sexual selection. We found that individuals in populations with greater genetic diversity had greater performance (metamorphic success, postmetamorphic survival, and postmetamorphic size) than individuals in populations with lower genetic diversity. Furthermore, we show that by mating with multiple males and thereby increasing genetic variation within a single clutch of offspring, females gain indirect fitness benefits in the absence of mate-choice. Our results show that when siblings are likely to interact, genetic variation among individuals can decrease competition for resources and generate substantial fitness benefits within a single generation. | 43 | yes | yes | NA | yes | yes |
| 44 | 2017 | 43004 | Semaan, MT; Dodd, RS | 2008 | Genetic variability and structure of the remnant natural populations of Cedrus libani (Pinaceae) of Lebanon | Cedrus libani of Lebanon is a valuable natural resource and the dominant species in its natural ecosystem. Intense and diverse anthropogenic pressures over historical times raised concerns about its genetic vigor and continued survival. Our investigation of the genetic diversity included samples from all remnant natural populations. Assessment of the genetic diversity using random amplified polymorphic DNA markers revealed the persistence of considerable variation distributed within populations with low population differentiation corroborated by Bayesian and analysis of molecular variance estimates (G(ST) = 0.07, Phi(ST) = 0.09). Individual assignment tests were carried out to investigate measures of gene flow. Inferences concluded that this natural heritage is not currently threatened by inbreeding or by random genetic drift. Correlation studies investigated possible effects of spatial distribution and environmental conditions on genetic structure. A climatic trend corresponding to a temperature-humidity gradient correlated significantly with the level of genetic diversity, while the edaphic variation did not. | 44 | no | NA | NA | no | no |
| 45 | 2017 | 43004 | Mainguy, J; Cote, SD; Coltman, DW | 2009 | Multilocus heterozygosity, parental relatedness and individual fitness components in a wild mountain goat, Oreamnos americanus population | Matings between relatives lead to a decrease in offspring genetic diversity which can reduce fitness, a phenomenon known as inbreeding depression. Because alpine ungulates generally live in small structured populations and often exhibit a polygynous mating system, they are susceptible to inbreeding. Here, we used marker-based measures of pairwise genetic relatedness and inbreeding to investigate the fitness consequences of matings between relatives in a long-term study population of mountain goats (Oreamnos americanus) at Caw Ridge, Alberta, Canada. We first assessed whether individuals avoided mating with kin by comparing actual and random mating pairs according to their estimated genetic relatedness, which was derived from 25 unlinked polymorphic microsatellite markers and reflected pedigree relatedness. We then examined whether individual multilocus heterozygosity H, used as a measure of inbreeding, was predicted by parental relatedness and associated with yearling survival and the annual probability of giving birth to a kid in adult females. Breeding pairs identified by genetic parentage analyses of offspring that survived to 1 year of age were less genetically related than expected under random matings. Parental relatedness was negatively correlated with offspring H, and more heterozygous yearlings had higher survival to 2 years of age. The probability of giving birth was not affected by H in adult females. Because kids that survived to yearling age were mainly produced by less genetically related parents, our results suggest that some individuals experienced inbreeding depression in early life. Future research will be required to quantify the levels of gene flow between different herds, and evaluate their effects on population genetic diversity and dynamics. | 45 | no | only one pop | NA | no | no |
| 46 | 2017 | 43004 | Boakes, EH; Wang, J; Amos, W | 2007 | An investigation of inbreeding depression and purging in captive pedigreed populations | We use regression models to investigate the effects of inbreeding in 119 zoo populations, encompassing 88 species of mammals, birds, reptiles and amphibians. Meta-analyses show that inbreeding depression for neonatal survival was significant across the 119 populations although the severity of inbreeding depression appears to vary among taxa. However, few predictors of a population’s response to inbreeding are found reliable. The models are most likely to detect inbreeding depression in large populations, that is, in populations in which their statistical power is maximised. Purging was found to be significant in 14 populations and a significant trend of purging was found across populations. The change in inbreeding depression due to purging averaged across the 119 populations is < 1%, however, suggesting that the fitness benefits of purging are rarely appreciable. The study re-emphasises the necessity to avoid inbreeding in captive breeding programmes and shows that purging cannot be relied upon to remove deleterious alleles from zoo populations. | 46 | no | meta-analysis om zoo-animals, inbreedning drepression on neonatal survival | NA | no | no |
| 47 | 2017 | 43004 | Calleri, DV; Reid, EM; Rosengaus, RB; Vargo, EL; Traniello, JFA | 2006 | Inbreeding and disease resistance in a social insect: effects of heterozygosity on immnnocompetence in the termite Zootermopsis angusticollis | Recent research has shown that low genetic variation in individuals can increase susceptibility to infection and group living may exacerbate pathogen transmission. In the eusocial diploid termites, cycles of outbreeding and inbreeding characterizing basal species can reduce genetic variation within nestmates during the life of a colony, but the relationship of genetic heterogeneity to disease resistance is poorly understood. Here we show that, one generation of inbreeding differentially affects the survivorship of isolated and grouped termites (Zootermopsis angusticollis) depending on the nature of immune challenge and treatment. Inbred and outbred isolated and grouped termites inoculated with a bacterial pathogen, exposed to a low dose of fungal pathogen or challenged with an implanted nylon monofilament had similar levels of immune defence. However, inbred grouped termites exposed to a relatively high concentration of fungal conidia had significantly greater mortality than outbred grouped termites. Inbred termites also had significantly higher cuticular microbial loads, presumably due to less effective grooming by nestmates. Genetic analyses showed that inbreeding significantly reduced heterozygosity and allelic diversity. Decreased heterozygosity thus appeared to increase disease susceptibility by affecting social behaviour or some other group-level process influencing infection control rather than affecting individual immune physiology. | 47 | no | inbreeding | NA | no | no |
| 48 | 2017 | 43004 | Brekke, P; Wang, JL; Bennett, PM; Cassey, P; Dawson, DA; Horsburgh, GJ; Ewen, JG | 2012 | Postcopulatory mechanisms of inbreeding avoidance in the island endemic hihi (Notiomystis cincta) | Avoiding genetic incompatibility resulting from inbreeding is thought to be one of the main drivers of mate choice, promiscuity, and sexual conflict. Inbreeding avoidance has been found across a wide range of taxa and is predicted to be adaptive when the costs of inbreeding outweigh the benefits. This study tests the inbreeding avoidance hypothesis at the precopulatory and postcopulatory stages in a natural population of the promiscuous endemic bird, the hihi. This species has high costs associated with inbreeding as it depresses offspring survival. We generate alternative predictions to explain the observed fertilization patterns based on the existence or absence of precopulatory and/or postcopulatory mechanisms of inbreeding avoidance. Nonrandom mating with respect to relatedness is found mainly at the postcopulatory stage. Interestingly, mating patterns appear opposed. There is a trend for females choosing more closely related social males than random, but postcopulatory patterns are biased toward less related extrapair males. This strategy suggests that at the precopulatory stage females may tolerate inbreeding as the costs of developing inbreeding avoidance may be high, especially in light of forced copulations, if natal dispersal is limited or if they gain inclusive fitness. However, as postcopulatory patterns are biased toward less-related individuals inclusive fitness explanations are unlikely. Postcopulatory patterns may arise if there are mechanisms such as sperm ejection or gametic compatibility such as sperm selection or biased fertility/mortality of offspring by related males. The observed patterns are likely to be an optimal compromise between the divergent selection pressures on each sex. | 48 | no | only one pop | NA | no | no |
| 49 | 2017 | 43004 | Tiira, K; Laurila, A; Peuhkuri, N; Piironen, J; Ranta, E; Primmer, CR | 2003 | Aggressiveness is associated with genetic diversity in landlocked salmon (Salmo salar) | The amount of intraindividual genetic variation has often been found to have profound effects on life history traits. However, studies concerning the relationship between behaviour and genetic diversity are scarce. Aggressiveness is an important component of competitive ability in juvenile salmonids affecting their later performance and survival. In this study, we used an experimental approach to test the prediction that juveniles with low estimated genetic diversity should be less aggressive than juveniles with high estimated genetic diversity in fry from a highly endangered population of land-locked salmon (Salmo salar). This was achieved by using a method enabling the accurate estimation of offspring genetic diversity based on parental microsatellite genotype data. This allowed us to create two groups of offspring expected to have high or low genetic diversity in which aggressive behaviour could be compared. Salmon fry with low estimated genetic diversity were significantly less aggressive than fry with high estimated genetic diversity. Closer analysis of the data suggested that this difference was due to differences in more costly acts of aggression. Our result may reflect a direct effect of genetic variation on a fitness-related trait, however, we cannot rule out an alternative explanation of allele-specific phenotype matching, where lowered aggression is expressed towards genetically more similar individuals. | 49 | no | NA | NA | no | no |
| 50 | 2017 | 43004 | Reid, JM; Arcese, P; Keller, LF; Elliott, KH; Sampson, L; Hasselquist, D | 2007 | Inbreeding effects on immune response in free-living song sparrows (Melospiza melodia) | The consequences of inbreeding for host immunity to parasitic infection have broad implications for the evolutionary and dynamical impacts of parasites on populations where inbreeding occurs. To rigorously assess the magnitude and the prevalence of inbreeding effects on immunity, multiple components of host immune response should be related to inbreeding coefficient (f) in free-living individuals. We used a pedigreed, free-living population of song sparrows (Melospiza melodia) to test whether individual responses to widely used experimental immune challenges varied consistently with f. The patagial swelling response to phytohaemagglutinin declined markedly with f in both females and males in both 2002 and 2003, although overall inbreeding depression was greater in males. The primary antibody response to tetanus toxoid declined with f in females but not in males in both 2004 and 2005. Primary antibody responses to diphtheria toxoid were low but tended to decline with f in 2004. Overall inbreeding depression did not solely reflect particularly strong immune responses in outbred offspring of immigrant-native pairings or weak responses in highly inbred individuals. These data indicate substantial and apparently sex-specific inbreeding effects on immune response, implying that inbred hosts may be relatively susceptible to parasitic infection to differing degrees in males and females. | 50 | no | only one pop | NA | no | no |
| 51 | 2017 | 43004 | Mcaliley, LR; Willis, RE; Ivanyi, C; Densmore, LD | 2016 | CAPTIVE BREEDING OF THE ENDANGERED SAN ESTEBAN CHUCKWALLA, SAUROMALUS VARIUS: EFFECTS OF A DECADE OF CAPTIVE BREEDING ON MAINTAINING GENETIC DIVERSITY | Increasingly, endangered vertebrate species are being maintained in captive breeding programs as a method of ensuring their survival. Despite these increasing numbers of captive breeding colonies, there have been few studies designed to examine the long-term success of these programs at maintaining genetic diversity. To elucidate the effectiveness of a captive breeding colony of the endangered species Sauromalus varius (the San Esteban Chuckwalla) at maintaining genetic variation over time, we employed seven polymorphic microsatellite loci. F-statistics and analysis of molecular variance strongly suggest that the colony is exhibiting genetic signs of inbreeding. Genetic variation within the colony has decreased by an average of 12.2% within the colony between the 2 y sampled with an average loss of 30.5% of genetic variation within offspring. This study provides an important test of the effectiveness of captive breeding colonies at maintaining genetic variation within a vertebrate group over time. | 51 | no | NA | NA | no | no |
| 52 | 2017 | 43004 | Ganz, HH; Ebert, D | 2010 | Benefits of host genetic diversity for resistance to infection depend on parasite diversity | Host populations with high genetic diversity are predicted to have lower levels of infection prevalence. This theory assumes that host genetic diversity results in variation in susceptibility and that parasites exhibit variation in infectivity. Empirical studies on the effects of host heterogeneity typically neglect the role of parasite diversity. We conducted three laboratory experiments designed to test if genetic variation in Daphnia magna populations and genetic variation in its parasites together influence the course of parasite spread after introduction. We found that a natural D. magna population exhibited variation in susceptibility to infection by three parasite species and had strong host clone parasite species interactions. There was no effect of host heterogeneity in experimental host populations (polycultures and monocultures) separately exposed to single strains of three parasite species. When we manipulated the genetic diversity of a single parasite species and exposed them to host monocultures and polycultures, we found that parasite prevalence increased with the number of parasite strains. Host monocultures exposed to several parasite strains had higher mean parasite prevalence and higher variance than polycultures. These results indicate that effect of host genetic diversity on the spread of infection depends on the level of genetic diversity in the parasite population. | 52 | yes | from thesis | NA | yes | yes |
| 53 | 2017 | 43004 | Van Coillie, S; Galbusera, P; Roeder, AD; Schempp, W; Stevens, JMG; Leus, K; Reinartz, G; Pereboom, Z | 2008 | Molecular paternity determination in captive bonobos and the impact of inbreeding on infant mortality | Inbreeding and the loss of genetic diversity may lower fitness and reduce the potential for a population to adapt to changing environments. In small populations, for example in captive populations or populations of endangered species, this can have considerable consequences for their survival. We investigated the effects of inbreeding on infant mortality in the world captive population of bonobos Pan paniscus. Using a combination of studbook data and high-quality pedigree data from genotyped individuals, inbreeding information was available for 142 captive-born individuals. For the determination of paternities that were unresolved in the studbook, nuclear microsatellite DNA was amplified from hair and blood samples using the Great Ape Kit and PowerPlex((R)) 16 System. In total, 54 bonobos (17 offspring and their putative parents) were genotyped at eight tetranucleotide repeat microsatellite loci. Inbreeding coefficients were calculated for each individual for whom paternity was confirmed by either studbook data or DNA analysis. We found significantly higher infant mortality in inbred offspring compared with non-inbred offspring, suggesting that inbreeding reduces infant survival in captive bonobos. In addition, we argue that the total magnitude of inbreeding depression is probably underestimated in this captive population. In conclusion, even though the breeding programme of captive bonobos is aimed at avoiding inbreeding, closely related individuals do occasionally produce offspring that do show inbreeding depression. There is, however, no indication that this currently threatens the long-time survival of the captive population of bonobos. | 53 | no | only one pop | NA | no | no |
| 54 | 2017 | 43004 | Blomqvist, D; Pauliny, A; Larsson, M; Flodin, LA | 2010 | Trapped in the extinction vortex? Strong genetic effects in a declining vertebrate population | Background: Inbreeding and loss of genetic diversity are expected to increase the extinction risk of small populations, but detailed tests in natural populations are scarce. We combine long-term population and fitness data with those from two types of molecular markers to examine the role of genetic effects in a declining metapopulation of southern dunlins Calidris alpina schinzii, an endangered shorebird. Results: The decline is associated with increased pairings between related individuals, including close inbreeding (as revealed by both field observations of parentage and molecular markers). Furthermore, reduced genetic diversity seems to affect individual fitness at several life stages. Higher genetic similarity between mates correlates negatively with the pair’s hatching success. Moreover, offspring produced by related parents are more homozygous and suffer from increased mortality during embryonic development and possibly also after hatching. Conclusions: Our results demonstrate strong genetic effects in a rapidly declining population, emphasizing the importance of genetic factors for the persistence of small populations. | 54 | no | individual level | NA | no | no |
| 55 | 2017 | 43004 | Ficetola, GF; Garner, TWJ; De Bernardi, F | 2007 | Genetic diversity, but not hatching success, is jointly affected by postglacial colonization and isolation in the threatened frog, Rana latastei | Both postglacial colonization and habitat fragmentation can reduce the genetic diversity of populations, which in turn can affect fitness. However, since these processes occur at different spatial and temporal scales, the consequences of either process may differ. To disentangle the relative role of isolation and postglacial colonization in determining genetic diversity and fitness, we studied microsatellite diversity of 295 individuals from 10 populations and measured the hatch rate of 218 clutches from eight populations of a threatened frog, R. latastei. The populations that were affected by fragmentation to a greater extent suffered higher embryo mortality and reduced hatch rate, while no effects of distance from glacial refugium on hatch rate were detected. Altogether, distance from glacial refugium and isolation explained > 90% of variation in genetic diversity. We found that the genetic diversity was lowest in populations both isolated and far from the glacial refugium, and that distance from refugium seems to have the primary role in determining genetic diversity. The relationship between genetic diversity and hatch rate was not significant. However, the proportion of genetic diversity lost through recent isolation had a significant, negative effect on fitness. It is possible that selection at least partially purged the negative effects of the ancestral loss of genetic diversity. | 55 | no | no good fitness measure | NA | no | no |
| 56 | 2017 | 43004 | Slaa, EJ; Chappell, P; Hughes, WOH | 2014 | Colony genetic diversity affects task performance in the red ant Myrmica rubra | High relatedness and low genetic diversity among individuals in a group is generally considered crucial to the evolution of cooperative behaviour. However, in about a third of social insect species, intracolonial genetic diversity is increased because of derived polyandry (multiple mating by queens) and/or polygyny (multiple reproductive queens). Several studies have shown that increased intracolonial genetic diversity can enhance task performance in honey bees, but evidence of such effect in other social insects is still lacking. Why increased genetic diversity has evolved in some, but not all species, is a fundamental question in sociobiology. In this study, we investigated the effect of intracolonial genetic diversity on the task of nest migration, using the facultatively polyandrous and polygynous red ant Myrmica rubra. Genetic diversity significantly affected migration speed, but its effects were context dependent. Migration speed correlated positively with genetic diversity in one experiment in which migrations were into a known nest site, due to quicker transfer of brood into the new nest once consensus was reached. However, in a another experiment in which migration included scouting for new nest sites, migration speed correlated negatively with genetic diversity, due to slower discovery of new nest sites and slower transfer of brood into the new nest. Our results show for the first time that genetic diversity affects task performance in a social insect other than the honeybee, but that it can produce contrasting effects under different conditions. | 56 | no | no good fitness measure | NA | no | no |
| 57 | 2017 | 43004 | Mitchell, J; Vitikainen, EIK; Wells, DA; Cant, MA; Nichols, HJ | 2017 | Heterozygosity but not inbreeding coefficient predicts parasite burdens in the banded mongoose | Inbreeding, reproduction between relatives, often impinges on the health and survival of resulting offspring. Such inbreeding depression may manifest itself through immunological costs as inbred individuals suffer increased propensity to disease, infection and parasites compared to outbred conspecifics. Here, we assess how the intestinal parasite loads of wild banded mongooses (Mungos mungo) vary with pedigree inbreeding coefficient (f) and standardized multi-locus heterozygosity. We find a significant association between increased heterozygosity and lower parasite loads; however, this correlation does not stand when considering f. Such findings may be explained by local genetic effects, linkage between genetic markers and genes influencing parasite burdens. Indeed, we find heterozygosity at certain loci to correlate with parasite load. Although these tentative local effects are lost following multiple test correction, they warrant future investigation to determine their strength and impact. We also suggest frequent inbreeding within banded mongooses may mean heterozygosity is a better predictor of inbreeding than pedigree f. This is because inbreeding facilitates linkage disequilibrium, increasing the chances of neutral markers representing genome-wide heterozygosity. Finally, neither f nor heterozygosity had a significant influence on the loads of two specific gastrointestinal parasites. Nevertheless, more heterozygous individuals benefited from reduced overall parasitic infection and genetic diversity appears to explain some variation in parasite burdens in the banded mongoose. | 57 | no | individual level | NA | no | no |
| 58 | 2017 | 43004 | Garcia-Fernandez, A; Iriondo, JM; Escudero, A | 2012 | Inbreeding at the edge: does inbreeding depression increase under more stressful conditions? | Edge populations are frequently small and subject to stressful conditions that may compromise their long-term viability. Inbreeding can play an important role in small populations by reducing genetic diversity, leading to the fixation of deleterious mutations and, finally, carrying populations to an extinction vortex through inbreeding depression. Although stressful conditions may enhance the intensity of inbreeding depression, evidence to date is inconclusive in marginal habitats. Local adaptation, promoting native genotypes, and gene flow, reducing allele fixation, are two factors that can have different effects on the intensity of inbreeding depression. Three populations of Silene ciliata distributed across an elevation gradient at the southernmost edge of the species distribution were used for this study. Several fitness components germination, survival and growth rate were compared between inbred seedlings and seedlings from within- and between-population outcrosses, both in the field and controlled conditions. Overall, inbred seedlings had lower fitness than outcrossed seedlings. For most of the variables analysed, similar inbreeding depression effects were found in all three populations, but, for seed weight and seedling survival curve, inbreeding depression was only found in the low altitude population. Similarly, inbreeding depression was more evident in the field than in controlled chamber conditions. Outcrosses between populations contributed to an increase in most fitness estimates and populations, suggesting that the benefits of reducing inbreeding depression overrode the potentially deleterious effects of disrupting local adaptation. Our results suggest that inbreeding depression plays an important role in the fitness of early life stages of Silene ciliata at its southernmost distribution limit, but only provided partial support to the hypothesis that stressful conditions enhance the expression of inbreeding depression. | 58 | maybe | any way of getting GD per population?= | inbreeding | no | no |
| 59 | 2017 | 43004 | Bilde, T; Lubin, Y; Smith, D; Schneider, JM; Maklakov, AA | 2005 | The transition to social inbred mating systems in spiders: role of inbreeding tolerance in a subsocial predecessor | The social spiders are unusual among cooperatively breeding animals in being highly inbred. In contrast, most other social organisms are outbred owing to inbreeding avoidance mechanism,,. The social spiders appear to originate from solitary subsocial ancestors, implying a transition from outbreeding to inbreeding mating systems. Such a transition may be constrained by inbreeding avoidance tactics or fitness loss due to inbreeding depression. We examined whether the mating system of a subsocial spider, in a genus with three social congeners, is likely to facilitate or hinder the transition to inbreeding social systems. Populations of subsocial Stegodyphus lineatus are substructured and spiders occur in patches, which may consist of kin groups. We investigated whether male mating dispersal prevents matings within kin groups in natural populations. Approximately half of the marked males that were recovered made short moves (< 5m) and mated within their natal patch. This potential for inbreeding was counterbalanced by a relatively high proportion of immigrant males. In mating experiments, we tested whether inbreeding actually results in lower offspring fitness. Two levels of inbreeding were tested: full sibling versus non-sib matings and matings of individuals within and between naturally occurring patches of spiders. Neither full siblings nor patch mates were discriminated against as mates. Sibling matings had no effect on direct fitness traits such as fecundity, hatching success, time to hatching and survival of the offspring, but negatively affected offspring growth rates and adult body size of both males and females. Neither direct nor indirect fitness measures differed significantly between within patch and between-patch pairs. We tested the relatedness between patch mates and nonpatch mates using DNA fingerprinting (TE-AFLP). Kinship explained 30% of the genetic variation among patches, confirming that patches are often composed of kin. Overall, we found limited male dispersal, lack of kin discrimination, and tolerance to low levels of inbreeding These results suggest a history of inbreeding which may reduce the frequency of deleterious recessive alleles in the population and promote the evolution of inbreeding tolerance. It is likely that the lack of inbreeding avoidance in subsocial predecessors has facilitated the transition to regular inbreeding social systems. | 59 | no | NA | NA | no | no |
| 60 | 2017 | 43004 | Corona, E; Chen, R; Sikora, M; Morgan, AA; Patel, CJ; Ramesh, A; Bustamante, CD; Butte, AJ | 2013 | Analysis of the Genetic Basis of Disease in the Context of Worldwide Human Relationships and Migration | Genetic diversity across different human populations can enhance understanding of the genetic basis of disease. We calculated the genetic risk of 102 diseases in 1,043 unrelated individuals across 51 populations of the Human Genome Diversity Panel. We found that genetic risk for type 2 diabetes and pancreatic cancer decreased as humans migrated toward East Asia. In addition, biliary liver cirrhosis, alopecia areata, bladder cancer, inflammatory bowel disease, membranous nephropathy, systemic lupus erythematosus, systemic sclerosis, ulcerative colitis, and vitiligo have undergone genetic risk differentiation. This analysis represents a large-scale attempt to characterize genetic risk differentiation in the context of migration. We anticipate that our findings will enable detailed analysis pertaining to the driving forces behind genetic risk differentiation. | 60 | no | NA | NA | no | no |
| 61 | 2017 | 43004 | Jolivet, C; Rogge, M; Degen, B | 2013 | Molecular and quantitative signatures of biparental inbreeding depression in the self-incompatible tree species Prunus avium | Genetic diversity strongly influences populations’ adaptability to changing environments and therefore survival. Sustainable forest management practices have multiple roles including conservation of genetic resources and timber production. In this study, we aimed at better understanding the variation in genetic diversity among adult and offspring individuals, and the effects of mating system on offspring survival and growth in wild cherry, Prunus avium. We analysed adult trees and open pollinated seed-families from three stands in Germany at eight microsatellite loci and one incompatibility system locus and conducted paternity analyses. Seed viability testing and seed sowing in a nursery allowed further testing for the effects of pollen donor diversity and genetic similarity between mates on the offspring performance at the seed and seedling stages. Our results were contrasting across stands. Loss of genetic diversity from adult to seedling stages and positive effect of mate diversity on offspring performance occurred in one stand only, whereas biparental inbreeding depression and significant decrease in fixation index from adults to seedlings was detected in two stands. We discussed the effects of stand genetic diversity on the magnitude of biparental inbreeding depression at several life-stages and its consequences on the management of genetic resources in P. avium. Heredity (2013) 110, 439-448; doi:10.1038/hdy.2012.103; published online 5 December 2012 | 61 | no | individual level | NA | no | no |
| 62 | 2017 | 43004 | Johnson, WE; Onorato, DP; Roelke, ME; Land, ED; Cunningham, M; Belden, RC; McBride, R; Jansen, D; Lotz, M; Shindle, D; Howard, J; Wildt, DE; Penfold, LM; Hostetler, JA; Oli, MK; O’Brien, SJ | 2010 | Genetic Restoration of the Florida Panther | The rediscovery of remnant Florida panthers (Puma concolor coryi) in southern Florida swamplands prompted a program to protect and stabilize the population. In 1995, conservation managers translocated eight female pumas (P. c. stanleyana) from Texas to increase depleted genetic diversity, improve population numbers, and reverse indications of inbreeding depression. We have assessed the demographic, population-genetic, and biomedical consequences of this restoration experiment and show that panther numbers increased threefold, genetic heterozygosity doubled, survival and fitness measures improved, and inbreeding correlates declined significantly. Although these results are encouraging, continued habitat loss, persistent inbreeding, infectious agents, and possible habitat saturation pose new dilemmas. This intensive management program illustrates the challenges of maintaining populations of large predators worldwide. | 62 | no | NA | NA | no | no |
| 63 | 2017 | 43004 | Wilson-Rich, N; Tarpy, DR; Starks, PT | 2012 | Within- and across-colony effects of hyperpolyandry on immune function and body condition in honey bees (Apis mellifera) | Honey bees (Apis mellifera) have become a model system for studies on the influence of genetic diversity on disease. Honey bee queens mate with a remarkably high number of males up to 29 in the current study from which they produce a colony of genetically diverse daughter workers. Recent evidence suggests a significant benefit of intracolony genetic diversity on disease resistance. Here, we explored the relationship between the level of genetic diversity and multiple physiological mechanisms of cellular and humoral immune defense (encapsulation response and phenoloxidase activity). We also investigated an effect of genetic diversity on a measure of body condition (fat body mass). While we predicted that mean colony phenoloxidase activity, encapsulation response, and fat body mass would show a positive relationship with increased intracolonial genetic diversity, we found no significant relationship between genetic diversity and these immune measures, and found no consistent effect on body condition. These results suggest that high genetic diversity as a result of extreme polyandry may have little bearing on the physiological mechanisms of immune function at naturally occurring mating levels in honey bees. (C) 2012 Elsevier Ltd. All rights reserved. | 63 | maybe | any fitness measure? | no fitness measure | no | no |
| 64 | 2017 | 43004 | Mikheyev, AS; Tin, MMY; Arora, J; Seeley, TD | 2015 | Museum samples reveal rapid evolution by wild honey bees exposed to a novel parasite | Understanding genetic changes caused by novel pathogens and parasites can reveal mechanisms of adaptation and genetic robustness. Using whole-genome sequencing of museum and modern specimens, we describe the genomic changes in a wild population of honey bees in North America following the introduction of the ectoparasitic mite, Varroa destructor. Even though colony density in the study population is the same today as in the past, a major loss of haplotypic diversity occurred, indicative of a drastic mitochondrial bottleneck, caused by massive colony mortality. In contrast, nuclear genetic diversity did not change, though hundreds of genes show signs of selection. The genetic diversity within each bee colony, particularly as a consequence of polyandry by queens, may enable preservation of genetic diversity even during population bottlenecks. These findings suggest that genetically diverse honey bee populations can recover from introduced diseases by evolving rapid tolerance, while maintaining much of the standing genetic variation. | 64 | no | NA | NA | no | no |
| 65 | 2017 | 43004 | Hartmann, SA; Schaefer, HM; Segelbacher, G | 2014 | Genetic depletion at adaptive but not neutral loci in an endangered bird species | Many endangered species suffer from the loss of genetic diversity, but some populations may be able to thrive even if genetically depleted. To investigate the underlying genetic processes of population bottlenecks, we apply an innovative approach for assessing genetic diversity in the last known population of the endangered Pale-headed Brushfinch (Atlapetes pallidiceps) in Ecuador. First, we measure genetic diversity at eleven neutral microsatellite loci and adaptive SNP variation in five Toll-like receptor (TLR) immune system genes. Bottleneck tests confirm genetic drift as the main force shaping genetic diversity in this species and indicate a 99% reduction in population size dating back several hundred years. Second, we compare contemporary microsatellite diversity with historic museum samples of A.pallidiceps, finding no change in genetic diversity. Third, we compare genetic diversity in the Pale-headed Brushfinch with two co-occurring-related brushfinch species (Atlapetes latinuchus, Buarremon torquatus), finding a reduction of up to 91% diversity in the immune system genes but not in microsatellites. High TLR diversity is linked to decreased survival probabilities in A.pallidiceps. Low TLR diversity is thus probably an adaptation to the specific selection regime within its currently very restricted distribution (approximately 200ha), but could severely restrict the adaptive potential of the species in the long run. Our study illustrates the importance of investigating both neutral and adaptive markers to assess the effect of population bottlenecks and for recommending specific management plans in endangered species. | 65 | no | only one pop | NA | no | no |
| 66 | 2017 | 43004 | Linde, CC; Smith, LM; Peakall, R | 2016 | Weeds, as ancillary hosts, pose disproportionate risk for virulent pathogen transfer to crops | Background: The outcome of the arms race between hosts and pathogens depends heavily on the interactions between their genetic diversity, population size and transmission ability. Theory predicts that genetically diverse hosts will select for higher virulence and more diverse pathogens than hosts with low genetic diversity. Cultivated hosts typically have lower genetic diversity and thus small effective population sizes, but can potentially harbour large pathogen population sizes. On the other hand, hosts, such as weeds, which are genetically more diverse and thus have larger effective population sizes, usually harbour smaller pathogen population sizes. Large pathogen population sizes may lead to more opportunities for mutation and hence more diverse pathogens. Here we test the predictions that pathogen neutral genetic diversity will increase with large pathogen population sizes and host diversity, whereas diversity under selection will increase with host diversity. We assessed and compared the diversity of a fungal pathogen, Rhynchosporium commune, on weedy barley grass (which have a large effective population size) and cultivated barley (low genetic diversity) using microsatellites, effector locus nip1 diversity and pathogen aggressiveness in order to assess the importance of weeds in the evolution of the neutral and selected diversity of pathogens. Results: The findings indicated that the large barley acreage and low host diversity maintains higher pathogen neutral genetic diversity and lower linkage disequilibrium, while the weed maintains more pathotypes and higher virulence diversity at nip1. Strong evidence for more pathogen migration from barley grass to barley suggests transmission of virulence from barley grass to barley is common. Conclusions: Pathogen census population size is a better predictor for neutral genetic diversity than host diversity. Despite maintaining a smaller pathogen census population size, barley grass acts as an important ancillary host to R. commune, harbouring highly virulent pathogen types capable of transmission to barley. Management of disease on crops must therefore include management of weedy ancillary hosts, which may harbour disproportionate supplies of virulent pathogen strains. | 66 | no | NA | NA | no | no |
| 67 | 2017 | 43004 | WAUTERS, LA; HUTCHINSON, Y; PARKIN, DT; DHONDT, AA | 1994 | THE EFFECTS OF HABITAT FRAGMENTATION ON DEMOGRAPHY AND ON THE LOSS OF GENETIC-VARIATION IN THE RED SQUIRREL | A major problem in conservation biology is the extent to which the loss of genetic variability in isolated populations reduces their chance of survival. We present data in which the loss of genetic diversity in small and isolated populations can be directly related to population dynamics. Genetic similarity in red squirrels is inversely correlated with population size. The loss of genetic variation and the lower population densities in isolated populations are both the result of reduced immigration. Our data suggest that population processes rather than genetic problems are the real threat to small squirrel populations. | 67 | maybe | any fitness measure? | NA | no | yes |
| 68 | 2017 | 43004 | Giese, AR; Hedrick, PW | 2003 | Genetic variation and resistance to a bacterial infection in the endangered Gila topminnow | We tested the association between host genetic diversity and resistance to a novel pathogen using controlled experiments to compare bacterially induced mortality among four populations of the endangered Gila topminnow (Poeciliopsis occidentalis) with different levels of genetic diversity, and between nine lines of inbred topminnows and their respective (non-inbred) source populations. We exposed fish in treatment groups to salinity shock and high concentrations of the bacterium Listonella (Vibrio) angitillarum, a causative agent of vibriosis in fish. Mortality associated with vibriosis (mortality) 1 to 6 days post-exposure differed among the populations, and between inbred and non-inbred samples. However, mortality was not associated with expected heterozygosity based on nlicrosatellite loci diversity, nor was it associated with expected or observed heterozygosity at a major histocompatibility complex (MHC) class II locus. Neither was mortality related to the presence or absence of specific MHC alleles. Additionally, the effect of severe inbreeding was to reduce mortality in the inbred fish relative to the non-inbred controls in every population. Taken in the context of other studies, we conclude that effects of genetic diversity may be context- and locus-specific, environmental stress may be an important determinant of host immunity, and genetic diversity should be used cautiously to predict the potential response of a population to a specific environmental challenge. | 68 | yes | four pop | inbreeding | no | no |
| 69 | 2017 | 43004 | Kittelson, PM; Wagenius, S; Nielsen, R; Qazi, S; Howe, M; Kiefer, G; Shaw, RG | 2015 | How functional traits, herbivory, and genetic diversity interact in Echinacea: implications for fragmented populations | Habitat fragmentation produces small, spatially isolated populations that promote inbreeding. Remnant populations often contain inbred and outbred individuals, but it is unclear how inbreeding relative to outbreeding affects the expression of functional traits and biotic interactions such as herbivory. We measured a suite of 12 functional traits and herbivore damage on three genotypic cross types in the prairie forb, Echinacea angustifolia: inbred, and outbred crosses resulting from matings within and between remnant populations. Inbreeding significantly affected the expression of all 12 functional traits that influence resource capture. Inbred individuals had consistently lower photosynthetic rates, water use efficiencies, specific leaf areas, and had higher trichome numbers, percent C, and percent N than outbred individuals. However, herbivore damage did not differ significantly among the cross types and was not correlated with other leaf functional traits. Leaf architecture and low physiological rates of the inbred compared to outbred individuals imply poorer capture or use of resources. Inbred plants also had lower survival and fitness relative to outbred plants. Our results show that inbreeding, a phenomenon predicted and observed to occur in fragmented populations, influences key functional traits such as plant structure, physiology and elemental composition. Because of their likely role in fitness of individuals and ecological dynamics plant functional traits can serve as a bridge between evolution and community or ecosystem ecology. | 69 | no | individual level | NA | no | no |
| 70 | 2017 | 43004 | Hadziabdic, D; Fitzpatrick, BM; Wang, X; Wadl, PA; Rinehart, TA; Ownley, BH; Windham, MT; Trigiano, RN | 2010 | Analysis of genetic diversity in flowering dogwood natural stands using microsatellites: the effects of dogwood anthracnose | Flowering dogwood (Cornus florida L.) populations recently have experienced severe declines caused by dogwood anthracnose. Mortality has ranged from 48 to 98%, raising the concern that genetic diversity has been reduced significantly. Microsatellite data were used to evaluate the level and distribution of genetic variation throughout much of the native range of the tree. Genetic variation in areas affected by anthracnose was as high as or higher than areas without die-offs. We found evidence of four widespread, spatially contiguous genetic clusters. However, there was little relationship between geographic distance and genetic difference. These observations suggest that high dispersal rates and large effective population sizes have so far prevented rapid loss of genetic diversity. The effects of anthracnose on demography and community structure are likely to be far more consequential than short-term genetic effects. | 70 | maybe | N populations/areas? | no fitness measure | no | no |
| 71 | 2017 | 43004 | Chang, CC; Smith, MD | 2012 | Invasion of an intact plant community: the role of population versus community level diversity | To improve the understanding of how native plant diversity influences invasion, we examined how population and community diversity may directly and indirectly be related to invasion in a natural field setting. Due to the large impact of the dominant C-4 grass species (Andropogon gerardii) on invasion resistance of tallgrass prairie, we hypothesized that genetic diversity and associated traits within a population of this species would be more strongly related to invasion than diversity or traits of the rest of the community. We added seeds of the exotic invasive C-4 grass, A. bladhii, to 1-m(2) plots in intact tallgrass prairie that varied in genetic diversity of A. gerardii and plant community diversity, but not species richness. We assessed relationships among genetic diversity and traits of A. gerardii, community diversity, community aggregated traits, resource availability, and early season establishment and late-season persistence of the invader using structural equation modeling (SEM). SEM models suggested that community diversity likely enhanced invasion indirectly through increasing community aggregated specific leaf area as a consequence of more favorable microclimatic conditions for seedling establishment. In contrast, neither population nor community diversity was directly or indirectly related to late season survival of invasive seedlings. Our research suggests that while much of diversity-invasion research has separately focused on the direct effects of genetic and species diversity, when taken together, we find that the role of both levels of diversity on invasion resistance may be more complex, whereby effects of diversity may be primarily indirect via traits and vary depending on the stage of invasion. | 71 | maybe | “N”“groups”“?” | no good fitness measure | no | no |
| 72 | 2017 | 43004 | Weeks, SC; Crosser, BR; Bennett, R; Gray, M; Zucker, N | 2000 | Maintenance of androdioecy in the freshwater shrimp, Eulimnadia texana: Estimates of inbreeding depression in two populations | “Androdioecy is an uncommon form of reproduction in which males coexist with hermaphrodites. Androdioecy is thought to be difficult to evolve in species that regularly inbreed. The freshwater shrimp Eulimnadia texana has recently been described as both androdioecious and highly selfing and is thus anomalous. Inbreeding depression is one factor that may maintain males in these populations. Here we examine the extent of”“late”" inbreeding depression (after sexual maturity) in these clam shrimp using two tests: (1) comparing the fitness of shrimp varying in their levels of individual heterozygosity from two natural populations that differ in overall genetic diversity | 72 | no | inbreeding ind | NA | no | no |
| 73 | 2017 | 43004 | Tarpy, DR | 2003 | Genetic diversity within honeybee colonies prevents severe infections and promotes colony growth | Multiple mating by social insect queens increases the genetic diversity among colony members, thereby reducing intracolony relatedness and lowering the potential inclusive fitness gains of altruistic workers. Increased genetic diversity may be adaptive, however, by reducing the prevalence of disease within a nest. Honeybees, whose queens have the highest levels of multiple mating among social insects, were investigated to determine whether genetic variation helps to prevent chronic infections. I instrumentally inseminated honeybee queens with semen that was either genetically similar (from one male) or genetically diverse (from multiple males), and then inoculated their colonies with spores of Ascosphaera apis, a fungal pathogen that kills developing brood. I show that genetically diverse colonies had a lower variance in disease prevalence than genetically similar colonies, which suggests that genetic diversity may benefit colonies by preventing severe infections. | 73 | yes | from thesis | NA | no | yes |
| 74 | 2017 | 43004 | Widdig, A; Muniz, L; Minkner, M; Barth, Y; Bley, S; Ruiz-Lambides, A; Junge, O; Mundry, R; Kulik, L | 2017 | Low incidence of inbreeding in a long-lived primate population isolated for 75 years | When close relatives mate, offspring are expected to suffer fitness consequences due to inbreeding depression. Inbreeding has previously been quantified in two ways: using a sufficiently large panel of markers or deep and complete pedigrees over several generations. However, the application of both approaches is still limited by the challenge of compiling such data for species with long generation times, such as primates. Here, we assess inbreeding in rhesus macaques living on Cayo Santiago (Puerto Rico), a population genetically isolated since 1938, but descendant of a large set of presumably unrelated founders. Using comprehensive genetic data, we calculated inbreeding coefficients (F) for 2669 individuals with complete three generation pedigrees and 609 individuals with complete four generation pedigrees. We found that 0.79 and 7.39% of individuals had an F > 0 when using data from three and four generation pedigrees, respectively. No evidence of an increase in inbreeding over the study period (up to 23 years) was found. Furthermore, the observed mean relatedness of breeding pairs differed significantly from the distribution of parental relatedness expected as simulated based on previous reproductive data, suggesting that kin generally avoid breeding with each other. Finally, inbreeding was not a predictor of early mortality measured as survival until weaning and sexual maturation, respectively. Our results remain consistent with three estimators of inbreeding (standardized heterozygosity, internal relatedness, and homozygosity by loci) using up to 42 highly polymorphic microsatellites for the same set of individuals. Together, our results demonstrate that close inbreeding may not be prevalent even in populations isolated over long periods when mechanisms of inbreeding avoidance can operate. Significance statement When close relatives mate, offspring may suffer from such inbreeding, e.g., via lower survival and/or fertility. Using (i) a large panel of genetic markers and (ii) complete three or four generation pedigrees, respectively, we show that incidences of inbreeding in a long-lived primate population are rare, even after genetic isolation for 75 years. Moreover, our simulations suggest that kin in our population generally avoid breeding with each other. Finally, the few inbred individuals detected in our large sample did not suffer from lower survival. Given that many animal species face dramatic habitat loss combined with critical population declines, our study provides important implications for conservation biology in general and for population management in particular. | 74 | no | only one pop | NA | no | no |
| 75 | 2017 | 43004 | Coutts, NJ; Harley, EH | 2009 | Comparative population genetics of the German shepherd dog in South Africa | Modern breeding practices strive to achieve distinctive phenotypic uniformity in breeds of dogs, but these strategies are associated with the inevitable loss of genetic diversity. Thus, in parallel with the morphological variation displayed by breeds, purebred dogs commonly express genetic defects as a result of the inbreeding associated with artificial selection and the reduction of selection against disease phenotypes. Microsatellite marker analyses of 15 polymorphic canine loci were used to investigate measures of genetic diversity and population differentiation within and between German-bred and South African-bred German shepherd dogs. These data were quantified by comparison with typically outbred mongrel or crossbred dogs. Both the imported and locally-bred German shepherd dogs exhibited similar levels of genetic diversity, The breed is characterised by only a moderate loss of genetic diversity relative to outbred dogs, despite originating from a single founding sire and experiencing extensive levels of inbreeding throughout the history of the breed. Non-significant population differentiation between the ancestral German and derived South African populations indicates sufficient contemporary gene flow between these populations, suggesting that migration resulting from the importation of breeding stock has mitigated the effects of random genetic drift and a population bottleneck caused by the original founder event in South Africa. Significant differentiation between the combined German shepherd dog population and the outbred dogs illustrates the effects of selection and genetic drift on the breed since its establishment just over 100 years ago. | 75 | no | domesticated animal | NA | no | no |
| 76 | 2017 | 43004 | Tuni, C; Goodacre, S; Bechsgaard, J; Bilde, T | 2012 | Moderate Multiple Parentage and Low Genetic Variation Reduces the Potential for Genetic Incompatibility Avoidance Despite High Risk of Inbreeding | Background: Polyandry is widespread throughout the animal kingdom. In the absence of direct benefits of mating with different males, the underlying basis for polyandry is enigmatic because it can carry considerable costs such as elevated exposure to sexual diseases, physical injury or other direct fitness costs. Such costs may be balanced by indirect genetic benefits to the offspring of polyandrous females. We investigated polyandry and patterns of parentage in the spider Stegodyphus lineatus. This species experiences relatively high levels of inbreeding as a result of its spatial population structure, philopatry and limited male mating dispersal. Polyandry may provide an opportunity for post mating inbreeding avoidance that reduces the risk of genetic incompatibilities arising from incestuous matings. However, multiple mating carries direct fitness costs to females suggesting that genetic benefits must be substantial to counter direct costs. Methodology/Principal Findings: Genetic parentage analyses in two populations from Israel and a Greek island, showed mixed-brood parentage in approximately 50% of the broods. The number of fathers ranged from 1-2 indicating low levels of multiple parentage and there was no evidence for paternity bias in mixed-broods from both populations. Microsatellite loci variation suggested limited genetic variation within populations, especially in the Greek island population. Relatedness estimates among females in the maternal generation and potentially interacting individuals were substantial indicating full-sib and half-sib relationships. Conclusions/Significance: Three lines of evidence indicate limited potential to obtain substantial genetic benefits in the form of reduced inbreeding. The relatively low frequency of multiple parentage together with low genetic variation among potential mates and the elevated risk of mating among related individuals as corroborated by our genetic data suggest that there are limited actual outbreeding opportunities for polyandrous females. Polyandry in S. lineatus is thus unlikely to be maintained through adaptive female choice. | 76 | no | no good fitness measure | NA | no | no |
| 77 | 2017 | 43004 | Chen, YY; Bao, ZX; Qu, Y; Li, W; Li, ZZ | 2014 | Genetic diversity and population structure of the medicinal orchid Gastrodia elata revealed by microsatellite analysis | The wild resources of Gastrodia elata are currently threatened with extinction due to over-harvesting because of their high medicinal value. Genetic diversity plays a key role in the survival of endangered orchid species. In this study we investigated the genetic pattern in eight microsatellite loci within eight G. elata populations from central China. Compared with the other orchids, G. elata showed a low level of genetic variation within populations (H-E = 0.356-0.622). The main factors responsible for the genetic pattern were the plant’s inbreeding system due to mating within clone patches, and the genetic bottlenecks and genetic drift caused by a long-history over-collecting. The significant heterozygote deficit was detected in all the populations. The F statistics calculated by different approaches consistently revealed a clear genetic differentiation among populations, contributing about 20% of the total gene diversity. The results are discussed in relation to both in situ and ex situ conservation efforts of the species. The populations with a high level of genetic diversity or with great genetic distinction were identified, which should be a high priority for conservation managers. (C) 2014 Elsevier Ltd. All rights reserved. | 77 | no | no good fitness measure | NA | no | no |
| 78 | 2017 | 43004 | Rogell, B; Thorngren, H; Laurila, A; Hoglund, J | 2010 | Fitness costs associated with low genetic variation are reduced in a harsher environment in amphibian island populations | A basic premise of conservation geneticists is that low levels of genetic variation are associated with fitness costs in terms of reduced survival and fecundity. These fitness costs may frequently vary with environmental factors and should increase under more stressful conditions. However, there is no consensus on how fitness costs associated with low genetic variation change under natural conditions in relation to the stressfulness of the environment. On the Swedish west coast, natterjack toad Bufo calamita populations show a strong population genetic structure and large variation in the amount of within-population genetic variation. We experimentally examined the survival of natterjack larvae from six populations with different genetic variation in three thermal environments corresponding to (a) the mean temperature of natural ponds (stable, laboratory), (b) a high temperature environment occurring in desiccating ponds (stable, laboratory) and (c) an outdoor treatment mimicking the natural, variable thermal conditions (fluctuating, semi-natural). We found that larvae in the outdoor treatment had poorer survival than larvae in the stable environments suggesting that the outdoor treatment was more stressful. Overall, populations with higher genetic variation had higher larval survival. However, a significant interaction between treatments and genetic variation indicated that fitness costs associated with low genetic variation were less severe in the outdoor treatment. Thus, we found no support for the hypothesis that fitness costs associated with low genetic variation increase under more stressful conditions. Our results suggest that natural thermal stress may mask fitness losses associated with low genetic variation in these populations. | 78 | yes | 6 pop | not pop level gd | yes | no |
| 79 | 2017 | 43004 | Liao, JC; Wang, Y; Zhao, LM; Fang, FJ; Liu, NF | 2013 | Influence of climatic factors on genetic diversity of midday gerbil (Meriones meridianus Pallas, 1773) | Environmental factors are considered important in shaping the genetic diversity of animal populations. Mitochondrial DNA sequences of cytochrome b gene (Cyt-b) of 264 midday gerbils (Meriones meridianus Pallas, 1773) from 21 populations were used to investigate the genetic structure and the relationship between genetic diversity and climatic factors. The results showed that the populations were divided into two clades by the Tian Shan Mountains and significant differentiation was found between most populations. The genetic diversity showed a significant relationship with the variation coefficients for sunshine hours and wind speed. The variation coefficients for annual mean temperature, precipitation, and wind speed were negatively related to nucleotide diversity. We conclude that environmental barriers have limited the gene flow between populations and have played a role on the observed phylogenetic pattern. Sunshine hours and wind speed have important effects on nucleotide diversity in midday gerbils, in terms of energy conservation, search for food in winter, reproduction, and mortality. Thus, unstable environments could lead to a decline in genetic diversity and disappearance of individuals that do not adapt to fluctuating environments. | 79 | no | NA | NA | no | no |
| 80 | 2017 | 43004 | Luo, MF; Pan, HJ | 2013 | MHC II DRB variation and trans-species polymorphism in the golden snub-nosed monkey (Rhinopithecus roxellana) | Genetic variation is generally believed to be important in studying endangered species’ adaptive potential. Early studies assessed genetic diversity using nearly neutral markers, such as microsatellite loci and mitochondrial DNA (mtDNA), which are very informative for phylogenetic and phylogeographic reconstructions. However, the variation at these loci cannot provide direct information on selective processes involving the interaction of individuals with their environment, or on the capability to resist continuously evolving pathogens and parasites. The importance of genetic diversity at informative adaptive markers, such as major histocompatibility complex (MHC) genes, is increasingly being realized, especially in endangered, isolated species. Small population size and isolation make the golden snub-nosed monkey (Rhinopithecus roxellana) particularly susceptible to genetic variation losses through inbreeding and restricted gene flow. In this study, we compared the genetic variation and population structure of microsatellites, mtDNA, and the most relevant adaptive region of the MHC II-DRB genes in the golden snub-nosed monkey. We examined three Chinese R. roxellana populations and found the same variation patterns in all gene regions, with the population from Shennongjia population, Hubei Province, showing the lowest polymorphism among three populations. Genetic drift that outweighed balancing selection and the founder effect in these populations may explain the similar genetic variation pattern found in these neutral and adaptive genes. | 80 | no | no good fitness measure | NA | no | no |
| 81 | 2017 | 43004 | Da Silva, A; Luikart, G; Yoccoz, NG; Cohas, A; Allaine, D | 2006 | Genetic diversity-fitness correlation revealed by microsatellite analyses in European alpine marmots (Marmota marmota) | “The relationship between individual genetic diversity and fitness-related traits are poorly understood in the wild. The availability of highly polymorphic molecular markers, such as microsatellites, has made research on this subject more feasible. We used three microsatellite-based measures of genetic diversity, individual heterozygosity H, mean d(2) and mean d(2) (outbreeding) to test for a relationship between individual genetic diversity and important fitness trait, juvenile survival, in a population of alpine marmots (Marmota marmota), after controlling for the effects of ecological, social and physiological parameters that potentially influence juvenile survival in marmots. Analyses were conducted on 158 juveniles, and revealed a positive association between juvenile survival and genetic diversity measured by mean H. No association was found with mean d(2) and with mean d(2) (outbreeding). This suggests a fitness disadvantage to less heterozygous juveniles. The genetic diversity-fitness correlation (GDFC) was somewhat stronger during years with poor environmental conditions (i.e. wet summers). The stressful environmental conditions of this high mountain population might enhance inbreeding depression and make this association between genetic diversity and fitness detectable. Moreover the mating system, allowing extra pair copulation by occasional immigrants, as well as close inbreeding, favours a wide range of individual genetic diversity (mean H ranges from 0.125 to 1), which also may have facilitated the detection of the GDFC. The results further suggest that the observed GDFC is likely to be explained by the”“local effect”" hypothesis rather than by the "“general effect”" hypothesis." | 81 | no | only one pop | NA | no | no |
| 82 | 2017 | 43004 | Reid, JM; Arcese, P; Keller, LF; Germain, RR; Duthie, AB; Losdat, S; Wolak, ME; Nietlisbach, P | 2015 | Quantifying inbreeding avoidance through extra-pair reproduction | Extra-pair reproduction is widely hypothesized to allow females to avoid inbreeding with related socially paired males. Consequently, numerous field studies have tested the key predictions that extra-pair offspring are less inbred than females’ alternative within-pair offspring, and that the probability of extra-pair reproduction increases with a female’s relatedness to her socially paired male. However, such studies rarely measure inbreeding or relatedness sufficiently precisely to detect subtle effects, or consider biases stemming from failure to observe inbred offspring that die during early development. Analyses of multigenerational song sparrow (Melospiza melodia) pedigree data showed that most females had opportunity to increase or decrease the coefficient of inbreeding of their offspring through extra-pair reproduction with neighboring males. In practice, observed extra-pair offspring had lower inbreeding coefficients than females’ within-pair offspring on average, while the probability of extra-pair reproduction increased substantially with the coefficient of kinship between a female and her socially paired male. However, simulations showed that such effects could simply reflect bias stemming from inbreeding depression in early offspring survival. The null hypothesis that extra-pair reproduction is random with respect to kinship therefore cannot be definitively rejected in song sparrows, and existing general evidence that females avoid inbreeding through extra-pair reproduction requires reevaluation given such biases. | 82 | no | NA | NA | no | no |
| 83 | 2017 | 43004 | Agudo, R; Carrete, M; Alcaide, M; Rico, C; Hiraldo, F; Donazar, JA | 2012 | Genetic diversity at neutral and adaptive loci determines individual fitness in a long-lived territorial bird | There is compelling evidence about the manifest effects of inbreeding depression on individual fitness and populations’ risk of extinction. The majority of studies addressing inbreeding depression on wild populations are generally based on indirect measures of inbreeding using neutral markers. However, the study of functional loci, such as genes of the major histocompatibility complex (MHC), is highly recommended. MHC genes constitute an essential component of the immune system of individuals, which is directly related to individual fitness and survival. In this study, we analyse heterozygosity fitness correlations of neutral and adaptive genetic variation (22 microsatellite loci and two loci of the MHC class II, respectively) with the age of recruitment and breeding success of a decimated and geographically isolated population of a long-lived territorial vulture. Our results indicate a negative correlation between neutral genetic diversity and age of recruitment, suggesting that inbreeding may be delaying reproduction. We also found a positive correlation between functional (MHC) genetic diversity and breeding success, together with a specific positive effect of the most frequent pair of cosegregating MHC alleles in the population. Globally, our findings demonstrate that genetic depauperation in small populations has a negative impact on the individual fitness, thus increasing the populations’ extinction risk. | 83 | no | NA | NA | no | no |
| 84 | 2017 | 43004 | MEROLA, M | 1994 | REASSESSMENT OF HOMOZYGOSITY ACID THE CASE FOR INBREEDING DEPRESSION IN THE CHEETAH, ACINONYX-JUBATUS - IMPLICATIONS FOR CONSERVATION | Preservation of genetic diversity within declining populations of endangered species is a major concern in the discipline of conservation biology. The endangered cheetah, Acinonyx jubatus, exhibits relatively little genetic variability (polymorphism = 0.02-0.04, heterozygosity = 0.0004-0.014). Since the discovery of the cheetah’s relative homozygosity, this species has been frequently cited as an example of one whose survival may be compromised fry the loss of genetic diversity. The cheetah’s genetic uniformity is generally believed to be the result of an historical population bottle neck followed by a high level of inbreeding. Evidence offered in support of this hypothesis includes the cheetah’s present low level of genetic variability and symptoms of inbreeding depression in captive populations. Using available data on fluctuating asymmetry and genetic variation in other carnivores, I question the assumption that the present level of genetic diversity in the cheetah is indicative of a loss of former variability. Carnivores exhibit significantly lower levels of genetic variation than other mammals, and several carnivores for which data are available exhibit lower levels of heterozygosity and polymorphism than the cheetah does. Measures of fluctuating asymmetry do not support the hypothesis that the cheetah is suffering an increased level of homozygosity due to genetic stress. Many of the phenotypic effects attributed to inbreeding depression, such as infertility, reduced litter sizes, and increased susceptibility to disease, are limited to captive individuals and may be explained as physiological or behavioral artifacts of captivity. In sum, the genetic constitution of the cheetah does not ap-Una reevaluacion de la homosigocidad y un argumento para la depresion de endocria en el Cheetah Acinonyx jubatus: Implicaciones para la conservacion | 84 | no | NA | NA | no | no |
| 85 | 2017 | 43004 | Cole, BJ; Wiernasz, DC | 1999 | The selective advantage of low relatedness | Relatedness within colonies of social Hymenoptera is often significantly lower than the outbred population maximum of 0.75, Several hypotheses address the widespread occurrence of Low relatedness, but none have measured the covariation of colony fitness and relatedness. In a polyandrous harvester ant, Pogonomyrmex occidentalis, average within-colony relatedness in the population is Low but highly variable among colonies, and relatedness is negatively correlated with colony growth rate. Differences in growth rate strongly influence survival and the onset of reproduction, leading to a 35-fold increase in fitness of fast-growing colonies, Benefits of a genetically diverse worker population may favor polyandry in this species. | 85 | yes | from thesis | no ok fitness measure, colony growth | no | no |
| 86 | 2017 | 43004 | Krummenacher, TS; Zschokke, S | 2007 | Inbreeding and outbreeding in African rhinoceros species | Effective breeding strategies in ex situ conservation require an optimal balance between inbreeding and out-breeding, as both can lead to a decrease in population fitness. Thus optimizing breeding strategies to maintain genetic diversity entails a profound knowledge of the actual situation (including conservation units). This study examines the consequences of inbreeding and outbreeding in captive populations of two threatened species, the white and the black rhinoceros (Ceratotherium simum and Diceros bicornis), based on data from recent studbooks (2004). We also assessed the conservation units of the black rhinoceros, whose classification into subspecies remains a matter of discussion. Theory predicts that juvenile mortality increases with increasing degree of inbreeding. We calculated inbreeding coefficients and examined possible correlations with juvenile mortality rates. To assess the conservation units of the black rhinoceros, we compared juvenile mortality rates of outbred animals with those of non-outbred animals and additionally performed a geographical distance analysis. With both approaches we aimed to draw breeding borderlines between putative conservation units to preserve genetic diversity. Our assessment of the current inbreeding situation based on records in international studbooks was severely limited by small sample sizes in both the white and the black rhinoceros. For the same reason we could not evaluate the conservation units in the black rhinoceros. Nonetheless, we conclude that inbreeding and outbreeding must be followed closely in both species, as their consequences can be severe. | 86 | no | inbreeding ind | NA | no | no |
| 87 | 2017 | 43004 | Vignieri, SN | 2010 | The Genetic Effects of Ecological Disturbance: Flooding in Jumping Mice | Pacific jumping mice (Zapus trinotatus) live in riparian habitats that are discontinuously distributed and subject to regular flooding. Both of these characteristics have a spatial component. Habitat-restricted dispersal frequently leads to spatial genetic structure among individuals, and flooding often imposes spatially specific mortality. Here I report that following a severe flood, an interaction between these factors resulted in an immediate reduction of genetic diversity and genetic divergence of the postflood population. Survival was spatially biased toward more closely related individuals, and this was propagated throughout the postflood population by changes in reproduction. Not only did the number of closely related breeding pairs increase, but so did the production of offspring by individuals. These changes precipitated strong genetic effects, including a reduction in observed heterozygosity, an increase in relatedness, a doubling of inbreeding levels, and significant genetic divergence from previous years. | 87 | no | individual level | NA | no | no |
| 88 | 2017 | 43004 | Gazal, S; Sahbatou, M; Babron, MC; Genin, E; Leutenegger, AL | 2015 | High level of inbreeding in final phase of 1000 Genomes Project | The 1000 Genomes Project provides a unique source of whole genome sequencing data for studies of human population genetics and human diseases. The last release of this project includes more than 2,500 sequenced individuals from 26 populations. Although relationships among individuals have been investigated in some of the populations, inbreeding has never been studied. In this article, we estimated the genomic inbreeding coefficient of each individual and found an unexpected high level of inbreeding in 1000 Genomes data: nearly a quarter of the individuals were inbred and around 4% of them had inbreeding coefficients similar or greater than the ones expected for first-cousin offspring. Inbred individuals were found in each of the 26 populations, with some populations showing proportions of inbred individuals above 50%. We also detected 227 previously unreported pairs of close relatives (up to and including first-cousins). Thus, we propose subsets of unrelated and outbred individuals, for use by the scientific community. In addition, because admixed populations are present in the 1000 Genomes Project, we performed simulations to study the robustness of inbreeding coefficient estimates in the presence of admixture. We found that our multi-point approach (FSuite) was quite robust to admixture, unlike single-point methods (PLINK). | 88 | no | individual level | NA | no | no |
| 89 | 2017 | 43004 | Kretzmann, MB; Gilmartin, WG; Meyer, A; Zegers, GP; Fain, SR; Taylor, BF; Costa, DP | 1997 | Low genetic variability in the Hawaiian monk seal | The Hawaiian monk seal (Monachus schauinslandi) is a critically endangered species that has failed to recover from human exploitation despite decades of protection and ongoing management efforts designed to increase population growth. The seals breed at five principal locations in the northwestern Hawaiian islands, and inter-island migration is limited. Genetic variation in this species is expected to be low due to a recent population bottleneck and probable inbreeding within small subpopulations. To test the hypothesis that small population size and strong site fidelity has led to low within-island genetic variability and significant between-island differentiation, we used two independent approaches to quantify genetic variation both within and among the principal subpopulations. Mitochondrial control region and tRNA gene sequences (359 base pairs) were obtained from 50 seals and revealed very low genetic diversity (0.6% variable sites), with no evidence of subpopulation differentiation. Multilocus DNA fingerprints from 22 individuals also indicated low genetic variation in at least some subpopulations (band-sharing values for ‘’unrelated’’ seals from the same island ranged from 49 to 73%). This method also provided preliminary evidence of population subdivision (F’(st) estimates of 0.20 and 0.13 for two adjacent island pairs). Translocations of seals among islands may therefore have the potential to relieve local inbreeding and possibly to reduce the total amount of variation preserved in the population. Genetic variation is only one of many factors that determine the ability of an endangered species to recover. Maintenance of existing genetic diversity, however, remains an important priority for conservation programs because of the possibility of increased disease resistance in more variable populations and the chance that inbreeding depression may only be manifest under adverse environmental conditions. | 89 | no | inbreeding ind | NA | no | no |
| 90 | 2017 | 43004 | Gonzalez-Garza, BI; Stow, A; Sanchez-Teyer, LF; Zapata-Perez, O | 2015 | Genetic variation, multiple paternity, and measures of reproductive success in the critically endangered hawksbill turtle (Eretmochelys imbricata) | The Yucatan Peninsula in Mexico contains some of the largest breeding groups of the globally distributed and critically endangered hawksbill turtle (Eretmochelys imbricata). An improved understanding of the breeding system of this species and how its genetic variation is structured among nesting areas is required before the threats to its survival can be properly evaluated. Here, we genotype 1195 hatchlings and 41 nesting females at 12 microsatellite loci to assess levels of multiple paternity, genetic variation and whether individual levels of homozygosity are associated with reproductive success. Of the 50 clutches analyzed, only 6% have multiple paternity. The distribution of pairwise relatedness among nesting localities (rookeries) was not random with elevated within-rookery relatedness, and declining relatedness with geographic distance indicating some natal philopatry. Although there was no strong evidence that particular rookeries had lost allelic variation via drift, younger turtles had significantly lower levels of genetic variation than older turtles, suggesting some loss of genetic variation. At present there is no indication that levels of genetic variation are associated with measures of reproductive success such as clutch size, hatching success, and frequency of infertile eggs. | 90 | no | inbreeding ind | NA | no | no |
| 91 | 2017 | 43004 | Parrish, RC; Penley, MJ; Morran, LT | 2016 | The Integral Role of Genetic Variation in the Evolution of Outcrossing in the Caenorhabditis elegans-Serratia marcescens Host-Parasite System | Outcrossing is predicted to facilitate more rapid adaptation than self-fertilization as a result of genetic exchange between genetically variable individuals. Such genetic exchange may increase the efficacy of selection by breaking down Hill-Robertson interference, as well as promoting the maintenance of within-lineage genetic diversity. Experimental studies have demonstrated the selective advantage of outcrossing in novel environments. Here, we assess the specific role of genetic variation in the evolution of outcrossing. We experimentally evolved genetically variable and inbred populations of mixed mating (outcrossing and self-fertilizing) Caenorhabditis elegans nematodes under novel ecological conditions-specifically the presence of the virulent parasite Serratia marcescens. Outcrossing rates increased in genetically variable host populations evolved in the presence of the parasite, whereas parasite exposure in inbred populations resulted in reduced rates of host outcrossing. The host populations with genetic variation also exhibited increased fitness in the presence of the parasite over eight generations, whereas inbred populations did not. This increase in fitness was primarily the result of adaptation to the parasite, rather than recovery from initial inbreeding depression. Therefore, the benefits of outcrossing were only manifested in the presence of genetic variation, and outcrossing was favored over self-fertilization as a result. As predicted, the benefits of outcrossing under novel ecological conditions are a product of genetic exchange between genetically diverse lineages.E91 | 91 | yes | any fitness measure? | inbreeding | no | no |
| 92 | 2017 | 43004 | Hansson, B; Richardson, DS | 2005 | Genetic variation in two endangered Acrocephalus species compared to a widespread congener: estimates based on functional and random loci | Substantial genetic variation is hypothesised to be necessary for the long-term survival of species. Therefore, a major aim in conservation is to maintain and restore variation in small and endangered populations. However, in most populations the amount of genetic variation and, thus, the potential threats posed by limited variation are unknown. In the present study, we assess genetic variation, both at 10 microsatellite loci and at the major histocompatibility complex (MHC), in three closely related Acrocephalus warbler species with contrasting demographies. We found that the recently bottlenecked, island endemic, Seychelles warbler (A. sechellensis; SW) has substantially reduced microsatellite and MHC diversity compared to the widespread great reed warbler (A. arundinaceus; GRW). In contrast, another endangered species with a small breeding range, the Basra reed warbler (A. griseldis; BRW), harboured as much variation as did the GRW. This suggests that significant genetic variation and, therefore, adaptive potential, remains in the BRW - a situation that should hold as long as its habitat and numbers are maintained. Our study is one of the first to assess genetic variation at both ‘non-critical’ microsatellite markers and ‘critical’ MHC loci within endangered species. The two types of loci provided a similar picture of the genetic variation in the species we studied, but this has not been the case in studies of some other species. Using a combination of specific functional loci and genome-wide random markers appears to be the best way to assess the threat posed by reduced genetic diversity. | 92 | no | no | NA | no | no |
| 93 | 2017 | 43004 | Keane, B; Creel, SR; Waser, PM | 1996 | No evidence of inbreeding avoidance or inbreeding depression in a social carnivore | Dispersal by young mammals away from their natal site is generally thought to reduce inbreeding, with its attendant negative fitness consequences. Genetic data from the dwarf mongoose, a pack-living carnivore common in African savannas, indicate that there are exceptions to this generalization. In dwarf mongoose populations in the Serengeti National Park, Tanzania, breeding pairs are commonly related, and close inbreeding has no measurable effect on offspring production or adult survival. Inbreeding occurs because average relatedness among potential mates within a pack is high, because mating patterns within the pack are random with respect to the relatedness of mates, and because dispersal does little to decrease the relatedness among mates. Young females are more likely to leave a pack when the dominant male is a close relative but are relatively infrequent dispersers. Young males emigrate at random with respect to the relatedness of the dominant female and tend to disperse to packs that contain genetically similar individuals. | 93 | no | NA | NA | no | no |
| 94 | 2017 | 43004 | Branco, CC; Pacheco, PR; Cabral, R; Vicente, AM; Mota-Vieira, L | 2008 | Genetic signature of the Sao Miguel Island population (Azores) assessed by 21 microsatellite loci | To study the genetic diversity of Sao Miguel’s population we compared 21 microsatellite loci in 204 individuals from Sao Miguel island and 103 individuals from mainland Portugal. The results show that Sao Miguel and mainland Portugal populations have an average gene diversity of 0.767 and 0.765, respectively. Allele frequencies of all markers are comparable to other European populations. This observation is corroborated by the genetic relationships analysis based on the NJ tree and principal component, where Sao Miguel is closely related to mainland Portugal. Overall, the data suggests that Sao Miguel does not show population structure and is outbred with high genetic diversity. Moreover, the characterization here described is crucial to predict and explain genotypes implicated in genetic diseases in the Azorean population. | 94 | no | No GD diff between pop | NA | no | no |
| 95 | 2017 | 43004 | Zhang, W; Luo, ZH; Zhao, M; Wu, H | 2015 | High genetic diversity in the endangered and narrowly distributed amphibian species Leptobrachium leishanense | Threatened species typically have a small or declining population size, which make them highly susceptible to loss of genetic diversity through genetic drift and inbreeding. Genetic diversity determines the evolutionary potential of a species; therefore, maintaining the genetic diversity of threatened species is essential for their conservation. In this study, we assessed the genetic diversity of the adaptive major histocompatibility complex (MHC) genes in an endangered and narrowly distributed amphibian species, Leptobrachium leishanense in Southwest China. We compared the genetic variation of MHC class I genes with that observed in neutral markers (5 microsatellite loci and cytochrome b gene) to elucidate the relative roles of genetic drift and natural selection in shaping the current MHC polymorphism in this species. We found a high level of genetic diversity in this population at both MHC and neutral markers compared with other threatened amphibian species. Historical positive selection was evident in the MHC class I genes. The higher allelic richness in MHC markers compared with that of microsatellite loci suggests that selection rather than genetic drift plays a prominent role in shaping the MHC variation pattern, as drift can affect all the genome in a similar way but selection directly targets MHC genes. Although demographic analysis revealed no recent bottleneck events in L. leishanense, additional population decline will accelerate the dangerous status for this species. We suggest that the conservation management of L. leishanense should concentrate on maximizing the retention of genetic diversity through preventing their continuous population decline. Protecting their living habitats and forbidding illegal hunting are the most important measures for conservation of L. leishanense. | 95 | no | NA | NA | no | no |
| 96 | 2017 | 43004 | Fitzgerald, TL; Shapter, FM; McDonald, S; Waters, DLE; Chivers, IH; Drenth, A; Nevo, E; Henry, RJ | 2011 | Genome diversity in wild grasses under environmental stress | Patterns of diversity distribution in the Isa defense locus in wild-barley populations suggest adaptive selection at this locus. The extent to which environmental selection may act at additional nuclear-encoded defense loci and within the whole chloroplast genome has now been examined by analyses in two grass species. Analysis of genetic diversity in wild barley (Hordeum spontaneum) defense genes revealed much greater variation in biotic stress-related genes than abiotic stress-related genes. Genetic diversity at the Isa defense locus in wild populations of weeping ricegrass [Microlaena stipoides (Labill.) R. Br.], a very distant wild-rice relative, was more diverse in samples from relatively hotter and drier environments, a phenomenon that reflects observations in wild barley populations. Whole-chloroplast genome sequences of bulked weeping ricegrass individuals sourced from contrasting environments showed higher levels of diversity in the drier environment in both coding and noncoding portions of the genome. Increased genetic diversity may be important in allowing plant populations to adapt to greater environmental variation in warmer and drier climatic conditions. | 96 | no | no good fitness measure | NA | no | no |
| 97 | 2017 | 43004 | Lane, JE; Boutin, S; Gunn, MR; Slate, J; Coltman, DW | 2007 | Genetic relatedness of mates does not predict patterns of parentage in North American red squirrels | Previously limited to laboratory studies, the deleterious effects of inbreeding are now being revealed in a number of wild systems. Female North American red squirrels, Tamiasciurus hudsonicus, show high levels of multimale mating and little to no overt precopulatory mate selection. We hypothesized that the negative repercussions of inbreeding should select for a female’s ability to select sperm from more distantly related males. Consequently, successful sires should be less genetically related to the female than are unsuccessful males. We tested this hypothesis using both an analysis of absolute success among all copulating males and also relative success of sires within multiply sired litters. Pairwise genetic relatedness and paternity were determined through molecular analysis of tissue samples collected from reproductive females, copulating males and resultant offspring. In contrast to other systems, we found no evidence that the genetic similarity of mates predicts patterns of parentage in red squirrels. Genetic relatedness did not predict whether a copulating male would sire any offspring, and relative success of sires within multiply sired litters was unrelated to their relatedness to the dam of the litter. Furthermore, selection for inbreeding avoidance mechanisms may be minimal, as there were no observable negative fitness repercussions to inbreeding. We detected no relationship between the genetic relatedness of an offspring’s parents and its neonatal mass, growth rate or survival to reproductive age. In red squirrels, we found no evidence of parentage patterns based on genetic similarity of mates, and this phenomenon may be less universal than previously thought. (c) 2007 The Association for the Study of Animal Behaviour. Published by Elsevier Ltd. All rights reserved. | 97 | no | NA | NA | no | no |
| 98 | 2017 | 43004 | Alcala, N; Streit, D; Goudet, J; Vuilleumier, S | 2013 | Peak and Persistent Excess of Genetic Diversity Following an Abrupt Migration Increase | Genetic diversity is essential for population survival and adaptation to changing environments. Demographic processes (e.g., bottleneck and expansion) and spatial structure (e.g., migration, number, and size of populations) are known to shape the patterns of the genetic diversity of populations. However, the impact of temporal changes in migration on genetic diversity has seldom been considered, although such events might be the norm. Indeed, during the millions of years of a species’ lifetime, repeated isolation and reconnection of populations occur. Geological and climatic events alternately isolate and reconnect habitats. We analytically document the dynamics of genetic diversity after an abrupt change in migration given the mutation rate and the number and sizes of the populations. We demonstrate that during transient dynamics, genetic diversity can reach unexpectedly high values that can be maintained over thousands of generations. We discuss the consequences of such processes for the evolution of species based on standing genetic variation and how they can affect the reconstruction of a population’s demographic and evolutionary history from genetic data. Our results also provide guidelines for the use of genetic data for the conservation of natural populations. | 98 | no | NA | NA | no | no |
| 99 | 2017 | 43004 | Luong, LT; Heath, BD; Polak, M | 2007 | Host inbreeding increases susceptibility to ectoparasitism | Inbreeding, which increases homozygosity throughout the genome by increasing the proportion of alleles that are identical by descent, is expected to compromise resistance against parasitism. Here, we demonstrate that host inbreeding increases susceptibility to ectoparasitism in a natural fruit fly (Drosophila nigrospiracula) - mite (Macrocheles subbadius) association, and that this effect depends on host genetic background. Moreover, flies generated from reciprocal crosses between susceptible inbred lines exhibited elevated levels of resistance similar to that in the mass-bred base population, confirming in reverse direction the causative link between expected heterozygosity and resistance. We also show that inbreeding reduces the host’s ability to sustain energetically expensive behaviours, and that host exhaustion dramatically increases susceptibility. These findings suggest that inbreeding depression for resistance results from an inability to sustain defensive behaviours because of compromised physiological competence. | 99 | no | individual level | NA | no | no |
| 100 | 2017 | 43004 | Jobet, E; Durand, P; Langand, J; Muller-Graf, CDM; Hugot, JP; Bougnoux, ME; Rivault, C; Cloarec, A; Morand, S | 2000 | Comparative genetic diversity of parasites and their hosts: population structure of an urban cockroach and its haplo-diploid parasite (oxyuroid nematode) | Few studies have investigated the genetic structure of both host and parasite populations at a level of populations and at a level of individuals. We investigated the genetic structure of the urban cockroach Blattella germanica and its oxyuroid parasite Blatticola blattae. Random amplified polymorphic DNA (RAPD) markers were used to quantify genetic diversity between and within four populations (from two cities in France) of the host and its parasite. Diversity based on phenotypic frequencies was calculated for each RAPD marker using Shannon-Wiener’s index. We used multivariate analyses to test the significance of genetic differentiation between host and parasite populations. Analysis of molecular variance was also used. Both methods gave similar results. Diversity between pairs of individuals was estimated by Nei & Li’s index. Genetic diversity was higher within host or parasite populations (80% and 82%, respectively, of explained diversity) than between host or parasite populations (20% and 18%, respectively, explained diversity). The genetic distances between pairs of parasite populations (or individuals) were not correlated with the genetic distances between the corresponding pairs of host populations (or individuals). | 100 | maybe | any pop fitness? | no difference gd pop | no | no |
| 101 | 2017 | 43004 | Jimenez, A; Weigelt, B; Santos-Guerra, A; Caujape-Castells, J; Fernandez-Palacios, JM; Conti, E | 2017 | Surviving in isolation: genetic variation, bottlenecks and reproductive strategies in the Canarian endemic Limonium macrophyllum (Plumbaginaceae) | Oceanic archipelagos are typically rich in endemic taxa, because they offer ideal conditions for diversification and speciation in isolation. One of the most remarkable evolutionary radiations on the Canary Islands comprises the 16 species included in Limonium subsection Nobiles, all of which are subject to diverse threats, and legally protected. Since many of them are single-island endemics limited to one or a few populations, there exists a risk that a loss of genetic variation might limit their long-term survival. In this study, we used eight newly developed microsatellite markers to characterize the levels of genetic variation and inbreeding in L. macrophyllum, a species endemic to the North-east of Tenerife that belongs to Limonium subsection Nobiles. We detected generally low levels of genetic variation over all populations (H (T) = 0.363), and substantial differentiation among populations (F (ST) = 0.188; R (ST) = 0.186) coupled with a negligible degree of inbreeding (F = 0.042). Obligate outcrossing may have maintained L. macrophyllum relatively unaffected by inbreeding despite the species’ limited dispersal ability and the genetic bottlenecks likely caused by a prolonged history of grazing. Although several factors still constitute a risk for the conservation of L. macrophyllum, the lack of inbreeding and the recent positive demographic trends observed in the populations of this species are factors that favour its future persistence. | 101 | no | no good fitness measure | NA | no | no |
| 102 | 2017 | 43004 | Hansson, B; Bensch, S; Hasselquist, D; Akesson, M | 2001 | Microsatellite diversity predicts recruitment of sibling great reed warblers | Inbreeding increases the level of homozygosity, which in turn might depress fitness. In addition, individuals having the same inbreeding coefficient (e.g. siblings) vary in homozygosity. The potential fitness effects of variation in homozygosity that is unrelated to the inbreeding coefficient have seldom been examined. Here, we present evidence from wild birds that genetic variation at five microsatellite loci predicts the recruitment success of siblings. Dyads of full-sibling great reed warblers (Acrocephalus arundinaceus), one individual of which became a recruit to the natal population while the other did not return, were selected for the analysis. Each dyad was matched for sex and size. Local recruitment is strongly lied to fitness in great reed warblers as the majority of offspring die before adulthood, philopatry predominates among surviving individuals and emigrants have lower lifetime fitness. Paired tests showed that recruited individuals had higher individual heterozygosity and higher genetic diversity, which was measured as the mean squared distance between microsatellite alleles (mean d(2)), than their non-recruited siblings. These relationships suggest that the microsatellite markers, which are generally assumed to be neutral, cosegregated with genes exhibiting genetic variation for fitness. | 102 | no | NA | NA | no | no |
| 103 | 2017 | 43004 | Scully, LR; Bidochka, MJ | 2006 | The host acts as a genetic bottleneck during serial infections: an insect-fungal model system | The genetic variation of a pathogen population is a pivotal component of pathogen evolution, having important implications for emerging diseases, nosocomial infections, and laboratory subculturing practices. Furthermore, it is undoubtedly altered during infection of a host. We address this issue using an insect-fungal model system to examine the influence of serial host passage on the genetic variation of a pathogen population. Using amplified fragment length polymorphism, a strain of the opportunistic fungus, Aspergillus flavus, showing initially 98% genetic similarity, was assessed for changes in genetic diversity during repeated passage through Galleria mellonella larvae and compared to that of a parallel population serially subcultured on artificial media. In two independent trials, the genetic diversity of the population passed through the insect dropped significantly, while the genetic variation of the population subcultured on media increased or remained unchanged. However, there were no changes in virulence or the production of protease or aflatoxin, indicating an apparent lack of selection. We suggest that the insect acted as a genetic bottleneck, reducing the genetic diversity of the A. flavus population. The ability of a host to produce a genetic bottleneck in a pathogen population impacts our understanding of emerging diseases, nosocomial infections, and laboratory subculturing practices. | 103 | no | no good fitness measure, N pop differnt in GD? | NA | no | no |
| 104 | 2017 | 43004 | Michaelides, SN; While, GM; Zajac, N; Aubret, F; Calsbeek, B; Sacchi, R; Zuffi, MAL; Uller, T | 2016 | Loss of genetic diversity and increased embryonic mortality in non-native lizard populations | Many populations are small and isolated with limited genetic variation and high risk of mating with close relatives. Inbreeding depression is suspected to contribute to extinction of wild populations, but the historical and demographic factors that contribute to reduced population viability are often difficult to tease apart. Replicated introduction events in non-native species can offer insights into this problem because they allow us to study how genetic variation and inbreeding depression are affected by demographic events (e.g. bottlenecks), genetic admixture and the extent and duration of isolation. Using detailed knowledge about the introduction history of 21 non-native populations of the wall lizard Podarcis muralis in England, we show greater loss of genetic diversity (estimated from microsatellite loci) in older populations and in populations from native regions of high diversity. Loss of genetic diversity was accompanied by higher embryonic mortality in non-native populations, suggesting that introduced populations are sufficiently inbred to jeopardize long-term viability. However, there was no statistical correlation between population-level genetic diversity and average embryonic mortality. Similarly, at the individual level, there was no correlation between female heterozygosity and clutch size, infertility or hatching success, or between embryo heterozygosity and mortality. We discuss these results in the context of human-mediated introductions and how the history of introductions can play a fundamental role in influencing individual and population fitness in non-native species. | 104 | maybe | inbreeding ind | inbreeding | no | no |
| 105 | 2017 | 43004 | Miller, EJ; Eldridge, MDB; Morris, KD; Zenger, KR; Herbert, CA | 2011 | Genetic consequences of isolation: island tammar wallaby (Macropus eugenii) populations and the conservation of threatened species | Isolation and restricted gene flow can lead to genetic deterioration in populations. Populations of many species are increasingly becoming fragmented due to human impacts and active management is required to prevent further extinctions. Islands provide an ideal location to protect species from many mainland threatening processes such as habitat loss and fragmentation, disease and competition/predation from introduced species. However their isolation and small population size renders them prone to loss of genetic diversity and to inbreeding. This study examined two endemic and one introduced population of tammar wallaby (Macropus eugenii) on three islands in the Houtman Abrolhos Archipelago, Western Australia: East Wallabi (EWI), West Wallabi (WWI) and North Islands (NI). Nine autosomal and four Y-linked microsatellite loci, and sequence data from the mitochondrial DNA (mtDNA) control region were used to examine the impact of long-term isolation (EWI and WWI) and small founder size (NI) on genetic diversity and inbreeding. This study found all three populations had low genetic diversity, high levels of effective inbreeding and increased frequency of morphological abnormalities. Isolation has also led to significant inter-population genetic differentiation. These results highlight the importance of incorporating genetic management strategies when utilising islands as refuges for declining mainland populations. | 105 | no | inbreeding | NA | no | no |
| 106 | 2017 | 43004 | Ruiz-Lopez, MJ; Monello, RJ; Gompper, ME; Eggert, LS | 2012 | The Effect and Relative Importance of Neutral Genetic Diversity for Predicting Parasitism Varies across Parasite Taxa | Understanding factors that determine heterogeneity in levels of parasitism across individuals is a major challenge in disease ecology. It is known that genetic makeup plays an important role in infection likelihood, but the mechanism remains unclear as does its relative importance when compared to other factors. We analyzed relationships between genetic diversity and macroparasites in outbred, free-ranging populations of raccoons (Procyon lotor). We measured heterozygosity at 14 microsatellite loci and modeled the effects of both multi-locus and single-locus heterozygosity on parasitism using an information theoretic approach and including non-genetic factors that are known to influence the likelihood of parasitism. The association of genetic diversity and parasitism, as well as the relative importance of genetic diversity, differed by parasitic group. Endoparasite species richness was better predicted by a model that included genetic diversity, with the more heterozygous hosts harboring fewer endoparasite species. Genetic diversity was also important in predicting abundance of replete ticks (Dermacentor variabilis). This association fit a curvilinear trend, with hosts that had either high or low levels of heterozygosity harboring fewer parasites than those with intermediate levels. In contrast, genetic diversity was not important in predicting abundance of non-replete ticks and lice (Trichodectes octomaculatus). No strong single-locus effects were observed for either endoparasites or replete ticks. Our results suggest that in outbred populations multi-locus diversity might be important for coping with parasitism. The differences in the relationships between heterozygosity and parasitism for the different parasites suggest that the role of genetic diversity varies with parasite-mediated selective pressures. | 106 | maybe | individual level? Gd of parasites? | no pop level | no | no |
| 107 | 2017 | 43004 | Whitehorn, PR; Tinsley, MC; Brown, MJF; Darvill, B; Goulson, D | 2011 | Genetic diversity, parasite prevalence and immunity in wild bumblebees | Inbreeding and a consequent loss of genetic diversity threaten small, isolated populations. One mechanism by which genetically impoverished populations may become extinct is through decreased immunocompetence and higher susceptibility to parasites. Here, we investigate the relationship between immunity and inbreeding in bumblebees, using Hebridean island populations of Bombus muscorum. We sampled nine populations and recorded parasite prevalence and measured two aspects of immunity: the encapsulation response and levels of phenoloxidase (PO). We found that prevalence of the gut parasite Crithidia bombi was higher in populations with lower genetic diversity. Neither measure of immune activity was correlated with genetic diversity. However, levels of PO declined with age and were also negatively correlated with parasite abundance. Our results suggest that as insect populations lose heterozygosity, the impact of parasitism will increase, pushing threatened populations closer to extinction. | 107 | yes | from thesis | NA | yes | no |
| 108 | 2017 | 43004 | Berenos, C; Wegner, KM; Schmid-Hempel, P | 2011 | Antagonistic coevolution with parasites maintains host genetic diversity: an experimental test | Genetic variation in natural populations is a prime prerequisite allowing populations to respond to selection, but is under constant threat from forces that tend to reduce it, such as genetic drift and many types of selection. Haldane emphasized the potential importance of parasites as a driving force of genetic diversity. His theory has been taken for granted ever since, but despite numerous studies showing correlations between genetic diversity and parasitism, Haldane’s hypothesis has rarely been tested experimentally for unambiguous support. We experimentally staged antagonistic coevolution between the host Tribolium castaneum and its natural microsporidian parasite, Nosema whitei, to test for the relative importance of two separate evolutionary forces (drift and parasite-induced selection) on the maintenance of genetic variation. Our results demonstrate that coevolution with parasites indeed counteracts drift as coevolving populations had significantly higher levels of heterozygosity and allelic diversity. Genetic drift remained a strong force, strongly reducing genetic variation and increasing genetic differentiation in small populations. To our surprise, differentiation between the evolving populations was smaller when they coevolved with parasites, suggesting parallel balancing selection. Hence, our results experimentally vindicate Haldane’s original hypothesis 60 years after its conception. | 108 | no | no gd diff between pop | NA | no | no |
| 109 | 2017 | 43004 | Jugran, AK; Bhatt, ID; Rawal, RS; Nandi, SK; Pande, V | 2013 | Patterns of morphological and genetic diversity of Valeriana jatamansi Jones in different habitats and altitudinal range of West Himalaya, India | Importance to know and understand diversity of Himalayan plants is increasingly recognized considering the fact that various natural and anthropogenic pressures might bring about serious influences to morphological and genetic diversity of the vegetation in the region. In this context, Valeriana jatamansi was investigated in detail, taking into account its importance in various Ayurvedic and modern medicines. Randomly selected mature plants from twenty five different populations (located between 1215 m to 2775 m as’) of V. jatamansi were analysed for their morphological attributes. Further, ISSR markers were used to detect genetic variation among 151 plants of selected 25 populations. Use of 20 primers yielded 125 reproducible polymorphic loci which were used to estimate different parameters of genetic diversity. These parameters were in turn applied to develop relationships with habitat types and altitude range. Significant variation (p<0.05) in above ground dry weight (AGDW) and below ground dry weight (BGDW) across the populations was observed. Nei’s genetic diversity index (He) ranged from 0.25 to 0.37 across the populations, with a mean of 0.31. Genetic diversity exhibited a decreasing trend with increasing altitude, and maximum diversity (He=0.325) was observed in the range of 1201-1500 m asl. Among the different habitat conditions, highest genetic diversity (He = 0.334; Pp = 84.38) was observed in grassland habitats while minimum in mixed forest habitats (He = 0.285; Pp = 72.433). The genetic diversity (He) had significant negative relationships with AGDW, BGDW and rhizome diameter (Pearson r=-0.359, -0.424 and -0.317, respectively; p<0.05). The genetic characterization of V. jatamansi from the western Himalaya by this study suggests influences of habitat types and the altitudinal range upon genetic diversity, and based on these proposals for conservation strategies in favour of the species are made. (C) 2012 Elsevier GmbH. All rights reserved. | 109 | no | no good fitness measure | NA | no | no |
| 110 | 2017 | 43004 | Li, QM; He, TH; Xu, ZF | 2005 | Genetic evaluation of the efficacy of in situ and ex situ conservation of Parashorea chinensis (Dipterocarpaceae) in southwestern China | The majority of research in genetic diversity yields recommendations rather than actual conservation achievements. We assessed the efficacy of actual in situ and ex situ efforts to conserve Parashorea chinensis (Dipterocarpaceae) against the background of the geographic pattern of genetic variation of this species. Samples from seven natural populations, including three in a nature reserve, and one ex situ conservation population were studied. Across the natural populations, 47.8% of RAPD loci were polymorphic; only 20.8% on average varied at the population level. Mean population genetic diversity was 0.787 within natural populations and 1.410 for the whole species. Significant genetic differentiation among regions and isolation by distance were present on larger scales (among regions). AMOVA revealed that the majority of the among-population variation occurred among regions rather than among populations within regions. Regression analysis, Mantel test, principal coordinates analysis, and cluster analysis consistently demonstrated increasing genetic isolation with increasing geographic distance. Genetic differentiation within the region was quite low compared to that among regions. Multilocus spatial autocorrelation analysis of these three populations revealed random distribution of genetic variation in two populations, but genetic clustering was detected in the third population. The ex situ conserved population contained a medium level of genetic variation compared with the seven natural populations; it contained 77.1% of the total genetic variation of this species and 91% of the moderate to high frequency RAPD fragments (f > 0.05). Exclusive bands were detected in natural populations, but none were found in the ex situ conserved population. The populations protected in the nature reserve contained most of the genetic variation of the whole species, with 81.4% of the total genetic variation and 95.7% of the fragments with moderate to high frequency (f > 0.05) of this species conserved. The results show that the ex situ conserved population does not contain enough genetic variation to meet the need of release in the future, and that more extensive ex situ sampling in natural populations TY, NP, HK, and MG is needed. The in situ conserved population contains representative genetic variation to maintain long-term survival and evolutionary processes of P. chinensis. | 110 | no | no good fitness measure | NA | no | no |
| 111 | 2017 | 43004 | Agashe, D; Falk, JJ; Bolnick, DI | 2011 | EFFECTS OF FOUNDING GENETIC VARIATION ON ADAPTATION TO A NOVEL RESOURCE | Population genetic theory predicts that adaptation in novel environments is enhanced by genetic variation for fitness. However, theory also predicts that under strong selection, demographic stochasticity can drive populations to extinction before they can adapt. We exposed wheat-adapted populations of the flour beetle (Tribolium castaneum) to a novel suboptimal corn resource, to test the effects of founding genetic variation on population decline and subsequent extinction or adaptation. As previously reported, genetically diverse populations were less likely to go extinct. Here, we show that among surviving populations, genetically diverse groups recovered faster after the initial population decline. Within two years, surviving populations significantly increased their fitness on corn via increased fecundity, increased egg survival, faster larval development, and higher rate of egg cannibalism. However, founding genetic variation only enhanced the increase in fecundity, despite existing genetic variation-and apparent lack of trade-offs-for egg survival and larval development time. Thus, during adaptation to novel habitats the positive impact of genetic variation may be restricted to only a few traits, although change in many life-history traits may be necessary to avoid extinction. Despite severe initial maladaptation and low population size, genetic diversity can thus overcome the predicted high extinction risk in new habitats. | 111 | maybe | egg survival ok fitness measure? | NA | yes | no |
| 112 | 2017 | 43004 | Kovach, RP; Gharrett, AJ; Tallmon, DA | 2013 | Temporal patterns of genetic variation in a salmon population undergoing rapid change in migration timing | Though genetic diversity is necessary for population persistence in rapidly changing environments, little is known about how climate-warming influences patterns of intra-population genetic variation. For a pink salmon population experiencing increasing temperatures, we used temporal genetic data (microsatellite=1993, 2001, 2009; allozyme=1979, 1981, 1983) to quantify the genetic effective population size (N-e) and genetic divergence due to differences in migration timing and to estimate whether these quantities have changed over time. We predicted that temporal trends toward earlier migration timing and a corresponding loss of phenotypic variation would decrease genetic divergence based on migration timing and N-e. We observed significant genetic divergence based on migration timing and genetic heterogeneity between early- and late-migrating fish. There was also some evidence for divergent selection between early- and late-migrating fish at circadian rhythm genes, but results varied over time. Estimates of N-e from multiple methods were large (>1200) and N-e/N-c generally exceeded 0.2. Despite shifts in migration timing and loss of phenotypic variation, there was no evidence for changes in within-population genetic divergence or N-e over the course of this study. These results suggest that in instances of population stability, genetic diversity may be resistant to climate-induced changes in migration timing. | 112 | no | only one pop | NA | no | no |
| 113 | 2017 | 43004 | HUGHES, KA | 1995 | THE EVOLUTIONARY GENETICS OF MALE LIFE-HISTORY CHARACTERS IN DROSOPHILA-MELANOGASTER | Alternative models of the maintenance of genetic variability, theories of life-history evolution, and theories of sexual selection and mate choice can be tested by measuring additive and nonadditive genetic variances of components of fitness. A quantitative genetic breeding design was used to produce estimates of genetic variances for male life-history traits in Drosophila melanogaster. Additive genetic covariances and correlations between traits were also estimated. Flies from a large, outbred, laboratory population were assayed for age-specific competitive mating ability, age-specific survivorship, body mass, and fertility. Variance-component analysis then allowed the decomposition of phenotypic variation into components associated with additive genetic, nonadditive genetic, and environmental variability. A comparison of dominance and additive components of genetic variation provides little support for an important role for balancing selection in maintaining genetic variance in this suite of traits. The results provide support for the mutation-accumulation theory, but not the antagonistic-pleiotropy theory of senescence. No evidence is found for the positive genetic correlations between mating success and offspring quality or quantity chat are predicted by ‘’good genes’’ models of sexual selection. Additive genetic coefficients of variation for life-history characters are larger than those for body weight. Finally, this set of male life-history characters exhibits a very low correspondence between estimates of genetic and phenotypic correlations. | 113 | no | NA | NA | no | no |
| 114 | 2017 | 43004 | Fisher, MC; Viney, ME | 1998 | The population genetic structure of the facultatively sexual parasitic nematode Strongyloides ratti in wild rats | We have investigated the population genetic structure of the parasitic nematode Strongyloides ratti in wild rats. In the UK, S. ratti reproduces predominantly by mitotic parthenogenesis, with sexual forms present at a rate of less than 1%. S. ratti was found to be a prevalent parasite and substantial genetic diversity was detected. Most rats were infected with a genotypic mixture of parasites. A hierarchical analysis of the genetic variation found in S. ratti sampled across Britain and Germany showed that 73.3% was explained by variation between parasites within individual hosts and 25.3% by variation between rats within sample sites. Only a small proportion (1.4%) of the total genetic variation was attributable to genetic subdivision between sample sites, suggesting that there is substantial gene flow between these sites. Most parasites sampled were found to exist in Hardy-Weinberg Equilibrium and this population genetic structure is discussed in view of the virtual absence of sexual reproduction. | 114 | no | Gd of parasote | NA | no | no |
| 115 | 2017 | 43004 | Janova, E; Futas, J; Klumplerova, M; Putnova, L; Vrtkova, I; Vyskocil, M; Frolkova, P; Horin, P | 2013 | Genetic diversity and conservation in a small endangered horse population | The Old Kladruber horses arose in the 17th century as a breed used for ceremonial purposes. Currently, grey and black coat colour varieties exist as two sub-populations with different recent breeding history. As the population underwent historical bottlenecks and intensive inbreeding, loss of genetic variation is considered as the major threat. Therefore, genetic diversity in neutral and non-neutral molecular markers was examined in the current nucleus population. Fifty microsatellites, 13 single nucleotide polymorphisms (SNPs) in immunity-related genes, three mutations in coat colour genes and one major histocompatibility (MHC-DRA) gene were studied for assessing genetic diversity after 15 years of conservation. The results were compared to values obtained in a similar study 13 years ago. The extent of genetic diversity of the current population was comparable to other breeds, despite its small size and isolation. The comparison between 1997 and 2010 did not show differences in the extent of genetic diversity and no loss of allele richness and/or heterozygosity was observed. Genetic differences identified between the black and grey sub-populations observed 13 years ago persisted. Deviations from the Hardy-Weinberg equilibrium found in 19 microsatellite loci and in five SNP loci are probably due to selective breeding. No differences between neutral and immunity-related markers were found. No changes in the frequencies of markers associated with two diseases, melanoma and insect bite hypersensitivity, were observed, due probably to the short interval of time between comparisons. It, thus, seems that, despite its small size, previous bottlenecks and inbreeding, the molecular variation of Old Kladruber horses is comparable to other horse breeds and that the current breeding policy does not compromise genetic variation of this endangered population. | 115 | no | only one pop | NA | no | no |
| 116 | 2017 | 43004 | Gonzalez-Varo, JP; Albaladejo, RG; Aparicio, A; Arroyo, J | 2010 | Linking genetic diversity, mating patterns and progeny performance in fragmented populations of a Mediterranean shrub | 1. The long-term persistence of fragmented plant populations is predicted to be threatened by a loss of genetic variability and increasing inbreeding, which might lower offspring fitness through inbreeding depression. Assessing plant progeny performance together with measurements of genetic diversity and mating patterns is therefore essential in the understanding of the role of the historical (i.e. genetic diversity in adults) and contemporary (i.e. current mating patterns) genetic effects of fragmentation on inbreeding depression, thus, on recruitment potential. 2. We evaluated genetic diversity, mating patterns and progeny performance at different life stages in seven populations of a Mediterranean shrub (myrtle Myrtus communis) that differed in size and degree of isolation (Large, Small-connected and Small-isolated populations). The study was conducted in the Guadalquivir Valley (SW Spain), a chronically and severely fragmented landscape characterized by c. 1% of woodland cover. 3. Parameters of genetic diversity (A(r), H(o) and H(e)) of adult plants were in general higher in the Large populations than in the two types of Small populations, which were similar. Outcrossing rates were higher in Small-connected populations (mean: t(m) = 0.62), intermediate in Large (t(m) = 0.35) and lower in Small-isolated populations (t(m) = 0.13), and were positively correlated with the genetic diversity of progenies. 4. Several measurements of progeny performance were higher in Small-connected populations, intermediate in Large and lower in Small-isolated populations, in particular those related with the quantity of viable seedlings produced (germination and survival). Outcrossing rates rather than the genetic diversity of adult plants were positively correlated with these measurements of progeny performance. 5. We thus conclude that contemporary mating patterns (outcrossing rates) have a more critical influence on progeny performance than either population fragmentation or the historical levels of genetic diversity. 6. Synthesis and applications. It may be possible to enhance either the fitness or certain levels of genetic diversity in progenies by promoting outcrossed matings in fragmented populations of self-compatible plant species. In our study species, this would be feasible either by controlling honeybee Apis mellifera hives or maintaining and/or enhancing landscape connectivity around small patches. | 116 | probably no | gd difference? | Allelic richness not corrected for sample size | no | no |
| 117 | 2017 | 43004 | Magalhaes, S; Fayard, J; Janssen, A; Carbonell, D; Olivieri, I | 2007 | Adaptation in a spider mite population after long-term evolution on a single host plant | Evolution in a single environment is expected to erode genetic variability, thereby precluding adaptation to novel environments. To test this, a large population of spider mites kept on cucumber for approximately 300 generations was used to establish populations on novel host plants (tomato or pepper), and changes in traits associated to adaptation were measured after 15 generations. Using a half-sib design, we investigated whether trait changes were related to genetic variation in the base population. Juvenile survival and fecundity exhibited genetic variation and increased in experimental populations on novel hosts. Conversely, no variation was detected for host choice and developmental time and these traits did not evolve. Longevity remained unchanged on novel hosts despite the presence of genetic variation, suggesting weak selection for this trait. Hence, patterns of evolutionary changes generally matched those of genetic variation, and changes in some traits were not hindered by long-term evolution in a constant environment. | 117 | no | gd of parasite | NA | no | no |
| 118 | 2017 | 43004 | Li, JZ; Absher, DM; Tang, H; Southwick, AM; Casto, AM; Ramachandran, S; Cann, HM; Barsh, GS; Feldman, M; Cavalli-Sforza, LL; Myers, RM | 2008 | Worldwide human relationships inferred from genome-wide patterns of variation | Human genetic diversity is shaped by both demographic and biological factors and has fundamental implications for understanding the genetic basis of diseases. We studied 938 unrelated individuals from 51 populations of the Human Genome Diversity Panel at 650,000 common single- nucleotide polymorphism loci. Individual ancestry and population substructure were detectable with very high resolution. The relationship between haplotype heterozygosity and geography was consistent with the hypothesis of a serial founder effect with a single origin in sub- Saharan Africa. In addition, we observed a pattern of ancestral allele frequency distributions that reflects variation in population dynamics among geographic regions. This data set allows the most comprehensive characterization to date of human genetic variation. | 118 | no | human | NA | no | no |
| 119 | 2017 | 43004 | Stanton, S; Honnay, O; Jacquemyn, H; Roldan-Ruiz, I | 2009 | A comparison of the population genetic structure of parasitic Viscum album from two landscapes differing in degree of fragmentation | Parasite populations do not necessarily conform to expected patterns of genetic diversity and structure. Parasitic plants may be more vulnerable to the negative consequences of landscape fragmentation because of their specialized life history strategies and dependence on host plants, which are themselves susceptible to genetic erosion and reduced fitness following habitat change. We used AFLP genetic markers to investigate the effects of habitat fragmentation on genetic diversity and structure within and among populations of hemiparasitic Viscum album. Comparing populations from two landscapes differing in the amount of forest fragmentation allowed us to directly quantify habitat fragmentation effects. Populations from both landscapes exhibited significant isolation-by-distance and sex ratios biased towards females. The less severely fragmented landscape had larger and less isolated populations, resulting in lower levels of population genetic structure (F(ST) = 0.05 vs. 0.09) and inbreeding (F(IS) = 0.13 vs. 0.27). Genetic differentiation between host-tree subpopulations was also higher in the more fragmented landscape. We found no significant differences in within-population gene diversity, percentage of polymorphic loci, or molecular variance between the two regions, nor did we find relationships between genetic diversity measures and germination success. Our results indicate that increasing habitat fragmentation negatively affects population genetic structure and levels of inbreeding in V. album, with the degree of isolation among populations exerting a stronger influence than forest patch size. | 119 | no | gd of parasite | NA | no | no |
| 120 | 2017 | 43004 | Valsecchi, E; Amos, W; Raga, JA; Podesta, M; Sherwin, W | 2004 | The effects of inbreeding on mortality during a morbillivirus outbreak in the Mediterranean striped dolphin (Stenella coeruleoalba) | Between 1990 and 1992, Mediterranean striped dolphins (Stenella coeruleoalba) suffered high mortality due to a morbillivirus epidemic. Ten highly variable microsatellite markers were used to assess the population structure of a sample of these stranded animals and to assess the genetic consequences of the epizootic on present stocks. We found little evidence of population structure within the Mediterranean, but distinct separation between this and the North Sea (Atlantic) population, the latter also showing greater genetic diversity. Using a genetic measure of inbreeding, we found that dolphins dying early in the outbreak were significantly more inbred than those dying later. Within 10 years of the end of the epidemic, the level of inbreeding among stranded dolphins had returned to its pre-outbreak levels. However, on average all stranded animals showed elevated levels of inbreeding, suggesting that animals dying from disease may venture towards the shore more than those dying of old age. Our results imply an important role for inbreeding in the dynamics of disease spread and that, in marine mammal research, caution should be exercised when inferring demographic parameters from stranded specimens. | 120 | no | inbreeding ind | NA | no | no |
| 121 | 2017 | 43004 | de Oliveira, EJF; Garcia, JE; Contel, EPB; Duarte, JMB | 2005 | Genetic structure of Blastocerus dichotomus populations in the Parana River basin (Brazil) based on protein variability | The population structure of 147 marsh deer (Blastocerus dichotomus) from three areas in the Parana River basin, Brazil, was studied by observing protein polymorphism at 17 loci. Six loci were polymorphic and 11 monomorphic. The proportion of polymorphic loci (P) was 35.29% and the average heterozygosity (H) was 6.31%. Wright’s F-ST indicated that only 4.9% of the total variation in allelic frequencies was due to genetic differences between the three groups. The high value of F-IS (0.246) indicated inbreeding in the marsh deer. Genetic distance values (D = 0.014-0.051) showed little divergence between the three areas. We suggest that probable mechanisms accounting for the genetic structure are female phylopatry and polygyny and also that inbreeding has resulted from decreasing areas of wetland leading to isolation, overhunting, and diseases transmitted by cattle. | 121 | no | no good fitness measure | NA | no | no |
| 122 | 2017 | 43004 | MacDougall-Shackleton, EA; Derryberry, EP; Foufopoulos, J; Dobson, AP; Hahn, TP | 2005 | Parasite-mediated heterozygote advantage in an outbred songbird population | Coevolution with parasites is thought to maintain genetic diversity in host populations. However, while there are sound theoretical reasons to expect heterozygosity and parasite resistance to be related, this pattern has generally been shown only in inbred laboratory and island populations. This leaves doubt as to whether parasite-mediated selection for genetic diversity is in fact a general process. Here we show that haematozoan parasite load is linked to two complementary measures of microsatellite variability in an outbred population of mountain white-crowned sparrows (Zonotrichia leucophrys oriantha) for which we know that parasites reduce fitness. Moreover, each of the genetic measures predicts a subtly different aspect of parasitism. Microsatellite heterozygosity is related to an individual’s risk of parasitism, and mean d(2) (a broader, more long-term measure of parental relatedness) to the severity of infection among parasitized individuals. | 122 | no | only one pop | NA | no | no |
| 123 | 2017 | 43004 | Akesson, M; Liberg, O; Sand, H; Wabakken, P; Bensch, S; Flagstad, O | 2016 | Genetic rescue in a severely inbred wolf population | Natural populations are becoming increasingly fragmented which is expected to affect their viability due to inbreeding depression, reduced genetic diversity and increased sensitivity to demographic and environmental stochasticity. In small and highly inbred populations, the introduction of only a few immigrants may increase vital rates significantly. However, very few studies have quantified the long-term success of immigrants and inbred individuals in natural populations. Following an episode of natural immigration to the isolated, severely inbred Scandinavian wolf (Canis lupus) population, we demonstrate significantly higher pairing and breeding success for offspring to immigrants compared to offspring from native, inbred pairs. We argue that inbreeding depression is the underlying mechanism for the profound difference in breeding success. Highly inbred wolves may have lower survival during natal dispersal as well as competitive disadvantage to find a partner. Our study is one of the first to quantify and compare the reproductive success of first-generation offspring from migrants vs. native, inbred individuals in a natural population. Indeed, our data demonstrate the profound impact single immigrants can have in small, inbred populations, and represent one of very few documented cases of genetic rescue in a population of large carnivores. | 123 | no | only one pop | NA | no | no |
| 124 | 2017 | 43004 | Solazzo, G; Moritz, RFA; Settele, J | 2014 | The social parasite Phengaris (Maculinea) nausithous affects genetic diversity within Myrmica rubra host ant colonies | Evolutionary theory predicts that high genetic variation maintains plasticity in a species’ response to parasite pressure. However, higher genetic diversity might also cause easier infiltration by social parasites, because odour diversity is high and nest-mate recognition poor. Here we test if the obligate myrmecophile Lycaenid butterfly Phengaris nausithous, a parasite of colonies of the highly polygynous ant Myrmica rubra causes local adaptation by enhancing genetic variance in parasitized versus non parasitized ant populations M. rubra colonies from six infested and three uninfested sites were assayed at five microsatellite loci to quantify genetic variation. Our results reveal isolation by distance and a significantly enhanced intracolonial variance due to the parasite pressure. | 124 | maybe | parasite drives GD of host colony? | NA | yes | yes |
| 125 | 2017 | 43004 | Blanchet, S; Rey, O; Berthier, P; Lek, S; Loot, G | 2009 | Evidence of parasite-mediated disruptive selection on genetic diversity in a wild fish population | Identifying the processes maintaining genetic variability in wild populations is a major concern in conservation and evolutionary biology. Parasite-mediated selection may strongly affect genetic variability in wild populations. The inbreeding depression theory predicts that directional selection imposed by parasites should act against the most inbred hosts, thus favouring genetic diversity in wild populations. We have tested this prediction by evaluating the strength and shape of the relationship between the load of a harmful fin-feeder ectoparasite (Tracheliastes polycolpus) and the genome-wide genetic diversity (i.e. heterozygosity measured at a set of 15 microsatellites) of its fish host, the rostrum dace (Leuciscus leuciscus). Contrary to expectation, we found a nonlinear relationship between host genetic diversity and ectoparasite load, with hosts that were either homozygous or heterozygous harbouring significantly fewer parasites than hosts with an intermediate level of heterozygosity. This relationship suggests that parasites could increase the variance of global heterozygosity in this host population through disruptive selection on genetic diversity. Moreover, when genetic diversity was measured at each locus separately, we found two very strong positive associations between host genetic diversity and the ectoparasite load. This latter result has three main implications: (i) genome-wide effect cannot alone explain the nonlinear relationship between global heterozygosity and ectoparasite load, (ii) negative non-additive allelic interactions (i.e. underdominance) may be a mechanism for resisting ectoparasite infection, and (iii) ectoparasites may favour homozygosity at some loci in this host population. | 125 | no | NA | NA | no | no |
| 126 | 2017 | 43004 | Biebach, I; Keller, LF | 2012 | Genetic variation depends more on admixture than number of founders in reintroduced Alpine ibex populations | Reintroductions and other conservation translocations have become increasingly important conservation tools, albeit with variable success. Genetic variation is one factor, which may influence reintroduction success. Genetic variation in reintroduced populations can be augmented by increasing the number of founders or by admixing animals from different source populations. At present there is no clear understanding of the relative importance of the two. Here we address this question by combining detailed demographic information about the reintroduction history of 40 Alpine ibex populations with genetic data from neutral markers, including coalescent-based estimates of the number of genetic founders. Number of genetic founders was a better predictor of present-day genetic variation than number of released founders, indicating that differential survival of founders can substantially affect the genetic variation of reintroduced populations. The degree of admixture in the founder group had about twice as much impact on genetic variation than the number of founders. Thus, to maintain genetic variation in reintroduced populations, releasing animals from different sources might be more important than releasing many animals from a single source. This even applies to cases such as the Alpine ibex where all individuals descended from a single ancestral population, and where the admixture was only between sub-populations created by the reintroduction program and thus between populations with relatively little genetic differentiation. (C) 2012 Elsevier Ltd. All rights reserved. | 126 | no | any gd and fitness of groups? | NA | no | no |
| 127 | 2017 | 43004 | Fajardo, CG; Vieira, FD; Felix, LP; Molina, WF | 2017 | Negligence in the Atlantic forest, northern Brazil: a case study of an endangered orchid | Currently, many Brazilian orchids are threatened with extinction resulting from habitat loss and intense harvesting pressure stemming from their value as ornamental plants. Therefore, the genetic diversity in remaining populations is fundamental to the survival of these species in natural environments. In order to inform conservation strategies, this study evaluated the genetic diversity and structure of Cattleya granulosa populations. The sample consisted of 151 individuals from 12 populations in the Atlantic Forest, northeastern Brazil, evaluated using 91 ISSR markers. Genetic variability was assessed through molecular variance, diversity indexes, clusters of genotypes through Bayesian analysis, and tests for genetic bottlenecks. From all polymorphic loci, genetic diversity (H-E) varied between 0.210 and 0.321 and the Shannon index ranged from 0.323 and 0.472. Significant genetic differentiation between populations (I broken vertical bar(ST) = 0.391; P < 0.0001) resulted in the division of the populations into five groups based on the log-likelihood Bayesian analysis. We found significant positive correlation between geographical and genetic distances between populations (r = 0.794; P = 0.017), indicating isolation by distance. Patterns of allelic diversity within populations suggest the occurrence of bottlenecks in most C. granulosa populations (n = 8). Therefore, in order to maintain the genetic diversity of the species, the conservation of spatially distant groups is necessary. | 127 | no | no good fitness measure | NA | no | no |
| 128 | 2017 | 43004 | Guha, S; Chakraborty, R | 2008 | Correlation analyses reveal a substantial influence of allelic gaps on the investigation of genetic diversity of modern human populations with microsatellites | High intra-population genetic diversity and multiple measures of genetic variability at STR loci are useful in inferring past evolutionary history. However, STRs, categorized by their repeat motif size, differ in a number of aspects, requiring separate analyses. We analyzed 783 STRs in 36 worldwide populations to examine marker suitability as well as correlations between various measurements, to evaluate the extent of genomic diversity present in modern human populations. The loci were grouped by type and analyzed separately for each population group. Genetic variation defined by gene diversity and allele size variance, shows different trends of variation across four types of STRs. Additionally, there is little variation of genetic diversity, but there is decreased allelic size variance with increasing repeat motifs. A poor correlation between genetic diversity and allelic size variance across loci in all groups for Di-STRs is probably caused by the presence of allelic size gaps. In contrast, allelic size variance, genetic diversity, and number of alleles are strongly correlated with both tri- and tetra-STRs. The positive correlation of allelic size variance and presence of gaps within the range of allelic sizes in Di-STRs alone explains these observations. An unexpected high imbalance index (beta) at Di-STRs due to high allelic size variance also supports this assertion. | 128 | no | NA | NA | no | no |
| 129 | 2017 | 43004 | Ciborowski, K; Jordan, WC; de Leaniz, CG; Consuegra, S | 2017 | Temporal and spatial instability in neutral and adaptive (MHC) genetic variation in marginal salmon populations | The role of marginal populations for the long-term maintenance of species’ genetic diversity and evolutionary potential is particularly timely in view of the range shifts caused by climate change. The Centre-Periphery hypothesis predicts that marginal populations should bear reduced genetic diversity and have low evolutionary potential. We analysed temporal stability at neutral microsatellite and adaptive MHC genetic variation over five decades in four marginal Atlantic salmon populations located at the southern limit of the species’ distribution with a complicated demographic history, which includes stocking with foreign and native salmon for at least 2 decades. We found a temporal increase in neutral genetic variation, as well as temporal instability in population structuring, highlighting the importance of temporal analyses in studies that examine the genetic diversity of peripheral populations at the margins of the species’ range, particularly in face of climate change. | 129 | no | NA | NA | no | no |
| 130 | 2017 | 43004 | Tan, CKW; Pizzari, T; Wigby, S | 2013 | PARENTAL AGE, GAMETIC AGE, AND INBREEDING INTERACT TO MODULATE OFFSPRING VIABILITY IN DROSOPHILA MELANOGASTER | In principle, parental relatedness, parental age, and the age of parental gametes can all influence offspring fitness through inbreeding depression and the parental effects of organismal and postmeiotic gametic senescence. However, little is known about the extent to which these factors interact and contribute to fitness variation. Here, we show that, in Drosophila melanogaster, offspring viability is strongly affected by a three-way interaction between parental relatedness, parental age, and gametic age at successive developmental stages. Overall egg-to-adult viability was lowest for offspring produced with old gametes of related, young parents. This overall effect was largely determined at the pupa-adult stage, although three-way interactions between parental relatedness, parental age and gametic age also explained variation in egg hatchability and larva-pupa survival. Controlling for the influence of parental and gametic age, we show that inbreeding depression is negligible for egg hatchability but significant at the larva-pupa and pupa-adult stages. At the pupa-adult stage, where offspring could be sexed, parental relatedness, parental age, and gametic age interacted differently in male and female offspring, with daughters suffering higher inbreeding depression than sons. Collectively, our results demonstrate that the architecture of offspring fitness is strongly influenced by a complex interaction between parental effects, inbreeding depression and offspring sex. | 130 | no | NA | NA | no | no |
| 131 | 2017 | 43004 | Strand, TM; Segelbacher, G; Quintela, M; Xiao, LY; Axelsson, T; Hoglund, J | 2012 | Can balancing selection on MHC loci counteract genetic drift in small fragmented populations of black grouse? | The ability of natural populations to adapt to new environmental conditions is crucial for their survival and partly determined by the standing genetic variation in each population. Populations with higher genetic diversity are more likely to contain individuals that are better adapted to new circumstances than populations with lower genetic diversity. Here, we use both neutral and major histocompatibility complex (MHC) markers to test whether small and highly fragmented populations hold lower genetic diversity than large ones. We use black grouse as it is distributed across Europe and found in populations with varying degrees of isolation and size. We sampled 11 different populations; five continuous, three isolated, and three small and isolated. We tested patterns of genetic variation in these populations using three different types of genetic markers: nine microsatellites and 21 single nucleotide polymorphisms (SNPs) which both were found to be neutral, and two functional MHC genes that are presumably under selection. The small isolated populations displayed significantly lower neutral genetic diversity compared to continuous populations. A similar trend, but not as pronounced, was found for genotypes at MHC class II loci. Populations were less divergent at MHC genes compared to neutral markers. Measures of genetic diversity and population genetic structure were positively correlated among microsatellites and SNPs, but none of them were correlated to MHC when comparing all populations. Our results suggest that balancing selection at MHC loci does not counteract the power of genetic drift when populations get small and fragmented. | 131 | no | NA | NA | no | no |
| 132 | 2017 | 43004 | Biagini, B; Imazio, S; Scienza, A; Failla, O; De Lorenzis, G | 2016 | Renewal of wild grapevine (Vitis vinifera L. subsp sylvestris (Gmelin) Hegi) populations through sexual pathway: Some Italian case studies | Wild Euro-Asiatic grapevine (Vitis vinifera L. subsp. sylvestris (Gmelin) Hegi) is an opportunistic liana with reproductive patterns characterized both by vegetative propagation and sexual reproduction. Vegetative propagation would ensure a rapid vine regeneration and land colonization, while sexual reproduction would assure genetic recombination and chromosome re-assortment crucial for evolution and survival of the species. The aim of this study was the analysis of the parentage relationships within and between fourteen Italian wild grapevine populations, in order to characterize the natural mating system of this taxon. Beside parentage relationship analyses, the estimation of genetic diversity, genetic distance and genetic differentiation were assessed. The populations showed a good extent of genetic diversity and low levels of genetic distance and isolation between them, indicating gene flow among sites. This result was supported by parentage relationships between different populations. Nevertheless, populations from North Italy showed the lowest genetic diversity, while internal relatedness and inbreeding estimation values were higher than the populations from Central and Southern Italy. One of the northern populations resulted particularly high in inbreeding intensity. The parentage relationship analysis pointed out in all sites a, sometimes active, sexual reproduction within populations. Seed dispersal by frugivorous vectors among neighbouring sites seemed less frequent and depending on specific environmental circumstances. (C) 2016 Elsevier GmbH. All rights reserved. | 132 | no | NA | NA | no | no |
| 133 | 2017 | 43004 | Berry, OF | 2006 | Inbreeding and promiscuity in the endangered grand skink | The inbreeding avoidance hypothesis predicts that organisms that often encounter relatives as potential mates should evolve behaviours to avoid incestuous matings. Avoidance behaviours have practical importance for small populations because deleterious genetic processes may be less imminent than otherwise expected from genetic models that assume random mating. I used genetic techniques to investigate the extent of inbreeding and inbreeding avoidance behaviours in rare lizards from southern New Zealand. Grand skinks, Oligosoma grande, live in small patchily distributed groups, and have low rates of inter-group dispersal (ca. 3-20% disperse). I used data from 15 microsatellite loci to test the hypothesis that adults are likely to encounter kin as potential mates and will inbreed. These data showed that adult skinks usually inhabited rock outcrops with adult relatives of the opposite sex - up to 35% of potential mates were of equivalent relatedness as half-sibs and 17% were equivalent to full sibs. However, skinks did not preferentially breed with less related mates, and 18.2% of matings were between individuals of equivalent relatedness as full-sibs. Instead, skinks mated with partners of all levels of relatedness, and were promiscuous - almost half of adult females and nearly three quarters of adult males reproduced with multiple partners. In addition, inbreeding had no effect on survival of offspring in their first year. Two other putative mechanisms of inbreeding avoidance, sex-biased and natal dispersal, were not pronounced in this species. This study adds to a growing list of species that inbreed despite the risks. | 133 | no | NA | NA | no | no |
| 134 | 2017 | 43004 | Tambarussi, EV; Boshier, D; Vencovsky, R; Freitas, MLM; Sebbenn, AM | 2017 | Inbreeding depression from selfing and mating between relatives in the Neotropical tree Cariniana legalis Mart. Kuntze | Selfing or mating between related individuals in self-compatible hermaphroditic tree species may lead to inbreeding depression (ID) due to homozygosis in recessive, identical by descent alleles. In general, studies of ID in tree species have been based on comparisons of selfed individuals (produced by controlled pollination) with outcrossed individuals for quantitative traits in progeny tests. However, this approach requires a long time to quantify the extent of ID. Thus, we used an approach based on genetic markers to estimate coancestry coefficients between assigned parents from paternity analysis in two populations of the Neotropical tree Cariniana legalis. Using this method, we were able to determine which seedlings in a nursery trial originated from; (i) outcrossing between un-related trees, (ii) mating between related trees and (iii) selfing. We detected a low selfing rate (< 10 %), but a substantial quantity of seedlings from mating between related parents (minimum of 35.7 %). In general, the outcrossed seedlings from unrelated parents exhibited significantly greater genetic diversity than those resulting from selfing and mating among relatives. The extent of ID varied among traits and populations. Outcrossed seedlings originating from unrelated trees generally showed greater survival than seedlings originating from selfing and related parents. Inbreeding depression was greater in the selfed seedlings than in those from mating among related parents. The results are discussed in terms of implications for genetic conservation, breeding and environmental restoration using the species. | 134 | no | NA | NA | no | no |
| 135 | 2017 | 43004 | Grueber, CE; Waters, JM; Jamieson, IG | 2011 | The imprecision of heterozygosity-fitness correlations hinders the detection of inbreeding and inbreeding depression in a threatened species | In nonpedigreed wild populations, inbreeding depression is often quantified through the use of heterozygosity-fitness correlations (HFCs), based on molecular estimates of relatedness. Although such correlations are typically interpreted as evidence of inbreeding depression, by assuming that the marker heterozygosity is a proxy for genome-wide heterozygosity, theory predicts that these relationships should be difficult to detect. Until now, the vast majority of empirical research in this area has been performed on generally outbred, nonbottlenecked populations, but differences in population genetic processes may limit extrapolation of results to threatened populations. Here, we present an analysis of HFCs, and their implications for the interpretation of inbreeding, in a free-ranging pedigreed population of a bottlenecked species: the endangered takahe (Porphyrio hochstetteri). Pedigree-based inbreeding depression has already been detected in this species. Using 23 microsatellite loci, we observed only weak evidence of the expected relationship between multilocus heterozygosity and fitness at individual life-history stages (such as survival to hatching and fledging), and parameter estimates were imprecise (had high error). Furthermore, our molecular data set could not accurately predict the inbreeding status of individuals (as ‘inbred’ or ‘outbred’, determined from pedigrees), nor could we show that the observed HFCs were the result of genome-wide identity disequilibrium. These results may be attributed to high variance in heterozygosity within inbreeding classes. This study is an empirical example from a free-ranging endangered species, suggesting that even relatively large numbers (> 20) of microsatellites may give poor precision for estimating individual genome-wide heterozygosity. We argue that pedigree methods remain the most effective method of quantifying inbreeding in wild populations, particularly those that have gone through severe bottlenecks. | 135 | no | NA | NA | no | no |
| 136 | 2017 | 43004 | Gibbs, HL; Chiucchi, JE | 2012 | Inbreeding, body condition, and heterozygosity-fitness correlations in isolated populations of the endangered eastern massasauga rattlesnake (Sistrurus c. catenatus) | Endangered species often occur in small populations that may have a greater risk of short-term extinction due to the negative effects of inbreeding depression. The eastern massasauga (Sistrurus c. catenatus) is an endangered rattlesnake that is found in isolated populations of varying size throughout its range. Here, we investigate whether variation in an indirect measure of individual fitness (relative body condition) can be explained by genome-wide levels of genetic variation (based on 19 microsatellite loci) and other factors. To do this, we use genetic and phenotypic data from individual snakes sampled from 14 populations throughout the species’ range. We tested for levels of inbreeding by comparing observed mean multi-locus heterozygosity (MLH) for each population (an estimate of average inbreeding) with the expected distribution under random mating. We then looked for evidence of heterozygosity-fitness correlations (HFCs) using a measures of individual MLH and relative body condition. In all but one population, observed MLH values are indistinguishable from those generated under a model of random mating implying low levels of inbreeding in most populations. There was significant variation in both mean MLH and mean body condition within and among populations but evidence for inbreeding depression was equivocal: in support, there were some high (but largely non-significant) HFCs effect sizes within a number populations, including one that showed significant evidence for both inbreeding and a HFC. Overall, however, there was no significant correlation between MLH and body condition across all populations after controlling for non-genetic factors such as sex, season of capture and year of capture. Our results suggest that among-population and individual differences in fitness (measured as body condition) in these snakes are better explained by short-term ecological factors rather than genetic mechanisms, but leave open the possibility that limited undetected effects of inbreeding depression are present. | 136 | no | NA | NA | no | no |
| 137 | 2017 | 43004 | Lifjeld, JT; Gohli, J; Johnsen, A | 2013 | PROMISCUITY, SEXUAL SELECTION, AND GENETIC DIVERSITY: A REPLY TO SPURGIN | We recently reported a positive association between female promiscuity and genetic diversity across passerine birds, and launched the hypothesis that female promiscuity acts as a balancing selection, pressure maintaining genetic diversity in populations (Gohli et al. ). Spurgin () questions both our analyses and interpretations. While we agree that the hypothesis needs more comprehensive empirical testing, we find his specific points of criticism unjustified. In a more general perspective, we call for a more explicit recognition of female mating preferences as mechanisms of selection in population genetics theory. | 137 | no | NA | NA | no | no |
| 138 | 2017 | 43004 | Zaghloul, MS; Hamrick, JL; Moustafa, AA; Kamel, WM; El-Ghareeb, R | 2006 | Genetic diversity within and among sinai populations of three Ballota species (Lamiaceae) | Ballota undulata, Ballota kaiseri, and Ballota saxatilis are very rare ( and endemic - B. kaiseri), threatened species growing in St. Catherine Protectorate, southern Sinai, Egypt. They are subjected to a number of threats that have caused populations to decline in both number and size. For the long-term survival of these species, an appropriate conservation strategy for the maintenance of their genetic variation should be developed. This study measures genetic diversity within and among populations of these Ballota species and determines the conservation implications of the results. The genetic analyses demonstrated that the three Ballota species maintain relatively high levels of genetic diversity ( H-e = 0.195-0.317) and that most of the their genetic diversity was found within populations ( G(ST) = 0.045 - 0.099). Indirect estimates of historical gene flow for B. undulata and B. saxatilis were relatively high ( Nm(W) = 5.25 and 3.37, respectively) but suggest that there is somewhat less gene movement among B. kaiseri populations ( Nm( W) = 2.29). The levels of genetic diversity maintained within populations of the three Ballota species indicate that an appropriate sampling design for ex situ safeguarding should capture the majority of the genetic diversity found within these taxa. | 138 | no | no good fitness measure | NA | no | no |
| 139 | 2017 | 43004 | Kojo, N; Higuchi, N; Minami, M; Ohnishi, N; Okada, A; Takatsuki, S; Tamate, HB | 2012 | Correlation between genetic diversity and neonatal weight of sika deer (Cervus nippon) fawns | “Genetic variations and their relationships to individual fitness have been the major topics in studies of ecology and evolution. The concept that a reduction in genetic variations leads to lower fecundity termed”“inbreeding depression”" has been predominantly supported by studies involving laboratory organisms, but has rarely been tested in wild populations. We therefore examined whether the individual genetic diversity among a population of wild sika deer (Cervus nippon) on Kinkazan Island, northern Japan, affected neonatal weight that is often considered as a fitness-related trait. First, the neonatal weights of fawns born during 1994-2004 were recorded, and 11 microsatellite loci were genotyped to obtain 5 different estimates of an individual’s genetic diversity. To test whether the genetic diversities of fawns correlated with their neonatal weights, multivariate analyses using a generalized linear mixed model were performed. The neonatal weights were significantly affected by standardized multilocus heterozygosity and mother’s body condition, as well as the interaction of these factors. Interestingly, the model indicated that neonatal weight was positively correlated with the genetic diversities of fawns only when the mothers’ body weight exceeded a certain level that varies according to their reproductive history." | 139 | no | only one pop | NA | no | no |
| 140 | 2017 | 43004 | Hadziabdic, D; Wang, XW; Wadl, PA; Rinehart, TA; Ownley, BH; Trigiano, RN | 2012 | Genetic diversity of flowering dogwood in the Great Smoky Mountains National Park | In the past three decades, flowering dogwood (Cornus florida) populations have experienced severe declines caused by dogwood anthracnose. Mortality has ranged from 48% to 98%, raising the concern that the genetic diversity of this native tree has been reduced significantly. In this study, we investigated levels of genetic diversity and population structure of flowering dogwood populations in the Great Smoky Mountains National Park (GSMNP). Understanding the factors influencing geographic distribution of genetic variation is one of the major concerns for preserving biodiversity and conservation of native populations. Eighteen microsatellite loci were used to evaluate the level and distribution of genetic variation of native flowering dogwood trees throughout the GSMNP. Significant genetic structure exists at both landscape and local levels. Two genetic clusters exist within the park and are separated by the main dividing ridges of the Great Smoky Mountains. The differentiation of the clusters is subtle, but statistically significant. Gene flow, evident through low-elevation corridors, indicates that nonrandom mating occurs between related individuals despite wide dispersal of seeds. Although high mortality rate and reduced fecundity caused by dogwood anthracnose severely affected native flowering dogwood populations throughout the entire GSMNP, this study confirmed that considerable genetic diversity still exists at the population level. It seems unlikely that recent demographic dynamics have resulted in a depletion of genetic variation. | 140 | no | NA | NA | no | no |
| 141 | 2017 | 43004 | Gooley, R; Hogg, CJ; Belov, K; Grueber, CE | 2017 | No evidence of inbreeding depression in a Tasmanian devil insurance population despite significant variation in inbreeding | Inbreeding depression occurs when inbred individuals experience reduced fitness as a result of reduced genome-wide heterozygosity. The Tasmanian devil faces extinction due to a contagious cancer, devil facial tumour disease (DFTD). An insurance metapopulation was established in 2006 to ensure the survival of the species and to be used as a source population for re-wilding and genetic rescue. The emergence of DFTD and the rapid decline of wild devil populations have rendered the species at risk of inbreeding depression. We used 33 microsatellite loci to (1) reconstruct a pedigree for the insurance population and (2) estimate genome-wide heterozygosity for 200 individuals. Using heterozygosity-fitness correlations, we investigated the effect of heterozygosity on six diverse fitness measures (ulna length, asymmetry, weight-at-weaning, testes volume, reproductive success and survival). Despite statistically significant evidence of variation in individual inbreeding in this population, we found no associations between inbreeding and any of our six fitness measurements. We propose that the benign environment in captivity may decrease the intensity of inbreeding depression, relative to the stressful conditions in the wild. Future work will need to measure fitness of released animals to facilitate translation of this data to the broader conservation management of the species in its native range. | 141 | no | only one pop | NA | no | no |
| 142 | 2017 | 43004 | Jackson, ND; Fahrig, L | 2011 | Relative effects of road mortality and decreased connectivity on population genetic diversity | “Roads can have two important effects on populations that impact genetic variation: reduced gene flow and reduced abundance. Reduced gene flow (”“barrier effects”“) due to road avoidance behavior or road mortality can lead to reduced genetic diversity because genetic drift is enhanced in fragmented populations. Road mortality can also reduce population abundance (”“depletion effects”“) whenever road-caused mortality outpaces recruitment, also lowering diversity even when barrier effects are inconsequential. Although roads are expected to affect both genetic diversity and fragmentation, most research focuses only on fragmentation. Furthermore, in studies that do investigate road effects on genetic diversity, correlations are usually attributed to barrier effects and little attention is paid to the potentially confounding influence of mortality-caused depletion effects. Here we investigate the relative importance of barrier and depletion effects on genetic diversity of populations separated by a road by performing coalescent simulations wherein these two road effects are varied independently. By simulating wide ranging rates of migration and population decline, we also determine how the importance of these forces changes depending on their relative magnitude. We show that the vast majority of potential variation in genetic diversity is governed by depletion (mortality) rather than barrier effects. We also show that unless migration is sufficiently high and population decline due to mortality is sufficiently low, increasing migration across roads will generally not recoup genetic variation lost due to road mortality. We argue that the genetic effects of road-mediated mortality have been underappreciated and should be more often considered before prioritizing road-mitigation measures. (C) 2011 Elsevier Ltd. All rights reserved.” | 142 | no | NA | NA | no | no |
| 143 | 2017 | 43004 | Lin, WZ; Chang, LH; Frere, CH; Zhou, RL; Chen, JL; Chen, X; Wu, YP | 2012 | Differentiated or not? An assessment of current knowledge of genetic structure of Sousa chinensis in China | Estimating the degree of isolation or gene flow is central to understanding population dynamics and conservation. In this study, we used both mitochondrial and nuclear markers to investigate the genetic structure of Indo-Pacific humpback dolphins (genus Sousa) in China. Contrary to previous photo-identification and genetic studies, we found no evidence for population genetic structure or inter-population gene flow across the geographical scale examined. Significant inbreeding, which seems to support genetic isolation to some extent, was within expectation as a result of low genetic diversity in a random-mating system. Overall, the Indo-Pacific humpback dolphins in China were characterized by low genetic diversity on both maternal and bi-parental markers. While significant steps have been taken towards implementing marine reserves, our study highlights the critical need for fine-scale genetic and ecological monitoring programs and a coordinated approach to secure the survival of Indo-Pacific humpback dolphins in Chinese waters. (C) 2012 Elsevier B.V. All rights reserved. | 143 | no | NA | NA | no | no |
| 144 | 2017 | 43004 | Machon, N; LeFranc, M; Bilger, I; Mazer, SJ; Sarr, A | 1997 | Allozyme variation in Ulmus species from France: Analysis of differentiation | In the present paper, the geographical structure of genetic variation in three French elm taxa is described using electrophoretic data. For three: species, Ulmus laevis, U. glabra and U. minor two kinds of analyses were performed. First, the genetic parameters (genetic diversity indices) of individuals sampled from naturally regenerating forest were compared to samples of cultivated ornamental trees. Secondly, when sample sizes were sufficient, the genetic parameters of trees sampled from different regions of France were compared to detect geographical differentiation. From these analyses, the ultimate aim was to offer recommendations concerning the conservation policies of the genetic resources of French elms. The heterozygosity of Ulmus x hollandica (the presumed hybrid between U. minor and U. glabra) was compared to its putative parent taxa to determine whether the hybrid harbours great levels of genetic variation. In spite of the ravaging effects of Dutch Elm disease in the past, all three species exhibit high levels of electrophoretic variation. The three species surveyed displayed similar levels of genetic diversity, proportions of polymorphic loci and levels of allelic diversity in trees harvested from naturally regenerated forests relative to cultivated ornamental trees. High levels of genetic diversity in U. minor within geographical regions of France were detected, with only moderate levels of genetic differentiation detected between regions. is not more heterozygous than either of its parent species, suggesting that extant representatives of this taxon are the result of past and ongoing backcrosses with the parental taxa. Ongoing efforts to preserve the genetic variation still present in French elms should take advantage of their high levels of electrophoretic variation and target genetically distinct, vegetatively reproducing genotypes in their natural environment. | 144 | no | NA | NA | no | no |
| 145 | 2017 | 43004 | Sanllorente, O; Hammond, RL; Ruano, F; Keller, L; Tinaut, A | 2010 | Extreme population differentiation in a vulnerable slavemaking ant with a fragmented distribution | Understanding levels of population differentiation and inbreeding are important issues in conservation biology, especially for social Hymenoptera with fragmented and small population sizes. Isolated populations are more vulnerable to genetic loss and extinction than those with extended continuous distributions. However, small populations are not always a consequence of a recent reduction of their habitat. Thus, determining the history of population isolation and current patterns of genetic variation of a species is crucial for its conservation. Rossomyrmex minuchae is a slave-making ant with patchy distribution in South Eastern Spain and is classified as vulnerable by the IUCN. In contrast, the other three known species of the genus are presumed to show more uniform distributions. Here we investigate the genetic diversity and population structure of R. minuchae and compare it with that found in two other species of the genus: R. anatolicus and R. quandratinodum. We conclude that although genetic diversity of R. minuchae is low, there is no evidence of a recent bottleneck, suggesting a gradual and natural fragmentation process. We also show extreme population differentiation at nuclear and mitochondrial markers, and isolation by distance at a local scale. Despite some evidence for inbreeding and low genetic variation within populations, we found almost no diploid males, a finding which contrasts with that expected in inbred Hymenoptera with single locus complementary sex determination. This could mean that sex is determined by another mechanism. We argue that continued low population size means that detrimental effects of inbreeding and low genetic variation are likely in the future. We suggest that a policy of artificial gene flow aimed at increasing within population variation is considered as a management option. | 145 | no | NA | NA | no | no |
| 146 | 2017 | 43004 | Jian, SG; Zhong, Y; Liu, N; Gao, ZZ; Wei, Q; Xie, ZH; Ren, H | 2006 | Genetic variation in the endangered endemic species Cycas fairylakea (Cycadaceae) in China and implications for conservation | Cycas fairylakea is an endangered endemic species in China. Genetic diversity within and among four natural populations of this species in China was investigated using amplified fragments length polymorphism (AFLP). A moderate to low level of intraspecific genetic diversity was detected in this species (at population level: P = 39.57 %, H-0 = 0.244; at species level: P = 60.22%, H-0 = 0.356). The among-population component accounted for, respectively, 25.7 and 31.5% of the genetic variation, according to AMOVA and Shannon’s index, indicating most of the genetic variation was found between individuals within populations. All four populations have opposite pyramid age structure, and few coning individuals, which is still decreasing. Possibly because of habitat degradation and environmental pollution, plant diseases and insect pests in the populations were extremely serious, suggesting that the main factors threatening the survival of C. fairylakea populations were not genetic variation, but human activities and the breeding system of this species. | 146 | no | NA | NA | no | no |
| 147 | 2017 | 43004 | Cobben, MMP; Verboom, J; Opdam, PFM; Hoekstra, RF; Jochem, R; Smulders, MJM | 2012 | Landscape prerequisites for the survival of a modelled metapopulation and its neutral genetic diversity are affected by climate change | In response to climate change a species may move, adapt, or go extinct. For the adaptability of a population its genetic diversity is essential, but climate change-induced range shifts can cause a loss of genetic diversity. We investigated how landscape structure affects the level and distribution of genetic diversity in metapopulations subject to climate change-induced range shifts. For this we used the spatially explicit, individual-based model METAPHOR which simulates metapopulation demography and genetics under different temperature increase scenarios. The results indicated that increasing total habitat area may enhance the maintenance of the genetic diversity in metapopulations while they are shifting their range under climate change. However, the results also showed that a high level of total habitat area did not prevent the populations in the newly colonised habitat area of being depleted of much of the original genetic diversity. We therefore conclude that enhancing landscape connectivity may lead to a delayed loss of genetic diversity in metapopulations under climate change, but that additional measures would be necessary to ensure its long-term conservation. Importantly, our simulations also show that a landscape which could be regarded as well-structured under stable climatic conditions, may be inferior for the conservation of genetic diversity during a range shift. This is important information for landscape management when developing strategies for the in situ conservation of genetic variation in natural populations under climate change. | 147 | no | NA | NA | no | no |
| 148 | 2017 | 43004 | Milot, E; Weimerskirch, H; Duchesne, P; Bernatchez, L | 2007 | Surviving with low genetic diversity: the case of albatrosses | Low genetic diversity is predicted to negatively impact species viability and has been a central concern for conservation. In contrast, the possibility that some species may thrive in spite of a relatively poor diversity has received little attention. The wandering and Amsterdam albatrosses (Diomedea exulans and Diomedea amsterdamensis) are long-lived seabirds standing at an extreme along the gradient of life strategies, having traits that may favour inbreeding and low genetic diversity. Divergence time of the two species is estimated at 0.84 Myr ago from cytochrome b data. We tested the hypothesis that both albatrosses inherited poor genetic diversity from their common ancestor. Within the wandering albatross, per cent polymorphic loci and expected heterozygosity at amplified fragment length polymorphisms were approximately one-third of the minimal values reported in other vertebrates. Genetic diversity in the Amsterdam albatross, which is recovering from a severe bottleneck, was about twice as low as in the wandering albatross. Simulations supported the hypothesis that genetic diversity in albatrosses was already depleted prior to their divergence. Given the generally high breeding success of these species, it is likely that they are not suffering much from their impoverished diversity. Whether albatrosses are unique in this regard is unknown, but they appear to challenge the classical view about the negative consequences of genetic depletion on species survival. | 148 | no | NA | NA | no | no |
| 149 | 2017 | 43004 | Schmidt, AM; Linksvayer, TA; Boomsma, JJ; Pedersen, JS | 2011 | No benefit in diversity? The effect of genetic variation on survival and disease resistance in a polygynous social insect | 0﻿1. Multiple mating by queens has been shown to enhance disease resistance in insect societies, because higher genetic diversity among nestmates improves collective immune defences or offers a certain level of herd immunity. However, it has remained ambiguous whether polygynous societies with large numbers of queens also benefit from increased genetic diversity. 2. We used one of the very few ant species that can be reared across generations, the pharaoh ant, Monomorium pharaonis Linnaeus, to create experimental colonies with two types of enhanced genetic diversity: (i) mixed workers from three divergent inbred lineages representing the ‘polygyny-equivalent’ of multiple mating by queens (i.e. increased between-worker variation); and (ii) uniform workers whose overall heterozygosity was increased by two subsequent generations of crossing between the same divergent inbred lineages (i.e. increased within-worker variation). 3. We found significant differences in worker survival among the three inbred lineages, with exposure to conidiospores of the fungal pathogen Beauveria bassiana causing significant mortality to the workers independently of their diversity type. Increased diversity did not improve the resistance to Beauveria. 4. Enhanced heterozygosity colonies had worker survival rates similar to the most resistant inbred lineage, whereas colonies with mixed workers from the three inbred lineages had lower worker and larval survival. Workers did not show any infection- avoidance behaviour. 5. Average larval survival appeared unaffected by the presence of conidiospores. It benefitted from increased heterozygosity but was reduced in mixed colonies independent of infection. This suggests that negative, but cryptic social interactions in mixed colonies may affect overall survival. 6. The present results do not provide evidence for or against a link between increased genetic variation and increased disease resistance in pharaoh ants, but show that colonies differ considerably in general survival. Thus, increasing the genetic diversity of pharaoh ant colonies may not provide survival advantages in the face of pathogen exposure, and polygyny and polyandry may not be directly comparable mechanisms for creating adaptive resistance towards pathogens. | 149 | yes | from thesis | NA | yes | yes |
| 150 | 2017 | 43004 | Perronne, R; Makowski, D; Goffaux, R; Montalent, P; Goldringer, I | 2017 | Temporal evolution of varietal, spatial and genetic diversity of bread wheat between 1980 and 2006 strongly depends upon agricultural regions in France | An in-depth analysis of the spatio-temporal evolution of crop diversity in agricultural landscapes is necessary to provide insights on how to improve the resilience of agroecosystems to climate instability and new pest pressures by increasing in situ crop diversity, while promoting a greater sustainability of agriculture. However, most of the indicators of crop diversity used in previous studies were based on ex situ genetic diversity, or on official lists of varieties or breeding programs, without taking acreage of the varieties actually grown in farmers’ fields into account. Acreage of varieties represent however a relevant information to assess the actual genetic vulnerability of a crop (genetic uniformity at landscape scale) cultivated in the considered regions over the period studied. To bridge this gap, we analyzed spatial varietal and genetic diversity of bread wheat in France - an important production area - over the 1980-2006 period at a yearly time step and a district scale, i.e. ‘departement’. We used H-T*, an indicator integrating both the spatial evenness of variety distribution and molecular genetic data, to finely examine the spatio-temporal evolution of spatially weighted bread wheat genetic diversity. We also computed several simpler and complementary indicators accounting for the number of varieties, the spatial varietal diversity and the genetic diversity, and we estimated the individual contribution of these simpler indicators to the spatio-temporal structure of the spatial genetic diversity H-T*. H-T\* values showed significant but non linear temporal evolutions between 1980 and 2006, these evolutions being besides spatially structured. Moreover, both spatial varietal diversity and genetic diversity partly explained these temporal changes of H-T\* values while the number of varieties appeared non-explanatory. Similarly to H-T\*, all indicators studied also showed substantial differences of temporal changes among major French agricultural regions, furthermore sometimes opposite for varietal and genetic diversity. As an example, in the north of France, varietal richness and spatial varietal diversity showed a substantial increase, suggesting the adoption of an increasing number of bread wheat varieties by farmers in this region. However, considering that genetic diversity showed a slight but continuous decrease over the recent period, this could suggest an increase in genetic similarity among bread wheat varieties chosen by farmers. Taken as a whole, our results suggest that several determinants of crop diversity, such as the shortening of crop sequences or the recurrence of epidemics of some pathogens, could differentially affect varietal choices of farmers and thus the spatial structuration of the temporal changes of crop diversity. Finally, our results question the dominant use of ex situ nationwide datasets to characterize the evolution of spatial genetic diversity as a proxy of genetic vulnerability of a crop to face with future changes. (C) 2016 Elsevier B.V. All rights reserved. | 150 | no | NA | NA | no | no |
| 151 | 2017 | 43005 | Wilson, GA; Nishi, JS; Elkin, BT; Strobeck, C | 2005 | Effects of a recent founding event and intrinsic population dynamics on genetic diversity in an ungulate population | Maintenance of genetic diversity has recently become a management goal for a number of species, due to its importance for present and future population viability. Genetic drift, primarily through differential reproductive success and inbreeding, can accelerate the loss of genetic diversity in recently recovered populations. We attempt to quantify the consequences of these factors on the genetic diversity contained in a small, recently founded wood bison (Bison bison athabascae) population by examining the genetic variation in this conservation herd, the calves born therein, and its large source population. The Hook Lake Wood Bison Recovery Project was initiated to found a disease-free herd of wood bison containing a representative amount of the genetic diversity present in the Wood Buffalo National Park metapopulation. Levels of diversity in the Hook Lake Wood Bison Recovery Project founders are higher than in previous salvage attempts. To examine the effects of differential reproductive success on this population, we monitored parentage of the calves born in the Hook Lake Wood Bison Recovery Project for 3 years since the founders reached sexual maturity. Two of the male founders sired over 90% of the offspring born in this population, which has led to a reduction in diversity in their calves. Monitoring of reproductive success, and incorporation of selective breeding strategies will be required to reduce the rate at which genetic diversity is lost from this small, isolated population. These steps should occur in other recovery projects, particularly when a small number of individuals are capable of dominating reproduction. | 151 | no | only one pop | NA | no | no |
| 152 | 2017 | 43005 | Mortlock, SA; Khatkar, MS; Williamson, P | 2016 | Comparative Analysis of Genome Diversity in Bullmastiff Dogs | Management and preservation of genomic diversity in dog breeds is a major objective for maintaining health. The present study was undertaken to characterise genomic diversity in Bullmastiff dogs using both genealogical and molecular analysis. Genealogical analysis of diversity was conducted using a database consisting of 16,378 Bullmastiff pedigrees from year 1980 to 2013. Additionally, a total of 188 Bullmastiff dogs were genotyped using the 170,000 SNP Illumina CanineHD Beadchip. Genealogical parameters revealed a mean inbreeding coefficient of 0.047; 142 total founders (f); an effective number of founders (f(e)) of 79; an effective number of ancestors (fa) of 62; and an effective population size of the reference population of 41. Genetic diversity and the degree of genome-wide homogeneity within the breed were also investigated using molecular data. Multiple-locus heterozygosity (MLH) was equal to 0.206; runs of homozygosity (ROH) as proportion of the genome, averaged 16.44%; effective population size was 29.1, with an average inbreeding coefficient of 0.035, all estimated using SNP Data. Fine-scale population structure was analysed using NETVIEW, a population analysis pipeline. Visualisation of the high definition network captured relationships among individuals within and between subpopulations. Effects of unequal founder use, and ancestral inbreeding and selection, were evident. While current levels of Bullmastiff heterozygosity, inbreeding and homozygosity are not unusual, a relatively small effective population size indicates that a breeding strategy to reduce the inbreeding rate may be beneficial. | 152 | no | domesticated animal | NA | no | no |
| 153 | 2017 | 43005 | Whitehorn, PR; Tinsley, MC; Brown, MJF; Darvill, B; Goulson, D | 2014 | Genetic diversity and parasite prevalence in two species of bumblebee | Many bumblebee species have been suffering from significant declines across their ranges in the Northern Hemisphere over the last few decades. The remaining populations of the rare species are now often isolated due to habitat fragmentation and have reduced levels of genetic diversity. The persistence of these populations may be threatened by inbreeding depression, which may result in a higher susceptibility to parasites. Here we investigate the relationship between genetic diversity and prevalence of the parasitic mite Locustacarus buchneri in bumblebees, using the previously-studied system of Bombus muscorum and Bombus jonellus in the Western Isles of Scotland. We recorded L. buchneri prevalence in 17 populations of B. muscorum and 13 populations of B. jonellus and related the results to levels of heterozygosity. For B. muscorum, we found that prevalence of the mite was higher in populations with lower genetic diversity but there was no such relationship in the more genetically diverse B. jonellus. In contrast to population-level measures of genetic diversity, the heterozygosity of individual bees was not correlated with infection status. We suggest population-level genetic homogeneity may facilitate parasite transmission and elevate prevalence, with potential consequences for population persistence. | 153 | yes | from thesis | NA | yes | no |
| 154 | 2017 | 43005 | Hughes, WOH; Boomsma, JJ | 2006 | Does genetic diversity hinder parasite evolution in social insect colonies? | Polyandry is often difficult to explain because benefits of the behaviour have proved elusive. In social insects, polyandry increases the genetic diversity of workers within a colony and this has been suggested to improve the resistance of the colony to disease. Here we examine the possible impact of host genetic diversity on parasite evolution by carrying out serial passages of a virulent fungal pathogen through leaf-cutting ant workers of known genotypes. Parasite virulence increased over the nine-generation span of the experiment while spore production decreased. The effect of host relatedness upon virulence appeared limited. However, parasites cycled through more genetically diverse hosts were more likely to go extinct during the experiment and parasites cycled through more genetically similar hosts had greater spore production. These results indicate that host genetic diversity may indeed hinder the ability of parasites to adapt while cycling within social insect colonies. | 154 | yes | from thesis | NA | no | yes |
| 155 | 2017 | 43005 | Hangelbroek, HH; Ouborg, NJ; Santamaria, L; Schwenk, K | 2002 | Clonal diversity and structure within a population of the pondweed Potamogeton pectinatus foraged by Bewick’s swans | Clonal diversity within plant populations is affected by factors that influence genet (clone) survival and seed recruitment, such as resource availability, disturbance, seed dispersal mechanism, propagule predation and the age of the population. Here we studied a population of Potamogeton pectinatus , a pseudo-annual aquatic macrophyte. Within populations reproduction appears to be mainly asexually through subterranean propagules (tubers), while recruitment via seeds is believed to be relatively unimportant. RAPD markers were used to analyse clonal diversity and genetic variation within the population. Ninety-seven genets were identified among 128 samples taken from eight plots. The proportion of distinguishable genets (0.76) and Simpson’s diversity index (0.99) exhibited high levels of clonal diversity compared to other clonal plants. According to an analysis of molecular variance (amova) most genetic variation occurred between individuals within plots (93-97%) rather than between plots (8-3%). These results imply that sexual reproduction plays an unexpectedly important role within the population. Nevertheless, autocorrelation statistics revealed a spatial genetic structure resulting from clonal growth. In contrast to genetic variation, clonal diversity was affected by several ecological factors. Water depth and silt content had direct negative effects on clonal diversity. Tuber predation by Bewick’s swans had an unexpected indirect negative effect on clonal diversity through reducing the tuber-bank biomass in spring, which on its turn was positively correlated to clonal diversity. The disturbance by swans, therefore, did not enhance seed recruitment and thus clonal diversity; on the contrary, heavily foraged areas are probably more prone to stochastic loss of genets leading to reduced clonal diversity. | 155 | no | only one pop | NA | no | no |
| 156 | 2017 | 43005 | SHYKOFF, JA; SCHMIDHEMPEL, P | 1991 | PARASITES AND THE ADVANTAGE OF GENETIC-VARIABILITY WITHIN SOCIAL INSECT COLONIES | Genetic variability within colonies of eusocial insects is often higher than expected if kin selection alone explains sociality. Parasites and pathogens have been proposed as selective agents maintaining genetic variability in populations and promoting polyandry in social insects. Using the natural system, bumble bees, Bombus terrestris, and their trypanosome parasites, Crithidia bombi, we find that hosts vary in susceptibility or parasites in infectiousness, and that parasite transmission in social groups correlates with genetic relatedness among hosts. Therefore parasite-mediated negative frequency-dependent selection could play an important role in structuring the genetic composition of social groups by counteracting kin selection for high relatedness. | 156 | yes | NA | NA | no | no |
| 157 | 2017 | 43005 | Frantz, AC; Proess, R; Burke, T; Schley, L | 2009 | A genetic assessment of the two remnant populations of the natterjack toad (Bufo calamita) in Luxembourg | The natterjack toad (Bufo calamita) has experienced a dramatic decline in Luxembourg over the last 100 years. Today, only two remnant populations are known. Here, we examine their genetic constitution at 10 microsatellite loci in order to assess the genetic risk from isolation and inbreeding to the species’ long-term survival in the country. Genetic diversity in both populations was relatively high, and we did not find evidence for inbreeding. However, the natterjack toads have experienced a recent reduction in their effective population sizes, and there was no evidence of recent gene flow between the two localities. The main short-term objective of conservation measures should be to increase population sizes by continuous safeguarding and management of the two sites. | 157 | maybe | any fitness measure? | no fitness measure | no | no |
| 158 | 2017 | 43005 | Dona, J; Moreno-Garcia, M; Criscione, CD; Serrano, D; Jovani, R | 2015 | Species mtDNA genetic diversity explained by infrapopulation size in a host-symbiont system | Understanding what shapes variation in genetic diversity among species remains a major challenge in evolutionary ecology, and it has been seldom studied in parasites and other host-symbiont systems. Here, we studied mtDNA variation in a host-symbiont non-model system: 418 individual feather mites from 17 feather mite species living on 17 different passerine bird species. We explored how a surrogate of census size, the median infrapopulation size (i.e., the median number of individual parasites per infected host individual), explains mtDNA genetic diversity. Feather mite species genetic diversity was positively correlated with mean infrapopulation size, explaining 34% of the variation. As expected from the biology of feather mites, we found bottleneck signatures for most of the species studied but, in particular, three species presented extremely low mtDNA diversity values given their infrapopulation size. Their star-like haplotype networks (in contrast with more reticulated networks for the other species) suggested that their low genetic diversity was the consequence of severe bottlenecks or selective sweeps. Our study shows for the first time that mtDNA diversity can be explained by infrapopulation sizes, and suggests that departures from this relationship could be informative of underlying ecological and evolutionary processes. | 158 | no | gd of parasite | NA | no | no |
| 159 | 2017 | 43005 | Bourgeois, L; Sylvester, A; Danka, R; Rinderer, T | 2008 | Comparison of microsatellite DNA diversity among commercial queen breeder stocks of Italian honey bees in the United States and Italy | Declines in the numbers of breeder honey bee queens and the concomitant loss of genetic diversity could potentially result in inbreeding, and increased susceptibility to pests and diseases. Genetic diversity of commercial Italian bee colonies in the United States and Italy was assessed using six variable microsatellite DNA loci. Worker bees were sampled from colonies of queen breeders in both countries (USA, n = 18; Italy, n = 24). Overall, allelic richness (mean alleles/locus), gene diversity (heterozygosity), and F-IS (inbreeding coefficient) did not differ between the two groups. A total of 48 alleles were present among all colonies. Sampled colonies from each country had a total of 38 alleles, although alleles were present that were unique to each group. There were a total of 10 unique alleles among USA bees and 10 among Italian bees. Estimates of the level of genetic differentiation based on different allele frequency patterns among the USA and Italian bees were measured with the population genetic parameter F-ST. These estimates showed that bees from the USA and Italy were measurably distinct relative to the frequencies of the microsatellite alleles present in samples from each country. Overall allelic diversity levels were sufficiently high indicating that inbreeding does not appear to be an immediate threat to existing honey bee populations. | 159 | no | no good fitness measure | NA | no | no |
| 160 | 2017 | 43005 | Kesaniemi, JE; Mustonen, M; Bostrom, C; Hansen, BW; Knott, KE | 2014 | Temporal genetic structure in a poecilogonous polychaete: the interplay of developmental mode and environmental stochasticity | Background: Temporal variation in the genetic structure of populations can be caused by multiple factors, including natural selection, stochastic environmental variation, migration, or genetic drift. In benthic marine species, the developmental mode of larvae may indicate a possibility for temporal genetic variation: species with dispersive planktonic larvae are expected to be more likely to show temporal genetic variation than species with benthic or brooded non-dispersive larvae, due to differences in larval mortality and dispersal ability. We examined temporal genetic structure in populations of Pygospio elegans, a poecilogonous polychaete with within-species variation in developmental mode. P. elegans produces either planktonic, benthic, or intermediate larvae, varying both among and within populations, providing a within-species test of the generality of a relationship between temporal genetic variation and larval developmental mode. Results: In contrast to our expectations, our microsatellite analyses of P. elegans revealed temporal genetic stability in the UK population with planktonic larvae, whereas there was variation indicative of drift in temporal samples of the populations from the Baltic Sea, which have predominantly benthic and intermediate larvae. We also detected temporal variation in relatedness within these populations. A large temporal shift in genetic structure was detected in a population from the Netherlands, having multiple developmental modes. This shift could have been caused by local extiction due to extreme environmental conditions and (re)colonization by planktonic larvae from neighboring populations. Conclusions: In our study of P. elegans, temporal genetic variation appears to be due to not only larval developmental mode, but also the stochastic environment of adults. Large temporal genetic shifts may be more likely in marine intertidal habitats (e.g. North Sea and Wadden Sea) which are more prone to environmental stochasticity than the sub-tidal Baltic habitats. Sub-tidal and/or brackish (less saline) habitats may support smaller P. elegans populations and these may be more susceptible to the effects of random genetic drift. Moreover, higher frequencies of asexual reproduction and the benthic larval developmental mode in these populations leads to higher relatedness and contributes to drift. Our results indicate that a general relationship between larval developmental mode and temporal genetic variation may not exist. | 160 | no | no good fitness measure | NA | no | no |
| 161 | 2017 | 43005 | Shete, S | 2003 | Uniformly minimum variance unbiased estimation of gene diversity | Gene diversity is an important measure of genetic variability in inbred populations. The survival of species in changing environments depends on, among other factors, the genetic variability of the population. In this communication, I have derived the uniformly minimum variance unbiased estimator of gene diversity. The proposed estimator of gene diversity does not assume that the inbreeding coefficient is known. I have also provided the approximate variance of this estimator according to Fisher’s method. In addition, I have developed a numerical resampling-based method for obtaining variances and confidence intervals based on the maximum likelihood estimator and the uniformly minimum variance unbiased estimator. Efficiency in estimation of the gene diversity based on these two estimators is discussed. In accordance with the simulation results, I found that the uniformly minimum variance estimator developed in this report is more accurate for estimation of gene diversity than the maximum likelihood estimator. | 161 | no | NA | NA | no | no |
| 162 | 2017 | 43005 | Moreno, E; Perez-Gonzalez, J; Carranza, J; Moya-Larano, J | 2015 | Better Fitness in Captive Cuvier’s Gazelle despite Inbreeding Increase: Evidence of Purging? | Captive breeding of endangered species often aims at preserving genetic diversity and to avoid the harmful effects of inbreeding. However, deleterious alleles causing inbreeding depression can be purged when inbreeding persists over several generations. Despite its great importance both for evolutionary biology and for captive breeding programmes, few studies have addressed whether and to which extent purging may occur. Here we undertake a longitudinal study with the largest captive population of Cuvier’s gazelle managed under a European Endangered Species Programme since 1975. Previous results in this population have shown that highly inbred mothers tend to produce more daughters, and this fact was used in 2006 to reach a more appropriate sex-ratio in this polygynous species by changing the pairing strategy (i.e., pairing some inbred females instead of keeping them as surplus individuals in the population). Here, by using studbook data we explore whether purging has occurred in the population by investigating whether after the change in pairing strategy a) inbreeding and homozygosity increased at the population level, b) fitness (survival) increased, and c) the relationship between inbreeding and juvenile survival, was positive. Consistent with the existence of purging, we found an increase in inbreeding coefficients, homozygosity and juvenile survival. In addition, we showed that in the course of the breeding programme the relationship between inbreeding and juvenile survival was not uniform but rather changed over time: it was negative in the early years, flat in the middle years and positive after the change in pairing strategy. We highlight that by allowing inbred individuals to mate in captive stocks we may favour sex-ratio bias towards females, a desirable managing strategy to reduce the surplus of males that force most zoos to use ethical culling and euthanizing management tools. We discuss these possibilities but also acknowledge that many other effects should be considered before implementing inbreeding and purging as elements in management decisions. | 162 | no | NA | NA | no | no |
| 163 | 2017 | 43005 | Kennington, WJ; Cadee, SA; Berry, O; Groth, DM; Johnson, MS; Melville-Smith, R | 2013 | Maintenance of genetic variation and panmixia in the commercially exploited western rock lobster (Panulirus cygnus) | Marine species with high fecundities and mortalities in the early life stages can have low effective population sizes, making them vulnerable to declines in genetic diversity when they are commercially harvested. Here, we compare levels of microsatellite and mitochondrial sequence variation in the western rock lobster (Panulirus cygnus) over a 14-year period to test whether genetic variation is being maintained. Panulirus cygnus is a strong candidate for loss of genetic variation because it is a highly fecund species that is likely to experience high variance in reproductive success due to an extended larval planktonic stage. It also supports one of the largest and most economically important fisheries in Australia, with landings of between 8,000 and 14,500 tons (similar to 70 % of the total legal-sized biomass) being harvested in some years. We found remarkably high levels of genetic variation in all samples and no evidence of a decline in genetic diversity over the time interval we studied. Furthermore, there was no evidence of a recent genetic bottleneck, and effective population size estimates based on single sample and temporal methods were infinitely large. Analysis of molecular variance indicated no significant population structure along 960 km of coastline or genetic differentiation among temporal samples. Our results support the view that P. cygnus is a single, panmictic population, and suggest genetic drift is not strong enough to reduce neutral genetic diversity in this species if current management practices and breeding stock sizes are maintained. | 163 | no | NA | NA | no | no |
| 164 | 2017 | 43005 | Franck, P; Koeniger, N; Lahner, G; Crewe, RM; Solignac, M | 2000 | Evolution of extreme polyandry: an estimate of mating frequency in two African honeybee subspecies, Apis mellifea monticola and A.m. scutellata | Mating frequencies in two related honeybee subspecies Apis mellifera monticola and A. in. scutellata were compared using a set of five microsatellite loci in order to discriminate among the major factors which have affected the evolution of extreme polyandry. We found that the queens of eight A. in. monticola colonies collected from an apiary in Malawi, mated 5-19 times producing an average relatedness among workers of 0.328 +/- 0.039. The six A. In. scutellata colonies collected from an apiary in South Africa displayed a significantly larger degree of polyandry, i.e. queens mated 10-25 times and the genetic relatedness among nestmates was 0.283 +/- 0.020. The difference in the degree of polyandry observed between the populations suggests that socially based selective pressures are not of primary importance in the evolution of extreme polyandry but that ecological selective pressures play a more significant role. However, exploring the relative contributions of these factors to the variation in levels of polyandry will require the use of colonies in which the level of polyandry is experimentally controlled. | 164 | maybe | no good fitness measure, N pop differnt in GD? | no fitness measure | no | no |
| 165 | 2017 | 43005 | Ellison, A; Allainguillaume, J; Girdwood, S; Pachebat, J; Peat, KM; Wright, P; Consuegra, S | 2012 | Maintaining functional major histocompatibility complex diversity under inbreeding: the case of a selfing vertebrate | Major histocompatibility complex (MHC) genes encode proteins that present pathogen-derived antigens to T-cells, initiating the adaptive immune response in vertebrates. Although populations with low MHC diversity tend to be more susceptible to pathogens, some bottlenecked populations persist and even increase in numbers despite low MHC diversity. Thus, the relative importance of MHC diversity versus genome-wide variability for the long-term viability of populations after bottlenecks and/or under high inbreeding is controversial. We tested the hypothesis that genome-wide inbreeding (estimated using microsatellites) should be more critical than MHC diversity alone in determining pathogen resistance in the self-fertilizing fish Kryptolebias marmoratus by analysing MHC diversity and parasite loads in natural and laboratory populations with different degrees of inbreeding. Both MHC and neutral diversities were lost after several generations of selfing, but we also found evidence of parasite selection acting on MHC diversity and of non-random loss of alleles, suggesting a possible selective advantage of those individuals with functionally divergent MHC, in accordance with the hypothesis of divergent allele advantage. Moreover, we found that parasite loads were better explained by including MHC diversity in the model than by genome-wide (microsatellites) heterozygosity alone. Our results suggest that immune-related overdominance could be the key in maintaining variables rates of selfing and outcrossing in K. marmoratus and other mixed-mating species. | 165 | no | NA | NA | no | no |
| 166 | 2017 | 43005 | Oliver, MK; Piertney, SB | 2012 | Selection Maintains MHC Diversity through a Natural Population Bottleneck | A perceived consequence of a population bottleneck is the erosion of genetic diversity and concomitant reduction in individual fitness and evolutionary potential. Although reduced genetic variation associated with demographic perturbation has been amply demonstrated for neutral molecular markers, the effective management of genetic resources in natural populations is hindered by a lack of understanding of how adaptive genetic variation will respond to population fluctuations, given these are affected by selection as well as drift. Here, we demonstrate that selection counters drift to maintain polymorphism at a major histocompatibility complex (MHC) locus through a population bottleneck in an inbred island population of water voles. Before and after the bottleneck, MHC allele frequencies were close to balancing selection equilibrium but became skewed by drift when the population size was critically low. MHC heterozygosity generally conformed to Hardy-Weinberg expectations except in one generation during the population recovery where there was a significant excess of heterozygous genotypes, which simulations ascribed to strong differential MHC-dependent survival. Low allelic diversity and highly skewed frequency distributions at microsatellite loci indicated potent genetic drift due to a strong founder affect and/or previous population bottlenecks. This study is a real-time examination of the predictions of fundamental evolutionary theory in low genetic diversity situations. The findings highlight that conservation efforts to maintain the genetic health and evolutionary potential of natural populations should consider the genetic basis for fitness-related traits, and how such adaptive genetic diversity will vary in response to both the demographic fluctuations and the effects of selection. | 166 | no | NA | NA | no | no |
| 167 | 2017 | 43005 | Hughes, WOH; Boomsma, JJ | 2004 | Genetic diversity and disease resistance in leaf-cutting ant societies | Multiple mating by females (polyandry) remains hard to explain because, while it has substantial costs, clear benefits have remained elusive. The problem is acute in the social insects because polyandry is probably particularly costly for females and most material benefits of the behavior are unlikely to apply. It has been suggested that a fitness benefit may arise from the more genetically diverse worker force that a polyandrous queen will produce. One leading hypothesis is that the increased genetic diversity of workers will improve a colony’s resistance to disease. We investigated this hypothesis using a polyandrous leaf-cutting ant and a virulent fungal parasite as our model system. At high doses of the parasite most patrilines within colonies were similarly susceptible, but a few showed greater resistance. At a low dose of the parasite there was more variation between patrilines in their resistance to the parasite. Such genetic variation is a key prerequisite for polyandry to result in increased disease resistance of colonies. The relatedness of two hosts did not appear to affect the transmission of the parasite between them, but this was most likely because the parasite tested was a virulent generalist that is adapted to transmit between distantly related hosts. The resistance to the parasite was compared between small groups of ants of either high or low genetic diversity. No difference was found at high doses of the parasite, but a significant improvement in resistance in high genetic diversity groups was found at a low dose of the parasite. That there is generic variation for disease resistance means that there is the potential for polyandry to produce more disease-resistant colonies. That this genetic variation can improve the resistance of groups even under the limited conditions tested suggests that polyandry may indeed produce colonies with improved resistance to disease. | 167 | yes | from thesis | NA | no | yes |
| 168 | 2017 | 43005 | Foitzik, S; Bauer, S; Laurent, S; Pennings, PS | 2009 | Genetic diversity, population structure and sex-biased dispersal in three co-evolving species | Genetic diversity and spatial structure of populations are important for antagonistic coevolution. We investigated genetic variation and population structure of three closely related European ant species: the social parasite Harpagoxenus sublaevis and its two host species Leptothorax acervorum and Leptothorax muscorum. We sampled populations in 12 countries and analysed eight microsatellite loci and an mtDNA sequence. We found high levels of genetic variation in all three species, only slightly less variation in the host L. muscorum. Using a newly introduced measure of differentiation (Jost’s D-est), we detected strong population structuring in all species and less male-biased dispersal than previously thought. We found no phylogeographic patterns that could give information on post-glacial colonization routes - northern populations are as variable as more southern populations. We conclude that conditions for Thompson’s geographic mosaic of coevolution are ideal in this system: all three species show ample genetic variation and strong population structure. | 168 | no | no good fitness measure, N pop differnt in GD? | NA | no | no |
| 169 | 2017 | 43005 | Ge, MK; Sun, ET; Jia, CN; Kong, DD; Jiang, YX | 2014 | Genetic diversity and differentiation of Lepidoglyphus destructor (Acari: Glycyphagidae) inferred from inter-simple sequence repeat (ISSR) fingerprinting | Lepidoglyphus destructor (Schrank) is a prominent and world-wide pest of stored grain. Currently, genetic diversity of L. destructor is still little known due to the limited number of studies. The objective of this investigation was to assess genetic diversity and differentiation of four populations of L. destructor from four geographic locations in China using inter-simple sequence repeat (ISSR) fingerprinting. A total of 58 DNA bands were tested, 47 of which were polymorphic and the percentage of polymorphic bands (PPB) was 81.03%. Nei’s gene diversity (He) and Shannon’s Information index (I) revealed that the genetic diversity was high. Further, the value of Gst was 0.5680, indicating a median level of genetic differentiation in these populations. At the same time, analysis of molecular variation (AMOVA) showed that 45.93% of variation can be identified within populations. The level of gene flow (Nm) was moderate. Cluster analysis suggested that genotypes isolated from the same locations displayed a higher genetic similarity than those from different ones, forming a specific clade according to their geographic locations. Nevertheless, there was no significant correlation between the genetic distance and geographic distance. | 169 | no | NA | NA | no | no |
| 170 | 2017 | 43005 | Dibattista, JD; Feldheim, KA; Gruber, SH; Hendry, AP | 2008 | Are indirect genetic benefits associated with polyandry? Testing predictions in a natural population of lemon sharks | Multiple mating has clear fitness benefits for males, but uncertain benefits and costs for females. We tested for indirect genetic benefits of polyandry in a natural population, by using data from a long-term genetic and demographic study of lemon sharks (Negaprion brevirostris) at Bimini, Bahamas. To do so, we followed the fates of individuals from six cohorts (450 age-0 and 254 age-1 fish) in relation to their individual level of genetic variation, and whether they were from polyandrous or monoandrous litters. We find that offspring from polyandrous litters did not have a greater genetic diversity or greater survival than did the offspring of monoandrous litters. We also find no evidence of positive associations between individual offspring genetic diversity metrics and our surrogate measure of fitness (i.e. survival). In fact, age-1 individuals with fewer heterozygous microsatellite loci and more genetically similar parents were more likely to survive to age-2. Thus, polyandry in female lemon sharks does not appear to be adaptive from the perspective of indirect genetic benefits to offspring. It may instead be the result of convenience polyandry, whereby females mate multiply to avoid harassment by males. Our inability to find indirect genetic benefits of polyandry despite detailed pedigree and survival information suggests the need for similar assessments in other natural populations. | 170 | no | no good fitness measure | NA | no | no |
| 171 | 2017 | 43005 | Aerts, R; Berecha, G; Gijbels, P; Hundera, K; Van Glabeke, S; Vandepitte, K; Muys, B; Roldan-Ruiz, I; Honnay, O | 2013 | Genetic variation and risks of introgression in the wild Coffea arabica gene pool in south-western Ethiopian montane rainforests | The montane rainforests of SW Ethiopia are the primary centre of diversity of Coffea arabica and the origin of all Arabica coffee cultivated worldwide. This wild gene pool is potentially threatened by forest fragmentation and degradation, and by introgressive hybridization with locally improved coffee varieties. We genotyped 703 coffee shrubs from unmanaged and managed coffee populations, using 24 microsatellite loci. Additionally, we genotyped 90 individuals representing 23 Ethiopian cultivars resistant to coffee berry disease (CBD). We determined population genetic diversity, genetic structure, and admixture of cultivar alleles in the in situ gene pool. We found strong genetic differentiation between managed and unmanaged coffee populations, but without significant differences in within-population genetic diversity. The widespread planting of coffee seedlings including CBD-resistant cultivars most likely offsets losses of genetic variation attributable to genetic drift and inbreeding. Mixing cultivars with original coffee genotypes, however, leaves ample opportunity for hybridization and replacement of the original coffee gene pool, which already shows signs of admixture. In situ conservation of the wild gene pool of C.arabica must therefore focus on limiting coffee production in the remaining wild populations, as intensification threatens the genetic integrity of the gene pool by exposing wild genotypes to cultivars. | 171 | maybe | any gd and fitness of groups? | no fitness measure | no | no |
| 172 | 2017 | 43005 | Jones, JM; Gibson, JP | 2011 | Population genetic diversity and structure within and among disjunct populations of Alnus maritima (seaside alder) using microsatellites | Small, isolated populations are prone to genetic drift and high levels of inbreeding that can threaten their long-term survival. Alnus maritima persists exclusively in three groups of small, highly disjunct, regional populations in the Delmarva Peninsula, Georgia, and Oklahoma. Trees in the three regions are recognized as separate subspecies. Microsatellite markers were used to measure fine-scale population genetic diversity and structure (1) within and among regions and (2) within and among populations in each region. Compared to a previous study utilizing allozymes, microsatellite data show higher levels of variation, lower levels of inbreeding, but similar levels of genetic differentiation among regions. Significant genetic differentiation was detected among regions and among distinct populations within regions. Genetic differentiation was significantly correlated with geographic distance among regional populations, but not among populations within regions. Populations, therefore, likely represent fragments of formerly extensive networks of populations that have decayed and retracted due to competition with other species better adapted to the shadier habitats of late-succession environments. The unique genetic features of populations within different regions should be considered as part of future conservation efforts. | 172 | no | NA | NA | no | no |
| 173 | 2017 | 43005 | Sackett, LC; Collinge, SK; Martin, AP | 2013 | Do pathogens reduce genetic diversity of their hosts? Variable effects of sylvatic plague in black-tailed prairie dogs | Introduced diseases can cause dramatic declines inand even the loss ofnatural populations. Extirpations may be followed by low recolonization rates, leading to inbreeding and a loss of genetic variation, with consequences on population viability. Conversely, extirpations may create vacant habitat patches that individuals from multiple source populations can colonize, potentially leading to an influx of variation. We tested these alternative hypotheses by sampling 15 colonies in a prairie dog metapopulation during 7years that encompassed an outbreak of sylvatic plague, providing the opportunity to monitor genetic diversity before, during and after the outbreak. Analysis of nine microsatellite loci revealed that within the metapopulation, there was no change in diversity. However, within extirpated colonies, patterns varied: In half of the colonies, allelic richness after recovery was less than the preplague conditions, and in the other half, richness was greater than the preplague conditions. Finally, analysis of variation within individuals revealed that prairie dogs present in recolonized colonies had higher heterozygosity than those present before plague. We confirmed plague survivorship in six founders; these individuals had significantly higher heterozygosity than expected by chance. Collectively, our results suggest that high immigration rates can maintain genetic variation at a regional scale despite simultaneous extirpations in spatially proximate populations. Thus, virulent diseases may increase genetic diversity of host populations by creating vacant habitats that allow an influx of genetic diversity. Furthermore, even highly virulent diseases may not eliminate individuals randomly; rather, they may selectively remove the most inbred individuals. | 173 | no | no good fitness measure | NA | no | no |
| 174 | 2017 | 43005 | Finke, E; Jetschke, G | 1999 | How inbreeding and outbreeding influence the risk of extinction - a genetically explicit model | We have developed a stochastic model to explore the common effect which genetics and demography have on the extinction risk of endangered populations. The dynamics is formulated as a Markovian birth and death process (in continuous time), whereby selection acts through different mortalities of each genotype. With the help of this model we are able to show how inbreeding and outbreeding can influence the genetic variability and the survival of a population. Whether inbreeding or outbreeding takes place depends on the specific mating system. In our model we consider positive assortative as well as disassortative mating. In the case of additive fitness we show that inbreeding reduces the extinction risk and the genetic variability. (C) 1999 Elsevier Science Inc. All rights reserved. | 174 | no | NA | NA | no | no |
| 175 | 2017 | 43005 | Schueler, S; Kapeller, S; Konrad, H; Geburek, T; Mengl, M; Bozzano, M; Koskela, J; Lefe’vre, F; Hubert, J; Kraigher, H; Longauer, R; Olrik, DC | 2013 | Adaptive genetic diversity of trees for forest conservation in a future climate: a case study on Norway spruce in Austria | Genetic resources of forest trees are considered as a key factor for the persistence of forest ecosystems because the ability of tree species to survive under changing climate depends strongly on their intraspecific variation in climate response. Therefore, utilizing available genetic variation in climate response and planting alternative provenances suitable for future climatic conditions is considered as an important adaptation measure for forestry. On the other hand, the distribution of adaptive genetic diversity of many tree species is still unknown and the predicted shift of ecological zones and species’ distribution may threaten forest genetic resources that are important for adaptation. Here, we use Norway spruce in Austria as a case study to demonstrate the genetic variation in climate response and to analyse the existing network of genetic conservation units for its effectiveness to safeguard the hotspots of adaptive and neutral genetic diversity of this species. An analysis of the climate response of 480 provenances, clustered into 9 groups of climatically similar provenances, revealed high variation among provenance groups. The most productive and promising provenance clusters for future climates originate from three regions that today depict the warmest and driest areas of the natural spruce distribution in Austria. Gap analysis of the Austrian genetic conservation units in the EUFGIS Portal suggests adequate coverage of the genetic hotspots in southern parts of Austria, but not in eastern and northern Austria. Therefore conservation measures and sustainable utilization of the valuable genetic resources in these regions need to be expanded to cover their high adaptive genetic variation and local adaptation to a warmer climate. The study shows that current conservation efforts need to be evaluated for their effectiveness to protect genetic resources that are important for the survival of trees in a future climate. | 175 | no | NA | NA | no | no |
| 176 | 2017 | 43005 | Altermatt, F; Ebert, D | 2008 | Genetic diversity of Daphnia magna populations enhances resistance to parasites | The diversity-disease hypothesis states that decreased genetic diversity in host populations increases the incidence of diseases caused by pathogens (= monoculture effect) and eventually influences ecosystem functioning. The monoculture effect is well-known from crop studies and may be partially specific to the artificial situation in agriculture. The effect received little attention in animal populations of different diversities. Compared with plants, animals are mobile and exhibiting social interactions. We followed the spread of a microsporidian parasite in semi-natural outdoor Daphnia magna populations of low and high genetic diversity. We used randomly, selected, naturally occurring host genotypes. Host populations of low diversity were initially monoclonal, while the host populations of high diversity started with 10 genotypes per replicate. We found that the parasite spread significantly better in host populations of low diversity compared with host populations of high diversity, independent of parasite diversity. The difference was visible over a 3-year period. Host genotypic diversity did not affect host population density. Our experiment demonstrated a monoculture effect in independently replicated semi-natural zooplankton populations, indicating that the monoculture effect may be relevant beyond agriculture. | 176 | yes | from thesis | NA | no | yes |
| 177 | 2017 | 43005 | SCHMIDHEMPEL, P | 1994 | INFECTION AND COLONY VARIABILITY IN SOCIAL INSECTS | The average relatedness among colony members in the social insects, such as bees, wasps and ants, is often low, contrary to the expectations of kin selection theory. Lower relatedness results from multiple mating by the queens (polyandry) or from the presence of more than one functional queen (polygyny). Among the proposed advantages for such mating systems, selection by parasites for within-colony genetic variability is discussed. Empirical studies of this problem are few, but several lines of evidence suggest a role for parasites, such that genetic diversity reduces the rate of within-group transmission. Theoretical considerations indicate that multiple mating is advantageous under conditions of low mating costs relative to parasite pressure and when intermediately sized colonies have a disproportionately large share of reproductive success in the population. In this view, mating strategies (as in polyandry) and strategies of female associations (as in polygyny) that lead to an increased genetic diversity among offspring are, at least in part, an instance of variance reduction in relation to parasitism. | 177 | maybe | any data from this study or just discussion? | no gd of host pop and fitness of host pop, just for discussion | no | no |
| 178 | 2017 | 43005 | Valimaki, K; Hinten, G; Hanski, I | 2007 | Inbreeding and competitive ability in the common shrew (Sorex araneus) | Common shrews (Sorex araneus) maintain a foraging territory for most of their immature life. Possessing a high-quality territory is vital for overwinter survival in the harsh boreal climate, and hence, competitive ability in territorial disputes is expected to be an important component of individual fitness. To test possible association between individual inbreeding and fitness, we used neutral arena trials to assess the competitive performance of young common shrews. The experiment involved pairs of individuals originating from small island populations, where breeding must often occur between related individuals, and from large outbred mainland populations. The percentage of neutral arena tests that an individual won was highly significantly explained by internal relatedness, a surrogate measure of individual inbreeding, measured using ten microsatellite markers. Body size, sex, learning, and population type (mainland vs island) made no significant contributions. Even a low level of individual inbreeding may lead to significant adverse consequences in multiple territorial contests, which may represent a significant cause of inbreeding depression in many wild vertebrate populations. | 178 | no | inbreeding ind | NA | no | no |
| 179 | 2017 | 43005 | Hacker, M; Kaib, M; Bagine, RKN; Epplen, JT; Brandl, R | 2005 | Unrelated queens coexist in colonies of the termite Macrotermes michaelseni | Relatedness increases the likelihood of cooperation within colonies of social insects. Polygyny, the coexistence of numerous reproductive females (queens) in a colony, is common in mature colonies of the termite Macrotermes michaelseni. In this species, polygyny results from pleometrosis and from several female alates that jointly found a new colony. To explain this phenomenon, it was suggested that only related females cooperate and survive during maturation of colonies. Using multilocus fingerprints as well as microsatellites, we showed that nestmate queens in mature colonies are unrelated. Furthermore, we found that all nestmate queens contributed to the production of steriles. Even in mature colonies, several matrilines of steriles coexist within a colony. Although genetic diversity within colonies may increase the likelihood of conflicts, high genetic diversity may be important for foraging, colony growth, and resistance to disease and parasites. | 179 | no | no good fitness measure | NA | no | no |
| 180 | 2017 | 43005 | Tishkoff, SA; Kidd, KK | 2004 | Implications of biogeography of human populations for ‘race’ and medicine | In this review, we focus on the biogeographical distribution of genetic variation and address whether or not populations cluster according to the popular concept of ‘race’. We show that racial classifications are inadequate descriptors of the distribution of genetic variation in our species. Although populations do cluster by broad geographic regions, which generally correspond to socially recognized races, the distribution of genetic variation is quasicontinuous in clinal patterns related to geography. The broad global pattern reflects the accumulation of genetic drift associated with a recent African origin of modern humans, followed by expansion out of Africa and across the rest of the globe. Because disease genes may be geographically restricted due to mutation, genetic drift, migration and natural selection, knowledge of individual ancestry will be important for biomedical studies. Identifiers based on race will often be insufficient. | 180 | no | NA | NA | no | no |
| 181 | 2017 | 43005 | Pearman, PB; Garner, TWJ | 2005 | Susceptibility of Italian agile frog populations to an emerging strain of Ranavirus parallels population genetic diversity | Western populations of the Italian agile frog (Rana latastei) experience widespread genetic depletion. Based on population genetic theory, molecular models of immunity and previous empirical studies, population genetic depletion predicts increased susceptibility of populations to emergent pathogens. We experimentally compared susceptibility of R. latastei populations upon exposure to an emerging strain of Ranavirus, frog virus 3 (FV3), using six populations spanning the geographical range and range of population genetic diversity found in nature. Our findings confirm this prediction, suggesting that the loss of genetic diversity accompanying range expansion and population isolation is coincident with increased mortality risk from an emergent pathogen. Loss of heterozygosity and escape from selection imposed by immunologically cross-reactive pathogens may potentially generate range-wide variation in disease resistance. | 181 | yes | from thesis | NA | no | yes |
| 182 | 2017 | 43005 | Androsiuk, P; Shimono, A; Westin, J; Lindgren, D; Fries, A; Wang, XR | 2013 | Genetic status of Norway spruce (Picea abies) breeding populations for northern Sweden | Efficient use of any breeding resources requires a good understanding of the genetic value of the founder breeding materials for predicting the gain and diversity in future generations. This study evaluates the distribution of genetic variation and level of relatedness among and within nine breeding populations of Norway spruce for Northern Sweden using nuclear microsatellite markers. A sample set of 456 individuals selected from 140 stands were genotyped with, 15 SSR loci. Over all loci each individual was identified with unique multilocus genotype. High genetic diversity (average H-e=0.820) and low population differentiation (F-ST = 0.0087) characterized this material. Although low in F-ST, the two northernmost populations were clustered as a distinct group diverged from the central populations. The population differentiation pattern corresponds well with the post glacial migration history of Norway spruce and the current gene flow and human activity in the region. The average inbreeding coefficient was 0.084 after removal loci with high frequency of null alleles. The estimated relatedness of the trees gathered in the breeding populations was very low (average kinship coefficient 0.0077) and not structured. The high genetic variation and low and not structured relatedness between individuals found in the breeding populations confirm that the Norway spruce breeding stock for northern Sweden represent valuable genetic resources for both long-term breeding and conservation programs. | 182 | no | no good fitness measure | NA | no | no |
| 183 | 2017 | 43005 | Latzel, V; Allan, E; Silveira, AB; Colot, V; Fischer, M; Bossdorf, O | 2013 | Epigenetic diversity increases the productivity and stability of plant populations | Biological diversity within species can be an important driver of population and ecosystem functioning. Until now, such within-species diversity effects have been attributed to underlying variation in DNA sequence. However, within-species differences, and thus potentially functional biodiversity, can also be created by epigenetic variation. Here, we show that epigenetic diversity increases the productivity and stability of plant populations. Epigenetically diverse populations of Arabidopsis thaliana produce up to 40% more biomass than epigenetically uniform populations. The positive epigenetic diversity effects are strongest when populations are grown together with competitors and infected with pathogens, and they seem to be partly driven by complementarity among epigenotypes. Our study has two implications: first, we may need to re-evaluate previous within-species diversity studies where some effects could reflect epigenetic diversity; second, we need to incorporate epigenetics into basic ecological research, by quantifying natural epigenetic diversity and testing for its ecological consequences across many different species. | 183 | maybe | any fitness measure? GD-measurement ok? | inbred pop | no | no |
| 184 | 2017 | 43005 | Sanchez-Mazas, A; Buhler, S; Nunes, JM | 2013 | A New HLA Map of Europe: Regional Genetic Variation and Its Implication for Peopling History, Disease-Association Studies and Tissue Transplantation | Objectives: HLA genes are highly polymorphic in human populations as a result of diversifying selection related to their immune function. However, HLA geographic variation worldwide suggests that demographic factors also shaped their evolution. We here analyzed in detail HLA genetic variation in Europe in order to identify signatures of migration history and/or natural selection. Methods: Relationships between HLA diversity and geography were analyzed at 7 loci through several approaches including linear regression on gene diversity and haplotype frequencies. Regional variation was also assessed on HLA multi-locus phenotypes through structure analysis. Deviation from neutrality was tested by resampling. Results: Geographic distance was a strong predictor of HLA variation at 5 loci (A, B, C, DRB1 and DPB1) in Europe, and latitude significantly shaped HLA gene diversity and haplotype frequencies. Whereas the main level of genetic diversity was found within populations, both HLA gene frequencies and phenotypic profiles revealed regional variation, Southeast Europe, Great Britain and Finland being the most distinctive. Effects of natural selection were suggested at the DQ loci. Conclusions: HLA regional variation was observed in Europe and can be related to population history, locus HLA-A providing by far the strongest signals. This new HLA map of Europe represents an invaluable reference for disease-association studies and tissue transplantation. (C) 2014 S. Karger AG, Basel | 184 | no | NA | NA | no | no |
| 185 | 2017 | 43005 | Hendricks, S; Epstein, B; Schonfeld, B; Wiench, C; Hamede, R; Jones, M; Storfer, A; Hohenlohe, P | 2017 | Conservation implications of limited genetic diversity and population structure in Tasmanian devils (Sarcophilus harrisii) | Tasmanian devils face a combination of threats to persistence, including devil facial tumor disease (DFTD), an epidemic transmissible cancer. We used RAD sequencing to investigate genome-wide patterns of genetic diversity and geographic population structure. Consistent with previous results, we found very low genetic diversity in the species as a whole, and we detected two broad genetic clusters occupying the northwestern portion of the range, and the central and eastern portions. However, these two groups overlap across a broad geographic area, and differentiation between them is modest ( = 0.1081). Our results refine the geographic extent of the zone of mixed ancestry and substructure within it, potentially informing management of genetic variation that existed in pre-diseased populations of the species. DFTD has spread across both genetic clusters, but recent evidence points to a genomic response to selection imposed by DFTD. Any allelic variation for resistance to DFTD may be able to spread across the devil population under selection by DFTD, and/or be present as standing variation in both genetic regions. | 185 | no | NA | NA | no | no |
| 186 | 2017 | 43005 | Hendrati, R; Byrne, M; Barbour, E; Plummer, J | 2011 | Effect of genetic relatedness among parents on gain in salt tolerance in progeny of crosses of Eucalyptus occidentalis | Genetic diversity of a Eucalyptus occidentalis breeding population screened for salt and waterlogging tolerance was examined using eight microsatellite loci. Mating using an immature style ‘one stop pollination’ method between parents was carried out to produce progeny for testing under 500 mM salt waterlogging. The effect of parental genetic distance on seed production and early seedling survival was examined and inheritance of salt/or waterlogging tolerance was assessed by testing performance of progeny in comparison to parents. Diversity was moderate among the nine provenances and the families, and most of the diversity was distributed within rather than between families. Genetic relationships showed no structure in relation to provenance indicating any adaptation to saline environments has not affected genetic similarity. Breeding for salt and water tolerance may be achieved without decline in genetic diversity. There was a significant correlation between capsule production and parental genetic distance and a positive trend between increasing parental genetic distance and increasing number of germinated seeds/capsule, and seedling survival at 2 weeks and 9 months. These trends indicate expression of inbreeding depression in crosses between genetically similar parents. Heritability values, under 500 mM salt-waterlogging treatment, indicated that height had moderate heritability (h(2) = 0.5). Crosses with the widest parental genetic distance produced progeny with considerable height increase above parents and this trend was evident even with moderate genetic distance indicating crossing at this level of differentiation may achieve optimum breeding gain. | 186 | no | NA | NA | no | no |
| 187 | 2017 | 43005 | Miyazaki, S; Yoshimura, M; Saiki, R; Hayashi, Y; Kitade, O; Tsuji, K; Maekawa, K | 2014 | Intracolonial genetic variation affects reproductive skew and colony productivity during colony foundation in a parthenogenetic termite | Background: In insect societies, intracolonial genetic variation is predicted to affect both colony efficiency and reproductive skew. However, because the effects of genetic variation on these two colony characteristics have been tested independently, it remains unclear whether they are affected by genetic variation independently or in a related manner. Here we test the effect of genetic variation on colony efficiency and reproductive skew in a rhinotermitid termite, Reticulitermes speratus, a species in which female-female pairs can facultatively found colonies. We established colonies using two types of female-female pairs: colonies founded by sisters (i.e., sister-pair colonies) and those founded by females from different colonies (i.e., unrelated-pair colonies). Colony growth and reproductive skew were then compared between the two types of incipient colonies. Results: At 15 months after colony foundation, unrelated-pair colonies were larger than sister-pair colonies, although the caste ratio between workers and nymphs, which were alternatively differentiated from young larvae, did not differ significantly. Microsatellite DNA analyses of both founders and their parthenogenetically produced offspring indicated that, in both sister-pair and unrelated-pair colonies, there was no significant skew in the production of eggs, larvae, workers and soldiers. Nymph production, however, was significantly more skewed in the sister-pair colonies than in unrelated-pair colonies. Because nymphs can develop into winged adults (alates) or nymphoid reproductives, they have a higher chance of direct reproduction than workers in this species. Conclusions: Our results support the idea that higher genetic variation among colony members could provide an increase in colony productivity, as shown in hymenopteran social insects. Moreover, this study suggests that low genetic variation (high relatedness) between founding females increases reproductive skew via one female preferentially channeling her relatives along the reproductive track. This study thus demonstrated that, in social insects, intracolonial genetic variation can simultaneously affect both colony efficiency and reproductive skew. | 187 | maybe | ""“colony growth”" ok Fitnessmeasure?" | no good fitness measure | no | no |
| 188 | 2017 | 43005 | Jara, L; Munoz, I; Cepero, A; Martin-Hernandez, R; Serrano, J; Higes, M; De la Rua, P | 2015 | Stable genetic diversity despite parasite and pathogen spread in honey bee colonies | In the last decades, the rapid spread of diseases, such as varroosis and nosemosis, associated with massive honey bee colonies mortality around the world has significantly decreased the number and size of honey bee populations and possibly their genetic diversity. Here, we compare the genetic diversity of Iberian honey bee colonies in two samplings performed in 2006 and 2010 in relation to the presence of the pathogenic agents Nosema apis, Nosema ceranae, and Varroa destructor in order to determine whether parasite and pathogen spread in honey bee colonies reflects changes in genetic diversity. We found that the genetic diversity remained similar, while the incidence of N. ceranae increased and the incidence of N. apis and V. destructor decreased slightly. These results indicate that the genetic diversity was not affected by the presence of these pathogenic agents in the analyzed period. However, the two groups of colonies with and without Nosema/Varroa detected showed significant genetic differentiation (G test). A detailed analysis of the allelic segregation of microsatellite loci in Nosema/Varroa-negative colonies and parasitized ones revealed two outlier loci related to genes involved in immune response. | 188 | maybe | gd difference? | gd mean of ind/pop not pop level | no | no |
| 189 | 2017 | 43005 | Caplins, SA; Gilbert, KJ; Ciotir, C; Roland, J; Matter, SF; Keyghobadi, N | 2014 | Landscape structure and the genetic effects of a population collapse | Both landscape structure and population size fluctuations influence population genetics. While independent effects of these factors on genetic patterns and processes are well studied, a key challenge is to understand their interaction, as populations are simultaneously exposed to habitat fragmentation and climatic changes that increase variability in population size. In a population network of an alpine butterfly, abundance declined 60-100% in 2003 because of low over-winter survival. Across the network, mean microsatellite genetic diversity did not change. However, patch connectivity and local severity of the collapse interacted to determine allelic richness change within populations, indicating that patch connectivity can mediate genetic response to a demographic collapse. The collapse strongly affected spatial genetic structure, leading to a breakdown of isolation-by-distance and loss of landscape genetic pattern. Our study reveals important interactions between landscape structure and temporal demographic variability on the genetic diversity and genetic differentiation of populations. Projected future changes to both landscape and climate may lead to loss of genetic variability from the studied populations, and selection acting on adaptive variation will likely occur within the context of an increasing influence of genetic drift. | 189 | no | only one pop | NA | no | no |
| 190 | 2017 | 43005 | Maebe, K; Meeus, I; Vray, S; Claeys, T; Dekoninck, W; Boeve, JL; Rasmont, P; Smagghe, G | 2016 | A century of temporal stability of genetic diversity in wild bumblebees | Since the 1950s, bumblebee (Bombus) species are showing a clear decline worldwide. Although many plausible drivers have been hypothesized, the cause(s) of this phenomenon remain debated. Here, genetic diversity in recent versus historical populations of bumblebee species was investigated by selecting four currently restricted and four currently widespread species. Specimens from five locations in Belgium were genotyped at 16 microsatellite loci, comparing historical specimens (1913-1915) with recent ones (2013-2015). Surprisingly, our results showed temporal stability of genetic diversity in the restricted species. Furthermore, both historical and recent populations of restricted species showed a significantly lower genetic diversity than found in populations of co-occurring widespread species. The difference in genetic diversity between species was thus already present before the alleged recent drivers of bumblebee decline could have acted (from the 1950’s). These results suggest that the alleged drivers are not directly linked with the genetic variation of currently declining bumblebee populations. A future sampling in the entire distribution range of these species will infer if the observed link between low genetic diversity and population distribution on the Belgium scale correlates with species decline on a global scale. | 190 | no | individual level | NA | no | no |
| 191 | 2017 | 43005 | Rosset, H; Keller, L; Chapuisat, M | 2005 | Experimental manipulation of colony genetic diversity had no effect on short-term task efficiency in the Argentine ant Linepithema humile | Genetic diversity might increase the performance of social groups by improving task efficiency or disease resistance, but direct experimental tests of these hypotheses are rare. We manipulated the level of genetic diversity in colonies of the Argentine ant Linepithema humile, and then recorded the short-term task efficiency of these experimental colonies. The efficiency of low and high genetic diversity colonies did not differ significantly for any of the following tasks: exploring a new territory, foraging, moving to a new nest site, or removing corpses. The tests were powerful enough to detect large effects, but may have failed to detect small differences. Indeed, observed effect sizes were generally small, except for the time to create a trail during nest emigration. In addition, genetic diversity had no statistically significant impact on the number of workers, males and females produced by the colony, but these tests had low power. Higher genetic diversity also did not result in lower variance in task efficiency and productivity. In contrast to genetic diversity, colony size was positively correlated with the efficiency at performing most tasks and with colony productivity. Altogether, these results suggest that genetic diversity does not strongly improve short-term task efficiency in L. humile, but that worker number is a key factor determining the success of this invasive species. | 191 | no | no good fitness measure | NA | no | no |
| 192 | 2017 | 43005 | FRANK, SA | 1993 | EVOLUTION OF HOST-PARASITE DIVERSITY | Hosts and parasites often have extensive genetic diversity for resistance and virulence (host range). Qualitative diversity occurs when the success of attack is an all-or-nothing response that varies according to the genotypes of the host and parasite. Quantitative diversity occurs when the success of attack is a graded response that depends on additive genetic variation in the host and parasite. Community diversity occurs when parasites vary in the success with which they can attack different host species, leading to a mixture of specialists and generalists. I developed a series of models that classify components of host-parasite interactions according to whether they cause stabilizing or disruptive selection for resistance and virulence. Stabilizing selection reduces diversity by favoring a single optimal phenotype. Disruptive selection creates diversity by favoring a mixture of widely separated phenotypes. The evolution of maximal resistance and virulence are opposed by one of three forces: metabolic costs, frequency dependence, or negative genetic correlations among beneficial traits. The models predict that qualitatively inherited resistance and virulence traits typically cause greater diversity than quantitatively inherited traits. However, each natural system is composed of many stabilizing factors that reduce diversity and disruptive factors that promote diversity. I advocate a style of modeling in which families of related assumptions are compared by their equilibrium properties, and general conclusions from equilibrium properties are tested by complete dynamical analysis. The comparison among models highlights the need for empirical studies that compare levels of diversity among related host-parasite systems. | 192 | no | NA | NA | no | no |
| 193 | 2017 | 43005 | Reber, A; Castella, G; Christe, P; Chapuisat, M | 2008 | Experimentally increased group diversity improves disease resistance in an ant species | A leading hypothesis linking parasites to social evolution is that more genetically diverse social groups better resist parasites. Moreover, group diversity can encompass factors other than genetic variation that may also influence disease resistance. Here, we tested whether group diversity improved disease resistance in an ant species with natural variation in colony queen number. We formed experimental groups of workers and challenged them with the fungal parasite Metarhizium anisopliae. Workers originating from monogynous colonies (headed by a single queen and with low genetic diversity) had higher survival than workers originating from polygynous ones, both in uninfected groups and in groups challenged with M. anisopliae. However, an experimental increase of group diversity by mixing workers originating from monogynous colonies strongly increased the survival of workers challenged with M. anisopliae, whereas it tended to decrease their survival in absence of infection. This experiment suggests that group diversity, be it genetic or environmental, improves the mean resistance of group members to the fungal infection, probably through the sharing of physiological or behavioural defences. | 193 | yes | of interest | NA | no | yes |
| 194 | 2017 | 43005 | Ginja, C; Gama, LT; Cortes, O; Delgado, JV; Dunner, S; Garcia, D; Landi, V; Martin-Burriel, I; Martinez-Martinez, A; Penedo, MCT; Rodellar, C; Zaragoza, P; Canon, J | 2013 | Analysis of conservation priorities of Iberoamerican cattle based on autosomal microsatellite markers | Background: Determining the value of livestock breeds is essential to define conservation priorities, manage genetic diversity and allocate funds. Within-and between-breed genetic diversity need to be assessed to preserve the highest intra-specific variability. Information on genetic diversity and risk status is still lacking for many Creole cattle breeds from the Americas, despite their distinct evolutionary trajectories and adaptation to extreme environmental conditions. Methods: A comprehensive genetic analysis of 67 Iberoamerican cattle breeds was carried out with 19 FAO recommended microsatellites to assess conservation priorities. Contributions to global diversity were investigated using alternative methods, with different weights given to the within-and between-breed components of genetic diversity. Information on Iberoamerican plus 15 worldwide cattle breeds was used to investigate the contribution of geographical breed groups to global genetic diversity. Results: Overall, Creole cattle breeds showed a high level of genetic diversity with the highest level found in breeds admixed with zebu cattle, which were clearly differentiated from all other breeds. Within-breed kinships revealed seven highly inbred Creole breeds for which measures are needed to avoid further genetic erosion. However, if contribution to heterozygosity was the only criterion considered, some of these breeds had the lowest priority for conservation decisions. The Weitzman approach prioritized highly differentiated breeds, such as Guabala, Romosinuano, Cr. Patagonico, Siboney and Caracu, while kinship-based methods prioritized mainly zebu-related breeds. With the combined approaches, breed ranking depended on the weights given to the within-and between-breed components of diversity. Overall, the Creole groups of breeds were generally assigned a higher priority for conservation than the European groups of breeds. Conclusions: Conservation priorities differed significantly according to the weight given to within-and between-breed genetic diversity. Thus, when establishing conservation programs, it is necessary to also take into account other features. Creole cattle and local isolated breeds retain a high level of genetic diversity. The development of sustainable breeding and crossbreeding programs for Creole breeds, and the added value resulting from their products should be taken into consideration to ensure their long-term survival. | 194 | no | domesticated animal | NA | no | no |
| 195 | 2017 | 43005 | Wisely, SM; Buskirk, SW; Fleming, MA; McDonald, DB; Ostrander, EA | 2002 | Genetic diversity and fitness in black-footed ferrets before and during a bottleneck | The black-footed ferret (Mustela nigripes) is an endangered North American carnivore that underwent a well-documented population bottleneck in the mid-1980s. To better understand the effects of a bottleneck on a free-ranging carnivore population, we used 24 microsatellite loci to compare genetic diversity before versus during the bottleneck, and compare the last wild population to two historical populations. We also compared genetic diversity in black-footed ferrets to that of two sibling species, the steppe polecat (Mustela eversmanni) and the European polecat (Mustela putorius). Black-footed ferrets during the bottleneck had less genetic diversity than steppe polecats. The three black-footed ferret populations were well differentiated (F-ST = 0.57 +/- 0.15; mean +/- SE). We attributed the decrease in genetic diversity in black-footed ferrets to localized extinction of these genetically distinct subpopulations and to the bottleneck in the surviving subpopulation. Although genetic diversity decreased, female fecundity and juvenile survival were not affected by the population bottleneck. | 195 | no | only one pop | NA | no | no |
| 196 | 2017 | 43005 | Woodworth, LM; Montgomery, ME; Briscoe, DA; Frankham, R | 2002 | Rapid genetic deterioration in captive populations: Causes and conservation implications | Many species require captive breeding to ensure their survival. The eventual aim of such programs is usually to reintroduce the species into the wild. Populations in captivity deteriorate due to inbreeding depression, loss of genetic diversity, accumulation of new deleterious mutations and genetic adaptations to captivity that are deleterious in the wild. However, there is little evidence on the magnitude of these problems. We evaluated changes in reproductive fitness in populations of Drosophila maintained under benign captive conditions for 50 generations with effective population sizes of 500 (2 replicates), 250 (3), 100 (4), 50 (6) and 25 (8). At generation 50, fitness in the benign captive conditions was reduced in small populations due to inbreeding depression and increased in some of the large populations due to modest genetic adaptation. When the populations were moved to `wild' conditions, all 23 populations showed a marked decline (64-86%percnt;) in reproductive fitness compared to controls. Reproductive fitness showed a curvilinear relationship with population size, the largest and smallest population size treatments being the worst. Genetic analyses indicated that inbreeding depression and genetic adaptation were responsible for the genetic deterioration in`wild’ fitness. Consequently, genetic deterioration in captivity is likely to be a major problem when long-term captive bred populations of endangered species are returned to the wild. A regime involving fragmentation of captive populations of endangered species is suggested to minimize the problems. | 196 | no | NA | NA | no | no |
| 197 | 2017 | 43005 | GROBLER, JP; VANDERBANK, FH | 1993 | GENETIC-VARIABILITY IN SOUTH-AFRICAN BLUE WILDEBEEST (CONNOCHAETES-TAURINUS) | 1. We used protein gel-electrophoresis to investigate genetic heterogeneity at 33 protein coding loci in a total of 46 blue wildebeest (C. taurinus) kept under different management regimes. 2. Average heterozygosity ranged from 2.14 to 4.3% and within-population differences accounted for 97.2% of total relative gene diversity. 3. Comparatively little divergence was found between animals sampled from populations with very diverse population sizes and management histories, with the largest genetic distance estimated between any two populations being only 0.0021. 4. We discuss our results with particular emphasis on the influence of management history on genetic diversity and divergence in C. taurinus. | 197 | no | NA | NA | no | no |
| 198 | 2017 | 43005 | Gasparini, C; Pilastro, A | 2011 | Cryptic female preference for genetically unrelated males is mediated by ovarian fluid in the guppy | As inbreeding is costly, it has been suggested that polyandry may evolve as a means to reduce the negative fitness consequences of mating with genetically related males. While several studies provide support for this hypothesis, evidence of pure post-copulatory mechanisms capable of biasing paternity towards genetically unrelated males is still lacking; yet these are necessary to support inbreeding avoidance models of polyandry evolution. Here we showed, by artificially inseminating a group of female guppies with an equal number of sperm from related (full-sib) and unrelated males, that sperm competition success of the former was 10 per cent lower, on average, than that of the unrelated male. The paternity bias towards unrelated males was not due to differential embryo survival, as the size of the brood produced by control females, which were artificially inseminated with the sperm of a single male, was not influenced by their relatedness with the male. Finally, we collected ovarian fluid (OF) from virgin females. Using computer-assisted sperm analysis, we found that sperm velocity, a predictor of sperm competition success in the guppy, was significantly lower when measured in a solution containing the OF from a sister as compared with that from an unrelated female. Our results suggest that sperm-OF interaction mediates sperm competition bias towards unrelated mates and highlight the role of post-copulatory mechanisms in reducing the cost of mating with relatives in polyandrous females. | 198 | no | NA | NA | no | no |
| 199 | 2017 | 43005 | Ellison, A; De Leaniz, CG; Consuegra, S | 2013 | Inbred and furious: negative association between aggression and genetic diversity in highly inbred fish | Aggressive behaviour plays an important role in securing resources, defending against predators and shaping social interactions. Although aggression can have positive effects on growth and reproductive success, it is also energetically costly and may increase injury and compromise survival. Individual genetic diversity has been positively associated with aggression, but the cause for such an association is not clear, and it might be related to the ability to recognize kin. To disentangle the relationships between genetic diversity, kinship and aggression, we quantified aggressive behaviour in a wild, self-fertilizing fish (Kryptolebias marmoratus) with naturally variable degrees of genetic diversity, relatedness and familiarity. We found that in contrast to captive fish, levels of aggression among wild K.marmoratus are positively associated with individual homozygosity, but not with relatedness or familiarity. We suggest that the higher aggression shown by homozygous fish could be related to better kin discrimination and may be facilitated by hermaphrodite competition for scarce males, given the fitness advantages provided by outcrossing in terms of parasite resistance. It seems likely that the relationship between aggression and genetic diversity is largely influenced by both the environment and population history. | 199 | no | NA | NA | no | no |
| 200 | 2017 | 43005 | Adeyemo, O; Omidiji, O | 2014 | SSR-based and carotenoid diversity assessment of tropical yellow endosperm maize inbred lines | Yellow endosperm maize can be used to reduce vitamin A deficiency among many pre-school children and women of reproductive age in sub-Saharan Africa. Assessment of the genetic diversity of tropical yellow endosperm maize inbred lines will have genetic gains in breeding design to develop lines with an enhanced level of provitamin A. We screened 122 tropical yellow endosperm maize inbred lines with 62 simple sequence repeat (SSR) markers and 51 SSR loci were polymorphic. We detected 190 alleles with an average of 3.72 alleles per locus, and polymorphic information content values among the inbred lines varied from 0.12 to 0.74 with an average of 0.43. Genetic distance (GD) values among all the pairs of the 122 inbred lines varied from 0.02 to 0.61 with an average of 0.41 for the SSR markers. The inbred lines exhibited a substantial level of genetic diversity. Genetic diversity was also evaluated using carotenoid data. The Euclidean GDs varied from 1.00 to 9.97 with an average of 3.81 for the carotenoid data. Cluster and principal coordinate analyses revealed clear separation of maize inbred lines into SSR-based groupings and carotenoid-based groupings. Cluster based on SSR markers were predominantly consistent with known pedigree data of the inbred lines. The correlation using Mantel’s test between carotenoid-based GD estimates and SSR marker-based GD estimates gave a low r value (-0.06). The grouping of lines will facilitate the selection of parental lines for making crosses to develop new lines with enhanced provitamin A content. | 200 | no | NA | NA | no | no |
| 201 | 2017 | 43005 | Dong, YS; Zhuang, BC; Zhao, LM; Sun, H; He, MY | 2001 | The genetic diversity of annual wild soybeans grown in China | Annual wild soybeans (Glycine soja), the ancestors of cultivated soybeans (G. max), are important sources of major genes for resistance to pests, diseases and environmental stresses. The study of their genetic diversity is invaluable for efficient utilization. conservation and management of germplasm collections. In this paper, the number of accessions. the variation of traits. the genetic diversity indexes (Shannon index) and the coefficient of variation were employed to study the geographical distribution of accessions, genetic diversity of characters and genetic diversity centers of annual wild soybean by statistical analysis of the database from the National Germplasm Evaluation Program of China. Most annual wild soybeans are distributed in Northeast China. and the number of accessions decreases from the Northeast to other directions in China. The genetic diversity indexes (Shannon index) were 0.49, 0.74. 0.02, 0.55, 1.45. 2.41, 1.27 and 1.89 for flower color. sootiness of seed coat. cotyledon color. pubescence color. hilum color. leaf shape, stem type and seed color, respectively. Coefficients of variation were 7.1%. 28.7%, 76.43% and 18.2% for protein content, oil content, 100-seed weight and days to maturity, respectively. Three genetic diversity centers, the Northeast. the Yellow River Valley and the Southeast Coasts of China, are proposed based on the geographical distribution of the number of accessions, genetic diversity and the multivariate variation coefficient. Based on these results and Vavilov’s theory of crop origination, two opposing possible models for the formation of the three centers are proposed, either these centers are independent of each other and the annual wild soybeans in these centers originated separately, or the Northeast center was the primary center for annual wild soybeans in China. while the Yellow River Valley center was derived from this primary center and served as the origin for the Southeast Coast center. | 201 | no | NA | NA | no | no |
| 202 | 2017 | 43005 | Reynolds, LK; Waycott, M; McGlathery, KJ; Orth, RJ; Zieman, JC | 2012 | Eelgrass restoration by seed maintains genetic diversity: case study from a coastal bay system | Genetic diversity is positively associated with plant fitness, stability, and the provision of ecosystem services. Preserving genetic diversity is therefore considered an important component of ecosystem restoration as well as a measure of its success. We examined the genetic diversity of restored Zostera marina meadows in a coastal bay system along the USA mid-Atlantic coast using microsatellite markers to compare donor and recipient meadows. We show that donor meadows in Chesapeake Bay have high genetic diversity and that this diversity is maintained in meadows restored with seeds in the Virginia coastal bays. No evidence of inbreeding depression was detected (F-IS-0.2 to 0) in either donor or recipient meadows, which is surprising because high levels of inbreeding were expected following the population contractions that occurred in Chesapeake Bay populations due to disease and heat stress. Additionally, there was no evidence for selection of genotypes at the restoration sites, suggesting that as long as donor sites are chosen carefully, issues that diminish fitness and survival such as heterosis or out-breeding depression can be avoided. A cluster analysis showed that, in addition to the Chesapeake Bay populations that acted as donors, the Virginia coastal bay populations shared a genetic signal with Chincoteague Bay populations, their closest neighbor to the north, suggesting that natural recruitment into the area may be occurring and augmenting restored populations. We hypothesize that the high genetic diversity in seagrasses restored using seeds rather than adult plants confers a greater level of ecosystem resilience to the restored meadows. | 202 | no | NA | NA | no | no |
| 203 | 2017 | 43005 | Bodmer, WF | 1997 | Genetic diversity and disease susceptibility | The range of genetic diversity within human populations is enormous. Genetic susceptibility to common chronic disease is a significant part of this genetic diversity, which also includes a variety of rare clear-cut inherited diseases. Modern DNA-based genomic analysis can now routinely lead to the identification of es involved in disease susceptibility, provides the basis for genetic counselling in affected families, and more widely for a genetically targeted approach to disease prevention. This naturally raises problems concerning the use of information on an individual’s decisions, but for employment, and health and life insurance. | 203 | no | NA | NA | no | no |
| 204 | 2017 | 43005 | Chan, CH; Robertson, HA; Saul, EK; Nia, LV; Vy, PL; Kong, XC; Zhao, YD; Chambers, GK | 2011 | Genetic variation in the kakerori (Pomarea dimidiata), an endangered endemic bird successfully recovering in the Cook Islands | The Cook Islands endemic kakerori (Pomarea dimidiata) underwent a severe population decline following the introduction of ship rats (Rattus rattus) in the late 1800s. By 1989, the sole population on Rarotonga consisted of 29 known birds. Subsequent intensive management efforts enabled this population to recover to around 250-300 birds in recent years. This study, using microsatellite and mitochondrial DNA markers, assesses the level of genetic diversity and the genetic structure of the contemporary kakerori population on Rarotonga. No mitochondrial control region and cytochrome b haplotype diversity was found in the 11 samples examined at each locus. In 81 samples genotyped at 7 polymorphic microsatellite loci, an average of 4 alleles per locus were found, with an average observed heterozygosity of 0.65. No subpopulation division was found in this population. There was no evidence of inbreeding, but genetic bottleneck tests showed that the population had indeed experienced a significant genetic bottleneck. Recovery of the kakerori was successful in the past two decades despite low genetic diversity in terms of allelic diversity. Our data suggested that low allelic diversity did not hamper population expansion and the continued survival of this species, however, longer-term effects are still possible. | 204 | no | NA | NA | no | no |
| 205 | 2017 | 43005 | Van Geert, A; Van Rossum, F; Triest, L | 2008 | Genetic diversity in adult and seedling populations of Primula vulgaris in a fragmented agricultural landscape | Habitat fragmentation is known to generally reduce the size of plant populations and increase their isolation, leading to genetic erosion and increased between-population genetic differentiation. In Flanders (northern Belgium) Primula vulgaris is very rare and declining. Populations have incurred strong fragmentation for the last decades and are now restricted to a few highly fragmented areas in an intensively used agricultural landscape. Previous studies showed that small populations of this long-lived perennial herb still maintained high levels of genetic variation and low genetic differentiation. This pattern can either indicate recent gene flow or represent historical variation. Therefore, we used polymorphic microsatellite loci to investigate genetic variation and structure in adult (which may still reflect historical variation) and seedling (recent generation, thus affected by current processes) life stages. The recent generation (seedlings) showed a significant loss of observed heterozygosity (H,,) together with lower expected heterozygosity (H,), a trend for higher inbreeding levels (F-IS) and higher differentiation (F-ST) between populations compared to the adult generation. This might result from (1) a reduction in effective population size, (2) higher inbreeding levels in the seedlings, (3) a higher survival of heterozygotes over time due to a higher fitness of heterozygotes (heterosis) and/or a lower fitness of homozygotes (inbreeding depression), (4) overlapping generations in the adult life stage, or (5) a lack of establishment of new (inbred) adults from seedlings due to degraded habitat conditions. Combining restoration of both habitat quality and gene flow between populations may be indispensable to ensure a sustainable. conservation of fragmented populations. | 205 | no | NA | NA | no | no |
| 206 | 2017 | 43005 | Blaha, M; Zurovcova, M; Kouba, A; Policar, T; Kozak, P | 2016 | Founder event and its effect on genetic variation in translocated populations of noble crayfish (Astacus astacus) | Establishing translocated populations is a common process to preserve and maintain genetic diversity of threatened species. In 2001, three translocated populations of noble crayfish (Astacus astacus) were established in the Czech Republic, founded by either adult or juvenile individuals from three particular source populations. We assessed genetic diversity at seven microsatellite loci after one decade (assumed three generations) from establishment. Although the translocated populations exhibited a slight but non-significant reduction in genetic diversity (A (R) = 2.2-5.0; H (O) = 0.11-0.31), the most striking result was generally very low genetic diversity in source populations (A (R) = 3.0-5.3; H (O) = 0.15-0.38). Similarly, a high degree of inbreeding (F (IS) = 0.36-0.60) demonstrates the nature of source populations, already affected by isolation and small size. In spite of that, based on the results of this study, the establishment of new translocated noble crayfish populations was successful, since there is no significant decline in genetic variability and all populations are still viable. Although source populations did not exhibit high genetic diversity, their distinctiveness makes them possible to use for conservation purposes. Continued monitoring is necessary to track the long-term progress of the translocation program, including other parameters describing the state of the population, such as the occurrence and frequency of diseases or morphological changes. | 206 | no | NA | NA | no | no |
| 207 | 2017 | 43005 | Feiner, ZS; DeWoody, JA; Breck, JE; Hook, TO | 2017 | Influences of multilocus heterozygosity on size during early life | Genetic diversity has been hypothesized to promote fitness of individuals and populations, but few studies have examined how genetic diversity varies with ontogeny. We examined patterns in population and individual genetic diversity and the effect of genetic diversity on individual fitness among life stages (adults and juveniles) and populations of captive yellow perch (Perca flavescens) stocked into two ponds and allowed to spawn naturally. Significant genetic structure developed between adults and offspring in a single generation, even as heterozygosity and allelic richness remained relatively constant. Heterozygosity had no effect on adult growth or survival, but was significantly and consistently positively related to offspring length throughout the first year of life in one pond but not the other. The largest individuals in the pond exhibiting this positive relationship were more outbred than averaged size individuals and also more closely related to one another than they were to average-sized individuals, suggesting potential heritability of body size or spawn timing effects. These results indicate that the influence of heterozygosity may be mediated through an interaction, likely viability selection, between ontogeny and environment that is most important during early life. In addition, populations may experience significant genetic change within a single generation in captive environments, even when allowed to reproduce naturally. Accounting for the dynamic influences of genetic diversity on early life fitness could lead to improved understanding of recruitment and population dynamics in both wild and captive populations. | 207 | no | individual level | NA | no | no |
| 208 | 2017 | 43005 | Valbuena-Urena, E; Soler-Membrives, A; Steinfartz, S; Alonso, M; Carbonell, F; Larios-Martin, R; Obon, E; Carranza, S | 2017 | Getting off to a good start? Genetic evaluation of the ex situ conservation project of the Critically Endangered Montseny brook newt (Calotriton arnoldi) | “Ex situ management strategies play an important role in the conservation of threatened species when the wild survival of the species cannot be ensured. Molecular markers have become an outstanding tool for the evaluation and management of captive breeding programs. Two main genetic objectives should be prioritized when planning breeding programs: the maintenance of maximum neutral genetic diversity, and to obtain”“selfsustaining’’ captive populations. In this study, we use 24 microsatellite loci to analyze and evaluate the genetic representativity of the initial phases of the captive breeding program of the Montseny brook newt, Calotriton arnoldi, an Iberian endemic listed as Critically Endangered. The results show that the initial captive stock has 74-78% of the alleles present in the wild populations, and captures roughly 93-95% of their total genetic diversity as observed in a previous study on wild newts, although it does not reach the desired 97.5%. Moreover, the percentage of unrelatedness among individuals does not exceed 95%. Therefore, we conclude that the genetic diversity of the captive stock should be improved by incorporating genetic material from unrelated wild newts. In recognition of the previously described significant genetic and morphological differentiation between eastern and western wild populations of C. arnoldi, we suggest maintaining two distinct breeding lines, and we do not recommend outbreeding between these lines. Our comparisons of genetic diversity estimates between real and distinct sample-sized simulated populations corroborated that a minimum of 20 individuals are needed for each captive population, in order to match the level of genetic diversity present in the wild populations. Thus, the current initial stock should be reinforced by adding wild specimens. The captive stock and subsequent cohorts should be monitored in order to preserve genetic variation. In order to avoid genetic adaptation to captivity, occasionally incorporating previously genotyped individuals from the wild into the captive populations is recommended.” | 208 | no | NA | NA | no | no |
| 209 | 2017 | 43005 | Garant, D; Dodson, JD; Bernatchez, L | 2005 | Offspring genetic diversity increases fitness of female Atlantic salmon (Salmo salar) | Inbreeding has negative effects on individual and population performances. Therefore, enhancement of offspring genetic diversity is believed to play a major role in shaping mating systems. However, no study has clearly separated the direct effect of having multiple partners from the indirect effect of having more outbred offspring on the resulting reproductive success of individuals in the wild. In this study, we report significant associations between both multiple mating and within-individual genetic diversity of offspring, and an increased reproductive success of wild female Atlantic salmon, Salmo salar. Specifically, we found that females with a higher number of mates also have more outbred offspring (within-individuals), and that both of these characteristics increased their reproductive success expressed in terms of offspring surviving when combining all freshwater juvenile stages. Our findings also indicate that determinants of fitness are different among sexes as within-individual offspring genetic diversity was not a strong predictor of male reproductive success, while the number of mates was important. We also show that females mated with more outbred males than on average, which potentially increased their chances of producing outbred offspring. These results therefore suggest that there could be more important indirect genetic benefits of multiple mating for females than for males. | 209 | no | individual level | NA | no | no |
| 210 | 2017 | 43005 | Edenhamn, P; Hoggren, M; Carlson, A | 2000 | Genetic diversity and fitness in peripheral and central populations of the European tree frog Hyla arborea | Genetic diversity is expected to decrease in small and isolated populations as a consequence of founder effects, bottlenecks, inbreeding and genetic drift. In this study we analyse temporal and spatial effects on genetic variation and progeny viability of the European tree frog (Hyla arborea) at two scales. First, the Swedish distribution has been isolated from the continental distribution for more than 8000 thousand years, and secondly, within Sweden, recent habitat alterations that have taken place during this century have increased isolation between local populations. Genetic variation and progeny survival in relation to isolation was studied within the entire Swedish distribution of the tree frog. Allozyme electrophoresis analysis of froglets, sampled across the Swedish distribution, revealed a low overall genetic variation (1.06 alleles/locus) at the protein level in comparison with continental populations (1.54-1.68 alleles/locus). However, egg hatchability (97 %) and early larval survival (95 %) were not lower than in other parts of the tree frog distribution or in other anuran species. Within the Swedish distribution, early larval survival was lower in isolated breeding ponds than in more central ones. However, no differences in genetic variation were found in relation to isolation. Polymorphism was detected only at a single locus, and was restricted geographically to the eastern part of the Swedish distribution. Bottlenecks due to climatic changes and fragmentation of suitable habitat (primarily natural pastures with ponds) are suggested as possible causes of the low genetic diversity of the Swedish tree frog population. | 210 | no | NA | NA | no | no |
| 211 | 2017 | 43005 | Montgomery, ME; Ballou, JD; Nurthen, RK; England, PR; Briscoe, DA; Frankham, R | 1997 | Minimizing kinship in captive breeding programs | Captive populations of endangered species are managed to preserve genetic diversity and retain reproductive fitness. Minimizing kinship (MK) has been predicted to maximize the retention of gene diversity in pedigreed populations with unequal founder representation. MK was compared with maximum avoidance of inbreeding (MAI) and random choice of parents (RAND) using Drosophila melanogaster. Forty replicate populations of each treatment were initiated with unequal founder representation and managed for four generations. MK retained significantly more gene diversity and allelic diversity based on six microsatellite loci and seven allozyme loci than MAI or RAND. Reproductive fitness under both benign and competitive conditions did not differ significantly among treatments. Of the methods considered, MK is currently the best available for the genetic management of captive populations. (C) 1997 Wiley-Liss, Inc. | 211 | no | NA | NA | no | no |
| 212 | 2017 | 43005 | Jansson, M; Stahl, I; Laikre, L | 2013 | mPed: a computer program for converting pedigree data to a format used by the PMx-software for conservation genetic analysis | There is a growing need for conservation genetic management of animal populations when individual relatedness data (pedigrees) are available. Such data can be used to monitor rates of inbreeding and loss of genetic diversity. Traditionally, pedigree analysis for conservation management has focused on zoo populations of threatened wild animals; available software has been developed in that context. Population Management x (PMx) is a free software for estimating genetic parameters including inbreeding, kinship, founder allele contribution and survival. PMx is an accessory program to the zoo studbook platform Single Population Analysis and Records Keeping System (SPARKS) and is not easily applied outside this platform, but such use is of interest for various domestic breeds or wild populations. We developed a converter program (mPed) for making pedigrees of any studbook format fitting the input requirements of PMx. mPed can be downloaded free at www.popgen.su.se/mped.php. | 212 | no | NA | NA | no | no |
| 213 | 2017 | 43005 | Wang, RH; Yu, YT; Zhao, JR; Shi, YS; Song, YC; Wang, TY; Li, Y | 2008 | Population structure and linkage disequilibrium of a mini core set of maize inbred lines in China | “Understanding genetic diversity, population structure, and the level and distribution of linkage disequilibrium (LD) in target populations are of great importance and the prerequisite for association mapping. In the present study, 145 genome-wide SSR markers were used to assess the genetic diversity, population structure, and LD of a set of 95 maize inbred lines which represented the Chinese maize inbred lines. Results showed that the population included a diverse genetic variation. A model-based population structure analysis subdivided the inbred lines into four subgroups that correspond to the four major empirical germplasm origins in China, i.e., Lancaster, Reid, Tangsipingtou and P. Among all of the inbred lines, 65.3% were assigned into the corresponding subgroups; others were assigned into a”“mixed”" subgroup. LD was significant at a 0.01 level between 63.89% of the SSR pairs in the entire sample and with a range of 18.75-40.28% in the subgroups. Among factors influencing LD, linkage was the major cause for LD of SSR loci. The results suggested that the population may be used in the detection of genome-wide SSR marker-phenotype association." | 213 | no | NA | NA | no | no |
| 214 | 2017 | 43005 | Ellis, JS; Knight, ME; Darvill, B; Goulson, D | 2006 | Extremely low effective population sizes, genetic structuring and reduced genetic diversity in a threatened bumblebee species, Bombus sylvarum (Hymenoptera : Apidae) | Habitat fragmentation may severely affect survival of social insect populations as the number of nests per population, not the number of individuals, represents population size, hence they may be particularly prone to loss of genetic diversity. Erosion of genetic diversity may be particularly significant among social Hymenoptera such as bumblebees (Bombus spp.), as this group may be susceptible to diploid male production, a suggested direct cost of inbreeding.Here, for the first time, we assess genetic diversity and population structuring of a threatened bumblebee species (Bombus sylvarum) which exists in highly fragmented habitat (rather than oceanic) islands. Effective population sizes, estimated from identified sisterhoods, were very low (range 21-72) suggesting that isolated populations will be vulnerable to loss of genetic variation through drift. Evidence of significant genetic structuring between populations (theta = 0.084) was found, but evidence of a bottleneck was detected in only one population. Comparison across highly fragmented UK populations and a continental population (where this species is more widespread) revealed significant differences in allelic richness attributable to a high degree of genetic diversity in the continental population. While not directly related to population size, this is perhaps explained by the high degree of isolation between UK populations relative to continental populations. We suggest that populations now existing on isolated habitat islands were probably linked by stepping-stone populations prior to recent habitat loss. | 214 | no | NA | NA | no | no |
| 215 | 2017 | 43005 | Ochoa, A; Wells, SA; West, G; Al-Smadi, M; Redondo, SA; Sexton, SR; Culver, M | 2016 | Can captive populations function as sources of genetic variation for reintroductions into the wild? A case study of the Arabian oryx from the Phoenix Zoo and the Shaumari Wildlife Reserve, Jordan | The Arabian oryx (Oryx leucoryx) historically ranged across the Arabian Peninsula and neighboring countries until its extirpation in 1972. In 1963-1964 a captive breeding program for this species was started at the Phoenix Zoo (PHX); it ultimately consisted of 11 animals that became known as the ‘World Herd’. In 1978-1979 a wild population was established at the Shaumari Wildlife Reserve (SWR), Jordan, with eight descendants from the World Herd and three individuals from Qatar. We described the mtDNA and nuclear genetic diversity and structure of PHX and SWR. We also determined the long-term demographic and genetic viability of these populations under different reciprocal translocation scenarios. PHX displayed a greater number of mtDNA haplotypes (n = 4) than SWR (n = 2). Additionally, PHX and SWR presented nuclear genetic diversities of = 2.88 vs. 2.75, = 0.469 vs. 0.387, and = 0.501 vs. 0.421, respectively. Although these populations showed no signs of inbreeding ( ae 0), they were highly differentiated ( = 0.580; P < 0.001). Migration between PHX and SWR (Nm = 1, 4, and 8 individuals/generation) increased their genetic diversity in the short-term and substantially reduced the probability of extinction in PHX during 25 generations. Under such scenarios, maximum genetic diversities were achieved in the first generations before the effects of genetic drift became predominant. Although captive populations can function as sources of genetic variation for reintroduction programs, we recommend promoting mutual and continuous gene flow with wild populations to ensure the long-term survival of this species. | 215 | no | NA | NA | no | no |
| 216 | 2017 | 43005 | Johnson, DW; Freiwald, J; Bernardi, G | 2016 | Genetic diversity affects the strength of population regulation in a marine fish | Variation is an essential feature of biological populations, yet much of ecological theory treats individuals as though they are identical. This simplifying assumption is often justified by the perception that variation among individuals does not have significant effects on the dynamics of whole populations. However, this perception may be skewed by a historic focus on studying single populations. A true evaluation of the extent to which among-individual variation affects the dynamics of populations requires the study of multiple populations. In this study, we examined variation in the dynamics of populations of a live-bearing, marine fish (black surfperch; Embiotoca jacksoni). In collaboration with an organization of citizen scientists (Reef Check California), we were able to examine the dynamics of eight populations that were distributed throughout similar to 700km of coastline, a distance that encompasses much of this species’ range. We hypothesized that genetic variation within a local population would be related to the intensity of competition and to the strength of population regulation. To test this hypothesis, we examined whether genetic diversity (measured by the diversity of mitochondrial DNA haplotypes) was related to the strength of population regulation. Low-diversity populations experienced strong density dependence in population growth rates and population sizes were regulated much more tightly than they were in high-diversity populations. Mechanisms that contributed to this pattern include links between genetic diversity, habitat use, and spatial crowding. On average, low-diversity populations used less of the available habitat and exhibited greater spatial clustering (and more intense competition) for a given level of density (measured at the scale of the reef). Although the populations we studied also varied with respect to exogenous characteristics (habitat complexity, densities of predators, and interspecific competitors), none of these characteristics was significantly related to the strength of population regulation. In contrast, an endogenous characteristic of the population (genetic diversity) explained 77% of the variation in the strength of population regulation (95% CI: 27-94%). Our results suggest that the genetic and phenotypic composition of populations can play a major role in their dynamics. | 216 | no | no good fitness measure | NA | no | no |
| 217 | 2017 | 43005 | Queiros, J; Vicente, J; Boadella, M; Gortazar, C; Alves, PC | 2014 | The impact of management practices and past demographic history on the genetic diversity of red deer (Cervus elaphus): an assessment of population and individual fitness | The influences of management practices and past demographic history on genetic diversity are of critical relevance to sustainable practices and the conservation of wildlife populations. The red deer (Cervus elaphus) is an interesting model species to address these questions because it has a wide geographical distribution and it has been intensively managed for humans in the last decades. In the present study, we have analyzed the impact of recent management practices on the genetic diversity of Iberian red deer populations and assessed the genetic variation effects on population and individual fitness-related traits. Four populations subjected to distinct management systems were selected: Cabaneros (CB) and Donana (DN), not hunted populations; Fraga/Caspe (FG/CP), open hunting area with very low or absent management; and PE, fenced private hunting estate founded 31 years ago through the introduction of deer of different origins. Ten microsatellites were amplified in a total of 172 individuals. Additionally, several fitness-related traits such as the presence of tuberculosis compatible lesions (TBCL), spleen weight (SW), and body length (BL) were estimated. We found a marked genetic variation and differentiation among populations, suggesting a strong population structure. In the fenced population, the introduction of genetically distinct animals has led to high genetic variability (no evidence of inbreeding) despite intensive management. Lower levels of genetic diversity were observed in two historically isolated natural populations (DN and FG/CP). The past demographic history of Iberian populations appears to be more relevant than the current management policy in shaping the genetic variability of natural populations. Population genetic diversity may correlate with life-history traits and disease susceptibility, which could compromise the conservation and management of these wildlife populations. Although no significant effects of individual genetic diversity (general and local effect hypotheses) were observed on TBCL, SW and BL, some single-locus effects had almost significant trends for the TBCL and SW traits.(c) 2013 The Linnean Society of London, Biological Journal of the Linnean Society, 2014, 111, 209-223. | 217 | yes | NA | No good data | yes | no |
| 218 | 2017 | 43005 | Meyer-Lucht, Y; Sommer, S | 2009 | Number of MHC alleles is related to parasite loads in natural populations of yellow necked mice, Apodemus flavicollis | Hypothesis: Low levels of immune gene variation (major histocompatibility complex) in it Population arc associated with increased parasite load and infection Intensity. Organism: Different Populations of the yellow necked mouse (Apodemus flavicollis). a common rodent in European deciduous and mixed forest habitats Methods: We assessed genetic diversity at selectively neutral, non-coding markers (microsatellites) and adaptive genetic variation at a functionally important part of the immune complex MHC (major histocompatibility complex). We investigated the load with gastrointestinal parasites non-invasively by faecal egg counts and assessed the influence of population genetic variation on parasite burden Results: Both neutral and adaptive genetic diversity differed between mice populations. We could not detect an effect of neutral genetic diversity on the parasite burden in a population Heterozygosity at the MFIC did not reveal an effect on the parasite burden either. However, we did identify significant effects of the number of different MHC alleles in a population oil parasite burden Mice populations with a large number of different MHC alleles displayed lower parasite loads than those populations with few different MHC alleles | 218 | yes | two types of GD- measure tested! | MHC | yes | no |
| 219 | 2017 | 43005 | POTTS, WK; MANNING, CJ; WAKELAND, EK | 1994 | THE ROLE OF INFECTIOUS-DISEASE, INBREEDING AND MATING PREFERENCES IN MAINTAINING MHC GENETIC DIVERSITY - AN EXPERIMENTAL TEST | In house mice, and probably most mammals, major histocompatibility complex (MHC) gene products influence both immune recognition and individual odours in an allele-specific fashion. Although it is generally assumed that some form of pathogen-driven balancing selection is responsible for the unprecedented genetic diversity of MHC genes, the MHC-based mating preferences observed in house mice are sufficient to account for the genetic diversity of MHC genes found in this and other vertebrates. These MHC disassortative mating preferences are completely consistent with the conventional view that pathogen-driven MHC heterozygote advantage operates on MHC genes. This is because such matings preferentially produce MHC-heterozygours progeny, which could enjoy enhanced disease resistance. However, such matings could also function to avoid genome-wide inbreeding. To discriminate between these two hypotheses we measured the fitness consequences of both experimentally manipulated levels of inbreeding and MHC homozygosity and heterozygosity in semi-natural populations of wild-derived house mice. We were able to measure a fitness decline associated with inbreeding, but were unable to detect fitness declines associated with MHC homozygosity. These data suggest that inbreeding avoidance may be the most important function of MHC-based mating preferences and therefore the fundamental selective force diversifying MHC genes in species with such mating patterns. Although controversial, this conclusion is consistent with the majority of the data from the inbreeding and immunological literature. | 219 | no | inbreeding | NA | no | no |
| 220 | 2017 | 43005 | Welsh, AB; Baerwald, MR; Friday, M; May, B | 2015 | The effect of multiple spawning events on cohort genetic diversity of lake sturgeon (Acipenser fulvescens) in the Kaministiquia River | Lake sturgeon Acipenser fulvescens populations have experienced declines throughout much of the Great Lakes. Understanding key demographic characteristics about lake sturgeon populations can help identify potential limiting factors to their recovery. Within a single spawning season, there may be multiple spawning events, which could affect genetic diversity of the resulting cohort. Our objective was to determine whether multiple discrete spawning events resulted in a larger effective number of breeders and higher genetic diversity. Larval samples were collected following the spawning periods in 2005 (n = 479) and 2006 (n = 279). In 2005, there were two discrete spawning events and a longer spawning season; in 2006, the spawning events were less discrete and the spawning season was shorter. Genetic samples from larval sturgeon were analyzed at 12 microsatellite loci. The effective number of breeders (N-b), genetic diversity (observed heterozygosity, expected heterozygosity, allelic richness, inbreeding coefficient), and relatedness were measured for each cohort. The effective population size (N-e) and genetic diversity were also measured in the adult population (n = 85). The larval cohorts had a high N-b (2005: 54; 2006: 73) relative to the N-e of the adult population (N-e = 28). Multiple spawning events did not result in more breeders, but did result in lower relatedness among the resulting offspring. Therefore, environmental factors should be maintained that encourage an extended spawning season, increasing the likelihood of multiple spawning events and decreasing the relatedness among individuals in the cohort. | 220 | no | no good fitness measure | NA | no | no |
| 221 | 2017 | 43005 | Gardner, MG; Schonrogge, K; Elmes, GW; Thomas, JA | 2007 | Increased genetic diversity as a defence against parasites is undermined by social parasites: Microdon mutabilis hoverflies infesting Formica lemani ant colonies | Genetic diversity can benefit social insects by providing variability in immune defences against parasites and pathogens. However, social parasites of ants infest colonies and not individuals, and for them a different relationship between genetic diversity and resistance may exist. Here, we investigate the genetic variation, assessed using up to 12 microsatellite loci, of workers in 91 Formica lemani colonies in relation to their infestation by the specialist social parasite Microdon mutabilis. At the main study site, workers in infested colonies exhibited lower relatedness and higher estimated queen numbers, on average, than uninfested ones. Additionally, estimated queen numbers were negatively correlated with estimated average numbers of mates per queen within infested colonies. At another site, infested colonies also exhibited significantly lower worker relatedness, and estimated queen numbers were comparable in trend. In contrast, in two populations of F. lemani where M. mutabilis was absent, relatedness within colonies was high (40 and 90% with R > 0.6). While high genetic variation can benefit social insects by increasing their resistance to pathogens, there may be a cost in the increased likelihood of infiltration by social parasites owing to greater variation in nestmate recognition cues. This study provides the first empirical test of this hypothesis. | 221 | yes | comparing uninfested with infested colonies | NA | no | yes |
| 222 | 2017 | 43005 | Chung, MY | 2009 | Lack of allozyme diversity in populations of the rare, endangered terrestrial orchids Tipularia japonica and Epipactis papillosa in Korea | Since high levels of genetic diversity may ensure long-term survival of a plant species, it is essential to preserve the genetic diversity of the species. Tipularia japonica and Epipactis papillosa are rare terrestrial orchids in southern Korea with fewer than 50 mature individuals in a population and southern Japan and considered to be threatened (endangered or vulunerable). To obtain knowledge of how the genetic variation of these species is partitioned within and among populations in Korea, I used enzyme electrophoresis to examine the genetic diversity of each eight known populations of the two species from South Korea. Twenty-three (E. papillosa) and 24 putative loci (T. japonica) resolved from 15 enzyme systems revealed no variation either within or among populations of each species (0.0% of the percentage of polymorphic loci, %P). Previous studies, in contrast, showed that their more widely distributed disjunct congeners T. discolor and E. helleborine harbored high allozyme-based genetic diversity within populations in eastern United States (%P = 75%) and in Denmark (%P = 73.6%), respectively. In theory, small population size leads to allelic fixation at many loci over generations within a population, resulting in population genetic divergence or differentiation. In this regard, the complete lack of genetic differences between conspecific populations of T. japonica and E. papillosa cannot be explained by genetic drift. Instead, the present allozyme data suggest that recent origin from the same genetically depauperate ancestral or source population could result in this observation. The current status of T. japonica and E. papillosa (rarity and lack of genetic variation) significantly threatens the long-term survival of the species in Korea. | 222 | no | only one pop | NA | no | no |
| 223 | 2017 | 43005 | Davis, MC; Novak, SJ; Hampikian, G | 2011 | Mitochondrial DNA Analysis of an Immigrant Basque Population: Loss of Diversity Due to Founder Effects | The Basques have a well-documented history of migration and settlement in the Americas, and they often retain cultural identity across generations. Numerous genetic studies have been carried out on European Basques; thus, immigrant Basques are an ideal population for investigating the genetic consequences of a recent human migration event. We have sampled 53 unrelated individuals with Basque ancestry in Boise, Idaho and determined the mitochondrial DNA (mtDNA) sequence variation of the first and second hypervariable regions. Thirty-six mtDNA haplotypes were detected in our sample. We found evidence of genetic changes consistent with founder effects, which is compatible with the known history of migration. Compared with the European Basque population, the immigrant Basques are significantly different in terms of haplogroup frequency distribution and diversity. They have a lower measure of weighted intralineage mean pairwise diversity (WIMP) and greater genetic distance from other European populations. These data indicate that this immigrant Basque population has experienced a reduction in genetic diversity compared with the putative source population. However, this loss of diversity is not detectable using indices of demographic history such as Tajima’s D and Fu’s F. This study represents the first description of mtDNA diversity in an immigrant Basque population, and our findings indicate that founder effects accompanying this relatively recent migration event have shaped the genetic diversity of this population. Am J Phys Anthropol 144:516-525, 2011. (C) 2010 Wiley-Liss, Inc. | 223 | no | NA | NA | no | no |
| 224 | 2017 | 43005 | Tagg, N; Innes, DJ; Doncaster, CP | 2005 | Outcomes of reciprocal invasions between genetically diverse and genetically uniform populations of Daphnia obtusa (Kurz) | Ecological theory predicts that genetic variation produced by sexual reproduction results in niche diversification and provides a competitive advantage both to facilitate invasion into genetically uniform asexual populations and to withstand invasion by asexual competitors. We tested the hypothesis that a large group of diverse clones of Daphnia obtusa has greater competitive advantage when invading into genetically uniform populations of this species than a smaller group with inherently less genetic diversity. We compared competitive outcomes to those of genetically uniform groups of small and large size invading into genetically diverse populations. Genetically diverse invaders of initially large group size increased their representation by more than those of initially small size; in contrast. genetically uniform invaders of initially large group size diminished on average by more than those of initially small size. These results demonstrate an advantage to the genetic variation produced by sexual reproduction, both in invasion and resisting invasion, which we attribute to competitive release experienced by individuals in genetically diverse populations. | 224 | no | no good fitness measure | NA | no | no |
| 225 | 2017 | 43005 | Wisely, SM; McDonald, DB; Buskirk, SW | 2003 | Evaluation of the genetic management of the endangered black-footed ferret (Mustela nigripes) | Empirical support for the genetic management strategies employed by captive breeding and reintroduction programs is scarce. We evaluated the genetic management plan for the highly endangered black-footed ferret (Mustela nigripes) developed by the American Zoo and Aquarium Associations (AZA) as a part of the species survival plan (SSP). We contrasted data collected from five microsatellite loci to predictions from a pedigree-based kinship matrix analysis of the captive black-footed ferret population. We compared genetic diversity among captive populations managed for continued captive breeding or reintroduction, and among wild-born individuals from two reintroduced populations. Microsatellite data gave an accurate but only moderately precise estimate of heterozygosity. Genetic diversity was similar in captive populations maintained for breeding and release, and it appears that the recovery program will achieve its goal of maintaining 80% of the genetic diversity of the founder population over 25 years. Wild-born individuals from reintroduced populations maintained genetic diversity and avoided close inbreeding. We detected small but measurable genetic differentiation between the reintroduced populations. The model of random mating predicted only slightly lower levels of heterozygosity retention compared to the SSP strategy. The random mating strategy may be a viable alternative for managing large, stable, captive populations such as that of the black-footed ferret. (C) 2003 Wiley-Liss, Inc. | 225 | no | NA | NA | no | no |
| 226 | 2017 | 43005 | van Leeuwen, E; O’Neill, S; Matthews, A; Raymond, B | 2015 | Making pathogens sociable: The emergence of high relatedness through limited host invasibility | Cooperation depends upon high relatedness, the high genetic similarity of interacting partners relative to the wider population. For pathogenic bacteria, which show diverse cooperative traits, the population processes that determine relatedness are poorly understood. Here, we explore whether within-host dynamics can produce high relatedness in the insect pathogen Bacillus thuringiensis. We study the effects of host/pathogen interactions on relatedness via a model of host invasion and fit parameters to competition experiments with marked strains. We show that invasibility is a key parameter for determining relatedness and experimentally demonstrate the emergence of high relatedness from well-mixed inocula. We find that a single infection cycle results in a bottleneck with a similar level of relatedness to those previously reported in the field. The bottlenecks that are a product of widespread barriers to infection can therefore produce the population structure required for the evolution of cooperative virulence. | 226 | no | gd of bacteria | NA | no | no |
| 227 | 2017 | 43005 | Sexton, AC; Howlett, BJ | 2004 | Microsatellite markers reveal genetic differentiation among populations of Sclerotinia sclerotiorum from Australian canola fields | Eight microsatellite markers were applied to 154 Sclerotinia sclerotiorum isolates from four Australian canola fields, to determine the extent of genetic variation and differentiation in populations of this pathogen. A total of 82 different haplotypes were identified and in each population many haplotypes were unique. Mycelial compatibility grouping, a phenotypic marker system controlled by multiple loci, was often associated with groups of identical or closely related microsatellite haplotypes. Genotypic diversity ranged from 36% to 80% of maximum in the four populations, and gene diversity ranged from 0.23 to 0.79. Genotypic disequilibrium analyses on each of the four populations suggested that both clonal and sexual reproduction contributed to population structure. Analyses based on genetic diversity and fixation indices demonstrated a moderate to high level of differentiation (R-ST=0.16-0.33, F-ST=0.18-0.23) between populations from New South Wales and those from Victoria. Despite this genetic diversity, most isolates did not vary in virulence on canola leaves. | 227 | no | gd of pathogen | NA | no | no |
| 228 | 2017 | 43005 | Shellman-Reeve, JS | 2001 | Genetic relatedness and partner preference in a monogamous, wood-dwelling termite | A mate preference study on adult dampwood termites, Zootermopsis nevadensis subspecies nuttingi, supports the hypothesis that mate-seeking adults avoid forming partnerships with nestmates. The observed proportion of nestmate pairs formed from all potential pairs was Significantly less than expected. In addition, fewer pairs formed when there was a higher availability of nestmates versus non-nestmates. Genetic relatedness estimates, obtained from multilocus fingerprints of overwintering pairs, showed that relatedness between partners was not significantly different from background relatednesses of members chosen randomly from the population. Thus, nest-founding reproductive adults typically do not breed incestuously in this wood-dwelling termite. Furthermore, relatedness estimates between adult breeding males and between adult breeding females coinhabiting each log showed that there was a low probability of genetic relatedness among adults of neighbouring pairs, indicating that adults are typically dispersing in ways that minimize contact with relatives. Relatedness between laboratory-reared full siblings produced by field-collected pairs showed that relatedness was not significantly higher than r=0.50, which is expected if sustained incestuous breeding does not occur, or if cycles of inbreeding occur with negligible frequency among breeding adults. These findings do not support arguments that selection favours inbreeding because it confers disease resistance in termites. They also indicate that the level of genetic relatedness between nest-founding adults and among siblings cannot, by itself, explain the high levels of altruism within colonies of this single-site, wood-dwelling termite. Ecological pressures and the benefits of group living, together with ordinary genetic relatedness characteristics of outbred families appear sufficient to explain the advantages of kin-based altruism in wood-dwelling termites like Zootermopsis (C) 2001 The Association for the Study of Animal Behaviour. | 228 | no | NA | NA | no | no |
| 229 | 2017 | 43005 | Elbers, JP; Clostio, RW; Taylor, SS | 2017 | Neutral Genetic Processes Influence MHC Evolution in Threatened Gopher Tortoises (Gopherus polyphemus) | Levels of adaptive genetic variation influence how species deal with environmental and ecological change, but these levels are frequently inferred using neutral genetic markers. Major histocompatibility complex (MHC) genes play a key role in the adaptive branch of the immune system and have been used extensively to estimate levels of adaptive genetic variation. Parts of the peptide binding region, sites where MHC molecules directly interact with pathogen and self-proteins, were sequenced from a MHC class I (95/441 tortoises) and class II (245/441 tortoises) gene in threatened and nonthreatened populations of gopher tortoises (Gopherus polyphemus), and adaptive genetic variation at MHC genes was compared to neutral genetic variation derived from 10 microsatellite loci (441 tortoises). Genetic diversity at the MHC class II locus and microsatellites was greater in populations in the nonthreatened portion of the gopher tortoise’s range (MHC class II difference in mean A = 8.11, A(R) = 0.79, HO = 0.51, and HE = 0.16; microsatellite difference in mean A = 1.05 and AR = 0.47). Only MHC class II sequences showed evidence of positive selection (d(N)/d(S) > 1, Z = 1.81, P = 0.04). Historical gene flow as estimated with Migrate-N was greater than recent migration estimated with BayesAss, suggesting that populations were better connected in the past when habitat was less fragmented. MHC genetic differentiation was correlated with microsatellite differentiation (Mantel r = 0.431, P = 0.001) suggesting neutral genetic processes are influencing MHC evolution, and advantageous MHC alleles could be lost due to genetic drift. | 229 | no | no good fitness measure | NA | no | no |
| 230 | 2017 | 43005 | Hawley, DM; Dhondt, KV; Dobson, AP; Grodio, JL; Hochachka, WM; Ley, DH; Osnas, EE; Schat, KA; Dhondt, AA | 2010 | Common garden experiment reveals pathogen isolate but no host genetic diversity effect on the dynamics of an emerging wildlife disease | Host genetic diversity can mediate pathogen resistance within and among populations. Here we test whether the lower prevalence of Mycoplasmal conjunctivitis in native North American house finch populations results from greater resistance to the causative agent, Mycoplasma gallisepticum (MG), than introduced, recently-bottlenecked populations that lack genetic diversity. In a common garden experiment, we challenged wild-caught western (native) and eastern (introduced) North American finches with a representative eastern or western MG isolate. Although introduced finches in our study had lower neutral genetic diversity than native finches, we found no support for a population-level genetic diversity effect on host resistance. Instead we detected strong support for isolate differences: the MG isolate circulating in western house finch populations produced lower virulence, but higher pathogen loads, in both native and introduced hosts. Our results indicate that contemporary differences in host genetic diversity likely do not explain the lower conjunctivitis prevalence in native house finches, but isolate-level differences in virulence may play an important role. | 230 | no | individual level | NA | no | no |
| 231 | 2017 | 43005 | van Baalen, M; Beekman, M | 2006 | The costs and benefits of genetic heterogeneity in resistance against parasites in social insects | The occurrence of polygyny and polyandry in social insects has long puzzled evolutionary biologists. If cooperation requires genetic relatedness, how do we explain the occurrence and maintenance of mechanisms that reduce the degree of relatedness among colony members? A much-discussed hypothesis states that genetically diverse colonies are more resistant to parasitism than homogenous colonies because genetic diversity reduces the spread of a disease within a colony. However, as we will argue in this note, a necessary condition for the parasite hypothesis is that genetically heterogeneous colonies have a larger suite of parasites that are capable of infecting them. This implicit relationship is important because it implies that even if the cost per infection is reduced, this may not be sufficient to offset the increased rate of acquiring infections. The advantages of genetic heterogeneity as a defense against parasites thus may not be as big as commonly thought. | 231 | no | no data, good for discussion maybe | NA | no | no |
| 232 | 2017 | 43005 | Kumar, A; Rai, U; Roka, B; Jha, AK; Reddy, PA | 2016 | Genetic assessment of captive red panda (Ailurus fulgens) population | Red panda (Ailurus fulgens) is threatened across its range by detrimental human activities and rapid habitat changes necessitating captive breeding programs in various zoos globally to save this flagship species from extinction. One of the ultimate aims of ex situ conservation is reintroduction of endangered animals into their natural habitats while maintaining 90 % of the founder genetic diversity. Advances in molecular genetics and microsatellite genotyping techniques make it possible to accurately estimate genetic diversity of captive animals of unknown ancestry. Here we assess genetic diversity of the red panda population in Padmaja Naidu Himalayan Zoological Park, Darjeeling, which plays a pivotal role in ex situ conservation of red panda in India. We generated microsatellite genotypes of fifteen red pandas with a set of fourteen loci. This population is genetically diverse with 68 % observed heterozygosity (H-O) and mean inbreeding (F-IS) coefficient of 0.05. However population viability analysis reveals that this population has a very low survival probability (< 2 %) and will rapidly loose its genetic diversity to 37 % mainly due to small population size and skewed male-biased sex ratio. Regular supplementation with a pair of adult individuals every five years will increase survival probability and genetic diversity to 99 and 61 % respectively and will also support future harvesting of individuals for reintroduction into the wild and exchange with other zoos. | 232 | no | zoo | NA | no | no |
| 233 | 2017 | 43005 | Osborne, AJ; Negro, SS; Chilvers, BL; Robertson, BC; Kennedy, MA; Gemmell, NJ | 2016 | Genetic Evidence of a Population Bottleneck and Inbreeding in the Endangered New Zealand Sea Lion, Phocarctos hookeri | The New Zealand sea lion (NZSL) is of high conservation concern due to its limited distribution and its declining population size. Historically, it occupied most of coastal New Zealand, but is now restricted to a few coastal sites in southern mainland New Zealand and the sub-Antarctic Islands. NZSLs have experienced a recent reduction in population size due to sealing in the 1900s, which is expected to have resulted in increased inbreeding and a loss of genetic variation, potentially reducing the evolutionary capacity of the species and negatively impacting on its long-term prospects for survival. We used 17 microsatellite loci, previously shown to have cross-species applications in pinnipeds, to determine locus-and population-specific statistics for 1205 NZSLs from 7 consecutive breeding seasons. We show that the NZSL population has a moderate level of genetic diversity in comparison to other pinnipeds. We provide genetic evidence for a population reduction, likely caused by historical sealing, and a measure of allele sharing/parental relatedness (internal relatedness) that is suggestive of increased inbreeding in pups that died during recent epizootic episodes. We hypothesize that population bottlenecks and nonrandom mating have impacted on the population genetic architecture of NZSLs, affecting its population recovery. | 233 | no | only one pop | NA | no | no |
| 234 | 2017 | 43005 | Atwater, DZ; Callaway, RM | 2015 | Testing the mechanisms of diversity-dependent overyielding in a grass species | Plant diversity enhances many ecosystem processes, including productivity, but these effects have been studied almost exclusively at the taxonomic scale of species. We explore the effect of intraspecific diversity on the productivity of a widespread and dominant grassland species using accessions collected from populations throughout its range. We found that increasing population/ecotype diversity of Pseudoroegneria spicata increased productivity to a similar degree as that reported for species diversity. However, we did not find evidence that overyielding was related to either resource depletion or to pathogenic soil fungi, two causes of overyielding in species-diverse communities. Instead, larger accessions overyielded at low diversity at the expense of smaller accessions, and small accessions overyielded through complementarity at all levels of diversity. Furthermore, overyielding was stronger for accessions from mesic environments, suggesting that local adaptation might predictably influence how plants respond to increases in diversity. This suggests that mass-based competition or other cryptic accession-specific processes had complex but important effects on overyielding. Our results indicate that the effects of diversity within a species can be substantial but that overyielding by intraspecifically diverse populations may not be through the same processes thought to cause overyielding in species diverse communities. | 234 | no | no good fitness measure | NA | no | no |
| 235 | 2017 | 43005 | Mauger, LA; Velez, E; Cherkiss, MS; Brien, ML; Mazzotti, FJ; Spotila, JR | 2017 | Conservation genetics of American crocodile, Crocodylus acutus, populations in Pacific Costa Rica | Maintaining genetic diversity is crucial for the survival and management of threatened and endangered species. In this study, we analyzed genetic diversity and population genetic structure at neutral loci in American crocodiles, Crocodylus acutus, from several areas (Parque Nacional Marino Las Baulas, Parque Nacional Santa Rosa, Parque Nacional Palo Verde, Rio Tarcoles, and Osa Conservation Area) in Pacific Costa Rica. We genotyped 184 individuals at nine microsatellite loci to describe the genetic diversity and conservation genetics between and among populations. No population was at Hardy-Weinberg Equilibrium (HWE) over all loci tested and a small to moderate amount of inbreeding was present. Populations along the Pacific coast had an average heterozygosity of 0.572 across all loci. All populations were significantly differentiated from each other with both FST and RST measures of population differentiation with a greater degree of molecular variance (81%) found within populations. Our results suggest C. acutus populations in Pacific Costa Rica were not panmictic with moderate levels of genetic diversity. An effective management plan that maintains the connectivity between clusters is critical to the success of C. acutus in Pacific Costa Rica. | 235 | no | no good fitness measure | NA | no | no |
| 236 | 2017 | 43005 | Jeong, H; Choi, BH; Eo, J; Kwon, YJ; Lee, HE; Choi, Y; Gim, JA; Kim, TH; Seong, HH; Lee, DH; Ha, JH; Han, KI; Kim, HS | 2014 | Statistical analysis and genetic diversity of three dog breeds using simple sequence repeats | There are more than 400 pure dog breeds developed through intentional artificial selection and purebred breeding. Purebred animals have higher risk of inbreeding depression and hereditary diseases. We investigated the genetic diversity and structure of three dog breeds in South Korea by using 12 microsatellite loci for one Korean native dog breed, Sapsaree, and two foreign breeds, German shepherd and Belgian Malinois. The mean allele number of nine loci across all dog breeds was 4.833, and the number of alleles per locus ranged from 2 to 8. The mean of expected and observed heterozygosity were 0.415 and 0.577, respectively. Sapsaree, Korean native dog, had higher level of genetic diversity than the foreign German shepherd and Belgian Malinois. The highest mean value of polymorphism information content was found in Sapsaree (0.480), followed by Belgian Malinois (0.373) and German shepherd (0.355). Pairwise genetic differentiation was estimated using fixation index F (ST). Sapsaree and German shepherd (F (ST) = 0.2536) and Sapsaree and Belgian Malinois (F (ST) = 0.2522) had very great genetic differentiation, while moderate level of genetic differentiation was observed between German shepherd and Belgian Malinois (F (ST) = 0.1003). These genetic information and structure of the three dog breeds will be effective in conservation and preservation of the genetic diversity of the three dog breeds. | 236 | no | domesticated animal | NA | no | no |
| 237 | 2017 | 43005 | Kolb, A; Durka, W | 2013 | Reduced genetic variation mainly affects early rather than late life-cycle stages | A large threat to biological diversity worldwide is habitat fragmentation, with negative effects on organisms arising as a result of changes in the abiotic or biotic environment and via changes in genetic processes. We examined genetic diversity, population structure and gene flow as well as the importance of genetic diversity for plant and population performance in 17 differently-sized populations of the locally rare, self-incompatible, perennial forest herb Phyteuma spicatum. Genetic variation, characterized by AFLPs, was low (mean gene diversity, H-e: 0.126) and generally increased with increasing population size. Bayesian analysis of population structure showed that populations located in the same forest patch or in close vicinity formed genetic clusters. Population differentiation conformed to a pattern of isolation by distance (IBD). Gene dispersal distances were higher when estimated within than among populations, suggesting that gene flow is more restricted on a landscape scale. Two seedling vital rates were influenced by population genetic parameters, while most late life-cycle stages and population growth rate were not affected. These results suggest that genetic load is purged early in the life cycle by selective mortality, which may buffer populations against the negative genetic effects of small population size. The overall low levels of genetic diversity, however, imply that populations are poorly equipped to respond to future changes in the environment. This is of special concern as short-distance gene dispersal indicates that colonization of new sites in response to putative changes in the environment is unlikely. (C) 2012 Elsevier Ltd. All rights reserved. | 237 | no | no good fitness measure | NA | no | no |
| 238 | 2017 | 43005 | Huchard, E; Baniel, A; Schliehe-Diecks, S; Kappeler, PM | 2013 | MHC-disassortative mate choice and inbreeding avoidance in a solitary primate | Sexual selection theory suggests that choice for partners carrying dissimilar genes at the major histocompatibility complex (MHC) may play a role in maintaining genetic variation in animal populations by limiting inbreeding or improving the immunity of future offspring. However, it is often difficult to establish whether the observed MHC dissimilarity among mates drives mate choice or represents a by-product of inbreeding avoidance based on MHC-independent cues. Here, we used 454-sequencing and a 10-year study of wild grey mouse lemurs (Microcebus murinus), small, solitary primates from western Madagascar, to compare the relative importance on the mate choice of two MHC class II genes, DRB and DQB, that are equally variable but display contrasting patterns of selection at the molecular level, with DRB under stronger diversifying selection. We further assessed the effect of the genetic relatedness and of the spatial distance among candidate mates on the detection of MHC-dependent mate choice. Our results reveal inbreeding avoidance, along with disassortative mate choice at DRB, but not at DQB. DRB-disassortative mate choice remains detectable after excluding all related dyads (characterized by a relatedness coefficient r > 0), but varies slightly with the spatial distance among candidate mates. These findings suggest that the observed deviations from random mate choice at MHC are driven by functionally important MHC genes (like DRB) rather than passively resulting from inbreeding avoidance and further emphasize the need for taking into account the spatial and genetic structure of the population in correlative tests of MHC-dependent mate choice. | 238 | no | mate choice | NA | no | no |
| 239 | 2017 | 43005 | Gao, LZ; Hong, SGDY | 2000 | Allozyme variation and population genetic structure of common wild rice Oryza rufipogon Griff. in China | “In order to determine the genetic diversity and genetic structure of populations in common wild rice Oryza rufipogon, an endangered species, allozyme diversity was analyzed using 22 loci in 607 individuals of 21 natural populations from the Guangxi, Guangdong, Hainan, Yunnan, Hunan, Jiangxi and Fujian provinces in China. The populations studied showed a moderate allozyme variability (A=1.33, P=22.7%, Ho=0.033 and He=0.068), which was relatively high for the genus Oryza The levels of genetic diversity for Guangxi and Guangdong were significantly higher than those for the other regions, and thus South China appeared to be the center of genetic diversity of O. rufipogon in China. A moderate genetic differentiation (F-ST=0.310, I=0.964) was found among the populations studied. Interestingly the pattern of population differentiation does not correspond to geographic distance. An estimate of the outcrossing rate (t=0.324) suggests that the species has a typical mixed-mating system. The deficit of heterozygotes (F=0.511) indicates that some inbreeding may have taken place in outcrossing asexual populations because of intra-clone outcrossing events and”“isolation by distance”" as a result of human disturbance. In order to predict the long-term genetic survival of fragmented populations, further studies on gene flow among the remaining populations and the genetic effects of fragmentation are proposed. Finally, some implications for the conservation of endangered species are suggested." | 239 | no | no good fitness measure | NA | no | no |
| 240 | 2017 | 43005 | Su, LN; Meng, HZ; Li, XC | 2015 | Genetic diversity and historical demography of the narrow-range endemic Alpine toad, Scutiger liupanensis, in the Liupan Mountains of central China | The genetic diversity and historical demography of the narrow-range endemic Alpine toad, Scutiger liupanensis, in the Liupanshan National Forest Park of central China were estimated using cytochrome b and cytochrome c oxidase subunit I (COI) from 85 individuals from five local populations. Both the haplotype diversity (Hd) and the nucleotide diversity (Pi) were very high. Phylogenetic analysis of the 63 haplotypes revealed two major clades, and an analysis of molecular variance attributed most of the variation to within populations. Mantel tests did not reveal an isolation by distance pattern of genetic divergence between populations, and SAMOVA showed no phylogeographic structure. The results of neutrality tests, mismatch distribution analyses, and allelic frequency spectra suggest that a sudden demographic expansion occurred, and that high genetic variation is beneficial to the survival and development of this species. | 240 | no | no good fitness measure | NA | no | no |
| 241 | 2017 | 43005 | Lan, Q; Shen, M; Garcia-Rossi, D; Chanock, S; Zheng, TZ; Berndt, SI; Puri, V; Li, GL; He, XZ; Welch, R; Zahm, SH; Zhang, LP; Zhang, YW; Smith, M; Wang, SS; Chiu, BCH; Linet, M; Hayes, R; Rothman, N; Yeager, M | 2007 | Genotype frequency and F-ST analysis of polymorphisms in immunoregulatory genes in Chinese and Caucasian populations | Selection and genetic drift can create genetic differences between populations. Cytokines and chemokines play an important role in both hematopoietic development and the inflammatory response. We compared the genotype frequencies of 45 SNPs in 30 cytokine and chemokine genes in two healthy Chinese populations and one Caucasian population. Several SNPs in IL4 had substantial genetic differentiation between the Chinese and Caucasian populations (F-ST similar to 0.40), and displayed a strikingly different haplotype distribution. To further characterize common genetic variation in worldwide populations at the IL4 locus, we genotyped 9 SNPs at the IL4 gene in the Human Diversity Panel’s (N=1056) individuals from 52 world geographic regions. We observed low haplotype diversity, yet strikingly different haplotype frequencies between non-African populations, which may indicate different selective pressures on the IL4 gene in different parts of the world. SNPs in CSF2, IL6, IL10, CTLA4, and CX3CR1 showed moderate genetic differentiation between the Chinese and Caucasian populations (0.15 < F-ST < 0.25). These results suggest that there is substantial genetic diversity in immune genes and exploration of SNP associations with immune-related diseases that vary in incidence across these two populations may be warranted. | 241 | no | no good fitness measure, N pop differnt in GD? | NA | no | no |
| 242 | 2017 | 43005 | Branco, CC; Mota-Vieira, L | 2005 | Surnames in the Azores: Analysis of the isonymy structure | Geographic isolation is a significant factor to consider when characterizing human populations. The knowledge of the genetic structure of isolated populations has been of great importance to disease-locus positioning and gene identification. To investigate the genetic structure of the Azorean population, we conducted a survey based on the frequencies of surnames listed in the 2001 telephone book. We calculated the following parameters: isonymy (I), the random component of inbreeding (F-ST), genetic diversity according to Fisher (alpha), Karlin-McGregor’s migration rate (v), and Nei’s distance. For the 1,271 subscribers and 163 different surnames, Graciosa island presented the lowest value of abundance of surnames (alpha = 15.75), suggesting great genetic isolation compared to the other eight islands. Migration, calculated on the basis of the diversity of surnames within islands, ranged from 0.2747 (Corvo island) to 0.0026 (Sao Miguel island), indicating that people migrated preferentially toward the economically more developed islands. The value of the random component of inbreeding obtained for the whole population (FST = 0.0039) indicates little genetic differentiation (Wright’s F-ST< 0.05). Moreover, isonymy similarity revealed using the UPGMA method shows three subclusters corresponding to the geographic distribution of the islands. | 242 | no | no good fitness measure | NA | no | no |
| 243 | 2017 | 43005 | Lee, HR; Kim, KS; Bang, IC | 2014 | Isolation and characterization of polymorphic microsatellite DNA markers for an endangered freshwater fish in Korea, the bull-head torrent catfish Liobagrus obesus | The bull-head torrent catfish Liobagrus obesus (family Amblycipitidae) is an endangered freshwater fish in Korea. There has been a rapid decline in its population, caused by anthropogenic activities. This may also lead to reduced genetic diversity, which can significantly affect survival. Thus, molecular markers are important to assess the level of genetic variation. We developed 12 microsatellite markers that yielded an average of 7.83 alleles per locus. The average observed and expected heterozygosities were 0.663 and 0.699, respectively. This study is valuable for protecting the endangered species L. obesus; investigations of genetic diversity and population structure will provide useful information for conservation measures. | 243 | no | individual level | NA | no | no |
| 244 | 2017 | 43005 | Oliehoek, PA; Bijma, P; van der Meijden, A | 2009 | History and structure of the closed pedigreed population of Icelandic Sheepdogs | Background: Dog breeds lose genetic diversity because of high selection pressure. Breeding policies aim to minimize kinship and therefore maintain genetic diversity. However, policies like mean kinship and optimal contributions, might be impractical. Cluster analysis of kinship can elucidate the population structure, since this method divides the population in clusters of related individuals. Kinship-based analyses have been carried out on the entire Icelandic Sheepdog population, a sheep-herding breed. Results: Analyses showed that despite increasing population size and deliberately transferring dogs, considerable genetic diversity has been lost. When cluster analysis was based on kinships calculated seven generation backwards, as performed in previous studies, results differ markedly from those based on calculations going back to the founder-population, and thus invalidate recommendations based on previous research. When calculated back to the founder-population, kinship-based clustering reveals the distribution of genetic diversity, similarly to strategies using mean kinship. Conclusion: Although the base population consisted of 36 Icelandic Sheepdog founders, the current diversity is equivalent to that of only 2.2 equally contributing founders with no loss of founder alleles in descendants. The maximum attainable diversity is 4.7, unlikely achievable in a non-supervised breeding population like the Icelandic Sheepdog. Cluster analysis of kinship coefficients can provide a supporting tool to assess the distribution of available genetic diversity for captive population management. | 244 | no | domesticated animal | NA | no | no |
| 245 | 2017 | 43005 | Kandil, OM; Abdelrahman, KA; Fahmy, HA; Mahmoud, MS; El Namaky, AH; Miller, JE | 2017 | Phylogenetic patterns of Haemonchus contortus and related trichostrongylid nematodes isolated from Egyptian sheep | Haemonchus contortus is a major parasite of small ruminants and its blood-feeding behaviour causes effects ranging from mild anaemia to death. Knowledge of the genetic variation within and among H. contortus populations can provide the foundation for understanding transmission patterns and aid in the control of haemonchosis. Adult male H. contortus were collected from three geographical regions in Egypt. The second internal transcribed spacer (ITS2) of nuclear ribosomal DNA was amplified using the polymerase chain reaction (PCR) and sequenced directly. The population genetic diversity and sequence variations were determined. Nucleotide sequence analyses revealed one genotype (ITS2) in all worms, without genetic differentiation. The similarity in population genetic diversity and genetic patterns observed among the three geographical regions could be attributed to possible movement between the sites. This is the first study of genetic variation in H. contortus in Egypt. The present results could have implications for the rapid characterization of H. contortus and other trichostrongyloid nematodes, and evaluation of the epidemiology of H. contortus in Egypt. | 245 | no | gd of pathogen | NA | no | no |
| 246 | 2017 | 43005 | Sefc, KM; Hermann, CM; Taborsky, B; Koblmuller, S | 2012 | Brood mixing and reduced polyandry in a maternally mouthbrooding cichlid with elevated among-breeder relatedness | Uniparental maternal brood care often coincides with multiple paternity and single maternity of broods, possibly reflecting benefits of polyandry and costs of uniparental care. Genetic data from the maternally mouthbrooding cichlid fish Simochromis pleurospilus revealed the opposite pattern low polyandry and allomaternal care. More than 70% of the investigated females had mated with a single male, and 14% of the females had unrelated fry in their broods. Broods with foreign fry were in the late stage of brood care, in which females guard free-swimming fry and recall the broods into their mouths for protection. With one exception, fostering females were related to their adopted fry at the level of first cousins (RQG > 0.12), but relatedness between fosters and adopted fry was not significantly higher than between fosters and fry tended by other females. Relatedness among breeders extended to the level of first-order relatives. Mean relatedness among contemporaneously breeding dams (RQG = 0.08) was significantly higher than among dams breeding in different seasons (RQG = -0.04), which suggests a temporal or spatial concentration of mouthbrooding relatives. Indeed, females sometimes brood in small groups. This behaviour may reduce brood predation but will increase the risk of brood mixing, which is possibly mitigated by low costs of brood care and indirect benefits accrued by relatedness among the breeders in the group. Remarkably, the apparent inbreeding potential did not give rise to bet-hedging polyandry or active avoidance of relatives, as half of the mated individuals were related at RQG > 0.13 and polyandry did not coincide with high within-pair relatedness. | 246 | no | no pop level | NA | no | no |
| 247 | 2017 | 43005 | Kaunisto, KM; Viitaniemi, HM; Leder, EH; Suhonen, J | 2013 | Association between host’s genetic diversity and parasite burden in damselflies | Recent research indicates that low genetic variation in individuals can increase susceptibility to parasite infection, yet evidence from natural invertebrate populations remains scarce. Here, we studied the relationship between genetic heterozygosity, measured as AFLP-based inbreeding coefficient f(AFLP), and gregarine parasite burden from eleven damselfly, Calopteryx splendens, populations. We found that in the studied populations, 5-92% of males were parasitized by endoparasitic gregarines (Apicomplexa: Actinocephalidae). Number of parasites ranged from none to 47 parasites per male, and parasites were highly aggregated in a few hosts. Mean individual f(AFLP) did not differ between populations. Moreover, we found a positive association between individual’s inbreeding coefficient and parasite burden. In other words, the more homozygous the individual, the more parasites it harbours. Thus, parasites are likely to pose strong selection pressure against inbreeding and homozygosity. Our results support the heterozygosity-fitness correlation hypothesis, which suggests the importance of heterozygosity for an individual’s pathogen resistance. | 247 | no | individual level | NA | no | no |
| 248 | 2017 | 43005 | Hoebe, PN; Stift, M; Holub, EB; Mable, BK | 2011 | The effect of mating system on growth of Arabidopsis lyrata in response to inoculation with the biotrophic parasite Albugo candida | The effects of variation in host reproductive systems on response to pathogens are not well understood. We inoculated individuals from outcrossing and inbreeding populations of North American Arabidopsis lyrata with Albugo candida (white blister rust) to test the effect of mating system and heterozygosity on disease response. We observed three host infection phenotypes, classified as fully resistant, partially resistant and fully susceptible. Overall, inbreeding populations had more susceptible and fewer partially resistant individuals than outcrossing populations, but the highest proportion of resistant individuals was found in two of the inbreeding populations. Mating system did not affect relative growth rate of inoculated plants, but there were strong effects of population and infection phenotype. We conclude that mating system per se does not determine the resistance of natural A. lyrata populations to infection by Albugo, but that the increased variability in responses among inbreeding populations may be due to reduced effective population size. | 248 | no | inbreeding | NA | no | no |
| 249 | 2017 | 43005 | Godoy, I; Vigilant, L; Perry, SE | 2016 | Inbreeding risk, avoidance and costs in a group-living primate, Cebus capucinus | Documenting inbreeding and its potential costs in wild populations is a complicated matter. Early infant death before genetic samples can be collected limits the ability of researchers to measure fitness costs, and pedigree information is necessary to accurately estimate relatedness between breeding individuals. Using data from 25 years of research from the Lomas Barbudal Capuchin Monkey Project, and a sample of 109 females that have given birth, we find that despite frequent co-residency of adult opposite-sexed individuals, capuchins produce offspring with close kin (i.e., related at the half-sibling level or higher) less often than would be expected in the absence of inbreeding avoidance. We do not find support for alternative, non-behavioral explanations for this pattern and thus argue for mate choice. Furthermore, we find evidence for fitness costs among inbred animals in the form of delayed female age at first birth but not significantly higher juvenile mortality. Further research is necessary in order to determine the mechanisms by which individuals develop sexual aversion to close kin. Through a combination of demographic records, maternal pedigrees, and genetically determined paternity, this study provides a detailed study of inbreeding and inbreeding avoidance in a well-studied mammal population. This study provides (1) evidence that capuchin monkeys avoid mating with close kin at both the level of parent-offspring and half sibling and (2) evidence of fitness costs to inbreeding in the form of delayed first age at reproduction. | 249 | no | inbreeding | NA | no | no |
| 250 | 2017 | 43005 | King, KC; Jokela, J; Lively, CM | 2011 | PARASITES, SEX, AND CLONAL DIVERSITY IN NATURAL SNAIL POPULATIONS | Under the Red Queen hypothesis, host-parasite coevolution selects against common host genotypes. Although this mechanism might underlie the persistence of sexual reproduction, it might also maintain high clonal diversity. Alternatively, clonal diversity might be maintained by multiple origins of parthenogens from conspecific sexuals, a feature in many animal groups. Herein, we addressed the maintenance of overall genetic diversity by coevolving parasites, as predicted by the Red Queen hypothesis. We specifically examined the contribution of parasites to host clonal diversity and the frequency of sexually reproducing individuals in natural stream populations of Potamopyrgus antipodarum snails. We also tested the alternative hypothesis that clonal diversity is maintained by the input of clones by mutation from sympatric sexuals. Clonal diversity and the frequency of sexual individuals were both positively related to infection frequency. Surprisingly, although clones are derived by mutation from sexual snails, parasites explained more of the genotypic variation among parthenogenetic subpopulations. Our findings thus highlight the importance of parasites as drivers of clonal diversity, as well as sex. | 250 | maybe | orignially thought to only include GD of parasite not host, double checked and found OK data | NA | yes | yes |
| 251 | 2017 | 43005 | Zhou, N; Shen, HD; Chen, C; Sun, BN; Zheng, P; Wang, CN | 2016 | “Genetic structure of Onchidium”“struma’’ ( Mollusca: Gastropoda: Eupulmonata) from the coastal area of China based on mtCO I” | The genetic diversity and population genetic structure of Onchidium struma were investigated using mitochondrial cytochrome c oxidase subunit I (CO I) gene sequences. A total of 240 individuals representing 10 collection sites from across a large portion of its known range were included in the analysis. Overall, 42 haplotypes were defined and 97 polymorphic sites were observed. The O. struma populations had high haplotype diversity (0.9280) and nucleotide diversity (0.0404). We inferred that the early maturity and extensive survival habitat led to high genetic diversity of O. struma populations in China. Bayesian analysis and SAMOVA analysis showed significant genetic differentiation among populations and all populations were divided into two groups, (HK and HN) versus (GY, DF, CX, CN, ND and XM). The Mantel test revealed no significant correlation between geographic distance and genetic distance (r=0.251; p=0.058). Restricted gene flow caused by a shorter term pelagic veliger stage and limited dispersal potential were inferred to result in genetic differentiation among populations based on nested analysis. HK population might be an invasive species by artificial transplantation. | 251 | maybe | any fitness measure? GD-measurement ok? | no good fitness measure | no | no |
| 252 | 2017 | 43005 | Silva, GF; Paixao, RDV; Queiroz, CB; Santana, MF; Souza, A; Sousa, NR; Hanada, RE; Gasparotto, L | 2014 | Genetic diversity of Mycosphaerella fijiensis in Brazil analyzed using an ERIC-PCR marker | The Enterobacterial repetitive intergenic consensus (ERIC) marker was used to analyze the genetic variability of Mycosphaerella fijiensis, the causative agent of Black Sigatoka disease in banana plants. A total of 123 isolates were used, which were divided into populations based on their original hosts and collection sites in Brazil. A total of 9 loci were amplified, 77.8% of which were found to be polymorphic. The genetic diversity found in the population was 0.20. Analysis of molecular variance (AMOVA) demonstrated that the highest level of genetic variation is within populations. Cluster analysis revealed three main groups in Brazil, with no correlation between geographic and genetic distance. | 252 | no | gd of pathogen | NA | no | no |
| 253 | 2017 | 43005 | Keeney, DB; King, TM; Rowe, DL; Poulin, R | 2009 | Contrasting mtDNA diversity and population structure in a direct-developing marine gastropod and its trematode parasites | The comparative genetic structure of hosts and their parasites has important implications for their coevolution, but has been investigated in relatively few systems. In this study, we analysed the genetic structure and diversity of the New Zealand intertidal snail Zeacumantus subcarinatus (n = 330) and two of its trematode parasites, Maritrema novaezealandensis (n = 269) and Philophthalmus sp. (n = 246), using cytochrome c oxidase subunit I gene (COI) sequences. Snails and trematodes were examined from 11 collection sites representing three regions on the South Island of New Zealand. Zeacumantus subcarinatus displayed low genetic diversity per geographic locality, strong genetic structure following an isolation by distance pattern, and low migration rates at the scale of the study. In contrast, M. novaezealandensis possessed high genetic diversity, genetic homogeneity among collection sites and high migration rates. Genetic diversity and migration rates were typically lower for Philophthalmus sp. compared to M. novaezealandensis and it displayed weak to moderate genetic structure. The observed patterns likely result from the limited dispersal ability of the direct developing snail and the utilization of bird definitive hosts by the trematodes. In addition, snails may occasionally experience long-distance dispersal. Discrepancies between trematode species may result from differences in their effective population sizes and/or life history traits. | 253 | maybe | any gd and fitness of groups? | NA | no | yes |
| 254 | 2017 | 43005 | Rauch, EM; Bar-Yam, Y | 2004 | Theory predicts the uneven distribution of genetic diversity within species | Global efforts to conserve species have been strongly influenced by the heterogeneous distribution of species diversity across the Earth. This is manifest in conservation efforts focused on diversity hotspots(1-3). The conservation of genetic diversity within an individual species(4,5) is an important factor in its survival in the face of environmental changes and disease(6,7). Here we show that diversity within species is also distributed unevenly. Using simple genealogical models, we show that genetic distinctiveness has a scale-free power law distribution. This property implies that a disproportionate fraction of the diversity is concentrated in small sub-populations, even when the population is well-mixed. Small groups are of such importance to overall population diversity that even without extrinsic perturbations, there are large fluctuations in diversity owing to extinctions of these small groups. We also show that diversity can be geographically non-uniform potentially including sharp boundaries between distantly related organisms - without extrinsic causes such as barriers to gene flow or past migration events. We obtained these results by studying the fundamental scaling properties of genealogical trees. Our theoretical results agree with field data from global samples of Pseudomonas bacteria. Contrary to previous studies(8), our results imply that diversity loss owing to severe extinction events is high, and focusing conservation efforts on highly distinctive groups can save much of the diversity. | 254 | no | NA | NA | no | no |
| 255 | 2017 | 43005 | Lie, HC; Simmons, LW; Rhodes, G | 2009 | Does Genetic Diversity Predict Health in Humans? | Genetic diversity, especially at genes important for immune functioning within the Major Histocompatibility Complex (MHC), has been associated with fitness-related traits, including disease resistance, in many species. Recently, genetic diversity has been associated with mate preferences in humans. Here we asked whether these preferences are adaptive in terms of obtaining healthier mates. We investigated whether genetic diversity (heterozygosity and standardized mean d(2)) at MHC and nonMHC microsatellite loci, predicted health in 153 individuals. Individuals with greater allelic diversity (d(2)) at nonMHC loci and at one MHC locus, linked to HLA-DRB1, reported fewer symptoms over a four-month period than individuals with lower d(2). In contrast, there were no associations between MHC or nonMHC heterozygosity and health. NonMHC-d(2) has previously been found to predict male preferences for female faces. Thus, the current findings suggest that nonMHC diversity may play a role in both natural and sexual selection acting on human populations. | 255 | no | NA | NA | no | no |
| 256 | 2017 | 43005 | Portnoy, DS; Hollenbeck, CM; Vidal, RR; Gold, JR | 2014 | A Comparison of Neutral and Immune Genetic Variation in Atlantic Salmon, Salmo salar L. in Chilean Aquaculture Facilities | Genetic diversity was assessed in samples of cultured Atlantic salmon, Salmo salar L., obtained from facilities in Chile between 2005 and 2010, a period of time during which the infectious pathogens Infectious Salmon Anemia (ISA) virus, Caligus rogercresseyi (sea lice), and Piscirickettsia salmonis (salmon rickettsial syndrome) were common. Two panels of microsatellite markers were utilized: one with microsatellites with no known gene associations (neutral) and one featuring microsatellites linked to putative immune-related genes (immune-related). Allelic richness and gene diversity across samples were significantly greater in neutral loci as compared to immune-related loci. Both diversity measures were homogeneous among samples for immune-related loci and heterogeneous among samples for neutral loci. Immune-related loci were identified as F-ST outliers in pairwise comparisons of samples at a 10-fold higher frequency than neutral loci. These results indicate that neutral and immune-related portions of the Atlantic salmon genome may have differed in response to the gauntlet of pathogens and that monitoring of specific, well characterized immune-related loci as well as neutral loci in cultured species could be useful when disease control and prevention is a goal. | 256 | no | cultivated fish | NA | no | no |
| 257 | 2017 | 43005 | Bruniche-Olsen, A; Burridge, CP; Austin, JJ; Jones, ME | 2013 | Disease induced changes in gene flow patterns among Tasmanian devil populations | “Infectious diseases of wildlife reduce population size and may erode genetic diversity, constituting an extinction threat. The Tasmanian devil (Sarcophilus harrisii) is threatened with extinction by an infectious cancer, the devil facial tumour disease (DFTD). In less than two decades, DFTD has caused a more than 85% overall population decline. We used ten polymorphic microsatellite loci to quantify the effects of this decline on genetic diversity, population differentiation, effective population size, and gene flow. Samples from 1999 and 2006 at five locations were analysed, three of which had been affected by DFTD during this time interval. Significant increases in inbreeding coefficient (f) and non-significant reductions in effective population size were observed for both diseased and non-diseased populations, and therefore there was no consistent effect of DFTD. There was significant but stable structuring of genetic variation among locations through time, although a dynamic”“source-sink”" relationship was evident for gene flow associated with disease-mediated changes in population densities. These changes in gene flow may have contributed to the maintenance of genetic diversity in disease-affected areas. Simulations suggest that the estimated population declines, although severe, have been insufficient to yield significant changes in genetic variation | 257 | no | inbreeding | NA | no | no |
| 258 | 2017 | 43005 | Sischo, DR; Price, MR; Pascua, MA; Hadfield, MG | 2016 | Genetic and Demographic Insights into the Decline of a Captive Population of the Endangered Hawaiian Tree Snail Achatinella fuscobasis (Achatinellinae) | Hawaiian tree snails in subfamily Achatinellinae have experienced drastic population declines and range reductions as a result of habitat destruction, overcollection, and introduced predators. In 1991,11 individuals from one of the last remaining populations of Achatinella fuscobasis, a federally listed species, were brought to the University of Hawai’i’s tree-snail captive-rearing facility. After increasing in population size to 441 snails in 2005, the population subsequently declined to only 120 snails by 2014, a 73% reduction. In this study, we assessed demographic and genetic changes that occurred in the captive-bred population of A. fuscobasis. Demographic data were compared to genetic data collected from 11 microsatellite markers for the F-1 to F-3 generations to investigate the role bottleneck effects such as loss of genetic diversity and inbreeding may have had on the population decline. Demographic measures such as juvenile survival and survival to maturity remained constant for the F-1 and F-2 generations, and significantly decreased in the F-3 generation. There was no difference in genetic diversity measures such as allelic richness, gene diversity, and observed heterozygosity among generations, but all generations were characterized by low genetic diversity and high inbreeding. The drastic population decline appears to have been due to stochastic demographic dynamics characteristic of small populations, with bottleneck effects that occurred before the founding of the ex situ population acting in synergy with factors such as disease. | 258 | no | only one pop | NA | no | no |
| 259 | 2017 | 43005 | Li, XY; Li, J; Zhao, J; Yang, F; Fu, QW; Liu, HS; Wang, DD; Yang, YC; Wang, RY | 2014 | SEQUENCE-RELATED AMPLIFIED POLYMORPHISM (SRAP) FOR STUDYING GENETIC DIVERSITY AND POPULATION STRUCTURE OF PLANTS AND OTHER LIVING ORGANISMS: A PROTOCOL | Genetic variation is widespread in living organisms. The accurate analysis of genetic variation and studies of population structures have significant implications for understanding the genetic traits, population genetics, molecular breeding and disease control programs in plants and animals. Sequence-related amplified polymorphism (SRAP) analysis has been widely used for studying genetic diversity and population structures of living organisms in the last decade. This molecular approach preferentially targets the open reading frame (ORF) regions of the genome with universal primers to screen the mutations across the whole genome, allowing to investigate genetic variations in the genome, and to develop new specific markers for species or population differentiation and molecular breeding. This article briefly outlined the applications of SRAP technique in plants and animals, and detailed described the protocol of this technique. It will make the usage of this approach conveniently and maneuverably, and further expand its applications to a wider range of living organisms. | 259 | no | NA | NA | no | no |
| 260 | 2017 | 43005 | Lee, JW; Simeoni, M; Burke, T; Hatchwell, BJ | 2010 | The consequences of winter flock demography for genetic structure and inbreeding risk in vinous-throated parrotbills, Paradoxornis webbianus | The adaptive significance of flocking behaviour has been intensively studied, especially among birds, but the demography and genetic structure of winter flocks is poorly understood, despite their importance for many biological processes. For three successive winters, we studied the demography and genetic structure of winter flocks in a small passerine, the vinous-throated parrotbill Paradoxornis webbianus. Our objectives were to determine the match between observed demography and the genetic structure of winter flocks, and to investigate the consequences of kin structure for the risk of inbreeding during the breeding season. The size of five main study flocks ranged from 60 to 120 individuals. The sex ratio of each flock did not deviate from parity and survival rates of adults were moderately low (36 and 17% in 2 years). Adults showed strong fidelity to a winter flock not only within a season but also between years. As expected from observational data, we found significant genetic differentiation and isolation by distance among males and females in winter flocks at a fine spatial scale. Likewise, relatedness among individuals within a winter flock was significantly greater than between flocks. Despite these demographic and genetic circumstances, the inbreeding rate was relatively low (4%). We conclude that fine-scale genetic structure occurs in the winter flocks of birds as a result of demographic processes without physical barriers. This genetic structure introduced a risk of inbreeding, but our evidence on whether individuals avoided incest was equivocal. Heredity (2010) 104, 472-481; doi:10.1038/hdy.2009.135; published online 7 October 2009 | 260 | no | inbreeding | NA | no | no |
| 261 | 2017 | 43005 | Juarez, RL; Schwartz, MK; Pilgrim, KL; Thompson, DJ; Tucker, SA; Smith, JB; Jenks, JA | 2016 | Assessing temporal genetic variation in a cougar population: influence of harvest and neighboring populations | The geography of the Black Hills region of South Dakota and Wyoming may limit connectivity for many species. For species with large energetic demands and large home ranges or species at low densities this can create viability concerns. Carnivores in this region, such as cougars (Puma concolor), have the additive effect of natural and human-induced mortality; this may act to decrease long-term viability. In this study we set out to explore genetic diversity among cougar populations in the Black Hills and surrounding areas. Specifically, our objectives were to first compare genetic variation and effective number of breeders of cougars in the Black Hills during three harvest regimes: pre (2003-2006), moderate (2007-2010), and heavy (2011-2013), to determine if harvest impacted genetic variation. Second, we compared genetic structure of the Black Hills cougar population with cougar populations in neighboring eastern Wyoming and North Dakota. Using 20 microsatellite loci, we conducted genetic analysis on DNA samples from cougars in the Black Hills (n = 675), North Dakota (n = 113), and eastern Wyoming (n = 62) collected from 2001-2013. Here we report that the Black Hills cougar population maintained genetic variation over the three time periods. Our substructure analysis suggests that the maintenance of genetic variation was due to immigration from eastern Wyoming and possibly North Dakota. | 261 | no | only one pop | NA | no | no |
| 262 | 2017 | 43005 | Hoggard, SJ; Wilson, PD; Beattie, AJ; Stow, AJ | 2013 | The Effectiveness of Antimicrobial Defenses Declines With Increasing Group Size and Genetic Similarity | The secretion of cuticular antimicrobial compounds is an important defensive mechanism for social insects and recent studies have demonstrated their role in the evolution of sociality. However, the factors that might affect their production and effectiveness are not well understood. For example, natural variation in colony size and genetic diversity are predicted to influence susceptibility to disease and, as a consequence, we hypothesized that colonies adjust by varying antimicrobial production. We examined the effects of colony size and genetic diversity on the antimicrobial properties of cuticular compounds in 10 colonies of the Australian paper wasp, Polistes humilis F. (Vespidae: Polistinae). The effectiveness of antimicrobial compounds produced by a colony was found to increase with genetic diversity but decrease with colony size. Further, genetic diversity appears to be the stronger factor in determining effectiveness of antimicrobial compounds and may compensate for larger group sizes. These data highlight a possible trade-off between increasing group size for work efficiency and maximizing relatedness for indirect fitness benefits, with disease risk being the underlying driving factor. | 262 | maybe | any gd and fitness of groups? | NA | yes | yes |
| 263 | 2017 | 43005 | Li, X; Zhang, SD; Yang, ZY; Song, KX; Yi, TS | 2013 | Conservation genetics and population diversity of Erigeron breviscapus (Asteraceae), an important Chinese herb | Erigeron breviscapus (Vant.) Hand.-Mazz. is an important Chinese herb in treating cerebral infarction and heart diseases, which is increasingly endangered because of over-harvesting and habitat fragmentation. We applied AFLP method to investigate its genetic diversity and population structure in order to make efficient conservation strategies. The results indicated a relatively low intraspecific genetic diversity and feeble genetic differentiation among populations, and no Evolutionarily Significant Units (ESUs) was detected. The relationships between population genetic diversity and populations’ distributions (latitude, longitude and altitude) were tested, and the genetic diversity of each population (H-POP) positively correlated with the populations’ distribution altitudes. The suitable germinating and growing conditions and less human disturbance at the higher latitude regions might be attributable for this phenomenon. For conservation purpose, an ex situ conservation measure for the populations of E. breviscapus with large size in higher altitude regions should be conserved in priority. (c) 2013 Elsevier Ltd. All rights reserved. | 263 | no | no good fitness measure | NA | no | no |
| 264 | 2017 | 43005 | Schreier, A; Stephenson, S; Rust, P; Young, S | 2015 | The case of the endangered Kootenai River white sturgeon (Acipenser transmontanus) highlights the importance of post-release genetic monitoring in captive and supportive breeding programs | The success of captive and supportive breeding programs is often determined by abundance criteria but it is also necessary to consider genetic characteristics of reintroduced or supplemented populations as genetic diversity loss can reduce population viability. Genetic analysis of the parent pools is often used to determine whether captive or supportive breeding programs conserve adequate levels of genetic diversity and maximize the effective population size (Ne). This practice assumes that released cohorts reflect the genetic characteristics of parents. Here we provide a case study of how post-release mortality can alter the genetic composition of released cohorts in a supportive breeding program for an endangered population of white sturgeon. Data from ongoing genetic monitoring of wild broodstock in the Kootenai River white sturgeon conservation aquaculture program are combined with multi-year post-release abundance monitoring of captive bred juveniles to reveal high variability in recapture among families. We found that genetic monitoring of broodstock used in supportive breeding overestimates genetic diversity conservation in most year classes due to differential post-release mortality among families. Ne was reduced in most year classes when post-release mortality was considered due to reduced parental representation in released cohorts. Although rarely performed, our results indicate that post-release genetic monitoring is necessary to accurately characterize the genetic composition of released cohorts altered by post-release mortality and should be considered when designing a captive or supportive breeding program. (C) 2015 Elsevier Ltd. All rights reserved. | 264 | no | only one pop | NA | no | no |
| 265 | 2017 | 43005 | McGreevy, TJ; Dabek, L; Husband, TP | 2011 | Genetic evaluation of the Association of Zoos and Aquariums Matschie’s tree Kangaroo (Dendrolagus matschiei) captive breeding program | Matschie’s tree kangaroo (Dendrolagus matschiei) is an endangered species that has been bred in captivity since the 1970s. In 1992, the Tree Kangaroo Species Survival Plan (R) (TKSSP) was established to coordinate the captive management of Association of Zoos and Aquariums (AZA) D. matschiei. The TKSSP makes annual breeding recommendations primarily based on the mean kinship (MK) strategy. Captive breeding programs often use the MK strategy to preserve genetic diversity in small populationsto avoid the negative consequences of inbreeding and retain their adaptive potential. The ability of a captive breeding program to retain the population’s genetic diversity over time can be evaluated by comparing the genetic diversity of the captive population to wild populations. We analyzed DNA extracted from blood and fecal samples from AZA (n = 71), captive (n = 28), and wild (n = 22) D. matschiei using eight microsatellite markers and sequenced the partial mitochondrial DNA control region gene. AZA D. matschiei had a similar expected heterozygosity (He = 0.595 +/- 0.184) compared with wild D. matschiei (He = 0.628 +/- 0.143), but they had different allelic frequencies (FST = 0.126; P<0.001). AZA D. matschiei haplotype diversity was almost two times lower than wild D. matschiei (). These data will assist management of AZA D. matschiei and serve as a baseline for AZA and wild D. matschiei genetic diversity values that could be used to monitor future changes in their genetic diversity. Zoo Biol 30:636646, 2011. (c) 2010 Wiley Periodicals, Inc. | 265 | no | zoo | NA | no | no |
| 266 | 2017 | 43005 | Gaggiotti, OE | 2003 | Genetic threats to population persistence | Human activities are having a devastating effect on the survival of natural populations. The reduction in population size and changes in the connectivity of populations due to human disturbances enhance the effect of demographic and genetic factors that can lead to population extinction. This article provides an overview of our current understanding of the role of genetic factors in the extinction of populations. The three primary genetic factors are loss of genetic variability, inbreeding depression, and accumulation of mildly deleterious mutations. The effects of these factors are discussed in the context of three different scenarios: isolated populations, local populations with immigration, and metapopulations. | 266 | no | NA | NA | no | no |
| 267 | 2017 | 43005 | Bitencourth, K; Voloch, CM; Serra-Freire, NM; Machado-Ferreira, E; Amorim, M; Gazeta, GS | 2016 | Analysis of Amblyomma sculptum haplotypes in an area endemic for Brazilian spotted fever | Amblyomma sculptum (Ixodida: Ixodidae) Berlese, 1888, a member of the Amblyomma cajennense complex, is the major vector of Brazilian spotted fever (BSF) in southeastern Brazil. In this study, the genetic diversity of A. sculptum populations in the state of Rio de Janeiro (RJ), Brazil, was investigated because genetic variability in tick populations may be related to vector competence. Samples of A. sculptum from 19 municipalities in 7 regions of RJ were subjected to DNA extraction, amplification and sequencing of D-loop, cytochrome oxidase II and 12S rDNA mitochondrial genes. These sequences were used to map the genetic diversity of this tick. Amblyomma sculptum populations are genetically diverse in RJ, especially in the South Centre and Highland regions. Few unique haplotypes were observed in all populations, and the majority of genetic variation found was among ticks within each population. Phylogenetic reconstruction reinforced the assumption that all the haplotypes identified in RJ belong to A. sculptum. However, some RJ haplotypes are closer to A. sculptum from Argentina than to A. sculptum from elsewhere in Brazil. In RJ, A. sculptum has high genetic diversity, although little genetic differentiation. Observations also indicated a high level of gene flow among the studied populations and no evidence of population structure according to region in RJ. | 267 | no | NA | NA | no | no |
| 268 | 2017 | 43005 | Thoss, M; Ilmonen, P; Penn, DJ | 2009 | Inbreeding does not reduce aggressiveness in wild male mice, Mus musculus | Inbreeding reduces quality and survival of offspring due to increased homozygosity and the expression of recessive deleterious mutations. However, there are only few studies examining how inbreeding affects behavior in adults. We aimed to replicate an earlier study in wild house mice by inducing a stress factor - infection with Salmonella. To examine whether less inbred males are more aggressive and have a higher probability to win brief encounters, we confronted full-sib inbred and outbred males in a neutral arena and recorded aggressive as well as defensive behaviors. Contrary to our expectations, any effects of inbreeding on aggressive and defensive behaviors were not dependent on infection status. Furthermore, neither infection treatment nor inbreeding affected the amount of aggressive and defensive behaviors displayed by males. Short-term aggression assays may be a useful tool for investigating certain aspects of aggressive behavior; however, long-term aggression assays might be more suitable to monitor all aspects of competitive ability and antagonistic interactions as well as effects of certain treatments on competitive ability and aggressiveness. These results may have important implications for opposed selection pressures arising from female choice and male-male competition. | 268 | no | inbreeding | NA | no | no |
| 269 | 2017 | 43005 | Woyciechowski, M; Krol, E | 2001 | Worker genetic diversity and infection by Nosema apis in honey bee colonies | The hypothesis that parasites and pathogens select for polyandry in eusocial Hymenoptera was tested, using the honey bee Apis mellifera and its microsporidian parasite Nosema apis. Five honey bee colonies with low and five with high worker generic diversity were infected with N. apis spores. At 54-56 days after inoculation, parasite spores in the workers’ midguts were counted to determine whether there was a greater variation of infection intensity (spore counts per worker) in high-diversity colonies than in low-diversity ones. In ail colonies there were two discrete sets of workers, with few or many parasite spores. To compare the variations of infection intensity between two colony groups, coefficients of variation were calculated for all workers examined, and for the slightly infected and strongly infected workers. The percentages of slightly infected workers in the low- and high-diversity groups were also compared. None of the comparisons between low- and high-diversity colonies showed significant differences, therefore no relation was found between honey bee workers’ genetic diversity and their infection with N. apis. | 269 | yes | from thesis | NA | no | yes |
| 270 | 2017 | 43005 | Vinson, A; Raboin, MJ | 2015 | A Practical Approach for Designing Breeding Groups to Maximize Genetic Diversity in a Large Colony of Captive Rhesus Macaques (Macaca mulatto) | Limited guidance is available on practical approaches for maintaining genetic diversity in large NHP colonies that support biomedical research, despite the fact that reduced diversity in these colonies is likely to compromise the application of findings in NHP to human disease. In particular, constraints related to simultaneously housing, breeding, and providing ongoing veterinary care for thousands of animals with a highly complex social structure creates unique challenges for genetic management in these colonies. Because the composition of new breeding groups is a critical component of genetic management, here we outline a 3-stage protocol for forming new breeding groups of NHP that is aimed at maximizing genetic diversity in the face of frequent restrictions on age, sex, and numbers of animals per breeding group. As an example application of this protocol, we describe optimal combinations of rhesus macaques from an analysis of candidate animals available for breeding in July 2013, selected from among the approximately 4000 macaques maintained at the Oregon National Primate Research Center. In addition, a simulation study to explore the genetic diversity in breeding groups formed by using this protocol, indicated an approximate 10-fold higher genome uniqueness, 50% lower mean kinship, and an 84-fold lower mean inbreeding coefficient among potential offspring within groups, when compared with a suboptimal group design. We conclude that this protocol provides a practical and effective approach to breeding group design for colony managers who want to prevent the loss of genetic diversity in large, semiisolated NHP colonies. | 270 | no | only one pop | NA | no | no |
| 271 | 2017 | 43005 | Wallberg, A; Han, F; Wellhagen, G; Dahle, B; Kawata, M; Haddad, N; Simoes, ZLP; Allsopp, MH; Kandemir, I; De la Rua, P; Pirk, CW; Webster, MT | 2014 | A worldwide survey of genome sequence variation provides insight into the evolutionary history of the honeybee Apis mellifera | The honeybee Apis mellifera has major ecological and economic importance. We analyze patterns of genetic variation at 8.3 million SNPs, identified by sequencing 140 honeybee genomes from a worldwide sample of 14 populations at a combined total depth of 634x. These data provide insight into the evolutionary history and genetic basis of local adaptation in this species. We find evidence that population sizes have fluctuated greatly, mirroring historical fluctuations in climate, although contemporary populations have high genetic diversity, indicating the absence of domestication bottlenecks. Levels of genetic variation are strongly shaped by natural selection and are highly correlated with patterns of gene expression and DNA methylation. We identify genomic signatures of local adaptation, which are enriched in genes expressed in workers and in immune system and sperm motility related genes that might underlie geographic variation in reproduction, dispersal and disease resistance. This study provides a framework for future investigations into responses to pathogens and climate change in honeybees. | 271 | no | no pop level | NA | no | no |
| 272 | 2017 | 43005 | Koh, KS; Matz, C; Tan, CH; Le, HL; Rice, SA; Marshall, DJ; Steinberg, PD; Kjelleberg, S | 2012 | Minimal increase in genetic diversity enhances predation resistance | The importance of species diversity to emergent, ecological properties of communities is increasingly appreciated, but the importance of within-species genetic diversity for analogous emergent properties of populations is only just becoming apparent. Here, the properties and effects of genetic variation on predation resistance in populations were assessed and the molecular mechanism underlying these emergent effects was investigated. Using biofilms of the ubiquitous bacterium Serratia marcescens, we tested the importance of genetic diversity in defending biofilms against protozoan grazing, a main source of mortality for bacteria in all natural ecosystems. S.marcescens biofilms established from wild-type cells produce heritable, stable variants, which when experimentally combined, persist as a diverse assemblage and are significantly more resistant to grazing than either wild type or variant biofilms grown in monoculture. This diversity effect is biofilm-specific, a result of either facilitation or resource partitioning among variants, with equivalent experiments using planktonic cultures and grazers resulting in dominance by a single resistant strain. The variants studied are all the result of single nucleotide polymorphisms in one regulatory gene suggesting that the benefits of genetic diversity in clonal biofilms can occur through remarkably minimal genetic change. The findings presented here provide a new insight on the integration of genetics and population ecology, in which diversity arising through minimal changes in genotype can have major ecological implications for natural populations. | 272 | probably yes | “Biofilm = one”“group”“?” | GD based on morphs | no | no |
| 273 | 2017 | 43005 | Johansson, M; Primmer, CR; Merila, J | 2007 | Does habitat fragmentation reduce fitness and adaptability? A case study of the common frog (Rana temporaria) | Studies examining the effects of anthropogenic habitat fragmentation on both neutral and adaptive genetic variability are still scarce. We compared tadpole fitness-related traits (viz. survival probability and body size) among populations of the common frog (Rana temporaria) from fragmented (F) and continuous (C) habitats that differed significantly in population sizes (C > F) and genetic diversity (C > F) in neutral genetic markers. Using data from common garden experiments, we found a significant positive relationship between the mean values of the fitness related traits and the amount of microsatellite variation in a given population. While genetic differentiation in neutral marker loci (F-ST) tended to be more pronounced in the fragmented than in the continuous habitat, genetic differentiation in quantitative traits (Q(ST)) exceeded that in neutral marker traits in the continuous habitat (i.e. Q(ST) > F-ST), but not in the fragmented habitat (i.e. Q(ST) approximate to F-ST). These results suggest that the impact of random genetic drift relative to natural selection was higher in the fragmented landscape where populations were small, and had lower genetic diversity and fitness as compared to populations in the more continuous landscape. The findings highlight the potential importance of habitat fragmentation in impairing future adaptive potential of natural populations. | 273 | yes | using survival as fitness measure, not body size | NA | no | yes |
| 274 | 2017 | 43005 | Ortego, J; Aparicio, JM; Calabuig, G; Cordero, PJ | 2007 | Risk of ectoparasitism and genetic diversity in a wild lesser kestrel population | Parasites and infectious diseases are major determinants of population dynamics and adaptive processes, imposing fitness costs to their hosts and promoting genetic variation in natural populations. In the present study, we evaluate the role of individual genetic diversity on risk of parasitism by feather lice Degeeriella rufa in a wild lesser kestrel population (Falco naumanni). Genetic diversity at 11 microsatellite loci was associated with risk of parasitism by feather lice, with more heterozygous individuals being less likely to be parasitized, and this effect was statistically independent of other nongenetic parameters (colony size, sex, location, and year) which were also associated with lice prevalence. This relationship was nonlinear, with low and consistent prevalences among individuals showing high levels of genetic diversity that increased markedly at low levels of individual heterozygosity. This result appeared to reflect a genome-wide effect, with no single locus contributing disproportionably to the observed effect. Thus, overall genetic variation, rather than linkage of markers to genes experiencing single-locus heterosis, seems to be the underlying mechanism determining the association between risk of parasitism and individual genetic diversity in the study host-parasite system. However, feather lice burden was not affected by individual heterozygosity; what suggest that differences in susceptibility, rather than variation in defences once the parasite has been established, may shape the observed pattern. Overall, our results highlight the role of individual genetic diversity on risk of parasitism in wild populations, what has both important evolutionary implications and major consequences for conservation research on the light of emerging infectious diseases that may endanger genetically depauperated populations. | 274 | no | individual level | NA | no | no |
| 275 | 2017 | 43005 | Tsangaras, K; Avila-Arcos, MC; Ishida, Y; Helgen, KM; Roca, AL; Greenwood, AD | 2012 | Historically low mitochondrial DNA diversity in koalas (Phascolarctos cinereus) | Background: The koala (Phascolarctos cinereus) is an arboreal marsupial that was historically widespread across eastern Australia until the end of the 19th century when it suffered a steep population decline. Hunting for the fur trade, habitat conversion, and disease contributed to a precipitous reduction in koala population size during the late 1800s and early 1900s. To examine the effects of these reductions in population size on koala genetic diversity, we sequenced part of the hypervariable region of mitochondrial DNA (mtDNA) in koala museum specimens collected in the 19th and 20th centuries, hypothesizing that the historical samples would exhibit greater genetic diversity. Results: The mtDNA haplotypes present in historical museum samples were identical to haplotypes found in modern koala populations, and no novel haplotypes were detected. Rarefaction analyses suggested that the mtDNA genetic diversity present in the museum samples was similar to that of modern koalas. Conclusions: Low mtDNA diversity may have been present in koala populations prior to recent population declines. When considering management strategies, low genetic diversity of the mtDNA hypervariable region may not indicate recent inbreeding or founder events but may reflect an older historical pattern for koalas. | 275 | no | no pop level | NA | no | no |
| 276 | 2017 | 43005 | Lloyd, MW; Burnett, RK; Engelhardt, KAM; Neel, MC | 2012 | Does genetic diversity of restored sites differ from natural sites? A comparison of Vallisneria americana (Hydrocharitaceae) populations within the Chesapeake Bay | The goal of ecological restoration is to re-establish self-sustaining ecosystems that will resist future perturbation without additional human input. We focus here on the re-establishment of submersed aquatic macrophyte beds in the restoration of the Chesapeake Bay estuary. Degraded environmental conditions are often to blame for poor bed establishment, but genetic factors could also be contributing to low survival. We quantified the effect of restoration practices on genetic diversity in the submersed aquatic plant species Vallisneria americana Michx. (Hydrocharitaceae) in the Chesapeake Bay. In 2007, we collected 440 shoots from 8 restored/natural site pairs and 4 restoration stock repositories, and genotyped those individuals at 10 microsatellite loci. Restoration practices do not appear to negatively impact genetic diversity, and basic measures of genetic diversity within restored sites overlap with natural sites. However, small population size of restored sites, significant inbreeding coefficients within 3 sites, and low overlap of allele composition among sites provide cause for concern. These problems are relatively minor, and we propose several corrections that would alleviate them altogether. Managers should be encouraged by our findings as well as the current state of the genetic diversity within V. americana restoration efforts. | 276 | no | no good fitness measure | NA | no | no |
| 277 | 2017 | 43005 | Annavi, G; Newman, C; Buesching, CD; Macdonald, DW; Burke, T; Dugdale, HL | 2014 | Heterozygosity-fitness correlations in a wild mammal population: accounting for parental and environmental effects | HFCs (heterozygosity-fitness correlations) measure the direct relationship between an individual’s genetic diversity and fitness. The effects of parental heterozygosity and the environment on HFCs are currently under-researched. We investigated these in a high-density U. K. population of European badgers (Meles meles), using a multimodel capture-mark-recapture framework and 35 microsatellite loci. We detected interannual variation in first-year, but not adult, survival probability. Adult females had higher annual survival probabilities than adult males. Cubs with more heterozygous fathers had higher first-year survival, but only in wetter summers; there was no relationship with individual or maternal heterozygosity. Moist soil conditions enhance badger food supply (earthworms), improving survival. In dryer years, higher indiscriminate mortality rates appear to mask differential heterozygosity-related survival effects. This paternal interaction was significant in the most supported model; however, the model-averaged estimate had a relative importance of 0.50 and overlapped zero slightly. First-year survival probabilities were not correlated with the inbreeding coefficient (f); however, small sample sizes limited the power to detect inbreeding depression. Correlations between individual heterozygosity and inbreeding were weak, in line with published meta-analyses showing that HFCs tend to be weak. We found support for general rather than local heterozygosity effects on first-year survival probability, and g2 indicated that our markers had power to detect inbreeding. We emphasize the importance of assessing how environmental stressors can influence the magnitude and direction of HFCs and of considering how parental genetic diversity can affect fitness-related traits, which could play an important role in the evolution of mate choice. | 277 | no | individual level | NA | no | no |
| 278 | 2017 | 43005 | Wang, PP; Song, XH; Zhang, HY | 2013 | High genetic variation among Aschersonia placenta (Clavicipitaceae) isolates from citrus orchards in China | Aschersonia placenta had been recognized as an important fungal pathogen of whiteflies. In recent years, natural occurrence of Aschersonia in whitefly populations was observed in many citrus orchards in the southern regions of China. We analyzed 60 A. placenta isolates obtained from Chinese citrus orchards, using inter-simple sequence repeats to examine the genetic diversity and to determine whether intraspecific variation is correlated with geographic origin. One hundred and fourteen fragments were generated from these isolates; 97% were polymorphic. The Nei’s gene diversity (H) was estimated to be 0.1748 within the populations (range 0.0974-0.2179) and 0.3057 at the species level. Analysis of molecular variance showed that the genetic variation was found mainly within populations (74.9%). The coefficient of gene differentiation (G(ST) = 0.4315) indicated that 56.85% of the genetic diversity resided within populations. The Mantel test revealed no significant correlation between the genetic distance and the corresponding geographical distance (r = 0.142 and P = 0.887); the unweighted pair-group method using arithmetic average clustering gave similar results. | 278 | no | no good fitness measure | NA | no | no |
| 279 | 2017 | 43005 | Evison, SEF; Fazio, G; Chappell, P; Foley, K; Jensen, AB; Hughes, WOH | 2013 | Host-parasite genotypic interactions in the honey bee: the dynamics of diversity | Parasites are thought to be a major driving force shaping genetic variation in their host, and are suggested to be a significant reason for the maintenance of sexual reproduction. A leading hypothesis for the occurrence of multiple mating (polyandry) in social insects is that the genetic diversity generated within-colonies through this behavior promotes disease resistance. This benefit is likely to be particularly significant when colonies are exposed to multiple species and strains of parasites, but host-parasite genotypic interactions in social insects are little known. We investigated this using honey bees, which are naturally polyandrous and consequently produce genetically diverse colonies containing multiple genotypes (patrilines), and which are also known to host multiple strains of various parasite species. We found that host genotypes differed significantly in their resistance to different strains of the obligate fungal parasite that causes chalkbrood disease, while genotypic variation in resistance to the facultative fungal parasite that causes stonebrood disease was less pronounced. Our results show that genetic variation in disease resistance depends in part on the parasite genotype, as well as species, with the latter most likely relating to differences in parasite life history and host-parasite coevolution. Our results suggest that the selection pressure from genetically diverse parasites might be an important driving force in the evolution of polyandry, a mechanism that generates significant genetic diversity in social insects. | 279 | no | no pop level | NA | no | no |
| 280 | 2017 | 43005 | Budd, C; Zimmer, E; Freeland, JR | 2015 | Conservation genetics of Magnolia acuminata, an endangered species in Canada: Can genetic diversity be maintained in fragmented, peripheral populations? | The genetic diversity of peripheral populations is potentially important to the future adaptive capacity of species, although may be difficult to predict. A large number of species-at-risk in Canada are at the northern edge of their distribution, and many of these live in fragmented habitat. We used nuclear and chloroplast markers to assess patterns of genetic diversity and differentiation within and among populations of Canadian Magnolia acuminata (Cucumber tree), an endangered species in Canada that extends as far north as the fragmented Carolinian forest in southern Ontario. We also compared the genetic composition of Canadian M. acuminata to populations sampled throughout its central distribution in the USA. We found a high proportion of shared microsatellite alleles, plus a single cpDNA haplotype, distributed throughout the entire M. acuminata range. We also found that despite occupying fragmented habitat at their range periphery, Canadian populations showed little reduction in genetic diversity relative to the USA populations, and we attribute this to effective historical dispersal in a long-lived, polyploid species. However, a combination of private alleles, genetic substructuring, and lower levels of genetic diversity in seedlings compared to mature trees, suggests that current levels of gene flow are relatively low among Canadian populations. Therefore, despite high levels of genetic diversity in Canadian M. acuminata, managers should be aware that without intervention, populations will likely become increasingly isolated and experience a reduction in genetic diversity which in turn may threaten their long-term survival in Canada. | 280 | no | no good fitness measure | NA | no | no |
| 281 | 2017 | 43005 | Turner, AK; Begon, M; Jackson, JA; Bradley, JE; Paterson, S | 2011 | Genetic Diversity in Cytokines Associated with Immune Variation and Resistance to Multiple Pathogens in a Natural Rodent Population | Pathogens are believed to drive genetic diversity at host loci involved in immunity to infectious disease. To date, studies exploring the genetic basis of pathogen resistance in the wild have focussed almost exclusively on genes of the Major Histocompatibility Complex (MHC); the role of genetic variation elsewhere in the genome as a basis for variation in pathogen resistance has rarely been explored in natural populations. Cytokines are signalling molecules with a role in many immunological and physiological processes. Here we use a natural population of field voles (Microtus agrestis) to examine how genetic diversity at a suite of cytokine and other immune loci impacts the immune response phenotype and resistance to several endemic pathogen species. By using linear models to first control for a range of non-genetic factors, we demonstrate strong effects of genetic variation at cytokine loci both on host immunological parameters and on resistance to multiple pathogens. These effects were primarily localized to three cytokine genes (Interleukin 1 beta (Il1b), Il2, and Il12b), rather than to other cytokines tested, or to membrane-bound, non-cytokine immune loci. The observed genetic effects were as great as for other intrinsic factors such as sex and body weight. Our results demonstrate that genetic diversity at cytokine loci is a novel and important source of individual variation in immune function and pathogen resistance in natural populations. The products of these loci are therefore likely to affect interactions between pathogens and help determine survival and reproductive success in natural populations. Our study also highlights the utility of wild rodents as a model of ecological immunology, to better understand the causes and consequences of variation in immune function in natural populations including humans. | 281 | no | only one pop | NA | no | no |
| 282 | 2017 | 43005 | Bussell, JD | 1999 | The distribution of random amplified polymorphic DNA (RAPD) diversity amongst populations of Isotoma petraea (Lobeliaceae) | RAPDs were generated from plants of six populations of Isotoma petraea F Muell. The species occurs on rock outcrops in southern and western Australia, with populations exhibiting different breeding systems, including complete autogamy, varying levels of outbreeding and complex hybridity. Non-metric multidimensional scaling (nMDS) analysis of the random amplified polymorphic DNA (RAPD) data set clearly resolved all populations. The Pigeon Pock population, which is home to both complex hybrid and structural homozygote plants, was divided into those two groups by the nMDS analysis. There was little diversity in highly autogamous populations, but levels were higher in the outbred Yackeyackine population. All complex hybrid populations and plants possessed numerous genetic system-specific RAPDs, some of which were shown to be held in fixed heterozygosity. Estimating GS, using RAPDs has been problematical due to their dominance, and analytical methods usually rely on knowledge of the selfing rate or assume Hardy-Weinberg equilibrium. This assumption does not hold when populations exhibit fixed heterozygosity, and an alternative method, Shannon’s Index, was used to partition genetic diversity. The distribution of genetic diversity fit expectations for an inbreeding species, with most of the variation (87.5%) occurring between populations. This compares to an average RAPD-based G(ST) of 59.6% for inbreeding species generally and 15.5% for outbreeding species. | 282 | no | no good fitness measure | NA | no | no |
| 283 | 2017 | 43005 | Fregene, MA; Suarez, M; Mkumbira, J; Kulembeka, H; Ndedya, E; Kulaya, A; Mitchel, S; Gullberg, U; Rosling, H; Dixon, AGO; Dean, R; Kresovich, S | 2003 | Simple sequence repeat marker diversity in cassava landraces: genetic diversity and differentiation in an asexually propagated crop | Cassava (Manihot esculenta) is an allogamous, vegetatively propagated, Neotropical crop that is also widely grown in tropical Africa and Southeast Asia. To elucidate genetic diversity and differentiation in the crop’s primary and secondary centers of diversity, and the forces shaping them, SSR marker variation was assessed at 67 loci in 283 accessions of cassava landraces from Africa (Tanzania and Nigeria) and the Neotropics (Brazil, Colombia, Peru, Venezuela, Guatemala, Mexico and Argentina). Average gene diversity (i.e., genetic diversity) was high in all countries, with an average heterozygosity of 0.5358 +/- 0.1184. Although the highest was found in Brazilian and Colombian accessions, genetic diversity in Neotropical and African materials is comparable. Despite the low level of differentiation [F-st(theta) = 0.091 +/- 0.005] found among country samples, sufficient genetic distance (1-proportion of shared alleles) existed between individual genotypes to separate African from Neotropical accessions and to reveal a more pronounced substructure in the African landraces. Forces shaping differences in allele frequency at SSR loci and possibly counterbalancing successive founder effects involve probably spontaneous recombination, as assessed by parent-offspring relationships, and farmer-selection for adaptation. | 283 | no | crop | NA | no | no |
| 284 | 2017 | 43005 | Piertney, SB; Webster, LMI | 2010 | Characterising functionally important and ecologically meaningful genetic diversity using a candidate gene approach | Over the past two decades the fields of molecular ecology and population genetics have been dominated by the use of putatively neutral DNA markers, primarily to resolve spatio-temporal patterns of genetic variation to inform our understanding of population structure, gene flow and pedigree. Recent emphasis in comparative functional genomics, however, has fuelled a resurgence of interest in functionally important genetic variation that underpins phenotypic traits of adaptive or ecological significance. It may prove a major challenge to transfer genomics information from classical model species to examine functional diversity in non-model species in natural populations, but already multiple gene-targeted candidate loci with major effect on phenotype and fitness have been identified. Here we briefly describe some of the research strategies used for isolating and characterising functional genetic diversity at candidate gene-targeted loci, and illustrate the efficacy of some of these approaches using our own studies on red grouse (Lagopus lagopus scoticus). We then review how candidate gene markers have been used to: (1) quantify genetic diversity among populations to identify those depauperate in genetic diversity and requiring specific management action; (2) identify the strength and mode of selection operating on individuals within natural populations; and (3) understand direct mechanistic links between allelic variation at single genes and variance in individual fitness. | 284 | no | NA | NA | no | no |
| 285 | 2017 | 43005 | Zhang, F; Deng, HW | 2010 | Confounding from cryptic relatedness in haplotype-based association studies | Cryptic relatedness was suggested to be an important source of confounding in population-based association studies (PBAS). The impact of cryptic relatedness on the performance of haplotype phase inference and haplotype-based association tests is not clear. In this study, we used the Hapmap genetic data to simulate a set of related samples. We evaluated the accuracy of haplotype phase inferred by PHASE 2.1 and calculated the power, type I error rates, accuracy and positive prediction value (PPV) of haplotype frequency-based association tests (HFAT) and haplotype similarity-based association tests (HSAT) under various scenarios, considering relatedness levels, disease models and sample sizes. Cryptic relatedness appeared to slightly increase the accuracy of haplotype phase inference. We observed significant negative effect of cryptic relatedness on the performance of HFAT and HSAT. Ignoring cryptic relatedness may increase spurious association results in haplotype-based PBAS. | 285 | no | NA | NA | no | no |
| 286 | 2017 | 43005 | Uzans, AJ; Lucas, Z; McLeod, BA; Frasier, TR | 2015 | Small Ne of the Isolated and Unmanaged Horse Population on Sable Island | “For small, isolated populations 2 common conservation concerns relate to genetic threats: inbreeding and negative consequences associated with loss of genetic diversity due to drift. Mitigating these threats often involves conservation actions that can be controversial, such as translocations or captive breeding programs. Although such actions have been successful in some situations, in others they have had undesirable outcomes. Here, we estimated the effective population size (N (e) ) of the Sable Island horses to assess the risk to this population of these genetic threats. We found surprising consistency of N (e) estimates across the 5 different methods used, with a mean of 48 effective individuals. This estimate falls below the 50 criterion of the”“50/500 rule,”" below which inbreeding depression is a concern for population viability. However, simulations and knowledge of population history indicate that this population is still in its early stages of approaching equilibrium between mutation, drift, and genetic diversity | 286 | no | only one pop | NA | no | no |
| 287 | 2017 | 43005 | IBRAHIM, KM; BARRETT, JA | 1991 | EVOLUTION OF MILDEW RESISTANCE IN A HYBRID BULK POPULATION OF BARLEY | Inbred lines derived from successive generations of a composite cross population of barley, CCV, by single seed descent were screened for variation in mildew resistance with the aim of investigating the effect of natural selection on the population. A significant increase in the frequency of plants resistant to the open-air mildew spores over 10 generations was observed. Tests of reaction to selected mildew isolates of known virulence genotypes showed a similar shift towards increased resistance to single and combined isolates. These results are discussed in relation to the effect of selection on inbreeding species and the usefulness of composite crosses as dynamic reservoirs of genetic diversity. | 287 | no | crop | NA | no | no |
| 288 | 2017 | 43005 | Schiemann, K; Tyler, T; Widen, B | 2000 | Allozyme diversity in relation to geographic distribution and population size in Lathyrus vernus (L.) Bernh. (Fabaceae) | Lathyrus vernus (L.) Bernh. is a diploid, long-lived perennial and insect-pollinated herb with no special adaptation to long-distance dispersal. It occurs on neutral soil in deciduous forests throughout western Eurasia. Due to specific habitat preferences, L. vernus has a fragmented distribution with isolated populations. We investigated allozyme variation at eleven loci in 20 populations of L. vernus from one geographically central region (the Czech Republic and the Slovak Republic) and two geographically marginal regions (southern and central Sweden) in the species present-day distribution. There was a clear differentiation between the three regions and the genetic distance between the populations was highly correlated with geographic distance. The total genetic diversity (H-T) was 0.354. The proportion of genetic diversity due to differentiation between regions, and to differentiation between populations within regions, accounted for 10% each. There was no difference in level of genetic diversity between the three regions. No significant difference in level of genetic diversity was found between small and large populations. The genetic diversity in L. vernus may either be a result of the long generation-time of the species or peculiarities in the post-glacial migration species, e.g, survival only in refugia far east of the sampled populations and/or migration as a continuous process not involving founder-events. | 288 | no | no good fitness measure, N pop differnt in GD? | NA | no | no |
| 289 | 2017 | 43005 | Nevo, E | 1998 | Molecular evolution and ecological stress at global, regional and local scales: The Israeli perspective | The enigma of genetic diversity and genome organization and evolution in nature has been investigated in plants and animals at the Institute of Evolution, University of Haifa, Israel, during the period 1974-1997. The major problem investigated was how much of the genetic diversity at single and multilocus structures is adaptive, processed by natural selection, and contributes to differences in fitness? Genetic diversity was found in different degrees among and within the numerous species tested at both the protein and DNA levels. Its organization in nature, at all scales, global, regional, and local, is nonrandom, heavily structured, frequently displays parallel trends in unrelated taxa, is positively correlated with abiotic and biotic ecological diversity and environmental stress, and is negatively correlated with population size. These results are inconsistent with the predictions of the neutral theory of molecular evolution and appear to be primarily driven by natural selection. Critical laboratory experiments using marine organisms show that inorganic and organic pollutants cause fast differential mortality, indicating that allozyme genotypes are selected by the environment. Theoretically, spatial and temporal ecological variation are of prime importance in maintaining genetic diversity in natural populations, particularly in dynamically cycling environments that can generate complex supercycles, T-cycles and chaotic-like behavior. This mode of multilocus dynamics exceeds the potential for maintaining genetic polymorphism under ordinary selection models. It may represent a novel evolutionary mechanism increasing genetic polymorphism over long time periods. Likewise, the genetic interaction between multiple species can drive the evolution of genetic diversity in nature. Modern molecular ecological-genetics and computational techniques of biomolecular sequence comparisons through ecological-genomics could highlight the origin, maintenance, abundance, structure and function of genetic polymorphisms and comparative genome evolution in dynamically changing environments. Critical studies are needed to identify, measure, and estimate the kind, degree and stage of operation of the major evolutionary forces, natural selection, migration, mutation, recombination, and genetic drift. J. Exp. Zool. 282:95-119, 1998. (C) 1998 Wiley-Liss, Inc. | 289 | no | NA | NA | no | no |
| 290 | 2017 | 43005 | Schmid-Hempel, P; Crozier, RH | 1999 | Polyandry versus polygyny versus parasites | Although social insect colonies are most easily conceptualized as consisting of a single, once-mated queen and her worker progeny, the number of queens per colony and the number of times queens mate varies broadly in ants and other social insects. Various hypotheses have been suggested for the resulting range of breeding systems and social organizations, respectively; one set of hypotheses relating to both queen number and mate number at the same time is a need for genetic variation, especially in relation to disease resistance. We here carry out a comparative analysis using phylogenetic information and, contrary to one non-phylogenetic previous study, we find that polyandry and polygyny are not significantly associated. However, the level of relatedness within colonies, a quantity affected by both polyandry and polygyny, is significantly associated with parasite loads: species with colonies with low relatedness levels have lower parasite loads. Given that, under the variance-reduction principle, selection on queens for mating frequency ought to continue even in polygynous colonies, we suggest that while parasite loads indeed seem to correlate with intra-colony genetic variability, the relationship to polyandry and polygyny may be complex and requires considerably more experimental investigation. | 290 | probably yes | Right topic and for discussion, but no data for analysis? | no data for analysis | no | no |
| 291 | 2017 | 43005 | Miller, HC; Allendorf, F; Daugherty, CH | 2010 | Genetic diversity and differentiation at MHC genes in island populations of tuatara (Sphenodon spp.) | Neutral genetic markers are commonly used to understand the effects of fragmentation and population bottlenecks on genetic variation in threatened species. Although neutral markers are useful for inferring population history, the analysis of functional genes is required to determine the significance of any observed geographical differences in variation. The genes of the major histocompatibility complex (MHC) are well-known examples of genes of adaptive significance and are particularly relevant to conservation because of their role in pathogen resistance. In this study, we survey diversity at MHC class I loci across a range of tuatara populations. We compare the levels of MHC variation with that observed at neutral microsatellite markers to determine the relative roles of balancing selection, diversifying selection and genetic drift in shaping patterns of MHC variation in isolated populations. In general, levels of MHC variation within tuatara populations are concordant with microsatellite variation. Tuatara populations are highly differentiated at MHC genes, particularly between the northern and Cook Strait regions, and a trend towards diversifying selection across populations was observed. However, overall our results indicate that population bottlenecks and isolation have a larger influence on patterns of MHC variation in tuatara populations than selection. | 291 | no | no good fitness measure | NA | no | no |
| 292 | 2017 | 43005 | Rorvik, KA; Pedersen, HC; Steen, JB | 1999 | An impact of genetic variation and predation on chick survival in willow ptarmigan Lagopus l. lagopus | The present study investigates the determinants of chick survival in willow ptarmigan Lagopus l. lagopus. Chick survival was negatively associated with genetic relatedness of mates, This may imply that more chicks die when genetic relatedness of mates is high, i.e. low chick heterozygosity at hatching. Hence, newly hatched chicks with low heterozygosity may have reduced viability and, therefore, might suffer higher mortality due to biotic/abiotic conditions. However, there was no association between the proportion of chicks with low heterozygosity and ambient temperature, but a highly significant association with predation pressure was found. Because newly hatched chicks are unable to maintain their body temperature even at normal ambient temperatures and because willow ptarmigan chicks start chirping when they get cold, it is suggested that chicks with low genetic variation may become more exposed to predation, It is proposed that genetic variation significantly affects viability of ptarmigan chicks, but that predation is the proximate cause of death. Consistent with several earlier studies, predation was related both to nesting females and to survival of the chicks. However, the present study extends these findings by suggesting a relationship between predation on one side and genetic constitution on the other, and that predation on nesting females, by reducing genetic variation among chicks at hatching, enhance chick mortality. | 292 | no | individual level | NA | no | no |
| 293 | 2017 | 43005 | Ennen, JR; Kreiser, BR; Qualls, CP | 2010 | LOW GENETIC DIVERSITY IN SEVERAL GOPHER TORTOISE (GOPHERUS POLYPHEMUS) POPULATIONS IN THE DESOTO NATIONAL FOREST, MISSISSIPPI | Gopherus polyphemus has experienced severe population declines, especially in the western portion of its range. As a consequence, G. polyphemus may have experienced population bottlenecks that resulted in a decrease in genetic diversity and all accumulation of deleterious alleles. The importance of genetic diversity has been well-documented for several fitness parameters (e.g., survival, disease resistance, growth and developmental rates, and developmental instability). Western populations of G. polyphemus in south Mississippi (USA) have lower hatching success (16.7-48%) than that found in eastern populations (67-97%). Even under laboratory conditions, approximately 40% of the eggs still failed to hatch, suggesting that intrinsic (egg quality) factors may be affecting development. Using nine microsatellite loci, we genotyped individuals from four populations in south Mississippi and one eastern population (Fort Benning, GA) and compared several genetic diversity indices (e.g., allelic richness, expected heterozygosity, and percent polymorphic loci) with published data from populations in the eastern portion of the range, such as populations east of the Mobile and Tombigbee rivers. We found significantly lower genetic diversity in the four Mississippi populations than in the eastern populations. However, these findings only demonstrate that these populations have low genetic diversity, and establishing any causal relationship between low genetic diversity (or other intrinsic factors; e.g., female condition) and reduced reproductive success should be further investigated. | 293 | no | no good fitness measure | NA | no | no |
| 294 | 2017 | 43005 | Miller, HC; Miller, KA; Daugherty, CH | 2008 | Reduced MHC variation in a threatened tuatara species | The relationship between neutral and adaptive genetic diversity is important to understand in assessing the implications of a population bottleneck. Fitness-related genes, such as those of the major histocompatibility complex (MHC), may be influenced by selection, and so retain diversity even when it is lost at neutral markers. We measured MHC class I variation in an archaic reptile species Sphenodon guntheri [North Brother Island (NBI) tuatara], which naturally occurs on one 4 ha island in Cook Strait, New Zealand, and has low levels of microsatellite diversity. MHC variation in S. guntheri was compared with microsatellite DNA variation, and with MHC variation in a large population of Sphenodon punctatus (Cook Strait tuatara) on Stephens Island. The NBI population shows significantly decreased levels of genetic diversity compared with the Stephens Island population. Only three different MHC sequences and three genotypes were found on NBI, compared with 15 sequences and 21 genotypes in a similar sample size from Stephens Island. Two sequences appear to be unique to the NBI population. The assortment of sequence variants into genotypes suggests strong gametic disequilibrium between two MHC class I loci in S. guntheri, and only two haplotypes that were present in Hardy-Weinberg proportions were identified. MHC diversity in NBI tuatara appears to be largely influenced by genetic drift, consistent with a recent population bottleneck. This may compromise the ability of this population to respond to novel disease threats. | 294 | no | no good fitness measure | NA | no | no |
| 295 | 2017 | 43005 | Paterson, S; Wilson, K; Pemberton, JM | 1998 | Major histocompatibility complex variation associated with juvenile survival and parasite resistance in a large unmanaged ungulate population (Ovis aries L.) | Antagonistic coevolution between hosts and parasites has been proposed as a mechanism maintaining genetic diversity in both host and parasite populations, In particular, the high levels of genetic diversity widely observed at the major histocompatibility complex (MHC) of vertebrate hosts are consistent with the hypothesis of parasite-driven balancing selection acting to maintain MHC genetic diversity, To date, however, empirical evidence in support of this hypothesis, especially from natural populations, has been lacking, A large unmanaged population of Soay sheep (Ovis aries L.) is used to investigate associations between MHC variation, juvenile survival, and parasite resistance. We show in an unmanaged, nonhuman population that allelic variation within the MHC is significantly associated with differences in both juvenile survival and resistance to intestinal nematodes, Certain MHC alleles are associated with low survivorship probabilities and high levels of parasitism or vies versa, We conclude that parasites ape likely to play a major role in the maintenance of MHC diversity In this population. | 295 | no | individual level | NA | no | no |
| 296 | 2017 | 43005 | Taylor, JE; Pacheco, MA; Bacon, DJ; Beg, MA; Machado, RL; Fairhurst, RM; Herrera, S; Kim, JY; Menard, D; Povoa, MM; Villegas, L; Mulyanto; Snounou, G; Cui, LW; Zeyrek, FY; Escalante, AA | 2013 | The Evolutionary History of Plasmodium vivax as Inferred from Mitochondrial Genomes: Parasite Genetic Diversity in the Americas | Plasmodium vivax is the most prevalent human malaria parasite in the Americas. Previous studies have contrasted the genetic diversity of parasite populations in the Americas with those in Asia and Oceania, concluding that New World populations exhibit low genetic diversity consistent with a recent introduction. Here we used an expanded sample of complete mitochondrial genome sequences to investigate the diversity of P. vivax in the Americas as well as in other continental populations. We show that the diversity of P. vivax in the Americas is comparable to that in Asia and Oceania, and we identify several divergent clades circulating in South America that may have resulted from independent introductions. In particular, we show that several haplotypes sampled in Venezuela and northeastern Brazil belong to a clade that diverged from the other P. vivax lineages at least 30,000 years ago, albeit not necessarily in the Americas. We propose that, unlike in Asia where human migration increases local genetic diversity, the combined effects of the geographical structure and the low incidence of vivax malaria in the Americas has resulted in patterns of low local but high regional genetic diversity. This could explain previous views that P. vivax in the Americas has low genetic diversity because these were based on studies carried out in limited areas. Further elucidation of the complex geographical pattern of P. vivax variation will be important both for diversity assessments of genes encoding candidate vaccine antigens and in the formulation of control and surveillance measures aimed at malaria elimination. | 296 | no | gd of parasite | NA | no | no |
| 297 | 2017 | 43005 | Xu, SX; Ju, JF; Zhou, XM; Wang, L; Zhou, KY; Yang, G | 2012 | Considerable MHC Diversity Suggests That the Functional Extinction of Baiji Is Not Related to Population Genetic Collapse | To further extend our understanding of the mechanism causing the current nearly extinct status of the baiji (Lipotes vexillifer), one of the most critically endangered species in the world, genetic diversity at the major histocompatibility complex (MHC) class II DRB locus was investigated in the baiji. Nine highly divergent DRB alleles were identified in 17 samples, with an average of 28.4 (13.2%) nucleotide difference and 16.7 (23.5%) amino acid difference between alleles. The unexpectedly high levels of DRB allelic diversity in the baiji may partly be attributable to its evolutionary adaptations to the freshwater environment which is regarded to have a higher parasite diversity compared to the marine environment. In addition, balancing selection was found to be the main mechanisms in generating sequence diversity at baiji DRB gene. Considerable sequence variation at the adaptive MHC genes despite of significant loss of neutral genetic variation in baiji genome might suggest that intense selection has overpowered random genetic drift as the main evolutionary forces, which further suggested that the critically endangered or nearly extinct status of the baiji is not an outcome of genetic collapse. | 297 | no | no good fitness measure | NA | no | no |
| 298 | 2017 | 43005 | Kaeuffer, R; Reale, D; Pontier, D; Chapuis, JL; Coltman, DW | 2008 | Local effects of inbreeding on embryo number and consequences for genetic diversity in Kerguelen mouflon | A classical paradigm in population genetics is that homozygosity or inbreeding affects individual fitness through increased disease susceptibility and mortality, and diminished breeding success. Using data from an insular population of mouflon (Ovis aries) founded by a single pair of individuals, we compare embryo number of ewes with different levels of inbreeding. Contrary to expectations, ewes with the highest levels of homozygosity showed the largest number of embryos. Using two different statistical approaches, we showed that this relationship is probably caused by heterozygosity at specific genes. The genetics of embryo number coupled with cyclic dynamics could play a central role in promoting genetic variation in this population. | 298 | no | inbreeding | NA | no | no |
| 299 | 2017 | 43005 | Lange, R; Gruber, B; Henle, K; Sarre, SD; Hoehn, M | 2013 | Mating system and intrapatch mobility delay inbreeding in fragmented populations of a gecko | Habitat fragmentation can cause high levels of inbreeding within populations by creating small isolated populations and therefore can affect population survival. While most genetic studies of fragmented systems focus on the impact of habitat fragmentation on interpatch patterns or processes, here we focus on inbreeding (avoidance) processes within patches. We used both genetic and mark-recapture data to study within-patch processes in fragmented and continuous populations of the gecko Oedura reticulata in the Western Australian Wheatbelt. Our results revealed that O. reticulata in fragments and continuous populations alike share a promiscuous mating system (involving both polygyny and polyandry) and exhibit no detectable difference in mean levels of relatedness. However, we also found that proportions of full-and half-sib matings were higher in the habitat fragments, subadults disperse further, and all demographic classes move further and more frequently. We contend that the net result of this combination of promiscuity and greater movement is that small isolated populations have been able to retain mean levels of relatedness similar to those in nearby continuous habitat despite fragmentation and close to complete isolation over generations. It is likely that these behavioral changes have ameliorated some of the genetic effects caused by habitat fragmentation, potentially delaying reductions in the fitness effects expected from fragmentation into small populations. | 299 | no | no good fitness measure | NA | no | no |
| 300 | 2017 | 43005 | Lee, GM; McGee, PA; Oldroyd, BP | 2013 | Variable virulence among isolates of Ascosphaera apis: testing the parasite-pathogen hypothesis for the evolution of polyandry in social insects | “The queens of many eusocial insect species are polyandrous. The evolution of polyandry from ancestral monoandry is intriguing because polyandry undermines the kin-selected benefits of high intracolonial relatedness that are understood to have been central to the evolution of eusociality. An accumulating body of evidence suggests that polyandry evolved from monoandry in part because genetically diverse colonies better resist infection by pathogens. However, a core assumption of the”“parasite-pathogen hypothesis”“, that there is variation in virulence among strains of pathogens, remains largely untested in vivo. Here, we demonstrate variation in virulence among isolates of Ascosphaera apis, the causative organism of chalkbrood disease in its honey bee (Apis mellifera) host. More importantly, we show a pathogen-host genotypic interaction for resistance and pathogenicity. Our findings therefore support the parasite-parasite hypothesis as a factor in the evolution of polyandry among eusocial insects.” | 300 | no | variance in vilulance of disease | NA | no | no |
| 301 | 2017 | 43006 | Hadian, J; Karami, A; Azizi, A; Khadivi-Khub, A | 2015 | Ubiquitous genetic diversity among and within wild populations of Satureja rechingeri assessed with ISSR markers | Genetic variation within and among populations is the basis for survival of the populations both in short and long term. Thus, studying the plant genetic diversity is essential for any conservation program. In this research, inter-simple sequence repeat (ISSR) was used to unravel the genetic variability and relationships across 75 wild individuals of Satureja rechingeri. Amplification of genomic DNA using 13 ISSR primers yielded 145 products, of which 115 products were polymorphic revealing 79.09 % polymorphism. The average polymorphism information content value obtained was 0.35. Furthermore, marker index (3.13) and resolving power (10.03) indicated that the ISSR marker was efficient revealing the genetic diversity of S. rechingeri. Averages of Shannon’s information index (I) and gene diversity (He) were 0.44 and 0.30, respectively. Analysis of molecular variance showed that the genetic variation was found mainly within populations (89 %), but variance among populations was only 11 %. The marker data indicated that the studied germplasm of S. rechingeri has a high level of diversity, and four reconstructed main clusters were identified by Bayesian analysis. The unweighted pair group method with arithmetic mean dendrogram grouped the samples by origin in most cases which was also confirmed by Bayesian analysis. These results have an important implication for S. rechingeri germplasm characterization, improvement, and conservation. | 301 | no | no pop level | NA | no | no |
| 302 | 2017 | 43006 | Charpentier, M; Setchell, JM; Prugnolle, F; Knapp, LA; Wickings, EJ; Peignot, P; Hossaert-McKey, M | 2005 | Genetic diversity and reproductive success in mandrills (Mandrillus sphinx) | Recent studies of wild animal populations have shown that estimators of neutral genetic diversity, such as mean heterozygosity, are often correlated with various fitness traits, such as survival, disease susceptibility, or reproductive success. We used two estimators of genetic diversity to explore the relationship between heterozygosity and reproductive success in male and female mandrills (Mandrillus sphinx) living in a semifree ranging setting in Gabon. Because social rank is known to influence reproductive success in both sexes, we also examined the correlation between genetic diversity and social rank in females, and acquisition of alpha status in males, as well as length of alpha male tenure. We found that heterozygous individuals showed greater reproductive success, with both females and males producing more offspring. However, heterozygosity influenced reproductive success only in dominant males, not in subordinates. Neither the acquisition of alpha status in males, nor social rank in females, was significantly correlated with heterozygosity, although more heterozygous alpha males showed longer tenure than homozygous ones. We also tested whether the benefits of greater genetic diversity were due mainly to a genome-wide effect of inbreeding depression or to heterosis at one or a few loci. Multilocus effects best explained the correlation between heterozygosity and reproductive success and tenure, indicating the occurrence of inbreeding depression in this mandrill colony. | 302 | no | no pop level | NA | no | no |
| 303 | 2017 | 43006 | Lachish, S; Miller, KJ; Storfer, A; Goldizen, AW; Jones, ME | 2011 | Evidence that disease-induced population decline changes genetic structure and alters dispersal patterns in the Tasmanian devil | Infectious disease has been shown to be a major cause of population declines in wild animals. However, there remains little empirical evidence on the genetic consequences of disease-mediated population declines, or how such perturbations might affect demographic processes such as dispersal. Devil facial tumour disease (DFTD) has resulted in the rapid decline of the Tasmanian devil, Sarcophilus harrisii, and threatens to cause extinction. Using 10 microsatellite DNA markers, we compared genetic diversity and structure before and after DFTD outbreaks in three Tasmanian devil populations to assess the genetic consequences of disease-induced population decline. We also used both genetic and demographic data to investigate dispersal patterns in Tasmanian devils along the east coast of Tasmania. We observed a significant increase in inbreeding (FIS pre/post-disease -0.030/0.012, P<0.05; relatedness pre/post-disease 0.011/0.038, P=0.06) in devil populations after just 2-3 generations of disease arrival, but no detectable change in genetic diversity. Furthermore, although there was no subdivision apparent among pre-disease populations (theta=0.005, 95% confidence interval (CI) -0.003 to 0.017), we found significant genetic differentiation among populations post-disease (theta=0.020, 0.010-0.027), apparently driven by a combination of selection and altered dispersal patterns of females in disease-affected populations. We also show that dispersal is male-biased in devils and that dispersal distances follow a typical leptokurtic distribution. Our results show that disease can result in genetic and demographic changes in host populations over few generations and short time scales. Ongoing management of Tasmanian devils must now attempt to maintain genetic variability in this species through actions designed to reverse the detrimental effects of inbreeding and subdivision in disease-affected populations. Heredity (2011) 106, 172-182; doi:10.1038/hdy.2010.17; published online 10 March 2010 | 303 | no | inbreeding and no fitness measure | NA | no | no |
| 304 | 2017 | 43006 | Duc, NM; Duy, VD; Xuan, BTT; Thang, BV; Ha, NTH; Tam, NM | 2016 | Genetic structure of the threatened Dipterocarpus costatus populations in lowland tropical rainforests of southern Vietnam | Dipterocarpus costatus is an endangered species restricted to the lowland forests of southern Vietnam. Habitat loss and over-exploitation of D. costatus wood are the major threats to this species. We investigated the level of genetic variability within and among populations of D. costatus in order to provide guidelines for the conservation, management, and restoration of this species to the Forest Protection Department, Vietnam. Nine microsatellite markers were used to analyze 114 samples from four populations representing the natural range of D. costatus in southeast Vietnam. We indicated the low allelic diversity (N-A = 2.3) and low genetic diversities with an average observed and expected heterozygosity of 0.130 and 0.151, respectively, in the lowland forests of southeast Vietnam. The low genetic diversity might be a consequence of inbreeding within the small and isolated populations of D. costatus owing to its habitat loss and over-exploitation. All populations deviated from Hardy-Weinberg equilibrium showing reduced heterozygosity. Alleles were lost from the populations by genetic drift. Genetic differentiation among populations was high (average pairwise F-ST = 0.405), indicating low gene flow (< 1) and isolated populations due to its destructed habitat and large geographical distances (P < 0.05) among populations. Heterozygosity excess tests (except of Bu Gia Map only under infinite allele model) were negative. The high genetic variation (62.7%) was found within populations. The STRUCTURE and neighbor joining tree results suggest strong differentiation among D. costatus populations, with the three genetic clusters, Phu Quoc, Tan Phu and Bu Gia Map, and Lo Go-Xa Mat due to habitat fragmentation and isolation. The threatened status of D. costatus was related to a lack of genetic diversity, with all its populations isolated in small forest patches. We recommend the establishment of an ex situ conservation site for D. costatus with a new big population comprising all genetic groups in order to enhance its survival under different environmental stresses. | 304 | no | no good fitness measure | NA | no | no |
| 305 | 2017 | 43006 | BURT, A; BELL, G | 1992 | Tests of sib diversification theories of outcrossing in Impatiens capensis: Effects of inbreeding and neighbour relatedness on production and infestation | Several models of the evolution of genetic systems posit very strong frequency-dependent selection acting on small spatial scales; in such circumstances a genetically diverse sibship outperforms a genetically uniform sibship, and genes for mixis may spread in a population. Such selection regimes may derive from resource limitation and/or parasite transmission. We describe a greenhouse experiment designed to test these ideas, using the annual herb Impatiens capensis. Plants were potted in pairs; the genetic variance within pots was manipulated by using progeny from either inbred or outcrossed parents and by using either full sibs or unrelated individuals. Treatment combinations designed to increase genetic diversity resulted in greater phenotypic variance in both morphology and production, though not in the density of spider mites or whiteflies. Despite evidence of resource limitation, there was no effect of genetic diversity on productivity, nor was there an effect on infestation. These results fail to support either the sib competition or the sib contagion theory of outcrossing. | 305 | yes | comparing full sibs och unrelated ind groups | “only two plants in each”“group”"" | no | no |
| 306 | 2017 | 43006 | Keller, LF; Jeffery, KJ; Arcese, P; Beaumont, MA; Hochachka, WM; Smith, JNM; Bruford, MW | 2001 | Immigration and the ephemerality of a natural population bottleneck: evidence from molecular markers | Population bottlenecks are often linked to explain low levels of genetic variation in natural populations, yet few studies have documented the direct genetic consequences of known bottlenecks in the wild. Empirical studies of natural population bottlenecks are therefore needed, because key assumptions of theoretical and laboratory studies of bottlenecks may not hold in the wild. Here we present microsatellite data fr om a severe bottleneck (95% mortality) in an insular population of song sparrows (Melospiza melodia). The major findings of our study are as follows: (i) The bottleneck reduced heterozygosity and allelic diversity nearly to neutral expectations, despite non-random survival of birds with respect to inbreeding and wing length. (ii) All measures of genetic diversity regained pre-bottleneck levels within two to three years of the crash. This rapid recovery was due to low levels of immigration. (iii) The rapid recovery occurred despite a coincident, strong increase in average inbreeding. These results show that immigration at levels that are hard to measure in most field studies can lead to qualitatively very different and genetic outcomes from those expected from mutations only. We suggest that future thee empirical work on bottlenecks and metapopulations should address the impact of immigration. | 306 | no | only one pop | NA | no | no |
| 307 | 2017 | 43006 | Vitikainen, E; Sundstrom, L | 2011 | Inbreeding and caste-specific variation in immune defence in the ant Formica exsecta | Social insects are a widespread and ecologically dominant group. Reproductive division of labour among the females in the colonies is a key trait for their success, but at the same time, it creates dense aggregations of relatives which may promote the spread of disease in the colonies. Hence, the appropriate regulation of immune defence is crucial for the well-being of a colony. Inbreeding may disturb this process through reduced resistance or by impairing the colony’s ability to regulate the responses. We tested the effect of inbreeding and the within-colony differences in the encapsulation response between the two female castes of the ant Formica exsecta. New reproductive females (gynes) born in more inbred colonies, and being more inbred themselves, showed an elevated immune response whereas inbreeding had no effect on worker response. Furthermore, the immune response exhibited by gynes was lower than that of workers and was not dependent on their body size whereas the worker response increased with body size. The elevated response is likely to reflect genetic stress caused by inbreeding, which in turn may compromise colony founding and longevity. Indeed, eliciting a high immune response in itself might not be adaptive. Our results show that the regulation of the expression of immunity differs between female castes despite their similar genetic make-up. | 307 | no | inbreeding | NA | no | no |
| 308 | 2017 | 43006 | Gaafar, ARZ; Al-Qurainy, F; Khan, S | 2014 | Assessment of genetic diversity in the endangered populations of Breonadia salicina (Rubiaceae) growing in The Kingdom of Saudi Arabia using inter-simple sequence repeat markers | Background: Breonadia salicina (Rubiaceae) is a critically endangered plant at the local scale native to southwestern Saudi Arabia. To understand the levels and partitioning of genetic variation across populations and geographical regions of this species, we assessed its genetic diversity using inter-simple sequence repeat (ISSR) markers. Results: Fourteen ISSR primers selected from 43 primers gave rise to 211 amplified loci, of which 68 were polymorphic. The percentage of polymorphic loci (PPL) at the population level ranged from 17.1 to 23.7%, with an average of 21.3%. Nei’s gene diversity (h) and Shannon’s information index (I) were 0.086 and 0.125, respectively. At the species level, PPL was 32.2%, while h and I were 0.116 and 0.172, respectively. A hierarchical analysis of molecular variance revealed a high level of genetic differentiation among populations (17% of total variance, P = 0.001), consistent with the gene differentiation coefficient (G(ST) = 0.256). Nevertheless, the evaluated genetic diversity was very low within populations; while relatively high among populations, levels were insufficient for long-term survival. Saudi Arabian accessions were also compared to accessions of a population from Yemen, where the species is more widespread. The Yemeni population also showed low genetic diversity but clustered separately. Conclusions: Breonadia salicina in Saudi Arabia is characterized by low within-population genetic diversity and high among-population genetic differentiation. Based on our findings, this locally endangered species is on the verge of local extinction. The species’ survival depends on successful implementation of suggested strategies for its long-term conservation. | 308 | no | no good fitness measure | NA | no | no |
| 309 | 2017 | 43006 | Li, JM; Jin, ZX | 2006 | High genetic differentiation revealed by RAPD analysis of narrowly endemic Sinocalycanthus chinensis Cheng et S.Y. Chang, an endangered species of China | Genetic diversity and genetic differentiation of narrowly endemic Sinocalycanthus chinensis Cheng et S.Y. Chang, an endangered species of China, were analyzed using random amplified polymorphic DNA (RAPD) markers. Totally, 165 stable and clearly scored RAPD bands were achieved from 12 primers. The genetic diversity of S. chinensis was high (P% = 68.84; h=0.2421 +/- 0.1978; I=0.3615 +/- 0.2789), whereas that at the population level was relatively low (P%=14.49; h = 0.0578 +/- 0.0167; I = 0.0843 +/- 10.0236). High genetic differentiation among populations was detected based on Shannon’s information index (0.7668), Nei’s gene diversity (0.7613) and AMOVA (0.8183). This might be explained by its survival in refugia during the last glaciation in southeastern China with origin from a widespread continental progenitor. The 10 populations from different geographical sites could be clustered into two groups. Low gene flow due to mixed mating system and anthropogenic activities likely played important roles in shaping the population genetic structure of the species. (c) 2006 Elsevier Ltd. All rights reserved. | 309 | no | no good fitness measure | NA | no | no |
| 310 | 2017 | 43006 | Hoffman, JI; Forcada, J; Amos, W | 2006 | No relationship between microsatellite variation and neonatal fitness in Antarctic fur seals, Arctocephalus gazella | Published studies of wild vertebrate populations have almost universally reported positive associations between genetic variation measured at microsatellite loci and fitness, creating the impression of ubiquity both in terms of the species and the traits involved. However, there is concern that this picture may be misleading because negative results frequently go unpublished. Here, we analyse the relationship between genotypic variation at nine highly variable microsatellite loci and neonatal fitness in 1070 Antarctic fur seal pups born at Bird Island, South Georgia. Despite our relatively large sample size, we find no significant association between three different measures of heterozygosity and two fitness traits, birth weight and survival. Furthermore, increasing genetic resolution by calculating parental relatedness also yields no association between genetic variation and fitness. Our findings are consistent with necropsy data showing that most pups die from starvation or trauma, conditions that are unlikely to be influenced strongly by genetic factors, particularly if the benefits of high heterozygosity are linked to immune-related genes. | 310 | no | no pop level | NA | no | no |
| 311 | 2017 | 43006 | Hitchings, SP; Beebee, TJC | 1997 | Genetic substructuring as a result of barriers to gene flow in urban Rana temporaria (common frog) populations: implications for biodiversity conservation | The ability to maintain small populations in quasi-natural settings is an issue of considerable importance in biodiversity conservation. The genetic structure of urban common frog (Rana temporaria) populations was determined by allozyme electrophoresis and used to evaluate the effects of restricted intersite migration. Despite the lack of any absolute barrier to movement between ponds, substantial genetic differentiation was found between sites separated by an average of only 2.3 km. Genetic distances between these town ponds correlated positively with geographical distances and were almost twice as great as those found between rural sites separated by an average of 41 km. Measures of genetic diversity and fitness were always lowest in the town, where the degree of subpopulation differentiation (F-ST = 0.388) was high. Population decline was not evident in the town, but molecular and fitness data indicated the presence of genetic drift and inbreeding depression. The long-term survival of artificially restricted populations, particularly of relatively sedentary species, may require molecular monitoring, if genetic diversity is not to be lost by chance when facets of the species niche prove to be poorly understood. | 311 | maybe | any fitness measure? GD-measurement ok? | not pop level gd | no | no |
| 312 | 2017 | 43006 | Frye, CT | 2005 | Persistence of allozyme and clonal diversity in an insular population of Cleistes bifaria (Fern.) Catling and Gregg (Orchidaceae) in Wilkes county, North Carolina | The genetics of small population size is of considerable interest to conservation biologists and land managers concerned with maintaining genetic diversity in populations of rare plants. In this paper, I present evidence obtained through starch gel electrophoresis of allozymes that an isolated population of Cleistes bifaria (Fern.) Gregg and Catling is multiclonal with levels of genetic diversity comparable to other taxa in the Orchidaceae. A total of 24 unique multilocus genotypes were resolved by eight alleles expressed by three polymorphic loci in a census of 64 aboveground stems. Levels of clonal diversity and evenness in the distribution of genotypes indicate that the population rapidly responds to favorable habitat conditions maintaining allozyme diversity despite repeated population contractions to few reproductive individuals. Sixty stems (35 flowering) had appeared in a newly created field after only five years with allozyme and clonal diversity mirroring that found in a long-existing habitat. The population genetic data obtained from allozymes plus population monitoring and knowledge of site history suggest that intrapopulation genetic variation is maintained within persistent individual clones that apparently survive underground during periods of adverse environmental conditions. | 312 | no | only one pop | NA | no | no |
| 313 | 2017 | 43006 | Dobelmann, J; Loope, KJ; Wilson-Rankin, E; Quinn, O; Baty, JW; Gruber, MAM; Lester, PJ | 2017 | Fitness in invasive social wasps: the role of variation in viral load, immune response and paternity in predicting nest size and reproductive output | Within any one habitat, the relative fitness of organisms in a population can vary substantially. Social insects like the common wasp are among the most successful invasive animals, but show enormous variation in nest size and other fitness-related traits. Some of this variation may be caused by pathogens such as viruses that can have serious consequences in social insects, which range from reduced productivity to colony death. Both individual immune responses and colony-level traits such as genetic diversity are likely to influence effects of pathogen infections on colony fitness. Here we investigate how infections with Kashmir Bee Virus (KBV), immune response and intracolony genetic diversity (due to queen polyandry) affect nest size in the invasive common wasp Vespula vulgaris. We show that KBV is highly prevalent in wasps and expression of antiviral immune genes is significantly increased with higher viral loads across individuals. Patriline membership within a nest did not influence KBV susceptibility or immune response. A permutational MANCOVA revealed that polyandry, viral load and expression of the immune gene Dicer were significant predictors of variation in nest size. High intracolony genetic diversity due to polyandry has previously been hypothesized to improve colony-level resistance to parasites and pathogens. Consistent with this hypothesis, we observed genetically diverse colonies to be significantly larger and to produce more queens, although this effect was not driven by the pathogen we investigated. Invasive wasps clearly suffer from pathogens and expend resources, as indicated here by elevated immune gene expression, toward reducing pathogen-impact on colony fitness. | 313 | maybe | any GD and fitness of groups that works? | NA | yes | yes |
| 314 | 2017 | 43006 | Wall, WA; Douglas, NA; Hoffmann, WA; Wentworth, TR; Gray, JB; Xiang, QYJ; Knaus, BK; Hohmann, MG | 2014 | Evidence of population bottleneck in Astragalus michauxii (Fabaceae), a narrow endemic of the southeastern United States | Genetic factors such as decreased genetic diversity and increased homozygosity can have detrimental effects on rare species, and may ultimately limit potential adaptation and exacerbate population declines. The Gulf and Atlantic Coastal Plain physiographic region has the second highest level of endemism in the continental USA, but habitat fragmentation and land use changes have resulted in catastrophic population declines for many species. Astragalus michauxii (Fabaceae) is an herbaceous plant endemic to the region that is considered vulnerable to extinction, with populations generally consisting of fewer than 20 individuals. We developed eight polymorphic microsatellites and genotyped 355 individuals from 24 populations. We characterized the population genetic diversity and structure, tested for evidence of past bottlenecks, and identified evidence of contemporary gene flow between populations. The mean ratios of the number of alleles to the allelic range (M ratio) across loci for A. michauxii populations were well below the threshold of 0.68 identified as indicative of a past genetic bottleneck. Genetic diversity estimates were similar across regions and populations, and comparable to other long-lived perennial species. Within-population genetic variation accounted for 92 % of the total genetic variation found in the species. Finally, there is evidence for contemporary gene flow among the populations in North Carolina. Although genetic factors can threaten rare species, maintaining habitats through prescribed burning, in concert with other interventions such as population augmentation or (re)introduction, are likely most critical to the long term survival of A. michauxii. | 314 | no | no good fitness measure | NA | no | no |
| 315 | 2017 | 43006 | Ovenden, JR; Peel, D; Street, R; Courtney, AJ; Hoyle, SD; Peel, SL; Podlich, H | 2007 | The genetic effective and adult census size of an Australian population of tiger prawns (Penaeus esculentus) | This study compares estimates of the census size of the spawning population with genetic estimates of effective current and long-term population size for an abundant and commercially important marine invertebrate, the brown tiger prawn (Penaeus esculentus). Our aim was to focus on the relationship between genetic effective and census size that may provide a source of information for viability analyses of naturally occurring populations. Samples were taken in 2001, 2002 and 2003 from a population on the east coast of Australia and temporal allelic variation was measured at eight polymorphic microsatellite loci. Moments-based and maximum-likelihood estimates of current genetic effective population size ranged from 797 to 1304. The mean long-term genetic effective population size was 9968. Although small for a large population, the effective population size estimates were above the threshold where genetic diversity is lost at neutral alleles through drift or inbreeding. Simulation studies correctly predicted that under these experimental conditions the genetic estimates would have non-infinite upper confidence limits and revealed they might be overestimates of the true size. We also show that estimates of mortality and variance in family size may be derived from data on average fecundity, current genetic effective and census spawning population size, assuming effective population size is equivalent to the number of breeders. This work confirms that it is feasible to obtain accurate estimates of current genetic effective population size for abundant Type III species using existing genetic marker technology. | 315 | no | only one pop | NA | no | no |
| 316 | 2017 | 43006 | Lopez-Uribe, MM; Appler, RH; Youngsteadt, E; Dunn, RR; Frank, SD; Tarpy, DR | 2017 | Higher immunocompetence is associated with higher genetic diversity in feral honey bee colonies (Apis mellifera) | Honey bees are the most important managed pollinators as they provide key ecosystem services for crop production worldwide. Recent losses of honey bee colonies in North America and Europe have demonstrated a need to develop strategies to improve their health and conserve their populations. Previously, we showed that feral honey bees-colonies that live in the wild without human assistance-exhibit higher levels of immunocompetence than managed colonies in North Carolina (USA). In a first attempt to investigate the underlying mechanisms of this difference in immune response, here we characterize the genetic composition of feral and managed honey bees using microsatellite markers. Our results reveal significant but small genetic differentiation between feral and managed honey bee colonies (phi(CT) = 0.047, P = 0.03) indicating admixture between these two groups. Higher genetic diversity was correlated with higher immune response in feral (P (MANOVA) = 0.011) but not managed bees, despite the fact that the latter group showed significantly higher average genetic diversity (P (ANCOVA) < 0.001). These findings suggest that genetic diversity is positively associated with immunocompetence in feral honey bee colonies, and that the benefits of genetic diversity are obscured in managed bees, perhaps as a result of artificial selection. We hypothesize that high genetic variability provides the raw material upon which natural selection acts and generates adaptive genotypes in unmanaged populations. Feral populations could be useful sources of genetic variation to use in breeding programs that aim to improve honey bee health. | 316 | maybe | any fitness measure? | no good fitness measure | no | no |
| 317 | 2017 | 43006 | Song, ZQ; Li, XF; Wang, HG; Wang, JH | 2010 | Genetic diversity and population structure of Salvia miltiorrhiza Bge in China revealed by ISSR and SRAP | Salvia miltiorrhiza Bge is a traditional Chinese medicinal herb used as an important drug to cure cardiovascular diseases. In this work, inter simple sequence repeats (ISSR) and sequence related amplified polymorphism (SRAP) markers, were applied to assess the level and pattern of genetic diversity in five important cultivated populations of S. miltiorrhiza. Among these populations, 120 bands were amplified by 5 ISSR primers, of which all were polymorphic, and 110 polymorphic bands (90.16%) were observed in 122 bands amplified by 6 SRAP primers. A high levels of genetic diversity at the species level was detected with Hs = 0.1951, 0.1927 respectively. Analysis of molecular variance revealed that a greater proportion of total genetic variation existed within populations (86.64 and 84.83% respectively) rather than among populations (13.36 and 15.17% respectively). Cluster analysis divided the five populations into two groups. The genetic relationships among populations have low correlation with their geographical distribution (Mantel test; r = 0.4870 and 0.5740 respectively). The study indicated that both ISSR and SRAP markers were effective and reliable for assessing the degree of genetic variation of S. miltiorrhiza. Our results suggested that random collecting, preserving and planting seeds without deliberate selection might be an efficient way to conserve genetic resources of medicinal plants. Their effective use was also discussed on the further breeding. | 317 | no | no good fitness measure | NA | no | no |
| 318 | 2017 | 43006 | Egizi, A; Fonseca, DM | 2015 | Ecological limits can obscure expansion history: patterns of genetic diversity in a temperate mosquito in Hawaii | Because biological invasions can be swift and are rarely examined immediately and/or followed over time, spatial genetic diversity analyses grounded in a well-developed body of theory are often used to reconstruct historical patterns of expansion. Unfortunately, the role of selection in shaping and potentially disrupting such reconstructions has seldom been examined. The mosquito Aedes japonicus japonicus is a temperate, cold-adapted species native to northern Japan that has recently established populations on the island of Hawaii. We used variation at seven microsatellite loci and one mitochondrial locus to examine Hawaiian populations collected in 2004, shortly after its first detection, and then in 2010-2011. Samples were collected along an elevational/temperature gradient, ranging from sea level to 1,200 m. Specimens collected near sea level in 2004 from the earliest detected population exhibited high genetic diversity. Contrary to expectations that diversity would decrease outward from the point of introduction, in 2010-2011 high elevation populations had the greatest genetic diversity, while low elevation populations (including those with high diversity in 2004) now had lower diversity and were significantly differentiated from each other, suggesting severe bottlenecks. We hypothesize that differential survival across temperatures at high versus low elevations has subverted the expected genetic signature of an expanding population. | 318 | no | no good fitness measure | NA | no | no |
| 319 | 2017 | 43006 | Bower, AD; Aitken, SN | 2011 | Changes in Genetic Diversity of Whitebark Pine (Pinus albicaulis Engelm.) Associated with Inbreeding and White Pine Blister Rust Infection | We investigated the association of inbreeding and infection by the introduced disease white pine blister rust (caused by the fungus Cronartium ribicola J. C. Fisch) with genetic diversity of whitebark pine (Pinus albicaulis Engelm.) by genetically comparing cohorts of different ages in natural stands. Isozyme analysis of bud tissue was used to estimate expected and observed heterozygosity (H(e) and H(o)), and Wright’s fixation index (F(is)) for three age cohorts (seedling, young, and mature), sampled from 14 sites in British Columbia, Oregon, Idaho, and Montana. Comparison of genetic diversity parameters among cohorts within a site was used to assess the extent and persistence of inbreeding with age, while comparisons of parameters among sites within a cohort were used to assess the impact of the disease on genetic diversity. Significant evidence of inbreeding (F(is) > 0) was found in all age cohorts. When sites were stratified by level of blister rust infection, differences in F(is) and H(o) among cohorts were only significant when level of infection was low. A significant negative association was found between level of blister rust infection and H(o) in the mature cohort. This suggests that when differential selection due to blister rust is weak, more heterozygous individuals may be favored; however, more homozygous individuals may have higher fitness under higher blister rust levels | 319 | no | inbreeding | NA | no | no |
| 320 | 2017 | 43006 | Palomares, F; Godoy, JA; Lopez-Bao, JV; Rodriguez, A; Roques, S; Casas-Marce, M; Revilla, E; Delibes, M | 2012 | Possible Extinction Vortex for a Population of Iberian Lynx on the Verge of Extirpation | Theory suggests that demographic and genetic traits deteriorate (i.e., fitness and genetic diversity decrease) when populations become small, and that such deterioration could precipitate positive feedback loops called extinction vortices. We examined whether demographic attributes and genetic traits have changed over time in one of the 2 remaining small populations of the highly endangered Iberian lynx (Lynx pardinus) in Donana, Spain. From 1983 to 2008, we recorded nontraumatic mortality rates, litter size, offspring survival, age at territory acquisition, and sex ratio. We combined these demographic attributes with measures of inbreeding and genetic diversity at neutral loci (microsatellites) and genes subjected to selection (major histocompatibility complex). Data on demographic traits were obtained through capture and radio tracking, checking dens during breeding, track surveys, and camera trapping. For genetic analyses, we obtained blood or tissue samples from captured or necropsied individuals or from museum specimens. Over time a female-biased sex ratio developed, age of territory acquisition decreased, mean litter size decreased, and rates of nontraumatic mortality increased, but there were no significant changes in overall mortality rates, standardized individual heterozygosity declined steadily, and allelic diversity of exon 2 of class II major histocompatibility complex DRB genes remained constant (2 allelic variants present in all individuals analyzed). Changes in sex ratio and age of territory acquisition may have resulted from demographic stochasticity, whereas changes in litter size and nontraumatic mortality may be related to observed increases in inbreeding. Concomitant deterioration of both demographic attributes and genetic traits is consistent with an extinction vortex. The co-occurrence, with or without interaction, of demographic and genetic deterioration may explain the lack of success of conservation efforts with the Donana population of Iberian lynx. | 320 | no | only two pop, inbred? No good fitnessmeasure | NA | no | no |
| 321 | 2017 | 43006 | Newman, D; Pilson, D | 1997 | Increased probability of extinction due to decreased genetic effective population size: Experimental populations of Clarkia pulchella | We established replicated experimental populations of the annual plant Clarkia pulchella to evaluate the existence of a causal relationship between loss of genetic variation and population survival probability. Two treatments differing in the relatedness of the founders, and thus in the genetic effective population size (N-e), were maintained as isolated populations in a natural environment. After three generations, the low N-e treatment had significantly lower germination and survival rates than did the high N-e treatment. These lower germination and survival rates led to decreased mean fitness in the low N-e populations: estimated mean fitness in the low N-e populations was only 21% of the estimated mean fitness in the high N-e populations. This inbreeding depression led to a reduction in population survival: at the conclusion of the experiment, 75% of the high N-e populations were still extant, whereas only 31% of the low N-e populations had survived. Decreased genetic effective population size, which leads to both inbreeding and the loss of alleles by genetic drift, increased the probability of population extinction over that expected from demographic and environmental stochasticity alone. This demonstrates that the genetic effective population size can strongly affect the probability of population persistence. | 321 | maybe | inbreeding of pop? | inbreeding | no | no |
| 322 | 2017 | 43006 | Chen, YS; Su, YC; Pan, W | 2016 | Effect of spatial constraints on Hardy-Weinberg equilibrium | Panmixia is a key issue in maintaining genetic diversity, which facilitates evolutionary potential during environmental changes. Additionally, conservation biologists suggest the importance of avoiding small or subdivided populations, which are prone to losing genetic diversity. In this paper, computer simulations were performed to the genetic drift of neutral alleles in random mating populations with or without spatial constraints by randomly choosing a mate among the closest neighbours. The results demonstrated that the number of generations required for the neutral allele to become homozygous (T-h) varied proportionally to the population size and also strongly correlated with spatial constraints. The average T-h for populations of the same size with spatial constraints was approximately one-and-a-half times longer than without constraints. With spatial constraints, homozygous population clusters formed, which reduced local diversity but preserved global diversity. Therefore, panmixia might be harmful in preserving the genetic diversity of an entire population. The results also suggested that the gene flow or gene exchange among the subdivided populations must be carefully processed to restrict diseases transmission or death during transportation and to monitor the genetic diversity. The application of this concept to similar systems, such as information transfer among peers, is also discussed. | 322 | no | NA | NA | no | no |
| 323 | 2017 | 43006 | Simons, ND; Wagner, RS; Lorenz, JG | 2013 | Genetic diversity of North American captive-born gorillas (Gorilla gorilla gorilla) | Western lowland gorillas (Gorilla gorilla gorilla) are designated as critically endangered and wild populations are dramatically declining as a result of habitat destruction, fragmentation, diseases (e.g., Ebola) and the illegal bushmeat trade. As wild populations continue to decline, the genetic management of the North American captive western lowland gorilla population will be an important component of the long-term conservation of the species. We genotyped 26 individuals from the North American captive gorilla collection at 11 autosomal microsatellite loci in order to compare levels of genetic diversity to wild populations, investigate genetic signatures of a population bottleneck and identify the genetic structure of the captive-born population. Captive gorillas had significantly higher levels of allelic diversity (t7=4.49, P=0.002) and heterozygosity (t7=4.15, P=0.004) than comparative wild populations, yet the population has lost significant allelic diversity while in captivity when compared to founders (t7=2.44, P=0.04). Analyses suggested no genetic evidence for a population bottleneck of the captive population. Genetic structure results supported the management of North American captive gorillas as a single population. Our results highlight the utility of genetic management approaches for endangered nonhuman primate species. | 323 | no | no good fitness measure, N pop differnt in GD? | NA | no | no |
| 324 | 2017 | 43006 | Kanthaswamy, S; Kou, A; Smith, DG | 2010 | Population Genetic Statistics from Rhesus Macaques (Macaca mulatta) in Three Different Housing Configurations at the California National Primate Research Center | This study analyzed the genetic composition of 3382 genetically characterized and pedigreed animals currently maintained under 3 different housing configurations at the California National Primate Research Center, including the indoor colony, outdoor ‘corn cribs,’ and half-acre field cages. Summary statistics based on 15 short tandem repeats strongly suggest significant effects of genetic drift, including the loss of allele diversity among the enclosures within the housing facilities even though gene flow among the different housing units is actively promoted by colony management. Management methods of selectively harvesting female macaques to prevent overrepresentation of one or only a few matrilines and cross-fostering 1-wk-old infants among breeding cages and corn cribs have been insufficient to prevent genetic subdivisions among the cages and corn cribs and to evenly distribute genetic diversity throughout the colony. In addition to promoting several colony management strategies recommended herein to effectively curb inbreeding and genetic differentiation, current attempts of infant cross-fostering and minimizing matriline fragmentation should be expanded. The inclusion of inbred or highly genetically homogeneous animals with diminished allele diversity in linkage and association studies will likely compromise the potential for identifying allele disease associations, whereas the inclusion of macaques from different geographic origins or their hybrids (or both) in experimental research confounds interpretations of phenotypic differences, due to inflation of the genetic contribution to phenotypic variance. | 324 | no | individual level | NA | no | no |
| 325 | 2017 | 43006 | Keller, LF | 1998 | Inbreeding and its fitness effects in an insular population of song sparrows (Melospiza melodia) | Inbreeding depression is thought to be a major factor affecting the evolution of mating systems and dispersal. While there is ample evidence for inbreeding depression in captivity, it has rarely been documented in natural populations. In this study, I examine data from a long-term demographic study of an insular population of song sparrows (Melospiza melodia) and present evidence for inbreeding depression. Forty-four percent of all matings on Mandarte Island, British Columbia, were among known relatives. Offspring of a full-sib mating (f = 0.25) experienced a reduction in annual survival rate of 17.5% on average. Over their lifetime, females with f = 0.25 produced 48% fewer young that reached independence from parental care. In contrast, male lifetime reproductive success was not affected by inbreeding. Reduced female lifetime reproductive success was mostly due to reduced hatching rates of the eggs of inbred females. Relatedness among the parents did not affect their reproductive success. Using data on survival from egg stage to breeding age, I estimated the average song sparrow egg on Mandarte Island to carry a minimum of 5.38 lethal equivalents (the number of deleterious genes whose cumulative effect is equivalent to one lethal); 2.88 of these lethal equivalents were expressed from egg stage to independence of parental care. This estimate is higher than most estimates reported for laboratory populations and lower than those reported for zoo populations. Hence, the costs of inbreeding in this population were substantial and slightly above those expected from laboratory studies. Variability in estimates of lethal equivalents among years showed that costs of inbreeding were not constant across years. | 325 | no | inbreeding | NA | no | no |
| 326 | 2017 | 43006 | Fraser, BA; Ramnarine, IW; Neff, BD | 2010 | TEMPORAL VARIATION AT THE MHC CLASS IIB IN WILD POPULATIONS OF THE GUPPY (POECILIA RETICULATA) | Understanding genetic diversity in natural populations is a fundamental objective of evolutionary biology. The immune genes of the major histocompatibility complex (MHC) are excellent candidates to study such diversity because they are highly polymorphic in populations. Although balancing selection may be responsible for maintaining diversity at these functionally important loci, temporal variation in selection pressure has rarely been examined. We examine temporal variation in MHC class IIB diversity in nine guppy (Poecilia reticulata) populations over two years. We found that five of the populations changed significantly more at the MHC than at neutral (microsatellite) loci as measured by F(ST), which suggests that the change at the MHC was due to selection and not neutral processes. Additionally, pairwise population differentiation measures at the MHC were higher in 2007 than in 2006, with the signature of selection changing from homogenizing to diversifying selection or neutral evolution. Interestingly, within the populations the magnitude of the change at the MHC between years was related to the change in the proportion of individuals infected by a common parasite, indicating a link between genetic structure and the parasite. Our data thereby implicate temporal variation in selective pressure as an important mechanism maintaining diversity at the MHC in wild populations. | 326 | maybe | any GD and fitness of groups that works? | NA | yes | yes |
| 327 | 2017 | 43006 | Tucker, KP; Hunter, ME; Bonde, RK; Austin, JD; Clark, AM; Beck, CA; McGuire, PM; Oli, MK | 2012 | Low genetic diversity and minimal population substructure in the endangered Florida manatee: implications for conservation | Species of management concern that have been affected by human activities typically are characterized by low genetic diversity, which can adversely affect their ability to adapt to environmental changes. We used 18 microsatellite markers to genotype 362 Florida manatees (Trichechus manatus latirostris), and investigated genetic diversity, population structure, and estimated genetically effective population size (Ne). The observed and expected heterozygosity and average number of alleles were 0.455 +/- 0.04, 0.479 +/- 0.04, and 4.77 +/- 0.51, respectively. All measures of Florida manatee genetic diversity were less than averages reported for placental mammals, including fragmented or nonideal populations. Overall estimates of differentiation were low, though significantly greater than zero, and analysis of molecular variance revealed that over 95% of the total variance was among individuals within predefined management units or among individuals along the coastal subpopulations, with only minor portions of variance explained by between group variance. Although genetic issues, as inferred by neutral genetic markers, appear not to be critical at present, the Florida manatee continues to face demographic challenges due to anthropogenic activities and stochastic factors such as red tides, oil spills, and disease outbreaks; these can further reduce genetic diversity of the manatee population. | 327 | no | no good fitness measure, N pop differnt in GD? | NA | no | no |
| 328 | 2017 | 43006 | Liu, GH; Luo, LM; Wang, B; Li, W; Song, ZP | 2006 | Comparison of genetic variation in populations of wild rice, Oryza rufipogon, plants and their soil seed banks | Wild rice, Oryza rufipogon, has endangered species conservation status and it is subject to in situ conservation in China. To understand the potential of the seed bank in species conservation and population restoration, this study compared the genetic diversity of O. rufipogon plants with that of its soil seed banks in two marshes. A total of 11 pairs of rice SSR primers were used and 9 were polymorphic. Allele frequencies of the seeds differed significantly from those of surface plants and varied between soil layers. Relatively more alleles and higher genetic diversity (H (e)) were found in plant populations, relative to seed banks. The numbers of germinable seeds and the level of genetic variation in seed banks decreased with the increasing of soil depth, indicating a rapid seed loss. Genetic differentiation was detected between sites and between plant and seed populations, as well as among seeds of different soil strata. Rapid seed loss, partly dormancy loss, and nonrandom seed mortality are discussed as the possible contributors to the pattern of reduced genetic variation within seed banks, compared to plants. These could also be responsible for the considerable genetic differentiation between populations. The seed population held about 72% of the total genetic variation of O. rufipogon in each marsh, indicating the potential of seed banks for restoring population variabilities if the plant populations were lost. | 328 | no | no good fitness measure | NA | no | no |
| 329 | 2017 | 43006 | Schaeff, CM; Kraus, SD; Brown, MW; Perkins, JS; Payne, R; White, BN | 1997 | Comparison of genetic variability of North and South Atlantic right whales (Eubalaena), using DNA fingerprinting | The North Atlantic right whale (Eubalaena glacialis) is the most endangered large whale in the world and shows few signs of recovery despite over 60 years of protection. In contrast, the South Atlantic right whale (E. australis), a closely related species, appears to be recovering successfully. Using DNA extracted from skin biopsy samples and two hypervariable minisatellite probes, we compared the levels of genetic diversity within the two populations. Our results revealed that unrelated North Atlantic (NA) right whales exhibit significantly less genetic variation than unrelated South Atlantic (SA) right whales (Jeffreys 33.15 (J33.15) probe: SNA-U-observed = 0.56 versus SSA-U-observed = 0.37, p < 0.01; M13 probe: SNA-U-observed = 0.74 versus SSA-U-observed = 0.46, P < 0.01). The level of band sharing among unrelated North Atlantic right whales was greater than that predicted for second-degree-related South Atlantic right whales (J33.15: SNA-U-observed = 0.56 versus SSA-2 degrees-expected = 0.53, p < 0.05; M13: SNA-U-observed = 0.74 versus SSA-2 degrees-expected = 0.60, p < 0.01), indicating that the northern animals have lost a substantial amount of genetic variability. Observed band sharing among North Atlantic first-degree relatives was lower than expected, based on band sharing among unrelated animals (J33.15: SNA-1 degrees-observed = 0.67 versus SNA-1 degrees-expected = 0.78, p < 0.01; M13: SNA-1 degrees-observed = 0.83 versus SNA-1 degrees-expected = 0.87, p = 0.15). This suggests that the matings between closely related Individuals that would have resulted in the higher band-sharing values were unsuccessful. These results, in conjunction with behavioral and population data which indicate that North Atlantic right whales may be suffering from reduced fertility, fecundity, and juvenile survivorship, support the hypothesis that inbreeding depression is influencing the recovery of this species. | 329 | no | two species comp | NA | no | no |
| 330 | 2017 | 43006 | Wernsdorfer, H; Caron, H; Gerber, S; Cornu, G; Rossi, V; Mortier, F; Gourlet-Fleury, S | 2011 | Relationships between demography and gene flow and their importance for the conservation of tree populations in tropical forests under selective felling regimes | Determining how tropical tree populations subject to selective felling (logging) pressure may be conserved is a crucial issue for forest management and studying this issue requires a comprehensive understanding of the relationships between population demography and gene flow. We used a simulation model, SELVA, to study (1) the relative impact of demographic factors (juvenile mortality, felling regime) and genetic factors (selfing, number and location of fathers, mating success) on long-term genetic diversity; and (2) the impact of different felling regimes on population size versus genetic diversity. Impact was measured by means of model sensitivity analyses. Juvenile mortality had the highest impact on the number of alleles and genotypes, and on the genetic distance between the original and final populations. Selfing had the greatest impact on observed heterozygote frequency and fixation index. Other factors and interactions had only minor effects. Overall, felling had a greater impact on population size than on genetic diversity. Interestingly, populations under relatively low felling pressure even had a somewhat lower fixation index than undisturbed populations (no felling). We conclude that demographic processes such as juvenile mortality should be modelled thoroughly to obtain reliable long-term predictions of genetic diversity. Mortality in selfed and outcrossed progenies should be modelled explicitly by taking inbreeding depression into account. The modelling of selfing based on population rate appeared to be oversimplifying and should account for inter-tree variation. Forest management should pay particular attention to the regeneration capacities of felled species. | 330 | no | no good fitness measure, N pop differnt in GD? | NA | no | no |
| 331 | 2017 | 43006 | Garte, S | 2003 | Locus-specific genetic diversity between human populations: An analysis of the literature | “The debate over classification of the human species according to racial or continental lines has involved reports on genetic differences in allele frequencies of a number of loci with important biomedical functions. Such differences are in contrast with the fact that, for human beings, intrapopulation genetic diversity is larger than that seen between populations. In an attempt to address the hypothesis that certain genes show high interpopulation diversity due to selective pressure, the literature was surveyed to quantify such diversity using Wrights Fst statistic. The gene-specific Fst values were then compared to pairwise population values of Fst taken over a large number of genes, which presumably reflect mostly neutral mechanisms of genetic diversity such as drift. The results showed that the majority of pairwise population values of Fst for over 30 genes of biomedical significance were either below or within the expected limits of Fst based on published values. These results do not support the idea that positive or diversifying natural selection plays an important role in increasing genetic diversity, even in genes that might be expected to be subject to selection pressure. Balancing selection, whereby the degree of genetic diversity is actually lower than that expected, appears to occur more frequently for these genes. The fact that allele frequency differences between populations might be”“statistically significant”" does not therefore necessarily imply a degree of genetic diversity greater than would be expected due to nonselective mechanisms. (C) 2003 Wiley-Liss, Inc." | 331 | no | human | NA | no | no |
| 332 | 2017 | 43006 | Chavez-Trevino, A; Canales-del-Castillo, R; Ruvalcaba-Ortega, I; Resendez-Perez, D; Gonzalez-Rojas, JI; Guzman-Velasco, A; Panjabi, AO | 2017 | Primer development for amplification of toll-like genes for the assessment of adaptive genetic diversity in vulnerable grassland bird species | Assessing genetic diversity in species of conservation concern is a critical parameter to implement management plans that may increase their viability. Traditionally, genetic diversity has been assessed using neutral genes; nevertheless, recent perspectives have proposed that genetic variation under natural selection (adaptive genes) is more representative of the adaptive potential of a population or species. Toll-like receptors (TLR) are an innate immune gene family that binds to Pathogen Associated Molecular Patterns. These receptors evolved under positive selection and by coevolution; therefore, they represent adaptive genetic diversity. We designed and tested six pairs of primers that amplify TLR genes in seven grassland species, one of the most vulnerable North American bird groups. Analysis of the sequences showed that all were polymorphic and evolved under positive selection, with the exception of TLR3, which evolved neutrally. These primers may constitute a useful tool for conservation studies for grassland sparrow species. | 332 | no | primer design | NA | no | no |
| 333 | 2017 | 43006 | Field, SG; Lange, M; Schulenburg, H; Velavan, TP; Michiels, NK | 2007 | Genetic diversity and parasite defense in a fragmented urban metapopulation of earthworms | Habitat fragmentation can seriously affect the population biology and persistence of small isolated populations. We evaluated the interplay between the patch size of natural habitats, genetic diversity, immunocompetence and parasite load in an urban metapopulation of the earthworm Lumbricus terrestris. Large-scale genetic analysis of earthworm populations was accomplished using newly established mtDNA markers in combination with single-strand conformation polymorphism haplotype screening. Our analysis yielded the following: (1) parasite load varied significantly among patches, but small versus large patches did not differ; a similar pattern was found for immunocompetence and genetic diversity; (2) in general, the urban metapopulation showed genetic structuring, which seems to be due to restricted gene flow among small and large patches; (3) the overall genetic variability of the patch has little or no effect on parasite load, as it was not significantly associated with parasite load; (4) small patches do not show molecular patterns associated with a general reduction in genetic diversity. These findings are in accordance with the view that among- and within-patch components of genetic diversity approach equilibrium values at differing rates and suggest that the date of isolation (i.e. patch age) could be too recent for the expected pattern between genetic diversity, immunocompetence and parasite load to become evident. Our study provides initial insights into the population genetics of one of the most economically important soil organisms and, as such, will prove valuable as a primer for future research regarding the human impact on soil environments. | 333 | yes | from thesis | NA | no | yes |
| 334 | 2017 | 43006 | Smith, AL; Landguth, EL; Bull, CM; Banks, SC; Gardner, MG; Driscoll, DA | 2016 | Dispersal responses override density effects on genetic diversity during post-disturbance succession | Dispersal fundamentally influences spatial population dynamics but little is known about dispersal variation in landscapes where spatial heterogeneity is generated predominantly by disturbance and succession. We tested the hypothesis that habitat succession following fire inhibits dispersal, leading to declines over time in genetic diversity in the early successional gecko Nephrurus stellatus. We combined a landscape genetics field study with a spatially explicit simulation experiment to determine whether successional patterns in genetic diversity were driven by habitat-mediated dispersal or demographic effects (declines in population density leading to genetic drift). Initial increases in genetic structure following fire were likely driven by direct mortality and rapid population expansion. Subsequent habitat succession increased resistance to gene flow and decreased dispersal and genetic diversity in N. stellatus. Simulated changes in population density alone did not reproduce these results. Habitat-mediated reductions in dispersal, combined with changes in population density, were essential to drive the field-observed patterns. Our study provides a framework for combining demographic, movement and genetic data with simulations to discover the relative influence of demography and dispersal on patterns of landscape genetic structure. Our results suggest that succession can inhibit connectivity among individuals, opening new avenues for understanding how disturbance regimes influence spatial population dynamics. | 334 | no | no good fitness measure | NA | no | no |
| 335 | 2017 | 43006 | Jorgensen, TH; Emerson, BC | 2008 | Functional variation in a disease resistance gene in populations of Arabidopsis thaliana | Analyses of functional genetic diversity in natural populations may provide important new insights into gene function and are necessary to understand the evolutionary processes maintaining diversity itself. The importance of including diversity within and between local populations in such studies is often ignored although many of the processes affecting genetic diversity act on this scale. Here we examine the molecular diversity in RPW8 (Recognition of Powdery Mildew), a gene conferring broad-spectrum resistance to powdery mildews in Arabidopsis thaliana stock-center accessions. Our eight UK study populations of the weedy A. thaliana were from locations judged to be subject to a minimum of anthropogenic disturbance and potentially long established. The majority of populations comprised considerable variation both in disease phenotype and RPW8 genotype. Although resistant individuals shared a major RPW8 genotype, no single allele was uniquely associated with resistance. It is concluded that RPW8 is an essential component of resistance to powdery mildews in A. thaliana, but not the only genetic factor involved in this process. No signature of selection was detected at RPW8 with a microsatellite multilocus test using an empirical null model. Unlike many previous studies of this model plant species, we found high levels of genetic diversity and relatively low differentiation (F-ST = 0.31) between populations at 14 microsatellite markers. This is judged to be due to our sampling being aimed at potentially long established populations and highlights the importance of population choice for studies of genetic diversity within this species. | 335 | no | no good fitness measure | NA | no | no |
| 336 | 2017 | 43006 | De Barba, M; Waits, LP; Garton, EO; Genovesi, P; Randi, E; Mustoni, A; Groff, C | 2010 | The power of genetic monitoring for studying demography, ecology and genetics of a reintroduced brown bear population | Genetic monitoring has rarely been used for wildlife translocations despite the potential benefits this approach offers, compared to traditional field-based methods. We applied genetic monitoring to the reintroduced brown bear population in northern Italy. From 2002 to 2008, 2781 hair and faecal samples collected noninvasively plus 12 samples obtained from captured or dead bears were used to follow the demographic and geographical expansion and changes in genetic composition. Individual genotypes were used to reconstruct the wild pedigree and revealed that the population increased rapidly, from nine founders to > 27 individuals in 2008 (lambda = 1.17-1.19). Spatial mapping of bear samples indicated that most bears were distributed in the region surrounding the translocation site; however, individual bears were found up to 163 km away. Genetic diversity in the population was high, with expected heterozygosity of 0.74-0.79 and allelic richness of 4.55-5.41. However, multi-year genetic monitoring data showed that mortality rates were elevated, immigration did not occur, one dominant male sired all cubs born from 2002 to 2005, genetic diversity declined, relatedness increased, inbreeding occurred, and the effective population size was extremely small (Ne = 3.03, ecological method). The comprehensive information collected through genetic monitoring is critical for implementing future conservation plans for the brown bear population in the Italian Alps. This study provides a model for other reintroduction programmes by demonstrating how genetic monitoring can be implemented to uncover aspects of the demography, ecology and genetics of small and reintroduced populations that will advance our understanding of the processes influencing their viability, evolution, and successful restoration. | 336 | no | only one pop | NA | no | no |
| 337 | 2017 | 43006 | Richter, SC; Nunziata, SO | 2014 | Survival to metamorphosis is positively related to genetic variability in a critically endangered amphibian species | Isolated small populations typically have reduced genetic variation and are more susceptible to local extinction. Examining the relationship between genetic variability and survival is important for conservation because it provides insight on evolutionary potential and population viability. We studied genetic associations of survival from egg to metamorphosis in the largest population of critically endangered dusky gopher frogs Lithobates sevosus. This approach allowed us to examine whether heterozygosity-fitness correlations (HFCs) existed in an r-selected species with large effect size, whereas previous research on HFCs has largely focused on k-selected species often with low effect sizes. Our objectives were to determine if genetic-fitness associations exist, if associations are a result of inbreeding and if natural selection against inbreeding has occurred. Specifically, we examined averaged population responses and HFC in relation to (1) mortality within egg clutches and (2) differential survival of offspring through metamorphosis. To do so, we used eight microsatellite DNA markers to compare genotypes across three life-history stages from a single year in the following temporal order: adults, eggs and metamorphs. We discovered a strong genetic association with survival in early life stages. In terms of average population responses, we documented a within-cohort decrease in FIS with life-history stage progression (adults: FIS=0.067; eggs: FIS=0.063; metamorphs: FIS=-0.134), high FST between eggs and metamorphs (FST=0.113), and a locus that had larger than expected FST. We found positive genetic-fitness associations for survival of egg clutches and for survival to metamorphosis. We believe that inbreeding is the primary cause; however, we also found evidence suggesting existence of purging selection against a deleterious mutant linked to a microsatellite locus. Survival of individuals with greater genetic variability should prolong persistence of this isolated population. Our study underscores the importance of species management focused on preservation of genetic diversity. | 337 | no | inbreeding | NA | no | no |
| 338 | 2017 | 43006 | Jolivet, C; Holtken, AM; Liesebach, H; Steiner, W; Degen, B | 2011 | Spatial genetic structure in wild cherry (Prunus avium L.): I. variation among natural populations of different density | Conservation of forest genetic resources requires intensive knowledge of the spatial arrangement of genetic diversity. In this study, we used four natural Prunus avium stands in Germany with contrasting for densities to understand patterns of spatial genetic structure. To this end, we genotyped adults and saplings at eight microsatellite markers, 54 AFLP loci and at the gametophytic incompatibility locus. We estimated levels of clonal propagation, spatial genetic structure and gene dispersal. High mortality occurred among young clonal individuals, as depicted by the lower clonal diversity in saplings. Contrasting levels of spatial genetic structure were observed among markers, ontogenic stages and populations. AFLP were more efficient for detecting spatial autocorrelation but did not allow us to differentiate low and high density populations, while high density populations showed substantially stronger spatial genetic structure at microsatellite loci. Furthermore, kinship decreased with tree age only in low density stands. We discuss the present results in terms of population history, pollen and seed dispersal and population density. Although conspecific density seems to be an interesting indicator of genetic diversity for conservation programmes, we still need to disentangle the relative influence of clonal propagation and density on the strength of spatial genetic structure. Simulation studies are needed to further address this question. | 338 | maybe | any GD and fitness of groups that works? | no good fitness measure | no | no |
| 339 | 2017 | 43006 | Kneeland, KM; Skoda, SR; Foster, JE | 2016 | Genetic variability of the stable fly assessed on a global scale using amplified fragment length polymorphism | The stable fly, Stomoxys calcitrans (L.) (Diptera: Muscidae), is a blood-feeding, economically important pest of animals and humans worldwide. Improved management strategies are essential and their development would benefit from studies on genetic diversity of stable flies. Especially if done on a global scale, such research could generate information necessary for the development and application of more efficient control methods. Herein we report on a genetic study of stable flies using amplified fragment length polymorphism, with samples of 10-40 individuals acquired from a total of 25 locations in the Nearctic, Neotropic, Palearctic, Afrotropic and Australasian biogeographical regions. We hypothesized that genetic differentiation would exist across geographical barriers. Although F-ST (0.33) was moderately high, the G(ST) (0.05; representing genetic diversity between individuals) was very low; N-m values (representing gene flow) were high (9.36). The mismatch distribution and tests of neutrality suggested population expansion, with no genetic differentiation between locations. The analysis of molecular variance (AMOVA) results showed the majority of genetic diversity was within groups. The mantel test showed no correlation between geographic and genetic distance; this strongly supports the AMOVA results. These results suggest that stable flies did not show genetic differentiation but are panmictic, with no evidence of isolation by distance or across geographical barriers. | 339 | no | no good fitness measure | NA | no | no |
| 340 | 2017 | 43006 | Lira, CF; Cardoso, SRS; Ferreira, PCG; Cardoso, MA; Provan, J | 2003 | Long-term population isolation in the endangered tropical tree species Caesalpinia echinata Lam. revealed by chloroplast microsatellites | Habitat fragmentation represents the single most serious threat to the survival of tropical ecosystems. In formulating strategies to counteract the detrimental effects of fragmentation, knowledge of the levels and patterns of genetic diversity within and between natural populations is vital to the establishment of any conservation programme. We utilized polymorphic chloroplast microsatellite markers to analyse genetic diversity in populations of the endangered tropical tree Caesalpinia echinata Lam. representing the entire extant range of the species. Levels of within-population diversity were low, with only two of seven populations studied displaying any variation. The vast majority of the genetic variation was partitioned between geographical regions (36%) and between populations within regions (55%). These levels of genetic structuring, coupled with a calculated pollen-to-seed flow ratio of approximate to 6.7:1, suggest that there has been little gene flow between the three major geographical regions over an extended period. Thus, the current tripartite distribution of the species is more consistent with the existence of separate glacial refugia, rather than reflecting any anthropogenic effects. | 340 | no | no good fitness measure | NA | no | no |
| 341 | 2017 | 43006 | Gizaw, S; Komen, H; Windig, JJ; Hanotte, O; van Arendonk, JAM | 2008 | Conservation priorities for Ethiopian sheep breeds combining threat status, breed merits and contributions to genetic diversity | Prioritizing livestock breeds for conservation needs to incorporate both genetic and non-genetic aspects important for the survival of the breeds. Here, we apply a maximum-utility-strategy to prioritize 14 traditional Ethiopian sheep breeds based on their threat status, contributions to farmer livelihoods (current breed merits) and contributions to genetic diversity. Contributions of the breeds to genetic diversity were quantified using Eding’s marker-estimated kinship approaches. Non-genetic aspects included threats (e. g. low population size, low preferences by farmers) and current merits (economic, ecological and cultural merits). Threat analysis identified eight of the 14 breeds as threatened. Analysis of current merits showed that sub-alpine and arid-lowland breeds contribute most to farmer livelihoods in comparison to other breeds. The highest contribution to the genetic diversity conserved was from the Simien breed. Simien showed high between-breed (low between-breed kinship = 0.04) as well as high within-breed diversity (low within-breed kinship = 0.09 and high H(E) = 0.73 and allelic richness = 6.83). We combined the results on threat status, current breed merits and contributions to genetic diversity to produce a ranking of the 14 breeds for conservation purposes. Our results balance the trade-offs between conserving breeds as insurance against future uncertainties and current sustainable utilization. The ranking of breeds provides a basis for conservation strategies for Ethiopian sheep and contributes to a regional or global conservation plan. | 341 | no | domesticated animal | NA | no | no |
| 342 | 2017 | 43006 | Margan, SH; Nurthen, RK; Montgomery, ME; Woodworth, LM; Lowe, EH; Briscoe, DA; Frankham, R | 1998 | Single large or several small? Population fragmentation in the captive management of endangered species | Captive populations of endangered species are typically maintained effectively as single random-mating populations by translocating individuals between institutions. Genetic, disease, and cost considerations, however, suggest that this may not be the optimal management strategy. Genetic theory predicts that a pooled population derived from several small isolated populations will have greater genetic diversity, less inbreeding, and less genetic adaptation to captivity than a single large population of equivalent total size, provided there are no population extinctions. These predictions were tested using populations of Drosophila with effective size comparisons of 50 vs. 2 x 25; 100 vs. 2 x 50 vs. 4 x 25, and 500 vs. 2 x 250 vs. 4 x 100 + 2 x 50 vs. 8 x 25 + 6 x 50. Populations were maintained at the indicated sizes as separate pedigreed populations for 50 generations. The several small treatments were subsequently pooled and maintained for eight to 10 generations prior to determination of fitness and evolutionary potential. Several small populations (pooled), when compared to single large populations of equivalent total size, were found to have lower average inbreeding coefficients, significantly higher reproductive fitness under competitive conditions, similar fitness under benign captive conditions, higher genetic diversity, and equivalent evolutionary potential. Trends favored the several small (pooled) populations in all comparisons at population sizes of 50 and 100. We recommend that endangered species in captivity be maintained as several small populations, with occasional exchange of genetic material. This has genetic benefits over current management both in captivity and especially for reintroductions, as well as reducing translocation costs and risks of disease transfer. (C) 1998 Wiley-Liss, Inc. | 342 | no | no good fitness measure | NA | no | no |
| 343 | 2017 | 43006 | Silva-Zolezzi, I; Hidalgo-Miranda, A; Estrada-Gil, J; Fernandez-Lopez, JC; Uribe-Figueroa, L; Contreras, A; Balam-Ortiz, E; del Bosque-Plata, L; Velazquez-Fernandez, D; Lara, C; Goya, R; Hernandez-Lemus, E; Davila, C; Barrientos, E; March, S; Jimenez-Sanchez, G | 2009 | Analysis of genomic diversity in Mexican Mestizo populations to develop genomic medicine in Mexico | Mexico is developing the basis for genomic medicine to improve healthcare of its population. The extensive study of genetic diversity and linkage disequilibrium structure of different populations has made it possible to develop tagging and imputation strategies to comprehensively analyze common genetic variation in association studies of complex diseases. We assessed the benefit of a Mexican haplotype map to improve identification of genes related to common diseases in the Mexican population. We evaluated genetic diversity, linkage disequilibrium patterns, and extent of haplotype sharing using genomewide data from Mexican Mestizos from regions with different histories of admixture and particular population dynamics. Ancestry was evaluated by including 1 Mexican Amerindian group and data from the HapMap. Our results provide evidence of genetic differences between Mexican subpopulations that should be considered in the design and analysis of association studies of complex diseases. In addition, these results support the notion that a haplotype map of the Mexican Mestizo population can reduce the number of tag SNPs required to characterize common genetic variation in this population. This is one of the first genomewide genotyping efforts of a recently admixed population in Latin America. | 343 | no | NA | NA | no | no |
| 344 | 2017 | 43006 | Sanchez-Mazas, A; Lemaitre, JF; Currat, M | 2012 | Distinct evolutionary strategies of human leucocyte antigen loci in pathogen-rich environments | Human leucocyte antigen (HLA) loci have a complex evolution where both stochastic (e. g. genetic drift) and deterministic (natural selection) forces are involved. Owing to their extraordinary level of polymorphism, HLA genes are useful markers for reconstructing human settlement history. However, HLA variation often deviates significantly from neutral expectations towards an excess of genetic diversity. Because HLA molecules play a crucial role in immunity, this observation is generally explained by pathogen-driven-balancing selection (PDBS). In this study, we investigate the PDBS model by analysing HLA allelic diversity on a large database of 535 populations in relation to pathogen richness. Our results confirm that geographical distances are excellent predictors of HLA genetic differentiation worldwide. We also find a significant positive correlation between genetic diversity and pathogen richness at two HLA class I loci (HLA-A and -B), as predicted by PDBS, and a significant negative correlation at one HLA class II locus (HLA-DQB1). Although these effects are weak, as shown by a loss of significance when populations submitted to rapid genetic drift are removed from the analysis, the inverse relationship between genetic diversity and pathogen richness at different loci indicates that HLA genes have adopted distinct evolutionary strategies to provide immune protection in pathogen-rich environments. | 344 | no | human | NA | no | no |
| 345 | 2017 | 43006 | Baer, B; Schmid-Hempel, P | 1999 | Experimental variation in polyandry affects parasite loads and fitness in a bumble-bee | In many species of animals, females typically mate with more than one male (polyandry). Some social insects carry this behaviour to extremes(1). For example, honeybee queens mate with ten to twenty (car even more) males on their nuptial flights(2). The reasons for this behaviour remain unknown, given the obvious costs of time, energy and exposure to predation. Several potential benefits of polyandry have been proposed(1,3,4), but none are well supported yet. Here we test the hypothesis that genetic diversity among a female’s offspring may offer some protection from parasitism(5-7). We artificially inseminated queens of a bumble-bee (Bombus terrestris L.) with sperm of either low or high genetic diversity. The resulting colonies were exposed to parasitism under field conditions. High-diversity colonies had fewer parasites and showed greater reproductive success, on average, than did low-diversity colonies. We suggest that female mating frequency may be influenced in part by parasites. | 345 | yes | from thesis | NA | no | yes |
| 346 | 2017 | 43006 | Kekkonen, J; Brommer, JE | 2015 | Reducing the loss of genetic diversity associated with assisted colonization-like introductions of animals | “Translocations, especially assisted colonizations, of animals are increasingly used as a conservation management tool. In many cases, however, limited funding and other logistic challenges limit the number of individuals available for translocation. In conservation genetics, small populations are predicted to rapidly lose genetic diversity which can deteriorate population survival. Thus, how worried should we be about the loss of genetic diversity when introducing small, isolated populations? Historical species introductions provide a means to assess these issues. Here we review 13 studies of”“assisted colonization-like”" introductions of animals, where only a small known number of founders established an isolated population without secondary contact to the source population. We test which factors could be important in retaining genetic diversity in these cases. In many cases, loss in heterozygosity (-12.1%) was detected, and more seriously the loss in allelic richness (-27.8 %). Number of founders seemed to have an effect but it also indicated that high population growth rate could help to retain genetic diversity, i.e. future management actions could be effective even with a limited number of founders if population growth would be enhanced. On the contrary, translocated organisms with longer generation times did not seem to retain more genetic diversity. We advocate that, where possible, future studies on translocated animals should report the loss of genetic diversity (both heterozygosity and allelic richness), which is essential for meta-analyses like this one for deepening our understanding of the genetic consequences of assisted colonization, and justifying management decisions [Current Zoology 61 (5): 827-834, 2015]." | 346 | no | NA | NA | no | no |
| 347 | 2017 | 43006 | Bouzid, W; Lek, S; Mace, M; Ben Hassine, O; Etienne, R; Legal, L; Loot, G | 2008 | Genetic diversity of Ligula intestinalis (Cestoda: Diphyllobothriidea) based on analysis of inter-simple sequence repeat markers | In order to investigate the genetic diversity of Ligula intestinalis populations, nine inter-simple sequence repeat (ISSR) markers were applied to populations from nine geographical areas around the world and 10 host species. The 110 loci selected from the ISSR patterns produced revealed high variability among the analysed samples, with a polymorphism of 100% and a global coefficient of gene differentiation estimated by Nei’s index (G(ST)) of 0.776. Major genetic differentiation was found to be correlated to five broad geographical regions (Europe, China, Canada, Australia and Algeria). Nevertheless, no significant genetic variation was found among European isolates, although they originated from disparate geographical localities and/or unrelated hosts. Classical classification methods: maximum parsimony and factorial correspondence analysis were compared with an advanced statistical method: the self-organizing map (SOM). The results demonstrated that the ISSR approach is rapid and inexpensive and provides reliable markers to assess genetic diversity of L. intestinalis. Furthermore, SOM artificial neuronal networks are considered to provide an efficient alternative tool for mapping the genetic structures of parasite populations. | 347 | no | gd of parasite | NA | no | no |
| 348 | 2017 | 43006 | Howells, EJ; Willis, BL; Bay, LK; van Oppen, MJH | 2013 | Spatial and temporal genetic structure of Symbiodinium populations within a common reef-building coral on the Great Barrier Reef | The dinoflagellate photosymbiont Symbiodinium plays a fundamental role in defining the physiological tolerances of coral holobionts, but little is known about the dynamics of these endosymbiotic populations on coral reefs. Sparse data indicate that Symbiodinium populations show limited spatial connectivity; however, no studies have investigated temporal dynamics for in hospite Symbiodinium populations following significant mortality and recruitment events in coral populations. We investigated the combined influences of spatial isolation and disturbance on the population dynamics of the generalist Symbiodinium type C2 (ITS1 rDNA) hosted by the scleractinian coral Acropora millepora in the central Great Barrier Reef. Using eight microsatellite markers, we genotyped Symbiodinium in a total of 401 coral colonies, which were sampled from seven sites across a 12-year period including during flood plume-induced coral bleaching. Genetic differentiation of Symbiodinium was greatest within sites, explaining 70-86% of the total genetic variation. An additional 9-27% of variation was explained by significant differentiation of populations among sites separated by 0.4-13km, which is consistent with low levels of dispersal via water movement and historical disturbance regimes. Sampling year accounted for 6-7% of total genetic variation and was related to significant coral mortality following severe bleaching in 1998 and a cyclone in 2006. Only 3% of the total genetic variation was related to coral bleaching status, reflecting generally small (8%) reductions in allelic diversity within bleached corals. This reduction probably reflected a loss of genotypes in hospite during bleaching, although no site-wide changes in genetic diversity were observed. Combined, our results indicate the importance of disturbance regimes acting together with limited oceanographic transport to determine the genetic composition of Symbiodinium types within reefs. | 348 | no | no good fitness measure | NA | no | no |
| 349 | 2017 | 43006 | Cheng, XY; Cheng, FX; Xu, RM; Xie, BY | 2008 | Genetic variation in the invasive process of Bursaphelenchus xylophilus (Aphelenchida : Aphelenchoididae) and its possible spread routes in China | Pinewood nematode (Bursaphelenchus xylophilus) is an invasive species that causes a destructive forest disease pine wilt disease. This disease has been prevalent in some countries in Asia since the 1970s. An amplified fragment length polymorphism survey was used to compare the genetic variation of native and invasive nematode populations in China and to examine the changes in genetic diversity during the invasion process. The genetic diversity of Chinese populations was slightly higher than that of American populations. Analysis of groups sampled from different invasive stages in China, showed that no obvious change in genetic diversity. Hence, genetic drift and founder effects are not obvious in the invasion process. Phylogenetic analysis showed that Chinese pinewood nematode populations were closer to Japanese populations than to American populations. On the basis of the genetic relationships among samples, two major invasion pathways in China are suggested. One is from Guangdong to Anhui and Zhejiang, and the other is from Guangdong to Jiangsu and then from Jiangsu to Hubei, Guizhong and Congqing. The results imply that it is important to reinforce both domestic and international quarantine to control the spread of pinewood nematode. | 349 | no | gd of pathogen | NA | no | no |
| 350 | 2017 | 43006 | Schad, J; Sommer, S; Ganzhorn, JU | 2004 | MHC variability of a small lemur in the littoral forest fragments of southeastern Madagascar | Habitat fragmentation inhibits gene flow between populations often resulting in a loss of genetic diversity with possible negative effects on fitness parameters. In vertebrates, growing evidence suggests that such genetic diversity is particularly important at the level of the major histocompatibility complex (MHC) because its gene products play an important role in immune functions. Diversity in the MHC is assumed to improve population viability. Here, we investigated the impact of forest fragmentation on the genetic variability of one of the functionally important parts of the MHC, DRB exon 2, of the endemic mouse lemur Microcebus murinus by comparing populations inhabiting two littoral forest fragments of different size in southeastern Madagascar. Twelve different alleles of DRB exon 2 were found in 145 individuals of M. murinus with high levels of sequence divergence between alleles. In both subpopulations, levels of genetic diversity were high, and the genetic analyses revealed only limited effects of fragmentation. Significantly more non-synonymous than synonymous substitutions were found in the functionally important antigen recognition and binding sites indicating selection processes maintaining MHC polymorphism. This is the first study on MHC variation in a free-ranging Malagasy lemur population. | 350 | no | no good fitness measure, N pop differnt in GD? | NA | no | no |
| 351 | 2017 | 43006 | Campanella, JJ; Bologna, PA; Smalley, JV; Rosenzweig, EB; Smith, SM | 2010 | Population Structure of Zostera marina (Eelgrass) on the Western Atlantic Coast Is Characterized by Poor Connectivity and Inbreeding | Zostera marina (eelgrass) can be found in the North Atlantic on the coast of Europe and on the east and west coasts of North America. Over the last 30 years, this once robust species has been reduced to sparse patchy populations due to disease and anthropogenic effects. In order to better understand the consequences of this devastation on the population genetics of the species, we have analyzed the population structure of western Atlantic Z. marina, employing microsatellite DNA polymorphisms. Although high fixation index values suggest moderate genetic differentiation among most of the Z. marina sites, population diversity was low. This lack of diversity was supported by a general dearth of observable heterozygotes in these sites; mean observed heterozygosity values (0.14-0.46) were lower than the mean expected heterozygosity values (0.57-0.81). Additionally, the mean FIS (coefficient of local inbreeding) values in these sites were positive, again indicating a surfeit of homozygotes. Allelic richness suggests that Chesapeake Bay has the greatest internal genetic diversity of the sites studied. Inbreeding seems prevalent in these American populations, suggesting possible reproductive fitness problems in the future. There is evidence of demographic bottlenecking and particularly low genetic diversity in Long Island. Northern Maine had the highest effective population size, suggesting a possible use in future restoration projects. | 351 | no | inbreeding | NA | no | no |
| 352 | 2017 | 43006 | Banaszek, A; Ziomek, J | 2012 | Genetic variation and effective population size in an isolated population of the common hamster, Cricetus cricetus | An isolated population of the common hamster, forming the western border of the species range in Poland was analysed by the use of 16 microsatellite loci and the mtDNA control region in two consecutive generations. The genetic diversity and the effective population size in this population are low. We found the evidence for ancient bottleneck in this population, but the results of tests for recent reduction of Ne were ambiguous. However, population functions properly i.e. it is in HW equilibrium, Fis and relatedness coefficients do not indicate inbreeding. It indicates that even isolated and small populations of the common hamster have good chance of survival on the condition of the protection and restoration of the habitats. Moreover, the sex-related differences in dispersal in the common hamster were demonstrated through the relatedness analysis. | 352 | no | only one pop | NA | no | no |
| 353 | 2017 | 43006 | Horreo, JL; Raab, R; Spakovszky, P; Alonso, JC | 2016 | Genetic structure of the threatened West-Pannonian population of Great Bustard (Otis tarda) | The genetic diversity, population structure and gene flow of the Great Bustards (Otis tarda) living in Austria-Slovakia-West Hungary (West-Pannonian region), one of the few populations of this globally threatened species that survives across the Palaearctic, has been assessed for the first time in this study. Fourteen recently developed microsatellite loci identified one single population in the study area, with high values of genetic diversity and gene flow between two different genetic subunits. One of these subunits (Heideboden) was recognized as a priority for conservation, as it could be crucial to maintain connectivity with the central Hungarian population and thus contribute to keeping contemporary genetic diversity. Current conservation efforts have been successful in saving this threatened population from extinction two decades ago, and should continue to guarantee its future survival. | 353 | no | only one pop | NA | no | no |
| 354 | 2017 | 43006 | Campbell, DR | 1997 | Genetic and environmental variation in life-history traits of a monocarpic perennial: A decade-long field experiment | Directional and stabilizing selection tend to deplete additive genetic variance. On the other hand, genetic variance in traits related to fitness could be retained through polygenic mutation, spatially varying selection, genotype-environment interaction, or antagonistic pleiotropy. Most estimates of genetic variance in fitness-related traits have come from laboratory studies, with few estimates of heritability made under natural conditions, particularly for longer lived organisms. Here I estimated additive genetic variance in life-history characters of a monocarpic herb, Ipomopsis aggregata, that lives for up to a decade. Experimental crosses yielded 229 full-sibships nested within 32 paternal half-sibships. More than 5000 offspring were planted as seeds into natural field sites and were followed in most cases through their entire life cycle. Survival showed substantial additive genetic variance (genetic coefficient of variation approximate to 54%). Small differences at seedling emergence were magnified over time, such that the genetic variability in survival was only detectable by tracking the success of offspring for several years starting from seed. In contrast to survival, reproductive traits such as flower number, seeds per flower, and age at flowering showed l’ittle or no genetic variability. Despite relatively high levels of additive genetic variation for some life history characters, high environmental variance in survival resulted in very low heritabilities (0-9%) for all of these characters. Maternal effects were evident in seed mass and remained strong throughout the lengthy vegetative period. No negative genetic correlations between major components of female fitness were detected. Mean corolla width for a paternal family was, however, negatively correlated with the finite rate of increase based on female fitness. That negative correlation could help to maintain additive genetic variance in the face of strong selection through male function for wide corollas. | 354 | no | individual level | NA | no | no |
| 355 | 2017 | 43006 | Joshi, A; Souframanien, J; Chand, R; Pawar, SE | 2006 | Genetic diversity study of Cercospora canescens (Ellis & Martin) isolates, the pathogen of Cercospora leaf spot in legumes | Genetic diversity was studied in eleven different isolates of Cercospora canescens (Ellis & Martin), the causative agent of Cercospora leaf spot in legumes. The isolates, which were obtained from different geographical locations, had different morphological and pigment production characteristics. The polymorphism at the molecular level was studied by random amplified polymorphic DNA (RAPD) marker technique and variation in the internal transcribed spacer (ITS) region of ribosomal DNA (rDNA). RAPD profiling clustered all the isolates into three clusters. Considerable genetic diversity was observed in the isolates from the same geographical location. rDNA analysis showed length variation in ITS of two isolates from mungbean, with one 600 bp band common to both. Restriction analysis could differentiate between the common 600 bp bands of the two isolates. The present study indicates that compared to restriction analysis of the ITS region, the RAPD technique is better suited for determining the genetic diversity and differentiation of C. canescens isolates. | 355 | no | gd of pathogen | NA | no | no |
| 356 | 2017 | 43006 | van de Wiel, CCM; Rajicic, TS; van Treuren, R; Dehmer, KJ; van der Linden, CG; van Hintum, TJL | 2010 | Distribution of genetic diversity in wild European populations of prickly lettuce (Lactuca serriola): implications for plant genetic resources management | Genetic variation in Lactuca serriola, the closest wild relative of cultivated lettuce, was studied across Europe from the Czech Republic to the United Kingdom, using three molecular marker systems, simple sequence repeat (SSR, microsatellites), AFLP and nucleotide-binding site (NUS) profiling. The ‘functional’ marker system NBS profiling, targeting disease resistance genes of the NBS/LRR family, did not show marked differences in genetic diversity parameters to the other systems. The autogamy of the species resulted in low observed heterozygosity and high population differentiation. Intra-population variation ranged from complete homogeneity to nearly complete heterogeneity. The highest genetic diversity was found in central Europe. The SSR results were compared to SSR variation screened earlier in the lettuce collection of the Centre for Genetic Resources, the Netherlands (CGN). In the UK, practically only a single SSR genotype was found. This genotype together with a few other common SSR genotypes comprised a large part of the plants sampled on the continent. Among the ten most frequent SSR genotypes observed, eight were already present in the CGN collection. Overall, the CGN collection appears to already have a fair representation of genetic variation from NW Europe. The results are discussed in relation to sampling strategies for improving genebank collections of crop wild relatives. | 356 | no | no good fitness measure | NA | no | no |
| 357 | 2017 | 43006 | Nunes, F; Norris, RD; Knowlton, N | 2009 | Implications of isolation and low genetic diversity in peripheral populations of an amphi-Atlantic coral | Limited dispersal and connectivity in marine organisms can have negative fitness effects in populations that are small and isolated, but reduced genetic exchange may also promote the potential for local adaptation. Here, we compare the levels of genetic diversity and connectivity in the coral Montastraea cavernosa among both central and peripheral populations throughout its range in the Atlantic. Genetic data from one mitochondrial and two nuclear loci in 191 individuals show that M. cavernosa is subdivided into three genetically distinct regions in the Atlantic: Caribbean-North Atlantic, Western South Atlantic (Brazil) and Eastern Tropical Atlantic (West Africa). Within each region, populations have similar allele frequencies and levels of genetic diversity; indeed, no significant differentiation was found between populations separated by as much as 3000 km, suggesting that this coral species has the ability to disperse over large distances. Gene flow within regions does not, however, translate into connectivity across the entire Atlantic. Instead, substantial differences in allele frequencies across regions suggest that genetic exchange is infrequent between the Caribbean, Brazil and West Africa. Furthermore, markedly lower levels of genetic diversity are observed in the Brazilian and West African populations. Genetic diversity and connectivity may contribute to the resilience of a coral population to disturbance. Isolated peripheral populations may be more vulnerable to human impacts, disease or climate change relative to those in the genetically diverse Caribbean-North Atlantic region. | 357 | no | no good fitness measure | NA | no | no |
| 358 | 2017 | 43006 | Waldeck, P; Andersson, M; Kilpi, M; Ost, M | 2008 | Spatial relatedness and brood parasitism in a female-philopatric bird population | The spatial structure of relatedness between individuals in a population can be crucial for social selection and evolution. Here we analyze a female alternative reproductive tactic, conspecific brood parasitism, in relation to spatial relatedness among females in a Baltic Sea population of the common eider Somateria mollissima. The role of relatedness in brood parasitism is debated: some models predict parasite avoidance of related hosts, others predict host-parasite relatedness. We estimate pairwise relatedness from protein fingerprinting of egg albumen in 156 nests, with pairwise nest distances ranging from 1 to 6 km. Relatedness increases significantly from the longest distances to an average of r approximate to 0.09 below 20 m. Brood parasitism is common, and average pairwise relatedness between host and parasite is estimated at 0.18-0.21. Parasites thus do not avoid relatives, and combined with the findings of a similar study in another eider population, the results show that mean host-parasite relatedness is higher than that among close neighbors. High host-parasite relatedness is therefore not an effect of natal philopatry alone; some other form of kin bias is also involved. Recognition and association between birth nest mates is a candidate mechanism for further study. | 358 | no | brood parasitism | NA | no | no |
| 359 | 2017 | 43006 | Ren, JS; Yu, YC; Gao, FY; Zeng, LH; Lu, XJ; Wu, XT; Yan, WG; Ren, GJ | 2013 | Application of resistance gene analog markers to analyses of genetic structure and diversity in rice | Plant disease resistance gene analog (RGA) markers were designed according to the conserved sequence of known RGAs and used to map resistance genes. We used genome-wide RGA markers for genetic analyses of structure and diversity in a global rice germplasm collection. Of the 472 RGA markers, 138 were polymorphic and these were applied to 178 entries selected from the USDA rice core collection. Results from the RGA markers were similar between two methods, UPGMA and STRUCTURE. Additionally, the results from RGA markers in our study were agreeable with those previously reported from SSR markers, including cluster of ancestral classification, genetic diversity estimates, genetic relatedness, and cluster of geographic origins. These results suggest that RGA markers are applicable for analyses of genetic structure and diversity in rice. However, unlike SSR markers, the RGA markers failed to differentiate temperate japonica, tropical japonica, and aromatic subgroups. The restricted way for developing RGA markers from the cDNA sequence might limit the polymorphism of RGA markers in the genome, thus limiting the discriminatory power in comparison with SSR markers. Genetic differentiation obtained using RGA markers may be useful for defining genetic diversity of a suite of random R genes in plants, as many studies show a differentiation of resistance to a wide array of pathogens. They could also help to characterize the genetic structure and geographic distribution in crops, including rice, wheat, barley, and banana. | 359 | no | NA | NA | no | no |
| 360 | 2017 | 43006 | Grueber, CE; Wallis, GP; Jamieson, IG | 2013 | Genetic drift outweighs natural selection at toll-like receptor (TLR) immunity loci in a re-introduced population of a threatened species | During population establishment, genetic drift can be the key driver of changes in genetic diversity, particularly while the population is small. However, natural selection can also play a role in shaping diversity at functionally important loci. We used a well-studied, re-introduced population of the threatened Stewart Island robin (N=722 pedigreed individuals) to determine whether selection shaped genetic diversity at innate immunity toll-like receptor (TLR) genes, over a 9-year period of population growth following establishment with 12 genetic founders. We found no evidence for selection operating with respect to TLR diversity on first-year overwinter survival for the majority of loci, genotypes and alleles studied. However, survival of individuals with TLR4(BE) genotype was significantly improved: these birds were less than half as likely to die prior to maturity compared with all other TLR4 genotypes. Furthermore, the population frequency of this genotype, at a two-fold excess over Hardy-Weinberg expectation, was increased by nonrandom mating. Near-complete sampling and full pedigree and reproductive data enabled us to eliminate other potential causes of these patterns including inbreeding, year effects, density dependence, selection on animals at earlier life history stages or genome-level association of the TLR4(E) allele with good genes’. However, comparison of observed levels of gene diversity to predictions under simulated genetic drift revealed results consistent with neutral expectations for all loci, including TLR4. Although selection favoured TLR4(BE) heterozygotes in this population, these effects were insufficient to outweigh genetic drift. This is the first empirical study to show that genetic drift can overwhelm natural selection in a wild population immediately following establishment. | 360 | no | only one pop | NA | no | no |
| 361 | 2017 | 43006 | Abedi, S; Darvishzadeh, R; Bernousi, I; Mandoulakani, BA; Maleki, HH; Shah, D | 2014 | Genetic variability of Orobanche aegyptiaca infesting tobacco in Iran by Bayesian analysis | Broomrapes (Orobanche L.) are holoparasitic plants, parasitizing roots of a wide range of host plants. In this study, genetic polymorphism among 44 Orobanche aegyptiaca Pers. individuals collected from different regions of northwest Iran was investigated using inter-simple sequence repeat (ISSR) markers. Two hundred-sixty one discernible bands were amplified using 20 ISSR primers which 245 (94%) was polymorphic, indicating considerable genetic variation among the examined individuals. The number of polymorphic bands per primer ranged from 4 to 17, averaging 12.25. UPGMA clustering using Jaccard’s similarity coefficients revealed six main groups. Genetic similarity coefficients varied from 0.71 (between individuals 23 and 27) to 0.34 (between 13 and 30). A model-based Bayesian approach subdivided 38 out of 44 broomrape genotypes into 2 genetic groups and the remaining ones were categorized as mixed genotypes based on Q values. According to an analysis of molecular variance, 99% of the total variation was partitioned within genetic groups. The results demonstrated the potential usefulness of ISSR markers for determination of genetic variation in O. aegyptiaca. Understanding the variability in broomrape is important when attempting to develop resistant host crops against this parasite. | 361 | no | gd of parasite | NA | no | no |
| 362 | 2017 | 43006 | Vandewoestijne, S; Schtickzelle, N; Baguette, M | 2008 | Positive correlation between genetic diversity and fitness in a large, well-connected metapopulation | Background: Theory predicts that lower dispersal, and associated gene flow, leads to decreased genetic diversity in small isolated populations, which generates adverse consequences for fitness, and subsequently for demography. Here we report for the first time this effect in a well-connected natural butterfly metapopulation with high population densities at the edge of its distribution range. Results: We demonstrate that: (1) lower genetic diversity was coupled to a sharp decrease in adult lifetime expectancy, a key component of individual fitness; (2) genetic diversity was positively correlated to the number of dispersing individuals (indicative of landscape functional connectivity) and adult population size; (3) parameters inferred from capture-recapture procedures (population size and dispersal events between patches) correlated much better with genetic diversity than estimates usually used as surrogates for population size (patch area and descriptors of habitat quality) and dispersal (structural connectivity index). Conclusion: Our results suggest that dispersal is a very important factor maintaining genetic diversity. Even at a very local spatial scale in a metapopulation consisting of large high-density populations interconnected by considerable dispersal rates, genetic diversity can be decreased and directly affect the fitness of individuals. From a biodiversity conservation perspective, this study clearly shows the benefits of both in-depth demographic and genetic analyses. Accordingly, to ensure the long-term survival of populations, conservation actions should not be blindly based on patch area and structural isolation. This result may be especially pertinent for species at their range margins, particularly in this era of rapid environmental change. | 362 | no | no good fitness measure | NA | no | no |
| 363 | 2017 | 43006 | Lopez, L; Retuerto, R; Roiloa, S; Santiso, X; Barreiro, R | 2015 | A multi-faceted approach for assessing evolutionary significant conservation units in the endangered Omphalodes littoralis subsp gallaecica (Boraginaceae) | Genetic diversity is now regarded as a key component of biodiversity and its assessment has become a frequent addition to conservation studies. However, due to practical limitations, most studies assess genetic variation using neutral markers while the variability of evolutionary relevant quantitative traits is typically overlooked. Here, we simultaneously assessed neutral and quantitative variation in an endangered plant to identify the mechanism behind their spatial arrangement and to propose conservation guidelines for maximizing mid- to long-term survival. Omphalodes littoralis subsp. gallaecica is a self-fertilizing therophyte with an extremely narrow and fragmented distribution. Regardless of the marker set (non-coding sequences of cpDNA or Amplified Fragment Length Polymorphism loci), the five extant populations of O. littoralis subsp. gallaecica showed minimal to none neutral genetic diversity and a lack of gene flow between them. Moreover, genetic structure was identical in samples collected on two consecutive years suggesting that the seed bank cannot buffer against genetic loss. High rates of self-fertilization together with a strongly fragmented distribution and recurrent bottlenecks seem the likely mechanisms that may have led to a dramatic loss of genetic variation in a classic scenario drawn by genetic drift. Despite the extremely narrow distribution range, reciprocal transplant experiments revealed that the populations differed in several quantitative traits and that these differences likely have a genetic basis. Nevertheless, the pattern of differences among populations did not fit the expectations of local adaptation. Instead, phenotypic variation seemed another outcome of genetic drift with important implications for conservation because each population should be designated as an independent evolutionary significant unit (ESU). Our study evidences the benefits of combining neutral markers with appropriate assessments of phenotype variation, and shows that even endemics with extremely narrow ranges can contain multiple conservation units. (C) 2014 Geobotanisches Institut ETH, Stiftung Ruebel. Published by Elsevier GmbH. All rights reserved. | 363 | no | no good fitness measure, N pop differnt in GD? | NA | no | no |
| 364 | 2017 | 43006 | Bodkin, JL; Ballachey, BE; Cronin, MA; Scribner, KT | 1999 | Population demographics and genetic diversity in remnant and translocated populations of sea otters | The effects of small population size on genetic diversity and subsequent population recovery are theoretically predicted, but few empirical data are available to describe those relations. We use data from four remnant and three translocated sea otter (Enhydra lutris) populations to examine relations among magnitude and duration of minimum population size, population growth rates, and genetic variation. Mitochondrial (mt)DNA haplotype diversity was correlated with the number of years at minimum population size (r(s) = -0.741, p = 0.038) and minimum population size, (r(s) = 0.709, p = 0.054). We found no relation between population growth and haplotype diversity, although growth was significantly greater in translocated than in remnant populations. Haplotype diversity in populations established from two sources was higher than in a population established from a single source and was higher than in the respective source populations. Haplotype frequencies in translocated populations of foundling sizes of 4 and 28 differed from expected, indicating genetic drift and differential reproduction between source populations, whereas haplotype frequencies in a translocated population with a founding size of 150 did not. Relations between population demographics and genetic characteristics suggest that genetic sampling of source and translocated populations can provide valuable inferences about translocations. | 364 | no | no good fitness measure | NA | no | no |
| 365 | 2017 | 43006 | Athrey, G; Lindsay, DL; Lance, RF; Leberg, PL | 2011 | Crumbling diversity: comparison of historical archived and contemporary natural populations indicate reduced genetic diversity and increasing genetic differentiation in the golden-cheeked warbler | Genetic viability of threatened and endangered species is of increasing concern with habitat loss and fragmentation. Valuable assessments of the genetic status of endangered species are difficult in most cases, where only single sample estimates are available. Using historical and contemporary samples, we assessed the impact of both historical and recent demographic changes on population genetics of the endangered golden-cheeked warbler, (Dendroica chrysoparia). Our study documents a steep decline in genetic diversity in an endangered species over a 100-year period, along with concurrent increase in genetic differentiation, and low contemporary effective sizes for all the populations we evaluated. While adding to the growing body of literature that describes the genetic impacts of habitat fragmentation, our study may also serve as an informative guide to future management of endangered species. Our study underlines the importance of long term population genetic monitoring in understanding the full extent of genetic changes in endangered species. | 365 | no | no good fitness measure, N pop differnt in GD? | NA | no | no |
| 366 | 2017 | 43006 | McGrath, JM; Derrico, CA; Yu, Y | 1999 | Genetic diversity in selected, historical US sugarbeet germplasm and Beta vulgaris ssp maritima | Diversity among sugar beet accessions released over the first 50 years of public breeding in the United States was examined to ascertain a baseline of genetic diversity and to gauge the effect of breeding on the loss or gain of diversity over this time period. Accessions were chosen as released germplasm from the major breeding stations contributing to the US germplasm pool and their presumed ancestors from Europe, including representatives for the wild forms Beta vulgaris ssp. maritima. Sixty nine polymorphic RAPD fragments were used for gene frequency analysis, and heterozygosity was determined within and among groups of accessions related either by breeding station or simply inherited agronomic characters for monogerm seed and restoration of fertility in a cytoplasmic male-sterile background. In general, heterozygosity within releases declined with time, but total genetic diversity in the US germplasm pool remained constant. Breeding for the agronomic characters had a marked influence in reducing diversity. | 366 | no | crop | NA | no | no |
| 367 | 2017 | 43006 | Blasco-Costa, I; Waters, JM; Poulin, R | 2012 | Swimming against the current: genetic structure, host mobility and the drift paradox in trematode parasites | Life-cycle characteristics and habitat processes can potentially interact to determine gene flow and genetic structuring of parasitic species. In this comparative study, we analysed the genetic structure of two freshwater trematode species with different life histories using cytochrome c oxidase I gene (COI) sequences and examined the effect of a unidirectional river current on their genetic diversity at 10 sites along the river. We found moderate genetic structure consistent with an isolation-by-distance pattern among subpopulations of Coitocaecum parvum but not in Stegodexamene anguillae. These contrasting parasite population structures were consistent with the relative dispersal abilities of their most mobile hosts (i.e. their definitive hosts). Genetic diversity decreased, as a likely consequence of unidirectional river flow, with increasing distance upstream in C. parvum, which utilizes a definitive host with only restricted mobility. The absence of such a pattern in S. anguillae suggests that unidirectional river flow affects parasite species differently depending on the dispersal abilities of their most mobile host. In conclusion, genetic structure, genetic diversity loss and drift are stronger in parasites whose most mobile hosts have low dispersal abilities and small home ranges. An additional prediction can be made for parasites under unidirectional drift: those parasites that stay longer in their benthic intermediate host or have more than one benthic intermediate hosts would have relatively high local recruitment and hence increased retention of upstream genetic diversity. | 367 | no | gd of parasite | NA | no | no |
| 368 | 2017 | 43006 | Purwandana, D; Ariefiandy, A; Imansyah, MJ; Ciofi, C; Forsyth, DM; Gormley, AM; Rudiharto, H; Seno, A; Fordham, DA; Gillespie, G; Jessop, TS | 2015 | Evaluating environmental, demographic and genetic effects on population-level survival in an island endemic | The population dynamics of island species are considered particularly sensitive to variation in environmental, demographic and/or genetic processes. However, few studies have attempted to evaluate the relative importance of these processes for key vital rates in island endemics. We integrated the results of long-term capture-mark-recapture analysis, prey surveys, habitat quality assessments and molecular analysis to determine the causes of variation in the survival rates of Komodo dragons Varanus komodoensis at 10 sites on four islands in Komodo National Park, Indonesia. Using open population capture-mark-recapture methods, we ranked competing models that considered environmental, ecological, genetic and demographic effects on site-specific Komodo dragon survival rates. Site-specific survival rates ranged from 0.49 (95% CI: 0.33-0.68) to 0.92 (0.79-0.97) in the 10 study sites. The three highest-ranked models (i.e. QAIC(c) < 2) explained approximate to 70% of variation in Komodo dragon survival rates and identified interactions between inbreeding coefficients, prey biomass density and habitat quality as important explanatory variables. There was evidence of additive effects from ecological and genetic (e.g. inbreeding) processes affecting Komodo dragon survival rates. Our results indicate that maintaining high ungulate prey biomass and habitat quality would enhance the persistence of Komodo dragon populations. Assisted gene flow may also increase the genetic and demographic viability of the smaller Komodo dragon populations. | 368 | maybe | any fitness measure? GD-measurement ok? | inbreeding and no good fitness measure | no | no |
| 369 | 2017 | 43006 | Nakahara, M; Tsubaki, Y | 2008 | Sperm mortality, insemination and fertilization in the damselfly Ischnura senegalensis: comparisons between wild and inbred populations | Inbreeding can have deleterious effects on individual or population fitness. To avoid fitness reduction, individuals may adopt behavioral or physiological mechanisms to reduce their investment in the production of offspring with genetically similar mates. We examined whether insemination by inbred males introduced more dead sperm than insemination by wild males by counting sperm in female Ischnura senegalensis (Rambur) sperm storage organs. If inbred males inseminated fewer or lower-quality sperm, females would avoid inferior sperm. Our results revealed three features of damselfly inbreeding: insemination failed in a larger proportion of inbred pairs than in wild pairs, inbred pairs showed significantly reduced fertility, and the numbers of live and dead sperm in an inbred female’s sperm storage organs did not differ from those in wild females. These results suggested that neither sperm quantity nor sperm quality was responsible for low fertility to a significant extent, but some kind of female quality, such as sperm usage or storing ability, was. Although inbred pairs had lower fertility, there were no significant differences between inbred and wild pairs in the total numbers of live or dead sperm. It thus seemed that female choice at the insemination stage was responsible for low fertility rather than sperm quantity or quality measured by live-to-dead ratio. | 369 | no | inbreeding | NA | no | no |
| 370 | 2017 | 43006 | Breed, MF; Ottewell, KM; Gardner, MG; Marklund, MHK; Stead, MG; Harris, JBC; Lowe, AJ | 2015 | Mating system and early viability resistance to habitat fragmentation in a bird-pollinated eucalypt | Habitat fragmentation has been shown to disrupt ecosystem processes such as plant-pollinator mutualisms. Consequently, mating patterns in remnant tree populations are expected to shift towards increased inbreeding and reduced pollen diversity, with fitness consequences for future generations. However, mating patterns and phenotypic assessments of open-pollinated progeny have rarely been combined in a single study. Here, we collected seeds from 37 Eucalyptus incrassata trees from contrasting stand densities following recent clearance in a single South Australian population (intact woodland = 12.6 trees ha(-1); isolated pasture = 1.7 trees ha(-1); population area = 10 km(2)). 649 progeny from these trees were genotyped at eight microsatellite loci. We estimated genetic diversity, spatial genetic structure, indirect contemporary pollen flow and mating patterns for adults older than the clearance events and open-pollinated progeny sired post-clearance. A proxy of early stage progeny viability was assessed in a common garden experiment. Density had no impact on mating patterns, adult and progeny genetic diversity or progeny growth, but was associated with increased mean pollen dispersal. Weak spatial genetic structure among adults suggests high historical gene flow. We observed preliminary evidence for inbreeding depression related to stress caused by fungal infection, but which was not associated with density. Higher observed heterozygosities in adults compared with progeny may relate to weak selection on progeny and lifetime-accumulated mortality of inbred adults. E. incrassata appears to be resistant to the negative mating pattern and fitness changes expected within fragmented landscapes. This pattern is likely explained by strong outcrossing and regular long-distance pollen flow. | 370 | no | inbreeding | NA | no | no |
| 371 | 2017 | 43006 | Cheng, BB; Zheng, YQ; Sun, QW | 2015 | Genetic diversity and population structure of Taxus cuspidata in the Changbai Mountains assessed by chloroplast DNA sequences and microsatellite markers | Taxus cuspidata, a tree species with high economic value because of its anticancer properties, is experiencing severe reduction in populations across its range in China. We examined one chloroplast DNA (cpDNA) region (petA-psbE) and 9 nuclear simple sequence repeats (SSRs) loci variations among seven populations in the Changbai Mountains of China to investigate the levels of genetic diversity and population structure. A moderate level of haplotype diversity (H-T = 0.625), low nuclear microsatellite diversity (H-E = 0.261 +/- 0.028), significant genetic differences (F-ST = 0.065) and substantial gene flow (Nm = 2.806) were observed. Most of the total genetic variation was partitioned within the population (87.8% and 94.0% for cpDNA and SSRs, respectively). Our haplotype identification permutation tests revealed that G(ST) > N-ST, indicating an absence of phylogeographic structure in T. cuspidata. Neither STRUCTURE nor UPGMA analyses showed any geographic pattern in T. cuspidata populations. By comparatively analyzing the genetic diversity and survival situation of T. cuspidata, our results provide a theoretical foundation for the resource protection, utilization cultivation and breeding of this valuable plant. (C) 2015 Elsevier Ltd. All rights reserved. | 371 | no | no good fitness measure | NA | no | no |
| 372 | 2017 | 43006 | Brunet, J; Zalapa, J; Guries, R | 2016 | Conservation of genetic diversity in slippery elm (Ulmus rubra) in Wisconsin despite the devastating impact of Dutch elm disease | Forest pest epidemics are responsible for many population declines reported in forest trees. While forest tree populations tend to be genetically diverse, in principle mortality resulting from disease could diminish that genetic diversity and alter the genetic structure of the remnant populations with consequences for the ability of a species to adapt to changing environments. Slippery elm (Ulmus rubra Muhl.) is a long-lived, wind-pollinated forest tree with a native range covering essentially all of eastern North America. Dutch elm disease (DED) caused by an introduced fungal pathogen (Ophiostoma ulmi) devastated North American elm populations, including slippery elm, beginning in the 1930s. Estimates of the numbers of elms lost to DED are unknown but range into the hundreds of millions of trees given their former abundance. In this study, the genotypes of 77 herbarium specimens collected between 1890 and 2004 in Wisconsin, and of 100 slippery elm trees from five wild Wisconsin populations, were characterized using 13 microsatellite loci. Levels of genetic diversity were compared between the herbarium specimens collected pre- and post-DED spread in Wisconsin. In addition, the levels of genetic diversity and degree of genetic differentiation were quantified in the five wild populations. The allelic diversity and expected levels of heterozygosity were similar between the pre- and post-DED herbarium specimens. The five wild populations were only slightly differentiated and no genetic bottleneck was detected for any population. At least in Wisconsin, slippery elm apparently has maintained levels of genetic diversity that could facilitate adaptation to future climatic and environmental changes. | 372 | no | no good fitness measure | NA | no | no |
| 373 | 2017 | 43006 | Mathias, RA; Taub, MA; Gignoux, CR; Fu, WQ; Musharoff, S; O’Connor, TD; Vergara, C; Torgerson, DG; Pino-Yanes, M; Shringarpure, SS; Huang, LL; Rafaels, N; Boorgula, MP; Johnston, HR; Ortega, VE; Levin, AM; Song, W; Torres, R; Padhukasahasram, B; Eng, C; Mejia-Mejia, DA; Ferguson, T; Qin, ZHS; Scott, AF; Yazdanbakhsh, M; Wilson, JG; Marrugo, J; Lange, LA; Kumar, R; Avila, PC; Williams, LK; Watson, H; Ware, LB; Olopade, C; Olopade, O; Oliveira, R; Ober, C; Nicolae, DL; Meyers, D; Mayorga, A; Knight-Madden, J; Hartert, T; Hansel, NN; Foreman, MG; Ford, JG; Faruque, MU; Dunston, GM; Caraballo, L; Burchard, EG; Bleecker, E; Araujo, MI; Herrera-Paz, EF; Gietzen, K; Grus, WE; Bamshad, M; Bustamante, CD; Kenny, EE; Hernandez, RD; Beaty, TH; Ruczinski, I; Akey, J; Barnes, KC | 2016 | A continuum of admixture in the Western Hemisphere revealed by the African Diaspora genome | The African Diaspora in the Western Hemisphere represents one of the largest forced migrations in history and had a profound impact on genetic diversity in modern populations. To date, the fine-scale population structure of descendants of the African Diaspora remains largely uncharacterized. Here we present genetic variation from deeply sequenced genomes of 642 individuals from North and South American, Caribbean and West African populations, substantially increasing the lexicon of human genomic variation and suggesting much variation remains to be discovered in African-admixed populations in the Americas. We summarize genetic variation in these populations, quantifying the post-colonial sex-biased European gene flow across multiple regions. Moreover, we refine estimates on the burden of deleterious variants carried across populations and how this varies with African ancestry. Our data are an important resource for empowering disease mapping studies in African-admixed individuals and will facilitate gene discovery for diseases disproportionately affecting individuals of African ancestry. | 373 | no | NA | NA | no | no |
| 374 | 2017 | 43006 | Marshall, HD; Langille, BL; Hann, CA; Whitney, HG | 2016 | Patterns of MHC-DRB1 polymorphism in a post-glacial island canid, the Newfoundland red fox (Vulpes vulpes deletrix), suggest balancing selection at species and population timescales | As the only native insular Newfoundland canid between the extinction of the wolf in the 1930s and the recent arrival of coyotes, the red fox (Vulpes vulpes deletrix Bangs 1898) poses interesting questions about genetic distinctiveness and the post-glacial colonization history of the island’s depauperate mammalian fauna. Here, we characterized genetic variability at the major histocompatibility complex (MHC) class II DR beta 1 domain (DRB1) locus in 28 red foxes from six sampling localities island-wide and compared it with mitochondrial control region (CR) diversity and DRB1 diversity in other canids. Our goals were to describe novel DRB1 alleles in a new canid population and to make inferences about the role of selection in maintaining their diversity. As in numerous studies of vertebrates, we found an order-of-magnitude higher nucleotide diversity at the DRB1 locus compared with the CR and significantly positive nonsynonymous-to-synonymous substitution ratios, indicative of selection in the distant past. Although the evidence is weaker, the Ewens-Watterson test of neutrality and the geographical distribution of variation compared with the CR suggest a role for selection over the evolutionary timescale of populations. We report the first genetic data from the DRB1 locus in the red fox and establish baseline information regarding immunogenetic variation in this island canid population which should inform continued investigations of population demography, adaptive genetic diversity, and wildlife disease in red foxes and related species. | 374 | no | no good fitness measure, N pop differnt in GD? | NA | no | no |
| 375 | 2017 | 43006 | Glass, WR; Walter, RP; Heath, DD; Mandrak, NE; Corkum, LD | 2015 | Genetic structure and diversity of spotted gar (Lepisosteus oculatus) at its northern range edge: implications for conservation | Understanding the spatial context of genetic variation for species at risk is important for effective management and long-term survival of the species. We use multilocus microsatellite data to investigate the population genetic structure of the spotted gar (Lepisosteus oculatus) across its northern range edge in Canada. We then compare these northern individuals with samples taken from the southern core of the species range. For the northern samples, significant genetic differentiation among groups of individuals forming two major genetically distinct populations, and as many as 7-9 smaller subpopulations, was recovered using hierarchical Bayesian assignment methods and non-equilibrial discriminant function analyses. Spatial genetic variation is present, particularly at higher hierarchical groupings; however, some population admixture at sites is evident and is indicative of dispersal and gene flow among some locations or shared ancestry. Gene flow estimates among populations and subpopulations is very low, ranging from essentially complete isolation to as high as 5 %-suggesting that mechanisms in addition to geographic isolation are operating to create genetic structure. In Lake Erie, the physical isolation of Point Pelee appears to confer distinct genetic differentiation for those populations and provide a source of genetic variation for Lake Erie proper when breaches to the barrier beach occur. Results indicate that the northern edge populations are distinct from southern populations and should be conserved to maintain the overall genetic diversity of this species. Additionally, the asymmetrical genetic connectivity among the Point Pelee and Rondeau Bay sites highlights the sensitivity of Point Pelee to environmental perturbation and habitat degradation. | 375 | no | no good fitness measure, N pop differnt in GD? | NA | no | no |
| 376 | 2017 | 43006 | Morozova, EV; Ryskov, AP; Semyenova, SK | 2002 | RAPD variation in two trematode species (Fasciola hepatica and Dicrocoelium dendriticum) from a single cattle population | The method of random DNA amplification by PCR with arbitrary primers (RAPD-PCR) was used for the description and estimation of genetic variation in two trematode species, Fasciola hepatica (n = 21) and Dicrocoelium dendriticum (n = 8). The studied trematodes were liver parasites of five cattle individuals belonging to the same herd. To study the F. hepatica population, five primers were selected, which revealed 230 RAPD markers in five samples of parasites isolated from five different host individuals. Using 87 RAPD markers, a comparison of variation was conducted between F. hepatica and D. dendriticum samples from the same host individual. Based on the estimates of RAPD variation for the individual samples of parasites collected from each of five host individuals and for the total F. hepatica population, standard indices of genetic similarity (S), diversity (H), polymorphism (P), and population subdivision (F(S)T) were calculated. From the indices of similarity in pairs (S), dendrograms were constructed, which reflect genetic relationship between the representatives of two species and between F. hepatica individuals isolated from the same or different host individuals. It was revealed that polymorphism level (P) varied within a range of 35.5 to 83.2% in the studied F. hepatica population and reached 95.1% in the studied D. dendriticum population. Two different trematode species that simultaneously parasitize the same host animal were characterized by similar estimates of polymorphism and genetic diversity and by similar topology of genetic similarity dendrograms. The degree of genetic similarity between F. hepatica and D. dendriticum was significantly lower (20%) than between five F. hepatica samples (41.4%) that formed two unequal clusters. Each of these clusters represents a heterogeneous group consisting of parasites collected from three or four host individuals. In the individual samples of parasites related to each of the studied host individuals, the indices of genetic similarity (S) and diversity (H) varied within a range of 43.3 to 64.8% and 25.1 to 56.6%, respectively. In the total F. hepatica sample, the estimates of intraspecific variation, the topology of dendrograms, and the F(S)T index (7.4%) indicate the absence of clear genetic differentiation between the samples of parasites isolated from different host individuals. Possible reasons for the high level of genetic variation in the studied trematode populations and the genetic consequences of host-parasite interaction are discussed. | 376 | no | gd of parasite | NA | no | no |
| 377 | 2017 | 43006 | Singh, P; Singh, M; Mastana, SS | 2002 | Genetics of apolipoprotein H (beta(2)-glycoprotein I) polymorphism in India | Background: Human apolipoprotein H (beta(2)-glycoprotein I, apoH, protein; APOH, gene) is a single-chain glycoprotein that has been implicated in several metabolic pathways, including lipid metabolism, coagulation and production of antiphospholipid antibodies and many disease phenotypes. The structural, molecular and genetic bases of APOH have been studied in detail but population studies, especially from the Indian subcontinent, are limited. Objective: This study seeks to enlarge our understanding of APOH genetic diversity in human populations from different regions and social groups of India. Also, we examine the level and extent of genetic variation at this locus in world populations and its utility as a population genetic marker. Subjects and methods: Blood samples from 1381 unrelated and randomly selected individuals were screened for APOH genetic polymorphism. Eleven populations from North India (Brahmins, Banias, Jat Sikhs, Khatris, Scheduled Castes, Lobanas and Rajputs), West India (Brahmin and Patels) and Central India (Brahmins and Baiga tribe) were studied for APOH polymorphism using isoelectric focusing. Allele frequencies were calculated by the gene counting method. The results were statistically evaluated using chi-square statistics for regional and ethnic variation. Genetic distances were computed on Indian populations to determine the population affinities. Correspondence analysis was used to assess ethnic variation in world populations. Results: An interesting and wide genetic variation at this locus was observed in Indian populations. The frequency distribution of three observed alleles ranged from 0.034 to 0.091 for APOH\* 1, 0.852 to 0.917 for APOH\* 2 and 0.027 to 0.075 for APOH*3. The world’s highest APOH* 2 allele frequency was observed in the Patel (0.917) caste group from West India. Conclusions: Overall, the observed variation at this locus in Indian populations is comparable to many Caucasian populations. An analysis of world populations showed that APOH is a useful genetic marker for population and anthropological studies. | 377 | no | human | NA | no | no |
| 378 | 2017 | 43006 | Luijten, SH; Dierick, A; Oostermeijer, JGB; Raijmann, LEL; Den Nijs, HCM | 2000 | Population size, genetic variation, and reproductive success in a rapidly declining, self-incompatible perennial (Arnica montana) in The Netherlands | Arnica montana is a rare and rapidly declining, self-incompatible plant species. In 26 populations in The Netherlands we investigated the relationship between population size and genetic variation using allozyme markers. Genetic variation was low in A. montana (H-e = 0.088). There were positive correlations between population size and the proportion of polymorphic loci, the number of effective alleles, and expected heterozygosity, but not with observed heterozygosity. There was a significantly positive correlation between population size and the inbreeding coefficient. Generally, small populations showed heterozygote excess, which decreased with increasing population size. Possibly, the heterozygous individuals in small populations are survivors from the formerly larger populations with relatively high fitness. The F statistics showed a moderately high level of differentiation among populations (F-ST = 0.140 +/- 0.02), implying a low level of gene flow. For three out of four allozyme loci, we found significant inbreeding (F-IS = 0.104 +/- 0.03). Only 14 of 26 populations were in Hardy-Weinberg equilibrium at all four polymorphic loci. In a subset of 14 populations of various size, rue investigated natural seed production and offspring fitness. Population size was positively coil-elated with seed set, seedling size, number of flowering stems and flowerheads, adult survival and total relative fitness, but not with the number of florets per flowerhead, germination rate, or the proportion of germination. Offspring performance in the greenhouse was not associated with genetic diversity measured on their mothers in the field We conclude that the fitness of small population is significantly reduced, but that there is as yet no evidence that this was caused by inbreeding Possibly, the self-incompatibility system of A montana has been effective in reducing selfing rates and inbreeding depression. | 378 | no | individual level | NA | no | no |
| 379 | 2017 | 43006 | Peng, Y; Zhang, XQ; Deng, YL; Ma, X | 2008 | Evaluation of genetic diversity in wild orchardgrass (Dactylis glomerata L.) based on AFLP markers | Orchardgrass (Dactylis glomerata L.) is a highly variable perennial forage grass, widely cultivated in all temperate and subtropical growing regions of the world. Despite its economic importance, there is a lack of information on the genetic diversity within and among populations. In order to assess DNA molecular genetic variations and formulate appropriate strategies for conservation and utilization of wild orchardgrass genetic resources available in China, mainly amplified fragment length polymorphism (AFLP) DNA profiling method was used to detect the genetic diversity and relatedness among 32 wild orchardgrass accessions collected in China and two in the USA. Using nine primer combinations, 400 DNA fragments were amplified, among which 322 (80.50%) were polymorphic. Genetic similarity coefficients (GSC) for 34 accessions ranged from 0.69 to 0.93, with an average of 0.81. Further examination of the different components of genetic variation by analysis of molecular variance (AMOVA) indicated that larger proportions of variability existed within ploidy levels (87.98%) and geographical regions (85.65%). Cluster analysis using the UPGMA approach seperated the studied accessions into six major clusters. On the basis of principal coordinate analyses (PCoA) on the genetic characteristics, the studied accessions could be grouped into three main clusters. All tetraploid (2n=4x=28) accessions originating from different regions were grouped into the same cluster whereas the diploid (2n=2x=14) accessions were grouped into other two clusters associated with their geographical distributions. The results revealed by AFLP markers were concordant with the morphological variability, agronomic traits and karyotype. The results also showed that comprehensive germplasm collection in major geographic regions and exploitation of the existing variation are required to widen the genetic base and sample the full extent of the available variation in breeding strategies for orchardgrass. | 379 | no | no good fitness measure | NA | no | no |
| 380 | 2017 | 43006 | Ben Slimen, H; Gedeon, CI; Hoffmann, IE; Suchentrunk, F | 2012 | Dwindling genetic diversity in European ground squirrels? | “The European ground squirrel (Spermophilus citellus) is endangered and in decline. Populations are increasingly fragmented, and only a coordinated conservation effort at the European level may guarantee its long-term survival. To obtain a general population genetic picture on a larger geographic scale, we screened 117 individuals from seven local populations in Hungary, Romania, and Austria for allelic variation at eleven microsatellite loci. We found a high (23.4%) proportion of private alleles, and a moderate to somewhat elevated level (15.27%) of partitioning of genetic diversity among populations, compared to that found in many other terrestrial mammals. Genetic variability was significantly higher than in earlier studied Czech populations that are considered genetically depleted, but significantly lower than in undisturbed populations of S. suslicus and S. brunneus, that are similar to the European ground squirrel in their ecological requirements, reproductive biology, and social organization. Genetic diversity was also lower than in most presumably”“undisturbed”" populations of other Sciurid species. This, together with the observed level and pattern of genetic differentiation among populations, such as no significant increase of genetic differentiation with geographic distance and similar variance of genetic differentiation between populations independent of geographic distance, indicated the prevalence of relatively strong drift effects for all populations. A Bayesian STRUCTURE analysis and a factorial correspondence analysis concordantly revealed a fairly complex genetic composition of local populations, but no major geographic trend in the pattern of the genetic composition. Overall, the results suggest disintegration of local colonies that might earlier have been more connected genetically. The STRUCTURE analysis also suggested anthropogenic translocations among single Hungarian populations. Our data on genetic diversity and its distribution do not object to such conservation measures. Translocation of individuals particularly from nearby populations may increase the chances of survival of small and isolated populations and counteract inbreeding at low densities. (C) 2011 Deutsche Gesellschaft fur Saugetierkunde. Published by Elsevier GmbH. All rights reserved." | 380 | no | no good fitness measure, N pop differnt in GD? | NA | no | no |
| 381 | 2017 | 43006 | Agrimonti, C; Bianchi, R; Bianchi, A; Ballero, M; Poli, F; Marmiroli, N | 2007 | Understanding biological conservation strategies: a molecular-genetic approach to the case of myrtle (Myrtus communis L.) in two Italian regions: Sardinia and Calabria | Myrtle (Myrtus communis L.), a shrub widespread in the Mediterranean area, is the only species belonging to the Myrtaceae family growing in Europe. The pharmacological and aromatic properties of myrtle have caused a growing interest in this plant. The use of myrtle as an aromatic plant is traditionally established in the Italian regions of Sardinia and Calabria, where it is subjected to an ecological stress due to the large biomass removal for liquor production and environmental damage. The reduction in size and number of wild populations is often related to a loss of genetic variation and reproductive potential. In this study fluorescent amplified fragment length polymorphisms (fAFLPs) were employed to assess the genetic variation within and among natural populations of myrtle from Sardinia and Calabria to gain new insights into their fitness and survival potential. AMOVA analysis indicated that genetic variation was greater within populations (51.86%) than among populations (16.99%), as previously reported for outcrossing species. A significant amount of variation (31.15%) was attributed to variation between Sardinia and Calabria population groups, suggesting a genotypic differentiation between the myrtle populations of these two regions. Intra-population genetic variation, assessed by estimating expected unbiased heterozygosity (H (E)), ranged from 0.0595 to 0.2595. These values resulted correlated with population extension (r = 0.918; P < 0.01) and with two reproductive parameters: seed germinability (r = 0.793; P < 0.01) and number of seeds per fruit (r = 0.631; P < 0.05). A moderate gene flow among Sardinia myrtle populations and Calabria populations (1.2719 and 1.0478, respectively) counteracts the low level of genetic variation observed in some populations and avoids their differentiation and isolation. | 381 | no | no good fitness measure | NA | no | no |
| 382 | 2017 | 43006 | Claw, KG; Tito, RY; Stone, AC; Verrelli, BC | 2010 | Haplotype Structure and Divergence at Human and Chimpanzee Serotonin Transporter and Receptor Genes: Implications for Behavioral Disorder Association Analyses | Genetic variation in the human serotonin system has long-been studied because of its functional consequences and links to various behavior-related disorders and it being routinely targeted in research and development for drug therapy. However, aside from clinical studies, little is known about this genetic diversity and how it differs within and between human populations with respect to haplotype structure, which can greatly impact phenotype association studies. In addition, no evolutionary approach among humans and other primates has examined how long- and short-term selective pressures explain existing serotonin variation. Here, we examine DNA sequence variation in natural population samples of 192 human and 40 chimpanzee chromosome sequences for the most commonly implicated similar to 38-kb serotonin transporter (SLC6A4) and similar to 63-kb serotonin 2A receptor (HTR2A) genes. Our comparative population genetic analyses find significant linkage disequilibrium associated with functionally relevant variants in humans, as well as geographic variation for these haplotypes, at both loci. In addition, although amino acid divergence is consistent with purifying selection, promoter and untranslated regions exhibit significantly high divergence in both species lineages. These evolutionary analyses imply that the serotonin system may have accumulated significant regulatory variation over both recent and ancient periods of time in both humans and chimpanzees. We discuss the implications of this variation for disease association studies and for the evolution of behavior-related phenotypes during the divergence of humans and our closest primate relatives. | 382 | no | human | NA | no | no |
| 383 | 2017 | 43006 | Solorzano, CD; Szalanski, AL; Owens, CB; Steelman, CD | 2010 | Genetic Diversity of Aedes vexans (Diptera, Culicidae) from New Orleans: Pre- and Post-Katrina | The floodwater mosquito Aedes vexans, a potential vector of West Nile virus, has a worldwide distribution that includes the continental United States and southern Canada. In order to determine the effect that Hurricane Katrina had on the temporal genetic variation of Ae. vexans from New Orleans, we compared genetic diversity of a portion of the mtDNA ND5 gene of mosquito specimens collected during 2005 (n = 99) with specimens from 2006 (n = 103), after the hurricane. Average haplotype diversity (Hd) was high (> 0.88) in 2005 and 2006 for both the parishes studied. It does not appear that Hurricane Katrina had any impact on genetic diversity, and despite the intense efforts to control mosquitoes in New Orleans, Ae. vexans has not undergone a population bottleneck. A bottleneck effect may be lacking because this species breeds outside the city and the adults migrate into the city. | 383 | no | NA | NA | no | no |
| 384 | 2017 | 43006 | Avitia, M; Escalante, AE; Rebollar, EA; Moreno-Letelier, A; Eguiarte, LE; Souza, V | 2014 | Population expansions shared among coexisting bacterial lineages are revealed by genetic evidence | Comparative population studies can help elucidate the influence of historical events upon current patterns of biodiversity among taxa that coexist in a given geographic area. In particular, comparative assessments derived from population genetics and coalescent theory have been used to investigate population dynamics of bacterial pathogens in order to understand disease epidemics. In contrast, and despite the ecological relevance of non-host associated and naturally occurring bacteria, there is little understanding of the processes determining their diversity. Here we analyzed the patterns of genetic diversity in coexisting populations of three genera of bacteria (Bacillus, Exiguobacterium, and Pseudomonas) that are abundant in the aquatic systems of the Cuatro Cienegas Basin, Mexico. We tested the hypothesis that a common habitat leaves a signature upon the genetic variation present in bacterial populations, independent of phylogenetic relationships. We used multilocus markers to assess genetic diversity and (1) performed comparative phylogenetic analyses, (2) described the genetic structure of bacterial populations, (3) calculated descriptive parameters of genetic diversity, (4) performed neutrality tests, and (5) conducted coalescent-based historical reconstructions. Our results show a trend of synchronic expansions across most populations independent of both lineage and sampling site. Thus, we provide empirical evidence supporting the analysis of coexisting bacterial lineages in natural environments to advance our understanding of bacterial evolution beyond medical or health-related microbes. | 384 | no | no good fitness measure, N pop differnt in GD? | NA | no | no |
| 385 | 2017 | 43006 | Michaelides, S; Cole, N; Funk, SM | 2015 | Translocation retains genetic diversity of a threatened endemic reptile in Mauritius | The island of Mauritius has experienced five reptile extinctions since the 1600s. Approximately half of the remaining herpetofauna has been restricted to offshore islets, resulting in small populations at high risk of extinction. Under the combined pressures of invasive species, habitat loss and fragmentation and climate change, translocations are considered a powerful tool in conservation of threatened and endangered species. The Bojer’s skink, Gongylomorphus bojerii, on the offshore island on Ilot Vacoas represents the remnant population of the species in the southeast of Mauritius. Given the geographic isolation and its genetic distinctiveness, individuals were translocated to the neighbouring island of Ile aux Fouquets in order to re-establish historical range, minimize extinction risk and maintain genetic variation within the species. Using fifteen microsatellite loci, we assessed the genetic structure of the population on Ilot Vacoas in relation to a northern offshore population (on Round Island) and evaluated the genetic consequences of the translocation. Results revealed that the population on Ilot Vacoas exhibits significantly lower levels of genetic variation and strong differentiation (F (ST) = 0.16) compared to the northern population. The inbreeding coefficient was low and no recent bottleneck event was detected from its genetic signature. The translocation on Ile aux Fouquets did not provide evidence of negative genetic effects. The newly established population retained much of the source’s genetic material, though the effective population size was found to be relatively small. These findings confirmed the importance of incorporating genetic management and continuous monitoring to detect changes in the long-term survival of translocated populations. | 385 | no | no good fitness measure, N pop differnt in GD? | NA | no | no |
| 386 | 2017 | 43006 | Rouger, R; Reichel, K; Malrieu, F; Masson, JP; Stoeckel, S | 2016 | Effects of complex life cycles on genetic diversity: cyclical parthenogenesis | Neutral patterns of population genetic diversity in species with complex life cycles are difficult to anticipate. Cyclical parthenogenesis (CP), in which organisms undergo several rounds of clonal reproduction followed by a sexual event, is one such life cycle. Many species, including crop pests (aphids), human parasites (trematodes) or models used in evolutionary science (Daphnia), are cyclical parthenogens. It is therefore crucial to understand the impact of such a life cycle on neutral genetic diversity. In this paper, we describe distributions of genetic diversity under conditions of CP with various clonal phase lengths. Using a Markov chain model of CP for a single locus and individual-based simulations for two loci, our analysis first demonstrates that strong departures from full sexuality are observed after only a few generations of clonality. The convergence towards predictions made under conditions of full clonality during the clonal phase depends on the balance between mutations and genetic drift. Second, the sexual event of CP usually resets the genetic diversity at a single locus towards predictions made under full sexuality. However, this single recombination event is insufficient to reshuffle gametic phases towards full-sexuality predictions. Finally, for similar levels of clonality, CP and acyclic partial clonality (wherein a fixed proportion of individuals are clonally produced within each generation) differentially affect the distribution of genetic diversity. Overall, this work provides solid predictions of neutral genetic diversity that may serve as a null model in detecting the action of common evolutionary or demographic processes in cyclical parthenogens (for example, selection or bottlenecks). | 386 | no | no good fitness measure, N pop differnt in GD? | NA | no | no |
| 387 | 2017 | 43006 | Barnaud, A; Houliston, GJ | 2010 | Population genetics of the threatened tree daisy Olearia gardneri (Asteraceae), conservation of a critically endangered species | “All known populations of the nationally critical tree daisy, Olearia gardneri, were studied using AFLP markers. With fewer than 160 individual plants, Olearia gardneri is the third-rarest tree in New Zealand and a good model with which to study evolutionary process in fragmented endangered plants. Genetic variation was at similar levels to other long-lived tree species in New Zealand and also as in other studies to date had poor correspondence between genetic and geographic distance. Genetic factors such as inbreeding depression and the loss of genetic diversity might lower fitness and have substantial consequences for evolution and survival of rare threatened plants. Due to the decline of this species in recent times it is imperative that conservation measures are undertaken, including revegetation. Despite considerable emphasis on”“eco-sourcing”" in plant recovery programmes there is strong evidence that this may not be the best strategy for O. gardneri due to breeding system and population size considerations." | 387 | no | inbreeding | NA | no | no |
| 388 | 2017 | 43006 | Rodriguez-Rodriguez, MD; Lopera-Barrero, NM; dos Santos, SCA; Vargas, L; Streit, DP; Ribeiro, RP | 2013 | GENETIC DIVERSITY OF Piaractus mesopotamicus (Characiformes: Characidae) BROODSTOCKS USING IN THE RESTOCKING PROGRAM OF TIETE RIVER, BRAZIL | The restocking programs are being used more frequently as methods of fish conservation. However, the reduction in the genetic diversity can affect survival of juveniles used in this programs and cause effects on the wild populations. The aim of this study was assess the genetic diversity in three broodstocks (BA, BB and BC) of pacu Piaractus mesopotamicus of a Hydropower plant in Sao Paulo - Brazil, using in restocking program of Tiete River. Nine RAPD primers were amplified used extracted DNA from 89 fin-clipping samples. Sixty-nine fragments were polymorphic, 15 had frequencies with significant differences (P<0.05), seven were excluded, and six were fixed fragments. High values for polymorphic fragments (47.83% to 71.01%) and Shannon index (0.270 to 0.424) were observed. Most of the genetic variation was found within the groups through the AMOVA analysis, which was confirmed by the results of the identity and genetic distance. Ancestry levels (F-ST) among the groups values indicated little and moderate genetic differentiation. The estimate of number of migrants by generation (Nm) indicated levels of gene flow. Moderate genetic divergence between groups (0.214 to 0.259) was observed. The results indicate high (BA and BB) and moderate (BC) variability within broodstocks and genetic differentiation among them. The fish stocks analyzed represent a genetic base that will allow the fish technicians to release juveniles without genetic risks to wild populations present in the river. These genetics procedures can be used as models for other migratory species. | 388 | no | no good fitness measure, N pop differnt in GD? | NA | no | no |
| 389 | 2017 | 43006 | Sunny, A; Monroy-Vilchis, O; Zarco-Gonzalez, MM; Mendoza-Martinez, GD; Martinez-Gomez, D | 2015 | Genetic diversity and genetic structure of an endemic Mexican Dusky Rattlesnake (Crotalus triseriatus) in a highly modified agricultural landscape: implications for conservation | It is necessary to determine genetic diversity of fragmented populations in highly modified landscapes to understand how populations respond to land-use change. This information will help guide future conservation and management strategies. We conducted a population genetic study on an endemic Mexican Dusky Rattlesnake (Crotalus triseriatus) in a highly modified landscape near the Toluca metropolitan area, in order to provide crucial information for the conservation of this species. There was medium levels of genetic diversity, with a few alleles and genotypes. We identified three genetically differentiated clusters, likely as a result of different habitat cover type. We also found evidence of an ancestral genetic bottleneck and medium values of effective population size. Inbreeding coefficients were low and there was a moderate gene flow. Our results can be used as a basis for future research and C. triseriatus conservation efforts, particularly considering that the Trans-Mexican Volcanic Belt is heavily impacted by destructive land-use practices. | 389 | no | no good fitness measure, N pop differnt in GD? | NA | no | no |
| 390 | 2017 | 43006 | Aylor, DL; Valdar, W; Foulds-Mathes, W; Buus, RJ; Verdugo, RA; Baric, RS; Ferris, MT; Frelinger, JA; Heise, M; Frieman, MB; Gralinski, LE; Bell, TA; Didion, JD; Hua, KJ; Nehrenberg, DL; Powell, CL; Steigerwalt, J; Xie, YY; Kelada, SNP; Collins, FS; Yang, IV; Schwartz, DA; Branstetter, LA; Chesler, EJ; Miller, DR; Spence, J; Liu, EY; McMillan, L; Sarkar, A; Wang, J; Wang, W; Zhang, Q; Broman, KW; Korstanje, R; Durrant, C; Mott, R; Iraqi, FA; Pomp, D; Threadgill, D; de Villena, FPM; Churchill, GA | 2011 | Genetic analysis of complex traits in the emerging Collaborative Cross | The Collaborative Cross (CC) is a mouse recombinant inbred strain panel that is being developed as a resource for mammalian systems genetics. Here we describe an experiment that uses partially inbred CC lines to evaluate the genetic properties and utility of this emerging resource. Genome-wide analysis of the incipient strains reveals high genetic diversity, balanced allele frequencies, and dense, evenly distributed recombination sites-all ideal qualities for a systems genetics resource. We map discrete, complex, and biomolecular traits and contrast two quantitative trait locus (QTL) mapping approaches. Analysis based on inferred haplotypes improves power, reduces false discovery, and provides information to identify and prioritize candidate genes that is unique to multifounder crosses like the CC. The number of expression QTLs discovered here exceeds all previous efforts at eQTL mapping in mice, and we map local eQTL at 1-Mb resolution. We demonstrate that the genetic diversity of the CC, which derives from random mixing of eight founder strains, results in high phenotypic diversity and enhances our ability to map causative loci underlying complex disease-related traits. | 390 | no | NA | NA | no | no |
| 391 | 2017 | 43006 | Luis, C; Cothran, EG; Oom, MDM | 2007 | Inbreeding and genetic structure in the endangered sorraia horse breed: Implications for its conservation and management | The Sorraia horse is a closed breed with reduced effective population size and considered in critical maintained risk status. The breed exists in 2 main breeding populations, one in Portugal and one in Germany, with a smaller population size. A set of 22 microsatellite loci was used to examine generic diversity and structure of the Sorraia horse breed and to compare individual inbreeding coefficient F, estimated from pedigree data, with individual heterozygosity and mean. The Sorraia horse shows lower levels of microsatellite diversity when compared with other horse breeds. Due to management strategies, there are clear differences in the genetic structure of the 2 main Sorraia horse populations. Individual heterozygosity was shown to be a good estimator, used together with or as an alternative to inbreeding coefficient, in predicting fitness and evaluating the inbreeding level of the Sorraia horse. The information gathered in this study, combined with information available from previous studies, offers an important and wide information base for the future development of an effective breeding management of the Sorraia horse in order to preserve this endangered breed. | 391 | no | domesticated animal | NA | no | no |
| 392 | 2017 | 43006 | Erfmeier, A; Hantsch, L; Bruelheide, H | 2013 | The Role of Propagule Pressure, Genetic Diversity and Microsite Availability for Senecio vernalis Invasion | Genetic diversity is supposed to support the colonization success of expanding species, in particular in situations where microsite availability is constrained. Addressing the role of genetic diversity in plant invasion experimentally requires its manipulation independent of propagule pressure. To assess the relative importance of these components for the invasion of Senecio vernalis, we created propagule mixtures of four levels of genotype diversity by combining seeds across remote populations, across proximate populations, within single populations and within seed families. In a first container experiment with constant Festuca rupicola density as matrix, genotype diversity was crossed with three levels of seed density. In a second experiment, we tested for effects of establishment limitation and genotype diversity by manipulating Festuca densities. Increasing genetic diversity had no effects on abundance and biomass of S. vernalis but positively affected the proportion of large individuals to small individuals. Mi+E394xtures composed from proximate populations had a significantly higher proportion of large individuals than mixtures composed from within seed families only. High propagule pressure increased emergence and establishment of S. vernalis but had no effect on individual growth performance. Establishment was favoured in containers with Festuca, but performance of surviving seedlings was higher in open soil treatments. For S. vernalis invasion, we found a shift in driving factors from density dependence to effects of genetic diversity across life stages. While initial abundance was mostly linked to the amount of seed input, genetic diversity, in contrast, affected later stages of colonization probably via sampling effects and seemed to contribute to filtering the genotypes that finally grew up. In consequence, when disentangling the mechanistic relationships of genetic diversity, seed density and microsite limitation in colonization of invasive plants, a clear differentiation between initial emergence and subsequent survival to juvenile and adult stages is required. | 392 | no | no good fitness measure | NA | no | no |
| 393 | 2017 | 43006 | Pennings, PS; Achenbach, A; Foitzik, S | 2011 | Similar evolutionary potentials in an obligate ant parasite and its two host species | The spatial structure of host-parasite coevolution is shaped by population structure and genetic diversity of the interacting species. We analysed these population genetic parameters in three related ant species: the parasitic slavemaking ant Protomognathus americanus and its two host species Temnothorax longispinosus and T. curvispinosus. We sampled throughout their range, genotyped ants on six to eight microsatellite loci and an MtDNA sequence and found high levels of genetic variation and strong population structure in all three species. Interestingly, the most abundant species and primary host, T. longispinosus, is characterized by less structure, but lower local genetic diversity. Generally, differences between the species were small, and we conclude that they have similar evolutionary potentials. The coevolutionary interaction between this social parasite and its hosts may therefore be less influenced by divergent evolutionary potentials, but rather by varying selection pressures. We employed different methods to quantify and compare genetic diversity and structure between species and genetic markers. We found that Jost D is well suited for these comparisons, as long as mutation rates between markers and species are similar. If this is not the case, for example, when using MtDNA and microsatellites to study sex-specific dispersal, model-based inference should be used instead of descriptive statistics (such as D or G(ST)). Using coalescent-based methods, we indeed found that males disperse much more than females, but this sex bias in dispersal differed between species. The findings of the different approaches with regard to genetic diversity and structure were in good accordance with each other. | 393 | no | no good fitness measure, N pop differnt in GD? | NA | no | no |
| 394 | 2017 | 43006 | Sun, XY; Kang, S; Zhang, YJ; Tan, XQ; Yu, YF; He, HY; Zhang, XY; Liu, YF; Wang, S; Sun, WX; Cai, L; Li, SJ | 2013 | Genetic Diversity and Population Structure of Rice Pathogen Ustilaginoidea virens in China | Rice false smut caused by the fungal pathogen Ustilaginoidea virens is becoming a destructive disease throughout major rice-growing countries. Information about its genetic diversity and population structure is essential for rice breeding and efficient control of the disease. This study compared the genome sequences of two U. virens isolates. Three SNP-rich genomic regions were identified as molecular markers that could be used to analyze the genetic diversity and population structure of U. virens in China. A total of 56 multilocus sequence types (haplotypes) were identified out of 162 representative isolates from 15 provinces covering five major rice-growing areas in China. However, the phylogeny, based on sequences at individual SNP-rich regions, strongly conflicted with each other and there were significant genetic differences between different geographical populations. Gene flow between the different geographical populations and genetic differentiation within each geographical population were also detected. In addition, genetic recombination and genetic isolation resulting from geographic separation was also found. | 394 | no | gd of pathogen | NA | no | no |
| 395 | 2017 | 43006 | Cheung, PPH; Watson, SJ; Choy, KT; Sia, SF; Wong, DDY; Poon, LLM; Kellam, P; Guan, Y; Peiris, JSM; Yen, HL | 2014 | Generation and characterization of influenza A viruses with altered polymerase fidelity | Genetic diversity of influenza A viruses (IAV) acquired through the error-prone RNA-dependent RNA polymerase (RdRP) or through genetic reassortment enables perpetuation of IAV in humans through epidemics or pandemics. Here, to assess the biological significance of genetic diversity acquired through RdRP, we characterize an IAV fidelity variant derived from passaging a seasonal H3N2 virus in the presence of ribavirin, a purine analogue that increases guanosine-to-adenosine mutations. We demonstrate that a single PB1-V43I mutation increases selectivity to guanosine in A/Wuhan/359/95 (H3N2) and A/Vietnam/1203/04 (H5N1) viruses. The H5N1 PB1-V43I-recombinant virus replicates to comparable titres as the wild-type virus in vitro or in the mouse lungs. However, a decrease in viral population diversity at day 3 post inoculation is associated with a tenfold reduced lethality and neurotropism in mice. Applying a fidelity variant with reduced mutational frequency, we provide direct experimental evidence for the role of genetic diversity in IAV pathogenesis. | 395 | no | gd of pathogen | NA | no | no |
| 396 | 2017 | 43006 | Gillet, F; Garrido, MTC; Blanc, F; Fournier-Chambrillon, C; Nemoz, M; Sourp, E; Vial-Novella, C; Zardoya, R; Aulagnier, S; Michaux, JR | 2017 | Evidence of fine-scale genetic structure for the endangered Pyrenean desman (Galemys pyrenaicus) in the French Pyrenees | The Pyrenean desman (Galemys pyrenaicus) is a small, semiaquatic mammal endemic to the Pyrenean Mountains and the northern half of the Iberian Peninsula where it lives in cold and well-oxygenated flowing mountain streams. This species is currently classified as vulnerable on the IUCN Red List and has been undergoing habitat loss and fragmentation for decades, inevitably impacting its distribution. A recent genetic study, based on mitochondrial and intronic sequences, showed that the genetic variability of the Pyrenean desman is very low in the Pyrenees. In this study, we investigated the potential existence of genetic structure and gene flow at a smaller scale using 24 polymorphic microsatellite loci. As the Pyrenean desman is a very elusive species, we supplemented our tissue sample collection with samples of feces collected in the French range of this species. We successfully identified 70 individuals based on 355 fecal samples. Bayesian analyses revealed 3 genetic and geographic clusters (1 eastern, 1 central, and 1 western, including 3 genetic subclusters), with origins tracing back only 200 years. These clusters were characterized by low levels of genetic diversity and high inbreeding coefficients. Although gene flow among clusters appeared to be limited, populations seem to have exchanged alleles recently. Therefore, connectivity between watersheds should be enhanced to maintain genetic diversity and potentially improve the long-term survival of the Pyrenean desman in France. | 396 | no | no pop level | NA | no | no |
| 397 | 2017 | 43006 | Smith, AL; Bull, CM; Gardner, MG; Driscoll, DA | 2014 | Life history influences how fire affects genetic diversity in two lizard species | Fire mosaics’ are often maintained in landscapes to promote successional diversity in vegetation with little understanding of how this will affect ecological processes in animal populations such as dispersal, social organization and re-establishment. To investigate these processes, we conducted a replicated, spatiotemporal landscape genetics study of two Australian woodland lizard species [Amphibolurus norrisi (Agamidae) and Ctenotus atlas (Scincidae)]. Agamids have a more complex social and territory structure than skinks, so fire might have a greater impact on their population structure and thus genetic diversity. Genetic diversity increased with time since fire in C.atlas and decreased with time since fire in A.norrisi. For C.atlas, this might reflect its increasing population size after fire, but we could not detect increased gene flow that would reduce the loss of genetic diversity through genetic drift. Using landscape resistance analyses, we found no evidence that postfire habitat succession or topography affected gene flow in either species and we were unable to distinguish between survival and immigration as modes of postfire re-establishment. In A.norrisi, we detected female-biased dispersal, likely reflecting its territorial social structure and polygynous mating system. The increased genetic diversity in A.norrisi in recently burnt habitat might reflect a temporary disruption of its territoriality and increased male dispersal, a hypothesis that was supported with a simulation experiment. Our results suggest that the effects of disturbance on genetic diversity will be stronger for species with territorial social organization. | 397 | no | no good fitness measure, N pop differnt in GD? | NA | no | no |
| 398 | 2017 | 43006 | Queney, G; Ferrand, N; Marchandeau, S; Azevedo, M; Mougel, F; Branco, M; Monnerot, M | 2000 | Absence of a genetic bottleneck in a wild rabbit (Oryctolagus cuniculus) population exposed to a severe viral epizootic | Infectious diseases and their demographic consequences are thought to influence the genetic diversity of populations. In Europe, during the last 50 years, the European rabbit (Oryctolagus cuniculus) has suffered two important viral epizootics: myxomatosis and rabbit viral haemorraghic disease (RVHD). Although mortality rates were very high, the impact of these diseases on genetic diversity has never been assessed directly. The subject of this paper is a wild rabbit population in France, which has been studied since the beginning of the 1980s. The first outbreak of RVHD occurred in 1995 and provoked a demographic crash. The population, sampled for the first time in 1982 and 1994, was sampled again at the end of 1996 to examine the impact of the epizootic on genetic diversity. In spite of the observed high mortality rate (approximate to 90%), analysis of 14 polymorphic loci (allozymes and microsatellites) showed no loss in genetic diversity after the epizootic. Determination of temporal changes in allele frequencies indicated that the population evolved under genetic drift. The temporal method of Waples demonstrated a significant decrease in the effective population size (N-e) correlated with the demographic crash due to the epizootic. However, the population had only been studied for two generations after the epizootic and the remnant population size probably stayed high enough (approximate to 50 individuals) to keep its genetic diversity at the precrash level. These results suggest that, contrary to what is usually thought and in spite of the subsequent high mortality rates, past epizootics (especially myxomatosis) may have had little effect on the genetic diversity of wild rabbit populations in Europe. | 398 | no | only one pop | NA | no | no |
| 399 | 2017 | 43006 | Calboli, FCF; Sampson, J; Fretwell, N; Balding, DJ | 2008 | Population structure and inbreeding from pedigree analysis of purebred dogs | Dogs are of increasing interest as models for human diseases, and many canine population-association studies are beginning to emerge. The choice of breeds for such studies should be informed by a knowledge of factors such as inbreeding, genetic diversity, and population structure, which are likely to depend on breed specific selective breeding patterns. To address the lack of such studies we have exploited one of the world’s most extensive resources for canine population-genetics studies: the United Kingdom (UK) Kennel Club registration database. We chose 10 representative breeds and analyzed their pedigrees since electronic records were established around 1970, corresponding to about eight generations before present. We find extremely inbred dogs in each breed except the greyhound and estimate an inbreeding effective population size between 40 and 80 for all but 2 breeds. For all but 3 breeds, >90% of unique genetic variants are lost over six generations, indicating a dramatic effect of breeding patterns on genetic diversity. We introduce a novel index T for measuring population structure directly from the pedigree and use it to identify subpopulations in several breeds. As well as informing the design of canine population genetics studies, our results have implications for breeding practices to enhance canine welfare. | 399 | no | domesticated animal | NA | no | no |
| 400 | 2017 | 43006 | KURDYLA, TM; GUTHRIE, PAI; MCDONALD, BA; APPEL, DN | 1995 | RFLPS IN MITOCHONDRIAL AND NUCLEAR-DNA INDICATE LOW-LEVELS OF GENETIC DIVERSITY IN THE OAK WILT PATHOGEN CERATOCYSTIS-FAGACEARUM | Genetic diversity in the oak wilt pathogen Ceratocystis fagacearum was assessed using restriction fragment length polymorphisms (RFLPs) of the mitochondrial DNA (mtDNA) and anonymous RFLP loci in the nuclear DNA (nuDNA). No genetic variation was detected in the mtDNA among 27 isolates sampled from a broad geographical area, Southern hybridization to 100 anonymous, random, nuDNA probes detected a low level of variation among nine of the isolates. Only 35 out of 437 probe-enzyme combinations detected RFLPs. Most of the RFLPs appeared to result from insertions and deletions of less than 200 bp. A composite multilocus haplotype based on hybridization to six anonymous probes could differentiate each of the nine isolates tested, suggesting that these probes may be useful for further studies of the population biology and epidemiology of this pathogen. Hypotheses are presented to account for the low level of genetic variation. | 400 | no | gd of pathogen | NA | no | no |
| 401 | 2017 | 43007 | Daleszczyk, K; Bunevich, AN | 2009 | Population viability analysis of European bison populations in Polish and Belarusian parts of Bialowieza Forest with and without gene exchange | European bison (Bison bonasus) became extinct in the wild at the beginning of the 20th century. The contemporary Lowland line of bison was founded by seven individuals that survived in captivity. The largest population of Lowland bison live in the Bialowieza Forest, but the forest and the population are divided by a border fence between Poland and Belarus. This fence is a barrier to the movement of ungulates. In this study, we used population genetic models to predict the future viability of the Polish and Belarusian bison populations. We determined the founder contribution, founder equivalent, mean inbreeding coefficient, mean kinship, and the proportion of genetic diversity retained in the Belarusian bison population. Although the founding group of the Belarusian population was larger than that of the Polish population, the latter had more favourable genetic parameters. We assessed inbreeding depression for fecundity in free-ranging European bison compared to captive individuals. Using population viability analysis (PVA) we modelled both bison populations with or without gene exchange, and with or without incorporating the kinship of the founders. When founder kinship was included, in both populations the mean number of alleles and the gene diversity retained within extant populations decreased substantially compared to PVA models in which founders were not related. The worst genetic parameters were obtained for the Belarusian population under a scenario in which the founders are related and gene exchange is lacking, which is the closest to the real situation. Creation of passages for animals by partial removal of the border fence would have a favourable effect on the genetic variation and viability of both bison populations, especially the Belarusian. (C) 2009 Elsevier Ltd. All rights reserved. | 401 | no | no good fitness measure, N pop differnt in GD? | NA | no | no |
| 402 | 2017 | 43007 | Born, C; Kjellberg, F; Chevallier, MH; Vignes, H; Dikangadissi, JT; Sanguie, J; Wickings, EJ; Hossaert-McKey, M | 2008 | Colonization processes and the maintenance of genetic diversity: insights from a pioneer rainforest tree, Aucoumea klaineana | Despite recurrent episodes of range expansion and contraction, forest trees often harbour high genetic diversity. Studies of temperate forest trees suggest that prolonged juvenile phase and high pollen flow are the main factors limiting founder effects. Here, we studied the local colonization process of a pioneer rainforest tree in central Africa, Aucoumea klaineana. We identified 87% of parents among trees up to 20-25 years old and could thus compare direct parentage structure data with classical population genetics estimators. In this species, genetic diversity was maintained during colonization. The absence of founder effects was explained by (i) local random mating and (ii) local recruitment, as we showed that 75% of the trees in the close neighbourhood participated in the recruitment of new saplings. Long-distance pollen flow contributed little to genetic diversity: pollen and seed dispersal was mainly within stand (128 and 118 m, respectively). Spatial genetic structure was explained by aggregated seed dispersal rather than by mother-offspring proximity as assumed in classical isolation-by-distance models. Hence, A. klaineana presents a genetic diversity pattern typical of forest trees but does not follow the classical rules by which this diversity is generally achieved. We suggest that while high local genetic variability is of general importance to forest tree survival, the proximate mechanisms by which it is achieved may follow very different scenarios. | 402 | no | no good fitness measure, N pop differnt in GD? | NA | no | no |
| 403 | 2017 | 43007 | Toth, EG; Vendramin, GG; Bagnoli, F; Cseke, K; Hohn, M | 2017 | High genetic diversity and distinct origin of recently fragmented Scots pine (Pinus sylvestris L.) populations along the Carpathians and the Pannonian Basin | Historical evolutionary events highly affect the modern-day genetic structure of natural populations. Scots pine (Pinus sylvestris L.), as a dominant tree species of the Eurasian taiga communities following the glacial cycles of the Pleistocene, has survived in small, scattered populations at the range limits of its south-eastern European distribution. In this study, we examined genetic relationships, genetic divergence and demographic history of peripheral populations from central-eastern Europe, the Carpathian Mountains and the Pannonian Basin. Four hundred twenty-one individuals from 20 populations were sampled and characterized with both nuclear and chloroplast simple sequence repeat (SSR) markers. Standard population genetic indices, the degree of genetic differentiation and spatial genetic structure were analysed. Our results revealed that peripheral Scots pine populations retained high genetic diversity despite the recently ongoing fragmentation and isolation of the persisting relict populations. Analysis of molecular variance (AMOVA) showed 7% among-population genetic differentiation, and there was no isolation by distance among the island-like occurrences. Genetic discontinuities with strong barriers (99-100% bootstrap support) were identified in the Carpathians. Based on both marker types, populations of the Western Carpathians were delimited from those inhabiting the Eastern Carpathians, and two main genetic lineages were traced that most probably originate from two main refugia. One refugium presumably existed in the region of the Eastern Alps with the Hungarian Plain, while the other was probably found in the Eastern Carpathians. These findings are supported by recent palynological records. The strongest genetic structure was revealed within the Romanian Carpathians on the basis of both marker types. With only some exceptions, no signs of recent bottlenecks or inbreeding were detected. However, Carpathian natural populations of Scots pine are highly fragmented and have a small census size, though they have not yet been affected by genetic erosion induced by isolation. | 403 | no | no good fitness measure, N pop differnt in GD? | NA | no | no |
| 404 | 2017 | 43007 | Peakall, R; Ebert, D; Scott, LJ; Meagher, PF; Offord, CA | 2003 | Comparative genetic study confirms exceptionally low genetic variation in the ancient and endangered relictual conifer, Wollemia nobilis (Araucariaceae) | The Wollemi pine, Wollemia nobilis (Araucariaceae), was discovered in 1994 as the only extant member of the genus, previously known only from the fossil record. With fewer than 100 trees known from an inaccessible canyon in southeastern Australia, it is one of the most endangered tree species in the world. We conducted a comparative population genetic survey at allozyme, amplified fragment length polymorphism (AFLP) and simple sequence repeat (SSR) loci in W. nobilis, Araucaria cunninghatnii and Agathis robusta - representatives of the two sister genera. No polymorphism was detected at 13 allozyme loci, more than 800 AFLP loci or the 20 SSR loci screened in W. nobilis. In Ag. robusta only one of 12 allozyme loci, five of 800 AFLP loci and none of the 15 SSR loci were variable. For A. cunninghamii, 10 of > 800 AFLP loci and five of 20 SSR loci were variable. Thus low genetic diversity characterizes all three species. While not ruling out the existence of genetic variation, we conclude that genetic diversity is exceptionally low in the Wollemi pine. To our knowledge this is the most extreme case known in plants. We conclude that the combination of small population effects, clonality and below-average genetic variation in the family are probable contributing factors to the low diversity. The exceptionally low genetic diversity of the Wollemi pine, combined with its known susceptibility to exotic fungal pathogens, reinforces current management policies of strict control of access to the pines and secrecy of the pine locations. | 404 | no | no pop level | NA | no | no |
| 405 | 2017 | 43007 | Ramirez, O; Altet, L; Ensenat, C; Vila, C; Sanchez, A; Ruiz, A | 2006 | Genetic assessment of the Iberian wolf Canis lupus signatus captive breeding program | The main goal of ex situ conservation programs is to improve the chances of long term survival of natural populations by founding and managing captive colonies that can serve as a source of individuals for future reintroductions or to reinforce existing populations. The degree in which a captive breeding program has captured the genetic diversity existing in the source wild population has seldom been evaluated. In this study we evaluate the genetic diversity in wild and captive populations of the Iberian wolf, Canis lupus signatus, in order to assess how much genetic diversity is being preserved in the ongoing ex situ conservation program for this subspecies. A sample of domestic dogs was also included in the analysis for comparison. Seventy-four wolves and 135 dogs were genotyped at 13 unlinked microsatellite loci. The results show that genetic diversity in Iberian wolves is comparable in magnitude to that of other wild populations of gray wolf. Both the wild and the captive Iberian wolf populations have a similarly high genetic diversity indicating that no substantial loss of diversity has occurred in the captive-breeding program. The effective number of founders of the program was estimated as similar to 16, suggesting that all founders in the studbook pedigree were genetically independent. Our results emphasize also the genetic divergence between wolves and domestic dogs and indicate that our set of 13 microsatellite loci provide a powerful diagnostic test to distinguish wolves, dogs and their hybrids. | 405 | no | no good fitness measure, N pop differnt in GD? | NA | no | no |
| 406 | 2017 | 43007 | Ruf, D; Mazzi, D; Dorn, S | 2010 | No kin discrimination in female mate choice of a parasitoid with complementary sex determination | Discrimination against kin as mates, via genetic or environmentally derived cues of relatedness, can prevent inbreeding and thus enhance individual fitness and promote population survival. Sex in the parasitoid wasp Cotesia glomerata L. (Hymenoptera: Braconidae) is determined by one locus with multiple alleles, a mechanism termed single-locus complementary sex determination (sl-CSD). Under sl-CSD, haploid individuals are males, whereas diploid individuals are females when heterozygous at the sex determination locus but males when homozygous. In species with sl-CSD, inbreeding leads to increased incidence of matings between individuals sharing an allele at the sex locus and thus to increased diploid male production. Diploid males cause an undesirable sex ratio distortion and can be of inferior fitness. To evade these deleterious effects, species with sl-CSD are expected to avoid inbreeding. We investigated whether C. glomerata females discriminate against close kin as mating partners. We performed a mate choice experiment, which allowed us to distinguish between kin discrimination based on the perception of phenotype-related cues and kin discrimination based on the perception of cues associated with the developmental environment. As kin discrimination is often mediated through cuticular hydrocarbons (CHCs), we additionally examined composition of the CHC profiles of males. We found no evidence for discrimination against related or familiar males nor for differences in the CHC profiles of males. These results indicate that kin discrimination is not a relevant inbreeding avoidance strategy in C. glomerata. | 406 | no | mate choice | NA | no | no |
| 407 | 2017 | 43007 | Goss, EM; Kreitman, M; Bergelson, J | 2005 | Genetic diversity, recombination and cryptic clades in Pseudomonas viridiflava infecting natural populations of Arabidopsis thaliana | Species-level genetic diversity and recombination in bacterial pathogens of wild plant populations have been nearly unexplored. Pseudomonas viridiflava is a common natural bacterial pathogen of Arabidopsis thaliana, for which pathogen defense genes and mechanisms are becoming increasing well known. The genetic variation contained within a worldwide sample of P. viridiflava collected from wild populations of A. thaliana was investigated using five genomic sequence fragments totaling 2.3 kb. Two distinct and deeply diverged clades were found within the P. viridiflava sample and in close proximity in multiple populations, each genetically diverse with synonymous variation as high as 9.3% in one of these clades. Within clades, there is evidence of frequent recombination within and between each sequenced locus and little geographic differentiation. Isolates from both clades were also found in a small sample of other herbaceous species in Midwest populations, indicating a possibly broad host range for P. viridiflava. The high levels of genetic variation and recombination together with a lack of geographic differentiation in this pathogen distinguish it from other bacterial plant pathogens for which intraspecific variation has been examined. | 407 | no | gd of parasite | NA | no | no |
| 408 | 2017 | 43007 | Deacon, NJ; Cavender-Bares, J | 2015 | Limited Pollen Dispersal Contributes to Population Genetic Structure but Not Local Adaptation in Quercus oleoides Forests of Costa Rica | Background 0pc?>Quercus oleoides Cham. and Schlect., tropical live oak, is a species of conservation importance in its southern range limit of northwestern Costa Rica. It occurs in high-density stands across a fragmented landscape spanning a contrasting elevation and precipitation gradient. We examined genetic diversity and spatial genetic structure in this geographically isolated and genetically distinct population. We characterized population genetic diversity at 11 nuclear microsatellite loci in 260 individuals from 13 sites. We monitored flowering time at 10 sites, and characterized the local environment in order to compare observed spatial genetic structure to hypotheses of isolation-by-distance and isolation-by-environment. Finally, we quantified pollen dispersal distances and tested for local adaptation through a reciprocal transplant experiment in order to experimentally address these hypotheses. Results High genetic diversity ismaintained in the population and the genetic variation is significantly structured among sampled sites. We identified 5 distinct genetic clusters and average pollen dispersal predominately occurred over short distances. Differences among sites in flowering phenology and environmental factors, however, were not strictly associated with genetic differentiation. Growth and survival of upland and lowland progeny in their native and foreign environments was expected to exhibit evidence of local adaptation due to the more extreme dry season in the lowlands. Seedlings planted in the lowland garden experienced much higher mortality than seedlings in the upland garden, but we did not identify evidence for local adaptation. Conclusion Overall, this study indicates that the Costa Rican Q. oleoides population has a rich population genetic history. Despite environmental heterogeneity and habitat fragmentation, isolation-by-distance and isolation-by-environment alone do not explain spatial genetic structure. These results add to studies of genetic structure by examining a common, tropical tree over multiple habitats and provide information for managers of a successional forest in a protected area. | 408 | no | no good fitness measure, N pop differnt in GD? | NA | no | no |
| 409 | 2017 | 43007 | Ramstad, KM; Colbourne, RM; Robertson, HA; Allendorf, FW; Daugherty, CH | 2013 | Genetic consequences of a century of protection: serial founder events and survival of the little spotted kiwi (Apteryx owenii) | We present the outcome of a century of post-bottleneck isolation of a long-lived species, the little spotted kiwi (Apteryx owenii, LSK) and demonstrate that profound genetic consequences can result from protecting few individuals in isolation. LSK were saved from extinction by translocation of five birds from South Island, New Zealand to Kapiti Island 100 years ago. The Kapiti population now numbers some 1200 birds and provides founders for new populations. We used 15 microsatellite loci to compare genetic variation among Kapiti LSK and the populations of Red Mercury, Tiritiri Matangi and Long Islands that were founded with birds from Kapiti. Two LSK native to D’Urville Island were also placed on Long Island. We found extremely low genetic variation and signatures of acute and recent genetic bottleneck effects in all four populations, indicating that LSK have survived multiple genetic bottlenecks. The Long Island population appears to have arisen from a single mating pair from Kapiti, suggesting there is no genetic contribution from D’Urville birds among extant LSK. The N-e/N-C ratio of Kapiti Island LSK (0.03) is exceptionally low for terrestrial vertebrates and suggests that genetic diversity might still be eroding in this population, despite its large census size. | 409 | no | no good fitness measure | NA | no | no |
| 410 | 2017 | 43007 | Waddington, SJ; Santorelli, LA; Ryan, FR; Hughes, WOH | 2010 | Genetic polyethism in leaf-cutting ants | Females mating with multiple males (polyandry) is taxonomically widespread but is hard to explain in many animals in which it has no obvious direct benefits. In some, of which social insects are the best example, it is suggested that females benefit from the increased genetic diversity of their offspring. Social insect colonies express division of labor, and if genotypes behave differently (genetic polyethism), then genetically diverse colonies may be fitter. However, unequivocal evidence of genetic polyethism is only known from honey bees. Here, we show that such a genetic influence on behavior is also present in the leaf-cutting ant Acromyrmex echinatior. In 2 of the 3 colonies examined, we found that the offspring of some fathers (patrilines) were more likely to engage in waste management, whereas other patrilines were more likely to engage in foraging. When we increased the stimulus for waste management, the representation of patrilines in the ants that responded was the same as normally engaged in waste management in 2 colonies but differed in the third colony. The leaf-cutting ant A. echinatior therefore shows genetic polyethism. Although other factors such as disease resistance may have also pla’yed a role in the evolution of polyandry, the results suggest that genetic polyethism may be widespread in social insects and potentially of general importance in the evolution of polyandry. | 410 | no | no good fitness measure | NA | no | no |
| 411 | 2017 | 43007 | Angelone, S | 2010 | Are differences in fitness traits related to genetic clusters? An empirical test on the European tree frog | Microsatellites are one of the preferred marker types to estimate the level of genetic diversity and subdivision in natural Populations of endangered species. Many studies thereby attempted to identify genetically defined management units for conservation by applying a variety of genetic clustering methods. It is not clear, however, whether genetic clusters inferred from neutral molecular markers reflect differences in fitness or adaptation. In this study, I conducted a common garden experiment on the endangered European tree frog (Hyla arborea) to clarify whether fitness-related traits of larval development differed between three genetic groups defined by Bayesian clustering analysis. I reared larvae under semi-natural conditions and measured growth and developmental rates as well as survival at five larval stages from eclosion to completion of metamorphosis. Nested general and generalized linear models showed significant cluster differences for several variables in terms of smaller growth rates and body sizes at early larval stages. These differences were, however, probably not linked to adaptive divergence among clusters but rather to neutral genetic processes in the breeding sites of one cluster, which were spatially isolated and subject of recent bottlenecks. Hence, a genetic load effect (e.g. inbreeding depression) may have acted on the populations of this particular cluster. I advise studies aiming to define management units for conservation to not only use genetic clustering methods but to complement their findings with experimental approaches on fitness-related traits. (C) 2009 Elsevier Ltd. All rights reserved. | 411 | no | No GD diff between pop | NA | no | no |
| 412 | 2017 | 43007 | Dybdahl, MF; Lively, CM | 1996 | The geography of coevolution: Comparative population structures for a snail and its trematode parasite | Gene flow and the genetic structure of host and parasite populations are critical to the coevolutionary process, including the conditions under which antagonistic coevolution favors sexual reproduction. Here we compare the genetic structures of different populations of a freshwater New Zealand snail (Potamopyrgus antipodarum) with its trematode parasite (Microphallus sp.) using allozyme frequency data. Allozyme variation among snail populations was found to be highly structured among lakes; but for the parasite there was little allozyme structure among Lake populations, suggesting much higher levels of parasite gene flow. The overall pattern of variation was confirmed with principal component analysis, which also showed that the organization of genetic differentiation for the snail (but not the parasite) was strongly related to the geographic arrangement of lakes. Some snail populations from different sides of the Alps near mountain passes were more similar to each other than to other snail populations on the same side of the Alps. Furthermore, genetic distances among parasite populations were correlated with the genetic distances among host populations, and genetic distances among both host and parasite populations were correlated with ‘’stepping-stone’’ distances among lakes. Hence, the host snail and its trematode parasite seem to be dispersing to adjacent lakes in a stepping-stone fashion, although parasite dispersal among lakes is clearly greater. High parasite gene Bow should help to continuously reintroduce genetic diversity within local populations where strong selection might otherwise isolate ‘’host races.’’ Parasite gene Bow can thereby facilitate the coevolutionary (Red Queen) dynamics that confer an advantage to sexual reproduction by restoring lost genetic variation. | 412 | maybe | any GD and fitness of groups that works? | no good fitness measure, gd of parasite not infection rates etc | no | no |
| 413 | 2017 | 43007 | Markert, JA; Grant, PR; Grant, BR; Keller, LF; Coombs, JL; Petren, K | 2004 | Neutral locus heterozygosity, inbreeding, and survival in Darwin’s ground finches (Geospiza fortis and G-scandens) | Comprehensive long-term studies of isolated populations provide valuable comparative data that may be used to evaluate different methods for quantifying the relationship between genetic diversity and fitness. Here, we report on data collected from large and well-characterized cohorts of the two numerically dominant species of Darwin’s finches on Isla Daphne Major, Galapagos, Ecuador - Geospiza fortis and G. scandens. Multilocus microsatellite (SSR) genetic diversity estimates (heterozygosity and (d) over bar (2)) and pedigree-based estimates of the inbreeding coefficient (f) were compared to each other and to two fitness components: lifespan and recruitment. In the larger sample of G. fortis, heterozygosity (H) was correlated with both fitness components, but no relationship was detected in the smaller sample of G. scandens. Analyses of the inbreeding coefficient detected highly significant relationships between f and recruitment, but no relationship between f and overall lifespan. The (d) over bar (2) statistic showed no relationship to either fitness component. When the two SSR-based estimators were compared to f, (d) over bar (2) was correlated with f in G. fortis in the predicted direction, while in G. scandens the relationship was positive. Multilocus heterozygosity was correlated with f in G. fortis but not in the G. scandens sample. A pedigree simulation demonstrated that the variation in true autozygosity can be large among individuals with the same level of inbreeding. This observation may supplement the interpretation of patterns relevant to the local (locus-specific) and general (genome-wide) effects hypotheses, which have been proposed to explain the mechanism responsible for associations between genetic diversity and fitness. | 413 | no | inbreeding | NA | no | no |
| 414 | 2017 | 43007 | Stronen, AV; Salmela, E; Baldursdottir, BK; Berg, P; Espelien, IS; Jarvi, K; Jensen, H; Kristensen, TN; Melis, C; Manenti, T; Lohi, H; Pertoldi, C | 2017 | Genetic rescue of an endangered domestic animal through outcrossing with closely related breeds: A case study of the Norwegian Lundehund | Genetic rescue, outcrossing with individuals from a related population, is used to augment genetic diversity in populations threatened by severe inbreeding and extinction. The endangered Norwegian Lundehund dog underwent at least two severe bottlenecks in the 1940s and 1960s that each left only five inbred dogs, and the approximately 1500 dogs remaining world-wide today appear to descend from only two individuals. The Lundehund has a high prevalence of a gastrointestinal disease, to which all remaining dogs may be predisposed. Outcrossing is currently performed with three Nordic Spitz breeds: Norwegian Buhund, Icelandic Sheepdog, and Norrbottenspets. Examination of single nucleotide polymorphism (SNP) genotypes based on 165K loci in 48 dogs from the four breeds revealed substantially lower genetic diversity for the Lundehund (H-E 0.035) than for other breeds (H-E 0.209-0.284). Analyses of genetic structure with > 15K linkage disequilibrium-pruned SNPs showed four distinct genetic clusters. Pairwise F-ST values between Lundehund and the candidate breeds were highest for Icelandic Sheepdog, followed by Buhund and Norrbottenspets. We assessed the presence of outlier loci among candidate breeds and examined flanking genome regions (1 megabase) for genes under possible selection to identify potential adaptive differences among breeds; outliers were observed in flanking regions of genes associated with key functions including the immune system, metabolism, cognition and physical development. We suggest crossbreeding with multiple breeds as the best strategy to increase genetic diversity for the Lundehund and to reduce the incidence of health problems. For this project, the three candidate breeds were first selected based on phenotypes and then subjected to genetic investigation. Because phenotypes are often paramount for domestic breed owners, such a strategy could provide a helpful approach for genetic rescue and restoration of other domestic populations at risk, by ensuring the involvement of owners, breeders and managers at the start of the project. | 414 | no | domesticated animal | NA | no | no |
| 415 | 2017 | 43007 | Chuluunbat, B; Charruau, P; Silbermayr, K; Khorloojav, T; Burger, PA | 2014 | Genetic diversity and population structure of Mongolian domestic Bactrian camels (Camelus bactrianus) | The tradition of animal husbandry in the context of a nomadic lifestyle has been of great significance in the Mongolian society. Both Bactrian camels and horses have been invaluable for the survival and development of human activities in the harsh arid environment of the Mongolian steppe. As camels offer unique and sustainable opportunities for livestock production in marginal agro-ecological zones, we investigated the current genetic diversity of three local Mongolian camel breeds and compared their levels of variation with common native Mongolian camels distributed throughout the country. Based on mitochondrial and nuclear markers, we found levels of genetic diversity in Mongolian populations similar to that reported for Chinese Bactrian camels and for dromedaries. Little differentiation was detected between single breeds, except for a small group originating from the northwestern Mongolian Altai. We found neither high inbreeding levels in the different breeds nor evidence for a population decline. Although the Mongolian camel census size has severely declined over the past 20 years, our analyses suggest that there still exists a stable population with adequate genetic variation for continued sustainable utilization. | 415 | no | domesticated animal | NA | no | no |
| 416 | 2017 | 43007 | Verdade, LM; Zucoloto, RB; Coutinho, LL | 2002 | Microgeographic variation in Caiman latirostris | In theory, geographic scale is related to genetic variation at the population level, whereas microgeographic scale may reveal intra-population structure such as social groups and families. In the present work, both levels of genetic variation in the broad-snouted caiman (Caiman latirostris) were evaluated in small wetlands associated with the Piracicaba River and some of its tributaries in the state of Sao Paulo, Brazil. Genetic variation was determined using microsatellite DNA markers originally developed for the American alligator (Alligator mississipiensis) and previously tested in pedigreed captive broad-snouted caimans. Using these markers, we were able to detect variability among individuals from different sites, even those within a small geographic distance. Genetic results suggest that the groups sampled at each site are composed predominantly of related individuals. A possible combination of high mortality and low natality rates results in a low number of successfully dispersed individuals per generation. Future studies using a recently constructed Caiman latirostris microsatellite library (Zucoloto et al., 2002) might help us to understand metapopulation processes that may be occurring within this species. (C) 2002 Wiley-Liss, Inc. | 416 | no | no good fitness measure, N pop differnt in GD? | NA | no | no |
| 417 | 2017 | 43007 | Schulte, RD; Makus, C; Schulenburg, H | 2013 | Host-parasite coevolution favours parasite genetic diversity and horizontal gene transfer | Host-parasite coevolution is predicted to favour genetic diversity and the underlying mechanisms (e.g. sexual reproduction and, more generally, genetic exchange), because diversity enhances the antagonists’ potential for rapid adaptation. To date, this prediction has mainly been tested and confirmed for the host. It should similarly apply to the parasite. Indeed, our previous work demonstrated that experimental coevolution between the nematode Caenorhabditis elegans and its microparasite Bacillus thuringiensis selects for genetic diversity in both antagonists. For the parasite, the previous analysis was based on plasmid-encoded toxin gene markers. Thus, it was restricted to a very small part of the bacterial genome and did not cover the main chromosome, which harbours a large variety of virulence factors. Here, we present new data for chromosomal gene markers of B. thuringiensis and combine this information with the previous results on plasmid-encoded toxins. Our new results demonstrate that, in comparison with the control treatment, coevolution with a host similarly leads to higher levels of genetic diversity in the bacterial chromosome, thus indicating the relevance of chromosomal genes for coevolution. Furthermore, the frequency of toxin gene gain is significantly elevated during coevolution, highlighting the importance of horizontal gene transfer as a diversity-generating mechanism. In conclusion, our study emphasizes the strong influence of antagonistic coevolution on parasite genetic diversity and gene exchange. | 417 | no | gd of parasite | NA | no | no |
| 418 | 2017 | 43007 | Tollefsrud, MM; Kissling, R; Gugerli, F; Johnsen, O; Skroppa, T; Cheddadi, R; van Der Knaap, WO; Latalowa, M; Terhurne-Berson, R; Litt, T; Geburek, T; Brochmann, C; Sperisen, C | 2008 | Genetic consequences of glacial survival and postglacial colonization in Norway spruce: combined analysis of mitochondrial DNA and fossil pollen | Norway spruce (Picea abies [L.] Karst.) is a broadly distributed European conifer tree whose history has been intensively studied by means of fossil records to infer the location of full-glacial refugia and the main routes of postglacial colonization. Here we use recently compiled fossil pollen data as a template to examine how past demographic events have influenced the species’ modern genetic diversity. Variation was assessed in the mitochondrial nad1 gene containing two minisatellite regions. Among the 369 populations (4876 trees) assayed, 28 mitochondrial variants were identified. The patterns of population subdivision superimposed on interpolated fossil pollen distributions indicate that survival in separate refugia and postglacial colonization has led to significant structuring of genetic variation in the southern range of the species. The populations in the northern range, on the other hand, showed a shallow genetic structure consistent with the fossil pollen data, suggesting that the vast northern range was colonized from a single refugium. Although the genetic diversity decreased away from the putative refugia, there were large differences between different colonization routes. In the Alps, the diversity decreased over short distances, probably as a result of population bottlenecks caused by the presence of competing tree species. In northern Europe, the diversity was maintained across large areas, corroborating fossil pollen data in suggesting that colonization took place at high population densities. The genetic diversity increased north of the Carpathians, probably as a result of admixture of expanding populations from two separate refugia. | 418 | no | no good fitness measure | NA | no | no |
| 419 | 2017 | 43007 | Meffert, LM; Mukana, N; Hicks, SK; Day, SB | 2005 | Testing alternative captive breeding strategies with the subsequent release into the wild | “We used the housefly (Musca domestica L.) as an experimental model to compare two strategies for the captive breeding of an endangered species: a strategy to minimize inbreeding and balance founder contributions (termed”“MAI”" for "“maximum avoidance of inbreeding”“) versus a scheme to select against less fit individuals (disregarding relatedness). By balancing the initial founder contributions, the MAI protocol was analogous to methods for minimizing kinship. in both breeding strategies, the population growth rate was limited to a maximum increase of 50% per generation. Five replicate populations, each starting with five male-female pairs, were subjected to five generations of captive breeding. Six generations of simulated”“release into the wild”" allowed ad lib breeding with less restrictive population growth potential, in either a benign or stressful environment (i.e., constant or variable temperature). Population size, fecundity, and fertility were assayed throughout the experiment, with juvenile-to-adult survival assayed in the second phase of the project. Allozyme assays determined the resultant inbreeding coefficients from the captive breeding schemes. The MAI breeding scheme resulted in significantly lower inbreeding coefficients and higher fitness, with qualitatively reduced extinction potential, most notable in the stressful environment. Spontaneous fitness rebounds suggested that the MAI strategy facilitated some form of purging of inbreeding depression effects. Importantly, the advantages of the MAI strategy were difficult to detect during the captive breeding phase, suggesting that the long-term advantages of the MAI approach could be underestimated in actual breeding programs. We concur with the common recommendation of maximum avoidance of inbreeding at least for systems with low reproductive potential." | 419 | no | no good fitness measure, N pop differnt in GD? | NA | no | no |
| 420 | 2017 | 43007 | Meagher, S | 1999 | Genetic diversity and Capillaria hepatica (Nematoda) prevalence in Michigan deer mouse populations | There have been few field tests of the hypothesis that homozygous populations are prone to high levels of disease. I tested for a negative correlation between genetic diversity and parasitism by estimating the allozyme heterozygosity, population density, and proportion of individuals infected by Capillaria hepatica (Nematoda) in nine Michigan populations of deer mice (Peromyscus maniculatus). Parasite prevalence was correlated negatively with heterozygosity when the effects of density were held constant, but was not correlated with population density after controlling for the effects of genetic diversity. These data support the prediction that inbred populations will be more susceptible to parasite infestations. | 420 | yes | from thesis | delete, not good gd | no | no |
| 421 | 2017 | 43007 | Saha, D; Rana, RS; Arya, L; Verma, M; Gowda, MVC; Upadhyaya, HD | 2017 | Genetic polymorphisms among and between blast disease resistant and susceptible finger millet, Eleusine coracana (L.) Gaertn | Fungal blast disease is one of the major constraints in finger millet production. Breeding for disease resistance in finger millet, needs characterization of genetic polymorphism among and between the resistant and susceptible genotypes. In total, 67 finger millet genotypes, which are resistant or susceptible to fungal blast disease, were analysed using sequence-related amplified polymorphism (SRAP) and simple sequence repeat (SSR) markers to assess genetic variations and select diverse parents. Twelve each of SRAP and SSR primers produced 95.1 and 93.1% polymorphic bands and grouped them into unweighted pair-group method with arithmetic average clusters. Two of the finger millet genotypes, IE 4709 (blast resistant) and INDAF 7 (susceptible) were distinguished as most diverse genotypes as parents. Several genotype-specific bands observed with SSR primers are potential in developing genotype-specific markers. A high genetic diversity within the resistant and susceptible genotypes, rather than between them, was revealed through Nei’s gene diversity (h) index and analysis of molecular variance. The finding helps us to understand the extent of genetic polymorphism between blast disease resistant and susceptible finger millet genotypes to exploit in resistance breeding programs. | 421 | no | no pop level | NA | no | no |
| 422 | 2017 | 43007 | Bruniche-Olsen, A; Jones, ME; Austin, JJ; Burridge, CP; Holland, BR | 2014 | Extensive population decline in the Tasmanian devil predates European settlement and devil facial tumour disease | The Tasmanian devil (Sarcophilus harrisii) was widespread in Australia during the Late Pleistocene but is now endemic to the island of Tasmania. Low genetic diversity combined with the spread of devil facial tumour disease have raised concerns for the species’ long-term survival. Here, we investigate the origin of low genetic diversity by inferring the species’ demographic history using temporal sampling with summary statistics, full-likelihood and approximate Bayesian computation methods. Our results show extensive population declines across Tasmania correlating with environmental changes around the last glacial maximum and following unstable climate related to increased ‘El Nino-Southern Oscillation’ activity. | 422 | no | no pop level | NA | no | no |
| 423 | 2017 | 43007 | Grubisha, LC; Cotty, PJ | 2009 | Twenty-four microsatellite markers for the aflatoxin-producing fungus Aspergillus flavus | Aspergillus flavus infects both plants and humans and contaminates diverse agricultural crops with aflatoxins, highly carcinogenic fungal metabolites. We describe 24 microsatellite markers developed to assess genetic diversity and recombination within and between three vegetative compatibility groups (VCGs) of Aspergillus flavus. These loci are polymorphic within at least one VCG or between VCGs. For loci polymorphic across all three VCGs, the number of alleles ranged from two to 19. These markers will be useful for genetic studies of this economically important pathogen. | 423 | no | gd of pathogen | NA | no | no |
| 424 | 2017 | 43007 | Xu, LJ; He, CF; Shen, C; Jiang, TL; Shi, LM; Sun, KP; Berquist, SW; Feng, JA | 2010 | Phylogeography and Population Genetic Structure of the Great Leaf-Nosed Bat (Hipposideros armiger) in China | The geographical patterns of the genetic structure of Hipposideros armiger in China were assessed by analyzing sequence variation in the mitochondrial DNA control region. Analysis of molecular variance revealed a very strong genetic structure among 5 regions in H. armiger. A neighbor-joining tree, haplotype network construction by TCS and multidimensional scaling plots all showed significant geographic differentiation among 5 regions. The high genetic structure detected in H. armiger could be a consequence of poor dispersal ability, local adaptation, or marked female philopatry. The lack of genetic structure among 3 regions separated by the Gaoligong Range and the Qiongzhou Strait could be due to incomplete lineage sorting. Our estimated times of divergence for H. armiger populations suggested a relatively recent split. The S Yunnan population with the highest genetic diversity and the Hainan population with the lowest genetic diversity should be equally given priority for conservation. Although H. armiger has been shown to carry viruses implicated in human disease, we find little evidence for population mixing. We thus suggest minimizing disturbance to bats’ roosting caves for minimizing the potential risk of virus transmission. | 424 | no | no good fitness measure | NA | no | no |
| 425 | 2017 | 43007 | Mattila, HR; Seeley, TD | 2007 | Genetic diversity in honey bee colonies enhances productivity and fitness | Honey bee queens mate with many males, creating numerous patrilines within colonies that are genetically distinct. The effects of genetic diversity on colony productivity and long-term fitness are unknown. We show that swarms from genetically diverse colonies (15 patrilines per colony) founded new colonies faster than swarms from genetically uniform colonies (1 patriline per colony). Accumulated differences in foraging rates, food storage, and population growth led to impressive boosts in the fitness (i.e., drone production and winter survival) of genetically diverse colonies. These results further our understanding of the origins of polyandry in honey bees and its benefits for colony performance. | 425 | yes | use winter survival as fitness | no data for analysis | no | no |
| 426 | 2017 | 43007 | BLOUIN, MS; DAME, JB; TARRANT, CA; COURTNEY, CH | 1992 | UNUSUAL POPULATION-GENETICS OF A PARASITIC NEMATODE - MTDNA VARIATION WITHIN AND AMONG POPULATIONS | Very little is known about the distribution of genetic variance within and among pop-ulations of parasitic helminths. In this study we used mitochondrial DNA (MtDNA) restriction fragment analysis to describe the Population genetic structure of Osteragia ostertagi, parasite of cattle, in the United States. Estimates of within-population mtDNA diversity are 5 to 10 times greater than typical estimates reported for species in other taxa. Although populations are genetically differentiated for a key life-history trait, greater than 98% of the total genetic diversity is partitioned within populations, and the geographic distribution of individual mtDNA haplotypes suggests high gene flow among populations. | 426 | no | gd of parasite | NA | no | no |
| 427 | 2017 | 43007 | Karaca, S; Cesuroglu, T; Karaca, M; Erge, S; Polimanti, R | 2015 | Genetic diversity of disease-associated loci in Turkish population | Many consortia and international projects have investigated the human genetic variation of a large number of ethno-geographic groups. However, populations with peculiar genetic features, such as the Turkish population, are still absent in publically available datasets. To explore the genetic predisposition to health-related traits of the Turkish population, we analyzed 34 genes associated with different health-related traits (for example, lipid metabolism, cardio-vascular diseases, hormone metabolism, cellular detoxification, aging and energy metabolism). We observed relevant differences between the Turkish population and populations with non-European ancestries (that is, Africa and East Asia) in some of the investigated genes (that is, AGT, APOE, CYP1B1, GNB3, IL10, IL6, LIPC and PON1). As most complex traits are highly polygenic, we developed polygenic scores associated with different health-related traits to explore the genetic diversity of the Turkish population with respect to other human groups. This approach showed significant differences between the Turkish population and populations with non-European ancestries, as well as between Turkish and Northern European individuals. This last finding is in agreement with the genetic structure of European and Middle East populations, and may also agree with epidemiological evidences about the health disparities of Turkish communities in Northern European countries. | 427 | no | human | NA | no | no |
| 428 | 2017 | 43007 | Vergeer, P; Rengelink, R; Copal, A; Ouborg, NJ | 2003 | The interacting effects of genetic variation, habitat quality and population size on performance of Succisa pratensis | 1 We studied the performance of 17 Dutch populations of the perennial Succisa pratensis , in relation to population size, genetic variation and habitat quality. We used a path-analytical model to analyse the possible relationships between these variables and performance. 2 Plants in smaller populations produced fewer seeds per flower head. Their seeds had lower germination rates and higher seedling mortality, and more seeds were dormant or non-viable. 3 Population size was also correlated with genetic measures. Small populations had higher inbreeding coefficients than large populations and observed heterozygosity was positively correlated with population size. The mean genetic diversity (expected heterozygosity) was relatively high (H(exp) = 0.42), but not correlated with population size. 4 Less eutrophic habitats appeared to support larger populations. High concentrations of NH(4) and NO(3) in the soil were significantly negatively correlated with population size. 5 Path-analysis showed that Succisa pratensis is vulnerable to habitat deterioration (eutrophication). Population size was strongly influenced by habitat quality. Reduced performance, however, was better explained by direct genetic effects and by habitat deterioration rather than by effects of population size per se . Both habitat quality and genetic effects are thus important for population persistence, even in the short term. The results suggest that there will be a continuing decline of the small populations, due to deteriorating habitat conditions, decreased genetic variation and a reduced reproductive capacity. | 428 | no | human | NA | no | no |
| 429 | 2017 | 43007 | Andersen, LW; Fog, K; Damgaard, C | 2004 | Habitat fragmentation causes bottlenecks and inbreeding in the European tree frog (Hyla arborea) | A genetic study of the European tree frog, Hyla arborea, in Denmark was undertaken to examine the population structure on mainland Jutland and the island of Lolland after a period of reduction in suitable habitat and population sizes. The two regions have experienced the same rate of habitat loss but fragmentation has been more severe on Lolland. Genetic variation based on 12 polymorphic DNA microsatellites was analysed in 494 tree frogs sampled from two ponds in Jutland and 10 ponds on Lolland. A significant overall deviation from Hardy-Weinberg expectations could be attributed to three ponds, all on Lolland. This was most probably caused by an inbreeding effect reducing fitness, which was supported by the observed significant negative correlation between larva survival and mean F-IS value and mean individual inbreeding coefficient. A significant reduction in genetic variation (bottleneck) was detected in most of the ponds on Lolland. Population-structure analysis suggested the existence of at least 11 genetically different populations, corresponding to most of the sampled population units. The results indicated that the populations were unique genetic units and could be used to illustrate the migration pattern between newly established ponds arisen either by natural colonization of tree frogs or by artificial introduction. A high degree of pond fidelity in the tree frogs was suggested. A severe fragmentation process reducing population size and fitness within some of the populations probably caused the significant reduction in genetic variation of tree frog populations on Lolland. | 429 | no | inbreeding | NA | no | no |
| 430 | 2017 | 43007 | Bauert, MR; Kalin, M; Baltisberger, M; Edwards, PJ | 1998 | No genetic variation detected within isolated relict populations of Saxifraga cernua in the Alps using RAPD markers | Genetic variation in seven relict populations of Saxifraga cernua from three regions of the Alps was investigated using RAPD (random amplified polymorphic DNA) markers. No variation, either within the populations or within the regions, could be demonstrated. Nevertheless, each alpine region was characterized by a unique RAPD phenotype. Absence of genetic variation in these relict populations is attributed to population bottlenecks and founder effects during or following the ice ages. Contrasting hypotheses about the history of these populations, either as survivors of the glacial period or as products of postglacial immigration, are discussed in the light of the data presented. | 430 | no | No GD diff between pop | NA | no | no |
| 431 | 2017 | 43007 | Lohr, JN; David, P; Haag, CR | 2014 | REDUCED LIFESPAN AND INCREASED AGEING DRIVEN BY GENETIC DRIFT IN SMALL POPULATIONS | Explaining the strong variation in lifespan among organisms remains a major challenge in evolutionary biology. Whereas previous work has concentrated mainly on differences in selection regimes and selection pressures, we hypothesize that differences in genetic drift may explain some of this variation. We develop a model to formalize this idea and show that the strong positive relationship between lifespan and genetic diversity predicted by this model indeed exists among populations of Daphnia magna, and that ageing is accelerated in small populations. Additional results suggest that this is due to increased drift in small populations rather than adaptation to environments favoring faster life histories. First, the correlation between genetic diversity and lifespan remains significant after statistical correction for potential environmental covariates. Second, no trade-offs are observed; rather, all investigated traits show clear signs of increased genetic load in the small populations. Third, hybrid vigor with respect to lifespan is observed in crosses between small but not between large populations. Together, these results suggest that the evolution of lifespan and ageing can be strongly affected by genetic drift, especially in small populations, and that variation in lifespan and ageing may often be nonadaptive, due to a strong contribution from mutation accumulation. | 431 | no | No GD diff between pop | NA | no | no |
| 432 | 2017 | 43007 | Wilson, AB; Whittington, CM; Bahr, A | 2014 | High intralocus variability and interlocus recombination promote immunological diversity in a minimal major histocompatibility system | Background: The genes of the major histocompatibility complex (MHC/MH) have attracted considerable scientific interest due to their exceptional levels of variability and important function as part of the adaptive immune system. Despite a large number of studies on MH class II diversity of both model and non-model organisms, most research has focused on patterns of genetic variability at individual loci, failing to capture the functional diversity of the biologically active dimeric molecule. Here, we take a systematic approach to the study of MH variation, analyzing patterns of genetic variation at MH class II alpha and II beta loci of the seahorse, which together form the immunologically active peptide binding cleft of the MH class II molecule. Results: The seahorse carries a minimal class II system, consisting of single copies of both MH class IIa and II beta, which are physically linked and inherited in a Mendelian fashion. Both genes are ubiquitously expressed and detectible in the brood pouch of male seahorses throughout pregnancy. Genetic variability of the two genes is high, dominated by non-synonymous variation concentrated in their peptide-binding regions. Coding variation outside these regions is negligible, a pattern thought to be driven by intra- and interlocus recombination. Despite the tight physical linkage of MH IIa and II beta loci, recombination has produced novel composite alleles, increasing functional diversity at sites responsible for antigen recognition. Conclusions: Antigen recognition by the adaptive immune system of the seahorse is enhanced by high variability at both MH class IIa and II beta loci. Strong positive selection on sites involved in pathogen recognition, coupled with high levels of intra- and interlocus recombination, produce a patchwork pattern of genetic variation driven by genetic hitchhiking. Studies focusing on variation at individual MH loci may unintentionally overlook an important component of ecologically relevant variation. | 432 | no | no good fitness measure, N pop differnt in GD? | NA | no | no |
| 433 | 2017 | 43007 | Braun, DC; Moore, JW; Candy, J; Bailey, RE | 2016 | Population diversity in salmon: linkages among response, genetic and life history diversity | Response diversity and asynchrony are important for stability and resilience of meta-populations, however little is known about the mechanisms that might drive such processes. In salmon populations, response diversity and asynchrony have been linked to the stability of their meta-populations and the fisheries that integrate across them. We examined how population diversity influenced response diversity and asynchrony in 42 populations of Chinook salmon from the Fraser River, British Columbia. We examined diversity in the survival responses to large-scale ocean climate variables for populations that differed in life history. Different life-histories responded differently to ocean environmental conditions. For instance, an increase of offshore temperature was associated with decreased survival for a population with ocean rearing juveniles but increased survival for a population with stream rearing juveniles. In a second analysis, we examined asynchrony in abundance between populations, which we then correlated with life history, spatial, and genetic diversity. Populations that were more genetically distant had the most different population dynamics. Collectively, these results suggest that fine-scale population diversity can contribute to the asynchrony and response diversity that underpins the stability of fisheries or metapopulation dynamics, and emphasize the need to manage and conserve this scale of population diversity. | 433 | maybe | any GD and fitness of groups that works? | no good GD and fitness of groups in main text (maybe gd in supp but no fitness any wat?) | no | no |
| 434 | 2017 | 43007 | Herbers, JM; Stuart, RJ | 1996 | Multiple queens in ant nests: Impact on genetic structure and inclusive fitness | Polygyny, the occurrence of multiple laying queens in colonies of social insects, presents a potential challenge to our understanding of evolution by kin selection. The ant species Leptothorax longispinosus is exceptionally useful for studying this problem, since populations vary in their frequency of polygyny and queen number had previously been linked to ecological factors of density of nest sites and overwinter survival rate. In addition, colony structure is complex, with nests undergoing fissions, fusions, and worker exchanges. Here we investigate genetic and spatial structure in three North American populations of this ant. Worker-worker relatedness remains high in these populations, primarily because queen-queen relatedness is likewise high; despite considerable genetic structure within nests, however, we found no evidence of spatiogenetic structure between nests. We conclude that the nest is the functional unit of selection for these populations. Across nests, there appears to be consistent, strong, stabilizing selection for intermediate levels of polygyny in two northern populations. Variation in queen number among populations and among nests within populations thus represents the outcome of a complex interplay between genetic and ecological factors. | 434 | no | no good fitness measure | NA | no | no |
| 435 | 2017 | 43007 | Du, HJ; Zheng, JS; Wu, M; Zhao, QZ; Wang, D | 2010 | High MHC DQB Variation and Asymmetric Allelic Distribution in the Endangered Yangtze Finless Porpoise, Neophocaena phocaenoides asiaeorientalis | The endangered Yangtze finless porpoise is found in the middle and lower reaches of the Yangtze River and its adjoining big lakes. To explore the major histocompatibility complex (MHC) genetic diversity and allelic distribution patterns across its range, we investigated variation at DQB exon 2. From 76 porpoises, we identified 18 DQB sequences. The freshwater Yangtze populations had much higher allelic diversity than marine populations. Among these freshwater populations, the middle-reach population had higher allelic diversity than the lower-reach population. The high DQB diversity level, relative to that of a neutral mtDNA locus, suggests that balancing selection is acting at the DQB gene and that rapid evolution and local positive selection play critical roles in generating and retaining high MHC diversity in the freshwater population. As the balancing selection might be driven by environmental pathogens, we suggest that maintaining MHC variation should be a high priority in the conservation and management of this endangered population, especially as an ex situ conservation strategy. | 435 | no | no good fitness measure, N pop differnt in GD? | NA | no | no |
| 436 | 2017 | 43007 | Riesgo, A; Blasco, G; Erwin, PM; Perez-Portela, R; Lopez-Legentil, S | 2014 | Optimization of 14 microsatellite loci in a Mediterranean demosponge subjected to population decimation, Ircinia fasciculata | The recovery potential of decimated populations of sponges will largely hinge on their populations’ size retrieval and their connectivity with conspecifics in unaffected locations. Here, we report on the development of microsatellite markers for estimation of the population connectivity and bottleneck and inbreeding signals in a Mediterranean sponge suffering from disease outbreaks, Ircinia fasciculata. From the 220,876 sequences obtained by genomic pyrosequencing, we isolated 14 polymorphic microsatellite loci and assessed the allelic variation of loci in 24 individuals from 2 populations in the Northwestern Mediterranean. The allele number per locus ranged from 3 to 11, observed heterozygosity from 0.68 to 0.73, and expected heterozygosity from 0.667 to 0.68. No significant linkage disequilibrium between pairs of loci was detected. The 14 markers developed here will be valuable tools for conservation strategies across the distributional range of this species allowing the detection of populations with large genetic diversity loss and high levels of inbreeding. | 436 | no | no good fitness measure, N pop differnt in GD? | NA | no | no |
| 437 | 2017 | 43007 | Le Gouar, PJ; Vallet, D; David, L; Bermejo, M; Gatti, S; Levrero, F; Petit, EJ; Menard, N | 2009 | How Ebola Impacts Genetics of Western Lowland Gorilla Populations | Background: Emerging infectious diseases in wildlife are major threats for both human health and biodiversity conservation. Infectious diseases can have serious consequences for the genetic diversity of populations, which could enhance the species’ extinction probability. The Ebola epizootic in western and central Africa induced more than 90% mortality in Western lowland gorilla population. Although mortality rates are very high, the impacts of Ebola on genetic diversity of Western lowland gorilla have never been assessed. Methodology/Principal Findings: We carried out long term studies of three populations of Western lowland gorilla in the Republic of the Congo (Odzala-Kokoua National Park, Lossi gorilla sanctuary both affected by Ebola and Lossi’s periphery not affected). Using 17 microsatellite loci, we compared genetic diversity and structure of the populations and estimate their effective size before and after Ebola outbreaks. Despite the effective size decline in both populations, we did not detect loss in genetic diversity after the epizootic. We revealed temporal changes in allele frequencies in the smallest population. Conclusions/Significance: Immigration and short time elapsed since outbreaks could explain the conservation of genetic diversity after the demographic crash. Temporal changes in allele frequencies could not be explained by genetic drift or random sampling. Immigration from genetically differentiated populations and a non random mortality induced by Ebola, i.e., selective pressure and cost of sociality, are alternative hypotheses. Understanding the influence of Ebola on gorilla genetic dynamics is of paramount importance for human health, primate evolution and conservation biology. | 437 | maybe | any difference in GD between groups before ebola? Any data on survuval/ebola within populations? | no good fitness measure | no | no |
| 438 | 2017 | 43007 | Coates, DJ; Tischler, G; McComb, JA | 2006 | Genetic variation and the mating system in the rare Acacia sciophanes compared with its common sister species Acacia anfractuosa (Mimosaceae) | Acacia sciophanes is an extremely rare and Critically Endangered species known from two small populations separated by less than 7 km. Specifically we aimed to investigate whether rarity in A. sciophanes is associated with decreased levels of genetic variation and increased levels of selfing by comparing patterns of genetic variation and mating system parameters with its widespread and common sister species A. anfractuosa. Fourteen polymorphic allozyme loci were used to assess genetic diversity with four of these used in the estimation of mating system parameters. At the species level A. sciophanes has lower allelic richness, polymorphism, observed heterozygosity and gene diversity than A. anfractuosa and significantly lower levels of gene diversity at the population level. Both species have a mixed mating system but the largest population of A. sciophanes has lower levels of outcrossing, higher correlated paternity and increased bi-parental inbreeding compared with two A. anfractuosa populations. However, both correlated paternity and bi-parental inbreeding appear to be at least partly influenced by population size regardless of the species. We suggest that A. sciophanes is likely to be an intrinsically rare species and that in particular the lower levels of genetic diversity and increased selfing are a feature of a species that has the ability to persist in a few localised small populations. Despite recent extensive habitat destruction our comparative study provided no clear evidence that such events have contributed to the lower genetic diversity and increased selfing in A. sciophanes and we believe its ability to exist in small populations may not only be an important factor in its survival as a rare species but also indicates that it may be less susceptible to the impacts of habitat loss and fragmentation. The key to this species conservation will be the maintenance of suitable habitat, particularly through improved fire regimes and control of invasive weeds, that will allow the two small populations to continue to persist in extremely restricted areas of remnant vegetation. | 438 | no | two species comp | NA | no | no |
| 439 | 2017 | 43007 | Agashe, D; Bolnick, DI | 2012 | Dietary niche and population dynamic feedbacks in a novel habitat | Population dynamics and resource use are often intricately connected via density-dependent intraspecific competition. However, experimental studies of concurrent change in population and resource use dynamics are scarce. In particular, the impact of factors such as genetic diversity, which can affect both population dynamics and competition, remains unexplored. Using stable isotope analysis and periodic population censuses, we quantified both diet and population dynamics in wheat-adapted Tribolium castaneum (flour beetle) populations provided with an additional novel resource (corn). Populations were initiated with different levels of genetic variation for traits relevant to population growth and resource use (e.g. fecundity and survival).We found that high population size decreased subsequent corn use, and high corn use in turn lowered population size. Surprisingly, we did not detect a significant effect of founding genetic variation on resource niche expansion, although genetic variation increased overall population size and stability. In contrast, dietary niche expansion decreased both population size and stability. Finally, larval and adult niche dynamics were uncorrelated, suggesting that various life stages perceive or respond differentially to intraspecific competition and resource availability. Our experiments indicate that population performance in a novel habitat depends on stage-specific interactions between resource use, standing genetic variation, and population size. | 439 | maybe | any gD and fitness of groups that works? | no good fitness measure | no | no |
| 440 | 2017 | 43007 | Exeler, N; Kratochwil, A; Hochkirch, A | 2010 | Does recent habitat fragmentation affect the population genetics of a heathland specialist, Andrena fuscipes (Hymenoptera: Andrenidae)? | Habitat fragmentation is believed to be a key threat to biodiversity as it decreases the probability of survival of populations, reduces gene flow among populations and increases the possibility of inbreeding and loss of genetic diversity within populations. Heathlands represent excellent systems to study fragmentation effects as the spatial and temporal course of fragmentation is well documented for these habitats. At the beginning of the nineteenth century, heathlands were widespread in northern Germany, but they became increasingly fragmented at the end of the nineteenth century until only few fragments had been left. As many insect species are strongly specialized on heathland habitats, they represent ideal study systems to test the genetic effects of such recent fragmentation processes. The solitary bee Andrena fuscipes is strongly specialized on heather (Calluna vulgaris) and, therefore, occurs exclusively in heathland habitats. The species is red-listed in Germany and other parts of Europe. Here, we present an analysis of the genetic structure of 12 populations of A. fuscipes using eight microsatellite loci. The populations showed little geographical structure and the degree of genetic differentiation was low. Compared to related bee species, inbreeding coefficients were relatively low and seem to be mainly affected by the bees’ solitary nesting behaviour. | 440 | no | no good fitness measure | NA | no | no |
| 441 | 2017 | 43007 | Vardo, AM; Schall, JJ | 2007 | Clonal diversity of a lizard malaria parasite, Plasmodium mexicanum, in its vertebrate host, the western fence lizard: role of variation in transmission intensity over time and space | Within the vertebrate host, infections of a malaria parasite (Plasmodium) could include a single genotype of cells (single-clone infections) or two to several genotypes (multiclone infections). Clonal diversity of infection plays an important role in the biology of the parasite, including its life history, virulence, and transmission. We determined the clonal diversity of Plasmodium mexicanum, a lizard malaria parasite at a study region in northern California, using variable microsatellite markers, the first such study for any malaria parasite of lizards or birds (the most common hosts for Plasmodium species). Multiclonal infections are common (50-88% of infections among samples), and measures of genetic diversity for the metapopulation (expected heterozygosity, number of alleles per locus, allele length variation, and effective population size) all indicated a substantial overall genetic diversity. Comparing years with high prevalence (1996-1998 = 25-32% lizards infected), and years with low prevalence (2001-2005 = 6-12%) found fewer alleles in samples taken from the low-prevalence years, but no reduction in overall diversity (H = 0.64-0.90 among loci). In most cases, rare alleles appeared to be lost as prevalence declined. For sites chronically experiencing low transmission intensity (prevalence similar to 1%), overall diversity was also high (H = 0.79-0.91), but there were fewer multiclonal infections. Theory predicts an apparent excess in expected heterozygosity follows a genetic bottleneck. Evidence for such a distortion in genetic diversity was observed after the drop in parasite prevalence under the infinite alleles mutation model but not for the stepwise mutation model. The results are similar to those reported for the human malaria parasite, Plasmodium falciparum, worldwide, and support the conclusion that malaria parasites maintain high genetic diversity in host populations despite the potential for loss in alleles during the transmission cycle or during periods/locations when transmission intensity is low. | 441 | no | no good fitness measure | NA | no | no |
| 442 | 2017 | 43007 | Sela, H; Cheng, JP; Jun, Y; Nevo, E; Fahima, T | 2009 | Divergent diversity patterns of NBS and LRR domains of resistance gene analogs in wild emmer wheat populations | Disease resistance (R) genes are intriguing in their evolution and diversity patterns because of their constant interactions with evolving pathogens. In this study, we demonstrate the use of resistance gene analog (RGA) markers to estimate genetic diversity among 13 populations (118 genotypes) of Triticum dicoccoides collected along a natural aridity gradient in Israel. The diversity patterns of 204 markers derived from two R-gene domains, nucleotide binding site (NBS) and leucine-rich repeat (LRR), were compared and contrasted. Diversity patterns of NBS domain markers differed significantly from those of the LRR domain. NBS markers showed higher between-population diversity (F(st) = 0.58), while LRR markers showed higher within-population diversity (F(st) = 0.35). Gene diversity (H(e)) values were twofold higher in the LRR domain than in the NBS domain (0.144 vs. 0.067). LRR H(e) values were correlated with precipitation in the spring (r = 0.8, p = 0.01), while NBS H(e) values showed no correlation with any ecogeographical variable. The evolutionary and applicative inferences of these findings are discussed. The current study demonstrates that RGA profiling is an excellent tool for studying diversity of R genes in natural plant populations. | 442 | no | no good fitness measure | NA | no | no |
| 443 | 2017 | 43007 | Contasti, AL; Tissier, EJ; Johnstone, JF; McLoughlin, PD | 2012 | Explaining Spatial Heterogeneity in Population Dynamics and Genetics from Spatial Variation in Resources for a Large Herbivore | Fine-scale spatial variation in genetic relatedness and inbreeding occur across continuous distributions of several populations of vertebrates; however, the basis of observed variation is often left untested. Here we test the hypothesis that prior observations of spatial patterns in genetics for an island population of feral horses (Sable Island, Canada) were the result of spatial variation in population dynamics, itself based in spatial heterogeneity in underlying habitat quality. In order to assess how genetic and population structuring related to habitat, we used hierarchical cluster analysis of water sources and an indicator analysis of the availability of important forage species to identify a longitudinal gradient in habitat quality along the length of Sable Island. We quantify a west-east gradient in access to fresh water and availability of two important food species to horses: sandwort, Honckenya peploides, and beach pea, Lathyrus japonicas. Accordingly, the population clusters into three groups that occupy different island segments (west, central, and east) that vary markedly in their local dynamics. Density, body condition, and survival and reproduction of adult females were highest in the west, followed by central and east areas. These results mirror a previous analysis of genetics, which showed that inbreeding levels are highest in the west (with outbreeding in the east), and that there are significant differences in fixation indices among groups of horses along the length of Sable Island. Our results suggest that inbreeding depression is not an important limiting factor to the horse population. We conclude that where habitat gradients exist, we can anticipate fine-scale heterogeneity in population dynamics and hence genetics. | 443 | maybe | any fitness measure? GD-measurement ok (no inbreeding?) | inbreeding and no good GD measure | no | no |
| 444 | 2017 | 43007 | Haridas, CV; Rajarshi, MB | 1997 | On computing worker-brood genetic relatedness in colonies of eusocial insects: a matrix model | We consider a matrix model to describe the population dynamics of the Indian paper wasp Ropalidia marginata, a eusocial insect species of peninsular India. We obtain the stable class distribution of this model and apply it to compute the worker-brood genetic relatedness of the colony. Our results are compared with those obtained by observational studies. Further, we study the effect of changes in turnover rates and survival rates on the genetic relatedness coefficient with the help of the theoretical formulae. | 444 | no | NA | NA | no | no |
| 445 | 2017 | 43007 | Dev, SA; Kjellberg, F; Hossaert-McKey, M; Borges, RM | 2011 | Fine-scale Population Genetic Structure of Two Dioecious Indian Keystone Species, Ficus hispida and Ficus exasperata (Moraceae) | Although Ficus (Moraceae) is a keystone plant genus in the tropics, providing resources to many frugivorous vertebrates, its population genetic structure, which is an important determinant of its long-term survival, has rarely been investigated. We examined the population genetic structure of two dioecious fig species (Ficus hispida and Ficus exasperata) in the Indian Western Ghats using co-dominant nuclear microsatellite markers. We found high levels of microsatellite genetic diversity in both species. The regression slopes between genetic relationship coefficients (f(ij)) and spatial distances were significantly negative in both species indicating that, on average, individuals in close spatial proximity were more likely to be related than individuals further apart. Mean parent-offspring distance (Sigma) calculated using these slopes was about 200 m in both species. This should be contrasted with the very long pollen dispersal distances documented for monoecious Ficus species. Nevertheless, overall population genetic diversity remained large suggesting immigrant gene flow. Further studies will be required to analyze broader scale patterns. | 445 | no | no good fitness measure, N pop differnt in GD? | NA | no | no |
| 446 | 2017 | 43007 | Scally, A; Yngvadottir, B; Xue, YL; Ayub, Q; Durbin, R; Tyler-Smith, C | 2013 | A Genome-Wide Survey of Genetic Variation in Gorillas Using Reduced Representation Sequencing | All non-human great apes are endangered in the wild, and it is therefore important to gain an understanding of their demography and genetic diversity. Whole genome assembly projects have provided an invaluable foundation for understanding genetics in all four genera, but to date genetic studies of multiple individuals within great ape species have largely been confined to mitochondrial DNA and a small number of other loci. Here, we present a genome-wide survey of genetic variation in gorillas using a reduced representation sequencing approach, focusing on the two lowland subspecies. We identify 3,006,670 polymorphic sites in 14 individuals: 12 western lowland gorillas (Gorilla gorilla gorilla) and 2 eastern lowland gorillas (Gorilla beringei graueri). We find that the two species are genetically distinct, based on levels of heterozygosity and patterns of allele sharing. Focusing on the western lowland population, we observe evidence for population substructure, and a deficit of rare genetic variants suggesting a recent episode of population contraction. In western lowland gorillas, there is an elevation of variation towards telomeres and centromeres on the chromosomal scale. On a finer scale, we find substantial variation in genetic diversity, including a marked reduction close to the major histocompatibility locus, perhaps indicative of recent strong selection there. These findings suggest that despite their maintaining an overall level of genetic diversity equal to or greater than that of humans, population decline, perhaps associated with disease, has been a significant factor in recent and long-term pressures on wild gorilla populations. | 446 | no | no good fitness measure, N pop differnt in GD? | NA | no | no |
| 447 | 2017 | 43007 | Caesar, S; Karlsson, M; Forsman, A | 2010 | Diversity and Relatedness Enhance Survival in Colour Polymorphic Grasshoppers | Evolutionary theory predicts that different resource utilization and behaviour by alternative phenotypes may reduce competition and enhance productivity and individual performance in polymorphic, as compared with monomorphic, groups of individuals. However, firm evidence that members of more heterogeneous groups benefit from enhanced survival has been scarce or lacking. Furthermore, benefits associated with phenotypic diversity may be counterbalanced by costs mediated by reduced relatedness, since closely related individuals typically are more similar. Pygmy grasshoppers (Tetrix subulata) are characterized by extensive polymorphism in colour pattern, morphology, behaviour and physiology. We studied experimental groups founded by different numbers of mothers and found that survival was higher in low than in high density, that survival peaked at intermediate colour morph diversity in high density, and that survival was independent of diversity in low density where competition was less intense. We further demonstrate that survival was enhanced by relatedness, as expected if antagonistic and competitive interactions are discriminately directed towards non-siblings. We therefore also performed behavioural observations and staged encounters which confirmed that individuals recognized and responded differently to siblings than to non-siblings. We conclude that negative effects associated with competition are less manifest in diverse groups, that there is conflicting selection for and against genetic diversity occurring simultaneously, and that diversity and relatedness may facilitate the productivity and ecological success of groups of interacting individuals. | 447 | no | from thesis but not to be included - plymorphism not guaranteed GD difference of groups! | NA | no | no |
| 448 | 2017 | 43007 | Frankham, R | 2008 | Genetic adaptation to captivity in species conservation programs | As wild environments are often inhospitable, many species have to be captive-bred to save them from extinction. In captivity, species adapt genetically to the captive environment and these genetic adaptations are overwhelmingly deleterious when populations are returned to wild environments. I review empirical evidence on (i) the genetic basis of adaptive changes in captivity, (ii) factors affecting the extent of genetic adaptation to captivity, and (iii) means for minimizing its deleterious impacts. Genetic adaptation to captivity is primarily due to rare alleles that in the wild were deleterious and partially recessive. The extent of adaptation to captivity depends upon selection intensity, genetic diversity, effective population size and number of generation in captivity, as predicted by quantitative genetic theory. Minimizing generations in captivity provides a highly effective means for minimizing genetic adaptation to captivity, but is not a practical option for most animal species. Population fragmentation and crossing replicate captive populations provide practical means for minimizing the deleterious effects of genetic adaptation to captivity upon populations reintroduced into the wild. Surprisingly, equalization of family sizes reduces the rate of genetic adaptation, but not the deleterious impacts upon reintroduced populations. Genetic adaptation to captivity is expected to have major effects on reintroduction success for species that have spent many generations in captivity. This issue deserves a much higher priority than it is currently receiving. | 448 | no | NA | NA | no | no |
| 449 | 2017 | 43007 | Savage, AE; Becker, CG; Zamudio, KR | 2015 | Linking genetic and environmental factors in amphibian disease risk | A central question in evolutionary biology is how interactions between organisms and the environment shape genetic differentiation. The pathogen Batrachochytrium dendrobatidis (Bd) has caused variable population declines in the lowland leopard frog (Lithobates yavapaiensis); thus, disease has potentially shaped, or been shaped by, host genetic diversity. Environmental factors can also influence both amphibian immunity and Bd virulence, confounding our ability to assess the genetic effects on disease dynamics. Here, we used genetics, pathogen dynamics, and environmental data to characterize L.yavapaiensis populations, estimate migration, and determine relative contributions of genetic and environmental factors in predicting Bd dynamics. We found that the two uninfected populations belonged to a single genetic deme, whereas each infected population was genetically unique. We detected an outlier locus that deviated from neutral expectations and was significantly correlated with mortality within populations. Across populations, only environmental variables predicted infection intensity, whereas environment and genetics predicted infection prevalence, and genetic diversity alone predicted mortality. At one locality with geothermally elevated water temperatures, migration estimates revealed source-sink dynamics that have likely prevented local adaptation. We conclude that integrating genetic and environmental variation among populations provides a better understanding of Bd spatial epidemiology, generating more effective conservation management strategies for mitigating amphibian declines. | 449 | yes | ok gd measurement? | Allelic richness not corrected for sample size | yes | no |
| 450 | 2017 | 43007 | MEUWISSEN, THE; WOOLLIAMS, JA | 1994 | EFFECTIVE SIZES OF LIVESTOCK POPULATIONS TO PREVENT A DECLINE IN FITNESS | In livestock populations, fitness may decrease due to inbreeding depression or as a negatively correlated response to artificial selection. On the other hand, fitness may increase due to natural selection. In the absence of a correlated response due to artificial selection, the critical population size at which the increase due to natural selection and the decrease due to inbreeding depression balance each other is approximately D/2 sigma(wa)(2), where D=the inbreeding depression of fitness with complete inbreeding, and sigma(wa)(2)=the additive genetic variance of fitness. This simple expression agrees well with results from transmission probability matrix methods. If fitness declines as a correlated negative response to artificial selection, then a large increase in the critical effective population size is needed. However, if the negative response is larger than the response to natural selection, a reduction in fitness cannot be prevented. From these results it is concluded that a negative correlation between artificial and natural selection should be avoided. Effective sizes to prevent a decline in fitness are usually larger than those which maximize genetic gain of overall efficiency, i.e., the former is a more stringent restriction on effective size. In the examples presented, effective sizes ranged from 31 to 250 animals per generation. | 450 | no | NA | NA | no | no |
| 451 | 2017 | 43008 | Reif, BP; Mathiasen, RL; Kenaley, SC; Allan, GJ | 2015 | Genetic Structure and Morphological Differentiation of Three Western North American Dwarf Mistletoes (Arceuthobium: Viscaceae) | Dwarf mistletoes (Arceuthobium spp., Viscaceae) are ecologically and economically important parasitic flowering plants. Despite detection of morphological and host specialization differences, ribosomal DNA and plastid sequence information have not resolved genetic differentiation among the dwarf mistletoes parasitizing white pines (Pinus subgenus Strobus) in the western U. S. A. We assessed the effectiveness of integrating amplified fragment length polymorphism and morphometric analyses in the delineation of three closely related and morphologically similar species of white pine dwarf mistletoes. Using amplified fragment length polymorphism data for 995 loci, we quantified intra- and inter-specific genetic variation, tested for geographic isolation-by-distance, and visualized genetic patterns using Bayesian-based structure analyses, neighbor-joining tree reconstruction, and principal coordinate analysis ordination. A total of 19 morphological characters were also analyzed using standard statistical methods and discriminant analysis. We found that 13% of the total genetic variation (p = 0.0001) was due to among-species differences, and species were distinguished by tree and ordination plots. Species also differed in estimates of genetic diversity and differentiation, and the degree of isolation-by-distance in genetic structuring. Amplified fragment length polymorphism and morphometric analyses both demonstrated that the dwarf mistletoes examined are well-differentiated genetically and morphologically, and therefore, should be considered distinct species. | 451 | no | three species | NA | no | no |
| 452 | 2017 | 43008 | Buckland, S; Cole, NC; Groombridge, JJ; Kupper, C; Burke, T; Dawson, DA; Gallagher, LE; Harris, S | 2014 | High Risks of Losing Genetic Diversity in an Endemic Mauritian Gecko: Implications for Conservation | Genetic structure can be a consequence of recent population fragmentation and isolation, or a remnant of historical localised adaptation. This poses a challenge for conservationists since misinterpreting patterns of genetic structure may lead to inappropriate management. Of 17 species of reptile originally found in Mauritius, only five survive on the main island. One of these, Phelsuma guimbeaui (lowland forest day gecko), is now restricted to 30 small isolated subpopulations following severe forest fragmentation and isolation due to human colonisation. We used 20 microsatellites in ten subpopulations and two mitochondrial DNA (mtDNA) markers in 13 subpopulations to: (i) assess genetic diversity, population structure and genetic differentiation of subpopulations; (ii) estimate effective population sizes and migration rates of subpopulations; and (iii) examine the phylogenetic relationships of haplotypes found in different subpopulations. Microsatellite data revealed significant population structure with high levels of genetic diversity and isolation by distance, substantial genetic differentiation and no migration between most subpopulations. MtDNA, however, showed no evidence of population structure, indicating that there was once a genetically panmictic population. Effective population sizes of ten subpopulations, based on microsatellite markers, were small, ranging from 44 to 167. Simulations suggested that the chance of survival and allelic diversity of some subpopulations will decrease dramatically over the next 50 years if no migration occurs. Our DNA-based evidence reveals an urgent need for a management plan for the conservation of P. guimbeaui. We identified 18 threatened and 12 viable subpopulations and discuss a range of management options that include translocation of threatened subpopulations to retain maximum allelic diversity, and habitat restoration and assisted migration to decrease genetic erosion and inbreeding for the viable subpopulations. | 452 | probably no | no good fitness measure, N pop differnt in GD? ( not inbreeding?) | no fitness measure | no | no |
| 453 | 2017 | 43008 | Mosseler, A; Major, JE; Rajora, OP | 2003 | Old-growth red spruce forests as reservoirs of genetic diversity and reproductive fitness | Old-growth forests are assumed to be potential reservoirs of genetic diversity for the dominant tree species, yet there is little empirical evidence for this assumption. Our aim was to characterize the relationship of stand traits, such as age, height and stem diameter, with the genetic and reproductive status of old-growth and older second-growth stands of red spruce (Picea rubens Sarg.) in eastern Canada. We found strong relationships between height growth (a fitness trait) and measures of genetic diversity based on allozyme analyses in red spruce. The negative relationship between height and the proportion of rare alleles suggests that high proportions of these rare alleles may be deleterious to growth performance. Latent genetic potential, however, showed a significant and positive relationship with height. Stand age was not correlated to height, but was correlated to seedling progeny height. In late-successional-species such as red spruce, age and size (e.g., height and stem diameter) relationships may be strongly influenced by local stand disturbance dynamics that determine availability of light, growing space, moisture and nutrients. In larger and older stands, age appeared to provide a good surrogate measure or indicator for genetic diversity and progeny height growth. However, in smaller and more isolated populations, these age and fitness relationships may be strongly influenced by the effects of inbreeding and genetic drift. Therefore, older populations or old-growth forests may represent superior seed sources, but only if they are also of sufficient size and structure (e.g., stem density and spatial family structure) to avoid the effects of inbreeding and genetic drift. Thus, larger and older forests appear to have an important evolutionary role as reservoirs of both genetic diversity and reproductive fitness. Given the rapid environmental changes anticipated (as a result of climate change, increasing population isolation through fragmentation, or following the introduction of exotic pests and diseases) these older populations of trees may have a valuable function in maintaining the adaptive potential of tree species. | 453 | no | no good fitness measure, N pop differnt in GD? | NA | no | no |
| 454 | 2017 | 43008 | Hernandez-Garcia, R; De la Rua, P; Serrano, J | 2009 | Mating frequency in Apis mellifera iberiensis queens | Honey bees exhibit high levels of polyandry. This mating behaviour provides high levels of genetic diversity within the colonies which increases colony productivity, queen fitness and resistance to diseases. We investigated the mating frequency in two Iberian populations of Apis mellifera iberiensis, one from northern Spain inhabiting the Cantabrian Mountains and showing west European (M) mitochondrial haplotypes, the other located in south-eastern Spain close to the Mediterranean Sea and bearing African (A) mitochondrial DNA. Queens of the northern apiaries mated with 8 - 25 drones (average 15.73 +/- 4.58), those of the south-eastern with 10 - 29 drones (average 18.92 +/- 5.07), a difference that was significant (p = 0.036). Genetic relatedness among workers was 0.30 +/- 0.02 in both populations. It is discussed whether climatic conditions and genetic background may influence honey bee queen mating behaviour. | 454 | probably no | no good fitness measure | no fitness measure | no | no |
| 455 | 2017 | 43008 | Morris, K; Austin, JJ; Belov, K | 2013 | Low major histocompatibility complex diversity in the Tasmanian devil predates European settlement and may explain susceptibility to disease epidemics | The Tasmanian devil (Sarcophilus harrisii) is at risk of extinction owing to the emergence of a contagious cancer known as devil facial tumour disease (DFTD). The emergence and spread of DFTD has been linked to low genetic diversity in the major histocompatibility complex (MHC). We examined MHC diversity in historical and ancient devils to determine whether loss of diversity is recent or predates European settlement in Australia. Our results reveal no additional diversity in historical Tasmanian samples. Mainland devils had common modern variants plus six new variants that are highly similar to existing alleles. We conclude that low MHC diversity has been a feature of devil populations since at least the Mid-Holocene and could explain their tumultuous history of population crashes. | 455 | no | no good fitness measure, N pop differnt in GD? | NA | no | no |
| 456 | 2017 | 43008 | Barari, H; Dalili, SA; Rezaii, SA | 2013 | POPULATION GENETIC STRUCTURE ANALYSIS OF SCLEROTINIA SCLEROTIORUM (LIB.) DE BARY FROM DIFFERENT HOST PLANT SPECIES IN NORTHERN IRAN | The genetic structure of 65 Sclerotinia sclerotiorum isolates representing 52 field populations from three provinces in northern Iran were analyzed with Mycalial Compatibility Groupings (MCGs) and five polymorphic microsatellite loci. In total, 44 haplotypes were detected with 25 allele polymorphisms. A high level of genetic diversity was observed in about 67.6% (with clonal fraction = 0.077 to 0.20) of the regional populations studies, and the Shannon diversity index (H-o) for the whole region was found to be 0.86 (H-tot). Partition of total diversity (H-tot) showed that 64% corresponded to a variation in diversity within the S. sclerotiorum populations. By mycelial compatibility grouping (MCG) tests, the isolates were classified into 39 groups of which 26 MCGs were individual. Molecular and phenotypic analyses results of all of the. isolates (except MCG4 and MCG23) were similar; however, the isolates in the MCG4 and MCG23 groups, with variable microsatellite haplotypes, were morphologically dissimilar. The results shown here were possibly due to high rates of outcrossing, as well as to the evolutionary potential within population of the pathogen in different locations in Iran. Nei’s genetic identity showed that populations from Golestan province and wild plants were the most diverse. | 456 | no | no good fitness measure | NA | no | no |
| 457 | 2017 | 43008 | Desender, K; Verdyck, P | 2001 | Geographic scaling and genetic differentiation in two highly mobile European saltmarsh beetles | Genetic structure and diversity are studied in two European saltmarsh beetles, Bembidion minimum and B. normannum, on a regional as well as a Western European scale. Results are based on allozymes, studied at four polymorphic loci for more than 1600 individuals from all remaining saltmarshes in Belgium and from a selection of European reference sites. Average gene diversity is not related to habitat or population size, but is larger in the more common B. minimum, in comparison to Atlantic samples of B. normannum. One Mediterranean sample of the latter species reveals a much higher diversity and suggests this region as the evolutionary centre of origin and/or as a possible glacial refugium of the species. Significant overall genetic structure is observed in the complete data of both species, with 2 to 6 % of the total genetic variation explained by differentiation between populations. Genetic differentiation in both species is significant at different geographic scales, with higher values at a larger scale. A Mantel-test (isolation by distance) between geographic and genetic distance is significant in B. normannum. Our results indicate that habitat fragmentation has not yet resulted in genetic erosion, probably because of the large population sizes of both species, even in very small saltmarshes. The observed genetic differentiation suggests that metapopulations at a relatively large geographic scale are still functional in these highly mobile species. Re-establishment of even small saltmarshes is suggested as a positive conservation measure for long term survival of these specialised ground beetles. | 457 | no | no good fitness measure | NA | no | no |
| 458 | 2017 | 43008 | Gorospe, KD; Karl, SA | 2015 | Depth as an Organizing Force in Pocillopora damicornis: Intra-Reef Genetic Architecture | Relative to terrestrial plants, and despite similarities in life history characteristics, the potential for corals to exhibit intra-reef local adaptation in the form of genetic differentiation along an environmental gradient has received little attention. The potential for natural selection to act on such small scales is likely increased by the ability of coral larval dispersal and settlement to be influenced by environmental cues. Here, we combine genetic, spatial, and environmental data for a single patch reef in K ne’ohe Bay, O’ahu, Hawai’i, USA in a landscape genetics framework to uncover environmental drivers of intra-reef genetic structuring. The genetic dataset consists of near-exhaustive sampling (n = 2352) of the coral, Pocillopora damicornis at our study site and six microsatellite genotypes. In addition, three environmental parameters - depth and two depth-independent temperature indices - were collected on a 4 m grid across 85 locations throughout the reef. We use ordinary kriging to spatially interpolate our environmental data and estimate the three environmental parameters for each colony. Partial Mantel tests indicate a significant correlation between genetic relatedness and depth while controlling for space. These results are also supported by multi-model inference. Furthermore, spatial Principle Component Analysis indicates a statistically significant genetic cline along a depth gradient. Binning the genetic dataset based on size-class revealed that the correlation between genetic relatedness and depth was significant for new recruits and increased for larger size classes, suggesting a possible role of larval habitat selection as well as selective mortality in structuring intra-reef genetic diversity. That both pre- and post-recruitment processes may be involved points to the adaptive role of larval habitat selection in increasing adult survival. The conservation importance of uncovering intra-reef patterns of genetic diversity is discussed. | 458 | no | no good fitness measure, N pop differnt in GD? | NA | no | no |
| 459 | 2017 | 43008 | Chadha, S; Gopalakrishna, T | 2005 | Genetic diversity of Indian isolates of rice blast pathogen (Magnaporthe grisea) using molecular markers | Magnaporthe grisea, the rice blast fungus is one of the main pathological threats to rice crop worldwide. The genetic relatedness and the probable mechanisms of genetic variation among the Indian isolates of rice blast pathogen were studied. A total of 171 polymorphic markers were scored using 33 selected random decamer primers. The isolates exhibited an overall polymorphism of about 64%. The similarity degree value for the isolates ranged from 0.76 to 0.92. The high polymorphism could be explained by natural and stress-induced transposition and horizontal gene transfer. Understanding the source of pathogen variation will aid in designing improved methods for management of the rice blast disease. | 459 | no | gd of pathogen | NA | no | no |
| 460 | 2017 | 43008 | Coates, DJ; McArthur, SL; Byrne, M | 2015 | Significant genetic diversity loss following pathogen driven population extinction in the rare endemic Banksia brownii (Proteaceae) | Rapid range-wide extinction of whole populations due to introduced disease would be expected to lead to significant loss of genetic diversity, with impacts being greater for a species that is geographically restricted and with disjunct populations. We investigated the genetic consequences of extirpation in the rare endemic woody shrub, Banksia brownii, following the introduction of the pathogen Phytophthora cinnamomi. B. brownii shows a pattern of disease driven population extinction typical of significant numbers of threatened plant species within southwestern Australia. Based on microsatellite genotyping of material from extinct (ex situ seed collections) and extant populations we estimated that 38.2% (gene diversity) and 36.7% (allelic richness) of total genetic diversity, based on contributions of within population variation and differentiation, have been lost from Banksia brownii due to P. cinnamomi. Furthermore, a loss of genetic diversity was reflected in the loss of 16 out of 55 and 6 out of 33 private alleles respectively from two geographically disjunct genetically distinct population groups. We found significant genetic structure within B. brownii with three geographically disjunct population assemblages that should be regarded as separate units for conservation. These findings have been important in developing reintroductions to disease free sites for each conservation unit. Crown Copyright (C) 2015 Published by Elsevier Ltd. All rights reserved. | 460 | no | no good fitness measure | NA | no | no |
| 461 | 2017 | 43008 | Clark, RW; Marchand, MN; Clifford, BJ; Stechert, R; Stephens, S | 2011 | Decline of an isolated timber rattlesnake (Crotalus horridus) population: Interactions between climate change, disease, and loss of genetic diversity | Extinction of populations from anthropogenic forces rarely has a single cause. Instead, population declines result from a variety of factors, including habitat loss, inbreeding depression, disease, and climate change. These impacts often have synergistic effects that can lead to rapid decline in isolated populations, but case studies documenting such processes are rare. Here, we describe the recent decline of the last known population of timber rattlesnakes (Crotalus horridus) in the state of New Hampshire. We used polymorphic nuclear DNA markers to compare genetic diversity of this population to other populations in the region that are not isolated. We also compare results from ongoing field monitoring of these populations. Genetic analyses reveal that the New Hampshire population lacks genetic diversity and exhibits signs of a recent bottleneck. New Hampshire snakes also exhibited high levels of morphological abnormalities (unique piebald coloration, amelanistic tongues) indicative of inbreeding depression. Furthermore, after a year with exceptionally high summer rainfall, a skin infection of unknown etiology caused significant mortality in the New Hampshire population, whereas other surveyed non-inbred populations were unaffected. This case study demonstrates how different anthropogenic impacts on natural environments can interact in unexpected ways to drive threatened populations toward extinction. (c) 2010 Elsevier Ltd. All rights reserved. | 461 | no | only one pop + inbreeding | NA | no | no |
| 462 | 2017 | 43008 | Soglia, D; Rossi, L; Cauvin, E; Citterio, C; Ferroglio, E; Maione, S; Meneguz, PG; Spalenza, V; Rasero, R; Sacchi, P | 2010 | Population genetic structure of Alpine chamois (Rupicapra r. rupicapra) in the Italian Alps | Analysis of the genetic diversity of the Alpine chamois in Italy was conducted using a pool of 26 microsatellite loci. A total of 209 animals were analyzed, representing six geographical populations from different location of the Southern slope of the Alps. Clear genetic differences have emerged between the sampled chamois groups. Some were consistent with an isolation-by-distance model. However, in parallel, other mechanisms intervened in areas that, in addition to being peripheral to the main alpine ridge, had suffered from recent bottlenecks. In such areas, genetic drift and a low rate of gene flow are likely explanations for the current genetic structure. | 462 | no | no good fitness measure, N pop differnt in GD? | NA | no | no |
| 463 | 2017 | 43008 | Larson, WA; Lisi, PJ; Seeb, JE; Seeb, LW; Schindler, DE | 2016 | Major histocompatibility complex diversity is positively associated with stream water temperatures in proximate populations of sockeye salmon | Local adaptation to heterogeneous environments generates population diversity within species, significantly increasing ecosystem stability and flows of ecosystem services. However, few studies have isolated the specific mechanisms that create and maintain this diversity. Here, we examined the relationship between water temperature in streams used for spawning and genetic diversity at a gene involved in immune function [the major histocompatibility complex (MHC)] in 14 populations of sockeye salmon (Oncorhynchus nerka) sampled across the Wood River basin in south-western Alaska. The largest influence on MHC diversity was lake basin, but we also found a significant positive correlation between average water temperature and MHC diversity. This positive relationship between temperature and MHC diversity appears to have been produced by natural selection at very local scales rather than neutral processes, as no correlation was observed between temperature and genetic diversity at 90 neutral markers. Additionally, no significant relationship was observed between temperature variability and MHC diversity. Although lake basin was the largest driver of differences in MHC diversity, our results also demonstrate that fine-scale differences in water temperature may generate variable selection regimes in populations that spawn in habitats separated by as little as 1km. Additionally, our results indicated that some populations may be reaching a maximum level of MHC diversity. We postulate that salmon from populations in warm streams may delay spawning until late summer to avoid thermal stress as well as the elevated levels of pathogen prevalence and virulence associated with warm temperatures earlier in the summer. | 463 | no | no good fitness measure, N pop differnt in GD? | NA | no | no |
| 464 | 2017 | 43008 | Becheler, R; Xhaard, C; Klein, EK; Hayden, KJ; Frey, P; De Mita, S; Halkett, F | 2016 | Genetic signatures of a range expansion in natura: when clones play leapfrog | The genetic consequences of range expansions have generally been investigated at wide geographical and temporal scales, long after the colonization event. A unique ecological system enabled us to both monitor the colonization dynamics and decipher the genetic footprints of expansion over a very short time period. Each year an epidemic of the poplar rust (Melampsora larici-populina) expands clonally and linearly along the Durance River, in the Alps. The colonization dynamics observed in 2004 showed two phases with different genetic outcomes. Upstream, fast colonization maintained high genetic diversity. Downstream, the colonization wave progressively faltered, diversity eroded, and differentiation increased, as expected under recurrent founder events. In line with the high dispersal abilities of rust pathogens, we provide evidence for leapfrog dispersal of clones. Our results thus emphasize the importance of colonization dynamics in shaping spatial genetic structure in the face of high gene flow. | 464 | no | no good fitness measure, N pop differnt in GD? | NA | no | no |
| 465 | 2017 | 43008 | POWER, AG | 1991 | VIRUS SPREAD AND VECTOR DYNAMICS IN GENETICALLY DIVERSE PLANT-POPULATIONS | Little is known about the influence of genetic diversity in plant populations on the dynamics of plant viruses, particularly those transmitted by insects. For these viruses, plant genetic diversity may affect virus incidence through impacts on the population dynamics of the vector insects or through impacts on vector feeding behavior, which determines transmission of the virus. This study was designed to explore the influence of plant genetic diversity on virus dispersal by aphid vectors and to examine the biological mechanisms responsible for that influence. In a set of field experiments using the aphid-transmitted barley yellow dwarf virus, I examined the influence of genetic diversity in oat (Avena sativa L.) populations on the spread of the virus and on the population dynamics and movement behavior of aphid vectors of the virus. Only at relatively high aphid abundance were the densities of aphid vectors influenced by plant genetic diversity. In one year out of three, densities of the oat-bird cherry aphid, Rhopalosiphum padi (L.), were significantly lower in the genetically diverse stand than in the genetically homogeneous stands. In no year were densities of the English grain aphid, Sitobion avenae (F.), influenced by the host-plant population. Despite these weak or absent effects on vector abundance, the incidence of the virus was consistently lower in the genetically diverse oat populations. Disease reduction in the diverse populations appears to depend upon changes in aphid movement behavior that affect the efficiency of virus transmission. Mark-release experiments with S. avenae demonstrated that movement rates were significantly higher and plant tenure times were significantly lower in the genetically diverse oat populations. Because the barley yellow dwarf virus requires several hours of aphid feeding for effective transmission, these reduced tenure times and increased travel time among plants led to a reduction of virus transmission. While plant genotype can clearly influence herbivorous insects dramatically, this study suggests that the effects on insects of genetic diversity per se in the host-plant population are likely to be subtle and not easily detected using standard field sampling techniques, except at high insect densities. Yet even at low vector densities, behavioral responses to plant genetic diversity can lead to significant effects on the spread of pathogens. | 465 | yes | crop | domesticated, oat | no | no |
| 466 | 2017 | 43008 | Yan, G; Severson, DW; Christensen, BM | 1997 | Costs and benefits of mosquito refractoriness to malaria parasites: Implications for genetic variability of mosquitoes and genetic control of malaria | The problem of fitness costs associated with host resistance to parasitism is related to the evolution of parasite virulence, population genetic diversity and the dynamics of host-parasite relationships, and proposed strategies for disease control through the genetic manipulation of mosquito vectors. Two Aedes aegypti populations, refractory and susceptible to Plasmodium gallinaceum, were previously selected from the Moyo-In-Dry strain (MOYO) through inbreeding (F = 0.5). Reproductive success and survivorship of the two populations were compared, and the influence of the parasite on mosquito fitness also was evaluated. Fitness components studied include fecundity, adult survivorship and egg-to-adult developmental time, blood-meal size, and adult body size. The refractory population has a significantly shorter egg-to-adult developmental time and a smaller body size, takes a smaller blood meal, and subsequently lays fewer eggs than the susceptible population. The mean longevity of the refractory population is significantly shorter than the susceptible population. Exposure to the parasite exhibited little effect on the survivorship and fecundity of either population. Several factors may contribute to the lower fitness of the refractory population, including founder effect, inbreeding depression, the effect of other uncharacterized genes linked to genes conferring refractoriness, and pleiotropic effects associated with these genes. The results are discussed in relation to the genetic diversity of natural mosquito populations and their implications for the genetic control of malaria. | 466 | probably no | two pop | only two pop | no | no |
| 467 | 2017 | 43008 | Pichler, FB; Baker, CS | 2000 | Loss of genetic diversity in the endemic Hector’s dolphin due to fisheries-related mortality | The endemic New Zealand Hector’s dolphin is considered the rarest species of marine dolphin with a total abundance of less than 4000. The species is listed as vulnerable because of fisheries-related mortality due to entanglement in set nets. The vulnerability of this species is further increased by its fidelity to local natal ranges and the genetic isolation of regional populations. Here we present evidence, based on 108 contemporary samples and 55 historical samples dating back to 1870, of a significant loss of mitochondrial DNA (mtDNA) diversity in two regional populations of Hector’s dolphin. The haplotype diversity (h) was calculated from sequences of a 206 bp fragment in the mtDNA control region, designed to identify 13 out of the 14 known maternal lineages. Over the last 20 years, the North Island population has been reduced from at least three lineages (h = 0.41) to a single lineage (h = 0; P < 0.05). Given its small size, reproductive isolation and reduced genetic diversity, this population is likely to become extinct. The diversity of the East Coast South Island population has declined significantly from h = 0.65 to h = 0.35 (p < 0.05). Based on trend analysis of the mtDNA diversity, we predict that the East Coast population will lose all mtDNA diversity within the next 20 years. This time-series of reduction in genetic variation provides independent evidence of the severity of population decline and habitat contraction resulting from fisheries and perhaps other human activities. | 467 | no | no good fitness measure, N pop differnt in GD? | NA | no | no |
| 468 | 2017 | 43008 | Strobel, HM; Alda, F; Sprehn, CG; Blum, MJ; Heins, DC | 2016 | Geographic and host-mediated population genetic structure in a cestode parasite of the three-spined stickleback | Comparative studies of genetic diversity and population structure can shed light on the ecological and evolutionary factors that influence host-parasite interactions. Here we examined whether geography, time and genetic variation in Alaskan three-spined stickleback (Gasterosteus aculeatus Linneaus) hosts shape the population genetic structure of the diphyllobothridean cestode parasite Schistocephalus solidus (Muller, 1776). Host lineages and haplotypes were identified by sequencing the mitochondrial cytochrome b gene, and host population structure was assessed by Bayesian clustering analysis of allelic variation at 11 microsatellite loci. Parasite population structure was characterized according to allelic variation at eight microsatellite loci. Mantel tests and canonical redundancy analysis were conducted to evaluate the proportion of parasite genetic variation attributable to time and geography vs. host lineage, haplotype, and genotypic cluster. Host and parasite population structure were largely discordant across the study area, probably reflecting differences in gene flow, environmental influences external to the host, and genomic admixture among host lineages. We found that geography explained the greatest proportion of parasite genetic variation, but that variation also reflects time, host lineage, and host haplotype. Associations with host haplotypes suggest that one parasite genotypic cluster exhibits a narrower host range, predominantly infecting the most common host haplotypes, whereas the other parasite cluster infects all haplotypes equally, including rare haplotypes. Although experimental infection trials might prove otherwise, distributional differences in hosts preferentially infected by S. solidus could underlie the observed pattern of population structure. (c) 2016 The Linnean Society of London, Biological Journal of the Linnean Society, 2016, 119, 381-396. | 468 | no | no good fitness measure | NA | no | no |
| 469 | 2017 | 43008 | Naciri-Graven, Y; Launey, S; Lebayon, N; Gerard, A; Baud, JP | 2000 | Influence of parentage upon growth in Ostrea edulis: evidence for inbreeding depression | Genetic variability for growth was analysed in three populations of Ostrea edulis, selected for resistance to the protozoan parasite Bonamia ostreae. This study was undertaken first to determine the potential for selection for growth in populations that have never been selected for this character, and second to estimate heterosis versus inbreeding depression. Growth was monitored in culture for 10 months. The selected populations (namely S85-G3, S89I-G2 and S89W-G2), their crossbred population and a control population were composed of full-sib families whose parents were already genotyped using five microsatellite markers. This genotyping allowed the estimation of genetic relatedness among pairs of parents. The parents’ relatedness was then correlated with the growth performance of their offspring within each of the three populations, and inbreeding depression was estimated. The population effect for growth was highly significant, with the crossbred population having the highest growth rate, followed by S89I-G2 and S89W-G2, S85-G3 and the control population. The within-populations family effect was also highly significant, indicating, as well as the high value for heritability at the family level (between 0.57 and 0.92), that a potential for a further selection for growth still exists within the three populations. Estimates of inbreeding depression (relative to the mean, for complete inbreeding) were high (1 for S891-G2, 0.44 for S89W-G2 and between 0.02 and 0.43 for S85-G3), which correlates with the apparent heterosis for growth observed in the crossbred population. These results are discussed in the context of the future management of the selected populations. | 469 | no | inbreeding | NA | no | no |
| 470 | 2017 | 43008 | Xue, YL; Prado-Martinez, J; Sudmant, PH; Narasimhan, V; Ayub, Q; Szpak, M; Frandsen, P; Chen, Y; Yngvadottir, B; Cooper, DN; de Manuel, M; Hernandez-Rodriguez, J; Lobon, I; Siegismund, HR; Pagani, L; Quail, MA; Hvilsom, C; Mudakikwa, A; Eichler, EE; Cranfield, MR; Marques-Bonet, T; Tyler-Smith, C; Scally, A | 2015 | Mountain gorilla genomes reveal the impact of long-term population decline and inbreeding | Mountain gorillas are an endangered great ape subspecies and a prominent focus for conservation, yet we know little about their genomic diversity and evolutionary past. We sequenced whole genomes from multiple wild individuals and compared the genomes of all four Gorilla subspecies. We found that the two eastern subspecies have experienced a prolonged population decline over the past 100,000 years, resulting in very low genetic diversity and an increased overall burden of deleterious variation. A further recent decline in the mountain gorilla population has led to extensive inbreeding, such that individuals are typically homozygous at 34% of their sequence, leading to the purging of severely deleterious recessive mutations from the population. We discuss the causes of their decline and the consequences for their future survival. | 470 | no | no good fitness measure, N pop differnt in GD? | NA | no | no |
| 471 | 2017 | 43008 | Mader, G; Castro, L; Bonatto, SL; de Freitas, LB | 2016 | Multiple introductions and gene flow in subtropical South American populations of the fireweed, Senecio madagascariensis (Asteraceae) | Non-indigenous plants exhibit different attributes that make them aggressive competitors with indigenous plants and serious threats to biodiversity. Senecio madagascariensis (fireweed, Asteraceae), a native from southern Africa, is a strong competitor in agricultural activities and has toxic alkaloids that may result in high cattle mortality. In Brazil, this weed was collected for the first time in 1995 and has since spread quickly throughout the Pampas region. To better understand the invasion of the fireweed in South America, we used a genetic characterization with internal transcribed spacer (ITS) and microsatellite markers. Based on the ITS data, the southern Brazil populations of S. madagascariensis shared genetic homology with samples taken from the Hawaiian Islands and South Africa. Microsatellite analysis showed the genetic diversity split in two clusters, perhaps intimating the independent introduction of each species into South America. Although fireweed was introduced recently in southern Brazil, the considerable levels of genetic diversity, gene flow, and inbreeding may indicate success in the species establishment in this environment. | 471 | no | no good fitness measure, N pop differnt in GD? | NA | no | no |
| 472 | 2017 | 43008 | Earnhardt, JM; Thompson, SD; Faust, LJ | 2009 | Extinction Risk Assessment for the Species Survival Plan (SSP (R)) Population of the Bali Mynah (Leucopsar rothschildi) | The Bali mynah Species Survival Plan (SSP (R)), an Association of Zoos and Aquariums program, strives to maintain the genetic and demographic health of its Population, avoid unplanned changes in size, and minimize the risk of Population extinction. The SSP Population meets current demographic and genetic objectives with a Population size of 209 birds at 61 institutions and 96%,, genetic diversity (GD) retained from the Source Population. However. participating institution have expressed concerns regarding space allocation, target Population size (TPS). breeding restrictions, inbreeding depression. and harvest in relation to future Population availability and viability. Based oil these factors. we assess five questions with a quantitative risk assessment, specifically a population viability analysis (PVA) using ZooRisk software. Using In individual-based stochastic model, we project potential population changes under different conditions (e.g. changes in TPS and genetic management) to identify the most effective management actions. Our projections indicate that Under current management conditions, population decline and extinction are Unlikely and that although GD will decline over 100 years the projected loss does not exceed levels acceptable to Population managers (less than 90% GD retained). Model simulations indicate that the combination of two genetic management strategies (i.e. priority breeding based oil mean kinship and inbreeding avoidance) benefits the retention of GD and reduces the accumulation of inbreeding. The Current TPS (250) is greater than necessary to minimize the risk of extinction for the SSP Population but any reduction in TPS must be accompanied by continued application of genetic management. If carefully planned, birds can be harvested for transfer to Bali for a reintroduction program without jeopardizing the SSP population . Zoo Biol 28:230-252, 2009. (C) 2009 Wiley-Liss. Inc. | 472 | no | NA | NA | no | no |
| 473 | 2017 | 43008 | Guinand, B; Page, KS; Burnham-Curtis, MK; Scribner, KT | 2012 | Genetic signatures of historical bottlenecks in sympatric lake trout (Salvelinus namaycush) morphotypes in Lake Superior | Humans have played a significant role in reducing levels of genetic diversity and differentiation of many teleost fishes, leading to homogenization across biological entities. We compare patterns of historical and contemporary genetic structure for three sympatric Great Lake’s lake trout (Salvelinus namaycush) morphs (lean, siscowet, and humper) that differ in patterns of habitat occupancy, susceptibility to overfishing and predation by invasive sea lamprey (Petromyzon marinus). Differential susceptibilities to overfishing and predation were expected to result in different impacts to levels of genetic diversity loss for each morphotype. Genetic data was collected for samples at three points in time: 1948 (pre-collapse), 1959 (collapse) and 1990s (current), corresponding to periods of intensive fishing, mortality due to lamprey and recovery, respectively. The lean morph preferentially targeted by the fishery and recognized as highly preyed upon by sea lamprey was more highly impacted genetically than other morphs, as evidenced by greater loss of genetic diversity first during the period of overfishing, then during the period of high sea lamprey abundance once the fishery collapsed. The siscowet morph also experienced genetic bottlenecks during the period of overfishing (pre-collapse period). Results indicate significant levels of genetic differentiation among morphs historically prior to declines in abundance and also among contemporary populations, suggesting that periods of population decline and resurgence in abundance and distribution did not result in loss of genetic distinctiveness among morphs. | 473 | no | no good fitness measure, N pop differnt in GD? | NA | no | no |
| 474 | 2017 | 43008 | Frantzen, MAJ; Ferguson, JWH; de Villiers, MS | 2001 | The conservation role of captive African wild dogs (Lycaon pictus) | Since 1954, several southern African institutions have established captive breeding programs to ensure the long-term survival of the African wild dog (Lycaon pictus). To aid this, a studbook was assembled to provide genetic and demographic information for the southern African captive populations, comprising the largest existing regional population of captive African wild dogs. These populations were investigated over three time frames: during 1985-1990, during 1991-1996 and populations alive in January 1997. The captive-breeding programme is successful with a positive population growth, a significant lowering of inbreeding and mean kinship and an increased genetic diversity. However, genetic variability levels appear lower and levels of inbreeding appear higher compared with wild populations. In addition, there have been no successful long-term re-introductions into the southern African wild using captive-bred dogs, mainly due to the lack of close collaboration between captive breeding and nature conservation institutions. The ultimate success of a conservation programme not only depends on proper demographic and genetic management of the captive population, but primarily on the successful collaboration of all scientific, captive breeding and conservation agencies involved. (C) 2001 Elsevier Science Ltd. All rights reserved. | 474 | no | no good fitness measure | NA | no | no |
| 475 | 2017 | 43008 | Aggarwal, RAK; Dixit, SP; Verma, NK; Mathew, S; Kumar, D; Ahlawat, SPS; Kumar, S; Kumar, Y | 2006 | Genetic diversity in Attappady breed of Indian goat as analyzed with microsatellite markers | Attappady goats are reared by tribal communities of Kerala (India) for meat and manure purposes. These goats have excellent immunity to most of the diseases and can survive under fairly simple and extensive production system with a low level of managerial skills. Genetic diversity within this breed was investigated using 25 microsatellite markers. The mean number of observed alleles per microsatellite marker was 11.08 and that of effective alleles was 5.48. The average observed and expected heterozygosity values were 0.605 and 0.779, respectively. The mean polymorphic information content (PIC) value (0.736) further reflected high level of polymorphism across the loci. Within population inbreeding estimate (F-is = 0.223) suggested moderate level of inbreeding. The study suggested that Attappady goats need appropriate genetic management for their conservation and improvement under harsh climatic conditions. The information generated in this study may further be utilized for studying differentiation and relationships among different Indian goat breeds. | 475 | no | domesticated animal | NA | no | no |
| 476 | 2017 | 43008 | Ogden, R; Shuttleworth, C; McEwing, R; Cesarini, S | 2005 | Genetic management of the red squirrel, Sciurus vulgaris: a practical approach to regional conservation | The progressive decline in red squirrel (Sciurus vulgaris) numbers in Wales has led to conservation and reintroduction projects being established on the island of Anglesey. The recovery of the island’s remnant wild population was initially successful, however concern remained over potential loss of genetic diversity resulting from an observed demographic bottleneck. We used mitochondrial DNA (mtDNA) control region sequences and six microsatellite loci to assess current levels of genetic variation in the population. Samples were monomorphic for control region sequences and a historic specimen from the same area carrying a different haplotype demonstrated a loss of mtDNA diversity during the last 20 years. Inclusion of other Welsh haplotypes indicated phylogeographic structure in the region, in contrast to previous UK studies. Genotyping results showed allelic diversity and heterozygosity to be less than 50% of that recorded in other UK populations, with strong evidence for a recent genetic bottleneck. A parallel reintroduction programme on Anglesey included genetic analysis of individuals during the selection of captive breeding pairs. We present analysis of sequence and microsatellite data, and subsequent management decisions taken to maximise diversity in the founder and F1 generations. Population and Habitat Viability Analysis applied to both populations modelled future levels of heterozygosity and allelic diversity. Supplementation of the remnant and reintroduced populations with translocated squirrels was simulated as a potential management tool; results support use of this strategy to reduce loss of diversity and increase survival. The limitations of applying conservation genetic theory within small-scale management projects are discussed. | 476 | no | only one pop | NA | no | no |
| 477 | 2017 | 43008 | Zhang, L; Huanqiong, N; Gapare, WJ; Dilln, SK; Li, X; Wu, HX | 2014 | Comparison of allelic diversity between native gene resource plantings and selections in open-pollinated progeny test of Pinus radiata D. Don | Genetic diversity within radiata pine first generation of open-pollinated selections (OPS) from the native resource stands was compared with that observed in native populations to monitor potential changes in genetic diversity during domestication. Genetic diversity was estimated using 58 single nucleotide polymorphisms (SNPs) from 8 expressed genes. Nucleotide diversity maintained in first generation of selections (OPS) (mean pi = 0.0036; mean theta(w) = 0.0058) was similar to that found within the native population material (mean pi = 0.0043; mean for theta(w) = 0.0065). Likewise, mean values for expected heterozygosity (HE) within and between native population material and OPS were similar (mean = 0.27 +/- 0.04) and not significantly different (P = 0.068). Also, the overall distribution of allele frequency classes was not significantly different between native population material and OPS. These results point to no evidence of loss of diversity in OPS due to artificial selection. One possible reason is that the domestication of the OPS is at a very early stage. Another may be that artificial selection in the OPS was based on tree growth and form, not wood properties. The genes selected in this study are mostly involved in cell wall formation, thus genetic diversity of these genes should remain stable between natural population and OPS, unless there was a significant sampling bias in the OPS. Although the SNP information suggests similarities among mainland populations, results from quantitative genetic studies found large provenance differences for growth-, morphological-, stem-form traits, and disease resistance. Determining the threshold at which genetic diversity levels will be significantly reduced during selection should help breeders to make informed decisions regarding the intensity of selection in managed breeding populations as well as gene resource populations. | 477 | no | no good fitness measure | NA | no | no |
| 478 | 2017 | 43008 | Albert, EM; Fernandez-Beaskoetxea, S; Godoy, JA; Tobler, U; Schmidt, BR; Bosch, J | 2015 | Genetic management of an amphibian population after a chytridiomycosis outbreak | An epidemic of the disease chytridiomycosis, caused by the pathogenic fungus Batrachochytrium dendrobatidis, induced a massive decline of populations of the common midwife toad (Alytes obstetricans) inhabiting the Pealara Massif (Guadarrama National Park, Central Spain) in the years 1997-2001. The disease outbreak caused the disappearance of about 90 % of populations, leaving only eight remnant breeding populations. In response to the disease-induced population decline, a captive breeding program was started in 2008. Populations were kept separate to minimize possible outbreeding depression. Here, we examined indices of genetic diversity and population structure in these remnant populations to inform future reintroductions. Analysis of ten microsatellite loci showed strong genetic structure between breeding sites suggesting little genetic exchange and relatively low global genetic diversity. In accordance with the demographic bottleneck observed in the last years we found strong evidence for a reduction in genetic diversity. Our results suggest that the captive breeding program should mix animals from multiple sites from the Guadarrama Mountain Range, but avoid the genetically most divergent populations. | 478 | no | no good fitness measure, N pop differnt in GD? | NA | no | no |
| 479 | 2017 | 43008 | Schrempf, A; Aron, S; Heinze, J | 2006 | Sex determination and inbreeding depression in an ant with regular sib-mating | Haplodiploidy is one of the most widespread mechanisms of sex determination in animals. In many Hymenoptera, including all hitherto investigated social species, diploid individuals, which are heterozygous at the sex locus, develop as females, whereas haploid, hemizygous individuals develop as males (single-locus complementary sex determination, sl-CSD). Inbreeding leads to homozygosity at the sex locus, resulting in the production of diploid males, which are usually sterile and constitute a considerable fitness cost. Nevertheless, regular inbreeding without diploid male production is known from several solitary wasps, suggesting that in these species sex is not determined by sl-CSD but alternative mechanisms. Here, we examine sex determination in an ant with regular inbreeding, Cardiocondyla obscurior. The almost complete absence of diploid males after 10 generations of brother-sister mating in the laboratory documents for the first time the absence of sl-CSD and CSD with two or a few unlinked sex loci in a species of social Hymenoptera. Queens, which mated with a brother, appeared to decrease the number of males in their brood, as expected from the relatedness relationships under inbreeding. In contrast, some colonies began to show signs of an inbreeding depression after several generations of sib-mating, such as shortened queen life span, higher brood mortality, and a shift to more male-biased sex ratios in some colonies, presumably due to lower insemination capability of sperm. | 479 | no | inbreeding | NA | no | no |
| 480 | 2017 | 43008 | Doeschl-Wilson, AB; Davidson, R; Conington, J; Roughsedge, T; Hutchings, MR; Villanueva, B | 2011 | Implications of Host Genetic Variation on the Risk and Prevalence of Infectious Diseases Transmitted Through the Environment | Previous studies have shown that host genetic heterogeneity in the response to infectious challenge can affect the emergence risk and the severity of diseases transmitted through direct contact between individuals. However, there is substantial uncertainty about the degree and direction of influence owing to different definitions of genetic variation, most of which are not in line with the current understanding of the genetic architecture of disease traits. Also, the relevance of previous results for diseases transmitted through environmental sources is unclear. In this article a compartmental genetic-epidemiological model was developed to quantify the impact of host genetic diversity on epidemiological characteristics of diseases transmitted through a contaminated environment. The model was parameterized for footrot in sheep. Genetic variation was defined through continuous distributions with varying shape and degree of dispersion for different disease traits. The model predicts a strong impact of genetic heterogeneity on the disease risk and its progression and severity, as well as on observable host phenotypes, when dispersion in key epidemiological parameters is high. The impact of host variation depends on the disease trait for which variation occurs and on environmental conditions affecting pathogen survival. In particular, compared to homogeneous populations with the same average susceptibility, disease risk and severity are substantially higher in populations containing a large proportion of highly susceptible individuals, and the differences are strongest when environmental contamination is low. The implications of our results for the recording and analysis of disease data and for predicting response to selection are discussed. | 480 | no | NA | NA | no | no |
| 481 | 2017 | 43008 | Piel, FB; Howes, RE; Nyangiri, OA; Moyes, CL; Williams, TN; Weatherall, DJ; Hay, SI | 2013 | Online Biomedical Resources for Malaria-Related Red Cell Disorders | Warnings about the expected increase of the global public health burden of malaria-related red cell disorders are accruing. Past and present epidemiological data are necessary to track spatial and temporal changes in the frequencies of these genetic disorders. A number of open access biomedical databases including data on malaria-related red cell disorders have been launched over the last two decades. Here, we review the content of these databases, most of which focus on genetic diversity, and we describe a new epidemiological resource developed by the Malaria Atlas Project. To tackle upcoming public health challenges, the integration of epidemiological and genetic data is important. As many countries are considering implementing national screening programs, strategies to make such data more accessible are also needed. | 481 | no | human | NA | no | no |
| 482 | 2017 | 43008 | Medugorac, I; Veit-Kensch, CE; Ramljak, J; Brka, M; Markovic, B; Stojanovic, S; Bytyqi, H; Kochoski, L; Kume, K; Grunenfelder, HP; Bennewitz, J; Forster, M | 2011 | Conservation priorities of genetic diversity in domesticated metapopulations: a study in taurine cattle breeds | We estimated neutral diversity of 21 European cattle breeds with 105 microsatellites. Nine of them resembled unselected Balkan Busa strains with diffuse breeding barriers and the 12 others were strongly differentiated, isolated breeds. Because of the impact of neutral genetic diversity on long-term population adaptive capacity, we discuss the long-term outcome of different conservation priorities in a subdivided metapopulation of the investigated cattle breeds. The optimal contribution to a pool of total genetic diversity allocated more than 95% of long-term relevant neutral diversity to virtually unselected strains of the Balkan Busa, while the maximization of total variance preferred inbred breeds. Current artificial selection methods, such as genomic selection sped up and a recovery of underestimated traits becomes quickly impossible. We emphasize that currently neutral and even deleterious alleles might be required for future genotypes in sustainable and efficient livestock breeding and production systems of a 21st century. We provide cumulative evidences that long-term survival relies on genetic complexity and complexity relies on allelic diversity. Our results suggest that virtually unselected, nonuniform strains harbor a crucial proportion of neutral diversity and should be conserved with high global priority. As one example, we suggest a cooperative maintenance of the nondifferentiated, highly fragmented, and fast vanishing metapopulation of Balkan Busa. | 482 | no | domesticated animal | NA | no | no |
| 483 | 2017 | 43008 | Browne, L; Karubian, J | 2016 | Frequency-dependent selection for rare genotypes promotes genetic diversity of a tropical palm | Negative frequency-dependent selection among species is a key driver of community diversity in natural systems, but the degree to which negative frequency-dependent selection shapes patterns of survival and genetic diversity within species is poorly understood. In a 5-year field experiment, we show that seedlings of a tropical palm with rare genotypes had a pronounced survival advantage over seedlings with common genotypes, with effect sizes comparable to that of light availability. This ‘rare genotype advantage’ led to an increase in population-wide genetic diversity among seedlings compared to null expectations, as predicted by negative frequency-dependent selection, and increased reproductive success in adult trees with rare genotypes. These results suggest that within-species negative frequency-dependent selection of genotypes can shape genetic variation on ecologically relevant timescales in natural systems and may be a key, overlooked source of non-random mortality for tropical plants. | 483 | no | individual level | NA | no | no |
| 484 | 2017 | 43008 | Khadivi-Khub, A; Shabanian, N; Alikhani, L; Rahmani, MS | 2015 | Genotypic analysis and population structure of Lebanon oak (Quercus libani G. Olivier) with molecular markers | Genetic diversity can play a key role in the survival of plant species. In the present study, the first data on genetic diversity and structure of nine populations in Quercus libani G. Olivier collected from northwest of Iran was provided using inter-simple sequence repeat (ISSR), interretrotransposon amplified polymorphism (IRAP), and start codon targeted (SCoT) markers. The proportions of polymorphic bands were 94.54, 97.96, and 90.86 %, detected by 18 ISSR, 10 IRAP, and 10 SCoT primers, respectively. The mean of Nei’s gene diversity index (H) among the populations by ISSR, IRAP, and SCoT was 0.16, 0.19, and 0.19, and Shannon diversity index (I) was 0.25, 0.29, and 0.28, respectively. Partitioning of genetic variability by analysis of molecular variance (AMOVA) according to each marker and combined data revealed that the intra-population genetic diversity was higher than among populations. Cophenetic correlation coefficient values were statistically significant between ISSR and SCoT, while it was low and insignificant between IRAP and ISSR and between IRAP and SCoT. The dendrogram obtained by each marker and combined dataset reflected geographic structure among most of the populations. Regarding the differentiation among the studied populations and the complexity in taxonomic situation within Q. libani, inter-specific crossings and/or the presence of more than one species within Q. libani is possible. This genetic characterization of Q. libani from Iran contributes to knowledge of the genetic structure of the species and is valuable to define strategies for conservation. | 484 | no | no good fitness measure | NA | no | no |
| 485 | 2017 | 43008 | Machado, CA; Ayala, FJ | 2001 | Nucleotide sequences provide evidence of genetic exchange among distantly related lineages of Trypanosoma cruzi | Simple phylogenetic tests were applied to a large data set of nucleotide sequences from two nuclear genes and a region of the mitochondrial genome of Trypanosoma cruzi,the agent of Chagas’ disease. Incongruent gene genealogies manifest genetic exchange among distantly related lineages of T. cruzi. Two widely distributed isoenzyme types of T. cruzi are hybrids, their genetic composition being the likely result of genetic exchange between two distantly related lineages. The data show that the reference strain for the T. cruzi genome project (CL Brener) is a hybrid. Well-supported gene genealogies show that mitochondrial and nuclear gene sequences from T. cruzi cluster, respectively, in three or four distinct clades that do not fully correspond to the two previously defined major lineages of T. cruzi. There is clear genetic differentiation among the major groups of sequences, but genetic diversity within each major group is low. We estimate that the major extant lineages of T. cruzi have diverged during the Miocene or early Pliocene (3-16 million years ago). | 485 | no | gd of pathogen | NA | no | no |
| 486 | 2017 | 43008 | Dobler, R; Kolliker, M | 2011 | Influence of weight asymmetry and kinship on siblicidal and cannibalistic behaviour in earwigs | In group-living or social species intraspecific predation and cannibalism can lead to substantial decreases in inclusive fitness because related individuals may encounter one another. Therefore, selection on the ability and accuracy of kin recognition to avoid losses in inclusive fitness is expected. Competition and relatedness are two key factors for the evolution of siblicide and cannibalism. However, our knowledge of the temporal dynamics and the accuracy of kin-mediated siblicide and cannibalism is scarce and experiments have usually focused on either relatedness (kin recognition) or weight asymmetry (dominance and competition) as independent components. In an experiment we set up mixed-kinship groups of three nymphs of the European earwig, Forficula auricularia Linnaeus, a gregarious and (sub-) social species, to investigate the combined effects of relatedness and weight asymmetry on the temporal dynamics of siblicide and cannibalism. We found that the temporal dynamics were jointly influenced by relatedness and weight asymmetry among nymphs. Weight asymmetry effects on siblicide and cannibalism were stronger between unrelated individuals than between related individuals as shown by a significant relatedness\*weight asymmetry interaction on survival patterns. The opportunity to choose between related and unrelated individuals did not enhance the accuracy of kin recognition but induced the above interaction between relatedness and weight asymmetry on cannibalistic outcomes. These results show that relatedness and weight affect siblicidal and cannibalistic behaviour, and that their combined effect depends on the social context. (C) 2011 The Association for the Study of Animal Behaviour. Published by Elsevier Ltd. All rights reserved. | 486 | no | no good fitness measure | NA | no | no |
| 487 | 2017 | 43008 | Yan, BQ; Li, ZZ; Huang, HW; Qin, L | 2007 | Genetic diversity and population differentiation of chestnut blight fungus, Cryphonectria parasitica, in China as revealed by RAPD | Seventeen Cryphonectria parasitica populations sampled from six regions in China were investigated using RAPD. Across all 169 isolates from the 17 populations evaluated, 52 of the 71 markers (73%) were polymorphic, total genetic diversity (h) was 0.1463, and Shannon’s index was 0.2312. Diversity within populations accounted for 74% of total genetic diversity, and genetic differentiation among populations was 0.26 (G (ST) = 0.26). Gene flow was 1.4 among the populations; higher gene flow was found among populations within regions and among regions [N-m (G(SR)) = 2.8 and N-m (G(RT)) = 3.5]. The unweighted pair group mean analysis (UPGMA) dendrogram revealed two distinct clusters: the northern China group and the southern China group. The spatial autocorrelation analysis revealed that the variation at most loci was randomly distributed and lacked spatial structure, but several loci and closer distances were spatially structured. Human activity and habitat could also be important factors affecting genetic structure among C. parasitica populations in China. Genetic diversity was highest in Southwest China, descending in an orderly fashion to Northeast China. This pattern indicated that Southwest China might be the center of origin of C. parasitica in China. The present study provides useful information for understanding the origin and spread of chestnut blight fungus in China and valuable data for formulating relevant strategies for controlling the disease in China. | 487 | no | Gd of parasote | NA | no | no |
| 488 | 2017 | 43008 | Lana, TG; Azevedo, JL; Pomella, AWV; Monteiro, RTR; Silva, CB; Araujo, WL | 2011 | Endophytic and pathogenic isolates of the cacao fungal pathogen Moniliophthora perniciosa (Tricholomataceae) are indistinguishable based on genetic and physiological analysis | We evaluated the genetic and physiological variability of Moniliophthora perniciosa obtained from healthy and diseased branches of cacao (Theobroma cacao) plants. The diversity of the isolates was evaluated by RAPD technique and by studies of virulence and exoenzyme production. The genetic variability of endophytic and pathogenic M. perniciosa was evaluated in association with pathogenicity assays. RAPD analysis showed eight genetic groups, which were not related to plant disease status (healthy versus diseased branches). Isolates from cacao were included in three groups, excluding isolates from other host plants. Pathogenicity and enzyme analysis showed that the virulence of the isolates is not related to exoenzyme production. This is the first evidence that M. perniciosa colonizes healthy parenchymatic tissues, showing that endophytic behavior may occur in this species. | 488 | no | gd of parasite | NA | no | no |
| 489 | 2017 | 43008 | Ozener, B; Graham, JH | 2014 | Growth and Fluctuating Asymmetry of Human Newborns: Influence of Inbreeding and Parental Education | Historically, medical concerns about the deleterious effects of closely inbred marriages have focused on the risk posed by recessive Mendelian disease, with much less attention to developmental instability. We studied the effects of inbreeding (first-cousin marriage) on growth and fluctuating asymmetry of 200 full-term infants (101 inbred and 99 outbred) whose parents were of similar socioeconomic status in Sivas Province, Turkey. In addition to differences in their mean inbreeding coefficients (f = 1/16 for first cousins and f < 1/1,024 for unrelated parents), the consanguineous parents were less well educated (3 years, on average for both husbands and wives). We measured weight, height, head circumference, and chest circumference of the newborns, as well as four bilateral traits (ear width, ear length, and second and fourth digit lengths). After taking education into account, none of the measures of size (weight, height, head circumference, and chest circumference) and fluctuating asymmetry differed between the inbred and outbred groups. Male children of well-educated parents, however, were larger and had less fluctuating asymmetry. Female children of well-educated parents weighed more than those of less well-educated parents, but were otherwise indistinguishable for height, head circumference, chest circumference, and fluctuating asymmetry. We conclude that inbreeding depression causes neither an increase in fluctuating asymmetry of full-term newborns, nor a decrease in body size. Unmeasured variables correlated with education appear to have an effect on fluctuating asymmetry and size of male children and only a weak effect on size (weight) of female children. Am J Phys Anthropol 153:45-51, 2014. (c) 2013 Wiley Periodicals, Inc. | 489 | no | human | NA | no | no |
| 490 | 2017 | 43008 | Eimes, JA; Bollmer, JL; Whittingham, LA; Johnson, JA; Van Oosterhout, C; Dunn, PO | 2011 | Rapid loss of MHC class II variation in a bottlenecked population is explained by drift and loss of copy number variation | Population bottlenecks may reduce genetic variation and potentially increase the risk of extinction. Here, we present the first study to use historic samples to analyse loss of variation at the major histocompatibility complex (MHC), which plays a central role in vertebrate disease resistance. Balancing selection acts on the MHC and could moderate the loss of variation expected from drift; however, in a Wisconsin population of greater prairie-chickens (Tympanuchus cupido), the number of MHC class II B alleles per individual declined by 44% following a population bottleneck, compared to a loss of only 8% at microsatellites. Simulations indicate that drift likely reduced MHC variation at the population level, as well as within individuals by reducing the number of gene copies per individual or by fixing the same alleles across multiple loci. These multiple effects of genetic drift on MHC variation could have important implications for immunity and fitness. | 490 | no | no good fitness measure, N pop differnt in GD? | NA | no | no |
| 491 | 2017 | 43008 | Lee, GM; Brown, MJF; Oldroyd, BP | 2013 | Inbred and outbred honey bees (Apis mellifera) have similar innate immune responses | Social insect colonies provide ideal conditions for the spread of pathogens. It has been proposed that the extreme polyandry and genetic diversity seen in the colonies of some eusocial insect species is central to a colony’s defence against disease. Indeed, empirically, colonies headed by polyandrous queens have lower incidence of pathogens than genetically uniform monoandrous colonies. The mechanisms of improved resistance in genetically diverse colonies could arise from the genetic diversity among worker genotypes or from increased innate immunity arising from heterozygosity at immune gene loci within individual workers. Here, we investigate the effects of heterozygosity on two components of the honey bee (Apis mellifera) innate immune system: encapsulation and phenoloxidase (PO) activity. No significant effect of heterozygosity on immune system activity was evident for either encapsulation or PO activity. Thus, we conclude that while encapsulation and PO activity are important components of the immune response, it seems that they do not underlie the positive effects of genetic diversity on parasite and pathogen resistance in honey bees. | 491 | no | no good fitness measure, N pop differnt in GD? (inbreeding?) | NA | no | no |
| 492 | 2017 | 43008 | Hayden, HL; Howlett, BJ | 2005 | Genetic structure of a population of the fungus Leptosphaeria maculans in a disease nursery of Brassica napus in Australia | Microsatellite, minisatellite and mating type markers were used to determine the genetic structure of the fungus Leptosphaeria maculans within a disease nursery, where Brassica napus lines were screened for resistance to blackleg disease under high inoculum pressure. Fungal isolates were collected from pseudothecia in infected stubble and pycnidia within cotyledon lesions on seedlings within the nursery. Genetic diversity was high with gene diversity at H=0.700 across four polymorphic loci, and genotypic diversity at D=0.9193. Among the 159 isolates analysed, 102 multilocus genotypes were identified. The even distribution of mating type idiomorphs MAT1-1 and MAT1-2 and gametic equilibrium within the population provided further evidence of random mating. Genetic diversity was distributed on a very fine scale in the disease nursery. I he majority of genetic diversity (67%) was distributed among conidia within a lesion or among ascospores from a piece of stubble, while the remainder (33%) was distributed within lesions on seedlings or different stubble pieces. There were no among-group differences between samples from stubble and seedlings. This is consistent with the low level of genetic differentiation between the ascospore and conidia samples (F-ST = 0.017) indicating that all isolates of L. maculans from the disease nursery most likely belong to one population, and that ascospores form the primary inoculum in the disease nursery. | 492 | no | NA | NA | no | no |
| 493 | 2017 | 43008 | Sayar-Turet, M; Dreisigacker, S; Braun, HJ; Hede, A; MacCormack, R; Boyd, LA | 2011 | Genetic variation within and between winter wheat genotypes from Turkey, Kazakhstan, and Europe as determined by nucleotide-binding-site profiling | The genetic diversity within wheat breeding programs across Turkey and Kazakhstan was compared with a selection of European cultivars that represented the genetic diversity across eight European countries and six decades of wheat breeding. To focus the measure of genetic diversity on that relevant to disease-resistant phenotypes, nucleotide-binding-site (NBS) profiling was used to detect polymorphisms associated with the NBS motifs found within the NBS - leucine-rich repeat (LRR) class of resistance (R) genes. Cereal-specific NBS primers, designed specifically to the conserved NBS motifs found within cereal R-genes, provided distinct NBS profiles. Although the genetic diversity associated with NBS motifs was only slightly higher within the Eastern wheat genotypes, the NBS profiles produced by Eastern and European wheat lines differed considerably. Structure analysis divided the wheat genotypes into four groups, which compared well with the origin of the wheat genotypes. The highest levels of genetic diversity were seen for the wheat genotypes from the Genetic Resource Collection held in Ankara, Turkey, as wheat genotypes within breeding programs were genetically more similar. The wheat genotypes from Kazakhstan were the most similar to the European cultivars, reflecting the significant number of eastern European cultivars used in the breeding program in Kazakhstan. In general, the NBS profiles suggested that NBS-LRR R-gene usage in winter wheat breeding in Turkey and Kazakhstan differed from that deployed in European cultivars. | 493 | no | crop | NA | no | no |
| 494 | 2017 | 43008 | Halbert, ND; Raudsepp, T; Chowdhary, BP; Derr, JN | 2004 | Conservation genetic analysis of the Texas State Bison Herd | The Texas State Bison Herd is directly descended from the herd assembled from 5 wild-caught bison by Charles Goodnight in the 1880s. In 1997, 36 bison were used to establish a herd at Caprock Canyons State Park. To aid in the development of a long-term genetic conservation plan for this population, we examined and analyzed allelic variation at 54 microsatellite loci representing each of the nuclear chromosomes in the bison genome. The current Texas State Bison Herd population exhibits low genetic diversity and heterozygosity levels compared with bison at Yellowstone National Park and Theodore Roosevelt National Park. Parentage analysis indicates that relatively few adults have contributed offspring in the last 5 years, leading to low effective population size estimations and a rapid increase in the average age of animals in the herd. The very limited number of original founders, multiple population bottlenecks over the last 120 years, and chronically small population size, coupled with genetic drift and inbreeding, have resulted in dangerously low levels of genetic diversity. This, in turn, has likely triggered demographic problems such as low recruitment and high calf mortality rates. Population viability analysis based on current population demography reveals that there is a 99% chance of extinction of the herd within the next 41 years. Based on these findings, the continued existence of this historically important bison population appears doubtful without the introduction of new genetic variation from another plains bison herd. | 494 | no | no pop level | NA | no | no |
| 495 | 2017 | 43008 | Collevatti, RG; Leite, KCE; de Miranda, GHB; Rodrigues, FHG | 2007 | Evidence of high inbreeding in a population of the endangered giant anteater, Myrmecophaga tridactyla (Myrmecophagidae), from Emas National Park, Brazil | We report the genetic structure, relatedness and mating structure of a population of the endangered giant anteater Myrmecophaga tridactyla Linnaeus, 1758 in the Emas National Park, Brazil, based on variability at five microsatellite loci. Additionally, we addressed the hypothesis that the M. tridactyla population studied has low levels of polymorphism and high levels of inbreeding and relatedness and that animals with overlapping home range are highly related. All five microsatellite loci displayed low levels of polymorphism and of expected and observed heterozygosity. The low level of polymorphism and high inbreeding showed by the population studied may be the outcome of high mortality and reduction in population size due to recurrent fire events in the Emas National Park, as reported in 1994. The reduction in population size may have led to a higher frequency of mating between closely related animals, augmented by the isolation of the population in the park because of the expansion of agricultural land and fragmentation of the Cerrado environment. The natural history of M. tridactyla and the phylopatric (sex-biased dispersal) behavior of females should increase the effects of isolation and bottlenecking, decreasing gene flow and increasing inbreeding. However, the low levels of polymorphism found in this population may simply be due to the natural history and evolution of M. tridactyla as reported for other species. The genetic structure and dynamics of this population needs to be investigated more profoundly in order to provide sound data for the design of conservation strategies for M. tridactyla in the Emas National Park. | 495 | no | inbreeding | NA | no | no |
| 496 | 2017 | 43008 | Jaatinen, K; Noreikiene, K; Merila, J; Ost, M | 2012 | Kin association during brood care in a facultatively social bird: active discrimination or by-product of partner choice and demography? | Intra-group relatedness does not necessarily imply kin selection, a leading explanation for social evolution. An overlooked mechanism for generating population genetic structure is variation in longevity and fecundity, referred to as individual quality, affecting kin structure and the potential for cooperation. Individual quality also affects choosiness in partner choice, a key process explaining cooperation through direct fitness benefits. Reproductive skew theory predicts that relatedness decreases with increasing group size, but this relationship could also arise because of quality-dependent demography and partner choice, without active kin association. We addressed whether brood-rearing eider (Somateria mollissima) females preferentially associated with kin using a 6-year data set with individuals genotyped at 19 microsatellite loci and tested whether relatedness decreased with increasing female group size. We also determined the relationship between local relatedness and indices of female age and body condition. We further examined whether the level of female intracoalition relatedness differed from background relatedness in any year. As predicted, median female intra-group relatedness decreased with increasing female group size. However, the proportion of related individuals increased with advancing female age, and older females prefer smaller brood-rearing coalitions, potentially producing a negative relationship between group size and relatedness. There were considerable annual fluctuations in the level of relatedness between coalition-forming females, and in 1 year this level exceeded that expected by random association. Thus, both passive and active mechanisms govern kin associations in brood-rearing eiders. Eiders apparently can discriminate between kin, but the benefits of doing so may vary over time. | 496 | no | no pop level | NA | no | no |
| 497 | 2017 | 43008 | Kark, S; Alkon, PU; Safriel, UN; Randi, E | 1999 | Conservation priorities for chukar partridge in Israel based on genetic diversity across an ecological gradient | Recent studies suggest that patterns of genetic diversity significantly influence the viability and persistence of local populations. Revealing and mapping spatial patterns of genetic diversity within species’ ranges may be vital when defining criteria and prioritizing areas for conservation. Chukar Partridges (Alectoris chukar) in Israel occur along a steep ecogeographical gradient extending from mesic Mediterranean zones in the north to steppe and desert regions in the south. To rest the hypothesis that the most genetically diverse populations within a species’ range occur within the ecotone, an area of transition between ecosystems where a sharp environmental gradient exists, we examined the allozyme diversity of chukars collected at five locations within the species’ continuous range in each of 2 years. Based on 32 allozyme loci, the genetic diversity of chukars increased significantly, along a gradient from population in Mediterranean regions to those at the ecotone in the northern Negev desert, despite close geographical proximity among populations Genetic diversity As estimated by percent polymorphic loci, observed and expected heterozygosity, and mean number of alleles was not homogeneous among sampling localities: single and multilocus Hardy-Weinberg and linkage disequilibria increased along the gradient toward the ecotone. Populations exhibited some isolation by distance effects in the face of substantial gene flow. We therefore recommend that higher conservation priority be assigned to the Mediterranean-Negev ecotone area. For Chukar Partridges, it supports the highest overall genetic diversity across ecological gradients. Rapid urbanization of Israeli landscapes, and management of chukars in Israel within a metapopulation context is an urgent requirement. | 497 | no | no good fitness measure, N pop differnt in GD? | NA | no | no |
| 498 | 2017 | 43008 | Monzon-Arguello, C; de Leaniz, CG; Gajardo, G; Consuegra, S | 2014 | Eco-immunology of fish invasions: the role of MHC variation | The relationship between invaders and the pathogens encountered in their new environment can have a large effect on invasion success. Invaders can become free from their natural pathogens and reallocate costly immune resources to growth and reproduction, thereby increasing invasion success. Release from enemies and relaxation of selective pressures could render newly founded populations more variable at immune-related genes, such as the major histocompatibility complex (MHC), particularly when they have different origins. Using rainbow and brown trout, two of the world’s most successful fish invaders, we tested the general hypothesis that invaders should display high intrapopulation immunogenetic diversity and interpopulation divergence, due to the interplay between genetic drift and successive waves of genetically divergent introductions. We analysed genetic diversity and signatures of selection at the MHC class II beta immune-related locus. In both species, MHC diversity (allelic richness and heterozygosity) for southern hemisphere populations was similar to values reported for populations at their native range. However, MHC functional diversity was limited, and population immunogenetic structuring weaker than that observed using neutral markers. Depleted MHC functional diversity could reflect a decrease in immune response, immune-related assortative mating or selection for resistance to newly encountered parasites. Given that the role of MHC diversity in the survival of these populations remains unclear, depleted functional diversity of invasive salmonids could compromise their long-term persistence. A better understanding of the eco-immunology of invaders may help in managing and preventing the impact of biological invasions, a major cause of loss of biodiversity worldwide. | 498 | no | no good fitness measure, N pop differnt in GD? | NA | no | no |
| 499 | 2017 | 43008 | Mueller, UG | 1996 | Life history and social evolution of the primitively eusocial bee Augochlorella striata (Hymenoptera: Halictidae) | Life history parameters of the primitively eusocial bee Augochlorella striata (Hymenoptera: Halictidae) are summarized for a population in central New York, and analyzed with respect to the evolutionary maintenance of eusociality in this species. High mortality (39%) of solitary foundresses and low mortality (12%) of social colonies indicate severe ecological constraints limiting independent (solitary) reproduction. Extreme relatedness asymmetries (3:1, due to singly-mated queens) and female-biased reproductive broods create genetic incentives favoring worker behavior (helping). In addition, workers appear to capitalize on relatedness asymmetries by biasing the sex ratio towards their more closely related sisters and away from their more distantly related brothers, because eusocial colonies (headed by a mother queen; relatedness asymmetry present) consistently produce more female-biased sex ratios than parasocial colonies (headed by a sister queen after queen supersedure; relatedness asymmetry absent). Both intrinsic factors inherent in the haplo-diploid system of sex determination (relatedness asymmetries in conjunction with female-biased sex ratios) and extrinsic factors (ecological constraints) therefore appear to modulate eusocial evolution in A. striata. | 499 | no | No GD diff between pop | NA | no | no |
| 500 | 2017 | 43008 | Lowe, JK; Maller, JB; Pe’er, I; Neale, BM; Salit, J; Kenny, EE; Shea, JL; Burkhardt, R; Smith, JG; Ji, WZ; Noel, M; Foo, JN; Blundell, ML; Skilling, V; Garcia, L; Sullivan, ML; Lee, HE; Labek, A; Ferdowsian, H; Auerbach, SB; Lifton, RP; Newton-Cheh, C; Breslow, JL; Stoffel, M; Daly, MJ; Altshuler, DM; Friedman, JM | 2009 | Genome-Wide Association Studies in an Isolated Founder Population from the Pacific Island of Kosrae | It has been argued that the limited genetic diversity and reduced allelic heterogeneity observed in isolated founder populations facilitates discovery of loci contributing to both Mendelian and complex disease. A strong founder effect, severe isolation, and substantial inbreeding have dramatically reduced genetic diversity in natives from the island of Kosrae, Federated States of Micronesia, who exhibit a high prevalence of obesity and other metabolic disorders. We hypothesized that genetic drift and possibly natural selection on Kosrae might have increased the frequency of previously rare genetic variants with relatively large effects, making these alleles readily detectable in genome-wide association analysis. However, mapping in large, inbred cohorts introduces analytic challenges, as extensive relatedness between subjects violates the assumptions of independence upon which traditional association test statistics are based. We performed genome-wide association analysis for 15 quantitative traits in 2,906 members of the Kosrae population, using novel approaches to manage the extreme relatedness in the sample. As positive controls, we observe association to known loci for plasma cholesterol, triglycerides, and C-reactive protein and to a compelling candidate loci for thyroid stimulating hormone and fasting plasma glucose. We show that our study is well powered to detect common alleles explaining >= 5% phenotypic variance. However, no such large effects were observed with genome-wide significance, arguing that even in such a severely inbred population, common alleles typically have modest effects. Finally, we show that a majority of common variants discovered in Caucasians have indistinguishable effect sizes on Kosrae, despite the major differences in population genetics and environment. | 500 | no | no good fitness measure, N pop differnt in GD? (inbreeding?) | NA | no | no |
| 501 | 2017 | 43008 | Patterson, N; Petersen, DC; van der Ross, RE; Sudoyo, H; Glashoff, RH; Marzuki, S; Reich, D; Hayes, VM | 2010 | Genetic structure of a unique admixed population: implications for medical research | Understanding human genetic structure has fundamental implications for understanding the evolution and impact of human diseases. In this study, we describe the complex genetic substructure of a unique and recently admixed population arising similar to 350 years ago as a direct result of European settlement in South Africa. Analysis was performed using over 900 000 genome-wide single nucleotide polymorphisms in 20 unrelated ancestry-informative marker selected Coloured individuals and made comparisons with historically predicted founder populations. We show that there is substantial genetic contribution from at least four distinct population groups: Europeans, South Asians, Indonesians and a population genetically close to the isiXhosa sub-Saharan Bantu. This is in good accord with the historical record. We briefly examine the implications of determining the genetic diversity of this population, not only for furthering understanding of human evolution out of Africa, but also for genome-wide association studies using admixture mapping. In conclusion, we define the genetic structure of a uniquely admixed population that holds great potential to advance genetic-based medical research. | 501 | no | human | NA | no | no |
| 502 | 2017 | 43008 | Dubey, S; Ursenbacher, S; Pellet, J; Fumagalli, L | 2009 | Genetic differentiation in two European tree frog (Hyla arborea) metapopulations in contrasted landscapes of western Switzerland | The survival of threatened species its the European tree frog (Hyla arborea) is strongly dependent on the genetic variability within populations. its well as gene flow between them,. In Switzerland. only two sectors in its western part still harbour metapopulations. The first is characterised by a very heterogeneous and Urbanized landscape. while the second is characterised by it uninterrupted array Of suitable habitats. In this study, six microsatellite loci were used it) establish levels of genetic differentiation among the populations from the two different location,,. The results, show that the metapopulations have (i) weak levels of genetic differentiation (F(ST) within metapopulation approximate to 0.04) (ii) no difference in levels of genetic structuring between them, (iii) significant (p = 0.019) differences in terms of genetic diversity (Hs) and observed heterozygozity (Ho), the metapopulation located in it disturbed landscape showing lower values. Our results suggest that even if the dispersal of H. arborea among contiguous ponds seems to be efficient in areas of heterogeneous landscape, a loss of genetic diversity call occur. | 502 | no | no good fitness measure | NA | no | no |
| 503 | 2017 | 43008 | Pecnerova, P; Diez-del-Molino, D; Vartanyan, S; Dalen, L | 2016 | Changes in variation at the MHC class II DQA locus during the final demise of the woolly mammoth | According to the nearly-neutral theory of evolution, the relative strengths of selection and drift shift in favour of drift at small population sizes. Numerous studies have analysed the effect of bottlenecks and small population sizes on genetic diversity in the MHC, which plays a central role in pathogen recognition and immune defense and is thus considered a model example for the study of adaptive evolution. However, to understand changes in genetic diversity at loci under selection, it is necessary to compare the genetic diversity of a population before and after the bottleneck. In this study, we analyse three fragments of the MHC DQA gene in woolly mammoth samples radiocarbon dated to before and after a well-documented bottleneck that took place about ten thousand years ago. Our results indicate a decrease in observed heterozygosity and number of alleles, suggesting that genetic drift had an impact on the variation on MHC. Based on coalescent simulations, we found no evidence of balancing selection maintaining MHC diversity during the Holocene. However, strong trans-species polymorphism among mammoths and elephants points to historical effects of balancing selection on the woolly mammoth lineage. | 503 | no | no good fitness measure, N pop differnt in GD? | NA | no | no |
| 504 | 2017 | 43008 | Li, J; Shen, H; Wang, H; Zhao, M; Luo, Z; Wu, H | 2016 | Diversifying selection is the dominant factor affecting the geographical variation of MHC class II genes in the Omei tree frog | Genes encoding the major histocompatibility complex (MHC) are excellent candidates for elucidating adaptive variation because of their essential role in immune function. Hypotheses for how genetic variations of MHC genes are maintained include balancing selection, diversifying selection and neutral events; however, which of these forces are dominant remains controversial. In this study, we determined the preliminary forces that shaped MHC geographical variation in Omei tree frog by comparing with neutral microsatellites. The results revealed high genetic diversity and significant population differentiation in MHC genes of this species. The differentiation patterns of MHC and microsatellites were uncorrelated. Stronger level of genetic differentiation in MHC genes compared with that in microsatellites (based on comparison in both genetic differentiation patterns and strength of isolation by distance patterns) indicates the effects of diversifying selection on the geographical variation of MHC genes. Considering the significant evidence of positive selection acting on antigen-binding sites, we presumed that selection pressures may be mainly from locally different pathogens when gene flow is restricted. The results of this study contribute to a more comprehensive understanding of the evolutionary forces that maintain MHC geographical variation among natural populations. | 504 | no | no pop level | NA | no | no |
| 505 | 2017 | 43008 | Paschke, M; Bernasconi, G; Schmid, B | 2005 | Effects of inbreeding and pollen donor provenance and diversity on offspring performance under environmental stress in the rare plant Cochlearia bavarica | Habitat degradation and loss can reduce size and genetic variability of natural populations, increasing individual homozygosity and average relatedness between individuals. While the resulting inbreeding depression may be reduced by natural selection under prevailing environmental conditions, it may increase again under environmental stress. To investigate the effect of environmental stress on offspring performance and the expression of inbreeding depression, we hand-pollinated maternal plants in small (< 100, n = 5) and large populations (> 400 flowering plants, n = 5) of the rare plant Cochlearia bavarica (Brassicaceae) and raised the offspring under experimentally manipulated water and light regimes (normal or reduced supply). In addition to considering natural variation in inbreeding levels due to population size, we manipulated pollen donor provenance and diversity. Maternal plants were pollinated with nine donors from a different population or with one or nine donors from the same population. One further inflorescence of each maternal plant was exposed to free pollination. Offspring growth and survival were monitored over 300 days. Offspring performance varied significantly among populations and maternal plants. Environmental stress interacted significantly with these factors. However, there was no general indication that offspring from small populations were more negatively affected. In seven out of 10 populations, offspring derived from between-population pollination performed better than offspring derived from within-population pollination. Also, in five out of 10 populations, average offspring size was higher after within-population pollination with nine than after pollination with one pollen donor. These results suggest low genetic diversity within C. bavarica populations, both smaller and larger ones. Interactions between environmental stress and pollination treatment indicated that using pollen donors from outside a population or increasing the number of pollen donors can reduce inbreeding depression, but that this beneficial effect is impaired under stressful conditions. (c) 2005 Gesellschaft fur Okologie. Published by Elsevier GmbH. All rights reserved. | 505 | no | inbreeding | NA | no | no |
| 506 | 2017 | 43008 | Branch, OH; Sutton, PL; Barnes, C; Castro, JC; Hussin, J; Awadalla, P; Hijar, G | 2011 | Plasmodium falciparum Genetic Diversity Maintained and Amplified Over 5 Years of a Low Transmission Endemic in the Peruvian Amazon | “Plasmodium falciparum entered into the Peruvian Amazon in 1994, sparking an epidemic between 1995 and 1998. Since 2000, there has been sustained low P. falciparum transmission. The Malaria Immunology and Genetics in the Amazon project has longitudinally followed members of the community of Zungarococha (N = 1,945, 4 villages) with active household and health center-based visits each year since 2003. We examined parasite population structure and traced the parasite genetic diversity temporally and spatially. We genotyped infections over 5 years (2003-2007) using 14 microsatellite (MS) markers scattered across ten different chromosomes. Despite low transmission, there was considerable genetic diversity, which we compared with other geographic regions. We detected 182 different haplotypes from 302 parasites in 217 infections. Structure v2.2 identified five clusters (subpopulations) of phylogenetically related clones. To consider genetic diversity on a more detailed level, we defined haplotype families (hapfams) by grouping haplotypes with three or less loci differences. We identified 34 different hapfams identified. The F(st) statistic and heterozygosity analysis showed the five clusters were maintained in each village throughout this time. A minimum spanning network (MSN), stratified by the year of detection, showed that haplotypes within hapfams had allele differences and haplotypes within a cluster definition were more separated in the later years (2006-2007). We modeled hapfam detection and loss, accounting for sample size and stochastic fluctuations in frequencies overtime. Principle component analysis of genetic variation revealed patterns of genetic structure with time rather than village. The population structure, genetic diversity, appearance/disappearance of the different haplotypes from 2003 to 2007 provides a genome-wide”“real-time”" perspective of P. falciparum parasites in a low transmission region." | 506 | no | gd of parasite | NA | no | no |
| 507 | 2017 | 43008 | van Oven, M; Hammerle, JM; van Schoor, M; Kushnick, G; Pennekamp, P; Zega, I; Lao, O; Brown, L; Kennerknecht, I; Kayser, M | 2011 | Unexpected Island Effects at an Extreme: Reduced Y Chromosome and Mitochondrial DNA Diversity in Nias | The amount of genetic diversity in a population is determined by demographic and selection events in its history. Human populations which exhibit greatly reduced overall genetic diversity, presumably resulting from severe bottlenecks or founder events, are particularly interesting, not least because of their potential to serve as valuable resources for health studies. Here, we present an unexpected case, the human population of Nias Island in Indonesia, that exhibits severely reduced Y chromosome (non-recombining portion of the Y chromosome [NRY]) and to a lesser extent also reduced mitochondrial DNA (mtDNA) diversity as compared with most other populations from the Asia/Oceania region. Our genetic data, collected from more than 400 individuals from across the island, suggest a strong previously undetected bottleneck or founder event in the human population history of Nias, more pronounced for males than for females, followed by subsequent genetic isolation. Our findings are unexpected given the island’s geographic proximity to the genetically highly diverse Southeast Asian world, as well as our previous knowledge about the human history of Nias. Furthermore, all NRY and virtually all mtDNA haplogroups observed in Nias can be attributed to the Austronesian expansion, in line with linguistic data, and in contrast with archaeological evidence for a pre-Austronesian occupation of Nias that, as we show here, left no significant genetic footprints in the contemporary population. Our work underlines the importance of human genetic diversity studies not only for a better understanding of human population history but also because of the potential relevance for genetic disease-mapping studies. | 507 | no | human | NA | no | no |
| 508 | 2017 | 43008 | Yan, GY; Chadee, DD; Severson, DW | 1998 | Evidence for genetic hitchhiking effect associated with insecticide resistance in Aedes aegypti | Information on genetic variation within and between populations is critical for understanding the evolutionary history of mosquito populations and disease epidemiology. Previous studies with Drosophila suggest that genetic variation of selectively neutral loci in a large fraction of genome may be constrained by fixation of advantageous mutations associated with hitchhiking effect. This study examined restriction fragment length polymorphisms of four natural Aedes aegypti mosquito populations from Trinidad and Tobago, at 16 loci. These populations have been subjected to organophosphate (OP) insecticide treatments for more than two decades, while dichlor-diphenyltrichlor (DDT) was the insecticide of choice prior to this period. We predicted that genes closely linked to the OP target loci would exhibit reduced genetic variation as a result of the hitchhiking effect associated with intensive OP insecticide selection. We also predicted that genetic variability of the genes conferring resistance to DDT and loci near the target site would be similar to other unlinked loci. As predicted, reduced genetic variation was found for loci in the general chromosomal region of a putative OP target site, and these loci generally exhibited larger F-ST values than other random loci. In contrast, the gene conferring resistance to DDT and its linked loci show polymorphisms and genetic differentiation similar to other random loci. The reduced genetic variability and apparent gene deletion in some regions of chromosome 1 likely reflect the hitchhiking effect associated with OP insecticide selection. | 508 | no | human | NA | no | no |
| 509 | 2017 | 43008 | Asad, HA; Meah, MB; Begum, SN; Khalil, MI; Rafii, MY; Latif, MA | 2015 | Study of genetic variation of eggplant cultivars by using RAPD-PCR molecular markers and the relationship with Phomopsis blight disease reaction | Disease susceptibility and genetic variability in 10 eggplant genotypes were studied after inoculating Phomopsis vexans under confined field conditions. Random amplified polymorphic DNA (RAPD) markers were used to assess genetic variation and relationships among eggplant genotypes. The disease index of leaves ranged 0.208-13.79%, while fruit infection ranged 2.15-42.76%. Two varieties, Dohazari G and Laffa S, were found to be susceptible, 6 were moderately resistant, 1 was moderately susceptible, and BAU Begun-1 was resistant to P. vexans. Amplification of genomic DNA by using 3 RAPD primers produced 20 bands: 14 (70%) were polymorphic and 6 (30%) were monomorphic. The highest intra-variety similarity indices values were found in ISD 006, Ishurdi L, Jessore L, and BAU Begun-1 (100%), while the lowest was in Dohazari G (90%). The lowest genetic distance (0.0513) and the highest genetic identity (0.9500) were observed between the ISD 006 and Ishurdi L combinations. A comparatively higher genetic distance (0.3724) and the lowest genetic identity (0.6891) were observed between the ISD 006 and Dohazari G combinations. A dendogram was constructed based on Nei’s genetic distance, which produced 2 main clusters of the genotypes - Cluster I: ISD 006, Ishurdi L, Marich begun L, BAU Begun-1, Marich begun S, and Chega and Cluster 2: Laffa S, Dohazari G, Jessore L, and Singhnath. Genetic variation and its relationship with disease susceptibility were assessed using RAPD markers, to develop disease-resistant varieties and improve eggplant crops. | 509 | no | crop | NA | no | no |
| 510 | 2017 | 43008 | Tensen, L; Groom, RJ; van Belkom, J; Davies-Mostert, HT; Marnewick, K; van Vuuren, BJ | 2016 | Genetic diversity and spatial genetic structure of African wild dogs (Lycaon pictus) in the Greater Limpopo transfrontier conservation area | The Greater Limpopo Transfrontier Conservation Area (GLTFCA) is one of the last refuges for the endangered African wild dog and hosts roughly one-tenth of the global population. Wild dogs in this area are currently threatened by human encroachment, habitat fragmentation and scarcity of suitable connecting habitat between protected areas. We derived genetic data from mitochondrial and nuclear markers to test the following hypotheses: (i) demographic declines in wild dogs have caused a loss of genetic variation, and (ii) Zimbabwean and South African populations in the GLTFCA have diverged due to the effects of isolation and genetic drift. Genetic patterns among five populations, taken with comparisons to known information, illustrate that allelic richness and heterozygosity have been lost over time, presumably due to effects of inbreeding and genetic drift. Genetic structuring has occurred due to low dispersal rates, which was most apparent between Kruger National Park and the Zimbabwean Lowveld. Immediate strategies to improve gene flow should focus on increasing the quality of habitat corridors between reserves in the GLTFCA and securing higher wild dog survival rates in unprotected areas, with human-mediated translocation only undertaken as a last resort. | 510 | no | no good fitness measure | NA | no | no |
| 511 | 2017 | 43008 | Rumbaugh, KP; Trivedi, U; Watters, C; Burton-Chellew, MN; Diggle, SP; West, SA | 2012 | Kin selection, quorum sensing and virulence in pathogenic bacteria | Bacterial growth and virulence often depends upon the cooperative release of extracellular factors excreted in response to quorum sensing (QS). We carried out an in vivo selection experiment in mice to examine how QS evolves in response to variation in relatedness (strain diversity), and the consequences for virulence. We started our experiment with two bacterial strains: a wild-type that both produces and responds to QS signal molecules, and a lasR (signal-blind) mutant that does not release extracellular factors in response to signal. We found that: (i) QS leads to greater growth within hosts; (ii) high relatedness favours the QS wild-type; and (iii) low relatedness favours the lasR mutant. Relatedness matters in our experiment because, at relatively low relatedness, the lasR mutant is able to exploit the extracellular factors produced by the cells that respond to QS, and hence increase in frequency. Furthermore, our results suggest that because a higher relatedness favours cooperative QS, and hence leads to higher growth, this will also lead to a higher virulence, giving a relationship between relatedness and virulence that is in the opposite direction to that usually predicted by virulence theory. | 511 | no | bacteria relatedness | NA | no | no |
| 512 | 2017 | 43008 | MAPPES, T; YLONEN, H; VIITALA, J | 1995 | HIGHER REPRODUCTIVE SUCCESS AMONG KIN GROUPS OF BANK VOLES (CLETHRIONOMYS-GLAREOLUS | According to the kin selection theory the degree of genetic relatedness affects the nature of intraspecific competition and it might promote cooperative and/or altruistic behavior between individuals. We examined kinship effects on reproductive success of territorial females and survival of juveniles in the bank voIe Clethrionomys glareolus. Four experimental populations were founded in large enclosures (0.5 ha): two consisted of five related (Related, R) and two of five mutually unrelated females (Unrelated, UR). Each population had five males of heterogeneous origin. Our earlier results showed that populations of the R grew twice as fast as the UR. Here we show that recruitment and offspring survival is connected to spacing behavior of mothers. Unrelated females who had their home ranges close to each other produced significantly fewer recruits than the related ones. Their home ranges overlapped significantly less and ranges were smaller if they were situated close to each other. Furthermore, the survival of juveniles declined with dispersal distance from the natal territory in populations of the UR. Trappabilities of juveniles were also lower among the young whose home ranges were close to unrelated females, indicating avoidance of mature females. Our results indicate that competition for space is more intense among unrelated neighbors, decreasing their reproductive success. Infanticide towards unrelated nestlings and/or juveniles is one possible mechanism decreasing survival and causing a lower recruitment of young. On the contrary, sharing of space among related neighbors did not seem to cause notable costs on their reproduction. Mature females allowed related young females to use their territory, but still, the young: did not acquire an area for their own reproduction, probably due to a mother-daughter conflict. The basic social structure of Clethrionomys populations, i.e., exclusive female territoriality, remained the same regardless of a high degree of relatedness. | 512 | probably no | no good fitness measure | no fitness measure | no | no |
| 513 | 2017 | 43008 | Simone-Finstrom, M; Foo, B; Tarpy, DR; Starks, PT | 2014 | Impact of Food Availability, Pathogen Exposure, and Genetic Diversity on Thermoregulation in Honey Bees (Apis mellifera) | Accurate thermoregulation in honey bees is crucial for colony survival. Multiple factors influence how colonies manage in-hive temperature, including genetic diversity. We explored the influence of genetic diversity on thermoregulatory behavior under three conditions: natural foraging, supplemental feeding, and exposure to the fungal pathogen shown to induce a social fever in honey bees. Our data suggest that (1) the degree of genetic diversity expected under normal conditions is not predictive of thermoregulatory stability, (2) the social fever response of honey bees is not a simple stimulus-response mechanism but appears to be influenced by ambient temperature conditions, and (3) a temperature-based circadian rhythm emerges under high nectar flow conditions. Taken together, these data suggest that a richer, context-dependent thermoregulatory system exists in honey bees than previously understood. | 513 | maybe | any gD and fitness of groups that works? | no good fitness measure | no | no |
| 514 | 2017 | 43008 | Li, RJ; Erpelding, JE | 2016 | Genetic diversity analysis of Gossypium arboreum germplasm accessions using genotyping-by-sequencing | The diploid cotton species Gossypium arboreum possesses many favorable agronomic traits such as drought tolerance and disease resistance, which can be utilized in the development of improved upland cotton cultivars. The USDA National Plant Germplasm System maintains more than 1600 G. arboreum accessions. Little information is available on the genetic diversity of the collection thereby limiting the utilization of this cotton species. The genetic diversity and population structure of the G. arboreum germplasm collection were assessed by genotyping-by-sequencing of 375 accessions. Using genome-wide single nucleotide polymorphism sequence data, two major clusters were inferred with 302 accessions in Cluster 1, 64 accessions in Cluster 2, and nine accessions unassigned due to their nearly equal membership to each cluster. These two clusters were further evaluated independently resulting in the identification of two sub-clusters for the 302 Cluster 1 accessions and three sub-clusters for the 64 Cluster 2 accessions. Low to moderate genetic diversity between clusters and sub-clusters were observed indicating a narrow genetic base. Cluster 2 accessions were more genetically diverse and the majority of the accessions in this cluster were landraces. In contrast, Cluster 1 is composed of varieties or breeding lines more recently added to the collection. The majority of the accessions had kinship values ranging from 0.6 to 0.8. Eight pairs of accessions were identified as potential redundancies due to their high kinship relatedness. The genetic diversity and genotype data from this study are essential to enhance germplasm utilization to identify genetically diverse accessions for the detection of quantitative trait loci associated with important traits that would benefit upland cotton improvement. | 514 | no | no good fitness measure, N pop differnt in GD? | NA | no | no |
| 515 | 2017 | 43008 | Agudo, R; Alcaide, M; Rico, C; Lemus, JA; Blanco, G; Hiraldo, F; Donazar, JA | 2011 | Major histocompatibility complex variation in insular populations of the Egyptian vulture: inferences about the roles of genetic drift and selection | Insular populations have attracted the attention of evolutionary biologists because of their morphological and ecological peculiarities with respect to their mainland counterparts. Founder effects and genetic drift are known to distribute neutral genetic variability in these demes. However, elucidating whether these evolutionary forces have also shaped adaptive variation is crucial to evaluate the real impact of reduced genetic variation in small populations. Genes of the major histocompatibility complex (MHC) are classical examples of evolutionarily relevant loci because of their well-known role in pathogen confrontation and clearance. In this study, we aim to disentangle the partial roles of genetic drift and natural selection in the spatial distribution of MHC variation in insular populations. To this end, we integrate the study of neutral (22 microsatellites and one mtDNA locus) and MHC class II variation in one mainland (Iberia) and two insular populations (Fuerteventura and Menorca) of the endangered Egyptian vulture (Neophron percnopterus). Overall, the distribution of the frequencies of individual MHC alleles (n = 17 alleles from two class II B loci) does not significantly depart from neutral expectations, which indicates a prominent role for genetic drift over selection. However, our results point towards an interesting co-evolution of gene duplicates that maintains different pairs of divergent alleles in strong linkage disequilibrium on islands. We hypothesize that the co-evolution of genes may counteract the loss of genetic diversity in insular demes, maximize antigen recognition capabilities when gene diversity is reduced, and promote the co-segregation of the most efficient allele combinations to cope with local pathogen communities. | 515 | no | no good fitness measure | NA | no | no |
| 516 | 2017 | 43008 | Lehman, N; Decker, DJ; Stewart, BS | 2004 | Divergent patterns of variation in major histocompatibility complex class II alleles among antarctic phocid pinnipeds | The 4 species of phocid pinnipeds that live in pack-ice habitats of the Antarctic have been relatively isolated from infectious diseases that are prevalent in mammals elsewhere. Consequently, patterns of genetic variability at key immune system loci in these seals might be primarily related to other selective factors correlative with interspecific differences in life history, demography, and ecological niches. To test that hypothesis, we investigated nucleotide-sequence variation in a 162-base pair region of DQalpha exon 2 in major histocompatibility complex (MHC) class II genes of Weddell seals (Leptonychotes weddellii), crabeater seals (Lobodon carcinophaga), Ross seals (Ommatophoca rossii), and leopard seals (Hydrurga leptonyx) from the Ross Sea of Antarctica. We found substantial differences in patterns of population genetic diversity among these seals. Crabeater seals were the most diverse, with 2 DQalpha loci, 39 distinct alleles among 30 seals, and at least 93% observed heterozygosity. Leopard seals were the least diverse, with only 1 allele detected in 13 seals. Weddell and Ross seals had intermediate diversity, with 11 alleles in 35 Weddell seals (89% observed heterozygosity) and 2 alleles in 42 Ross seals (21% observed heterozygosity). These patterns are congruent with extant knowledge of interspecific variation in life histories, population biology, and ecological niches of these species and consistent with previously reported patterns of microsatellite genetic variability. Moreover, our findings suggest that responses to past evolutionary pressures have differed in each species and that interspecific variation in future responses to introduced infectious diseases also may vary substantially. | 516 | no | no pop level | NA | no | no |
| 517 | 2017 | 43008 | LeBas, NR | 2002 | Mate choice, genetic incompatibility, and outbreeding in the ornate dragon lizard, Ctenophorus ornatus | There has been recent interest in the role genetic incompatibility may play in mate or sperm choice. One source of incompatibility may be too similar or disparate genomes. An isolated population of the ornate dragon lizard, Ctenophorus ornatus, was followed over a breeding season and parentage assigned to the offspring using microsatellites. It was found that the adults in the population had an eight per cent chance of mating with a relative. I examined whether C. ornatus mate (or fertilize their eggs) with respect to genetic similarity. There was no difference in a female’s relatedness to the male in whose territory she resided and her average relatedness to all other males. Neither was there a difference in the relatedness of the male that sired a female’s offspring and the female’s average relatedness to all other males. There was no evidence of a cost to mating with a more genetically similar mate, because offspring survival was not influenced by degree of inbreeding or outbreeding. However, females that were more outbred had fewer offspring survive. In this small population there are two possible explanations for the decreased fitness associated with outbreeding. First, the species may have an evolutionary history of inbreeding and thus may be susceptible to outbreeding depression. Second, as fitter individuals produce more offspring, these offspring have an increased probability of breeding with relatives, leading to an indirect relationship between fitness and outbreeding. | 517 | no | only one pop | NA | no | no |
| 518 | 2017 | 43008 | Zhang, FT; Duan, YJ; Cao, SM; Wang, JW; Tan, DQ | 2012 | High genetic diversity in population of Lepturichthys fimbriata from the Yangtze River revealed by microsatellite DNA analysis | Lepturichthys fimbriata (Gunther) is one of the benthic and rock-attached fish species that is typically found in torrential flows of the upper reaches of the Yangtze River in China. Several dams in the Yangtze River (the Ertan Dam, the Three Gorges Dam, the Gezhouba Dam, the Xiluodu Dam and the Xiangjiaba Dam (the latter two dams are under construction)) may have significant effects on the habitat and spawning behaviors of L. fimbriata, and could ultimately threaten the survival of this fish. We studied the population genetic diversity of L. fimbriata samples collected at three sites within the Yangtze River and one of its tributaries, the Yalong River. Genetic diversity patterns were determined by analyzing genetic data from 14 polymorphic microsatellite loci isolated in Jinshaia sinensis (Sauvage et Dabry). A high genetic diversity among these L. fimbriata local populations was indicated by the number of microsatellite alleles (A) and the expected heterozygosity. No reductions of genetic diversity in any L. fimbriata population were observed. However, significant population differentiations were observed among three local populations by pairwise comparisons (P<0.001). We deduced that L. fimbriata local populations were not small ones. In addition, the habitat behaviors of rock-attachment and possible residence of L. fimbriata could account for the genetic differences found in local populations. | 518 | no | No GD diff between pop | NA | no | no |
| 519 | 2017 | 43008 | Uphyrkina, O; Miquelle, D; Quigley, H; Driscoll, C; O’Brien, SJ | 2002 | Conservation genetics of the Far Eastern leopard (Panthera pardus orientalis) | The Far Eastern or Amur leopard (Panthera pardus orientalis) survives today as a tiny relict population of 25-40 individuals in the Russian Far East. The population descends from a 19th-century northeastern Asian subspecies whose range extended over southeastern Russia, the Korean peninsula, and northeastern China. A molecular genetic survey of nuclear microsatellite and mitochondrial DNA (mtDNA) sequence variation validates subspecies distinctiveness but also reveals a markedly reduced level of genetic variation. The, amount of genetic diversity measured is the lowest among leopard subspecies and is comparable to the genetically depleted Florida panther and Asiatic lion populations. When considered in the context of nonphysiological perils that threaten small populations (e.g., chance mortality, poaching, climatic extremes, and infectious disease), the genetic and demographic data indicate a critically diminished wild population under severe threat of extinction. An established captive population of P. p. orientalis displays much higher diversity than the wild population sample, but nearly all captive individuals are derived from a history of genetic admixture with the adjacent Chinese subspecies, P. p. japonensis. The conservation management implications of potential restoration/augmentation of the wild population with immigrants from the captive population are discussed. | 519 | no | no pop level | NA | no | no |
| 520 | 2017 | 43008 | Rowntree, JK; Zytynska, SE; Frantz, L; Hurst, B; Johnson, A; Preziosi, RF | 2014 | The genetics of indirect ecological effects-plant parasites and aphid herbivores | When parasitic plants and aphid herbivores share a host, both direct and indirect ecological effects (IEEs) can influence evolutionary processes. We used a hemiparasitic plant (Rhinanthus minor), a grass host (Hordeum vulgare) and a cereal aphid (Sitobion avenae) to investigate the genetics of IEEs between the aphid and the parasitic plant, and looked to see how these might affect or be influenced by the genetic diversity of the host plants. Survival of R. minor depended on the parasite’s population of origin, the genotypes of the aphids sharing the host and the genetic diversity in the host plant community. Hence the indirect effects of the aphids on the parasitic plants depended on the genetic environment of the system. Here, we show that genetic variation can be important in determining the outcome of IEEs. Therefore, IEEs have the potential to influence evolutionary processes and the continuity of species interactions over time. | 520 | no | no pop level | NA | no | no |
| 521 | 2017 | 43008 | Annan, Z; Durand, P; Ayala, FJ; Arnathau, C; Awono-Ambene, P; Simard, F; Razakandrainibe, FG; Koella, JC; Fontenille, D; Renaud, F | 2007 | Population genetic structure of Plasmodium falciparum in the two main African vectors, Anopheles gambiale and Anopheles funestus | We investigated patterns of genetic diversity of Plasmodium falciparum associated with its two main African vectors: Anopheles gambiae and Anopheles funestus. We dissected 10,296 wild-caught mosquitoes from three tropical sites, two in Cameroon (Simbock and Tibati, separated by 320 km) and one in Kenya (Rota, > 2,000 km from the other two sites). We assayed seven microsatellite loci in 746 oocysts from 183 infected mosquito guts. Genetic polymorphism was very high in parasites isolated from both vector species. The expected heterozygosity (H-E) was 0.79 in both species; the observed heterozygosities (H-O) were 0.32 in A. funestus and 0.42 in A. gambiae, indicating considerable inbreeding within both vector species. Mean selfing (s) between genetically identical gametes was s = 0.33. Differences in the rate of inbreeding were statistically insignificant among sites and between the two vector species. As expected, because of the high rate of inbreeding, linkage disequilibrium was very high; it was significant for all 21 loci pairs in A. gambiae and for 15 of 21 pairs in A. funestus, although only two pairwise comparisons were between loci on the same chromosome. Overall, the genetic population structure of A falciparum, as evaluated by F statistics, was predominantly clonal rather than panmictic, a population structure that facilitates the spread of antimalarial drug and vaccine resistance and thus may impair the effectiveness of malaria control efforts. | 521 | no | gd of parasite | NA | no | no |
| 522 | 2017 | 43008 | Zhang, FT; Tan, DQ | 2010 | Genetic diversity in population of largemouth bronze gudgeon (Coreius guichenoti Sauvage et Dabry) from Yangtze River determined by microsatellite DNA analysis | Largemouth bronze gudgeon (Coreius guichenoti Sauvage et Dabry 1874), one of the endemic fish species in the upper reaches of the Yangtze River in China, is a benthic and potamodromous fish that is typically found in rivers with torrential flow. Three dams in the Yangtze River, Ertan Dam, Three Gorges Dam and Gezhouba Dam, may have had vital impacts on the habitat and spawning behaviors of largemouth bronze gudgeon, and could ultimately threaten the survival of this fish. We studied the population genetic diversity of C. guichenoti samples collected at seven sites (JH, GLP, BX, HJ, MD, SDP and XB) within the Yangtze River and one of its tributaries, the Yalong River. Genetic diversity patterns were determined by analyzing genetic data from 11 polymorphic microsatellite loci. A high genetic diversity among these largemouth bronze gudgeon populations was indicated by the number of microsatellite alleles (A) and the expected heterozygosity (HE). No significant population variation occurred among GLP, BX, HJ and MD populations, but dramatic population differentiation was observed among JH and XB, two dam-blocked populations, versus other populations. Tests for bottlenecks did not indicate recent dramatic population declines and concurrent losses of genetic diversity in any largemouth bronze gudgeon populations. To the contrary, we found that dams accelerated the population differentiation of this fish. | 522 | no | no good fitness measure | NA | no | no |
| 523 | 2017 | 43008 | Pinheiro, LR; Rabbani, ARC; da Silva, AVC; Ledo, AD; Pereira, KLG; Diniz, LEC | 2012 | Genetic diversity and population structure in the Brazilian Cattleya labiata (Orchidaceae) using RAPD and ISSR markers | Brazilian orchids are currently threatened with extinction due to habitat loss and, because of their high ornamental value, intense collecting pressure. Genetic diversity can play a key role in the survival of endangered orchid species. Here we provide the first data on genetic diversity and structure of wild populations in the genus Cattleya, in particular C. labiata, using random amplified polymorphic DNA (RAPD) and intersimple sequence repeat (ISSR) markers. We studied 130 individuals, 117 belonging to Cattleya labiata and 13 from 10 other species in the same genus. Data generated from 12 ISSR and 12 RAPD primers were used to determine genetic variability via a model-based Bayesian procedure (Structure) and molecular variance analysis. In addition, Shannon index, genetic diversity and Jaccard coefficients were also estimated. The marker data indicated that C. labiata has a high level of polymorphism, and five reconstructed populations were identified by Structure. The unweighted pair group method with arithmetic mean dendrogram did not group the samples by origin, which was also confirmed by Bayesian analysis, demonstrating the complex genetic structure of C. labiata. Other Cattleya species showed no relationship with any C. labiata sample. This genetic characterization of Cattleya from northeast Brazil contributes to knowledge of the genetic structure of the species and can be used to define strategies for conservation and breeding programmes. | 523 | no | no pop level | NA | no | no |
| 524 | 2017 | 43008 | Gordo, I; Campos, PRA | 2007 | Patterns of genetic variation in populations of infectious agents | Background: The analysis of genetic variation in populations of infectious agents may help us understand their epidemiology and evolution. Here we study a model for assessing the levels and patterns of genetic diversity in populations of infectious agents. The population is structured into many small subpopulations, which correspond to their hosts, that are connected according to a specific type of contact network. We considered different types of networks, including fully connected networks and scale free networks, which have been considered as a model that captures some properties of real contact networks. Infectious agents transmit between hosts, through migration, where they grow and mutate until elimination by the host immune system. Results: We show how our model is closely related to the classical SIS model in epidemiology and find that: depending on the relation between the rate at which infectious agents are eliminated by the immune system and the within host effective population size, genetic diversity increases with R-0 or peaks at intermediate R-0 levels; patterns of genetic diversity in this model are in general similar to those expected under the standard neutral model, but in a scale free network and for low values of R-0 a distortion in the neutral mutation frequency spectrum can be observed; highly connected hosts (hubs in the network) show patterns of diversity different from poorly connected individuals, namely higher levels of genetic variation, lower levels of genetic differentiation and larger values of Tajima’s D. Conclusion: We have found that levels of genetic variability in the population of infectious agents can be predicted by simple analytical approximations, and exhibit two distinct scenarios which are met according to the relation between the rate of drift and the rate at which infectious agents are eliminated. In one scenario the diversity is an increasing function of the level of transmission and in a second scenario it is peaked around intermediate levels of transmission. This is independent of the type of host contact structure. Furthermore for low values of R-0, very heterogeneous host contact structures lead to lower levels of diversity. | 524 | no | gd of pathogen | NA | no | no |
| 525 | 2017 | 43008 | Schmitt, C; Garant, D; Doyon, K; Bousquet, N; Gaudreau, L; Belisle, M; Pelletier, F | 2017 | Patterns of Diversity and Spatial Variability of beta-Defensin Innate Immune Genes in a Declining Wild Population of Tree Swallows | Assessing the genetic variation and distribution of immune genes across heterogeneous environmental conditions in wild species is essential to further our understanding of the role of pathogen pressure and potential resistance or prevalence in hosts. Researchers have recently investigated beta-defensin genes in the wild, because their variability suggests that they may play an important role in innate host defense. This study investigated the variation occurring at 6 innate immune genes of the beta-defensin family in a declining population of tree swallows (Tachycineta bicolor) in southern Quebec, Canada (N = 160). We found that all 6 genes showed synonymous and nonsynonymous single nucleotide polymorphisms (SNPs) within the exon coding for the mature peptide. These results indicated that this group of genes was diverse in tree swallows. Our results suggested a potential interaction of this group of genes with fluctuating pathogen diversity, however, we found no sign of positive or negative selection. We assessed whether or not the distribution of genetic diversity of beta-defensin genes in our study population differed between 2 regions that strongly differ in their level of agricultural intensification. Adults are highly philopatric to their breeding sites and their immunological responses differ between these 2 regions. However, we found little evidence that the level and distribution of genetic variability differed between these heterogeneous environmental conditions. Further studies should aim to assess the link between genetic diversity of beta-defensin genes and fitness-related traits in wild populations. | 525 | no | no pop level | NA | no | no |
| 526 | 2017 | 43008 | Nielsen, RK; Pertoldi, C; Loeschcke, V | 2007 | Genetic evaluation of the captive breeding program of the Persian wild ass | During the last century, many species have become endangered and conservation in terms of captive breeding has been crucial for their survival. Classical management of captive species is based on recorded genealogies. However, if pedigrees are incomplete or inaccurate, it can bias the interpretation of the results obtained from analyses based on such data. In this investigation, 12 microsatellite loci were investigated to evaluate the studbook information of the critically endangered Persian wild ass, Equus heinionus onager. Relatedness and inbreeding coefficients were calculated in order to compare the same coefficients estimated from the recorded studbook. A significant correlation between coefficients obtained by microsatellites and the studbook validates the recorded studbook as a reasonable tool for future genetic management. Furthermore, a Bayesian-based method divided the captive onager population into four subgroups that indicate departure from random mating, and thus minor rotation of animals between zoos. Lastly, analyses for inferring past demographic changes revealed a gradual population decline and inbreeding over several generations. This may indicate a low genetic load in captive onagers as a consequence of some degree ofpurging. Consequently, the risk of inbreeding depression should currently be minimal in the captive breeding program. Therefore, it is recommended to increase the connectivity between the four subgroups of onagers in order to reduce the risk of demographic and genetic stochasticity. This study underlines the importance of using molecular markers to evaluate genetic management of captive breeding programs. | 526 | no | no pop level | NA | no | no |
| 527 | 2017 | 43008 | Gurdasani, D; Carstensen, T; Tekola-Ayele, F; Pagani, L; Tachmazidou, I; Hatzikotoulas, K; Karthikeyan, S; Iles, L; Pollard, MO; Choudhury, A; Ritchie, GS; Xue, YL; Asimit, J; Nsubuga, RN; Young, EH; Pomilla, C; Kivinen, K; Rockett, K; Kamali, A; Doumatey, AP; Asiki, G; Seeley, J; Sisay-Joof, F; Jallow, M; Tollman, S; Mekonnen, E; Ekong, R; Oljira, T; Bradman, N; Bojang, K; Ramsay, M; Adeyemo, A; Bekele, E; Motala, A; Norris, SA; Pirie, F; Kaleebu, P; Kwiatkowski, D; Tyler-Smith, C; Rotimi, C; Zeggini, E; Sandhu, MS | 2015 | The African Genome Variation Project shapes medical genetics in Africa | Given the importance of Africa to studies of human origins and disease susceptibility, detailed characterization of African genetic diversity is needed. The African Genome Variation Project provides a resource with which to design, implement and interpret genomic studies in sub-Saharan Africa and worldwide. The African Genome Variation Project represents dense genotypes from 1,481 individuals and whole-genome sequences from 320 individuals across sub-Saharan Africa. Using this resource, we find novel evidence of complex, regionally distinct hunter-gatherer and Eurasian admixture across sub-Saharan Africa. We identify new loci under selection, including loci related to malaria susceptibility and hypertension. We show that modern imputation panels (sets of reference genotypes from which unobserved or missing genotypes in study sets can be inferred) can identify association signals at highly differentiated loci across populations in sub-Saharan Africa. Using whole-genome sequencing, we demonstrate further improvements in imputation accuracy, strengthening the case for large-scale sequencing efforts of diverse African haplotypes. Finally, we present an efficient genotype array design capturing common genetic variation in Africa. | 527 | no | human | NA | no | no |
| 528 | 2017 | 43008 | Kerstes, NAG; Wegner, KM | 2011 | The effect of inbreeding and outcrossing of Tribolium castaneum on resistance to the parasite Nosema whitei | Background: The microsporidian Nosema whitei is a natural parasite of the red flour beetle, Tribolium castaneum. The results of a previous study showed that, during co-evolution of the two species in the laboratory, host populations maintained elevated levels of heterozygosity. Hypothesis: Heterozygote advantage accounts for the maintenance of high levels of host heterozygosity during co-evolution with the parasite. Reduced heterozygosity of the beetle will lead to a decrease in resistance against parasite infection. Methods: In two experiments, we tested for (a) the effect of inbreeding and (b) the effect of inbreeding and outcrossing on the beetle’s resistance to infection, in relation to effects on egg hatching success, development time, and reproductive success. Results: Inbreeding reduced egg hatching success, prolonged development time, and resulted in lower reproductive success. Outcrossing shortened development time, while we did not find evidence for heterosis for reproductive success. We were unable to detect an effect of changes in heterozygosity on overall resistance to parasitism. The effect of inbreeding on development time did, however, influence parasite-induced mortality profiles: the prolonged development time, resulting from inbreeding, led to higher mortality in earlier developmental stages, but left the overall mortality rate unchanged. Hence, we conclude that heterozygosity is not a principal determinant of the beetle’s resistance to infection by N. whitei. | 528 | no | inbreeding | NA | no | no |
| 529 | 2017 | 43008 | PAUL, REL; PACKER, MJ; WALMSLEY, M; LAGOG, M; RANFORDCARTWRIGHT, LC; PARU, R; DAY, KP | 1995 | MATING PATTERNS IN MALARIA PARASITE POPULATIONS OF PAPUA-NEW-GUINEA | Description of the genetic structure of malaria parasite populations is central to an understanding of the spread of multiple-locus drug and vaccine resistance. The Plasmodium falciparum mating patterns from Madang, Papua New Guinea, where intense transmission of malaria occurs, are described here. A high degree of inbreeding occurs in the absence of detectable linkage disequilibrium. This contrasts with other studies, indicating that the genetic structure of malaria parasite populations is neither clonal nor panmictic but will vary according to the transmission characteristics of the region. | 529 | no | gd of parasite | NA | no | no |
| 530 | 2017 | 43008 | McCluskey, EM; Mockford, SW; Sands, K; Herman, TB; Johnson, G; Gonser, RA | 2016 | Population Genetic Structure of Blanding’s Turtles (Emydoidea blandingii) in New York | The conservation and management of Blanding’s Turtles (Emydoidea blandingii) has attracted considerable attention in recent years as the species is recognized as being at risk over much of its current range. We used seven variable microsatellites to examine five populations from Ontario (St. Lawrence River valley) to southeastern New York (Dutchess County) to assess genetic diversity and estimate gene flow, genetic drift, and the roles of distance and topography in dispersal of Blanding’s Turtles. Three peripheral populations in the St. Lawrence River valley exhibited low levels of differentiation (F-ST < 0.07); past or current gene flow between these adjoining populations has likely been facilitated by the St. Lawrence River. A disjunct population in Dutchess County was divergent from other populations but showed no signs of loss of genetic diversity. A recently discovered population in Saratoga County displayed low levels of genetic diversity, possibly indicating that it has been isolated for some time. The St. Lawrence River valley populations appear to have sufficient habitat corridors to have maintained gene flow after the Wisconsin glaciation. This may not reflect current connectivity, however, and conservation measures should be aimed at maintaining connectivity and decreasing road mortality. Management plans for the Dutchess County population should consider maintaining connectivity among subpopulations a priority to avoid losing unique alleles to genetic drift. The Saratoga area should be investigated for other pockets of individuals that could potentially contribute to overall genetic diversity. The discontinuous distribution of this species within New York, coupled with regional differences in genetic variation, highlight the importance of maintaining all extant populations. | 530 | no | no good fitness measure | NA | no | no |
| 531 | 2017 | 43008 | Hu, LP; Huang, XT; Sun, Y; Mao, JX; Wang, S; Wang, CD; Bao, ZM | 2015 | Molecular genetic analysis of heterosis in interspecific hybrids of Argopecten purpuratus x A. irradians irradians | Argopecten purpuratus and Argopecten irradians irradians hybridization was successfully performed and the hybrid offspring displayed apparent heterosis in growth traits. To better understand the genetic basis of heterosis, the genomic composition and genetic variation of the hybrids were analyzed with amplified fragment length polymorphism (AFLP) and simple sequence repeat (SSR) markers. Seven of eight universal SSR primers displayed polymorphism in the hybrids and their parental groups, and hybrids inherited both parental genotypes at each locus. Using five primer combinations in AFLP analysis, 433 loci were amplified in the hybrids and their parental groups. The frequency of polymorphisms was 88.22%. F1 hybrids inherited 88.11 and 92.88% of AFLP bands from their parents. Some loci did not follow Mendelian Law, including 48 loci in parents that were lost, and 11 new loci that were amplified in the hybrids. The parameters of Nei’s gene diversity, Shannon’s Information index, genetic distance, and molecular variance between groups were calculated. The genetic differentiation between two hybrid groups (0.253) was smaller than that between hybrids and their parents (0.554 to 0.645), and was especially smaller than that between two parental groups (0.769). The high genetic similarity (0.9347) and low genetic differentiation (0.2531) between two hybrid groups suggests that these hybrid groups were genetically very close. Heterozygosities of hybrid groups were higher than those of parental groups, indicating that the hybrids had increased genetic diversity. | 531 | no | hybrids | NA | no | no |
| 532 | 2017 | 43008 | Willis, K | 2001 | Unpedigreed populations and worst-case scenarios | “A population in which parentage information is not recorded is considered a pedigree”“black hole”" in terms of genetic management. Integration of animals from such an unpedigreed population into a genetically managed population is often accomplished by assuming a worst-case scenario and recording all animals as highly interrelated (e.g., full siblings). The assumption that a worst-case scenario is appropriate under a wide variety of circumstances is evaluated. It was determined that the genetic costs of overestimating relationships among animals from an unpedigreed population are always greater than the genetic costs of underestimating relationships by the same amount. This demonstrates that worst-case scenarios, which are by definition intentional overestimates, are not generally appropriate. A method of obtaining an estimate that will yield the lowest overall genetic cost using the possible maximum and minimum levels of relatedness among animals from the unpedigreed population is presented along with two methods for implementation. Finally, for cases in which the estimated average relatedness among animals from an unpedigreed population is low, it is recommended that different analytical studbook datasets be used for calculation of inbreeding coefficients and mean kinship coefficients. This strategy would allow close estimation of average relatedness without risking inadvertent pairing of highly related animals from the unpedigreed population. Zoo Biol 20:305-314, 2001. (C) 2001 Wiley-Liss, Inc." | 532 | no | NA | NA | no | no |
| 533 | 2017 | 43008 | Dionne, M; Miller, KM; Dodson, JJ; Caron, F; Bernatchez, L | 2007 | Clinal variation in mhc diversity with temperature: Evidence for the role of host-pathogen interaction on local adaptation in Atlantic salmon | In vertebrates, variability at genes of the Major Histocompatibility Complex (MHC) represents an important adaptation for pathogen resistance, whereby high allelic diversity confers resistance to a greater number of pathogens. Pathogens can maintain diversifying selection pressure on their host’s immune system that can vary in intensity based on pathogen richness, pathogen virulence, and length of the cohabitation period, which tend to increase with temperature. In this study, we tested the hypothesis that genetic diversity of MHC increases with temperature along a latitudinal gradient in response to pathogen selective pressure in the wild. A total of 1549 Atlantic salmon from 34 rivers were sampled between 46 degrees N and 58 degrees N in Eastern Canada. The results supported our working hypothesis. In contrast to the overall pattern observed at microsatellites, MHC class II allelic diversity increased with temperature, thus creating a latitudinal gradient. The observed temperature gradient was more pronounced for MHC amino acids of the peptide-binding region (PBR), a region that specifically binds to pathogens, than for the non-PBR. For the subset of rivers analyzed for bacterial diversity, MHC amino acid diversity of the PBR also increased significantly with bacterial diversity in each river. A comparison of the relative influence of temperature and bacterial diversity revealed that the latter could have a predominant role on MHC PBR variability. However, temperature was also identified as an important selective agent maintaining MHC diversity in the wild. Based on the bacteria results and given the putative role of temperature in shaping large-scale patterns of pathogen diversity and virulence, bacterial diversity is a plausible selection mechanism explaining the observed association between temperature and MHC variability. Therefore, we propose that genetic diversity at MHC class II represents local adaptation to cope with pathogen diversity in rivers associated with different thermal regimes. This study illuminates the link between selection pressure from the environment, host immune adaptation, and the large-scale genetic population structure for a nonmodel vertebrate in the wild. | 533 | no | no good fitness measure, N pop differnt in GD? | NA | no | no |
| 534 | 2017 | 43008 | Ibanez, B; Cervantes, I; Gutierrez, JP; Goyache, F; Moreno, E | 2014 | Estimates of direct and indirect effects for early juvenile survival in captive populations maintained for conservation purposes: the case of Cuvier’s gazelle | Together with the avoidance of any negative impact of inbreeding, preservation of genetic variability for life-history traits that could undergo future selective pressure is a major issue in endangered species management programmes. However, most of these programmes ignore that, apart from the direct action of genes on such traits, parents, as contributors of offspring environment, can influence offspring performance through indirect parental effects (when parental genotype and phenotype exerts environmental influences on offspring phenotype independently of additive genetic effects). Using quantitative genetic models, we estimated the additive genetic variance for juvenile survival in a population of the endangered Cuvier’s gazelle kept in captivity since 1975. The dataset analyzed included performance recording for 700 calves and a total pedigree of 740 individuals. Results indicated that in this population juvenile survival harbors significant additive genetic variance. The estimates of heritability obtained were in general moderate (0.115-0.457) and not affected by the inclusion of inbreeding in the models. Maternal genetic contribution to juvenile survival seems to be of major importance in this gazelle’s population as well. Indirect genetic and indirect environmental effects assigned to mothers (i.e., maternal genetic and maternal permanent environmental effects) roughly explain a quarter of the total variance estimated for the trait analyzed. These findings have major evolutionary consequences for the species as show that offspring phenotypes can evolve strictly through changes in the environment provided by mothers. They are also relevant for the captive breeding programme of the species. To take into account, the contribution that mothers have on offspring phenotype through indirect genetic effects when designing pairing strategies might serve to identify those females with better ability to recruit, and, additionally, to predict reliable responses to selection in the captive population. | 534 | no | only one pop | NA | no | no |
| 535 | 2017 | 43008 | Mendoza, A; Ng, J; Bales, KL; Mendoza, SP; George, DA; Smith, DG; Kanthaswamy, S | 2015 | Population genetics of the California National Primate Research Center’s (CNPRC) captive Callicebus cupreus colony | The California National Primate Research Center maintains a small colony of titi monkeys (Callicebus cupreus) for behavioral studies. While short tandem repeat (STR) markers are critical for the genetic management of the center’s rhesus macaque (Macaca mulatta) breeding colony, STRs are not used for this purpose in the maintenance of the center’s titi monkey colony. Consequently, the genetic structure of this titi monkey population has not been characterized. A lack of highly informative genetic markers in titi monkeys has also resulted in scant knowledge of the species’ genetic variation in the wild. The purpose of this study was to develop a panel of highly polymorphic titi monkey STRs using a cross-species polymerase chain reaction (PCR) amplification protocol that could be used for the genetic management of the titi monkey colony. We screened 16 STR primer pairs and selected those that generated robust and reproducible polymorphic amplicons. Loci that were found to be highly polymorphic, very likely to be useful for parentage verification, pedigree assessment, and studying titi monkey population genetics, were validated using Hardy-Weinberg equilibrium and linkage disequilibrium analyses. The genetic data generated in this study were also used to assess directly the impact on the colony’s genetic diversity of a recent adenovirus outbreak. While the adenovirus epizootic disease caused significant mortality (19 deaths among the 65 colony animals), our results suggest that the disease exhibited little or no influence on the overall genetic diversity of the colony. | 535 | no | no good fitness measure, N pop differnt in GD? (inbreeding?) | NA | no | no |
| 536 | 2017 | 43008 | Fourcade, Y; Keiss, O; Richardson, DS; Secondi, J | 2014 | Continental-scale patterns of pathogen prevalence: a case study on the corncrake | Pathogen infections can represent a substantial threat to wild populations, especially those already limited in size. To determine how much variation in the pathogens observed among fragmented populations is caused by ecological factors, one needs to examine systems where host genetic diversity is consistent among the populations, thus controlling for any potentially confounding genetic effects. Here, we report geographic variation in haemosporidian infection among European populations of corncrake. This species now occurs in fragmented populations, but there is little genetic structure and equally high levels of genetic diversity among these populations. We observed a longitudinal gradient of prevalence from western to Eastern Europe negatively correlated with national agricultural yield, but positively correlated with corncrake census population sizes when only the most widespread lineage is considered. This likely reveals a possible impact of local agriculture intensity, which reduced host population densities in Western Europe and, potentially, insect vector abundance, thus reducing the transmission of pathogens. We conclude that in the corncrake system, where metapopulation dynamics resulted in variations in local census population sizes, but not in the genetic impoverishment of these populations, anthropogenic activity has led to a reduction in host populations and pathogen prevalence. | 536 | no | gd of parasite | NA | no | no |
| 537 | 2017 | 43008 | Beaty, TH; Fallin, MD; Hetmanski, JB; McIntosh, I; Chong, SS; Ingersoll, R; Sheng, X; Chakraborty, R; Scottt, AF | 2005 | Haplotype diversity in 11 candidate genes across four populations | “Analysis of haplotypes based on multiple single-nucleotide polymorphisms (SNP) is becoming common for both candidate gene and fine-mapping studies. Before embarking on studies of haplotypes from genetically distinct populations, however, it is important to consider variation both in linkage disequilibrium (LD) and in haplotype frequencies within and across populations, as both vary. Such diversity will influence the choice of”“tagging”" SNPs for candidate gene or whole-genome association studies because some markers will not be polymorphic in all samples and some haplotypes will be poorly represented or completely absent. Here we analyze 11 genes, originally chosen as candidate genes for oral clefts, where multiple markers were genotyped on individuals from four populations. Estimated haplotype frequencies, measures of pairwise LD, and genetic diversity were computed for 135 European-Americans, 57 Chinese-Singaporeans, 45 Malay-Singaporeans, and 46 Indian-Singaporeans. Patterns of pairwise LD were compared across these four populations and haplotype frequencies were used to assess genetic variation. Although these populations are fairly similar in allele frequencies and overall patterns of LD, both haplotype frequencies and genetic diversity varied significantly across populations. Such haplotype diversity has implications for designing studies of association involving samples from genetically distinct populations." | 537 | no | human | NA | no | no |
| 538 | 2017 | 43008 | Saarela, J; Finnas, F | 2010 | Mortality Variation by Birth Region and Ethnicity: An Illustration Based on the Finnish Population Register | Previous research has shown that the persistent regional mortality differences in Finland cannot be attributed to sociodemographic factors, health behaviors, or living conditions at young age. Using longitudinal population register data from Finland, we analyze the effect of birth region and ethnicity on mortality risks in men age 65-74 years and women age 75-84 years. Mortality from ischemic heart disease in particular is found to be highly influenced by these proxies for people’s ancestry. The results are consistent with findings from population genetic and medical research, which says that genetic diversity is great across major regions of Finland and that this diversity may underlie variation in disease susceptibility. We argue that the regional mortality variation might reflect geographic clustering of hereditary factors, enforced by Finland’s specific population development. We illustrate that large-scale population register data without genetic information or biomarkers can be useful for understanding cause-specific mortality within a national population. | 538 | no | human | NA | no | no |
| 539 | 2017 | 43008 | Pearson, RD; Amato, R; Auburn, S; Miotto, O; Almagro-Garcia, J; Amaratunga, C; Suon, S; Mao, S; Noviyanti, R; Trimarsanto, H; Marfurt, J; Anstey, NM; William, T; Boni, MF; Dolecek, C; Tran, HT; White, NJ; Michon, P; Siba, P; Tavul, L; Harrison, G; Barry, A; Mueller, I; Ferreira, MU; Karunaweera, N; Randrianarivelojosia, M; Gao, Q; Hubbart, C; Hart, L; Jeffery, B; Drury, E; Mead, D; Kekre, M; Campino, S; Manske, M; Cornelius, VJ; MacInnis, B; Rockett, KA; Miles, A; Rayner, JC; Fairhurst, RM; Nosten, F; Price, RN; Kwiatkowski, DP | 2016 | Genomic analysis of local variation and recent evolution in Plasmodium vivax | The widespread distribution and relapsing nature of Plasmodium vivax infection present major challenges for the elimination of malaria. To characterize the genetic diversity of this parasite in individual infections and across the population, we performed deep genome sequencing of > 200 clinical samples collected across the Asia-Pacific region and analyzed data on > 300,000 SNPs and nine regions of the genome with large copy number variations. Individual infections showed complex patterns of genetic structure, with variation not only in the number of dominant clones but also in their level of relatedness and inbreeding. At the population level, we observed strong signals of recent evolutionary selection both in known drug resistance genes and at new loci, and these varied markedly between geographical locations. These findings demonstrate a dynamic landscape of local evolutionary adaptation in the parasite population and provide a foundation for genomic surveillance to guide effective strategies for control and elimination of P. vivax. | 539 | no | gd of parasite | NA | no | no |
| 540 | 2017 | 43008 | Leclaire, S; van Dongen, WFD; Voccia, S; Merkling, T; Ducamp, C; Hatch, SA; Blanchard, P; Danchin, E; Wagner, RH | 2014 | Preen secretions encode information on MHC similarity in certain sex-dyads in a monogamous seabird | Animals are known to select mates to maximize the genetic diversity of their offspring in order to achieve immunity against a broader range of pathogens. Although several bird species preferentially mate with partners that are dissimilar at the major histocompatibility complex (MHC), it remains unknown whether they can use olfactory cues to assess MHC similarity with potential partners. Here we combined gas chromatography data with genetic similarity indices based on MHC to test whether similarity in preen secretion chemicals correlated with MHC relatedness in the black-legged kittiwake (Rissa tridactyla), a species that preferentially mates with genetically dissimilar partners. We found that similarity in preen secretion chemicals was positively correlated with MHC relatedness in male-male and male-female dyads. This study provides the first evidence that preen secretion chemicals can encode information on MHC relatedness and suggests that odor-based mechanisms of MHC-related mate choice may occur in birds. | 540 | no | no pop level | NA | no | no |
| 541 | 2017 | 43008 | Volkman, SK; Barry, AE; Lyons, EJ; Nielsen, KM; Thomas, SM; Choi, M; Thakore, SS; Day, KP; Wirth, DF; Hartl, DL | 2001 | Recent origin of Plasmodium falciparum from a single progenitor | Genetic variability of Plasmodium falciparum underlies its transmission success and thwarts efforts to control disease caused by this parasite. Genetic variation in antigenic, drug resistance, and pathogenesis determinants is abundant, consistent with an ancient origin of P. falciparum, whereas DNA variation at silent (synonymous) sites in coding sequences appears virtually absent, consistent with a recent origin of the parasite. To resolve this paradox, we analyzed introns and demonstrated that these are deficient in single-nucleotide polymorphisms, as are synonymous sites in coding regions. These data establish the recent origin of P. falciparum and further provide an explanation for the abundant diversity observed in antigen and other selected genes. | 541 | no | gd of parasite | NA | no | no |
| 542 | 2017 | 43008 | Larmuseau, MHD; Boon, N; Vanderheyden, N; Van Geystelen, A; Larmuseau, HFM; Matthys, K; De Clercq, W; Decorte, R | 2015 | High Y-chromosomal diversity and low relatedness between paternal lineages on a communal scale in the Western European Low Countries during the surname establishment | There is limited knowledge on the biological relatedness between citizens and on the demographical dynamics within villages, towns and cities in pre-17th century Western Europe. By combining Y-chromosomal genotypes, in-depth genealogies and surname data in a strict genetic genealogical approach, it is possible to provide insights into the genetic diversity and the relatedness between indigenous paternal lineages within a particular community at the time of the surname adoption. To obtain these insights, six Flemish communities were selected in this study based on the differences in geography and historical development. After rigorous selection of appropriate DNA donors, low relatedness between Y chromosomes of different surnames was found within each community, although there is co-occurrence of these surnames in each community since the start of the surname adoption between the 14th and 15th century. Next, the high communal diversity in Y-chromosomal lineages was comparable with the regional diversity across Flanders at that time. Moreover, clinal distributions of particular Y-chromosomal lineages between the communities were observed according to the clinal distributions earlier observed across the Flemish regions and Western Europe. No significant indication for genetic differences between communities with distinct historical development was found in the analysis. These genetic results provide relevant information for studies in historical sciences, archaeology, forensic genetics and genealogy. | 542 | no | human | NA | no | no |
| 543 | 2017 | 43008 | Grogan, KE; McGinnis, GJ; Sauther, ML; Cuozzo, FP; Drea, CM | 2016 | Next-generation genotyping of hypervariable loci in many individuals of a non-model species: technical and theoretical implications | Background: Across species, diversity at the Major Histocompatibility Complex (MHC) is critical to disease resistance and population health; however, use of MHC diversity to quantify the genetic health of populations has been hampered by the extreme variation found in MHC genes. Next generation sequencing (NGS) technology generates sufficient data to genotype even the most diverse species, but workflows for distinguishing artifacts from alleles are still under development. We used NGS to evaluate the MHC diversity of over 300 captive and wild ring-tailed lemurs (Lemur catta: Primates: Mammalia). We modified a published workflow to address errors that arise from deep sequencing individuals and tested for evidence of selection at the most diverse MHC genes. Results: In addition to evaluating the accuracy of 454 Titanium and Ion Torrent PGM for genotyping large populations at hypervariable genes, we suggested modifications to improve current methods of allele calling. Using these modifications, we genotyped 302 out of 319 individuals, obtaining an average sequencing depth of over 1000 reads per amplicon. We identified 55 MHC-DRB alleles, 51 of which were previously undescribed, and provide the first sequences of five additional MHC genes: DOA, DOB, DPA, DQA, and DRA. The additional five MHC genes had one or two alleles each with little sequence variation; however, the 55 MHC-DRB alleles showed a high dN/dS ratio and trans-species polymorphism, indicating a history of positive selection. Because each individual possessed 1-7 MHC-DRB alleles, we suggest that ring-tailed lemurs have four, putatively functional, MHC-DRB copies. Conclusions: In the future, accurate genotyping methods for NGS data will be critical to assessing genetic variation in non-model species. We recommend that future NGS studies increase the proportion of replicated samples, both within and across platforms, particularly for hypervariable genes like the MHC. Quantifying MHC diversity within non-model species is the first step to assessing the relationship of genetic diversity at functional loci to individual fitness and population viability. Owing to MHC-DRB diversity and copy number, ring-tailed lemurs may serve as an ideal model for estimating the interaction between genetic diversity, fitness, and environment, especially regarding endangered species. | 543 | no | no good fitness measure, N pop differnt in GD? | NA | no | no |
| 544 | 2017 | 43008 | Montesinos, A; Tonsor, SJ; Alonso-Blanco, C; Pico, FX | 2009 | Demographic and Genetic Patterns of Variation among Populations of Arabidopsis thaliana from Contrasting Native Environments | Background: Understanding the relationship between environment and genetics requires the integration of knowledge on the demographic behavior of natural populations. However, the demographic performance and genetic composition of Arabidopsis thaliana populations in the species’ native environments remain largely uncharacterized. This information, in combination with the advances on the study of gene function, will improve our understanding on the genetic mechanisms underlying adaptive evolution in A. thaliana. Methodology/Principal Findings: We report the extent of environmental, demographic, and genetic variation among 10 A. thaliana populations from Mediterranean (coastal) and Pyrenean (montane) native environments in northeast Spain. Geographic, climatic, landscape, and soil data were compared. Demographic traits, including the dynamics of the soil seed bank and the attributes of aboveground individuals followed over a complete season, were also analyzed. Genetic data based on genome-wide SNP markers were used to describe genetic diversity, differentiation, and structure. Coastal and montane populations significantly differed in terms of environmental, demographic, and genetic characteristics. Montane populations, at higher altitude and farther from the sea, are exposed to colder winters and prolonged spring moisture compared to coastal populations. Montane populations showed stronger secondary seed dormancy, higher seedling/juvenile mortality in winter, and initiated flowering later than coastal populations. Montane and coastal regions were genetically differentiated, montane populations bearing lower genetic diversity than coastal ones. No significant isolation-by-distance pattern and no shared multilocus genotypes among populations were detected. Conclusions/Significance: Between-region variation in climatic patterns can account for differences in demographic traits, such as secondary seed dormancy, plant mortality, and recruitment, between coastal and montane A. thaliana populations. In addition, differences in plant mortality can partly account for differences in the genetic composition of coastal and montane populations. This study shows how the interplay between variation in environmental, demographic, and genetic parameters may operate in natural A. thaliana populations. | 544 | yes | are the comparisions of pop ok? | gd not corrected for sample size | no | no |
| 545 | 2017 | 43008 | Jorgensen, MH; Elameen, A; Hofman, N; Klemsdal, S; Malaval, S; Fjellheim, S | 2016 | What’s the meaning of local? Using molecular markers to define seed transfer zones for ecological restoration in Norway | According to the Norwegian Diversity Act, practitioners of restoration in Norway are instructed to use seed mixtures of local provenance. However, there are no guidelines for how local seed should be selected. In this study, we use genetic variation in a set of alpine species (Agrostis mertensii, Avenella flexuosa, Carex bigelowii, Festuca ovina, Poa alpina and Scorzoneroides autumnalis) to define seed transfer zones to reduce confusion about the definition of ` local seeds’. The species selected for the study are common in all parts of Norway and suitable for commercial seed production. The sampling covered the entire alpine region (720 populations per species, 3-15 individuals per population). We characterised genetic diversity using amplified fragment length polymorphisms. We identified different spatial genetic diversity structures in the species, most likely related to differences in reproductive strategies, phylogeographic factors and geographic distribution. Based on results from all species, we suggest four general seed transfer zones for alpine Norway. This is likely more conservative than needed for all species, given that no species show more than two genetic groups. Even so, the approach is practical as four seed mixtures will serve the need for restoration of vegetation in alpine regions in Norway. | 545 | no | no pop level | NA | no | no |
| 546 | 2017 | 43008 | Zhang, MJ; Chen, YP; Yuan, JH; Meng, QC | 2015 | Development of Genomic SSR Markers and Analysis of Genetic Diversity of 40 Haploid Isolates of Ustilago maydis in China | Ustilago maydis (DC) Corda is an important pathogen of maize (Zea mays L.) in China. The poorly defined genetic diversity of this pathogen hinders efforts to better predict and complement resistance of maize in the breeding programs. In this study, we developed 2196 SSR markers in U. maydis, of which 122 were successfully experimentally validated and used to analyze genetic diversity and population structure of the 40 U. maydis isolates. In total, these markers generated 488 alleles with an average of 4.0 alleles per marker. The PIC values ranged from 0.094 to 0.811 with an average of 0.479. The averaged genetic similarity was 0.462 among all isolates. Cluster analysis and population structure analysis both classified the 40 isolates into six different groups. Group classification showed no clear relationship with mating type locus ‘a’ or the geographical origin of the studied isolates. This study demonstrates a unique genetic diversity among U. maydis haplotypes in corn production areas in China. (C) 2015 Friends Science Publishers | 546 | no | gd of pathogen | NA | no | no |
| 547 | 2017 | 43008 | Fang, XL; Finnegan, PM; Barbetti, MJ | 2013 | Wide Variation in Virulence and Genetic Diversity of Binucleate Rhizoctonia Isolates Associated with Root Rot of Strawberry in Western Australia | Strawberry (Fragariaxananassa) is one of the most important berry crops in the world. Root rot of strawberry caused by Rhizoctonia spp. is a serious threat to commercial strawberry production worldwide. However, there is no information on the genetic diversity and phylogenetic status of Rhizoctonia spp. associated with root rot of strawberry in Australia. To address this, a total of 96 Rhizoctonia spp. isolates recovered from diseased strawberry plants in Western Australia were characterized for their nuclear condition, virulence, genetic diversity and phylogenetic status. All the isolates were found to be binucleate Rhizoctonia (BNR). Sixty-five of the 96 BNR isolates were pathogenic on strawberry, but with wide variation in virulence, with 25 isolates having high virulence. Sequence analysis of the internal transcribed spacers of the ribosomal DNA separated the 65 pathogenic BNR isolates into six distinct clades. The sequence analysis also separated reference BNR isolates from strawberry or other crops across the world into clades that correspond to their respective anastomosis group (AG). Some of the pathogenic BNR isolates from this study were embedded in the clades for AG-A, AG-K and AG-I, while other isolates formed clades that were sister to the clades specific for AG-G, AG-B, AG-I and AG-C. There was no significant association between genetic diversity and virulence of these BNR isolates. This study demonstrates that pathogenic BNR isolates associated with root rot of strawberry in Western Australia have wide genetic diversity, and highlights new genetic groups not previously found to be associated with root rot of strawberry in the world (e. g., AG-B) or in Australia (e. g., AG-G). The wide variation in virulence and genetic diversity identified in this study will be of high value for strawberry breeding programs in selecting, developing and deploying new cultivars with resistance to these multi-genetic groups of BNR. | 547 | no | crop | NA | no | no |
| 548 | 2017 | 43008 | Volkman, SK; Sabeti, PC; DeCaprio, D; Neafsey, DE; Schaffner, SF; Milner, DA; Daily, JP; Sarr, O; Ndiaye, D; Ndir, O; Mboup, S; Duraisingh, MT; Lukens, A; Derr, A; Stange-Thomann, N; Waggoner, S; Onofrio, R; Ziaugra, L; Mauceli, E; Gnerre, S; Jaffe, DB; Zainoun, J; Wiegand, RC; Birren, BW; Hartl, DL; Galagan, JE; Lander, ES; Wirth, DF | 2007 | A genome-wide map of diversity in Plasmodium falciparum | Genetic variation allows the malaria parasite Plasmodium falciparum to overcome chemotherapeutic agents, vaccines and vector control strategies and remain a leading cause of global morbidity and mortality(1). Here we describe an initial survey of genetic variation across the P. falciparum genome. We performed extensive sequencing of 16 geographically diverse parasites and identified 46,937 SNPs, demonstrating rich diversity among P. falciparum parasites (pi=1.16 x 10(-3)) and strong correlation with gene function. We identified multiple regions with signatures of selective sweeps in drug-resistant parasites, including a previously unidentified 160-kb region with extremely low polymorphism in pyrimethamine-resistant parasites. We further characterized 54 worldwide isolates by genotyping SNPs across 20 genomic regions. These data begin to define population structure among African, Asian and American groups and illustrate the degree of linkage disequilibrium, which extends over relatively short distances in African parasites but over longer distances in Asian parasites. We provide an initial map of genetic diversity in P. falciparum and demonstrate its potential utility in identifying genes subject to recent natural selection and in understanding the population genetics of this parasite. | 548 | no | gd of parasite | NA | no | no |
| 549 | 2017 | 43008 | Zhang, YN; Liu, BY; Dong, L; Jiang, HX; Wang, X; Wang, XM; Song, CZ; Li, XM; Li, SH | 2017 | High genetic diversity and absence of phylogeographic structure in breeding populations of vulnerable Saundersilarus saundersi (Charadriiformes: Laridae) in Bohai Bay, China | Genetic diversity is an important consideration in developing effective conservation strategies for threatened species. The Saunders’s Gull (Saundersilarus saundersi) is a globally vulnerable species, which breeds in coastal wetlands in eastern China and western South Korea. Approximately 85% of the world population breeds at three sites along Bohai Bay in eastern China: Nanxiaohe in Liaohekou National Nature Reserve of Liaoning Province, and Yiqianer and Dawenliu in Yellow River Delta National Nature Reserve of Shandong Province. We examined mitochondrial and nuclear markers to detect genetic diversity and phylogeographic structure of the three Bohai Bay subpopulations. Unexpectedly high genetic diversity was observed in both mitochondrial and nuclear markers for every subpopulation. There was no evidence of phylogeographic structure among the three breeding subpopulations, indicating high levels of gene flow in the recent past, possibly continuing into the present day. These results reveal high levels of genetic diversity and evolutionary potential among the current populations of S. saundersi in Bohai Bay. If timely conservation measures are implemented, this gull species would has excellent prospects for long-term survival. | 549 | no | no good fitness measure, N pop differnt in GD? | NA | no | no |
| 550 | 2017 | 43008 | SCHMID, B | 1994 | EFFECTS OF GENETIC DIVERSITY IN EXPERIMENTAL STANDS OF SOLIDAGO-ALTISSIMA - EVIDENCE FOR THE POTENTIAL ROLE OF PATHOGENS AS SELECTIVE AGENTS IN PLANT-POPULATIONS | 1 Pathogens are assumed to maintain high genetic diversity and the need for sexual reproduction in plants by continually reducing the fitness of genotypes that become dominant in a population. If this were true, a mixed stand should perform better than the average pure stand of the same plant genotypes. To test this hypothesis I deliberately created thirty-two 16-shoot stands of different genetic diversity of Solidago altissima, a plant species which in the field can form mono- or polyclonal patches, and allowed natural infestations by the mildew Erysiphe cichoracearum to occur. 2 Preliminary experiments revealed significant genetic variation in the source population of S. altissima with regard to pathogen level. However, the pattern of this variation changed between years, indicating that different strains of E. cichoracearum were dominant at different times. 3 In the main experiment pathogen levels at the beginning tended to be higher in more diverse than in less diverse plant stands. As the growing season progressed, levels of E. cichoracearum became higher in the genetically less diverse than in the genetically more diverse stands. 4 Despite the effects on pathogen level, genetic diversity did not significantly influence mean plant performance per stand (height growth and biomass at harvest). However, individual plant performance and pathogen level were positively correlated with each other at low levels of E. cichoracearum and negatively correlated at higher levels. Small plants possibly were in a phenological condition of low susceptibility to pathogen attack. The proportion of above-ground biomass allocated to leaves steadily decreased with increasing pathogen level. 5 The present study suggests that small-scale genetic diversity in the perennial plant Solidago altissima can influence pathogen levels which in turn may affect plant performance and ultimately fitness. I suggest that selection pressures exerted by plant pathogens may play a major role not only in the maintenance of sexual reproduction but also in the maintenance of lateral clonal growth that leads to a mixing of genotypes in polyclonal patches within plant populations and communities. | 550 | no | No GD diff between pop | NA | no | no |
| 551 | 2017 | 43008 | Dynes, TL; De Roode, JC; Lyons, JI; Berry, JA; Delaplane, KS; Brosi, BJ | 2017 | Fine scale population genetic structure of Varroa destructor, an ectoparasitic mite of the honey bee (Apis mellifera) | Varroa destructor is an obligate ectoparasitic mite and the most important biotic threat currently facing honey bees (Apis mellifera). We used neutral microsatellites to analyze previously unreported fine scale population structure of V. destructor, a species characterized by extreme lack of genetic diversity owing to multiple bottleneck events, haplodiploidy, and primarily brother-sister matings. Our results surprisingly indicate that detectable hierarchical genetic variation exists between apiaries, between colonies within an apiary, and even within colonies. This finding of within-colony parasite diversity provides empirical evidence that the spread of V. destructor is not accomplished solely by vertical transmission but that horizontal transmission (natural or human-mediated) must occur regularly. | 551 | no | gd of parasite | NA | no | no |
| 552 | 2017 | 43009 | Angelone-Alasaad, S; Biebach, I; Perez, JM; Soriguer, RC; Granados, JE | 2017 | Molecular Analyses Reveal Unexpected Genetic Structure in Iberian Ibex Populations | Background Genetic differentiation in historically connected populations could be the result of genetic drift or adaptation, two processes that imply a need for differing strategies in population management. The aim of our study was to use neutral genetic markers to characterize C. pyrenaica populations genetically and examine results in terms of (i) demographic history, (ii) subspecific classification and (iii) the implications for the management of Iberian ibex. Methodology/Principal Findings We used 30 neutral microsatellite markers from 333 Iberian ibex to explore genetic diversity in the three main Iberian ibex populations in Spain corresponding to the two persisting subspecies (victoria and hispanica). Our molecular analyses detected recent genetic bottlenecks in all the studied populations, a finding that coincides with the documented demographic decline in C. pyrenaica in recent decades. Genetic divergence between the two C. pyrenaica subspecies (hispanica and victoriae) was substantial (FST between 0.39 and 0.47). Unexpectedly, we found similarly high genetic differentiation between two populations (Sierra Nevada and Maestrazgo) belonging to the subspecies hispanica. The genetic pattern identified in our study could be the result of strong genetic drift due to the severe genetic bottlenecks in the studied populations, caused in turn by the progressive destruction of natural habitat, disease epidemics and/or uncontrolled hunting. Conclusions Previous Capra pyrenaica conservation decision-making was based on the clear distinction between the two subspecies (victoriae and hispanica); yet our paper raises questions about the usefulness for conservation plans of the distinction between these subspecies. | 552 | no | no good fitness measure | NA | no | no |
| 553 | 2017 | 43009 | Kanthaswamy; Ng, J; Penedo, MCT; Ward, T; Smith, DG; Ha, JC | 2012 | Population Genetics of the Washington National Primate Research Center’s (WaNPRC) Captive Pigtailed Macaque (Macaca nemestrina) Population | Pigtailed macaques (Macaca nemestrina) provide an important model for biomedical research on human disease and for studying the evolution of primate behavior. The genetic structure of captive populations of pigtailed macaques is not as well described as that of captive rhesus (M. mulatta) or cynomolgus (M. fascicularis) macaques. The Washington National Primate Research Center houses the largest captive colony of pigtailed macaques located in several different housing facilities. Based on genotypes of 18 microsatellite (short tandem repeat [STR]) loci, these pigtailed macaques are more genetically diverse than captive rhesus macaques and exhibit relatively low levels of inbreeding. Colony genetic management facilitates the maintenance of genetic variability without compromising production goals of a breeding facility. The periodic introduction of new founders from specific sources to separate housing facilities at different times influenced the colony’s genetic structure over time and space markedly but did not alter its genetic diversity significantly. Changes in genetic structure over time were predominantly due to the inclusion of animals from the Yerkes National Primate Research Center in the original colony and after 2005. Strategies to equalize founder representation in the colony have maximized the representation of the founders genomes in the extant population. Were exchange of animals among the facilities increased, further differentiation could be avoided. The use of highly differentiated animals may confound interpretations of phenotypic differences due to the inflation of the genetic contribution to phenotypic variance of heritable traits. Am. J. Primatol. 74:1017-1027, 2012. (c) 2012 Wiley Periodicals, Inc. | 553 | no | no good fitness measure, N pop differnt in GD? | NA | no | no |
| 554 | 2017 | 43009 | Mondol, S; Karanth, KU; Ramakrishnan, U | 2009 | Why the Indian Subcontinent Holds the Key to Global Tiger Recovery | With only similar to 3,000 wild individuals surviving restricted to just 7% of their historical range, tigers are now a globally threatened species. Therefore, conservation efforts must prioritize regions that harbor more tigers, as well try to capture most of the remaining genetic variation and habitat diversity. Only such prioritization based on demographic, genetic, and ecological considerations can ensure species recovery and retention of evolutionary flexibility in the face of ongoing global changes. Although scientific understanding of ecological and demographic aspects of extant wild tiger populations has improved recently, little is known about their genetic composition and variability. We sampled 73 individual tigers from 28 reserves spread across a diversity of habitats in the Indian subcontinent to obtain 1,263 bp of mitochondrial DNA and 10 microsatellite loci. Our analyses reveals that Indian tigers retain more than half of the extant genetic diversity in the species. Coalescent simulations attribute this high genetic diversity to a historically large population size of about 58,200 tigers for peninsular India south of the Gangetic plains. Furthermore, our analyses indicate a precipitous, possibly human-induced population crash similar to 200 years ago in India, which is in concordance with historical records. Our results suggest that only 1.7% (with an upper limit of 13% and a lower limit of 0.2%) of tiger numbers in historical times remain now. In the global conservation context our results suggest that, based on genetic, demographic, and ecological considerations, the Indian subcontinent holds the key to global survival and recovery of wild tigers. | 554 | no | no good fitness measure, N pop differnt in GD? | NA | no | no |
| 555 | 2017 | 43009 | Boraks, A; Broders, KD | 2016 | Population genetic diversity of the rare hardwood butternut (Juglans cinerea) in the northeastern USA | Populations of butternut tree (Juglans cinerea) have undergone range-wide extirpation. A fungal pathogen, Ophiognomonia clavigignenti-juglandacearum, of unknown origin has been recognized as the causal factor. This population collapse has allowed for observations of a broadleaf hardwood in rapid decline. This study made use of six neutral microsatellite markers to describe the present genetic diversity of butternut in the northeastern USA. Our results indicated weak population differentiation (F-ST = 0.084), further supported by an absence of regional genetic structure. Despite reports of high mortality rates, genetic analysis revealed no sign of a recent bottleneck. Population statistics and Bayesian analysis indicated significant historical gene flow among butternut populations of the northeast. Attention should be given to genetic differences between upland and riparian habitat as riparian populations appear to contain greater allele diversity. | 555 | no | no good fitness measure | NA | no | no |
| 556 | 2017 | 43009 | Naegele, RP; Boyle, S; Quesada-Ocampo, LM; Hausbeck, MK | 2014 | Genetic Diversity, Population Structure, and Resistance to Phytophthora capsici of a Worldwide Collection of Eggplant Germplasm | Eggplant (Solanum melongena L.) is an important solanaceous crop with high phenotypic diversity and moderate genotypic diversity. Ninety-nine genotypes of eggplant germplasm (species (S. melongena, S. incanum, S. linnaeanum and S. gilo), landraces and heirloom cultivars) from 32 countries and five continents were evaluated for genetic diversity, population structure, fruit shape, and disease resistance to Phytophthora fruit rot. Fruits from each line were measured for fruit shape and evaluated for resistance to two Phytophthora capsici isolates seven days post inoculation. Only one accession (PI 413784) was completely resistant to both isolates evaluated. Partial resistance to Phytophthora fruit rot was found in accessions from all four eggplant species evaluated in this study. Genetic diversity and population structure were assessed using 22 polymorphic simple sequence repeats (SSRs). The polymorphism information content (PIC) for the population was moderate (0.49) in the population. Genetic analyses using the program STRUCTURE indicated the existence of four genetic clusters within the eggplant collection. Population structure was detected when eggplant lines were grouped by species, continent of origin, country of origin, fruit shape and disease resistance. | 556 | no | crop | NA | no | no |
| 557 | 2017 | 43009 | Gong, J; Wu, WW; Ran, ML; Wang, XW; Liu, W; Wan, Z; Yao, LM; Li, RY | 2016 | Population differentiation and genetic diversity of Trichophyton rubrum as revealed by highly discriminatory microsatellites | Little is known regarding the population information of Trichophyton rubrum due to a lack of strains with clear sampling information and molecular markers with high discriminatory power. In the present study, we developed a set of microsatellite markers that have a cumulative discriminatory power was 0.993. Using these microsatellites loci, 243 strains T. rubrum that had clear sampling information were analysed. Three genetic diversity indices (Shannon’s Information Index, Nei’s unbiased gene diversity and allelic richness) were shown to be related to the human population size of the sampling city rather than mean annual temperature or humidity. Population structure analyses revealed that T. rubrum can be separated into two clusters. AMOVA results indicated that genetic variation was more significant between these two clusters than among geographical populations. Our work is the first to reveal population information of T. rubrum using highly discriminatory molecular markers, and suggest that T. rubrum populations in cities with larger population size might have better adaptability due to higher genetic diversity under selective pressures (such as antifungal agents). (C) 2016 Elsevier Inc. All rights reserved. | 557 | no | human | NA | no | no |
| 558 | 2017 | 43009 | Garrido, M; Perez-Mellado, V | 2014 | Assessing factors involved in determining fluctuating asymmetry in four insular populations of the Balearic lizard Podarcis lilfordi | In animals, developmental stability is frequently assessed by the level of fluctuating asymmetry. Several environmental and genetic stress factors can increase the developmental instability in a population.. Anyhow, the use of fluctuating asymmetry as a measurement of developmental instability and its relationship to other measurements of genetic and environmental stress, remain controversial. We studied this subject in the Balearic lizard, Podarcis lilfordi, and examined four populations inhabiting different coastal islets of Minorca (Balearic Islands, Spain). These populations show clear ecological and genetic differences that allowed us to study factors potentially determining the level of fluctuating asymmetry. We examined the following predictions: (1) fluctuating asymmetry will increase on smaller islands; (2) fluctuating asymmetry will increase on islands with a higher lizard density; (3) fluctuating asymmetry will increase on islands with more heavily parasitized lizards; and (4) populations with higher inbreeding levels would be more susceptible to environmental stress. Our results would partially support the fourth prediction of a higher fluctuating asymmetry in populations with lower genetic variability and, consequently, a higher inbreeding level. Probably, other environmental factors modulate this relationship. | 558 | no | no good fitness measure, N pop differnt in GD? (inbreeding?) | NA | no | no |
| 559 | 2017 | 43009 | Yoshida, Y; Honjo, M; Kitamoto, N; Ohsawa, R | 2009 | Reconsideration for conservation units of wild Primula sieboldii in Japan based on adaptive diversity and molecular genetic diversity | Primula sieboldii E. Morren is a perennial clonal herb that is widely distributed in Japan, but In danger of extinction in the wild. In a previous Study, we revealed the genetic diversity or the species using chloroplast and nuclear DNA and used this information to define conservation units. However, we lacked information on adaptive genetic diversity, which is important for long-term Survival and, thus, for the definition of conservation units. In order to identify adaptive traits that showed adaptive differentiation among Populations, we Studied the genetic variation in six quantitative traits within and among populations for 3 years in a common garden Using 110 genets from five natural Populations from three regions of Japan. The number of days to bud initiation was adaptive quantitative trait for which the degree of genetic differentiation among Populations (Q(ST)) was considerably larger than that in eight microsatellite markers (F(ST)). The relationship between this trait and environmental factors revealed that the number of days to bud initiation was negatively correlated, with the mean temperature during the growing period at each habitat. This suggests that adaptive differentiation in the delay before bud initiation Was caused by selective pressure resulting from temperature differences among habitats. Our results Suggest that based on adaptive diversity and neutral genetic diversity, the Saitama population represents a new conservation unit. | 559 | no | no good fitness measure | NA | no | no |
| 560 | 2017 | 43009 | Dalton, DL; Vermaak, E; Roelofse, M; Kotze, A | 2016 | Diversity in the Toll-Like Receptor Genes of the African Penguin (Spheniscus demersus) | The African penguin, Spheniscus demersus, is listed as Endangered by the IUCN Red List of Threatened Species due to the drastic reduction in population numbers over the last 20 years. To date, the only studies on immunogenetic variation in penguins have been conducted on the major histocompatibility complex (MHC) genes. It was shown in humans that up to half of the genetic variability in immune responses to pathogens are located in non-MHC genes. Toll-like receptors (TLRs) are now increasingly being studied in a variety of taxa as a broader approach to determine functional genetic diversity. In this study, we confirm low genetic diversity in the innate immune region of African penguins similar to that observed in New Zealand robin that has undergone several severe population bottlenecks. Single nucleotide polymorphism (SNP) diversity across TLRs varied between ex situ and in situ penguins with the number of non-synonymous alterations in ex situ populations (n = 14) being reduced in comparison to in situ populations (n = 16). Maintaining adaptive diversity is of vital importance in the assurance populations as these animals may potentially be used in the future for re-introductions. Therefore, this study provides essential data on immune gene diversity in penguins and will assist in providing an additional monitoring tool for African penguin in the wild, as well as to monitor diversity in ex situ populations and to ensure that diversity found in the in situ populations are captured in the assurance populations. | 560 | no | no good fitness measure, N pop differnt in GD? | NA | no | no |
| 561 | 2017 | 43009 | Mastana, SS; Papiha, SS | 2001 | D1S80 distribution in world populations with new data from the UK and the Indian sub-continent | Background: Highly polymorphic genetic markers including variable number of tandem repeats (VNTRs), amplified fragment length polymorphisms (AMP-FLPs) and short tandem repeats (STRs) have been used successfully in disease diagnostics, forensics, paternity analysis and population diversity studies. The D1S80 locus has been extensively investigated in many populations but studies on the UK and Indian subcontinent populations are limited. Aim: This study aims to enlarge our understanding of genetic variation at the D1S80 locus in the populations of the UK and the Indian subcontinent. Also, the spectrum of genetic variation at this locus in world populations is analysed. Subjects and methods: Six geographically and ethnically diverse populations were genotyped for the D1S80 locus using the polymerase chain reaction (PCR) technique. Two UK populations were from the East Midlands and North East England, while Brahmins, Parsis, Sinhalese and Moors represented the Indian subcontinent populations. In addition, allele frequency data of the present study were compared with 78 world populations using different methods of multivariate analyses to document level and extent of genetic diversity. Results: All study populations were in Hardy-Weinberg equilibrium. A trimodal distribution (alleles 18, 24 and 31) was observed in four populations (North East England, East Midlands, Brahmins and Parsis). The Sinhalese and the Moors had different trimodal distributions. The overall heterozygosity and the level of variation are comparable to many Caucasian populations. Multivariate analyses (correspondence analysis and multidimensional scaling analysis) provided similar results in differentiation of major ethnic population groups. Conclusions: Since D1S80 variation shows considerable homogeneity within a given ethnic group, but marked variation among them, it is a useful anthropological marker for the differentiation of these populations. | 561 | no | human | NA | no | no |
| 562 | 2017 | 43009 | Wegner, KM; Reusch, TBH; Kalbe, M | 2003 | Multiple parasites are driving major histocompatibility complex polymorphism in the wild | Parasite mediated selection may result in arms races between host defence and parasite virulence. In particular, simultaneous infections from multiple parasite species should cause diversification (i.e. balancing selection) in resistance genes both at the population and the individual level. Here, we tested these ideas in highly polymorphic major histocompatibility complex (MHC) genes from three-spined sticklebacks (Gasterosteus aculeatus L.). In eight natural populations, parasite diversity (15 different species), and MHC class IIB diversity varied strongly between habitat types (lakes vs. rivers vs. estuaries) with lowest values in rivers. Partial correlation analysis revealed an influence of parasite diversity on MHC class IIB variation whereas general genetic diversity assessed at seven microsatellite loci was not significantly correlated with parasite diversity. Within individual fish, intermediate, rather than maximal allele numbers were associated with minimal parasite load, supporting theoretical models of self-reactive T-cell elimination. The optimal individual diversity matched those values female fish try to achieve in their offspring by mate choice. We thus present correlative evidence supporting the ‘allele counting’ strategy for optimizing the immunocompetence in stickleback offspring. | 562 | maybe | any gD and fitness of groups that works? | Allelic richness not corrected for sample size and pop level | no | no |
| 563 | 2017 | 43009 | Ashby, B; King, KC | 2015 | Diversity and the maintenance of sex by parasites | The Red Queen hypothesis (RQH) predicts that parasite-mediated selection will maintain sexual individuals in the face of competition from asexual lineages. The prediction is that sexual individuals will be difficult targets for coevolving parasites if they give rise to more genetically diverse offspring than asexual lineages. However, increasing host genetic diversity is known to suppress parasite spread, which could provide a short-term advantage to clonal lineages and lead to the extinction of sex. We test these ideas using a stochastic individual-based model. We find that if parasites are readily transmissible, then sex is most likely to be maintained when host diversity is high, in agreement with the RQH. If transmission rates are lower, however, we find that sexual populations are most likely to persist for intermediate levels of diversity. Our findings thus highlight the importance of genetic diversity and its impact on epidemiological dynamics for the maintenance of sex by parasites. | 563 | no | individual level | NA | no | no |
| 564 | 2017 | 43009 | Gau, RD; Merz, U; Falloon, RE; Brunner, PC | 2013 | Global Genetics and Invasion History of the Potato Powdery Scab Pathogen, Spongospora subterranea f.sp subterranea | “Spongospora subterranea f. sp. subterranea (Sss) causes two diseases on potato (Solanum tuberosum), lesions on tubers and galls on roots, which are economically important worldwide. Knowledge of global genetic diversity and population structure of pathogens is essential for disease management including resistance breeding. A combination of microsatellite and DNA sequence data was used to investigate the structure and invasion history of Sss. South American populations (four countries, 132 samples) were consistently more diverse than those from all other regions (15 countries, 566 samples), in agreement with the hypothesis that Sss originated in South America where potato was domesticated. A substantial genetic differenciation was found between root and tuber-derived samples from South America. Estimates of past and recent gene flow suggested that Sss was probably introduced from South America into Europe. Subsequently, Europe is likely to have been the recent source of migrants of the pathogen, acting as a”“bridgehead”" for further global dissemination. Quarantine measures must continue to be focussed on maintaining low global genetic diversity and avoiding exchange of genetic material between the native and introduced regions. Nevertheless, the current low global genetic diversity of Sss allows potato breeders to select for resistance, which is likely to be durable." | 564 | no | gd of pathogen | NA | no | no |
| 565 | 2017 | 43009 | Wu, ZH; Shi, JS; Xi, ML; Jiang, FX; Deng, MW; Dayanandan, S | 2015 | Inter-Simple Sequence Repeat Data Reveals High Genetic Diversity in Wild Populations of the Narrowly Distributed Endemic Lilium regale in the Minjiang River Valley of China | Lilium regale E. H. Wilson is endemic to a narrow geographic area in the Minjiang River valley in southwestern China, and is considered an important germplasm for breeding commercially valuable lily varieties, due to its vigorous growth, resistance to diseases and tolerance for low moisture. We analyzed the genetic diversity of eight populations of L. regale sampled across the entire natural distribution range of the species using Inter-Simple Sequence Repeat markers. The genetic diversity (expected heterozygosity=0.3356) was higher than those reported for other narrowly distributed endemic plants. The levels of inbreeding (F-st = 0.1897) were low, and most of the genetic variability was found to be within (80.91%) than amongpopulations (19.09%). An indirect estimate of historical levels of gene flow (N-m = 1.0678) indicated high levels of gene flow among populations. The eight analyzed populations clustered into three genetically distinct groups. Based on these results, we recommend conservation of large populations representing these three genetically distinct groups. | 565 | no | no good fitness measure | NA | no | no |
| 566 | 2017 | 43009 | Helantera, H; Aehle, O; Roux, M; Heinze, J; d’Ettorre, P | 2013 | Family-based guilds in the ant Pachycondyla inversa | High relatedness promotes the evolution of sociality because potentially costly cooperative behaviours are directed towards kin. However, societies, such as those of social insects, also benefit from genetic diversity, e.g. through enhanced disease resistance and division of labour. Effects of genetic diversity have been investigated in a few complex eusocial species. Here, we show that genetically based division of labour may also be important in ‘simple societies’, with fewer individuals and limited morphological caste differentiation. The ponerine ant Pachycondyla inversa has small colonies, headed by several unrelated queens. We show that nest-mate workers from different matrilines engage in different tasks, have distinct chemical profiles and associate preferentially with kin in the nest, while queens and brood stay together. This suggests that genetically based division of labour may precede the evolution of complex eusociality and facilitate the existence of low relatedness societies functioning as associations of distinct families that mutually benefit from group living. | 566 | no | no good fitness measure, N pop differnt in GD? | NA | no | no |
| 567 | 2017 | 43009 | Lau, Q; Jaratlerdsiri, W; Griffith, JE; Gongora, J; Higgins, DP | 2014 | MHC class II diversity of koala (Phascolarctos cinereus) populations across their range | Major histocompatibility complex class II (MHCII) genes code for proteins that bind and present antigenic peptides and trigger the adaptive immune response. We present a broad geographical study of MHCII DA beta 1 (DAB) and DB beta 1 (DBB) variants of the koala (Phascolarctos cinereus; n = 191) from 12 populations across eastern Australia, with a total of 13 DAB and 7 DBB variants found. We identified greater MHCII variation and, possibly, additional gene copies in koala populations in the north (Queensland and New South Wales) relative to the south (Victoria), confirmed by STRUCTURE analyses and genetic differentiation using analysis of molecular variance. The higher MHCII diversity in the north relative to south could potentially be attributed to (i) significant founder effect in Victorian populations linked to historical translocation of bottlenecked koala populations and (ii) increased pathogen-driven balancing selection and/or local genetic drift in the north. Low MHCII genetic diversity in koalas from the south could reduce their potential response to disease, although the three DAB variants found in the south had substantial sequence divergence between variants. This study assessing MHCII diversity in the koala with historical translocations in some populations contributes to understanding the effects of population translocations on functional genetic diversity. | 567 | no | no good fitness measure | NA | no | no |
| 568 | 2017 | 43009 | Kolleck, J; Yang, M; Zinner, D; Roos, C | 2013 | Genetic Diversity in Endangered Guizhou Snub-Nosed Monkeys (Rhinopithecus brelichi): Contrasting Results from Microsatellite and Mitochondrial DNA Data | To evaluate the conservation status of a species or population it is necessary to gain insight into its ecological requirements, reproduction, genetic population structure, and overall genetic diversity. In our study we examined the genetic diversity of Rhinopithecus brelichi by analyzing microsatellite data and compared them with already existing data derived from mitochondrial DNA, which revealed that R. brelichi exhibits the lowest mitochondrial diversity of all so far studied Rhinopithecus species. In contrast, the genetic diversity of nuclear DNA is high and comparable to other Rhinopithecus species, i.e. the examined microsatellite loci are similarly highly polymorphic as in other species of the genus. An explanation for these differences in mitochondrial and nuclear genetic diversity could be a male biased dispersal. Females most likely stay within their natal band and males migrate between bands, thus mitochondrial DNA will not be exchanged between bands but nuclear DNA via males. A Bayesian Skyline Plot based on mitochondrial DNA sequences shows a strong decrease of the female effective population size (N-ef) starting about 3,500 to 4,000 years ago, which concurs with the increasing human population in the area and respective expansion of agriculture. Given that we found no indication for a loss of nuclear DNA diversity in R. brelichi it seems that this factor does not represent the most prominent conservation threat for the long-term survival of the species. Conservation efforts should therefore focus more on immediate threats such as development of tourism and habitat destruction. | 568 | no | NA | NA | no | no |
| 569 | 2017 | 43009 | de Vere, N; Jongejans, E; Plowman, A; Williams, E | 2009 | Population size and habitat quality affect genetic diversity and fitness in the clonal herb Cirsium dissectum | Remaining populations of plant species in fragmented landscapes are threatened by declining habitat quality and reduced genetic diversity, but the interactions of these major factors are rarely studied together for species conservation. In this study, the interactions between population size, habitat quality, genetic diversity and fitness were investigated in 22 populations of the clonal herb Cirsium dissectum throughout the British Isles. Regression analysis was used to identify significant factors, and a structural equation model was developed to illustrate and integrate these interactions. It was found that smaller populations (measured as the total number of plants) had lower genetic diversity (proportion of polymorphic loci), and that reduced genetic diversity (allelic richness) had a negative impact on the survival of seedlings grown under standard conditions. Habitat quality also had a large effect on C. dissectum. Unmanaged sites with tall vegetation, no bare soil and higher nutrient levels had smaller populations of C. dissectum, but flowering was promoted. Flowering was suppressed in heavily grazed sites with short vegetation. Higher levels of bare soil and phosphorus both had a positive relationship with genetic diversity, but probably for distinctly different reasons: bare soil provides safe sites for establishment, whilst phosphorus may promote flowering and improve seed germination. In order to conserve C. dissectum, management needs to maintain site heterogeneity so that C. dissectum can flower and establishment gaps are still available for seedlings; when either component is reduced, negative feedbacks through reduced genetic diversity and individual fitness can be expected. This study therefore highlights the importance of considering both conservation genetics and habitat quality in the conservation of plant species. | 569 | maybe | fintess and gd ok? | NA | no | yes |
| 570 | 2017 | 43009 | Taylor, HR; Kardos, MD; Ramstad, KM; Allendorf, FW | 2015 | Valid estimates of individual inbreeding coefficients from marker-based pedigrees are not feasible in wild populations with low allelic diversity | Pedigrees are frequently recommended for estimating inbreeding coefficients (F (PED) ), but are error-prone due to missing behavioural data in wild populations. Genetic marker-based pedigrees have been suggested as a remedy to this problem, but their accuracy depends on the number and polymorphism of loci available, and the completeness of population sampling. We used simulations to examine how accuracy of marker-based pedigrees varies with number of loci and sampling regime when allelic diversity is low (2.2-4 alleles per locus in founders), as is often the case in threatened species. We also examined the impact of pedigree errors on the validity of F (PED) estimated from marker-based pedigrees. Our results indicate that accurate parentage assignments are only feasible if genotypes are available for all individuals that ever existed in the population, and that accuracy does not improve past 40 loci. Errors in marker-based pedigrees resulted in underestimation of F (PED) by up to 27 % and overestimation of the variance in F (PED) by up to 182 %. At least 80 % pedigree accuracy was required to produce unbiased estimates of F (PED) , which were still highly imprecise. Given the degree of sampling required, it is not currently feasible to measure inbreeding in wild populations of threatened species with a pedigree based solely on microsatellite data. Resources may be better directed towards developing more robust genetic tools (whole genome sequencing and large SNP panels) to facilitate direct estimation of inbreeding coefficients without a pedigree. Where this is not possible, long-term monitoring projects will be required to accurately estimate inbreeding coefficients via a combination of behavioural and genetic data. | 570 | no | NA | NA | no | no |
| 571 | 2017 | 43009 | Bearoff, F; Case, LK; Krementsov, DN; Wall, EH; Saligrama, N; Blankenhorn, EP; Teuscher, C | 2015 | Identification of Genetic Determinants of the Sexual Dimorphism in CNS Autoimmunity | Multiple sclerosis (MS) is a debilitating chronic inflammatory disease of the nervous system that affects approximately 2.3 million individuals worldwide, with higher prevalence in females, and a strong genetic component. While over 200 MS susceptibility loci have been identified in GWAS, the underlying mechanisms whereby they contribute to disease susceptibility remains ill-defined. Forward genetics approaches using conventional laboratory mouse strains are useful in identifying and functionally dissecting genes controlling disease- relevant phenotypes, but are hindered by the limited genetic diversity represented in such strains. To address this, we have combined the powerful chromosome substitution (consomic) strain approach with the genetic diversity of a wild-derived inbred mouse strain. Using experimental allergic encephalomyelitis (EAE), a mouse model of MS, we evaluated genetic control of disease course among a panel of 26 consomic strains of mice inheriting chromosomes from the wild-derived PWD strain on the C57BL/6J background, which models the genetic diversity seen in human populations. Nineteen linkages on 18 chromosomes were found to harbor loci controlling EAE. Of these 19 linkages, six were male-specific, four were female-specific, and nine were non-sex-specific, consistent with a differential genetic control of disease course between males and females. An MS-GWAS candidate-driven bioinformatic analysis using orthologous genes linked to EAE course identified sex-specific and non-sex-specific gene networks underlying disease pathogenesis. An analysis of sex hormone regulation of genes within these networks identified several key molecules, prominently including the MAP kinase family, known hormone-dependent regulators of sex differences in EAE course. Importantly, our results provide the framework by which consomic mouse strains with overall genome-wide genetic diversity, approximating that seen in humans, can be used as a rapid and powerful tool for modeling the genetic architecture of MS. Moreover, our data represent the first step towards mechanistic dissection of genetic control of sexual dimorphism in CNS autoimmunity. | 571 | no | human | NA | no | no |
| 572 | 2017 | 43009 | Narain, RB; Lalithambika, S; Kamble, ST | 2015 | Genetic Variability and Geographic Diversity of the Common Bed Bug (Hemiptera: Cimicidae) Populations from the Midwest Using Microsatellite Markers | With the recent global resurgence of the bed bugs (Cimex lectularius L.), there is a need to better understand its biology, ecology, and ability to establish populations. Bed bugs are domestic pests that feed mainly on mammalian blood. Although bed bugs have not been implicated as vectors of pathogens, their biting activity inflicts severe insomnia and allergic reactions. Moreover, they have recently developed resistance to various insecticides, which requires further molecular research to determine genetic variation and appropriate interventions. Population dynamics, including genetic differentiation and genetic distance of 10 populations from the Midwest were analyzed in this study. The bed bug samples collected by pest control companies were genotyped using eight species-specific microsatellite markers. Results showed all eight markers were polymorphic, with 8-16 alleles per locus, suggesting high genetic diversity. The F-ST values were >0.25, signifying pronounced genetic differentiation. The G-test results also indicated high genetic differentiation among populations. The frequency of the most common allele across all eight loci was 0.42. The coefficient of relatedness between each of the populations was >0.5, indicative of sibling or parent-offspring relationships, while the F-IS and its confidence interval values were statistically insignificant within the populations tested. The populations departed from Hardy-Weinberg equilibrium, possibly because of high heterozygosity. The genetic distance analysis using a neighbor-joining tree showed that the populations from Kansas City, MO, were genetically separate from most of those from Nebraska, indicating a geographic pattern of genetic structure. Our study demonstrated the effectiveness of using microsatellite markers to study bed bugs population structure, thereby improving our understanding of bed bug population dynamics in the Midwest. Overall, this study showed a high genetic diversity and identified several new alleles in the bed bug populations in the Midwest. | 572 | no | no good fitness measure | NA | no | no |
| 573 | 2017 | 43009 | Schuster, AC; Herde, A; Mazzoni, CJ; Eccard, JA; Sommer, S | 2016 | Evidence for selection maintaining MHC diversity in a rodent species despite strong density fluctuations | Strong spatiotemporal variation in population size often leads to reduced genetic diversity limiting the adaptive potential of individual populations. Key genes of adaptive variation are encoded by the immune genes of the major histocompatibility complex (MHC) playing an essential role in parasite resistance. How MHC variation persists in rodent populations that regularly experience population bottlenecks remains an important topic in evolutionary genetics. We analysed the consequences of strong population fluctuations on MHC class II DRB exon 2 diversity in two distant common vole (Microtus arvalis) populations in three consecutive years using a high-throughput sequencing approach. In 143 individuals, we detected 25 nucleotide alleles translating into 14 unique amino acid MHC alleles belonging to at least three loci. Thus, the overall allelic diversity and amino acid distance among the remaining MHC alleles, used as a surrogate for the range of pathogenic antigens that can be presented to T-cells, are still remarkably high. Both study populations did not show significant population differentiation between years, but significant differences were found between sites. We concluded that selection processes seem to be strong enough to maintain moderate levels of MHC diversity in our study populations outcompeting genetic drift, as the same MHC alleles were conserved between years. Differences in allele frequencies between populations might be the outcome of different local parasite pressures and/or genetic drift. Further understanding of how pathogens vary across space and time will be crucial to further elucidate the mechanisms maintaining MHC diversity in cyclic populations. | 573 | no | no good fitness measure | NA | no | no |
| 574 | 2017 | 43009 | Gonzalez-Quevedo, C; Spurgin, LG; Illera, JC; Richardson, DS | 2015 | Drift, not selection, shapes toll-like receptor variation among oceanic island populations | Understanding the relative role of different evolutionary forces in shaping the level and distribution of functional genetic diversity among natural populations is a key issue in evolutionary and conservation biology. To do so accurately genetic data must be analysed in conjunction with an unambiguous understanding of the historical processes that have acted upon the populations. Here, we focused on diversity at toll-like receptor (TLR) loci, which play a key role in the vertebrate innate immune system and, therefore, are expected to be under pathogen-mediated selection. We assessed TLR variation within and among 13 island populations (grouped into three archipelagos) of Berthelot’s pipit, Anthus berthelotii, for which detailed population history has previously been ascertained. We also compared the variation observed with that found in its widespread sister species, the tawny pipit, Anthus campestris. We found strong evidence for positive selection at specific codons in TLR1LA, TLR3 and TLR4. Despite this, we found that at the allele frequency level, demographic history has played the major role in shaping patterns of TLR variation in Berthelot’s pipit. Levels of diversity and differentiation within and across archipelagos at all TLR loci corresponded very closely with neutral microsatellite variation and with the severity of the bottlenecks that occurred during colonization. Our study shows that despite the importance of TLRs in combating pathogens, demography can be the main driver of immune gene variation within and across populations, resulting in patterns of functional variation that can persist over evolutionary timescales. | 574 | no | no good fitness measure, N pop differnt in GD? | NA | no | no |
| 575 | 2017 | 43009 | Adetunji, I; Willems, G; Tschoep, H; Burkholz, A; Barnes, S; Boer, M; Malosetti, M; Horemans, S; van Eeuwijk, F | 2014 | Genetic diversity and linkage disequilibrium analysis in elite sugar beet breeding lines and wild beet accessions | Key message Linkage disequilibrium decay in sugar beet is strongly affected by the breeding history, and varies extensively between and along chromosomes, allowing identification of known and unknown signatures of selection. Genetic diversity and linkage disequilibrium (LD) patterns were investigated in 233 elite sugar beet breeding lines and 91 wild beet accessions, using 454 single nucleotide polymorphisms (SNPs) and 418 SNPs, respectively. Principal coordinate analysis suggested the existence of three groups of germplasm, corresponding to the wild beets, the seed parent and the pollen parent breeding pool. LD was investigated in each of these groups, with and without correction for genetic relatedness. Without correction for genetic relatedness, in the pollen as well as the seed parent pool, LD persisted beyond 50 centiMorgan (cM) on four (2, 3, 4 and 5) and three chromosomes (2, 4 and 6), respectively; after correction for genetic relatedness, LD decayed after < 6 cM on all chromosomes in both pools. In the wild beet accessions, there was a strong LD decay: on average LD disappeared after 1 cM when LD was calculated with a correction for genetic relatedness. Persistence of LD was not only observed between distant SNPs on the same chromosome, but also between SNPs on different chromosomes. Regions on chromosomes 3 and 4 that harbor disease resistance and monogermy loci showed strong genetic differentiation between the pollen and seed parent pools. Other regions, on chromosomes 8 and 9, for which no a priori information was available with respect to their contribution to the phenotype, still contributed to clustering of lines in the elite breeding material. | 575 | no | crop | NA | no | no |
| 576 | 2017 | 43009 | Rodriguez-Quilon, I; Santos-del-Blanco, L; Serra-Varela, MJ; Koskela, J; Gonzalez-Martinez, SC; Alia, R | 2016 | Capturing neutral and adaptive genetic diversity for conservation in a highly structured tree species | Preserving intraspecific genetic diversity is essential for long-term forest sustainability in a climate change scenario. Despite that, genetic information is largely neglected in conservation planning, and how conservation units should be defined is still heatedly debated. Here, we use maritime pine (Pinus pinaster Ait.), an outcrossing long-lived tree with a highly fragmented distribution in the Mediterranean biodiversity hotspot, to prove the importance of accounting for genetic variation, of both neutral molecular markers and quantitative traits, to define useful conservation units. Six gene pools associated to distinct evolutionary histories were identified within the species using 12 microsatellites and 266 single nucleotide polymorphisms (SNPs). In addition, height and survival standing variation, their genetic control, and plasticity were assessed in a multisite clonal common garden experiment (16 544 trees). We found high levels of quantitative genetic differentiation within previously defined neutral gene pools. Subsequent cluster analysis and post hoc trait distribution comparisons allowed us to define 10 genetically homogeneous population groups with high evolutionary potential. They constitute the minimum number of units to be represented in a maritime pine dynamic conservation program. Our results uphold that the identification of conservation units below the species level should account for key neutral and adaptive components of genetic diversity, especially in species with strong population structure and complex evolutionary histories. The environmental zonation approach currently used by the pan-European genetic conservation strategy for forest trees would be largely improved by gradually integrating molecular and quantitative trait information, as data become available. | 576 | no | no good fitness measure, N pop differnt in GD? | NA | no | no |
| 577 | 2017 | 43009 | Luvesuto, E; de Freitas, PD; Galetti, PM | 2007 | Genetic variation in a closed line of the white shrimp Litopenaeus vannamei (Penaeidae) | The culture of the marine shrimp Litopenaeus vannamei has recently boosted the Brazilian shrimp industry. However, it is well known that selection methods based solely on phenotypic characteristics, a reduced number of breeders and the practice of inbreeding may promote a significant raise in the genetic similarity of the captive populations, leading to greater disease susceptibility and impairing both the growth and final size of the shrimps. We used four microsatellite loci to investigate genetic variation in three generations (F-5, F-6 and F-7) of a closed and reared L. vannamei lineage. Although an accentuated heterozygosis deficit was detected, we also observed that the captive propagation of this lineage did not lead to a significant loss of genetic variability over the three generations studied. One possible reason for this is that the breeding conditions of this lineage were good enough to prevent any significant loss of genetic variability. However, three generations may have been insufficient to produce detectable changes in genetic frequencies in the loci studied. Alternatively, the microsatellite loci may have been non-neutral (biased) and related to the conditions in which the shrimps were kept, resulting in a similar allele pool in respect to these four microsatellites over the three generations studied. Any generalizations regarding microsatellite variation in closed shrimp lines may thus be incomplete and should be carefully analyzed. | 577 | no | no good fitness measure, N pop differnt in GD? | NA | no | no |
| 578 | 2017 | 43009 | Dufresnes, C; Wassef, J; Ghali, K; Brelsford, A; Stock, M; Lymberakis, P; Crnobrnja-Isailovic, J; Perrin, N | 2013 | Conservation phylogeography: does historical diversity contribute to regional vulnerability in European tree frogs (Hyla arborea)? | Documenting and preserving the genetic diversity of populations, which conditions their long-term survival, have become a major issue in conservation biology. The loss of diversity often documented in declining populations is usually assumed to result from human disturbances; however, historical biogeographic events, otherwise known to strongly impact diversity, are rarely considered in this context. We apply a multilocus phylogeographic study to investigate the late-Quaternary history of a tree frog (Hyla arborea) with declining populations in the northern and western part of its distribution range. Mitochondrial and nuclear polymorphisms reveal high genetic diversity in the Balkan Peninsula, with a spatial structure moulded by the last glaciations. While two of the main refugial lineages remained limited to the Balkans (Adriatic coast, southern Balkans), a third one expanded to recolonize Northern and Western Europe, loosing much of its diversity in the process. Our findings show that mobile and a priori homogeneous taxa may also display substructure within glacial refugia (refugia within refugia’) and emphasize the importance of the Balkans as a major European biodiversity centre. Moreover, the distribution of diversity roughly coincides with regional conservation situations, consistent with the idea that historically impoverished genetic diversity may interact with anthropogenic disturbances, and increase the vulnerability of populations. Phylogeographic models seem important to fully appreciate the risks of local declines and inform conservation strategies. | 578 | no | no good fitness measure, N pop differnt in GD? | NA | no | no |
| 579 | 2017 | 43009 | Luquet, E; Garner, TW; Lena, JP; Bruel, C; Joly, P; Lengagne, T; Grolet, O; Plenet, S | 2012 | GENETIC EROSION IN WILD POPULATIONS MAKES RESISTANCE TO A PATHOGEN MORE COSTLY | Populations that have suffered from genetic erosion are expected to exhibit reduced average trait values or decreased variation in adaptive traits when experiencing periodic or emergent stressors such as infectious disease. Genetic erosion may consequentially modify the ability of a potential host population to cope with infectious disease emergence. We experimentally investigate this relationship between genetic variability and host response to exposure to an infectious agent both in terms of susceptibility to infection and indirect parasite-mediated responses that also impact fitness. We hypothesized that the deleterious consequences of exposure to the pathogen (Batrachochytrium dendrobatidis) would be more severe for tadpoles descended from European treefrog (Hyla arborea) populations lacking genetic variability. Although all exposed tadpoles lacked detectable infection, we detected this relationship for some indirect host responses, predominantly in genetically depleted animals, as well as an interaction between genetic variability and pathogen dose on life span during the postmetamorphic period. Lack of infection and a decreased mass and postmetamorphic life span in low genetic diversity tadpoles lead us to conclude that genetic erosion, while not affecting the ability to mount effective resistance strategies, also erodes the capacity to invest in resistance, increased tadpole growth rate, and metamorphosis relatively simultaneously. | 579 | probably no | No GD diff between pop | No GD diff between pop | no | no |
| 580 | 2017 | 43009 | Guicking, D; Finke, L; Wittich, M; Pfeiffer, I; Veith, M; Geske, C; Braukmann, U; Weising, K; Neubeck, C | 2017 | Conservation genetics of Bombina v. variegata (Anura: Bombinatoridae) in northern Hesse, Germany | We report the genetic diversity and population structure of the Yellow-bellied Toad (Bombina variegata) at the northern edge of its distribution range in northern Hesse, Germany. A total of 281 samples from 20 populations were analysed, using six polymorphic nuclear microsatellite markers (ncSSRs). Moderate genetic diversity (H-e = 0.37-0.59) was detected within these B. variegata populations. We found evidence of a distinctive population structure, with populations at the edge of the study area showing lower degrees of diversity and higher degrees of isolation than more central populations. No genetic differentiation was found between populations from the middle Fulda and Werra river valleys, suggesting that fragmentation, e.g., by the A(4) highway that separates the two river valleys, has not yet had genetic consequences. Furthermore, indications of an isolation-by-distance pattern was found, suggestive of restricted gene flow between the studied populations. To ensure the long-term survival of the Yellow-bellied Toad in northern Hesse, we recommend continuous management efforts that focus on the reconnection of isolated populations and continuation of demographic population monitoring supplemented by population genetic analyses. | 580 | no | no good fitness measure | NA | no | no |
| 581 | 2017 | 43009 | Coltman, DW; Bowen, WD; Wright, JM | 1998 | Birth weight and neonatal survival of harbour seal pups ape positively correlated with genetic variation measured by microsatellites | We examined the relations between fitness-related traits of wild harbour seal (Phoca vitulina) pups with microsatellite heterozygosity, and with a measure of genomic diversity based on the mean squared distance between microsatellite alleles within an individual, mean d(2). Birth weight was positively influenced by maternal age, pup sex, and either mean d(2) or individual heterozygosity in separate multiple regression models. The association of birth weight with mean d(2) was stronger than that with heterozygosity, however. The factors maternal age, pup sex, and mean d(2) combined to account for 36.8% of the variation in birth weight, with mean d(2) accounting for the greatest explanatory power (52.3% of the variance explained). Pups which survived until weaning had significantly higher mean d(2) than pups which died, independent of birth weight. These effects are consistent with heterosis resulting from recent population mixing, and/or inbreeding depression in this population. Mean d(2) thus provides (i) a better measure of individual genetic variability than heterozygosity for microsatellite data; and (ii) a convenient tool for assessing the effects of inbreeding and outbreeding in natural populations. | 581 | no | individual level | NA | no | no |
| 582 | 2017 | 43009 | Ooi, K; Yahara, T | 1999 | Genetic variation of geminiviruses: comparison between sexual and asexual host plant populations | One of the most promising hypotheses for the evolution of sex is that sexual reproduction is advantageous because it increases the rate of adaptive evolution in response to parasites. To investigate this advantage of sex, we compared genetic variation of geminiviruses infecting sexual and asexual populations of Eupatorium (Asteraceae). The infection frequency was 37.5% in the sexual population and 87.8% in the asexual population. The lower infection frequency in the sexual population might be the result of higher genetic diversity of host plants. If geminiviruses have diverged to counter defence systems of genetically variable hosts, genetic diversity of viruses is expected to be higher in sexual host populations than in asexual host populations. To test this expectation, we used single-strand conformation polymorphism (SSCP) analysis to examine genetic diversity of the geminiviruses in a DNA region containing the open-reading frame (ORF) C4 gene, which is known to function as a host range determinant. As predicted, higher genetic diversity of viruses was observed in the sexual population: three SSCP types were found in the asexual population while six types were found in the sexual population. Sequencing of the polymerase chain reaction (PCR) products revealed further genetic diversity. Phylogenetic analysis of the sequences showed that the SSCP types belonged to four different clades. Several SSCP types from the same clade were found in the sexual population, whereas the asexual population included only one SSCP type from each clade. Amino acid replacements of ORF C4 are suggested to be accelerated in the sexual population. This evidence supports the hypothesis that sexual reproduction is advantageous as a defence against epidemic disease. | 582 | probably no | only two pop | only two pop | no | no |
| 583 | 2017 | 43009 | Lorenzini, R; Posillico, M; Lovari, S; Petrella, A | 2004 | Non-invasive genotyping of the endangered Apennine brown bear: a case study not to let one’s hair down | Individual identification was assessed for 30 brown bears (Ursus arctos) from the endangered Apennine population in central Italy, mostly through non-invasive sampling. Shed hairs were used as a source of DNA to obtain single genotype profiling at 12 microsatellite loci. Average expected heterozygosity (0.438) and mean number of alleles per locus (2.2) showed that the level of genetic diversity was one of the lowest recorded for brown bear populations across their European and North American ranges. A large portion of the original genetic variation may have been lost through random genetic drift during the recent period of isolation. The level of genetic variability proved sufficient to identify the bears individually by using nine microsatellite loci. The probability that two animals shared by chance the same multilocus genotype was estimated to be 1 in 100. Despite the recent history of small population size, the average relatedness indicated that the majority of individuals are not first-order relatives. Simulated paternity tests showed that a 12-loci genotype may be necessary to assign paternity with an 80-95% confidence level. Lack of genetic diversity, as well as the maintenance of an adequately effective number, may seriously jeopardise the long-term survival of this population. Furthermore, high mortality rates, poaching and encroachment by human activities represent immediate, urgent concerns. A population increase through enforced protection and habitat restoration is recommended. Presently, restocking with bears from other source populations should be discouraged to avoid genetic extinction. Non-invasive sampling provided reliable population and individual genetic data. Microsatellite genotyping proved a valuable genetic tagging method and a feasible alternative to conventional field counts for the brown bear in central Italy. | 583 | no | no good fitness measure, N pop differnt in GD? | NA | no | no |
| 584 | 2017 | 43009 | Goossens, B; Sharma, R; Othman, N; Kun-Rodrigues, C; Sakong, R; Ancrenaz, M; Ambu, LN; Jue, NK; O’Neill, RJ; Bruford, MW; Chikhi, L | 2016 | Habitat fragmentation and genetic diversity in natural populations of the Bornean elephant: Implications for conservation | The Bornean elephant population in Sabah, with only 2000 individuals, is currently mainly restricted to a limited number of forest reserves. The main threats to the species’ survival are population fragmentation and isolation of the existing herds. To support and help monitor future conservation and management measures, we assessed the genetic diversity and population structure of Bornean elephants using mitochondrial DNA, microsatellites and single nucleotide polymorphisms. Our results confirmed a previously reported lack of mitochondrial control region diversity, characterized by a single widespread haplotype. However, we found low but significant degree of genetic differentiation among populations and marked variation in genetic diversity with the other two types of markers among Bornean elephants. Microsatellite data showed that Bornean elephants from the Lower Kinabatangan and North Kinabatangan ranges are differentiated and perhaps isolated from the main elephant populations located in the Central Forest and Tabin Wildlife Reserve. The pairwise F-ST values between these sites ranged from 0.08 to 0.14 (p < 0.001). Data from these markers also indicate that the Bornean elephant populations from Lower Kinabatangan Wildlife Sanctuary and North Kinabatangan (Deramakot Forest Reserve) possess higher levels of genetic variation compared to the elephant populations from other areas. Our results suggest that (i) Bornean elephants probably derive from a very small female population, (ii) they rarely disperse across current human-dominated landscapes that separate forest fragments, and (iii) forest fragments are predominantly comprised of populations that are already undergoing genetic drift. To maintain the current levels of genetic diversity in fragmented habitats, conservation of the Bornean elephants should aim at securing connectivity between spatially distinct populations. (C) 2016 Elsevier Ltd. All rights reserved. | 584 | no | no good fitness measure, N pop differnt in GD? | NA | no | no |
| 585 | 2017 | 43009 | Sutton, JT; Robertson, BC; Grueber, CE; Stanton, JAL; Jamieson, IG | 2013 | Characterization of MHC class II B polymorphism in bottlenecked New Zealand saddlebacks reveals low levels of genetic diversity | The major histocompatibility complex (MHC) is integral to the vertebrate adaptive immune system. Characterizing diversity at functional MHC genes is invaluable for elucidating patterns of adaptive variation in wild populations, and is particularly interesting in species of conservation concern, which may suffer from reduced genetic diversity and compromised disease resilience. Here, we use next generation sequencing to investigate MHC class II B (MHCIIB) diversity in two sister taxa of New Zealand birds: South Island saddleback (SIS), Philesturnus carunculatus, and North Island saddleback (NIS), Philesturnus rufusater. These two species represent a passerine family outside the more extensively studied Passerida infraorder, and both have experienced historic bottlenecks. We examined exon 2 sequence data from populations that represent the majority of genetic diversity remaining in each species. A high level of locus co-amplification was detected, with from 1 to 4 and 3 to 12 putative alleles per individual for South and North Island birds, respectively. We found strong evidence for historic balancing selection in peptide-binding regions of putative alleles, and we identified a cluster combining non-classical loci and pseudogene sequences from both species, although no sequences were shared between the species. Fewer total alleles and fewer alleles per bird in SIS may be a consequence of their more severe bottleneck history; however, overall nucleotide diversity was similar between the species. Our characterization of MHCIIB diversity in two closely related species of New Zealand saddlebacks provides an important step in understanding the mechanisms shaping MHC diversity in wild, bottlenecked populations. | 585 | no | two species comp | NA | no | no |
| 586 | 2017 | 43009 | Zhang, XJ; Kanthaswamy, S; Trask, JS; Ng, J; Oldt, RF; Mankowski, JL; Adams, RJ; Smith, DG | 2017 | Genetic Characterization of a Captive Colony of Pigtailed Macaques (Macaca nemestrina) | Effective colony management is critical to guarantee the availability of captive NHP as subjects for biomedical research. Pigtailed macaques (Macaca nemestrina) are an important model for the study of human and nonhuman primate diseases and behavior. Johns Hopkins University hosts one of the largest captive colonies of pigtailed macaques in the United States. In this study, we used 56 single-nucleotide polymorphisms (SNP) to characterize this population of pigtailed macaques, understand their population structure, and assess the effectiveness of their colony management. The results demonstrate that the colony has maintained a high level of genetic diversity, with no loss of heterozygosity since its origin, and low levels of inbreeding and genetic subdivision. | 586 | no | no good fitness measure, N pop differnt in GD? | NA | no | no |
| 587 | 2017 | 43009 | Foitzik, S; Stratz, M; Heinze, J | 2003 | Ecology, life history and resource allocation in the ant, Leptothorax nylanderi | We aimed at identifying the causal basis of previously shown interrelations between demographic and genetic colony structure, ecological factors and split sex ratios in the ant, Leptothorax nylanderi . Colony-level variation in sex allocation was only partly explained by annual fluctuations during eight study years and by resource availability as indicated by sexual production of colonies. Allocation ratios were highly male-biased in dense populations with ephemeral nest sites and high frequencies of colonies containing several unrelated matrilines. Field observations and experimental manipulations showed that nest site limitation leads to such heterogeneous colonies. Laboratory experiments demonstrated that genetic heterogeneity directly causes male-biased investment, although relatedness asymmetry is not influenced by invasion of unrelated queens. The influence of genetic composition on allocation strategies might either be explained by negative feedback mechanisms connected with habitat saturation or by a lower efficiency of heterogeneous colonies. Our results thus demonstrate which factors other than variation in relatedness asymmetry can explain split sex ratios in ants. An empirical test of a model on reproductive allocation revealed on-going queen-worker conflict over colony growth and sexual reproduction. Workers controlled reproductive allocation, but queen-worker conflict ceased in large colonies with a high survival rate. | 587 | probably no | no good fitness measure, N pop differnt in GD? | no fitness measure | no | no |
| 588 | 2017 | 43009 | Le Page, S; Sepil, I; Flintham, E; Pizzari, T; Carazo, P; Wigby, S | 2017 | Male relatedness and familiarity are required to modulate male-induced harm to females in Drosophila | Males compete over mating and fertilization, and often harm females in the process. Inclusive fitness theory predicts that increasing relatedness within groups of males may relax competition and discourage male harm of females as males gain indirect benefits. Recent studies in Drosophila melanogaster are consistent with these predictions, and have found that within-group male relatedness increases female fitness, though others have found no effects. Importantly, these studies did not fully disentangle male genetic relatedness from larval familiarity, so the extent to which modulation of harm to females is explained by male familiarity remains unclear. Here we performed a fully factorial design, isolating the effects of male relatedness and larval familiarity on female harm. While we found no differences in male courtship or aggression, there was a significant interaction between male genetic relatedness and familiarity on female reproduction and survival. Relatedness among males increased female lifespan, reproductive lifespan and overall reproductive success, but only when males were familiar. By showing that both male relatedness and larval familiarity are required to modulate female harm, these findings reconcile previous studies, shedding light on the potential role of indirect fitness effects on sexual conflict and the mechanisms underpinning kin recognition in fly populations. | 588 | no | no pop level | NA | no | no |
| 589 | 2017 | 43009 | White, KL; Eason, DK; Jamieson, IG; Robertson, BC | 2015 | Evidence of inbreeding depression in the critically endangered parrot, the kakapo | Decreased genome-wide heterozygosity of inbred individuals can result in reduced survival and reproductive fitness (i.e. inbreeding depression). However, showing such heterozygosity-fitness correlations in endangered species, especially those that are already genetically impoverished, has proven to be difficult. New Zealand’s kakapo (Strigops habroptilus) is a critically endangered, flightless parrot that now survives only on islands free of introduced predators. The recent population bottleneck of 51 individuals, lek mating system (where only females care for eggs and young) and the fact that all but one of the population’s founders came from the same insular population, render them particularly susceptible to inbreeding depression. The present study uses 25 microsatellite loci to derive estimates of relatedness to investigate heterozygosity-fitness correlations in kakapo. After accounting for the effects of co-variables, there was no evidence that male heterozygosity affected variation in egg fertility, but there was evidence that female heterozygosity affected fecundity (i.e. clutch size) and hatching success; that is, more homozygous females lay smaller clutches and had lower hatching success. The present study detected significant heterozygosity-fitness correlations and demonstrates that inbreeding is a contributing factor towards relatively low hatching success, which in turn reduces population growth in this highly endangered, flightless parrot. | 589 | no | inbreeding | NA | no | no |
| 590 | 2017 | 43009 | Reid, BN; Peery, MZ | 2014 | Land use patterns skew sex ratios, decrease genetic diversity and trump the effects of recent climate change in an endangered turtle | AimChanges in both land use and climate can cause population declines and species extinctions, but the relative effects of these two stressors are often dependent on species-specific life histories. Turtles are highly threatened as a taxonomic group and are particularly sensitive to environmental change due to their life history, habitat preferences and physiology. We used both sex data and genetic diversity across a landscape-scale gradient in land use intensity and recent climate change to characterize the relative effects of these factors on three turtle species (including the endangered Blanding’s turtle, Emydoidea blandingii) with different life histories and thus different vulnerabilities to environmental change. LocationWisconsin, USA. MethodsTurtles were captured and sampled for genetic analyses at 18 sites. Sampled individuals were genotyped for species-specific sets of microsatellite markers, and population-level genetic diversity was assessed using rarefied allelic richness and heterozygosity. The relative effects of road density, public land area and recent climate change on genetic diversity and sex ratio were evaluated using regression methods and model-selection criteria. ResultsIncreased land use intensity was associated with male-skewed sex ratios and decreased genetic diversity in E.blandingii but not co-occurring species. Recent climate change did not explain variability in either sex ratio or genetic diversity for any of the three species examined. Main conclusionsChanging land use has multiple impacts on turtle populations that are modulated by species-specific life history traits. Extensive terrestrial movements associated with nesting in E. blandingii increase female vulnerability to road mortality, which in turn has led to declines in genetic diversity. The predicted effects of recent climate change may have been dampened by behavioural plasticity. We suggest that the disproportionate effect of land use on E. blandingii warrants greater legal protection for this species. | 590 | no | no good fitness measure, N pop differnt in GD? | NA | no | no |
| 591 | 2017 | 43009 | Gompper, ME; Monello, RJ; Eggert, LS | 2011 | Genetic variability and viral seroconversion in an outcrossing vertebrate population | Inverse correlations between genetic variability and parasitism are important concerns for conservation biologists. We examined correlations between neutral genetic variability and the presence of antibodies to canine distemper virus (CDV) and feline parvovirus (FPV) in a free-ranging population of raccoons. Over 3 years there was a strong relationship between age and seroprevalence rates. Most young animals were seronegative to CDV and FPV, but the oldest age class was greater than 80 per cent seropositive to both viruses. CDV-seropositive animals had greater heterozygosity and lower measures of inbreeding compared with CDV-seronegative animals. This relationship was strongest among the youngest animals and did not occur during a 1 year CDV epidemic. In contrast, FPV-seropositive animals only had significantly lower measures of inbreeding in 1 year, perhaps because FPV-associated mortality is relatively low or primarily occurs among very young individuals that were under-represented in our sampling. These results suggest that even in large outcrossing populations, animals with lower heterozygosity and higher measures of inbreeding are less likely to successfully mount an immune response when challenged by highly pathogenic parasites. | 591 | no | no pop level | NA | no | no |
| 592 | 2017 | 43009 | Song, XY; Zhang, P; Huang, K; Chen, D; Guo, ST; Qi, XG; He, G; Pan, RL; Li, BG | 2016 | The influence of positive selection and trans-species evolution on DPB diversity in the golden snub-nosed monkeys (Rhinopithecus roxellana) | Genetic variation plays a significant role in the adaptive potential of the endangered species. The variation at major histocompatibility complex (MHC) genes can offer valuable information on selective pressure related to natural selection and environmental adaptation, particularly the ability of a host to continuously resist evolving parasites. Thus, the genetic polymorphism on exon 2 of the MHC DPB1 gene in the golden snub-nosed monkeys (Rhinopithecus roxellana) was specifically analyzed. The results show that the 6 Rhro-DPB1 alleles identified from 87 individuals exhibit positive selection and trans-species polymorphism. The results also imply that although the populations of the species have experienced dramatic reduction and severe habitat fragmentation in recent Chinese history, balancing selection still maintains relatively consistent, with moderate DPB1 polymorphism. Thus, the study provides valuable information and evidence in developing effective strategies and tactics for genetic health and population size expansion of the species. It also offers strong genetic background for further studies on other primate species, particularly those in Rhinopithecus-a further endeavor that would result in fully understanding the MHC genetic information of the Asian colobines. | 592 | no | no good fitness measure, N pop differnt in GD? | NA | no | no |
| 593 | 2017 | 43009 | Bichet, C; Moodley, Y; Penn, DJ; Sorci, G; Garnier, S | 2015 | Genetic structure in insular and mainland populations of house sparrows (Passer domesticus) and their hemosporidian parasites | Small and isolated populations usually exhibit low levels of genetic variability, and thus, they are expected to have a lower capacity to adapt to changes in environmental conditions, such as exposure to pathogens and parasites. Comparing the genetic variability of selectively neutral versus functional loci allows one to assess the evolutionary history of populations and their future evolutionary potential. The genes of the major histocompatibility complex (MHC) control immune recognition of parasites, and their unusually high diversity is genes which is likely driven by parasite-mediated balancing selection. Here, we examined diversity and differentiation of neutral microsatellite loci and functional MHC class I genes in house sparrows (Passer domesticus), living in six insular and six mainland populations, and we aimed to determine whether their diversity or differentiation correlates with the diversity and the prevalence of infection of hemosporidian parasites. We found that island bird populations tended to have lower neutral genetic variability, whereas MHC variability gene was similar between island and mainland populations. Similarly, island populations tended to show greater genetic differentiation than mainland populations, especially at microsatellite markers. The maintenance of MHC genetic diversity and its less marked structure in the island populations could be attributed to balancing-selection. The greater MHC differentiation among populations was negatively correlated with similarity in blood parasites (prevalence and diversity of parasite strains) between populations. Even at low prevalence and small geographical scale, haemosporidian parasites might contribute to structure the variability of immune genes among populations of hosts. | 593 | maybe | any gD and fitness of groups that works? | NA | yes | yes |
| 594 | 2017 | 43009 | Simpson, S; Blampied, N; Peniche, G; Dozieres, A; Blackett, T; Coleman, S; Cornish, N; Groombridge, JJ | 2013 | Genetic structure of introduced populations: 120-year-old DNA footprint of historic introduction in an insular small mammal population | Wildlife populations have been introduced to new areas by people for centuries, but this human-mediated movement can disrupt natural patterns of genetic structure by altering patterns of gene flow. Insular populations are particularly prone to these influences due to limited opportunities for natural dispersal onto islands. Consequently, understanding how genetic patterns develop in island populations is important, particularly given that islands are frequently havens for protected wildlife. We examined the evolutionary origins and extent of genetic structure within the introduced island population of red squirrels (Sciurus vulgaris) on the Channel Island of Jersey using mitochondrial DNA (mtDNA) control region sequence and nuclear microsatellite genotypes. Our findings reveal two different genetic origins and a genetic architecture reflective of the introductions 120 years ago. Genetic structure is marked within the maternally inherited mtDNA, indicating slow dispersal of female squirrels. However, nuclear markers detected only weak genetic structure, indicating substantially greater male dispersal. Data from both mitochondrial and nuclear markers support historic records that squirrels from England were introduced to the west of the island and those from mainland Europe to the east. Although some level of dispersal and introgression across the island between the two introductions is evident, there has not yet been sufficient gene flow to erase this historic genetic footprint. We also investigated if inbreeding has contributed to high observed levels of disease, but found no association. Genetic footprints of introductions can persist for considerable periods of time and beyond traditional timeframes of wildlife management. | 594 | no | no good fitness measure, N pop differnt in GD? | NA | no | no |
| 595 | 2017 | 43009 | Savova, V; Chun, S; Sohail, M; McCole, RB; Witwicki, R; Gai, LS; Lenz, TL; Wu, CT; Sunyaev, SR; Gimelbrant, AA | 2016 | Genes with monoallelic expression contribute disproportionately to genetic diversity in humans | An unexpectedly large number of human autosomal genes are subject to monoallelic expression (MAE). Our analysis of 4,227 such genes uncovers surprisingly high genetic variation across human populations. This increased diversity is unlikely to reflect relaxed purifying selection. Remarkably, MAE genes exhibit an elevated recombination rate and an increased density of hypermutable sequence contexts. However, these factors do not fully account for the increased diversity. We find that the elevated nucleotide diversity of MAE genes is also associated with greater allelic age: variants in these genes tend to be older and are enriched in polymorphisms shared by Neanderthals and chimpanzees. Both synonymous and nonsynonymous alleles of MAE genes have elevated average population frequencies. We also observed strong enrichment of the MAE signature among genes reported to evolve under balancing selection. We propose that an important biological function of widespread MAE might be the generation of cell-to-cell heterogeneity; the increased genetic variation contributes to this heterogeneity. | 595 | no | human | NA | no | no |
| 596 | 2017 | 43009 | Cohen, S; Tirindelli, J; Gomez-Chiarri, M; Nacci, D | 2006 | Functional implications of major histocompatibility (MH) variation using estuarine fish populations | Recently, there has been a dramatic expansion of studies of major histocompatibility complex (MHC) variation aimed at discovering functional differences in immunity across wild populations of diverse vertebrate species. Some species with relatively low genetic diversity or under strong directional selection by pathogens have revealed fascinating cases of MHC allelic disease linkage. More generally in genetically diverse species, however, these linkages may be hard to find. In this paper, we review approaches for assessing functional variation in MHC and discuss their potential use for discovering smaller-scale intraspecific spatial and temporal patterns of MHC variation. Then, we describe and illustrate an approach using the structural model to produce a population composite of variation in antigen-binding regions by mapping population-specific substitutions onto functional regions of the molecule. We are producing models of variation in major histocompatibility (MH) loci for populations of non-migratory fish (killifish, Fundulus heteroclitus) resident at sites that vary dramatically in environmental quality. We discuss the goal of relating MH population variation to functional differences in disease susceptibility such as those inferred by observations of parasitic infection and direct measurement of bacterial challenges in the laboratory. Our study has focused on relatively well-studied killifish populations, including those resident in a highly disturbed, chemically contaminated estuary and nearby less contaminated sites. Population-specific genetic changes at MHC antigen-binding loci are described, and evidence relevant to functional implications of these changes is reviewed. Population-specific patterns of variation in antigen-binding regions in combination with a range of assessments of immune function will provide a powerful new approach to reveal functional changes in MHC. | 596 | no | NA | NA | no | no |
| 597 | 2017 | 43009 | McLeod, BA; Burns, LE; Frasier, TR; Broders, HG | 2015 | Effect of oceanic straits on gene flow in the recently endangered little brown bat (Myotis lucifugus) in maritime Canada: implications for the spread of white-nose syndrome | White-nose syndrome is rapidly spreading in eastern North America, causing mass mortality of hibernating bats. We characterized levels of genetic diversity and population structure of the little brown bat (Myotis lucifugus (Le Conte, 1831)) in eastern Canada to infer the extent to which oceanic straits may be barriers to movement. To quantify metrics of gene flow and infer movement dynamics, we genotyped 679 M. lucifugus at nine nuclear microsatellites (nDNA) and sequenced a portion of the mitochondrial DNA (mtDNA). We found high levels of genetic diversity and little population structure, with approximate to 13-fold higher differentiation of mtDNA than nDNA markers, suggesting that structuring patterns largely result from female philopatry. Discriminant analysis of principle components suggested that the subtle underlying structure was not concordant with sampling site. Regional differentiation (FST, Dest, Mantel test residuals) is mostly consistent with genetic isolation by distance. However, samples from Newfoundland showed genetic differentiation over and above the effects of distance, lower levels of genetic diversity, and less genetic connectivity with other sampled regions. Despite this, oceanic straits in the Gulf of Saint Lawrence do not appear to create an impenetrable barrier to movement, therefore it may be possible for white-nose syndrome to spread to Newfoundland. | 597 | no | no good fitness measure, N pop differnt in GD? | NA | no | no |
| 598 | 2017 | 43009 | Sielezniew, M; Rutkowski, R | 2012 | Population isolation rather than ecological variation explains the genetic structure of endangered myrmecophilous butterfly Phengaris (=Maculinea) arion | Genetic variation of the globally threatened obligatorily myrmecophilous Large Blue butterfly Phengaris (Maculinea) arion (Lepidoptera) was studied, using six microsatellite markers, in a country where its decline is dramatic (Poland). Material was collected on 13 sites showing considerable ecological variation as far as biotope, larval food plant and host ants of the butterfly were concerned. Genetic variability, estimated in terms of number of alleles and heterozygosity, was the lowest in the most isolated populations. However on sites localized in areas where suitable biotopes were extensive and interconnected, P. arion still held relatively high genetic diversity. Pairwise F (ST) values indicated small and moderate differentiation among samples (F (ST) = 0.01-0.15), with the exceptions of two isolated localities (0.20). We did not find clear evidence of isolation by distance. The presence of four or five genetic clusters was indicated. Analysis of the membership of each individual to each cluster showed that the vast majority of individuals from three isolated populations were clustered in three separate genetic groups. The most distinct population was the one, which had been found to be specialized towards Myrmica lobicornis in previous studies. Individuals from the remaining populations could not be clustered in separate genetic groups, however some dominance of different clusters in geographical regions was observed. Some portion of the population’s genetic variability could be explained by geographical distribution, however the percentage of variation, explaining the differences between two main regions (S and NE Poland), was very low. We conclude that the main factor shaping the current genetic structure of P. arion in Poland is the recent isolation of populations related to habitat fragmentation but local ecological specializations may be also a potential factor. Therefore the necessity of activities aiming to halt the further reduction of genetic variability, as well as the monitoring of priority populations (e.g. those belonging to unique host races), should be emphasized in future action plans in Central Europe. | 598 | no | no good fitness measure | NA | no | no |
| 599 | 2017 | 43009 | Talbot, B; Vonhof, MJ; Broders, HG; Fenton, B; Keyghobadi, N | 2016 | Range-wide genetic structure and demographic history in the bat ectoparasite Cimex adjunctus | Background: Evolutionary histories of parasite and host populations are intimately linked such that their spatial genetic structures may be correlated. While these processes have been relatively well studied in specialist parasites and their hosts, less is known about the ecological and evolutionary consequences of relationships between generalist ectoparasites and their hosts. The aim of this study was to investigate the genetic structure and demographic history of a bat ectoparasite, Cimex adjunctus, whose host affinity is weak but the biology of the potential hosts have been well studied. This ectoparasite has been hypothesized to rely on its hosts for dispersal due to its low inherent dispersal potential. Here we describe genetic diversity and demographic history in C. adjunctus through most of its range in North America. We investigated variation at the cytochrome c oxidase 1 mitochondrial gene and nine microsatellite markers, and tested the prediction that genetic diversity in C. adjunctus is spatially structured. We also tested the prediction that demographic history in C. adjunctus is characterized by range and demographic expansion as a consequence of post-Pleistocene climate warming. Results: We found stronger spatial structuring of genetic diversity in C. adjunctus than has been quantified in two of its hosts, but contrast in amount of variation explained by host association with different genetic markers (i.e., nuclear vs mitochondrial DNA). Also, C. adjunctus’ history is not primarily characterized by demographic and range expansion, as is the case with two of its key hosts. Conclusions: Our study shows different patterns of genetic structure and demographic history in C. adjunctus than have been detected in two of its key hosts. Our results suggest an effect of a loose parasite-host relationship and anti-parasitism strategies on genetic structure and post-Pleistocene recovery of population size. | 599 | no | gd of parasite | NA | no | no |
| 600 | 2017 | 43009 | Zhou, YB; Yang, MX; Zhao, GM; Wei, JG; Jiang, QW | 2007 | Oncomelania hupensis (Gastropoda : Rissooidea), intermediate host of Schistosoma japonicum in China: Genetics and molecular phylogeny based on amplified fragment length polymorphisms | Schistosomiasis japonica is one of the most serious parasitic diseases in China. Subspecies of the pornatiopsid snail species Oncomelania hupensis transmit the human blood fluke Schistosoma japonicum, the parasite causing the disease. In at least one study involving the subspecies Oncomelania hupensis robertsoni, there was no clear phylogenetic concordance between the mitochondrial and nuclear data, and it was strongly recommended that future studies incorporate more data from nuclear loci in order to better understand phylogeography and host-parasite coevolution (Wilke et al., 2006). In this paper, we explore genetic diversity based AFLP data involving 25 populations throughout mainland China. AFLP is a DNA fingerprinting technique that detects the polymorphism of the whole genome without prior knowledge of the nucleotide sequence, with the high degree of reproducibility and small amount of template DNA needed. Our AFLP data show that: (1) the genetic diversity within an O. hupensis population increases gradually with snail sample size, and when the sample size is more than 30 individuals, the genetic variation within one O. hupensis population trends to stabilization. (2) There is high intra-population genetic variation on the mainland of China, and these intra-population genetic variations from different areas differ significantly. (3) The considerable genetic differentiation occurs throughout China. (4) The genetic variation among populations of O. h. hupensis is higher than that of O. h. robertsoni. (5) The patterns of genetic differentiation are basically consistent with geographical distribution of snail populations. Our results are similar to the results of allozymes and COI gene sequences, excluding a snail population from Guangxi Province (Gx-1), indicating that there are three distinct subspecies in mainland China, namely O. h. hupensis, O. h. robertsoni, and O. h. tangi. However, our results do not support that the snail population from Guangxi Province belongs to O. h. hupensis. | 600 | no | no good fitness measure, N pop differnt in GD? | NA | no | no |
| 601 | 2017 | 43009 | Menescal, LA; Goncalves, EC; Silva, A; Ferrari, SF; Schneider, MPC | 2009 | Genetic Diversity of Red-Bellied Titis (Callicebus moloch) from Eastern Amazonia Based on Microsatellite Markers | The titi monkeys (Callicebus spp.) are a large, diverse genus of platyrrhines, widely distributed in tropical South America. The genetic variability of these monkeys is still relatively poorly known, especially at the population level. In the present study, four heterologous microsatellite markers were used to investigate genetic diversity in 23 individuals from a wild population of red-bellied titis (Callicebus moloch) in eastern Amazonia. An unexpectedly low level of diversity was found. The average number of alleles was 8.75 (range: 5-15), and the average heterozygosity was 0.33 (range: 0.09-0.65). This preliminary information suggests a reduction of the potential for long-term survival of the population and indicates the putative necessity of implementation of a species conservation program. | 601 | no | no pop level | NA | no | no |
| 602 | 2017 | 43009 | Halbert, ND; Grant, WE; Derr, JN | 2005 | Genetic and demographic consequences of importing animals into a small population: a simulation model of the Texas State Bison Herd (USA) | The extant 40 bison (Bison bison) constituting the Texas State Bison Herd (TSBH; USA) are directly and exclusively descended from a bison herd assembled by Charles Goodnight in the 1880s, representing a historically and genetically valuable resource. The population currently suffers from low genetic variation, low heterozygosity, high calf mortality, and low natality rates compared with other closed bison populations. Population viability analysis using the VORTEX program previously indicated a 99% chance of population extinction within the next 41 years [J. Mamm. 85 (2004) in press]. We developed a stochastic simulation model to evaluate the genetic and demographic consequences of various management scenarios for the TSBH using genotypic data from 51 microsatellite loci and demographic information recorded over a 6-year period. Our results reveal that without the introduction of new genetic variation, approximately 37% of the representative microsatellite loci will become fixed as the TSBH continues to lose genetic variation at a staggering rate of 30-40% within the next 50 years. Furthermore, if the current trends in natality and mortality rates continue, our model indicates the TSBH faces a 99% chance of extinction in the next 51 years. With the importation of unrelated male bison into the TSBH, and under the assumption of increased fitness, the probability of population survival in the next 100 years increases to 100%, and the population will reach the approximate carrying capacity of 200 bison in 15-16 years. Furthermore, our model predicts increases in genetic diversity and heterozygosity of 24.7-48.4% and 17.5-36.5%, respectively, in the next 100 years following the addition of new genetic variation. We conclude that the importation of bison into the TSBH is necessary to prevent extinction and ensure long-term population survival. (C) 2004 Elsevier B.V. All fights reserved. | 602 | no | no good fitness measure, N pop differnt in GD? | NA | no | no |
| 603 | 2017 | 43009 | Wickert, E; de Goes, A; de Souza, A; Lemos, EGD | 2012 | Genetic Diversity and Population Differentiation of the Causal Agent of Citrus Black Spot in Brazil | One of the most important diseases that affect sweet orange orchards in Brazil is the Citrus Black Spot that is caused by the fungus Guignardia citricarpa. This disease causes irreparable losses due to the premature falling of fruit, as well as its severe effects on the epidermis of ripe fruit that renders them unacceptable at the fresh fruit markets. Despite the fact that the fungus and the disease are well studied, little is known about the genetic diversity and the structure of the fungi populations in Brazilian orchards. The objective of this work was study the genetic diversity and population differentiation of G. citricarpa associated with four sweet orange varieties in two geographic locations using DNA sequence of ITS1-5.8S-ITS2 region from fungi isolates. We observed that different populations are closely related and present little genetic structure according to varieties and geographic places with the highest genetic diversity distributed among isolates of the same populations. The same haplotypes were sampled in different populations from the same and different orange varieties and from similar and different origins. If new and pathogenic fungi would become resistant to fungicides, the observed genetic structure could rapidly spread this new form from one population to others. | 603 | no | gd of pathogen | NA | no | no |
| 604 | 2017 | 43009 | Reeves, PA; Panella, LW; Richards, CM | 2012 | Retention of agronomically important variation in germplasm core collections: implications for allele mining | “The primary targets of allele mining efforts are loci of agronomic importance. Agronomic loci typically exhibit patterns of allelic diversity that are consistent with a history of natural or artificial selection. Natural or artificial selection causes the distribution of genetic diversity at such loci to deviate substantially from the pattern found at neutral loci. The germplasm utilized for allele mining should contain maximum allelic variation at loci of interest, in the smallest possible number of samples. We show that the popular core collection assembly procedure”“M”" (marker allele richness), which leverages variation at neutral loci, performs worse than random assembly for retaining variation at a locus of agronomic importance in sugar beet (Beta vulgaris L. subsp. vulgaris) that is under selection. We present a corrected procedure ("“M+”“) that outperforms M. An extensive coalescent simulation was performed to demonstrate more generally the retention of neutral versus selected allelic variation in core subsets assembled with M+. A negative correlation in level of allelic diversity between neutral and selected loci was observed in 42% of simulated data sets. When core collection assembly is guided by neutral marker loci, as is the current common practice, enhanced allelic variation at agronomically important loci should not necessarily be expected.” | 604 | no | no good fitness measure, N pop differnt in GD? | NA | no | no |
| 605 | 2017 | 43009 | Mestre, A; Butlin, RK; Kelso, WE; Romaire, R; Bonvillain, CP; Monros, JS; Mesquita-Joanes, F | 2016 | Contrasting patterns of genetic diversity and spatial structure in an invasive symbiont-host association | Do host invaders and their associated symbiont co-invaders have different genetic responses to the same invasion process? To answer this question, we compared genetic patterns of native and exotic populations of an invasive symbiont-host association. This is an approach applied by very few studies, of which most are based on parasites with complex life cycles. We used the mitochondrial genetic marker cytochrome oxidase subunit I (COI) to investigate a non-parasitic freshwater ectosymbiont with direct life-cycle, low host specificity and well-documented invasion history. The study system was the crayfish Procambarus clarkii and its commensal ostracod Ankylocythere sinuosa, sampled in native (N American) and exotic (European) ranges. Results of analyses indicated: (1) higher genetic diversity in the symbiont than its host; (2) genetic diversity loss in the exotic range for both species, but less pronounced in the symbiont; (3) native populations genetically structured in space, with stronger patterns in the symbiont and (4) loss of spatial genetic structure in the exotic range in both species. The combination of historical, demographic and genetic data supports a higher genetic diversity of source populations and a higher propagule size that allowed the symbiont to overcome founder effects better than its host co-invader. Thus, the symbiont might be endowed with a higher adaptive potential to new hosts or off-host environmental pressures expected in the invasive range. We highlight the usefulness of this relatively unexplored kind of symbiont-host systems in the invasion context to test important ecological and evolutionary questions. | 605 | no | gd of pathogen | NA | no | no |
| 606 | 2017 | 43009 | Talbot, B; Garant, D; Paquette, SR; Mainguy, J; Pelletier, F | 2013 | Genetic structure and diversity among rabid and nonrabid raccoons | Population genetics approaches are becoming widely used to assess the propagation potential of wildlife diseases. Such studies are often conducted on uninfected hosts because acute zoonotic diseases pose significant health risks to humans and infected hosts are thus more difficult to obtain. Predictions of disease spread potential assume that infected and uninfected individuals exhibit similar movement behaviours and genotypes. However, some diseases, such as rabies, might enhance individual’s dispersal behaviour. Thus, if rabid animals are mostly long-distance migrants, prediction of rabies spread potential based on genetic analyses of uninfected animals might be misleading. Here, we genotyped 84 rabid and 113 nonrabid raccoons (Procyon lotor) to assess whether a difference in genetic structure pattern could be detected between these groups in a rabies epizootic area in southern Quebec, Canada. We also assessed whether genetic diversity differs among rabid and nonrabid raccoons by comparing expected heterozygosity, allelic richness, and inbreeding coefficient of the 2 groups. Finally, we tested for possible associations between rabies infection and microsatellite genotypes. We found no evidence of genetic structuring and no difference in genetic diversity among nonrabid and rabid raccoons. We also have limited evidence for a link between genotype and infection status. Our results thus suggest that the genetic structure of nonrabid raccoons is representative of that found in infected individuals and is thus effective to infer rabies propagation patterns in the wild. | 606 | no | no pop level | NA | no | no |
| 607 | 2017 | 43009 | Hao, CY; Perretant, MR; Choulet, F; Wang, LF; Paux, E; Sourdille, P; Zhang, XY; Feuillet, C; Balfourier, F | 2010 | Genetic diversity and linkage disequilibrium studies on a 3.1-Mb genomic region of chromosome 3B in European and Asian bread wheat (Triticum aestivum L.) populations | Genetic diversity and linkage disequilibrium (LD) were investigated in 376 Asian and European accessions of bread wheat (Triticum aestivum L.). After a first and rapid screening about diversity and genetic structure at the whole genome scale using 70 simple sequence repeats (SSRs), we focused on a sequenced contig (ctg954) of 3.1 Mb located on the short arm of chromosome 3B of cv. Chinese Spring, using 32 SSRs and 10 single nucleotide polymorphisms. This contig is part of a multiple fungal resistance region. Mean polymorphism information content value on the 32 SSRs was slightly higher in the Asian genepool (0.396) than that for the European (0.329) pool. Compared with results at the whole genome scale, data from this 3.1-Mb region indicated similar trends in genetic diversity indices between both genepools. Population structure and molecular variance analyses demonstrated significant genetic differentiation and geographical subdivision in both groups of accessions. Concerning LD at the contig level, the European population had a significantly higher mean r (2) value (0.23) than the Asian population (0.18), indicating a stronger LD in the European material. With a mean of 1 marker every 74 kb, the resolution reached here allowed to perform a detailed comparative analysis of the LD and genetic diversity along the complete 3.1-Mb region in both genepools. A sliding-window approach revealed some interesting regions of the contig where LD is increasing when genetic diversity is decreasing. This study provides an in-depth understanding of molecular population genetics in European and Asian wheat gene pools, and prospects for association mapping of important sources of fungal disease resistance. | 607 | no | no good fitness measure, N pop differnt in GD? | NA | no | no |
| 608 | 2017 | 43009 | Ellison, A; Wright, P; Taylor, DS; Cooper, C; Regan, K; Currie, S; Consuegra, S | 2012 | Environmental diel variation, parasite loads, and local population structuring of a mixed-mating mangrove fish | Genetic variation within populations depends on population size, spatial structuring, and environmental variation, but is also influenced by mating system. Mangroves are some of the most productive and threatened ecosystems on earth and harbor a large proportion of species with mixed-mating (self-fertilization and outcrossing). Understanding population structuring in mixed-mating species is critical for conserving and managing these complex ecosystems. Kryptolebias marmoratus is a unique mixed-mating vertebrate inhabiting mangrove swamps under highly variable tidal regimes and environmental conditions. We hypothesized that geographical isolation and ecological pressures influence outcrossing rates and genetic diversity, and ultimately determine the local population structuring of K. marmoratus. By comparing genetic variation at 32 microsatellites, diel fluctuations of environmental parameters, and parasite loads among four locations with different degrees of isolation, we found significant differences in genetic diversity and genotypic composition but little evidence of isolation by distance. Locations also differed in environmental diel fluctuation and parasite composition. Our results suggest that mating system, influenced by environmental instability and parasites, underpins local population structuring of K. marmoratus. More generally, we discuss how the conservation of selfing species inhabiting mangroves and other biodiversity hotspots may benefit from knowledge of mating strategies and population structuring at small spatial scales. | 608 | yes | how many pop? | “sent email, effect size is found in”“Best of both worlds? Association between outcrossing and parasite loads in a selfing fish”"" | yes | no |
| 609 | 2017 | 43009 | Tarazona-Santos, E; Tishkoff, SA | 2005 | Divergent patterns of linkage disequilibrium and haplotype structure across global populations at the interleukin-13 (IL13) locus | Interleukin- 13 ( IL- 13) is a cytokine involved in Th2 immune response, which plays a role in susceptibility to infection by extracellular parasites as well as complex diseases of the immune system such as asthma and allergies. To determine the pattern of genetic diversity at the IL13 gene, we sequenced 3950 bp encompassing the IL13 gene and its promoter in 264 chromosomes from individuals originating from East and West Africa, Europe, China and South America. Thirty- one single-nucleotide polymorphisms ( SNPs) arranged in 88 haplotypes were indentified, including the nonsynonymous substitution Arg130Gln in exon 4, which differs in frequency across ethnic groups. We show that genetic diversity and linkage disequilibrium ( LD) are not evenly distributed across the gene and that sites in the 50 and 30 regions of the gene show strong differentiation among continental groups. We observe a divergent pattern of haplotype variation and LD across geographic regions and we identify a set of htSNPs that will be useful for functional genetic association studies of complex disease. We use several statistical tests to distinguish the effects of natural selection and demographic history on patterns of genetic diversity at the IL13 locus. | 609 | no | NA | NA | no | no |
| 610 | 2017 | 43009 | Randall, DA; Pollinger, JP; Wayne, RK; Tallents, LA; Johnson, PJ; Macdonald, DW | 2007 | Inbreeding is reduced by female-biased dispersal and mating behavior in Ethiopian wolves | Molecular tools have enabled wildlife researchers to obtain accurate information on the kinship, mating behavior, and dispersal of individuals. We genotyped 192 Ethiopian wolves (n = 29 packs) in the Bale Mountains for 17 microsatellite loci to 1) elucidate kinship within and between packs, 2) assess parentage of pups, and 3) evaluate whether inbreeding is avoided by dispersal and/or mating behavior. Mean pairwise relatedness within packs (R = 0.39) was significantly greater than that estimated from random assignment of individuals to packs. However, breeding pairs were most often unrelated, suggesting that female-biased dispersal reduces inbreeding. We assigned maternity to 49 pups and paternity to 47 pups (n = 12 litters) rising a combination of exclusion, likelihood analyses (using CERVUS software), and sibship reconstruction. Multiple paternity occurred in 33% of litters; extrapack paternity accounted for 28% of all resolved paternities, occurring in 50% of litters. We found no evidence that extrapack copulations reduce inbreeding; however, more detailed analyses may elucidate the effect of recent population declines and demographic disturbances due to recurring disease outbreaks. The adaptive advantages of female-biased dispersal and the observed mating system are discussed in relation to Ethiopian wolf sociobiology and ecology. | 610 | no | inbreeding | NA | no | no |
| 611 | 2017 | 43009 | Luo, WB; Callaway, RM; Atwater, DZ | 2016 | Intraspecific diversity buffers the inhibitory effects of soil biota | Plant community productivity can increase with increasing intraspecific genotypic diversity. Previous studies have attributed the genetic diversity-productivity pattern to differential resource use among genotypes, as many studies have found for species. But here we ask whether suppression of productivity at low intraspecific diversity by soil biota might also drive a positive diversity-productivity relationship. In a previous study, we manipulated genetic diversity by varying the number of Pseudoroegneria accessions growing together in common garden plots, and used soil from that experiment to evaluate soil feedbacks. The total biomass of P. spicata plants grown in unsterilized soil increased with accession richness, specifically when comparing soil that had contained plants from 3-accessions to soil that had contained plants from either 8 or 12 population accessions. Furthermore, soil from high-richness (8 or 12-accession) plots drove neutral feedbacks, whereas soil in the 3-accession plots (3) drove negative feedbacks. However, within each level of richness, there was no relationship between relative yield and feedback. Our results suggest that soil biota might play an integral role in the emerging understanding of the relationship between intraspecific diversity and ecosystem productivity. | 611 | no | No GD diff between pop | NA | no | no |
| 612 | 2017 | 43009 | Cook-Patton, SC; Hastings, AP; Agrawal, AA | 2017 | Genotypic diversity mitigates negative effects of density on plant performance: a field experiment and life cycle analysis of common evening primrose Oenothera biennis | 1. Genotypic diversity in plant populations is known to enhance plant performance and ecosystem function. Nonetheless, the effect of genotypic diversity has rarely been examined across a population’s lifecycle despite the expectation that changing conditions, such as population density, will alter the benefits of diversity. 2. We simultaneously manipulated a component of genotypic diversity (richness, the number of genotypes) and density of common evening primrose Oenothera biennis to address the consequences for herbivory and lifetime fitness in a 2-year field experiment that spanned seed germination to lifetime fruit production. We genotyped >1100 seedlings with microsatellite DNA markers to determine realized diversity and density in plots sown with O. biennis seeds. Our design achieved quantitative variation in plant density and diversity, with one to 44 individuals established in field plots and two to eight genotypes per polyculture plot (based on microsatellite analysis of reproductive plants). 3. We found a strong interaction between seed density and genetic diversity, with germination and establishment being 24% higher in genetic polycultures than monocultures, but only at low seed density. At high seed density, the opposite pattern emerged, with polycultures having 12% fewer individuals established than monocultures. Initial effects of emergence on plot density persisted through to the fruiting stage. 4. Higher plant densities resulted in increased mortality, decreased probability of reproduction, decreased plant height and lower levels of lifetime fruit production per plant. Increasing genotypic diversity increased the probability of reproduction overall and showed a significant interaction with plant density mitigating the negative effects of high density on individual height and lifetime fruit production. 5. Synthesis. Plant density and genotypic diversity interacted from the very early stages of seed germination and establishment of Oenothera biennis. This effect persisted over the 2-year life cycle of plants, and genotypic diversity buffered against the negative fitness consequences of high plant density. These results imply a dynamic interplay between the long-held paradigm of density effects in plant ecology and the genetic structure of populations. | 612 | probably yes | any fitness (survival) of groups of different gd? | NA | yes | yes |
| 613 | 2017 | 43009 | Zhigileva, ON; Ozhirelev, VV; Stepanova, TF; Moiseenko, TI | 2014 | Population structure of Opisthorchis felineus (Trematoda) and its second intermediate hosts - cyprinid fishes in the Ob-Irtysh focus of opisthorchiasis, based on allozyme data | Genetic variability of West Siberian populations of Opisthorchis felineus and two species of cyprinid fish, its second intermediate hosts, was studied by isozyme analysis. Low levels of allozyme variation and genetic differentiation in O. felineus from the Ob-Irtysh focus of opisthorchiasis were detected. The proportion of polymorphic loci was 21.1 %, the average observed heterozygosity (Hobs) was 0.008, and expected heterozygosity (Hexp) was 0.052. For most loci in O. felineus deficit of heterozygotes (FIS = 0.7424) was observed. A comparison of population genetic structure of fish and parasites showed they were not congruent. Estimates of genetic differentiation of the parasite were smaller than for the fish - its intermediate host. Migration and population structure of the second intermediate hosts do not play an important role in formation of the population-genetic structure of O. felineus in the Ob-Irtysh focus of opisthorchiasis. | 613 | no | no good fitness measure | NA | no | no |
| 614 | 2017 | 43009 | Velavan, TP; Schulenburg, H; Michiels, NK | 2007 | Development and characterization of novel microsatellite markers for the common earthworm (Lumbricus terrestris L.) | We developed and characterized 10 highly polymorphic microsatellite loci from an SSR-enriched genomic DNA library of the common earthworm (Lumbricus terrestris L). Characterization of these loci using 32 individuals revealed high levels of genetic diversity, five to 18 alleles per locus and a high observed and expected heterozygosity. These loci will be used for paternity analysis and population genetic studies of the co-evolution between L. terrestris and its parasites. | 614 | no | no good fitness measure | NA | no | no |
| 615 | 2017 | 43009 | Zhang, ZY; Cashins, S; Philips, A; Burridge, CP | 2014 | Significant population genetic structuring but a lack of phylogeographic structuring in the endemic Tasmanian tree frog (Litoria burrowsae) | Conservation of frogs is of global concern, owing to declines resulting from habitat destruction, global climate change, and disease. Knowledge of genetic variation in frog species is therefore desirable for the identification of management units. Here we surveyed mitochondrial DNA sequence variation in the Tasmanian endemic hylid frog Litoria burrowsae, which is infected by chytrid fungus, Batrachochytrium dendrobatidis, and may be declining. Neither phylogeographic structure nor deep phylogenetic divergence was detected in the species, although its populations were highly differentiated with respect to haplotype frequencies. The low-haplotype diversity in L. burrowsae suggests a recent bottleneck in the species, and population genetic structuring may reflect isolation by distance as well as founder effects associated with range expansion. Three putative management units were identified that require verification based on nuclear DNA variation and adaptation to local environments. | 615 | no | no good fitness measure | NA | no | no |
| 616 | 2017 | 43009 | Ayele, TB; Gailing, O; Finkeldey, R | 2011 | Assessment and integration of genetic, morphological and demographic variation in Hagenia abyssinica (Bruce) JF Gmel to guide its conservation | Assessing and integrating genetic, morphological and demographic variation is instrumental for planning conservation, tree improvement and domestication programs. We discuss the variation of the gravely endangered tropical tree species Hagenia abyssinica in Ethiopia with regard to its morphological and genetic traits as well as its population demography in a conservation context. An analysis of variance (ANOVA) revealed a highly significant differentiation among 22 natural populations of H. abyssinica in all quantitative morphological traits assessed. Multivariate and univariate taxonomic distances of leaf traits among populations are not correlated with the corresponding genetic distances at AFLP markers (multivariate r=-0.03484 at p=0.3926), showing that the genetic differentiation at AFLPs is not associated with the morphological differences among populations. Chloroplast microsatellite data allowed us to identify lineages and to reconstruct population history, while the AFLP data enabled us to identify populations of high genetic diversity. A weighted-score population prioritisation matrix (WPPM) that integrates genetic, morphological and demographic criteria was developed and used for the first time to prioritise populations for conservation and domestication. Action is needed to launch conservation and domestication programs of H. abyssinica to ensure the long-term survival of the species and to boost its economic and ecological value. (C) 2010 Elsevier GmbH. All rights reserved. | 616 | no | no good fitness measure | NA | no | no |
| 617 | 2017 | 43009 | Choi, Y; Wijsman, EM; Weir, BS | 2009 | Case-Control Association Testing in the Presence of Unknown Relationships | Genome-wide association studies result in inflated false-positive results when unrecognized cryptic relatedness exists. A number of methods have been proposed for testing association between markers and disease with a correction for known pedigree-based relationships. However, in most case-control studies, relationships are generally unknown, yet the design is predicated on the assumption of at least ancestral relatedness among cases. Here, we focus on adjusting cryptic relatedness when the genealogy of the sample is unknown, particularly in the context of samples from isolated populations where cryptic relatedness may be problematic. We estimate cryptic relatedness using maximum-likelihood methods and use a corrected chi(2) test with estimated kinship coefficients for testing in the context of unknown cryptic relatedness. Estimated kinship coefficients characterize precisely the relatedness between truly related people, but are biased for unrelated pairs. The proposed test substantially reduces spurious positive results, producing a uniform null distribution of P-values. Especially with missing pedigree information, estimated kinship coefficients can still be used to correct non-independence among individuals. The corrected test was applied to real data sets from genetic isolates and created a distribution of P-value that was close to uniform. Thus, the proposed test corrects the non-uniform distribution of P-values obtained with the uncorrected test and illustrates the advantage of the approach on real data. Genet. Epidemiol. 33:668-678, 2009. (C) 2009 Wiley-Liss, Inc. | 617 | no | NA | NA | no | no |
| 618 | 2017 | 43009 | Simone-Finstrom, M; Walz, M; Tarpy, DR | 2016 | Genetic diversity confers colony-level benefits due to individual immunity | Several costs and benefits arise as a consequence of eusociality and group-living. With increasing group size, spread of disease among nest-mates poses selective pressure on both individual immunity and group-level mechanisms of disease resistance (social immunity). Another factor known to influence colony-level expression of disease is intracolony genetic diversity, which in honeybees (Apis mellifera) is a direct function of the number of mates of the queen. Colonies headed by queens with higher mating numbers have less variable infections of decreased intensity, though the underlying mechanisms remain unclear. By pathogen-challenging larvae in vitro, we decoupled larval immune response from mechanisms of social immunity. Our results show that baseline immunity and degree of immune response do not vary with genetic diversity. However, intracolony variance in antimicrobial peptide production after pathogen challenge decreases with increasing genetic diversity. This reduction in variability of the larval immune response could drive the mitigation of disease observed in genetically diverse colonies. | 618 | probably no | no good fitness measure | no good fitness measure | no | no |
| 619 | 2017 | 43009 | Dizkirici, A; Kaya, Z; Guren, HE; Budak, H | 2010 | Barley germplasms developed for scald disease resistance exhibited a high level of genetic diversity based on SRAP markers | The objective of this study was to assess the genetic diversity and genetic relationships among 59 Turkish barley (Hordeum vulgare L.) germplasm lines (maintained for scald disease resistance breeding) using sequence-related amplified polymorphism (SRAP) markers. Seventeen SRAP primer combinations produced 83 polymorphic markers, with a mean polymorphism of 73.5%. The dendrogram created based on Nei’s unweighted-pair group method using arithmetic average (UPGMA) indicated that there were 4 main clusters, which was supported by principle component analysis (PCA). Cluster I primarily included only scald-resistant germplasm lines, while clusters III and IV consisted of only scald-sensitive lines, but cluster 11 had both scald-resistant and scald-sensitive barley germplasm lines. The coefficients of genetic similarity among the genotypes ranged from 0.58 to 0.96, with a cophenetic correlation (r = 0.71) suggesting that the cluster analysis moderately represented the similarity matrix. The results indicate that a large amount of the genetic diversity present could be of great use in the development of future scald-resistant barley lines. | 619 | no | crop | NA | no | no |
| 620 | 2017 | 43009 | Ortego, J; Cordero, PJ; Aparicio, JM; Calabuig, G | 2007 | No relationship between individual genetic diversity and prevalence of avian malaria in a migratory kestrel | Insight into the genetic basis of malaria resistance is crucial for understanding the consequences of this parasite group on animal populations. Here, we analyse the relationship between genotypic variation at 11 highly variable microsatellite loci and prevalence of three different lineages of avian malaria, two Plasmodium (RTSR1, LK6) and one Haemoproteus (LK2), in a wild population of the endangered lesser kestrel (Falco naumanni). Although we used a large sample size (584 typed individuals), we did not find any significant association between the prevalence of the studied parasite lineages and individual genetic diversity. Although our data set is large, the 11 neutral markers typed may have had low power to detect such association, in part because of the low parasite prevalence observed (less than 5% of infected birds). However, the fact that we have detected previous correlations between genetic diversity and other traits (ectoparasitism risk, fecundity) in the study population using the same panel of neutral markers and lower sample sizes suggests that other factors could underlie the absence of such a similar correlation with avian malaria. Differences in the genetics of the studied traits and in their particular basis of inbreeding depression (dominance vs. overdominance) may have led to malaria prevalence, but not other traits, being uncoupled with individual genetic diversity. Also, we cannot discard the possibility that the absence of association was a consequence of a low pathogenic effect of these particular malaria lineages on our lesser kestrel population, and thus we should not expect the evolution of genetic resistance against these parasites. | 620 | no | individual level | NA | no | no |
| 621 | 2017 | 43009 | Hellmair, M; Kinziger, AP | 2014 | Increased Extinction Potential of Insular Fish Populations with Reduced Life History Variation and Low Genetic Diversity | Theoretical work has shown that reduced phenotypic heterogeneity leads to population instability and can increase extinction potential, yet few examples exist of natural populations that illustrate how varying levels expressed diversity may influence population persistence, particularly during periods of stochastic environmental fluctuation. In this study, we assess levels of expressed variation and genetic diversity among demographically independent populations of tidewater goby (Eucyclogobius newberryi), show that reductions in both factors typically coincide, and describe how low levels of diversity contribute to the extinction risk of these isolated populations. We illustrate that, for this annual species, continuous reproduction is a safeguard against reproductive failure by any one population segment, as natural, stochastically driven salinity increases frequently result in high mortality among juvenile individuals. Several study populations deviated from the natural pattern of year-round reproduction typical for the species, rendering those with severely truncated reproductive periods vulnerable to extinction in the event of environmental fluctuation. In contrast, demographically diverse populations are more likely to persist through such periods through the continuous presence of adults with broader physiological tolerance to abrupt salinity changes. Notably, we found a significant correlation between genetic diversity and demographic variation in the study populations, which could be the result of population stressors that restrict both of these diversity measures simultaneously, or suggestive of a causative relationship between these population characteristics. These findings demonstrate the importance of biocomplexity at the population level, and assert that the maintenance of diversity contributes to population resilience and conservation of this endangered species. | 621 | no | no good fitness measure | NA | no | no |
| 622 | 2017 | 43009 | Delmotte, F; Chen, WJ; Richard-Cervera, S; Greif, C; Papura, D; Giresse, X; Mondor-Genson, G; Corio-Costet, MF | 2006 | Microsatellite DNA markers for Plasmopara viticola, the causal agent of downy mildew of grapes | Microsatellite loci were isolated from Plasmopara viticola (Oomycetes), the causal agent of downy mildew of grape, one of the most damaging fungal diseases of grapevine worldwide. Seven polymorphic loci were obtained from an enriched partial genomic library. A low genetic diversity was observed at all loci, with a mean observed allele number of 3.75 and an observed heterozygosity ranging from 0.074 to 0.547. Cross-amplification tests on three closely related taxa indicated that two loci could be used in other Oomycetes species. These microsatellite loci were proved to be useful for population genetic analysis. | 622 | no | gd of pathogen | NA | no | no |
| 623 | 2017 | 43009 | McKenzie, GW; Abbott, J; Zhou, HT; Fang, QA; Merrick, N; Forrest, RH; Sedcole, JR; Hickford, JG | 2010 | Genetic diversity of selected genes that are potentially economically important in feral sheep of New Zealand | Background: Feral sheep are considered to be a source of genetic variation that has been lost from their domestic counterparts through selection. Methods: This study investigates variation in the genes KRTAP1-1, KRT33, ADRB3 and DQA2 in Merino-like feral sheep populations from New Zealand and its offshore islands. These genes have previously been shown to influence wool, lamb survival and animal health. Results: All the genes were polymorphic, but no new allele was identified in the feral populations. In some of these populations, allele frequencies differed from those observed in commercial Merino sheep and other breeds found in New Zealand. Heterozygosity levels were comparable to those observed in other studies on feral sheep. Our results suggest that some of the feral populations may have been either inbred or outbred over the duration of their apparent isolation. Conclusion: The variation described here allows us to draw some conclusions about the likely genetic origin of the populations and selective pressures that may have acted upon them, but they do not appear to be a source of new genetic material, at least for these four genes. | 623 | no | domesticated animal | NA | no | no |
| 624 | 2017 | 43009 | Michailidou, S; Kalivas, A; Ganopoulos, I; Stea, E; Michailidis, G; Tsaftaris, A; Argiriou, A | 2014 | A multi-farm assessment of Greek black pig genetic diversity using microsatellite molecular markers | Local breeds are important for the maintenance of genetic diversity and future food security. Nowadays, the worldwide distribution of pigs is dominated by a few breeds, tending towards a severe loss of pig biodiversity. Thus, it is critical to maintain distinct populations of pig breeds. The Greek black pig, a breed raised locally and known for the high quality of its meat for cured products, is the only traditional indigenous pig breed reared in Greece. We investigated the genetic diversity, based on microsatellite analysis, of the Greek black pig and evaluated its genetic uniqueness. One hundred and three pigs from 12 Greek farms were analyzed using 11 microsatellites. The total number of alleles amounted to 135, with a mean number of alleles per locus of 12.27, ranging between 10 and 16 alleles. The observed heterozygosity ranged from 0.363 to 0.825 per locus. The expected heterozygosity ranged from 0.471 to 0.707. The inbreeding coefficient ranged from -0.329 to 0.229. We conclude that the Greek black pig, despite its low population size, has a high degree of genetic variability, which will be useful for breeding programs aimed at maintaining long-term survival of this ancient breed. | 624 | no | domesticated animal | NA | no | no |
| 625 | 2017 | 43009 | Kawuki, RS; Herselman, L; Labuschagne, MT; Nzuki, I; Ralimanana, I; Bidiaka, M; Kanyange, MC; Gashaka, G; Masumba, E; Mkamilo, G; Gethi, J; Wanjala, B; Zacarias, A; Madabula, F; Ferguson, ME | 2013 | Genetic diversity of cassava (Manihot esculenta Crantz) landraces and cultivars from southern, eastern and central Africa | Studies to quantify genetic variation in cassava germplasm, available within the national breeding programmes in Africa, have been limited. Here, we report on the nature and extent of genetic variation that exists within 1401 cassava varieties from seven countries: Tanzania (270 genotypes); Uganda (268); Kenya (234); Rwanda (184); Democratic Republic of Congo (DRC; 177); Madagascar (186); Mozambique (82). The vast majority of these genotypes do not exist within a formal germplasm conservation initiative and were derived from farmers’ fields and National Agricultural Research Systems breeding programmes. Genotypes were assayed using 26 simple sequence repeat markers. Moderate genetic variation was observed with evidence of a genetic bottleneck in the region. Some differentiation was observed among countries in both cultivars and landraces. Euclidean distance revealed the pivotal position of Tanzanian landraces in the region, and STRUCTURE analysis revealed subtle and fairly complex relationships among cultivars and among landraces and cultivars analysed together. This is likely to reflect original germplasm introductions, gene flow including farmer exchanges, disease pandemics, past breeding programmes and the introduction of cultivars from the International Institute of Tropical Agriculture - Nigeria. Information generated from this study will be useful to justify and guide a regional cassava genetic resource conservation strategy, to identify gaps in cassava diversity in the region and to guide breeding strategies. | 625 | no | crop | NA | no | no |
| 626 | 2017 | 43009 | Munoz-Amatriain, M; Xiong, YW; Schmitt, MR; Bilgic, H; Budde, AD; Chao, SAM; Smith, KP; Muehlbauer, GJ | 2010 | Transcriptome analysis of a barley breeding program examines gene expression diversity and reveals target genes for malting quality improvement | “Background: Advanced cycle breeding utilizes crosses among elite lines and is a successful method to develop new inbreds. However, it results in a reduction in genetic diversity within the breeding population. The development of malting barley varieties requires the adherence to a narrow malting quality profile and thus the use of advanced cycle breeding strategies. Although attention has been focused on diversity in gene expression and its association with genetic diversity, there are no studies performed in a single breeding program examining the implications that consecutive cycles of breeding have on gene expression variation and identifying the variability still available for future improvement. Results: Fifteen lines representing the historically important six-rowed malting barley breeding program of the University of Minnesota were genotyped with 1,524 SNPs, phenotypically examined for six malting quality traits, and analyzed for transcript accumulation during germination using the Barley1 GeneChip array. Significant correlation was detected between genetic and transcript-level variation. We observed a reduction in both genetic and gene expression diversity through the breeding process, although the expression of many genes have not been fixed. A high number of quality-related genes whose expression was fixed during the breeding process was identified, indicating that much of the diversity reduction was associated with the improvement of the complex phenotype”“malting quality”“, the main goal of the University of Minnesota breeding program. We also identified 49 differentially expressed genes between the most recent lines of the program that were correlated with one or more of the six primary malting quality traits. These genes constitute potential targets for the improvement of malting quality within the breeding program. Conclusions: The present study shows the repercussion of advanced cycle breeding on gene expression diversity within an important barley breeding program. A reduction in gene expression diversity was detected, although there is diversity still present after forty years of breeding that can exploited for future crop improvement. In addition, the identification of candidate genes for enhancing malting quality may be used to optimize the selection of targets for further improvements in this economically important phenotype.” | 626 | no | crop | NA | no | no |
| 627 | 2017 | 43009 | Riquet, F; Le Cam, S; Fonteneau, E; Viard, F | 2016 | Moderate genetic drift is driven by extreme recruitment events in the invasive mollusk Crepidula fornicata | Effective population size (Ne) is a measure of genetic drift and is thus a central parameter in evolution, conservation genetics and invasion biology. Interestingly, in native marine species, Ne is typically several orders of magnitude lower than the census size. This pattern has often been explained by high fecundity, variation in reproductive success and pronounced early mortality, resulting in genetic drift across generations. Data documenting genetic drift and/or N-e in marine invasive species are, however, still scarce. We examined the importance of genetic drift in the invasive species Crepidula fornicata by genotyping 681 juveniles sampled during each annual recruitment peak over nine consecutive years in the Bay of Morlaix (Brittany, France). Observed variations in genetic diversity were partially explained by variation in recruitment intensity. In addition, substantial temporal genetic differentiation was documented (that is, genetic drift), and was attributed to nonrandom variance in the reproductive success of different breeding groups across years in the study species. Using a set of single-sample and temporal estimators for N-e, we estimated N-e to be three or four orders of magnitude smaller than the census size (N-c). On one hand, this reduction in N-e relative to N-c appeared congruent with, although slight higher than, values commonly observed in native marine species. Particular life-history traits of this invasive species may play an important role in buffering genetic drift. On the other hand, N-e still remained far below N-c, hence, possibly reducing the efficiency of selection effects. | 627 | no | no good fitness measure | NA | no | no |
| 628 | 2017 | 43009 | Rajora, OP; Rahman, MH; Buchert, GP; Dancik, BP | 2000 | Microsatellite DNA analysis of genetic effects of harvesting in old-growth eastern white pine (Pinus strobus) in Ontario, Canada | Microsatellite DNA markers from 13 simple sequence repeat (SSR) loci were used to compare genetic diversity between preharvest pristine and postharvest residual gene pools of two adjacent virgin, old-growth (approximate to 250 years) stands of eastern white pine (Pinus strobus L.) in Ontario. There was concurrence in genetic diversity changes in the postharvest gene pools of the two stands. The total and mean numbers of alleles detected in each stand were reduced by approximate to 26% after tree density reductions of approximate to 75%. Approximately 18 and 21% of the low-frequency (0.25 > P greater than or equal to 0.01) alleles and 76 and 92% of the rare (P < 0.01) alleles were lost from residual stands A and B, respectively, after harvesting. Multilocus gametic diversity was reduced by 38 and 85% and genotype additivity by approximate to 50% in the residual stands after harvesting. Latent genetic potential of each stand was reduced by approximate to 40%. Although heterozygosity was reduced (1-5%) in the postharvest residual stands, the reductions were not substantial and not comparable to those using other genetic diversity measures. The reductions in genetic diversity measures were slightly higher than those theoretically expected in postbottleneck populations according to drift theory. In the absence of substantial gene migration that could ameliorate the genetic losses, the ability of the postharvest white pine gene pools to adapt to changing environmental and disease conditions may have been compromised. The microsatellite DNA results for genetic effects of harvesting in old-growth eastern white pine stands were similar to those that we reported earlier from allozyme analysis (Buchert et al. 1997). The results indicate that silvicultural practices should ensure that the gene pools of remaining pristine old-growth stands are reconstituted in the regenerating stands. | 628 | no | no good fitness measure | NA | no | no |
| 629 | 2017 | 43009 | Kelly, JK; Williamson, S; Orive, ME; Smith, MS; Holt, RD | 2003 | Linking dynamical and population genetic models of persistent viral infection | This article develops a theoretical framework to link dynamical and population genetic models of persistent viral infection. This linkage is useful because, while the dynamical and population genetic theories have developed independently, the biological processes they describe are completely interrelated. Parameters of the dynamical models are important determinants of evolutionary processes such as natural selection and genetic drift. We develop analytical methods, based on coupled differential equations and Markov chain theory, to predict the accumulation of genetic diversity within the viral population as a function of dynamical parameters. These methods are first applied to the standard model of viral dynamics and then generalized to consider the infection of multiple host cell types by the viral population. Each cell type is characterized by specific parameter values. Inclusion of multiple cell types increases the likelihood of persistent infection and can increase the amount of genetic diversity within the viral population. However, the overall rate of gene sequence evolution may actually be reduced. | 629 | no | NA | NA | no | no |
| 630 | 2017 | 43009 | Lopez-Pujol, J; Font, J; Simon, J; Blanche, C | 2007 | Can the preservation of historical relicts permit the conservation of endangered plant species? The case of Silene sennenii (Caryophyllaceae) | Allozyme electrophoresis was used to measure levels and distribution of genetic diversity in the critically endangered (CR) Silene sennenii (Caryophyllaceae), a narrow endemic plant species found in northeastern Catalonia (Spain). At present, only 5 populations remain, containing no more than 5,000 individuals, which are subjected to several human pressures such as habitat fragmentation and land use changes. Interestingly, the largest population is located in and around a fortress built in the XVIII century, a circumstance which might facilitated its preservation until now. From the 21 satisfactorily interpreted loci, low levels of genetic variation were detected (P = 20.9, A = 1.31 and H (e) = 0.071), which may be related to small population size, isolation, and fragmentation of extant populations. Moderate to high levels of inbreeding were also found, probably as consequence of the population’s genetic structuring. The conservation of the population located in the fortress would allow the preservation of all the alleles detected at species level; nevertheless, conservation of other populations, coupled with the inclusion of S. sennenii in the Spanish Catalogue of Endangered Species, would also be desirable in order to ensure the long-term survival of the species. | 630 | no | no good fitness measure, N pop differnt in GD? | NA | no | no |
| 631 | 2017 | 43009 | Brasileiro, BP; Silva, SA; Souza, DR; Santos, PA; Oliveira, RS; Lyra, DH | 2013 | Genetic diversity and selection gain in the physic nut (Jatropha curcas) | The use of efficient breeding methods depends on knowledge of genetic control of traits to be improved. We estimated genetic parameters, selection gain, and genetic diversity in physic nut half-sib families, in order to provide information for breeding programs of this important biofuel species. The progeny test included 20 half-sib families in 4 blocks and 10 plants per plot. The mean progeny heritability values were: 50% for number of bunches, 47% for number of fruits, 35% for number of seeds, 6% for stem diameter, 26% for number of primary branches, 14% for number of secondary branches, 66% for plant height, and 25% for survival of the plants, demonstrating good potential for early selection in plant height, number of branches, and number of fruits per plant. In the analysis of genetic diversity, genotypes were divided into 4 groups. Genotypes 18, 19, 20, and 8 clustered together and presented the highest means for the vegetative characters and production. Lower means were observed in the 17, 12, 13, and 9 genotypes from the same group. We detected genetic variability in this population, with high heritability estimates and accuracy, demonstrating the possibility of obtaining genetic gains for vegetative characters and production at 24 months after planting. | 631 | no | no good fitness measure, N pop differnt in GD? | NA | no | no |
| 632 | 2017 | 43009 | Karafet, TM; Bulayeva, KB; Bulayev, OA; Gurgenova, F; Omarova, J; Yepiskoposyan, L; Savina, OV; Veeramah, KR; Hammer, MF | 2015 | Extensive genome-wide autozygosity in the population isolates of Daghestan | Isolated populations are valuable resources for mapping disease genes, as inbreeding increases genome-wide homozygosity and enhances the ability to map disease alleles on a genetically uniform background within a relatively homogenous environment. The populations of Daghestan are thought to have resided in the Caucasus Mountains for hundreds of generations and are characterized by a high prevalence of certain complex diseases. To explore the extent to which their unique population history led to increased levels of inbreeding, we genotyped >550 000 autosomal single-nucleotide polymorphisms (SNPs) in a set of 14 population isolates speaking Nakh-Daghestanian (ND) languages. The ND-speaking populations showed greatly elevated coefficients of inbreeding, very high numbers and long lengths of Runs of Homozygosity, and elevated linkage disequilibrium compared with surrounding groups from the Caucasus, the Near East, Europe, Central and South Asia. These results are consistent with the hypothesis that most ND-speaking groups descend from a common ancestral population that fragmented into a series of genetic isolates in the Daghestanian highlands. They have subsequently maintained a long-term small effective population size as a result of constant inbreeding and very low levels of gene flow. Given these findings, Daghestanian population isolates are likely to be useful for mapping genes associated with complex diseases. | 632 | no | NA | NA | no | no |
| 633 | 2017 | 43009 | Byrne, PG; Whiting, MJ | 2011 | Effects of simultaneous polyandry on offspring fitness in an African tree frog | In externally fertilizing animals in which females mate with multiple males at the same time (simultaneous polyandry), the possibility that females accrue genetic benefits that improve offspring viability remains largely unexamined. Here, we investigate whether simultaneous polyandry influences offspring fitness in a wild population of the Grey Foam Nest Treefrog Chiromantis xerampelina. Simultaneous polyandry in this frog is the most extreme reported for any vertebrate, with more than 90% of females mating with 10 or more males during the deposition of a single clutch. We compared growth (using age and size at metamorphosis as proxies) and survival of offspring produced by females that naturally mated with either 1 male (monandrous females) or 10-12 males (polyandrous females). Polyandry did not influence size or age at metamorphosis, but we found that offspring from polyandrous matings had both significantly higher mean survival and reduced variance in offspring survival. These findings implicate a genetic mechanism, but targeted cross-classified breeding experiments that control for both maternal and material effects will be required to conclusively determine whether elevated offspring viability is linked to enhanced genetic diversity, intrinsic sire effects, or genetic compatibility. Irrespective of the causation, the findings provide the first evidence that naturally formed polyandrous matings have increased offspring viability in an anuran amphibian. | 633 | maybe | were the offspring in groups? | no good data comp | yes | yes |
| 634 | 2017 | 43009 | Ellis, JR; McCauley, DE | 2009 | Phenotypic differentiation in fitness related traits between populations of an extremely rare sunflower: Conservation management of isolated populations | Knowledge of the genetic and demographic consequences of rarity is crucial when evaluating the effects of habitat loss and fragmentation on population viability, and for creating management plans in rare plant species. Reduction in population size and in the number of populations can lead to decreased genetic diversity and increased inbreeding. Genetic diversity is often correlated with fitness and is frequently used to identify populations of greatest conservation concern, or those that may be good candidates for ex situ conservation programs. However, an association between these factors is not always clear, and crossing studies evaluating whether there is phenotypic differentiation among populations in fitness related traits can inform managers of suffering populations or good sources for ex situ materials. Crossing studies can also evaluate the potential for genetic rescue to boost fitness in suffering populations. To address these questions, we conducted two generations of controlled crosses between populations of the extremely rare and fragmented sunflower, Helianthus verticillatus. We measured achene viability, germination, survival, and pollen viability (F(1) only) in 176 F(1) and 159 F(2) families. The populations were differentiated with respect to phenotypic fitness measures with one population having significantly lower achene viability and germination. Also, the potential for genetic rescue was observed as gene flow into the less fit population resulted in higher fitness measures in both the F(1) and F(2). Results are discussed with respect to the importance of combining genetic marker data with crosses and the implications for conservation in disjunct populations of rare species. (C) 2009 Elsevier Ltd. All rights reserved. | 634 | no | No GD diff between pop | NA | no | no |
| 635 | 2017 | 43009 | Oliveira, PRR; Costa, MC; Silveira, LF; Francisco, MR | 2016 | Genetic Guidelines for Captive Breeding and Reintroductions of the Endangered Black-Fronted Piping Guan, Aburria jacutinga (Galliformes, Cracidae), an Atlantic Forest Endemic | The survival of a number of birds rely on captive breeding and reintroduction into the wild, but captive populations are often small and can be exposed to the negative effects of inbreeding and genetic drift. Then, managers are concerned not only with producing as much offspring as possible, but also with the retention of the maximum genetic variability within and between populations. The Black-fronted Piping Guan, Aburria jacutinga, is an endangered cracid endemic to the Atlantic Forest of southeastern South America. Because of its conservation status and functional importance, a captive breeding program started independently, mainly in three aviaries, in the decade of 1980. Although they have supplied animals for reintroductions, genetic variability aspects have never been considered. Here we addressed levels of genetic variability within and between these aviaries. Bayesian clustering analyses revealed two lineages. Inbreeding was not detected, although we found evidences for a recent bottleneck in one of the aviaries. Then, our main management recommendations are: i) reintroducing the species in areas where it has been extinct is more prudent than supplementing natural populations, as it could involve risks of disrupting local adaptive complexes; ii) as far as inbreeding can be avoided, the captive groups should be managed separately to minimize adaptation to captivity; iii) crossbreedings in pre-release generations could improve reintroduction success; and iv) a studbook should be implemented. As populations of Black-fronted Piping Guan from conservation units are progressively declining, these captive genetic repositories may gain importance in a near future. (C) 2016 Wiley Periodicals, Inc. | 635 | no | no good fitness measure, N pop differnt in GD? | NA | no | no |
| 636 | 2017 | 43009 | Wright, B; Morris, K; Grueber, CE; Willet, CE; Gooley, R; Hogg, CJ; O’Meally, D; Hamede, R; Jones, M; Wade, C; Belov, K | 2015 | Development of a SNP-based assay for measuring genetic diversity in the Tasmanian devil insurance population | Background: The Tasmanian devil (Sarcophilus harrisii) has undergone a recent, drastic population decline due to the highly contagious devil facial tumor disease. The tumor is one of only two naturally occurring transmissible cancers and is almost inevitably fatal. In 2006 a disease-free insurance population was established to ensure that the Tasmanian devil is protected from extinction. The insurance program is dependent upon preserving as much wild genetic diversity as possible to maximize the success of subsequent reintroductions to the wild. Accurate genotypic data is vital to the success of the program to ensure that loss of genetic diversity does not occur in captivity. Until recently, microsatellite markers have been used to study devil population genetics, however as genetic diversity is low in the devil and potentially decreasing in the captive population, a more sensitive genotyping assay is required. Methods: Utilising the devil reference genome and whole genome re-sequencing data, we have identified polymorphic regions for use in a custom genotyping assay. These regions were amplified using PCR and sequenced on the Illumina MiSeq platform to refine a set a markers to genotype the Tasmanian devil insurance population. Results: We have developed a set of single nucleotide polymorphic (SNP) markers, assayed by amplicon sequencing, that provide a high-throughput method for monitoring genetic diversity and assessing familial relationships among devils. To date we have used a total of 267 unique SNPs within both putatively neutral and functional loci to genotype 305 individuals in the Tasmanian devil insurance population. We have used these data to assess genetic diversity in the population as well as resolve the parentage of 21 offspring. Conclusions: Our molecular data has been incorporated with studbook management practices to provide more accurate pedigree information and to inform breeding recommendations. The assay will continue to be used to monitor the genetic diversity of the insurance population of Tasmanian devils with the aim of reducing inbreeding and maximizing success of reintroductions to the wild. | 636 | no | no good fitness measure, N pop differnt in GD? | NA | no | no |
| 637 | 2017 | 43009 | Qutob, N; Balloux, F; Raj, T; Liu, H; de Proce, SM; Trowsdale, J; Manica, A | 2012 | Signatures of historical demography and pathogen richness on MHC class I genes | The extreme polymorphism of MHC class I has been argued to be driven by balancing selection from pathogens, with the prediction that populations exposed to a wider variety of diseases should have higher diversity. We assembled a global database of allotype frequencies for MHC class I genes and investigated possible drivers of genetic diversity, measured in different ways. We first looked for a decline in diversity with distance from Africa (a consequence of drift during human expansions) and then investigated the link with pathogen richness once the effect of drift had been corrected for. Using heterozygosity, we recovered a clear decline in diversity from Africa and confirmed the positive relationship between genetic diversity and pathogen richness for all three classical MHC class I genes. However, when we considered a sequence-based measure of genetic diversity, the correlation with geographic distance from Africa vanished for HLA-C, and the correlations with pathogen richness for the three MHC class I genes were much weaker. HLA-C is known to consist of two functional classes of allotypes (classified with respect to the 80th residue), which interact with different KIR receptors. While this separation provided some improvement in the fit between genetic diversity and distance from Africa for one class, much clearer and consistent patterns were recovered when we used the 90th residue to separate HLA-C allotypes into two new classes. This suggests that this residue, which is also involved in the binding of KIR, might have had an important evolutionary role that has been overlooked. | 637 | no | human | NA | no | no |
| 638 | 2017 | 43009 | Edge, MD; Rosenberg, NA | 2015 | A General Model of the Relationship between the Apportionment of Human Genetic Diversity and the Apportionment of Human Phenotypic Diversity | Models that examine genetic differences between populations alongside a genotype-phenotype map can provide insight about phenotypic variation among groups. We generalize a simple model of a completely heritable, additive, selectively neutral quantitative trait to examine the relationship between single- locus genetic differentiation and phenotypic differentiation on quantitative traits. In agreement with similar efforts using different models, we show that the expected degree to which two groups differ on a neutral quantitative trait is not strongly affected by the number of genetic loci that influence the trait: neutral trait differences are expected to have a magnitude comparable to the genetic differences at a single neutral locus. We discuss this result with respect to population differences in disease phenotypes, arguing that although neutral genetic differences between populations can contribute to specific differences between populations in health outcomes, systematic patterns of difference that run in the same direction for many genetically independent health conditions are unlikely to be explained by neutral genetic differentiation. | 638 | no | human | NA | no | no |
| 639 | 2017 | 43009 | Neumann, P; Mortiz, RFA | 2000 | Testing genetic variance hypotheses for the evolution of polyandry in the honeybee (Apis mellifera L.) | Colony size, honey yields and colony levels of infestation with Varroa jacobsoni of 30 queenright honeybee (Apis mellifera) colonies with naturally mated queens were evaluated over a two-year period. Workers taken from each colony were genotyped at four DNA-microsatellite loci to determine the level of polyandry. All queens mated with more than 10 drones (mean number of observed patrilines =17.7+/-5.23). We found significant correlations between colony size and honey yield and between colony sizes of two subsequent years. Analyses of variance revealed a strong impact of the breeding lines on the tested phenotypic traits. The impact of polyandry on colony honey yields was weak (p<0.05, not significant when applying a Bonferroni adjustment) and 8% of the phenotype was determined by the effect of polyandry. The contribution of polyandry to colony size (0.25%) or levels of infestation with Varroa jacobsoni (0.09%) was even weaker in both test years. Likewise, we could not find any averaging effect of polyandry on the honey yield, size nor parasite load of honeybee colonies. Our data set does not resolve the question, whether polyandry and genetic diversity causes more productive colonial phenotypes. If colony level selection is an evolutionary force for polyandry, the effects are hard to detect in man-kept colonies headed by naturally mated queens. | 639 | yes | from thesis | NA | no | yes |
| 640 | 2017 | 43009 | Vieira, FD; Fajardo, CG; de Souza, AM; Reis, CAF; de Carvalho, D | 2012 | Fine-scale genetic dynamics of a dominant neotropical tree in the threatened Brazilian Atlantic Rainforest | We present a case study of the relationship between spatial genetic structure (SGS) and age structure in Protium spruceanum (Burseraceae), an insect-pollinated, mass-fruiting, and secondary bird-dispersed tree, as determined through variation in allozyme loci. Using ten polymorphic loci, we investigated spatial and temporal patterns of a genetic structure within a 40 m x 60 m plot in a small (1.0 ha) fragment of Atlantic Rainforest to investigate the processes shaping the distribution of genetic diversity. Individuals (n = 345) from seedlings to adults were grouped and analyzed in four diameter classes. The results showed a high average level of genetic diversity (H (e) = 0.438), but genetic diversity parameters did not vary significantly among cohorts. The spatial distribution pattern analysis of individuals showed significant levels of aggregation among small- and medium-diameter classes and random distribution among the highest diameter class, likely due to processes of competitive thinning. There was an association between demographic and SGS at short distances (less than 10 m) which is likely the consequence of restricted seed dispersal. The degree of SGS decreased across small- to large-diameter classes. We inferred that limited seed dispersal and subsequent density-dependent mortality from the family clusters are responsible for the observed changes in fine-scale SGS across different demographic classes. | 640 | no | no pop level | NA | no | no |
| 641 | 2017 | 43009 | Roberts, DG; Forrest, CN; Denham, AJ; Ayre, DJ | 2013 | Microsatellite markers for vulnerable Australian aridzone Acacias | Several Australian arid zone Acacia species are under threat because of decades of fruiting and recruitment failure that may reflect the loss of genetic diversity within small and isolated populations. We developed primers for eight microsatellite loci for Acacia carneorum and Acacia loderi. We detected high levels of clonality in each of two stands of A. carneorum (1 and 2 genets). In contrast, one stand of A. loderi was wholly clonal (1 genet), while in a second there were 30 unique genotypes. These loci allow assessment of the genetic diversity and connectedness of populations, the relative contribution of asexual reproduction to genotypic diversity and population structure, and use of paternity analysis to identify sires of seed within populations known to have set seed in past decades. This type of information may provide a basis for a recovery plan based on ‘genetic rescue’. | 641 | no | no good fitness measure, N pop differnt in GD? | NA | no | no |
| 642 | 2017 | 43009 | Smitz, N; Cornelis, D; Chardonnet, P; Caron, A; de Garine-Wichatitsky, M; Jori, F; Mouton, A; Latinne, A; Pigneur, LM; Melletti, M; Kanapeckas, KL; Marescaux, J; Pereira, CL; Michaux, J | 2014 | Genetic structure of fragmented southern populations of African Cape buffalo (Syncerus caffer caffer) | “Background: African wildlife experienced a reduction in population size and geographical distribution over the last millennium, particularly since the 19th century as a result of human demographic expansion, wildlife overexploitation, habitat degradation and cattle-borne diseases. In many areas, ungulate populations are now largely confined within a network of loosely connected protected areas. These metapopulations face gene flow restriction and run the risk of genetic diversity erosion. In this context, we assessed the”“genetic health”" of free ranging southern African Cape buffalo populations (S.c. caffer) and investigated the origins of their current genetic structure. The analyses were based on 264 samples from 6 southern African countries that were genotyped for 14 autosomal and 3 Y-chromosomal microsatellites. Results: The analyses differentiated three significant genetic clusters, hereafter referred to as Northern (N), Central (C) and Southern (S) clusters. The results suggest that splitting of the N and C clusters occurred around 6000 to 8400 years ago. Both N and C clusters displayed high genetic diversity (mean allelic richness (A(r)) of 7.217, average genetic diversity over loci of 0.594, mean private alleles (P-a) of 11), low differentiation, and an absence of an inbreeding depression signal (mean F-IS = 0.037). The third (S) cluster, a tiny population enclosed within a small isolated protected area, likely originated from a more recent isolation and experienced genetic drift (F-IS = 0.062, mean A(r) = 6.160, P-a = 2). This study also highlighted the impact of translocations between clusters on the genetic structure of several African buffalo populations. Lower differentiation estimates were observed between C and N sampling localities that experienced translocation over the last century. Conclusions: We showed that the current genetic structure of southern African Cape buffalo populations results from both ancient and recent processes. The splitting time of N and C clusters suggests that the current pattern results from human-induced factors and/or from the aridification process that occurred during the Holocene period. The more recent S cluster genetic drift probably results of processes that occurred over the last centuries (habitat fragmentation, diseases). Management practices of African buffalo populations should consider the micro-evolutionary changes highlighted in the present study." | 642 | no | no good fitness measure, N pop differnt in GD? | NA | no | no |
| 643 | 2017 | 43009 | Street, GT; Lotufo, GR; Montagna, PA; Fleeger, JW | 1998 | Reduced genetic diversity in a meiobenthic copepod exposed to a xenobiotic | Populations that encounter a novel environment can lose genetic diversity. Intense, directional selection can affect reproduction and lead to a gene pool dominated by relatively few genotypes. A loss of haplotype diversity (h) in the mitochondrial genome in populations of meiobenthic, harpacticoid copepods around offshore oil platforms has been linked to lethal and sublethal responses to factors proximal to offshore oil platforms. However, it was not possible to determine if the effects were related directly to increased concentrations of contaminants produced by drilling at the platforms. An experiment was designed to test whether populations of Nitocra lacustris Schmankevitsch (Copepoda: Harpacticoida) would lose haplotype diversity over 3 generations following exposure to sediment-associated hydrocarbons. Haplotype diversity was estimated by comparing restriction fragment length polymorphisms generated from mitochondrial DNA amplified from individual copepods. An additional component of the study was to determine the effects of population size on estimates of diversity. Haplotype diversity could be underestimated by using too few individuals, because random genetic drift in very small populations could cause lower diversity even in the absence of strong selection. The relationship between sample size and h was determined by randomly sub-sampling, at different sample sizes, a large data set collected for two copepod species. Cultures treated with phenanthrene, a polycyclic aromatic hydrocarbon, experienced lower adult survival, fewer surviving offspring per female, and lower haplotype diversity relative to control cultures. After the first generation, mortality declined, but larval survival and haplotype diversity did not change in subsequent generations. Diversity was slightly underestimated due to assaying too small a number of individuals, but could not account for all of the differences between treatments. Despite mortality, populations in culture were not reduced to the point where random drift seemed to have a significant effect. These results support the hypothesis that loss of mtDNA diversity in naturally occurring populations of Harpacticoida may serve as a marker for negative effects of exposure to xenobiotic contaminants. (C) 1998 Elsevier Science B.V. | 643 | no | no good fitness measure, N pop differnt in GD? | NA | no | no |
| 644 | 2017 | 43009 | Kittles, RA; Bergen, AW; Urbanek, M; Virkkunen, M; Linnoila, M; Goldman, D; Long, JC | 1999 | Autosomal, mitochondrial, and Y chromosome DNA variation in Finland: Evidence for a male-specific bottleneck | The high prevalence of rare genetic diseases in Finland has been attributed to a founder effect some 2,000 years ago. However, this hypothesis has not been supported from mtDNA sequence and autosomal microsatellite data which indicate high levels of gene diversity. Here we have identified genetic evidence for a population bottleneck by examining variable microsatellite loci on the nonrecombining portion of Y chromosomes from Finland and four populations from Europe and the Americas. Sequence data from segment I of the control region (HVS-1) of mtDNA (360 bases) and 20 autosomal dinucleotide repeat markers were also analyzed. Partitions of genetic variance within and between populations revealed significant levels of Y-chromosome differentiation between populations. Phylogenetic and diversity analyses revealed divergent Finnish Y-haplotype clades and significantly lower Y-haplotype diversity among Finns as compared to other populations. Surprisingly, Finnish Y-haplotype diversity was even lower than the Native American populations, These results provide support for the Finnish bottleneck hypothesis. Evidence for two separate founding Finnish Y-chromosome lineages was also observed from the Y-chromosome phylogeny, A limited number of closely related founding males may have contributed to the low number of paternal lineages in the Finnish population. In contrast, high levels of genetic diversity for mtDNA and autosomal STRs may be the result of sex-biased gene flow and recent immigration to urban areas from established internal isolates within Finland. Published 1999 Wiley-Liss, Inc. | 644 | no | human | NA | no | no |
| 645 | 2017 | 43009 | Potter, KM; Jetton, RM; Dvorak, WS; Hipkins, VD; Rhea, R; Whittier, WA | 2012 | Widespread inbreeding and unexpected geographic patterns of genetic variation in eastern hemlock (Tsuga canadensis), an imperiled North American conifer | Eastern hemlock (Tsuga canadensis [L.] Carr.) is an ecologically important tree species experiencing severe mortality across much of its eastern North American distribution, caused by infestation of the exotic hemlock woolly adelgid (Adelges tsugae Annand). To guide gene conservation strategies for this imperiled conifer, we conducted a range-wide genetic variation study for eastern hemlock, amplifying 13 highly polymorphic nuclear microsatellite loci in 1,180 trees across 60 populations. The results demonstrate that eastern hemlock exhibits moderate inbreeding, possibly a signature of a prehistoric decline associated with a widespread insect outbreak. Contrary to expectations, populations in formerly glaciated regions are not less genetically diverse than in the putative southern refugial region. As expected, peripheral disjunct populations are less genetically diverse than main-range populations, but some are highly genetically differentiated or contain unique alleles. Spatially explicit Bayesian clustering analyses suggest that three or four Pleistocene glacial refuges may have existed in the Southeastern United States, with a main post-glacial movement into the Northeast and the Great Lakes region. Efforts to conserve eastern hemlock genetic material should emphasize the capture of broad adaptability that occurs across the geographic range of the species, as well as genetic variability within regions with the highest allelic richness and heterozygosity, such as the Southern Appalachians and New England, and within disjunct populations that are genetically distinct. Much genetic variation exists in areas both infested and uninfested by the adelgid. | 645 | no | inbreeding | NA | no | no |
| 646 | 2017 | 43009 | Shiina, T; Tanaka, K; Katsuyama, Y; Otabe, K; Sakamoto, K; Kurata, M; Nomura, M; Yamanaka, H; Nakagawa, H; Inoko, H; Ota, M | 2010 | Mitochondrial DNA Diversity among Three Subpopulations of Cynomolgus Macaques (Macaca fascicularis) Originating from the Indochinese Region | The cynomolgus macaque (Macaca fascicularis) has emerged as an important experimental animal model for biomedical research in various domains, necessitating the more extensive characterization of the genetic backgrounds influencing the macaque’s response to drugs and sensitivity to experimental disease. The diversity of the variable mitochondrial DNA (mtDNA) D-loop region has been analyzed phylogenetically among geographically isolated populations or within subdivisions of the same regional population. However, the genetic differences among several substructures originating from a common population have not yet been investigated. By sequencing fragments of the mtDNA D-loop region from two subpopulations from the Indochinese region (Cambodian-Chinese and Vietnamese) along with two native Indonesian and Filipino populations, we identified 87 mtDNA D-loop haplotypes, of which 67 are new. The phylogenetic relationship suggests that the Indochinese haplotypes are intermingled in comparison to the distinct divergence of the Indonesian and Filipino lineages. The subpopulations were shown by estimation of evolutionary divergence and Wright’s F-statistic (Fst) to have little genetic differentiation. Altogether, the subpopulations may be used in biomedical research, even though a slight difference is observed in haplotype frequencies among them. Therefore, genetic diversity analyses will be necessary for the elucidation of genetic differences among the populations, as well as to obtain a better understanding of genetic diversity for biomedical research. This will involve the selection of macaques and the monitoring of genetic heterogeneity among and within breeding facilities. | 646 | no | no good fitness measure, N pop differnt in GD? | NA | no | no |
| 647 | 2017 | 43009 | Yates, WR; Johnson, C; McKee, P; Cannon-Albright, LA | 2013 | Genetic Analysis of Low BMI Phenotype in the Utah Population Database | The low body mass index (BMI) phenotype of less than 18.5 has been linked to medical and psychological morbidity as well as increased mortality risk. Although genetic factors have been shown to influence BMI across the entire BMI, the contribution of genetic factors to the low BMI phenotype is unclear. We hypothesized genetic factors would contribute to risk of a low BMI phenotype. To test this hypothesis, we conducted a genealogy data analysis using height and weight measurements from driver’s license data from the Utah Population Data Base. The Genealogical Index of Familiality (GIF) test and relative risk in relatives were used to examine evidence for excess relatedness among individuals with the low BMI phenotype. The overall GIF test for excess relatedness in the low BMI phenotype showed a significant excess over expected (GIF 4.47 for all cases versus 4.10 for controls, overall empirical p-value<0.001). The significant excess relatedness was still observed when close relationships were ignored, supporting a specific genetic contribution rather than only a family environmental effect. This study supports a specific genetic contribution in the risk for the low BMI phenotype. Better understanding of the genetic contribution to low BMI holds promise for weight regulation and potentially for novel strategies in the treatment of leanness and obesity. | 647 | no | human | NA | no | no |
| 648 | 2017 | 43009 | Sasaki, A; Hamilton, WD; Ubeda, F | 2002 | Clone mixtures and a pacemaker: new facets of Red-Queen theory and ecology | Host-parasite antagonistic interaction has been proposed as a potential agent to promote genetic polymorphism and to favour sex against asex, despite its twofold cost in reproduction. However, the host-parasite gene-for-gene dynamics often produce unstable cycles that tend to destroy genetic diversity. Here, we examine such diversity destroying coevolutionary dynamics of host and parasite, which is coupled through local or global migration, or both, between demes in a metapopulation structure. We show that, with global migration in the island model, peculiar out-of-phase islands spontaneously arise in the cluster of islands converging to a global synchrony. Such asynchrony induced by the ‘pacemaker islands’ serves to restore genetic variation. With increasing fraction of local migration, spots of asynchrony are converted into loci or foci of spiral and target patterns, whose rotating arms then cover the majority of demes. A multi-locus analogue of the model reproduces the same tendency toward asynchrony, and the condition arises for an advantage of asexual clones over their sexual counterpart when enough genetic diversity is maintained through metapopulation storage-migration serves as a cheap alternative to sex. | 648 | no | NA | NA | no | no |
| 649 | 2017 | 43009 | Sullivan, AP; de Manuel, M; Marques-Bonet, T; Perry, GH | 2017 | An evolutionary medicine perspective on Neandertal extinction | “The Eurasian sympatry of Neandertals and anatomically modern humans beginning at least 45,000 years ago and possibly lasting for more than 5000 years has sparked immense anthropological interest into the factors that potentially contributed to Neandertal extinction. Among many different hypotheses, the”“differential pathogen resistance”" extinction model posits that Neandertals were disproportionately affected by exposure to novel infectious diseases that were transmitted during the period of spatiotemporal sympatry with modern humans. Comparisons of new archaic hominin paleogenome sequences with modern human genomes have confirmed a history of genetic admixture and thus direct contact between humans and Neandertals. Analyses of these data have also shown that Neandertal nuclear genome genetic diversity was likely considerably lower than that of the Eurasian anatomically modern humans with whom they came into contact, perhaps leaving Neandertal innate immune systems relatively more susceptible to novel pathogens. In this study, we compared levels of genetic diversity in genes for which genetic variation is hypothesized to benefit pathogen defense among Neandertals and African, European, and Asian modern humans, using available exome sequencing data (three individuals, or six chromosomes, per population). We observed that Neandertals had only 31-39% as many non synonymous (amino acid changing) polymorphisms across 73 innate immune system genes compared to modern human populations. We also found that Neandertal genetic diversity was relatively low in an unbiased set of balancing selection candidate genes for primates, those genes with the highest 1% genetic diversity genome-wide in non-human hominoids (apes). In contrast, Neandertals had similar or higher levels of genetic diversity than humans in 12 major histocompatibility complex (MHC) genes. Thus, while Neandertals may have been relatively more susceptible to some novel pathogens and differential pathogen resistance could be considered as one potential contributing factor in their extinction, the expectations of this model are not universally met. (C) 2017 Elsevier Ltd. All rights reserved." | 649 | no | human | NA | no | no |
| 650 | 2017 | 43009 | Buhler, S; Nunes, JM; Nicoloso, G; Tiercy, JM; Sanchez-Mazas, A | 2012 | The Heterogeneous HLA Genetic Makeup of the Swiss Population | This study aims at investigating the HLA molecular variation across Switzerland in order to determine possible regional differences, which would be highly relevant to several purposes: optimizing donor recruitment strategies in hematopoietic stem cell transplantation (HSCT), providing reliable reference data in HLA and disease association studies, and understanding the population genetic background(s) of this culturally heterogeneous country. HLA molecular data of more than 20,000 HSCT donors from 9-13 recruitment centers of the whole country were analyzed. Allele and haplotype frequencies were estimated by using new computer tools adapted to the heterogeneity and ambiguity of the data. Nonparametric and resampling statistical tests were performed to assess Hardy-Weinberg equilibrium, selective neutrality and linkage disequilibrium among different loci, both in each recruitment center and in the whole national registry. Genetic variation was explored through genetic distance and hierarchical analysis of variance taking into account both geographic and linguistic subdivisions in Switzerland. The results indicate a heterogeneous genetic makeup of the Swiss population: first, allele frequencies estimated on the whole national registry strongly deviate from Hardy-Weinberg equilibrium, by contrast with the results obtained for individual centers; second, a pronounced differentiation is observed for Ticino, Graubunden, and, to a lesser extent, Wallis, suggesting that the Alps represent(ed) a barrier to gene flow; finally, although cultural (linguistic) boundaries do not represent a main genetic differentiation factor in Switzerland, the genetic relatedness between population from south-eastern Switzerland and Italy agrees with historical and linguistic data. Overall, this study justifies the maintenance of a decentralized donor recruitment structure in Switzerland allowing increasing the genetic diversity of the national-and hence global-donor registry. It also indicates that HLA data of local donor recruitment centers can be used as reference data in both epidemiological and population genetic studies focusing on the genetic history of present European populations. | 650 | no | human | NA | no | no |
| 651 | 2017 | 43009 | Myles, S; Boyko, AR; Owens, CL; Brown, PJ; Grassi, F; Aradhya, MK; Prins, B; Reynolds, A; Chia, JM; Ware, D; Bustamante, CD; Buckler, ES | 2011 | Genetic structure and domestication history of the grape | The grape is one of the earliest domesticated fruit crops and, since antiquity, it has been widely cultivated and prized for its fruit and wine. Here, we characterize genome-wide patterns of genetic variation in over 1,000 samples of the domesticated grape, Vitis vinifera subsp. vinifera, and its wild relative, V. vinifera subsp. sylvestris from the US Department of Agriculture grape germ-plasm collection. We find support for a Near East origin of vinifera and present evidence of introgression from local sylvestris as the grape moved into Europe. High levels of genetic diversity and rapid linkage disequilibrium (LD) decay have been maintained in vinifera, which is consistent with a weak domestication bottleneck followed by thousands of years of widespread vegetative propagation. The considerable genetic diversity within vinifera, however, is contained within a complex network of close pedigree relationships that has been generated by crosses among elite cultivars. We show that first-degree relationships are rare between wine and table grapes and among grapes from geographically distant regions. Our results suggest that although substantial genetic diversity has been maintained in the grape subsequent to domestication, there has been a limited exploration of this diversity. We propose that the adoption of vegetative propagation was a double-edged sword: Although it provided a benefit by ensuring true breeding cultivars, it also discouraged the generation of unique cultivars through crosses. The grape currently faces severe pathogen pressures, and the long-term sustainability of the grape and wine industries will rely on the exploitation of the grape’s tremendous natural genetic diversity. | 651 | no | crop | NA | no | no |
| 652 | 2017 | 43009 | Hedenas, L; Bisang, I | 2015 | Infraspecific diversity in a spore-dispersed species with limited distribution range | Infraspecific genetic diversity is generally underestimated in biodiversity assessments. We use haplotype patterns based on the nuclear gpd and plastid rpl16 to examine whether genetic diversity is related to geography, gender, habitat and/or frequency of sexual reproduction in Drepanocladus lycopodioides. This moss is restricted to western Eurasian semi-natural and natural habitats and decreases in frequency in many parts of Europe. Haplotype diversity differs among three regional populations (oland, Gotland, Stockholm archipelago) in its South Swedish Baltic core distribution area and is positively affected by patch size. The regional populations differ from each other genetically and haplotype variation is unequally partitioned between the genders, most likely a result of immigration history and limited equalizing effects of sexual reproduction. In a Holocene context, the regional populations of oland and Gotland display evidence of a decrease or bottleneck, and immigration into both regions. We suggest that higher haplotype diversity in Gotland and oland and lack of migration to the Stockholm archipelago may be an effect of longer colonization history of the former, and niche pre-emption in the natural habitats of the latter. The Baltic population is representative for the genetically relatively homogeneous European population, and D. lycopodioides displays low ITS variation compared with other European mosses. In a conservation context it is relevant that the three regional populations are not interchangeable. It is of concern that three populations within the species’ core distribution are vulnerable. Our results strongly suggest that infraspecific genetic diversity must be considered in the management of the species at the continental level. | 652 | no | no good fitness measure, N pop differnt in GD? | NA | no | no |
| 653 | 2017 | 43010 | Riesgo, A; Perez-Portela, R; Pita, L; Blasco, G; Erwin, PM; Lopez-Legentil, S | 2016 | Population structure and connectivity in the Mediterranean sponge Ircinia fasciculata are affected by mass mortalities and hybridization | Recent episodes of mass mortalities in the Mediterranean Sea have been reported for the closely related marine sponges Ircinia fasciculata and Ircinia variabilis that live in sympatry. In this context, the assessment of the genetic diversity, bottlenecks and connectivity of these sponges has become urgent in order to evaluate the potential effects of mass mortalities on their latitudinal range. Our study aims to establish (1) the genetic structure, connectivity and signs of bottlenecks across the populations of I. fasciculata and (2) the hybridization levels between I. fasciculata and I. variabilis. To accomplish the first objective, 194 individuals of I. fasciculata from 12 locations across the Mediterranean were genotyped at 14 microsatellite loci. For the second objective, mitochondrial cytochrome c oxidase subunit I sequences of 16 individuals from both species were analyzed along with genotypes at 12 microsatellite loci of 40 individuals coexisting in 3 Mediterranean populations. We detected strong genetic structure along the Mediterranean for I. fasciculata, with high levels of inbreeding in all locations and bottleneck signs in most locations. Oceanographic barriers like the Almeria-Oran front, North-Balearic front and the Ligurian-Thyrrenian barrier seem to be impeding gene flow for I. fasciculata, adding population divergence to the pattern of isolation by distance derived from the low dispersal abilities of sponge larvae. Hybridization between both species occurred in some populations that might be increasing genetic diversity and somewhat palliating the genetic loss caused by population decimation in I. fasciculata. | 653 | no | inbreeding | NA | no | no |
| 654 | 2017 | 43010 | Goossens, B; Funk, SM; Vidal, C; Latour, S; Jamart, A; Ancrenaz, M; Wickings, EJ; Tutin, CEG; Bruford, MW | 2002 | Measuring genetic diversity in translocation programmes: principles and application to a chimpanzee release project | Logging and poaching have dramatically reduced chimpanzee density and distribution in the Republic of Congo. Most chimpanzee translocations attempted in the past failed because a number of biological and non-biological factors can influence success. Biological considerations include knowledge of behaviour, disease, habitat requirements and genetics. We critically review genetic considerations in pre- and post-release phases of translocations and apply them to a welfare-based chimpanzee release project in the Republic of Congo which aimed to re-establish orphan chimpanzees in the wild with a native chimpanzee population. We analyze genetic diversity and relatedness in released animals and devise a genotyping strategy for monitoring of released individuals and their future offspring. Fifty-nine confiscated chimpanzees from different areas of the Republic of Congo were typed for 20 microsatellites using plucked hair as the DNA source. Genetic diversity was high, with an average expected heterozygosity of 81%, and three to 18 alleles per locus. Between 1996 and 1998, 19 individuals were released, and genetic analysis showed that these are unrelated (mean r +/- jack-knifed SD = -0.014 +/- 0.001). Using F(ST) and population admixture analysis, we identified population structure in wild chimpanzees. For long-term genetic monitoring of released and native chimpanzees, we identified a set of six informative markers, which are easy to score using basic techniques. | 654 | no | no good fitness measure, N pop differnt in GD? | NA | no | no |
| 655 | 2017 | 43010 | Oyler-McCance, SJ; Ransler, FA; Berkman, LK; Quinn, TW | 2007 | A rangewide population genetic study of trumpeter swans | For management purposes, the range of naturally occurring trumpeter swans (Cygnus buccinator) has been divided into two populations, the Pacific Coast Population (PP) and the Rocky Mountain Population (RMP). Little is known about the distribution of genetic variation across the species’ range despite increasing pressure to make difficult management decisions regarding the two populations and flocks within them. To address this issue, we used rapidly evolving genetic markers (mitochondrial DNA sequence and 17 nuclear microsatellite loci) to elucidate the underlying genetic structure of the species. Data from both markers revealed a significant difference between the PP and RMP with the Yukon Territory as a likely area of overlap. Additionally, we found that the two populations have somewhat similar levels of genetic diversity (PP is slightly higher) suggesting that the PP underwent a population bottleneck similar to a well-documented one in the RMP. Both genetic structure and diversity results reveal that the Tri-State flock, a suspected unique, non-migratory flock, is not genetically different from the Canadian flock of the RMP and need not be treated as a unique population from a genetic standpoint. Finally, trumpeter swans appear to have much lower mitochondrial DNA variability than other waterfowl studied thus far which may suggest a previous, species-wide bottleneck. | 655 | no | No GD diff between pop | NA | no | no |
| 656 | 2017 | 43010 | Ferrer, ES; Garcia-Navas, V; Sanz, JJ; Ortego, J | 2014 | Individual genetic diversity and probability of infection by avian malaria parasites in blue tits (Cyanistes caeruleus) | Understanding the importance of host genetic diversity for coping with parasites and infectious diseases is a long-standing goal in evolutionary biology. Here, we study the association between probability of infection by avian malaria (Plasmodium relictum) and individual genetic diversity in three blue tit (Cyanistes caeruleus) populations that strongly differ in prevalence of this parasite. For this purpose, we screened avian malaria infections and genotyped 789 blue tits across 26 microsatellite markers. We used two different arrays of markers: 14 loci classified as neutral and 12 loci classified as putatively functional. We found a significant relationship between probability of infection and host genetic diversity estimated at the subset of neutral markers that was not explained by strong local effects and did not differ among the studied populations. This relationship was not linear, and probability of infection increased up to values of homozygosity by locus (HL) around 0.15, reached a plateau at values of HL from 0.15 to 0.40 and finally declined among a small proportion of highly homozygous individuals (HL>0.4). We did not find evidence for significant identity disequilibrium, which may have resulted from a low variance of inbreeding in the study populations and/or the small power of our set of markers to detect it. A combination of subtle positive and negative local effects and/or a saturation threshold in the association between probability of infection and host genetic diversity in combination with increased resistance to parasites in highly homozygous individuals may explain the observed negative quadratic relationship. Overall, our study highlights that parasites play an important role in shaping host genetic variation and suggests that the use of large sets of neutral markers may be more appropriate for the study of heterozygosity-fitness correlations. | 656 | no | individual level | NA | no | no |
| 657 | 2017 | 43010 | Banks, SC; Dubach, J; Viggers, KL; Lindenmayer, DB | 2010 | Adult survival and microsatellite diversity in possums: effects of major histocompatibility complex-linked microsatellite diversity but not multilocus inbreeding estimators | Adult survival is perhaps the fitness parameter most important to population growth in long-lived species. Intrinsic and extrinsic covariates of survival are therefore likely to be important drivers of population dynamics. We used long-term mark-recapture data to identify genetic, individual and environmental covariates of local survival in a natural population of mountain brushtail possums (Trichosurus cunninghami). Rainfall and intra-individual diversity at microsatellite DNA markers were associated with increased local survival of adults and juveniles. We contrasted the performance of several microsatellite heterozygosity measures, incl’uding internal relatedness (IR), homozygosity by loci (HL) and the mean multilocus estimate of the squared difference in microsatellite allele sizes within an individual (mean d (2)). However, the strongest effect on survival was not associated with multilocus microsatellite diversity (which would indicate a genome-wide inbreeding effect), but a subset of two loci. This included a major histocompatibility complex (MHC)-linked marker and a putatively neutral microsatellite locus. For both loci, diversity measures incorporating allele size information had stronger associations with survival than measures based on heterozygosity, whether or not allele frequency information was included (such as IR). Increased survival was apparent among heterozygotes at the MHC-linked locus, but the benefits of heterozygosity to survival were reduced in heterozygotes with larger differences in allele size. The effect of heterozygosity on fitness-related traits was supported by data on endoparasites in a subset of the individuals studied in this population. There was no apparent density dependence in survival, nor an effect of sex, age or immigrant status. Our findings suggest that in the apparent absence of inbreeding, variation at specific loci can generate strong associations between fitness and diversity at linked markers. | 657 | no | individual level | NA | no | no |
| 658 | 2017 | 43010 | Karaca, S; Karaca, M; Cesuroglu, T; Erge, S; Polimanti, R | 2015 | GSTM1, GSTP1, and GSTT1 Genetic Variability in Turkish and Worldwide Populations | ObjectiveGlutathione S-transferase (GST) variants have been widely investigated to better understand their role in several pathologic conditions. To our knowledge, no data about these genetic polymorphisms within the Turkish population are currently available. The aim of this study was to analyze GSTM1 positive/null, GSTT1 positive/null, GSTP1*I105V (rs1695), and GSTP1*A114V (rs1138272) variants in the general Turkish population, to provide information about its genetic diversity, and predisposition to GST-related diseases. MethodsGenotyping was performed in 500 Turkish individuals using the Sequenom MassARRAY platform. A comparative analysis was executed using the data from the HapMap and Human Genome Diversity Projects (HGDP). Sequence variation was deeply explored using the Phase 1 data of the 1,000 Genomes Project. ResultsThe variability of GSTM1, GSTT1, and GSTP1 polymorphisms in the Turkish population was similar to that observed in Central Asian, European, and Middle Eastern populations. The high linkage disequilibrium between GSTP1*I105V and GSTP1*A114V in these populations may have a confounding effect on GSTP1 genetic association studies. In analyzing GSTM1, GSTT1, and GSTP1 sequence variation, we observed other common functional variants that may be candidates for associated studies of diseases related to GST genes (e.g., cancer, cardiovascular disease, and allergy). ConclusionsThis study provides novel data about GSTM1 positive/null, GSTT1 positive/null, GSTP1*I105V, and GSTP1*A114V variants in the Turkish population, and other functional variants that may affect GSTM1, GSTT1, and GSTP1 functions among worldwide populations. This information can assist in the design of future genetic association studies investigating oxidative stress-related diseases. Am. J. Hum. Biol. 27:310-316, 2015. (c) 2014 Wiley Periodicals, Inc. | 658 | no | human | NA | no | no |
| 659 | 2017 | 43010 | Diaz, V; Ferrer, E | 2003 | Genetic variation of populations of Pinus oocarpa revealed by resistance gene analog polymorphism (RGAP) | Primers based on conserved motifs of plant resistance genes were used to generate multilocus molecular markers - referred to as resistance gene analog polymorphisms (RGAPs) - in Pinus oocarpa subsp. oocarpa. Ten populations from three regions of Nicaragua were analyzed with 53 RGAPs. The aim of this study was to determine the levels of within- and between-population diversity with this kind of marker, and to compare estimates with previously obtained results based on RAPD and AFLP. All populations showed high levels of diversity. G(ST) values and the analysis of molecular variance (AMOVA) revealed most. variation to be within populations, although significant differences between populations and regions were also detected. This pattern of genetic diversity was similar to that obtained for RAPD and AFLP, which suggests that variation at RGAP loci as detected in this work is mostly influence by nonselective forces. | 659 | no | no good fitness measure, N pop differnt in GD? | NA | no | no |
| 660 | 2017 | 43010 | Moges, AD; Admassu, B; Belew, D; Yesuf, M; Njuguna, J; Kyalo, M; Ghimire, SR | 2016 | Development of Microsatellite Markers and Analysis of Genetic Diversity and Population Structure of Colletotrichum gloeosporioides from Ethiopia | Twenty three polymorphic microsatellite markers were developed for citrus plant pathogenic fungus, Colletotrichum gloeosporioides, and were used to analyze genetic diversity and population structure of 163 isolates from four different geographical regions of Ethiopia. These loci produced a total of 118 alleles with an average of 5.13 alleles per microsatellite marker. The polymorphic information content values ranged from 0.104 to 0.597 with an average of 0.371. The average observed heterozygosity across all loci varied from 0.046 to 0.058. The gene diversity among the loci ranged from 0.106 to 0.664. Unweighted Neighbor-joining and population structure analysis grouped these 163 isolates into three major groups. The clusters were not according to the geographic origin of the isolates. Analysis of molecular variance showed 85% of the total variation within populations and only 5% among populations. There was low genetic differentiation in the total populations (FST = 0.049) as evidenced by high level of gene flow estimate (N-m = 4.8 per generation) among populations. The results show that Ethiopian C. gloeosporioides populations are generally characterized by a low level of genetic diversity. The newly developed microsatellite markers were useful in analyzing the genetic diversity and population structure of the C. gloeosporioides populations. Information obtained from this study could be useful as a base to design strategies for better management of leaf and fruit spot disease of citrus in Ethiopia. | 660 | no | gd of pathogen | NA | no | no |
| 661 | 2017 | 43010 | Clark, RW; Brown, WS; Stechert, R; Zamudio, KR | 2010 | Roads, Interrupted Dispersal, and Genetic Diversity in Timber Rattlesnakes | Anthropogenic habitat modification often creates barriers to animal movement, transforming formerly contiguous habitat into a patchwork of habitat islands with low connectivity. Roadways are a feature of most landscapes that can act as barriers or filters to migration among local populations. Even small and recently constructed roads can have a significant impact on population genetic structure of some species, but not others. We developed a research approach that combines fine-scale molecular genetics with behavioral and ecological data to understand the impacts of roads on population structure and connectivity. We used microsatellite markers to characterize genetic variation within and among populations of timber rattlesnakes (Crotalus horridus) occupying communal hibernacula (dens) in regions bisected by roadways. We examined the impact of roads on seasonal migration, genetic diversity, and gene flow among populations. Snakes in hibernacula isolated by roads had significantly lower genetic diversity and higher genetic differentiation than snakes in hibernacula in contiguous habitat. Genetic-assignment analyses revealed that interruption to seasonal migration was the mechanism underlying these patterns. Our results underscore the sizeable impact of roads on this species, despite their relatively recent construction at our study sites (7 to 10 generations of rattlesnakes), the utility of population genetics for studies of road ecology, and the need for mitigating effects of roads. | 661 | no | no good fitness measure | NA | no | no |
| 662 | 2017 | 43010 | Lounnas, M; Vazquez, AA; Alda, P; Sartori, K; Pointier, JP; David, P; Hurtrez-Bousses, S | 2017 | Isolation, characterization and population-genetic analysis of microsatellite loci in the freshwater snail Galba cubensis (Lymnaeidae) | The freshwater snail Galba cubensis (Pfeiffer, 1839) has a large distribution in the Americas. Despite being an intermediate host of Fasciola hepatica-the trematode causing fasciolosis in livestock and humans-its population genetics have never been studied. We isolated and characterized 15 microsatellite loci in G. cubensis to evaluate its genetic diversity, population-genetic structure and mating system. We tested the microsatellite loci in 359 individuals from 13 populations of G. cubensis from Cuba, Guadeloupe, Martinique, Puerto Rico, Venezuela, Colombia and Ecuador. We also tested cross-amplification in three closely related species: G. truncatula, G. viator and G. neotropica. We found that G. cubensis has a similar population structure to other selfing lymnaeids that live in temporary habitats: low genetic diversity, large departure from Hardy-Weinberg equilibrium, marked population structure and high selfing rate. We found that seven and six loci amplified in G. truncatula and G. viator, respectively, and that all 15 loci amplified in G. neotropica. This last finding suggests a close relatedness between G. cubensis and G. neotropica, probably being conspecific and synonymous. This new set of microsatellite markers will be a useful tool to study the genetic diversity of this snail species across a large geographical range and, consequently, to understand the emergence and re-emergence of fasciolosis in the Americas. | 662 | no | no good fitness measure, N pop differnt in GD? | NA | no | no |
| 663 | 2017 | 43010 | Rueppell, O; Meier, S; Deutsch, R | 2012 | Multiple Mating But Not Recombination Causes Quantitative Increase in Offspring Genetic Diversity for Varying Genetic Architectures | Explaining the evolution of sex and recombination is particularly intriguing for some species of eusocial insects because they display exceptionally high mating frequencies and genomic recombination rates. Explanations for both phenomena are based on the notion that both increase colony genetic diversity, with demonstrated benefits for colony disease resistance and division of labor. However, the relative contributions of mating number and recombination rate to colony genetic diversity have never been simultaneously assessed. Our study simulates colonies, assuming different mating numbers, recombination rates, and genetic architectures, to assess their worker genotypic diversity. The number of loci has a strong negative effect on genotypic diversity when the allelic effects are inversely scaled to locus number. In contrast, dominance, epistasis, lethal effects, or limiting the allelic diversity at each locus does not significantly affect the model outcomes. Mating number increases colony genotypic variance and lowers variation among colonies with quickly diminishing returns. Genomic recombination rate does not affect intra-and inter-colonial genotypic variance, regardless of mating frequency and genetic architecture. Recombination slightly increases the genotypic range of colonies and more strongly the number of workers with unique allele combinations across all loci. Overall, our study contradicts the argument that the exceptionally high recombination rates cause a quantitative increase in offspring genotypic diversity across one generation. Alternative explanations for the evolution of high recombination rates in social insects are therefore needed. Short-term benefits are central to most explanations of the evolution of multiple mating and high recombination rates in social insects but our results also apply to other species. | 663 | no | no good fitness measure, N pop differnt in GD? | NA | no | no |
| 664 | 2017 | 43010 | De Rosas, ARP; Segura, EL; Garcia, BA | 2007 | Microsatellite analysis of genetic structure in natural Triatoma infestans (Hemiptera : Reduviidae) populations from Argentina: its implication in assessing the effectiveness of Chagas’ disease vector control programmes | The genetic structure in populations of the Chagas’ disease vector Triatoma infestans was examined. Comparisons of the levels of genetic variability in populations of this species from areas with different periods since last insecticide treatment and from areas that never received treatment were also carried out. A total of 598 insects from 19 populations were typed for 10 polymorphic microsatellite loci. The average observed and expected heterozygosities ranged from 0.186 to 0.625 and from 0.173 to 0.787, respectively. Genetic drift and limited gene flow appear to have generated a substantial degree of genetic differentiation among the populations of T. infestans. Departures from Hardy-Weinberg expectations due to an excess of homozygotes suggested the presence of null alleles and population subdivision. Microgeographical analysis supports the existence of subdivision in T. infestans populations. Levels of genetic diversity in the majority of the populations of T. infestans from insecticide-treated localities were similar or higher than those detected in populations from areas without treatment. Since the populations of T. infestans are subdivided, a population bottleneck would result in independent genetic drift effects that could randomly preserve different combinations of alleles in each subpopulation. These events followed by a rapid population growth could have preserved high levels of genetic diversity. This study supports the hypothesis of vector population recovery from survivors of the insecticide-treated areas and therefore highlights the value of population genetic analyses in assessing the effectiveness of Chagas’ disease vector control programmes. | 664 | maybe | any fitness (survival) of groups of different gd? | no good fitness measure | no | no |
| 665 | 2017 | 43010 | Mtunguja, MK; Ranjan, A; Laswai, HS; Muzanila, Y; Ndunguru, J; Sinha, NR | 2017 | Genetic diversity of farmer-preferred cassava landraces in Tanzania based on morphological descriptors and single nucleotide polymorphisms | Cassava germplasm collection is important for the preservation of genetic variability, allowing the development of improved cultivars with desirable traits such as drought and disease tolerance, better starch quality and yield. Therefore, the assessment of diversity in cassava germplasm maintained by farmers is important for maintaining biodiversity and crop improvement. Herein, we report genetic diversity relationships of 52 farmer-preferred cassava landraces from the eastern zone of Tanzania based on morphological descriptors and single nucleotide polymorphisms (SNPs). Cluster analysis was performed for both morphological traits (genetic distance 1.18-0.15) and SNPs (genetic distance 0.078-0.002). The analysis revealed that there were a total of 17,393 variant positions, and that several of the SNPs were distributed across all the chromosomes. The abundance of SNP varied remarkably among the 18 cassava chromosomes, with chromosome 2 having the highest number of SNPs (1335) and chromosome 18 having the lowest number of SNPs (734). The power of SNPs in distinguishing morphologically similar landraces was shown. Both analyses did not group landraces according to geographical locations, suggesting that farmers were moving cassava germplasm to different areas. Their diversity was mainly due to adaptation and preferential selection by farmers. This further implied that within a geographical location, the cultivars were more diverse and there was no misnaming of cassava cultivars by farmers. The collection revealed a wide range of genetic diversity, and represented a valuable resource for trait improvement, allowing the capture of farmer-preferred traits in future cassava breeding programmes. | 665 | no | crop | NA | no | no |
| 666 | 2017 | 43010 | Olech, W; Perzanowski, K | 2002 | A genetic background for reintroduction program of the European bison (Bison bonasus) in the Carpathians | The European bison, extirpated from the Carpathian Mountains over 200 years ago, was reintroduced to the Bieszczady Mountains in the 1960/1970s in two small, isolated herds, and is now threatened by high inbreeding and low genetic variability A new program of re-establishing viable populations in the Carpathians is based on the genetic analysis of formerly released animals, using the European Bison Pedigree Book. Calculating founder contributions, founder genome survival, founder genome equivalent, inbreeding coefficient and mean kinship allows the identification of under-represented or missing founders. Since genetic variability is much higher among bison in captivity and, in Poland, the Lowland-Caucasian line is represented only in those free-ranging herds, the reintroduction will be based on animals from foreign breeding centres. Bison will be released into existing herds, and planned new introduction sites, to facilitate natural gene exchange in the future. The same approach will be recommended for planned introductions in the Slovakian and Romanian Carpathians. (C) 2002 Elsevier Science Ltd. All rights reserved. | 666 | no | no good fitness measure, N pop differnt in GD? | NA | no | no |
| 667 | 2017 | 43010 | Sharma, M; Nagavardhini, A; Thudi, M; Ghosh, R; Pande, S; Varshney, RK | 2014 | Development of DArT markers and assessment of diversity in Fusarium oxysporum f. sp ciceris, wilt pathogen of chickpea (Cicer arietinum L.) | Background: Fusarium oxysporum f. sp. ciceris (Foc), the causal agent of Fusarium wilt of chickpea is highly variable and frequent recurrence of virulent forms have affected chickpea production and exhausted valuable genetic resources. The severity and yield losses of Fusarium wilt differ from place to place owing to existence of physiological races among isolates. Diversity study of fungal population associated with a disease plays a major role in understanding and devising better disease control strategies. The advantages of using molecular markers to understand the distribution of genetic diversity in Foc populations is well understood. The recent development of Diversity Arrays Technology (DArT) offers new possibilities to study the diversity in pathogen population. In this study, we developed DArT markers for Foc population, analysed the genetic diversity existing within and among Foc isolates, compared the genotypic and phenotypic diversity and infer the race scenario of Foc in India. Results: We report the successful development of DArT markers for Foc and their utility in genotyping of Foc collections representing five chickpea growing agro-ecological zones of India. The DArT arrays revealed a total 1,813 polymorphic markers with an average genotyping call rate of 91.16% and a scoring reproducibility of 100%. Cluster analysis, principal coordinate analysis and population structure indicated that the different isolates of Foc were partially classified based on geographical source. Diversity in Foc population was compared with the phenotypic variability and it was found that DArT markers were able to group the isolates consistent with its virulence group. A number of race-specific unique and rare alleles were also detected. Conclusion: The present study generated significant information in terms of pathogenic and genetic diversity of Foc which could be used further for development and deployment of region-specific resistant cultivars of chickpea. The DArT markers were proved to be a powerful diagnostic tool to study the genotypic diversity in Foc. The high number of DArT markers allowed a greater resolution of genetic differences among isolates and enabled us to examine the extent of diversity in the Foc population present in India, as well as provided support to know the changing race scenario in Foc population. | 667 | no | gd of pathogen | NA | no | no |
| 668 | 2017 | 43010 | Abbott, WGH; Winship, IM; Gane, EJ; Finau, SA; Munn, SR; Tukuitonga, CF | 2006 | Genetic diversity and linkage disequilibrium in the polynesian population of Niue Island | Isolated populations that recently have been derived from small homogeneous groups of founders should have low genetic diversity and high levels of linkage disequilibrium and should be ideal for mapping ancestral polymorphisms that influence complex genetic disease susceptibility. Populations that fulfill these criteria have been difficult to identify. We have been looking for Polynesian populations with these characteristics, because Polynesians have high rates of complex genetic diseases. In Niue Islanders all ancestral female (mitochondrial HSV1 sequence) and 90.4% of ancestral male (Y-chromosome haplogroup) lineages are of Southeast Asian origin. The frequency of European Y-chromosome haplogroups is 7.2%. The diversities of mitochondrial HSV1 sequences (h = 0.18 +/- 0.05) and Y-chromosome haplogroups (h = 0.18 +/- 0.05) are lower than values published for any other population. Ten autosomal microsatellites spaced over 5.8 cM show low allele numbers in Niue Islanders relative to Europeans (55 vs. 88 total alleles, respectively) and a modest reduction in heterozygous loci (0.71 +/- 0.02 vs. 0.78 +/- 0.02, p = 0.04). The higher linkage disequilibrium (d(2)) between these loci in Niue Islanders relative to Europeans (p = 0.001) is negatively correlated (r = -0.47, p = 0.01) with genetic distance. In summary, Niue Islanders are genetically isolated and have a homogeneous Southeast Asian ancestry. They have reduced autosomal genetic diversity and high levels of linkage disequilibrium that are consistent with the influence of genetic drift mechanisms, such as a founder effect or bottlenecks. High-powered linkage disequilibrium studies designed to map ancestral polymorphisms that influence complex genetic disease susceptibility may be feasible in this population. | 668 | no | human | NA | no | no |
| 669 | 2017 | 43010 | Prugnolle, F; Durand, P; Neel, C; Ollomo, B; Ayala, FJ; Arnathau, C; Etienne, L; Mpoudi-Ngole, E; Nkoghe, D; Leroy, E; Delaporte, E; Peeters, M; Renaud, F | 2010 | African great apes are natural hosts of multiple related malaria species, including Plasmodium falciparum | Plasmodium reichenowi, a chimpanzee parasite, was until very recently the only known close relative of Plasmodium falciparum, the most virulent agent of human malaria. Recently, Plasmodium gaboni, another closely related chimpanzee parasite, was discovered, suggesting that the diversity of Plasmodium circulating in great apes in Africa might have been underestimated. It was also recently shown that P. reichenowi is a geographically widespread and genetically diverse chimpanzee parasite and that the world diversity of P. falciparum is fully included within the much broader genetic diversity of P. reichenowi. The evidence indicates that all extant populations of P. falciparum originated from P. reichenowi, likely by a single transfer from chimpanzees. In this work, we have studied the diversity of Plasmodium species infecting chimpanzees and gorillas in Central Africa (Cameroon and Gabon) from both wild-living and captive animals. The studies in wild apes used noninvasive sampling methods. We confirm the presence of P. reichenowi and P. gaboni in wild chimpanzees. Moreover, our results reveal the existence of an unexpected genetic diversity of Plasmodium lineages circulating in gorillas. We show that gorillas are naturally infected by two related lineages of parasites that have not been described previously, herein referred to as Plasmodium GorA and P. GorB, but also by P. falciparum, a species previously considered as strictly human specific. The continuously increasing contacts between humans and primate populations raise concerns about further reciprocal host transfers of these pathogens. | 669 | no | gd of parasite | NA | no | no |
| 670 | 2017 | 43010 | Liu, JJ; Sniezko, R; Murray, M; Wang, N; Chen, H; Zamany, A; Sturrock, RN; Savin, D; Kegley, A | 2016 | Genetic Diversity and Population Structure of Whitebark Pine (Pinus albicaulis Engelm.) in Western North America | Whitebark pine (WBP, Pinus albicaulis Engelm.) is an endangered conifer species due to heavy mortality from white pine blister rust (WPBR, caused by Cronartium ribicola) and mountain pine beetle (Dendroctonus ponderosae). Information about genetic diversity and population structure is of fundamental importance for its conservation and restoration. However, current knowledge on the genetic constitution and genomic variation is still limited for WBP. In this study, an integrated genomics approach was applied to characterize seed collections from WBP breeding programs in western North America. RNA-seq analysis was used for de novo assembly of the WBP needle transcriptome, which contains 97,447 protein-coding transcripts. Within the transcriptome, single nucleotide polymorphisms (SNPs) were discovered, and more than 22,000 of them were non-synonymous SNPs (ns-SNPs). Following the annotation of genes with ns-SNPs, 216 ns-SNPs within candidate genes with putative functions in disease resistance and plant defense were selected to design SNP arrays for high-throughput genotyping. Among these SNP loci, 71 were highly polymorphic, with sufficient variation to identify a unique genotype for each of the 371 individuals originating from British Columbia (Canada), Oregon and Washington (USA). A clear genetic differentiation was evident among seed families. Analyses of genetic spatial patterns revealed varying degrees of diversity and the existence of several genetic subgroups in the WBP breeding populations. Genetic components were associated with geographic variables and phenotypic rating of WPBR disease severity across landscapes, which may facilitate further identification of WBP genotypes and gene alleles contributing to local adaptation and quantitative resistance to WPBR. The WBP genomic resources developed here provide an invaluable tool for further studies and for exploitation and utilization of the genetic diversity preserved within this endangered conifer and other five-needle pines. | 670 | no | no good fitness measure, N pop differnt in GD? | NA | no | no |
| 671 | 2017 | 43011 | Ross-Ibarra, J; Wright, SI; Foxe, JP; Kawabe, A; DeRose-Wilson, L; Gos, G; Charlesworth, D; Gaut, BS | 2008 | Patterns of Polymorphism and Demographic History in Natural Populations of Arabidopsis lyrata | Background: Many of the processes affecting genetic diversity act on local populations. However, studies of plant nucleotide diversity have largely ignored local sampling, making it difficult to infer the demographic history of populations and to assess the importance of local adaptation. Arabidopsis lyrata, a self-incompatible, perennial species with a circumpolar distribution, is an excellent model system in which to study the roles of demographic history and local adaptation in patterning genetic variation. Principal Findings: We studied nucleotide diversity in six natural populations of Arabidopsis lyrata, using 77 loci sampled from 140 chromosomes. The six populations were highly differentiated, with a median FST of 0.52, and STRUCTURE analysis revealed no evidence of admixed individuals. Average within-population diversity varied among populations, with the highest diversity found in a German population; this population harbors 3-fold higher levels of silent diversity than worldwide samples of A. thaliana. All A. lyrata populations also yielded positive values of Tajima’s D. We estimated a demographic model for these populations, finding evidence of population divergence over the past 19,000 to 47,000 years involving non-equilibrium demographic events that reduced the effective size of most populations. Finally, we used the inferred demographic model to perform an initial test for local adaptation and identified several genes, including the flowering time gene FCA and a disease resistance locus, as candidates for local adaptation events. Conclusions: Our results underscore the importance of population-specific, non-equilibrium demographic processes in patterning diversity within A. lyrata. Moreover, our extensive dataset provides an important resource for future molecular population genetic studies of local adaptation in A. lyrata. | 671 | no | no good fitness measure | NA | no | no |
| 672 | 2017 | 43011 | Cunha, C; Doadrio, I; Abrantes, J; Coelho, MM | 2011 | The evolutionary history of the allopolyploid Squalius alburnoides (Cyprinidae) complex in the northern Iberian Peninsula | Understanding the population structure, population dynamics and processes that give rise to polyploidy and helps to maintain it is central to our knowledge of the evolution of asexual vertebrates. Previous studies revealed high genetic diversity and several reproductive pathways in the southern populations of the Squalius alburnoides hybrid complex. In contrast, lower genetic variability and the associated limited chance of introducing new genetic combinations may threaten the survival of the northern Mondego populations. We analysed the genetic diversity and structure of nine populations of S. alburnoides in the Iberian Peninsula using microsatellite loci to provide further insights on the evolutionary history of this complex. Special attention was given to the less-studied northern populations (Mondego and Douro basins). Marked population structure, a high frequency of private alleles and a high diversity of some biotypes in the Douro basin indicate that some northern populations may not be at high risk of extinction, contrary to what was expected. The genetic diversity found in the northern Douro populations contradicts the general trend of remarkable genetic impoverishment northwards that occurs in other species and regions. The results indicate the possible existence of a glacial refugium in the Rabacal River, corroborating findings in other species of this region. Historical events seem to have affected the geographical patterns of genetic variability found among and within the northern and southern populations of this complex and contributed to different patterns of genome composition. Therefore, historical events might have a major role in the long-term persistence of some polyploid hybrid taxa. Heredity (2011) 106, 100-112; doi:10.1038/hdy.2010.70; published online 9 June 2010 | 672 | no | no good fitness measure | NA | no | no |
| 673 | 2017 | 43011 | Knafler, GJ; Grueber, CE; Sutton, JT; Jamieson, IG | 2017 | Differential patterns of diversity at microsatellite, MHC, and TLR loci in bottlenecked South Island saddleback populations | For populations that experienced historical population bottlenecks, subsequent contemporary bottlenecks may continue to degrade genetic diversity at loci that are still variable. However, it is currently unclear how different types of contemporary bottlenecks may affect diversity at functional versus neutral loci. In this study, we examine genetic diversity of microsatellite, major histocompatibility complex (MHC), and toll-like receptor (TLR) loci of three New Zealand South Island saddleback (Philesturnus carunculatus) populations: Big Island, Kaimohu Island and Motuara Island. One population, Motuara, experienced two sets of contemporary bottlenecks (translocation event followed by a disease-outbreak and population crash) and was sampled in three different years. For all populations and sampling periods, we compare patterns of diversity across the three sets of loci to infer the effects of contemporary bottlenecks. We found that translocation-induced bottlenecks resulted in greater losses of microsatellite than MHC diversity, whereas temporal (encompassing a potential disease-induced bottleneck) fluctuations in diversity were greater for MHC markers than microsatellite loci. Both translocation- and disease-induced contemporary bottlenecks affected TLR diversity to a greater extent than diversity of either microsatellites or MHC. We found significant temporal changes in the frequencies of two TLR1LA alleles on Motuara Island (coincident with the population crash), but no further subsequent change. These alleles differ by an amino acid variant (associated with the leucine-rich repeat region of TLR1LA). Since TLR1LA may initiate an innate immune response in the presence of haemosporidia parasites, it is possible that a selection event has contributed to the observed patterns at TLR1LA. Our results indicate that patterns of genetic diversity loss at microsatellite, MHC and TLR loci are not equally affected by contemporary population bottlenecks, and may be influenced by bottleneck type. | 673 | no | no good fitness measure | NA | no | no |
| 674 | 2017 | 43011 | Lodh, N; Rizzo, DM; Kerans, BL; McGinnis, S; Fytilis, N; Stevens, L | 2015 | If you’ve seen one worm, have you seen them all? Spatial, community, and genetic variability of tubificid communities in Montana | Genetic studies are recognized increasingly as important for understanding naturally occurring disease dynamics and are used to predict host genetic diversity and coevolutionary processes and to identify species composition in ecological communities. Tubifex tubifex, the definitive host of the whirling disease parasite Myxobolus cerebralis, comprises 6 known lineages that vary widely in parasite susceptibility. We used 16S ribosomal DNA (16S rDNA) to identify relationships among genetic variability of 3 oligochaete genera (T. tubifex, Rhyacodrilus spp., and Ilyodrilus spp.; Oligochaeta: Tubificidae), oligochaete assemblage composition, and the presence of whirling disease in 9 locations across 4 watersheds in Montana, USA. We assessed genetic variability among 183 tubificid worms from locations classified as positive or negative for whirling disease based on 5 to 8 y of monitoring by the Montana Department of Fish, Wildlife, and Parks. Within genera, we found 2 groups of T. tubifex (lineages I and III), 2 groups of Rhyacodrilus spp., and 4 groups of Ilyodrilus spp., possibly suggesting cryptic species. The maximum genetic variability within taxa was relatively high (similar to 10% sequence divergence) for all 3 genera, but haplotype diversity within groups with >5% sequence divergence was greater for Ilyodrilus spp. (0.719) than for Tubifex spp. (0.246) and Rhyacodrilus spp. (0.143). The variation was nonrandomly distributed over the landscape. Oligochaete genetic composition was more similar among locations in the same watershed than among locations with or without whirling disease. Thus, oligochaete assemblage composition did not appear to be related to the presence of the disease at this watershed spatial scale. | 674 | no | no pop level | NA | no | no |
| 675 | 2017 | 43011 | Branco, CC; Mota-Vieira, LM | 2003 | Population structure of Sao Miguel Island, Azores: A surname study | The knowledge of a population structure may constitute a powerful tool for mapping genes underlying susceptibility to Mendelian and complex diseases. To obtain a better understanding of the population structure of Sao Miguel Island (Azorean Archipelago, Portugal), we carried out a Surname survey using the surnames listed in the most recent telephone book (2001). We identified 1315 different surnames in a total of 27,621 subscribers. The frequency of the different surnames was used to calculate the following parameters: isonymy (I), random component of inbreeding (F-ST), genetic diversity according to Fisher (alpha), migration rate according to Karlin-McGregor (v) and Nei’s genetic distance. Eleven localities were selected, according to population size and geographic distribution, for analysis using the above parameters. Our results show that 51% of Salga’s population and 52% of Sete Cidades’s population are represented by six and eight surnames, respectively. These figures demonstrate the effective isolation of these two small places, which are located at opposite ends of Sao Miguel Island. Salga, Achada, and Sete Cidades present the lowest values of Fisher’s alpha, indicating less genetic diversity. In contrast, the capital, Ponta Delgada, presents the highest value of alpha (78.13), indicating more genetic diversity. Our data indicate that the clustering of the localities corresponds to the geographic features of the island, where localities close together tend to share similar surnames. In conclusion, the population of Sao Miguel is relatively homogeneous and may constitute an ideal model for genetic mapping studies. | 675 | no | human | NA | no | no |
| 676 | 2017 | 43011 | Dias, G; Beltran, JF; Tejedo, M; Benitez, M; Miras, EG; Ferrand, N; Goncalves, H | 2015 | Limited gene flow and high genetic diversity in the threatened Betic midwife toad (Alytes dickhilleni): evolutionary and conservation implications | Habitat fragmentation may involve a loss of genetic diversity and increments the vulnerability to species persistence. It could be a particular issue when coupled with other negative factors as the predicted climatic changes and the emergence of infectious diseases. In Southern Iberian Peninsula several endemic amphibian species have confined and fragmented distributions, including the Betic midwife toad Alytes dickhilleni. Herein, we present the first range-wide assessment of genetic diversity and structure in this species, using mitochondrial and microsatellite data. A mitochondrial fragment of the ND4 gene was amplified for 65 individuals and a set of 20 microsatellite loci, specifically developed for this species, was genotyped for 490 individuals from several sampling sites distributed across the species entire range. While both markers revealed high genetic diversity, only for microsatellites a marked genetic substructure was apparent. Our results evidence low levels of gene flow, suggesting the persistence of the species in fragmented habitats for several generations and a very limited connectivity between most of mountain ranges. The high diversity within A. dickhilleni populations could help to respond to the emergence of new diseases and to the predicted effects of climatic changes in Southeastern Iberian Peninsula. We hypothesize that the lack of gene flow is due to the absence of available breeding habitats and recommend that future management efforts of A. dickhilleni include the creation and maintenance of aquatic breeding habitats in a way that most of genetic diversity is preserved. | 676 | no | no good fitness measure, N pop differnt in GD? | NA | no | no |
| 677 | 2017 | 43011 | Nishat, S; Hamim, I; Khalil, MI; Ali, MA; Hossain, MA; Meah, MB; Islam, MR | 2015 | Genetic diversity of the bacterial wilt pathogen Ralstonia solanacearum using a RAPD marker | Bacterial wilt caused by Ralstonia solanacearum is a destructive disease of many economically important crop species. A significant variation in wilt incidence and severity in eggplant and potato was observed among the growing areas surveyed. R. solanacearum isolates obtained both from eggplant and potato belong to biovar III, while isolates from eggplant belong to race 1 and isolates obtained from potato belong to race 3. Random amplified polymorphic DNA (RAPD) technique was used as a tool for assessing genetic variation and relationship among seven isolate groups of R. solanacearum viz., RsB-1, RsB-2, RsB-3, RsP-1, RsP-2, RsP-3 and RsP-4, consisting in a total of 28 isolates. Out of the RAPD markers used, amplification with four decamer primers produced 70 bands with sizes ranging from 100 to 1400 bp. Out of 70 bands, 68 bands (97.06%) were polymorphic and two bands (2.94%) were monomorphic amongst the seven R. solanacearum isolates group. The Unweighted Pair Group Method of Arithmetic Means (UPGMA) dendrogram constructed from Nei’s genetic distance produced two main clusters of the seven isolates of R. solanacearum. The isolates RsB-1, RsB-2, RsB-3 and R-4 grouped in cluster I, while RsP-2, RsP-3 and RsP-4 grouped in cluster IL The highest intra-variety similarity index (S-i) was found in RsB-1 isolate (86.35%) and the lowest one in RsP-2 (56.59%). The results indicated that relatively higher and lower levels of genetic variation were found in R5P-3 and RsB-3, respectively. The coefficient of gene differentiation (G(st)) was 0.5487, reflecting the existence of a high level of genetic variations among seven isolates of R. solanacearum. Comparatively higher genetic distance (0.4293) and lower genetic identity (0.6510) were observed between RsB-2 and RsP-4 combinations. The lowest genetic distance (0.0357) and highest genetic identity (0.9650) were found in RsB-1 vs. RsB-2 pair. Thus, RAPD offers a potentially simple, rapid and reliable method to evaluate genetic diversity analysis in R. solanacearum. (C) 2015 Academie des sciences. Published by Elsevier Masson SAS. All rights reserved. | 677 | no | gd of pathogen | NA | no | no |
| 678 | 2017 | 43011 | Vieira, P; Burgermeister, W; Mota, M; Metge, K; Silva, G | 2007 | Lack of genetic variation of Bursaphelenchus xylophilus in Portugal revealed by RAPD-PCR analyses | Random Amplified Polymorphic DNA (RAPD-PCR) technique was used to assess the level of genetic variability and genetic relationships among 24 Portuguese isolates of pinewood nematode, Bursaphelenchus xylophilus. The isolates represent the main infested areas of Portugal. Two additional isolates of B. xylophilus representing North America and East Asia were included, and B. mucronatus was used as out-group. Twenty-eight random primers generated a total of 640 DNA fragments. The Nei and Li similarity index revealed a high genetic similarity among the Portuguese isolates (above 90%). Hierarchical cluster analysis was performed to illustrate the relatedness among the isolates. No indication for separate groups among the Portuguese isolates was obtained, and the low level of genetic diversity strongly suggests that they were dispersed recently from a single introduction. The lack of apparent relationship between the genetic and the geographic matrices of the Portuguese isolates limits the use of this technique for following recent pathways of distribution. Genetic distance of the Portuguese isolates towards an isolate from China was much lower as compared to an isolate from the USA. This confirmed previous results suggesting an East Asian origin of the Portuguese B. xylophilus. | 678 | no | gd of pathogen | NA | no | no |
| 679 | 2017 | 43011 | Morris, KM; Wright, B; Grueber, CE; Hogg, C; Belov, K | 2015 | Lack of genetic diversity across diverse immune genes in an endangered mammal, the Tasmanian devil (Sarcophilus harrisii) | The Tasmanian devil (Sarcophilus harrisii) is threatened with extinction due to the spread of devil facial tumour disease. Polymorphisms in immune genes can provide adaptive potential to resist diseases. Previous studies in diversity at immune loci in wild species have almost exclusively focused on genes of the major histocompatibility complex (MHC); however, these genes only account for a fraction of immune gene diversity. Devils lack diversity at functionally important immunity loci, including MHC and Toll-like receptor genes. Whether there are polymorphisms at devil immune genes outside these two families is unknown. Here, we identify polymorphisms in a wide range of key immune genes, and develop assays to type single nucleotide polymorphisms (SNPs) within a subset of these genes. A total of 167 immune genes were examined, including cytokines, chemokines and natural killer cell receptors. Using genome-level data from ten devils, SNPs within coding regions, introns and 10kb flanking genes of interest were identified. We found low polymorphism across 167 immune genes examined bioinformatically using whole-genome data. From this data, we developed long amplicon assays to target nine genes. These amplicons were sequenced in 29-220 devils and found to contain 78 SNPs, including eight SNPS within exons. Despite the extreme paucity of genetic diversity within these genes, signatures of balancing selection were exhibited by one chemokine gene, suggesting that remaining diversity may hold adaptive potential. The low functional diversity may leave devils highly vulnerable to infectious disease, and therefore, monitoring and preserving remaining diversity will be critical for the long-term management of this species. Examining genetic variation in diverse immune genes should be a priority for threatened wildlife species. This study can act as a model for broad-scale immunogenetic diversity analysis in threatened species. | 679 | no | no good fitness measure, N pop differnt in GD? | NA | no | no |
| 680 | 2017 | 43011 | Scharis, I; Amundin, M | 2015 | Cross-fostering in gray wolves (Canis lupus lupus) | Cross-fostering in canids, with captive-bred pups introduced into endangered wild populations, might aid conservation efforts by increasing genetic diversity and lowering the risk of inbreeding depression. The gray wolf (Canis lupus lupus) population in Scandinavia suffers from severe inbreeding due to a narrow genetic base and geographical isolation. This study aimed at evaluating the method to cross-foster wolf pups from zoo-born to zoo-born litters. The following was assessed: female initial acceptance of foster pups, growth rate in relation to age difference between foster pups and pups in recipient litters and survival over the first 33 weeks. The study included four litters added by two foster pups in each. The age differences between the foster pups and the recipient litters were 2-8 days. After augmentation, all four females accepted the foster pups, demonstrated by her moving the entire litter to a new den site. Growth rate was dependent on the age difference of the pups in the foster litters, with a considerably slower growth rate in the 8 days younger pups. However, these pups later appeared to be at no disadvantage. Foster pups had a higher survival rate than females’ pups, however, the causes of death were probably not kin or non-kin related. The results indicate that cross-fostering works in gray wolves and that this might be a plausible way to increase genetic variation in the wild population. Zoo Biol. 34:217-222, 2015. (c) 2015 Wiley Periodicals Inc. | 680 | no | no pop level | NA | no | no |
| 681 | 2017 | 43011 | Provan, J; Wilson, S; Portig, AA; Maggs, CA | 2008 | The importance of reproductive strategies in population genetic approaches to conservation: an example from the marine angiosperm genus Zostera | Knowledge of the levels of genetic diversity maintained in natural populations can play a central role in conservation programmes, particularly in threatened habitats or species. Fluctuations in population size can lead to loss of variation and, consequently, increase the risk of extinction. We have examined whether such a genetic bottleneck has occurred in populations of two species in the seagrass genus Zostera, which are believed to have been affected by an outbreak of wasting disease at the start of the last century. A test for heterozygote excess at five nuclear microsatellite loci did not suggest the occurrence of a genetic bottleneck, but analysis of seven chloroplast microsatellite loci and sequence data from two regions did suggest a bottleneck in the chloroplast genome. Extremely low levels of between-population diversity suggest that all subpopulations can be treated as a single management unit for each species. Comparable levels of nuclear genetic diversity were found in the three populations of the primarily sexual Zostera marina var. angustifolia studied but a wider range of within-population diversity was found in Zostera noltii, which displays both. sexual and vegetative reproductive strategies. This may be due to an increase in sexual recruitment due to localised fresh water inflow into the study site near to the most diverse population. Such populations should be prioritised as source material for any replanting or remediation due to natural or anthropogenic loss of Zostera beds in the area. | 681 | no | no good fitness measure, N pop differnt in GD? | NA | no | no |
| 682 | 2017 | 43011 | KONIG, B | 1994 | FITNESS EFFECTS OF COMMUNAL REARING IN-HOUSE MICE - THE ROLE OF RELATEDNESS VERSUS FAMILIARITY | Newborn female house mice, Mus domesticus, were cross-fostered to analyse the influence of relatedness and familiarity during juvenile development on subsequent reproductive success during communal rearing of young. The reproductive behaviour of polygynously mated pairs of full-sisters reared apart (unfamiliar and related) and of genetically unrelated females reared together (familiar, unrelated) was analysed over a standardized ‘lifespan’ of 120 days after the females were given access to a male compared with that of sisters reared together (familiar, related) and unrelated females reared apart (unfamiliar, unrelated). Familiarity with a same-sex partner (irrespective of relatedness) improved a female’s probability of successfully reproducing and the probability that both partners weaned young (egalitarian reproduction). In groups with unfamiliar partners, the female that weighed less at mating suffered a higher probability of being reproductively suppressed in a despotic relationship. Furthermore, familiarity significantly improved offspring survival until weaning compared with that in social groups with previously unfamiliar females. Consequently, a female living with a familiar partner had a significantly higher lifetime reproductive success than a female with an unfamiliar partner. By choosing a familiar group member a female had a high probability of communally rearing young with a relative, owing to the basic family structure of house mouse demes. However, despite the paramount importance of familiarity with a partner for reproductive success, some data indicated that females discriminated between familiar related and familiar unrelated partners. Familiar sisters reached higher scores than familiar unrelated females in almost all fitness components and weaned the highest number and biomass of offspring within their lifespan of all groups analysed. | 682 | no | no good fitness measure | NA | no | no |
| 683 | 2017 | 43011 | Acevedo-Whitehouse, K; Petetti, L; Duignan, P; Castinel, A | 2009 | Hookworm infection, anaemia and genetic variability of the New Zealand sea lion | Hookworms are intestinal blood-feeding nematodes that parasitize and cause high levels of mortality in a wide range of mammals, including otariid pinnipeds. Recently, an empirical study showed that inbreeding (assessed by individual measures of multi-locus heterozygosity) is associated with hookworm-related mortality of California sea lions. If inbreeding increases susceptibility to hookworms, effects would expectedly be stronger in small, fragmented populations. We tested this assumption in the New Zealand sea lion, a threatened otariid that has low levels of genetic variability and high hookworm infection rates. Using a panel of 22 microsatellites, we found that average allelic diversity (5.9) and mean heterozygosity (0.72) were higher than expected for a small population with restricted breeding, and we found no evidence of an association between genetic variability and hookworm resistance. However, similar to what was observed for the California sea lion, homozygosity at a single locus explained the occurrence of anaemia and thrombocytopenia in hookworm-infected pups (generalized linear model, F = 11.81, p < 0.001) and the effect was apparently driven by a particular allele (odds ratio 34.95%; CI: 7.12-162.41; p < 0.00001). Our study offers further evidence that these haematophagus parasites exert selective pressure on otariid blood-clotting processes. | 683 | no | gd of parasite | NA | no | no |
| 684 | 2017 | 43011 | Cohuet, A; Krishnakumar, S; Simard, F; Morlais, I; Koutsos, A; Fontenille, D; Mindrinos, M; Kafatos, FC | 2008 | SNP discovery and molecular evolution in Anopheles gambiae, with special emphasis on innate immune system | Background: Anopheles innate immunity affects Plasmodium development and is a potential target of innovative malaria control strategies. The extent and distribution of nucleotide diversity in immunity genes might provide insights into the evolutionary forces that condition pathogen-vector interactions. The discovery of polymorphisms is an essential step towards association studies of susceptibility to infection. Results: We sequenced coding fragments of 72 immune related genes in natural populations of Anopheles gambiae and of 37 randomly chosen genes to provide a background measure of genetic diversity across the genome. Mean nucleotide diversity (pi) was 0.0092 in the A. gambiae S form, 0.0076 in the M form and 0.0064 in A. arabiensis. Within each species, no statistically significant differences in mean nucleotide diversity were detected between immune related and non immune related genes. Strong purifying selection was detected in genes of both categories, presumably reflecting strong functional constraints. Conclusion: Our results suggest similar patterns and rates of molecular evolution in immune and non-immune genes in A. gambiae. The 3,214 Single Nucleotide Polymorphisms (SNPs) that we identified are the first large set of Anopheles SNPs from fresh, field-collected material and are relevant markers for future phenotype-association studies. | 684 | no | NA | NA | no | no |
| 685 | 2017 | 43011 | ELLEGREN, H; HARTMAN, G; JOHANSSON, M; ANDERSSON, L | 1993 | MAJOR HISTOCOMPATIBILITY COMPLEX MONOMORPHISM AND LOW-LEVELS OF DNA-FINGERPRINTING VARIABILITY IN A REINTRODUCED AND RAPIDLY EXPANDING POPULATION OF BEAVERS | Less of genetic variation due to population bottlenecks may be a severe threat for the survival of endangered species. Assessment and maintenance of genetic variability are thus crucial for conservation programs related to endangered populations. Scandinavian beavers went through an extensive bottleneck during the last century due to over-hunting. In Sweden the species became extirpated but in Norway extinction was avoided by legal protection. Following reintroductions of small numbers of remaining Norwegian animals in 1922-1939, the Swedish population has increased tremendously, now harboring 100,000 animals. We show here that this viable population of beavers possesses extremely low levels of genetic variability at DNA fingerprinting loci and monomorphism at major histocompatibility complex (MHC) class I and class II loci. A similar pattern was also evident among Norwegian beavers but low levels of genetic variability were not a characteristic of the species since Russian conspecifics displayed substantial DNA fingerprinting polymorphism. However, the Russian animals were monomorphic at MHC loci, indicating that the European beaver is exceptional in its low level of MHC variability. The results demonstrate that a conservation program can be successful despite low levels of genetic variation in the founder population. | 685 | no | no good fitness measure, N pop differnt in GD? | NA | no | no |
| 686 | 2017 | 43011 | Valbuena-Urena, E; Soler-Membrives, A; Steinfartz, S; Orozco-terWengel, P; Carranza, S | 2017 | No signs of inbreeding despite long-term isolation and habitat fragmentation in the critically endangered Montseny brook newt (Calotriton arnoldi) | Endemic species with restricted geographic ranges potentially suffer the highest risk of extinction. If these species are further fragmented into genetically isolated subpopulations, the risk of extinction is elevated. Habitat fragmentation is generally considered to have negative effects on species survival, despite some evidence for neutral or even positive effects. Typically, non-negative effects are ignored by conservation biology. The Montseny brook newt (Calotriton arnoldi) has one of the smallest distribution ranges of any European amphibian (8 km(2)) and is considered critically endangered by the International Union for Conservation of Nature. Here we apply molecular markers to analyze its population structure and find that habitat fragmentation owing to a natural barrier has resulted in strong genetic division of populations into two sectors, with no detectable migration between sites. Although effective population size estimates suggest low values for all populations, we found low levels of inbreeding and relatedness between individuals within populations. Moreover, C. arnoldi displays similar levels of genetic diversity to its sister species Calotriton asper, from which it separated around 1.5 million years ago and which has a much larger distribution range. Our extensive study shows that natural habitat fragmentation does not result in negative genetic effects, such as the loss of genetic diversity and inbreeding on an evolutionary timescale. We hypothesize that species in such conditions may evolve strategies (for example, special mating preferences) to mitigate the effects of small population sizes. However, it should be stressed that the influence of natural habitat fragmentation on an evolutionary timescale should not be conflated with anthropogenic habitat loss or degradation when considering conservation strategies. | 686 | no | inbreeding | NA | no | no |
| 687 | 2017 | 43011 | Hawley, DM; Briggs, J; Dhondt, AA; Lovette, IJ | 2008 | Reconciling molecular signatures across markers: mitochondrial DNA confirms founder effect in invasive North American house finches (Carpodacus mexicanus) | Well-characterized species introductions provide opportunities to compare the genetic signatures of known founder effects across classes of molecular markers. The release of small numbers of house finches (Carpodacus mexicanus) into the eastern United States in the 1940s led to substantial interest in the effects of this introduction on genetic diversity in this now abundant species, an issue that has been highlighted by a recent Mycoplasma disease epidemic that most intensively affects the introduced and potentially genetically depauperate house finch populations. Previous studies comparing genetic diversity levels in native and introduced house finch populations produced seemingly disparate results: comparisons based on amplified fragment length polymorphism, RFLP mtDNA, and allozyme markers found essentially equivalent levels of diversity in eastern and western populations, whereas microsatellite markers showed clear reductions in diversity in the introduced populations. Here we employ sequence variation at the ND2 mtDNA locus to further compare levels of diversity between the four native and five introduced house finch populations that were previously examined in the microsatellite study. We found substantially lower ND2 haplotype richness and diversity across all introduced populations of house finches. The majority of sequence variation (78%) was detected within subpopulations, with the remainder (22%) explained by the historical status of each population (native or introduced). Our results are consistent with previous microsatellite evidence for a founder effect during the introduction of eastern house finches, and suggest that the mtDNA founder effect was particularly severe, likely owing to a male-biased sex ratio at the time of introduction coupled with the lower effective population size of clonally inherited markers. We discuss how the inconsistencies between past studies of house finch diversity can inform the usefulness of distinct marker sets for detecting molecular signatures of founder events. | 687 | no | no good fitness measure, N pop differnt in GD? | NA | no | no |
| 688 | 2017 | 43011 | Garcia-Obregon, S; Alfonso-Sanchez, MA; Perez-Miranda, AM; Gomez-Perez, L; de Pancorbo, MM; Pena, JA | 2010 | Genetic variability in autochthonous Basques from Guipuzcoa: a view from MHC microsatellites | P>Five short tandem repeats (STRs) located at human chromosome 6 were analysed in 97 autochthonous Basques from Guipuzcoa (northern Spain), with the aim of assessing the genetic relationships of Basques at a European scale, based on the variability of the major histocompatibility complex (MHC) region, and comparing the phylogenetic information obtained from STRs, and from HLA class I genes (HLA-A and HLA-B) for the same set of European populations. The integrative approach was focused on D6S265 and D6S2792, according to availability of population databases. F-ST genetic distances obtained from STRs and from HLA loci were very similar, thereby describing a comparable pattern of genetic structuring among the European populations. These findings were supported by results of the Mantel test of matrix correspondence (r = 0.796, P = 0.0022) and by significant correlations between the first two F-ST eigenvectors of STRs and HLA genes. Coinciding with previous phylogenetic studies, Basques showed substantial genetic differentiation within the European context, probably as a result of the impact of random genetic drift and high inbreeding levels for extended periods of isolation even from adjacent populations. Analysis of the geographical distribution of the allele frequencies revealed a great number of latitudinal frequency clines in both the MHC STRs and the HLA class I genes, which supports the notion of the post-glacial resettlement of Europe being a crucial factor in the genetic make-up of Europeans. Our results indicate that analysing the genetic variability of MHC microsatellites could be a suitable strategy in evaluating the role of evolutionary forces such as natural selection (because of genetic hitchhiking effect), genetic drift and gene flow in the maintenance of polymorphism at the MHC region, because STRs can efficiently complement the genetic information obtained from HLA genes. | 688 | no | human | NA | no | no |
| 689 | 2017 | 43011 | Li, J; Lao, XZ; Zhang, C; Tian, L; Lu, DS; Xu, SH | 2014 | Increased genetic diversity of ADME genes in African Americans compared with their putative ancestral source populations and implications for Pharmacogenomics | Background: African Americans have been treated as a representative population for African ancestry for many purposes, including pharmacogenomic studies. However, the contribution of European ancestry is expected to result in considerable differences in the genetic architecture of African American individuals compared with an African genome. In particular, the genetic admixture influences the genomic diversity of drug metabolism-related genes, and may cause high heterogeneity of drug responses in admixed populations such as African Americans. Results: The genomic ancestry information of African-American (ASW) samples was obtained from data of the 1000 Genomes Project, and local ancestral components were also extracted for 32 core genes and 252 extended genes, which are associated with drug absorption, distribution, metabolism, and excretion (ADME) genes. As expected, the global genetic diversity pattern in ASW was determined by the contributions of its putative ancestral source populations, and the whole profiles of ADME genes in ASW are much closer to those in YRI than in CEU. However, we observed much higher diversity in some functionally important ADME genes in ASW than either CEU or YRI, which could be a result of either genetic drift or natural selection, and we identified some signatures of the latter. We analyzed the clinically relevant polymorphic alleles and haplotypes, and found that 28 functional mutations (including 3 missense, 3 splice, and 22 regulator sites) exhibited significantly higher differentiation between the three populations. Conclusions: Analysis of the genetic diversity of ADME genes showed differentiation between admixed population and its ancestral source populations. In particular, the different genetic diversity between ASW and YRI indicated that the ethnic differences in pharmacogenomic studies are broadly existed despite that African ancestry is dominant in Africans Americans. This study should advance our understanding of the genetic basis of the drug response heterogeneity between populations, especially in the case of population admixture, and have significant implications for evaluating potential inter-population heterogeneity in drug treatment effects. | 689 | no | human | NA | no | no |
| 690 | 2017 | 43011 | Wahbe, TR; Ritland, C; Bunnell, FL; Ritland, K | 2005 | Population genetic structure of tailed frogs (Ascaphus truei) in clearcut and old-growth stream habitats in south coastal British Columbia | “Ascaphus truei Stejneger, 1899 relies on cool, fast-flowing, forested mountain streams, which receive little protection from logging activities. During recovery of post-logging habitats, local extirpation of Ascaphus is a concern because their recolonization may be slow. In British Columbia, Oregon, and California, coastal Ascaphus populations are designated as”“at risk”“. To infer fragmentation impacts from patterns of genetic structure, we used RAPD molecular markers and compared population genetic structures of Ascaphus larvae in an old growth with an adjacent clearcut stream located in coastal British Columbia. In the clearcut, larvae were less genetically diverse than in the old growth and exhibited no relationship between physical distance and genetic relatedness, whereas in the old growth, genetic similarity decreased with physical distance. Patterns of gene flow between the clearcut and old-growth streams were significantly different. The clearcut population went through a bottleneck/founding event, but also exhibited greater dispersal. The frogs were perhaps searching for new habitat (which would lower isolation by distance) and suffering mortality (which would decrease diversity). Most problems in conservation will require genetic and ecological data, and future research should aim to incorporate methodologies from both fields.” | 690 | no | no good fitness measure | NA | no | no |
| 691 | 2017 | 43011 | Chen, WK; Swartz, JD; Rush, LJ; Alvarez, CE | 2009 | Mapping DNA structural variation in dogs | DNA structural variation (SV) comprises a major portion of genetic diversity, but its biological impact is unclear. We propose that the genetic history and extraordinary phenotypic variation of dogs make them an ideal mammal in which to study the effects of SV on biology and disease. The hundreds of existing dog breeds were created by selection of extreme morphological and behavioral traits. And along with those traits, each breed carries increased risk for different diseases. We used array CGH to create the first map of DNA copy number variation (CNV) or SV in dogs. The extent of this variation, and some of the gene classes affected, are similar to those of mice and humans. Most canine CNVs affect genes, including disease and candidate disease genes, and are thus likely to be functional. We identified many CNVs that may be breed or breed class specific. Cluster analysis of CNV regions showed that dog breeds tend to group according to breed classes. Our combined findings suggest many CNVs are (1) in linkage disequilibrium with flanking sequence, and (2) associated with breed-specific traits. We discuss how a catalog of structural variation in dogs will accelerate the identification of the genetic basis of canine traits and diseases, beginning with the use of whole genome association and candidate-CNV/gene approaches. | 691 | no | domesticated animal | NA | no | no |
| 692 | 2017 | 43011 | Qian, J; He, TH; Song, ZP; Lu, BR | 2005 | Genetic evaluation of in situ conserved and reintroduced populations of wild rice (Oryza rufipogon : Poaceae) in China | We evaluated the genetic consequences and efficiency of conservation practices in Oryza rufipogon using microsatellite DNA markers. Spatial autocorrelation analysis from 12 microsatellite loci revealed that microsatellite alleles were exclusively distributed it? patches within the population, indicating that large populations were unlikely to be homogeneous. At? in situ conserved stand of O. rufipogon, which has been protected by a concrete wall from a large population, captured only 67.9% of the total genetic variation of the previous large population. The concrete wall was built to protect the wild rice. but it acted more as a physical barrier to gene exchanges between the two sides. An assignment test revealed only 11.1% putative seed exchanges across the wall. A reintroduced population was found to be genetically very diverse. About 76.3% of the total genetic variation detected in other populations was captured in this reintroduced population. and 24.8% of the total genetic variation in this population was not found in other populations. These results display two important findings for conservation of O. rufipogon. First, conserving one part of a large population of O. rufipogon will not preserve an adequate sample of the genetic variability, since populations are not homogeneous, and genotype distribution varies among localities. Second, a reintroduced population is not genetically depauperate, but it is too early to assess its long-term survival. | 692 | no | no good fitness measure, N pop differnt in GD? | NA | no | no |
| 693 | 2017 | 43011 | Benson, JF; Mahoney, PJ; Sikich, JA; Serieys, LEK; Pollinger, JP; Ernest, HB; Riley, SPD | 2016 | Interactions between demography, genetics, and landscape connectivity increase extinction probability for a small population of large carnivores in a major metropolitan area | The extinction vortex is a theoretical model describing the process by which extinction risk is elevated in small, isolated populations owing to interactions between environmental, demographic, and genetic factors. However, empirical demonstrations of these interactions have been elusive. We modelled the dynamics of a small mountain lion population isolated by anthropogenic barriers in greater Los Angeles, California, to evaluate the influence of demographic, genetic, and landscape factors on extinction probability. The population exhibited strong survival and reproduction, and the model predicted stable median population growth and a 15% probability of extinction over 50 years in the absence of inbreeding depression. However, our model also predicted the population will lose 40-57% of its heterozygosity in 50 years. When we reduced demographic parameters proportional to reductions documented in another wild population of mountain lions that experienced inbreeding depression, extinction probability rose to 99.7%. Simulating greater landscape connectivity by increasing immigration to greater than or equal to one migrant per generation appears sufficient to largely maintain genetic diversity and reduce extinction probability. We provide empirical support for the central tenet of the extinction vortex as interactions between genetics and demography greatly increased extinction probability relative to the risk from demographic and environmental stochasticity alone. Our modelling approach realistically integrates demographic and genetic data to provide a comprehensive assessment of factors threatening small populations. | 693 | no | no good fitness measure, N pop differnt in GD? | NA | no | no |
| 694 | 2017 | 43011 | Steiner, CC; Charter, SJ; Goddard, N; Davis, H; Brandt, M; Houck, ML; Ryder, OA | 2015 | Chromosomal variation and perinatal mortality in San Diego zoo Soemmerring’s gazelles | Chromosomal translocations play a fundamental role in the evolution and speciation of antelopes (Antilopinae, Bovidae), with several species exhibiting polymorphism for centric fusions. For the past 35 years, the San Diego Zoo Global (SDZG) captive population of Soemmerring’s gazelles has revealed complex karyotypes resulting from chromosomal translocations with diploid numbers ranging from 34 to 39. Poor reproductive performance of this species in captivity and elevated mortality the first month of life (perinatal) has been attributed to this chromosomal dynamism. We have extended the studies of karyotypic variation in the SDZG Soemmerring’s gazelle population and analyzed the effect of chromosomal and genetic variation upon perinatal mortality. Karyotypes from 149 captive Soemmerring’s gazelles were evaluated revealing two unreported autosomal combinations, now constituting a total of 15 distinct karyotypes for the 3 Robertsonian centric fusions originally described for this population. Among SDZG founders, distinct chromosomal variation and nuclear and mitochondrial genetic structure were detected corresponding to the institution of origin of the founders. Low levels of genetic distance and nucleotide diversity among individuals, in addition to high relatedness values, suggested that outbreeding is less of a concern than inbreeding for maintaining a sustainable captive population. Finally, analysis of karyotypes of offspring born into the SDZG Soemmerring’s gazelle herds, in conjunction with the maternal karyotype showed association of chromosomal makeup with perinatal mortality. This supports the importance of continuing cytogenetic screening efforts, particularly to evaluate the presence of deleterious chromosomal rearrangements in stillborns. Zoo Biol. 34:374-384, 2015. (c) 2015 Wiley Periodicals Inc. | 694 | no | no good fitness measure, N pop differnt in GD? | NA | no | no |
| 695 | 2017 | 43011 | Keane, B; Ross, S; Crist, TO; Solomon, NG | 2015 | Fine-scale spatial patterns of genetic relatedness among resident adult prairie voles | Characterizing the spatial arrangement of related individuals within populations can convey information about opportunities for the evolution of kin-selected social behaviors, the potential for inbreeding, and the geographic distribution of genetic variation. Prairie voles (Microtus ochrogaster) are socially monogamous rodents that sometimes breed cooperatively. Individuals of both sexes are highly philopatric, and among natal dispersers, the average dispersal distance is about 30 m. Such limited natal dispersal can result in the spatial clustering of kin and we used microsatellite data to estimate genetic relatedness among resident adult prairie voles in 2 natural populations to test the hypothesis that limited natal dispersal of male and female prairie voles results in the spatial clustering of kin. Spatial autocorrelation analyses of nest residency and microsatellite data indicated that proximate same-sex adult residents of both sexes were significantly more related than more spatially distant resident same-sex adults in Kansas. In Indiana, adult female voles residing less than 20 m apart were also significantly more related than more spatially distant resident adult females but spatial clustering of kin was not detected among resident adult males. The spatial clustering of kin indicates that opportunities for kin-selected behaviors exist in both populations, especially among females. Differences in the patterns of spatial genetic structure among resident males between the Kansas and Indiana populations may be due to population differences in factors such as demography and mating system, as well as in the extent of natal philopatry. | 695 | no | no good fitness measure, N pop differnt in GD? | NA | no | no |
| 696 | 2017 | 43011 | Samuels, DC; Wang, J; Ye, F; He, J; Levinson, RT; Sheng, QH; Zhao, SL; Capra, JA; Shyr, Y; Zheng, W; Guo, Y | 2016 | Heterozygosity Ratio, a Robust Global Genomic Measure of Autozygosity and Its Association with Height and Disease Risk | Greater genetic variability in an individual is protective against recessive disease. However, existing quantifications of autozygosity, such as runs of homozygosity (ROH), have proved highly sensitive to genotyping density and have yielded inconclusive results about the relationship of diversity and disease risk. Using genotyping data from three data sets with >43,000 subjects, we demonstrated that an alternative approach to quantifying genetic variability, the heterozygosity ratio, is a robust measure of diversity and is positively associated with the nondisease trait height and several disease phenotypes in subjects of European ancestry. The heterozygosity ratio is the number of heterozygous sites in an individual divided by the number of nonreference homozygous sites and is strongly affected by the degree of genetic admixture of the population and varies across human populations. Unlike quantifications of ROH, the heterozygosity ratio is not sensitive to the density of genotyping performed. Our results establish the heterozygosity ratio as a powerful new statistic for exploring the patterns and phenotypic effects of different levels of genetic variation in populations. | 696 | no | individual level | NA | no | no |
| 697 | 2017 | 43011 | Couchoux, C; Seppa, P; Van Nouhuys, S | 2016 | Strong dispersal in a parasitoid wasp overwhelms habitat fragmentation and host population dynamics | The population dynamics of a parasite depend on species traits, host dynamics and the environment. Those dynamics are reflected in the genetic structure of the population. Habitat fragmentation has a greater impact on parasites than on their hosts because resource distribution is increasingly fragmented for species at higher trophic levels. This could lead to either more or less genetic structure than the host, depending on the relative dispersal rates of species. We examined the spatial genetic structure of the parasitoid wasp Hyposoter horticola, and how it was influenced by dispersal, host population dynamics and habitat fragmentation. The host, the Glanville fritillary butterfly, lives as a metapopulation in a fragmented landscape in the Aland Islands, Finland. We collected wasps throughout the 50 by 70 km archipelago and determined the genetic diversity, spatial population structure and genetic differentiation using 14 neutral DNA microsatellite loci. We compared the genetic structure of the wasp with that of the host butterfly using published genetic data collected over the shared landscape. Using maternity assignment, we also identified full-siblings among the sampled parasitoids to estimate the dispersal range of individual females. We found that because the parasitoid is dispersive, it has low genetic structure, is not very sensitive to habitat fragmentation and has less spatial genetic structure than its butterfly host. The wasp is sensitive to regional rather than local host dynamics, and there is a geographic mosaic landscape for antagonistic co-evolution of host resistance and parasite virulence. | 697 | no | gd of parasite | NA | no | no |
| 698 | 2017 | 43011 | Gilroy, D; van Oosterhout, C; Komdeur, J; Richardson, DS | 2016 | Avian beta-defensin variation in bottlenecked populations: the Seychelles warbler and other congeners | beta-defensins are important components of the vertebrate innate immune system responsible for encoding a variety of anti-microbial peptides. Pathogen-mediated selection is thought to act on immune genes and potentially maintain allelic variation in the face of genetic drift. The Seychelles warbler, Acrocephalus sechellensis, is an endemic passerine that underwent a recent bottleneck in its last remaining population, resulting in a considerable reduction in genome-wide variation. We genotyped avian beta-defensin (AvBD) genes in contemporary (2000-2008) and museum samples (1876-1940) of the Seychelles warbler to investigate whether immunogenetic variation was lost through this bottleneck, and examined AvBD variation across four other Acrocephalus species with varying demographic histories. No variation was detected at four of the six AvBD loci screened in the post-bottleneck population of Seychelles warbler, but two silent nucleotide polymorphisms were identified at AvBD8 and one potentially functional amino-acid variation was observed at AvBD11. Variation in the Seychelles warbler was significantly lower than in the mainland migratory congeneric species investigated, but it similar to that found in other bottlenecked species. In addition, screening AvBD7 in 15 museum specimens of Seychelles warblers sampled prior to the bottleneck (1877-1905) revealed that this locus possessed two alleles previously, compared to the single allele in the contemporary population. Overall, the results show that little AvBD variation remains in the Seychelles warbler, probably as a result of having low AvBD diversity historically rather than the loss of variation due to drift associated with past demographic history. Given the limited pathogen fauna, this lack of variation at the AvBD loci may currently not pose a problem for this isolate population of Seychelles warblers, but it may be detrimental to the species’ long-term survival if new pathogens reach the population in the future. | 698 | no | No GD diff between pop | NA | no | no |
| 699 | 2017 | 43011 | Carazo, P; Perry, JC; Johnson, F; Pizzari, T; Wigby, S | 2015 | Related male Drosophila melanogaster reared together as larvae fight less and sire longer lived daughters | Competition over access to reproductive opportunities can lead males to harm females. However, recent work has shown that, in Drosophila melanogaster, male competition and male harm of females are both reduced under conditions simulating male-specific population viscosity (i.e., in groups where males are related and reared with each other as larvae). Here, we seek to replicate these findings and investigate whether male population viscosity can have repercussions for the fitness of offspring in the next generation. We show that groups of unrelated-unfamiliar (i.e., unrelated individuals raised apart) males fight more intensely than groups of related-familiar males (i.e., full siblings raised together as larvae), supporting previous findings, and that exposure to a female is required to trigger these differential patterns of male-male competition. Importantly, we show that differences in male-male competition can be associated with transgenerational effects: the daughters of females exposed to unrelated-unfamiliar males suffered higher mortality than the daughters of females exposed to related-familiar males. Collectively, these results suggest that population structure (i.e., variation in the relatedness and/or larval familiarity of local male groups) can modulate male-male competition with important transgenerational consequences. | 699 | probably no | no good fitness measure, N pop differnt in GD? | no good fitness measure | no | no |
| 700 | 2017 | 43011 | SPITZE, K | 1991 | CHAOBORUS PREDATION AND LIFE-HISTORY EVOLUTION IN DAPHNIA-PULEX - TEMPORAL PATTERN OF POPULATION DIVERSITY, FITNESS, AND MEAN-LIFE HISTORY | The effect of predation by the aquatic dipteran larva Chaoborus americanus on genetic diversity and life-history evolution in the cladoceran Daphnia pulex was investigated in large replicate laboratory populations. Instantaneous daily loss rates of clonal diversity and genetic variance for fitness indicate that 93-99% of initial genetic diversity can be removed from populations during the 8-12 generations of clonal reproduction that occur each year in natural populations. In the absence of predation, the principal evolved changes in mean population life history were smaller immature body size and increased and earlier fecundity. In the presence of size-selective Chaoborus predation, populations evolved toward larger body size and increased and earlier reproduction. The difference between these two trajectories is an estimate of the direct additive effect of Chaoborus predation. This effect was manifested as evolution toward larger body size with a trend toward earlier and increased reproduction. | 700 | no | no good fitness measure | NA | no | no |
| 701 | 2017 | 43011 | Suwannakut, S; Boucias, DG; Wiwat, C | 2005 | Genotypic analysis of Nomuraea rileyi collected from various noctuid hosts | The genetic diversity of 79 Nomuraea rileyi isolates from various lepidopteran hosts in Asia, North America, and South America was evaluated using amplified fragment length polymorphism (AFLP) analysis. Cluster analysis separated the X rileyi isolates into two major groups and seven subgroups. The resulting dendrogram generally classified the N. rileyi isolates based on insect host and geographical region. The haplotypic diversity index of N. rileyi subpopulations from each location and host origin was ranging from 0.8788 to 1.000. However, analysis of molecular variance (AMOVA) demonstrated no significant differences (p = 0.3421) among N. rileyi isolates from different continents. Whereas the genetic variation among the N. rileyi populations from the different host insects within each continent was significantly different (p < 0.0001). (c) 2005 Elsevier Inc. All rights reserved. | 701 | no | gd of pathogen | NA | no | no |
| 702 | 2017 | 43011 | Jacquemyn, H; Roldan-Ruiz, I; Honnay, O | 2010 | Evidence for demographic bottlenecks and limited gene flow leading to low genetic diversity in a rare thistle | European calcareous grasslands have decreased dramatically in area and number during the last two centuries. As a result, many populations of calcareous grassland species are confined to small and isolated fragments, where their long-term survival is to some extent uncertain. Recently, several restoration projects have been initiated to enlarge the current grassland area in order to maintain the exceptionally high species richness. However, from a genetic point of view, the success of these restoration measures is not necessarily guaranteed, as strong historical decreases in population size and limited gene flow may have led to low genetic diversity through genetic bottlenecks and drift. In this study, we investigated genetic diversity and structure of 16 populations of the calcareous grassland specialist Cirsium acaule in a severely fragmented landscape in south-western Belgium. The overall distribution of this species in the study area was significantly and positively related to patch area, suggesting that small patches do not allow survival of this species. Both allelic richness and genetic diversity were significantly and positively related to population size. Estimation of observed and expected gene diversity provided evidence for population bottlenecks in the history of not less than 31% of all sampled populations. Reconstruction of the historical land use showed that patch area decline in populations that went through a recent bottleneck was significantly larger than that in populations that showed no evidence of a bottleneck. Assignment analyses showed low migration rates, suggesting that replenishment of lost alleles through gene flow is highly unlikely. Overall, our results indicate that in the absence of gene flow strong decreases in calcareous grassland area may have long-lasting effects on genetic diversity of plant populations and may hamper the success of restoration projects that simply aim at restoring initial habitat conditions or enlarging population fragments, as indicated by the fact that none of the recently restored areas has been occupied by C. acaule. | 702 | no | no good fitness measure | NA | no | no |
| 703 | 2017 | 43011 | Parks, A; Jenkins, M; Ostry, M; Zhao, P; Woeste, K | 2014 | Biotic and abiotic factors affecting the genetic structure and diversity of butternut in the southern Appalachian Mountains, USA | The abundance of butternut (Juglans cinerea L.) trees has severely declined rangewide over the past 50 years. An important factor in the decline is butternut canker, a disease caused by the fungus Ophiognomonia clavigigenti-juglandacearum, which has left the remaining butternuts isolated and sparsely distributed. To manage the remaining populations effectively, information regarding how butternut’s population genetic structure is affected by environmental and historical factors is needed. In this study, we assessed genetic structure and diversity of 161 butternut trees from 19 adjacent watersheds in the southern portion of butternut’s range using 12 microsatellite markers. We assessed the genetic diversity and genetic differentiation among trees grouped at various spatial scales. Our goal was to use historical abundance and land use data for these watersheds, which are now all a part of the Great Smoky Mountains National Park (GSMNP), to understand the ecological and evolutionary forces that challenge the conservation and management of butternut. In general, butternuts within the 19 neighboring watersheds were all part of one continuous population, with gene flow throughout. Significant genetic differentiation was detected between some groups of trees, but the differentiation was quite small and may not represent an ecologically significant distinction. The mean heterozygosity in all watersheds remained high, despite extensive mortality. Overall, genetic diversity and rare alleles were evenly distributed across all watersheds, with some variability in subpopulations containing butternut-Japanese walnut hybrids (Juglans x bixbyi or buarts). These results indicate that management of this species should focus on protection from future hybridization with Japanese walnut, promotion of regeneration, and persistence of all remaining butternut trees, which still retain high levels of genetic diversity. | 703 | no | no good fitness measure, N pop differnt in GD? | NA | no | no |
| 704 | 2017 | 43011 | Johnson, CH; Woollacott, RM | 2010 | Larval settlement preference maximizes genetic mixing in an inbreeding population of a simultaneous hermaphrodite (Bugula stolonifera, Bryozoa) | Conspecific aggregations in terrestrial and aquatic organisms can have a significant effect on an individual’s survival, growth and reproductive fitness, particularly if these aggregations are composed of closely related individuals. Such aggregations can form passively, as a consequence of dispersal, or actively, as a consequence of kin recognition. In this study, we investigated the genetic composition of individuals in conspecific aggregations in the simultaneous hermaphroditic marine bryozoan Bugula stolonifera. Conspecific larvae routinely metamorphose on adult colonies; the possibility that larvae select or avoid their maternal colony was investigated utilizing 10 newly developed polymorphic microsatellite loci. Adult colonies were collected from Eel Pond, Woods Hole, Massachusetts and inspected for the presence of attached individuals. Adult colonies and their attached individuals were genotyped and compared to assess genetic relatedness within and among these groups relative to the overall genetic variability of the sampling site. Overall, the population of B. stolonifera at this site was found to be outside Hardy-Weinberg equilibrium because of significant levels of inbreeding. No significant genetic differentiation, however, was found between any groups, documenting that a group containing an adult colony and its attached individuals had as much genetic variability as was found for the entire sampling site. Parentage-exclusion analyses showed that the vast majority of attached individuals (>93%) could not have derived from the colony on which they were attached. Kinship analyses showed that the majority of attached individuals (approximate to 63%) shared less than a half-sibling relationship. These results suggest that a colony’s nearest neighbours are not composed of siblings, and thus, larval settlement preference can maximize outcrossing in this inbreeding population. | 704 | no | no good fitness measure, N pop differnt in GD? (inbreeding?) | NA | no | no |
| 705 | 2017 | 43011 | Reunova, GD; Kats, IL; Muzarok, TI; Zhuravlev, YN | 2010 | Polymorphism of RAPD, ISSR and AFLP markers of the Panax ginseng C. A. Meyer (Araliaceae) genome | The genus Panax (Araliaceae) is world-famous because many its members have important medicinal properties. Panax ginseng C. A. Meyer is more popular than other species of the genus because remedies prepared from this plant stimulate immunity, help to prevent diseases, and have antistress effects. In addition, the ginseng root extract is traditionally used as a means against aging. At present, this species is found in the wild only in Primorsky krai, Russia, but its populations are extremely exhausted and need to be restored. In this study, effectiveness of molecular DNA markers in detecting genetic variation and differentiation of the ginseng populations was tested. Genetic variation of ginseng, identified using RAPD (P = 4%; H (pop) = 0.0130) and ISSR (P = 9.3%; H (pop) = 0.0139) markers was low. The AFLP\* approach, according to which amplicons are separated in polyacrylamide gel and visualized by means of silver staining, showed somewhat higher variability (P = 21.8%; H (pop) = 0.0509), while its effectiveness in population differentiation was as low as that of RAPD and ISSR. The AFLP\*\* technique, which included analysis of the fragments using genetic analyzer, revealed high genetic diversity of ginseng (P = 94.4%; H (pop) = 0.3246). All populations examined using the AFLP\*\* markers were statistically significantly differentiated based on the AMOVA results. Our result suggest effectiveness of AFLP\*\* markers for characterization of the genetic structure and genetic relationships of the ginseng populations. These markers are recommended for use in large-scale population genetic studies of this species to develop measures of its conservation. | 705 | no | no good fitness measure, N pop differnt in GD? | NA | no | no |
| 706 | 2017 | 43011 | Dias, LAD; Kageyama, PY | 1997 | Multivariate genetic divergence and hybrid performance of cacao (Theobroma cacao L) | Genetic distances among cacao cultivars were calculated through multivariate analysis, using the D-2 statistic, to examine racial group classification and to assess heterotic hybrids. A 5 x 5 complete diallel was evaluated. Over a five-year period (1986-1990), five cultivars of the S-1 generation, pertaining to the Lower Amazon Forastero and Trinitario racial groups and 20 crosses between the corresponding So parents were analyzed, based upon five yield components - number of healthy and collected fruits per plant (NHFP and NCFP), wet seed weight per plant and per fruit (WSWP and WSWF), and percentage of diseased fruits per plant (PDFP). The diversity analysis suggested a close relationship between the Trinitario and Lower Amazon Forastero groups. A correlation coefficient (r) was calculated to determine the association between genetic diversity and heterosis. Genetic distance of parents by D-2 was found to be linearly related to average performance of hybrids for WSWP and WSWF (r = 0.68, P < 0.05 and r = 0.76, P < 0.05, respectively). The heterotic performance for the same components was also correlated with D-2, both with r = 0.66 (P < 0.05). A relationship between genetic divergence and combining ability effects was suggested because the most divergent cultivar exhibited a high general combining ability, generating the best performing hybrids. Results indicated that genetic diversity estimates can be useful in selecting parents for crosses and in assessing relationships among cacao racial groups. | 706 | no | no good fitness measure | NA | no | no |
| 707 | 2017 | 43011 | Duffy, KJ; Fay, MF; Smith, RJ; Stout, JC | 2011 | POPULATION GENETICS AND CONSERVATION OF THE SMALL WHITE ORCHID, PSEUDORCHIS ALBIDA, IN IRELAND | Genetic diversity can play a key role in the survival of endangered species. Both the number and size of populations of a species can affect genetic diversity and may be reduced by fragmentation of natural habitats. Here we examine the effects of a low number of populations and small population sizes on the population genetics of the nationally endangered and red-listed orchid Psettdorchis albida. To determine genetic variability within and among populations of P. albida, we employed AFLP and four plastid microsatellite (SSR) markers. In addition, we compared the SSR data from Irish P. albida to the closely related P. straminea. AFLP markers showed that P. albida has a high level of polymorphism and that significant differentiation occurred both within and among populations, with increasing population genetic isolation through distance. However, SSR markers revealed no variation, but did show that all Irish individuals had the same alleles as P. straminea rather than P. albida. Further work is required to assess the taxonomic status of P. albida/straminea in Western Europe. Irish populations of P. albida s.l. require immediate conservation attention due to their decreasing number and low numbers of individuals within populations. | 707 | no | no good fitness measure, N pop differnt in GD? | NA | no | no |
| 708 | 2017 | 43011 | Miura, O; Kanaya, G; Nakai, S; Itoh, H; Chiba, S; Makino, W; Nishimura, T; Kojima, S; Urabe, J | 2017 | Ecological and genetic impact of the 2011 Tohoku Earthquake Tsunami on intertidal mud snails | Natural disturbances often destroy local populations and can considerably affect the genetic properties of these populations. The 2011 Tohoku Earthquake Tsunami greatly damaged local populations of various coastal organisms, including the mud snail Batillaria attramentaria, which was an abundant macroinvertebrate on the tidal flats in the Tohoku region. To evaluate the impact of the tsunami on the ecology and population genetic properties of these snails, we monitored the density, shell size, and microsatellite DNA variation of B. attramentaria for more than ten years (2005-2015) throughout the disturbance event. We found that the density of snails declined immediately after the tsunami. Bayesian inference of the genetically effective population size (Ne) demonstrated that the Ne declined by 60-99% at the study sites exposed to the tsunami. However, we found that their genetic diversity was not significantly reduced after the tsunami. The maintenance of genetic diversity is essential for long-term survival of local populations, and thus, the observed genetic robustness could play a key role in the persistence of snail populations in this region which has been devastated by similar tsunamis every 500-800 years. Our findings have significant implications for understanding the sustainability of populations damaged by natural disturbances. | 708 | no | no good fitness measure | NA | no | no |
| 709 | 2017 | 43011 | Wagenius, S; Hangelbroek, HH; Ridley, CE; Shaw, RG | 2010 | BIPARENTAL INBREEDING AND INTERREMNANT MATING IN A PERENNIAL PRAIRIE PLANT: FITNESS CONSEQUENCES FOR PROGENY IN THEIR FIRST EIGHT YEARS | Despite fundamental importance to population dynamics, mating system evolution, and conservation management, the fitness consequences of breeding patterns in natural settings are rarely directly and rigorously evaluated. We experimentally crossed Echinacea angustifolia, a widespread, perennial prairie plant undergoing radical changes in distribution and abundance due to habitat fragmentation. We quantified the effects of both biparental inbreeding and crossing between remnant populations on progeny survival and reproduction in the field over the first eight years. Lifetime fitness is notoriously difficult to assess particularly for iteroparous species because of the long sequence and episodic nature of selection events. Even with fitness data in hand, analysis is typically plagued by nonnormal distributions of overall fitness that violate the assumptions of the usual parametric statistical approaches. We applied aster modeling, which integrates the measurements of separate, sequential, nonnormally distributed annual fitness components, and estimated current biparental inbreeding depression at 68% in progeny of sibling-mating. The effect of between-remnant crossing on fitness was negligible. Given that relatedness among individuals in remnant populations is already high and dispersal very limited, inbreeding depression may profoundly affect future dynamics and persistence of these populations, as well as their genetic composition. | 709 | no | inbreeding | NA | no | no |
| 710 | 2017 | 43011 | Lyon, R; Correll, J; Feng, C; Bluhm, B; Shrestha, S; Shi, A; Lamour, K | 2016 | Population Structure of Peronospora effusa in the Southwestern United States | Peronospora effusa is an obligate pathogen that causes downy mildew on spinach and is considered the most economically important disease of spinach. The objective of the current research was to assess genetic diversity of known historical races and isolates collected in 2014 from production fields in Yuma, Arizona and Salinas Valley, California. Candidate neutral single nucleotide polymorphisms (SNPs) were identified by comparing sequence data from reference isolates of known races of the pathogen collected in 2009 and 2010. Genotypes were assessed using targeted sequencing on genomic DNA extracted directly from infected plant tissue. Genotyping 26 historical and 167 contemporary samples at 46 SNP loci revealed 82 unique multi-locus genotypes. The unique genotypes clustered into five groups and the majority of isolates collected in 2014 were genetically closely related, regardless of source location. The historical samples, representing several races, showed greater genetic differentiation. Overall, the SNP data indicate much of the genotypic variation found within fields was produced during asexual development, whereas overall genetic diversity may be influenced by sexual recombination on broader geographical and temporal scales. | 710 | no | gd of pathogen | NA | no | no |
| 711 | 2017 | 43011 | Koren, OG; Yatsunskaya, MS; Nakonechnaya, OV | 2012 | Low level of allozyme polymorphism in relict aquatic plants of the Far East Nelumbo komarovii Grossh. and Euryale ferox Salisb. | Using allozyme analysis, genetic variation of two relict aquatic plants from Primorsky krai, Komarov lotus (Nelumbo komarovii Grossh.) and Gorgon plant (Euryale ferox Salisb.), was examined. The absence of allozyme variation in the Primorye populations of Nelumbo komarovii along with low polymorphism level in the population of Euryale ferox (P (95) = 7.69; A = 1.07; H (o) = 0.072; H (e) = 0.038) was demonstrated. Since the data for the species examined are reported for the first time ever, the phenotypes and genetic interpretation of the enzyme systems tested are presented. The isoenzyme profiles of N. komarovii were compared with the data reported for N. nucifera from China. The absence of allozyme variation in N. komarovii, along with extremely low level of variation revealed for E. ferox, is discussed in association with the evolutionary histories of these species, their dispersal after the Pleistocene-Holocene cooling, and survival on this territory in range boundaries. | 711 | no | no good fitness measure, N pop differnt in GD? | NA | no | no |
| 712 | 2017 | 43011 | Ansell, SW; Stenoien, HK; Grundmann, M; Schneider, H; Hemp, A; Bauer, N; Russell, SJ; Vogel, JC | 2010 | Population structure and historical biogeography of European Arabidopsis lyrata | Understanding the natural history of model organisms is important for the effective use of their genomic resourses. Arabidopsis lyrata has emerged as a useful plant for studying ecological and evolutionary genetics, based on its extensive natural variation, sequenced genome and close relationship to A. thaliana. We studied genetic diversity across the entire range of European Arabidopsis lyrata ssp. petraea, in order to explore how population history has influenced population structure. We sampled multiple populations from each region, using nuclear and chloroplast genome markers, and combined population genetic and phylogeographic approaches. Within-population diversity is substantial for nuclear allozyme markers (mean P = 0.610, A(e) = 1.580, H-e = 0.277) and significantly partitioned among populations (F-ST = 0.271). The Northern populations have modestly increased inbreeding (F-IS = 0.163 verses F-IS = 0.093), but retain comparable diversity to central European populations. Bottlenecks are common among central and northern Europe populations, indicating recent demographic history as a dominant factor in structuring the European diversity. Although the genetic structure was detected at all geographic scales, two clear differentiated units covering northern and central European areas (F-CT = 0.155) were identified by Bayesian analysis and supported by regional pairwise F-CT calculations. A highly similar geographic pattern was observed from the distribution of chloroplast haplotypes, with the dominant northern haplotypes absent from central Europe. We conclude A. l. petraea’s cold-tolerance and preference for disturbed habitats enabled glacial survival between the alpine and Nordic glaciers in central Europe and an additional cryptic refugium. While German populations are probable peri-glacial leftovers, Eastern Austrian populations have diversity patterns possibly compatible with longer-term survival. Heredity (2010) 105, 543-553; doi:10.1038/hdy.2010.10; published online 17 February 2010 | 712 | no | inbreeding | NA | no | no |
| 713 | 2017 | 43011 | Olano-Marin, J; Mueller, JC; Kempenaers, B | 2011 | Heterozygosity and survival in blue tits (Cyanistes caeruleus): contrasting effects of presumably functional and neutral loci | The relationship between genetic diversity and fitness has important implications in evolutionary and conservation biology. This relationship has been widely investigated at the individual level in studies of heterozygosity-fitness correlations (HFC). General effects caused by inbreeding and/or local effects at single loci have been used as explanations of HFC, but the debate about the causes of HFC in open, natural populations is still ongoing. Study designs that control for variation in the inbreeding level of the individuals, and knowledge on the function and location of the markers used to measure heterozygosity, are fundamental to understand the causes of HFC. Here we investigated correlations between individual heterozygosity and estimates of survival at different life-history stages in an open population of blue tits (Cyanistes caeruleus). For survival at the embryo, nestling and fledgling stage, we used a full-sibling approach, i.e. we controlled for the level of inbreeding. We genotyped 1496 individuals with 79 microsatellites mapped across 25 chromosomes in the zebra finch (Taeniopygia guttata) that were classified either as potentially functional (58 loci) or as neutral (21 loci). We found different effects of standardized multilocus heterozygosity (SH): SHfunctional had a negative effect on the probability of hatching and local recruitment of females, whereas SHneutral had a positive effect on adult survival. The negative effects of functional loci are better explained by local effects, whereas the positive effects of neutral markers could reflect inbreeding effects in the population. Our results highlight the importance of considering the characteristics of the markers used in HFC studies and confirm the mixed effects of heterozygosity in different contexts (e.g. sex and life-history stage). | 713 | no | inbreeding | NA | no | no |
| 714 | 2017 | 43011 | TRANVIK, L; SJOGREN, M; BENGTSSON, G | 1994 | ALLOZYME POLYMORPHISM AND PROTEIN PROFILE IN ORCHESELLA-BIFASCIATA (COLLEMBOLA) - INDICATIVE OF EXTENDED METAL POLLUTION | Allozyme variation and protein profiles of Orchesella bifasciata (Collembola) populations collected from a range of metal polluted forest soils were compared using starch gel and polyacrylamide electrophoresis. The purpose of the work was to examine whether simple biochemical expressions for genetic differences can be used to distinguish individuals collected at a metal polluted field site from those collected at an unpolluted site. Significant differences were observed between populations in allele and genotype frequencies and in survival rates, but there was no significant correlation between the genetic differences and the metal concentration in the litter. The absence of consistent effects of metals on the allozyme and protein pattern may be explained by a high degree of genetic exchange between the populations, selection pressures other than metals dominating the genetic variation among populations or simply an absence of selection at the studied, or at any linked loci. The genetic homogeneity among populations was high, and the value of Nei’s genetic distance ranged from 0.001 to 0.016. Calculations of genetic divergence (F-ST) indicate that less than 2% of the total variation in O. bifasciata was due to genetic differences between populations. If this value is translated to a number of migrants, the populations may exchange 10-50 individuals per generation. Among the 32 identified proteins two (120 and 189 kDa) were detected at a significantly higher frequency in individuals from the metal polluted soil. Classical stress proteins in the range of 60-80 kDa were not detected. It is suggested that the resolution of one-dimensional gel electrophoresis is too low to distinguish a resistant population from a non-resistant without prior experimental exposure to high metal concentrations. | 714 | no | no good fitness measure, N pop differnt in GD? | NA | no | no |
| 715 | 2017 | 43011 | Zhang, M; He, HX | 2013 | Parasite-mediated selection of major histocompatibility complex variability in wild brandt’s voles (Lasiopodomys brandtii) from Inner Mongolia, China | Background: Genes of the major histocompatibility complex (MHC) exhibit high levels of variability, which is believed to have arisen through pathogen-mediated selection. We investigated the relationship between parasite load and genetic diversity at selectively neutral, non-coding markers (microsatellites) and adaptive genetic variation at a functionally important part of the MHC in six independent natural populations of Brandt’s voles (Lasiopodomys brandtii) from two regions of the Xilingol Grassland area of Inner Mongolia. Results: Two-hundred and fifty-two voles were screened for gastrointestinal parasites, and were assessed for genetic variation. Parasite screening was done through non-invasive fecal egg counts, while allelic diversity was determined via single-stranded conformation polymorphism and DNA sequencing. We detected eight distinct helminth egg morphotypes. A total of 10 microsatellite loci were genotyped and 19 unique MHC class II B alleles were isolated. The rate of nonsynonymous substitutions (d(N)) exceeded the rate of synonymous substitutions (d(S)) at putative antigen binding sites of DRB. Neutral and adaptive genetic diversity differed between the six vole populations. To test the main pathogen-driven selection hypotheses for the maintenance of host MHC diversity and parasite species-specific co-evolutionary effects, multivariate approaches (generalized linear mixed models) were used to test for associations between the MHC class II DRB genotype and infections with nematodes. We found no evidence for heterozygote advantage, and overall heterozygosity was lower than expected in the MHC alleles. We identified an association between the parasite load and specific MHC alleles in the voles, and this pattern varied between geographic regions. Conclusions: The results suggest that MHC variability in Brandt’s voles is maintained by rare allele advantage and fluctuating selection, but the data failed to show any heterozygote advantage effect. Our results add to a growing body of evidence showing that the mode and relative strength of pathogen-driven selection acting on MHC diversity varies within specific wild populations. In addition, our study contributes to the understanding of what maintains MHC diversity, of host-pathogen coevolution and of how genetic diversity is maintained in voles. | 715 | yes | fintess and gd ok? | MHC | yes | no |
| 716 | 2017 | 43011 | Baer, B; Schmid-Hempel, P | 2001 | Unexpected consequences of polyandry for parasitism and fitness in the bumblebee, Bombus terrestris | Multiple mating by females (polyandry) is taxonomically widespread but the evolution of such behaviors is not clearly understood given potential costs of polyandry such as time, energy, or predation risk. The genetic variability versus parasites hypothesis predicts a reduction of parasitism due to increased genetic variability among offspring and an associated fitness gain. We tested this hypothesis with a field experiment in the bumblebee, Bombus terrestris L. Worker heterogeneity within the colony was experimentally altered by artificially inseminating queens with sperm from one male, four brothers, two males, or four unrelated males, We found genetic variability to be effective, because intensity and prevalence of the most common parasite, Crithidia bombi, a trypanosome, decreased with increasing levels of colony heterogeneity. Fitness differed between treatments but did not increase in a simple way, with increasing genetic heterogeneity among colony workers. Instead, fitness followed a U-shaped function with a minimum for small amounts of genetic heterogeneity. We therefore suggest that polyandry also induces a cost, perhaps as a result of the social structure within the colony. In evolutionary terms, singly mated females appear to be constrained by an adaptive valley that needs to be crossed before high degrees of mating frequency can be reached. This may help to explain why B. terrestris and most other social insects are often monandrous. | 716 | yes | from thesis | NA | no | yes |
| 717 | 2017 | 43011 | Lloret, F; Garcia, C | 2016 | Inbreeding and neighbouring vegetation drive drought-induced die-off within juniper populations | Drought-induced episodes of forest die-off are being reported world-wide, and recent evidence shows that increases in temperature due to climate change are contributing to this decline. Several ecological mechanisms have been invoked at the plant level to explain the vulnerability of trees to this widespread drought-induced mortality. Apart from ecological factors, some genetic factors might also influence population responses to disturbances: it has been reported, for example, that genetic diversity enhances population resilience through genotypic complementarity, whereas genetic relatedness between conspecific individuals exerts a negative influence on variations in fecundity. However, we lack empirical data that prove any functional relationship between levels of individual inbreeding or kinship and patterns of drought-induced forest die-off resulting from field conditions. Here, we investigated the role of ecological, phenotypic and genetic factors in determining the response of trees to prolonged drought conditions. We focused on populations of the small tree Juniperus phoenicea L. that recently experienced severe drought-induced die-off in semi-arid woodlands from central Spain. We correlated mean individual inbreeding (f) and mean kinship between individuals within a plot (f(ij)) with remaining green plant canopy and crop size. We found that f was negatively correlated with plant performance, whereas f(ij) was not. Healthier canopy also tended to correspond with large plants and neighbourhoods dominated by trees and shrubs, suggesting a role for facilitative interactions and microsite heterogeneity. Accordingly, the probability of severe damage increased linearly with f, and individuals growing in open habitats with f values > 0<bold></bold>625 only had 15% chance of remaining unaffected after a period of prolonged drought. We show that both vegetative and reproductive responses to prolonged drought episodes are determined by multiple factors, including individual inbreeding. Therefore, any fuller understanding of phenomena such as drought-induced forest die-off needs to incorporate the contribution of individual genetic variations in conjunction with plant-plant interactions. | 717 | no | inbreeding | NA | no | no |
| 718 | 2017 | 43011 | Crawford, JE; Bischoff, E; Garnier, T; Gneme, A; Eiglmeier, K; Holm, I; Riehle, MM; Guelbeogo, WM; Sagnon, N; Lazzaro, BP; Vernick, KD | 2012 | Evidence for Population-Specific Positive Selection on Immune Genes of Anopheles gambiae | Host-pathogen interactions can be powerful drivers of adaptive evolution, shaping the patterns of molecular variation at the genes involved. In this study, we sequenced alleles from 28 immune-related loci in wild samples of multiple genetic subpopulations of the African malaria mosquito Anopheles gambiae, obtaining unprecedented sample sizes and providing the first opportunity to contrast patterns of molecular evolution at immune-related loci in the recently discovered GOUNDRY population to those of the indoor-resting M and S molecular forms. In contrast to previous studies that focused on immune genes identified in laboratory studies, we centered our analysis on genes that fall within a quantitative trait locus associated with resistance to Plasmodium falciparum in natural populations of A. gambiae. Analyses of haplotypic and genetic diversity at these 28 loci revealed striking differences among populations in levels of genetic diversity and allele frequencies in coding sequence. Putative signals of positive selection were identified at 11 loci, but only one was shared by two subgroups of A. gambiae. Striking patterns of linkage disequilibrium were observed at several loci. We discuss these results with respect to ecological differences among these strata as well as potential implications for disease transmission. | 718 | no | no good fitness measure, N pop differnt in GD? | NA | no | no |
| 719 | 2017 | 43011 | VANDERBANK, FH; SWART, MKI; FERREIRA, JT | 1992 | GENETIC-VARIATION IN 9 RAINBOW-TROUT (ONCORHYNCHUS-MYKISS) POPULATIONS IN SOUTH-AFRICA | 1. Twenty-six protein coding loci in nine populations of rainbow trout, from different localities in South Africa, were analyzed by starch and polyacrylamide gel electrophoresis to estimate the amount of genetic diversity present. 2. Heterozygosity ranged between 0.0445 and 0.0745. 3. The relatively high heterozygosity levels are probably due to the historical mixing of strains, and the differences in heterozygosity indicate that the populations originate from different sources, each with different genetical characteristics. 4. Differences in phenotypic characters (i.e. growth) seem to relate to genotypic variation, whilst food conversion rate and survival performance results did not seem to relate to genotypic variation. | 719 | no | no good fitness measure | NA | no | no |
| 720 | 2017 | 43011 | Kim, J; Hanotte, O; Mwai, OA; Dessie, T; Bashir, S; Diallo, B; Agaba, M; Kim, K; Kwak, W; Sung, S; Seo, M; Jeong, H; Kwon, T; Taye, M; Song, KD; Lim, D; Cho, S; Lee, HJ; Yoon, D; Oh, SJ; Kemp, S; Lee, HK; Kim, H | 2017 | The genome landscape of indigenous African cattle | Background: The history of African indigenous cattle and their adaptation to environmental and human selection pressure is at the root of their remarkable diversity. Characterization of this diversity is an essential step towards understanding the genomic basis of productivity and adaptation to survival under African farming systems. Results: We analyze patterns of African cattle genetic variation by sequencing 48 genomes from five indigenous populations and comparing them to the genomes of 53 commercial taurine breeds. We find the highest genetic diversity among African zebu and sanga cattle. Our search for genomic regions under selection reveals signatures of selection for environmental adaptive traits. In particular, we identify signatures of selection including genes and/or pathways controlling anemia and feeding behavior in the trypanotolerant N’Dama, coat color and horn development in Ankole, and heat tolerance and tick resistance across African cattle especially in zebu breeds. Conclusions: Our findings unravel at the genome-wide level, the unique adaptive diversity of African cattle while emphasizing the opportunities for sustainable improvement of livestock productivity on the continent. | 720 | no | domesticated animal | NA | no | no |
| 721 | 2017 | 43011 | Cervantes, I; Gutierrez, JP; Meuwissen, THE | 2016 | Response to selection while maximizing genetic variance in small populations | Background: Rare breeds represent a valuable resource for future market demands. These populations are usually well-adapted, but their low census compromises the genetic diversity and future of these breeds. Since improvement of a breed for commercial traits may also confer higher probabilities of survival for the breed, it is important to achieve good responses to artificial selection. Therefore, efficient genetic management of these populations is essential to ensure that they respond adequately to genetic selection in possible future artificial selection scenarios. Scenarios that maximize the maximum genetic variance in a unique population could be a valuable option. The aim of this work was to study the effect of the maximization of genetic variance to increase selection response and improve the capacity of a population to adapt to a new environment/production system. Results: We simulated a random scenario (A), a full-sib scenario (B), a scenario applying the maximum variance total (MVT) method (C), a MVT scenario with a restriction on increases in average inbreeding (D), a MVT scenario with a restriction on average individual increases in inbreeding (E), and a minimum coancestry scenario (F). Twenty replicates of each scenario were simulated for 100 generations, followed by 10 generations of selection. Effective population size was used to monitor the outcomes of these scenarios. Although the best response to selection was achieved in scenarios B and C, they were discarded because they are unpractical. Scenario A was also discarded because of its low response to selection. Scenario D yielded less response to selection and a smaller effective population size than scenario E, for which response to selection was higher during early generations because of the moderately structured population. In scenario F, response to selection was slightly higher than in Scenario E in the last generations. Conclusions: Application of MVT with a restriction on individual increases in inbreeding resulted in the largest response to selection during early generations, but if inbreeding depression is a concern, a minimum coancestry scenario is then a valuable alternative, in particular for a long-term response to selection. | 721 | no | NA | NA | no | no |
| 722 | 2017 | 43011 | Barrett, LG; Thrall, PH; Burdon, JJ; Nicotra, AB; Linde, CC | 2008 | Population structure and diversity in sexual and asexual populations of the pathogenic fungus Melampsora lini | Many pathogens undergo both sexual and asexual reproduction to varying degrees, yet the ecological, genetic and evolutionary consequences of different reproductive strategies remain poorly understood. Here we investigate the population genetic structure of wild populations of the plant pathogen Melampsora lini on its host Linum marginale, using amplified fragment length polymorphism (AFLP) markers, two genes underlying pathogen virulence, and phenotypic variation in virulence. In Australia, M. lini occurs as two genetically and geographically divergent lineages (AA and AB), one of which is completely asexual (AB), and the other able to reproduce both clonally and sexually (AA). To quantify the genetic and evolutionary consequences of these different life histories, we sampled five populations in each of two biogeographical regions. Analysis of AFLP data obtained for 275 isolates revealed largely disjunct geographical distributions for the two different lineages, low genetic diversity within lineages, and strong genetic structure among populations within each region. We also detected significant divergence among populations for both Avr genes and virulence phenotypes, although generally these values were lower than those obtained with AFLP markers. Furthermore, isolates belonging to lineage AA collectively harboured significantly higher genotypic and phenotypic diversity than lineage AB isolates. Together these results illustrate the important roles of reproductive modes and geographical structure in the generation and maintenance of virulence diversity in populations of M. lini. | 722 | no | gd of pathogen | NA | no | no |
| 723 | 2017 | 43011 | Banks, SC; Lorin, T; Shaw, RE; McBurney, L; Blair, D; Blyton, MDJ; Smith, AL; Pierson, JC; Lindenmayer, DB | 2015 | Fine-scale refuges can buffer demographic and genetic processes against short-term climatic variation and disturbance: a 22-year case study of an arboreal marsupial | Ecological disturbance and climate are key drivers of temporal dynamics in the demography and genetic diversity of natural populations. Microscale refuges are known to buffer species’ persistence against environmental change, but the effects of such refuges on demographic and genetic patterns in response to short-term environmental variation are poorly understood. We quantified demographic and genetic responses of mountain brushtail possums (Trichosurus cunninghami) to rainfall variability (1992-2013) and to a major wildfire. We hypothesized that there would be underlying differences in demographic and genetic processes between an unburnt mesic refuge and a topographically exposed zone that was burnt in 2009. Fire caused a 2-year decrease in survival in the burnt zone, but the population grew after the fire due to immigration, leading to increased expected heterozygosity. We documented a fire-related behavioural shift, where the rate of movement by individuals in the unburnt refuge to the burnt zone decreased after fire. Irrespective of the fire, there were long-term differences in demographic and genetic parameters between the mesic/unburnt refuge and the nonmesic/burnt zone. Survival was high and unaffected by rainfall in the refuge, but lower and rainfall-dependent in the nonmesic zone. Net movement of individuals was directional, from the mesic refuge to the nonmesic zone, suggesting fine-scale source-sink dynamics. There were higher expected heterozygosity (H-E) and temporal genetic stability in the refuge, but lower H-E and marked temporal genetic structure in the exposed habitat, consistent with reduced generational overlap caused by elevated mortality and immigration. Thus, fine-scale refuges can mediate the short-term demographic and genetic effects of climate and ecological disturbance. | 723 | no | no good fitness measure, N pop differnt in GD? | NA | no | no |
| 724 | 2017 | 43011 | LAMBIN, X; KREBS, CJ | 1991 | CAN CHANGES IN FEMALE RELATEDNESS INFLUENCE MICROTINE POPULATION-DYNAMICS | We present a model relating fluctuations in density of vole and lemming populations to changes in the degree of relatedness among females. We propose that a high degree of relatedness prior to the spring decline causes more overlap between female home ranges and leads to a high density of breeding females in spring and vole outbreak densities in summer. Low relatedness in spring leads to competition for territories between females and to a severe spring decline. We suggest that severe mortality owing to predators or reduced mortality resulting from favorable environmental conditions that allow winter breeding may influence the genetic structure of a population and thus the intensity of competition for space among females. The predictions of our model are opposite to those of the Charnov-Finerty (1980) model. Charnov and Finerty predicted that the degree of relatedness would be inversely correlated with population density and that a decline in degree of relatedness at high density causes population crashes. We predict that relatedness fluctuates seasonally and is greatest at high density owing to the philopatry of juvenile females. We also predict that declines in relatedness are due to predation and immigration during the non-breeding season. | 724 | no | no pop level | NA | no | no |
| 725 | 2017 | 43011 | Hoarau, G; Boon, E; Jongma, DN; Ferber, S; Palsson, J; Van der Veer, HW; Rijnsdorp, AD; Stam, WT; Olsen, JL | 2005 | Low effective population size and evidence for inbreeding in an overexploited flatfish, plaice (Pleuronectes platessa L.) | Overexploitation and subsequent collapse of major worldwide fisheries has made it clear that marine stocks are not inexhaustible. Unfortunately, the perception remains that marine fishes are resilient to large population reductions, as even a commercially ‘collapsed’ stock will still consist of millions of individuals. Coupled with this notion is the idea that fisheries can, therefore, have little effect on the genetic diversity of stocks. We used DNA from archived otoliths collected between 1924 and 1972 together with 2002 juvenile’s tissue to estimate effective population size (Ne) in plaice (Pleuronectes platessa). Ne was estimated at 20 000 in the North Sea and 2000 in Iceland. These values are five orders of magnitude smaller than the estimated census size for the two locations. Populations examined between 1924 and 1960 were in Hardy-Weinberg equilibrium, whereas populations examined after approximately 1970 were not. Extensive testing was performed to rule out genotyping artefacts and Wahlund effects. The significant heterozygote deficiencies found from 1970 onward were attributed to inbreeding. The emergence of inbreeding between 1950 and 1970 coincides with the increase in fishing mortality after World War II. Although the biological mechanisms remain speculative, our demonstration of inbreeding signals the need for understanding the social and mating behaviour in commercially important fishes. | 725 | no | inbreeding | NA | no | no |
| 726 | 2017 | 43011 | Dalton, DL; Vermaak, E; Smit-Robinson, HA; Kotze, A | 2016 | Lack of diversity at innate immunity Toll-like receptor genes in the Critically Endangered White-winged Flufftail (Sarothrura ayresi) | The White-winged Flufftail (Sarothrura ayresi) population is listed as globally Critically Endangered. White-winged Flufftails are only known to occur, with any regularity, in the high-altitude wetlands of South Africa and Ethiopia. Threats to the species include the limited number of suitable breeding sites in Ethiopia and severe habitat degradation and loss both in Ethiopia and South Africa. Toll-like receptors (TLRs) are increasingly being studied in a variety of taxa as a broader approach to determine functional genetic diversity. In this study, we confirm low genetic diversity in the innate immune regions of the White-winged Flufftail similar to that observed in other bird species that have undergone population bottlenecks. Low TLR diversity in White-winged Flufftail indicates that this species is more likely to be threatened by changes to the environment that would potentially expose the species to new diseases. Thus, conservation efforts should be directed towards maintaining pristine habitat for White-winged Flufftail in its current distribution range. To date, no studies on immunogenetic variation in White-winged Flufftail have been conducted and to our knowledge, this is the first study of TLR genetic diversity in a critically endangered species. | 726 | no | no good fitness measure, N pop differnt in GD? | NA | no | no |
| 727 | 2017 | 43011 | Zhong, XX; Feng, DD; Yu, H; Kong, LF; Li, Q | 2016 | Genetic Variation and Breeding Signature in Mass Selection Lines of the Pacific Oyster (Crassostrea gigas) Assessed by SNP Markers | In breeding industries, a challenging problem is how to keep genetic diversity over generations. To investigate genetic variation and identify breeding signatures in mass selected lines of Pacific oyster (Crassostrea gigas), three sixth-generation selected lines and four wild populations were assessed using 103 single nucleotide polymorphism (SNP) markers. The genetic diversity data indicated that the selected lines exhibited a significant reduction in the observed heterozygosity and observed number of alleles per locus compared with the wild populations (P <= 0.05), indicating the selected lines tended to lose genetic diversity contrasted with the wild populations. The unweighted pair-group method with arithmetic mean (UPGMA) analysis showed that the wild populations and selected lines were not separated into two groups. Using four outlier tests, a total of 17 loci were found under selection at two levels. The global outlier detection suggested that 4 common outlier loci were subject to selection using both the hierarchical island model and Bayesian likelihood approaches. At regional level, 3 SNPs were detected as outlier using at least two outlier tests and one outlier SNP (CgSNP309) was overlapped in the two wild-selected population comparisons. The candidate outlier SNPs provide valuable resources for future association studies in C. gigas. | 727 | no | no good fitness measure | NA | no | no |
| 728 | 2017 | 43011 | Mounce, H; Raisin, C; Leonard, D; Wickenden, H; Swinnerton, K; Groombridge, J | 2015 | Spatial genetic architecture of the critically-endangered Maui Parrotbill (Pseudonestor xanthophrys): management considerations for reintroduction strategies | Conservation translocations are an important tool to circumvent extinctions on oceanic islands. A thorough understanding of all components of a species’ biology, including genetic diversity and structure, can maximize their likelihood of success. The Maui Parrotbill (Pseudonestor xanthophrys) is an endangered Hawaiian honeycreeper endemic to the island of Maui. With a population of approximately 500 individuals restricted to 50 km(2) of habitat, this species is at high risk of extinction. Using nuclear and mitochondrial DNA, this study quantified the levels of genetic diversity and structure in wild and captive parrotbill populations, and compared these genetic patterns to those observed within levels of contemporary and historical nuclear diversity derived from 100-year old museum samples. Substantial differences in the effective population sizes estimated between contemporary and historical parrotbill populations highlight the impact that introduced disease had on this species just before the turn of the century. Contemporary parrotbill diversity was low (global F (st) = 0.056), and there has been a 96 % reduction in genetic effective population size between contemporary and historical samples. This should not eliminate a conservation translocation (or reintroduction) as a viable recovery option. Measures of population differentiation (pairwise F (st) and R (st) ) between different sections of the current population on either side of the Koolau Gap suggest that current genetic structure may be the result of this topographic barrier to gene flow. These data can enable the design of a conservation translocation strategy that is tailored to the patterns of genetic structure across the species’ range. | 728 | no | no good fitness measure, N pop differnt in GD? | NA | no | no |
| 729 | 2017 | 43011 | Henn, BM; Cavalli-Sforza, LL; Feldman, MW | 2012 | The great human expansion | “Genetic and paleoanthropological evidence is in accord that today’s human population is the result of a great demic (demographic and geographic) expansion that began approximately 45,000 to 60,000 y ago in Africa and rapidly resulted in human occupation of almost all of the Earth’s habitable regions. Genomic data from contemporary humans suggest that this expansion was accompanied by a continuous loss of genetic diversity, a result of what is called the”“serial founder effect.”" In addition to genomic data, the serial founder effect model is now supported by the genetics of human parasites, morphology, and linguistics. This particular population history gave rise to the two defining features of genetic variation in humans: genomes from the substructured populations of Africa retain an exceptional number of unique variants, and there is a dramatic reduction in genetic diversity within populations living outside of Africa. These two patterns are relevant for medical genetic studies mapping genotypes to phenotypes and for inferring the power of natural selection in human history. It should be appreciated that the initial expansion and subsequent serial founder effect were determined by demographic and sociocultural factors associated with hunter-gatherer populations. How do we reconcile this major demic expansion with the population stability that followed for thousands years until the inventions of agriculture? We review advances in understanding the genetic diversity within Africa and the great human expansion out of Africa and offer hypotheses that can help to establish a more synthetic view of modern human evolution." | 729 | no | human | NA | no | no |
| 730 | 2017 | 43011 | POTTS, WK; MANNING, CJ; WAKELAND, EK | 1991 | MATING PATTERNS IN SEMINATURAL POPULATIONS OF MICE INFLUENCED BY MHC GENOTYPE | BECAUSE of the central role of major histocompatibility complex (MHC) genes in immune recognition 1-3, it is often assumed that parasite-driven selection maintains the unprecedented genetic diversity of these genes 4-7. But associations between MHC genotype and specific infectious diseases have been difficult to identify 8,9 with a few exceptions such as Marek’s disease 10 and malaria 11. Alternatively, MHC-related reproductive mechanisms such as selective abortion 12-15 and mating preferences 16,17 could be responsible for the diversity. To determine both the nature and strength of selection operating on MHC genes by we have studied components of selection in seminatural populations of mice (Mus musculus domesticus). Here we assess MHC-related patterns of reproduction and early (preweaning) mortality by analysing 1,139 progeny born in nine populations, and 662 progeny from laboratory matings. Reproductive mechanisms, primarily mating preferences, result in 27% fewer MHC-homozygous offspring than expected from random mating. MHC genotype had no detectable influence on neonatal (preweaning) mortality. These mating preferences are strong enough to account for most of the MHC genetic diversity found in natural populations of Mus. | 730 | no | no good fitness measure, N pop differnt in GD? | NA | no | no |
| 731 | 2017 | 43011 | Kubinak, JL; Cornwall, DH; Hasenkrug, KJ; Adler, FR; Potts, WK | 2015 | Serial infection of diverse host (Mus) genotypes rapidly impedes pathogen fitness and virulence | Reduced genetic variation among hosts may favour the emergence of virulent infectious diseases by enhancing pathogen replication and its associated virulence due to adaptation to a limited set of host genotypes. Here, we test this hypothesis using experimental evolution of a mouse-specific retroviral pathogen, Friend virus (FV) complex. We demonstrate rapid fitness (i.e. viral titre) and virulence increases when FV complex serially infects a series of inbred mice representing the same genotype, but not when infecting a diverse array of inbred mouse strains modelling the diversity in natural host populations. Additionally, a single infection of a different host genotype was sufficient to constrain the emergence of a high fitness/high virulence FV complex phenotype in these experiments. The potent inhibition of viral fitness and virulence was associated with an observed loss of the defective retroviral genome (spleen focus-forming virus), whose presence exacerbates infection and drives disease in susceptible mice. Results from our experiments provide an important first step in understanding how genetic variation among vertebrate hosts influences pathogen evolution and suggests that serial exposure to different genotypes within a single host species may act as a constraint on pathogen adaptation that prohibits the emergence of more virulent infections. From a practical perspective, these results have implications for low-diversity host populations such as endangered species and domestic animals. | 731 | maybe | any fitness of groups and of different gd? Not individual level? | no good GD and fitness of groups in main text | no | no |
| 732 | 2017 | 43011 | da Silva, AT; Penna, JCV; Goulart, LR; dos Santos, MA; Arantes, NE | 2000 | Genetic variability among and within races of Heterodera glycines Ichinohe assessed by RAPD markers | Heterodera glycines Ichinohe, the soybean cyst nematode (SCN) was detected in Brazil in 1992 and since then it has been causing losses to Brazilian soybean crop yields. SCN populations have great genetic diversity which makes it difficult to manage this disease. The objectives of this research were to investigate the genetic variation of 16 SCN populations sampled in middle western and southeastern Brazil, utilizing random amplified polymorphic DNA (RAPD) techniques, and to establish useful and specific RAPD markers for SCN race 3. RAPD markers demonstrated genetic variability among and within SCN populations, and they could be used for monitoring nematode population dynamics. The OPA-07 primer was a reliable molecular marker for race 3, while electrophoretic profile analysis of DNA fragments amplified with OPA-10 primer detected slight variation within those populations identified as race 3. The SCN population from Chapadao do Ceu, GO - sample 2 - was the most genetically distant from the other populations. | 732 | no | gd of parasite | NA | no | no |
| 733 | 2017 | 43011 | Latutrie, M; Merian, P; Picq, S; Bergeron, Y; Tremblay, F | 2015 | The effects of genetic diversity, climate and defoliation events on trembling aspen growth performance across Canada | Tree genetic makeup may provide an important control of growth dynamics; however, no studies have previously attempted to evaluate its effects in natural trembling aspen stands. In this study, we examined the relative contribution of genetics (i.e. clonal diversity, observed heterozygosity) and environmental conditions (i.e. insects, climate) on aspen growth as represented by mean inter-tree correlation (RBAR), tree basal area increment (TBAI) and inter-annual growth variability (MS). We sampled 440 trees in 22 even-aged natural stands dominated by aspen along an east-west continental gradient of decreasing annual precipitation in the Canadian boreal forest. Linear and mixed-effect models tested the relationships between tree growth, genetics and environmental factors. We showed that clonal diversity and number of years with forest tent caterpillar (FTC) defoliation (N-FTC) reduced and increased the level of growth synchronicity (RBAR), respectively. Clonal diversity explained 30 % of variation in RBAR among sites. TBAI was positively influenced by high moisture conditions while NFTC and climate explained the variation in MS among trees for each site. No genetic effect could explain either TBAI or the MS variation. Climate and NFTC drive annual growth variability in trembling aspen at stand and subcontinental scales. Tree genetic makeup contributed to these dynamics, the annual growth dynamics of multi-clonal stands being less homogeneous than those of monoclonal stands. Maintaining diverse aspen stands may ensure a wider range of growth responses to environmental variability, which in turn may help maintain resilience of aspen stands under future climate. | 733 | no | no good fitness measure | NA | no | no |
| 734 | 2017 | 43011 | Robinson, SJ; Walrath, RD; VanDeelen, TR; VerCauteren, KC | 2012 | Genetic Measures Confirm Familial Relationships and Strengthen Study Design | Social structure and behavioral interactions between individuals shape basic biological processes, such as breeding; foraging and predator avoidance; movement and dispersal; and disease transmission. We used a targeted trapping strategy to capture kin groups of white-tailed deer (Odocoileus virginianus) during 2007 and 2008 in Sandhill Wildlife Research Area, Wisconsin, USA, in order to observe social behaviors. Because inferring family relationships from observation of behavior is subjective, we usedmeasures of genetic relatedness and parentage assignment tests to determine that our capture strategy was efficient for capturing related pairs (78% of groups contained >= 1 dyad of related animals). The results of our genetic tests verified that study animals were related; therefore, our capture strategy was successful and the assumptions of the research design were met. This demonstrates both the utility of a targeted sampling approach, and the importance of genetic techniques to verify relationships among animals, especially when kin association forms a basis for further biological study or management action. Published 2012. This article is a U.S. Government work and is in the public domain in the USA. | 734 | no | no good fitness measure | NA | no | no |
| 735 | 2017 | 43011 | Hedrick, PW; Peterson, RO; Vucetich, LM; Adams, JR; Vucetich, JA | 2014 | Genetic rescue in Isle Royale wolves: genetic analysis and the collapse of the population | While genetic rescue is known to benefit population viability, the duration of that benefit is poorly understood. We document what appears to be the waning benefit of genetic rescue after approximately 2-3 generations for the wolf population in Isle Royale National Park. The fitness benefit of genetic rescue declined because of inbreeding and population abundance declined when the inbred individuals exhibited low reproduction and survival. Only detailed studies of other cases will reveal what aspects of these dynamics represent general features of genetic rescue. We also present evidence indicating that numerous past immigration events have likely gone undetected. This finding is of particular significance because the Isle Royale wolf population has maintained good population viability for decades even though it was small and thought to be isolated from the mainland population of wolves. Past gene flow also suggests that human-assisted gene flow is necessary to conserve the ecosystem services associated with predation, since climate warming has reduced the frequency of ice bridges and with it the only opportunity for unassisted gene flow. | 735 | no | only one pop | NA | no | no |
| 736 | 2017 | 43011 | Yule, KM; Koop, JAH; Alexandre, NM; Johnston, LR; Whiteman, NK | 2016 | Population structure of a vector-borne plant parasite | Parasites are among the most diverse groups of life on Earth, yet complex natural histories often preclude studies of their speciation processes. The biology of parasitic plants facilitates in situ collection of data on both genetic structure and the mechanisms responsible for that structure. Here, we studied the role of mating, dispersal and establishment in host race formation of a parasitic plant. We investigated the population genetics of a vector-borne desert mistletoe (Phoradendron californicum) across two legume host tree species (Senegalia greggii and Prosopis velutina) in the Sonoran desert using microsatellites. Consistent with host race formation, we found strong host-associated genetic structure in sympatry, little genetic variation due to geographic site and weak isolation by distance. We hypothesize that genetic differentiation results from differences in the timing of mistletoe flowering by host species, as we found initial flowering date of individual mistletoes correlated with genetic ancestry. Hybrids with intermediate ancestry were detected genetically. Individuals likely resulting from recent, successful establishment events following dispersal between the host species were detected at frequencies similar to hybrids between host races. Therefore, barriers to gene flow between the host races may have been stronger at mating than at dispersal. We also found higher inbreeding and within-host individual relatedness values for mistletoes on the more rare and isolated host species (S. greggii). Our study spanned spatial scales to address how interactions with both vectors and hosts influence parasitic plant structure with implications for parasite virulence evolution and speciation. | 736 | no | gd of parasite | NA | no | no |
| 737 | 2017 | 43011 | Hernandez-Salmeron, JE; Prieto-Barajas, CM; Valencia-Cantero, E; Moreno-Hagelsieb, G; Santoyo, G | 2014 | Isolation and characterization of genetic variability in bacteria with beta-hemolytic and antifungal activity isolated from the rhizosphere of Medicago truncatula plants | In the present study, we analyzed the frequency of hemolytic and antifungal activities in bacterial isolates from the rhizosphere of Medicago truncatula plants. Of the 2000 bacterial colonies, 96 showed beta-hemolytic activities (frequency, 4.8 x 10(-2)). Hemolytic isolates were analyzed for their genetic diversity by using random amplification of polymorphic DNA, yielding 88 haplotypes. The similarity coefficient of Nei and Li showed a polymorphic diversity ranging from 0.3 to 1. Additionally, 8 of the hemolytic isolates showed antifungal activity toward plant pathogens, Diaporthe phaseolorum, Colletotrichum acutatum, Rhizoctonia solani, and Fusarium oxysporum. The 16S ribosomal sequencing analysis showed that antagonistic bacterial isolates corresponded to Bacillus subtilis (UM15, UM33, UM42, UM49, UM52, and UM91), Bacillus pumilus (UM24), and Bacillus licheniformis (UM88). The present results revealed a higher genetic diversity among hemolytic isolates compared to that of isolates with antifungal action. | 737 | no | gd of pathogen | NA | no | no |
| 738 | 2017 | 43011 | Ernest, HB; Vickers, TW; Morrison, SA; Buchalski, MR; Boyce, WM | 2014 | Fractured Genetic Connectivity Threatens a Southern California Puma (Puma concolor) Population | Pumas (Puma concolor; also known as mountain lions and cougars) in southern California live among a burgeoning human population of roughly 20 million people. Yet little is known of the consequences of attendant habitat loss and fragmentation, and human-caused puma mortality to puma population viability and genetic diversity. We examined genetic status of pumas in coastal mountains within the Peninsular Ranges south of Los Angeles, in San Diego, Riverside, and Orange counties. The Santa Ana Mountains are bounded by urbanization to the west, north, and east, and are separated from the eastern Peninsular Ranges to the southeast by a ten lane interstate highway (I-15). We analyzed DNA samples from 97 pumas sampled between 2001 and 2012. Genotypic data for forty-six microsatellite loci revealed that pumas sampled in the Santa Ana Mountains (n = 42) displayed lower genetic diversity than pumas from nearly every other region in California tested (n = 257), including those living in the Peninsular Ranges immediately to the east across I-15 (n = 55). Santa Ana Mountains pumas had high average pairwise relatedness, high individual internal relatedness, a low estimated effective population size, and strong evidence of a bottleneck and isolation from other populations in California. These and ecological findings provide clear evidence that Santa Ana Mountains pumas have been experiencing genetic impacts related to barriers to gene flow, and are a warning signal to wildlife managers and land use planners that mitigation efforts will be needed to stem further genetic and demographic decay in the Santa Ana Mountains puma population. | 738 | no | no good fitness measure | NA | no | no |
| 739 | 2017 | 43011 | ELY, JJ; MANIS, GS; KEELING, ME; STONE, WH | 1994 | MAINTENANCE OF GENETIC-VARIABILITY IN A SPECIFIC PATHOGEN-FREE BREEDING COLONY | We used 18 genetic loci including blood groups, isozymes, and a serum protein to evaluate our efforts to preserve genetic variability in a specific pathogen-free (SPF) colony of rhesus monkeys. We compared genetic variability in the SPF population to the virally contaminated, non-SPF population from which it was derived. There was no change in the average gene diversity between the SPF and non-SPF populations. However, gene diversity at blood group and locus increased significantly in the SPF population, while blood groupM locus showed an insignificant trend toward decreased gene diversity Allele frequencies changed significantly at blood group and locus, although no alleles were lost from the population. We hypothesized that this change was due to extensive overreproducticsn by a small number of founder males that possessed the initially rare allele, Q(1). There was no evidence that this change was associated with genes involved in viral infection. | 739 | no | only one pop | NA | no | no |
| 740 | 2017 | 43011 | Adoukonou-Sagbadja, H; Dansi, A; Vodouhe, R; Akpagana, K | 2006 | Indigenous knowledge and traditional conservation of fonio millet (Digitaria exilis, Digitaria iburua) in Togo | Fonio millet (Digitaria exilis Stapf, Digitaria iburua Stapf) is known in Togo far several centuries and has played a strategic role in the household food security mainly in rural areas. Using Participatory Research Appraisal (PRA) tools and techniques, 55 villages randomly selected in the two production zones of Togo were surveyed to document the ethnobotanical and indigenous knowledge related to its production, diversity, use and conservation. For all of the ethnic groups involved in fonio production in Togo (Akposso and Akebou in the south; Losso-Nawda, Lamba, Tamberma, Tchokossi and Gangan in the north), the crop has a high sociocultural value. The fonio genetic diversity being managed by the farmers seems important as 42 landraces were recorded. Two lines of origin that contributed to this current genetic diversity (local domestication or introduction from neighbouring countries) were reported by the farmers. Cooking qualities, growth cycle, colour and size of the grains are the main criteria used by farmers to describe varieties. According to farmers, fonio production and diversity are being regressing because of several constraints of which the most important are lack of adequate harvest, threshing and processing technologies and development of pests and diseases. The important ethnobotanical and indigenous data recorded will be useful in accessing the genetic diversity of the crop in Togo and in defining appropriate strategies for its conservation on farm. | 740 | no | NA | NA | no | no |
| 741 | 2017 | 43011 | Janecka, JE; Tewes, ME; Davis, IA; Haines, AM; Caso, A; Blankenship, TL; Honeycutt, RL | 2016 | Genetic differences in the response to landscape fragmentation by a habitat generalist, the bobcat, and a habitat specialist, the ocelot | The ecology of a species strongly influences genetic variation and population structure. This interaction has important conservation implications because taxa with low dispersal capability and inability to use different habitats are more susceptible to anthropogenic stressors. Ocelots (Leopardus pardalis albescens) and bobcats (Lynx rufus texensis) are sympatric in Texas and northeastern Mexico; however, their ecology and conservation status are markedly different. We used 10 microsatellite loci and a 397-bp segment of the mitochondrial control region to examine how historical and ecological differences in these two species have influenced current patterns of genetic diversity in a landscape heavily altered by anthropogenic activities. Substantially higher genetic diversity (heterozygosity and haplotype diversity) and population connectivity was observed for bobcats in comparison to ocelots. The level of divergence among proximate ocelot populations (< 30 km) was greater than between bobcat populations separated by > 100 km. Ocelot populations in the US have never recovered from reductions experienced during the twentieth century, and their low genetic variation and substantial isolation are exacerbated by strong preference for dense native thornshrub and avoidance of open habitat. In contrast, despite continued legal harvesting and frequent road-related mortality, bobcats have maintained wide distribution, high abundance, and population connectivity. Our study illustrates that sympatric species with a similar niche can still have sufficient ecological differences to alter their response to anthropogenic change. Sensitive species, such as the ocelot, require additional conservation actions to sustain populations. Ecological differences among species occupying a similar guild are important to consider when developing conservation plans. | 741 | no | no good fitness measure, N pop differnt in GD? | NA | no | no |
| 742 | 2017 | 43012 | Radford, AD; Dawson, S; Ryvar, R; Coyne, K; Johnson, DR; Cox, MB; Acke, EFJ; Addie, DD; Gaskell, RM | 2003 | High genetic diversity of the immunodominant region of the feline calicivirus capsid gene in endemically infected cat colonies | Feline calicivirus (FCV) is an important pathogen of domestic cats. In this study, we have determined the genetic diversity of FCV within four geographically separate colonies of endemically infected cats by sequencing the immunodominant and variable region E of the capsid gene. Comparison of isolates between colonies and between unrelated published sequences gave nucleotide distance values of 26-35% and 22-40%, respectively and suggested each colony was infected with a distinct virus strain. Comparison of isolates within individual endemically infected colonies showed nucleotide distance variability of 0-16%. This was greater than distances previously reported for epidemiologically related isolates from cases of acute disease (0-5%) and was consistent with the evolution of FCV from a single distinct ancestor sequence in each colony. The pattern of nucleotide substitutions generating the observed intra-colony diversity was associated with strong evidence for positive selection acting on immunodominant regions of the FCV capsid protein. We suggest that endemically infected colonies of cats may be important generators of genetic diversity for FCV and that this may ultimately lead to the generation of new strains. | 742 | no | gd of parasite | NA | no | no |
| 743 | 2017 | 43012 | CallegariJacques, SM; Salzano, FM; Weimer, TA; Franco, MHLP; Mestriner, MA; Hutz, MH; Schuler, L | 1996 | The Wai Wai Indians of South America: History and genetics | Demographic, medical and genetic information was obtained in a population of Carib-speaking Wai Wai Indians living in northern Brazil. At present mortality is low and Fertility moderate, with a low variance in offspring number in completed families. Mobility is high, but about two-thirds of the unions are endogamic. Malaria is the main health problem. Phenotype and allele frequencies were obtained for 27 protein genetic systems. Comparison with six other Carib groups indicates closest affinities with another Amazonian tribe, the Arara. Quantification of the intra- and interpopulation genetic diversity in these seven populations indicate that the variation within groups is only slightly lower than the variation between groups. The level of Carib interpopulation diversity, on the other hand, does not differ significantly from that found in 11 Tupi-speaking populations. In accordance with their history of intermarriage with groups which speak slightly different languages, and consider themselves as distinct, the Wai Wai are clearly more diversified at the intrapopulation level than at least three of the six Carib populations with which they were compared. | 743 | no | human | NA | no | no |
| 744 | 2017 | 43012 | Pigliucci, M; Pollard, H; Cruzan, MB | 2003 | Comparative studies of evolutionary responses to light environments in Arabidopsis | In this article, we compare the reaction norms to foliage shade (changes in light quality, spatially fine-grained environmental variation) and photoperiod (day length, spatially coarse-grained environmental variation) in several haplotypes of Arabidopsis thaliana from Scandinavia. We found that both across-environment means and phenotypic plasticities evolved continuously and very rapidly within this group. Both character means and trait plasticities were highly integrated, in part as predicted by the adaptive plasticity hypothesis for response to foliage shade (the so-called shade-avoidance syndrome). We found that a significant but small fraction of the variance in across-treatment trait means and plasticities in response to one environmental factor is explained by variation of the same traits in response to the other factor. Genetic relatedness based on chloroplast DNA sequence variation among haplotypes was not associated with variation in across-treatment character means or their plasticities, suggesting that evolution of these characters has occurred on a local geographic scale via reticulation (outcrossing) among maternal lines rather than by the differential survival of selfing lineages. | 744 | no | no good fitness measure, N pop differnt in GD? | NA | no | no |
| 745 | 2017 | 43012 | Abu Choudhury, M; Lott, WB; Banu, S; Cheng, AY; Teo, YY; Ong, RTH; Aaskov, J | 2015 | Nature and Extent of Genetic Diversity of Dengue Viruses Determined by 454 Pyrosequencing | Dengue virus (DENV) populations are characteristically highly diverse. Regular lineage extinction and replacement is an important dynamic DENV feature, and most DENV lineage turnover events are associated with increased incidence of disease. The role of genetic diversity in DENV lineage extinctions is not understood. We investigated the nature and extent of genetic diversity in the envelope (E) gene of DENV serotype 1 representing different lineages histories. A region of the DENV genome spanning the E gene was amplified and sequenced by Roche/454 pyrosequencing. The pyrosequencing results identified distinct sub-populations (haplotypes) for each DENV-1 E gene. A phylogenetic tree was constructed with the consensus DENV-1 E gene nucleotide sequences, and the sequences of each constructed haplotype showed that the haplotypes segregated with the Sanger consensus sequence of the population from which they were drawn. Haplotypes determined through pyrosequencing identified a recombinant DENV genome that could not be identified through Sanger sequencing. Nucleotide level sequence diversities of DENV-1 populations determined from SNP analysis were very low, estimated from 0.009-0.01. There were also no stop codon, frameshift or non-frameshift mutations observed in the E genes of any lineage. No significant correlations between the accumulation of deleterious mutations or increasing genetic diversity and lineage extinction were observed (p>0.5). Although our hypothesis that accumulation of deleterious mutations over time led to the extinction and replacement of DENV lineages was ultimately not supported by the data, our data does highlight the significant technical issues that must be resolved in the way in which population diversity is measured for DENV and other viruses. The results provide an insight into the within-population genetic structure and diversity of DENV-1 populations. | 745 | no | gd of pathogen | NA | no | no |
| 746 | 2017 | 43012 | Ferrucho, RL; Zala, M; Zhang, Z; Cubeta, MA; Garcia-Dominguez, C; Ceresini, PC | 2009 | Highly polymorphic in silico-derived microsatellite loci in the potato-infecting fungal pathogen Rhizoctonia solani anastomosis group 3 from the Colombian Andes | Fourteen polymorphic microsatellite DNA markers derived from the draft genome sequence of Rhizoctonia solani anastomosis group 3 (AG-3), strain Rhs 1AP, were designed and characterized from the potato-infecting soil fungus R. solani AG-3. All loci were polymorphic in two field populations collected from Solanum tuberosum and S. phureja in the Colombian Andes. The total number of alleles per locus ranged from two to seven, while gene diversity (expected heterozygosity) varied from 0.11 to 0.81. Considering the variable levels of genetic diversity observed, these markers should be useful for population genetic analyses of this important dikaryotic fungal pathogen on a global scale. | 746 | no | gd of pathogen | NA | no | no |
| 747 | 2017 | 43012 | Bol’shakov, VN; Moiseenko, TI | 2009 | Anthropogenic Evolution of Animals: Facts and Their Interpretation | Problems concerning microevolutionary transformations in animal populations are considered. It is shown that genetic variation is the main factor providing the basis for adaptation to environmental changes, including toxic pollution. The selection pressure of a toxic factor gives an advantage in survival to more resistant genotypes in animal populations, which eventually leads to the reduction of their genetic diversity and potential for adaptation to other natural or anthropogenic stress factors. Microevolutionary transformations follow the pattern of r-selection, i.e., occur in favor of smaller, earlier maturing individuals capable of expending a greater proportion of their energy resources for reproduction. | 747 | no | NA | NA | no | no |
| 748 | 2017 | 43012 | Aldrich-Wolfe, L; Travers, S; Nelson, BD | 2015 | Genetic Variation of Sclerotinia sclerotiorum from Multiple Crops in the North Central United States | Sclerotinia sclerotiorum is an important pathogen of numerous crops in the North Central region of the United States. The objective of this study was to examine the genetic diversity of 145 isolates of the pathogen from multiple hosts in the region. Mycelial compatibility groups (MCG) and microsatellite haplotypes were determined and analyzed for standard estimates of population genetic diversity and the importance of host and distance for genetic variation was examined. MCG tests indicated there were 49 different MCGs in the population and 52 unique microsatellite haplotypes were identified. There was an association between MCG and haplotype such that isolates belonging to the same MCG either shared identical haplotypes or differed at no more than 2 of the 12 polymorphic loci. For the majority of isolates, there was a one-to-one correspondence between MCG and haplotype. Eleven MCGs shared haplotypes. A single haplotype was found to be prevalent throughout the region. The majority of genetic variation in the isolate collection was found within rather than among host crops, suggesting little genetic divergence of S. sclerotiorum among hosts. There was only weak evidence of isolation by distance. Pairwise population comparisons among isolates from canola, dry bean, soybean and sunflower suggested that gene flow between host-populations is more common for some crops than others. Analysis of linkage disequilibrium in the isolates from the four major crops indicated primarily clonal reproduction, but also evidence of genetic recombination for isolates from canola and sunflower. Accordingly, genetic diversity was highest for populations from canola and sunflower. Distribution of microsatellite haplotypes across the study region strongly suggest that specific haplotypes of S. sclerotiorum are often found on multiple crops, movement of individual haplotypes among crops is common and host identity is not a barrier to gene flow for S. sclerotiorum in the north central United States. | 748 | no | gd of pathogen | NA | no | no |
| 749 | 2017 | 43012 | Wamalwa, EN; Muoma, J; Wekesa, C | 2016 | Genetic Diversity of Cowpea (Vigna unguiculata (L.) Walp.) Accession in Kenya Gene Bank Based on Simple Sequence Repeat Markers | Increased agricultural production is an urgent issue. Projected global population is 9 million people by mid of this century. Estimation projects death of 1 million people for lack of food quality (micronutrient deficit) and quantity ( protein deficit). Majority of these people will be living in developing countries. Other global challenges include shrinking cultivable lands, salinity, and flooding due to climate changes, new emerging pathogens, and pests. These affect crop production. Furthermore, they are major threats to crop genetic resources and food security. Genetic diversity in cultivated crops indicates gene pool richness. It is the greatest resource for plant breeders to select lines that enhance food security. This study was conducted by Masinde Muliro University to evaluate genetic diversity in 19 cowpea accessions from Kenya national gene bank. Accessions clustered into two major groups. High divergence was observed between accessions from Ethiopia and Australia and those from Western Kenya. Upper Volta accessions were closely related to those from Western Kenya. Low variation was observed between accessions from Eastern and Rift Valley than those from Western and Coastal regions of Kenya. Diversity obtained in this study can further be exploited for the improvement of cowpea in Kenya as a measure of food security. | 749 | no | crop | NA | no | no |
| 750 | 2017 | 43012 | Stoen, OG; Bellemain, E; Saebo, S; Swenson, JE | 2005 | Kin-related spatial structure in brown bears Ursus arctos | Kin-related social structure may influence reproductive success and survival and, hence, the dynamics of populations. It has been documented in many gregarious animal populations, but few solitary species. Using molecular methods and field data we tested: (1) whether kin-related spatial structure exists in the brown bear (Ursus arctos), which is a solitary carnivore, (2) whether home ranges of adult female kin overlap more than those of nonkin, and (3) whether multigenerational matrilinear assemblages, i.e., aggregated related females, are formed. Pairwise genetic relatedness between adult (5 years and older) female dyads declined significantly with geographic distance, whereas this was not the case for male-male dyads or opposite sex dyads. The amount of overlap of multiannual home ranges was positively associated with relatedness among adult females. This structure within matrilines is probably due to kin recognition. Plotting of multiannual home-range centers of adult females revealed formation of two types of matrilines, matrilinear assemblages exclusively using an area and dispersed matrilines spread over larger geographic areas. The variation in matrilinear structure might be due to differences in competitive abilities among females and habitat limitations. The influence of kin-related spatial structure on inclusive fitness needs to be clarified in solitary mammals. | 750 | no | no good fitness measure | NA | no | no |
| 751 | 2017 | 43012 | Curik, I; Zechner, P; Solkner, J; Achmann, R; Bodo, I; Dovc, P; Kavar, T; Marti, E; Brem, G | 2003 | Inbreeding, microsatellite heterozygosity, and morphological traits in Lipizzan horses | While the negative effects of inbreeding and reduced heterozygosity on fecundity and survival are well established, only a few investigations have been carried out concerning their influence on morphological traits. This topic is of particular interest for a small and closed population such as the Lipizzan horse. Thus, 27 morphological traits were measured in 360 Lipizzan mares and were regressed on the individual inbreeding coefficients, as well as on the individual heterozygosity and mean squared distances (mean d(2)) between microsatellite alleles within an individual. Both individual heterozygosity and mean d(2) were based on 17 microsatellite loci dispersed over 14 chromosomes. The results obtained by multivariate analysis reveal significant effects of stud (P < .0001), age at measurement (P < .0001), and mean d(2) (P = .0143). In univariate analyses, significant associations were obtained between length of pastern-hindlimbs and inbreeding coefficient (P < .01), length of cannons-hindlimb and mean d(2) (P < .01), and length of neck and mean d(2) (P < .001). After adjustment of single-test P values for multiple tests (Hochberg’s step-up Bonferroni method), only the association of the length of neck and mean d(2) remained significant (P = .0213). Thus, no overall large effects of inbreeding, microsatellite heterozygosity, and mean d(2) on morphological traits were observed in the Lipizzan horse. | 751 | no | inbreeding | NA | no | no |
| 752 | 2017 | 43012 | Sepil, I; Lachish, S; Sheldon, BC | 2013 | Mhc-linked survival and lifetime reproductive success in a wild population of great tits | Major histocompatibility complex (Mhc) genes are frequently used as a model for adaptive genetic diversity. Although associations between Mhc and disease resistance are frequently documented, little is known about the fitness consequences of Mhc variation in wild populations. Further, most work to date has involved testing associations between Mhc genotypes and fitness components. However, the functional diversity of the Mhc, and hence the mechanism by which selection on Mhc acts, depends on how genotypes map to the functional properties of Mhc molecules. Here, we test three hypotheses that relate Mhc diversity to fitness: (i) the maximal diversity hypothesis, (ii) the optimal diversity hypothesis and (iii) effect of specific Mhc types. We combine markrecapture methods with analysis of long-term breeding data to investigate the effects of Mhc class I functional diversity (Mhc supertypes) on individual fitness in a wild great tit (Parus major) population. We found that the presence of three different Mhc supertypes was associated with three different components of individual fitness: survival, annual recruitment and lifetime reproductive success (LRS). Great tits possessing Mhc supertype 3 experienced higher survival rates than those that did not, whereas individuals with Mhc supertype 6 experienced higher LRS and were more likely to recruit offspring each year. Conversely, great tits that possessed Mhc supertype 5 had reduced LRS. We found no evidence for a selective advantage of Mhc diversity, in terms of either maximal or optimal supertype diversity. Our results support the suggestion that specific Mhc types are an important determinant of individual fitness. | 752 | no | no pop level | NA | no | no |
| 753 | 2017 | 43012 | Parker, RW; Paige, KN; De Vries, AL | 2002 | Genetic variation among populations of the Antarctic toothfish: evolutionary insights and implications for conservation | Commercial fishing is having an increasingly negative impact on marine biodiversity, with over 70% of the world’s fish stocks being fully exploited and. in many cases, overexploited. On top of this, the Commission for the Conservation of Antarctic Marine Living Resources (CCAMLR) has granted commercial fishing permits in the most remote marine environment on earth. the high-latitude Southern Ocean. The primary target of these new commercial fishing ventures is the large pelagic piscivorous predator, the Antarctic toothfish (Dissostichus mawsoni). Unfortunately, little information is available on the demography, genetics, or life history of this large fish. Without such information we have little idea as to the effects of commercial fishing on the population structure and survival of this species. In this study. we focus on patterns of genetic diversity within and between geographically disparate populations of the Antarctic toothfish, using randomly amplified polymorphic DNA markers. Results of our study showed high levels of genetic similarity within and between populations. Despite high levels of genetic similarity, genetic analyses detected significant population structure, including fixed differences among populations, a significant fixation index (Fst) and between-population differentiation via a Mantel test. From a conservation perspective, low levels of genetic diversity may be indicative of relatively small populations that would not be able to withstand heavy commercial fishing pressures. Given that there is evidence for significant genetic structure, it will be important to manage these fisheries in a manner that will help prevent the loss of unique genetic variation from regional overfishing. | 753 | no | no good fitness measure, N pop differnt in GD? | NA | no | no |
| 754 | 2017 | 43012 | Mastana, S; Lee, D; Singh, PP; Singh, M | 2003 | Molecular genetic variation in the East Midlands, England: Analysis of VNTR, STR and Alu insertion/deletion polymorphisms | Background: Short tandem repeats (STRs) and variable number of tandem repeats (VNTRs) have been used successfully in disease analysis and studies of human evolution and population genetic diversity. However DNA-based comprehensive population genetic studies of the East Midlands, England are limited. Subjects and methods: To enlarge our understanding of genetic variation in the East Midlands, a study was conducted on five regional populations: north-west Derbyshire, north-east Derbyshire, south Derbyshire, Nottinghamshire and Leicestershire. Blood samples were collected from donors whose ancestors had lived in the region for at least three generations. Seven VNTRs (MS1 (D1S7), MS31 (D7S21), MS43A (D12S11) and YNH24 (D2S44), D1S80, APOB, YNZ22 (D17S5)), six STRs (HumTHO1, HumVWA31A, HumF13A01, HumFESFPS, HumCSF1PO, HumTPOX) and six Alu insertion/deletion polymorphisms (TPA25, ACE, PV92, F13B, APO, D1) were analysed in approximately 500 individuals. Allele or bin frequencies were calculated using gene counting and fixed bin methods. The chi-square method and exact tests were used to assess Hardy-Weinberg equilibrium. Genetic distances were calculated using Nei’s D-A method and correspondence analysis was used to assess population affinities. Results: The overall pattern of allele frequencies was similar to many European and UK populations for a number of genetic systems. Overall heterogeneity was observed for five loci: MS43A, MS31, HumF13A01, HumFESFPS and HumTHO1. Twenty-three of 190 pairwise population comparisons were also statistically significant at the 5% level. Average molecular genetic system heterozygosity was 1.5 times higher than observed with conventional blood group systems. G(ST) values for molecular systems were also higher than conventional systems (0.012 vs 0.005) and suggest a low to moderate level of differentiation. Conclusion: The allele frequency spectrum and inter-population comparisons show that there is significant genetic variation in the five contiguous regional populations of the East Midlands. Some of this variation may be due to local geographical barriers, genetic drift and possibly the settlement patterns of Continental European invaders. | 754 | no | human | NA | no | no |
| 755 | 2017 | 43012 | Sim, SC; Robbins, MD; Chilcott, C; Zhu, T; Francis, DM | 2009 | Oligonucleotide array discovery of polymorphisms in cultivated tomato (Solanum lycopersicum L.) reveals patterns of SNP variation associated with breeding | Background: Cultivated tomato (Solanum lycopersicum L.) has narrow genetic diversity that makes it difficult to identify polymorphisms between elite germplasm. We explored array-based single feature polymorphism (SFP) discovery as a high-throughput approach for marker development in cultivated tomato. Results: Three varieties, FL7600 (fresh-market), OH9242 (processing), and PI114490 (cherry) were used as a source of genomic DNA for hybridization to oligonucleotide arrays. Identification of SFPs was based on outlier detection using regression analysis of normalized hybridization data within a probe set for each gene. A subset of 189 putative SFPs was sequenced for validation. The rate of validation depended on the desired level of significance (alpha) used to define the confidence interval (CI), and ranged from 76% for polymorphisms identified at alpha <= 10(-6) to 60% for those identified at alpha <= 10(-2). Validation percentage reached a plateau between alpha <= 10(-4) and alpha <= 10(-7), but failure to identify known SFPs (Type II error) increased dramatically at alpha <= 10(-6). Trough sequence validation, we identified 279 SNPs and 27 InDels in 111 loci. Sixty loci contained >= 2 SNPs per locus. We used a subset of validated SNPs for genetic diversity analysis of 92 tomato varieties and accessions. Pairwise estimation of theta (Fst) suggested significant differentiation between collections of fresh-market, processing, vintage, Latin American (landrace), and S. pimpinellifolium accessions. The fresh-market and processing groups displayed high genetic diversity relative to vintage and landrace groups. Furthermore, the patterns of SNP variation indicated that domestication and early breeding practices have led to progressive genetic bottlenecks while modern breeding practices have reintroduced genetic variation into the crop from wild species. Finally, we examined the ratio of nonsynonymous (Ka) to synonymous substitutions (Ks) for 20 loci with multiple SNPs (>= 4 per locus). Six of 20 loci showed ratios of Ka/Ks >= 0.9. Conclusion: Array-based SFP discovery was an efficient method to identify a large number of molecular markers for genetics and breeding in elite tomato germplasm. Patterns of sequence variation across five major tomato groups provided insight into to the effect of human selection on genetic variation. | 755 | no | crop | NA | no | no |
| 756 | 2017 | 43012 | Eyre-Walker, YC; Eyre-Walker, A | 2014 | The Role of Mutation Rate Variation and Genetic Diversity in the Architecture of Human Disease | Background: We have investigated the role that the mutation rate and the structure of genetic variation at a locus play in determining whether a gene is involved in disease. We predict that the mutation rate and its genetic diversity should be higher in genes associated with disease, unless all genes that could cause disease have already been identified. Results: Consistent with our predictions we find that genes associated with Mendelian and complex disease are substantially longer than non-disease genes. However, we find that both Mendelian and complex disease genes are found in regions of the genome with relatively low mutation rates, as inferred from intron divergence between humans and chimpanzees, and they are predicted to have similar rates of non-synonymous mutation as other genes. Finally, we find that disease genes are in regions of significantly elevated genetic diversity, even when variation in the rate of mutation is controlled for. The effect is small nevertheless. Conclusions: Our results suggest that gene length contributes to whether a gene is associated with disease. However, the mutation rate and the genetic architecture of the locus appear to play only a minor role in determining whether a gene is associated with disease. | 756 | no | gd of pathogen | NA | no | no |
| 757 | 2017 | 43012 | Zuccherato, LW; Schneider, S; Tarazona-Santos, E; Hardwick, RJ; Berg, DE; Bogle, H; Gouveia, MH; Machado, LR; Machado, M; Rodrigues-Soares, F; Soares-Souza, GB; Togni, DL; Zamudio, R; Gilman, RH; Duarte, D; Hollox, EJ; Rodrigues, MR | 2017 | Population genetics of immune-related multilocus copy number variation in Native Americans | While multiallelic copy number variation (mCNV) loci are a major component of genomic variation, quantifying the individual copy number of a locus and defining genotypes is challenging. Few methods exist to study how mCNV genetic diversity is apportioned within and between populations (i.e. to define the population genetic structure of mCNV). These inferences are critical in populations with a small effective size, such as Amerindians, that may not fit the Hardy-Weinberg model due to inbreeding, assortative mating, population subdivision, natural selection or a combination of these evolutionary factors. We propose a likelihood-based method that simultaneously infers mCNV allele frequencies and the population structure parameter f, which quantifies the departure of homozygosity from the Hardy-Weinberg expectation. This method is implemented in the freely available software CNVice, which also infers individual genotypes using information from both the population and from trios, if available. We studied the population genetics of five immune-related mCNV loci associated with complex diseases (beta-defensins, CCL3L1/CCL4L1, FCGR3A, FCGR3B and FCGR2C) in 12 traditional Native American populations and found that the population structure parameters inferred for these mCNVs are comparable to but lower than those for single nucleotide polymorphisms studied in the same populations. | 757 | no | human | NA | no | no |
| 758 | 2017 | 43012 | Mattila, HR; Burke, KM; Seeley, TD | 2008 | Genetic diversity within honeybee colonies increases signal production by waggle-dancing foragers | Recent work has demonstrated considerable benefits of intracolonial genetic diversity for the productivity of honeybee colonies: single-patriline colonies have depressed foraging rates, smaller food stores and slower weight gain relative to multiple-patriline colonies. We explored whether differences in the use of foraging-related communication behaviour (waggle dances and shaking signals) underlie differences in foraging effort of genetically diverse and genetically uniform colonies. We created three pairs of colonies; each pair had one colony headed by a multiply mated queen (inseminated by 15 drones) and one colony headed by a singly mated queen. For each pair, we monitored the production of foraging-related signals over the course of 3 days. Foragers in genetically diverse colonies had substantially more information available to them about food resources than foragers in uniform colonies. On average, in genetically diverse colonies compared with genetically uniform colonies, 36% more waggle dances were identified daily, dancers performed 62% more waggle runs per dance, foragers reported food discoveries that were farther from the nest and 91% more shaking signals were exchanged among workers each morning prior to foraging. Extreme polyandry by honeybee queens enhances the production of worker-worker communication signals that facilitate the swift discovery and exploitation of food resources. | 758 | no | no good fitness measure | NA | no | no |
| 759 | 2017 | 43012 | Neveu, H; Hafen, T; Zimmermann, E; Rumpler, Y | 1998 | Comparison of the genetic diversity of wild and captive groups of Microcebus murinus using the random amplified polymorphic DNA method | Continued survival of most animal species depends on population management and active protection. It is generally agreed that, in order to avoid extinction of endangered species, ex situ and in situ conservation must be developed in tandem. However, even though many recommendations have been put forward to promote the survival of captive populations, some rapidly become extinct due to loss of genetic diversity (drift effect). Genetic markers, such as random amplified polymorphic DNA (RAPD) markers, can be applied to rapid testing of many individuals. They also permit analysis of very small amounts of DNA, when small species such as mouse lemurs (Microcebus) are to be tested. Using RAPD markers, we compare genetic diversity in four captive groups of Microcebus murinus to that in a sample of 70 wild mouse lemurs. Following the principles of Mendelian inheritance, each amplified fragment of DNA may be considered as a ‘locus’ (or an amplifying site). The series of bands amplified by a particular primer in any individual is referred to as the individual’s ‘profile’. We tested 5 primers, or, in the above terms, we studied 98 different ‘loci’. Results showed that the captive groups had lost genetic information with respect to the wild sample. Among the four captive groups, the loss of genetic diversity varied according to their number of founders and/or the management of their captive reproduction. Our study of polymorphism permitted us to establish tools for the genetic management of captive breeding, and for the determination of paternity which frequently give better results than behavioural studies; and simulation of introductions or departures of individuals in one very monomorphic group permitted estimation of future increases in its genetic diversity. | 759 | no | no good fitness measure, N pop differnt in GD? | NA | no | no |
| 760 | 2017 | 43012 | Kosinski, I; Stefanowicz-Hajduk, J; Ochocka, R | 2009 | DEMOGRAPHIC VERSUS GENETIC (RAPD) VARIATION BETWEEN AND WITHIN TWO POPULATIONS OF THE CLONAL PLANT PARIS QUADRIFOLIA L. (LILIACEAE) | The two populations of Paris quadrifolia L. were studied in isolated habitats in a currently fragmented landscape. Both populations were located in deciduous forests, the first (A) - on fresh mineral and acidic soil, and the second (B) - on wet organogenic, less acidic and more fertile soil. We hypothesized that genetic variation should be higher within population of more fecund plants, and that genetic distance between the two populations that occupy different isolated habitats in a fragmented landscape should be high. Demographic characteristics of populations were studied in the 2000-2005 period. In patches of both populations, 22 permanent plots measuring 1m(2) each were designated. For molecular testing 41 samples from both populations were selected. The share of generative shoots was higher in the population A than population B (0.35 and 0.20, respectively). However, the fecundity of ramets expressed as the number of seeds in the ripe fruit was lower in A than in the B population (15 versus 21 seeds). The germination ability was significantly higher for the seeds from A than from the B population (79% versus 44%). The survival of the juveniles was high in both populations (54 and 76%). The Random Amplified Polymorphic DNA (RAPD) analysis with the application of five primers permitted identifying 91 loci. The estimation of genetic diversity was based on polymorphic loci, the share of which was average 44%. Nei’s gene diversity (h) was higher in the A than B population (0.28 versus 0.22). The genetic diversity between the populations was not large (G(ST) = 0.14). Clonal diversity was very high, G/N ratio 1, and cluster analysis showed intermingling between samples from both the populations. There were quite a small genetic distance (D = 0.10), and a rather high level of gene flow (Nm = 1.51) between the populations from currently isolated habitats. The obtained results indicate that the genetic diversity was lower within population of more fecund plants from more productive habitat. | 760 | no | no good fitness measure | NA | no | no |
| 761 | 2017 | 43012 | Tarpy, DR; Seeley, TD | 2006 | Lower disease infections in honeybee (Apis mellifera) colonies headed by polyandrous vs monandrous queens | We studied the relationship between genetic diversity and disease susceptibility in honeybee colonies living under natural conditions. To do so, we created colonies in which each queen was artificially inseminated with sperm from either one or ten drones. Of the 20 colonies studied, 80% showed at least one brood disease. We found strong differences between the two types of colonies in the infection intensity of chalkbrood and in the total intensity of all brood diseases (chalkbrood, sacbrood, American foulbrood, and European foulbrood) with both variables lower for the colonies with higher genetic diversity. Our findings demonstrate that disease can be an important factor in the ecology of honeybee colonies and they provide strong support for the disease hypothesis for the evolution of polyandry by social insect queens. | 761 | yes | from thesis | NA | no | yes |
| 762 | 2017 | 43012 | Song, K; Elston, RC | 2003 | Tests for a disease-susceptibility locus allowing for an inbreeding coefficient (F) | We begin by discussing the false positive test results that arise because of cryptic relatedness and population substructure when testing a disease susceptibility locus. We extend and evaluate the Hardy - Weinberg disequilibrium (HWD) method, allowing for an inbreeding coefficient ( F) in a similar way that Devlin and Roeder (1999) allowed for inbreeding in a case-control study. Then we compare the HWD measure and the common direct measure of linkage disequilibrium, both when there is no population substructure (F = 0) and when there is population substructure (F not equal 0), for a single marker. The HWD test statistic gives rise to false positives caused by population stratification. These false positives can be controlled by adjusting the test statistic for the amount of variance inflation caused by the inbreeding coefficient (F). The power loss for the HWD test that arises when controlling for population structure is much less than that which arises for the common direct measure of linkage disequilibrium. However, in the multiplicative model, the HWD test has virtually no power even when allowing for non-zero F. | 762 | no | no good fitness measure, N pop differnt in GD? (inbreeding?) | NA | no | no |
| 763 | 2017 | 43012 | Mastana, SS | 2014 | Unity in diversity: an overview of the genomic anthropology of India | Context: India is considered a treasure for geneticists and evolutionary biologists due to its vast human diversity, consisting of more than 4500 anthropologically well-defined populations (castes, tribes and religious groups). Each population differs in terms of endogamy, language, culture, physical features, geographic and climatic position and genetic architecture. These factors contributed to India-specific genetic variations which may be responsible for various common diseases in India and its migratory populations. As a result, interpretations of the origins and affinities of Indian populations as well as health and disease conditions require complex and sophisticated genetic analysis. Evidence of ancient human dispersals and settlements is preserved in the genome of Indian inhabitants and this has been extensively analysed in conventional and genomic analyses. Objective and methods: Using genomic analyses of STRs and Alu on a set of populations, this study estimates the level and extent of genetic variation and its implications. Results: The results show that Indian populations have a higher level of unique genetic diversity which is structured by many social processes and geographical attributes of the country. Conclusion: This overview highlights the need to study the anthropological structure and evolutionary history of Indian populations while designing genomic and epigenomic investigations. | 763 | no | human | NA | no | no |
| 764 | 2017 | 43012 | Das, B; Satapathy, T; Kar, SK; Hazra, RK | 2014 | Genetic Structure and Wolbachia Genotyping in Naturally Occurring Populations of Aedes albopictus across Contiguous Landscapes of Orissa, India | Background: Aedes albopictus has recently been implicated as a major vector in the emergence of dengue and chikungunya in several parts of India, like Orissa, which is gradually gaining endemicity for arboviral diseases. Ae. albopictus is further known to be naturally infected with Wolbachia (maternally inherited bacterium), which causes cytoplasmic incompatibility (CI) in mosquitoes leading to sperm-egg incompatibility inducing the death of embryo. Knowledge of genetic diversity of Ae. albopictus, along with revealing the type of Wolbachia infection in Ae. albopictus is important to explore the genetic and biological characteristics of Ae. albopictus, prior to exploring the uses of CI-based vector control strategies. In this study, we assessed the population genetic structure and the pattern of Wolbachia infection in Ae. albopictus mosquitoes of Orissa. Methods and Results: Ae. albopictus mosquitoes were collected from 15 districts representing the four physiographical regions of Orissa from 2010-2012, analyzed for genetic variability at seven microsatellite loci and genotyped for Wolbachia strain detection using wsp gene primers. Most microsatellite markers were successfully amplified and were polymorphic, showing moderate genetic structure among all geographic populations (F-ST = 0.088). Genetic diversity was high (F-ST = 0.168) in Coastal Plains populations when compared with other populations, which was also evident from cluster analyses that showed most Coastal Plains populations consisted of a separate genetic cluster. Genotyping analyses revealed that Wolbachia-infected Ae. albopictus field populations of Orissa were mostly superinfected with wAlbA and wAlbB strains. Wolbachia superinfection was more pronounced in the Coastal Plain populations. Conclusion: High genetic structure and Wolbachia superinfection, observed in the Coastal Plain populations of Orissa suggested it to be genetically and biologically more unique than other populations, and hence could influence their vectorial attributes. Such high genetic diversity observed among Coastal Plains populations could be attributed to multiple introductions of Ae. albopictus in this region. | 764 | no | no good fitness measure, N pop differnt in GD? | NA | no | no |
| 765 | 2017 | 43012 | Vlcek, J; Hoeck, PEA; Keller, LF; Wayhart, JP; Dolinova, I; Stefka, J | 2016 | Balancing selection and genetic drift create unusual patterns of MHCII variation in Galapagos mockingbirds | The extracellular subunit of the major histocompatibility complex MHCII plays an important role in the recognition of pathogens and the initiation of the adaptive immune response of vertebrates. It is widely accepted that pathogen-mediated selection in combination with neutral micro-evolutionary forces (e.g. genetic drift) shape the diversity of MHCII, but it has proved difficult to determine the relative effects of these forces. We evaluated the effect of genetic drift and balancing selection on MHCII diversity in 12 small populations of Galapagos mockingbirds belonging to four different species, and one larger population of the Northern mockingbird from the continental USA. After genotyping MHCII loci by high-throughput sequencing, we applied a correlational approach to explore the relationships between MHCII diversity and population size by proxy of island size. As expected when drift predominates, we found a positive effect of population size on the number of MHCII alleles present in a population. However, the number of MHCII alleles per individual and number of supertypes were not correlated with population size. This discrepancy points to an interesting feature of MHCII diversity dynamics: some levels of diversity might be shaped by genetic drift while others are independent and possibly maintained by balancing selection. | 765 | no | no good fitness measure, N pop differnt in GD? | NA | no | no |
| 766 | 2017 | 43012 | Christie, MR; Knowles, LL | 2015 | Habitat corridors facilitate genetic resilience irrespective of species dispersal abilities or population sizes | Corridors are frequently proposed to connect patches of habitat that have become isolated due to human-mediated alterations to the landscape. While it is understood that corridors can facilitate dispersal between patches, it remains unknown whether corridors can mitigate the negative genetic effects for entire communities modified by habitat fragmentation. These negative genetic effects, which include reduced genetic diversity, limit the potential for populations to respond to selective agents such as disease epidemics and global climate change. We provide clear evidence from a forward-time, agent-based model (ABM) that corridors can facilitate genetic resilience in fragmented habitats across a broad range of species dispersal abilities and population sizes. Our results demonstrate that even modest increases in corridor width decreased the genetic differentiation between patches and increased the genetic diversity and effective population size within patches. Furthermore, we document a trade-off between corridor quality and corridor design whereby populations connected by high-quality habitat (i.e., low corridor mortality) are more resilient to suboptimal corridor design (e.g., long and narrow corridors). The ABM also revealed that species interactions can play a greater role than corridor design in shaping the genetic responses of populations to corridors. These results demonstrate how corridors can provide long-term conservation benefits that extend beyond targeted taxa and scale up to entire communities irrespective of species dispersal abilities or population sizes. | 766 | no | no good fitness measure, N pop differnt in GD? | NA | no | no |
| 767 | 2017 | 43012 | Boles, BR; Thoendel, M; Singh, PK | 2004 | “Self-generated diversity produces”“insurance effects”" in biofilm communities" | “Diversity generally protects communities from unstable environmental conditions. This principle, known as the”“insurance hypothesis,”" has been tested in many different ecosystems. Here we show that the opportunistic pathogen Pseudomonas aeruginosa undergoes extensive genetic diversification during short-term growth in biofilm communities. The induced genetic changes are produced by a recA-dependent mechanism and affect multiple traits, including the behavior of the bacteria in biofilms. Some biofilm-derived variants exhibit an increased ability to disseminate, whereas others manifest accelerated biofilm formation. Furthermore, the presence of these functionally diverse bacteria increases the ability of biofilms to resist an environmental stress. These findings suggest that self-generated diversity in biofilms provides a form of biological insurance that can safeguard the community in the face of adverse conditions." | 767 | no | no good fitness measure, N pop differnt in GD? | NA | no | no |
| 768 | 2017 | 43012 | Boles, BR; Singh, PK | 2008 | Endogenous oxidative stress produces diversity and adaptability in biofilm communities | Many bacterial species are capable of biofilm growth, in which cells live and replicate within multicellular community groups. Recent work shows that biofilm growth by a wide variety of bacterial species can generate genetic diversity in microbial populations. This finding is significant because the presence of diverse sub-populations can extend the range of conditions in which communities can thrive. Here, we used biofilms formed by the pathogen Pseudomonas aeruginosa to investigate how this population diversity is produced. We found that some cells within biofilms incur double-stranded DNA breaks caused by endogenous oxidative stress. Genetic variants then result when breaks are repaired by a mutagenic mechanism involving recombinatorial DNA repair genes. We hypothesized that the mutations produced could promote the adaptation of biofilm communities to changing conditions in addition to generating diversity. To test this idea, we exposed biofilms to an antibiotic and found that the oxidative stress-break repair mechanism increased the emergence of antibiotic-resistant bacteria. The diversity and adaptability produced by this mechanism could help biofilm communities survive in harsh environments. | 768 | no | no good fitness measure, N pop differnt in GD? | NA | no | no |
| 769 | 2017 | 43012 | Ujvari, B; Madsen, T; Kotenko, T; Olsson, M; Shine, R; Wittzell, H | 2002 | Low genetic diversity threatens imminent extinction for the Hungarian meadow viper (Vipera ursinii rakosiensis) | Meadow vipers (Vipera ursinii) are small venomous snakes whose range in Hungary has been greatly fragmented by anthropogenic habitat disturbance (especially, agriculture). We obtained DNA from a total of eight Hungarian snakes. Genetic variability at the major histocompatibility (Mhc) class I loci was much lower for these snakes than for specimens from two large Ukrainian populations. Within two Hungarian populations for which we had multiple individuals, band-sharing indices were 100 and 84.6% (versus 63.3 and 57% for the Ukraine populations). The Ukrainian snakes also displayed more RFLP fragments than the Hungarian vipers (mean 13.7 versus 9.0, respectively). In combination with reports of birth deformities, chromosomal abnormalities and low juvenile survival, these data strongly suggest that the Hungarian vipers are experiencing inbreeding depression. Genetic diversity is still present in the Hungarian vipers but among rather than within populations. Given the very low numbers of animals, the only feasible strategy to increase the genetic diversity and to save the Hungarian vipers from extinction is to implement a captive breeding program based on genetically screened animals. (C) 2002 Elsevier Science Ltd. All rights reserved. | 769 | no | no pop level | NA | no | no |
| 770 | 2017 | 43012 | Stassen, HH; Hoffman, K; Scharfetter, C | 2003 | Similarity by state/descent and genetic vector spaces: analysis of a longitudinal family study | “Using the genome-wide screening data of the Framingham Heart Study ( 394 nuclear families, 1328 genotyped subjects, 397 marker loci) we have quantified the underlying genetic diversity through high-dimensional genetic feature vectors and constructed a genetic vector space for the analysis of population substructure. Adaptive clustering procedures led to three major subgroups that were regarded as being related to”“biological”" ethnicity and that included more than 70% of the subjects. Based on these subgroups we addressed the question of ethnicity- related and ethnicity-independent risk factors for coronary heart disease ( CHD). To this end, we relied upon hypertension as an endophenotype of CHD and applied a multivariate sib-pair method in order to search for oligogenic marker configurations for which the sib-sib similarities deviated from the parent-offspring similarities. Indeed, the latter similarities are always "“0.5”" irrespective of the affection status of parents and offspring. Loci with significant contributions to the oligogenic marker configuration constituted a CHD-specific genetic vector space. We found several ethnicity-independent signals. One signal on chromosome 8 may relate to the CYP11B1/CYP11B2 genes." | 770 | no | NA | NA | no | no |
| 771 | 2017 | 43012 | Conte, R; Nodari, RO; Vencovsky, R; dos Reis, MS | 2003 | Genetic diversity and recruitment of the tropical palm, Euterpe edulis Mart., in a natural population from the Brazilian Atlantic Forest | The genetic diversity and recruitment of plants of heart-of-palm tree (Euterpe edulis Mart.) were investigated in a natural population located in Southern Brazil. Five categories of plants, from seedlings to adults, were analysed using 16 allozymic loci. The results showed an average population level of genetic diversity (H-e = 0.278) greater than the average of plant species already studied. The recruitment process of E. edulis is related to its genetic characteristics. A significant increase in the heterozygote frequency towards the adult stages was observed at three loci (Pgdh-2, G6pdh-1 and Mdh-1). This suggests the possible action of natural selection in promoting such heterozygote increase. In the same way, a linear increase in allele frequencies was observed at four loci (Prx-3, Prx-4, Pgdh-2 and G6pdh-1), indicating that recruitment is also related to a greater survival of individuals that are carriers of certain alleles. The maintenance of high diversity levels, as well as the increase in the heterozygote frequency, are positive aspects for in situ conservation. Furthermore, an increase in heterozygote frequency is favourable to the management of the species, since the maintenance of a stock of reproductive individuals with high heterozygosity levels favours the maintenance of the population dynamic and structure. | 771 | no | only one pop | NA | no | no |
| 772 | 2017 | 43012 | Chen, SF; Li, MW; Hou, RF; Liao, WB; Zhou, RC; Fan, Q | 2014 | Low genetic diversity and weak population differentiation in Firmiana danxiaensis, a tree species endemic to Danxia landform in northern Guangdong, China | Danxia landform, a unique type of petrographic geomorphology, sporadically occurs in southern China. There are about 400 rare or threatened plants and animals in this landform, but little information on genetic aspects of these species has been available. Of them, Firmiana danxiaensis is a dominant tree species endemic to Mount Danxia, a typical Danxia landform in northern Guangdong, China. In this study, we examined genetic diversity and population structure of E danxiaensis, in comparison with two other narrowly distributed Firmiana species, Firmiana hainanensis and Firmiana kwangsiensis, which grow in non-Danxia-landform regions. Nine microsatellite markers were used to investigate genetic diversity and differentiation in a total of 256 individuals from nine populations of E danxiaensis, three of E hainanensis, and one of E kwangsiensis. The results showed that E danxiaensis had the lowest genetic diversity (H-E = 0.364 +/- 0.019) among the three species, but weak population structure similar to another Firmiana species (F-ST = 0.042 and 0.020, respectively). This low genetic diversity observed in E danxiaensis is possibly due to confined natural distribution range and strong selective pressure in soils of Danxia landform, which may also be true for other endemic species in Danxia landform. For the long term survival of these endemic species, habitat conservation and anthropogenic introduction to similar habitats such as the adjacent Danxia landform areas should be two major strategies. (C) 2014 Elsevier Ltd. All rights reserved. | 772 | no | comparing species | NA | no | no |
| 773 | 2017 | 43012 | Sork, VL; Smouse, PE | 2006 | Genetic analysis of landscape connectivity in tree populations | Genetic connectivity in plant populations is determined by gene movement within and among populations. When populations become genetically isolated, they are at risk of loss of genetic diversity that is critical to the long-term survival of populations. Anthropogenic landscape change and habitat fragmentation have become so pervasive that they may threaten the genetic connectivity of many plant species. The theoretical consequences of such changes are generally understood, but it is not immediately apparent how concerned we should be for real organisms, distributed across real landscapes. Our goals here are to describe how one can study gene movement of both pollen and seeds in the context of changing landscapes and to explain what we have learned so far. In the first part, we will cover methods of describing pollen movement and then review evidence for the impact of fragmentation in terms of both the level of pollen flow into populations and the genetic diversity of the resulting progeny. In the second part, we will describe methods for contemporary seed movement, and describe findings about gene flow and genetic diversity resulting from seed movement. Evidence for pollen flow suggests high connectivity, but it appears that seed dispersal into fragments may create genetic bottlenecks due to limited seed sources. Future work should address the interaction of pollen and seed flow and attention needs to be paid to both gene flow and the diversity of the incoming gene pool. Moreover, if future work is to model the impact of changing landscapes on propagule movement, with all of its ensuing consequences for genetic connectivity and demographic processes, we will need an effective integration of population genetics and landscape ecology. | 773 | no | no good fitness measure, N pop differnt in GD? | NA | no | no |
| 774 | 2017 | 43012 | Prochazka, P; Bellinvia, E; Fainova, D; Hajkova, P; Elhalah, A; Alomari, K | 2008 | Immigration as a possible rescue of a reduced population of a long-distant migratory bird: Reed warblers in the Azraq Oasis, Jordan | After a population bottleneck, genetic drift can result in sudden and dramatic changes in allele frequencies that occur independently of selection. In such instances, many beneficial adaptations may be eliminated even if the population later increases again. Conversely, immigration may successfully counter the adverse effect of reduced population size, bringing new or lost alleles and replenishing thus genetic diversity of a reduced population. Extensive groundwater abstraction from the Azraq Oasis led to a considerable deterioration of the unique wetland ecosystem during the 1980s and most of the aquatic vegetation consequently died off in the early 1990s. At that time, numbers of local reed warblers (Acrocephalus scirpaceus) dramatically dropped to a few pairs. The numbers slightly increased again after wetland restoration efforts were begun. To infer possible consequences of the population collapse on genetic diversity, we genotyped 40 local breeders at 10 polymorphic microsatellite loci. After 15 years from the crash, the population showed no evidence for past genetic bottlenecks. The absence of reduced genetic diversity suggests that the population has largely benefited front gene flow. The study shows how immigration may sustain the viability of fragmented populations of migratory birds after restoration of their former breeding habitat. (c) 2008 Elsevier Ltd. All rights reserved. | 774 | no | only one pop | NA | no | no |
| 775 | 2017 | 43012 | Mboup, M; Leconte, M; Gautier, A; Wan, AM; Chen, W; de Vallavieille-Pope, C; Enjalbert, J | 2009 | Evidence of genetic recombination in wheat yellow rust populations of a Chinese oversummering area | Wheat yellow rust (Puccinia striiformis f.sp. tritici) (PST) has been described as a strongly clonal species in both European and Australian populations, with very limited molecular diversity but rapidly evolving virulences. Contrastingly, marked genetic diversity has been reported in Chinese PST populations. To test whether such variability could originate from oversummering areas, we assessed the diversity of virulence and molecular markers (AFLP and SSR) using 412 PST isolates from the highlands of Tianshui county in Gansu province. Very marked phenotypic and genotypic diversity (38% and 89%, respectively) was found. No genetic structure dependent on the sites sampled (Fst = 0.004) or altitude distribution (Fst = 0.0098) was detected, indicating important gene flow at the county scale. This study also revealed genetic recombination between molecular markers and thus strongly suggests the existence of a sexual or parasexual cycle in PST in Tianshui county. The observations of higher rates of sexual spore production in genotypes originating from Tianshui are the very first elements suggestive of the existence of a sexual cycle in this species. Crown Copyright (C) 2009 Published by Elsevier Inc. All rights reserved. | 775 | no | gd of pathogen | NA | no | no |
| 776 | 2017 | 43012 | Espindola, A; Alvarez, N | 2011 | Comparative Phylogeography in a Specific and Obligate Pollination Antagonism | In specific and obligate interactions the nature and abundance of a given species can have important effects on the survival and population dynamics of associated organisms. In a phylogeographic framework, we therefore expect that the fates of organisms interacting specifically are also tightly interrelated. Here we investigate such a scenario by analyzing the genetic structures of species interacting in an obligate plant-insect pollination lure-and-trap antagonism, involving Arum maculatum (Araceae) and its specific psychodid (Diptera) visitors Psychoda phalaenoides and Psycha grisescens. Because the interaction is asymmetric (i.e., only the plant depends on the insect), we expect the genetic structure of the plant to be related with the historical pollinator availability, yielding incongruent phylogeographic patterns between the interacting organisms. Using insect mtDNA sequences and plant AFLP genome fingerprinting, we inferred the large-scale phylogeographies of each species and the distribution of genetic diversities throughout the sampled range, and evaluated the congruence in their respective genetic structures using hierarchical analyses of molecular variances (AMOVA). Because the composition of pollinator species varies in Europe, we also examined its association with the spatial genetic structure of the plant. Our findings indicate that while the plant presents a spatially well-defined genetic structure, this is not the case in the insects. Patterns of genetic diversities also show dissimilar distributions among the three interacting species. Phylogeographic histories of the plant and its pollinating insects are thus not congruent, a result that would indicate that plant and insect lineages do not share the same glacial and postglacial histories. However, the genetic structure of the plant can, at least partially, be explained by the type of pollinators available at a regional scale. Differences in life-history traits of available pollinators might therefore have influenced the genetic structure of the plant, the dependent organism, in this antagonistic interaction. | 776 | no | no pop level | NA | no | no |
| 777 | 2017 | 43012 | Kang, HM; Zaitlen, NA; Wade, CM; Kirby, A; Heckerman, D; Daly, MJ; Eskin, E | 2008 | Efficient control of population structure in model organism association mapping | Genomewide association mapping in model organisms such as inbred mouse strains is a promising approach for the identification of risk factors related to human diseases. However, genetic association studies in inbred model organisms are confronted by the problem of complex population structure among strains. This induces inflated false positive rates, which cannot be corrected using standard approaches applied in human association studies such as genomic control or structured association. Recent studies demonstrated that mixed models successfully correct for the genetic relatedness in association mapping in maize and Arabidopsis panel data sets. However, the currently available mixed-model methods suffer froth computational inefficiency. In this article, we propose a new method, efficient mixed-model association (EMMA), which corrects for population structure and genetic relatedness in model organism association mapping. Our method takes advantage of the specific nature of the optimization problem in applying mixed models for association snapping, which allows us to substantially increase the computational speed and reliability of the results. We applied EMMA to in silico whole-genome association mapping of inbred mouse strains involving hundreds of thousands of SNPs, in addition to Arabidopsis and maize data sets. We also performed extensive simulation studies to estimate the statistical power of EMMA under various SNP effects, varying degrees of population structure, and differing numbers of multiple measurements per strain. Despite the fruited power of inbred mouse association mapping due to the limited number of available inbred strains, we are able to identify significantly associated SNPs, which fall into known QTL or genes identified through previous studies while avoiding an inflation of false positives. An R package implementation and webserver of our EMMA method are publicly available. | 777 | no | no good fitness measure, N pop differnt in GD? | NA | no | no |
| 778 | 2017 | 43012 | Gagnaire, PA; Albert, V; Jonsson, B; Bernatchez, L | 2009 | Natural selection influences AFLP intraspecific genetic variability and introgression patterns in Atlantic eels | Investigating patterns of genetic variation in hybridizing species provides an opportunity to understand the impact of natural selection on intraspecific genetic variability and interspecific gene exchange. The Atlantic eels Anguilla rostrata and A. anguilla each occupy a large heterogeneous habitat upon which natural selection could differentially shape genetic variation. They also produce viable hybrids only found in Iceland. However, the possible footprint of natural selection on patterns of genetic variation within species and introgressive hybridization in Icelandic eels has never been assessed. We revisited amplified fragment length polymorphism data collected previously using population genomics and admixture analyses to test if (i) genetic variation could be influenced by non-neutral mechanisms at both the intra- and interspecific levels, and if (ii) selection could shape the spatio-temporal distribution of Icelandic hybrids. We first found candidate loci for directional selection within both species. Spatial distributions of allelic frequencies displayed by some of these loci were possibly related with the geographical patterns of life-history traits in A. rostrata, and could have been shaped by natural selection associated with an environmental gradient along European coasts in A. anguilla. Second, we identified outlier loci at the interspecific level. Non-neutral introgression was strongly suggested for some of these loci. We detected a locus at which typical A. rostrata allele hardly crossed the species genetic barrier, whereas three other loci showed accelerated patterns of introgression into A. anguilla in Iceland. Moreover, the level of introgression at these three loci increased from the glass eel to the yellow eel stage, supporting the hypothesis that differential survival of admixed genotypes partly explains the spatio-temporal pattern of hybrid abundance previously documented in Iceland. | 778 | no | hybrids | NA | no | no |
| 779 | 2017 | 43012 | Shafer, ABA; Fan, CW; Cote, SD; Coltman, DW | 2012 | (Lack of) Genetic Diversity in Immune Genes Predates Glacial Isolation in the North American Mountain Goat (Oreamnos americanus) | The major histocompatibility complex (MHC) plays an important role in an organism’s ability to respond to pathogens. Immunogenetic diversity is advantageous as it permits the recognition of more external antigens. For this reason, MHC and immune gene variation are considered a barometer for the genetic health of wild populations. Mountain goats (Oreamnos americanus) were previously shown to have little variation at the MHC Class II Oram-DRB locus, which was attributed to population bottlenecks during the last glacial maximum (LGM). In this paper, we extended the analysis of immunogenetic variability in mountain goats to 5 genes representing the 3 classes of MHC gene (Class I OLA, Class II DRA and DRB, and Class III TNF-alpha) and the natural resistance-associated macrophage protein. We sequenced approximately 3000 bp from 31 individuals sampled across the range of mountain goats and found very low levels of diversity (1-3 polymorphic sites per gene) with the exception of the Class I Oram-OLA gene. Oram-OLA was nearly 30 times more diverse than the other immune genes and appears to represent a source of increased immunogenetic diversity. This diversity may be attributed to multiple loci, mediated by pathogen exposure, or potentially influenced by social factors. The distribution of SNPs was not associated with refugial history, suggesting that the current distribution of immunogenetic diversity was present prior to the LGM. These data suggest that although they have low levels of diversity at the 4 of 5 immune loci, mountain goats may be better equipped for future climate oscillations and pathogen exposure than previously thought. | 779 | no | no pop level | NA | no | no |
| 780 | 2017 | 43012 | Neafsey, DE; Schaffner, SF; Volkman, SK; Park, D; Montgomery, P; Milner, DA; Lukens, A; Rosen, D; Daniels, R; Houde, N; Cortese, JF; Tyndall, E; Gates, C; Stange-Thomann, N; Sarr, O; Ndiaye, D; Ndir, O; Mboup, S; Ferreira, MU; Moraes, SD; Dash, AP; Chitnis, CE; Wiegand, RC; Hartl, DL; Birren, BW; Lander, ES; Sabeti, PC; Wirth, DF | 2008 | Genome-wide SNP genotyping highlights the role of natural selection in Plasmodium falciparum population divergence | Background: The malaria parasite Plasmodium falciparum exhibits abundant genetic diversity, and this diversity is key to its success as a pathogen. Previous efforts to study genetic diversity in P. falciparum have begun to elucidate the demographic history of the species, as well as patterns of population structure and patterns of linkage disequilibrium within its genome. Such studies will be greatly enhanced by new genomic tools and recent large-scale efforts to map genomic variation. To that end, we have developed a high throughput single nucleotide polymorphism (SNP) genotyping platform for P. falciparum. Results: Using an Affymetrix 3,000 SNP assay array, we found roughly half the assays (1,638) yielded high quality, 100% accurate genotyping calls for both major and minor SNP alleles. Genotype data from 76 global isolates confirm significant genetic differentiation among continental populations and varying levels of SNP diversity and linkage disequilibrium according to geographic location and local epidemiological factors. We further discovered that nonsynonymous and silent (synonymous or noncoding) SNPs differ with respect to within-population diversity, interpopulation differentiation, and the degree to which allele frequencies are correlated between populations. Conclusions: The distinct population profile of nonsynonymous variants indicates that natural selection has a significant influence on genomic diversity in P. falciparum, and that many of these changes may reflect functional variants deserving of follow-up study. Our analysis demonstrates the potential for new high-throughput genotyping technologies to enhance studies of population structure, natural selection, and ultimately enable genome-wide association studies in P. falciparum to find genes underlying key phenotypic traits. | 780 | no | no good fitness measure, N pop differnt in GD? | NA | no | no |
| 781 | 2017 | 43012 | Morran, LT; Cappy, BJ; Anderson, JL; Phillips, PC | 2009 | SEXUAL PARTNERS FOR THE STRESSED: FACULTATIVE OUTCROSSING IN THE SELF-FERTILIZING NEMATODE CAENORHABDITIS ELEGANS | Sexual reproduction shuffles genetic variation, potentially enhancing the evolutionary response to environmental change. Many asexual organisms respond to stress by generating facultative sexual reproduction, presumably as a means of escaping the trap of low genetic diversity. Self-fertilizing organisms are subject to similar genetic limitations: the consistent loss of genetic diversity within lineages restricts the production of variation through recombination. Selfing organisms may therefore benefit from a similar shift in mating strategy during periods of stress. We determined the effects of environmental stress via starvation and passage through the stress-resistant dauer stage on mating system dynamics of Caenorhabditis elegans, which reproduces predominantly through self-fertilization but is capable of outcrossing in the presence of males. Starvation elevated male frequencies in a strain-specific manner through differential male survival during dauer exposure and increased outcrossing rates after dauer exposure. In the most responsive strain, the mating system changed from predominantly selfing to almost exclusively outcrossing. Like facultative sex in asexual organisms, facultative outcrossing in C. elegans may periodically facilitate adaptation under stress. Such a shift in reproductive strategy should have a major impact on evolutionary change within these populations and may be a previously unrecognized feature of other highly selfing organisms. | 781 | no | no good fitness measure, N pop differnt in GD? | NA | no | no |
| 782 | 2017 | 43012 | Crozier, RH; Fjerdingstad, EJ | 2001 | Polyandry in social Hymenoptera - disunity in diversity? | Multiple mating by queens occurs in many species of social Hymenoptera despite its likely costs. Hypotheses to explain multiple mating include a need for more sperm than provided by a single male, the convergence of queen and worker sex-allocation optima and various genetic diversity hypotheses. For some species the sperm need hypothesis fails since queens retain only a single male’s worth of sperm. In other cases, sperm store does increase with the number of matings. Similarly for the sex-allocation and genetic diversity hypotheses, data from some species are in support, those from others are not. Comparative analysis reveals a negative correlation between level of genetic diversity (of which queen mate number is an important determinant) and parasite load; findings in the best-studied case are complex: monandry and higher levels of polyandry are each selectively favored over moderate polyandry. Out of 14 identifiable hypotheses five are judged most useful for future work. Unfortunately, the search for a simple unitary model to explain all cases seems futile. A model encompassing all of these factors is desirable for studies on single species, but would be complex. Comparative analyses remain desirable, but should encompass the likelihood that different factors predominate in different groups. | 782 | probably no | comparing different studies? Interestingb for discussion! | no good group comp | yes | no |
| 783 | 2017 | 43012 | Bishop, JM; O’Ryan, C; Jarvis, JUM | 2007 | Social common mole-rats enhance outbreeding via extra-pair mating | Females in many species engage in matings with males that are not their social mates. These matings are predicted to increase offspring heterozygosity and fitness, and thereby prevent the deleterious effects of inbreeding. We tested this hypothesis in a cooperative breeding mammal, the common mole-rat Cryptomys hottentotus hottentotus. Laboratory-based studies suggested a system of strict social monogamy, while recent molecular studies indicate extensive extra-pair paternity despite colonies being founded by an outbred pair. Our data show that extra-pair and within-colony breeding males differed significantly in relatedness to breeding females, suggesting that females may gain genetic benefits from breeding with non-resident males. Extra-colony male mating success was not based on heterozygosity criteria at microsatellite loci; however, litters sired by extra-colony males exhibited increased heterozygosity. While we do not have the data that refute a relationship between individual levels of inbreeding (H(s)) and fitness, we propose that a combination of both male and female factors most likely explain the adaptive significance of extra-pair mating whereby common mole-rats maximize offspring fitness by detecting genetic compatibility with extra-pair mates at other key loci, but it is not known which sex controls these matings. | 783 | no | no good fitness measure, N pop differnt in GD? | NA | no | no |
| 784 | 2017 | 43012 | Ellis, SA; Hammond, JA | 2014 | The Functional Significance of Cattle Major Histocompatibility Complex Class I Genetic Diversity | Current concerns about food security highlight the importance of maintaining productive and disease-resistant livestock populations. Major histocompatibility complex (MHC) class I genes have a central role in immunity. A high level of diversity in these genes allows populations to survive despite exposure to rapidly evolving pathogens. This review aims to describe the key features of MHC class I genetic diversity in cattle and to discuss their role in disease resistance. Discussion centers on data derived from the cattle genome sequence and studies addressing MHC class I gene expression and function. The impact of intensive selection on MHC diversity is also considered. A high level of complexity in MHC class I genes and functionally related gene families is revealed. This highlights the need for increased efforts to determine key genetic components that govern cattle immune responses to disease, which is increasingly important in the face of changing human and environmental demands. | 784 | no | domesticated animal | NA | no | no |
| 785 | 2017 | 43012 | Canestrelli, D; Bisconti, R; Sacco, F; Nascetti, G | 2014 | What triggers the rising of an intraspecific biodiversity hotspot? Hints from the agile frog | Hotspots of genetic diversity are regions of utmost importance for species survival and conservation, and their intimate link with the geographic location of glacial refugia has been well established. Nonetheless, the microevolutionary processes underlying the generation of hotspots in such regions have only recently become a fervent field of research. We investigated the phylogeographic and population genetic structure of the agile frog, Rana dalmatina, within its putative refugium in peninsular Italy. We found this region to harbour far more diversity, phylogeographic structure, and lineages of ancient origin than that by the rest of the species’ range in Europe. This pattern appeared to be well explained by climate-driven microevolutionary processes that occurred during both glacial and interglacial epochs. Therefore, the inferred evolutionary history of R. dalmatina in Italy supports a view of glacial refugia as ‘factories’ rather than as repositories of genetic diversity, with significant implications for conservation strategies for hotspots. | 785 | no | no good fitness measure | NA | no | no |
| 786 | 2017 | 43012 | Nikzad, S; Tan, SG; Yien, CYS; Ng, J; Alitheen, NB; Khan, R; Rovie-Ryan, JJ; Valdiani, A; Khajeaian, P; Kanthaswamy, S | 2014 | Genetic diversity and population structure of long-tailed macaque (Macaca fascicularis) populations in Peninsular Malaysia | BackgroundThe genetic diversity and structure of long-tailed macaques (Macaca fascicularis) in Peninsular Malaysia, a widely used non-human primate species in biomedical research, have not been thoroughly characterized. MethodsThirteen sites of wild populations of long-tailed macaques representing six states were sampled and analyzed with 18 STR markers. ResultsThe Sunggala and Penang Island populations showed the highest genetic diversity estimates, while the Jerejak Island population was the most genetically discrete due to isolation from the mainland shelf. Concordant with pairwise F-st estimates, STRUCTURE analyses of the seven PCA-correlated clusters revealed low to moderate differentiation among the sampling sites. No association between geographic and genetic distances exists, suggesting that the study sites, including island study sites, are genetically if not geographically contiguous. ConclusionsThe status of the genetic structure and composition of long-tailed macaque populations require further scrutiny to develop this species as an important animal model in biomedical research. | 786 | no | no good fitness measure | NA | no | no |
| 787 | 2017 | 43012 | Brown, SM; Harrisson, KA; Clarke, RH; Bennett, AF; Sunnucks, P | 2013 | Limited Population Structure, Genetic Drift and Bottlenecks Characterise an Endangered Bird Species in a Dynamic, Fire-Prone Ecosystem | Fire is a major disturbance process in many ecosystems world-wide, resulting in spatially and temporally dynamic landscapes. For populations occupying such environments, fire-induced landscape change is likely to influence population processes, and genetic patterns and structure among populations. The Mallee Emu-wren Stipiturus mallee is an endangered passerine whose global distribution is confined to fire-prone, semi-arid mallee shrublands in south-eastern Australia. This species, with poor capacity for dispersal, has undergone a precipitous reduction in distribution and numbers in recent decades. We used genetic analyses of 11 length-variable, nuclear loci to examine population structure and processes within this species, across its global range. Populations of the Mallee Emu-wren exhibited a low to moderate level of genetic diversity, and evidence of bottlenecks and genetic drift. Bayesian clustering methods revealed weak genetic population structure across the species’ range. The direct effects of large fires, together with associated changes in the spatial and temporal patterns of suitable habitat, have the potential to cause population bottlenecks, serial local extinctions and subsequent recolonisation, all of which may interact to erode and homogenise genetic diversity in this species. Movement among temporally and spatially shifting habitat, appears to maintain long-term genetic connectivity. A plausible explanation for the observed genetic patterns is that, following extensive fires, recolonisation exceeds in-situ survival as the primary driver of population recovery in this species. These findings suggest that dynamic, fire-dominated landscapes can drive genetic homogenisation of populations of species with low-mobility and specialised habitat that otherwise would be expected to show strongly structured populations. Such effects must be considered when formulating management actions to conserve species in fire-prone systems. | 787 | no | no good fitness measure, N pop differnt in GD? | NA | no | no |
| 788 | 2017 | 43012 | Zhao, M; Li, CL; Zhang, W; Wang, H; Luo, ZH; Gu, Q; Gu, ZR; Liao, CL; Wu, H | 2016 | Male pursuit of higher reproductive success drives female polyandry in the Omei treefrog | Female polyandry and corresponding multiple paternity have been detected in many species. Several hypotheses, such as the fertility guarantee hypothesis, the good gene hypothesis, the genetic compatibility hypothesis and the genetic diversity hypothesis, have been proposed to explain the evolution of female polyandry. Anuran amphibians are widely observed to breed in polyandrous spawning groups; however, few studies have evaluated multiple paternity in these species or the evolutionary mechanisms underlying polyandry. In this study, we addressed these questions pertaining to polyandry in the Omei treefrog, Rhacophorus omeimontis. Eight microsatellite markers of R. omeimontis were used to determine the patterns of paternity. Paternity analyses revealed that offspring were multiply sired in a majority (79.07%) of the polyandrous spawning groups. A male’s reproductive output during a breeding season was greatly affected by the mating rate, revealing that male fitness benefited from multiple copulations. By contrast, no evidence supported the fertility guarantee hypothesis that polyandry increases the fertility rate of females or the compatible/good gene hypotheses that polyandrous females produce more compatible or superior offspring. The increased allelic diversity observed in polyandrous clutches is probably a nonadaptive by-product of multiple mating. In conclusion, males join as many copulations as possible to increase their reproductive success, regardless of whether their mate has already been in amplexus with other males. Females may gain no benefits from polyandry but are incapable of preventing superfluous males from joining the mating group due to the physical characteristics and breeding behaviour of anurans. Thus, in R. omeimontis, male pursuit of higher reproductive success is the primary explanation for female polyandry. (C) 2015 The Association for the Study of Animal Behaviour. Published by Elsevier Ltd. All rights reserved. | 788 | no | no good fitness measure, N pop differnt in GD? | NA | no | no |
| 789 | 2017 | 43012 | Sajantila, A; Salem, AH; Savolainen, P; Bauer, K; Gierig, C; Paabo, S | 1996 | Paternal and maternal DNA lineages reveal a bottleneck in the founding of the Finnish population | An analysis of Y-chromosomal haplotypes in several European populations reveals an almost monomorphic pattern in the Finns, whereas Y-chromosomal diversity is significantly higher in other populations. Furthermore, analyses of nucleotide positions in the mitochondrial control region that evolve slowly show a decrease in genetic diversity in Finns. Thus, relatively few men and women have contributed the genetic lineages that today survive in the Finnish population. This is likely to have caused the so-called ‘’Finnish disease heritage’’-i.e., the occurrence of several genetic diseases in the Finnish population that are rare elsewhere. A preliminary analysis of the mitochondrial mutations that have accumulated subsequent to the bottleneck suggests that it occurred about 4000 years ago, presumably when populations using agriculture and animal husbandry arrived in Finland. | 789 | no | human | NA | no | no |
| 790 | 2017 | 43012 | Bellemain, E; Nawaz, MA; Valentini, A; Swenson, JE; Taberlet, P | 2007 | Genetic tracking of the brown bear in northern Pakistan and implications for conservation | Asian bears face major threats due to the impact of human activities as well as a critical lack of knowledge about their status, distribution and needs for survival. Once abundant in northern Pakistan, the Himalayan brown bear (Ursus arctos isabellinus) has been exterminated in most of its former distribution range. It presently occurs sparsely, in small populations, the Deosai National Park supporting the largest isolate. This decline might imply a reduction in genetic diversity, compromising the survival of the population. Using a combination of fecal DNA analysis and field data, our study aimed at assessing the size and genetic status of the Deosai population and give guidelines for its conservation and management. Using fecal genetic analysis, we estimated the population to be 40-50 bears, which compares well with the field census of 38 bears. The northern Pakistani brown bear population may have undergone an approximate 200-300-fold decrease during the last thousand years, probably due to glaciations and the influence of growing human population. However, in spite of the presence of a bottleneck genetic signature, the Deosai population has a moderate level of genetic diversity and is not at immediate risk of inbreeding depression. Gene flow might exist with adjacent populations. We recommend careful monitoring of this population in the future both with field observations and genetic analyses, including sampling of adjacent populations to assess incoming gene flow. The connectivity with adjacent populations in Pakistan and India will be of prime importance for the longterm survival of Deosai bears. (c) 2006 Elsevier Ltd. All rights reserved. | 790 | no | only one pop | NA | no | no |
| 791 | 2017 | 43012 | Cureton, JC; Janis, M; Lutterschmidt, WI; Randle, CP; Ruthven, DC; Deaton, R | 2014 | Effects of urbanization on genetic diversity, gene flow, and population structure in the ornate box turtle (Terrapene ornata) | Large-scale declines in North American box turtle (Terrapene spp.) populations have been attributed to habitat fragmentation as a result of urbanization. We compared microsatellite markers and mitochondrial control region sequences of Ornate Box Turtles (Terrapene ornata) in two populations (a natural and an urban habitat) to test two hypotheses. We hypothesized that urban populations of T. ornata experience genetic bottlenecks due to road mortality and habitat fragmentation, and that roadways represent a barrier to gene flow among turtle populations, resulting in increased fragmentation of gene pools. Both populations shared similar allelic diversity and observed heterozygosity, with eight and seven of twelve microsatellite loci exhibiting heterozygote deficiency in the natural and urban populations, respectively. The number of mitochondrial control region haplotypes in the urban population was nearly four times that of the natural population, although only one haplotype occurred in appreciable frequency in both populations. We did not detect conclusive evidence of a recent genetic bottleneck in the urban population, thereby rejecting our first hypothesis. We detected weak differentiation among populations on opposing sides of a large highway, but did not detect any evidence of population structure, thereby rejecting our second hypothesis. This study indicates that a population of T. ornata with moderate road mortality currently has high genetic diversity, moderate inbreeding, and displays some evidence of genetic differentiation, but no conclusive evidence of recent genetic bottlenecks or unique genetic clustering. We suggest this is primarily due to the species long generation time and is a positive aspect of their life-history. | 791 | no | no good fitness measure, N pop differnt in GD? | NA | no | no |
| 792 | 2017 | 43012 | WIDEN, B; ANDERSSON, S | 1993 | QUANTITATIVE GENETICS OF LIFE-HISTORY AND MORPHOLOGY IN A RARE PLANT, SENECIO-INTEGRIFOLIUS | A paternal half-sib analysis was performed to estimate the narrow-sense heritability for 35 morphological and life-history characters in two populations of Senecio integrifolius, a rare and threatened plant in Sweden. The two populations differed greatly in size, the small one consisting of separate patches and the large one having a more or less continuous distribution. In addition to significant population differences in the mean of 26 characters, we found slightly higher heritabilities in the small and patchy population (average 0.49) than in the large and continuous population (average 0.43). Overall, the small population displayed significant additive genetic variation for a larger number of characters than the large population (19 vs. 14). Paternal (genetic) effects were consistent in two different environments; the ranking of sire means was similar despite a drastic reduction in light intensity, and few characters had a significant sire x environment interaction. Genetic correlations were also found between different characters measured in the same environment, although most of these were in the ‘favourable’ direction. Population differences in genetic parameters may reflect contrasting selection regimens at the two sites, although genetic drift may also be important. That more genetic variability was detected in the small population where plants occur in small and isolated patches indicates that the spatial structure has great effects on the retention of heritable variation, and that demographic and environmental factors are more important in the long-term survival of S. integrifolius. This is the first study that relates quantitative genetic variation to population size in a rare and threatened plant. | 792 | no | no good fitness measure, N pop differnt in GD? | NA | no | no |
| 793 | 2017 | 43012 | Woo, C; Kang, I; Yun, H; Dirisala, VP; Kim, N; Um, SJ; Kim, J; Lee, HT; Lee, JH; Park, C | 2005 | Molecular cloning and analysis of allelic variation of the SLA-2 gene in Korean native pigs | Genes consisting of major histocompatibility complex (MHC) are highly polymorphic. Understanding the genetic variation can provide important information on estimation of disease resistance and susceptibility, evaluation of the success rate of tissue transplantation or estimation of genetic diversity in populations. There has been no report on allelic variation of SLA genes for Korean native pigs. Therefore, we cloned and analyzed a MHC class I gene, SLA-2, from Korean native pigs. We found three new SLA-2 alleles from five Korean native pigs, suggesting the genetic diversity of the Korean native pig is significantly reduced. The allelic types were very unique in two cases, named SLA-2*kn01 and SLA-2*kn02, proposing as new groups to SLA nomenclature committee. SLA-2*kn03 was grouped with the previously proposed SLA-2*06 group, although SLA-2\*kn03 has nucleotide difference in five different places in the 1095 nucleotides of the SLA-2 coding region. These three alleles represent the first SLA-2 alleles analyzed at the level of the complete cDNA from any Asian native pigs. Therefore, it will be interesting to evaluate the genetic relationship between the SLA-2 genes of Korean native pigs and other Asian pig breeds. This study also provides evidence that the Korean native pig population still maintains the genetic characteristics of a unique pig breed despite of concerns on intermingling of foreign genes from modern pig breeds due to introduction of Western breeds for meat animal production. | 793 | no | no good fitness measure, N pop differnt in GD? | NA | no | no |
| 794 | 2017 | 43012 | Ahmed, S; Olsen, JE; Herrero-Fresno, A | 2017 | The genetic diversity of commensal Escherichia coli strains isolated from non antimicrobial treated pigs varies according to age group | This is the first report on the genetic diversity of commensal E. coli from pigs reared in an antibiotic free production system and belonging to different age groups. The study investigated the genetic diversity and relationship of 900 randomly collected commensal E. coli strains from non-antimicrobial treated pigs assigned to five different age groups in a Danish farm. Fifty-two unique REP profiles were detected suggesting a high degree of diversity. The number of strains per pig ranged from two to 13. The highest and the lowest degree of diversity were found in the early weaners group (Shannon diversity index, H’of 2.22) and piglets (H’of 1.46) respectively. The REP profiles, R1, R7 and R28, were the most frequently observed in all age groups. E. co/istrains representing each REP profile and additional strains associated with the dominant profiles were subjected to PFGE and were assigned to 67 different genotypes. Whole genome sequence analysis of 52 isolates leading to unique REP profiles identified a high level of sequence variation. Six and six strains were assigned to sequence type ST10 and sequence type ST58, respectively. Virulence and antimicrobial resistance genes, as well as, genes associated with mobile genetic elements were commonly found among these commensal E. coli strains. Interestingly, strains yielding the three most common REP profiles clustered together in the SNPs phylogenetic tree, and such strains may represent the archetypal commensal E. coli in Danish pigs. | 794 | no | no good fitness measure, N pop differnt in GD? | NA | no | no |
| 795 | 2017 | 43012 | Mitton, JB; Latta, RG; Rehfeldt, GE | 1997 | The pattern of inbreeding in Washoe pine and survival of inbred progeny under optimal environmental conditions | Four enzyme polymorphisms were used to estimate the proportion of inbred offspring among seedlings of Washoe pine (Pinus washoensis MASON and STOCKWELL) grown in a shadehouse. Seeds from 10 trees in each of 4 natural populations were collected in the field, germinated in a green house, and grown for 2 years in a shade house at Moscow, ID. Progeny arrays were analyzed from each population. Among all populations pooled, the multilocus outcrossing rate estimate was t(m) = 0.862. The multilocus outcrossing rate exceeded the average of the single locus outcrossing rate by about 0.07 in the pooled data, indicating some degree of mating between related individuals. In addition, the correlation of paternal parentage was r = 0.07, indicating approximately 14% of progeny pairs in an array were full sibs. Thus, the number of pollen parents in each array was limited. While the seedlings exhibited a significant degree of inbreeding, the genotypes inferred for the maternal seed trees had an inbreeding coefficient of F-is = -0.037, significantly lower than the inbreeding coefficient predicted from the mating system (F-is = 0.11). These results indicate that the inbred genotypes detected in the seedlings persist when trees are cultivated under optimal environmental conditions, but are eliminated in natural populations. Single-locus estimates of F in the pooled sample of seed trees were consistently negative, with a mean of F = -0.10, indicating that selection favored heterozygous trees in the pool of outcrossed genotypes. | 795 | no | no good fitness measure, N pop differnt in GD? (inbreeding?) | NA | no | no |
| 796 | 2017 | 43012 | Lachapelle, J; Bell, G | 2012 | Evolutionary Rescue Of Sexual And Asexual Populations In A Deteriorating Environment | The environmental change experienced by many contemporary populations of organisms poses a serious risk to their survival. From the theory of evolutionary rescue, we predict that the combination of sex and genetic diversity should increase the probability of survival by increasing variation and thereby the probability of generating a type that can tolerate the stressful environment. We tested this prediction by comparing experimental populations of Chlamydomonas reinhardtii that differ in sexuality and in the initial amount of genetic diversity. The lines were serially propagated in an environment where the level of stress caused by salt increased over time from fresh water to the limits of marine conditions. In the long term, the combination of high diversity and obligate sexuality was most effective in supporting evolutionary rescue. Most of the adaptation to high-salt environments in the obligate sexual-high diversity lines had occurred by midway through the experiment, indicating that positive genetic correlations of adaptation to lethal stress with adaptation to sublethal stress greatly increased the probability of evolutionary rescue. The evolutionary rescue events observed in this study provide evidence that major shifts in ways of life can arise within short time frames through the action of natural selection in sexual populations. | 796 | probably yes | NA | discard | yes | no |
| 797 | 2017 | 43012 | Oddou-Muratorio, S; Davi, H | 2014 | Simulating local adaptation to climate of forest trees with a Physio-Demo-Genetics model | One challenge of evolutionary ecology is to predict the rate and mechanisms of population adaptation to environmental variations. The variations in most life history traits are shaped both by individual genotypic and by environmental variation. Forest trees exhibit high levels of genetic diversity, large population sizes, and gene flow, and they also show a high level of plasticity for life history traits. We developed a new Physio-Demo-Genetics model (denoted PDG) coupling (i) a physiological module simulating individual tree responses to the environment; (ii) a demographic module simulating tree survival, reproduction, and pollen and seed dispersal; and (iii) a quantitative genetics module controlling the heritability of key life history traits. We used this model to investigate the plastic and genetic components of the variations in the timing of budburst (TBB) along an elevational gradient of Fagus sylvatica (the European beech). We used a repeated 5years climatic sequence to show that five generations of natural selection were sufficient to develop nonmonotonic genetic differentiation in the TBB along the local climatic gradient but also that plastic variation among different elevations and years was higher than genetic variation. PDG complements theoretical models and provides testable predictions to understand the adaptive potential of tree populations. | 797 | maybe | any fitness of groups and of different gd ok? | no gd of host pop and fitness of host pop | no | no |
| 798 | 2017 | 43012 | Mattila, HR; Seeley, TD | 2010 | Promiscuous honeybee queens generate colonies with a critical minority of waggle-dancing foragers | Honeybees present a paradox that is unusual among the social Hymenoptera: extremely promiscuous queens generate colonies of nonreproducing workers who cooperate to rear reproductives with whom they share limited kinship. Extreme polyandry, which lowers relatedness but creates within-colony genetic diversity, produces substantial fitness benefits for honeybee queens and their colonies because of increased disease resistance and workforce productivity. However, the way that these increases are generated by individuals in genetically diverse colonies remains a mystery. We assayed the foraging and dancing performances of workers in multiple-patriline and single-patriline colonies to discover how within-colony genetic diversity, conferred to colonies by polyandrous queens, gives rise to a more productive foraging effort. We also determined whether the initiation by foragers of waggle-dance signaling in response to an increasing sucrose stimulus (their dance response thresholds) was linked to patriline membership. Per capita, foragers in multiple-patriline colonies visited a food source more often and advertised it with more waggle-dance signals than foragers from single-patriline colonies, although there was variability among multiple-patriline colonies in the strength of this difference. High-participation patrilines emerged within multiple-patriline colonies, but their more numerous foragers and dancers were neither more active per capita nor lower-threshold dancers than their counterparts from low-participation patrilines. Our results demonstrate that extreme polyandry does not enhance recruitment effort through the introduction of low-dance-threshold, high-activity workers into a colony’s population. Rather, genetic diversity is critical for injecting into a colony’s workforce social facilitators who are more likely to become engaged in foraging-related activities, so boosting the production of dance signals and a colony’s responsiveness to profitable food sources. | 798 | no | no good fitness measure | NA | no | no |
| 799 | 2017 | 43012 | Weber, DS; Stewart, BS; Lehman, N | 2004 | Genetic consequences of a severe population bottleneck in the Guadalupe fur seal (Arctocephalus townsendi) | Population bottlenecks may lead to diminished genetic variability and correlative effects on fitness. The Guadalupe fur seal was nearly exterminated by commercial sealers during the late 18th and early 19th centuries. To determine the genetic consequences of this population bottleneck, we compared the variation at a 181 bp section of the mitochondrial DNA (mtDNA) control region from the bones of 26 prebottleneck fur seals versus variation in the extant population. We found 25 different mtDNA genotypes in the prebottleneck fur seals and only 7 genotypes among 32 extant fur seals, including only one of the ancient genotypes. These data demonstrate a substantial loss of genetic variability correlating with the recent population bottleneck. We also found from several genetic measures that the prehistoric population of Guadalupe fur seals was robust and that it had been increasing at some time during the late prehistoric period. Continued recovery of this species may, however, owe more to more immediate nongenetic factors, such as poaching and local availability of food resources during the breeding season and consequent effects on pup survival, than on the reduced genetic variability. | 799 | no | no good fitness measure, N pop differnt in GD? | NA | no | no |
| 800 | 2017 | 43012 | Vetriventhan, M; Upadhyaya, HD; Anandakumar, CR; Senthilvel, S; Parzies, HK; Bharathi, A; Varshney, RK; Gowda, CLL | 2012 | Assessing genetic diversity, allelic richness and genetic relationship among races in ICRISAT foxtail millet core collection | Foxtail millet (Setaria italica (L.) P. Beauv.) is an ideal crop for changing climate and food habits of peoples due to its short duration, high photosynthetic efficiency, nutritional richness and fair resistance to pest and diseases. However, foxtail millet yields are low mainly due to the lack of effort for its improvement and the lack of proper utilization of existing genetic variability. To enhance the use of diverse germplasm in breeding programmes, a core collection in foxtail millet consisting of 155 accessions was established. Core collection accessions were fingerprinted using 84 markers (81 simple sequence repeats (SSRs) and three Expressed Sequence Tag (EST)-SSRs). Our results showed the presence of greater molecular diversity in the foxtail millet core collection. The 84 markers detected a total of 1356 alleles with an average of 16.14 alleles (4-35) per locus. Of these, 368 were rare alleles, 906 common alleles and 82 the most frequent alleles. Sixty-one unique alleles that were specific to a particular accession and useful for germplasm identification were also detected. In this study, the genetic diversity of foxtail millet was fairly correlated well with racial classification, and the race Indica showed a greater genetic distance from the races Maxima and Moharia. The pairwise estimate of dissimilarity was > 0.50 except in 123 out of 11,935 pairs which indicated a greater genetic variability. Two hundred and fifty pairs of genetically most diverse accessions were identified. This large molecular variation observed in the core collection could be utilized effectively by breeders or researchers for the selection of diverse parents for breeding cultivars and the development of mapping populations. | 800 | no | crop | NA | no | no |
| 801 | 2017 | 43012 | Costa, F; Richardson, JL; Dion, K; Mariani, C; Pertile, AC; Burak, MK; Childs, JE; Ko, AI; Caccone, A | 2016 | Multiple Paternity in the Norway Rat, Rattus norvegicus, from Urban Slums in Salvador, Brazil | The Norway rat, Rattus norvegicus, is one of the most important pest species globally and the main reservoir of leptospires causing human leptospirosis in the urban slums of tropical regions. Rodent control is a frequent strategy in those settings to prevent the disease but rapid growth from residual populations and immigration limit the long-term effectiveness of interventions. To characterize the breeding ecology of R. norvegicus and provide needed information for the level of genetic mixing, which can help identify inter-connected eradication units, we estimated the occurrence of multiple paternity, distances between mothers and sires, and inbreeding in rats from urban slum habitat in Salvador, Brazil. We genotyped 9 pregnant females, their 66 offspring, and 371 males at 16 microsatellite loci. Multiple paternity was observed in 22% (2/9) of the study litters. Of the 12 sires that contributed to the 9 litters, we identified 5 (42%) of those sires among our genotyped males. Related males were captured in close proximity to pregnant females (the mean inter-parent trapping distance per litter was 70 m, +/- 58 m SD). Levels of relatedness between mother-sire pairs were higher than expected and significantly higher than relatedness between all females and non-sire males. Our findings indicate multiple paternity is common, inbreeding is apparent, and that mother-sire dyads occur in close proximity within the study area. This information is relevant to improve the spatial definition of the eradication units that may enhance the effectiveness of rodent management programs aimed at preventing human leptospirosis. High levels of inbreeding may also be a sign that eradication efforts are successful. | 801 | no | no good fitness measure, N pop differnt in GD? | NA | no | no |
| 802 | 2017 | 43012 | Monty, A; Bizoux, JP; Escarre, J; Mahy, G | 2013 | Rapid Plant Invasion in Distinct Climates Involves Different Sources of Phenotypic Variation | “When exotic species spread over novel environments, their phenotype will depend on a combination of different processes, including phenotypic plasticity (PP), local adaptation (LA), environmental maternal effects (EME) and genetic drift (GD). Few attempts have been made to simultaneously address the importance of those processes in plant invasion. The present study uses the well-documented invasion history of Senecio inaequidens (Asteraceae) in southern France, where it was introduced at a single wool-processing site. It gradually invaded the Mediterranean coast and the Pyrenean Mountains, which have noticeably different climates. We used seeds from Pyrenean and Mediterranean populations, as well as populations from the first introduction area, to explore the phenotypic variation related to climatic variation. A reciprocal sowing experiment was performed with gardens under Mediterranean and Pyrenean climates. We analyzed climatic phenotypic variation in germination, growth, reproduction, leaf physiology and survival. Genetic structure in the studied invasion area was characterized using AFLP. We found consistent genetic differentiation in growth traits but no home-site advantage, so weak support for LA to climate. In contrast, genetic differentiation showed a relationship with colonization history. PP in response to climate was observed for most traits, and it played an important role in leaf trait variation. EME mediated by seed mass influenced all but leaf traits in a Pyrenean climate. Heavier, earlier-germinating seeds produced larger individuals that produced more flower heads throughout the growing season. However, in the Mediterranean garden, seed mass only influenced the germination rate. The results show that phenotypic variation in response to climate depends on various ecological and evolutionary processes associated with geographical zone and life history traits. Seeing the relative importance of EME and GD, we argue that a”“local adaptation vs. phenotypic plasticity”" approach is therefore not sufficient to fully understand what shapes phenotypic variation and genetic architecture of invasive populations." | 802 | no | no good fitness measure, N pop differnt in GD? | NA | no | no |
| 803 | 2017 | 43012 | Kellner, R; Hanschke, C; Begerow, D | 2014 | Patterns of Variation at Ustilago maydis Virulence Clusters 2A and 19A Largely Reflect the Demographic History of Its Populations | The maintenance of an intimate interaction between plant-biotrophic fungi and their hosts over evolutionary times involves strong selection and adaptative evolution of virulence-related genes. The highly specialised maize pathogen Ustilago maydis is assigned with a high evolutionary capability to overcome host resistances due to its high rates of sexual recombination, large population sizes and long distance dispersal. Unlike most studied fungus-plant interactions, the U. maydis - Zea mays pathosystem lacks a typical gene-for-gene interaction. It exerts a large set of secreted fungal virulence factors that are mostly organised in gene clusters. Their contribution to virulence has been experimentally demonstrated but their genetic diversity within U. maydis remains poorly understood. Here, we report on the intraspecific diversity of 34 potential virulence factor genes of U. maydis. We analysed their sequence polymorphisms in 17 isolates of U. maydis from Europe, North and Latin America. We focused on gene cluster 2A, associated with virulence attenuation, cluster 19A that is crucial for virulence, and the cluster-independent effector gene pep1. Although higher compared to four house-keeping genes, the overall levels of intraspecific genetic variation of virulence clusters 2A and 19A, and pep1 are remarkably low and commensurate to the levels of 14 studied non-virulence genes. In addition, each gene is present in all studied isolates and synteny in cluster 2A is conserved. Furthermore, 7 out of 34 virulence genes contain either no polymorphisms or only synonymous substitutions among all isolates. However, genetic variation of clusters 2A and 19A each resolve the large scale population structure of U. maydis indicating subpopulations with decreased gene flow. Hence, the genetic diversity of these virulence-related genes largely reflect the demographic history of U. maydis populations. | 803 | no | gd of pathogen | NA | no | no |
| 804 | 2017 | 43012 | Dharmarajan, G; Beasley, JC; Beatty, WS; Olson, ZH; Fike, JA; Rhodes, OE | 2016 | Genetic co-structuring in host-parasite systems: Empirical data from raccoons and raccoon ticks | Many aspects of parasite biology critically depend on their hosts, and understanding how host-parasite populations are co-structured can help improve our understanding of the ecology of parasites, their hosts, and host-parasite interactions. This study utilized genetic data collected from raccoons (Procyon lotor), and a specialist parasite, the raccoon tick (Ixodes texanus), to test for genetic co-structuring of host-parasite populations at both landscape and host scales. At the landscape scale, our analyses revealed a significant correlation between genetic and geographic distance matrices (i.e., isolation by distance) in ticks, but not their hosts. While there are several mechanisms that could lead to a stronger pattern of isolation by distance in tick vs. raccoon datasets, our analyses suggest that at least one reason for the above pattern is the substantial increase in statistical power (due to the approximate to 8-fold increase in sample size) afforded by sampling parasites. Host-scale analyses indicated higher relatedness between ticks sampled from related vs. unrelated raccoons trapped within the same habitat patch, a pattern likely driven by increased contact rates between related hosts. By utilizing fine-scale genetic data from both parasites and hosts, our analyses help improve our understanding of epidemiology and host ecology. | 804 | no | no good fitness measure, N pop differnt in GD? | NA | no | no |
| 805 | 2017 | 43012 | Winternitz, JC; Minchey, SG; Garamszegi, LZ; Huang, S; Stephens, PR; Altizer, S | 2013 | Sexual selection explains more functional variation in the mammalian major histocompatibility complex than parasitism | Understanding drivers of genetic diversity at the major histocompatibility complex (MHC) is vitally important for predicting how vertebrate immune defence might respond to future selection pressures and for preserving immunogenetic diversity in declining populations. Parasite-mediated selection is believed to be the major selective force generating MHC polymorphism, and while MHC-based mating preferences also exist for multiple species including humans, the general importance of mate choice is debated. To investigate the contributions of parasitism and sexual selection in explaining among-species variation in MHC diversity, we applied comparative methods and meta-analysis across 112 mammal species, including carnivores, bats, primates, rodents and ungulates. We tested whether MHC diversity increased with parasite richness and relative testes size (as an indicator of the potential for mate choice), while controlling for phylogenetic autocorrelation, neutral mutation rate and confounding ecological variables. We found that MHC nucleotide diversity increased with parasite richness for bats and ungulates but decreased with parasite richness for carnivores. By contrast, nucleotide diversity increased with relative testes size for all taxa. This study provides support for both parasite-mediated and sexual selection in shaping functional MHC polymorphism across mammals, and importantly, suggests that sexual selection could have a more general role than previously thought. | 805 | no | no good fitness measure, N pop differnt in GD? | NA | no | no |
| 806 | 2017 | 43012 | Miller, SM; Harper, CK; Bloomer, P; Hofmeyr, J; Funston, PJ | 2015 | Fenced and Fragmented: Conservation Value of Managed Metapopulations | Population fragmentation is threatening biodiversity worldwide. Species that once roamed vast areas are increasingly being conserved in small, isolated areas. Modern management approaches must adapt to ensure the continued survival and conservation value of these populations. In South Africa, a managed metapopulation approach has been adopted for several large carnivore species, all protected in isolated, relatively small, reserves that are fenced. As far as possible these approaches are based on natural metapopulation structures. In this network, over the past 25 years, African lions (Panthera leo) were reintroduced into 44 fenced reserves with little attention given to maintaining genetic diversity. To examine the situation, we investigated the current genetic provenance and diversity of these lions. We found that overall genetic diversity was similar to that in a large national park, and included a mixture of four different southern African evolutionarily significant units (ESUs). This mixing of ESUs, while not ideal, provides a unique opportunity to study the impact of mixing ESUs over the long term. We propose a strategic managed metapopulation plan to ensure the maintenance of genetic diversity and improve the long-term conservation value of these lions. This managed metapopulation approach could be applied to other species under similar ecological constraints around the globe. | 806 | no | no good fitness measure, N pop differnt in GD? | NA | no | no |
| 807 | 2017 | 43012 | Bull, JK; Heurich, M; Saveljev, AP; Schmidt, K; Fickel, J; Forster, DW | 2016 | The effect of reintroductions on the genetic variability in Eurasian lynx populations: the cases of Bohemian-Bavarian and Vosges-Palatinian populations | Over the past similar to 40 years, several attempts were made to reintroduce Eurasian lynx to suitable habitat within their former distribution range in Western Europe. In general, limited numbers of individuals have been released to establish new populations. To evaluate the effects of reintroductions on the genetic status of lynx populations we used 12 microsatellite loci to study lynx populations in the Bohemian-Bavarian and Vosges-Palatinian forests. Compared with autochthonous lynx populations, these two reintroduced populations displayed reduced genetic diversity, particularly the Vosges-Palatinian population. Our genetic data provide further evidence to support the status of ‘endangered’ and ‘critically endangered’ for the Bohemian-Bavarian and Vosges-Palatinian populations, respectively. Regarding conservation management, we highlight the need to limit poaching, and advocate additional translocations to bolster genetic variability. | 807 | no | no good fitness measure, N pop differnt in GD? | NA | no | no |
| 808 | 2017 | 43012 | Simkova, A; Ottova, E; Morand, S | 2006 | MHC variability, life-traits and parasite diversity of European cyprinid fish | Potential links between fish life history traits, an immune investment as measured by variability of the MHC genes and parasitism were analysed in 14 species of cyprinid fish. The hypothesis of the diversity of MHC genes being driven by high parasite diversity, i.e. species richness, was tested and a potential relationship between the MHC diversity and fish life-history traits including adult mortality rate, fecundity, longevity and maturity was investigated. Molecular techniques (SSCP and sequencing) were applied to analyse the MHC nucleotide diversity of the exons 2 and 3 of DAB genes belonging to the MHC class IIB. The comparative analyses, using phylogenetic independent contrasts, revealed a negative relationship between parasite species richness and adult fish mortality rate. We also found a positive relationship between nucleotide diversity of the exon 2 and parasite species richness. Our results suggest that fish species, of which populations are exposed to high parasite pressure, in terms of high parasite species richness, maintain a high genetic diversity of the exon 2 of the MHC genes (presenting the peptide binding regions), allowing them to decrease their natural mortality rate. | 808 | maybe | fitness and gd pop level? | 14 fish species, no gd measure per pop and species | no | no |
| 809 | 2017 | 43012 | Lancaster, ML; Arnould, JPY; Kirkwood, R | 2010 | Genetic status of an endemic marine mammal, the Australian fur seal, following historical harvesting | Genetic variation, and the way in which it is partitioned among populations, has implications for a species’ survival and evolutionary potential. Such information is particularly important for the successful conservation and management of species that have experienced past human impacts and potential losses of genetic diversity. Overharvesting of the Australian fur seal Arctocephalus pusillus doriferus in the 18th and 19th centuries resulted in severe population reductions and elimination of an estimated 17 of 26 colonies. Currently, the subspecies is recovering and c. 20 000 pups are produced annually at 13 colony sites, most of which are situated in Bass Strait in south-eastern Australia. Genetic analysis of samples collected from pups captured at nine colonies revealed no difference in allelic diversity or heterozygosity at five microsatellite loci and no differences in haplotype diversity within a 344 bp region of the mitochondrial DNA control region. There was some evidence for isolation by distance but the program structure predicted a single cluster of individuals. Gene flow among colonies appears to be substantial at present, indicating that the Australian fur seal is currently a single, panmictic unit. | 809 | no | no good fitness measure, N pop differnt in GD? | NA | no | no |
| 810 | 2017 | 43012 | Fabuel, E; Barragan, C; Silio, L; Rodriguez, MC; Toro, MA | 2004 | Analysis of genetic diversity and conservation priorities in Iberian pigs based on microsatellite markers | The Iberian breed is the most important pig population of the Mediterranean type. The genetic structure of two strains (Torbiscal and Guadyerbas) and three varieties (Retinto, Entrepelado and Lampino) of this breed was studied using 173 pigs genotyped for 36 microsatellites. In addition, 40 pigs of the related Duroc breed were also analysed. In the 1960s, the Iberian breed’s numbers were severely reduced by disease, due to economic change and to crossbreeding. Varieties are in danger of disappearance or blending. A new conservation strategy is required. An analysis was performed that allows us to ascertain the loss or gain of genetic diversity if one or several subpopulations are removed. The results are compared with those using the Weitzman method. The two methodologies produce conservation priorities that are completely different, the reason being that the Weitzman method does not take into account the within-population genetic diversity. We apply optimal contribution theory and a new procedure for cluster analysis, and discuss their value in the general framework of the problems of setting of priorities and tactics for the conservation of genetic resources. | 810 | no | domesticated animal | NA | no | no |
| 811 | 2017 | 43012 | Shivaprakash, KN; Ramesha, BT; Shaanker, RU; Dayanandan, S; Ravikanth, G | 2014 | Genetic Structure, Diversity and Long Term Viability of a Medicinal Plant, Nothapodytes nimmoniana Graham. (Icacinaceae), in Protected and Non-Protected Areas in the Western Ghats Biodiversity Hotspot | Background and Question: The harvesting of medicinal plants from wild sources is escalating in many parts of the world, compromising the long-term survival of natural populations of medicinally important plants and sustainability of sources of raw material to meet pharmaceutical industry needs. Although protected areas are considered to play a central role in conservation of plant genetic resources, the effectiveness of protected areas for maintaining medicinal plant populations subject to intense harvesting pressure remain largely unknown. We conducted genetic and demographic studies of Nothapodytes nimmoniana Graham, one of the extensively harvested medicinal plant species in the Western Ghats biodiversity hotspot, India to assess the effectiveness of protected areas in long-term maintenance of economically important plant species. Methodology/Principal Findings: The analysis of adults and seedlings of N. nimmoniana in four protected and four non-protected areas using 7 nuclear microsatellite loci revealed that populations that are distributed within protected areas are subject to lower levels of harvesting and maintain higher genetic diversity (H-e=0.816, H-o=0.607, A=18.857) than populations in adjoining non-protected areas (H-e=0.781, H-o=0.511, A=15.571). Furthermore, seedlings in protected areas had significantly higher observed heterozygosity (H-o=0.630) and private alleles as compared to seedlings in adjoining non-protected areas (H-o=0.426). Most populations revealed signatures of recent genetic bottleneck. The prediction of long-term maintenance of genetic diversity using BOTTLESIM indicated that current population sizes of the species are not sufficient to maintain 90% of present genetic diversity for next 100 years. Conclusions/Significance: Overall, these results highlight the need for establishing more protected areas encompassing a large number of adult plants in the Western Ghats to conserve genetic diversity of economically and medicinally important plant species. | 811 | no | no good fitness measure | NA | no | no |
| 812 | 2017 | 43012 | Ugelvig, LV; Kronauer, DJC; Schrempf, A; Heinze, J; Cremer, S | 2010 | Rapid anti-pathogen response in ant societies relies on high genetic diversity | Social organisms are constantly exposed to infectious agents via physical contact with conspecifics. While previous work has shown that disease susceptibility at the individual and group level is influenced by genetic diversity within and between group members, it remains poorly understood how group-level resistance to pathogens relates directly to individual physiology, defence behaviour and social interactions. We investigated the effects of high versus low genetic diversity on both the individual and collective disease defences in the ant Cardiocondyla obscurior. We compared the antiseptic behaviours (grooming and hygienic behaviour) of workers from genetically homogeneous and diverse colonies after exposure of their brood to the entomopathogenic fungus Metarhizium anisopliae. While workers from diverse colonies performed intensive allogrooming and quickly removed larvae covered with live fungal spores from the nest, workers from homogeneous colonies only removed sick larvae late after infection. This difference was not caused by a reduced repertoire of antiseptic behaviours or a generally decreased brood care activity in ants from homogeneous colonies. Our data instead suggest that reduced genetic diversity compromises the ability of Cardiocondyla colonies to quickly detect or react to the presence of pathogenic fungal spores before an infection is established, thereby affecting the dynamics of social immunity in the colony. | 812 | maybe | no good fitness measure? | NA | yes | yes |
| 813 | 2017 | 43012 | Fisher, MC; Bosch, J; Yin, Z; Stead, DA; Walker, J; Selway, L; Brown, AJP; Walker, LA; Gow, NAR; Stajich, JE; Garner, TWJ | 2009 | Proteomic and phenotypic profiling of the amphibian pathogen Batrachochytrium dendrobatidis shows that genotype is linked to virulence | Population genetics of the amphibian pathogen Batrachochytrium dendrobatidis (Bd) show that isolates are highly related and globally homogenous, data that are consistent with the recent epidemic spread of a previously endemic organism. Highly related isolates are predicted to be functionally similar due to low levels of heritable genetic diversity. To test this hypothesis, we took a global panel of Bd isolates and measured (i) the genetic relatedness among isolates, (ii) proteomic profiles of isolates, (iii) the susceptibility of isolates to the antifungal drug caspofungin, (iv) the variation among isolates in growth and phenotypic characteristics, and (v) the virulence of isolates against the European common toad Bufo bufo. Our results show (i) genotypic differentiation among isolates, (ii) proteomic differentiation among isolates, (iii) no significant differences in susceptibility to caspofungin, (iv) differentiation in growth and phenotypic/morphological characters, and (v) differential virulence in B. bufo. Specifically, our data show that Bd isolates can be profiled by their genotypic and proteomic characteristics, as well as by the size of their sporangia. Bd genotypic and phenotypic distance matrices are significantly correlated, showing that less-related isolates are more biologically unique. Mass spectrometry has identified a set of candidate genes associated with inter-isolate variation. Our data show that, despite its rapid global emergence, Bd isolates are not identical and differ in several important characters that are linked to virulence. We argue that future studies need to clarify the mechanism(s) and rate at which Bd is evolving, and the impact that such variation has on the host-pathogen dynamic. | 813 | no | gd of pathogen | NA | no | no |
| 814 | 2017 | 43012 | Rodriguez-Rodriguez, MA; Gasca-Pineda, J; Medellin, RA; Eguiarte, LE | 2015 | Analysis of genetic diversity of bighorn sheep (Ovis canadensis) from Mexican populations | The current distribution of the bighorn sheep in Mexico represents a reduced proportion of its original area. Previous population genetics studies conducted in Mexico have only included data from Tiburon Island in the Gulf of California and few individuals from the continent. The aim of this article was to describe aspects of the population genetics of Mexican bighorn sheep in order to aid in the management and conservation of the species. We analyzed 117 samples from the states of Sonora and Baja California Sur using 91 intersimple sequence repeat loci. Our results indicated that the Mexican samples of bighorn sheep have relatively low levels of genetic diversity (H approximate to 0.26) and low genetic differentiation (theta approximate to 0.07) that may be the result of the recent colonization and origin of the populations in Mexico. The individuals from Southern Baja California are genetically different from the Sonoran sample, but this genetic differentiation is low, perhaps due to the low levels of genetic variation of the Mexican populations. The results obtained in this study are relevant for population management of the bighorn sheep in Mexico in order to design translocation plans and management strategies to maintain genetic diversity and, in consequence, the health and future survival of the populations. | 814 | no | no good fitness measure, N pop differnt in GD? | NA | no | no |
| 815 | 2017 | 43012 | Hammerly, SC; de la Cerda, DA; Bailey, H; Johnson, JA | 2016 | A pedigree gone bad: increased offspring survival after using DNA-based relatedness to minimize inbreeding in a captive population | Captive breeding programs often use a pedigree to identify breeding pairs that maintain genetic diversity and limit inbreeding. However, unintentional breeding of closely related individuals can occur when errors exist in the pedigree and may subsequently result in inbreeding depression. In this study, a DNA-based approach was used to identify parentage assignment errors in the captive pedigree of the critically endangered Attwater’s prairie-chicken Tympanuchus cupido attwateri, and tested whether clutch survival increased using DNA-relatedness values for assigning breeding pairs instead of the pedigree and mean kinship. Parentage assignment error was observed in each year surveyed ranging from 2.4 to 7.3%. After correcting identified errors prior to the 2013 breeding season, 11 of 38 assigned pedigree-based breeding pairs still possessed DNA-based relatedness coefficients (r(DNA)) >= 0.125 suggesting that additional errors remained in the pedigree. Two approaches were used to prevent breeding among close relatives in 2013 and 2014. Assigned pedigree-based breeding pairs in 2013 that possessed r(DNA) >= 0.125 were reassigned unrelated breeding partners, while all individuals in 2014 were used to identify the minimum overall r(DNA) for the breeding population without reference to the pedigree other than to verify founder representation. Both years resulted in a significant reduction in mean parental relatedness among chicks (P < 0.001) and a significant increase in the proportion of the clutch surviving to 5 weeks post-hatch (P <= 0.006) compared to 2012 when breeding pairs were assigned using only the pedigree. No significant difference in the proportion of the clutch surviving was observed between 2013 and 2014 (P > 0.300). These results have important implications for the captive management of endangered species, and highlight the importance of periodically evaluating for pedigree errors. To what degree pedigree errors limit fitness in other endangered species captive populations deserves further attention. | 815 | no | inbreeding | NA | no | no |
| 816 | 2017 | 43012 | Bjorndal, KA; Bolten, AB | 2008 | Annual variation in source contributions to a mixed stock: implications for quantifying connectivity | Connectivity among populations of highly migratory species is an area of active research and is often quantified with genetic markers. We determined mitochondrial DNA (mtDNA) sequences in 350 green turtles, Chelonia mydas, in 10 annual samples over a 12-year period from an aggregation of immature green turtles in the southern Bahamas. We found significant temporal structuring in haplotype frequencies among years for all turtles and for recruits. These significant differences were reflected in substantial variation in the relative contributions from different rookeries among years estimated by a Bayesian hierarchical model. Because this foraging aggregation has been the subject of a demographic study for over 30 years, we were able to determine that, among the three potential causes of temporal structuring-differential recruitment, mortality and emigration-recruitment accounts for most of this variation. We found that estimates of connectivity and genetic diversity in sea turtle populations are affected by the level of temporal variation reported here. More studies on the extent of temporal variation in composition of mixed stocks of other migratory species are needed to determine how this affects measures of connectivity. | 816 | no | no good fitness measure, N pop differnt in GD? | NA | no | no |
| 817 | 2017 | 43012 | Schwartz, TS; Karl, SA | 2005 | Population and conservation genetics of the gopher tortoise (Gopherus polyphemus) | The gopher tortoise (Gopherus polyphemus) is an important member of the sandhill, longleaf pine, and scrub ecosystems in the southeastern United States. Even though it is currently protected throughout its range, tortoise populations continue to decline. We assessed genetic diversity at nine microsatellite loci in 300 individuals from 21 locations throughout Florida and southern Georgia. Tortoise populations are clearly subdivided into at least eight genetic assemblages with an F-ST = 0.24 +/- 0.11. Furthermore, we found indications of anthropogenic effects in the form of population bottlenecks in five populations and putative admixture in four. From these data, we recommend that the populations be managed to maintain existing genetic structure without further isolation of populations and the establishment of a holistic database to include genetic and demographic information useful for relocation and management purposes. | 817 | no | no good fitness measure | NA | no | no |
| 818 | 2017 | 43012 | Chapman, NC; Lim, J; Oldroyd, BP | 2008 | Population genetics of commercial and feral honey bees in Western Australia | Due to the introduction of exotic honey bee (Apis mellifera L.) diseases in the eastern states, the borders of the state of Western Australia were closed to the import of bees for breeding and other purposes >25 yr ago. to provide genetically improved stock for the industry, a closed population breeding program was established that now provides stock for the majority of Western Australian beekeepers. Given concerns that inbreeding may have resulted from the closed population breeding structure, we assessed the genetic diversity within and between the breeding lines by using microsatellite and mitochondrial markers. We found that the breeding population still maintains considerable genetic diversity, despite 25 yr of selective breeding. We also investigated the genetic distance of the closed population breeding program to that of beekeepers outside of the program, and the feral Western Australian honey bee population. The feral population is genetically distinct from the closed population, but not from the genetic stock maintained by beekeepers outside of the program. The honey bees of Western Australia show three mitotypes, originating from two subspecies: Apis mellifera ligustica (mitotypes C1 and M7b) and Apis mellifera iberica (mitotype M6). Only mitotypes C1 and M6 are present in the commercial populations. The feral population contains all three mitotypes. | 818 | no | no good fitness measure, N pop differnt in GD? | NA | no | no |
| 819 | 2017 | 43012 | Mastana, S; Singh, PP | 2002 | Population genetic study of the STR loci (HUMCSF1PO, HUMTPOX, HUMTHO1, HUMLPL, HUMF13AO1, HUMF13B, HSFESFPS and HUMVWA) in North Indians | Background: Highly polymorphic genetic markers like short tandem repeats (STRs) have been used successfully in disease analysis and studies of human evolution and population genetic diversity. However, DNA-based population genetic studies of Indian populations are limited. Subjects and Methods: To enlarge our understanding, of genetic variation in Indian populations, a population genetic study was carried out on Jat Sikh (Punjab, North India) individuals (n = 150) using a battery of the STR loci. The STR loci analysed by means of PCR amplification followed by electrophoresis and silver staining included HUMCSF1PO, HUMTPOX, HUMTHO1, HUMLPL, HUMF13A01, HUMF13B, HUMFESFPS and HUMVWA loci. Results: The overall pattern of allele frequencies was similar to many Caucasian and Indian populations and heterozygosity varied from 65% (HUMLPL) to 85% (HUMVWA). For all eight loci, no deviations from the Hardy-Weinberg equilibrium hypothesis were detected. Significant differences were observed between Jat Sikhs and African, Chinese and Indian tribes. The mean exclusion probability ranged from 35% to 70%, and the power of discrimination from 81% to 93%, indicating the potential of these loci for forensic and paternity investigations. Conclusion: The allele frequency spectrum, heterozygosity, probability of exclusion, match probability and discrimination probability estimates show interesting variation and suggest the usefulness of these loci for anthropogenetic, paternity and forensic investigations in Indian populations. | 819 | no | human | NA | no | no |
| 820 | 2017 | 43012 | Shinya, R; Takeuchi, Y; Ichimura, K; Takemoto, S; Futai, K | 2012 | Establishment of a set of inbred strains of the pine wood nematode, Bursaphelenchus xylophilus (Aphelenchida: Aphelenchoididae), and evidence of their varying levels of virulence | Pine wilt disease (PWD) caused by the pine wood nematode, Bursaphelenchus xylophilus (Steiner and Buhrer) Nickle, has become a worldwide problem. The pathogenic mechanism of PWD continues to remain controversial, which in part may be attributed to the lack of universal materials of B. xylophilus with a high genetic purity. The intrinsic high genetic diversity in B. xylophilus isolates/populations must be a fatal obstacle for performing forward genetics and other molecular approaches to controlling them. We conducted a series of successive full-sib mating of conventional isolates of B. xylophilus to establish a set of inbred strains. Using DNA markers, we also determined their genetic diversity and biological characteristics, such as virulence and reproductive ability. Consequently, the newly established strains yielded a higher genetic purity than the conventional isolates and showed varying virulence despite sharing a common ancestor. The significance of this study lies not only in establishing a set of inbred strains of B. xylophilus with the certification of their purity but also in demonstrating that avirulent strain(s) with a genotype similar to the virulent strains can be obtained by simple successive full-sib mating. This technique is one of the most powerful tools for elucidating the pathogenic mechanism(s) of PWD. | 820 | no | gd of pathogen | NA | no | no |
| 821 | 2017 | 43012 | Hemond, EM; Vollmer, SV | 2010 | Genetic Diversity and Connectivity in the Threatened Staghorn Coral (Acropora cervicornis) in Florida | Over the past three decades, populations of the dominant shallow water Caribbean corals, Acropora cervicornis and A. palmata, have been devastated by white-band disease (WBD), resulting in the listing of both species as threatened under the U.S. Endangered Species Act. A key to conserving these threatened corals is understanding how their populations are genetically interconnected throughout the greater Caribbean. Genetic research has demonstrated that gene flow is regionally restricted across the Caribbean in both species. Yet, despite being an important site of coral reef research, little genetic data has been available for the Florida Acropora, especially for the staghorn coral, A. cervicornis. In this study, we present new mitochondrial DNA sequence data from 52 A. cervicornis individuals from 22 sites spread across the upper and lower Florida Keys, which suggest that Florida’s A. cervicornis populations are highly genetically interconnected (F(ST)=-0.081). Comparison between Florida and existing mtDNA data from six regional Caribbean populations indicates that Florida possesses high levels of standing genetic diversity (h = 0.824) relative to the rest of the greater Caribbean (h = 0.701 +/- 0.043). We find that the contemporary level of gene flow across the greater Caribbean, including Florida, is restricted (Phi(CT) = 0.117), but evidence from shared haplotypes suggests the Western Caribbean has historically been a source of genetic variation for Florida. Despite the current patchiness of A. cervicornis in Florida, the relatively high genetic diversity and connectivity within Florida suggest that this population may have sufficient genetic variation to be viable and resilient to environmental perturbation and disease. Limited genetic exchange across regional populations of the greater Caribbean, including Florida, indicates that conservation efforts for A. cervicornis should focus on maintaining and managing populations locally rather than relying on larval inputs from elsewhere. | 821 | no | no good fitness measure | NA | no | no |
| 822 | 2017 | 43012 | Stiver, JR; Apa, AD; Remington, TE; Gibson, RM | 2008 | Polygyny and female breeding failure reduce effective population size in the lekking Gunnison sage-grouse | Populations with small effective sizes are at risk for inbreeding depression and loss of adaptive potential. Variance in reproductive success is one of several factors reducing effective population size (N-e) below the actual population size (N). Here, we investigate the effects of polygynous (skewed) mating and variation in female breeding success on the effective size of a small population of the Gunnison sage-grouse (Centrocercus minimus), a ground nesting bird with a lek mating system. During a two-year field study, we recorded attendance of marked birds at leks, male mating success, the reproductive success of radio-tagged females, and annual survival. We developed simulations to estimate the distribution of male reproductive success. Using these data, we estimated population size ((N) over cap) and effective population size N-e for the study population. We also simulated the effects of population size, skewed vs. random mating, and female breeding failure on N-e. In our study population, the standardized variance in seasonal reproductive success was almost as high in females as in males, primarily due to a high rate of nest failure (73%). Estimated N-e (42) was 19% of (N) over cap in our population, below the level at which inbreeding depression is observed in captive breeding studies. A high hatching failure rate (28%) was also consistent with ongoing inbreeding depression. In the simulations, N-e was reduced by skewed male mating success, especially at larger population sizes, and by female breeding failure. Extrapolation of our results suggests that six of the seven extant populations of this species may have effective sizes low enough to induce inbreeding depression and hence that translocations may be needed to supplement genetic diversity. (C) 2007 Elsevier Ltd. All rights reserved. | 822 | no | no good fitness measure, N pop differnt in GD? | NA | no | no |
| 823 | 2017 | 43012 | Irzykowska, L; Werner, M; Bocianowski, J; Karolewski, Z; Fruzynska-Jozwiak, D | 2013 | Genetic variation of horse chestnut and red horse chestnut and trees susceptibility to Erysiphe flexuosa and Cameraria ohridella | Trees of Aesculus sp. are often present in European landscapes. Common horse chestnut and red horse chestnut are seriously damaged by the fungus Erysiphe flexuosa as well as the pest Cameraria ohridella. Both pathogen and pest have spread throughout Europe. A genetic background of trees resistance still remains unknown. The present study was undertaken to estimate genetic variation of A. hippocastanum and A.xcarnea grown in urban greens and their susceptibility to powdery mildew and horse chestnut leafminer. According to obtained results both species were infected by E. flexuosa in a similar degree but there were significant differences in susceptibility of particular trees (ANOVA). C. ohridella damaged only A. hippocastanum. The correlation between age of trees and degree of infestation by pathogen and pest was not observed. The significant genetic variability between two Aesculus species was revealed by the analysis of molecular variance (AMOVA) where both the intra- and the inter-species variation were found to be significant. It was shown that 73.0% of the genetic variance was contributed by the differentiation between A. hippocastanum and A.xcarnea, whereas 27.0% was partitioned within species. The medium level of genetic diversity of Aesculus spp. was determined using SRAP and RAPD analyses. The mean value of genetic similarity was equal to 0.55 for common horse chestnut and 0.98 for red horse chestnut. Among 40 polymorphic SRAP and RAPD markers 17 were associated with degree of leaf damage caused by C. ohridella. | 823 | no | no good fitness measure, N pop differnt in GD? | NA | no | no |
| 824 | 2017 | 43012 | Rawlings-Goss, RA; Campbell, MC; Tishkoff, SA | 2014 | Global population-specific variation in miRNA associated with cancer risk and clinical biomarkers | Background: MiRNA expression profiling is being actively investigated as a clinical biomarker and diagnostic tool to detect multiple cancer types and stages as well as other complex diseases. Initial investigations, however, have not comprehensively taken into account genetic variability affecting miRNA expression and/or function in populations of different ethnic backgrounds. Therefore, more complete surveys of miRNA genetic variability are needed to assess global patterns of miRNA variation within and between diverse human populations and their effect on clinically relevant miRNA genes. Methods: Genetic variation in 1524 miRNA genes was examined using whole genome sequencing (60x coverage) in a panel of 69 unrelated individuals from 14 global populations, including European, Asian and African populations. Results: We identified 33 previously undescribed miRNA variants, and 31 miRNA containing variants that are globally population-differentiated in frequency between African and non-African populations (PD-miRNA). The top 1% of PD-miRNA were significantly enriched for regulation of genes involved in glucose/insulin metabolism and cell division (p < 10(-7)), most significantly the mitosis pathway, which is strongly linked to cancer onset. Overall, we identify 7 PD-miRNAs that are currently implicated as cancer biomarkers or diagnostics: hsa-mir-202, hsa-mir-423, hsa-mir-196a-2, hsa-mir-520h, hsa-mir-647, hsa-mir-943, and hsa-mir-1908. Notably, hsa-mir-202, a potential breast cancer biomarker, was found to show significantly high allele frequency differentiation at SNP rs12355840, which is known to affect miRNA expression levels in vivo and subsequently breast cancer mortality. Conclusion: MiRNA expression profiles represent a promising new category of disease biomarkers. However, population specific genetic variation can affect the prevalence and baseline expression of these miRNAs in diverse populations. Consequently, miRNA genetic and expression level variation among ethnic groups may be contributing in part to health disparities observed in multiple forms of cancer, specifically breast cancer, and will be an essential consideration when assessing the utility of miRNA biomarkers for the clinic. | 824 | no | human | NA | no | no |
| 825 | 2017 | 43012 | Hughes, KA; Houde, AE; Price, AC; Rodd, FH | 2013 | Mating advantage for rare males in wild guppy populations | To understand the processes that maintain genetic diversity is a long-standing challenge in evolutionary biology, with implications for predicting disease resistance, response to environmental change, and population persistence(1-3). Simple population genetic models are not sufficient to explain the high levels of genetic diversity sometimes observed in ecologically important traits(2). In guppies (Poecilia reticulata), male colour pattern is both diverse and heritable, and is arguably one of the most extreme examples of morphological polymorphism known(4,5). Negative frequency-dependent selection (NFDS), a form of selection in which genotypes are favoured when they are rare(6), can potentially maintain such extensive polymorphism, but few experimental studies have confirmed its operation in nature(7,8). Here we use highly replicated experimental manipulations of natural populations to show that males with rare colour patterns have higher reproductive fitness, demonstrating NFDS mediated by sexual selection. Rare males acquired more mates and sired more offspring compared to common males and, as previously reported, had higher rates of survival(8). Orange colour, implicated in other studies of sexual selection in guppies, did predict male reproductive success, but only in one of three populations. These data support the hypothesis that NFDS maintains diversity in the colour patterns of male guppies through two selective agents, mates and predators. Similar field-based manipulations of genotype frequencies could provide a powerful approach to reveal the underlying ecological and behavioural mechanisms that maintain genetic and phenotypic diversity. | 825 | no | no good fitness measure, N pop differnt in GD? | NA | no | no |
| 826 | 2017 | 43012 | Franklin, MT; Ritland, CE; Myers, JH; Cory, JS | 2012 | Multiple Mating and Family Structure of the Western Tent Caterpillar, Malacosoma californicum pluviale: Impact on Disease Resistance | Background: Levels of genetic diversity can strongly influence the dynamics and evolutionary changes of natural populations. Survival and disease resistance have been linked to levels of genetic diversity in eusocial insects, yet these relationships remain untested in gregarious insects where disease transmission can be high and selection for resistance is likely to be strong. Methodology/Principal Findings: Here we use 8 microsatellite loci to examine genetic variation in 12 families of western tent caterpillars, Malacosoma californicum pluviale from four different island populations to determine the relationship of genetic variability to survival and disease resistance. In addition these genetic markers were used to elucidate the population structure of western tent caterpillars. Multiple paternity was revealed by microsatellite markers, with the number of sires estimated to range from one to three per family (mean +/- SE = 1.92 +/- 0.23). Observed heterozygosity (H-O) of families was not associated to the resistance of families to a nucleopolyhedrovirus (NPV) (r = 0.161, F-1,F-12 = 0.271, P = 0.614), a major cause of mortality in high-density populations, but was positively associated with larval survival (r = 0.635, F-1,F-10 = 5.412, P = 0.048). Genetic differentiation among the families was high (F-ST = 0.269, P<0.0001), and families from the same island were as differentiated as were families from other islands. Conclusion/Significance: We have been able to describe and characterize 8 microsatellite loci, which demonstrate patterns of variation within and between families of western tent caterpillars. We have discovered an association between larval survival and family-level heterozygosity that may be relevant to the population dynamics of this cyclic forest lepidopteran, and this will be the topic of future work. | 826 | probably yes | ok gd and fitness measurement? | NA | yes | yes |
| 827 | 2017 | 43012 | Booth, W; Balvin, O; Vargo, EL; Vilimova, J; Schal, C | 2015 | Host association drives genetic divergence in the bed bug, Cimex lectularius | Genetic differentiation may exist among sympatric populations of a species due to long-term associations with alternative hosts (i.e. host-associated differentiation). While host-associated differentiation has been documented in several phytophagus insects, there are far fewer cases known in animal parasites. The bed bug, Cimex lectularius, a wingless insect, represents a potential model organism for elucidating the processes involved in host-associated differentiation in animal parasites with relatively limited mobility. In conjunction with the expansion of modern humans from Africa into Eurasia, it has been speculated that bed bugs extended their host range from bats to humans in their shared cave domiciles throughout Eurasia. C.lectularius that associate with humans have a cosmopolitan distribution, whereas those associated with bats occur across Europe, often in human-built structures. We assessed genetic structure and gene flow within and among populations collected in association with each host using mtDNA, microsatellite loci and knock-down resistance gene variants. Both nuclear and mitochondrial data support a lack of significant contemporary gene flow between host-specific populations. Within locations human-associated bed bug populations exhibit limited genetic diversity and elevated levels of inbreeding, likely due to human-mediated movement, infrequent additional introduction events per infestation, and pest control. In contrast, populations within bat roosts exhibit higher genetic diversity and lower levels of relatedness, suggesting populations are stable with temporal fluctuations due to host dispersal and bug mortality. In concert with previously published evidence of morphological and behavioural differentiation, the genetic data presented here suggest C.lectularius is currently undergoing lineage divergence through host association. | 827 | no | gd of pathogen | NA | no | no |
| 828 | 2017 | 43012 | Lallias, D; Boudry, P; Lapegue, S; King, JW; Beaumont, AR | 2010 | Strategies for the retention of high genetic variability in European flat oyster (Ostrea edulis) restoration programmes | The native European flat oyster Ostrea edulis is listed in the OSPAR Convention for the Protection of the Marine Environment of the North-East Atlantic (species and habitat protection) and in the UK Biodiversity Action Plan. Once extremely abundant in the nineteenth century, European stocks of O. edulis have declined during the twentieth century to rare, small, localised populations due to overexploitation, habitat degradation and, most recently, the parasitic disease bonamiosis. Selective breeding programmes for resistance to bonamiosis have been initiated in France and Ireland. High genetic diversity and bonamiosis-resistance would be important features of any sustainable restoration programmes for O. edulis. Oysters were sampled across Europe from four hatchery sources, four pond-cultured sources and four wild, but managed fisheries and were genotyped at five microsatellite loci. Hatchery-produced populations from small numbers of broodstock showed a significant loss of genetic diversity relative to wild populations and pedigree reconstruction revealed that they were each composed of a single large full-sib family and several small full-sib families. This extremely low effective population size highlights the variance in reproductive success among the potential breeders. Pond-cultured oysters were intermediate in genetic diversity and effective population size between hatchery and wild populations. Controlled hatchery production allows the development of bonamiosis-resistant strains, but at the expense of genetic diversity. Large scale pond culture on the other hand can provide a good level of genetic diversity. A mixture of these two approaches is required to ensure a healthy and sustainable restoration programme for O. edulis in Europe. | 828 | no | no good fitness measure, N pop differnt in GD? | NA | no | no |
| 829 | 2017 | 43012 | Ejsmond, MJ; Radwan, J; Wilson, AB | 2014 | Sexual selection and the evolutionary dynamics of the major histocompatibility complex | The genes of the major histocompatibility complex (MHC) are a key component of the adaptive immune system and among the most variable loci in the vertebrate genome. Pathogen-mediated natural selection and MHC-based disassortative mating are both thought to structure MHC polymorphism, but their effects have proven difficult to discriminate in natural systems. Using the first model of MHC dynamics incorporating both survival and reproduction, we demonstrate that natural and sexual selection produce distinctive signatures of MHC allelic diversity with critical implications for understanding host-pathogen dynamics. While natural selection produces the Red Queen dynamics characteristic of host-parasite interactions, disassortative mating stabilizes allele frequencies, damping major fluctuations in dominant alleles and protecting functional variants against drift. This subtle difference generates a complex interaction between MHC allelic diversity and population size. In small populations, the stabilizing effects of sexual selection moderate the effects of drift, whereas pathogen-mediated selection accelerates the loss of functionally important genetic diversity. Natural selection enhances MHC allelic variation in larger populations, with the highest levels of diversity generated by the combined action of pathogen-mediated selection and disassortative mating. MHC-based sexual selection may help to explain how functionally important genetic variation can be maintained in populations of conservation concern. | 829 | no | no good fitness measure, N pop differnt in GD? | NA | no | no |
| 830 | 2017 | 43012 | Nakamura, M; Kaneko, S; Isagi, Y; Hata, K; Sone, K | 2009 | Development of microsatellite markers for Pasania edulis (Makino) Makino, one of the dominant species of lucidphyllous forests in southern Kyushu, Japan | Pasania edulis (Makino) Makino is a Fagaceous tree species endemic to Japan and one of the dominant species in lucidphyllous forests in southern Kyushu, Japan. Recently, P. edulis was attacked by ambrosia beetles, Platypus quereivorus (Murayama) and mass mortality occurred in several areas in southern Kyushu. We isolated and characterized ten microsatellite loci in this species to reveal the genetic structure and gene flow. The number of alleles ranged from 3 to 11 and expected heterozygosities from 0.3761 to 0.8346. The markers described here will be useful for investigating the genetic diversity, genetic structure and gene flow, and planning for conservation of viable population of P. edulis. | 830 | no | no good fitness measure, N pop differnt in GD? | NA | no | no |
| 831 | 2017 | 43012 | SASTRY, JG; RAMAKRISHNA, W; SIVARAMAKRISHNAN, S; THAKUR, RP; GUPTA, VS; RANJEKAR, PK | 1995 | DNA-FINGERPRINTING DETECTS GENETIC-VARIABILITY IN THE PEARL-MILLET DOWNY MILDEW PATHOGEN (SCLEROSPORA-GRAMINICOLA) | Genetic variability in six host genotype-specific pathotypes of pearl millet downy mildew pathogen S. graminicola was studied at the molecular level using mini- and micro-satellites. Our results indicated that microsatellites (GAA)(6), (GACA)(4), and especially (GATA)(4) were quite informative and showed high levels of polymorphism among the pathotypes. The six pathotypes could be classified into five groups based on the cluster analysis of their genetic similarities, thereby confirming the existence of distinct host genotype-specific virulence in S. graminicola pathotypes. We demonstrate, for the first time, the use of DNA fingerprinting to detect genetic variation in downy mildew fungus of pearl millet. | 831 | no | gd of pathogen | NA | no | no |
| 832 | 2017 | 43012 | Nogueiro, I; Teixeira, J; Amorim, A; Gusmao, L; Alvarez, L | 2015 | Echoes from Sepharad: signatures on the maternal gene pool of crypto-Jewish descendants | The majority of genetic studies on Jewish populations have been focused on Ashkenazim, and genetic data from the Sephardic original source, the Iberian Peninsula, are particularly scarce. Regarding the mitochondrial genome, the available information is limited to a single Portuguese village, Belmonte, where just two different lineages (a single one corresponding to 93.3%) were found in 30 individuals. Aiming at disclosing the ancestral maternal background of the Portuguese Jewry, we enlarged the sampling to other crypto-Jewish descendants in the Braganc, a district (NE Portugal). Fifty-seven complete mtDNA genomes were newly sequenced and - in contrast with Belmonte - a high level of diversity was found, with five haplogroups (HV0b, N1, T2b11, T2e and U2e) being putatively identified as Sephardic founding lineages. Therefore - in sharp contrast with Belmonte - these communities have managed to escape the expected inbreeding effects caused by centuries of religious repression and have kept a significant proportion of the Sephardic founder gene pool. This deeper analysis of the surviving Sephardic maternal lineages allowed a much more comprehensive and detailed perspective on the origins and survival of the Sephardic genetic heritage. In line with previously published results on Sephardic paternal lineages, our findings also show a surprising resistance to the erosion of genetic diversity in the maternal lineages. | 832 | no | human | NA | no | no |
| 833 | 2017 | 43012 | Souza-Firmino, TS; Alevi, KCC; Pereira, LLV; Souza, ERS; Junior, FCS; Banho, CA; Carmo, GO; Itoyama, MM | 2015 | High genetic variability and polychromatism in Pachycoris torridus (Heteroptera: Scutelleridae) | The stink bug Pachycoris torridus is listed among the most polyphagous insects in the world and it is a major pest of diverse crops, in particular the physic nut Jatropha curcas, which is used as a raw material for biodiesel production. A peculiar characteristic of this species is its high phenotypic variability, a characteristic that makes identification difficult: P. torridus has been described as a new species eight times. Thus, to aid in identification, genetic characterization of this insect was performed. We verified that, due to the high genetic variability of P. torridus, several genetic patterns exist that result in the same phenotype. | 833 | no | no good fitness measure, N pop differnt in GD? | NA | no | no |
| 834 | 2017 | 43012 | Hadziabdic, D; Vito, LM; Windham, MT; Pscheidt, JW; Trigiano, RN; Kolarik, M | 2014 | Genetic differentiation and spatial structure of Geosmithia morbida, the causal agent of thousand cankers disease in black walnut (Juglans nigra) | The main objectives of this study were to evaluate genetic composition of Geosmithia morbida populations in the native range of black walnut and provide a better understanding regarding demography of the pathogen. The fungus G. morbida, and the walnut twig beetle, Pityophthorus juglandis, have been associated with a disease complex of black walnut (Juglans nigra) known as thousand cankers disease (TCD). The disease is manifested as branch dieback and canopy loss, eventually resulting in tree death. In 2010, the disease was detected in black walnut in Tennessee, and subsequently in Virginia and Pennsylvania in 2011 and North Carolina in 2012. These were the first incidences of TCD east of Colorado, where the disease has been established for more than a decade on indigenous walnut species. A genetic diversity and population structure study of 62 G. morbida isolates from Tennessee, Pennsylvania, North Carolina and Oregon was completed using 15 polymorphic microsatellite loci. The results revealed high haploid genetic diversity among seven G. morbida populations with evidence of gene flow, and significant differentiation among two identified genetic clusters. There was a significant correlation between geographic and genetic distance. Understanding the genetic composition and demography of G. morbida can provide valuable insight into recognizing factors affecting the persistence and spread of an invasive pathogen, disease progression, and future infestation predictions. Overall, these data support the hypotheses of two separate, highly diverse pathogen introductions into the native range of black walnut. | 834 | no | gd of pathogen | NA | no | no |
| 835 | 2017 | 43012 | Drury, DW; Dapper, AL; Siniard, DJ; Zentner, GE; Wade, MJ | 2017 | CRISPR/Cas9 gene drives in genetically variable and nonrandomly mating wild populations | Synthetic gene drives based on CRISPR/Cas9 have the potential to control, alter, or suppress populations of crop pests and disease vectors, but it is unclear how they will function in wild populations. Using genetic data from four populations of the flour beetle Tribolium castaneum, we show that most populations harbor genetic variants in Cas9 target sites, some of which would render them immune to drive (ITD). We show that even a rare ITD allele can reduce or eliminate the efficacy of a CRISPR/Cas9-based synthetic gene drive. This effect is equivalent to and accentuated by mild inbreeding, which is a characteristic of many disease-vectoring arthropods. We conclude that designing such drives will require characterization of genetic variability and the mating system within and among targeted populations. | 835 | no | no good fitness measure, N pop differnt in GD? | NA | no | no |
| 836 | 2017 | 43012 | da Silva, MJF; Godinho, R; Casanova, C; Minhos, T; Sa, R; Bruford, MW | 2014 | Assessing the impact of hunting pressure on population structure of Guinea baboons (Papio papio) in Guinea-Bissau | Guinea baboons are heavily hunted for bushmeat consumption in Guinea-Bissau. We investigated whether hunting-driven mortality has affected population structure in this generalist primate using two genetic markers. Sampling was conducted in protected areas separated by anthropogenic landscape features. We predicted significant genetic differentiation between samples and investigated whether genetic discontinuities in the data were concordant with the location of human infrastructures. Genetic diversity was not significantly reduced when compared with a neighbouring population in Senegal and we inferred historically female-biased dispersal and recent contact between localities. Evidence was found for a contact zone between genetically differentiated populations where gene-flow is unidirectional, admixed individuals are at a higher proportion and individuals differentiated for both genetic markers co-exist within the same social units. Genetic discontinuities were, however, unrelated to anthropogenic dispersal barriers and we could not explain the existence of a contact zone by geographic distance, habitat type or the effect of social structure. We propose that hunting practices have affected the population structure by increasing dispersal distances, facilitating contact between previously separated gene pools within social groups. We suggest that hunting-related density sinks found in areas where the quality of the habitat remains adequate could precipitate the immigration of genetically distinct individuals from distant populations. Alternatively, migrants found in protected areas might be avoiding hunters, in locations they may perceive as less disturbed. This study suggests that hunting practices must be considered when investigating genetic patterns in primates and underlines the utility of molecular approaches to detect population perturbations due to bushmeat hunting. | 836 | no | no good fitness measure, N pop differnt in GD? | NA | no | no |
| 837 | 2017 | 43012 | Galavemi, M; Caniglia, R; Milanesi, P; Lapalombella, S; Fabbri, E; Randi, E | 2016 | Choosy Wolves? Heterozygote Advantage But No Evidence of MHC-Based Disassortative Mating | A variety of nonrandom mate choice strategies, including disassortative mating, are used by vertebrate species to avoid inbreeding, maintain heterozygosity and increase fitness. Disassortative mating may be mediated by the major histocompatibility complex (MHC), an important gene cluster controlling immune responses to pathogens. We investigated the patterns of mate choice in 26 wild-living breeding pairs of gray wolf (Canis lupus) that were identified through noninvasive genetic methods and genotyped at 3 MHC class II and 12 autosomal microsatellite (STR) loci. We tested for deviations from random mating and evaluated the covariance of genetic variables at functional and STR markers with fitness proxies deduced from pedigree reconstructions. Results did not show evidences of MHC-based disassortative mating. Rather we found a higher peptide similarity between mates at MHC loci as compared with random expectations. Fitness values were positively correlated with heterozygosity of the breeders at both MHC and STR loci, whereas they decreased with relatedness at STRs. These findings may indicate fitness advantages for breeders that, while avoiding highly related mates, are more similar at the MHC and have high levels of heterozygosity overall. Such a pattern of MHC-assortative mating may reflect local coadaptation of the breeders, while a reduction in genetic diversity may be balanced by heterozygote advantages. | 837 | no | no good fitness measure, N pop differnt in GD? | NA | no | no |
| 838 | 2017 | 43012 | Andersson, M; Waldeck, P | 2007 | Host-parasite kinship in a female-philopatric bird population: evidence from relatedness trend analysis | Conspecific brood parasitism (CBP), an alternative reproductive tactic where some females lay eggs in the nests of other females of the same species, occurs in many animals with egg care. It is particularly common in waterfowl, for reasons that are debated. Many waterfowl females nest near their birthplace, making it likely that some local females are relatives. We analyse brood parasitism in a Hudson Bay population of common eiders, testing predictions from two alternative hypotheses on the role of relatedness in CBP. Some models predict host-parasite relatedness, others predict that parasites avoid close relatives as hosts. To distinguish between the alternatives, we use a novel approach, where the relatedness of host-parasite pairs is tested against the spatial population trend in pairwise relatedness. We estimate parasitism, nest take-over and relatedness with protein fingerprinting and bandsharing analysis of egg albumen, nondestructively sampled from each new egg in the nest throughout the laying period. The results refute the hypothesis that parasites avoid laying eggs in the nests of related hosts, and corroborate the alternative of host-parasite relatedness. With an estimated r of 0.12-0.14, females laying eggs in the same nest are on average closer kin than nesting neighbour females. Absence of a population trend in female pairwise relatedness vs. distance implies that host-parasite relatedness is not only an effect of strong natal philopatry: some additional form of kin bias is also involved. | 838 | no | brood parasitism | NA | no | no |
| 839 | 2017 | 43012 | Clauss, MJ; Mitchell-Olds, T | 2006 | Population genetic structure of Arabidopsis lyrata in Europe | Population genetic theory predicts that the self-incompatible and perennial herb, Arabidopsis lyrata, will have a genetic structure that differs from the self-fertilizing, annual Arabidopsis thaliana. We quantified the genetic structure for eight populations of A. lyrata ssp. petraea in historically nonglaciated regions of central Europe. Analysis of 20 microsatellite loci for 344 individuals demonstrated that, in accordance with predictions, diploid populations had high genome-wide heterozygosity (H-O = 0.48; H-E = 0.52), high within-population diversity (83% of total) compatible with mutation-drift equilibrium, and moderate differentiation among populations (F-ST = 0.17). Within a single population, the vast majority of genetic variability (92%) was found at the smallest spatial scale (< 3 m). Although there was no evidence of biparental inbreeding or clonal propagation at this scale (F-IS = 0.003), significant fine-scale spatial autocorrelation indicated localized gene flow presumably due to gravity dispersed seeds (Sp = 0.018). Limited gene flow between isolated population clusters (regions) separated by hundreds of kilometres has given rise to an isolation by distance pattern of diversification, with low, but significant, differentiation among regions (F-ST = 0.05). The maintenance of geographically widespread polymorphisms and uniformly high diversity throughout central Europe is consistent with periglacial survival of A. lyrata ssp. petraea north of the Alps in steppe-tundra habitats during the last glacial maximum. As expected of northern and previously glaciated localities, A. lyrata in Iceland was genetically less diverse and highly differentiated from central Europe (H-E = 0.37; F-ST = 0.27). | 839 | no | no good fitness measure, N pop differnt in GD? | NA | no | no |
| 840 | 2017 | 43012 | Hawkins, AJS; Day, AJ | 1999 | Metabolic interrelations underlying the physiological and evolutionary advantages of genetic diversity | Past findings have established how the faster growth, greater reproductive output and/or longer survival that are associated with heterosis and genomic diversity measured as multi-locus heterozygosity stem from slower intensities with which proteins are renewed and replaced (= protein turnover), Slower turnover results in lower energy requirements and reduced metabolic sensitivity to environmental change, representing a mechanistic basis for evolutionary consequences of genetic polymorphism. To determine the genetic and functional basis of differences in whole-body protein turnover, we have begun to resolve different proteolytic pathways, searching for genetic polymorphisms with a direct effect upon proteolysis, and assessing the metabolic and physiological consequences of those genetic influences in the mussel Mytilus edulis, Our recent work has established the physiological importance of lysosomal enzymes under normal conditions of basal proteolysis, and shown that associated effects on energy flux may vary according to functional differences between separate enzymes. Data are presented here which compare metabolic consequences of polymorphism in the lysosomal aminopeptidases Lap-1 and Lap-2, Findings establish that metabolic and phenotypic effects of genetic polymorphism result directly from genetic variation at the loci coding for these peptidases, rather than from linked loci. They also illustrate the complexity of interrelations that ultimately influence the evolutionary consequences of genomic diversity, including associated influences of both Lap-1 and Lap-2 on energy requirements and animal condition. We impress that energy requirements for protein turnover may represent a functional basis for epistasis, including associations whereby advantages of genetic polymorphism are greatest at loci that code for enzymes acting in both protein catabolism and energy provision. | 840 | no | no good fitness measure, N pop differnt in GD? | NA | no | no |
| 841 | 2017 | 43012 | Desai, SD; Currie, RW | 2015 | Genetic diversity within honey bee colonies affects pathogen load and relative virus levels in honey bees, Apis mellifera L | The evolution of polyandry is one of most highly debated topics in sociobiology. One hypothesis suggests genetic diversity increases resistance against the wide range of parasites and diseases affecting colonies. We investigated effects of manipulating genetic diversity on colony population size, fall Varroa population, Varroa and bee mortality rates, virus prevalence and concentration, and prevalence of Nosema apis Z. and Nosema ceranae F. Sister queens selected for resistance to Varroa were inseminated with either mixed semen from 12 drone sources (genetically diverse colonies, GDC) or single drone inseminated from 12 drone sources (genetically similar colonies, GSC), and colonies from these queens were compared to unrelated queens that were open-mated. When exposed to parasites and pathogens, open-mated colonies (OMC) and GDCs had larger worker populations before winter than GSCs. The bees of GDCs had higher Varroa mortality rates than those of GSCs in the field study. Within the instrumentally inseminated treatments, three pathogens occurred at lower prevalence (acute bee paralysis virus (ABPV) and N. ceranae) or concentrations (deformed wing virus, DWV) in GDCs than in GSCs. Both GDCs and GSCs that had been selected for resistance to Varroa had lower DWV concentration in spring when compared to unselected OMCs. Colonies inseminated by multiple drones and having greater within-colony genetic diversity showed reduced prevalence, concentrations in about a quarter of the pathogens assayed relative to genetically uniform colonies, and the reverse pattern was never observed. | 841 | yes | from thesis | NA | yes | yes |
| 842 | 2017 | 43012 | Tan, QH; Li, WL; Vandin, F | 2017 | Disease-Concordant Twins Empower Genetic Association Studies | Genome-wide association studies with moderate sample sizes are underpowered, especially when testing SNP alleles with low allele counts, a situation that may lead to high frequency of false-positive results and lack of replication in independent studies. Related individuals, such as twin pairs concordant for a disease, should confer increased power in genetic association analysis because of their genetic relatedness. We conducted a computer simulation study to explore the power advantage of the disease-concordant twin design, which uses singletons from disease-concordant twin pairs as cases and ordinary healthy samples as controls. We examined the power gain of the twin-based design for various scenarios (i.e., cases from monozygotic and dizygotic twin pairs concordant for a disease) and compared the power with the ordinary case-control design with cases collected from the unrelated patient population. Simulation was done by assigning various allele frequencies and allelic relative risks for different mode of genetic inheritance. In general, for achieving a power estimate of 80%, the sample sizes needed for dizygotic and monozygotic twin cases were one half and one fourth of the sample size of an ordinary case-control design, with variations depending on genetic mode. Importantly, the enriched power for dizygotic twins also applies to disease-concordant sibling pairs, which largely extends the application of the concordant twin design. Overall, our simulation revealed a high value of disease-concordant twins in genetic association studies and encourages the use of genetically related individuals for highly efficiently identifying both common and rare genetic variants underlying human complex diseases without increasing laboratory cost. | 842 | no | no good fitness measure, N pop differnt in GD? | NA | no | no |
| 843 | 2017 | 43012 | Bux, H; Rasheed, A; Mangrio, SM; Abro, SA; Shah, SJA; Ashraf, M; Chen, XM | 2012 | Comparative Virulence and Molecular Diversity of Stripe Rust (Puccinia striiformis f. sp tritici) Collections from Pakistan and United States | Information on virulence and molecular diversity of Puccinia striiformis f. sp. tritici (Pst) is a pre-requisite for mitigating the substantial yield losses caused by the stripe rust pathogen in Pakistan, the United States and other countries of the world. This study was undertaken to analyze both virulence and molecular diversity of 46 Pst isolates from Pakistan, in comparison with 9 US isolates. Avirulence to stripe rust resistance genes Yr5, Yr15 and YrSP was common among all tested isolates. Isolates from Pakistan had low virulence frequency for differentials carrying Yr2, (Yr10, YrMor) and (Yr2, Yr4a, YrYam). Clustering based on virulence data grouped contemporary isolates together and revealed high genetic diversity among pathotypes of both countries. Molecular analysis using sequence tagged site (STS) and microsatellites markers revealed high diversity based on polymorphic information contents (PIC)and marker index (MI) which was higher for SSRs (0.78 & 39.51, respectively) than STS markers(0.04 & 0.29, respectively). Dendrogram based on molecular marker data grouped together contemporary patbotypes indicating genetic similarity. Pathotypes belonging to both countries clustered together indicating common ancestry. Furthermore, very low correlation (r=0.08) was observed between virulence and molecular diversity showing independence in both trends of diversity. The depicted virulence pattern of Pst isolates will guide in devising cultivar improvement efforts for stripe rust resistance. (C) 2012 Friends Science Publishers | 843 | no | gd of pathogen | NA | no | no |
| 844 | 2017 | 43012 | Jones, ME; Paetkau, D; Geffen, E; Moritz, C | 2003 | Microsatellites for the Tasmanian devil (Sarcophilus laniarius) | The Tasmanian devil (Sarcophilus laniarius), a medium-sized predator/scavenger, is the largest member of the short-lived carnivorous marsupial Family Dasyuridae. Now restricted to Tasmania, populations are impacted by habitat clearance and anthropogenic mortality and genetic studies could be of value in informing levels of genetic diversity, mating system, dispersal and the effects of natural and anthropogenic landscape features on gene flow. Microsatellite markers were isolated from a partial, size-selected genomic library that was enriched for microsatellite sequences. Primer pairs were developed for 11 polymorphic dinucleotide microsatellite loci that conform with Hardy-Weinberg equilibrium and reveal moderate genetic variability across the species range. | 844 | no | no good fitness measure, N pop differnt in GD? | NA | no | no |
| 845 | 2017 | 43012 | Cooper, AM; Miller, LM; Kapuscinski, AR | 2010 | Conservation of population structure and genetic diversity under captive breeding of remnant coaster brook trout (Salvelinus fontinalis) populations | Rebuilding wild populations often involves captive broodstocks derived from small, remnant populations. We measured a hatchery program’s ability to conserve genetic diversity when founding captive broodstocks from such populations. Migratory coaster brook trout were extirpated from most of their historic range in US waters of Lake Superior and were proposed for listing under the Endangered Species Act. Two captive broodstocks, one with 19 founders and another with 99 founders, were established to rebuild US populations. We used microsatellite markers to examine genetic variation in source populations and early hatchery generations. Broodstocks retained the strong differentiation found between source populations; however, one founder, with a low probability of belonging to either source population, sired 5.7% of F(1) progeny. We found small changes in within-population genetic variation across successive wild and hatchery generations of broodstocks. Evaluation of stage-specific survivorship indicated that equalizing family sizes of embryos produced modest gains in the effective number of breeders, and that survival in the hatchery was nearly random across families. Our study demonstrates the value of genetic monitoring during initial stages of hatchery programs for small and declining populations. | 845 | no | no good fitness measure, N pop differnt in GD? | NA | no | no |
| 846 | 2017 | 43012 | Arias, D; Gonzalez, M; Prada, F; Ayala-Diaz, I; Montoya, C; Daza, E; Romero, HM | 2015 | Genetic and phenotypic diversity of natural American oil palm (Elaeis oleifera (HBK) Cortes) accessions | Elaeis oleifera has become a valuable genetic resource for the production of interspecific hybrids of E. oleifera (H.B.K) Cortes x Elaeis guineensis Jacq. to address plant disease problems affecting oil palm cultivation. In this study, we evaluated the phenotypic and genetic diversity of accessions of E. oleifera from different countries in South America using morpho-agronomic traits and microsatellite markers (Simple Sequence Repeats, SSRs). Analyses of variance for yield and bunch components demonstrated statistically significant differences among countries and geographical regions for several of the traits evaluated. SSR marker analyses revealed high genetic diversity (H-T=0.797) and the presence of specific alleles by each country of origin from E. oleifera. The clustering topology obtained showed four distinguishable E. oleifera groups, which matched the geographical distribution of the accessions and thus exhibited a high genetic differentiation (G(ST)=0.512) and a low gene flow N-m=0.238 among countries. The results enabled us to demonstrate that E. oleifera has a specific genetic structure and a phenotypic variability with different characteristics between origins, and the accessions from each country of origin contributed to the increase in total genetic diversity. A core collection was defined based on the accuracy of the classification of individuals with respect to their country of origin. The information reported in this study will be of great interest to oil palm researchers because new strategies for breeding programs can be developed based on these advances. | 846 | no | no good fitness measure, N pop differnt in GD? | NA | no | no |
| 847 | 2017 | 43012 | Fussi, B; Konnert, M | 2014 | Genetic analysis of European common ash (Fraxinus excelsior L.) populations affected by ash dieback | European common ash is an important component of mixed forest ecosystems in Bavaria and is considered a valuable tree species under climate change. The first aim of the present study was to assess the genetic diversity within and between ash populations in Bavaria in comparison with neighboring regions. Because ash stands have been heavily attacked by ash shoot disease in the last few years, the second aim of the study was to detect genetic differences between susceptible and less susceptible trees (trees with defoliation up to 30%) within populations. Altogether 41 populations were investigated using nuclear and chloroplast microsatellites. The results showed high genetic variation within and high genetic differentiation between ash populations. Higher chloroplast microsatellite variation was detected instead populations from south-eastern Bavaria compared to other regions. The comparison of susceptible and less susceptible groups of individuals within each population revealed medium to high genetic differences in some cases. For the observed heterozygosity, higher values were found for the group of less susceptible trees compared to the group of all trees or to the group of susceptible trees within populations. This could be a first indication that individuals with a higher heterozygosity might be able to withstand ash dieback in a better way compared to homozygotic individuals. Within the group of less susceptible trees a relatively wide genetic base exists whereupon a future breeding programme can be built. Thus it is of utmost importance for the species to maintain less affected trees in order to keep the genetic potential for future adaptation processes within ash populations, seed production and regeneration. | 847 | probably yes | ok gd difference of pop? any fitness measure? | no good fitness measure of pop of different GD | no | no |
| 848 | 2017 | 43012 | Giovambattista, G; Takeshima, SN; Ripoli, MV; Matsumoto, Y; Franco, LAA; Saito, H; Onuma, M; Aida, Y | 2013 | Characterization of bovine MHC DRB3 diversity in Latin American Creole cattle breeds | In cattle, bovine leukocyte antigens (BoLAs) have been extensively used as markers for diseases and immunological traits. However, none of the highly adapted Latin American Creole breeds have been characterized for BoLA gene polymorphism by high resolution typing methods. In this work, we sequenced exon 2 of the BoLA class II DRB3 gene from 179 cattle (113 Bolivian Yacumeno cattle and 66 Colombian Harton del Valle cattle breeds) using a polymerase chain reaction sequence-based typing (PCR-SBT) method. We identified 36 previously reported alleles and three novel alleles. Thirty-five (32 reported and three new) and 24 alleles (22 reported and two new) were detected in Yacumeno and Harton del Valle breeds, respectively. Interestingly, Latin American Creole cattle showed a high degree of gene diversity despite their small population sizes, and 10 alleles including three new alleles were found only in these two Creole breeds. We next compared the degree of genetic variability at the population and sequence levels and the genetic distance in the two breeds with those previously reported in five other breeds: Holstein, Japanese Shorthorn, Japanese Black, Jersey, and Hanwoo. Both Creole breeds presented gene diversity higher than 0.90, a nutleotide diversity higher than 0.07, and mean number of pairwise differences higher than 19, indicating that Creole cattle had similar genetic diversity at BoLA-DRB3 to the other breeds. A neutrality test showed that the high degree of genetic variability may be maintained by balancing selection. The F-ST index and the exact G test showed significant differences across all cattle populations (F-ST = 0.0478; p<0.001). Results from the principal components analysis and the phylogenetic tree showed that Yacumeno and Harton del Valle breeds were closely related to each other. Collectively, our results suggest that the high level of genetic diversity could be explained by the multiple origins of the Creole germplasm (European, African and Indicus), and this diversity might be maintained by balancing selection. (C) 2013 Elsevier B.V. All rights reserved. | 848 | no | domesticated animal | NA | no | no |
| 849 | 2017 | 43012 | Colson, KE; White, KS; Hundertmark, KJ | 2016 | Parturition site selection in moose (Alces alces): evidence for social structure | Female natal philopatry has often been implicated as an important factor in moose (Alces alces) home range formation, with many populations showing behavioral evidence of sympatric home ranges among related individuals. However, previous genetic studies have failed to detect genetic subpopulation structure, leading to questions as to whether philopatry is a significant factor contributing to intra-and inter-population genetic structure. Here, we examine calving location data from radiomarked individuals (n = 110) collected in 2 separate populations in Berners Bay and Gustavus, Alaska, to examine the extent to which genetic structure originating from philopatry is evident at fine spatial scales. When populations were combined, their overall relatedness correlogram was significant (P < 0.001), with mean r = 0.079 between 0 km and 1.5 km (P = 0.079). Additionally, 13% of the population shows significantly positive relatedness to their 4 nearest neighbors, with an overall average r = 0.19 of those focal individuals to their neighbors. We suggest that habitat structure, especially linear habitats (i.e., river valleys), or habitat bounded with barriers to dispersal, may be a factor in promoting the development of this structure. This study presents the 1st known evidence for fine-scale social genetic structure in moose and natal philopatry to calving locations in moose. In the context of natal philopatry within cervids, these findings identify several cervid-wide commonalities. | 849 | no | no good fitness measure, N pop differnt in GD? | NA | no | no |
| 850 | 2017 | 43012 | Peters, JL; Sonsthagen, SA; Lavretsky, P; Rezsutek, M; Johnson, WP; McCracken, KG | 2014 | Interspecific hybridization contributes to high genetic diversity and apparent effective population size in an endemic population of mottled ducks (Anas fulvigula maculosa) | Under drift-mutation equilibrium, genetic diversity is expected to be correlated with effective population size (N (e) ). Changes in population size and gene flow are two important processes that can cause populations to deviate from this expected relationship. In this study, we used DNA sequences from six independent loci to examine the influence of these processes on standing genetic diversity in endemic mottled ducks (Anas fulvigula) and geographically widespread mallards (A. platyrhynchos), two species known to hybridize. Mottled ducks have an estimated census size that is about two orders-of-magnitude smaller than that of mallards, yet these two species have similar levels of genetic diversity, especially at nuclear DNA. Coalescent analyses suggest that a population expansion in the mallard at least partly explains this discrepancy, but the mottled duck harbors higher genetic diversity and apparent N (e) than expected for its census size even after accounting for a population decline. Incorporating gene flow into the model, however, reduced the estimated N (e) of mottled ducks to 33 % of the equilibrium N (e) and yielded an estimated N (e) consistent with census size. We also examined the utility of these loci to distinguish among mallards, mottled ducks, and their hybrids. Most putatively pure individuals were correctly assigned to species, but the power for detecting hybrids was low. Although hybridization with mallards potentially poses a conservation threat to mottled ducks by creating a risk of extinction by hybridization, introgression of mallard alleles has helped maintain high genetic diversity in mottled ducks and might be important for the adaptability and survival of this species. | 850 | no | hybrids | NA | no | no |
| 851 | 2017 | 43013 | Dagan, Y; Liljeroos, K; Jokela, J; Ben-Ami, F | 2013 | Clonal diversity driven by parasitism in a freshwater snail | One explanation for the widespread abundance of sexual reproduction is the advantage that genetically diverse sexual lineages have under strong pressure from virulent coevolving parasites. Such parasites are believed to track common asexual host genotypes, resulting in negative frequency-dependent selection that counterbalances the population growth-rate advantage of asexuals in comparison with sexuals. In the face of genetically diverse asexual lineages, this advantage of sexual reproduction might be eroded, and instead sexual populations would be replaced by diverse assemblages of clonal lineages. We investigated whether parasite-mediated selection promotes clonal diversity in 22 natural populations of the freshwater snail Melanoides tuberculata. We found that infection prevalence explains the observed variation in the clonal diversity of M.tuberculata populations, whereas no such relationship was found between infection prevalence and male frequency. Clonal diversity and male frequency were independent of snail population density. Incorporating ecological factors such as presence/absence of fish, habitat geography and habitat type did not improve the predictive power of regression models. Approximately 11% of the clonal snail genotypes were shared among 2-4 populations, creating a web of 17 interconnected populations. Taken together, our study suggests that parasite-mediated selection coupled with host dispersal ecology promotes clonal diversity. This, in return, may erode the advantage of sexual reproduction in M.tuberculata populations. | 851 | yes | ok gd measurement? | NA | yes | yes |
| 852 | 2017 | 43013 | Hughes, WOH; Bot, ANM; Boomsma, JJ | 2010 | Caste-specific expression of genetic variation in the size of antibiotic-producing glands of leaf-cutting ants | Social insect castes represent some of the most spectacular examples of phenotypic plasticity, with each caste being associated with different environmental conditions during their life. Here we examine the level of genetic variation in different castes of two polyandrous species of Acromyrmex leaf-cutting ant for the antibiotic-producing metapleural gland, which has a major role in defence against parasites. Gland size increases allometrically. The small workers that play the main role in disease defence have relatively large glands compared with larger workers, while the glands of gynes are substantially larger than those of any workers, for their body size. The gland size of large workers varies significantly between patrilines in both Acromyrmex echinatior and Acromyrmex octospinosus. We also examined small workers and gynes in A. echinatior, again finding genetic variation in gland size in these castes. There were significant positive relationships between the gland sizes of patrilines in the different castes, indicating that the genetic mechanism underpinning the patriline variation has remained similar across phenotypes. The level of expressed genetic variation decreased from small workers to large workers to gynes. This is consistent with the hypothesis that there is individual selection on disease defence in founding queens and colony-level selection on disease defence in the worker castes. | 852 | no | no good fitness measure | NA | no | no |
| 853 | 2017 | 43013 | Arnau, G; Bhattacharjee, R; Sheela, MN; Chair, H; Malapa, R; Lebot, V; Abraham, K; Perrier, X; Petro, D; Penet, L; Pavis, C | 2017 | Understanding the genetic diversity and population structure of yam (Dioscorea alata L.) using microsatellite markers | Yams (Dioscorea sp.) are staple food crops for millions of people in tropical and subtropical regions. Dioscorea alata, also known as greater yam, is one of the major cultivated species and most widely distributed throughout the tropics. Despite its economic and cultural importance, very little is known about its origin, diversity and genetics. As a consequence, breeding efforts for resistance to its main disease, anthracnose, have been fairly limited. The objective of this study was to contribute to the understanding of D. alata genetic diversity by genotyping 384 accessions from different geographical regions (South Pacific, Asia, Africa and the Caribbean), using 24 microsatellite markers. Diversity structuration was assessed via Principal Coordinate Analysis, UPGMA analysis and the Bayesian approach implemented in STRUCTURE. Our results revealed the existence of a wide genetic diversity and a significant structuring associated with geographic origin, ploidy levels and morpho-agronomic characteristics. Seventeen major groups of genetically close cultivars have been identified, including eleven groups of diploid cultivars, four groups of triploids and two groups of tetraploids. STRUCTURE revealed the existence of six populations in the diploid genetic pool and a few admixed cultivars. These results will be very useful for rationalizing D. alata genetic resources in breeding programs across different regions and for improving germplasm conservation methods. | 853 | no | crop | NA | no | no |
| 854 | 2017 | 43013 | Cuesta, S; Guzman, C; Alvarez, JB | 2015 | Molecular characterization of novel LMW-i glutenin subunit genes from Triticum urartu Thum. ex Gandil. | A high level of genetic diversity was found in LMW-i genes from Triticum urartu, resulting in detection of 11 novel alleles. The variability detected could affect gluten quality. Low-molecular weight glutenin subunits are important in determining the viscoelastic properties of wheat dough. Triticum urartu Thum. ex Gandil., which is related to the A genome of polyploid wheat, has been shown as a good source of variation for these subunits. The present study evaluated the variability of LMW-i genes in this species. High polymorphism was found in the sequences analysed and resulted in the detection of 11 novel alleles, classified into two sets (Group-I and -II) showing unique SNPs and InDels. Both groups were associated with Glu-A3-1 genes from common wheat. In general, deduced proteins from Group-II genes possessed a higher proportion of glutamine and proline, which has been previously suggested to be related with good quality. Moreover, there were other changes compared to common wheat. This novel variation could affect dough quality. Additional epitopes for celiac disease were also detected, suggesting that these subunits could be highly reactive. The results showed that T. urartu could be an important source of genetic variability for LMW-i genes that could enlarge the genetic pool of modern wheat. | 854 | no | no good fitness measure, N pop differnt in GD? | NA | no | no |
| 855 | 2017 | 43013 | Ruarus, SP; Paquette, SR; Hale, K; Ritchie, PA | 2011 | Genetic diversity and population viability in translocated North Island saddleback (Philesturnus rufusater) populations at Zealandia Karori Sanctuary and Kapiti Island | Genetic variation in two translocated populations of North Island saddleback (Philesturnus rufusater) on Kapiti Island and at Zealandia was investigated using five microsatellite loci and compared with the source populations in the Hauraki Gulf. Although the absolute number of alleles in the two populations was low (3 alleles per locus), both populations carried all the alleles found in their immediate source populations, but lacked one rare allele found in only one individual from the original remnant population on Hen Island. Overall heterozygosity was high and inbreeding coefficients were low. Population viability analyses showed that these populations will likely reach carrying capacity by the middle of this decade, and genetic simulations predicted that they should retain between 90% (Kapiti) and 95% (Zealandia) of the heterozygosity of their sources. The difference between the two populations is most likely due to the prolonged post-translocation bottleneck on Kapiti when rats were still present on the island. While our results suggest that additional top-up translocations would be unnecessary and unwarranted at this time, further work on potentially selected loci or inbreeding depression could justify this decision to be revisited. | 855 | no | no good fitness measure, N pop differnt in GD? | NA | no | no |
| 856 | 2017 | 43013 | Townsend, AK; Clark, AB; McGowan, KJ | 2010 | Direct Benefits and Genetic Costs of Extrapair Paternity for Female American Crows (Corvus brachyrhynchos) | The idea that extrapair paternity (EPP) in birds is part of a mixed reproductive strategy driven primarily by females is controversial. In cooperatively breeding American crows, we compared predictions of four female benefits hypotheses-the genetic diversity, good genes, genetic compatibility, and direct benefits hypotheses-to our predictions if EPP was primarily male driven. We found that genetically diverse broods were not more successful, extrapair young were not in better condition and did not have a higher survival probability, and, contrary to prediction, offspring sired by within-group extrapair males were more inbred than within-pair offspring. There was evidence of direct benefits, however: provisioning rate and number of surviving offspring were higher in groups containing within-group extrapair sires. Females therefore derived no apparent benefits from extragroup extrapair males but both direct benefits and genetic costs from within-group extrapair males. We suggest that males and females both influence the distribution of EPP in this system. | 856 | yes | ok gd measurement? | no ok group gd and fitness | no | no |
| 857 | 2017 | 43013 | Grueber, CE; Jamieson, IG | 2013 | Primers for amplification of innate immunity toll-like receptor loci in threatened birds of the Apterygiformes, Gruiformes, Psittaciformes and Passeriformes | Assaying diversity at functional genomic regions, such as those of the immune system, allows us to test hypotheses about processes that determine the distribution of genetic diversity in threatened populations, and the fitness consequences of those distributions. Toll-like receptors (TLR) are a family of genes responsible for initiating innate and acquired immune responses to a diversity of pathogens. We provide 30 new primers, which, along with cross-species application of published primers, amplify TLR gene sequences in nine bird species of conservation concern in New Zealand. By including one member each of Apterygiformes and Gruiformes, two members of Psittaciformes, and five members of Passeriformes, our data significantly expand the number of avian species for which TLR sequences are available, and facilitates study of these genes in a greater diversity of taxa. | 857 | no | no good fitness measure, N pop differnt in GD? | NA | no | no |
| 858 | 2017 | 43013 | Mora-Vicente, S; Caujape-Castells, J; de Paz, JMP; Febles-Hernandez, R; Malo, JE | 2009 | Isozyme diversity in some Canarian woody endemisms of the genus Echium L. (Boraginaceae) | Oceanic archipelagos are considered hot-spots of biodiversity because they harbor unique biota with a high level of endemicity. However, the endemic biodiversity of oceanic islands is very vulnerable to extinction. In recent decades, intensive exploitation of these territories and human-mediated introduction of alien species have posed unprecedented threats to the long-term survival of the endemic contingent. The very limited population genetic information available until now for the 28 Canarian endemic Echium taxa has hindered the development of conservation strategies for the ca. 25% of them that are under threat. In this paper, we analyze the levels and distribution of genetic isozyme diversity in 23 natural populations of three endangered endemics of restricted distribution (E. acanthocarpum, CR; E. onosmifolium ssp. spectabile, EN; and E. callithyrsum, VU), and two endemics of wide distribution and in principle free of threat (E. decaisnei and E. onosmifolium). Our results reveal high levels of genetic variability in all these taxa that have plausibly been reached despite a predominance of selfing. They also point out a high incidence of inbreeding in the reproductive dynamics of populations and suggest the potential value of hybridisation processes in shaping the genetic makeup of these species. Among-population differentiation, as estimated by Gst, and genetic distances within taxa are low overall, but they do support the current taxonomic separation between the two subspecies of E. onosmifolium and do not furnish evidence that the current status of the endangered species may be attributed to genetic factors. We use the genetic parameters to suggest some guidelines to help implement a conservation strategy of these taxa. | 858 | no | no good fitness measure | NA | no | no |
| 859 | 2017 | 43013 | Li, JQ; Wang, Y; Lv, L; Wang, PC; Zhang, ZW | 2016 | No facultative manipulation of offspring sex ratio in relation to parental genetic characteristics in a bird with sex-specific heterozygosity-fitness correlation | Sex allocation theory predicts that parents should adjust their investment between female and male offspring when the fitness return of producing them differs. Genome-wide heterozygosity is related to many aspects of individual fitness, and an individual’s heterozygosity is usually determined by the genetic characteristics of its parents. When heterozygosity affects fitness differently for female and male offspring, parents may be expected to manipulate offspring sex ratio based on their own genetic characteristics. We examined whether parental genetic characteristics affected primary offspring sex ratios in a small passerine bird, the black-throated tit (Aegithalos concinnus). We found that offspring heterozygosity was significantly related to the heterozygosity of parents and the relatedness between parents. Importantly, genetic heterozygosity had a significantly negative effect on the survival of male but not female black-throated tits, implying higher variance in fitness in males than in females regarding the genetic heterozygosity inherited from parents. This sex-specific effect of heterozygosity on fitness provides black-throated tit parents with a potential reason to adjust offspring sex ratio in relation to their own genetic characteristics. However, although we found a seasonal decline of offspring sex ratio (proportion of males), offspring sex ratio was associated with neither the relatedness between parents nor the heterozygosity of parents. The results suggest that at least based on current data, there is a lack of evidence that black-throated tit parents manipulate offspring sex ratio with respect to their own genetic characteristics. The correlation between genome-wide heterozygosity and individual fitness has been extensively studied, but its potential role in mediating parental manipulation of offspring sex ratio has received attention only recently. If heterozygosity affects fitness differently for the two sexes of offspring, parents are likely to adjust their investment between female and male offspring based on their own genetic characteristics. This is because their own genetic characteristics determine their offspring’s heterozygosity and therefore fitness returns to parents. Existing studies have provided support to this hypothesized manipulation of offspring ratio, but studies in the area remain scarce. Our study shows that parents of a bird species do not manipulate offspring sex ratio with respect to their own genetic characteristics, even when there is a sex-specific heterozygosity-fitness correlation. | 859 | no | individual level | NA | no | no |
| 860 | 2017 | 43013 | Miller, BF; DeYoung, RW; Campbell, TA; Laseter, BR; Ford, WM; Miller, KV | 2010 | Fine-scale genetic and social structuring in a central Appalachian white-tailed deer herd | Spatial genetic structure in white-tailed deer (Odocoileus virginianus) has been examined at regional scales, but genetic markers with the resolution to detect fine-scale patterns have appeared only recently. We used a panel of microsatellite DNA markers, radiotelemetry data, and visual observations of marked deer to study fine-scale social and genetic structure in a high-density population of white-tailed deer (12-20 deer/km(2)). We collected genetic data on 229 adult females, 102 of which were assigned to 28 social groups. Our results were consistent with the conceptual model of white-tailed deer social structure, where philopatric females form social groups composed of related individuals. Within-group relatedness values approached the expected value for 1st cousins (R = 0.103, SE = 0.033), but individuals among groups (R = 0.014, SE = 0.003) and overall (R = -0.009, SE = 0.003) were unrelated. Fixation indices revealed a significant departure from equilibrium values among social groups (F(ST) = 0.076, SE = 0.007) and an excess of heterozygotes within groups (F(ls) = 0.050, SE = 0.018), consistent with theoretical expectations for mammal populations characterized by female philopatry and a polygynous mating system. Analyses of spatial autocorrelation indicated genetic structuring occurred at a very fine spatial scale, where pairs of adult females within 1 km were genetically nonindependent. The occurrence of fine-scale genetic and social structure has implications for the ecology and management of white-tailed deer, including habitat use and resource competition, offspring sex allocation theories, disease transmission, and the consideration of social behaviors in management. DOI: 10.1644/09-MAMM-A-258.1. | 860 | no | no good fitness measure | NA | no | no |
| 861 | 2017 | 43013 | Liaubet, L; Lobjois, V; Faraut, T; Tircazes, A; Benne, F; Iannuccelli, N; Pires, J; Glenisson, J; Robic, A; Le Roy, P; SanCristobal, M; Cherel, P | 2011 | Genetic variability of transcript abundance in pig peri-mortem skeletal muscle: eQTL localized genes involved in stress response, cell death, muscle disorders and metabolism | Background: The genetics of transcript-level variation is an exciting field that has recently given rise to many studies. Genetical genomics studies have mainly focused on cell lines, blood cells or adipose tissues, from human clinical samples or mice inbred lines. Few eQTL studies have focused on animal tissues sampled from outbred populations to reflect natural genetic variation of gene expression levels in animals. In this work, we analyzed gene expression in a whole tissue, pig skeletal muscle sampled from individuals from a half sib F2 family shortly after slaughtering. Results: QTL detection on transcriptome measurements was performed on a family structured population. The analysis identified 335 eQTLs affecting the expression of 272 transcripts. The ontologic annotation of these eQTLs revealed an over-representation of genes encoding proteins involved in processes that are expected to be induced during muscle development and metabolism, cell morphology, assembly and organization and also in stress response and apoptosis. A gene functional network approach was used to evidence existing biological relationships between all the genes whose expression levels are influenced by eQTLs. eQTLs localization revealed a significant clustered organization of about half the genes located on segments of chromosome 1, 2, 10, 13, 16, and 18. Finally, the combined expression and genetic approaches pointed to putative cis-drivers of gene expression programs in skeletal muscle as COQ4 (SSC1), LOC100513192 (SSC18) where both the gene transcription unit and the eQTL affecting its expression level were shown to be localized in the same genomic region. This suggests cis-causing genetic polymorphims affecting gene expression levels, with (e. g. COQ4) or without (e. g. LOC100513192) potential pleiotropic effects that affect the expression of other genes (cluster of trans-eQTLs). Conclusion: Genetic analysis of transcription levels revealed dependence among molecular phenotypes as being affected by variation at the same loci. We observed the genetic variation of molecular phenotypes in a specific situation of cellular stress thus contributing to a better description of muscle physiologic response. In turn, this suggests that large amounts of genetic variation, mediated through transcriptional networks, can drive transient cell response phenotypes and contribute to organismal adaptative potential. | 861 | no | no good fitness measure, N pop differnt in GD? | NA | no | no |
| 862 | 2017 | 43013 | Vardo-Zalik, AM; Schall, JJ | 2009 | Clonal diversity alters the infection dynamics of a malaria parasite (Plasmodium mexicanum) in its vertebrate host | Ecological and evolutionary theory predicts that genetic diversity of microparasites within infected hosts will influence the parasite replication rate, parasitemia, transmission strategy, and virulence. We manipulated clonal diversity (number of genotypes) of the malaria parasite, Plasmodium mexicanum, in its natural lizard host and measured important features of the infection dynamics, the first such study for any natural Plasmodium host association. Hosts harboring either a single P. mexicanum clone or various combinations of clones (scored via three microsatellite markers) were established. Production of asexually replicating stages (meronts) and maximal meront parasitemia did not differ by clonal diversity, nor did timing of first production of transmission stages (gametocytes). However, mean rate of gametocyte increase and maximal gametocyte parasitemia were greater for hosts with mixed-clone infections. Characteristics of infections were more variable in hosts with mixed-clone infections than with single-clone infections except for first production of gametocytes. One or more of the parasite reproductive traits were extreme in 20 of 52 hosts with mixed-clone infections. This was not associated with specific clones, but diversity itself. The overall pattern from studies of clonal diversity for human, rodent, and now reptile malaria parasites confirms that the genetic diversity of infections in the vertebrate host is of central importance for the ecology of Plasmodium. | 862 | no | gd of parasite | NA | no | no |
| 863 | 2017 | 43013 | Ozawa, H; Watanabe, A; Uchiyama, K; Saito, Y; Ide, Y | 2013 | Influence of Long-Distance Seed Dispersal on the Genetic Diversity of Seed Rain in Fragmented Pinus densiflora Populations Relative to Pollen-Mediated Gene Flow | Long-distance dispersal (LDD) of seeds has a critical impact on species survival in patchy landscapes. However, relative to pollen dispersal, empirical data on how seed LDD affects genetic diversity in fragmented populations have been poorly reported. Thus, we attempted to indirectly evaluate the influence of seed LDD by estimating maternal and paternal inbreeding in the seed rain of fragmented 8 Pinus densiflora populations. In total, the sample size was 458 seeds and 306 adult trees. Inbreeding was estimated by common parentage analysis to evaluate gene flow within populations and by sibship reconstruction analysis to estimate gene flow within and among populations. In the parentage analysis, the observed probability that sampled seeds had the same parents within populations was significantly larger than the expected probability in many populations. This result suggested that gene dispersal was limited to within populations. In the sibship reconstruction, many donors both within and among populations appeared to contribute to sampled seeds. Significant differences in sibling ratios were not detected between paternity and maternity. These results suggested that seed-mediated gene flow and pollen-mediated gene flow from outside population contributed some extent to high genetic diversity of the seed rain (H-E > 0.854). We emphasize that pine seeds may have excellent potential for gene exchange within and among populations. | 863 | no | no good fitness measure, N pop differnt in GD? | NA | no | no |
| 864 | 2017 | 43013 | Pujolar, JM; Vincenzi, S; Zane, L; Jesensek, D; De Leo, GA; Crivelli, AJ | 2011 | The Effect of Recurrent Floods on Genetic Composition of Marble Trout Populations | A changing global climate can threaten the diversity of species and ecosystems. We explore the consequences of catastrophic disturbances in determining the evolutionary and demographic histories of secluded marble trout populations in Slovenian streams subjected to weather extremes, in particular recurrent flash floods and debris flows causing massive mortalities. Using microsatellite data, a pattern of extreme genetic differentiation was found among populations (global F-ST of 0.716), which exceeds the highest values reported in freshwater fish. All locations showed low levels of genetic diversity as evidenced by low heterozygosities and a mean of only 2 alleles per locus, with few or no rare alleles. Many loci showed a discontinuous allele distribution, with missing alleles across the allele size range, suggestive of a population contraction. Accordingly, bottleneck episodes were inferred for all samples with a reduction in population size of 3-4 orders of magnitude. The reduced level of genetic diversity observed in all populations implies a strong impact of genetic drift, and suggests that along with limited gene flow, genetic differentiation might have been exacerbated by recurrent mortalities likely caused by flash flood and debris flows. Due to its low evolutionary potential the species might fail to cope with an intensification and altered frequency of flash flood events predicted to occur with climate change. | 864 | no | no good fitness measure, N pop differnt in GD? | NA | no | no |
| 865 | 2017 | 43013 | Hoffman, JI; Simpson, F; David, P; Rijks, JM; Kuiken, T; Thorne, MAS; Lacy, RC; Dasmahapatra, KK | 2014 | High-throughput sequencing reveals inbreeding depression in a natural population | Proxy measures of genome-wide heterozygosity based on approximately 10 microsatellites have been used to uncover heterozygosity fitness correlations (HFCs) for a wealth of important fitness traits in natural populations. However, effect sizes are typically very small and the underlying mechanisms remain contentious, as a handful of markers usually provides little power to detect inbreeding. We therefore used restriction site associated DNA (RAD) sequencing to accurately estimate genome-wide heterozygosity, an approach transferrable to any organism. As a proof of concept, we first RAD sequenced oldfield mice (Peromyscus polionotus) from a known pedigree, finding strong concordance between the inbreeding coefficient and heterozygosity measured at 13,198 single-nucleotide polymorphisms (SNPs). When applied to a natural population of harbor seals (Phoca vitulina), a weak HFC for parasite infection based on 27 microsatellites strengthened considerably with 14,585 SNPs, the deviance explained by heterozygosity increasing almost fivefold to a remarkable 49%. These findings arguably provide the strongest evidence to date of an HFC being due to inbreeding depression in a natural population lacking a pedigree. They also suggest that under some circumstances heterozygosity may explain far more variation in fitness than previously envisaged. | 865 | no | inbreeding | NA | no | no |
| 866 | 2017 | 43013 | Prasad, P; Bhardwaj, SC; Gangwar, OP; Kumar, S; Khan, H; Kumar, S; Rawal, HC; Sharma, TR | 2017 | Population differentiation of wheat leaf rust fungus Puccinia triticina in South Asia | Leaf or brown rust caused by Puccinia triticina (Pt) is one of the most important diseases of wheat. Among the rusts, it is the most ubiquitous in all the wheatgrowing regions and causes considerable yield loss. Microsatellite marker-based genotyping and virulence-based phenotyping of 48 pathotypes of Pt was performed. The pathotypes exhibit low virulence frequencies for Indian leaf rust differentials Lr24, Lr9, Lr10, Lr19, Lr28 and Lr9. Using avirulence/virulence formula six major clusters of pathotypes were observed, revealing high degree of phenotypic variation. Molecular analysis performed using SSR markers showed high genetic diversity among the pathotypes, and grouped them in seven major clusters. The percentage of polymorphic loci ranged from 17.95 to 84.62, Nei’s gene diversity from 0.07 to 0.32 and Shannon’s information index from 0.11 to 0.47. Analysis of molecular variance revealed significantly high genetic variation within Pt population. Mantel’s Z test proved low positive correlation (r = 0.28) between virulence and molecular diversity, suggesting independent nature of the duo. These findings offer valuable information for framing suitable disease management strategies through appropriate region-specific gene deployment and improve the understanding of the population biology and evolution of Pt in the Indian subcontinent. | 866 | no | gd of pathogen | NA | no | no |
| 867 | 2017 | 43013 | Zhang, F; Su, B; Zhang, YP; Jin, L | 2007 | Genetic studies of human diversity in East Asia | East Asia is one of the most important regions for studying evolution and genetic diversity of human populations. Recognizing the relevance of characterizing the genetic diversity and structure of East Asian populations for understanding their genetic history and designing and interpreting genetic studies of human diseases, in recent years researchers in China have made substantial efforts to collect samples and generate data especially for markers on Y chromosomes and mtDNA. The hallmark of these efforts is the discovery and confirmation of consistent distinction between northern and southern East Asian populations at genetic markers across the genome. With the confirmation of an African origin for East Asian populations and the observation of a dominating impact of the gene flow entering East Asia from the south in early human settlement, interpretation of the north-south division in this context poses the challenge to the field. Other areas of interest that have been studied include the gene flow between East Asia and its neighbouring regions (i.e. Central Asia, the Sub-continent, America and the Pacific Islands), the origin of Sino-Tibetan populations and expansion of the Chinese. | 867 | no | human | NA | no | no |
| 868 | 2017 | 43013 | Ahmed, MMM; Sheikh, A; Mutwakil, MHZ; Saini, KS; Alsulaimany, FAS; El Hanafy, AA; Sabir, JSM | 2016 | COMPARATIVE ANALYSIS OF ATP6 MITOCHONDRIAL GENE DIVERSITY IN ARABIAN AND NON-ARABIAN HORSE BREEDS | Arabian horse breeds are famous for their strength, disease resistance and endurance. Originating from Arabian Peninsula, their progeny is considered among the best horse breeds of the world. Maternally inherited mitochondrial genome represents high genetic diversity in modern horse population. ATP6 gene of mt DNA from 46 horse samples of Middle Eastern Arabian, Western Arabian, mixed (hybrid) Arabians and non-Arabians were sequenced and analyzed to assess the genetic diversity and phylogenetic relationships among them. We have found nine haplotypes in our study. Totally, 99-nucleotide base substitutions were observed with seven variables, which accounted for six transitions and one transversion. Four single nucleotide polymorphismswereobserved in our study. Arabian horse breeds showed high diversity and shared many haplotypes among the population. The observed haplotype diversity and the average evolutionary divergence over all the sequence pairs were 0.8141 and 0.007 respectively. In addition, these datasets may also be useful for strain genotyping, data conservation, effective breeding and individual breed selection for desirable traits. | 868 | no | domesticated animal | NA | no | no |
| 869 | 2017 | 43013 | Hitchings, SP; Beebee, TJC | 1998 | Loss of genetic diversity and fitness in Common Toad (Bufo bufo) populations isolated by inimical habitat | Measures of genetic diversity (including heterozygosity), survival and developmental homeostasis were found to be significantly lower in small, urban populations of the Common Toad (Bufo bufo) than in larger, rural populations of the same region. The autecology and genetic analysis of this relatively sedentary species suggested that the causal mechanism was genetic drift, arising from barriers to migration created by urban development. The pre-metamorphic survival of larvae cultured in identical conditions increased positively with the mean number of alleles at a locus and the percentage of polymorphic loci. Observed heterozygosity in urban garden and rural populations was correlated inversely with the number of observed physical abnormalities (used as st measure of developmental homeostasis) in the developing tadpoles. Genetic distances between town sites of mean 2.2 km separation were significantly higher than those between rural sites of mean 37 km separation. Genetic data were based on allozyme analysis of 27 loci in 8 urban and 4 rural populations. A subset of these sites (3 urban, 2 rural) were also assessed at 3 minisatellite loci and a positive correlation found between the average number of alleles per locus detected by the two methods. Estimates of Nei’s 1972 genetic distance, derived separately from the DNA and protein data, were not, however, correlated. The reduction in genetic diversity and fitness observed in these urban toads provides an example of the effect on population persistence that longer term depletion in numbers and habitat fragmentation can have in the wider environment. | 869 | probably yes | no good comparisons of groups, can’t rule out if difference in survival of pop is due to pop size or heterozygosity? | not pop level gd | no | no |
| 870 | 2017 | 43013 | Bucheli, E; Gautschi, B; Shykoff, JA | 2001 | Differences in population structure of the anther smut fungus Microbotryum violaceum on two closely related host species, Silene latifolia and S. dioica | We investigated the genetic population structure of the sexually transmitted plant pathogen, the fungus Microbotryum violaceum, on the two closely related host species Silene latifolia and S. dioica using microsatellite markers. We found strong deviations from Hardy-Weinberg expectations, with significant heterozygote deficiency in almost all populations. Fungal strains from the two host species were differentiated, and these host races differed in amount of variation within populations and differentiation among populations. Anther smut from S. latifolia harboured significantly less microsatellite diversity and were more genetically differentiated from each other than those from S. dioica. Small effective population sizes, rapid population turnover, and less gene flow among populations could lead to this higher population differentiation and lower within population genetic diversity for anther smut populations on S. latifolia than on S. dioica. These results are in concordance with host ecology because S. latifolia grows in more disturbed habitats than S. dioica and may provide a shorter-lived host environment. | 870 | no | gd of pathogen | NA | no | no |
| 871 | 2017 | 43013 | Qiu, JB; Sun, JT; Yu, MZ; Xu, JH; Shi, JR | 2016 | Temporal dynamics, population characterization and mycotoxins accumulation of Fusarium graminearum in Eastern China | Trichothecene genotype composition, mycotoxin production, genetic diversity, and population structure were analyzed, using 185 Fusarium strains collected from wheat (Triticum aestivum L.) throughout the Jiangsu province during 1976, 1983, 1998, 2006, and 2014. The results showed that 3-acetyldeoxynivalenol (3ADON) was consistently the predominant type in this region over 40 years, and the nivalenol (NIV) type has emerged since 1998. Long-term rotation of wheat and rice (Oryza sativa L.), rather than fungicide application, crop fitness, or weather conditions, might be the main cause of this phenomenon. The genetic diversity results from two toxin synthetic genes, Pks4 and Tri10, and variable number of tandem repeat (VNTR) markers revealed the largest variance within the population in 1998, which was also the year with the highest production of mycotoxins. Population differentiation analysis indicated that major temporal population comparisons from the same area were not significantly differentiated. Our results showed that dominant species could maintain genetic stability for a long time, and Pks4 would be of utility in genetic and population studies. | 871 | no | crop | NA | no | no |
| 872 | 2017 | 43013 | Spear, SF; Peterson, CR; Matocq, MD; Storfer, A | 2006 | Molecular evidence for historical and recent population size reductions of tiger salamanders (Ambystoma tigrinum) in Yellowstone National Park | Population declines caused by natural and anthropogenic factors can quickly erode genetic diversity in natural populations. In this study, we examined genetic variation within 10 tiger salamander populations across northern Yellowstone National Park in Wyoming and Montana, USA using eight microsatellite loci. We tested for the genetic signature of population decline using heterozygosity excess, shifts in allele frequencies, and low ratios of allelic number to allelic size range (M-ratios). We found different results among the three tests. All 10 populations had low M-ratios, five had shifts in allele frequencies and only two had significant heterozygosity excesses. These results support theoretical expectations of different temporal signatures among bottleneck tests and suggest that both historical fish stocking, recent, sustained drought, and possibly an emerging amphibian disease have contributed to declines in effective population size. | 872 | no | no good fitness measure, N pop differnt in GD? | NA | no | no |
| 873 | 2017 | 43013 | NEVO, E; FILIPPUCCI, MG; BEILES, A | 1994 | GENETIC POLYMORPHISMS IN SUBTERRANEAN MAMMALS (SPALAX-EHRENBERGI SUPERSPECIES) IN THE NEAR-EAST REVISITED - PATTERNS AND THEORY | Allozyme diversity in the superspecies Spalax ehrenbergi has been revisited by studying 36 gene loci in 241 subterranean mole rats from 22 populations and nine chromosomal species, four from Turkey (2n = 52E (east), 52W (west), 56 and 58), four from Israel(2n = 52, 54, 58 and 60), and one from Egypt (2n = 60). The following results were indicated. (1) Genetic patterns: 11 of the 36 loci analysed (30.5 per cent) were monomorphic across the range, fifteen (41.7 per cent) were weakly polymorphic and the remaining 10 loci (27.8 per cent) were strongly polymorphic. (2) Heterozygosity: the average H was 0.051, range 0.00-0.098. In Israel, H increased with aridity and climatic unpredictability towards the northern Negev Desert, and was remarkably high in small steppic semi-isolates and desert isolates. (3) Species discrimination: some of the S. ehrenbergi species can be discriminated qualitatively. (4) Genetic distances (D): between species these values averaged 0.077, range 0.001-0.269, with the highest D between the ancestor Turkish and descendant Israeli and Egyptian species. The phylogenetic tree supports the Turkish origin of the Israeli Spalax ehrenbergi species, and the recent speciation of the Egyptian Spalax. (5) Genetic diversity is mostly (58 per cent) within populations. (6) Allozyme correlates: allozyme diversity was significantly correlated with the external physical (both climatic and edaphic) and biotic (parasite infection and plant cover) environment. (7) Spatial autocorrelation of allozyme frequencies suggests that migration is not influential. (8) Gametic phase disequilibria were significant in four out of five species tested, and were associated with climatic and edaphic factors. These results support the environmental selection hypothesis of genetic diversity including the niche-width variation hypothesis in space and time. Natural selection appears to play a major role in genetic differentiation of proteins in adaptive radiation and speciation. | 873 | no | no good fitness measure | NA | no | no |
| 874 | 2017 | 43013 | Hoisington, D; Khairallah, M; Reeves, T; Ribaut, JV; Skovmand, B; Taba, S; Warburton, M | 1999 | Plant genetic resources: What can they contribute toward increased crop productivity? | “To feed a world population growing by up to 160 people per minute, with >90% of them in developing countries, will require an astonishing increase in food production. Forecasts call for wheat to become the most important cereal in the world, with maize close behind; together, these crops will account for approximate to 80% of developing countries’ cereal import requirements. Access to a range of genetic diversity is critical to the success of breeding programs. The global effort to assemble, document, and utilize these resources is enormous, and the genetic diversity in the collections is critical to the world’s fight against hunger. The introgression of genes that reduced plant height and increased disease and viral resistance in wheat provided the foundation for the”“Green Revolution”" and demonstrated the tremendous impact that genetic resources can have on production. Wheat hybrids and synthetics may provide the yield increases needed in the future. A wild relative of maize, Tripsacum, represents an untapped genetic resource for abiotic and biotic stress resistance and for apomixis, a trait that could provide developing world farmers access to hybrid technology. Ownership of genetic resources and genes must be resolved to ensure global access to these critical resources. The application of molecular and genetic engineering technologies enhances the use of genetic resources. The effective and complementary use of all of our technological tools and resources will be required for meeting the challenge posed by the world’s expanding demand for food." | 874 | no | NA | NA | no | no |
| 875 | 2017 | 43013 | Razakandrainibe, FG; Durand, P; Koella, JC; De Meeus, T; Rousset, F; Ayala, FJ; Renaud, F | 2005 | Clonal population structure of the malaria agent Plasmodium falciparum in high-infection regions | “The population genetic structure of Plasmodium falciparum, the agent of malignant malaria, has been shown to be predominantly”“clonal”" (i.e., highly inbred) in regions of low infectivity | 875 | no | gd of parasite | NA | no | no |
| 876 | 2017 | 43013 | Meyer-Lucht, Y; Otten, C; Puttker, T; Pardini, R; Metzger, JP; Sommer, S | 2010 | Variety matters: adaptive genetic diversity and parasite load in two mouse opossums from the Brazilian Atlantic forest | The adaptive potential of a species to a changing environment and in disease defence is primarily based on genetic variation. Immune genes, such as genes of the major histocompatibility complex (MHC), may thereby be of particular importance. In marsupials, however, there is very little knowledge about natural levels and functional importance of MHC polymorphism, despite their key role in the mammalian evolution. In a previous study, we discovered remarkable differences in the MHC class II diversity between two species of mouse opossums (Gracilinanus microtarsus, Marmosops incanus) from the Brazilian Atlantic forest, which is one of the most endangered hotspots for biodiversity conservation. Since the main forces in generating MHC diversity are assumed to be pathogens, we investigated in this study gastrointestinal parasite burden and functional associations between the individual MHC constitution and parasite load. We tested two contrasting scenarios, which might explain differences in MHC diversity between species. We predicted that a species with low MHC diversity would either be under relaxed selection pressure by low parasite diversity (‘Evolutionary equilibrium’ scenario), or there was a recent loss in MHC diversity leading to a lack of resistance alleles and increased parasite burden (‘Unbalanced situation’ scenario). In both species it became apparent that the MHC class II is functionally important in defence against gastrointestinal helminths, which was shown here for the first time in marsupials. On the population level, parasite diversity did not markedly differ between the two host species. However, we did observe considerable differences in the individual parasite load (parasite prevalence and infection intensity): while M. incanus revealed low MHC DAB diversity and high parasite load, G. microtarsus showed a tenfold higher population wide MHC DAB diversity and lower parasite burden. These results support the second scenario of an unbalanced situation. | 876 | no | two species comp | NA | no | no |
| 877 | 2017 | 43013 | Xie, BY; Cheng, XY; Shi, J; Zhang, QW; Dai, SM; Cheng, FX; Luo, YQ | 2009 | Mechanisms of invasive population establishment and spread of pinewood nematodes in China | This paper summarizes the results of our study of the pinewood nematode (Bursaphelenchus xylophilus). By population genetic analysis, it was determined that there was no genetic bottle caused by the founder effect and genetic drift in the Chinese invasive population. Multiple invasions with large amounts of nematodes from different sources led to rich genetic diversity in the invasive population. Keeping high genetic diversity in the invasive process may be one of the genetic mechanisms in its successful invasion. By testing interspecies competition, it was shown that, with high fecundity and a strong competitive ability, B. xylophilus outcompeted the native species B. mucronatus in the natural ecosystem during the invasion process. Competitive displacement may be one of the ecological mechanisms of B. xylophilus’s invasion. In addition, an unequal interspecific hybridization with introgress was in favor of the invasive species which also accelerated the replacement of B. mucronatus by B. xylophilus. The structures, functions and evolutions of a few important genes that are closely related to the ecological adaptation of pinewood nematodes were studied to explore the molecular mechanism of its ecological adaptations. Further, the resistance and resilience mechanism of the pine ecosystem invaded by pinewood nematodes was also investigated. The results of these studies uncovered a portion of the genetic and ecological mechanisms of PWN’s successful invasion and laid a foundation for further study to obtain a comprehensive interpretation of the mechanisms of the nematode invasion. The results also provided a scientific basis for effectively controlling the occurrence and spread of pine wilt disease which is caused by nematodes. Various aspects requiring further investigation are considered. | 877 | no | gd of parasite | NA | no | no |
| 878 | 2017 | 43013 | Weber, DS; Stewart, BS; Schienman, J; Lehman, N | 2004 | Major histocompatibility complex variation at three class II loci in the northern elephant seal | Northern elephant seals were hunted to near extinction in the 19th century, yet have recovered remarkably and now number around 175 000. We surveyed 110 seals for single-strand conformation polymorphism (SSCP) and sequence variation at three major histocompatibility (MHC) class II loci (DQA, DQB and DRB) to evaluate the genetic consequences of the population bottleneck at these loci vs. other well-studied genes. We found very few alleles at each MHC locus, significant variation among breeding sites for the DQA locus, and linkage disequilibrium between the DQB and DRB loci. Northern elephant seals are evidently inbred, although there is as yet no evidence of correlative reductions in fitness. | 878 | no | no good fitness measure, N pop differnt in GD? | NA | no | no |
| 879 | 2017 | 43013 | Hannonen, M; Helantera, H; Sundstrom, L | 2004 | Habitat age, breeding system and kinship in the ant Formica fusca | In polygyne ants (multiple queens per colony) factors that affect the distribution and survival of queens may play a key role in shaping the population-wide mating system and colony kin structure. The aim of this paper was to study the breeding system in two populations of different age in the facultatively polygyne ant Formica fusca. Both the observed numbers of queens, and the relatedness patterns among queens, workers and colony fathers were compared in two adjacent populations (ages 17 years and > 100 years) in Southern Finland. The results showed that both the mating system and colony kin structure differed between the study populations. In the old population the relatedness among workers, queens and colony fathers was high. The queens were also related to their mates, resulting in significant inbreeding in workers, but not in queens. Finally, the number of queens per colony fluctuated between years, suggesting queen turnover, and nest-mate queens shared their reproduction unequally (reproductive skew). In the younger population relatedness among queens and workers was lower than in the old population, and the colony fathers were unrelated. Furthermore, inbreeding was absent, and no conclusive evidence was found for reproductive skew among nest-mate queens. Finally, the number of queens per colony appeared more stable between years, although queen turnover occurred also in this population. The observed differences in dispersal and mating behaviour are discussed in the light of a potential connection between population age and habitat saturation. | 879 | no | no good fitness measure | NA | no | no |
| 880 | 2017 | 43013 | Mokhtar-Jamai, K; Pascual, M; Ledoux, JB; Coma, R; Feral, JP; Garrabou, J; Aurelle, D | 2011 | From global to local genetic structuring in the red gorgonian Paramuricea clavata: the interplay between oceanographic conditions and limited larval dispersal | Defining the scale of connectivity among marine populations and identifying the barriers to gene flow are tasks of fundamental importance for understanding the genetic structure of populations and for the design of marine reserves. Here, we investigated the population genetic structure at three spatial scales of the red gorgonian Paramuricea clavata (Cnidaria, Octocorallia), a key species dwelling in the coralligenous assemblages of the Mediterranean Sea. Colonies of P. clavata were collected from 39 locations across the Mediterranean Sea from Morocco to Turkey and analysed using microsatellite loci. Within three regions (Medes, Marseille and North Corsica), sampling was obtained from multiple locations and at different depths. Three different approaches (measures of genetic differentiation, Bayesian clustering and spatially explicit maximum-difference algorithm) were used to determine the pattern of genetic structure. We identified genetic breaks in the spatial distribution of genetic diversity, which were concordant with oceanographic conditions in the Mediterranean Sea. We revealed a high level of genetic differentiation among populations and a pattern of isolation by distance across the studied area and within the three regions, underlining short effective larval dispersal in this species. We observed genetic differentiation among populations in the same locality dwelling at different depths, which may be explained by local oceanographic conditions and which may allow a process of local adaptation of the populations to their environment. We discuss the implications of our results for the conservation of the species, which is exposed to various threats. | 880 | no | no good fitness measure | NA | no | no |
| 881 | 2017 | 43013 | Brotherstone, S; Goddard, M | 2005 | Artificial selection and maintenance of genetic variance in the global dairy cow population | Genetic improvement of dairy cows, which has increased the milk yield of cows in the UK by 1200 kg per lactation in 12 years, is an excellent example of the application of quantitative genetics to agriculture. The most important traits of dairy cattle are expressed only in females, but the main opportunity for selection is in males. Despite this, genetic improvement was achieved by the invention of a new statistical methodology, called ‘best linear unbiased prediction’ to estimate the breeding value of bulls. Intense selection of the best bulls, combined with the worldwide use of these bulls through artificial insemination and frozen semen, has created a global population and caused concern that the genetic variation available in the future will be reduced. Maintenance of genetic variation and long-term genetic gains would be aided by rational payment systems, use of crossbreeding where profitable, inclusion of all economically important traits in the breeding objective, recognition of genotype by environment interactions and the use of selection algorithms that balance estimated breeding value against the average relationship among the selected animals. Fortunately, all of these things are happening to some degree. | 881 | no | domesticated animal | NA | no | no |
| 882 | 2017 | 43013 | Booth, RE; Grime, JP | 2003 | Effects of genetic impoverishment on plant community diversity | 1 Established individuals removed at random from populations of 11 long-lived herbaceous species coexisting in a local area of ancient limestone pasture at Cress-brookdale in North Derbyshire were subjected to clonal propagation to produce stocks of genetically identical individuals sufficient to create 36 model communities identical in species composition but widely contrasted in genetic diversity. 2 Three levels of genetic diversity were imposed. In one treatment, all individuals of each species were genetically unique. The second contained four randomly selected genotypes of each species. In the third, there was no genetic diversity in any of the species but each community contained a unique combination of genotypes. 3 Over a period of 5 years the communities were allowed to develop in microcosms containing natural rendzina soil and exposed to a standardized regime of simulated grazing and trampling. The treatments were maintained by the removal of flowers, immature seed-heads and seedlings originating from the seed-bank and seed rain. Point quadrat surveys were used to monitor changes in species composition and diversity in the three experimental treatments. 4 During the experiment a distinction rapidly developed between five canopy dominants and five subordinates, a process that caused the vegetation structure to closely resemble that occurring at Cressbrookdale. 5 A gradual loss of species diversity occurred in all three treatments but by the end of the fifth growing season species diversity was higher in the most genetically diverse communities. 6 Ordination of the 36 communities at intervals over a 5-year period revealed a gradual convergence in the species composition of the 4-genotype and 16-genotype communities and this effect was more strongly developed in the latter. A comparable process was not observed in the 1-genotype communities, suggesting that interaction between particular genotypes of different species in local neighbourhoods may be an essential part of the mechanism that determines the predictable composition of a mature pasture community. 7 It is concluded that, under the conditions of this experiment, genetic diversity within component species reduced the rate at which species diversity declined. The relative importance in this effect of factors such as greater disease resistance and moderated competitive interactions remains uncertain. | 882 | no | no good fitness measure | NA | no | no |
| 883 | 2017 | 43013 | Gonzalez-Ferrer, S; Pineda-Bernal, L; Delgado-Luengo, W; Villalobos-Cabrera, H | 2004 | Medical genetics in Zulia, a state of Venezuela | Zulia is a state located in the northwest of Venezuela. Congenital malformations, deformities and chromosomal anomalies are the second cause of infant and neonatal mortality. There are seven public and private groups providing genetic services, the most important of which, the Medical Genetic Unit at the Zulia University was created in 1973. So far, this unit has provided genetic services to 12,000 families, and has been responsible for undergraduate and postgraduate education in human and medical genetics. Prenatal diagnosis is performed at the Unit and a private practice group, the most frequent referral reason being advanced maternal age. The most frequent genetic diseases in the state are Huntington’s disease, sickle cell anemia, neural tube defects and Down’s syndrome. Research in genetics includes the clinical, epidemiological and molecular characterization of hereditary diseases, cancer, reproductive problems and genetic diversity. Other public groups are conducting research on dementias, including Alzheimer’s disease, and on the genotoxic effects of environmental pollutants. Copyright (c) 2004 S. Karger AG, Basel. | 883 | no | human | NA | no | no |
| 884 | 2017 | 43013 | van Schaik, J; Kerth, G; Bruyndonckx, N; Christe, P | 2014 | The effect of host social system on parasite population genetic structure: comparative population genetics of two ectoparasitic mites and their bat hosts | Background: The population genetic structure of a parasite, and consequently its ability to adapt to a given host, is strongly linked to its own life history as well as the life history of its host. While the effects of parasite life history on their population genetic structure have received some attention, the effect of host social system has remained largely unstudied. In this study, we investigated the population genetic structure of two closely related parasitic mite species (Spinturnix myoti and Spinturnix bechsteini) with very similar life histories. Their respective hosts, the greater mouse-eared bat (Myotis myotis) and the Bechstein’s bat (Myotisbechsteinii) have social systems that differ in several substantial features, such as group size, mating system and dispersal patterns. Results: We found that the two mite species have strongly differing population genetic structures. In S. myoti we found high levels of genetic diversity and very little pairwise differentiation, whereas in S. bechsteini we observed much less diversity, strongly differentiated populations and strong temporal turnover. These differences are likely to be the result of the differences in genetic drift and dispersal opportunities afforded to the two parasites by the different social systems of their hosts. Conclusions: Our results suggest that host social system can strongly influence parasite population structure. As a result, the evolutionary potential of these two parasites with very similar life histories also differs, thereby affecting the risk and evolutionary pressure exerted by each parasite on its host. | 884 | no | gd of parasite | NA | no | no |
| 885 | 2017 | 43013 | Sallaberry-Pincheira, N; Gonzalez-Acuna, D; Padilla, P; Dantas, GPM; Luna-Jorquera, G; Frere, E; Valdes-Velasquez, A; Vianna, JA | 2016 | Contrasting patterns of selection between MHC I and II across populations of Humboldt and Magellanic penguins | The evolutionary and adaptive potential of populations or species facing an emerging infectious disease depends on their genetic diversity in genes, such as the major histocompatibility complex (MHC). In birds, MHC class I deals predominantly with intracellular infections (e.g., viruses) and MHC class II with extracellular infections (e.g., bacteria). Therefore, patterns of MHC I and II diversity may differ between species and across populations of species depending on the relative effect of local and global environmental selective pressures, genetic drift, and gene flow. We hypothesize that high gene flow among populations of Humboldt and Magellanic penguins limits local adaptation in MHC I and MHC II, and signatures of selection differ between markers, locations, and species. We evaluated the MHC I and II diversity using 454 next-generation sequencing of 100 Humboldt and 75 Magellanic penguins from seven different breeding colonies. Higher genetic diversity was observed in MHC I than MHC II for both species, explained by more than one MHC I loci identified. Large population sizes, high gene flow, and/or similar selection pressures maintain diversity but limit local adaptation in MHC I. A pattern of isolation by distance was observed for MHC II for Humboldt penguin suggesting local adaptation, mainly on the northernmost studied locality. Furthermore, trans species alleles were found due to a recent speciation for the genus or convergent evolution. High MHC I and MHC II gene diversity described is extremely advantageous for the long term survival of the species. | 885 | no | no good fitness measure, N pop differnt in GD? | NA | no | no |
| 886 | 2017 | 43013 | Lanes, ECM; Motoike, SY; Kuki, KN; Resende, MDV; Caixeta, ET | 2016 | Mating System and Genetic Composition of the Macaw Palm (Acrocomia aculeata): Implications for Breeding and Genetic Conservation Programs | Acrocomia aculeata (Arecaceae), a palm endemic to South and Central America, is a potential oil crop. Knowledge of the mating system of this species is limited to its reproductive biology and to studies using molecular markers. The present study analyzed genetic diversity between its developmental stages and determined its prevailing mating system in order to support genetic conservation and breeding programs. We tested 9 microsatellite markers in 27 mother trees (adult plants) and 157 offspring (juvenile plants) from the southeastern region of Brazil. Heterozygosity levels differed between the 2 studied life stages, as indicated by the fixation index of adult and juvenile trees, suggesting that selection against homozygotes occurs during the plant life cycle. The mating system parameters analyzed indicate that A. aculeata is predominantly outcrossing (allogamous). However, its low levels of selfing suggest that there is individual variation with regard to self-incompatibility, which can be a survival strategy in isolated or fragmented habitats. Deviations in variance effective size were detected because of high mating rates among relatives and correlated matings. These findings indicate that the main source of inbreeding results from biparental inbreeding in the population and that the progenies are predominantly composed of full-sibs. The information provided by this study on the ecology and reproduction dynamics of A. aculeata should be useful to both breeding and genetic conservation programs, allowing the development of more precise mathematical models and the estimation of the appropriate number of mother trees for seed collection. | 886 | no | individual level | NA | no | no |
| 887 | 2017 | 43013 | Huyghe, JR; Fransen, E; Hannula, S; Van Laer, L; Van Eyken, E; Maki-Torkko, E; Aikio, P; Sorri, M; Huentelman, MJ; Van Camp, G | 2011 | A genome-wide analysis of population structure in the Finnish Saami with implications for genetic association studies | The understanding of patterns of genetic variation within and among human populations is a prerequisite for successful genetic association mapping studies of complex diseases and traits. Some populations are more favorable for association mapping studies than others. The Saami from northern Scandinavia and the Kola Peninsula represent a population isolate that, among European populations, has been less extensively sampled, despite some early interest for association mapping studies. In this paper, we report the results of a first genome-wide SNP-based study of genetic population structure in the Finnish Saami. Using data from the HapMap and the human genome diversity project (HGDP-CEPH) and recently developed statistical methods, we studied individual genetic ancestry. We quantified genetic differentiation between the Saami population and the HGDP-CEPH populations by calculating pair-wise F(ST) statistics and by characterizing identity-by-state sharing for pair-wise population comparisons. This study affirms an east Asian contribution to the predominantly European-derived Saami gene pool. Using model-based individual ancestry analysis, the median estimated percentage of the genome with east Asian ancestry was 6% (first and third quartiles: 5 and 8%, respectively). We found that genetic similarity between population pairs roughly correlated with geographic distance. Among the European HGDP-CEPH populations, F(ST) was smallest for the comparison with the Russians (F(ST)=0.0098), and estimates for the other population comparisons ranged from 0.0129 to 0.0263. Our analysis also revealed fine-scale substructure within the Finnish Saami and warns against the confounding effects of both hidden population structure and undocumented relatedness in genetic association studies of isolated populations. European Journal of Human Genetics (2011) 19, 347-352; doi:10.1038/ejhg.2010.179; published online 8 December 2010 | 887 | no | human | NA | no | no |
| 888 | 2017 | 43013 | Charbonnel, N; Angers, B; Rasatavonjizay, R; Bremond, P; Jarne, P | 2002 | Evolutionary aspects of the metapopulation dynamics of Biomphataria pfeifferi, the intermediate host of Schistosoma mansoni | Combining genetic and demographic data is a powerful approach to study adaptation process and evolutionary forces acting in natural populations. We focus on the freshwater snail Biomphalaria pfeifferi, the intermediate host of Schistosoma mansoni. Twenty-one populations sampled in the south of Madagascar were genotyped at six microsatellite loci. Demographic parameters and parasitic prevalence were estimated monthly over the year preceding the genetic sampling. Our results indicate that populations experience recurrent bottlenecks and size fluctuations, which strongly depresses the genetic diversity within population. The recolonization of depleted sites involves genetically differentiated immigrants. We detected frequent migration events along rivers and rare migration events between watersheds. This explains the high level of differentiation observed among populations. The negative regression observed between the prevalence of S. mansoni and the genetic diversity of B. pfeifferi populations indicates that host consanguinity may affect prevalence through the genetic mechanisms involved in resistance. Coevolutionary outcomes are also influenced by the relative migration rates of snails and flukes, but the parasite local adaptation may be prevented by rare long distance dispersal in snails and the phylogeographical patterns of colonization of both hosts and snails. | 888 | yes | NA | not pop level gd | no | no |
| 889 | 2017 | 43013 | Oldroyd, BP; Clifton, MJ; Wongsiri, S; Rinderer, TE; Sylvester, HA; Crozier, RH | 1997 | Polyandry in the genus Apis, particularly Apis andreniformis | Using four polymorphic microsatellite loci, we found that four Apis andreniformis queens collected in Thailand each mated at least 10-20 times, producing an average relatedness, g(ww), of workers of 0.30 +/- 0.007, and an average effective number of matings of 9.1 +/- 2.2. The degrees of polyandry and intracolonial genetic relatedness in A. andreniformis are similar to those in A. mellifera, slightly more than in A. por err, and up to 6 times less than in A. dorsata. We argue that while presently favoured hypotheses for the evolution of polyandry in monogynous social insects may adequately explain the evolution of up to five or six matings, they are inadequate to explain the extreme polyandry (10-60 matings) observed in Apis. One alternative possibility is that colony fitness is a non-additive function of the fitness of individual subfamilies. Such behavioral over-dominance may mean that queen fitness is increased by high levels of polyandry, which increase the probability of desirable combinations of worker genotypes occurring in one colony. The special attributes of honey bees which may lead to behavioral over-dominance include colony aggregation (which may increase the incidence of disease), and frequent long-distance migration. | 889 | no | no good fitness measure, N pop differnt in GD? | NA | no | no |
| 890 | 2017 | 43013 | Vander Mijnsbrugge, K; Bischoff, A; Smith, B | 2010 | A question of origin: Where and how to collect seed for ecological restoration | Native plant species are routinely planted or sown in ecological restoration projects, but successful establishment and survival depend on where and how seeds are collected. Research suggests that it is important to use locally adapted seeds. Local populations often show a home-site advantage and non-local genotypes may be maladapted to local environmental conditions. Furthermore, intraspecific hybridisation of local and non-local genotypes may have a negative impact on the genetic structure of local populations via mechanisms such as outbreeding depression. Many species show a strong small-scale genetic differentiation between different habitats so that matching habitats of the restoration and donor site can be more important than minimizing geographical separation. It is a challenge to identify appropriate seed sources because strong small-scale population differentiation makes it difficult to delineate geographically defined seed zones to which seed exchange should be limited. Moreover, it is important to consider the genetic diversity of introduced material because it may be crucial to avoid genetic bottlenecks, inbreeding depression and poor establishment of plant populations. Repeated propagation in stock, which is often required to obtain a sufficient amount of seeds, can further reduce genetic diversity and may select for particular genotypes. Negative impacts of improper seed choice for nursery planting stock may become detectable only after many years, especially in long-lived and slow growing plants. Although scientific information on many species remains limited, the increasing demand for translocation of seed means that mandatory regulations are necessary. Guidelines should prescribe a specification of seed provenance, a record of genetic diversity of wild collections and rules for subsequent processing such as direct transfer and propagation of stock or seed orchards. We use a literature review to evaluate current legislation and to develop recommendations for herbaceous and woody species. | 890 | no | no good fitness measure, N pop differnt in GD? | NA | no | no |
| 891 | 2017 | 43013 | Gelatt, TS; Davis, CS; Stirling, I; Siniff, DB; Strobeck, C; Delisle, I | 2010 | History and fate of a small isolated population of Weddell seals at White Island, Antarctica | Weddell seals (Leptonychotes weddellii Lesson) at White Island, Antarctica form a small, completely enclosed, natural population hypothesized to be of recent origin, likely founded by individuals from nearby Erebus Bay. This population constitutes an ideal model to document a founder event and ensuing genetic drift, with implications for conservation. Here we combined historical accounts, census and tagging data since the late 1960s, and genetic data (41 microsatellite loci and mitochondrial DNA sequences) from 84 individuals representing nearly all individuals present between 1990 and 2000 to investigate the history of the founding of the White Island population, document its population dynamics and evaluate possible future threats. We fully resolved parental relationships over three overlapping generations. Cytonuclear disequilibrium among the first generation suggested that it comprised the direct descendants of a founding group. We estimated that the White Island population was founded by a small group of individuals that accessed the island during a brief break in the surrounding sea ice in the mid-1950s, consistent with historical accounts. Direct and indirect methods of calculating effective population size were highly congruent and suggested a minimum founding group consisting of three females and two males. The White Island population showed altered reproductive dynamics compared to Erebus Bay, including highly skewed sex ratio, documented inbred mating events, and the oldest known reproducing Weddell seals. A comparison with the putative source population showed that the White Island population has an effective inbreeding coefficient (F (e)) of 0.29. Based on a pedigree analysis including the hypothesized founding group, 86% of the individuals for whom parents were known had inbreeding coefficients ranging 0.09-0.31. This high level of inbreeding was correlated with reduced pup survival. Seals at White Island therefore face the combined effects of low genetic variability, lack of immigration, and inbreeding depression. Ultimately, this study provides evidence of the effects of natural isolation on a large, long-lived vertebrate and can provide clues to the potential effects of anthropogenic-caused isolation of similar taxa. | 891 | no | only one pop | NA | no | no |
| 892 | 2017 | 43013 | Bjorklund, M; Ruiz, I; Senar, JC | 2010 | Genetic differentiation in the urban habitat: the great tits (Parus major) of the parks of Barcelona city | The increase of urban areas has led to a fragmentation of habitats for many forest-living species. Man-made parks might be a solution, but they can also act as sinks that are unable to maintain themselves without immigration from natural areas. Alternatively, parks might act as true metapopulations with extinctions and colonizations. In both cases, we can expect genetic variation to be reduced in the parks compared to the natural habitat. A third alternative is that the parks have sufficient reproduction to maintain themselves. To test these hypotheses, we analysed the pattern of genetic variation in the great tit (Parus major) in 12 parks in central Barcelona, and in an adjacent forest population using microsatellites. Genetic variation was not lower in the parks compared to the forest population, but larger, and gene flow was higher from the town to the forest compared to vice versa. We found a significant genetic differentiation among the parks, with a structure that only partly reflected the geographic position of the parks. Relatedness among individuals within parks was higher than expected by chance, although we found no evidence of kin groups. Assignment tests suggest that some parks are acting as net donors of individuals to other parks. (C) 2010 The Linnean Society of London, Biological Journal of the Linnean Society, 2010, 99, 9-19. | 892 | no | no good fitness measure, N pop differnt in GD? | NA | no | no |
| 893 | 2017 | 43013 | Bhutta, WM; Shahzad, A; Akhtar, J; Ibrahim, M | 2005 | Assessment of genetic divergence among wheat (Triticum aestivum) genotypes using random amplified polymorphic DNA (RAPD) analysis | The degree of genetic divergence was estimated in 7 wheat (Triticum aestivum L.) genotypes from diverse locations of Pakistan using Random Amplified Polymorphic DNA (RAPD) methodology. A total of 160 DNA fragments were amplified with 20 random decamer primers with an average of 8 bands per primer. Genetic similarity matrix ranged from 84.0% to 93.0%, which indicated a narrow genetic base among the genotypes. The maximum similarity, 93.0%, was observed between WC-65 and SARC-1. The local variety, PARC-N2, showed the lowest similarity with the exotic types studied. It is suggested that RAPD analysis can be used for the characterization and grouping of wheat genotypes and the results will be helpful to wheat breeders. | 893 | no | crop | NA | no | no |
| 894 | 2017 | 43013 | Souter, P; Willis, BL; Bay, LK; Caley, MJ; Muirhead, A; van Oppen, MJH | 2010 | Location and disturbance affect population genetic structure in four coral species of the genus Acropora on the Great Barrier Reef | The impact of a mass bleaching event on temporal and spatial population genetic structure in 4 scleractinian coral species in the Acropora aspera group was studied around the Palm Islands in the central Great Barrier Reef. Species status of sympatric populations of 2 of the 4 species, A. millepora and A. spathulata, was confirmed by the population genetic data; these species have recently been separated based on morphological and breeding characters. Spatial analyses of population samples from 2004 detected differences in the level of gene flow among locations. No significant genetic differentiation was inferred between conspecific populations at Orpheus and Pelorus Islands, which are both located in the northern part of the island group and separated by similar to 1000 m. In contrast, all populations at Fantome Island were genetically differentiated, despite this island being located only 11 km south. Sampling of A. millepora and A. pulchra in the year prior to the 1998 mass bleaching event enabled a temporal comparison across this event. The genetic composition of these populations changed between 1997 and 2004, but patterns of genetic differentiation among locations were similar in 1997 and 2004. Extensive mortality of these species following the 1998 bleaching event did not cause an apparent reduction in genetic diversity and identical multi-locus genotypes were encountered in both temporal samples, suggesting that re-growth of surviving genotypes contributed to the recovery of these populations. Comparisons among the 4 study species revealed lower genetic diversity in A. papillare, consistent with its low abundance throughout its distributional range. | 894 | no | no good fitness measure, N pop differnt in GD? | NA | no | no |
| 895 | 2017 | 43013 | Sampson, JF; Byrne, M | 2016 | Assessing genetic structure in a rare clonal eucalypt as a basis for augmentation and introduction translocations | Genetics can provide information that is critical for planning translocations for conservation, such as levels of diversity and divergence of target and source populations. For clonal plants, assessing population characteristics (size, diversity, mortality, gene flow) that influence conservation values also requires identification of different genetic individuals. We used 12 microsatellite markers to guide germplasm source recommendations for augmentation and introduction translocations to conserve the critically endangered Eucalyptus cuprea that occurs in fragmented populations in the semi-arid shrublands of Western Australia. Ramet clumps with identical multilocus genotypes were identified in all populations but clonal richness (R = 0 - 0.86) and heterogeneity (D = 0 - 0.98) varied among populations. Genetic diversity was low to moderate in all populations (mean H (o) = 0.61, mean A = 3.78) and did not differ significantly between localities. There was evidence of inbreeding in some populations but outcrossing (t (m) = 0.495) in the small number of families available for study (N = 4) and genotypic diversity of the larger extant populations suggest the generation of novel genotypes is a component of the reproductive strategy. Most diversity was within populations and differentiation among populations was moderate (F (ST) = 0.100) suggesting mixing of source population for translocation is unlikely to lead to outbreeding depression. Principal Co-ordinate and Bayesian analyses indicated the Northern population is distinct from Central/Southern populations. We recommend use of mixed germplasm to conserve the moderate diversity characterising larger remnant populations and to enable the production of recombinants through sexual reproduction. But given seed availability and the distinction of the Northern population, an initial precautionary approach to a translocation proposed for south of the geographical range may be to source germplasm from the Central/Southern locality. | 895 | no | no good fitness measure, N pop differnt in GD? | NA | no | no |
| 896 | 2017 | 43013 | Neel, JV | 2000 | Some ethical issues at the population level raised by ‘soft’ eugenics, euphenics, and isogenics | It is argued that at the population level there are three central genetic developments raising ethical issues. The first is the emergence of ‘soft’ eugenics, due primarily to the increasing ability to detect carriers of genetic diseases, to monitor their pregnancies, and to provide the option to abort a fetus predisposed to major genetic disease. The second development is the recognition of the extent to which many serious diseases of adult life are due to a disturbance of ancient genetic homeostatic mechanisms due to changing life style, raising the question of whether a society that increasingly pays the medical bills should attempt to impose healthier standards of living on its members. Such an attempt at ‘euphenics’ may be thought of as the antithesis to eugenics. The third development relates to recognition of the need to regulate the size of the earth’s population to numbers that can be indefinitely sustained; this regulation in a fashion (isogenic) that will preserve existing genetic diversity. | 896 | no | NA | NA | no | no |
| 897 | 2017 | 43013 | He, YF; Wang, JW; Blanchet, S; Lek, S | 2012 | Genetic structure of an endangered endemic fish (Gobiocypris rarus) in the upper Yangtze River | The goal of this study was to examine the genetic diversity and structure of wild populations of rare minnow (Gobiocypris rams) in the upper Yangtze River. Individuals from nine locations were sampled and genotyped at eight microsatellite markers. Populations differed from each other on allelic frequency, although there were no significant differences on other genetic diversity indices. Our results further suggested low to moderate levels of population differentiation (F-ST: 0.0130-0.1537). Specifically, two distinct genetic clusters (C1 and C2) were found, which may be highly correlated with the evolution of water systems. In addition, a weak but significant isolation-by-distance pattern was best explained by riparian distance through man-made channels. It suggested that fish might use man-made channels for gene flow in history, although no significant recent migration event was found. Therefore, this study highlights the need to consider the genetic specificities of G. rams for sustaining long-term survival of this species. (C) 2012 Elsevier Ltd. All rights reserved. | 897 | no | no good fitness measure, N pop differnt in GD? | NA | no | no |
| 898 | 2017 | 43013 | Elias, BA; Shipley, LA; McCusker, S; Sayler, RD; Jobnson, TR | 2013 | Effects of genetic management on reproduction, growth, and survival in captive endangered pygmy rabbits (Brachylagus idahoensis) | A quarter of all lagomorph species worldwide are threatened with extinction. Captive breeding programs, such as that developed for the Columbia Basin (CB) pygmy rabbit (Brachylagus idahoensis), sometimes are implemented as emergency conservation measures to restore small, genetically distinct populations. However, small source populations also may have low genetic diversity, which may influence attributes related to fitness, including growth, survival, and reproduction. We used mixed-effects regression models to explore the influence of genetic pedigree (% CB) on pairing success, growth, and survival during the 10-year captive breeding program at Washington State University, which included controlled pairings and outbreeding with pygmy rabbits from Idaho. Pairing success, juvenile growth, and juvenile survival declined with increasing CB pedigree of 1 or both parents, suggesting inbreeding depression among the small number of related founders. Demographic variables such as age, sex, and previous pregnancies, and environmental variables such as month and temperature at birth also were associated with production of pygmy rabbits. Our study illustrates the difficulty of retaining a unique genome of a small source population while simultaneously producing enough rabbits for restoration into natural habitat as part of endangered species recovery programs. | 898 | no | breeding model | NA | no | no |
| 899 | 2017 | 43013 | Stefani, F; Aquaro, G; Azzurro, E; Colorni, A; Galli, P | 2012 | Patterns of genetic variation of a Lessepsian parasite | Genetic studies of Lessepsian species often demonstrate the absence of a genetic bottleneck in a wide plethora of taxa, from plants to fish, but information regarding the genetic responses of their parasites in the newly colonized ecosystems is still lacking. This study compared genetic diversity of Red Sea (Eilat, Nabq), Suez canal (Ismailia) and Mediterranean (Rhodes, Tel Aviv) populations of the Monogenoidea Glyphidohaptor plectocirra by sequencing a portion of the mitochondrial CoxI gene. Despite evidence of a slight decrease in the genetic diversity of Mediterranean populations, a simulation analysis based on coalescent theory demonstrated the absence of significant bottlenecks, but there was directional selection along a cline moving further from the Suez canal. The absence of bottlenecks was congruent with that described for G. plectocirra hosts Siganus rivulatus and Siganus luridus, and reflected a common history of high propagule pressure during initial colonization, and constant or repeated gene flow from the Red Sea to the Mediterranean area. However, directional selection was peculiar to the parasites and likely originated from parasite genotype x environment interactions. Finally, an anisotropic contribution of Red Sea populations to the Lessepsian invasion was demonstrated. | 899 | no | gd of parasite | NA | no | no |
| 900 | 2017 | 43013 | Chebon, LJ; Ngalah, BS; Ingasia, LA; Juma, DW; Muiruri, P; Cheruiyot, J; Opot, B; Mbuba, E; Imbuga, M; Akala, HM; Bulimo, W; Andagalu, B; Kamau, E | 2016 | Genetically Determined Response to Artemisinin Treatment in Western Kenyan Plasmodium falciparum Parasites | Genetically determined artemisinin resistance in Plasmodium falciparum has been described in Southeast Asia. The relevance of recently described Kelch 13-propellermutations for artemisinin resistance in Sub-Saharan Africa parasites is still unknown. Southeast Asia parasites have low genetic diversity compared to Sub-Saharan Africa, where parasites are highly genetically diverse. This study attempted to elucidate whether genetics provides a basis for discovering molecular markers in response to artemisinin drug treatment in P. falciparum in Kenya. The genetic diversity of parasites collected pre- and post-introduction of artemisinin combination therapy (ACT) in western Kenya was determined. A panel of 12 microsatellites and 91 single nucleotide polymorphisms (SNPs) distributed across the P. falciparum genome were genotyped. Parasite clearance rates were obtained for the post-ACT parasites. The 12 microsatellites were highly polymorphic with post-ACT parasites being significantly more diverse compared to pre- ACT (p < 0.0001). The median clearance half-life was 2.55 hours for the post-ACT parasites. Based on SNP analysis, 15 of 90 post-ACT parasites were single-clone infections. Analysis revealed 3 SNPs that might have some causal association with parasite clearance rates. Further, genetic analysis using Bayesian tree revealed parasites with similar clearance phenotypes were more closely genetically related. With further studies, SNPs described here and genetically determined response to artemisinin treatment might be useful in tracking artemisinin resistance in Kenya. | 900 | no | gd of parasite | NA | no | no |
| 901 | 2017 | 43013 | Hildner, KK; Soule, ME | 2004 | Relationship between the energetic cost of burrowing and genetic variability among populations of the pocket gopher, T bottae: does physiological fitness correlate with genetic variability? | Many studies have reported relationships between genetic variability and fitness characters in invertebrates, but there is a paucity of such studies in mammals. Here, we use a statistically powerful paired sampling design to test whether the metabolic cost of burrowing, an important physiological trait in the pocket gopher, Thomomys bottae, correlates with genetic variability. Three pairs of pocket gopher populations were used, with each pair selected from a different subspecies and comprising one high genetic variability and one low genetic variability population. Genetic variability was measured using average allozyme heterozygosity and two measures of DNA fingerprint band sharing. In addition, the cost of burrowing for individuals from each population was determined from the oxygen consumption per gram of body mass per unit of work performed. Our results indicate that the cost of burrowing was significantly higher in populations with lower genetic variability (3-way ANCOVA, P=0.0150); mass-adjusted cost of burrowing in the low variability populations averaged 0.57 +/- 0.24 ml O-2 g(-1) kgm(-1) and that in the high variability populations averaged 0.42 +/- 0.19 ml o(2) g(-1) kgm(-1). The magnitude of the population differences in cost of burrowing was associated with the magnitude of difference in genetic variability. We conclude that population differences in genetic variability are reflected in physiological fitness differences for a trait that is essential to gopher survival. | 901 | no | no good fitness measure | NA | no | no |
| 902 | 2017 | 43013 | Tzika, AC; Remy, C; Gibson, R; Milinkovitch, MC | 2009 | Molecular genetic analysis of a captive-breeding program: the vulnerable endemic Jamaican yellow boa | “The endemic Jamaican boa (or”“yellow boa”“, Epicrates subflavus) is a vulnerable species of the Caribbean biodiversity hotspot whose natural populations greatly declined mainly due to predation by introduced species, human persecution, and habitat destruction. A captive breeding program was initiated in 1976 and rationalized in 2002 by the establishment of a European Endangered Species Program. During the last 30 years, more than 600 offspring, of which 80 are still alive today, have been produced and distributed among European host institutions and privates. Here, using nine nuclear microsatellite loci and a fragment of the mitochondrial cytochrome b gene, we (i) determine the natural population from which the founders originate, (ii) identify parental allocation errors and ambiguities in the studbook, and (iii) assess the genetic diversity and estimate levels of inbreeding of the current captive population based on loss of alleles, variance in reproductive success, and relatedness among individuals. Combining measures of relatedness derived from multilocus genotypes with practical parameters such as age of animals and localization of host institutions, we propose mating groups that would maximize genetic diversity in the captive population of the Jamaican boa. Our analyses provide guidance for a more efficient breeding program that, in turn, could be used as the starting point of a repatriation program to increase the probability of the species long-term survival.” | 902 | no | no good fitness measure, N pop differnt in GD? | NA | no | no |
| 903 | 2017 | 43013 | May, S; Zeisset, I; Beebee, TJC | 2011 | Larval fitness and immunogenetic diversity in chytrid-infected and uninfected natterjack toad (Bufo calamita) populations | Chytrid fungus Batrachochytrium dendrobatidis (Bd), an emerging disease, has been decimating amphibian populations around the world for several decades. We quantified aspects of larval fitness, adaptive (major histocompatibility complex) diversity and neutral (microsatellite) diversity in natterjack toad (Bufo calamita) populations in two regions of north-west England. Toads in region one had no evidence of chytrid infection, whereas in region two there was a substantial prevalence of Bd. Larval fitness (growth rate, time to metamorphosis and survival) of B. calamita did not differ between the regions. Genetic diversity at microsatellite loci was much higher in the infected than in the uninfected region, but the converse was true of MHC diversity indicating that genetic drift was unlikely to explain the differences in MHC between the regions. Furthermore, MHC allele frequencies varied significantly between Bd-infected and uninfected populations. Microsatellite diversity was not a robust indicator of larval fitness in these toad populations while MHC genotype frequencies varied in a way that was consistent with directional selection in response to pathogen prevalence. The acquired immune defences may therefore play an important role in determining the susceptibility of amphibian species to chytridiomycosis. | 903 | yes | gd difference? | just two pop | yes | no |
| 904 | 2017 | 43013 | Bearoff, F; del Rio, R; Case, LK; Dragon, JA; Nguyen-Vu, T; Lin, CY; Blankenhorn, EP; Teuscher, C; Krementsov, DN | 2016 | Natural genetic variation profoundly regulates gene expression in immune cells and dictates susceptibility to CNS autoimmunity | Regulation of gene expression in immune cells is known to be under genetic control, and likely contributes to susceptibility to autoimmune diseases such as multiple sclerosis (MS). How this occurs in concert across multiple immune cell types is poorly understood. Using a mouse model that harnesses the genetic diversity of wild-derived mice, more accurately reflecting genetically diverse human populations, we provide an extensive characterization of the genetic regulation of gene expression in five different naive immune cell types relevant to MS. The immune cell transcriptome is shown to be under profound genetic control, exhibiting diverse patterns: global, cell-specific and sex-specific. Bioinformatic analysis of the genetically controlled transcript networks reveals reduced cell type specificity and inflammatory activity in wild-derived PWD/PhJ mice, compared with the conventional laboratory strain C57BL/6J. Additionally, candidate MS-GWAS (genome-wide association study candidate genes for MS susceptibility) genes were significantly enriched among transcripts overrepresented in C57BL/6J cells compared with PWD. These expression level differences correlate with robust differences in susceptibility to experimental autoimmune encephalomyelitis, the principal model of MS, and skewing of the encephalitogenic T-cell responses. Taken together, our results provide functional insights into the genetic regulation of the immune transcriptome, and shed light on how this in turn contributes to susceptibility to autoimmune disease. | 904 | no | no good fitness measure, N pop differnt in GD? | NA | no | no |
| 905 | 2017 | 43013 | Ballingall, KT; McIntyre, A; Lin, ZZ; Timmerman, N; Matthysen, E; Lurz, PW; Melville, L; Wallace, A; Meredith, AL; Romeo, C; Wauters, LA; Sainsbury, AW; McInnes, CJ | 2016 | Limited diversity associated with duplicated class II MHC-DRB genes in the red squirrel population in the United Kingdom compared with continental Europe | The red squirrel (Sciurus vulgaris) population in the United Kingdom has declined over the last century and is now on the UK endangered species list. This is the result of competition from the eastern grey squirrel (S. carolinensis) which was introduced in the 19th century. However, recent evidence suggests that the rate of population decline is enhanced by squirrelpox disease, caused by a viral infection carried asymptomatically by grey squirrels but to which red squirrels are highly susceptible. Population genetic diversity provides some resilience to rapidly evolving or exotic pathogens. There is currently no data on genetic diversity of extant UK squirrel populations with respect to genes involved in disease resistance. Diversity is highest at loci involved in the immune response including genes clustered within the major histocompatibility complex (MHC). Using the class II DRB locus as a marker for diversity across the MHC region we genotyped 110 red squirrels from locations in the UK and continental Europe. Twenty-four Scvu-DRB alleles at two functional loci; Scvu-DRB1 and Scvu-DRB2, were identified. High levels of diversity were identified at both loci in the continental populations. In contrast, no diversity was observed at the Scvu-DRB2 locus in the mainland UK population while a high level of homozygosity was observed at the Scvu-DRB1 locus. The red squirrel population in the UK appears to lack the extensive MHC diversity associated with continental populations, a feature which may have contributed to their rapid decline. | 905 | no | No GD diff between pop | NA | no | no |
| 906 | 2017 | 43013 | Seltmann, P; Hensen, I; Renison, D; Wesche, K; Ploch, S; Duenas, JR; Cocucci, A; Jung, K | 2009 | Biparental inbreeding depression, genetic relatedness and progeny vigour in a wind-pollinated treeline species in Argentina | Spatially restricted gene flow and resulting spatial genetic structure are generally considered as being the primary controlling factors in the dynamics of biparental inbreeding depression in a wide range of plant species. However, wind-pollinated angiosperm trees have not been studied adequately in this respect. The present study analyses the relationships among parental genetic similarity, outcrossing distances, progeny vigour and mortality in Polylepis australis (Rosaceae), a wind-pollinated treeline species endemic to Argentina. We investigated whether spatial genetic structuring occurs in anthropogenically fragmented P. australis woodlands of the Crdoba Mountains. We also performed a controlled crossing experiment using pollen collected from different distances. Genetic variability (using RAPD-PCR), vigour (N-metabolism capacity) and mortality of the resulting progeny were contrasted with progeny from unmanipulated flowers. We found a continuous decrease in parental genetic similarity with spatial distance among mates and an increase in N-metabolism capacity in the progeny produced from pollen at increasing distances, as well as a very high mortality of seedlings resulting from short-distance crosses. Additionally, our results suggest that there is still fragment connectivity in P. australis through long-distance pollen-mediated gene flow. | 906 | no | inbreeding | NA | no | no |
| 907 | 2017 | 43013 | Kajdacsi, B; Costa, F; Hyseni, C; Porter, F; Brown, J; Rodrigues, G; Farias, H; Reis, MG; Childs, JE; Ko, AI; Caccone, A | 2013 | Urban population genetics of slum-dwelling rats (Rattus norvegicus) in Salvador, Brazil | Throughout the developing world, urban centres with sprawling slum settlements are rapidly expanding and invading previously forested ecosystems. Slum communities are characterized by untended refuse, open sewers and overgrown vegetation, which promote rodent infestation. Norway rats (Rattus norvegicus) are reservoirs for epidemic transmission of many zoonotic pathogens of public health importance. Understanding the population ecology of R.norvegicus is essential to formulate effective rodent control strategies, as this knowledge aids estimation of the temporal stability and spatial connectivity of populations. We screened for genetic variation, characterized the population genetic structure and evaluated the extent and patterns of gene flow in the urban landscape using 17 microsatellite loci in 146 rats from nine sites in the city of Salvador, Brazil. These sites were divided between three neighbourhoods within the city spaced an average of 2.7km apart. Surprisingly, we detected very little relatedness among animals trapped at the same site and found high levels of genetic diversity, as well as structuring across small geographical distances. Most F-ST comparisons among sites were statistically significant, including sites <400m apart. Bayesian analyses grouped the samples in three genetic clusters, each associated with distinct sampling sites from different neighbourhoods or valleys within neighbourhoods. These data indicate the existence of complex genetic structure in R.norvegicus in Salvador, linked to the heterogeneous urban landscape. Future rodent control measures need to take into account the spatial and temporal linkage of rat populations in Salvador, as revealed by genetic data, to develop informed eradication strategies. | 907 | no | no good fitness measure, N pop differnt in GD? | NA | no | no |
| 908 | 2017 | 43013 | Giammarioli, M; Rossi, E; Casciari, C; Bazzucchi, M; Claudia, T; De Mia, GM | 2015 | Genetic characterization of border disease virus (BDV) isolates from small ruminants in Italy | Border disease virus (BDV) belongs to the Pestivirus genus in the family Flaviviridae. Genetic analyses of pestiviruses that have been isolated from sheep and goat have led to the proposal that BDV isolates can be phylogenetically segregated into at least seven clusters, subtypes BDV-1 to BDV-7. In order to investigate the genetic heterogeneity of small ruminant pestivirus isolates in Italy, a selection of 50-UTR sequences from isolates that were collected from clinical specimens between 2002 and 2014 was analysed. Phylogenetic reconstructions indicated that the BDV-positive samples clustered within the BDV-1, BDV-3, BDV-5, and BDV-7 groups. These results suggested high genetic diversity within the Italian BDV field isolates. The phylogenetic analysis indicated the first evidence of BDV-1 and BDV-5 circulation in Italy. The marked diversity of the pestivirus isolates might reflect the sheep trade with foreign countries. | 908 | no | no good fitness measure, N pop differnt in GD? | NA | no | no |
| 909 | 2017 | 43013 | Patricelli, D; Sielezniew, M; Ponikwicka-Tyszko, D; Ratkiewicz, M; Bonelli, S; Barbero, F; Witek, M; Bus, MM; Rutkowski, R; Balletto, E | 2013 | Contrasting genetic structure of rear edge and continuous range populations of a parasitic butterfly infected by Wolbachia | Background: Climatic oscillations are among the long-term factors shaping the molecular features of animals and plants and it is generally supposed that the rear edges (i.e., the low-latitude limits of distribution of any given specialised species) situated closer to glacial refugia are vital long-term stores of genetic diversity. In the present study, we compared the genetic structure of several populations of an endangered and obligate myrmecophilous butterfly (Maculinea arion) from two distinct and geographically distant parts of its European distribution (i.e., Italy and Poland), which fully represent the ecological and morphological variation occurring across the continent. Results: We sequenced the COI mitochondrial DNA gene (the ‘barcoding gene’) and the EF-1 alpha nuclear gene and found substantial genetic differentiation among M. arion Italian populations in both markers. Eleven mtDNA haplotypes were present in Italy. In contrast, almost no mtDNA polymorphisms was found in the Polish M. arion populations, where genetic differentiation at the nuclear gene was low to moderate. Interestingly, the within-population diversity levels in the EF-1 alpha gene observed in Italy and in Poland were comparable. The genetic data did not support any subspecies divisions or any ecological specialisations. All of the populations studied were infected with a single strain of Wolbachia and our screening suggested 100% prevalence of the bacterium. Conclusions: Differences in the genetic structure of M. arion observed in Italy and in Poland may be explained by the rear edge theory. Although we were not able to pinpoint any specific evolutionarily significant units, we suggest that the Italian peninsula should be considered as a region of special conservation concern and one that is important for maintaining the genetic diversity of M. arion in Europe. The observed pattern of mtDNA differentiation among the populations could not be explained by an endosymbiotic infection. | 909 | no | No GD diff between pop | NA | no | no |
| 910 | 2017 | 43013 | Chernenko, A; Helantera, H; Sundstrom, L | 2012 | Colony kin structure and queen recruitment in the ant Formica fusca (Hymenoptera: Formicidae) | The acceptance of extra queens in social insects changes colony kin structure and may reduce the inclusive fitness of workers. Nonetheless, colonies do not always reject intruding queens, although more so in colonies with high rather than low relatedness among colony members (e.g., mono- and polygyne colonies, respectively). This begs the question to what extent initially accepted queens come to reproduce in the colony and whether the outcome depends on colony kin structure regardless of the number of queens actually present in the colony. Here we test whether related daughter queens are more amenable for adoption than unrelated non-nestmate queens, whether acceptance is contingent on within-colony relatedness, and whether resident queens are favoured over new ones. We compared adoption and over-wintering survival of young queens of the facultatively polygyne black ant Formica fusca and found that nearly 100% of the introduced queens were killed or died within four months, whereas nearly all resident queens were retained regardless of colony kin structure. Contrary to our expectations based on inclusive fitness arguments, related daughter queens were not more eligible for adoption than unrelated prospective adoptees. Although colony kin structure had no effect on the tendency to adopt additional queens, dead queens were more often dismembered and thus presumably killed in high-relatedness than low-relatedness colonies. | 910 | no | no good fitness measure, N pop differnt in GD? | NA | no | no |
| 911 | 2017 | 43013 | Tyagi, S; Pande, V; Das, A | 2014 | New insights into the evolutionary history of Plasmodium falciparum from mitochondrial genome sequence analyses of Indian isolates | Estimating genetic diversity and inferring the evolutionary history of Plasmodium falciparum could be helpful in understanding origin and spread of virulent and drug-resistant forms of the malaria pathogen and therefore contribute to malaria control programme. Genetic diversity of the whole mitochondrial (mt) genome of P.falciparum sampled across the major distribution ranges had been reported, but no Indian P.falciparum isolate had been analysed so far, even though India is highly endemic to P.falciparum malaria. We have sequenced the whole mt genome of 44 Indian field isolates and utilized published data set of 96 genome sequences to present global genetic diversity and to revisit the evolutionary history of P.falciparum. Indian P.falciparum presents high genetic diversity with several characteristics of ancestral populations and shares many of the genetic features with African and to some extent Papua New Guinean (PNG) isolates. Similar to African isolates, Indian P.falciparum populations have maintained high effective population size and undergone rapid expansion in the past with oldest time to the most recent common ancestor (TMRCA). Interestingly, one of the four single nucleotide polymorphisms (SNPs) that differentiates P.falciparum from P.falciparum-like isolates (infecting non-human primates in Africa) was found to be segregating in five Indian P.falciparum isolates. This SNP was in tight linkage with other two novel SNPs that were found exclusively in these five Indian isolates. The results on the mt genome sequence analyses of Indian isolates on the whole add to the current understanding on the evolutionary history of P.falciparum. | 911 | no | gd of parasite | NA | no | no |
| 912 | 2017 | 43013 | Grobler, JP; Mokgalong, NM | 2002 | Patterns of genetic heterogeneity in Neutraclinostomum intermedialis (Digenea : Clinostomatidae): geographical and temporal considerations | Approximately 300 individuals of Neutraclinostomum intermedialis from three localities in the Northern Province of South Africa were screened using allozyme electrophoresis. The aim was to determine genetic diversity and divergence within and between populations.;The within population heterozygosity values (H = 0.5-3.0 %) were generally higher than the value of 0.7 % previously estimated for this species from a single population. The mean F-ST value of 0.69 indicated marked structuring among the populations studied. Genetic distances (Nei 1972) between populations ranged from 0-0.09 and the clustering of populations closely reflected their geographical distribution and linkage to specific river systems. One locus of malate dehydrogenase, E.C.1.1.1.37 (MDH-2), provided a fixed allelic difference between populations from two river systems (the Limpopo and Olifants river systems). We discuss patterns of geographical genetic diversity in N. intermedialis, with reference to the constraints of habitat and host specificity. | 912 | no | gd of parasite | NA | no | no |
| 913 | 2017 | 43013 | Colson, KE; Brinkman, TJ; Person, DK; Hundertmark, KJ | 2013 | Fine-scale social and spatial genetic structure in Sitka black-tailed deer | The spatial extent of Sitka black-tailed deer (Odocoileus hemionus sitkensis) populations below the regional scale is relatively unknown, as is dispersal between populations. Here, we use noninvasive samples to genotype 221 Sitka black-tailed deer in three watersheds on Prince of Wales Island, Alaska, separated by a maximum of 44 km, using traditional and spatial genetic approaches. We find that despite geographic proximity, multiple lines of evidence suggest fine-scale genetic structure among the three study sites. The 2 most geographically distant watersheds differed significantly in genetic composition, suggesting an isolation-by-distance pattern. Within study sites, deer exhibited spatial genetic structure within a radius of 1,000 m. Based on a reduced sample of known-sex individuals, females exhibited positive spatial genetic structure within a radius of 500 m but males showed no structure. Moreover, females were more likely to be related to their 5 nearest female neighbors, regardless of distance, than were males. Evidence indicates dispersal by both sexes although it may be more common, or dispersal distances are greater, in males. Nonetheless, analysis of assignment indices and comparison of sex-specific correlograms found no evidence of sex-biased dispersal between watersheds. Patterns of spatial relatedness and connectivity suggest limited dispersal among Sitka black-tailed deer, creating genetic structure on a fine spatial scale, perhaps as small as the watershed. | 913 | no | no good fitness measure, N pop differnt in GD? | NA | no | no |
| 914 | 2017 | 43013 | BARBAULT, R | 1995 | BIODIVERSITY DYNAMICS - FROM POPULATION AND COMMUNITY ECOLOGY APPROACHES TO A LANDSCAPE ECOLOGY POINT-OF-VIEW | Species richness and species diversity are classic concepts in ecology. What is new in the science of biodiversity after the Convention on Biological Diversity is: (1) that the emphasis has moved from the species to the ecosystem; (2) that the functional significance of biodiversity has been stressed. Thus, population and community ecology along with landscape ecology, should offer the best theoretical framework to analyse what can be called ‘biodiversity dynamics’. Some promising pathways and areas are emphasized and the very concept of functional diversity is discussed. Species richness, genetic variability and extinction probability are closely linked with landscape traits such as habitat diversity, structural heterogeneity, patch dynamics and perturbations. Thus, it is suggested that landscape ecology hold a central role, since it will allow the response to biodiversity issues in the framework of environmental heterogeneity and patchiness. | 914 | no | no good fitness measure, N pop differnt in GD? | NA | no | no |
| 915 | 2017 | 43013 | Maciel, DB; de Medeiros, LV; de Medeiros, VV; Leao, MPC; Camargo, LEA; de Oliveira, NT | 2010 | Amplification of the cap20 Pathogenicity Gene and Genetic Characterization Using Different Markers Molecular in Colletotrichum gloeosporioides Isolates | Studies were performed to analyze the genetic characterization using RFLP-ITS and Intron (primer E11) markers and the amplification of the cap20 pathogenicity gene by PCR in Colletotrichum gloeosporioides isolates of different hosts plant. The genetic variability was accessed using RFLP- ITS and Intron markers and grouping by UPGMA method. Primers to cap20 gene were constructed using selected sequences of the GenBank (National Center of Biotechnology Information, http://www.ncbi.nlm.nih.gov) with the Primer 3 program. The dendrograms analysis showed that the RFLP-ITS marker was more informative to separate the Colletotrichum sp, and that primer E11 demonstrated greater genetic diversity. The amplification of the DNA of the Colletotrichum isolates to the cap20 gene with primers P1 and P2 indicated that this gene could present variations into C. gloeosporioides related with the host, and also that it was present in other Colletotrichum sp. | 915 | no | no good fitness measure, N pop differnt in GD? | NA | no | no |
| 916 | 2017 | 43013 | Smith, DR; Hagen, RH | 1996 | Population structure and interdemic selection in the cooperative spider Anelosimus eximius | The degree of relatedness among interacting individuals helps determine the fitness consequences of particular behaviors, whereas the partitioning (and amount) of genetic variation among and within groups controls the level at which selection will act most effectively. Three criteria are considered necessary for selection to act at the group or interdemic level: high rate of group initiation/extinction; differential survival and reproduction among groups; and highly subdivided population structure. The first two criteria have been demonstrated by earlier studies of Anelosimus eximius colonies. This study employs hierarchical analysis of allozyme polymorphisms to demonstrate the third criterion, subdivided population structure. Anelosimus eximius were collected from Suriname, Panama, Ecuador, Peru and Trinidad. Seven of 40 scorable enzyme loci revealed variation; 4 of these were polymorphic within colonies or regions. Expected heterozygosities were low, ranging from 0 (Ecuador, Peru) to similar to 0.03 (Suriname). For each polymorphic locus, hierarchical F-statistics were used to partition overall genetic variation into among-region (or among-population; F-rt), among-colony (F-sr), and within-colony (F-is) components. Samples from Suriname (43 colonies, 4 local populations) were the most informative; lack of scorable variation limited the inferences that could be drawn from other regions. A. eximius colonies are highly inbred: negative estimates of F-is imply very small effective colony sizes (similar to 6.5 for Suriname samples). By contrast, estimates of F-sr were very high: the mean for Suriname samples was 0.890, indicating neglibible gene flow among established colonies. Inbreeding within colonies, and genetic differentiation among colonies are consistent with demographic and behavioral observations of A. eximius. We suggest that interdemic selection is probable in this species and other cooperative spiders with this type of social system, and that mutual tolerance and absence of nest-mate recognition, as well as female-biased sex ratios, may have arisen by interdemic selection. | 916 | no | no good fitness measure | NA | no | no |
| 917 | 2017 | 43013 | Sanchez-Buso, L; Coscolla, M; Pinto-Carbo, M; Catalan, V; Gonzalez-Candelas, F | 2013 | Genetic Characterization of Legionella pneumophila Isolated from a Common Watershed in Comunidad Valenciana, Spain | Legionella pneumophila infects humans to produce legionellosis and Pontiac fever only from environmental sources. In order to establish control measures and study the sources of outbreaks it is essential to know extent and distribution of strain variants of this bacterium in the environment. Sporadic and outbreak-related cases of legionellosis have been historically frequent in the Comunidad Valenciana region (CV, Spain), with a high prevalence in its Southeastern-most part (BV). Environmental investigations for the detection of Legionella pneumophila are performed in this area routinely. We present a population genetics study of 87 L. pneumophila strains isolated in 13 different localities of the BV area irrigated from the same watershed and compare them to a dataset of 46 strains isolated in different points of the whole CV. Our goal was to compare environmental genetic variation at two different geographic scales, at county and regional levels. Genetic diversity, recombination and population structure were analyzed with Sequence-Based Typing data and three intergenic regions. The results obtained reveal a low, but detectable, level of genetic differentiation between both datasets, mainly, but not only, attributed to the occurrence of unusual variants of the neuA locus present in the BV populations. This differentiation is still detectable when the 10 loci considered are analyzed independently, despite the relatively high incidence of the most common genetic variant in this species, sequence type 1 (ST-1). However, when the genetic data are considered without their associated geographic information, four major groups could be inferred at the genetic level which did not show any correlation with sampling locations. The overall results indicate that the population structure of these environmental samples results from the joint action of a global, widespread ST-1 along with genetic differentiation at shorter geographic distances, which in this case are related to the common watershed for the BV localities. | 917 | no | gd of pathogen | NA | no | no |
| 918 | 2017 | 43013 | Hadas, L; Hermon, D; Boldo, A; Arieli, G; Gafny, R; King, R; Bar-Gal, GK | 2015 | Wild Gazelles of the Southern Levant: Genetic Profiling Defines New Conservation Priorities | The mountain gazelle (Gazella gazelle), Dorcas gazelle (Gazella Dorcas) and acacia gazelle (Gazella arabica acacia) were historically abundant in the southern Levant, and more specifically in Israel. Anthropogenic and natural changes have caused a rapid decline in gazelle populations, raising concerns about their conservation status and future survival. The genetic profile of 111 wild gazelles from Israel was determined based on three regions of mitochondrial DNA (control region, Cytochrome b and 12S ribosomal RNA) and nine nuclear microsatellite markers. Genetic analysis of the mountain gazelle population, the largest known population of this rare species, revealed adequate diversity levels and gene flow between subpopulations. Nevertheless, ongoing habitat degradation and other human effects, such as poaching, suggest the need for drastic measures to prevent species extinction. Dorcas gazelles in Israel displayed inbreeding within subpopulations while still maintaining considerable genetic diversity overall. This stable population, represented by a distinctive genetic profile, is fragmented and isolated from its relatives in neighboring localities. Based on the genetic profile of a newly sampled subpopulation in Israel, we provide an alternative hypothesis for the historic dispersal of Dorcas gazelle, from the Southern Levant to northern Africa. The small acacia gazelle population was closest to gazelles from the Farasan Islands of Saudi Arabia, based on mitochondrial markers. The two populations did not share haplotypes, suggesting that these two populations may be the last remnant wild gazelles of this species worldwide. Only a dozen acacia gazelles survive in Israel, and urgent steps are needed to ensure the survival of this genetically distinctive lineage. The genetic assessments of our study recognize new conservation priorities for each gazelle species in the Southern Levant. | 918 | no | no good fitness measure, N pop differnt in GD? | NA | no | no |
| 919 | 2017 | 43013 | Hamner, RM; Pichler, FB; Heimeier, D; Constantine, R; Baker, CS | 2012 | Genetic differentiation and limited gene flow among fragmented populations of New Zealand endemic Hector’s and Maui’s dolphins | Gene flow among small fragmented populations is critical for maintaining genetic diversity, and therefore the evolutionary potential of a species. Concern for two New Zealand endemic subspecies, the Hector’s (Cephalorhynchus hectori hectori) and Maui’s (C. h. maui) dolphins, arises from their low abundance, slow rate of reproduction, and susceptibility to fisheries-related mortality. Our work examined genetic differentiation and migration between the subspecies and among regional and local Hector’s dolphin populations using mitochondrial (mt) DNA and microsatellite genotypes from 438 samples. Results confirmed earlier reports of a single unique mtDNA control region haplotype fixed in the Maui’s dolphin, and provided new evidence of reproductive isolation from Hector’s dolphins (9-locus microsatellite F (ST) = 0.167, P < 0.001). Independent evolutionary trajectories were also supported for Hector’s dolphin populations of the East Coast, West Coast, Te Waewae Bay and Toetoe Bay. Low asymmetrical migration rates were found among several Hector’s dolphin populations and assignment tests identified five Hector’s dolphins likely to have a migrant father from another regional population. There appears to be sufficient step-wise gene flow to maintain genetic diversity within the East and West Coasts; however, the two local South Coast populations exhibited a high degree of differentiation given their close proximity (similar to 100 km). To maintain the evolutionary potential and long-term survival of both subspecies, genetic monitoring and conservation management must focus on maintaining corridors to preserve gene flow and prevent further population fragmentation and loss of genetic diversity, in addition to maintaining local population abundances. | 919 | no | no good fitness measure, N pop differnt in GD? | NA | no | no |
| 920 | 2017 | 43013 | Gross, A; Hosoya, T; Queloz, V | 2014 | Population structure of the invasive forest pathogen Hymenoscyphus pseudoalbidus | Understanding the genetic diversity and structure of invasive pathogens in source and in introduced areas is crucial to the revelation of hidden biological features of an organism, to the reconstruction of the course of invasions and to the establishment of effective control measures. Hymenoscyphus pseudoalbidus (anamorph: Chalara fraxinea) is an invasive and highly destructive fungal pathogen found on common ash Fraxinus excelsior in Europe and is native to East Asia. To gain insights into its dispersal mechanisms and history of invasion, we used microsatellite markers and characterized the genetic structure and diversity of H.pseudoalbidus populations at three spatial levels: (i) between Europe and Japan, (ii) in Europe and (iii) at the epidemic’s front in Switzerland. Phylogenetic and network analysis demonstrated that individuals from both regions are conspecific. However, populations from Japan harboured a higher genetic diversity and were genetically differentiated from European ones. No evident population structure was found among the 1208 European strains using Bayesian and multivariate clustering analysis. Only the distribution of genetic diversity in space, pairwise population differentiation (GST) and the spatial analysis of principal components revealed a faint geographical pattern around Europe. A significant allele deficiency in most European populations pointed to a recent genetic bottleneck, whereas no pattern of isolation by distance was found. Our data suggest that H.pseudoalbidus was introduced just once by at least two individuals. The potential source region of H.pseudoalbidus is vast, and further investigations are required for a more accurate localization of the source population. | 920 | no | gd of pathogen | NA | no | no |
| 921 | 2017 | 43013 | Wilson, PJ; Grewal, S; Rodgers, A; Rempel, R; Saquet, J; Hristienko, H; Burrows, F; Peterson, R; White, BN | 2003 | Genetic variation and population structure of moose (Alces alces) at neutral and functional DNA loci | Genetic variation was examined for moose (Alces alces) from Riding Mountain, Isle Royale, and Pukaskwa national parks; northwestern, Nipigon, northeastern, and central Ontario; New Brunswick; and Newfoundland. The national parks were identified as maintaining potentially different local selection pressures due to the absence of hunting and the presence or absence of the parasite Parelaphostrongylus tenuis. Genetic variation was estimated using neutral DNA markers, assessed by multilocus DNA fingerprinting and five microsatellite loci, and the functional antigen binding region (ARS) (exon 2) of the major histocompatibility complex (MHC) gene DRB. There was discordance in the allelic diversity observed at the neutral loci compared with the MHC DRB locus in a number of populations. Ontario populations demonstrated higher levels of variability at the neutral loci and relatively low levels at the DRB locus. Conversely, the Isle Royale population has the lowest genetic variability, consistent with a historic small founding event, at the neutral DNA markers and relatively high variability at the MHC gene. Relatively high levels of genetic variation at the DRB locus were observed in protected park populations concomitant with the absence of white-tailed deer (Odocoileus virginianus) or the parasite P. tenuis and an absence of hunting. Gene flow was observed among the neighboring geographic regions within Ontario, including Pukaskwa National Park, with evidence of isolation-by-distance among more distant regions within Ontario. The discordant patterns between DNA markers suggest that neutral DNA markers may not accurately reflect adaptive variation present at functional loci. | 921 | no | no good fitness measure | NA | no | no |
| 922 | 2017 | 43013 | Jones, CM; Brown, MJF | 2014 | Parasites and genetic diversity in an invasive bumblebee | Biological invasions are facilitated by the global transportation of species and climate change. Given that invasions may cause ecological and economic damage and pose a major threat to biodiversity, understanding the mechanisms behind invasion success is essential. Both the release of non-native populations from natural enemies, such as parasites, and the genetic diversity of these populations may play key roles in their invasion success. We investigated the roles of parasite communities, through enemy release and parasite acquisition, and genetic diversity in the invasion success of the non-native bumblebee, Bombus hypnorum, in the United Kingdom. The invasive B.hypnorum had higher parasite prevalence than most, or all native congeners for two high-impact parasites, probably due to higher susceptibility and parasite acquisition. Consequently parasites had a higher impact on B.hypnorum queens’ survival and colony-founding success than on native species. Bombus hypnorum also had lower functional genetic diversity at the sex-determining locus than native species. Higher parasite prevalence and lower genetic diversity have not prevented the rapid invasion of the United Kingdom by B.hypnorum. These data may inform our understanding of similar invasions by commercial bumblebees around the world. This study suggests that concerns about parasite impacts on the small founding populations common to re-introduction and translocation programs may be less important than currently believed. | 922 | no | comparing species | NA | no | no |
| 923 | 2017 | 43013 | Palomares-Rius, JE; Tsai, IJ; Karim, N; Akiba, M; Kato, T; Maruyama, H; Takeuchi, Y; Kikuchi, T | 2015 | Genome-wide variation in the pinewood nematode Bursaphelenchus xylophilus and its relationship with pathogenic traits | Background: Bursaphelenchus xylophilus is an emerging pathogenic nematode that is responsible for a devastating epidemic of pine wilt disease across Asia and Europe. In this study, we report the first genome-wide variation analysis of the nematode with an aim to obtain a full picture of its diversity. Methods: We sequenced six key B. xylophilus strains using Illumina HiSeq sequencer. All the strains were isolated in Japan and have been widely used in previous studies. Detection of genomic variations were done by mapping the reads to the reference genome. Results: Over 3 Mb of genetic variations, accounting for 4.1 % of the total genome, were detected as single nucleotide polymorphisms or small indels, suggesting multiple introductions of this invaded species from its native area into the country. The high level of genetic diversity of the pine wood nematode was related to its pathogenicity and ecological trait differences. Moreover, we identified a gene set affected by genomic variation, and functional annotation of those genes indicated that some of them had potential roles in pathogenesis. Conclusions: This study provides an important resource for understanding the population structure, pathogenicity and evolutionary ecology of the nematode, and further analysis based on this study with geographically diverse B. xylophilus populations will greatly accelerate our understanding of the complex evolutionary/epidemic history of this emerging pathogen. | 923 | no | gd of parasite | NA | no | no |
| 924 | 2017 | 43013 | PAMILO, P | 1991 | LIFE-SPAN OF QUEENS IN THE ANT FORMICA-EXSECTA | The ant Formica exsecta commonly has two types of colonies: either polygynous and polydomous or monogynous and monodomous. The longevity of queens was studied in monogynous colonies in southern Finland by indirect methods using genetic markers; these data were also used to estimate the number of matings and queen replacement. The average genetic relatedness among worker nest mates was 0.72. Taking inbreeding into account (the inbreeding coefficient was F = 0.16), this value agrees with the assumption that 40 % of the queens mated with one male and 60 % with two males. The distribution of genotypes within colonies remained stable in successive years, indicating that queen replacement did not occur or was extremely rare. This means that the life span of nests reflects directly the life span of the queens. Eleven of the 16 nests found in 1979 were still alive ten years later. This corresponds to an annual mortality of 3.7 % and a mean life span of 27 years. A total of 57 colonies were mapped in the population over a period of ten years. Averaging over the years, the annual mortality was estimated to be 4.9 %. This represents a mean life span of 20 years if mortality was independent of age. | 924 | probably yes | ok gd measurement and pop level comp? | NA | no | no |
| 925 | 2017 | 43013 | Gordo, I; Gomes, MGM; Reis, DG; Campos, PRA | 2009 | Genetic Diversity in the SIR Model of Pathogen Evolution | We introduce a model for assessing the levels and patterns of genetic diversity in pathogen populations, whose epidemiology follows a susceptible-infected-recovered model (SIR). We model the population of pathogens as a metapopulation composed of subpopulations (infected hosts), where pathogens replicate and mutate. Hosts transmit pathogens to uninfected hosts. We show that the level of pathogen variation is well predicted by analytical expressions, such that pathogen neutral molecular variation is bounded by the level of infection and increases with the duration of infection. We then introduce selection in the model and study the invasion probability of a new pathogenic strain whose fitness (R(0)(1+s)) is higher than the fitness of the resident strain (R0). We show that this invasion probability is given by the relative increment in R(0) of the new pathogen (s). By analyzing the patterns of genetic diversity in this framework, we identify the molecular signatures during the replacement and compare these with those observed in sequences of influenza A. | 925 | no | gd of pathogen | NA | no | no |
| 926 | 2017 | 43013 | Puurtinen, M; Knott, KE; Suonpaa, S; van Ooik, T; Kaitala, V | 2004 | Genetic variability and drift load in populations of an aquatic snail | Population genetic theory predicts that in small populations, random genetic drift will fix and accumulate slightly deleterious mutations, resulting in reduced reproductive output. This genetic load due to random drift (i.e., drift load) can increase the extinction risk of small populations. We studied the relationship between genetic variability (indicator of past population size) and reproductive output in eight isolated, natural populations of the hermaphroditic snail Lymnaea stagnalis. In a common laboratory environment, snails from populations with the lowest genetic variability mature slower and have lower fecundity than snails from genetically more variable populations. This result suggests that past small population size has resulted in increased drift load, as predicted. The relationship between genetic variability and reproductive output is independent of the amount of nonrandom mating within populations. However, reproductive output and the current density of snails in the populations were not correlated. Instead, data from the natural populations suggest that trematode parasites may determine, at least in part, population densities of the snails. | 926 | no | no good fitness measure | NA | no | no |
| 927 | 2017 | 43013 | MacLeod, A; Tait, A; Turner, CMR | 2001 | The population genetics of Trypanosoma brucei and the origin of human infectivity | The African trypanosome, Trypanosoma brucei, is a zoonotic parasite transmitted by tsetse flies. Two of the three subspecies, T brucei gambiense and T b. rhodesiense, cause sleeping sickness in humans whereas the third subspecies, T b. brucei, is not infective to humans. We propose that the key to understanding genetic relationships within this species is the analysis of gene flow to determine the importance of genetic exchange within populations and the relatedness of populations. T. brucei parasites undergo genetic exchange when present in infections of mixed genotypes in tsetse flies in the laboratory although this is not an obligatory process. Infections of mixed genotype are surprisingly common in field isolates from tsetse flies such that there is opportunity for genetic exchange to occur. Population genetic analyses, taking into account geographical and host species of origin, show, that genetic exchange occurs sufficiently frequently in the field to be an important determinant of genetic diversity I except,where particular clones have acquired the ability to infect humans. Thus, T brucei populations have an ‘epidemic’ genetic structure, but the better-characterized human-infective populations have a ‘clonal’ structure. Remarkably, the ability to infect humans appears to have arisen on multiple occasions in different geographical locations in sub-Saharan Africa. Our data indicate that the classical subspecies terminology for T. brucei is genetically inappropriate. It is an implicit assumption in most infectious disease biology that when a zoonotic pathogen acquires the capability to infect humans, it does so once and then spreads through the human population from that single-source event. For at least one major pathogen in tropical medicine, T. brucei, this assumption is invalid. | 927 | no | gd of parasite | NA | no | no |
| 928 | 2017 | 43013 | Byrne, PG; Roberts, JD | 2000 | Does multiple paternity improve fitness of the frog Crinia georgiana? | In the Australian myobatrachid frog Crinia georgiana simultaneous polyandry occurs in about half of all matings, which leads to multiple paternity, but reduced fertilization success and occasional female mortality. Multiple paternity may provide benefits to females that compensate for these costs, for example, through enhanced genetic diversity of a clutch. In nature, embryos and tadpoles of C. georgiana develop in shallow, temporary pools and may be exposed to fluctuating water levels and the risk of desiccation between rain events. Fertilization by genetically diverse sires may act as a bet hedge against these conditions. To evaluate this hypothesis, females were artificially mated with one or two males in the held and eggs and larvae reared in the laboratory under constant or fluctuating developmental conditions. Experiment 1 exposed embryos from single- and multiple-paternity clutches to conditions where eggs were completely covered during development or eggs sat in air on a moist substrate. Experiment 2 exposed freshly hatched larvae from single- and multiple-paternity clutches to constant wet conditions, where larvae were completely covered, or fluctuating wet conditions, where larvae ranged from being completely submersed to partially exposed over a 13- day cycle. We measured mean performance and best performance as alternate measures of genetic benefits. There were no effects of paternity on percent survival, to hatching, time to hatching, body size at hatching, percent survival to metamorphosis, time to metamorphosis, or body size at metamorphosis. We also analyzed variance within clutches as a measure of genetic diversity. Again there were no predictable effects of multiple paternity. Polyandry does not appear to provide any genetic benefits that compensate for the high costs of polyandry in this species. | 928 | probably no | no pop level? | NA | no | yes |
| 929 | 2017 | 43013 | Chang, HH; Dordel, J; Donker, T; Worby, CJ; Feil, EJ; Hanage, WP; Bentley, SD; Huang, SS; Lipsitch, M | 2016 | Identifying the effect of patient sharing on between-hospital genetic differentiation of methicillin-resistant Staphylococcus aureus | Background: Methicillin-resistant Staphylococcus aureus (MRSA) is one of the most common healthcare-associated pathogens. To examine the role of inter-hospital patient sharing on MRSA transmission, a previous study collected 2,214 samples from 30 hospitals in Orange County, California and showed by spa typing that genetic differentiation decreased significantly with increased patient sharing. In the current study, we focused on the 986 samples with spa type t008 from the same population. Methods: We used genome sequencing to determine the effect of patient sharing on genetic differentiation between hospitals. Genetic differentiation was measured by between-hospital genetic diversity, F-ST, and the proportion of nearly identical isolates between hospitals. Results: Surprisingly, we found very similar genetic diversity within and between hospitals, and no significant association between patient sharing and genetic differentiation measured by F-ST. However, in contrast to F-ST, there was a significant association between patient sharing and the proportion of nearly identical isolates between hospitals. We propose that the proportion of nearly identical isolates is more powerful at determining transmission dynamics than traditional estimators of genetic differentiation (F-ST) when gene flow between populations is high, since it is more responsive to recent transmission events. Our hypothesis was supported by the results from coalescent simulations. Conclusions: Our results suggested that there was a high level of gene flow between hospitals facilitated by patient sharing, and that the proportion of nearly identical isolates is more sensitive to population structure than F-ST when gene flow is high. | 929 | no | NA | NA | no | no |
| 930 | 2017 | 43013 | de Cara, MAR; Villanueva, B; Toro, MA; Fernandez, J | 2013 | Using genomic tools to maintain diversity and fitness in conservation programmes | Conservation programmes aim at maximizing the survival probability of populations, by minimizing the loss of genetic diversity, which allows populations to adapt to changes, and controlling inbreeding increases. The best known strategy to achieve these goals is optimizing the contributions of the parents to minimize global coancestry in their offspring. Results on neutral scenarios showed that management based on molecular coancestry could maintain more diversity than management based on genealogical coancestry when a large number of markers were available. However, if the population has deleterious mutations, managing using optimal contributions can lead to a decrease in fitness, especially using molecular coancestry, because both beneficial and harmful alleles are maintained, compromising the long-term viability of the population. We introduce here two strategies to avoid this problem: The first one uses molecular coancestry calculated removing markers with low minor allele frequencies, as they could be linked to selected loci. The second one uses a coancestry based on segments of identity by descent, which measures the proportion of genome segments shared by two individuals because of a common ancestor. We compare these strategies under two contrasting mutational models of fitness effects, one assuming many mutations of small effect and another with few mutations of large effect. Using markers at intermediate frequencies maintains a larger fitness than using all markers, but leads to maintaining less diversity. Using the segment-based coancestry provides a compromise solution between maintaining diversity and fitness, especially when the population has some inbreeding load. | 930 | no | no good fitness measure, N pop differnt in GD? | NA | no | no |
| 931 | 2017 | 43013 | Zein-Eddine, R; Djuikwo-Teukeng, FF; Al-Jawhari, M; Senghor, B; Huyse, T; Dreyfuss, G | 2014 | Phylogeny of seven Bulinus species originating from endemic areas in three African countries, in relation to the human blood fluke Schistosoma haematobium | Background: Snails species belonging to the genus Bulinus (Planorbidae) serve as intermediate host for flukes belonging to the genus Schistosoma (Digenea, Platyhelminthes). Despite its importance in the transmission of these parasites, the evolutionary history of this genus is still obscure. In the present study, we used the partial mitochondrial cytochrome oxidase subunit I (cox1) gene, and the nuclear ribosomal ITS, 18S and 28S genes to investigate the haplotype diversity and phylogeny of seven Bulinus species originating from three endemic countries in Africa (Cameroon, Senegal and Egypt). Results: The cox1 region showed much more variation than the ribosomal markers within Bulinus sequences. High levels of genetic diversity were detected at all loci in the seven studied species, with clear segregation between individuals and appearance of different haplotypes, even within same species from the same locality. Sequences clustered into two lineages; (A) groups Bulinus truncatus, B. tropicus, B. globosus and B. umbilicatus; while (B) groups B. forskalii, B. senegalensis and B. camerunensis. Interesting patterns emerge regarding schistosome susceptibility: Bulinus species with lower genetic diversity are predicted to have higher infection prevalence than those with greater diversity in host susceptibility. Conclusion: The results reported in this study are very important since a detailed understanding of the population genetic structure of Bulinus is essential to understand the epidemiology of many schistosome parasites. | 931 | no | no good fitness measure, N pop differnt in GD? | NA | no | no |
| 932 | 2017 | 43013 | Martinez-Ibarra, JA; Barcenas-Ortega, NM; Nogueda-Torres, B; Ramirez-Vallejo, P; Rodriguez-Lopez, MH | 2003 | Genetic variability among populations of Triatoma longipennis, vector of Chagas disease in Western Mexico | Enzyme polymorphism in Triatoma longipennis Usinger, one of the most important vectors of Chagas disease in Mexico, was analized using starch gel electrophoresis. Seven geographic locations were sampled in order to determine gene flow among populations and to characterize intraspecific differences. Of 18 enzymes assayed, three (ES, MDH, and ME) were successfully resolved and then used to score genetic variation. ES was used to differentiate between populations. Both polymorphism and heterozygosity indicated genetic variability in the populations studied. Gene flow between some populations was found to be high. This finding and the low genetic distance between populations indicate similarity among most of the nearby localities, suggesting an important epidemiological threat. | 932 | no | no good fitness measure | NA | no | no |
| 933 | 2017 | 43013 | Zhu, Y; Wan, QH; Yu, B; Ge, YF; Fang, SG | 2013 | Patterns of genetic differentiation at MHC class I genes and microsatellites identify conservation units in the giant panda | Background: Evaluating patterns of genetic variation is important to identify conservation units (i.e., evolutionarily significant units [ESUs], management units [MUs], and adaptive units [AUs]) in endangered species. While neutral markers could be used to infer population history, their application in the estimation of adaptive variation is limited. The capacity to adapt to various environments is vital for the long-term survival of endangered species. Hence, analysis of adaptive loci, such as the major histocompatibility complex (MHC) genes, is critical for conservation genetics studies. Here, we investigated 4 classical MHC class I genes (Aime-C, Aime-F, Aime-I, and Aime-L) and 8 microsatellites to infer patterns of genetic variation in the giant panda (Ailuropoda melanoleuca) and to further define conservation units. Results: Overall, we identified 24 haplotypes (9 for Aime-C, 1 for Aime-F, 7 for Aime-I, and 7 for Aime-L) from 218 individuals obtained from 6 populations of giant panda. We found that the Xiaoxiangling population had the highest genetic variation at microsatellites among the 6 giant panda populations and higher genetic variation at Aime-MHC class I genes than other larger populations (Qinling, Qionglai, and Minshan populations). Differentiation index (FST)-based phylogenetic and Bayesian clustering analyses for Aime MHC I and microsatellite loci both supported that most populations were highly differentiated. The Qinling population was the most genetically differentiated. Conclusions: The giant panda showed a relatively higher level of genetic diversity at MHC class I genes compared with endangered felids. Using all of the loci, we found that the 6 giant panda populations fell into 2 ESUs: Qinling and non-Qinling populations. We defined 3 MUs based on microsatellites: Qinling, Minshan-Qionglai, and Daxiangling-Xiaoxiangling-Liangshan. We also recommended 3 possible AUs based on MHC loci: Qinling, Minshan Qionglai, and Daxiangling-Xiaoxiangling-Liangshan. Furthermore, we recommend that a captive breeding program be considered for the Qinling panda population. | 933 | no | no good fitness measure | NA | no | no |
| 934 | 2017 | 43013 | Zeisset, I; Beebee, TJC | 2014 | Drift Rather than Selection Dominates MHC Class II Allelic Diversity Patterns at the Biogeographical Range Scale in Natterjack Toads Bufo calamita | Study of major histocompatibility complex (MHC) loci has gained great popularity in recent years, partly due to their function in protecting vertebrates from infections. This is of particular interest in amphibians on account of major threats many species face from emergent diseases such as chytridiomycosis. In this study we compare levels of diversity in an expressed MHC class II locus with neutral genetic diversity at microsatellite loci in natterjack toad (Bufo (Epidalea) calamita) populations across the whole of the species’ biogeographical range. Variation at both classes of loci was high in the glacial refugium areas (REF) and much lower in postglacial expansion areas (PGE), especially in range edge populations. Although there was clear evidence that the MHC locus was influenced by positive selection in the past, congruence with the neutral markers suggested that historical demographic events were the main force shaping MHC variation in the PGE area. Both neutral and adaptive genetic variation declined with distance from glacial refugia. Nevertheless, there were also some indications from differential isolation by distance and allele abundance patterns that weak effects of selection have been superimposed on the main drift effect in the PGE zone. | 934 | no | no good fitness measure | NA | no | no |
| 935 | 2017 | 43013 | Kang, ES; Ha, SM; Ko, HC; Yu, HJ; Chae, WB | 2016 | Reproductive traits and molecular evidence related to the global distribution of cultivated radish (Raphanus sativus L.) | Reproductive characteristics frequently vary geographically, and reproductive success is critical for survival and local adaption in cultivated species. To document such variation in radish (Raphanus sativus L.), we examined reproductive traits and neutral genetic markers in 64 wild and landrace accessions varying in geographic origin. Flowering time tended to increase with latitude of origin, with southeastern Asian accessions (the lowest latitude) flowering earliest. Accessions from eastern Asia, which encompasses the greatest latitudinal range (18A degrees and 54A degrees N) of the regions we examined, exhibited the greatest variation in flowering time, clustering into a single group according to neutral genetic markers. Cluster analysis of genetic data divided the radish accessions into European and Asian groups. These groups were further subdivided into six, and two subgroups of Europe could be distinguished by root skin color. Although the accessions tended to cluster by geographic origin, those from central, southern, and western Asia clustered with both European and Asian accessions. Our results suggest that artificial selection of flowering time as well as root skin color has played important roles in local adaptation and increases in the genetic diversity of radish landraces in different geographic regions. | 935 | no | no good fitness measure, N pop differnt in GD? | NA | no | no |
| 936 | 2017 | 43013 | Yang, RC | 2017 | Genome-wide estimation of heritability and its functional components for flowering, defense, ionomics, and developmental traits in a geographically diverse population of Arabidopsis thaliana | Narrow-sense heritability (portion of the total phenotypic variation attributable to additive genetic effect, h(2)) is a critical parameter in plant breeding and genetics, but its estimation is difficult for populations with unknown pedigree information. This study applied a marker-based linear mixed model (LMM) analysis to estimate narrow-sense heritability and its seven functional components corresponding to SNPs in coding and noncoding regions for each of 107 flowering, defense, ionomics, and developmental traits in an Arabidopsis (Arabidopsis thaliana) population of 199 inbred lines with unknown genetic relatedness. Genetic relationship matrix (GRM) based on 214 051 SNPs and component GRMs based on seven subsets of SNPs were computed for LMM estimation of h(2) and functional components contributing to h(2), respectively. The h(2) estimates for flowering traits were higher than those for defense, ionomics, and developmental traits, supporting a general view that the fitness-related traits have lower heritabilities than other traits. The function component owing to SNPs in coding (exon) regions was the least contributor to h(2). Our LMM analysis provides an opportunity to gain a comprehensive view on heritability and its functional components for populations with unknown structure but with genome-wide DNA markers. | 936 | no | no good fitness measure, N pop differnt in GD? | NA | no | no |
| 937 | 2017 | 43013 | Julca, I; Droby, S; Sela, N; Marcet-Houben, M; Gabaldon, T | 2016 | Contrasting Genomic Diversity in Two Closely Related Postharvest Pathogens: Penicillium digitatum and Penicillium expansum | Penicillium digitatum and Penicillium expansum are two closely related fungal plant pathogens causing green and blue mold in harvested fruit, respectively. The two species differ in their host specificity, being P. digitatum restricted to citrus fruits and P. expansum able to infect a wide range of fruits after harvest. Although host-specific Penicillium species have been found to have a smaller gene content, it is so far unclear whether these different host specificities impact genome variation at the intraspecific level. Here we assessed genome variation across four P. digitatum and seven P. expansum isolates from geographically distant regions. Our results show very high similarity (average 0.06 SNPs [single nucleotide polymorphism] per kb) between globally distributed isolates of P. digitatum pointing to a recent expansion of a single lineage. This low level of genetic variation found in our samples contrasts with the higher genetic variability observed in the similarly distributed P. expansum isolates (2.44 SNPs per kb). Patterns of polymorphism in P. expansum indicate that recombination exists between genetically diverged strains. Consistent with the existence of sexual recombination and heterothallism, which was unknown for this species, we identified the two alternative mating types in different P. expansum isolates. Patterns of polymorphism in P. digitatum indicate a recent clonal population expansion of a single lineage that has reached worldwide distribution. We suggest that the contrasting patterns of genomic variation between the two species reflect underlying differences in population dynamics related with host specificities and related agricultural practices. It should be noted, however, that this results should be confirmed with a larger sampling of strains, as new strains may broaden the diversity so far found in P. digitatum. | 937 | no | gd of pathogen | NA | no | no |
| 938 | 2017 | 43013 | Acevedo-Whitehouse, K; Spraker, TR; Lyons, E; Melin, SR; Gulland, F; Delong, RL; Amos, W | 2006 | Contrasting effects of heterozygosity on survival and hookworm resistance in California sea lion pups | Low genetic heterozygosity is associated with loss of fitness in many natural populations. However, it remains unclear whether the mechanism is related to general (i.e. inbreeding) or local effects, in particular from a subset of loci lying close to genes under balancing selection. Here we analyse involving heterozygosity-fitness correlations on neonatal survival of California sea lions and on susceptibility to hookworm (Uncinaria spp.) infection, the single most important cause of pup mortality. We show that regardless of differences in hookworm burden, homozygosity is a key predictor of hookworm-related lesions, with no single locus contributing disproportionately. Conversely, the subsequent occurrence of anaemia due to blood loss in infected pups is overwhelmingly associated with homozygosity at one particular locus, all other loci showing no pattern. Our results suggest contrasting genetic mechanisms underlying two pathologies related to the same pathogen. First, relatively inbred pups are less able to expel hookworms and prevent their attachment to the intestinal mucosa, possibly due to a weakened immune response. In contrast, infected pups that are homozygous for a gene near to microsatellite Hg4.2 are strongly predisposed to anaemia. As yet, this gene is unknown, but could plausibly be involved in the blood-coagulation cascade. Taken together, these results suggest that pathogenic burden alone may not be the main factor regulating pathogen-related mortality in natural populations. Our study could have important implications for the conservation of small, isolated or threatened populations, particularly when they are at a risk of facing pathogenic challenges. | 938 | no | No GD diff between pop | NA | no | no |
| 939 | 2017 | 43013 | Lesbarreres, D; Primmer, CR; Laurila, A; Merila, J | 2005 | Environmental and population dependency of genetic variability-fitness correlations in Rana temporaria | Considerable effort has been invested in studying the relationship between fitness and genetic variability. While evidence exists both for and against positive genetic variability-fitness correlations (GFC), the possible environment and population-dependency of GFCs has seldom been tested. We investigated GFCs in common frog (Rana temporaria) tadpoles reared under different temperatures and feeding regimes in four replicate populations. Genetic variability in eight microsatellite loci in 238 parents was used to estimate heterozygosity (H) and mean expected d(2) in 158-sibships (4515 offspring). Generalized linear mixed model analyses of offspring fitness traits (survival to metamorphosis, developmental and growth rate) revealed that offspring survival probability was positively correlated with H, and that relationships were similar in all four populations tested. However, significant interaction between other genetic variability measures (d(2), relatedness) and treatment conditions indicated that GFCs were detectable in some, but not in all environments. Interestingly, GFCs between survival and both heterozygosity and relatedness were most pronounced in stressful environments (i.e. limited food). Developmental and growth rates were significantly associated with d(2) but less with H and relatedness. Furthermore, many of these GFCs were population-specific. These results suggest - in line with the contention that expression of inbreeding depression can be environment dependent - that GFCs can also be highly sensitive to the environmental conditions under which they are measured. The results further suggest that the observed positive correlation between H and survival probability is likely to be explainable by the ‘general’, rather than by the ‘local’ or ‘direct’ effect hypotheses. | 939 | yes | ok gd measurement? | not pop level gd | no | no |
| 940 | 2017 | 43013 | Noble, CW; Bono, JM; Pigage, HK; Hale, DW; Pigage, JC | 2016 | FINE-SCALE GENETIC STRUCTURE IN FEMALE MULE DEER (ODOCOILEUS HEMIONUS) | Fine-scale genetic structure in animal populations can have important consequences for evolutionary processes and can influence conservation and management decisions. Cervids often live in matrilineal social groups, and this spatial grouping can create fine-scale genetic structure among females. We used DNA and radio-location data at Fort Carson Military Reservation in south central Colorado, USA, to determine whether female mule deer (Odocoileus hemionus) living in close proximity were more likely to be related. Spatial data were obtained over an 18-month period using data transmitted from GPS collars. Average positions for each animal were correlated with relatedness estimates calculated using 7 microsatellite loci. We found significant spatial autocorrelation for females at distances <= 1000 m, which suggests that females were frequently philopatric. In addition, females appeared to occasionally disperse over relatively longer distances, as we found evidence of related females separated by distances up to 28,000 m. Fine-scale genetic structure may have important implications for managing chronic wasting disease, which is relatively common at this site. | 940 | no | no good fitness measure, N pop differnt in GD? | NA | no | no |
| 941 | 2017 | 43013 | De La Fuente, GN; Murray, SC; Isakeit, T; Park, YS; Yan, Y; Warburton, ML; Kolomiets, MV | 2013 | Characterization of Genetic Diversity and Linkage Disequilibrium of ZmLOX4 and ZmLOX5 Loci in Maize | Maize (Zea mays L.) lipoxygenases (ZmLOXs) are well recognized as important players in plant defense against pathogens, especially in cross kingdom lipid communication with pathogenic fungi. This study is among the first to investigate genetic diversity at important gene paralogs ZmLOX4 and ZmLOX5. Sequencing of these genes in 400 diverse maize lines showed little genetic diversity and low linkage disequilibrium in the two genes. Importantly, we identified one inbred line in which ZmLOX5 has a disrupted open reading frame, a line missing ZmLOX5, and five lines with a duplication of ZmLOX5. Tajima’s D test suggests that both ZmLOX4 and ZmLOX5 have been under neutral selection. Further investigation of haplotype data revealed that within the ZmLOX family members only ZmLOX12, a monocot specific ZmLOX, showed strong linkage disequilibrium that extends further than expected in maize. Linkage disequilibrium patterns at these loci of interest are crucial for future candidate gene association mapping studies. ZmLOX4 and ZmLOX5 mutations and copy number variants are under further investigation for crop improvement. | 941 | no | no good fitness measure, N pop differnt in GD? | NA | no | no |
| 942 | 2017 | 43013 | Baidu-Forson, JJ; Hodgkin, T; Jones, M | 2012 | Introduction to special issue on agricultural biodiversity, ecosystems and environment linkages in Africa | “This paper introduces the contents of a special issue of Agriculture, Ecosystems and Environment dedicated to”“Agrobiodiversity & Africa”“. Placed within the context of the scientific literature on agricultural biodiversity, its contribution to sustainable production and its importance in Africa, this special issue starts off with background articles which reviewed major agroecosystems in Africa, descriptions of their biodiversity resources, management, limitations and concerns. The follow-up scientific papers covered major components of biodiversity for agriculture namely soil biota, species diversity and germplasm diversity. The soil biota papers report research and findings on arbuscular mycorrhizal fungi in the rhizosphere, termite and bacterial communities and evidence on the influence of their diversity on crop production. The crop species diversity papers provide evidence on the role of the diversity in influencing soil microbial properties as well as managing pest and diseases in crop production. Scientific evidence on the role and interactions of germplasm diversity and provenance with the environment are also presented. (c) 2012 Elsevier B.V. All rights reserved.” | 942 | no | no good fitness measure, N pop differnt in GD? | NA | no | no |
| 943 | 2017 | 43013 | Auge, H; Neuffer, B; Erlinghagen, F; Grupe, R; Brandl, R | 2001 | Demographic and random amplified polymorphic DNA analyses reveal high levels of genetic diversity in a clonal violet | We performed demographic and molecular investigations on woodland populations of the clonal herb Viola riviniana in central Germany. We investigated the pattern of seedling recruitment, the amount of genotypic (clonal) variation and the partitioning of genetic variation among and within populations. Our demographic study was carried out in six violet populations of different ages and habitat conditions. Ii revealed that repeated seedling recruitment takes place in all of these populations, and that clonal propagation is accompanied by high ramet mortality. Our molecular investigations were performed on a subset of three of these six violet populations. Random amplified polymorphic DNA analyses using six primers yielded 45 scorable bands that were used to identify multilocus genotypes, i.e. putative clones. Consistent with our demographic results and independent of population age, we found a large genotypic diversity with a mean proportion of distinguishable genotypes of 0.93 and a mean Simpson’s diversity index of 0.99. Using AMOVA we found a strong genetic differentiation among these violet populations with a Phi (ST), value of 0.41. We suggest that a high selfing rate, limited gene flow due to short seed dispersal distances and drift due to founder effects are responsible for this pattern. Although Viola riviniana is a clonal plant, traits associated with sexual reproduction rather than clonality per se are moulding the pattern of genetic variation in this species. | 943 | probably yes | ok gd and fitness measurement? | sent email, no fitness measure from pop from author | no | no |
| 944 | 2017 | 43013 | Benson, JF; Hostetler, JA; Onorato, DP; Johnson, WE; Roelke, ME; O’Brien, SJ; Jansen, D; Oli, MK | 2011 | Intentional genetic introgression influences survival of adults and subadults in a small, inbred felid population | 1. Inbreeding and low genetic diversity can cause reductions in individual fitness and increase extinction risk in animal populations. Intentional introgression, achieved by releasing genetically diverse individuals into inbred populations, has been used as a conservation tool to improve demographic performance in endangered populations. 2. By the 1980s, Florida panthers (Puma concolor coryi) had been reduced to a small, inbred population that appeared to be on the brink of extinction. In 1995, female pumas from Texas (P. c. stanleyana) were released in occupied panther range as part of an intentional introgression programme to restore genetic variability and improve demographic performance of panthers. 3. We used 25 years (1981-2006) of continuous radiotelemetry and genetic data to estimate and model subadult and adult panther survival and cause-specific mortality to provide rigorous sex and age class-specific survival estimates and evaluate the effect of the introgression programme on these parameters. 4. Genetic ancestry influenced annual survival of subadults and adults after introgression, as F-1 generation admixed panthers ((s) over cap = 0 98) survived better than pre-introgression type panthers ((s) over cap = 0 77) and other admixed individuals ((s) over cap = 0 82). Furthermore, heterozygosity was higher for admixed panthers relative to pre-introgression type panthers and positively influenced survival. 5. Our results are consistent with hybrid vigour; however, extrinsic factors such as low density of males in some areas of panther range may also have contributed to higher survival of F-1 panthers. Regardless, improved survival of F-1 subadults and adults likely contributed to the numerical increase in panthers following introgression, and our results indicate that intentional admixture, achieved here by releasing individuals from another population, appears to have been successful in improving demographic performance in this highly endangered population. | 944 | no | inbreeding | NA | no | no |
| 945 | 2017 | 43013 | Goicoechea, AS; Carnese, FR; Caratini, AL; Avena, S; Salaberry, M; Salzano, FM | 2000 | Demography, genetic diversity, and population relationships among Argentinean Mapuche Indians | Fertility, mortality and migration data from four Mapuche Indian communities located along a 215-km NE-SW linear area in the Province of Rio Negro, Argentina, were collated with genetic information furnished by nine blood group systems and by mtDNA haplogroups. The demographic and genetic data indicated a clear dichotomy, which split the four populations into two groups of two. Differing degrees of non-Indian exchanges was probably the main determining factor for this separation. Total genetic variability was very similar in all groups, and the interpopulational variability accounted for only 10% of the total variability. A low prevalence of the Diego(a) antigen among the Mapuche was confirmed. The fact that significant genetic heterogeneity and population clusters were found in such a small territorial region attests to the sensitivity of demographic and genetic approaches in unraveling human history. | 945 | no | human | NA | no | no |
| 946 | 2017 | 43013 | Wu, JY; Guo, H; Wang, HM; Yi, GH; Zhou, LM; He, XW; Zhang, Y; Xu, JP | 2017 | Multilocus sequence analyses reveal extensive diversity and multiple origins of fluconazole resistance in Candida tropicalis from tropical China | Candida tropicalis is among the most prevalent human pathogenic yeast species, second only to C. albicans in certain geographic regions such as East Asia and Brazil. However, compared to C. albicans, relatively little is known about the patterns of genetic variation in C. tropicalis. This study analyzed the genetic diversity and relationships among isolates of C. tropicalis from the southern Chinese island of Hainan. A total of 116 isolates were obtained from seven geographic regions located across the Island. For each isolate, a total of 2677 bp from six gene loci were sequenced and 79 (2.96%) polymorphic nucleotide sites were found in our sample. Comparisons with strains reported from other parts of the world identified significant novel diversities in Hainan, including an average of six novel sequences (with a range 1 to 14) per locus and 80 novel diploid sequence types. Most of the genetic variation was found within individual strains and there was abundant evidence for gene flow among the seven geographic locations within Hainan. Interestingly, our analyses identified no significant correlation between the diploid sequence types at the six loci and fluconazole susceptibility, consistent with multiple origins of fluconazole resistance in the Hainan population of C. tropicalis. | 946 | no | gd of pathogen | NA | no | no |
| 947 | 2017 | 43013 | Huang, WJ; Li, CX; ; Zhou, Y; Li, PX; Hu, B; ; ; Fang, JQ; Wang, YM | 2006 | High resolution linkage disequilibrium and haplotype maps for the genes in the centromeric region of chromosome 15 in Tibetans and comparisons with Han population | Genetic variations and their functional implications have been one of the focuses in recent genome research. With the release of the HapMap by the International Consortium, and the availability of the ultra-high-volume genotyping platform, it will soon be possible to use genome-wide association approach to identify genetic variations responsible for complex traits/diseases. While the power of this approach is generally agreed, it is a debated issue as to how much population difference should be exploited, and how best it should be applied. To address this issue we have sequenced 7 genes in the centromeric region of chromosome 15, investigated their SNPs, SNP frequencies, tagSNPs, LD structures, and haplotypes in 50 Tibetan subjects, and compared them with those from the Han population. Genetic diversities between the two populations were also quantified. Our results show that the overall genetic variation between the two populations is very little, but there are differences, primarily in allele frequencies, which is a dominating factor for haplotypes and tagSNPs. In general Tibetans have longer LD and less diversity in the region studied. These data provide genetic evidence for the close relationship between the two populations, and support the idea that all populations are fundamentally the same, but also indicate population variations, particularly in allele frequency, should be taken into account in complex traits/diseases analysis. Data obtained in this investigation not only help us understand the genome region, but also provide road maps for variation study in the genes/region in Tibetan population. | 947 | no | human | NA | no | no |
| 948 | 2017 | 43013 | Steinfartz, S; Glaberman, S; Lanterbecq, D; Marquez, C; Rassmann, K; Caccone, A | 2007 | Genetic Impact of a Severe El Nino Event on Galapagos Marine Iguanas (Amblyrhynchus cristatus) | The El Nino-Southern Oscillation (ENSO) is a major source of climatic disturbance, impacting the dynamics of ecosystems worldwide. Recent models predict that human-generated rises in green-house gas levels will cause an increase in the strength and frequency of El Nino warming events in the next several decades, highlighting the need to understand the potential biological consequences of increased ENSO activity. Studies have focused on the ecological and demographic implications of El Nino in a range of organisms, but there have been few systematic attempts to measure the impact of these processes on genetic diversity in populations. Here, we evaluate whether the 1997-1998 El Nino altered the genetic composition of Galapagos marine iguana populations from eleven islands, some of which experienced mortality rates of up to 90% as a result of El Nino warming. Specifically, we measured the temporal variation in microsatellite allele frequencies and mitochondrial DNA diversity (mtDNA) in samples collected before (1991/1993) and after (2004) the El Nino event. Based on microsatellite data, only one island (Marchena) showed signatures of a genetic bottleneck, where the harmonic mean of the effective population size (N(e)) was estimated to be less than 50 individuals during the period between samplings. Substantial decreases in mtDNA variation between time points were observed in populations from just two islands (Marchena and Genovesa). Our results suggests that, for the majority of islands, a single, intense El Nino event did not reduce marine iguana populations to the point where substantial neutral genetic diversity was lost. In the case of Marchena, simultaneous changes to both nuclear and mitochondrial DNA variation may also be the result of a volcanic eruption on the island in 1991. Therefore, studies that seek to evaluate the genetic impact of El Nino must also consider the confounding or potentially synergistic effect of other environmental and biological forces shaping populations. | 948 | no | NA | NA | no | no |
| 949 | 2017 | 43013 | Medri, C; Ruas, EA; Medri, ME; Ruas, CF; Sayhun, S; Medri, PS; Silva, DCG; Bianchini, E; Ruas, PM | 2011 | Genetic diversity and flooding survival in Aegiphila sellowiana (Lamiaceae), a typical tree species from upland riparian forests | Saplings of Aegiphila sellowiana were submitted to flooding and analysis of genetic diversity in order to investigate flooding tolerance as well as its genetic determination. This response is important because it means that some lines could be planted in degraded riparian areas. Leaves were sampled from each plant, and they were submitted to different flooding periods. Mortality of saplings was 40, 80, 50, 53.3, 33.3, and 33.3% in flooding for 15, 18, 25, 50, 80 days, and flooding for 50 days followed by re-aeration for 30 days, respectively. From the total number of flooded plants, 46.7% died in the first seven days of treatment, while 53.3% survived the flooding. The percentage of polymorphic loci (P(p)), Nei’s genetic diversity (H) and the Shannon index (I) were slightly higher for the group that survived the stress of flooding (surviving: P(p) (%) = 67.48, H = 0.184, I = 0.287; not surviving: P(p) (%) = 66.67, H = 0.165, I = 0.261). Analysis of molecular variance showed that 5.88% of the genetic variability was due to the differences between groups of plants surviving and not surviving flooding, while 94.12% was due to genetic differences between individuals within these groups. Similar results were obtained by principal coordinate analysis. Based on these results, we can assume the existence of environment-specific genotypes and the genetic determination of flooding tolerance in A. sellowiana. Thus, some lines of A. sellowiana could be used in the reforestation of riparian habitats, especially in uplands along riverbanks. | 949 | no | no good fitness measure, N pop differnt in GD? | NA | no | no |
| 950 | 2017 | 43013 | Costa, JT; Ross, KG | 2003 | Fitness effects of group merging in a social insect | Animal social groups often consist of non-relatives, a condition that arises in many cases because of group merging. Although indirect fitness contributions are reduced in such groups compared with those in groups composed of close kin, the genetic-heterogeneity hypothesis suggests that these groups may benefit from increased intracolony genetic variation, which may boost group performance through increased task efficiency or parasite resistance. We confirm one prediction of the task-efficiency explanation by demonstrating a genetic basis for task thresholds of socially important behaviours in eastern tent caterpillars. However, we found no evidence that the expanded range of task thresholds in mixed colonies translates into improved individual or colony performance in the field. By contrast, increased group size, a less commonly considered correlate of group mixing, was found to enhance individual fitness through its effects on larval growth. We conclude that fitness benefits offsetting the dilution of relatedness in heterogeneous social groups may often stem from augmented group size rather than increased genotypic diversity. | 950 | no | no good fitness measure | NA | no | no |
| 951 | 2017 | 43013 | Voight, BF; Pritchard, JK | 2005 | Confounding from cryptic relatedness in case-control association studies | “Case-control association studies are widely used in the search for genetic variants that contribute to human diseases. It has long been known that such studies may suffer from high rates of false positives if there is unrecognized population structure. It is perhaps less widely appreciated that so-called”“cryptic relatedness”" (i.e., kinship among the cases or controls that is not known to the investigator) might also potentially inflate the false positive rate. Until now there has been little work to assess how serious this problem is likely to be in practice. In this paper, we develop a formal model of cryptic relatedness, and study its impact on association studies. We provide simple expressions that predict the extent of confounding due to cryptic relatedness. Surprisingly, these expressions are functions of directly observable parameters. Our analytical results show that, for well-designed studies in outbred populations, the degree of confounding due to cryptic relatedness will usually be negligible. However, in contrast, studies where there is a sampling bias toward collecting relatives may indeed suffer from excessive rates of false positives. Furthermore, cryptic relatedness may be a serious concern in founder populations that have grown rapidly and recently from a small size. As an example, we analyze the impact of excess relatedness among cases for six phenotypes measured in the Hutterite population." | 951 | no | no good fitness measure, N pop differnt in GD? | NA | no | no |
| 952 | 2017 | 43013 | Gu, XF; Dong, YH; Yao, HH; Zhou, XL; Qi, XY; Lin, ZH | 2015 | Microsatellite marker analysis reveals the distinction between the north and south groups of hard clam (Meretrix meretrix) in China | Meretrix meretrix is one of the important commercial bivalves in China. A total of 198 individual clams were collected from 5 locations characteristic of the clam’s 5 main natural habitats in China, that is, Shandong, Jiangsu, Fujian, Guangdong, and Guangxi. Ten polymorphic microsatellite markers were selected to examine the genetic diversity and identify genetic differences between the 5 populations. A total of 183 alleles across 10 loci were detected in the individual clams. The observed heterozygosity and expected heterozygosity ranged from 0.197 to 0.7026 and from 0.6264 to 0.9408, respectively. The genetic diversity within samples was high (8.6-11.2 alleles per locus, observed heterozygosity = 0.25-0.875 and expected heterozygosity = 0.6848-0.9259). Most of the genotype distributions significantly deviated from Hardy-Weinberg equilibrium. Genetic structure analysis showed that the 5 populations could be divided into 2 groups, the north and south groups. Neighbor-joining analysis revealed a clear distinction between the north group (Shandong and Jiangsu) and the south group (Fujian, Guangdong, and Guangxi). Locus MM1031 was used to distinguish between groups. Our results can be used for population identification and crossbreeding of Meretrix meretrix. | 952 | no | no good fitness measure | NA | no | no |
| 953 | 2017 | 43013 | Malvezzi, AJ; Murray, CS; Feldheim, KA; DiBattista, JD; Garant, D; Gobler, CJ; Chapman, DD; Baumann, H | 2015 | A quantitative genetic approach to assess the evolutionary potential of a coastal marine fish to ocean acidification | Assessing the potential of marine organisms to adapt genetically to increasing oceanic CO2 levels requires proxies such as heritability of fitness-related traits under ocean acidification (OA). We applied a quantitative genetic method to derive the first heritability estimate of survival under elevated CO2 conditions in a metazoan. Specifically, we reared offspring, selected from a wild coastal fish population (Atlantic silverside, Menidia menidia), at high CO2 conditions (similar to 2300atm) from fertilization to 15days posthatch, which significantly reduced survival compared to controls. Perished and surviving offspring were quantitatively sampled and genotyped along with their parents, using eight polymorphic microsatellite loci, to reconstruct a parent-offspring pedigree and estimate variance components. Genetically related individuals were phenotypically more similar (i.e., survived similarly long at elevated CO2 conditions) than unrelated individuals, which translated into a significantly nonzero heritability (0.20 +/- 0.07). The contribution of maternal effects was surprisingly small (0.05 +/- 0.04) and nonsignificant. Survival among replicates was positively correlated with genetic diversity, particularly with observed heterozygosity. We conclude that early life survival of M.menidia under high CO2 levels has a significant additive genetic component that could elicit an evolutionary response to OA, depending on the strength and direction of future selection. | 953 | maybe | groups or ind? | not pop level gd | yes | no |
| 954 | 2017 | 43013 | Rasic, G; Schama, R; Powell, R; Maciel-de Freitas, R; Endersby-Harshman, NM; Filipovic, I; Sylvestre, G; Maspero, RC; Hoffmann, AA | 2015 | Contrasting genetic structure between mitochondrial and nuclear markers in the dengue fever mosquito from Rio de Janeiro: implications for vector control | Dengue is the most prevalent global arboviral disease that affects over 300million people every year. Brazil has the highest number of dengue cases in the world, with the most severe epidemics in the city of Rio de Janeiro (Rio). The effective control of dengue is critically dependent on the knowledge of population genetic structuring in the primary dengue vector, the mosquito Aedesaegypti. We analyzed mitochondrial and nuclear genomewide single nucleotide polymorphism markers generated via Restriction-site Associated DNA sequencing, as well as traditional microsatellite markers in Ae.aegypti from Rio. We found four divergent mitochondrial lineages and a strong spatial structuring of mitochondrial variation, in contrast to the overall nuclear homogeneity across Rio. Despite a low overall differentiation in the nuclear genome, we detected strong spatial structure for variation in over 20 genes that have a significantly altered expression in response to insecticides, xenobiotics, and pathogens, including the novel biocontrol agent Wolbachia. Our results indicate that high genetic diversity, spatially unconstrained admixing likely mediated by male dispersal, along with locally heterogeneous genetic variation that could affect insecticide resistance and mosquito vectorial capacity, set limits to the effectiveness of measures to control dengue fever in Rio. | 954 | no | NA | NA | no | no |
| 955 | 2017 | 43013 | Poteaux, C; Baubet, E; Kaminski, G; Brandt, S; Dobson, FS; Baudoin, C | 2009 | Socio-genetic structure and mating system of a wild boar population | Wild boars Sus scrofa have a social organization based on female groups that can include several generations of adults and offspring, and are thus likely matrilineal. However, little is known about the degree of relatedness between animals living in such groups or occupying the same core area of spatial activity. Also, polygynous male mating combined with matrilineal female groups can have strong influences on the genetic structure of populations. We used microsatellite genotyping combined with behavioral data to investigate the fine-scale population genetic structure and the mating system of wild boars in a multi-year study at Chateauvillain-Arc-en-Barrois (France). According to spatial genetic autocorrelation, females in spatial proximity were significantly inter-related. However, we found that numerous males contributed to the next generation, even within the same social group. Based on our genetic data and behavioral observations, wild boars in this population appear to have a low level of polygyny associated with matrilineal female groups, and infrequent multiple paternity. Mortality due to hunting may facilitate the breakup of what historically has been a more predominantly polygynous mating system, and likely accelerates the turnover of adults within the matrilineal groups. | 955 | no | no good fitness measure | NA | no | no |
| 956 | 2017 | 43014 | Karns, R; Zhang, G; Sun, GY; Indugula, SR; Cheng, H; Havas-Augustin, D; Novokmet, N; Rudan, D; Durakovic, Z; Missoni, S; Chakraborty, R; Rudan, P; Deka, R | 2012 | Genome-Wide Association of Serum Uric Acid Concentration: Replication of Sequence Variants in an Island Population of the Adriatic Coast of Croatia | A genome-wide association study of serum uric acid (SUA) laevels was performed in a relatively isolated population of European descent from an island of the Adriatic coast of Croatia. The study sample included 532 unrelated and 768 related individuals from 235 pedigrees. Inflation due to relatedness was controlled by using genomic control. Genetic association was assessed with 2,241,249 single nucleotide polymorphisms (SNPs) in 1300 samples after adjusting for age and gender. Our study replicated four previously reported SUA loci (SLC2A9, ABCG2, RREB1, and SLC22A12). The strongest association was found with a SNP in SLC2A9 (rs13129697, P= 2.33 x 10(-19)), which exhibited significant gender-specific effects, 35.76 mu mol/L (P= 2.11 x 10(-19)) in females and 19.58 mu mol/L (P= 5.40 x 10(-5)) in males. Within this region of high linkage disequilibrium, we also detected a strong association with a nonsynonymous SNP, rs16890979 (P= 2.24 x 10(-17)), a putative causal variant for SUA variation. In addition, we identified several novel loci suggestive of association with uric acid levels (SEMA5A, TMEM18, SLC28A2, and ODZ2), although the P-values (P < 5 x 10(-6)) did not reach the threshold of genome-wide significance. Together, these findings provide further confirmation of previously reported uric-acid-related genetic variants and highlight suggestive new loci for additional investigation. | 956 | no | no good fitness measure, N pop differnt in GD? | NA | no | no |
| 957 | 2017 | 43014 | Da Silva, A; Gaillard, JM; Yoccoz, NG; Hewison, AJM; Galan, M; Coulson, T; Allaine, D; Vial, L; Delorme, D; Van Laere, G; Klein, F; Luikart, G | 2009 | HETEROZYGOSITY-FITNESS CORRELATIONS REVEALED BY NEUTRAL AND CANDIDATE GENE MARKERS IN ROE DEER FROM A LONG-TERM STUDY | “Heterozygosity-fitness correlations (HFCs) are increasingly reported but the underlying mechanisms causing HFCs are generally poorly understood. Here, we test for HFCs in roe deer (Capreolus capreolus) using 22 neutral microsatellites widely distributed in the genome and four microsatellites in genes that are potentially under selection. Juvenile survival was used as a proxy for individual fitness in a population that has been intensively studied for 30 years in northeastern France. For 222 juveniles, we computed two measures of genetic diversity: individual heterozygosity (H), and mean d(2) (relatedness of parental genomes). We found a relationship between genetic diversity and fitness both for the 22 neutral markers and two candidate genes: IGF1 (Insulin-like Growth Factor I) and NRAMP (natural resistance-associated macrophage protein). Statistical evidence and the size of genetic effects on juvenile survival were comparable to those reported for early development and cohort variation, suggesting a substantial influence of genetic components on fitness in this roe deer population. For the 22 neutral microsatellites, a correlation with fitness was revealed for mean d(2), but not for H, suggesting a possible outbreeding advantage. This heterosis effect could have been favored by introduction of genetically distant (Hungarian) roe deer to the population in recent times and, possibly, by the structuring of the population into distinct clans. The locus-specific correlations with fitness may be driven by growth rate advantages and resistance to diseases known to exist in the studied population. Our analyses of neutral and candidate gene markers both suggest that the observed HFCs are likely mainly due to linkage with dominant or overdominant loci that affect fitness (”“local”" effect) rather than to a genome-wide relationship with homozygosity due to inbreeding ("“general”" effect)." | 957 | no | no pop level? | NA | no | no |
| 958 | 2017 | 43014 | Melzer, MJ; Borth, WB; Sether, DM; Ferreira, S; Gonsalves, D; Hu, JS | 2010 | Genetic diversity and evidence for recent modular recombination in Hawaiian Citrus tristeza virus | The Hawaiian Islands are home to a widespread and diverse population of Citrus tristeza virus (CTV), an economically important pathogen of citrus. In this study, we quantified the genetic diversity of two CTV genes and determined the complete genomic sequence for two strains of Hawaiian CTV. The nucleotide diversity was estimated to be 0.0565 +/- A 0.0022 for the coat protein (CP) gene (n = 137) and 0.0822 +/- A 0.0033 for the p23 gene (n = 30). The genome size and organization of CTV strains HA18-9 and HA16-5 were similar to other fully sequenced strains of CTV. The 3’-terminal halves of their genomes were nearly identical (98.5% nucleotide identity), whereas the 5’-terminal halves were more distantly related (72.3% nucleotide identity), suggesting a possible recombination event. Closer examination of strain HA16-5 indicated that it arose through recent recombination between the movement module of an HA18-9 genotype, and the replication module of an undescribed CTV genotype. | 958 | no | gd of pathogen | NA | no | no |
| 959 | 2017 | 43014 | Lane-deGraaf, KE; Amish, SJ; Gardipee, F; Jolles, A; Luikart, G; Ezenwa, VO | 2015 | Signatures of natural and unnatural selection: evidence from an immune system gene in African buffalo | Pathogens often have negative effects on wildlife populations, and disease management strategies are important for mitigating opportunities for pathogen transmission. Bovine tuberculosis (Mycobacterium bovis; BTB) is widespread among African buffalo (Syncerus caffer) populations in southern Africa, and strategies for managing this disease vary. In two high profile parks, Kruger National Park (KNP) and Hluhluwe-iMfolozi Park (HIP), BTB is either not actively managed (KNP) or managed using a test-and-cull program (HIP). Exploiting this variation in management tactics, we investigated potential evolutionary consequences of BTB and BTB management on buffalo by examining genetic diversity at IFNG, a locus which codes for interferon gamma, a signaling molecule vital in the immune response to BTB. Both heterozygosity and allelic richness were significantly and positively correlated with chromosomal distance from IFNG in KNP, suggesting that directional selection is acting on IFNG among buffalo in this park. While we did not see the same reduction in genetic variation around IFNG in HIP, we found evidence of a recent bottleneck, which might have eroded this signature due to genome-wide reductions in diversity. In KNP, alleles at IFNG were in significant gametic disequilibrium at both short and long chromosomal distances, but no statistically significant gametic disequilibrium was associated with IFNG in HIP. When, we compared genetic diversity between culled and non-culled subsets of HIP animals, we also found that individuals in the culled group had more rare alleles than those in the non-culled group, and that these rare alleles occurred at higher frequency. The observed excess of rare alleles in culled buffalo and the patterns of gametic disequilibrium in HIP suggest that management may be eroding immunogenetic diversity, disrupting haplotype associations in this population. Taken together, our results suggest that both infectious diseases and disease management strategies can influence host genetic diversity with important evolutionary consequences. | 959 | no | No GD diff between pop | NA | no | no |
| 960 | 2017 | 43014 | Bennett, JS; Jolley, KA; Sparling, PF; Saunders, NJ; Hart, CA; Feavers, IM; Maiden, MCJ | 2007 | Species status of Neisseria gonorrhoeae: evolutionary and epidemiological inferences from multilocus sequence typing | Background: Various typing methods have been developed for Neisseria gonorrhoeae, but none provide the combination of discrimination, reproducibility, portability, and genetic inference that allows the analysis of all aspects of the epidemiology of this pathogen from a single data set. Multilocus sequence typing (MLST) has been used successfully to characterize the related organisms Neisseria meningitidis and Neisseria lactamica. Here, the same seven locus Neisseria scheme was used to characterize a diverse collection of N. gonorrhoeae isolates to investigate whether this method would allow differentiation among isolates, and to distinguish these three species. Results: A total of 149 gonococcal isolates were typed and submitted to the Neisseria MLST database. Although relatively few (27) polymorphisms were detected among the seven MLST loci, a total of 66 unique allele combinations ( sequence types, STs), were observed, a number comparable to that seen among isolate collections of the more diverse meningococcus. Patterns of genetic variation were consistent with high levels of recombination generating this diversity. There was no evidence for geographical structuring among the isolates examined, with isolates collected in Liverpool, UK, showing levels of diversity similar to a global collection of isolates. There was, however, evidence that populations of N. meningitidis, N. gonorrhoeae and N. lactamica were distinct, with little support for frequent genetic recombination among these species, with the sequences from the gdh locus alone grouping the species into distinct clusters. Conclusion: The seven loci Neisseria MLST scheme was readily adapted to N. gonorrhoeae isolates, providing a highly discriminatory typing method. In addition, these data permitted phylogenetic and population genetic inferences to be made, including direct comparisons with N. meningitidis and N. lactamica. Examination of these data demonstrated that alleles were rarely shared among the three species. Analysis of variation at a single locus, gdh, provided a rapid means of identifying misclassified isolates and determining whether mixed cultures were present. | 960 | no | no good fitness measure, N pop differnt in GD? | NA | no | no |
| 961 | 2017 | 43014 | Miller, MP; Vincent, ER | 2008 | Rapid natural selection for resistance to an introduced parasite of rainbow trout | Introduced species and infectious diseases both independently pose challenges for the preservation of existing biodiversity. However, native species or disease hosts are by no means ‘unarmed’ when faced with novel environmental challenges, provided that adequate adaptive genetic variation exists to mount effective evolutionary responses. In this study, we examined the consequences of the recently introduced parasite and causative agent of whirling disease (Myxobolus cerebralis) in a wild rainbow trout (Oncorhynchus mykiss) population from Harrison Lake, Montana (USA). Consistent with the parasite’s age-specific effects, juvenile rainbow trout recruitment into Harrison Lake was substantially reduced following parasite detection in 1995. However, experimental data suggest that natural selection has rapidly reduced whirling disease susceptibility within the population over time. The rapid observed temporal change in resistance patterns argues that the standing genetic variation for parasite resistance facilitated this process. Our findings ultimately underscore the importance of preserving genetic diversity to ensure that species of economic importance or of conservation concern have maximal chances for persistence in future changing environments. | 961 | no | only one pop | NA | no | no |
| 962 | 2017 | 43014 | Tarpy, DR; vanEngelsdorp, D; Pettis, JS | 2013 | Genetic diversity affects colony survivorship in commercial honey bee colonies | Honey bee (Apis mellifera) queens mate with unusually high numbers of males (average of approximately 12 drones), although there is much variation among queens. One main consequence of such extreme polyandry is an increased diversity of worker genotypes within a colony, which has been shown empirically to confer significant adaptive advantages that result in higher colony productivity and survival. Moreover, honey bees are the primary insect pollinators used in modern commercial production agriculture, and their populations have been in decline worldwide. Here, we compare the mating frequencies of queens, and therefore, intracolony genetic diversity, in three commercial beekeeping operations to determine how they correlate with various measures of colony health and productivity, particularly the likelihood of queen supersedure and colony survival in functional, intensively managed beehives. We found the average effective paternity frequency (m (e) ) of this population of honey bee queens to be 13.6 +/- 6.76, which was not significantly different between colonies that superseded their queen and those that did not. However, colonies that were less genetically diverse (headed by queens with m (e) a parts per thousand currency signaEuro parts per thousand 7.0) were 2.86 times more likely to die by the end of the study when compared to colonies that were more genetically diverse (headed by queens with m (e) > 7.0). The stark contrast in colony survival based on increased genetic diversity suggests that there are important tangible benefits of increased queen mating number in managed honey bees, although the exact mechanism(s) that govern these benefits have not been fully elucidated. | 962 | yes | from thesis | NA | yes | yes |
| 963 | 2017 | 43014 | Haase, M; Bisenberger, A | 2003 | Allozymic differentiation in the land snail Arianta arbustorum (Stylommatophora, Helicidae): historical inferences | The intraspecific genetic differentiation depends to a large part on historical factors. The mode of dispersal is an important determinant of the genetic diversity especially of newly founded populations. Fast long-distance colonization of formerly unsuitable habitats results in reduced genetic diversity and lineages originating from different glacial refugia form well-defined hybrid zones, where they meet. Slow dispersers, on the other hand, preserve their refugial genetic diversity. Paleontological findings and a previous analysis of mtDNA suggest that the land snail Arianta arbustorum , which today is widely spread across Europe, has survived the Pleistocene glaciations within the boundaries of permafrost. In the present paper, we asked, whether the differentiation of nuclear markers, i.e. allozymes, supports these results. We investigated the allozymic differentiation at 15 polymorphic loci in 14 Alpine and one English populations of the land snail A. arbustorum . Various tests of genetic population differentiation yielded ambiguous results. Allele frequencies differed significantly across populations and all but five pairwise F (ST) values were significant. Genetic distances between populations were comparatively high. On the other hand, an exact test of population differentiation found no differences and the assignment of individuals to their populations of origin was poor. This indicates that allozymic differentiation is constrained. Whether these constraints are of natural or methodological nature remains open. Matrices of genetic and geographical distances were correlated only after removal of the English population from the analysis. Similarly, with one exception F (ST) values increased as the number of regions included in the calculations increased. Thus, on a regional scale, we largely observed a pattern of isolation by distance, which does no longer hold on a larger scale. This is well in accordance with the previous findings based on mtDNA. Mitochondrial lineages were widely distributed across Europe and overlapped extensively. Considering the low potential for dispersal of a land snail, the patchy distribution of lineages, and the high genetic diversity also found in the present study suggests that A. arbustorum has survived the Pleistocene within the boundaries of permafrost. | 963 | no | no good fitness measure | NA | no | no |
| 964 | 2017 | 43014 | Czerwinski, SA; Williams, JT; Demerath, EW; Towne, B; Siervogel, RM; Blangero, J | 2001 | Does accounting for mitochondrial genetic variation improve the fit of genetic models? | We describe a simple variance component model for estimating the effect of mitochondrial DNA (mtDNA) inheritance on quantitative trait variation. The model is applied to quantitative trait Q5 in the simulated general population data from Genetic Analysis Workshop (GAW) 12. Although the mitochondrial effect on Q5 is small (5,3%) and the power of the method to detect the effect is correspondingly low, analysis over the available population replicates demonstrates that the effect of maternal relatedness can be detected and estimated accurately. (C) 2001 Wiley-Liss, Inc. | 964 | no | NA | NA | no | no |
| 965 | 2017 | 43014 | Tang, M; Yu, FH; Jin, XB; Ge, S | 2010 | HIGH GENETIC DIVERSITY IN THE NATURALLY RARE PLANT TAIHANGIA RUPESTRIS YU ET LI (ROSACEAE) DWELLING ONLY CLIFF FACES | Cliffs are the primary habitat of many rare and endangered plant species, but few studies have investigated the genetic diversity of these species. Taihangia rupestris Yu et Li (Rosaceae) is the only species of the genus Taihangia, which occurs exclusively in small crevices on the faces of north-facing vertical cliffs at altitudes ranging from 600 to 1500 m above sea level. It is a perennial herb endemic to the southern part of the Taihang Mountains in northern China. We sampled fresh leaves from 11 to 38 individuals of T rupestris in each of eight cliff-face populations located in the provinces of Hebei, Shanxi and Henan in China. The leaves were dried by silica gel and DNA was extracted. We then assessed the genetic variability within and among the eight populations of T rupestris using random amplified polymorphic DNA (RAPD). Within the 150 plants sampled, 81.2% of the 117 RAPD markers detected were polymorphic, and Nei’s diversity (H) was on average 0.21 in the eight populations and 0.28 in the species as a whole. The grouping of the eight populations by clustering analysis agreed with their pattern of geographical distribution and with the separation of the species into two varieties (T rupestris var. ciliata and T rupestris var. rupestris). Genetic distances (Phi st) were significantly correlated with geographic distances. Although significant genetic differentiation existed between groups (varieties) and among populations, a high proportion (65.4%) of the total genetic variation was maintained within populations. Therefore, high genetic diversity is preserved in the cliff-dwelling populations, and in situ protection of T rupestris should focus on the protection of the habitat of both varieties, which may be of particular importance for the long-term survival of this species. | 965 | no | no good fitness measure, N pop differnt in GD? | NA | no | no |
| 966 | 2017 | 43014 | Izzah, NK; Lee, J; Jayakodi, M; Perumal, S; Jin, M; Park, BS; Ahn, K; Yang, TJ | 2014 | Transcriptome sequencing of two parental lines of cabbage (Brassica oleracea L. var. capitata L.) and construction of an EST-based genetic map | Background: Expressed sequence tag (EST)-based markers are preferred because they reflect transcribed portions of the genome. We report the development of simple sequence repeat (SSR) and single nucleotide polymorphism (SNP) markers derived from transcriptome sequences in cabbage, and their utility for map construction. Results: Transcriptome sequences were obtained from two cabbage parental lines, C1184 and C1234, which are susceptible and resistant to black rot disease, respectively, using the 454 platform. A total of 92,255 and 127,522 reads were generated and clustered into 34,688 and 40,947 unigenes, respectively. We identified 2,405 SSR motifs from the unigenes of the black rot-resistant parent C1234. Trinucleotide motifs were the most abundant (66.15%) among the repeat motifs. In addition, 1,167 SNPs were detected between the two parental lines. A total of 937 EST-based SSR and 97 SNP-based dCAPS markers were designed and used for detection of polymorphism between parents. Using an F-2 population, we built a genetic map comprising 265 loci, and consisting of 98 EST-based SSRs, 21 SNP-based dCAPS, 55 IBP markers derived from B. rapa genome sequence and 91 public SSRs, distributed on nine linkage groups spanning a total of 1,331.88 cM with an average distance of 5.03 cM between adjacent loci. The parental lines used in this study are elite breeding lines with little genetic diversity; therefore, the markers that mapped in our genetic map will have broad spectrum utility. Conclusions: This genetic map provides additional genetic information to the existing B. oleracea map. Moreover, the new set of EST-based SSR and dCAPS markers developed herein is a valuable resource for genetic studies and will facilitate cabbage breeding. Additionally, this study demonstrates the usefulness of NGS transcriptomes for the development of genetic maps even with little genetic diversity in the mapping population. | 966 | no | no good fitness measure | NA | no | no |
| 967 | 2017 | 43014 | Nemli, S; Kutlu, B; Tanyolac, B | 2015 | Determination of the population structure of common bean (Phaseolus vulgaris L.) accessions using lipoxygenase and resistance gene analog markers | The common bean (Phaseolus vulgaris L.) is an important food legume throughout the world. Because of the conservation across different plant species, it is possible to evaluate the degree of genetic diversity in the common bean using gene-based marker techniques. The lipoxygenase (LOX) and resistance gene analog (RGA) genes play an important role in the response to biotic and abiotic stresses. Eighty-six common bean accessions were genotyped using gene-based LOX and RGA markers. The total number of polymorphic bands ranged from 193 for LOX to 17 for RGA markers. We detected considerable diversity with a mean of 8.7 alleles per primer for the LOX analysis. For the RGA markers, the number of alleles per polymorphic locus varied from 1 to 4 with an average allele number of 2.8. The genetic similarity between the accessions based on the LOX and RGA markers ranged from 0.12 to 0.55. Using STRUCTURE, 3 groups were revealed among the accessions. The results of this study should provide valuable information for future studies on the genetic diversity of common bean accessions and for association mapping studies examining the relationships between the genotypic and phenotypic traits related to the stress response. (C) 2015 Elsevier Ltd. All rights reserved. | 967 | no | no good fitness measure, N pop differnt in GD? | NA | no | no |
| 968 | 2017 | 43014 | Vorburger, C; Rhyner, N; Hartikainen, H; Jokela, J | 2014 | A set of new and cross-amplifying microsatellite loci for conservation genetics of the endangered stone crayfish (Austropotamobius torrentium) | Like other indigenous crayfish species in Europe, the stone crayfish (Austropotamobius torrentium) is endangered due to habitat degradation, competition with invasive American crayfish and a deadly disease (crayfish plague) vectored by these invaders. To study the genetic diversity and connectivity of the remnant stone crayfish populations, eight new microsatellite loci were developed and supplemented by four cross-amplifying loci developed for related species. These twelve loci were tested for polymorphism in 130 stone crayfish from five streams in Switzerland. Variability was low with 1-7 alleles per locus, but the markers revealed strong genetic differentiation among streams. Use of these microsatellites will provide important information for the conservation and management of this endangered species. | 968 | no | no good fitness measure, N pop differnt in GD? | NA | no | no |
| 969 | 2017 | 43014 | Thach, T; Ali, S; de Vallavieille-Pope, C; Justesen, AF; Hovmoller, MS | 2016 | Worldwide population structure of the wheat rust fungus Puccinia striiformis in the past | Puccinia striiformis is a basidiomycete causing yellow rust on wheat. The availability of historic samples of this pathogen from the ‘Stubbs collection’ enabled us to investigate past population structure and temporal dynamics on a global scale. A set of 212 single genotype urediniospore isolates, representing samples collected from five continents between 1958 and 1991, were genotyped using 19 polymorphic microsatellite markers. The population genetic analyses revealed the existence of seven genetic groups in the past worldwide P. striiformis population. This genetic grouping generally corresponded with geographical sample origin except for the Middle East, where six of the seven genetic groups were represented. The presence of many genetic groups in the Middle Eastern population reflected a low differentiation from the populations in East Africa (F-ST = 0.052) and in South Asia (F-ST = 0.064). A high diversity and recombinant population structure was observed in China and South Asia, while a clonal population structure was observed in NW Europe, East Africa and the Mediterranean region. The high genetic diversity in the Himalayan region supported recent studies suggesting a putative center of diversity for P. striiformis in this area. Four of the 89 multilocus genotypes detected were resampled in different geographical regions suggesting long-distance migration in the past. Comparison of the past populations with more recent ones, represented by 309 isolates mainly collected between 2001 and 2009, revealed temporal divergence for all populations except for Northwest Europe. Overall, we observed a clear subdivision within the worldwide population structure of P. striiformis and migration in the past. (C) 2015 Elsevier Inc. All rights reserved. | 969 | no | gd of pathogen | NA | no | no |
| 970 | 2017 | 43014 | Li, W; Feng, YX; Sun, HY; Deng, YY; Yu, HS; Chen, HG | 2014 | Analysis of simple sequence repeats in the Gaeumannomyces graminis var. tritici genome and the development of microsatellite markers | Understanding the genetic structure of Gaeumannomyces graminis var. tritici is essential for the establishment of efficient disease control strategies. It is becoming clear that microsatellites, or simple sequence repeats (SSRs), play an important role in genome organization and phenotypic diversity, and are a large source of genetic markers for population genetics and meiotic maps. In this study, we examined the G. graminis var. tritici genome (1) to analyze its pattern of SSRs, (2) to compare it with other plant pathogenic filamentous fungi, such as Magnaporthe oryzae and M. poae, and (3) to identify new polymorphic SSR markers for genetic diversity. The G. graminis var. tritici genome was rich in SSRs; a total 13,650 SSRs have been identified with mononucleotides being the most common motifs. In coding regions, the densities of tri- and hexanucleotides were significantly higher than in noncoding regions. The di-, tri-, tetra, penta, and hexanucleotide repeats in the G. graminis var. tritici genome were more abundant than the same repeats in M. oryzae and M. poae. From 115 devised primers, 39 SSRs are polymorphic with G. graminis var. tritici isolates, and 8 primers were randomly selected to analyze 116 isolates from China. The number of alleles varied from 2 to 7 and the expected heterozygosity (He) from 0.499 to 0.837. In conclusion, SSRs developed in this study were highly polymorphic, and our analysis indicated that G. graminis var. tritici is a species with high genetic diversity. The results provide a pioneering report for several applications, such as the assessment of population structure and genetic diversity of G. graminis var. tritici. | 970 | no | gd of pathogen | NA | no | no |
| 971 | 2017 | 43014 | FRANK, SA | 1991 | ECOLOGICAL AND GENETIC MODELS OF HOST PATHOGEN COEVOLUTION | A model is presented to analyse the forces that maintain genetic polymorphism in interactions between host plants and their pathogens. Genetic variability in hosts occurs for specific resistance to different pathogen races and variability in pathogens occurs for specific virulence to different host races. The model tracks both fluctuating population sizes and changing gene frequencies. Analyses over a range of parameters show that ecological and demographic factors, such as birth and death rates, often have a more profound effect on the amount of polymorphism than genetic parameters, such as the pleiotropic costs of resistance and virulence associated with different alleles. A series of simple measures are proposed to predict the amount of genetic polymorphism expected in particular host-pathogen interactions. These measures can be used to develop and test a comparative theory of genetic polymorphism in host-pathogen coevolution. | 971 | no | NA | NA | no | no |
| 972 | 2017 | 43014 | Wiernasz, DC; Hines, J; Parker, DG; Cole, BJ | 2008 | Mating for variety increases foraging activity in the harvester ant, Pogonomyrmex occidentalis | Multiple mating by females characterizes most insect species, but is relatively uncommon in social insects. Females may mate with multiple mates because they experience the direct benefits of increased survival or fecundity, to acquire high quality mates, or to lower the risk of reduced fecundity by mating with incompatible males. We used the extensive natural variation in mating frequency in the western harvester ant, Pogonomyrmex occidentalis, to test the hypothesis that increased mating by the queen leads to an increase in colony performance. Colonies with greater genetic diversity began to forage earlier in the day and foraged for longer time periods. The workers which initiated foraging were a nonrandom subset of the genotypes present in the colony. We used a statistical approach to correctly predict the direction and magnitude of the correlation between genetic diversity and colony foraging activity. | 972 | no | no good fitness measure | NA | no | no |
| 973 | 2017 | 43014 | Lax, P; Duenas, JCR; Gardenal, CN; Doucet, ME | 2004 | Genetic variability estimated with RAPD-PCR markers in two populations of Heterodera glycines Ichinohe, 1952 (Nematoda : Heteroderidae) from Argentina | Knowledge of genetic variability within and between phytophagous nematode populations is important for the selection of suitable control strategies. The nematode Heterodera glycines is the most important pathogen of soybean in many producer countries, causing significant economic losses. The levels of variability were analysed in two populations of the nematode from Argentina, using Random Amplified Polymorphic DNA markers in order to evaluate the genetic population structure of this species. DNA was extracted from single females of race I and race 3 populations. Five primers were selected and 50 consistent fragments were considered for further analysis. Populations studied showed high levels of genetic diversity. Most of the total variation detected in allele frequencies was attributed to variability among individuals of the same population (83%) rather than inter-population differences (17%). None of the populations demonstrated exclusive bands. However, 68% of fragments showed significant differences in their allele frequencies. This study revealed an important degree of genetic differentiation between both populations, probably as a consequence of limited gene flow between them or because each population was under different management practices at its site of origin. Results herein represent the first analysis of genetic structure in H. glycines populations using RAPD-PCR technique. | 973 | no | gd of pathogen | NA | no | no |
| 974 | 2017 | 43014 | Loope, KJ; Lopez-Osorio, F; Dvorak, L | 2017 | Convergent Reversion to Single Mating in a Wasp Social Parasite | While eusociality arose in species with single-mating females, multiple mating by queens has evolved repeatedly across the social ants, bees, and wasps. Understanding the benefits and costs of multiple mating of queens is important because polyandry results in reduced relatedness between siblings, reducing kin-selected benefits of helping while also selecting for secondary social traits that reduce intracolony conflict. The leading hypothesis for the benefits of polyandry in social insects emphasizes advantages of a genetically diverse workforce. Workerless social parasite species (inquilines) provide a unique opportunity to test this hypothesis, since they are derived from social ancestors but do not produce workers of their own. Such parasites are thus predicted to evolve single mating because they would experience the costs of multiple mating but not the benefits if such benefits accrue through the production of a genetically diverse group of workers. Here we show that the workerless social parasite Dolichovespula arctica, a derived parasite of wasps, has reverted to obligate single mating from a facultatively polyandrous ancestor, mirroring a similar reversion from obligate polyandry to approximate monandry in a social parasite of fungus-farming ants. This finding and a comparison with two other cases where inquilinism did not induce reversal to monandry support the hypothesis that facultative polyandry can be costly and may be maintained by benefits of a genetically diverse workforce. | 974 | no | no good fitness measure, N pop differnt in GD? | NA | no | no |
| 975 | 2017 | 43014 | Fiumera, AC; Parker, PG; Fuerst, PA | 2000 | Effective population size and maintenance of genetic diversity in captive-bred populations of a Lake Victoria Cichlid | We used microsatellite DNA markers to investigate the maintenance of genetic diversity within and between samples of subpopulations (spanning five captive-bred generations) of the haplochromine cichlid Prognathochromis perrieri. The subpopulations are maintained as part of the Lake Victoria Cichlid species survival plan. Changes in the frequencies of 24 alleles, over four polymorphic loci, were used to estimate effective population size (N-e). Point estimates of N-e ranged frp, 2.5 to 7.7 individuals and were significantly smaller than the actual census size (N-obs) for all subpopulations (32-243 individuals per generation), with the corresponding conservative N-e/N-obs ratios ranging from 0.01 to 0.12. Approximately 19% of the initial alleles were lost within the first four generations of captive breeding. Between-generation comparisons of expected heterozygosity showed significant losses ranging from 6% to 12% per generation. Seven private alleles were observed in the last sampled generation of four subpopulations, and analysis of population structure by F-ST indicated that approximately 33% of the total genetic diversity is maintained between the subpopulations from different institutions. To reduce the loss of genetic variation, we recommend that offspring production be equalized by periodically removing dominant males, which will encourage reproduction by additional males. Consideration should also be given to encouraging more institutions to maintain populations, because a significant fraction of the genetic variation exists as among-population differences resulting from random differentiation among subpopulations. | 975 | no | no good fitness measure, N pop differnt in GD? | NA | no | no |
| 976 | 2017 | 43014 | Singh, AK; Rai, VP; Chand, R; Singh, RP; Singh, MN | 2013 | Genetic diversity studies and identification of SSR markers associated with Fusarium wilt (Fusarium udum) resistance in cultivated pigeonpea (Cajanus cajan) | Genetic diversity and identification of simple sequence repeat markers correlated with Fusarium wilt resistance was performed in a set of 36 elite cultivated pigeonpea genotypes differing in levels of resistance to Fusarium wilt. Twenty-four polymorphic sequence repeat markers were screened across these genotypes, and amplified a total of 59 alleles with an average high polymorphic information content value of 0.52. Cluster analysis, done by UPGMA and PCA, grouped the 36 pigeonpea genotypes into two main clusters according to their Fusarium wilt reaction. Based on the Kruskal-Wallis ANOVA and simple regression analysis, six simple sequence repeat markers were found to be significantly associated with Fusarium wilt resistance. The phenotypic variation explained by these markers ranged from 23.7 to 56.4%. The present study helps in finding out feasibility of prescreened SSR markers to be used in genetic diversity analysis and their potential association with disease resistance. | 976 | no | crop | NA | no | no |
| 977 | 2017 | 43014 | Rampersad, K; Ramdial, H; Rampersad, SN | 2016 | Utility of internally transcribed spacer region of rDNA (ITS) and -tubulin gene sequences to infer genetic diversity and migration patterns of Colletotrichum truncatum infecting Capsicum spp. | Anthracnose is among the most economically important diseases affecting pepper (Capsicum spp.) production in the tropics and subtropics. Of the three species of Colletotrichum implicated as causal agents of pepper anthracnose, C.truncatum is considered to be the most destructive in agro-ecosystems worldwide. However, the genetic variation and the migration potential of C.truncatum infecting pepper are not known. Five populations were selected for study and a two-locus (internally transcribed spacer region, ITS1-5.8S-ITS2, and -tubulin, -TUB) sequence data set was generated and used in the analyses. Sequences of the ITS region were less informative than -tubulin gene sequences based on comparisons of DNA polymorphism indices. Trinidad had the highest genetic diversity and also had the largest effective population size in pairwise comparisons with the other populations. The Trinidad population also demonstrated significant genetic differentiation from the other populations. AMOVA and STRUCTURE analyses both suggested significant genetic variation within populations more so than among populations. A consensus Maximum Likelihood tree based on -TUB gene sequences revealed very little intraspecific diversity for all isolates except for Trinidad. Two clades consisting solely of Trinidad isolates may have diverged earlier than the other isolates. There was also evidence of directional migration among the five populations. These findings may have a direct impact on the development of integrated disease management strategies to control C.truncatum infection in pepper. | 977 | no | gd of pathogen | NA | no | no |
| 978 | 2017 | 43014 | Pujol, B; Mckey, D | 2006 | Size asymmetry in intraspecific competition and the density-dependence of inbreeding depression in a natural plant population: a case study in cassava (Manihot esculenta Crantz, Euphorbiaceae) | The effects of competition on the genetic composition of natural populations are not well understood. We combined demography and molecular genetics to study how intraspecific competition affects microevolution in cohorts of volunteer plants of cassava (Manihot esculenta) originating from seeds in slash-and-burn fields of Palikur Amerindians in French Guiana. In this clonally propagated crop, genotypic diversity is enhanced by the incorporation of volunteer plants into farmers’ stocks of clonal propagules. Mortality of volunteer plants was density-dependent. Furthermore, the size asymmetry of intraspecific competition increased with local clustering of plants. Size of plants was correlated with their multilocus heterozygosity, and stronger size-dependence of survival in clusters of plants, compared with solitary plants, increased the magnitude of inbreeding depression when competition was severe. The density-dependence of inbreeding depression of volunteer plants helps explain the high heterozygosity of volunteers that survive to harvest time and thus become candidates for clonal propagation. This effect could help favour the maintenance of sex in this ‘vegetatively’ propagated crop plant. | 978 | no | no good fitness measure, N pop differnt in GD? | NA | no | no |
| 979 | 2017 | 43014 | Wang, LS; Hranilovic, D; Wang, K; Lindquist, IE; Yurcaba, L; Petkovic, ZB; Gidaya, N; Jernej, B; Hakonarson, H; Bucan, M | 2010 | Population-based study of genetic variation in individuals with autism spectrum disorders from Croatia | Background: Genome-wide studies on autism spectrum disorders ( ASDs) have mostly focused on large-scale population samples, but examination of rare variations in isolated populations may provide additional insights into the disease pathogenesis. Methods: As a first step in the genetic analysis of ASD in Croatia, we characterized genetic variation in a sample of 103 subjects with ASD and 203 control individuals, who were genotyped using the Illumina HumanHap550 BeadChip. We analyzed the genetic diversity of the Croatian population and its relationship to other populations, the degree of relatedness via Runs of Homozygosity ( ROHs), and the distribution of large (>500 Kb) copy number variations. Results: Combining the Croatian cohort with several previously published populations in the FastME analysis ( an alternative to Neighbor Joining) revealed that Croatian subjects cluster, as expected, with Southern Europeans; in addition, individuals from the same geographic region within Europe cluster together. Whereas Croatian subjects could be separated from a sample of healthy control subjects of European origin from North America, Croatian ASD cases and controls are well mixed. A comparison of runs of homozygosity indicated that the number and the median length of regions of homozygosity are higher for ASD subjects than for controls ( p = 6 x 10(-3)). Furthermore, analysis of copy number variants found a higher frequency of large chromosomal rearrangements (>2 Mb) in ASD cases ( 5/103) than in ethnically matched control subjects ( 1/197, p = 0.019). Conclusions: Our findings illustrate the remarkable utility of high-density genotype data for subjects from a limited geographic area in dissecting genetic heterogeneity with respect to population and disease related variation. | 979 | no | human | NA | no | no |
| 980 | 2017 | 43014 | de Rosas, ARP; Segura, EL; Fichera, L; Garcia, BA | 2008 | Macrogeographic and microgeographic genetic structure of the Chagas’ disease vector Triatoma infestans (Hemiptera : Reduviidae) from Catamarca, Argentina | The genetic structure in populations of the Chagas’ disease vector Triatoma infestans from six localities belonging to areas under the same insecticide treatment conditions of Catamarca province (Argentina) was examined at macrogeographical and microgeographical scales. A total of 238 insects were typed for 10 polymorphic microsatellite loci. The average observed and expected heterozygosities ranged from 0.319 to 0.549 and from 0.389 to 0.689, respectively. The present results confirm that populations of T. infestans are highly structured. Spatial genetic structure was detectable at macrogeographical and microgeographical levels. Comparisons of the levels of genetic variability between two temporal samples were carried out to assess the impact of the insecticide treatment. The genetic diversity of the population was not significantly affected after insecticide use since different genetic parameters (allele number, observed and expected heterozygosities) remained stable. However, loss of low frequency alleles and not previously found alleles were detected. The effective population size (Ne) estimated was substantially lower in the second temporal sample than in the first; nevertheless, it is possible that the size of the remnant population after insecticide treatment was still large enough to retain the genetic diversity. Very few individuals did not belong to the local T. infestans populations as determined by assignment analyses, suggesting a low level of immigration in the population. The results of the assignment and first-generation migrant tests suggest male-biased dispersal at microgeographical level. | 980 | no | no good fitness measure, N pop differnt in GD? | NA | no | no |
| 981 | 2017 | 43014 | Baird, HP; Miller, KJ; Stark, JS | 2011 | Evidence of hidden biodiversity, ongoing speciation and diverse patterns of genetic structure in giant Antarctic amphipods | Recent molecular research on Antarctic benthic organisms has challenged traditional taxonomic classifications, suggesting that our current perceptions of Antarctic biodiversity and species distributions must be thoroughly revised. Furthermore, genetic differentiation at the intraspecific level remains poorly understood, particularly in eastern Antarctica. We addressed these issues using DNA sequence data for two sibling amphipod species that could be collected on a circum-Antarctic scale: Eusirus perdentatus and Eusirus giganteus. Haplotype networks and Bayesian phylogenies based on mitochondrial (COI, CytB) and nuclear (ITS2) DNA provided strong evidence of multiple cryptic species of Eusirus, with several occurring in sympatry and at least one likely to have a true circum- Antarctic distribution. Within species, gene flow was often highly restricted, consistent with a brooding life history and in some cases suggestive of current or future allopatric speciation. Patterns of genetic structure were not always predictable: one cryptic species showed preliminary evidence of high genetic differentiation across similar to 150 km in eastern Antarctica (F-ST > 0.47, P < 0.01), yet another was remarkably homogenous across similar to 5000 km (F-ST = 0.00, P = 1.00). Genetic diversity also varied among cryptic species, independent of sample size (pi = 0.00-0.99). These results indicate several hidden levels of genetic complexity in these Antarctic amphipods that are neither apparent from previous taxonomic or ecological studies nor predictable from their life history. Such genetic diversity and structure may reflect different modes of survival for Antarctic benthic organisms during historic glacial cycles, and/or subsequent re-establishment of populations on the shelf, and highlight our misunderstanding of Antarctic marine species diversity. | 981 | no | comparing species | NA | no | no |
| 982 | 2017 | 43014 | Hawley, DM; Hanley, D; Dhondt, AA; Lovette, IJ | 2006 | Molecular evidence for a founder effect in invasive house finch (Carpodacus mexicanus) populations experiencing an emergent disease epidemic | The impact of founder events on levels of genetic variation in natural populations remains a topic of significant interest. Well-documented introductions provide a valuable opportunity to examine how founder events influence genetic diversity in invasive species. House finches (Carpodacus mexicanus) are passerine birds native to western North America, with the large eastern North American population derived from a small number of captive individuals released in the 1940s. Previous comparisons using amplified fragment length polymorphism (AFLP) markers found equivalent levels of diversity in eastern and western populations, suggesting that any genetic effects of the founder event were ameliorated by the rapid growth of the newly established population. We used an alternative marker system, 10 highly polymorphic microsatellites, to compare levels of genetic diversity between four native and five introduced house finch populations. In contrast to the AFLP comparisons, we found significantly lower allelic richness and heterozygosity in introduced populations across all loci. Three out of five introduced populations showed significant reductions in the ratio of the number of alleles to the allele size range, a within-population characteristic of recent bottlenecks. Finally, native and introduced populations showed significant pairwise differences in allele frequencies in every case, with stronger isolation by distance within the introduced than native range. Overall, our results provide compelling molecular evidence for a founder effect during the introduction of eastern house finches that reduced diversity levels at polymorphic microsatellite loci and may have contributed to the emergence of the Mycoplasma epidemic which recently swept the eastern range of this species. | 982 | no | no good fitness measure | NA | no | no |
| 983 | 2017 | 43014 | Vanpe, C; Kjellander, P; Gaillard, JM; Cosson, JF; Galan, M; Hewison, AJM | 2009 | Multiple paternity occurs with low frequency in the territorial roe deer, Capreolus capreolus | An explanation for female multiple mating when males offer no material benefits but sperm remains elusive, largely because of a lack of empirical support for the genetic benefits hypothesis. We used 21 microsatellite markers to test for multiple paternities among 88 litters of roe deer, Capreolus capreolus, and to investigate the inbreeding avoidance hypothesis as a potential mechanism for the evolution of female multiple mating. From paternity analyses, we found that 13.5% of polytocous litters were sired by more than one male. We also found that a half-sib relationship was more likely than a full-sib relationship for 20.5% of all litters. This is the first report of multiple paternities in a territorial ungulate species. In support of the inbreeding avoidance hypothesis, we found that parents who were strongly related produced offspring with lower individual heterozygosity that survived less well during their first summer than fawns with unrelated parents. In addition, fawns from multiple paternity litters survived their first summer better than fawns from single paternity litters. However, it remains unclear whether all female multiple paternity events in this species are provoked by an initial consanguineous mating. (C) 2009 The Linnean Society of London, Biological Journal of the Linnean Society 2009, 97, 128-139. | 983 | yes | litter gd ok? | NA | no | yes |
| 984 | 2017 | 43014 | Marden, JH; Mangan, SA; Peterson, MP; Wafula, E; Fescemyer, HW; Der, JP; dePamphilis, CW; Comita, LS | 2017 | Ecological genomics of tropical trees: how local population size and allelic diversity of resistance genes relate to immune responses, cosusceptibility to pathogens, and negative density dependence | In tropical forests, rarer species show increased sensitivity to species-specific soil pathogens and more negative effects of conspecific density on seedling survival (NDD). These patterns suggest a connection between ecology and immunity, perhaps because small population size disproportionately reduces genetic diversity of hyperdiverse loci such as immunity genes. In an experiment examining seedling roots from six species in one tropical tree community, we found that smaller populations have reduced amino acid diversity in pathogen resistance (R) genes but not the transcriptome in general. Normalized R gene amino acid diversity varied with local abundance and prior measures of differences in sensitivity to conspecific soil and NDD. After exposure to live soil, species with lower R gene diversity had reduced defence gene induction, more cosusceptibility of maternal cohorts to colonization by potentially pathogenic fungi, reduced root growth arrest (an R gene-mediated response) and their root-associated fungi showed lower induction of self-defence (antioxidants). Local abundance was not related to the ability to induce immune responses when pathogen recognition was bypassed by application of salicylic acid, a phytohormone that activates defence responses downstream of R gene signalling. These initial results support the hypothesis that smaller local tree populations have reduced R gene diversity and recognition-dependent immune responses, along with greater cosusceptibility to species-specific pathogens that may facilitate disease transmission and NDD. Locallyrare species may be less able to increase their equilibrium abundance without genetic boosts to defencevia immigration of novel R gene alleles from a larger and more diverse regional population. | 984 | no | no good fitness measure, N pop differnt in GD? | NA | no | no |
| 985 | 2017 | 43014 | Herrero-Medrano, JM; Megens, HJ; Crooijmans, RP; Abellaneda, JM; Ramis, G | 2013 | Farm-by-farm analysis of microsatellite, mtDNA and SNP genotype data reveals inbreeding and crossbreeding as threats to the survival of a native Spanish pig breed | The Chato Murciano (CM), a pig breed from the Murcia region in the southeastern region of Spain, is a good model for endangered livestock populations. The remaining populations are bred on approximately 15 small farms, and no herdbook exists. To assess the genetic threats to the integrity and survival of the CM breed, and to aid in designing a conservation program, three genetic marker systems microsatellites, SNPs and mtDNA were applied across the majority of the total breeding stock. In addition, mtDNA and SNPs were genotyped in breeds that likely contributed genetically to the current CM gene pool. The analyses revealed the levels of genetic diversity within the range of other European local breeds (He=0.53). However, when the eight farms that rear at least 10 CM pigs were independently analyzed, high levels of inbreeding were found in some. Despite the evidence for recent crossbreeding with commercial breeds on a few farms, the entire breeding stock remains readily identifiable as CM, facilitating the design of traceability assays. The genetic management of the breed is consistent with farm size, farm owner and presence of other pig breeds on the farm, demonstrating the highly ad hoc nature of current CM breeding. The results of genetic diversity and substructure of the entire breed, as well as admixture and crossbreeding obtained in the present study, provide a benchmark to develop future conservation strategies. Furthermore, this study demonstrates that identifying farm-based practices and farm-based breeding stocks can aid in the design of a sustainable breeding program for minority breeds. | 985 | no | domesticated animal | NA | no | no |
| 986 | 2017 | 43014 | Wilson, A; Fenton, B; Malloch, G; Boag, B; Hubbard, S; Begg, G | 2016 | Urbanisation versus agriculture: a comparison of local genetic diversity and gene flow between wood mouse Apodemus sylvaticus populations in human-modified landscapes | Urbanisation and agriculture dramatically modify the landscapes available for use by wildlife, affecting key aspects of their ecology such as survival, foraging, predation, competition and reproductive success. Relatively little is known about the effects of urbanisation and agriculture on the genetic structure, gene flow and genetic diversity of wild species. Here, landscape genetic techniques were applied to compare local genetic diversity and gene flow between wood mouse populations in urban and arable landscapes. Using nine microsatellite markers, individuals were genotyped from six arable and seven urban sample sites. Inter-population genetic differentiation was significantly greater in urban than arable habitat, while allele richness, private allele richness and heterozygosity were higher for arable sample sites, with varying degrees of significance. These suggest that urban habitat was sufficiently fragmented to limit gene flow. To test the effect of landscape features on gene flow, several cost-distance measures were generated. Overland distance and Euclidean distance correlated best with inter-population genetic differentiation in arable habitat, whereas distances that accommodated differences in habitat quality better explained differentiation in urban habitat. There was no evidence that margins adjacent to roads, rivers or railways facilitated gene flow. Together, the results indicate that urban landscapes expose wood mice to greater fragmentation in habitat quality than arable areas, leading to greater population isolation that is not mitigated by the presence of dispersal corridors. | 986 | no | no good fitness measure | NA | no | no |
| 987 | 2017 | 43014 | Decroocq, S; Cornille, A; Tricon, D; Babayeva, S; Chague, A; Eyquard, JP; Karychev, R; Dolgikh, S; Kostritsyna, T; Liu, S; Liu, WS; Geng, WJ; Liao, K; Asma, BM; Akparov, Z; Giraud, T; Decroocq, V | 2016 | New insights into the history of domesticated and wild apricots and its contribution to Plum pox virus resistance | Studying domesticated species and their wild relatives allows understanding of the mechanisms of population divergence and adaptation, and identifying valuable genetic resources. Apricot is an important fruit in the Northern hemisphere, where it is threatened by the Plum pox virus (PPV), causing the sharka disease. The histories of apricot domestication and of its resistance to sharka are however still poorly understood. We used 18 microsatellite markers to genotype a collection of 230 wild trees from Central Asia and 142 cultivated apricots as representatives of the worldwide cultivated apricot germplasm; we also performed experimental PPV inoculation tests. The genetic markers revealed highest levels of diversity in Central Asian and Chinese wild and cultivated apricots, confirming an origin in this region. In cultivated apricots, Chinese accessions were differentiated from more Western accessions, while cultivated apricots were differentiated from wild apricots. An approximate Bayesian approach indicated that apricots likely underwent two independent domestication events, with bottlenecks, from the same wild population. Central Asian native apricots exhibited genetic subdivision and high frequency of resistance to sharka. Altogether, our results contribute to the understanding of the domestication history of cultivated apricot and point to valuable genetic diversity in the extant genetic resources of wild apricots. | 987 | no | no good fitness measure, N pop differnt in GD? | NA | no | no |
| 988 | 2017 | 43014 | Leignel, V; Humbert, JF | 2001 | Mitochondrial DNA variation in benzimidazole-resistant and -susceptible populations of the small ruminant parasite Teladorsagia circumcincta | The genetic diversity of the mtDNA ND4 gene in 11 Teladorsagia circumcincta populations from France and Morocco was assessed by sequencing. Some of these nematode populations were resistant to benzimidazole (BZ) anthelmintics, while others were susceptible. The nucleotide diversity in all populations studied was very high, probably due to a high mutation rate in nematodes, but there was no significant difference between them. This suggests that no strong, recurrent bottlenecks occur during the acquisition of BZ resistance by a worm population. The conservation of genetic variations during the acquisition of BZ resistance is probably due to the fact that anthelmintic treatments do not kill all the susceptible adult worms and to the presence of numerous free-living larvae that are not submitted to this anthelmintic pressure. There was no genetic subdivision between worm populations on a small geographical scale (less than 200 km), but significant F(ST)s were found on a larger geographical scale. This kind of subdivision cannot be explained by different genetic flows between populations because all these populations were isolated from each other. This subdivision is probably due to the breeding management practices and the large size of these worm populations, which limit genetic drift. | 988 | no | no good fitness measure, N pop differnt in GD? | NA | no | no |
| 989 | 2017 | 43014 | Montana, L; Caniglia, R; Galaverni, M; Fabbri, E; Ahmed, A; Bolfikova, BC; Czarnomska, SD; Galov, A; Godinho, R; Hindrikson, M; Hulva, P; Jedrzejewska, B; Jelencic, M; Kutal, M; Saarma, U; Skrbinsek, T; Randi, E | 2017 | Combining phylogenetic and demographic inferences to assess the origin of the genetic diversity in an isolated wolf population | The survival of isolated small populations is threatened by both demographic and genetic factors. Large carnivores declined for centuries in most of Europe due to habitat changes, overhunting of their natural prey and direct persecution. However, the current rewilding trends are driving many carnivore populations to expand again, possibly reverting the erosion of their genetic diversity. In this study we reassessed the extent and origin of the genetic variation of the Italian wolf population, which is expanding after centuries of decline and isolation. We genotyped wolves from Italy and other nine populations at four mtDNA regions (control-region, ATP6, COIII and ND4) and 39 autosomal microsatellites. Results of phylogenetic analyses and assignment procedures confirmed in the Italian wolves a second private mtDNA haplotype, which belongs to a haplogroup distributed mostly in southern Europe. Coalescent analyses showed that the unique mtDNA haplotypes in the Italian wolves likely originated during the late Pleistocene. ABC simulations concordantly showed that the extant wolf populations in Italy and in south-western Europe started to be isolated and declined right after the last glacial maximum. Thus, the standing genetic variation in the Italian wolves principally results from the historical isolation south of the Alps. | 989 | no | no good fitness measure, N pop differnt in GD? | NA | no | no |
| 990 | 2017 | 43014 | Parsons, PA | 1996 | Conservation strategies: Adaptation to stress and the preservation of genetic diversity | The level of genetic diversity in free-living populations is not normally restrictive for conservation, since it tends to be enhanced in stressed outlier populations. At the physiological level, this enhancement is supported by the favouring of heterozygotes, especially when energy demands needed to adapt to stress are high. Therefore ecophysiological considerations are important for conservation strategies, whereby survival depends upon the metabolic potential of organisms to counter the energy cost of sti ess in their environments. while abiotic stresses are primary, biotic stresses, in particular competition, can be consolidated into this model as second-order effects. Irrespective of levels of genetic diversity, any species can be incorporated into this approach to conservation. I therefore regard the monitoring of stress response traits to be primary to the preservation of genetic diversity in developing conservation strategies. In arriving at this conclusion, Fisher’s 1930 discussions of the environment and consequences for adaptation, as presented in the The Genetical Theory of Natural Selection, play an initiating role. (C) 1996 The Linnean Society of London | 990 | no | NA | NA | no | no |
| 991 | 2017 | 43014 | Gaddis, KD; Zukin, HL; Dieterich, IA; Braker, E; Sork, VL | 2014 | Effect of clonal reproduction on genetic structure in Pentaclethra macroloba (Fabaceae: Mimosoideae) | The existence of monodominant forests on well-drained soils in tropical regions has been widely reported. Such forests most likely result from a combination of both ecological and evolutionary factors. Under conditions of high seed and seedling mortality, vegetative reproduction could create a reproductive advantage leading to forest dominance, and profoundly affect the distribution of genetic variation in a clonal species. We investigated these effects in a low diversity forest site in Northeastern Costa Rica dominated by the species Pentaclethra macroloba, which sprouts from the root mass of fallen trees and from snapped trunks. We examined the population structure of juvenile R macroloba growing in different soil types and across an elevational gradient. Using seven molecular markers, we genotyped 173 juvenile P. macroloba from 18 plots (six plots in seasonally inundated swamps, and 12 plots in upland non-swamp) spanning 50-300m in elevation at La Selva Biological Station and the adjacent Reserva Ecologica Bijagual in Northeastern Costa Rica. We answered two specific questions: (1) How extensive is clonal reproduction? and (2) what is the distribution of genetic diversity and structure?. We found that clonal reproduction occurred exclusively within inundated swamp areas. However, there was no significant difference between genetic diversity measures in swamp and non-swamp plots, which were both generally low when compared with other tropical forest species. Genetic structure was significant across all plots (F-ST=0.109). However, genetic structure among swamp plots (F-ST=0.128) was higher than among non-swamp upland plots (F-ST=0.093). Additionally, spatial autocorrelation among individuals within non-swamp upland plots was significant from the 25 to 100m spatial scale, but not within swamp plots. The degree of overall genetic structure we found in R macroloba is high for a tropical forest tree. The incidence of clonal reproduction is a contributing factor in genetic differentiation, but the high structure among plots without clonal reproduction indicates that other factors contribute as well. | 991 | no | no good fitness measure | NA | no | no |
| 992 | 2017 | 43014 | Santos, ESL; Cerqueira-Silva, CBM; Mori, GM; Ahnert, D; Mello, DLN; Pires, JL; Correa, RX; de Souza, AP | 2015 | Genetic Structure and Molecular Diversity of Cacao Plants Established as Local Varieties for More than Two Centuries: The Genetic History of Cacao Plantations in Bahia, Brazil | Bahia is the most important cacao-producing state in Brazil, which is currently the sixth-largest country worldwide to produce cacao seeds. In the eighteenth century, the Comum, Para and Maranhao varieties of cacao were introduced into southern Bahia, and their descendants, which are called ‘Bahian cacao’ or local Bahian varieties, have been cultivated for over 200 years. Comum plants have been used to start plantations in African countries and extended as far as countries in South Asia and Oceania. In Brazil, two sets of clones selected from Bahian varieties and their mutants, the Agronomic Institute of East (SIAL) and Bahian Cacao Institute (SIC) series, represent the diversity of Bahian cacao in germplasm banks. Because the genetic diversity of Bahian varieties, which is essential for breeding programs, remains unknown, the objective of this work was to assess the genetic structure and diversity of local Bahian varieties collected from farms and germplasm banks. To this end, 30 simple sequence repeat (SSR) markers were used to genotype 279 cacao plants from germplasm and local farms. The results facilitated the identification of 219 cacao plants of Bahian origin, and 51 of these were SIAL or SIC clones. Bahian cacao showed low genetic diversity. It could be verified that SIC and SIAL clones do not represent the true diversity of Bahian cacao, with the greatest amount of diversity found in cacao trees on the farms. Thus, a core collection to aid in prioritizing the plants to be sampled for Bahian cacao diversity is suggested. These results provide information that can be used to conserve Bahian cacao plants and applied in breeding programs to obtain more productive Bahian cacao with superior quality and tolerance to major diseases in tropical cacao plantations worldwide. | 992 | no | crop | NA | no | no |
| 993 | 2017 | 43014 | Cavanagh, CR; Chao, SM; Wang, SC; Huang, BE; Stephen, S; Kiani, S; Forrest, K; Saintenac, C; Brown-Guedira, GL; Akhunova, A; See, D; Bai, GH; Pumphrey, M; Tomar, L; Wong, DB; Kong, S; Reynolds, M; da Silva, ML; Bockelman, H; Talbert, L; Anderson, JA; Dreisigacker, S; Baenziger, S; Carter, A; Korzun, V; Morrell, PL; Dubcovsky, J; Morell, MK; Sorrells, ME; Hayden, MJ; Akhunov, E | 2013 | Genome-wide comparative diversity uncovers multiple targets of selection for improvement in hexaploid wheat landraces and cultivars | Domesticated crops experience strong human-mediated selection aimed at developing high-yielding varieties adapted to local conditions. To detect regions of the wheat genome subject to selection during improvement, we developed a high-throughput array to interrogate 9,000 gene-associated single-nucleotide polymorphisms (SNP) in a worldwide sample of 2,994 accessions of hexaploid wheat including landraces and modern cultivars. Using a SNP-based diversity map we characterized the impact of crop improvement on genomic and geographic patterns of genetic diversity. We found evidence of a small population bottleneck and extensive use of ancestral variation often traceable to founders of cultivars from diverse geographic regions. Analyzing genetic differentiation among populations and the extent of haplotype sharing, we identified allelic variants subjected to selection during improvement. Selective sweeps were found around genes involved in the regulation of flowering time and phenology. An introgression of a wild relative-derived gene conferring resistance to a fungal pathogen was detected by haplotype-based analysis. Comparing selective sweeps identified in different populations, we show that selection likely acts on distinct targets or multiple functionally equivalent alleles in different portions of the geographic range of wheat. The majority of the selected alleles were present at low frequency in local populations, suggesting either weak selection pressure or temporal variation in the targets of directional selection during breeding probably associated with changing agricultural practices or environmental conditions. The developed SNP chip and map of genetic variation provide a resource for advancing wheat breeding and supporting future population genomic and genome-wide association studies in wheat. | 993 | no | crop | NA | no | no |
| 994 | 2017 | 43014 | Souza, EA; Camargo, OA; Pinto, JMA | 2010 | Sexual recombination in Colletotrichum lindemuthianum occurs on a fine scale | Glomerella cingulata f. sp phaseoli is the sexual phase of the fungus Colletotrichum lindemuthianum, the causal agent of common bean anthracnose. This fungus is of great concern, because it causes large economic losses in common bean crops. RAPD markers of five populations of G. cingulata f. sp phaseoli from two Brazilian states were analyzed to determine if this population possesses the sexual reproductive potential to generate the genetic variation that is observed in this phytopathogen. We identified 128 polymorphic bands, amplified by 28 random primers. The estimates of genetic similarity in this analysis ranged from 0.43 to 1.00, and the dendrogram generated from analysis of all genotypes displayed five principal groups, coinciding with the five populations. Genetic differentiation was observed between the populations (G(ST) = 0.6455); 69% of the overall observed genetic variation was between individual populations and 31% of the variance was within the sub-populations. We identified significant levels of linkage disequilibrium in all populations. However, the values of the disequilibrium ranged from low to moderate, indicating that this pathogen maintains a genetic structure consistent with sexual reproduction. The mean contribution of sexual reproduction was determined by comparison of the amplitudes of genetic similarity of isolates from sexual and asexual phases. These results support the hypothesis that recombination plays an important role in determining the amplitude of variability in this pathogen population and that this determination occurs on a fine scale. | 994 | no | gd of pathogen | NA | no | no |
| 995 | 2017 | 43014 | Forsman, A | 2014 | Effects of genotypic and phenotypic variation on establishment are important for conservation, invasion, and infection biology | There is abundant evidence that the probability of successful establishment in novel environments increases with number of individuals in founder groups and with number of repeated introductions. Theory posits that the genotypic and phenotypic variation among individuals should also be important, but few studies have examined whether founder diversity influences establishment independent of propagule pressure, nor whether the effect is model or context dependent. I summarize the results of 18 experimental studies and report on a metaanalysis that provides strong evidence that higher levels of genotypic and phenotypic diversity in founder groups increase establishment success in plants and animals. The effect of diversity is stronger in experiments carried out under natural conditions in the wild than under seminatural or standardized laboratory conditions. The realization that genetic and phenotypic variation is key to successful establishment may improve the outcome of reintroduction and translocation programs used to vitalize or restore declining and extinct populations. Founder diversity may also improve the ability of invasive species to establish and subsequently spread in environments outside of their native community, and enhance the ability of pathogens and parasites to colonize and invade the environment constituted by their hosts. It is argued that exchange of ideas, methodological approaches, and insights of the role of diversity for establishment in different contexts may further our knowledge, vitalize future research, and improve management plans in different disciplines. | 995 | probably no | meta-analysis, read this | no data | yes | no |
| 996 | 2017 | 43014 | Collevatti, RG; Hay, JD | 2011 | Kin structure and genotype-dependent mortality: a study using the neotropical tree Caryocar brasiliense | P>1. In many species, density-dependent processes regulate population size through size- and neighbourhood-dependent mortality. 2. If a kin structure exists in a particular population, mortality may be spatially clustered due to enhanced competition among relatives. 3. Demography, kin structure and genetic diversity, based on 10 microsatellite loci, of a local population of the Neotropical tree Caryocar brasiliense were studied for 23 years. Overall population growth was static (lambda = 1.0) during this time, but some time intervals showed negative growth. 4. Mortality was spatially clustered and negatively correlated with spatial distance among pairs of individuals at distances lower than c. 13 m. Probability of death was related to individual genotypes, and seedlings and juveniles with lower proportion of heterozygous loci had the highest probability of mortality. 5. Kinship was significantly related to spatial distance among pairs of individuals at distance lower than 10 m and kinship structure did not change along the life stages, but the inbreeding coefficient was not significantly different from zero in adults. 6. Synthesis. We hypothesize that genetic clustering in C. brasiliense dissipates over time due to more intense competition among relatives. Thus, the kin structure in C. brasiliense is highly important in determining mortality patterns, driving the spatial pattern in plant recruitment. | 996 | probably no | No GD diff between pop | no differfence in GD of groups | no | no |
| 997 | 2017 | 43014 | Rojas-Mendez, KJ; Penaloza-Ramirez, JM; Rocha-Ramirez, V; Cortes-Palomec, A; McCauley, RA; Oyama, K | 2017 | Massive Extraction of the Orchid Laelia speciosa (HBK) Schltr. for Trading in Local Markets Affect Its Population Genetic Structure in a Fragmented Landscape in Central Mexico | Laelia speciosa is an orchid species listed as threatened of extinction in the Mexican standard NOM-059. Wild populations of L speciosa have been declining due to fragmentation of its habitat and massive extraction for trading in local markets in Mexico. In this study, we aimed to evaluate the evolutionary potential of L. speciosa within a fragmented landscape of ca. 4000 km 2 in the Cuitzeo basin, State of Michoacan. We sampled 15 populations throughout the Cuitzeo basin and amplified eight nuclear microsatellite markers to assess genetic diversity, structure, and connectivity and to test for evidence of recent bottlenecks. Surprisingly, L. speciosa populations showed high genetic diversity, with values ranging from moderate to high compared with those reported for other orchid species. Also, the analysis of molecular variance and R-ST results indicated the existence of low genetic differentiation, favored by its cross pollination habit which facilitates the maintenance of gene flow and that have been observed in other orchid species. Wright’s within-population inbreeding (F-IS) was positive in all cases, denoting a heterozygosity deficit, with moderate-to-high values. Fragmentation may also lead to inbreeding due to either increased self-fertilization or mating between related individuals within remnant fragments. The L. speciosa populations examined showed evidence suggesting that some populations had recently gone through a bottleneck. We also observed that all the L. speciosa populations had a moderate effective population size. The history of L. speciosa in the Cuitzeo basin suggests that both fragmented and non-fragmented populations may have been recently subject to moderate reductions in effective population size, large enough to affect their allelic diversity, FIS, but not their HE. Such reductions may have been caused by episodic environmental fluctuations or resulted from the recent founding of some of the populations. The effective population size can be used as an indicator of habitat quality, and this was confirmed for the L. speciosa populations, which have undergone a drastic decline due to environmental changes, habitat destruction, and illegal collection. The ultimate goals of conservation are to ensure the continuous survival of populations and maintain their evolutionary potential by preserving natural levels of genetic diversity. Great efforts should be made to preserve this species’ extant populations and their habitats to prevent further population reductions and preserve its overall genetic basis. Collection of this orchid should be banned and robust legal protection measures should be enforced through local authorities. | 997 | no | no good fitness measure, N pop differnt in GD? | NA | no | no |
| 998 | 2017 | 43014 | Lillie, M; Grueber, CE; Sutton, JT; Howitt, R; Bishop, PJ; Gleeson, D; Belov, K | 2015 | Selection on MHC class II supertypes in the New Zealand endemic Hochstetter’s frog | “Background: The New Zealand native frogs, family Leiopelmatidae, are among the most archaic in the world. Leiopelma hochstetteri (Hochstetter’s frog) is a small, semi-aquatic frog with numerous, fragmented populations scattered across New Zealand’s North Island. We characterized a major histocompatibility complex (MHC) class II B gene ( DAB) in L. hochstetteri from a spleen transcriptome, and then compared its diversity to neutral microsatellite markers to assess the adaptive genetic diversity of five populations (”“evolutionarily significant units”“, ESUs). Results: L. hochstetteri possessed very high MHC diversity, with 74 DAB alleles characterized. Extremely high differentiation was observed at the DAB locus, with only two alleles shared between populations, a pattern that was not reflected in the microsatellites. Clustering analysis on putative peptide binding residues of the DAB alleles indicated four functional supertypes, all of which were represented in 4 of 5 populations, albeit at different frequencies. Otawa was an exception to these observations, with only two DAB alleles present. Conclusions: This study of MHC diversity highlights extreme population differentiation at this functional locus. Supertype differentiation was high among populations, suggesting spatial and/or temporal variation in selection pressures. Low DAB diversity in Otawa may limit this population’s adaptive potential to future pathogenic challenges.” | 998 | no | No GD diff between pop | NA | no | no |
| 999 | 2017 | 43014 | Kizito, EB; Chiwona-Karltun, L; Egwang, T; Fregene, M; Westerbergh, A | 2007 | Genetic diversity and variety composition of cassava on small-scale farms in Uganda: an interdisciplinary study using genetic markers and farmer interviews | Cassava is a tropical crop and grown for its tuberous starchy roots. In Africa it is mainly cultivated by small-scale farmers who observe, select and name their cassava varieties based on morphology, food, social and economic interest. Here we have used an interdisciplinary approach involving farmer interviews, genetic markers and morphological descriptors to study the composition of cassava varieties on small-scale farms in 11 villages located in three districts in Uganda, the genetic structure within and between these varieties and their morphology. The composition of local, newly introduced and improved varieties differed widely between villages and districts. The Ugandan farmers in our study seemed to adopt improved varieties to a greater extent when there was a nearby market, prevalence of disease epidemics and good extension service. We found considerable genetic variation both within and between cassava varieties though the variation was larger between varieties. However, most local and improved varieties showed predominating genotypes at many loci. Accessions of commonly grown varieties meeting farmers’ preferences could therefore be selected and implemented in future breeding programmes involving development, dissemination and adoption. The like-named varieties in different villages were genetically similar, demonstrating farmers’ ability to differentiate and maintain the same variety over large areas. However, some varieties with different names in different villages showed both genetic and morphological similarity, suggesting that farmers may rename plants when they are introduced into their fields. The large differences found in variety and genetic composition between villages and districts in Uganda may be a result of the diverse needs and growing conditions characteristic for traditional farming system. This suggests that efforts to conserve and increase the genetic diversity in farmers’ fields will require policies tailored to each area. | 999 | no | crop | NA | no | no |
| 1000 | 2017 | 43014 | Velando, A; Barros, A; Moran, P | 2015 | Heterozygosity-fitness correlations in a declining seabird population | Loss of genetic diversity is thought to lead to increased risk of extinction in endangered populations due to decreasing fitness of homozygous individuals. Here, we evaluated the presence of inbreeding depression in a long-lived seabird, the European shag (Phalacrocorax aristotelis), after a severe decline in population size by nearly 70%. During three reproductive seasons, 85 breeders were captured and genotyped at seven microsatellite loci. Nest sites were monitored during the breeding season to estimate reproductive success as the number of chicks surviving to full-size-grown per nest. Captured birds were tagged with a ring with an individual code, and resighting data were collected during 7-year period. We found a strong effect of multilocus heterozygosity on female reproductive performance, and a significant, although weaker, effect on breeder survival. However, our matrix population model suggests that this relatively small effect of genetic diversity on breeder survival may have a profound effect on fitness. This highlights the importance of integrating life history consequences in HFC studies. Importantly, heterozygosity was correlated across loci, suggesting that genomewide effects, rather than single loci, are responsible for the observed HFCs. Overall, the HFCs are a worrying symptom of genetic erosion in this declining population. Many long-lived species are prone to extinction, and future studies should evaluate the magnitude of fitness impact of genetic deterioration on key population parameters, such as survival of breeders. | 1000 | no | inbreeding | NA | no | no |
| 1001 | 2017 | 43014 | Mkare, TK; van Vuuren, BJ; Teske, PR | 2017 | Conservation implications of significant population differentiation in an endangered estuarine seahorse | The spatial distribution of a species’ genetic diversity can provide insights into underlying evolutionary, ecological and environmental processes, and can contribute information towards the delineation of conservation units. The Knysna seahorse, Hippocampus capensis, is endangered and occurs in only three estuaries on the warm-temperate south coast of South Africa: Knsyna, Keurbooms and Swartvlei. Population sizes in the latter two estuaries have been very small for a prolonged period of time, and the populations residing in them may thus benefit from translocations as a means of increasing population sizes and possibly also genetic diversity. However, information on whether these three estuaries constitute distinct conservation units that warrant separate management is presently lacking. Here, we used genetic information from mitochondrial (control region) and nuclear microsatellite loci to assess the genetic diversity and spatial structure across the three estuaries, and also whether translocations should be included in the management plan for the Knysna seahorse. Although each population had a unique combination of alleles, and clustering methods identified the Swartvlei Estuary as being distinct from the others, levels of genetic admixture were high, and there was no evidence for reciprocal monophyly that would indicate that each estuary has a unique demographic history. On these grounds, we suggest recognising the three populations as a single evolutionarily significant unit (ESU), and encourage translocations between them to ensure the species’ long-term survival. | 1001 | no | no good fitness measure, N pop differnt in GD? | NA | no | no |
| 1002 | 2017 | 43014 | Sapoukhina, N; Durel, CE; Le Cam, B | 2009 | Spatial deployment of gene-for-gene resistance governs evolution and spread of pathogen populations | We formulate a spatially realistic population-genetic model for ascertaining the synergetic effect between genetic and spatial composition of the host population on the pathogen spread reinforced by evolutionary processes. We show that spatial arrangement of host genotypes is crucial to the efficacy of host genetic diversification. In particular, the reductive effect of multigenic resistance on the pathogen density can be produced by a random patterning of monogenic resistances. Random patterns can reduce both density and genetic diversity of the pathogen population and delay invasion promoted by sexual recombination. By contrast, patchy distributions diversify pathogen population and, hence, reduce the efficacy of resistance genes. The proposed approach provides theoretical support for studying fast emergence and spread of novel pathogen genotypes carrying multiple virulence genes. It has a practical applicability to design innovative strategies for the most appropriate deployment of plant resistance genes. | 1002 | no | no good fitness measure, N pop differnt in GD? | NA | no | no |
| 1003 | 2017 | 43014 | Yang, WY; Novembre, J; Eskin, E; Halperin, E | 2012 | A model-based approach for analysis of spatial structure in genetic data | Characterizing genetic diversity within and between populations has broad applications in studies of human disease and evolution. We propose a new approach, spatial ancestry analysis, for the modeling of genotypes in two-or three-dimensional space. In spatial ancestry analysis (SPA), we explicitly model the spatial distribution of each SNP by assigning an allele frequency as a continuous function in geographic space. We show that the explicit modeling of the allele frequency allows individuals to be localized on the map on the basis of their genetic information alone. We apply our SPA method to a European and a worldwide population genetic variation data set and identify SNPs showing large gradients in allele frequency, and we suggest these as candidate regions under selection. These regions include SNPs in the well-characterized LCT region, as well as at loci including FOXP2, OCA2 and LRP1B. | 1003 | no | no good fitness measure, N pop differnt in GD? | NA | no | no |
| 1004 | 2017 | 43014 | Teatero, S; Ramoutar, E; McGeer, A; Li, AM; Melano, RG; Wasserscheid, J; Dewar, K; Fittipaldi, N | 2016 | Clonal Complex 17 Group B Streptococcus strains causing invasive disease in neonates and adults originate from the same genetic pool | A significant proportion of group B Streptococcus (GBS) neonatal disease, particularly late-onset disease, is associated with strains of serotype III, clonal complex (CC) 17. CC17 strains also cause invasive infections in adults. Little is known about the phylogenetic relationships of isolates recovered from neonatal and adult CC17 invasive infections. We performed whole-genome-based phylogenetic analysis of 93 temporally and geographically matched CC17 strains isolated from both neonatal and adult invasive infections in the metropolitan region of Toronto/Peel, Canada. We also mined the whole-genome data to reveal mobile genetic elements carrying antimicrobial resistance genes. We discovered that CC17 GBS strains causing neonatal and adult invasive disease are interspersed and cluster tightly in a phylogenetic tree, signifying that they are derived from the same genetic pool. We identified limited variation due to recombination in the core CC17 genome. We describe that loss of Pilus Island 1 and acquisition of different mobile genetic elements carrying determinants of antimicrobial resistance contribute to CC17 genetic diversity. Acquisition of some of these mobile genetic elements appears to correlate with clonal expansion of the strains that possess them. Our results provide a genome-wide portrait of the population structure and evolution of a major disease-causing clone of an opportunistic pathogen. | 1004 | no | no good fitness measure, N pop differnt in GD? | NA | no | no |
| 1005 | 2017 | 43014 | Prittinen, K; Pusenius, J; Tahvanainen, J; Rousi, M; Heinonen, J; Roininen, H | 2006 | Herbivory modifies the genetic structure of birch populations | We studied the effects of selective herbivory on genetic diversity in seedling populations of silver birch (Betula pendula), a light-demanding pioneer tree species. The experimental populations consisted of 20 families competing with each other in dense stands. The seedling stands were randomly assigned to insect and vole exposure and fertilization treatments. The study covered the first two growing seasons, during which considerable thinning occurred within the experimental populations. We compared the observed mortality to a simulated control representing random mortality. Our results show that mortality resulted in decreased diversity compared to the simulated control. When insect and vole herbivory and nutrient enrichment treatments were compared to each other, taking into account differences in mortality due to the treatments, vole herbivory led to increased diversity among the tallest seedlings (i.e. canopy layer). This is noteworthy, since the canopy layer includes those with the best prospects of surviving in competition for light. When mortality was accounted for, 2-year natural exposure to insects had no effect on diversity; but cluster analysis showed that insect herbivory changed the genetic composition of the experimental populations. Our results demonstrate the importance of herbivores in shaping the genetic structure of plant populations during the thinning phase. | 1005 | no | no good fitness measure, N pop differnt in GD? | NA | no | no |
| 1006 | 2017 | 43014 | Jung, J; Han, H; Ryu, SH; Kim, W | 2010 | Amplified fragment length polymorphism analysis and genetic variation of the pinewood nematode Bursaphelenchus xylophilus in South Korea | The pinewood nematode Bursaphelenchus xylophilus causes pine wilt disease and is a serious economic concern for the forest industry of South Korea. To achieve effective control with limited resources, it is necessary to clarify the transmission routes and mechanisms of dispersal of this organism. Highly polymorphic and easy-to-use molecular markers can be used for investigating this aspect. In this study, we evaluated the usefulness of amplified fragment length polymorphisms (AFLPs) for investigating the genetic variations of B. xylophilus and related individuals from China. Japan, and South Korea. The AFLP patterns obtained in our study were similar to the microsatellite patterns reported in a previous study; our AFLP patterns indicated high genetic variability and cryptic genetic structure, but did not indicate any peculiar geographic structure. Moreover, the genetic distances between individuals suggested that the Korean population was affected to a greater extent by the Chinese population than the Japanese population. Further, the gene flow among the related species appeared to be limited; however, there may be also the possibility of genetic introgression among species. These results confirm the usefulness of AFLPs for understanding the epidemiology of pine wilt disease, thereby contributing to the effective control of this disease. | 1006 | no | gd of parasite | NA | no | no |
| 1007 | 2017 | 43014 | Gorla, E; Cozzi, MC; Roman-Ponce, SI; Lopez, FJR; Vega-Murillo, VE; Cerolini, S; Bagnato, A; Strillacci, MG | 2017 | Genomic variability in Mexican chicken population using copy number variants | Background: Copy number variations are genome polymorphism that influence phenotypic variation and are an important source of genetic variation in populations. The aim of this study was to investigate genetic variability in the Mexican Creole chicken population using CNVs. Results: The Hidden Markov Model of the PennCNV software detected a total of 1924 CNVs in the genome of the 256 samples processed with Axiom (R) Genome-Wide Chicken Genotyping Array (Affymetrix). The mapped CNVs comprised 1538 gains and 386 losses, resulting at population level in 1216 CNV regions (CNVRs), of which 959 gains, 226 losses and 31 complex (i.e. containing both losses and gains). The CNVRs covered a total of 47 Mb of the whole genome sequence length, corresponding to 5.12% of the chicken galGal4 autosome assembly. Conclusions: This study allowed a deep insight into the structural variation in the genome of unselected Mexican chicken population, which up to now has not been genetically characterized. The genomic study disclosed that the population, even if presenting extreme morphological variation, cannot be organized in differentiated genetic subpopulations. Finally this study provides a chicken CNV map based on the 600 K SNP chip array jointly with a genome-wide gene copy number estimates in a native unselected for more than 500 years chicken population. | 1007 | no | no good fitness measure | NA | no | no |
| 1008 | 2017 | 43014 | Das, A; Bajaj, R; Mohanty, S; Swain, V | 2007 | Genetic, diversity and evolutionary history of Plasmodium falciparum and P-vivax | Understanding the genetic diversity and evolutionary history of the malaria parasite and how it is genetically distinct in different regions of the genome and populations, may open up new avenues to population-specific malaria control measures. The two principal human malaria parasites, Plasmodium falciparum and P. vivax, seem to be very different in origin and in phylogenetic resemblance to other species of Plasmodium. Further, the mortality and morbidity that these two parasites express are quite different, so also the percentage of nucleotide composition in their respective genomes. However, the net genetic diversity at the molecular level (in different independent genes and in both nuclear and mitochondrial genomes) seems to have very little differences, with P. vivax at a slightly higher scale. Whereas African populations of P. falciparum are highly diverse at the DNA level among other populations, high genetic diversity was found in Asian P. vivax; clearly depicting that P. falciparum has possibly originated in Africa and P. vivax in Asia. Furthermore, the findings of a comparably higher genomic diversity in P. vivax corroborate to earlier hypothesis of being older than P. falciparum and also a close genetic affinity with the malaria parasite species of Asian monkeys. On the other hand, P. falciparum is genetically much similar to the chimpanzee malaria parasite, P. reichenowi. The advancements in genomics and parallel statistical analyses of the DNA sequence data so far have succeeded in revealing new evolutionary information on the parasite genes and populations and generate renewed hopes for effective and common vaccines for both forms of malaria. | 1008 | no | gd of parasite | NA | no | no |
| 1009 | 2017 | 43014 | Sexton, JP; McKay, JK; Sala, A | 2002 | Plasticity and genetic diversity may allow saltcedar to invade cold climates in North America | Two major mechanisms have been proposed to explain the ability of introduced populations to colonize over large habitat gradients, despite significant population bottlenecks during introduction: (1) Broad environmental tolerance-successful invaders possess life history traits that confer superior colonizing ability and/or phenotypic plasticity, allowing acclimation to a wide range of habitats. (2) Local adaptation-successful invaders rapidly adapt to local selective pressures. However, even with bottlenecks, many introduced species exhibit surprisingly high levels of genetic variation and thus the potential for evolutionary increases in invasive traits and plasticity. Here we assess the invasive potential of Tamarix ramosissima, by examining the degree of genetic differentiation within and among populations from the latitudinal extremes of its introduced range. Using growth chamber experiments we examined ecologically important variation in seedlings, both in trait means and their reaction norms across temperature environments. Although we found no genetic variation for gas exchange traits, within or among populations, we did find significant genetic variation for growth traits, both in the trait means and in the degree of plasticity in these traits. Northern ecotypes invested more in roots relative to southern ecotypes but only under low temperatures. Both ecotypes increased shoot investment in warm temperatures. Increased root investment in cold temperatures by northern ecotypes may increase their first winter survival. Genetic differences in seedling root investment may contribute to the ability of this species to successfully tolerate and invade a broader latitudinal range. Our data support a model in which both plasticity and adaptive evolution can contribute to the invasive potential of introduced species. | 1009 | no | no good fitness measure | NA | no | no |
| 1010 | 2017 | 43014 | Jones, FA; Hubbell, SP | 2006 | Demographic spatial genetic structure of the Neotropical tree, Jacaranda copaia | We used genotypes from six microsatellite loci and demographic data from a large mapped forest plot to study changes in spatial genetic structure across demographic stages, from seed rain to seedlings, juveniles, and adult diameter classes in the Neotropical tree, Jacaranda copaia. In pairwise comparisons of genetic differentiation among demographic classes, only seedlings were significantly differentiated from the other diameter classes; F-ST values ranged from 0.006 to 0.009. Furthermore, only seedlings showed homozygote excess suggesting biparental inbreeding in the large diameter reproductive adults. We found very low levels of relatedness in the first distance class of trees, 1-26 cm diameter (F-ij = 0.011). However, there was a 5- to 10-fold rise in relatedness in the smallest distance class, from the smallest to the largest tree diameter classes (F-ij = 0.110 for individuals > 56 cm diameter). A variety of non-mutually exclusive mechanisms have been invoked perviously to explain such a pattern, including natural selection, history, or nonequilibrium population dynamics. The long-term demographic data available for this species allow us to evaluate these mechanisms. Jacaranda is a fast-growing, light-demanding species with low recruitment rates and high mortality rates in the smaller diameter classes. It successfully regenerates only in large light gaps, which occur infrequently and stochastically in space and time. These factors contribute to the nonequilibrium population dynamics and observed low genetic structure in the small size classes. We conclude that the pattern of spatial genetic transitions in Jacaranda is consistent with overlapping related generations and strong but infrequent periods of high recruitment, followed by long periods of population decline. | 1010 | no | no good fitness measure, N pop differnt in GD? | NA | no | no |
| 1011 | 2017 | 43014 | Jackson, CR; Groom, RJ; Jordan, NR; McNutt, JW | 2017 | The effect of relatedness and pack size on territory overlap in African wild dogs | Background: Spacing patterns mediate competitive interactions between conspecifics, ultimately increasing fitness. The degree of territorial overlap between neighbouring African wild dog (Lycaon pictus) packs varies greatly, yet the role of factors potentially affecting the degree of overlap, such as relatedness and pack size, remain unclear. We used movement data from 21 wild dog packs to calculate the extent of territory overlap (20 dyads). Results: On average, unrelated neighbouring packs had low levels of overlap restricted to the peripheral regions of their 95% utilisation kernels. Related neighbours had significantly greater levels of peripheral overlap. Only one unrelated dyad included overlap between 75%-75% kernels, but no 50%-50% kernels overlapped. However, eight of 12 related dyads overlapped between their respective 75% kernels and six between the frequented 50% kernels. Overlap between these more frequented kernels confers a heightened likelihood of encounter, as the mean utilisation intensity per unit area within the 50% kernels was 4.93 times greater than in the 95% kernels, and 2.34 times greater than in the 75% kernels. Related packs spent significantly more time in their 95% kernel overlap zones than did unrelated packs. Pack size appeared to have little effect on overlap between related dyads, yet among unrelated neighbours larger packs tended to overlap more onto smaller packs’ territories. However, the true effect is unclear given that the model’s confidence intervals overlapped zero. Conclusions: Evidence suggests that costly intraspecific aggression is greatly reduced between related packs. Consequently, the tendency for dispersing individuals to establish territories alongside relatives, where intensively utilised portions of ranges regularly overlap, may extend kin selection and inclusive fitness benefits from the intra-pack to inter-pack level. This natural spacing system can affect survival parameters and the carrying capacity of protected areas, having important management implications for intensively managed populations of this endangered species. | 1011 | no | no good fitness measure, N pop differnt in GD? | NA | no | no |
| 1012 | 2017 | 43014 | Bittner, TD; Hajek, AE; Haavik, L; Allison, J; Nahrung, H | 2017 | Multiple introductions of Sirex noctilio (Hymenoptera: Siricidae) in northeastern North America based on microsatellite genotypes, and implications for biological control | The invasive woodwasp Sirex noctilio (Hymenoptera: Siricidae) has been moved from Eurasia into regions in the Southern Hemisphere, where extensive tree mortality has occurred in pines (Pinus spp.) introduced for forestry. More recently this woodwasp was found in northeastern North America, where pines are native, and it is a species of concern due to the economic importance of pines. Understanding the genetic diversity of North American S. noctilio points to new areas of inquiry, particularly regarding the ability of parasitic nematodes to sterilize woodwasps, which could provide control methods in the US and/or Canada. We investigated the genetic diversity of 924 S. noctilio from nine populations from New York and Pennsylvania (US), Ontario (CA), and Queensland (AU) using nine microsatellite loci. To avoid inflating the number of populations estimated by Bayesian inference, we measured the full-sibling relationships of woodwasps within 13 trees and removed all but one member of each full-sib family from the genetic analysis, resulting in a final sample size of 741 S. noctilio. Within a tree, on average 39% of woodwasps did not have a full sibling, and there were 5.6 families with at least two full-sibling members per tree. The mean family size across trees was 1.9 when single offspring (i.e., no full siblings) were included. Given the short time span since invasion, variation within North American S. noctilio is likely due to differences among founding genotypes. Genetic analyses support the hypothesis that at least two separate introductions occurred. Within North America, genetic distance measures were greatest between a site in southwestern Ontario and all other sites, suggesting that this population could represent a separate introduction event. Two methods of Bayesian clustering also support this idea; they detected 4 or 5 distinct genetic clusters with little admixture between the southwestern Ontario site and other North American populations. The wasps from Australia, where biological control with nematodes has been successful, showed low genetic diversity and clustered with the southwestern Ontario population in one out of two Bayesian analyses. Within the Ontario subset of samples, high woodwasp activity level (i.e., attack and mortality of trees) was associated with one genetic cluster more strongly than another. Population variation should be taken into account in studies of S. noctilio spread and management within North America. | 1012 | no | gd of pathogen | NA | no | no |
| 1013 | 2017 | 43014 | Prado-Martinez, J; Sudmant, PH; Kidd, JM; Li, H; Kelley, JL; Lorente-Galdos, B; Veeramah, KR; Woerner, AE; O’Connor, TD; Santpere, G; Cagan, A; Theunert, C; Casals, F; Laayouni, H; Munch, K; Hobolth, A; Halager, AE; Malig, M; Hernandez-Rodriguez, J; Hernando-Herraez, I; Prufer, K; Pybus, M; Johnstone, L; Lachmann, M; Alkan, C; Twigg, D; Petit, N; Baker, C; Hormozdiari, F; Fernandez-Callejo, M; Dabad, M; Wilson, ML; Stevison, L; Camprubi, C; Carvalho, T; Ruiz-Herrera, A; Vives, L; Mele, M; Abello, T; Kondova, I; Bontrop, RE; Pusey, A; Lankester, F; Kiyang, JA; Bergl, RA; Lonsdorf, E; Myers, S; Ventura, M; Gagneux, P; Comas, D; Siegismund, H; Blanc, J; Agueda-Calpena, L; Gut, M; Fulton, L; Tishkoff, SA; Mullikin, JC; Wilson, RK; Gut, IG; Gonder, MK; Ryder, OA; Hahn, BH; Navarro, A; Akey, JM; Bertranpetit, J; Reich, D; Mailund, T; Schierup, MH; Hvilsom, C; Andres, AM; Wall, JD; Bustamante, CD; Hammer, MF; Eichler, EE; Marques-Bonet, T | 2013 | Great ape genetic diversity and population history | Most great ape genetic variation remains uncharacterized(1,2); however, its study is critical for understanding population history(3-6), recombination(7), selection(8) and susceptibility to disease(9,10). Here we sequence to high coverage a total of 79 wild-and captive-born individuals representing all six great ape species and seven subspecies and report 88.8 million single nucleotide polymorphisms. Our analysis provides support for genetically distinct populations within each species, signals of gene flow, and the split of common chimpanzees into two distinct groups: Nigeria-Cameroon/western and central/eastern populations. We find extensive inbreeding in almost all wild populations, with eastern gorillas being the most extreme. Inferred effective population sizes have varied radically over time in different lineages and this appears to have a profound effect on the genetic diversity at, or close to, genes in almost all species. We discover and assign 1,982 loss-of-function variants throughout the human and great ape lineages, determining that the rate of gene loss has not been different in the human branch compared to other internal branches in the great ape phylogeny. This comprehensive catalogue of great ape genome diversity provides a framework for understanding evolution and a resource for more effective management of wild and captive great ape populations. | 1013 | no | no good fitness measure, N pop differnt in GD? | NA | no | no |
| 1014 | 2017 | 43014 | Hamner, RM; Constantine, R; Oremus, M; Stanley, M; Brown, P; Baker, CS | 2014 | Long-range movement by Hector’s dolphins provides potential genetic enhancement for critically endangered Maui’s dolphin | For endangered populations with low genetic diversity, low levels of immigration could lead to genetic rescue, reducing the risk of inbreeding depression and enhancing chances of long-term species survival. Our genetic monitoring of Maui’s dolphins revealed the first contemporary dispersal of their sister subspecies, Hector’s dolphin, from New Zealand’s South Island into the Maui’s dolphin distribution along similar to 300km of the North Island’s northwest coast. From 2010 to 2012, 44 individuals were sampled within the Maui’s dolphin distribution, four of which were genetically identified as Hector’s dolphins (two living females, one dead female, one dead male). We also report two Hector’s dolphins (one dead female neonate, one living male) sampled along the North Island’s southwest coast, outside the presumed range of either subspecies. Together, these records demonstrate long-distance dispersal by Hector’s dolphins (400km) and the possibility of an unsampled Hector’s dolphin population along the southwest coast of the North Island. Although two living Hector’s dolphins were found in association with Maui’s dolphins, there is currently no evidence of interbreeding between the subspecies. These results highlight the value of genetic monitoring for subspecies lacking distinctive physical appearances as such discoveries are not detected by other means, but have important conservation implications. | 1014 | no | no good fitness measure, N pop differnt in GD? | NA | no | no |
| 1015 | 2017 | 43014 | Blanquer, A; Uriz, MJ | 2011 | Living Together Apart: The Hidden Genetic Diversity of Sponge Populations | Intraorganism genetic stability is assumed in most organisms. However, here we show for the first time intraorganism genetic heterogeneity in natural populations of marine sponges. A total of 36 different multilocus genotypes (MLGs) were detected in 13 individuals of Scopalina lophyropoda sampled at 4 distant points within each sponge. All genotypes (showing a mosaic distribution), were transmitted to the progeny, thus contributing to the high genetic diversity and low clonality reported for this species, although its populations are small and structured and show high fission rates. There did not seem to be intraindividual genotype conflicts; on the contrary, chimeric individuals are expected to show low mortality thanks to the differential mortality of their different MLGs. This novel mechanism may also counterbalance genetic constraints in other benthic invertebrate species. The presence of sponge chimerism also suggests that results from previous population genetics studies could have been misinterpreted. | 1015 | no | no good fitness measure, N pop differnt in GD? | NA | no | no |
| 1016 | 2017 | 43014 | Papura, D; Van-Helden, M; Giresse, X; Salar, P; Danet, JL; Foissac, X; Malembic-Maher, S | 2007 | “Genetic structure of Scaphoideus titanus populations and genetic diversity of the epidemic strains of”“flavescence doree”" phytoplasma: the situation in France" | “The genetic diversity and structure of the Scaphoideus titanus leafhopper populations and of the”“flavescence doree”" phytoplasma (FDp) they carry, was studied in parallel in southern French vineyards. Seven polymorphic microsatellite loci were used to genotype S. titanus individuals sampled in 24 healthy or FDp-infected vineyards. For each insect, FDp detection and typing was performed by amplifying and sequencing the FDp map gene. A low level of population genetic differentiation (F-ST <= 0.114) was found in S. titanus French populations. It suggests that long-distance gene flows occur due to migration between populations. In addition, it can be hypothesized that passive transport of grapevine planting material carrying eggs could play a role in homogenizing S. titanus population genetic structure. No significant correlation was found between the genetic structure of the French S. titanus populations and the distribution of the different strain types they carry." | 1016 | no | no good fitness measure, N pop differnt in GD? | NA | no | no |
| 1017 | 2017 | 43014 | Migliore, J; Baumel, A; Juin, M; Fady, B; Roig, A; Duong, N; Medail, F | 2013 | Surviving in Mountain Climate Refugia: New Insights from the Genetic Diversity and Structure of the Relict Shrub Myrtus nivellei (Myrtaceae) in the Sahara Desert | The identification of past glacial refugia has become a key topic for conservation under environmental change, since they contribute importantly to shaping current patterns of biodiversity. However, little attention has been paid so far to interglacial refugia despite their key role for the survival of relict species currently occurring in climate refugia. Here, we focus on the genetic consequences of range contraction on the relict populations of the evergreen shrub Myrtus nivellei, endemic in the Saharan mountains since at least the end of the last Green Sahara period, around 5.5 ka B.P. Multilocus genotypes (nuclear microsatellites and AFLP) were obtained from 215 individuals collected from 23 wadis (temporary rivers) in the three main mountain ranges in southern Algeria (the Hoggar, Tassili n’Ajjer and Tassili n’Immidir ranges). Identical genotypes were found in several plants growing far apart within the same wadis, a pattern taken as evidence of clonality. Multivariate analyses and Bayesian clustering revealed that genetic diversity was mainly structured among the mountain ranges, while low isolation by distance was observed within each mountain range. The range contraction induced by the last episode of aridification has likely increased the genetic isolation of the populations of M. nivellei, without greatly affecting the genetic diversity of the species as a whole. The pattern of genetic diversity observed here suggests that high connectivity may have prevailed during humid periods, which is consistent with recent paleoenvironmental reconstructions. | 1017 | no | no good fitness measure, N pop differnt in GD? | NA | no | no |
| 1018 | 2017 | 43014 | Strunk, KE; Amann, V; Threadgill, DW | 2004 | Phenotypic variation resulting from a deficiency of epidermal growth factor receptor in mice is caused by extensive genetic heterogeneity that can be genetically and moleculaidy partitioned | The timing of lethality caused by homozygosity for a null allele of the epidermal growth factor receptor (Egfr(tm1Mag)) in mice is strongly dependent on genetic background. Initial attempts to genetically map background modifiers using Swiss-derived, outbred CD-1 mice were unsuccessful. To investigate the genetic architecture contributing to survival of Egfr(tm1Mag) homozygous embryos, the genetic variability segregating within the outbred population was partitioned by surveying viability of Egfr(tm1Mag) mutants using intercrosses between 129S6/SvEvTAC-Egfr(tm1Mag) and nine Swiss-derived, inbred strains: ALR/LtJ, ALS/LtJ, APN, APS, ICR/HaRos, NOD/LtJ, NON/LtJ, SJL/J, and SWR/J The observations showed that these strains support varying levels of survival of Egfr(tm1Mag) homozygous embryos, suggesting that genetic heterogeneity within the CD-1 stock contributed to the original lack of Egfr(tm1Mag) modifier detection. Similar to the Swiss-derived intercrosses, nine congenic strains, derived from 129S6/SvEvTAC, AKR/J, APN, BALB/cJ, BTBR-T+ tf/tf, C3H/HeJ, C57BL/6J, DBA/2J, and nrB/NJ inbred backgrounds, also supported varying levels of survival of Egfr(tm1Mag) mutants. By intercrossing the congenic lines to create hybrid F-1 embryos, different genetic backgrounds were found to have complementary modifiers. Analysis of the congenic lines argues against heterosis of outbred backgrounds contributing to Egfr(tm1Mag) phenotypic variability. A detailed analysis of the crosses suggests that modifiers function at three distinct stages of development. One class of modifiers supports survival of Egfr(tm1Mag) homozygous embryos to mid-gestation, another class supports development through the mid-gestation transition from yolk-sac to placental-derived nutrient sources, and a third class supports survival through later stages of gestation. Data from microarray analysis using RNA from wildtype and Egfr(tm1Mag) mutant placentas support the existence of extensive genetic heterogeneity and suggest that it can be molecularly partitioned. This method should be generally useful to partition heterogeneity contributing to other complex traits. | 1018 | no | No GD diff between pop | NA | no | no |
| 1019 | 2017 | 43014 | Zakour, MK; Bienefeld, K | 2014 | Basic considerations in the development of breeding plans for honey bees, illustrated by data on the native Syrian honey bee (Apis mellifera syriaca) | “The principles of valuing biodiversity are well understood, but methodologies are not yet adequate for enhancing the diversity of honey bee genetic resources. Genetic diversity is generally assumed to be an important barrier against changing environmental conditions, yet some indigenous honey bee breeds are at risk of becoming extinct. The causes of this often stem from development policies in developing countries in the Middle East and other parts of the world, which have largely ignored well-adapted local breeds. The focus instead has been on the introduction of”“improved”“, exotic ones. Hence, one potential way to ensure survival and promote conservation is by establishing selection programmes that improve efficiency and behaviour in endangered breeds, whilst maintaining their environmental adaptation. However, to develop appropriate genetic models and breeding strategies, genetic aspects and population peculiarities of honey bees must be taken into account. This study aimed at outlining all information needed to plan an efficient honey bee breeding programme, and illustrate this with the example of the endangered Syrian honey bee (Apis mellifera syriaca).” | 1019 | no | no good fitness measure, N pop differnt in GD? | NA | no | no |
| 1020 | 2017 | 43014 | Rottstock, T; Kummer, V; Fischer, M; Joshi, J | 2017 | Rapid transgenerational effects in Knautia arvensis in response to plant community diversity | 1. Plant species persistence in natural communities requires coping with biotic and abiotic challenges. These challenges also depend on plant community composition and diversity. Over time, biodiversity effects have been shown to be strengthened via increasing species complementarity in mixtures. Little is known, however, whether differences in community diversity and composition induce rapid transgenerational phenotypic adaptive differentiation during community assembly. We expect altered plant-plant and other biotic interactions (mutualists or antagonists) in high vs. low diverse communities to affect immediate within-and between-species trait differentiations due to competition for light and nutrients. 2. Three years after the initiation of a large-scale, long-term biodiversity experiment in Jena, Germany, we tested for effects of varying experimental plant community diversity (1-60 plant species; one to four plant functional groups) and composition (with or without legumes and/or grasses) on phenotypic differentiation and variation of the tall herb Knautia arvensis. We measured reproduction at different diversity levels in the Jena Experiment (residents hereafter) and, in an additional common garden experiment without competition, recorded subsequent offspring performance (i.e. growth, reproductive success and susceptibility to powdery mildew) to test for differentiation in phenotypic expression and variability. 3. We observed phenotypic differences among diversity levels with reduced fecundity of K. arvensis residents in more diverse communities. In the next generation grown under common garden conditions, offspring from high-diversity plots showed reduced growth (i.e. height) and lower reproduction (i.e. fewer infructescences), but increased phenotypic trait variability (e.g. in leaf width and powdery mildew presence) and also tended to be less susceptible to powdery mildew infection. 4. Community composition also affected Knautia parents and offspring. In the presence of legumes, resident plants produced more seeds (increased fecundity); however, germination rate of those seeds was reduced at an early seedling stage (reduced fertility). 5. Synthesis. We conclude that rapid transgenerational effects of community diversity and composition on both mean and variation of phenotypic traits among offspring exist. In addition to heritable variation, environmentally induced epigenetic and/or maternal processes matter for early plant community assembly and may also determine future species coexistence and community stability. | 1020 | no | no good fitness measure, N pop differnt in GD? | NA | no | no |
| 1021 | 2017 | 43014 | Kumar, A; Mishra, P; Singh, SC; Sundaresan, V | 2014 | Efficiency of ISSR and RAPD markers in genetic divergence analysis and conservation management of Justicia adhatoda L., a medicinal plant | Genetic variation within and among population is the basis for survival of the population both in short and long term. Thus, studying the plant genetic diversity is essential for any conservation program. Indigenous medicinal plants like Justicia adhatoda L. which are facing high rate of depletion from the wild population need immediate attention. DNA-based dominant molecular marker techniques, random amplification of polymorphic DNA (RAPD) and inter-simple sequence repeat (ISSR) were used to unravel the genetic variability and relationships across thirty-two wild accessions of J. adhatoda L., a valuable medicinal shrub widespread throughout the tropical regions of Southeast Asia. Amplification of genomic DNA using 38 primers (18 RAPD and 20 ISSR) yielded 434 products, of which 404 products were polymorphic revealing 93.11 % polymorphism. The average polymorphic information content value obtained with RAPD and ISSR markers was 0.25 and 0.24, respectively. Marker index (RAPD = 3.94; ISSR = 3.53) and resolving power (RAPD = 4.24; ISSR = 3.94) indicate that the RAPD markers were relatively more efficient than the ISSR assay revealing the genetic diversity of J. adhatoda. The Shannon diversity index obtained with RAPD and ISSR markers was 0.40 and 0.38, respectively. The similarity coefficient ranged from 0.26 to 0.89, 0.33 to 0.93 and 0.31 to 0.90 with RAPD, ISSR and combined UPGMA dendrogram, respectively. PCA derived on the basis of pooled data of both the markers illustrated that the first three principal coordinate components accounted 79.27 % of the genetic similarity variance. The mantel test between two Jaccard’s similarity matrices gave r = 0.901, showing the fit correlation between ISSR- and RAPD-based similarities. Based on the results, ex-situ methods may be the most suitable and efficient measure for long-term conservation. | 1021 | no | no good fitness measure, N pop differnt in GD? | NA | no | no |
| 1022 | 2017 | 43014 | Price, MR; Hadfield, MG | 2014 | Population Genetics and the Effects of a Severe Bottleneck in an Ex Situ Population of Critically Endangered Hawaiian Tree Snails | As wild populations decline, ex situ propagation provides a potential bank of genetic diversity and a hedge against extinction. These programs are unlikely to succeed if captive populations do not recover from the severe bottleneck imposed when they are founded with a limited number of individuals from remnant populations. In small captive populations allelic richness may be lost due to genetic drift, leading to a decline in fitness. Wild populations of the Hawaiian tree snail Achatinella lila, a hermaphroditic snail with a long life history, have declined precipitously due to introduced predators and other human impacts. A captive population initially thrived after its founding with seven snails, exceeding 600 captive individuals in 2009, but drastically declined in the last five years. Measures of fitness were examined from 2,018 captive snails that died between 1998 and 2012, and compared with genotypic data for six microsatellite loci from a subset of these deceased snails (N=335), as well as live captive snails (N=198) and wild snails (N=92). Surprisingly, the inbreeding coefficient (F-is) declined over time in the captive population, and is now approaching values observed in the 2013 wild population, despite a significant decrease in allelic richness. However, adult annual survival and fecundity significantly declined in the second generation. These measures of fitness were positively correlated with heterozygosity. Snails with higher measures of heterozygosity had more offspring, and third generation offspring with higher measures of heterozygosity were more likely to reach maturity. These results highlight the importance of maintaining genetic diversity in captive populations, particularly those initiated with a small number of individuals from wild remnant populations. Genetic rescue may allow for an increase in genetic diversity in the captive population, as measures of heterozygosity and rarified allelic richness were higher in wild tree snails. | 1022 | no | no good fitness measure, N pop differnt in GD? | NA | no | no |
| 1023 | 2017 | 43014 | Nelson, WA; McCauley, E; Wrona, FJ | 2005 | Stage-structured cycles promote genetic diversity in a predator-prey system of Daphnia and algae | Competition theory predicts that population fluctuations can promote genetic diversity when combined with density-dependent selection(1,2). However, this stabilizing mechanism has rarely been tested, and was recently rejected as an explanation for maintaining diversity in natural populations of the freshwater herbivore Daphnia pulex(3). The primary limitation of competition theory is its failure to account for the alternative types of population cycles that are caused by size- or stage-dependent population vital rates - even though such structure both explains the fluctuating dynamics of many species(4) and may alter the outcome of competition(5). Here we provide the first experimental test of whether alternative types of cycles affect natural selection in predator - prey systems. Using competing Daphnia genotypes, we show that internally generated, stage-structured cycles substantially reduce the magnitude of selection ( thereby contributing to the maintenance of genetic diversity), whereas externally forced cycles show rapid competitive exclusion. The change in selection is ecologically significant, spanning the observed range in natural populations(3). We argue that structured cycles reduce selection through a combination of stalled juvenile development and stage-specific mortality. This potentially general fitness-equalizing mechanism may reduce the need for strong stabilizing mechanisms to explain the maintenance of genetic diversity in natural systems. | 1023 | no | no good fitness measure, N pop differnt in GD? | NA | no | no |
| 1024 | 2017 | 43014 | Heath, SC; Gut, IG; Brennan, P; McKay, JD; Bencko, V; Fabianova, E; Foretova, L; Georges, M; Janout, V; Kabesch, M; Krokan, HE; Elvestad, MB; Lissowska, J; Mates, D; Rudnai, P; Skorpen, F; Schreiber, S; Soria, JM; Syvanen, AC; Meneton, P; Hercberg, S; Galan, P; Szeszenia-Dabrowska, N; Zaridze, D; Genin, E; Cardon, LR; Lathrop, M | 2008 | Investigation of the fine structure of European populations with applications to disease association studies | An investigation into fine-scale European population structure was carried out using high-density genetic variation on nearly 6000 individuals originating from across Europe. The individuals were collected as control samples and were genotyped with more than 300 000 SNPs in genome-wide association studies using the Illumina Infinium platform. A major East-West gradient from Russian (Moscow) samples to Spanish samples was identified as the first principal component (PC) of the genetic diversity. The second PC identified a North-South gradient from Norway and Sweden to Romania and Spain. Variation of frequencies at markers in three separate genomic regions, surrounding LCT, HLA and HERC2, were strongly associated with this gradient. The next 18 PCs also accounted for a significant proportion of genetic diversity observed in the sample. We present a method to predict the ethnic origin of samples by comparing the sample genotypes with those from a reference set of samples of known origin. These predictions can be performed using just summary information on the known samples, and individual genotype data are not required. We discuss issues raised by these data and analyses for association studies including the matching of case-only cohorts to appropriate pre-collected control samples for genome-wide association studies. | 1024 | no | NA | NA | no | no |
| 1025 | 2017 | 43014 | Cui, J; Cheng, YY; Belov, K | 2015 | Diversity in the Toll-like receptor genes of the Tasmanian devil (Sarcophilus harrisii) | The Tasmanian devil is an endangered marsupial species that has survived several historical bottlenecks and now has low genetic diversity. Here we characterize the Toll-like receptor (TLR) genes and their diversity in the Tasmanian devil. TLRs are a key innate immune gene family found in all animals. Ten TLR genes were identified in the Tasmanian devil genome. Unusually low levels of diversity were found in 25 devils from across Tasmania. We found two alleles at TLR2, TLR3 and TLR6. The other seven genes were monomorphic. The insurance population, which safeguards the species from extinction, has successfully managed to capture all of these TLR alleles, but concerns remain for the long-term survival of this species. | 1025 | no | no good fitness measure, N pop differnt in GD? | NA | no | no |
| 1026 | 2017 | 43014 | Li, P; Cao, S; Dai, YL; Li, XL; Xu, DF; Guo, M; Pan, YM; Gao, ZM | 2012 | Genetic diversity of Phytophthora capsici (Pythiaceae) isolates in Anhui Province of China based on ISSR-PCR markers | Phytophthora capsici is a plant pathogenic oomycete that damages numerous crops worldwide. Consequently, interest in research on the genetic structure of this species has grown in recent decades. However, there is little information about P. capsici in eastern China. We investigated the genetic diversity of P. capsici isolates from three large regions of Anhui Province in eastern China based on ISSR-PCR technology. Thirteen random primers were screened and used to amplify DNA from 51 samples. We obtained 158 reproducible ISSR fragments, of which 90% were polymorphic, revealing a high degree of polymorphism among the isolates. Genetic similarity coefficients among all the isolates ranged from 0.56 to 0.94, with a mean of 0.84 based on the ISSR data, indicating a high level of genetic variation in these P. capsici isolates. Cluster analysis using UPGMA indicated that the Anhui isolates were divided into seven groups according to the DNA fingerprints, although there was no correlation between the ISSR group and geographic origin. Isolates from the same location showed no clustering based on the year of sampling. AMOVA partitioned variability among (13.6%) and within populations (86.4%). The gene flow among populations ranged from 2.804 to 4.937, with a mean of 3.545, indicating highly frequent gene exchange. Genetic distances and genetic differentiation were negatively correlated with geographic distances. These results lead us to suggest that this pathogen has considerable evolutionary potential, which will enable it to adapt to and overcome management strategies over time. | 1026 | no | gd of pathogen | NA | no | no |
| 1027 | 2017 | 43014 | Gozalo, AS; Elkins, WR; Lambert, LE; Stock, F; Thomas, ML; Woodward, RA | 2016 | Genetic diversity of Klebsiella pneumoniae isolates during an outbreak in a non-human primate research colony | BackgroundKlebsiella pneumoniae can be a serious pathogen in non-human primates, particularly Neotropical monkeys. MethodsDuring a K.pneumoniae outbreak in an owl monkey research colony, 13 K.pneumoniae isolates were DNA fingerprinted by automated repetitive extragenic palindromic-polymerase chain reaction and the profiles compared to isolates obtained from other non-human primate species during the same time period and isolates from previous outbreaks. ResultsEleven different types of K.pneumoniae were circulating in the owl monkey colony at the time of the outbreak. When comparing owl monkey isolates relatedness to previous colony outbreak isolates and squirrel monkey and capuchin monkey isolates, all were different. ConclusionsThese results agree with recent reports where K.pneumoniae nosocomial isolates in hospital settings can have high genetic diversity, and multiple strains can be circulating simultaneously. This potential genetic diversity should be considered when designing strategies for controlling K.pneumoniae outbreaks in captive non-human primate colonies. | 1027 | no | NA | NA | no | no |
| 1028 | 2017 | 43014 | Kempf, F; De Meeus, T; Arnathau, C; Degeilh, B; Mccoy, KD | 2009 | Assortative Pairing in Ixodes ricinus (Acari: Ixodidae), the European Vector of Lyme Borreliosis | In sexual organisms, the way in which gamets aossociate call greatly influence the maintenance of genetic variation, the structure of this valiation in space, and ultimately organismal evolution, Based oil patterns of genetic structure previously found, we explicitly tested whether adults of the sheep tick Ixodes ricinus pair according to their genetic relatedness. We sampled tick pairs from the vegetation in four and genotyped individual ticks at seven microsatellite loci. Based on this data. we observed highly significant assortative mating in two of the four locations. a pattern that could not be accounted for by it spatial autocorrelation in the distribution of related ticks. One explanation for these observations may be the existence of local host associations that develop independently in different populations. Assortative mating in I. ricinus win have clear consequences for its through processes of adaptation and transmission, may significantly alter the epidemiological patterns of the pathogens it carries, including the Lyme disease agent tests win flow be required to examine them mechanisms leading to this pattern and its epidemiological consequences. | 1028 | no | NA | NA | no | no |
| 1029 | 2017 | 43014 | Schmitt, T; Cizek, O; Konvicka, M | 2005 | Genetics of a butterfly relocation: large, small and introduced populations of the mountain endemic Erebia epiphron silesiana | Species specific levels of genetic diversity are necessary for the viability of populations. Erebia epiphron (Lepidoptera: Satyrinae) is a relic species in the Sudetan Mountains, Czech Republic. These populations represent a suitable study object to test the population genetic effects of large, small and introduced populations by means of allozyme electrophoresis. A large continuous and viable population (>100,000 individuals) shows a significantly higher genetic diversity than a small isolated and declining population (ca. 5000 individuals), which most probably was founded during the Little Ice Age in the 17th and 18th centuries and became isolated afterwards. In 1932 and 1933, 50 females were transferred from the large population to the Krkonose Mountains, where they established a viable and quickly expanding population. The number of transferred individuals was sufficient to transfer most of the allozyme diversity of the large natural population. Due to the higher elevation of the Krkonose in comparison to the native range, this allocation might safeguard the survival of the Czech race of E epiphron even under conditions of global warming. (C) 2004 Elsevier Ltd. All rights reserved. | 1029 | no | no good fitness measure | NA | no | no |
| 1030 | 2017 | 43014 | Xie, DF; Zhang, L; Hu, HY; Guo, XL; He, XJ | 2016 | Fragmented habitat drives significant genetic divergence in the Chinese endemic plant, Urophysa henryi (Ranuculaceae) | Urophysa henryi (Oliv.) Ulbr., endemic to China with small populations, is known as a medicinal plant. In this study, ISSR markers were used to assess the genetic diversity and population structure throughout its entire distribution areas. Twelve primers revealed high genetic diversity at the species level, (PPB = 95.6%; H = 0.3441; I = 0.5111), as well as high level of genetic differentiation (FsT = 0.659, p < 0.001; GsT = 0.677) and restricted gene flow (Nm = 0.239) among populations. According to the UPGMA and PCoA analysis, the 9 populations were clustered into three main groups, which were roughly in accordance with their geographical regions. In addition, a significant correlation between the genetic difference and geographic distances among populations was detected from the IBD analysis (r = 0:516, p = 0.003). These results indicated that the habitat heterogeneity and physical barriers play important roles in the modern distribution pattern and population divergence of U. henryi. However, human activities have posed serious threat to its living environment and continued survival. It is necessary to adopt some measures to restrict anthropogenic disturbances and preserve the existing populations. (C) 2016 Elsevier Ltd. All rights reserved. | 1030 | no | no good fitness measure | NA | no | no |
| 1031 | 2017 | 43014 | Winkler, M; Tribsch, A; Paun, O; Englisch, T; Schonswetter, P | 2010 | Pleistocene distribution range shifts were accompanied by breeding system divergence within Hornungia alpina (Brassicaceae) in the Alps | Impact of glacial history on the phylogeography of silicate-dwelling plants of the European Alps has been particularly well studied, whereas virtually no data are available for species growing on different bedrock types, as for Homungia alpina. Bayesian clustering of AFLP data only partly support the distinction of three subspecies as morphologically defined. Whereas the phylogeographical N-group corresponds to subsp. alpina, the congruence of the SW-group and SE-group with subsp. brevicaulis, and subsp. austroalpina. respectively, is limited. High levels of rarity and genetic diversity in the N-group suggest Pleistocene survival along the outer margin of the Alpine arc. For subsp. brevicaulis we suggest a single origin from a refugium in the Southwestern Alps, whereas subsp. austroalpina might have originated twice in the Southern and Southeastern Alps. Different levels of genetic diversity and partitioning of genetic variation indicate a divergence in breeding system, which is corroborated by pollinator exclusion experiments revealing self-incompatibility in the N-group and autonomous selfing in the SE-group. (C) 2009 Elsevier Inc. All rights reserved. | 1031 | no | NA | NA | no | no |
| 1032 | 2017 | 43014 | Fox, M; Sear, R; Beise, J; Ragsdale, G; Voland, E; Knapp, LA | 2010 | Grandma plays favourites: X-chromosome relatedness and sex-specific childhood mortality | Biologists use genetic relatedness between family members to explain the evolution of many behavioural and developmental traits in humans, including altruism, kin investment and longevity. Women’s postmenopausal longevity in particular is linked to genetic relatedness between family members. According to the ‘grandmother hypothesis’, post-menopausal women can increase their genetic contribution to future generations by increasing the survivorship of their grandchildren. While some demographic studies have found evidence for this, others have found little support for it. Here, we re-model the predictions of the grandmother hypothesis by examining the genetic relatedness between grandmothers and grandchildren. We use this new model to re-evaluate the grandmother effect in seven previously studied human populations. Boys and girls differ in the per cent of genes they share with maternal versus paternal grandmothers because of differences in X-chromosome inheritance. Here, we demonstrate a relationship between X-chromosome inheritance and grandchild mortality in the presence of a grandmother. With this sex-specific and X-chromosome approach to interpreting mortality rates, we provide a new perspective on the prevailing theory for the evolution of human female longevity. This approach yields more consistent support for the grandmother hypothesis, and has implications for the study of human evolution. | 1032 | no | human | NA | no | no |
| 1033 | 2017 | 43014 | Elhassan, N; Gebremeskel, EI; Elnour, MA; Isabirye, D; Okello, J; Hussien, A; Kwiatksowski, D; Hirbo, J; Tishkoff, S; Ibrahim, ME | 2014 | The Episode of Genetic Drift Defining the Migration of Humans out of Africa Is Derived from a Large East African Population Size | Human genetic variation particularly in Africa is still poorly understood. This is despite a consensus on the large African effective population size compared to populations from other continents. Based on sequencing of the mitochondrial Cytochrome C Oxidase subunit II (MT-CO2), and genome wide microsatellite data we observe evidence suggesting the effective size (Ne) of humans to be larger than the current estimates, with a foci of increased genetic diversity in east Africa, and a population size of east Africans being at least 2-6 fold larger than other populations. Both phylogenetic and network analysis indicate that east Africans possess more ancestral lineages in comparison to various continental populations placing them at the root of the human evolutionary tree. Our results also affirm east Africa as the likely spot from which migration towards Asia has taken place. The study reflects the spectacular level of sequence variation within east Africans in comparison to the global sample, and appeals for further studies that may contribute towards filling the existing gaps in the database. The implication of these data to current genomic research, as well as the need to carry out defined studies of human genetic variation that includes more African populations; particularly east Africans is paramount. | 1033 | no | human | NA | no | no |
| 1034 | 2017 | 43014 | Zeisset, I; Beebee, TJC | 2010 | Larval fitness, microsatellite diversity and MHC class II diversity in common frog (Rana temporaria) populations | There is increasing interest in using functional rather than neutral loci to assess the genetic health of wild populations. We compared growth and survival of urban and rural frog larvae under three temperature regimes, to vary stress levels, and measured genetic diversities at nine microsatellites and at one major histocompatibility complex ( MHC) class II locus. We found no significant differences between urban and rural frogs in larval survivorship, nor in microsatellite and MHC diversities. However, mean larval growth rates were significantly higher ( by 4.5-18.9% according to temperature) in urban compared with rural populations. Microsatellite and MHC diversities were uncorrelated, though pairwise inter-site F(ST) estimates based on the two types of loci were correlated. MHC F(ST) estimates averaged about twice as high as those based on microsatellites. There was no evidence of isolation by distance with either marker. There were no correlations at the population level between any larval fitness attribute (growth rate or survival) and any diversity estimate (microsatellite or MHC). There was, however, a weak correlation at the sibship level between mean growth rate and microsatellite expected heterozygosity. MHC alleles varied among dying larvae and survivors, and in one case between urban and rural sites, with one allele being under-represented in dying larvae and one allele only occurring in the rural sites. Drift was probably the primary cause of genetic population structure at both types of loci. The use of functional loci to assess population genetic health should focus on the roles of specific alleles as well as overall diversity. Heredity (2010) 104, 423-430; doi:10.1038/hdy.2009.157; published online 18 November 2009 | 1034 | probably yes | ok gd measurement and pop level comp? | no good % survival measure, no big difference GD of pop and corrected for sample size | yes | no |
| 1035 | 2017 | 43014 | Tarr, CL; Conant, S; Fleischer, RC | 1998 | Founder events and variation at microsatellite loci in an insular passerine bird, the Laysan finch (Telespiza cantans) | Historically documented founder events provide opportunities to assess the effects of population size reductions on genetic variation, but the actual magnitude of genetic change can be measured only when direct comparisons can be made to the source or ancestral population. We assayed variation at nine microsatellite loci in the translocated population of the Laysan finch (Telespiza cantans) at Pearl and Hermes reef (PHR), and compared the level of variation to that in the source population pn Laysan Island. Heterogeneity in allele frequencies was highly significant at eight of the nine loci, primarily as a result of fluctuations in allele frequencies in the three PHR populations. Intra- and interpopulational measures of genetic diversity generally matched predictions based on the well-documented history of three islet populations at PHR: significantly lower numbers of alleles and polymorphic loci, as well as higher pairwise F(ST) values and genetic distance, were observed for the two populations that underwent severe size reductions. Changes in heterozygosity at single loci were unpredictable, as both significant increases and decreases were observed in founder populations. A significant excess of heterozygotes was found in two populations and was highly significant over all four finch populations (P < 0.003). Estimates of effective population size from temporal changes in heterozygosity and allele frequencies were very small (N(e) less than or equal to 30) as a result of the founding events and the constraints of islet area on population numbers. We concluded that the PHR population is not adequate as a secondary genetic reserve for T. cantans, and an alternative refuge needs to be established. | 1035 | no | no good fitness measure, N pop differnt in GD? | NA | no | no |
| 1036 | 2017 | 43014 | Lockyear, KM; Waddell, WT; Goodrowe, KL; MacDonald, SE | 2009 | Retrospective Investigation of Captive Red Wolf Reproductive Success in Relation to Age and Inbreeding | The critically endangered red wolf (Canis rufus) has been subject to a strictly managed captive breeding program For three decades. A retrospective demographic analysis of the captive population was performed based on data from the red wolf studbook. Data analyses revealed a decrease in the effective population size relative to the total population size. and changes in age structure and inbreeding coefficients over time. To varying degrees, the probability Of successful breeding and litter sizes declined in association with increasing dam age mid sire inbreeding coefficients. Neonate survival also declined with increasing dam age. Recent changes in strategies regarding breed-pair recommendations have resulted in moderate increases in reproductive success. Zoo Biol 28:214-229, 2009. (C) 2009 Wiley-Liss. Inc. | 1036 | no | NA | NA | no | no |
| 1037 | 2017 | 43014 | Veeramah, KR; Tonjes, A; Kovacs, P; Gross, A; Wegmann, D; Geary, P; Gasperikova, D; Klimes, I; Scholz, M; Novembre, J; Stumvoll, M | 2011 | Genetic variation in the Sorbs of eastern Germany in the context of broader European genetic diversity | Population isolates have long been of interest to genetic epidemiologists because of their potential to increase power to detect disease-causing genetic variants. The Sorbs of Germany are considered as cultural and linguistic isolates and have recently been the focus of disease association mapping efforts. They are thought to have settled in their present location in eastern Germany after a westward migration from a largely Slavic-speaking territory during the Middle Ages. To examine Sorbian genetic diversity within the context of other European populations, we analyzed genotype data for over 30 000 autosomal single-nucleotide polymorphisms from over 200 Sorbs individuals. We compare the Sorbs with other European individuals, including samples from population isolates. Despite their geographical proximity to German speakers, the Sorbs showed greatest genetic similarity to Polish and Czech individuals, consistent with the linguistic proximity of Sorbian to other West Slavic languages. The Sorbs also showed evidence of subtle levels of genetic isolation in comparison with samples from non-isolated European populations. The level of genetic isolation was less than that observed for the Sardinians and French Basque, who were clear outliers on multiple measures of isolation. The finding of the Sorbs as only a minor genetic isolate demonstrates the need to genetically characterize putative population isolates, as they possess a wide range of levels of isolation because of their different demographic histories. European Journal of Human Genetics (2011) 19, 995-1001; doi: 10.1038/ejhg.2011.65; published online 11 May 2011 | 1037 | no | NA | NA | no | no |
| 1038 | 2017 | 43014 | Salgueiro, F; Caron, H; De Souza, MIF; Kremer, A; Margis, R | 2005 | Characterization of nuclear microsatellite loci in South American Araucariaceae species | The Araucariaceae family has only two species in South America: Araucaria angustifolia and Araucaria araucana. Both species are mainly used for timber and have been over-exploited in the past. Currently, they are found as fragmented populations and are classified under the International Union for Conservation of Nature and Natural Resources (IUCN) guidelines as vulnerable species. Population fragmentation may seriously affect the genetic diversity of these two species of Araucariaceae and can consequently lead to decreased survival. To better understand the genetic structure of these South American Araucaria species, eight nuclear microsatellites are reported: six new microsatellites loci developed based on a membrane enrichment procedure and two microsatellites loci transferred from the related species, Araucaria cunninghamii. | 1038 | no | no good fitness measure, N pop differnt in GD? | NA | no | no |
| 1039 | 2017 | 43014 | Gandour, M; Hessini, K; Abdelly, C | 2008 | Understanding the population genetic structure of coastal species (Cakile maritima): seed dispersal and the role of sea currents in determining population structure | The nature and extent of long-distance seed dispersal are currently poorly understood, largely due to the inherent difficulty in detecting such a phenomenon. Genetic methods provide one of the few general approaches that offer the potential to accurately address this issue. Phenotypic and allozymic approaches were applied to characterize inter-population seed dispersal of the sea rocket (Cakile maritima, Brassicaceae), a glabrous and succulent annual herb. Genetic variation was assessed on 360 individuals sampled from nine populations. Genetic diversity across populations was high, 37 % of which was represented by Qst and 16 % by Fst. When genetic distances were used to construct the UPGMA dendrogram, populations were clustered into three groups at the 90 % similarity level. The pattern of clustering can be explained by examining the direction of sea currents around Tunisian coasts. We have shown in this study that C. maritima seeds can survive up to 4 months immersion in sea water and up to I year of floating in sea water; therefore, seed dispersal between populations is possible both in terms of seed survival and current patterns. | 1039 | no | no good fitness measure, N pop differnt in GD? | NA | no | no |
| 1040 | 2017 | 43014 | Tantrawatpan, C; Saijuntha, W; Pilab, W; Sakdakham, K; Pasorn, P; Thanonkeo, S; Thiha; Satrawaha, R; Petney, T | 2011 | Genetic differentiation among populations of Brachytrupes portentosus (Lichtenstein 1796) (Orthoptera: Gryllidae) in Thailand and the Lao PDR: the Mekong River as a biogeographic barrier | The Mekong River is known to act as a boundary between a number of terrestrial and freshwater species, including various parasites and their intermediate hosts as well as endangered mammal species. Little information is available, however, on the genetic differentiation between terrestrial invertebrates to the east and the west of this wide river. The genetic diversity among eight natural populations of Brachytrupes portentosus (Lichtenstein, 1796) (Orthoptera: Gryllidae) collected from Thailand and the Lao People’s Democratic Republic (PDR) were analyzed by multilocus enzyme electrophoresis. The allelic profiles of 20 enzymes encoding 23 loci were analyzed. An average of 41% fixed differences was detected between the populations from Thailand and Lao PDR, which are separated by the Mekong River. The percent fixed differences ranged between 4% and 26% within the populations from Thailand and between 4% and 22% within the populations from Lao PDR. A phenogram shows that the eight populations fell into two major clusters based on the Thai and Lao sampling sites. The genetic distance between the samples within Thailand and within Lao PDR was related to the distances between sampling areas. The genetic variability between populations of this cricket indicates that genetic relationships are influenced by a natural barrier as well as by the geographical distance between these allopatric populations. | 1040 | no | no good fitness measure, N pop differnt in GD? | NA | no | no |
| 1041 | 2017 | 43014 | Sabater-Lleal, M; Soria, JM; Bertranpetit, J; Almasy, L; Blangero, J; Fontcuberta, J; Calafell, F | 2006 | Human F7 sequence is split into three deep clades that are related to FVII plasma levels | It is widely accepted that FVII levels are strongly, consistently, and independently related to cardiovascular risk. These levels are influenced by genetic and environmental factors. Among the genetic factors, only a limited number of polymorphisms in the F7 gene have been reported, and they explain only a small proportion of the genetic variability. Recently, we have accomplished the complete dissection of the F7 quantitative trait locus responsible for all of the genetic variability observed in FVII levels. Now, we present the thorough study of the haplotype organization of F7 DNA sequence variation among individuals and the evolutionary processes that produced this variation, by sequencing 15 kb of genomic DNA sequence from the F7 locus in 40 unrelated individual (80 chromosomes) from the genetic analysis of idiopathic thrombophilia (GAIT) project as well as four non-human primate species. Our study revealed 49 polymorphisms, of which 39 SNPs were further considered. Genotyping of these DNA variations in the whole family-based GAIT sample helped resolve linkage phases, and a total of 37 distinct haplotypes were identified. Tajima’s D was significantly positive in this sample, suggesting balancing selection. This parameter was a reflection of the phylogenetic structure of F7 haplotype, which was deeply split into three well-supported clades or haplogroups, suggesting that functional differences among F7 variants do not depend on a few single-site variations. Moreover, haplogroup 2 was associated with high FVII levels and haplogroup 3 with low levels. In this study, we have for the first time established a clear relation between genotypic variability structure and phenotypic variability of a particular quantitative trait involved in a complex disease. | 1041 | no | human | NA | no | no |
| 1042 | 2017 | 43014 | Herrmann, HW; Pozarowski, KM; Ochoa, A; Schuett, GW | 2017 | An interstate highway affects gene flow in a top reptilian predator (Crotalus atrox) of the Sonoran Desert | “Roads can substantially impact the population connectivity of a wide range of terrestrial vertebrates, often resulting in loss of genetic diversity and an increase of spatial genetic structure. We studied the Western Diamond-backed Rattlesnake (Crotalus atrox), a large and abundant venomous predator, to test the hypothesis that a large and relatively new roadway in Arizona (Interstate Highway I-10) is a barrier that impacts gene flow and population genetics via habitat fragmentation. Based on 72 C. atrox sampled from three specific sampling sites (”“subpopulations”“) on both the west and east corridors of I-10, we used 30 nuclear microsatellite DNA loci and three mitochondrial DNA genes (2615 bp) to assess genetic diversity and structure, estimate effective population size (N (e) ), and describe patterns of gene flow. We found no evidence for loss of genetic diversity or a decrease in N (e) between the three subpopulations. Our microsatellite analysis showed that two subpopulations in close proximity (4 km), but separated by I-10, showed greater levels of genetic differentiation than two subpopulations that were separated by a greater distance (7 km) and not by I-10 or any other obvious barriers. Mitochondrial DNA analyses showed no significant genetic differentiation nor any indication of historically impeded gene flow. Tajima’s D and mismatch distribution tests revealed that demographic expansion is occurring in the overall population (all three subpopulations). Bayesian clustering and spatial genetic autocorrelation analyses of microsatellite data showed resistance to gene flow at the approximate location of I-10. Simulations that investigated gene flow between the subpopulations (with and without a highway barrier present) were consistent with our molecular results. We conclude that I-10 has reduced gene flow in a population of an important reptilian predator of the Sonoran Desert in southern Arizona and make conservation recommendations for reversing this trend.” | 1042 | no | no good fitness measure, N pop differnt in GD? | NA | no | no |
| 1043 | 2017 | 43014 | Tyler, T | 2002 | Geographical distribution of allozyme variation in relation to post-glacial history in Carex digitata, a widespread European woodland sedge | Aim To investigate the distribution, and broad-scale geographical patterns, of variation, in the widespread boreo-nemoral woodland herb Carex digitata throughout its native European range. To interpret the revealed geographical pattern of variation in terms of glacial survival, post-glacial migration and inter-regional gene-flow. Location The whole of Europe divided into twenty-five geographical regions. Methods Genetic variation at nine polymorphic allozyme loci was analysed in 10-25 individuals from 66 populations from throughout the European range of C. digitata . Allele frequencies were calculated both at the level of populations and at the level of geographical regions, and these frequencies were used to calculate the Cavalli-Sforza chord distance (CSCD). CSCD between neighbouring regions were presented on geographical maps. CSCD at both the population and the regional level were subjected to UPGMA cluster analysis. Standard measures of genetic diversity were calculated and divided into within population, region and species components. Results Most alleles had a scattered distribution throughout, but several alleles were mainly found in Fennoscandia and regional allelic richness was the highest here. Cluster analysis on the level of populations did not recover any geographical structure. However, genetic distances between regions, each consisting of 1-4 populations, revealed a clear geographical pattern. Genetic distances were low between (1) Scandinavian and British regions and (2) between Mediterranean regions, moderate between Central European regions and high between far-east European and Caucasian regions. Main conclusions A post-glacial scenario involving independent glacial survivals in south-eastern European Russia, the Caucasian Mountains, the Mediterranean area and central Europe is proposed. Northern Europe (i.e. Fennoscandia) appears to have been colonized through many independent long-distance dispersals from different extra-Fennoscandian populations. High regional population densities in Fennoscandia are assumed to have facilitated accumulation of genetic variation and inter-regional gene-flow as compared with more southern populations which are generally of restricted size and appear to have been mutually isolated and subjected to strong genetic drift. | 1043 | no | no good fitness measure | NA | no | no |
| 1044 | 2017 | 43014 | Larson, S; Jameson, R; Bodkin, J; Staedler, M; Bentzen, P | 2002 | Microsatellite DNA and mitochondrial DNA variation in remnant and translocated sea otter (Enhydra lutris) populations | All existing sea otter (Enhydra lutris) populations have suffered at least 1, and in some cases 2, population bottlenecks. The 1st occurred during the 18th and 19th centuries as a result of commercial hunting that eliminated sea otters from much their native range and reduced surviving populations to small remnants. The 2nd bottleneck occurred when small numbers of otters were reintroduced, via translocation, to areas where the species had been eliminated. We examined genetic variation at 7 microsatellite loci and the mitochondrial DNA (mtDNA) control region in 3 remnant populations, Amchitka Island (Aleutian Islands, Alaska), central coastal California, and Prince William Sound (Alaska), and in 2 reintroduced populations, southeast Alaska and Washington, that were founded with transplants from Amchitka, and in the case of southeast Alaska, individuals from Prince William Sound as well. We found no evidence of reduced genetic diversity in translocated populations. Average expected microsatellite heterozygosities (H,) were similar in all populations (range, 0.40-0.47), and mtDNA haplotype diversities were higher in reintroduced populations (0.51 for both Washington and southeast Alaska) than in remnant populations ((X) over bar = 0.35; range, 0.18-0.45). The levels of genetic diversity we observed within sea otter populations were relatively low when compared with other mammals and are thought to be the result of fur trade exploitation. | 1044 | no | no good fitness measure, N pop differnt in GD? | NA | no | no |
| 1045 | 2017 | 43014 | Royal, CDM; Dunston, GM | 2004 | Changing the paradigm from ‘race’ to human genome variation | Knowledge from the Human Genome Project and research on human genome variation increasingly challenges the applicability of the term ‘race’ to human population groups, raising questions about the validity of inferences made about ‘race’ in the biomedical and scientific literature. Despite the acknowledged contradictions in contemporary science, population-based genetic variation is continually used to explain differences in health between ‘racial’ and ‘ethnic’ groups. In this commentary we posit that resolution of apparent paradoxes in relating biology to ‘race’ and genetics requires thinking ‘outside of the box’. | 1045 | no | human | NA | no | no |
| 1046 | 2017 | 43014 | Forsman, A; Wennersten, L; Karlsson, M; Caesar, S | 2012 | Variation in founder groups promotes establishment success in the wild | Environmental changes currently pose severe threats to biodiversity, and reintroductions and translocations are increasingly used to protect declining populations and species from extinction. Theory predicts that establishment success should be higher for more variable groups of dissimilar individuals. To test this ‘diversity promotes establishment’ hypothesis, we introduced colour polymorphic pygmy grasshoppers (Tetrix subulata) to different sites in the wild. The number of descendants found at the release sites the subsequent year increased with increasing number of colour morphs in the founder group, and variation in founder groups also positively affected colour morph diversity in the established populations. Since colour morphs differ in morphology, physiology, behaviour, reproductive life history and types of niche used, these findings demonstrate that variation among individuals in functionally important traits promotes establishment success under natural conditions, and further indicate that founder diversity may contribute to evolutionary rescue and increased population persistence. | 1046 | no | no good Gd measurement | NA | no | no |
| 1047 | 2017 | 43014 | Maugeri, N; Radhakrishnan, J; Knight, JC | 2010 | Genetic determinants of HSP70 gene expression following heat shock | The regulation of heat shock protein expression is of significant physiological and pathophysiological significance. Here we show that genetic diversity is an important determinant of heat shock protein 70 expression involving local, likely cis-acting, polymorphisms. We define DNA sequence variation for the highly homologous HSPA1A and HSPA1B genes in the major histocompatibility complex on chromosome 6p21 and establish quantitative and specific assays for determining transcript abundance. We show for lymphoblastoid cell lines established from individuals of African ancestry that following heat shock, expression of HSPA1B is associated with rs400547 (P 3.88 x 10(-8)) and linked single nucleotide polymorphisms (SNPs) located 62-93 kb telomeric to HSPA1B. This association was found to explain 31 and 29% of the variance in HSPA1B expression following heat shock or in resting cells, respectively. The associated SNPs show marked variation in minor allele frequency among populations, being more common in individuals of African ancestry, and are located in a region showing population-specific haplotypic block structure. The work illustrates how analysis of a heritable induced expression phenotype can be highly informative in defining functionally important genetic variation. | 1047 | no | NA | NA | no | no |
| 1048 | 2017 | 43016 | Gillingham, MAF; Bechet, A; Courtiol, A; Rendon-Martos, M; Amat, JA; Samraoui, B; Onmus, O; Sommer, S; Cezilly, F | 2017 | Very high MHC Class IIB diversity without spatial differentiation in the mediterranean population of greater Flamingos | Background: Selective pressure from pathogens is thought to shape the allelic diversity of major histocompatibility complex (MHC) genes in vertebrates. In particular, both local adaptation to pathogens and gene flow are thought to explain a large part of the intraspecific variation observed in MHC allelic diversity. To date, however, evidence that adaptation to locally prevalent pathogens maintains MHC variation is limited to species with limited dispersal and, hence, reduced gene flow. On the one hand high gene flow can disrupt local adaptation in species with high dispersal rates, on the other hand such species are much more likely to experience spatial variation in pathogen pressure, suggesting that there may be intense pathogen mediated selection pressure operating across breeding sites in panmictic species. Such pathogen mediated selection pressure operating across breeding sites should therefore be sufficient to maintain high MHC diversity in high dispersing species in the absence of local adaptation mechanisms. We used the Greater Flamingo, Phoenicopterus roseus, a long-lived colonial bird showing a homogeneous genetic structure of neutral markers at the scale of the Mediterranean region, to test the prediction that higher MHC allelic diversity with no population structure should occur in large panmictic populations of long-distance dispersing birds than in other resident species. Results: We assessed the level of allelic diversity at the MHC Class IIB exon 2 from 116 individuals born in four different breeding colonies of Greater Flamingo in the Mediterranean region. We found one of the highest allelic diversity ( 109 alleles, 2 loci) of any non-passerine avian species investigated so far relative to the number of individuals and loci genotyped. There was no evidence of population structure between the four major Mediterranean breeding colonies. Conclusion: Our results suggest that local adaptation at MHC Class IIB in Greater Flamingos is constrained by high gene flow and high MHC diversity appears to be maintained by population wide pathogen-mediated selection rather than local pathogen-mediated selection. Further understanding of how pathogens vary across space and time will be crucial to further elucidate the mechanisms maintaining MHC diversity in species with large panmictic populations and high dispersal rates. | 1048 | no | no good fitness measure, N pop differnt in GD? | NA | no | no |
| 1049 | 2017 | 43016 | Moeller, DA; Tiffin, P | 2005 | Genetic diversity and the evolutionary history of plant immunity genes in two species of Zea | Plant pathogenesis-related genes (PR genes) code for enzymes, enzyme inhibitors, and other peptides that confer resistance to pathogens and herbivores. Although several PR genes have been the subject of molecular population genetic analyses, a general understanding of their long-term evolutionary dynamics remains incomplete. Here we analyze sequence data from 17 PR genes from two closely related teosinte species of central Mexico. In addition to testing whether patterns of diversity at individual loci depart from expectations under a neutral model, we compared patterns of diversity at defense genes, as a class, to nondefense genes. In Zea diploperennis, the majority of defense genes have patterns of diversity consistent with neutral expectations while at least two genes showed evidence of recent positive selection consistent with arms-race models of antagonistic coevolution. In Zea mays ssp. parviglumis, by contrast, analyses of both defense and nondefense genes revealed strong and consistent departures from the neutral model, suggestive of nonequilibrium population dynamics or population structure. Nevertheless, we found a significant excess of replacement polymorphism in defense genes compared to nondefense genes. Although we cannot exclude relaxed selective constraint as an explanation, our results are consistent with temporally variable (transient or episodic) selection or geographically variable selection acting on parviglumis defense genes. The different patterns of diversity found in the two Zea species may be explained by parviglumis’ greater distribution and population structure together with geographic variation in selection. | 1049 | no | no good fitness measure, N pop differnt in GD? | NA | no | no |
| 1050 | 2017 | 43016 | Jordano, P; Godoy, JA | 2000 | RAPD variation and population genetic structure in Prunus mahaleb (Rosaceae), an animal-dispersed tree | We examined the patterns of random amplified polymorphic DNA (RAPD) variation among seven Prunus mahaleb (Rosaceae) populations extending over approximate to 100 km(2) to examine local differentiation in relation to spatial isolation due to both geographical distance and differences in elevation. No less than 51.4% of the RAPD loci were polymorphic, but very few were fixed and among-population variation accounted for 16.46% of variation in RAPD patterns. Mean gene diversity was 0.1441, with mean Nei’s genetic diversity for individual populations ranging between 0.089 and 0.149. Mean G(ST) value across loci was 0.1935 (range, 0.0162-0.4685), giving an average estimate for Nm of 1.191. These results suggest extensive gene flow among populations, but higher G(ST) and lower Nm values relative to other outcrossing. woody species with endozoochorous dispersal, also suggest a process of isolation by distance. The combined effect of both geographical and elevation distances and nonoverlapping flowering and fruiting phenophases on the G(ST) matrix was partially significant, revealing only marginal isolation of the P. mahaleb populations. The matrix correlation between estimated Nm values among populations and the geographical + elevation distance matrices (r = -0.4623, P = 0.07), suggests a marginal trend for more isolated populations to exchange less immigrants. Long-distance seed dispersal by efficient medium-sized frugivorous birds and mammals is most likely associated to the high levels of within-population genetic diversity. However, vicariance factors and demographic bottlenecks (high postdispersal seed and seedling mortality) explain comparatively high levels of local differentiation. | 1050 | probably no | any fitness (survival) of groups of different gd? | no good fitness measure of pop of different GD | no | no |
| 1051 | 2017 | 43016 | Finger, A; Kettle, CJ; Kaiser-Bunbury, CN; Valentin, T; Mougal, J; Ghazoul, J | 2012 | Forest fragmentation genetics in a formerly widespread island endemic tree: Vateriopsis seychellarum (Dipterocarpaceae) | Habitat fragmentation and changed land use have seriously reduced population size in many tropical forest tree species. Formerly widespread species with limited gene flow may be particularly vulnerable to the negative genetic effects of forest fragmentation and small population size. Vateriopsis seychellarum (Dipterocarpaceae) is a formerly widespread canopy tree of the Seychelles, but is now reduced to 132 adult individuals distributed in eleven sites. Using ten microsatellite loci, a genetic inventory of all adult trees and a sample of 317 progeny, we demonstrate that despite its restricted range, overall genetic diversity was relatively high (HE: 0.56). The juvenile cohort, however, had significantly lower allelic richness (adults RS: 3.91; juveniles RS: 2.83) and observed heterozygosity than adult trees (adults HO: 0.62; juveniles HO: 0.48). Rare alleles were fewer and kinship between individuals was stronger in juveniles. Significant fine-scale spatial genetic structure was observed in remnant adults, and parentage analysis indicated that more than 90% of sampled progeny disperse <25 m and pollen dispersed <50 m. The molecular data confirmed that two populations were derived entirely from self-fertilized offspring from a single surviving mother tree. These populations produce viable offspring. Despite this extreme genetic bottleneck, self-compatibility may provide V. seychellarum with some resistance to the genetic consequences of habitat fragmentation, at least in the short term. We discuss our findings in the context of other rare and threatened dipterocarp species which are vulnerable to miss-management of genetic resources and population fragmentation. | 1051 | no | no pop level | NA | no | no |
| 1052 | 2017 | 43016 | Lin, GH; Ci, HX; Thirgood, SJ; Zhang, TZ; Su, JP | 2010 | GENETIC VARIATION AND MOLECULAR EVOLUTION OF ENDANGERED KOZLOV’S PIKA (OCHOTONA KOSLOWI BUCHNER) BASED ON MITOCHONDRIAL CYTOCHROME B GENE | Kozlov’s pika is a rare and endangered lagomorph species with a limited distribution in the southern Kunlun Mountains in western China. Because of its endangered status, Kozlov’s pika is considered a priority species for research and conservation action. Genetic variation and molecular evolution of the Kozlov’s pika were studied based on a total of 14 individuals from four locations along the eastern boundary between Xinjiang and Tibet province (35.20-36.48 degrees N, 86.08-83.04 degrees E) on extremely high elevation (usually over 4800 m a.s.l.). The density of local populations was about 3-4 per ha, living in a typical alpine desert grassland habitat. The complete mitochondrial cytochrome b (cytb) gene was amplified and sequenced. Based on the cytb gene sequences the genetic variation and molecular evolution were analyzed. Unexpected high haplotype diversity (0.956 +/- 0.045) but low nucleotide diversity (0.00537 +/- 0.00126) was found, indicating past demographic expansion. Significant partitioning of variance (P <0.01) among populations (46.7%), and within populations (53.3%), indicating low level of genetic differentiations among local populations. Our results gave an optimistic survival status of Kozlov’s pika at the genetic level. Bayes Empirical Bayes analysis with model M2a and M8 detected three positively selected amino acid sites at the significance level of 0.05. The mutant types with either or both of the mutations aspartic acid to asparagine and glutamic acid to lysine had higher isoelectric point values. We suggested these mutant types might have biological significance to help individuals to adapt to the extremely high elevation habitats. | 1052 | no | no good fitness measure, N pop differnt in GD? | NA | no | no |
| 1053 | 2017 | 43016 | Consuegra, S; de Leaniz, CG | 2008 | MHC-mediated mate choice increases parasite resistance in salmon | Natural (parasite-driven) and sexual selection are thought to maintain high polymorphism in the genes of the major histocompatibility complex (MHC), but support for a link between mate choice, MHC variation and increased parasite resistance is circumstantial. We compared MHC diversity and Anisakis loads among anadromous Atlantic salmon (Salmo salar L.) returning to four rivers to spawn, which had originated from natural spawning (parents allowed to mate freely) or artificial crosses (parents deprived from the potential benefits of mate choice). We found that the offspring of artificially bred salmon had higher parasite loads and were almost four times more likely to be infected than free-mating salmon, despite having similar levels of MHC diversity. Moreover, the offspring of wild salmon were more MHC dissimilar than the offspring of artificially crossed salmon, and uninfected fish were more dissimilar for MHC than infected fish. Thus, our results suggest a link between disassortative mating and offspring benefits and indicate that MHC-mediated mate choice and natural (parasite-driven) selection act in combination to maintain MHC diversity, and hence fitness. Therefore, artificial breeding programmes that negate the potential genetic benefits of mate choice may result in inherently inferior offspring, regardless of population size, rearing conditions or genetic diversity. | 1053 | no | No GD diff between pop | NA | no | no |
| 1054 | 2017 | 43016 | Muller, LAH; Mccusker, JH | 2009 | Microsatellite analysis of genetic diversity among clinical and nonclinical Saccharomyces cerevisiae isolates suggests heterozygote advantage in clinical environments | The genetic structure of a global sample of 170 clinical and nonclinical Saccharomyces cerevisiae isolates was analysed using 12 microsatellite markers. High levels of genetic diversity were revealed both among the clinical and among the nonclinical S. cerevisiae isolates without significant differentiation between these two groups of isolates, rendering a single origin of pathogenic isolates unlikely. This suggests that S. cerevisiae is a true opportunistic pathogen, with a diversity of unrelated genetic backgrounds able to cause infections in humans, and that the ability of S. cerevisiae isolates to cause infections is likely due to a combination of their phenotypic plasticity and the immune system status of the exposed individuals. As was previously reported for bread, beer and wine strains and for environmental S. cerevisiae isolates, the microsatellite genotypes indicated ploidy level variation, from possibly haploid up to tetraploid, among clinical S. cerevisiae isolates. However, rather than haploid, sporulation proficiency and spore viability data indicated that most S. cerevisiae isolates that were mono-allelic at all examined microsatellite loci were likely homothallic and self-diploidized. Interestingly, the proportion of heterozygous clinical isolates was found to be significantly higher than the proportion of heterozygous nonclinical isolates, suggesting a selective advantage of heterozygous S. cerevisiae yeasts in clinical environments. | 1054 | no | gd of pathogen | NA | no | no |
| 1055 | 2017 | 43016 | Broquet, T; Jaquiery, J; Perrin, N | 2009 | OPPORTUNITY FOR SEXUAL SELECTION AND EFFECTIVE POPULATION SIZE IN THE LEK-BREEDING EUROPEAN TREEFROG (HYLA ARBOREA). | Sexual selection in lek-breeding species might drastically lower male effective population size, with potentially important consequences for evolutionary and conservation biology. Using field-monitoring and parental-assignment methods, we analyzed sex-specific variances in breeding success in a population of European treefrogs, to (1) help understanding the dynamics of genetic variance at sex-specific loci, and (2) better quantify the risk posed by genetic drift in this species locally endangered by habitat fragmentation. The variance in male mating success turned out to be markedly lower than values obtained from other amphibian species with polygamous mating systems. The ratio of effective breeding size to census breeding size was only slightly lower in males (0.44) than in females (0.57), in line with the patterns of genetic diversity previously reported from H. arborea sex chromosomes. Combining our results with data on age at maturity and adult survival, we show that the negative effect of the mating system is furthermore compensated by the effect of delayed maturity, so that the estimated instantaneous effective size broadly corresponded to census breeding size. We conclude that the lek-breeding system of treefrogs impacts only weakly the patterns of genetic diversity on sex-linked genes and the ability of natural populations to resist genetic drift. | 1055 | no | no good fitness measure, N pop differnt in GD? | NA | no | no |
| 1056 | 2017 | 43016 | Mayer, F; Bjorklund, N; Wallen, J; Langstrom, B; Cassel-Lundhagen, A | 2014 | Mitochondrial DNA haplotypes indicate two postglacial re-colonization routes of the spruce bark beetle Ips typographus through northern Europe to Scandinavia | Species in northern Europe re-colonized the region after the last glacial maximum via several routes, which could have lingering signatures in current intraspecific trait variation. The spruce bark beetle, Ips typographus, occurs across Europe, and biological differences have been found between southern and northern Scandinavian populations. However, the postglacial history of I. typographus in Scandinavia has not been previously studied at a fine geographical scale. Therefore, we collected specimens across northern Europe and analysed the genetic variation in a quite large mitochondrial fragment (698 bp). A high genetic diversity was found in some of the most northern populations, in the Baltic States, Gotland and central Europe. Detected genetic and phylogeographic structures suggest that I. typographus re-colonized Scandinavia via two pathways, one from the northeast and one from the south. These findings are consistent with the re-colonization history of its host plant, Picea abies. However, we observed low haplotype and nucleotide diversity in southern Scandinavian populations of I. typographus, indicating that (unlike P. abies) it did not disperse across the Baltic Sea in multiple events. Further, the divergence among Scandinavian populations was shallow, conflicting with a scenario where I. typographus expanded concurrently with its host plant from a ‘cryptic refugium’ in the northwest. | 1056 | no | no good fitness measure | NA | no | no |
| 1057 | 2017 | 43016 | Kretzmann, M; Mentzer, L; DiGiovanni, R; Leslie, MS; Amato, G | 2006 | Microsatellite diversity and fitness in stranded juvenile harp seals (Phoca groenlandica) | A positive relationship between genetic diversity at neutral markers and juvenile survival has been demonstrated for many vertebrate populations, although the correlation is typically weak and the explanation for it remains controversial. We assessed variation at 9-12 microsatellite loci in 65 juvenile harp seals (Phoca groenlandica) that stranded in poor condition around Long Island, NY, from 2001 to 2004. Compared with seals that died, surviving individuals had slightly higher measures of mean d(2), which reflects the size difference between alleles within an individual and provides an index of outbreeding. In contrast, there were no significant differences between survivors and nonsurvivors in heterozygosity or estimates of internal relatedness. This pattern is attributed to the fact that these microsatellite markers were exceptionally variable in this species (9-22 alleles per locus), and all individuals were heterozygous at most loci. Under these circumstances, mean d(2) may provide a powerful measure for assessing diversity-fitness correlations. | 1057 | no | no pop level | NA | no | no |
| 1058 | 2017 | 43016 | Puurtinen, M; Hytonen, M; Knott, KE; Taskinen, J; Nissinen, K; Kaitala, V | 2004 | The effects of mating system and genetic variability on susceptibility to trematode parasites in a freshwater snail, Lymnaea stagnalis | The amount and distribution of genetic variability in host populations can have significant effects on the outcome of host-parasite interactions. We studied the effect of mating system and genetic variability on susceptibility of Lymnaea stagnalis snails to trematode parasites. Mating system of snails from eight populations differing in the amount of genetic variability was manipulated, and self- and cross-fertilized offspring were exposed to naturally occurring trematode parasites in a controlled lake experiment. Susceptibility of snails varied between populations, but mating-system treatment did not have a significant effect. Heterozygosity of snails was negatively correlated with the probability of trematode infection, however, suggesting that parasitic diseases may pose a serious threat to populations lacking genetic variability. | 1058 | yes | “from thesis, changed fromm”“prob yes”" to "“yes”"" | NA | no | no |
| 1059 | 2017 | 43016 | Branham, SE; Levi, A; Farnham, MW; Wechter, WP | 2017 | A GBS-SNP-based linkage map and quantitative trait loci (QTL) associated with resistance to Fusarium oxysporum f. sp niveum race 2 identified in Citrullus lanatus var. citroides | A major QTL for resistance to Fusarium oxysporum f. sp. niveum race 2 was mapped to a narrow 1.2 Mb interval using a high-density GBS-SNP linkage map, the first map of Citrullus lanatus var. citroides. Fusarium wilt, a fungal disease caused by Fusarium oxysporum f. sp. niveum (Fon), devastates watermelon crop production worldwide. Several races, which are differentiated by host range, of the pathogen exist. Resistance to Fon race 2, a particularly virulent strain prevalent in the United States, does not exist in edible cultivars of the sweet cultivated watermelon Citrullus lanatus var. lanatus (Cll) and has been well described in a few plant introductions of the wild subspecies of watermelon, C. lanatus var. citroides (Clc). Clc provides a vital source of genetic diversity, as well as resistance to numerous diseases. Unfortunately, both genetic diversity and disease resistance are lacking in Cll due to the narrow genetic base. Despite the importance of Clc to continued watermelon improvement, intra-variety genetic studies are lacking. Here, we present the first Clc genetic linkage map, generated with 2495 single nucleotide polymorphisms developed through genotyping-by-sequencing, and use it to identify quantitative trait loci associated with Fon race 2 resistance. Multiple QTL mapping in a Clc F-2:3 population (N = 173) identified one major and four minor QTL. The major QTL explained 43% of the variation in Fon race 2 resistance and was delimited to a 1.2-Mb interval on chromosome 9, a region spanning 44 genes. | 1059 | no | crop | NA | no | no |
| 1060 | 2017 | 43016 | Hoffman, EA; Kovacs, JL; Goodisman, MAD | 2008 | Genetic structure and breeding system in a social wasp and its social parasite | Background: Social insects dominate ecological communities because of their sophisticated group behaviors. However, the intricate behaviors of social insects may be exploited by social parasites, which manipulate insect societies for their own benefit. Interactions between social parasites and their hosts lead to unusual coevolutionary dynamics that ultimately affect the breeding systems and population structures of both species. This study represents one of the first attempts to understand the population and colony genetic structure of a parasite and its host in a social wasp system. Results: We used DNA microsatellite markers to investigate gene flow, genetic variation, and mating behavior of the facultative social parasite Vespula squamosa and its primary host, V. maculifrons. Our analyses of genetic variability uncovered that both species possessed similar amounts of genetic variation and failed to show genetic structure over the sampling area. Our analysis of mating system of V. maculifrons and V. squamosa revealed high levels of polyandry and no evidence for inbreeding in the two species. Moreover, we found no significant differences between estimates of worker relatedness in this study and a previous investigation conducted over two decades ago, suggesting that the selective pressures operating on queen mate number have remained constant. Finally, the distribution of queen mate number in both species deviated from simple expectations suggesting that mate number may be under stabilizing selection. Conclusion: The general biology of V. squamosa has not changed substantially from that of a typical, nonparasitic Vespula wasp. For example, population sizes of the host and its parasite appear to be similar, in contrast to other social parasites, which often display lower population sizes than their hosts. In addition, parasitism has not caused the mating behavior of V. squamosa queens to deviate from the high levels of multiple mating that typify Vespula wasps. This stands in contrast to some socially parasitic ants, which revert to mating with few males. Overall, the general similarity of the genetic structure of V. maculifrons and V. squamosa presumably reflects the fact that V. squamosa is still capable of independent colony founding and thus reflects an intermediate stage in the evolution of social parasitism. | 1060 | no | no good fitness measure, N pop differnt in GD, focus on the parasite in paper | NA | no | no |
| 1061 | 2017 | 43016 | Sabovljevic, M; Frahm, JP | 2011 | Genetic diversity of the relict moss Rhytidium rugosum (Hypnales) in Europe inferred from the ITS region (nrDNA) | Post-glacial survival, potential migration routes, genetic diversity and phylogeography of the boreal moss species Rhytidium rugosum have been studied. This species is considered to be one of glacial relics of the wide but scattered Holarctic range. According to molecular data sampling from the selected European, American and Asian populations high genetic diversity of this species is present, even if this species is mostly sterile and produced sex organs extremely rarely and spread mostly asexually. Analysing the internal transcribed spacer (ITS) of the nuclear ribosomal DNA, it can be concluded that the populations of this species survived glaciations in various places in Europe and settled and re-settled present range space in various times from various refuges. | 1061 | no | no good fitness measure | NA | no | no |
| 1062 | 2017 | 43016 | Lu, PL; Han, XW; Qi, J; Yang, JG; Wijeratne, AJ; Li, T; Ma, H | 2012 | Analysis of Arabidopsis genome-wide variations before and after meiosis and meiotic recombination by resequencing Landsberg erecta and all four products of a single meiosis | Meiotic recombination, including crossovers (COs) and gene conversions (GCs), impacts natural variation and is an important evolutionary force. COs increase genetic diversity by redistributing existing variation, whereas GCs can alter allelic frequency. Here, we sequenced Arabidopsis Landsberg erecta (Ler) and two sets of all four meiotic products from a Columbia (Col)/Ler hybrid to investigate genome-wide variation and meiotic recombination at nucleotide resolution. Comparing Ler and Col sequences uncovered 349,171 Single Nucleotide Polymorphisms (SNPs), 58,085 small and 2315 large insertions/deletions (indels), with highly correlated genome-wide distributions of SNPs, and small indels. A total of 943 genes have at least 10 nonsynonymous substitutions in protein-coding regions, with enrichment for disease-resistance genes. Another 316 genes are affected by large indels, including 130 genes with complete deletion of coding regions in Ler. Using the Arabidopsis qrtl mutant, two sets of four meiotic products were generated and analyzed by sequencing for meiotic recombination, representing the first tetrad analysis with whole-genome sequencing in a nonfungal species. We detected 18 COs, six of which had an associated GC event, and four GCs without COs (NCOs), and revealed that Arabidopsis GCs are likely fewer and with shorter tracts than those in yeast. Meiotic recombination and chromosome assortment events dramatically redistributed genome variation in meiotic products, contributing to population diversity. In particular, meiosis provides a rapid mechanism to generate copy-number variation (CNV) of sequences that have different chromosomal positions in Col and Ler. | 1062 | no | no good fitness measure, N pop differnt in GD? | NA | no | no |
| 1063 | 2017 | 43016 | Gross, A; Tonjes, A; Kovacs, P; Veeramah, KR; Ahnert, P; Roshyara, NR; Gieger, C; Rueckert, IM; Loeffler, M; Stoneking, M; Wichmann, HE; Novembre, J; Stumvoll, M; Scholz, M | 2011 | Population-genetic comparison of the Sorbian isolate population in Germany with the German KORA population using genome-wide SNP arrays | Background: The Sorbs are an ethnic minority in Germany with putative genetic isolation, making the population interesting for disease mapping. A sample of N = 977 Sorbs is currently analysed in several genome-wide meta-analyses. Since genetic differences between populations are a major confounding factor in genetic meta-analyses, we compare the Sorbs with the German outbred population of the KORA F3 study (N = 1644) and other publically available European HapMap populations by population genetic means. We also aim to separate effects of over-sampling of families in the Sorbs sample from effects of genetic isolation and compare the power of genetic association studies between the samples. Results: The degree of relatedness was significantly higher in the Sorbs. Principal components analysis revealed a west to east clustering of KORA individuals born in Germany, KORA individuals born in Poland or Czech Republic, Half-Sorbs (less than four Sorbian grandparents) and Full-Sorbs. The Sorbs cluster is nearest to the cluster of KORA individuals born in Poland. The number of rare SNPs is significantly higher in the Sorbs sample. FST between KORA and Sorbs is an order of magnitude higher than between different regions in Germany. Compared to the other populations, Sorbs show a higher proportion of individuals with runs of homozygosity between 2.5 Mb and 5 Mb. Linkage disequilibrium (LD) at longer range is also slightly increased but this has no effect on the power of association studies. Oversampling of families in the Sorbs sample causes detectable bias regarding higher FST values and higher LD but the effect is an order of magnitude smaller than the observed differences between KORA and Sorbs. Relatedness in the Sorbs also influenced the power of uncorrected association analyses. Conclusions: Sorbs show signs of genetic isolation which cannot be explained by over-sampling of relatives, but the effects are moderate in size. The Slavonic origin of the Sorbs is still genetically detectable. Regarding LD structure, a clear advantage for genome-wide association studies cannot be deduced. The significant amount of cryptic relatedness in the Sorbs sample results in inflated variances of Beta-estimators which should be considered in genetic association analyses. | 1063 | no | human | NA | no | no |
| 1064 | 2017 | 43016 | Loiseau, C; Zoorob, R; Robert, A; Chastel, O; Julliard, R; Sorci, G | 2011 | Plasmodium relictum infection and MHC diversity in the house sparrow (Passer domesticus) | Antagonistic coevolution between hosts and parasites has been proposed as a mechanism maintaining genetic diversity in both host and parasite populations. In particular, the high level of genetic diversity usually observed at the major histocompatibility complex (MHC) is generally thought to be maintained by parasite-driven selection. Among the possible ways through which parasites can maintain MHC diversity, diversifying selection has received relatively less attention. This hypothesis is based on the idea that parasites exert spatially variable selection pressures because of heterogeneity in parasite genetic structure, abundance or virulence. Variable selection pressures should select for different host allelic lineages resulting in population-specific associations between MHC alleles and risk of infection. In this study, we took advantage of a large survey of avian malaria in 13 populations of the house sparrow (Passer domesticus) to test this hypothesis. We found that (i) several MHC alleles were either associated with increased or decreased risk to be infected with Plasmodium relictum, (ii) the effects were population specific, and (iii) some alleles had antagonistic effects across populations. Overall, these results support the hypothesis that diversifying selection in space can maintain MHC variation and suggest a pattern of local adaptation where MHC alleles are selected at the local host population level. | 1064 | probably no | no good fitness measure, N pop differnt in GD, focus on the parasite in paper | gd mean of ind/pop not pop level/not corrected for pop sample size | yes | no |
| 1065 | 2017 | 43016 | Berthouly-Salazar, C; Thevenon, S; Van, TN; Nguyen, BT; Pham, LD; Chi, CV; Maillard, JC | 2012 | Uncontrolled admixture and loss of genetic diversity in a local Vietnamese pig breed | The expansion of intensive livestock production systems in developing countries has increased the introduction of highly productive exotic breeds facilitating indiscriminate crossbreeding with local breeds. In this study, we set out to investigate the genetic status of the Vietnamese Black H’mong pig breed by evaluating (1) genetic diversity and (2) introgression from exotic breeds. Two exotic breeds, namely Landrace and Yorkshire used for crossbreeding, and the H’mong pig population from Ha Giang (HG) province were investigated using microsatellite markers. Within the province, three phenotypes were observed: a White, a Spotted and a Black phenotype. Genetic differentiation between phenotypes was low (0.5-6.1%). The White phenotypes showed intermediate admixture values between exotic breeds and the Black HG population (0.53), indicating a crossbreed status. Management practices were used to predict the rate of private diversity loss due to exotic gene introgressions. After 60 generations, 100% of Black private alleles will be lost. This loss is accelerated if the admixture rate is increased but can be slowed down if the mortality rate (e.g., recruitment rate) is decreased. Our study showed that a large number of markers are needed for accurately identifying hybrid classes for closely related populations. While our estimate of admixture still seems underestimated, genetic erosion can occur very fast even through indiscriminate crossbreeding. | 1065 | no | domesticated animal | NA | no | no |
| 1066 | 2017 | 43016 | Morton, ES | 2009 | The Function of Multiple Mating by Female Promethea Moths, Callosamia promethea (Drury) (Lepidoptera: Saturniidae) | Promethea (Callosomia promethea) and tulip tree silk moths (C. angulifera) were compared under semi-natural conditions for the presence of polyandry. Promethea were polyandrous, the first documentation for a saturniid moth, whereas tulip tree moths were monandrous. Experiments showed that polyandrous and monandrous promethea females achieved similar egg fertility, but polyandrous females laid 10% more eggs, a significant difference (P < 0.05). This difference was found in five sibling groups, whose larvae were reared on the same individual food plant. Higher fecundity for polyandrous promethea females was not related to female size, duration of copulation, egg size and number, number of days in laying period or sibling group. Egg size declined later in the laying period for all females. Small females laid more eggs earlier in the laying period than large females. A separate mask/recapture study showed that male promethea also mate multiple times (polygyny) and distinguish virgin from nonvirgin females in mating preference. The diurnally mating promethea may grain increased genetic variability and/or possibly seminal gifts from polyandry. Monandry in totally nocturnal saturniid moths may result from a time conflict between egg laying and mating, which overrides the benefits of polyandry. If polyandry increases fecundity, it is predicted to occur in other diurnally mating saturniids. | 1066 | no | no pop level | NA | no | no |
| 1067 | 2017 | 43016 | Smetham, G; Ades, P; Ford, R | 2008 | Development of microsatellite markers for the grapevine fungal pathogen Phaeomoniella chlamydospora | Twenty polymorphic microsatellite markers from microsatellite-enriched genomic DNA of the grapevine fungal pathogen, Phaeomoniella chlamydospora, were developed and characterized. The markers were used to genotype isolates from Australia and from Europe/Eurasia. The number of alleles per locus ranged from two to 11. Gene diversity per locus ranged from 0.08 to 0.63 among Australian isolates, and from 0.2 to 0.77 among isolates from Europe/Eurasia, demonstrating the suitability of these markers for population genetic studies of P. chlamydospora. Eighteen of the 20 markers also amplified a product in the closely related Phaeoacremonium aleophilum. | 1067 | no | gd of pathogen | NA | no | no |
| 1068 | 2017 | 43016 | Lopez-Pujol, J; Martinell, MC; Masso, S; Blanche, C; Saez, L | 2013 | The ‘paradigm of extremes’: extremely low genetic diversity in an extremely narrow endemic species, Coristospermum huteri (Umbelliferae) | Low levels of genetic diversity in endemic species are generally attributable to the small size of their populations. This lack of genetic variability will, predictably, be more evident in those species that occur in only one or a very few localities with a total population consisting of a few dozen individuals, or sometimes fewer (i.e. ‘extremely narrow endemics’, ENEs). We used allozyme electrophoresis to survey the genetic variability of Coristospermum huteri, an endemic species from the island of Majorca (Balearic Islands, W. Mediterranean Basin) with a single natural population of about 100 individuals. As expected, allozyme variability was virtually nil for this species (P = 8.3 %, A = 1.08, H (e) = 0.022), which seems to be a general rule for ENEs (mean H (e) = 0.057). A founder effect associated with a dispersal event from the continent is probably behind the lack of genetic diversity in this highly threatened species. Preservation of the mountain summit where the plant is found (Puig Major) is essential for the survival of C. huteri, and would also guarantee the conservation of other ENEs and rare and threatened species. | 1068 | no | no good fitness measure, N pop differnt in GD, focus on the parasite in paper | NA | no | no |
| 1069 | 2017 | 43016 | Newhouse, DJ; Balakrishnan, CN | 2015 | High major histocompatibility complex class I polymorphism despite bottlenecks in wild and domesticated populations of the zebra finch (Taeniopygia guttata) | Background: Two subspecies of zebra finch, Taeniopygia guttata castanotis and T. g. guttata are native to Australia and the Lesser Sunda Islands, respectively. The Australian subspecies has been domesticated and is now an important model system for research. Both the Lesser Sundan subspecies and domesticated Australian zebra finches have undergone population bottlenecks in their history, and previous analyses using neutral markers have reported reduced neutral genetic diversity in these populations. Here we characterize patterns of variation in the third exon of the highly variable major histocompatibility complex (MHC) class I a chain. As a benchmark for neutral divergence, we also report the first mitochondrial NADH dehydrogenase 2 (ND2) sequences in this important model system. Results: Despite natural and human-mediated population bottlenecks, we find that high MHC class I polymorphism persists across all populations. As expected, we find higher levels of nucleotide diversity in the MHC locus relative to neutral loci, and strong evidence of positive selection acting on important residues forming the peptide-binding region (PBR). Clear population differentiation of MHC allele frequencies is also evident, and this may be due to adaptation to new habitats and associated pathogens and/or genetic drift. Whereas the MHC Class I locus shows broad haplotype sharing across populations, ND2 is the first locus surveyed to date to show reciprocal monophyly of the two subspecies. Conclusions: Despite genetic bottlenecks and genetic drift, all surveyed zebra finch populations have maintained high MHC Class I diversity. The diversity at the MHC Class I locus in the Lesser Sundan subspecies contrasts sharply with the lack of diversity in previously examined neutral loci, and may thus be a result of selection acting to maintain polymorphism. Given uncertainty in historical population demography, however, it is difficult to rule out neutral processes in maintaining the observed diversity. The surveyed populations also differ in MHC Class I allele frequencies, and future studies are needed to assess whether these changes result in functional immune differences. | 1069 | no | no good fitness measure | NA | no | no |
| 1070 | 2017 | 43016 | Campbell, H; Carothers, AD; Rudan, I; Hayward, C; Biloglav, Z; Barac, L; Pericic, M; Janicijevic, B; Smolej-Narancic, N; Polasek, O; Kolcic, I; Weber, JL; Hastie, ND; Rudan, P; Wright, AF | 2007 | Effects of genome-wide heterozygosity on a range of biomedically relevant human quantitative traits | The dramatic changes in human population structure over the last 200 years have resulted in significant levels of outbreeding, which, in turn, is predicted to lead to increased levels of individual genetic diversity (genome-wide heterozygosity, h). To investigate possible effects of these large demographic changes on global health, we studied the effect of h, measured as relative heterozygosity, h(R), on 15 disease-related traits in four groups of individuals with widely differing ancestral histories (ranging from outbred to inbred) from the Dalmatian islands in Croatia. Higher levels of h(R), estimated using 1184 STR/indel markers, were found in the outbred group (P < 0.0001) and were associated with lower blood pressure (BP) and total/LDL cholesterol (P = 0.01 and 0.01, respectively) after controlling for other factors, with BP showing a strong sex effect (males P > 0.5 and females P = 0.002). These findings, if replicated, suggest that h(R) be considered as a genetic risk factor in genetic epidemiological studies on common disease traits. They are consistent with the well-known effects of heterosis (hybrid vigour) described when outcrossing animals and plants. Outbreeding resulting from urbanization and migration from traditional population subgroups may be leading to increasing h(R) and may have beneficial effects on a range of traits associated with human health and disease. Other traits, such as age at menarche, IQ and lifespan, which have been changing during the decades of urbanization, may also have been influenced by demographic factors. | 1070 | no | human | NA | no | no |
| 1071 | 2017 | 43016 | Amat, ME; Silvertown, J; Vargas, P | 2013 | Strong Spatial Genetic Structure Reduces Reproductive Success in the Critically Endangered Plant Genus Pseudomisopates | Clonal growth can be a double-edged sword for endangered species, because the short-term insurance against extinction may incur a longer-term hazard of creating small inbred populations with low fecundity. In the present study, we quantify the advantages and disadvantages of clonal growth regarding the fitness of the central Iberian monotypic endangered genus Pseudomisopates. Preliminary studies showed that the species is self-incompatible and exhibits extensive clonal growth with plants flowering profusely. However, seeds at many sites seemed to be unviable, and no seedlings have been observed in the field. A fully replicated nested sampling design (n = 100) was conducted to explore genetic (using seven SSR loci) and environmental factors potentially affecting seed viability, such as: 1) clonal and genetic diversity, 2) spatial genetic structure, and 3) environmental factors (shrub cover and grazing). Generalized Linear Mixed Models were fitted relating genetic and environmental variables to reproductive variables (seed viability and flower display). Our results indicate that the relatively low genotypic diversity of the population (PD = 0.23), as quantified by SSRs, and the strong spatial genetic structure observed are congruent with intense clonal growth. This clonal growth is enhanced by unfavorable environmental conditions, such as canopy closure and grazing. Under these circumstances, both flower display and mate availability decrease, thus hindering sexual reproduction. Indeed, a mixed reproductive system (clonal and sexual) to escape environmental stochasticity is crucial for the survival of Pseudomisopates, a species inhabiting a disturbance-prone ecosystem. | 1071 | no | no good fitness measure, N pop differnt in GD? | NA | no | no |
| 1072 | 2017 | 43016 | Gsell, AS; Domis, LNDS; Verhoeven, KJF; van Donk, E; Ibelings, BW | 2013 | Chytrid epidemics may increase genetic diversity of a diatom spring-bloom | Contrary to expectation, populations of clonal organisms are often genetically highly diverse. In phytoplankton, this diversity is maintained throughout periods of high population growth (that is, blooms), even though competitive exclusion among genotypes should hypothetically lead to the dominance of a few superior genotypes. Genotype-specific parasitism may be one mechanism that helps maintain such high-genotypic diversity of clonal organisms. Here, we present a comparison of population genetic similarity by estimating the beta-dispersion among genotypes of early and peak bloom populations of the diatom Asterionella formosa for three spring-blooms under high or low parasite pressure. The Asterionella population showed greater beta-dispersion at peak bloom than early bloom in the 2 years with high parasite pressure, whereas the within group dispersion did not change under low parasite pressure. Our findings support that high prevalence parasitism can promote genetic diversification of natural populations of clonal hosts. | 1072 | maybe | what do they mean with high and low parasite preasure, natural? | no gd of host pop and fitness of host pop | no | no |
| 1073 | 2017 | 43016 | Kobayashi, Y; Achaz, G; Telschow, A | 2011 | Effect of parasitic sex-ratio distorters on host gene frequencies in a mainland-island context | It was previously argued that infection by parasitic sex-ratio distorters can enhance both random genetic drift and genetic influx from outside the population. However, these two enhancement effects have been studied independently. Here, we study the equilibrium frequencies of alleles (neutral and selected) in a mainland-island scenario where both genetic drift and genetic influx are enhanced due to infection by a cytoplasmic feminizing element. Interestingly, our model reveals that at neutral loci, the two effects almost exactly cancel each other out, such that infection has only a very minor effect on the equilibrium frequency distributions of alleles. At selected loci, in contrast, the two effects are unbalanced and infection has conspicuous effects. Despite the cryptic effects of infection at neutral loci, we demonstrate that temporally spaced data can be used to evaluate the effect of infection on genetic drift and that on gene flow separately. | 1073 | no | NA | NA | no | no |
| 1074 | 2017 | 43016 | Bjorklund, M; Arrendal, J | 2008 | Demo-genetic analysis of a recovering population of otters in Central Sweden | We performed a demo-genetic population viability analysis on a recovering population of otters Lutra lutra in Central Sweden, using data on population size, survival and genetic data from microsatellites. Population data were obtained from genotyping faeces. At present, the size and genetic variability of the population is increasing. We found that survival to first reproduction was the most crucial demographic parameter, and that even slight changes downward in this parameter, might lead to a declining population trajectory. Human factors that can affect mortality are traffic, fishing equipment and traps, and we argue that efforts to minimize road kills by means of safe passages as well as careful fishing efforts in streams and lakes would reduce the risk of extinction. In general, even though the population is now growing and has no inbreeding problem, its small abundance could make it vulnerable to chance events and environmental perturbations. | 1074 | no | No GD diff between pop | NA | no | no |
| 1075 | 2017 | 43016 | Liu, XB; Etienne, RS; Liang, MX; Wang, YF; Yu, SX | 2015 | Experimental evidence for an intraspecific Janzen-Connell effect mediated by soil biota | The negative effect of soil pathogens on seedling survival varies considerably among conspecific individuals, but the underlying mechanisms are largely unknown. For variation between heterospecifics, a common explanation is the Janzen-Connell effect: negative density dependence in survival due to specialized pathogens aggregating on common hosts. We test whether an intraspecific Janzen-Connell effect exists, i.e., whether the survival chances of one population’s seedlings surrounded by a different conspecific population increase with genetic difference, spatial distance, and trait dissimilarity between them. In a shade-house experiment, we grew seedlings of five populations of each of two subtropical tree species (Castanopsis fissa and Canarium album) for which we measured genetic distance using intersimple sequence repeat (ISSR) analysis and eight common traits/characters, and we treated them with soil material or soil biota filtrate collected from different populations. We found that the relative survival rate increased with increasing dissimilarity measured by spatial distance, genetic distance, and trait differences between the seedling and the population around which the soil was collected. This effect disappeared after soil sterilization. Our results provide evidence that genetic variation, trait similarity, and spatial distance can explain intraspecific variation in plant-soil biotic interactions and suggest that limiting similarity also occurs at the intraspecific level. | 1075 | maybe | two species? How is gd? | no good gd measure, genetic distance population rather than GD diversity differece | no | no |
| 1076 | 2017 | 43016 | Sirvio, A; Gadau, J; Rueppell, O; Lamatsch, D; Boomsma, JJ; Pamilo, P; Page, RE | 2006 | High recombination frequency creates genotypic diversity in colonies of the leaf-cutting ant Acromyrmex echinatior | Honeybees are known to have genetically diverse colonies because queens mate with many males and the recombination rate is extremely high. Genetic diversity among social insect workers has been hypothesized to improve general performance of large and complex colonies, but this idea has not been tested in other social insects. Here, we present a linkage map and an estimate of the recombination rate for Acromyrmex echinatior, a leaf-cutting ant that resembles the honeybee in having multiple mating of queens and colonies of approximately the same size. A map of 145 AFLP markers in 22 linkage groups yielded a total recombinational size of 2076 cM and an inferred recombination rate of 161 kb cM(-1) (or 6.2 cM Mb(-1)). This estimate is lower than in the honeybee but, as far as the mapping criteria can be compared, higher than in any other insect mapped so far. Earlier studies on A. echinatior have demonstrated that variation in division of labour and pathogen resistance has a genetic component and that genotypic diversity among workers may thus give colonies of this leaf-cutting ant a functional advantage. The present result is therefore consistent with the hypothesis that complex social life can select for an increased recombination rate through effects on genotypic diversity and colony performance. | 1076 | no | no good fitness measure | NA | no | no |
| 1077 | 2017 | 43016 | Carson, EW; Turner, TF; Saltzgiver, MJ; Adams, D; Kesner, BR; Marsh, PC; Pilger, TJ; Dowling, TE | 2016 | Retention of Ancestral Genetic Variation Across Life-Stages of an Endangered, Long-Lived Iteroparous Fish | “As with many endangered, long-lived iteroparous fishes, survival of razorback sucker depends on a management strategy that circumvents recruitment failure that results from predation by nonnative fishes. In Lake Mohave, AZ-NV, management of razorback sucker centers on capture of larvae spawned in the lake, rearing them in off-channel habitats, and subsequent release (”“repatriation”") to the lake when adults are sufficiently large to resist predation. The effects of this strategy on genetic diversity, however, remained uncertain. After correction for differences in sample size among groups, metrics of mitochondrial DNA (mtDNA | 1077 | no | no good fitness measure | NA | no | no |
| 1078 | 2017 | 43016 | Gonzalez, AV; Santelices, B | 2017 | Frequency of chimerism in populations of the kelp Lessonia spicata in central Chile | “Chimerism occurs when two genetically distinct conspecific individuals fuse together generating a single entity. Coalescence and chimerism in red seaweeds has been positively related to an increase in body size, and the consequent reduction in susceptibility to mortality factors, thus increasing survival, reproductive potential and tolerance to stress in contrast to genetically homogeneous organisms. In addition, they showed that a particular pattern of post-fusion growth maintains higher genetic diversity and chimerism in the holdfast but homogenous axes. In Chilean kelps (brown seaweeds), intraorganismal genetic heterogeneity (IGH) and holdfast coalescence has been described in previous research, but the extent of chimerism in wild populations and the patterns of distribution of the genetically heterogeneous thallus zone have scarcely been studied. Since kelps are under continuous harvesting, with enormous social, ecological and economic importance, natural chimerism can be considered a priceless in-situ reservoir of natural genetic resources and variability. In this study, we therefore examined the frequency of IGH and chimerism in three harvested populations of Lessonia spicata. We then evaluated whether chimeric wild-type holdfasts show higher genetic diversity than erect axes (stipe and lamina) and explored the impact of this on the traditional estimation of genetic diversity at the population level. We found a high frequency of IGH (60 - 100%) and chimerism (33.3 - 86.7%), varying according to the studied population. We evidenced that chimerism occurs mostly in holdfasts, exhibiting heterogeneous tissues, whereas stipes and lamina were more homogeneous, generating a vertical gradient of allele and genotype abundance as well as divergence, constituting the first time”" within- plant "" genetic patterns have been reported in kelps. This is very different from the chimeric patterns described in land plants and animals. Finally, we evidenced that IGH affected genetic differentiation among populations, showed lower levels of FST index when we compared holdfast than lamina samples. In the light of this, future studies should evaluate the significance of chimeric holdfasts in their ability to increase kelps resilience, improve restoration and ecosystem service." | 1078 | no | no good fitness measure, N pop differnt in GD? | NA | no | no |
| 1079 | 2017 | 43016 | Sutton, JT; Castro, I; Robertson, BC; Tompkins, DM; Stanton, JAL; Jamieson, IG | 2016 | MHC genetic diversity and avian malaria prevalence in Mokoia Island saddlebacks | Studies of wildlife populations have identified associations between disease resistance and diversity at genes of the major histocompatibility complex (MHC), which are involved with adaptive immunity. We compare MHC class II B (MHCIIB) and microsatellite genetic diversity in a population of New Zealand passerine birds, North Island saddlebacks on Mokoia Island, that was also tested for avian malaria. Prevalence of infection was low, and we found no conclusive evidence that infection status was linked to particular MHCIIB alleles. Individual-level MHCIIB nucleotide diversity, and the number of alleles per bird were both lower in infected compared with uninfected samples; however, neither trend was statistically significant. Genetic structure was observed in the MHCIIB dataset, suggesting that selective forces are shaping MHCIIB diversity in this population. Future research would benefit from long-term monitoring of allele frequencies and larger sample sizes where possible. Additionally, including disease intensity data (i.e. the degree of infection in an individual) alongside prevalence data (i.e. presence/absence of infection in an individual) may enhance the ability to detect phenotype-genotype associations in future research. | 1079 | no | only one pop | NA | no | no |
| 1080 | 2017 | 43016 | de Carvalho, LP; Farias, FJC; Rodrigues, JIS; Suassuna, ND; Teodoro, PE | 2017 | Genetic diversity among exotic cotton accessions as for qualitative and quantitative traits | Studying genetic diversity among a group of genotypes is important in genetic breeding because identifying hybrid combinations of greater heterotic effect also increases the chance of obtaining plants with favorable allele combinations in an intra-population selection program. The objective of this study was to compare different types of long and extra-long staple cotton and their genetic diversity in relation to the fiber traits and some agronomic traits in order to grant breeding programs. Diversity analysis among 29 cotton accessions based on qualitative and quantitative traits and joint including qualitative and quantitative traits was performed. Analysis based on qualitative and quantitative traits and joint met the accessions in three, two, and three groups, respectively. The cross between genotypes Giza 59 and Pima unknown was the most promising to generate segregating populations, comprising simultaneously resistance (based on molecular markers) to blue disease and bacterial blight, partial resistance to root-knot nematode, smaller size, in addition to good fiber characteristics. These populations can be used in recurrent selection programs as donors of alleles for development of long-staple cotton genotypes. | 1080 | no | no good fitness measure, N pop differnt in GD? | NA | no | no |
| 1081 | 2017 | 43016 | Lopes, MS; Mendonca, D; Bettencourt, SX; Borba, AR; Melo, C; Baptista, C; Machado, AD | 2014 | Genetic diversity of an Azorean endemic and endangered plant species inferred from inter-simple sequence repeat markers | Knowledge of the levels and distribution of genetic diversity is important for designing conservation strategies for threatened and endangered species so as to guarantee sustainable survival of populations and to preserve their evolutionary potential. Picconia azorica is a valuable Azorean endemic species recently classified as endangered. To contribute with information useful for the establishment of conservation programmes, the genetic variability and differentiation among 230 samples from 11 populations collected in three Azorean islands was accessed with eight inter-simple sequence repeat markers. A total of 64 polymorphic loci were detected. The majority of genetic variability was found within populations and no genetic structure was detected between populations and between islands. Also the coefficient of genetic differentiation and the level of gene flow indicate that geographical distances do not act as barriers for gene flow. In order to ensure the survival of populations in situ and ex situ management practices should be considered, including artificial propagation through the use of plant tissue culture techniques, not only for the restoration of habitat but also for the sustainable use of its valuable wood. | 1081 | no | no good fitness measure | NA | no | no |
| 1082 | 2017 | 43016 | Hoban, SM; Borkowski, DS; Brosi, SL; McCleary, TS; Thompson, LM; McLachlan, JS; Pereira, MA; Schlarbaum, SE; Romero-Severson, J | 2010 | Range-wide distribution of genetic diversity in the North American tree Juglans cinerea: a product of range shifts, not ecological marginality or recent population decline | The spatial distribution of genetic diversity is a product of recent and historical ecological processes, as well as anthropogenic activities. A current challenge in population and conservation genetics is to disentangle the relative effects of these processes, as a first step in predicting population response to future environmental change. In this investigation, we compare the influence of contemporary population decline, contemporary ecological marginality and postglacial range shifts. Using classical model comparison procedures and Bayesian methods, we have identified postglacial range shift as the clear determinant of genetic diversity, differentiation and bottlenecks in 29 populations of butternut, Juglans cinerea L., a North American outcrossing forest tree. Although butternut has experienced dramatic 20th century decline because of an introduced fungal pathogen, our analysis indicates that recent population decline has had less genetic impact than postglacial recolonization history. Location within the range edge vs. the range core also failed to account for the observed patterns of diversity and differentiation. Our results suggest that the genetic impact of large-scale recent population losses in forest trees should be considered in the light of Pleistocene-era large-scale range shifts that may have had long-term genetic consequences. The data also suggest that the population dynamics and life history of wind-pollinated forest trees may provide a buffer against steep population declines of short duration, a result having important implications for habitat management efforts, ex situ conservation sampling and population viability analysis. | 1082 | no | no good fitness measure, N pop differnt in GD, focus on the parasite in paper | NA | no | no |
| 1083 | 2017 | 43016 | de Bakker, DM; Meesters, EHWG; van Bleijswijk, JDL; Luttikhuizen, PC; Breeuwer, HJAJ; Becking, LE | 2016 | Population Genetic Structure, Abundance, and Health Status of Two Dominant Benthic Species in the Saba Bank National Park, Caribbean Netherlands: Montastraea cavernosa and Xestospongia muta | Saba Bank, a submerged atoll in the Caribbean Sea with an area of 2,200 km2, has attained international conservation status due to the rich diversity of species that reside on the bank. In order to assess the role of Saba Bank as a potential reservoir of diversity for the surrounding reefs, we examined the population genetic structure, abundance and health status of two prominent benthic species, the coral Montastraea cavernosa and the sponge Xestospongia muta. Sequence data were collected from 34 colonies of M. cavernosa (nDNA ITS-15.8S-ITS2; 892 bp) and 68 X. muta sponges (mtDNA I3-M11 partition of COI; 544 bp) on Saba Bank and around Saba Island, and compared with published data across the wider Caribbean. Our data indicate that there is genetic connectivity between populations on Saba Bank and the nearby Saba Island as well as multiple locations in the wider Caribbean, ranging in distance from 100s-1000s km. The genetic diversity of Saba Bank populations of M. cavernosa (p = 0.055) and X. muta (p = 0.0010) was comparable to those in other regions in the western Atlantic. Densities and health status were determined along 11 transects of 50 m(2) along the south-eastern rim of Saba Bank. The densities of M. cavernosa (0.27 ind.m(-2), 95% CI: 0.12-0.52) were average, while the densities of X. muta (0.09 ind. m(-2), 95% CI: 0.02-0.32) were generally higher with respect to other Caribbean locations. No disease or bleaching was present in any of the specimens of the coral M. cavernosa, however, we did observe partial tissue loss (77.9% of samples) as well as overgrowth (48.1%), predominantly by cyanobacteria. In contrast, the majority of observed X. muta (83.5%) showed signs of presumed bleaching. The combined results of apparent gene flow among populations on Saba Bank and surrounding reefs, the high abundance and unique genetic diversity, indicate that Saba Bank could function as an important buffer for the region. Either as a natural source of larvae to replenish genetic diversity or as a storehouse of diversity that can be utilized if needed for restoration practices. | 1083 | no | no good fitness measure, N pop differnt in GD? | NA | no | no |
| 1084 | 2017 | 43016 | Hooftman, DAP; Billeter, RC; Schmid, B; Diemer, M | 2004 | Genetic effects of habitat fragmentation on common species of Swiss fen meadows | The area of Caricion davallianae alliance in Switzerland has been considerably reduced and fragmented during the last 150 years. We assessed the genetic variability, inbreeding level, and among-population differentiation of two common habitat-specific plant species, Carex davalliana SM. and Succisa pratensis Moench, in 18 Caricion davallianae fen meadows subjected to fragmentation. We used a spatial field design of fen systems (six systems total), each consisting of one large habitat island and two small habitat islands. We used allozyme electrophoresis to derive standard genetic parameters (A, P, H-O, H-E, F-IS, F-ST). In Carex we identified a consistently lower A in isolated habitat islands; furthermore, H-E was lower in small habitat islands than in large habitat islands. In Succisa we identified a lower H-O in small habitat islands than in larger ones. Small habitat islands were marginally significantly differentiated (F-ST)from large islands for Succisa. For both species, no effects were evident for F-IS; therefore, we argue that genetic drift rather than inbreeding is the main cause of the observed differences. The genetic structure of Carex and Succisa in small habitat islands differed from that in large habitat islands, but differences were small. It appears that the observed differences in genetic variability among fen meadows correspond to observed differences in fitness and demographic traits. We show that habitat fragmentation affects not only the rare species in an ecosystem but also reduces the survival probabilities of common species. One of the main goals of conservation should be to mitigate fragmentation of natural habitats in order to increase population sizes and connectivity. | 1084 | no | no good fitness measure | NA | no | no |
| 1085 | 2017 | 43016 | Alves, DMT; Pereira, RW; Leal-Bertioli, SCM; Moretzsohn, MC; Guimaraes, PM; Bertioli, DJ | 2008 | Development and use of single nucleotide polymorphism markers for candidate resistance genes in wild peanuts (Arachis spp) | The cultivated peanut (Arachis hypogaea L.) is an allotetraploid of recent origin, with an AABB genome and low genetic diversity. Perhaps because of its limited genetic diversity, this species lacks resistance to a number of important pests and diseases. In contrast, wild species of Arachis are genetically diverse and are rich sources of disease resistance genes. Consequently, a study of wild peanut relatives is attractive from two points of view: to help understand peanut genetics and to characterize wild alleles that could confer disease resistance. With this in mind, a diploid population from a cross between two wild peanut relatives was developed, in order to make a dense genetic map that could serve as a reference for peanut genetics and in order to characterize the regions of the Arachis genome that code for disease resistance. We tested two methods for developing and genotyping single nucleotide polymorphisms in candidate genes for disease resistance; one is based on single-base primer extension methods and the other is based on amplification refractory mutation system-polymerase chain reaction. We found single-base pair extension to be an efficient method, suitable for high-throughput, single-nucleotide polymorphism mapping; it allowed us to locate five candidate genes for resistance on our genetic map. | 1085 | no | no good fitness measure, N pop differnt in GD, focus on the parasite in paper | NA | no | no |
| 1086 | 2017 | 43016 | Kovacs, A; Ben-Jacob, N; Tayem, H; Halperin, E; Iraqi, FA; Gophna, U | 2011 | Genotype Is a Stronger Determinant than Sex of the Mouse Gut Microbiota | The mammalian gut microbiota is considered to be determined mostly by diet, while the effect of genotype is still controversial. Here, we examined the effect of genotype on the gut microbiota in normal populations, exhibiting only natural polymorphisms, and evaluated this effect in comparison to the effect of sex. DNA fingerprinting approaches were used to profile the gut microbiota of eight different recombinant inbred mouse lines of the collaborative cross consortium, whose level of genetic diversity mimics that of a natural human population. Analyses based on automated ribosomal internal transcribed spacer analysis demonstrated significant higher similarity of the gut microbiota composition within mouse lines than between them or within same-gender groups. Thus, genetic background significantly impacts the microbiota composition and is a stronger determinant than gender. These findings imply that genetic polymorphisms help shape the intestinal microbiota of mammals and consequently could affect host susceptibility to diseases. | 1086 | no | no good fitness measure, N pop differnt in GD, focus on the parasite in paper | NA | no | no |
| 1087 | 2017 | 43016 | Wright, FA; Sullivan, PF; Brooks, AI; Zou, F; Sun, W; Xia, K; Madar, V; Jansen, R; Chung, WI; Zhou, YH; Abdellaoui, A; Batista, S; Butler, C; Chen, G; Chen, TH; D’Ambrosiol, D; Gallins, P; Ha, MJ; Hottenga, JJ; Huang, SP; Kattenberg, M; Kochar, J; Middeldorp, CM; Qui, A; Shabalinn, A; Tischfield, J; Todd, L; Tzeng, JY; van Grootheest, G; Vink, JM; Wang, Q; Wang, W; Wang, WB; Willemsen, G; Smit, JH; de Geus, EJ; Yin, ZY; Penninx, BWJH; Boomsma, DI | 2014 | Heritability and genomics of gene expression in peripheral blood | We assessed gene expression profiles in 2,752 twins, using a classic twin design to quantify expression heritability and quantitative trait loci (eQTLs) in peripheral blood. The most highly heritable genes (similar to 777) were grouped into distinct expression clusters, enriched in gene-poor regions, associated with specific gene function or ontology classes, and strongly associated with disease designation. The design enabled a comparison of twin-based heritability to estimates based on dizygotic identity-by-descent sharing and distant genetic relatedness. Consideration of sampling variation suggests that previous heritability estimates have been upwardly biased. Genotyping of 2,494 twins enabled powerful identification of eQTLs, which we further examined in a replication set of 1,895 unrelated subjects. A large number of non-redundant local eQTLs (6,756) met replication criteria, whereas a relatively small number of distant eQTLs (165) met quality control and replication standards. Our results provide a new resource toward understanding the genetic control of transcription. | 1087 | no | human | NA | no | no |
| 1088 | 2017 | 43016 | Winternitz, JC; Wares, JP; Yabsley, MJ; Altizer, S | 2014 | Wild cyclic voles maintain high neutral and MHC diversity without strong evidence for parasite-mediated selection | The major histocompatibility complex (MHC) is an important component of vertebrate immune defense involved with self/nonself recognition and disease susceptibility. The high variability of genes of the MHC is thought to arise from both parasite-mediated and sexual selection. An outstanding question involves the degree to which balancing selection can oppose genetic drift to maintain high MHC diversity in the face of population bottlenecks. To address this question we examined genetic diversity and population structure at neutral (microsatellite) and MHC genes in montane voles [Microtus montanus (Peale, 1848)] subject to high amplitude population fluctuations, and compared these to measures of infection by common gastrointestinal parasites. We found high neutral and MHC allelic variability, indicating low impacts of genetic drift despite large fluctuations in population size. Greater MHC diversity did not predict lower parasite richness or infection by the two most common endoparasites (cestodes and coccidian protozoa), as might be expected if genotypic composition confers resistance to infection. One specific MHC allele predicted lower cestode intensity, but we found no other associations between MHC and infection measures. Neutral heterozygosity was positively associated with total parasite richness, possibly owing to greater parasite tolerance among heterozygous relative to more inbred hosts. Overall, these results suggest that factors beyond the parasites examined here, such as high inter-patch migration, mate choice, gene conversion or other infectious agents, are likely maintaining the high levels of MHC diversity observed in wild montane voles. | 1088 | maybe | pop level? | NA | yes | yes |
| 1089 | 2017 | 43016 | Xue, C; Raveendran, M; Harris, RA; Fawcett, GL; Liu, XM; White, S; Dandouli, M; Deiros, DR; Below, JE; Salerno, W; Cox, L; Fan, GP; Ferguson, B; Horvath, J; Johnson, Z; Kanthaswamy, S; Kubisch, HM; Liu, DH; Platt, M; Smith, DG; Sun, BH; Vallenderi, EJ; Wang, F; Wiseman, RW; Chen, R; Muzny, DM; Gibbs, RA; Yu, FL; Rogers, J | 2016 | The population genomics of rhesus macaques (Macaca mulatta) based on whole-genome sequences | Rhesus macaques (Macaca mulatta) are the most widely used nonhuman primate in biomedical research, have the largest natural geographic distribution of any nonhuman primate, and have been the focus of much evolutionary and behavioral investigation. Consequently, rhesus macaques are one of the most thoroughly studied nonhuman primate species. However, little is known about genome-wide genetic variation in this species. A detailed understanding of extant genomic variation among rhesus macaques has implications for the use of this species as a model for studies of human health and disease, as well as for evolutionary population genomics. Whole-genome sequencing analysis of 133 rhesus macaques revealed more than 43.7 million single-nucleotide variants, including thousands predicted to alter protein sequences, transcript splicing, and transcription factor binding sites. Rhesus macaques exhibit 2.5-fold higher overall nucleotide diversity and slightly elevated putative functional variation compared with humans. This functional variation in macaques provides opportunities for analyses of coding and noncoding variation, and its cellular consequences. Despite modestly higher levels of nonsynonymous variation in the macaques, the estimated distribution of fitness effects and the ratio of nonsynonymous to synonymous variants suggest that purifying selection has had stronger effects in rhesus macaques than in humans. Demographic reconstructions indicate this species has experienced a consistently large but fluctuating population size. Overall, the results presented here provide new insights into the population genomics of nonhuman primates and expand genomic information directly relevant to primate models of human disease. | 1089 | no | no good fitness measure, N pop differnt in GD? | NA | no | no |
| 1090 | 2017 | 43016 | Taris, N; Ernande, B; McCombie, H; Boudry, P | 2006 | Phenotypic and genetic consequences of size selection at the larval stage in the Pacific oyster (Crassostrea gigas) | The life histories of oysters in the genus Crassostrea, like those of most marine bivalves, are typified by high fecundity and low survival in nature. Rearing conditions in hatcheries however ensure optimized density, diet, and temperature. Hatcheries are becoming increasingly important for the production of juveniles in aquaculture, and their culture practices often include culling of slow growing larvae to reduce and synchronize the time taken to reach settlement. Because previous studies have found substantial genetic variation for early life developmental traits in Crassostrea gigas, these culling practices are likely to cause highly different selective pressures in hatcheries from those in the natural environment. We studied the phenotypic and genetic impact of such culling practices in a factorial cross between 10 males and 3 females subjected to progressive culling of the smallest 50% of larvae, compared with a non-culled control. Measurements were made on larval growth, survival, time taken to attain pediveliger stage and settlement success. Culling had a larger effect on the variance of these larval traits than on their means. The larvae in culled cultures were approximately 10% larger than those in controls, whereas the coefficient of variation was reduced by 30-40%. Culling also reduced the mean time to settlement by 12% and its variance by 55%. Using a multiplexed set of microsatellite markers to trace parentage, we also estimated the variance in reproductive success in a controlled experiment to quantify the consequences of intensive hatchery rearing practices. We also focused on changes in effective population size and genetic structure over time (and developmental stages). Our results show a loss of genetic diversity following removal of the smallest larvae by culling, as well as temporally varying genetic structure of the larval population. This supports the existence of genetic variability in early life developmental traits in C. gigas. Culling in hatcheries, like size-related selective pressures in the wild, are likely to have a significant genetic impact, through their effects on the timing of settlement. (c) 2006 Elsevier B.V. All rights reserved. | 1090 | no | no good fitness measure, N pop differnt in GD? | NA | no | no |
| 1091 | 2017 | 43016 | Beltran, S; Cezilly, F; Boissier, J | 2008 | Genetic Dissimilarity between Mates, but Not Male Heterozygosity, Influences Divorce in Schistosomes | Background: Correlational studies strongly suggest that both genetic similarity and heterozygosity can influence female mate choice. However, the influence of each variable has usually been tested independently, although similarity and heterozygosity might be correlated. We experimentally determined the relative influence of genetic similarity and heterozygosity in divorce and re-mating in the monogamous endoparasite Schistosoma mansoni. Methodology/Principal Findings: We performed sequential infections of vertebrate hosts with controlled larval populations of parasites, where sex and individual genetic diversity and similarity were predetermined before infection. Divorce rate increased significantly when females were given the opportunity to increase genetic dissimilarity through re-mating with a new partner, independently of the intensity of male-male competition. We found however no evidence for females attempting to maximize the level of heterozygosity of their reproductive partner through divorce. Conclusions/Significance: Female preference for genetically dissimilar males should result in more heterozygous offspring. Because genetic heterozygosity might partly determine the ability of parasites to counter host resistance, adaptive divorce could be an important factor in the evolutionary arms race between schistosomes and their hosts. | 1091 | no | NA | NA | no | no |
| 1092 | 2017 | 43016 | Fei, HX; Henderson, G | 2003 | Comparative study of incipient colony development in the Formosan subterranean termite, Coptotermes formosanus Shiraki (Isoptera, Rhinotermitidae) | The Formosan subterranean termite, Coptotermes formosanus Shiraki is the most destructive, difficult to control and economically important species of termite in the southern United States. At present, no information is available on the genetic relatedness of primary Formosan subterranean termite reproductives that establish new colonies. Information on survivorship and fitness components of primary reproductives from different sibships (sibling or nonsibling) is helpful to our understanding of biological and ecological characteristics of different breeding generations in C formosanus. The present study examined the effects of sibship and colony origin on growth and mortality of incipient colonies of C. formosanus. Seven stock colonies of C. formosanus were collected in 1996 through 1997 in New Orleans and Lake Charles, La, USA. A total of 338 incipient colonies of sibling pairs or nonsibling pairs of C.formosanus were set up. The study indicated that mate relatedness significantly affected mortality and fitness. Nonsibling mates suffered significantly higher mortality than sibling mates originated from New Orleans. However, the decreased success of outbred mates was offset by an increased fecundity compared to inbred colonies over time. Both sibling- and nonsibling-founded colonies from Lake Charles had a significantly higher survival rate than did colonies from New Orleans. Colonies from Lake Charles also produced a significantly higher number of larvae/workers than colonies from New Orleans. The mismatch of habits by mates from different locations and the potential for greater disease risks may be associated with higher mortality in outbred pairs. However, heterozygous offspring of outbred pairs probably have increased genetic variation, which provides greater adaptation potential, thus making the colony more robust in the face of environmental fluctuations. | 1092 | no | no good gd measurement, inbred lines? | NA | no | no |
| 1093 | 2017 | 43016 | LAIKRE, L; RYMAN, N; THOMPSON, EA | 1993 | HEREDITARY BLINDNESS IN A CAPTIVE WOLF (CANIS-LUPUS) POPULATION - FREQUENCY REDUCTION OF A DELETERIOUS ALLELE IN RELATION TO GENE CONSERVATION | Numerous cases of hereditary diseases and disorders have been reported in wild animals bred in captivity, but little attention has been paid to the particular genetic management problems that arise when such defects occur These problems include the obstacle of eliminating the deleterious allele(s) without contemporary loss of genetic variability. In this paper we use the statistical methods of pedigree analysis to address questions regarding a previously presumed hereditary form of blindness observed in a captive wolf population bred for conservation purposes in Scandinavian zoos. The most likely mode of inheritance coincides with an autosomal recessive allele with either a full penetrance or a reduced penetrance of 0.6 (depending on the reliability of studbook records). Using these two models of inheritance we calculate the probability of carrying the blindness allele for each living animal. Analysis of the effect of remOving high-probability carriers on founder allele survival and level of inbreeding demonstrates that the frequency of the deleterious allele can be significantly reduced without seriously affecting founder allele survival or current degree of inbreeding in the wolf population. | 1093 | no | no good fitness measure, N pop differnt in GD, focus on the parasite in paper | NA | no | no |
| 1094 | 2017 | 43016 | Khan, A; Shaik, JS; Behnke, M; Wang, QL; Dubey, JP; Lorenzi, HA; Ajioka, JW; Rosenthal, BM; Sibley, LD | 2014 | NextGen sequencing reveals short double crossovers contribute disproportionately to genetic diversity in Toxoplasma gondii | Background: Toxoplasma gondii is a widespread protozoan parasite of animals that causes zoonotic disease in humans. Three clonal variants predominate in North America and Europe, while South American strains are genetically diverse, and undergo more frequent recombination. All three northern clonal variants share a monomorphic version of chromosome Ia (ChrIa), which is also found in unrelated, but successful southern lineages. Although this pattern could reflect a selective advantage, it might also arise from non-Mendelian segregation during meiosis. To understand the inheritance of ChrIa, we performed a genetic cross between the northern clonal type 2 ME49 strain and a divergent southern type 10 strain called VAND, which harbors a divergent ChrIa. Results: NextGen sequencing of haploid F1 progeny was used to generate a genetic map revealing a low level of conventional recombination, with an unexpectedly high frequency of short, double crossovers. Notably, both the monomorphic and divergent versions of ChrIa were isolated with equal frequency. As well, ChrIa showed no evidence of being a sex chromosome, of harboring an inversion, or distorting patterns of segregation. Although VAND was unable to self fertilize in the cat, it underwent successful out-crossing with ME49 and hybrid survival was strongly associated with inheritance of ChrIII from ME49 and ChrIb from VAND. Conclusions: Our findings suggest that the successful spread of the monomorphic ChrIa in the wild has not been driven by meiotic drive or related processes, but rather is due to a fitness advantage. As well, the high frequency of short double crossovers is expected to greatly increase genetic diversity among progeny from genetic crosses, thereby providing an unexpected and likely important source of diversity. | 1094 | no | gd of parasite | NA | no | no |
| 1095 | 2017 | 43016 | Goulding, TC; Cohen, CS | 2014 | Phylogeography of a marine acanthocephalan: lack of cryptic diversity in a cosmopolitan parasite of mole crabs | Aim Little is known about phylogeography and cryptic diversity of parasites in the marine environment. The acanthocephalan Profilicollis altmani parasitizes intermediate hosts that are broadly distributed around the Americas and final hosts that are highly motile. We investigated the spatial genetic structure of this acanthocephalan found in three species of Emerita crabs: (1) to test whether land masses serve as biogeographic barriers promoting ocean basin divergence among parasite lineages or species; and (2) to test whether the distribution of parasite species matches the distribution of different crab host species. Location The Pacific, Atlantic and Gulf coasts of the USA, and the Pacific coast of Panama and Chile. Methods Sequences of cytochrome c oxidase subunit I (COI) and ribosomal internal transcribed spacers (ITS) were obtained from 204 acanthocephalans. Parasites were sampled from crabs in 15 sampling localities. These sequences were analysed with coalescent-based methods and other population genetic analyses to infer phylogeographic patterns. Results Haplotype diversity for COI sequences was high (0.96) among parasites sampled, but nucleotide diversity was low (0.071) and there was no distinct geographic pattern between regions. Pairwise genetic distances were generally low, although there was a degree of population structure between oceans. Sequence comparisons showing an excess of low divergence alleles and a bimodal mismatch distribution provide evidence of either past selective events or demographic expansions. No variation was observed in the ITS sequences. Main conclusions The lack of geographic patterning in haplotype diversity of this parasite indicates that gene flow is probably occurring between ocean basins. In addition, the low genetic diversity suggests that the acanthocephalan parasitizing E. analoga in Chile is conspecific to the species found parasitizing several Emerita species along the coasts of North America, and is thus a cosmopolitan parasite that is most likely dispersed long distances by marine birds that serve as definitive hosts. | 1095 | no | gd of parasite | NA | no | no |
| 1096 | 2017 | 43016 | Rowe, C; Ittiprasert, W; Patterson, C; Eliff, C; Page, K; Bandoni, S; Wilke, T; Minchella, D; Lewis, F; Knight, M | 2003 | Use of microsatellite variation and RAPD-PCR to assess genetic polymorphism in Biomphalaria glabrata snails from a single locale in a schistosomiasis endemic area | Genetic variation was assessed in Biomphalaria glabrata snails using variations in microsatellite loci and by RAPD-PCR analysis. Populations of snails examined were field-collected isolates from a small pond in a schistosomiasis endemic region in Brazil, after standard conditions were developed for analyzing snails from two laboratory-maintained stocks. The analyses were performed using a total of 60 microsatellite primer sets and, for RAPD-PCR, a total of 19 random primers. We show that genetic diversity can readily be detected by both molecular methods among the field-collected snails from this small site. In addition, RAPD-PCR bands that were found in another study to segregate with parasite resistance were not detected in any of the field-collected snails analyzed. | 1096 | no | no good fitness measure | NA | no | no |
| 1097 | 2017 | 43016 | Mlynarek, JJ; Iserbyt, A; Nagel, L; Forbes, MR | 2015 | Differential Water Mite Parasitism, Phenoloxidase Activity, and Resistance to Mites Are Unrelated across Pairs of Related Damselfly Species | “Related host species often demonstrate differences in prevalence and/or intensity of infection by particular parasite species, as well as different levels of resistance to those parasites. The mechanisms underlying this interspecific variation in parasitism and resistance expression are not well understood. Surprisingly, few researchers have assessed relations between actual levels of parasitism and resistance to parasites seen in nature across multiple host species. The main goal of this study was to determine whether interspecific variation in resistance against ectoparasitic larval water mites either was predictive of interspecific variation in parasitism for ten closely related species of damselflies (grouped into five”“species pairs”“), or was predicted by interspecific variation in a commonly used measure of innate immunity (total Phenoloxidase or potential PO activity). Two of five species pairs had interspecific differences in proportions of individuals resisting larval Arrenurus water mites, only one of five species pairs had species differences in prevalence of larval Arrenurus water mites, and another two of five species pairs showed species differences in mean PO activity. Within the two species pairs where species differed in proportion of individuals resisting mites the species with the higher proportion did not have correspondingly higher PO activity levels. Furthermore, the proportion of individuals resisting mites mirrored prevalence of parasitism in only one species pair. There was no interspecific variation in median intensity of mite infestation within any species pair. We conclude that a species’ relative ability to resist particular parasites does not explain interspecific variation in parasitism within species pairs and that neither resistance nor parasitism is reflected by interspecific variation in total PO or potential PO activity.” | 1097 | no | No GD diff between pop | NA | no | no |
| 1098 | 2017 | 43016 | Larcombe, L; Rempel, JD; Dembinski, I; Tinckam, K; Rigatto, C; Nickerson, P | 2005 | Differential cytokine genotype frequencies among Canadian Aboriginal and Caucasian populations | Genetic diversity related to the human immune response is a key factor in individual and population survival throughout human history. Population diversity in disease susceptibility and resistance have been identified and linked to differences in cytokine mRNA and protein expression levels. Polymorphisms in the regulatory regions of cytokine genes can influence gene transcription levels and they have been associated with susceptibility to, and/or severity of, autoimmune disorders such as rheumatoid arthritis, meningococcus and sepsis. It is reported here that in two study populations, Canadian Aboriginal individuals have a higher frequency of cytokine single-nucleotide polymorphisms favouring a low production of TNFalpha, IFNgamma and IL-10 and high production of IL-6 as compared to a Caucasian population. We postulate that the evolution of this unique cytokine genotype profile may be linked to the Aboriginal adaptation to selection pressures related to an environment in which helminthic, parasitic and fungal infections predominated. | 1098 | no | NA | NA | no | no |
| 1099 | 2017 | 43016 | Brown, EW; Mammel, MK; LeClerc, JE; Cebula, TA | 2003 | Limited boundaries for extensive horizontal gene transfer among Salmonella pathogens | Recombination is thought to be rare within Salmonella, as evidenced by absence of gene transfer among SARC strains that represent the broad genetic diversity of the eight primary subspecies of this common facultative intracellular pathogen. We adopted a phylogenetic approach to assess recombination within the mutS gene of 70 SARB strains, a genetically homogeneous population of Salmonella enterica subspecies I strains, which have in common the ability to infect warm-blooded animals. We report here that SARB strains show evidence for widespread recombinational exchange in contrast to results obtained with strains exhibiting species-level genetic variation. Besides extensive allele shuffling, SARB strains showed notably larger recombinagenic patch sizes for mutS (at least approximate to1.1 kb) than previously reported for S. enterica SARC strains. Explaining these experimental dichotomies provides important insight for understanding microbial evolution, because they suggest likely ecologic and genetic barriers that limit extensive gene transfer in the feral setting. | 1099 | no | gd of pathogen | NA | no | no |
| 1100 | 2017 | 43016 | Kjaer, ED; McKinney, LV; Nielsen, LR; Hansen, LN; Hansen, JK | 2012 | Adaptive potential of ash (Fraxinus excelsior) populations against the novel emerging pathogen Hymenoscyphus pseudoalbidus | An emerging infectious pathogen Hymenoscyphus pseudoalbidus has spread across much of Europe within recent years causing devastating damage on European common ash trees (Fraxinus excelsior) and associated plant communities. The present study demonstrates the presence of additive genetic variation in susceptibility of natural F. excelsior populations to the new invasive disease. We observe high levels of additive variation in the degree of susceptibility with relatively low influence of environmental factors (narrow-sense heritability = 0.370.52). Most native trees are found to be highly susceptible, and we estimate that only around 1% has the potential of producing offspring with expected crown damage of <10% under the present disease pressure. The results suggest that the presence of additive genetic diversity in natural F. excelsior populations can confer the species with important ability to recover, but that low resistance within natural European populations is to be expected because of a low frequency of the hypo-sensitive trees. Large effective population sizes will be required to avoid genetic bottlenecks. The role of artificial selection and breeding for protection of the species is discussed based on the findings. | 1100 | no | No GD diff between pop | NA | no | no |
| 1101 | 2017 | 43016 | Giron, D; Dunn, DW; Hardy, ICW; Strand, MR | 2004 | Aggression by polyembryonic wasp soldiers correlates with kinship but not resource competition | Kin selection theory predicts that individuals will show less aggression and more altruism towards relatives(1,2). However, recent theoretical developments suggest that with limited dispersal, competition between relatives can override the effects of relatedness(3-9). The predicted and opposing influences of relatedness and competition are difficult to approach experimentally because conditions that increase average relatedness among individuals also tend to increase competition. Polyembryonic wasps in the family Encyrtidae are parasites whose eggs undergo clonal division to produce large broods(10). These insects have also evolved a caste system: some embryos in a clone develop into reproductive larvae that mature into adults, whereas others develop into sterile soldier larvae that defend siblings from competitors(11-14). In a brood from a single egg, reproductive altruism by soldiers reflects clone-level allocation to defence at the cost of reproduction, with no conflict between individuals. When multiple eggs are laid into a host, inter-clone conflicts of interest arise. Here we report that soldier aggression in Copidosoma floridanum is inversely related to the genetic relatedness of competitors but shows no correlation with the level of resource competition. | 1101 | no | no good fitness measure | NA | no | no |
| 1102 | 2017 | 43016 | Slothouber Galbreath, JGM; Smith, JE; Becnel, JJ; Butlin, RK; Dunn, AM | 2010 | Reduction in post-invasion genetic diversity in Crangonyx pseudogracilis (Amphipoda: Crustacea): a genetic bottleneck or the work of hitchhiking vertically transmitted microparasites? | Parasites can strongly influence the success of biological invasions. However, as invading hosts and parasites may be derived from a small subset of genotypes in the native range, it is important to examine the distribution and invasion of parasites in the context of host population genetics. We demonstrate that invasive European populations of the North American Crangonyx pseudogracilis have experienced a reduction in post-invasion genetic diversity. We predict that vertically transmitted parasites may evade the stochastic processes and selective pressures leading to enemy release. As microsporidia may be vertically or horizontally transmitted, we compared the diversity of these microparasites in the native and invasive ranges of the host. In contrast to the reduction in host genetic diversity, we find no evidence for enemy release from microsporidian parasites in the invasive populations. Indeed, a single, vertically transmitted, microsporidian sex ratio distorter dominates the microsporidian parasite assemblage in the invasive range and appears to have invaded with the host. We propose that overproduction of female offspring as a result of parasitic sex ratio distortion may facilitate host invasion success. We also propose that a selective sweep resulting from the increase in infected individuals during the establishment may have contributed to the reduction in genetic diversity in invasive Crangonyx pseudogracilis populations. | 1102 | maybe | any fitness (survival) of groups of different gd? | no good gd measure of host pop | no | no |
| 1103 | 2017 | 43016 | Ma, Q; Du, YJ; Chen, N; Zhang, LY; Li, JH; Fu, CX | NA | Phylogeography of Davidia involucrata (Davidiaceae) Inferred from cpDNA Haplotypes and nSSR Data | “Davidia involucrata Baill. (Davidiaceae), commonly known as”“Chinese dove tree”", is a rare and endangered species endemic to China. Little is known about the genetic structure and population history of D. involucrata. Both cpDNA and nSSR markers showed high genetic diversity among D. involucrata populations (H-S = 0.717 | 1103 | no | no good fitness measure | NA | no | no |
| 1104 | 2017 | 43016 | Ficetola, GF; De Bernardi, F | 2009 | Offspring size and survival in the frog Rana latastei: from among-population to within-clutch variation | Egg size is considered to be a major maternal effect for offspring in oviparous organisms. It has profound consequences on fitness, and differences in egg size are viewed as plastic responses to environmental variability. However, it is difficult to identify the effect of egg size per se because egg size can covary with genetic features of the mother and with other nongenetic factors. We analysed the relationship between offspring starting size (i.e. a proxy of egg size) and larval survival in the frog Rana latastei. We analysed this relationship: (1) among five populations at different altitudes; (2) among clutches laid from different females; and (3) among siblings within clutches, to evaluate the effect of starting size. We observed differences among populations for offspring size, but starting size was not related to altitude or genetic diversity. Mortality was higher in populations and families with small average starting size; however, among siblings, the relationship between starting size and mortality was not verified. The relationship observed among clutches may therefore be caused by covariation between egg size and other effects. This suggests that the covariation between egg size and other effects can result in apparent relationships between egg size and fitness-related traits. Proximate and ultimate factors can cause the phenotypic variation of hatchlings in the wild, and key traits can be related to this variation, but the underlying causes require further investigation. (C) 2009 The Linnean Society of London, Biological Journal of the Linnean Society, 2009, 97, 845-853. | 1104 | probably yes | ok gd measurement and pop level comp? | no good fitness measure, not % survival | no | no |
| 1105 | 2017 | 43016 | Hausman, CE; Bertke, MM; Jaeger, JF; Rocha, OJ | 2014 | Genetic structure of green ash (Fraxinus pennsylvanica): implications for the establishment of ex situ conservation protocols in light of the invasion of the emerald ash borer | The USA is experiencing a prolific invasion of the wood-boring emerald ash borer, Agrilus planipennis. Native to Asia, this beetle completes its life cycle on ash trees and results in nearly complete mortality of all infested trees. In the present study, we examined the levels of genetic diversity and differentiation among eight populations of Fraxinus pennsylvanica (green ash) using five polymorphic microsatellite loci. Genetic information was used to design guidelines for the establishment of a seed collection sampling strategy to conserve the genetic diversity of ash trees. We found high levels of genetic diversity, as indicated by the allelic richness, both across the populations (16.4 +/- 5.18 alleles per locus) and within them (8.03 +/- 1.21 alleles per locus). The expected and observed heterozygosity was also high (0.805 +/- 0.38 and 0.908 +/- 0.04, respectively), and there was moderate genetic differentiation among the populations (F-ST = 0.083) with members of these eight populations grouped into three distinct clusters. We examined the relationship between the number of individuals sampled and the number of alleles captured in a random sample taken from a population of 10,000 individuals. Only sample sizes of 100 individuals captured most of the alleles (average = 78.74 alleles), but only seven of 50 samples effectively captured all the 82 alleles. Smaller samples did not capture all alleles. A probabilistic model was used to determine an optimal sampling strategy, and it was concluded that a collection of 200 seeds from each of five mother trees would have the highest likelihood of capturing all alleles in a population. | 1105 | no | no good fitness measure | NA | no | no |
| 1106 | 2017 | 43016 | Valdiani, A; Kadir, MA; Saad, MS; Talei, D; Tan, SG | 2012 | Intra-specific hybridization: Generator of genetic diversification and heterosis in Andrographis paniculata Nees. A bridge from extinction to survival | Andrographis paniculata (AP) has been stated as a low-diverse, endangered and red-listed plant species. Self-pollinated mating system, being an introduced species and experiencing a bottleneck as well as over exploitation cause such a consequence. Inter and intra-specific hybridizations have been suggested as essential techniques for generating genetic diversity. To test the effect of intra-specific hybridization on diversification and heterosis of AP, seven accessions were outcrossed manually in all 21 possible combinations. Three types of markers including morphological, phytochemical and RAPD markers were employed to evaluate the mentioned hypothesis. The results revealed that hybridization acted as a powerful engine for diversification of AP as it caused heterotic expression of the studied traits, simultaneously. Initially, it seems that additive and non-additive gene effects both can be considered as the genetic basis of heterosis in AP for the investigated traits. Agronomic and morphological traits were differentiated from each other, while positive heterosis was recorded mainly for agronomic traits but not for the morphological traits. Intra-specific hybridization increased the genetic diversity in AP population. Nevertheless, a part of this variation could also be attributed to the negative heterosis. The current exploration demonstrated the first ever conducted manual intra-specific hybridization among AP accessions in a mass scale. However, the 17 RAPD primers produced a monomorph pattern, but perhaps increasing the number of markers can feature a new genetic profile in this plant. (C) 2012 Elsevier B.V. All rights reserved. | 1106 | no | no good fitness measure, N pop differnt in GD? | NA | no | no |
| 1107 | 2017 | 43016 | Diallo, K; Gamougam, K; Daugla, DM; Harrison, OB; Bray, JE; Caugant, DA; Lucidarme, J; Trotter, CL; Hassan-King, M; Stuart, JM; Manigart, O; Greenwood, BM; Maiden, MCJ | NA | Hierarchical genomic analysis of carried and invasive serogroup A Neisseria meningitidis during the 2011 epidemic in Chad | “Background: Serogroup A Neisseria meningitidis (NmA) was the cause of the 2011 meningitis epidemics in Chad. This bacterium, often carried asymptomatically, is considered to be an”“accidental pathogen”" | 1107 | no | gd of pathogen | NA | no | no |
| 1108 | 2017 | 43016 | Koskella, B; Giraud, T; Hood, ME | 2006 | Pathogen relatedness affects the prevalence of within-host competition | Although the evolutionary consequences of within-host competition among pathogens have been examined extensively, there exists a critical gap in our understanding of factors determining the prevalence of multiple infections. Here we examine the effects of relatedness among strains of the anther-smut pathogen Microbotryum violaceum on the probability of multiple infection in its host, Silene latifolia, after sequential inoculations. We found a significantly higher probability of multiple infection when interacting strains were more closely related, suggesting mechanisms of competitive exclusion that are conditional on genotypic characteristics of the strains involved. Pathogen relatedness therefore determines the prevalence of multiple infection in addition to its outcome, with important consequences for our understanding of virulence evolution and pathogen population structure and diversity. | 1108 | no | no good fitness measure, N pop differnt in GD? | NA | no | no |
| 1109 | 2017 | 43016 | West, SA; Buckling, A | 2003 | Cooperation, virulence and siderophore production in bacterial parasites | Kin selection theory predicts that the damage to a host resulting from parasite infection (parasite virulence) will be negatively correlated to the relatedness between parasites within the host. This occurs because a lower relatedness leads to greater competition for host resources, which favours rapid growth to achieve greater relative success within the host, and that higher parasite growth rate leads to higher virulence. We show that a biological feature of bacterial infections can lead to the opposite prediction: a positive correlation between relatedness and virulence. This occurs because a high relatedness can favour greater (cooperative) production of molecules that scavenge iron (siderophores), which results in higher growth rates and virulence. More generally, the same underlying idea can predict a positive relationship between relatedness and virulence in any case where parasites can cooperate to increase their growth rate; other examples include immune suppression and the production of biofilms to aid colonization. | 1109 | no | gd of parasite | NA | no | no |
| 1110 | 2017 | 43016 | CHEN, P; KIDSON, C; LAVIN, M | 1993 | EVIDENCE OF DIFFERENT COMPLEMENTATION GROUPS AMONGST HUMAN GENETIC-DISORDERS CHARACTERIZED BY RADIOSENSITIVITY | The genetic diversity of a clinically heterogeneous group of ionizing radiation-sensitive human mutants has been examined. In this group, the relationship between ataxia telangiectasia (A-T), Alzheimer’s disease (AD) and Down’s syndrome (DS) was studied, on the basis of their cellular radiosensitivity. Cell-fusion analysis was used to determine the presence of different complementation groups. In a series of 4A-T, 5AD and 4DS cell lines, 8 complementation groups were documented. These findings suggest that this group of primary neuronal degenerative disorders might have some overlap in their genetic defects. | 1110 | no | human | NA | no | no |
| 1111 | 2017 | 43016 | Berthier, K; Leippert, F; Fumagalli, L; Arlettaz, R | 2012 | Massive Nest-Box Supplementation Boosts Fecundity, Survival and Even Immigration without Altering Mating and Reproductive Behaviour in a Rapidly Recovered Bird Population | Habitat restoration measures may result in artificially high breeding density, for instance when nest-boxes saturate the environment, which can negatively impact species’ demography. Potential risks include changes in mating and reproductive behaviour such as increased extra-pair paternity, conspecific brood parasitism, and polygyny. Under particular cicumstances, these mechanisms may disrupt reproduction, with populations dragged into an extinction vortex. With the use of nuclear microsatellite markers, we investigated the occurrence of these potentially negative effects in a recovered population of a rare secondary cavity-nesting farmland bird of Central Europe, the hoopoe (Upupa epops). High intensity farming in the study area has resulted in a total eradication of cavity trees, depriving hoopoes from breeding sites. An intensive nest-box campaign rectified this problem, resulting in a spectacular population recovery within a few years only. There was some concern, however, that the new, high artificially-induced breeding density might alter hoopoe mating and reproductive behaviour. As the species underwent a serious demographic bottleneck in the 1970-1990s, we also used the microsatellite markers to reconstitute the demo-genetic history of the population, looking in particular for signs of genetic erosion. We found i) a low occurrence of extra-pair paternity, polygyny and conspecific brood parasitism, ii) a high level of neutral genetic diversity (mean number of alleles and expected heterozygosity per locus: 13.8 and 83%, respectively) and, iii) evidence for genetic connectivity through recent immigration of individuals from well differentiated populations. The recent increase in breeding density did thus not induce so far any noticeable detrimental changes in mating and reproductive behaviour. The demographic bottleneck undergone by the population in the 1970s-1990s was furthermore not accompanied by any significant drop in neutral genetic diversity. Finally, genetic data converged with a concomitant demographic study to evidence that immigration strongly contributed to local population recovery. | 1111 | no | no good fitness measure, N pop differnt in GD? | NA | no | no |
| 1112 | 2017 | 43016 | Marsden, CD; Wayne, RK; Mable, BK | 2012 | Inferring the ancestry of African wild dogs that returned to the Serengeti-Mara | An endangered population of African wild dogs (Lycaon pictus) disappeared from the Serengeti-Mara area in 1991. The reasons for the extinction are not well understood, but disease was implicated in the disappearance. In 2001, wild dogs naturally re-established themselves in the region. We conducted genetic profiling on samples collected prior and subsequent to this event, as well as samples from three geographically close populations, to determine the potential source of colonisers. Contrary to expectations, we found no evidence of re-colonisation from these nearby wild dog populations. Rather, our analyses suggest that the re-established animals are primarily derived from the same genetic population as the pre-extinction animals, indicating that wild dogs are likely to have persisted in the Serengeti-Mara after 1991. We also detected some migrants that could be derived from genetically distinct populations outside the recovery area. Overall, we did not detect a decline in genetic diversity at either neutral microsatellites or major histocompatibility complex loci, indicating that the supposed disappearance of wild dogs in the Serengeti-Mara did not substantially impact genetic variation of the population. | 1112 | no | no good fitness measure, N pop differnt in GD? | NA | no | no |
| 1113 | 2017 | 43016 | Freeman, AR; Meghen, CM; MacHugh, DE; Loftus, RT; Achukwi, MD; Bado, A; Sauveroche, B; Bradley, DG | 2004 | Admixture and diversity in West African cattle populations | We present a population genetic analysis of microsatellite variation in 16 West African cattle populations. West Africa represents a unique juxtaposition of different climatic and ecological zones in a relatively small geographical area. While more humid coastal regions are inhabited by the tsetse fly, a vector which spreads trypanosomiasis among cattle, the disease is not transmitted in the drier areas outside this zone. This is the most thorough study of genetic diversity in cattle within this area, which contains genetically important trypanotolerant Bos taurus breeds. Genetic relationships among the many breeds are examined and levels of diversity are assessed. Admixture levels were determined using a variety of methods. Ancestry informative or population-associated alleles (PAAs) were selected using populations from India, the Near East and Europe. Multivariate analysis, the ADMIX program and model-based Bayesian admixture analysis approaches were also employed. These analyses reveal the direct impact of ecological factors and the profound effect of admixture on the cattle of this region. They also highlight the importance of efforts to prevent further dilution of African taurine breeds by B. indicus cattle. | 1113 | no | domesticated animal | NA | no | no |
| 1114 | 2017 | 43016 | Cizkova, D; Javurkova, V; Champagnon, J; Kreisinger, J | 2012 | Duck’s not dead: Does restocking with captive bred individuals affect the genetic integrity of wild mallard (Anas platyrhynchos) population? | The genetic integrity of natural populations can be threatened through large-scale introduction of farmed stocks with different genetic or geographic origin. Huge numbers of farm-reared mallard (Anas platyrhynchos, Anatidae) have been introduced into the wild in many European countries since 1970. Czech breeding facilities currently produce around 200-300,000 ducks annually, exceeding wild numbers by around 10 times. Such facilities, however, were founded with hybrid ducks from outside the Czech Republic. Three types of DNA markers, two neutral (14 microsatellite DNA loci and the mitochondrial DNA control region) and one under selection (MHC class I locus), were used to genotype mallards from six Czech breeding facilities (n = 131) and seven wild nesting localities (n = 139). We found marked genetic divergence between wild and captive-bred populations, the latter having significantly lower genetic diversity. Released captive-bred mallards were integrated into breeding wild population through hybridization mediated by high frequency nesting. Overall, our data suggest that release of captive-bred individuals threatens the genetic integrity of wild population. Massive restocking may also be undesirable as regards public health. Waterfowl are known reservoirs of transmittable pathogens and large-scale restocking could alter immune defence gene frequencies in wild population. We propose the establishment of a national genetic monitoring programme for breeding facilities. (c) 2012 Published by Elsevier Ltd. | 1114 | no | no good fitness measure, N pop differnt in GD? | NA | no | no |
| 1115 | 2017 | 43016 | Das, B; Sengupta, S; Prasad, M; Ghose, TK | 2014 | Genetic diversity of the conserved motifs of six bacterial leaf blight resistance genes in a set of rice landraces | Background: Bacterial leaf blight (BLB) caused by the vascular pathogen Xanthomonas oryzae pv. oryzae (Xoo) is one of the most serious diseases leading to crop failure in rice growing countries. A total of 37 resistance genes against Xoo has been identified in rice. Of these, ten BLB resistance genes have been mapped on rice chromosomes, while 6 have been cloned, sequenced and characterized. Diversity analysis at the resistance gene level of this disease is scanty, and the landraces from West Bengal and North Eastern states of India have received little attention so far. The objective of this study was to assess the genetic diversity at conserved domains of 6 BLB resistance genes in a set of 22 rice accessions including landraces and check genotypes collected from the states of Assam, Nagaland, Mizoram and West Bengal. Results: In this study 34 pairs of primers were designed from conserved domains of 6 BLB resistance genes; Xa1, xa5, Xa21, Xa21(A1), Xa26 and Xa27. The designed primer pairs were used to generate PCR based polymorphic DNA profiles to detect and elucidate the genetic diversity of the six genes in the 22 diverse rice accessions of known disease phenotype. A total of 140 alleles were identified including 41 rare and 26 null alleles. The average polymorphism information content (PIC) value was 0.56/primer pair. The DNA profiles identified each of the rice landraces unequivocally. The amplified polymorphic DNA bands were used to calculate genetic similarity of the rice landraces in all possible pair combinations. The similarity among the rice accessions ranged from 18% to 89% and the dendrogram produced from the similarity values was divided into 2 major clusters. The conserved domains identified within the sequenced rare alleles include Leucine-Rich Repeat, BED-type zinc finger domain, sugar transferase domain and the domain of the carbohydrate esterase 4 superfamily. Conclusions: This study revealed high genetic diversity at conserved domains of six BLB resistance genes in a set of 22 rice accessions. The inclusion of more genotypes from remote ecological niches and hotspots holds promise for identification of further genetic diversity at the BLB resistance genes. | 1115 | no | crop | NA | no | no |
| 1116 | 2017 | 43016 | Reding, DM; Freed, LA; Cann, RL; Fleischer, RC | 2010 | Spatial and temporal patterns of genetic diversity in an endangered Hawaiian honeycreeper, the Hawaii Akepa (Loxops coccineus coccineus) | As a result of disease, habitat destruction, and other anthropogenic factors, the Hawaii Akepa (Loxops coccineus coccineus) currently occupies <10% of its original range and exists in five disjunct populations, raising concerns about what effect such reduction and fragmentation has had on the connectivity and diversity of Akepa populations. In this study, we used both historical and contemporary samples to assess genetic diversity and structure in this endangered Hawaiian honeycreeper. We generated sequence data from two mtDNA regions (ND2, control region) and two nuclear introns for contemporary samples representing three of the five current populations. We also generated control region sequence data for museum specimens collected over 100 years ago from throughout the historical range of the bird. Results indicate that despite recent declines and fragmentation, genetic diversity has not been lost. We detected a modest level of genetic differentiation, which followed a combined pattern of isolation-by-barriers and isolation-by-distance, across the historical range of Akepa. The similarly low level of differentiation observed between the contemporary populations indicates that not much divergence, if any, has occurred post-fragmentation. Rather, the present structure seen likely reflects the historical pattern of distribution. Ironically, this declining species exhibits the genetic signal of an expanding population, demonstrating that earlier demographic events are outweighing the effects of recent changes in population size, and genetic estimates of Ne, though crude, suggest Hawaii Akepa were at least an order of magnitude more abundant prior to the decline. | 1116 | no | no good fitness measure, N pop differnt in GD? | NA | no | no |
| 1117 | 2017 | 43016 | Sonsthagen, SA; Jay, CV; Fischbach, AS; Sage, GK; Talbot, SL | 2012 | Spatial genetic structure and asymmetrical gene flow within the Pacific walrus | Pacific walruses (Odohenus rosmarus divergens) occupying shelf waters of Pacific Arctic seas migrate during spring and summer from 3 breeding areas in the Bering Sea to form sexually segregated nonbreeding aggregations. We assessed genetic relationships among 2 putative breeding populations and 6 nonbreeding aggregations. Analyses of mitochondrial DNA (mtDNA) control region sequence data suggest that males are distinct among breeding populations (Phi(ST) = 0.051), and between the eastern Chukchi and other nonbreeding aggregations (Phi(ST) = 0.336-0.449). Nonbreeding female aggregations were genetically distinct across marker types (microsatellite F-ST = 0.019; mtDNA Phi(ST) = 0.313), as was eastern Chukchi and all other nonbreeding aggregations (microsatellite F-ST = 0.019-0.035; mtDNA Phi(ST) = 0.386-0.389). Gene flow estimates are asymmetrical from St. Lawrence Island into the southeastern Bering breeding population for both sexes. Partitioning of haplotype frequencies among breeding populations suggests that individuals exhibit some degree of philopatry, although weak. High levels of genetic differentiation among eastern Chukchi and all other nonbreeding aggregations, but considerably lower genetic differentiation between breeding populations, suggest that at least I genetically distinct breeding population remained unsampled. Limited genetic structure at microsatellite loci between assayed breeding areas can emerge from several processes, including male-mediated gene flow, or population admixture following a decrease in census size (i.e., due to commercial harvest during 1880-1950s) and subsequent recovery. Nevertheless, high levels of genetic diversity in the Pacific walrus, which withstood prolonged decreases in census numbers with little impact on neutral genetic diversity, may reflect resiliency in the face of past environmental challenges. | 1117 | no | no good fitness measure, N pop differnt in GD? | NA | no | no |
| 1118 | 2017 | 43016 | Couce, A; Rodriguez-Rojas, A; Blazquez, J | 2016 | Determinants of Genetic Diversity of Spontaneous Drug Resistance in Bacteria | Any pathogen population sufficiently large is expected to harbor spontaneous drug-resistant mutants, often responsible for disease relapse after antibiotic therapy. It is seldom appreciated, however, that while larger populations harbor more mutants, the abundance distribution of these mutants is expected to be markedly uneven. This is because a larger population size allows early mutants to expand for longer, exacerbating their predominance in the final mutant subpopulation. Here, we investigate the extent to which this reduction in evenness can constrain the genetic diversity of spontaneous drug resistance in bacteria. Combining theory and experiments, we show that even small variations in growth rate between resistant mutants and the wild type result in orders-of-magnitude differences in genetic diversity. Indeed, only a slight fitness advantage for the mutant is enough to keep diversity low and independent of population size. These results have important clinical implications. Genetic diversity at antibiotic resistance loci can determine a population’s capacity to cope with future challenges (i.e., second-line therapy). We thus revealed an unanticipated way in which the fitness effects of antibiotic resistance can affect the evolvability of pathogens surviving a drug-induced bottleneck. This insight will assist in the fight against multidrug-resistant microbes, as well as contribute to theories aimed at predicting cancer evolution. | 1118 | no | no good fitness measure, N pop differnt in GD? | NA | no | no |
| 1119 | 2017 | 43016 | Peixoto, RF; Creste, S; Landell, MGA; Nunes, DS; Sanguino, A; Campos, MF; Vencovsky, R; Tambarussi, EV; Figueira, A | 2014 | Genetic diversity among Puccinia melanocephala isolates from Brazil assessed using simple sequence repeat markers | Brown rust (causal agent Puccinia melanocephala) is an important sugarcane disease that is responsible for large losses in yield worldwide. Despite its importance, little is known regarding the genetic diversity of this pathogen in the main Brazilian sugarcane cultivation areas. In this study, we characterized the genetic diversity of 34 P. melanocephala isolates from 4 Brazilian states using loci identified from an enriched simple sequence repeat (SSR) library. The aggressiveness of 3 isolates from major sugarcane cultivation areas was evaluated by inoculating an intermediately resistant and a susceptible cultivar. From the enriched library, 16 SSR-specific primers were developed, which produced scorable alleles. Of these, 4 loci were polymorphic and 12 were monomorphic for all isolates evaluated. The molecular characterization of the 34 isolates of P. melanocephala conducted using 16 SSR loci revealed the existence of low genetic variability among the isolates. The average estimated genetic distance was 0.12. Phenetic analysis based on Nei’s genetic distance clustered the isolates into 2 major groups. Groups I and II included 18 and 14 isolates, respectively, and both groups contained isolates from all 4 geographic regions studied. Two isolates did not cluster with these groups. It was not possible to obtain clusters according to location or state of origin. Analysis of disease severity data revealed that the isolates did not show significant differences in aggressiveness between regions. | 1119 | no | gd of pathogen | NA | no | no |
| 1120 | 2017 | 43016 | Rizvi, A; Ashraf, M; Ghafoor, A | 2013 | Genetic Divergence for Seedling Traits in Tomato (Solanum lycopersicum) | Three hundred and eighty genotypes of tomato were investigated for genetic diversity for nine seedling traits and considerable genetic variation was observed for all the traits except pubescence. Only two genotypes (19901 and 6836-9) were glabrous, whereas all others had hair on the hypocotyl. Eight clusters were observed based on k-means clustering with average distance ranging from 0.47 (cluster 6) to 0.79 (cluster 8). Some of the discrete traits did not exhibit variation within individual cluster that could be one of the bases for clustering pattern. The scatter diagram partially indicated the clustering pattern and the clusters 3, 4 and 5 intermixed. Sub clustering of individual clusters revealed 5 sub clusters of cluster 1, three of cluster 2 and 5 in each case, six in cluster 3, 4 and 7 and cluster 6 and 8 had 4 each The cluster 2, 3, 4 and 6 joined at higher genetic linkage with induction of single genotype in the cluster 2, 4 and 6, whereas in cluster 3, two main groups joined at higher distance including one group comprising of the 41 genotypes and the second comprising 19 genotypes, which joined at 80% linkage distance. The data on seedling traits along with other data will be utilized for establishment of core set and the genotypes with maximum genetic distance from individual clusters along with representative samples from low distance groups are likely to be chosen for core set. (C) 2013 Friends Science Publishers | 1120 | no | crop | NA | no | no |
| 1121 | 2017 | 43016 | Al-Hamidhi, S; Tageldin, MH; Weir, W; Al-Fahdi, A; Johnson, EH; Bobade, P; Alqamashoui, B; Beja-Pereira, A; Thompson, J; Kinnaird, J; Shiels, B; Tait, A; Babiker, H | 2015 | Genetic Diversity and Population Structure of Theileria annulata in Oman | Background Theileriosis, caused by a number of species within the genus Theileria, is a common disease of livestock in Oman. It is a major constraint to the development of the livestock industry due to a high rate of morbidity and mortality in both cattle and sheep. Since little is currently known about the genetic diversity of the parasites causing theileriosis in Oman, the present study was designed to address this issue with specific regard to T. annulata in cattle. Methods Blood samples were collected from cattle from four geographically distinct regions in Oman for genetic analysis of the Theileria annulata population. Ten genetic markers (micro- and mini-satellites) representing all four chromosomes of T. annulata were applied to these samples using a combination of PCR amplification and fragment analysis. The resultant genetic data was analysed to provide a first insight into the structure of the T. annulata population in Oman. Results We applied ten micro-and mini-satellite markers to a total of 310 samples obtained from different regions (174 [56%] from Dhofar, 68 [22%] from Dhira, 44 [14.5%] from Batinah and 24 [8%] from Sharqia). A high degree of allelic diversity was observed among the four parasite populations. Expected heterozygosity for each site ranged from 0.816 to 0.854. A high multiplicity of infection was observed in individual hosts, with an average of 3.3 to 3.4 alleles per locus, in samples derived from Batinah, Dhofar and Sharqia regions. In samples from Dhira region, an average of 2.9 alleles per locus was observed. Mild but statistically significant linkage disequilibrium between pairs of markers was observed in populations from three of the four regions. In contrast, when the analysis was performed at farm level, no significant linkage disequilibrium was observed. Finally, no significant genetic differentiation was seen between the four populations, with most pair-wise F-ST values being less than 0.03. Slightly higher FST values (G(ST)’ = 0.075, theta=0.07) were detected when the data for T. annulata parasites in Oman was compared with that previously generated for Turkey and Tunisia. Conclusion Genetic analyses of T. annulata samples representing four geographical regions in Oman revealed a high level of genetic diversity in the parasite population. There was little evidence of genetic differentiation between parasites from different regions, and a high level of genetic diversity was maintained within each sub-population. These findings are consistent with a high parasite transmission rate and frequent movement of animals between different regions in Oman. | 1121 | no | gd of pathogen | NA | no | no |
| 1122 | 2017 | 43016 | Miller, W; Hayes, VM; Ratan, A; Petersen, DC; Wittekindt, NE; Miller, J; Walenz, B; Knight, J; Qi, J; Zhao, FQ; Wang, QY; Bedoya-Reina, OC; Katiyar, N; Tomsho, LP; Kasson, LM; Hardie, RA; Woodbridge, P; Tindall, EA; Bertelsen, MF; Dixon, D; Pyecroft, S; Helgen, KM; Lesk, AM; Pringle, TH; Patterson, N; Zhang, Y; Kreiss, A; Woods, GM; Jones, ME; Schuster, SC | 2011 | Genetic diversity and population structure of the endangered marsupial Sarcophilus harrisii (Tasmanian devil) | The Tasmanian devil (Sarcophilus harrisii) is threatened with extinction because of a contagious cancer known as Devil Facial Tumor Disease. The inability to mount an immune response and to reject these tumors might be caused by a lack of genetic diversity within a dwindling population. Here we report a whole-genome analysis of two animals originating from extreme northwest and southeast Tasmania, the maximal geographic spread, together with the genome from a tumor taken from one of them. A 3.3-Gb de novo assembly of the sequence data from two complementary next-generation sequencing platforms was used to identify 1 million polymorphic genomic positions, roughly one-quarter of the number observed between two genetically distant human genomes. Analysis of 14 complete mitochondrial genomes from current and museum specimens, as well as mitochondrial and nuclear SNP markers in 175 animals, suggests that the observed low genetic diversity in today’s population preceded the Devil Facial Tumor Disease disease outbreak by at least 100 y. Using a genetically characterized breeding stock based on the genome sequence will enable preservation of the extant genetic diversity in future Tasmanian devil populations. | 1122 | no | no good fitness measure, N pop differnt in GD? | NA | no | no |
| 1123 | 2017 | 43016 | Katariya, L; Ramesh, PB; Gopalappa, T; Borges, RM | 2017 | Sex and diversity: The mutualistic and parasitic fungi of a fungus-growing termite differ in genetic diversity and reproductive strategy | Sex in symbionts is useful in creating beneficial traits. However, the resultant decrease in genetic relatedness between symbionts can be potentially detrimental for mutualistic interactions such as those between fungus-growing termites and the cultivated fungi in the genus Termitomyces, where the termite host should try to increase intra-nest symbiont relatedness to increase crop productivity. Any parasites of this mutualism such as the fungi in the sub-genus Pseudoxylaria may also use sex to generate variation to counter evolving host defensive mechanisms. Using molecular phylogenetic tools, we found within-nest genetic homogeneity in Termitomyces species but not in Pseudoxylaria species associated with the fungus-growing termite Odontotermes obesus. There was lower OTU but higher genotypic diversity (within the most abundant OTU) in the genus Termitomyces than in the sub-genus Pseudoxylaria. Additionally, population genetics methods suggest a sexual population structure for Termitomyces species and clonal propagation for Pseudoxylaria species. This is the first study to investigate the population genetics of the symbiotic fungi associated with the genus Odontotermes from India. (C) 2016 Elsevier Ltd and British Mycological Society. All rights reserved. | 1123 | no | no good fitness measure, N pop differnt in GD, focus on the parasite in paper | NA | no | no |
| 1124 | 2017 | 43016 | Xia, S; Fan, ZX; Zhang, XY; Jie, C; Zhang, XJ; Yue, BS | 2016 | Molecular polymorphism of MHC-DRB gene and genetic diversity analysis of captive forest musk deer (Moschus berezovskii) | The major histocompatibility complex (MHC) is one of the most important elements in immune system for nearly all vertebrates, and is thought to be essential for an organism to recognize foreign molecules. In this study, we investigated the MHC variation in 51 forest musk deer (Moschus berezovskii) collected from three captive populations in Sichuan Province, China. Seventeen haplotypes were isolated from the 51 samples. A total of 51 mutation sites were identified and yielded a nucleotide diversity of 0.056. These haplotype sequences possessed 83 putative amino acid sites, including 24 PBR sites (peptide binding region) and 59 non-PBR sites. Out of 24 PBR sites, 15 codons showed variation (0.625), while 12 codons showed variation (0.203) in 59 non-PBR sites. Non-synonymous substitutions primarily occurred in PBR, with analyses suggesting that the Mobe-DRB gene had undergone strong positive selection during their evolution. Compared with that of some other endangered species, the forest musk deer had relatively high level of MHC diversity. Our results suggested that the MHC diversity characteristic of captive forest musk deer populations might be not responsible for their high morbidity of abscess disease. (C) 2016 Elsevier Ltd. All rights reserved. | 1124 | no | no good fitness measure, N pop differnt in GD, focus on the parasite in paper | NA | no | no |
| 1125 | 2017 | 43016 | ROSENGAUS, RB; TRANIELLO, JFA | 1993 | DISEASE RISK AS A COST OF OUTBREEDING IN THE TERMITE ZOOTERMOPSIS-ANGUSTICOLLIS | The effect of the sibship of primary reproductives on mate mortality and the survivorship and growth of incipient colonies was studied in the dampwood termite Zootermopsis angusticollis. Males and females paired with nonsibling mates had higher mortality during the first 10-40 days after pairing, although male and female reproductives showed similar patterns of mortality after colony establishment. The source of mortality appeared to be fungal and/or bacterial pathogens. There were no overall differences in the number of eggs and larvae produced by sibling and nonsibling pairs, and no differences in colony size and biomass 4 years after colony establishment. We therefore could not identify any negative effect of inbreeding in the early phases of colony development. Our results suggest that the risk of exposure to pathogens and the ability of termites to locally adapt to disease could influence the genetic identity of primary reproductives and the extent of inbreeding in termite populations. | 1125 | no | inbreeding | NA | no | no |
| 1126 | 2017 | 43016 | Ramirez, JD; Llewellyn, MS | 2014 | Reproductive clonality in protozoan pathogens-truth or artefact? | The debate around the frequency and importance of genetic exchange in parasitic protozoa is now several decades old. Recently, fresh assertions have been made that predominant clonal evolution explains the population structures of several key protozoan pathogens. Here, we present an alternative perspective. On the assumption that much apparent clonality may be an artefact of inadequate sampling and study design, we review current research to define why sex might be so difficult to detect in protozoan parasite populations. In doing so, we contrast laboratory models of genetic exchange in parasitic protozoa with natural patterns of genetic diversity and consider the fitness advantage of sex at different evolutionary scales. We discuss approaches to improve the accuracy of efforts to characterize genetic exchange in the field. We also examine the implications of the first population genomic studies for the debate around sex and clonality in parasitic protozoa and discuss caveats for the future. | 1126 | no | no good fitness measure, N pop differnt in GD? | NA | no | no |
| 1127 | 2017 | 43016 | Dutech, C; Barres, B; Bridier, J; Robin, C; Milgroom, MG; Ravigne, V | 2012 | The chestnut blight fungus world tour: successive introduction events from diverse origins in an invasive plant fungal pathogen | Clonal expansion has been observed in several invasive fungal plant pathogens colonizing new areas, raising the question of the origin of clonal lineages. Using microsatellite markers, we retraced the evolutionary history of introduction of the chestnut blight fungus, Cryphonectria parasitica, in North America and western Europe. Combining discriminant analysis of principal components and approximate Bayesian computation analysis, we showed that several introduction events from genetically differentiated source populations have occurred in both invaded areas. In addition, a low signal of genetic recombination among different source populations was suggested in North America. Finally, two genetic lineages were present in both invaded areas as well as in the native areas, suggesting the existence of genetic lineages with a high capacity to establish in diverse environments and host species. This study confirmed the importance of multiple introductions, but questioned the role of genetic admixture in the success of introduction of a fungal plant pathogen. | 1127 | no | gd of pathogen | NA | no | no |
| 1128 | 2017 | 43016 | Zwick, ME; Kiley, MP; Stewart, AC; Mateczun, A; Read, TD | 2008 | Genotyping of Bacillus cereus Strains by Microarray-Based Resequencing | “The ability to distinguish microbial pathogens from closely related but nonpathogenic strains is key to understanding the population biology of these organisms. In this regard, Bacillus anthracis, the bacterium that causes inhalational anthrax, is of interest because it is closely related and often difficult to distinguish from other members of the B. cereus group that can cause diverse diseases. We employed custom-designed resequencing arrays (RAs) based on the genome sequence of Bacillus anthracis to generate 422 kb of genomic sequence from a panel of 41 Bacillus cereus sensu lato strains. Here we show that RAs represent a”“one reaction’’ genotyping technology with the ability to discriminate between highly similar B. anthracis isolates and more divergent strains of the B. cereus s.l. Clade 1. Our data show that RAs can be an efficient genotyping technology for pre-screening the genetic diversity of large strain collections to selected the best candidates for whole genome sequencing.” | 1128 | no | gd of pathogen | NA | no | no |
| 1129 | 2017 | 43016 | Goncalves, H; Maia-Carvalho, B; Sousa-Neves, T; Garcia-Paris, M; Sequeira, F; Ferrand, N; Martinez-Solano, I | 2015 | Multi locus phylogeography of the common midwife toad, Alytes obstetricans (Anura, Alytidae): Contrasting patterns of lineage diversification and genetic structure in the Iberian refugium | “Recent investigations on the evolutionary history of the common midwife toad (Alytes obstetricans) revealed high levels of geographically structured genetic diversity but also a situation where delineation of major historical lineages and resolution of their relationships are much more complex than previously thought. We studied sequence variation in one mitochondrial and four nuclear genes throughout the entire distribution range of all recognized A. obstetricans subspecies to infer the evolutionary processes that shaped current patterns of genetic diversity and population subdivision. We found six divergent, geographically structured mtDNA haplogroups diagnosing population lineages, and varying levels of admixture in nuclear markers. Given the timeframe inferred for the splits between major lineages, the climatic and environmental changes that occurred during the Pleistocene seem to have shaped the diversification history of A. obstetricans. Survival of populations in allopatric refugia through the Ice Ages supports the generality of the”“refugia-within-refugia”" scenario for the Iberian Peninsula. However, lineages corresponding to subspecies A. o. almogavarii, A. o. pertinax, A. o. obstetricans, and A. o. boscai responded differently to Pleistocene climatic oscillations after diverging from a common ancestor. Alytes o. obstetricans expanded northward from a northern Iberian refugium through the western Pyrenees, leaving a signal of contrasting patterns of genetic diversity, with a single mtDNA haplotype north of the Pyrenees from SW France to Germany. Both A. o. pertinax and A. o. boscai are widespread and genetically diverse in Iberia, the latter comprising two divergent lineages with a long independent history. Finally, A. o. almogavarii is mostly restricted to the north-eastern corner of Iberia north of the Ebro river, with additional populations in a small region in south-eastern France. This taxon exhibits unparalleled levels of genetic diversity and little haplotype sharing with other lineages, suggesting a process of incipient speciation. (C) 2015 Elsevier Inc. All rights reserved." | 1129 | no | no good fitness measure, N pop differnt in GD? | NA | no | no |
| 1130 | 2017 | 43016 | Anacleto, O; Garcia-Cortes, LA; Lipschutz-Powell, D; Woolliams, JA; Doeschl-Wilson, AB | 2015 | A Novel Statistical Model to Estimate Host Genetic Effects Affecting Disease Transmission | There is increasing recognition that genetic diversity can affect the spread of diseases, potentially affecting plant and livestock disease control as well as the emergence of human disease outbreaks. Nevertheless, even though computational tools can guide the control of infectious diseases, few epidemiological models can simultaneously accommodate the inherent individual heterogeneity in multiple infectious disease traits influencing disease transmission, such as the frequently modeled propensity to become infected and infectivity, which describes the host ability to transmit the infection to susceptible individuals. Furthermore, current quantitative genetic models fail to fully capture the heritable variation in host infectivity, mainly because they cannot accommodate the nonlinear infection dynamics underlying epidemiological data. We present in this article a novel statistical model and an inference method to estimate genetic parameters associated with both host susceptibility and infectivity. Our methodology combines quantitative genetic models of social interactions with stochastic processes to model the random, nonlinear, and dynamic nature of infections and uses adaptive Bayesian computational techniques to estimate the model parameters. Results using simulated epidemic data show that our model can accurately estimate heritabilities and genetic risks not only of susceptibility but also of infectivity, therefore exploring a trait whose heritable variation is currently ignored in disease genetics and can greatly influence the spread of infectious diseases. Our proposed methodology offers potential impacts in areas such as livestock disease control through selective breeding and also in predicting and controlling the emergence of disease outbreaks in human populations. | 1130 | no | no good fitness measure, N pop differnt in GD? | NA | no | no |
| 1131 | 2017 | 43016 | Andersson, M | 2001 | Relatedness and the evolution of conspecific brood parasitism | In conspecific brood parasitism (CBP), a parasitic female takes advantage of the parental care performed by a host female by laying eggs in the nest of the host. The host female raises the offspring of the parasitic female as well as her own. In species where local females are related, direct costs for the host might be more than compensated for by gains in inclusive fitness through increased reproduction of a related parasite, but the role of relatedness in CBP is debated. This inclusive-fitness model of parasitism, structured as a game between host and parasite, suggests that both females can gain inclusive fitness and that host-parasite relatedness can therefore facilitate the evolution of CBP. Crucial assumptions are that there is kin discrimination and a potential for host resistance to parasitism by unrelated females but close relatives are accepted. The cost of parasitism in terms of reduced clutch size or offspring survival for the host must not be large; otherwise, parasitism will reduce her inclusive fitness. Therefore, if these costs are high, it does not benefit a host to accept a parasite, even if the parasite is closely related. The secondary female may still have higher fitness from parasitism, but if the costs are high, she should parasitize an unrelated host, not a relative. This requires that the reduction in parasite success that a host can cause by resistance is not too large; otherwise, it will be better for the secondary female to parasitize an accepting related host or to nest solitarily. For these reasons, host-parasite relatedness is most likely to occur in animals where costs of being parasitized are low and host resistance can markedly reduce the success of an unrelated parasite. When costs are higher, parasitism of unrelated hosts may be better, and if host resistance strongly reduces parasite success, solitary breeding is preferable. In some cases, CBP is directly advantageous for the host, and it may sometimes evolve in close connection with cooperative breeding, which is also considered in the model. Some but not all empirical results support these ideas, and more detailed studies of behavior, relatedness, and reproduction of host and parasite are needed for critical tests. | 1131 | no | brood parasitism | NA | no | no |
| 1132 | 2017 | 43016 | Branchiccela, B; Arredondo, D; Higes, M; Invernizzi, C; Martin-Hernandez, R; Tomasco, I; Zunino, P; Antunez, K | 2017 | Characterization of Nosema ceranae Genetic Variants from Different Geographic Origins | In recent years, large-scale colony losses of honey bees (Apis mellifera) have been reported and the infection with the microsporidia Nosema ceranae has been involved. However, the effect of N. ceranae at the colony level and its role in colony losses vary in different geographic areas. This difference may be related to the presence of multiple N. ceranae genetic variants resulting in different biological consequences. In this study, we analyzed the genetic diversity of 75 N. ceranae samples obtained from 13 countries and Hawaii through inter-sequence single repetition (ISSR) and evaluated if two of these genetic variants triggered different immune responses when infecting Apis mellifera iberiensis. The genetic diversity analysis showed that 41% of the samples had the same DNA amplification pattern, including samples from most European countries except Spain, while the remaining samples showed high variability. Infection assays were performed to analyze the infection levels and the immune response of bees infected with N. ceranae from Spain and Uruguay. The infected bees presented similar infection levels, and both isolates downregulated the expression of abaecin, confirming the ability of the microsporidia to depress the immune response. Only N. ceranae from Uruguay downregulated the expression level of imd compared to control bees. On the other hand, both genetic variants triggered different expression levels of lysozyme. As imd and lysozyme play important roles in the response to pathogens, these results could reflect differences in the biological consequences of N. ceranae variants in A. mellifera infection. | 1132 | no | gd of parasite | NA | no | no |
| 1133 | 2017 | 43016 | Bray, SM; Mulle, JG; Dodd, AF; Pulver, AE; Wooding, S; Warren, ST | 2010 | Signatures of founder effects, admixture, and selection in the Ashkenazi Jewish population | The Ashkenazi Jewish (AJ) population has long been viewed as a genetic isolate, yet it is still unclear how population bottlenecks, admixture, or positive selection contribute to its genetic structure. Here we analyzed a large AJ cohort and found higher linkage disequilibrium (LD) and identity-by-descent relative to Europeans, as expected for an isolate. However, paradoxically we also found higher genetic diversity, a sign of an older or more admixed population but not of a long-term isolate. Recent reports have reaffirmed that the AJ population has a common Middle Eastern origin with other Jewish Diaspora populations, but also suggest that the AJ population, compared with other Jews, has had the most European admixture. Our analysis indeed revealed higher European admixture than predicted from previous Y-chromosome analyses. Moreover, we also show that admixture directly correlates with high LD, suggesting that admixture has increased both genetic diversity and LD in the AJ population. Additionally, we applied extended haplotype tests to determine whether positive selection can account for the level of AJ-prevalent diseases. We identified genomic regions under selection that account for lactose and alcohol tolerance, and although we found evidence for positive selection at some AJ-prevalent disease loci, the higher incidence of the majority of these diseases is likely the result of genetic drift following a bottleneck. Thus, the AJ population shows evidence of past founding events; however, admixture and selection have also strongly influenced its current genetic makeup. | 1133 | no | human | NA | no | no |
| 1134 | 2017 | 43016 | Xu, P; Wu, XH; Wang, BG; Liu, YH; Qin, DH; Ehlers, JD; Close, TJ; Hu, TT; Lu, ZF; Li, GJ | 2010 | Development and polymorphism of Vigna unguiculata ssp unguiculata microsatellite markers used for phylogenetic analysis in asparagus bean (Vigna unguiculata ssp sesquipedialis (L.) Verdc.) | Asparagus bean (V. unguiculata ssp. sesquipedialis), a specific form of cowpea (V. unguiculata L. Walp.), is cultivated as a vegetable crop throughout eastern and southern Asia for its tender long pods. Little is known about the genetic relationship between asparagus bean and the broader species, particularly the dominant ssp. unguiculata. We report here the development and transferability of simple sequence repeat (SSR) markers, over 40% of which are EST-derived, from ssp. unguiculata to asparagus bean and the use of a subset of the polymorphic markers to assess the genetic diversity of asparagus bean cultivars from diverse geographic origins across China. A total of 410 EST derived SSR (eSSR) markers and 600 SSR markers derived from cowpea genespace sequences (GSS) were developed, with a cross-subspecies transferability of 100% and 98.5%, respectively. In a recombinant inbred line population of asparagus bean, a 1:1 segregation was observed for most loci. Principal coordinate analysis (PCA) and phylogenetic clustering based on 62 alleles detected by 14 polymorphic SSR markers distinguished ssp. unguiculata and sesquipedialis into separate groups. Improved asparagus bean cultivars in China generally have a narrow genetic basis compared with landraces varieties. This suggests that asparagus bean breeding programs need to consider utilizing landrace germplasm to enhance genetic variability and ensure long-term gains from selection and reduce genetic vulnerability to pathogen/pest epidemics. Because of their transferability across subspecies, the SSR markers described in this study could be effectively employed in cross-subspecies trait introgression breeding from ssp. unguiculata to sesquipedialis. | 1134 | no | crop | NA | no | no |
| 1135 | 2017 | 43016 | Dyer, GA; Lopez-Feldman, A; Yunez-Naude, A; Taylor, JE | 2014 | Genetic erosion in maize’s center of origin | Crop genetic diversity is an indispensable resource for farmers and professional breeders responding to changing climate, pests, and diseases. Anecdotal appraisals in centers of crop origin have suggested serious threats to this diversity for over half a century. However, a nationwide inventory recently found all maize races previously described for Mexico, including some formerly considered nearly extinct. A flurry of social studies seems to confirm that farmers maintain considerable diversity. Here, we compare estimates of maize diversity from case studies over the past 15 y with nationally and regionally representative matched longitudinal data from farmers across rural Mexico. Our findings reveal an increasing bias in inferences based on case study results and widespread loss of diversity. Cross-sectional, case study data suggest that farm-level richness has increased by 0.04 y(-1) nationwide; however, direct estimates using matched longitudinal data reveal that richness dropped -0.04 y(-1) between 2002 and 2007, from 1.43 to 1.22 varieties per farm. Varietal losses occurred across regions and altitudinal zones, and regardless of farm turnover within the sector. Extinction of local maize populations may not have resulted in an immediate loss of alleles, but low varietal richness and changes in maize’s metapopulation dynamics may prevent farmers from accessing germplasm suitable to a rapidly changing climate. Declining yields could then lead farmers to leave the sector and result in a further loss of diversity. Similarities in research approaches across crops suggest that methodological biases could conceal a loss of diversity at other centers of crop origin. | 1135 | no | crop | NA | no | no |
| 1136 | 2017 | 43016 | Rhone, B; Remoue, C; Galic, N; Goldringer, I; Bonnin, I | 2008 | Insight into the genetic bases of climatic adaptation in experimentally evolving wheat populations | Experimental populations evolving under natural selection represent an interesting tool to study genetic bases of adaptation. Evolution of genes possibly involved in adaptive response can be followed together with the corresponding phenotypic traits. Using experimental populations of hexaploid wheat, we studied the evolution of flowering time, a major adaptive trait that synchronizes the initiation of reproduction and the occurrence of favourable environmental conditions. During 12 generations, three populations were grown in contrasted environments (Vervins North France, Le Moulon near Paris, Toulouse South France) under the influence of natural selection, drift, mutation and recombination. Evolution of diversity at the major gene VRN-1 involved in wheat vernalization response has been analysed jointly with earliness estimated in controlled conditions. Whatever the population, rapid phenotypic changes as well as parallel genotypic variations were observed in the first seven generations, probably as the result of selection acting on this major gene which explains 80% of the trait variation overall. Different allelic combinations at physically unlinked copies of VRN-1 located on distinct genomes (A, B and D) were selected between populations. As theoretically expected, due to population differentiation, a high level of genetic diversity was maintained overall in generation 12. Surprisingly, in two populations out of three, the emergence of new alleles by mutation or migration, coupled with temporal variable selection or frequency-dependent selection, allowed to maintain within-population diversity despite local genetic drift and natural selection. This result may plead for an evolutionary approach of wheat genetic resource conservation. | 1136 | no | no good fitness measure | NA | no | no |
| 1137 | 2017 | 43016 | Thanwisai, A; Tandhavanant, S; Saiprom, N; Waterfield, NR; Long, PK; Bode, HB; Peacock, SJ; Chantratita, N | 2012 | Diversity of Xenorhabdus and Photorhabdus spp. and Their Symbiotic Entomopathogenic Nematodes from Thailand | Xenorhabdus and Photorhabdus spp. are bacterial symbionts of entomopathogenic nematodes (EPNs). In this study, we isolated and characterized Xenorhabdus and Photorhabdus spp. from across Thailand together with their associated nematode symbionts, and characterized their phylogenetic diversity. EPNs were isolated from soil samples using a Galleria-baiting technique. Bacteria from EPNs were cultured and genotyped based on recA sequence. The nematodes were identified based on sequences of 28S rDNA and internal transcribed spacer regions. A total of 795 soil samples were collected from 159 sites in 13 provinces across Thailand. A total of 126 EPNs isolated from samples taken from 10 provinces were positive for Xenorhabdus (n = 69) or Photorhabdus spp. (n = 57). Phylogenetic analysis separated the 69 Xenorhabdus isolates into 4 groups. Groups 1, 2 and 3 consisting of 52, 13 and 1 isolates related to X. stockiae, and group 4 consisting of 3 isolates related to X. miraniensis. The EPN host for isolates related to X. stockiae was S. websteri, and for X. miraniensis was S. khoisanae. The Photorhabdus species were identified as P. luminescens (n = 56) and P. asymbiotica (n = 1). Phylogenenic analysis divided P. luminescens into five groups. Groups 1 and 2 consisted of 45 and 8 isolates defined as subspecies hainanensis and akhurstii, respectively. One isolate was related to hainanensis and akhurstii, two isolates were related to laumondii, and one isolate was the pathogenic species P. asymbiotica subsp. australis. H. indica was the major EPN host for Photorhabdus. This study reveals the genetic diversity of Xenorhabdus and Photorhabdus spp. and describes new associations between EPNs and their bacterial symbionts in Thailand. | 1137 | no | no good fitness measure, N pop differnt in GD? | NA | no | no |
| 1138 | 2017 | 43016 | Nikbakht, G; Esmailnejad, A | 2015 | Chicken major histocompatibility complex polymorphism and its association with production traits | Major histocompatibility complex (MHC) is the best characterized genetic region controlling disease resistance and immune responses in chicken. MHC genes are also involved in various non-immune functions such as productive traits and reproductive success. The genetic diversity of MHC in an Iranian indigenous chicken (Khorasan) was studied, and association of the MHC alleles with production traits was determined. The MHC polymorphism was ascertained by genotyping the LEI0258 microsatellite locus by PCR-based fragment analysis. LEI0258 microsatellite marker is a genetic indicator for MHC, which is located on microchromosome 16 and strongly associated with serologically defined MHC haplotypes. A total of 25 different LEI0258 alleles (185-493 bp) and 76 genotypes were identified in 313 chickens. An allele of 361 bp had the highest frequency (26.44 %), and alleles of 207 and 262 bp had the lowest (0.16 %). High level of heterozygosity (87 %) and good genotype frequency fit to the Hardy-Weinberg equilibrium was observed in this population (P = 0.238). The association study also revealed a significant influence of MHC alleles on body weight, egg weight, egg laying intensity, and weight of sexual maturity in Khorasan population (P < 0.05). The information obtained from this study indicates a high MHC genetic diversity and the association of MHC alleles with important production traits in Khorasan chicken. These data would be applicable in designing breeding and genetic resource conservation for indigenous chicken populations. | 1138 | no | no good fitness measure, N pop differnt in GD? | NA | no | no |
| 1139 | 2017 | 43016 | Pringle, JM; Blakeslee, AMH; Byers, JE; Roman, J | 2011 | Asymmetric dispersal allows an upstream region to control population structure throughout a species’ range | In a single well-mixed population, equally abundant neutral alleles are equally likely to persist. However, in spatially complex populations structured by an asymmetric dispersal mechanism, such as a coastal population where larvae are predominantly moved downstream by currents, the eventual frequency of neutral haplotypes will depend on their initial spatial location. In our study of the progression of two spatially separate, genetically distinct introductions of the European green crab (Carcinus maenas) along the coast of eastern North America, we captured this process in action. We documented the shift of the genetic cline in this species over 8 y, and here we detail how the upstream haplotypes are beginning to dominate the system. This quantification of an evolving genetic boundary in a coastal system demonstrates that novel genetic alleles or haplotypes that arise or are introduced into upstream retention zones (regions whose export of larvae is not balanced by import from elsewhere) will increase in frequency in the entire system. This phenomenon should be widespread when there is asymmetrical dispersal, in the oceans or on land, suggesting that the upstream edge of a species’ range can influence genetic diversity throughout its distribution. Efforts to protect the upstream edge of an asymmetrically dispersing species’ range are vital to conserving genetic diversity in the species. | 1139 | no | no good fitness measure, N pop differnt in GD? | NA | no | no |
| 1140 | 2017 | 43016 | Gow, JL; Noble, LR; Rollinson, D; Tchuente, LAT; Jones, CS | 2007 | Contrasting temporal dynamics and spatial patterns of population genetic structure correlate with differences in demography and habitat between two closely-related African freshwater snails | The relationship between habitat stability, demography, and population genetic structure was explored by comparing temporal microsatellite variability spanning a decade in two closely-related hermaphroditic freshwater snails from Cameroon, Bulinus forskalii and Bulinus camerunensis. Although both species show similar levels of preferential selfing, microsatellite analysis revealed significantly greater allelic richness and gene diversity in populations of the highly endemic B. camerunensis compared to those of the geographically-widespread B. forskalii. Additionally, B. camerunensis populations showed significantly lower spatial genetic differentiation, higher dispersal rates, and greater temporal stability compared to B. forskalii populations over a similar spatial scale. This suggests that a more stable demography and greater gene flow account for the elevated genetic diversity observed in this geographically-restricted snail. This contrasts sharply with a metapopulation model (which includes extinction/contraction, recolonization/expansion, and passive dispersal) invoked to account for population structuring in B. forskalii. As intermediate hosts for medically important schistosome parasites, these findings have ramifications for determining the scale at which local adaptation may occur in the coevolution of these snails and their parasites. (c) 2007 The Linnean Society of London. | 1140 | no | no good fitness measure | NA | no | no |
| 1141 | 2017 | 43016 | Wielgoss, S; Sanetra, M; Meyer, A; Wirth, T | 2007 | Isolation and characterization of short tandem repeats in an invasive swimbladder nematode, parasitic in Atlantic freshwater eels, Anguillicola crassus | We describe the isolation and characterization of seven polymorphic short tandem repeats (STR) for the eel parasite Anguillicola crassus. This invasive swimbladder nematode endemic in East Asia was recently introduced into Europe. The number of alleles for each STR ranged from 13 to 39 per locus with observed heterozygosities between 0.49 and 0.98. The Taiwanese population displayed higher genetic diversity compared to the Irish sample, an observation consistent with the Asian biogeographical origin of the nematode. Availability of the reported STR will facilitate the investigation of the population genetic structure with regard to multiple invasions. | 1141 | no | gd of parasite | NA | no | no |
| 1142 | 2017 | 43016 | Maltagliati, F | 2002 | Genetic monitoring of brackish-water populations: the Mediterranean toothcarp Aphanius fasciatus (Cyprinodontidae) as a model | The measurement of genetic variability and assessment of population genetic losses are important components of environmental management programs. Twenty-three natural populations of the Mediterranean brackish-water toothcarp Aphanius fasciatus were investigated using different statistical approaches based on genetic data at 13 polymorphic allozyme loci, In general, no differences between values of within-population genetic variability estimates occurred. The Wilcoxon sign-rank test for heterozygosity excess due to a recent bottleneck was conducted on the array of populations, In addition, a qualitative descriptor of allele frequency distribution was used to infer bottlenecks. Only populations from the Orbetello lagoon and La Salina at Elba Island revealed significant heterozygosity excess under both the infinite allele model (IAM) and stepwise mutation model (SMM). A recent dystrophic crisis may account for the genetic loss detected in the population of A. fasciatus from the Orbetello lagoon, whereas exceptionally high predation pressure and/or the increased mortality rate created by local hydrological conditions could be responsible for the bottleneck in the population at La Salina. Tests for bottlenecks have proved effective tools for genetic monitoring of A. fasciatus populations. | 1142 | no | no good fitness measure | NA | no | no |
| 1143 | 2017 | 43016 | Raboin, LM; Selvi, A; Oliveira, KM; Paulet, F; Calatayud, C; Zapater, MF; Brottier, P; Luzaran, R; Garsmeur, O; Carlier, J; D’Hont, A | 2007 | Evidence for the dispersal of a unique lineage from Asia to America and Africa in the sugarcane fungal pathogen Ustilago scitaminea | The basidiomycete Ustilago scitaminea Sydow, which causes sugarcane smut disease, has been spreading throughout Africa and America since the 1940s. The genetic diversity and structure of different populations of this fungus worldwide was investigated using microsatellites. A total of 142 single-teliospore were isolated from 77 distinct whips (sori) collected in 15 countries worldwide. Mycelium culture derived from on generation of setting of these single teliospores were analysed for their polymorphisms at 17 microsatellite loci. All these strains but one were homozygous at all loci, indicating that selfing is likely the predominant reproductive mode of U. scitaminea. The genetic diversity of either American or African U. scitaminea populations was found to be extremely low and all strains belong to a single lineage. This lineage was also found in some populations of Asia, where most U. scitaminea genetic diversity was detected, suggesting that this fungal species originated from this region. The strong founder effect observed in U. scitaminea African and American populations suggests that the fungus migrated from Asia to other continents on rare occasions through movement of infected plant material. (c) 2006 Elsevier Inc. All rights reserved. | 1143 | no | gd of pathogen | NA | no | no |
| 1144 | 2017 | 43016 | Del-Toro-Sanchez, CL; Villasenor-Alvarado, S; Zurita-Martinez, F; Castellanos-Hernandez, OA; Rodriguez-Sahagun, A; Torres-Moran, MI; Rojas-Bravo, D; Gutierrez-Lomeli, M | 2013 | OPTIMIZATION OF DNA ISOLATION AND PCR PROTOCOL FOR ANALYSIS AND EVALUATION OF GENETIC DIVERSITY OF THE MEDICINAL PLANT, ANEMOPSIS CALIFORNICA USING RAPD | Anemopsis californica is a perennial herbaceous plant that has been utilized as a medicinal plant for the treatment of various diseases. The present work was carried out with the objective of optimizing a method of extraction of the genomic DNA of A. californica and a PCR protocol and later to evaluate the existing genetic diversity among the genotypes deriving from different origins. For DNA extraction, we tested four procedures: with the CTA B-2 protocol, we obtained the highest yield (61.5 +/- 2.2 mu g DNA/g of leaf tissues) and the best quality (A(260/280) 1.83 +/- 0.022). To estimate genetic variability, we utilized the randomly amplified polymorphism DNA (RAPD) technique, employing 20 oligonucleotides, of which only 18 generated reproducible banding patterns, producing 123 polymorphic bands generated, thus obtaining a polymorphism rate of 93.93% among the genotypes analyzed. The Jaccard similarity coefficient generated a variation ranging from 0.325-0.921, indicating a high level of genetic variation among the studied genotypes. An Unweighted pair-group method with arithmetic mean (UPGMA) group analysis indicated six distinct groups. The present optimized method for DNA isolation and RAPD protocol may serve as an efficient tool for further molecular studies. | 1144 | no | no good fitness measure | NA | no | no |
| 1145 | 2017 | 43016 | Butaud, JF; Rives, F; Verhaegen, D; Bouvet, JM | 2005 | Phylogeography of Eastern Polynesian sandalwood (Santalum insulare), an endangered tree species from the Pacific: a study based on chloroplast microsatellites | Aim Patterns of genetic variation within forest species are poorly documented in island ecosystems. The distribution of molecular variation for Santalum insulare, an endangered tree species endemic to the islands of eastern Polynesia, was analysed using chloroplast microsatellite markers. The aims were to quantify the genetic diversity; to assess the genetic structure; and to analyse the geographical distribution of the diversity within and between archipelagoes. The ultimate goal was to pre-define evolutionary significant units (ESUs) for conservation and restoration programmes of this species, which constitutes a natural resource on small, isolated islands. Location Eleven populations, each representative of one island, covering most of the natural occurrence of S. insulare were sampled: five populations from the Marquesas Archipelago; three from the Society Archipelago; and three from the Cook-Austral Archipelago. These South Pacific islands are known for their high degree of plant endemism, and for their human occupation by Polynesian migrations. The extensive exploitation of sandalwood by Europeans nearly 200 years ago for its fragrant heartwood, used overseas in incense, carving and essential oil production for perfume, has dramatically reduced the population size of this species. Methods We used chloroplast microsatellites, which provide useful information in phylogeographical forest tree analyses. They are maternally inherited in most angiosperms and present high polymorphism. Among the 499 individuals sampled, 345 were genotyped successfully. Classical models of population genetics were used to assess diversity parameters and phylogenetic relationships between populations. Results Four microsatellite primers showed 16 alleles and their combinations provided 17 chlorotypes, of which four exhibited a frequency > 10% in the total population. The gene diversity index was high for the total population (H-e = 0.82) and varied among archipelagoes from H-e = 0.40 to 0.67. Genetic structure is characterized by high levels of differentiation between archipelagoes (36% of total variation) and between islands, but differentiation between islands varied according to archipelago. The relationship between genetic and geographical distance confirms the low gene flow between archipelagoes. The minimum spanning tree of chlorotypes exhibits three clusters corresponding to the geographical distribution in the three main archipelagoes. Main conclusions The high level of diversity within the species was explained by an ancient presence on and around the hotspot traces currently occupied by young islands. Diversity in the species has enabled survival in a range of habitats. Relationships between islands show that the Cook-Austral chlorotype cluster constitutes a link between the Marquesas and the Society Islands. This can be explained by the evolution of the island systems over millions of years, and extinction of intermediary populations on the Tuamotu Islands following subsidence there. Based on the unrooted neighbour-joining tree and on the genetic structure, we propose four ESUs to guide the conservation and population restoration of Polynesian Sandalwood: the Society Archipelago; the Marquesas Archipelago; Raivavae Island; and Rapa Island. | 1145 | no | no good fitness measure | NA | no | no |
| 1146 | 2017 | 43016 | Rieux, A; De Bellaire, LD; Zapater, MF; Ravigne, V; Carlier, J | 2013 | Recent range expansion and agricultural landscape heterogeneity have only minimal effect on the spatial genetic structure of the plant pathogenic fungus Mycosphaerella fijiensis | Understanding how geographical and environmental features affect genetic variation at both the population and individual levels is crucial in biology, especially in the case of pathogens. However, distinguishing between these factors and the effects of historical range expansion on spatial genetic structure remains challenging. In the present study, we investigated the case of Mycosphaerella fijiensis-a plant pathogenic fungus that has recently colonized an agricultural landscape characterized by the presence of potential barriers to gene flow, including several commercial plantations in which disease control practises such as the use of fungicides are applied frequently, and low host density areas. We first genotyped 300 isolates sampled at a global scale on untreated plants in two dimensions over a 50 x 80-km area. Using two different clustering algorithms, no genetic structure was detected in the studied area, suggesting expansion of large populations and/or no influence of potential barriers. Second, we investigated the potential effect of disease control practises on M. fijiensis diversity by comparing populations sampled in commercial vs food-crop plantations. At this local scale, we detected significantly higher allelic richness inside commercial plantations compared with the surrounding food-crop plantation populations. Analysis of molecular variance indicated that 99% of the total genetic variance occurred within populations. We discuss the suggestion that high population size and/or high migration rate between populations might be responsible for the absence of any effect of disease control practises on genetic diversity and differentiation. Heredity (2013) 110, 29-38; doi:10.1038/hdy.2012.55; published online 19 September 2012 | 1146 | no | gd of pathogen | NA | no | no |
| 1147 | 2017 | 43016 | Ramos, E; Doumatey, A; Elkahloun, AG; Shriner, D; Huang, H; Chen, G; Zhou, J; McLeod, H; Adeyemo, A; Rotimi, CN | 2014 | Pharmacogenomics, ancestry and clinical decision making for global populations | Pharmacogenomically relevant markers of drug response and adverse drug reactions are known to vary in frequency across populations. We examined minor allele frequencies (MAFs), genetic diversity (F-ST) and population structure of 1156 genetic variants (including 42 clinically actionable variants) in 212 genes involved in drug absorption, distribution, metabolism and excretion (ADME) in 19 populations (n=1478). There was wide population differentiation in these ADME variants, reflected in the range of mean MAF (Delta MAF) and F-ST. The largest mean Delta MAF was observed in African ancestry populations (0.10) and the smallest mean AMAF in East Asian ancestry populations (0.04). MAFs ranged widely, for example, from 0.93 for single-nucleotide polymorphism (SNP) rs9923231, which influences warfarin dosing to 0.01 for SNP rs3918290 associated with capecitabine metabolism. ADME genetic variants show marked variation between and within continental groupings of populations. Enlarging the scope of pharmacogenomics research to include multiple global populations can improve the evidence base for clinical translation to benefit all peoples. | 1147 | no | NA | NA | no | no |
| 1148 | 2017 | 43016 | Martinez-Fresno, M; Henriques-Gil, N; Arana, P | 2008 | Mitochondrial DNA sequence variability in red-legged partridge, Alectoris rufa, Spanish populations and the origins of genetic contamination from A-chukar | The analysis of 135 mitochondrial D-loop sequences of the Iberian autochthonous red-legged partridge (Alectoris rufa) from wild population hunting bags from various locations and fowl runs in Spain yielded 37 different haplotypes. Among these, three haplotypes correspond to chukar partridges (Alectoris chukar), indicating genetic introgression from birds illegally introduced for restocking: three individuals carrying such haplotypes where found in natural populations, one appeared among those sampled on a mass reproduction farm and the remaining 10 in another fowl-run. The geographical origin of the contaminating chukar haplotypes could be assigned to the most easterly area of the chukar partridge geographical distribution in China. Molecular diversity parameters in the A. rufa samples indicate a considerable amount of genetic variation. Phi(ST) showed significant differences among populations that are not explained by geographical distance alone. Particularly, one northern population (Palencia) shows a certain degree of genetic differentiation that could reflect a previously suggested subspecies division. | 1148 | no | NA | NA | no | no |
| 1149 | 2017 | 43016 | Rabeling, C; Love, CN; Lance, SL; Jones, KL; Pierce, NE; Bacci, M | 2014 | Development of twenty-one polymorphic microsatellite markers for the fungus-growing ant, Mycocepurus goeldii (Formicidae: Attini), using Illumina paired-end genomic sequencing | Obligate social parasites, or inquilines, exploit the colonies of free-living social species and evolved at least 80 times in ants alone. Most species of the highly specialized inquiline social parasites are rare, only known from one or very few, geographically isolated populations, and the sexual offspring of most inquiline species mates inside the maternal colony. Therefore, inquiline populations are believed to be small and genetically homogeneous due to inbreeding. To comparatively study the genetic diversity of the socially parasitic fungus-growing ant, Mycocepurus castrator, and its only known host species, Mycocepurus goeldii, and to infer the parasite’s conservation status, we developed 21 microsatellite markers for the host species, M. goeldii, and evaluated whether these markers cross-amplify in the social parasite, M. castrator. We isolated and characterized a total of 21 microsatellite loci for M. goeldii. The loci were screened for 24 individuals from geographically distant and genetically divergent populations in Brazil. The number of alleles per locus ranged from 18 to 4, the observed heterozygosity ranged from 0.25 to 0.636, and the probability of identity values ranged from 0.011 to 0.146. Preliminary analyses show that these markers cross amplify in the closely related social parasite species M. castrator. These newly developed loci provide tools for studying the genetic diversity and the evolution of social parasitism in the Mycocepurus host-parasite system. | 1149 | no | no good fitness measure, N pop differnt in GD? | NA | no | no |
| 1150 | 2017 | 43016 | Devillard, S; Santin-Janin, H; Say, L; Pontier, D | 2011 | Linking genetic diversity and temporal fluctuations in population abundance of the introduced feral cat (Felis silvestris catus) on the Kerguelen archipelago | Linking temporal variations of genetic diversity, including allelic richness and heterozygosity, and spatio-temporal fluctuations in population abundance has emerged as an important tool for understanding demographic and evolutionary processes in natural populations. This so-called genetic monitoring was conducted across 12 consecutive years (19962007) at three sites for the feral cat, introduced onto the Kerguelen archipelago fifty years ago. Temporal changes in allelic richness and heterozygosity at 18 microsatellite DNA loci were compared with temporal changes in the adult population abundance index, obtained by typical demographic monitoring. No association was found at the island spatial scale, but we observed an association between genetic diversity and adult population indices from year to year within each study site. More particularly, the magnitude of successive increases or decreases in the adult population abundance index appeared to be the major factor linking the trajectories of genetic diversity and adult population abundance indices. Natal dispersal and/or local recruitment, both facilitated by high juvenile survival when the adult population size is small, is proposed as the major demographic processes contributing to such an observed pattern. Finally, we suggested avoiding the use of the harmonic mean as an estimator of long-term population size to study the relationships between demographic fluctuations and heterozygosity in populations characterized by strong multiannual density fluctuations. | 1150 | no | no good fitness measure, N pop differnt in GD? | NA | no | no |
| 1151 | 2017 | 43016 | Liu, RH; Hu, B; Li, QB; Jing, XY; Zhong, C; Chang, Y; Liao, QJ; Lam, MF; Leung, JCK; Lai, KN; Wang, YM | 2015 | Novel genes and variants associated with IgA nephropathy by co-segregating with the disease phenotypes in 10 IgAN families | Background: Previously, a large proportion of the genetic components predisposing individuals to IgA nephropathy (IgAN) have been unidentified. Familial IgAN is enriched with genetic variations predisposing individuals to the disease. Whole exome sequencing is an effective way to explore disease-causing genes and gene variants. Methods: We performed exome sequencing on the probands from each of ten IgAN families, and on one of the unaffected member from 7 of the families. Sanger sequencing, bioinformatics and co-segregation analysis were performed for all available family members to detect deleterious genetic variation. The relatedness of the families was tested by haplotype analyses. Results: Six deleterious variants in 4 genes were observed to be associated with IgA nephropathy by co-segregating with the disease phenotypes in study families. MYCT1 p.Asp22Glufs*34 was associated with IgAN by co-segregating with its phenotypes in families 2, 7, and 9; DEFA4 p.Ala8Pro, p.Ala8Val, c.172 + 1G>T co-segregated in families 1, 2, and 3; ZNF543 p.Pro226Ala co-segregated in families 3, 5, and 6 and CARD8 p.Val98Lysfs*26 co-segregated in families 7 and 8. Among these genes, MYCT1, CARD8 and ZNF543 are novel. Our haplotype analyses showed that families in which the same variation(s) were co-segregating with IgAN were unrelated, except for DEFA4. Of the families carrying DEFA4, families 2 and 3 were possibly related, but not family 1, indicating that common genes/variations in these families were not due to the same founder. Inter-familial sharing of different co-segregating genes was also observed, demonstrating the polygenic nature of this disease. Conclusions: We discovered 6 deleterious variants in 4 genes associated with familial IgAN. These genes are good candidate genes that appear to be causally related to IgAN and warrant further study. (C) 2015 Elsevier B.V. All rights reserved. | 1151 | no | no good fitness measure, N pop differnt in GD? | NA | no | no |
| 1152 | 2017 | 43016 | Vergilino, R; Leung, C; Angers, B | 2016 | Inconsistent phylogeographic pattern between a sperm dependent fish and its host: in situ hybridization vs dispersal | Background: Co-dispersal of sperm-dependent hybrids and their sexual relatives is expected to result in consistent spatial patterns between assemblages of hybrids and genetic structure of parental species. However, local hybridization events may blur this signal as assemblages could be organized under different connectivity constraints. This study aims at testing the hypothesis of local hybridization events by comparing the assemblage of hybrid fish Chrosomus eos-neogaeus to the genetic diversity of one of its parental species, Chrosomus eos. Results: An extensive survey performed on a total of 132 sites located in two regions of Southern Quebec (West-Qc and East-Qc) revealed a distinct organization of hybrid lineages. One of the six hybrid lineages detected in West-Qc is widespread throughout this region resulting in a low a-diversity (1.38) and beta-diversity (4.35). On the other hand, 36 hybrid lineages were detected in East-Qc and displayed narrow geographic distributions leading to a high a-diversity (2.30) and beta-diversity (15.68). In addition, the C. eos multilocus haplotype of several of these hybrids is assigned to their respective sympatric C. eos population. Finally, contrasting with hybrids, the paternal species C. eos displayed a higher rho(ST) in West-Qc (0.2300) than in East-Qc (0.0734). Conclusion: The unusually high diversity of hybrid lineages in East-Qc as well as the spatial organization and the close genetic relationship with C. eos sympatric populations support the hypothesis that multiple hybridization events occurred in situ. These findings coupled to the near absence of the maternal species Chrosomus neogeaus suggest that the decline of this species could be the trigger event at the origin of the high rates of spontaneous hybridization in this region. | 1152 | no | no good fitness measure, N pop differnt in GD? | NA | no | no |
| 1153 | 2017 | 43016 | Ackermann, AA; Panunzi, LG; Cosentino, RO; Sanchez, DO; Aguero, F | 2012 | A genomic scale map of genetic diversity in Trypanosoma cruzi | Background: Trypanosoma cruzi, the causal agent of Chagas Disease, affects more than 16 million people in Latin America. The clinical outcome of the disease results from a complex interplay between environmental factors and the genetic background of both the human host and the parasite. However, knowledge of the genetic diversity of the parasite, is currently limited to a number of highly studied loci. The availability of a number of genomes from different evolutionary lineages of T. cruzi provides an unprecedented opportunity to look at the genetic diversity of the parasite at a genomic scale. Results: Using a bioinformatic strategy, we have clustered T. cruzi sequence data available in the public domain and obtained multiple sequence alignments in which one or two alleles from the reference CL-Brener were included. These data covers 4 major evolutionary lineages (DTUs): TcI, TcII, TcIII, and the hybrid TcVI. Using these set of alignments we have identified 288,957 high quality single nucleotide polymorphisms and 1,480 indels. In a reduced re-sequencing study we were able to validate similar to 97% of high-quality SNPs identified in 47 loci. Analysis of how these changes affect encoded protein products showed a 0.77 ratio of synonymous to non-synonymous changes in the T. cruzi genome. We observed 113 changes that introduce or remove a stop codon, some causing significant functional changes, and a number of tri-allelic and tetra-allelic SNPs that could be exploited in strain typing assays. Based on an analysis of the observed nucleotide diversity we show that the T. cruzi genome contains a core set of genes that are under apparent purifying selection. Interestingly, orthologs of known druggable targets show statistically significant lower nucleotide diversity values. Conclusions: This study provides the first look at the genetic diversity of T. cruzi at a genomic scale. The analysis covers an estimated similar to 60% of the genetic diversity present in the population, providing an essential resource for future studies on the development of new drugs and diagnostics, for Chagas Disease. These data is available through the TcSNP database (http://snps.tcruzi.org). | 1153 | no | human | NA | no | no |
| 1154 | 2017 | 43016 | Olsson, M; Pagel, M; Shine, R; Madsen, T; Doums, C; Gullberg, A; Tegelstrom, H | 1999 | Sperm choice and sperm competition: suggestions for field and laboratory studies | “The recent explosion of research on sperm competition, and the emerging idea that females may control paternity of their offspring via”“sperm choice”“, raise many methodological questions. One central issue is the role of inbreeding-induced mortality of offspring. We present a theoretical model which makes the relationship between parental relatedness and offspring mortality explicit, and discuss its applicability as a null model of offspring mortality in studies of sperm competition/sperm choice. We also discuss the extent to which partner relatedness may be overlooked in studies of behavioural ecology in natural populations, and the extent to which a failure to mimic crucial aspects of sperm competition scenarios in the wild may limit our ability to make evolutionary inferences from laboratory experiments.” | 1154 | no | no good fitness measure, N pop differnt in GD? | NA | no | no |
| 1155 | 2017 | 43016 | Ruas, EA; Ruas, CF; Medri, PS; Medri, C; Medri, ME; Bianchini, E; Pimenta, JA; Rodrigues, LA; Ruas, PM | 2011 | Anatomy and genetic diversity of two populations of Schinus terebinthifolius (Anacardiaceae) from the Tibagi River basin in Parana, Brazil | were significantly higher in plants from one population. Genetic analysis using DNA samples obtained from both populations showed a moderate degree of genetic variation between populations (13.7%); most of the variation was found within populations (86.3%). These results show that for conservation purposes and management of degraded areas, both populations should be preserved and could be used in programs that intend to recompose riparian forests.Knowledge of the effects of flooding on plant survival is relevant for the efficiency of management and conservation programs. Schinus terebinthifolius is a tree of economic and ecological importance that is common in northeast Brazil. Flooding tolerance and genetic variation were investigated in two riparian populations of S. terebinthifolius distributed along two different ecological regions of the Tibagi River basin. Flooding tolerance was evaluated through the investigation of young plants, submitted to different flooding intensities to examine the morphological and anatomical responses to this stress. The growth rate of S. terebinthifolius was not affected by flooding, but total submersion proved to be lethal for 100% of the plants. Morphological alterations such as hypertrophied lenticels were observed in both populations and lenticel openings | 1155 | no | no good fitness measure, N pop differnt in GD? | NA | no | no |
| 1156 | 2017 | 43016 | Sudmant, PH; Kitzman, JO; Antonacci, F; Alkan, C; Malig, M; Tsalenko, A; Sampas, N; Bruhn, L; Shendure, J; Eichler, EE | 2010 | Diversity of Human Copy Number Variation and Multicopy Genes | “Copy number variants affect both disease and normal phenotypic variation, but those lying within heavily duplicated, highly identical sequence have been difficult to assay. By analyzing short-read mapping depth for 159 human genomes, we demonstrated accurate estimation of absolute copy number for duplications as small as 1.9 kilobase pairs, ranging from 0 to 48 copies. We identified 4.1 million”“singly unique nucleotide”" positions informative in distinguishing specific copies and used them to genotype the copy and content of specific paralogs within highly duplicated gene families. These data identify human-specific expansions in genes associated with brain development, reveal extensive population genetic diversity, and detect signatures consistent with gene conversion in the human species. Our approach makes similar to 1000 genes accessible to genetic studies of disease association." | 1156 | no | human | NA | no | no |
| 1157 | 2017 | 43016 | Carputo, D; Alioto, D; Aversano, R; Garramone, R; Miraglia, V; Villano, C; Frusciante, L | 2013 | Genetic diversity among potato species as revealed by phenotypic resistances and SSR markers | The evolutionary diversity of wild potato species makes them excellent materials for improving the narrow genetic basis of the cultivated potato Solanum tuberosum. Understanding their genetic diversity is important not only to choose the best parents for breeding, but also to design proper crossing schemes and selection strategies. The objectives of this study were to determine the resistance response to Ralstonia solanacearum, Potato virus Y and low temperatures of 21 clones of 12 potato species, and to determine their genetic diversity through simple sequence repeat (SSR) markers. Sources of resistance have been found for all the investigated traits, with high resistance variability not only between but also within species. Combined resistances were also identified, with positive implications for efficient breeding. SSR analysis allowed the detection of 12 loci and 46 alleles across all genotypes, with an average value of 3.8 alleles per locus. Both unique and rare alleles useful for marker-assisted selection were found. SSR-based cluster analysis revealed that resistant genotypes were distributed among all clusters, suggesting that genetically different resistant genotypes were identified. The information obtained in this study is discussed from a breeding perspective. | 1157 | no | no good fitness measure, N pop differnt in GD? | NA | no | no |
| 1158 | 2017 | 43016 | Hoban, SM; Schlarbaum, SE; Brosi, SL; Romero-Severson, J | 2012 | A rare case of natural regeneration in butternut, a threatened forest tree, is parent and space limited | The genetic consequences of natural in situ recovery for rare or threatened species are not as well understood as the impact of population bottlenecks, fragmentation and admixture, particularly the mechanisms by which genetic diversity is lost or preserved as populations recover. Here we examine how mating patterns, dispersal and ecologically constrained regeneration influences genetic diversity and kinship in a naturally regenerating population of a threatened temperate forest tree, Juglans cinerea L. (butternut). Butternut regeneration is now rare throughout the native range due to the butternut canker, a lethal fungal disease from Asia, and land use changes. In this study of one of the only known regenerating patches large enough for kinship and parentage analysis, we used 12 microsatellite markers, direct and inferred parentage analyses and Bayesian clustering of 152 trees to show that natural regeneration at this site resulted in loss of allele richness due to a small number of parents, most of which are spatially proximal to the regenerants. Of the 116 potential parents tested, one contributed 20.8 % and the top four contributed 71.1 % of the gametes in 36 regenerants. Parent-parent and parent-offspring distances revealed limited pollen and seed dispersal (< 100 m). Regenerants were highly related and spatially clustered in sibling groups. Proximity to the regenerating patch was the most significant factor in parental success. Our results suggest that in situ regeneration of forest trees with limited propagule dispersal and specific site requirements may be insufficient to preserve native genetic diversity in protected areas with few suitable sites. | 1158 | no | No GD diff between pop | NA | no | no |
| 1159 | 2017 | 43016 | Amills, M; Jimenez, N; Jordana, J; Riccardi, A; Fernandez-Arias, A; Guiral, J; Bouzat, JL; Folch, J; Sanchez, A | 2004 | Low diversity in the major histocompatibility complex class II DRB1 gene of the Spanish ibex, Capra pyrenaica | During the last two centuries, the Spanish ibex (Capra pyrenaica) has shown a significant demographic decline as a result of the progressive destruction of its natural habitat, disease epidemics, and uncontrolled hunting. Partial sequencing of the class II MHC DRB1 gene revealed that the Spanish ibex has remarkably low levels of genetic variation at this locus, with only six different DRB1 alleles and an observed heterozygosity of 0.429 - 0.579. The rates of nonsynonymous vs synonymous substitutions were significantly different in the peptide-binding region (d(N)/d(S) = 5.347, P = 0.002), a feature that indicates that the DRB1 gene is under positive selection. A phylogenetic analysis of the Spanish ibex and a set of domestic goat DRB1 alleles revealed that the reported sequences represent four major allelic lineages. The limited allelic repertoire of the DRB1 gene in the Spanish ibex is likely the direct result of the recent history of population bottlenecks and marked demographic decline of this species. A genetic survey of 13 microsatellite loci was consistent with this idea. The Spanish ibex subspecies C. p. hispanica and C. p. victoriae consistently showed considerably lower levels of microsatellite heterozygosity (H-o = 0.184 - 0.231) and allelic diversity ( mean number of alleles per locus = 2 - 2.4) than those reported in other wild ruminants. This study demonstrates the significance of both natural selection and the demographic history of populations in determining patterns of genetic variation at MHC loci. In addition, our results emphasize the importance of locally adapted populations for the preservation of genetic diversity. | 1159 | no | no good fitness measure, N pop differnt in GD? | NA | no | no |
| 1160 | 2017 | 43016 | Del Real-Monroy, M; Ortega, J | 2017 | Spatial distribution of microsatellite and MHC-DRB exon 2 gene variability in the Jamaican fruit bat (Artibeus jamaicensis)in Mexico | Genetic diversity is essential to the evolutionary and adaptive potential of a species. Several empirical genetic studies have highlighted the importance of considering both neutral and adaptive genetic variation when characterizing microevolutionary dynamics. Genes at the major histocompatibility complex (MHC) have become excellent models for researching adaptive variation and natural selection, because of the crucial role they play against pathogens. The Jamaican fruit bat, Artibeus jamaicensis, is one of the most common and well-studied Neotropical mammals and is characterized by generalist feeding habits, high dispersal capability, and abundant populations. Fifteen localities of Artibeus jamaicensis were genetically assessed using ten neutral microsatellites and one expressed MHC class II locus (DRB) in order to detect footprints of balancing selection. Extensive polymorphism was found at both markers. Overall, 161 alleles were identified at DRB exon 2 gene, and 315 at microsatellites. The observed and expected heterozygosity averaged over all localities (+/- SD) was 0.756 +/- 0.15 and 0.885 +/- 0.11, respectively, with all localities in Hardy-Weinberg equilibrium. Pairwise genetic differentiation estimates were generally significant, but the overall differentiation was lower at DRB gene (F-st = 0.039) than at microsatellites (F-st = 0.154). We detected significant isolation by distance at microsatellite loci, but not at DRB exon 2 gene. STRUCTURE and BAPS analyses detected a population genetic structure made up of five defined clusters. Our results suggest that balancing selection has maintained the allele frequencies of DRB exon 2 gene across the distribution of A. jamaicensis in Mexico. (C) 2016 Deutsche Gesellschaft far Saugetierkunde. Published by Elsevier GmbH. All rights reserved. | 1160 | no | no good fitness measure | NA | no | no |
| 1161 | 2017 | 43016 | Israel, DA; Salama, N; Krishna, U; Rieger, UM; Atherton, JC; Falkow, S; Peek, RM | 2001 | Helicobacter pylori genetic diversity within the gastric niche of a single human host | Isolates of the gastric pathogen Helicobacter pylori harvested from different individuals are highly polymorphic. Strain variation also has been observed within a single host. To more fully ascertain the extent of H. pylori genetic diversity within the ecological niche of its natural host, we harvested additional isolates of the sequenced H. pylori strain J99 from its human source patient after a 6-year interval. Randomly amplified polymorphic DNA PCR and DNA sequencing of four unlinked loci indicated that these isolates were closely related to the original strain. In contrast, microarray analysis revealed differences in genetic content among all of the isolates that were not detected by randomly amplified polymorphic DNA PCR or sequence analysis. Several ORFs from loci scattered throughout the chromosome in the archival strain did not hybridize with DNA from the recent strains, including multiple ORFs within the J99 plasticity zone. In addition, DNA from the recent isolates hybridized with probes for ORFs specific for the other fully sequenced H. pylori strain 26695, including a putative traG homolog. Among the additional J99 isolates, patterns of genetic diversity were distinct both when compared with each other and to the original prototype isolate. These results indicate that within an apparently homogeneous population, as determined by macroscale comparison and nucleotide sequence analysis, remarkable genetic differences exist among single-colony isolates of H. pylori. Direct evidence that H. pylori has the capacity to lose and possibly acquire exogenous DNA is consistent with a model of continuous microevolution within its cognate host. | 1161 | no | NA | NA | no | no |
| 1162 | 2017 | 43016 | Ching, A; Caldwell, KS; Jung, M; Dolan, M; Smith, OS; Tingey, S; Morgante, M; Rafalski, AJ | 2002 | SNP frequency, haplotype structure and linkage disequilibrium in elite maize inbred lines | Background: Recent studies of ancestral maize populations indicate that linkage disequilibrium tends to dissipate rapidly, sometimes within 100 bp. We set out to examine the linkage disequilibrium and diversity in maize elite inbred lines, which have been subject to population bottlenecks and intense selection by breeders. Such population events are expected to increase the amount of linkage disequilibrium, but reduce diversity. The results of this study will inform the design of genetic association studies. Results: We examined the frequency and distribution of DNA polymorphisms at 18 maize genes in 36 maize inbreds, chosen to represent most of the genetic diversity in U.S. elite maize breeding pool. The frequency of nucleotide changes is high, on average one polymorphism per 31 bp in non-coding regions and 1 polymorphism per 124 bp in coding regions. Insertions and deletions are frequent in non-coding regions (1 per 85 bp), but rare in coding regions. A small number (2-8) of distinct and highly diverse haplotypes can be distinguished at all loci examined. Within genes, SNP loci comprising the haplotypes are in linkage disequilibrium with each other. Conclusions: No decline of linkage disequilibrium within a few hundred base pairs was found in the elite maize germplasm. This finding, as well as the small number of haplotypes, relative to neutral expectation, is consistent with the effects of breeding-induced bottlenecks and selection on the elite germplasm pool. The genetic distance between haplotypes is large, indicative of an ancient gene pool and of possible interspecific hybridization events in maize ancestry. | 1162 | no | inbreeding | NA | no | no |
| 1163 | 2017 | 43016 | Gurgul, A; Jasielczuk, I; Szmatola, T; Pawlina, K; Zabek, T; Zukowski, K; Bugno-Poniewierska, M | 2015 | Genome-wide characteristics of copy number variation in Polish Holstein and Polish Red cattle using SNP genotyping assay | Copy number variation (CNV), which results from deletions or amplifications of large fragments of genomic DNA, is widespread in mammalian genomes and apart from its potential pathogenic effect it is considered as a source of natural genetic diversity. In cattle populations, this kind of genetic variability remains still insufficiently elucidated and studies focusing on the detection of new structural genomic variants in different cattle populations may contribute to a better understanding of cattle breeds’ diversity and genetic basis of production traits. In this study, by using BovineSNP50 assay and cnvPartition algorithm we identified CNVs in two different cattle breeds: Holstein (859 animals) and Polish Red (301). In Holstein cattle we found 648 CNVs which could be reduced to 91 non-redundant variable genomic regions (CNVRs) covering in total 168.6 Mb of the genomic sequence. In Polish Red cattle we detected 62 CNVs, localized in 37 variable regions encompassing 22.3 Mb of the sequence, corresponding to 0.89 % of the autosomal genome. Within the regions we identified 1,192 unique RefSeq genes which are engaged in a variety of biological processes. High concordance of the regions’ distribution was found between the studied breeds, however copy number variants seemed to be more common in Holstein cattle. About 26 % of the regions described in this study could be classified as newly identified. The results of this study will broaden the knowledge of CNVs in genomes of cattle of different breeds and will provide foundations for further research aiming to identify a relationship between this type of genetic variation and phenotypic traits. | 1163 | no | domesticated animal | NA | no | no |
| 1164 | 2017 | 43016 | Ghawar, W; Attia, H; Bettaieb, J; Yazidi, R; Laouini, D; Ben Salah, A | 2014 | Genotype Profile of Leishmania major Strains Isolated from Tunisian Rodent Reservoir Hosts Revealed by Multilocus Microsatellite Typing | Zoonotic cutaneous leishmaniasis (ZCL) caused by Leishmania (L.) major parasites represents a major health problem with a large spectrum of clinical manifestations. Psammomys (P.) obesus and Meriones (M.) shawi represent the most important host reservoirs of these parasites in Tunisia. We already reported that infection prevalence is different between these two rodent species. We aimed in this work to evaluate the importance of genetic diversity in L. major parasites isolated from different proven and suspected reservoirs for ZCL. Using the multilocus microsatellites typing (MLMT), we analyzed the genetic diversity among strains isolated from (i) P. obesus (n = 31), (ii) M. shawi (n = 8) and (iii) Mustela nivalis (n = 1), captured in Sidi Bouzid, an endemic region for ZCL located in the Center of Tunisia. Studied strains present a new homogeneous genotype profile so far as all tested markers and showed no polymorphism regardless of the parasite host-reservoir origin. This lack of genetic diversity among these L. major isolates is the first genetic information on strains isolated from Leishmania reservoirs hosts in Tunisia. This result indicates that rodent hosts are unlikely to exert a selective pressure on parasites and stresses on the similarity of geographic and ecological features in this study area. Overall, these results increase our knowledge among rodent reservoir hosts and L. major parasites interaction. | 1164 | no | no good fitness measure, N pop differnt in GD, focus on the parasite in paper | NA | no | no |
| 1165 | 2017 | 43016 | Markus, B; Alshafee, I; Birk, OS | 2014 | Deciphering the fine-structure of tribal admixture in the Bedouin population using genomic data | The Bedouin Israeli population is highly inbred and structured with a very high prevalence of recessive diseases. Many studies in the past two decades focused on linkage analysis in large, multiple consanguineous pedigrees of this population. The advent of high-throughput technologies motivated researchers to search for rare variants shared between smaller pedigrees, integrating data from clinically similar yet seemingly non-related sporadic cases. However, such analyses are challenging because, without pedigree data, there is no prior knowledge regarding possible relatedness between the sporadic cases. Here, we describe models and techniques for the study of relationships between pedigrees and use them for the inference of tribal co-ancestry, delineating the complex social interactions between different tribes in the Negev Bedouins of southern Israel. Through our analysis, we differentiate between tribes that share many yet small genomic segments because of co-ancestry versus tribes that share larger segments because of recent admixture. The emergent pattern is well correlated with the prevalence of rare mutations in the different tribes. Tribes that do not intermarry, mostly because of social restrictions, hold private mutations, whereas tribes that do intermarry demonstrate a genetic flow of mutations between them. Thus, social structure within an inbred community can be delineated through genomic data, with implications to genetic counseling and genetic mapping. | 1165 | no | no good fitness measure, N pop differnt in GD? | NA | no | no |
| 1166 | 2017 | 43016 | Lively, CM | 2010 | The Effect of Host Genetic Diversity on Disease Spread | Host genetic diversity is thought to reduce the likelihood that disease will spread in natural populations. In this study, I present an epidemiological model for the intrinsic rate of spread (R(0)) for an infectious disease. The results show that the average value for R(0) ((R) over bar (0)) is inversely related to the number of host genotypes in the population (G), assuming that each host genotype is susceptible to a different parasite genotype. Specifically, for large host populations, (R) over bar (0) is equal to B/G, where B is the number of infectious propagules produced by each infection that contact a different host. The results also suggest that virulent, single-strain infections, which initially spread in genetically diverse host populations, would quickly die out when the parasite depresses the frequency of susceptible hosts below 1/B. These results are consistent with empirical studies showing that genetically diverse host populations suffer less from pathogens and parasites. | 1166 | no | NA | NA | no | no |
| 1167 | 2017 | 43016 | Decaestecker, E; De Gersem, H; Michalakis, Y; Raeymaekers, JAM | 2013 | Damped long-term host-parasite Red Queen coevolutionary dynamics: a reflection of dilution effects? | An increase in biological diversity leads to a greater stability of ecosystem properties. For host-parasite interactions, this is illustrated by the dilution effect’: a negative correlation between host biodiversity and disease risk. We show that a similar mechanism might stabilise host-parasite dynamics at a lower level of diversity, i.e. at the level of genetic diversity within host species. A long-term time shift experiment, based on a historical reconstruction of a Daphnia-parasite coevolution, reveals infectivity cycles with more stable amplitude in experienced than in naive hosts. Coevolutionary models incorporating an increase in host allelic diversity over time explain the detected asymmetry. The accumulation of resistance alleles creates an opportunity for the host to stabilise Red Queen dynamics. It leads to a larger arsenal enhancing the host performance in its coevolution with the parasite in which it takes all the running both antagonists can do to keep in the same place’. | 1167 | no | No GD diff between pop | NA | no | no |
| 1168 | 2017 | 43016 | Stewart, B; Rozen, DE | 2012 | GENETIC VARIATION FOR ANTIBIOTIC PERSISTENCE IN ESCHERICHIA COLI | Bacterial persistence describes a heterogeneous response to antibiotics in clonal populations of bacteria due to phenotypic variation within the population, with a small proportion of cells surviving treatment even at very high concentrations of drug. The aim of this study was to determine whether different natural isolates of Escherichia coli, selected at random from a collection representing the spectrum of genetic diversity in the species, generate different fractions of persister cells. Despite comparable minimum inhibitory concentrations (MICs) to the antibiotics between the different strains, highly significant variation was observed in persister fractions following exposure to ampicillin, streptomycinm, or norfloxacin. Survival following treatment with one drug did not, however, correlate with survival against another. Finally, using competition assays we quantify fitness benefits of persistence. These results show that different strains of E. coli vary markedly in their response to antibiotics despite comparable genetic susceptibility and indicate different mechanisms of evolved persistence to different antibiotics. | 1168 | no | no good Gd measurement | NA | no | no |
| 1169 | 2017 | 43016 | Carvalho, AA; Cardoso, LL; Nogueira, HS; Menezes, EV; Xavier, MAS; Barreto, NAP; Fernandes, LF; Xavier, AREO | 2016 | Characterization and molecular epidemiology of extensively prevalent nosocomial isolates of drug-resistant Acinetobacter spp | Acinetobacter sp isolates deserve special attention once they have emerged globally in healthcare institutions because they display numerous intrinsic and acquired drug-resistance mechanisms. This study assessed the antibiotic susceptibility profile, the presence of the genetic marker bla(OXA-23), and the clonal relationship among 34 nosocomial isolates of Acinetobacter spp obtained at a hospital in southeastern Brazil. Antibiotic sensitivity analysis was performed by the standard disc-diffusion method. All isolates were found to be extensively resistant to several drugs, but sensitive to polymyxin B. A polymerase chain reaction (PCR) assay was used to detect the bla(OXA-23) gene, which is associated with carbapenem resistance. The genetic profile and the clonal relationship among isolates were analyzed via enterobacterial repetitive intergenic consensus (ERIC)-PCR. The Acinetobacter spp were divided into four groups with 22 distinct genetic subgroups. ERIC-PCR analysis revealed the genetic diversity among isolates, which, despite having a heterogeneous profile, displayed 100% clonality among 56% (19/34) of them. | 1169 | no | No GD diff between pop | NA | no | no |
| 1170 | 2017 | 43016 | Pogorzelec, M; Glebocka, K; Hawrylak-Nowak, B; Bronowicka-Mielniczuk, U | 2015 | Assessment of chosen reproductive cycle processes and genetic diversity of Salix myrtilloides L. in wetlands of Polesie Lubelskie: the prospects of its survival in the region | Salix myrtilloides L. is an endangered species whose western limit of range runs through Poland. The main aim of the study was to increase the knowledge on the ecology and biology of S. myrtilloides populations in the Polesie Lubelskie region (Eastern Poland) in order to create an effective protection program. An 80% decrease in its population was found in this area. Our study was conducted to identify the mechanisms responsible for the process of withdrawal of this species from its natural stands by determining whether the processes of generative reproduction (pollen viability and germinability, seed germination ability and dynamics) in the populations occur properly and by characterizing within- and among-population genetic diversity of S. myrtilloides, using ISSR (Inter-Simple Sequence Repeat) primers. The results confirmed that S. myrtilloides pollen viability was high (84.17 +/- 8.67), and so were the seed germination ability (76% of the total number of sown seeds/24 h) and dynamics. The within-population genetic diversity was quite high for this species and the inter-population genetic variation was of medium value (Phi(PT)=0.148). The condition of two populations, their genetic diversity and sex ratio as well as the correctness of the investigated reproductive cycle stages altogether give prospects for their survival. It seems to be very important to start conservation involving the reintroduction and reconstruction of S. myrtilloides populations in the Polesie Lubelskie region. The proposed method for restoration of S. myrtilloides resources would be possible if ex situ cultivation and in vitro methods were used. | 1170 | no | no good fitness measure, N pop differnt in GD? | NA | no | no |
| 1171 | 2017 | 43016 | Hoelzel, AR | 1999 | Impact of population bottlenecks on genetic variation and the importance of life-history; a case study of the northern elephant seal | This paper reviews some of the important factors related to the impact of population bottlenecks, using the northern elephant seal (Mirounga angustirostrus) as a case study for illustration. The northern elephant seal was hunted extensively in the 19th century and forced through a bottleneck of approximately 10-20 seals. All measures of molecular genetic variation show current levels for the northern elephant seal to be low. Levels of generic variation were compared with expectations based on a simulation model that recapitulates demographic growth, based on age-specific data oil reproduction and mortality. Predictions from the simulation model are then presented to illustrate the importance of differences in life-history strategy and skewed reproductive success. Either high reproductive skew (e.g. polygyny) or a low growth rate in a population can increase the impact of a bottleneck on molecular variation. Severe population bottlenecks can also disrupt aspects of developmental stability and thereby increase the fluctuating asymmetry and variability of quantitative traits. A comparison of skulls collected before and after the bottleneck showed this to have occurred for some elephant seal quantitative characters. (C) 1999 The Linnean Society of London. | 1171 | no | no good fitness measure, N pop differnt in GD? | NA | no | no |
| 1172 | 2017 | 43016 | Renzette, N; Kowalik, TF; Jensen, JD | 2016 | On the relative roles of background selection and genetic hitchhiking in shaping human cytomegalovirus genetic diversity | A central focus of population genetics has been examining the contribution of selective and neutral processes in shaping patterns of intraspecies diversity. In terms of selection specifically, surveys of higher organisms have shown considerable variation in the relative contributions of background selection and genetic hitchhiking in shaping the distribution of polymorphisms, although these analyses have rarely been extended to bacteria and viruses. Here, we study the evolution of a ubiquitous, viral pathogen, human cytomegalovirus (HCMV), by analysing the relationship among intraspecies diversity, interspecies divergence and rates of recombination. We show that there is a strong correlation between diversity and divergence, consistent with expectations of neutral evolution. However, after correcting for divergence, there remains a significant correlation between intraspecies diversity and recombination rates, with additional analyses suggesting that this correlation is largely due to the effects of background selection. In addition, a small number of loci, centred on long noncoding RNAs, also show evidence of selective sweeps. These data suggest that HCMV evolution is dominated by neutral mechanisms as well as background selection, expanding our understanding of linked selection to a novel class of organisms. | 1172 | no | human | NA | no | no |
| 1173 | 2017 | 43016 | Lieutenant-Gosselin, M; Bernatchez, L | 2006 | Local heterozygosity-fitness correlations with global positive effects on fitness in threespine stickleback | The complex interactions between genetic diversity and evolution have important implications in many biological areas including conservation, speciation, and mate choice. A common way to study these interactions is to look at heterozygosity-fitness correlations (HFCs). Until recently, HFCs based on noncoding markers were believed to result primarily from global inbreeding effects. However, accumulating theoretical and empirical evidence shows that HFCs may often result from genes being linked to the markers used (local effect). Moreover, local effect HFCs could differ from global inbreeding effects in their direction and occurrence. Consequently, the investigation of the structure and consequences of local HFCs is emerging as a new important goal in evolutionary biology. In this study of a wild threespine stickleback (Gasterosteus aculeatus) population, we first tested the presence of significant positive or negative local effects of heterozygosity at 30 microsatellites loci on five fitness components: survival, mating success, territoriality, length, and body condition. Then, we evaluated the direction and shape of total impact of local HFCs, and estimated the magnitude of the impacts on fitness using regression coefficients and selection differentials. We found that multilocus heterozygosity was not a reliable estimator of individual inbreeding coefficient, which supported the relevance of single-locus based analyses. Highly significant and temporally stable local HFCs were observed. These were mainly positive, but negative effects of heterozygosity were also found. Strong and opposite effects of heterozygosity are probably present in many populations, but may be blurred in HFC analyses looking for global effects only. In this population, both negative and positive HFCs are apparently driving mate preference by females, which is likely to contribute to the maintenance of both additive and nonadditive genetic variance. | 1173 | no | No GD diff between pop | NA | no | no |
| 1174 | 2017 | 43016 | Gaudeul, M; Gardner, MF; Thomas, P; Ennos, RA; Hollingsworth, PM | 2014 | Evolutionary dynamics of emblematic Araucaria species (Araucariaceae) in New Caledonia: nuclear and chloroplast markers suggest recent diversification, introgression, and a tight link between genetics and geography within species | Background: New Caledonia harbours a highly diverse and endemic flora, and 13 (out of the 19 worldwide) species of Araucaria are endemic to this territory. Their phylogenetic relationships remain largely unresolved. Using nuclear microsatellites and chloroplast DNA sequencing, we focused on five closely related Araucaria species to investigate among-species relationships and the distribution of within-species genetic diversity across New Caledonia. Results: The species could be clearly distinguished here, except A. montana and A. laubenfelsii that were not differentiated and, at most, form a genetic cline. Given their apparent morphological and ecological similarity, we suggested that these two species may be considered as a single evolutionary unit. We observed cases of nuclear admixture and incongruence between nuclear and chloroplast data, probably explained by introgression and shared ancestral polymorphism. Ancient hybridization was evidenced between A. biramulata and A. laubenfelsii in Mt Do, and is strongly suspected between A. biramulata and A. rulei in Mt Tonta. In both cases, extensive asymmetrical backcrossing eliminated the influence of one parent in the nuclear DNA composition. Shared ancestral polymorphism was also observed for cpDNA, suggesting that species diverged recently, have large effective sizes and/or that cpDNA experienced slow rates of molecular evolution. Within-species genetic structure was pronounced, probably because of low gene flow and significant inbreeding, and appeared clearly influenced by geography. This may be due to survival in distinct refugia during Quaternary climatic oscillations. Conclusions: The study species probably diverged recently and/or are characterized by a slow rate of cpDNA sequence evolution, and introgression is strongly suspected. Within-species genetic structure is tightly linked with geography. We underline the conservation implications of our results, and highlight several perspectives. | 1174 | no | no good fitness measure, N pop differnt in GD? | NA | no | no |
| 1175 | 2017 | 43016 | Fernandez-de-Mera, IG; Vicente, J; Perez de la Lastra, JM; Mangold, AJ; Naranjo, V; Fierro, Y; de la Fuente, J; Gortazar, C | 2009 | Reduced major histocompatibility complex class II polymorphism in a hunter-managed isolated Iberian red deer population | The major histocompatibility complex (MHC) contains the most variable functional genes described in vertebrates. Individuals from natural populations deal constantly with a diverse range of pathogens and the polymorphism at MHC loci is what determines the diversity of foreign antigens that the host immune system can recognize. Polymorphism at individual loci may result in variable MHC class II (MHC-II) haplotypes. We characterized for the first time, the allelic diversity at the second DRB locus of the MHC-II in the Iberian red deer Cervus elaphus hispanicus. The studied population was sampled from a fenced estate that has been managed for hunting purposes and may provide information of the effect of game management on the genetic diversity of this species. Deer presented high levels of variation at MHC-II DRB-2 with 18 different alleles detected in 94 individuals. However, a significant heterozygous deficiency was found for MHC-II DRB-2 locus (92.5% of individuals only amplified one allele), whereas genotype frequencies at three neutral microsatellite loci were according to Hardy-Weinberg equilibrium, with heterozygosity over 50%. The analysis of control Iberian red deer from different geographic locations identified two expressed DRB-2 loci with a high degree of heterozygosis. The annual diversity index of MHC-II DRB-2 alleles significantly decreased along the 16-year study period, which was confirmed with losses in microsatellite markers. Although we cannot exclude positive non-assortative mating and/or substructured breeding (Wahlund effect) within our reference population, such unexpected apparent homozygosity at MHC DRB-2 loci is suggestive of null alleles occurring at our population. The observed pattern could be the result of a founder effect in this recently established population. Nonetheless, the loss of MHC-II DRB-2 allele diversity could reflect the effects of inbreeding in this fenced population managed for hunting. These findings support the importance of immunogenetic studies to assess management decisions, especially in isolated ungulate populations. | 1175 | no | no good fitness measure, N pop differnt in GD? | NA | no | no |
| 1176 | 2017 | 43016 | Tyagi, S; Das, A | 2015 | Mitochondrial population genomic analyses reveal population structure and demography of Indian Plasmodium falciparum | Inference on the genetic diversity of Plasmodium falciparum populations could help in better management of malaria. A very recent study with mitochondrial (mt) genomes in global P. falciparum had revealed interesting evolutionary genetic patterns of Indian isolates in comparison to global ones. However, no population genetic study using the whole mt genome sequences of P. falciparum isolates collected in the entire distribution range in India has yet been performed. We herewith have analyzed 85 whole mtgenomes (48 already published and 37 entirely new) sampled from eight differentially endemic Indian locations to estimate genetic diversity and infer population structure and historical demography of Indian P. falciparum. We found 19 novel Indianspecific Single Nucleotide Polymorphisms (SNPs) and 22 novel haplotypes segregating in Indian P. falciparum. Accordingly, high haplotype and nucleotide diversities were detected in Indian P. fakiparum in comparison to many other global isolates. Indian P. fakiparum populations were found to be moderately sub-structured with four different genetic clusters. Interestingly, group-of local populations aggregate to form each cluster; while samples from Jharkhand and Odisha formed a single cluster, P. falciparum isolates from Asom formed an independent one. Similarly, Surat, Bilaspur and Betul formed a single cluster and Goa and Mangalore formed another. Interestingly, P. falciparum isolates from the two later populations were significantly genetically differentiated from isolates collected in other six Indian locations. Signature of historical population expansion was evident in five population samples, and the onset of expansion event was found to be very similar to African P. fakiparum. In agreement with the previous finding, the estimated Time to Most Recent Common Ancestor (TMRCA) and the effective population size were high in Indian P. falciparum. All these genetic features of Indian P. falciparum with high mt genome diversity are somehow similar to Africa, but quite different from other Asian population samples. (C) 2015 Elsevier B.V. and Mitochondria Research Society. All rights reserved. | 1176 | no | gd of parasite | NA | no | no |
| 1177 | 2017 | 43016 | Chung, MY; Park, CW; Chung, MG | 2007 | Extremely low levels of allozyme variation in southern Korean populations of the two rare and endangered lithophytic or epiphytic Bulbophyllum drymoglossum and Sarcanthus scolopendrifolius (Orchidaceae): implications for conservation | Allozyme variation was investigated in two local populations of Bulbophyllum drymoglossum and three populations Sarcanthus scolopendrifolius, two rare and endangered lithophytes and epiphytes from South Korea. Genetic diversity was extremely low within populations (mean H-e = 0.011 for B. drymoglossum; 0.002 for S. scolopendrifolius). Among the putative screened 21 loci, we found only one polymorphic locus for each species. Only one polymorphic locus, detected just one population of each species, revealed significantly high degree of population differentiation between and among populations (F-ST = 0.253 for B. drymoglossum and F-ST = 0.899 for S. scolopendrifolius). These results suggest that genetic drift (consequence of a very small effective population size), coupled with a limited gene flow would play a major role in shaping population genetics of these species in South Korea. The current status of both species (small population sizes, spatially isolated populations, and highly localized habitats) in addition to the extremely low levels of genetic diversity and reckless collection of endangered orchids by plant sellers, significantly threaten the long-term survival of these species in Korea. Conservation of the two species requires both in situ strategies, by introducing of genets to increase effective population sizes by minimizing adverse effects (e.g., outbreeding depression and genetic swamping by non-native genotypes), and ex situ strategies, such as collection of genets from clonal ramets. | 1177 | no | no good fitness measure, N pop differnt in GD? | NA | no | no |
| 1178 | 2017 | 43016 | Belda-Baillie, CA; Sison, M; Silvestre, V; Villamor, K; Monje, V; Gomez, ED; Baillie, BK | 1999 | Evidence for changing symbiotic algae in juvenile tridacnids | This study investigated the effects of different clonal strains of Symbiodinium sp. (zooxanthellae) on clam growth and survival, while monitoring the persistence of the induced symbioses in outdoor tanks and in the held using allozyme and random amplified polymorphic DNA (RAPD) analyses. Aposymbiotic clam larvae that were inoculated with homologous zooxanthellae (cultured or freshly isolated from the same host species) or heterologous zooxanthellae (cultured from different host species) had significantly-different survival rates at harvest (3 months post-spawning) with small growth differences. The improved survival rates in juvenile Hippopus hippopus (heterologous infection) and Tridacna gigas (homologous infection) were maintained through 3 months onshore and 3 months offshore. However, isozyme and RAPD analyses of re-isolated zooxanthellae after 3, 6, and 9 months revealed a high genetic diversity of symbionts (ca. 99% variation in 200 re-isolates) from individual hosts, within and between treatments. Furthermore, the genetic patterns of the re-isolated algae following clonal culture were different from those of the introduced clones, which, in contrast, retained their unique genetic patterns over many culture generations in the laboratory. These results demonstrate that the subsequently-established symbiont populations in juvenile clams were not clonal. The allozyme and RAPD techniques detected individual genetic differences in clam symbionts, but not differences between algal taxa. The presence of significant survival trends suggests possible differences between subsequently-established dominant symbiont taxa, which were uncultureable or undetectable using these genetic markers. The implications of this symbiont diversity in giant clams are discussed. (C) 1999 Elsevier Science B.V. All rights reserved. | 1178 | no | gd difference? | NA | no | no |
| 1179 | 2017 | 43016 | Falahati-Anbaran, M; Lundemo, S; Ansell, SW; Stenoien, HK | 2014 | Contrasting Patterns of Genetic Structuring in Natural Populations of Arabidopsis lyrata Subsp petraea across Different Regions in Northern Europe | Level and partitioning of genetic diversity is expected to vary between contrasting habitats, reflecting differences in strength of ecological and evolutionary processes. Therefore, it is necessary to consider processes acting on different time scales when trying to explain diversity patterns in different parts of species’ distributions. To explore how historical and contemporary factors jointly may influence patterns of genetic diversity and population differentiation, we compared genetic composition in the perennial herb Arabidopsis lyrata ssp. petraea from the northernmost parts of its distribution range on Iceland to that previously documented in Scandinavia. Leaf tissue and soil were sampled from ten Icelandic populations of A. lyrata. Seedlings were grown from soil samples, and tissue from above-ground and seed bank individuals were genotyped with 21 microsatellite markers. Seed bank density in Icelandic populations was low but not significantly different from that observed in Norwegian populations. While within-population genetic diversity was relatively high on Iceland (H-E = 0.35), among-population differentiation was low (F-ST = 0.10) compared to Norwegian and Swedish populations. Population differentiation was positively associated with geographical distance in both Iceland and Scandinavia, but the strength of this relationship varied between regions. Although topography and a larger distribution range may explain the higher differentiation between mountainous Norwegian relative to lowland populations in Sweden, these factors cannot explain the lower differentiation in Icelandic compared to Swedish populations. We propose that low genetic differentiation among Icelandic populations is not caused by differences in connectivity, but is rather due to large historical effective population sizes. Thus, rather than contemporary processes, historical factors such as survival of Icelandic lineages in northern refugia during the last glacial period may have contributed to the observed pattern. | 1179 | no | no good fitness measure | NA | no | no |
| 1180 | 2017 | 43016 | Martinez-Camblor, P; Corral, N; Vicente, D | 2011 | Statistical comparison of the genetic sequence type diversity of invasive Neisseria meningitidis isolates in northern Spain (1997-2008) | A secondary effect derived from the mass vaccination campaigns against meningococcal disease could be the increase in the genetic sequence type variability of the Neisseria meningitidis. The study and measurement of diversity (or/and richness) is an old problem from Ecology. However, although there exist a huge number of diversity indices, they often do not have satisfactory statistical properties and their use in inference is limited or null. In this paper, the ratio of the number of different observed species (S) to the number of observed individuals (d(N)=S/N) is analyzed from both non-parametric and parametric approaches. The two methodologies are applied to the problem to know whether the genetic diversity of the invasive Neisseria meningitidis is equal or not along the last twelve years (1997-2008) in a region of northern Spain (Basque Country) where two mass immunization campaigns have been carried out during this period. (C) 2011 Elsevier B.V. All rights reserved. | 1180 | no | NA | NA | no | no |
| 1181 | 2017 | 43016 | Brown, KH; Dobrinski, KP; Lee, AS; Gokcumen, O; Mills, RE; Shi, XH; Chong, WWS; Chen, JYH; Yoo, P; David, S; Peterson, SM; Raj, T; Choy, KW; Stranger, BE; Williamson, RE; Zon, LI; Freeman, JL; Lee, C | 2012 | Extensive genetic diversity and substructuring among zebrafish strains revealed through copy number variant analysis | Copy number variants (CNVs) represent a substantial source of genomic variation in vertebrates and have been associated with numerous human diseases. Despite this, the extent of CNVs in the zebrafish, an important model for human disease, remains unknown. Using 80 zebrafish genomes, representing three commonly used laboratory strains and one native population, we constructed a genome-wide, high-resolution CNV map for the zebrafish comprising 6,080 CNV elements and encompassing 14.6% of the zebrafish reference genome. This amount of copy number variation is four times that previously observed in other vertebrates, including humans. Moreover, 69% of the CNV elements exhibited strain specificity, with the highest number observed for Tubingen. This variation likely arose, in part, from Tubingen’s large founding size and composite population origin. Additional population genetic studies also provided important insight into the origins and substructure of these commonly used laboratory strains. This extensive variation among and within zebrafish strains may have functional effects that impact phenotype and, if not properly addressed, such extensive levels of germ-line variation and population substructure in this commonly used model organism can potentially confound studies intended for translation to human diseases. | 1181 | no | no good fitness measure, N pop differnt in GD? | NA | no | no |
| 1182 | 2017 | 43016 | Rougeron, V; De Meeus, T; Hide, M; Waleckx, E; Bermudez, H; Arevalo, J; Llanos-Cuentas, A; Dujardin, JC; De Doncker, S; Le Ray, D; Ayala, FJ; Banuls, AL | 2009 | Extreme inbreeding in Leishmania braziliensis | Leishmania species of the subgenus Viannia and especially Leishmania braziliensis are responsible for a large proportion of New World leishmaniasis cases. The reproductive mode of Leishmania species has often been assumed to be predominantly clonal, but remains unsettled. We have investigated the genetic polymorphism at 12 microsatellite loci on 124 human strains of Leishmania braziliensis from 2 countries, Peru and Bolivia. There is substantial genetic diversity, with an average of 12.4 +/- 4.4 alleles per locus. There is linkage disequilibrium at a genome-wide scale, as well as a substantial heterozygote deficit (more than 50% the expected value from Hardy-Weinberg equilibrium), which indicates high levels of inbreeding. These observations are inconsistent with a strictly clonal model of reproduction, which implies excess heterozygosity. Moreover, there is large genetic heterogeneity between populations within countries (Wahlund effect), which evinces a strong population structure at a microgeographic scale. Our findings are compatible with the existence of population foci at a microgeographic scale, where clonality alternates with sexuality of an endogamic nature, with possible occasional recombination events between individuals of different genotypes. These findings provide key clues on the ecology and transmission patterns of Leishmania parasites. | 1182 | no | inbreeding | NA | no | no |
| 1183 | 2017 | 43016 | Crowhurst, RS; Faries, KM; Collantes, J; Briggler, JT; Koppelman, JB; Eggert, LS | 2011 | Genetic relationships of hellbenders in the Ozark highlands of Missouri and conservation implications for the Ozark subspecies (Cryptobranchus alleganiensis bishopi) | The hellbender (Cryptobranchus alleganiensis) is an obligately aquatic salamander that is in decline due to habitat loss and disease. Two subspecies of hellbender have been described based on morphological characteristics: C. a. alleganiensis (eastern subspecies) and C. a. bishopi (Ozark hellbender). Current conservation strategies include captive propagation for restorative releases even though information regarding the current levels of genetic variability and structure within populations is not sufficient to effectively plan for conservation of the genetic diversity of the species. To investigate patterns of population structure in the hellbender, we genotyped 276 hellbenders from eight Missouri River drainages, representing both subspecies. Our results showed low levels of within-drainage diversity but strong population structure among rivers, and three distinct genetic clusters. F (ST) values ranged from 0.00 to 0.61 and averaged 0.40. Our results confirmed previous reports that C. a. bishopi and C. a. alleganiensis are genetically distinct, but also revealed an equidistant relationship between two groups within C. a. bishopi and all populations of C. a. alleganiensis. Current subspecies delineations do not accurately incorporate genetic structure, and for conservation purposes, these three groups should be considered evolutionarily significant units. | 1183 | no | no good fitness measure | NA | no | no |
| 1184 | 2017 | 43016 | Thompson, JA; Stow, AJ; Raftos, DA | 2017 | Lack of genetic introgression between wild and selectively bred Sydney rock oysters Saccostrea glomerata | Sydney rock oysters Saccostrea glomerata are among the most important estuarine species on the eastern coast of Australia and also the basis of a major aquaculture industry. The industry now largely relies on Sydney rock oysters that have been selectively bred for fast growth and disease resistance. Selectively bred S. glomerata are currently farmed in estuaries that also sustain wild populations of Sydney rock oysters, providing the opportunity for interbreeding. This has led to concern that gene flow from farmed, selectively bred oysters could alter the genetic variability of wild oyster populations. Here, we use next-generation genotype-by-sequencing to test for genetic introgression between wild and farmed, selectively bred (B2 line) Sydney rock oysters from 2 sites in the Georges River, NSW. Strong genetic partitioning was identified between the wild and selectively bred populations. There was no evidence of gene flow in the form of introgression, even though selectively bred B2 oysters have been farmed in the Georges River since the early 1990s. Contrary to our expectations, we also found significantly higher levels of genetic diversity and heterozygosity in the selectively bred population relative to the wild population. The relatively low level of genetic diversity that we detected in wild oysters may be particularly relevant to the adaptive capacity of this species in the wild. | 1184 | no | no good fitness measure | NA | no | no |
| 1185 | 2017 | 43016 | Ferriol, M; Pichot, C; Lefevre, F | 2011 | Variation of selfing rate and inbreeding depression among individuals and across generations within an admixed Cedrus population | We investigated the variation and short-term evolution of the selfing rate and inbreeding depression (ID) across three generations within a cedar forest that was established from admixture ca 1860. The mean selfing rate was 9.5%, ranging from 0 to 48% among 20 seed trees (estimated from paternally inherited chloroplast DNA). We computed the probability of selfing for each seed and we investigated ID by comparing selfed and outcrossed seeds within progenies, thus avoiding maternal effects. In all progenies, the germination rate was high (88-100%) and seedling mortality was low (0-12%). The germination dynamics differed significantly between selfed and outcrossed seeds within progenies in the founder gene pool but not in the following generations. This transient effect of selfing could be attributed to epistatic interactions in the original admixture. Regarding the seedling growth traits, the ID was low but significant: 8 and 6% for height and diameter growth, respectively. These rates did not vary among generations, suggesting minor gene effects. At this early stage, outcrossed seedlings outcompeted their selfed relatives, but not necessarily other selfed seedlings from other progenies. Thus, purging these slightly deleterious genes may only occur through within-family selection. Processes that maintain a high level of genetic diversity for fitness-related traits among progenies also reduce the efficiency of purging this part of the genetic load. Heredity (2011) 106, 146-157; doi:10.1038/hdy.2010.45; published online 28 April 2010 | 1185 | no | inbreeding | NA | no | no |
| 1186 | 2017 | 43016 | Krzeminska, U; Wilson, R; Song, BK; Seneviratne, S; Akhteruzzaman, S; Gruszczynska, J; Swiderek, W; Huy, TS; Austin, CM; Rahman, S | 2016 | Genetic diversity of native and introduced populations of the invasive house crow (Corvus splendens) in Asia and Africa | The common house crow (Corvus splendens) is one of the best known and most wide spread species of the family Corvidae. It is a successful invasive species able to exploit urban environments, well removed from its natural distribution. It is considered a pest as it attains high population densities, can cause serious economic losses and has many adverse effects on native fauna and flora, including predation, competitive displacement and disease transmission. Little genetic research on the house crow has been undertaken so we have only a limited understanding of its natural genetic population structure and invasion history. In this study, we employ microsatellite and mitochondrial DNA markers to assess genetic diversity, phylogeography and population structure of C. splendens within its native range represented by Sri Lanka and Bangladesh and introduced range represented by Malaysia, Singapore, Kenya and South Africa. We found high levels of genetic diversity in some of the invasive populations for which multiple invasions are proposed. The lowest genetic diversity was found for the intentionally introduced population in Selangor, Malaysia. Sri Lanka is a possible source population for Malaysia Selangor consistent with a documented introduction over 100 years ago, with port cities within the introduced range revealing possible presence of migrants from other unsampled locations. We demonstrate the power of the approach of using multiple molecular markers to untangle patterns of invasion, provide insights into population structure and phylogeographic relationships and illustrate how historical processes may have contributed to making this species such a successful invader. | 1186 | no | no good fitness measure | NA | no | no |
| 1187 | 2017 | 43016 | Chan, CW; Sakihama, N; Tachibana, SI; Idris, ZM; Lum, JK; Tanabe, K; Kaneko, A | 2015 | Plasmodium vivax and Plasmodium falciparum at the Crossroads of Exchange among Islands in Vanuatu: Implications for Malaria Elimination Strategies | Understanding the transmission and movement of Plasmodium parasites is crucial for malaria elimination and prevention of resurgence. Located at the limit of malaria transmission in the Pacific, Vanuatu is an ideal candidate for elimination programs due to low endemicity and the isolated nature of its island setting. We analyzed the variation in the merozoite surface protein 1 (msp1) and the circumsporozoite protein (csp) of P. falciparum and P. vivax populations to examine the patterns of gene flow and population structures among seven sites on five islands in Vanuatu. Genetic diversity was in general higher in P. vivax than P. falciparum from the same site. In P. vivax, high genetic diversity was likely maintained by greater extent of gene flow among sites and among islands. Consistent with the different patterns of gene flow, the proportion of genetic variance found among islands was substantially higher in P. falciparum (28.81-31.23%) than in P. vivax (-0.53-3.99%). Our data suggest that the current island-by-island malaria elimination strategy in Vanuatu, while adequate for P. falciparum elimination, might need to be complemented with more centrally integrated measures to control P. vivax movement across islands. | 1187 | no | gd of parasite | NA | no | no |
| 1188 | 2017 | 43016 | Landry, C; Garant, D; Duchesne, P; Bernatchez, L | 2001 | ‘Good genes as heterozygosity’: the major histocompatibility complex and mate choice in Atlantic salmon (Salmo salar | According to the theory of mate choice based on heterozygosity, mates should choose each other in order to increase the heterozygosity of their offspring. In this study, we tested the ‘good genes as heterozygosity’ hypothesis of mate choice by documenting the mating patterns of wild Atlantic salmon (Salmo salar) using both major histocompatibility complex (MHC) and microsatellite loci. Specifically, we tested the null hypotheses that mate choice in Atlantic salmon is not dependent on the relatedness between potential partners or on the MHC: similarity between mates. Three parameters were assessed: (i) the number of shared alleles between partners (x and y) at the MHC (M-xy), (ii) the MHC amino-acid genotypic distance between mates’ genotypes (AA(xy)), and (iii) genetic relatedness between mates (r(xy)). We found that Atlantic salmon choose their mates in order to increase the heterozygosity of their offspring at the MHC and, more specifically, at the peptide-binding region, presumably in order to provide them with better defence against parasites and pathogens. This was supported by a significant difference between the observed and expected AA(xy) (p=0.0486). Furthermore, mate choice was not a mechanism of overall inbreeding a avoidance as genetic relatedness supported a random mating scheme (P = 0.445). This study provides the first evidence that MHC genes influence mate choice in fish. | 1188 | no | no good fitness measure, N pop differnt in GD? | NA | no | no |
| 1189 | 2017 | 43016 | Fiorito, G; Di Gaetano, C; Guarrera, S; Rosa, F; Feldman, MW; Piazza, A; Matullo, G | 2016 | The Italian genome reflects the history of Europe and the Mediterranean basin | Recent scientific literature has highlighted the relevance of population genetic studies both for disease association mapping in admixed populations and for understanding the history of human migrations. Deeper insight into the history of the Italian population is critical for understanding the peopling of Europe. Because of its crucial position at the centre of the Mediterranean basin, the Italian peninsula has experienced a complex history of colonization and migration whose genetic signatures are still present in contemporary Italians. In this study, we investigated genomic variation in the Italian population using 2.5 million single-nucleotide polymorphisms in a sample of more than 300 unrelated Italian subjects with well-defined geographical origins. We combined several analytical approaches to interpret genome-wide data on 1272 individuals from European, Middle Eastern, and North African populations. We detected three major ancestral components contributing different proportions across the Italian peninsula, and signatures of continuous gene flow within Italy, which have produced remarkable genetic variability among contemporary Italians. In addition, we have extracted novel details about the Italian population’s ancestry, identifying the genetic signatures of major historical events in Europe and the Mediterranean basin from the Neolithic (e.g., peopling of Sardinia) to recent times (e.g., ‘barbarian invasion’ of Northern and Central Italy). These results are valuable for further genetic, epidemiological and forensic studies in Italy and in Europe. | 1189 | no | human | NA | no | no |
| 1190 | 2017 | 43016 | Talbot, B; Garant, D; Paquette, SR; Mainguy, J; Pelletier, F | 2012 | Lack of Genetic Structure and Female-Specific Effect of Dispersal Barriers in a Rabies Vector, the Striped Skunk (Mephitis mephitis) | Evaluating the permeability of potential barriers to movement, dispersal and gene exchanges can help describe spreading patterns of wildlife diseases. Here, we used landscape genetics methods to assess the genetic structure of the striped skunk (Mephitis mephitis), which is a frequent vector of rabies, a lethal zoonosis of great concern for public health. Our main objective was to identify landscape elements shaping the genetic structure of this species in Southern Quebec, Canada, in an area where the raccoon rabies variant has been detected. We hypothesised that geographic distance and landscape barriers, such as highways and major rivers, would modulate genetic structure. We genotyped a total of 289 individuals sampled across a large area (22,000 km(2)) at nice microsatellite loci. Genetic structure analyses identified a single genetic cluster in the study area. Major rivers and highways, however, influenced the genetic relatedness among sampled individuals. Sex-specific analyses revealed that rivers significantly limited dispersal only for females while highways only had marginal effects. Rivers and highways did not significantly affect male dispersal. These results support the contention that female skunks are more philopatric than males. Overall, our results suggest that the effects of major rivers and highways on dispersal are sex-specific and rather weak and are thus unlikely to prevent the spread of rabies within and among striped skunk populations. | 1190 | no | no good fitness measure, N pop differnt in GD? | NA | no | no |
| 1191 | 2017 | 43016 | Campanella, JJ; Bologna, PAX; Smith, SM; Rosenzweig, EB; Smalley, JV | 2010 | Zostera marina population genetics in Barnegat Bay, New Jersey, and implications for grass bed restoration | Within Barnegat Bay, New Jersey, Zostera marina populations have declined by 62% over the last 20 years, and restoration efforts have met with mixed success. We have completed a microsatellite-based genetic investigation of eight populations of Z. marina within Barnegat Bay to determine whether the genetic stock origins of the plants used in management projects may affect restoration success. Additionally, we assessed the genetic diversity of Z. marina in Barnegat Bay to better understand its population structure. Clonal diversity ranged from 0.70 to 0.95 for the populations studied. Individually, Barnegat Bay populations are not genetically diverse, and there is also little divergence among populations. The Atlantic populations had mean Hobs values (0.20-0.34) that were far lower than the Hexp values (0.69-0.83). Also, the F (IS) values in all of the eastern populations indicate a surfeit of homozygotes over heterozygotes, suggesting a low degree of outcrossing in the Barnegat Bay populations. Six of the ten populations studied (Ham Island, Manahawkin Bay, Shelter Island, Marsh Elder, Harvey Cedar Sedge, and Long Island) show evidence of historical bottlenecks. Mean estimated F (ST) values would suggest that most alleles are undergoing moderate genetic differentiation, with values that range from 0.06 to 0.13. Oyster Creek and Sedge Island demonstrate the largest estimated effective population sizes and may be the most appropriate populations for use in future eelgrass restoration projects. | 1191 | no | no good fitness measure | NA | no | no |
| 1192 | 2017 | 43016 | Boufana, B; Lett, W; Lahmar, S; Griffiths, A; Jenkins, DJ; Buishi, I; Engliez, SA; Alrefadi, MA; Eljaki, AA; Elmestiri, FM; Reyes, MM; Pointing, S; Al-Hindi, A; Torgerson, PR; Okamoto, M; Craig, PS | 2015 | Canine echinococcosis: genetic diversity of Echinococcus granulosus sensu stricto (s.s.) from definitive hosts | Canids, particularly dogs, constitute the major source of cystic echinococcosis (CE) infection to humans, with the majority of cases being caused by Echinococcus granulosus (G1 genotype). Canine echinococcosis is an asymptomatic disease caused by adult tapeworms of E. granulosus sensu lato (s.l.). Information on the population structure and genetic variation of adult E. granulosus is limited. Using sequenced data of the mitochondrial cytochrome c oxidase subunit 1 (cox1) we examined the genetic diversity and population structure of adult tapeworms of E. granulosus (G1 genotype) from canid definitive hosts originating from various geographical regions and compared it to that reported for the larval metacestode stage from sheep and human hosts. Echinococcus granulosus (s.s) was identified from adult tapeworm isolates from Kenya, Libya, Tunisia, Australia, China, Kazakhstan, United Kingdom and Peru, including the first known molecular confirmation from Gaza and the Falkland Islands. Haplotype analysis showed a star-shaped network with a centrally positioned common haplotype previously described for the metacestode stage from sheep and humans, and the neutrality indices indicated population expansion. Low Fst values suggested that populations of adult E. granulosus were not genetically differentiated. Haplotype and nucleotide diversities for E. granulosus isolates from sheep and human origin were twice as high as those reported from canid hosts. This may be related to self-fertilization of E. granulosus and/or to the longevity of the parasite in the respective intermediate and definitive hosts. Improved nuclear single loci are required to investigate the discrepancies in genetic variation seen in this study. | 1192 | no | gd of pathogen | NA | no | no |
| 1193 | 2017 | 43016 | Drygala, F; Korablev, N; Ansorge, H; Fickel, J; Isomursu, M; Elmeros, M; Kowalczyk, R; Baltrunaite, L; Balciauskas, L; Saarma, U; Schulze, C; Borkenhagen, P; Frantz, AC | 2016 | Homogenous Population Genetic Structure of the Non-Native Raccoon Dog (Nyctereutes procyonoides) in Europe as a Result of Rapid Population Expansion | The extent of gene flow during the range expansion of non-native species influences the amount of genetic diversity retained in expanding populations. Here, we analyse the population genetic structure of the raccoon dog (Nyctereutes procyonoides) in north-eastern and central Europe. This invasive species is of management concern because it is highly susceptible to fox rabies and an important secondary host of the virus. We hypothesized that the large number of introduced animals and the species’ dispersal capabilities led to high population connectivity and maintenance of genetic diversity throughout the invaded range. We genotyped 332 tissue samples from seven European countries using 16 microsatellite loci. Different algorithms identified three genetic clusters corresponding to Finland, Denmark and a large ‘central’ population that reached from introduction areas in western Russia to northern Germany. Cluster assignments provided evidence of long-distance dispersal. The results of an Approximate Bayesian Computation analysis supported a scenario of equal effective population sizes among different pre-defined populations in the large central cluster. Our results are in line with strong gene flow and secondary admixture between neighbouring demes leading to reduced genetic structuring, probably a result of its fairly rapid population expansion after introduction. The results presented here are remarkable in the sense that we identified a homogenous genetic cluster inhabiting an area stretching over more than 1500km. They are also relevant for disease management, as in the event of a significant rabies outbreak, there is a great risk of a rapid virus spread among raccoon dog populations. | 1193 | no | no good fitness measure | NA | no | no |
| 1194 | 2017 | 43016 | REED, JM; WALTERS, JR; EMIGH, TE; SEAMAN, DE | 1993 | EFFECTIVE POPULATION-SIZE IN RED-COCKADED WOODPECKERS - POPULATION AND MODEL DIFFERENCES | Loss of genetic variability in isolated populations is an important issue for conservation biology. Most studies involve only a single population of a given species and a single method of estimating rate of loss. Here we present analyses for three different Red-cockaded Woodpecker (Picoides borealis) populations from different geographic regions. We compare two different models for estimating the expected rate of loss of genetic variability, and test their sensitivity to model parameters. We found that the simpler model (Reed et al. 1988) consistently estimated a greater rate of loss of genetic variability from a population than did the Emigh and Pollak (1979) model. The ratio of effective population size (which describes the expected rate of loss of genetic variability) to breeder population size varied widely among Red-cockaded Woodpecker populations due to geographic variation in demography. For this species, estimates of effective size were extremely sensitive to survival parameters, but not to the probability of breeding or reproductive success. Sensitivity was sufficient that error in estimating survival rates in the field could easily mask true population differences in effective size. Our results indicate that accurate and precise demographic data are prerequisites to determining effective population size for this species using genetic models, and that a single estimate of rate of loss of genetic variability is not valid across populations. | 1194 | maybe | any fitness (survival) of groups of different gd? | no gd of host pop and fitness of host pop | no | no |
| 1195 | 2017 | 43016 | Stanford, AM; Ray, GJ; Forbes, V; Mustafa, L | 2013 | Population genetics and reintroduction performance of Solanum conocarpum, an endemic shrub of St. John, US Virgin Islands | Numerous Virgin Islands plants have become rare or extinct over the last 200 years due to cultivation, development, and introduction of exotic species. St. John endemic Solanum conocarpum (Solanaceae) has fewer than 200 known individuals currently extant in the wild. DNA fingerprinting with RAPD PCR was used to assess the population genetics of S. conocarpum and congener S. polygamum. In addition, controlled crosses were performed to determine whether the plant was self-incompatible. Finally, seedlings were outplanted and monitored to (1) restore small populations and (2) compare performance of individuals from the largest and smallest populations of the species. Population genetic analysis revealed that species level diversity in S. conocarpum remains high, but that virtually all diversity is confined to a single population. The crosses performed indicated that the species is completely self-incompatible. While seedlings from the smallest population performed poorly in the greenhouse by comparison with plants from the large population with higher genetic diversity, both groups performed equally well in the field over a three-year period. The species’ high level of overall diversity and ability to survive even when diversity is reduced suggest that prospects for conservation are good. In order to ensure the survival of the species, all populations should be brought up to sizes large enough to allow outcrossing, and ex-situ conservation should be employed. | 1195 | probably no | any fitness (survival) of groups of different gd? | no fitness measure pop | no | no |
| 1196 | 2017 | 43016 | Meloni, M; Perini, D; Binelli, G | 2007 | The distribution of genetic variation in Norway spruce (Picea abies Karst.) populations in the western Alps | Aim In order to look for a possible centre of survival for the Norway spruce (Picea abies Karst.) in the south-western Alps, six natural populations of this area were investigated by means of genetic markers in order to assess the degree and the distribution of genetic diversity within the species. Location Western and South-western Alps. Methods Populations were genotyped using seven simple sequence repeat (SSR) markers. Basic population genetics parameters were estimated and the amount of genetic differentiation calculated. Results A large amount of variability was found (0.59 < H, < 0.67); genetic differentiation as measured by F-ST, was 0.05, close to other similar studies; no isolation by distance was detected by a Mantel test. Analysis of molecular variance confirmed a high degree of variability within populations and a low degree of variability among populations. Finally, the number of populations from which those observed could have arisen was estimated by Bayesian analysis. Main conclusions The results presented here suggest that the present populations derive their genetic make-up from three inferred clusters. The possible existence in this area of a relict/refuge population during the last glaciation is discussed. | 1196 | no | no good fitness measure | NA | no | no |
| 1197 | 2017 | 43016 | Katzerke, A; Neumann, P; Pirk, CWW; Bliss, P; Moritz, RFA | 2006 | Seasonal nestmate recognition in the ant Formica exsecta | Under favorable conditions, the mound-building ant Formica exsecta may form polydomous colonies and can establish large nest aggregations. The lack of worker aggression towards nonnestmate conspecifics is a typical behavioral feature in such social organization, allowing for a free flux of individuals among nests. However, this mutual worker toleration may vary over the seasons and on spatial scales. We studied spatio-temporal variation of worker-worker aggression within and among nests of a polydomous F. exsecta population. In addition, we determined inter- and intracolony genetic relatedness by microsatellite DNA genotyping and assessed its effect on nestmate recognition. We found significant differences in the frequency of worker exchange among nests between spring, summer, and autumn. Moreover, we found significant seasonal variation in the level of aggression among workers of different nests. Aggression levels significantly correlated with spatial distance between nests in spring, but neither in summer nor in autumn. Multiple regression analysis revealed a stronger effect of spatial distances rather than genetic relatedness on aggressive behavior. Because nestmate discrimination disappeared over the season, the higher aggression in spring is most plausibly explained by cue intermixing during hibernation. | 1197 | no | no good fitness measure | NA | no | no |
| 1198 | 2017 | 43016 | Beadell, JS; Atkins, C; Cashion, E; Jonker, M; Fleischer, RC | 2007 | Immunological Change in a Parasite-Impoverished Environment: Divergent Signals from Four Island Taxa | Dramatic declines of native Hawaiian avifauna due to the human-mediated emergence of avian malaria and pox prompted an examination of whether island taxa share a common altered immunological signature, potentially driven by reduced genetic diversity and reduced exposure to parasites. We tested this hypothesis by characterizing parasite prevalence, genetic diversity and three measures of immune response in two recently-introduced species (Neochmia temporalis and Zosterops lateralis) and two island endemics (Acrocephalus aequinoctialis and A. rimitarae) and then comparing the results to those observed in closely-related mainland counterparts. The prevalence of blood parasites was significantly lower in 3 of 4 island taxa, due in part to the absence of certain parasite lineages represented in mainland populations. Indices of genetic diversity were unchanged in the island population of N. temporalis; however, allelic richness was significantly lower in the island population of Z. lateralis while both allelic richness and heterozygosity were significantly reduced in the two island-endemic species examined. Although parasite prevalence and genetic diversity generally conformed to expectations for an island system, we did not find evidence for a pattern of uniformly altered immune responses in island taxa, even amongst endemic taxa with the longest residence times. The island population of Z. lateralis exhibited a significantly reduced inflammatory cell-mediated response while levels of natural antibodies remained unchanged for this and the other recently introduced island taxon. In contrast, the island endemic A. rimitarae exhibited a significantly increased inflammatory response as well as higher levels of natural antibodies and complement. These measures were unchanged or lower in A. aequinoctialis. We suggest that small differences in the pathogenic landscape and the stochastic history of mutation and genetic drift are likely to be important in shaping the unique immunological profiles of small isolated populations. Consequently, predicting the impact of introduced disease on the many other endemic faunas of the remote Pacific will remain a challenge. | 1198 | maybe | any fitness of groups of different gd? | too small difference GD of the two populations/species | no | no |
| 1199 | 2017 | 43016 | Conson, ARO; Ruas, EA; Vieira, BG; Rodrigues, LA; Costa, BF; Bianchini, E; Prioli, AJ; Ruas, CD; Ruas, PM | 2013 | Genetic structure of the Atlantic Rainforest tree species Luehea divaricata (Malvaceae) | The Atlantic Rain Forest is one of the most important Brazilian biomes and a hotspot for biodiversity that is characterized by its high level of endemism, where new species are still being described. Luehea divaricata (Malvaceae) is commonly found in riparian forests areas of the Atlantic forest. Because of the importance of this species in reforestation programs, we used nine pairs of microsatellite loci to study the genetic variability of this species along its distribution area and verify if fragmentation is compromising the survival of these populations. A total of 50 alleles were obtained with an average observed and expected heterozygosity of 0.53 and 0.67, respectively. Seven of the nine populations studied showed a heterozygosity deficit. Most of the genetic diversity was found within populations; while the level of genetic differentiation was moderated (6.84) between populations. Different levels of gene flow between the populations were detected. Positive and significant values of Fis were found for seven populations. The signal test for excess of heterozygosity indicated that a recent genetic bottleneck occurred in the fragmented populations. The dendrogram constructed by the UPGMA method revealed the formation of seven clusters, which was confirmed by the Bayesian analysis for number of K clusters. The presence of several pairs of loci in linkage disequilibrium confirms that these populations experienced a loss of genetic diversity caused by genetic drift. The results showed that it is necessary to develop management strategies for the conservation of these populations of L. divaricata as the viability of the next generations are severely compromised. | 1199 | no | no good fitness measure | NA | no | no |
| 1200 | 2017 | 43016 | O’Dushlaine, CT; Morris, D; Moskvina, V; Gill, M; Corvin, A; Wilson, JF; Cavalleri, GL | 2010 | Population structure and genome-wide patterns of variation in Ireland and Britain | Located off the northwestern coast of the European mainland, Britain and Ireland were among the last regions of Europe to be colonized by modern humans after the last glacial maximum. Further, the geographical location of Britain, and in particular of Ireland, is such that the impact of historical migration has been minimal. Genetic diversity studies applying the Y chromosome and mitochondrial systems have indicated reduced diversity and an increased population structure across Britain and Ireland relative to the European mainland. Such characteristics would have implications for genetic mapping studies of complex disease. We set out to further our understanding of the genetic architecture of the region from the perspective of (i) population structure, (ii) linkage disequilibrium (LD), (iii) homozygosity and (iv) haplotype diversity (HD). Analysis was conducted on 3654 individuals from Ireland, Britain (with regional sampling in Scotland), Bulgaria, Portugal, Sweden and the Utah HapMap collection. Our results indicate a subtle but clear genetic structure across Britain and Ireland, although levels of structure were reduced in comparison with average cross-European structure. We observed slightly elevated levels of LD and homozygosity in the Irish population compared with neighbouring European populations. We also report on a cline of HD across Europe with greatest levels in southern populations and lowest levels in Ireland and Scotland. These results are consistent with our understanding of the population history of Europe and promote Ireland and Scotland as relatively homogenous resources for genetic mapping of rare variants. European Journal of Human Genetics (2010) 18, 1248-1254; doi: 10.1038/ejhg.2010.87; published online 23 June 2010 | 1200 | no | human | NA | no | no |
| 1201 | 2017 | 43016 | Turchetto, C; Segatto, ALA; Mader, G; Rodrigues, DM; Bonatto, SL; Freitas, LB | 2016 | High levels of genetic diversity and population structure in an endemic and rare species: implications for conservation | The analysis of genetic structure and variability of isolated species is of critical importance in evaluating whether stochastic or human-caused factors are affecting rare species. Low genetic diversity compromises the ability of populations to evolve and reduces their chances of survival under environmental changes. Petunia secreta, a rare and endemic species, is an annual and heliophilous herb that is bee-pollinated and easily recognizable by its purple and salverform corolla. It was described as a new species of the Petunia genus in 2005. Few individuals of P. secreta have been observed in nature and little is known about this species. All the natural populations of P. secreta that were found were studied using 15 microsatellite loci, two intergenic plastid sequences and morphological traits. Statistical analysis was performed to describe the genetic diversity of this rare species and the results compared with those of more widespread and frequent Petunia species from the same geographic area to understand whether factors associated with population size could affect rare species of this genus. The results showed that despite its rarity, P. secreta presented high genetic diversity that was equivalent to or even higher than that of widespread Petunia species. It was shown that this species is divided into two evolutionary lineages, and the genetic differentiation indices between them and other congeneric species presented different patterns. The major risk to P. secreta maintenance is its rarity, suggesting the necessity of a preservation programme and more biological and evolutionary studies that handle the two evolutionary lineages independently. | 1201 | no | no good fitness measure | NA | no | no |
| 1202 | 2017 | 43016 | Hong, Y; Kim, KS; Lee, H; Min, MS | 2013 | Population genetic study of the raccoon dog (Nyctereutes procyonoides) in South Korea using newly developed 12 micro satellite markers | The raccoon dog (Nyctereutes procyonoides) is distributed from southeastern Siberia to northern Vietnam, including Korea and Japan, as well as Europe. In Korea, most of its predators and competitors are extinct, which has resulted in rapid growth of the raccoon dog population. This population increase has raised concerns about its role in the ecosystem and the zoonotic transfer of various contagious diseases, and thus an effective method of raccoon dog population control in Korea is required. To investigate the genetic diversity and structure of raccoon dog populations, 12 polymorphic microsatellite loci were identified and characterized. These novel microsatellite markers were employed to obtain basic population genetic parameters for 104 N. procyonoides specimens from five locations in South Korea. The mean allele number of 12 loci across samples was 8.7, and the number of alleles per locus ranged 2-13. Mean expected and observed heterozygosities were 0.723 and 0.619, respectively. Genetic differentiation, estimated by pairwise F-ST, was significant for all population pairs excepting Seoul/Gyeonggi and Gangwon pair, with a moderate level of genetic differentiation for all the population pairs (mean F-ST = 0.054), but little differentiation between Seoul/Gyeonggi and Gangwon (F-ST = 0.024). Bayesian-based clustering analysis predicted that Korean raccoon dog population is composed of four distinct genetic subpopulations. These genetic information and structure of raccoon dog will be very useful to prevent spreading infectious diseases. | 1202 | no | no good fitness measure | NA | no | no |
| 1203 | 2017 | 43016 | Kim, ES; Zaya, DN; Fant, JB; Ashley, MV | 2015 | Genetic factors accelerate demographic decline in rare Asclepias species | We investigated the role of genetic, reproductive and demographic factors in the decline of two co-occurring milkweeds, Asclepias lanuginosa and A. viridiflora, in fragmented populations in Illinois and Wisconsin. Asclepias lanuginosa flowers but does not set seed while seed set is regularly observed in A. viridiflora. We used microsatellite genotyping to determine the extent of clonal growth, genetic diversity, and genetic structure in nine populations of A. lanuginosa and five populations of A. viridiflora. Microsatellite genotyping revealed extremely high clonality in A. lanuginosa; only 32 multilocus genotypes occurred among more than 300 ramets, compared to 118 multilocus genotypes among 124 ramets for A. viridiflora. Four A. lanuginosa populations were monoclonal. While we found no evidence for inbreeding, A. lanuginosa had significantly lower expected heterozygosity and a lower mean number of effective alleles than A. viridiflora. Population viability analysis (PVA) conducted at one site indicated a high probability of persistence, although the population was comprised of only two clones. Because PVA does not distinguish between ramets and genets, results should be interpreted with caution when conducted on highly clonal species. A nearly complete shift in the mode of reproduction, from sexual to asexual, appears to be the most immediate threat to survival of A. lanuginosa in these fragmented prairie remnants. Conservation management strategies should include actions to increase clonal diversity in remnant populations. | 1203 | no | no good fitness measure | NA | no | no |
| 1204 | 2017 | 43016 | Frank, HK; Frishkoff, LO; Mendenhall, CD; Daily, GC; Hadly, EA | 2017 | Phylogeny, Traits, and Biodiversity of a Neotropical Bat Assemblage: Close Relatives Show Similar Responses to Local Deforestation | If species’ evolutionary pasts predetermine their responses to evolutionarily novel stressors, then phylogeny could predict species survival in an increasingly human-dominated world. To understand the role of phylogenetic relatedness in structuring responses to rapid environmental change, we focused on assemblages of Neotropical bats, an ecologically diverse and functionally important group. We examined how taxonomic and phylogenetic diversity shift between tropical forest and farmland. We then explored the importance of evolutionary history by ascertaining whether close relatives share similar responses to environmental change and which species traits might mediate these trends. We analyzed a 5-year data set (5,011 captures) from 18 sites in a countryside landscape in southern Costa Rica using statistical models that account and correct for imperfect detection of species across sites, spatial autocorrelation, and consideration of spatial scale. Taxonomic and phylogenetic diversity decreased with deforestation, and assemblages became more phylogenetically clustered. Species’ responses to deforestation were strongly phylogenetically correlated. Body mass and absolute wing loading explained a substantial portion of species variation in species’ habitat preferences, likely related to these traits’ influence on maneuverability in cluttered forest environments. Our findings highlight the role that evolutionary history plays in determining which species will survive human impacts and the need to consider diversity metrics, evolutionary history, and traits together when making predictions about species persistence for conservation or ecosystem functioning. | 1204 | no | no good fitness measure, N pop differnt in GD? | NA | no | no |
| 1205 | 2017 | 43016 | Barrett, TL; Farina, A; Barrett, GW | 2009 | Aesthetic landscapes: an emergent component in sustaining societies | A revival in the concept of sustainability is appreciated as Earth’s human population continues to increase and its related global concerns in disease ecology, energy resource management, environmental literacy, food production, genetic diversity, and landscape vitality continue to magnify. Sustain is defined within this paper as to keep in existence or to supply with resources or necessities to prevent from falling below a given threshold of health or vitality. Barrett et al. (Bioscience 47:531-535, 1997) illustrated how seven (7) processes (behaviour, development, diversity, energetics, evolution, integration, regulation) transcend eleven (11) levels of ecological organization, ranging from the ecosphere to the cellular. Comprehension of how these processes transcend all levels of ecological organization allow programs and initiatives (e.g. preserving biotic diversity) to be defined by informed incentive, rather than regulatory mandate, within societal systems. We describe how the integration of an eighth transcending process-aesthetics-is essential in the approach to and managing of market and nonmarket capital necessary in sustaining societies. | 1205 | no | human | NA | no | no |
| 1206 | 2017 | 43016 | Van Oosten, AR; Heylen, DJA; Jordaens, K; Backeljau, T; Matthysen, E | 2014 | Population genetic structure of the tree-hole tick Ixodes arboricola (Acari: Ixodidae) at different spatial scales | The endophilic tick Ixodes arboricola infests cavity-nesting birds, and its dispersal strongly depends on the movements of its host. Population genetic structure of I. arboricola was studied with seven polymorphic microsatellite markers. We collected 268 ticks from 76 nest boxes in four woodlots near Antwerp, Belgium. These nest boxes are mainly used by the principal hosts of I. arboricola, the great tit Parus major and the blue tit Cyanistes caeruleus. As these birds typically return to the same cavity for roosting or breeding, ticks within nest boxes were expected to be highly related, and tick populations were expected to be spatially structured among woodlots and among nest boxes within woodlots. In line with the expectations, genetic population structure was found among woodlots and among nest boxes within woodlots. Surprisingly, there was considerable genetic variation among ticks within nest boxes. This could be explained by continuous gene flow from ticks from nearby tree holes, yet this remains to be tested. A pairwise relatedness analysis conducted for all pairs of ticks within nest boxes showed that relatedness among larvae was much higher than among later instars, which suggests that larvae are the most important instar for tick dispersal. Overall, tick populations at the studied spatial scale are not as differentiated as predicted, which may influence the scale at which host-parasite evolution occurs. | 1206 | no | gd of parasite | NA | no | no |
| 1207 | 2017 | 43016 | Iacolina, L; Scandura, M; Bongi, P; Apollonio, M | 2009 | NONKIN ASSOCIATIONS IN WILD BOAR SOCIAL UNITS | We investigated the social organization of wild boars (Sits scrofa) using genetic and spatial data from a study population in Tuscany, Italy. In total, 120 wild boars of different sexes and age classes were captured and monitored from 2002 to 2006. All of them were genetically analyzed by using 10 polymorphic microsatellites (H(E) = 0.693, k = 6.6) and a matrix of pairwise relatedness was calculated. In addition, a reference sample of fully related individuals was created by genotyping 11 adult females and their fetuses (n = 56). Spatial data were gathered for 65 animals that had been fitted with either radiocollars or ear transmitters. Sixteen social units were identified by capture data and confirmed by observations and telemetry. A correlation between interindividual spatial distance and relatedness was observed only in summer-early autumn and seemed to be associated to the presence of piglets. The prediction of matrilinearity in wild boar social units was not confirmed, because a low degree of relatedness among boars was observed within groups. Aggregations of unrelated adult females (with their litters) were detected in the study population. The high turnover in the population due to human-caused mortality seems to be the main factor responsible for this altered social structure. Accordingly, we suggest that the observed social organization would result from grouping of unrelated survivors that is promoted by the presence of wolves in the area. | 1207 | no | no good fitness measure, N pop differnt in GD? | NA | no | no |
| 1208 | 2017 | 43016 | Royo, AA; Ristau, TE | 2013 | Stochastic and deterministic processes regulate spatio-temporal variation in seed bank diversity | Question Seed banks often serve as reservoirs of taxonomic and genetic diversity that buffer plant populations and influence post-disturbance vegetation trajectories; yet evaluating their importance requires understanding how their composition varies within and across spatial and temporal scales (- and -diversity). Shifts in seed bank diversity are strongly governed by the deterministic role of differential seed longevities. Nevertheless, spatio-temporal variability in propagule depletion from and recruitment into seed banks may alter both - and -diversity. Here, we ask to what degree deterministic seed exhaustion, stochasticity in recruitment and mortality, or both, shape - and -diversity? Location Temperate hardwood forest stands of varying ages in northern Pennsylvania, USA. Methods We surveyed the seed bank and herbaceous vegetation communities at 39 sites and examined whether the species richness, abundance and composition of either community differs among and within sites of increasing age ranging from 43 to 106yr old. We explored how -diversity (species richness) and abundance (percentage cover, seed density) varied across the chronosequence age using regression analyses. We analysed differences in -diversity (community composition) using permutational multivariate analyses (i.e. PERMANOVA, PERMDISP). Finally, we tested whether community composition of the herbaceous layer and seed bank communities exhibited nestedness, where nestedness refers to the degree to which less species-rich sites are a non-random subset of more species-rich sites. Results We found seed bank -diversity and abundance consistently declined across a gradient of increasingly older sites. Moreover, nestedness analyses indicated species composition at older sites represented a subset of the species found at younger sites characterized by species with persistent seeds. Nevertheless, seed bank communities demonstrated divergent compositional trajectories, whereby older sites were increasingly dissimilar, not only from younger sites, but also from each other. Conclusions Our results suggest that even in assemblages strongly structured by deterministic forces, such as seed banks, minor stochastic differences in colonization and extinction events may increase -diversity over time. Therefore, we argue deterministic and stochastic processes are complementary factors governing post-disturbance turnover in species assemblages, and suggest that seed banks contribute to species persistence and overall forest community diversity across space and time. | 1208 | no | multiple species diversity | NA | no | no |
| 1209 | 2017 | 43017 | Mitchell, SE; Rogers, ES; Little, TJ; Read, AF | 2005 | Host-parasite and genotype-by-environment interactions: Temperature modifies potential for selection by a sterilizing pathogen | Parasite-mediated selection is potentially of great importance in modulating genetic diversity. Genetic variation for resistance, the fuel for natural selection, appears to be common in host-parasite interactions, but responses to selection are rarely observed. In the present study, we tested whether environmental variation could mediate infection and determine evolutionary outcomes. Temperature was shown to dramatically alter the potential for parasite-mediated selection in two independent laboratory infection experiments at four temperatures. The bacterial parasite, Pasteuria rainosa, was extremely virulent at 20degreesC and 25degreesC, sterilizing its host, Daphnia magna, so that females often never produced a single brood. However, at 10degreesC and 15degreesC, the host-parasite interaction was much more benign, as nearly all females produced broods before becoming sterile. This association between virulence and temperature alone could stabilize coexistence and lead to the maintenance of diversity, because it would weaken parasite-mediated selection during parts of the season. Additionally, highly significant genotype-by-environment interactions were found, with changes in clone rank order for infection rates at different temperatures. Our results clearly show that the outcome of parasite-mediated selection in this system is strongly context dependent. | 1209 | no | No GD diff between pop | NA | no | no |
| 1210 | 2017 | 43017 | Tayeh, A; Estoup, A; Laugier, G; Loiseau, A; Turgeon, J; Toepfer, S; Facon, B | 2012 | Evolution in biocontrol strains: insight from the harlequin ladybird Harmonia axyridis | After being used as a biocontrol agent against aphids for decades without harmful consequences, the Asian harlequin ladybird Harmonia axyridis has suddenly become an invasive pest on a worldwide scale. We investigate the impact of captive breeding on several traits of this ladybird such as genetic diversity, fecundity, survival and pathogen resistance. We conducted an experiment in the laboratory to compare the fecundity and the susceptibility to the entomopathogenic fungus Beauveria bassiana of wild and biocontrol adults of H. axyridis. We compiled these new findings with already published data. Altogether, our findings suggest that mass rearing of biological control agents may strongly impact genetic diversity and life-history traits. We discuss how such changes may subsequently affect the fitness of biological control strains in natural environments. | 1210 | no | No GD diff between pop | NA | no | no |
| 1211 | 2017 | 43017 | Fontaine, MC; Austerlitz, F; Giraud, T; Labbe, F; Papura, D; Richard-Cervera, S; Delmotte, F | 2013 | Genetic signature of a range expansion and leap-frog event after the recent invasion of Europe by the grapevine downy mildew pathogen Plasmopara viticola | Biologic invasions can have important ecological, economic and social consequences, particularly when they involve the introduction and spread of plant invasive pathogens, as they can threaten natural ecosystems and jeopardize the production of human food. Examples include the grapevine downy mildew, caused by the oomycete Plasmopara viticola, an invasive species native to North America, introduced into Europe in the 1870s. We investigated the introduction and spread of this invasive pathogen, by analysing its genetic structure and diversity in a large sample from European vineyards. Populations of P.viticola across Europe displayed little genetic diversity, consistent with the occurrence of a bottleneck at the time of introduction. Bayesian coalescent analyses revealed a clear population expansion signal in the genetic data. We detected a weak, but significant, continental-wide population structure, with two geographically and genetically distinct clusters in Western and Eastern European vineyards. Approximate Bayesian computation, analyses of clines of genetic diversity and of isolation-by-distance patterns provided evidence for a wave of colonization moving in an easterly direction across Europe. This is consistent with historical reports, first mentioning the introduction of the disease in Bordeaux vineyards (France) and sub-sequently documenting its rapid spread across Europe. This initial introduction in the west was probably followed by a leap-frog’ event into Eastern Europe, leading to the formation of the two genetic clusters we detected. This study shows that recent population genetics methods within the Bayesian and coalescence frameworks are extremely powerful for increasing our understanding of pathogen population dynamics and invasion histories. | 1211 | no | gd of pathogen | NA | no | no |
| 1212 | 2017 | 43017 | Cui, J; Frankham, GJ; Johnson, RN; Polkinghorne, A; Timms, P; O’Meally, D; Cheng, YY; Belov, K | 2015 | SNP Marker Discovery in Koala TLR Genes | Toll-like receptors (TLRs) play a crucial role in the early defence against invading pathogens, yet our understanding of TLRs in marsupial immunity is limited. Here, we describe the characterisation of nine TLRs from a koala immune tissue transcriptome and one TLR from a draft sequence of the koala genome and the subsequent development of an assay to study genetic diversity in these genes. We surveyed genetic diversity in 20 koalas from New South Wales, Australia and showed that one gene, TLR10 is monomorphic, while the other nine TLR genes have between two and 12 alleles. 40 SNPs (16 non-synonymous) were identified across the ten TLR genes. These markers provide a springboard to future studies on innate immunity in the koala, a species under threat from two major infectious diseases. | 1212 | no | no good fitness measure, N pop differnt in GD? | NA | no | no |
| 1213 | 2017 | 43017 | Lijavetzky, D; Cabezas, JA; Ibanez, A; Rodriguez, V; Martinez-Zapater, JM | 2007 | High throughput SNP discovery and genotyping in grapevine (Vitis vinifera L.) by combining a re-sequencing approach and SNPlex technology | Background: Single-nucleotide polymorphisms SNPs) are the most abundant type of DNA sequence polymorphisms. Their higher availability and stability when compared to simple sequence repeats SSRs) provide enhanced possibilities for genetic and breeding applications such as cultivar identification, construction of genetic maps, the assessment of genetic diversity, the detection of genotype/phenotype associations, or marker-assisted breeding. In addition, the efficiency of these activities can be improved thanks to the ease with which SNP genotyping can be automated. Expressed sequence tags EST) sequencing projects in grapevine are allowing for the in silico detection of multiple putative sequence polymorphisms within and among a reduced number of cultivars. In parallel, the sequence of the grapevine cultivar Pinot Noir is also providing thousands of polymorphisms present in this highly heterozygous genome. Still the general application of those SNPs requires further validation since their use could be restricted to those specific genotypes. Results: In order to develop a large SNP set of wide application in grapevine we followed a systematic re-sequencing approach in a group of II grape genotypes corresponding to ancient unrelated cultivars as well as wild plants. Using this approach, we have sequenced 230 gene fragments, what represents the analysis of over I Mb of grape DNA sequence. This analysis has allowed the discovery of 1573 SNPs with an average of one SNP every 64 bp one SNP every 47 bp in ;lnon-coding regions and every 69 bp in coding regions). Nucleotide diversity in grape pi = 0.0051) was found to be similar to values observed in highly polymorphic plant species such as maize. The average number of haplotypes per gene sequence was estimated as six, with three haplotypes representing over 83% of the analyzed sequences. Short-range linkage disequilibrium LD) studies within the analyzed sequences indicate the existence of a rapid decay of LD within the selected grapevine genotypes. To validate the use of the detected polymorphisms in genetic mapping, cultivar identification and genetic diversity studies we have used the SNPlex(TM) genotyping technology in a sample of grapevine genotypes and segregating progenies. Conclusion: These results provide accurate values for nucleotide diversity in coding sequences and a first estimate of short-range LD in grapevine. Using SNPlex(TM) genotyping we have shown the application of a set of discovered SNPs as molecular markers for cultivar identification, linkage mapping and genetic diversity studies. Thus, the combination a highly efficient re-sequencing approach and the SNPlex(TM) high throughput genotyping technology provide a powerful tool for grapevine genetic analysis. | 1213 | no | no good fitness measure | NA | no | no |
| 1214 | 2017 | 43017 | SHYKOFF, JA; SCHMIDHEMPEL, P | 1991 | Genetic relatedness and eusociality parasite mediated selection on the genetic composition of groups | Contrary to the expectations of kin selection theory, intracolony relatedness in eusocial insects is often low. We examined the idea that associations of low relatedness (high genetic variability) may be advantageous because of negative frequency-dependent selection on common host phenotypes by rapidly evolving parasites and pathogens. Using the natural host-parasite system of the bumble bee Bombus terrestris and its intestinal trypanosome Crithidia bombi, we investigated the transmission properties of parasites in host groups. Within naturally infested nests and in artificially constructed groups of workers, prevalence of infestation increased with time of exposure (Table 1). The susceptibility of isolated groups of workers to the parasites to which they were exposed differed with identity and natural infestation of their nest of origin (Table 2). In addition, those workers that were related to the individual introducing an infection to their group were more likely to become infested than were unrelated workers (Table 3). Although the bumble bee workers in experimental boxes appeared to differ in behavior toward kin and non-kin, making more physical contacts with kin, we found no discernible relationship between number of physical contacts and prevalence of infestation in a group. Therefore, we conclude that differences in parasite transmission reflected interactions among different host and parasite phenotypes. This system thus demonstrates the factors necessary for negative frequency-dependent selection by parasites on common host phenotypes - variability for susceptibility and infectiousness in host and parasite populations and similarity for these traits among related individuals. If, as we show here, high genetic relatedness within groups enhances parasite transmission, kin directed altruism may increase the risk of contracting parasites and infectious diseases. Therefore, parasites and pathogens may be an important force moderating the genetic structure of social groups. | 1214 | yes | from thesis | NA | no | yes |
| 1215 | 2017 | 43017 | Mathers, A; Smith, RP; Cahill, B; Lubelczyk, C; Elias, SP; Lacombe, E; Morris, SR; Vary, CP; Parent, CE; Rand, PW | 2011 | Strain diversity of Borrelia burgdorferi in ticks dispersed in North America by migratory birds | The role of migratory birds in the dispersal of Ixodes scapularis ticks in the northeastern U. S. is well established and is presumed to be a major factor in the expansion of the geographic risk for Lyme disease. Population genetic studies of B. burgdorferi sensu stricto, the agent of Lyme disease in this region, consistently reveal the local presence of as many as 15 distinct strain types as designated by major groups of the ospC surface lipoprotein. Recent evidence suggests such strain diversity is adaptive to the diverse vertebrate hosts that maintain enzootic infection. How this strain diversity is established in emergent areas is unknown. To determine whether similar strain diversity is present in ticks imported by birds, we examined B. burgdorferi strains in I. scapularis ticks removed from migrants at an isolated island site. Tick mid-guts were cultured and isolates underwent DNA amplification with primers targeting ospC. Amplicons were separated by gel electrophoresis and sequenced. One hundred thirty-seven nymphal ticks obtained from 68 birds resulted in 24 isolates of B. burgdorferi representing eight ospC major groups. Bird-derived ticks contain diverse strain types of B. burgdorferi, including strain types associated with invasive Lyme disease. Birds and the ticks that feed on them may introduce a diversity of strains of the agent of Lyme disease to emergent areas. Journal of Vector Ecology 36 (1): 24-29. 2011. | 1215 | no | gd of pathogen | NA | no | no |
| 1216 | 2017 | 43017 | Brankovic, S; Cvjeticanin, S | 2016 | ANTHROPOGENETIC VARIABILITY IN GROUPS OF CHILDREN FROM REGULAR AND SPECIAL SCHOOLS FROM DIFFERENT LOCALITIES IN SERBIA | This population-genetic study compares morpholophysiological and genetic variability in five control groups of individuals (children from five regular schools, N=996) with children from that many special schools (N=736) from Serbia, by using a test of determination of homozygously recessive characteristics in humans (HRC-test). Genetic homozygosity degree showed not only statistically significant difference between the mean values obtained for two groups of studied samples (control group 6.95 +/- 0.07; children from special schools 8.63 +/- 0.08 HRCs, out of 30 analyzed characteristics), but also differences in the type of distribution, as well as the presence of specific combinations of such traits. Results of comparisons done in different places (Kraljevo, Nis, Vranje, Leskovac, Pirot) showed the same tendency-the increase of genetic homozygosity and relative decrease of variability in samples of children from special schools. The number of HRCs among individuals from control groups varied from 2 to 15/30, and from 3 to 16/30 among children from special schools. It is possible that increased recessive homozygosity present in the group of children from special schools leads to increase of genetic loads, what may cause easier expression of some physiological and mental abilities that children from special schools have. A great individual variation in amount of genetic homozygosity that exists among human individuals may influence their potentials for different kinds of adaptation, including their mental abilities, physical capacities or resistance to different diseases. | 1216 | no | human | NA | no | no |
| 1217 | 2017 | 43017 | Aikio, S; Pakkasmaa, S | 2003 | Relatedness and competitive asymmetry - implications for growth and population dynamics | The members of natural populations often differ in size and relatedness to each other, which may affect the division of limited resources and have consequences on reproductive success and population dynamics. We modeled seasonal growth and dynamics in populations composed of different types of relatives (full-sibs, half-sibs and non-related individuals) under the continuum of competitive scenarios between complete symmetry and asymmetry. Growth was assumed logistic in proportion to individual biomass and the size-differences were weighted by the relatedness of individuals. The symmetric component of competition was experienced by all individuals in proportion to their biomass, whereas the asymmetric component was individual-specific, and influenced only by the individuals larger than the focal individual. Relatedness decreased and competitive asymmetry increased the variability of individual biomasses. Mortality of the smallest individuals and the size threshold of reproduction decreased population density. Population dynamics were stable when there was no size threshold for reproduction but the presence of the threshold led to cyclic dynamics under low competitive asymmetry. The effects of the threshold were greater among related than unrelated individuals. The results suggest that individual differences and the asymmetry of competition can greatly affect population dynamics. Full symmetry of competition may be evolutionarily unstable in populations of related individuals as it may increase the probability of extinction due to demographic stochasticity. | 1217 | no | no good fitness measure | NA | no | no |
| 1218 | 2017 | 43017 | Barshis, DJ; Sotka, EE; Kelly, RP; Sivasundar, A; Menge, BA; Barth, JA; Palumbi, SR | 2011 | Coastal upwelling is linked to temporal genetic variability in the acorn barnacle Balanus glandula | Dispersal and recruitment are central processes that shape the geographic and temporal distributions of populations of marine organisms. However, significant variability in factors such as reproductive output, larval transport, survival, and settlement success can alter the genetic identity of recruits from year to year. We designed a temporal and spatial sampling protocol to test for genetic heterogeneity among adults and recruits from multiple time points along a similar to 400 km stretch of the Oregon (USA) coastline. In total, 2824 adult and recruiting Balanus glandula were sampled between 2001 and 2008 from 9 sites spanning the Oregon coast. Consistent with previous studies, we observed high mitochondrial DNA diversity at the cytochrome oxidase I locus (884 unique haplotypes) and little to no spatial genetic population structure among the 9 sites (Phi(ST) = 0.00026, p = 0.170). However, subtle but significant temporal shifts in genetic composition were observed among year classes (Phi(ST) = 0.00071, p = 0.035), and spatial Phi(ST) varied from year to year. These temporal shifts in genetic structure were correlated with yearly differences in the strength of coastal upwelling (p = 0.002), with greater population structure observed in years with weaker upwelling. Higher levels of barnacle settlement were also observed in years with weaker upwelling (p < 0.001). These data suggest the hypothesis that low upwelling intensity maintains more local larvae close to shore, thereby shaping the genetic structure and settlement rate of recruitment year classes. | 1218 | no | No GD diff between pop | NA | no | no |
| 1219 | 2017 | 43017 | Cameron, SA; Lim, HC; Lozier, JD; Duennes, MA; Thorp, R | 2016 | Test of the invasive pathogen hypothesis of bumble bee decline in North America | Emergent fungal diseases are critical factors in global biodiversity declines. The fungal pathogen Nosema bombi was recently found to be widespread in declining species of North American bumble bees (Bombus), with circumstantial evidence suggesting an exotic introduction from Europe. This interpretation has been hampered by a lack of knowledge of global genetic variation, geographic origin, and changing prevalence patterns of N. bombi in declining North American populations. Thus, the temporal and spatial emergence of N. bombi and its potential role in bumble bee decline remain speculative. We analyze Nosema prevalence and genetic variation in the United States and Europe from 1980, before an alleged introduction in the early 1990s, to 2011, extracting Nosema DNA from Bombus natural history collection specimens from across this time period. Nosema bombi prevalence increased significantly from low detectable frequency in the 1980s to significantly higher frequency in the mid-to late-1990s, corresponding to a period of reported massive infectious outbreak of N. bombi in commercial bumble bee rearing stocks in North America. Despite the increased frequency, we find no conclusive evidence of an exotic N. bombi origin based on genetic analysis of global Nosema populations; the widespread Nosema strain found currently in declining United States bumble bees was present in the United States before commercial colony trade. Notably, the US N. bombi is not detectably different from that found predominantly throughout Western Europe, with both regions characterized by low genetic diversity compared with high levels of diversity found in Asia, where commercial bee breeding activities are low or nonexistent. | 1219 | maybe | gd difference? | no gd of host pop and fitness of host pop | no | no |
| 1220 | 2017 | 43017 | Bylebyl, K; Poschlod, P; Reisch, C | 2008 | Genetic variation of Eryngium campestre L. (Apiaceae) in Central Europe | In Germany, Eryngium campestre is restricted to dry habitats along the rivers Rhine and Elbe and to a few areas in Central Germany. This distribution pattern is usually regarded as a typical pattern of postglacial immigration. In the present study, we investigated whether these two geographically distinct distribution areas are genetically differentiated and whether conclusions can be drawn regarding colonization history. To analyse the phylogeographic structure of E. campestre in Central Europe, 278 individuals from 29 populations within Germany and from further reference populations within Europe were analysed. We applied amplified fragment length polymorphisms to examine their genetic relatedness. Our analyses revealed three groups: a Mediterranean group additionally including two Rhine populations; a Rhine-Main group which further includes the westernmost population from the central German dry area; and one group which includes all eastern populations. Our results show that the two geographically distinct areas are genetically differentiated. As genetic diversity within the Elbe populations is very low, we conclude that this area, which was strongly affected through the late glacial maximum, was colonized relatively recently. High genetic diversity in the Rhine populations indicates a contact zone where lineages of different origin met. This would imply that today’s patterns of genetic variation were caused through glacial range contractions and expansions. The present study is one of the first studies that deal with the postglacial distribution pattern of a dry grassland plant species in Central Europe and the results suggest that a survival of E. campestre at least during the Dryas cold stage might be possible. | 1220 | no | no good fitness measure | NA | no | no |
| 1221 | 2017 | 43017 | Gonzalez-Martin, A; Gorostiza, A; Regalado-Liu, L; Arroyo-Pena, S; Tirado, S; Nuno-Arana, I; Rubi-Castellanos, R; Sandoval, K; Coble, MD; Rangel-Villalobos, H | 2015 | Demographic History of Indigenous Populations in Mesoamerica Based on mtDNA Sequence Data | The genetic characterization of Native American groups provides insights into their history and demographic events. We sequenced the mitochondrial D-loop region (control region) of 520 samples from eight Mexican indigenous groups. In addition to an analysis of the genetic diversity, structure and genetic relationship between 28 Native American populations, we applied Bayesian skyline methodology for a deeper insight into the history of Mesoamerica. AMOVA tests applying cultural, linguistic and geographic criteria were performed. MDS plots showed a central cluster of Oaxaca and Maya populations, whereas those from the North and West were located on the periphery. Demographic reconstruction indicates higher values of the effective number of breeding females (Nef) in Central Mesoamerica during the Preclassic period, whereas this pattern moves toward the Classic period for groups in the North and West. Conversely, Nef minimum values are distributed either in the Lithic period (i.e. founder effects) or in recent periods (i.e. population declines). The Mesomerican regions showed differences in population fluctuation as indicated by the maximum Inter-Generational Rate (IGRmax): i) Center-South from the lithic period until the Preclassic; ii) West from the beginning of the Preclassic period until early Classic; iii) North characterized by a wide range of temporal variation from the Lithic to the Preclassic. Our findings are consistent with the genetic variations observed between central, South and Southeast Mesoamerica and the North-West region that are related to differences in genetic drift, structure, and temporal survival strategies (agriculture versus hunter-gathering, respectively). Interestingly, although the European contact had a major negative | 1221 | no | NA | NA | no | no |
| 1222 | 2017 | 43017 | Dara, SK; McGuire, MR; Ulloa, M; Kaya, HK | 2008 | Evaluation and molecular characterization of Beauveria bassiana for the control of the glassy-winged sharpshooter (Homoptera : Cicadellidae) in California | The glassy-winged sharpshooter, Homalodisca coagulata (Say), is an important pest on grapes, citrus, almonds and other commercial crops in California as it is a vector of Xylella fastidiosa Wells, a bacterium that causes Pierce’s disease in grapes, citrus variegated chlorosis, almond leaf scorch and other plant diseases. Various entomopathogenic fungi isolated from natural infections of H. coagulata, its habitats and other insect hosts were evaluated against this insect vector. Based on these studies, 3 isolates of the hyphomycetous fungus, Beauveria bassiana (Balsamo) Vuillemin, were selected for further evaluation. Two of these were California isolates, one each from the three-cornered alfalfa hopper, Spissistilus festinus (Say), and soil from H. coagulata habitat, and the third was a Texas isolate from natural infections of H. coagulata. All three were similar in their virulence to H. coagulata under laboratory conditions. The genetic relatedness of the B. bassiana isolates also was compared using single sequence repeat (SSR) markers which showed genetic diversity of this species based on the source of the isolate. Some isolates were 4x more infectious than others demonstrating that virulence of B. bassiana is not necessarily associated with their genetic relatedness. | 1222 | no | gd of pathogen | NA | no | no |
| 1223 | 2017 | 43017 | SAITO, Y | 1995 | CLINAL VARIATION IN MALE-TO-MALE ANTAGONISM AND WEAPONRY IN A SUBSOCIAL MITE | Male aggressiveness is highly variable among populations of a subsocial spider mite that occurs throughout Japan. The average level of aggressiveness is positively correlated with mean winter temperature and with the relative size among males of leg I, which is used as a weapon in this species. The relatedness of males within nests is influenced by rates of overwinter survival, with high survivorship leading to low relatedness, increased aggressiveness, and larger legs I. Within this species, variation in the intensity of sexual selection may therefore be influenced both by natural selection and by kin selection. | 1223 | probably yes | ok gd and fitness measurement? | no good gd measure and fitness measure | no | no |
| 1224 | 2017 | 43017 | Namroud, MC; Tremblay, F; Bergeron, Y | 2005 | Temporal variation in quaking aspen (Populus tremuloides) genetic and clonal structures in the mixedwood boreal forest of eastern Canada | Two sites that burned in 1847 (H) and 1823 (1) in the mixedwood boreal forest in Quebec were selected to follow aspen genetic and clonal diversity over time. At each site, three cohorts were identified by core dating, and about 30 trees per cohort were randomly selected to compare tree genotypes using four microsatellite loci. The first cohorts were of post-fire origin (large disturbance), while the second and third cohorts were promoted by gap disturbances. These gaps were created by the natural mortality of post-fire aspen trees and a spruce budworm outbreak that attacked the coniferous species. Expected heterozygosity ranged from 0.37 to 0.72 across cohorts and averaged 0.66 and 0.54 in H and 1, respectively. More than 99% and 96% of the genetic variability existed within cohorts, respectively. Genotypic diversity was high in all cohorts, and most genets were unique. Only two clones suckered for three successive cohorts, indicating little selection for specific genets to dominate aspen stands with time. High genetic and clonal diversity changed slightly between post-fire and gap disturbance cohorts. Apical dominance might have favoured the suckering of genets that existed in the post-fire cohorts but that were later eliminated by natural mortality. | 1224 | no | no good fitness measure | NA | no | no |
| 1225 | 2017 | 43017 | Brandt, JR; Brandt, AL; Ammer, FK; Roca, AL; Serfass, TL | 2014 | Impact of Population Expansion on Genetic Diversity and Structure of River Otters (Lontra canadensis) in Central North America | Populations of North American river otters (Lontra canadensis) declined throughout large portions of the continent during the early 1900s due to habitat degradation and unregulated trapping. River otters had been extirpated in North Dakota (ND), but the Red River Valley has since been recolonized, with potential source populations including the neighboring states of Minnesota or South Dakota, or the Canadian province of Manitoba (MB). We genotyped 9 microsatellite loci in 121 samples to determine the source population of river otters in the Red River Valley of ND, as well as to assess population structure and diversity of river otters in central North America. Overall, genetic diversity was high, with an average observed heterozygosity of 0.58. Genetic differentiation was low (F-ST < 0.05) between river otters in ND and those of Minnesota, suggesting that eastern ND was recolonized by river otters from Minnesota. River otters from MB were genetically distinct from all other sampled populations. Low genetic differentiation (F-ST 0.044) between South Dakota and Louisiana (LA) suggested that reintroductions using LA stock were successful. The genetic distinctiveness of river otters from different geographic regions should be considered when deciding on source populations for future translocations. | 1225 | no | No GD diff between pop | NA | no | no |
| 1226 | 2017 | 43017 | Gu, XB; Zhu, JY; Jian, KL; Wang, BJ; Peng, XR; Yang, GY; Wang, T; Zhong, ZJ; Peng, KY | 2016 | Absence of population genetic structure in Heterakis gallinarum of chicken from Sichuan, inferred from mitochondrial cytochrome c oxidase subunit I gene | Population genetics information provides a foundation for understanding the transmission and epidemiology of parasite and, therefore, may be used to assist in the control of parasitosis. However, limited available sequence information in Heterakis gallinarum has greatly impeded the study in this area. In this study, we first investigated the genetic variability and genetic structure of H. gallinarum. The 1325 bp fragments of the mitochondrial COX1 gene were amplified in 56 isolates of H. gallinarum from seven different geographical regions in Sichuan province, China. The 56 sequences were classified into 22 haplotypes (H1-H22). The values of haplotype diversity (0.712) and nucleotide diversity (0.00158) in Sichuan population indicate a rapid expansion occurred from a relatively small, short-term effective population in the past. The haplotype network formed a distribution around H1 in a star-like topology, and the haplotypes did not cluster according to their geographical location. Similar conclusions could be made from MP phylogenetic tree. The F-st value (F-st<0.16965) and AMOVA analysis revealed that no significant genetic differentiation was observed among the seven different geographical populations. Neutrality tests (Tajima’s D and Fu’s Fs) and mismatch analysis indicated that H. gallinarum experienced a population expansion in the past. Our results indicated that H. gallinarum experienced a rapid population expansion in the past, and there was a low genetic diversity and an absence of population structure across the population. | 1226 | no | gd of pathogen | NA | no | no |
| 1227 | 2017 | 43017 | Beatty, GE; Brown, JA; Cassidy, EM; Finlay, CMV; McKendrick, L; Montgomery, WI; Reid, N; Tosh, DG; Provan, J | 2015 | Lack of genetic structure and evidence for long-distance dispersal in ash (Fraxinus excelsior) populations under threat from an emergent fungal pathogen: implications for restorative planting | Genetic analysis on populations of European ash (Fraxinus excelsior) throughout Ireland was carried out to determine the levels and patterns of genetic diversity in naturally seeded trees in ash woodlands and hedgerows, with the aim of informing conservation and replanting strategies in the face of potential loss of trees as a result of ash dieback. Samples from 33 sites across Northern Ireland and three sites in the Republic of Ireland were genotyped for eight nuclear and ten chloroplast microsatellites. Levels of diversity were (h)igh (mean A(R)=10.53; mean H-O=0.709; mean H-E=0.765) and were similar to those in Great Britain and continental Europe, whilst levels of population genetic differentiation based on nuclear microsatellites were extremely low ((Phi) over bar (ST)=0.0131). Levels of inbreeding (mean F-IS=0.067) were significantly lower than those reported for populations from Great Britain. Fine-scale analysis of seed dispersal indicated potential for dispersal over hundreds of metres. Our results suggest that ash woodlands across Ireland could be treated as a single management unit, and thus native material from anywhere in Ireland could be used as a source for replanting. In addition, high potential for dispersal has implications for recolonization processes post-ash dieback (Chalara fraxinea) infection, and could aid in our assessment of the capacity of ash to shift its range in response to global climate change. | 1227 | no | no good fitness measure | NA | no | no |
| 1228 | 2017 | 43017 | Konvicka, M; Mihaly, CV; Rakosy, L; Benes, J; Schmitt, T | 2014 | Survival of cold-adapted species in isolated mountains: the population genetics of the Sudeten ringlet, Erebia sudetica sudetica, in the Jesenik Mts., Czech Republic | Relic populations of cold-adapted species, trapped in isolated mountain pockets within the temperate zone, are predicted to suffer considerably due to ongoing climate warming. The butterfly Erebia sudetica sudetica is an example restricted to the Eastern Sudety Mts. Here, the butterfly forms permanent populations on subalpine tall-herb grasslands, but also occupies woodland clearings and hay meadows at lower altitudes. We assume differences among the genetic diversities of the populations due to differences in the temporal continuity of these habitats. Therefore, 17 allozyme loci were analysed for 276 individuals from 13 different localities (six tall-herb stands, two meadows, five forest clearings) in the Jesenik Mts. with a maximum distance of 20 km among them. We obtained a significantly higher genetic diversity for the subalpine populations than for the forest clearing populations. The genetic differentiation among the forest clearing populations was higher than among the subalpine ones. They also showed a significant isolation-by-distance system. These findings support the idea that the lower-elevation populations might have been founded by more than one dispersal event from the subalpine sites, but also secondary colonisations and gene flow in the forest belt. Due to founder effects and possibly further subsequent bottlenecks, these forest clearing populations did not harbour the entire genetic diversity of the taxon. Therefore, conservation actions should focus on the subalpine tall-herb formation. | 1228 | no | no good fitness measure | NA | no | no |
| 1229 | 2017 | 43017 | Schultz, JK | 2011 | Population Genetics of the Monk Seals (Genus Monachus): A Review | Monk seals (genus Monachus) are among the most endangered mammals in the world, with all species sharing a similar history of anthropogenic overexploitation. Reductions in population abundance have been accompanied by the loss of genetic diversity in Mediterranean (M. monachus) and Hawaiian (M. schauinslandi) monk seals. Both species are characterized by extremely low variability at all genetic markers tested to date, including microsatellite loci, the mitochondrial control region, and major histocompatibility complex class I genes. Genetic variation is partitioned differently in the two species. The Hawaiian monk seal does not exhibit spatial population structure throughout its range in the Hawaiian Archipelago (F-ST = 0.00); therefore, it is unlikely that the translocation of monk seals will result in genetic incompatibilities. In contrast, Eastern Mediterranean and Western Saharan M. monachus populations are reproductively isolated (F-ST = 0.56), though the distribution of alleles likely signifies a once contiguous range sundered by the extirpation of geographically intermediate subpopulations. Given the magnitude of genetic differentiation, moving Mediterranean monk seals between eastern and western populations could result in reduced overall fitness; additional data is required to assess the risk of moving monk seals within a population (e. g., the Eastern Mediterranean). Recent advances in genomics will facilitate future investigation into the reproductive success of both species and guide managers in their quest to recover Mediterranean and Hawaiian monk seal populations. | 1229 | no | no good fitness measure | NA | no | no |
| 1230 | 2017 | 43017 | Roscher, C; Schumacher, J; Foitzik, O; Schulze, ED | 2007 | Resistance to rust fungi in Lolium perenne depends on within-species variation and performance of the host species in grasslands of different plant diversity | The hypothesis that plant species diversity and genetic variation of the host species decrease the severity of plant diseases is supported by studies of agricultural systems, but experimental evidence from more complex systems is scarce. In an experiment with grassland communities of varying species richness (1, 2, 4, 8, 16, and 60 species) and functional group richness (1, 2, 3, and 4 functional groups), we used different cultivars of Lolium perenne (perennial ryegrass) to study effects of biodiversity and cultivar identity on the occurrence and severity of foliar fungal diseases caused by Puccinia coronata (crown rust) and P. graminis (stem rust). Cultivar monocultures of perennial ryegrass revealed strong differences in pathogen susceptibility among these cultivars. Disease intensity caused by both rust fungi decreased significantly with growing species richness of species mixtures. The response to the diversity gradient was related to the decreased density and size of the host individuals with increasing species richness. The occurrence of other grass species known to be possible hosts of the pathogens in the experimental mixtures did not promote disease intensity in L. perenne, indicating that there was a high host specificity of pathogen strains. Differences in pathogen susceptibility among perennial ryegrass cultivars persisted independent of diversity treatment, host density and host individual size, but resulted in a cultivar- specific pattern of changes in pathogen infestation across the species-richness gradient. Our study provided evidence that within-species variation in pathogen susceptibility and competitive interactions of the host species with the environment, as caused by species diversity treatments, are key determinants of the occurrence and severity of fungal diseases. | 1230 | no | multiple species diversity | NA | no | no |
| 1231 | 2017 | 43017 | Lopez, Z; Cardenas, L; Runil, F; Gonzalez, MT | 2015 | Contrasting definitive hosts as determinants of the genetic structure in a parasite with complex life cycle along the south-eastern Pacific | The spatial genetic structure (and gene flow) of parasites with complex life cycles, such as digeneans, has been attributed mainly to the dispersion ability of the most mobile host, which most often corresponds to the definitive host (DH). In this study, we compared the genetic structure and diversity of adult Neolebouria georgenascimentoi in two fish species (DHs) that are extensively distributed along the south-eastern Pacific (SEP). The analysis was based on the cytochrome oxidase subunit I gene sequences of parasites collected between 23 degrees S and 45 degrees S. In total, 202 sequences of N.georgenascimentoi in Pinguipes chilensis isolated from nine sites and 136 sequences of Prolatilus jugularis from five sites were analysed. Our results showed that N.georgenascimentoi is a species complex that includes three different parasite species; however, in this study, only lineage 1 and 2 found in P.chilensis and P.jugularis, respectively, were studied because they are widely distributed along the coastline. Lineage 1 parasites had two common haplotypes with wide distribution and unique haplotypes in northern sites. Lineage 2 had only one common haplotype with wide distribution and a large number of unique haplotypes with greater genetic diversity. Both lineages have experienced recent population expansion. Only lineage 1 exhibited a genetic structure that was mainly associated with a biogeographical break at approximately 30 degrees S along the SEP. Our finding suggests that host access to different prey (=intermediate hosts) could affect the genetic structure of the parasite complex discovered here. Consequently, difference between these patterns suggests that factors other than DH dispersal are involved in the genetic structure of autogenic parasites. | 1231 | no | gd of parasite | NA | no | no |
| 1232 | 2017 | 43017 | Shrestha, MK; Golan-Goldhirsh, A; Ward, D | 2002 | Population genetic structure and the conservation of isolated populations of Acacia raddiana in the Negev Desert | There is much concern over the high mortality of many populations of Acacia raddiana, a keystone tree species in the Negev desert of Israel. We used random amplified polymorphic DNA (RAPD) to assess patterns of genetic variation within and among 12 populations of A. raddiana from the Arava (Syrian-African Rift) valley and western Negev. A high level of genetic polymorphism was recorded within populations. An analysis of molecular variance (AMOVA) showed that about 59.4% of total genetic variance occurred among populations, which is considerably greater population differentiation than that recorded for other outbreeding species. Cluster and principal coordinates analyses and AMOVA indicate that the western Negev and Arava valley populations are highly differentiated. We suggest that there may have been two invasions of A. raddiana into Israel: one across the northern Sinai/Gaza Strip area into the western Negev, with some plants reaching the Dead Sea and a second invasion across the southern part of the Sinai peninsula, or even from Saudi Arabia, up to the Arava valley. From the conservation point of view, each population should be conserved separately because they are genetically highly differentiated and loss of any one population would lead to a dramatic loss of genetic variation. The mixing of genetically distinct populations may give rise to outbreeding depression (particularly because of GXE interactions). An obvious first step to the maintenance of this species’ genetic diversity is the separate management of the western Negev and Arava valley populations because of their different evolutionary histories. (C) 2002 Elsevier Science Ltd. All rights reserved. | 1232 | no | no good fitness measure | NA | no | no |
| 1233 | 2017 | 43017 | Wang, L; Wang, ZK; Chen, JB; Liu, CY; Zhu, WL; Wang, LY; Meng, LH | 2015 | De Novo Transcriptome Assembly and Development of Novel Microsatellite Markers for the Traditional Chinese Medicinal Herb, Veratrilla baillonii Franch (Gentianaceae) | Veratrilla baillonii Franch is an important Chinese medicinal herb for treating liver-related diseases, which has been over-collected in the recent decades. However, the effective conservation and related population genetic study has been hindered because of the lack of genome sequences and genetic markers in the natural population. We have conducted RNA-seq on V. baillonii. We performed de novo assembly of these data to characterize the V. baillonii transcriptome, resulting in 133,019 contigs with size >200 bp. These contigs were annotated using the NCBI nonredundant database and Gene Ontology (GO) terms. From these contigs, we developed novel microsatellite simple sequence repeat (SSR) markers, identifying a total of 40,885 SSRs. SSRs with repeat motifs of 1-4 bp (mono-, di-, tri-, and tetranucleotides) accounted for 99.8% of all SSRs, with mononucleotide repeats most common, followed by dinucleotide (16.2%) and trinucleotide repeats (14.7%). We selected 151 SSRs for experimental validation, of which 74 were confirmed by polymerase chain reaction. Fourteen SSRs were determined to be polymorphic by screening 40 individuals from six distant populations. The number of alleles per locus ranged from two to four, and the expected heterozygosity varied from 0.2637 to 0.8571, suggesting that these SSR markers are highly polymorphic and effective for further genetic analysis in the nature population. In addition, we explored the genetic structure of V. baillonii using five SSRs in four geographic populations and found that the identified genotypes were clustered into two phylogenetic clades: the Mekong River clade and Jinsha River clade. This result indicates that these two regions may harbor highly divergent genetic lineages and enriched genetic diversity. The de novo transcriptome sequences and new SSR markers discovered by this study provide an initial step for understanding the population genetics of V. baillonii, and a valuable resource for effective conservation management. | 1233 | no | no good fitness measure | NA | no | no |
| 1234 | 2017 | 43017 | Siemens, DH; Roy, BA | 2005 | Tests for parasite-mediated frequency-dependent selection in natural populations of an asexual plant species | Genetic variation in plant populations for resistance to pathogens and herbivores might be maintained by parasite-mediated negative frequency-dependent selection (FDS). But it is difficult to observe the time-lagged oscillations between host and parasite genotypes that should result from FDS. To evaluate the potential for FDS, we tested for local adaptation of parasites to common clones, the role of host genetic diversity in resistance to parasites, and genetic correlations among fitness, parasitism, and the frequency of host clones. We studied three populations of Arabis holboellii, a short-lived apomictic (asexual by seed) plant attacked by rust fungi and insect herbivores. To estimate clone frequency, we used polymorphic allozyme markers on 200 individuals in each population in 1990 and in 2000. We also recorded levels of parasitism and host fitness (fruit production). Only the rust fungi showed evidence for local host adaptation; they usually increased in incidence as a function of clone frequency, and they tracked temporal change in clone frequency. In further support of FDS, parasitism was lower in populations with higher genetic diversity. However, total parasitism (herbivory and disease combined) decreased as host clone frequency and fitness increased. Thus, although the highly virulent rust pathogen showed potential for driving the cycles that result from FDS, this apparently does not occur in the populations studied because the host clones were also attacked by herbivores. | 1234 | probably yes | gd difference? | no good gd measure of host pop | no | yes |
| 1235 | 2017 | 43017 | ROY, BA | 1993 | PATTERNS OF RUST INFECTION AS A FUNCTION OF HOST GENETIC DIVERSITY AND HOST DENSITY IN NATURAL-POPULATIONS OF THE APOMICTIC CRUCIFER, ARABIS-HOLBOELLII | It is often assumed that genetic diversity contributes to reduced disease incidence in natural plant populations. However, little is known about the genetic structure of natural populations affected by disease. Here I present data from three apomictic (asexual) populations of Arabis holboellii infected by the rusts Puccinia monoica and P. thlaspeos. An average of 300 host individuals per population were genotyped (using seven variable allozyme loci) and scored for disease presence. Arabis holboellii populations are genetically diverse; the number of clones detected per population ranged from 6 to 27. There was substantial variation in frequency of host clones within and among sites, and significant variation among clones in susceptibility to the different rusts. Contrary to predictions based on frequency-dependent selection theory there was not a consistent positive relationship between clone frequency and disease incidence within any of the populations (Spearman’s r = -0.096, P > 0.5). In addition, clonally diverse populations did not necessarily have decreased disease incidence. The population with the lowest overall (both pathogens combined) disease incidence (7.5 +/- 1.9%) had the smallest number of clones (6), the lowest spatial variability, and the highest Arabis density. By comparison, another population had 22 clones, high spatial variability, low Arabis density and significantly more disease overall (16.8 +/- 2.7%). Although this study does not eliminate the possibility of frequency-dependent pathogen attack in these populations, the results suggest that it is likely to be weak or intermittent. | 1235 | yes | from thesis | crop | no | no |
| 1236 | 2017 | 43017 | Tzika, AC; Rosa, SFP; Fabiani, A; Snell, HL; Snell, HM; Marquez, C; Tapia, W; Rassmann, K; Gentile, G; Milinkovitch, MC | 2008 | Population genetics of Galapagos land iguana (genus Conolophus) remnant populations | The Galapagos land iguanas (genus Conolophus) have faced significant anthropogenic disturbances since the 17th century, leading to severe reduction of some populations and the extinction of others. Conservation activities, including the repatriation of captive-bred animals to depleted areas, have been ongoing since the late 1970s, but genetic information has not been extensively incorporated. Here we use nine species-specific microsatellite loci of 703 land iguanas from the six islands where the species occur today to characterize the genetic diversity within, and the levels of genetic differentiation among, current populations as well as test previous hypotheses about accidental translocations associated with early conservation efforts. Our analyses indicate that (i) five populations of iguanas represent distinct conservation units (one of them being the recently discovered rosada form) and could warrant species status, (ii) some individuals from North Seymour previously assumed to be from the natural Baltra population appear related to both Isabela and Santa Cruz populations, and (iii) the five different management units exhibit considerably different levels of intrapopulation genetic diversity, with the Plaza Sur and Santa Fe populations particularly low. Although the initial captive breeding programmes, coupled with intensive efforts to eradicate introduced species, saved several land iguana populations from extinction, our molecular results provide objective data for improving continuing in situ species survival plans and population management for this spectacular and emblematic reptile. | 1236 | no | no good fitness measure | NA | no | no |
| 1237 | 2017 | 43017 | Charbonnel, N; Angers, B; Rasatavonjizay, R; Bremond, P; Debain, C; Jarne, P | 2002 | The influence of mating system, demography, parasites and colonization on the population structure of Biomphalaria pfeifferi in Madagascar | Current evolutionary forces and historical processes interact to shape the distribution of neutral genetic variability within and among populations. Focusing on the genetics of recently introduced organisms offers a good opportunity to understand the relative importance of these factors. This study concerns variation at 8 polymorphic microsatellite loci in 30 populations of Biomphalaria pfeifferi. The sampling area spans most of the species’ range in Madagascar where it was probably introduced recently. Extremely low variation was found within all populations studied, which may partly result from high selfing rates. However, this cannot account for the variance of variation across populations, which is better explained by habitat openness (that reflects environmental stochasticity), the prevalence of the parasitic trematode Schistosoma mansoni and historical demography (colonization and subsequent bottlenecks). Large global differentiation was also observed, suggesting that current gene flow among populations is limited to small distances, within watersheds and to few individuals. Our data set also allowed us to test several hypotheses regarding colonization, based on bottleneck and admixture tests. The observed pattern requires at least two independent introductions from slightly differentiated genetic sources in the western part of Madagascar. Another introduction, from a very different genetic origin, should also be postulated to explain the genetic composition of eastern populations. That this introduction occurred recently suggests that the colonization of Madagascar by B. pfeifferi is an ongoing process. | 1237 | maybe | gd difference? | no good gd measure of host pop | no | no |
| 1238 | 2017 | 43017 | Vander Wal, E; Paquet, PC; Andres, JA | 2012 | Influence of landscape and social interactions on transmission of disease in a social cervid | The mechanisms of pathogen transmission are often social behaviours. These occur at local scales and are affected by landscape-scale population structure. Host populations frequently exist in patchy and isolated environments that create a continuum of genetic and social familiarity. Such variability has an important multispatial effect on pathogen spread. We assessed elk dispersal (i.e. likelihood of interdeme pathogen transmission) through spatially explicit genetic analyses. At a landscape scale, the elk population was composed of one cluster within a southeast-to-northwest cline spanning three spatially discrete subpopulations of elk across two protected areas in Manitoba (Canada). Genetic data are consistent with spatial variability in apparent prevalence of bovine tuberculosis (TB) in elk. Given the existing population structure, between-subpopulation spread of disease because of elk dispersal is unlikely. Furthermore, to better understand the risk of spread and distribution of the TB, we used a combination of close-contact logging biotelemetry and genetic data, which highlights how social intercourse may affect pathogen transmission. Our results indicate that close-contact interaction rate and duration did not covary with genetic relatedness. Thus, direct elk-to-elk transmission of disease is unlikely to be constrained to related individuals. That social intercourse in elk is not limited to familial groups provides some evidence pathogen transmission may be density-dependent. We show that the combination of landscape-scale genetics, relatedness and local-scale social behaviours is a promising approach to understand and predict landscape-level pathogen transmission within our system and within all social ungulate systems affected by transmissible diseases. | 1238 | no | no good fitness measure, N pop differnt in GD? | NA | no | no |
| 1239 | 2017 | 43017 | Harrill, AH; Watkins, PB; Su, S; Ross, PK; Harbourt, DE; Stylianou, IM; Boorman, GA; Russo, MW; Sackler, RS; Harris, SC; Smith, PC; Tennant, R; Bogue, M; Paigen, K; Harris, C; Contractor, T; Wiltshire, T; Rusyn, I; Threadgill, DW | 2009 | Mouse population-guided resequencing reveals that variants in CD44 contribute to acetaminophen-induced liver injury in humans | Interindividual variability in response to chemicals and drugs is a common regulatory concern. It is assumed that xenobiotic-induced adverse reactions have a strong genetic basis, but many mechanism-based investigations have not been successful in identifying susceptible individuals. While recent advances in pharmacogenetics of adverse drug reactions show promise, the small size of the populations susceptible to important adverse events limits the utility of whole-genome association studies conducted entirely in humans. We present a strategy to identify genetic polymorphisms that may underlie susceptibility to adverse drug reactions. First, in a cohort of healthy adults who received the maximum recommended dose of acetaminophen (4 g/d x 7d), we confirm that about one third of subjects develop elevations in serum alanine aminotransferase, indicative of liver injury. To identify the genetic basis for this susceptibility, a panel of 36 inbred mouse strains was used to model genetic diversity. Mice were treated with 300 mg/kg or a range of additional acetaminophen doses, and the extent of liver injury was quantified. We then employed whole-genome association analysis and targeted sequencing to determine that polymorphisms in Ly86, Cd44, Cd59a, and Capn8 correlate strongly with liver injury and demonstrated that dose-curves vary with background. Finally, we demonstrated that variation in the orthologous human gene, CD44, is associated with susceptibility to acetaminophen in two independent cohorts. Our results indicate a role for CD44 in modulation of susceptibility to acetaminophen hepatotoxicity. These studies demonstrate that a diverse mouse population can be used to understand and predict adverse toxicity in heterogeneous human populations through guided resequencing. | 1239 | no | No GD diff between pop | NA | no | no |
| 1240 | 2017 | 43017 | Rhone, B; Raquin, AL; Goldringer, I | 2007 | Strong linkage disequilibrium near the selected Yr17 resistance gene in a wheat experimental population | Dynamic management (DM) is a method of genetic resources conservation that aims at maintaining evolutionary process in subdivided populations cultivated in contrasted environments. Such populations are often submitted to strong natural selection as it was the case for experimental wheat populations maintained under DM. Understanding impacts of selection on genetic diversity around selected genes is necessary for the middle-term maintenance of genetic variability in DM populations. Evolution of diversity at six neutral markers located near the yellow rust resistance gene Yr17 has been studied for the parental lines and for generations 1, 5, 10 and 17 in one of the DM populations. Yr17 provided complete resistance to yellow rust in France until 1997 and thus was suspected to be under strong selection. The gene is located on a fragment introgressed in winter wheat from a wild species. The presence of the gene was estimated using a marker closely related to the gene. We showed that the Yr17 gene has been selected between generations 5 and 10. Generally, selection tends to reduce diversity around selected genes, generating linkage disequilibrium (LD) between a gene and adjacent markers. Here, the major effect of the Yr17 gene selection was a reduction of multilocus diversity and the maintenance of strong pre-existing LD in the zone surrounding the gene for a distance of 20 cM. As expected, the presence of the exogenous introgression was responsible for restrictions to recombination which contributed to the maintenance of strong correlations between loci. However, we found a noticeable number of recombinations around the gene indicating a progressive incorporation of the fragment into the wheat genome. | 1240 | no | no good fitness measure, N pop differnt in GD? | NA | no | no |
| 1241 | 2017 | 43017 | Irzykowska, L; Weber, Z; Bocianowski, J | 2012 | Comparison of Claviceps purpurea populations originated from experimental plots or fields of rye | The phytopathogenic ascomycete Claviceps purpurea causes the ergot - serious disease of rye and grasses. Its sclerotia containing toxic ergot alkaloids decrease a quality of cereal grain. The fungus infects young, unfertilized ovaries of the hosts. Due to the very short time in which infection can occur, growth rate of mycelium can play some role in the infection process. Resistance genes to C. purpurea have not been found so far. We estimate the mycelial growth rate and the genetic diversity of C. purpurea 45 isolates originated from rye plots in plant breeding stations with 47 isolates obtained from rye fields (AMOVA). To assess DNA polymorphism PCR-based methods were used. To our knowledge, this was the first application of SRAP marker system on Claviceps genus study. Among 92 isolates significant differences in growth rate were revealed. Some isolates grew two times faster than others what can increase their chance to infect flowers of rye. High genetic intraspecies variation was revealed. Four molecular markers were associated with the growth rate. The population originated from experimental plots displayed higher genetic variation than those from rye fields possibly due to genotypes variety of host plants in plant breeding stations. | 1241 | no | gd of pathogen | NA | no | no |
| 1242 | 2017 | 43017 | JUDSON, OP | 1995 | PRESERVING GENES - A MODEL OF THE MAINTENANCE OF GENETIC-VARIATION IN A METAPOPULATION UNDER FREQUENCY-DEPENDENT SELECTION | Understanding how genetic variability is maintained in natural populations is of both theoretical and practical interest. In particular, the subdivision of populations into demes linked by low levels of migration has been suggested to play an important role. But the maintenance of genetic variation in populations is also often linked to the maintenance of sexual reproduction: any force that acts to maintain sex should also act to maintain variation. One theory for the maintenance of sex, the Red Queen, states that sex and variation are maintained by antagonistic coevolutionary interactions - especially those between hosts and their harmful parasites - that give rise to negative frequency-dependent selection. In this paper I present a model to examine the relationships between population subdivision, negative frequency-dependent selection due to parasites, the maintenance of sex, and the preservation of alleles from fixation. The results show strong interactions between migration rates, negative frequency-dependent selection, and the maintenance of variability for sexual and asexual populations. | 1242 | no | no good fitness measure, N pop differnt in GD? | NA | no | no |
| 1243 | 2017 | 43017 | Lek, M; Karczewski, KJ; Minikel, EV; Samocha, KE; Banks, E; Fennell, T; O’Donnell-Luria, AH; Ware, JS; Hill, AJ; Cummings, BB; Tukiainen, T; Birnbaum, DP; Kosmicki, JA; Duncan, LE; Estrada, K; Zhao, FM; Zou, J; Pierce-Hollman, E; Berghout, J; Cooper, DN; Deflaux, N; DePristo, M; Do, R; Flannick, J; Fromer, M; Gauthier, L; Goldstein, J; Gupta, N; Howrigan, D; Kiezun, A; Kurki, MI; Moonshine, AL; Natarajan, P; Orozeo, L; Peloso, GM; Poplin, R; Rivas, MA; Ruano-Rubio, V; Rose, SA; Ruderfer, DM; Shakir, K; Stenson, PD; Stevens, C; Thomas, BP; Tiao, G; Tusie-Luna, MT; Weisburd, B; Won, HH; Yu, DM; Altshuler, DM; Ardissino, D; Boehnke, M; Danesh, J; Donnelly, S; Elosua, R; Florez, JC; Gabriel, SB; Getz, G; Glatt, SJ; Hultman, CM; Kathiresan, S; Laakso, M; NcCarroll, S; McCarthy, MI; McGovern, D; McPherson, R; Neale, BM; Palotie, A; Purcell, SM; Saleheen, D; Scharf, JM; Sklar, P; Sullivan, PF; Tuomilehto, J; Tsuang, MT; Watkins, HC; Wilson, JG; Daly, MJ; MacArthur, DG | 2016 | Analysis of protein-coding genetic variation in 60,706 humans | Large-scale reference data sets of human genetic variation are critical for the medical and functional interpretation of DNA sequence changes. Here we describe the aggregation and analysis of high-quality exome (protein-coding region) DNA sequence data for 60,706 individuals of diverse ancestries generated as part of the Exome Aggregation Consortium (ExAC). This catalogue of human genetic diversity contains an average of one variant every eight bases of the exome, and provides direct evidence for the presence of widespread mutational recurrence. We have used this catalogue to calculate objective metrics of pathogenicity for sequence variants, and to identify genes subject to strong selection against various classes of mutation; identifying 3,230 genes with near-complete depletion of predicted protein-truncating variants, with 72% of these genes having no currently established human disease phenotype. Finally, we demonstrate that these data can be used for the efficient filtering of candidate disease-causing variants, and for the discovery of human ‘knockout’ variants in protein-coding genes. | 1243 | no | human | NA | no | no |
| 1244 | 2017 | 43017 | HARTL, GB; MARKOWSKI, J; SWIATECKI, A; JANISZEWSKI, T; WILLING, R | 1992 | STUDIES ON THE EUROPEAN HARE .43. GENETIC DIVERSITY IN THE POLISH BROWN HARE LEPUS-EUROPAEUS PALLAS, 1778 - IMPLICATIONS FOR CONSERVATION AND MANAGEMENT | A total of 193 brown hares, collected from 7 sampling sites in Poland during 1986 - 1990 were examined for genetic variability and differentiation at 39 presumptive isozyme loci by means of horizontal starch gel electrophoresis. Values of polymorphism (mean P = 0.180, SD 0.039) and average heterozygosity (mean H = 0.047, SD 0.006) were similar to those detected in previous studies on the population genetics of the brown hare. Relative (G(ST) = 0.041) and absolute (mean D/Nei, 1978/ = 0.0012, SD 0.0013) genetic differentiation among populations were very low, which fits well to the high number of migrant individuals per generation (Nm = 12.7), estimated using the private allele method of Slatkin (1985). Average heterozygosity was examined for associations with geographical distribution, the year of culling, population density, age, sex, body weight and health status, whereby a better survival of heterozygous femals could be detected. According to our results, the present decline of the brown hare is not due to genetic depletion. However, once population sizes drop below a critical threshold, a pronounced inbreeding depression can be expected. | 1244 | maybe | pop level? | no fitness measure pop | no | no |
| 1245 | 2017 | 43017 | Lundholm, N; Ribeiro, S; Godhe, A; Nielsen, LR; Ellegaard, M | 2017 | Exploring the impact of multidecadal environmental changes on the population genetic structure of a marine primary producer | Many marine protists form resting stages that can remain viable in coastal sediments for several decades. Their long-term survival offers the possibility to explore the impact of changes in environmental conditions on population dynamics over multidecadal time scales. Resting stages of the phototrophic dinoflagellate Pentapharsodinium dalei were isolated and germinated from five layers in dated sediment cores from Koljo fjord, Sweden, spanning ca. 1910-2006. This fjord has, during the last century, experienced environmental fluctuations linked to hydrographic variability mainly driven by the North Atlantic Oscillation. Population genetic analyses based on six microsatellite markers revealed high genetic diversity and suggested that samples belonged to two clusters of subpopulations that have persisted for nearly a century. We observed sub-population shifts coinciding with changes in hydrographic conditions. The large degree of genetic diversity and the potential for both fluctuation and recovery over longer time scales documented here, may help to explain the long-term success of aquatic protists that form resting stages. | 1245 | no | no good fitness measure, N pop differnt in GD? | NA | no | no |
| 1246 | 2017 | 43017 | Moreira, RG; McCauley, RA; Cortes-Palomec, AC; Fernandes, GW; Oyama, K | 2010 | Spatial genetic structure of Coccoloba cereifera (Polygonaceae), a critically endangered microendemic species of Brazilian rupestrian fields | Coccoloba cereifera (Polygonaceae) is an extremely rare endemic shrub found exclusively in the rupestrian fields of Serra do Cip, southeastern, Brazil. We assessed the genetic diversity and structure across the single occurrence area of C. cereifera. The genetic variation at 13 microsatellite loci was estimated from 139 individuals sampled in nine patches. The number of alleles per locus varied from two to ten; the expected and observed heterozygosity ranged from 0.324 to 0.566 and 0.337 to 0.529, respectively. Microsatellites detected low but statistically significant levels of differentiation among patches (F (ST) = 0.123, R (ST) = 0.105), whereas Mantel test results showed a weak but significant pattern of isolation by distance (r (2) = 0.31, P < 0.002). Bayesian clustering indicated two subdivisions connected via admixture. Habitat heterogeneity across the drainage basin of the Rio Indequic, is likely limiting gene flow within patches of the geographically restricted population. While there is currently no evidence for a direct genetic risk to species survival, the apparent natural segregation occurring within the species could be exacerbated by future land use changes and the influx of alien species which could lead to demographic reductions in population size leading to a reduction in genetic diversity and an increase in population subdivision. We suggest that maintaining the integrity of the habitat within the small range of the species and continued monitoring of the effects of alien species would be the wisest use of management resources. | 1246 | no | no good fitness measure | NA | no | no |
| 1247 | 2017 | 43017 | Audigeos, D; Buonamici, A; Belkadi, L; Rymer, P; Boshier, D; Scotti-Saintagne, C; Vendramin, GG; Scotti, I | 2010 | Aquaporins in the wild: natural genetic diversity and selective pressure in the PIP gene family in five Neotropical tree species | Background: Tropical trees undergo severe stress through seasonal drought and flooding, and the ability of these species to respond may be a major factor in their survival in tropical ecosystems, particularly in relation to global climate change. Aquaporins are involved in the regulation of water flow and have been shown to be involved in drought response; they may therefore play a major adaptive role in these species. We describe genetic diversity in the PIP sub-family of the widespread gene family of Aquaporins in five Neotropical tree species covering four botanical families. Results: PIP Aquaporin subfamily genes were isolated, and their DNA sequence polymorphisms characterised in natural populations. Sequence data were analysed with statistical tests of standard neutral equilibrium and demographic scenarios simulated to compare with the observed results. Chloroplast SSRs were also used to test demographic transitions. Most gene fragments are highly polymorphic and display signatures of balancing selection or bottlenecks; chloroplast SSR markers have significant statistics that do not conform to expectations for population bottlenecks. Although not incompatible with a purely demographic scenario, the combination of all tests tends to favour a selective interpretation of extant gene diversity. Conclusions: Tropical tree PIP genes may generally undergo balancing selection, which may maintain high levels of genetic diversity at these loci. Genetic variation at PIP genes may represent a response to variable environmental conditions. | 1247 | no | no good fitness measure, N pop differnt in GD? | NA | no | no |
| 1248 | 2017 | 43017 | Pernin, P; Cariou, ML | 1997 | Evidence for clonal structure of natural populations of free-living amoebae of the genus Naegleria | Genetic diversity of natural populations of the two species Naegleria gruberi and N. australiensis collected at the same time in the same area was studied using multilocus enzyme electrophoresis (MLEE analysis). Reference strains and related species were also analysed for comparison. Five loci were found to be polymorphic and allowed identification of 19 different multilocus genotypes among 67 isolates - eight for N. gruberi and 11 for N. australiensis - none being common to the two species. Large departures from Hardy-Weinberg equilibrium were found at all the loci, with significant heterozygote deficiencies in most cases. The two species exhibited strong linkage disequilibrium and the predominance of a few multilocus genotypes within each species was observed. These lines of evidence strongly suggest non-random association of alleles, leading to the conclusion that N. gruberi and N. australiensis have predominantly clonal genetic structures in the wild. Populations consist of a few major clones with some closely related ones - a structure found for several parasitic protozoans and bacteria. This contrasts with the panmictic structure found for N. lovaniensis, another species of this genus, and suggests that closely related species within the genus have evolved to have different reproductive strategies. | 1248 | no | no good fitness measure | NA | no | no |
| 1249 | 2017 | 43017 | Malinowski, J; Goodloe, R; Brown-Gentry, K; Crawford, DC | 2015 | Cryptic relatedness in epidemiologic collections accessed for genetic association studies: experiences from the Epidemiologic Architecture for Genes Linked to Environment (EAGLE) study and the National Health and Nutrition Examination Surveys (NHANES) | “Epidemiologic collections have been a major resource for genotype-phenotype studies of complex disease given their large sample size, racial/ethnic diversity, and breadth and depth of phenotypes, traits, and exposures. A major disadvantage of these collections is they often survey households and communities without collecting extensive pedigree data. Failure to account for substantial relatedness can lead to inflated estimates and spurious associations. To examine the extent of cryptic relatedness in an epidemiologic collection, we as the Epidemiologic Architecture for Genes Linked to Environment (EAGLE) study accessed the National Health and Nutrition Examination Surveys (NHANES) linked to DNA samples (”“Genetic NHANES”“) from NHANES III and NHANES 1999-2002. NHANES are population-based cross-sectional surveys conducted by the National Center for Health Statistics at the Centers for Disease Control and Prevention. Genome-wide genetic data is not yet available in NHANES, and current data use agreements prohibit the generation of GWAS-level data in NHANES samples due issues in maintaining confidentiality among other ethical concerns. To date, only hundreds of single nucleotide polymorphisms (SNPs) genotyped in a variety of candidate genes are available for analysis in NHANES. We performed identity-by-descent (IBD) estimates in three self identified subpopulations of Genetic NHANES (non Hispanic white, non Hispanic black, and Mexican American) using PLINK software to identify potential familial relationships from presumed unrelated subjects. We then compared the PLINK-identified relationships to those identified by an alternative method implemented in Kinship based /Nference for Genome-wide association studies (KING). Overall, both methods identified familial relationships in NHANES III and NHANES 1999-2002 for all three subpopulations, but little concordance was observed between the two methods due in major part to the limited SNP data available in Genetic NHANES. Despite the lack of genome-wide data, our results suggest the presence of cryptic relatedness in this epidemiologic collection and highlight the limitations of restricted datasets such as NHANES in the context of modern day genetic epidemiology studies.” | 1249 | no | human | NA | no | no |
| 1250 | 2017 | 43017 | Jackson, ND; Fahrig, L | 2014 | Landscape context affects genetic diversity at a much larger spatial extent than population abundance | “Regional landscape context influences the fate of local populations, yet the spatial extent of this influence (called the”“scale of effect”“) is difficult to predict. Thus, a major problem for conservation management is to understand the factors governing the scale of effect such that landscape structure surrounding a focal area is measured and managed at the biologically relevant spatial scale. One unresolved question is whether and how scale of effect may depend on the population response measured (e.g., abundance vs. presence/absence). If scales of effect differ across population outcomes of a given species, management based on one outcome may compromise another, further complicating conservation decision making. Here we used an individual-based simulation model to investigate how scales of effect of landscapes that vary in the amount and fragmentation of habitat differ among three population responses (local abundance, presence/absence, and genetic diversity). We also explored how the population response measured affects the relative importance of habitat amount and fragmentation in shaping local populations, and how dispersal distance mediates the magnitude and spatial scale of these effects. We found that the spatial scale most strongly influencing local populations depended on the outcome measured and was predicted to be small for abundance, medium-sized for presence/absence, and large for genetic diversity. Increasing spatial scales likely resulted from increasing temporal scales over which outcomes were regulated (with local genetic diversity being regulated over the largest number of generations). Thus, multiple generations of dispersal and gene flow linked local population patterns to regional population size. The effects of habitat amount dominated the effects of fragmentation for all three outcomes. Increased dispersal distance strongly reduced abundance, but not presence/absence or genetic diversity. Our results suggest that managing protected species at spatial scales based on population abundance data may ignore broader landscape effects on population genetic diversity and persistence, lending support to the importance of managing large buffers surrounding areas of conservation concern.” | 1250 | no | NA | NA | no | no |
| 1251 | 2017 | 43017 | Liu, N; Liu, ZL; Gong, GS; Zhang, M; Wang, X; Zhou, Y; Qi, XB; Chen, HB; Yang, JZ; Luo, PG; Yang, CP | 2015 | Virulence Structure of Blumeria graminis f. sp tritici and Its Genetic Diversity by ISSR and SRAP Profiling Analyses | Blumeria graminis f. sp. tritici, which causes wheat powdery mildew, is an obligate biotrophic pathogen that can easily genetically adapt to its host plant. Understanding the virulence structure of and genetic variations in this pathogen is essential for disease control and for breeding resistance to wheat powdery mildew. This study investigated 17 pathogenic populations in Sichuan, China and classified 109 isolates into two distinct groups based on pathogenicity analysis: high virulence (HV, 92 isolates) and low virulence (LV, 17 isolates). Populations from Yibin (Southern region), Xichang (Western region), and Meishan (Middle region) showed lower virulence frequencies than populations from other regions. Many of the previously known resistance genes did not confer resistance in this study. The resistance gene Pm21 displayed an immune response to pathogenic challenge with all populations in Sichuan, and Pm13, Pm5b, Pm2+6, and PmXBD maintained resistance. AMOVA revealed significantly higher levels of variation within populations and lower levels of variation among populations within regions. High levels of gene flow were detected among populations in the four regions. Closely related populations within each region were distinguished by cluster analyses using ISSR and SRAP alleles. Both ISSR and SRAP allele profiling analyses revealed high levels of genetic diversity among pathogenic populations in Sichuan. Although ISSR and SRAP profiling analysis showed similar resolutions, the SRAP alleles appeared to be more informative. We did not detect any significant association between these alleles and the virulence or pathogenicity of the pathogen. Our results suggest that ISSR and SRAP alleles are more efficient for the characterization of small or closely related populations versus distantly related populations. | 1251 | no | gd of pathogen | NA | no | no |
| 1252 | 2017 | 43017 | Ramey, AM; Ely, CR; Schmutz, JA; Pearce, JM; Heard, DJ | 2012 | Molecular Detection of Hematozoa Infections in Tundra Swans Relative to Migration Patterns and Ecological Conditions at Breeding Grounds | Tundra swans (Cygnus columbianus) are broadly distributed in North America, use a wide variety of habitats, and exhibit diverse migration strategies. We investigated patterns of hematozoa infection in three populations of tundra swans that breed in Alaska using satellite tracking to infer host movement and molecular techniques to assess the prevalence and genetic diversity of parasites. We evaluated whether migratory patterns and environmental conditions at breeding areas explain the prevalence of blood parasites in migratory birds by contrasting the fit of competing models formulated in an occupancy modeling framework and calculating the detection probability of the top model using Akaike Information Criterion (AIC). We described genetic diversity of blood parasites in each population of swans by calculating the number of unique parasite haplotypes observed. Blood parasite infection was significantly different between populations of Alaska tundra swans, with the highest estimated prevalence occurring among birds occupying breeding areas with lower mean daily wind speeds and higher daily summer temperatures. Models including covariates of wind speed and temperature during summer months at breeding grounds better predicted hematozoa prevalence than those that included annual migration distance or duration. Genetic diversity of blood parasites in populations of tundra swans appeared to be relative to hematozoa prevalence. Our results suggest ecological conditions at breeding grounds may explain differences of hematozoa infection among populations of tundra swans that breed in Alaska. | 1252 | no | No GD diff between pop | NA | no | no |
| 1253 | 2017 | 43017 | Maquia, I; Ribeiro, NS; Silva, V; Bessa, F; Goulao, LF; Ribeiro, AI | 2013 | Genetic diversity of Brachystegia boehmii Taub. and Burkea africana Hook. f. across a fire gradient in Niassa National Reserve, northern Mozambique | Miombo woodlands constitute the most important type of vegetation in southern Africa, covering about 70% of the Zambezian phytoregion. This ecosystem, dominated by the genera Brachystegia, Julbernardia and Isoberlinia, has an immeasurable socio-economic and environmental value, playing a key role in formal and informal economies and in energy, water and carbon balances. Anthropogenic fires represent one of the major threats, compromising the stability of miombo. In this study we report on the usefulness of ISSR markers to assess, for the first time, the genetic diversity in two typical miombo species, Brachystegia boehmii Taub. and Burkea africana Hook. f. across a fire gradient in the Niassa National Reserve (NNR). According to our data, ISSR seem to be a suitable molecular marker’s system for biodiversity studies in both species, generating high levels of polymorphisms coupled with a convenient resolving power. The results point to a link between fire-tolerance and genetic diversity, as judged by the higher diversity levels observed in B. africana (fire-tolerant) and by the evolutive fire response of B. boehmii. Although fire differentially affects the biodiversity in each species, in general, the overall genetic diversity was high and their survival does not seem to be compromised by the frequency of fires, agreeing with the fact that NNR is one of the least disturbed areas of deciduous miombo. (C) 2013 Elsevier Ltd. All rights reserved. | 1253 | no | no good fitness measure | NA | no | no |
| 1254 | 2017 | 43017 | Urdaneta, L; Lal, A; Barnabe, C; Oury, B; Goldman, I; Ayala, FJ; Tibayrenc, M | 2001 | Evidence for clonal propagation in natural isolates of Plasmodium falciparum from Venezuela | “We have analyzed 75 isolates of Plasmodium falciparum. collected in Venezuela during both the dry (November) and rainy (May-July) seasons, with a range of genetic markers including antigen genes and 14 random amplified polymorphic DNA (RAPD) primers. Thirteen P, falciparum stocks from Kenya and four other Plasmodium species are included in the analysis for comparison. Cross-hybridization shows that the 14 RAPD primers reveal 14 separate regions of the parasite’s genome. The P, falciparum isolates are a monophyletic clade, significantly different from the other Plasmodium species. We identify three RAPD characters that could be useful as”“tags”" for rapid species identification, The Venezuelan genotypes fall into two discrete genetic subdivisions associated with either the dry or the rainy season | 1254 | no | gd of parasite | NA | no | no |
| 1255 | 2017 | 43017 | Takeuchi, T; Takahashi, J; Kiyoshi, T; Nomura, T; Tsubaki, Y | 2015 | Genetic differentiation in the endangered myrmecophilous butterfly Niphanda fusca: a comparison of natural and secondary habitats | Niphanda fusca is an endangered myrmecophilous butterfly inhabiting environments at early stages of succession. Most of its habitats are places where succession is prevented by human activity. In some places, however, N. fusca lives in natural semi-bare areas, such as cliffs in mountains or grasslands around volcanos. We investigate the genetic structure of N. fusca in Japan and South Korea to address two questions. (1) Are populations in natural environments genetically different from those in secondary environments? and (2) Do populations in natural environments possess greater genetic diversity than those in secondary environments? The AMOVA results indicated that the populations in natural environments and those in secondary environments were differentiated to some extent; however, more than 80 % of genetic variation was found to occur within the same habitat type and within each population. We found no differences in genetic diversity between populations in the two environments. At present, we have not found a strong reason to consider populations in the two environments as different evolutionarily significant units. We think it is practical to conserve populations in natural environments at first, because in this case we need not manage habitats to protect N. fusca. We have only to inhibit habitat destruction. In contrast, in order to conserve populations in secondary environments, we would have to continue managing the habitats. This is far more difficult than inhibiting habitat destruction. | 1255 | no | no good fitness measure, N pop differnt in GD? | NA | no | no |
| 1256 | 2017 | 43017 | Jones, MW; McParland, TL; Hutchings, JA; Danzmann, RG | 2001 | Low genetic variability in lake populations of brook trout (Salvelinus fontinalis): A consequence of exploitation? | Previous studies have found lower levels of genetic variation in lake than stream populations of brook trout (Salvelinus fontinalis). We test the generality of this observation by examining whether brook trout genetic variation at 10 allozyme loci differed within and among 9 pairs of lake and adjacent stream populations. With one exception, we found that lake populations had lower heterozygosity than their adjacent stream populations. Although the lakes in this study are small and some have had documented fish mortality events, no association was found between lake size characteristics and the degree of difference in heterozygosity between lakes and their adjacent stream populations. There were, however, negative associations between metrics of fishing mortality and the difference in heterozygosity between lakes and their adjacent stream populations. The greater the estimated fishing pressure on lake-dwelling trout, the greater the reduction in heterozygosity in those populations relative to their adjacent stream populations. We interpret our findings to suggest that intensive fishing pressure can significantly reduce genetic variation. Managers should therefore prevent human-induced mortality at any indication of a large natural mortality event to allow populations to increase in size as rapidly as possible following a decline. | 1256 | no | no good fitness measure | NA | no | no |
| 1257 | 2017 | 43017 | May, A; Hazelhurst, S; Li, YL; Norris, SA; Govind, N; Tikly, M; Hon, C; Johnson, KJ; Hartmann, N; Staedtler, F; Ramsay, M | 2013 | Genetic diversity in black South Africans from Soweto | Background: Due to the unparalleled genetic diversity of its peoples, Africa is attracting growing research attention. Several African populations have been assessed in global initiatives such as the International HapMap and 1000 Genomes Projects. Notably excluded, however, is the southern Africa region, which is inhabited predominantly by southeastern Bantu-speakers, currently suffering under the dual burden of infectious and non-communicable diseases. Limited reference data for these individuals hampers medical research and prevents thorough understanding of the underlying population substructure. Here, we present the most detailed exploration, to date, of genetic diversity in 94 unrelated southeastern Bantu-speaking South Africans, resident in urban Soweto (Johannesburg). Results: Participants were typed for similar to 4.3 million SNPs using the Illumina Omni5 beadchip. PCA and ADMIXTURE plots were used to compare the observed variation with that seen in selected populations worldwide. Results indicated that Sowetans, and other southeastern Bantu-speakers, are a clearly distinct group from other African populations previously investigated, reflecting a unique genetic history with small, but significant contributions from diverse sources. To assess the suitability of our sample as representative of Sowetans, we compared our results to participants in a larger rheumatoid arthritis case-control study. The control group showed good clustering with our sample, but among the cases were individuals who demonstrated notable admixture. Conclusions: Sowetan population structure appears unique compared to other black Africans, and may have clinical implications. Our data represent a suitable reference set for southeastern Bantu-speakers, on par with a HapMap type reference population, and constitute a prelude to the Southern African Human Genome Programme. | 1257 | no | human | NA | no | no |
| 1258 | 2017 | 43017 | Rinderer, TE; Stelzer, JA; Oldroyd, BP; Tingek, S | 1998 | Levels of polyandry and intracolonial genetic relationships in Apis koschevnikovi | Adults of five Apis koschevnikovi colonies from Sabah, Malaysia (Borneo) were collected into alcohol and within two days, transferred to liquid nitrogen. DNA was extracted, amplified with four, or in one case six, microsatellite primer pairs or primers for a sequence tagged site, and genotypes for these loci were determined for 40 to 100 workers per colony. Four colonies were monogynous, one colony had worker daughters of two, probably highly related, queens. For the monogynous colonies, the queen genotypes and the number and genotypes of males mated by each queen was inferred from the worker genotypes. The queens mated with 10 to 32 drones. The average number of observed matings was 16.3 +/- 10.5 and the average number of effective matings was 10.5 +/- 8.4. The within-colony genetic relatedness was 0.31 +/- 0.03. The fitness advantages of genetic variance are discussed. | 1258 | no | no good fitness measure | NA | no | no |
| 1259 | 2017 | 43017 | Franklin, D; Cardini, A; Oxnard, CE | 2010 | A Geometric Morphometric Approach to the Quantification of Population Variation in Sub-Saharan African Crania | We report here on new data examining cranial variation in 18 modern human sub-Saharan African populations. Previously, we investigated variation within southern Africa; we now extend our analyses to include a series of Central, East, and West African crania, to further knowledge of the relationships between, and variation and regional morphological patterning in, those populations. The sample comprises 377 male individuals; the three-dimensional coordinates of 96 landmarks are analyzed using Procrustes-based methods. Interpopulation variation is examined by calculating shape distances between groups, which are compared using resampling statistics and parametric tests. Phenotypic variance, as a proxy for genetic variance, is measured and compared across populations. Principal components and cluster analyses are employed to explore relationships between the populations. Shape differences are visualized using three-dimensional rendered models. Observed disparity patterns imply a mix of differences and similarities across populations, with no apparent support for genetic bottlenecks, which is likely a consequence of migrations that may have influenced differences in cranial form; supporting data are found in recent molecular studies. The Pygmy sample had the most distinctive cranial morphology; characteristically small in size with marked prognathism. These features characterized, although less strongly, the neighboring Bateke, and are possibly related to similar selective pressures in conjunction with interbreeding. Small cranial size is also involved in the considerable distinctiveness of the San and Khoikhoi. The statistical procedures applied in this study afford a powerful and robust means of quantifying and visualizing the magnitude and pattern of cranial variation between sub-Saharan African populations. Am. J. Hum. Biol. 22:23-35, 2010. (C) 2009 Wiley-Liss, Inc. | 1259 | no | human | NA | no | no |
| 1260 | 2017 | 43017 | Brock, PM; Goodman, SJ; Hall, AJ; Cruz, M; Acevedo-Whitehouse, K | 2015 | Context-dependent associations between heterozygosity and immune variation in a wild carnivore | Background: A multitude of correlations between heterozygosity and fitness proxies associated with disease have been reported from wild populations, but the genetic basis of these associations is unresolved. We used a longitudinal dataset on wild Galapagos sea lions (Zalophus wollebaeki) to develop a relatively new perspective on this problem, by testing for associations between heterozygosity and immune variation across age classes and between ecological contexts. Results: Homozygosity by locus was negatively correlated with serum immunoglobulin G production in pups (0-3 months of age), suggesting that reduced genetic diversity has a detrimental influence on the early development of immune defence in the Galapagos sea lion. In addition, homozygosity by locus was positively correlated with total circulating leukocyte concentration in juveniles (6-24 months of age), but only in a colony subject to the anthropogenic environmental impacts of development, pollution and introduced species, which suggests that reduced genetic diversity influences mature immune system activity in circumstances of high antigen exposure. Conclusions: These findings demonstrate the environmental context-dependency of the phenotypic expression of immune variation, which is implicit in the theory of ecoimmunology, but which has been rarely demonstrated in the wild. They also indicate that heterozygosity may be linked to the maintenance of heterogeneity in mammalian immune system development and response to infection, adding to the body of evidence on the nature of the mechanistic link between heterozygosity and fitness. | 1260 | no | no pop level? | NA | no | no |
| 1261 | 2017 | 43017 | Faria, PJ; Guedes, NMR; Yamashita, C; Martuscelli, P; Miyaki, CY | 2008 | Genetic variation and population structure of the endangered Hyacinth Macaw (Anodorhynchus hyacinthinus): implications for conservation | The Hyacinth Macaw (Anodorhynchus hyacinthinus) is one of 14 endangered species in the family Psittacidae occurring in Brazil, with an estimated total population of 6,500 specimens. We used nuclear molecular markers (single locus minisatellites and microsatellites) and 472 bp of the mitochondrial DNA control region to characterize levels of genetic variability in this species and to assess the degree of gene flow among three nesting sites in Brazil (Pantanal do Abobral, Pantanal de Miranda and Piaui). The origin of five apprehended specimens was also investigated. The results suggest that, in comparison to other species of parrots, Hyacinth Macaws possess relatively lower genetic variation and that individuals from two different localities within the Pantanal (Abobral and Miranda) belong to a unique interbreeding population and are genetically distinct at nuclear level from birds from the state of Piaui. The analyses of the five apprehended birds suggest that the Pantanal is not the source of birds for illegal trade, but their precise origin could not be assigned. The low genetic variability detected in the Hyacinth Macaw does not seem to pose a threat to the survival of this species. Nevertheless, habitat destruction and nest poaching are the most important factors negatively affecting their populations in the wild. The observed genetic structure emphasizes the need of protection of Hyacinth Macaws from different regions in order to maintain the genetic diversity of this species. | 1261 | no | no good fitness measure, N pop differnt in GD? | NA | no | no |
| 1262 | 2017 | 43017 | Tennessen, JA; Blouin, MS | 2008 | Balancing Selection at a Frog Antimicrobial Peptide Locus: Fluctuating Immune Effector Alleles? | Balancing selection is common on many defense genes, but it has rarely been reported for immune effector proteins such as antimicrobial peptides (AMPs). We describe genetic diversity at a brevinin-1 AMP locus in three species of leopard frogs (Rana pipiens, Rana blairi, and Rana palustris). Several highly divergent allelic lineages are segregating at this locus. That this unusual pattern results from balancing selection is demonstrated by multiple lines of evidence, including a ratio of nonsynonymous/synonymous polymorphism significantly higher than 1, the ZnS test, incongruence between the number of segregating sites and haplotype diversity, and significant Tajima’s D values. Our data are more consistent with a model of fluctuating selection in which alleles change frequencies over time than with a model of stable balancing selection such as overdominance. Evidence for fluctuating selection includes skewed allele frequencies, low levels of synonymous variation, nonneutral values of Tajima’s D within allelic lineages, an inverse relationship between the frequency of an allelic lineage and its degree of polymorphism, and divergent allele frequencies among populations. AMP loci could be important sites of adaptive genetic diversity, with consequences for host-pathogen coevolution and the ability of species to resist disease epidemics. | 1262 | no | no good fitness measure, N pop differnt in GD? | NA | no | no |
| 1263 | 2017 | 43017 | Saavedra-Sotelo, NC; Calderon-Aguilera, LE; Reyes-Bonilla, H; Paz-Garcia, DA; Lopez-Perez, RA; Cupul-Magana, A; Cruz-Barraza, JA; Rocha-Olivares, A | 2013 | Testing the genetic predictions of a biogeographical model in a dominant endemic Eastern Pacific coral (Porites panamensis) using a genetic seascape approach | The coral fauna of the Eastern Tropical Pacific (ETP) is depauperate and peripheral; hence, it has drawn attention to the factors allowing its survival. Here, we use a genetic seascape approach and ecological niche modeling to unravel the environmental factors correlating with the genetic variation of Porites panamensis, a hermatypic coral endemic to the ETP. Specifically, we test if levels of diversity and connectivity are higher among abundant than among depauperate populations, as expected by a geographically relaxed version of the Abundant Center Hypothesis (rel-ACH). Unlike the original ACH, referring to a geographical center of distribution of maximal abundance, the rel-ACH refers only to a center of maximum abundance, irrespective of its geographic position. The patterns of relative abundance of P. panamensis in the Mexican Pacific revealed that northern populations from Baja California represent its center of abundance; and southern depauperate populations along the continental margin are peripheral relative to it. Genetic patterns of diversity and structure of nuclear DNA sequences (ribosomal DNA and a single copy open reading frame) and five alloenzymatic loci partially agreed with rel-ACH predictions. We found higher diversity levels in peninsular populations and significant differentiation between peninsular and continental colonies. In addition, continental populations showed higher levels of differentiation and lower connectivity than peninsular populations in the absence of isolation by distance in each region. Some discrepancies with model expectations may relate to the influence of significant habitat discontinuities in the face of limited dispersal potential. Environmental data analyses and niche modeling allowed us to identify temperature, water clarity, and substrate availability as the main factors correlating with patterns of abundance, genetic diversity, and structure, which may hold the key to the survival of P. panamensis in the face of widespread environmental degradation. | 1263 | no | no good fitness measure | NA | no | no |
| 1264 | 2017 | 43017 | Brown, JA; Beatty, GE; Montgomery, WI; Provan, J | 2016 | Broad-scale genetic homogeneity in natural populations of common hazel (Corylus avellana) in Ireland | Hazel (Corylus avellana) has been a key species in European woodlands throughout the Holocene (10 KYA-present). Like many tree species, it is increasingly under threat from climate change, habitat loss and fragmentation, invasive species and emergent pathogens. As knowledge of the genetic structure of natural populations of trees is vital for managing these threats, as well as an essential basis for selection of material for replanting and restocking, we analysed levels and patterns of genetic diversity in the species at a range of spatial scales using high-resolution microsatellite markers. Our findings indicate that hazel populations exhibit high levels of genetic diversity along with low levels of population differentiation, suggesting extensive gene flow. Fine-scale genetic structuring was observed in some of the woodlands studied, probably resulting from restricted dispersal of the heavy nuts produced by the species. This, coupled with higher levels of pollen-mediated gene flow, resulted in a weak but significant pattern of isolation by distance. These results suggest that replanting following potential loss of hazel populations may not necessarily require the use of material from the same locality and mirror findings in other broadleaved tree species from the same area. | 1264 | no | no good fitness measure, N pop differnt in GD? | NA | no | no |
| 1265 | 2017 | 43017 | Kiesow, AM; Monroe, EM; Britten, HB | 2012 | Genetic structure of the arboreal squirrels (Glaucomys sabrinus and Tamiasciurus hudsonicus) in the North American Black Hills | We selected two isolated mammalian populations, the Black Hills northern flying squirrel (Glaucomys sabrinus (Shaw, 1801)) and red squirrel (Tamiasciurus hudsonicus (Erxleben, 1777)), to elucidate their genetic structure. We trapped both squirrels from 2005 to 2007, in three regions of the Black Hills, differing in geology and vegetation, to collect ear samples for genetic analyses. Microsatellite loci (northern flying (9) and red squirrel (13)) were used to examine genetic structure. Data analyses estimated genetic variability, substructure, and gene flow. Northern flying and red squirrel populations have allelic diversity and observed heterozygosity similar to other isolated populations. Each species shows weak substructure from STRUCTURE and GENELAND analyses, suggesting squirrel movements may be inhibited by topography or unsuitable habitat. Recent gene flow estimates from BAYESASS indicate that both species experience some within population gene flow and red squirrels may be more structured than northern flying squirrels because of lower migration rates. Concordant patterns of genetic structure in northern flying and red squirrels indicate that other species’ movements in the Black Hills may be affected by topography and habitat. Because their habitat is isolated in the Black Hills, management practices and conservation measures are recommended to promote viability and survival of each species. | 1265 | no | no good fitness measure | NA | no | no |
| 1266 | 2017 | 43017 | Zhang, NZ; Xu, Y; Huang, SY; Zhou, DH; Wang, RA; Zhu, XQ | 2014 | Sequence Variation in Toxoplasma gondii rop17 Gene among Strains from Different Hosts and Geographical Locations | Genetic diversity of T. gondii is a concern of many studies, due to the biological and epidemiological diversity of this parasite. The present study examined sequence variation in rhoptry protein 17 (ROP17) gene among T. gondii isolates from different hosts and geographical regions. The rop17 gene was amplified and sequenced from 10 T. gondii strains, and phylogenetic relationship among these T. gondii strains was reconstructed using maximum parsimony (MP), neighbor-joining (NJ), and maximum likelihood (ML) analyses. The partial rop17 gene sequences were 1375 bp in length and A+T contents varied from 49.45% to 50.11% among all examined T. gondii strains. Sequence analysis identified 33 variable nucleotide positions (2.1%), 16 of which were identified as transitions. Phylogeny reconstruction based on rop17 gene data revealed two major clusters which could readily distinguish Type I and Type II strains. Analyses of sequence variations in nucleotides and amino acids among these strains revealed high ratio of nonsynonymous to synonymous polymorphisms (>1), indicating that rop17 shows signs of positive selection. This study demonstrated the existence of slightly high sequence variability in the rop17 gene sequences among T. gondii strains from different hosts and geographical regions, suggesting that rop17 gene may represent a new genetic marker for population genetic studies of T. gondii isolates. | 1266 | no | gd of parasite | NA | no | no |
| 1267 | 2017 | 43017 | Lal, MM; Southgate, PC; Jerry, DR; Bosserelle, C; Zenger, KR | 2017 | Swept away: ocean currents and seascape features influence genetic structure across the 18,000 Km Indo-Pacific distribution of a marine invertebrate, the black-lip pearl oyster Pinctada margaritifera | Background: Genetic structure in many widely-distributed broadcast spawning marine invertebrates remains poorly understood, posing substantial challenges for their fishery management, conservation and aquaculture. Under the Core-Periphery Hypothesis (CPH), genetic diversity is expected to be highest at the centre of a species’ distribution, progressively decreasing with increased differentiation towards outer range limits, as populations become increasingly isolated, fragmented and locally adapted. The unique life history characteristics of many marine invertebrates such as high dispersal rates, stochastic survival and variable recruitment are also likely to influence how populations are organised. To examine the microevolutionary forces influencing population structure, connectivity and adaptive variation in a highly-dispersive bivalve, populations of the black-lip pearl oyster Pinctada margaritifera were examined across its similar to 18,000 km Indo-Pacific distribution. Results: Analyses utilising 9,624 genome-wide SNPs and 580 oysters, discovered differing patterns of significant and substantial broad-scale genetic structure between the Indian and Pacific Ocean basins. Indian Ocean populations were markedly divergent (F-st = 0.2534-0.4177, p < 0.001), compared to Pacific Ocean oysters, where basin-wide gene flow was much higher (F-st = 0.0007-0.1090, p < 0.001). Partitioning of genetic diversity (hierarchical AMOVA) attributed 18.1% of variance between ocean basins, whereas greater proportions were resolved within samples and populations (45.8% and 35.7% respectively). Visualisation of population structure at selectively neutral loci resolved three and five discrete genetic clusters for the Indian and Pacific Oceans respectively. Evaluation of genetic structure at adaptive loci for Pacific populations (89 SNPs under directional selection; Fst = 0.1012-0.4371, FDR = 0.05), revealed five clusters identical to those detected at neutral SNPs, suggesting environmental heterogeneity within the Pacific. Patterns of structure and connectivity were supported by Mantel tests of isolation by distance (IBD) and independent hydrodynamic particle dispersal simulations. Conclusions: It is evident that genetic structure and connectivity across the natural range of P. margaritifera is highly complex, and produced by the interaction of ocean currents, IBD and seascape features at a broad scale, together with habitat geomorphology and local adaptation at regional levels. Overall population organisation is far more elaborate than generalised CPH predictions, however valuable insights for regional fishery management, and a greater understanding of range-wide genetic structure in a highly-dispersive marine invertebrate have been gained. | 1267 | no | no good fitness measure | NA | no | no |
| 1268 | 2017 | 43017 | Sommerhalder, RJ; McDonald, BA; Zhan, JS | 2007 | Concordant evolution of mitochondrial and nuclear genomes in the wheat pathogen Phaeosphaeria nodorum | We compared patterns of mitochondrial restriction fragment length polymorphism (RFLP) diversity with patterns of nuclear RFLP diversity to investigate the effects of selection, gene flow, and sexual reproduction on the population genetic structure and evolutionary history of the wheat pathogen Phaeosphaeria nodorum. A total of 315 fungal isolates from Texas, Oregon, and Switzerland were analyzed using seven nuclear RFLP probes that hybridized to discrete loci and purified mitochondrial DNA that hybridized to the entire mtDNA genome. Forty-two different mitochondrial haplotypes and 298 different nuclear haplotypes were detected. The two most frequent mtDNA haplotypes were present in every population and represented 32% of all isolates. High levels of gene flow, low levels of population subdivision, no evidence for either host specificity or cyto-nuclear disequilibrium were inferred from the analysis of both genomes. The concordance in estimates of these population genetic parameters from both genomes suggests that the two genomes experienced similar degrees of migration, genetic drift and selection. (c) 2007 Elsevier Inc. All rights reserved. | 1268 | no | gd of pathogen | NA | no | no |
| 1269 | 2017 | 43017 | Pinho, C; Kaliontzopoulou, A; Harris, DJ; Ferrand, N | 2011 | Recent evolutionary history of the Iberian endemic lizards Podarcis bocagei (Seoane, 1884) and Podarcis carbonelli Perez-Mellado, 1981 (Squamata: Lacertidae) revealed by allozyme and microsatellite markers | Podarcis bocagei and Podarcis carbonelli are two species of wall lizards endemic to the western Iberian Peninsula. A detailed phylogeographical study based on mitochondrial DNA (mtDNA) variation has shown that they responded differently to the Quaternary climatic oscillations. These differences have been attributed to their distribution patterns: P. bocagei is distributed in the north of the Peninsula and in a continuous fashion, whereas P. carbonelli has a more southern and fragmented distribution. In this study, we assessed whether nuclear markers reveal similar evolutionary patterns to those inferred from mtDNA variation. We studied a battery of allozyme and microsatellite loci in a geographically representative set of individuals from both species. For each species we evaluated overall levels of differentiation, patterns of geographical variation in genetic diversity, genetic relationships amongst localities, and applied model-based individual multilocus genotype clustering approaches to detect hidden population structure. Our results for P. bocagei are highly concordant with the phylogeographical scenario inferred from mtDNA variation: we found very low levels of population differentiation, consistent with survival in a single glacial refugium, and detected signatures of a rapid demographic and geographical expansion. The analyses of nuclear markers furthermore helped to identify a probable refugial area, as well as expansion routes. Additionally, in concordance with observations based on mtDNA variation, a low level of population differentiation was observed in P. carbonelli, but this was significantly higher than in P. bocagei. However, the geographical basis for differentiation in P. carbonelli is highly inconsistent between mtDNA and nuclear markers, suggesting a complex, albeit recent, history of fragmentation. A recent reduction of this species’ distribution has probably erased the signatures of glacial isolation and post-glacial expansion that are normally found in other Iberian species, suggesting that the currently observed pattern of genetic differentiation in this species was shaped more by recent genetic drift than by the Pleistocene climatic oscillations. (C) 2011 The Linnean Society of London, Zoological Journal of the Linnean Society, 2011, 162, 184-200. doi: 10.1111/j.1096-3642.2010.00669.x | 1269 | no | no good fitness measure, N pop differnt in GD? | NA | no | no |
| 1270 | 2017 | 43017 | Bilic, I; Jaskulska, B; Souillard, R; Liebhart, D; Hess, M | 2014 | Multi-Locus Typing of Histomonas meleagridis Isolates Demonstrates the Existence of Two Different Genotypes | “Histomonas meleagridis is the aetiological agent of histomonosis or”“blackhead disease”“. Histomonosis is of special importance today, because there is no effective treatment to prevent its occurrence with considerable losses for the poultry industry. Despite its importance only a few molecular studies have yet been performed to investigate the degree of genetic diversity between different isolates of this parasite. In the present study a collection of well defined samples, previously shown positive for the DNA of H. meleagridis, was used to investigate genetic relatedness of the parasite. Samples originated from 25 turkey flocks collected in France between 2007 and 2010. Additionally, diagnostic samples, collected at our Clinic in Vienna, from different European countries and Azerbaijan, during 2010 to 2013 were included in the analyses. For the analysis three different genetic loci were analyzed: 18S rRNA, alpha-actinin1 and rpb1 genes. To amplify partial sequences of alpha-actinin1 and rpb1 genes, primers specifically targeting H. meleagridis were designed. Following PCR, the sequences of 18S rRNA, alpha-actinin1 and rpb1 loci were analyzed. Phylogenetic analyses demonstrated separation of H. meleagridis isolates in two different clusters. The majority of isolates grouped within the cluster 1 and originated from different European countries. The cluster 2 was rare and predominantly found in samples originating from France. Considering that the genetic variability of clusters can be seen as two distinct genetic types we propose the term genotype instead of cluster.” | 1270 | no | no good fitness measure, N pop differnt in GD? | NA | no | no |
| 1271 | 2017 | 43017 | Hansen, AA; Merrison, J; Nornberg, P; Lomstein, BA; Finster, K | 2005 | Activity and stability of a complex bacterial soil community under simulated Martian conditions | A simulation experiment with a complex bacterial soil community in a Mars simulation chamber was performed to determine the effect of Martian conditions on community activity, stability and survival. At three different depths in the soil core short-term effects of Martian conditions with and without ultraviolet (UV) exposure corresponding to 8 Martian Sol were compared. Community metabolic activities and functional diversity, measured as glucose respiration and versatility in substrate utilization, respectively, decreased after UV exposure, whereas they remained unaffected by Martian conditions without UV exposure. In contrast, the numbers of culturable bacteria and the genetic diversity were unaffected by the simulated Martian conditions both with and without UV exposure. The genetic diversity of the soil community and of the colonies grown on agar plates were evaluated by denaturant gradient gel electrophoresis (DGGE) on DNA extracts. Desiccation of the soil prior to experimentation affected the functional diversity by decreasing the versatility in substrate utilization. The natural dominance of endospores and Gram-positive bacteria in the investigated Mars-analogue soil may explain the limited effect of the Mars incubations on the survival and community structure. Our results suggest that UV radiation and desiccation are major selecting factors on bacterial functional diversity in terrestrial bacterial communities incubated under simulated Martian conditions. Furthermore, these results suggest that forward contamination of Mars is a matter of great concern in future space missions. | 1271 | no | no good fitness measure | NA | no | no |
| 1272 | 2017 | 43017 | Tullu, A; Diederichsen, A; Suvorova, G; Vandenberg, A | 2011 | Genetic and genomic resources of lentil: status, use and prospects | Extensive collections of lentil germplasm now exist in various genebanks around the world. This germplasm including wild Lens species has been used in plant introduction strategies and in efforts to widen the potential sources of increasing genetic diversity in the breeding programmes of lentil. Improved techniques are emerging to overcome hybridization barriers between species and as a result, interspecific hybrids have been successfully obtained between species. Several interspecific recombinant inbred line populations have been developed. Selected and backcrossed lentil lines are currently in advanced yield trial stages, and desirable traits such as yield, disease resistance and agronomic traits have been incorporated into cultivated lentil especially from Lens ervoides, generating a wider spectrum of variability. Secondly, further expansion of the overall pool of germplasm and examination of allelic variation at the nucleotide level will benefit lentil-breeding programmes by augmenting phenotype-based variation to further advance cultivar development. Genomic resources for lentil are limited now, but this situation is changing rapidly as the cost of genotyping has declined. As a result, two successive expressed sequence tags (EST) projects were undertaken under the NAPGEN EST project initiative (http://www.nrc-cnrc.gc.ca/eng/programs/pbi/plant-products/napgen/.htm) and an Agricultural Development Fund project initiative. We emphasize that creation of intraspecific and interspecific genetic populations, genetic maps, association maps, quantitative trait loci and marker-assisted selection technologies for implementation in the breeding programme will enhance deployment of genes responsible for traits of interest. The economical use of genomic technologies for use in germplasm resource management and genetic improvement is on the near horizon. | 1272 | no | no good fitness measure, N pop differnt in GD? | NA | no | no |
| 1273 | 2017 | 43017 | Burokiene, D; Prospero, S; Jung, E; Marciulyniene, D; Moosbrugger, K; Norkute, G; Rigling, D; Lygis, V; Schoebel, CN | 2015 | Genetic population structure of the invasive ash dieback pathogen Hymenoscyphus fraxineus in its expanding range | Introduced plant pathogens are increasingly recognized as a major threat to biodiversity and ecosystem functioning. One such pathogen, the causal agent of the devastating ash dieback in Europe, Hymenoscyphus fraxineus, was most likely introduced into Europe from eastern Asia in the 1990s. To investigate the genetic population structure of this invasive fungus at the epidemic disease front (Switzerland) and in the post-epidemic phase (Lithuania), a total of 847 H. fraxineus isolates were genotyped at 11 microsatellite loci. Among these isolates, 244 multilocus genotypes were found in five post-epidemic subpopulations (367 isolates) of the fungus and 263 in five epidemic subpopulations (480 isolates). No genetic differentiation was found between isolates recovered from bark lesions and fallen leaf petioles, which suggests that all H. fraxineus genotypes have the potential to induce bark infections on living trees and to survive saprophytically. Moreover, no genetic differentiation and no difference in genetic diversity were detected between the epidemic and post-epidemic populations. The entire genetic diversity present in the original founding populations in north-eastern Europe seems to have been transmitted to the epidemic disease front. Nonetheless, gene flow among post-epidemic subpopulations occurs slightly more random than among epidemic subpopulations. Furthermore, the probability of correct assignment of a particular H. fraxineus genotype to its subpopulation of origin was greater in Switzerland than in Lithuania. These two analyses point to weak founder effects at the disease front. | 1273 | no | gd of pathogen | NA | no | no |
| 1274 | 2017 | 43017 | Minot, S; Melo, MB; Li, F; Lu, D; Niedelman, W; Levine, SS; Saeij, JPJ | 2012 | Admixture and recombination among Toxoplasma gondii lineages explain global genome diversity | Toxoplasma gondii is a highly successful protozoan parasite that infects all warm-blooded animals and causes severe disease in immunocompromised and immune-naive humans. It has an unusual global population structure: In North America and Europe, isolated strains fall predominantly into four largely clonal lineages, but in South America there is great genetic diversity and the North American clonal lineages are rarely found. Genetic variation between Toxoplasma strains determines differences in virulence, modulation of host-signaling pathways, growth, dissemination, and disease severity in mice and likely in humans. Most studies on Toxoplasma genetic variation have focused on either a few loci in many strains or low-resolution genome analysis of three clonal lineages. We use whole-genome sequencing to identify a large number of SNPs between 10 Toxoplasma strains from Europe and North and South America. These were used to identify haplotype blocks (genomic regions) shared between strains and construct a Toxoplasma haplotype map. Additional SNP analysis of RNA-sequencing data of 26 Toxoplasma strains, representing global diversity, allowed us to construct a comprehensive genealogy for Toxoplasma gondii that incorporates sexual recombination. These data show that most current isolates are recent recombinants and cannot be easily grouped into a limited number of haplogroups. A complex picture emerges in which some genomic regions have not been recently exchanged between any strains, and others recently spread from one strain to many others. | 1274 | no | gd of parasite | NA | no | no |
| 1275 | 2017 | 43017 | Karakas, O; Gurel, F; Uncuoglu, AA | 2010 | Exploiting a wheat EST database to assess genetic diversity | Expressed sequence tag (EST) markers have been used to assess variety and genetic diversity in wheat (Triticum aestivum). In this study, 1549 ESTs from wheat infested with yellow rust were used to examine the genetic diversity of six susceptible and resistant wheat cultivars. The aim of using these cultivars was to improve the competitiveness of public wheat breeding programs through the intensive use of modern, particularly marker-assisted, selection technologies. The F 2 individuals derived from cultivar crosses were screened for resistance to yellow rust at the seedling stage in greenhouses and adult stage in the field to identify DNA markers genetically linked to resistance. Five hundred and sixty ESTs were assembled into 136 contigs and 989 singletons. BlastX search results showed that 39 (29%) contigs and 96 (10%) singletons were homologous to wheat genes. The database-matched contigs and singletons were assigned to eight functional groups related to protein synthesis, photosynthesis, metabolism and energy, stress proteins, transporter proteins, protein breakdown and recycling, cell growth and division and reactive oxygen scavengers. PCR analyses with primers based on the contigs and singletons showed that the most polymorphic functional categories were photosynthesis (contigs) and metabolism and energy (singletons). EST analysis revealed considerable genetic variability among the Turkish wheat cultivars resistant and susceptible to yellow rust disease and allowed calculation of the mean genetic distance between cultivars, with the greatest similarity (0.725) being between Harmankaya99 and SOnmez2001, and the lowest (0.622) between Aytin98 and Izgi01. | 1275 | no | crop | NA | no | no |
| 1276 | 2017 | 43017 | Redon, R; Ishikawa, S; Fitch, KR; Feuk, L; Perry, GH; Andrews, TD; Fiegler, H; Shapero, MH; Carson, AR; Chen, WW; Cho, EK; Dallaire, S; Freeman, JL; Gonzalez, JR; Gratacos, M; Huang, J; Kalaitzopoulos, D; Komura, D; MacDonald, JR; Marshall, CR; Mei, R; Montgomery, L; Nishimura, K; Okamura, K; Shen, F; Somerville, MJ; Tchinda, J; Valsesia, A; Woodwark, C; Yang, FT; Zhang, JJ; Zerjal, T; Zhang, J; Armengol, L; Conrad, DF; Estivill, X; Tyler-Smith, C; Carter, NP; Aburatani, H; Lee, C; Jones, KW; Scherer, SW; Hurles, ME | 2006 | Global variation in copy number in the human genome | Copy number variation (CNV) of DNA sequences is functionally significant but has yet to be fully ascertained. We have constructed a first-generation CNV map of the human genome through the study of 270 individuals from four populations with ancestry in Europe, Africa or Asia (the HapMap collection). DNA from these individuals was screened for CNV using two complementary technologies: single-nucleotide polymorphism (SNP) genotyping arrays, and clone-based comparative genomic hybridization. A total of 1,447 copy number variable regions (CNVRs), which can encompass overlapping or adjacent gains or losses, covering 360 megabases (12% of the genome) were identified in these populations. These CNVRs contained hundreds of genes, disease loci, functional elements and segmental duplications. Notably, the CNVRs encompassed more nucleotide content per genome than SNPs, underscoring the importance of CNV in genetic diversity and evolution. The data obtained delineate linkage disequilibrium patterns for many CNVs, and reveal marked variation in copy number among populations. We also demonstrate the utility of this resource for genetic disease studies. | 1276 | no | human | NA | no | no |
| 1277 | 2017 | 43017 | Lesbarreres, D; Primmer, CR; Lode, T; Merila, J | 2006 | The effects of 20 years of highway presence on the genetic structure of Rana dalmatina populations | Habitat destruction and fragmentation caused by highways can negatively affect animal populations, but a better understanding of the effects of highways on population genetic structure is still needed to improve conservation plans in urbanized landscapes. We investigated the degree of genetic variability and differentiation within and among seven Rana dalmatina populations located far from highly trafficked roads (non-fragmented populations) and four populations sampled on both sides of a major highway (fragmented populations). The degree of population subdivision was significantly higher among fragmented (F-ST = 0.238) as compared to non-fragmented populations (F-ST= 0.022). Furthermore, in the four fragmented populations, significantly lower allelic richness as compared to non-fragmented populations was observed. Together with potential high levels of road mortality leading to smaller population size, these results suggest that separation by highways not only has reduced the genetic diversity and polymorphism in local populations over two decades, but also has resulted in a higher degree of population differentiation, most likely due to genetic drift. | 1277 | no | no good fitness measure, N pop differnt in GD? | NA | no | no |
| 1278 | 2017 | 43017 | Booth, W; Montgomery, WI; Prodohl, PA | 2007 | Polyandry by wood mice in natural populations | Multiple paternity was investigated for the first time in natural populations of the wood mouse Apodemus sylvaticus. Thirteen females and their respective litters sampled within distinct habitats, seasons and years were screened for eight microsatellite loci. Allelic variation was compared with a dataset comprising 307 adult mice collected from the same source populations as pregnant females. Multiple paternity was unambiguously identified in seven litters (53.8%). In each case, a minimum of two or three male parents were involved. Populations of A. sylvaticus inhabiting the northern latitudes of the species range are characterized by annual cycles of abundance during which numbers can fluctuate by several orders of magnitude. Hence, the discovery of multiple paternity within litters sampled between May and July (high and low densities, respectively) in all years suggests that polyandry maximizes genetic diversity of the litter and, hence, survival of some of the offspring through such cycles. The results indicate that polyandry is a common mode of reproduction within wild populations of A. sylvaticus. | 1278 | no | no good fitness measure | NA | no | no |
| 1279 | 2017 | 43017 | Tiffin, P; Hacker, R; Gaut, BS | 2004 | Population genetic evidence for rapid changes in intraspecific diversity and allelic cycling of a specialist defense gene in Zea | Two patterns of plant defense gene evolution are emerging from molecular population genetic surveys. One is that specialist defenses experience stronger selection than generalist defenses. The second is that specialist defenses are more likely to be subject to balancing selection, i.e., evolve in a manner consistent with balanced-polymorphism or trench-warfare models of host-parasite coevolution. Because most of the data of specialist defenses come from Arabidopsis thaliana, we examined the genetic diversity and evolutionary history of three defense genes in two outcrossing species, the autotetraploid Zea perennis and its most closely related extant relative the diploid Z. diploperennis. Intraspecific diversity at two generalist defenses, the protease inhibitors wip1 and mpi, were consistent with a neutral model. Like previously studied genes in these taxa, wip1 and mpi harbored similar levels of diversity in Z. diploperennis and Z. perennis. In contrast, the specialist defense hm2 showed strong although distinctly different departures from a neutral model in the two species. Z. diploperennis appears to have experienced a strong and recent selective sweep. Using a rejection-sampling coalescent method, we estimate the strength of selection on Z. diploperennis hm2 to be similar to3.0%, which is approximately equal to the strength of selection on tb1 during maize domestication. Z. perennis hm2 harbors three highly diverged alleles, two of which are found at high frequency. The distinctly different patterns of diversity may be due to differences in the phase of host-parasite coevolutionary cycles, although higher hm2 diversity in Z. perennis may also reflect reduced efficacy of selection in the autotetraploid relative to its diploid relative. | 1279 | no | no good fitness measure, N pop differnt in GD? | NA | no | no |
| 1280 | 2017 | 43017 | Perez-Morales, R; Mendez-Ramirez, I; Castro-Hernandez, C; Martinez-Ramirez, OC; Gonsebatt, ME; Rubio, J | 2011 | Polymorphisms associated with the risk of lung cancer in a healthy Mexican Mestizo population: Application of the additive model for cancer | Lung cancer is the leading cause of cancer mortality in Mexico and worldwide. In the past decade, there has been an increase in the number of lung cancer cases in young people, which suggests an important role for genetic background in the etiology of this disease. In this study, we genetically characterized 16 polymorphisms in 12 low penetrance genes (AhR, CYP1A1, CYP2E1, EPHX1, GSTM1, GSTT1, GSTPI, XRCC1, ERCC2, MGMT, CCND1 and TP53) in 382 healthy Mexican Mestizos as the first step in elucidating the genetic structure of this population and identifying high risk individuals. All of the genotypes analyzed were in Hardy-Weinberg equilibrium, but different degrees of linkage were observed for polymorphisms in the CYP1A1 and EPHX1 genes. The genetic variability of this population was distributed in six clusters that were defined based on their genetic characteristics. The use of a polygenic model to assess the additive effect of low penetrance risk alleles identified combinations of risk genotypes that could be useful in predicting a predisposition to lung cancer. Estimation of the level of genetic susceptibility showed that the individual calculated risk value (iCRV) ranged from 1 to 16, with a higher iCRV indicating a greater genetic susceptibility to lung cancer. | 1280 | no | human | NA | no | no |
| 1281 | 2017 | 43017 | Suefuji, M; Heinze, J | 2015 | Degenerate slave-makers, but nevertheless slave-makers? Host worker relatedness in the ant Myrmoxenus kraussei | Socially parasitic ants of the formicoxenine genus Myrmoxenus exhibit considerable diversity in colony structure and life history. While some species are active slave-makers with many workers and others are workerless murder-parasites,’ Myrmoxenus kraussei is considered as a degenerate slave-maker’ because of its very low worker numbers. Here, we document that Temnothorax recedens host workers in single colonies of M. kraussei from Lago di Garda, Italy, exhibit significantly more genetic diversity than workers in unparasitized colonies. This raises the possibility that, despite its low worker numbers, M. kraussei may actively engage in slave raids in nature. | 1281 | probably yes | ok gd and fitness measurement? | no good fitness measurement (colony parasitism)? | no | no |
| 1282 | 2017 | 43017 | Gaur, A; Shailaja, K; Singh, A; Arunabala, V; Satyarebala, B; Singh, L | 2006 | Twenty polymorphic microsatellite markers in the Asiatic lion (Panthera leo persica) | The Asiatic lion (Panthera leo persica) is driven to a single habitat in Gir forests in India for its survival. In order to devise adequate conservation and management strategies for this critically endangered species, it is important to characterize its genetic diversity and understand its population structure. Here we report twenty microsatellite loci, in addition to seven reported earlier, from the genome of a pure Asiatic lion. The microsatellite loci described here will provide potentially useful markers for the assessment of genetic variability in the only existing wild population of the Asiatic lions and other big cat species. | 1282 | no | no pop level? | NA | no | no |
| 1283 | 2017 | 43017 | Sarris, PF; Trantas, EA; Mpalantinaki, E; Ververidis, F; Goumas, DE | 2012 | Pseudomonas viridiflava, a Multi Host Plant Pathogen with Significant Genetic Variation at the Molecular Level | The pectinolytic species Pseudomonas viridiflava has a wide host range among plants, causing foliar and stem necrotic lesions and basal stem and root rots. However, little is known about the molecular evolution of this species. In this study we investigated the intraspecies genetic variation of P. viridiflava amongst local (Cretan), as well as international isolates of the pathogen. The genetic and phenotypic variability were investigated by molecular fingerprinting (rep-PCR) and partial sequencing of three housekeeping genes (gyrB, rpoD and rpoB), and by biochemical and pathogenicity profiling. The biochemical tests and pathogenicity profiling did not reveal any variability among the isolates studied. However, the molecular fingerprinting patterns and housekeeping gene sequences clearly differentiated them. In a broader phylogenetic comparison of housekeeping gene sequences deposited in GenBank, significant genetic variability at the molecular level was found between isolates of P. viridiflava originated from different host species as well as among isolates from the same host. Our results provide a basis for more comprehensive understanding of the biology, sources and shifts in genetic diversity and evolution of P. viridiflava populations and should support the development of molecular identification tools and epidemiological studies in diseases caused by this species. | 1283 | no | gd of pathogen | NA | no | no |
| 1284 | 2017 | 43017 | DEAN, M; STEPHENS, JC; WINKLER, C; LOMB, DA; RAMSBURG, M; BOAZE, R; STEWART, C; CHARBONNEAU, L; GOLDMAN, D; ALBAUGH, BJ; GOEDERT, JJ; BEASLEY, RP; HWANG, LY; BUCHBINDER, S; WEEDON, M; JOHNSON, PA; EICHELBERGER, M; OBRIEN, SJ | 1994 | POLYMORPHIC ADMIXTURE TYPING IN HUMAN ETHNIC POPULATIONS | A panel of 257 RFLP loci was selected on the basis of high heterozygosity in Caucasian DNA surveys and equivalent spacing throughout the human genome. Probes from each locus were used in a Southern blot survey of allele frequency distribution for four human ethnic groups: Caucasian, African American, Asian (Chinese), and American Indian (Cheyenne). Nearly all RFLP loci were polymorphic in each group, albeit with a broad range of differing allele frequencies (delta). The distribution of frequency differences (delta values) was used for three purposes: (1) to provide estimates for genetic distance (differentiation) among these ethnic groups, (2) to revisit with a large data set the proportion of human genetic variation attributable to differentiation within ethnic groups, and (3) to identify loci with high delta values between recently admired populations of use in mapping by admixture linkage disequilibrium (MALD). Although most markers display significant allele frequency differences between ethnic groups, the overall genetic distances between ethnic groups were small(.066-.098), and <10% of the measured overall molecular genetic diversity in these human samples can be attributed to ‘’racial’’ differentiation. The median 6 values for pairwise comparisons between groups fell between .15 and .20, permitting identification of highly informative RFLP loci for MALD disease association studies. | 1284 | no | human | NA | no | no |
| 1285 | 2017 | 43017 | Horner, AA; Hoffman, EA; Tye, MR; Hether, TD; Savage, AE | 2017 | Cryptic chytridiomycosis linked to climate and genetic variation in amphibian populations of the southeastern United States | North American amphibians have recently been impacted by two major emerging pathogens, the fungus Batrachochytrium dendrobatidis (Bd) and iridoviruses in the genus Ranavirus (Rv). Environmental factors and host genetics may play important roles in disease dynamics, but few studies incorporate both of these components into their analyses. Here, we investigated the role of environmental and genetic factors in driving Bd and Rv infection prevalence and severity in a biodiversity hot spot, the southeastern United States. We used quantitative PCR to characterize Bd and Rv dynamics in natural populations of three amphibian species: Notophthalmus perstriatus, Hyla squirella and Pseudacris ornata. We combined pathogen data, genetic diversity metrics generated from neutral markers, and environmental variables into general linear models to evaluate how these factors impact infectious disease dynamics. Occurrence, prevalence and intensity of Bd and Rv varied across species and populations, but only one species, Pseudacris ornata, harbored high Bd intensities in the majority of sampled populations. Genetic diversity and climate variables both predicted Bd prevalence, whereas climatic variables alone predicted infection intensity. We conclude that Bd is more abundant in the southeastern United States than previously thought and that genetic and environmental factors are both important for predicting amphibian pathogen dynamics. Incorporating both genetic and environmental information into conservation plans for amphibians is necessary for the development of more effective management strategies to mitigate the impact of emerging infectious diseases. | 1285 | no | gd of pathogen | NA | no | no |
| 1286 | 2017 | 43017 | Mattison, SM; Brown, MJ; Floyd, B; Feldman, MW | 2015 | Adoption Does Not Increase the Risk of Mortality among Taiwanese Girls in a Longitudinal Analysis | Adopted children often experience health and well-being disadvantages compared to biological children remaining in their natal households. The degree of genetic relatedness is thought to mediate the level of parental investment in children, leading to poorer outcomes of biologically unrelated children. We explore whether mortality is related to adoption in a historical Taiwanese population where adoption rarely occurred among kin. Using Cox proportional hazards models in which adoption is included as a time-dependent covariate, we show that adoption of girls does not increase the risk of mortality, as previously suggested; in fact, it is either protective or neutral with respect to mortality. These results suggest that socio-structural variables may produce positive outcomes for adopted children, even compared to biological children who remain in the care of their parents. | 1286 | no | human | NA | no | no |
| 1287 | 2017 | 43017 | Herraez, DL; Bauchet, M; Tang, K; Theunert, C; Pugach, I; Li, J; Nandineni, MR; Gross, A; Scholz, M; Stoneking, M | 2009 | Genetic Variation and Recent Positive Selection in Worldwide Human Populations: Evidence from Nearly 1 Million SNPs | Background: Genome-wide scans of hundreds of thousands of single-nucleotide polymorphisms (SNPs) have resulted in the identification of new susceptibility variants to common diseases and are providing new insights into the genetic structure and relationships of human populations. Moreover, genome-wide data can be used to search for signals of recent positive selection, thereby providing new insights into the genetic adaptations that occurred as modern humans spread out of Africa and around the world. Methodology: We genotyped approximately 500,000 SNPs in 255 individuals (5 individuals from each of 51 worldwide populations) from the Human Genome Diversity Panel (HGDP-CEPH). When merged with non-overlapping SNPs typed previously in 250 of these same individuals, the resulting data consist of over 950,000 SNPs. We then analyzed the genetic relationships and ancestry of individuals without assigning them to populations, and we also identified candidate regions of recent positive selection at both the population and regional (continental) level. Conclusions: Our analyses both confirm and extend previous studies; in particular, we highlight the impact of various dispersals, and the role of substructure in Africa, on human genetic diversity. We also identified several novel candidate regions for recent positive selection, and a gene ontology (GO) analysis identified several GO groups that were significantly enriched for such candidate genes, including immunity and defense related genes, sensory perception genes, membrane proteins, signal receptors, lipid binding/metabolism genes, and genes involved in the nervous system. Among the novel candidate genes identified are two genes involved in the thyroid hormone pathway that show signals of selection in African Pygmies that may be related to their short stature. | 1287 | no | human | NA | no | no |
| 1288 | 2017 | 43017 | Craul, M; Chikhi, L; Sousa, V; Olivieri, GL; Rabesandratana, A; Zimmermann, E; Radespiel, U | 2009 | Influence of forest fragmentation on an endangered large-bodied lemur in northwestern Madagascar | Madagascar’s diverse and mostly endemic fauna and flora suffer from recent landscape changes that are primarily caused by high levels of human activities. The loss and fragmentation of forest habitats are well known consequences of human activities. In this study, we investigate the effects of forest fragmentation on presence, abundance and genetic diversity in a larger-bodied lemur species, Lepilemur edwardsi, in northwestern Madagascar. In addition, we characterized the genetic differentiation among populations and demographic changes. We found L. edwardsi at only 13 (76.5%) of 17 visited sites, I I of which were situated in the Ankarafantsika National Park (ANP). We captured between two and 17 individuals per site. We sequenced the mtDNA d-loop of all samples and genotyped 14 microsatellite loci in two exemplary populations for demographic analyses. A negative influence on forest fragmentation could be detected, since the fragments had a lower genetic diversity than sites in the ANP. Genetic differentiation between populations ranged from low to high but was almost always significant. A typical pattern of isolation-by-distance could not be detected and the data could rather be interpreted as results of random genetic drift. The data furthermore revealed signals of a demographic collapse of about two orders of magnitude in the two exemplary sites. This decline probably started during the last few hundred years of intensified human disturbances and population growth. Given the results of this study, urgent conservation actions are needed and should concentrate on an effective protection of the few remaining populations in order to ensure the long-term survival of L. edwardsi. (C) 2009 Elsevier Ltd. All rights reserved. | 1288 | no | no good fitness measure | NA | no | no |
| 1289 | 2017 | 43017 | Huang, MH; Dornhaus, A | 2008 | A meta-analysis of ant social parasitism: host characteristics of different parasitism types and a test of Emery’s rule | 1. In ant social parasitism, the process by which parasite-host systems evolved and the types of invasion mechanisms parasites use are being debated. Emery’s rule, for example, states that social parasites are the closest relatives to their hosts. The present study uses previously published data to test whether Emery’s rule applies equally to all parasitism types (i.e. xenobiosis, temporary, dulosis, and inquilinism). In addition, this study also investigates other links between parasite-host relatedness and host biology, which has implications for understanding the invasion mechanisms used by certain parasites. 2. We find that xenobiotic parasites typically use distantly-related host species that are of at least medium colony size. Temporary parasites often have multiple host species that are very closely related to the parasite and hosts with medium-size colonies. Dulotic parasites frequently have multiple host species that are slightly less related and of any size. Lastly, inquiline parasites tend to have a single, very closely related, host species with medium-size colonies. 3. Parasites tend to be more closely related to host species if they have a single host species or when the host has a large colony size. In contrast, parasites with multiple host species or hosts of small colony size tend to be less related to their hosts. 4. This study is the first to examine trends in ant social parasitism across all known parasite species. Our meta-analysis shows that Emery’s rule applies to inquilinism and temporary parasitism, but not to dulosis and xenobiosis. Our results also suggest that both parasitism type and parasite-host relatedness predict the number of hosts and host colony size. It may be that a chemical mimicry mechanism allows invasion of large host colonies, but requires close relatedness of parasite and host, and concentration on a single host species. | 1289 | no | NA | NA | no | no |
| 1290 | 2017 | 43017 | Ascunce, MS; Toups, MA; Kassu, G; Fane, J; Scholl, K; Reed, DL | 2013 | Nuclear Genetic Diversity in Human Lice (Pediculus humanus) Reveals Continental Differences and High Inbreeding among Worldwide Populations | Understanding the evolution of parasites is important to both basic and applied evolutionary biology. Knowledge of the genetic structure of parasite populations is critical for our ability to predict how an infection can spread through a host population and for the design of effective control methods. However, very little is known about the genetic structure of most human parasites, including the human louse (Pediculus humanus). This species is composed of two ecotypes: the head louse (Pediculus humanus capitis De Geer), and the clothing (body) louse (Pediculus humanus humanus Linnaeus). Hundreds of millions of head louse infestations affect children every year, and this number is on the rise, in part because of increased resistance to insecticides. Clothing lice affect mostly homeless and refugee-camp populations and although they are less prevalent than head lice, the medical consequences are more severe because they vector deadly bacterial pathogens. In this study we present the first assessment of the genetic structure of human louse populations by analyzing the nuclear genetic variation at 15 newly developed microsatellite loci in 93 human lice from 11 sites in four world regions. Both ecotypes showed heterozygote deficits relative to Hardy-Weinberg equilibrium and high inbreeding values, an expected pattern given their parasitic life history. Bayesian clustering analyses assigned lice to four distinct genetic clusters that were geographically structured. The low levels of gene flow among louse populations suggested that the evolution of insecticide resistance in lice would most likely be affected by local selection pressures, underscoring the importance of tailoring control strategies to population-specific genetic makeup and evolutionary history. Our panel of microsatellite markers provides powerful data to investigate not only ecological and evolutionary processes in lice, but also those in their human hosts because of the long-term coevolutionary association between lice and humans. | 1290 | no | gd of parasite | NA | no | no |
| 1291 | 2017 | 43017 | Maside, X; Gomez-Moracho, T; Jara, L; Martin-Hernandez, R; De la Rua, P; Higes, M; Bartolome, C | 2015 | Population Genetics of Nosema apis and Nosema ceranae: One Host (Apis mellifera) and Two Different Histories | Two microsporidians are known to infect honey bees: Nosema apis and Nosema ceranae. Whereas population genetics data for the latter have been released in the last few years, such information is still missing for N. apis. Here we analyze the patterns of nucleotide polymorphism at three single-copy loci (PTP2, PTP3 and RPB1) in a collection of Apis mellifera isolates from all over the world, naturally infected either with N. apis (N = 22) or N. ceranae (N = 23), to provide new insights into the genetic diversity, demography and evolution of N. apis, as well as to compare them with evidence from N. ceranae. Neutral variation in N. apis and N. ceranae is of the order of 1%. This amount of diversity suggests that there is no substantial differentiation between the genetic content of the two nuclei present in these parasites, and evidence for genetic recombination provides a putative mechanism for the flow of genetic information between chromosomes. The analysis of the frequency spectrum of neutral variants reveals a significant surplus of low frequency variants, particularly in N. ceranae, and suggests that the populations of the two pathogens are not in mutation-drift equilibrium and that they have experienced a population expansion. Most of the variation in both species occurs within honey bee colonies (between 62%-90% of the total genetic variance), although in N. apis there is evidence for differentiation between parasites isolated from distinct A. mellifera lineages (20%-34% of the total variance), specifically between those collected from lineages A and C (or M). This scenario is consistent with a long-term host-parasite relationship and contrasts with the lack of differentiation observed among host-lineages in N. ceranae (<4% of the variance), which suggests that the spread of this emergent pathogen throughout the A. mellifera worldwide population is a recent event. | 1291 | no | gd of parasite | NA | no | no |
| 1292 | 2017 | 43017 | Chwedorzewska, KJ; Bednarek, PT | 2012 | Genetic and epigenetic variation in a cosmopolitan grass Poa annua from Antarctic and Polish populations | Poa annua L. is the only non-native vascular plant that was successfully established in the maritime Antarctic. This project aimed to determine the amount of genetic and epigenetic variation within and between two populations of P. annua, one from South Shetland Is. (Antarctic) and the other one from Central Europe. We applied two AFLP marker systems, using endonucleases that recognised the same restriction site but differed in their sensitivity towards methylation. The Antarctic population differed from the Polish one both at the genetic and epigenetic levels. Genetic variability in the Antarctic population was lower than in the Polish one. Some loci in the Antarctic population showed signs of selection. The difference between Polish and Antarctic populations might be due to a weak bottleneck effect followed by population expansion. Using only epigenetic markers, the Antarctic population exhibited increased variation level compared to the Polish one. These may have resulted from plastic responses to environmental factors and could be associated with survival in extreme conditions. | 1292 | no | no good fitness measure | NA | no | no |
| 1293 | 2017 | 43017 | Jaatinen, K; Jaari, S; O’Hara, RB; Ost, M; Merila, J | 2009 | Relatedness and spatial proximity as determinants of host-parasite interactions in the brood parasitic Barrow’s goldeneye (Bucephala islandica) | Recent studies, which have found evidence for kin-biased egg donation, have sparked interest in re-assessing the parasitic nature of conspecific brood parasitism (CBP). Since host-parasite kinship is essential for mutual benefits to arise from CBP, we explored the role of relatedness in determining the behaviour of conspecific nest parasites and their hosts in nesting female Barrow’s goldeneyes (Bucephala islandica), a duck in which CBP is common. The results revealed that the amount of parasitism increased with host-parasite relatedness, the effect of which was independent of geographical proximity of host and parasite nests. Proximity per se was also positively associated with the amount of parasitism. Furthermore, while hosts appeared to reduce their clutch size as a response to the presence of parasitic eggs, the magnitude of host clutch reduction also tended to increase with increasing relatedness to the parasite. Hence, our results indicate that both relatedness and spatial proximity are important determinants of CBP, and that host clutch reduction may be an adaptation to nest parasitism, modulated by host-parasite relatedness. Taken together, the results provide a demonstration that relatedness influences host and parasite behaviour in Barrow’s goldeneyes, resulting in kin-biased egg donation. | 1293 | no | brood parasitism | NA | no | no |
| 1294 | 2017 | 43017 | Scandura, M; Iacolina, L; Capitani, C; Gazzola, A; Mattioli, L; Apollonio, M | 2011 | Fine-scale genetic structure suggests low levels of short-range gene flow in a wolf population of the Italian Apennines | We investigated local gene flow in a high-density wolf (Canis lupus) population of the Italian Apennines, where no effective barrier to wolf dispersal was present. From 1998 to 2004 we examined wolf carcasses and non-invasively collected samples, focusing on three mountain districts, separated by two valleys, where wolf packs showed high spatial stability. Using nine autosomal microsatellites we successfully genotyped 177 samples, achieving the identification of 74 wolves. Genetic relatedness steeply decreased with increasing distance between sampling areas, thus suggesting that short-distance interpack migration is infrequent in this population. In addition, no individual from a central pack under intensive monitoring was sampled in the range of the surrounding packs over a 4-year period. The limited short-distance gene flow resulted in a cryptic genetic structure, which was revealed by Bayesian analysis. A different genetic cluster was found in each of the three mountain areas, and a small proportion of first-generation immigrants was detected. Overall, the present study suggests that local genetic differentiation in Italian wolves might arise from high spatial stability of packs and can be favoured by a combination of long-range dispersal, the attitude to mate between unrelated individuals and a high young mortality rate. | 1294 | no | only one pop | NA | no | no |
| 1295 | 2017 | 43017 | Khan, A; Tian, L; Zhang, C; Yuan, K; Xu, SH | 2016 | Genetic diversity and natural selection footprints of the glycine amidinotransferase gene in various human populations | The glycine amidinotransferase gene (GATM) plays a vital role in energy metabolism in muscle tissues and is associated with multiple clinically important phenotypes. However, the genetic diversity of the GATM gene remains poorly understood within and between human populations. Here we analyzed the 1,000 Genomes Project data through population genetics approaches and observed significant genetic diversity across the GATM gene among various continental human populations. We observed considerable variations in GATM allele frequencies and haplotype composition among different populations. Substantial genetic differences were observed between East Asian and European populations (F-ST = 0.56). In addition, the frequency of a distinct major GATM haplotype in these groups was congruent with population-wide diversity at this locus. Furthermore, we identified GATM as the top differentiated gene compared to the other statin drug response-associated genes. Composite multiple analyses identified signatures of positive selection at the GATM locus, which was estimated to have occurred around 850 generations ago in European populations. As GATM catalyzes the key step of creatine biosynthesis involved in energy metabolism, we speculate that the European prehistorical demographic transition from hunter-gatherer to farming cultures was the driving force of selection that fulfilled creatine-based metabolic requirement of the populations. | 1295 | no | human | NA | no | no |
| 1296 | 2017 | 43017 | Castella, G; Christe, P; Chapuisat, M | 2010 | Covariation between colony social structure and immune defences of workers in the ant Formica selysi | Several ant species vary in the number of queens per colony, yet the causes and consequences of this variation remain poorly understood. In previous experiments, we found that Formica selysi workers originating from multiple-queen (=polygyne) colonies had a lower resistance to a fungal pathogen than workers originating from single-queen (=monogyne) colonies. In contrast, group diversity improved disease resistance in experimental colonies. This discrepancy between field and experimental colonies suggested that variation in social structure in the field had antagonistic effects on worker resistance, possibly through a down-regulation of the immune system balancing the positive effect of genetic diversity. Here, we examined if workers originating from field colonies with alternative social structure differed in three major components of their immune system. We found that workers from polygyne colonies had a lower bacterial growth inhibitory activity than workers from monogyne colonies. In contrast, workers from the two types of colonies did not differ significantly in bacterial cell wall lytic activity and prophenoloxidase activity. Overall, the presence of multiple queens in a colony correlated with a slight reduction in one inducible component of the immune system of individual workers. This reduced level of immune defence might explain the lower resistance of workers originating from polygyne colonies despite the positive effect of genetic diversity. More generally, these results indicate that social changes at the group level can modulate individual immune defences. | 1296 | yes | fitness and gd pop level? | excluded because measure immune response | yes | no |
| 1297 | 2017 | 43017 | Puttamuk, T; Zhou, LJ; Thaveechai, N; Zhang, SA; Armstrong, CM; Duan, YP | 2014 | Genetic Diversity of Candidatus Liberibacter asiaticus Based on Two Hypervariable Effector Genes in Thailand | Huanglongbing (HLB), also known as citrus greening, is one of the most destructive diseases of citrus worldwide. HLB is associated with three species of ‘Candidatus Liberibacter’ with ‘Ca. L. asiaticus’ (Las) being the most widely distributed around the world, and the only species detected in Thailand. To understand the genetic diversity of Las bacteria in Thailand, we evaluated two closely-related effector genes, lasA(I) and lasA(II), found within the Las prophages from 239 infected citrus and 55 infected psyllid samples collected from different provinces in Thailand. The results indicated that most of the Las-infected samples collected from Thailand contained at least one prophage sequence with 48.29% containing prophage 1 (FP1), 63.26% containing prophage 2 (FP2), and 19.38% containing both prophages. Interestingly, FP2 was found to be the predominant population in Las-infected citrus samples while Las-infected psyllids contained primarily FP1. The multiple banding patterns that resulted from amplification of lasAI imply extensive variation exists within the full and partial repeat sequence while the single band from lasAII indicates a low amount of variation within the repeat sequence. Phylogenetic analysis of Las-infected samples from 22 provinces in Thailand suggested that the bacterial pathogen may have been introduced to Thailand from China and the Philippines. This is the first report evaluating the genetic variation of a large population of Ca. L. asiaticus infected samples in Thailand using the two effector genes from Las prophage regions. | 1297 | no | gd of pathogen | NA | no | no |
| 1298 | 2017 | 43017 | Krehenwinkel, H; Tautz, D | 2013 | Northern range expansion of European populations of the wasp spider Argiope bruennichi is associated with global warmingcorrelated genetic admixture and population-specific temperature adaptations | Poleward range expansions are observed for an increasing number of species, which may be an effect of global warming during the past decades. However, it is still not clear in how far these expansions reflect simple geographical shifts of species ranges, or whether new genetic adaptations play a role as well. Here, we analyse the expansion of the wasp spider Argiope bruennichi into Northern Europe during the last century. We have used a range-wide sampling of contemporary populations and historical specimens from museums to trace the phylogeography and genetic changes associated with the range shift. Based on the analysis of mitochondrial, microsatellite and SNP markers, we observe a higher level of genetic diversity in the expanding populations, apparently due to admixture of formerly isolated lineages. Using reciprocal transplant experiments for testing overwintering tolerance, as well as temperature preference and tolerance tests in the laboratory, we find that the invading spiders have possibly shifted their temperature niche. This may be a key adaptation for survival in Northern latitudes. The museum samples allow a reconstruction of the invasion’s genetic history. A first, small-scale range shift started around 1930, in parallel with the onset of global warming. A more massive invasion of Northern Europe associated with genetic admixture and morphological changes occurred in later decades. We suggest that the latter range expansion into far Northern latitudes may be a consequence of the admixture that provided the genetic material for adaptations to new environmental regimes. Hence, global warming could have facilitated the initial admixture of populations and this resulted in genetic lineages with new habitat preferences. | 1298 | no | no good fitness measure, N pop differnt in GD? | NA | no | no |
| 1299 | 2017 | 43017 | Jaatinen, K; Lehtonen, J; Kokko, H | 2011 | Strategy selection under conspecific brood parasitism: an integrative modeling approach | Conspecific brood parasitism (CBP), where females lay eggs in nests of conspecifics, is taxonomically widespread. Following recent calls to consider CBP in a more integrative manner, we explore breeding strategies related to CBP by using a model of competing strategies that incorporates variation in individual quality, relatedness, recognition of own versus parasite-laid eggs, as well as the costs of egg laying and postlaying care. Our model creates 5 main conclusions. First, variation in individual quality plays a central role in shaping breeding strategies. Second, kinship plays a central role in the evolution of CBP. Third, egg recognition ability may affect the prevalence of parasitism: If hosts recognize parasitic eggs, relatedness between host and parasite facilitates CBP. Fourth, the relative costs of egg laying and postlaying care play a so far underestimated role in determining the prevalence of parasitism. Fifth, natal philopatry may lead to a reduction in productivity. To sum up, our theoretical study combines factors known to affect both breeding in general and CBP in particular and shows that these factors can explain a large proportion of the variation in CBP strategies found in the wild and therefore facilitates the understanding of the mechanisms shaping these strategies. | 1299 | no | brood parasitism | NA | no | no |
| 1300 | 2017 | 43017 | Mainguy, J; Worley, K; Cote, SD; Coltman, DW | 2007 | Low MHC DRB class II diversity in the mountain goat: past bottlenecks and possible role of pathogens and parasites | Major histocompatibility complex (MHC) genes are the most polymorphic in vertebrates and code for molecules playing a central role in pathogen resistance. We studied levels of MHC DRB class II diversity in a long-term study population of mountain goats (Oreamnos americanus) at Caw Ridge, Alberta, and two other populations from British Columbia, Canada. Only two alleles were found among the three populations sampled. The Caw Ridge population was fixed for one of the two MHC DRB alleles, but this lack of variation did not appear to have affected it negatively because the population doubled over two decades and had no history of any apparent infectious diseases. Past population bottlenecks during Pleistocene glaciations are thought to have been the main factor contributing to the low levels of MHC diversity in mountain goats, a hypothesis supported by our previous work reporting low polymorphism at neutral loci. Additionally, the limited MHC variability in mountain goats may be related to its northern distribution as we found that allelic diversity at MHC DRB class II in wild ungulates decreases with increasing latitude, possibly as a result of low parasite diversity at high latitudes. The low MHC variation in mountain goats and other northern ungulates such as muskoxen (Ovibos moschatus) may expose these species to population outbreaks that could be generated by introduced pathogens or northward shifts in the distribution of pathogens with global climate warming. | 1300 | no | only one pop | NA | no | no |
| 1301 | 2017 | 43017 | Fausser, JL; Rabarivola, C; Meier, B; Hahn, T; Rumpler, Y | 2000 | Genetic comparison between different populations of Eulemur macaco flavifrons in northwest Madagascar using RAPD markers | Eulemur macaco flavifrons, the Sclater’s black lemur, is a critically endangered subspecies of northwest Madagascar, which is not yet protected by any reserve. In order to study the feasibility of creating such a reserve, an area of outstanding biological importance was selected in the region of Maromandia-Sahamalaza, which is probably the only remaining place which would permit the long-term survival of the Sclater’s black lemur. To determine if genetic management is needed for the Sahamalaza black lemur population, its genetic variability was estimated with random amplified polymorphic DNA (RAPD) markers and compared with other populations. These comparisons demonstrate that the Sahamalaza black lemurs have a genetic variability equivalent to those in other areas. Thus, we conclude that no genetic management is required at the present time. (C) 2000 Wiley-Liss, Inc. | 1301 | no | no good fitness measure, N pop differnt in GD? | NA | no | no |
| 1302 | 2017 | 43017 | Glatston, AR | 2001 | Relevance of studbook data to the successful captive management of grey mouse lemurs | This paper illustrates the importance of a well-managed studbook to the long-term captive management of an exotic species: Microcebus murinus. Analysis of the studbook provides an insight into the genetic diversity and demographic stability of the registered population. It also yields invaluable data on patterns of fertility and mortality occurring under the prevalent management conditions. This information is equally relevant to research and to captive management I established a European studbook far the grey mouse lemur in 1994. It contains data on individuals in toes and research establishments Analyses of the data do not bode well for future of the European mouse lemur population. Although the number of individuals would seem to be sufficient to form a sound basis for future management, the population has inadequate genetic variability Furthermore, inbreeding also seems to have had a negative impact on the reproductive output of the population. Thus, immediate action needs to be taken if a viable mouse lemur population is to be maintained in Europe. | 1302 | no | no good fitness measure, N pop differnt in GD? | NA | no | no |
| 1303 | 2017 | 43017 | Van Putten, WF; Biere, A; Van Damme, JMM | 2005 | Host-related genetic differentiation in the anther smut fungus Microbotryum violaceum in sympatric, parapatric and allopatric populations of two host species Silene latifolia and S-dioica | We investigated genetic diversity in West European populations of the fungal pathogen Microbotryum violaceum in sympatric, parapatric and allopatric populations of the host species Silene latifolia and S. dioica, using four polymorphic microsatellite loci. In allopatric host populations, the fungus was highly differentiated by host species, exhibiting high values of F-ST and R-ST, and revealed clear and distinct host races. In sympatric and parapatric populations we found significant population differentiation as well, except for one sympatric population in which the two host species grew truly intermingled. The mean number of alleles per locus for isolates from each of the host species was significantly higher in sympatric/parapatric than in allopatric populations. This suggests that either gene flow between host races in sympatry, or in case of less neutral loci, selection in a more heterogeneous host environment can increase the level of genetic variation in each of the demes. The observed pattern of host-related genetic differentiation among these geographically spread populations suggest a long-term divergence between these host races. In sympatric host populations, both host races presumably come in secondary contact, and host-specific alleles are exchanged depending on the amount of fungal gene flow. | 1303 | no | no good fitness measure, N pop differnt in GD? | NA | no | no |
| 1304 | 2017 | 43017 | Ishibashi, Y; Oi, T; Arimoto, I; Fujii, T; Mamiya, K; Nishi, N; Sawada, S; Tado, H; Yamada, T | 2017 | Loss of allelic diversity in the MHC class II DQB gene in western populations of the Japanese black bear Ursus thibetanus japonicus | In Japan, the black bear, Ursus thibetanus, is distributed on Honshu and Shikoku Islands. Most populations in western Japan declined considerably during the twentieth century, but a few populations are now rebounding due to conservation efforts. Here, we examined the sequence variation in the second exon of the major histocompatibility complex class II DQB gene (270 bp), which is critical for pathogen recognition. We measured variation within six populations in western Japan, including two threatened populations in the Chugoku region on Honshu and one on Shikoku. Eight sequence variants were observed among the examined bears (n = 417), and two to eight variants were retained within populations. Our samples, collected in 2001-2013, retained a smaller number of sequence variants in each population compared with the allelic diversity in an earlier study that examined the same gene and used samples collected mainly during the last century. Many rare variants that were observed previously and may have been maintained by balancing selection have disappeared from recent populations. Although the earlier study suggested a loss of genetic diversity in western Japan, the present study shows that further loss of rare variants has occurred, probably due to genetic drift during the end of the last century. | 1304 | no | no good fitness measure | NA | no | no |
| 1305 | 2017 | 43017 | Allender, CJ; Clancy, KM; Degomez, TE; McMillin, JD; Woolbright, SA; Keim, P; Wagner, DM | 2008 | Lack of genetic differentiation in aggressive and secondary bark beetles (Coleoptera : Curculionidae, Scolytinae) from Arizona | Bark beetles (Coleoptera: Curculionidae, Scolytinae) play an important role as disturbance agents in ponderosa pine (Pinus ponderosa Douglas ex Lawson) forests of Arizona. However, from 2001 to 2003, elevated bark beetle activity caused unprecedented levels of ponderosa pine mortality. A better understanding of the population structure of these species will facilitate analysis of their dispersal patterns and improve management strategies. Here, we use fluorescently labeled amplified fragment length polymorphism (fAFLP) analysis to resolve genetic variation among and within sampling locations in northcentral Arizona of Ips pini (Say), Dendroctonus brevicomis LeConte, and D. frontalis Zimmermann. We generated genetic fingerprints for > 500 beetle specimens and analyzed genetic diversity. For all species, gene flow estimates among sampling locations were high, and significant population subdivision was not discernible across a large portion of ponderosa pine forests in Arizona. However, a weak relationship was detected with L pini population structure and elevation. Because of the lack of genetic differentiation detected throughout the large study area, our findings suggest these insects are capable of long distance dispersal and exhibit a high degree of gene flow across abroad region. We conclude that our results are consistent with strong dispersal patterns and large population sizes of all three species. | 1305 | no | no good fitness measure | NA | no | no |
| 1306 | 2017 | 43017 | Munwes, I; Geffen, E; Roll, U; Friedmann, A; Daya, A; Tikochinski, Y; Gafny, S | 2010 | The change in genetic diversity down the core-edge gradient in the eastern spadefoot toad (Pelobates syriacus) | Several hypotheses are available to predict change in genetic diversity when approaching peripheral populations. We used the eastern spadefoot toad in Israel as a model system to examine these hypotheses using population genetics analyses and network theory. Our results contradicted most of the predictions from the ‘abundant centre’ model, that edge populations should have lower density and lower genetic diversity than core populations. Furthermore, dispersal rate between core and peripheral populations is expected to be asymmetric, mostly directed outwards from the core population, but we did not detect such a trend. Our data did not support the hypothesis of no change or a non-linear change in genetic diversity towards the range edge. However, our results did fit the Fisher (The Genetical Theory of Natural Selection, Clarendon Press, Oxford, 1930) hypothesis, which predicts increase in genetic variability from core to edge of distribution. We attributed this finding to the much harsher climatic and abiotic conditions at the edge, which must be tolerated over generations by both tadpoles and post-metamorphic individuals in this region. Finally, our results have significant conservation implications for the survival of this species in Israel, where it is critically endangered. We identified two distinct communities, which are genetically linked through two specific rain pools in the Upper Galilee. Details on the spatial subdivision of this species are cardinal for future management and restoration of temporary wetlands in Israel. | 1306 | no | no good fitness measure, N pop differnt in GD? | NA | no | no |
| 1307 | 2017 | 43017 | Lawler, RR | 2011 | Historical demography of a wild lemur population (Propithecus verreauxi) in southwest Madagascar | The human colonization of Madagascar is associated with the extinction of numerous lemur species. However, the degree to which humans have negatively influenced the historical population dynamics of extant lemur species is not well understood. This study employs genetic and demographic analyses to estimate demographic parameters relating to the historical population dynamics of a wild lemur population, Verreaux’s sifaka (Propithecus verreauxi). The genetic analyses are used to determine whether this population experienced a historically recent (i.e., within the last 2000 years) population bottleneck, as well as to estimate the historical population growth rate and the timing of any changes in population size in the past. In addition, a retrospective demographic analysis is used to determine sources of variation and covariation in the sifaka life cycle and how variation in life-cycle transitions contributes to variation in population growth rate. The genetic analyses indicate that the sifaka population did not experience a recent population bottleneck; however, the historical population growth rate was negative, indicating that the ancestral population size was much larger than the current size. The timing of the ancestral population decline has a point estimate of 2300 years ago, but with large credible intervals: 3611-1736 years ago. This point estimate corresponds with the first evidence for human arrival to Madagascar. Climatic variation has also likely influenced past (and current) population dynamics due to stochastic annual rainfall patterns and climatic desiccation, the latter of which began in southwestern Madagascar around 4000 years ago. Variation in the survival of 2-year-old animals as well as large adult females makes the largest contribution to variation in population growth rate. In the absence of more explicit models pertaining to historical population dynamics, it is difficult to attribute the negative population growth rate of this species solely to a single factor (e.g., hunting, habitat destruction). | 1307 | no | only one pop | NA | no | no |
| 1308 | 2017 | 43017 | Abd Razak, MRM; Sastu, UR; Norahmad, NA; Abdul-Karim, A; Muhammad, A; Muniandy, PK; Jelip, J; Rundi, C; Imwong, M; Mudin, RN; Abdullah, NR | 2016 | Genetic Diversity of Plasmodium falciparum Populations in Malaria Declining Areas of Sabah, East Malaysia | Malaysia has a national goal to eliminate malaria by 2020. Understanding the genetic diversity of malaria parasites in residual transmission foci can provide invaluable information which may inform the intervention strategies used to reach elimination targets. This study was conducted to determine the genetic diversity level of P. falciparum isolates in malaria residual foci areas of Sabah. Malaria active case detection was conducted in Kalabakan and Kota Marudu. All individuals in the study sites were screened for malaria infection by rapid diagnostic test. Blood from P. falciparum-infected individuals were collected on filter paper prior to DNA extraction. Genotyping was performed using merozoite surface protein-1 (MSP-1), merozoite surface protein-2 (MSP-2), glutamate rich protein (GLURP) and 10 neutral microsatellite loci markers. The size of alleles, multiplicity of infection (MOI), mean number of alleles (Na), expected heterozygosity (He), linkage disequilibrium (LD) and genetic differentiation (F-ST) were determined. In Kalabakan, the MSP-1 and MSP-2 alleles were predominantly K1 and FC27 family types, respectively. The GLURP genotype VI (751-800 bp) was predominant. The MOI for MSP-1 and MSP-2 were 1.65 and 1.20, respectively. The Na per microsatellite locus was 1.70. The He values for MSP-1, MSP-2, GLURP and neutral microsatellites were 0.17, 0.37, 0.70 and 0.33, respectively. In Kota Marudu, the MSP-1 and MSP-2 alleles were predominantly MAD20 and 3D7 family types, respectively. The GLURP genotype IV (651-700 bp) was predominant. The MOI for both MSP-1 and MSP-2 was 1.05. The Na per microsatellite locus was 3.60. The He values for MSP-1, MSP-2, GLURP and neutral microsatellites were 0.24, 0.25, 0.69 and 0.30, respectively. A significant LD was observed in Kalabakan (0.495, p<0.01) and Kota Marudu P. falciparum populations (0.601, p<0.01). High genetic differentiation between Kalabakan and Kota Marudu P. falciparum populations was observed (F-ST = 0.532). The genetic data from the present study highlighted the limited diversity and contrasting genetic pattern of P. falciparum populations in the malaria declining areas of Sabah. | 1308 | no | gd of parasite | NA | no | no |
| 1309 | 2017 | 43017 | Bauchet, G; Grenier, S; Samson, N; Bonnet, J; Grivet, L; Causse, M | 2017 | Use of modern tomato breeding germplasm for deciphering the genetic control of agronomical traits by Genome Wide Association study | Key message A panel of 300 tomato accessions including breeding materials was built and characterized with > 11,000 SNP. A population structure in six subgroups was identified. Strong heterogeneity in linkage disequilibrium and recombination landscape among groups and chromosomes was shown. GWAS identified several associations for fruit weight, earliness and plant growth. Genome-wide association studies (GWAS) have become a method of choice in quantitative trait dissection. First limited to highly polymorphic and outcrossing species, it is now applied in horticultural crops, notably in tomato. Until now GWAS in tomato has been performed on panels of heirloom and wild accessions. Using modern breeding materials would be of direct interest for breeding purpose. To implement GWAS on a large panel of 300 tomato accessions including 168 breeding lines, this study assessed the genetic diversity and linkage disequilibrium decay and revealed the population structure and performed GWA experiment. Genetic diversity and population structure analyses were based on molecular markers (> 11,000 SNP) covering the whole genome. Six genetic subgroups were revealed and associated to traits of agronomical interest, such as fruit weight and disease resistance. Estimates of linkage disequilibrium highlighted the heterogeneity of its decay among genetic subgroups. Haplotype definition allowed a fine characterization of the groups and their recombination landscape revealing the patterns of admixture along the genome. Selection footprints showed results in congruence with introgressions. Taken together, all these elements refined our knowledge of the genetic material included in this panel and allowed the identification of several associations for fruit weight, plant growth and earliness, deciphering the genetic architecture of these complex traits and identifying several new loci useful for tomato breeding. | 1309 | no | crop | NA | no | no |
| 1310 | 2017 | 43017 | Cottam, EM; Thebaud, G; Wadsworth, J; Gloster, J; Mansley, L; Paton, DJ; King, DP; Haydon, DT | 2008 | Integrating genetic and epidemiological data to determine transmission pathways of foot-and-mouth disease virus | Estimating detailed transmission trees that reflect the relationships between infected individuals or populations during a disease outbreak often provides valuable insights into both the nature of disease transmission and the overall dynamics of the underlying epidemiological process. These trees may be based on epidemiological data that relate to the timing of infection and infectiousness, or genetic data that show the genetic relatedness of pathogens isolated from infected individuals. Genetic data are becoming increasingly important in the estimation of transmission trees of viral pathogens due to their inherently high mutation rate. Here, we propose a maximum-likelihood approach that allows epidemiological and genetic data to be combined within the same analysis to infer probable transmission trees. We apply this approach to data from 20 farms infected during the 2001 UK foot-and-mouth disease outbreak, using complete viral genome sequences from each infected farm and information on when farms were first estimated to have developed clinical disease and when livestock on these farms were culled. Incorporating known infection links due to animal movement prior to imposition of the national movement ban results in the reduction of the number of trees from 41 472 that are consistent with the genetic data to 1728, of which just 4 represent more than 95% of the total likelihood calculated using a model that accounts for the epidemiological data. These trees differ in several ways from those constructed prior to the availability of genetic data. | 1310 | no | NA | NA | no | no |
| 1311 | 2017 | 43017 | Guerier, AS; Bishop, JM; Crawford, SJ; Schmidt-Kuntzel, A; Stratford, KJ | 2012 | Parentage analysis in a managed free ranging population of southern white rhinoceros: genetic diversity, pedigrees and management | Small populations are vulnerable to the consequences of breeding within closed groups as the loss of genetic variability can lead to inbreeding depression. Here, we use microsatellite genotypes to assess variability and parentage within a small, managed population of southern white rhinoceros in northern Namibia. Tissue samples gathered from either a modified biopsy darting technique or ear notches allowed us to obtain genotypic data for all individuals in the population. As expected for this species, genetic variability in the population was relatively low (overall H (obs) 0.45). In combination with detailed management records for the period 1993-2009, we were able to assign both parents for all 23 offspring. Only one calf of seven in the F-2 generation arose from father-daughter inbreeding within the population. Our analysis revealed that paternity was initially dominated by a single founder bull siring 10 of 13 calves over 9 years; paradoxically, the other founder bull was selected for removal based on observations suggesting he was behaviourally dominant and therefore the likely sire of most calves. We also found that young introduced bulls were breeding successfully within 6 months of their arrival, well before having established their home ranges. We argue that in order to optimally manage and conserve the southern African white rhinoceros meta-population it is essential to have accurate pedigree information and genetic data for all individuals in the numerous small populations that are key to the survival of the species. | 1311 | no | only one pop | NA | no | no |
| 1312 | 2017 | 43017 | Pohjamo, M; Korpelainen, H; Kalinauskaite, N | 2008 | Restricted gene flow in the clonal hepatic Trichocolea tomentella in fragmented landscapes | We studied the genetic diversity, gene flow and population structure among 18 populations of the clonal bryophyte Trichocolea tomentella located in Finland, Lithuania, the UK and Canada using DNA fingerprinting methods. T. tomentella is a habitat-limited, unisexual hepatic, which occupies spring and mesic habitats in woodland. The relatively small populations are increasingly fragmented with a high risk for extinction for extrinsic reasons. The presence of relatively high levels of genetic diversity regardless of population size highlights the role of even small remnant populations as important sources of genetic diversity in T tomentella. The long-term accumulation of genotypes and somatic mutations may explain the observed levels of diversity. Gene flow among populations seems to be infrequent indicating dispersal limitation also on the relatively small spatial scale. Colonization within populations is not affected by isolation by distance suggesting the occurrence of random short-range dispersal of detached vegetative fragments. The population structure study confirmed the low mortality rates of shoots indicating a long life span of the clones in favourable conditions. Efficient ramet production by branching is likely to operate against interspecific competition. To conclude, T tomentella appears to persist well in undisturbed habitats due to clonal regeneration, although restricted dispersal capacity is likely to prevent successful (re-)colonization in the potential habitat patches of recovering forest landscapes. The implications of the results for conservation are introduced. (C) 2008 Elsevier Ltd. All rights reserved. | 1312 | no | no good fitness measure, N pop differnt in GD? | NA | no | no |
| 1313 | 2017 | 43017 | Monteiro, SS; Vingada, JV; Lopez, A; Pierce, GJ; Ferreira, M; Brownlow, A; Mikkelsen, B; Niemeyer, M; Deaville, RJ; Eira, C; Piertney, S | 2016 | Major Histocompatibility Complex (MHC) class II sequence polymorphism in long-finned pilot whale (Globicephala melas) from the North Atlantic | Determining how intra-specific genetic diversity is apportioned among natural populations is essential for detecting local adaptation and identifying populations with inherently low levels of extant diversity which may become a conservation concern. Sequence polymorphism at two adaptive loci (MHC DRA and DQB) was investigated in long-finned pilot whales (Globicephala melas) from four regions in the North Atlantic and compared with previous data from New Zealand (South Pacific). Three alleles were resolved at each locus, with trans-species allele sharing and higher levels of non-synonymous to synonymous substitution, especially in the DQB locus. Overall nucleotide diversities of 0.49 +/- 0.38% and 4.60 +/- 2.39% were identified for the DRA and DQB loci, respectively, which are relatively low for MHC loci in the North Atlantic, but comparable to levels previously described in New Zealand (South Pacific). There were significant differences in allele frequencies within the North Atlantic and between the North Atlantic and New Zealand. Patterns of diversity and divergence are consistent with the long-term effects of balancing selection operating on the MHC loci, potentially mediated through the effects of host-parasite coevolution. Differences in allele frequency may reflect variation in pathogen communities, coupled with the effects of differential drift and gene flow. | 1313 | no | no good fitness measure | NA | no | no |
| 1314 | 2017 | 43017 | Trask, AE; Bignal, EM; McCracken, DI; Piertney, SB; Reid, JM | 2017 | Estimating demographic contributions to effective population size in an age-structured wild population experiencing environmental and demographic stochasticity | 1. A population’s effective size (N-e) is a key parameter that shapes rates of inbreeding and loss of genetic diversity, thereby influencing evolutionary processes and population viability. However, estimating Ne, and identifying key demographic mechanisms that underlie the N-e to census population size (N) ratio, remains challenging, especially for small populations with overlapping generations and substantial environmental and demographic stochasticity and hence dynamic age-structure. 2.A sophisticated demographic method of estimating N-e/N, which uses Fisher’s reproductive value to account for dynamic age-structure, has been formulated. However, this method requires detailed individual-and population-level data on sex-and age-specific reproduction and survival, and has rarely been implemented. 3.Here, we use the reproductive value method and detailed demographic data to estimate N-e/N for a small and apparently isolated red-billed chough (Pyrrhocorax pyrrhocorax) population of high conservation concern. We additionally calculated two single-sample molecular genetic estimates of N-e to corroborate the demographic estimate and examine evidence for unobserved immigration and gene flow. 4. The demographic estimate of N-e/N was 0.21, reflecting a high total demographic variance (sigma(2)(dg)) of 0.71. Females and males made similar overall contributions to sigma(2)(dg). However, contributions varied among sex-age classes, with greater contributions from 3 year-old females than males, but greater contributions from = 5 year-old males than females. 5. The demographic estimate of N-e was similar to 30, suggesting that rates of increase of inbreeding and loss of genetic variation per generation will be relatively high. Molecular genetic estimates of N-e computed from linkage disequilibrium and approximate Bayesian computation were approximately 50 and 30, respectively, providing no evidence of substantial unobserved immigration which could bias demographic estimates of Ne. 6. Our analyses identify key sex-age classes contributing to demographic variance and thus decreasing N-e/N in a small age-structured population inhabiting a variable environment. They thereby demonstrate how assessments of N-e can incorporate stochastic sex-and age-specific demography and elucidate key demographic processes affecting a population’s evolutionary trajectory and viability. Furthermore, our analyses show that Ne for the focal chough population is critically small, implying that management to re-establish genetic connectivity may be required to ensure population viability. | 1314 | no | only one pop | NA | no | no |
| 1315 | 2017 | 43017 | Akst, EP; Boersma, PD; Fleischer, RC | 2002 | A comparison of genetic diversity between the Galapagos Penguin and the Magellanic Penguin | The Galapagos Penguin (Spheniscus mendiculus) is a United States federally listed endangered species with populations on the Galapagos Islands of Fernandina and Isabela. Although the waters around the islands are normally productive, low productivity during El Nino years results in high adult penguin mortality and low recruitment in following years. We used microsatellite markers developed. for Spheniscus penguins to study the long term genetic effects of serial bottleneck events in the Galapagos Penguin, and compared this variation to that of its congener, the Magellanic penguin (Spheniscus magellanicus). The observed heterozygosity for the Galapagos Penguin was 3%, significantly lower than the 46% heterozygosity of the Magellanic Penguin. This low level of heterozygosity is directly related to its low effective population size. While this population has survived long term, presumably without high levels of genetic variation, we feel that the greater frequency of El Nino events, coupled with increased human impacts such as introduced disease, oil discharge, and competition with fisheries, may put the species in particular danger of extinction. | 1315 | no | two species comp | NA | no | no |
| 1316 | 2017 | 43017 | Breen, AL; Murray, DF; Olson, MS | 2012 | Genetic consequences of glacial survival: the late Quaternary history of balsam poplar (Populus balsamifera L.) in North America | Aim Beringia, the unglaciated region encompassing the former Bering land bridge, as well as the land between the Lena and Mackenzie rivers, is recognized as an important refugium for arctic plants during the last ice age. Compelling palaeobotanical evidence also supports the presence of small populations of boreal trees within Beringia during the Last Glacial Maximum. The occurrence of balsam poplar (Populus balsamifera) in Beringia provides a unique opportunity to assess the implications of persistence in a refugium on present-day genetic diversity for this boreal tree species. Location North America. Methods We sequenced three variable non-coding regions of the chloroplast genome (cpDNA) from 40 widely distributed populations of balsam poplar across its North American range. We assessed patterns of genetic diversity, geographic structure and historical demography between glaciated and unglaciated regions of the balsam poplar’s range. We also utilized a coalescent model to test for divergence between regions. Results Levels of genetic diversity were consistently greater for populations at the southern margin (theta(W) = 0.00122) than in the central (theta(W) = 0.00086) or northern (theta(W) = 0.00034) regions of the current distribution of balsam poplar, and diversity decreased with increasing latitude (R-2 = 0.49, P < 0.01). We detected low, but significant, structure (FCT = 0.05, P = 0.05), among regions of P. balsamifera’s distribution. The cpDNA genealogy was shallow, however, showing an absence of highly differentiated chloroplast haplotypes. Coalescent analyses supported a model of divergence between the southern ice margin and the northern unglaciated region of balsam poplar’s distribution, but analyses of other regional comparisons did not converge. Main conclusions The palaeobotanical record supports the presence of a Beringian refugium for balsam poplar, but we were unable to definitively identify the presence of known refugial populations based on genetic data alone. Balsam poplar populations from Beringia are not a significant reservoir of cpDNA diversity today. Unique alleles that may have been present in the small, isolated populations that survived within Beringia were probably lost through genetic drift or swamped by post-glacial, northward migration from populations south of the ice sheets. | 1316 | no | no good fitness measure | NA | no | no |
| 1317 | 2017 | 43017 | Leggett, HC; El Mouden, C; Wild, G; West, S | 2012 | Promiscuity and the evolution of cooperative breeding | Empirical data suggest that low levels of promiscuity have played a key role in the evolution of cooperative breeding and eusociality. However, from a theoretical perspective, low levels of promiscuity can favour dispersal away from the natal patch, and have been argued to select against cooperation in a way that cannot be explained by inclusive fitness theory. Here, we use an inclusive fitness approach to model selection to stay and help in a simple patch-structured population, with strict density dependence, where helping increases the survival of the breeder on the patch. Our model predicts that the level of promiscuity has either no influence or a slightly positive influence on selection for helping. This prediction is driven by the fact that, in our model, staying to help leads to increased competition between relatives for the breeding position-when promiscuity is low (and relatedness is high), the best way to aid relatives is by dispersing to avoid competing with them. Furthermore, we found the same results with an individual-based simulation, showing that this is not an area where inclusive fitness theory ‘gets it wrong’. We suggest that our predicted influence of promiscuity is sensitive to biological assumptions, and that if a possibly more biologically relevant scenario were examined, where helping provided fecundity benefits and there was not strict density dependence, then low levels of promiscuity would favour helping, as has been observed empirically. | 1317 | no | no good fitness measure, N pop differnt in GD? | NA | no | no |
| 1318 | 2017 | 43017 | Shimogiri, T; Nishida, N; Kudo, M; Niwa, K; Nishibori, M; Kinoshita, K; Okamoto, S; Maeda, Y; Tokunaga, K; Yasue, H | 2012 | Genetic relationships between Japanese native and commercial breeds using 70 chicken autosomal SNP genotypes by the DigiTag2 assay | Recently, single nucleotide polymorphisms (SNPs) have been used to identify genes or genomic regions responsible for economic traits, including genetic diseases in domestic animals, and to examine genetic diversity of populations. In this study, we genotyped 70 chicken autosomal SNPs using DigiTag2 assay to understand the genetic structure of the Japanese native chicken breeds Satsumadori and Ingie, and the relationship of these breeds with other established breeds, Rhode Island Red (RIR), commercial broiler and layer. Five breeds, each consisting of approximately 20 chickens, were subjected to the assay, revealing the following: Average expected heterozygosities of broiler, Satsumadori, RIR, layer and Ingie were 0.265, 0.254, 0.244, 0.179 and 0.176, respectively. Phylogenetic analysis using the concatenated 70 autosomal SNP genotypes distinguished all chickens and formed clusters of chickens belonging to the respective breeds. In addition, the 2-D scatter plot of the first two principal components was consistent with the phylogenic tree. Taken together with the pairwise Fst distances, broiler and RIR were closely positioned near each other, while Ingie was positioned far from the other breeds. Structure analysis revealed that the probable number of genetic clusters (K) was six and four with maximum likelihood and ?K values, respectively. The clustering with maximum likelihood revealed that, in addition to the clustering of the other five breeds, the Satsumadori was subdivided into two genetic clusters. The clustering with ?K value indicated that the broiler and Rhode Island Red were assigned to the same genetic cluster. | 1318 | no | domesticated animal | NA | no | no |
| 1319 | 2017 | 43017 | Serrano, M; Calvo, JH; Martinez, M; Marcos-Carcavilla, A; Cuevas, J; Gonzalez, C; Jurado, JJ; de Tejada, PD | 2009 | Microsatellite based genetic diversity and population structure of the endangered Spanish Guadarrama goat breed | Background: Assessing genetic biodiversity and population structure of minor breeds through the information provided by neutral molecular markers, allows determination of their extinction risk and to design strategies for their management and conservation. Analysis of microsatellite loci is known to be highly informative in the reconstruction of the historical processes underlying the evolution and differentiation of animal populations. Guadarrama goat is a threatened Spanish breed which actual census (2008) consists of 3057 females and 203 males distributed in 22 populations more or less isolated. The aim of this work is to study the genetic status of this breed through the analysis of molecular data from 10 microsatellites typed in historic and actual live animals. Results: The mean expected heterozygosity across loci within populations ranged from 0.62 to 0.77. Genetic differentiation measures were moderate, with a mean FST of 0.074, GST of 0.081 and RST of 0.085. Percentages of variation among and within populations were 7.5 and 92.5, respectively. Bayesian clustering analyses pointed out a population subdivision in 16 clusters, however, no correlation between geographical distances and genetic differences was found. Management factors such as the limited exchange of animals between farmers ( estimated gene flow Nm = 3.08) mostly due to sanitary and social constraints could be the major causes affecting Guadarrama goat population subdivision. Conclusion: Genetic diversity measures revealed a good status of biodiversity in the Guadarrama goat breed. Since diseases are the first cause affecting the census in this breed, population subdivision would be an advantage for its conservation. However, to maintain private alleles present at low frequencies in such small populations minimizing the inbreeding rate, it would necessitate some mating designs of animals carrying such alleles among populations. The systematic use of molecular markers will facilitate the comprehensive management of these populations, which in combination with the actual breeding program to increase milk yield, will constitute a good strategy to preserve the breed. | 1319 | no | domesticated animal | NA | no | no |
| 1320 | 2017 | 43017 | Vos, M; Didelot, X | 2009 | A comparison of homologous recombination rates in bacteria and archaea | It is a standard practice to test for the signature of homologous recombination in studies examining the genetic diversity of bacterial populations. Although it has emerged that homologous recombination rates can vary widely between species, comparing the results from different studies is made difficult by the diversity of estimation methods used. Here, Multi Locus Sequence Typing (MLST) datasets from a wide variety of bacteria and archaea are analyzed using the ClonalFrame method. This enables a direct comparison between species and allows for a first exploration of the question whether phylogeny or ecology is the primary determinant of homologous recombination rate. | 1320 | no | no good fitness measure, N pop differnt in GD? | NA | no | no |
| 1321 | 2017 | 43017 | Nash, D; Nair, S; Mayxay, M; Newton, PN; Guthmann, JP; Nosten, F; Anderson, TJC | 2005 | Selection strength and hitchhiking around two anti-malarial resistance genes | Neutral mutations may hitchhike to high frequency when they are situated close to sites under positive selection, generating local reductions in genetic diversity. This process is thought to be an important determinant of levels of genomic variation in natural populations. The size of genome regions affected by genetic hitchhiking is expected to be dependent on the strength of selection, but there is little empirical data supporting this prediction. Here, we compare microsatellite variation around two drug resistance genes (chloroquine resistance transporter (pfcrt), chromosome 7, and dihydrofolate reductase (dhfr), chromosome 4) in malaria parasite populations exposed to strong (Thailand) or weak selection (Laos) by anti-malarial drugs. In each population, we examined the point mutations underlying resistance and length variation at 22 (chromosome 4) or 25 (chromosome 7) microsatellite markers across these chromosomes. All parasites from Thailand carried the K76T mutation in pfcrt conferring resistance to chloroquine (CQ) and 2-4 mutations in dhfr conferring resistance to pyrimethamine. By contrast, we found both wild-type and resistant alleles at both genes in Laos. There were dramatic differences in the extent of hitchhiking in the two countries. The size of genome regions affected was smaller in Laos than in Thailand. We observed significant reduction in variation relative to sensitive parasites for 34-64 kb (2-4 cM) in Laos on chromosome 4, compared with 98-137 kb (6-8 cM) in Thailand. Similarly, on chromosome 7, we observed reduced variation for 34-69 kb (2-4 cM) around pfcrt in Laos, but for 195-268 kb (11-16 cM) in Thailand. Reduction in genetic variation was also less extreme in Laos than in Thailand. Most loci were monomorphic in a 12 kb region surrounding both genes on resistant chromosomes from Thailand, whereas in Laos, even loci immediately proximal to selective sites showed some variation on resistant chromosomes. Finally, linkage disequilibrium (LD) decayed more rapidly around resistant pfcrt and dhfr alleles from Laos than from Thailand. These results demonstrate that different realizations of the same selective sweeps may vary considerably in size and shape, in a manner broadly consistent with selection history. From a practical perspective, genomic regions containing resistance genes may be most effectively located by genome-wide association in populations exposed to strong drug selection. However, the lower levels of LD surrounding resistance alleles in populations under weak selection may simplify identification of functional mutations. | 1321 | no | gd of parasite | NA | no | no |
| 1322 | 2017 | 43017 | Morlais, I; Nsango, SE; Toussile, W; Abate, L; Annan, Z; Tchioffo, MT; Cohuet, A; Awono-Ambene, PH; Fontenille, D; Rousset, F; Berry, A | 2015 | Plasmodium falciparum Mating Patterns and Mosquito Infectivity of Natural Isolates of Gametocytes | Plasmodium falciparum infections in malaria endemic areas often harbor multiple clones of parasites. However, the transmission success of the different genotypes within the mosquito vector has remained elusive so far. The genetic diversity of malaria parasites was measured by using microsatellite markers in gametocyte isolates from 125 asymptomatic carriers. For a subset of 49 carriers, the dynamics of co-infecting genotypes was followed until their development within salivary glands. Also, individual oocysts from midguts infected with blood from 9 donors were genotyped to assess mating patterns. Multiplicity of infection (MOI) was high both in gametocyte isolates and sporozoite populations, reaching up to 10 genotypes. Gametocyte isolates with multiple genotypes gave rise to lower infection prevalence and intensity. Fluctuations of genotype number occurred during the development within the mosquito and sub-patent genotypes, not detected in gametocyte isolates, were identified in the vector salivary glands. The inbreeding coefficient Fis was positively correlated to the oocyst loads, suggesting that P. falciparum parasites use different reproductive strategies according to the genotypes present in the gametocyte isolate. The number of parasite clones within an infection affects the transmission success and the mosquito has an important role in maintaining P. falciparum genetic diversity. Our results emphasize the crucial importance of discriminating between the different genotypes within an infection when studying the A. gambiae natural resistance to P. falciparum, and the need to monitor parasite diversity in areas where malaria control interventions are implemented. | 1322 | no | gd of parasite | NA | no | no |
| 1323 | 2017 | 43017 | Nielsen, CR; Semel, B; Sherman, PW; Westneat, DF; Parker, PG | 2006 | Host-parasite relatedness in wood ducks: patterns of kinship and parasite success | We investigated the role of kinship in intraspecific nest parasitism of wood ducks (Aix sponsa). Among waterfowl, female philopatry creates the potential for female relatives to nest in proximity. Costs of intraspecific nest parasitism to host females may be reduced if parasites lay eggs with kin. However, previous observations of marked wood ducks indicated that females avoided parasitizing clutch mates or the female that incubated them. To further examine the role of kinship, we determined the genotypes of 27 host-parasite pairs at five microsatellite loci. Average relatedness between hosts and all females laying parasitic eggs was only 0.04 +/- 0.03. Parasites appeared to choose hosts randomly with respect to kinship from among females with nests in the neighborhood and those within the entire study area. However, host relatedness to the parasite with the greatest number of young leaving the nest was 0.11 +/- 0.03, which was greater than expected if eggs were accepted randomly from neighboring females or from females present on the entire study area (p = .03 and p = .02, respectively). These patterns may reflect parasitism of randomly selected nests followed by differential acceptance by hosts, differential hatching success of related parasites (e.g., due to greater laying synchrony), or a mixture of parasitic strategies, one with a focus on related hosts and the other on unrelated hosts. Genetic data revealed that social relationships did not always reflect true relatedness and that success of primary parasites was associated with kinship to hosts. | 1323 | no | no good fitness measure, N pop differnt in GD? | NA | no | no |
| 1324 | 2017 | 43017 | Bauer, MM; Reed, KM | 2011 | Extended sequence of the turkey MHC B-locus and sequence variation in the highly polymorphic B-G loci | Genetic variation in the major histocompatibility complex (MHC) is directly correlated to differences in disease resistance. Immunity is greatly dependent on highly polymorphic genes in the MHC, such as class I, class II, and class III complement genes. Preliminary studies of wild turkey populations show extreme polymorphisms in a family of genes exclusive to the avian MHC, the class IV or B-G genes. Significance of this variation is unclear as there are few and conflicting studies of the expression of these genes. Confounding understanding of B-G variation is the lack of a complete delineation of the number of loci in the turkey genome. Direct 454 sequencing of a clone from the CHORI-260 BAC library was used to extend the turkey MHC B-locus sequence, identifying five additional complete B-locus genes including two B-G loci. Sequences of the new B-G genes were compared with those of other turkey gene (BG1-3) and sequences available for other galliformes. Phylogenetic analysis shows species-specific gene evolution supporting a birth-death model of evolution for the B-G gene family. Analysis of variation within the signal peptide sequence (exon 1) found two clusters of polymorphism among the turkey B-G genes. Resequencing of exon 1 in a diverse sample including wild, heritage, and commercial turkeys confirmed multiple alleles at each B-G gene. Future studies aim to correlate B-G variation with group and individual immunological differences. | 1324 | no | no good fitness measure, N pop differnt in GD? | NA | no | no |
| 1325 | 2017 | 43017 | Geml, J; Kauff, F; Brochmann, C; Taylor, DL | 2010 | Surviving climate changes: high genetic diversity and transoceanic gene flow in two arctic-alpine lichens, Flavocetraria cucullata and F-nivalis (Parmeliaceae, Ascomycota) | Aim We examined genetic structure and long-distance gene flow in two lichenized ascomycetes, Flavocetraria cucullata and Flavocetraria nivalis, which are widespread in arctic and alpine tundra. Location Circumpolar North. Methods DNA sequences were obtained for 90 specimens (49 for F. cucullata and 41 for F. nivalis) collected from various locations in Europe, Asia and North America. Sequences of the nuclear internal transcribed spacer (ITS) + 5.8S ribosomal subunit gene region were generated for 89 samples, and supplemented by beta-tubulin (BTUB) and translation elongation factor 1-alpha gene (EF1) sequences for a subset of F. cucullata specimens. Phylogenetic, nonparametric permutation methods and coalescent analyses were used to assess population divergence and to estimate the extent and direction of migration among continents. Results Both F. cucullata and F. nivalis were monophyletic, supporting their morphology-based delimitation, and had high and moderately high intraspecific genetic diversity, respectively. Clades within each species contained specimens from both North America and Eurasia. We found only weak genetic differentiation among North American and Eurasian populations, and evidence for moderate to high transoceanic gene flow. Main conclusions Our results suggest that both F. cucullata and F. nivalis have been able to migrate over large distances in response to climatic fluctuations. The high genetic diversity observed in the Arctic indicates long-term survival at high latitudes, whereas the estimated migration rates and weak geographic population structure suggest a continuing long-distance gene flow between continents that has prevented pronounced genetic differentiation. The mode of long-distance dispersal is unknown, but wind dispersal of conidia and/or ascospores is probably important in the open arctic landscapes. The high genetic diversity and efficient long-distance dispersal capability of F. cucullata and F. nivalis suggest that these species, and perhaps other arctic lichens as well, will be able to track their potential niche in the changing Arctic. | 1325 | no | no good fitness measure, N pop differnt in GD? | NA | no | no |
| 1326 | 2017 | 43017 | Bean, K; Amos, W; Pomeroy, PP; Twiss, SD; Coulson, TN; Boyd, IL | 2004 | Patterns of parental relatedness and pup survival in the grey seal (Halichoerus grypus) | Levels of parental relatedness can affect offspring survival and susceptibility to disease. We investigated parental relatedness of live and dead Halichoerus grypus pups between and within island populations and between possible causes of mortality. Nine microsatellites were used to calculate internal relatedness (IR) and standardized mean d(2). We find that pups with higher than average levels of IR have significantly lower survival and that this varied between island populations and that certain loci contributed to the effect more than others. Although, there were no significant differences between causes of mortality, peritonitis, infection and stillborn had the highest levels of IR. These results provide evidence that parental relatedness is an important determinant of pre-weaning pup survival in the grey seal and that this may vary with cause of mortality given a larger sample size. | 1326 | no | individual level | NA | no | no |
| 1327 | 2017 | 43017 | Sabater-Lleal, M; Almasy, L; Martinez-Marchan, E; Martinez-Sanchez, E; Souto, R; Blangero, J; Souto, J; Fontcuberta, J; Soria, JM | 2006 | Genetic architecture of the F7 gene in a Spanish population: implication for mapping complex diseases and for functional assays | Delineating the genetic variability of loci coding for complex diseases helps to understand the individual variation in disease susceptibility and drug response. We present the allelic architecture of the F7 gene. This gene is the major determinant of FVII plasma levels, and these plasma levels constitute an important intermediate risk factor for cardiovascular disease. As part of the Genetic Analysis of Idiopathic Thrombophila Project, we completely re-sequenced the F7 locus (promoter, exons, introns, and 3’-untranslated region) in 40 unrelated individuals. We found 49 polymorphisms with only two amino acid changes suggesting that regulatory non-coding and intronic variants are responsible for the FVII variability. These results are important for mapping susceptibility alleles of complex diseases, because differences in pair-wise linkage disequilibrium patterns between DNA variants and haplotype frequency distributions may help to detect disease-associated alleles. In addition, we present the results of an in silico search that established genomic comparisons among different species. In conclusion, our study of the F7 DNA sequence variations is an example of a strategy for analyzing the genetic architecture of a quantitative trait locus. Furthermore, it provides a model for future analyses of genetic factors that contribute to the susceptibility of complex diseases in humans. | 1327 | no | human | NA | no | no |
| 1328 | 2017 | 43017 | Paris, M; Boyer, S; Bonin, A; Collado, A; David, JP; Despres, L | 2010 | Genome scan in the mosquito Aedes rusticus: population structure and detection of positive selection after insecticide treatment | Identification of genes involved in local adaptation is particularly challenging for species functioning as a network of interconnected populations undergoing frequent extinctions-recolonizations, because populations are submitted to contrasted evolutionary pressures. Using amplified fragment length polymorphism markers, population genetic structure of the mosquito Aedes rusticus was analysed in five geographical areas of the French Rhone-Alpes region. We included a number of sites that were treated with the bio-insecticide Bacillus thuringiensis israelensis (Bti) for more than 15 years. Analysis of molecular variance revealed that most of the genetic variability was found within populations (96%), with no significant variation among geographical areas, although variation among populations within areas (4%) was significant. The global genetic differentiation index F(ST) was low (0.0366 +/- 0.167). However, pairwise F(ST) values were significant and no isolation-by-distance at the regional level was observed, suggesting a metapopulation structure in this species. Bti-treatment had no effect on genetic structure and on within-population genetic diversity. Potential signatures of positive selection associated with Bti-treatment were detected for five loci, even though toxicological bioassays performed on field-collected larvae showed no significant difference in mortality between Bti-treated and nontreated sites. The difficulty of detecting moderate resistance in field-collected larvae together with possible differential persistence of toxins in the environment may explain our inability to detect a toxicological response to Bti in treated sites. The evidence for positive selection occurring at several genomic regions suggests a first step towards Bti resistance in natural mosquito populations treated with this bio-insecticide. Furthermore, this signal was detectable using genomic tools before any toxicological evidence for resistance could be identified. | 1328 | no | no good fitness measure | NA | no | no |
| 1329 | 2017 | 43017 | Liu, JJ; Hammett, C | 2014 | Development of novel polymorphic microsatellite markers by technology of next generation sequencing in western white pine | The invasive fungal pathogen Cronartium ribicola has seriously damaged five-needle pines in North America. Genetic diversity assessment using DNA markers among natural populations will benefit conservation and restoration programs. This report presents characterization of the first set of microsatellite markers developed by analyzing a reference transcriptome de novo assembled from RNA-seq data and expressed sequence tags in western white pine (Pinus monticola). A total of 1,948 microsatellite loci were detected in 1,876 contigs. Forty-six loci were verified to be polymorphic, with allele number per locus ranging from 2 to 12. The mean observed and expected heterozygosity per locus was measured as 0.671 and 0.485, respectively. These microsatellite markers will be useful for characterizing genetic diversity and gene-flow among natural stands and population structure among seed families for conservation of this conifer species across western North America. | 1329 | no | no good fitness measure, N pop differnt in GD? | NA | no | no |
| 1330 | 2017 | 43017 | Jacob, T; Jebasingh, T; Venugopal, MN; Usha, R | 2003 | High genetic diversity in the coat protein and 3 ’ untranslated regions among geographical isolates of Cardamom mosaic virus from south India | A survey was conducted to study the biological and genetic diversity of Cardamom mosaic virus (CdMV) that causes the most widespread disease in the cardamom growing area in the Western Ghats of south India. Six distinct subgroups were derived based on their symptomatology and host range from the sixty isolates collected. The serological variability between the virus isolates was analysed by ELISA and Western blotting. The 3’ terminal region consisting of the coat protein (CP) coding sequence and 3’ untranslated region (3’UTR) was cloned and sequenced from seven isolates. Sequence comparisons revealed considerable genetic diversity among the isolates in their CP and 3’UTR, making CdMV one of the highly variable members of Potyviridae. The possible occurrence of recombination between the isolates and the movement of the virus in the cardamom tract of south India are discussed. | 1330 | no | gd of pathogen | NA | no | no |
| 1331 | 2017 | 43017 | Ben Halim, N; Nagara, M; Regnault, B; Hsouna, S; Lasram, K; Kefi, R; Azaiez, H; Khemira, L; Saidane, R; Ben Ammar, S; Besbes, G; Weil, D; Petit, C; Abdelhak, S; Romdhane, L | 2015 | Estimation of Recent and Ancient Inbreeding in a Small Endogamous Tunisian Community Through Genomic Runs of Homozygosity | Runs of homozygosity (ROHs) are extended genomic regions of homozygous genotypes that record populations’ mating patterns in the past. We performed microarray genotyping on 15 individuals from a small isolated Tunisian community. We estimated the individual and population genome-wide level of homozygosity from data on ROH above 0.5 Mb in length. We found a high average number of ROH per individual (48.2). The smallest ROH category (0.5-1.49 Mb) represents 0.93% of the whole genome, while medium-size (1.5-4.99 Mb) and long-size ROH (>= 5 Mb) cover 1.18% and 0.95%, respectively. We found that genealogical individual inbreeding coefficients (Fped) based on three-to four-generation pedigrees are not reliable indicators of the current proportion of genome-wide homozygosity inferred from ROH (FROH) either for 0.5 or 1.5 Mb ROH length thresholds, while identity-by-descent sharing is a function of shared coancestry. This study emphasizes the effect of reproductive isolation and a prolonged practice of consanguinity that limits the genetic heterogeneity. It also provides evidence of both recent and ancient parental relatedness contribution to the current level of genome-wide homozygosity in the studied population. These findings may be useful for evaluation of long-term effects of inbreeding on human health and for future applications of ROHs in identifying recessive susceptibility genes. | 1331 | no | inbreeding | NA | no | no |
| 1332 | 2017 | 43017 | Chen, JH; Huang, CL; Lai, YL; Chang, CT; Liao, PC; Hwang, SY; Sun, CW | 2017 | Postglacial range expansion and the role of ecological factors in driving adaptive evolution of Musa basjoo var. formosana | Genetic variation evolves during postglacial range expansion of a species and is important for adapting to varied environmental conditions. It is crucial for the future survival of a species. We investigate the nuclear DNA sequence variation to provide evidence of postglacial range expansion of Musa basjoo var. formosana, a wild banana species, and test for adaptive evolution of amplified fragment length polymorphic (AFLP) loci underlying local adaptation in association with environmental variables. Postglacial range expansion was suggested by phylogeographical analyses based on sequence variation of the second intron of copper zinc superoxide dismutase 2 gene. Two glacial refugia were inferred by the average F-ST parameter (mean F-ST of a population against the remaining populations). Using variation partitioning by redundancy analysis, we found a significant amount of explained AFLP variation attributed to environmental and spatially-structured environmental effects. By combining genome scan methods and multiple univariate logistic regression, four AFLP loci were found to be strongly associated with environmental variables, including temperature, precipitation, soil moisture, wet days, and surface coverage activity representing vegetation greenness. These environmental variables may have played various roles as ecological drivers for adaptive evolution of M. basjoo var. formosana during range expansion after the last glacial maximum. | 1332 | no | no good fitness measure | NA | no | no |
| 1333 | 2017 | 43017 | Barbujani, G; Belle, EMS | 2006 | Genomic boundaries between human populations | Different authors disagree on whether human genome variation should be described as continuous or discontinuous; in the latter case, by attributing an individual’s genotype to one genetic cluster, one would also obtain information on the individual’s genome in general. An analysis of 377 microsatellites of the CEPH human diversity panel was interpreted as evidence that most genotypes cluster into one of five distinct groups, approximately corresponding to continents, which were proposed by some authors as the major biological subdivisions of humankind. Here we analyse the same dataset by a specific numerical method, designed to detect genomic boundaries, i.e. zones of increased change in maps of genomic variation. We show that statistically significant boundaries can be described between groups of populations, but different clusters are identified, depending on the assumptions of the model. In addition, these clusters do not correspond to the clusters inferred from previous analyses of the same or of other polymorphisms. We conclude that it is indeed possible to cluster genotypes according to geography, but no study so far identified unambiguously anything that can be regarded as a major genetic subdivision of humankind, and hence discontinuous models of human diversity are unsupported by data. Copyright (c) 2006 S. Karger AG, Basel. | 1333 | no | human | NA | no | no |
| 1334 | 2017 | 43017 | Dercole, F; Della Rossa, F | 2017 | A deterministic eco-genetic model for the short-term evolution of exploited fish stocks | Eco-genetic models describe the contemporary evolution of quantitative genetic traits by integrating the key ecological aspects with the modes of inheritance. Because of the flexibility in incorporating physiological, ecological, and genetic detail, eco-genetic models are typically individual-based and stochastic. Here we propose a deterministic eco-genetic population model to study the evolution of maturation schedules in exploited fish stocks. It is the first deterministic model addressing the evolution of the probabilistic maturation reaction norm (PMRN) the genetic trait recognized to control maturation in fish species. The PMRN is the probability of interseasonal maturation as a function of age and size of juvenile individuals. Being independent of the growth trajectory followed by the individual, it can be considered a non-plastic phenotype, under limited change in temperature and climate. We consider the continuous age and size structures of the fish stock and explicitly represent foraging on a single food resource and mating under 1:1 sex ratio. Inheritance follows a single-locus-two-alleles Mendelian rule to limit the number of genotypes. We adapt the standard escalator boxcar train method for the simulation of physiologically structured population models to the case of two-dimensional age distributions mature individuals being also distributed according to the age at maturation. The model simulations qualitatively reproduce empirical trends, specifically the genotypic redistribution toward early-maturing types under increasing exploitation and the eventual collapse of the stock. Interestingly, increased effort targeted at large sizes seems to erode the genetic diversity, an effect missed by stochastic models that might serve as warning to fishery managers and policy makers. (C) 2016 Elsevier B.V. All rights reserved. | 1334 | no | NA | NA | no | no |
| 1335 | 2017 | 43017 | Grossen, C; Keller, L; Biebach, I; Croll, D | 2014 | Introgression from Domestic Goat Generated Variation at the Major Histocompatibility Complex of Alpine Ibex | The major histocompatibility complex (MHC) is a crucial component of the vertebrate immune system and shows extremely high levels of genetic polymorphism. The extraordinary genetic variation is thought to be ancient polymorphisms maintained by balancing selection. However, introgression from related species was recently proposed as an additional mechanism. Here we provide evidence for introgression at the MHC in Alpine ibex (Capra ibex ibex). At a usually very polymorphic MHC exon involved in pathogen recognition (DRB exon 2), Alpine ibex carried only two alleles. We found that one of these DRB alleles is identical to a DRB allele of domestic goats (Capra aegagrus hircus). We sequenced 2489 bp of the coding and non-coding regions of the DRB gene and found that Alpine ibex homozygous for the goat-type DRB exon 2 allele showed nearly identical sequences (99.8%) to a breed of domestic goats. Using Sanger and RAD sequencing, microsatellite and SNP chip data, we show that the chromosomal region containing the goat-type DRB allele has a signature of recent introgression in Alpine ibex. A region of approximately 750 kb including the DRB locus showed high rates of heterozygosity in individuals carrying one copy of the goat-type DRB allele. These individuals shared SNP alleles both with domestic goats and other Alpine ibex. In a survey of four Alpine ibex populations, we found that the region surrounding the DRB allele shows strong linkage disequilibria, strong sequence clustering and low diversity among haplotypes carrying the goat-type allele. Introgression at the MHC is likely adaptive and introgression critically increased MHC DRB diversity in the genetically impoverished Alpine ibex. Our finding contradicts the long-standing view that genetic variability at the MHC is solely a consequence of ancient trans-species polymorphism. Introgression is likely an underappreciated source of genetic diversity at the MHC and other loci under balancing selection. | 1335 | no | domesticated animal | NA | no | no |
| 1336 | 2017 | 43017 | Rodriguez-Quilon, I; Santos-del-Blanco, L; Grivet, D; Jaramillo-Correa, JP; Majada, J; Vendramin, GG; Alia, R; Gonzalez-Martinez, SC | 2015 | Local effects drive heterozygosity-fitness correlations in an outcrossing long-lived tree | Heterozygosity-fitness correlations (HFCs) have been used to understand the complex interactions between inbreeding, genetic diversity and evolution. Although frequently reported for decades, evidence for HFCs was often based on underpowered studies or inappropriate methods, and hence their underlying mechanisms are still under debate. Here, we used 6100 genome-wide single nucleotide polymorphisms (SNPs) to test for general and local effect HFCs in maritime pine (Pinus pinaster Ait.), an iconic Mediterranean forest tree. Survival was used as a fitness proxy, and HFCs were assessed at a four-site common garden under contrasting environmental conditions (total of 16 288 trees). We found no significant correlations between genome-wide heterozygosity and fitness at any location, despite variation in inbreeding explaining a substantial proportion of the total variance for survival. However, four SNPs (including two non-synonymous mutations) were involved in significant associations with survival, in particular in the common gardens with higher environmental stress, as shown by a novel heterozygosity-fitness association test at the species-wide level. Fitness effects of SNPs involved in significant HFCs were stable across maritime pine gene pools naturally growing in distinct environments. These results led us to dismiss the general effect hypothesis and suggested a significant role of heterozygosity in specific candidate genes for increasing fitness in maritime pine. Our study highlights the importance of considering the species evolutionary and demographic history and different spatial scales and testing environments when assessing and interpreting HFCs. | 1336 | no | no pop level? | NA | no | no |
| 1337 | 2017 | 43017 | Santonastaso, T; Lighten, J; van Oosterhout, C; Jones, KL; Foufopoulos, J; Anthony, NM | 2017 | The effects of historical fragmentation on major histocompatibility complex class II beta and microsatellite variation in the Aegean island reptile, Podarcis erhardii | The major histocompatibility complex (MHC) plays a key role in disease resistance and is the most polymorphic gene region in vertebrates. Although habitat fragmentation is predicted to lead to a loss in MHC variation through drift, the impact of other evolutionary forces may counter this effect. Here we assess the impact of selection, drift, migration, and recombination on MHC class II and microsatellite variability in 14 island populations of the Aegean wall lizard Podarcis erhardii. Lizards were sampled from islands within the Cyclades (Greece) formed by rising sea levels as the last glacial maximum approximately 20,000 before present. Bathymetric data were used to determine the area and age of each island, allowing us to infer the corresponding magnitude and timing of genetic bottlenecks associated with island formation. Both MHC and microsatellite variation were positively associated with island area, supporting the hypothesis that drift governs neutral and adaptive variation in this system. However, MHC but not microsatellite variability declined significantly with island age. This discrepancy is likely due to the fact that microsatellites attain mutation-drift equilibrium more rapidly than MHC. Although we detected signals of balancing selection, recombination and migration, the effects of these evolutionary processes appeared negligible relative to drift. This study demonstrates how land bridge islands can provide novel insights into the impact of historical fragmentation on genetic diversity as well as help disentangle the effects of different evolutionary forces on neutral and adaptive diversity. | 1337 | no | no good fitness measure | NA | no | no |
| 1338 | 2017 | 43017 | Brooks, AW; Kohl, KD; Brucker, RM; van Opstal, EJ; Bordenstein, SR | 2016 | Phylosymbiosis: Relationships and Functional Effects of Microbial Communities across Host Evolutionary History | Phylosymbiosis was recently proposed to describe the eco-evolutionary pattern, whereby the ecological relatedness of host-associated microbial communities parallels the phylogeny of related host species. Here, we test the prevalence of phylosymbiosis and its functional significance under highly controlled conditions by characterizing the microbiota of 24 animal species from four different groups (Peromyscus deer mice, Drosophila flies, mosquitoes, and Nasonia wasps), and we reevaluate the phylosymbiotic relationships of seven species of wild hominids. We demonstrate three key findings. First, intraspecific microbiota variation is consistently less than interspecific microbiota variation, and microbiota-based models predict host species origin with high accuracy across the dataset. Interestingly, the age of host clade divergence positively associates with the degree of microbial community distinguishability between species within the host clades, spanning recent host speciation events (similar to 1 million y ago) to more distantly related host genera (similar to 108 million y ago). Second, topological congruence analyses of each group’s complete phylogeny and microbiota dendrogram reveal significant degrees of phylosymbiosis, irrespective of host clade age or taxonomy. Third, consistent with selection on host-microbiota interactions driving phylosymbiosis, there are survival and performance reductions when interspecific microbiota transplants are conducted between closely related and divergent host species pairs. Overall, these findings indicate that the composition and functional effects of an animal’s microbial community can be closely allied with host evolution, even across wide-ranging timescales and diverse animal systems reared under controlled conditions. | 1338 | no | NA | NA | no | no |
| 1339 | 2017 | 43017 | Elderkin, CL; Christian, AD; Metcalfe-Smith, JL; Berg, DJ | 2008 | Population genetics and phylogeography of freshwater mussels in North America, Elliptio dilatata and Actinonaias ligamentina (Bivalvia : Unionidae) | Extrinsic and intrinsic forces combined shape the population structure of every species differently. Freshwater mussels are obligate parasites to a host fish during a juvenile stage (glochidia). Elliptio dilatata (ED) and Actinonaias ligamentina (AL) are co-occurring freshwater mussel taxa with similar North American distribution and share some potential host fish. Using mitochondrial DNA, we determined the genotypes of 190 + individuals from collection sites in at least two tributaries in the Lake Erie and Ohio River watersheds, along with the Ouachita and Strawberry rivers in the southeast. Both species had followed a stepping-stone model of dispersal, with greater pairwise genetic structure among collection sites of ED. Also, phylogeographical analysis for ED found significant geographical structuring of haplotype diversity. Overall, within-population variation increased significantly from north to south, with low genetic diversity in the Strawberry River. We calculated significant among-population structure for both species (ED: Phi(ST) = 0.62, P < 0.001; AL: Phi(ST) = 0.16, P < 0.001). Genetic analysis identified the Ouachita River as an area of significant reproductive isolation for both species. Results for AL indicated dispersal into northern areas from two genetically distinct glacial refugia, where results for ED indicated dispersal followed by low gene flow in northern areas. The conservation strategies for mussels that co-occur in the same ‘bed’ could be species specific. Species such as ED have management units on the population scale, where AL has a more homogeneous genetic structure across its range. | 1339 | no | gd of parasite | NA | no | no |
| 1340 | 2017 | 43017 | Engelbrecht, CJB; Harrington, TC; Steimel, J; Capretti, P | 2004 | Genetic variation in eastern North American and putatively introduced populations of Ceratocystis fimbriata f. platani | The plant pathogenic fungus Ceratocystis fimbriata f. platani attacks Platanus species (London plane, oriental plane and American sycamore) and has killed tens of thousands of plantation trees and street trees in the eastern United States, southern Europe and Modesto, California. Nuclear and mitochondrial DNA fingerprints and alleles of eight polymorphic microsatellite markers of isolates of C. fimbriata from these regions delineated major differences in gene diversities. The 33 isolates from the eastern United States had a moderate degree of gene diversity, and unique genotypes were found at each of seven collection sites. Fingerprints of 27 isolates from 21 collection sites in southern Europe were identical with each other; microsatellite markers were monomorphic within the European population, except that three isolates differed at one locus each, due perhaps to recent mutations. The genetic variability of C. fimbriata f. platani in the eastern United States suggests that the fungus is indigenous to this region. The genetic homogeneity of the fungus in Europe suggests that this population has gone through a recent genetic bottleneck, perhaps from the introduction of a single genotype. This supports the hypothesis that the pathogen was introduced to Europe through Naples, Italy during World War II on infected crating material from the eastern United States. The Californian population may also have resulted from introduction of one or a few related genotypes because it, too, had a single nuclear and mitochondrial genotype and limited variation in microsatellite alleles. | 1340 | no | gd of pathogen | NA | no | no |
| 1341 | 2017 | 43017 | Sindicic, M; Polanc, P; Gomercic, T; Jelencic, M; Huber, D; Trontelj, P; Skrbinsek, T | 2013 | Genetic data confirm critical status of the reintroduced Dinaric population of Eurasian lynx | Eurasian lynx (Lynx lynx) reintroduction to the Dinaric Mountains is considered one of the most successful reintroductions of a large predator. Six reintroduced animals founded the population, which rapidly expanded from Slovenia, through Croatia, and all the way to Bosnia and Herzegovina. However, a decrease of the population size has been observed during the last 10-15 years. Considering that possible inbreeding depression would be additive to threats like poaching, traffic mortality and prey base depletion, another extinction of this species from the Dinaric Mountains is a real possibility. We analyzed 204 samples collected between 1979 and 2010 using twenty microsatellite loci and 900-bp mitochondrial DNA control region sequence to evaluate conservation genetics aspects of this endangered population. Both markers confirmed low genetic variability of the Dinaric lynx population, and considerable effective inbreeding (0.3) compared to the source Carpathian population. Our analysis of effective population size and microsatellite variability supported field observations of decreasing population number. As a natural recolonization is a very remote possibility, we recommend population augmentation from a large source population. | 1341 | no | no good fitness measure | NA | no | no |
| 1342 | 2017 | 43017 | Springbett, AJ; MacKenzie, K; Woolliams, JA; Bishop, SC | 2003 | The contribution of genetic diversity to the spread of infectious diseases in livestock populations | This article uses stochastic simulations with a compartmental epidemic model to quantify the impact of genetic diversity within animal populations on the transmission of infectious disease. Genetic diversity is defined by the number of distinct genotypes in the population conferring resistance to microparasitic (e.g., viral or bacterial) infections. Scenarios include homogeneous populations and populations composed of few (finite-locus model) or many (infinitesimal model) genotypes. Genetic heterogeneity has no impact upon the expected value of the basic reproductive ratio (the primary description of the transmission of infection) but affects the variability of this parameter. Consequently, increasing genetic heterogeneity is associated with an increased probability of minor epidemics and decreased probabilities of both major (catastrophic) epidemics and no epidemics. Additionally, heterogeneity per se is associated with a breakdown in the expected relationship between the basic reproductive ratio and epidemic severity, which has been developed for homogeneous populations, with increasing heterogeneity generally resulting in fewer infected animals than expected. Furthermore, increased heterogeneity is associated with decreased disease-dependent mortality in major epidemics and a complex trend toward decreased duration of these epidemics. In summary, more heterogeneous populations are not expected to suffer fewer epidemics on average, but are less likely to suffer catastrophic epidemics. | 1342 | no | NA | NA | no | no |
| 1343 | 2017 | 43017 | Lane, A; Cheng, YY; Wright, B; Hamede, R; Levan, L; Jones, M; Ujvari, B; Belov, K | 2012 | New Insights into the Role of MHC Diversity in Devil Facial Tumour Disease | Background: Devil facial tumour disease (DFTD) is a fatal contagious cancer that has decimated Tasmanian devil populations. The tumour has spread without invoking immune responses, possibly due to low levels of Major Histocompatibility Complex (MHC) diversity in Tasmanian devils. Animals from a region in north-western Tasmania have lower infection rates than those in the east of the state. This area is a genetic transition zone between sub-populations, with individuals from north-western Tasmania displaying greater diversity than eastern devils at MHC genes, primarily through MHC class I gene copy number variation. Here we test the hypothesis that animals that remain healthy and tumour free show predictable differences at MHC loci compared to animals that develop the disease. Methodology/Principal Findings: We compared MHC class I sequences in 29 healthy and 22 diseased Tasmanian devils from West Pencil Pine, a population in north-western Tasmania exhibiting reduced disease impacts of DFTD. Amplified alleles were assigned to four loci, Saha-UA, Saha-UB, Saha-UC and Saha-UD based on recently obtained genomic sequence data. Copy number variation (caused by a deletion) at Saha-UA was confirmed using a PCR assay. No association between the frequency of this deletion and disease status was identified. All individuals had alleles at Saha-UD, disproving theories of disease susceptibility relating to copy number variation at this locus. Genetic variation between the two sub-groups (healthy and diseased) was also compared using eight MHC-linked microsatellite markers. No significant differences were identified in allele frequency, however differences were noted in the genotype frequencies of two microsatellites located near non-antigen presenting genes within the MHC. Conclusions/Significance: We did not find predictable differences in MHC class I copy number variation to account for differences in susceptibility to DFTD. Genotypic data was equivocal but indentified genomic areas for further study. | 1343 | no | No GD diff between pop | NA | no | no |
| 1344 | 2017 | 43017 | Salmier, A; de Thoisy, B; Crouau-Roy, B; Lacoste, V; Lavergne, A | 2016 | Spatial pattern of genetic diversity and selection in the MHC class II DRB of three Neotropical bat species | Background: Although bats are natural reservoirs of many pathogens, few studies have been conducted on the genetic variation and detection of selection in major histocompatibility complex (MHC) genes. These genes are critical for resistance and susceptibility to diseases, and host-pathogen interactions are major determinants of their extensive polymorphism. Here we examined spatial patterns of diversity of the expressed MHC class II DRB gene of three sympatric Neotropical bats, Carollia perspicillata and Desmodus rotundus (Phyllostomidae), and Molossus molossus (Molossidae), all of which use the same environments (e.g., forests, edge habitats, urban areas). Comparison with neutral marker (mtDNA D-loop) diversity was performed at the same time. Results: Twenty-three DRB alleles were identified in 19 C. perspicillata, 30 alleles in 35 D. rotundus and 20 alleles in 28 M. molossus. The occurrence of multiple DRB loci was found for the two Phyllostomidae species. The DRB polymorphism was high in all sampling sites and different signatures of positive selection were detected depending on the environment. The patterns of DRB diversity were similar to those of neutral markers for C. perspicillata and M. molossus. In contrast, these patterns were different for D. rotundus for which a geographical structure was highlighted. A heterozygote advantage was also identified for this species. No recombination or gene conversion event was found and phylogenetic relationships showed a trans-species mode of evolution in the Phyllostomids. Conclusions: This study of MHC diversity demonstrated the strength of the environment and contrasting pathogen pressures in shaping DRB diversity. Differences between positively selected sites identified in bat species highlighted the potential role of gut microbiota in shaping immune responses. Furthermore, multiple geographic origins and/or population admixtures observed in C. perspicillata and M. molossus populations acted as an additional force in shaping DRB diversity. In contrast, DRB diversity of D. rotundus was shaped by environment rather than demographic history. | 1344 | no | no good fitness measure, N pop differnt in GD? | NA | no | no |
| 1345 | 2017 | 43017 | Guidolin, AS; Fresia, P; Consoli, FL | 2014 | The Genetic Structure of an Invasive Pest, the Asian Citrus Psyllid Diaphorina citri (Hemiptera: Liviidae) | The Asian citrus psyllid Diaphorina citri is currently the major threat to the citrus industry as it is the vector of Candidatus Liberibacter, the causal agent of huanglongbing disease (HLB). D. citri is native to Asia and now colonizes the Americas. Although it has been known in some countries for a long time, invasion routes remain undetermined. There are no efficient control methods for the HLB despite the intensive management tools currently in use. We investigated the genetic variability and structure of populations of D. citri to aid in the decision making processes toward sustainable management of this species/disease. We employed different methods to quantify and compare the genetic diversity and structure of D. citri populations among 36 localities in Brazil, using an almost complete sequence of the cytochrome oxidase I (COI) gene. Our analyses led to the identification of two geographically and genetically structured groups. The indices of molecular diversity pointed to a recent population expansion, and we discuss the role of multiple invasion events in this scenario. We also argue that such genetic diversity and population structure may have implications for the best management strategies to be adopted for controlling this psyllid and/or the disease it vectors in Brazil. | 1345 | no | gd of pathogen | NA | no | no |
| 1346 | 2017 | 43017 | Vega-Ramos, KL; Uvalle-Bueno, JX; Gomez-Leyva, JF | 2013 | Molecular Variability among Isolates of Fusarium oxysporum Associated with Root Rot Disease of Agave tequilana | In this study, 115 isolates of Fusarium oxysporum from roots of Agave tequilana Weber cv azul plants and soil in commercial plantations in western Mexico were characterized using morphological and molecular methods. Genetic analyses of monosporic isolates included restriction enzyme analysis of rDNA (ARDRA) using HaeIII and HinfI, and genetic diversity was determined using Box-PCR molecular markers. Box-PCR analysis generated 14 groups. The groups correlated highly with the geographic location of the isolate and sample type. These results demonstrate the usefulness of ARDRA and Box-PCR techniques in the molecular characterization of the Fusarium genus for the discrimination of pathogenic isolates. | 1346 | no | gd of pathogen | NA | no | no |
| 1347 | 2017 | 43017 | Piaggio, AJ; Russell, AL; Osorio, IA; Ramirez, AJ; Fischer, JW; Neuwald, JL; Tibbels, AE; Lecuona, L; McCracken, GF | 2017 | Genetic demography at the leading edge of the distribution of a rabies virus vector | The common vampire bat, Desmodus rotundus, ranges from South America into northern Mexico in North America. This sanguivorous species of bat feeds primarily on medium to large-sized mammals and is known to rely on livestock as primary prey. Each year, there are hotspot areas of D. rotundus-specific rabies virus outbreaks that lead to the deaths of livestock and economic losses. Based on incidental captures in our study area, which is an area of high cattle mortality from D. rotundus transmitted rabies, it appears that D. rotundus are being caught regularly in areas and elevations where they previously were thought to be uncommon. Our goal was to investigate demographic processes and genetic diversity at the north eastern edge of the range of D. rotundus in Mexico. We generated control region sequences (441bp) and 12-locus microsatellite genotypes for 602 individuals of D. rotundus. These data were analyzed using network analyses, Bayesian clustering approaches, and standard population genetic statistical analyses. Our results demonstrate panmixia across our sampling area with low genetic diversity, low population differentiation, loss of intermediate frequency alleles at microsatellite loci, and very low mtDNA haplotype diversity with all haplotypes being very closely related. Our study also revealed strong signals of population expansion. These results follow predictions from the leading-edge model of expanding populations and supports conclusions from another study that climate change may allow this species to find suitable habitat within the U.S. border. | 1347 | no | no good fitness measure, N pop differnt in GD? | NA | no | no |
| 1348 | 2017 | 43017 | Munzbergova, Z; Plackova, I | 2010 | Seed mass and population characteristics interact to determine performance of Scorzonera hispanica under common garden conditions | Many recent studies deal with within-species variation in seed mass and its consequences for plant growth The possibility to explicitly separate the effects of population characteristics and seed mass are, however, usually hindered by availability of seeds of different size from different populations We examined the effect of seed mass on germination, establishment, growth, and flowering of Scorzonera inspanica, a perennial herb. We used seeds of different masses from 20 populations, which differed greatly in size, habitat conditions, and genetic diversity We selected seeds of the same range of seed masses from each population to ensure that all the variation in seed mass is represented within population The results show, that at the population level, seed mass was enhanced only by habitat quality estimated using Beals index Mass of seeds affected plant germination, but not subsequent growth. Growth of the plants was affected by genetic diversity of the populations There were also several significant interactions between the effects of seed mass and genetic properties of the populations Results of this study show that the effect of seed mass on plant germination and growth interacts with characteristics of the original population This indicates that conclusions on the effect of seed mass derived using data from one or few populations only may not be representative A range of populations should be examined before drawing conclusions on the seed mass-plant performance relationship (C) 2010 Elsevier GmbH All rights reserved | 1348 | no | no good fitness measure | NA | no | no |
| 1349 | 2017 | 43017 | Pelin, A; Moteshareie, H; Sak, B; Selman, M; Naor, A; Eyahpaise, ME; Farinelli, L; Golshani, A; Kvac, M; Corradi, N | 2016 | The genome of an Encephalitozoon cuniculi type III strain reveals insights into the genetic diversity and mode of reproduction of a ubiquitous vertebrate pathogen | Encephalitozoon cuniculi is a model microsporidian species with a mononucleate nucleus and a genome that has been extensively studied. To date, analyses of genome diversity have revealed the existence of four genotypes in E. cuniculi (EcI, II, III and IV). Genome sequences are available for EcI, II and III, and are all very divergent, possibly diploid and genetically homogeneous. The mechanisms that cause low genetic diversity in E. cuniculi (for example, selfing, inbreeding or a combination of both), as well as the degree of genetic variation in their natural populations, have been hard to assess because genome data have been so far gathered from laboratory-propagated strains. In this study, we aim to tackle this issue by analyzing the complete genome sequence of a natural strain of E. cuniculi isolated in 2013 from a steppe lemming. The strain belongs to the EcIII genotype and has been designated EcIII-L. The EcIII-L genome sequence harbors genomic features intermediate to known genomes of II and III lab strains, and we provide primers that differentiate the three E. cuniculi genotypes using a single PCR. Surprisingly, the EcIII-L genome is also highly homogeneous, harbors signatures of heterozygosity and also one strain-specific single-nucleotide polymorphism (SNP) that introduces a stop codon in a key meiosis gene, Spo11. Functional analyses using a heterologous system demonstrate that this SNP leads to a deficient meiosis in a model fungus. This indicates that EcIII-L meiotic machinery may be presently broken. Overall, our findings reveal previously unsuspected genome diversity in E. cuniculi, some of which appears to affect genes of primary importance for the biology of this pathogen. | 1349 | no | gd of pathogen | NA | no | no |
| 1350 | 2017 | 43017 | Ciobanu, DC; Day, AE; Nagy, A; Wales, R; Rothschild, MF; Plastow, GS | 2001 | Genetic variation in two conserved local Romanian pig breeds using type 1 DNA markers | Analysis of the genetic variation of an endangered population is an important component for the success of conservation. Animals from two local Romanian pig breeds, the Mangalitsa and Bazna, were analysed for variation at a number of genetic loci using PCR-based DNA tests. Polymorphism was assessed at loci which 1) are known to cause phenotypic variation, 2) are potentially involved in trait differences or 3) are putative candidate genes. The traits considered are disease resistance, growth, coat colour, meat quality and prolificacy. Even though the populations are small and the markers are limited to specific genes, we found significant differences in five of the ten characterised loci. In some cases the observed allele frequencies were interesting in relation to gene function and the phenotype of the breed. These breeds are part of a conservation programme in Romania and marker information may be useful in preserving a representative gene pool in the populations. The use of polymorphisms in type 1 (gene) markers may be a useful complement to analysis based on anonymous markers. | 1350 | no | domesticated animal | NA | no | no |
| 1351 | 2017 | 43017 | Brown, JA; Beatty, GE; Finlay, CMV; Montgomery, WI; Tosh, DG; Provan, J | 2016 | Genetic analyses reveal high levels of seed and pollen flow in hawthorn (Crataegus monogyna Jacq.), a key component of hedgerows | “Hedgerows represent important components of agri-environment landscapes that are increasingly coming under threat from climate change, emergent diseases, invasive species and land use change. Given that population genetic data can be used to inform best-practice management strategies for woodland and hedgerow tree species, we carried out a study on hawthorn (Crataegus monogyna Jacq.), a key component of hedgerows, on a regional basis using a combination of nuclear and chloroplast microsatellite markers. We found that levels of genetic diversity were high and comparable to, or slightly higher than, other tree species from the same region. Levels of population differentiation for both sets of markers, however, were extremely low, suggesting extensive gene flow via both seed and pollen. These findings suggest that a holistic approach to woodland management, one which does not necessarily rely on the concept of”“seed zones”" previously suggested, but which also takes into account populations with high and/or rare chloroplast (i.e. seed-specific) genetic variation, might be the best approach to restocking and replanting." | 1351 | no | no good fitness measure, N pop differnt in GD? | NA | no | no |
| 1352 | 2017 | 43017 | Lao, O; Dupanloup, I; Barbujani, G; Bertranpetit, J; Calafell, F | 2008 | The mediterranean paradox for susceptibility factors in coronary heart disease extends to genetics | “The incidence of coronary heart disease (CHD) shows a North to South gradient in Europe. We tested whether that gradient could be accounted for by the distribution of putative susceptibility genotypes. We correlated the published frequencies of susceptibility genotypes for the genes most often associated with CHD (ACE, AGT, APOE, F2, F5, MTHFR, PON1, and SERPINE1) with the incidence of the disease, controlling for the effects of smoking, systolic pressure, total cholesterol, and body-mass index. In three polymorphisms a negative correlation between the incidence of CHD and the frequency of a suceptibility genotype was observed. For ACE this correlation was significantly negative even when discounting classical susceptibility factors. This suggests that some alleles described as susceptibility factors cannot account for disease incidence at the population level. A genetic component must be added to the”“Mediterranean paradox”“: genetic variants deemed to be risk factors for CHD show a geographical pattern uncorrelated with the disease incidence. This pattern can be understood from the history of populations which has shaped the genetic diversity of the European populations in North-South clines, similar to what is observed for CHD incidence, which will tend to create spurious correlations with polymorphisms related, or not related, to the disease.” | 1352 | no | human | NA | no | no |
| 1353 | 2017 | 43017 | Diao, YZ; Zhang, C; Xu, JP; Lin, D; Liu, L; Mtung’e, OG; Liu, XL | 2015 | Genetic differentiation and recombination among geographic populations of the fungal pathogen Colletotrichum truncatum from chili peppers in China | Colletotrichum truncatum is an extremely important fungal pathogen. It can cause diseases both in humans and in over 460 plant species. However, little is known about its genetic diversity within and among populations. One of the major plant hosts of C.truncatum is pepper, and China is one of the main pepper-producing countries in the world. Here, we propose the hypotheses that geography has a major influence on the relationships among populations of C.truncatum in China and that infections in different populations need to be managed differently. To test these hypotheses, we obtained and analyzed 266 C.truncatum isolates from 13 regions representing the main pepper-growing areas throughout China. The analysis based on nine microsatellite markers identified high intrapopulation genetic diversity, evidence of sexual recombination, and geographic differentiation. The genetic differentiation was positively correlated with geographic distance, with the southern and northern China populations grouped in two distinct clusters. Interestingly, isolates collected from the pepper-breeding center harbored the most private alleles. The results suggest that the geographic populations of C.truncatum on peppers in China are genetically differentiated and should be managed accordingly. Our study also provides a solid foundation from which to further explore the global genetic epidemiology of C.truncatum in both plants and humans. | 1353 | no | gd of pathogen | NA | no | no |
| 1354 | 2017 | 43017 | Michael, CA; Gillings, MR; Holmes, AJ; Hughes, L; Andrew, NR; Holley, MP; Stokes, HW | 2004 | Mobile gene cassettes: A fundamental resource for bacterial evolution | Horizontal gene transfer increases genetic diversity in prokaryotes to a degree not allowed by the limitations of reproduction by binary fission. The integron/gene cassette system is one of the most recently characterized examples of a system that facilitates horizontal gene transfer. This system, discovered in the context of multidrug resistance, is recognized in a clinical context for its role in allowing pathogens to adapt to the widespread use of antibiotics. Recent studies suggest that gene cassettes are common and encode functions relevant to many adaptive traits. To estimate the diversity of mobile cassettes in a natural environment, a molecular technique was developed to provide representative distributions of cassette populations at points within a sampling area. Subsequently, statistical methods analogous to those used for calculating species diversity were employed to assess the diversity of gene cassettes within the sample area in addition to gaining an estimate of cassette pool size. Results indicated that the number of cassettes within a 5 x 10-m sample area was large and that the overall mobile cassette metagenome was likely to be orders of magnitude larger again. Accordingly, gene cassettes appear to be capable of mobilizing a significant genetic resource and consequently have a substantial impact on bacterial adaptability. | 1354 | no | gd of pathogen | NA | no | no |
| 1355 | 2017 | 43017 | Calazans, SH; Walters, LJ; Fernandes, FC; Ferreira, CEL; Hoffman, EA | 2017 | Genetic structure provides insights into the geographic origins and temporal change in the invasive charru mussel (Sururu) in the southeastern United States | In 2004, Mytella charruana (d’Orbigny, 1842) (Mollusca: Bivalvia: Mytilidae) became established along the coast of the southeastern United States (SE-US). Using mitochondrial DNA sequencing (cytochrome c oxidase subunit I), we compared genetic variation throughout its native range in South America to its invasive range in the SE-US. Samples from the SE-US were collected in 2006 and 2010 enabling a temporal comparison to evaluate possible genetic changes of the invasive population. We addressed two questions. First, what are the potential source populations (or geographic regions) for the SE-US invasion? Second, how has genetic diversity changed between the two sampling periods within the SE-US? We identified a total of 72 haplotypes, 64 of which were isolated to geographic sites and only 8 were shared among sites. The highly structured native range provides insight into the origin of invasive populations where our results suggest that the introduced SE-US population originated from multiple source populations with the Panama region as the primary source. Additionally, our results indicate that genetic composition of the non-native populations was unchanged between the two sampling periods. Mytella charruana exhibit a significant pattern of genetic structure among natural populations, owing to biogeographic barriers that limit natural dispersal, and an ability to persist in novel habitats, owing to a suite of life-history characters that favor survival under variable conditions. Overall, this study explains why M. charruana may become an increasing threat to locations founded by anthropogenic transportation. | 1355 | no | no good fitness measure | NA | no | no |
| 1356 | 2017 | 43017 | Ebert, D; Altermatt, F; Lass, S | 2007 | A short term benefit for outcrossing in a Daphnia metapopulation in relation to parasitism | Because host parasite interactions are often specific to the host and parasite genotype, it may be important whether a host reproduces by selfing or outcrossing. The latter is associated with higher genetic diversity among the offspring and may reduce parasite success. Here, we test whether outbred offspring of Daphnia magna have an advantage over selfed offspring in the presence of a parasite transmitted from mothers to offspring. Using outdoor mesocosms, we set up monoclonal and polyclonal host populations of D. magna infected with a prevalence of 100% with the horizontally and vertically transmitted microsporidian parasite Octosporea bayeri. These populations diapaused after sexual reproduction and hatchlings were screened for signs of O. bayeri. Parasite prevalence was 98.9% for hatchlings from the monoclonal treatment, but only 85.2% among the hatchlings from the polyclonal populations, indicating a short-term benefit for outbreeding. This bene. t occurs, we hypothesize, not owing to inbreeding depression, but because the vertically transmitted parasite is less able to establish itself in the relatively new genetic environment of the outbred offspring, as compared to the more stable environment when transmitted to selfed offspring. To quantify the fitness consequences of this 14% prevalence difference, we studied the within-season epidemiology of O. bayeri, using an epidemiological model. We then examined whether descendants of outbred offspring produce more resting eggs than the descendants of selfed offspring. The data and our model show that Daphnia which are uninfected at the beginning of the growth season have a large advantage when the entire season is considered. Our data support the Red Queen hypothesis which states that in the presence of coevolving parasites, outbreeding is favoured in the host. | 1356 | no | no good Gd measurement | NA | no | no |
| 1357 | 2017 | 43017 | Suarez-Garcia, C; de Paz, JP; Febles, R; Caujape-Castells, J | 2009 | Genetic diversity and floral dimorphism in Limonium dendroides (Plumbaginaceae), a woody Canarian species on the way of extinction | We combined reproductive and allozyme data to assess the levels and structuring of genetic variation and propose conservation guidelines in Limonium dendroides, a critically endangered Canarian endemic Plumbaginaceae featuring the floral and pollen pap/cob dimorphisms associated with the heteromorphic diallelic self-incompatibility system described for this family. Although seed germination has been reported in greenhouse conditions, the detection of individuals of only one morph in all wild subpopulations surveyed explains the extremely limited seed production and recruitment in nature. The geographical proximity and genetic closeness between some subpopulations, together with absence of inbreeding depression symptoms, and a higher allozyme variation in the cultured or reintroduced offspring than in their parental wild subpopulations indicate the viability of occasional compatible matings, thereby suggesting that even low levels of gene flow could mitigate the deleterious effects of fragmentation on subpopulation survival. However, our overall results indicate that L. dendroides is in a critical conservation situation where the utter scarcity of compatible mates within the subpopulations, radically low subpopulation sizes, poor inter-subpopulation gene flow, impoverished genetic variation, herbivore grazing, and the extreme habitat topography have overridden the reproductive capabilities of the species. According to our results, once morph types in all wild and cultured specimens can be determined, inducing fertile crosses through mixed reinforcements is advisable only in the most extremely isolated and small subpopulations (Argaga and Guarimiar) using individuals from the nearest ones (Azadoe and Palmarejo), whilst non-mixed reinforcements seem viable in the remaining subpopulations. Only when the subpopulations attain higher seed production and recruitment rates will it be adequate to collect seeds for their storage at a germplasm bank facility. | 1357 | no | no good Gd measurement | NA | no | no |
| 1358 | 2017 | 43017 | Bulayeva, KB; Glatt, SJ; Bulayev, OA; Pavlova, TA; Tsuang, MT | 2007 | Genome-wide linkage scan of schizophrenia: A cross-isolate study | “Genetic isolates are exceptional resources for the detection of susceptibility genes for complex diseases because of the potential reduction in genetic and clinical heterogeneity. However, the outcome of these mapping efforts is dependent upon the demographic history of a given isolated population, with the most significant factors being a constant population size, the number of generations since founding, and the pathogenic loci and their allele frequencies among founders. Here we employed a cross-isolate genome-wide multipoint linkage study design using uniform genetic and clinical methods in four Daghestan ethnically and demographically diverse isolates with an aggregation of schizophrenia. Our previous population-genetics study showed that Daghestan has an extremely high genetic diversity between ethnic populations and a low genetic diversity within them. The isolates selected for this study include some with more than 200 and some with fewer than 100 generations of demographical history since their founding. Updated clinical data using DSM-IV criteria showed between-isolate differences in aggregation of distinct types of schizophrenia: one of the isolates had a predominant aggregation of disorganized schizophrenia, while the other three had predominantly paranoid schizophrenia. The summarized cross-isolate results indicated prominent within and between-isolate differences in clinical and genetic heterogeneity: the most ancient isolates have roughly twofold fewer incidences of distinct clinical phenotypes and fewer linked genomic regions compared to the demographically younger isolates, which exhibit higher clinical and genetic heterogeneity. Affected individuals in the demographically ancient isolate of ethnic Dargins (No. 6022) who suffered from disorganized schizophrenia showed the highest linkage evidence at 17p11-p12 (LOD = 3.73), while isolates with a predominant aggregation of paranoid schizophrenia (Nos. 6005, 6011, and 6034) showed the highest linkage evidence at 22q11(LOD= 3.0 and 4.4). The unified clinical, genomic, and statistical design we used enabled us to separate the linked and unlinked pedigrees in an unbiased fashion for each genomic location. Overall maximized heterogeneity lod scores for the combined pedigrees ranging from 3.5 to 8.7 were found at 2p24, 10q26, 11q23, 12q24, 17p11-p12, 22q11, and 22q13. The cross-isolate homogeneity in linkage patterns may be ascribed to an identical-by-descent”“metahaplotype”" block with pathogenic loci derived from the Daghestan ethnic groups’ common ancestral metapopulation, while the cross-isolate differences may reflect differences in gene drift and recombination events in the history of local isolates. The results obtained support the notion that mapping genes of any complex disease (e.g., schizophrenia) in demographically older genetic isolates may be more time and cost effective in comparison with demographically younger isolates, especially in genetically heterogeneous outbred populations, due to higher clinical and genetic homogeneity of the primary isolates. A study at higher genotyping density across the regions of interest and fluorescence in situ hybridization analyses are currently under-way. (c) 2006 Elsevier Inc. All rights reserved." | 1358 | no | human | NA | no | no |
| 1359 | 2017 | 43017 | Boerner, M; Hoffman, JI; Amos, W; Chakarov, N; Kruger, O | 2013 | No correlation between multi-locus heterozygosity and fitness in the common buzzard despite heterozygote advantage for plumage colour | Correlations between heterozygosity and fitness are frequently found but rarely well understood. Fitness can be affected by single loci of large effect which correlate with neutral markers via linkage disequilibrium, or as a result of variation in genome-wide heterozygosity following inbreeding. We explored these alternatives in the common buzzard, a raptor species in which three colour morphs differ in their lifetime reproductive success. Using 18 polymorphic microsatellite loci, we evaluated potential genetic differences among the morphs which may lead to subpopulation structuring and tested for correlations between three fitness-related traits and heterozygosity, both genome wide and at each locus separately. Despite their assortative mating pattern, the buzzard morphs were found to be genetically undifferentiated. Multilocus heterozygosity was only found to be correlated with a single fitness-related trait, infection with the blood parasite, Leucocytozoon buteonis, and this was via interactions with vole abundance and age. One locus also showed a significant relationship with blood parasite infection and ectoparasite infestation. The vicinity of this locus contains two genes, one of which is potentially implicated in the immune system of birds. We conclude that genome-wide heterozygosity is unlikely to be a major determinant of parasite burden and body condition in the polymorphic common buzzard. | 1359 | no | no good fitness measure | NA | no | no |
| 1360 | 2017 | 43017 | Orjuela-Sanchez, P; Karunaweera, ND; da Silva-Nunes, M; da Silva, NS; Scopel, KKG; Goncalves, RM; Amaratunga, C; Sa, JM; Socheat, D; Fairhust, RM; Gunawardena, S; Thavakodirasah, T; Galapaththy, GLN; Abeysinghe, R; Kawamoto, F; Wirth, DF; Ferreira, MU | 2010 | Single-nucleotide polymorphism, linkage disequilibrium and geographic structure in the malaria parasite Plasmodium vivax: prospects for genome-wide association studies | Background: The ideal malaria parasite populations for initial mapping of genomic regions contributing to phenotypes such as drug resistance and virulence, through genome-wide association studies, are those with high genetic diversity, allowing for numerous informative markers, and rare meiotic recombination, allowing for strong linkage disequilibrium (LD) between markers and phenotype-determining loci. However, levels of genetic diversity and LD in field populations of the major human malaria parasite P. vivax remain little characterized. Results: We examined single-nucleotide polymorphisms (SNPs) and LD patterns across a 100-kb chromosome segment of P. vivax in 238 field isolates from areas of low to moderate malaria endemicity in South America and Asia, where LD tends to be more extensive than in holoendemic populations, and in two monkey-adapted strains (Salvador-I, from El Salvador, and Belem, from Brazil). We found varying levels of SNP diversity and LD across populations, with the highest diversity and strongest LD in the area of lowest malaria transmission. We found several clusters of contiguous markers with rare meiotic recombination and characterized a relatively conserved haplotype structure among populations, suggesting the existence of recombination hotspots in the genome region analyzed. Both silent and nonsynonymous SNPs revealed substantial between-population differentiation, which accounted for similar to 40% of the overall genetic diversity observed. Although parasites clustered according to their continental origin, we found evidence for substructure within the Brazilian population of P. vivax. We also explored between-population differentiation patterns revealed by loci putatively affected by natural selection and found marked geographic variation in frequencies of nucleotide substitutions at the pvmdr-1 locus, putatively associated with drug resistance. Conclusion: These findings support the feasibility of genome-wide association studies in carefully selected populations of P. vivax, using relatively low densities of markers, but underscore the risk of false positives caused by population structure at both local and regional levels. | 1360 | no | gd of parasite | NA | no | no |
| 1361 | 2017 | 43017 | Ansari, TH; Bertozzi, T; Miller, RD; Gardner, MG | 2015 | MHC in a monogamous lizard - Characterization of class I MHC genes in the Australian skink Tiliqua rugosa | The major histocompatibility complex (MHC) is a highly variable region of vertebrate genomes that encodes cellular proteins involved in the immune response. In addition to the benefits of MHC research in understanding the genetic basis of host resistance to disease, the MHC is an ideal candidate for studying genetic diversity under strong natural selection. However, the MHC of many non-model vertebrate taxa are poorly characterized, hindering an understanding of disease resistance and its application to conservation genetics in these groups. Squamates (lizards and snakes) remain particularly underrepresented despite their being the most diverse order of non-avian sauropsids. We characterized MHC class I sequence diversity from an Australian skink, the sleepy lizard (Tiliqua rugosa), using both cDNA and genomic sequence data and also present genomic class I sequences from the related skinks Tiliqua adelaidensis and Egernia stokesii. Phylogenetic analysis of Tiliqua and other published sqamate MHC class I sequences suggest that MHC diverged very early in Tiliqua compared with the other studied squamates. We identified at least 4 classical MHC class I loci in T rugosa and also shared polymorphism among T rugosa, T. adelaidensis and E. stokesii in the sequences encoding peptide-binding alpha 1 and alpha 2 domains. (C) 2015 Elsevier Ltd. All rights reserved. | 1361 | no | no good fitness measure, N pop differnt in GD? | NA | no | no |
| 1362 | 2017 | 43017 | GEYER, CJ; RYDER, OA; CHEMNICK, LG; THOMPSON, EA | 1993 | ANALYSIS OF RELATEDNESS IN THE CALIFORNIA CONDORS, FROM DNA FINGERPRINTS | Genealogical relationships between extant individuals are an important criterion in the development of a genetic management plan for an endangered species. Where survival is ensured by captive breeding, the genealogy of current individuals is often available, but relationships among founders from the wild may be almost completely unknown. Genetic data can provide information on these unknown relationships, but the inference of genealogical structure from genetic data is not straightforward, especially where the available data are multilocus DNA fingerprints. Here we use a new model to analyze the DNA fingerprint data available for the California condor population and to make inferences as to the structure of relationships among the founder individuals. We show that inferences can be made on the basis of these fingerprint data. The inferences made have implications for the genetic management of the species. | 1362 | no | no good fitness measure, N pop differnt in GD? | NA | no | no |
| 1363 | 2017 | 43017 | Cakic, S; Mojsilovic, M; Mihaljica, D; Milutinovic, M; Petrovic, A; Tomanovic, S | 2014 | MOLECULAR CHARACTERIZATION OF COI GENE OF IXODES RICINUS (LINNAEUS, 1758) FROM SERBIA | The Ixodes ricinus tick is common in the central part of the Balkan Peninsula. It is a vector of pathogenic agents causing diseases in humans and animals. Little is known about the genetic structure of I. ricinus in this region. We have investigated intraspecific variability of the COI gene among I. ricinus ticks collected from different regions of Serbia, and the correlation between the various types of habitat and genetic variability of ticks. The obtained COI gene sequences are the first barcoding sequences of I. ricinus ticks collected at localities in Serbia. Intraspecific variability of these COI gene sequences was very low, and there was no correlation between the various types of habitat and genetic variability of ticks. Samples from isolated localities (canyon/gorge) showed no genetic differentiations from the majority of samples from open areas. | 1363 | no | gd of parasite | NA | no | no |
| 1364 | 2017 | 43017 | Shultz, AJ; Baker, AJ; Hill, GE; Nolan, PM; Edwards, SV | 2016 | SNPs across time and space: population genomic signatures of founder events and epizootics in the House Finch (Haemorhous mexicanus) | Identifying genomic signatures of natural selection can be challenging against a background of demographic changes such as bottlenecks and population expansions. Here, we disentangle the effects of demography from selection in the House Finch (Haemorhous mexicanus) using samples collected before and after a pathogen-induced selection event. Using ddRADseq, we genotyped over 18,000 SNPs across the-genome in native pre-epizootic western US birds, introduced birds from Hawaii and the eastern United States, post-epizootic eastern birds, and western birds sampled across a similar time span. We found 14% and 7% reductions in nucleotide diversity, respectively, in Hawaiian and pre-epizootic eastern birds relative to pre-epizootic western birds, as well as elevated levels of linkage disequilibrium and other signatures of founder events. Despite finding numerous significant frequency shifts (outlier loci) between pre-epizootic native and introduced populations, we found no signal of reduced genetic diversity, elevated linkage disequilibrium, or outlier loci as a result of the epizootic. Simulations demonstrate that the proportion of outliers associated with founder events could be explained by genetic drift. This rare view of genetic evolution across time in an invasive species provides direct evidence that demographic shifts like founder events have genetic consequences more widespread across the genome than natural selection. | 1364 | no | no good fitness measure, N pop differnt in GD? | NA | no | no |
| 1365 | 2017 | 43017 | Francuski, L; Matic, I; Ludoski, J; Milankov, V | 2011 | Temporal patterns of genetic and phenotypic variation in the epidemiologically important drone fly, Eristalis tenax | Eristalis tenax L. (Diptera: Syrphidae) is commonly known as the drone fly (adult) or rat-tailed maggot (immature). Both adults and immature stages are identified as potential mechanical vectors of mycobacterial pathogens, and early-stage maggots cause accidental myiasis. We compared four samples from Mount Fruska Gora, Serbia, with the aim of obtaining insights into the temporal variations and sexual dimorphism in the species. This integrative approach was based on allozyme loci, morphometric wing parameters (shape and size) and abdominal colour patterns. Consistent sexual dimorphism was observed, indicating that male specimens had lighter abdomens and smaller and narrower wings than females. The distribution of genetic diversity at polymorphic loci indicated genetic divergence among collection dates. Landmark-based geometric morphometrics revealed, contrary to the lack of divergence in wing size, significant wing shape variation throughout the year. In addition, temporal changes in the frequencies of the abdominal patterns observed are likely to relate to the biology of the species and ecological factors in the locality. Hence, the present study expands our knowledge of the genetic diversity and phenotypic plasticity of E. tenax. The quantification of such variability represents a step towards the evaluation of the adaptive potential of this species of medical and epidemiological importance. | 1365 | no | no good fitness measure, N pop differnt in GD? | NA | no | no |
| 1366 | 2017 | 43017 | de Lucio, A; Amor-Aramendia, A; Bailo, B; Saugar, JM; Anegagrie, M; Arroyo, A; Loez-Quintana, B; Zewdie, D; Ayehubizu, Z; Yizengaw, E; Abera, B; Yimer, M; Mulu, W; Hailu, T; Herrador, Z; Fuentes, I; Carmena, D | 2016 | Prevalence and Genetic Diversity of Giardia duodenalis and Cryptosporidium spp. among School Children in a Rural Area of the Amhara Region, North-West Ethiopia | Backgroud Giardia duodenalis and Cryptosporidium spp. are enteric protozoan causing gastrointestinal illness in humans and animals. Giardiasis and cryptosporidiosis are not formally considered as neglected tropical diseases, but belong to the group of poverty-related infectious diseases that impair the development and socio-economic potential of infected individuals in developing countries. Methods We report here the prevalence and genetic diversity of G. duodenalis and Cryptosporidium spp. in children attending rural primary schools in the Bahir Dar district of the Amhara Region, Ethiopia. Stool samples were collected from 393 children and analysed by molecular methods. G. duodenalis was detected by real-time PCR, and the assemblages and sub-assemblages were determined by multilocus sequence-based genotyping of the glutamate dehydrogenase and beta-giardin genes of the parasite. Detection and identification of Cryptosporidium species was carried out by sequencing of a partial fragment of the small-subunit ribosomal RNA gene. Principal Findings The PCR-based prevalences of G. duodenalis and Cryptosporidium spp. were 55.0% (216/393) and 4.6% (18/393), respectively. A total of 78 G. duodenalis isolates were successfully characterized, revealing the presence of sub-assemblages AII (10.3%), BIII (28.2%), and BIV (32.0%). Discordant typing results AII/AIII and BIII/BIV were identified in 7.7% and 15.4% of the isolates, respectively. An additional five (6.4%) isolates were assigned to assemblage B. No mixed infections of assemblages A+B were found. Extensive genetic variation at the nucleotide level was observed within assemblage B (but no within assemblage A), resulting in the identification of a large number of sub-types. Cryptosporidium diversity was demonstrated by the occurrence of C. hominis, C. parvum, and C. viatorum in the population under study. Conclusions Our data suggest an epidemiological scenario with an elevated transmission intensity of a wide range of G. duodenalis genetic variants. Importantly, the elevated degree of genetic diversity observed within assemblage B is consistent with the occurrence of intra-assemblage recombination in G. duodenalis. | 1366 | no | human | NA | no | no |
| 1367 | 2017 | 43017 | Di Fiore, A | 2003 | Molecular genetic approaches to the study of primate behavior, social organization, and reproduction | “In the past several decades, the development of novel molecular techniques and the advent of noninvasive DNA sampling, coupled with the ease and speed with which molecular analyses can now be performed, have made it possible for primatologists to directly examine the fitness effects of individual behavior and to explore how variation in behavior and social systems influences primate population genetic structure. This review describes the theoretical connections between individual behavior and primate social systems on the one hand and population genetic structure on the other, discusses the kinds of molecular markers typically employed in genetic studies of primates, and summarizes what primatologists have learned from molecular studies over the past few decades about dispersal patterns, mating systems, reproductive strategies, and the influence of kinship on social behavior. Several important conclusions can be drawn from this overview. First, genetic data confirm that, in many species, male dominance rank and fitness are positively related, at least over the short term, though this relationship need not simply be a reflection of male-male contest competition over mates. More importantly, genetic research reveals the significance of female choice in determining male reproductive success, and documents the efficacy of alternative mating tactics among males. Second, genetic data suggest that the presumed importance of kinship in structuring primate social relationships needs to be evaluated further, at least for some taxa such as chimpanzees in which demographic factors may be more important than relatedness. I conclude this paper by offering several suggestions of additional ways in which molecular techniques might be employed in behavioral and ecological studies of primates (e.g., for conducting,”“molecular censuses”" of unhabituated populations, for studying disease and host-parasite interactions, or for tracking seed fate in studies of seed dispersal) and by providing a brief introduction to the burgeoning field of nonhuman primate behavioral genetics. (C) 2003 Wiley-Liss, Inc." | 1367 | no | no good fitness measure, N pop differnt in GD? | NA | no | no |
| 1368 | 2017 | 43017 | Lehmann, T; Weetman, D; Huestis, DL; Yaro, AS; Kassogue, Y; Diallo, M; Donnelly, MJ; Dao, A | 2017 | Tracing the origin of the early wet-season Anopheles coluzzii in the Sahel | In arid environments, the source of the malaria mosquito populations that re-establish soon after first rains remains a puzzle and alternative explanations have been proposed. Using genetic data, we evaluated whether the early rainy season (RS) population of Anopheles coluzzii is descended from the preceding late RS generation at the same locality, consistent with dry season (DS) dormancy (aestivation), or from migrants from distant locations. Distinct predictions derived from these two hypotheses were assessed, based on variation in 738 SNPs in eleven A.coluzzii samples, including seven samples spanning 2years in a Sahelian village. As predicted by the local origin under aestivation hypothesis, temporal samples from the late RS and those collected after the first rain of the following RS were clustered together, while larger genetic distances were found among samples spanning the RS. Likewise, multilocus genotype composition of samples from the end of the RS was similar across samples until the following RS, unlike samples that spanned the RS. Consistent with reproductive arrest during the DS, no genetic drift was detected between samples taken over that period, despite encompassing extreme population minima, whereas it was detected between samples spanning the RS. Accordingly, the variance in allele frequency increased with time over the RS, but not over the DS. However, not all the results agreed with aestivation. Large genetic distances separated samples taken a year apart, and during the first year, within-sample genetic diversity declined and increased back during the late RS, suggesting a bottleneck followed by migration. The decline of genetic diversity followed by a mass distribution of insecticide-treated nets was accompanied by a reduced mosquito density and a rise in the mutation conferring resistance to pyrethroids, indicating a bottleneck due to insecticidal selection. Overall, our results support aestivation in A.coluzzii during the DS that is accompanied by long-distance migration in the late RS. | 1368 | no | no good fitness measure, N pop differnt in GD? | NA | no | no |
| 1369 | 2017 | 43017 | Tian, W; Boggs, DA; Uko, G; Essiet, A; Inyama, M; Banjoko, B; Adewold, T; Ding, WZ; Mohseni, M; Fritz, R; Chen, DF; Palmer, LJ; Fraser, PA | 2003 | MICA, HLA-B haplotypic variation in five population groups of sub-Saharan African ancestry | The human major histocompatibility complex (MHC) class I chain-related gene A (MICA), located 46 kb centromeric to HLA-B, encodes a stress-inducible protein, which is a ligand for the NKG2D receptor. In addition to its primary role in immune surveillance, data suggest that MICA is involved in the immune response to transplants and in susceptibility to some diseases. In this study, 152 subjects from the Yoruba (n=74), Efik (n=32), and Igbo (n=46) tribes of southern Nigeria, 39 nationwide African-American stem cell donors, and 60 African-American individuals residing in the metropolitan Boston area were studied for MICA, HLA-B allelic variation, haplotypic diversity, and linkage disequilibrium (LD). MICA and HLA-B exhibited a high degree of genetic diversity among the populations studied. In particular, MICA allele and HLA-B-MICA haplotype frequencies and LD in the Efik and Igbo tribes were significantly different from the other study groups. HLA-B and MICA loci demonstrated significant global LD in all five populations (P-values <0.00001). LD also varied in a haplotype-specific manner. A novel MICA allele was detected in the Boston population. These findings are important from an anthropologic perspective, and will inform future HLA-linked disease association studies in related ethnic groups of African-derived ancestry. | 1369 | no | human | NA | no | no |
| 1370 | 2017 | 43017 | Fjerdingstad, EJ; Boomsma, JJ; Thoren, P | 1998 | Multiple paternity in the leafcutter ant Atta colombica - a microsatellite DNA study | An evolutionary understanding of the causes, correlates and consequences of multiple paternity in eusocial Hymenoptera (all ants, some bees and some wasps) relies on accurate estimation of this parameter at the species, population and colony level. We developed dinucleotide microsatellite DNA markers in order to study in detail the degree of multiple paternity in a population of the monogynous Panamanian leafcutter ant Atra colombica. These microsatellite markers were highly polymorphic such that nondetection caused by finite allelic diversity was very low (0.016). Hence, accurate information on the patterns of multiple paternity could be obtained. Genetic analysis revealed that in 33 out of 36 colonies two or more males had contributed genetically to the offspring. The mean number of fathers per colony was 2.6 and the mean effective paternity was 2.31. These are the highest values of effective paternity yet reported fur any ant species. We examined the patterns of paternity frequency in young and old colonies to test if within-colony genetic diversity is related to colony survival, but found no support for any relationship. Our results confirm previous nongenetic studies showing high levels of multiple mating by queens of higher leafcutter ants. We discuss our findings in relation to known patterns of polyandry and paternity in other eusocial Hymenoptera. | 1370 | no | no good fitness measure | NA | no | no |
| 1371 | 2017 | 43017 | Atterby, H; Allnutt, TR; MacNicoll, AD; Jones, EP; Smith, GC | 2015 | Population genetic structure of the red fox (Vulpes vulpes) in the UK | The red fox (Vulpes vulpes) is common and widely distributed within the UK. It is a carrier or potential carrier of numerous zoonotic diseases. Despite this, there are no published reports on the population genetics of foxes in Britain. In this study, we aim to provide an insight into recent historical movement of foxes within Britain, as well as a current assessment of the genetic diversity and gene flow within British populations. We used 14 microsatellite markers to analyse 501 red fox samples originating from England, southern Scotland and northern France. High genetic diversity was evident within the sample set as a whole and limited population genetic structure was present in British samples analysed. Notably, STRUCTURE analysis found support of four population clusters, one of which grouped two southern England sampling areas with the nearby French samples from Calais, indicating recent (post-formation of the Channel) mixing of British and French populations. This may coincide with reports of large-scale translocations of foxes into Britain during the nineteenth century for sport hunting. Other STRUCTURE populations may be related to geographic features or to cultural practices such as fox hunting. In addition, the two British urban populations analysed showed some degree of differentiation from their local rural counterparts. | 1371 | no | no good fitness measure | NA | no | no |
| 1372 | 2017 | 43017 | Koepfli, C; Timinao, L; Antao, T; Barry, AE; Siba, P; Mueller, I; Felger, I | 2013 | A Large Plasmodium vivax Reservoir and Little Population Structure in the South Pacific | Introduction: The importance of Plasmodium vivax in malaria elimination is increasingly being recognized, yet little is known about its population size and population genetic structure in the South Pacific, an area that is the focus of intensified malaria control. Methods: We have genotyped 13 microsatellite markers in 295 P. vivax isolates from four geographically distinct sites in Papua New Guinea (PNG) and one site from Solomon Islands, representing different transmission intensities. Results: Diversity was very high with expected heterozygosity values ranging from 0.62 to 0.98 for the different markers. Effective population size was high (12’872 to 19’533 per site). In PNG population structuring was limited with moderate levels of genetic differentiation. F-ST values (adjusted for high diversity of markers) were 0.14-0.15. Slightly higher levels were observed between PNG populations and Solomon Islands (F-ST = 0.16). Conclusions: Low levels of population structure despite geographical barriers to transmission are in sharp contrast to results from regions of low P. vivax endemicity. Prior to intensification of malaria control programs in the study area, parasite diversity and effective population size remained high. | 1372 | no | gd of parasite | NA | no | no |
| 1373 | 2017 | 43017 | Ittiprasert, W; Rowe, C; Patterson, C; Miller, A; Raghavan, N; Bandoni, S; Lewis, F; Knight, M | 2003 | Assessment of genetic heterogeneity within laboratory-maintained Schistosoma mansoni-resistant stocks of Biomphalaria glabrata snails by RAPD-PCR | Random amplified polymorphic DNA (RAPD)-PCR analysis was used to assess the extent of genetic diversity within two laboratory-maintained Schistosoma mansoni-resistant stocks of Biomphalaria glabrata (10-R2 and BS-90). Both stocks routinely serve as parents in crosses with susceptible snails for studying the genetics of parasite resistance in the snail host. Genomic DNA was isolated from individual adult 10-R2 and BS-90 snails. From RAPD-PCR conducted with 16 anonymous primers, no polymorphisms were detected within the BS-90 stock, whereas 13 primers revealed considerable intrastrain variations showing different sized bands among the 10-R2 snails. The polymorphisms in the 10-R2 stock allowed us to identify three distinct groups (Types 1, 2 and 3) within these snails. Random screening of individual 10-R2 snails revealed that, of the three distinct types, Types 1 and 2 snails were found at similar frequencies (approximately 45%), whereas 10% fell into the third group (Type 3). The identification of genetic variants within the 10-R2 stock demonstrates the need for careful assessment of the existence of diverse forms in this stock prior to conducting genetic crosses with these snails. | 1373 | no | NA | NA | no | no |
| 1374 | 2017 | 43017 | Wang, N; Wang, JH; Hu, DD; Zhong, XQ; Jiang, ZR; Yang, AG; Deng, SJ; Guo, L; Tsering, DW; Wang, SX; Gu, XB; Peng, XR; Yang, GY | 2015 | Genetic variability of Echinococcus granulosus based on the mitochondrial 16S ribosomal RNA gene | Echinococcus granulosus is the etiological agent of cystic echinococcosis, a major zoonotic disease of both humans and animals. In this study, we assessed genetic variability and genetic structure of E. granulosus in the Tibet plateau, using the complete mitochondrial 16 S ribosomal RNA gene for the first time. We collected and sequenced 62 isolates of E. granulosus from 3 populations in the Tibet plateau. A BLAST analysis indicated that 61 isolates belonged to E. granulosus sensu stricto (genotypes G1-G3), while one isolate belonged to E. canadensis (genotype G6). We detected 16 haplotypes with a haplotype network revealing a star-like expansion, with the most common haplotype occupying the center of the network. Haplotype diversity and nucleotide diversity were low, while negative values were observed for Tajima’s D and Fu’s Fs. AMOVA results and Fst values revealed that the three geographic populations were not genetically differentiated. Our results suggest that a population bottleneck or population expansion has occurred in the past, and that this explains the low genetic variability of E. granulosus in the Tibet Plateau. | 1374 | no | gd of pathogen | NA | no | no |
| 1375 | 2017 | 43017 | Single, RM; Martin, MP; Gao, XJ; Meyer, DG; Yeager, M; Kidd, JR; Kidd, KK; Carrington, M | 2007 | Global diversity and evidence for coevolution of KIR and HLA | The killer immunoglobulin- like receptor ( KIR) gene cluster shows extensive genetic diversity, as do the HLA class I loci, which encode ligands for KIR molecules. We genotyped 1,642 individuals from 30 geographically distinct populations to examine population- level evidence for coevolution of these two functionally related but unlinked gene clusters. We observed strong negative correlations between the presence of activating KIR genes and their corresponding HLA ligand groups across populations, especially KIR3DS1 and its putative HLA- B Bw4-80I ligands ( r=- 0.66, P 0.038). In contrast, we observed weak positive relationships between the various inhibitory KIR genes and their ligands. We observed a negative correlation between distance from East Africa and frequency of activating KIR genes and their corresponding ligands, suggesting a balance between selection on HLA and KIR loci. Most KIR- HLA genetic association studies indicate a primary influence of activating KIR- HLA genotypes in disease risk(1,2); concomitantly, activating receptor- ligand pairs in this study show the strongest signature of coevolution of these two complex genetic systems as compared with inhibitory receptor- ligand pairs. | 1375 | no | no good Gd measurement host pop | NA | no | no |
| 1376 | 2017 | 43017 | Augusto, DG; Zehnder-Alves, L; Pincerati, MR; Martin, MP; Carrington, M; Petzl-Erler, ML | 2012 | Diversity of the KIR gene cluster in an urban Brazilian population | The activity of natural killer cells depends on the balance between activating and inhibitory signals coming from their receptors. Among these are the killer cell immunoglobulin-like receptors (KIR) that recognize specific HLA class I allotypes. Here we characterized KIR genetic diversity and their HLA ligands in the population of Curitiba, Parana State (n = 164), and compared it with other worldwide populations. The distribution of 2DL4 alleles was also analyzed. The Curitiba population did not differ significantly from European and Euro-descendant populations, but as an admixed population showed higher genetic diversity. We found 27 KIR profiles, many of them uncommon in European populations, in agreement with the elevated historically recent gene flow in the study population. The frequencies of KIR genes and their respective HLA ligands were distributed independently and none of the analyzed individuals lacked functional KIR-HLA ligand combinations. KIR gene frequencies of 33 worldwide populations were consistent with geographic and ethnic distribution, in agreement with demography being the major factor shaping the observed gene content diversity of the KIR locus. | 1376 | no | no good Gd measurement host pop | NA | no | no |
| 1377 | 2017 | 43017 | Lott, TJ; Fundyga, RE; Kuykendall, RJ; Arnold, J | 2005 | The human commensal yeast, Candida albicans, has an ancient origin | Candida albicans, the primary causative agent of candidiasis, is a ubiquitous member of the human flora and is capable of causing severe invasive disease. Despite its importance as a human pathogen, little is known concerning those factors creating and maintaining genetic diversity within the species and how extant strains reflect their evolutionary history. Based on nucleotide polymorphism frequencies, we estimated the time to a most recent common ancestor for the species to be about 3-16 million years, with variation due to molecular clock calibration. As C. albicans genotypes have broad geographic associations, this suggests that the origins of DNA sequence variation in extant populations coincided with early hominid evolution. This is consistent with an emerging view of a genetically complex organism that is able to survive under host immunity as an obligate commensal species. Published by Elsevier Inc. | 1377 | no | no good fitness measure, N pop differnt in GD? | NA | no | no |
| 1378 | 2017 | 43017 | Dakouri, A; McCallum, BD; Cloutier, S | 2014 | Haplotype diversity and evolutionary history of the Lr34 locus of wheat | Leaf rust caused by Puccinia triticina is a major disease of wheat, and genetic resistance remains the best strategy for managing it. The resistance gene Lr34 has been key in the genetic management of wheat leaf rust worldwide. However, little is known about the geo-genetic diversity, history and origin of this unique gene. This study was conducted to provide a comprehensive analysis of the genetic diversity at the Lr34 locus of a world wheat germplasm collection. A total of 52 alleles were detected for the 10 Lr34 markers. On the basis of the Lr34-specific markers, the world collection was divided into five major haplotypes (H), of which H1 was consistently associated with the resistance phenotype Lr34+. Phenotypic data confirmed the susceptible phenotypes of H2, H3 and H4 and the susceptible or intermediate phenotype of H5. SNP12 (C/T) was the only mutation differentiating the resistant haplotype from the susceptible ones. Combined analysis of the 10 markers resulted in dividing the major haplotypes into 118 different sub-haplotypes. Structure and clustering analyses grouped them into two main clusters and seven sub-clusters. Variance between the main clusters represented the largest proportion of the total variation. H2, the only haplotype found in Aegilops tauschii, is the ancestral haplotype and H1 (Lr34+) likely arose after the advent of hexaploid wheat. Analysis of geographical distribution showed that H1 was more frequent in the Asian germplasm while H2 dominated the European germplasm. Lr34, a gain-of-function mutation, is hypothesized to have originated in Asia. | 1378 | no | gd of pathogen | NA | no | no |
| 1379 | 2017 | 43017 | O’Fallon, B | 2011 | Two optimal mutation rates in obligate pathogens subject to deleterious mutation | Pathogen species with high mutation rates are likely to accumulate deleterious mutations that reduce their reproductive potential within the host. By altering the within-host growth rate of the pathogen, the deleterious mutation load has the potential to affect epidemiological properties such as prevalence, mean pathogen load, and the mean duration of infections: Here, I examine an epidemiological model that allows for multiple segregating mutations that affect within-host replication efficiency. The model demonstrates a complex range of outcomes depending on pathogen mutation rate, including two distinct, widely separated mutation rates associated with high pathogen prevalence. The low mutation rate prevalence peak is associated with small amounts of genetic diversity within the pathogen population, relatively stable prevalence and infection dynamics, and genetic variation partitioned between hosts. The high mutation rate peak is characterized by considerable genetic diversity both within and between hosts, relatively frequent invasions by more virulent types, and is qualitatively similar to an RNA virus quasispecies. The two prevalence peaks are separated by a valley where natural selection favors evolution toward the optimal within-host state, which is associated with high virulence and relatively rapid host mortality. Both chronic and acute infections are examined using stochastic forward simulations. (C) 2011 Elsevier Ltd. All rights reserved. | 1379 | no | gd of pathogen | NA | no | no |
| 1380 | 2017 | 43017 | Han, HJ; Wen, HL; Zhao, L; Liu, JW; Luo, LM; Zhou, CM; Qin, XR; Zhu, YL; Zheng, XX; Yu, XJ | 2017 | Novel Bartonella Species in Insectivorous Bats, Northern China | Bartonella species are emerging human pathogens. Bats are known to carry diverse Bartonella species, some of which are capable of infecting humans. However, as the second largest mammalian group by a number of species, the role of bats as the reservoirs of Bartonella species is not fully explored, in term of their species diversity and worldwide distribution. China, especially Northern China, harbors a number of endemic insectivorous bat species; however, to our knowledge, there are not yet studies about Bartonella in bats in China. The aim of the study was to investigate the prevalence and genetic diversity of Bartonella species in bats in Northern China. Bartonella species were detected by PCR amplification of gltA gene in 25.2% ( 27/107) bats in Mengyin County, Shandong Province of China, including 1/3 Rhinolophus ferrumequinum, 2/10 Rhinolophus pusillus, 9/16 Myotis fimbriatus, 1/5 Myotis ricketti, 14/58 Myotis pequinius. Phylogenetic analysis showed that Bartonella species detected in bats in this study clustered into ten groups, and some might be novel Bartonella species. An association between Bartonella species and bat species was demonstrated and co-infection with different Bartonella species in a single bat was also observed. Our findings expanded our knowledge on the genetic diversity of Bartonella in bats, and shed light on the ecology of bat-borne Bartonella species. | 1380 | no | gd of pathogen | NA | no | no |
| 1381 | 2017 | 43017 | Pelikan, S; Rogstad, SH | 2013 | NEWGARDEN: a computer program to model the population dynamics and genetics of establishing and fragmented plant populations | Conservation of plants often involves isolated in situ or ex situ protective restoration or supplemental plantings. Plant preservation also frequently involves maintenance of recently fragmented populations. The effects of the spatial patterning of the initial target species individuals in such situations on future population growth and genetic diversity maintenance are commonly difficult to predict, especially given that plants have varying life history characteristics. NEWGARDEN allows users to create a virtual preserve or fragment of specified size, place initial individuals at designated locations (the program is spatially explicit using a grid system), and follow population development stemming from bouts of mating influenced by user-specified life history characteristics (e.g., genetic diversity of the initial individuals; age-specific mortality; age-specific reproduction; gene dispersal distances; etc.). Output, which can be reported for the entire population data set and/or specified regions of the preserve or fragments after each bout of mating, includes total population and individual newest cohort data on the number of individuals, total number of founding alleles retained, observed heterozygosity, expected heterozygosity, and F-IT. Use of the program is illustrated with a NEWGARDEN analysis of the effects of varying founder pattern and gene dispersal distance on population growth and genetic diversity for an annual plant. | 1381 | no | NA | NA | no | no |
| 1382 | 2017 | 43017 | Montarry, J; Andrivon, D; Glais, I; Corbiere, R; Mialdea, G; Delmotte, F | 2010 | Microsatellite markers reveal two admixed genetic groups and an ongoing displacement within the French population of the invasive plant pathogen Phytophthora infestans | Potato late blight is an example of a re-emerging disease of plants. Phytophthora infestans was first introduced into Europe during the 19th century, where it caused the Irish potato famine. During the 20th century several additional introduction events have been suspected, especially in the mid-70s due to the import of large quantities of potato needed after the shortage caused by drought in 1976. Here, we investigate the genetic population structure of Phytophthora infestans, at the first stages of a recent invasion process in France. A total of 220 isolates was collected from 20 commercial fields of the potato susceptible cultivar Bintje, during two consecutive years (2004 and 2005). Clustering analyses based on eight recently developed microsatellite markers reveal that French P. infestans populations are made of two differentiated genetic clusters of isolates (F(ST) = 0.19). This result suggests multiple introductions of P. infestans into France, either through the introduction of a composite population of isolates or through the successive introduction of isolates having differentiated genetic backgrounds. Both clusters identified have a strong clonal structure and are similar regarding genetic diversity and mating type composition. The maintenance of differentiation between the two genetic clusters should result from the low or non-existent contribution of sexual reproduction in French P. infestans populations. | 1382 | no | gd of pathogen | NA | no | no |
| 1383 | 2017 | 43017 | Clotuche, G; Navajas, M; Mailleux, AC; Hance, T | 2013 | Reaching the Ball or Missing the Flight? Collective Dispersal in the Two-Spotted Spider Mite Tetranychus urticae | The two-spotted spider mite is a worldwide phytophagous pest displaying a peculiar dispersal. At high density, when plants are exhausted, individuals gather at the plant apex to form a collective silk-ball. This structure can be dispersed by wind or phoresy. Individuals initiating the ball are enclosed in the centre and have a high risk to die. For the first time, the ultimate and proximate mechanisms leading to this group dispersal are examined. To explore if a particular mite genotype was involved in the ball formation, plants were infested with individuals of different genetic background. After the silk-ball formation, the mites in the ball and those remaining on the plant were collected and genotyped. The balls were harvested after 4h and 24h to determine the role of timing between the formation and dispersal on the mortality of mites. Mites do not segregate according to their degree of relatedness, stage, or sex. Mites parallel humans using public transportation: they climb up in the ball whatever their genetic background. Silk-balls composed of unrelated individuals may help avoiding inbreeding when colonizing a new plant. Our results also emphasize the importance of an adequate timing for efficient dispersal between the time spent between ball formation and dispersal. | 1383 | no | NA | NA | no | no |
| 1384 | 2017 | 43017 | GarciaMoreno, J; Matocq, MD; Roy, MS; Geffen, E; Wayne, RK | 1996 | Relationships and genetic purity of the endangered Mexican wolf based on analysis of microsatellite loci | The Mexican wolf (Canis lupus baileyi), and endangered subspecies of gray wolf, was native to parts of Mexico and the southwestern United States. Currently, only a few individuals, if any, exist in the wild, so planned reintroduction programs must use captive-raised wolves. In only one captive population, however, designated the certified lineage, are all the founders (n = 4) known to be obtained from a wild population of Mexican wolves. Two captive populations were founded from individuals of uncertain ancestry and have not been included in the species survival plan. To preserve genetic diversity and reduce inbreeding so that fitness will be maintained, it would be desirable to include these two captive populations in the breeding program if it could be shown that they were derived from a wild population similar to the certified lineage. We compared allele frequencies of 10 hypervariable microsatellite loci in Mexican gray wolves with those found in a sample of 42 domestic dogs. 151 northern gray wolves, and 142 coyotes to determine if uncertified Mexican wolves had specific markers from these animals. We analyzed pairwise genetic distance measures to demonstrate that the three captive populations of Mexican gray wolves were closely related to each other and distinct from dogs and northern gray wolves. The three captive populations are genetically more similar to each other than to any other population of dog or wolf-like canid, and they shared alleles that were rare in other canids. The genetic distance between them is similar to that between closely spaced populations of northern gray wolves. As a group, moreover, they are the most genetically distinct population of North American gray wolf. Therefor, the three captive populations could potentially be interbred to augment the genetic diversity of the certified lineage. Source individuals for reintroduction should be derived from the captive Mexican wolf population rather than populations of captive or wild northern gray wolves. | 1384 | no | no good fitness measure, N pop differnt in GD? | NA | no | no |
| 1385 | 2017 | 43017 | Helgason, A; Nicholson, G; Stefansson, K; Donnelly, P | 2003 | A reassessment of genetic diversity in icelanders: Strong evidence from multiple loci for relative homogeneity caused by genetic drift | There has been some controversy in the literature concerning whether Icelanders are genetically homogenous or heterogeneous relative to other European populations. We reassess this question in the light of large data sets spanning 83 autosomal SNP loci, 14 serogenetic loci, 6622 Y-chromosomes and 3214 sequences from mtDNA hypervariable segments 1 and 2 (HVS1 and HVS2). Our results strongly support the hypothesis that genetic drift, with a consequent loss of variation, has had a greater impact on Icelanders than most other Europeans. We also analyse 7245 HVS1 sequences from 2-3 European populations. In line with other studies, we observe a deficit of rare HVS1 haplotypes and an excess of intermediate frequency haplotypes in Icelanders compared to most European populations, with some measures of genetic diversity indicating relative heterogeneity and others indicating relative homogeneity of Icelanders. Simulations indicate that genetic drift, and not admixture (as proposed by Arnason, 2003) is the most likely cause of the atypical Icelandic HVS1 frequency spectrum. These simulations reveal that gene diversity (heterozygosity) and mean pairwise differences are largely insensitive to events in recent population history, while statistics based on the number of haplotypes or segregating sites are much more sensitive. Overall, our analyses strongly indicate that the Icelandic gene pool is less heterogeneous than those of most other European populations. | 1385 | no | human | NA | no | no |
| 1386 | 2017 | 43017 | Maharramov, J; Meeus, I; Maebe, K; Arbetman, M; Morales, C; Graystock, P; Hughes, WOH; Plischuk, S; Lange, CE; de Graaf, DC; Zapata, N; de la Rosa, JJP; Murray, TE; Brown, MJF; Smagghe, G | 2013 | Genetic Variability of the Neogregarine Apicystis bombi, an Etiological Agent of an Emergent Bumblebee Disease | The worldwide spread of diseases is considered a major threat to biodiversity and a possible driver of the decline of pollinator populations, particularly when novel species or strains of parasites emerge. Previous studies have suggested that populations of introduced European honeybee (Apis mellifera) and bumblebee species (Bombus terrestris and Bombus ruderatus) in Argentina share the neogregarine parasite Apicystis bombi with the native bumblebee (Bombus dahlbomii). In this study we investigated whether A. bombi is acting as an emergent parasite in the non-native populations. Specifically, we asked whether A. bombi, recently identified in Argentina, was introduced by European, non-native bees. Using ITS1 and ITS2 to assess the parasite’s intraspecific genetic variation in bees from Argentina and Europe, we found a largely unstructured parasite population, with only 15% of the genetic variation being explained by geographic location. The most abundant haplotype in Argentina (found in all 9 specimens of non-native species) was identical to the most abundant haplotype in Europe (found in 6 out of 8 specimens). Similarly, there was no evidence of structuring by host species, with this factor explaining only 17% of the genetic variation. Interestingly, parasites in native Bombus ephippiatus from Mexico were genetically distant from the Argentine and European samples, suggesting that sufficient variability does exist in the ITS region to identify continent-level genetic structure in the parasite. Thus, the data suggest that A. bombi from Argentina and Europe share a common, relatively recent origin. Although our data did not provide information on the direction of transfer, the absence of genetic structure across space and host species suggests that A. bombi may be acting as an emergent infectious disease across bee taxa and continents. | 1386 | no | no good fitness measure, N pop differnt in GD? | NA | no | no |
| 1387 | 2017 | 43017 | WALLS, SC; BLAUSTEIN, AR | 1994 | DOES KINSHIP INFLUENCE DENSITY-DEPENDENCE IN A LARVAL SALAMANDER | We examined the effects of variation in larval density, genetic relatedness and their interaction in the marbled salamander, Ambystoma opacum. In a factorial experiment, we reared larvae in artificial ponds at low and high densities and in groups comprised of either a single sibship (all siblings) or an equal mixture of six different sibships. After 150 d (before the onset of metamorphosis), we measured the proportion of larvae surviving, body size (snout-vent length and mass), and the size distribution of larvae in each pond. High initial densities significantly reduced body mass, but had no significant effect on larval snout-vent length, the size distributions of individuals, or survival. Neither variation in genetic relatedness (single vs mixed sibship groups), nor its interaction with initial density, influenced larval performance. Because larval survival was independent of initial density, we also assessed whether body size variables were primarily a function of the final number of survivors in each pond. Simple linear regression analyses revealed that larval snout-vent length and mass were negatively related to final density, whereas the skew in the size distributions of individuals increased with increasing final density. Separate regression analyses for groups of siblings vs groups of mixed relatedness revealed no significant differences in the regression slopes and intercepts for these two groups, thus indicating that both estimated the same regression population. Therefore, kinship did not appear to influence density-dependent larval performance in the environment of our artificial ponds. Our results contrast with evidence of kinship effects on behavior under laboratory conditions for this and other species of Ambystoma and illustrate the context-dependency With which kin recognition may operate under variable experimental conditions. | 1387 | yes | of interest | NA | no | yes |
| 1388 | 2017 | 43017 | Kocijan, I; Galov, A; Cetkovic, H; Kusak, J; Gomercic, T; Huber, D | 2011 | Genetic diversity of Dinaric brown bears (Ursus arctos) in Croatia with implications for bear conservation in Europe | Brown bears have lost most of their range on the European continent. The remaining western populations are small, isolated and highly endangered. The Dinaric-Pindos brown bear population is the western-most stable population and the fourth largest in Europe. It has been recognized as a potential source for recolonization of populations whose survival is at risk. Indeed, several translocations of Dinaric bears to Italy, Austria and France have recently been made. Despite the importance of the Dinaric bear population, its genetic status remains poorly understood. Using tissue samples from 156 hunted or accidentally killed Dinaric bears in Croatia, this study analysed genetic diversity at 12 microsatellite loci, as well as population structure and past reductions in size. In addition, a subset of 59 samples was used to assess diversity of the mitochondrial DNA control region. The results indicate that Dinaric bears have high nuclear genetic diversity, as compared to other extant brown bear populations, despite genetic evidence of a bottleneck caused by past persecutions. However, haplotype diversity was low, probably as a result of male-biased dispersal and female philopatry. Not surprisingly, no evidence of population sub-structure was found using nuclear markers, as the bear habitat has remained continuous and the highway network has been built only recently. Management should focus on maintaining habitat connectivity and keeping the effective population size as large as possible. In addition, when removing individuals, care should be taken not to further deplete the population of rare haplotypes. A coordinated transboundary management of the entire Dinaric-Pindos brown bear population should be a priority for its long-term conservation. (C) 2010 Deutsche Gesellschaft fur Saugetierkunde. Published by Elsevier GmbH. All rights reserved. | 1388 | no | no good fitness measure | NA | no | no |
| 1389 | 2017 | 43017 | JARVI, SI; GEE, GF; MILLER, MM; BRILES, WE | 1995 | A COMPLEX ALLOANTIGEN SYSTEM IN FLORIDA-SANDHILL-CRANES, GRUS-CANADENSIS-PRATENSIS - EVIDENCE FOR THE MAJOR HISTOCOMPATIBILITY (B)-SYSTEM | The B blood group system constitutes the major histocompatibility complex (Mhc) in birds. The Mhc is a cluster of genes largely devoted to the processing and presentation of antigen. The Mhc is highly polymorphic in many species and, thus, useful in the evaluation of genetic diversity for fitness traits within populations of a variety of animals. Correlations found between particular Mhc haplotypes and resistance to certain diseases emphasize the importance of understanding the functional significance of diversity of the Mhc, particularly in species threatened with extinction. As part of studies focused on genetic diversity in wild birds, serological techniques were used to define a highly polymorphic alloantigen system in seven families of Florida sandhill cranes (Grus canadensis pratensis). The results of analyses with antisera produced within the crane families and with chicken Mhc antigen-specific reagents revealed a single major alloantigen system that is likely the Mhc of the Florida sandhill crane, Preliminary experiments indicate that these crane alloantisera will provide a means of defining the Mhc in other species of cranes. | 1389 | no | no good Gd measurement | NA | no | no |
| 1390 | 2017 | 43017 | Anamthawat-Jonsson, K | 2001 | Genetic and genomic relationships in Leymus Hochst. | Genetic and genomic relationships among three taxonomically related species of Leymus, northern European L. arenarius (octoploid, 2n = 56), northern American/Pacific L. mollis (tetraploid, 2n = 28) and central Eurasian L. racemosus (tetraploid, 2n = 28), were examined using molecular and cytogenetic methods. The amplified fragment length polymorphism (AFLP) analysis clearly differentiated Icelandic populations of L. arenarius from Alaskan populations of L. mollis. The former group is more genetically homogeneous than the latter. Leymus arenarius in Iceland has a common gene pool and a relatively recent origin. The Alaskan L. mollis, on the other hand, is probably a glacial Survival that has accumulated high level of genetic variation and has differentiated into subspecies. Analysis of the 18S-26S ribosomal genes, by restriction fragment length polymorphism (RFLP) and fluorescence in situ hybridization (FISH), revealed a very close relationship between the octoploid northern European L. arenarius and the tetraploid Eurasian L. racemosus, such that the former could have originated from the latter, probably via interspecific hybridization. Leymus-specific DNA sequences were isolated and used for analyzing genetic relatedness among five Leymus species and four Psathyrostachys species. The RFLP analysis of retrotransposon sequence pLm44 and ribosomal clone pTa71 clearly revealed a close relationship between these two genera, i.e. higher variation was found within genera than between them. The results support the previous notion that Leymus is autopolyploid having all genomes being designated N-s as in Psathyrostachys, but a major taxonomic revision of this group would require analysis of more species. | 1390 | no | no good fitness measure, N pop differnt in GD? | NA | no | no |
| 1391 | 2017 | 43017 | Tognazzo, M; Schmid-Hempel, R; Schmid-Hempel, P | 2012 | Probing Mixed-Genotype Infections II: High Multiplicity in Natural Infections of the Trypanosomatid, Crithidia bombi, in Its Host, Bombus spp | Mixed-genotype infections have major consequences for many essential elements of host-parasite interactions. With genetic exchange between co-infecting parasite genotypes increased diversity among parasite offspring and the emergence of novel genotypes from infected hosts is possible. We here investigated mixed-genotype infections using the host, Bombus spp. and its trypanosome parasite Crithidia bombi as our study case. The natural infections of C. bombi were genotyped with a novel method for a representative sample of workers and spring queens in Switzerland. We found that around 60% of all infected hosts showed mixed-genotype infections with an average of 2.47 +/- 0.22 (S.E.) and 3.65 +/- 1.02 genotypes per worker or queen, respectively. Queens, however, harboured up to 29 different genotypes. Based on the genotypes of co-infecting strains, these could be putatively assigned to either ‘primary’ and ‘derived’ genotypes - the latter resulting from genetic exchange among the primary genotypes. High genetic relatedness among co-infecting derived but not primary genotypes supported this scenario. Co-infection in queens seems to be a major driver for the diversity of genotypes circulating in host populations.Citation: Tognazzo M, Schmid-Hempel R, Schmid-Hempel P (2012) Probing Mixed-Genotype Infections II: High Multiplicity in Natural Infections of the Trypanosomatid, Crithidia bombi, in Its Host, Bombus spp. PLoS ONE 7(11): e49137. doi:10.1371/journal.pone. 0049137 | 1391 | no | gd of pathogen | NA | no | no |
| 1392 | 2017 | 43017 | Redman, E; Grillo, V; Saunders, G; Packard, E; Jackson, F; Berriman, M; Gilleard, JS | 2008 | Genetics of Mating and Sex Determination in the Parasitic Nematode Haemonchus contortus | Genetic analysis of parasitic nematodes has been a neglected area of research and the basic genetics of this important group of pathogens are poorly understood. Haemonchus contortus is one of the most economically significant livestock parasites workwide and is a key experimental model for the strongylid nematode group that includes many important human and animal pathogens. We have Undertaken a study of the genetics and the mode of mating of this parasite Using microsatellite markers. Inheritance studies with antosomal markers demonstrated obligate dioecious sexual reproduction and polyandrous mating that. are reported here for the first time in a parasitic helminth and provide the parasite with a mechanism of increasing genetic diversity. The karyotype of the H. contortus, MHco3(ISE) isolate was determined as 2n = 11 or 12. We have developed a panel of microsatellite markers that are tightly linked on the X chromosome and have used them to determine the sex chromosomal karyotype as XO male and XX female. Haplotype analysis using the X-chromosomal markers also demonstrated polyandry, independent of the autosomal marker analysis, and enabled a more direct estimate of the number of male parental genotypes contributing to each brood. This work provides a basis for future forward genetic analysis on H. contortus and related parasitic nematodes. | 1392 | no | gd of parasite | NA | no | no |
| 1393 | 2017 | 43017 | Shumway, CA | 1999 | A neglected science: applying behavior to aquatic conservation | Behavioral theories, insights, and techniques are too frequently ignored by conservation biologists. Yet an animal’s survival and reproductive success clearly depend on its behavior. Using examples from marine, freshwater, and terrestrial realms, I assert that behavioral information is invaluable in five conservation areas: (1) managing wild species (e.g., designing marine reserves; reducing animal-human conflicts; understanding and managing species’ responses to human-induced environmental stress such as fishing, introduced species, and chemical, visual, and acoustic pollution); (2) actively reversing the decline of imperiled wild species (e.g., reducing by catch by improving selectivity of fishing gear; re-establishing breeding populations and boosting reproduction); (3) assessing biodiversity (e.g., modeling population viability; censusing and monitoring populations and species); (4) captive breeding and reintroduction programs (e.g., minimizing loss of valuable phenotypes; teaching or maintaining valuable survival skills); and (5) changing human behavior in resource exploitation (e.g., using principles from social psychology). Both realized and potential applications to fishes are stressed. Finally, behavioral diversity, a valuable but neglected element of biodiversity, needs to be explicitly conserved to maintain diverse populations. Arguments are presented that the conservation of species diversity and genetic diversity alone does not necessarily protect important behavioral diversity. The maintenance of both individual and population variability may be essential for the preservation of a species. | 1393 | no | no good fitness measure, N pop differnt in GD? | NA | no | no |
| 1394 | 2017 | 43017 | Harrison, F; Buckling, A | 2007 | High relatedness selects against hypermutability in bacterial metapopulations | Mutation rate and cooperation have important ecological and evolutionary consequences and, moreover, can affect pathogen virulence. While hypermutability accelerates adaptation to novel environments, hypermutable lineages (‘mutators’) are selected against in well-adapted populations. Using the model organism Pseudomonas aeruginosa, we previously demonstrated a further potential disadvantage to hypermutability, namely, that it can accelerate the breakdown of cooperation. We now investigate how this property of mutators can affect their persistence in metapopulations. Mutator and wild-type bacteria were competed for 250 generations in globally competing metapopulations, imposing conditions of high or low intra-deme relatedness. High relatedness favours cooperating groups, so we predicted that mutators should achieve lower equilibrium frequencies under high relatedness than under low relatedness. This was observed in our study. Consistent with our hypothesis, there was a positive correlation between mean mutator and cheat frequencies. We conclude that when dense population growth requires cooperation, and when cooperation is favoured ( high relatedness), demes containing high frequencies of mutators are likely to be selected against because they also contain high frequencies of non-cooperating cheats. We have also identified conditions where mutator lineages are likely to dominate metapopulations; namely, when low relatedness reduces kin selection for cooperation. These results may help to explain clinical distributions of mutator bacteria. | 1394 | no | no good fitness measure | NA | no | no |
| 1395 | 2017 | 43017 | Dussex, N; Rawlence, NJ; Robertson, BC | 2015 | Ancient and Contemporary DNA Reveal a Pre-Human Decline but No Population Bottleneck Associated with Recent Human Persecution in the Kea (Nestor notabilis) | The impact of population bottlenecks is an important factor to consider when assessing species survival. Population declines can considerably limit the evolutionary potential of species and make them more susceptible to stochastic events. New Zealand has a well documented history of decline of endemic avifauna related to human colonization. Here, we investigate the genetic effects of a recent population decline in the endangered kea (Nestor notabilis). Kea have undergone a long-lasting persecution between the late 1800s to 1970s where an estimated 150,000 kea were culled under a governmental bounty scheme. Kea now number 1,000-5,000 individuals in the wild and it is likely that the recent population decline may have reduced the genetic diversity of the species. Comparison of contemporary (n = 410), historical (n = 15) and fossil samples (n = 4) showed a loss of mitochondrial diversity since the end of the last glaciation (Otiran Glacial) but no loss of overall genetic diversity associated with the cull. Microsatellite data indicated a recent bottleneck for only one population and a range-wide decline in N-e dating back some 300 - 6,000 years ago, a period predating European arrival in NZ. These results suggest that despite a recent human persecution, kea might have experienced a large population decline before stabilizing in numbers prior to human settlement of New Zealand in response to Holocene changes in habitat distribution. Our study therefore highlights the need to understand the respective effects of climate change and human activities on endangered species dynamics when proposing conservation guidelines. | 1395 | no | no good fitness measure, N pop differnt in GD? | NA | no | no |
| 1396 | 2017 | 43017 | Gowda, M; Zhao, Y; Wuerschum, T; Longin, CFH; Miedaner, T; Ebmeyer, E; Schachschneider, R; Kazman, E; Schacht, J; Martinant, JP; Mette, MF; Reif, JC | 2014 | Relatedness severely impacts accuracy of marker-assisted selection for disease resistance in hybrid wheat | The accuracy of genomic selection depends on the relatedness between the members of the set in which marker effects are estimated based on evaluation data and the types for which performance is predicted. Here, we investigate the impact of relatedness on the performance of marker-assisted selection for fungal disease resistance in hybrid wheat. A large and diverse mapping population of 1739 elite European winter wheat inbred lines and hybrids was evaluated for powdery mildew, leaf rust and stripe rust resistance in multi-location field trials and fingerprinted with 9 k and 90 k SNP arrays. Comparison of the accuracies of prediction achieved with data sets from the two marker arrays revealed a crucial role for a sufficiently high marker density in genome-wide association mapping. Cross-validation studies using test sets with varying degrees of relationship to the corresponding estimation sets revealed that close relatedness leads to a substantial increase in the proportion of total genotypic variance explained by the identified QTL and consequently to an overoptimistic judgment of the precision of marker-assisted selection. | 1396 | no | hybrid | NA | no | no |
| 1397 | 2017 | 43017 | Eggert, F; Muller-Ruchholtz, W; Ferstl, R | 1998 | Olfactory cues associated with the major histocompatibility complex | Besides its immunological function of self/non-self discrimination the major histocompatibility complex (MHC) has been recognized as a possible source of individual specific body odors. Dating back to speculations on the role of the extraordinary polymorphism of the MHC as background of an individual chemosensory identity and to early observations of MHC-dependent mate choice in inbred strains of mice, systematic experimental studies revealed a first evidence for H-2 related body odors in this species. Meanwhile a large number of animal studies with rodents and a series of field studies and experiments with humans have extended our knowledge of MHC-related odor signals and substantiated the hypothesis of immunogenetic associated odortypes. These results suggest that the most prominent feature of the MHC, its extraordinary genetic diversity, seems in part to be selectively maintained by behavioral mechanisms which operate in contemporary natural populations. The high degree of heterozygosity found in natural populations of most species seems to be promoted by non-disease-based selection such as mating preferences and selective block of pregnancy. | 1397 | no | NA | NA | no | no |
| 1398 | 2017 | 43017 | Janson, EM; Peeden, ER; Stireman, JO; Abbot, P | 2010 | Symbiont-mediated phenotypic variation without co-evolution in an insect-fungus association | Recent studies have shown that symbionts can be a source of adaptive phenotypic variation for their hosts. It is assumed that co-evolution between hosts and symbionts underlies these ecologically significant phenotypic traits. We tested this assumption in the ectosymbiotic fungal associate of the gall midge Asteromyia carbonifera. Phylogenetic analysis placed the fungal symbiont within a monophyletic clade formed by Botryosphaeria dothidea, a typically free-living (i.e. not associated with an insect host) plant pathogen. Symbiont isolates from four divergent midge lineages demonstrated none of the patterns common to heritable microbial symbioses, including parallel diversification with their hosts, substitution rate acceleration, or A+T nucleotide bias. Amplified fragment length polymorphism genotyping of the symbiont revealed that within-lineage genetic diversity was not clustered along host population lines. Culture-based experiments demonstrated that the symbiont-mediated variation in gall phenotype is not borne out in the absence of the midge. This study shows that symbionts can be important players in phenotypic variation for their hosts, even in the absence of a co-evolutionary association. | 1398 | no | no good fitness measure, N pop differnt in GD? | NA | no | no |
| 1399 | 2017 | 43017 | Schonswetter, P; Popp, M; Brochmann, C | 2006 | Central Asian origin of and strong genetic differentiation among populations of the rare and disjunct Carex atrofusca (Cyperaceae) in the Alps | Aim Carex atrofusca has an arctic-alpine distribution in the Northern Hemisphere, with only a few, disjunct localities known in the European Alps. These alpine populations are declining in number and size. In contrast, C. atrofusca has a wide circumpolar distribution range and is abundant in large parts of the Arctic. The degree of genetic differentiation of the alpine populations and their importance for the conservation of the intraspecific genetic variation of the species is unknown. Location Eurasia and Greenland, with emphasis on the European Alps. Methods We applied amplified fragment length polymorphism (AFLP) fingerprinting and sequences of chloroplast DNA to determine the position of the alpine populations in a circumpolar phylogeography of C. atrofusca and to unravel the patterns of genetic diversity and differentiation within the Alps. Results Two distinct major groups were detected in a neighbour-joining analysis of AFLP data and in parsimony analysis of chloroplast DNA sequences: one consisting of the populations from Siberia and Greenland and one consisting of all European populations as sister to the populations from Central Asia. Within Europe, the populations from the Tatra Mountains and those from Scotland and Scandinavia formed two well-supported groups, whereas the alpine populations did not constitute a group of their own. The genetic variation in the Alps was almost completely partitioned among the populations, and the populations were almost invariable. Main conclusions The alpine populations possibly originated due to immigration from Central Asia. The strong differentiation among them suggests that genetic drift has been strongly acting on the populations, either as a consequence of founder events during colonization or due to subsequent reduction of population sizes during warm stages of the Holocene. | 1399 | no | no good fitness measure | NA | no | no |
| 1400 | 2017 | 43017 | Hess, CM; Wang, ZS; Edwards, SV | 2007 | Evolutionary genetics of Carpodacus mexicanus, a recently colonized host of a bacterial pathogen, Mycoplasma gallisepticum | We present molecular data documenting how introduction to the eastern United States and an epizootic involving a bacterial pathogen has affected the genetic diversity of house finches, a cardueline songbird. Population bottlenecks during introduction can cause loss of genetic variation and may negatively affect a population’s ability to adapt to novel stressors such as disease. Although a genome-wide survey using Amplified Fragment Length Polymorphism (AFLP) markers suggests little loss of genetic diversity in introduced populations, an epizootic of bacterial Mycoplasma has nonetheless caused dramatic declines in the eastern US population. Sequence analysis of a candidate gene for pathogen resistance in the Major Histocompatibity Complex (MHC) in pre- and post-epizootic population samples reveals allele frequency shifts since introduction of the pathogen, but similar shifts are also observed in control populations not exposed to the bacteria, and in a neutral non-coding locus. Expression studies using a novel subtractive hybridization approach indicate decreased expression of the class 11 MHC locus upon exposure to Mycoplasma, a pattern also seen in MHC class I loci in mice infected with cytomegalovirus and consistent with manipulation of the finch immune system by Mycoplasma. These results will be further expanded using experimental studies as well as examination of evolution of the pathogen genome itself. | 1400 | no | gd of pathogen | NA | no | no |
| 1401 | 2017 | 43015 | Staehlin, BM; Fant, JB | 2015 | Climate Change Impacts on Seedling Establishment for a Threatened Endemic Thistle, Cirsium pitcheri | A study was conducted to assess the effects of predicted climate change on seedling emergence, growth, and survival for the federally listed threatened species Cirsium pitcheri in the region proximal to Chicago, Illinois, U.S.A. Three geographically distant extant populations that could potentially serve as donors to re- introduced Illinois populations were tested to determine the influence of local adaptation on seed response to climate scenarios at two future time points. For all three populations, temperature was shown to be the most critical factor impacting future growth. All populations performed worst at the 2095 temperature for four measured growth metrics: length of longest adult leaf, number of adult leaves, dry shoot mass, and dry root mass. Growth performance at the 2095 conditions did not correlate with climate familiarity but did reflect the genetic diversity of the parent population. Predicted changes in average precipitation did not produce a significant effect on any growth metric, possibly reflecting a limitation in the study conditions. Seedling emergence and survival were tied to seed mass, a trait influenced by maternal effects and correlated with the level of inbreeding depression in the parent population. These results suggest managing populations to maintain high genetic diversity could be an important factor in tolerating temperature stress associated with climate change. | 1401 | no | no good fitness measure | NA | no | no |
| 1402 | 2017 | 43015 | Pujolar, JM; Bevacqua, D; Capoccioni, F; Ciccotti, E; De Leo, GA; Zane, L | 2011 | No apparent genetic bottleneck in the demographically declining European eel using molecular genetics and forward-time simulations | The stock of the European eel is considered to be outside safe biological limits, following a dramatic demographic decline in recent decades (90-99% drop) that involves a large number of factors including overfishing, contaminants and environmental fluctuations. The aim of the present study is to estimate the effective population size of the European eel and the possible existence of a genetic bottleneck, which is expected during or after a severe demographic crash. Using a panel of 22 EST-derived microsatellite loci, we found no evidence for a genetic bottleneck in the European eel as our data showed moderate to high levels of genetic diversity, no loss of allele size range or rare alleles, and a stationary population with growth values not statistically different from zero, which is confirmed by finding comparable value of short-term and long-term effective population size. Our results suggest that the observed demographic decline in the European eel did not entail a genetic decline of the same magnitude. Forward-time simulations confirmed that large exploited marine fish populations can undergo genetic bottleneck episodes and experience a loss of genetic variability. Simulations indicated that the failure to pick up the signal of a genetic bottleneck in the European eel is not due to lack of power. Although anthropogenic factors lowered the continental stock biomass, the observation of a stable genetic effective population size suggests that the eel crash was not due to a reduction in spawning stock abundance. Alternatively, we propose that overfishing, pollution and/or parasites might have affected individual fitness and fecundity, leading to an impoverished spawning stock that may fail to produce enough good quality eggs. A reduced reproduction success due to poor quality of the spawners may be exacerbated by oceanic processes inducing changes in primary production in the Sargasso Sea and/or pathway of transport across the Atlantic Ocean leading to a higher larval mortality. | 1402 | no | no good fitness measure | NA | no | no |
| 1403 | 2017 | 43015 | Hu, XS; Yeh, FC; Hu, Y; Deng, LT; Ennos, RA; Chen, XY | 2017 | High mutation rates explain low population genetic divergence at copy-number-variable loci in Homo sapiens | Copy-number-variable (CNV) loci differ from single nucleotide polymorphic (SNP) sites in size, mutation rate, and mechanisms of maintenance in natural populations. It is therefore hypothesized that population genetic divergence at CNV loci will differ from that found at SNP sites. Here, we test this hypothesis by analysing 856 CNV loci from the genomes of 1184 healthy individuals from 11 HapMap populations with a wide range of ancestry. The results show that population genetic divergence at the CNV loci is generally more than three times lower than at genome-wide SNP sites. Populations generally exhibit very small genetic divergence (G(st) = 0.05 +/- 0.049). The smallest divergence is among African populations (G(st) = 0.0081 +/- 0.0025), with increased divergence among non-African populations (G(st) = 0.0217 +/- 0.0109) and then among African and non-African populations (G(st) = 0.0324 +/- 0.0064). Genetic diversity is high in African populations (similar to 0.13), low in Asian populations (similar to 0.11), and intermediate in the remaining 11 populations. Few significant linkage disequilibria (LDs) occur between the genome-wide CNV loci. Patterns of gametic and zygotic LDs indicate the absence of epistasis among CNV loci. Mutation rate is about twice as large as the migration rate in the nonAfrican populations, suggesting that the high mutation rates play dominant roles in producing the low population genetic divergence at CNV loci. | 1403 | no | human | NA | no | no |
| 1404 | 2017 | 43015 | Namouchi, A; Didelot, X; Schock, U; Gicquel, B; Rocha, EPC | 2012 | After the bottleneck: Genome-wide diversification of the Mycobacterium tuberculosis complex by mutation, recombination, and natural selection | Many of the most virulent bacterial pathogens show low genetic diversity and sexual isolation. Accordingly, Mycobacterium tuberculosis, the deadliest human pathogen, is thought to be clonal and evolve by genetic drift. Yet, its genome shows few of the concomitant signs of genome degradation. We analyzed 24 genomes and found an excess of genetic diversity in regions encoding key adaptive functions including the type VII secretion system and the ancient horizontally transferred virulence-related regions. Four different approaches showed evident signs of recombination in M. tuberculosis. Recombination tracts add a high density of polymorphisms, and many are thus predicted to arise from outside the clade. Some of these tracts match Mycobacterium canettii sequences. Recombination introduced an excess of non-synonymous diversity in general and even more in genes expected to be under positive or diversifying selection, e.g., cell wall component genes. Mutations leading to non-synonymous SNPs are effectively purged in MTBC., which shows dominance of purifying selection. MTBC mutation bias toward AT nucleotides is not compensated by biased gene conversion, suggesting the action of natural selection also on synonymous changes. Together, all of these observations point to a strong imprint of recombination and selection in the genome affecting both non-synonymous and synonymous positions. Hence, contrary to some other pathogens and previous proposals concerning M. tuberculosis, this lineage may have come out of its ancestral bottleneck as a very successful pathogen that is rapidly diversifying by the action of mutation, recombination, and natural selection. | 1404 | no | gd of pathogen | NA | no | no |
| 1405 | 2017 | 43015 | Simard, F; Lehmann, T; Lemasson, JJ; Diatta, M; Fontenille, D | 2000 | Persistence of Anopheles arabiensis during the severe dry season conditions in Senegal: an indirect approach using microsatellite loci | Variation at nine microsatellite loci was investigated to understand how Anopheles arabiensis populations survive the dry season in the sahelian region of Senegal. Low estimates of genetic differentiation (F-ST = 0.012, R-ST = 0.009) between two populations, 250 km apart, suggested extensive gene flow across this distance. Despite extreme seasonal fluctuation in abundance with dry season minima in which mosquitoes virtually disappeared, allele frequencies remained stable over time in the village of Barkedji from August 1994 to December 1997 (including four rainy seasons and three dry seasons). The effective population size (Ne) was estimated to be 601 with 95% CI (281, 1592), providing strong evidence against annual bottlenecks. Differences in measures of genetic diversity and linkage disequilibrium between the dry and the rainy seasons were not detected. These results suggest that despite extreme minima in local density, An. arabiensis maintains large permanent deme spread out over large area. | 1405 | no | No GD diff between pop | NA | no | no |
| 1406 | 2017 | 43015 | LaBonte, NR; Ostry, ME; Ross-Davis, A; Woeste, KE | 2015 | Estimating heritability of disease resistance and factors that contribute to long-term survival in butternut (Juglans cinerea L.) | For most wild species affected by exotic pests or pathogens, the relative importance of heritable genetic differences in determining apparent variation in disease resistance is unknown. This is true in particular for butternut, a North American hardwood affected by butternut canker disease and undergoing demographic contraction. Little is known about site effects on butternut decline, in part because long-term monitoring data are lacking. We collected detailed disease phenotypes and multilocus microsatellite genotypes for all surviving individuals in a large natural population of butternut in 2003 (n=302) and 2012 (n=113). Two analytical methods, correlations between pairwise phenotypic similarity and pairwise relatedness, and estimation of among-family variance, both indicated weak heritability of disease-related traits and no heritability for overall tree health in the population. Additionally, an analysis of spatial data collected in 2001 (n=341) and 2012 (n=113) demonstrated that drier, upland sites contribute to increased likelihood of survival. We conclude that genetic differences among wild butternut individuals contributed little to observed variance in survival over 10 years but fine-scale site differences were useful predictors of butternut mortality. | 1406 | no | no good fitness measure, N pop differnt in GD? | NA | no | no |
| 1407 | 2017 | 43015 | Zeng, QQ; He, K; Sun, DD; Ma, MY; Ge, YF; Fang, SG; Wan, QH | 2016 | Balancing selection and recombination as evolutionary forces caused population genetic variations in golden pheasant MHC class I genes | Background: The major histocompatibility complex (MHC) genes are vital partners in the acquired immune processes of vertebrates. MHC diversity may be directly associated with population resistance to infectious pathogens. Here, we screened for polymorphisms in exons 2 and 3 of the IA1 and IA2 genes in 12 golden pheasant populations across the Chinese mainland to characterize their genetic variation levels, to understand the effects of historical positive selection and recombination in shaping class I diversity, and to investigate the genetic structure of wild golden pheasant populations. Results: Among 339 individual pheasants, we identified 14 IA1 alleles in exon 2 (IA1-E2), 11 IA1-E3 alleles, 27 IA2-E2 alleles, and 28 IA2-E3 alleles. The non-synonymous substitution rate was significantly greater than the synonymous substitution rate at sequences in the IA2 gene encoding putative peptide-binding sites but not in the IA1 gene; we also found more positively selected sites in IA2 than in IA1. Frequent recombination events resulted in at least 9 recombinant IA2 alleles, in accordance with the intermingling pattern of the phylogenetic tree. Although some IA alleles are widely shared among studied populations, large variation occurs in the number of IA alleles across these populations. Allele frequency analysis across 2 IA loci showed low levels of genetic differentiation among populations on small geographic scales; however, significant genetic differentiation was observed between pheasants from the northern and southern regions of the Yangtze River. Both STRUCTURE analysis and F-statistic (F-ST) value comparison classified those populations into 2 major groups: the northern region of the Yangtze River (NYR) and the southern region of the Yangtze River (SYR). Conclusions: More extensive polymorphisms in IA2 than IA1 indicate that IA2 has undergone much stronger positive-selection pressure during evolution. Moreover, the recombination events detected between the genes and the intermingled phylogenetic pattern indicate that interlocus recombination accounts for much of the allelic variation in IA2. Analysis of the population differentiation implied that homogenous balancing selection plays an important part in maintaining an even distribution of MHC variations. The natural barrier of the Yangtze River and heterogeneous balancing selection might help shape the NYR-SYR genetic structure in golden pheasants. | 1407 | no | no good fitness measure | NA | no | no |
| 1408 | 2017 | 43015 | Green, JP; Cant, MA; Field, J | 2014 | Using social parasitism to test reproductive skew models in a primitively eusocial wasp | Remarkable variation exists in the distribution of reproduction (skew) among members of cooperatively breeding groups, both within and between species. Reproductive skew theory has provided an important framework for understanding this variation. In the primitively eusocial Hymenoptera, two models have been routinely tested: concessions models, which assume complete control of reproduction by a dominant individual, and tug-of-war models, which assume on-going competition among group members over reproduction. Current data provide little support for either model, but uncertainty about the ability of individuals to detect genetic relatedness and difficulties in identifying traits conferring competitive ability mean that the relative importance of concessions versus tug-of-war remains unresolved. Here, we suggest that the use of social parasitism to generate meaningful variation in key social variables represents a valuable opportunity to explore the mechanisms underpinning reproductive skew within the social Hymenoptera. We present a direct test of concessions and tug-of-war models in the paper wasp Polistes dominulus by exploiting pronounced changes in relatedness and power structures that occur following replacement of the dominant by a congeneric social parasite. Comparisons of skew in parasitized and unparasitized colonies are consistent with a tug-of-war over reproduction within P. dominulus groups, but provide no evidence for reproductive concessions. | 1408 | no | No GD diff between pop | NA | no | no |
| 1409 | 2017 | 43015 | Chung, EK; Yang, Y; Rennebohm, RM; Lokki, ML; Higgins, GC; Jones, KN; Zhou, B; Blanchong, CA; Yu, CY | 2002 | Genetic sophistication of human complement components C4A and C4B and RP-C4-CYP21-TNX (RCCX) modules in the major histocompatibility complex | Human populations are endowed with a sophisticated genetic diversity of complement C4 and its flanking genes RP, CYP21, and TNX in the RCCX modules of the major histocompatibility complex class III region. We applied definitive techniques to elucidate (a) the complement C4 polymorphisms in gene sizes, gene numbers, and protein isotypes and (b) their gene orders. Several intriguing features are unraveled, including (1) a trimodular RCCX haplotype with three long C4 genes expressing C4A protein only, (2) two trimodular haplotypes with two long (L) and one short (S) C4 genes organized in LSL configurations, (3) a quadrimodular haplotype with four C4 genes organized in a SLSL configuration, and (4) another quadrimodular structure, with four long C4 genes (LLLL), that has the human leukocyte antigen haplotype that is identical to ancestral haplotype 7.2 in the Japanese population. Long-range PCR and PsbAI-RFLP analyses conclusively revealed that the short genes from the LSL and SLSL haplotypes are C4A. In four informative families, an astonishingly complex pattern of genetic diversity for RCCX haplotypes with one, two, three and four C4 genes is demonstrated; each C4 gene may be long or short, encoding a C4A or C4B protein. Such diversity may be related to different intrinsic strengths among humans to defend against infections and susceptibilities to autoimmune diseases. | 1409 | no | human | NA | no | no |
| 1410 | 2017 | 43015 | Turner, TF; Osborne, MJ; Moyer, GR; Benavides, MA; Alo, D | 2006 | Life history and environmental variation interact to determine effective population to census size ratio | Successful recovery and sustainability of threatened and exploited species depends in part on retention and maintenance of genetic diversity. Theory indicates that genetic diversity is lost at a rate inversely proportional to the genetically effective population size (N-e), which is roughly equal to one-half the adult census size (N) in many organisms. However, Ne has been reported to be up to five orders of magnitude lower than N in species with life histories that result in type III survivorship (high fecundity, but heavy mortality in early life stages, e.g. bony fishes), prompting speculation that low values of Ne may be a general feature of such organisms despite sometimes vast abundances. Here, we compared Ne and the ratio N-e/N across three ecologically similar fish species from the arid southwestern United States, all with type III life histories but with differing expectations of egg and larval survivorship that correlate with the degree of human-imposed habitat fragmentation. Our study indicates that type III life history may be necessary, but this alone is insufficient to account for extraordinarily low values of N-e/N. Rather, life history interacts with environmentally imposed mortality to determine the rate and magnitude of change in genetic diversity in these desert fish species. | 1410 | no | multiple species comp | NA | no | no |
| 1411 | 2017 | 43015 | Ferreira, LFR; Duarte, KMR; Gomes, LH; Carvalho, RS; Leal, GA; Aguiar, MM; Armas, RD; Tavares, FCA | 2012 | Genetic diversity of polysporic isolates of Moniliophthora perniciosa (Tricholomataceae) | The causal agent of witches’ broom disease, Moniliophthora perniciosa is a hemibiotrophic and endemic fungus of the Amazon basin and the most important cocoa disease in Brazil. The purpose of this study was to analyze the genetic diversity of polysporic isolates of M. perniciosa to evaluate the adaptation of the pathogen from different Brazilian regions and its association with different hosts. Polysporic isolates obtained previously in potato dextrose agar cultures of M. perniciosa from different Brazilian states and different hosts (Theobroma cacao, Solanum cernuum, S. paniculatum, S. lycocarpum, Solanum sp, and others) were analyzed by somatic compatibility grouping where the mycelium interactions were distinguished after 4-8 weeks of confrontation between the different isolates of M. perniciosa based on the precipitation line in the transition zone and by protein electrophoresis through SDS-PAGE. The diversity of polysporic isolates of M. perniciosa was grouped according to geographic proximity and respective hosts. The great genetic diversity of M. perniciosa strains from different Brazilian states and hosts favored adaptation in unusual environments and dissemination at long distances generating new biotypes. | 1411 | no | gd of pathogen | NA | no | no |
| 1412 | 2017 | 43015 | Pischedda, A; Friberg, U; Stewart, AD; Miller, PM; Rice, WR | 2015 | Sexual selection has minimal impact on effective population sizes in species with high rates of random offspring mortality: An empirical demonstration using fitness distributions | The effective population size (N-e) is a fundamental parameter in population genetics that influences the rate of loss of genetic diversity. Sexual selection has the potential to reduce N-e by causing the sex-specific distributions of individuals that successfully reproduce to diverge. To empirically estimate the effect of sexual selection on N-e, we obtained fitness distributions for males and females from an outbred, laboratory-adapted population of Drosophila melanogaster. We observed strong sexual selection in this population (the variance in male reproductive success was approximate to 14 times higher than that for females), but found that sexual selection had only a modest effect on N-e, which was 75% of the census size. This occurs because the substantial random offspring mortality in this population diminishes the effects of sexual selection on N-e, a result that necessarily applies to other high fecundity species. The inclusion of this random offspring mortality creates a scaling effect that reduces the variance/mean ratios for male and female reproductive success and causes them to converge. Our results demonstrate that measuring reproductive success without considering offspring mortality can underestimate N-e and overestimate the genetic consequences of sexual selection. Similarly, comparing genetic diversity among different genomic components may fail to detect strong sexual selection. | 1412 | no | no good fitness measure, N pop differnt in GD? | NA | no | no |
| 1413 | 2017 | 43015 | Keyghobadi, N; LaPointe, D; Fleischer, RC; Fonseca, DM | 2006 | Fine-scale population genetic structure of a wildlife disease vector: the southern house mosquito on the island of Hawaii | The southern house mosquito, Culex quinquefasciatus, is a widespread tropical and subtropical disease vector. In the Hawaiian Islands, where it was introduced accidentally almost two centuries ago, it is considered the primary vector of avian malaria and pox. Avian malaria in particular has contributed to the extinction and endangerment of Hawaii’s native avifauna, and has altered the altitudinal distribution of native bird populations. We examined the population genetic structure of Cx. quinquefasciatus on the island of Hawaii at a smaller spatial scale than has previously been attempted, with particular emphasis on the effects of elevation on population genetic structure. We found significant genetic differentiation among populations and patterns of isolation by distance within the island. Elevation per se did not have a limiting effect on gene flow; however, there was significantly lower genetic diversity among populations at mid elevations compared to those at low elevations. A recent sample taken from just above the predicted upper altitudinal distribution of Cx. quinquefasciatus on the island of Hawaii was confirmed as being a temporary summer population and appeared to consist of individuals from more than one source population. Our results indicate effects of elevation gradients on genetic structure that are consistent with known effects of elevation on population dynamics of this disease vector. | 1413 | no | no good fitness measure, N pop differnt in GD? | NA | no | no |
| 1414 | 2017 | 43015 | Tarpy, DR; Fletcher, DJC | 1998 | Effects of relatedness on queen competition within honey bee colonies | The influence of relatedness on the pre-and post-emergent survival of honey bee queens was investigated. Workers did not preferentially rear sisters over non-siblings under conditions of natural queen replacement. After queen emergence, however, there was a significant effect of a queen’s relatedness to the workers on her survivorship during fights with rival queens. The mechanism of this bias towards related queens is unknown, and several hypotheses are discussed. The difference in post-emergent survivability suggests that kin selection may operate during competition among adult queens at this crucial stage of honey bee reproduction. (C) 1998 The Association for the Study of Animal Behaviour. | 1414 | no | no good fitness measure | NA | no | no |
| 1415 | 2017 | 43015 | McCoy, KD; Beis, P; Barbosa, A; Cuervo, JJ; Fraser, WR; Gonzalez-Solis, J; Jourdain, E; Poisbleau, M; Quillfeldt, P; Leger, E; Dietrich, M | 2012 | Population genetic structure and colonisation of the western Antarctic Peninsula by the seabird tick Ixodes uriae | Recent observations on the western Antarctic Peninsula have suggested that changing climatic conditions may be increasing pressure on breeding seabirds due to higher exploitation rates by the tick Ixodes uriae. Using data from 8 microsatellite markers and ticks from 6 Pygoscelis spp. colonies, we employed a population genetics approach to specifically test the hypothesis that I. uriae is expanding south-westward along the peninsula from the Subantarctic region. Contrary to expectations, tick genetic diversity was high within all colonies, and no remaining signal of colonisation events was evident. Although significant geographic genetic structure occurred among ticks from different colonies, these ectoparasites tended to belong to 2 major genetic groups, one found principally in south-western locations (Palmer Station area) and the other in more north-eastern areas (South Shetland Islands). More central colonies showed a mixture of ticks from each genetic group, suggesting that this area represents a hybridisation zone of ticks from 2 distinct origins. A subsequent clustering analysis, including ticks from 2 Subantarctic locations, did not reveal the source population for the northern peninsula group. Overall, our data refute the hypothesis of a recent south-westward expansion of I. uriae along the peninsula and suggest that this tick has been present at more southern latitudes for an extended period of time. Further studies on the distribution and genetic characteristics of this ectoparasite around Antarctica are now required to better understand the colonisation process and predict how changing environmental conditions may affect its presence and diversity in seabird colonies. | 1415 | no | gd of parasite | NA | no | no |
| 1416 | 2017 | 43015 | Thomas, VP; Fudali, SL; Schaff, JE; Liu, QL; Scholl, EH; Opperman, CH; Bird, DM; Williamson, VM | 2012 | A Sequence-Anchored Linkage Map of the Plant-Parasitic Nematode Meloidogyne hapla Reveals Exceptionally High Genome-Wide Recombination | Root-knot nematodes (Meloidogyne spp.) cause major yield losses to many of the world’s crops, but efforts to understand how these pests recognize and interact with their hosts have been hampered by a lack of genetic resources. Starting with progeny of a cross between inbred strains (VW8 and VW9) of Meloidogyne hapla that differed in host range and behavioral traits, we exploited the novel, facultative meiotic parthenogenic reproductive mode of this species to produce a genetic linkage map. Molecular markers were derived from SNPs identified between the sequenced and annotated VW9 genome and de novo sequence of VW8. Genotypes were assessed in 183 F2 lines. The colinearity of the genetic and physical maps supported the veracity of both. Analysis of local crossover intervals revealed that the average recombination rate is exceptionally high compared with that in other metazoans. In addition, F2 lines are largely homozygous for markers flanking crossover points, and thus resemble recombinant inbred lines. We suggest that the unusually high recombination rate may be an adaptation to generate within-population genetic diversity in this organism. This work presents the most comprehensive linkage map of a parasitic nematode to date and, together with genomic and transcript sequence resources, empowers M. hapla as a tractable model. Alongside the molecular map, these progeny lines can be used for analyses of genome organization and the inheritance of phenotypic traits that have key functions in modulating parasitism, behavior, and survival and for the eventual identification of the responsible genes. | 1416 | no | gd of pathogen | NA | no | no |
| 1417 | 2017 | 43015 | Choukri, F; Chakib, A; Himmich, H; Raissi, H; Caillat-Zucman, S | 2002 | HLA class I polymorphism in a Moroccan population from Casablanca | We have studied the distribution of HLA-A and -B alleles and haplotypes by sequence-specific primer amplification in a sample of 100 unrelated healthy individuals belonging to both Berber and Arabic-speaking groups from the region of Casablanca in Morocco. Among the 17 HLA-A and 23 HLA-B alleles observed, the most frequent were HLA-A2 (21%),-A1 (11%),-A3 (10%), -B44 (11.4%), -B50 (9.9%), -B5(8.5%) and -B35 (6.5%). Six two-locus haplotypes were observed with a frequency above 5%: A2-B50 (9.6%), A23-B44 (7.4%), A2-B15 (6.4%), A68-B39 (5.3%), A1-B51 (5.3%) and A68-B44 (4.3%). Our data confirm that, on the basis of genetic distances, the majority of present-day North Africans from Morocco are closely related to Berbers and also to Iberians. They cluster apart from Middle-Eastern Mediterranean populations, and show greater genetic distances to Eastern and other Mediterranean populations. This study will serve as a reference for further anthropological studies, as well as studies of HLA and disease associations. | 1417 | no | human | NA | no | no |
| 1418 | 2017 | 43015 | Aggarwal, RK; Velavan, TP; Udaykumar, D; Hendre, PS; Shanker, K; Choudhury, BC; Singh, L | 2004 | Development and characterization of novel microsatellite markers from the olive ridley sea turtle (Lepidochelys olivacea) | Olive ridley turtles, although widely distributed globally and in Indian coastal waters, have undergone declines in recent years due to anthropogenic factors, particularly fishery-related mortality. Assessment of genetic variability in existing populations is critical to the development of effective conservation strategies. Here we describe the development of six highly polymorphic microsatellite loci from a simple sequence repeat-enriched genomic DNA library of olive ridley turtle. Characterization of five of these loci using 83 individual olive ridley turtles revealed eight to 24 alleles per locus, high observed and expected heterozygosity values and broad cross-species amplifications. The sixth microsatellite was found to be monomorphic in the olive ridley samples but was polymorphic in two related marine turtle species. These microsatellites thus provide efficient genetic markers to understand the population structure, phylogeography and species relationships of olive ridley and other marine turtle species. | 1418 | no | no good fitness measure, N pop differnt in GD? | NA | no | no |
| 1419 | 2017 | 43015 | Rasheed, SB; Boots, M; Frantz, AC; Butlin, RK | 2013 | Population structure of the mosquito Aedes aegypti (Stegomyia aegypti) in Pakistan | Eleven microsatellite markers were used to determine the genetic population structure and spread of Aedes aegypti (Stegomyia aegypti) (Diptera: Culicidae) in Pakistan using mosquitoes collected from 13 different cities. There is a single genetic cluster of Ae. aegypti in Pakistan with a pattern of isolation by distance within the population. The low level of isolation by distance suggests the long-range passive dispersal of this mosquito, which may be facilitated by the tyre trade in Pakistan. A decrease in genetic diversity from south to north suggests a recent spread of this mosquito from Karachi. A strong negative correlation between genetic distance and the quality of road connections shows that populations in cities connected by better road networks are less differentiated, which suggests the human-aided passive dispersal of Ae. aegypti in Pakistan. Dispersal on a large spatial scale may facilitate the strategy of introducing transgenic Ae. aegypti or intracellular bacteria such as Wolbachia to control the spread of dengue disease in Pakistan, but it also emphasizes the need for simple measures to control container breeding sites. | 1419 | no | no good fitness measure, N pop differnt in GD? | NA | no | no |
| 1420 | 2017 | 43015 | Higashino, A; Sakate, R; Kameoka, Y; Takahashi, I; Hirata, M; Tanuma, R; Masui, T; Yasutomi, Y; Osada, N | 2012 | Whole-genome sequencing and analysis of the Malaysian cynomolgus macaque (Macaca fascicularis) genome | Background: The genetic background of the cynomolgus macaque (Macaca fascicularis) is made complex by the high genetic diversity, population structure, and gene introgression from the closely related rhesus macaque (Macaca mulatta). Herein we report the whole-genome sequence of a Malaysian cynomolgus macaque male with more than 40-fold coverage, which was determined using a resequencing method based on the Indian rhesus macaque genome. Results: We identified approximately 9.7 million single nucleotide variants (SNVs) between the Malaysian cynomolgus and the Indian rhesus macaque genomes. Compared with humans, a smaller nonsynonymous/synonymous SNV ratio in the cynomolgus macaque suggests more effective removal of slightly deleterious mutations. Comparison of two cynomolgus (Malaysian and Vietnamese) and two rhesus (Indian and Chinese) macaque genomes, including previously published macaque genomes, suggests that Indochinese cynomolgus macaques have been more affected by gene introgression from rhesus macaques. We further identified 60 nonsynonymous SNVs that completely differentiated the cynomolgus and rhesus macaque genomes, and that could be important candidate variants for determining species-specific responses to drugs and pathogens. The demographic inference using the genome sequence data revealed that Malaysian cynomolgus macaques have experienced at least three population bottlenecks. Conclusions: This list of whole-genome SNVs will be useful for many future applications, such as an array-based genotyping system for macaque individuals. High-quality whole-genome sequencing of the cynomolgus macaque genome may aid studies on finding genetic differences that are responsible for phenotypic diversity in macaques and may help control genetic backgrounds among individuals. | 1420 | no | no good fitness measure, N pop differnt in GD? | NA | no | no |
| 1421 | 2017 | 43015 | Rollinson, N; Keith, DM; Houde, ALS; Debes, PV; Mcbride, MC; Hutchings, JA | 2014 | Risk Assessment of Inbreeding and Outbreeding Depression in a Captive-Breeding Program | Captive-breeding programs can be implemented to preserve the genetic diversity of endangered populations such that the controlled release of captive-bred individuals into the wild may promote recovery. A common difficulty, however, is that programs are founded with limited wild broodstock, and inbreeding can become increasingly difficult to avoid with successive generations in captivity. Program managers must choose between maintaining the genetic purity of populations, at the risk of inbreeding depression, or interbreeding populations, at the risk of outbreeding depression. We evaluate these relative risks in a captive-breeding program for 3 endangered populations of Atlantic salmon (Salmo salar). In each of 2 years, we released juvenile F-1 and F-2 interpopulation hybrids, backcrosses, as well as inbred and noninbred within-population crosstypes into 9 wild streams. Juvenile size and survival was quantified in each year. Few crosstype effects were observed, but interestingly, the relative fitness consequences of inbreeding and outbreeding varied from year to year. Temporal variation in environmental quality might have driven some of these annual differences, by exacerbating the importance of maternal effects on juvenile fitness in a year of low environmental quality and by affecting the severity of inbreeding depression differently in different years. Nonetheless, inbreeding was more consistently associated with a negative effect on fitness, whereas the consequences of outbreeding were less predictable. Considering the challenges associated with a sound risk assessment in the wild and given that the effect of inbreeding on fitness is relatively predictable, we suggest that risk can be weighted more strongly in terms of the probable outcome of outbreeding. Factors such as genetic similarities between populations and the number of generations in isolation can sometimes be used to assess outbreeding risk, in lieu of experimentation. Evaluacion del Riesgo de Depresion por Endogamia y Exogamia en un Programa de Reproduccion en Cautiverio Resumen Los programas de reproduccion en cautiverio pueden ser implementados para preservar la diversidad genetica de las poblaciones en peligro, de tal forma que la liberacion controlada de los individuos criados en cautiverio a la vida libre puede promover la recuperacion. Sin embargo, una dificultad comun es que los programas se encuentran dentro del limitado capital de reproduccion silvestre, y la endogamia puede volverse cada vez mas dificil de evitar con generaciones sucesivas en cautiverio. Los directores del programa deben elegir entre mantener la pureza genetica de las poblaciones, con el riesgo de una depresion endogamica, o reproducir entre poblaciones, con el riesgo de una depresion exogamica. Evaluamos estos riesgos relativos en un programa de reproduccion en cautiverio para tres poblaciones en peligro de salmon del Atlantico (Salmo salar). Cada 2 anos, liberamos juveniles hibridos inter-poblacionales F-1 y F-2, retrocruzamientos, asi como cruzas endogamicas y no endogamicas entre poblaciones en nueve arroyos silvestres. El tamano juvenil y la supervivencia se cuantificaron cada ano. Se observaron pocos efectos de cruza, pero interesantemente, las consecuencias de la adaptabilidad relativa de la endogamia y exogamia variaron ano con ano. La variacion temporal en la calidad ambiental pudo ser conductora en alguna de estas diferencias anuales, al exacerbar la importancia de los efectos maternales sobre la aptitud juvenil en un ano de baja calidad ambiental y al afectar diferentemente la severidad de la depresion endogamica en anos diferentes. Sin embargo, la endogamia fue asociada constantemente con un efecto negativo sobre la adaptabilidad, mientras que las consecuencias de la exogamia fueron menos predecibles. Considerando los obstaculos asociados con un estudio concreto de riesgo en vida silvestre y dado que el efecto de la endogamia sobre la aptitud es relativamente predecible, sugerimos que el riesgo puede considerarse mas fuerte en terminos de un resultado probable de la exogamia. Factores como las similitudes geneticas entre poblaciones y el numero de generaciones en aislamiento puede usarse algunas veces para estudiar el riesgo de la exogamia, en lugar de la experimentacion. | 1421 | no | inbreeding | NA | no | no |
| 1422 | 2017 | 43015 | Faeth, SH; Shochat, E | 2010 | Inherited microbial symbionts increase herbivore abundances and alter arthropod diversity on a native grass | Some microbial symbionts of plants are maternally inherited and thus functionally increase genetic and phenotypic variation within plant populations. This variation, coupled with that of the host plant and environment, may alter abundances, diversity, and trophic structure of associated plant and animal communities. Fungal endophytes in the genus Neotyphodium are vertically transmitted, asexual microbial symbionts of grasses that remain asymptomatic and rely upon their hosts for resources and transmission via seeds, often providing benefits to their hosts, including protection against herbivores. Endophyte infections may influence associated arthropod communities in agronomic grasses, but the long-term effects of endophytes and variation in host genotype and resource availability on arthropod communities in native grass populations are unknown. We conducted a long-term field experiment with four maternal genotypes of an infected (E+) native grass (Festuca arizonica) from whence the endophyte was experimentally removed (E-) and water availability was controlled, to test the effects of infection, plant genotype, and resources on abundances, biomass, diversity (richness and evenness), and trophic structure of the arthropod community. Generally, E+ grasses harbored more arthropods, including more herbivores, predators, and detritivores, suggesting that the effects of endophytes cascaded upward through trophic levels in terms of abundances, at least in early ontogeny of the host. That E+ plants harbored more herbivorous insects than E- plants suggests that infection does not increase but instead decreases resistance to herbivores, contrary to prevailing concepts of endophytes as defensive mutualists. Infection did not alter overall species richness of the arthropod community or richness of herbivores but reduced natural enemy richness, especially that of parasites, and increased richness of detritivores. Reduced richness and shifts in evenness of natural enemies on E+ plants suggest that endophytes may disproportionately affect diversity at higher trophic levels and may partially explain increases in abundances of herbivorous insects on E+ plants. Biomass of predators, detritivores, and omnivores increased on plants with supplemented water, and arthropod and herbivore biomass varied by plant genotype. Symbiont-mediated phenotypic variation interacts with variation from plant genotype and environmental factors to alter arthropod abundances and diversity, and these effects shift with ontogeny of the host. | 1422 | no | no good fitness measure, N pop differnt in GD? | NA | no | no |
| 1423 | 2017 | 43015 | Janecka, JE; Tewes, ME; Laack, LL; Caso, A; Grassman, LI; Haines, AM; Shindle, DB; Davis, BW; Murphy, WJ; Honeycutt, RL | 2011 | Reduced genetic diversity and isolation of remnant ocelot populations occupying a severely fragmented landscape in southern Texas | The ocelot Leopardus pardalis has become a conservation priority in the US as a result of severe population decline and loss of habitat during the 20th century. Only two small populations remain in this country. Their short-term viability is threatened by the disappearance of dense thornshrub communities, human-caused mortality and demographic stochasticity. The influence these factors have on ocelot persistence must be considered to develop effective conservation initiatives. We therefore examined neutral genetic diversity and connectivity among ocelots in the US and northeastern Mexico using 25 autosomal microsatellites and a 395-bp segment of the mitochondrial control region. Genetic variation was lowest in the population occurring on Laguna Atascosa National Wildlife Refuge, Texas (autosomal microsatellite HE=0.399 and mtDNA-haplotype diversity=0) and highest in northeastern Mexico (0.637 and 0.73, respectively), while intermediate on private lands in Willacy County, Texas (0.553 and 0.252, respectively). Significant genetic differentiation between the two Texas populations was observed, despite their close proximity (similar to 30 km). Both populations were also significantly divergent from northeastern Mexico. The absence of any detectable gene flow implies that the human modified landscape of the Lower Rio Grande Valley in southern Texas acts as a strong barrier to ocelot movement, disrupting metapopulation dynamics and contributing to loss of diversity. As a consequence, continued genetic erosion among the Texas populations is expected. The lack of movement through the fragmented landscape also suggests it is unlikely ocelots will recolonize unoccupied habitat patches along the Lower Rio Grande and the delta interior where agriculture and urban land uses predominate. The continued rapid development will exacerbate this problem. These factors threaten the persistence of the Texas populations and limit their recovery. Translocations are necessary to link ocelot populations in the US. | 1423 | no | no good fitness measure | NA | no | no |
| 1424 | 2017 | 43015 | Sasmal, I; Jenks, JA; Waits, LP; Gonda, MG; Schroeder, GM; Datta, S | 2013 | Genetic diversity in a reintroduced swift fox population | Swift fox (Vulpes velox) were historically distributed in southwestern South Dakota including the region surrounding Badlands National Park (BNP). The species declined during the mid-1800s, largely due to habitat loss and poisoning targeted at wolves (Canis lupus) and coyotes (Canis latrans). Only a small population of swift foxes near Ardmore, which is located in Fall River County, South Dakota, persisted. In 2003, a reintroduction program was initiated at BNP with swift foxes translocated from Colorado and Wyoming. Foxes released in the years 2003, 2004 and 2005 were translocated from Colorado (BNP-Colorado) whereas in 2006, released foxes were translocated from Wyoming (BNP-Wyoming). Our objective was to evaluate genetic diversity and structure of the restored swift fox population in the area surrounding BNP compared to source fox populations in an area of Colorado and Wyoming, as well as the local swift fox population neighboring BNP near Ardmore in Fall River County, South Dakota. A total of 400 swift foxes (28 released in 2003, 28 released in 2004, 26 released in 2005, 26 released in 2006, 252 wild-born foxes, 40 individual foxes from the Ardmore area of South Dakota) was genotyped using twelve microsatellite loci. We report mean gene diversity values of 0.778 (SD = 0.156) for the BNP-Colorado population, 0.753 (SD = 0.165) for the BNP-Wyoming population, 0.751 (SD = 0.171) for the BNP population, and 0.730 (SD = 0.166) for the Fall River population. We also obtained F-st values ranging from 0.014 to 0.029 for pair-wise comparisons of fox populations (BNP, Fall River, BNP-Wyoming, BNP-Colorado). We conclude that the reintroduced fox population around BNP has high genetic diversity comparable to its source populations in Colorado and Wyoming. Although genetic diversity indicates that the reintroduction was successful, additional time is necessary to fully evaluate long-term genetic maintenance and interconnectivity among these populations. | 1424 | no | no good fitness measure | NA | no | no |
| 1425 | 2017 | 43015 | Delaplane, KS; Pietravalle, S; Brown, MA; Budge, GE | 2015 | Honey Bee Colonies Headed by Hyperpolyandrous Queens Have Improved Brood Rearing Efficiency and Lower Infestation Rates of Parasitic Varroa Mites | A honey bee queen mates on wing with an average of 12 males and stores their sperm to produce progeny of mixed paternity. The degree of a queen’s polyandry is positively associated with measures of her colony’s fitness, and observed distributions of mating number are evolutionary optima balancing risks of mating flights against benefits to the colony. Effective mating numbers as high as 40 have been documented, begging the question of the upper bounds of this behavior that can be expected to confer colony benefit. In this study we used instrumental insemination to create three classes of queens with exaggerated range of polyandry- 15, 30, or 60 drones. Colonies headed by queens inseminated with 30 or 60 drones produced more brood per bee and had a lower proportion of samples positive for Varroa destructor mites than colonies whose queens were inseminated with 15 drones, suggesting benefits of polyandry at rates higher than those normally obtaining in nature. Our results are consistent with two hypotheses that posit conditions that reward such high expressions of polyandry: (1) a queen may mate with many males in order to promote beneficial non-additive genetic interactions among subfamilies, and (2) a queen may mate with many males in order to capture a large number of rare alleles that regulate resistance to pathogens and parasites in a breeding population. Our results are unique for identifying the highest levels of polyandry yet detected that confer colony-level benefit and for showing a benefit of polyandry in particular toward the parasitic mite V. destructor. | 1425 | yes | ok fitness and gd? | no differfence in GD of groups, Relatedness from number of males: Relatedness from number of males, 15 = 0.266667, 30 = 0.2583333, 60 = 0.2541667 | no | no |
| 1426 | 2017 | 43015 | Larroque, J; Ruette, S; Vandel, JM; Queney, G; Devillard, S | 2016 | Age and sex-dependent effects of landscape cover and trapping on the spatial genetic structure of the stone marten (Martes foina) | “Maintenance of genetic variation is of critical importance for wild populations since low levels limit the species’ ability to respond to different threats (diseases, predators, environmental changes) in both the long and the short term. Human activities could impact the genetic variation of wild species in multiple ways, including via fragmentation and harvesting. We used an individual-based landscape genetics approach to describe the impact of landscape elements and trapping pressure on the spatial genetic structure of a large sample (n = 370) of the stone marten (Martes foina) in central-eastern France (Bresse). An analysis of isolation-by-resistance using a causal modeling approach showed an influence of landscape cover and/or trapping pressure on gene flow according to age and sex class. Overall, the connectivity in the study area is provided mainly by vegetation cover, while roads and open areas partially impede it. Unexpectedly for this”“urban adapter”" species, buildings could reduce gene flow. We also emphasized the sex-dependent effect of trapping on gene flow. Genetic differentiation in males was influenced by trapping pressure and landscape structure while only the latter influenced genetic differentiation in females. A stronger isolation by distance in males than in females suggested that at the scale of the study area, males are more exposed to trapping pressure, which reduces effective dispersal. Overall, the combination of both landscape and trapping costs might create an ‘ecological trap’ that could disrupt gene flow, leading to a north-south division in the study area." | 1426 | no | no good fitness measure, N pop differnt in GD? | NA | no | no |
| 1427 | 2017 | 43015 | Nims, BD; Vargas, FH; Merkel, J; Parker, PG | 2008 | Low genetic diversity and lack of population structure in the endangered Galapagos penguin (Spheniscus mendiculus) | Long-term monitoring of the endangered Galapagos penguin (Spheniscus mendiculus) has indicated poor reproductive periods and severe population fluctuations in association with El Ni (n) over tildeo - Southern Oscillation events. An earlier mark and recapture study indicated that adults exhibit some degree of breeding-site and mate fidelity, and that juveniles potentially move more frequently than adults; however, the extent to which migrants and gene flow occur between islands within the Galapagos archipelago is largely unknown. This study tested the hypothesis that geographic isolation and adult breeding philopatry has led to a degree of genetic differentiation between island subpopulations within the archipelago. We examined the genetic diversity within and among different subpopulations and the extent to which gene flow occurs between island subpopulations. Estimates of allelic richness and gene diversity were not significantly different between subpopulations. Tests to detect genetic heterogeneity failed to reject the H-0 of no difference in allele frequencies for chi-square (P = 0.28) and Fisher’s exact test (P = 0.19). All pairwise values of the F-ST variant theta were not significant, while a power analysis revealed a > 99% probability of detecting a biologically true F-ST of 0.05. Migration estimates in BAYESASS(+) suggest symmetrical gene flow throughout the species’ distribution. Our results indicate a low level of genetic diversity throughout the population and a seemingly high level of gene flow between subpopulations. We argue that the Galapagos penguin should be managed as one panmictic population and we discuss the risk of disease threats in the archipelago. | 1427 | no | no good fitness measure, N pop differnt in GD? | NA | no | no |
| 1428 | 2017 | 43015 | de Leon, JH; Jones, WA | 2005 | Genetic differentiation among geographic populations of Gonatocerus ashmeadi, the predominant egg parasitoid of the glassy-winged sharpshooter, Homalodisca coagulata | The aim of genetically comparing different populations of the same species of natural enemies is to identify the strain that is most adapted to the environment where it will be released. In the present study, Inter-Simple Sequence Repeat-Polymerase Chain Reaction (ISSR-PCR) was utilized to estimate the population genetic structure of Gonatocerus ashmeadi (Girault) (Hymenoptera: Mymaridae), the predominant egg parasitoid of Homalodisca coagulata (Say) (Homoptera: Cicadellidae), the glassy-winged sharpshooter. Six populations from throughout the U. S. and a population from Argentina identified as near G. ashmeadi were analyzed. Four populations (California; San Antonio, Texas; Weslaco, Texas [WTX- 2]; and Florida) were field collected and two (Louisiana and Weslaco, Texas [WTX- 1]) were reared. Three ISSR-PCR reactions were pooled to generate 41 polymorphic markers among the six U.S. populations. Nei’s expected heterozygosity values (h), including the reared population from Louisiana, were high (9.01-14.3%) for all populations, except for a reared population from WTX-1 (2.9%). The total genetic diversity value (Ht) for the field populations was high (23%). Interestingly, the Florida population that was collected from one egg mass (siblings) generated the greatest number of polymorphic markers (20) and was observed with the highest gene diversity value (14.3%). All populations, except WTX-2 generated population-specific markers. Comparison of genetic differentiation estimates, which evaluate the degree of genetic subdivision, demonstrated good agreement between G(ST) and. values, 0.38 and 0.50, respectively for field populations, and 0.44 and 0.50, respectively for all populations. Genetic divergence (D) indicated that the WTX-1 population was the most differentiated. Average D results from the Argentina population support the taxonomic data that it is a different species. The present results estimate the population genetic structure of G. ashmeadi, demonstrating genetic divergence and restricted gene flow (Nm = 0.83) among populations. These results are of interest to the Pierce’s disease/glassy-winged sharpshooter biological control program because the key to successful biological control may not be in another species, but instead in different geographic races or biotypes. | 1428 | no | gd of parasite | NA | no | no |
| 1429 | 2017 | 43015 | Gomez-Diaz, E; Morris-Pocock, JA; Gonzalez-Solis, J; McCoy, KD | 2012 | Trans-oceanic host dispersal explains high seabird tick diversity on Cape Verde islands | Parasites represent ideal models for unravelling biogeographic patterns and mechanisms of diversification on islands. Both host-mediated dispersal and within-island adaptation can shape parasite island assemblages. In this study, we examined patterns of genetic diversity and structure of Ornithodoros seabird ticks within the Cape Verde Archipelago in relation to their global phylogeography. Contrary to expectations, ticks from multiple, geographically distant clades mixed within the archipelago. Trans-oceanic colonization via host movements probably explains high local tick diversity, contrasting with previous research that suggests little large-scale dispersal in these birds. Although host specificity was not obvious at a global scale, host-associated genetic structure was found within Cape Verde colonies, indicating that post-colonization adaptation to specific hosts probably occurs. These results highlight the role of host metapopulation dynamics in the evolutionary ecology and epidemiology of avian parasites and pathogens. | 1429 | no | gd of parasite | NA | no | no |
| 1430 | 2017 | 43015 | Lozier, JD; Zayed, A | 2017 | Bee conservation in the age of genomics | Many wild and managed bee pollinators have experienced population declines over the past several decades, and molecular and population genetic tools have been valuable in understanding conservation threats across the bee tree of life. Emerging genomic tools have the potential to improve classical applications of conservation genetics, such as assessing species status, and quantifying genetic diversity, gene flow and effective population sizes. Genomic tools can also revolutionize novel research in bee conservation and management, including the identification of loci underlying adaptive and economically desirable traits, such as those involved in disease susceptibility, responses to multiple environmental stressors, and even discovering and understanding the hidden diversity of beneficial microorganisms associated with bees. In this perspective, we provide a survey of some of the ways genomic tools can be applied to bee conservation to bridge the gap between basic and applied genomics research. | 1430 | no | no good fitness measure | NA | no | no |
| 1431 | 2017 | 43015 | Lehmann, L; Perrin, N | 2006 | On metapopulation resistance to drift and extinction | The spatial configuration of metapopulations (numbers, sizes, and localization of patches) affects their ability to resist demographic extinction and genetic drift, but sometimes with opposite effects. Small and isolated patches, for instance, contribute marginally to demography but may play a large role in genetics by maintaining a sizeable amount of genetic variance among demes. In source-sink systems, similarly, connectivity may be beneficial in terms of effective size, but detrimental in terms of survival, by lowering the reproductive value of source populations.. How to reconcile these opposite effects? Here we propose an analytical framework that integrates fixation time (ability to resist genetic drift) and extinction time (ability to resist demographic extinction) into a single index of resistance, measuring the ability of a metapopulation to maintain its demo-genetic integrity. We then illustrate with numerical examples how conflicting demands may be resolved. | 1431 | no | No GD diff between pop | NA | no | no |
| 1432 | 2017 | 43015 | Swanson, BL; Lyons, AC; Bouzat, JL | 2014 | Distribution, prevalence and host specificity of avian malaria parasites across the breeding range of the migratory lark sparrow (Chondestes grammacus) | The lark sparrow (Chondestes grammacus) is a ground-nesting passerine that breeds across much of the central North American steppe and sand barrens. Through genotyping and sequencing of avian malaria parasites we examined levels of malaria prevalence and determined the distribution of Haemoproteus and Plasmodium lineages across the breeding range of the lark sparrow. Analysis of 365 birds collected from five breeding locations revealed relatively high levels of malaria prevalence in adults (80 %) and juveniles (46 %), with infections being primarily of Haemoproteus (91 % of sequenced samples). Levels of genetic diversity and genetic structure of malaria parasites with respect to the avian host populations revealed distinct patterns for Haemoproteus and Plasmodium, most likely as a result of their distinct life histories, host specificity, and transmission vectors. With the exception of one common Haemoproteus haplotype detected in all populations, all other haplotypes were either population-specific or shared by two to three populations. A hierarchical analysis of molecular variance of Haemoproteus sequences revealed that 15-18 % of the genetic variation can be explained by differences among host populations/locations (p < 0.001). In contrast to the regional patterns of genetic differentiation detected for the lark sparrow populations, Haemoproteus parasites showed high levels of population-specific variation and no significant differences among regions, which suggests that the population dynamics of the parasites may be driven by evolutionary processes operating at small spatial scales (e.g., at the level of host populations). These results highlight the potential effects of host population structure on the demographic and evolutionary dynamics of parasites. | 1432 | no | No GD diff between pop | NA | no | no |
| 1433 | 2017 | 43015 | Bucheli, E; Leuchtmann, A | 1996 | Evidence for genetic differentiation between choke-inducing and asymptomatic strains of the Epichloe grass endophyte from Brachypodium sylvaticum | Life cycle and breeding system variation in Epichloe grass endophytes (choke disease) is tightly linked to the degree of stroma formation. It is not known whether this variation results from differences in host resistance, fungal virulence, or environmental conditions. We found genetic differentiation between 173 asymptomatic (NS) and 93 stromata-forming (S) Epichloe strains isolated from one grass species, Brachypodium sylvaticum, based on 13 presumed allozyme loci, of which six were variable. The fungal strains originated from 10 sites in Switzerland, three sites of which were represented by both NS and S subpopulations. In total, 19 allozyme genotypes, that were nonrandomly distributed among S and NS were detected. Genetic variation measured as G(ST) between S and NS strains isolated from the same site ranged from 0.73 to 0.98. Clonality, measured as linkage disequilibrium at one site, was significant in the NS subpopulation (P much less than 0.001), but not in the S subpopulation (P = 0.21), implying asexual reproduction by NS strains as well as successful horizontal transmission of S strains. Since all seeds are usually infected vegetatively, horizontal transmission implies the occurrence of multiple host infections. Altogether, these results provide indirect evidence that NS and S strains do not belong to one panmictic population and that differentiation patterns of stroma formation found in nature are due to genetic differences among fungi in associations with their host plants. We discuss the direction of evolution of disease expression in this system. The distribution of genetic variability suggests that the asymptomatic strains were derived from stromata-forming populations. | 1433 | no | no good fitness measure, N pop differnt in GD? | NA | no | no |
| 1434 | 2017 | 43015 | Anderholm, S; Waldeck, P; Van der Jeugd, HP; Marshall, RC; Larsson, K; Andersson, M | 2009 | Colony kin structure and host-parasite relatedness in the barnacle goose | Conspecific brood parasitism (CBP), females laying eggs in the nest of other ‘host’ females of the same species, is a common alternative reproductive tactic among birds. For hosts there are likely costs of incubating and rearing foreign offspring, but costs may be low in species with precocial chicks such as waterfowl, among which CBP is common. Waterfowl show strong female natal philopatry, and spatial relatedness among females may influence the evolution of CBP. Here we investigate fine-scale kin structure in a Baltic colony of barnacle geese, Branta leucopsis, estimating female spatial relatedness using protein fingerprints of egg albumen, and testing the performance of this estimator in known mother-daughter pairs. Relatedness was significantly higher between neighbour females (nesting < 40 metres from each other) than between females nesting farther apart, but there was no further distance trend in relatedness. This pattern may be explained by earlier observations of females nesting close to their mother or brood sisters, even when far from the birth nest. Hosts and parasites were on average not more closely related than neighbour females. In 25 of 35 sampled parasitized nests, parasitic eggs were laid after the host female finished laying, too late to develop and hatch. Timely parasites, laying eggs in the host’s laying sequence, had similar relatedness to hosts as that between neighbours. Females laying late parasitic eggs tended to be less related to the host, but not significantly so. Our results suggest that CBP in barnacle geese might represent different tactical life-history responses. | 1434 | no | brood parasitism | NA | no | no |
| 1435 | 2017 | 43015 | Pruvost, O; Magne, M; Boyer, K; Leduc, A; Tourterel, C; Drevet, C; Ravigne, V; Gagnevin, L; Guerin, F; Chiroleu, F; Koebnik, R; Verdier, V; Verniere, C | 2014 | A MLVA Genotyping Scheme for Global Surveillance of the Citrus Pathogen Xanthomonas citri pv. citri Suggests a Worldwide Geographical Expansion of a Single Genetic Lineage | MultiLocus Variable number of tandem repeat Analysis (MLVA) has been extensively used to examine epidemiological and evolutionary issues on monomorphic human pathogenic bacteria, but not on bacterial plant pathogens of agricultural importance albeit such tools would improve our understanding of their epidemiology, as well as of the history of epidemics on a global scale. Xanthomonas citri pv. citri is a quarantine organism in several countries and a major threat for the citrus industry worldwide. We screened the genomes of Xanthomonas citri pv. citri strain IAPAR 306 and of phylogenetically related xanthomonads for tandem repeats. From these in silico data, an optimized MLVA scheme was developed to assess the global diversity of this monomorphic bacterium. Thirty-one minisatellite loci (MLVA-31) were selected to assess the genetic structure of 129 strains representative of the worldwide pathological and genetic diversity of X. citri pv. citri. Based on Discriminant Analysis of Principal Components (DAPC), four pathotype-specific clusters were defined. DAPC cluster 1 comprised strains that were implicated in the major geographical expansion of X. citri pv. citri during the 20th century. A subset of 12 loci (MLVA-12) resolved 89% of the total diversity and matched the genetic structure revealed by MLVA-31. MLVA-12 is proposed for routine epidemiological identification of X. citri pv. citri, whereas MLVA-31 is proposed for phylogenetic and population genetics studies. MLVA-31 represents an opportunity for international X. citri pv. citri genotyping and data sharing. The MLVA-31 data generated in this study was deposited in the Xanthomonas citri genotyping database (http://www.biopred.net/MLVA/). | 1435 | no | NA | NA | no | no |
| 1436 | 2017 | 43015 | Li, WB; Song, QJ; Brlansky, RH; Hartung, JS | 2007 | Genetic diversity of citrus bacterial canker pathogens preserved in herbarium specimens | Citrus bacterial canker (CBC) caused by Xanthomonas axonopodis pv. citri (Xac) was first documented in India and Java in the mid 19th century. Since that time, the known distribution of the disease has steadily increased. Concurrent with the dispersion of the pathogen, the diversity of described strains continues to increase, with novel strains appearing in Saudi Arabia, Iran, and Florida in the last decade. Herbarium specimens of infected plants provide an historical record documenting both the geographic distribution and genetic diversity of the pathogen in the past. However, no method was available to assess the genetic diversity within these herbarium samples. We have developed a method, insertion event scanning (IES), and applied the method to characterize the diversity present within CEIC populations documented as herbarium specimens over the past century. IES is based on the specific amplification of junction fragments that define insertion events. The potential for IES in current forensic applications is demonstrated by finding an exact match of pathogen genotypes preserved in herbarium specimens from Japan and Florida, demonstrating the source of the original outbreak of citrus canker in Florida in 1911. IES is a very sensitive technique for differentiating bacterial strains and can be applied to any of the several hundred bacteria for which full genomic sequence data are available. | 1436 | no | gd of pathogen | NA | no | no |
| 1437 | 2017 | 43015 | Paaby, AB; Schmidt, PS | 2008 | Functional Significance of Allelic Variation at methuselah, an Aging Gene in Drosophila | Background: Longevity and age-specific patterns of mortality are complex traits that vary within and among taxa. Multiple candidate genes for aging have been identified in model systems by extended longevity mutant phenotypes, including the G-protein coupled receptor methuselah (mth) in D. melanogaster. These genes offer important insights into the mechanisms of lifespan determination and have been major targets of interest in the biology of aging. However, it is largely unknown whether these genes contribute to genetic variance for lifespan in natural populations, and consequently contribute to lifespan evolution. Methodology/Principle Findings: For a gene to contribute to genetic variance for a particular trait, it must meet two criteria: natural allelic variation and functional differences among variants. Previous work showed that mth varies significantly among wild populations; here we assess the functional significance of wild-derived mth alleles on lifespan, fecundity and stress resistance using a quantitative complementation scheme. Our results demonstrate that mth alleles segregating in nature have a functional effect on all three traits. Conclusions/Significance: These results suggest that allelic variation at mth contributes to observed differences in lifespan and correlated phenotypes in natural populations, and that evaluation of genetic diversity at candidate genes for aging can be a fruitful approach to identifying loci contributing to lifespan evolution. | 1437 | no | No GD diff between pop | NA | no | no |
| 1438 | 2017 | 43015 | Esko, T; Mezzavilla, M; Nelis, M; Borel, C; Debniak, T; Jakkula, E; Julia, A; Karachanak, S; Khrunin, A; Kisfali, P; Krulisova, V; Kucinskiene, ZA; Rehnstrom, K; Traglia, M; Nikitina-Zake, L; Zimprich, F; Antonarakis, SE; Estivill, X; Glavac, D; Gut, I; Klovins, J; Krawczak, M; Kucinskas, V; Lathrop, M; Macek, M; Marsal, S; Meitinger, T; Melegh, B; Limborska, S; Lubinski, J; Paolotie, A; Schreiber, S; Toncheva, D; Toniolo, D; Wichmann, HE; Zimprich, A; Metspalu, M; Gasparini, P; Metspalu, A; D’Adamo, P | 2013 | Genetic characterization of northeastern Italian population isolates in the context of broader European genetic diversity | Population genetic studies on European populations have highlighted Italy as one of genetically most diverse regions. This is possibly due to the country’s complex demographic history and large variability in terrain throughout the territory. This is the reason why Italy is enriched for population isolates, Sardinia being the best-known example. As the population isolates have a great potential in disease-causing genetic variants identification, we aimed to genetically characterize a region from northeastern Italy, which is known for isolated communities. Total of 1310 samples, collected from six geographically isolated villages, were genotyped at > 4145 000 single-nucleotide polymorphism positions. Newly genotyped data were analyzed jointly with the available genome-wide data sets of individuals of European descent, including several population isolates. Despite the linguistic differences and geographical isolation the village populations still show the greatest genetic similarity to other Italian samples. The genetic isolation and small effective population size of the village populations is manifested by higher levels of genomic homozygosity and elevated linkage disequilibrium. These estimates become even more striking when the detected substructure is taken into account. The observed level of genetic isolation in Friuli-Venezia Giulia region is more extreme according to several measures of isolation compared with Sardinians, French Basques and northern Finns, thus proving the status of an isolate. | 1438 | no | human | NA | no | no |
| 1439 | 2017 | 43015 | Schweinsberg, M; Weiss, LC; Striewski, S; Tollrian, R; Lampert, KP | 2015 | More than one genotype: how common is intracolonial genetic variability in scleractinian corals? | In recent years, a few colonial marine invertebrates have shown intracolonial genetic variability, a previously unreported phenomenon. Intracolonial genetic variability describes the occurrence of more than a single genotype within an individual colony. This variability can be traced back to two underlying processes: chimerism and mosaicism. Chimerism is the fusion of two or more individuals, whereas mosaicism mostly derives from somatic cell mutations. Until now, it remained unclear to what degree the ecologically important group of hermatypic (reef building) corals might be affected. We investigate the occurrence of intracolonial genetic variability in five scleractinian corals: Acropora florida, Acropora hyacinthus, Acropora sarmentosa, Pocillopora species complex and Porites australiensis. The main focus was to test different genera for the phenomenon via microsatellite markers and to distinguish which underlying process caused the genetic heterogeneity. Our results show that intracolonial genetic variability was common (between 46.6% for A.sarmentosa and 23.8% for P.species complex) in all tested corals. The main process was mosaicism (69 cases of 222 tested colonies), but at least one chimera existed in every species. This suggests that intracolonial genetic variability is widespread in scleractinian corals and could challenge the view of a coral colony as an individual and therefore a unit of selection. However, it might also hold potential for colony survival under rapidly changing environmental conditions. | 1439 | no | no good fitness measure | NA | no | no |
| 1440 | 2017 | 43015 | Lawrence, BA; Kaye, TN | 2011 | Reintroduction of Castilleja levisecta: Effects of Ecological Similarity, Source Population Genetics, and Habitat Quality | A suite of ecological and genetic factors are likely to contribute to reintroduction performance. Potential factors include the ecological similarity between seed source and introduction site, population size and genetic diversity of seed sources, and the habitat quality of the introduction site. We conducted common garden experiments with golden paintbrush (Castilleja levisecta), an endangered species from the Pacific Northwest, U.S.A., in order to test hypotheses about reintroduction performance and to provide management recommendations. Ten common gardens, each composed of C. levisecta individuals grown from seed from six of the remaining populations, were planted into field conditions and monitored during two growing seasons. Plant community characteristics were important predictors of observed variation in C. levisecta performance. Exotic species-cover at common garden sites was associated with a reduction in performance of first-year C. levisecta transplants, while survival to the second growing season increased with increasing similarity in plant functional groups between source and common garden sites. Although measures of genetic diversity, population size, and geographic distance are often used to make conservation decisions during species recovery, here they were poor predictors of C. levisecta performance and establishment. We recommend choosing material for reintroduction from ecologically similar habitats, rather than those most proximate geographically, and selecting recovery sites with low exotic species abundance. | 1440 | no | no good fitness measure, N pop differnt in GD? | NA | no | no |
| 1441 | 2017 | 43015 | Fahima, T; Roder, MS; Grama, A; Nevo, E | 1998 | Microsatellite DNA polymorphism divergence in Triticum dicoccoides accessions highly resistant to yellow rust | Stripe rust (yellow rust), caused by Puccinia striiformis f. sp. tritici, is one of the most important diseases of wheat throughout the world. Wild emmer wheat, Triticum dicoccoides, the progenitor of cultivated wheat, was found to be a valuable source for novel stripe-rust-resistance genes. The objective of the present study was to estimate the extent of genetic diversity among the wild emmer wheat accessions, previously identified as highly resistant to stripe rust, in order to select suitable parents for genetic-mapping studies. Twenty three wheat microsatellite (WMS) markers were used to detect DNA polymorphism among 21 accessions of T. dicoccoides, which included 19 resistant and two susceptible accessions originating mainly from the center of origin and diversity in the Upper Galilee and Hermon Mountain in northern Israel. In addition, two Triticum durum and one Triticum aestivum lines were also included in the analysis. The 23 WMS markers used were located on 23 chromosome arms, representing all 14 chromosomes of genomes A and B of wheat, and revealed a total of 230 alleles. The number of alleles ranged from 5 to 18, with an average of ten alleles per WMS. Genetic dissimilarity values between genotypes, calculated by the WMS-derived data, were used to produce a dendrogram of the relationships among accessions using the unweighted pair-group method with arithmetic averages (UPGMA). The results showed that all of the wild emmer wheat accessions could be distinguished. Most of the resulting groups were strongly related to the ecogeographical origin of the accessions, indicating that the genetic diversity of T. dicoccoides is correlated with geographic distribution. The three major groups were the Rosh Pinna group (north of the Sea of Galilee), the Mount Hermon group (north of the Golan Heights) and Mount Kena’an group (Upper Galilee). The genetic similarity (GS) of the 21 T. dicoccoides accessions based on WMS results averaged 0.31. As expected, the T. durum and T. aestivum lines were grouped separately from the T. dicoccoides accessions. The results obtained suggest that a relatively small number of microsatellites can be used for the estimation of genetic diversity in wild material of T. dicoccoides. These results will be useful in the identification of suitable parents for the development of mapping populations for tagging yellow-rust resistance genes derived from T. dicoccoides. Furthermore, future work could test the adaptive evolutionary significance of microsatellites in natural populations of wild emmer wheat. | 1441 | no | no good fitness measure, N pop differnt in GD? | NA | no | no |
| 1442 | 2017 | 43015 | Francis, SS; Plucinski, MM; Wallace, AD; Riley, LW | 2016 | Genotyping Oral Commensal Bacteria to Predict Social Contact and Structure | Social network structure is a fundamental determinant of human health, from infectious to chronic diseases. However, quantitative and unbiased approaches to measuring social network structure are lacking. We hypothesized that genetic relatedness of oral commensal bacteria could be used to infer social contact between humans, just as genetic relatedness of pathogens can be used to determine transmission chains of pathogens. We used a traditional, questionnaire survey-based method to characterize the contact network of the School of Public Health at a large research university. We then collected saliva from a subset of individuals to analyze their oral microflora using a modified deep sequencing multilocus sequence typing (MLST) procedure. We examined micro-evolutionary changes in the S. viridans group to uncover transmission patterns reflecting social network structure. We amplified seven housekeeping gene loci from the Streptococcus viridans group, a group of ubiquitous commensal bacteria, and sequenced the PCR products using next-generation sequencing. By comparing the generated S. viridans reads between pairs of individuals, we reconstructed the social network of the sampled individuals and compared it to the network derived from the questionnaire survey-based method. The genetic relatedness significantly (p-value < 0.001) correlated with social distance in the questionnaire-based network, and the reconstructed network closely matched the network derived from the questionnaire survey-based method. Oral commensal bacterial are thus likely transmitted through routine physical contact or shared environment. Their genetic relatedness can be used to represent a combination of social contact and shared physical space, therefore reconstructing networks of contact. This study provides the first step in developing a method to measure direct social contact based on commensal organism genotyping, potentially capable of unmasking hidden social networks that contribute to pathogen transmission. | 1442 | no | NA | NA | no | no |
| 1443 | 2017 | 43015 | Reques, R; Tejedo, M | 1997 | Reaction norms for metamorphic traits in natterjack toads to larval density and pond duration | “The evolution of environmentally-induced changes in phenotype or reaction norm implies both the existence at some time of genetic variation within a population for that plasticity measured by the presence of genotype x environment interaction (G x E), and that phenotypic variation affects fitness. Otherwise, the genetic structure of polygenic traits may restrict the evolution of the reaction norm by the lack of independent evolution of a given trait in different environments or by genetic trade-offs with other traits that affect fitness. In this paper, we analyze the existence of G x E in metamorphic traits to two environmental factors, larval density and pond duration in a factorial experiment with Bufo calamita tadpoles in semi-natural conditions and in the laboratory. Results showed no plastic temporal response in metamorphosis to pond durability at low larval density. The rank of genotypes did not change across different hydroperiods, implying a high genetic correlation that may constrain the evolution of the reaction norm. At high larval density a significant G x E interaction was found, suggesting the potential for the evolution of the reaction norm. A sibship (#1) attained the presumed”“optimal”" reaction norm by accelerating developmental rate in short duration ponds and delaying it in longer ponds. This could be translated in fitness by an increment in metamorphic survival and size at metamorphosis in short and long ponds respectively with respect to non-plastic sibships. However, genetic variability for plasticity suggests that optimal reaction norm for developmental rates may be variable and hard to achieve in the heterogeneous pond environment. Mass at metamorphosis was not plastic across different pond durations but decreased at high larval density. Significant adaptive plasticity for growth rates appeared in environments that differed drastically in level of crowding conditions, both in the field and in the laboratory. The fact that survival of juveniles metamorphosed at high density ponds was a monotonic function of metamorphic size, implies that response to selection may occur in this population of natterjacks and that genetic variability in plasticity may be a reliable mechanism maintaining adaptive genetic Variation in growth rates in the highly variable pond environment." | 1443 | no | No GD diff between pop | NA | no | no |
| 1444 | 2017 | 43015 | Ellegren, H; Savolainen, P; Rosen, B | 1996 | The genetical history of an isolated population of the endangered grey wolf Canis lupus: A study of nuclear and mitochondrial polymorphisms | The grey wolf was thought to have been exterminated in the Scandinavian peninsula when the sudden appearance of a few animals in southern Sweden was reported in 1980. These wolves founded a new Swedish population which currently numbers at least 25 individuals, one of the world’s smallest populations of the species. The sudden occurrence of the founder animals caused speculation that these had not appeared by ‘natural’ means but rather were Swedish zoo animals deliberately released by man. To analyse if this was the case and to elucidate the genetic status bi; this small and isolated population, we assessed nuclear and mitochondrial (mt) genetic variability in wild and captive grey wolves, using microsatellite typing and sequence analysis of the mtDNA D-loop. The new population was found to be monomorphic for a mtDNA haplotype which also was present in the Swedish zoo population. A total of four different mtDNA haplotypes were found among all captive and wild wolves (including two animals from an occasional establishment of a few wolves in northern Sweden in the late 1970s), with a maximum sequence divergence of 3.1 %. Despite the mtDNA congruence, animals from the zoo population could most likely be excluded as founders for the wild population since the latter group of animals displayed several unique microsatellite alleles (i.e. alleles not found in the zoo population). Moreover, a phylogenetic analysis of individual wolves, using microsatellite allele sharing as distance measure, placed all wild animals on a branch separated from that of the captive animals. The average degree of nuclear variability as well as allelic diversity was similar in the wild and the captive populations, respectively, but was lower than that reported for North-American populations of grey wolves. Polymorphism has declined in wild wolves born in recent years suggesting that this small population is currently suffering from a loss of genetic variability due to inbreeding. Inbreeding depression is documented in captive wolves and the long-term survival of the wild Swedish population may therefore depend on immigration of animals from Russia. This study illustrates the usefulness of microsatellites for dissecting close genetic relationships and for addressing the genetic status of individuals. | 1444 | no | no good fitness measure, N pop differnt in GD? | NA | no | no |
| 1445 | 2017 | 43015 | Wattier, RA; Haine, ER; Beguet, J; Martin, G; Bollache, L; Musko, IB; Platvoet, D; Rigaud, T | 2007 | No genetic bottleneck or associated microparasite loss in invasive populations of a freshwater amphipod | Understanding what factors determine the success of an invasive species in its adopted range is crucial from an evolutionary ecology point of view, because it can provide insights into which biological characteristics are required for survival in varied environmental conditions. Successful establishment may depend on both maintaining genetic diversity, which will allow the species to evolve and/or adapt to new environments, and the presence or absence of natural enemies such as parasites. We tested these two hypotheses by studying populations of the amphipod crustacean Dikerogammarus villosus. This Ponto-Caspian invader has rapidly and successfully invaded western Europe and threatens macroinvertebrate biodiversity in its adopted ranges. It is a unique system to study since both its colonisation history and its geographic origins are well-known. Using samples from the whole geographic range of the invasion route, and using four molecular markers, we found no evidence for genetic bottlenecks during the invasion of D. villosus in western Europe, despite slight variations in allelic proportions according to spatio-temporal subdivisions of our dataset. In addition, we analysed the prevalence and diversity of parasites across its native and adopted range. We found no macro-parasites, and no significant parasite loss of microsporidian parasites during the invasive process. Our data suggest that D. villosus invasion was either massive, or recurrent, or both, allowing a parasitic cortege to follow the host. The maintenance of genetic diversity may have contributed to its success, including the variation in.resistance in the face of the natural enemies. | 1445 | no | No GD diff between pop | NA | no | no |
| 1446 | 2017 | 43015 | MCCULLOUGH, JM; BARTON, EY | 1991 | RELATEDNESS AND MORTALITY RISK DURING A CRISIS YEAR - PLYMOUTH COLONY, 1620-1621 | To test the association of relatedness with risk of death during a demographic crisis, individual relatedness values were compared with mortality histories of the 103 passengers on the 1620 voyage of the Mayflower to Plymouth. Fifty-three (51.5%) died during the first winter due to malnutrition, disease, and lack of preparedness. There was no bias in proportion dying by sex or social class, but children survived in higher proportion (74.2%) than adults (37.5%) (t = 3.75; p < 0.001), and if 1 parent survived the winter, the children all lived. Of the 16 children without at least 1 surviving parent, 8 died (t = 3.87; p < 0.01), but the presence of other relatives had no effect on mortality risk. Family size did not affect survival probability of either parents or children. Survivors had a significantly higher mean level of total relatedness, S(t), (0.870; s.d., 0.694) than non-survivors (0.429; s.d., 0.515) (t = 3.61; p < 0.001) and this finding was also true for subsamples of males, females, Saints, and Strangers, but not children, hired hands, or servants. Using discriminant function and multiple regression analysis, survival was best predicted by level of summed relatedness to survivors, S(s), to decedents, S(d), and age; however, only 71.8% of the sample was classified to correct survival category. Percent survivorship and relatedness were associated in simple linear fashion for the entire sample as tested by regression analysis, using either Spearman’s or Pearson’s methods. Thus, relatedness was directly associated with probability of survival, but the presence of different levels of relatedness for different social and demographic categories suggests that other factors also influence the outcome. | 1446 | no | human | NA | no | no |
| 1447 | 2017 | 43015 | DeMay, SM; Becker, PA; Rachlow, JL; Waits, LP | 2017 | Genetic monitoring of an endangered species recovery: demographic and genetic trends for reintroduced pygmy rabbits (Brachylagus idahoensis) | Monitoring demographic and genetic parameters of reintroduced populations of endangered species is essential for evaluating and informing conservation strategies to maximize the chances of a successful recovery. We applied noninvasive genetic monitoring using fecal DNA to evaluate demographic and genetic parameters as they relate to the recovery of the endangered Columbia Basin pygmy rabbit (Brachylagus idahoensis) in central Washington, United States, during the initial 3 years of a renewed reintroduction effort. We quantified post-release dispersal, apparent survival, and reproduction in the wild, and monitored the genetic diversity and composition of the released cohorts, wild surviving population, and breeding population held in large enclosures. During this study, we reintroduced 1,206 pygmy rabbits into the wild, and detected a total of 176 individuals living on or near the release area during surveys the following winters. Median dispersal distance of juveniles was 776 m, compared to 471 m for adults, and dispersal distances for juveniles decreased for rabbits released later in the release season. Survival of juveniles differed across years and was positively associated with release date, release weight (a surrogate for age), and heterozygosity. Survival of adults was similarly influenced by release day, with some evidence for a positive effect of heterozygosity. Only 14 wild-born individuals were detected during the study. Genetic monitoring was an effective way to evaluate the demographic and genetic status of the reintroduced population within a limited study area, to inform changes to the conservation strategy, and to generate a data set to address long-term research and recovery goals. | 1447 | no | No GD diff between pop | NA | no | no |
| 1448 | 2017 | 43015 | Russo, LM; Abdeltawab, NF; O’Brien, AD; Kotb, M; Melton-Celsa, AR | 2015 | Mapping of genetic loci that modulate differential colonization by Escherichia coli O157:H7 TUV86-2 in advanced recombinant inbred BXD mice | Background: Shiga toxin (Stx)-producing E. coli (STEC) are responsible for foodborne outbreaks that can result in severe human disease. During an outbreak, differential disease outcomes are observed after infection with the same STEC strain. One question of particular interest is why some infected people resolve infection after hemorrhagic colitis whereas others progress to the hemolytic uremic syndrome (HUS). Host age and infection dose have been implicated; however, these parameters do not appear to fully account for all of the observed variation in disease severity. Therefore, we hypothesized that additional host genetic factors may play a role in progression to HUS. Methods and Results: To mimic the genetic diversity in the human response to infection by STEC, we measured the capacity of an O157:H7 outbreak isolate to colonize mouse strains from the advanced recombinant inbred (ARI) BXD panel. We first infected the BXD parental strains C57BL/6 J (B6) and DBA/2 J (D2) with either 86-24 (Stx2a+) or TUV86-2, an Stx2a-negative isogenic mutant. Colonization levels were determined in an intact commensal flora (ICF) infection model. We found a significant difference in colonization levels between the parental B6 and D2 strains after infection with TUV86-2 but not with 86-24. This observation suggested that a host factor that may be masked by Stx2a affects O157: H7 colonization in some genetic backgrounds. We then determined the TUV86-2 colonization levels of 24 BXD strains in the ICF model. We identified several quantitative trait loci (QTL) associated with variation in colonization by correlation analyses. We found a highly significant QTL on proximal chromosome 9 (12.5-26.7 Mb) that strongly predicts variation in colonization levels and accounts for 15-20 % of variance. Linkage, polymorphism and co-citation analyses of the mapped region revealed 36 candidate genes within the QTL, and we identified five genes that are most likely responsible for the differential colonization. Conclusions: The identification of the QTL on chromosome 9 supports our hypothesis that individual genetic makeup affects the level of colonization after infection with STEC O157: H7. | 1448 | no | NA | NA | no | no |
| 1449 | 2017 | 43015 | Anderson, MZ; Gerstein, AC; Wigen, L; Baller, JA; Berman, J | 2014 | Silencing Is Noisy: Population and Cell Level Noise in Telomere-Adjacent Genes Is Dependent on Telomere Position and Sir2 | Cell-to-cell gene expression noise is thought to be an important mechanism for generating phenotypic diversity. Furthermore, telomeric regions are major sites for gene amplification, which is thought to drive genetic diversity. Here we found that individual subtelomeric TLO genes exhibit increased variation in transcript and protein levels at both the cell-to-cell level as well as at the population-level. The cell-to-cell variation, termed Telomere-Adjacent Gene Expression Noise (TAGEN) was largely intrinsic noise and was dependent upon genome position: noise was reduced when a TLO gene was expressed at an ectopic internal locus and noise was elevated when a non-telomeric gene was expressed at a telomere-adjacent locus. This position-dependent TAGEN also was dependent on Sir2p, an NAD(+)-dependent histone deacetylase. Finally, we found that telomere silencing and TAGEN are tightly linked and regulated in cis: selection for either silencing or activation of a TLO-adjacent URA3 gene resulted in reduced noise at the neighboring TLO but not at other TLO genes. This provides experimental support to computational predictions that the ability to shift between silent and active chromatin states has a major effect on cell-to-cell noise. Furthermore, it demonstrates that these shifts affect the degree of expression variation at each telomere individually. | 1449 | no | NA | NA | no | no |
| 1450 | 2017 | 43015 | Jiang, DM; Peng, S; Wu, ZJ; Cheng, ZM; Li, SF | 2009 | Genetic diversity and phylogenetic analysis of Australian Grapevine Viroid (AGVd) isolated from different grapevines in China | Australian grapevine viroid (AGVd) is found in only three countries in the world. Here, the genetic diversity and phylogenetic relationships of AGVd isolates from three different grape varieties (Thomson Seedless, Jingchuan and Zaoyu) in China were studied. A hundred of independent cDNA clones from each of the three isolates, in total of 300, were sequenced. We identified 29 sequence variants including two predominant ones in Thomson Seedless, and 48 each including a unique predominant one in Jingchuan and Zaoyu. In silico structure analysis revealed that base changes were clustered in the left terminal domain of the predicted secondary structure in all three isolates. Further, these changes were shown to affect their secondary structures to varying degrees. Genetic diversity and phylogenetic analysis of four predominant sequence variants from this study, plus four others from Australia and Tunisia, revealed obvious regional disparity and variety-specificity in AGVd. | 1450 | no | NA | NA | no | no |
| 1451 | 2017 | 43015 | Criscione, CD; Blouin, MS | 2006 | Minimal selfing, few clones, and no among-host genetic structure in a hermaphroditic parasite with asexual larval propagation | Little is known about actual mating systems in natural populations of parasites or about what constitutes the limits of a parasite deme. These parameters are interesting because they affect levels of genetic diversity, opportunities for local adaptation, and other evolutionary processes. We expect that transmission dynamics and the distribution of parasites among hosts should have a large effect on mating systems and demic structure, but currently we have mostly speculation and very few data. For example, infrapopulations (all the parasites in a single host) should behave as demes if parasite offspring are transmitted as a clump from host to host over several generations. However, if offspring are well mixed, then the parasite component population (all the parasites among a host population) would function as the deme. Similarly, low mean intensities or a high proportion of worms in single infections should increase the selfing rate. For species having an asexual amplification stage, transmission between intermediate and definitive (final) hosts will control the variance in clonal reproductive success, which in turn could have a large influence on effective sizes and rates of inbreeding. We examined demic structure, selfing rates, and the variance in clonal reproductive success in natural populations of Plagioporus shawi, a hermaphroditic trematode that parasitizes salmon. Overall levels of genetic diversity were very high. An a posteriori inference of population structure overwhelmingly supports the component population as the deme, rather than individual infrapopulations. Only a single pair of 597 adult individuals was identified as clones. Thus, the variance in clonal reproductive success was almost zero. Despite being hermaphroditic, P. shawi appears to be almost entirely outcrossing. Genetic estimates of selfing (< 5%) were in accordance with the proportion of parasites from single infections. Thus, it appears that individual flukes outcross whenever possible and only resort to selfing when alone. Finally, our data support the hypothesis that aquatic transmission and the use of several intermediate hosts promotes high genetic diversity and well-mixed infrapopulations. | 1451 | no | gd of parasite | NA | no | no |
| 1452 | 2017 | 43015 | Kyle, CJ; Rico, Y; Castillo, S; Srithayakumar, V; Cullingham, CI; White, BN; Pond, BA | 2014 | Spatial patterns of neutral and functional genetic variations reveal patterns of local adaptation in raccoon (Procyon lotor) populations exposed to raccoon rabies | Local adaptation is necessary for population survival and depends on the interplay between responses to selective forces and demographic processes that introduce or retain adaptive and maladaptive attributes. Host-parasite systems are dynamic, varying in space and time, where both host and parasites must adapt to their ever-changing environment in order to survive. We investigated patterns of local adaptation in raccoon populations with varying temporal exposure to the raccoon rabies virus (RRV). RRV infects approximately 85% of the population when epizootic and has been presumed to be completely lethal once contracted; however, disease challenge experiments and varying spatial patterns of RRV spread suggest some level of immunity may exist. We first assessed patterns of local adaptation in raccoon populations along the eastern seaboard of North America by contrasting spatial patterns of neutral (microsatellite loci) and functional, major histocompatibility complex (MHC) genetic diversity and structure. We explored variation of MHC allele frequencies in the light of temporal population exposure to RRV (0-60years) and specific RRV strains in infected raccoons. Our results revealed high levels of MHC variation (66 DRB exon 2 alleles) and pronounced genetic structure relative to neutral microsatellite loci, indicative of local adaptation. We found a positive association linking MHC genetic diversity and temporal RRV exposure, but no association with susceptibility and resistance to RRV strains. These results have implications for landscape epidemiology studies seeking to predict the spread of RRV and present an example of how population demographics influence the degree to which populations adapt to local selective pressures. | 1452 | no | no good fitness measure | NA | no | no |
| 1453 | 2017 | 43015 | Hitchman, RB; Hodgson, DJ; King, LA; Hails, RS; Cory, JS; Possee, RD | 2007 | Host mediated selection of pathogen genotypes as a mechanism for the maintenance of baculovirus diversity in the field | The genetic diversity of many DNA Virus Populations in nature is unknown, but for those that have been studied it has been found to be relatively high. This is particularly true for baculoviruses, a family of large double-stranded DNA viruses that infect the larval stages of insects. Why there should be Such heterogeneity within these Virus Populations is puzzling and what Sustains it is still unknown. It has long been recognized that some baculoviruses have a relatively wide host range, but the effect of different host species on the genotypic structure of a baculovirus population has received little attention. We provide evidence that infection of different insect species can influence the genetic diversity of a Panolis flammea nucleopolyhedrovirus (PaflNPV) Population, isolated from the pine beauty moth. Variable regions of the PaflNPV genome were sequenced and novel ORF’s were identified on each of the enlarged fragments. The roles of these orfs and the implications of their presence or absence within different genotypes are discussed. The variable fragments were also labelled with P-32 and used as polymorphic genetic markers of genotype abundance. The proportion of polymorphic loci changed after passage in different insect species and this varied among species, suggesting a role for host selection of pathogen genotypes in the field as a mechanism for maintaining genetic diversity. These results have wide-ranging implications for understanding the ecology of insect-virus interactions in the natural environment and the evolution of baculovirus life history strategies. (c) 2006 Elsevier Inc. All rights reserved. | 1453 | no | gd of pathogen | NA | no | no |
| 1454 | 2017 | 43015 | Yan, G; Christensen, BM; Severson, DW | 1997 | Comparisons of genetic variability and genome structure among mosquito strains selected for refractoriness to a malaria parasite | Restriction fragment length polymorphism (RFLP) markers were used to evaluate Aedes aegypti genome structure and genetic variability within and between substrains selected for different levers of refractoriness to the malaria parasite, Plasmodium gallinaceum, The MOYO-R substrain was previously selected for complete refractoriness and the MOYO-IS substrain for intermediate susceptibility from the Moyo-In-Dry (MOYO) strain by selective inbreeding (F = 0.5), Eighteen mapped RFLP markers were used to provide coverage of the mosquito genome, The two substrains showed reduced genetic diversity compared with the MOYO strain, including significant reductions in mean heterozygosity, number of alleles per locus, and proportion of polymorphic loci, Genetic differentiation between the two substrains was statistically significant, as reflected by differences in allele frequencies, Significant pairwise linkage disequilibrium among the RFLP loci was detected in all three strains, most evidently in the MOYO strain, This is surprising because the RFLP loci examined are separated by large map distances, and therefore linkage disequilibrium should decay to zero after many generations of laboratory culture, Our hypothesis to explain this phenomena is that lack of recombination, or low recombination rates in some regions of the A. aegypti genome, is a result of chromosome inversions, Finally, we used graphical genotyping, wherein whole genome genotypic information for individual mosquitoes is represented in a simple graphic format, to illustrate genome structure and allelic variation within and among the mosquito strains, Our analysis revealed an apparent chromosomal deletion on chromosome 3 for some individuals in the MOYO strain and MOYO-IS substrain. | 1454 | no | NA | NA | no | no |
| 1455 | 2017 | 43015 | Langille, BL; O’Leary, KE; Whitney, HG; Marshall, HD | 2014 | Mitochondrial DNA diversity and phylogeography of insular Newfoundland red foxes (Vulpes vulpes deletrix) | As the only native insular Newfoundland, Canada, canid from the extinction of the wolf in the 1930s to the recent arrival of coyotes, the red fox (Vulpes vulpes deletrix) poses interesting questions about genetic distinctiveness and the postglacial colonization history of the island’s depauperate mammalian fauna. Here, we characterize genetic variability at the mitochondrial control region in 189 foxes from the island of Newfoundland, Prince Edward Island, and Labrador. We identified 8 haplotypes (3 new to this study) defined by 11 polymorphic sites, with an average pairwise sequence divergence of similar to 0.003 and haplotypic diversity of 0.56 among localities. A pairwise distribution of control region sequence differences, rho estimate of divergence time, and tests of neutrality (Fu’s F-S and Tajima’s D) are weakly consistent with a population expansion similar to 9,000 years ago, correlating with retreat of glacial ice from the region. Haplotype composition reflects primarily the Eastern subclade of Aubry and colleagues and supports Aubry’s 2-refugia hypothesis that indigenous red foxes in North America are derived from disparate refugia isolated during the Wisconsinan glaciation. Haplotype identity and pattern of population differentiation suggest recolonization of the island of Newfoundland via a northern glacial refugium via Quebec or Labrador rather than an Atlantic or southern route, but provide no mitochondrial genetic evidence to support differentiation of this population of foxes along subspecies lines. We establish a baseline for continued investigations of population demography, genetic structure, and adaptive genetic diversity in island Newfoundland red foxes, a population of interest from both ecological and wildlife disease perspectives. | 1455 | no | no good fitness measure | NA | no | no |
| 1456 | 2017 | 43015 | Davis, JL; Childers, DL; Kuhn, DN | 1999 | Clonal variation in a Florida Bay Thalassia testudinum meadow: molecular genetic assessment of population structure | Thalassia testudinum (Banks ex Konig) is an important primary producer in nearshore tropical marine ecosystems. In the past several years, mass mortality of T. testudinum populations within the Florida Bay estuary has highlighted the need to know more about levels of clonal variation and the spatial arrangement of genets within populations of this species. Historically, most of the reproduction within this species was thought to be through clonal growth, and populations have been assumed to exhibit little genetic diversity. We used DNA fingerprinting techniques and spatially hierarchical sampling to search for genetically distinct plants within T. testudinum meadows in Rabbit Key Basin, Florida Bay, USA. We detected 22 genetically distinct individuals out of 74 samples. Multiple genetic individuals were detected over small spatial scales (<0.25 m). Analysis of molecular variance (AMOVA) detected significant levels of variation at both the largest and smallest spatial scales studied. This result suggested that genets of this species generally do not grow in discreet monoclonal patches and that most genets are not large enough to span the entire basin (similar to 5 km). Further, analysis of genetic distances between phenotypes suggests a pattern of isolation by distance, with individuals that were nearer in space being more genetically similar. GIS analysis of seagrass density over the period from 1985 to 1994, combined with the clonal diversity data presented here, suggest seedlings may play a significant role in colonization of bare patches. These results imply that the role of sexual reproduction in population structure within this species is greater than previously thought. | 1456 | no | no good fitness measure, N pop differnt in GD? | NA | no | no |
| 1457 | 2017 | 43015 | David-Palma, M; Libkind, D; Sampaio, JP | 2014 | Global distribution, diversity hot spots and niche transitions of an astaxanthin-producing eukaryotic microbe | Microbes establish very diverse but still poorly understood associations with other microscopic or macroscopic organisms that do not follow the more conventional modes of competition or mutualism. Phaffia rhodozyma, an orange-coloured yeast that produces the biotechnologically relevant carotenoid astaxanthin, exhibits a Holarctic association with birch trees in temperate forests that contrasts with the more recent finding of a South American population associated with Nothofagus (southern beech) and with stromata of its biotrophic fungal parasite Cyttaria spp. We investigated whether the association of Phaffia with Nothofagus-Cyttaria could be expanded to Australasia, the other region of the world where Nothofagus are endemic, studied the genetic structure of populations representing the known worldwide distribution of Phaffia and analysed the evolution of the association with tree hosts. The phylogenetic analysis revealed that Phaffia diversity in Australasia is much higher than in other regions of the globe and that two endemic and markedly divergent lineages seem to represent new species. The observed genetic diversity correlates with host tree genera rather than with geography, which suggests that adaptation to the different niches is driving population structure in this yeast. The high genetic diversity and endemism in Australasia indicate that the genus evolved in this region and that the association with Nothofagus is the ancestral tree association. Estimates of the divergence times of Phaffia lineages point to splits that are much more recent than the break-up of Gondwana, supporting that long-distance dispersal rather than vicariance is responsible for observed distribution of P.rhodozyma. | 1457 | no | no good fitness measure, N pop differnt in GD? | NA | no | no |
| 1458 | 2017 | 43015 | Abadio, AKR; Lima, SS; Santana, MF; Salomao, TMF; Sartorato, A; Mizubuti, ESG; Araujo, EF; de Queiroz, MV | 2012 | Genetic diversity analysis of isolates of the fungal bean pathogen Pseudocercospora griseola from central and southern Brazil | Planting resistant varieties is the most effective control measure against the angular leaf spot of dry beans, a fungal disease caused by Pseudocercospora griseola. However, dry bean varieties with durable resistance are not easily obtained. Knowledge about the genetic variability of the pathogen population is key for the success of dry bean breeding programs aimed at developing resistant materials, but finding suitable operationally simple and genetically accurate markers is not an easy task. The aim of this study was to assess the suitability of the ISSR-PCR technique to quantify the genetic variability of P. griseola isolates. Total DNA of 27 P. griseola isolates from Goias, Minas Gerais, Espirito Santo, and Parana States was extracted and amplified using specific primers for ISSR. Using cluster analysis, 27 genotypes were identified. The ISSR-PCR technique was suitable for assessing intraspecific variability of P. griseola. The ISSR-PCR marker was found to be highly sensitive to genetic variation and can aid in elucidating the genetic structure of the population of this plant pathogen as a support tool for the dry bean breeding programs. | 1458 | no | gd of pathogen | NA | no | no |
| 1459 | 2017 | 43015 | Hu, YB; Qi, DW; Wang, HJ; Wei, FW | 2010 | Genetic evidence of recent population contraction in the southernmost population of giant pandas | Anthropogenic habitat loss and fragmentation have been implicated in the endangerment and extinction of many species. Here we assess genetic variation and demographic history in the southernmost population of giant pandas (Ailuropoda melanoleuca) that continues to be threatened by habitat degradation and fragmentation, using noninvasive genetic sampling, mitochondrial control region sequence and 12 microsatellite loci. Compared to other giant panda populations, this population has medium-level genetic diversity based on the measure of both mitochondrial and nuclear markers. Mitochondrial DNA-based demographic analyses revealed that no historical population expansion or contraction has occurred, indicating a relatively stable population size. However, a Bayesian-coalescent method based on the observed allele distribution and allele frequencies of microsatellite clearly did detect, quantify and date a recent decrease in population size. Overall, the results indicate that a population contraction in the order of 95-96% has taken place over the last 910-999 years and is most likely due to anthropogenic habitat loss. These findings highlight the need for a greater focus on habitat protection and restoration for the long-term survival of this giant panda population. | 1459 | no | no good fitness measure | NA | no | no |
| 1460 | 2017 | 43015 | Jadwiszczak, KA; Banaszek, A; Jablonska, E; Sozinov, OV | 2011 | Could Betula humilis have survived the last glaciation at a current margin of its distribution? Testing the hypothesis of a glacial refugium using nuclear microsatellites | The probability of population extinction seems to differ within the species range. Populations occupying former glacial refugia could harbor substantial genetic resources, hence they should be less prone to extirpation. It was hypothesized that the shrub birch Betula humilis could have survived the Last Glacial Maximum (LGM) at the current southwestern margin of its range. Using ten nuclear microsatellites, we studied genetic variation within and between 18 localities of B. humilis situated in marginal and subcentral areas. Six marginal populations were located in areas covered by an ice sheet during the LGM, and the remaining samples came from unglaciated areas. Analysis of private allele frequencies as well as hierarchical AMOVAs conducted for geographical regions, marginal versus central populations, and glaciated versus unglaciated areas did not confirm the hypothesis of glacial isolate of the shrub birch in southeastern Poland. On the other hand, very high genetic variation in some localities in northeastern Poland was found. Survival in periglacial areas followed by broad-fronted colonization or the existence of an admixture zone of phylogeographic lineages was proposed to explain this phenomenon. | 1460 | no | no good fitness measure | NA | no | no |
| 1461 | 2017 | 43015 | Li, QM; Xu, ZF; He, TH | 2002 | Ex situ genetic conservation of endangered Vatica guangxiensis (Dipterocarpaceae) in China | RAPD polymorphisms were applied to check the efficiency of ex situ genetic conservation of endangered Vatica guangxiensis X. L. Mo. endemic to southwestern China. Low level of genetic variation was revealed in three remaining natural populations. Twenty random primers, each with 10 base pairs, generated 231 bands with 53.68% being polymorphic, and with an average of 32.46% being polymorphic in each natural population. Strong Population differentiation was revealed by AMOVA (analysis of molecular variance) and Gst value was 0.3764. The population ML ex situ conserved in the Xishuangbanna Tropical Botanical Garden contained an intermediate genetic variation compared with natural Populations. with 30.74% bands being polymorphic. Of the total 231 bands generated in V. guangxiensis, 204 bands were also detected in population M L, indicating that 88.31% of the total genetic variations of this species were conserved in ex situ population. If only the alleles with moderate to high frequency (P>0.05) were considered, 204 out of 209 bands (97.61%) occurred in ex situ population M L. RAPD analysis also detected one exclusive band in natural population NS, and five in natural population NP, three of these exclusive bands were generated in every samples of natural population (NP), and other three had moderate to high frequencies. While none of these exclusive bands were detected in ex situ conserved population ML. Our conclusions Lire that the ex situ conserved population ML contains representative genetic variation to maintain long-term survival and evolutionary process of V. guangxiensis, and that more extensive ex situ sampling in natural population NS and NP is needed to conserve more exclusive alleles in ex situ population. The tropical area in the Botanical Garden would play a more important role in the ex situ conservation of rare and endangered plants. (C) 2002 Elsevier Science Ltd. All rights reserved. | 1461 | no | no good fitness measure, N pop differnt in GD? | NA | no | no |
| 1462 | 2017 | 43015 | Mantooth, K; Hadziabdic, D; Boggess, S; Windham, M; Miller, S; Cai, GH; Spatafora, J; Zhang, N; Staton, M; Ownley, B; Trigiano, R | 2017 | Confirmation of independent introductions of an exotic plant pathogen of Cornus species, Discula destructiva, on the east and west coasts of North America | Cornus florida (flowering dogwood) and C. nuttallii (Pacific dogwood) are North American native tree species that belong to the big-bracted group of dogwoods. Cornus species are highly valued for their ornamental characteristics, and have fruits that contain high fat content for animals. Also, they are an important understory tree in natural forests. Dogwood anthracnose, caused by Discula destructiva, was observed in the late 1970s on the east and west coasts of the United States and by 1991 had quickly spread throughout most of the native ranges of C. florida and C. nuttalli. We investigated the genetic diversity and population structure of 93 D. destructiva isolates using 47 microsatellite loci developed from the sequenced genome of the type strain of D. destructiva. Clone-corrected data indicated low genetic diversity and the presence of four genetic clusters that corresponded to two major geographic areas, the eastern United States and the Pacific Northwest, and to the two collection time periods when the isolates were collected (pre- and post-1993). Linkage disequilibrium was present in five out of six subpopulations, suggesting that the fungus only reproduced asexually. Evidence of population bottlenecks was indicated across four identified genetic clusters, and was probably the result of the limited number of founding individuals on both coasts. These results support the hypothesis that D. destructiva is an exotic pathogen with independent introductions on the east and west coasts of North America. We also tested the cross-amplification of these microsatellite primers to other Discula species. Genomic DNA from 17 isolates of four other Discula species and two isolates of Juglanconis species (formerly Melanconis species) were amplified by 17 of 47 primer pairs. These primers may be useful for investigating the genetic diversity and population structure of these Discula species. | 1462 | no | gd of pathogen | NA | no | no |
| 1463 | 2017 | 43015 | Satow, S; Satoh, T; Hirota, T | 2013 | Colony fusion in a parthenogenetic ant, Pristomyrmex punctatus | In the ant Pristomyrmex punctatus Smith (Hymenoptera: Formicidae), all young workers lay a small number of eggs parthenogenetically. Some colonies consist of monoclonal individuals that provide high inclusive fitness, according to the kin selection theory. However, in some populations, a majority of the colonies contain multiple lineages. Intracolonial genetic variation of parthenogenetic ants cannot be explained by the multiple mating of single founderesses or by the foundation of a colony by multiple foundresses, which are the usual causes of genetically diverse colonies in social insects. Here, we hypothesized that the fusion of established colonies might facilitate the formation of multiclonal colonies. Colony fusion decreases indirect benefits because of the reduction in intracolonial relatedness. However, when suitable nesting places for overwintering are scarce, colony fusion provides a strategy for the survival of colonies. Here, ants derived from different colonies were allowed to encounter one another in a container with just one nesting place. Initially, high aggression was observed; however, after several days, no aggression was observed and the ants shared the nest. When the fused colonies were allowed to transfer to two alternative nests, ants from different colonies occupied the same nest. This study highlights the importance of limiting the number of nesting places in order to understand the genetic diversity of parthenogenetic ant colonies. | 1463 | no | no good fitness measure | NA | no | no |
| 1464 | 2017 | 43015 | Baums, IB; Miller, MW; Hellberg, ME | 2006 | Geographic variation in clonal structure in a reef-building Caribbean coral, Acropora palmata | Species that build the physical structure of ecosystems often reproduce clonally, both in terrestrial (e.g., grasses, trees) and marine (e.g., corals, seagrasses) environments. The degree of clonality may vary over a species’ range in accordance with the relative success of sexual and asexual recruitment. High genotypic (clonal) diversity of structural species may promote the species diversity and resilience of ecosystems in the face of environmental extremes. Conversely, low genotypic diversity may indicate an asexual strategy to maintain resources and genetic variation during population decline. Here, we use microsatellite markers to assess geographic variation in clonality in the coral Acropora palmata sampled from 26 reefs in eight regions spanning its tropical western Atlantic range (n=751). Caribbean-wide, the ratio (+/- SD) of genets (N-g) to sampled ramets (N) was 0.51 +/- 0.28. Within reefs (30-70 m) and among reefs (10-100 km) within regions, clonal structure varied from being predominantly asexual (N-g/N approaching 0) to purely sexual (N-g/N=1). However, two genetically isolated regions (western and eastern Caribbean) differed in clonal structure: genotypically depauperate populations (N-g/N=0.43 +/- 0.31) with lower densities (0.13 +/- 0.08 colonies/m(2)) characterized the western region, while denser (0.30 +/- 0.21 colonies/m(2)), genotypically rich stands (N-g/N=0.64 +/- 0.17) typified the eastern Caribbean. Genotypic richness (standardized to sample size; N-g/N) and genotypic diversity (G(o)/G(e)) were negatively related to colony density within each province (r(2)=0.49-0.66, P < 0.001), indicating that dense stands have higher rates of asexual recruitment than less dense populations. Asexual recruitment was not correlated with large-scale disturbance history or abundance of large colonies (potential fragment sources) but was negatively correlated with shelf area (r(2)=0.57, P < 0.01). We argue that sexual recruitment is more prevalent in the eastern range of A. palmata than the west, and that these geographic differences in the contribution of reproductive modes to population structure may be related to habitat characteristics. The two populations of the threatened A. palmata differ fundamentally in reproductive character and may respond differently to environmental change. | 1464 | no | no good fitness measure | NA | no | no |
| 1465 | 2017 | 43015 | Polimanti, R; Piacentini, S; Iorio, A; De Angelis, F; Kozlov, A; Novelletto, A; Fuciarelli, M | 2015 | Haplotype differences for copy number variants in the 22q11.23 region among human populations: a pigmentation-based model for selective pressure | Two gene clusters are tightly linked in a narrow region of chromosome 22q11.23: the macrophage migration inhibitory factor (MIF) gene family and the glutathione S-transferase theta class. Within 120 kb in this region, two 30-kb deletions reach high frequencies in human populations. This gives rise to four haplotypic arrangements, which modulate the number of genes in both families. The variable patterns of linkage disequilibrium (LD) between these copy number variants (CNVs) in diverse human populations remain poorly understood. We analyzed 2469 individuals belonging to 27 human populations with different ethnic origins. Then we correlated the genetic variability of 22q11.23 CNVs with environmental variables. We confirmed an increasing strength of LD from Africa to Asia and to Europe. Further, we highlighted strongly significant correlations between the frequency of one of the haplotypes and pigmentation-related variables: skin color (R-2 = 0.675, P<0.001), distance from the equator (R-2 = 0.454, P<0.001), UVA radiation (R-2 = 0.439, P<0.001), and UVB radiation (R-2 = 0.313, P = 0.002). The fact that all MIF-related genes are retained on this haplotype and the evidences gleaned from experimental systems seem to agree with the role of MIF-related genes in melanogenesis. As such, we propose a model that explains the geographic and ethnic distribution of 22q11.23 CNVs among human populations, assuming that MIF-related gene dosage could be associated with adaptation to low UV radiation. | 1465 | no | human | NA | no | no |
| 1466 | 2017 | 43015 | Jolley, KA; Wilson, DJ; Kriz, P; Mcvean, G; Maiden, MCJ | 2005 | The influence of mutation, recombination, population history, and selection on patterns of genetic diversity in Neisseria meningitidis | “Patterns of genetic diversity within populations of human pathogens, shaped by the ecology of host-microbe interactions, contain important information about the epidemiological history of infectious disease. Exploiting this information, however, requires a systematic approach that distinguishes the genetic signal generated by epidemiological processes from the effects of other forces, such as recombination, mutation, and population history. Here, a variety of quantitative techniques were employed to investigate multilocus sequence information from isolate collections of Neisseria meningitidis, a major cause of meningitis and septicemia world wide. This allowed quantitative evaluation of alternative explanations for the observed population structure. A coalescent-based approach was employed to estimate the rate of mutation, the rate of recombination, and the size distribution of recombination fragments from samples from disease-associated and carried meningococci obtained in the Czech Republic in 1993 and a global collection of disease-associated isolates collected globally from 1937 to 1996. The parameter estimates were used to reject a model in which genetic structure arose by chance in small populations, and analysis of molecular variation showed that geographically restricted gene flow was unlikely to be the cause of the genetic structure. The genetic differentiation between disease and carriage isolate collections indicated that, whereas certain genotypes were overrepresented among the disease-isolate collections (the”“hyperinvasive”" lineages), disease-associated and carried meningococci exhibited remarkably little differentiation at the level of individual nucleotide polymorphisms. In combination, these results indicated the repeated action of natural selection on meningococcal populations, possibly arising from the coevolutionary dynamic of host-pathogen interactions." | 1466 | no | NA | NA | no | no |
| 1467 | 2017 | 43015 | Lorenzon, P; Clobert, J; Massot, M | 2001 | The contribution of phenotypic plasticity to adaptation in Lacerta vivipara | Correlation between intraspecific phenotypic variability and variation of environmental conditions could reflect adaptation. Different phenotypes may result from differential expression of a genotype in different environments (phenotypic plasticity) or from expression of different genotypes (genetic diversity). Populations of Lacerta vivipara exhibit larger adult body length, lower age at maturity, higher fecundity, and smaller neonatal size in humid habitats compared to dry habitats. We conducted reciprocal transplants of juvenile L. vivipara to test for the genetic or plastic origin of this variation. We captured gravid females from four populations that differed in the relative humidity of their habitats, and during the last 2 to 4 weeks of gestation, we manipulated heat and water availability under laboratory conditions. Juveniles were released into the different populations and families were divided to compare growth rate and survival of half-sibs in two environments. Growth rate and survival were assessed using capture-recapture techniques. Growth rate was plastic in response to postnatal. conditions and did not differ between populations of origin. Survival differed between populations of origin, partially because of differences in neonatal body length. The response of juvenile body length and body condition to selection in the different habitats was affected by the population of origin. This result cannot be simply interpreted in terms of adaptation; however, phenotypic plasticity of fecundity or juvenile size most probably resulted in adaptive reproductive strategies. Adaptation to the habitat by means of genetic specialization was not detected. Further investigation is needed to discriminate between genetic and long-term maternal effects. | 1467 | no | NA | NA | no | no |
| 1468 | 2017 | 43015 | Barbosa, CEA; Misiewicz, TM; Fine, PVA; Costa, FRC | 2013 | Plant Ontogeny, Spatial Distance, and Soil Type Influence Patterns of Relatedness in a Common Amazonian Tree | The formation of spatial genetic structure (SGS) may originate from different patterns of seed deposition in the landscape, and is mostly determined by seed dispersal limitation. After dispersal, mechanisms such as filtering by environmental factors or attack by herbivores/pathogens throughout plant development stages, and potentially either disrupt or intensify SGS patterns. We investigated how the genotype of Protium subserratum (Burseraceae), a common tree species in the Ducke Reserve, Brazil, is distributed across the landscape. We used seven microsatellite markers to assess the SGS among plants at different life stages and in different environments. By quantifying the patterns of relatedness among plants of different sizes, we inferred the ontogenetic stage in which SGS changes occurred, and compared these effects across soil types. Relatedness among seedlings decreased when distance between seedlings increased, especially for the youngest seedlings. However, this trend was not continued by older plants, as relatedness values were higher among neighboring individuals of the juvenile and adult size class. Contrasting relatedness patterns between seedlings and larger individuals suggests a trade-off between the negative effects of being near closely-related adults (e. g. due to herbivore and pathogen attack) and the advantage of being in a site favorable to establishment. We also found that soil texture strongly influenced density-dependence patterns, as young seedlings in clay soils were more related to each other than were seedlings in bottomland sandy soils, suggesting that the mechanisms that create and maintain patterns of SGS within a population may interact with environmental heterogeneity. | 1468 | no | no good fitness measure | NA | no | no |
| 1469 | 2017 | 43015 | Botigue, LR; Henn, BM; Gravel, S; Maples, BK; Gignoux, CR; Corona, E; Atzmon, G; Burns, E; Ostrer, H; Flores, C; Bertranpetit, J; Comas, D; Bustamante, CD | 2013 | Gene flow from North Africa contributes to differential human genetic diversity in southern Europe | Human genetic diversity in southern Europe is higher than in other regions of the continent. This difference has been attributed to postglacial expansions, the demic diffusion of agriculture from the Near East, and gene flow from Africa. Using SNP data from 2,099 individuals in 43 populations, we show that estimates of recent shared ancestry between Europe and Africa are substantially increased when gene flow from North Africans, rather than Sub-Saharan Africans, is considered. The gradient of North African ancestry accounts for previous observations of low levels of sharing with Sub-Saharan Africa and is independent of recent gene flow from the Near East. The source of genetic diversity in southern Europe has important biomedical implications; we find that most disease risk alleles from genome-wide association studies follow expected patterns of divergence between Europe and North Africa, with the principal exception of multiple sclerosis. | 1469 | no | human | NA | no | no |
| 1470 | 2017 | 43015 | Drees, C; De Vries, H; Hardtle, W; Matern, A; Persigehl, M; Assmann, T | 2011 | Genetic erosion in a stenotopic heathland ground beetle (Coleoptera: Carabidae): a matter of habitat size? | Two centuries ago large areas of north-west Europe were covered by coherent heathlands which hosted numerous specialized species. Changes in land use made heathlands fragmented and rare, consequently, they are in the focus of nature conservation efforts today. But how large should remaining heathland patches be in order to secure the survival of populations of specialized species? We investigated the genetic diversity at five allozyme loci of Poecilus lepidus, a flightless and stenotopic heathland ground beetle. 29 populations from differently sized heathland patches in north-west Germany were analyzed. Results show a weak but significant genetic differentiation and no evidence for isolation by distance or other patterns of spatial autocorrelation. Linear regression analysis revealed significant relationships between patch size, allelic richness, number of alleles and expected heterozygosity. These findings are explained by severe habitat fragmentation together with strong fluctuations in population size which have been reported for this species in the past. To conserve the vast majority of the species’ genetic diversity for a period of 100 years we suggest to maintain heathland patches of at least 50 ha in size. | 1470 | no | no good fitness measure | NA | no | no |
| 1471 | 2017 | 43015 | Perez, G; Slippers, B; Wingfield, BD; Hunter, GC; Wingfield, MJ | 2010 | Micro- and macrospatial scale analyses illustrates mixed mating strategies and extensive geneflow in populations of an invasive haploid pathogen | Sexual reproduction in fungi involves either a single individual (selfing) or two individuals (outcrossing). To investigate the roles that these two strategies play in the establishment of an invasive alien pathogen, the Eucalyptus leaf-infecting fungus, Teratosphaeria (Mycosphaerella) nubilosa was studied. Specifically, the genetic diversity of the pathogen was investigated at micro and macrospatial scales. Interestingly, while data obtained at microspatial scales show clearly that selfing is the main reproductive strategy, at macrospatial scales the population genetic structure was consistent with a genetically outcrossing organism. Additional analyses were performed to explore these apparently discordant results at different spatial scales and to quantify the contribution of selfing vs. outcrossing to the genotypic diversity. The results clearly show that the fungus has a mixed mating strategy. While selfing is the predominant form of mating, outcrosses must have occurred in the pathogen that increased the genotypic diversity of the fungus over time. This mating strategy, coupled with the high levels of geneflow between distant populations of the pathogen, has created an even distribution of maximum diversity from the smallest (leaf) to largest scales (>500 km), which will make breeding for resistance difficult. These data illustrate the evolutionary potential and danger of the introduction of multiple genotypes of a potentially outcrossing pathogen, especially when it has a high dispersal potential. | 1471 | no | gd of pathogen | NA | no | no |
| 1472 | 2017 | 43015 | Ortius-Lechner, D; Maile, R; Morgan, ED; Petersen, HC; Boomsma, JJ | 2003 | Lack of patriline-specific differences in chemical composition of the metapleural gland secretion in Acromyrmex octospinosus | Multiple queen-mating (polyandry) in social insects increases the genetic variability among worker offspring, which may enhance colony survival, social productivity and defence against parasites. The unique and complex symbiosis of leaf-cutting ants with a clonal mutualistic fungus makes this social system particularly vulnerable to contamination by pathogenic and unwanted saprophytic fungi and bacteria. Proper defence against such threats requires effective and flexible chemical defence mechanisms. A prime candidate for providing such defences is the meta-pleural gland secretion, which is known to have broad antibiotic properties. Here we use the leaf-cutting ant Acromyrmex octospinosus to specifically test the hypothesis that genetically more diverse worker-offspring produce a more variable spectrum of metapleural gland compounds. We used DNA microsatellite markers to assign workers from two colonies to the six most common patrilines in each colony, and have analysed the degree to which the observed variance in the quantitative chemical composition of the metapleural gland secretion can be explained by genetic differences among patrilines. We found a marginally significant patriline-effect on the overall variability of metapleural gland compounds in one colony, but could not detect such effect in the other colony. We discuss a number of possible reasons why the genetic variance component for quantitative variation in metapleural gland secretion may be low. | 1472 | no | no good fitness measure | NA | no | no |
| 1473 | 2017 | 43015 | Chen, JM; Zhao, SY; Liao, YY; Gichira, AW; Gituru, RW; Wang, QF | 2015 | Chloroplast DNA phylogeographic analysis reveals significant spatial genetic structure of the relictual tree Davidia involucrata (Davidiaceae) | “The dove tree, Davidia involucrata Baill. (Davidiaceae), is a relict species endemic to China. Human activities and the strict requirements for seedling recruitments have led to the decline in number of individuals in extant natural populations of this species. In order to provide information for the design of conservation management strategies for D. involucrata, we investigated the phylogeographical pattern of this relict tree. Our sampling included 256 individuals from 32 natural populations of D. involucrata in China and we sequenced six chloroplast DNA (cpDNA) non-coding regions. We distinguished a total of 13 different cpDNA haplotypes. From the cpDNA variation in D. involucrata, we found a very high level of regional differentiation (F (ST) = 0.812) and a strong phylogeographical pattern (N (ST) = 0.996 > G (ST) = 0.981, P < 0.05). Phylogenetic analysis reveals three main cpDNA haplotype lineages and four population groups. The split between these geographical groups can be dated back from the late Pliocene to early Pleistocene. Non-overlapping distribution of chloroplast haplotypes and high genetic differentiation among four distinct geographical groups suggest that D. involucrata probably survives in four separate glacial refugia. Our findings have an important implication for conservation of its genetic diversity. The deduced glacial survival areas for D. involucrata should be recognized as four”“evolutionary significant units”" and be considered as separate targets in conserving its genetic diversity." | 1473 | no | no good fitness measure | NA | no | no |
| 1474 | 2017 | 43015 | Quinteiro, J; Manent, P; Perez-Dieguez, L; Gonzalez, JA; Almeida, C; Lopes, E; Araujo, R; Carreira, GP; Rey-Mendez, M; Gonzalez-Henriquez, N | 2015 | Phylogeography of a Marine Insular Endemic in the Atlantic Macaronesia: The Azorean Barnacle, Megabalanus azoricus (Pilsbry, 1916) | The Azorean barnacle, Megabalanus azoricus (Pilsbry, 1916), is a Macaronesian endemic whose obscure taxonomy and the unknown relationships among forms inhabiting isolated Northern Atlantic oceanic islands is investigated by means of molecular analysis herein. Mitochondrial data from the 16S rRNA and COX1 genes support its current species status, tropical ancestry, and the taxonomic homogeneity throughout its distribution range. In contrast, at the intraspecific level and based on control region sequences, we detected an overall low level of genetic diversity and three divergent lineages. The haplogroups a and. were sampled in the Azores, Madeira, Canary, and Cabo Verde archipelagos; whereas haplogroup beta was absent from Cabo Verde. Consequently, population analysis suggested a differentiation of the Cabo Verde population with respect to the genetically homogenous northern archipelagos generated by current oceanographic barriers. Furthermore, haplogroup a, beta, and. demographic expansions occurred during the interglacial periods MIS5 (130 Kya - thousands years ago -), MIS3 (60 Kya), and MIS7 (240 Kya), respectively. The evolutionary origin of these lineages is related to its survival in the stable southern refugia and its demographic expansion dynamics are associated with the glacial-interglacial cycles. This phylogeographic pattern suggests the occurrence of genetic discontinuity informative to the delimitation of an informally defined biogeographic entity, Macaronesia, and its generation by processes that delineate genetic diversity of marine taxa in this area. | 1474 | no | no good fitness measure | NA | no | no |
| 1475 | 2017 | 43015 | Poysa, H; Paasivaara, A; Lindblom, K; Rutila, J; Sorjonen, J | 2014 | Co-parasites preferentially lay with kin and in safe neighbourhoods: experimental evidence from goldeneye ducks | Conspecific brood parasitism (CBP) is an alternative reproductive tactic in several animal taxa. Recently the role of kinship in CBP has been in focus, and some studies have demonstrated high host-parasite relatedness in avian CBP systems. However, high natal and breeding site fidelity of hosts and parasites complicates the interpretation of previous observational findings, and the mechanisms leading to high host-parasite relatedness remain unknown. Some recent findings suggest that broadening the scope of the study of interactions may cast light on these mechanisms. We studied relatedness between parasites laying in the same nest (co-parasites) and factors possibly driving relatedness patterns between co-parasites in the common goldeneye, Bucephala clangula. Based on a field experiment, controlling for site fidelity and host role, we report here that both relatedness and safety of the nest site, and their interaction, affected the likelihood of two females engaging in co-parasitism. At the population level, parasites indeed seemed to lay eggs preferentially with kin. Analyses at a finer spatial scale revealed that high relatedness between co-parasites was not due to the philopatry effect only. Parasites engaging in co-parasitism also laid in safer neighbourhoods than parasites that did not engage in co-parasitism; the number of nondepredated nesting attempts the previous year was higher for the parasites engaging in co-parasitism. However, the interaction between relatedness and safety of the nest site suggests that co-parasitism at dangerous lakes was more likely to involve relatives. Our results provide the first experimental evidence that nest predation risk and interaction between related parasites are associated with kin-biased co-parasitism in a CBP system. (C) 2014 The Association for the Study of Animal Behaviour. Published by Elsevier Ltd. All rights reserved. | 1475 | no | brood parasitism | NA | no | no |
| 1476 | 2017 | 43015 | Sheng, HY; See, DR; Murray, TD | 2012 | Mapping QTL for resistance to eyespot of wheat in Aegilops longissima | Eyespot is an economically important disease of wheat caused by the soilborne fungi Oculimacula yallundae and O. acuformis. These pathogens infect and colonize the stem base, which results in lodging of diseased plants and reduced grain yield. Disease resistant cultivars are the most desirable control method, but resistance genes are limited in the wheat gene pool. Some accessions of the wheat wild relative Aegilops longissima are resistant to eyespot, but nothing is known about the genetic control of resistance. A recombinant inbred line population was developed from the cross PI 542196 (R) x PI 330486 (S) to map the resistance genes and better understand resistance in Ae. longissima. A genetic linkage map of the S-l genome was constructed with 169 wheat microsatellite markers covering 1261.3 cM in 7 groups. F-5 lines (189) were tested for reaction to O. yallundae and four QTL were detected in chromosomes 1S(l), 3S(l), 5S(l), and 7S(l). These QTL explained 44 % of the total phenotypic variation in reaction to eyespot based on GUS scores and 63 % for visual disease ratings. These results demonstrate that genetic control of O. yallundae resistance in Ae. longissima is polygenic. This is the first report of multiple QTL conferring resistance to eyespot in Ae. longissima. Markers cfd6, wmc597, wmc415, and cfd2 are tightly linked to Q.Pch.wsu-1S (l) , Q.Pch.wsu-3S (l) , Q.Pch.wsu-5S (l) , and Q.Pch.wsu-7S (l) , respectively. These markers may be useful in marker-assisted selection for transferring resistance genes to wheat to increase the effectiveness of resistance and broaden the genetic diversity of eyespot resistance. | 1476 | no | NA | NA | no | no |
| 1477 | 2017 | 43015 | Bourgeois, AL; Rinderer, TE; Sylvester, HA; Holloway, B; Oldroyd, BP | 2012 | Patterns of Apis mellifera infestation by Nosema ceranae support the parasite hypothesis for the evolution of extreme polyandry in eusocial insects | We investigated the relationship between infestation levels of Nosema ceranae and patriline membership by sampling individual worker bees from five colonies from both Russian and Italian lineages. Individual workers were tested for N. ceranae infestation level using qPCR, and then genotyped to determine their patriline membership. Levels of N. ceranae infestation differed significantly between lineages and colonies for both Russian and Italian workers. Patriline-based variance was evident only among the Russian workers. There was substantial variation in N. ceranae levels among Italian workers, ranging from 0 to 2 x 10(9) Nosema/bee, but this variation was unrelated to patriline membership. The results for Russian honey bees are congruent with predictions derived from the parasite hypothesis for the evolution of polyandry-patrilinial variance in parasite tolerance contributes to colony level resistance by reducing the probability of catastrophic failure that might occur if a colony was genetically homogeneous. | 1477 | maybe | gd difference? | no gd of host pop and fitness of host pop | no | no |
| 1478 | 2017 | 43015 | Morehouse, EA; James, TY; Ganley, ARD; Vilgalys, R; Berger, L; Murphy, PJ; Longcore, JE | 2003 | Multilocus sequence typing suggests the chytrid pathogen of amphibians is a recently emerged clone | Chytridiomycosis is a recently identified fungal disease associated with global population declines of frogs. Although the fungus, Batrachochytrium dendrobatidis , is considered an emerging pathogen, little is known about its population genetics, including the origin of the current epidemic and how this relates to the dispersal ability of the fungus. In this study, we use multilocus sequence typing to examine genetic diversity and relationships among 35 fungal strains from North America, Africa and Australia. Only five variable nucleotide positions were detected among 10 loci (5918 bp). This low level of genetic variation is consistent with the description of B. dendrobatidis as a recently emerged disease agent. Fixed (i.e. 100%) or nearly fixed frequencies of heterozygous genotypes at two loci suggested that B. dendrobatidis is diploid and primarily reproduces clonally. In contrast to the lack of nucleotide polymorphism, electrophoretic karyotyping of multiple strains demonstrated a number of chromosome length polymorphisms. | 1478 | no | gd of pathogen | NA | no | no |
| 1479 | 2017 | 43015 | Long, CR; Tessanne, KJ; Golding, MC | 2010 | Applications of RNA interference-based gene silencing in animal agriculture | Classical genetic selection, recently aided by genomic selection tools, has been successful in achieving remarkable progress in livestock improvement. However, genetic selection has led to decreased genetic diversity and, in some cases, acquisition of undesirable traits. In order to meet the increased demands of our expanding population, new technologies and practices must be developed that contend with zoonotic and animal disease, environmental impacts of large farming operations and the increased food and fibre production needed to feed and clothe our society. Future increases in productivity may be dependent upon the acquisition of genetic traits not currently encoded by the genomes of animals used in standard agricultural practice, thus making classical genetic selection impossible. Genetic engineering of livestock is commonly used to produce pharmaceuticals or to impart enhanced production characteristics to animals, but has also demonstrated its usefulness in producing animals with disease resistance. However, significant challenges remain because it has been more difficult to produce animals in which specific genes have been removed. It is now possible to modify livestock genomes to block expression of endogenous and exogenous genes (such as those expressed following virus infection). In the present review, we discuss mechanisms of silencing gene expression via the biology of RNA interference (RNAi), the technology of activating the RNAi pathway and the application of this technology to enhance livestock production through increased production efficiency and prevention of disease. An increased demand for sustainable food production is at the forefront of scientific challenges and RNAi technology will undoubtedly play a key role. | 1479 | no | NA | NA | no | no |
| 1480 | 2017 | 43015 | Kosch, TA; Eimes, JA; Didinger, C; Brannelly, LA; Waldman, B; Berger, L; Skerratt, LF | 2017 | Characterization of MHC class IA in the endangered southern corroboree frog | Southern corroboree frogs (Pseudophryne corroboree) have declined to near extinction in the wild after the emergence of the amphibian chytrid fungus Batrachochytrium dendrobatidis in southeastern Australia in the 1980s. A major captive breeding and reintroduction program is underway to preserve this iconic species, but improving resistance to B. dendrobatidis would help the wild population to be self-sustaining. Using 3’ and 5’ rapid amplification of complementary DNA ends (RACE), we characterized the major histocompatibility complex (MHC) class IA locus in this species. We then used sequences generated from RACE to design primers to amplify the peptide-binding region (PBR) of this functional genetic marker. Finally, we analysed the diversity, phylogeny, and selection patterns of PBR sequences from four P. corroboree populations and compared this with other amphibian species. We found moderately high MHC class IA genetic diversity in this species and evidence of strong positive and purifying selection at sites that are associated with putative PBR pockets in other species, indicating that this gene region may be under selection for resistance to Bd. Future studies should focus on identifying alleles associated with Bd resistance in P. corroboree by performing a Bd laboratory challenge study to confirm the functional importance of our genetic findings and explore their use in artificial selection or genetic engineering to increase resistance to chytridiomycosis. | 1480 | no | no good fitness measure | NA | no | no |
| 1481 | 2017 | 43015 | Cosme, S; Cuevas, HE; Zhang, D; Oleksyk, TK; Irish, BM | 2016 | Genetic diversity of naturalized cacao (Theobroma cacao L.) in Puerto Rico | Identification of genetically diverse cacao with disease resistance, high productivity, and desirable organoleptic traits is vitally important to the agricultural crop’s long-term sustainability. Environmental changes, pests, and diseases as well as nation’s sovereign property rights have led to a decrease in accessibility and exchange of germplasm of interest. Having been introduced during colonial times, naturalized cacao in Puerto Rico could serve as an unexplored source of genetic diversity in improvement programs. An island-wide survey was carried out to identify naturalized trees and to determine their genetic associations to reference cacao accessions. Samples were genotyped with Expressed Sequence Tag-derived single nucleotide polymorphism (SNP) markers. Principal coordinate, cluster, and population structure analysis using the genotype data for both local and reference samples assigned individuals into five distinct genetic backgrounds: Criollo, Trinitario, Amelonado, Upper Amazon Forastero (UAF), and Nacional. Puerto Rican cacao fit into four (Criollo, Trinitario, Amelonado and UAF) of the five genetic backgrounds, being mainly composed of individuals of Criollo ancestry. Based on historical evidence, cacao of Criollo background was probably brought to Puerto Rico from Venezuela and/or Central America during colonial times. Trinitario, Amelonado, and UAF genetic backgrounds are most likely products of more modern introductions. Genotyping cacao in Puerto Rico provides information on the history and possible origin of the naturalized trees on the island. In addition, the assessment has allowed the targeting of material for incorporation and long-term conservation filling gaps in the existing collection and providing new germplasm to be evaluated for agronomic performance. | 1481 | no | no good fitness measure | NA | no | no |
| 1482 | 2017 | 43015 | Santra, DK; Singh, G; Kaiser, WJ; Gupta, VS; Ranjekar, PK; Muehlbauer, FJ | 2001 | Molecular analysis of Ascochyta rabiei (Pass.) Labr., the pathogen of ascochyta blight in chickpea | Genetic diversity in Ascochyta rabiei (Pass.) Labr., the causative agent of ascochyta blight of chickpea, was determined using 37 Indian, five American (USA), three Syrian, and two Pakistani isolates. A total of 48 polymorphic RAPD markers were scored for each isolate and the data used for cluster analysis. Most of the isolates clustered in the dendrogram essentially according to geographic origin. Based on the two major clusters A and B, Indian isolates were grouped into two categories, type-A and type-B. Isolates of A. rabiei within the Punjab state were more diverse than isolates from other states in northwestern India. A DNA marker (ubc756(1.6 kb)), specific to Indian isolates was identified. This is the first report of a molecular diversity analysis of Indian isolates of A. rabiei. The information may assist Indian chickpea breeders in the proper deployment of blight-resistant cultivars and in disease management. | 1482 | no | gd of pathogen | NA | no | no |
| 1483 | 2017 | 43015 | Grueber, CE; Wallis, GP; King, TM; Jamieson, IG | 2012 | Variation at Innate Immunity Toll-Like Receptor Genes in a Bottlenecked Population of a New Zealand Robin | Toll-like receptors (TLRs) are an ancient family of genes encoding transmembrane proteins that bind pathogen-specific molecules and initiate both innate and adaptive aspects of the immune response. Our goal was to determine whether these genes show sufficient genetic diversity in a bottlenecked population to be a useful addition or alternative to the more commonly employed major histocompatibility complex (MHC) genotyping in a conservation genetics context. We amplified all known avian TLR genes in a severely bottlenecked population of New Zealand’s Stewart Island robin (Petroica australis rakiura), for which reduced microsatellite diversity was previously observed. We genotyped 17-24 birds from a reintroduced island population (including the 12 founders) for nine genes, seven of which were polymorphic. We observed a total of 24 single-nucleotide polymorphisms overall, 15 of which were non-synonymous, representing up to five amino-acid variants at a locus. One locus (TLR1LB) showed evidence of past directional selection. Results also confirmed a passerine duplication of TLR7. The levels of TLR diversity that we observe are sufficient to justify their further use in addressing conservation genetic questions, even in bottlenecked populations. | 1483 | no | only one pop | NA | no | no |
| 1484 | 2017 | 43015 | Branco, CC; Pacheco, PR; Cabrol, E; Cabral, R; Vicente, AM; Mota-Vieira, L | 2009 | Linkage disequilibrium and diversity for three genomic regions in Azoreans and mainland Portuguese | Studies on linkage disequilibrium (LD) across the genome and populations have been used in recent years with the main objective of improving gene mapping of complex traits. Here, we characterize the patterns of genetic diversity of HLA loci and evaluate LD (D’) extent in three genomic regions: Xq13.3, NRY and HLA. In addition, we examine the distribution of DXS1225-DXS8082 haplotype diversity in Azoreans and mainland Portuguese. Allele distribution has demonstrated that the Sao Miguel population is genetically very diverse; haplotype analysis revealed 100% discriminatory power for X- and Y-markers and 94.3% for HLA markers. Standardized multiallelic D’ in these three genomic regions shows values lower than 0.33, thereby suggesting there is no extensive LD in the Sao Miguel population. Data regarding the distribution of DXS1225-DXS8082 haplotypes indicate that there are no significant differences among all the populations studied, ( Azorean geographical groups, the Azores archipelago and mainland Portugal). Moreover, in these as well as in other European populations, the most frequent DXS1225-DXS8082 haplotype is 210-219. Even though Sao Miguel islanders and Azoreans do not constitute isolated populations and show LD for only very short physical distances, certain characteristics, such as the absence of genetic structure, the same environment and the possibility of constructing extensive pedigrees through church and civil records, offer an opportunity for dissecting the genetic background of complex diseases in these populations. | 1484 | no | NA | NA | no | no |
| 1485 | 2017 | 43015 | Dussex, N; Robertson, BC; Salis, AT; Kalinin, A; Best, H; Gemmell, NJ | 2016 | Low Spatial Genetic Differentiation Associated with Rapid Recolonization in the New Zealand Fur Seal Arctocephalus forsteri | Population declines resulting from anthropogenic activities are of major consequence for the long-term survival of species because the resulting loss of genetic diversity can lead to extinction via the effects of inbreeding depression, fixation of deleterious mutations, and loss of adaptive potential. Otariid pinnipeds have been exploited commercially to near extinction with some species showing higher demographic resilience and recolonization potential than others. The New Zealand fur seal (NZFS) was heavily impacted by commercial sealing between the late 18th and early 19th centuries, but has recolonized its former range in southern Australia. The species has also recolonized its former range in New Zealand, yet little is known about the pattern of recolonization. Here, we first used 11 microsatellite markers (n = 383) to investigate the contemporary population structure and dispersal patterns in the NZFS (Arctocephalus forsteri). Secondly, we model postsealing recolonization with 1 additional mtDNA cytochrome b (n = 261) marker. Our data identified 3 genetic clusters: an Australian, a subantarctic, and a New Zealand one, with a weak and probably transient subdivision within the latter cluster. Demographic history scenarios supported a recolonization of the New Zealand coastline from remote west coast colonies, which is consistent with contemporary gene flow and with the species’ high resilience. The present data suggest the management of distinct genetic units in the North and South of New Zealand along a genetic gradient. Assignment of individuals to their colony of origin was limited (32%) with the present data indicating the current microsatellite markers are unlikely sufficient to assign fisheries bycatch of NZFSs to colonies. | 1485 | no | no good fitness measure | NA | no | no |
| 1486 | 2017 | 43015 | Honorio, ICG; Bertoni, BW; Telles, MPD; Braga, RD; Franca, SD; Coppede, JD; Correa, VSC; Diniz, JAF; Pereira, AMS | 2017 | Genetic and chemical diversity of Uncaria tomentosa (Willd. ex. Schult.) DC. in the Brazilian Amazon | Uncaria tomentosa (Willd. ex Schult.) DC., a plant native to the Amazon region, is used widely in popular medicine and by the pharmaceutical industry because of its anti-inflammatory activity. However, the survival of this species is endangered by deforestation and indiscriminate collection, and a preservation plan is urgently required. The objectives of this study were to determine the genetic and chemical variability between and within eight populations of U. tomentosa from the Brazilian states of Acre, Para and Amapa, and to investigate possible correlations between genetic and geographical distances, and between geographical distances or altitude and the accumulation of bioactive oxindole alkaloids. Three sequence-related amplified polymorphism (SRAP) markers were employed to fingerprint genomic DNA, and the amounts of mitraphylline and isomitraphylline in leaf samples were established by high-performance liquid chromatography. Although significant divergence existed between the tested populations (F-ST = 0.246), the largest genetic diversity and the highest percentage of polymorphism (95.68%) was found within the population from Mancio Lima, Acre. Gene flow was considered rather limited (Nm = 1.57), and no correlations between genetic and geographical distances were detected, suggesting that population structure followed an island model. Accumulations of mitraphylline and isomitraphylline varied in the range 32.94 to 0.57 and 3.75 to 0.36 mg g(-1) dry weight, respectively. The concentration of isomitraphylline was positively influenced by altitude, such that the population collected at the site with the highest elevation (Tarauaca, Acre) exhibited the greatest alkaloid content. SRAP markers were very efficient in fingerprinting genomic DNA from U. tomentosa populations and clearly showed that genetic variability within populations was greater than between populations. A conservation and management plan should prioritize the creation of germplasm banks to prevent the loss of existing genetic variability, particularly within alkaloid-rich populations such as those of Tarauaca | 1486 | no | no good fitness measure | NA | no | no |
| 1487 | 2017 | 43015 | Regidor-Cerrillo, J; Diez-Fuertes, F; Garcia-Culebras, A; Moore, DP; Gonzalez-Warleta, M; Cuevas, C; Schares, G; Katzer, F; Pedraza-Diaz, S; Mezo, M; Ortega-Mora, LM | 2013 | Genetic Diversity and Geographic Population Structure of Bovine Neospora caninum Determined by Microsatellite Genotyping Analysis | The cyst-forming protozoan parasite Neospora caninum is one of the main causes of bovine abortion worldwide and is of great economic importance in the cattle industry. Recent studies have revealed extensive genetic variation among N. caninum isolates based on microsatellite sequences (MSs). MSs may be suitable molecular markers for inferring the diversity of parasite populations, molecular epidemiology and the basis for phenotypic variations in N. caninum, which have been poorly defined. In this study, we evaluated nine MS markers using a panel of 11 N. caninum-derived reference isolates from around the world and 96 N. caninum bovine clinical samples and one ovine clinical sample collected from four countries on two continents, including Spain, Argentina, Germany and Scotland, over a 10-year period. These markers were used as molecular tools to investigate the genetic diversity, geographic distribution and population structure of N. caninum. Multilocus microsatellite genotyping based on 7 loci demonstrated high levels of genetic diversity in the samples from all of the different countries, with 96 microsatellite multilocus genotypes (MLGs) identified from 108 N. caninum samples. Geographic sub-structuring was present in the country populations according to pairwise F-ST. Principal component analysis (PCA) and Neighbor Joining tree topologies also suggested MLG segregation partially associated with geographical origin. An analysis of the MLG relationships, using eBURST, confirmed that the close genetic relationship observed between the Spanish and Argentinean populations may be the result of parasite migration (i.e., the introduction of novel MLGs from Spain to South America) due to cattle movement. The eBURST relationships also revealed genetically different clusters associated with the abortion. The presence of linkage disequilibrium, the co-existence of specific MLGs to individual farms and eBURST MLG relationships suggest a predominant clonal propagation for Spanish N. caninum MLGs in cattle. | 1487 | no | NA | NA | no | no |
| 1488 | 2017 | 43015 | Spear, SF; Crisafulli, CM; Storfer, A | 2012 | Genetic structure among coastal tailed frog populations at Mount St. Helens is moderated by post-disturbance management | “Catastrophic disturbances often provide”“natural laboratories”" that allow for greater understanding of ecological processes and response of natural populations. The 1980 eruption of the Mount St. Helens volcano in Washington, USA, provided a unique opportunity to test biotic effects of a large-scale stochastic disturbance, as well as the influence of post-disturbance management. Despite severe alteration of nearly 600 km 2 of habitat, coastal tailed frogs (Ascaphus truei) were found within a portion of the blast area five years after eruption. We investigated the genetic source of recolonization within the blast area and tested whether post-eruption salvage logging and subsequent tree planting influenced tailed frog movement patterns. Our results support widespread recolonization across the blast area from multiple sources, as all sites are grouped into one genetic cluster. Landscape genetic models suggest that gene flow through the unmanaged portion of the blast area is influenced only by distance between sites and the frost-free period (r(2) = 0.74). In contrast, gene flow pathways within the blast area where salvage logging and replanting occurred post-eruption are strongly limited (r(2) = 0.83) by the physiologically important variables of heat load and precipitation. These data suggest that the lack of understory and coarse wood (downed and standing dead tree boles) refugia in salvaged areas may leave frogs more susceptible to desiccation and mortality than those frogs moving through the naturally regenerated area. Simulated populations based on the landscape genetic models show an increase in the inbreeding coefficient in the managed area relative to the unmanaged blast area. In sum, we show surprising resilience of an amphibian species to a catastrophic disturbance, and we suggest that, at least for this species, naturally regenerating habitat may better maintain long-term genetic diversity of populations than actively managed habitat." | 1488 | no | no good fitness measure | NA | no | no |
| 1489 | 2017 | 43015 | Hunt, HV; Ansell, SW; Russell, SJ; Schneider, H; Vogel, JC | 2009 | Genetic diversity and phylogeography in two diploid ferns, Asplenium fontanum subsp fontanum and A-petrarchae subsp bivalens, in the western Mediterranean | Asplenium fontanum subsp. fontanum and A. petrarchae subsp. bivalens are diploid rock ferns of limestone outcrops of the western Mediterranean region. Asplenium fontanum subsp. fontanum occurs from Valencia through northeastern Spain to the Alpes-Maritimes and Swiss Jura. Asplenium petrarchae subsp. bivalens occurs only on Majorca, in Valencia and possibly in southern Spain. We analysed allozyme and chloroplast genetic marker diversity in 75 populations of A. fontanum subsp. fontanum and 12 populations of A. petrarchae subsp. bivalens sampled from across their respective ranges. The two species show similar levels of species and population genetic diversity to one another and to other diploid European Asplenium taxa. Both are predominantly outbreeding, as indicated by F(IS) = 0.108 and 0.167 respectively. Substantial between-population differentiation results largely from differentiation between regions. Isolation by distance operates over limited geographic ranges, up to 50 km. In A. fontanum subsp. fontanum, the major geographical differentiation between Valencia and the rest of the taxon range probably represents an ancient range fragmentation. A less pronounced differentiation divides populations in the SW from those in the NE of the range, with evidence for a biogeographic link between the eastern Pyrenees and southeastern France. High diversity in the Pyrenees may either represent ancient population differentiation, or a suture zone. In A. petrarchae subsp. bivalens, populations on Majorca exhibit a subset of the genetic diversity present in Valencia, although the two regions are strongly differentiated by differing allele frequencies. Dispersal from the mainland may have founded Majorcan populations, although a role for in situ island survival cannot be excluded. | 1489 | no | no good fitness measure | NA | no | no |
| 1490 | 2017 | 43015 | Poulsen, NA; Nielsen, EE; Schierup, MH; Loeschcke, V; Gronkjaer, P | 2006 | Long-term stability and effective population size in North Sea and Baltic Sea cod (Gadus morhua) | DNA from archived otoliths was used to explore the temporal stability of the genetic composition of two cod populations, the Moray Firth (North Sea) sampled in 1965 and 2002, and the Bornholm Basin (Baltic Sea) sampled in 1928 and 1997. We found no significant changes in the allele frequencies for the Moray Firth population, while subtle but significant genetic changes over time were detected for the Bornholm Basin population. Estimates of the effective population size (N-e) generally exceeded 500 for both populations when employing a number of varieties of the temporal genetic method. However, confidence intervals were very wide and N-e’s most likely range in the thousands. There was no apparent loss of genetic variability and no evidence of a genetic bottleneck for either of the populations. Calculations of the expected levels of genetic variability under different scenarios of N-e showed that the number of alleles commonly reported at microsatellite loci in Atlantic cod is best explained by N-e’s exceeding thousand. Recent fishery-induced bottlenecks can, however, not be ruled out as an explanation for the apparent discrepancy between high levels of variability and recently reported estimates of N-e << 1000. From life history traits and estimates of survival rates in the wild, we evaluate the compatibility of the species’ biology and extremely low N-e/N ratios. Our data suggest that very small N-e’s are not likely to be of general concern for cod populations and, accordingly, most populations do not face any severe threat of losing evolutionary potential due to genetic drift. | 1490 | no | no good fitness measure, N pop differnt in GD? | NA | no | no |
| 1491 | 2017 | 43015 | Johnson, JA; Tingay, RE; Culver, M; Hailer, F; Clarke, ML; Mindell, DP | 2009 | Long-term survival despite low genetic diversity in the critically endangered Madagascar fish-eagle | The critically endangered Madagascar fish-eagle (Haliaeetus vociferoides) is considered to be one of the rarest birds of prey globally and at significant risk of extinction. In the most recent census, only 222 adult individuals were recorded with an estimated total breeding population of no more than 100-120 pairs. Here, levels of Madagascar fish-eagle population genetic diversity based on 47 microsatellite loci were compared with its sister species, the African fish-eagle (Haliaeetus vocifer), and 16 of these loci were also characterized in the white-tailed eagle (Haliaeetus albicilla) and the bald eagle (Haliaeetus leucocephalus). Overall, extremely low genetic diversity was observed in the Madagascar fish-eagle compared to other surveyed Haliaeetus species. Determining whether this low diversity is the result of a recent bottleneck or a more historic event has important implications for their conservation. Using a Bayesian coalescent-based method, we show that Madagascar fish-eagles have maintained a small effective population size for hundreds to thousands of years and that its low level of neutral genetic diversity is not the result of a recent bottleneck. Therefore, efforts made to prevent Madagascar fish-eagle extinction should place high priority on maintenance of habitat requirements and reducing direct and indirect human persecution. Given the current rate of deforestation in Madagascar, we further recommend that the population be expanded to occupy a larger geographical distribution. This will help the population persist when exposed to stochastic factors (e.g. climate and disease) that may threaten a species consisting of only 200 adult individuals while inhabiting a rapidly changing landscape. | 1491 | no | no good fitness measure, N pop differnt in GD? | NA | no | no |
| 1492 | 2017 | 43015 | Marsh, DM; Page, RB; Hanlon, TJ; Corritone, R; Little, EC; Seifert, DE; Cabe, PR | 2008 | Effects of roads on patterns of genetic differentiation in red-backed salamanders, Plethodon cinereus | Roads can fragment animal populations by reducing gene flow, which can lead to drift and the loss of genetic diversity. One of the principle signatures of reduced gene flow is increased genetic differentiation in isolated populations, and evidence that roads contribute to such differentiation has been reported for several species. We used microsatellites to examine whether six roads led to increased genetic differentiation in red-backed salamanders (Plethodon cinereus). These six roads included one divided interstate highway, one undivided four-lane highway, and four secondary roads. We found that the genetic distance between plots that were bisected by the interstate highway was significantly greater than the genetic distance between equidistant plots on the same side of the highway. However, for the five smaller roads, plots across the road were no more genetically distinct than were plots on the same side of the road. Bayesian clustering methods also supported both of these findings. The optimal clustering of plots for the interstate highway consisted of two clusters that corresponded to the two sides of highway. For the other five sites, the optimal grouping consisted of a single cluster containing all of the plots. Our findings suggest that gene flow across very large roads is rare and that bisected red-backed salamander populations are likely to diverge from one another. For smaller roads, our results imply that the indirect effects of roads on genetic population structure are probably less of a pressing concern for terrestrial salamanders than are the direct effects of mortality and habitat alteration. | 1492 | no | no good fitness measure | NA | no | no |
| 1493 | 2017 | 43015 | Rudnick, JA; Katzner, TE; Bragin, EA; Rhodes, OE; Dewoody, JA | 2005 | Using naturally shed feathers for individual identification, genetic parentage analyses, and population monitoring in an endangered Eastern imperial eagle (Aquila heliaca) population from Kazakhstan | Genetic analyses on noninvasively collected samples have revolutionized how populations are monitored. Most noninvasive monitoring studies have used hair or scat for individual identification of elusive mammals, but here we utilize naturally shed feathers. The Eastern imperial eagle (EIE) is a species of conservation concern throughout Central Asia and, like most raptors, EIEs are inherently challenging to study because adults are difficult to capture and band using conventional techniques. Over 6 years, we noninvasively collected hundreds of adult feathers and directly sampled EIE chicks at a national nature reserve in Kazakhstan. All samples were genetically sexed and genotyped at a suite of microsatellite loci. Genetically profiled adult feathers identified and monitored the presence of individual eagles over time, enabling us to address a variety of issues related to the biology, demography, and conservation of EIEs. Specifically, we characterized (i) the genetic mating system, (ii) relatedness among mated pairs, (iii) chick sex ratios, and (iv) annual turnover in an adult breeding population. We show that EIEs are genetically monogamous and furthermore, there is no apparent relatedness-based system of mate choice (e.g. inbreeding avoidance). Results indicate that annual adult EIE survivorship (84%) is lower than expected for a long-lived raptor, but initial analyses suggest the current reproductive rate at our study site is sufficient to maintain a stable breeding population. The pristine habitat at our study site supports an EIE population that is probably the most demographically robust in the world; thus, our results caution that populations in marginal habitats may not be self-sustaining. | 1493 | no | No GD diff between pop | NA | no | no |
| 1494 | 2017 | 43015 | Newman, SJ; Easteal, S | 2015 | A New Metric of Inclusive Fitness Predicts the Human Mortality Profile | Biological species have evolved characteristic patterns of age-specific mortality across their life spans. If these mortality profiles are shaped by natural selection they should reflect underlying variation in the fitness effect of mortality with age. Direct fitness models, however, do not accurately predict the mortality profiles of many species. For several species, including humans, mortality rates vary considerably before and after reproductive ages, during life-stages when no variation in direct fitness is possible. Variation in mortality rates at these ages may reflect indirect effects of natural selection acting through kin. To test this possibility we developed a new two-variable measure of inclusive fitness, which we term the extended genomic output or EGO. Using EGO, we estimate the inclusive fitness effect of mortality at different ages in a small hunter-gatherer population with a typical human mortality profile. EGO in this population predicts 90% of the variation in age-specific mortality. This result represents the first empirical measurement of inclusive fitness of a trait in any species. It shows that the pattern of human survival can largely be explained by variation in the inclusive fitness cost of mortality at different ages. More generally, our approach can be used to estimate the inclusive fitness of any trait or genotype from population data on birth dates and relatedness. | 1494 | no | human | NA | no | no |
| 1495 | 2017 | 43015 | Keller, I; Largiader, CR | 2003 | Recent habitat fragmentation caused by major roads leads to reduction of gene flow and loss of genetic variability in ground beetles | Although habitat fragmentation is suspected to jeopardize the long-term survival of many species, few data are available on its impact on the genetic variability of invertebrates. We assess the genetic population structure of the flightless ground beetle Carabus violaceus L., 1758 in a Swiss forest, which is divided into several fragments by a highway and two main roads. Eight samples were collected from different forest fragments and analysed at six microsatellite loci. The largest genetic differentiation was observed between samples separated by roads and in particular by the highway. The number of roads between sites explained 44% of the variance in pairwise F-ST estimates, whereas the age of the road and the geographical distance between locations were not significant factors. Furthermore, a comparison of allelic richness showed that the genetic variability in a small forest fragment isolated by the highway was significantly lower than in the rest of the study area. These findings strongly support the hypothesis that large roads are absolute barriers to gene flow in C. violaceus, which may lead to a loss of genetic variability in fragmented populations. | 1495 | no | NA | NA | no | no |
| 1496 | 2017 | 43015 | Zhang, YB; Li, X; Zhang, F; Wang, DM; Yu, J | 2012 | A Preliminary Study of Copy Number Variation in Tibetans | “Genetic features of Tibetans have been broadly investigated, but the properties of copy number variation (CNV) have not been well examined. To get a preliminary view of CNV in Tibetans, we scanned 29 Tibetan genomes with the Illumina Human-1 M high-resolution genotyping microarray and identified 139 putative copy number variable regions (CNVRs), consisting of 70 deletions, 61 duplications, and 8 multi-allelic loci. Thirty-four of the 139 CNVRs showed differential allele frequencies versus other East-Asian populations, with P values < 0.0001. These results indicated a distinct pattern of CNVR allele frequency distribution in Tibetans. The Tibetan CNVRs are enriched for genes in the disease class of human reproduction (such as genes from the DAZ, BPY2, CDY, and HLA-DQ and -DR gene clusters) and biological process categories of”“response to DNA damage stimulus”" and "“DNA repair”" (such as RAD51, RAD52, and MRE11A). These genes are related to the adaptive traits of high infant birth weight and darker skin tone of Tibetans, and may be attributed to recent local adaptation. Our results provide a different view of genetic diversity in Tibetans and new insights into their high-altitude adaptation." | 1496 | no | human | NA | no | no |
| 1497 | 2017 | 43015 | Singru, R; Sivaramakrishnan, S; Thakur, RP; Gupta, VS; Ranjekar, PK | 2003 | Detection of genetic variability in pearl millet downy mildew (Sclerospora graminicola) by AFLP | Downy mildew, caused by Sclerospora graminicola, is an economically important disease of pearl millet in the semiarid regions of Asia and Africa. Amplified restriction fragment length polymorphism (AFLP) was used to detect the extent of genomic variation among 19 fungal isolates from different cultivars of pearl millet grown in various regions of India. Fourteen AFLP primer combinations produced 184 polymorphic bands. An unweighted pair-group method of averages cluster analysis represented by dendrogram and principal coordinate analysis separated the mildew collections into four distinct groups. Isolates having characteristic opposite mating abilities, geographic relatedness, virulence, common host cultivars, and changes through asexual generations reflected heterogeneity of the pathogen. The use of AFLP to detect genetic variation is particularly important in selecting mildew isolates to screen breeding material for identification of resistant millet and monitoring changes in S. graminicola in relation to changes in host for effective disease management. | 1497 | no | gd of pathogen | NA | no | no |
| 1498 | 2017 | 43015 | Hadziabdic, D; Wadl, PA; Staton, ME; Klingeman, WE; Moulton, JK; Pscheidt, JW; Wiggins, GJ; Grant, JF; Lambdin, PL; Windham, MT; Faccoli, M; Merten, PR; Trigiano, RN | 2015 | Development of microsatellite loci in Pityophthorus juglandis, a vector of thousand cankers disease in Juglans spp. | Using next-generation sequencing, 18 microsatellite loci were developed and characterized for walnut twig beetle, Pityophthorus juglandis, a vector of thousand cankers disease (TCD) affecting Juglans spp. Although all Juglans species are susceptible to TCD infection, native populations of J. nigra and J. cinerea, which is endangered in Canada, are most susceptible and threatened by habitat loss. Novel primers amplified di-, tri-, and tetra nucleotide repeats and detected 4-14 alleles per locus. Averaged observed and expected heterozygosity was 0.22 and 0.67, respectively. Our results indicate that P. juglandis microsatellite loci can be used to investigate genetic diversity and population structure of this vector across a widespread geography. These markers will be useful tools for evaluating genetic structure of P. juglandis population outbreaks and developing appropriate conservation strategies. Microsatellite loci obtained in this study can also be utilized to determine relationships of P. juglandis to other closely related Pityophthorus spp. | 1498 | no | no good fitness measure, N pop differnt in GD? | NA | no | no |
| 1499 | 2017 | 43015 | Wallace, RG; Fitch, WM | 2008 | Influenza A H5N1 Immigration Is Filtered Out at Some International Borders | Background: Geographic spread of highly pathogenic influenza A H5N1, the bird flu strain, appears a necessary condition for accelerating the evolution of a related human-to-human infection. As H5N1 spreads the virus diversifies in response to the variety of socioecological environments encountered, increasing the chance a human infection emerges. Genetic phylogenies have for the most part provided only qualitative evidence that localities differ in H5N1 diversity. For the first time H5N1 variation is quantified across geographic space. Methodology and Principal Findings: We constructed a statistical phylogeography of 481 H5N1 hemagglutinin genetic sequences from samples collected across 28 Eurasian and African localities through 2006. The MigraPhyla protocol showed southern China was a source of multiple H5N1 strains. Nested clade analysis indicated H5N1 was widely dispersed across southern China by both limited dispersal and long distance colonization. The UniFrac metric, a measure of shared phylogenetic history, grouped H5N1 from Indonesia, Japan, Thailand and Vietnam with those from southeastern Chinese provinces engaged in intensive international trade. Finally, H5N1’s accumulative phylogenetic diversity was greatest in southern China and declined beyond. The gradient was interrupted by areas of greater and lesser phylogenetic dispersion, indicating H5N1 migration was restricted at some geopolitical borders. Thailand and Vietnam, just south of China, showed significant phylogenetic clustering, suggesting newly invasive H5N1 strains have been repeatedly filtered out at their northern borders even as both countries suffered recurring outbreaks of endemic strains. In contrast, Japan, while successful in controlling outbreaks, has been subjected to multiple introductions of the virus. Conclusions: The analysis demonstrates phylogenies can provide local health officials with more than hypotheses about relatedness. Pathogen dispersal, the functional relationships among disease ecologies across localities, and the efficacy of control efforts can also be inferred, all from viral genetic sequences alone. | 1499 | no | gd of pathogen | NA | no | no |
| 1500 | 2017 | 43015 | Jagannathan, S; Bradley, RK | 2016 | Translational plasticity facilitates the accumulation of nonsense genetic variants in the human population | Genetic variants that disrupt protein-coding DNA are ubiquitous in the human population, with about 100 such loss-of function variants per individual. While most loss-of-function variants are rare, a subset have risen to high frequency and occur in a homozygous state in healthy individuals. It is unknown why these common variants are well tolerated, even though some affect essential genes implicated in Mendelian disease. Here, we combine genomic, proteomic, and biochemical data to demonstrate that many common nonsense variants do not ablate protein production from their host genes. We provide computational and experimental evidence for diverse mechanisms of gene rescue, including alternative splicing, stop codon readthrough, alternative translation initiation, and C-terminal truncation. Our results suggest a molecular explanation for the mild fitness costs of many common nonsense variants and indicate that translational plasticity plays a prominent role in shaping human genetic diversity. | 1500 | no | human | NA | no | no |
| 1501 | 2017 | 43019 | Paez, DJ; Fleming-Davies, AE; Dwyer, G | 2015 | Effects of pathogen exposure on life-history variation in the gypsymoth (Lymantria dispar) | Investment in host defences against pathogens may lead to trade-offs with host fecundity. When such trade-offs arise from genetic correlations, rates of phenotypic change by natural selection may be affected. However, genetic correlations between host survival and fecundity are rarely quantified. To understand trade-offs between immune responses to baculovirus exposure and fecundity in the gypsy moth (Lymantria dispar), we estimated genetic correlations between survival probability and traits related to fecundity, such as pupal weight. In addition, we tested whether different virus isolates have different effects on male and female pupal weight. To estimate genetic correlations, we exposed individuals of known relatedness to a single baculovirus isolate. To then evaluate the effect of virus isolate on pupal weight, we exposed a single gypsy moth strain to 16 baculovirus isolates. We found a negative genetic correlation between survival and pupal weight. In addition, virus exposure caused late-pupating females to be identical in weight to males, whereas unexposed females were 2-3 times as large as unexposed males. Finally, we found that female pupal weight is a quadratic function of host mortality across virus isolates, which is likely due to trade-offs and compensatory growth processes acting at high and low mortality levels, respectively. Overall, our results suggest that fecundity costs may strongly affect the response to selection for disease resistance. In nature, baculoviruses contribute to the regulation of gypsy moth outbreaks, as pathogens often do in forest-defoliating insects. We therefore argue that trade-offs between host life-history traits may help explain outbreak dynamics. | 1501 | no | No GD diff between pop | NA | no | no |
| 1502 | 2017 | 43019 | Laroque, A; Min-Oo, G; Tam, M; Radovanovic, I; Stevenson, MM; Gros, P | 2012 | Genetic control of susceptibility to infection with Plasmodium chabaudi chabaudi AS in inbred mouse strains | To identify genetic effects modulating the blood stage replication of the malarial parasite, we phenotyped a group of 25 inbred mouse strains for susceptibility to Plasmodium chabaudi chabaudi AS infection (peak parasitemia, survival). A broad spectrum of responses was observed, with strains such as C57BL/6J being the most resistant (low parasitemia, 100% survival) and strains such as NZW/LacJ and C3HeB/FeJ being extremely susceptible (very high parasitemia and uniform lethality). A number of strains showed intermediate phenotypes and gender-specific effects, suggestive of rich genetic diversity in response to malaria in inbred strains. An F2 progeny was generated from SM/J (susceptible) and C57BL/6J (resistant) parental strains, and was phenotyped for susceptibility to P. chabaudi chabaudi AS. A whole-genome scan in these animals identified the Char1 locus (LOD = 7.40) on chromosome 9 as a key regulator of parasite density and pointed to a conserved 0.4-Mb haplotype at Char1 that segregates with susceptibility/resistance to infection. In addition, a second locus was detected in [SM/J x C57BL/6J] F2 mice on the X chromosome (LOD = 4.26), which was given the temporary designation Char11. These studies identify a conserved role of Char1 in regulating response to malaria in inbred mouse strains, and provide a prioritized 0.4-Mb interval for the search of positional candidates. Genes and Immunity (2012) 13, 155-163; doi:10.1038/gene.2011.67; published online 6 October 2011 | 1502 | no | no good fitness measure, N pop differnt in GD? (inbreeding?) | NA | no | no |
| 1503 | 2017 | 43019 | Nyakaana, S | 2007 | Microgeographical genetic structure of forest robusta coffee (Coffea canephora, Pierre), in Kibale National Park, Uganda | Genetic variation within and between forest coffee (Coffea canephora, Pierre) populations sampled from five localities in Kibale National Park was assessed using six polymorphic microsatellite loci. In a total sample of 113 individuals, 53 alleles were observed across all loci in all populations while the total number of alleles across loci in each population ranged from fifteen to forty-five. Significant genetic differentiation was observed between all populations in all pair-wise population comparisons (F-ST = 0.041-0.284; P < 0.01). A high proportion of private alleles was observed across all loci with some of them occurring at relatively high frequencies. The implications of these findings for the future survival of the species are discussed. | 1503 | no | no good fitness measure | NA | no | no |
| 1504 | 2017 | 43019 | Bickel, TO; Bruhl, CA; Gadau, JR; Holldobler, B; Linsenmair, K | 2006 | Influence of habitat fragmentation on the genetic variability in leaf litter ant populations in tropical rainforests of Sabah, Borneo | Two ant species, Odontomachus rixosus and Pheidole annexus, were studied in the tropical rainforests of Sabah, Malaysia, North Borneo, to analyze the impact of habitat fragmentation on the genetic diversity and population structure of ant populations using RAPD-fingerprinting. Ants were sampled in a contiguous (43,800 ha) and three patches of primary rainforests of varying size (4294, 146 and 20 ha) that were fragmented about 40 years ago. We found a decrease in genetic variability for both species in the fragmented populations compared to the contiguous. Genetic distances between populations resembled the geographical arrangement of populations and can be explained by an effect of isolation by distance. A high degree in population subdivision suggests a lack of meta-population dynamics due to a shortage of gene flow between populations, possibly the result of the high degree of habitat isolation by oil palm plantations. Although the results of this study are limited due to low replication this is the first data on genetic patterns of insect populations in fragmented rainforests and should be seen as starting point for future research. The value of small to medium sized protection areas for conservation needs to be carefully evaluated in the context of this study, as even relatively large areas (4294 ha) may not prevent the critical loss of genetic variability and guarantee long-term survival of organisms. | 1504 | no | no good fitness measure | NA | no | no |
| 1505 | 2017 | 43019 | Manunza, A; Amills, M; Noce, A; Cabrera, B; Zidi, A; Eghbalsaied, S; de Albornoz, EC; Portell, M; Mercade, A; Sanchez, A; Balteanu, V | 2016 | Romanian wild boars and Mangalitza pigs have a European ancestry and harbour genetic signatures compatible with past population bottlenecks | We aimed to analyse the genetic diversity of Romanian wild boars and to compare it with that from other wild boar and pig populations from Europe and Asia. Partial sequencing of the mitochondrial encoded cytochrome b (MT-CYB) gene from 36 Romanian wild boars and 36 domestic pigs (Mangalitza, Bazna and Vietnamese breeds) showed that the diversity of Romanian wild boars and Mangalitza pigs is fairly reduced, and that most of the members of these two populations share a common MT-CYB haplotype. Besides, in strong contrast with the Bazna animals, Romanian wild boars and Mangalitza swine did not carry Asian variants at the MT-CYB locus. The autosomal genotyping of 18 Romanian wild boars with the Illumina Porcine SNP60 BeadChip revealed that their genetic background is fundamentally European, even though signs of a potential Near Eastern ancestry (similar to 25%) were detectable at K = 4 (the most significant number of clusters), but not at higher K-values. Admixture analysis also showed that two wild boars are of a hybrid origin, which could be explained by the mating of feral animals with domestic pigs. Finally, a number of Romanian wild boars displayed long runs of homozygosity, an observation that is consistent with the occurrence of past population bottlenecks and the raise of inbreeding possibly due to overhunting or to the outbreak of infectious diseases. | 1505 | no | no good fitness measure | NA | no | no |
| 1506 | 2017 | 43019 | Khajavi, M; Zhou, Y; Birsner, AE; Bazinet, L; Di Sant, AR; Schiffer, AJ; Rogers, MS; Krishnaji, ST; Hu, B; Nguyen, V; Zon, L; D’Amato, RJ | 2017 | Identification of Padi2 as a novel angiogenesis-regulating gene by genome association studies in mice | Recent findings indicate that growth factor-driven angiogenesis is markedly influenced by genetic variation. This variation in angiogenic responsiveness may alter the susceptibility to a number of angiogenesis-dependent diseases. Here, we utilized the genetic diversity available in common inbred mouse strains to identify the loci and candidate genes responsible for differences in angiogenic response. The corneal micropocket neovascularization assay was performed on 42 different inbred mouse strains using basic fibroblast growth factor (bFGF) pellets. We performed a genome-wide association study utilizing efficient mixed-model association (EMMA) mapping using the induced vessel area from all strains. Our analysis yielded five loci with genome-wide significance on chromosomes 4, 8, 11, 15 and 16. We further refined the mapping on chromosome 4 within a haplotype block containing multiple candidate genes. These genes were evaluated by expression analysis in corneas of various inbred strains and in vitro functional assays in human microvascular endothelial cells (HMVECs). Of these, we found the expression of peptidyl arginine deiminase type II (Padi2), known to be involved in metabolic pathways, to have a strong correlation with a haplotype shared by multiple high angiogenic strains. In addition, inhibition of Padi2 demonstrated a dosage-dependent effect in HMVECs. To investigate its role in vivo, we knocked down Padi2 in transgenic kdrl: zsGreen zebrafish embryos using morpholinos. These embryos had disrupted vessel formation compared to control siblings. The impaired vascular pattern was partially rescued by human PADI2 mRNA, providing evidence for the specificity of the morphant phenotype. Taken together, our study is the first to indicate the potential role of Padi2 as an angiogenesis-regulating gene. The characterization of Padi2 and other genes in associated pathways may provide new understanding of angiogenesis regulation and novel targets for diagnosis and treatment of a wide variety of angiogenesisdependent diseases. | 1506 | no | no good fitness measure, N pop differnt in GD? (inbreeding?) | NA | no | no |
| 1507 | 2017 | 43019 | Moreno, CR; Lantier, F; Berthon, P; Gautier-Bouchardon, AV; Boivin, R; Lantier, I; Brunel, JC; Weisbecker, JL; Francois, D; Bouix, J; Elsen, JM | 2003 | Genetic parameters for resistance to the Salmonella abortusovis vaccinal strain Rv6 in sheep | An experimental population (1216 lambs from 30 sires) of the Inra401 sheep was created in an Inra flock to allow QTL detection for susceptibility to Salmonella infection, wool and carcass traits. The Inra401 is a sheep composite line developed from two breeds: Berrichon du Cher and Romanov. At 113 days of age on average, the lambs were inoculated intravenously with 10(8) Salmonella abortusovis Rv6 (vaccinal strain). They were slaughtered 10 days after the inoculation. Several traits were measured at inoculation and/or slaughtering to estimate the genetic resistance of the lambs to Salmonella infection: specific IgM and IgG1 antibody titres, body weight loss, spleen and pre-scapular node weights and counts of viable Salmonella persisting in these organs. This paper presents a quantitative analysis of the genetic variability of the traits related to salmonellosis susceptibility. The heritabilities of the traits varied between 0.10 and 0.64 (significantly different from zero). Thus, in sheep as well as in other species, the determinism of resistance to Salmonella infection is under genetic control. Moreover, the correlations between the traits are in agreement with the known immune mechanisms. The genetic variability observed should help QTL detection. | 1507 | no | domesticated animal | NA | no | no |
| 1508 | 2017 | 43019 | Frantz, AC; Heddergott, M; Lang, J; Schulze, C; Ansorge, H; Runge, M; Braune, S; Michler, FU; Wittstatt, U; Hoffmann, L; Hohmann, U; Michler, BA; Van den Berge, K; Horsburgh, GJ | 2013 | Limited mitochondrial DNA diversity is indicative of a small number of founders of the German raccoon (Procyon lotor) population | The raccoon (Procyon lotor) has successfully invaded central Europe, despite the population apparently having been founded by a small number of individuals in two distinct populations in Germany. The ecological success of the invasion has been explained by raccoons being an adaptable, truly omnivorous species. However, the German raccoon population might have a larger number of founders and be more genetically diverse than assumed, as accidental or deliberate releases of household pets or individuals from zoos are relatively common. In the present study, we sequenced a 550-base-pair long fragment of the mitochondrial control region in 193 raccoons from Germany and neighbouring countries. We only identified six different haplotypes; of which, five were limited to Germany. Our results support the notions that the population was founded by a small number of females and that the German raccoons originate from two separate release events in central and eastern Germany. Additionally, however, we provide evidence for the presence of a distinct population in Saxony, eastern Germany. Further studies using different genetic markers are necessary to gain additional information on genetic diversity and population genetic structure. | 1508 | no | no good fitness measure, N pop differnt in GD? | NA | no | no |
| 1509 | 2017 | 43019 | Li, SB; Li, B; Cheng, C; Xiong, ZJ; Liu, QB; Lai, JH; Carey, HV; Zhang, Q; Zheng, HB; Wei, SG; Zhang, HB; Chang, L; Liu, SP; Zhang, SX; Yu, B; Zeng, XF; Hou, Y; Nie, WH; Guo, YM; Chen, T; Han, JQ; Wang, J; Wang, J; Chen, C; Liu, JK; Stambrook, PJ; Xu, M; Zhang, GJ; Gilbert, MTP; Yang, HM; Jarvis, ED; Yu, J; Yan, JQ | 2014 | Genomic signatures of near-extinction and rebirth of the crested ibis and other endangered bird species | Background Nearly a quarter of all avian species is either threatened or nearly threatened. Of these, 73 species are currently being rescued from going extinct in wildlife sanctuaries. One of the previously most critically-endangered is the crested ibis, Nipponia nippon. Once widespread across North-East Asia, by 1981 only seven individuals from two breeding pairs remained in the wild. The recovering crested ibis populations thus provide an excellent example for conservation genomics since every individual bird has been recruited for genomic and demographic studies. Results Using high-quality genome sequences of multiple crested ibis individuals, its thriving cohabitant, the little egret, Egretta garzetta, and the recently sequenced genomes of 41 other avian species that are under various degrees of survival threats, including the bald eagle, we carry out comparative analyses for genomic signatures of near extinction events in association with environmental and behavioral attributes of species. We confirm that both loss of genetic diversity and enrichment of deleterious mutations of protein-coding genes contribute to the major genetic defects of the endangered species. We further identify that genetic inbreeding and loss-of-function genes in the crested ibis may all constitute genetic susceptibility to other factors including long-term climate change, over-hunting, and agrochemical overuse. We also establish a genome-wide DNA identification platform for molecular breeding and conservation practices, to facilitate sustainable recovery of endangered species. Conclusions These findings demonstrate common genomic signatures of population decline across avian species and pave a way for further effort in saving endangered species and enhancing conservation genomic efforts. | 1509 | no | no good fitness measure, N pop differnt in GD? | NA | no | no |
| 1510 | 2017 | 43019 | Segarra-Moragues, JG; Catalan, P | 2003 | Life history variation between species of the relictual genus Borderea (Dioscoreaceae): phylogeography, genetic diversity, and population genetic structure assessed by RAPD markers | The genus Borderea consists of two species, B. pyrenaica and B. chouardii, taxa which have been previously considered as conspecific due to their overall close morphology. These two sole species of the rare genus of Dioscoreaceae are endemic to the Pyrenees (Spain, France). This mountain range likely operated as a refugium for these plants during the last glaciations. B. chouardii is only known from a single population in the Spanish Prepyrenees and has been classified as at risk of extinction in the Red List of Endangered Species (IUCN); B. pyrenaica shows a narrow distribution range in the central Pyrenees and Prepyrenees. We analysed genetic variation, population structure and differentiation in these two taxa using RAPD markers. Our study was conducted on the same seven populations for which very low levels of genetic differentiation were detected previously through allozyme analysis. By contrast, high levels of genetic variability were detected through the RAPD hypervariable markers. Twelve RAPD primers produced 112 distinct bands in the 397 surveyed individuals, totalling 395 different RAPD phenotypes. Only four bands were monomorphic across all samples of Borderea, whereas 21 of the polymorphic bands were species-specific (20 for B. chouardii, and one for B. pyrenaica). The largest genetic distances were those between the B. chouardii and the B. pyrenaica phenotypes. An analysis of molecular variance showed greater variance between groups (B. chouardii vs. B. pyrenaica, 76.08%) than within groups (3.60%). RAPD band specificity, phenotypic distances, and the partitioning of variance all support the taxonomic separation of the two species. Statistical evaluation of within- and among-population RAPD genetic variability in B. pyrenaica showed that genetic variability was higher within populations (>80%) than among them. No clear pattern of RAPD differentiation could be observed among the six studied populations of this taxon though slight differences in genetic diversity could be observed in the more isolated Prepyrenean populations compared with the more widespread Pyrenean ones. These results suggest a recent post-glacial origin of the present B. pyrenaica populations. (C) 2003 The Linnean Society of London. | 1510 | no | no good fitness measure | NA | no | no |
| 1511 | 2017 | 43019 | Corander, J; Connor, TR; O’Dwyer, CA; Kroll, JS; Hanage, WP | 2012 | Population structure in the Neisseria, and the biological significance of fuzzy species | Phenotypic and genetic variation in bacteria can take bewilderingly complex forms even within a single genus. One of the most intriguing examples of this is the genus Neisseria, which comprises both pathogens and commensals colonizing a variety of body sites and host species, and causing a range of disease. Complex relatedness among both named species and previously identified lineages of Neisseria makes it challenging to study their evolution. Using the largest publicly available collection of bacterial sequence data in combination with a population genetic analysis and experiment, we probe the contribution of inter-species recombination to neisserial population structure, and specifically whether it is more common in some strains than others. We identify hybrid groups of strains containing sequences typical of more than one species. These groups of strains, typical of a fuzzy species, appear to have experienced elevated rates of inter-species recombination estimated by population genetic analysis and further supported by transformation experiments. In particular, strains of the pathogen Neisseria meningitidis in the fuzzy species boundary appear to follow a different lifestyle, which may have considerable biological implications concerning distribution of novel resistance elements and meningococcal vaccine development. Despite the strong evidence for negligible geographical barriers to gene flow within the population, exchange of genetic material still shows directionality among named species in a non-uniform manner. | 1511 | no | no good fitness measure, N pop differnt in GD? | NA | no | no |
| 1512 | 2017 | 43019 | Kapranas, A; Maher, AMD; Griffin, CT | 2016 | Higher relatedness mitigates mortality in a nematode with lethal male fighting | According to kin selection theory, individuals show less aggression towards their relatives. Limited dispersal promotes interactions among relatives but also increases competition among them. The evolution of cooperation in viscous populations has been subject of mainly theoretical exploration. We investigated the influence of relatedness on aggression in males of entomopathogenic nematode Steinernema longicaudum that engage in lethal fighting. In a series of invitro experiments, we found that both competitor male group size and relatedness influence male mortality rates. Higher relatedness led to progressively lower rates of male mortality. In experimentally infected insects, wherein large numbers of males and females interact, the proportion of dead and paralysed (=terminally injured) males was higher when infection was established by infective juveniles originating from a mixture of three lines than in those infected by a single line. The results collectively show that Steinernema longicaudum males recognize their kin and consequently male mortality rates are lower in groups consisting of more related males. Furthermore, this monotonic negative relationship between aggression and relatedness suggests that kin selection benefits are still substantial even under extreme competition. Our experiments also suggest that kin recognition in entomopathogenic nematodes has a genetic basis rather than being strictly based on environmental cues. We discuss our findings within the theoretical context of the evolution of altruistic/cooperative behaviour in structured populations. | 1512 | maybe | maybe | NA | yes | yes |
| 1513 | 2017 | 43019 | Launey, S; Barre, M; Gerard, A; Naciri-Graven, Y | 2001 | Population bottleneck and effective size in Bonamia ostreae-resistant populations of Ostrea edulis as inferred by microsatellite markers | Genetic variability at five microsatellite loci was analysed in three hatchery-propagated populations of the flat oyster, Ostrea edulis. These populations were part of a selection programme for resistance to the protozoan parasite Bonamia ostreae and were produced by mass spawns, without control of the genealogy. Evidence for population bottlenecks and inbreeding was sought. A reduction in the number of alleles, mainly due to the loss of rare alleles, was observed in all selected populations, relative to the natural population from which they were derived. Heterozygote excesses were observed in two populations, and were attributed to substructuring of the population into a small number of families. Pedigree reconstruction showed that these two populations were produced by at most two spawning events involving a limited number of parents. Most individuals within these populations are half or full-sib, as shown by relatedness coefficients. The occurrence of population bottlenecks was supported by estimates of effective number of breeders derived by three methods: temporal variance in allelic frequencies, heterozygote excess, and a new method based on reduction in the number of alleles. The estimates from the different methods were consistent. The evidence for bottleneck and small effective number of breeders are expected to lead to increasing inbreeding, and have important consequences for the future management of the three O. edulis selected populations. | 1513 | no | no good fitness measure, N pop differnt in GD? | NA | no | no |
| 1514 | 2017 | 43019 | Alarcon, M; Vargas, P; Saez, L; Molero, J; Aldasoro, JJ | 2012 | Genetic diversity of mountain plants: Two migration episodes of Mediterranean Erodium (Geraniaceae) | This paper examines the phylogeny of Erodium subsect. Petraea, a group of six morphologically and genetically very similar species from the mountains of the western Mediterranean. Combined trnL-F-ITS analysis was unable to determine the phylogenetic relationships of these species owing to sequence similarity. AFLP fragment analysis showed different populations to cluster in six closely related phylo-groups that partially coincided with morphological species. In the Iberian Peninsula, high temperatures during interstadial periods probably impeded the survival of these species at low altitudes, and their populations may have been forced to migrate northward within Iberia or remain isolated on high mountains. AFLP variation suggests that this might have led to their differentiation into groups and speciation during interglacials, but it probably also provided the basis for recurrent recolonisations and the mixing of neighbouring populations at the last glacial maxima. The genetic diversity of the two Erodium lineages suggests two migration episodes took place from southern Iberia towards the north, with one lineage migrating via western Iberia and the other via eastern Iberia. The patterns of genetic diversity observed in populations of 56 European species (27 genera) leads to the hypothesis that disparate proportions of unique polymorphic fragments are the result of the evolutionary histories of their mountain populations irrespective of the currently recognised species. (C) 2012 Elsevier Inc. All rights reserved. | 1514 | no | no good fitness measure | NA | no | no |
| 1515 | 2017 | 43019 | Murria, C; Rugenski, AT; Whiles, MR; Vogler, AP | 2015 | Long-term isolation and endemicity of Neotropical aquatic insects limit the community responses to recent amphibian decline | AimNeotropical highland streams have shown diminished ecosystem functioning after amphibian extirpation infected by the chytrid fungus Batrachochytrium dendrobatidis. The loss of amphibians could affect communities of aquatic insects co-occurring in these streams in various ways. We examined patterns of species and genetic diversity of these communities and their evolutionary history along the chytrid expansion gradient to elucidate potential community responses. LocationSix streams over a 320-km transect in Panama affected by chytrid expansion from west to east for up to 14years, and two apparently chytrid-free streams in the east. MethodsPatterns of - and -diversity were investigated at three hierarchical levels: genus, species and haplotypes. Genus identification was based on morphology, and putative species were inferred by grouping the DNA barcodes (749 cox1 sequences) with the GMYC method on all collected individuals of Ephemeroptera, Trichoptera, Coleoptera and Plecoptera. ResultsA total of 96 genera in 43 families (9 orders) of insects were encountered. Genus-level -diversity was higher in the easternmost streams, possibly due to a separate biogeographical history, whereas -diversity was constant along the chytrid expansion gradient. Community DNA barcoding resulted in 426 cox1 haplotypes and 154 putative species, most of them limited to single sites. High -diversity along the gradient at both species and haplotype levels argues against community homogenization by migration in the wake of amphibian declines. In contrast, phylo–diversity was low, indicating community similarity at deep levels. Main conclusionsAquatic insect communities in this region are influenced by long-term limited dispersion that generated high endemicity. The pattern persists mostly unperturbed after disease-driven amphibian declines; hence, if indeed insects fill the niches vacated by tadpoles, they would originate from local communities rather than immigration. Given the unique evolutionary history and physical isolation of local assemblages, the ecosystem deterioration carries the risk of losing unique diversity. | 1515 | no | no pop level? | NA | no | no |
| 1516 | 2017 | 43019 | Chancerel, E; Lamy, JB; Lesur, I; Noirot, C; Klopp, C; Ehrenmann, F; Boury, C; Le Provost, G; Label, P; Lalanne, C; Leger, V; Salin, F; Gion, JM; Plomion, C | 2013 | High-density linkage mapping in a pine tree reveals a genomic region associated with inbreeding depression and provides clues to the extent and distribution of meiotic recombination | Background: The availability of a large expressed sequence tags (EST) resource and recent advances in high-throughput genotyping technology have made it possible to develop highly multiplexed SNP arrays for multi-objective genetic applications, including the construction of meiotic maps. Such approaches are particularly useful in species with a large genome size, precluding the use of whole-genome shotgun assembly with current technologies. Results: In this study, a 12 k-SNP genotyping array was developed for maritime pine from an extensive EST resource assembled into a unigene set. The offspring of three-generation outbred and inbred mapping pedigrees were then genotyped. The inbred pedigree consisted of a classical F2 population resulting from the selfing of a single inter-provenance (Landes x Corsica) hybrid tree, whereas the outbred pedigree (G2) resulted from a controlled cross of two intra-provenance (Landes x Landes) hybrid trees. This resulted in the generation of three linkage maps based on SNP markers: one from the parental genotype of the F2 population (1,131 markers in 1,708 centimorgan (cM)), and one for each parent of the G2 population (1,015 and 1,110 markers in 1,447 and 1,425 cM for the female and male parents, respectively). A comparison of segregation patterns in the progeny obtained from the two types of mating (inbreeding and outbreeding) led to the identification of a chromosomal region carrying an embryo viability locus with a semi-lethal allele. Following selfing and segregation, zygote mortality resulted in a deficit of Corsican homozygous genotypes in the F2 population. This dataset was also used to study the extent and distribution of meiotic recombination along the length of the chromosomes and the effect of sex and/or genetic background on recombination. The genetic background of trees in which meiotic recombination occurred was found to have a significant effect on the frequency of recombination. Furthermore, only a small proportion of the recombination hot-and cold-spots were common to all three genotypes, suggesting that the spatial pattern of recombination was genetically variable. Conclusion: This study led to the development of classical genomic tools for this ecologically and economically important species. It also identified a chromosomal region bearing a semi-lethal recessive allele and demonstrated the genetic variability of recombination rate over the genome. | 1516 | no | inbreeding | NA | no | no |
| 1517 | 2017 | 43019 | Guha, S; Rosenfeld, JA; Malhotra, AK; Lee, AT; Gregersen, PK; Kane, JM; Pe’er, I; Darvasi, A; Lencz, T | 2012 | Implications for health and disease in the genetic signature of the Ashkenazi Jewish population | Background: Relatively small, reproductively isolated populations with reduced genetic diversity may have advantages for genomewide association mapping in disease genetics. The Ashkenazi Jewish population represents a unique population for study based on its recent (< 1,000 year) history of a limited number of founders, population bottlenecks and tradition of marriage within the community. We genotyped more than 1,300 Ashkenazi Jewish healthy volunteers from the Hebrew University Genetic Resource with the Illumina HumanOmni1-Quad platform. Comparison of the genotyping data with that of neighboring European and Asian populations enabled the Ashkenazi Jewish-specific component of the variance to be characterized with respect to disease-relevant alleles and pathways. Results: Using clustering, principal components, and pairwise genetic distance as converging approaches, we identified an Ashkenazi Jewish-specific genetic signature that differentiated these subjects from both European and Middle Eastern samples. Most notably, gene ontology analysis of the Ashkenazi Jewish genetic signature revealed an enrichment of genes functioning in transepithelial chloride transport, such as CFTR, and in equilibrioception, potentially shedding light on cystic fibrosis, Usher syndrome and other diseases over-represented in the Ashkenazi Jewish population. Results also impact risk profiles for autoimmune and metabolic disorders in this population. Finally, residual intra-Ashkenazi population structure was minimal, primarily determined by class 1 MHC alleles, and not related to host country of origin. Conclusions: The Ashkenazi Jewish population is of potential utility in disease-mapping studies due to its relative homogeneity and distinct genomic signature. Results suggest that Ashkenazi-associated disease genes may be components of population-specific genomic differences in key functional pathways. | 1517 | no | human | NA | no | no |
| 1518 | 2017 | 43019 | Donati, C; Hiller, NL; Tettelin, H; Muzzi, A; Croucher, NJ; Angiuoli, SV; Oggioni, M; Hotopp, JCD; Hu, FZ; Riley, DR; Covacci, A; Mitchell, TJ; Bentley, SD; Kilian, M; Ehrlich, GD; Rappuoli, R; Moxon, ER; Masignani, V | 2010 | Structure and dynamics of the pan-genome of Streptococcus pneumoniae and closely related species | Background: Streptococcus pneumoniae is one of the most important causes of microbial diseases in humans. The genomes of 44 diverse strains of S. pneumoniae were analyzed and compared with strains of non-pathogenic streptococci of the Mitis group. Results: Despite evidence of extensive recombination, the S. pneumoniae phylogenetic tree revealed six major lineages. With the exception of serotype 1, the tree correlated poorly with capsular serotype, geographical site of isolation and disease outcome. The distribution of dispensable genes - genes present in more than one strain but not in all strains - was consistent with phylogeny, although horizontal gene transfer events attenuated this correlation in the case of ancient lineages. Homologous recombination, involving short stretches of DNA, was the dominant evolutionary process of the core genome of S. pneumoniae. Genetic exchange occurred both within and across the borders of the species, and S. mitis was the main reservoir of genetic diversity of S. pneumoniae. The pan-genome size of S. pneumoniae increased logarithmically with the number of strains and linearly with the number of polymorphic sites of the sampled genomes, suggesting that acquired genes accumulate proportionately to the age of clones. Most genes associated with pathogenicity were shared by all S. pneumoniae strains, but were also present in S. mitis, S. oralis and S. infantis, indicating that these genes are not sufficient to determine virulence. Conclusions: Genetic exchange with related species sharing the same ecological niche is the main mechanism of evolution of S. pneumoniae. The open pan-genome guarantees the species a quick and economical response to diverse environments. | 1518 | no | gd of pathogen | NA | no | no |
| 1519 | 2017 | 43019 | Padhi, A; Moore, AT; Brown, MB; Foster, JE; Pfeffer, M; Brown, CR | 2011 | Isolation by distance explains genetic structure of Buggy Creek virus, a bird-associated arbovirus | Many of the arthropod-borne viruses (arboviruses) show extensive genetic variability and are widely distributed over large geographic areas. Understanding how virus genetic structure varies in space may yield insight into how these pathogens are adapted to and dispersed by different hosts or vectors, the relative importance of mutation, drift, or selection in generating genetic variability, and where and when epidemics or epizootics are most likely to occur. However, because most arboviruses tend to be sampled opportunistically and often cannot be isolated in large numbers at a given locale, surprisingly little is known about their spatial genetic structure on the local scale at which host/vector/virus interactions typically occur. Here, we examine fine-scale spatial structure of two sympatric lineages of Buggy Creek virus (BCRV, Togaviridae), an alphavirus transmitted by the ectoparasitic swallow bug (Oeciacus vicarius) to colonially nesting cliff swallows (Petrochelidon pyrrhonota) and invasive house sparrows (Passer domesticus) in North America. Data from 377 BCRV isolates at cliff swallow colony sites in western Nebraska showed that both virus lineages were geographically structured. Most haplotypes were detected at a single colony or were shared among nearby colonies, and pair-wise genetic distance increased significantly with geographic distance between colony sites. Genetic structure of both lineages is consistent with isolation by distance. Sites with the most genetically distinct BCRV isolates were occupied by large numbers of house sparrows, suggesting that concentrations of invasive sparrows may represent foci for evolutionary change in BCRV. Our results show that bird-associated arboviruses can show genetic substructure over short geographic distances. | 1519 | no | gd of pathogen | NA | no | no |
| 1520 | 2017 | 43019 | Luna-Marin, KP; Angulo-Silva, VM; Hernandez-Torres, J; Ruiz-Garcia, M | 2017 | Genetic Relationships and Spatial Genetic Structure Among Populations of Rhodnius prolixus (Hemiptera: Reduviidae) in Colombia and Venezuela Based on Mitochondrial Cytochrome-b Sequences | One hundred twenty Rhodnius prolixus (Stal) (Hemiptera: Reduviidae) specimens from 6 Colombian Departments and 1 Venezuelan State had 594-bp of the mitochondrial cytochrome-b gene sequenced to improve the understanding of evolutionary processes that shape the main vector of Chagas disease. The levels of genetic diversity for this species were low-medium with reference to other bugs. The genetic heterogeneity among the populations was very limited which means there has been extensive gene flow and/or very recent split processes. The overall sample as well as some individual populations showed evidence of recent population expansions (with the exception of Arauca, which yielded evidence of a bottleneck for a mismatch distribution). This expansion (11,000 or 2000-25,000 year ago depending of two procedures employed) coincides with the ending of the last intense glacial conditions during the Pleistocene and the beginning of the Holocene that had a warmer and wetter climate. Some of our autocorrelation analyses (AIDA and Genetic Landscape Interpolation Analysis) indicated local patches of high genetic similarity but no globally significant spatial structure. We did show an original haplotype distributed throughout the entirety of the geographical area studied. | 1520 | no | no good fitness measure | NA | no | no |
| 1521 | 2017 | 43019 | Mc Ginty, SE; Lehmann, L; Brown, SP; Rankin, DJ | 2013 | The interplay between relatedness and horizontal gene transfer drives the evolution of plasmid-carried public goods | Plasmids carry a wide range of genes that are often involved in bacterial social behaviour. The question of why such genes are frequently mobile has received increasing attention. Here, we use an explicit population genetic approach to model the evolution of plasmid-borne bacterial public goods production. Our findings highlight the importance of both transmission and relatedness as factors driving the evolution of plasmid-borne public goods production. We partition the effects of plasmid transfer of social traits into those of infectivity and the effect of increased relatedness. Our results demonstrate that, owing to its effect on relatedness, plasmid mobility increases the invasion and stability of public goods, in a way not seen in individually beneficial traits. In addition, we show that plasmid transfer increases relatedness when public goods production is rare but this effect declines when production is common, with both scenarios leading to an increase in the frequency of plasmid-borne public goods. Plasmids remain important vectors for the spread of social genes involved in bacterial virulence thus an understanding of their dynamics is highly relevant from a public health perspective. | 1521 | no | NA | NA | no | no |
| 1522 | 2017 | 43019 | PAMILO, P; SUNDSTROM, L; FORTELIUS, W; ROSENGREN, R | 1994 | DIPLOID MALES AND COLONY-LEVEL SELECTION IN FORMICA ANTS | It is suggested that the evolution of polyandry by social hymenopteran queens is caused by colony-level selection, either because polyandry affects the distribution of non-functional diploid males in colonies (the load hypothesis) or because it increases the genetic diversity of the worker force (the diversity hypothesis). Diploid males that arise from fertilized eggs that are homozygous at the sex-determining locus (or loci) are inviable or infertile. Models of the load hypothesis analysed in this study suggest that slow growth and high mortality of colonies with diploid males favour single mating by queens. The longer the period of colony growth (the period with selective differences) and the heavier the mortality, the stronger is the selection for monandry. The load hypothesis also predicts an association between monogyny and monandry. In contrast, the diversity hypothesis predicts an association between monogyny and polyandry, as multiple mating offers a way by which a monogynous colony could increase its genetic heterogeneity. Up to 10% of all males are diploid in species and populations of Formica ants with highly polygynous colonies (F. aquilonia, F. polyctena, F. truncorum). No diploid males were found in two mainly monogynous species (F. exsecta, F. pratensis) which also have a high level of monandry. This agrees with the prediction of the load hypothesis. A surprisingly high frequency of nests in three other species (F. rufa, F. lugubris, F. truncorum) with monogynous/weakly polygynous colonies produce diploid males, although the frequency varies among their populations. In extreme cases half of the diploid sexuals within a colony develop into males. Diploid males have been observed only at the time of normal sexual production. It seems that at other times they are eliminated at early developmental stages, so as to minimize the load on the colony. | 1522 | no | no good fitness measure, N pop differnt in GD? | NA | no | no |
| 1523 | 2017 | 43019 | Fonseca, DM; Campbell, S; Crans, WJ; Mogi, M; Miyagi, I; Toma, T; Bullians, M; Andreadis, TG; Berry, RL; Pagac, B; Sardelis, MR; Wilkerson, RC | 2001 | Aedes (Finlaya) japonicus (Diptera : Culicidae), a newly recognized mosquito in the United States: Analyses of genetic variation in the United States and putative source populations | Introduction of potential disease vectors into a new geographic area poses health risks to local human, livestock, and wildlife populations. It is therefore important to gain understanding of the dynamics of these invasions, in particular its sources, modes of spread after the introduction, and vectorial potential. We studied the population genetics of Aedes (Finlaya) japonicus japonicus (Theobald), an Asian mosquito that was recognized for the first time in the United States in 1998. We examined patterns of genetic diversity using random amplified polymorphic DNA and sequences of ND4 of mtDNA by comparing samples from populations spanning the range of this mosquito in Japan (six samples) and the United States (nine samples) as well as specimens intercepted in New Zealand in 1999. We found geographically differentiated populations in Japan, indicating limited gene now even on small spatial scales. In the United States, we found evidence of significant genetic differentiation between samples from New York, Connecticut, and New Jersey and those from mid-Pennsylvania and Maryland. We were unable to pinpoint the source location(s) in Japan, although some of the U.S, samples are genetically close to samples from south Honshu and western Kyushu. Further studies should include samples from Korean populations. Distinct genetic signatures in U.S. populations undergoing expansion suggest the possibility of local increases in genetic diversity if and where they meet. | 1523 | no | no good fitness measure | NA | no | no |
| 1524 | 2017 | 43019 | Zhou, ZM; McCann, A; Litrup, E; Murphy, R; Cormican, M; Fanning, S; Brown, D; Guttman, DS; Brisse, S; Achtman, M | 2013 | Neutral Genomic Microevolution of a Recently Emerged Pathogen, Salmonella enterica Serovar Agona | Salmonella enterica serovar Agona has caused multiple food-borne outbreaks of gastroenteritis since it was first isolated in 1952. We analyzed the genomes of 73 isolates from global sources, comparing five distinct outbreaks with sporadic infections as well as food contamination and the environment. Agona consists of three lineages with minimal mutational diversity: only 846 single nucleotide polymorphisms (SNPs) have accumulated in the non-repetitive, core genome since Agona evolved in 1932 and subsequently underwent a major population expansion in the 1960s. Homologous recombination with other serovars of S. enterica imported 42 recombinational tracts (360 kb) in 5/143 nodes within the genealogy, which resulted in 3,164 additional SNPs. In contrast to this paucity of genetic diversity, Agona is highly diverse according to pulsed-field gel electrophoresis (PFGE), which is used to assign isolates to outbreaks. PFGE diversity reflects a highly dynamic accessory genome associated with the gain or loss (indels) of 51 bacteriophages, 10 plasmids, and 6 integrative conjugational elements (ICE/IMEs), but did not correlate uniquely with outbreaks. Unlike the core genome, indels occurred repeatedly in independent nodes (homoplasies), resulting in inaccurate PFGE genealogies. The accessory genome contained only few cargo genes relevant to infection, other than antibiotic resistance. Thus, most of the genetic diversity within this recently emerged pathogen reflects changes in the accessory genome, or is due to recombination, but these changes seemed to reflect neutral processes rather than Darwinian selection. Each outbreak was caused by an independent clade, without universal, outbreak-associated genomic features, and none of the variable genes in the pan-genome seemed to be associated with an ability to cause outbreaks. | 1524 | no | NA | NA | no | no |
| 1525 | 2017 | 43019 | Haridas, CV; Rajarshi, MB | 2001 | A stochastic model for evolution of sociality in insects | We study population biology of eusocial insects such as Ropalidia marginata through a stochastic model based on random (matrix) difference equations, This facilitates a study of dynamics of such populations when the survival and other rates vary randomly over time, The worker-brood relatedness, which is a function of the underlying population structure, can be used to explain theories on the evolution of altruism, The effect of demographic parameters and the queen takeover probabilities on the worker-brood relatedness has been studied. Based on the proposed model, we simulate insect colonies where queens are replaced. Simulation results help us to study the effect of various factors on the worker-brood relatedness. Further, we study two estimators of the worker-brood relatedness and suggest procedures for estimating their standard errors, Approximate confidence intervals for the same can be constructed with the help of these results. (C) 2001 Academic Press. | 1525 | no | no good fitness measure | NA | no | no |
| 1526 | 2017 | 43019 | San-Jose, LM; Penalver-Alcazar, M; Mila, B; Gonzalez-Jimena, V; Fitze, PS | 2014 | Cumulative frequency-dependent selective episodes allow for rapid morph cycles and rock-paper-scissors dynamics in species with overlapping generations | Rock-paper-scissors (RPS) dynamics, which maintain genetic polymorphisms over time through negative frequency-dependent (FD) selection, can evolve in short-lived species with no generational overlap, where they produce rapid morph frequency cycles. However, most species have overlapping generations and thus, rapid RPS dynamics are thought to require stronger FD selection, the existence of which yet needs to be proved. Here, we experimentally demonstrate that two cumulative selective episodes, FD sexual selection reinforced by FD selection on offspring survival, generate sufficiently strong selection to generate rapid morph frequency cycles in the European common lizard Zootoca vivipara, a multi-annual species with major generational overlap. These findings show that the conditions required for the evolution of RPS games are fulfilled by almost all species exhibiting genetic polymorphisms and suggest that RPS games may be responsible for the maintenance of genetic diversity in a wide range of species. | 1526 | no | No GD diff between pop | NA | no | no |
| 1527 | 2017 | 43019 | He, TH; Krauss, SL; Lamont, BB; Miller, BP; Enright, NJ | 2004 | Long-distance seed dispersal in a metapopulation of Banksia hookeriana inferred from a population allocation analysis of amplified fragment length polymorphism data | There is currently a poor understanding of the nature and extent of long-distance seed dispersal, largely due to the inherent difficulty of detection. New statistical approaches and molecular markers offer the potential to accurately address this issue. A log-likelihood population allocation test (AFLPOP) was applied to a plant metapopulation to characterize interpopulation seed dispersal. Banksia hookeriana is a fire-killed shrub, restricted to sandy dune crests in fire-prone shrublands of the Eneabba sandplain, southwest Australia. Population genetic variation was assessed for 221 individuals sampled from 21 adjacent dune-crest populations of B. hookeriana using amplified fragment length polymorphism. Genetic diversity was high, with 175 of 183 (96%) amplified fragment length polymorphism markers polymorphic. Of the total genetic diversity, 8% was partitioned among populations by AMOVA and F-ST. There was no relationship between genetic diversity within populations and population demographic parameters such as population size and sample size. A population allocation test on these data unambiguously assigned 177 of 221 (80.1%) individuals to a single population. Of these, 171 (77.4% of total) were assigned to the population from which they were sampled and 6 (2.7% of total) were assigned to a known population other than the one from which they were sampled. A further 9 (4.1% of total) were assigned to outside the sampled metapopulation area, and 35 individuals (15.8%) could not be assigned unambiguously to any particular population. These results suggest that both the extent [15 of 221 (6.8%) individuals originating from a population other than the one in which they occur] and distance (1.6 to > 2.5 km), of seed dispersal between dune-crest populations is greater than expected from previous studies. The extent of long-distance interpopulation seed dispersal observed provides a basis for explaining the survival of populations of the fire-killed B. hookeriana in a landscape experiencing frequent fire, where local extinctions and recolonizations may be a regular occurrence. | 1527 | no | no good fitness measure | NA | no | no |
| 1528 | 2017 | 43019 | Hansen, PL; Holmstrup, M; Bayley, M; Simonsen, V | 2006 | Low genetic variation for Dendrobaena octaedra from Greenland compared to populations from Europe and North America: Refuge or selection? | The genetic relationship of 345 specimens of the parthenogenetic lumbricid Dendrobaena octaedra from Greenland, Canada and Europe were analysed by means of isozymes. The results showed that populations from Greenland were markedly different from Canadian and European populations, suggesting that dispersal between Greenland and the continents has been much more restricted in the past than dispersal between North America and Europe. This observation supports to the notion that Greenland populations have persisted for a long period and perhaps have survived the Last glacial period in ice-free refugia. A highly significant positive correlation was seen between diversity measured either as mean haptoid diversity or clonal. diversity and mean temperature of the annual coldest month. These results indicate that temperature might cause selection in colder climates or that sexual processes in D. dendroboeno could have been active recently on an evolutionary time scale. (c) 2006 Elsevier GmbH. All rights reserved. | 1528 | no | no good fitness measure, N pop differnt in GD? | NA | no | no |
| 1529 | 2017 | 43019 | Zander, M; Patel, DA; Van de Wouw, A; Lai, KT; Lorenc, MT; Campbell, E; Hayward, A; Edwards, D; Raman, H; Batley, J | 2013 | Identifying genetic diversity of avirulence genes in Leptosphaeria maculans using whole genome sequencing | Next generation sequencing technology allows rapid re-sequencing of individuals, as well as the discovery of single nucleotide polymorphisms (SNPs), for genomic diversity and evolutionary analyses. By sequencing two isolates of the fungal plant pathogen Leptosphaeria maculans, the causal agent of blackleg disease in Brassica crops, we have generated a resource of over 76 million sequence reads aligned to the reference genome. We identified over 21,000 SNPs with an overall SNP frequency of one SNP every 2,065 bp. Sequence validation of a selection of these SNPs in additional isolates collected throughout Australia indicates a high degree of polymorphism in the Australian population. In preliminary phylogenetic analysis, isolates from Western Australia clustered together and those collected from Brassica juncea stubble were identical. These SNPs provide a novel marker resource to study the genetic diversity of this pathogen. We demonstrate that re-sequencing provides a method of validating previously characterised SNPs and analysing differences in important genes, such as the disease related avirulence genes of L. maculans. Understanding the genetic characteristics of this devastating pathogen is vital in developing long-term solutions to managing blackleg disease in Brassica crops. | 1529 | no | gd of pathogen | NA | no | no |
| 1530 | 2017 | 43019 | Gosselin, L; Jobidon, R; Bernier, L | 1999 | Genetic variability and structure of Canadian populations of Chondrostereum purpureum, a potential biophytocide | Genetic diversity was studied in four Canadian ecological populations, each corresponding to a Canadian ecozone, of Chondrostereum purpureum, including 93 isolates of various host origin. Pseudo-allelic frequencies were estimated at each of 22 putative RAPD loci by scoring for presence or absence of amplicons in haploid mycelial cultures. The analysis of the hierarchical population structure did not reveal any trend with regard to ecological or host origin. Total gene diversity (H(T)dagger = 0.288) was mostly attributable to diversity within populations (H(S)dagger = 0.269). In addition, the AMOVA analysis detected most of the molecular variability within subpopulations (89.3%; P < 0.001), whereas a significant (7.3%; P = 0.001) proportion of the gene diversity was found among subpopulations, within ecozones. The results indicate that C. purpureum is a highly heterogeneous pathogen with a continuously distributed population across Canada (G(ST)dagger = 0.048), and underscore the importance of considering the population structure in the process of its registration as a microbial control agent. A genotype isolated in either central or eastern Canada (ecozones 2, 4 and 5 populations) and selected for its potential as a biophytocide should be considered indigenous in any of these regions of intended use. | 1530 | no | gd of pathogen | NA | no | no |
| 1531 | 2017 | 43019 | Aguirre-Obando, OA; Bona, ACD; Duque, IE; Navarro-Silva, MA | 2015 | Insecticide resistance and genetic variability in natural populations of Aedes (Stegomyia) aegypti (Diptera: Culicidae) from Colombia | Mosquito control prevails as the most efficient method to protect humans from the dengue virus, despite recent efforts to find a vaccine for this disease. We evaluated insecticide resistance and genetic variability in natural populations of Aedes aegypti (Linnaeus, 1762) from Colombia. This is the first Colombian study examining kdr mutations and population structure. Bioassays with larvae of three mosquito populations (Armenia, Calarca and Montenegro) were performed according to the World Health Organization (WHO) guidelines, using Temephos. For the analysis of the Val1016lle mutation and genetic diversity, we sampled recently-emerged adults from four mosquito populations (Armenia, Calarca, Montenegro and Barcelona). Following the WHO protocol, bioassays implemented with larvae showed resistance to Temephos in mosquito populations from Armenia (77% +/- 2) and Calarca (62% +/- 14), and an incipient altered susceptibility at Montenegro (88% +/- 8). The RR95 of mosquito populations ranged from 3.7 (Montenegro) to 6.0 (Calarca). The Val1016lle mutation analysis of 107 genotyped samples indicates that 94% of the specimens were homozygous for the wild allele (1016Val) and 6% were heterozygous (Val1016lle). The 1016lle allele was not found in Barcelona. Genetic variability analysis found three mitochondrial lineages with low genetic diversity and gene flow. In comparison with haplotypes from the American continent, those from this study suggest connections with Mexican and North American populations. These results confirm that a continuous monitoring and managing program of A. aegypti resistance in the state of Quindio is required. | 1531 | no | no good fitness measure | NA | no | no |
| 1532 | 2017 | 43019 | Landi, M; Queller, DC; Turillazzi, S; Strassmann, JE | 2003 | Low relatedness and frequent queen turnover in the stenogastrine wasp Eustenogaster fraterna favor the life insurance over the haplodiploid hypothesis for the origin of eusociality | The origin of sociality is best studied in taxa with rudimentary social development, like the stenogastrine wasp, Eustenogaster fraterna. Our study of colony structure and relatedness in this species found very small colonies averaging only 2.7 adult females, including a single mated, reproductive female. Microsatellite genotyping showed that adult females were related to each other but not generally as full sisters (r = 0.427 +/- 0.131, 95 % confidence interval). Microsatellite genotyping showed that sociality is not favored by the haplodiploid hypothesis, because relatedness of unmated females to female brood is low (r = 0.210 0.171, 95 % confidence interval), far lower than that among sisters in either haplodiploid or diploid species. Relatedness of unmated females to female brood is significantly lower than that of mated females to female brood (r = 0.374 0.266). Mated females are also significantly more related to the male brood (r = 0.871 +/- 0.168) than are unmated females (r = 0.588 +/- 0.339), suggesting that unmated helpers do not generally produce sons. These results argue against an important role for exceptionally high relatedness in the origin of eusociality. One quarter of the brood could not have been the progeny of any collected female, suggesting high rates of queen turnover. In all, 7/17 nests had some brood that could not be assigned to existing adult females. These high adult mortality rates result in direct advantages to helpers in the form of colony inheritance, and indirect advantages via life insurance benefits. | 1532 | probably no | no good fitness measure | no fitness measure pop | no | no |
| 1533 | 2017 | 43019 | Lipowsky, A; Schmid, B; Roscher, C | 2011 | Selection for monoculture and mixture genotypes in a biodiversity experiment | “Studies in experimental grasslands have shown variation in plant individual performance in response to neighbourhood diversity. To which extent these responses are due to phenotypic plasticity or genetic variation is largely unknown. We collected seed families of five herbaceous species (Cirsiwn oleraceum, Crepis biennis, Plantago lanceolata, Plantago media and Rumex acetosa) in monocultures and 60-species mixtures five years after establishment and replanted or transplanted the offspring into the same monocultures and 60-species mixtures. In all five species the actual environment significantly affected plant survival, growth and performance in terms of shoot biomass and investment into reproduction, indicating stronger competition for light and different levels of herbivory in mixtures as compared with monocultures. Effects of the original environment were smaller and less consistent, but indicated differential selection in monocultures vs. mixtures. The interaction between actual and original environment, corresponding to the”“home”" vs. "“away”" comparison, was rarely significant, yet this was providing a first sign of local adaptation. We conclude that, for the investigated plant species, more than five growing seasons in monocultures or mixtures would be needed to better demonstrate the selection of genotypes specifically adapted to monocultures or mixtures. A faster local adaptation may have been prevented by the ability of these species to respond to variation in neighbourhood diversity to a large degree via phenotypic plasticity and other factors." | 1533 | no | multiple species diversity | NA | no | no |
| 1534 | 2017 | 43019 | Khiari, S; Boussaid, M; Messaoud, C | 2015 | Genetic diversity and population structure in natural populations of Tunisian Azarole (Crataegus azarolus L. var. aronia L.) assessed by microsatellite markers | Azarole tree (Crataegus azarolus var. aronia) is a medicinal plant largely reported to possess a wide range of pharmacological properties. In Tunisia, the species grows wild in different bioclimates and geographic areas. It occurs in small scattered populations in continuously distributed habitats. In order to elaborate efficient conservation strategies of the species, nine SSR loci were used to estimate the genetic diversity and population structure of nine populations growing wild in different bioclimatic zones extending from the upper semiarid to the lower humid. A relatively high level of genetic diversity within population was detected (Na = 2.889, Ne = 2.137, P = 65.43% and He = 0.386). The level of genetic diversity varied according to bioclimate. A low rate of heterozygotes was observed for all populations. The deficit of heterozygotes was more important for populations belonging to the upper semi-arid bioclimate. A significant differentiation among all populations (F-ST = 0.255; Phi(ST) = 0.297), and among populations within bioclimatic stages (Phi(SC) = 0.288) were revealed. The Mantel test showed a lack of significant correlation between F-ST values and geographical distances among populations. The UPGMA and the Bayesian analyses did not show a relationship between population clustering and their eco-geographic appurtenances. Considering these results as well as the current situation of all populations and their endangered habitats, the conservation of this medicinal species is required. (C) 2015 Elsevier Ltd. All rights reserved. | 1534 | no | no good fitness measure | NA | no | no |
| 1535 | 2017 | 43019 | Sepil, I; Radersma, R; Santure, AW; De Cauwer, I; Slate, J; Sheldon, BC | 2015 | No evidence for MHC class I-based disassortative mating in a wild population of great tits | Genes of the major histocompatibility complex (MHC) are regarded as a potentially important target of mate choice due to the fitness benefits that may be conferred to the offspring. According to the complementary genes hypothesis, females mate with MHC dissimilar males to enhance the immunocompetence of their offspring or to avoid inbreeding depression. Here, we investigate whether selection favours a preference for maximally dissimilar or optimally dissimilar MHC class I types, based on MHC genotypes, average amino acid distances and the functional properties of the antigen-binding sites (MHC supertypes); and whether MHC type dissimilarity predicts relatedness between mates in a wild great tit population. In particular, we explore the role that MHC class I plays in female mate choice decisions while controlling for relatedness and spatial population structure, and examine the reproductive fitness consequences of MHC compatibility between mates. We find no evidence for the hypotheses that females select mates on the basis of either maximal or optimal MHC class I dissimilarity. A weak correlation between MHC supertype sharing and relatedness suggests that MHC dissimilarity at functional variants may not provide an effective index of relatedness. Moreover, the reproductive success of pairs did not vary with MHC dissimilarity. Our results provide no support for the suggestion that selection favours, or that mate choice realizes, a preference for complimentary MHC types. | 1535 | no | no pop level? | NA | no | no |
| 1536 | 2017 | 43019 | Polimanti, R; Carboni, C; Baesso, I; Piacentini, S; Iorio, A; De Stefano, GF; Fuciarelli, M | 2013 | Genetic variability of glutathione S-transferase enzymes in human populations: Functional inter-ethnic differences in detoxification systems | Glutathione S-Transferase enzymes (GSTs) constitute the principal Phase II superfamily which plays a key role in cellular detoxification and in other biological processes. Studies of GSTs have revealed that genetic polymorphisms are present in these enzymes and that some of these are Loss-of-Function (LoF) variants, which affect enzymatic functions and are related to different aspects of human health. The aim of this study was to analyze functional genetic differences in GST enzymes among human populations. Attention was focused on LoF polymorphisms of GSTA1, GSTM1, GSTO1, GSTO2, GSTP1 and GSTT1 genes. These LoF variants were analyzed in 668 individuals belonging to six human groups with different ethnic backgrounds: Amhara and Oromo from Ethiopia; Colorado and Cayapa Amerindians and African Ecuadorians from Ecuador; and one sample from central Italy. The HapMap database was used to compare our data with reference populations and to analyze the haplotype and Linkage Disequilibrium diversity in different ethnic groups. Our results highlighted that ethnicity strongly affects the genetic variability of GST enzymes. In particular, GST haplotypes/variants with functional impact showed significant differences in human populations, according to their ethnic background. These data underline that human populations have different structures in detoxification genes, suggesting that these ethnic differences influence disease risk or response to drugs and therefore have implications for genetic association studies involving GST enzymes. In conclusion, our investigation provides data about the distribution of important LoF variants in GST genes in human populations. This information may be useful for designing and interpreting genetic association studies. (C) 2012 Elsevier B.V. All rights reserved. | 1536 | no | human | NA | no | no |
| 1537 | 2017 | 43019 | Gladieux, P; Zhang, XG; Afoufa-Bastien, D; Sanhueza, RMV; Sbaghi, M; Le Cam, B | 2008 | On the Origin and Spread of the Scab Disease of Apple: Out of Central Asia | Venturia inaequalis is an ascomycete fungus responsible for apple scab, a disease that has invaded almost all apple growing regions worldwide, with the corresponding adverse effects on apple production. Monitoring and predicting the effectiveness of intervention strategies require knowledge of the origin, introduction pathways, and population biology of pathogen populations. Analysis of the variation of genetic markers using the inferential framework of population genetics offers the potential to retrieve this information. Methodology/Principal Findings. Here, we present a population genetic analysis of microsatellite variation in 1,273 strains of V. inaequalis representing 28 orchard samples from seven regions in five continents. Analysis of molecular variance revealed that most of the variation (88%) was distributed within localities, which is consistent with extensive historical migrations of the fungus among and within regions. Despite this shallow population structure, clustering analyses partitioned the data set into separate groups corresponding roughly to geography, indicating that each region hosts a distinct population of the fungus. Comparison of the levels of variability among populations, along with coalescent analyses of migration models and estimates of genetic distances, was consistent with a scenario in which the fungus emerged in Central Asia, where apple was domesticated, before its introduction into Europe and, more recently, into other continents with the expansion of apple growing. Across the novel range, levels of variability pointed to multiple introductions and all populations displayed signatures of significant post-introduction increases in population size. Most populations exhibited high genotypic diversity and random association of alleles across loci, indicating recombination both in native and introduced areas. Conclusions/Significance. Venturia inaequalis is a model of invasive phytopathogenic fungus that has now reached the ultimate stage of the invasion process with a broad geographic distribution and well-established populations displaying high genetic variability, regular sexual reproduction, and demographic expansion. | 1537 | no | gd of pathogen | NA | no | no |
| 1538 | 2017 | 43019 | Magle, SB; Samuel, MD; Van Deelen, TR; Robinson, SJ; Mathews, NE | 2013 | Evaluating Spatial Overlap and Relatedness of White-tailed Deer in a Chronic Wasting Disease Management Zone | Wildlife disease transmission, at a local scale, can occur from interactions between infected and susceptible conspecifics or from a contaminated environment. Thus, the degree of spatial overlap and rate of contact among deer is likely to impact both direct and indirect transmission of infectious diseases such chronic wasting disease (CWD) or bovine tuberculosis. We identified a strong relationship between degree of spatial overlap (volume of intersection) and genetic relatedness for female white-tailed deer in Wisconsin’s area of highest CWD prevalence. We used volume of intersection as a surrogate for contact rates between deer and concluded that related deer are more likely to have contact, which may drive disease transmission dynamics. In addition, we found that age of deer influences overlap, with fawns exhibiting the highest degree of overlap with other deer. Our results further support the finding that female social groups have higher contact among related deer which can result in transmission of infectious diseases. We suggest that control of large social groups comprised of closely related deer may be an effective strategy in slowing the transmission of infectious pathogens, and CWD in particular. | 1538 | no | no good fitness measure, N pop differnt in GD? | NA | no | no |
| 1539 | 2017 | 43019 | Kloek, AT; van Setten, J; van der Ende, A; Bots, ML; Asselbergs, FW; Seron, MV; Brouwer, MC; van de Beek, D; Ferwerda, B | 2016 | Exome Array Analysis of Susceptibility to Pneumococcal Meningitis | Host genetic variability may contribute to susceptibility of bacterial meningitis, but which genes contribute to the susceptibility to this complex disease remains undefined. We performed a genetic association study in 469 community-acquired pneumococcal meningitis cases and 2072 population-based controls from the Utrecht Health Project in order to find genetic variants associated with pneumococcal meningitis susceptibility. A HumanExome BeadChip was used to genotype 102,097 SNPs in the collected DNA samples. Associations were tested with the Fisher exact test. None of the genetic variants tested reached Bonferroni corrected significance (p-value < 5 x 10(-7)). Our strongest signals associated with susceptibility to pneumococcal meningitis were rs139064549 on chromosome 1 in the COL11A1 gene (p = 1.51 x 10(-6); G allele OR 3.21 [95% CI 2.05-5.02]) and rs9309464 in the EXOC6B gene on chromosome 2 (p = 6.01 x 10(-5); G allele OR 0.66 [95% CI 0.54-0.81]). The sequence kernel association test (SKAT) tests for associations between multiple variants in a gene region and pneumococcal meningitis susceptibility yielded one significant associated gene namely COL11A1 (p = 1.03 x 10(-7)). Replication studies are needed to validate these results. If replicated, the functionality of these genetic variations should be further studied to identify by which means they influence the pathophysiology of pneumococcal meningitis. | 1539 | no | human | NA | no | no |
| 1540 | 2017 | 43019 | Cammen, K; Hoffman, JI; Knapp, LA; Harwood, J; Amos, W | 2011 | Geographic variation of the major histocompatibility complex in Eastern Atlantic grey seals (Halichoerus grypus) | Pathogen-driven balancing selection maintains high genetic diversity in many vertebrates, particularly in the major histocompatibility complex (MHC) immune system gene family, which is often associated with disease susceptibility. In large natural populations where subpopulations face different pathogen pressures, the MHC should show greater genetic differentiation within a species than neutral markers. We examined genetic diversity at the MHC-DQB locus and nine putatively neutral microsatellite markers in grey seals (Halichoerus grypus) from eight United Kingdom (UK) colonies, the Faeroe Islands and Sable Island, Canada. Five DQB alleles were identified in grey seals, which varied in prevalence across the grey seal range. Among the seal colonies, significant differences in DQB allele and haplotype frequencies and in average DQB heterozygosity were observed. Additionally, the DQB gene exhibited greater differentiation among colonies compared with neutral markers, yet a weaker pattern of isolation by distance (IBD). After correcting for the underlying IBD pattern, subpopulations breeding in similar habitats were more similar to one another in DQB allele frequencies than populations breeding in different habitats, but the same did not hold true for microsatellites, suggesting that habitat-specific pathogen pressure influences MHC evolution. Overall, the data are consistent with selection at MHC-DQB loci in grey seals with both varying selective pressures and geographic population structure appearing to influence the DQB genetic composition of breeding colonies. | 1540 | no | no good fitness measure | NA | no | no |
| 1541 | 2017 | 43019 | Barnes, I; Wingfield, MJ; Carbone, I; Kirisits, T; Wingfield, BD | 2014 | Population structure and diversity of an invasive pine needle pathogen reflects anthropogenic activity | Dothistroma septosporum is a haploid fungal pathogen that causes a serious needle blight disease of pines, particularly as an invasive alien species on Pinus radiata in the Southern Hemisphere. During the course of the last two decades, the pathogen has also incited unexpected epidemics on native and non-native pine hosts in the Northern Hemisphere. Although the biology and ecology of the pathogen has been well documented, there is a distinct lack of knowledge regarding its movement or genetic diversity in many of the countries where it is found. In this study we determined the global population diversity and structure of 458 isolates of D.septosporum from 14 countries on six continents using microsatellite markers. Populations of the pathogen in the Northern Hemisphere, where pines are native, displayed high genetic diversities and included both mating types. Most of the populations from Europe showed evidence for random mating, little population differentiation and gene flow between countries. Populations in North America (USA) and Asia (Bhutan) were genetically distinct but migration between these continents and Europe was evident. In the Southern Hemisphere, the population structure and diversity of D.septosporum reflected the anthropogenic history of the introduction and establishment of plantation forestry, particularly with Pinus radiata. Three introductory lineages in the Southern Hemisphere were observed. Countries in Africa, that have had the longest history of pine introductions, displayed the greatest diversity in the pathogen population, indicating multiple introductions. More recent introductions have occurred separately in South America and Australasia where the pathogen population is currently reproducing clonally due to the presence of only one mating type. | 1541 | no | gd of pathogen | NA | no | no |
| 1542 | 2017 | 43019 | Zheng, WM; Liu, F; Kang, ZS; Chen, SY; Li, ZQ; Wu, LR | 2001 | AFLP fingerprinting of Chinese epidemic strains of Puccinia striiformis f. sp tritici | Amplified fragment length polymorphism (AFLP) was used to fingerprint the epidemic strains CY25, CY27, CY28, CY29, CY30, CY31, Hy3, Hy7, Sy13 and a mutant strain WV-4 of P. striiformis f. sp. tritici, the pathogen of wheat stripe rust. The results showed that (i) genetic diversity existed in the pathogen populations, and based on it a dendrogram of these strains was constructed by unweighted pair-group mean average to demonstrate the relationships of the tested strains; (ii) no significant correlation between virulence of the pathogens and the polymorphism of DNA fingerprints was found; (iii) AFLP fingerprints showed higher polymorphism than that of the virulence variation; (iv) several new pathotypes identified might evolve independently of the reference strains identified before. | 1542 | no | gd of pathogen | NA | no | no |
| 1543 | 2017 | 43019 | Sun, JF; Huang, Y; Huang, HQ; Liang, P; Wang, XY; Mao, Q; Men, JT; Chen, WJ; Deng, CH; Zhou, CH; Lv, XL; Zhou, JJ; Zhang, F; Li, R; Tian, YL; Lei, HL; Liang, C; Hu, XC; Xu, J; Li, XR; XinbingYu | 2013 | Low Divergence of Clonorchis sinensis in China Based on Multilocus Analysis | Clonorchis sinensis, an ancient parasite that infects a number of piscivorous mammals, attracts significant public health interest due to zoonotic exposure risks in Asia. The available studies are insufficient to reflect the prevalence, geographic distribution, and intraspecific genetic diversity of C. sinensis in endemic areas. Here, a multilocus analysis based on eight genes (ITS1, act, tub, ef-1 alpha, cox1, cox3, nad4 and nad5 [4.986 kb]) was employed to explore the intra-species genetic construction of C. sinensis in China. Two hundred and fifty-six C. sinensis isolates were obtained from environmental reservoirs from 17 provinces of China. A total of 254 recognized Multilocus Types (MSTs) showed high diversity among these isolates using multilocus analysis. The comparison analysis of nuclear and mitochondrial phylogeny supports separate clusters in a nuclear dendrogram. Genetic differentiation analysis of three clusters (A, B, and C) showed low divergence within populations. Most isolates from clusters B and C are geographically limited to central China, while cluster A is extraordinarily genetically diverse. Further genetic analyses between different geographic distributions, water bodies and hosts support the low population divergence. The latter haplotype analyses were consistent with the phylogenetic and genetic differentiation results. A recombination network based on concatenated sequences showed a concentrated linkage recombination population in cox1, cox3, nad4 and nad5, with spatial structuring in ITS1. Coupled with the history record and archaeological evidence of C. sinensis infection in mummified desiccated feces, these data point to an ancient origin of C. sinensis in China. In conclusion, we present a likely phylogenetic structure of the C. sinensis population in mainland China, highlighting its possible tendency for biogeographic expansion. Meanwhile, ITS1 was found to be an effective marker for tracking C. sinensis infection worldwide. Thus, the present study improves our understanding of the global epidemiology and evolution of C. sinensis. | 1543 | no | gd of parasite | NA | no | no |
| 1544 | 2017 | 43019 | Silva, GF; Santos, VS; Sousa, NR; Hanada, RE; Gasparotto, L | 2016 | Virulence and genetic diversity among isolates of Mycosphaerella fijiensis in two regions of Brazil | Black sigatoka, caused by the fungus Mycosphaerella fijiensis (anamorphic stage: Paracercospora fijiensis), was first detected in Brazil in early 1998 in the Benjamin Constant and Tabatinga municipalities in the State of Amazonas, near to where the borders of Brazil, Colombia, and Peru converge. Understanding how cultivars react to the pathogen, and characterizing the genetic variability of isolates from two distant and distinct banana-producing regions, are important for determining the virulence of M. fijiensis. In the present study, the genetic diversity of 22 M. fijiensis isolates was assessed using simple sequence repeats (SSR) markers, and their virulence was determined following inoculation on three different banana tree cultivars. All 22 isolates caused symptoms of the disease in the Maca and Prata Comum cultivars 45 days after inoculation, and at least two virulence groups were identified for the Maca and Prata Comum cultivars. For the D’Angola cultivars, two virulence groups were observed only after 60 days post-inoculation, and three of the isolates were not virulent. Using SSR markers, the isolates from two different regions of Brazil were placed into two genetic groups, both genetically distant from the Mf 138 isolate collected in Leticia, Colombia. There was no evidence of correlation between the virulence groups and the genetic diversity groups. These results demonstrate variability in virulence between isolates as measured by the severity of black sigatoka in the analyzed cultivars. | 1544 | no | gd of pathogen | NA | no | no |
| 1545 | 2017 | 43019 | Souza, ASMDE; Del Lama, SN; Mino, CI | 2013 | Conspecific Brood Parasitism in the White-faced Ibis Plegadis chihi (Aves: Pelecaniformes) Revealed by Microsatellites’ Based Kinship-Reconstruction | The white-faced ibis Plegadis chihi Vieillot, 1817 (Pelecaniformes: Threskiornithidae) is a socially monogamous colonially breeding bird in which behavioral and ecological observations suggest the occurrence of conspecific brood parasitism (CBP). We inferred aspects of the genetic mating system of P. chihi in nature, using a genetic approach in the absence of parental information. We used five heterologous microsatellite loci and a multiple-step methodological approach to infer kinship patterns among 104 pairs of nestlings sampled inside 80 nests in a breeding colony from southern Brazil. The estimated effective population size was 69 white-faced ibises (95% CI: 5098), enough to ensure long-term population survival. Kinship patterns were identified for 38% of the analyzed pairs: 60% of the diagnosed pairs were identified as full-siblings, 2.5% as half-siblings and 37.5% as unrelated individuals. CBP could explain the presence of unrelated nestlings within broods, in agreement with available non-genetic evidence. The presence of half-siblings within broods could indicate extra-pair paternity. Results suggest that a non-strictly monogamous genetic mating system may be present in the white-faced ibis. This study is the first molecular approach to better characterize the reproductive behavior of P. chihi in the wild. Our findings set the stage for further research to investigate the possible causes and consequences of alternative reproductive strategies in this species. J. Exp. Zool. 319A:277284, 2013. (c) 2013 (c) 2013 Wiley Periodicals, Inc. | 1545 | no | brood parasitism | NA | no | no |
| 1546 | 2017 | 43019 | Bell, KC; Calhoun, KL; Hoberg, EP; Demboski, JR; Cook, JA | 2016 | Temporal and spatial mosaics: deep host association and shallow geographic drivers shape genetic structure in a widespread pinworm, Rauschtineria eutamii (Nematoda: Oxyuridae) | Climate and host demographic cycling often shape both parasite genetic diversity and host distributions, processes that transcend a history of strict host-parasite association. We explored host associations and histories based on an evaluation of mitochondrial and nuclear sequences to reveal the underlying history and genetic structure of a pinworm, Rauschtineria eutamii, infecting ten species of western North American chipmunks (Rodentia: Tamias, subgenus Neotamias). Rauschtineria eutamii contains divergent lineages influenced by the diversity of hosts and variation across the complex topography of western North America. We recovered six reciprocally monophyletic R. eutamii mitochondrial clades, largely supported by a multilocus concordance tree, exhibiting divergence levels comparable with intraspecific variation reported for other nematodes. Phylogenetic relationships among pinworm clades suggest that R. eutamii colonized an ancestral lineage of western chipmunks and lineages persisted during historical isolation in diverging Neotamias species or species groups. Pinworm diversification, however, is incongruent and asynchronous relative to host diversification. Secondarily, patterns of shallow divergence were shaped by geography through events of episodic colonization reflecting an interaction of taxon pulses and ecological fitting among assemblages in recurrent sympatry. Pinworms occasionally infect geographically proximal host species; however, host switching may be unstable or ephemeral, as there is no signal of host switching in the deeper history of R. eutamii. (c) 2016 The Linnean Society of London, Biological Journal of the Linnean Society, 2016, 119, 397-413. | 1546 | no | no good fitness measure, N pop differnt in GD? | NA | no | no |
| 1547 | 2017 | 43019 | Hudson, MA; Young, RP; Jackson, JD; Orozco-terWengel, P; Martin, L; James, A; Sulton, M; Garcia, G; Griffiths, RA; Thomas, R; Magin, C; Bruford, MW; Cunningham, AA | 2016 | Dynamics and genetics of a disease-driven species decline to near extinction: lessons for conservation | Amphibian chytridiomycosis has caused precipitous declines in hundreds of species worldwide. By tracking mountain chicken (Leptodactylus fallax) populations before, during and after the emergence of chytridiomycosis, we quantified the real-time species level impacts of this disease. We report a range-wide species decline amongst the fastest ever recorded, with a loss of over 85% of the population in fewer than 18 months on Dominica and near extinction on Montserrat. Genetic diversity declined in the wild, but emergency measures to establish a captive assurance population captured a representative sample of genetic diversity from Montserrat. If the Convention on Biological Diversity’s targets are to be met, it is important to evaluate the reasons why they appear consistently unattainable. The emergence of chytridiomycosis in the mountain chicken was predictable, but the decline could not be prevented. There is an urgent need to build mitigation capacity where amphibians are at risk from chytridiomycosis. | 1547 | no | no good fitness measure, N pop differnt in GD? | NA | no | no |
| 1548 | 2017 | 43019 | Ariff, A; Wise, MJ; Kahler, CM; Tay, CY; Peters, F; Perkins, TT; Chang, BJ | 2015 | Novel Moraxella catarrhalis prophages display hyperconserved non-structural genes despite their genomic diversity | Background: Moraxella catarrhalis is an important pathogen that often causes otitis media in children, a disease that is not currently vaccine preventable. Asymptomatic colonisation of the human upper respiratory tract is common and lack of clearance by the immune system is likely due to the emergence of seroresistant genetic lineages. No active bacteriophages or prophages have been described in this species. This study was undertaken to identify and categorise prophages in M. catarrhalis, their genetic diversity and the relationship of such diversity with the host-species phylogeny. Results: This study presents a comparative analysis of 32 putative prophages identified in 95 phylogenetically variable, newly sequenced M. catarrhalis genomes. The prophages were genotypically classified into four diverse clades. The genetic synteny of each clade is similar to the group 1 phage family Siphoviridae, however, they form genotypic clusters that are distinct from other members of this family. No core genetic sequences exist across the 32 prophages despite clades 2, 3, and 4 sharing the most sequence identity. The analysis of non-structural prophage genes (coding the integrase, and terminase), and portal gene showed that the respective genes were identical for clades 2, 3, and 4, but unique for clade 1. Empirical analysis calculated that these genes are unexpectedly hyperconserved, under purifying selection, suggesting a tightly regulated functional role. As such, it is improbable that the prophages are decaying remnants but stable components of a fluctuating, flexible and unpredictable system ultimately maintained by functional constraints on non-structural and packaging genes. Additionally, the plate encoding genes were well conserved across all four prophage clades, and the tail fibre genes, commonly responsible for receptor recognition, were clustered into three major groups distributed across the prophage clades. A pan-genome of 283,622 bp was identified, and the prophages were mapped onto the diverse M. catarrhalis multi-locus sequence type (MLST) backbone. Conclusion: This study has provided the first evidence of putatively mobile prophages in M. catarrhalis, identifying a diverse and fluctuating system dependent on the hyperconservation of a few key, non-structural genes. Some prophages harbour virulence-related genes, and potentially influence the physiology and virulence of M. catarrhalis. Importantly our data will provide supporting information on the identification of novel prophages in other species by adding greater weight to the identification of non-structural genes. | 1548 | no | gd of pathogen | NA | no | no |
| 1549 | 2017 | 43019 | Ray, JW; King, RB; Duvall, MR; Robinson, JW; Jaeger, CP; Dreslik, MJ; Swanson, BJ; Mulkerin, D | 2013 | Genetic Analysis and Captive Breeding Program Design for the Eastern Massasauga Sistrurus catenatus catenatus | The eastern massasauga Sistrurus catenatus catenatus is a declining species for which a captive breeding program was established in 2006. To effectively manage wild and captive populations, an understanding of genetic diversity within the species is necessary. We analyzed mitochondrial DNA sequences of 186 individuals: 109 wild snakes from 34 U. S. and Canadian counties and districts, all 52 breeding program members (23 of known and 29 of unknown origin), 18 other captives of unknown origin, and 7 outgroup representatives of desert massasauga S. c. edwardsii, and western massasauga, S. c. tergeminus. Statistical parsimony, maximum likelihood, and maximum parsimony analyses all identified eastern massasaugas as divergent from western and desert massasaugas. We found 18 different haplotypes among eastern massasaugas, comprising three geographically and genetically differentiated NADH dehydrogenase II (ND2) subunits that potentially reflect post-Pleistocene range expansion from unglaciated into formerly glaciated regions. Snakes of unknown origin could all be assigned unambiguously to these ND2 subunits. To maintain natural genetic variation, preserve diversity in captive lineages, and allow future augmentation or reintroduction, the Association of Zoos and Aquariums is managing these three geographic ND2 subunits separately within the Eastern Massasauga Species Survival Plan breeding program. | 1549 | no | no good fitness measure, N pop differnt in GD? | NA | no | no |
| 1550 | 2017 | 43019 | Routh, A; Domitrovic, T; Johnson, JE | 2012 | Host RNAs, including transposons, are encapsidated by a eukaryotic single-stranded RNA virus | Next-generation sequencing is a valuable tool in our growing understanding of the genetic diversity of viral populations. Using this technology, we have investigated the RNA content of a purified nonenveloped single-stranded RNA virus, flock house virus (FHV). We have also investigated the RNA content of virus-like particles (VLPs) of FHV and the related Nudaurelia capensis omega virus. VLPs predominantly package ribosomal RNA and transcripts of their baculoviral expression vectors. In addition, we find that 5.3% of the packaged RNAs are transposable elements derived from the Sf21 genome. This observation may be important when considering the therapeutic use of VLPs. We find that authentic FHV virions also package a variety of host RNAs, accounting for 1% of the packaged nucleic acid. Significant quantities of host messenger RNAs, ribosomal RNA, noncoding RNAs, and transposable elements are readily detected. The packaging of these host RNAs elicits the possibility of horizontal gene transfer between eukaryotic hosts that share a viral pathogen. We conclude that the genetic content of nonenveloped RNA viruses is variable, not just by genome mutation, but also in the diversity of RNA transcripts that are packaged. | 1550 | no | gd of pathogen | NA | no | no |
| 1551 | 2017 | 43019 | Alo, D; Turner, TF | 2005 | Effects of habitat fragmentation on effective population size in the endangered Rio Grande silvery minnow | We assessed spatial and temporal patterns of genetic diversity to evaluate effects of river fragmentation on remnant populations of the federally endangered Rio Grande silvery minnow (Hybognathus amarus). Analysis of microsatellite and mitochondrial DNA detected little spatial genetic structure over the current geographic range, consistent with high gene flow despite fragmentation by dams. Maximum-likelihood analysis of temporal genetic data indicated, however, that present-day effective population size (N-eV) of the largest extant population of this species was 78 and the ratio of effective size to adult numbers (N-eV/N) was similar to 0.001 during the study period (1999 to 2001). Coalescent-based analytical methods provided an estimate of historical (river fragmentation was completed in 1975) effective size (N-el) that ranged between 10(5) and 10(6). We propose that disparity between contemporary and historical estimates of N-e and low contemporary N-e/N result from recent changes in demography related to river fragmentation. Rio Grande silvery minnows produce pelagic eggs and larvae subject to downstream transport through diversion dams. This life-history feature results in heavy losses of yearly reproductive effort to emigration and mortality, and extremely large variance in reproductive success among individuals and spawning localities. Interaction of pelagic early life history and river fragmentation has altered demographic and genetic dynamics of remnant populations and reduced N-e to critically low values over ecological time. | 1551 | no | no good fitness measure, N pop differnt in GD? | NA | no | no |
| 1552 | 2017 | 43019 | Atzmon, G; Hao, L; Pe’er, I; Velez, C; Pearlman, A; Palamara, PF; Morrow, B; Friedman, E; Oddoux, C; Burns, E; Ostrer, H | 2010 | Abraham’s Children in the Genome Era: Major Jewish Diaspora Populations Comprise Distinct Genetic Clusters with Shared Middle Eastern Ancestry | For more than a century, Jews and non-Jews alike have tried to define the relatedness of contemporary Jewish people. Previous genetic studies of blood group and serum markers suggested that Jewish groups had Middle Eastern origin with greater genetic similarity between paired Jewish populations. However, these and successor studies of monoallelic Y chromosomal and mitochondrial genetic markers did not resolve the issues of within and between-group Jewish genetic identity. Here, genome-wide analysis of seven Jewish groups (Iranian, Iraqi, Syrian, Italian, Turkish, Greek, and Ashkenazi) and comparison with non-Jewish groups demonstrated distinctive Jewish population clusters, each with shared Middle Eastern ancestry, proximity to contemporary Middle Eastern populations, and variable degrees of European and North African admixture. Two major groups were identified by principal component, phylogenetic, and identity by descent (IBD) analysis: Middle Eastern Jews and European/Syrian Jews. The IBD segment sharing and the proximity of European Jews to each other and to southern European populations suggested similar origins for European Jewry and refuted large-scale genetic contributions of Central and Eastern European and Slavic populations to the formation of Ashkenazi Jewry. Rapid decay of IBD in Ashkenazi Jewish genomes was consistent with a severe bottleneck followed by large expansion, such as occurred with the so-called demographic miracle of population expansion from 50,000 people at the beginning of the 15(th) century to 5,000,000 people at the beginning of the 19(th) century. Thus, this study demonstrates that European/Syrian and Middle Eastern Jews represent a series of geographical isolates or clusters woven together by shared IBD genetic threads. | 1552 | no | human | NA | no | no |
| 1553 | 2017 | 43019 | Scholz, HC; Muhldorter, K; Shilton, C; Benedict, S; Whatmore, AM; Blom, J; Eisenberg, T | 2016 | The Change of a Medically Important Genus: Worldwide Occurrence of Genetically Diverse Novel Brucella Species in Exotic Frogs | The genus Brucella comprises various species of both veterinary and human medical importance. All species are genetically highly related to each other, sharing intra-species average nucleotide identities (ANI) of >99%. Infections occur among various warm-blooded animal species, marine mammals, and humans. Until recently, amphibians had not been recognized as a host for Brucella. In this study, however, we show that novel Brucella species are distributed among exotic frogs worldwide. Comparative recA gene analysis of 36 frog isolates from various continents and different frog species revealed an unexpected high genetic diversity, not observed among classical Brucella species. In phylogenetic reconstructions the isolates consequently formed various clusters and grouped together with atypical more distantly related brucellae, like B. inopinata, strain BO2, and Australian isolates from rodents, some of which were isolated as human pathogens. Of one frog isolate (10RB9215) the genome sequence was determined. Comparative genome analysis of this isolate and the classical Brucella species revealed additional genetic material, absent from classical Brucella species but present in Ochrobactrum, the closest genetic neighbor of Brucella, and in other soil associated genera of the Alphaproteobacteria. The presence of gene clusters encoding for additional metabolic functions, flanked by tRNAs and mobile genetic elements, as well as by bacteriophages is suggestive for a different ecology compared to classical Brucella species. Furthermore it suggests that amphibian isolates may represent a link between free living soil saprophytes and the pathogenic Brucella with a preferred intracellular habitat. We therefore assume that brucellae from frogs have a reservoir in soil and, in contrast to classical brucellae, undergo extensive horizontal gene transfer. | 1553 | no | no good fitness measure, N pop differnt in GD? | NA | no | no |
| 1554 | 2017 | 43019 | Louy, D; Habel, JC; Schmitt, T; Assmann, T; Meyer, M; Muller, P | 2007 | Strongly diverging population genetic patterns of three skipper species: the role of habitat fragmentation and dispersal ability | The fragmentation of landscapes has an important impact on the conservation of biodiversity, and the genetic diversity is an important factor for a populations viability, influenced by the landscape structure. However, different species with differing ecological demands react rather different on the same landscape pattern. To address this feature, we studied three skipper species with differing habitat requirements (Lulworth Skipper Thymelicus acteon: a habitat specialist with low dispersal ability, Small Skipper Thymelicus sylvestris: a habitat generalist with low dispersal ability, Essex Skipper Thymelicus lineola: a habitat generalist with higher dispersal ability). We analysed 18 allozyme loci for 1,063 individuals in our western German study region with adjoining areas in Luxembourg and north-eastern France. The genetic diversity of all three species were intermediate in comparison with other butterfly species. The F-ST was relatively high for T. acteon (5.1%), low for T. sylvestris (1.6%) and not significant for T. lineola. Isolation by distance analyses revealed a significant correlation for T. sylvestris explaining 20.3% of its differentiation, but no such structure was found for the two other species. Most likely, the high dispersal ability of T. lineola in comparison with T. sylvestris leads to a more or less panmictic structure and hence impedes isolation by distance. On the other hand, the isolation of the populations of T. acteon seems to be so strict that the populations develop independently. Although no general genetic impoverishing was observed for the endangered T. acteon, small populations had significantly lower genetic diversities than big populations, and therefore the high degree of isolation among populations might threaten its local and regional survival. | 1554 | no | no good fitness measure | NA | no | no |
| 1555 | 2017 | 43019 | Aguiar, LMS; Brito, D; Machado, RB | 2010 | Do current vampire bat (Desmodus rotundus) population control practices pose a threat to Dekeyser’s nectar bat’s (Lonchophylla dekeyseri) long-term persistence in the Cerrado? | The Cerrado is rapidly losing space to agriculture, pastures and urbanization. Current management practices to control rabies outbreaks through the eradication of vampire bat populations may put other bat species in peril. Our objective is to evaluate if the current vampire bat population control practices could pose a threat to Lonchophylla dekeyseri’s persistence, an endemic bat of the Cerrado. We used the VORTEX program to model different vampire bat management scenarios, causing low (25%), medium (50%) or high (75%) incidental mortality to L. dekeyseri populations. Inbreeding depression has been identified as a threat to the species, therefore we also modeled scenarios evaluating such effects. Results show that current vampire bat management practices have serious impacts on populations of L. dekeyseri. In all cases marked declines in population sizes were observed (even when there was no decline in survival probabilities). For medium and high incidental mortality management scenarios, we also observed decreases in survival probability and in genetic diversity. In those scenarios evaluating vampire bat management and inbreeding depression together, the models suggest that such interaction results in more pronounced declines. Habitat loss and fragmentation in the Cerrado are severe threats and have already negatively impacted L. dekeyseri. Unfortunately, if currentpopulation control practices dealing with vampire bats are not changed, inappropriate rabies management may be the coup de grace to the long-term persistence of this species. | 1555 | no | no good fitness measure, N pop differnt in GD? | NA | no | no |
| 1556 | 2017 | 43019 | Valadas, V; Laranjo, M; Barbosa, P; Espada, M; Mota, M; Oliveira, S | 2012 | The pine wood nematode, Bursaphelenchus xylophilus, in Portugal: possible introductions and spread routes of a serious biological invasion revealed by molecular methods | The pine wood nematode (PWN), Bursaphelenchus xylophilus, the causal agent of pine wilt disease (PWD), is a major world-wide pathogen and pest of pine, with impacts on forest health, natural ecosystem stability and international trade. In Portugal, PWN was first diagnosed in 1999, the first occurrence also for Europe. The disease was recently detected on the island of Madeira and in northern Spain. In an attempt to search for more reliable and robust molecular markers that enable the study of intraspecific variability of B. xylophilus from different geographic locations, the intergenic spacer (IGS) region of the 5S rRNA gene and inter-simple sequence repeats (ISSR) analysis were used to determine the genetic relationships among 43 B. xylophilus isolates from Portugal, China, Japan, South Korea and USA. IGS sequence analysis showed that this region can only be used to establish interspecific relationships, since no differences were detected among Portuguese isolates from different geographic locations. Fingerprints obtained with ISSR show high genetic variability among Portuguese isolates, except for the ones obtained prior to 2008. The ISSR dendrogram suggests the spread of the disease inside continental Portugal and to Madeira. Until 2008, B. xylophilus populations found in continental Portugal showed low genetic diversity, pointing to a single introduction, probably from Asia, whereas recent populations from continental Portugal (2009-2010) and Madeira show high genetic diversity, suggesting multiple introductions from different origins. | 1556 | no | gd of pathogen | NA | no | no |
| 1557 | 2017 | 43019 | Wang, IN; Hu, CC; Lee, CW; Yen, SM; Yeh, WB; Hsu, YH; Lin, NS | 2014 | Genetic Diversity and Evolution of Satellite RNAs Associated with the Bamboo Mosaic Virus | Satellite RNAs (satRNAs) are subviral agents that depend on cognate helper viruses for genome replication and encapsidation. Their negative impacts on helper viruses have been exploited to control plant viral diseases. SatBaMV is a commonly found satRNA associated with Bamboo mosaic virus (BaMV) that infects diverse bamboo species in the field. To investigate the genetic diversity and evolution of satRNAs, we examined seven satBaMV populations derived from five bamboo species and cultivars from Taiwan, China, and India and one from the greenhouse. We found 3 distinct clades among the seven populations. Clade I is consisted of all satBaMV isolates, except for those from Dendrocalamus latiflorus in Taiwan and Bambusa vulgaris in India, which belong to Clades II and III, respectively. Interestingly, nucleotide diversity was lower for Clade I than II and III. However, the nucleotide diversity did not seem to depend on bamboo species or geographic location. Our population genetic analyses revealed the presence of excessive low-frequency polymorphic sites, which suggests that the satBaMV population was under purifying selection and/or population expansion. Further analysis of P20, the only satBaMV gene that encodes a non-structural protein involved in the long-distance movement of satBaMV, showed evidence of purifying selection. Taken together, our results suggest that purifying selection against defective P20 protein is responsible at least in part for the evolution of the satBaMV genome. | 1557 | no | gd of pathogen | NA | no | no |
| 1558 | 2017 | 43019 | Hukowska-Szematowicz, B; Tokarz-Deptula, B; Deptula, W | 2013 | Analysis of genetic variability and phylogenetic analysis of selected Czech and French strains of rabbit haemorrhagic disease virus (RHDV) | The objective of this study was to analyse the genetic variability and phylogenetic analysis of six strains of rabbit haemorrhagic disease virus (RHDV), including four Czech strains (CAMPV-351, CAMPV-561, CAMPV-562, CAMPV-558) and two French strains (Fr-1, Fr-2), on the basis of a fragment of the VP60 capsid structural protein-coding gene N-terminal region. The results of our own studies were compared to 26 RHDV strains obtained from GenBank. The analysis of the genetic variability of six RHDV strains indicated that the CAMPV-561 strain is the most genetically variable. Less variable were the Fr-1 and Fr-2 strains, while the least variable was CAMPV-351. In turn, the genetic distance among the six analysed strains and 26 strains obtained from GenBank was the greatest for CAMPV-351 and Whn/China [11.3 % according to the observed divergence (OD) method and 12.2 % according to the maximum likelihood (ML) method], while it was the lowest for CAMPV-351 and FRG (0.8 % in both the OD and ML methods). In turn, the scale of the genetic distances among the six analysed strains and five RHDVa strains (99-05, NY-01, Whn/China, Triptis, Iowa2000) ranged from 9.3-10.3 % in the OD method to 10.3-13.7 % in the ML method. The image of phylogenetic dependencies generated for the strains analysed and those obtained from GenBank revealed their distribution to be in five genetic groups (G1-G5), whereas the analysed strains were included in genetic groups 2 and 3. | 1558 | no | gd of pathogen | NA | no | no |
| 1559 | 2017 | 43019 | Paterson, S; Viney, ME | 2003 | Functional consequences of genetic diversity in Strongyloides ratti infections | Parasitic nematodes show levels of genetic diversity comparable to other taxa, but the functional consequences of this are not understood. Thus, a large body of theoretical work highlights the potential consequences of parasite genetic diversity for the epidemiology of parasite infections and its possible implications for the evolution of host and parasite populations. However, few relevant empirical data are available from parasites in general and none from parasitic nematodes in particular. Here, we test two hypotheses. First, that different parasitic nematode genotypes vary in life-history traits, such as survivorship and fecundity, which may cause variation in infection dynamics. Second, that different parasitic nematode genotypes interact within the host (either directly or via the host immune system) to increase the mean reproductive output of mixed-genotype infections compared with single-genotype infections. We test these hypotheses in laboratory infections using genetically homogeneous lines of Strongyloides ratti. We find that nematode genotypes do vary in their survivorship and fecundity and, consequently, in their dynamics of infection. However, we find little evidence of interactions between genotypes within hosts under a variety of trickle- and single-infected infection regimes. | 1559 | no | gd of parasite | NA | no | no |
| 1560 | 2017 | 43019 | Wootton, JC; Feng, XR; Ferdig, MT; Cooper, RA; Mu, JB; Baruch, DI; Magill, AJ; Su, XZ | 2002 | Genetic diversity and chloroquine selective sweeps in Plasmodium falciparum | Widespread use of antimalarial agents can profoundly influence the evolution of the human malaria parasite Plasmodium falciparum. Recent selective sweeps for drug-resistant genotypes may have restricted the genetic diversity of this parasite, resembling effects attributed in current debates(1-4) to a historic population bottleneck. Chloroquine-resistant (CQR) parasites were initially reported about 45 years ago from two foci in southeast Asia and South America(5), but the number of CQR founder mutations and the impact of chlorquine on parasite genomes worldwide have been difficult to evaluate. Using 342 highly polymorphic microsatellite markers from a genetic map(6), here we show that the level of genetic diversity varies substantially among different regions of the parasite genome, revealing extensive linkage disequilibrium surrounding the key CQR gene pfcrt(7) and at least four CQR founder events. This disequilibrium and its decay rate in the pfcrt-flanking region are consistent with strong directional selective sweeps occurring over only similar to20-80 sexual generations, especially a single resistant pfcrt haplotype spreading to very high frequencies throughout most of Asia and Africa. The presence of linkage disequilibrium provides a basis for mapping genes under drug selection in P. falciparum. | 1560 | no | gd of parasite | NA | no | no |
| 1561 | 2017 | 43019 | De Chiara, M; Hood, D; Muzzi, A; Pickard, DJ; Perkins, T; Pizza, M; Dougan, G; Rappuoli, R; Moxon, ER; Soriani, M; Donati, C | 2014 | Genome sequencing of disease and carriage isolates of nontypeable Haemophilus influenzae identifies discrete population structure | One of the main hurdles for the development of an effective and broadly protective vaccine against nonencapsulated isolates of Haemophilus influenzae (NTHi) lies in the genetic diversity of the species, which renders extremely difficult the identification of cross-protective candidate antigens. To assess whether a population structure of NTHi could be defined, we performed genome sequencing of a collection of diverse clinical isolates representative of both carriage and disease and of the diversity of the natural population. Analysis of the distribution of polymorphic sites in the core genome and of the composition of the accessory genome defined distinct evolutionary clades and supported a predominantly clonal evolution of NTHi, with the majority of genetic information transmitted vertically within lineages. A correlation between the population structure and the presence of selected surface-associated proteins and lipooligosaccharide structure, known to contribute to virulence, was found. This high-resolution, genome-based population structure of NTHi provides the foundation to obtain a better understanding, of NTHi adaptation to the host as well as its commensal and virulence behavior, that could facilitate intervention strategies against disease caused by this important human pathogen. | 1561 | no | gd of pathogen | NA | no | no |
| 1562 | 2017 | 43019 | Udupa, SM; Weigand, F; Saxena, MC; Kahl, G | 1998 | Genotyping with RAPD and microsatellite markers resolves pathotype diversity in the ascochyta blight pathogen of chickpea | The poor definition of variation in the ascochyta blight fungus (Ascochyta rabiei) has historically hindered breeding for resistance to the chickpea (Cicer ar arietinum L,) blight disease in West Asia and North Africa. We have employed 14 RAPD markers and an oligonucleotide probe complementary to the microsatellite sequence (GATA)(4) to construct a genotype-specific DNA fragment profile from periodically sampled Syrian field isolates of this fungus. By using conventional pathogenicity tests and genome analysis with RAPD and microsatellite markers, we demonstrated that the DNA markers distinguish variability within and among the major pathotypes of A. rabiei and resolved each pathotypes into several genotypes. The genetic diversity estimate based on DNA marker analysis within pathotypes was highest for the least-aggressive pathotype (pathotype I), followed by the aggressive (pathotype II) and the most-aggressive pathotype (pathotype III). The pair-wise genetic distance estimated for all the isolates varied from 0.00 to 0.39, indicating a range from a clonal to a diverse relationship. On the basis of genome analysis, and information on the spatial and temporal distribution of the pathogen, a general picture of A. rabiei evolution in Syria is proposed. | 1562 | no | gd of pathogen | NA | no | no |
| 1563 | 2017 | 43019 | Soubeyrand, S; Tollenaere, C; Haon-Lasportes, E; Laine, AL | 2014 | Regression-Based Ranking of Pathogen Strains with Respect to Their Contribution to Natural Epidemics | Genetic variation in pathogen populations may be an important factor driving heterogeneity in disease dynamics within their host populations. However, to date, we understand poorly how genetic diversity in diseases impact on epidemiological dynamics because data and tools required to answer this questions are lacking. Here, we combine pathogen genetic data with epidemiological monitoring of disease progression, and introduce a statistical exploratory method to investigate differences among pathogen strains in their performance in the field. The method exploits epidemiological data providing a measure of disease progress in time and space, and genetic data indicating the relative spatial patterns of the sampled pathogen strains. Applying this method allows to assign ranks to the pathogen strains with respect to their contributions to natural epidemics and to assess the significance of the ranking. This method was first tested on simulated data, including data obtained from an original, stochastic, multi-strain epidemic model. It was then applied to epidemiological and genetic data collected during one natural epidemic of powdery mildew occurring in its wild host population. Based on the simulation study, we conclude that the method can achieve its aim of ranking pathogen strains if the sampling effort is sufficient. For powdery mildew data, the method indicated that one of the sampled strains tends to have a higher fitness than the four other sampled strains, highlighting the importance of strain diversity for disease dynamics. Our approach allowing the comparison of pathogen strains in natural epidemic is complementary to the classical practice of using experimental infections in controlled conditions to estimate fitness of different pathogen strains. Our statistical tool, implemented in the R package StrainRanking, is mainly based on regression and does not rely on mechanistic assumptions on the pathogen dynamics. Thus, the method can be applied to a wide range of pathogens. | 1563 | no | no good fitness measure, N pop differnt in GD? | NA | no | no |
| 1564 | 2017 | 43019 | Canal, D; Serrano, D; Potti, J | 2014 | Exploring Heterozygosity-Survival Correlations in a Wild Songbird Population: Contrasting Effects between Juvenile and Adult Stages | The relationship between genetic diversity and fitness, a major issue in evolutionary and conservation biology, is expected to be stronger in traits affected by many loci and those directly influencing fitness. Here we explore the influence of heterozygosity measured at 15 neutral markers on individual survival, one of the most important parameters determining individual fitness. We followed individual survival up to recruitment and during subsequent adult life of 863 fledgling pied flycatchers born in two consecutive breeding seasons. Mark-recapture analyses showed that individual heterozygosity did not influence juvenile or adult survival. In contrast, the genetic relatedness of parents was negatively associated with the offspring’s survival during the adult life, but this effect was not apparent in the juvenile (from fledgling to recruitment) stage. Stochastic factors experienced during the first year of life in this long-distance migratory species may have swamped a relationship between heterozygosity and survival up to recruitment. | 1564 | no | individual level | NA | no | no |
| 1565 | 2017 | 43019 | Sahebzadeh, N; NurAzura, A; Lau, WH; Mardan, M; Ali, MA; Tan, SG | 2013 | Genetic structure of Malaysian Apis dorsata aggregations in Marang district (Terengganu) | Apis dorsata is a major pollinator in the rainforests of Peninsular Malaysia, which is threatened by frequent harvesting via the removal of whole comb nests from trees during the harvesting season. There is an urgent need for conservation of this species and the long-term survival of tropical lowland forests. In this paper, the genetic relatedness of A. dorsata aggregations are addressed with a preliminary study on local populations in Malaysia. The analysis of 30 single locus DNA microsatellite markers on three aggregations at intra-aggregation level showed that the queens of different nests within an aggregation were significantly different and not related as mother-daughter (P > 0.063). At inter-aggregation level, the results demonstrated that there was no significant relatedness between the aggregations (P > 0.05) separated by a distance of 0.5 km. The results showed that adjacent aggregations were genetically different and had enough time for establishing the colonies to produce honey. | 1565 | no | no good fitness measure | NA | no | no |
| 1566 | 2017 | 43019 | LABEDA, DP | 1992 | DNA-DNA HYBRIDIZATION IN THE SYSTEMATICS OF STREPTOMYCES | The DNA relatedness among strains in several different phenotypically defined Streptomyces species clusters was evaluated. It was found that the data from DNA-relatedness studies do not necessarily agree with the clustering generated using numerical taxonomic techniques. A study of the morphologically heterogeneous ‘S. cyaneus’ cluster showed that morphological criteria traditionally used to classify and identify Streptomyces species still have value, since strains in DNA-relatedness cluster groups were also similar morphologically (i.e., they had similar spore color, surface properties, and sporophore morphology). An evaluation of DNA relatedness among strains in the S. violaceusniger and S. lavendulae clusters indicated that, if anything, the genus is underspeciated, based on the number of single-member clusters observed. A study of strains of the sweet potato pathogen, S. ipomoea, collected in various locations in the United States and Japan indicated, not surprisingly, that all of the strains belong to the same species. | 1566 | no | NA | NA | no | no |
| 1567 | 2017 | 43019 | Gschwind, AR; Singh, A; Certa, U; Reymond, A; Heckel, T | 2017 | Diversity and regulatory impact of copy number variation in the primate Macaca fascicularis | Background: Copy number variations (CNVs) are a significant source of genetic diversity and commonly found in mammalian genomes. We have generated a genome-wide CNV map for Cynomolgus monkeys (Macaca fascicularis). This crab-eating macaque is the closest animal model to humans that is used in biomedical research. Results: We show that Cynomolgus monkey CNVs are in general much smaller in size than gene loci and are specific to the population of origin. Genome-wide expression data from five vitally important organs demonstrates that CNVs in close proximity to transcription start sites associate strongly with expression changes. Among these eQTL genes we find an overrepresentation of genes involved in metabolism, receptor activity, and transcription. Conclusion: These results provide evidence that CNVs shape tissue transcriptomes in monkey populations, potentially offering an adaptive advantage. We suggest that this genetic diversity should be taken into account when using Cynomolgus macaques as models. | 1567 | no | no pop level? | NA | no | no |
| 1568 | 2017 | 43019 | Ordon, F; Schiemann, A; Friedt, W | 1997 | Assessment of the genetic relatedness of barley accessions (Hordeum vulgare sl) resistant to soil-borne mosaic-inducing viruses (BaMMV, BaYMV, BaYMV-2) using RAPDs | Thirty-six Hordeum vulgare varieties and 12 H. spontaneum germplasms originating from different parts of the world and showing different reactions to the barley yellow mosaic virus complex (BaMMV, BaYMV, BaYMV-2) were analyzed for genetic similarity using RAPDs. On the basis of an analysis of 20 selected RAPD-primers corresponding to 544 bands genetic similarity according to-Nei and Li (1979) was estimated to be between 0.685 and 0.964. Associations between the 48 genotypes were calculated using UPGMA-clustering and principal coordinate analysis. By applying these methods we were able to separate H. spontaneum accessions from H. vulgare varieties, and within these groups all the genotypes were clustered correctly according to their origin. Consequently, RAPD analysis can be considered a very useful and efficient tool for the fast estimation of genetic relationships in barley. The correlation between genetic similarity with respect to German varieties and adaptation of exotic barley varieties to German growing conditions is discussed. | 1568 | no | no good fitness measure, N pop differnt in GD? | NA | no | no |
| 1569 | 2017 | 43019 | Hornoy, B; Pavy, N; Gerardi, S; Beaulieu, J; Bousquet, J | 2015 | Genetic Adaptation to Climate in White Spruce Involves Small to Moderate Allele Frequency Shifts in Functionally Diverse Genes | Understanding the genetic basis of adaptation to climate is of paramount importance for preserving and managing genetic diversity in plants in a context of climate change. Yet, this objective has been addressed mainly in short-lived model species. Thus, expanding knowledge to nonmodel species with contrasting life histories, such as forest trees, appears necessary. To uncover the genetic basis of adaptation to climate in the widely distributed boreal conifer white spruce (Picea glauca), an environmental association study was conducted using 11,085 single nucleotide polymorphisms representing 7,819 genes, that is, approximately a quarter of the transcriptome. Linear and quadratic regressions controlling for isolation-by-distance, and the Random Forest algorithm, identified several dozen genes putatively under selection, among which 43 showed strongest signals along temperature and precipitation gradients. Most of them were related to temperature. Small to moderate shifts in allele frequencies were observed. Genes involved encompassed a wide variety of functions and processes, some of them being likely important for plant survival under biotic and abiotic environmental stresses according to expression data. Literature mining and sequence comparison also highlighted conserved sequences and functions with angiosperm homologs. Our results are consistent with theoretical predictions that local adaptation involves genes with small frequency shifts when selection is recent and gene flow among populations is high. Accordingly, genetic adaptation to climate in P. glauca appears to be complex, involving many independent and interacting gene functions, biochemical pathways, and processes. From an applied perspective, these results shall lead to specific functional/association studies in conifers and to the development of markers useful for the conservation of genetic resources. | 1569 | no | no good fitness measure | NA | no | no |
| 1570 | 2017 | 43019 | Stukenbrock, EH; Banke, S; McDonald, BA | 2006 | Global migration patterns in the fungal wheat pathogen Phaeosphaeria nodorum | The global migration patterns of the fungal wheat pathogen Phaeosphaeria nodorum were analysed using 12 microsatellite loci. Analysis of 693 isolates from nine populations indicated that the population structure of P. nodorum is characterized by high levels of genetic diversity and a low degree of subdivision between continents. To determine whether genetic similarity of populations was a result of recent divergence or extensive gene flow, the microsatellite data were analysed using an isolation-with-migration model. We found that the continental P. nodorum populations diverged recently, but that enough migration occurred to reduce population differentiation. The migration patterns of the pathogen indicate that immigrants originated mainly from populations in Europe, China and North America. | 1570 | no | gd of pathogen | NA | no | no |
| 1571 | 2017 | 43019 | Poysa, H; Paasivaara, A | 2016 | Nest predation risk, but not demography, drives dynamics of conspecific brood parasitism | Conspecific brood parasitism (CBP) is an alternative reproductive tactic in several animal taxa. Although various behavioral aspects related to CBP have been studied in several species, understanding spatial and temporal dynamics of CBP and its drivers is still limited. We studied roles of nest predation risk and demography as possible drivers of dynamics of CBP in common goldeneyes (Bucephala clangula), a cavity-nesting duck. We provided decoy nests for parasitic laying in an experimental setting of 15 lakes for 7 consecutive years irrespective of local nest predation, being thus able to control for effects of predation-determined host nest availability. Individual parasites were recognized using protein fingerprints from egg albumen of parasitic eggs laid in the decoy nests. We found considerable spatial and temporal variation in the frequency of CBP within the experimental setting. Variation in CBP was driven by nest predation risk: the rate of CBP tracked the number of nonpredated nesting attempts at the lakes during the previous year. Neither variation in lake-specific number of potential homing first-time breeding females (i.e., demography) nor variation in lake-specific number of nesting females present explained the variation in lake-specific frequency of CBP. Our findings provide evidence that parasitically laying females pursue a genuine and flexible safety-seeking tactic in nest selection and that nest predation risk drives spatial and temporal dynamics of CBP. | 1571 | no | brood parasitism | NA | no | no |
| 1572 | 2017 | 43019 | Cobble, KR; Califf, KJ; Stone, NE; Shuey, MM; Birdsell, DN; Colman, RE; Schupp, JM; Aziz, M; Van Andel, R; Rocke, TE; Wagner, DM; Busch, JD | 2016 | Genetic variation at the MHC DRB1 locus is similar across Gunnison’s prairie dog (Cynomys gunnisoni) colonies regardless of plague history | Yersinia pestis was introduced to North America around 1900 and leads to nearly 100% mortality in prairie dog (Cynomys spp.) colonies during epizootic events, which suggests this pathogen may exert a strong selective force. We characterized genetic diversity at an MHC class II locus (DRB1) in Gunnison’s prairie dog (C. gunnisoni) and quantified population genetic structure at the DRB1 versus 12 microsatellite loci in three large Arizona colonies. Two colonies, Seligman (SE) and Espee Ranch (ES), have experienced multiple plague-related die-offs in recent years, whereas plague has never been documented at Aubrey Valley (AV). We found fairly low allelic diversity at the DRB1 locus, with one allele (DRB1*01) at high frequency (0.67-0.87) in all colonies. Two other DRB1 alleles appear to be trans-species polymorphisms shared with the black-tailed prairie dog (C. ludovicianus), indicating that these alleles have been maintained across evolutionary time frames. Estimates of genetic differentiation were generally lower at the MHC locus (F-ST = 0.033) than at microsatellite markers (F-ST = 0.098). The reduced differentiation at DRB1 may indicate that selection has been important for shaping variation at MHC loci, regardless of the presence or absence of plague in recent decades. However, genetic drift has probably also influenced the DRB1 locus because its level of differentiation was not different from that of microsatellites in an F-ST outlier analysis. We then compared specific MHC alleles to plague survivorship in 60 C. gunnisoni that had been experimentally infected with Y. pestis. We found that survival was greater in individuals that carried at least one copy of the most common allele (DRB1* 01) compared to those that did not (60% vs. 20%). Although the sample sizes of these two groups were unbalanced, this result suggests the possibility that this MHC class II locus, or a nearby linked gene, could play a role in plague survival. | 1572 | no | no pop level? | NA | no | no |
| 1573 | 2017 | 43019 | Bjornerfeldt, S; Hailer, F; Nord, M; Vila, C | 2008 | Assortative mating and fragmentation within dog breeds | Background: There are around 400 internationally recognized dog breeds in the world today, with a remarkable diversity in size, shape, color and behavior. Breeds are considered to be uniform groups with similar physical characteristics, shaped by selection rooted in human preferences. This has led to a large genetic difference between breeds and a large extent of linkage disequilibrium within breeds. These characteristics are important for association mapping of candidate genes for diseases and therefore make dogs ideal models for gene mapping of human disorders. However, genetic uniformity within breeds may not always be the case. We studied patterns of genetic diversity within 164 poodles and compared it to 133 dogs from eight other breeds. Results: Our analyses revealed strong population structure within poodles, with differences among some poodle groups as pronounced as those among other well-recognized breeds. Pedigree analysis going three generations back in time confirmed that subgroups within poodles result from assortative mating imposed by breed standards as well as breeder preferences. Matings have not taken place at random or within traditionally identified size classes in poodles. Instead, a novel set of five poodle groups was identified, defined by combinations of size and color, which is not officially recognized by the kennel clubs. Patterns of genetic diversity in other breeds suggest that assortative mating leading to fragmentation may be a common feature within many dog breeds. Conclusion: The genetic structure observed in poodles is the result of local mating patterns, implying that breed fragmentation may be different in different countries. Such pronounced structuring within dog breeds can increase the power of association mapping studies, but also represents a serious problem if ignored. In dog breeding, individuals are selected on the basis of morphology, behaviour, working or show purposes, as well as geographic population structure. The same processes which have historically | 1573 | no | domesticated animal | NA | no | no |
| 1574 | 2017 | 43019 | Le Jossec, M; Wambach, T; Labuda, D; Sinnett, D; Levy, E | 2004 | Genetic diversity patterns in the SR-BI/II locus can be explained by a recent selective sweep | The human scavenger receptor class B type I (SR-BI and splice variant SR-BII) plays a central role in HDL cholesterol metabolism and represents a candidate gene for a number of related diseases. We examined the genetic diversity of its coding and flanking regions in a sample of 178 chromosomes from individuals of European, African, East Asian (including Southeast Asian), Middle-Eastern as well as Amerindian descent. Nine of the 14 polymorphisms observed are new. Four of the five variants causing amino acid replacements, G2S, S229G, R484W, and G499R, are likely to affect protein structure and function. SR-BI/BII diversity is partitioned among 19 haplotypes; all but one interconnected by single mutation or a recombination event. Such tight haplotype network and the unusual geographic partitioning of this diversity, high not only in Africa but in East Asia as well, suggests its recent origin and possible effect of selection. Coalescent analysis infers a relatively short time to the most recent common ancestor and points to population expansion in Africa and East Asia. These two continents differ significantly in pairwise F-ST values, differing as well from a single cluster formed by Europe, Middle East and America. In the context of findings for similarly analyzed other loci, we propose that a selective sweep at the origin of modem human populations could explain the low level of ancestral SR-BI/ II diversity. The unusually deep split between Africa and Asia, well beyond the Upper Paleolithic when inferred under neutrality, is consistent with subsequent geographical and demographic expansion favoring the accumulation of new variants, especially in groups characterized by large effective population sizes, such as Asians and Africans. The relevance of such partitioning of SR-BI/II diversity remains to be investigated in genetic epidemiological studies which can be guided by the present findings. | 1574 | no | human | NA | no | no |
| 1575 | 2017 | 43019 | Nieberding, C; Morand, S; Libois, R; Michaux, JR | 2006 | Parasites and the island syndrome: the colonization of the western Mediterranean islands by Heligmosomoides polygyrus (Dujardin, 1845) | Aim Populations of free-living vertebrates on islands frequently differ from their mainland counterparts by a series of changes in morphometric, life-history, behavioural, physiological and genetic traits, collectively referred to as the ‘island syndrome’. It is not known, however, whether the ‘island syndrome’ also affects parasitic organisms. The present study establishes the colonization pattern of the Mediterranean islands by the nematode Heligmosomoides polygyrus, a direct and specific parasite of rodent hosts of the Apodemus genus, and evaluates the effects of island colonization by this species on two components of the island syndrome: the loss of genetic diversity and the enlargement of the ecological niche. Location Heligmosomoides polygyrus was sampled on seven western Mediterranean islands - Corsica, Crete, Elba, Majorca, Minorca, Sardinia and Sicily - as well as in 20 continental locations covering the Mediterranean basin. Methods The mitochondrial cytochrome b gene (690 base pairs) was sequenced in 166 adult H. polygyrus individuals sampled in the 27 continental and island locations. Phylogenetic reconstructions in distance, parsimony, maximum likelihood and Bayesian posterior probabilities were carried out on the whole cytochrome b gene data set. The levels of nucleotide, haplotype and genetic divergence (Kimura two-parameter distance estimator) diversities were estimated in each island population and in the various continental lineages. Results Phylogenetic reconstructions show that the mainland origins of H. polygyrus were continental Spain for the Balearic Islands (Majorca, Minorca), northern Italy for the Tyrrhenian Islands (Corsica, Sardinia, Elba), southern Italy for Sicily, and the Balkan region for Crete. A comparison of island H. polygyrus populations with their mainland source populations revealed two characteristic components of the island syndrome in this parasite. First, island H. polygyrus populations display a significant loss of genetic diversity, which is related (r(2) = 0.73) to the distance separating the island from the mainland source region. Second, H. polygyrus exhibits a niche enlargement following insularization. Indeed, H. polygyrus in Corsica is present in both A. sylvaticus and Mus musculus domesticus, while mainland H. polygyrus populations are present exclusively in Apodemus hosts. Main conclusions Our results show that H. polygyrus has undergone a loss of genetic diversity and a niche (host) enlargement following colonization of the western Mediterranean islands. To our knowledge, this study provides the first evidence for components of the ‘island syndrome’ in a parasitic nematode species. | 1575 | no | gd of pathogen | NA | no | no |
| 1576 | 2017 | 43019 | Powers, DA; Schulte, PM | 1998 | Evolutionary adaptations of gene structure and expression in natural populations in relation to a changing environment: A multidisciplinary approach to address the million-year saga of a small fish | We have used an experimentally based strategy to address molecular mechanisms underlying adaptation in Fundulus heteroclitus. In an attempt to falsify the hypothesis that selection is a major driving force in the maintenance of genetic diversity, we employed a multidisciplinary approach including allelic isozyme and mtDNA phylogeography, kinetic analyses of allelic isozymes, analysis of variation in coding and regulatory DNA sequences, metabolic biochemistry, organismal physiology, and selection experiments. Observed differences in gene structure and expression led us to make testable predictions about differences in metabolic flux, whole organism performance, and differential survival between allotypes. We have shown that variation in the lactate dehydrogenase-B (Ldh-B) protein results in differences in physiological function and is correlated with differences in survival at high temperatures. Recent work has investigated the role of variation in Ldh-B expression. There are differences in the levels of Ldh-B protein, mRNA, and transcription rate. We have addressed the mechanisms responsible for differences in transcription rate by a combination of sequence comparison; DNase I footprinting, and functional analyses both in vitro and in vivo. We have shown that variation in the regulatory sequence of Ldh-B is responsible for the differences in transcription rate between populations and that the patterns of variation are inconsistent with a neutral model of molecular evolution. This functional differentiation, coupled with departures from neutral expectations, suggests that natural selection has acted on the regulation of Ldh-B. This article illustrates the value of a multidisciplinary approach in addressing problems in gene structure, expression, and evolutionary adaptation. J. Exp. Zool. 282:71-94, 1998. (C) 1998 Wiley-Liss, Inc. | 1576 | probably no | no good fitness measure, N pop differnt in GD? | no fitness measure pop | no | no |
| 1577 | 2017 | 43019 | Medina, IL; Gomes, CB; Correa, VR; Mattos, VS; Castagnone-Sereno, P; Carneiro, RMDG | 2017 | Genetic diversity of Meloidogyne spp. parasitising potato in Brazil and aggressiveness of M. javanica populations on susceptible cultivars | Root-knot nematodes (Meloidogyne spp.) significantly impact potato production worldwide and in Brazil they are considered one of the most important group of nematodes affecting potatoes. The objectives of this study were to survey Meloidogyne spp. associated with potatoes in Brazil, determine their genetic diversity and assess the aggressiveness of M. javanica on two susceptible potato cultivars. Fifty-seven root-knot nematode populations were identified using esterase phenotyping, including Meloidogyne javanica, M. incognita, M. arenaria and M. ethiopica. Overall, root-knot nematodes were present in ca 43% of sampled sites, in which M. javanica was the most prevalent species, and the phenotypes Est J3, J2a and J2 occurred in 91.2, 6.7 and 2.1% of the positive samples, respectively. Other species, such as M. incognita, M. arenaria and M. ethiopica, were found less frequently and occurred at rates of 6.4, 4.3 and 2.1% of the samples, respectively. Sometimes, M. javanica was found in mixtures with other root-knot nematodes in ca 10.6% of sites containing Meloidogyne. After confirming the identification of 17 isolates of M. javanica and one isolate each of M. incognita, M. arenaria and M. ethiopica by SCAR markers, the populations were used to infer their genetic diversity using RAPD markers. Results revealed low intraspecifc genetic diversity among isolates (13.9%) for M. javanica. Similarly, M. javanica sub-populations (J2a) clustered together (81% of bootstrap), indicating subtle variation from typical J3 populations. The aggressiveness of four populations of M. javanica from different Brazilian states on two susceptible potato cultivars was tested under glasshouse conditions. Results indicated differences in aggressiveness among these populations and showed that potato disease was proportional to nematode reproduction factor. | 1577 | no | gd of pathogen | NA | no | no |
| 1578 | 2017 | 43019 | Richmond, JQ; Wood, DA; Stanford, JW; Fisher, RN | 2015 | Testing for multiple invasion routes and source populations for the invasive brown treesnake (Boiga irregularis) on Guam: implications for pest management | The brown treesnake (Boiga irregularis) population on the Pacific island of Guam has reached iconic status as one of the most destructive invasive species of modern times, yet no published works have used genetic data to identify a source population. We used DNA sequence data from multiple genetic markers and coalescent-based phylogenetic methods to place the Guam population within the broader phylogeographic context of B. irregularis across its native range and tested whether patterns of genetic variation on the island are consistent with one or multiple introductions from different source populations. We also modeled a series of demographic scenarios that differed in the effective size and duration of a population bottleneck immediately following the invasion on Guam, and measured the fit of these simulations to the observed data using approximate Bayesian computation. Our results exclude the possibility of serial introductions from different source populations, and instead verify a single origin from the Admiralty Archipelago off the north coast of Papua New Guinea. This finding is consistent with the hypothesis that B. irregularis was accidentally transported to Guam during military relocation efforts at the end of World War II. Demographic model comparisons suggest that multiple snakes were transported to Guam from the source locality, but that fewer than 10 individuals could be responsible for establishing the population. Our results also provide evidence that low genetic diversity stemming from the founder event has not been a hindrance to the ecological success of B. irregularis on Guam, and at the same time offers a unique ‘genetic opening’ to manage snake density using classical biological approaches. | 1578 | no | no good fitness measure, N pop differnt in GD? | NA | no | no |
| 1579 | 2017 | 43019 | Nair, S; Nash, D; Sudimack, D; Jaidee, A; Barends, M; Uhlemann, AC; Krishna, S; Nosten, F; Anderson, TJC | 2007 | Recurrent gene amplification and soft selective sweeps during evolution of multidrug resistance in malaria parasites | “When selection is strong and beneficial alleles have a single origin, local reductions in genetic diversity are expected. However, when beneficial alleles have multiple origins or were segregating in the population prior to a change in selection regime, the impact on genetic diversity may be less clear. We describe an example of such a”“soft”" selective sweep in the malaria parasite Plasmodium falciparum that involves adaptive genome rearrangements. Amplification in copy number of genome regions containing the pfmdr1 gene on chromosome 5 confer resistance to mefloquine and spread rapidly in the 1990s. Using flanking microsatellite data and real-time polymerase chain reaction determination of copy number, we show that 5-15 independent amplification events have occurred in parasites on the Thailand/Burma border. The amplified genome regions (amplicons) range in size from 14.7 to 49 kb and contain 2-11 genes, with 2-4 copies arranged in tandem. To examine the impact of drug selection on flanking variation, we genotyped 48 microsatellites on chromosome 5 in 326 parasites from a single Thai location. Diversity was reduced in a 170- to 250-kb (10-15 cM) region of chromosomes containing multiple copies of pfmdr1, consistent with hitchhiking resulting from the rapid recent spread of selected chromosomes. However, diversity immediately flanking pfmdr1 is reduced by only 42% on chromosomes bearing multiple amplicons relative to chromosomes carrying a single copy. We highlight 2 features of these results: 1) All amplicon break points occur in monomeric A/T tracts (9-45 bp). Given the abundance of these tracts in P. falciparum, we expect that duplications will occur frequently at multiple genomic locations and have been underestimated as drivers of phenotypic evolution in this pathogen. 2) The signature left by the spread of amplified genome segments is broad, but results in only limited reduction in diversity. If such "“soft”" sweeps are common in nature, statistical methods based on diversity reduction may be inefficient at detecting evidence for selection in genome-wide marker screens. This may be particularly likely when mutation rate is high, as appears to be the case for gene duplications, and in pathogen populations where effective population sizes are typically very large." | 1579 | no | gd of parasite | NA | no | no |
| 1580 | 2017 | 43019 | Reynolds, MH; Weiser, E; Jamieson, I; Hatfield, JS | 2013 | Demographic Variation, Reintroduction, and Persistence of an Island Duck (Anas laysanensis) | Population variation in life history can be important for predicting successful establishment and persistence of reintroduced populations of endangered species. The Laysan duck (Anas laysanensis) is an endangered bird native to the Hawaiian Archipelago that was extirpated from most islands after the introduction of mammalian predators. Laysan ducks were restricted to a single remote island, Laysan Island (4.1km(2)), for nearly 150 years. Since the species is not known to disperse between distant Hawaiian Islands today, 42 wild birds from Laysan Island were translocated to another mammalian predator-free low-lying atoll (Midway Atoll; 6.0km(2)) to reduce extinction risk. We explored how variation in demography influences establishment and longer-term retention of genetic diversity (rare alleles) for reintroductions of this species. We observed dramatic differences in population growth between the source (=1.18) and reintroduced (=3.28) population. The number of eggs hatched at Midway Atoll was greater than at Laysan Island, however, we found no difference in hatching success (proportion of clutch hatched) between populations. Adult females produced 3 times as many fledglings per breeding year on Midway Atoll compared to Laysan Island. We estimated population abundance of both populations until 2010 and applied a Gompertz model with a Bayesian approach to infer density dependence, process variation, observation error, and carrying capacity for the Laysan Island and Midway Atoll populations. The carrying capacity from the Gompertz model for Midway Atoll (K=883 +/- 210 SD) was estimated to be greater than that of Laysan Island (K=598 +/- 76 SD). Translocations with small numbers of founders and no immigration can create population bottlenecks, leading to loss of genetic variation over time, and potentially reducing the reintroduced population’s viability or its potential to serve as a source for future translocations. Therefore, we also assessed the probability of retaining rare alleles in an isolated reintroduced Laysan duck population using life history parameters observed from the Laysan Island and Midway Atoll populations; we concluded that additional founders are needed under scenarios using demographic estimates from both Laysan Island and Midway Atoll to retain either 90% or 95% of source population genetic diversity. (c) 2013 The Wildlife Society. | 1580 | no | no good fitness measure | NA | no | no |
| 1581 | 2017 | 43019 | FRANK, SA | 1993 | COEVOLUTIONARY GENETICS OF PLANTS AND PATHOGENS | The genetic polymorphism maintained by host-pathogen coevolution is analysed in a multilocus model. The model assumes gene-for-gene interactions of the type commonly observed between host plants and their fungal pathogens. Unstable (epidemic) systems maintain more resistance genes, fewer virulence genes, and less overall genetic diversity than stable (endemic) diseases. The stability of the system depends primarily on demographic parameters, such as the pathogen’s intrinsic rate of increase, rather than genetic parameters, such as the costs of resistance and virulence. At equilibrium the model predicts that the number of resistance alleles in each host plant follows a binomial distribution that depends on the cost to the pathogen for carrying virulence alleles. Similarly, the number of virulence alleles in each pathogen spore follows a binomial distribution that depends on one minus the cost to the host for carrying resistance alleles. Data from wild populations match the predicted binomial distributions. | 1581 | no | no good fitness measure, N pop differnt in GD? | NA | no | no |
| 1582 | 2017 | 43019 | Teacher, AGF; Garner, TWJ; Nichols, RA | 2009 | Population genetic patterns suggest a behavioural change in wild common frogs (Rana temporaria) following disease outbreaks (Ranavirus) | We use 14 microsatellite loci to investigate the impact of a viral disease (Ranavirus) on the population genetic structure of wild common frogs (Rana temporaria). Populations with a history of Ranavirus mortalities (and 83% declines in the number of frogs) were compared with populations with no history of infection. Infected ponds showed significantly elevated F(IS) (homozygote excess), significantly reduced relatedness, and no detectable effect on allelic richness. We hypothesize that the elevated F(IS) and reduced relatedness are consequences of assortative mating, and that allelic richness is maintained by immigration from nearby populations. Simulations indicate that the elevated F(IS) cannot be explained by population size reductions, but can indeed be explained by assortative mating (even if a mate choice locus is unlinked to the genetic markers). While the majority of studies consider demographic outcomes following disease outbreaks, our results indicate that emerging infectious diseases could also result in behavioural changes. | 1582 | maybe | ok gd and fitness measurement? | NA | no | yes |
| 1583 | 2017 | 43019 | Aparicio, A; Zuki, S; Pastorino, M; Martinez-Meier, A; Gallo, L | 2012 | Heritable variation in the survival of seedlings from Patagonian cypress marginal xeric populations coping with drought and extreme cold | The rear edges of tree species have begun to be perceived as highly valuable for genetic resources conservation and management. In view of expected climatic changes, the responses of trees at their xeric limits may largely be determined by their capacity to cope with augmented environmental variance. We assess the heritability of early survival of Patagonian cypress in two common-garden field tests with contrasting summer water deficits, comprising 140 and 163 open-pollinated families from 10 marginal xeric populations. The first experiment underwent less rigorous conditions than the average mesic, Mediterranean climatic conditions, which were sufficient to reveal additive genetic effects of summer drought on seedling survival. The second trial suffered strong summer water-deficit stress and a winter extreme cold event. In this harsher environment, the heritabilities of survival under summer water-deficit stress were high in all the populations (h (2) = 0.84 on average), while the heritabilities of seasonal, extreme cold survival were moderate or even nil (h (2) = 0.28 on average). We did not find evidence of genetic differentiation among populations in their capabilities to survive droughts and cold extremes. Our results indicate that even when climatic changes were strong enough to cause the extinction of the most threatened populations, heritable variation for traits underlying drought and cold tolerances may allow the marginal xeric edge of cypress to persist under augmented environmental variance, without losing overall genetic diversity. | 1583 | probably no | no good gd measure | no good gd measure of host pop | no | no |
| 1584 | 2017 | 43019 | Mann, AN; O’Reilly-Wapstra, JM; Iason, GR; Sanson, G; Davies, NW; Tilyard, P; Williams, D; Potts, BM | 2012 | Mammalian herbivores reveal marked genetic divergence among populations of an endangered plant species | Quantitative genetically based traits in dominant and keystone tree species can have extended effects on other biota and also on ecosystem processes. This has direct implications for managed plant systems, where choice of genetic stock in conservation or commercial plantings will affect the ecological and evolutionary trajectory of the associated biotic communities. Hence an understanding of genetic variation in quantitative traits, especially those that relate directly to fitness, should be incorporated into the management of species. In plants, quantitative traits such as foliar defences that mediate the complexity of biotic interactions (e.g. herbivory), may be key fitness traits to consider in the management of gene pools of species that are of high conservation value. In this paper we examine the interactions of an endangered eucalypt species, Eucalyptus morrisbyi and a marsupial herbivore, the common brushtail possum Trichosurus vulpecula. We investigate the genetic variability of resistance of plants sourced from two populations and genetic variability in foliage defences as key quantitative traits that may be essential for survival of this eucalypt species. Trichosurus vulpecula detect clear genetic divergence in the two E. morrisbyi populations as evidenced by their browsing preferences in the field. In addition, trees from the more susceptible population (Calverts Hill) suffered fitness consequences with lower flowering than trees from the more resistant population (Risdon Hills). Field feeding preferences were confirmed in captive feeding trials arguing differences were due to foliar attributes consistent with the genetic-based differences observed in key chemical and physical foliage traits. Biotic interactions such as herbivory may affect populations of rare plant species. Results of this study highlight the need to understand the degree of genetic differentiation of resistance to herbivores and in the quantitative traits mediating these interactions in species of high conservation value, as these traits affect the adaptive potential of populations. | 1584 | no | no good fitness measure | NA | no | no |
| 1585 | 2017 | 43019 | Ley, AC; Hardy, OJ | 2016 | Spatially limited clonality and pollen and seed dispersal in a characteristic climber of Central African rain forests: Haumania danckelmaniana (Marantaceae) | Gene dispersal and clonality are important aspects of plant evolution affecting the spatial genetic structure (SGS) and the long-term survival of species. In the tropics these parameters have mostly been investigated in trees and some herbs, but rarely in climbers which frequently: (1) show clonal growth leading to a patchy distribution pattern similar to that of understory herbs; and (2) flower in the canopy where they may have access to long-distance dispersal like canopy trees. We thus hypothesize for climbers an intermediate genetic structure between herbs and trees. The study aims at assessing breeding system and spatial extent of clonality and gene dispersal in Haumania danckelmaniana (Marantaceae), a common climber in the tropical rain forests from western Central Africa. In eastern Cameroon, 330 ramets were sampled at three spatial scales and genotyped at seven microsatellite loci. Clonality was moderate (clonal extend: 15-25 m, clonal diversity 0.4-0.65) indicating the importance of recruitment from seeds at this locality. The low inbreeding (FIS) suggested predominant outcrossing. The rate of decay of the relatedness between individuals with distance indicated limited gene dispersal distance (rg = 9-50 m, neighborhood sizes Nb = 23-67) in accordance with narrowly gravity dispersed seeds and restricted pollen transfer distance in densely flowering populations. The marked SGS (Sp = 0.011-0.026) was similar to that reported in tropical trees, but might increase with augmented clonality as in many herbs, especially under more severe disturbance regimes. | 1585 | no | no good fitness measure, N pop differnt in GD? | NA | no | no |
| 1586 | 2017 | 43019 | Stranger, BE; Montgomery, SB; Dimas, AS; Parts, L; Stegle, O; Ingle, CE; Sekowska, M; Smith, GD; Evans, D; Gutierrez-Arcelus, M; Price, A; Raj, T; Nisbett, J; Nica, AC; Beazley, C; Durbin, R; Deloukas, P; Dermitzakis, ET | 2012 | Patterns of Cis Regulatory Variation in Diverse Human Populations | The genetic basis of gene expression variation has long been studied with the aim to understand the landscape of regulatory variants, but also more recently to assist in the interpretation and elucidation of disease signals. To date, many studies have looked in specific tissues and population-based samples, but there has been limited assessment of the degree of inter-population variability in regulatory variation. We analyzed genome-wide gene expression in lymphoblastoid cell lines from a total of 726 individuals from 8 global populations from the HapMap3 project and correlated gene expression levels with HapMap3 SNPs located in cis to the genes. We describe the influence of ancestry on gene expression levels within and between these diverse human populations and uncover a non-negligible impact on global patterns of gene expression. We further dissect the specific functional pathways differentiated between populations. We also identify 5,691 expression quantitative trait loci (eQTLs) after controlling for both non-genetic factors and population admixture and observe that half of the cis-eQTLs are replicated in one or more of the populations. We highlight patterns of eQTL-sharing between populations, which are partially determined by population genetic relatedness, and discover significant sharing of eQTL effects between Asians, European-admixed, and African subpopulations. Specifically, we observe that both the effect size and the direction of effect for eQTLs are highly conserved across populations. We observe an increasing proximity of eQTLs toward the transcription start site as sharing of eQTLs among populations increases, highlighting that variants close to TSS have stronger effects and therefore are more likely to be detected across a wider panel of populations. Together these results offer a unique picture and resource of the degree of differentiation among human populations in functional regulatory variation and provide an estimate for the transferability of complex trait variants across populations. | 1586 | no | human | NA | no | no |
| 1587 | 2017 | 43019 | Lacy, RC; Alaks, G; Walsh, A | 2013 | Evolution of Peromyscus leucopus Mice in Response to a Captive Environment | Many wildlife species are propagated in captivity as models for behavioral, physiological, and genetic research or to provide assurance populations to protect threatened species. However, very little is known about how animals evolve in the novel environment of captivity. The histories of most laboratory strains are poorly documented, and protected populations of wildlife species are usually too small and too short-term to allow robust statistical analysis. To document the evolutionary change in captive breeding programs, we monitored reproduction and behavior across 18 generations in six experimental populations of Peromyscus leucopus mice started from a common set of 20 wild-caught founders. The mice were propagated under three breeding protocols: a strategy to retain maximal genetic diversity, artificial selection against stereotypic behaviors that were hypothesized to reflect poor adaptation to captivity, and random bred controls. Two replicates were maintained with each protocol, and inter-replicate crosses at generations 19 and 20 were used to reverse accumulated inbreeding. We found that one of the stereotypic behaviors (repetitive flipping) was positively associated with reproductive fitness, while the other (gnawing) was relatively invariant. Selection to reduce these stereotypic behaviors caused marked reduction in reproduction, and populations not under artificial selection to reduce these behaviors responded with large increases in flipping. In non-selected populations, there was rapid evolution toward much higher proportion of pairs breeding and more rapid conception. Litter size, pup survival, and weaning mass all declined slowly, to the extent that would be predicted based on inbreeding depression. Inter-crossing between replicate populations reversed these declines in fitness components but did not reverse the changes in behavior or the accelerated breeding. These findings indicate that adaptation to captivity can be rapid, affecting reproductive patterns and behaviors, even under breeding protocols designed to minimize the rate of genetic change due to random drift and inadvertent selection. | 1587 | maybe | no good fitness measure, N pop differnt in GD? | inbred pop | no | no |
| 1588 | 2017 | 43019 | Olbermann, P; Josenhans, C; Moodley, Y; Uhr, M; Stamer, C; Vauterin, M; Suerbaum, S; Achtman, M; Linz, B | 2010 | A Global Overview of the Genetic and Functional Diversity in the Helicobacter pylori cag Pathogenicity Island | The Helicobacter pylori cag pathogenicity island (cagPAI) encodes a type IV secretion system. Humans infected with cagPAI-carrying H. pylori are at increased risk for sequelae such as gastric cancer. Housekeeping genes in H. pylori show considerable genetic diversity; but the diversity of virulence factors such as the cagPAI, which transports the bacterial oncogene CagA into host cells, has not been systematically investigated. Here we compared the complete cagPAI sequences for 38 representative isolates from all known H. pylori biogeographic populations. Their gene content and gene order were highly conserved. The phylogeny of most cagPAI genes was similar to that of housekeeping genes, indicating that the cagPAI was probably acquired only once by H. pylori, and its genetic diversity reflects the isolation by distance that has shaped this bacterial species since modern humans migrated out of Africa. Most isolates induced IL-8 release in gastric epithelial cells, indicating that the function of the Cag secretion system has been conserved despite some genetic rearrangements. More than one third of cagPAI genes, in particular those encoding cell-surface exposed proteins, showed signatures of diversifying (Darwinian) selection at more than 5% of codons. Several unknown gene products predicted to be under Darwinian selection are also likely to be secreted proteins (e.g. HP0522, HP0535). One of these, HP0535, is predicted to code for either a new secreted candidate effector protein or a protein which interacts with CagA because it contains two genetic lineages, similar to cagA. Our study provides a resource that can guide future research on the biological roles and host interactions of cagPAI proteins, including several whose function is still unknown. | 1588 | no | human | NA | no | no |
| 1589 | 2017 | 43019 | Hagedorn, M; Spindler, R | 2014 | The Reality, Use and Potential for Cryopreservation of Coral Reefs | Throughout the world coral reefs are being degraded at unprecedented rates. Locally, reefs are damaged by pollution, nutrient overload and sedimentation from out-dated land-use, fishing and mining practices. Globally, increased greenhouse gases are warming and acidifying oceans, making corals more susceptible to stress, bleaching and newly emerging diseases. The coupling of climate change impacts and local anthropogenic stressors has caused a widespread and well-recognized reef crisis. Although in situ conservation practices, such as the establishment and enforcement of marine protected areas, reduce these stressors and may help slow the loss of genetic diversity on reefs, the global effects of climate change will continue to cause population declines. Gamete cryopreservation has already acted as an effective insurance policy to maintain the genetic diversity of many wildlife species, but has only just begun to be explored for coral. Already we have had a great deal of success with cryopreserving sperm and larval cells from a variety of coral species. Building on this success, we have now begun to establish genetic banks using frozen samples, to help offset these threats to the Great Barrier Reef and other areas. | 1589 | no | no good fitness measure | NA | no | no |
| 1590 | 2017 | 43019 | Ang, A; Srivasthan, A; Md-Zain, BM; Ismail, MRB; Meier, R | 2012 | LOW GENETIC VARIABILITY IN THE RECOVERING URBAN BANDED LEAF MONKEY POPULATION OF SINGAPORE | The banded leaf monkey (Presbytis femoralis femoralis) is critically endangered in Singapore and affected by widespread deforestation in southern Peninsular Malaysia. The Singapore population has recovered from a low of 15-20 to more than 40 individuals, but prior to our study it was unclear how severely the past bottleneck had depleted the genetic diversity of the population. Here, we provide the first analysis of the genetic variability based on seven samples (ca. 20% of population) collected over two years of fieldwork. We find only two haplotypes that differ only in one variable site for the hypervariable region I (HV-I) of the mitochondrial d-loop. Compared to available population-level data for other colobines (proboscis monkey, Yunnan snub-nosed monkey, Sichuan snub-nosed monkey, Angolan black and white colobus), the banded leaf monkey population in Singapore has the lowest number and the most similar haplotypes. This low genetic variability is the next challenge for the conservation of the population. Protected habitats in prospering urban environment may become important sanctuaries for endangered species, but reintroductions may have to be considered in order to restore genetic variability that was lost during past bottlenecks. | 1590 | no | only one pop | NA | no | no |
| 1591 | 2017 | 43019 | Gradish, AE; Keyghobadi, N; Otis, GW | 2015 | Population genetic structure and genetic diversity of the threatened White Mountain arctic butterfly (Oeneis melissa semidea) | The White Mountain arctic butterfly [WMA; Oeneis melissa semidea (Say)] is endemic to the alpine zone of Mts. Washington and Jefferson, New Hampshire, USA, and because of its small and declining population size, it is considered threatened. White Mountain arctic adults occur only within four alpine meadows, and it has been suggested that dispersal, and hence gene flow, may be restricted among these meadow subpopulations. Furthermore, although the WMA likely is biennial (i.e., requires 2 years for development) like all other species of Oeneis, adults emerge annually. Thus the WMA population may be further structured into two allochronic cohorts, reproductively isolated by their asynchronous adult emergence in either even- or odd-numbered years. We assessed the spatial (among meadows) and temporal (between even- and odd-year cohorts) genetic structure and diversity of the WMA using mtDNA and AFLP markers generated from non-lethally sampled wing and leg tissue. We found no evidence for restricted gene flow among meadows. AFLPs indicated weak differentiation between alternate year cohorts; however, it remains unclear whether this resulted from allochronic reproductive isolation or genetic drift. Despite the WMA’s small population size and isolation, levels of AFLP genetic diversity were generally high. Rather than focusing on factors related to population connectivity and adult dispersal, our results suggest that management efforts for the WMA should instead focus explicitly on factors affecting recruitment and mortality. | 1591 | no | no good fitness measure | NA | no | no |
| 1592 | 2017 | 43019 | Du, Y; Zheng, XX; Yao, YT; Lin, LH | 2015 | Development of microsatellite markers in the rice paddy snake Enhydris plumbea (Colubridae) | “The rice paddy snake (also known as Boie’s mud snake) Enhydris plumbea is a viviparous colubrid snake. This is a widely distributed species found from the Andaman Islands (India) and Myanmar eastwards across the Southeast Asian Peninsula and southern China southwards into Indonesia; and it is present on most of the Indonesian islands and eastwards to Sulawesi and the Moluccas. The snake has been overexploited by local people for traditional medicine to treat skin diseases, and is listed on the”“Lists of terrestrial wildlife under state protection, which are beneficial or of important economic or scientific value”" promulgated by China’s State Forestry Administration in 2000. Knowledge of population structure and genetic diversity is crucial for effective wildlife preservation. Specific molecular markers such as mitochondrial DNA and microsatellites are useful tools for achieving this goal. Here, eight polymorphic microsatellite loci were isolated from an enriched genomic library of E. plumbea. One hundred and twenty individuals were collected from Wenzhou population in Zhejiang, China. These markers revealed a high degree of genetic diversity (4-8 alleles per locus) and heterozygosity (H (O) ranged from 0.148 to 0.789, and H (E) ranged from 0.347 to 0.766). No locus exhibited significant deviations from Hardy-Weinberg equilibrium. There was no evidence of linkage disequilibrium among pairs of loci. These loci will be useful for future study of population structure, genetic diversity and conservation strategy design of E. plumbea." | 1592 | no | no good fitness measure | NA | no | no |
| 1593 | 2017 | 43019 | Schenk, A; Obbard, ME; Kovacs, KM | 1998 | Genetic relatedness and home-range overlap among female black bears (Ursus americanus) in northern Ontario, Canada | The degree of philopatry exhibited by females in an unhunted black bear (Ursus americanus) population occupying the Chapleau Crown Game Preserve in northern Ontario was examined. A truncated kernel estimator was used to identify home-range use. Pairs of adult females were categorized as having home ranges that had moderate overlap or low overlap or were adjacent and non-overlapping or non-adjacent and non-overlapping. Females had low overlap with 6.4 other females, on average, and moderate overlap with 1.5 females. The degree of philopatry was assessed using two methods, each of which was used in an attempt to examine home-range overlap and average genetic relatedness. Relatedness among bears was determined from DNA fingerprints, using an alkaline phosphatase labelled multilocus probe and chemiluminescence detection. The first method involved choosing the 3 oldest females in the region to represent potential matriarchs, and all neighbouring females were identified (n = 8, 8, and 11). DNA fingerprints from each matriarch were compared with those of her neighbours. Average band-sharing coefficients and relatedness estimates within the groups did not reveal patterns of close kinship. The second method involved band-sharing comparisons among pairs of females from each of the 4 home-range categories (n = 12, 57, 80, and 21). Again, no relationship between spatial proximity and average genetic relatedness (range 0.032-0.120) was suggested. The extensive home-range overlap exhibited by this population is not a consequence of natal philopatric tendencies. | 1593 | no | no good fitness measure | NA | no | no |
| 1594 | 2017 | 43019 | Yang, X; Charlebois, P; Gnerre, S; Coole, MG; Lennon, NJ; Levin, JZ; Qu, J; Ryan, EM; Zody, MC; Henn, MR | 2012 | De novo assembly of highly diverse viral populations | Background: Extensive genetic diversity in viral populations within infected hosts and the divergence of variants from existing reference genomes impede the analysis of deep viral sequencing data. A de novo population consensus assembly is valuable both as a single linear representation of the population and as a backbone on which intra-host variants can be accurately mapped. The availability of consensus assemblies and robustly mapped variants are crucial to the genetic study of viral disease progression, transmission dynamics, and viral evolution. Existing de novo assembly techniques fail to robustly assemble ultra-deep sequence data from genetically heterogeneous populations such as viruses into full-length genomes due to the presence of extensive genetic variability, contaminants, and variable sequence coverage. Results: We present VICUNA, a de novo assembly algorithm suitable for generating consensus assemblies from genetically heterogeneous populations. We demonstrate its effectiveness on Dengue, Human Immunodeficiency and West Nile viral populations, representing a range of intra-host diversity. Compared to state-of-the-art assemblers designed for haploid or diploid systems, VICUNA recovers full-length consensus and captures insertion/deletion polymorphisms in diverse samples. Final assemblies maintain a high base calling accuracy. VICUNA program is publicly available at: http://www.broadinstitute.org/scientific-community/science/projects/viral-genomics/viral-genomics-analysis-software. Conclusions: We developed VICUNA, a publicly available software tool, that enables consensus assembly of ultra-deep sequence derived from diverse viral populations. While VICUNA was developed for the analysis of viral populations, its application to other heterogeneous sequence data sets such as metagenomic or tumor cell population samples may prove beneficial in these fields of research. | 1594 | no | NA | NA | no | no |
| 1595 | 2017 | 43019 | Boman, S; Grapputo, A; Lindstrom, L; Lyytinen, A; Mappes, J | 2008 | Quantitative genetic approach for assessing invasiveness: geographic and genetic variation in life-history traits | Predicting the spread of invasive species is a challenge for modern ecology. Although many invasive species undergo genetic bottlenecks during introduction to new areas resulting in a loss of genetic diversity, successful invaders manage to flourish in novel environments either because of pre-adaptations or because important traits contain adaptive variation enabling rapid adaptation to changing conditions. To predict and understand invasion success, it is crucial to analyse these features. We assessed the potential of a well-known invader, the Colorado potato beetle (Leptinotarsa decemlineata), to expand north of its current range in Europe. A short growing season and harsh overwintering conditions are apparent limiting factors for this species’ range. By rearing full-sib families from four geographically distinct populations (Russia, Estonia, Poland, Italy) at two fluctuating temperature regimes, we investigated (a) possible differences in survival, development time, and body size among populations and (b) the amount of adaptive variation within populations in these traits. All populations were able to complete their development in cooler conditions than in their current range. A significant genotype-environment interaction for development time and body size suggests the presence of adaptive genetic variation, indicating potential to adapt to cooler conditions. The northernmost population had the highest survival rates and fastest development times on both temperature regimes, suggesting pre-adaptation to cooler temperatures. Other populations had minor differences in development times. Interestingly, this species lacks the classical trade-off between body size and development time which could have contributed to its invasion potential. This study demonstrates the importance of considering both ecological and evolutionary aspects when assessing invasion risk. | 1595 | no | No GD diff between pop | NA | no | no |
| 1596 | 2017 | 43019 | Ballard, JWO; Puslednik, L; Wolff, JN; Russell, RC | 2009 | Variation Under Nature: A Sesquicentennial DNA Barcoding Perspective | “Darwin’s classic reference to the”“tangled bank”" acknowledges the living biodiversity. Traditionally, biodiversity assessment has been the realm of the classical taxonomist. However, times have changed swiftly and now biodiversity may be assessed by high throughput sequencing of short regions of DNA. Commonly this is called DNA "“barcoding”“. In this study, we investigate the genetic diversity within and between four morphological mosquito species that are vectors of medically important viral pathogens. We obtained 627 bp of sequence information from the N-terminus of the mitochondrially encoded cytochrome c oxidase I (COI) gene from 12 Aedes vigilax and 10 Ae. camptorhynchus representing five localities in Australia. We also downloaded GenBank COI sequence data from 18 Ae. albopictus and 14 Ae. aegypti. Maximum likelihood phylogenetic analyses recovered the four distinct morphological species, as well as a distinct lineage within what is currently described as Ae. vigilax. We termed this lineage ‘A’. Intra- and interspecific variation quantified for Ae. vigilax and Ae. vigilax lineage ‘A’ suggest that this morphospecies may include distinct genetic or cryptic species. Ae. aegypti also had high intraspecific variation, however, we concluded that this is most likely due to the large effective population size of this species and its broad distribution through the tropics. Phylogenetic analyses and analyses of genetic diversity at COI suggests that Ae. camptorhynchus and Ae. albopictus are robust species. The results of this study highlight the efficiency, utility and ease of barcoding. The study also documents specific limitations including the importance of taxon sampling and the necessity for this tool to be used in conjunction with additional taxonomic techniques, ecological studies and physiological data.” | 1596 | no | no good fitness measure, N pop differnt in GD? | NA | no | no |
| 1597 | 2017 | 43019 | Suo, C; Xu, HY; Khor, CC; Ong, RTH; Sim, X; Chen, JM; Tay, WT; Sim, KS; Zeng, YX; Zhang, XJ; Liu, JJ; Tai, ES; Wong, TY; Chia, KS; Teo, YY | 2012 | Natural positive selection and north-south genetic diversity in East Asia | Recent reports have identified a north-south cline in genetic variation in East and South-East Asia, but these studies have not formally explored the basis of these clinical differences. Understanding the origins of these variations may provide valuable insights in tracking down the functional variants in genomic regions identified by genetic association studies. Here we investigate the genetic basis of these differences with genome-wide data from the HapMap, the Human Genome Diversity Project and the Singapore Genome Variation Project. We implemented four bioinformatic measures to discover genomic regions that are considerably differentiated either between two Han Chinese populations in the north and south of China, or across 22 populations in East and South-East Asia. These measures prioritized genomic stretches with: (i) regional differences in the allelic spectrum for SNPs common to the two Han Chinese populations; (ii) differential evidence of positive selection between the two populations as quantified by integrated haplotype score (iHS) and cross-population extended haplotype homozygosity (XP-EHH); (iii) significant correlation between allele frequencies and geographical latitudes of the 22 populations. We also explored the extent of linkage disequilibrium variations in these regions, which is important in combining genetic association studies from North and South Chinese. Two of the regions that emerged are found in HLA class I and II, suggesting that the HLA imputation panel from the HapMap may not be directly applicable to every Chinese sample. This has important implications to autoimmune studies that plan to impute the classical HLA alleles to fine map the SNP association signals. European Journal of Human Genetics (2012) 20, 102-110; doi:10.1038/ejhg.2011.139; published online 27 July 2011 | 1597 | no | NA | NA | no | no |
| 1598 | 2017 | 43019 | Yu, JM; Pressoir, G; Briggs, WH; Bi, IV; Yamasaki, M; Doebley, JF; McMullen, MD; Gaut, BS; Nielsen, DM; Holland, JB; Kresovich, S; Buckler, ES | 2006 | A unified mixed-model method for association mapping that accounts for multiple levels of relatedness | As population structure can result in spurious associations, it has constrained the use of association studies in human and plant genetics. Association mapping, however, holds great promise if true signals of functional association can be separated from the vast number of false signals generated by population structure(1,2). We have developed a unified mixed-model approach to account for multiple levels of relatedness simultaneously as detected by random genetic markers. We applied this new approach to two samples: a family-based sample of 14 human families, for quantitative gene expression dissection, and a sample of 277 diverse maize inbred lines with complex familial relationships and population structure, for quantitative trait dissection. Our method demonstrates improved control of both type I and type II error rates over other methods. As this new method crosses the boundary between family-based and structured association samples, it provides a powerful complement to currently available methods for association mapping. | 1598 | no | human | NA | no | no |
| 1599 | 2017 | 43019 | Pastor, T; Garza, JC; Allen, P; Amos, W; Aguilar, A | 2004 | Low genetic variability in the highly endangered Mediterranean monk seal | Genetic variability is an important component in the ability of populations to adapt in the face of environmental change. Here we report the first description of nuclear genetic variability in the only remaining sizable colony of the Mediterranean monk seal (Monachus monachus), located at Cap Blanc (Western Sahara, Mauritania), whose estimated size during the study period (1994-May 1997) was about 320 individuals. We tested 42 microsatellite loci isolated from five pinniped species in a sample of 52 pups. Three loci failed to give any product, and of the remaining 39, only 15 were polymorphic, with a maximum of 3 alleles detected. Three loci appeared to be X-linked. No departures from Hardy-Weinberg equilibrium were detected and no genetic structure was found between the two nursing caves currently occupied by the seals. Several analytical methods show that, as a consequence of a severe bottleneck, the population has suffered a decrease in genetic variability over the last few centuries. | 1599 | no | only one pop | NA | no | no |
| 1600 | 2017 | 43019 | Wang, JH; Wang, N; Hu, DD; Zhong, XQ; Wang, SX; Gu, XB; Peng, XR; Yang, GY | 2014 | Genetic Diversity of Echinococcus granulosus in Southwest China Determined by the Mitochondrial NADH Dehydrogenase Subunit 2 Gene | We evaluated genetic diversity and structure of Echinococcus granulosus by analyzing the complete mitochondrial NADH dehydrogenase subunit 2 (ND2) gene in 51 isolates of E. granulosus sensu stricto metacestodes collected at three locations in this region. We detected 19 haplotypes, which formed a distinct clade with the standard sheep strain (G1). Hence, all 51 isolates were identified as E. granulosus sensu stricto (G1-G3). Genetic relationships among haplotypes were not associated with geographical divisions, and fixation indices (Fst) among sampling localities were low. Hence, regional populations of E. granulosus in the southwest China are not differentiated, as gene flow among them remains high. This information is important for formulating unified region-wide prevention and control measures. We found large negative Fu’s Fs and Tajima’s D values and a unimodal mismatch distribution, indicating that the population has undergone a demographic expansion. We observed high genetic diversity among the E. granulosus s. s. isolates, indicating that the parasite population in this important bioregion is genetically robust and likely to survive and spread. The data from this study will prove valuable for future studies focusing on improving diagnosis and prevention methods and developing robust control strategies. | 1600 | no | no pop level? | NA | no | no |
| 1601 | 2017 | 43019 | Wang, JN; Fernandez-Pavia, SP; Larsen, MM; Garay-Serrano, E; Gregorio-Cipriano, R; Rodriguez-Alvarado, G; Grunwald, NJ; Goss, EM | 2017 | High levels of diversity and population structure in the potato late blight pathogen at the Mexico centre of origin | Globally destructive crop pathogens often emerge by migrating out of their native ranges. These pathogens are often diverse at their centre of origin and may exhibit adaptive variation in the invaded range via multiple introductions from different source populations. However, source populations are generally unidentified or poorly studied compared to invasive populations. Phytophthora infestans, the causal agent of late blight, is one of the most costly pathogens of potato and tomato worldwide. Mexico is the centre of origin and diversity of P.infestans and migration events out of Mexico have enormously impacted disease dynamics in North America and Europe. The debate over the origin of the pathogen, and population studies of P.infestans in Mexico, has focused on the Toluca Valley, whereas neighbouring regions have been little studied. We examined the population structure of P.infestans across central Mexico, including samples from Michoacan, Tlaxcala and Toluca. We found high levels of diversity consistent with sexual reproduction in Michoacan and Tlaxcala and population subdivision that was strongly associated with geographic region. We determined that population structure in central Mexico has contributed to diversity in introduced populations based on relatedness of U.S. clonal lineages to Mexican isolates from different regions. Our results suggest that P.infestans exists as a metapopulation in central Mexico, and this population structure could be contributing to the repeated re-emergence of P.infestans in the United States and elsewhere. | 1601 | no | gd of pathogen | NA | no | no |
| 1602 | 2017 | 43020 | Athey, TBT; Teatero, S; Takamatsu, D; Wasserscheid, J; Dewar, K; Gottschalk, M; Fittipaldi, N | 2016 | Population Structure and Antimicrobial Resistance Profiles of Streptococcus suis Serotype 2 Sequence Type 25 Strains | Strains of serotype 2 Streptococcus suis are responsible for swine and human infections. Different serotype 2 genetic backgrounds have been defined using multilocus sequence typing (MLST). However, little is known about the genetic diversity within each MLST sequence type (ST). Here, we used whole-genome sequencing to test the hypothesis that S. suis serotype 2 strains of the ST25 lineage are genetically heterogeneous. We evaluated 51 serotype 2 ST25 S. suis strains isolated from diseased pigs and humans in Canada, the United States of America, and Thailand. Whole-genome sequencing revealed numerous large-scale rearrangements in the ST25 genome, compared to the genomes of ST1 and ST28 S. suis strains, which result, among other changes, in disruption of a pilus island locus. We report that recombination and lateral gene transfer contribute to ST25 genetic diversity. Phylogenetic analysis identified two main and distinct Thai and North American clades grouping most strains investigated. These clades also possessed distinct patterns of antimicrobial resistance genes, which correlated with acquisition of different integrative and conjugative elements (ICEs). Some of these ICEs were found to be integrated at a recombination hot spot, previously identified as the site of integration of the 89K pathogenicity island in serotype 2 ST7 S. suis strains. Our results highlight the limitations of MLST for phylogenetic analysis of S. suis, and the importance of lateral gene transfer and recombination as drivers of diversity in this swine pathogen and zoonotic agent. | 1602 | no | gd of pathogen | NA | no | no |
| 1603 | 2017 | 43020 | Bollmer, JL; Hull, JM; Ernest, HB; Sarasola, JH; Parker, PG | 2011 | Reduced MHC and neutral variation in the Galapagos hawk, an island endemic | Background: Genes at the major histocompatibility complex (MHC) are known for high levels of polymorphism maintained by balancing selection. In small or bottlenecked populations, however, genetic drift may be strong enough to overwhelm the effect of balancing selection, resulting in reduced MHC variability. In this study we investigated MHC evolution in two recently diverged bird species: the endemic Galapagos hawk (Buteo galapagoensis), which occurs in small, isolated island populations, and its widespread mainland relative, the Swainson’s hawk (B. swainsoni). Results: We amplified at least two MHC class II B gene copies in each species. We recovered only three different sequences from 32 Galapagos hawks, while we amplified 20 unique sequences in 20 Swainson’s hawks. Most of the sequences clustered into two groups in a phylogenetic network, with one group likely representing pseudogenes or nonclassical loci. Neutral genetic diversity at 17 microsatellite loci was also reduced in the Galapagos hawk compared to the Swainson’s hawk. Conclusions: The corresponding loss in neutral diversity suggests that the reduced variability present at Galapagos hawk MHC class II B genes compared to the Swainson’s hawk is primarily due to a founder event followed by ongoing genetic drift in small populations. However, purifying selection could also explain the low number of MHC alleles present. This lack of variation at genes involved in the adaptive immune response could be cause for concern should novel diseases reach the archipelago. | 1603 | no | no good fitness measure, N pop differnt in GD? | NA | no | no |
| 1604 | 2017 | 43020 | Puscas, M; Choler, P; Tribsch, A; Gielly, L; Rioux, D; Gaudeul, M; Taberlet, P | 2008 | Post-glacial history of the dominant alpine sedge Carex curvula in the European Alpine System inferred from nuclear and chloroplast markers | The alpine sedge Carex curvula ssp. curvula is a clonal, dominant graminoid found in the European Alps, the Carpathians, the Pyrenees and in some of the Balkan Mountains. It is a late-successional species of acidophilous alpine meadows that occurs on sites that were covered by ice during the last glacial maximum (LGM). By applying the amplified fragment length polymorphism (AFLP) fingerprinting and chloroplast DNA (cpDNA) sequencing, we attempted to identify the recolonization routes followed by the species after the last ice retreat. We relied on the genetic diversity of 37 populations covering the entire distributional range of the species. As a wind-pollinated species, C. curvula is characterized by a low level of population genetic differentiation. Nuclear and chloroplast data both support the hypothesis of a long-term separation of Eastern (Balkans and Carpathians) and Western (Alps and Pyrenees) lineages. In the Alps, a continuum of genetic depauperation from the east to the west may be related to a recolonization wave originating in the eastern-most parts of the chain, where the main glacial refugium was likely located. The Pyrenean populations are nested within the western Alps group and show a low level of genetic diversity, probably due to recent long-distance colonization. In contrast to the Alps, we found no phylogeographical structure in the Carpathians. The combination of reduced ice extension during the Wurm period and the presence of large areas of siliceous substrate at suitable elevation suggest that in contrast to populations in the Alps, the species in the Carpathians underwent a local vertical migration rather than extinction and recolonization over long distance. | 1604 | no | no good fitness measure | NA | no | no |
| 1605 | 2017 | 43020 | Albrecht, E; Zhang, DP; Mays, AD; Saftner, RA; Stommel, JR | 2012 | Genetic diversity in Capsicum baccatum is significantly influenced by its ecogeographical distribution | Background: The exotic pepper species Capsicum baccatum, also known as the aji or Peruvian hot pepper, is comprised of wild and domesticated botanical forms. The species is a valuable source of new genes useful for improving fruit quality and disease resistance in C. annuum sweet bell and hot chile pepper. However, relatively little research has been conducted to characterize the species, thus limiting its utilization. The structure of genetic diversity in a plant germplasm collection is significantly influenced by its ecogeographical distribution. Together with DNA fingerprints derived from AFLP markers, we evaluated variation in fruit and plant morphology of plants collected across the species native range in South America and evaluated these characters in combination with the unique geography, climate and ecology at different sites where plants originated. Results: The present study mapped the ecogeographic distribution, analyzed the spatial genetic structure, and assessed the relationship between the spatial genetic pattern and the variation of morphological traits in a diverse C. baccatum germplasm collection spanning the species distribution. A combined diversity analysis was carried out on the USDA-ARS C. baccatum germplasm collection using data from GIS, morphological traits and AFLP markers. The results demonstrate that the C. baccatum collection covers wide geographic areas and is adapted to divergent ecological conditions in South America ranging from cool Andean highland to Amazonia rainforest. A high level of morphological diversity was evident in the collection, with fruit weight the leading variable. The fruit weight distribution pattern was compatible to AFLP-based clustering analysis for the collection. A significant spatial structure was observed in the C. baccatum gene pool. Division of the domesticated germplasm into two major regional groups (Western and Eastern) was further supported by the pattern of spatial population structure. Conclusions: The results reported improve our understanding of the combined effects of geography, ecology and human intervention on organization of the C. baccatum genepool. The results will facilitate utilization of C. baccatum for crop improvement and species conservation by providing a framework for efficient germplasm collection management and guidance for future plant acquisitions. | 1605 | no | no good fitness measure, N pop differnt in GD? | NA | no | no |
| 1606 | 2017 | 43020 | Acosta, JL; Hernandez-Mondragon, AC; Correa-Acosta, LC; Cazanas-Padilla, SN; Chavez-Florencio, B; Ramirez-Vega, EY; Monge-Cazares, T; Aguilar-Salinas, CA; Tusie-Luna, T; del Bosque-Plata, L | 2016 | Rare intronic variants of TCF7L2 arising by selective sweeps in an indigenous population from Mexico | Background: Genetic variations of the TCF7L2 gene are associated with the development of Type 2 diabetes (T2D). The associated mutations have demonstrated an adaptive role in some human populations, but no studies have determined the impact of evolutionary forces on genetic diversity in indigenous populations from Mexico. Here, we sequenced and analyzed the variation of the TCF7L2 gene in three Amerindian populations and compared the results with whole-exon-sequencing of Mestizo populations from Sigma and the 1000 Genomes Project to assess the roles of selection and recombination in diversity. Results: The diversity in the indigenous populations was biased to intronic regions. Most of the variation was low frequency. Only mutations rs77961654 and rs61724286 were located on exon 15. We did not observe variation in intronic region 4-6 in any of the three indigenous populations. In addition, we identified peaks of selective sweeps in the mestizo samples from the Sigma Project within this region. By replicating the analysis of association with T2D between case-controls from the Sigma Project, we determined that T2D was most highly associated with the rs7903146 risk allele and to a lesser extent with the other six variants. All associated markers were located in intronic region 4-6, and their r(2) values of linkage disequilibrium were significantly higher in the Mexican population than in Africans from the 1000 Genomes Project. We observed reticulations in both the haplotypes network analysis from seven marker associates and the neighborNet tree based on 6061 markers in the TCF7L2 gene identified from all samples of the 1000 Genomes Project. Finally, we identified two recombination hotspots in the upstream region and 3’ end of the TCF7L2 gene. Conclusions: The lack of diversity in intronic region 4-6 in Indigenous populations could be an effect of selective sweeps generated by the selection of neighboring rare variants at T2D-associated mutations. The survivors’ variants make the intronic region 4-6 the area of the greatest population differentiation within the TCF7L2 gene. The abundance of selective peak sweeps in the downstream region of the TCF7L2 gene suggests that the TCF7L2 gene is part of a region that is in constant recombination between populations. | 1606 | no | human | NA | no | no |
| 1607 | 2017 | 43020 | Trask, AE; Bignal, EM; McCracken, DI; Monaghan, P; Piertney, SB; Reid, JM | 2016 | Evidence of the phenotypic expression of a lethal recessive allele under inbreeding in a wild population of conservation concern | Deleterious recessive alleles that are masked in outbred populations are predicted to be expressed in small, inbred populations, reducing both individual fitness and population viability. However, there are few definitive examples of phenotypic expression of lethal recessive alleles under inbreeding conditions in wild populations. Studies that demonstrate the action of such alleles, and infer their distribution and dynamics, are required to understand their potential impact on population viability and inform management responses. The Scottish population of red-billed choughs (Pyrrhocorax pyrrhocorax), which currently totals <60 breeding pairs and is of major conservation concern, has recently been affected by lethal blindness in nestlings. We used family data to show that the pattern of occurrence of blindness within and across affected families that produced blind nestlings was exactly 025, matching that expected given a single-locus autosomal lethal recessive allele. Furthermore, the observed distribution of blind nestlings within affected families did not differ from that expected given Mendelian inheritance of such an allele. Relatedness estimates showed that individuals from affected families were not more closely related to each other than they were to individuals from unaffected families that did not produce blind nestlings. Blind individuals tended to be less heterozygous than non-blind individuals, as expected if blindness was caused by the expression of a recessive allele under inbreeding. However, there was no difference in the variance in heterozygosity estimates, suggesting that some blind individuals were relatively outbred. These results suggest carriers of the blindness allele may be widely distributed across contemporary families rather than restricted to a single family lineage, implying that the allele has persisted across multiple generations. Blindness occurred at low frequency (affecting 16% of observed nestlings since 1981). However, affected families had larger initial brood sizes than unaffected families. Such high fecundity of carriers of a lethal recessive allele might reflect overdominance, potentially reducing purging and increasing allele persistence probability. We thereby demonstrate the phenotypic expression of a lethal recessive allele in a wild population of conservation concern, and provide a general framework for inferring allele distribution and persistence and informing management responses. | 1607 | no | inbreeding | NA | no | no |
| 1608 | 2017 | 43020 | Cullingham, CI; Nakada, SM; Merrill, EH; Bollinger, TK; Pybus, MJ; Coltman, DW | 2011 | Multiscale population genetic analysis of mule deer (Odocoileus hemionus hemionus) in western Canada sheds new light on the spread of chronic wasting disease | To successfully manage wildlife diseases, it is necessary to understand factors that influence spread. One approach is to analyze host movement and social structure, as these behaviors can be associated with the probability of transmission. Some populations of mule deer (Odocoileus hemionus hemionus (Rafinesque, 1817)) in western Canada are infected with chronic wasting disease (CWD), a transmissible and fatal neurodegenerative disease. We used population analysis of spatial genetic structure of mule deer at broad and local scales to understand factors that influence spread. We genotyped 2535 mule deer sampled from Alberta, Saskatchewan, and portions of British Columbia using 16 microsatellite loci. We found weak genetic structure at broad spatial scales (overall F(ST) = 0.008) that was well defined by geographic distance, indicating the risk of CWD spread from the focus of infection will decline gradually with increasing distance, but there are no barriers to the spread over time. At the local scale of approximately 2 km, elevated relatedness among CWD-infected individuals suggests transmission rates within social groups. Sex-biased spatial autocorrelation in genetic relatedness also indicates that female philopatry underlies the social structure, and therefore transmission among relatives is potentially driving local disease persistence. | 1608 | no | no good fitness measure | NA | no | no |
| 1609 | 2017 | 43020 | Miretti, MM; Ferro, JA; Lara, MA; Contel, EPB | 2001 | Restriction fragment length polymorphism (RFLP) in Exon 2 of the BoLA-DRB3 gene in South American cattle | The Bola-DRB3 gene participates in the development of the immune response and is highly polymorphic. For these reasons, it has been a candidate gene in studies of the genetic basis of disease resistance and in population genetic analysis. South American native cattle breeds have been widely replaced by improved exotic breeds leading to a loss of genetic resources. In particular South American native breeds have high levels of fertility and disease resistance. This work describes genetic variability in the BoLA-DRB3 gene in native (Caracu, Pantaneiro, Argentinean Creole) and exotic (Holstein, Jersey, Nelore, Gir) cattle breeds in Brazil and Argentina. PCR-RFLP alleles were identified by combining the restriction patterns for the BoLA-DRB3.2 locus obtained with RsaI, BstY, and HaeIII restriction enzymes. Allelic frequencies and deviations from the Hardy-Weinberg equilibrium were also calculated. Analysis of the 24 BoLA-DRB3 PCR-RFLP alleles identified showed differences in the allele distributions among breeds. | 1609 | no | domesticated animal | NA | no | no |
| 1610 | 2017 | 43020 | Simon, B; Cenis, JL; De La Rua, P | 2007 | Distribution patterns of the Q and B biotypes of Bemisia tabaci in the Mediterranean Basin based on microsatellite variation | At least five of the biotypes described in the Bemisia tabaci (Gennadius) (Homoptera: Aleyrodidae) complex are known to be present in the Mediterranean Basin area. Only two of them, however, are economically relevant, that is, biotypes B and Q. Biological and genetic differences between the two biotypes have been well studied, but less is known about their patterns of genetic variation and population structure. To address these issues, a study was undertaken based on variation at six microsatellite loci among a subset of nine B. tabaci populations (five belonging to the Q and four to the B biotype). The data obtained show that (i) these loci showed considerable polymorphism in the Q and B biotypes populations although the presence of null alleles can obscure the picture; (ii) the Iberian-Q, Canarian-Q, and Egyptian-B populations exhibit heterozygosity excess as a result of bottleneck events; (iii) the low genetic differentiation between the Israeli, Iberian Peninsula, and Italian populations suggest that these populations share a common gene pool; (iv) the genetic distances between the Canarian-Q population and the geographically close population from Morocco indicates spatial isolation and a limited gene flow; and finally (v) the microsatellite data for the B populations indicate that the whiteflies from Egypt and Israel have a close phylogenetic relationship, but the source of these biotype B invasions into the Mediterranean area remains unknown. | 1610 | no | no good fitness measure | NA | no | no |
| 1611 | 2017 | 43020 | Pereira, F; Moreira, C; Fonseca, L; van Asch, B; Mota, M; Abrantes, I; Amorim, A | 2013 | New Insights into the Phylogeny and Worldwide Dispersion of Two Closely Related Nematode Species, Bursaphelenchus xylophilus and Bursaphelenchus mucronatus | The pinewood nematode, Bursaphelenchus xylophilus, is one of the greatest threats to coniferous forests worldwide, causing severe ecological damage and economic loss. The biology of B. xylophilus is similar to that of its closest relative, B. mucronatus, as both species share food resources and insect vectors, and have very similar morphological characteristics, although little pathogenicity to conifers has been associated with B. mucronatus. Using both nuclear and mitochondrial DNA markers, we show that B. xylophilus and B. mucronatus form distinct phylogenetic groups with contrasting phylogeographic patterns. B. xylophilus presents lower levels of intraspecific diversity than B. mucronatus, as expected for a species that evolved relatively recently through geographical or reproductive isolation. Genetic diversity was particularly low in recently colonised areas, such as in southwestern Europe. By contrast, B. mucronatus displays high levels of genetic diversity and two well-differentiated clades in both mitochondrial and nuclear DNA phylogenies. The lack of correlation between genetic and geographic distances in B. mucronatus suggests intense gene flow among distant regions, a phenomenon that may have remained unnoticed due to the reduced pathogenicity of the species. Overall, our findings suggest that B. xylophilus and B. mucronatus have different demographic histories despite their morphological resemblance and ecological overlap. These results suggest that Bursaphelenchus species are a valuable model for understanding the dispersion of invasive species and the risks posed to native biodiversity and ecosystems. | 1611 | no | gd of pathogen | NA | no | no |
| 1612 | 2017 | 43020 | Zegeye, H; Rasheed, A; Makdis, F; Badebo, A; Ogbonnaya, FC | 2014 | Genome-Wide Association Mapping for Seedling and Adult Plant Resistance to Stripe Rust in Synthetic Hexaploid Wheat | Use of genetic diversity from related wild and domesticated species has made a significant contribution to improving wheat productivity. Synthetic hexaploid wheats (SHWs) exhibit natural genetic variation for resistance and/or tolerance to biotic and abiotic stresses. Stripe rust caused by (Puccinia striiformis f. sp. tritici; Pst), is an important disease of wheat worldwide. To characterise loci conferring resistance to stripe rust in SHWs, we conducted a genome-wide association study (GWAS) with a panel of 181 SHWs using the wheat 9K SNP iSelect array. The SHWs were evaluated for their response to the prevailing races of Pst at the seedling and adult plant stages, the latter in replicated field trials at two sites in Ethiopia in 2011. About 28% of the SHWs exhibited immunity at the seedling stage while 56% and 83% were resistant to Pst at the adult plant stage at Meraro and Arsi Robe, respectively. A total of 27 SNPs in nine genomic regions (1BS, 2AS, 2BL, 3BL, 3DL, 5A, 5BL, 6DS and 7A) were linked with resistance to Pst at the seedling stage, while 38 SNPs on 18 genomic regions were associated with resistance at the adult plant stage. Six genomic regions were commonly detected at both locations using a mixed linear model corrected for population structure, kinship relatedness and adjusted for false discovery rate (FDR). The loci on chromosome regions 1AS, 3DL, 6DS and 7AL appeared to be novel QTL; our results confirm that resynthesized wheat involving its progenitor species is a rich source of new stripe (yellow) rust resistance that may be useful in choosing SHWs and incorporating diverse yellow rust (YR) resistance loci into locally adapted wheat cultivars. | 1612 | no | crop | NA | no | no |
| 1613 | 2017 | 43020 | Oleksa, A; Chybicki, IJ; Gawronski, R; Svensson, GP; Burczyk, J | 2013 | Isolation by distance in saproxylic beetles may increase with niche specialization | Species confined to temporally stable habitats are usually susceptible to habitat fragmentation, as living in long-lasting habitats is predicted to constrain evolution of dispersal ability. In Europe, saproxylic invertebrates associated with tree hollows are currently threatened due to the severe fragmentation of their habitat, but data on the population genetic consequences of such habitat decline are still scarce. By employing AFLP markers, we compared the spatial genetic structure of two ecologically and taxonomically related beetle species, Osmoderma barnabita and Protaetia marmorata (Cetoniidae). Both species are exclusively associated with tree hollows, but O. barnabita has a more restricted host preferences compared to P. marmorata. Analyses of spatial autocorrelation showed, in line with the predicted low dispersal potential of these saproxylic beetles, that both species are characterized by a strong kinship structure, which was more pronounced in the specialist O. barnabita than in the generalist P. marmorata. Individuals of both species sampled within single trees showed high relatedness (a parts per thousand 0.50 in O. barnabita and a parts per thousand 0.15 in P. marmorata). Interestingly, groups of pheromone-emitting O. barnabita males sampled on the same tree trunk were found to be full brothers. Whether this result can be explained by kin selection to increase attraction of conspecific females for mating or by severe inbreeding of beetles within individual tree hollows needs further study. Although our studied populations were significantly inbred, our results suggest that the dispersal ability of Osmoderma beetles may be one order of magnitude greater than suggested by previous dispersal studies and acceptable levels of habitat fragmentation for metapopulation survival may be bigger than previously thought. | 1613 | no | no good fitness measure | NA | no | no |
| 1614 | 2017 | 43020 | Sim, SC; Van Deynze, A; Stoffel, K; Douches, DS; Zarka, D; Ganal, MW; Chetelat, RT; Hutton, SF; Scott, JW; Gardner, RG; Panthee, DR; Mutschler, M; Myers, JR; Francis, DM | 2012 | High-Density SNP Genotyping of Tomato (Solanum lycopersicum L.) Reveals Patterns of Genetic Variation Due to Breeding | The effects of selection on genome variation were investigated and visualized in tomato using a high-density single nucleotide polymorphism (SNP) array. 7,720 SNPs were genotyped on a collection of 426 tomato accessions (410 inbreds and 16 hybrids) and over 97% of the markers were polymorphic in the entire collection. Principal component analysis (PCA) and pairwise estimates of F-st supported that the inbred accessions represented seven sub-populations including processing, large-fruited fresh market, large-fruited vintage, cultivated cherry, landrace, wild cherry, and S. pimpinellifolium. Further divisions were found within both the contemporary processing and fresh market sub-populations. These sub-populations showed higher levels of genetic diversity relative to the vintage sub-population. The array provided a large number of polymorphic SNP markers across each sub-population, ranging from 3,159 in the vintage accessions to 6,234 in the cultivated cherry accessions. Visualization of minor allele frequency revealed regions of the genome that distinguished three representative sub-populations of cultivated tomato (processing, fresh market, and vintage), particularly on chromosomes 2, 4, 5, 6, and 11. The PCA loadings and F-st outlier analysis between these three sub-populations identified a large number of candidate loci under positive selection on chromosomes 4, 5, and 11. The extent of linkage disequilibrium (LD) was examined within each chromosome for these sub-populations. LD decay varied between chromosomes and sub-populations, with large differences reflective of breeding history. For example, on chromosome 11, decay occurred over 0.8 cM for processing accessions and over 19.7 cM for fresh market accessions. The observed SNP variation and LD decay suggest that different patterns of genetic variation in cultivated tomato are due to introgression from wild species and selection for market specialization. | 1614 | no | crop | NA | no | no |
| 1615 | 2017 | 43020 | Sovic, MG; Carstens, BC; Gibbs, HL | 2016 | Genetic diversity in migratory bats: Results from RADseq data for three tree bat species at an Ohio windfarm | Genetic analyses can identify the scale at which wildlife species are impacted by human activities, and provide demographic information useful for management. Here, we use thousands of nuclear DNA genetic loci to assess whether genetic structure occurs within Lasiurus cinereus (Hoary Bat), L. borealis (Red Bat), and Lasionycteris noctivagans (Silver-Haired Bat) bats found at a wind turbine site in Ohio, and to also estimate demographic parameters in each of these three groups. Our specific goals are to: 1) demonstrate the feasibility of isolating RADseq loci from these tree bat species, 2) test for genetic structure within each species, including any structure that may be associated with time (migration period), and 3) use coalescent-based modeling approaches to estimate genetically-effective population sizes and patterns of population size changes over evolutionary timescales. Thousands of loci were successfully genotyped for each species, demonstrating the value of RADseq for generating polymorphic loci for population genetic analyses in these bats. There was no evidence for genetic differentiation between groups of samples collected at different times throughout spring and fall migration, suggesting that individuals from each species found at the wind facility are from single panmictic populations. Estimates of present-day effective population sizes varied across species, but were consistently large, on the order of 10(5)-10(6). All populations show evidence of expansions that date to the Pleistocene. These results, along with recent work also suggesting limited genetic structure in bats across North America, argue that additional biomarker systems such as stable-isotopes or trace elements should be investigated as alternative and/or complementary approaches to genetics for sourcing individuals collected at single wind farm sites. | 1615 | no | no good fitness measure, N pop differnt in GD? | NA | no | no |
| 1616 | 2017 | 43020 | Feder, J; Blech, I; Ovadia, O; Amar, S; Wainstein, J; Raz, I; Dadon, S; Arking, DE; Glaser, B; Mishmar, D | 2008 | Differences in mtDNA haplogroup distribution among 3 Jewish populations alter susceptibility to T2DM complications | Background: Recent genome-wide association studies searching for candidate susceptibility loci for common complex diseases such as type 2 diabetes mellitus (T2DM) and its common complications have uncovered novel disease-associated genes. Nevertheless these large-scale population screens often overlook the tremendous variation in the mitochondrial genome (mtDNA) and its involvement in complex disorders. Results: We have analyzed the mitochondrial DNA (mtDNA) genetic variability in Ashkenazi (Ash), Sephardic (Seph) and North African (NAF) Jewish populations (total n = 1179). Our analysis showed significant differences (p < 0.001) in the distribution of mtDNA genetic backgrounds (haplogroups) among the studied populations. To test whether these differences alter the pattern of disease susceptibility, we have screened our three Jewish populations for an association of mtDNA genetic haplogroups with T2DM complications. Our results identified population-specific susceptibility factors of which the best example is the Ashkenazi Jewish specific haplogroup N1b1, having an apparent protective effect against T2DM complications in Ash (p = 0.006), being absent in the NAF population and under-represented in the Seph population. We have generated and analyzed whole mtDNA sequences from the disease associated haplogroups revealing mutations in highly conserved positions that are good candidates to explain the phenotypic effect of these genetic backgrounds. Conclusion: Our findings support the possibility that recent bottleneck events leading to over-representation of minor mtDNA alleles in specific genetic isolates, could result in population-specific susceptibility loci to complex disorders. | 1616 | no | human | NA | no | no |
| 1617 | 2017 | 43020 | Perez, G; Slippers, B; Wingfield, MJ; Wingfield, BD; Carnegie, AJ; Burgess, TI | 2012 | Cryptic species, native populations and biological invasions by a eucalypt forest pathogen | Human-associated introduction of pathogens and consequent invasions is very evident in areas where no related organisms existed before. In areas where related but distinct populations or closely related cryptic species already exist, the invasion process is much harder to unravel. In this study, the population structure of the Eucalyptus leaf pathogen Teratosphaeria nubilosa was studied within its native range in Australia, including both commercial plantations and native forests. A collection of 521 isolates from across its distribution was characterized using eight microsatellite loci, resulting in 112 multilocus haplotypes (MLHs). Multivariate and Bayesian analyses of the population conducted in structure revealed three genetically isolated groups (A, B and C), with no evidence for recombination or hybridization among groups, even when they co-occur in the same plantation. DNA sequence data of the ITS (n = 32), beta-tubulin (n = 32) and 27 anonymous loci (n = 16) were consistent with microsatellite data in suggesting that T. nubilosa should be considered as a species complex. Patterns of genetic diversity provided evidence of biological invasions by the pathogen within Australia in the states of Western Australia and New South Wales and helped unravel the pattern of invasion beyond Australia into New Zealand, Brazil and Uruguay. No significant genetic differences in pathogen populations collected in native forests and commercial plantations were observed. This emphasizes the importance of sanitation in the acquisition of nursery stock for the establishment of commercial plantations. | 1617 | no | gd of pathogen | NA | no | no |
| 1618 | 2017 | 43020 | Rutkowski, R; Sielezniew, M; Szostak, A | 2009 | Contrasting levels of polymorphism in cross-amplified microsatellites in two endangered xerothermophilous, obligatorily myrmecophilous, butterflies of the genus Phengaris (Maculinea) (Lepidoptera: Lycaenidae) | “We analysed the polymorphism of cross-amplified microsatellite loci in two endangered butterflies of the genus Phengaris, which inhabit warm grasslands. Specimens of P. arion and P. ‘rebeli’ collected in Poland showed contrasting levels of variability in the investigated loci. All six tested microsatellites were highly variable in P. arion, whereas in P. ‘rebeli’ one locus was monomorphic and the other four loci showed a rather low level of polymorphism. Genetic differentiation was small or moderate among P. arion populations and high among P. ‘rebeli’ populations. We hypothesise that these differences reflect: (1) the relatively high genetic variability of P. arion compared with other members of the genus, indicated by previous studies on allozymes; (2) the small, in the majority of cases, genetic differentiation among populations of P. arion, which suggests recent gene flow; (3) a decrease in the genetic variability and increased isolation of peripheral populations of P. ‘rebeli’. In both species, the occurrence of”“null”" alleles was suggested for some loci, which should be taken into consideration in future studies of the population structure. Additional analysis performed on the corrected frequency of alleles indicated that "“null”" alleles constitute only a minor problem when estimating genetic differentiation, using F(ST) and AMOVA, however they strongly influence estimates of heterozygosity and F(IS), and inferences about the number of genetic groups and assignment of individuals, especially in the case of ‘P. rebeli’." | 1618 | no | no good fitness measure | NA | no | no |
| 1619 | 2017 | 43020 | Bridges, CM; Semlitsch, RD | 2001 | Genetic variation in insecticide tolerance in a population of southern leopard frogs (Rana sphenocephala): Implications for amphibian conservation | Currently, conservation efforts are devoted to determining the extent and the causes of the decline of many amphibian species worldwide. Human impacts frequently degrade amphibian habitat and have been implicated in many declines. Because genetic variance is critical in determining the persistence of a species in a changing environment, we examined the amount of genetic variability present in a single population for tolerance to an environmental stressor. We examined the amount of genetic variability among full- and half-sib families in a single population of southern leopard frogs (Rana sphenocephala) with respect to their tolerance to lethal concentrations of the agricultural chemical, carbaryl. Analysis of time-to-death data indicated significant differences among full-sib families and suggests a large amount of variability present in the responses to this environmental stressor. Significant differences in responses among half-sib families indicated that there is additive genetic variance. These data suggest that this population may have the ability to adapt to environmental stressors. It is possible that declines of amphibian populations in the western United States may be attributed to low genetic variability resulting from limited migration among populations and small population sizes. | 1619 | probably no | no good fitness measure, N pop differnt in GD? | no good comp between high and low gd | no | no |
| 1620 | 2017 | 43020 | Navarro, P; Vitart, V; Hayward, C; Tenesa, A; Zgaga, L; Juricic, D; Polasek, O; Hastie, ND; Rudan, I; Campbell, H; Wright, AE; Haley, CS; Knott, SA | 2010 | Genetic Comparison of a Croatian Isolate and CEPH European Founders | Human isolates have been postulated as a good resource for the identification of QTL due to reduced genetic diversity and a more homogeneous environment. Isolates may also have increased linkage disequilibrium (LD) due to small effective population size and, either loss or increase in frequency of alleles that are rare in the general population from which they originate. Here we investigate the difference in allele and genotype frequencies, LD and homozygous tracts between an isolate-several villages from the island of Vis in Croatia-and an outbred population of European origin: the Hapmap CEPH founders. Using the HumanHap300 v1 Genotyping BeadChip, we show that our population does not differ greatly from the reference CEU outbred population despite having a slightly higher proportion of monomorphic loci, a slightly higher long-range LD, and a greater proportion of individuals with long homozygous tracts. We conclude that genotyping arrays should perform equally well in our isolate as in outbred European populations for disease mapping studies ally that SNP-trait associations discovered in our well-characterized Croatian isolate should be valid in the general European population from which they descend. Genet. Epidemiol. 34:140-145, 2010. (C) 2009 Wiley-Liss, Inc. | 1620 | no | human | NA | no | no |
| 1621 | 2017 | 43020 | Lyu, ZY; Zhi, JR; Zhou, YF; Meng, ZH; Yue, WB | 2016 | Genetic diversity and origin of Dendrothrips minowai (Thysanoptera: Thripidae) in Guizhou, China | In this study, we explored the population genetics of Dendrothrips minowai Priesner, one of the most destructive horticultural pests, in Guizhou Province of China. We have analyzed the data of mitochondrial cytochrome c oxidose subunit (COI) gene sequences of D. minowai from 27 populations sampled over two years in Guizhou. The results confirmed that the thrips retained moderate levels of genetic diversity (including number of haplotypes, haplotype diversity and nucleotide diversity). Three population groups could be identified according to spatial analysis of molecular variance (SAMOVA). A similar tendency was found according to phylogenetic trees. The AMOVA revealed that >52% and 38% of variation was attributed to groups and individuals within populations, respectively. Gene flow among populations, estimated using the coalescent method, was relatively high (M = 196.8-825.4). Star-like networks and the demographic analysis (including neutrality testing and mismatch distribution) indicated that thrips underwent a recent population expansion in Guizhou and began to diverge in the Late Pleistocene. The results provided evidence that the eastern region of Guizhou was the source area for the species.(C) 2016 Korean Society of Applied Entomology, Taiwan Entomological Society and Malaysian Plant Protection Society. Published by Elsevier B.V. All rights reserved. | 1621 | no | gd of pathogen | NA | no | no |
| 1622 | 2017 | 43020 | Scorrano, G; Lelli, R; Martinez-Labarga, C; Scano, G; Contini, I; Hafez, HS; Rudan, P; Rickards, O | 2016 | Variability and distribution of COL1A2 (type I collagen) polymorphisms in the central-eastern Mediterranean Basin | Background: The most abundant of the collagen protein family, type I collagen is encoded by the COL1A2 gene. The COL1A2 restriction fragment length polymorphisms (RFLPs) EcoRI, RsaI and MspI in samples from several different central-eastern Mediterranean populations were analysed and found to be potentially informative anthropogenetic markers. Aim: The objective was to define the genetic variability of COL1A2 in the central-eastern Mediterranean and to shed light on its genetic distribution in human groups over a wide geographic area. Subjects and methods: PCR-RFLP analysis of EcoRI, RsaI and MspI polymorphisms of the COL1A2 gene was performed on oral swab and blood samples from 308 individuals from the central-eastern Mediterranean Basin. The genetic similarities among these groups and other populations described in the literature were investigated through correspondence analysis. Results: Single-marker data and haplotype frequencies seemed to suggest a genetic homogeneity within the European populations, whereas a certain degree of differentiation was noted for the Egyptians and the Turks. Conclusions: The genetic variability in the central-eastern Mediterranean area is probably a result of the geographical barrier of the Mediterranean Sea, which separated European and African populations over time. | 1622 | no | human | NA | no | no |
| 1623 | 2017 | 43020 | Ehlers, BK; David, P; Damgaard, CF; Lenormand, T | 2016 | Competitor relatedness, indirect soil effects and plant coexistence | How species coexist is a central question in ecology. Niche differentiation can prevent two species from excluding each other by competition. However, this interaction can vary in space because of internal factors such as intraspecific genetic variation, or external ones such as the presence of a third species. While these effects have been repeatedly observed, their joint effect on the outcome of competition has rarely been considered. We experimentally investigated how a dominant ecosystem engineer (the shrub Thymus vulgaris) affects interactions between two co-occurring and closely related annuals (Medicago minima and M.rigidula). We ask first whether the outcome of their interaction depends on the genetic identity of the competitors, and in particular whether intraspecific interaction differs between genetically related versus unrelated conspecifics. Secondly, we ask whether coexistence of the two species is modified by the presence of thyme plants altering local soil conditions. We grew genotypes collected in nature of both annual species in inter- and intraspecific competition experiments using soil collected directly underneath thyme plant (thyme soil) and soil collected away from thyme plants (no-thyme soil). We found that thyme soil affected interactions in a way that shifted the species’ rank. Crucial for this result was the division of intraspecific competition between genetically related and unrelated individuals. Intraspecific competition was significantly less detrimental to kin than to unrelated conspecifics. In highly structured plant populations, such effects could favour local dominance of a patch by one species. However, this kin effect occurred for each species only in its preferred soil (thyme vs. no thyme), promoting a stabilizing coexistence mechanism at the landscape level.Synthesis. This study shows that the importance of intraspecific competition relative to interspecific competition can be highly dependent on the genotype identity of intraspecific competitors and of the local environment in which the interaction occurs. The local environment can itself be modified by the presence of a third species in the community. These results emphasize how, in order to understand the overall problem of species coexistence, it can be insightful to divide this into smaller local-scale problems of coexistence. | 1623 | no | multiple species diversity | NA | no | no |
| 1624 | 2017 | 43020 | Nakao, R; Kitagawa, T | 2015 | Differences in the behavior and ecology of wild type medaka (Oryzias latipes complex) and an orange commercial variety (himedaka) | Genetic disturbance in wild populations of medaka (Oryzias latipes complex) has been mainly caused by the introduction of the orange-red commercial variety medaka (himedaka) in Japan. To examine whether survival, reproduction, and species recognition would be influenced by this difference in body coloration, we conducted three laboratory experiments (predatory pressure, mate choice, schooling behavior) using wild type medaka and himedaka. In the predation experiment using dark chub (Candidia temminckii) as a predator, himedaka were predated upon more often than wild type medaka. However, individuals did not choose mates or select schooling groups based on himedaka or wild type medaka phenotypes. The results indicate that himedaka receive higher predation pressure but are able to easily mate with wild type medaka in a natural environment. To conserve the genetic diversity of wild medaka populations, we need to control the risk of genetic disturbance caused by himedaka. J. Exp. Zool. 323A: 349-358, 2015. (c) 2015 Wiley Periodicals, Inc. | 1624 | no | no good fitness measure, N pop differnt in GD? | NA | no | no |
| 1625 | 2017 | 43020 | Gracia, E; Ortego, J; Godoy, JA; Perez-Garcia, JM; Blanco, G; Delgado, MD; Penteriani, V; Almodovar, I; Botella, F; Sanchez-Zapata, JA | 2015 | Genetic Signatures of Demographic Changes in an Avian Top Predator during the Last Century: Bottlenecks and Expansions of the Eurasian Eagle Owl in the Iberian Peninsula | The study of the demographic history of species can help to understand the negative impact of recent population declines in organisms of conservation concern. Here, we use neutral molecular markers to explore the genetic consequences of the recent population decline and posterior recovery of the Eurasian eagle owl (Bubo bubo) in the Iberian Peninsula. During the last century, the species was the object of extermination programs, suffering direct persecution by hunters until the 70’s. Moreover, during the last decades the eagle owl was severely impacted by increased mortality due to electrocution and the decline of its main prey species, the European rabbit (Oryctolagus cuniculus). In recent times, the decrease of direct persecution and the implementation of some conservation schemes have allowed the species’ demographic recovery. Yet, it remains unknown to which extent the past population decline and the later expansion have influenced the current species’ pattern of genetic diversity. We used eight microsatellite markers to genotype 235 eagle owls from ten Spanish subpopulations and analyse the presence of genetic signatures attributable to the recent population fluctuations experienced by the species. We found moderate levels of differentiation among the studied subpopulations and Bayesian analyses revealed the existence of three genetic clusters that grouped subpopulations from central, south-western and southeastern Spain. The observed genetic structure could have resulted from recent human-induced population fragmentation, a patchy distribution of prey populations and/or the philopatric behaviour and habitat selection of the species. We detected an old population bottleneck, which occurred approximately 10,000 years ago, and significant signatures of recent demographic expansions. However, we did not find genetic signatures for a recent bottleneck, which may indicate that population declines were not severe enough to leave detectable signals on the species genetic makeup or that such signals have been eroded by the rapid demographic recovery experienced by the species in recent years. | 1625 | no | no good fitness measure | NA | no | no |
| 1626 | 2017 | 43020 | Frary, VJ; Duchamp, J; Maehr, DS; Larkin, JL | 2011 | Density and distribution of a colonizing front of the American black bear Ursus americanus | Effective management of small expanding populations is aided by the availability of reliable estimates of distribution, as well as by demographic characteristics such as population density, genetic diversity and sex ratio. The range of the black bear Ursus americanus in the southeastern United States is expanding to include areas from which it has been extirpated for more than a century. Lack of baseline demographic data in recently reoccupied areas leaves little information on which to base emerging management needs. We estimated the current extent of expansion at the colonizing front of a black bear population in the central Appalachian Mountains and identified landscape-scale habitat characteristics affecting the expansion. In 2007, we genotyped hair samples collected throughout a 8,205-km(2) area at six microsatellite loci to identify individual black bears and estimate genetic diversity. We used capture-recapture and occupancy analyses to estimate density and distribution of black bears in our study area. Our results suggest that black bears were not uniformly distributed, but were localized to high elevations and protected public conservation lands. Limited availability of high elevations to the west, north and northeast of our study area may limit further expansion. Despite a limited distribution and low estimated population density (7.51 bears/100 km(2)), genetic diversity at genotyped loci was high (mean H-o=0.81). Until the population grows further, the small number of individuals in the region may be sensitive to management practices that result in mortalities, especially to females. Our research exemplifies the utility of remote genetic sampling to estimate population demographics of wide-ranging mammals throughout a large study area, particularly where private land ownership hinders intensive study. | 1626 | no | no good fitness measure, N pop differnt in GD? | NA | no | no |
| 1627 | 2017 | 43020 | Laroche, J; Durand, JD | 2004 | Genetic structure of fragmented populations of a threatened endemic percid of the Rhone river: Zingel asper | Zingel asper is an endemic percid of the Rhone basin considered to be critically endangered. This species was continuously distributed throughout the Rhone in 1900, but today only occupies 17% of its initial area. In the present study, five microsatellite loci were used to assess the level of genetic variability within and among populations localized in different sub-basins. Contrasting results were obtained for the three main populations from the Rhone. A reduced allelic diversity was observed for the two populations displaying the lowest patch sizes (length of the river system occupied); of these, a recent genetic bottleneck was detected for the population showing a particularly low density. However, the third population was characterized by a relatively large spatial extent, high local fish concentrations and an allelic diversity that was twice as high and associated with an equilibrium between mutation and drift. Thus, this population shows an apparently better evolutionary potential for long-term survival. Since 1930, a marked fragmentation of the whole Rhone system has appeared, related to the development of dams, and we assume that the significant genetic differentiation detected between the populations could mainly reflect the impact of this fragmentation. The high turnover of the Z. asper populations, and the major role of dispersal in population persistence (highlighted in a recent population dynamics study), indeed suggest that the differentiation observed could mainly have arisen from habitat fragmentation in recent history. | 1627 | no | no good fitness measure | NA | no | no |
| 1628 | 2017 | 43020 | Jerlstrom-Hultqvist, J; Franzen, O; Ankarklev, J; Xu, FF; Nohynkova, E; Andersson, JO; Svard, SG; Andersson, B | 2010 | Genome analysis and comparative genomics of a Giardia intestinalis assemblage E isolate | Background: Giardia intestinalis is a protozoan parasite that causes diarrhea in a wide range of mammalian species. To further understand the genetic diversity between the Giardia intestinalis species, we have performed genome sequencing and analysis of a wild-type Giardia intestinalis sample from the assemblage E group, isolated from a pig. Results: We identified 5012 protein coding genes, the majority of which are conserved compared to the previously sequenced genomes of the WB and GS strains in terms of microsynteny and sequence identity. Despite this, there is an unexpectedly large number of chromosomal rearrangements and several smaller structural changes that are present in all chromosomes. Novel members of the VSP, NEK Kinase and HCMP gene families were identified, which may reveal possible mechanisms for host specificity and new avenues for antigenic variation. We used comparative genomics of the three diverse Giardia intestinalis isolates P15, GS and WB to define a core proteome for this species complex and to identify lineage-specific genes. Extensive analyses of polymorphisms in the core proteome of Giardia revealed differential rates of divergence among cellular processes. Conclusions: Our results indicate that despite a well conserved core of genes there is significant genome variation between Giardia isolates, both in terms of gene content, gene polymorphisms, structural chromosomal variations and surface molecule repertoires. This study improves the annotation of the Giardia genomes and enables the identification of functionally important variation. | 1628 | no | gd of parasite | NA | no | no |
| 1629 | 2017 | 43020 | Callahan, CM; Rowe, CA; Ryel, RJ; Shaw, JD; Madritch, MD; Mock, KE | 2013 | Continental-scale assessment of genetic diversity and population structure in quaking aspen (Populus tremuloides) | Aim Quaking aspen (Populus tremuloides) has the largest natural distribution of any tree native to North America. The primary objectives of this study were to characterize range-wide genetic diversity and genetic structuring in quaking aspen, and to assess the influence of glacial history and rear-edge dynamics. Location North America. Methods Using a sample set representing the full longitudinal and latitudinal extent of the species’ distribution, we examined geographical patterns of genetic diversity and structuring using 8 nuclear microsatellite loci in 794 individuals from 30 sampling sites. Results Two major genetic clusters were identified across the range: a southwestern cluster and a northern cluster. The south-western cluster, which included two subclusters, was bounded approximately by the Continental Divide to the east and the southern extent of the ice sheet at the Last Glacial Maximum to the north. Subclusters were not detected in the northern cluster, despite its continent-wide distribution. Genetic distance was significantly correlated with geographical distance in the south-western but not the northern cluster, and allelic richness was significantly lower in south-western sampling sites compared with northern sampling sites. Population structuring was low overall, but elevated in the south-western cluster. Main conclusions Aspen populations in the south-western portion of the range are consistent with expectations for a historically stable edge, with low within-population diversity, significant geographical population structuring, and little evidence of northward expansion. Structuring within the southwestern cluster may result from distinct gene pools separated during the Pleistocene and reunited following glacial retreat, similar to patterns found in other forest tree species in the western USA. In aspen, populations in the southwestern portion of the species range are thought to be at particularly high risk of mortality with climate change. Our findings suggest that these same populations may be disproportionately valuable in terms of both evolutionary potential and conservation value. | 1629 | no | no good fitness measure | NA | no | no |
| 1630 | 2017 | 43020 | Evison, SEF; Fazio, G; Chappell, P; Foley, K; Jensen, AB; Hughes, WOH | 2016 | Innate expression of antimicrobial peptides does not explain genotypic diversity in resistance to fungal brood parasites in the honey bee | The genetic basis of host resistance to parasites is a fundamental aspect of host-parasite co-evolution, yet the precise mechanisms often remain unclear. Here, we follow on from a previous study on the genetically mediated variation in resistance to two common fungal brood parasites that cause chalkbrood and stonebrood in the honey bee. We assessed whether genetically mediated variation in resistance can be explained by the baseline immunocompetence of different larval genotypes by correlating the constitutive expression of two key immune genes with the observed level of resistance of each larval genotype to four different fungal brood parasites. We found significant variation between larval genotypes in the constitutive expression of abaecin but not defensin 2, but despite a suggestion of negative correlations between gene expression and resistance level in older larvae, there was no consistent evidence that baseline abaecin expression is a relevant predictor of resistance to these parasites. These results suggest that the constitutive expression of abaecin appears to have a genetic basis in honey bee larvae but that mechanisms other than innate expression of antimicrobial peptides might be more important in defence against the specific fungal brood parasites assessed here. | 1630 | no | no good fitness measure, N pop differnt in GD? | NA | no | no |
| 1631 | 2017 | 43020 | Santos, LM; Gama, RA; Eiras, AE; Fonseca, CG | 2010 | Genetic differences based on AFLP markers in the mosquito species Anopheles darlingi collected in versus near houses in the region of Porto Velho, RO, Brazil | Anopheles darlingi is the most important malaria vector in Central and South America. After a dramatic reduction of malaria cases in the whole Brazilian territory, with the lowest abundance being attained by 1970, the disease resurged in the Amazon region, where it is now a great public health concern. Consequently, better knowledge about vector species became urgent. We examined the genetic diversity and population structure of A. darlingi, sampled in four localities in the State of Rondonia, Brazil, using 139 amplified fragment length polymorphism marker loci. In each locality, samples were collected in two environments: a peri-domicile one (in the balconies of houses) and an extra-domicile environment (about 15 m from the house). Estimates of expected heterozygosity, Shannon diversity index and percentage of polymorphic loci showed medium to high values, with the samples from the areas closer to Porto Velho exhibiting the smallest values. There was evidence of small population differences, evaluated by F-st, genetic distance and analysis of molecular variance. Comparison between peri- and extra-domicile samples showed greater values of F-st and genetic distance than between pairs of localities, indicating influence of environmental conditions on the genetics of populations. | 1631 | no | no good fitness measure | NA | no | no |
| 1632 | 2017 | 43020 | Sauter, J; Solloch, UV; Giani, AS; Hofmann, JA; Schmidt, AH | 2016 | Simulation shows that HLA-matched stem cell donors can remain unidentified in donor searches | The heterogeneous nature of HLA information in real-life stem cell donor registries may hamper unrelated donor searches. It is even possible that fully HLA-matched donors with incomplete HLA information are not identified. In our simulation study, we estimated the probability of these unnecessarily failed donor searches. For that purpose, we carried out donor searches in several virtual donor registries. The registries differed by size, composition with respect to HLA typing levels, and genetic diversity. When up to three virtual HLA typing requests were allowed within donor searches, the share of unnecessarily failed donor searches ranged from 1.19% to 4.13%, thus indicating that non-identification of completely HLA-matched stem cell donors is a problem of practical relevance. The following donor registry characteristics were positively correlated with the share of unnecessarily failed donor searches: large registry size, high genetic diversity, and, most strongly correlated, large fraction of registered donors with incomplete HLA typing. Increasing the number of virtual HLA typing requests within donor searches up to ten had a smaller effect. It follows that the problem of donor non-identification can be substantially reduced by complete high-resolution HLA typing of potential donors. | 1632 | no | NA | NA | no | no |
| 1633 | 2017 | 43020 | Azuma, N; Miranda, RM; Goshima, S; Abe, S | 2015 | Phylogeography of Neptune whelk (Neptunea arthritica) suggests sex-biased impact of tributyltin pollution and overfishing around northern Japan | The Neptune whelk, Neptunea arthritica, is a sublittoral snail from Pacific waters that is a food resource and supports a commercially important fishery in northern Japan. This species showed a severe decline during the 1970s and 1980s, possibly because of overfishing, imposex caused by tributyltin (TBT) pollution and parasite infection. In the present study, we investigated genetic variation among the populations of N. arthritica from eight localities in northern Japan, including Hokkaido and Aomori, using a mitochondrial DNA (mtDNA) marker, a partial sequence of the cytochrome c oxidase subunit I (COI) gene. We also addressed the evolutionary history of N. arthritica and human impact on the population genetic profiles of this species. The parsimony network showed 14 COI haplotypes separated into two groups (Groups A and B), with an intermediate haplotype connecting both groups. Among eight populations, six were fixed for only one or two haplotypes, and no geographic-genetic correlation was found; they were probably affected by random drift. These results contrasted with those from previous microsatellite analysis, which indicated that geographic structure was the result of restricted gene flow between populations. Our results suggested that N. arthritica diverged into Groups A and B during the Pliocene; however, recent TBT pollution and size-selective fishing pressure have reduced genetic diversity and concealed the natural population structure. The present study also suggested that human impact may cause longstanding and possibly irreversible modification of ecosystems, particularly for species forming discrete and relatively small local populations, such as N. arthritica. Thus, the combined use of mtDNA and microsatellite genetic data provides a powerful tool to investigate the health of biodiversity in molluscs. | 1633 | no | no good fitness measure | NA | no | no |
| 1634 | 2017 | 43020 | Oomen, RA; Hutchings, JA | 2015 | Variation in spawning time promotes genetic variability in population responses to environmental change in a marine fish | The level of phenotypic plasticity displayed within a population (i.e. the slope of the reaction norm) reflects the short-term response of a population to environmental change, while variation in reaction norm slopes among populations reflects spatial variation in these responses. Thus far, studies of thermal reaction norm variation have focused on geographically driven adaptation among different latitudes, altitudes or habitats. Yet, thermal variability is a function of both space and time. For organisms that reproduce at different times of year, such variation has the potential to promote adaptive variability in thermal responses for critical early life stages. Using common-garden experiments, we examined the spatial scale of genetic variation in thermal plasticity for early life-history traits among five populations of endangered Atlantic cod (Gadus morhua) that spawn at different times of year. Patterns of plasticity for larval growth and survival suggest that population responses to climate change will differ substantially, with increasing water temperatures posing a considerably greater threat to autumn-spawning cod than to those that spawn in winter or spring. Adaptation to seasonal cooling or warming experienced during the larval stage is suggested as a possible cause. Furthermore, populations that experience relatively cold temperatures during early life might be more sensitive to changes in temperature. Substantial divergence in adaptive traits was evident at a smaller spatial scale than has previously been shown for a marine fish with no apparent physical barriers to gene flow (similar to 200 km). Our findings highlight the need to consider the impact of intraspecific variation in reproductive timing on thermal adaptation when forecasting the effects of climate change on animal populations. | 1634 | no | No GD diff between pop | NA | no | no |
| 1635 | 2017 | 43020 | Yasukochi, Y; Kurosaki, T; Yoneda, M; Koike, H; Satta, Y | 2012 | MHC class II DQB diversity in the Japanese black bear, Ursus thibetanus japonicus | Background: The major histocompatibility complex (MHC) genes are one of the most important genetic systems in the vertebrate immune response. The diversity of MHC genes may directly influence the survival of individuals against infectious disease. However, there has been no investigation of MHC diversity in the Asiatic black bear (Ursus thibetanus). Here, we analyzed 270-bp nucleotide sequences of the entire exon 2 region of the MHC DQB gene by using 188 samples from the Japanese black bear (Ursus thibetanus japonicus) from 12 local populations. Results: Among 185 of 188 samples, we identified 44 MHC variants that encoded 31 different amino acid sequences (allotypes) and one putative pseudogene. The phylogenetic analysis suggests that MHC variants detected from the Japanese black bear are derived from the DQB locus. One of the 31 DQB allotypes, Urth-DQB*01, was found to be common to all local populations. Moreover, this allotype was shared between the black bear on the Asian continent and the Japanese black bear, suggesting that Urth-DQB*01 might have been maintained in the ancestral black bear population for at least 300,000 years. Our findings, from calculating the ratio of non-synonymous to synonymous substitutions, indicate that balancing selection has maintained genetic variation of peptide-binding residues at the DQB locus of the Japanese black bear. From examination of genotype frequencies among local populations, we observed a considerably lower level of observed heterozygosity than expected. Conclusions: The low level of observed heterozygosity suggests that genetic drift reduced DQB diversity in the Japanese black bear due to a bottleneck event at the population or species level. The decline of DQB diversity might have been accelerated by the loss of rare variants that have been maintained by negative frequency-dependent selection. Nevertheless, DQB diversity of the black bear appears to be relatively high compared with some other endangered mammalian species. This result suggests that the Japanese black bears may also retain more potential resistance against pathogens than other endangered mammalian species. To prevent further decline of potential resistance against pathogens, a conservation policy for the Japanese black bear should be designed to maintain MHC rare variants in each local population. | 1635 | no | no good fitness measure, N pop differnt in GD? | NA | no | no |
| 1636 | 2017 | 43020 | Whitlock, R; Grime, JP; Booth, R; Burke, T | 2007 | The role of genotypic diversity in determining grassland community structure under constant environmental conditions | 1. A recent experiment varied the genetic diversity of model grassland communities under standardized soil and management conditions and at constant initial species diversity. After 5 years’ growth, genetically diverse communities retained more species diversity and became more similar in species composition than genetically impoverished communities. 2. Here we present the results of further investigation within this experimental system. We proposed that two mechanisms - the first invoking genetically determined and constant differences in plant phenotypes and the second invoking genotype-environment interactions - could each underpin these results. This mechanistic framework was used as a tool to interpret our findings. 3. We used inter-simple sequence repeat (ISSR) DNA markers to confirm which of the individuals of six study species initially included in the model communities were unique genotypes. We then used the molecular markers to assess the survival and abundance of each genotype at the end of the 5-year experimental period. 4. The DNA marker data were used to create, for the first time, a genotype abundance hierarchy describing the structure of a community at the level of genotypes. This abundance hierarchy revealed wide variation in the abundance of genotypes within species, and large overlaps in the performance of the genotypes of different species. 5. Each genotype achieved a consistent level of abundance within genetically diverse communities, which differed from that attained by other genotypes of the same species. The abundance hierarchy of genotypes within species also showed consistency across communities differing in their initial level of genetic diversity, such that species abundance in genetically impoverished communities could be predicted, in part, by genotypic identity. 6. Three species (including two canopy-dominants) experienced shifts in their community-level genotype abundance hierarchies that were consistent with an increased influence of genotype-environment interactions in genetically impoverished communities. 7. Our results indicate that under relatively constant environmental conditions the species abundance structure of plant communities can in part be predicted from the genotypic composition of their component populations. Genotype-environment interactions also appear to shape the structure of communities under such conditions, although further experiments are needed to clarify the magnitude and mechanism of these effects. | 1636 | no | multiple species diversity | NA | no | no |
| 1637 | 2017 | 43020 | Reece, SE; Drew, DR; Gardner, A | 2008 | Sex ratio adjustment and kin discrimination in malaria parasites | Malaria parasites and related Apicomplexans are the causative agents of the some of the most serious infectious diseases of humans, companion animals, livestock and wildlife. These parasites must undergo sexual reproduction to transmit from vertebrate hosts to vectors, and their sex ratios are consistently female- biased. Sex allocation theory, a cornerstone of evolutionary biology, is remarkably successful at explaining female- biased sex ratios in multicellular taxa, but has proved controversial when applied to malaria parasites. Here we show that, as predicted by theory, sex ratio is an important fitness- determining trait and Plasmodium chabaudi parasites adjust their sex allocation in response to the presence of unrelated conspecifics. This suggests that P. chabaudi parasites use kin discrimination to evaluate the genetic diversity of their infections, and they adjust their behaviour in response to environmental cues. Malaria parasites provide a novel way to test evolutionary theory, and support the generality and power of a darwinian approach. | 1637 | no | gd of parasite | NA | no | no |
| 1638 | 2017 | 43020 | Winter, SMJ; Shelp, BJ; Anderson, TR; Welacky, TW; Rajcan, I | 2007 | QTL associated with horizontal resistance to soybean cyst nematode in Glycine soja PI464925B | Soybean cyst nematode (Heterodera glycines Ichinohe; SCN) is the primary disease responsible for yield loss of soybean [Glycine max (L.) Merr.]. Resistant cultivars are an effective management tool; however, the sources currently available have common resistant genes. Glycine soja Sieb. and Zucc., the wild ancestor of domesticated soybean, represents a diverse germplasm pool with known SCN resistance. The objectives of this research were to: (1) determine the genetic variation and inheritance of SCN resistance in a G. max (‘S08-80’) G. soja (PI464925B) F (4:5) recombinant inbred line (RIL) population; and (2) identify and evaluate quantitative trait loci (QTL) associated with SCN resistance. Transgressive segregation for resistance was observed, although neither parent was resistant to the Chatham and Ruthven SCN isolates. Broad sense heritability was 0.81 for the Ruthven and 0.91 for the Chatham isolate. Root dry weight was a significant covariate that influenced cyst counts. One RIL [female index (FI) = 5.2 +/- 1.11] was identified as resistant to the Chatham isolate (FI < 10). Seventeen and three RILs infected with Chatham and Ruthven isolates, respectively, had mean adjusted cyst counts of zero. Unique and novel QTL, which derived resistance from G. soja, were identified on linkage groups I, K, and O, and individually explained 8, 7 and 5% (LOD = 2.1-2.7) of the total phenotypic variation, respectively. Significant epistatic interactions were found between pairs of SSR markers that individually may or may not have been associated with SCN resistance, which explained between 10 and 15% of the total phenotypic variation. Best-fit regression models explained 21 and 31% of the total phenotypic variation in the RIL population to the Chatham and Ruthven isolates, respectively. The results of this study help to improve the understanding of the genetic control of SCN resistance in soybean caused by minor genes resulting in horizontal resistance. The incorporation of the novel resistance QTL from G. soja could increase the durability of SCN-resistance in soybean cultivars, especially if major gene resistance breaks down. | 1638 | no | crop | NA | no | no |
| 1639 | 2017 | 43020 | Seeley, TD; Tarpy, DR | 2007 | Queen promiscuity lowers disease within honeybee colonies | Most species of social insects have singly mated queens, but in some species each queen mates with numerous males to create a colony with a genetically diverse worker force. The adaptive significance of polyandry by social insect queens remains an evolutionary puzzle. Using the honeybee (Apis mellifera), we tested the hypothesis that polyandry improves a colony’s resistance to disease. We established colonies headed by queens that had been artificially inseminated by either one or 10 drones. Later, we inoculated these colonies with spores of Paenibacillus larvae, the bacterium that causes a highly virulent disease of honeybee larvae (American foulbrood). We found that, on average, colonies headed by multiple-drone inseminated queens had markedly lower disease intensity and higher colony strength at the end of the summer relative to colonies headed by single-drone inseminated queens. These findings support the hypothesis that polyandry by social insect queens is an adaptation to counter disease within their colonies. | 1639 | yes | from thesis | NA | no | yes |
| 1640 | 2017 | 43020 | Westergaard, KB; Alsos, IG; Engelskjon, T; Flatberg, KI; Brochmann, C | 2011 | Trans-Atlantic genetic uniformity in the rare snowbed sedge Carex rufina | The red-listed, amphi-Atlantic sedge Carex rufina is highly specialized to certain alpine snowbeds, and threatened by current changes in snow cover duration and moisture conditions. Here we address its range-wide genetic diversity, history, and conservation using amplified fragment length polymorphisms (AFLPs). Despite extensive primer testing, we detected very low overall diversity (4.1% polymorphic markers). Only a single AFLP phenotype was found throughout Norway and across the Atlantic to Iceland and Greenland, while another was found in Canada, suggesting glacial survival in one East and one West Atlantic refugium. East Atlantic C. rufina has probably been heavily bottlenecked in a small refugium, possibly situated within the maximum limits of the ice sheets. Its lack of diversity is likely maintained through local clonal growth causing longevity of genotypes. Habitat availability appears as the main limiting factor for C. rufina, and its currently occupied habitats need to be preserved to ensure its long-time survival. | 1640 | no | no good fitness measure | NA | no | no |
| 1641 | 2017 | 43020 | Dharmarajan, G; Beasley, JC; Fike, JA; Rhodes, OE | 2014 | Effects of landscape, demographic and behavioral factors on kin structure: testing ecological predictions in a mesopredator with high dispersal capability | Kin structure, the spatial aggregation of related individuals, impacts many processes important to conservation (e.g. inbreeding), and patterns of kin structure could be impacted by human-mediated habitat fragmentation and loss. While kin structure is expected to increase with habitat fragmentation (reduced connectivity), the effects of habitat loss (reduced resource availability) remain unclear. Disentangling the effects of habitat fragmentation and loss is challenging because they usually are spatially correlated, and because most species are negatively affected by both processes. Raccoons Procyon lotor is a model species to test how habitat loss affects kin structure because, although relatively unaffected by habitat fragmentation (because of high dispersal ability), they are negatively affected by habitat loss (forest-related resources being important for female reproductive success). To elucidate the causes and consequences of kin structure in raccoons, we utilized genetic and demographic data collected from 998 individuals trapped from 27 spatially distinct habitat patches (local populations) situated in an agricultural ecosystem. Our results highlight an important, but often ignored fact: structural connectivity does not necessarily predict functional dispersal patterns in natural populations. Thus, in raccoons, local populations with low kin structure were associated with high landscape disturbance (i.e. high levels of habitat loss and low connectivity), and were characterized by demographic instability (i.e. high immigration, emigration and/or mortality). Alternatively, local populations exhibiting high kin structure were associated with low landscape disturbance and high demographic stability (i.e. high natality and philopatry). We propose that such increased philopatry in resource-rich patches could lead to a functional isolation (isolation by resource’) exacerbating the negative effects of landscape modification because of isolation by distance and/or resistance, especially in species with low dispersal capability. Our results also indicate that high levels of genetic diversity may be associated with low (rather than high) patch quality because populations in such patches could be composed predominantly of (unrelated) immigrants. | 1641 | probably no | ok gd and fitness measurement? | no fitness measure pop | no | no |
| 1642 | 2017 | 43020 | Kotowska, AM; Cahill, JF; Keddie, BA | 2010 | Plant genetic diversity yields increased plant productivity and herbivore performance | P>1. Plant genotypic diversity has important consequences for a variety of ecosystem processes, yet empirical evidence for its effects on productivity, one of the most fundamental of these processes, is lacking. In addition, the performance of insect herbivores in response to high genotypic diversity is unknown, despite previous work demonstrating differential herbivore performance among plant genotypes. 2. We manipulated genotypic diversity of the annual plant Arabidopsis thaliana in both the presence and absence of the generalist herbivore Trichoplusia ni under semi-natural growth conditions. We used nine genotypes (eight ecotypes and one mutant) of A. thaliana known to differ widely in functional traits. Productivity and insect biomass were measured in monocultures and mixtures of all nine genotypes grown at multiple fertilization levels and planting densities. 3. In both the absence and presence of herbivores, genotypic diversity increased plant productivity and survival. This effect was, for the most part, independent of fertility or density. Sampling or selection effects did not appear to be wholly responsible for these results as all genotypes were maintained in equal proportion and no single genotype became dominant for the duration of the experiment. 4. High diversity increased T. ni biomass and survival in all treatments. Insect biomass was positively, but not tightly, correlated to plant biomass, indicating that the higher herbivore performance observed in genotypic mixtures was only partially due to higher productivity. 5.Synthesis. Our data support the idea that even within a single plant species, genotypic diversity can exert strong influences on both the producer and herbivore communities. The exact mechanisms responsible for these effects and the relative importance of genotypic diversity in natural communities warrant further investigation. | 1642 | yes | ok gd measurement and pop level comp? | NA | yes | yes |
| 1643 | 2017 | 43020 | Acevedo-Whitehouse, K; Vicente, J; Gortazar, C; Hofle, U; Fernandez-De-Mera, IG; Amos, W | 2005 | Genetic resistance to bovine tuberculosis in the Iberian wild boar | Bovine tuberculosis (bTB) is an important re-emerging zoonotic disease, causing major economic losses and constraining international trade of animals and their products. Despite eradication programmes, some countries continue to encounter outbreaks, mainly due to wildlife acting as primary hosts or reservoirs. While the genetic component of tuberculosis in humans and cattle is well documented, the role of genetic factors as modulators of bTB resistance remains unclear for natural populations. To address this issue, we investigated the relative contribution of host genetic variability to susceptibility to bTB infection and disease progression in wild boars from southern Spain. We found that genetic heterozygosity is an important predictor of bTB, not only modulating resistance to infection but also influencing containment of disease progression in infected individuals. Our results provide further evidence that host genetic variability plays a central role in natural populations. Testing each marker separately reveals evidence of both general and single-locus associative effects on bTB and several loci reveal high homology to regions of the genome with known immune function. Our results may prove to be crucial for understanding outbreaks of bTB in wildlife that could potentially affect domestic livestock and humans. | 1643 | no | No GD diff between pop | NA | no | no |
| 1644 | 2017 | 43020 | Roberts, KE; Evison, SEF; Baer, B; Hughes, WOH | 2015 | The cost of promiscuity: sexual transmission of Nosema microsporidian parasites in polyandrous honey bees | Multiple mating (and insemination) by females with different males, polyandry, is widespread across animals, due to material and/or genetic benefits for females. It reaches particularly high levels in some social insects, in which queens can produce significantly fitter colonies by being polyandrous. It is therefore a paradox that two thirds of eusocial hymenopteran insects appear to be exclusively monandrous, in spite of the fitness benefits that polyandry could provide. One possible cost of polyandry could be sexually transmitted parasites, but evidence for these in social insects is extremely limited. Here we show that two different species of Nosema microsporidian parasites can transmit sexually in the honey bee Apis mellifera. Honey bee males that are infected by the parasite have Nosema spores in their semen, and queens artificially inseminated with either Nosema spores or the semen of Nosema-infected males became infected by the parasite. The emergent and more virulent N. ceranae achieved much higher rates of infection following insemination than did N. apis. The results provide the first quantitative evidence of a sexually transmitted disease (STD) in social insects, indicating that STDs may represent a potential cost of polyandry in social insects. | 1644 | no | no good fitness measure, N pop differnt in GD? | NA | no | no |
| 1645 | 2017 | 43020 | Collin, H; Burri, R; Comtesse, F; Fumagalli, L | 2013 | Combining molecular evolution and environmental genomics to unravel adaptive processes of MHC class IIB diversity in European minnows (Phoxinus phoxinus) | Host-pathogen interactions are a major evolutionary force promoting local adaptation. Genes of the major histocompatibility complex (MHC) represent unique candidates to investigate evolutionary processes driving local adaptation to parasite communities. The present study aimed at identifying the relative roles of neutral and adaptive processes driving the evolution of MHC class IIB (MHCIIB) genes in natural populations of European minnows (Phoxinus phoxinus). To this end, we isolated and genotyped exon 2 of two MHCIIB gene duplicates (DAB1 and DAB3) and 1665 amplified fragment length polymorphism (AFLP) markers in nine populations, and characterized local bacterial communities by 16S rDNA barcoding using 454 amplicon sequencing. Both MHCIIB loci exhibited signs of historical balancing selection. Whereas genetic differentiation exceeded that of neutral markers at both loci, the populations’ genetic diversities were positively correlated with local pathogen diversities only at DAB3. Overall, our results suggest pathogen-mediated local adaptation in European minnows at both MHCIIB loci. While at DAB1 selection appears to favor different alleles among populations, this is only partially the case in DAB3, which appears to be locally adapted to pathogen communities in terms of genetic diversity. These results provide new insights into the importance of host-pathogen interactions in driving local adaptation in the European minnow, and highlight that the importance of adaptive processes driving MHCIIB gene evolution may differ among duplicates within species, presumably as a consequence of alternative selective regimes or different genomic context. | 1645 | maybe | any fitness of groups and of different gd ok? | not pop level gd | yes | no |
| 1646 | 2017 | 43020 | Nandi, T; Holden, MTG; Didelot, X; Mehershahi, K; Boddey, JA; Beacham, I; Peak, I; Harting, J; Baybayan, P; Guo, Y; Wang, S; How, LC; Sim, B; Essex-Lopresti, A; Sarkar-Tyson, M; Nelson, M; Smither, S; Ong, C; Aw, LT; Hoon, CH; Michell, S; Studholme, DJ; Titball, R; Chen, SL; Parkhill, J; Tan, P | 2015 | Burkholderia pseudomallei sequencing identifies genomic clades with distinct recombination, accessory, and epigenetic profiles | Burkholderia pseudomallei (Bp) is the causative agent of the infectious disease melioidosis. To investigate population diversity, recombination, and horizontal gene transfer in closely related Bp isolates, we performed whole-genome sequencing (WGS) on 106 clinical, animal, and environmental strains from a restricted Asian locale. Whole-genome phylogenies resolved multiple genomic clades of Bp, largely congruent with multilocus sequence typing (MLST). We discovered widespread recombination in the Bp core genome, involving hundreds of regions associated with multiple haplotypes. Highly recombinant regions exhibited functional enrichments that may contribute to virulence. We observed clade-specific patterns of recombination and accessory gene exchange, and provide evidence that this is likely due to ongoing recombination between clade members. Reciprocally, interclade exchanges were rarely observed, suggesting mechanisms restricting gene flow between clades. Interrogation of accessory elements revealed that each clade harbored a distinct complement of restriction-modification (RM) systems, predicted to cause clade-specific patterns of DNA methylation. Using methylome sequencing, we confirmed that representative strains from separate clades indeed exhibit distinct methylation profiles. Finally, using an E. coli system, we demonstrate that Bp RM systems can inhibit uptake of non-self DNA. Our data suggest that RM systems borne on mobile elements, besides preventing foreign DNA invasion, may also contribute to limiting exchanges of genetic material between individuals of the same species. Genomic clades may thus represent functional units of genetic isolation in Bp, modulating intraspecies genetic diversity. | 1646 | no | gd of pathogen | NA | no | no |
| 1647 | 2017 | 43020 | Reis, NR; Fregonezi, MN; Peracchi, AL; Rossaneis, BK | 2012 | Metapopulation in bats of Southern Brazil | The purpose was to show that displacements, promoters of genetic diversity in metapopulations, increase the probability of survival of bat species adapted to medium and long-distance flights. Samples were taken in four forest fragments, distributed in three municipalities in northern Parana, and the maximum distance between the studied areas is 20 km. A monthly sampling was performed for each fragment, for the period of July 2008 to June 2009. We used eight nets for collection which remained open during the first four hours of the night, totalling 192 hours during a year of study. The marking occurred from October 2008 to March 2009 and was accomplished through the use of anodised metal rings of four different colours. One hundred and fifty individuals were banded and since the first capture, four displacements were recorded. After five months of collecting and marking, one Carollia perspicillata was found three km away. Two Artibeus lituratus were recorded about 20 km from the marking place: the first one after 22 months and the second one after 24 months. Additionally, one Platyrrhinus lineatus was captured at about 20 km, after 26 months. As they moved around over considerable distances and are not monogamous, they mate with females of other fragments, exchanging genes and reducing or even avoiding inbreeding. Thus, populations of bats have the ability to increase genetic diversity in metapopulations, provided by displacements between the forest fragments. Species that behave like this are not vulnerable to isolation. | 1647 | no | no good fitness measure, N pop differnt in GD? | NA | no | no |
| 1648 | 2017 | 43020 | Keating, BJ; Tischfield, S; Murray, SS; Bhangale, T; Price, TS; Glessner, JT; Galver, L; Barrett, JC; Grant, SFA; Farlow, DN; Chandrupatla, HR; Hansen, M; Ajmal, S; Papanicolaou, GJ; Guo, YR; Li, MY; DerOhannessian, S; de Bakker, PIW; Bailey, SD; Montpetit, A; Edmondson, AC; Taylor, K; Gai, XW; Wang, SS; Fornage, M; Shaikh, T; Groop, L; Boehnke, M; Hall, AS; Hattersley, AT; Frackelton, E; Patterson, N; Chiang, CWK; Kim, CE; Fabsitz, RR; Ouwehand, W; Price, AL; Munroe, P; Caulfield, M; Drake, T; Boerwinkle, E; Reich, D; Whitehead, AS; Cappola, TP; Samani, NJ; Lusis, AJ; Schadt, E; Wilson, JG; Koenig, W; McCarthy, MI; Kathiresan, S; Gabriel, SB; Hakonarson, H; Anand, SS; Reilly, M; Engert, JC; Nickerson, DA; Rader, DJ; Hirschhorn, JN; FitzGerald, GA | 2008 | Concept, Design and Implementation of a Cardiovascular Gene-Centric 50 K SNP Array for Large-Scale Genomic Association Studies | “A wealth of genetic associations for cardiovascular and metabolic phenotypes in humans has been accumulating over the last decade, in particular a large number of loci derived from recent genome wide association studies (GWAS). True complex disease-associated loci often exert modest effects, so their delineation currently requires integration of diverse phenotypic data from large studies to ensure robust meta-analyses. We have designed a gene-centric 50 K single nucleotide polymorphism (SNP) array to assess potentially relevant loci across a range of cardiovascular, metabolic and inflammatory syndromes. The array utilizes a”“cosmopolitan’’ tagging approach to capture the genetic diversity across similar to 2,000 loci in populations represented in the HapMap and SeattleSNPs projects. The array content is informed by GWAS of vascular and inflammatory disease, expression quantitative trait loci implicated in atherosclerosis, pathway based approaches and comprehensive literature searching. The custom flexibility of the array platform facilitated interrogation of loci at differing stringencies, according to a gene prioritization strategy that allows saturation of high priority loci with a greater density of markers than the existing GWAS tools, particularly in African HapMap samples. We also demonstrate that the IBC array can be used to complement GWAS, increasing coverage in high priority CVD-related loci across all major HapMap populations. DNA from over 200,000 extensively phenotyped individuals will be genotyped with this array with a significant portion of the generated data being released into the academic domain facilitating in silico replication attempts, analyses of rare variants and cross-cohort meta-analyses in diverse populations. These datasets will also facilitate more robust secondary analyses, such as explorations with alternative genetic models, epistasis and gene-environment interactions.” | 1648 | no | NA | NA | no | no |
| 1649 | 2017 | 43020 | Rico, Y; Morris-Pocock, J; Zigouris, J; Nocera, JJ; Kyle, CJ | 2015 | Lack of Spatial Immunogenetic Structure among Wolverine (Gulo gulo) Populations Suggestive of Broad Scale Balancing Selection | Elucidating the adaptive genetic potential of wildlife populations to environmental selective pressures is fundamental for species conservation. Genes of the major histocompatibility complex (MHC) are highly polymorphic, and play a key role in the adaptive immune response against pathogens. MHC polymorphism has been linked to balancing selection or heterogeneous selection promoting local adaptation. However, spatial patterns of MHC polymorphism are also influenced by gene flow and drift. Wolverines are highly vagile, inhabiting varied ecoregions that include boreal forest, taiga, tundra, and high alpine ecosystems. Here, we investigated the immunogenetic variation of wolverines in Canada as a surrogate for identifying local adaptation by contrasting the genetic structure at MHC relative to the structure at 11 neutral microsatellites to account for gene flow and drift. Evidence of historical positive selection was detected at MHC using maximum likelihood codonbased methods. Bayesian and multivariate cluster analyses revealed weaker population genetic differentiation at MHC relative to the increasing microsatellite genetic structure towards the eastern wolverine distribution. Mantel correlations of MHC against geographical distances showed no pattern of isolation by distance (IBD: r = -0.03, p = 0.9), whereas for microsatellites we found a relatively strong and significant IBD (r = 0.54, p = 0.01). Moreover, we found a significant correlation between microsatellite allelic richness and the mean number of MHC alleles, but we did not observe low MHC diversity in small populations. Overall these results suggest that MHC polymorphism has been influenced primarily by balancing selection and to a lesser extent by neutral processes such as genetic drift, with no clear evidence for local adaptation. This study contributes to our understanding of how vulnerable populations of wolverines may respond to selective pressures across their range. | 1649 | no | no good fitness measure | NA | no | no |
| 1650 | 2017 | 43020 | Zhang, LF; Thomas, JC; Miragaia, M; Bouchami, O; Chaves, F; d’Azevedo, PA; Aanensen, DM; de Lencastre, H; Gray, BM; Robinson, DA | 2013 | Multilocus Sequence Typing and Further Genetic Characterization of the Enigmatic Pathogen, Staphylococcus hominis | Staphylococcus hominis is a commensal resident of human skin and an opportunistic pathogen. The species is subdivided into two subspecies, S. hominis subsp. hominis and S. hominis subsp. novobiosepticus, which are difficult to distinguish. To investigate the evolution and epidemiology of S. hominis, a total of 108 isolates collected from 10 countries over 40 years were characterized by classical phenotypic methods and genetic methods. One nonsynonymous mutation in gyrB, scored with a novel SNP typing assay, had a perfect association with the novobiocin-resistant phenotype. A multilocus sequence typing (MLST) scheme was developed from six housekeeping gene fragments, and revealed relatively high levels of genetic diversity and a significant impact of recombination on S. hominis population structure. Among the 40 sequence types (STs) identified by MLST, three STs (ST2, ST16 and ST23) were S. hominis subsp. novobiosepticus, and they distinguished between isolates from different outbreaks, whereas 37 other STs were S. hominis subsp. hominis, one of which was widely disseminated (ST1). A modified PCR assay was developed to detect the presence of ccrAB4 from the SCCmec genetic element. S. hominis subsp. novobiosepticus isolates were oxacillin-resistant and carriers of specific components of SCCmec (mecA class A, ccrAB3, ccrAB4, ccrC), whereas S. hominis subsp. hominis included both oxacillin-sensitive and -resistant isolates and a more diverse array of SCCmec components. Surprisingly, phylogenetic analyses indicated that S. hominis subsp. novobiosepticus may be a polyphyletic and, hence, artificial taxon. In summary, these results revealed the genetic diversity of S. hominis, the identities of outbreak-causing clones, and the evolutionary relationships between subspecies and clones. The pathogenic lifestyle attributed to S. hominis subsp. novobiosepticus may have originated on more than one occasion. | 1650 | no | gd of pathogen | NA | no | no |
| 1651 | 2017 | 43020 | Sharpe, SA; White, AD; Sibley, L; Gleeson, F; Hall, GA; Basaraba, RJ; McIntyre, A; Clark, SO; Gooch, K; Marsh, PD; Williams, A; Dennis, MJ | 2017 | An aerosol challenge model of tuberculosis in Mauritian cynomolgus macaques | Background New interventions for tuberculosis are urgently needed. Non-human primate (NHP) models provide the most relevant pre-clinical models of human disease and play a critical role in vaccine development. Models utilising Asian cynomolgus macaque populations are well established but the restricted genetic diversity of the Mauritian cynomolgus macaques may be of added value. Methods Mauritian cynomolgus macaques were exposed to a range of doses of M. tuberculosis delivered by aerosol, and the outcome was assessed using clinical, imaging and pathology based measures. Results All macaques developed characteristic clinical signs and disease features of tuberculosis (TB). Disease burden and the ability to control disease were dependent on exposure dose. Mauritian cynomolgus macaques showed less variation in pulmonary disease burden and total gross pathology scores within exposure dose groups than either Indian rhesus macaques or Chinese cynomolgus macaques Conclusions The genetic homogeneity of Mauritian cynomolgus macaques makes them a potentially useful model of human tuberculosis. | 1651 | no | NA | NA | no | no |
| 1652 | 2017 | 43020 | Neafsey, DE; Galinsky, K; Jiang, RHY; Young, L; Sykes, SM; Saif, S; Gujja, S; Goldberg, JM; Young, S; Zeng, QD; Chapman, SB; Dash, AP; Anvikar, AR; Sutton, PL; Birren, BW; Escalante, AA; Barnwell, JW; Carlton, JM | 2012 | The malaria parasite Plasmodium vivax exhibits greater genetic diversity than Plasmodium falciparum | We sequenced and annotated the genomes of four P. vivax strains collected from disparate geographic locations, tripling the number of genome sequences available for this understudied parasite and providing the first genome-wide perspective of global variability in this species. We observe approximately twice as much SNP diversity among these isolates as we do among a comparable collection of isolates of P. falciparum, a malaria-causing parasite that results in higher mortality. This indicates a distinct history of global colonization and/or a more stable demographic history for P. vivax relative to P. falciparum, which is thought to have undergone a recent population bottleneck. The SNP diversity, as well as additional microsatellite and gene family variability, suggests a capacity for greater functional variation in the global population of P. vivax. These findings warrant a deeper survey of variation in P. vivax to equip disease interventions targeting the distinctive biology of this neglected but major pathogen. | 1652 | no | gd of parasite | NA | no | no |
| 1653 | 2017 | 43020 | Miladinovic, D; Taski-Ajdukovic, K; Nagl, N; Kovacevic, B; Dimitrijevic, A; Imerovski, I; Miladinovic, J; Dusanic, N; Balalic, I | 2014 | GENETIC DIVERSITY OF WILD SUNFLOWER (Helianthus sp.) ACCESSIONS WITH DIFFERENT TOLERANCE TO MID-STALK WHITE ROT | Random amplified polymorphic DNA (RAPD) markers were used to detect polymorphism among accessions of wild sunflower species Helianthus maximiliani, Helianthus tuberosus, Helianthus mollis and Helianthus rigidus with different tolerance to mid-stalk white rot and selection of potential markers for different levels of tolerance to this disease. Estimates of genetic variation showed that genetic diversity was equally distributed between Helianthus species and within them. Cluster analysis corresponded to the phylogenetic relations within the genus Helianthus. The results obtained by principal coordinates analysis (PCoA), where the first two principal coordinates accounted for 83.7% of total variation, perfectly coincided with the results of cluster analysis. Contingency coefficient significance test showed that most of the used primers generated bands associated with some level of tolerance or susceptibility to mid-stalk white rot. Furthermore, contingency analysis showed that primer C12 generated bands associated with resistance (100%) to mid-stalk white rot both in H. mollis and in all accessions, while primer X18 generated bands significantly associated with high tolerance (75%) in H. rigidus, H. mollis as well as in all tested accessions. The C15-600 bp locus was found to be significantly associated with high tolerance (75%) in all accessions, and medium tolerance (50%) in H. mollis. | 1653 | no | no pop level? | NA | no | no |
| 1654 | 2017 | 43020 | Fowler, EV; Houlden, BA; Hoeben, P; Timms, P | 2000 | Genetic diversity and gene flow among southeastern Queensland koalas (Phascolarctos cinereus) | Habitat fragmentation and destruction associated with the rapid urban and rural development of southeast Queensland presents an immediate threat to the survival of koala populations within this region. A sensitive method combining heteroduplex analysis (HDA) with temperature gradient gel electrophoresis (TGGE) was optimized to detect within-species variation in a mitochondrial DNA (mtDNA) control-region fragment, approximate to 670 bp in length, from the koala. Eight different haplotypes were characterized in koalas, of which four were novel. Analysis of mtDNA diversity in 96 koalas from five populations in southeast Queensland revealed that the number of haplotypes in a single population ranged from one to five, with an average within-population haplotype diversity of 0.379 +/- 0.016, and nucleotide diversity of 0.22 +/- 0.001%. Nucleotide divergence between populations averaged 0.09 +/- 0.001% and ranged from 0.00 to 0.14%. Significant genetic heterogeneity was observed among most populations, suggesting that koala populations may be spatially structured along matrilines, although this may not be universal. The limited distribution of the central phylogenetic haplotype suggested the possibility of historical population bottlenecks north of the Gold Coast, while the presence of two highly divergent haplotypes at the Moreton site may indicate the occurrence of one or more undocumented translocation events into this area. | 1654 | no | no good fitness measure | NA | no | no |
| 1655 | 2017 | 43020 | Delatte, H; Baudin, R; Becker, N; Girard, AL; Ramatoulaye Sidebe, T; Lett, JM; Reynaud, B | 2015 | Species and endosymbiont diversity of Bemisia tabaci (Homoptera: Aleyrodidae) on vegetable crops in Senegal | Bemisia tabaci-transmitted geminiviruses are one of the major threats on cassava and vegetable crops in Africa. However, to date, few studies are available on the diversity of B. tabaci and their associated endosymbionts in Africa. More than 28 species have been described in the complex of B. tabaci cryptic species; among them, 2 are invasive pests worldwide: MED and MEAM1. In order to assess the species diversity of B. tabaci in vegetable crops in Senegal, several samplings in different localities, hosts and seasons were collected and analyzed with nuclear (microsatellite) and mitochondrial (COI) markers. The bacterial endosymbiont community was also studied for each sample. Two species were detected: MED Q1 and MEAM1 B. Patterns of MED Q1 (dominance on most of the samples and sites, highest nuclear and mitochondrial diversity and broader secondary endosymbiont community: Hamiltonella, Cardinium, Wolbachia and Rickettsia), point toward a predominant resident begomovirus vector group for MED Q1 on market gardening crops. Furthermore, the lower prevalence of the second species MEAM1 B, its lower nuclear and mitochondrial diversity and a narrower secondary endosymbiont community (Hamiltonella/Rickettsia), indicate that this genetic group is exotic and results from a recent invasion in this area. | 1655 | no | NA | NA | no | no |
| 1656 | 2017 | 43020 | Yang, WL; Whitea, B; Spicer, EK; Weinstein, BL; Hildebrandt, JD | 2004 | Complex haplotype structure of the human GNAS gene identifies a recombination hotspot centred on a single nucleotide polymorphism widely used in association studies | The alpha subunit of the heterotrimeric G protein Gs (Gsalpha) is involved in numerous physiological processes and is a primary determinant of cellular responses to extracellular signals. Genetic variations in the Gsalpha gene may play an important role in complex diseases and drug responses. To characterize the genetic diversity in this locus, we resequenced exons and flanking introns of the gene in 44 genomic samples and analysed the haplotype structure of the gene in an additional 50 African-Americans and 50 Caucasians. Significant differences in allele frequency for nearly all the genotyped single nucleotide polymorphism (SNPs) were detected between the two ethnic groups. Linkage disequilibrium (LD) analysis of this locus revealed two haplotype blocks characterized by strong LD and reduced haplotype diversity, especially in Caucasians. Between the two blocks is a narrow (approximately 3 kb) recombination hotspot centred on exons 4 and 5, and a widely used genetic marker in association studies in this region (rs7121) was in linkage equilibrium with the rest of the gene. The haplotype structure of the GNAS locus warrants reevaluation of previous association studies that used marker rs7121 and affects choice of SNP markers to be used in future studies of this locus. (C) 2004 Lippincott Williams Wilkins. | 1656 | no | human | NA | no | no |
| 1657 | 2017 | 43020 | Isagi, Y; Tateno, R; Matsuki, Y; Hirao, A; Watanabe, S; Shibata, M | 2007 | Genetic and reproductive consequences of forest fragmentation for populations of Magnolia obovata | In order to evaluate the consequences of forest fragmentation on populations of Magnolia obovata, we compared genetic diversity and reproductive characteristics at two nearby sites, one conserved and one fragmented. The genetic diversity between adults trees of the different sites was not significantly different. However, saplings in the conserved site showed a significantly higher genetic diversity than both adult trees in the conserved site and saplings in the fragmented sites; this was found to be the result of the larger gene flow into the conserved site. The density of the adult trees was significantly related to all of the reproductive traits analyzed (fertilization of ovules, insect attack to seeds, ovules that developed into seeds and outcrossing at the stage of seeds) at both sites. At both sites, fertilization of ovules and insect attack on seeds were positively correlated to adult tree density while outcrossing rate was negatively correlated to adult tree density. The fertilization of ovules and outcrossing were more dependent on adult tree density in the fragmented site than in the conserved site. The probability of ovules developing into outcrossed seeds showed a negative correlation with adult tree density at both sites, indicating the advantage of low density for this species and possibly implying a resilience to habitat fragmentation. A two-generation-analysis did not identify significant differences between sites in terms of the structure of the pollen pool and the number of pollen donors. Although fragmentation affected reproductive characteristics, the effect on seedling establishment and subsequent survival remains to be determined. Proposals for future studies that will assist in the development of management strategies for forests suffering fragmentation are made. | 1657 | no | no good fitness measure | NA | no | no |
| 1658 | 2017 | 43020 | Leonova, IN; Badaeva, ED; Orlovskaya, OA; Roder, MS; Khotyleva, LV; Salina, EA; Shumny, VK | 2013 | Comparative characteristic of Triticum aestivum/Triticum durum and Triticum aestivum/Triticum dicoccum hybrid lines by genomic composition and resistance to fungal diseases under different environmental conditions | The genetic diversity of common wheat hybrid lines Triticum aestivum/Triticum durum and Triticum aestivum/Triticum dicoccum (2n = 42, F6-7) using chromosome-specific microsatellite (SSR) markers and C-banding of chromosomes was studied. Cluster analysis of data obtained by 42 SSR markers indicated that the hybrid lines can be broken into three groups according to their origin. There were two cases of complete genetic similarity between lines 183(2)-2/184(1)-6 and 208-3/213-1, which were obtained using common wheat as the parental plants. In cross combinations, when the stabilization of the nuclear genome of hexaploid lines occurred against a background of the cytoplasmic genome of tetraploid wheats, there was a high level of divergence between sister lines, in some cases exceeding 50%. The evaluation of the degree of susceptibility of the lines to powdery mildew, leaf and stem rust, and septoria leaf blotch was performed under different environmental conditions. It was shown that resistance to powdery mildew and leaf rust significantly depended on the region where assays were conducted. An evaluation of the field data showed that the lines 195-3, 196-1, and 221-1 with T. durum genetic material displayed complex resistance to fungal pathogens in Western Siberia and the Republic of Belarus. For lines 195-3 and 196-1, one shows a possible contribution of chromosomes 4B and 5B in the formation of complex resistance to diseases. Hybrid lines with complex resistance can be used to expand the genetic diversity of modern common wheat cultivars for genes of immunity. | 1658 | no | NA | NA | no | no |
| 1659 | 2017 | 43020 | Panova-Noeva, M; Arnold, N; Hermanns, MI; Prochaska, JH; Schulz, A; Spronk, HM; Binder, H; Pfeiffer, N; Beutel, M; Blankenberg, S; Zeller, T; Lotz, J; Munzel, T; Lackner, KJ; ten Cate, H; Wild, PS | 2017 | Mean Platelet Volume and Arterial Stiffness - Clinical Relationship and Common Genetic Variability | Vessel wall stiffening is an important clinical parameter, but it is unknown whether platelets, key elements in the pathogenesis of arterial thrombosis, are associated with arterial stiffness. The present studies sought to determine whether mean platelet volume (MPV), a potential marker of platelet activation, is linked to vascular elasticity as assessed by the augmentation index (AIx), in 15,010 individuals from the population-based Gutenberg Health Study. Multivariable analysis showed that MPV in both males (beta 0.776; 95thCI [0.250;1.16]; p = 0.0024) and females (beta 0.881[0.328;1.43]; p = 0.0018) is strongly associated with AIx. Individuals with MPV and AIx above the sex-specific medians had worse survival. Association analysis between MPV-related genetic variants and arterial stiffness identified four genetic variants in males and one in females related with AIx. Cox regression analysis for mortality identified one of these joint genetic variants close to ring finger protein 145 gene (RNF145, rs10076782) linked with increased mortality (hazard ratio 2.02; 95thCI [1.35;3.02]; p = 0.00061). Thus, these population-based data demonstrate a close relation between platelet volume as a potential marker of platelet activation and arterial stiffness in both sexes. Further research is warranted to further elucidate the mechanisms underlying larger platelets’ role in arterial stiffening including the role of shared common genetics. | 1659 | no | NA | NA | no | no |
| 1660 | 2017 | 43020 | Ludvico, L; Slifer, S; Massey, K; Mosman, K; Mohamed, C; Stone, W; Keeling, M; Bernacky, B; Dyke, B | 2001 | Demography and pedigree structure of an SPF colony of rhesus monkeys (Macaca mulatta) | The SPF rhesus colony at the M.D. Anderson Cancer Center in Bastrop, Texas, was analyzed with the aim of determining the demographic and genetic effects of stringent selection for virus-free breeders, permanent quarantine, continued surveillance, and culling of animals that show evidence of viral infection. The analysis shows minimal effects on population viability and loss of genetic variability in comparison with the traditionally managed (non-SPF) portion of the population. Am. J. Primatol. 53:155-165, 2001. (C) 2001 Wiley-Liss, Inc. | 1660 | no | no good fitness measure, N pop differnt in GD? | NA | no | no |
| 1661 | 2017 | 43020 | Fouchet, D; Le Pendu, J; Guitton, JS; Guiserix, M; Marchandeau, S; Pontier, D | 2009 | Evolution of microparasites in spatially and genetically structured host populations: The example of RHDV infecting rabbits | Several studies have shown that classical results of microparasite evolution could not extend to the case where the host species shows an important spatial structure. Rabbit haemorrhagic disease virus (RHDV), responsible for rabbit haemorrhagic disease (RHD), which recently emerged in rabbits, has strains within a wide range of virulence, thus providing an interesting example of competition between strains infecting a host species with a metapopulation structure. In addition, rabbits may show a genetic diversity regarding RHDV susceptibility. In the present paper we use the example of the rabbit-RHDV interaction to study the competition between strains of a same microparasite in a host population that is both spatially and genetically structured. Using metapopulation models we show that the evolution of the microparasite is guided by a trade-off between its capacity to invade subpopulations potentially infected by other strains and its capacity to persist within the subpopulation. In such a context, host genetic diversity acts by reducing the number of hosts susceptible to each strain, often favouring more persistent-and generally less virulent-strains. We also show that even in a stochastic context where host genes regularly go locally extinct, the microparasite pressure helps maintain the genetic diversity in the long term while reinforcing gene loss risk in the short term. Finally, we study how different demographic and epiderniologic parameters affect the coevolution between the rabbit and RHDV. (C) 2008 Elsevier Ltd. All rights reserved. | 1661 | no | No GD diff between pop | NA | no | no |
| 1662 | 2017 | 43020 | Taylor, BL; Rojas-Bracho, L | 1999 | Examining the risk of inbreeding depression in a naturally rare cetacean, the vaquita (Phocoena sinus) | “Uncertainty about the magnitude of various risks facing endangered species can paralyze conservation action. The vaquita is a naturally rare porpoise that has declined to the low hundreds of individuals because of gillnet mortality over the past 57 years. No variability in mitochondrial DNA (mtDNA) was found in vaquitas (n = 43). Because reducing gillnet mortality will require strong conservation action, the question was raised whether vaquitas are doomed because of inbreeding depression and whether, therefore, mitigation efforts would be futile. We use simulations to investigate the”“doom hypothesis”" by first asking whether the current level of genetic variability results from the recent decline or from historical factors. If fixation was historical then deleterious alleles could have been selected out of vaquitas over thousands of years, reducing concerns about inbreeding depression. Simulations showed that fixation most likely resulted from historical rather than recent loss. Of 1,000 simulations done at plausible abundances and mutation rates, 247 (84.3%) fixed before and 46 (15.7%) fixed during the recent decline. Fixation correlates with historical abundance, making it more likely that because vaquitas are fixed, they are also a naturally rare species. However, because studies on purging deleterious alleles have nor shown purging to be universally beneficial we also examine the doom hypothesis using data on the response to inbreeding of a wide variety of captive animals. Responses are so variable that the doom hypothesis cannot be affirmed. We further explore whether more data from vaquitas would lead to conclusive results and found that the data required, such as the adult survival rate, will be impossible to obtain. We conclude that because the doom hypothesis cannot be affirmed this risk factor should not delay conservation actions." | 1662 | no | inbreeding | NA | no | no |
| 1663 | 2017 | 43020 | Oliver, M; Piertney, S | 2010 | Beyond splitting hares and rabbiting on about major histocompatibility complex complexity | The genes of the major histocompatibility complex (MHC) have become the target of choice for studies wishing to examine adaptively important genetic diversity in natural populations. Within Molecular Ecology alone, there have been 71 papers on aspects of MHC evolution over the past few years, with an increasing year on year trend. This focus on the MHC is partly driven by the hypothesized links between MHC gene dynamics and ecologically interesting and relevant traits, such as mate choice and host-parasite interactions. However, an ability to pin down the evolutionary causes and ecological consequences of MHC variation in natural populations has proven challenging and has been hampered by the very issue that is attractive about MHC genes - their high levels of diversity. Linking high levels of MHC diversity to ecological factors in inherently complex natural populations requires a level of experimental design and analytical rigour that is extremely difficult to achieve owing to a plethora of potentially confounding and interacting variables. In this issue of Molecular Ecology, Smith et al. (2010) elegantly overcome the challenge of detecting complex interactions in complex systems by using an intricate analytical approach to demonstrate a role for MHC in the reproductive ability of a natural population of the European hare Lepus europaeus (Fig. 1). Also in this issue, Oppelt et al. (2010) demonstrate a role for MHC variation in determining levels of hepatic coccidian infection in the European rabbit Oryctolagus cuniculus (Fig. 2). | 1663 | no | no good fitness measure, N pop differnt in GD? | NA | no | no |
| 1664 | 2017 | 43020 | Lyytinen, A; Lindstrom, L; Mappes, J | 2008 | Genetic variation in growth and development time under two selection regimes in Leptinotarsa decemlineata | It is possible to predict the potential range of a species on the basis of its ecological characteristics and those of the invaded ecosystem. The existence of genetic variation indicates a species’ potential to respond to new environmental conditions, thus facilitating its success as an invader. Accordingly, evolutionary and ecological approaches are needed to identify the factors explaining both species’ range and their potential to invade new areas. We combined these two approaches and studied whether genetic variation in life-history traits under abiotic (temperature) and biotic (host plant) selection pressures contributes to the potential range expansion of Leptinotarsa decemlineata Say (Coleoptera: Chrysomelidae). We reared full-sib families of L. decemlineata from the current northernmost European population at 15 degrees C (temperature below that in the current range) and 25 degrees C (optimal temperature) and on three potato varieties. We monitored development time, adult weight and larval-to-adult survival, and estimated the amount of heritable variance. The development time and adult weight of progenies were more variable between than within families. Thus, there was genetic variability in traits relevant to the ability to adapt to a colder environment (i.e., accelerate development and become heavier) allowing range expansion further north in Europe, even though low temperature increased beetle mortality. Temperature strongly affected all traits measured. Potato variety, in turn, did not strongly affect beetles’ performance. Beetle ability to adapt to a cool environment was further enhanced by the fact that size was not constrained by fast development. The results showed that beetle populations possess genetic variation allowing a response to temperature and thus have the evolutionary potential to adapt and spread beyond their current range. | 1664 | no | NA | NA | no | no |
| 1665 | 2017 | 43020 | Vandebona, H; Gunasekera, MB; Goonesekere, NCW; Kodikara, DS; Alahakoon, J; Ratnasooriya, WD | 2002 | DNA fingerprints of the asian elephant in Sri Lanka, Elephas maximus maximus, using multilocus probe 33.15 (Jeffreys) | The elephant in Sri Lanka, with a population estimated to be about 4,000 today, is threatened by the loss of its jungle habitat due to human activity centred around the development programmes of the country. The wild elephant population is fragmented, and restricted to a few isolated small habitats in protected areas. The maintenance of a high degree of genetic diversity within elephant subpopulations is vital for its long-term survival. Monitoring the degree of genetic heterogeneity in the geographical subpopulations of elephant is therefore an essential pre-requisite for this purpose. Although the maternal lineage of the elephant population in Sri Lanka has been examined previously, nuclear DNA has not been examined to gather complementary information. The aim of this study was to develop the methodology to obtain nuclear DNA fingerprints of the elephant with the multilocus human DNA probe 33.15 (Jeffreys), and to evaluate the probe to study the degree of genetic heterogeneity in the elephant population. An unique DNA fingerprint was obtained for each elephant DNA sample under the optimized conditions of hybridization (61degreesC in buffer containing 0.25 M sodium phosphate, 7% SDS, 1% BSA, 6% PEG, 10 mM EDTA and 0.25% dried milk, 100 ng/15 ml probe) developed during the study. A pair-wise comparison of DNA banding patterns of 8 individuals in the 3-23 kb region revealed a mean band sharing coefficient of 0.307 + 0.085. A total of 57 variable banding positions were observed and the mean number of bands per individual was 19 +/- 2.14. The calculated heterozygosity values for the unrelated and related individuals were 0.73, and 0.57 (mean) respectively. The study clearly demonstrated that the 33.15 probe is highly suitable to monitor the degree of genetic heterogeneity in the geographical subpopulations of the Asian elephant. | 1665 | no | no good fitness measure, N pop differnt in GD? | NA | no | no |
| 1666 | 2017 | 43020 | Hyman, BC; Whipple, LE | 1996 | Application of mitochondrial DNA polymorphism to Meloidogyne molecular population biology | Recent advances in molecular biology have enabled the genotyping of individual nematodes, facilitating the analysis of genetic variability within and among plant-pathogenic nematode isolates. This review first describes representative examples of how RFLP, RAPD, AFLP, and DNA sequence analysis have been employed to describe populations of several phytonematodes, including the pinewood, burrowing, root-knot, and cyst nematodes. The second portion of this paper evaluates the utility of a size-variable mitochondrial DNA locus to examine the generic structure of Meloidogyne isolates using two alternate methodologies, variable number tandem repeat (VNTR) and repeat associated polymorphism (RAP) analysis. VNTR analysis has revealed genetic variation among individual nematodes, whereas RAP may provide useful markers for species and population differentiation. | 1666 | no | gd of parasite | NA | no | no |
| 1667 | 2017 | 43020 | Norman, PJ; Hollenbach, JA; Nemat-Gorgani, N; Guethlein, LA; Hilton, HG; Pando, MJ; Koram, KA; Riley, EM; Abi-Rached, L; Parham, P | 2013 | Co-evolution of Human Leukocyte Antigen (HLA) Class I Ligands with Killer-Cell Immunoglobulin-Like Receptors (KIR) in a Genetically Diverse Population of Sub-Saharan Africans | Interactions between HLA class I molecules and killer-cell immunoglobulin-like receptors (KIR) control natural killer cell (NK) functions in immunity and reproduction. Encoded by genes on different chromosomes, these polymorphic ligands and receptors correlate highly with disease resistance and susceptibility. Although studied at low-resolution in many populations, high-resolution analysis of combinatorial diversity of HLA class I and KIR is limited to Asian and Amerindian populations with low genetic diversity. At the other end of the spectrum is the West African population investigated here: we studied 235 individuals, including 104 mother-child pairs, from the Ga-Adangbe of Ghana. This population has a rich diversity of 175 KIR variants forming 208 KIR haplotypes, and 81 HLA-A, -B and -C variants forming 190 HLA class I haplotypes. Each individual we studied has a unique compound genotype of HLA class I and KIR, forming 1-14 functional ligand-receptor interactions. Maintaining this exceptionally high polymorphism is balancing selection. The centromeric region of the KIR locus, encoding HLA-C receptors, is highly diverse whereas the telomeric region encoding Bw4-specific KIR3DL1, lacks diversity in Africans. Present in the Ga-Adangbe are high frequencies of Bw4-bearing HLA-B*53:01 and Bw4-lacking HLA-B*35:01, which otherwise are identical. Balancing selection at key residues maintains numerous HLA-B allotypes having and lacking Bw4, and also those of stronger and weaker interaction with LILRB1, a KIR-related receptor. Correspondingly, there is a balance at key residues of KIR3DL1 that modulate its level of cell-surface expression. Thus, capacity to interact with NK cells synergizes with peptide binding diversity to drive HLA-B allele frequency distribution. These features of KIR and HLA are consistent with ongoing co-evolution and selection imposed by a pathogen endemic to West Africa. Because of the prevalence of malaria in the Ga-Adangbe and previous associations of cerebral malaria with HLA-B\* 53: 01 and KIR, Plasmodium falciparum is a candidate pathogen. | 1667 | no | human | NA | no | no |
| 1668 | 2017 | 43020 | Larno, V; Laroche, J; Launey, S; Flammarion, P; Devaux, A | 2001 | Responses of chub (Leuciscus cephalus) populations to chemical stress, assessed by genetic markers, DNA damage and cytochrome P4501A induction | Indicators of effects at the population level (genetic variation using allozymes) and early indicators of pollution (EROD activity and DNA strand break formation) were analysed in chub (Leuciscus cephalus) living in weakly and heavily contaminated stations of the Rhone River watershed. The genetic erosion was mainly detected in a fish population living in a contaminated small river system, through modifications in allelic and genotypic frequencies for PGM-2 locus and could be linked to a genetic bottleneck and to the reduced gene flow from upstream unable to maintain or restore the genetic diversity. In a contaminated large river system, the genetic diversity for PGM-2 and other loci was maintained and was probably the consequence of a high gene flow from upstream, linked to a sustained drift of larvae and juveniles in the system. A convergent increase of the frequency of the 90 allele at PGM-2 was observed in two contaminated stations compared with the reference station, this trend being confirmed on a more extensive geographic scale over the Rhone River basin. A high level of EROD activity was detected in both contaminated sites but only the fish in the large river system showed a significant DNA damage level compared to the reference population. The low DNA damage level and high hepato-somatic ratio characterized the impacted population of the small river system and could be associated to a chronic high-level exposure of fish to pollutants which selected individuals exhibiting a high level of DNA damage repair. In the two contaminated systems, some genotypes at the PGM-2 and EST-2 loci showed a low level of DNA damage and/or a high EROD activity and may be considered as being tolerant to pollutants. A higher tolerance of the most heterozygous fish was also detected in the contaminated large system and confirmed that a high level of heterozygosity may be necessary for survival in such a system. | 1668 | no | no good fitness measure, N pop differnt in GD? | NA | no | no |
| 1669 | 2017 | 43020 | Gaskin, JF; Schwarzlander, M; Williams, L; Gerber, E; Hinz, HL | 2012 | Minimal genetic diversity in the facultatively outcrossing perennial pepperweed (Lepidium latifolium) invasion | Perennial pepperweed (Lepidium latifolium L.) is a Eurasian plant species that is invasive in North America. The invasion often forms large, dense monocultural stands. We investigated the genetic diversity along transects in dense populations in the western USA using Amplified Fragment Length Polymorphisms. We also analyzed transect collections from the native Eurasian range for comparison. In addition, we conducted crossing studies to determine possible modes of seed production (sexual outcrossing vs. self-fertilization vs. apomixis). In our study of seed production we determined that self-fertilization and outcrossing both produce germinable seed in perennial pepperweed. Genetic diversity in the USA was unexpectedly low, with only three genotypes in 388 plants, and those three had genetic similarity of a parts per thousand yen98%. Up to 97% of the plants from Turkey and Russia transects were unique genotypes, while < 4% of USA plants in a transect were unique. This lack of diversity in the USA samples suggests that perennial pepperweed, despite its success as an invader, is not well-positioned to adapt to new selective pressures, or to recruit pre-adapted genotypes that may vary in resistance or tolerance to disease or herbivory. Because 99% of the USA plants were genetically identical, we were unable to determine if increases in stand size were due to spread by rhizomes or seed derived from outcrossing between genetically identical parents or self-fertilization, as each of these methods produces shoots genetically identical to parental plants. | 1669 | no | no good fitness measure | NA | no | no |
| 1670 | 2017 | 43020 | Gomez, D; Richardson, C; Thery, M; Lengagne, T; Lena, JP; Plenet, S; Joly, P | 2011 | Multimodal signals in male European treefrog (Hyla arborea) and the influence of population isolation on signal expression | In nocturnal treefrogs, mate choice implies the use of acoustic and visual signals. Multimodality is suspected to have evolved for either information redundancy or information complementariness. It is essential to explore multimodality in a natural context to understand the selection pressures operating on the signals. In the present study, we investigated calling and coloration in relation to male biometry and condition in four populations of European treefrog (Hyla arborea) varying in size and genetic isolation. We compared the signal intensity between core and satellite populations to estimate the impact of genetic diversity on male secondary sexual traits. The results obtained show important regional variations in both traits, likely as a result of local adaptations. Call and coloration are weakly correlated within an individual, implying that these traits likely convey different information about the signaller’s identity or quality, thus supporting the hypothesis of complementariness of multiple messages. By contrast to the experimental evidence, we find that call and coloration are not related to male condition (as estimated by the residual of mass over size), suggesting that the condition-dependence of these traits may be mediated by complex mechanisms not accurately reflected by the chosen estimator. Finally, male call and colour phenotypes present no robust pattern of variation with isolation status, probably because of variation in local selective pressures and in history of population dynamics. (C) 2011 The Linnean Society of London, Biological Journal of the Linnean Society, 2011, 103, 633-647. | 1670 | no | no good fitness measure | NA | no | no |
| 1671 | 2017 | 43020 | Arndt, A; Smith, MJ | 1998 | Genetic diversity and population structure in two species of sea cucumber: differing patterns according to mode of development | The population structure of two species of sea cucumber was examined based on mitochondrial DNA sequence analysis. Cucumaria miniata, a species with pelagic nonfeeding larvae lasting less than 2 weeks, and C. pseudocurata, a brooding species lacking a pelagic phase, both occur over similar wide ranges of the northeastern Pacific between Alaska and California. No significant genetic structuring was observed among C. miniata samples with 95% of the observed nucleotide variance attributable to that within population samples. Conversely, only 3.4% of the observed variance was attributed to that within C. pseudocurata population samples, with sampling sites typically greater than 100 km apart. At a finer scale, two C. pseudocurata population samples taken 5 km apart were not statistically different. A significant genetic disjunction was observed among populations of the brooding species, but not in species with pelagic larvae, north of Vancouver Island, Canada, corresponding to the splitting of the California and Alaska currents. Given the observed high levels of genetic diversity in northern samples, this genetic break indicates survival in northern, as well as southern, Pleistocene refugia. These results clearly demonstrate the effects that changes in life-history patterns can have on dispersal, population structure, and the potential for speciation events. | 1671 | no | no good fitness measure | NA | no | no |
| 1672 | 2017 | 43020 | Bart, JM; Breyer I; Gottstein, B; Romig, T; Piarroux, R | 2003 | Development of molecular tools to explore genetic diversity in Echinococcus multilocularis | In order to identify risk factors for the occurrence of alveolar echinococcosis on both regional and local levels in Europe, studies have to be performed to describe factors influencing the infection pressure to the human population, with special emphasis on the changing epidemiological situation in transmitting animals. However, risk of infection may not only be determined by the abundance of, and contact with, parasitic eggs, but also by genetically fixed differences in pathogenicity between parasite strains. Therefore, typing of isolates collected from different areas in foxes, as well as in humans presenting with various lesions, should lead to a better understanding of the putative role of the genetic, diversity of Echinococcus multilocularis. In addition, if such differences would correlate with the region of origin, a valuable tool would be available to trace sources of infection. Unfortunately, to date, with the genetic markers currently used, very limited variation have been detected within the world-wide population of E. multilocularis. Consequently, the differences, in terms of prevalence, pathogenicity or susceptibility to chemotherapy, observed in humans in the three main foci (Europe, Asia and North America) could not be correlated with the putative genetic typing of the parasite. Development of highly sensitive molecular tools which can detect genetic variations in small populations, would be useful for tracking the spatial and temporal dynamics of the infection of humans. With regard to results obtained with other pathogens, such as Leishmania, Candida, Schistosoma, microsatellite analysis and single nucleotide polymorphisms (SNPs) assessed by single-stranded conformational polymorphism or Constant Denaturant Capillary Electrophoresis (CDCE) may prove their relevance and their power of discrimination and finally allow for a distinction between closely-related genotypes. | 1672 | no | human | NA | no | no |
| 1673 | 2017 | 43020 | Campbell, MC; Ranciaro, A; Zinshteyn, D; Rawlings-Goss, R; Hirbo, J; Thompson, S; Woldemeskel, D; Froment, A; Rucker, JB; Omar, SA; Bodo, JM; Nyambo, T; Belay, G; Drayna, D; Breslin, PAS; Tishkoff, SA | 2014 | Origin and Differential Selection of Allelic Variation at TAS2R16 Associated with Salicin Bitter Taste Sensitivity in Africa | “Bitter taste perception influences human nutrition and health, and the genetic variation underlying this trait may play a role in disease susceptibility. To better understand the genetic architecture and patterns of phenotypic variability of bitter taste perception, we sequenced a 996 bp region, encompassing the coding exon of TAS2R16, a bitter taste receptor gene, in 595 individuals from 74 African populations and in 94 non-Africans from 11 populations. We also performed genotype-phenotype association analyses of threshold levels of sensitivity to salicin, a bitter anti-inflammatory compound, in 296 individuals from Central and East Africa. In addition, we characterized TAS2R16 mutants in vitro to investigate the effects of polymorphic loci identified at this locus on receptor function. Here, we report striking signatures of positive selection, including significant Fay and Wu’s H statistics predominantly in East Africa, indicating strong local adaptation and greater genetic structure among African populations than expected under neutrality. Furthermore, we observed a”“star-like”" phylogeny for haplotypes with the derived allele at polymorphic site 516 associated with increased bitter taste perception that is consistent with a model of selection for "“high-sensitivity”" variation. In contrast, haplotypes carrying the "“low-sensitivity”" ancestral allele at site 516 showed evidence of strong purifying selection. We also demonstrated, for the first time, the functional effect of nonsynonymous variation at site 516 on salicin phenotypic variance in vivo in diverse Africans and showed that most other nonsynonymous substitutions have weak or no effect on cell surface expression in vitro, suggesting that one main polymorphism at TAS2R16 influences salicin recognition. Additionally, we detected geographic differences in levels of bitter taste perception in Africa not previously reported and infer an East African origin for high salicin sensitivity in human populations." | 1673 | no | human | NA | no | no |
| 1674 | 2017 | 43020 | Branca, A; Paape, TD; Zhou, P; Briskine, R; Farmer, AD; Mudge, J; Bharti, AK; Woodward, JE; May, GD; Gentzbittel, L; Ben, C; Denny, R; Sadowsky, MJ; Ronfort, J; Bataillon, T; Young, ND; Tiffin, P | 2011 | Whole-genome nucleotide diversity, recombination, and linkage disequilibrium in the model legume Medicago truncatula | Medicago truncatula is a model for investigating legume genetics, including the genetics and evolution of legume-rhizobia symbiosis. We used whole-genome sequence data to identify and characterize sequence polymorphisms and linkage disequilibrium (LD) in a diverse collection of 26 M. truncatula accessions. Our analyses reveal that M. truncatula harbors both higher diversity and less LD than soybean (Glycine max) and exhibits patterns of LD and recombination similar to Arabidopsis thaliana. The population-scaled recombination rate is approximately one-third of the mutation rate, consistent with expectations for a species with a high selfing rate. Linkage disequilibrium, however, is not extensive, and therefore, the low recombination rate is likely not a major constraint to adaptation. Nucleotide diversity in 100-kb windows was negatively correlated with gene density, which is expected if diversity is shaped by selection acting against slightly deleterious mutations. Among putative coding regions, members of four gene families harbor significantly higher diversity than the genome-wide average. Three of these families are involved in resistance against pathogens; one of these families, the nodule-specific, cysteine-rich gene family, is specific to the galegoid legumes and is involved in control of rhizobial differentiation. The more than 3 million SNPs that we detected, approximately one-half of which are present in more than one accession, are a valuable resource for genome-wide association mapping of genes responsible for phenotypic diversity in legumes, especially traits associated with symbiosis and nodulation. | 1674 | no | no good fitness measure, N pop differnt in GD? | NA | no | no |
| 1675 | 2017 | 43020 | Sarwar, N; Ashfaq, S; Akhtar, KP; Jamil, FF | 2013 | BIOLOGICAL PATHOTYPING AND RAPD ANALYSIS OF ASCOCHYTA RABIEI, FROM VARIOUS CHICKPEA GROWING AREAS OF PAKISTAN | Blight disease caused by the fungus Ascochyta rabiei is considered to be the major constraint to chickpea production. Twenty-one isolates of A. rabiei were obtained from blighted samples collected from major chickpea growing areas in Pakistan during 2006-08. Biological pathotyping on a set of chickpea differentials indicated that four isolates were highly virulent; two were less virulent, while rest of the isolates was moderately virulent. RAPD assay was used to investigate the genetic diversity among the isolates. Twenty primers with random nucleotide sequences were tested. A total of 49 bands were polymorphic. Dendrogram constructed from RAPD data indicated that there was a high genetic variability among the isolates collected from different locations and were grouped into three main clusters. | 1675 | no | crop | NA | no | no |
| 1676 | 2017 | 43020 | Oklander, LI; Kowalewski, MM; Corach, D | 2010 | Genetic Consequences of Habitat Fragmentation in Black-and-Gold Howler (Alouatta caraya) Populations from Northern Argentina | Human-induced habitat fragmentation might seriously affect behavioural patterns and the survival of species whose ecological requirements strongly depend on specific environmental conditions. We compared the genetic structure and dispersal patterns of 2 populations of Alouatta caraya (Plathyrrhini, Atelidae) to understand how habitat reduction and fragmentation affect gene flow in this species. We sampled individuals from 7 groups living in continuous forest (CF, n = 46, 22 males and 24 females), and 11 groups that inhabit a fragmented forest (FF, n = 50, 24 males and 26 females). FST values based on 11 microsatellite loci showed a recent genetic differentiation among groups in the FF. In contrast, the CF showed no differentiation among groups. Further, FST values between sexes, as well as kinship relationships, also exhibited differences between habitats. In the CF, both males and females disperse, leading to nondifferentiated groups composed of adults that are not close relatives. Conversely, in the FF, some groups are differentiated, males disperse more than females, and groups are composed of closely related adult females. Our results suggest that habitat fragmentation modifies the dispersal patterns of black-and-gold howlers. These differences between habitats may reflect a reduced gene flow, providing genetic evidence that suggests that habitat fragmentation severely limits the howler’s ability to disperse. An increasing level of isolation due to uncontrolled deforestation may cause similar loss of genetic diversity on other arboreal primates, and nonprimates that depend on forest continuity to disperse, reducing their abilities to cope with environmental changes. | 1676 | no | no good fitness measure | NA | no | no |
| 1677 | 2017 | 43020 | Wu, YY; He, JB; Li, AH; Fang, NY; He, WW; Dang, LL; Zeng, GY; Huang, J; Bao, YM; Zhang, HS | 2016 | Population structure analysis and association mapping of blast resistance in indica rice (Oryza sativa L.) landraces | Rice blast caused by Magnaporthe oryzae is one of the most devastating rice diseases worldwide. To understand the genetic diversity of indica landrace accessions and identify simple sequence repeat (SSR) markers that are associated with blast resistance, a population of 276 indica landraces from across the world was constructed. This population was then used to evaluate the blast-resistance phenotype through artificial inoculation under controlled conditions in 2012 and 2013. The genetic diversity and association of the population with resistance were analyzed by examining the phenotype for 160 SSR markers distributed on 12 rice chromosomes. The 276 accessions were classified into seven groups using model- and distance-based cluster analyses. Associations between SSR markers and blast resistance showed that 26 SSR markers were significantly associated with blast resistance in 2012 and 2013 (P < 0.01) and that the phenotypic variation ranged from 2.68 to 13.11%. Nineteen of the markers associated with blast resistance were located in regions where genes or quantitative trait loci (QTLs) have been previously reported, and seven were newly identified in this study. These results indicate that marker-trait association has potential advantages over classical linkage analysis and QTL mapping, and that these markers could be used for marker-assisted selection in rice blast-resistance-breeding programs. | 1677 | no | NA | NA | no | no |
| 1678 | 2017 | 43020 | SERA, WE; GAINES, MS | 1994 | The Effect of Relatedness on Spacing Behavior and Fitness of Female Prairie Voles | Numerous behavioral hypotheses have been suggested to explain population cycles in microtine rodents. Charnov and Finerty (1980) proposed that preferential behavior toward relatives drives population cycles through kin selection. Their hypothesis makes three assumptions: (1) individuals behaviorally discriminate kin from non-kin, (2) kin associate, and (3) kin benefit from this association. These assumptions were tested in female prairie voles (Microtus ochrogaster) in eastern Kansas. An equal number of laboratory-born females and males were released into 12 fenced enclosures; 6 enclosures contained groups of littermate sisters and 6 contained groups of unrelated females. Space use, reproductive success, and survival were monitored during two replications of the experiment (Fall 1989 and Spring 1990). Female survival and reproductive success were not affected by relatedness, although more young were born in the Spring than in the Fall. Home range size and overlap were greater for females in related groups than those in unrelated groups. Home range size also was larger in the Fall than in the Spring. Prairie vole sisters associated spatially, but our measurements showed no fitness benefits conferred by these associations. Although the kin-selection hypothesis could not be demonstrated in this experimental population, relatedness in local neighborhoods may have consequences for small mammal population ecology. | 1678 | yes | ok gd measurement and pop level comp? | NA | no | yes |
| 1679 | 2017 | 43020 | Meunier, C; Tirard, C; Hurtrez-Bousses, S; Durand, P; Bargues, MD; Mas-Coma, S; Pointier, JP; Jourdane, J; Renaud, F | 2001 | Lack of molluscan host diversity and the transmission of an emerging parasitic disease in Bolivia | Fasciolosis is a re-emerging parasitic disease that affects an increasing number of people in developing countries. The most severe endemic affects the Bolivian Altiplano, where the liver fluke (Fasciola hepatica) and its hermaphroditic snail host, Lymnaea truncatula, have been introduced from Europe. To achieve a better understanding of the epidemiological situation and the consequences of the colonization event of this invasive species, genetic analysis of Bolivian snail populations was needed. Here we compare the genetic diversity and population structure of snail samples from the Bolivian Altiplano with samples from the Old World at six polymorphic microsatellite loci. Whereas some variability exists in the snail populations from the Old World, we observe only a single genotype of L. truncatula in the Bolivian Altiplano. We discuss the possible explanations for such a reduction in genetic variability, and, given the high natural parasitism pressures exerted on the snail populations, we discuss the relevance of this result for host-parasite interactions. | 1679 | no | no good fitness measure | NA | no | no |
| 1680 | 2017 | 43020 | Zhang, B; Fang, SG; Xi, YM | 2006 | Major histocompatibility complex variation in the endangered crested ibis Nipponia nippon and implications for reintroduction | The major histocompatibility complex (MHC), with its extraordinary levels of genetic variation, is thought to be an essential aspect of the ability of an organism to recognize different parasites and pathogens. It has also been proposed to regulate reproductive processes in many aspects. Here we examine the genetic variation of the second exon of the MHC class II B genes of the crested ibis, an endangered species known to descend from just two breeding pairs rediscovered in 1981. Only five alleles are identified by single-strand conformation polymorphism (SSCP) analysis of 36 samples taken from both wild and captive populations, and a comparatively low level of divergence between MHC alleles is observed. We suggest that representative sampling of individuals with most of the different MHC allele genotypes to constitute a founder population, together with the monitoring of the pathogen status of candidate sites before release, is of great importance for raising the success rate of reintroduction for the crested ibis. | 1680 | no | no good fitness measure, N pop differnt in GD? | NA | no | no |
| 1681 | 2017 | 43020 | Cheng, F; Yang, JM; Zhang, D; Hu, DF; Chu, HJ; Cao, J; Jiang, F; Bu, L; Li, K | 2014 | Isolation and characterization of microsatellite loci from Gasterophilus pecorum (Diptera: Gasterophilidae) | Gasterophilus pecorum (Diptera: Gasterophilidae) is the most damaging parasites in Przewalaski’s horses that were re-wilded in Kalamaili, Xinjiang, China. In this study, 18 polymorphic microsatellite loci were isolated for the first time from G. pecorum. The number of alleles per locus ranged from 3 to 19 and the mean number of alleles was 10.78. Mean observed and expected heterozygosity were 0.5935 and 0.7751, respectively. These polymorphic markers will be powerful tools for assessment of parentage analysis, genetic diversity and population structure of this species. | 1681 | no | no good fitness measure | NA | no | no |
| 1682 | 2017 | 43020 | Liu, MS; Deora, R; Doulatov, SR; Gingery, M; Eiserling, FA; Preston, A; Maskell, DJ; Simons, RW; Cotter, PA; Parkhill, J; Miller, JF | 2002 | Reverse transcriptase-mediated tropism switching in Bordetella bacteriophage | Host-pathogen interactions are often driven by mechanisms that promote genetic variability. We have identified a group of temperate bacteriophages that generate diversity in a gene, designated mtd (major tropism determinant), which specifies tropism for receptor molecules on host Bordetella species. Tropism switching is the result of a template-dependent, reverse transcriptase-mediated process that introduces nucleotide substitutions at defined locations within mtd. This cassette-based mechanism is capable of providing a vast repertoire of potential ligand-receptor interactions. | 1682 | no | NA | NA | no | no |
| 1683 | 2017 | 43020 | Pittman, SE; King, TL; Faurby, S; Dorcas, ME | 2011 | Demographic and genetic status of an isolated population of bog turtles (Glyptemys muhlenbergii): implications for managing small populations of long-lived animals | In this study, we sought to determine the population stability and genetic diversity of one isolated population of the federally-threatened bog turtle (Glyptemys muhlenbergii) in North Carolina. Using capture-recapture data, we estimated adult survival and population growth rate from 1992 to 2007. We found that the population decreased from an estimated 36 adult turtles in 1994 to approximately 11 adult turtles in 2007. We found a constant adult survival of 0.893 (SE = 0.018, 95% confidence interval, 0.853-0.924) between 1992 and 2007. Using 18 microsatellite markers, we compared the genetic status of this population with five other bog turtle populations. The target population displayed allelic richness (4.8 +/- 0.5) and observed heterozygosity (0.619 +/- 0.064) within the range of the other bog turtle populations. Coalescent analysis of population growth rate, effective population size, and timing of population structuring event also indicated the genetics of the target population were comparable to the other populations studied. Estimates of effective population size were a proportion of the census size in all populations except the target population, in which the effective population size was larger than the census size (30 turtles vs. 11 turtles). We attribute the high genetic diversity in the target population to the presence of multiple generations of old turtles. This study illustrates that the demographic status of populations of long-lived species may not be reflected genetically if a decline occurred recently. Consequently, the genetic integrity of populations of long-lived animals experiencing rapid demographic bottlenecks may be preserved through conservation efforts effective in addressing demographic problems. | 1683 | no | only one pop | NA | no | no |
| 1684 | 2017 | 43020 | Mullins, J; McDevitt, AD; Kowalczyk, R; Ruczynska, I; Gorny, M; Wojcik, JM | 2014 | The influence of habitat structure on genetic differentiation in red fox populations in north-eastern Poland | The red fox (Vulpes vulpes) has the widest global distribution among terrestrial carnivore species, occupying most of the Northern Hemisphere in its native range. Because it carries diseases that can be transmitted to humans and domestic animals, it is important to gather information about their movements and dispersal in their natural habitat but it is difficult to do so at a broad scale with trapping and telemetry. In this study, we have described the genetic diversity and structure of red fox populations in six areas of north-eastern Poland, based on samples collected from 2002-2003. We tested 22 microsatellite loci isolated from the dog and the red fox genome to select a panel of nine polymorphic loci suitable for this study. Genetic differentiation between the six studied populations was low to moderate and analysis in Structure revealed a panmictic population in the region. Spatial autocorrelation among all individuals showed a pattern of decreasing relatedness with increasing distance and this was not significantly negative until 93 km, indicating a pattern of isolation-by-distance over a large area. However, there was no correlation between genetic distance and either Euclidean distance or least-cost path distance at the population level. There was a significant relationship between genetic distance and the proportion of large forests and water along the Euclidean distances. These types of habitats may influence dispersal paths taken by red foxes, which is useful information in terms of wildlife disease management. | 1684 | no | no good fitness measure | NA | no | no |
| 1685 | 2017 | 43020 | Kence, M; Oskay, D; Giray, T; Kence, A | 2013 | Honey bee colonies from different races show variation in defenses against the varroa mite in a ‘common garden’ | Abstract Honey bee [Apis mellifera L. (Hymenoptera: Apidae)] genetic diversity may be the key to responding to novel health challenges faced by this important pollinator. In this study, we first compared colonies of four honey bee races, A. m. anatoliaca, A. m. carnica, A. m. caucasica, and A. m. syriaca from Turkey, with respect to honey storage, bee population size, and defenses against varroa. The mite Varroa destructor Anderson & Trueman (Acari: Varroidae) is an important pest of honey bee colonies. There are genetic correlates with two main defenses of bees against this parasite: hygienic behavior, or removing infested brood, and grooming, which involves shaking and swiping off mites and biting them. In the second part of this study, we examined the relationship of these two types of defenses, hygiene and grooming, and their correlation with infestation rates in 32 genetically diverse colonies in a ‘common garden’ apiary. Mite biting was found to be negatively correlated with mite infestation levels. | 1685 | no | No GD diff between pop | NA | no | no |
| 1686 | 2017 | 43020 | Riehl, C; Strong, MJ | 2015 | Social living without kin discrimination: experimental evidence from a communally breeding bird | In many cooperative animal societies, individuals can recognize their relatives and preferentially direct helping behaviors towards them. However, the ability to learn kin recognition cues may be constrained in societies with low relatedness, since group membership alone is not a reliable proxy for kinship. Here, we examine kin discrimination in the greater ani (Crotophaga major), a communally nesting bird in which several unrelated males and females reproduce in a single, shared nest and provide parental care to the mixed clutch of young. Each adult, therefore, is closely related to some nestlings in the clutch and unrelated to others. Food is limited and starvation is a significant cause of nestling mortality, suggesting that adults should increase their fitness by preferentially feeding their own offspring in the mixed clutch. To test this hypothesis, we cross-fostered broods of nestlings between pairs of nests, such that none of the nestlings in the manipulated nests were related to any of the adults feeding them. We found no evidence that adult greater anis discriminate between their own and unrelated nestlings: adults at cross-fostered groups fed nestlings at the same rates as adults at control (sham-manipulated) nests, and rates of nestling starvation were equal at cross-fostered and control nests. These results suggest that adult greater anis do not recognize their own nestlings, and they are consistent with the hypothesis that genetically encoded markers for kin recognition are rare in birds. | 1686 | no | no good fitness measure, N pop differnt in GD? | NA | no | no |
| 1687 | 2017 | 43020 | Dailianis, T; Tsigenopoulos, CS; Dounas, C; Voultsiadou, E | 2011 | Genetic diversity of the imperilled bath sponge Spongia officinalis Linnaeus, 1759 across the Mediterranean Sea: patterns of population differentiation and implications for taxonomy and conservation | The Mediterranean bath sponge Spongia officinalis is an iconic species with high socioeconomic value and precarious future owing to unregulated harvesting, mortality incidents and lack of established knowledge regarding its ecology. This study aims to assess genetic diversity and population structure of the species at different geographical scales throughout its distribution. For this purpose, 11 locations in the Eastern Mediterranean (Aegean Sea), Western Mediterranean (Provence coast) and the Strait of Gibraltar were sampled; specimens were analysed using partial mitochondrial cytochrome oxidase subunit I (COI) sequences, along with a set of eight microsatellite loci. According to our results (i) no genetic differentiation exists among the acknowledged Mediterranean morphotypes, and hence, S. officinalis can be viewed as a single, morphologically variable species; (ii) a notable divergence was recorded in the Gibraltar region, indicating the possible existence of a cryptic species; (iii) restriction to gene flow was evidenced between the Aegean Sea and Provence giving two well-defined regional clusters, thus suggesting the existence of a phylogeographic break between the two systems; (iv) low levels of genetic structure, not correlated to geographical distance, were observed inside geographical sectors, implying mechanisms (natural or anthropogenic) that enhance dispersal and gene flow have promoted population connectivity; (v) the genetic diversity of S. officinalis is maintained high in most studied locations despite pressure from harvesting and the influence of devastating epidemics. These findings provide a basis towards the effective conservation and management of the species. | 1687 | no | no good fitness measure | NA | no | no |
| 1688 | 2017 | 43020 | Danielewski, JA; Garland, SM; McCloskey, J; Hillman, RJ; Tabrizi, SN | 2013 | Human Papillomavirus Type 6 and 11 Genetic Variants Found in 71 Oral and Anogenital Epithelial Samples from Australia | Genetic variation of 49 human papillomavirus (HPV) 6 and 22 HPV11 isolates from recurrent respiratory papillomatosis (RRP) (n = 17), genital warts (n = 43), anal cancer (n = 6) and cervical neoplasia cells (n = 5), was determined by sequencing the long control region (LCR) and the E6 and E7 genes. Comparative analysis of genetic variability was examined to determine whether different disease states resulting from HPV6 or HPV11 infection cluster into distinct variant groups. Sequence variation analysis of HPV6 revealed that isolates cluster into variants within previously described HPV6 lineages, with the majority (65%) clustering to HPV6 sublineage B1 across the three genomic regions examined. Overall 72 HPV6 and 25 HPV11 single nucleotide variations, insertions and deletions were observed within samples examined. In addition, missense alterations were observed in the E6/E7 genes for 6 HPV6 and 5 HPV11 variants. No nucleotide variations were identified in any isolates at the four E2 binding sites for HPV6 or HPV11, nor were any isolates found to be identical to the HPV6 lineage A or HPV11 sublineage A1 reference genomes. Overall, a high degree of sequence conservation was observed between isolates across each of the regions investigated for both HPV6 and HPV11. Genetic variants identified a slight association with HPV6 and anogenital lesions (p = 0.04). This study provides important information on the genetic diversity of circulating HPV 6 and HPV11 variants within the Australian population and supports the observation that the majority of HPV6 isolates cluster to the HPV6 sublineage B1 with anogenital lesions demonstrating an association with this sublineage (p = 0.02). Comparative analysis of Australian isolates for both HPV6 and HPV11 to those from other geographical regions based on the LCR revealed a high degree of sequence similarity throughout the world, confirming previous observations that there are no geographically specific variants for these HPV types. | 1688 | no | human | NA | no | no |
| 1689 | 2017 | 43020 | Riffaut, L; McCoy, KD; Tirard, C; Friesen, VL; Boulinier, T | 2005 | Population genetics of the common guillemot Uria aalge in the North Atlantic: geographic impact of oil spills | The population genetic structure of a species can be an important conservation tool informing us about the potential for genetic loss and the capacity for species recovery. Depending on the nature of population subdivision, it can also provide a means for assessing the source population of dead or injured individuals of unknown geographic origin. This type of information can be particularly useful in instances of large-scale environmental accidents, such as oil spills. Following the wreck of the ‘Erika’ oil tanker in the Bay of Biscay in December 1999, more than 80 000 seabirds were washed ashore along the west coast of France. The most heavily affected species (80% of all birds) was the common guillemot Uria aalge, a widespread long-lived colonial seabird. In an attempt to evaluate the ‘true’ geographic extent of this accident, we carried out population genetic analyses using 6 microsatellite markers on samples from 22 breeding colonies in the North Atlantic and on individuals collected after the oil spill. A pattern of isolation by distance was detected among common guillemot populations, but populations were only weakly structured, even at large spatial scales. The low level of genetic differentiation between colonies prevented clear assignments of oiled birds to their population of origin using only the genetic information. The weak genetic structure suggests that little genetic variability was lost during the oil spill and implies a high potential for population recovery via dispersal. However, current gene flow among extant colonies may overestimate the capacity of locally extinct colonies to recover due to the behavioural processes involved in recruitment. Clearly, the mortality of tens of thousands of high-latitude seabirds due to oil pollution warrants ongoing scientific scrutiny and conservation effort. | 1689 | no | no good fitness measure | NA | no | no |
| 1690 | 2017 | 43020 | Lagrue, C; Poulin, R; Keeney, DB | 2009 | EFFECTS OF CLONALITY IN MULTIPLE INFECTIONS ON THE LIFE-HISTORY STRATEGY OF THE TREMATODE COITOCAECUM PARVUM IN ITS AMPHIPOD INTERMEDIATE HOST | Theoretical models predict that genetic relatedness affects the competition within and between parasite clonal groups sharing a common host. Here, we studied natural and experimental multiple infections of the trematode Coitocaecum parvum in its intermediate host. We focused on the effects of clonality on the life-history strategy of parasites competing for resources. Coitocaecum parvum can either delay maturation until its amphipod host is ingested by a definitive host, or adopt a progenetic strategy and reproduce inside the amphipod. Within a common host, clonal parasites were more likely to adopt identical life-history strategies than different genetic clones, both in natural and experimental infections. However, when timing of infection and other factors were controlled experimentally, parasites sharing a host were likely to adopt identical strategies regardless of their clonal identity, although pairs of clones were more likely to adopt progenesis than pairs of nonclones. The asymmetries in relative size and egg production between coinfecting parasites adopting the same life-history strategy were slightly, but not significantly, higher between different clones than identical clones. Our results suggest that the dynamics of competition between coinfecting parasites, although influenced by numerous external factors, is also modulated by genetic relatedness among parasites. | 1690 | no | No GD diff between pop | NA | no | no |
| 1691 | 2017 | 43020 | Velo-Anton, G; Rodriguez, D; Savage, AE; Parra-Olea, G; Lips, KR; Zamudio, KR | 2012 | Amphibian-killing fungus loses genetic diversity as it spreads across the New World | Emergent infectious diseases are a severe threat to global biodiversity, thus conservation biologists need to understand the emergence, spread, and evolution of pathogens to identify factors driving disease outbreaks. Amphibian chytridiomycosis is a recently emerged amphibian disease caused by the fungus Batrachochytrium dendrobatidis (Bd) that has led to species extinctions and declines worldwide. The spatio-temporal dynamics of pathogen occurrence and disease outbreaks, and comparative genomic analyses of global Bd strains, support the spreading pathogen hypothesis (SPH) with pandemics occurring after introduction of Bd into naive host populations. Here we used population genetics of the amphibian-killing fungus to test for genetic consequences of pathogen spread. Our population genetic analyses are consistent with the spread of Bd from North to Central America, based on low genetic diversity, reduction in heterozygosity, and increased allele fixation in Bd from recently infected populations. Our findings confirm the spread of Bd in the New World, and indicate that future conservation efforts should focus on: (i) functional consequences (such as changes in pathogenicity) of these genetic changes, and (ii) public education and restrictions on wildlife trade to help slow spread of the pathogen at the invasion front. (C) 2011 Elsevier Ltd. All rights reserved. | 1691 | no | gd of pathogen | NA | no | no |
| 1692 | 2017 | 43020 | Hapuarachchi, HC; Koo, C; Kek, R; Xu, H; Lai, YL; Liu, L; Kok, SY; Shi, Y; Chuen, RLT; Lee, KS; Maurer-Stroh, S; Ng, LC | 2016 | Intra-epidemic evolutionary dynamics of a Dengue virus type 1 population reveal mutant spectra that correlate with disease transmission | Dengue virus (DENV) is currently the most prevalent mosquito-borne viral pathogen. DENVs naturally exist as highly heterogeneous populations. Even though the descriptions on DENV diversity are plentiful, only a few studies have narrated the dynamics of intra-epidemic virus diversity at a fine scale. Such accounts are important to decipher the reciprocal relationship between viral evolutionary dynamics and disease transmission that shape dengue epidemiology. In the current study, we present a micro-scale genetic analysis of a monophyletic lineage of DENV-1 genotype III (epidemic lineage) detected from November 2012 to May 2014. The lineage was involved in an unprecedented dengue epidemic in Singapore during 2013-2014. Our findings showed that the epidemic lineage was an ensemble of mutants (variants) originated from an initial mixed viral population. The composition of mutant spectrum was dynamic and positively correlated with case load. The close interaction between viral evolution and transmission intensity indicated that tracking genetic diversity through time is potentially a useful tool to infer DENV transmission dynamics and thereby, to assess the epidemic risk in a disease control perspective. Moreover, such information is salient to understand the viral basis of clinical outcome and immune response variations that is imperative to effective vaccine design. | 1692 | no | gd of pathogen | NA | no | no |
| 1693 | 2017 | 43020 | Pava, Z; Noviyanti, R; Handayuni, I; Trimarsanto, H; Trianty, L; Burdam, FH; Kenangalem, E; Utami, RAS; Tirta, YK; Coutrier, F; Poespoprodjo, JR; Price, RN; Marfurt, J; Auburn, S | 2017 | Genetic micro-epidemiology of malaria in Papua Indonesia: Extensive P-vivax diversity and a distinct subpopulation of asymptomatic P-falciparum infections | Background Genetic analyses of Plasmodium have potential to inform on transmission dynamics, but few studies have evaluated this on a local spatial scale. We used microsatellite genotyping to characterise the micro-epidemiology of P. vivax and P. falciparum diversity to inform malaria control strategies in Timika, Papua Indonesia. Methods Genotyping was undertaken on 713 sympatric P. falciparum and P. vivax isolates from a cross-sectional household survey and clinical studies conducted in Timika. Standard population genetic measures were applied, and the data was compared to published data from Kalimantan, Bangka, Sumba and West Timor. Results Higher diversity (H-E = 0.847 vs 0.625; p = 0.017) and polyclonality (46.2% vs 16.5%, p<0.001) were observed in P. vivax versus P. falciparum. Distinct P. falciparum substructure was observed, with two subpopulations, K1 and K2. K1 was comprised solely of asymptomatic infections and displayed greater relatedness to isolates from Sumba than to K2, possibly reflecting imported infections. Conclusions The results demonstrate the greater refractoriness of P. vivax versus P. falciparum to control measures, and risk of distinct parasite subpopulations persisting in the community undetected by passive surveillance. These findings highlight the need for complimentary new surveillance strategies to identify transmission patterns that cannot be detected with traditional malariometric methods. | 1693 | no | gd of parasite | NA | no | no |
| 1694 | 2017 | 43020 | Breurec, S; Criscuolo, A; Diancourt, L; Rendueles, O; Vandenbogaert, M; Passet, V; Caro, V; Rocha, EPC; Touchon, M; Brisse, S | 2016 | Genomic epidemiology and global diversity of the emerging bacterial pathogen Elizabethkingia anophelis | Elizabethkingia anophelis is an emerging pathogen involved in human infections and outbreaks in distinct world regions. We investigated the phylogenetic relationships and pathogenesis-associated genomic features of two neonatal meningitis isolates isolated 5 years apart from one hospital in Central African Republic and compared them with Elizabethkingia from other regions and sources. Average nucleotide identity firmly confirmed that E. anophelis, E. meningoseptica and E. miricola represent demarcated genomic species. A core genome multilocus sequence typing scheme, broadly applicable to Elizabethkingia species, was developed and made publicly available (http://bigsdb.pasteur.fr/elizabethkingia). Phylogenetic analysis revealed distinct E. anophelis sublineages and demonstrated high genetic relatedness between the African isolates, compatible with persistence of the strain in the hospital environment. CRISPR spacer variation between the African isolates was mirrored by the presence of a large mobile genetic element. The pan-genome of E. anophelis comprised 6,880 gene families, underlining genomic heterogeneity of this species. African isolates carried unique resistance genes acquired by horizontal transfer. We demonstrated the presence of extensive variation of the capsular polysaccharide synthesis gene cluster in E. anophelis. Our results demonstrate the dynamic evolution of this emerging pathogen and the power of genomic approaches for Elizabethkingia identification, population biology and epidemiology. | 1694 | no | gd of pathogen | NA | no | no |
| 1695 | 2017 | 43020 | Schulke, O; Wenzel, S; Ostner, J | 2013 | Paternal Relatedness Predicts the Strength of Social Bonds among Female Rhesus Macaques | Forming strong, equitable, and enduring social bonds with a few individuals in a group carries adaptive benefits in terms of increased longevity, offspring survival and paternity success in birds and mammals, including humans. These recent insights generated a new interest in the factors creating variation in the strength of social relationships. Whether and how animals discriminate paternal kin from non-kin and bias their social behavior accordingly is being debated. This study explores the relative importance of dominance rank, age, maternal and paternal relatedness in shaping dyadic affiliative relationships in a group of 30 captive rhesus macaque females. The strength of social relationships, measured by the composite sociality index from observational data, was independently predicted in GLMMs by both maternal and paternal relatedness as well as rank similarity. In addition, social bonds between paternal half-sisters were stronger than between distantly related kin suggesting that females biased their affiliative effort towards paternal relatives. Despite identical relatedness coefficients bonds between mothers and their daughters were three times as strong as those between full sisters. Together these results add to the growing body of evidence for paternal kin biases in affiliative behavior and highlight that females prefer descendent over lateral kin. | 1695 | no | no good fitness measure, N pop differnt in GD? | NA | no | no |
| 1696 | 2017 | 43020 | Joshi, MB; Rout, PK; Mandal, AK; Tyler-Smith, C; Singh, L; Thangaraj, K | 2004 | Phylogeography and origin of Indian domestic goats | The Indian subcontinent contains 20 well-characterized goat breeds, which vary in their genetic potential for the production of milk, meat, and fibre; disease resistance; heat tolerance; and fecundity. Indian goats make up 20% of the world’s goat population, but there has been no extensive study of these economically important animals. Therefore, we have undertaken the present investigation of 363 goats belonging to 10 different breeds from different geographic regions of India using mtDNA sequence data from the HVRI region. We find evidence for population structure and novel lineages in Indian goats and cannot reconcile the genetic diversity found within the major lineage with domestication starting 10,000 years ago from a single mtDNA ancestor. Thus, we propose a more complex origin for domestic goats. | 1696 | no | domesticated animal | NA | no | no |
| 1697 | 2017 | 43020 | Meindl, C; Brune, V; Listl, D; Poschlod, P; Reisch, C | 2016 | Survival and postglacial immigration of the steppe plant Scorzonera purpurea to Central Europe | Temperate grasslands belong to the most diverse plant communities of Central Europe. However, there is still a lack of information about glacial refugia and migration processes of herbaceous grassland and especially steppe species in Central Europe. Therefore, we analyzed the survival and postglacial immigration of Scorzonera purpurea to Central Europe. We investigated 348 individuals from 37 populations in Europe using amplified fragment length polymorphisms and chloroplast microsatellite analyses. Our study revealed two major genetic groups along the European distribution range consisting of western populations on the one hand and closely related central and (south)eastern populations on the other hand. Genetic variation was highest within populations from the Pannonian basin and decreased toward Western and Central Europe. Our study gives evidence for the long-term survival of S. purpurea in Western Europe and the postglacial immigration from the southeastern parts of Europe, maybe by domestic livestock of migrating farmers during the Neolithic age to Central Europe. Immigration presumably followed two routes from Pannonia along the river Danube into Southern Germany and from Pannonia along the northern border of the Carpathians to Northern Germany. In Central Germany, the different genetic lineages may have met and formed contact zones. Our data show that steppe species may both have survived in and immigrated to Western and Central Europe. Further and more detailed studies on other steppe species are, therefore, needed to investigate the origin of these rare and often endangered species more generally. | 1697 | no | no good fitness measure | NA | no | no |
| 1698 | 2017 | 43020 | Obbard, DJ; Jiggins, FM; Bradshaw, NJ; Little, TJ | 2011 | Recent and Recurrent Selective Sweeps of the Antiviral RNAi Gene Argonaute-2 in Three Species of Drosophila | Antagonistic host-parasite interactions can drive rapid adaptive evolution in genes of the immune system, and such arms races may be an important force shaping polymorphism in the genome. The RNA interference pathway gene Argonaute-2 (AGO2) is a key component of antiviral defense in Drosophila, and we have previously shown that genes in this pathway experience unusually high rates of adaptive substitution. Here we study patterns of genetic variation in a 100-kbp region around AGO2 in three different species of Drosophila. Our data suggest that recent independent selective sweeps in AGO2 have reduced genetic variation across a region of more than 50 kbp in Drosophila melanogaster, D. simulans, and D. yakuba, and we estimate that selection has fixed adaptive substitutions in this gene every 30-100 thousand years. The strongest signal of recent selection is evident in D. simulans, where we estimate that the most recent selective sweep involved an allele with a selective advantage of the order of 0.5-1% and occurred roughly 13-60 Kya. To evaluate the potential consequences of the recent substitutions on the structure and function of AGO2, we used fold-recognition and homology-based modeling to derive a structural model for the Drosophila protein, and this suggests that recent substitutions in D. simulans are overrepresented at the protein surface. In summary, our results show that selection by parasites can consistently target the same genes in multiple species, resulting in areas of the genome that have markedly reduced genetic diversity. | 1698 | no | no good fitness measure, N pop differnt in GD? | NA | no | no |
| 1699 | 2017 | 43020 | Moore, RM; Pinel, T; Zhao, JH; March, R; Jawaid, A | 2005 | Selecting cases from nuclear families for case-control association analysis | We examine the efficiency of a number of schemes to select cases from nuclear families for case-control association analysis using the Genetic Analysis Workshop 14 simulated dataset. We show that with this simulated dataset comparing all affected siblings with unrelated controls is considerably more powerful than all of the other approaches considered. We find that the test statistic is increased by almost 3-fold compared to the next best sampling schemes of selecting all affected sibs only from families with affected parents (AF(aff)), one affected sib with most evidence of allele-sharing from each family (SF), and all affected sibs from families with evidence for linkage (AF(L)). We consider accounting for biological relatedness of samples in the association analysis to maintain the correct type I error. We also discuss the relative efficiencies of increasing the ratio of unrelated cases to controls, methods to confirm associations and issues to consider when applying our conclusions to other complex disease datasets. | 1699 | no | no good fitness measure, N pop differnt in GD? | NA | no | no |
| 1700 | 2017 | 43020 | Bratkowski, J; Pirk, CWW; Neumann, P; Wilde, J | 2012 | Genotypic diversity in queenless honey bee colonies reduces fitness | Honey bee queens mate with many males. The resulting genotypic diversity appears to enhance fitness of queenright colonies (those colonies with a reproductive queen present) which is difficult to measure, because measures of long-term fitness include successful matings of produced male sexuals (drones) and number of surviving swarms. The fitness of queenless colonies is, however, limited to worker-produced adult drones until natural colony death. Here we test the impact of genotypic diversity on fitness of queenless honey bee colonies, which were headed by queens inseminated with one, 10 and 20 drones or naturally mated. The data show that genetically diverse queenless colonies (20 subfamilies per colony) produced fewer adult drones, had a delayed onset of worker-derived drone flight activity, and a lower efficacy in drone production / per day colony life span compared to all other groups. Our data suggest that genotypic diversity may reduce fitness of queenless honey bee colonies, probably due to reproductive conflicts among subfamilies after queenloss. | 1700 | probably yes | ok fitness measurement? | no ok fitness measure | no | no |
| 1701 | 2017 | 43020 | Paupy, C; Brengues, C; Kamgang, B; Herve, JP; Fontenille, D; Simard, F | 2008 | Gene flow between domestic and sylvan populations of Aedes aegypti (Diptera : Culicidae) in North Cameroon | Polymorphisms at eight microsatellite loci and a fragment of the mitochondrial DNA (mtDNA) -ND4 gene were surveyed in Aedes aegypti (L.) (Diptera: Culicidae) populations collected from six localities in North Cameroon, with emphasis on comparing domestic versus sylvan populations. The microsatellites revealed significant genetic differentiation among sylvan populations, with mean F-ST = 0.066. Domestic collections were genetically homogeneous (mean F-ST = 0.012). No pattern of isolation by distance was detected, and one of highest levels of genetic differentiation was estimated between populations sampled a few kilometers apart, each in a distinctly different ecological environment (F-ST = 0.076). Analyses of mtDNA-ND4 polymorphisms and divergence between the two neighboring populations revealed increased genetic diversity within the domestic population, with molecular signatures suggesting recent demographic expansion, whereas a single haplotype was observed in the sylvan sample. These data suggest reduced gene How between sylvan and domestic Ae. aegypti populations in North Cameroon, reminiscent of the situation for Ae. aegypti in Kenya in East Africa. | 1701 | no | no good fitness measure | NA | no | no |
| 1702 | 2017 | 43020 | Rodriguez-Perez, MA; Nunez-Gonzalez, CA; Lizarazo-Ortega, C; Sanchez-Varela, A; Wooten, MC; Unnasch, TR | 2006 | Analysis of genetic variation in ribosomal DNA internal transcribed spacer and the NADH dehydrogenase subunit 4 mitochondrial genes of the onchocerciasis vector Simulium ochraceum | Onchocerciasis is a serious disease vectored by black flies in the genus Simulium that are infected with the filarial parasite Onchocerca volvulus. In the Americas, black flies of the Simulium ochraceum s.l. species complex are important vectors of this parasite. Cytological studies have suggested that this species complex consists of at least three cytotypes that inhabit distinct habitats. In this study, the NADH dehydrogenase subunit four (ND4) and internal transcribed spacer (ITS) of the ribosomal RNA gene cluster were used to explore the degree of genetic diversity among S. ochraceum s.l. populations found in the three O. volvulus foci in Mexico. Both sequence regions were found to exhibit intra- and interpopulation variation. Four different ND4 alleles were found among the populations examined. Similarly, variation was noted in the ITS domain sequences within and among populations. Variation within the ITS sequence was primarily confined to a complex micro-satellite locus. Four ITS length variants were observed, two of which were only seen in flies collected from the onchocerciasis focus in northern Chiapas. These data suggest that the ND4 and ITS sequences may prove to be useful markers for exploring interactions within and among the S. ochraceum s.l. populations in Mexico. | 1702 | no | no good fitness measure, N pop differnt in GD? | NA | no | no |
| 1703 | 2017 | 43020 | Razavi, M; Hughes, GR | 2004 | Microsatellite markers provide evidence for sexual reproduction of Mycosphaerella graminicola in Saskatchewan | This study examined the genetic structure of a Saskatchewan population of Mycosphaerella graminicola, cause of the foliar disease Septoria tritici blotch of wheat. Such knowledge is valuable for understanding the evolutionary potential of this pathogen and for developing control strategies based on host resistance. Nine pairs of single-locus microsatellite primers were used to analyze the genomic DNA of 90 isolates of M. graminicola that were collected using a hierarchical sampling procedure from different locations, leaves, and lesions within a wheat field near Saskatoon. Allelic series at eight different loci were detected. The number of alleles per locus ranged from one to five with an average of three alleles per locus. Genetic diversity values ranged from 0.04 to 0.67. Partitioning the total genetic variability into within- and among-location components revealed that 88% of the genetic variability occurred within locations, i.e., within areas of 1 m(2), but relatively little variability occurred among locations. Low variability among locations and a high degree of variability within locations would result if the primary source of inoculum was airborne ascospores, which would be dispersed uniformly within the field. This finding was confirmed by gametic disequilibrium analysis and suggests that the sexual reproduction of M. graminicola occurs in Saskatchewan. | 1703 | no | gd of pathogen | NA | no | no |
| 1704 | 2017 | 43020 | Tarpy, DR; Page, RE | 2002 | Sex determination and the evolution of polyandry in honey bees (Apis mellifera) | “Many hypotheses attempt to explain why queens of social insects mate multiply. We tested the sex locus hypothesis for the evolution of polyandry in honey bees (Apis mellifera). A queen may produce infertile, diploid males that reduce the viability of worker brood and, presumably, adversely affect colony fitness. Polyandry reduces the variance in diploid male production within a colony and may increase queen fitness if there are non-linear costs associated with brood viability, specifically if the relationship between brood viability and colony fitness is concave. We instrumentally inseminated queens with three of their own brothers to vary brood viability from 50% to 100% among colonies. We measured the colonies during three stages of their development: (1) colony initiation and growth, (2) winter survival, and (3) spring reproduction. We found significant relationships between brood viability and most colony measures during the growth phase of colonies, but the data were too variable to distinguish significant non-linear effects. However, there was a significant step function of brood viability on winter survival, such that all colonies above 72% brood viability survived the winter but only 37.5% of the colonies below 72% viability survived. We discuss the significance of this and other”“genetic diversity”" hypotheses for the evolution of polyandry." | 1704 | yes | ok gd measurement and pop level comp? | no differfence in GD of groups | no | no |
| 1705 | 2017 | 43020 | Field, J; Cronin, A; Bridge, C | 2006 | Future fitness and helping in social queues | Helpers in primitively eusocial and cooperatively breeding animal societies forfeit their own reproduction to rear the offspring of a queen or breeding pair, but may eventually attain breeding status themselves. Kin selection(1) provides a widely accepted theoretical framework for understanding these societies, but differences in genetic relatedness do not explain a universal societal feature: the huge variation between individuals in helping effort(2-10). An alternative explanation for this variation lies in a fundamental trade-off faced by helpers: by working harder, they increase the indirect component of their fitness, but simultaneously decrease their own future survival and fecundity(2,4,8). Here, we show that individuals work less hard when they stand to lose more future fitness through working. We experimentally manipulated two components of future fitness in social queues of hover wasps (Stenogastrinae): a helper’s chance of inheriting an egg-laying position, and the workforce available to rear her offspring should she inherit. After each manipulation, helpers increased or decreased their effort as appropriate to the change in expected future fitness that they experienced. Although helping provides significant indirect fitness benefits for hover wasps(11), our study shows that variation in the costs associated with helping is the major determinant of helping effort. | 1705 | no | no good fitness measure, N pop differnt in GD? | NA | no | no |
| 1706 | 2017 | 43020 | Peixouto, YS; Braganca, CAD; Andrade, WB; Ferreira, CF; Haddad, F; Oliveira, SAS; Brito, FSD; Miller, RNG; Amorim, EP | 2015 | Estimation of genetic structure of a Mycosphaerella musicola population using inter-simple sequence repeat markers | Among the diseases affecting banana (Musa sp), yellow Sigatoka, caused by the fungal pathogen Mycosphaerella musicola Leach, is considered one of the most important in Brazil, causing losses throughout the year. Understanding the genetic structure of pathogen populations will provide insight into the life history of pathogens, including the evolutionary processes occurring in agrosystems. Tools for estimating the possible emergence of pathogen variants with altered pathogenicity, virulence, or aggressiveness, as well as resistance to systemic fungicides, can also be developed from such data. The objective of this study was to analyze the genetic diversity and population genetics of M. musicola in the main banana-producing regions in Brazil. A total of 83 isolates collected from different banana cultivars in the Brazilian states of Bahia, Rio Grande do Norte, and Minas Gerais were evaluated using inter-simple sequence repeat markers. High variability was detected between the isolates, and 85.5% of the haplotypes were singletons in the populations. The highest source of genetic diversity (97.22%) was attributed to variations within populations. Bayesian cluster analysis revealed the presence of 2 probable ancestral groups, however, showed no relationship to population structure in terms of collection site, state of origin, or cultivar. Similarly, we detected noevidence of genetic recombination between individuals within different states, indicating that asexual cycles play a major role in M. musicola reproduction and that long-distance dispersal of the pathogen is the main factor contributing to the lack of population structure in the fungus. | 1706 | no | gd of pathogen | NA | no | no |
| 1707 | 2017 | 43020 | Karbassi, E; Monte, E; Chapski, DJ; Lopez, R; Garrido, MR; Kim, J; Wisniewski, N; Rau, CD; Wang, JJ; Weiss, JN; Wang, YB; Lusis, AJ; Vondriska, TM | 2016 | Relationship of disease-associated gene expression to cardiac phenotype is buffered by genetic diversity and chromatin regulation | Expression of a cohort of disease-associated genes, some of which are active in fetal myocardium, is considered a hallmark of transcriptional change in cardiac hypertrophy models. How this transcriptome remodeling is affected by the common genetic variation present in populations is unknown. We examined the role of genetics, as well as contributions of chromatin proteins, to regulate cardiac gene expression and heart failure susceptibility. We examined gene expression in 84 genetically distinct inbred strains of control and isoproterenol-treated mice, which exhibited varying degrees of disease. Unexpectedly, fetal gene expression was not correlated with hypertrophic phenotypes. Unbiased modeling identified 74 predictors of heart mass after isoproterenol-induced stress, but these predictors did not enrich for any cardiac pathways. However, expanded analysis of fetal genes and chromatin remodelers as groups correlated significantly with individual systemic phenotypes. Yet, cardiac transcription factors and genes shown by gain-/loss-of-function studies to contribute to hypertrophic signaling did not correlate with cardiac mass or function in disease. Because the relationship between gene expression and phenotype was strain specific, we examined genetic contribution to expression. Strikingly, strains with similar transcriptomes in the basal heart did not cluster together in the isoproterenol state, providing comprehensive evidence that there are different genetic contributors to physiological and pathological gene expression. Furthermore, the divergence in transcriptome similarity versus genetic similarity between strains is organ specific and genome-wide, suggesting chromatin is a critical buffer between genetics and gene expression. | 1707 | no | NA | NA | no | no |
| 1708 | 2017 | 43020 | Tarr, CL; Fleischer, RC | 1999 | Population boundaries and genetic diversity in the endangered Mariana crow (Corvus kubaryi) | The Mariana crow (Corvus kubaryi) is an endangered species that is restricted to the islands of Guam and Rota in the Mariana archipelago. Predation by the introduced brown tree snake (Boiga irregularis) has decimated bird populations on Guam, and the crow population there is the last wild remnant of the endemic forest avifauna. The population on Guam is critically endangered and, despite intensive management, the population has continued to decline. Additional management options include intermixing the Guam and Rota populations, but such options are best evaluated within a population genetics framework. We used three types of molecular markers to assay genetic variation in the Mariana crow: mitochondrial DNA (mtDNA) sequences, minisatellites and microsatellites. The two populations could be differentiated by mtDNA sequencing and they differed in allele frequencies at nuclear markers. Thus, the populations could be designated as evolutionarily significant units. However, the Guam population is genetically more diverse than the Rota population, and its survival probability if managed separately is very low. All markers did indicate that the two populations are closely related and separated by a shallow genealogical division. Intermixing the populations is justified by two rationales. First, the apparent population differences may result from recent human activities. Second, a greater amount of genetic information may be preserved by joint management. The translocation of birds from Rota to Guam has begun, but strategies that will ensure maintenance of the variation in the Guam population warrant further exploration. | 1708 | no | no good fitness measure | NA | no | no |
| 1709 | 2017 | 43020 | Hoffman, JI; Boyd, IL; Amos, W | 2004 | Exploring the relationship between parental relatedness and male reproductive success in the antarctic fur seal Arctocephalus gazella | Recent genetic studies of natural populations have shown that heterozygosity and other genetic estimates of parental relatedness correlate with a wide variety of fitness traits, from juvenile survival and parasite resistance to male reproductive success. Many of these traits involve health and survival, where the underlying mechanism may involve changes in the effectiveness of the immune system. However, for traits such as reproductive success, the likely mechanisms remain less obvious. In this paper, we examine the relationship between heterozygosity and a range of traits that contribute to male reproductive success, including time spent on territories and competitiveness. Our analysis is based on observational and genetic data from eight consecutive breeding seasons at a colony of the Antarctic fur seal. Arctocephalus gazella. Overall, male reproductive Success was found to correlate strongly with internal relatedness (IR, a form of heterozygosity). When different components of success were analyzed, we found that IR correlates independently with reproductive longevity, time spent ashore, and competitive ability per unit mating opportunity on the Study beach, with more heterozygous males being more successful. Behavioral observations were sufficiently detailed to allow examination of how daily mean IR values for males present on the beach varied within seasons and from year to year. Again, significant variation was found both among and within seasons, with more homozygous males appearing less able to hold territories in poor seasons when pup production is low and, within a season. at both the start of the season and to some extent around the peak of female estrus. Finally, we tested whether the benefits of high heterozygosity are due mainly to a genomewide effect (e.g. inbreeding depression) or to single locus heterosis by asking whether the relationship between IR and male success was robust to the removal of any single locus or to any pair of loci. Since the relationship remained significant in all cases, we favor a multilocus explanation for the effects we report. | 1709 | no | no good fitness measure, N pop differnt in GD? | NA | no | no |
| 1710 | 2017 | 43020 | Parsons, J; Matthews, W; Iorizzo, M; Roberts, P; Simon, P | 2015 | Meloidogyne incognita nematode resistance QTL in carrot | Root-knot nematodes (Meloidogyne spp.) are major pests attacking carrots (Daucus carota) worldwide, causing galling and forking of the storage roots, rendering them unacceptable for market. Genetic resistance could significantly reduce the need for broad-spectrum soil fumigants in carrot production. In this study, genetic resistance to Meloidogyne incognita was mapped. Three diverse sources of resistance, from Syria (HM), Europe (SFF) and South America (Br1091), were identified. Two F-2 mapping populations were developed using these parents, (Br1091 x HM1) and (SFF x HM2), as well as a segregating population derived from the self-pollination of a HM plant (HM3). Analysis revealed four QTLs conditioning resistance in Br1091 x HM1, three in SFF x HM2, and three in HM3. A consensus genetic map of the three populations revealed five non-overlapping QTLs for M. incognita resistance, one each on carrot chromosomes 1, 2, 4, 8, and 9. One QTL was present in all three populations, in the same region of chromosome 8 as Mj-1 which imparts resistance to M. javanica. | 1710 | no | no good fitness measure, N pop differnt in GD? | NA | no | no |
| 1711 | 2017 | 43020 | Cameron, SA; Lozier, JD; Strange, JP; Koch, JB; Cordes, N; Solter, LF; Griswold, TL | 2011 | Patterns of widespread decline in North American bumble bees | Bumble bees (Bombus) are vitally important pollinators of wild plants and agricultural crops worldwide. Fragmentary observations, however, have suggested population declines in several North American species. Despite rising concern over these observations in the United States, highlighted in a recent National Academy of Sciences report, a national assessment of the geographic scope and possible causal factors of bumble bee decline is lacking. Here, we report results of a 3-y interdisciplinary study of changing distributions, population genetic structure, and levels of pathogen infection in bumble bee populations across the United States. We compare current and historical distributions of eight species, compiling a database of >73,000 museum records for comparison with data from intensive nationwide surveys of >16,000 specimens. We show that the relative abundances of four species have declined by up to 96% and that their surveyed geographic ranges have contracted by 23-87%, some within the last 20 y. We also show that declining populations have significantly higher infection levels of the microsporidian pathogen Nosema bombi and lower genetic diversity compared with co-occurring populations of the stable (nondeclining) species. Higher pathogen prevalence and reduced genetic diversity are, thus, realistic predictors of these alarming patterns of decline in North America, although cause and effect remain uncertain. | 1711 | maybe | species comp? | species level comp, ind level gd measure | yes | no |
| 1712 | 2017 | 43020 | Haselkorn, TS; Markow, TA; Moran, NA | 2009 | Multiple introductions of the Spiroplasma bacterial endosymbiont into Drosophila | Bacterial endosymbionts are common in insects and can have dramatic effects on their host’s evolution. So far, the only heritable symbionts found in Drosophila have been Wolbachia and Spiroplasma. While the incidence and effects of Wolbachia have been studied extensively, the prevalence and significance of Spiroplasma infections in Drosophila are less clear. These small, gram-positive, helical bacteria infect a diverse array of plant and arthropod hosts, conferring a variety of fitness effects. Male-killing Spiroplasma are known from certain Drosophila species; however, in others, Spiroplasma appear not to affect sex ratio. Previous studies have identified different Spiroplasma haplotypes in Drosophila populations, although no extensive surveys have yet been reported. We used a multilocus sequence analysis to reconstruct a robust Spiroplasma endosymbiont phylogeny, assess genetic diversity, and look for evidence of recombination. Six loci were sequenced from over 65 Spiroplasma-infected individuals from nine different Drosophila species. Analysis of these sequences reveals at least five separate introductions of four phylogenetically distinct Spiroplasma haplotypes, indicating that more extensive sampling will likely reveal an even greater Spiroplasma endosymbiont diversity. Patterns of variation in Drosophila mitochondrial haplotypes in Spiroplasma-infected and uninfected flies imply imperfect vertical transmission in host populations and possible horizontal transmission. | 1712 | no | NA | NA | no | no |
| 1713 | 2017 | 43020 | van Houte, S; Ekroth, AKE; Broniewski, JM; Chabas, H; Ashby, B; Bondy-Denomy, J; Gandon, S; Boots, M; Paterson, S; Buckling, A; Westra, ER | 2016 | The diversity-generating benefits of a prokaryotic adaptive immune system | Prokaryotic CRISPR-Cas adaptive immune systems insert spacers derived from viruses and other parasitic DNA elements into CRISPR loci to provide sequence-specific immunity(1,2). This frequently results in high within-population spacer diversity(3-6), but it is unclear if and why this is important. Here we show that, as a result of this spacer diversity, viruses can no longer evolve to overcome CRISPR-Cas by point mutation, which results in rapid virus extinction. This effect arises from synergy between spacer diversity and the high specificity of infection, which greatly increases overall population resistance. We propose that the resulting short-lived nature of CRISPR-dependent bacteria-virus coevolution has provided strong selection for the evolution of sophisticated virus-encoded anti-CRISPR mechanisms(7). | 1713 | yes | from thesis | NA | yes | yes |
| 1714 | 2017 | 43020 | Madore, AM; Houde, L; Vezina, H; Vohl, MC; Perusse, L; Mior, N; Connelly, PW; Laberge, C; Gaudet, D; Laprise, C | 2007 | Contribution of hierarchical clustering techniques to the modeling of the geographic distribution of genetic polymorphisms associated with chronic inflammatory diseases in the quebec population | Objectives: The purpose of this project was to evaluate the potential of the downward hierarchical clustering analysis (DHCA) for studying genetic heterogeneity, i.e. differences in allele frequency in subpopulations, such as the 15 public health regions of the province of Quebec (Canada). Methods: The study relied on an anonymized sample of 1,680 individuals who had participated in the Quebec Heart Health Survey in 1990-1991. The genotyping of 11 variants in 8 candidate genes known to be involved in chronic inflammatory diseases, namely asthma and cardiovascular diseases, was performed using the amplification refractory mutation system and restriction fragment length polymorphism techniques. Only variants showing an allelic frequency >2% in the Quebec Heart Health Survey (n = 8) were selected. DHCA techniques were then applied to model the geographical distribution of these 8 genetic variants in 15 Quebec public health regions and to study genetic heterogeneity. Results: The DHCA allowed to group public health regions and gene variants on the basis of genetic variability. For both asthma and cardiovascular diseases, 3 significant clusters of public health regions and I cluster of gene variants were identified. Discussion: This study suggests that DHCA might be useful in studying genetic heterogeneity at the population level and for public health activities. Copyright (c) 2007 S. Karger AG, Basel. | 1714 | no | human | NA | no | no |
| 1715 | 2017 | 43020 | Pespeni, MH; Garfield, DA; Manier, MK; Palumbi, SR | 2012 | Genome-wide polymorphisms show unexpected targets of natural selection | Natural selection can act on all the expressed genes of an individual, leaving signatures of genetic differentiation or diversity at many loci across the genome. New power to assay these genome-wide effects of selection comes from associating multi-locus patterns of polymorphism with gene expression and function. Here, we performed one of the first genome-wide surveys in a marine species, comparing purple sea urchins, Strongylocentrotus purpuratus, from two distant locations along the species’ wide latitudinal range. We examined 9112 polymorphic loci from upstream non-coding and coding regions of genes for signatures of selection with respect to gene function and tissue-and ontogenetic gene expression. We found that genetic differentiation (F-ST) varied significantly across functional gene classes. The strongest enrichment occurred in the upstream regions of E3 ligase genes, enzymes known to regulate protein abundance during development and environmental stress. We found enrichment for high heterozygosity in genes directly involved in immune response, particularly NALP genes, which mediate pro-inflammatory signals during bacterial infection. We also found higher heterozygosity in immune genes in the southern population, where disease incidence and pathogen diversity are greater. Similar to the major histocompatibility complex in mammals, balancing selection may enhance genetic diversity in the innate immune system genes of this invertebrate. Overall, our results show that how genome-wide polymorphism data coupled with growing databases on gene function and expression can combine to detect otherwise hidden signals of selection in natural populations. | 1715 | no | no good fitness measure, N pop differnt in GD? | NA | no | no |
| 1716 | 2017 | 43020 | Pepperell, C; Hoeppner, VH; Lipatov, M; Wobeser, W; Schoolnik, GK; Feldman, MW | 2010 | Bacterial Genetic Signatures of Human Social Phenomena among M-tuberculosis from an Aboriginal Canadian Population | Despite a widespread global distribution and highly variable disease phenotype, there is little DNA sequence diversity among isolates of Mycobacterium tuberculosis. In addition, many regional population genetic surveys have revealed a stereotypical structure in which a single clone, lineage, or clade makes up the majority of the population. It is often assumed that dominant clones are highly adapted, that is, the overall structure of M. tuberculosis populations is the result of positive selection. In order to test this assumption, we analyzed genetic data from extant populations of bacteria circulating in Aboriginal communities in Saskatchewan, Canada. Demographic parameters of the bacterial population were estimated from archival epidemiological data collected over similar to 130 years since the onset of epidemic tuberculosis in the host communities. Bacterial genetic data were tested against neutral theory expectations and the local evolutionary history of M. tuberculosis investigated by phylogenetic analysis. Our findings are not consistent with positive selection on the bacterial population. Instead, we uncovered founder effects persisting over decades and barriers to gene flow within the bacterial population. Simulation experiments suggested that a combination of these neutral influences could result in the stereotypical structure of M. tuberculosis populations. Some aspects of population structure were suggestive of background selection, and data were on the whole consistent with combined effects of population bottlenecks, subdivision, and background selection. Neutral phenomena, namely, bottlenecks and partitions within populations, are prominent influences on the evolution of M. tuberculosis and likely contribute to restricted genetic diversity observed within this species. Given these influences, a complex evolutionary model will be required to define the relative fitness of different M. tuberculosis lineages and, ultimately, to uncover the genetic basis for its success as a pathogen. | 1716 | no | NA | NA | no | no |
| 1717 | 2017 | 43020 | Atkinson, RJ; Brown, GS; Stone, GN | 2003 | Skewed sex ratios and multiple founding in galls of the oak apple gall wasp Biorhiza pallida | 1. The gall wasp Biorhiza pallida (Hymenoptera: Cynipidae) reproduces by cyclical parthenogenesis. The adults of the sexual generation develop within galls (oak apples) that contain many larval cells. 2. Folliot [(1964) Annales Des Sciences Naturelles: Zoologie, 12, 407-564] found asexual generation females to be of three reproductive types. Androphores produce only sons, gynophores produce only daughters, and gynandrophores produce both sons and daughters. In nature, most oak apples give rise to either only males or only females but a proportion produces both sexes. These mixed-sex galls could result either from eggs laid by one or more gynandrophores or from eggs laid by androphores and gynophores developing within a single gall (multiple founding). 3. Here the frequency of mixed- and single-sex galls was quantified, and morphological and genetic analyses were carried out on the adults emerging from 10 galls to determine the frequency of multiple founding in B. pallida. 4. Seventy-five per cent of 627 galls yielded only one sex. The majority of the remaining 25% had a highly skewed sex ratio. Low genetic variation in B. pallida limited the application of allozyme-based genetic techniques, however seven of the 10 galls analysed in detail, including mixed-sex galls, appeared to have been multiply founded. Contributions by the different foundresses in multiply founded galls were highly skewed. 5. The significance of multiple founding is discussed in the light of possible adaptive scenarios (reduction of parasitoid-induced mortality, avoidance of local stochastic extinction and inbreeding) and possible competition for oviposition sites. | 1717 | no | no good fitness measure, N pop differnt in GD? | NA | no | no |
| 1718 | 2017 | 43020 | Stefka, J; Hoeck, PEA; Keller, LF; Smith, VS | 2011 | A hitchhikers guide to the Galapagos: co-phylogeography of Galapagos mockingbirds and their parasites | Background: Parasites are evolutionary hitchhikers whose phylogenies often track the evolutionary history of their hosts. Incongruence in the evolutionary history of closely associated lineages can be explained through a variety of possible events including host switching and host independent speciation. However, in recently diverged lineages stochastic population processes, such as retention of ancestral polymorphism or secondary contact, can also explain discordant genealogies, even in fully co-speciating taxa. The relatively simple biogeographic arrangement of the Galapagos archipelago, compared with mainland biomes, provides a framework to identify stochastic and evolutionary informative components of genealogic data in these recently diverged organisms. Results: Mitochondrial DNA sequences were obtained for four species of Galapagos mockingbirds and three sympatric species of ectoparasites - two louse and one mite species. These data were complemented with nuclear EF1 alpha sequences in selected samples of parasites and with information from microsatellite loci in the mockingbirds. Mitochondrial sequence data revealed differences in population genetic diversity between all taxa and varying degrees of topological congruence between host and parasite lineages. A very low level of genetic variability and lack of congruence was found in one of the louse parasites, which was excluded from subsequent joint analysis of mitochondrial data. The reconciled multi-species tree obtained from the analysis is congruent with both the nuclear data and the geological history of the islands. Conclusions: The gene genealogies of Galapagos mockingbirds and two of their ectoparasites show strong phylogeographic correlations, with instances of incongruence mostly explained by ancestral genetic polymorphism. A third parasite genealogy shows low levels of genetic diversity and little evidence of co-phylogeny with their hosts. These differences can mostly be explained by variation in life-history characteristics, primarily host specificity and dispersal capabilities. We show that pooling genetic data from organisms living in close ecological association reveals a more accurate phylogeographic history for these taxa. Our results have implications for the conservation and taxonomy of Galapagos mockingbirds and their parasites. | 1718 | no | gd of parasite | NA | no | no |
| 1719 | 2017 | 43020 | Mathiasen, P; Premoli, AC | 2010 | Out in the cold: genetic variation of Nothofagus pumilio (Nothofagaceae) provides evidence for latitudinally distinct evolutionary histories in austral South America | Nothofagus pumilio is the dominant and almost ubiquitous tree species in mountainous environments of temperate South America. We used two types of molecular markers (cpDNA and isozymes) to evaluate the effects of the Paleogene paleogeography of Patagonia and more recent climatic oscillations of the Neogene on such cold-tolerant species’ genetic makeup. Phylogeographic analysis on sequences of three cpDNA non-coding regions at 85 populations yielded two latitudinally disjunct monophyletic clades north and south of c. 42 degrees S containing 11 and three haplotypes, respectively. This indicates a long-lasting vicariant event due to the presence of an extended open paleobasin at mid latitudes of Patagonia. Also distribution patterns of cpDNA haplotypes suggest regional spread following stepping-stone models using pre-Cenozoic mountains as corridors. Comparable genetic diversity measured along 41 sampled populations using seven polymorphic isozyme loci provides evidence of local persistence and spread from multiple ice-free locations. In addition, significantly higher heterozygosity and allelic richness at high latitudes, i.e. in areas of larger glacial extent, suggest survival in large and isolated refugia. While, higher cpDNA diversity in lower latitudes reflects the complex orogeny that historically isolated northern populations, lower isozyme diversity and reduced F(ST) values provide evidence of local glacial survival in numerous small locales. Therefore, current genetic structure of N. pumilio is the result of regional processes which took place during the Tertiary that were enhanced by contemporary local effects of drift and isolation in response to Quaternary climatic cycles. | 1719 | no | no good fitness measure, N pop differnt in GD? | NA | no | no |
| 1720 | 2017 | 43020 | Barbosa, TCS; Sibov, ST; Telles, MPC; Soares, TN | 2010 | Genetic characterization of natural populations of the medicinal plant Palicourea coriacea (Rubiaceae) with molecular markers | “Palicourea coriacea (Rubiaceae) is a herbaceous, perennial species typical of the Cerrado; it is popularly known as”“douradinha”", because of its yellow flowers. It is utilized in popular medicine, mainly for the treatment of renal diseases. We used RAPD markers to evaluate the genetic structure of nine natural populations of P. coriacea, totaling 168 individuals, collected in the States of Goias and Bahia. This species showed a high level of genetic diversity, with He values varying between 0.259 and 0.338, with an overall mean of 0.296. Analysis by AMOVA revealed that 23% of the total variability was between populations and 77% was within populations. The estimate of apparent gene flow (Nm) was 0.83. Analyses of the fixation index (f) using a Bayesian approach yielded a mean value of 0.98, suggesting that this is an autogamous species. Analyses of genetic divergence and spatial pattern of the populations utilizing theta(B) and Phi(ST) matrices, pair to pair, revealed no correlation between geographic distance and genetic distance | 1720 | no | no good fitness measure | NA | no | no |
| 1721 | 2017 | 43020 | Dhaliwal, MS; Singh, M; Singh, K; Cheema, DS | 2011 | Genetic diversity analysis and DNA fingerprinting of elite genetic stocks of tomato using SSR markers | Thirty genetic stocks of tomato (Solanum lycopersicum L.) were subjected to genetic diversity analysis and DNA fingerprinting using SSR markers. The stocks included male sterile lines (both pollen abortive and functional types); non-ripening mutants; disease (leaf curl virus, late blight and early blight) and nematode resistant lines; temperature stress (both low and high); and salt tolerant lines. Of the 25 primers used, 21 showed polymorphism and amplified 60 alleles with an average of 2.86 alleles per locus. Of these alleles, 21 were polymorphic and the rest were monomorphic. The PIC values for 21 primers obtained varied from 0.06 for SSR128 to 0.68 for SSR565, with an average PIC value to be 0.43. The greater number of repeat units and longer SSRs tend to have higher PIC values. Based on the PIC values and number of alleles amplified, the primer SSR565 was found to be more informative in the present set of genotypes. Similarity coefficient between any two genotypes estimated based on DNA amplification by SSR primers varied from 0.18 to 0.94. The lowest similarity coefficient (0.18) observed between genotypes belonging to the cultivated species lycopersicum and the wild species pimpinellifolium confirmed their differentiation at the species level. Many of the cultivated types were found to have fairly narrow genetic base. UPGMA revealed that SSR markers were helpful in differentiating the genotypes on the basis of horticultural and genetic factors. However, grouping of the 30 genetic stocks was independent of their geographic distribution. Based on the DNA fingerprints, it was possible to differentiate 23 of the 30 genotypes screened. | 1721 | no | crop | NA | no | no |
| 1722 | 2017 | 43020 | Clement, JAJ; Magalon, H; Glais, I; Jacquot, E; Andrivon, D | 2012 | To Be or Not to Be Solitary: Phytophthora infestans’ Dilemma for Optimizing its Reproductive Fitness in Multiple Infections | The success of parasitic life lies in an optimal exploitation of the host to satisfy key functions directly involved in reproductive fitness. Resource availability generally decreases over time with host mortality, but also during multiple infections, where different strains of parasite share host resources. During multiple infections, the number of parasite strains and their genetic relatedness are known to influence their reproductive rates. Using infections of the potato plant Solanum tuberosum with the parasite Phytophthora infestans, we set up an experimental design to separate dose effects (double-vs. single-site infections) from genetic relatedness (different vs. identical genotypes) on the reproductive fitness of competing parasite genotypes. We showed the existence of two basic response patterns - increase or decrease in reproductive fitness in multiple infections-depending on the parasite genotype. In all cases, the intensity of the response of any genotype depended on the genotype of the competing strain. This diversity of responses to multiple infections is probably maintained by the fluctuating frequencies of multiple infections in nature, arising from variations in disease pressure over the course of an epidemic and between successive epidemics. It allows a rapid response of parasitic populations to changing environments, which are particularly intense in agricultural systems. | 1722 | no | gd of pathogen | NA | no | no |
| 1723 | 2017 | 43020 | Faria, VG; Martins, NE; Paulo, T; Teixeira, L; Sucena, E; Magalhaes, S | 2015 | Evolution of Drosophila resistance against different pathogens and infection routes entails no detectable maintenance costs | Pathogens exert a strong selective pressure on hosts, entailing host adaptation to infection. This adaptation often affects negatively other fitness-related traits. Such trade-offs may underlie the maintenance of genetic diversity for pathogen resistance. Trade-offs can be tested with experimental evolution of host populations adapting to parasites, using two approaches: (1) measuring changes in immunocompetence in relaxed-selection lines and (2) comparing life-history traits of evolved and control lines in pathogen-free environments. Here, we used both approaches to examine trade-offs in Drosophila melanogaster populations evolving for over 30 generations under infection with Drosophila C Virus or the bacterium Pseudomonas entomophila, the latter through different routes. We find that resistance is maintained after up to 30 generations of relaxed selection. Moreover, no differences in several classical life-history traits between control and evolved populations were found in pathogen-free environments, even under stresses such as desiccation, nutrient limitation, and high densities. Hence, we did not detect any maintenance costs associated with resistance to pathogens. We hypothesize that extremely high selection pressures commonly used lead to the disproportionate expression of costs relative to their actual occurrence in natural systems. Still, the maintenance of genetic variation for pathogen resistance calls for an explanation. | 1723 | no | No GD diff between pop | NA | no | no |
| 1724 | 2017 | 43020 | Trontti, K; Aron, S; Sundstrom, L | 2006 | The genetic population structure of the ant Plagiolepis xene-implications for genetic vulnerability of obligate social parasites | Obligatory social parasites, such as ant species that need colonies of other ant species for reproduction, are rare and many of them are classified as vulnerable. This is especially the case with highly adapted permanent inquilines that are specialised on one or a few host species. Their rarity may be due to reduced dispersal abilities, as a result of reduced body size, altered wing morphology, and curtailed nuptial flight, eventually leading to inbreeding. Furthermore, the host populations may differ in their ability to resist the parasite, yet the conditions of successful parasite invasion are largely unknown. Here we investigated the population structure of the inquiline ant Plagiolepis xene and its host P. pygmaea, using microsatellite data. Genetic differentiation, inbreeding, the effective population size and nest kin structure were analysed. We found that populations of P. xene are established by a single or at most a few individuals, and that the populations were genetically highly differentiated. However, within individual host populations the parasite is able to maintain panmixia, although data on the host suggests that the local distribution of the parasite also follows patterns of substructuring in the host population. Altogether our results suggest that inquiline parasite populations are genetically highly vulnerable. | 1724 | probably no | No GD diff between pop | no fitness measure | no | no |
| 1725 | 2017 | 43020 | Demanche, C; Deville, M; Michaux, J; Barriel, V; Pincon, C; Aliouat-Denis, CM; Pottier, M; Noel, C; Viscogliosi, E; Aliouat, E; Dei-Cas, E; Morand, S; Guillot, J | 2015 | What Do Pneumocystis Organisms Tell Us about the Phylogeography of Their Hosts? The Case of the Woodmouse Apodemus sylvaticus in Continental Europe and Western Mediterranean Islands | Pneumocystis fungi represent a highly diversified biological group with numerous species, which display a strong host-specificity suggesting a long co-speciation process. In the present study, the presence and genetic diversity of Pneumocystis organisms was investigated in 203 lung samples from woodmice (Apodemus sylvaticus) collected on western continental Europe and Mediterranean islands. The presence of Pneumocystis DNA was assessed by nested PCR at both large and small mitochondrial subunit (mtLSU and mtSSU) rRNA loci. Direct sequencing of nested PCR products demonstrated a very high variability among woodmouse-derived Pneumocystisorganisms with a total number of 30 distinct combined mtLSU and mtSSU sequence types. However, the genetic divergence among these sequence types was very low (up to 3.87%) and the presence of several Pneumocystis species within Apodemus sylvaticus was considered unlikely. The analysis of the genetic structure of woodmouse-derived Pneumocystis revealed two distinct groups. The first one comprised Pneumocystis from woodmice collected in continental Spain, France and Balearic islands. The second one included Pneumocystis from woodmice collected in continental Italy, Corsica and Sicily. These two genetic groups were in accordance with the two lineages currently described within the host species Apodemus sylvaticus. Pneumocystis organisms are emerging as powerful tools for phylogeographic studies in mammals. | 1725 | no | no good fitness measure, N pop differnt in GD? | NA | no | no |
| 1726 | 2017 | 43020 | Chakarov, N; Linke, B; Boerner, M; Goesmann, A; Kruger, O; Hoffman, JI | 2015 | Apparent vector-mediated parent-to-offspring transmission in an avian malaria-like parasite | Parasite transmission strategies strongly impact host-parasite co-evolution and virulence. However, studies of vector-borne parasites such as avian malaria have neglected the potential effects of host relatedness on the exchange of parasites. To test whether extended parental care in the presence of vectors increases the probability of transmission from parents to offspring, we used high-throughput sequencing to develop microsatellites for malaria-like Leucocytozoon parasites of a wild raptor population. We show that host siblings carry genetically more similar parasites than unrelated chicks both within and across years. Moreover, chicks of mothers of the same plumage morph carried more similar parasites than nestlings whose mothers were of different morphs, consistent with matrilineal transmission of morph-specific parasite strains. Ours is the first evidence of an association between host relatedness and parasite genetic similarity, consistent with vector-mediated parent-to-offspring transmission. The conditions for such quasi-vertical’ transmission may be common and could suppress the evolution of pathogen virulence. | 1726 | no | no good fitness measure, N pop differnt in GD? | NA | no | no |
| 1727 | 2017 | 43020 | Duffy, MA; Sivars-Becker, L | 2007 | Rapid evolution and ecological host-parasite dynamics | Traditionally, the termination of parasite epidemics has been attributed to ecological causes: namely, the depletion of susceptible hosts as a result of mortality or acquired immunity. Here, we suggest that epidemics can also end because of rapid host evolution. Focusing on a particular host-parasite system, Daphnia dentifera and its parasite Metschnikowia bicuspidata, we show that Daphnia from lakes with recent epidemics were more resistant to infection and had less variance in susceptibility than Daphnia from lakes without recent epidemics. However, our studies revealed little evidence for genetic variation in infectivity or virulence in Metschnikowia. Incorporating the observed genetic variation in host susceptibility into an epidemiological model parameterized for this system reveals that rapid evolution can explain the termination of epidemics on time scales matching what occurs in lake populations. Thus, not only does our study provide rare evidence for parasite-mediated selection in natural populations, it also suggests that rapid evolution has important effects on short-term host-parasite dynamics. | 1727 | no | no good fitness measure, N pop differnt in GD? | NA | no | no |
| 1728 | 2017 | 43020 | Ahlstrom, C; Barkema, HW; Stevenson, K; Zadoks, RN; Biek, R; Kao, R; Trewby, H; Haupstein, D; Kelton, DF; Fecteau, G; Labrecque, O; Keefe, GP; McKenna, SLB; De Buck, J | 2015 | Limitations of variable number of tandem repeat typing identified through whole genome sequencing of Mycobacterium avium subsp paratuberculosis on a national and herd level | “Background: Mycobacterium avium subsp. paratuberculosis (MAP), the causative bacterium of Johne’s disease in dairy cattle, is widespread in the Canadian dairy industry and has significant economic and animal welfare implications. An understanding of the population dynamics of MAP can be used to identify introduction events, improve control efforts and target transmission pathways, although this requires an adequate understanding of MAP diversity and distribution between herds and across the country. Whole genome sequencing (WGS) offers a detailed assessment of the SNP-level diversity and genetic relationship of isolates, whereas several molecular typing techniques used to investigate the molecular epidemiology of MAP, such as variable number of tandem repeat (VNTR) typing, target relatively unstable repetitive elements in the genome that may be too unpredictable to draw accurate conclusions. The objective of this study was to evaluate the diversity of bovine MAP isolates in Canadian dairy herds using WGS and then determine if VNTR typing can distinguish truly related and unrelated isolates. Results: Phylogenetic analysis based on 3,039 SNPs identified through WGS of 124 MAP isolates identified eight genetically distinct subtypes in dairy herds from seven Canadian provinces, with the dominant type including over 80% of MAP isolates. VNTR typing of 527 MAP isolates identified 12 types, including”“bison type”" isolates, from seven different herds. At a national level, MAP isolates differed from each other by 1-2 to 239-240 SNPs, regardless of whether they belonged to the same or different VNTR types. A herd-level analysis of MAP isolates demonstrated that VNTR typing may both over-estimate and under-estimate the relatedness of MAP isolates found within a single herd. Conclusions: The presence of multiple MAP subtypes in Canada suggests multiple introductions into the country including what has now become one dominant type, an important finding for Johne’s disease control. VNTR typing often failed to identify closely and distantly related isolates, limiting the applicability of using this typing scheme to study the molecular epidemiology of MAP at a national and herd-level." | 1728 | no | NA | NA | no | no |
| 1729 | 2017 | 43020 | Warne, B; Harkins, CP; Harris, SR; Vatsiou, A; Stanley-Wall, N; Parkhill, J; Peacock, SJ; Palmer, T; Holden, MTG | 2016 | The Ess/Type VII secretion system of Staphylococcus aureus shows unexpected genetic diversity | Background: Type VII protein secretion (T7SS) is a specialised system for excreting extracellular proteins across bacterial cell membranes and has been associated with virulence in Staphylococcus aureus. The genetic diversity of the ess locus, which encodes the T7SS, and the functions of proteins encoded within it are poorly understood. Results: We used whole genome sequence data from 153 isolates representative of the diversity of the species to investigate the genetic variability of T7SS across S. aureus. The ess loci were found to comprise of four distinct modules based on gene content and relative conservation. Modules 1 and 4, comprising of the 5’ and 3’ modules of the ess locus, contained the most conserved clusters of genes across the species. Module 1 contained genes encoding the secreted protein EsxA, and the EsaAB and EssAB components of the T7SS machinery, and Module 4 contained two functionally uncharacterized conserved membrane proteins. Across the species four variants of Module 2 were identified containing the essC gene, each of which was associated with a specific group of downstream genes. The most diverse module of the ess locus was Module 3 comprising a highly variable arrangement of hypothetical proteins. RNA-Seq was performed on representatives of the four Module 2 variants and demonstrated strain-specific differences in the levels of transcription in the conserved Module 1 components and transcriptional linkage Module 2, and provided evidence of the expression of genes the variable regions of the ess loci. Conclusions: The ess locus of S. aureus exhibits modularity and organisational variation across the species and transcriptional variation. In silico analysis of ess loci encoded hypothetical proteins identified potential novel secreted substrates for the T7SS. The considerable variety in operon arrangement between otherwise closely related isolates provides strong evidence for recombination at this locus. Comparison of these recombination regions with each other, and with the genomes of other Staphylococcal species, failed to identify evidence of intra- and inter-species recombination, however the analysis identified a novel T7SS in another pathogenic staphylococci, Staphylococcus lugdunensis. | 1729 | no | NA | NA | no | no |
| 1730 | 2017 | 43020 | Jerome, CA; Ford, BA | 2002 | Comparative population structure and genetic diversity of Arceuthobium americanum (Viscaceae) and its Pinus host species: insight into host-parasite evolution in parasitic angiosperms | In a recent study we revealed that the parasitic angiosperm Arceuthobium americanum is comprised of three distinct genetic races, each associated with a different host in regions of allopatry. In order to assess the role of host identity and geographical isolation on race formation in A. americanum, we compared the genetic population structure of this parasite with that of its three principal hosts, Pinus banksiana, Pinus contorta var. latifolia and Pinus contorta var. murrayana. Despite the fact that A. americanum was divided into three genetic races, hosts were divided into only two genetic groups: (i) Pinus banksiana and hybrids, and (ii) P. contorta var. latifolia and var. murrayana. These findings suggest that factors such as geographical isolation and adaptation to different environmental conditions are important for race formation in the absence of host-driven selection pressures. To assess factors impacting population structure at the fine-scale, genetic and geographical distance matrices of host and parasite were compared within A. americanum races. The lack of a relationship between genetic and geographical distance matrices suggests that isolation-by-distance plays a negligible role at this level. The effect of geographical isolation may have been diminished because of the influence of factors such as random seed dispersal by animal vectors or adaptation to nongeographically patterned environmental conditions. Host-parasite interactions might also have impacted the fine-scale structure of A. americanum because the parasite and host were found to have similar patterns of gene flow. | 1730 | no | no good fitness measure, N pop differnt in GD? | NA | no | no |
| 1731 | 2017 | 43020 | Atterby, H; Aegerter, JN; Smith, GC; Conyers, CM; Allnutt, TR; Ruedi, M; MacNicoll, AD | 2010 | Population genetic structure of the Daubenton’s bat (Myotis daubentonii) in western Europe and the associated occurrence of rabies | The Daubenton’s bat is widespread and common in the UK and countries bordering the English Channel and North Sea. European bat lyssavirus 2 (EBLV-2), a rabies virus, has been detected in Daubenton’s bats in the UK and continental Europe. Investigating the relatedness of colonies and gene flow between these regions would allow regional estimates of the movement of Daubenton’s bats and thus the potential for disease transmission. The genetic structure of the Daubenton’s bat in western Europe was investigated by analysing variability at eight microsatellite loci. Genetic diversity was found to be high at all sites (H (E) = 0.73-0.84), with little differentiation between bats sampled in the UK and continental Europe. Mantel tests indicated a significant correlation between geographic distance and pair-wise F (ST) (P = 0.000), between colonies sampled in Scotland and northern England. However, this was not continuous throughout the sampled range, with evidence of panmixia within the area sampled in continental Europe. Assignment tests show no evidence that the (potential) EBLV-2 sero-positive and virus positive bats were more likely to have originated from the continental rather than UK populations. There is no sufficient significant genetic differentiation amongst most UK and continental colonies to conclude that EBLV-2 is maintained in the UK by immigration. Results show that it is likely to be maintained at a low endemic level within the UK. The relative genetic uniformity of UK and continental populations implies that there is no migration barrier to EBLV-2, between these regions. | 1731 | no | no good fitness measure | NA | no | no |
| 1732 | 2017 | 43020 | Dybdahl, MF; Jokela, J; Delph, LF; Koskella, B; Lively, CM | 2008 | Hybrid Fitness in a Locally Adapted Parasite | The parasite (Red Queen) hypothesis for the maintenance of sexual reproduction and genetic diversity assumes that host-parasite interactions result from tight genetic specificity. Hence, hybridization between divergent parasite populations would be expected to disrupt adaptive gene combinations, leading to reduced infectivity on exposure to parental sympatric hosts, as long as gene effects are nonadditive. In contrast, hybridization would not cause reduced infectivity on allopatric hosts unless the divergent parasite populations possess alleles that are intrinsically incompatible when they are combined. In three different experiments, we compared the infectivity of locally adapted parasite (trematode) populations with that of F 1 hybrid parasites when exposed to host (snail) populations that were sympatric to one of the two parasite populations. We tested for intrinsic genetic incompatibilities in two experiments by including one host population that was allopatric to both parasite populations. As predicted, when the target host populations were sympatric to the parasite populations, the hybrids were significantly less infective than the parental average, while hybrid parasites on allopatric hosts were not, thereby ruling out intrinsic genetic incompatibilities. The results are consistent with nonadditive gene effects and tightly specific host-driven selection underlying parasite divergence, as envisioned by coevolutionary theory and the Red Queen hypothesis. | 1732 | no | NA | NA | no | no |
| 1733 | 2017 | 43020 | Lack, JB; Nichols, RD; Wilson, GM; Van Den Bussche, RA | 2011 | Genetic Signature of Reproductive Manipulation in the Phylogeography of the Bat Fly, Trichobius major | The bat fly (Trichobius major) is a blood-feeding ectoparasite of the cave myotis (Myotis velifer). A recent mitochondrial DNA (mtDNA) study examining population structure of T. major in the South Central United States detected a single haplotype from all individuals examined (N = 48 from 12 different caves), representing one of only a few known examples of such widespread mtDNA uniformity. We examined nuclear genetic diversity using amplified fragment length polymorphism and detected high levels of nuclear genetic diversity in all populations sampled. Amplified fragment length polymorphism analyses indicated significant levels of gene flow among caves > 700 km apart, suggesting the absence of mtDNA diversity in T. major is the result of a selective sweep, not a demographic event (i.e., a recent bottleneck). One mechanism by which mtDNA sweeps occur in arthropods is through bacterial parasites that manipulate host reproduction and mtDNA inheritance. We used PCR to test for the presence of all known reproductive parasites and detected a widespread infection (91.33% infection rate) of T. major with a novel Arsenophonus bacterium, as well as the infection of 2 individuals (1.16% infection rate) with a novel strain of Rickettsia. We discuss the implications for T. major phylogeography and the necessity of a bigenomic approach in arthropod population genetics. | 1733 | no | no good fitness measure | NA | no | no |
| 1734 | 2017 | 43020 | Ivy, JA; Lacy, RC | 2012 | A Comparison of Strategies for Selecting Breeding Pairs to Maximize Genetic Diversity Retention in Managed Populations | “Captive breeding programs aim to maintain populations that are demographically self-sustaining and genetically healthy. It has been well documented that the best way for managed breeding programs to retain gene diversity (GD) and limit inbreeding is to select breeding pairs that minimize a population’s average kinship. We used a series of computer simulations to test 4 methods of minimizing average kinship across a variety of scenarios with varying generation lengths, mortality rates, reproductive rates, and rates of breeding pair success.”“Static MK Selection’’ and”“Dynamic MK Selection’’ are 2 methods for iteratively selecting genetically underrepresented individuals for breeding, whereas”“Ranked MK Selection’’ and”“Simultaneous MK Selection’’ are 2 methods for concurrently selecting the group of breeding individuals that produce offspring with the lowest average kinship. For populations with discrete generations (24 tested scenarios), we found that the Simultaneous and Ranked MK Selection methods were generally the best, nearly equivalent methods for selecting breeding pairs that retained GD and limited inbreeding. For populations with overlapping generations (198 tested scenarios), we found that Dynamic MK Selection was the most robust method for selecting breeding pairs. We used these results to provide guidelines for identifying which method of minimizing average kinship was most appropriate for various breeding program scenarios.” | 1734 | no | no good fitness measure, N pop differnt in GD? | NA | no | no |
| 1735 | 2017 | 43020 | Werblow, A; Klimpel, S; Bolius, S; Dorresteijn, AWC; Sauer, J; Melaun, C | 2014 | Population Structure and Distribution Patterns of the Sibling Mosquito Species Culex pipiens and Culex torrentium (Diptera: Culicidae) Reveal Different Evolutionary Paths | Nowadays a number of endemic mosquito species are known to possess vector abilities for various diseases, as e. g. the sibling species Culex pipiens and Culex torrentium. Due to their morphological similarity, ecology, distribution and vector abilities, knowledge about these species’ population structure is essential. Culicidae from 25 different sampling sites were collected from March till October 2012. All analyses were performed with aligned cox1 sequences with a total length of 658 bp. Population structure as well as distribution patterns of both species were analysed using molecular methods and different statistical tests like distance based redundancy analysis (dbDRA), analysis of molecular variances (AMOVA) or McDonald & Kreitman test and Tajima’s D. Within both species, we could show a genetic variability among the cox1 fragment. The construction of haplotype networks revealed one dominating haplotype for Cx. pipiens, widely distributed within Germany and a more homogeneous pattern for Cx. torrentium. The low genetic differences within Cx. pipiens could be a result of an infection with Wolbachia which can induce a sweep through populations by passively taking the also maternally inherited mtDNA through the population, thereby reducing the mitochondrial diversity as an outcome of reproductive incompatibility. Pairwise population genetic differentiation (F-ST) ranged significantly from moderate to very great between populations of Cx. pipiens and Cx. torrentium. Analyses of molecular variances revealed for both species that the main genetic variability exists within the populations (Cx. pipiens [88.38%]; Cx. torrentium [66.54%]). Based on a distance based redundancy analysis geographical origin explained a small but significant part of the species’ genetic variation. Overall, the results confirm that Cx. pipiens and Cx. torrentium underlie different factors regarding their mitochondrial differentiation, which could be a result of endosymbiosis, dispersal between nearly located populations or human introduction. | 1735 | no | no good fitness measure | NA | no | no |
| 1736 | 2017 | 43020 | Hirsch, BT; Prange, S; Hauver, SA; Gehrt, SD | 2013 | Genetic relatedness does not predict racoon social network structure | Social assortativity, preferentially associating with certain individuals, is a widespread behaviour among a diverse range of taxa. Animals often choose to associate with other individuals based on characteristics such as sex, age, body size, rank and genetic relatedness. These preferences can scale up to shape the overall social structure of an animal group or population. We investigated possible factors that might shape the social network structure of common racoons, Procyon lotor, in a high-density urban population in Cook County, Illinois, U. S. A. Racoon associations were recorded using proximity detecting radiocollars that recorded when individuals came within 1-1.5 m of each other. In addition, dyadic measures of home range overlap and genetic relatedness were calculated for all individuals included in our study. We used multiple regression quadratic assignment procedures to determine what factors influenced the structure of racoon association networks. The only variable that positively influenced racoon social structure was male-male homophily, which is consistent with previous studies that documented frequent social interactions between adult male racoons. Genetic relatedness had no effect on racoon social networks and there was no evidence that males or females preferentially associated with close relatives, despite the presence of kin in the population. This pattern, that kinship does not play a significant role in shaping social structure, is strikingly unusual among mammals and is not consistent with many socioecological models. Although racoon individuals showed strong social partner preferences, it is unclear what factors drove these choices. This unpredictability in partner choice shaped the structure of the racoon social networks and has important implications for disease transfer in this widespread animal vector. (C) 2012 The Association for the Study of Animal Behaviour. Published by Elsevier Ltd. All rights reserved. | 1736 | no | no good fitness measure | NA | no | no |
| 1737 | 2017 | 43020 | Reber, A; Chapuisat, M | 2012 | Diversity, prevalence and virulence of fungal entomopathogens in colonies of the ant Formica selysi | The richness of the parasitic community associated with social insect colonies has rarely been investigated. Moreover, understanding how hosts and pathogens interact in nature is important to interpret results from laboratory experiments. Here, we assessed the diversity, prevalence and virulence of fungal entomopathogens present around and within colonies of the ant Formica selysi. We detected eight fungal species known to be entomopathogenic in soil sampled from the habitat of ants. Six of these entomopathogens were found in active nests, abandoned nests, and corpses from dump piles or live ants. A systematic search for the presence of three generalist fungal entomopathogens in ant colonies revealed a large variation in their prevalence. The most common of the three pathogens, Paecilomyces lilacinus, was detected in 44% of the colonies. Beauveria bassiana occurred in 17% of the colonies, often in association with P. lilacinus, whereas we did not detect Metarhizium brunneum (formerly M. anisopliae) in active colonies. The three fungal species caused significant mortality to experimentally challenged ants, but varied in their degree of virulence. There was a high level of genetic diversity within B. bassiana isolates, which delineated three genetic strains that also differed significantly in their virulence. Overall, our study indicates that the ants encounter a diversity of fungal entomopathogens in their natural habitat. Moreover, some generalist pathogens vary greatly in their virulence and prevalence in ant colonies, which calls for further studies on the specificity of the interactions between the ant hosts and their fungal pathogens. | 1737 | no | No GD diff between pop | NA | no | no |
| 1738 | 2017 | 43020 | Hadziabdic, D; Wadl, PA; Vito, LM; Boggess, SL; Scheffler, BE; Windham, MT; Trigiano, RN | 2012 | Development and characterization of sixteen microsatellite loci for Geosmithia morbida, the causal agent of thousand canker disease in black walnut (Juglans nigra) | Sixteen polymorphic microsatellite loci were identified from the fungal pathogen Geosmithia morbida. Loci were characterized for 13 different isolates collected in 2010 from symptomatic black walnut trees in Tennessee. A total of 77 loci were tested and 16 of those were optimized, screened and selected for diversity studies of G. morbida. Number of alleles per locus ranged from 3 to 8. These microsatellite loci will be useful for rapid disease diagnostic, population genetic analyses on a global scale as well as further epidemiological studies of G. morbida. | 1738 | no | gd of pathogen | NA | no | no |
| 1739 | 2017 | 43020 | Stevens, L; Monroy, MC; Rodas, AG; Hicks, RM; Lucero, DE; Lyons, LA; Dorn, PL | 2015 | Migration and Gene Flow Among Domestic Populations of the Chagas Insect Vector Triatoma dimidiata (Hemiptera: Reduviidae) Detected by Microsatellite Loci | Triatoma dimidiata (Latreille, 1811) is the most abundant and significant insect vector of the parasite Trypanosoma cruzi in Central America, and particularly in Guatemala. Tr. cruzi is the causative agent of Chagas disease, and successful disease control requires understanding the geographic distribution and degree of migration of vectors such as T. dimidiata that frequently re-infest houses within months following insecticide application. The population genetic structure of T. dimidiata collected from six villages in southern Guatemala was studied to gain insight into the migration patterns of the insects in this region where populations are largely domestic. This study provided insight into the likelihood of eliminating T. dimidiata by pesticide application as has been observed in some areas for other domestic triatomines such as Triatoma infestans. Genotypes of microsatellite loci for 178 insects from six villages were found to represent five genetic clusters using a Bayesian Markov Chain Monte Carlo method. Individual clusters were found in multiple villages, with multiple clusters in the same house. Although migration occurred, there was statistically significant genetic differentiation among villages (F-RT = 0.05) and high genetic differentiation among houses within villages (F-SR = 0.11). Relatedness of insects within houses varied from 0 to 0.25, i.e., from unrelated to half-sibs. The results suggest that T. dimidiata in southern Guatemala moves between houses and villages often enough that recolonization is likely, implying the use of insecticides alone is not sufficient for effective control of Chagas disease in this region and more sustainable solutions are required. | 1739 | no | no good fitness measure | NA | no | no |
| 1740 | 2017 | 43020 | Fisher, MC; Koenig, GL; White, TJ; San-Blas, G; Negroni, R; Alvarez, IG; Wanke, B; Taylor, JW | 2001 | Biogeographic range expansion into South America by Coccidioides immitis mirrors New World patterns of human migration | Long-distance population dispersal leaves its characteristic signature in genomes, namely, reduced diversity and increased linkage between genetic markers. This signature enables historical patterns of range expansion to be traced. Herein, we use microsatellite loci from the human pathogen Coccidioides immitis to show that genetic diversity in this fungus is geographically partitioned throughout North America. in contrast, analyses of South American C. immitis show that this population is genetically depauperate and was founded from a single North American population centered in Texas. Variances of allele distributions show that South American C. immitis have undergone rapid population growth, consistent with an epidemic increase in postcolonization population size. Herein, we estimate the introduction into South America to have occurred within the last 9,000-140,000 years. This range increase parallels that of Home sapiens. Because of known associations between Amerindians and this fungus, we suggest that the colonization of South America by C. immitis represents a relatively recent and rapid codispersal of a host and its pathogen. | 1740 | no | human | NA | no | no |
| 1741 | 2017 | 43020 | Brisson, D; Dykhuizen, DE | 2004 | ospC diversity in Borrelia burgdorferi: Different hosts are different niches | The outer surface protein C (ospC) locus of the Lyme disease bacterium, Borrelia burgdorferi, is at least an order of magnitude more variable than other genes in the species. This variation is classified into 22 ospC major groups, 15 of which are found in the northeastern United States. The frequency distributions of ospC within populations suggest that this locus is under balancing selection. In multiple-niche polymorphism, a type of balancing selection, diversity within a population can be maintained when the environment is heterogeneous and no one genotype has the highest fitness in all environments. Genetically different individuals within vertebrate species and different vertebrate species constitute diverse environments for B. burgdorferi. We examined four important host species of B. burgdorferi and found that the strains that infected each species had different sets of ospC major groups. We found no variation among conspecific hosts in the ospC major groups of their infecting strains. These results suggest multiple niches create balancing selection at the ospC locus. | 1741 | no | no good fitness measure, N pop differnt in GD? | NA | no | no |
| 1742 | 2017 | 43020 | Konig, G; Blanco, C; Knowles, NJ; Palma, EL; Maradei, E; Piccone, ME | 2001 | Phylogenetic analysis of foot-and-mouth disease viruses isolated in Argentina | We have analysed complete or partial VP1 sequences of 31 foot-and-mouth disease (FMD) viruses belonging to serotypes A, O and C to determine the genetic relatedness of field strains of FMD virus (FMDV) that have circulated in Argentina between 1961 and 1994. Phylogenetic analysis, which also included 15 previously published Argentinean sequences and six reference strains, revealed that (i) FMD type A strains showed the highest genetic heterogeneity and could be divided into five lineages with a sequence divergence of 0.9-18.5% between strains (ii) most of the FMD type O viruses grouped in two clusters (within cluster sequence divergence ranging from 0.2% to 6.0%) circulating in Argentina since the early 1960s, and (iii) FMD type C viruses were grouped in two clusters with a 13.4% nucleotide sequence divergence between each cluster. The availability of sequence data for many more field isolates from the region will enable us to understand the genetic relationships between FMDV strains and to rapidly trace the source of an FMD outbreak for epidemiological surveillance. | 1742 | no | gd of pathogen | NA | no | no |
| 1743 | 2017 | 43020 | Veillet, S; Filippi, MC; Gallais, A | 1996 | Combined genetic analysis of partial blast resistance in an upland rice population and recurrent selection for line and hybrid values | The CNA-IRAT 5 upland rice population has been improved for 4 years by recurrent selection for blast resistance in Brazil. In order to predict the efficiency of recurrent selection in different test systems and to compare the relative advantage of hybrids versus pure line breeding, a combined genetic analysis of partial blast resistance in the CNA-IRAT 5 population was undertaken. A three-level hierarchical design in inbreeding and a factorial design were derived from the base population. Partial blast resistance of lines and hybrids was evaluated in the greenhouse and in the field by inoculation with one virulent blast isolate. The means and genetic variances of the hybrids and lines were estimated. Genetic advance by recurrent selection was predicted from estimates of variance components. The inheritance of partial blast resistance was mainly additive but non-additive effects were detected at both levels of means and variances. Mean heterosis ranged from 4%-8% for lesion size and lesion density to 10-12% for leaf and panicle resistance. High dominance or homozygous dominance variances relative to additive variance and negative covariance between additive and homozygous dominance effects were estimated. A low frequency of favourable alleles for partial resistance would explain the observed organisation of genetic variability in the base population. Recurrent selection will efficiently improve partial blast resistance of the CNA-IRAT 5 population. Genetic advance for line or hybrid values was expected to be higher testing doubled haploid lines than S1 lines, or than general combining ability. Two components of partial resistance assessed in the greenhouse, lesion size and lesion density, could be used as indirect selection criteria to improve field resistance. On the whole, hybrid breeding for partial blast resistance appeared to be slightly more advantageous than pure line breeding. | 1743 | no | crop | NA | no | no |
| 1744 | 2017 | 43020 | Hedrick, PW; Parker, KM; Gutierrez-Espeleta, GA; Rattink, A; Lievers, K | 2000 | Major histocompatibility complex variation in the Arabian oryx | In the 1960s, the Arabian oryx was one of the most endangered species in the world, extinct in the wild and surviving in only a few captive herds. The present day population of over 2000 descends from a small number of founders and may have restricted genetic variation for important adaptive genes. We have examined the amount of genetic variation for a class II gene in the major histocompatibility complex thought to be the most important genetic basis for pathogen resistance in vertebrates. We found three very divergent alleles, which on average, differed by 24 nucleotides and 15 amino acids in the 236-bp fragment we examined. Using single-strand conformation polymorphism, we found that in a sample of 57 animals, the alleles were in Hardy-Weinberg proportions, although one allele was found only in four heterozygous individuals. The average heterozygosity for the 22 amino acid positions involved in antigen binding was 0.165, three times as high as that for the 56 amino acids not involved with antigen binding. Because the three alleles have such divergent sequences, it is likely that they may recognize peptides from quite different pathogens. As a result, maintenance of these variants should be considered as a goal in the captive breeding program of the Arabian oryx. | 1744 | no | no good fitness measure, N pop differnt in GD? | NA | no | no |
| 1745 | 2017 | 43020 | Paull, JG; Chalmers, KJ; Karakousis, A; Kretschmer, JM; Manning, S; Langridge, P | 1998 | Genetic diversity in Australian wheat varieties and breeding material based on RFLP data | Restriction fragment length polymorphisms (RFLPs) have been used to characterise the genetic diversity of wheat (Triticum aestivum) germplasm, One hundred and twenty-four accessions comprising all major Australian wheat varieties and lines important for breeding purposes were assayed for RFLPs with clones of known genetic location and selected to give uniform genome coverage. The objectives of this study were to determine RFLP-based genetic similarity between accessions and to derive associations between agronomically significant traits and RFLP phenotypes. Ninety-eight probes screened against genomic DNA digested with five restriction endonucleases detected a total of 1968 polymorphic fragments, Genetic similarity (GS) calculated from the RFLP data ranged from 0.004 to 0.409 between accessions, with a mean of 0.18. Cluster analysis based on GS estimates produced four groupings that were generally consistent with available pedigree information, Comparisons of the RFLP phenotypes of accessions containing disease resistance genes present on introgressed alien segments enabled the identification of specific alleles characteristic of these regions. Associations were derived for a range of stem-rust, leaf-rust and yellow-rust resistance genes, These results suggest that RFLP analysis can be used for the characterisation and grouping of elite breeding material of wheat and RFLP profiling can identify chromosome segments associated with agronomic traits. | 1745 | no | crop | NA | no | no |
| 1746 | 2017 | 43020 | Sturup, M; Nash, DR; Hughes, WOH; Boomsma, JJ | 2014 | Sperm mixing in the polyandrous leaf-cutting ant Acromyrmex echinatior | The insemination of queens by sperm from multiple males (polyandry) has evolved in a number of eusocial insect lineages despite the likely costs of the behavior. The selective advantages in terms of colony fitness must therefore also be significant and there is now good evidence that polyandry increases genetic variation among workers, thereby improving the efficiency of division of labor, resistance against disease, and diluting the impact of genetically incompatible matings. However, these advantages will only be maximized if the sperm of initially discrete ejaculates are mixed when stored in queen spermathecae and used for egg fertilization in a fair raffle. Remarkably, however, very few studies have addressed the level of sperm mixing in social insects. Here we analyzed sperm use over time in the highly polyandrous leaf-cutting ant Acromyrmex echinatior. We genotyped cohorts of workers produced either 2months apart or up to over a year apart, and batches of eggs laid up to over 2years apart, and tested whether fluctuations in patriline distributions deviated from random. We show that the representation of father males in both egg and worker cohorts does not change over time, consistent with obligatorily polyandrous queens maximizing their fitness when workers are as genetically diverse as possible. | 1746 | no | no good fitness measure | NA | no | no |
| 1747 | 2017 | 43020 | Aerts, R; Geeraert, L; Berecha, G; Hundera, K; Muys, B; De Kort, H; Honnay, O | 2017 | Conserving wild Arabica coffee: Emerging threats and opportunities | Climate change and emerging pests and diseases are posing important challenges to global crop productivity, including that of Arabica coffee. The genetic basis of commercially used Arabica coffee cultivars is extremely narrow, and it is uncertain how much genetic diversity is present in ex situ collections. Conserving the wild Arabica coffee gene pool and its evolutionary potential present in the montane forests of SW Ethiopia is thus critically important for maintaining coffee yield and yield stability worldwide. Globally, coffee agroforestry helps to conserve forest cover and forest biodiversity that cannot persist in open agricultural landscapes, but the conservation of the wild Arabica coffee gene pool requires other priorities than those that are usually set for conserving forest biodiversity in mixed tropical landscapes. We show how forest loss and degradation, coffee management, in particular production intensification, and the introduction of cultivars, are threatening the genetic integrity of these wild populations. We propose an active land sparing approach based on strict land use zoning to conserve the genetic resources and the in situ evolutionary potential of Arabica coffee and discuss the major challenges including the development of access and benefit sharing mechanisms for ensuring long-term support to conservation. (C) 2016 Elsevier B.V. All rights reserved. | 1747 | no | crop | NA | no | no |
| 1748 | 2017 | 43020 | TIXIERBOICHARD, M; DURAND, L; MORISSON, M; RICARD, F; COQUERELLE, G | 1994 | COMPARATIVE-ANALYSIS OF AVIAN-LEUKOSIS VIRUS-RELATED ENDOGENOUS VIRAL GENES IN EXPERIMENTAL STRAINS OF THE DOMESTIC CHICKEN | DNA sequences related to avian leukosis virus (ev genes) were identified in the genome of chickens from 6 experimental strains including 3 randombred lines (Wyandotte, Fayoumi and White Leghorn), 2 divergently selected Rhode Island Red lines and a synthetic broiler line. Digestion of genomic DNA with Sad and BamHI enzymes and hybridization with either RAV-2 sequences or an LTR-gag probe revealed 66 different Sad bands, 53 of which could be associated with particular BamHI bands such that the ev loci could be defined at the molecular level. The distribution patterns were very strain-specific. Average band frequency and heterozygosity level could be correlated with the genetic history of the lines and their inbreeding level which varied from 0.04 to 0.28. Within-family segregation did not show any allelic relationship between retroviral insertions that were considered to be 2 genes. The Fayoumi line showed the largest variation in ev number per bird and contained a few birds free of any endogenous viral sequences, designated ev0. The broiler line showed the highest ev number. Very few ev loci were shared by different lines; only ev6 was found in all strains. Comparison with the established classification in White Leghorns was not straightforward and further analysis of DNA polymorphism and viral expression is needed. | 1748 | no | domesticated animal | NA | no | no |
| 1749 | 2017 | 43020 | Yao, G; Zhu, Y; Wan, QH; Fang, SG | 2015 | Major histocompatibility complex class II genetic variation in forest musk deer (Moschus berezovskii) in China | The major histocompatibility complex (MHC) plays an important role in the immune system of vertebrates. We used the second exon of four MHC class II genes (DRA, DQA1, DQA2 and DRB3) to assess the overall MHC variation in forest musk deer (Moschus berezovskii). We also compared the MHC variation in captive and wild populations. We observed 22 alleles at four loci (four at DRA, four at DQA1, four at DQA2 and 10 at DRB3), 15 of which were newly identified alleles. Results suggest that forest musk deer maintain relatively high MHC variation, which may result from balancing selection. Moreover, considerable diversity was observed at the DRA locus. We found a high frequency of Mobe-DRA\* 02, Mobe-DQA1\* 01 and Mobe-DQA2\* 05 alleles, which may be important for pathogen resistance. A Ewens-Watterson test showed that the DRB3 locus in the wild population had experienced recent balancing selection. We detected a small divergence at the DRA locus, suggesting the effect of weak positive selection on the DRA gene. Alternatively, this locus may be young and not yet adapted a wide spectrum of alleles for pathogen resistance. The significant heterozygosity deficit observed at the DQA1 and DRB3 loci in the captive population and at all four loci in the wild population may be the result of a population bottleneck. Additionally, MHC genetic diversity was higher in the wild population than in the captive, suggesting that the wild population may have the ability to respond to a wider range of pathogens. | 1749 | no | no good fitness measure | NA | no | no |
| 1750 | 2017 | 43020 | Abdelkrim, J; Pascal, M; Samadi, S | 2007 | Establishing causes of eradication failure based on genetics: Case study of ship rat eradication in Ste. Anne archipelago | Determining the causes of a failed eradication of a pest species is important because it enables an argued adjustment of the methodologies used and the improvement of the protocols for future attempts. We examined how molecular monitoring can help distinguish between the two main reasons for an eradication failure (i.e., survival of some individuals vs. recolonization after eradication). We investigated genetic variation in seven microsatellite loci in ship rat (Rattus rattus) populations from four islets off the Martinique coast (French Caribbean). In 1999 an eradication attempt was conducted on the four islets. Three years later rats were observed again on two of them. We compared the genetic signatures of the populations before and after the eradication attempt. On one of the islands, the new rat population was likely a subset of the pre-eradication population. A weak genetic differentiation was found between them, with almost no new alleles observed in the new population and moderate F-ST values (0.15). Moreover, assignment procedures clustered the two populations together. In contrast, on the other islet, many new alleles were observed after the eradication attempt, resulting in an increase in genetic diversity (from 2.57 to 3.57 mean number of alleles per locus) and strong F-ST values (0.39). Moreover, genetic clustering clearly separated the two samples (i.e., before and after the eradication attempt) in two different populations. Thus, to achieve long-term eradication on these islets, it seems necessary to redevelop the eradication procedure to avoid individuals surviving and to prevent reinvasion, probably from the mainland, by installing permanent trapping and poisoning devices and conducting regular monitoring. We strongly encourage wildlife managers conducting eradication campaigns to integrate molecular biological tools in their protocols, which can be done easily for most common invasive species. | 1750 | no | no good fitness measure | NA | no | no |
| 1751 | 2017 | 43020 | Goodisman, MAD; Ross, KG | 1997 | Relationship of queen number and queen relatedness in multiple-queen colonies of the fire ant Solenopsis invicta | 1. The relationship between the number of queens per nest and their relatedness was examined in the polygyne (multiple queens per nest) social form of the fire ant Solenopsis invicta. 2. No significant relationship between these two variables was found. Moreover, the overall average relatedness among nestmate queens was not significantly different from zero. 3. These findings indicate that polygyne nests of S. invicta do not represent closed societies. Furthermore, they are consistent with continual acceptance of non-nestmate queens throughout a colony’s lifetime. This strategy differs from the expectation of kin selection theory that only related queens should he accepted as new reproductives within polygyne colonies. 4. The postulated acceptance of non-nestmate queens is associated with a decrease in the number of parasites, predators and diseases and a concomitant increase in the density of nests in introduced populations, suggesting that the permissive environment experienced by introduced fire ants may have decreased the relative importance of kinship as a stabilizing factor in the evolution of polygyny. | 1751 | probably yes | ok gd measurement and pop level comp? | no ok fitness measure | no | no |
| 1752 | 2017 | 43021 | Belov, K | 2011 | The role of the Major Histocompatibility Complex in the spread of contagious cancers | Major Histocompatibility Complex (MHC) genes play a key role in immune response to infectious diseases, immunosurveillance, and self/nonself recognition. Matching MHC alleles is critical for organ transplantation, while changes in the MHC profile of tumour cells allow effective evasion of the immune response. Two unique cancers have exploited these features to become transmissible. In this review I discuss the functional role of MHC molecules in the emergence and evolution of Devil Facial Tumour Disease (DFTD) and Canine Transmissible Venereal Tumour (CTVT). High levels of genetic diversity at MHC genes play a critical role in protecting populations of vertebrate species from contagious cancer. However, species that have undergone genetic bottlenecks and have lost diversity at MHC genes are at risk of transmissible tumours. Moreover, evolution and selection for tumour variants capable of evading the immune response allow contagious cancers to cross MHC barriers. Transmissible cancers are rare but they can provide unique insights into the genetics and immunology of tumours and organ transplants. | 1752 | no | no good fitness measure, N pop differnt in GD? | NA | no | no |
| 1753 | 2017 | 43021 | Kmentova, N; Gelnar, M; Mendlov, M; Van Steenberge, M; Koblmuller, S; Vanhove, MPM | 2016 | Reduced host-specificity in a parasite infecting non-littoral Lake Tanganyika cichlids evidenced by intraspecific morphological and genetic diversity | Lake Tanganyika is well-known for its high species-richness and rapid radiation processes. Its assemblage of cichlid fishes recently gained momentum as a framework to study parasite ecology and evolution. It offers a rare chance to investigate the influence of a deepwater lifestyle in a freshwater fish-parasite system. Our study represents the first investigation of parasite intraspecific genetic structure related to host specificity in the lake. It focused on the monogenean flatworm Cichlidogyrus casuarinus infecting deepwater cichlids belonging to Bathybates and Hemibates. Morphological examination of C. casuarinus had previously suggested a broad host range, while the lake’s other Cichlidogyrus species are usually host specific. However, ongoing speciation or cryptic diversity could not be excluded. To distinguish between these hypotheses, we analysed intraspecific diversity of C. casuarinus. Monogeneans from nearly all representatives of the host genera were examined using morphometrics, geomorphometrics and genetics. We confirmed the low host-specificity of C. casuarinus based on morphology and nuclear DNA. Yet, intraspecific variation of sclerotized structures was observed. Nevertheless, the highly variable mitochondrial DNA indicated recent population expansion, but no ongoing parasite speciation, confirming, for the first time in freshwater, reduced parasite host specificity in the deepwater realm, probably an adaptation to low host availability. | 1753 | no | no good fitness measure, N pop differnt in GD? | NA | no | no |
| 1754 | 2017 | 43021 | Ficetola, GF; De Bernardi, F | 2005 | Supplementation or in situ conservation? Evidence of local adaptation in the Italian agile frog Rana latastei and consequences for the management of populations | Relocation of endangered species can be an effective conservation tool if it does not mix populations that represent significant intraspecific variation. The threatened Italian agile frog, Rana latastei, has small populations with low genetic diversity: translocation has been proposed to improve the likelihood of survival of populations. Using a common environment experiment and field surveys, we investigated whether there were differences in larval growth and developmental rate between foothill and lowland R. latastei populations, to evaluate if they are evolutionarily significant units. In nature, the colder climate of the foothills causes delayed metamorphosis. Conversely, in a common environment, larvae from foothill populations show faster growth and development. We did not find a significant egg-size related maternal effect or any differences in size at metamorphosis. We hypothesise that counter-gradient selection promoted fast growing phenotypes in a cold environment, where low temperatures slow down larval development. Foothill populations, despite being only a small geographical distance away from lowland populations, seem to be adapted to a colder climate and represent an evolutionarily significant unit. Different populations should, therefore, be managed independently, avoiding translocation. We suggest that evolutionary divergence between populations should be verified prior to planning relocation programmes, to prevent the risk of genetic homogenisation. | 1754 | no | No GD diff between pop | NA | no | no |
| 1755 | 2017 | 43021 | Brusentsov, II; Katokhin, AV; Brusentsova, IV; Shekhovtsov, SV; Borovikov, SN; Goncharenko, GG; Lider, LA; Romashov, BV; Rusinek, OT; Shibitov, SK; Suleymanov, MM; Yevtushenko, AV; Mordvinov, VA | 2013 | Low Genetic Diversity in Wide-Spread Eurasian Liver Fluke Opisthorchis felineus Suggests Special Demographic History of This Trematode Species | Opisthorchis felineus or Siberian liver fluke is a trematode parasite (Opisthorchiidae) that infects the hepato-biliary system of humans and other mammals. Despite its public health significance, this wide-spread Eurasian species is one of the most poorly studied human liver flukes and nothing is known about its population genetic structure and demographic history. In this paper, we attempt to fill this gap for the first time and to explore the genetic diversity in O. felineus populations from Eastern Europe (Ukraine, European part of Russia), Northern Asia (Siberia) and Central Asia (Northern Kazakhstan). Analysis of marker DNA fragments from O. felineus mitochondrial cytochrome c oxidase subunit 1 and 3 (cox1, cox3) and nuclear rDNA internal transcribed spacer 1 (ITS1) sequences revealed that genetic diversity is very low across the large geographic range of this species. Microevolutionary processes in populations of trematodes may well be influenced by their peculiar biology. Nevertheless, we suggest that lack of population genetics structure observed in O. felineus can be primarily explained by the Pleistocene glacial events and subsequent sudden population growth from a very limited group of founders. Rapid range expansion of O. felineus through Asian and European territories after severe bottleneck points to a high dispersal potential of this trematode species. | 1755 | no | gd of pathogen | NA | no | no |
| 1756 | 2017 | 43021 | Mussmann, SM; Douglas, MR; Anthonysamy, WJB; Davis, MA; Simpson, SA; Louis, W; Douglas, ME | 2017 | Genetic rescue, the greater prairie chicken and the problem of conservation reliance in the Anthropocene | A central question in conservation is how best to manage biodiversity, despite human domination of global processes (= Anthropocene). Common responses (i.e. translocations, genetic rescue) forestall potential extirpations, yet have an uncertain duration. A textbook example is the greater prairie chicken (GRPC: Tympanuchus cupido pinnatus), where translocations (1992-1998) seemingly rescued genetically depauperate Illinois populations. We re-evaluated this situation after two decades by genotyping 21 microsatellite loci from 1831 shed feathers across six leks in two counties over 4 years (2010-2013). Low migration rates (less than 1%) established each county as demographically independent, but with declining-population estimates (4 year average N = 79). Leks were genetically similar and significantly bottlenecked, with low effective population sizes (average N-e = 13.1; 4 year N-e/N = 0.166). Genetic structure was defined by 12 significantly different family groups, with relatedness r = 0.31 > half-sib r = 0.25. Average heterozygosity, indicating short-term survival, did not differ among contemporary, pre-and post-translocated populations, whereas allelic diversity did. Our results, the natural history of GRPC (i.e. few leks, male dominance hierarchies) and its controlled immigration suggest demographic expansion rather than genetic rescue. Legal protection under the endangered species act (ESA) may enhance recovery, but could exacerbate political-economic concerns on how best to manage ‘conservation-reliant’ species, for which GRPC is now an exemplar. | 1756 | no | no good fitness measure | NA | no | no |
| 1757 | 2017 | 43021 | Procaccini, G; Piazzi, L | 2001 | Genetic polymorphism and transplantation success in the mediterranean seagrass Posidonia oceanica | We studied the role of genetic variability of donor beds in establishing transplantation criteria for the Mediterranean seagrass Posidonia oceanica. Horizontal rhizomes, collected from three geographically distinct populations, were transplanted into a common bed at a highly human-impacted locality. The transplantation site was located near one of the donor populations. After three years, the shoots collected in the population adjacent to the transplanting site showed the lowest growth performance. Genetic variability, assessed through the analysis of hypervariable microsatellite regions, and growth performance followed a similar trend. The shoots growing and branching at the highest rate were those collected from populations with the highest heterozygosity values, despite greater geographic distance. No genetic differences were found between the transplanted shoots and shoots from donor meadows, as expected due to the low rate of sexual reproduction in P. oceanica and the short time that had passed since the transplants. The problem of affecting the local gene pool by the introduction of foreign genotypes could arise, but introduction of new alleles could balance the degradation of genetic variability caused by human impact. In general our study suggests that the genetic variability of source material is an important aspect to consider in the development of seagrass restoration strategies. | 1757 | no | no good fitness measure, N pop differnt in GD? | NA | no | no |
| 1758 | 2017 | 43021 | Rich, SM; Leendertz, FH; Xu, G; LeBreton, M; Djoko, CF; Aminake, MN; Takang, EE; Diffo, JLD; Pike, BL; Rosenthal, BM; Formenty, P; Boesch, C; Ayala, FJ; Wolfe, ND | 2009 | The origin of malignant malaria | Plasmodium falciparum, the causative agent of malignant malaria, is among the most severe human infectious diseases. The closest known relative of P. falciparum is a chimpanzee parasite, Plasmodium reichenowi, of which one single isolate was previously known. The co-speciation hypothesis suggests that both parasites evolved separately from a common ancestor over the last 5-7 million years, in parallel with the divergence of their hosts, the hominin and chimpanzee lineages. Genetic analysis of eight new isolates of P. reichenowi, from wild and wild-born captive chimpanzees in Cameroon and Cote d’Ivoire, shows that P. reichenowi is a geographically widespread and genetically diverse chimpanzee parasite. The genetic lineage comprising the totality of global P. falciparum is fully included within the much broader genetic diversity of P. reichenowi. This finding is inconsistent with the co-speciation hypothesis. Phylogenetic analysis indicates that all extant P. falciparum populations originated from P. reichenowi, likely by a single host transfer, which may have occurred as early as 2-3 million years ago, or as recently as 10,000 years ago. The evolutionary history of this relationship may be explained by two critical genetic mutations. First, inactivation of the CMAH gene in the human lineage rendered human ancestors unable to generate the sialic acid Neu5Gc from its precursor Neu5Ac, and likely made humans resistant to P. reichenowi. More recently, mutations in the dominant invasion receptor EBA 175 in the P. falciparum lineage provided the parasite with preference for the overabundant Neu5Ac precursor, accounting for its extreme human pathogenicity. | 1758 | no | gd of parasite | NA | no | no |
| 1759 | 2017 | 43021 | Ciborowski, KL; Consuegra, S; de Leaniz, CG; Wang, J; Beaumont, MA; Jordan, WC | 2007 | Stocking may increase mitochondrial DNA diversity but fails to halt the decline of endangered Atlantic salmon populations | Over the last 50 years, Spanish Atlantic salmon (Salmo salar) populations have been in decline. In order to bolster these populations, rivers were stocked with fish of northern European origin during the period 1974-1996, probably also introducing the furunculosis-inducing pathogen, Aeromonas salmonicida. Here we assess the relative importance of processes influencing mitochondrial (mt)DNA variability in these populations from 1948 to 2002. Genetic material collected over this period from four rivers in northern Spain (Cantabria) was used to detect variability at the mtDNA ND1 gene. Before stocking, a single haplotype was found at high frequency (0.980). Following stocking, haplotype diversity (h) increased in all rivers (mean h before stocking was 0.041, and 0.245 afterwards). These increases were due principally to the dramatic increase in frequency of a previously very low frequency haplotype, reported at higher frequencies in northern European populations proximate to those used to stock Cantabrian rivers. Genetic structuring increased after stocking: among-river differentiation was low before stocking (1950s/1960s Phi(ST) = -0.00296-0.00284), increasing considerably at the height of stocking (1980s Phi(ST) = 0.18932) and decreasing post-stocking (1990s/2002 Phi(ST) = 0.04934-0.03852). Gene flow from stocked fish therefore seems to have had a substantial role in increasing mtDNA variability. Additionally, we found significant differentiation between individuals that had probably died from infectious disease and apparently healthy, angled fish, suggesting a possible role for pathogen-driven selection of mtDNA variation. Our results suggest that stocking with non-native fish may increase genetic diversity in the short term, but may not reverse population declines. | 1759 | probably no | no good fitness measure | no fitness measure | no | no |
| 1760 | 2017 | 43021 | Pfeifer, SP | 2017 | The Demographic and Adaptive History of the African Green Monkey | Relatively little is known about the evolutionary history of the African green monkey (genus Chlorocebus) due to the lack of sampled polymorphism data fromwild populations. Yet, this characterization of genetic diversity is not only critical for a better understanding of their own history, but also for human biomedical research given that they are one of the most widely used primate models. Here, I analyze the demographic and selective history of the African green monkey, utilizing one of the most comprehensive catalogs of wild genetic diversity to date, consisting of 1,795,643 autosomal single nucleotide polymorphisms in 25 individuals, representing all five major populations: C. a. aethiops, C. a. cynosurus, C. a. pygerythrus, C. a. sabaeus, and C. a tantalus. Assuming a mutation rate of 5.9 x 10(-9) per base pair per generation and a generation time of 8.5 years, divergence time estimates range from 523 to 621 kya for the basal split of C. a. aethiops from the other four populations. Importantly, the resulting tree characterizing the relationship and split-times between these populations differs significantly from that presented in the original genome paper, owing to their neglect of within-population variation when calculating between population-divergence. In addition, I find that the demographic history of all five populations is well explained by a model of population fragmentation and isolation, rather than novel colonization events. Finally, utilizing these demographic models as a null, I investigate the selective history of the populations, identifying candidate regions potentially related to adaptation in response to pathogen exposure. | 1760 | no | no good fitness measure, N pop differnt in GD? | NA | no | no |
| 1761 | 2017 | 43021 | Primmer, CR; Aho, T; Piironen, J; Estoup, A; Cornuet, JM; Ranta, E | 1999 | Microsatellite analysis of hatchery stocks and natural populations of Arctic charr, Salvelinas alpinus, from the Nordic region: implications for conservation | Semi-automated fluorescent genotyping of eight polymorphic microsatellite loci was used to assess the level of genetic diversity and population differentiation in Nordic stocks of non-anadromous Arctic charr, Salvelinus alpinus, of both wild and hatchery origin. Highly significant genetic heterogeneity was detected globally across wild and/or hatchery populations and even between populations from lakes separated by as little as six kilometres. The overall level of genetic differentiation among wild populations (F(ST) = 0.360) was substantially higher than that observed between populations of anadromous Arctic charr in Canada using microsatellite data. Cavalli-Sforza and Edward’s chord distance was used to construct a neighbour-joining tree and three population clusters were supported with relatively high bootstrap values which included the populations from north-west, north-east and southern Finland respectively. Use of Paetkau et al’s individual assignment rest further supported the strong differentiation of most populations as well as their classification into the three predicted geographical areas. No significant difference in average allele number or heterozygosity was observed between populations of wild and hatchery origin. However, the effects of hatchery rearing were revealed by a strong increase in the number of deviations from Hardy-Weinberg equilibrium as well as of linkage disequilibrium events in the hatchery stocks compared to natural populations. The population from Lake Saimaa; in south-cast Finland, is completely reliant upon aquaculture assistance for its survival. The broodstocks of this population exhibited particularly low levels of genetic variability. Although the hatchery stocks of this population suffer from increased egg and alevin mortality and disease susceptibility, it remains to be determined if this is due directly to a lack of genetic variation as some abundant unstocked natural populations possessed similarly low levels of microsatellite variability. | 1761 | no | no good fitness measure | NA | no | no |
| 1762 | 2017 | 43021 | Berg, JJ; Coop, G | 2014 | A Population Genetic Signal of Polygenic Adaptation | Adaptation in response to selection on polygenic phenotypes may occur via subtle allele frequencies shifts at many loci. Current population genomic techniques are not well posed to identify such signals. In the past decade, detailed knowledge about the specific loci underlying polygenic traits has begun to emerge from genome-wide association studies (GWAS). Here we combine this knowledge from GWAS with robust population genetic modeling to identify traits that may have been influenced by local adaptation. We exploit the fact that GWAS provide an estimate of the additive effect size of many loci to estimate the mean additive genetic value for a given phenotype across many populations as simple weighted sums of allele frequencies. We use a general model of neutral genetic value drift for an arbitrary number of populations with an arbitrary relatedness structure. Based on this model, we develop methods for detecting unusually strong correlations between genetic values and specific environmental variables, as well as a generalization of Q(ST)/F-ST comparisons to test for over-dispersion of genetic values among populations. Finally we lay out a framework to identify the individual populations or groups of populations that contribute to the signal of overdispersion. These tests have considerably greater power than their single locus equivalents due to the fact that they look for positive covariance between like effect alleles, and also significantly outperform methods that do not account for population structure. We apply our tests to the Human Genome Diversity Panel (HGDP) dataset using GWAS data for height, skin pigmentation, type 2 diabetes, body mass index, and two inflammatory bowel disease datasets. This analysis uncovers a number of putative signals of local adaptation, and we discuss the biological interpretation and caveats of these results. | 1762 | no | NA | NA | no | no |
| 1763 | 2017 | 43021 | Karamanlidis, AA; Gaughran, S; Aguilar, A; Dendrinos, P; Huber, D; Pires, R; Schultz, J; Skrbinsek, T; Amato, G | 2016 | Shaping species conservation strategies using mtDNA analysis: The case of the elusive Mediterranean monk seal (Monachus monachus) | Halting biodiversity loss is one of the major conservation challenges of our time and science-based conservation actions are required to safeguard the survival of endangered species. However the establishment of effective conservation strategies may be hampered by inherent difficulties of studying elusive animals. We used analysis of control region sequences to obtain baseline information on the genetic diversity and population structure and history of the elusive and critically endangered Mediterranean monk seal that will help define an effective conservation strategy for the species. We analyzed 165 samples collected throughout the entire extant range of the species and identified 5 haplotypes. Based on levels of genetic diversity (haplotypic diversity: 0.03; variable sites: 0.6%) the Mediterranean monk seal appears to be one of the most genetically depauperate mammals on Earth. We identified three genetically distinct monk seal subpopulations: one in the north Atlantic [Cabo Blanco vs. Aegean Sea (F-ST = 0.733; P = 0.000); Cabo Blanco vs. Ionian Sea (F-ST = 0.925; P = 0.000)] and two in the Mediterranean, one in the Ionian and another one in the Aegean Sea (Ionian vs. Aegean Sea F-ST = 0:577; P = 0.000). Results indicate a recent divergence and short evolutionary history of the extant Mediterranean monk seal subpopulations. Based on the results we recommend continuation of the monitoring efforts for the species and systematic collection of genetic samples and storage in dedicated sample banks. On a management level we argue that, based on genetic evidence, it is justified to manage the Atlantic and Mediterranean monk seal subpopulations as two separate management units. In Greece, the existence of two subpopulations should guide efforts for the establishment of a network of protected areas and identify the monitoring of habitat availability and suitability as an important conservation priority. (C) 2015 Elsevier Ltd. All rights reserved. | 1763 | no | no good fitness measure, N pop differnt in GD? | NA | no | no |
| 1764 | 2017 | 43021 | Dostal, P; Allan, E; Dawson, W; van Kleunen, M; Bartish, I; Fischer, M | 2013 | Enemy damage of exotic plant species is similar to that of natives and increases with productivity | 1. In their colonized ranges, exotic plants may be released from some of the herbivores or pathogens of their home ranges but these can be replaced by novel enemies. It is of basic and practical interest to understand which characteristics of invaded communities control accumulation of the new pests. Key questions are whether enemy load on exotic species is smaller than on native competitors as suggested by the enemy release hypothesis (ERH) and whether this difference is most pronounced in resource-rich habitats as predicted by the resource-enemy release hypothesis (R-ERH). 2. In 72 populations of 12 exotic invasive species, we scored all visible above-ground damage morphotypes caused by herbivores and fungal pathogens. In addition, we quantified levels of leaf herbivory and fruit damage. We then assessed whether variation in damage diversity and levels was explained by habitat fertility, by relatedness between exotic species and the native community or rather by native species diversity. 3. In a second part of the study, we also tested the ERH and the R-ERH by comparing damage of plants in 28 pairs of co-occurring native and exotic populations, representing nine congeneric pairs of native and exotic species. 4. In the first part of the study, diversity of damage morphotypes and damage levels of exotic populations were greater in resource-rich habitats. Co-occurrence of closely related, native species in the community significantly increased the probability of fruit damage. Herbivory on exotics was less likely in communities with high phylogenetic diversity. 5. In the second part of the study, exotic and native congeneric populations incurred similar damage diversity and levels, irrespective of whether they co-occurred in nutrient-poor or nutrient-rich habitats. 6. Synthesis. We identified habitat productivity as a major community factor affecting accumulation of enemy damage by exotic populations. Similar damage levels in exotic and native congeneric populations, even in species pairs from fertile habitats, suggest that the enemy release hypothesis or the R-ERH cannot always explain the invasiveness of introduced species. | 1764 | no | multiple species diversity | NA | no | no |
| 1765 | 2017 | 43021 | Habel, JC; Finger, A; Schmitt, T; Neve, G | 2011 | Survival of the endangered butterfly Lycaena helle in a fragmented environment: Genetic analyses over 15 years | Temporal changes in allele frequencies are often assumed in studies addressing the history of populations affected by different anthropogenic and natural impacts at different time scales. Yet, few studies directly compare the genetic composition of populations over time spans of more than 10 years. Therefore, to test the genetic effects of 15 years of population isolation in the butterfly Lycaena helle, we analysed 472 individuals from 27 samples, of which nine were collected in 1991 and 18 in 2006. Sampling was performed in five mountain regions (Pyrenees, Massif Central, Jura, Vosges and Ardennes). Genetic analyses were performed using five polymorphic microsatellites. Old and new samples of identical or neighbouring populations revealed similar genetic differentiations among these five mountain regions. A comparatively strong genetic differentiation among populations combined with a high amount of private alleles for each mountain area was detected, but mountain-specific alleles were in most cases identical in 1991 and 2006. Nevertheless, the obtained data also indicate moderate changes between 1991 and 2006 in the species’ genetic structure - genetic differentiation among local populations increased marginally and allele frequencies showed corresponding modifications. A significant decline in genetic diversity was not detectable, and nine private alleles exclusive to a single mountain region were only detected in samples from the year 1991, whereas eleven were only observed in the individuals collected in 2006. These observations might indicate the results of genetic drift within isolated populations.Zusammenfassung Anderungen von Allelfrequenzen wurden haufig als Effekte athropogener und naturlicher Einflusse auf Populationen diskutiert. Jedoch wurde bislang in nur wenigen Studien ein direkter Vergleich der genetischen Struktur von Populationen uber mehrere Jahre vorgenommen. Daher wurden die genetischen Effekte andauernder Isolation uber einen Zeitraum von 15 Jahren an 472 Individuen der Schmetterlingsart Lycaena helle an 27 Populationen untersucht, wovon neun 1991 und 18 2006 beprobt wurden. Die Probenahme wurde in funf voneinander getrennten Bergregionen (Pyrenaen, Massif Central, Jura, Vogesen und Ardennen) durchgefuhrt. Genetische Analysen wurden auf der Grundlage funf polymorpher Mikrosatelliten vorgenommen. Alte und neue Proben identischer oder benachbarter Populationen zeigen ubereinstimmende genetische Strukturen zwischen diesen funf Bergregionen. Eine vergleichbar starke genetische Differenzierung zwischen diesen Populationen sowie ein hoher Anteil privater Allele wurden fur die entsprechenden Bergregionen nachgewiesen. Die Daten zeigen eine langsame Veranderung der genetischen Struktur uber den Zeitraum von 1991 bis 2006: die genetische Differenzierung zwischen lokalen Populationen stieg leicht an, und die Allelfrequenzen veranderten sich. Ein signifikanter Ruckgang genetischer Diversitat konnte nicht festgestellt werden. Neun private Allele konnten ausschlie ss lich im Jahr 1991 und elf ausschlie ss lich im Jahr 2006 nachgewiesen werden. Diese Beobachtungen konnten das Ergebnis genetischer Drift innerhalb dieser isolierten Populationen sein. | 1765 | no | no good fitness measure | NA | no | no |
| 1766 | 2017 | 43021 | Thonhauser, KE; Raveh, S; Thoss, M; Penn, DJ | 2016 | Does multiple paternity influence offspring disease resistance? | It has been suggested that polyandry allows females to increase offspring genetic diversity and reduce the prevalence and susceptibility of their offspring to infectious diseases. We tested this hypothesis in wild-derived house mice (Mus musculus) by experimentally infecting the offspring from 15 single-and 15 multiple-sired litters with two different strains of a mouse pathogen (Salmonella Typhimurium) and compared their ability to control infection. We found a high variation in individual infection resistance (measured with pathogen loads) and significant differences among families, suggesting genetic effects on Salmonella resistance, but we found no difference in prevalence or infection resistance between single-vs. multiple-sired litters. We found a significant sex difference in infection resistance, but surprisingly, males were more resistant to infection than females. Also, infection resistance was correlated with weight loss during infection, although only for females, indicating that susceptibility to infection had more harmful health consequences for females than for males. To our knowledge, our findings provide the first evidence for sex-dependent resistance to Salmonella infection in house mice. Our results do not support the hypothesis that multiple-sired litters are more likely to survive infection than single-sired litters; however, as we explain, additional studies are required before ruling out this hypothesis. | 1766 | yes | ok gd measurement and pop level comp? | NA | yes | yes |
| 1767 | 2017 | 43021 | Huang, XY; Yang, QL; Yuan, JH; Liu, LX; Sun, WY; Jiang, YD; Zhao, SG; Zhang, SW; Huang, WZ; Gun, SB | 2016 | Effect of Genetic Diversity in Swine Leukocyte Antigen-DRA Gene on Piglet Diarrhea | The swine leukocyte antigens (SLAs) are the multigene families related to immune responses. Little is known about the effect of the DRA gene on diarrheal disease. This study reported the genetic diversity of the DRA gene in exons 1, 3 and 4 in 290 Chinese Yantai black pigs. No variation was identified in exon 3. In exon 1, three genotypes and two alleles were identified, generated by two single nucleotide polymorphisms (SNPs). In exon 4, there were eight genotypes and five alleles containing seven SNPs were detected with four SNPs being novel SNPs. The low polymorphism found in swine DRA is consistent with the concept that the DRA gene is highly conserved among all mammalian species. Statistical analyses indicated that the genotypes of exon 1 were not significantly associated with piglet diarrhea (p > 0.05); however, genotypes C4C4 (1.80 +/- 0.33) and A(4)E(4) (1.66 +/- 0.25) of exon 4 were significantly susceptible to diarrhea (p < 0.01). These indicate that the particular genotypes of the DRA gene are susceptible to diarrheal disease, which provides valuable information for disease-resistance breeding in swine. | 1767 | no | no good fitness measure, N pop differnt in GD? | NA | no | no |
| 1768 | 2017 | 43021 | Cussens, J; Bartlett, M; Jones, EM; Sheehan, NA | 2013 | Maximum Likelihood Pedigree Reconstruction Using Integer Linear Programming | Large population biobanks of unrelated individuals have been highly successful in detecting common genetic variants affecting diseases of public health concern. However, they lack the statistical power to detect more modest gene-gene and gene-environment interaction effects or the effects of rare variants for which related individuals are ideally required. In reality, most large population studies will undoubtedly contain sets of undeclared relatives, or pedigrees. Although a crude measure of relatedness might sometimes suffice, having a good estimate of the true pedigree would be much more informative if this could be obtained efficiently. Relatives are more likely to share longer haplotypes around disease susceptibility loci and are hence biologically more informative for rare variants than unrelated cases and controls. Distant relatives are arguably more useful for detecting variants with small effects because they are less likely to share masking environmental effects. Moreover, the identification of relatives enables appropriate adjustments of statistical analyses that typically assume unrelatedness. We propose to exploit an integer linear programming optimisation approach to pedigree learning, which is adapted to find valid pedigrees by imposing appropriate constraints. Our method is not restricted to small pedigrees and is guaranteed to return a maximum likelihood pedigree. With additional constraints, we can also search for multiple high-probability pedigrees and thus account for the inherent uncertainty in any particular pedigree reconstruction. The true pedigree is found very quickly by comparison with other methods when all individuals are observed. Extensions to more complex problems seem feasible. | 1768 | no | NA | NA | no | no |
| 1769 | 2017 | 43021 | Ascunce, MS; Fane, J; Kassu, G; Toloza, AC; Picollo, MI; Gonzalez-Oliver, A; Reed, DL | 2013 | Mitochondrial Diversity in Human Head Louse Populations Across the Americas | Anthropological studies suggest that the genetic makeup of human populations in the Americas is the result of diverse processes including the initial colonization of the continent by the first people plus post-1492 European migrations. Because of the recent nature of some of these events, understanding the geographical origin of American human diversity is challenging. However, human parasites have faster evolutionary rates and larger population sizes allowing them to maintain greater levels of genetic diversity than their hosts. Thus, we can use human parasites to provide insights into some aspects of human evolution that may be unclear from direct evidence. In this study, we analyzed mitochondrial DNA (mtDNA) sequences from 450 head lice in the Americas. Haplotypes clustered into two well-supported haplogroups, known as A and B. Haplogroup frequencies differ significantly among North, Central and South America. Within each haplogroup, we found evidence of demographic expansions around 16,000 and 20,000 years ago, which correspond broadly with those estimated for Native Americans. The parallel timing of demographic expansions of human lice and Native Americans plus the contrasting pattern between the distribution of haplogroups A and B through the Americas suggests that human lice can provide additional evidence about the human colonization of the New World. (C) 2013 Wiley Periodicals, Inc. | 1769 | no | gd of parasite | NA | no | no |
| 1770 | 2017 | 43021 | Bailey, AL; Lauck, M; Weiler, A; Sibley, SD; Dinis, JM; Bergman, Z; Nelson, CW; Correll, M; Gleicher, M; Hyeroba, D; Tumukunde, A; Weny, G; Chapman, C; Kuhn, JH; Hughes, AL; Friedrich, TC; Goldberg, TL; O’Connor, DH | 2014 | High Genetic Diversity and Adaptive Potential of Two Simian Hemorrhagic Fever Viruses in a Wild Primate Population | Key biological properties such as high genetic diversity and high evolutionary rate enhance the potential of certain RNA viruses to adapt and emerge. Identifying viruses with these properties in their natural hosts could dramatically improve disease forecasting and surveillance. Recently, we discovered two novel members of the viral family Arteriviridae: simian hemorrhagic fever virus (SHFV)-krc1 and SHFV-krc2, infecting a single wild red colobus (Procolobus rufomitratus tephrosceles) in Kibale National Park, Uganda. Nearly nothing is known about the biological properties of SHFVs in nature, although the SHFV type strain, SHFV-LVR, has caused devastating outbreaks of viral hemorrhagic fever in captive macaques. Here we detected SHFV-krc1 and SHFV-krc2 in 40% and 47% of 60 wild red colobus tested, respectively. We found viral loads in excess of 10(6)-10(7) RNA copies per milliliter of blood plasma for each of these viruses. SHFV-krc1 and SHFV-krc2 also showed high genetic diversity at both the inter-and intra-host levels. Analyses of synonymous and non-synonymous nucleotide diversity across viral genomes revealed patterns suggestive of positive selection in SHFV open reading frames (ORF) 5 (SHFV-krc2 only) and 7 (SHFV-krc1 and SHFV-krc2). Thus, these viruses share several important properties with some of the most rapidly evolving, emergent RNA viruses. | 1770 | no | gd of parasite | NA | no | no |
| 1771 | 2017 | 43021 | Massonnet, B; Simon, JC; Weisser, WW | 2002 | Metapopulation structure of the specialized herbivore Macrosiphoniella tanacetaria (Homoptera, Aphididae) | We investigated population dynamics, genetic diversity and spatial structure in the aphid species Macrosiphoniella tanacetaria, a specialist herbivore feeding on tansy, Tanacetum vulgare. Tansy plants (genets) consist of many shoots (ramets), and genets are grouped in sites. Thus, aphids feeding on tansy can cluster at the level of ramets, genets and sites. We studied aphid population dynamics in 1997 and 2001 and found that within sites: (i) at any time, aphids used only a fraction of the available ramets and genets; (ii) at the level of ramets, most aphid colonies survived only one week; (iii) at the level of genets, mean survival time was less than 4 weeks; and (iv) colonization and extinction events occurred throughout the season. We sampled aphids in seven sites in the Alsace region, France (4-45 km apart) and two sites in Germany in 1999 to study genetic structure within and between populations. Genetic analyses using nine microsatellite loci showed that: (i) genotypic variability was high, (ii) none of the populations was in Hardy-Weinberg equilibrium, (iii) heterozygote deficits and linkage disequilibria were frequent, and (iv) all populations were genetically differentiated, even at a small geographical scale. Renewed sampling of the Alsace sites in 2001 showed that three populations had become extinct and significant genetic changes had occurred in the remaining four populations. The frequencies of extinction and colonization events at several spatial scales suggest a hierarchical metapopulation structure for M. tanacetaria. Frequent population turnover and drift are likely causes for the genetic differentiation of M. tanacetaria populations. | 1771 | no | no good fitness measure | NA | no | no |
| 1772 | 2017 | 43021 | Shen, YJ; Guan, LH; Wang, DQ; Gan, XN | 2016 | DNA barcoding and evaluation of genetic diversity in Cyprinidae fish in the midstream of the Yangtze River | The Yangtze River is the longest river in China and is divided into upstream and mid-downstream regions by the Three Gorges (the natural barriers of the Yangtze River), resulting in a complex distribution of fish. Dramatic changes to habitat environments may ultimately threaten fish survival; thus, it is necessary to evaluate the genetic diversity and propose protective measures. Species identification is the most significant task in many fields of biological research and in conservation efforts. DNA barcoding, which constitutes the analysis of a short fragment of the mitochondrial cytochrome c oxidase subunit I (COI) sequence, has been widely used for species identification. In this study, we collected 561 COI barcode sequences from 35 fish from the midstream of the Yangtze River. The intraspecific distances of all species were below 2% (with the exception of Acheilognathus macropterus and Hemibarbus maculatus). Nevertheless, all species could be unambiguously identified from the trees, barcoding gaps and taxonomic resolution ratio values. Furthermore, the COI barcode diversity was found to be low (<= 0.5%), with the exception of H. maculatus (0.87%), A. macropterus (2.02%) and Saurogobio dabryi (0.82%). No or few shared haplotypes were detected between the upstream and downstream populations for ten species with overall nucleotide diversities greater than 0.00%, which indicated the likelihood of significant population genetic structuring. Our analyses indicated that DNA barcoding is an effective tool for the identification of cyprinidae fish in the midstream of the Yangtze River. It is vital that some protective measures be taken immediately because of the low COI barcode diversity. | 1772 | no | no good fitness measure, N pop differnt in GD? | NA | no | no |
| 1773 | 2017 | 43021 | Lallawmsanga; Passari, AK; Mishra, VK; Leo, VV; Singh, BP; Meyyappan, GV; Gupta, VK; Uthandi, S; Upadhyay, RC | 2016 | Antimicrobial Potential, Identification and Phylogenetic Affiliation of Wild Mushrooms from Two Sub-Tropical Semi-Evergreen Indian Forest Ecosystems | The diversity of wild mushrooms was investigated from two protected forest areas in India and 231 mushroom specimens were morphologically identified. Among them, 76 isolates were screened for their antimicrobial potential against seven bacterial and fungal pathogens. Out of 76 isolates, 45 isolates which displayed significant antimicrobial activities were identified using ITS rRNA gene amplification and subsequently phylogenetically characterized using random amplified polymorphic DNA (RAPD) and inter-simple sequence repeat (ISSR) markers. Sequencing of the ITS rRNA region classified the isolates into 16 genera belonging to 11 families. In total, 11 RAPD and 10 ISSR primers were selected to evaluate genetic diversity based on their banding profile produced. In total 337 RAPD and 312 ISSR bands were detected, among which percentage of polymorphism ranges from 34.2% to 78.8% and 38.6% to 92.4% by using RAPD and ISSR primers respectively. Unweighted Pair-Group Method with Arithmetic Mean (UPGMA) trees of selected two methods were structured similarly, grouping the 46 isolates into two clusters which clearly showed a significant genetic distance among the different strains of wild mushroom, with an similarity coefficient ranges from 0.58 to 1.00 and 0.59 to 1.00 with RAPD and ISSR analysis respectively. This reporthas highlighted both DTR and MNP forests provide a habitat for diverse macrofungal species, therefore having the potential to be used for the discovery of antimicrobials. The report has also demonstrated that both RAPD and ISSR could efficiently differentiate wild mushrooms and could thus be considered as efficient markers for surveying genetic diversity. Additionally, selected six wild edible mushroom strains (Schizophyllum commune BPSM01, Panusgiganteus BPSM27, Pleurotussp. BPSM34, Lentinussp. BPSM37, Pleurotusdjamor BPSM41 and Lentinula sp. BPSM45) were analysed for their nutritional (proteins, carbohydrates, fat and ash content), antioxidant potential. The present findings also suggested that the wild edible mushroom strains do not have only nutritional values but also can be used as an accessible source of natural antioxidants. | 1773 | no | no good fitness measure | NA | no | no |
| 1774 | 2017 | 43021 | Morgan, JAT; Dejong, RJ; Adeoye, GO; Ansa, EDO; Barbosa, CS; Bremond, P; Cesari, IM; Charbonnel, N; Correa, LR; Coulibaly, G; D’Andrea, PS; De Souza, CP; Doenhoff, MJ; File, S; Idris, MA; Incani, RN; Jarne, P; Karanja, DMS; Kazibwe, F; Kpikpi, J; Lwambo, NJS; Mabaye, A; Magalhaes, LA; Makundi, A; Mone, H; Mouahid, G; Muchemi, GM; Mungai, BN; Sene, M; Southgate, V; Tchuente, LAT; Theron, A; Yousif, F; Magalhaes, EMZ; Mkoji, GM; Loker, ES; Loker, ES | 2005 | Origin and diversification of the human parasite Schistosoma mansoni | Schistosoma mansoni is the most widespread of the human-infecting schistosomes, present in 54 countries, predominantly in Africa, but also in Madagascar, the Arabian Peninsula, and the Neotropics. Adult-stage parasites that infect humans are also occasionally recovered from baboons, rodents, and other mammals. Larval stages of the parasite are dependent upon certain species of freshwater snails in the genus Biomphalaria, which largely determine the parasite’s geographical range. How S. mansoni genetic diversity is distributed geographically and among isolates using different hosts has never been examined with DNA sequence data. Here we describe the global phylogeography of S. mansoni using more than 2500 bp of mitochondrial DNA (mtDNA) from 143 parasites collected in 53 geographically widespread localities. Considerable within-species mtDNA diversity was found, with 85 unique haplotypes grouping into five distinct lineages. Geographical separation, and not host use, appears to be the most important factor in the diversification of the parasite. East African specimens showed a remarkable amount of variation, comprising three clades and basal members of a fourth, strongly suggesting an East African origin for the parasite 0.30-0.43 million years ago, a time frame that follows the arrival of its snail host. Less but still substantial variation was found in the rest of Africa. A recent colonization of the New World is supported by finding only seven closely related New World haplotypes which have West African affinities. All Brazilian isolates have nearly identical mtDNA haplotypes, suggesting a founder effect from the establishment and spread of the parasite in this large country. | 1774 | no | gd of parasite | NA | no | no |
| 1775 | 2017 | 43021 | Carretero-Pinzon, X; Defler, TR; McAlpine, CA; Rhodes, JR | 2016 | What do we know about the effect of patch size on primate species across life history traits? | Habitat loss and fragmentation are significant threats for primate species worldwide. However, few attempts have been made to look for general patterns in primate responses to habitat loss and fragmentation, or whether these may be associated with species’ traits. We conducted a review of published literature on effects of patch size to quantify the effect of a reduction in this predictor on primates, and to determine whether these effects depend on species’ traits. The effects of patch size on seven response variables (density, parasite prevalence and diversity, presence, genetic diversity, time spent feeding, resting and movement), were extracted from 135 papers and compared across six species traits (diet specialisation, social structure, body size, home range size, group size and dispersal ability). We found that density, parasitic prevalence and diversity and time spent feeding were positively associated with a reduction in patch size, while species’ presence and genetic diversity were negatively associated. Time spent resting and moving did not show clear patterns. We found little evidence that the effect of patch size varies consistently with traits. This study provides important evidence for the consistent effect of patch size on a range of factors that influence the dynamics of primate populations. However, there is a need to move beyond quantifying patch size effects alone and to quantify the effects of changes occurring at broader landscape scales. This would allow more holistic primate conservation strategies to be developed across whole landscapes rather than being focussed on the management of individual patches. | 1775 | probably no | parasite prevalience - of groups of different GD - possible from here? | no good fitness measure of pop of different GD | no | no |
| 1776 | 2017 | 43021 | Dalmon, A; Halkett, F; Granier, M; Delatte, H; Peterschmitt, M | 2008 | Genetic structure of the invasive pest Bemisia tabaci: evidence of limited but persistent genetic differentiation in glasshouse populations | The geographic range of plant pests can be modified by the use of glasshouses. Bemisia tabaci, originating from warm to hot climates, has been shown to be a complex of distinct genetic groups with very limited gene flow. The genetic structure of this pest was studied in glasshouses in southern France, a region beyond the northern limit of its open-field development area in Europe. Seven microsatellite loci were scored in 22 populations sampled from various regions over 3 years. Two genetic groups were distinguished using a Bayesian clustering method and were assigned to the so-called biotypes B and Q using the gene sequence of cytochrome oxidase 1 (CO1). All but one population corresponded to biotype Q, even though only biotype B was previously reported. Despite the enclosed environment of glasshouses and their expected isolation due to low outdoor survival during the winter, only limited differentiation among biotype Q glasshouses was observed. A single sample site was notable for a decrease in expected heterozygosity and the mean number of alleles over the years. The lack of spatial genetic structure among biotype Q populations was indicative of a recent colonization event combined with large dispersal at all spatial scales. This migration pattern of biotype Q populations was further supported by additional CO1 sequences, since individuals from France, Asia and America exhibited 100% nucleotide identity. The evolution of genetic diversity observed in glasshouses in France is part of the worldwide invasion of biotype Q, which is discussed in light of human activities. | 1776 | no | no good fitness measure | NA | no | no |
| 1777 | 2017 | 43021 | GIRMAN, DJ; WAYNE, RK; KAT, PW; MILLS, MGL; GINSBERG, JR; BORNER, M; WILSON, V; FANSHAWE, JH; FITZGIBBON, C; LAU, LM | 1993 | MOLECULAR-GENETIC AND MORPHOLOGICAL ANALYSES OF THE AFRICAN WILD DOG (LYCAON-PICTUS) | African wild dog populations have declined precipitously during the last 100 years in eastern Africa. The possible causes of this decline include a reduction in prey abundance and habitat; disease; and loss of genetic variability accompanied by inbreeding depression. We examined the levels of genetic variability and distinctiveness among populations of African wild dogs using mitochondrial DNA (mtDNA) restriction site and sequence analyses and multivariate analysis of cranial and dental measurements. Our results indicate that the genetic variability of eastern African wild dog populations is comparable to that of southern Africa and similar to levels of variability found in other large canids. Southern and eastern populations of wild dogs show about 1% divergence in mtDNA sequence and form two monophyletic assemblages containing three mtDNA genotypes each. No genotypes are shared between the two regions. With one exception, all wild dogs examined from toes had southern African genotypes. Morphological analysis supports the distinction of eastern and southern African wild dog populations, and we suggest they should be considered separate subspecies. An eastern African wild dog breeding program should be initiated to ensure preservation of the eastern African form and to slow the loss of genetic variability that, while not yet apparent, will inevitably occur if wild populations continue to decline. Finally, we examined the phylogenetic relationships of wild dogs to other wolf-like canids through analysis of 736 base pairs (bp) of cytochrome b sequence and showed wild dogs to belong to a phylogenetically distinct lineage of the wolf-like canids. | 1777 | no | no good fitness measure | NA | no | no |
| 1778 | 2017 | 43021 | Handley, LJL; Estoup, A; Evans, DM; Thomas, CE; Lombaert, E; Facon, B; Aebi, A; Roy, HE | 2011 | Ecological genetics of invasive alien species | There is growing realisation that integrating genetics and ecology is critical in the context of biological invasions, since the two are explicitly linked. So far, the focus of ecological genetics of invasive alien species (IAS) has been on determining the sources and routes of invasions, and the genetic make-up of founding populations, which is critical for defining and testing ecological and evolutionary hypotheses. However an ecological genetics approach can be extended to investigate questions about invasion success and impacts on native, recipient species. Here, we discuss recent progress in the field, provide overviews of recent methodological advances, and highlight areas that we believe are of particular interest for future research. First, we discuss the main insights from studies that have inferred source populations and invasion routes using molecular genetic data, with particular focus on the role of genetic diversity, adaptation and admixture in invasion success. Second, we consider how genetic tools can lead to a better understanding of patterns of dispersal, which is critical to predicting the spread of invasive species, and how studying invasions can shed light on the evolution of dispersal. Finally, we explore the potential for combining molecular genetic data and ecological network modelling to investigate community interactions such as those between predator and prey, and host and parasite. We conclude that invasions are excellent model systems for understanding the role of natural selection in shaping phenotypes and that an ecological genetics approach offers great potential for addressing fundamental questions in invasion biology. | 1778 | no | no good fitness measure, N pop differnt in GD? | NA | no | no |
| 1779 | 2017 | 43021 | Ziegler, A; Bohossian, N; Diego, VP; Yao, C | 2014 | Genetic Prediction in the Genetic Analysis Workshop 18 Sequencing Data | High-throughput sequencing data can be used to predict phenotypes from genotypes, and this corresponds to establishing a prognostic model. In extended pedigrees the relatedness of subjects provides additional information so that genetic values, fixed or random genetic components, and heritability can be estimated. At the Genetic Analysis Workshop 18, the working group on genetic prediction dealt with both establishing a prognostic model and, in one contribution, comparing standard logistic regression with robust logistic regression in a sample of unrelated affected or unaffected individuals. Results of both logistic regression approaches were similar. All other contributions to this group used extended family data, in general using the quantitative trait blood pressure. The individual contributions varied in several important aspects, such as the estimation of the kinship matrix and the estimation method. Contributors chose various approaches for model validation, including different versions of cross-validation or within-family validation. Within-family validation included model building in the upper generations and validation in later generations. The choice of the statistical model and the computational algorithm had substantial effects on computation time. If decorrelation approaches were applied, the computational burden was substantially reduced. Some software packages estimated negative eigenvalues, although eigenvalues of correlation matrices should be non-negative. Most statistical models and software packages have been developed for experimental crosses and planned breeding programs. With their specialized pedigree structures, they are not sufficiently flexible to accommodate the variability of human pedigrees in general, and improved implementations are required. (C) 2014 Wiley Periodicals, Inc. | 1779 | no | no good fitness measure, N pop differnt in GD? | NA | no | no |
| 1780 | 2017 | 43021 | Zhu, XF; Feng, T; Tayo, BO; Liang, JJ; Young, JH; Franceschini, N; Smith, JA; Yanek, LR; Sun, YV; Edwards, TL; Chen, W; Nalls, M; Fox, E; Sale, M; Bottinger, E; Rotimi, C; Liu, YM; McKnight, B; Liu, K; Arnett, DK; Chakravati, A; Cooper, RS; Redline, S | 2015 | Meta-analysis of Correlated Traits via Summary Statistics from GWASs with an Application in Hypertension | Genome-wide association studies (GWASs) have identified many genetic variants underlying complex traits. Many detected genetic loci harbor variants that associate with multiple-even distinct-traits. Most current analysis approaches focus on single traits, even though the final results from multiple traits are evaluated together. Such approaches miss the opportunity to systemically integrate the phenome-wide data available for genetic association analysis. In this study, we propose a general approach that can integrate association evidence from summary statistics of multiple traits, either correlated, independent, continuous, or binary traits, which might come from the same or different studies. We allow for trait heterogeneity effects. Population structure and cryptic relatedness can also be controlled. Our simulations suggest that the proposed method has improved statistical power over single-trait analysis in most of the cases we studied. We applied our method to the Continental Origins and Genetic Epidemiology Network (COGENT) African ancestry samples for three blood pressure traits and identified four loci (CHIC2, HOXA-EVX1, IGFBP1/IGFBP3, and CDH17; p < 5.0 x 10(-8)) associated with hypertension-related traits that were missed by a single-trait analysis in the original report. Six additional loci with suggestive association evidence (p < 5.0 x 10(-7)) were also observed, including CACNA1D and WNT3. Our study strongly suggests that analyzing multiple phenotypes can improve statistical power and that such analysis can be executed with the summary statistics from GWASs. Our method also provides a way to study a cross phenotype (CP) association by using summary statistics from GWASs of multiple phenotypes. | 1780 | no | no good fitness measure, N pop differnt in GD? | NA | no | no |
| 1781 | 2017 | 43021 | Langeloh, L; Behrmann-Godel, J; Seppala, O | 2017 | Natural selection on immune defense: A field experiment | Predicting the evolution of phenotypic traits requires an understanding of natural selection on them. Despite its indispensability in the fight against parasites, selection on host immune defense has remained understudied. Theory predicts immune traits to be under stabilizing selection due to associated trade-offs with other fitness-related traits. Empirical studies, however, report mainly positive directional selection. This discrepancy could be caused by low phenotypic variation in the examined individuals and/or variation in host resource level that confounds trade-offs in empirical studies. In a field experiment where we maintained Lymnaea stagnalis snails individually in cages in a lake, we investigated phenotypic selection on two immune defense traits, phenoloxidase (PO)-like activity and antibacterial activity, in hemolymph. We used a diverse laboratory population and manipulated snail resource level by limiting their food supply. For six weeks, we followed immune activity, growth, and two fitness components, survival and fecundity of snails. We found that PO-like activity and growth were under stabilizing selection, while antibacterial activity was under positive directional selection. Selection on immune traits was mainly driven by variation in survival. The form of selection on immune defense apparently depends on the particular trait, possibly due to its importance for countering the present parasite community. | 1781 | no | no good fitness measure, N pop differnt in GD? | NA | no | no |
| 1782 | 2017 | 43021 | Stassen, HH; Scharfetter, C | 2001 | Oligogenic approaches to the predisposition of asthma in ethnically diverse populations | “Using the genome-wide screening data of the Collaborative Study on the Genetics of Asthma (CSGA) (226 families, 1,461 genotyped subjects, 323 marker loci) and Hutterite studies (129 families, 690 genotyped subjects, 365 marker loci), we applied a genetic similarity function in order to quantify the inter-individual genetic distances d(x(i),x(j)) between feature vectors x(i), xj made up by the allelic patterns of subjects i, j with respect to loci l(1), l(2), …,, l(n). Based on this similarity function, we structurally decomposed the genetic diversity of the CSGA population in order to address the question of ethnicity-related asthma vulnerability for genetically homogenous CSGA subgroups. The question of ethnicity-independent asthma vulnerability was investigated with all CSGA families as training and the Hutterite families as replication samples. We evaluated the between-sib similarities, which were expected to deviate from”“0.5”" in affected sib pairs if the region of interest contained markers close to disease-causing genes. The reference value 0.5 was derived by determining the parent-offspring similarities, which are always 0.5, irrespective of the affection status of parents and offspring. We found 18 vulnerability loci on chromosomes 1, 3, 4, 5, 6, 8, 12, 13, and 14, which were remarkably reproducible in the CSGA and the Hutterite data and constituted an ethnicity-independent oligogenic model. ((C))2001 Wiley-Liss, Inc." | 1782 | no | NA | NA | no | no |
| 1783 | 2017 | 43021 | Lee, KE; Seddon, JM; Corley, SW; Ellis, WAH; Johnston, SD; de Villiers, DL; Preece, HJ; Carrick, FN | 2010 | Genetic variation and structuring in the threatened koala populations of Southeast Queensland | Habitat fragmentation can act to cause reproductive isolation between conspecifics and undermine species’ persistence, though most studies have reported the genetic condition of populations that have already declined to a very small size. We examined genetic diversity within the vulnerable, declining koala (Phascolarctos cinereus) population in Southeast Queensland, Australia to determine the genetic impact of ongoing threatening processes. Five hundred and twelve koalas from ten Southeast Queensland Local Government Areas on the mainland and one island were genotyped at six polymorphic microsatellite loci. Based on Bayesian cluster analysis incorporating spatial data, the regional koala population was subdivided into six clusters, with location of major roads and rivers appearing to be consistent with being barriers to gene flow. The distribution of mtDNA control region haplotypes identified distinct coastal and inland clades suggesting that historically there was gene flow between koalas along the coast (though little interchange between coastal and inland animals). In contrast, koalas from the Koala Coast (Brisbane City, Logan City and Redland Shire) were shown by microsatellite analysis to be genetically distinct from adjacent areas. It is likely, therefore, that more recent reductions in population size and restricted gene flow through urbanisation have contributed to the genetic differentiation of koalas in the Koala Coast region. | 1783 | no | no good fitness measure | NA | no | no |
| 1784 | 2017 | 43021 | Bauduer, F; Degioanni, A; Dutour, O | 2009 | Medical Genetic Polymorphisms as Markers of Evolutionary Forces Within the Human Genome: Hypotheses Focusing on Natural Selection in the Basque Population | Natural selection, drift, and gene flow are the three major evolutionary forces at the origin of genetic diversity among human populations. To further explore these mechanisms, we present an innovative approach using various medical genetic markers and focusing on the Basque population. From this study we can confirm the important role of drift in this endogamous human group and can report some disorders related to founder effects. Most important, the peculiar distribution of various polymorphisms, such as blood group O, factor V Leiden, DF508, C282Y, and CCR5 D32 mutations, which are implicated in resistance to infection, hemostasis, or iron conservation, could be interpreted as an adaptive profile. Multidisciplinary data have shown that the Neolithic period arrived significantly later in this southwestern corner of Europe. We hypothesize that the long-lasting Paleolithic mode of life, especially regarding nutrition and microbial exposure, was at the origin of this selective pressure within this population of ancient local ancestry. This approach could open new avenues in the field of population genetics. | 1784 | no | human | NA | no | no |
| 1785 | 2017 | 43021 | Isagi, Y; Saito, D; Kawaguchi, H; Tateno, R; Watanabe, S | 2007 | Effective pollen dispersal is enhanced by the genetic structure of an Aesculus turbinata population | 1. Studies using highly informative genetic markers (e.g. microsatellites) have revealed effective movement of genes through pollen grains from more distant sources into offspring than those inferred on the basis of physical pollen movement alone. In order to identify the factors that cause such effective pollen movement in tree populations, analyses of the genetic structure of reproductive trees and parentage analyses for seedlings and saplings were conducted in a 110-ha population of Aesculus turbinata, a tall deciduous tree species that is pollinated by insects and has large seeds that are dispersed by gravity and small mammals. 2. By analysing genotypes of seed coats, which are identical to those of the seed parents, and of leaves from seedlings, pollen donors and seed parents were identified for seedlings. Differences in size and dispersal mode between pollen and seeds caused a significant difference in dispersal distances. Reflecting the restricted seed dispersal (25.3 m on average), there was significant positive relatedness between reproductive trees standing within 150 m of each other. 3. The percentage of self-pollinated offspring decreased from 8.3% at the seedling stage to 0.56% at the sapling stage. The disappearance of out-crossed individuals during the stage between seedling and sapling seemed to be associated with the spatial distance separating their parents: the proportion of juvenile trees that died was higher for those that originated from out-crosses between adults standing within 100 m of each other, where positive relatedness was found between adult trees. Consequently, the deaths of individuals arising from self-pollination events and from crosses between reproductive trees that were spatially adjacent caused longer effective pollen movement for the offspring that survived. The distance between the two parents of a tree, which indicates the distance of effective pollen movement, increased from 179.9 m for seedlings to 288.9 m for saplings. 4. The genetic structure of the adult population of A. turbinata affected the survival of offspring through biparental inbreeding depression, and, then, removal of individuals resulting from crosses between related adults in turn promoted effective pollination over longer distances. | 1785 | no | only one pop | NA | no | no |
| 1786 | 2017 | 43021 | Kapoor, A; Mehta, N; Esper, F; Poljsak-Prijatelj, M; Quan, PL; Qaisar, N; Delwart, E; Lipkin, WI | 2010 | Identification and Characterization of a New Bocavirus Species in Gorillas | A novel parvovirus, provisionally named Gorilla Bocavirus species 1 (GBoV1), was identified in four stool samples from Western gorillas (Gorilla gorilla) with acute enteritis. The complete genomic sequence of the new parvovirus revealed three open reading frames (ORFs) with an organization similar to that of known bocaviruses. Phylogenetic analysis using complete capsid and non structural (NS) gene sequence suggested that the new parvovirus is most closely related to human bocaviruses (HBoV). However, the NS ORF is more similar in length to the NS ORF found in canine minute virus and bovine parvovirus than in HBoV. Comparative genetic analysis using GBoV and HBoV genomes enabled characterization of unique splice donor and acceptor sites that appear to be highly conserved among all four HBoV species, and provided evidence for expression of two different NS proteins in all primate bocaviruses. GBoV is the first non-human primate bocavirus identified and provides new insights into the genetic diversity and evolution of this highly prevalent and recently discovered group of parvoviruses. | 1786 | no | gd of pathogen | NA | no | no |
| 1787 | 2017 | 43021 | Hermisson, J; Pennings, PS | 2017 | Soft sweeps and beyond: understanding the patterns and probabilities of selection footprints under rapid adaptation | 1. The tempo and mode of adaptive evolution determine how natural selection shapes patterns of genetic diversity in DNA polymorphism data. While slow mutation-limited adaptation leads to classical footprints of ‘hard’ selective sweeps, these patterns are different when adaptation responds quickly to a novel selection pressure, acting either on standing genetic variation or on recurrent new mutation. In the past decade, corresponding footprints of ‘soft’ selective sweeps have been described both in theoretical models and in empirical data. 2. Here, we summarize the key theoretical concepts and contrast model predictions with observed patterns in Drosophila, humans, and microbes. 3. Evidence in all cases shows that ‘soft’ patterns of rapid adaptation are frequent. However, theory and data also point to a role of complex adaptive histories in rapid evolution. 4. While existing theory allows for important implications on the tempo and mode of the adaptive process, complex footprints observed in data are, as yet, insufficiently covered by models. They call for in-depth empirical study and further model development. | 1787 | no | no good fitness measure, N pop differnt in GD? | NA | no | no |
| 1788 | 2017 | 43021 | Kanzi, AM; Wingfield, BD; Steenkamp, ET; Naidoo, S; van der Merwe, NA | 2016 | Intron Derived Size Polymorphism in the Mitochondrial Genomes of Closely Related Chrysoporthe Species | In this study, the complete mitochondrial (mt) genomes of Chrysoporthe austroafricana (190,834 bp), C. cubensis (89,084 bp) and C. deuterocubensis (124,412 bp) were determined. Additionally, the mitochondrial genome of another member of the Cryphonectriaceae, namely Cryphonectria parasitica (158,902 bp), was retrieved and annotated for comparative purposes. These genomes showed high levels of synteny, especially in regions including genes involved in oxidative phosphorylation and electron transfer, unique open reading frames (uORFs), ribosomal RNAs (rRNAs) and transfer RNAs (tRNAs), as well as intron positions. Comparative analyses revealed signatures of duplication events, intron number and length variation, and varying intronic ORFs which highlighted the genetic diversity of mt genomes among the Cryphonectriaceae. These mt genomes showed remarkable size polymorphism. The size polymorphism in the mt genomes of these closely related Chrysoporthe species was attributed to the varying number and length of introns, coding sequences and to a lesser extent, intergenic sequences. Compared to publicly available fungal mt genomes, the C. austroafricana mt genome is the second largest in the Ascomycetes thus far. | 1788 | no | no good fitness measure, N pop differnt in GD? | NA | no | no |
| 1789 | 2017 | 43021 | Pemberton, TJ; Absher, D; Feldman, MW; Myers, RM; Rosenberg, NA; Li, JZ | 2012 | Genomic Patterns of Homozygosity in Worldwide Human Populations | Genome-wide patterns of homozygosity runs and their variation across individuals provide a valuable and often untapped resource for studying human genetic diversity and evolutionary history. Using genotype data at 577,489 autosomal SNPs, we employed a likelihood-based approach to identify runs of homozygosity (ROH) in 1,839 individuals representing 64 worldwide populations, classifying them by length into three classes-short, intermediate, and long-with a model-based clustering algorithm. For each class, the number and total length of ROH per individual show considerable variation across individuals and populations. The total lengths of short and intermediate ROH per individual increase with the distance of a population from East Africa, in agreement with similar patterns previously observed for locus-wise homozygosity and linkage disequilibrium. By contrast, total lengths of long ROH show large interindividual variations that probably reflect recent inbreeding patterns, with higher values occurring more often in populations with known high frequencies of consanguineous unions. Across the genome, distributions of ROH are not uniform, and they have distinctive continental patterns. ROH frequencies across the genome are correlated with local genomic variables such as recombination rate, as well as with signals of recent positive selection. In addition, long ROH are more frequent in genomic regions harboring genes associated with autosomal-dominant diseases than in regions not implicated in Mendelian diseases. These results provide insight into the way in which homozygosity patterns are produced, and they generate baseline homozygosity patterns that can be used to aid homozygosity mapping of genes associated with recessive diseases. | 1789 | no | human | NA | no | no |
| 1790 | 2017 | 43021 | Pearson, SK; Godfrey, SS; Schwensow, N; Bull, CM; Gardner, MG | 2017 | Genes and Group Membership Predict Gidgee Skink (Egernia stokesii) Reproductive Pairs | Due to their role in mate choice, disease resistance and kin recognition, genes of the major histocompatibility complex (MHC) are good candidates for investigating genetic-based mate choice. MHC-based mate choice is context dependent and influenced by many factors including social structure. Social structure diversity makes the Egernia group of lizards suitable for comparative studies of MHC-based mate choice. We investigated mate choice in the gidgee skink (Egernia stokesii), a lizard that exhibits high levels of social group and spatial stability. Group membership was incorporated into tests of the good genes as heterozygosity and compatible genes hypotheses for adaptive (MHC) and neutral (microsatellite) genetic diversity (n = 47 individuals genotyped). Females were more likely to pair with a male with higher MHC diversity and with whom they had a lower degree of microsatellite relatedness. Males were more likely to pair with a female with higher microsatellite heterozygosity and with whom they shared a lower proportion of MHC alleles. Lizards were more likely to mate with an individual from within, rather than outside, their social group, which confirmed earlier findings for this species and indicated mate choice had already largely occurred prior to either social group formation or acceptance of an individual into an existing group. Thus, a combination of genes and group membership, rather than group membership alone, predicted mate choice in this species. This work will contribute to an enhanced understanding of squamate group formation and a deeper understanding of the evolution of sociality within all vertebrates. | 1790 | no | no good fitness measure, N pop differnt in GD? | NA | no | no |
| 1791 | 2017 | 43021 | Yu, TL; Deng, XH; Busam, M; Song, Y | 2017 | Does relatedness influence the intensity of competition in Bufo gargarizans minshanicus tadpoles? | Kin selection theory predicts that mechanisms should evolve to reduce kin competition when this maximizes inclusive fitness. In this study, we tested these predictions by investigating the effects of relatedness on fitness-related metamorphic traits (e.g., length of larval period, size at metamorphosis, body condition and survival rate). We did this in a laboratory experiment by exposing individuals of Bufo gargarizans minshanicus to competition with full-sibling or non-sibling larvae. Although tadpoles seemed to grow slightly better in environments when their competition consisted of fullsiblings than when their competitors consisted of non-siblings, these effects of relatedness on the growth and development were not significant. Therefore, we suggest that interference competition may be equally intense in in full-sibling and non-sibling groups. | 1791 | yes | no good gd measure host pop | Group size 2: minimum 3! | no | no |
| 1792 | 2017 | 43021 | WILKIE, TM; GILBERT, DJ; OLSEN, AS; CHEN, XN; AMATRUDA, TT; KORENBERG, JR; TRASK, BJ; DEJONG, P; REED, RR; SIMON, MI; JENKINS, NA; COPELAND, NG | 1992 | EVOLUTION OF THE MAMMALIAN G-PROTEIN ALPHA-SUBUNIT MULTIGENE FAMILY | Heterotrimeric guanine nucleotide binding proteins (G proteins) transduce extracellular signals received by transmembrane receptors to effector proteins. The multigene family of G protein alpha-subunits, which interact with receptors and effectors, exhibit a high level of sequence diversity. In mammals, 15 G-alpha subunit genes can be grouped by sequence and functional similarities into four classes. We have determined the murine chromosomal locations of all 15 G-alpha subunit genes using an interspecific backcross derived from crosses of C57BL/6J and Mus spretus mice. These data, in combination with mapping studies in humans, have provided insight into the events responsible for generating the genetic diversity found in the mammalian alpha-subunit genes and a framework for elucidating the role of the G-alpha subunits in disease. | 1792 | no | no good fitness measure, N pop differnt in GD? | NA | no | no |
| 1793 | 2017 | 43021 | Wickert, E; Lemos, EGD; Kishi, LT; de Souza, A; de Goes, A | 2012 | “Genetic Diversity and Population Differentiation of Guignardia mangiferae from”“Tahiti”" Acid Lime" | “Among the citrus plants,”“Tahiti”" acid lime is known as a host of G. mangiferae fungi. This species is considered endophytic for citrus plants and is easily isolated from asymptomatic fruits and leaves. G. mangiferae is genetically related and sometimes confused with G. citricarpa which causes Citrus Black Spot (CBS). "“Tahiti”" acid lime is one of the few species that means to be resistant to this disease because it does not present symptoms. Despite the fact that it is commonly found in citric plants, little is known about the populations of G. mangiferae associated with these plants. Hence, the objective of this work was to gain insights about the genetic diversity of the G. mangiferae populations that colonize "“Tahiti”" acid limes by sequencing cistron ITS1-5.8S-ITS2. It was verified that "“Tahiti”" acid lime plants are hosts of G. mangiferae and also of G. citricarpa, without presenting symptoms of CBS. Populations of G. mangiferae present low-to-moderate genetic diversity and show little-to-moderate levels of population differentiation. As gene flow was detected among the studied populations and they share haplotypes, it is possible that all populations, from citrus plants and also from the other known hosts of this fungus, belong to one great panmictic population." | 1793 | no | no good fitness measure | NA | no | no |
| 1794 | 2017 | 43021 | Mallez, S; Castagnone, C; Espada, M; Vieira, P; Eisenback, JD; Mota, M; Guillemaud, T; Castagnone-Sereno, P | 2013 | First Insights into the Genetic Diversity of the Pinewood Nematode in Its Native Area Using New Polymorphic Microsatellite Loci | The pinewood nematode, Bursaphelenchus xylophilus, native to North America, is the causative agent of pine wilt disease and among the most important invasive forest pests in the East-Asian countries, such as Japan and China. Since 1999, it has been found in Europe in the Iberian Peninsula, where it also causes significant damage. In a previous study, 94 pairs of microsatellite primers have been identified in silico in the pinewood nematode genome. In the present study, specific PCR amplifications and polymorphism tests to validate these loci were performed and 17 microsatellite loci that were suitable for routine analysis of B. xylophilus genetic diversity were selected. The polymorphism of these markers was evaluated on nematodes from four field origins and one laboratory collection strain, all originate from the native area. The number of alleles and the expected heterozygosity varied between 2 and 11 and between 0.039 and 0.777, respectively. First insights into the population genetic structure of B. xylophilus were obtained using clustering and multivariate methods on the genotypes obtained from the field samples. The results showed that the pinewood nematode genetic diversity is spatially structured at the scale of the pine tree and probably at larger scales. The role of dispersal by the insect vector versus human activities in shaping this structure is discussed. | 1794 | no | gd of parasite | NA | no | no |
| 1795 | 2017 | 43021 | Shirasawa, K; Isobe, S; Hirakawa, H; Asamizu, E; Fukuoka, H; Just, D; Rothan, C; Sasamoto, S; Fujishiro, T; Kishida, Y; Kohara, M; Tsuruoka, H; Wada, T; Nakamura, Y; Sato, S; Tabata, S | 2010 | SNP Discovery and Linkage Map Construction in Cultivated Tomato | Few intraspecific genetic linkage maps have been reported for cultivated tomato, mainly because genetic diversity within Solanum lycopersicum is much less than that between tomato species. Single nucleotide polymorphisms (SNPs), the most abundant source of genomic variation, are the most promising source of polymorphisms for the construction of linkage maps for closely related intraspecific lines. In this study, we developed SNP markers based on expressed sequence tags for the construction of intraspecific linkage maps in tomato. Out of the 5607 SNP positions detected through in silico analysis, 1536 were selected for high-throughput genotyping of two mapping populations derived from crosses between ‘Micro-Tom’ and either ‘Ailsa Craig’ or ‘M82’. A total of 1137 markers, including 793 out of the 1338 successfully genotyped SNPs, along with 344 simple sequence repeat and intronic polymorphism markers, were mapped onto two linkage maps, which covered 1467.8 and 1422.7 cM, respectively. The SNP markers developed were then screened against cultivated tomato lines in order to estimate the transferability of these SNPs to other breeding materials. The molecular markers and linkage maps represent a milestone in the genomics and genetics, and are the first step toward molecular breeding of cultivated tomato. Information on the DNA markers, linkage maps, and SNP genotypes for these tomato lines is available at http://www.kazusa.or.jp/tomato/. | 1795 | no | crop | NA | no | no |
| 1796 | 2017 | 43021 | Hersch, M; Peter, B; Kang, HM; Schupfer, F; Abriel, H; Pedrazzini, T; Eskin, E; Beckmann, JS; Bergmann, S; Maurer, F | 2012 | Mapping Genetic Variants Associated with Beta-Adrenergic Responses in Inbred Mice | beta-blockers and beta-agonists are primarily used to treat cardiovascular diseases. Inter-individual variability in response to both drug classes is well recognized, yet the identity and relative contribution of the genetic players involved are poorly understood. This work is the first genome-wide association study (GWAS) addressing the values and susceptibility of cardiovascular-related traits to a selective beta(1)-blocker, Atenolol (ate), and a beta-agonist, Isoproterenol (iso). The phenotypic dataset consisted of 27 highly heritable traits, each measured across 22 inbred mouse strains and four pharmacological conditions. The genotypic panel comprised 79922 informative SNPs of the mouse HapMap resource. Associations were mapped by Efficient Mixed Model Association (EMMA), a method that corrects for the population structure and genetic relatedness of the various strains. A total of 205 separate genome-wide scans were analyzed. The most significant hits include three candidate loci related to cardiac and body weight, three loci for electrocardiographic (ECG) values, two loci for the susceptibility of atrial weight index to iso, four loci for the susceptibility of systolic blood pressure (SBP) to perturbations of the beta-adrenergic system, and one locus for the responsiveness of QTc (p<10(-8)). An additional 60 loci were suggestive for one or the other of the 27 traits, while 46 others were suggestive for one or the other drug effects (p<10(-6)). Most hits tagged unexpected regions, yet at least two loci for the susceptibility of SBP to beta-adrenergic drugs pointed at members of the hypothalamic-pituitary-thyroid axis. Loci for cardiac-related traits were preferentially enriched in genes expressed in the heart, while 23% of the testable loci were replicated with datasets of the Mouse Phenome Database (MPD). Altogether these data and validation tests indicate that the mapped loci are relevant to the traits and responses studied. | 1796 | no | inbreeding | NA | no | no |
| 1797 | 2017 | 43021 | Ramo, A; Monteagudo, LV; Del Cacho, E; Sanchez-Acedo, C; Quilez, J | 2016 | Intra-Species Genetic Diversity and Clonal Structure of Cryptosporidium parvum in Sheep Farms in a Confined Geographical Area in Northeastern Spain | A multilocus fragment typing approach including eleven variable-number tandem-repeat (VNTR) loci and the GP60 gene was used to investigate the intra-farm and intra-host genetic diversity of Cryptosporidium parvum in sheep farms in a confined area in northeastern Spain. Genomic DNA samples of 113 C. parvum isolates from diarrheic pre-weaned lambs collected in 49 meat-type sheep farms were analyzed. Loci exhibited various degrees of polymorphism, the finding of 7-9 alleles in the four most variable and discriminatory markers (ML2, Cgd6\_5400, Cgd6\_3940, and GP60) being remarkable. The combination of alleles at the twelve loci identified a total of 74 multilocus subtypes (MLTs) and provided a Hunter-Gaston discriminatory index of 0.988 (95% CI, 0.979-0.996). The finding that most MLTs (n = 64) were unique to individual farms evidenced that cryptosporidial infection is mainly transmitted within sheep flocks, with herd-to-herd transmission playing a secondary role. Limited intra-host variability was found, since only five isolates were genotypically mixed. In contrast, a significant intra-farm genetic diversity was seen, with the presence of multiple MLTs on more than a half of the farms (28/46), suggesting frequent mutations or genetic exchange through recombination. Comparison with a previous study in calves in northern Spain using the same 12-loci typing approach showed differences in the identity of major alleles at most loci, with a single MLT being shared between lambs and calves. Analysis of evolutionary descent by the algorithm eBURST indicated a high degree of genetic divergence, with over 41% MLTs appearing as singletons along with a high number of clonal complexes, most of them linking only two MLTs. Bayesian Structure analysis and F statistics also revealed the genetic remoteness of most C. parvum isolates and no ancestral population size was chosen. Linkage analysis evidenced a prevalent pattern of clonality within the parasite population. | 1797 | no | domesticated animal | NA | no | no |
| 1798 | 2017 | 43021 | Cheddadi, R; Fady, B; Francois, L; Hajar, L; Suc, JP; Huang, K; Demarteau, M; Vendramin, GG; Ortu, E | 2009 | Putative glacial refugia of Cedrus atlantica deduced from Quaternary pollen records and modern genetic diversity | Aim To investigate the impact of past environmental changes on Cedrus atlantica and its current genetic diversity, and to predict its future distribution. Location Morocco, Algeria and Tunisia. Methods Eleven fossil pollen records from these three countries were used to locate putative glacial refugia and to reconstruct past climate changes. A mechanistic vegetation distribution model was used to simulate the distribution of C. atlantica in the year 2100. In addition, a genetic survey was carried out on modern Moroccan C. atlantica. Results Pollen records indicate that Cedrus was present during the last glacial period, probably in scattered refugia, in Tunisia, Algeria and Morocco. In the Tunisian and Algerian sites, cedar expanded during the late glacial and the early Holocene, then disappeared after c. 8000 yr bp. Reconstructed mean annual precipitation and January temperature show that the last glacial period in Morocco was cooler by 10-15 degrees C and drier by c. 300-400 mm year(-1) than the climate today. Modern chloroplast microsatellites of 15 C. atlantica populations in Morocco confirm the presence of multiple refugia and indicate that cedar recolonized the Moroccan mountains fairly recently. Model simulation indicates that by the year 2100 the potential distribution of C. atlantica will be much restricted with a potential survival area located in the High Atlas. Main conclusions Environmental changes in northern Africa since the last glacial period have had an impact on the geographical distribution of C. atlantica and on its modern genetic diversity. It is possible that by the end of this century C. atlantica may be unable to survive in its present-day locations. To preserve the species, we suggest that seedlings from modern C. atlantica populations located in the High Atlas mountains, where a high genetic diversity is found, be transplanted into the western High Atlas. | 1798 | no | no good fitness measure | NA | no | no |
| 1799 | 2017 | 43021 | Naegele, RP; Mitchell, J; Hausbeck, MK | 2016 | Genetic Diversity, Population Structure, and Heritability of Fruit Traits in Capsicum annuum | Cultivated pepper (Capsicum annuum) is a phenotypically diverse species grown throughout the world. Wild and landrace peppers are typically small-fruited and pungent, but contain many important traits such as insect and disease resistance. Cultivated peppers vary dramatically in size, shape, pungency, and color, and often lack resistance traits. Fruit characteristics (e.g. shape and pericarp thickness) are major determinants for cultivar selection, and their association with disease susceptibility can reduce breeding efficacy. This study evaluated a diverse collection of peppers for mature fruit phenotypic traits, correlation among fruit traits and Phytophthora fruit rot resistance, genetic diversity, population structure, and trait broad sense heritability. Significant differences within all fruit phenotype categories were detected among pepper lines. Fruit from Europe had the thickest pericarp, and fruit from Ecuador had the thinnest. For fruit shape index, fruit from Africa had the highest index, while fruit from Europe had the lowest. Five genetic clusters were detected in the pepper population and were significantly associated with fruit thickness, end shape, and fruit shape index. The genetic differentiation between clusters ranged from little to very great differentiation when grouped by the predefined categories. Broad sense heritability for fruit traits ranged from 0.56 (shoulder height) to 0.98 (pericarp thickness). When correlations among fruit phenotypes and fruit disease were evaluated, fruit shape index was negatively correlated with pericarp thickness, and positively correlated with fruit perimeter. Pepper fruit pericarp, perimeter, and width had a slight positive correlation with Phytophthora fruit rot, whereas fruit shape index had a slight negative correlation. | 1799 | no | crop | NA | no | no |
| 1800 | 2017 | 43021 | Hofinger, BJ; Russell, JR; Bass, CG; Baldwin, T; Dos Reis, M; Hedley, PE; Li, YD; Macaulay, M; Waugh, R; Hammond-Kosack, KE; Kanyuka, K | 2011 | An exceptionally high nucleotide and haplotype diversity and a signature of positive selection for the eIF4E resistance gene in barley are revealed by allele mining and phylogenetic analyses of natural populations | In barley, the eukaryotic translation initiation factor 4E (eIF4E) gene situated on chromosome 3H is recognized as an important source of resistance to the bymoviruses Barley yellow mosaic virus and Barley mild mosaic virus. In modern barley cultivars, two recessive eIF4E alleles, rym4 and rym5, confer different isolate-specific resistances. In this study, the sequence of eIF4E was analysed in 1090 barley landraces and noncurrent cultivars originating from 84 countries. An exceptionally high nucleotide diversity was evident in the coding sequence of eIF4E but not in either the adjacent MCT-1 gene or the sequence-related eIF(iso)4E gene situated on chromosome 1H. Surprisingly, all nucleotide polymorphisms detected in the coding sequence of eIF4E resulted in amino acid changes. A total of 47 eIF4E haplotypes were identified, and phylogenetic analysis using maximum likelihood provided evidence of strong positive selection acting on this barley gene. The majority of eIF4E haplotypes were found to be specific to distinct geographic regions. Furthermore, the eI4FE haplotype diversity (uh) was found to be considerably higher in East Asia, whereas SNP genotyping identified a comparatively low degree of genome-wide genetic diversity in 16 of 17 tested accessions (each carrying a different eIF4E haplotype) from this same region. In addition, selection statistic calculations using coalescent simulations showed evidence of non-neutral variation for eIF4E in several geographic regions, including East Asia, the region with a long history of the bymovirus-induced yellow mosaic disease. Together these findings suggest that eIF4E may play a role in barley adaptation to local habitats. | 1800 | no | crop | NA | no | no |
| 1801 | 2017 | 43021 | Sanz, AI; Fraile, A; Gallego, JM; Malpica, JM; Garcia-Arenal, F | 1999 | Genetic variability of natural populations of cotton leaf curl geminivirus, a single-stranded DNA virus | Reports on the genetic variability and evolution of natural populations of DNA viruses are scarce in comparison with the abundant information on the variability of RNA viruses. Geminiviruses are plant viruses with circular ssDNA genomes that are replicated by the host plans DNA polymerases. Whitefly-transmitted geminiviruses (WTG) are the agents of important diseases of crop plants and best exemplify emerging plant viruses. In this report we have analyzed the genetic diversity of cotton leaf curl geminivirus (CLCuV), a typical emerging WTG. No genetic differentiation was observed between isolates from different host plant species or geographic regions. Thus, the analyzed isolates represented a unique, undifferentiated population. Genetic variability, estimated as nucleotide diversities at synonymous positions in open reading frames (ORFs) for the ACI (=replication) protein and coat protein (CP = AV1), was very high, exceeding the values reported for different genes in several plant and animal RNA viruses. This was unexpected in a virus that uses the DNA replication machinery of its eukaryotic host. Diversities at nonsynonymous positions, on the other band, indicated that variability may be constrained in the genome of CLCuV. The ratio of nonsynonymous-to-synonymous substitutions varied for the different ORFs: they were higher for CP than for ACI and lower still for the AC4 and AV2 ORFs which overlap AC1 and CP ORFs, respectively. Analysis of nucleotide diversities at synonymous and nonsynonymous positions of the AC4 and;AV2 ORFs suggest that their evolution is constrained by AC1 and CP, respectively. Data suggest that AC4 and AV2 are new genes that may have originated by overprinting on the preexistent ACI and CP genes. Evidence for recombination was found for the AC1 and CP ORFs and for the noncoding intergenic region (IR). Data indicate that the origin of replication is a major recombination point in the IR, but not the only one. Analyses of the TR also suggest that recombinants may be frequent in the population and that recombination may have an important role in the generation of CLCuV variability. | 1801 | no | gd of pathogen | NA | no | no |
| 1802 | 2017 | 43021 | Gonzalez-Wevar, C; Salinas, P; Hune, M; Segovia, N; Vargas-Chacoff, L; Oda, E; Poulin, E | 2015 | Contrasting Genetic Structure and Diversity of Galaxias maculatus (Jenyns, 1848) Along the Chilean Coast: Stock Identification for Fishery Management | “Galaxias maculatus (Pisces: Galaxiidae) commonly known as”“puye”" has a disjunct distribution along the Southern Hemisphere including landlocked and migratory populations at latitudes over 30 degrees S in South America, Australia, Tasmania, and New Zealand. Chilean artisanal fishery of G. maculatus has become less important as a resource due to multiple factors including overexploitation, pollution, introduction of predators, and competitors. At the same time, the current conservation status of the species in Chile is still uncertain. Here, we used mtDNA control region sequences (925 bp) to investigate main patterns of genetic diversity and structure in populations from 2 biogeographic areas along the Chilean coast. Extremely high levels of genetic diversity characterize the species, suggesting a low amount of influence of the last glacial cycle over its demography compared with other studies in freshwater and marine South American fishes. However, we recognized contrasting genetic patterns between the Intermediate Area (between 30 degrees S and 42 degrees S) and the Magellanic Province (between 42 degrees S and 56 degrees S). On the one hand, over a narrow geographical range (<200 km) each Intermediate Area estuarine population constitutes a different genetic unit. On the other hand, the Magellanic populations of the species exhibited low levels of differentiation in an area extending for more than 500 km. Such differences may be a consequence of different coastal configurations, oceanographic regimes, and Quaternary glacial histories. Finally, our results support the existence of different stock units for G. maculatus and this information should be integrated in future management strategies and aquaculture programs for this species." | 1802 | no | no good fitness measure | NA | no | no |
| 1803 | 2017 | 43021 | Su, CL; Khan, A; Zhou, P; Majumdar, D; Ajzenberg, D; Darde, ML; Zhu, XQ; Ajioka, JW; Rosenthal, BM; Dubey, JP; Sibley, LD | 2012 | Globally diverse Toxoplasma gondii isolates comprise six major clades originating from a small number of distinct ancestral lineages | Marked phenotypic variation characterizes isolates of Toxoplasma gondii, a ubiquitous zoonotic parasite that serves as an important experimental model for studying apicomplexan parasites. Progress in identifying the heritable basis for clinically and epidemiologically significant differences requires a robust system for describing and interpreting evolutionary subdivisions in this prevalent pathogen. To develop such a system, we have examined more than 950 isolates collected from around the world and genotyped them using three independent sets of polymorphic DNA markers, sampling 30 loci distributed across all nuclear chromosomes as well as the plastid genome. Our studies reveal a biphasic pattern consisting of regions in the Northern Hemisphere where a few, highly clonal and abundant lineages predominate; elsewhere, and especially in portions of South America are characterized by a diverse assemblage of less common genotypes that show greater evidence of recombination. Clustering methods were used to organize the marked genetic diversity of 138 unique genotypes into 15 haplogroups that collectively define six major clades. Analysis of gene flow indicates that a small number of ancestral lineages gave rise to the existing diversity through a process of limited admixture. Identification of reference strains for these major groups should facilitate future studies on comparative genomics and identification of genes that control important biological phenotypes including pathogenesis and transmission. | 1803 | no | gd of pathogen | NA | no | no |
| 1804 | 2017 | 43021 | Huchard, E; Knapp, LA; Wang, JL; Raymond, M; Cowlishaw, G | 2010 | MHC, mate choice and heterozygote advantage in a wild social primate | Preferences for mates carrying dissimilar genes at the major histocompatibility complex (MHC) may help animals increase offspring pathogen resistance or avoid inbreeding. Such preferences have been reported across a range of vertebrates, but have rarely been investigated in social species other than humans. We investigated mate choice and MHC dynamics in wild baboons (Papio ursinus). MHC Class II DRB genes and 16 microsatellite loci were genotyped across six groups (199 individuals). Based on the survey of a key segment of the gene-rich MHC, we found no evidence of mate choice for MHC dissimilarity, diversity or rare MHC genotypes. First, MHC dissimilarity did not differ from random expectation either between parents of the same offspring or between immigrant males and females from the same troop. Second, female reproductive success was not influenced by MHC diversity or genotype frequency. Third, population genetic structure analysis revealed equally high genotypic differentiation among troops, and comparable excess heterozygosity within troops for juveniles, at both Mhc-DRB and neutral loci. Nevertheless, the age structure of Mhc-DRB heterozygosity suggested higher longevity for heterozygotes, which should favour preferences for MHC dissimilarity. We propose that high levels of within-group outbreeding, resulting from group-living and sex-biased dispersal, might weaken selection for MHC-disassortative mate choice. | 1804 | no | no good fitness measure | NA | no | no |
| 1805 | 2017 | 43021 | Wilson, DJ; Gabriel, E; Leatherbarrow, AJ; Cheesbrough, J; Gee, S; Bolton, E; Fox, A; Hart, CA; Diggle, PJ; Fearnhead, P | 2009 | Rapid Evolution and the Importance of Recombination to the Gastroenteric Pathogen Campylobacter jejuni | Responsible for the majority of bacterial gastroenteritis in the developed world, Campylobacter jejuni is a pervasive pathogen of humans and animals, but its evolution is obscure. In this paper, we exploit contemporary genetic diversity and empirical evidence to piece together the evolutionary history of C. jejuni and quantify its evolutionary potential. Our combined population genetics-phylogenetics approach reveals a surprising picture. Campylobacter jejuni is a rapidly evolving species, subject to intense purifying selection that purges 60% of novel variation, but possessing a massive evolutionary potential. The low mutation rate is offset by a large effective population size so that a mutation at any site can occur somewhere in the population within the space of a week. Recombination has a fundamental role, generating diversity at twice the rate of de novo mutation, and facilitating gene flow between C. jejuni and its sister species Campylobacter coli. We attempt to calibrate the rate of molecular evolution in C. jejuni based solely on within-species variation. The rates we obtain are up to 1,000 times faster than conventional estimates, placing the C. jejuni-C. coli split at the time of the Neolithic revolution. We weigh the plausibility of such recent bacterial evolution against alternative explanations and discuss the evidence required to settle the issue. | 1805 | no | gd of pathogen | NA | no | no |
| 1806 | 2017 | 43021 | Jiang, Y; Zhao, Y; Rodemann, B; Plieske, J; Kollers, S; Korzun, V; Ebmeyer, E; Argillier, O; Hinze, M; Ling, J; Roder, MS; Ganal, MW; Mette, MF; Reif, JC | 2015 | Potential and limits to unravel the genetic architecture and predict the variation of Fusarium head blight resistance in European winter wheat (Triticum aestivum L.) | Genome-wide mapping approaches in diverse populations are powerful tools to unravel the genetic architecture of complex traits. The main goals of our study were to investigate the potential and limits to unravel the genetic architecture and to identify the factors determining the accuracy of prediction of the genotypic variation of Fusarium head blight (FHB) resistance in wheat (Triticum aestivum L.) based on data collected with a diverse panel of 372 European varieties. The wheat lines were phenotyped in multi-location field trials for FHB resistance and genotyped with 782 simple sequence repeat (SSR) markers, and 9k and 90k single-nucleotide polymorphism (SNP) arrays. We applied genome-wide association mapping in combination with fivefold crossvalidations and observed surprisingly high accuracies of prediction for marker-assisted selection based on the detected quantitative trait loci (QTLs). Using a random sample of markers not selected for marker-trait associations revealed only a slight decrease in prediction accuracy compared with marker-based selection exploiting the QTL information. The same picture was confirmed in a simulation study, suggesting that relatedness is a main driver of the accuracy of prediction in marker-assisted selection of FHB resistance. When the accuracy of prediction of three genomic selection models was contrasted for the three marker data sets, no significant differences in accuracies among marker platforms and genomic selection models were observed. Marker density impacted the accuracy of prediction only marginally. Consequently, genomic selection of FHB resistance can be implemented most cost-efficiently based on low-to medium-density SNP arrays. | 1806 | no | crop | NA | no | no |
| 1807 | 2017 | 43021 | Genovart, M; Oro, D; Juste, J; Bertorelle, G | 2007 | What genetics tell us about the conservation of the critically endangered Balearic shearwater? | The Balearic shearwater Puffinus rnauretanicus is one of the most critically endangered seabirds in the world. The species is endemic to the Balearic archipelago, and conservation concerns are the low number of breeding pairs, the low adult survival, and the possible hybridization with a sibling species, the morphologically smaller Yelkouan shearwater (P. yelkouan). We sampled almost the entire breeding range of the species and analyzed the genetic variation at two mitochondrial DNA regions. No genetic evidence of population decline was found. Despite the observed philopatry, we detected a weak population structure mainly due to connectivity among colonies higher than expected, but also to a Pleistocene demographic expansion. Some colonies showed a high imbalance between immigration and emigration rates, suggesting spatial heterogeneity in patch quality. Genetic evidence of maternal introgression from the sibling species was reinforced, but almost only in a peripheral colony and not followed, at least to date, by the spread of the introgressed mtDNA lineages. Morphometric differences were not correlated with mtDNA haplotypes and introgression. is probably due to a secondary contact between the two species several generations ago. Overall, results suggested that the very recent demographic decline in this critically endangered species has not yet decreased its genetic variability, and connectivity found among most colonies should help to reduce species extinction risk. Spreading of introgression. should be monitored, but the species is not jeopardized at the moment by genetic factors and the major conservation actions should concentrate at enhancing adult survival. (C) 2007 Elsevier Ltd. All rights reserved. | 1807 | no | no good fitness measure | NA | no | no |
| 1808 | 2017 | 43021 | Gardner, M; Bertranpetit, J; Comas, D | 2008 | Worldwide Genetic Variation in Dopamine and Serotonin Pathway Genes: Implications for Association Studies | The dopamine and serotonin systems are two of the most important neurotransmitter pathways in the human nervous system and their roles in controlling behavior and mental status are well accepted. Genes from both systems have been widely implicated in psychiatric and behavioral disorders, with numerous reports of associations and almost equally as numerous reports of the failure to replicate a previous finding of association. We investigate a set of 21 dopamine and serotonin genes commonly tested for association with psychiatric disease in a set of 39 worldwide populations representing global genetic diversity to see whether the failure to replicate findings of association maybe explained by population based differences in allele frequencies and linkage disequilibrium (LD) in this gene set. We present results demonstrating a surprising homogeneity of the allele frequencies across worldwide populations in these genes. LD both for populations within continent groupings and across continental regions also showed a remarkable similarity. These findings taken together suggest that ethnic differences in these parameters are not major generators of artifacts in genetic association studies of psychiatric disorders with genes from this set. Therefore, factors other than ethnic differences in genetic variation may explain the discrepancies reported among genetic association studies with this set of genes to date. The transferability of tagSNPs defined in the HapMap populations to other worldwide populations was also investigated and found to be high. A list of tagSNPs per gene and continental region is proposed providing a guide for future association studies with these genes. (C) 2008 Wiley-Liss, Inc. | 1808 | no | NA | NA | no | no |
| 1809 | 2017 | 43021 | Pope, TR | 1998 | Effects of demographic change on group kin structure and gene dynamics of populations of red howling monkeys | Four populations of red howling monkeys (Alouatta seniculus) in different states of population growth were studied to examine how changes in intrasexual competition and kin availability influence mating patterns and population genetic structure. The demography of each population was monitored for 5 years. Genetic variance within and between groups and coefficients of relatedness ((r) over bar) were estimated from allozyme loci. Advantages of cooperating with kin over non-kin changed with different demographic circumstances for each sex, such that social groups were more likely to be kin groups when population density was approaching or at carrying capacity than when it was low. In populations growing rapidly through new social group formation, genetic differentiation between groups was low. Genetic differentiation increased as populations approached carrying capacity and groups matured socially (i.e., (r) over bar increased). That trend was reversed as (r) over bar within group breeding subsets (males and females, respectively) increased, thereby decreasing (r) over bar between males and females and reducing group (r) over bar. This was enhanced when inbreeding within groups was followed by transfer of pods of related males, which occurred in a saturated habitat and in a declining population experiencing high mortality of adult females. Heterozygosity within groups in excess of predicted values was increased due to Lineage effects as groups matured and as (r) over bar within breeding subsets increased. It also increased when population growth occurred through immigration from genetically differentiated demes, and when groups fused during population decline. There were large intergroup differences in mortality during population decline, which was accompanied by social instability that resulted in increased intergroup transfer of both sexes, thereby resorting survivors throughout remaining breeding groups. Conditions favoring intergroup selection thus decreased during periods of population growth when selection was relaxed and group (r) over bar was low, increased during periods of stasis when habitats were saturated and group (r) over bar was highest, and were most prominent during periods of population decline when differences in group fitness should have the most pronounced consequences. | 1809 | no | no good fitness measure | NA | no | no |
| 1810 | 2017 | 43021 | Warburton, EM; Khokhlova, IS; Kiefer, D; Krasnov, BR | 2017 | Morphological asymmetry and habitat quality: using fleas and their rodent hosts as a novel experimental system | Morphological asymmetry is widely used to measure developmental instability and higher levels of asymmetry often correlate with decreased mating success, increased inbreeding, increased stress and decreased habitat quality. We studied asymmetry and relationships between asymmetry and host identity in two flea species, host generalist Xenopsylla ramesis and host specialist Parapulex chephrenis, and asked: (1) what the level of asymmetry was in their femurs and tibiae; (2) which type of asymmetry predominates; and (3) whether fleas that fed on host species distantly related to their principal host species produced offspring that exhibited greater asymmetry compared with offspring of fleas that fed on their principal host species. We found fluctuating asymmetry in femurs and tibiae of X. ramesis and in the tibiae of P. chephrenis as well as significantly left-handed directional asymmetry in the femurs of P. chephrenis. Host species identity significantly impacted asymmetry in leg segments of P. chephrenis but not in those of X. ramesis. Offspring asymmetry increased when mother fleas fed on a host that was distantly related to the principal host. Fleas parasitizing multiple host species might compensate for developmental instability when utilizing a novel host species; therefore, host-switching events in host-specific parasites could be constrained by the relatedness between a novel and a principal host species. | 1810 | no | gd of parasite | NA | no | no |
| 1811 | 2017 | 43021 | Hubbard, A; Lewis, CM; Yoshida, K; Ramirez-Gonzalez, RH; de Vallavieille-Pope, C; Thomas, J; Kamoun, S; Bayles, R; Uauy, C; Saunders, DGO | 2015 | Field pathogenomics reveals the emergence of a diverse wheat yellow rust population | Background: Emerging and re-emerging pathogens imperil public health and global food security. Responding to these threats requires improved surveillance and diagnostic systems. Despite their potential, genomic tools have not been readily applied to emerging or re-emerging plant pathogens such as the wheat yellow (stripe) rust pathogen Puccinia striiformis f. sp. tritici (PST). This is due largely to the obligate parasitic nature of PST, as culturing PST isolates for DNA extraction remains slow and tedious. Results: To counteract the limitations associated with culturing PST, we developed and applied a field pathogenomics approach by transcriptome sequencing infected wheat leaves collected from the field in 2013. This enabled us to rapidly gain insights into this emerging pathogen population. We found that the PST population across the United Kingdom (UK) underwent a major shift in recent years. Population genetic structure analyses revealed four distinct lineages that correlated to the phenotypic groups determined through traditional pathology-based virulence assays. Furthermore, the genetic diversity between members of a single population cluster for all 2013 PST field samples was much higher than that displayed by historical UK isolates, revealing a more diverse population of PST. Conclusions: Our field pathogenomics approach uncovered a dramatic shift in the PST population in the UK, likely due to a recent introduction of a diverse set of exotic PST lineages. The methodology described herein accelerates genetic analysis of pathogen populations and circumvents the difficulties associated with obligate plant pathogens. In principle, this strategy can be widely applied to a variety of plant pathogens. | 1811 | no | gd of pathogen | NA | no | no |
| 1812 | 2017 | 43021 | Gonzalez-Quevedo, C; Phillips, K; Spurgin, L; Richardson, D | 2015 | 454 screening of individual MHC variation in an endemic island passerine | Genes of the major histocompatibility complex (MHC) code for receptors that are central to the adaptive immune response of vertebrates. These genes are therefore important genetic markers with which to study adaptive genetic variation in the wild. Next-generation sequencing (NGS) has increasingly been used in the last decade to genotype the MHC. However, NGS methods are highly prone to sequencing errors, and although several methodologies have been proposed to deal with this, until recently there have been no standard guidelines for the validation of putative MHC alleles. In this study, we used the 454 NGS platform to screen MHC class I exon 3 variation in a population of the island endemic Berthelot’s pipit (Anthus berthelotii). We were able to characterise MHC genotypes across 309 individuals with high levels of repeatability. We were also able to determine alleles that had low amplification efficiencies, whose identification within individuals may thus be less reliable. At the population level we found lower levels of MHC diversity in Berthelot’s pipit than in its widespread continental sister species the tawny pipit (Anthus campestris), and observed trans-species polymorphism. Using the sequence data, we identified signatures of gene conversion and evidence of maintenance of functionally divergent alleles in Berthelot’s pipit. We also detected positive selection at 10 codons. The present study therefore shows that we have an efficient method for screening individual MHC variation across large datasets in Berthelot’s pipit, and provides data that can be used in future studies investigating spatio-temporal patterns and scales of selection on the MHC. | 1812 | no | only one pop | NA | no | no |
| 1813 | 2017 | 43021 | Kemmett, K; Humphrey, T; Rushton, S; Close, A; Wigley, P; Williams, NJ | 2013 | A Longitudinal Study Simultaneously Exploring the Carriage of APEC Virulence Associated Genes and the Molecular Epidemiology of Faecal and Systemic E. coli in Commercial Broiler Chickens | Colibacillosis is an economically important syndromic disease of poultry caused by extra-intestinal avian pathogenic Escherichia coli (APEC) but the pathotype remains poorly defined. Combinations of virulence-associated genes (VAGs) have aided APEC identification. The intestinal microbiota is a potential APEC reservoir. Broiler chickens are selectively bred for fast, uniform growth. Here we simultaneously investigate intestinal E. coli VAG carriage in apparently healthy birds and characterise systemic E. coli from diseased broiler chickens from the same flocks. Four flocks were sampled longitudinally from chick placement until slaughter. Phylogrouping, macro-restriction pulsed-field gel electrophoresis (PFGE) and multilocus sequence typing (MLST) were performed on an isolate subset from one flock to investigate the population structure of faecal and systemic E. coli. Early in production, VAG carriage among chick intestinal E. coli populations was diverse (average Simpson’s D value = 0.73); 24.05% of intestinal E. coli (n = 160) from 1 day old chicks were carrying >= 5 VAGs. Generalised Linear models demonstrated VAG prevalence in potential APEC populations declined with age; 1% of E. coli carrying >= 5 VAGs at slaughter and demonstrated high strain diversity. A variety of VAG profiles and high strain diversity were observed among systemic E. coli. Thirty three new MLST sequence types were identified among 50 isolates and a new sequence type representing 22.2% (ST-2999) of the systemic population was found, differing from the pre-defined pathogenic ST-117 at a single locus. For the first time, this study takes a longitudinal approach to unravelling the APEC paradigm. Our findings, supported by other studies, highlight the difficulty in defining the APEC pathotype. Here we report a high genetic diversity among systemic E. coli between and within diseased broilers, harbouring diverse VAG profiles rather than single and/or highly related pathogenic clones suggesting host susceptibility in broilers plays an important role in APEC pathogenesis. | 1813 | no | gd of pathogen | NA | no | no |
| 1814 | 2017 | 43021 | Nunney, L; Yuan, XL; Bromley, R; Hartung, J; Montero-Astua, M; Moreira, L; Ortiz, B; Stouthamer, R | 2010 | Population Genomic Analysis of a Bacterial Plant Pathogen: Novel Insight into the Origin of Pierce’s Disease of Grapevine in the US | Invasive diseases present an increasing problem worldwide; however, genomic techniques are now available to investigate the timing and geographical origin of such introductions. We employed genomic techniques to demonstrate that the bacterial pathogen causing Pierce’s disease of grapevine (PD) is not native to the US as previously assumed, but descended from a single genotype introduced from Central America. PD has posed a serious threat to the US wine industry ever since its first outbreak in Anaheim, California in the 1880s and continues to inhibit grape cultivation in a large area of the country. It is caused by infection of xylem vessels by the bacterium Xylella fastidiosa subsp. fastidiosa, a genetically distinct subspecies at least 15,000 years old. We present five independent kinds of evidence that strongly support our invasion hypothesis: 1) a genome-wide lack of genetic variability in X. fastidiosa subsp. fastidiosa found in the US, consistent with a recent common ancestor; 2) evidence for historical allopatry of the North American subspecies X. fastidiosa subsp. multiplex and X. fastidiosa subsp. fastidiosa; 3) evidence that X. fastidiosa subsp. fastidiosa evolved in a more tropical climate than X. fastidiosa subsp. multiplex; 4) much greater genetic variability in the proposed source population in Central America, variation within which the US genotypes are phylogenetically nested; and 5) the circumstantial evidence of importation of known hosts (coffee plants) from Central America directly into southern California just prior to the first known outbreak of the disease. The lack of genetic variation in X. fastidiosa subsp. fastidiosa in the US suggests that preventing additional introductions is important since new genetic variation may undermine PD control measures, or may lead to infection of other crop plants through the creation of novel genotypes via inter-subspecific recombination. In general, geographically mixing of previously isolated subspecies should be avoided. | 1814 | no | gd of pathogen | NA | no | no |
| 1815 | 2017 | 43021 | Bennett, SN; Drummond, AJ; Kapan, DD; Suchard, MA; Munoz-Jordan, JL; Pybus, OG; Holmes, EC; Gubler, DJ | 2010 | Epidemic Dynamics Revealed in Dengue Evolution | Dengue is an emerging tropical disease infecting tens of millions of people annually. A febrile illness with potentially severe hemorrhagic manifestations, dengue is caused by mosquito-borne viruses (DENV-1 to -4) that are maintained in endemic transmission in large urban centers of the tropics with periodic epidemic cycles at 3- to 5-year intervals. Puerto Rico ( PR), a major population center in the Caribbean, has experienced increasingly severe epidemics since multiple dengue serotypes were introduced beginning in the late 1970s. We document the phylodynamics of DENV-4 between 1981 and 1998, a period of dramatic ecological expansion during which evolutionary change also occurs. The timescale of viral evolution is sufficiently short that viral transmission dynamics can be elucidated from genetic diversity data. Specifically, by combining virus sequence data with confirmed case counts in PR over these two decades, we show that the pattern of cyclic epidemics is strongly correlated with coalescent estimates of effective population size that have been estimated from sampled virus sequences using Bayesian Markov Chain Monte Carlo methods. Thus, we show that the observed epidemiologic dynamics are correlated with similar fluctuations in diversity, including severe interepidemic reductions in genetic diversity compatible with population bottlenecks that may greatly impact DENV evolutionary dynamics. Mean effective population sizes based on genetic data appear to increase prior to isolation counts, suggesting a potential bias in the latter and justifying more active surveillance of DENV activity. Our analysis explicitly integrates epidemiologic and sequence data in a joint model that could be used to further explore transmission models of infectious disease. | 1815 | no | gd of pathogen | NA | no | no |
| 1816 | 2017 | 43021 | Wildschutte, H; Wolfe, DM; Tamewitz, A; Lawrence, JG | 2004 | Protozoan predation, diversifying selection, and the evolution of antigenic diversity in Salmonella | Extensive population-level genetic variability at the Salmonella rfb locus, which encodes enzymes responsible for synthesis of the O-antigen polysaccharicle, is thought to have arisen through frequency-dependent selection (FDS) by means of exposure of this pathogen to host immune systems. The FDS hypothesis works well for pathogens such as Haemophilus influenzae and Neisseria meningitis, which alter the composition of their O-antigens during the course of bloodborne infections. In contrast, Salmonella remains resident in epithelial cells or macrophages during infection and does not have phase variability in its O-antigen. More importantly, Salmonella shows host-serovar specificity, whereby strains bearing certain O-antigens cause disease primarily in specific hosts; this behavior is inconsistent with FDS providing selection for the origin or maintenance of extensive polymorphism at the rfb locus. Alternatively, selective pressure may originate from the host intestinal environment itself, wherein diversifying selection mediated by protozoan predation allows for the continued existence of Salmonella able to avoid consumption by host-specific protozoa. This selective pressure would result in high population-level diversity at the Salmonella rfb locus without phase variation. We show here that intestinal protozoa recognize antigenically diverse Salmonella with different efficiencies and demonstrate that differences solely in the O-antigen are sufficient to allow for prey discrimination. Combined with observations of the differential distributions of both serotypes of bacterial species and their protozoan predators among environments, our data provides a framework for the evolution of high genetic diversity at the rfb locus and host-specific pathogenicity in Salmonella. | 1816 | no | gd of pathogen | NA | no | no |
| 1817 | 2017 | 43021 | Twiddy, SS; Holmes, EC; Rambaut, A | 2003 | Inferring the rate and time-scale of dengue virus evolution | Dengue is often referred to as an emerging disease because of the rapid increases in incidence and prevalence that have been observed in recent decades. To understand the rate at which genetic diversification occurs in dengue virus and to infer the time-scale of its evolution, we employed a maximum likelihood method that uses information about times of virus sampling to estimate the rate of molecular evolution in a large number of viral envelope (E) gene sequences and to place bounds around the dates of appearance of all serotypes and specific genotypes. Our analysis reveals that dengue virus generally evolves according to a molecular clock, although some serotype-specific and genotype-specific rate differences were observed, and that its origin is more recent than previously suggested, with the virus appealing approximately 1000 years ago. Furthermore, we estimate that the zoonotic transfer of dengue from sylvatic (monkey) to sustained human transmission occurred between 125 and 320 years ago, that the current global genetic diversity in the four serotypes of dengue virus only appeared during the past century, and that the recent rise in genetic diversity can be loosely correlated both to human activities such as population growth, urbanization, and mass transport and to the emergence of dengue hemorrhagic fever as a major disease problem. | 1817 | no | gd of pathogen | NA | no | no |
| 1818 | 2017 | 43021 | Bataille, A; Cunningham, AA; Cruz, M; Cedeno, V; Goodman, SJ | 2010 | Seasonal effects and fine-scale population dynamics of Aedes taeniorhynchus, a major disease vector in the Galapagos Islands | Characterization of the fine-scale population dynamics of the mosquito Aedes taeniorhynchus is needed to improve our understanding of its role as a disease vector in the Galapagos Islands. We used microsatellite data to assess the genetic structure of coastal and highland mosquito populations and patterns of gene flow between the two habitats through time on Santa Cruz Island. In addition, we assessed possible associations of mosquito abundance and genetic diversity with environmental variables. The coastal and highland mosquito populations were highly differentiated from each other all year round, with some gene flow detected only during periods of increased precipitation. The results support the hypothesis that selection arising from ecological differences between habitats is driving adaptation and divergence in A. taeniorhynchus, and maintaining long-term genetic differentiation of the populations against gene flow. The highland and lowland populations may constitute an example of incipient speciation in progress. Highland populations were characterized by lower observed heterozygosity and allelic richness, suggesting a founder effect and/or lower breeding site availability in the highlands. A lack of reduction in genetic diversity over time in highland populations suggests that they survive dry periods as dormant eggs. Association between mosquito abundance and precipitation was strong in the highlands, whereas tide height was the main factor affecting mosquito abundance on the coast. Our findings suggests differences in the infection dynamics of mosquito-borne parasites in the highlands compared to the coast, and a higher risk of mosquito-driven disease spread across these habitats during periods of increased precipitation. | 1818 | no | no good fitness measure | NA | no | no |
| 1819 | 2017 | 43021 | Zhang, XH; Liu, YJ; Wu, BL; Sun, BL; Chen, F; Ji, J; Ma, JY; Xie, QM | 2013 | Phylogenetic and molecular characterization of chicken anemia virus in southern China from 2011 to 2012 | Chicken anemia virus (CAV) is an important pathogen that causes severe immunosuppression in young chickens. We have characterized 13 CAVs isolated from different commercial farms in southern China between 2011 and 2012. We discovered 92 variable residues compared to 37 other CAV complete genome sequences from other parts of the world listed in GenBank; these residues have not been previously observed. All of the Chinese CAV genomes that were characterized in this study had a glutamine at position 394, a hallmark of highly pathogenic CAVs. We also discovered that intra-group genetic recombination plays a role in generating genetic diversity in natural populations of CAV. The GD-J-12 isolate was a possible recombinant between GD-C-12 and GD-M-12 in the genomic region that encompassed both the coding and non-coding regions. | 1819 | no | gd of pathogen | NA | no | no |
| 1820 | 2017 | 43021 | Contreras-Cubas, C; Sanchez-Hernandez, BE; Garcia-Ortiz, H; Martinez-Hernandez, A; Barajas-Olmos, F; Cid, M; Mendoza-Caamal, EC; Centeno-Cruz, F; Ortiz-Cruz, G; Jimenez-Lopez, JC; Cordova, EJ; Salas-Bautista, EG; Saldana-Alvarez, Y; Fernandez-Lopez, JC; Mutchinick, OM; Orozco, L | 2016 | Heterogenous Distribution of MTHFR Gene Variants among Mestizos and Diverse Amerindian Groups from Mexico | Methylenetetrahydrofolate reductase (MTHFR) is a key enzyme in folate metabolism. Folate deficiency has been related to several conditions, including neural tube defects (NTDs) and cardiovascular diseases. Hence, MTHFR genetic variants have been studied worldwide, particularly the C677T and A1298C. We genotyped the C677T and A1298C MTHFR polymorphisms in Mexican Amerindians (MAs), from the largest sample included in a genetic study (n = 2026, from 62 ethnic groups), and in a geographically-matched Mexican Mestizo population (MEZ, n = 638). The 677T allele was most frequent in Mexican individuals, particularly in MAs. The frequency of this allele in both MAs and MEZs was clearly enriched in the South region of the country, followed by the Central East and South East regions. In contrast, the frequency of the 1298C risk allele in Mexicans was one of the lowest in the world. Both in MAs and MEZs the variants 677T and 1298C displayed opposite allele frequency gradients from southern to northern Mexico. Our findings suggest that in Mestizos the 677T allele was derived from Amerindians while the 1298C allele was a European contribution. Some subgroups showed an allele frequency distribution that highlighted their genetic diversity. Notably, the distribution of the frequency of the 677T allele was consistent with that of the high incidence of NTDs reported in MEZ. | 1820 | no | NA | NA | no | no |
| 1821 | 2017 | 43021 | Su, WL; Sieberts, SK; Kleinhanz, RR; Lux, K; Millstein, J; Molony, C; Schadt, EE | 2010 | Assessing the prospects of genome-wide association studies performed in inbred mice | The remarkable success in mapping genes linked to a number of disease traits using genome-wide association studies (GWAS) in human cohorts has renewed interest in applying this same technique in model organisms such as inbred laboratory mice. Unlike humans, however, the limited genetic diversity in the ancestry of laboratory mice combined with selection pressure over the past decades have yielded an intricate population genetic structure that can complicate the results obtained from association studies. This problem is further exacerbated by the small number of strains typically used in such studies where multiple spurious associations arise as a result of random chance. We sought to empirically assess the viability of GWAS in inbred mice using hundreds of expression traits for which the true location of the expression quantitative trait locus was known a priori. We then measured transcript abundance levels for these expression traits in 16 classical and 3 wild-derived inbred strains and carried out a genome-wide association scan, demonstrating the low statistical power of such studies and empirically estimating the large extent to which allelic association of transcripts gives rise to spurious associations. We provide evidence illustrating that in a large fraction of cases, the marker with the most significant p values fails to map to the location of the true eQTL. Finally, we provide experimental support for hundreds of traits, and that combining linkage analysis with association mapping provides significant increases in statistical power over a stand-alone GWAS as well as significantly higher mapping resolution than either study alone. | 1821 | no | NA | NA | no | no |
| 1822 | 2017 | 43021 | Fabre, A; Balakishiyeva, G; Ember, I; Omar, A; Acs, Z; Kolber, M; Kauzner, L; Della Bartola, M; Danet, JL; Foissac, X | 2011 | StAMP encoding the antigenic membrane protein of stolbur phytoplasma is useful for molecular epidemiology | The antigenic membrane protein of stolbur phytoplasma has been cloned and characterized. The expression of StAMP in Escherichia coli produced a 16 kDa peptide recognized by an anti-stolbur monoclonal antibody. Stamp is submitted to a positive diversifying selection pressure (Fabre et al., 2011). The genetic diversity of stamp was evaluated among a collection of stolbur phytoplasma strains representative of the tuf and secY genetic diversity of stolbur phytoplasmas in the Euro-Mediterranean basin. Most of the French, Italian and Croatian strains clustered on the same phylogenetic branch (tuf-type b cluster I). A second branch of the phylogenetic tree corresponded to strains of central and Eastern Europe (tuf-type b cluster II), while a third branch grouped strains of the east of the Mediterranean basin (Greece, Serbia, Lebanon, and Azerbaijan). Strains of the tiff-type a genotype clustered together in an independent monophyletic branch of the stamp phylogenetic tree. In conclusion, stamp variability seems to be correlated to geographical origin in the case of the tot-type b strains. | 1822 | no | no good fitness measure, N pop differnt in GD? | NA | no | no |
| 1823 | 2017 | 43021 | Campbell, G; Noble, LR; Rollinson, D; Southgate, VR; Webster, JP; Jones, CS | 2010 | Low genetic diversity in a snail intermediate host (Biomphalaria pfeifferi Krass, 1848) and schistosomiasis transmission in the Senegal River Basin | Population genetic perturbations of intermediate hosts, often a consequence of human pressure on environmental resources, can precipitate unexpectedly severe disease outbreaks. Such disturbances are set to become increasingly common following range changes concomitant with climate shifts, dwindling natural resources and major infrastructure changes such as hydroprojects. Construction of the Diama dam in the Senegal River Basin (SRB) reduced river salinity, enabling the freshwater snail intermediate host Biomphalaria pfeifferi to rapidly expand its distribution. A serious public health problem ensued, with an epidemic of intestinal schistosomiasis occurring in the previously schistosome-free Richard-Toll region within 2 years. The current study aimed to assess the population variability of B. pfeifferi in the SRB, and speculate upon its subsequent impact on host-parasite interactions following such engineered ecological change. Genetic variation at nine polymorphic microsatellite loci revealed little population differentiation in SRB snails compared with those from natural habitats in Zimbabwe, where Schistosoma mansoni transmission is much lower. ‘Open’ SRB habitats are associated with greater water contact, smaller population sizes and less genetic diversity, with sites downstream of Richard-Toll showing greater inter- and intrapopulation variation, concomitant with less frequent human contact. These observations may be explained by rapid expansion into pristine habitat selecting for high fecundity genotypes at the expense of schistosome resistance, presenting S. mansoni with genetically homogenous highly fecund susceptible populations around the focal point, promoting development of a highly compatible host-parasite relationship. Longitudinal study of such systems may prove important in predicting public health risks engendered by future environmental engineering projects. | 1823 | no | NA | NA | no | no |
| 1824 | 2017 | 43021 | Lounnas, M; Correa, AC; Vazquez, AA; Dia, A; Escobar, JS; Nicot, A; Arenas, J; Ayaqui, R; Dubois, MP; Gimenez, T; Gutierrez, A; Gonzalez-Ramirez, C; Noya, O; Prepelitchi, L; Uribe, N; Wisnivesky-Colli, C; Yong, M; David, P; Loker, ES; Jarne, P; Pointier, JP; Hurtrez-Bousses, S | 2017 | Self-fertilization, long-distance flash invasion and biogeography shape the population structure of Pseudosuccinea columella at the worldwide scale | Population genetic studies are efficient for inferring the invasion history based on a comparison of native and invasive populations, especially when conducted at species scale. An expected outcome in invasive populations is variability loss, and this is especially true in self-fertilizing species. We here focus on the self-fertilizing Pseudosuccinea columella, an invasive hermaphroditic freshwater snail that has greatly expanded its geographic distribution and that acts as intermediate host of Fasciola hepatica, the causative agent of human and veterinary fasciolosis. We evaluated the distribution of genetic diversity at the largest geographic scale analysed to date in this species by surveying 80 populations collected during 16 years from 14 countries, using eight nuclear microsatellites and two mitochondrial genes. As expected, populations from North America, the putative origin area, were strongly structured by selfing and history and harboured much more genetic variability than invasive populations. We found high selfing rates (when it was possible to infer it), none-to-low genetic variability and strong population structure in most invasive populations. Strikingly, we found a unique genotype/haplotype in populations from eight invaded regions sampled all over the world. Moreover, snail populations resistant to infection by the parasite are genetically distinct from susceptible populations. Our results are compatible with repeated introductions in South America and flash worldwide invasion by this unique genotype/haplotype. Our study illustrates the population genetic consequences of biological invasion in a highly selfing species at very large geographic scale. We discuss how such a large-scale flash invasion may affect the spread of fasciolosis. | 1824 | no | no good fitness measure | NA | no | no |
| 1825 | 2017 | 43021 | Muller, R; Seeland, A; Jagodzinski, LS; Diogo, JB; Nowak, C; Oehlmann, J | 2012 | Simulated climate change conditions unveil the toxic potential of the fungicide pyrimethanil on the midge Chironomus riparius: a multigeneration experiment | Although it has been suggested that temperature increase may alter the toxic potential of environmental pollutants, few studies have investigated the potential risk of chemical stressors for wildlife under Global Climate Change (GCC) impact. We applied a bifactorial multigeneration study in order to test if GCC conditions alter the effects of low pesticide concentrations on life history and genetic diversity of the aquatic model organism Chironomus riparius. Experimental populations of the species were chronically exposed to a low concentration of the fungicide pyrimethanil (half of the no-observed-adverse-effect concentration: NOAEC/2) under two dynamic present-day temperature simulations (11.0-22.7 degrees C; 14.0-25.2 degrees C) and one future scenario (16.5-28.1 degrees C). During the 140-day multigeneration study, survival, emergence, reproduction, population growth, and genetic diversity of C. riparius were analyzed. Our results reveal that high temperature and pyrimethanil act synergistically on the midge C. riparius. In simulated present-day scenarios, a NOAEC/2 of pyrimethanil as derived from a life-cycle toxicity test provoked only slight-to-moderate beneficial or adverse effects on C. riparius. In contrast, exposure to a NOAEC/2 concentration of pyrimethanil at a thermal situation likely for a summer under GCC conditions uncovered adverse effects on mortality and population growth rate. In addition, genetic diversity was considerably reduced by pyrimethanil in the future scenario, but only slightly under current climatic conditions. Our multigeneration study under near-natural (climatic) conditions indicates that not only the impact of climate change, but also low concentrations of pesticides may pose a reasonable risk for aquatic insects in future. | 1825 | no | No GD diff between pop | NA | no | no |
| 1826 | 2017 | 43021 | Horne, BD; Camp, NJ | 2004 | Principal component analysis for selection of optimal SNP-sets that capture intragenic genetic variation | Candidate gene association studies often utilize one single nucleotide polymorphism (SNP) for analysis, with an initial report typically not being replicated by subsequent studies. The failure to replicate may result from incomplete or poor identification of disease-related variants or haplotypes, possibly due to naive SNP selection. A method for identification of linkage disequilibrium (LD) groups and selection of SNPs that capture sufficient intra-genic genetic diversity is described. We assume all SNPs with minor allele frequency above a pre-determined frequency have been identified. Principal component analysis (PCA) is applied to evaluate multivariate SNP correlations to infer groups of SNPs in LD (LD-groups) and to establish an optimal set of group-tagging SNPs (gtSNPs) that provide the most comprehensive coverage of intragenic diversity while minimizing the resources necessary to perform an informative association analysis. This PCA method differs from haplotype block (HB) and haplotype-tagging SNP (htSNP) methods, in that an LD-group of SNPs need not be a contiguous DNA fragment. Results of the PCA method compared well with existing htSNP methods while also providing advantages over those methods, including an indication of the optimal number of SNPs needed. Further, evaluation of the method over multiple replicates of simulated data indicated PCA to be a robust method for SNP selection. Our findings suggest that PCA may be a powerful tool for establishing an optimal SNP set that maximizes the amount of genetic variation captured for a candidate gene using a minimal number of SNPs. Genet Epidemiol 26:11-21, 2004. (C) 2003 Wiley-Liss, Inc. | 1826 | no | no good fitness measure | NA | no | no |
| 1827 | 2017 | 43021 | Tian, EW; Nason, JD; Machado, CA; Zheng, LN; Yu, H; Kjellberg, F | 2015 | Lack of genetic isolation by distance, similar genetic structuring but different demographic histories in a fig-pollinating wasp mutualism | Historical abiotic factors such as climatic oscillations and extreme climatic events as well as biotic factors have shaped the structuring of species’ genetic diversity. In obligate species-specific mutualisms, the biogeographic histories of the interacting species are tightly linked. This could be particularly true for nuclear genes in the Ficus-pollinating wasp mutualistic association as the insects disperse pollen from their natal tree. In this study, we compare spatial genetic structure of plant and pollinator for the Ficus hirta-Valisia javana association throughout southeast China including Hainan Island, for both nuclear and cytoplasmic markers. We show that dispersal of the insect leads to plant and insect presenting similar signatures of lack of genetic isolation by distance for nuclear genes on the continent over a distance of 1000km. But we also show that the demographic histories of plant and insect are strikingly different. This is in agreement with extreme climatic events leading to transient regional extinctions of the insects, associated with local survival of the plants. We also observe evidence of genetic differentiation for both wasps and fig-tree between the continent and Hainan Island, although the Qiongzhou Strait is only on average 30km wide, suggesting that geographic isolation by itself has not been sufficient to generate this differentiation. Hence, our results suggest that in highly dispersive mutualistic systems, isolation-by-dispersal limitation across a geographic barrier could be supplemented by isolation by adaptation, and maybe by coevolution, allowing further genetic divergence. In such systems, species may frequently be composed of a single population. | 1827 | no | no good fitness measure, N pop differnt in GD? | NA | no | no |
| 1828 | 2017 | 43021 | Pujolar, JM; Maes, GE; Vancoillie, C; Volckaert, FAM | 2006 | Environmental stress and life-stage dependence on the detection of heterozygosity-fitness correlations in the European eel, Anguilla anguilla | Heterozygosity-fitness correlations (HFCs) have been reported in populations of many species, although HFCs can clearly vary across species, conspecific populations, temporal samples, and sexes. We studied (i) the temporal stability of the association between genetic variation and growth rate (length and mass increase) and (ii) the influence of genetic variability on survival in the European eel (Anguilla anguilla Q. HFCs were assessed using genotypes from 10 allozyme and 6 microsatellite markers in 22-month-old experimental individuals. The results were compared with those of a previous study carried out in 12-month-old individuals, in which more heterozygous individuals showed a significantly faster growth rate. In contrast, 22-month-old individuals showed no evidence that genetic variability was correlated with growth rate. Additionally, heterozygous individuals did not show a higher survival rate compared with more homozygous individuals after either handling stress or parasite infection. The decrease in HFCs over time is consistent with the general prediction that differences in growth and survival among individuals are maximal early in life and in our case most likely due to the relaxation of environmental conditions related to population-density effects. Alternatively, the decline in HFCs could be attributed to either ontogenetic variance in gene activity between 12- and 22-month-old individuals or differential mortality leaving only the largest individuals. | 1828 | no | no good fitness measure, N pop differnt in GD? | NA | no | no |
| 1829 | 2017 | 43021 | Hughes, AL; Hughes, MAK | 2007 | Coding sequence polymorphism in avian mitochondrial genomes reflects population histories | Nucleotide sequence diversity at mitochondrial protein-coding loci from 72 species of birds from different geographical regions was analysed in order to test the hypothesis that temperate zone species show population genetic effects of past glaciation. Temperate zone species showed reduced nucleotide diversity in comparison to tropical mainland species, suggesting that the latter have long-term effective population sizes due to population bottleneck effects during the most recent glaciation. This hypothesis was further supported by evidence of an unusually high estimated rate of population growth in species breeding in North America and wintering in the New World tropics (Nearctic migrants), consistent with population expansion after a bottleneck. Nearctic migrants also showed evidence of an abundance of rare nonsynonymous (amino acid-altering) polymorphisms, a pattern suggesting that slightly deleterious polymorphisms drifted to high frequencies during a bottleneck and are now being eliminated by selection. Because the shape of the North American land mass limited the area available for refugia during glaciation, the bottleneck effects are predicted to have been particularly strong in Nearctic migrants, and this prediction was supported. The reduced genetic diversity of Nearctic migrants provides an additional basis for concern for the survival of these species, which are threatened by loss of habitat in the winter range and by introduced disease. | 1829 | no | no good fitness measure, N pop differnt in GD? | NA | no | no |
| 1830 | 2017 | 43021 | McQuillan, PB; Ek, CJ | 1997 | A biogeographical analysis of the Tasmanian endemic Ptunarra brown butterfly, Oreixenica ptunarra Couchman (Lepidoptera: Nymphalidae: Satyrinae) | Considerable geographic variation occurs in the Tasmanian endemic butterfly, Oreixenica ptunarra, and there is a high correlation between clusters of morphological characters and ecological factors, especially climate and elevation. Evidence is presented for the existence of a longitudinal dine in phenotypic characters of wing pattern and size, which is unrelated to the modest amount of variation in the male genitalia (a possible surrogate for genetic variability). Butterflies from warmer, less cloudy eastern Tasmania are larger and less dark in colour than those from the west, culminating in the small dark populations of the north-west. This suggests selection for efficiency in thermoregulation as climatic conditions become more marginal for adult activity from east to west. The prevailing subspecies classification does not fully reflect the range of variation in this species. Conservation strategies that aim to guarantee the survival of the collective phenotype of O. ptunarra based on this taxonomy are therefore misinformed. The north-west populations are disjunct geographically and in features of phenotype, but are not especially discrete in the morphology of the male genitalia. We propose that the subspecies angeli Couchman and roonina Couchman be reduced to synonymy with nominotypical ptunarra Couchman, and a new subspecies should be recognised to incorporate populations from the montane grasslands of northwestern Tasmania. | 1830 | no | no good fitness measure, N pop differnt in GD? | NA | no | no |
| 1831 | 2017 | 43021 | Baling, M; Stuart-Fox, D; Brunton, DH; Dale, J | 2016 | Habitat suitability for conservation translocation: The importance of considering camouflage in cryptic species | Habitat suitability is a critical aspect for the successful establishment of a translocated population. Past studies have identified multiple factors that contribute to habitat suitability, including resource availability, presence of invasive species, landscape connectivity and climate. However, visual camouflage - coloration that conceals individuals from predators or prey - is another important ecological requirement that has been largely overlooked. We provide a case study to illustrate how color patterns of a prey species can change from that of the source site following translocation. Shore skinks (Oligosoma smithi) were moved from a coastal sand dune ecosystem to an offshore island beach that differed in substrate and color. Within one year following release, the translocated population’s color pattern variation had reduced to mostly one pattern type. The high match in color patterns between the skinks and release site (including a new substrate type) may have contributed to the observed color pattern shift. This reduced variation in color pattern implies that not all founders survived, potentially decreasing the genetic diversity in the population. Our study highlights the importance of considering a species’ camouflage requirements when selecting habitat for release, not only to maximize founder survival and establishment success, but also to maintain phenotypic and genotypic diversity in the long-term. (C) 2016 Elsevier Ltd. All rights reserved. | 1831 | no | No GD diff between pop | NA | no | no |
| 1832 | 2017 | 43021 | Evans, TA | 1999 | Kin recognition in a social spider | Social spiders accept immigrant spiders into their kin-based groups, suggesting that spiders cannot recognize kin and may lose inclusive fitness benefits. A field and two laboratory experiments on Diaea ergandros, a social crab spider, demonstrated that younger and older instar D. ergandros do discriminate siblings, but potential benefits were variable and not equally distributed. First, proportional survival was greater in large groups regardless of the within-group relatedness, so accepting immigrants increases probability of group survival (although relatedness was more important among smaller groups). Second, juvenile D. ergandros ate unrelated spiders instead of siblings when starved, so immigrants might represent a food reserve in times of food shortage. Third, subadult resident, sibling females cannibalized unrelated, immigrant females and their brothers instead of immigrant males when starved, suggesting that subadult female spiders may maximize outbreeding opportunities. These benefits provide selective pressure for groups to accept immigrants, but as benefits are realized differentially, conflict and cooperation will exist within spider groups similar to that shown in other group-living taxa. | 1832 | maybe | ok gd measurement and pop level comp? | discarded, cannibalism as mortality | no | no |
| 1833 | 2017 | 43021 | Muwanika, VB; Kock, R; Masembe, C; Siegismund, HR | 2012 | Genetic diversity in the desert warthog (Phacochoerus aethiopicus delameri) population of eastern Africa | Genetic variability is an important component in the ability of populations to evolve and adapt to changing environmental conditions and consequently for their long-term survival. Here we report the first description of both nuclear loci and mitochondrial control region sequence variability in a population of the desert warthog (Phacochoerus aethiopicus) sampled from 12 localities in its natural range in eastern Africa. From the total sample (30 individuals), at the six microsatellite loci that were analysed, a total of 43 alleles was observed averaging seven alleles per locus. Expected heterozygosity (H-E) per locus was high, ranging from 0.53 to 0.87. At the mitochondrial loci, nucleotide diversity was low (pi = 0.12%) with two unique haplotypes observed from the 19 individuals that amplified successfully. The diversity indices observed in the desert warthog are comparable to those previously reported for the closely related but widespread species, the common warthog (Phacochoerus africanus). These results suggest that the desert warthog is not genetically depauperate despite the rinderpest epidemic of the 1880s that eliminated it from most of its natural range. | 1833 | no | no good fitness measure | NA | no | no |
| 1834 | 2017 | 43021 | Zhuang, Z; Gusev, A; Cho, J; Pe’er, I | 2012 | Detecting Identity by Descent and Homozygosity Mapping in Whole-Exome Sequencing Data | The detection of genetic segments of Identical by Descent (IBD) in Genome-Wide Association Studies has proven successful in pinpointing genetic relatedness between reportedly unrelated individuals and leveraging such regions to shortlist candidate genes. These techniques depend on high-density genotyping arrays and their effectiveness in diverse sequence data is largely unknown. Due to decreasing costs and increasing effectiveness of high throughput techniques for whole-exome sequencing, an influx of exome sequencing data has become available. Studies using exomes and IBD-detection methods within known pedigrees have shown that IBD can be useful in finding hidden genetic candidates where known relatives are available. We set out to examine the viability of using IBD-detection in whole exome sequencing data in population-wide studies. In doing so, we extend GERMLINE, a method to detect IBD from exome sequencing data by finding small slices of matching alleles between pairs of individuals and extending them into full IBD segments. This algorithm allows for efficient population-wide detection in dense data. We apply this algorithm to a cohort of Crohn’s Disease cases where whole-exome and GWAS array data is available. We confirm that GWAS-based detected segments are highly accurate and predictive of underlying shared variation. Where segments inferred from GWAS are expected to be of high accuracy, we compare exome-based detection accuracy of multiple detection strategies. We find detection accuracy to be prohibitively low in all assessments, both in terms of segment sensitivity and specificity. Even after isolating relatively long segments beyond 10cM, exome-based detection continued to offer poor specificity/sensitivity tradeoffs. We hypothesize that the variable coverage and platform biases of exome capture account for this decreased accuracy and look toward whole genome sequencing data as a higher quality source for detecting population-wide IBD. | 1834 | no | no good fitness measure, N pop differnt in GD? | NA | no | no |
| 1835 | 2017 | 43021 | De Wit, P; Rogers-Bennett, L; Kudela, RM; Palumbi, SR | 2014 | Forensic genomics as a novel tool for identifying the causes of mass mortality events | Toxic spills, hypoxia, disease outbreaks and toxin-producing algal blooms are all possible causes of mass mortality events, but in many cases it can be difficult to pinpoint the cause of death. Here we present a new approach that we name ‘forensic genomics’, combining field surveys, toxin testing and genomic scans. Forensic genomics queries allele frequencies of surviving animals for signatures of agents causing mass mortality and, where genetic diversity is high, is uniquely suited to identify natural selection in action. As a proof of concept, we use this approach to investigate the causes of an invertebrate mass mortality event, and its genetic effects on an abalone population. Our results support that a harmful algal bloom producing a yessotoxin was a major causative agent to the event. | 1835 | no | NA | NA | no | no |
| 1836 | 2017 | 43021 | Bennett, KL; Shija, F; Linton, YM; Misinzo, G; Kaddumukasa, M; Djouaka, R; Anyaele, O; Harris, A; Irish, S; Hlaing, T; Prakash, A; Lutwama, J; Walton, C | 2016 | Historical environmental change in Africa drives divergence and admixture of Aedes aegypti mosquitoes: a precursor to successful worldwide colonization? | Increasing globalization has promoted the spread of exotic species, including disease vectors. Understanding the evolutionary processes involved in such colonizations is both of intrinsic biological interest and important to predict and mitigate future disease risks. The Aedes aegypti mosquito is a major vector of dengue, chikungunya and Zika, the worldwide spread of which has been facilitated by Ae. aegypti’s adaption to human-modified environments. Understanding the evolutionary processes involved in this invasion requires characterization of the genetic make-up of the source population (s). The application of approximate Bayesian computation (ABC) to sequence data from four nuclear and one mitochondrial marker revealed that African populations of Ae. aegypti best fit a demographic model of lineage diversification, historical admixture and recent population structuring. As ancestral Ae. aegypti were dependent on forests, this population history is consistent with the effects of forest fragmentation and expansion driven by Pleistocene climatic change. Alternatively, or additionally, historical human movement across the continent may have facilitated their recent spread and mixing. ABC analysis and haplotype networks support earlier inferences of a single out-of-Africa colonization event, while a cline of decreasing genetic diversity indicates that Ae. aegypti moved first from Africa to the Americas and then to Asia. ABC analysis was unable to verify this colonization route, possibly because the genetic signal of admixture obscures the true colonization pathway. By increasing genetic diversity and forming novel allelic combinations, divergence and historical admixture within Africa could have provided the adaptive potential needed for the successful worldwide spread of Ae. aegypti. | 1836 | no | no good fitness measure, N pop differnt in GD? | NA | no | no |
| 1837 | 2017 | 43021 | Berghout, J; Higgins, S; Loucoubar, C; Sakuntabhai, A; Kain, KC; Gros, P | 2012 | Genetic diversity in human erythrocyte pyruvate kinase | Previously, we have shown that pyruvate kinase, liver and red cell isoform (PKLR) deficiency protects mice in vivo against blood-stage malaria, and observed that reduced PKLR function protects human erythrocytes against Plasmodium falciparum replication ex vivo. Here, we have sequenced the human PKLR gene in 387 individuals from malaria-endemic and other regions in order to assess genetic variability in different geographical regions and ethnic groups. Rich genetic diversity was detected in PKLR, including 59 single-nucleotide polymorphisms and several loss-of-function variants (frequency 1.5%). Haplotype distribution and allele frequency varied considerably with geography. Neutrality testing suggested positive selection of the genein the sub-Saharan African and Pakistan populations. It is possible that such positive selection involves the malarial parasite. Genes and Immunity (2012) 13, 98-102; doi:10.1038/gene.2011.54; published online 11 August 2011 | 1837 | no | human | NA | no | no |
| 1838 | 2017 | 43021 | Gaunt, ER; Jansen, RR; Poovorawan, Y; Templeton, KE; Toms, GL; Simmonds, P | 2011 | Molecular Epidemiology and Evolution of Human Respiratory Syncytial Virus and Human Metapneumovirus | Human respiratory syncytial virus (HRSV) and human metapneumovirus (HMPV) are ubiquitous respiratory pathogens of the Pneumovirinae subfamily of the Paramyxoviridae. Two major surface antigens are expressed by both viruses; the highly conserved fusion (F) protein, and the extremely diverse attachment (G) glycoprotein. Both viruses comprise two genetic groups, A and B. Circulation frequencies of the two genetic groups fluctuate for both viruses, giving rise to frequently observed switching of the predominantly circulating group. Nucleotide sequence data for the F and G gene regions of HRSV and HMPV variants from the UK, the Netherlands, Bangkok and data available from Genbank were used to identify clades of both viruses. Several contemporary circulating clades of HRSV and HMPV were identified by phylogenetic reconstructions. The molecular epidemiology and evolutionary dynamics of clades were modelled in parallel. Times of origin were determined and positively selected sites were identified. Sustained circulation of contemporary clades of both viruses for decades and their global dissemination demonstrated that switching of the predominant genetic group did not arise through the emergence of novel lineages each respiratory season, but through the fluctuating circulation frequencies of pre-existing lineages which undergo proliferative and eclipse phases. An abundance of sites were identified as positively selected within the G protein but not the F protein of both viruses. For HRSV, these were discordant with previously identified residues under selection, suggesting the virus can evade immune responses by generating diversity at multiple sites within linear epitopes. For both viruses, different sites were identified as positively selected between genetic groups. | 1838 | no | human | NA | no | no |
| 1839 | 2017 | 43021 | Tzedakis, PC; Emerson, BC; Hewitt, GM | 2013 | Cryptic or mystic? Glacial tree refugia in northern Europe | Here, we examine the evidence for tree refugia in northern Europe during the Late Pleniglacial (LPG) interval of maximum tree-range contraction. Our review highlights the often equivocal nature of genetic data and a tendency to overestimate potential tree distributions due to warm climate-model bias, and also reveals a convergence of macrofossil and pollen evidence. What emerges is the absence of temperate trees north of 45 degrees N and a west-east (W-E) asymmetry in boreal tree distribution, with a treeless Western Europe north of 46 degrees N, while restricted boreal populations persisted in Eastern Europe up to 49 degrees N, and higher latitudes east of the Fennoscandian ice-sheet. These results have implications for current thinking on European genetic diversity patterns, species migration capacity, and conservation strategies. | 1839 | no | no good fitness measure, N pop differnt in GD? | NA | no | no |
| 1840 | 2017 | 43021 | Bernardo, AN; Ma, HX; Zhang, DD; Bai, GH | 2012 | Single nucleotide polymorphism in wheat chromosome region harboring Fhb1 for Fusarium head blight resistance | Fusarium head blight (FHB) is a destructive disease that reduces wheat grain yield and quality. To date, the quantitative trait locus on 3BS (Fhb1) from Sumai 3 has shown the largest effect on FHB resistance. Single nucleotide polymorphism (SNP) is the most common form of genetic variation and is suitable for high-throughput marker-assisted selection (MAS). We analyzed SNPs derived from 23 wheat expressed sequence tags (ESTs) that previously mapped near Fhb1 on chromosome 3BS. Using 71 Ning 7840/Clark BC7F7 recombinant inbred lines and the single-base extension method, we mapped seven SNP markers between Xgwm533 and Xgwm493, flanking markers for Fhb1. Five of the SNPs explained 45-54% of the phenotypic variation for FHB resistance. Haplotype analysis of 63 wheat accessions from eight countries based on SNPs in EST sequences, simple sequence repeats, and sequence tagged sites in the Fhb1 region identified four major groups: (1) US-Clark, (2) Asian, (3) US-Ernie, and (4) Chinese Spring. The Asian group consisted of Chinese and Japanese accessions that carry Fhb1 and could be differentiated from other groups by marker Xsnp3BS-11. All Sumai 3-related accessions formed a subgroup within the Asian group and could be sorted out by Xsnp3BS-8. The SNP markers identified in this study should be useful for MAS of Fhb1 and fine mapping to facilitate cloning of the Fhb1 resistance gene. | 1840 | no | crop | NA | no | no |
| 1841 | 2017 | 43021 | Marroni, F; Pichler, I; De Grandi, A; Volpato, CB; Vogl, FD; Pinggera, GK; Bailey-Wilson, JE; Pramstaller, PP | 2006 | Population isolates in south Tyrol and their value for genetic dissection of complex diseases | The study of genetic isolates is a promising approach for the study of complex genetic traits. The small and constant population size, lack of migration, and multiple relationships between individuals in the isolate population could reduce the genetic diversity, and lead to increased levels of linkage disequilibrium (LD). We studied the extent of LD on Xq13 in six population isolates from South Tyrol in the Eastern Italian Alps. We found different levels of LD in our study samples, probably reflecting their degrees of isolation and their demographic histories. The highest values were obtained in Val Gardena (ranking among the highest levels of LD in Europe) and in Stelvio, which qualified as a microisolate according to historical information, and biodemographic and genealogical criteria. Phylogenetic analysis revealed that the two Ladin-speaking populations are genetically distant from each other, and from their German-speaking neighbours, and are characterized by a smaller effective population size than the neighbouring valleys. These peculiar characteristics suggest that South Tyrol could be a unique resource for the study of complex diseases, showing all the characteristics of isolated populations with the advantage of including, in a fairly homogeneous environment, two genetically differentiated sub-populations. This could allow investigators to gain an insight into the contribution of genetic heterogeneity in complex diseases. | 1841 | no | NA | NA | no | no |
| 1842 | 2017 | 43021 | Carpi, G; Walter, KS; Ben Mamoun, C; Krause, PJ; Kitchen, A; Lepore, TJ; Dwivedi, A; Cornillot, E; Caccone, A; Diuk-Wasser, MA | 2016 | Babesia microti from humans and ticks hold a genomic signature of strong population structure in the United States | Background: Babesia microti is an emerging tick-borne apicomplexan parasite with increasing geographic range and incidence in the United States. The rapid expansion of B. microti into its current distribution in the northeastern USA has been due to the range expansion of the tick vector, Ixodes scapularis, upon which the causative agent is dependent for transmission to humans. Results: To reconstruct the history of B. microti in the continental USA and clarify the evolutionary origin of human strains, we used multiplexed hybrid capture of 25 B. microti isolates obtained from I. scapularis and human blood. Despite low genomic variation compared with other Apicomplexa, B. microti was strongly structured into three highly differentiated genetic clusters in the northeastern USA. Bayesian analyses of the apicoplast genomes suggest that the origin of the current diversity of B. microti in northeastern USA dates back 46 thousand years with a signature of recent population expansion in the last 1000 years. Human-derived samples belonged to two rarely intermixing clusters, raising the possibility of highly divergent infectious phenotypes in humans. Conclusions: Our results validate the multiplexed hybrid capture strategy for characterizing genome-wide diversity and relatedness of B. microti from ticks and humans. We find strong population structure in B. microti samples from the Northeast indicating potential barriers to gene flow. | 1842 | no | NA | NA | no | no |
| 1843 | 2017 | 43021 | Ottewell, KM; Donnellan, SC; Paton, DC | 2010 | Evaluating the Demographic, Reproductive, and Genetic Value of Eucalypt Paddock Trees for Woodland Restoration in Agricultural Landscapes | “Eucalypt woodlands have been extensively cleared in southern Australia for agricultural production and the scattered trees that remain (”“paddock trees”“) are at risk of extinction without conservation action to lift recruitment rates. Here we assessed the reproductive, demographic, and genetic structure of trees from two vegetation types (paddock vs. intact vegetation fragments) for each of two species of eucalypts to characterize post-clearance population structure and to evaluate paddock trees as seed sources for revegetation. Paddock trees of both species were structurally larger than trees in vegetation fragments, but seedlings and saplings were completely absent in paddocks due to ongoing agricultural land use. Eucalyptus leucoxylon paddock trees had high mortality, but overall paddock trees were not in poorer condition than fragment trees. E. leucoxylon paddock trees had lower reproductive output than trees in fragments, and Eucalyptus camaldulensis paddock trees had higher. Levels of genetic diversity of adult trees were very similar in the two vegetation types, and there was no genetic differentiation between paddock trees and remnant vegetation across our survey sites. These results suggest that paddock trees represent a potential source of seed to contribute to genetically diverse woodland restoration plantings, though further trials will be required to evaluate seedling survival on agriculturally modified land and under predicted future climate conditions.” | 1843 | no | no good fitness measure, N pop differnt in GD? | NA | no | no |
| 1844 | 2017 | 43021 | Chesler, EJ; Miller, DR; Branstetter, LR; Galloway, LD; Jackson, BL; Philip, VM; Voy, BH; Culiat, CT; Threadgill, DW; Williams, RW; Churchill, GA; Johnson, DK; Manly, KF | 2008 | The Collaborative Cross at Oak Ridge National Laboratory: developing a powerful resource for systems genetics | Complex traits and disease comorbidity in humans and in model organisms are the result of naturally occurring polymorphisms that interact with each other and with the environment. To ensure the availability of resources needed to investigate biomolecular networks and systems-level phenotypes underlying complex traits, we have initiated breeding of a new genetic reference population of mice, the Collaborative Cross. This population has been designed to optimally support systems genetics analysis. Its novel and important features include a high level of genetic diversity, a large population size to ensure sufficient power in high-dimensional studies, and high mapping precision through accumulation of independent recombination events. Implementation of the Collaborative Cross has been ongoing at the Oak Ridge National Laboratory (ORNL) since May 2005. Production has been systematically managed using a software-assisted breeding program with fully traceable lineages, performed in a controlled environment. Currently, there are 650 lines in production, and close to 200 lines are now beyond their seventh generation of inbreeding. Retired breeders enter a high-throughput phenotyping protocol and DNA samples are banked for analyses of recombination history, allele drift and loss, and population structure. Herein we present a progress report of the Collaborative Cross breeding program at ORNL and a description of the kinds of investigations that this resource will support. | 1844 | no | no good fitness measure, N pop differnt in GD? | NA | no | no |
| 1845 | 2017 | 43021 | Miller, HC; Conrad, AM; Barker, SC; Daugherty, CH | 2007 | Distribution and phylogenetic analyses of an endangered tick, Amblyomma sphenodonti | In this study we investigate the geographic distribution, genetic diversity, and phylogenetic relationships of an endangered tick, Amblyomma sphenodonti (Family Ixodidae). Amblyomma sphenodonti and its host, the tuatara (Sphenodon), are found only on small offshore islands around New Zealand. Our results show that Amblyomma sphenodonti has a more severely restricted geographic distribution than its host, as it was found on only eight of 28 islands (four out of 12 island groups) where tuatara still live. The prevalence of A. sphenodonti is likely to have been affected by low host density and fluctuations in host population size as tuatara populations became isolated on offshore islands. Analysis of A. sphenodonti cytochrome oxidase 1 (CO1) sequences indicated a lack of gene flow between islands, with fixed differences in CO1 sequences between islands, but almost no genetic diversity within island populations. A similar phylogenetic pattern to that observed in tuatara mtDNA was observed, indicating co-evolution of two species, at least since the Pleistocene. Phylogenetic analysis using 18S rRNA sequences suggest that A. sphenodonti is not closely related to other Amblyomma species, and that a separate genus for this species may be warranted. However, data from other ixodid ticks are required before the distinctiveness of A. sphenodonti can be confirmed and the phylogenetic relationships among ixodid ticks fully understood. | 1845 | no | gd of pathogen | NA | no | no |
| 1846 | 2017 | 43021 | Gupta, R; Ratan, A; Rajesh, C; Chen, R; Kim, HL; Burhans, R; Miller, W; Santhosh, S; Davuluri, RV; Butte, AJ; Schuster, SC; Seshagiri, S; Thomas, G | 2012 | Sequencing and analysis of a South Asian-Indian personal genome | Background: With over 1.3 billion people, India is estimated to contain three times more genetic diversity than does Europe. Next-generation sequencing technologies have facilitated the understanding of diversity by enabling whole genome sequencing at greater speed and lower cost. While genomes from people of European and Asian descent have been sequenced, only recently has a single male genome from the Indian subcontinent been published at sufficient depth and coverage. In this study we have sequenced and analyzed the genome of a South Asian Indian female (SAIF) from the Indian state of Kerala. Results: We identified over 3.4 million SNPs in this genome including over 89,873 private variations. Comparison of the SAIF genome with several published personal genomes revealed that this individual shared similar to 50% of the SNPs with each of these genomes. Analysis of the SAIF mitochondrial genome showed that it was closely related to the U1 haplogroup which has been previously observed in Kerala. We assessed the SAIF genome for SNPs with health and disease consequences and found that the individual was at a higher risk for multiple sclerosis and a few other diseases. In analyzing SNPs that modulate drug response, we found a variation that predicts a favorable response to metformin, a drug used to treat diabetes. SNPs predictive of adverse reaction to warfarin indicated that the SAIF individual is not at risk for bleeding if treated with typical doses of warfarin. In addition, we report the presence of several additional SNPs of medical relevance. Conclusions: This is the first study to report the complete whole genome sequence of a female from the state of Kerala in India. The availability of this complete genome and variants will further aid studies aimed at understanding genetic diversity, identifying clinically relevant changes and assessing disease burden in the Indian population. | 1846 | no | human | NA | no | no |
| 1847 | 2017 | 43021 | Schrider, DR; Kern, AD | 2014 | Discovering Functional DNA Elements Using Population Genomic Information: A Proof of Concept Using Human mtDNA | Identifying the complete set of functional elements within the human genome would be a windfall for multiple areas of biological research including medicine, molecular biology, and evolution. Complete knowledge of function would aid in the prioritization of loci when searching for the genetic bases of disease or adaptive phenotypes. Because mutations that disrupt function are disfavored by natural selection, purifying selection leaves a detectable signature within functional elements; accordingly, this signal has been exploited for over a decade through the use of genomic comparisons of distantly related species. While this is so, the functional complement of the genome changes extensively across time and between lineages; therefore, evidence of the current action of purifying selection in humans is essential. Because the removal of deleterious mutations by natural selection also reduces within-species genetic diversity within functional loci, dense population genetic data have the potential to reveal genomic elements that are currently functional. Here, we assess the potential of this approach by examining an ultradeep sample of human mitochondrial genomes (n = 16,411). We show that the high density of polymorphism in this data set precisely delineates regions experiencing purifying selection. Furthermore, we show that the number of segregating alleles at a site is strongly correlated with its divergence across species after accounting for known mutational biases in human mitochondrial DNA (rho = 0.51; P < 2.2 x 10(-16)). These two measures track one another at a remarkably fine scale acrossmany loci-a correlation that is purely the result of natural selection. Our results demonstrate that genetic variation has the potential to reveal with surprising precision which regions in the genome are currently performing important functions and likely to have deleterious fitness effects when mutated. As more complete human genomes are sequenced, similar power to reveal purifying selection may be achievable in the human nuclear genome. | 1847 | no | human | NA | no | no |
| 1848 | 2017 | 43021 | Jones, ME; Jarman, PJ; Lees, CM; Hesterman, H; Hamede, RK; Mooney, NJ; Mann, D; Pukk, CE; Bergfeld, J; McCallum, H | 2007 | Conservation management of tasmanian devils in the context of an emerging, extinction-threatening disease: Devil facial tumor disease | An emerging infectious facial cancer threatens Tasmanian devils with extinction. The disease is likely to occur across the range of the devil within 5 years. This urgent time frame requires management options that can be implemented immediately: the establishment of insurance populations, in captivity, wild-living on islands, and aiming for eradication in areas that can be isolated. The long-term options of the spontaneous or assisted evolution of resistance or development of a field-deliverable vaccine are unlikely to be available in time. The disease’s characteristic allograft transmission through intimate contact simplifies isolation of insurance populations and breaking transmission in suppression trials. Better knowledge of contact matrices in wild devils will help focus timing and demographic targets of removals. A metapopulation approach is needed that integrates captive and wild-living island and peninsula (disease suppression) populations to minimize the loss of genetic diversity over 50 years until either extinction and reintroduction can occur, resistance evolves or a field-deliverable vaccine is developed. Given the importance of the insurance populations and the low genetic diversity of devils, a conservative target for retention of 95% genetic diversity is recommended. Encouraging preliminary results of the first disease-suppression trial on a large peninsula show fewer late stage tumors and no apparent population decline. Limiting geographic spread or suppressing the disease on a broadscale are both unlikely to be feasible. Since the synergy of devil decline and impending fox establishment could have devastating consequences for Tasmanian wildlife, it is crucial to manage the dynamics of new and old predator species together. | 1848 | no | no good fitness measure, N pop differnt in GD? | NA | no | no |
| 1849 | 2017 | 43021 | Maillard, JC; Kiem, PV; Tung, N; Thu, NV; Berthouly, C; Libeau, G; Kwiatek, O | 2008 | Examples of Probable Host-Pathogen Co-adaptation/Co-evolution in Isolated Farmed Animal Populations in the Mountainous Regions of North Vietnam | In Vietnam, for a number of specific geographical and historical reasons, the mountainous areas have preserved an exceptional diversity of wild and domestic animal species of high socioeconomic interest. This endemic genetic diversity fosters a rapid response to environmental change in mostly isolated local communities and, in particular, fosters the constant adaptation of ecosystems common to humans and farmed and wild animal populations and pathogens. During a 2-year study carried out in several mountainous regions of North Vietnam near the Chinese border, we surveyed 1697 breeders in 249 villages and gathered 5815 biological samples among the four main domesticated species of food animals: chickens, cattle, buffaloes, and goats. Serological analyses were carried out by ELISA on 726 sera in order to assess the prevalence of antibodies specific to two major diseases suspected to be present in the region: avian influenza (AI) and peste des petits ruminants (PPR). The results reported here reveal the presence of antibodies specific to AI, but not the H5N1 highly pathogenic strain, and the presence of antibodies specific to PPR, confirming that this disease, never previously described in Southeast Asia, is present in this region, with no mortality and little or no evidence of clinical cases. These are probably situations of co-evolutive epidemiological equilibrium between pathogen populations, which may have lost their virulence, and animal populations that have acquired genetic resistances over generations, either naturally or through genetic introgression from related wild species better adapted to such pathogens. These results suggest the need for more research, both short-term and, more globally, long-term. | 1849 | no | domesticated animal | NA | no | no |
| 1850 | 2017 | 43021 | Carpi, G; Kitchen, A; Kim, HL; Ratan, A; Drautz-Moses, DI; McGraw, JJ; Kazimirova, M; Rizzoli, A; Schuster, SC | 2016 | Mitogenomes reveal diversity of the European Lyme borreliosis vector Ixodes ricinus in Italy | “In Europe, the Ixodes ricinus tick is the most important vector of the etiological agents of Lyme borreliosis and several other emerging tick-borne diseases. Because tick-borne pathogens are dependent on their vectors for transmission, understanding the vector population structure is crucial to inform public health research of pathogen dynamics and spread. However, the population structure and dynamics of this important vector species are not well understood as most genetic studies utilize short mitochondrial and nuclear sequences with little diversity. Herein we obtained and analyzed complete mitochondrial genome (hereafter”“mitogenome”“) sequences to better understand the genetic diversity and the population structure of I. ricinus from two long-standing tick-borne disease foci in northern Italy. Complete mitogenomes of 23 I. ricinus ticks were sequenced at high coverage. Out of 23 mitogenome sequences we identified 17 unique haplotypes composed of 244 segregating sites. Phylogenetic reconstruction using 18 complete mitogenome sequences revealed the coexistence of four highly divergent I. ricinus maternal lineages despite the narrow spatial scale over which these samples were obtained (100 km). Notably, the estimated coalescence time of the 18 mitogenome haplotypes is 427 thousand years ago (95% HPD 330, 540). This divergence between I. ricinus lineages is consistent with the mitochondrial diversity of other arthropod vector species and indicates that long-term I. ricinus populations may have been less structured and larger than previously thought. Thus, this study suggests that a rapid and accurate retrieval of full mitochondrial genomes from this disease vector enables fine-resolution studies of tick intraspecies genetic relationships, population differentiation, and demographic history. (C) 2016 Elsevier Inc. All rights reserved.” | 1850 | no | gd of pathogen | NA | no | no |
| 1851 | 2017 | 43021 | Cabrera-Brandt, MA; Verdugo, JA; Ramirez, CC; Lacroze, JP; Sauge, MH; Figueroa, CC | 2015 | Intra-specific variation of behavioral signals in suppressing plant defenses in the green peach aphid Myzus persicae, feeding on the resistant wild peach Prunus davidiana | The green peach aphid, Myzus persicae (Sulzer), is a generalist insect herbivore capable of using a wide range of cultivated plants and weeds. In this laboratory study, we examined the inter-clonal variation in M. persicae’s ability to exploit the wild peach Prunus davidiana, a source of genetic resistance to aphids used by peach breeding programs. An initial screening of a set of sexually reproducing clones collected from commercial orchards planted with susceptible varieties found significant genetic variation in aphid survival on P. davidiana. Comparison of two clones (Fr2 and Fr12) found marked differences in colony sizes achieved. A detailed analysis of probing and feeding behavior showed that the clone exhibiting the highest performance on P. davidiana (Fr2) initiated probing earlier than the lower performing clone (Fr12). Periods of non-probing were also significantly shorter for this clone. Finally, Fr2 produced more and longer events of watery saliva injection into sieve elements. We discuss these results in terms of host plant adaptation by aphids and their capacity to overcome plant-resistance genes. | 1851 | no | no good fitness measure, N pop differnt in GD? | NA | no | no |
| 1852 | 2017 | 43021 | Lucht, JM; Mauch-Mani, B; Steiner, HY; Metraux, JP; Ryals, J; Hohn, B | 2002 | Pathogen stress increases somatic recombination frequency in Arabidopsis | Evolution is based on genetic variability and subsequent phenotypic selection. Mechanisms that modulate the rate of mutation according to environmental cues, and thus control the balance between genetic stability and flexibility, might provide a distinct evolutionary advantage(1-4). Stress-induced mutations stimulated by unfavorable environments, and possible mechanisms for their induction, have been described for several organisms(2-4), but research in this area has mainly focused on microorganisms. We have analyzed the influence of adverse environmental conditions on the genetic stability of the higher plant Arabidopsis thaliana. Here we show that a biotic stress factor-attack by the oomycete pathogen Peronospora parasitica can stimulate somatic recombination in Arabidopsis. The same effect was observed when plant pathogen-defense mechanisms were activated by the chemicals 2,6-dichloroisonicotinic acid (INA) or benzothiadiazole (BTH), or by a mutation (cim3). Together with previous studies of recombination induced by abiotic factors, these findings suggest that increased somatic recombination is a general stress response in plants. The increased genetic flexibility might facilitate evolutionary adaptation of plant populations to stressful environments. | 1852 | no | No GD diff between pop | NA | no | no |
| 1853 | 2017 | 43021 | Garcia, PE; Winkler, M; Flatscher, R; Sonnleitner, M; Krejcikova, J; Suda, J; Hulber, K; Schneeweiss, GM; Schonswetter, P | 2012 | Extensive range persistence in peripheral and interior refugia characterizes Pleistocene range dynamics in a widespread Alpine plant species (Senecio carniolicus, Asteraceae) | Recent evidence suggests that survival of arctic-alpine organisms in peripheral or interior glacial refugia are not mutually exclusive and may both be involved in shaping an organisms Pleistocene history, yet potentially at different time levels. Here, we test this hypothesis in a high-mountain plant (diploid lineage of Senecio carniolicus, Asteraceae) from the Eastern European Alps, in which patterns of morphological variation and current habitat requirements suggest survival in both types of refugia. To this end, we used AFLPs, nuclear and plastid DNA sequences and analysed them, among others, within a graph theoretic framework and using novel Bayesian methods of phylogeographic inference. On the basis of patterns of genetic diversity, occurrence of rare markers, distribution of distinct genetic lineages and patterns of range connectivity both interior refugia in the formerly strongly glaciated central Alps and peripheral refugia along the southern margin of the Alps were identified. The presence of refugia congruently inferred by markers resolving at different time levels suggests that these refugia acted as such throughout several glacial cycles. The high degree of range persistence together with gradual range expansion, which contrasts with the extent of range shifts implied for other Alpine species, is likely responsible for incipient lineage differentiation evident from the genetic data. Replacing a simplistic peripheral vs. interior refugia dualism by more complex models involving both types of refugia and considering different time levels will help identifying common phylogeographic patterns with respect to, for instance, location of refugia and colonization routes and elucidating their underlying genetic and/or ecological causes. | 1853 | no | no good fitness measure, N pop differnt in GD? | NA | no | no |
| 1854 | 2017 | 43021 | Gowda, M; Shirke, MD; Mahesh, HB; Chandarana, P; Rajamani, A; Chattoo, BB | 2015 | Genome analysis of rice-blast fungus Magnaporthe oryzae field isolates from southern India | The Indian subcontinent is the center of origin and diversity for rice (Oryza sativa L.). The O. sativa ssp. indica is a major food crop grown in India, which occupies the first and second position in area and production, respectively. Blast disease caused by Magnaporthe oryzae is a major constraint to rice production. Here, we report the analysis of genome architecture and sequence variation of two field isolates, B157 and MG01, of the blast fungus from southern India. The 40 Mb genome of B157 and 43 Mb genome of MG01 contained 11,344 and 11,733 predicted genes, respectively. Genomic comparisons unveiled a large set of SNPs and several isolate specific genes in the Indian blast isolates. Avr genes were analyzed in several sequenced Magnaporthe strains; this analysis revealed the presence of Avr-Pizt and Avr-Ace1 genes in all the sequenced isolates. Availability of whole genomes of field isolates from India will contribute to global efforts to understand genetic diversity of M. oryzae population and to track the emergence of virulent pathotypes. (C) 2015 The Authors. Published by Elsevier Inc. | 1854 | no | gd of pathogen | NA | no | no |
| 1855 | 2017 | 43021 | Britto, FB; Mendes, DSF; Ogawa, M; Cintra, IHA; Diniz, FM | 2011 | Single primer-based DNA amplification as a suitable and low-cost tool for assessing genetic diversity in mangrove crabs | We used single primer-based DNA markers to assess genetic variability of the mangrove crab, Ucides cordatus, collected from four different localities from Para to Santa Catarina States in Brazil (almost 5000 km distant). Five primers were chosen based on the consistency of the amplified bands and the polymorphism of each locus. A total of 78 loci were amplified in 76 samples; high polymorphism rates were detected in the entire sample (80.8%) and within each locality (73.5-79.5%). Analysis of molecular variance demonstrates significant differences between localities (P < 0.001); however, the Phi(ST) value (0.078) indicates a low level of genetic differentiation, which suggests that U. cordatus larvae can spread over large distances. The variation was distributed among the samples, and most of it was attributed to differences among individuals within localities. Cluster analysis, based on the Jaccard similarity coefficient, and the Mantel test gave similar results to the analysis of molecular variance data. Despite the low level of population structuring, these markers could be used for studying U. cordatus diversity, due to the high level of polymorphism. | 1855 | no | no good fitness measure | NA | no | no |
| 1856 | 2017 | 43021 | Zanetti, D; Carreras-Torres, R; Esteban, E; Via, M; Moral, P | 2015 | Potential Signals of Natural Selection in the Top Risk Loci for Coronary Artery Disease: 9p21 and 10q11 | Background Coronary artery disease (CAD) is a complex disease and the leading cause of death in the world. Populations of different ancestry do not always share the same risk markers. Natural selective processes may be the cause of some of the population differences detected for specific risk mutations. Objective In this study, 384 single nucleotide polymorphisms (SNPs) located in four genomic regions associated with CAD (1p13, 1q41, 9p21 and 10q11) are analysed in a set of 19 populations from Europe, Middle East and North Africa and also in Asian and African samples from the 1000 Genomes Project. The aim of this survey is to explore for the first time whether the genetic variability in these genomic regions is better explained by demography or by natural selection. Results The results indicate significant differences in the structure of genetic variation and in the LD patterns among populations that probably explain the population disparities found in markers of susceptibility to CAD. Conclusions The results are consistent with potential signature of positive selection in the 9p21 region and of balancing selection in the 9p21 and 10q11. Specifically, in Europe three CAD risk markers in the 9p21 region (rs9632884, rs1537371 and rs1333042) show consistent signals of positive selection. The results of this study are consistent with a potential selective role of CAD in the configuration of genetic diversity in current human populations. | 1856 | no | NA | NA | no | no |
| 1857 | 2017 | 43021 | McLaren, PJ; Fellay, J; Telenti, A | 2013 | European Genetic Diversity and Susceptibility to Pathogens | Infectious diseases, both in their endemic and epidemic forms, have shaped the human genome. Ecology has also contributed to geographically constrained pressures on human populations. There are now multiple examples of population-specific genetic variants that modulate susceptibility to infection several of which have been observed solely in Europeans. The pathogen genome also mutates and adapts to individuals and common alleles in populations. The current understanding has benefited from genome-wide association studies as well as from rapid progress in the genetic characterization of Mendelian immunodeficiencies that are defined by susceptibility to specific pathogens. It is expected that current efforts to characterize rare human genetic variants will contribute to the understanding of severe manifestations of common infections in European and other human groups. (C) 2014 S. Karger AG, Basel | 1857 | no | human | NA | no | no |
| 1858 | 2017 | 43021 | Qiu, WG; Dykhuizen, DE; Acosta, MS; Luft, BJ | 2002 | Geographic uniformity of the Lyme disease spirochete (Borrelia burgdorferi) and its shared history with tick vector (Ixodes scapularis) in the northeastern United States | “Over 80% of reported cases of Lyme disease in the United States occur in coastal regions of northeastern and mid-Atlantic states. The genetic structure of the Lyme disease spirochete (Borrelia burgdorferi) and its main tick vector (Ixodes scapularis) was studied concurrently and comparatively by sampling natural populations of I. scapularis ticks along the East Coast from 1996 to 1998. Borrelia is genetically highly diverse at the outer surface Protein ospC. Since Borrelia is highly clonal, the ospC alleles call be used to define clones. A newly designed reverse line blotting (RLB) assay shows that LIP to 10 Borrelia clones can infect a single tick. The clone frequencies in Borrelia populations are the same across the Northeast. On the other hand, L scapularis populations show strong regional divergence (among northeastern, mid-Atlantic, and southern states) as well as local differentiation. The high genetic diversity within Borrelia populations and the disparity in the genetic structure between Borrelia and its Lick vector are likely consequences of strong balancing selection on local Borrelia clones. Demographically, both Borrelia and L scapularis populations, in the Northeast show the characteristics of a species that has recently expanded front a population bottleneck. Major geological and ecological events, such as the List glacial maximum (18,000 years ago) and the modern-day expansion of tick habitats, are likely causes of the observed”“founder effects”" for the two organisms in the Northeast. We therefore conclude that the genetic structure of B. burgdorferi has been intimately shaped by the natural history, of its main vector, the northern lineage of L scalpularis ticks." | 1858 | no | gd of pathogen | NA | no | no |
| 1859 | 2017 | 43021 | Kwon, SJ; Brown, AF; Hu, J; McGee, R; Watt, C; Kisha, T; Timmerman-Vaughan, G; Grusak, M; McPhee, KE; Coyne, CJ | 2012 | Genetic diversity, population structure and genome-wide marker-trait association analysis emphasizing seed nutrients of the USDA pea (Pisum sativum L.) core collection | Genetic diversity, population structure and genome-wide marker-trait association analysis was conducted for the USDA pea (Pisum sativum L.) core collection. The core collection contained 285 accessions with diverse phenotypes and geographic origins. The 137 DNA markers included 102 polymorphic fragments amplified by 15 microsatellite primer pairs, 36 RAPD loci and one SCAR (sequence characterized amplified region) marker. The 49 phenotypic traits fall into the categories of seed macro- and micro-nutrients, disease resistance, agronomic traits and seed characteristics. Genetic diversity, population structure and marker-trait association were analyzed with the software packages PowerMarker, STUCTURE and TASSEL, respectively. A great amount of variation was revealed by the DNA markers at the molecular level. Identified were three sub-populations that constituted 56.1%, 13.0% and 30.9%, respectively, of the USDA Pisum core collection. The first sub-population is comprised of all cultivated pea varieties and landraces; the second of wild P. sativum ssp. elatius and abyssinicum and the accessions from the Asian highland (Afghanistan, India, Pakistan, China and Nepal); while the third is an admixture containing alleles from the first and second sub-populations. This structure was achieved using a stringent cutoff point of 15% admixture (q-value 85%) of the collection. Significant marker-trait associations were identified among certain markers with eight mineral nutrient concentrations in seed and other important phenotypic traits. Fifteen pairs of associations were at the significant levels of P a parts per thousand currency sign 0.01 when tested using the three statistical models. These markers will be useful in marker-assisted selection to breed pea cultivars with desirable agronomic traits and end-user qualities. | 1859 | no | no good fitness measure, N pop differnt in GD? | NA | no | no |
| 1860 | 2017 | 43021 | Gong, J; Sun, QP; Xue, F; Fang, SG; Wan, QH | 2013 | Molecular Characterization of the Major Histocompatibility Complex Class Ia Gene in the Black-Spotted Frog, Pelophylax nigromaculata | The genes of the major histocompatibility complex (MHC) are attractive candidates for investigating the link between adaptive variation and individual fitness. We improved rapid amplification of cDNA ends to obtain the whole coding sequence of the MHC class Ia gene of the black-spotted frog (Pelophylax nigromaculata), the most common amphibian in China. We also used genome walking to characterize the partial introns adjacent to exon 3 of the MHC Ia gene. Based on the sequences obtained, we designed locus-specific primers to investigate the molecular polymorphisms of this species in southeast China. The MHC class Ia gene showed a high level of genetic diversity, indicating that this species retains a relatively high potential for survival, despite a population decline among frog species in general and many other amphibians. | 1860 | no | no good fitness measure | NA | no | no |
| 1861 | 2017 | 43021 | Melosik, I; Ziomek, J; Winnicka, K; Eichert, U | 2016 | Genetic diversity and extinction risk in a small, declining Polish common hamster (Cricetus cricetus) population | Losses of common hamster populations (Cricetus cricetus) have become a major conservation issue in Poland and Europe. The species’ range has declined by approximately 75% since 1971 in Poland. We aimed to retrace a recent history and to characterize the genetic status of a small, declining Polish population to develop the best management strategy for this isolated population. By combining field observations with genetic information from 13 microsatellite markers genotyped in 45 individuals and a population viability analysis, we computed the projected time to extinction using models with varying migration rates, first-year mortality (FYM), and supplementation. Environmental changes in recent years have negatively affected the size of the population (burrow density dropped from 5.5 burrows per hectare to 0.6). The population is spatially structured in demes, with each deme having a small effective population size. The population exhibits an overall deficiency of heterozygosity. Restricted gene flow (m ranging from 0.01 to 0.05) induces inbreeding and spatial clustering of closely related individuals. This study encompasses the effect of deme supplementation as the best strategy to increase the population’s median time to extinction (MTE), gene diversity, and the number of alleles. Varying FYM does not greatly affect these parameters when demes are not supplemented, but it becomes more important when supplementation is occurring. A 5% increase in migration rates does not influence the parameters. Based on the deterministic scenarios, a positive growth rate is observed when FYM is reduced. This study contributes to ongoing efforts to ensure the persistence of the endangered C. cricetus. (C) 2016 Deutsche Gesellschaft fur Saugetierkunde. Published by Elsevier GmbH. All rights reserved. | 1861 | no | no good fitness measure, N pop differnt in GD? | NA | no | no |
| 1862 | 2017 | 43021 | Fernandez-Mazuecos, M; Jimenez-Mejias, P; Rotllan-Puig, X; Vargas, P | 2014 | Narrow endemics to Mediterranean islands: Moderate genetic diversity but narrow climatic niche of the ancient, critically endangered Naufraga (Apiaceae) | Narrow endemics constitute the cornerstone of Mediterranean plant diversity. Naufraga balearica (Apiaceae) is a critically endangered, extremely narrow endemic plant from the western Mediterranean island of Majorca. Because the species belongs to a monotypic genus, N. balearica was hypothesized to be a palaeoendemism. Here we conducted phylogenetic dating, population genetic and climatic niche analyses in order to understand the evolutionary history and conservation perspectives of this flagship species. Phylogenetic dating analysis of nuclear and plastid DNA sequences revealed a late Miocene to early Pliocene divergence between Naufraga and its sister genus Apium, supporting the palaeoendemic status of the former. Amplified fragment length polymorphism (AFLP) markers and plastid DNA sequences of the five Naufraga populations revealed moderate genetic diversity. This diversity is in line with that of other palaeoendemisms from western Mediterranean islands, as revealed by a comparison with 22 other narrow endemic species from this region. Despite the fact that all Naufraga populations are located at a maximum distance of 10 km in a straight line, a strikingly strong population differentiation was found for AFLP markers, which is explained by long-term isolation likely related to short-range pollination and dispersal strategies of the species. While the species is not genetically impoverished, species distribution modelling and microclimatic monitoring revealed that narrow ecological requirements underlie the current extreme rarity of Naufraga and may jeopardize its long-term survival. Our results indicate that a multidisciplinary approach provides powerful tools to develop conservation strategies for evolutionarily singular lineages. (C) 2014 Geobotanisches Institut ETH, Stiftung Ruebel. Published by Elsevier GmbH. All rights reserved. | 1862 | no | no good fitness measure | NA | no | no |
| 1863 | 2017 | 43021 | Gloria-Soria, A; Ayala, D; Bheecarry, A; Calderon-Arguedas, O; Chadee, DD; Chiappero, M; Coetzee, M; Bin Elahee, K; Fernandez-Salas, I; Kamal, HA; Kamgang, B; Khater, EIM; Kramer, LD; Kramer, V; Lopez-Solis, A; Lutomiah, J; Martins, A; Micieli, MV; Paupy, C; Ponlawat, A; Rahola, N; Rasheed, SB; Richardson, JB; Saleh, AA; Sanchez-Casas, RM; Seixas, G; Sousa, CA; Tabachnick, WJ; Troyo, A; Powell, JR | 2016 | Global genetic diversity of Aedes aegypti | Mosquitoes, especially Aedes aegypti, are becoming important models for studying invasion biology. We characterized genetic variation at 12 microsatellite loci in 79 populations of Ae. aegypti from 30 countries in six continents, and used them to infer historical and modern patterns of invasion. Our results support the two subspecies Ae. aegypti formosus and Ae. aegypti aegypti as genetically distinct units. Ae. aegypti aegypti populations outside Africa are derived from ancestral African populations and are monophyletic. The two subspecies co-occur in both East Africa (Kenya) and West Africa (Senegal). In rural/forest settings (Rabai District of Kenya), the two subspecies remain genetically distinct, whereas in urban settings, they introgress freely. Populations outside Africa are highly genetically structured likely due to a combination of recent founder effects, discrete discontinuous habitats and low migration rates. Ancestral populations in sub-Saharan Africa are less genetically structured, as are the populations in Asia. Introduction of Ae. aegypti to the New World coinciding with trans-Atlantic shipping in the 16th to 18th centuries was followed by its introduction to Asia in the late 19th century from the New World or from now extinct populations in the Mediterranean Basin. Aedes mascarensis is a genetically distinct sister species to Ae. aegypti s.l. This study provides a reference database of genetic diversity that can be used to determine the likely origin of new introductions that occur regularly for this invasive species. The genetic uniqueness of many populations and regions has important implications for attempts to control Ae. aegypti, especially for the methods using genetic modification of populations. | 1863 | no | no good fitness measure | NA | no | no |
| 1864 | 2017 | 43021 | Robinson, SJ; Samuel, MD; Johnson, CJ; Adams, M; McKenzie, DI | 2012 | Emerging prion disease drives host selection in a wildlife population | Infectious diseases are increasingly recognized as an important force driving population dynamics, conservation biology, and natural selection in wildlife populations. Infectious agents have been implicated in the decline of small or endangered populations and may act to constrain population size, distribution, growth rates, or migration patterns. Further, diseases may provide selective pressures that shape the genetic diversity of populations or species. Thus, understanding disease dynamics and selective pressures from pathogens is crucial to understanding population processes, managing wildlife diseases, and conserving biological diversity. There is ample evidence that variation in the prion protein gene (PRNP) impacts host susceptibility to prion diseases. Still, little is known about how genetic differences might influence natural selection within wildlife populations. Here we link genetic variation with differential susceptibility of white-tailed deer to chronic wasting disease (CWD), with implications for fitness and disease-driven genetic selection. We developed a single nucleotide polymorphism (SNP) assay to efficiently genotype deer at the locus of interest (in the 96th codon of the PRNP gene). Then, using a Bayesian modeling approach, we found that the more susceptible genotype had over four times greater risk of CWD infection; and, once infected, deer with the resistant genotype survived 49% longer (8.25 more months). We used these epidemiological parameters in a multi-stage population matrix model to evaluate relative fitness based on genotype-specific population growth rates. The differences in disease infection and mortality rates allowed genetically resistant deer to achieve higher population growth and obtain a long-term fitness advantage, which translated into a selection coefficient of over 1% favoring the CWD-resistant genotype. This selective pressure suggests that the resistant allele could become dominant in the population within an evolutionarily short time frame. Our work provides a rare example of a quantifiable disease-driven selection process in a wildlife population, demonstrating the potential for infectious diseases to alter host populations. This will have direct bearing on the epidemiology, dynamics, and future trends in CWD transmission and spread. Understanding genotype-specific epidemiology will improve predictive models and inform management strategies for CWD-affected cervid populations. | 1864 | no | individual level | NA | no | no |
| 1865 | 2017 | 43021 | Zhai, JC; Liu, WS; Yin, YJ; Xia, YL; Li, HP | 2017 | Analysis on Genetic Diversity of Reindeer (Rangifer tarandus) in the Greater Khingan Mountains Using Microsatellite Markers | The only population of reindeer (Rangifer tarandus) in China, herded extensively by the Ewenki people, is the most southern population in the world. Genetic diversity plays a key role in the survival of endangered reindeer. To systematically understand the genetic variability of reindeer in China, 163 individuals from 8 populations were analyzed using 11 microsatellite loci. A total of 85 alleles were detected and the average number of alleles per locus was 7.7. The observed heterozygosity and expected heterozygosity ranged from 0.3736 to 0.5299 and from 0.6491 to 0.7608. Hardy-Weinberg equilibrium analysis indicated that a deficiency of heterozygotes existed in all eight populations. Both the FST and AMOVA analyses showed a low level of genetic differentiation among populations. UPGMA dendrogram revealed that population SYL formed one cluster, separating from the other populations. Then the GWQ and YSH populations formed another cluster and clustered with the BDX, BLJY, DML, DW and MLYS populations. Increasing the current exchange rate of reindeer among different populations and establishing natural reserve may be the effective approaches to conserve the fragile reindeer populations in China. | 1865 | no | no good fitness measure | NA | no | no |
| 1866 | 2017 | 43021 | Bakhoum, MT; Fall, M; Fall, AG; Bellis, GA; Gottlieb, Y; Labuschagne, K; Venter, GJ; Diop, M; Mall, I; Seck, MT; Allene, X; Diarra, M; Gardes, L; Bouyer, J; Delecolle, JC; Balenghien, T; Garros, C | 2013 | First Record of Culicoides oxystoma Kieffer and Diversity of Species within the Schultzei Group of Culicoides Latreille (Diptera: Ceratopogonidae) Biting Midges in Senegal | The Schultzei group of Culicoides Latreille (Diptera: Ceratopogonidae) is distributed throughout Africa to northern Asia and Australasia and includes several potential vector species of livestock pathogens. The taxonomy of the species belonging to this species group is confounded by the wide geographical distribution and morphological variation exhibited by many species. In this work, morphological and molecular approaches were combined to assess the taxonomic validity of the species and morphological variants of the Schultzei group found in Senegal by comparing their genetic diversity with that of specimens from other geographical regions. The species list for Senegal was updated with four species: Culicoides kingi, C. oxystoma, C. enderleini and C. nevilli being recorded. This is the first record of C. oxystoma from Africa south of Sahara, and its genetic relationship with samples from Israel, Japan and Australia is presented. This work provides a basis for ecological studies of the seasonal and spatial dynamics of species of this species group that will contribute to better understanding of the epidemiology of the viruses they transmit. | 1866 | no | no good fitness measure, N pop differnt in GD? | NA | no | no |
| 1867 | 2017 | 43021 | Thrall, PH; Encinas-Viso, F; Hoebee, SE; Young, AG | 2014 | Life history mediates mate limitation and population viability in self-incompatible plant species | Genetically controlled self-incompatibility systems represent links between genetic diversity and plant demography with the potential to directly impact on population dynamics. We use an individual-based spatial simulation to investigate the demographic and genetic consequences of different self-incompatibility systems for plants that vary in reproductive capacity and lifespan. The results support the idea that, in the absence of inbreeding effects, populations of self-incompatible species will often be smaller and less viable than self-compatible species, particularly for shorter-lived organisms or where potential fecundity is low. At high ovule production and low mortality, self-incompatible and self-compatible species are demographically similar, thus self-incompatibility does not automatically lead to reduced mate availability or population viability. Overall, sporophytic codominant self-incompatibility was more limiting than gametophytic or sporophytic dominant systems, which generally behaved in a similar fashion. Under a narrow range of conditions, the sporophytic dominant system maintained marginally greater mate availability owing to the production of S locus homozygotes. While self-incompatibility reduces population size and persistence for a broad range of conditions, the actual number of S alleles, beyond that required for reproduction, is important for only a subset of life histories. For these situations, results suggest that addition of new S alleles may result in significant demographic rescue. | 1867 | no | no good fitness measure, N pop differnt in GD? | NA | no | no |
| 1868 | 2017 | 43021 | Andersen, DH; Pertoldi, C; Loeschcke, V; Cavicchi, S; Scali, V | 2008 | The impact of genetic parental distance on developmental stability and fitness in Drosophila buzzatii | Measures of genetic parental distances (GPD) based on microsatellite loci (D(2) and IR), have been suggested to be better correlated with fitness than individual heterozygosity (H), as they contain information about past events of inbreeding or admixture. We investigated if GPD increased with increasing genetic divergence between parental populations in Drosophila buzzatii and if the measures indicate past events of admixture. Further we evaluated the relationship between GPD, fitness and fluctuating asymmetry (FA) of size and shape. We investigated three populations of Drosophila buzzati, from Argentina, Europe and Australia. From these populations two intraspecific hybridisation lines were made; one between the Argentinean and European populations, which have been separated 200 years and one between the populations from Argentina and Australia, which have been separated 80 years. By doing this we obtained hybrid progeny having different levels of GPD. We found that D(2) and H can be used as indicators of admixture when comparing hybrid individuals with their parentals. IR was not informative. Our results does not exclude the presence of genetic fitness correlations (GFC) over individuals with a broad fitness range from populations in equilibrium, but we doubt the presence of GFC using GPD measures in admixed populations. Shape FA could be a relevant measure for fitness, however, only when comparing populations, not at individual level. | 1868 | no | no good fitness measure, N pop differnt in GD? | NA | no | no |
| 1869 | 2017 | 43021 | Kirin, M; McQuillan, R; Franklin, CS; Campbell, H; McKeigue, PM; Wilson, JF | 2010 | Genomic Runs of Homozygosity Record Population History and Consanguinity | The human genome is characterised by many runs of homozygous genotypes, where identical haplotypes were inherited from each parent. The length of each run is determined partly by the number of generations since the common ancestor: offspring of cousin marriages have long runs of homozygosity (ROH), while the numerous shorter tracts relate to shared ancestry tens and hundreds of generations ago. Human populations have experienced a wide range of demographic histories and hold diverse cultural attitudes to consanguinity. In a global population dataset, genome-wide analysis of long and shorter ROH allows categorisation of the mainly indigenous populations sampled here into four major groups in which the majority of the population are inferred to have: (a) recent parental relatedness (south and west Asians); (b) shared parental ancestry arising hundreds to thousands of years ago through long term isolation and restricted effective population size (N-e), but little recent inbreeding (Oceanians); (c) both ancient and recent parental relatedness (Native Americans); and (d) only the background level of shared ancestry relating to continental N-e (predominantly urban Europeans and East Asians; lowest of all in sub-Saharan African agriculturalists), and the occasional cryptically inbred individual. Moreover, individuals can be positioned along axes representing this demographic historic space. Long runs of homozygosity are therefore a globally widespread and under-appreciated characteristic of our genomes, which record past consanguinity and population isolation and provide a distinctive record of the demographic history of an individual’s ancestors. Individual ROH measures will also allow quantification of the disease risk arising from polygenic recessive effects. | 1869 | no | human | NA | no | no |
| 1870 | 2017 | 43021 | Mestre, O; Luo, T; Dos Vultos, T; Kremer, K; Murray, A; Namouchi, A; Jackson, C; Rauzier, J; Bifani, P; Warren, R; Rasolofo, V; Mei, JA; Gao, QA; Gicquel, B | 2011 | Phylogeny of Mycobacterium tuberculosis Beijing Strains Constructed from Polymorphisms in Genes Involved in DNA Replication, Recombination and Repair | Background: The Beijing family is a successful group of M. tuberculosis strains, often associated with drug resistance and widely distributed throughout the world. Polymorphic genetic markers have been used to type particular M. tuberculosis strains. We recently identified a group of polymorphic DNA repair replication and recombination (3R) genes. It was shown that evolution of M. tuberculosis complex strains can be studied using 3R SNPs and a high-resolution tool for strain discrimination was developed. Here we investigated the genetic diversity and propose a phylogeny for Beijing strains by analyzing polymorphisms in 3R genes. Methodology/Principal Findings: A group of 3R genes was sequenced in a collection of Beijing strains from different geographic origins. Sequence analysis and comparison with the ones of non-Beijing strains identified several SNPs. These SNPs were used to type a larger collection of Beijing strains and allowed identification of 26 different sequence types for which a phylogeny was constructed. Phylogenetic relationships established by sequence types were in agreement with evolutionary pathways suggested by other genetic markers, such as Large Sequence Polymorphisms (LSPs). A recent Beijing genotype (Bmyc10), which included 60% of strains from distinct parts of the world, appeared to be predominant. Conclusions/Significance: We found SNPs in 3R genes associated with the Beijing family, which enabled discrimination of different groups and the proposal of a phylogeny. The Beijing family can be divided into different groups characterized by particular genetic polymorphisms that may reflect pathogenic features. These SNPs are new, potential genetic markers that may contribute to better understand the success of the Beijing family. | 1870 | no | gd of pathogen | NA | no | no |
| 1871 | 2017 | 43021 | Volkman, SK; Lozovsky, E; Barry, AE; Bedford, T; Bethke, L; Myrick, A; Day, KP; Hartl, DL; Wirth, DF; Sawyer, SA | 2007 | Genomic heterogeneity in the density of noncoding single-nucleotide and microsatellite polyrnorphisms in Plasmodium falciparum | The density and distribution of single-nucleotide polymorphisms (SNPs) across the genome has important implications for linkage disequilibrium mapping and association Studies, and the level of simple-sequence microsatellite polymorphisms has important implications for the use of of oligonucleotide hybridization methods to genotype SNPs. To assess the density of these types of polymorphisms in P. falciparum, we sampled introns and noncoding DNA upstream and downstream of coding regions among a variety of geographically diverse parasites. Across 36,229 base pairs of noncoding sequence representing 41 genetic loci, a total of 307 polymorphisms including 248 polymorphic microsatellites and 39 SNPs were identified. We found a significant excess of microsatellite polymorphisms having a repeat unit length of one or two, compared to those with longer repeat lengths, as well as a nonrandom distribution of SNP polymorphisms. Almost half of the SNPs localized to only three of the 41 genetic loci sampled. Furthermore, we find significant differences in the frequency of polymorphisins across the two chromosomes (2 and 3) examined most extensively, with an excess of SNPs and a surplus of polymorphic microsatellites on chromosome 3 as compared to chromosome 2 (P=0.0001). Furthermore, at some individual genetic loci we also find a nonrandom distribution of polymorphisnis between coding and flanking noncoding sequences, where completely monomorphic regions may flank highly polymorphic genes. These data, combined with our previous findings of nonrandom distribution of SNPs across chromosome 2, Suggest that the Plasmodium 1 genome may be a mosaic with regard to genetic diversity, containing chromosomal regions that are highly polymorphic falciparum interspersed with regions that are much less polymorphic. (c) 2006 Elsevier B.V All rights reserved. | 1871 | no | gd of parasite | NA | no | no |
| 1872 | 2017 | 43021 | Nagylaki, T | 2015 | Dying on the way: The influence of migrational mortality on neutral models of spatial variation | Migrational mortality is introduced into the classical Malecot model for migration, mutation, and random genetic drift. To assess the influence of mortality, its effect on the backward migration rates and on the probabilities of identity in allelic state are studied. Perhaps surprisingly, some of the former may increase, but as is intuitive, their sum always decreases. As expected, in the island model, mortality does not change the migration pattern, but it decreases the migration rate. Furthermore, it decreases the expected heterozygosity, but increases the genetic diversity and differentiation. The circular habitat and the unbounded, linear stepping-stone model also illustrate the general results. Arbitrary migration is also analyzed. If migration is sufficiently weak, then mortality diminishes every migration rate; it decreases the expected heterozygosity and the genetic similarity between demes. In the strong-migration limit, mortality may raise or lower the probability of identity in state. Perhaps unexpectedly, under mild and reasonable biological assumptions, mortality does not alter the diffusion limit of the probabilities of identity. (C) 2014 Elsevier Inc. All rights reserved. | 1872 | no | No GD diff between pop | NA | no | no |
| 1873 | 2017 | 43021 | Williams, D; Overbaugh, J | 2004 | A real-time PCR-based method to independently sample single simian immunodeficiency virus genomes from macaques with a range of viral loads | The generation of a diverse population of viral variants is a hallmark of simian immunodeficiency virus (SIV) infection. In order to address what role this diversity plays in disease progression, accurate sampling of the viral population is necessary. However, traditional PCR-based methods often rely on amplification of multiple genomes in one reaction, leading to resampling of viral genomes and potential errors in the estimations of viral diversity, especially when sequences from only one or a small number of PCRs are examined and/or viral copy number is low. Here we describe a method to amplify one viral envelope gene per PCR, thereby avoiding resampling. For this purpose we developed a highly accurate real-time PCR method to quantify SIV copy number, then used a single SIV template in a sensitive, high-fidelity full-length envelope PCR. Using this method, we have estimated the intra-animal viral diversity for a cohort of five pig-tailed macaques (Macaca nemestrina) infected with SIVMne variants, which displayed a broad range of viral loads at setpoint. | 1873 | no | NA | NA | no | no |
| 1874 | 2017 | 43021 | Haddad-Boubaker, S; Bigarre, L; Bouzgarou, N; Megdich, A; Baud, M; Cabon, J; Ben Chehida, N | 2013 | Molecular epidemiology of betanodaviruses isolated from sea bass and sea bream cultured along the Tunisian coasts | Viral nervous necrosis (VNN) is a serious viral disease affecting farmed sea bass (Dicentrarchus labrax). Only scarce molecular data are available on the disease-causing betanodavirus populations in Tunisia. Therefore, we carried out the first molecular survey of betanodaviruses in farmed sea bass and sea bream (Sparus aurata) along the Tunisian coasts. Among 81 samples from five farms, 20 tested positive with RT-PCR, not only in clinical cases but also in asymptomatic fish before and during outbreaks. Positive fish were found in all farms, except in one farm investigated in the south of Tunisia. Sequencing the fragments of both genomic components (RNA1 and RNA2) in 16 isolates revealed that the Tunisian viruses were related to the red-spotted grouper nervous necrosis virus (RGNNV) genotype. Furthermore, the newly sequenced isolates were generally highly related to one another suggesting a recent common ancestor. They also showed high identities with other isolates obtained from wild fishes in the Mediterranean, but were slightly more divergent from strains recently obtained from farmed fishes in the Mediterranean. The poor genetic diversity of the viral population along the Tunisian coasts is striking. One hypothesis is that it is the result of the maintenance of a homogenous genetic pool among infected wild fish, groupers for instance and subsequent dissemination to farmed fish over the seasons. | 1874 | no | gd of pathogen | NA | no | no |
| 1875 | 2017 | 43021 | Loehr, J; Worley, K; Grapputo, A; Carey, J; Veitch, A; Coltman, DW | 2006 | Evidence for cryptic glacial refugia from North American mountain sheep mitochondrial DNA | The separation of populations by ice sheets into large refugia can account for much of the genetic diversity found in present day populations. The evolutionary implications of small glacial refugia have not been as thoroughly explored. To examine refugial origins of North American mountain sheep Ovis spp., we analyzed a 604 bp portion of the mitochondrial DNA (mtDNA) control region from 223 O. dalli and O. canadensis. Major refugia were identified in eastern Beringia and southern North America, and we found evidence for two smaller refugia situated between the Laurentide and Cordilleran glaciers. Our results are the first to demonstrate support for survival of any organism in the latter two refugia. These refugia also appear to have conserved a genetic signal that confirms past hybridization of O. dalli and O. canadensis. | 1875 | no | No GD diff between pop | NA | no | no |
| 1876 | 2017 | 43021 | Cole, BJ; Smith, AA; Huber, ZJ; Wiernasz, DC | 2010 | The structure of foraging activity in colonies of the harvester ant, Pogonomyrmex occidentalis | The timing of activity by desert dwelling poikilotherms can be critical to survival. In the western harvester ant, colonies that have higher levels of genetic diversity forage for longer time periods in the morning than colonies with less diversity. We determined whether the advantage of early foraging colonies was consistent by examining foraging behavior at other times of day and year. We used a combination of activity monitoring and temperature measurement at the nest entrance to quantify foraging activity during the morning and evening summer foraging periods in both June and August. The duration of morning and evening foraging was significantly positively correlated both within and across seasons-some colonies have a consistent advantage in foraging. The temperature range over which colonies foraged was also consistent across time, suggesting that intercolony differences are a consequence of variation in the thermal ranges/preferences of the colony’s workers. The duration of foraging during this study was correlated with the duration of foraging measured 6 years earlier, suggesting that it is an aspect of colony phenotype. | 1876 | no | no good fitness measure | NA | no | no |
| 1877 | 2017 | 43021 | Papa, R; Acosta, J; Delgado-Salinas, A; Gepts, P | 2005 | A genome-wide analysis of differentiation between wild and domesticated Phaseolus vulgaris from Mesoamerica | Lack of introgression or divergent selection may be responsible for the maintenance of phenotypic differences between sympatric populations of crops and their wild progenitors. To distinguish between these hypotheses, amplified fragment length polymorphism markers were located on a molecular linkage map of Phaseolus vulgaris relative to genes for the domestication syndrome and other traits. Diversity for these same markers was then analyzed in two samples of wild and domesticated populations from Mesoamerica. Differentiation between wild and domesticated populations was significantly higher in parapatric and allopatric populations compared to sympatric populations. It was also significantly higher near genes for domestication compared to those away from these genes. Concurrently, the differences in genetic diversity between wild and domesticated populations were strongest around such genes. These data suggest that selection in the presence of introgression appears to be a major evolutionary factor maintaining the identity of wild and domesticated populations in sympatric situations. Furthermore, alleles from domesticated populations appear to have displaced alleles in sympatric wild populations, thus leading to a reduction in genetic diversity in such populations. These results also provide a possible experimental framework for assessing the long-term risk of transgene escape and the targeting of transgenes inside the genome to minimize the survival of these transgenes into wild populations following introduction by gene flow. | 1877 | no | no good fitness measure | NA | no | no |
| 1878 | 2017 | 43021 | van Hooft, WF; Groen, AF; Prins, HHT | 2000 | Microsatellite analysis of genetic diversity in African buffalo (Syncerus caffer) populations throughout Africa | Genetic diversity in nine African buffalo (Syncerus caffer) populations throughout Africa was analysed with 14 microsatellites to study the effects of rinderpest epidemics and habitat fragmentation during the 20th century. A gradient of declining expected heterozygosity was observed among populations in Save Valley Conservancy (Zimbabwe), and northern and southern Kruger National Park (South Africa). This was explained by a high mortality in northern Kruger National Park during the rinderpest pandemic at the end of the 19th century followed by recolonization from neighbouring populations, resulting in intermediate heterozygosity levels in northern Kruger National Park. In other populations expected heterozygosity was very high, indicating that rinderpest and recent habitat fragmentation had a limited effect on genetic diversity. From expected heterozygosity, estimates of long-term effective population size were derived. Migration rates among populations in eastern and southern Africa were very high, as shown by a weak isolation by distance and significant correlation in allele frequencies between populations. However, there were indications that dry habitats could limit migration. Genetic distances within buffalo in central Africa were relatively large, supporting their status as distinct subspecies. Finally, it was observed that the higher polymorphic microsatellites were less sensitive at detecting isolation by distance and differences in N-e, which may be a result of the high mutation pressure at these loci. | 1878 | no | no good fitness measure, N pop differnt in GD? (inbreeding?) | NA | no | no |
| 1879 | 2017 | 43021 | Pan, D; Mionetto, A; Calero, N; Reynoso, MM; Torres, A; Bettucci, L | 2016 | Population genetic analysis and trichothecene profiling of Fusarium graminearum from wheat in Uruguay | Fusarium graminearum sensu stricto (F. graminearum s.s.) is the major causal agent of Fusarium head blight of wheat worldwide, and contaminates grains with trichothecene mycotoxins that cause serious threats to food safety and animal health. An important aspect of managing this pathogen and reducing mycotoxin contamination of wheat is knowledge regarding its population genetics. Therefore, isolates of F. graminearum s. s. from the major wheat-growing region of Uruguay were analyzed by amplified fragment length polymorphism assays, PCR genotyping, and chemical analysis of trichothecene production. Of the 102 isolates identified as having the 15-ADON genotype via PCR genotyping, all were DON producers, but only 41 strains were also 15-ADON producers, as determined by chemical analysis. The populations were genotypically diverse but genetically similar, with significant genetic exchange occurring between them. Analysis of molecular variance indicated that most of the genetic variability resulted from differences between isolates within populations. Multilocus linkage disequilibrium analysis suggested that the isolates had a panmictic population genetic structure and that there is significant recombination occurs in F. graminearum s.s. In conclusion, tour findings provide the first detailed description of the genetic structure and trichothecene production of populations of F. graminearum s. s. from Uruguay, and expands our understanding of the agroecology of F. graminearum and of the correlation between genotypes and trichothecene chemotypes. | 1879 | no | gd of pathogen | NA | no | no |
| 1880 | 2017 | 43021 | Makkonen, J; Jussila, J; Kokko, H | 2012 | The diversity of the pathogenic Oomycete (Aphanomyces astaci) chitinase genes within the genotypes indicate adaptation to its hosts | The aim of this work was to evaluate the genetic diversity of the crayfish plague pathogen Aphanomyces astaci (Oomycete) among different isolates and genotypes. Partial chitinase genes were cloned and sequenced from 28 A. astaci isolates including four of the five previously identified RAPD (random amplification of polymorphic DNA)-genotypes. The cloned chitinase sequences (n = 176) formed three main groups, CHI1. CHI2 and CHI3, with the CHI2 group then further divided into three subgroups, CHI2A, CHI2B and CHI2C. Some of these chitinases were specific for certain genotypes of A. astaci, as CHI2B and CHI2C were only found from the As-genotype and CHI3 from the Ps-genotypes of A. astaci. Highest diversity rate was observed in the CHI2 group, while the CHI3 group specific for Ps-genotypes was highly homologous. Based on our chitinase data, As- and Pc-genotypes seem to be related, while the two Ps-genotypes were identical to each other, but considerably different from the genotypes As and Pc. These are the first genotype specific differences that are located in the coding region of the chitinase gene of A. astaci and the differences observed here also enable the genotyping of A. astaci. The diversity observed here can also reflect differences in the epidemiological properties of the different genotypes. (C) 2012 Elsevier Inc. All rights reserved. | 1880 | no | gd of pathogen | NA | no | no |
| 1881 | 2017 | 43021 | Elberse, KEM; van de Pol, I; Witteveen, S; van der Heide, HGJ; Schot, CS; van Dijk, A; van der Ende, A; Schouls, LM | 2011 | Population Structure of Invasive Streptococcus pneumoniae in the Netherlands in the Pre-Vaccination Era Assessed by MLVA and Capsular Sequence Typing | The introduction of nationwide pneumococcal vaccination may lead to serotype replacement and the emergence of new variants that have expanded their genetic repertoire through recombination. To monitor alterations in the pneumococcal population structure, we have developed and utilized Capsular Sequence Typing (CST) in addition to Multiple-Locus Variable number tandem repeat Analysis (MLVA). To assess the serotype of each isolate CST was used. Based on the determination of the partial sequence of the capsular wzh gene, this method assigns a capsular type of an isolate within a single PCR reaction using multiple primersets. The genetic background of pneumococcal isolates was assessed by MLVA. MLVA and CST were used to create a snapshot of the Dutch pneumococcal population causing invasive disease before the introduction of the 7-valent pneumococcal conjugate vaccine in the Netherlands in 2006. A total of 1154 clinical isolates collected and serotyped by the Netherlands Reference Laboratory for Bacterial Meningitis were included in the snapshot. The CST was successful in discriminating most serotypes present in our collection. MLVA demonstrated that isolates belonging to some serotypes had a relatively high genetic diversity whilst other serotypes had a very homogeneous genetic background. MLVA and CST appear to be valuable tools to determine the population structure of pneumococcal isolates and are useful in monitoring the effects of pneumococcal vaccination. | 1881 | no | NA | NA | no | no |
| 1882 | 2017 | 43021 | Knight, S; Uh, HW; Martinez, M | 2009 | Summary of Contributions to GAW Group 15: Family-Based Samples Are Useful in Identifying Common Polymorphisms Associated with Complex Traits | Traditionally, family-based samples have been used for genetic analyses of single-gene traits caused by rare but highly penetrant risk variants. The utility of family-based genetic data for analyzing common complex traits is unclear and contains numerous challenges. To assess the utility as well as to address these challenges, members of Genetic Analysis Workshop 16 Group 15 analyzed Framingham Heart Study data using family-based designs ranging from parent-offspring trios to large pedigrees. We investigated different methods including traditional linkage tests, family-based association tests, and population-based tests that correct for relatedness between subjects, and tests to detect parent-of-origin effects. The analyses presented an assortment of positive findings. One contribution found increased power to detect epistatic effects through linkage using ascertainment of sibships based on extreme quantitative values or presence of disease associated with the quantitative value. Another contribution found four single-nucleotide polymorphisms (SNPs) showing a maternal effect, two SNPs with an imprinting effect, and one SNP having both effects on a binary high blood pressure trait. Finally, three contributions illustrated the advantage of using population-based methods to detect association to complex binary or quantitative traits. Our findings highlight the contribution of family-based samples to the genetic dissection of complex traits. Genet. Epidemiol. 33 (Suppl. 1):S99-S104, 2009. (C) 2009 Wiley-Liss, Inc. | 1882 | no | NA | NA | no | no |
| 1883 | 2017 | 43021 | Liu, RZ; Ma, MH; Cui, MY; Dong, Z; Wang, XL; Zhang, W; Yang, MH; Yu, SY | 2014 | Effects of Tumor Necrosis Factor-beta (TNF-beta) 252A > G Polymorphism on the Development of Migraine: A Meta-Analysis | “Background and Objective: Genetic factors including TNF-beta have been considered as important components in the aetiology of migraine. Many studies have investigated the association between TNF-beta 252A>G polymorphism and migraine risk, with debatable results. This study was designed to examine whether the TNF-beta 252A>G polymorphism confers genetic susceptibility to migraine in diverse populations. Method: Studies eligible for this meta-analysis were searched in the PubMed, Embase, and CNKI by using the keywords”"tumor necrosis factor’‘, ""TNF’‘, ""252A>G’‘, ""rs909253’‘, ""polymorphism’‘, ""polymorphisms’‘, ""variant’‘, ""SNP’‘, combined with "“migraine’’ or”“migraine with aura (MA)’’ or”"migraine without aura (MO)’’. Pooled ORs and 95% CI were appropriately calculated using the fixed-effect model. Results: We finally included a total of seven studies, providing 5 557 migraineurs and 20 543 unrelated healthy controls. Meta-analysis results showed no statistical evidence of a significant association between TNF-beta 252A>G polymorphism and overall migraine risk. Stratified analyses by type of migraine and gender revealed similar results. Interestingly, an OR with 95% CI representing an increased migraine risk was indicated in Asians under the recessive model (GG vs. AG + AA: OR, 1.38 | 1883 | no | no data | NA | no | no |
| 1884 | 2017 | 43021 | Han, LD; Abney, M | 2013 | Using identity by descent estimation with dense genotype data to detect positive selection | Identification of genomic loci and segments that are identical by descent (IBD) allows inference on problems such as relatedness detection, IBD disease mapping, heritability estimation and detection of recent or ongoing positive selection. Here, employing a novel statistical method, we use IBD to find signals of selection in the Maasai from Kinyawa, Kenya (MKK). In doing so, we demonstrate the advantage of statistical tools that can probabilistically estimate IBD sharing without having to thin genotype data because of linkage disequilibrium (LD), and that allow for both inbreeding and more than one allele to be shared IBD. We use our novel method, GIBDLD, to estimate IBD sharing between all pairs of individuals at all genotyped SNPs in the MKK, and, by looking for genomic regions showing excess IBD sharing in unrelated pairs, find loci that are known to have undergone recent selection (eg, the LCT gene and the HLA region) as well as many novel loci. Intriguingly, those loci that show the highest amount of excess IBD, with the exception of HLA, also show a substantial number of unrelated pairs sharing all four of their alleles IBD. In contrast to other IBD detection methods, GIBDLD provides accurate probabilistic estimates at each locus for all nine possible IBD sharing states between a pair of individuals, thus allowing for consanguinity, while also modeling LD, thus removing the need to thin SNPs. These characteristics will prove valuable for those doing genetic studies, and estimating IBD, in the wide variety of human populations. European Journal of Human Genetics (2013) 21, 205-211; doi:10.1038/ejhg.2012.148; published online 11 July 2012 | 1884 | no | human | NA | no | no |
| 1885 | 2017 | 43021 | Casjens, SR; Mongodin, EF; Qiu, WG; Luft, BJ; Schutzer, SE; Gilcrease, EB; Huang, WM; Vujadinovic, M; Aron, JK; Vargas, LC; Freeman, S; Radune, D; Weidman, JF; Dimitrov, GI; Khouri, HM; Sosa, JE; Halpin, RA; Dunn, JJ; Fraser, CM | 2012 | Genome Stability of Lyme Disease Spirochetes: Comparative Genomics of Borrelia burgdorferi Plasmids | Lyme disease is the most common tick-borne human illness in North America. In order to understand the molecular pathogenesis, natural diversity, population structure and epizootic spread of the North American Lyme agent, Borrelia burgdorferi sensu stricto, a much better understanding of the natural diversity of its genome will be required. Towards this end we present a comparative analysis of the nucleotide sequences of the numerous plasmids of B. burgdorferi isolates B31, N40, JD1 and 297. These strains were chosen because they include the three most commonly studied laboratory strains, and because they represent different major genetic lineages and so are informative regarding the genetic diversity and evolution of this organism. A unique feature of Borrelia genomes is that they carry a large number of linear and circular plasmids, and this work shows that strains N40, JD1, 297 and B31 carry related but non-identical sets of 16, 20, 19 and 21 plasmids, respectively, that comprise 33-40% of their genomes. We deduce that there are at least 28 plasmid compatibility types among the four strains. The B. burgdorferi similar to 900 Kbp linear chromosomes are evolutionarily exceptionally stable, except for a short <= 20 Kbp plasmid-like section at the right end. A few of the plasmids, including the linear lp54 and circular cp26, are also very stable. We show here that the other plasmids, especially the linear ones, are considerably more variable. Nearly all of the linear plasmids have undergone one or more substantial inter-plasmid rearrangements since their last common ancestor. In spite of these rearrangements and differences in plasmid contents, the overall gene complement of the different isolates has remained relatively constant. | 1885 | no | gd of pathogen | NA | no | no |
| 1886 | 2017 | 43021 | Simionatto, S; da Veiga Lima-Rosa, CA; Binneck, E; Ravazzolo, AP; Canal, CW | 2006 | Characterization and phylogenetic analysis of Brazilian chicken anaemia virus | Chicken anaemia virus (CAV) was detected by a Nested-PCR assay in field samples from different regions of Brazil. The 539 bp amplified fragments of vp1 gene from 44 field samples were sequenced and 10 new nucleotide sequences of CAV were observed. These sequences were phylogenetically analysed by Mega2 using neighbour joining distance methods with 1000 bootstrap replications. Phylogenetic analysis did not show correlation between CAV pathology pattern and genetic groups. The 10 nucleotide sequences of the Brazilian samples were also analysed together with 30 sequences of CAV strains previously described from other countries. The genetic variability observed was not related to the geographical distribution. Amino acid substitutions were detected at 9 positions of the Brazilian sequences and two of them had not been observed before, R-65 replacing the Q residue and F-98 replacing Y residue. | 1886 | no | gd of pathogen | NA | no | no |
| 1887 | 2017 | 43021 | Sevini, F; Yao, DY; Lomartire, L; Barbieri, A; Vianello, D; Ferri, G; Moretti, E; Dasso, MC; Garagnani, P; Pettener, D; Franceschi, C; Luiselli, D; Franceschi, ZA | 2013 | Analysis of Population Substructure in Two Sympatric Populations of Gran Chaco, Argentina | Sub-population structure and intricate kinship dynamics might introduce biases in molecular anthropology studies and could invalidate the efforts to understand diseases in highly admixed populations. In order to clarify the previously observed distribution pattern and morbidity of Chagas disease in Gran Chaco, Argentina, we studied two populations (Wichi and Criollos) recruited following an innovative bio-cultural model considering their complex cultural interactions. By reconstructing the genetic background and the structure of these two culturally different populations, the pattern of admixture, the correspondence between genealogical and genetic relationships, this integrated perspective had the power to validate data and to link the gap usually relying on a singular discipline. Although Wichi and Criollos share the same area, these sympatric populations are differentiated from the genetic point of view as revealed by Non Recombinant Y Chromosome genotyping resulting in significantly high Fst values and in a lower genetic variability in the Wichi population. Surprisingly, the Amerindian and the European components emerged with comparable amounts (20%) among Criollos and Wichi respectively. The detailed analysis of mitochondrial DNA showed that the two populations have as much as 87% of private haplotypes. Moreover, from the maternal perspective, despite a common Amerindian origin, an Andean and an Amazonian component emerged in Criollos and in Wichi respectively. Our approach allowed us to highlight that quite frequently there is a discrepancy between self-reported and genetic kinship. Indeed, if self-reported identity and kinship are usually utilized in population genetics as a reliable proxy for genetic identity and parental relationship, in our model populations appear to be the result not only and not simply of the genetic background but also of complex cultural determinants. This integrated approach paves the way to a rigorous reconstruction of demographic and cultural history as well as of bioancestry and propensity to diseases of Wichi and Criollos. | 1887 | no | NA | NA | no | no |
| 1888 | 2017 | 43021 | Behm, A; Becker, A; Dorflinger, H; Franke, A; Kleinschmit, J; Melchior, GH; Muhs, HJ; Schmitt, HP; Stephan, BR; Tabel, U; Weisgerber, H; Widmaier, T | 1997 | Concept for the conservation of forest genetic resources in the Federal Republic of Germany | The aim of the ‘’Concept for the Conservation of Forest Genetic Resources in the Federal Republic of Germany’’ is to: estimate the extent of danger to the genetic resources of our tree and shrub species; propose preventive measures for the maintenance of genetic variability of these species; consider proposals for the organisational realization of this programme and give a cost-estimate. The programme will help to reduce genetic losses due to anthropogenic environmental loads (immissions). but a basic precondition for this is a reduction of environmental load. The mandate of the working party for the elaboration of this concept was derived from a resolution made at a meeting held on 10.1.1985 between representatives of the Federal Ministry for Food, Agriculture and Forestry and the Federal States. The political importance of the project is emphasized by the resolution of the Bundesrat (Upper House of Parliament) made on 13.2.1985 concerning measures for conserving genetic diversity of forest tree species and the second edition of the Federal Government’s action programme ‘’Save the Forests’‘. The latter states that the Federal Government identifies the conservation of natural genetic resources to be of major importance and that it will try to establish a forest genebank. The working party aims to compile all existing measures that either directly or indirectly aid the conservation of forest genetic resources, and to work out a framework, including cost-estimates, for conserving forest genetic resources. Because of the immobility and the longevity of tree and shrub species, high genetic variation is the longterm base for adaptability and thus for survival of these species. For biological, economical and ethical reasons, forestry depends on the maintenance of high genetic diversity. The forests and therefore the forest genetic resources were already endangered by the clearing of forests for cultivation and other diverse interventions into forest ecosystems. The survey of the damages caused by forest decline has shown that serious gene losses from anthropogenic environmental loads are continuing. The influence of existing legal regulations on the conservation of forest genetic resources was evaluated. The Act on Forestry Seed and Planting Stock (Bundesgesetzblatt 1979) demands that forests be maintained because of their economic and environmental importance; the provision of habitat for other species is particularly important. Consequently, the high genetic variability of forests has to be maintained. However, no regulations exist for the realization of this aim either in the Federal Forest Law or in the respective laws of the states. In addition, the legal regulations concerning forest reproductive material and nature conservation do not offer such an instrumentation. Existing measures within the Federal Republic which directly serve gene conservation are concentrated in public organisations such as Federal Research Institutes and State Forest Administrations with their research institutes and seed extractories. There is little activity in the private sector Direct activities include: conservation of breeding material within the framework of breeding programmes; collection of provenances, families and clones in field tests and clonal archives; genebanks of seed, pollen and tissue cultures. Up to now, these conservation measures have been funded from the budgets of the research institutes, but these are insufficient for the additional activities which are needed. The criteria for the selection of material’‘worthy of conservation’’ are for example: selected or comparable populations covered by the Act on Forestry Seed and Planting Stock (Bundesgesetzblatt, 1979), populations under specific ecological conditions, marginal populations and the ‘’necessity for conservation’’ which results from the degree of current damage or from the rarity of the material. Conservation is necessary for: 18 tree species and the genus Populus as covered by the Act on Forestry Seed and Planting Stock (Bundesgesetzblatt, 1979); 29 species not under the law, but with importance for forestry; 10 indigenous and introduced tree species important from a regional view; and 37 indigenous shrub species. The measures for conservation depend on the biology, the developmental stage of the material, the technical feasability and on the costs. The following can be applied with different prospects for success depending on the tree species: conservation of stands; natural regeneration; sowing and planting in situ and ex situ; seedling and clonal seed orchards; clone collections; conservation of seed, pollen, plants, parts of plants including tissue, and conservation by macro-and microvegetative propagation. The individual measures are evaluated. In addition, the total work necessary in forest genetic conservation is presented. The current situation is discussed. The fields to be covered in the frame of the programme can only be achieved by close cooperation between the Federal and State Forest Administrations and their institutions. The following principles for cooperation are proposed: Activities for conservation of forest genetic resources are pel formed by the Federal Forest Research Centre and the State Forest Research Institutes including the respective seed extractories. These institutions have the professional capacity, the direct connection to forestry, the necessary technical facilities and the scientific background. In addition, they cover the interests of the States. The selection of genetic resources to be conserved will be coordinated and the institutions responsible for the conservation of forest genetic resources in the Federal Republic of Germany will regularly exchange information about incoming and outgoing gene resource material. To minimize the risk of loss, samples for ex situ conservation (seed, pollen, plants, parts of plants) will be stored at least at 2 locations. Research necessary for gene conservation occurs in the institutes of the forestry faculties at the universities, and the respective federal and state institutes in close coordination. Independent of the constitutional responsibilities for the single measures, Federal Government and States will coordinate the activities for conservation of forest genetic resources. It is proposed to give the responsibility for the coordination of the activities between Federal Government and States to the ‘’Federal and State Working Group on Conservation of Forest Genetic Resources’’. The Federal Government will maintain responsibility for its legal obligations, collective representation, resource orientated research and the safeguarding of important inter national relationships. Division of duties between Federal and States will be according to Article 30 of the constitution. For the further procedures it is recommended that the programme should be discussed at the conference of Federal and State Ministers for Agriculture and Forestry with the aim to reach an agreement for cooperation in the field of conservation of forest genetic resources. The forest administrations and the Federal and State Research Institutes should be charged with implementing the programme. In this paper, facts are presented, possibilities outlined and urgent recommendations given from a professional view. The realization of the necessary measures for the conservation of forest genetic resources needs the political decision. Forests are ecologically and economically an important stabilizing factor in the Federal Republic of Germany. The forest genetic resources therefore have to be saved; actions for their conservation have to be started without delay. The present state of the conservation programme is described. | 1888 | no | NA | NA | no | no |
| 1889 | 2017 | 43021 | Poulsen, M; Cafaro, M; Boomsma, JJ; Currie, CR | 2005 | Specificity of the mutualistic association between actinomycete bacteria and two sympatric species of Acromyrmex leaf-cutting ants | Acromyrmex leaf-cutting ants maintain two highly specialized, vertically transmitted mutualistic ectosymbionts: basidiomycete fungi that are cultivated for food in underground gardens and actinomycete Pseudonocardia bacteria that are reared on the cuticle to produce antibiotics that suppress the growth of Escovopsis parasites of the fungus garden. Mutualism stability has been hypothesized to benefit from genetic uniformity of symbionts, as multiple coexisting strains are expected to compete and, thus, reduce the benefit of the symbiosis. However, the Pseudonocardia symbionts are likely to be involved in Red-Queen-like antagonistic co-evolution with Escovopsis so that multiple strains per host might be favoured by selection provided the cost of competition between bacterial strains is low. We examined the genetic uniformity of the Pseudonocardia symbionts of two sympatric species of Acromyrmex ants by comparing partial sequences of the nuclear Elongation Factor-Tu gene. We find no genetic variation in Pseudonocardia symbionts among nest mate workers, neither in Acromyrmex octospinosus, where colonies are founded by a single queen, nor in Acromyrmex echinatior, where mixing of bacterial lineages might happen when unrelated queens cofound a colony. We further show that the two ant species maintain the same pool of Pseudonocardia symbionts, indicating that horizontal transmission occasionally occurs, and that this pool consists of two distinct clades of closely related Pseudonocardia strains. Our finding that individual colonies cultivate a single actinomycete strain is in agreement with predictions from evolutionary theory on host-symbiont conflict over symbiont mixing, but indicates that there may be constraints on the effectiveness of the bacterial symbionts on an evolutionary timescale. | 1889 | no | gd of parasite | NA | no | no |
| 1890 | 2017 | 43021 | Arruda, MP; Morielle-Versute, E | 2008 | Cytogenetic and random amplified polymorphic DNA analysis of Leptodactylus species from rural and urban environments (Anura, Amphibia) | Cytogenetic and random amplified polymorphic DNA analyses carried out in the species Leptodactylus podicipinus, L. ocellatus, L. labyrinthicus, and L. fuscus from rural and urban habitats of the northwest region of Sao Paulo State, Brazil, showed that the karyotypes (2n = 22), constitutive heterochromatin distribution and nucleolus organizer region (NOR) location did not differ between the populations from the two environments. The in situ hybridization with an rDNA probe confirmed the location of the NORs on chromosome 8 revealing an in tandem duplication of that region in one of the chromosomes of L. fuscus. DAPI showed that part of the C-band-positive heterochromatin is rich in AT, including that in the proximity the NORs in L. podicipinus and L. ocellatus. The molecular analyses showed that the two populations (urban and rural) of L. podicipinus and L. fuscus are similar from a genetic point of view. The urban and rural populations of species L. ocellatus and L. labyrinthicus showed differences in genetic structures, probably due to urbanization which interferes with the dispersion of those frogs. The marked differences observed between the two populations of L. ocellatus can be representing the cryptic condition of the species. Unweighted pair-group method of analysis and genetic distance analysis detected the genetic proximity between L. ocellatus and L. fuscus. The results indicate that there was no reduction in the genetic diversity in the populations from the urban environment; however, the survival of these frogs would not be guaranteed in the case of an increase in human impact especially for populations of L. labyrinthicus and L. ocellatus. | 1890 | no | no good fitness measure, N pop differnt in GD? | NA | no | no |
| 1891 | 2017 | 43021 | Huck, S; Budel, B; Kadereit, JW; Printzen, C | 2009 | Range-wide phylogeography of the European temperate-montane herbaceous plant Meum athamanticum Jacq.: evidence for periglacial persistence | Aim The aim of this study is to analyse the genetic population structure of Meum athamanticum Jacq. in order to explore the alternative hypotheses (1) that the central and northern highland populations are the result of post-glacial recolonization from southern refugia, and the disjunct distribution of M. athamanticum can be explained by modern ecological conditions, or (2) that extant populations north of the Alps and Pyrenees persisted in situ during glacial periods. Location Europe. Methods Variation of amplified fragment length polymorphisms (AFLPs) was analysed for 23 populations from the entire range of the species. We used band-based approaches and methods based on allele frequencies to measure genetic diversity and to identify population structure. Results Our analyses reveal a north-south differentiation within M. athamanticum. High levels of genetic diversity, as well as private fragments, are found in populations both north and south of the Alps. Differentiation among populations is lower in the northern than in the southern population group, and significant isolation-by-distance is found only in the latter group. Main conclusions Our results indicate that M. athamanticum survived the last ice age in multiple refugia throughout its contemporary range and did not expand into areas north of the Alps from southern refugia. We found evidence that regional-scale migration in northern, formerly periglacial, parts of the species range has resulted in the intermingling of populations. In contrast, southern populations are characterized by long-term isolation. The south-west Alps represent an area where immigration and mixing of populations from northern and southern refugia appears to have taken place. | 1891 | no | no good fitness measure | NA | no | no |
| 1892 | 2017 | 43021 | Sasaki, AA; Fernandes, GF; Rodrigues, AM; Lima, FM; Marini, MM; Feitosa, LD; Teixeira, MD; Felipe, MSS; da Silveira, JF; de Camargo, ZP | 2014 | Chromosomal Polymorphism in the Sporothrix schenckii Complex | Sporotrichosis is a polymorphic disease caused by a complex of thermodimorphic fungi including S. brasiliensis, S. schenckii sensu stricto (s. str.), S. globosa and S. luriei. Humans and animals can acquire the disease through traumatic inoculation of propagules into the subcutaneous tissue. Despite the importance of sporotrichosis as a disease that can take epidemic proportions there are just a few studies dealing with genetic polymorphisms and genomic architecture of these pathogens. The main objective of this study was to investigate chromosomal polymorphisms and genomic organization among different isolates in the S. schenckii complex. We used pulsed field gel electrophoresis (PFGE) to separate chromosomal fragments of isolated DNA, followed by probe hybridization. Nine loci (beta-tubulin, calmodulin, catalase, chitin synthase 1, Internal Transcribed Spacer, Pho85 cyclin-dependent kinase, protein kinase C Ss-2, G protein a subunit and topoisomerase II) were mapped onto chromosomal bands of Brazilian isolates of S. schenckii s. str. and S. brasiliensis. Our results revealed the presence of intra and interspecies polymorphisms in chromosome number and size. The gene hybridization analysis showed that closely related species in phylogenetic analysis had similar genetic organizations, mostly due to identification of synteny groups in chromosomal bands of similar sizes. Our results bring new insights into the genetic diversity and genome organization among pathogenic species in the Sporothrix schenckii complex. | 1892 | no | NA | NA | no | no |
| 1893 | 2017 | 43021 | Tellier, A; Brown, JKM | 2011 | Spatial heterogeneity, frequency-dependent selection and polymorphism in host-parasite interactions | Background: Genomic and pathology analysis has revealed enormous diversity in genes involved in disease, including those encoding host resistance and parasite effectors (also known in plant pathology as avirulence genes). It has been proposed that such variation may persist when an organism exists in a spatially structured metapopulation, following the geographic mosaic of coevolution. Here, we study gene-for-gene relationships governing the outcome of plant-parasite interactions in a spatially structured system and, in particular, investigate the population genetic processes which maintain balanced polymorphism in both species. Results: Following previous theory on the effect of heterogeneous environments on maintenance of polymorphism, we analysed a model with two demes in which the demes have different environments and are coupled by gene flow. Environmental variation is manifested by different coefficients of natural selection, the costs to the host of resistance and to the parasite of virulence, the cost to the host of being diseased and the cost to an avirulent parasite of unsuccessfully attacking a resistant host. We show that migration generates negative direct frequency-dependent selection, a condition for maintenance of stable polymorphism in each deme. Balanced polymorphism occurs preferentially if there is heterogeneity for costs of resistance and virulence alleles among populations and to a lesser extent if there is variation in the cost to the host of being diseased. We show that the four fitness costs control the natural frequency of oscillation of host resistance and parasite avirulence alleles. If demes have different costs, their frequencies of oscillation differ and when coupled by gene flow, there is amplitude death of the oscillations in each deme. Numerical simulations show that for a multiple deme island model, costs of resistance and virulence need not to be present in each deme for stable polymorphism to occur. Conclusions: Our theoretical results confirm the importance of empirical studies for measuring the environmental heterogeneity for genetic costs of resistance and virulence alleles. We suggest that such studies should be developed to investigate the generality of this mechanism for the long-term maintenance of genetic diversity at host and parasite genes. | 1893 | no | NA | NA | no | no |
| 1894 | 2017 | 43021 | Duffy, MA; Brassil, CE; Hall, SR; Tessier, AJ; Caceres, CE; Conner, JK | 2008 | Parasite-mediated disruptive selection in a natural Daphnia population | Background: A mismatch has emerged between models and data of host-parasite evolution. Theory readily predicts that parasites can promote host diversity through mechanisms such as disruptive selection. Yet, despite these predictions, empirical evidence for parasite-mediated increases in host diversity remains surprisingly scant. Results: Here, we document parasite-mediated disruptive selection on a natural Daphnia population during a parasite epidemic. The mean susceptibility of clones collected from the population before and after the epidemic did not differ, but clonal variance and broad-sense heritability of post-epidemic clones were significantly greater, indicating disruptive selection and rapid evolution. A maximum likelihood method that we developed for detecting selection on natural populations also suggests disruptive selection during the epidemic: the distribution of susceptibilities in the population shifted from unimodal prior to the epidemic to bimodal after the epidemic. Interestingly, this same bimodal distribution was retained after a generation of sexual reproduction. Conclusion: These results provide rare empirical support for parasite-driven increases in host genetic diversity, and suggest that this increase can occur rapidly. | 1894 | probably no | no good fitness measure, N pop differnt in GD? | only one pop | no | no |
| 1895 | 2017 | 43021 | Azzag, N; Haddad, N; Durand, B; Petit, E; Ammouche, A; Chomel, B; Boulouis, HJ | 2012 | Population Structure of Bartonella henselae in Algerian Urban Stray Cats | Whole blood samples from 211 stray cats from Algiers, Algeria, were cultured to detect the presence of Bartonella species and to evaluate the genetic diversity of B. henselae strains by multiple locus VNTR analysis (MLVA). Bartonella henselae was the only species isolated from 36 (17%) of 211 cats. B. henselae genotype I was the predominant genotype (64%). MLVA typing of 259 strains from 30 bacteremic cats revealed 52 different profiles as compared to only 3 profiles using MLST. Of these 52 profiles, 48 (92.3%) were identified for the first time. One-third of the cats harbored one MLVA profile only. As there was a correlation between the age of cats and the number of MLVA profiles, we hypothesized that the single profile in these cats was the profile of the initial infecting strain. Two-third of the cats harbored 2 to 6 MLVA profiles simultaneously. The similarity of MLVA profiles obtained from the same cat, neighbor-joining clustering and structure-neighbor clustering indicate that such a diversity likely results from two different mechanisms occurring either independently or simultaneously: independent infections and genetic drift from a primary strain. | 1895 | no | gd of parasite | NA | no | no |
| 1896 | 2017 | 43021 | Huang, L; Jiang, HF; Ren, XP; Chen, YN; Xiao, YJ; Zhao, XY; Tang, M; Huang, JQ; Upadhyaya, HD; Liao, BS | 2012 | Abundant Microsatellite Diversity and Oil Content in Wild Arachis Species | The peanut (Arachis hypogaea) is an important oil crop. Breeding for high oil content is becoming increasingly important. Wild Arachis species have been reported to harbor genes for many valuable traits that may enable the improvement of cultivated Arachis hypogaea, such as resistance to pests and disease. However, only limited information is available on variation in oil content. In the present study, a collection of 72 wild Arachis accessions representing 19 species and 3 cultivated peanut accessions were genotyped using 136 genome-wide SSR markers and phenotyped for oil content over three growing seasons. The wild Arachis accessions showed abundant diversity across the 19 species. A. duranensis exhibited the highest diversity, with a Shannon-Weaver diversity index of 0.35. A total of 129 unique alleles were detected in the species studied. A. rigonii exhibited the largest number of unique alleles (75), indicating that this species is highly differentiated. AMOVA and genetic distance analyses confirmed the genetic differentiation between the wild Arachis species. The majority of SSR alleles were detected exclusively in the wild species and not in A. hypogaea, indicating that directional selection or the hitchhiking effect has played an important role in the domestication of the cultivated peanut. The 75 accessions were grouped into three clusters based on population structure and phylogenic analysis, consistent with their taxonomic sections, species and genome types. A. villosa and A. batizocoi were grouped with A. hypogaea, suggesting the close relationship between these two diploid wild species and the cultivated peanut. Considerable phenotypic variation in oil content was observed among different sections and species. Nine alleles were identified as associated with oil content based on association analysis, of these, three alleles were associated with higher oil content but were absent in the cultivated peanut. The results demonstrated that there is great potential to increase the oil content in A. hypogaea by using the wild Arachis germplasm. | 1896 | no | multiple species diversity | NA | no | no |
| 1897 | 2017 | 43021 | Polimanti, R; Piacentini, S; Fuciarelli, M | 2011 | HapMap-based study of human soluble glutathione S-transferase enzymes: the role of natural selection in shaping the single nucleotide polymorphism diversity of xenobiotic-metabolizing genes | Objective Glutathione S-transferase enzymes (GSTs; EC: 2.5.1.18) constitute the principal phase II superfamily, which plays a key role in cellular detoxification. GST genes are organized in chromosomal clusters; most of these genes are polymorphic, mainly due to single nucleotide substitutions. Different studies proved significant interethnic differences in GST allelic frequencies but, at present, the role of natural selection in human genetic variability of GSTs is poorly understood. The aim of this study is to investigate the role of natural selection in shaping single nucleotide polymorphism (SNP) diversity of soluble GST genes. Methods Using the HapMap database, we analyzed the population differences in the soluble GST genes using the phasing data from unrelated individuals shared among 11 populations in the International HapMap project. A F(st)-based selection test was applied to HapMap data to detect soluble GST loci under selection. Results Comparisons between GST gene polymorphisms among HapMap populations highlight that ethnicity is an influencing factor of GST genetic variability. By applying a genome scan based on F-statistics, we identified nine SNPs that present F-coefficients significantly more different than those expected under neutrality (rs2239892, rs3814309, rs7483, rs1571858, rs929166, rs11807, rs4715344, rs4715354, and rs3734431). Conclusion Our study confirms that GST gene variation reflects human demographic history, but it also demonstrates that natural selection could shape the genetic profile of some GST SNPs. Moreover, the identification of human genome regions and targets of natural selection may have detected candidate genes for complex diseases. In analyzing the literature, we provide complex disease hypothesis (male infertility, embryotoxicity) for the identified GST SNPs. Pharmacogenetics and Genomics 21:665-672 (C) 2011 Wolters Kluwer Health \| Lippincott Williams & Wilkins. | 1897 | no | human | NA | no | no |
| 1898 | 2017 | 43021 | Narang, A; Jha, P; Rawat, V; Mukhopadhayay, A; Dash, D; Basu, A; Mukerji, M | 2011 | Recent Admixture in an Indian Population of African Ancestry | Identification and study of genetic variation in recently admixed populations not only provides insight into historical population events but also is a powerful approach for mapping disease loci. We studied a population (OG-W-IP) that is of African-Indian origin and has resided in the western part of India for 500 years; members of this population are believed to be descendants of the Bantu-speaking population of Africa. We have carried out this study by using a set of 18,534 autosomal markers common between Indian, CEPH-HGDP, and Hap Map populations. Principal-components analysis clearly revealed that the African-Indian population derives its ancestry from Bantu-speaking west-African as well as Indo-European-speaking north and northwest Indian population(s). STRUCTURE and ADMIXTURE analyses show that, overall, the OG-W-IPs derive 58.7%, of their genomic ancestry from their African past and have very little inter-individual ancestry variation (8.4%). The extent of linkage disequilibrium also reveals that the admixture event has been recent. Functional annotation of genes encompassing the ancestry-informative markers that are closer in allele frequency to the Indian ancestral population revealed significant enrichment of biological processes, such as ion-channel activity, and cadherins. We briefly examine the implications of determining the genetic diversity of this population, which could provide opportunities for studies involving admixture mapping. | 1898 | no | human | NA | no | no |
| 1899 | 2017 | 43021 | WAYNE, RK; LEHMAN, N; GIRMAN, D; GOGAN, PJP; GILBERT, DA; HANSEN, K; PETERSON, RO; SEAL, US; EISENHAWER, A; MECH, LD; KRUMENAKER, RJ | 1991 | CONSERVATION GENETICS OF THE ENDANGERED ISLE-ROYALE GRAY WOLF | The small group of wolves on Isle Royale has been studied for over three decades as a model of the relationship between large carnivores and their prey. During the last ten years the population declined from 50 individuals to as few as 12 individuals. The causes of this decline may be food shortages, disease, or reduced genetic variability. We address the issues of genetic variability and relationships of Isle Royale wolves using allozyme electrophoresis, mtDNA restriction-site analysis, and multilocus hypervariable minisatellite DNA analysis (genetic fingerprinting). Our results indicate that approximately 50% of the allozyme heterozygosity has been lost in the island population, a decline similar to that expected if no immigration had occurred from the mainland. The genetic fingerprinting data indicate that the seven sampled Isle Royale wolves are as similar as captive populations of siblings. Surprisingly, the Isle Royale wolves have an mtDNA genotype that is very rare on the mainland, being found in only one of 144 mainland wolves. This suggests that the remaining Isle Royale wolves are probably derived from a single female founder. | 1899 | no | only one pop | NA | no | no |
| 1900 | 2017 | 43021 | La Riviere, M; Roumagnac, M; Garrabou, J; Bally, M | 2013 | Transient Shifts in Bacterial Communities Associated with the Temperate Gorgonian Paramuricea clavata in the Northwestern Mediterranean Sea | Background: Bacterial communities that are associated with tropical reef-forming corals are being increasingly recognized for their role in host physiology and health. However, little is known about the microbial diversity of the communities associated with temperate gorgonian corals, even though these communities are key structural components of the ecosystem. In the Northwestern Mediterranean Sea, gorgonians undergo recurrent mass mortalities, but the potential relationship between these events and the structure of the associated bacterial communities remains unexplored. Because microbial assemblages may contribute to the overall health and disease resistance of their host, a detailed baseline of the associated bacterial diversity is required to better understand the functioning of the gorgonian holobiont. Methodology/Principal Findings: The bacterial diversity associated with the gorgonian Paramuricea clavata was determined using denaturing gradient gel electrophoresis, terminal-restriction fragment length polymorphism and the construction of clone libraries of the bacterial 16S ribosomal DNA. Three study sites were monitored for 4 years to assess the variability of communities associated with healthy colonies. Bacterial assemblages were highly dominated by one Hahellaceae-related ribotype and exhibited low diversity. While this pattern was mostly conserved through space and time, in summer 2007, a deep shift in microbiota structure toward increased bacterial diversity and the transient disappearance of Hahellaceae was observed. Conclusion/Significance: This is the first spatiotemporal study to investigate the bacterial diversity associated with a temperate shallow gorgonian. Our data revealed an established relationship between P. clavata and a specific bacterial group within the Oceanospirillales. These results suggest a potential symbiotic role of Hahellaceae in the host-microbe association, as recently suggested for tropical corals. However, a transient imbalance in bacterial associations can be tolerated by the holobiont without apparent symptoms of disease. The subsequent restoration of the Hahellaceae-dominated community is indicative of the specificity and resilience of the bacteria associated with the gorgonian host. | 1900 | no | no good fitness measure, N pop differnt in GD? | NA | no | no |
| 1901 | 2017 | 43022 | Laurin-Lemay, S; Angers, B; Benrey, B; Brodeur, J | 2013 | Inconsistent genetic structure among members of a multitrophic system: did bruchid parasitoids (Horismenus spp.) escape the effects of bean domestication? | Anthropogenic range expansion and cultural practices have modified the distribution, abundance and genetic diversity of domesticated organisms, thereby altering multitrophic assemblages through space and time. The putative Mesoamerican domestication centre of the common bean, Phaseolus vulgaris L., in Mexico allows investigating the effects of plant domestication on the genetic structure of members of a multitrophic system. The aim of this study was to compare the evolutionary history of Horismenus parasitoids (Hymenoptera: Eulophidae) to those of their bruchid beetle hosts (Coleoptera: Bruchidae) and their domesticated host plant (P. vulgaris), in the context of traditional agriculture in Mexico. We analyzed the population genetic structure of four Horismenus species in Mexico using mitochondrial COI haplotype data. The two most abundant parasitoid species were Horismenus depressus and Horismenus missouriensis. Horismenus missouriensis were infected by Wolbachia endosymbionts and had little to no population differentiation (F-ST = 0.06). We suspect the mitochondrial history of H. missouriensis to be blurred by Wolbachia, because differentiation among infected vs. non-infected individuals exists (F-ST = 0.11). Populations of H. depressus were found to be highly differentiated (F-ST = 0.34), but the genetic structuring could not be explained by tested spatial components. We then compared the genetic structure observed in this parasitoid species to previously published studies on bruchid beetles and their host plants. Despite extensive human-mediated migration and likely population homogenization of its two Acanthoscelides bruchid beetle hosts, H. depressus populations are structured like its host plant, by a recent dispersal from a diverse ancestral gene pool. Distinct evolutionary dynamics may explain inconsistent patterns among trophic levels. Parasitoids likely migrate from wild bean populations and are poorly adapted to bean storage conditions similar to their bruchid beetle hosts. Integrating several trophic levels to the study of evolutionary history has proven to be fruitful in detecting different ecological responses to human-mediated disturbances and host parasite interactions. | 1901 | no | no good fitness measure, N pop differnt in GD? | NA | no | no |
| 1902 | 2017 | 43022 | Cerutti, MC; Citterio, CV; Bazzocchi, C; Epis, S; D’Amelio, S; Ferrari, N; Lanfranchi, P | 2010 | Genetic variability of Haemonchus contortus (Nematoda: Trichostrongyloidea) in alpine ruminant host species | Genetic variability of the ovine parasite Haemonchus contortus from the Alpine area was investigated using mitochondrial DNA (nd4 gene), internal transcribed spacers 1 and 2 and microsatellites, in order to assess whether cross-transmission between domestic and wild ruminants occurs. The dataset was composed of 78 individual adult male H. contortus collected from chamois (Rupicapra r. rupicapra), roe deer (Capreolus capreolus), alpine ibex (Capra ibex ibex), domestic goat (Capra hircus) and sheep (Ovis aries) from different alpine areas. The data obtained show low host specificity and high genetic variation within H. contortus populations. The analyses indicate the presence of two mitochondrial haplotype clusters among host species and the absence of cryptic parasite species, confirming H. contortus as a generalist nematode and suggesting that parasite transmission between populations of domestic and wild ruminants normally occurs. | 1902 | no | gd of parasite | NA | no | no |
| 1903 | 2017 | 43022 | Brandt, M; Fischer-Blass, B; Heinze, J; Foitzik, S | 2007 | Population structure and the co-evolution between social parasites and their hosts | Co-evolutionary trajectories of host-parasite interactions are strongly affected by the antagonists’ evolutionary potential, which in turn depends on population sizes as well as levels of recombination, mutation, and gene flow. Under similar selection pressures, the opponent with the higher evolutionary rate is expected to lead the co-evolutionary arms race and to develop local adaptations. Here, we use mitochondrial DNA sequence data and microsatellite markers to assess the amount of genetic variability and levels of gene flow in two host-parasite systems, each consisting of an ant social parasite - the European slavemaker Harpagoxenus sublaevis and the North American slavemaker Protomognathus americanus - and its two main host species. Our population genetic analyses revealed limited gene flow between individual populations of both host and parasite species, allowing for a geographic mosaic of co-evolution. In a between-system comparison, we found less genetic variability and more pronounced structure in Europe, where previous behavioural studies demonstrated strong local adaptation. Within the European host-parasite system, the larger host species Leptothorax acervorum exhibited higher levels of both genetic variability and gene flow, and previous field data showed that it is less affected by the social parasite H. sublaevis than the smaller host Leptothorax muscorum, which has genetically depleted and isolated populations. In North America, the parasite P. americanus showed higher levels of gene flow between sites, but overall less genetic diversity than its hyper-variable main host species, Temnothorax longispinosus. Interestingly, recent ecological and chemical studies demonstrated adaptation of P. americanus to local host populations, indicating the importance of migration in co-evolutionary interactions. | 1903 | no | no good fitness measure | NA | no | no |
| 1904 | 2017 | 43022 | Liu, G; Liu, YX; Ali, T; Ferreri, M; Gao, J; Chen, W; Yin, JH; Su, JL; Fanning, S; Han, B | 2015 | Molecular and Phenotypic Characterization of Aerococcus viridans Associated with Subclinical Bovine Mastitis | Aerococcus viridans is a wide spread bacterium in the environment and clinically this organism is associated with different diseases in animals and humans. However, the geno- and phenotypic characterization of A. viridans associated with bovine mastitis has not yet been reported. The objectives of this study were to investigate the genetic and phenotypic diversity of A. viridans isolates using three different molecular methods including 16S rRNA gene sequencing, pulsed-field gel electrophoresis and random amplified polymorphic DNA (RAPD) along with biochemical tests, including antimicrobial susceptibility test. In total, 60 A. viridans strains were cultured from dairy herds presenting with subclinical mastitis. The results of biochemical tests revealed that most of the isolates (75.0%) were accurately identified by API Rapid 20 Strep system and the majority of A. viridans strains (96.7%) were found to be catalase negative, while two (3.3%) isolates were weakly positive. All isolates were resistant to trimethoprim-sulfamethoxazole, followed by streptomycin (96.7%), tetracycline (65.0%) and clindamycin (56.7%) by minimum inhibition concentration-determining broth microdilution technique. As compared to the sequence of 16S rRNA gene, both PFGE and RAPD showed their capacities to discriminate the intra-species diversity of A. viridans. Furthermore, most of the isolates obtained from the same herd or region belonged to the same major RAPD group, which indicated that RAPD is an appropriate assay for tracking the origins of isolates and epidemiological studies of A. viridans. This is a novel approach to use three molecular techniques and to compare their efficiency regarding the genetic diversity of A. viridans. The data suggest that A. viridans associated with subclinical mastitis has a considerable phenotypic and genotypic diversity. | 1904 | no | gd of pathogen | NA | no | no |
| 1905 | 2017 | 43022 | Ceresini, PC; Shew, HD; James, TY; Vilgalys, RJ; Cubeta, MA | 2007 | Phylogeography of the Solanaceae-infecting Basidiomycota fungus Rhizoctonia solani AG-3 based on sequence analysis of two nuclear DNA loci | Background: The soil fungus Rhizoctonia solani anastomosis group 3 (AG-3) is an important pathogen of cultivated plants in the family Solanaceae. Isolates of R. solani AG-3 are taxonomically related based on the composition of cellular fatty acids, phylogenetic analysis of nuclear ribosomal DNA (rDNA) and beta-tubulin gene sequences, and somatic hyphal interactions. Despite the close genetic relationship among isolates of R. solani AG-3, field populations from potato and tobacco exhibit comparative differences in their disease biology, dispersal ecology, host specialization, genetic diversity and population structure. However, little information is available on how field populations of R. solani AG-3 on potato and tobacco are shaped by population genetic processes. In this study, two field populations of R. solani AG-3 from potato in North Carolina (NC) and the Northern USA; and two field populations from tobacco in NC and Southern Brazil were examined using sequence analysis of two cloned regions of nuclear DNA (pP42F and pP89). Results: Populations of R. solani AG-3 from potato were genetically diverse with a high frequency of heterozygosity, while limited or no genetic diversity was observed within the highly homozygous tobacco populations from NC and Brazil. Except for one isolate (TBR24), all NC and Brazilian isolates from tobacco shared the same alleles. No alleles were shared between potato and tobacco populations of R. solani AG-3, indicating no gene flow between them. To infer historical events that influenced current geographical patterns observed for populations of R. solani AG-3 from potato, we performed an analysis of molecular variance (AMOVA) and a nested clade analysis (NCA). Population differentiation was detected for locus pP89 (Phi(ST) = 0.257, significant at P < 0.05) but not for locus pP42F (Phi(ST) = 0.034, not significant). Results based on NCA of the pP89 locus suggest that historical restricted gene flow is a plausible explanation for the geographical association of clades. Coalescent-based simulations of genealogical relationships between populations of R. solani AG-3 from potato and tobacco were used to estimate the amount and directionality of historical migration patterns in time, and the ages of mutations of populations. Low rates of historical movement of genes were observed between the potato and tobacco populations of R. solani AG-3. Conclusion: The two sisters populations of the basidiomycete fungus R. solani AG-3 from potato and tobacco represent two genetically distinct and historically divergent lineages that have probably evolved within the range of their particular related Solanaceae hosts as sympatric species. | 1905 | no | gd of pathogen | NA | no | no |
| 1906 | 2017 | 43022 | Hume, JCC; Hamilton, H; Lee, KL; Lehmann, T | 2011 | Susceptibility of Anopheles stephensi to Plasmodium gallinaceum: A Trait of the Mosquito, the Parasite, and the Environment | “Background: Vector susceptibility to Plasmodium infection is treated primarily as a vector trait, although it is a composite trait expressing the joint occurrence of the parasite and the vector with genetic contributions of both. A comprehensive approach to assess the specific contribution of genetic and environmental variation on”“vector susceptibility”" is lacking. Here we developed and implemented a simple scheme to assess the specific contributions of the vector, the parasite, and the environment to "“vector susceptibility.”" To the best of our knowledge this is the first study that employs such an approach. Methodology/Principal Findings: We conducted selection experiments on the vector (while holding the parasite "“constant”“) and on the parasite (while holding the vector”“constant”“) to estimate the genetic contributions of the mosquito and the parasite to the susceptibility of Anopheles stephensi to Plasmodium gallinaceum. We separately estimated the realized heritability of (i) susceptibility to parasite infection by the mosquito vector and (ii) parasite compatibility (transmissibility) with the vector while controlling the other. The heritabilities of vector and the parasite were higher for the prevalence, i. e., fraction of infected mosquitoes, than the corresponding heritabilities of parasite load, i. e., the number of oocysts per mosquito. Conclusions: The vector’s genetics (heritability) comprised 67% of”“vector susceptibility”" measured by the prevalence of mosquitoes infected with P. gallinaceum oocysts, whereas the specific contribution of parasite genetics (heritability) to this trait was only 5%. Our parasite source might possess minimal genetic diversity, which could explain its low heritability (and the high value of the vector). Notably, the environment contributed 28%. These estimates are relevant only to the particular system under study, but this experimental design could be useful for other parasite-host systems. The prospects and limitations of the genetic manipulation of vector populations to render the vector resistant to the parasite are better considered on the basis of this framework." | 1906 | no | no good fitness measure, N pop differnt in GD? | NA | no | no |
| 1907 | 2017 | 43022 | Bihlmeyer, NA; Brody, JA; Smith, AV; Lunetta, KL; Nalls, M; Smith, JA; Tanaka, T; Davies, G; Yu, L; Mirza, SS; Teumer, A; Coresh, J; Pankow, JS; Franceschini, N; Scaria, A; Oshima, J; Psaty, BM; Gudnason, V; Eiriksdottir, G; Harris, TB; Li, HY; Karasik, D; Kiel, DP; Garcia, M; Liu, YM; Faul, JD; Kardia, SLR; Zhao, W; Ferrucci, L; Allerhand, M; Liewald, DC; Redmond, P; Starr, JM; De Jager, PL; Evans, DA; Direk, N; Ikram, MA; Uitterlinden, A; Homuth, G; Lorbeer, R; Grabe, HJ; Launer, L; Murabito, JM; Singleton, AB; Weir, DR; Bandinelli, S; Deary, IJ; Bennett, DA; Tiemeier, H; Kocher, T; Lumley, T; Arking, DE | 2014 | Genetic diversity is a predictor of mortality in humans | Background: It has been well-established, both by population genetics theory and direct observation in many organisms, that increased genetic diversity provides a survival advantage. However, given the limitations of both sample size and genome-wide metrics, this hypothesis has not been comprehensively tested in human populations. Moreover, the presence of numerous segregating small effect alleles that influence traits that directly impact health directly raises the question as to whether global measures of genomic variation are themselves associated with human health and disease. Results: We performed a meta-analysis of 17 cohorts followed prospectively, with a combined sample size of 46,716 individuals, including a total of 15,234 deaths. We find a significant association between increased heterozygosity and survival (P = 0.03). We estimate that within a single population, every standard deviation of heterozygosity an individual has over the mean decreases that person’s risk of death by 1.57%. Conclusions: This effect was consistent between European and African ancestry cohorts, men and women, and major causes of death (cancer and cardiovascular disease), demonstrating the broad positive impact of genomic diversity on human survival. | 1907 | no | human | NA | no | no |
| 1908 | 2017 | 43022 | Santonastaso, TT; Dubach, J; Hauver, SA; Graser, WH; Gehrt, SD | 2012 | Microsatellite analysis of raccoon (Procyon lotor) population structure across an extensive metropolitan landscape | Understanding population structure can lend insight into the spread of animal-borne disease, and the effects of anthropogenic land use on habitat. Raccoons are highly adaptive to human land development and can persist in a wide range of habitat types, making them ideal subjects for investigating the level of population structure in a highly fragmented area. A total of 323 raccoons were livetrapped from 7 locations encompassing 3 distinct habitat types (agriculture, urban forest preserves, and residential) across the Chicago metropolitan region (maximum distance between 2 sites was 128 km). Genetic analyses of 14 microsatellite loci indicate that although raccoon populations across the region share up to 50% of the allelic diversity, they segregated into at least 2 distinct subpopulations, dividing the Chicago metropolitan region into northern and southern groups with further structure occurring within these larger groups. Incorporating sample sites between the identified north south groups may provide greater resolution as to where this split occurs. Although there is evidence of population structure between all sample sites, migrant analysis suggests there is enough gene flow to preserve genetic diversity throughout the population. | 1908 | no | no good fitness measure, N pop differnt in GD? | NA | no | no |
| 1909 | 2017 | 43022 | Balasubramanian, V; Selvarajan, R | 2014 | Genetic diversity and recombination analysis in the coat protein gene of Banana bract mosaic virus | Banana bract mosaic virus (BBrMV), a member of the genus Potyvirus, family Potyviridae, is the causal agent of the bract mosaic disease (BBrMD) that causes serious yield losses in banana and plantain in India and the Philippines. In this study, global genetic diversity and molecular evolution of BBrMV based on the capsid protein (CP) gene were investigated. Multiple alignments of CP gene of 49 BBrMV isolates showed nucleotide (nt) and amino acid (aa) identity of 79-100 and 80-100 %, respectively. Phylogenetic analysis revealed that except two Indians isolates (TN14 and TN16), all isolates clustered together. Eleven recombination events were detected using Recombination Detection Program. Codon-based maximum-likelihood methods revealed that most of the codons in the CP gene were under negative or neutral selection except for codons 28, 43, and 92 which were under positive selection. Gene flow between BBrMV populations of banana and cardamom was relatively frequent but not between two different populations of banana infecting isolates identified in this study. This is the first report on genetic diversity, and evolution of BBrMV isolates based on recombination and phylogenetic analysis in India. | 1909 | no | gd of pathogen | NA | no | no |
| 1910 | 2017 | 43022 | Fernandez-Garcia, MD; Kebe, O; Fall, AD; Ndiaye, K | 2017 | Identification and molecular characterization of non-polio enteroviruses from children with acute flaccid paralysis in West Africa, 2013-2014 | Besides polioviruses, non-polio enteroviruses (NPEVs) may also be associated with acute flaccid paralysis (AFP). Because poliomyelitis is on the verge of eradication, more attention should be paid to study NPEVs from non-polio AFP cases and their epidemic patterns. In West African countries the epidemiology of NPEVs remains largely unexplored. We investigated the genetic diversity, frequency, circulation patterns, and molecular epidemiology of NPEVs in seven West African countries by analyzing retrospectively a panel of 3195 stool samples from children with AFP collected through routine poliomyelitis surveillance activities between 2013 and 2014. VP1 sequencing and typing on 201 isolates revealed 39 NPEV types corresponding to EV-A (6.9%), EV-B (90.5%), EV-C (2%) and EV-D (0.5%) species. Echoviruses were isolated most frequently with 138 cases (68.6%), followed by coxsackievirus group B with 35 cases (17.4%). No single NPEV type was remarkably dominant. Interestingly, several rarely described types with limited detection worldwide were identified (EVA76, EVA119, EVB75, EVB77, EVB97, EVC99, CVA20, CVA21 and EVD94). This study demonstrates the extensive diversity and diverse circulation patterns of NPEVs from AFP surveillance and highlights the need to formulate effective long-term strategies to monitor NPEV circulations in West Africa. | 1910 | no | gd of pathogen | NA | no | no |
| 1911 | 2017 | 43022 | Sanna, D; Pala, M; Cossu, P; Dedola, GL; Melis, S; Fresu, G; Morelli, L; Obinu, D; Tonolo, G; Secchi, G; Triunfo, R; Lorenz, JG; Scheinfeldt, L; Torroni, A; Robledo, R; Francalacci, P | 2011 | Mendelian breeding units versus standard sampling strategies: Mitochondrial DNA variation in southwest Sardinia | We report a sampling strategy based on Mendelian Breeding Units (MBUs), representing an interbreeding group of individuals sharing a common gene pool. The identification of MBUs is crucial for case-control experimental design in association studies. The aim of this work was to evaluate the possible existence of bias in terms of genetic variability and haplogroup frequencies in the MBU sample, due to severe sample selection. In order to reach this goal, the MBU sampling strategy was compared to a standard selection of individuals according to their surname and place of birth. We analysed mitochondrial DNA variation (first hypervariable segment and coding region) in unrelated healthy subjects from two different areas of Sardinia: the area around the town of Cabras and the western Campidano area. No statistically significant differences were observed when the two sampling methods were compared, indicating that the stringent sample selection needed to establish a MBU does not alter original genetic variability and haplogroup distribution. Therefore, the MBU sampling strategy can be considered a useful tool in association studies of complex traits. | 1911 | no | no good fitness measure, N pop differnt in GD? | NA | no | no |
| 1912 | 2017 | 43022 | Martinez, RT; Baudouin, L; Berger, A; Dollet, M | 2010 | Characterization of the genetic diversity of the Tall coconut (Cocos nucifera L.) in the Dominican Republic using microsatellite (SSR) markers | The predominant coconut variety cultivated in the Dominican Republic is a local Tall, known as criollo. It was never characterized genetically. The Malayan Dwarf and its hybrid with the local Tall are also present. Thirteen accessions, representing nine localities, are planted in a collection at the Instituto Dominicano de Investigaciones Agropecuarias y Forestales (IDIAF). We explored genetic diversity in 114 individuals from this collection. The main aim was to detect possible relationship with resistant varieties to coconut lethal yellowing (LY) disease. Contrarily to what happened in other Caribbean countries, LY never became an epidemic in the Dominican Republic. Thirteen simple sequence repeats markers from a kit dedicated to coconut diversity were used. In addition to diversity parameters, we used Bayesian assignment tests and cluster analysis to determine its population structure and its relationship with other coconut populations. The criollo coconut proved to be a typical Indo-Atlantic variety and is probably highly susceptible to the usual LY pathogens. Local conditions and the nature of the local phytoplasma strain probably explain the particular epidemiology of LY in the Dominican Republic. As a cross-pollinating variety, the criollo presents polymorphism within a population, but there is little if any variation among populations. The marker study confirmed the hybrid status of each member of two accessions and, thus, the reliability of the sampling. | 1912 | no | no good fitness measure, N pop differnt in GD? | NA | no | no |
| 1913 | 2017 | 43022 | Gautschi, B; Widmer, A; Joshi, J; Koella, JC | 2002 | Increased frequency of scale anomalies and loss of genetic variation in serially bottlenecked populations of the dice snake, Natrix tessellata | Documented demographic bottlenecks resulting from introductions of the dice snake to several lakes in Switzerland provide a rare opportunity to study the effect of serial bottlenecks on the genetic properties of Natrix tessellata populations. We investigated two introduced populations using information derived from eight microsatellite markers. Both introduced populations had significantly reduced levels of allelic diversity relative to non-bottlenecked populations. The severity of the bottlenecks was underlined by the significant reduction in observed and expected heterozygosity. The loss of allelic diversity and observed heterozygosity was stronger in the serially bottlenecked population than in the population that was bottlenecked only once. From previous studies, scale anomalies were known to be more common in introduced populations relative to native populations. We investigated whether the higher occurrence of scale anomalies in introduced populations is associated with individual heterozygosity and mean genomic diversity d(2). We found a significant relationship between the occurrence of scale anomalies and individual heterozygosity but no significant relationship between scale anomalies and the microsatellite specific measurement, d(2), was found. Because of their known history, introduced populations in Switzerland may serve as a model to demonstrate the effect of severe population bottlenecks on genetic variability and developmental stability in N. tessellata. The results therefore help to device strategies for the management and protection of endangered natural N. tessellata populations. | 1913 | no | no good fitness measure, N pop differnt in GD? | NA | no | no |
| 1914 | 2017 | 43022 | Brunhoff, C; Yoccoz, NG; Ims, RA; Jaarola, M | 2006 | Glacial survival or late glacial colonization? Phylogeography of the root vole (Microtus oeconomus) in north-west Norway | Aim It has been proposed that the root vole subspecies, Microtus oeconomus finmarchicus, survived the last glacial period on islands on the north-west coast of Norway. The Norwegian island of Andoya may have constituted the only site with permanent ice-free conditions. Geological surveys and fossil finds from Andoya demonstrate that survival throughout the last glacial maximum was probably possible for some plants and animals. In this study we aim to infer the recent evolutionary history of Norwegian root vole populations and to evaluate the glacial survival hypothesis. Methods DNA sequence variation in the mitochondrial cytochrome b gene was studied in 46 root voles from 19 localities. Location Northern Fennoscandia and north-west Russia with a focus on islands on the north-west coast of Norway. Results The phylogeographical analyses revealed two North European phylogroups labelled ‘Andoya’ and ‘Fennoscandia’. The Andoya phylogroup contained root voles from the Norwegian islands of Andoya, Ringvassoya and Reinoya and two localities in north-west Russia. The Fennoscandian phylogroup encompassed root voles from the three Norwegian islands of Kvaloya, Hakoya and Arnoya and the remaining specimens from Norway, northern Sweden and Finland. Nucleotide diversity within the Andoya and Fennoscandian phylogroups was similar, ranging from 0.5% to 0.7%. Main conclusions Both our genetic data and previously published morphological data are consistent with in situ glacial survival of root voles on Andoya during the last glacial maximum. However, the level of genetic diversity observed in the extant island populations, the past periods of severe climatic conditions on Andoya and the ecology of the root vole are somewhat difficult to reconcile with this model. A biogeographical scenario involving late glacial recolonization along the northern coasts of Russia and Norway therefore represents a viable alternative. Our results demonstrate that complex recolonization and extinction histories can generate intricate phylogeographical patterns and relatively high levels of genetic variation in northern populations. | 1914 | no | No GD diff between pop | NA | no | no |
| 1915 | 2017 | 43022 | Aardema, ML; von Loewenich, FD | 2015 | Varying influences of selection and demography in host-adapted populations of the tick-transmitted bacterium, Anaplasma phagocytophilum | Background: The host range of a pathogenic bacterial strain likely influences its effective population size, which in turn affects the efficacy of selection. Transmission between competent hosts may occur more frequently for host generalists than for specialists. This could allow higher bacterial population densities to persist within an ecological community and increase the efficacy of selection in these populations. Conversely, specialist strains may be better adapted to their hosts and consequently achieve greater within-host population densities, with corresponding increases in selection efficacy. To assess these different hypotheses, we examined the effective population sizes of three strains of the bacterium Anaplasma phagocytophilum and categorized the varying roles of selection and demography on patterns of genetic diversity and divergence in these populations. A. phagocytophilum is a tick-transmitted, obligately intracellular pathogen. Strains of A. phagocytophilum display varying degrees of host specialization, making this a good species for exploring questions regarding host range, effective population size and selection efficacy. Results: We found that a roe deer specialist harbored the most genetic diversity of the three A. phagocytophilum strains and correspondingly had the largest effective population size. Another strain that is ecologically specialized on rodents and insectivores had the smallest effective population size. However, these mammalian hosts are distantly related evolutionarily. The third strain, a host generalist, was intermediate in its effective population size between the other two strains. Evolutionary constraint on non-synonymous sites was pervasive in all three strains, although some slightly deleterious mutations may also be segregating in these populations. We additionally found evidence of genome-wide selective sweeps in the generalist strain, whereas signals of repeated bottlenecks were detected in the strain with the smallest effective population size. Conclusions: A. phagocytophilum is a diverse bacterial species that differs among distinct strains in its effective population size, as well as how genetic diversity and divergence have been influenced by selection and demographic changes. In this species, host specialization may facilitate increased population growth and allow more opportunities for selection to act. These results provide insights into how host range has influenced evolutionary patterns of strain divergence in an emerging zoonotic bacterium. | 1915 | no | gd of pathogen | NA | no | no |
| 1916 | 2017 | 43024 | Barres, B; Dutech, C; Andrieux, A; Halkett, F; Frey, P | 2012 | Exploring the role of asexual multiplication in poplar rust epidemics: impact on diversity and genetic structure | Fungal plant pathogens, especially rust fungi (Pucciniales), are well known for their complex life cycles, which include phases of sexual and asexual reproduction. The effect of asexual multiplication on population genetic diversity has been investigated in the poplar rust fungus Melampsora larici-populina using a nested hierarchical sampling scheme. Four hierarchical levels were considered: leaf, twig, tree and site. Both cultivated and wild poplar stands were sampled at two time points at the start and end of rust epidemics. A total of 641 fungal isolates was analysed using nine microsatellite markers. This study revealed that the genetic signature of asexual multiplication in the wild poplar stand was seen only at lower hierarchical levels (leaf and twig). Moreover, we observed an erosion of clonal structure through time, with an increase in both gene and genotypic diversity. New genotypes contributed to host infection over time, which demonstrates the importance of allo-infection in the epidemic process in this host-pathogen system. Compared with the wild stands, the nearly lack of detection of clonal structure in the cultivated stands reflects the higher infection level on cultivated poplars. More generally, this genetic analysis illustrates the utility of population genetics approach for elucidating the proportion of asexual reproduction in the multiplication of isolates during an epidemic, and for proper quantification of asexual dispersal in plant pathogens. | 1916 | no | no good fitness measure, N pop differnt in GD? | NA | no | no |
| 1917 | 2017 | 43024 | Moraga, D; Tanguy, A | 1997 | EFFECTS OF ANTHROPOGENIC FACTORS ON GENETIC DIVERSITY IN THE MARINE BIVALVE CRASSOSTREA GIGAS: SEARCH FOR GENETIC MARKERS | The effects of various pollutants including heavy metals, pesticides and organic contaminant on the genetic structure of the marine bivalve Crassostrea gigas were studied as part of an environmental biomonitoring project. This research was carried out on two natural oyster populations from the French Atlantic coast. Results indicate a differential survival of allozyme genotypes for the populations which depends on the pollutant tested. The sensitivity of allozymes to environmental stress through differential mortality reflects the adaptive nature of the surviving individuals. Moreover, it supports the hypothesis that allozymes could be used as genetic indicators in marine bivalves. Our results revealed that the six studied enzyme loci (Aat-2, Ak, Pgdh, Cap-1, Pgi and Pgm) involved in the physiological processes were affected by the pollutants and can therefore be considered as potential genetic indicators. | 1917 | no | no good fitness measure, N pop differnt in GD? | NA | no | no |
| 1918 | 2017 | 43024 | Guzinski, J; Bull, CM; Donnellan, SC; Gardner, MG | 2009 | Molecular genetic data provide support for a model of transmission dynamics in an Australian reptile tick, Bothriocroton hydrosauri | Bothriocroton hydrosauri is a three-host ixodid tick that infests large reptiles in southeastern Australia, where its most common host is a large scincid lizard Tiliqua rugosa. Based on previous ecological and behavioural studies of this system, we propose a ‘ripple’ model of tick population dynamics, where only a few female ticks succeed in producing surviving offspring. These females then are the centres of ripples of their progeny spreading into the broader landscape. The model predicts higher relatedness among larvae than among nymphs or adults on a host, and significant spatial autocorrelation in larvae extending further than for the later life stages. The model also predicts that adult ticks are likely to encounter related partners and that this will generate inbreeding within the population. We tested those predictions using nine polymorphic microsatellite loci on a sample of 848 ticks (464 larvae, 140 nymphs and 244 adults) collected from 98 lizard hosts from near Bundey Bore Station in South Australia. Our data support the predictions and indicate that the dynamics of transmission among hosts play an important role in parasite population structure. | 1918 | no | gd of parasite | NA | no | no |
| 1919 | 2017 | 43024 | Leo, AE; Ford, R; Linde, CC | 2015 | Genetic homogeneity of a recently introduced pathogen of chickpea, Ascochyta rabiei, to Australia | The study examined the genetic structure and potential for adaption to host genotype of Ascochyta rabiei, a major necrotrophic fungal pathogen of chickpea. For this, A. rabiei populations derived from six major chickpea growing regions in Australia were characterized using 20 polymorphic microsatellite markers. The overall gene (H = 0.094) and genotypic (D = 0.80) diversities among the entire population were low, indicating the establishment of a recent founder population. Since, no significant genetic differentiation was detected among growing regions, subsequent anthropogenic dispersal was proposed, mainly through seed movement. The highest genotypic diversity and allelic richness was detected at Kingsford, South Australia, thought to be one of the sites of industry establishment in the 1970s and hence the centre of introduction. Despite assessing 206 isolates collected in 2010 from host genotypes with differential disease responses, no significant co-occurrence of fungal haplotype with host genotype was detected. Rather a single haplotype that accounted for 70 % of the total isolates assessed was detected on all host genotypes assessed and from all regions. Therefore, we propose that up until 2010, host reaction was not a major influence on the Australian A. rabiei population structure. Additionally, the detection of a single mating type only, MAT1-2 indicated asexual reproduction, further influencing low haplotype diversity and resulting in a population comprising of multiple clones with relatively few haplotypes compared to populations in other continents. | 1919 | no | gd of pathogen | NA | no | no |
| 1920 | 2017 | 43024 | Zuniga, J; Yu, N; Barquera, R; Alosco, S; Ohashi, M; Lebedeva, T; Acuna-Alonzo, V; Yunis, M; Granados-Montiel, J; Cruz-Lagunas, A; Vargas-Alarcon, G; Rodriguez-Reyna, TS; Fernandez-Vina, M; Granados, J; Yunis, EJ | 2013 | HLA Class I and Class II Conserved Extended Haplotypes and Their Fragments or Blocks in Mexicans: Implications for the Study of Genetic Diversity in Admixed Populations | Major histocompatibility complex (MHC) genes are highly polymorphic and informative in disease association, transplantation, and population genetics studies with particular importance in the understanding of human population diversity and evolution. The aim of this study was to describe the HLA diversity in Mexican admixed individuals. We studied the polymorphism of MHC class I (HLA-A, -B, -C), and class II (HLA-DRB1, -DQB1) genes using high-resolution sequence based typing (SBT) method and we structured the blocks and conserved extended haplotypes (CEHs) in 234 non-related admixed Mexican individuals (468 haplotypes) by a maximum likelihood method. We found that HLA blocks and CEHs are primarily from Amerindian and Caucasian origin, with smaller participation of African and recent Asian ancestry, demonstrating a great diversity of HLA blocks and CEHs in Mexicans from the central area of Mexico. We also analyzed the degree of admixture in this group using short tandem repeats (STRs) and HLA-B that correlated with the frequency of most probable ancestral HLA-C/-B and -DRB1/-DQB1 blocks and CEHs. Our results contribute to the analysis of the diversity and ancestral contribution of HLA class I and HLA class II alleles and haplotypes of Mexican admixed individuals from Mexico City. This work will help as a reference to improve future studies in Mexicans regarding allotransplantation, immune responses and disease associations. | 1920 | no | human | NA | no | no |
| 1921 | 2017 | 43024 | Malembic-Maher, S; Salar, P; Vergnes, D; Foissac, X | 2007 | “Detection and diversity of”“flavescence doree”" - related phytoplasmas in alders surrounding infected vineyards in Aquitaine (France)" | “Alder yellows is a disease caused by phytoplasmas (AlYp) genetically related to the”“flavescence doree”" grapevine phytoplasmas (FDp). To better document the genetic diversity of AlYp, alders surrounding infected vineyards were collected in Aquitaine, France. Sequence typing performed on the map gene (on 674 bp) revealed variability among AlYp isolates accounting for up to 13 Single Nucleotide Polymorphism. Phylogenetic analyses demonstrated that AlYp do not constitute a homogenous group, but are rather distributed in different clusters which comprise FDp isolates. The map gene sequence of some AlYp isolates was found identical to that of the FD1 group while other AlYp isolates clearly belong to the homogenous FD2 group. These data and published knowledge about AlY epidemiology and AlYp ecology are in agreement with existing or past exchanges between the vineyard and the wild compartment." | 1921 | no | gd of pathogen | NA | no | no |
| 1922 | 2017 | 43024 | Litholdo, CG; Gomes, EV; Lobo, M; Nasser, LCB; Petrofeza, S | 2011 | Genetic diversity and mycelial compatibility groups of the plant-pathogenic fungus Sclerotinia sclerotiorum in Brazil | The genetic variability of 40 Sclerotinia sclerotiorum isolates from various fields widely distributed throughout Brazil and different host crops was analyzed using RAPD markers and mycelial compatibility groupings (MCGs). The isolates were characterized using 16 random primers of the OPERON series, which produced 121 DNA fragments. UPGMA cluster analysis using Jaccard’s genetic distance and MCGs allowed separation of the isolates into three clusters, with similarity indices of 68.2, 61.8, and 61.8%, and five MCGs. The haplotypes obtained with RAPD markers provided very characteristic groupings of S. sclerotiorum isolates according to MCG, but did not show any relationship with geographic origin or host type. Furthermore, analysis of molecular variance demonstrated that 99.1% of the observed variation was a result of genetic differences between individuals; the host culture did not have a significant effect. This is the first report of high level variability of S. sclerotiorum in Brazil based on the study of isolates of wide geographical origin, supported by RAPD markers and MCGs. These results endorse the prevalence of sexual reproduction in tropical and subtropical regions in contrast to clonal reproduction in temperate regions. | 1922 | no | gd of pathogen | NA | no | no |
| 1923 | 2017 | 43024 | Ludoski, J; Djurakic, M; Pastor, B; Martinez-Sanchez, AI; Rojo, S; Milankov, V | 2014 | Phenotypic variation of the housefly, Musca domestica: amounts and patterns of wing shape asymmetry in wild populations and laboratory colonies | Musca domestica L. (Diptera: Muscidae) is a vector of a range variety of pathogens infecting humans and animals. During a year, housefly experiences serial population bottlenecks resulted in reduction of genetic diversity. Population structure has also been subjected to different selection regimes created by insect control programs and pest management. Both environmental and genetic disturbances can affect developmental stability, which is often reflected in morphological traits as asymmetry. Since developmental stability is of great adaptive importance, the aim of this study was to examine fluctuating asymmetry (FA), as a measure of developmental instability, in both wild populations and laboratory colonies of M. domestica. The amount and pattern of wing shape FA was compared among samples within each of two groups (laboratory and wild) and between groups. Firstly, the amount of FA does not differ significantly among samples within the group and neither does it differ between groups. Regarding the mean shape of FA, contrary to non-significant difference within the wild population group and among some colonies, the significant difference between groups was found. These results suggest that the laboratory colonies and wild samples differ in buffering mechanisms to perturbations during development. Hence, inbreeding and stochastic processes, mechanisms dominating in the laboratory-bred samples contributed to significant changes in FA of wing shape. Secondly, general patterns of left-right displacements of landmarks across both studied sample groups are consistent. Observed consistent direction of FA implies high degrees of wing integration. Thus, our findings shed light on developmental buffering processes important for population persistence in the environmental change and genetic stress influence on M. domestica. | 1923 | no | no good fitness measure, N pop differnt in GD? | NA | no | no |
| 1924 | 2017 | 43024 | Sahl, JW; Caporaso, JG; Rasko, DA; Keim, P | 2014 | The large-scale blast score ratio (LS-BSR) pipeline: a method to rapidly compare genetic content between bacterial genomes | Background. As whole genome sequence data from bacterial isolates becomes cheaper to generate, computational methods are needed to correlate sequence data with biological observations. Here we present the large-scale BLAST score ratio (LS-BSR) pipeline, which rapidly compares the genetic content of hundreds to thousands of bacterial genomes, and returns a matrix that describes the relatedness of all coding sequences (CDSs) in all genomes surveyed. This matrix can be easily parsed in order to identify genetic relationships between bacterial genomes. Although pipelines have been published that group peptides by sequence similarity, no other software performs the rapid, large-scale, full-genome comparative analyses carried out by LS-BSR. Results. To demonstrate the utility of the method, the LS-BSR pipeline was tested on 96 Escherichia coli and Shigella genomes; the pipeline ran in 163 min using 16 processors, which is a greater than 7-fold speedup compared to using a single processor. The BSR values for each CDS, which indicate a relative level of relatedness, were then mapped to each genome on an independent core genome single nucleotide polymorphism (SNP) based phylogeny. Comparisons were then used to identify clade specific CDS markers and validate the LS-BSR pipeline based on molecular markers that delineate between classical E. coli pathogenic variant (pathovar) designations. Scalability tests demonstrated that the LS-BSR pipeline can process 1,000 E. coli genomes in 27-57 h, depending upon the alignment method, using 16 processors. Conclusions. LS-BSR is an open-source, parallel implementation of the BSR algorithm, enabling rapid comparison of the genetic content of large numbers of genomes. The results of the pipeline can be used to identify specific markers between user-defined phylogenetic groups, and to identify the loss and/or acquisition of genetic information between bacterial isolates. Taxa-specific genetic markers can then be translated into clinical diagnostics, or can be used to identify broadly conserved putative therapeutic candidates. | 1924 | no | no good fitness measure, N pop differnt in GD? | NA | no | no |
| 1925 | 2017 | 43024 | Tsumori, Y; Ndounga, M; Sunahara, T; Hayashida, N; Inoue, M; Nakazawa, S; Casimiro, P; Isozumi, R; Uemura, H; Tanabe, K; Kaneko, O; Culleton, R | 2011 | Plasmodium falciparum: Differential Selection of Drug Resistance Alleles in Contiguous Urban and Peri-Urban Areas of Brazzaville, Republic of Congo | The African continent is currently experiencing rapid population growth, with rising urbanization increasing the percentage of the population living in large towns and cities. We studied the impact of the degree of urbanization on the population genetics of Plasmodium falciparum in urban and peri-urban areas in and around the city of Brazzaville, Republic of Congo. This field setting, which incorporates local health centers situated in areas of varying urbanization, is of interest as it allows the characterization of malaria parasites from areas where the human, parasite, and mosquito populations are shared, but where differences in the degree of urbanization (leading to dramatic differences in transmission intensity) cause the pattern of malaria transmission to differ greatly. We have investigated how these differences in transmission intensity affect parasite genetic diversity, including the amount of genetic polymorphism in each area, the degree of linkage disequilibrium within the populations, and the prevalence and frequency of drug resistance markers. To determine parasite population structure, heterozygosity and linkage disequilibrium, we typed eight microsatellite markers and performed haplotype analysis of the msp1 gene by PCR. Mutations known to be associated with resistance to the antimalarial drugs chloroquine and pyrimethamine were determined by sequencing the relevant portions of the crt and dhfr genes, respectively. We found that parasite genetic diversity was comparable between the two sites, with high levels of polymorphism being maintained in both areas despite dramatic differences in transmission intensity. Crucially, we found that the frequencies of genetic markers of drug resistance against pyrimethamine and chloroquine differed significantly between the sites, indicative of differing selection pressures in the two areas. | 1925 | no | gd of parasite | NA | no | no |
| 1926 | 2017 | 43024 | Lopez, G; Lopez-Parra, M; Garrote, G; Fernandez, L; Rey-Wamba, T; Arenas-Rojas, R; Garcia-Tardio, M; Ruiz, G; Zorrilla, I; Moral, M; Simon, MA | 2014 | Evaluating mortality rates and causalities in a critically endangered felid across its whole distribution range | The conservation of endangered species requires accurate data, and knowledge of cause-specific mortality rates is one of the most important issues. In recent years, conservation programs for the critically endangered Iberian lynx Lynx pardinus have been developed on the basis of mortality data derived 30 years ago from the small Donana population. Thus, there is an urgent need for an update of mortality rates and causes in both populations (Sierra Morena and Donana). Here we use radio-tracking information from the whole range of the Iberian lynx to quantify mortality rates and identify their causes. Between 2006 and 2011, we radio-tagged 78 Iberian lynxes from its two remaining populations (39 from Sierra Morena and 39 from Donana). Mortality events were evaluated to identify causes, and cause-specific annual mortality rates (AMR) were obtained using the nonparametric cumulative incidence function estimator. Overall, AMR was estimated at 0.16 +/- 0.05 (0.19 +/- 0.09 in Sierra Morena and 0.12 +/- 0.07 in Donana). Disease was the main cause of mortality both for the whole population and the Donana population. Poaching was the main cause of mortality in Sierra Morena. Our results suggest that the best strategy for conserving this species is to focus action on decreasing the fatal effect of disease and poaching. Given the possible existence of an underlying inbreeding-mediated immunosuppression, genetic management aimed at increasing the genetic diversity of this population is also recommended. | 1926 | no | No GD diff between pop | NA | no | no |
| 1927 | 2017 | 43024 | Xu, Z; Ali, Z; Hou, X; Li, H; Yi, JX; Abbasi, PA | 2013 | Characterization of Chinese eggplant isolates of the fungal pathogen Verticillium dahliae from different geographic origins | Verticillium dahliae is a fungal pathogen that causes wilt disease in a wide range of host plants. Characterization of virulence, morphological, and molecular variations among V. dahliae isolates from different geographic origins is essential for any breeding program aimed at producing plant cultivars resistant to this disease. We characterized virulence variation among V. dahliae isolates from Chinese cultivated eggplant grown in Northeast China by pathogenicity testing on susceptible, moderately resistant, and resistant eggplant accessions in a glasshouse using a root-dipping method of infection. These isolates were also characterized for morphological features based on growth on potato dextrose agar media and for genetic variation based on ISSR markers. All 12 isolates were divided into three pathotypes based on the virulence, i.e., strong, moderate, or weak type. Three isolates were categorized as defoliating pathotypes, with strong virulence, and the rest of the isolates were categorized as non-defoliating pathotypes, with moderate to weak virulence. The eggplant isolates were classified into three morphological types or morphotypes, hypha, hypha-sclerotia, and sclerotia; no significant correlations were detected between pathotypes and morphotypes or geographic origins. ISSR fingerprinting indicated genetic diversity among isolates, ranging from 0.26 to 0.69. Specific fingerprint types were not correlated with either pathotype or morphotype. However, ISSR analyses did reveal two clusters in which the isolates in each share the same or neighboring geographic origins. | 1927 | no | crop | NA | no | no |
| 1928 | 2017 | 43024 | Bohrer, G; Nathan, R; Volis, S | 2005 | Effects of long-distance dispersal for metapopulation survival and genetic structure at ecological time and spatial scales | 1 Long-distance dispersal (LDD) of seeds by wind plays an important role in population survival and structure, especially in naturally patchy or human-fragmented metapopulations. However, no study has tested its effects using a realistic dispersal kernel in a metapopulation context with explicit spatial structure and local extinctions. 2 We incorporated such kernels into a newly proposed simulation model, which combines within-patch (population) demographic processes and a simplified maternally inherited single-locus, two-allele genetic make-up of the populations. As a test case, we modelled a typical conservation scenario of Aleppo pine (Pinus halepensis) populations. 3 The effects of LDD were rather diverse and depended on initial population conditions and local extinction rates. LDD increased metapopulation survival at intermediate local-extinction probabilities. LDD helped maintain higher total genetic variability in populations that were initially drifted, but facilitated random genetic loss through drift in initially ‘well mixed’ populations. LDD prevented population differentiation in low extinction rates but increased it at intermediate to high extinction rates. 4 Our results suggest that LDD has broader evolutionary implications and would be selected for in populations facing intermediate local-extinction pressures. Our modelling approach provides a strong tool to test the effects of LDD on metapopulation survival and genetic variability and to identify the parameters to which such effects are most sensitive, in ecological and conservational scenarios. | 1928 | no | No GD diff between pop | NA | no | no |
| 1929 | 2017 | 43024 | Farley, RA; McNeilly, T | 2000 | Diversity and divergence in Cistus salvifolius (L.) populations from contrasting habitats | Cistus salvifolius L. is a widespread Mediterranean shrub, occurring over a wide range of environments. Given the degree of habitat differentiation, and geographic isolation of some populations, adaptation to local conditions and hence population divergence might be expected to have occurred. To test this hypothesis morphology and allozyme diversity was measured in 13 populations collected from contrasting habitats around the Mediterranean. Leaf morphology (length, width and petiole length) and internode length varied widely between populations. Leaf width and internode length were negatively correlated with longitude, and leaf length was negatively correlated with mean rainfall. All populations were polymorphic at all allozyme loci studied, and no populations showed significant difference between levels of expected and observed heterozygotes. Allelic diversity (H-s) within populations was high, and populations from the more extreme sites showed no decrease in diversity or predominance of rare genotypes, suggesting there is little selection for characters favouring survival in local conditions. Some populations from highly contrasting habitats, in terms of rainfall, appeared to be genetically similar. However, there were differences between some populations, in areas less than 1 km apart, which have similar geography and climate. Results suggest that the C. salvifolius populations examined may not be as adapted to local environmental conditions as expected. Periodic fires, gene flow, and environmental heterogeneity may all help maintain genetic diversity and hinder adaptation. | 1929 | no | no good fitness measure | NA | no | no |
| 1930 | 2017 | 43024 | Ruch, J; Heinrich, L; Bilde, T; Schneider, JM | 2009 | Relatedness facilitates cooperation in the subsocial spider, Stegodyphus tentoriicola | Background: Cooperative hunting and foraging in spiders is rare and prone to cheating such that the actions of selfish individuals negatively affect the whole group. The resulting social dilemma may be mitigated by kin selection since related individuals lose indirect fitness benefits by acting selfishly. Indeed, cooperation with genetic kin reduces the disadvantages of within-group competition in the subsocial spider Stegodyphus lineatus, supporting the hypothesis that high relatedness is an important pre-adaptation in the transition to sociality in spiders. In this study we examined the consequences of group size and relatedness on cooperative feeding in the subsocial spider S. tentoriicola, a species suggested to be at the transition to permanent sociality. Results: We formed groups of 3 and 6 spiders that were either siblings or non-siblings. We found that increasing group size negatively affected feeding efficiency but that these negative effects were reduced in sib-groups. Sib groups were more likely to feed cooperatively and all group members grew more homogenously than groups of unrelated spiders. The measured differences did not translate into differential growth or mortality during the experimental period of 8 weeks. Conclusion: The combination of our results with those from previous studies indicates that the conflict between individual interests and group interests may be reduced by nepotism and that the latter promote the maintenance of the social community. | 1930 | no | no good fitness measure | NA | no | no |
| 1931 | 2017 | 43024 | Vera, M; Diez-del-Molino, D; Garcia-Marin, JL | 2016 | Genomic survey provides insights into the evolutionary changes that occurred during European expansion of the invasive mosquitofish (Gambusia holbrooki) | Biological invasions rank among the main global threats for biodiversity. The Eastern mosquitofish (Gambusia holbrooki) is considered one of the 100 world worst invasive species due to its high adaptation capability to new environments. Using the restriction-site-associated DNA tags (RADtags), introduced European locations were compared against native US mosquitofish populations to analyse genomic changes that occurred during invasive process of European locations. After filtering, 7724 RADtags containing only one SNP were retained for population studies. Comparative genomics indicated that 186 of these RADtags matched sequences in the transcriptome of Xyphophorus maculatus, the most closely related genome available. Genomic analyses showed that invasive populations show high reductions in diversity. Further, analysesof population structuring based on these data are concordant with previous analyses based on microsatellites. It is concluded that during the invasion process genetic drift was the main evolutionary force affecting patterns of diversity and population structure. While recognizing that positive selection could be masked by the strong drift during founder events, adaptive processes were evidenced in a reduced number of RADtags (<2%), with only one of these in a putative coding region. Surprisingly, balancing selection was detected in several coding RADtags, suggesting that the preservation of polymorphism in specific genes could be more important than the average population diversity for the population maintenance at any location, particularly for the survival of introduced populations. | 1931 | no | no good fitness measure | NA | no | no |
| 1932 | 2017 | 43024 | McEvoy, BP; Lind, JM; Wang, ET; Moyzis, RK; Visscher, PM; Pellekaan, SMV; Wilton, AN | 2010 | Whole-Genome Genetic Diversity in a Sample of Australians with Deep Aboriginal Ancestry | Australia was probably settled soon after modern humans left Africa, but details of this ancient migration are not well understood. Debate centers on whether the Pleistocene Sahul continent (composed of New Guinea, Australia, and Tasmania) was first settled by a single wave followed by regional divergence into Aboriginal Australian and New Guinean populations (common origin) or whether different parts of the continent were initially populated independently. Australia has been the subject of relatively few DNA studies even though understanding regional variation in genomic structure and diversity will be important if disease-association mapping methods are to be successfully evaluated and applied across populations. We report on a genome-wide investigation of Australian Aboriginal SNP diversity in a sample of participants from the Riverine region. The phylogenetic relationship of these Aboriginal Australians to a range of other global populations demonstrates a deep common origin with Papuan New Guineans and Melanesians, with little evidence of substantial later migration until the very recent arrival of European colonists. The study provides valuable and robust insights into an early and important phase of human colonization of the globe. A broader survey of Australia, including diverse geographic sample populations, will be required to fully appreciate the continent’s unique population history and consequent genetic heritage, as well as the importance of both to the understanding of health issues. | 1932 | no | human | NA | no | no |
| 1933 | 2017 | 43024 | Li, M; Liu, T; Wang, B; Han, CG; Li, DW; Yu, JL | 2008 | Phylogenetic analysis of Beet necrotic yellow vein virus isolates from China | A survey detected Beet necrotic yellow vein virus (BNYVV) infection in six Chinese sugar-beet-growing regions. To study the diversity of virus isolates among the regions, nucleotide sequences of four proteins, namely CP, p25, p31, and p26, were determined and the amino acid sequences thus deduced were analyzed using sequence alignments and the phylogenetic method, respectively. Amino acid sequence analysis of CP revealed A-type isolates in Harbin, Hohhot, Baotou, Wuwei, and Jiuquan and B-type isolates in Hohhot and Changji; no Chinese isolate was found to cluster with European P-type isolates. Chinese p25 proteins clustered into three groups with seven tetrad motifs (positions 67-70). Of the seven, the tetrad ASHG has not been reported previously. Most Chinese p31 proteins clustered in p31-2 group, diverging from European p31 proteins. Isolates containing RNA 5 occurred in most of the investigated regions and were associated with both A-and B-types. Phylogenetic analyses of these four proteins showed complex patterns of genetic diversity among these Chinese isolates and indicated that the isolates of China and Japan were more closely related and may have a common origin. | 1933 | no | gd of pathogen | NA | no | no |
| 1934 | 2017 | 43024 | Schmid-Hempel, P; Puhr, K; Kruger, N; Reber, C; Schmid-Hempel, R | 1999 | Dynamic and genetic consequences of variation in horizontal transmission for a microparasitic infection | Transmission to a new host is a critical step in the life cycle of a parasite. Variation in the characteristics of the transmission process, for example, due to host demography, is assumed to select for different variants of the parasite. We have experimentally tested how variation in the time to transmission (early or late after infection) and exposure to adverse conditions outside the host (immediate or delayed contact with new host) interact to determine the success of the infection in the next host, using the trypanosome Crithidia bombi infecting its bumblebee host, Bombus terrestris. These two experimentally manageable steps mimic the processes of within- and among-host selection for the parasite. We found that early transmission led to higher infection success in the next host as did immediate contact with the new host. However, there was no interaction between the two parameters as would be expected if early-transmitted variants, resulting from rapid multiplication within the host, would be less adapted to the conditions encountered during the between-host transfer or infection of the next host. Furthermore, typing the genetic variability of the parasites with microsatellites showed that the four different transmission routes of our experiment selected for different degrees of allelic diversity of the infecting parasite populations. The results support the idea that variation in the transmission process selects for different genotypic variants of the parasite. At the same time, the relationship of allelic diversity with infection intensity suggested that the coinfection model of May and Nowak (1995) maybe appropriate, where each parasite is able to infect and multiply independent of others within the same host. | 1934 | no | no good fitness measure, N pop differnt in GD? | NA | no | no |
| 1935 | 2017 | 43024 | Miao, M; Yang, ZQ; Patch, H; Huang, YM; Escalante, AA; Cui, LW | 2012 | Plasmodium vivax populations revisited: mitochondrial genomes of temperate strains in Asia suggest ancient population expansion | Background: Plasmodium vivax is the most widely distributed human malaria parasite outside of Africa, and its range extends well into the temperate zones. Previous studies provided evidence for vivax population differentiation, but temperate vivax parasites were not well represented in these analyses. Here we address this deficit by using complete mitochondrial (mt) genome sequences to elucidate the broad genetic diversity and population structure of P. vivax from temperate regions in East and Southeast Asia. Results: From the complete mtDNA sequences of 99 clinical samples collected in China, Myanmar and Korea, a total of 30 different haplotypes were identified from 26 polymorphic sites. Significant differentiation between different East and Southeast Asian parasite populations was observed except for the comparison between populations from Korea and southern China. Haplotype patterns and structure diversity analysis showed coexistence of two different groups in East Asia, which were genetically related to the Southeast Asian population and Myanmar population, respectively. The demographic history of P. vivax, examined using neutrality tests and mismatch distribution analyses, revealed population expansion events across the entire P. vivax range and the Myanmar population. Bayesian skyline analysis further supported the occurrence of ancient P. vivax population expansion. Conclusions: This study provided further resolution of the population structure and evolution of P. vivax, especially in temperate/warm-temperate endemic areas of Asia. The results revealed divergence of the P. vivax populations in temperate regions of China and Korea from other populations. Multiple analyses confirmed ancient population expansion of this parasite. The extensive genetic diversity of the P. vivax populations is consistent with phenotypic plasticity of the parasites, which has implications for malaria control. | 1935 | no | gd of pathogen | NA | no | no |
| 1936 | 2017 | 43024 | Walker, FM; Foster, JT; Drees, KP; Chambers, CL | 2014 | Spotted bat (Euderma maculatum) microsatellite discovery using illumina sequencing | The spotted bat (Euderma maculatum) is a rarely-encountered species for which behavior and population attributes are largely unknown. Using next-generation sequencing, we identified and characterized 17 microsatellite loci, which were screened for 31 individuals from northern Arizona. Allelic diversity, observed heterozygosity, and power of discrimination were high (N-A: 5-8 alleles per locus; H-O: 0.55-0.90; P (ID) : 1.2 x 10(-15)). All loci were in HWE, there was no evidence of null alleles or linkage disequilibrium, and five loci amplified and were variable in another Vespertillionid (Eptesicus fuscus). We will use these loci to evaluate gene flow and genetic diversity across the range of the spotted bat and determine population size in northern Arizona. The latter information is important to resource managers, who attempt to set mortality thresholds for bats at wind energy facilities in this region. | 1936 | no | no good fitness measure | NA | no | no |
| 1937 | 2017 | 43024 | Lane, AB; Clarke, DJ | 2012 | Genome instability: Does genetic diversity amplification drive tumorigenesis? | Recent data show that catastrophic events during one cell cycle can cause massive genome damage producing viable clones with unstable genomes. This is in contrast with the traditional view that tumorigenesis requires a long-term process in which mutations gradually accumulate over decades. These sudden events are likely to result in a large increase in genomic diversity within a relatively short time, providing the opportunity for selective advantages to be gained by a subset of cells within a population. This genetic diversity amplification, arising from a single aberrant cell cycle, may drive a population conversion from benign to malignant. However, there is likely a period of relative genome stability during the clonal expansion of tumors this may provide an opportunity for therapeutic intervention, especially if mechanisms that limit tolerance of aneuploidy are exploited. | 1937 | no | no good fitness measure | NA | no | no |
| 1938 | 2017 | 43024 | Ahuja, MR | 2011 | Strategies for conservation of germplasm in endemic redwoods in the face of climate change: a review | This study reviews the various conservation strategies applied to the four redwood species, namely coast redwood (Sequoia sempervirens), Sierra redwood or giant sequoia (Sequoiadendron giganteum), dawn redwood (Metasequoia glyptostroboides) and South American redwood or alerce (Fitzroya cupressoides), which are endemic in the USA, China and South America, respectively. All four redwood genera belong to the family Cupressaceae; they are monospecific, share a number of common phenotypic traits, including red wood, and are threatened in their native ranges due to human activity and a changing climate. Therefore, the management objective should be to conserve representative populations of the native species with as much genetic diversity as possible for their future survival. Those representative populations exhibiting relatively high levels of genetic diversity should be selected for germplasm preservation and monitored during the conservation phase by using molecular markers. In situ and ex situ strategies for the preservation of germplasm of the redwoods are discussed in this study. A holistic in situ gene conservation strategy calls for the regeneration of a large number of diverse redwood genotypes that exhibit adequate levels of neutral and adaptive genetic variability, by generative and vegetative methods for their preservation and maintenance in their endemic locations. At the same time, it would be desirable to conserve the redwoods in new ex situ reserves, away from their endemic locations with similar as well as different environmental conditions for testing their growth and survival capacities. In addition, other ex situ strategies involving biotechnological approaches for preservation of seeds, tissues, pollen and DNA in genebanks should also be fully exploited in the face of global climate change. | 1938 | no | no good fitness measure, N pop differnt in GD? | NA | no | no |
| 1939 | 2017 | 43024 | Pfanzelt, S; Albach, DC; von Hagen, KB | 2017 | Tabula rasa in the Patagonian Channels? The phylogeography of Oreobolus obtusangulus (Cyperaceae) | The extent of the Pleistocene glaciations in the Patagonian Channel region (southwesternmost South America) and their impact on the vegetation there are largely unknown. Whether the regional flora was wiped out completely (tabula rasa) or survived in ice-free pockets (in situ survival) is still an open question. The molecular imprint of either scenario should still be visible in extant populations. Therefore, DNA sequence data of Oreobolus obtusangulus Gaudich. (Cyperaceae) were analysed. This species is an abundant constituent of Patagonian cushion peat bogs, one of the Patagonian Channel region’s major vegetation types. Three hundred and eighty-four individuals from 48 populations were sequenced for two chloroplast (ycf3-psaA and trnQ(UUG)-psbK intergenic spacers) and 14 nuclear loci containing simple sequence repeats (SSRs; microsatellites). Phylogenetic reconstructions and the geographic distribution of genetic diversity revealed that the species was split into three main lineages whose general distributions comprise three separate major regions, that is, south-central Chile, Fuego-Patagonia and the East Patagonian Andes, which probably constitute glacial refugia. Postglacial migration fronts formed a suture zone with high levels of genetic diversity in the Northwest Patagonian Andes, where remnants of a supposedly ancestral lineage were also found to be locally restricted to a single population (Huinay). The heavily glaciated Patagonian Channels were likely recolonized from the northwest, and partly from the south. Although the westernmost Patagonian Channel population (Estero Bachem) harboured private SSR alleles (singletons) and showed slightly elevated genetic diversity, it remained unclear whether this population actually survived in situ. This study helps fill a major gap in reconstructing the Pleistocene vegetation history of West and Andean Patagonia. | 1939 | no | no good fitness measure | NA | no | no |
| 1940 | 2017 | 43024 | Oliver, MK; Telfer, S; Piertney, SB | 2009 | Major histocompatibility complex (MHC) heterozygote superiority to natural multi-parasite infections in the water vole (Arvicola terrestris) | The fundamental role of the major histocompatibility complex (MHC) in immune recognition has led to a general consensus that the characteristically high levels of functional polymorphism at MHC genes is maintained by balancing selection operating through host-parasite coevolution. However, the actual mechanism by which selection operates is unclear. Two hypotheses have been proposed: overdominance (or heterozygote superiority) and negative frequency-dependent selection. Evidence for these hypotheses was evaluated by examining MHC-parasite relationships in an island population of water voles (Arvicola terrestris). Generalized linear mixed models were used to examine whether individual variation at an MHC class II DRB locus explained variation in the individual burdens of five different parasites. MHC genotype explained a significant amount of variation in the burden of gamasid mites, fleas (Megabothris walkeri) and nymphs of sheep ticks (Ixodes ricinus). Additionally, MHC heterozygotes were simultaneously co-infected by fewer parasite types than homozygotes. In each case where an MHC-dependent effect on parasite burden was resolved, the heterozygote genotype was associated with fewer parasites, and the heterozygote outperformed each homozygote in two of three cases, suggesting an overall superiority against parasitism for MHC heterozygote genotypes. This is the first demonstration of MHC heterozygote superiority against multiple parasites in a natural population, a mechanism that could help maintain high levels of functional MHC genetic diversity in natural populations. | 1940 | no | individual level | NA | no | no |
| 1941 | 2017 | 43024 | Gompert, Z; Forister, ML; Fordyce, JA; Nice, CC | 2008 | Widespread mito-nuclear discordance with evidence for introgressive hybridization and selective sweeps in Lycaeides | We investigated the extent and potential cause(s) of mitochondrial introgression within the polytypic North American Lycaeides species complex (Lepidoptera). By comparing population genetic structure based on mitochondrial DNA (COI and COII) and nuclear DNA (251 polymorphic amplified fragment length polymorphism markers), we detected substantial mito-nuclear discordance, primarily involving a single mitochondrial haplotype (h01), which is likely due to mitochondrial introgression between differentiated Lycaeides populations and/or species. We detected reduced mitochondrial genetic diversity relative to nuclear genetic diversity in populations where mitochondrial haplotype h01 occurs, suggesting that the spread of this haplotype was facilitated by selection. We found no evidence that haplotype h01 is associated with increased fitness (in terms of survival to eclosion, fresh adult weight, and adult longevity) in a polymorphic Lycaeides melissa population. However, we did find a positive association between mitochondrial haplotype h01 and infection by the endoparasitic bacterium Wolbachia in one out of three lineages tested. Linkage disequilibrium between mitochondrial haplotype h01 and Wolbachia infection status may have resulted in indirect selection favouring the spread of haplotype h01 in at least one lineage of North American Lycaeides. These results illustrate the potential for introgressive hybridization to produce substantial mito-nuclear discordance and demonstrate that an individual’s mitochondrial and nuclear genome may have strikingly different evolutionary histories resulting from non-neutral processes and intrinsic differences in the inheritance and biology of these genomes. | 1941 | no | No GD diff between pop | NA | no | no |
| 1942 | 2017 | 43024 | Mino, CI; Del Lama, SN | 2007 | Genetic structure in Brazilian breeding colonies of the Roseate Spoonbill (Platalea ajaja, aves : threskiornithidae) | Roseate Spoonbills (Platalea ajaja, Linnaeus) are wading birds present in two of the most important Brazilian wetlands: the Pantanal wetlands and Rio Grande do Sul marshes. Natural populations of these species have not been previously studied with variable nuclear molecular markers. In order to support decision making regarding the management and conservation of these populations, we estimated and characterized the distribution of genetic variability among five Brazilian breeding colonies. The average observed heterozygosity in Brazilian Roseate Spoonbill populations (Ho=0.575) did not differ significantly from the value determined in a U. S. wild-caught sample of 15 individuals, using data generated by the same set of microsatellite loci. Considering that the U. S. population underwent a recent reduction in size, we discuss this result supposing that the U. S. population was not genetically affected or that both populations had suffered a bottleneck. Global F-ST indicated the lack of genetic differentiation among colonies, indicating the occurrence of past and/or present gene flow among them. Analysis of molecular variance revealed that most of the genetic variation is distributed within the colonies. Results are explained by a recent origin of colonies or by high levels of gene flow. Management decisions should take into consideration the fact that, even in the presence of high genetic exchange, ecological adaptations to different environments are important for species survival. | 1942 | no | no good fitness measure | NA | no | no |
| 1943 | 2017 | 43024 | Sawyer, SL; Mukherjee, N; Pakstis, AJ; Feuk, L; Kidd, JR; Brookes, AJ; Kidd, KK | 2005 | Linkage disequilibrium patterns vary substantially among populations | A major initiative to create a global human haplotype map has recently been launched as a tool to improve the efficiency of disease gene mapping. The ‘HapMap’ project will study common variants in depth in four ( and to a lesser degree in up to 12) populations to catalogue haplotypes that are expected to be common to all populations. A hope of the ‘HapMap’ project is that much of the genome occurs in regions of limited diversity such that only a few of the SNPs in each region will capture the diversity and be relevant around the world. In order to explore the implications of studying only a limited number of populations, we have analyzed linkage disequilibrium (LD) patterns of three 175 - 320 kb genomic regions in 16 diverse populations with an emphasis on African and European populations. Analyses of these three genomic regions provide empiric demonstration of marked differences in frequencies of the same few haplotypes, resulting in differences in the amount of LD and very different sets of haplotype frequencies. These results highlight the distinction between the statistical concept of LD and the biological reality of haplotypes and their frequencies. The significant quantitative and qualitative variation in LD among populations, even for populations within a geographic region, emphasizes the importance of studying diverse populations in the HapMap project to assure broad applicability of the results. | 1943 | no | human | NA | no | no |
| 1944 | 2017 | 43024 | Barcelos, QL; Souza, EA; Silva, KJDE | 2011 | Vegetative compatibility and genetic analysis of Colletotrichum lindemuthianum isolates from Brazil | The causal agent of common bean anthracnose, Colletotrichum lindemuthianum, has considerable genetic and pathogenic variability, which makes the development of resistant cultivars difficult. We examined variability within and between Brazilian pathotypes of C. lindemuthianum through the identification of vegetative compatibility groups (VCGs) and by RAPD analysis. Two hundred and ninety-five nit mutants were obtained from 47 isolates of various pathotypes of the fungus collected from different regions, host cultivars and years. In complementation tests, 45 VCGs were identified. Eighteen RAPD primers were employed in the molecular analyses, producing 111 polymorphic bands. Estimates of genetic similarities, determined from the Sorence-Dice coefficient, ranged from 0.42 to 0.97; the dendrogram obtained by cluster analysis revealed 18 groups of isolates. RAPD and VCG markers presented high genotypic diversity. The number of significant associations (P = 0.05) between RAPD, VCG and pathogenicity markers ranged from 0 (VCG) to 80% (pathogenicity). The test of multilocus association (r(d)) for RAPD markers was significantly different from zero (P < 0.001), suggesting linkage disequilibrium. However, the results for VCG markers show the presence of recombination mechanisms. In conclusion, RAPD markers and VCGs were useful for detecting genetic variability among isolates of C. lindemuthianum. We found considerable diversity among isolates from the same geographic origin within a short interval; this suggests rapid evolution. There is a need for further studies to elucidate the population structure of this pathogen in agro-ecosystems. | 1944 | no | gd of pathogen | NA | no | no |
| 1945 | 2017 | 43024 | OBRIEN, TG; ROBINSON, JG | 1991 | ALLOMATERNAL CARE BY FEMALE WEDGE-CAPPED CAPUCHIN MONKEYS - EFFECTS OF AGE, RANK AND RELATEDNESS | We examined patterns of infant care and the relative importance of female age, rank and relatedness to allomaternal care for a wild population of wedge-capped capuchin monkeys, Cebus olivaceus in Venezuela. Mothers interacted primarily with their own infants throughout the study. Infant age affected the timing and type of allomaternal interaction; investigation occurred primarily in the first 3 months of life, carrying was dominant during the second 3 months, and association occurred primarily in the third 3 months. The onset and level of allomaternal care assured a high level of infant care as maternal care was declining, suggesting that allomaternal care is an important component of infant survival. The relative importance of female age, rank and relatedness varied for different behaviour. Relatedness was the most consistently important effect. Sibling females participated in allomaternal care almost 4 times more often than other females and were especially active in carrying and associating with infants. Female rank was an important effect when the coefficient of relatedness was less than 0.5: high-ranking females participated in allomaternal care more than low-ranking females. Old juveniles and nulliparous adults interacted with infants more than young juveniles and parous adults, but the effect of female age was relatively unimportant. Allomaternal nursing may represent a form of reciprocal altruism. Allomaternal nursing supplemented maternal nursing and was unrelated to kinship or rank of female. | 1945 | no | no good fitness measure, N pop differnt in GD? | NA | no | no |
| 1946 | 2017 | 43024 | Ballian, D; Belletti, P; Ferrazzini, D; Bogunic, F; Kajba, D | 2010 | Genetic variability of Pedunculate Oak (Quercus robur L.) in Bosnia and Herzegovina | Background and Purpose: The objective of this research was to determine variability of some remaining populations and groups of trees of pedunculate oak (Quercus robur L.) at the molecular-genetic level in Bosnia and Herzegovina. The analysis was made by means of highly polymorphic nuclear microsatellite markers, which were used in some earlier research involving pedunculate oak embryos. The obtained results will provide guidelines for proper management with and regeneration of pedunculate oak forests, and for their re-introduction. In particular, this research will contribute to further improvement and conservation of pedunculate oak using in situ and ex situ methods. Material and Methods: This study analyzes molecular genetic variability of 12 natural populations of pedunculate oak (Quercus robur L.) in Bosnia and Herzegovina. Molecular genetic variability was analyzed on the basis of DNA by means of the following four nuclear microsatellite markers: ssrOpZAG1/5, ssrQpZAG9, ssrQpZAG36 and ssrQpZAG108. The analysis included a total of 108 alleles. Results and Conclusion: Considerable differences were observed in the frequencies between populations. The same differences were confirmed with analysis of other parameters, such as the effective number of alleles, the fixation index and genetic differentiation. It should be pointed out that this research confirmed higher allele heterozygosis in the populations in relation to pedunculate oak populations in Western Europe. This result suggests that the investigated populations have not lost much of their genetic potential for adaptation. This could be attributed to the vicinity of the studied populations to their glacial refugium. This is the reason for their higher resistance and sturdiness in comparison with the populations of western and central Europe. They posses more genetic variability despite the fact that they had for centuries suffered a strong anthropogenic influence, which brought them to their survival limit and the verge of extinction. The obtained results will allow us to preserve the genetic structure of this valuable species in Bosnia and Herzegovina. | 1946 | no | no good fitness measure | NA | no | no |
| 1947 | 2017 | 43024 | Fang, Z; Eule-Nashoba, A; Powers, C; Kono, TY; Takuno, S; Morrell, PL; Smith, KP | 2013 | Comparative Analyses Identify the Contributions of Exotic Donors to Disease Resistance in a Barley Experimental Population | Introgression of novel genetic variation into breeding populations is frequently required to facilitate response to new abiotic or biotic pressure. This is particularly true for the introduction of host pathogen resistance in plant breeding. However, the number and genomic location of loci contributed by donor parents are often unknown, complicating efforts to recover desired agronomic phenotypes. We examined allele frequency differentiation in an experimental barley breeding population subject to introgression and subsequent selection for Fusarium head blight resistance. Allele frequency differentiation between the experimental population and the base population identified three primary genomic regions putatively subject to selection for resistance. All three genomic regions have been previously identified by quantitative trait locus (QTL) and association mapping. Based on the degree of identity-by-state relative to donor parents, putative donors of resistance alleles were also identified. The successful application of comparative population genetic approaches in this barley breeding experiment suggests that the approach could be applied to other breeding populations that have undergone defined breeding and selection histories, with the potential to provide valuable information for genetic improvement. | 1947 | no | crop | NA | no | no |
| 1948 | 2017 | 43024 | Campanella, JJ; Bologna, PAX; Smalley, JV; Avila, DN; Lee, KN; Areche, EC; Slavin, LJ | 2013 | An analysis of the population genetics of restored Zostera marina plantings in Barnegat Bay, New Jersey | Within Barnegat Bay, New Jersey, eelgrass (Zostera marina) populations have declined by 62 % over the last 20 years. To better understand the consequences of this devastation, we have previously employed microsatellite DNA polymorphisms to analyze the population structure of Z. marina within Barnegat Bay, as well as along the eastern United States seaboard. We have restored populations of Z. marina in Barnegat Bay over the last 10 years to help assess the best planting conditions and ecotypes that might be used in long-term restoration strategies. In this study, we examined the genetic health of the restored populations compared to that of the donor eelgrass populations within the bay. Using microsatellites, we can identify which parental founding ecotypes survived the restoration process over multiple generations. The frequency of observed heterozygotes, although higher than in the natural populations, still indicates reduced levels of diversity and connectivity. The inbreeding frequency is high in the restored populations, but lower than what is seen in the native populations. All restored populations have effective population values > 50, suggesting a high probability of survival in the short term. | 1948 | no | no good fitness measure | NA | no | no |
| 1949 | 2017 | 43024 | Ruiz-Garcia, M; Montilla, M; Nicholls, SO; Angarita, L; Alvarez, D | 2000 | Genetic relationships and spatial genetic structure among clonal stocks of Trypanosoma cruzi in Colombia | Genetic variability in the protozoan causative agent of Chagas’ disease, Trypanosoma cruzi, has been analysed in some Latin American countries; Brazil, Bolivia, Chile and Paraguay. Although Colombia is a country displaying enormous biological diversity, few studies have been conducted from the perspective of the population genetics of Trypanosoma cruzi. This study was carried out using 23 Colombian stocks of this protozoan, analysed for 13 isoenzyme loci. The main population genetic results were: (1) Colombia is one of the distribution areas where T. cruzi appears to have the highest genetic variability and heterogeneity in Latin America; (2) the Pgm locus was found in fixed heterozygosis, supporting the presence of diploidy in this organism; (3) the absence of segregating genotypes and the absence of Hardy-Weinberg equilibrium support the view of the existence of a clonal structure as claimed by Tibayrenc and Ayala. Nevertheless, one characteristic of clonal structure, that of over-representation of some identical zymodemes in vast areas of varying environmental conditions, was not found in Colombia. In this country, a strong spatial autocorrelation, with a classic structure of regional patches, was observed. | 1949 | no | gd of pathogen | NA | no | no |
| 1950 | 2017 | 43024 | Kirby, R; Alldredge, MW; Pauli, JN | 2017 | Environmental, not individual, factors drive markers of biological aging in black bears | Aging negatively affects individual survival and reproduction; consequently, characterizing the factors behind aging can enhance our understanding of fitness in wild populations. The drivers of biological age are diverse, but often related to factors like chronological age or sex of the individual. Recently, however, environmental factors have been shown to strongly influence biological age. To explore the relative importance of these influences on biological aging in a free-ranging and long-lived vertebrate, we quantified the length of telomeres-highly conserved DNA sequences that cap the ends of eukaryotic chromosomes and a useful molecular marker of biological age-for black bears sampled throughout Colorado, and measured a variety of environmental variables (habitat productivity, human development, latitude, elevation) and individual characteristics (age, sex, body size, genetic relatedness). Our extensive sampling of bears (n = 245) revealed no relationships between telomere length and any individual characteristics. Instead, we found a broad-scale latitudinal pattern in telomere length, with bears in northern Colorado possessing shorter telomeres. Our results suggest that environmental characteristics overwhelm individual ones in determining biological aging for this large carnivore. | 1950 | no | no good fitness measure | NA | no | no |
| 1951 | 2017 | 43024 | Fraser, BA; Neff, BD | 2010 | Parasite mediated homogenizing selection at the MHC in guppies | Understanding the selective forces influencing genetic diversity is a fundamental goal of evolutionary ecology. The genes of the major histocompatibility complex (MHC) play a key role in the adaptive immune response of vertebrates and thus provide an excellent opportunity to examine the agents of selection on a functionally important gene. Here we examine the genetic architecture of the MHC class IIB genes in 10 wild populations of guppies (Poecilia reticulata) in Northern Trinidad. We have previously shown that these populations are significantly less diverged at the class IIB locus than expected based on neutral (microsatellite) loci. We now survey infection by Gyrodactylus turnbulli and G. bullatarudis, common parasitic worms that infect guppies, as a potential agent of homogenizing selection. We used a genetic algorithm to partition both additive and non-additive genetic effects of the five most common MHC allele types as well as a rare allele category. Although we found no evidence for non-additive effects, across the populations we found that one allele type (the a-type) had a significant negative additive effect on parasite load. Thus, individuals who had more copies of the a-type allele were infected with fewer gyrodactylus than individuals with fewer copies of the allele. These results not only link parasite infection with MHC genotype, they provide a mechanism of homogenizing selection across these otherwise disparate populations. | 1951 | probably no | no fitness pop level | no pop level fitness | no | no |
| 1952 | 2017 | 43024 | Brate, J; Logares, R; Berney, C; Ree, DK; Klaveness, D; Jakobsen, KS; Shalchian-Tabrizi, K | 2010 | Freshwater Perkinsea and marine-freshwater colonizations revealed by pyrosequencing and phylogeny of environmental rDNA | Protist parasites are ecologically important, as they can have great impact on host population dynamics and functioning of entire ecosystems. Nevertheless, little is known about their prevalence in aquatic habitats. Here, we investigate the diversity and distributional patterns of the protist parasites Perkinsus and Parvilucifera (Perkinsea). Our approach included 454 pyrosequencing of the 18S rDNA gene obtained from a high-altitude lake (Lake Finsevatn, Norway) and phylogenetic analyses of all publicly available sequences related to Perkinsea. The applied PCR primers target a 450 bp region that encompass the variable V4 region of the 18S rDNA gene and have been optimized for the Titanium upgrade of the 454 technology. Nearly 5000 sequences longer than 150 bp were recovered from nearly all eukaryotic supergroups, and of those, 13 unique sequences were affiliated to Perkinsea. Thus, our new strategy for 454 amplicon sequencing was able to recover a large diversity of distantly related eukaryotes and previously unknown species of Perkinsea. In addition, we identified 40 Perkinsea sequences in GenBank generated by other recent diversity surveys. Importantly, phylogenetic analyses of these sequences identified 17 habitat-specific marine and freshwater clades (PERK 1-17). Hence, only a few successful transitions between these habitats have taken place over the entire history of Perkinsea, suggesting that the boundary between marine and fresh waters may constitute a barrier to cross-colonizations for intracellular parasites. The ISME Journal (2010) 4, 1144-1153; doi: 10.1038/ismej.2010.39; published online 15 April 2010 | 1952 | no | gd of parasite | NA | no | no |
| 1953 | 2017 | 43024 | Tsompana, M; Abad, J; Purugganan, M; Moyer, JW | 2005 | The molecular population genetics of the Tomato spotted wilt virus (TSWV) genome | RNA viruses are characterized by high genetic variability resulting in rapid adaptation to new or resistant hosts. Research for plant RNA virus genetic structure and its variability has been relatively scarce compared to abundant research done for human and animal RNA viruses. Here, we utilized a molecular population genetic framework to characterize the evolution of a highly pathogenic plant RNA virus [Tomato spotted wilt virus (TSWV), Tospovirus, Bunyaviridae]. Data from genes encoding five viral proteins were used for phylogenetic analysis, and for estimation of population parameters, subpopulation differentiation, recombination, divergence between Tospovirus species, and selective constraints on the TSWV genome. Our analysis has defined the geographical structure of TSWV, attributed possibly to founder effects. Also, we identify positive selection favouring divergence between Tospovirus species. At the species level, purifying selection has acted to preserve protein function, although certain amino acids appear to be under positive selection. This analysis provides demonstration of population structuring and species-wide population expansions in a multisegmented plant RNA virus, using sequence-based molecular population genetic analyses. It also identifies specific amino acid sites subject to selection within Bunyaviridae and estimates the level of genetic heterogeneity of a highly pathogenic plant RNA virus. The study of the variability of TSWV populations lays the foundation in the development of strategies for the control of other viral diseases in floral crops. | 1953 | no | gd of pathogen | NA | no | no |
| 1954 | 2017 | 43024 | Cohnstaedt, LW; Caceres, AC; Beati, L; Munstermann, LE | 2012 | The Population Structure of Lutzomyia verrucarum (Diptera: Psycodidae), a Bartonella bacilliformis and Leishmania peruviana Vector in Peru | The population genetic structure of Lutzomyia verrucarum (Townsend), a sand fly disease vector of Carrion’s disease and cutaneous leishmaniasis in the Peruvian Andes, was characterized by sequencing 653 bp of cytochrome b and 1,125 bp of the NADH dehydrogenase subunit 4 genes of its mitochondrial genome. DNA sequence variation within and between valleys was compared in a sample of 220 sand flies from three valleys (Purisima, Huaylas, and Conchucos) and five departments (Amazonas, Cajamarca, Piura, Lima, and Huancavelica). Gene network and phylogenetic analyses indicated a high similarity of haplotypes collected within a single valley (0-0.52% nucleotide divergence). Flies from each valley had unique genotypes not shared with specimens from other valleys or from more distant regions (0.8-3.1% nucleotide divergence). Mountain ranges and geographic distance appear to have impeded migration (N(m) = <0.18) between valleys and separated populations into discrete genetic units. | 1954 | no | NA | NA | no | no |
| 1955 | 2017 | 43024 | Oyler-McCance, SJ; St John, J; Taylor, SE; Apa, AD; Quinn, TW | 2005 | Population genetics of Gunnison sage-grouse: Implications for management | The newly described Gunnison sage-grouse (Centrocercus minimus) is a species of concern for management because of marked declines in distribution and abundance due to the loss and fragmentation of sagebrush habitat. This has caused remaining populations to be unusually small and isolated. We utilized mitochondrial DNA sequence data and data from 8 nuclear microsatellites to assess the extent of population subdivision among Gunnison sage-grouse populations in southwestern Colorado and southeastern Utah, USA. We found a high degree of population structure and low amounts of gene flow among all pairs of populations except the geographically adjacent Gunnison and Curecanti populations. Population structure for Gunnison sage-grouse was significantly higher than has been reported for greater sage-grouse (C. urophasianus). Further, we documented low levels of genetic diversity in some populations (particularly Dove Creek/Monticello and Pinon Mesa with an average of only 3.00 and 2.13 alleles per locus respectively) indicating that translocations from larger, more genetically diverse populations may be warranted. Bayesian analysis identified 3 potential migrants (involving San Miguel, Dove Creek/Monticello, Crawford, and Curecanti). Further, this analysis showed that 4 individuals from Cerro/Cimarron were more closely related to birds from San Miguel than to its geographically closer neighbors Gunnison and Curecanti. This suggests the Cerro/Cimarron area may act as a stepping stone for gene flow between San Miguel and Gunnison and that habitat restoration and protection in areas between these 2 basins should be a priority in an attempt to facilitate natural movement among these populations. Conservation plans should include monitoring and maintaining genetic diversity, preventing future habitat loss and fragmentation, enhancing existing habitat, and restoring converted sagebrush communities. | 1955 | no | no good fitness measure | NA | no | no |
| 1956 | 2017 | 43024 | Huang, HH; Su, M; Li, XY; Li, H; Tian, DP; Gao, YX; Guo, YB | 2010 | Y-Chromosome Evidence for Common Ancestry of Three Chinese Populations with a High Risk of Esophageal Cancer | High rates of esophageal cancer (EC) are found in people of the Henan Taihang Mountain, Fujian Minnan, and Chaoshan regions of China. Historical records describe great waves of populations migrating from north-central China (the Henan and Shanxi Hans) through coastal Fujian Province to the Chaoshan plain. Although these regions are geographically distant, we hypothesized that EC high-risk populations in these three areas could share a common ancestry. Accordingly, we used 16 East Asian-specific Y-chromosome biallelic markers (single nucleotide polymorphisms; Y-SNPs) and six Y-chromosome short tandem repeat (Y-STR) loci to infer the origin of the EC high-risk Chaoshan population (CSP) and the genetic relationship between the CSP and the EC high-risk Henan Taihang Mountain population (HTMP) and Fujian population (FJP). The predominant haplogroups in these three populations are O3*, O3e*, and O3e1, with no significant difference between the populations in the frequency of these genotypes. Frequency distribution and principal component analysis revealed that the CSP is closely related to the HTMP and FJP, even though the former is geographically nearer to other populations (Guangfu and Hakka clans). The FJP is between the CSP and HTMP in the principal component plot. The CSP, FJP and HTMP are more closely related to Chinese Hans than to minorities, except Manchu Chinese, and are descendants of Sino-Tibetans, not Baiyues. Correlation analysis, hierarchical clustering analysis, and phylogenetic analysis (neighbor-joining tree) all support close genetic relatedness among the CSP, FJP and HTMP. The network for haplogroup O3 (including O3*, O3e* and O3e1) showed that the HTMP have highest STR haplotype diversity, suggesting that the HTMP may be a progenitor population for the CSP and FJP. These findings support the potentially important role of shared ancestry in understanding more about the genetic susceptibility in EC etiology in high-risk populations and have implications for determining the molecular basis of this disease. | 1956 | no | no good fitness measure, N pop differnt in GD? | NA | no | no |
| 1957 | 2017 | 43024 | Altermatt, F; Hottinger, J; Ebert, D | 2007 | Parasites promote host gene flow in a metapopulation | Local adaptation is a powerful mechanism to maintain genetic diversity in subdivided populations. It counteracts the homogenizing effect of gene flow because immigrants have an inferior fitness in the new habitat. This picture may be reversed in host populations where parasites influence the success of immigrating hosts. Here we report two experiments testing whether parasite abundance and genetic background influences the success of host migration among pools in a Daphnia magna metapopulation. In 22 natural populations of D. magna, immigrant hosts were found to be on average more successful when the resident populations experienced high prevalences of a local microsporidian parasite. We then determined whether this success is due to parasitism per se, or the genetic background of the parasites. In a common garden competition experiment, we found that parasites reduced the fitness of their local hosts relatively more than the fitness of allopatric host genotypes. Our experiments are consistent with theoretical predictions based on coevolutionary host-parasite models in metapopulations. A direct consequence of the observed mechanism is an elevated effective migration rate for the host in the metapopulation. | 1957 | no | no good fitness measure, N pop differnt in GD? | NA | no | no |
| 1958 | 2017 | 43024 | Galvani, AP; Coleman, RM; Ferguson, NM | 2003 | The maintenance of sex in parasites | The maintenance of sex is an unresolved paradox in evolutionary biology, given the inherent twofold fitness advantage for asexuals. Parasitic helminths offer a unique opportunity to address this enigma. Parasites that can create novel antigenic strains are able to escape pre-existing host immunity. Viruses produce diversity through mutation with rapid clonal proliferation. The long generation times of helminth parasites prevent them from adopting this strategy. Instead, we argue that sexual reproduction enables parasitic helminths to rapidly generate strain diversity. We use both a stochastic, individual-based model and a simple analytical model to assess the selective value of sexual versus asexual reproduction in helminth parasites. We demonstrate that sexual reproduction can more easily produce and maintain strain diversity than asexual reproduction for long-lived parasites. We also show that sexual parasite populations are resistant to invasion by rare asexual mutants. These results are robust to high levels of cross-immunity between strains. We suggest that the enhancement of strain diversity, despite stochastic extinction of strains, may be critical to the evolutionary success of sex in long-lived parasites. | 1958 | no | NA | NA | no | no |
| 1959 | 2017 | 43024 | Zueter, AM; Rahman, ZA; Yean, CY; Harun, A | 2015 | Brief communication genotyping of Burkholderia pseudomallei revealed high genetic variability among isolates from a single population group | Burkholderia pseudomallei is a soil dwelling Gram-negative bacteria predominates in Southeast Asia zone and the tropical part of Australia. Genetic diversity has been explored among various populations and environments worldwide. To date, little data is available on MLST profiling of clinical B. pseudomallei isolates in peninsular Malaysia. In this brief report, thirteen culture positive B. pseudomallei cases collected from a single population of Terengganu state in the Western Peninsular Malaysia and were confirmed by In-house TTS1-PCR. Isolates were subjected for multi-locus sequence typing (MLST) to explore their genotypic diversity and to investigate for possible clonal clustering of a certain sequence type. Patient’s clinical information was examined to investigate for clinical correlation among the different genotypes. In spite of small sample set, MLST results indicated predictive results; considerable genotypic diversity, predominance and novelty among B. pseudomallei collected over a single geographically-located population in Malaysia. Massive genotypic heterogeneity was observed; 8 different sequence types with predominance of sequence type 54 and discovery of two novel sequence types. However, no clear pathogenomic or organ tropism clonal relationships were predicted. | 1959 | no | NA | NA | no | no |
| 1960 | 2017 | 43024 | Volkova, PA; Schanzer, IA; Soubani, E; Meschersky, IG; Widen, B | 2016 | Phylogeography of the European rock rose Helianthemum nummularium s.l. (Cistaceae): western richness and eastern poverty | Helianthemum nummularium s.l. is a young, morphologically diverse species distributed from western Europe to the Caucasus and the Southern Urals in the east. We analysed the rps16-trnK plastid intergenic spacer sequences from 85 localities covering most of the range of H. nummularium. Thirteen haplotypes were very unevenly distributed throughout the range of the species, and exhibited a strong phylogeographic signal. The results confirm range expansions of H. nummularium from Mediterranean refugia northwards, but also show the major role of eastern European (the Caucasus and the Southern Urals) refugia in rapid postglacial colonization of east, north and central Europe. The plastid haplotypes form distinct clades, one representing an eastern European lineage with few haplotypes and the other representing a western European lineage with many haplotypes. Parallel to this split in haplotype diversity is the pronounced differentiation in morphological variation displayed by the taxa found in west and east Europe. We discuss the role of topography in generating differences in morphological and genetic diversity between these two groups. We also discuss the taxonomical status of Helianthemum arcticum, which is regarded as an endangered local endemic of the Kola Peninsula. Helianthemum arcticum appears to represent an outlying peripheral population of H. nummularium preserved since the last postglacial major range expansion of this species, and bears the same plastid haplotype as the bulk of east and north European populations. | 1960 | no | no good fitness measure | NA | no | no |
| 1961 | 2017 | 43024 | Cattan-Toupance, I; Michalakis, Y; Neema, C | 1998 | Genetic structure of wild bean populations in their South-Andean centre of origin | The genetic structure of wild common bean populations was studied in the South-Andean centre of origin of the species. Plants were collected from 21 populations in Argentina and genetic variability was assessed for molecular and resistance markers. Polymorphism was weak for phaseolin, the major seed-storage protein, and for RAPD markers, while a high level of polymorphism was observed for resistance to anthracnose, one of the most important diseases of common bean. For the three traits, within-population variability was important and represented between 43.6% and 67.5% of the total variation. Although among-population differentiation was significant for all the traits, no correlation was found between the population distances calculated from RAPDs and resistance. These results indicate that pathogen selection pressure may be an important factor influencing the distribution of variability within and among host plant populations. | 1961 | no | no good fitness measure | NA | no | no |
| 1962 | 2017 | 43024 | Rigaud, T; Rousset, F | 1996 | What generates the diversity of Wolbachia - Arthropod interactions? | Wolbachia are strictly endocellular, vertically transmitted bacteria associated with insects and crustaceans. This group of parasites modify their hosts’ reproduction so as to increase their own fitness. This paper reviews the variability of these parasitic alterations and their consequences for host biology and populations. Wolbachia induce cytoplasmic incompatibility (a characteristic apparently specific to Wolbachia) in several insects and one isopod crustacean; parthenogenesis (thelytoky) in haplo-diploid insects; feminization in various isopods. The consequences of these phenomena on speciation, population dynamics and genetic polymorphism are discussed. The variability of the mechanisms of host sex determination is one important factor responsible for the diversity of Wolbachia-host interactions. However, parasite characteristics, such as the capacity to disturb host mitosis, and the ability to be horizontally transferred between hosts, also appear to play a role in this diversity. | 1962 | no | gd of parasite | NA | no | no |
| 1963 | 2017 | 43024 | Craddock, TP; Zumla, AM; Ollier, WER; Chintu, CZ; Muyinda, GP; Lancaster, FC; Boylston, AW | 2000 | Predominance of one T-cell antigen receptor BV haplotype in African populations | The human T-cell antigen receptor (TCR) is the counter-receptor for the HLA/peptide complex displayed on the surface of antigen-presenting cells. It confers antigen specificity on T lymphocytes and therefore plays a central role in pathogen recognition and host response. The most frequently used form of the TCR is a heterodimer composed of variable alpha and beta chains. We investigated allele frequencies for four variable-region gene segments of the beta chain (2S1, 3S1, 8S3, and 15S1) in 146 Caucasians and 165 Africans. The results reveal significant unexpected differences between the two populations for allele frequencies, phenotypes, genotypes, and haplotypes. Among Caucasians. there are 43 phenotypes, whereas there are 31 among the Africans studied. There are 17 haplotypes in the Caucasian sample but only 10 in Africans. This loss of diversity is largely due to the high frequency of one haplotype in the African sample which represents 65% of the informative chromosomes. At least one copy of this haplotype is present in 90% of informative individuals. As a result, 29% of Africans are homozygous for the common haplotype. Less genetic diversity at TCRBV is unexpected, since Africans usually show greater genetic diversity than other ethnic groups. For example, there are approximately twice as many HLA haplotypes in Africans compared to Caucasians. Homozygosity is also unexpected because it reduces the number of TCR variants available to recognize HLA pathogen-derived peptide complexes. | 1963 | no | human | NA | no | no |
| 1964 | 2017 | 43024 | Alvarez-Presas, M; Mateos, E; Vila-Farre, M; Sluys, R; Riutort, M | 2012 | Evidence for the persistence of the land planarian species Microplana terrestris (Muller, 1774) (Platyhelminthes, Tricladida) in microrefugia during the Last Glacial Maximum in the northern section of the Iberian Peninsula | The land planarian species Microplana terrestris (Muller, 1774), shows a wide distribution in the north of the Iberian Peninsula, where mature humid forests can be found. Since most terrestrial planarians require the presence and good condition of wet forests to survive, a parallel evolution of the taxon and its habitat might be expected. Performing molecular analyses (mitochondrial cytochrome oxidase I and nuclear ITS-1 genes) we estimated the demography and biogeographic history of the species in that region. Our results show the species to present levels of genetic diversity likely originating before the Pleistocene. However, it presents a genetic structure that presumably resulted from its survival in various refuges during the Pleistocene glacial cycles. The two main genetic groups, present on the Iberian Peninsula, seem to have different origins: the western one being of Iberian origin, while the eastern group may have been the result of a re-colonization from the north. In both cases, their biogeographical history mirrors their habitat range movements, reinforcing the phylogeographical hypothesis put forward for its preferred habitat, i.e. humid forests. (C) 2012 Elsevier Inc. All rights reserved. | 1964 | no | no good fitness measure | NA | no | no |
| 1965 | 2017 | 43024 | Davenport, ER; Cusanovich, DA; Michelini, K; Barreiro, LB; Ober, C; Gilad, Y | 2015 | Genome-Wide Association Studies of the Human Gut Microbiota | The bacterial composition of the human fecal microbiome is influenced by many lifestyle factors, notably diet. It is less clear, however, what role host genetics plays in dictating the composition of bacteria living in the gut. In this study, we examined the association of similar to 200K host genotypes with the relative abundance of fecal bacterial taxa in a founder population, the Hutterites, during two seasons (n = 91 summer, n = 93 winter, n = 57 individuals collected in both). These individuals live and eat communally, minimizing variation due to environmental exposures, including diet, which could potentially mask small genetic effects. Using a GWAS approach that takes into account the relatedness between subjects, we identified at least 8 bacterial taxa whose abundances were associated with single nucleotide polymorphisms in the host genome in each season (at genome-wide FDR of 20%). For example, we identified an association between a taxon known to affect obesity )genus Akkermansia) and a variant near PLD1, a gene previously associated with body mass index. Moreover, we replicate a previously reported association from a quantitative trait locus (QTL) mapping study of fecal microbiome abundance in mice (genus Lactococcus, rs3747113, P = 3.13 x 10(-7)). Finally, based on the significance distribution of the associated microbiome QTLs in our study with respect to chromatin accessibility profiles, we identified tissues in which host genetic variation may be acting to influence bacterial abundance in the gut. | 1965 | no | human | NA | no | no |
| 1966 | 2017 | 43024 | Hard, JJ; Mills, LS; Peek, JM | 2006 | Genetic implications of reduced survival of male red deer Cervus elaphus under harvest | We use simple, multivariate evolutionary models to evaluate the short-term potential for size-selective harvest to reduce genetic variability and alter life history in cervids. These genetic effects limit sustainable levels of harvest of the animals because they determine how changes in sex ratio, generation length and traits contributing to fitness influence population growth rate and local adaptation. Our analysis of harvest-mediated adaptive evolution employs a genetic approach that parameterizes models with empirical data obtained from European red deer Cervus elaphus. The analysis indicates that harvest, if sufficiently high to reduce the breeding ratio of males to females to below about 15: 100, can reduce effective population size to a level that threatens adaptive potential. The reduction in effective size is realized through decreases in both sex ratio of breeders and the age of breeding males. Predicted selective effects of harvest on body size indicates a weak potential to alter most life-history traits over 10 generations under two harvest scenarios; the patterns suggest that current modes of harvest are unlikely to produce substantial life-history changes in red deer over 10 or fewer generations unless the genetic influences on red deer traits are considerably higher than those predicted here. Nevertheless, male reproductive success is expected to decline detectably if male harvest rate is sufficiently high (> 30%). Collectively, our results imply that harvest methods should permit higher post-hunt male:female ratios (18: 100 or higher) and ensure that a sufficient number of larger, older males survive the breeding season. The capacity of selective harvest to alter demography and life history depends heavily on the genetic covariance structure underlying variation in these traits, information that is unknown for many red deer populations. Prudent harvest management should therefore implement and monitor approaches to hunting that aim to conserve life-history variation; meanwhile, use of less selective methods can reduce the risk to long-term adaptive potential and may permit higher sustainable harvest rates. | 1966 | no | no good fitness measure, N pop differnt in GD? | NA | no | no |
| 1967 | 2017 | 43024 | Campino, S; Auburn, S; Kivinen, K; Zongo, I; Ouedraogo, JB; Mangano, V; Djimde, A; Doumbo, OK; Kiara, SM; Nzila, A; Borrmann, S; Marsh, K; Michon, P; Mueller, I; Siba, P; Jiang, HY; Su, XZ; Amaratunga, C; Socheat, D; Fairhurst, RM; Imwong, M; Anderson, T; Nosten, F; White, NJ; Gwilliam, R; Deloukas, P; MacInnis, B; Newbold, CI; Rockett, K; Clark, TG; Kwiatkowski, DP | 2011 | Population Genetic Analysis of Plasmodium falciparum Parasites Using a Customized Illumina GoldenGate Genotyping Assay | The diversity in the Plasmodium falciparum genome can be used to explore parasite population dynamics, with practical applications to malaria control. The ability to identify the geographic origin and trace the migratory patterns of parasites with clinically important phenotypes such as drug resistance is particularly relevant. With increasing single-nucleotide polymorphism (SNP) discovery from ongoing Plasmodium genome sequencing projects, a demand for high SNP and sample throughput genotyping platforms for large-scale population genetic studies is required. Low parasitaemias and multiple clone infections present a number of challenges to genotyping P. falciparum. We addressed some of these issues using a custom 384-SNP Illumina GoldenGate assay on P. falciparum DNA from laboratory clones (long-term cultured adapted parasite clones), short-term cultured parasite isolates and clinical (non-cultured isolates) samples from East and West Africa, Southeast Asia and Oceania. Eighty percent of the SNPs (n = 306) produced reliable genotype calls on samples containing as little as 2 ng of total genomic DNA and on whole genome amplified DNA. Analysis of artificial mixtures of laboratory clones demonstrated high genotype calling specificity and moderate sensitivity to call minor frequency alleles. Clear resolution of geographically distinct populations was demonstrated using Principal Components Analysis (PCA), and global patterns of population genetic diversity were consistent with previous reports. These results validate the utility of the platform in performing population genetic studies of P. falciparum. | 1967 | no | gd of parasite | NA | no | no |
| 1968 | 2017 | 43024 | Romano, CM; Lauck, M; Salvador, FS; Lima, CR; Villas-Boas, LS; Araujo, ESA; Levi, JE; Pannuti, CS; O’Connor, D; Kallas, EG | 2013 | Inter- and Intra-Host Viral Diversity in a Large Seasonal DENV2 Outbreak | Background: High genetic diversity at both inter-and intra-host level are hallmarks of RNA viruses due to the error-prone nature of their genome replication. Several groups have evaluated the extent of viral variability using different RNA virus deep sequencing methods. Although much of this effort has been dedicated to pathogens that cause chronic infections in humans, few studies investigated arthropod-borne, acute viral infections. Methods and Principal Findings: We deep sequenced the complete genome of ten DENV2 isolates from representative classical and severe cases sampled in a large outbreak in Brazil using two different approaches. Analysis of the consensus genomes confirmed the larger extent of the 2010 epidemic in comparison to a previous epidemic caused by the same viruses in another city two years before (genetic distance = 0.002 and 0.0008 respectively). Analysis of viral populations within the host revealed a high level of conservation. After excluding homopolymer regions of 454/Roche generated sequences, we found 10 to 44 variable sites per genome population at a frequency of >1%, resulting in very low intra-host genetic diversity. While up to 60% of all variable sites at intra-host level were non-synonymous changes, only 10% of inter-host variability resulted from non-synonymous mutations, indicative of purifying selection at the population level. Conclusions and Significance: Despite the error-prone nature of RNA-dependent RNA-polymerase, dengue viruses maintain low levels of intra-host variability. | 1968 | no | gd of pathogen | NA | no | no |
| 1969 | 2017 | 43024 | Magalhaes, V; Abrantes, J; Munoz-Pajares, AJ; Esteves, PJ | 2015 | Genetic diversity comparison of the DQA gene in European rabbit (Oryctolagus cuniculus) populations | The European rabbit (Oryctolagus cuniculus) natural populations within the species native region, the Iberian Peninsula, are considered a reservoir of genetic diversity. Indeed, the Iberia was a Pleistocene refuge to the species and currently two subspecies are found in the peninsula (Oryctolagus cuniculus cuniculus and Oryctolagus cuniculus algirus). The genes of the major histocompatibility complex (MHC) have been substantially studied in wild populations due to their exceptional variability, believed to be pathogen driven. They play an important function as part of the adaptive immune system affecting the individual fitness and population viability. In this study, the MHC variability was assessed by analysing the exon 2 of the DQA gene in several European rabbit populations from Portugal, Spain and France and in domestic breeds. Twenty-eight DQA alleles were detected, among which 18 are described for the first time. The Iberian rabbit populations are well differentiated from the French population and domestic breeds. The Iberian populations retained the higher allelic diversity with the domestic breeds harbouring the lowest; in contrast, the DQA nucleotide diversity was higher in the French population. Signatures of positive selection were detected in four codons which are putative peptide-binding sites and have been previously detected in other mammals. The evolutionary relationships showed instances of trans-species polymorphism. Overall, our results suggest that the DQA in European rabbits is evolving under selection and genetic drift. | 1969 | no | no good fitness measure | NA | no | no |
| 1970 | 2017 | 43024 | Oliveira, ALD; Da Silva, D; Zanotti-Magalhaes, EM; Abdel-Hamid, AZ; Ribeiro-Paes, JT | 2008 | Schistosome/mollusk: genetic compatibility | Schistosomiasis remains one of the most prevalent parasitic infections and has significant economic and public health consequences in many developing countries. Economic development and improvement in standard of living in these countries are dependent on the elimination of this odious disease. For the control of Schistosomiasis, understanding the host/parasite association is important, since the host parasite relationship is often complex and since questions remain concerning the susceptibility of snails to infection by respective trematodes and their specificity and suitability as hosts for continued parasite development. Thus, the long term aim of this research is to learn more about the genetic basis of the snail/parasite relationship with the hope of finding novel ways to disrupt the transmission of this disease. In the current research, genetic variability among susceptible and resistant strains within and between Biomphalaria glabrata and B. tenagophila was investigated using RAPD-PCR. The results indicate great genetic variations within the two snail species using three different primers (intrapopulational variations), while specimens from the same snail species showed few individual differences between the susceptible and resistant strains (interpopulational variation). | 1970 | no | gd of parasite | NA | no | no |
| 1971 | 2017 | 43024 | Van Tyne, D; Park, DJ; Schaffner, SF; Neafsey, DE; Angelino, E; Cortese, JF; Barnes, KG; Rosen, DM; Lukens, AK; Daniels, RF; Milner, DA; Johnson, CA; Shlyakhter, I; Grossman, SR; Becker, JS; Yamins, D; Karlsson, EK; Ndiaye, D; Sarr, O; Mboup, S; Happi, C; Furlotte, NA; Eskin, E; Kang, HM; Hartl, DL; Birren, BW; Wiegand, RC; Lander, ES; Wirth, DF; Volkman, SK; Sabeti, PC | 2011 | Identification and Functional Validation of the Novel Antimalarial Resistance Locus PF10\_0355 in Plasmodium falciparum | The Plasmodium falciparum parasite’s ability to adapt to environmental pressures, such as the human immune system and antimalarial drugs, makes malaria an enduring burden to public health. Understanding the genetic basis of these adaptations is critical to intervening successfully against malaria. To that end, we created a high-density genotyping array that assays over 17,000 single nucleotide polymorphisms (similar to 1 SNP/kb), and applied it to 57 culture-adapted parasites from three continents. We characterized genome-wide genetic diversity within and between populations and identified numerous loci with signals of natural selection, suggesting their role in recent adaptation. In addition, we performed a genome-wide association study (GWAS), searching for loci correlated with resistance to thirteen antimalarials; we detected both known and novel resistance loci, including a new halofantrine resistance locus, PF10\_0355. Through functional testing we demonstrated that PF10\_0355 overexpression decreases sensitivity to halofantrine, mefloquine, and lumefantrine, but not to structurally unrelated antimalarials, and that increased gene copy number mediates resistance. Our GWAS and follow-on functional validation demonstrate the potential of genome-wide studies to elucidate functionally important loci in the malaria parasite genome. | 1971 | no | gd of parasite | NA | no | no |
| 1972 | 2017 | 43024 | Queiroz, CB; Miranda, EC; Hanada, RE; Sousa, NR; Gasparotto, L; Soares, MA; Silva, GF | 2013 | Distribution of mating-type alleles and M13 PCR markers in the black leaf spot fungus Mycosphaerella fijiensis of bananas in Brazil | The fungus Mycosphaerella fijiensis is the causative agent of black sigatoka, which is one of the most destructive diseases of banana plants. Infection with this pathogen results in underdeveloped fruit, with no commercial value. We analyzed the distribution of the M. fijiensis mating-type system and its genetic variability using M13 phage DNA markers. We found a 1:1 distribution of mating-type alleles, indicating MAT1-1 and MAT1-2 idiomorphs. A polymorphism analysis using three different primers for M13 markers showed that only the M13 minisatellite primers generated polymorphic products. We then utilized this polymorphism to characterize 40 isolates from various Brazilian states. The largest genetic distances were found between isolates from the same location and between isolates from different parts of the country. Therefore, there was no correlation between the genetic similarity and the geographic origin of the isolates. The M13 marker was used to generate genetic fingerprints for five isolates; these fingerprints were compared with the band profiles obtained from inter-simple sequence repeat (UBC861) and inter-retrotransposon amplified polymorphism analyses. We found that the M13 marker was more effective than the other two markers for differentiating these isolates. | 1972 | no | gd of pathogen | NA | no | no |
| 1973 | 2017 | 43024 | Patarro, TD; Guirado, MM; Ravazzi, LM; Bicudo, HEMD | 2013 | Genetic structure of Aedes aegypti populations determined using pairwise comparisons | The biological characteristics of Aedes aegypti (Diptera, Culicidae), which is a vector of dengue and yellow fever, make this organism a good model for studying population structure and the events that may influence it under the effect of human activity. We assessed the genetic variability of five A. aegypti populations using RAPD-PCR technique and six primers. Four populations were from Brazil and one was from the USA. A total of 165 polymorphic DNA loci were generated. Considering the six primers and the five populations, the mean value of inter-population genetic diversity (Gst) was 0.277, which is considered high according to the Wright classification. However, pairwise comparisons of the populations gave variable Gst values ranging from 0.044 to 0.289. This variation followed the population’s geographic distance to some extent but was also influenced by human activity. The lowest Gst values were obtained in the comparison of populations from cities with intensive commercial and medical contacts. These mosquito populations were previously classified as insecticide resistant, susceptible, or with decreased susceptibility; this parameter apparently had an effect on the Gst values obtained in the pairwise comparisons. | 1973 | no | No GD diff between pop | NA | no | no |
| 1974 | 2017 | 43024 | Fernando, P; Polet, G; Foead, N; Ng, LS; Pastorini, J; Melnick, DJ | 2006 | Genetic diversity, phylogeny and conservation of the Javan rhinoceros (Rhinoceros sondaicus) | With a total population of less than 60 individuals limited to two locations, the Javan rhinoceros is perhaps the most endangered large mammal on earth. Although species specific information is crucial to its conservation, its precarious status, habitat inaccessibility, and behavioral adaptations pose major obstacles to its study. Here we report on the first genetic analysis of the two extant populations, in Ujung Kulon, Indonesia, and Cat Tien, Vietnam, and discuss their conservation. As its critically endangered status precluded invasive sampling, we extracted DNA from dung, amplifying and sequencing segments of the mtDNA 12S rRNA gene and the non-coding D-loop. Divergence between Javan rhinos from Ujung Kulon and Cat Tien was similar to that between recognized subspecies of African rhinos, and exceeded that between Sumatran rhinos. The Ujung Kulon and Cat Tien populations represent separate Evolutionary Significant Units, advocating independent management. However, given the precariousness of the Cat Tien population, demographic considerations may override genetic issues in the short term. Genetic diversity of Javan rhinos was low and population expansion in the immediate future will be critical for its survival. | 1974 | no | no good fitness measure | NA | no | no |
| 1975 | 2017 | 43024 | Ledoux, JB; Mokhtar-Jamai, K; Roby, C; Feral, JP; Garrabou, J; Aurelle, D | 2010 | Genetic survey of shallow populations of the Mediterranean red coral [Corallium rubrum (Linnaeus, 1758)]: new insights into evolutionary processes shaping nuclear diversity and implications for conservation | Combined action from over-harvesting and recent mass mortality events potentially linked to ongoing climate changes has led to new concerns for the conservation of shallow populations (5-60 m) of Corallium rubrum, an octocorallian that is mainly found in the Mediterranean Sea. The present study was designed to analyse population structure and relationships at different spatial scales (from 10s of meters to 100s of kilometres) with a focus on dispersal pattern. We also performed the first analysis of the distribution of genetic diversity using a comparative approach between regional-clusters and samples. Forty populations dwelling in four distinct regions between 14 and 60 m in depth were genotyped using 10 microsatellites. Our main results indicate (i) a generalized pair-sample differentiation combined with a weak structure between regional-clusters; (ii) the occurrence of isolation by distance at the global scale, but also within two of the three analysed regional-clusters; (iii) a high level of genetic diversity over the surveyed area with a heterogeneous distribution from regional-cluster to sample levels. The evolutionary consequences of these results are discussed and their management implications are provided. | 1975 | no | no good fitness measure | NA | no | no |
| 1976 | 2017 | 43024 | Myers, AJ; Gibbs, JR; AWebster, J; Rohrer, K; Zhao, A; Marlowe, L; Kaleem, M; Leung, D; Bryden, L; Nath, P; Zismann, VL; Joshipura, K; Huentelman, MJ; Hu-Lince, D; Coon, KD; Craig, DW; Pearson, JV; Holmans, P; Heward, CB; Reiman, EM; Stephan, D; Hardy, J | 2007 | A survey of genetic human cortical gene expression | It is widely assumed that genetic differences in gene expression underpin much of the difference among individuals and many of the quantitative traits of interest to geneticists. Despite this, there has been little work on genetic variability in human gene expression and almost none in the human brain, because tools for assessing this genetic variability have not been available. Now, with whole-genome SNP genotyping arrays and whole-transcriptome expression arrays, such experiments have become feasible. We have carried out whole-genome genotyping and expression analysis on a series of 193 neuropathologically normal human brain samples using the Affymetrix GeneChip Human Mapping 500K Array Set and Illumina HumanRefseq-8 Expression BeadChip platforms. Here we present data showing that 58% of the transcriptome is cortically expressed in at least 5% of our samples and that of these cortically expressed transcripts, 21% have expression profiles that correlate with their genotype. These genetic-expression effects should be useful in determining the underlying biology of associations with common diseases of the human brain and in guiding the analysis of the genomic regions involved in the control of normal gene expression. | 1976 | no | human | NA | no | no |
| 1977 | 2017 | 43024 | Emebiri, L; Michael, P; Moody, D; Ogbonnaya, F; Black, C | 2009 | Pyramiding QTLs to improve malting quality in barley: gains in phenotype and genetic diversity | The ability of barley (Hordeum vulgare L.) breeders to deliver germplasm that combine elite malt quality characteristics, disease resistances, and important agronomic traits has been greatly enhanced by the use of molecular marker technologies. These technologies facilitate the rapid transfer of desirable traits from diverse, elite, germplasm into locally adapted varieties. This present study sought to obtain an additive genetic effect by combining favourable alleles associated with the malting quality of two elite donor parents (Harrington and Morex) such that the resultant progeny would possess quality superior to either parent. Analysis of genetic diversity, based on whole-genome profiling with 700 DArT markers, showed clear separation of the BC(6)F(1)-derived doubled haploid lines from existing malting barley germplasm, indicating they represent a distinctly different source population for genetic improvement. Micro-malting quality results of the BC-derived lines showed substantial quality improvements, compared with the recurrent parent. Malt extract levels were increased by 1.5-2.0%, while diastase levels increased from approximately 320 WKE to 400-460 WKE. Similarly, alpha-amylase levels were increased from 160 units to between 218 and 251 units, and wort viscosities lowered from 1.90 cps to 1.72-1.82 cps. Other quality improvements include increases in beta-glucanase levels from 375 to between 447 and 512 units, and reductions in wort beta-glucan levels by 30-60%. Whilst the genetic gains compared to the recurrent parent were impressive, quality of the derived lines were largely equivalent to the levels now available in the recently released varieties, Buloke and Flagship. In a few cases, the backcross-derived lines also showed similarities to the original donors, Harrington and Morex, but in none of the cases did quality of these lines exceed those of either Harrington or Morex. | 1977 | no | crop | NA | no | no |
| 1978 | 2017 | 43024 | Hendrickson, SL; Mayer, GC; Wallen, EP; Quigley, K | 2000 | Genetic variability and geographic structure of three subspecies of tigers (Panthera tigris) based on MHC class I variation | Genetic variation at the major histocompatibility (MHC) class I loci was examined in captive and wild samples of three subspecies of tiger (Bengal, Panthera tigris tigris; Siberian, P. t. altaica; Sumatran, P. t. sumatrae) by Southern blot analysis using the domestic cat probe FLA 24. All captive populations showed at least moderate variability compared to other felids. Captive tigers were, on average, significantly more variable than wild tigers, but this was not consistent among subspecies. Significant geographic genetic population structure was found at the MHC class I loci among both captive and wild tigers. Phylogenetic analysis of both captive and wild tigers placed Sumatran tigers basally. In a combined phylogenetic analysis, captive and wild tigers of the same subspecies occurred near one another, indicating that the structure of the captive populations reflects that of the wild populations. | 1978 | no | no good fitness measure | NA | no | no |
| 1979 | 2017 | 43024 | Cheng, YC; Kao, WHL; Mitchell, BD; O’Connell, JR; Shen, HQ; McArdle, PF; Gibson, Q; Ryan, KA; Shuldiner, AR; Pollin, TI | 2009 | Genome-Wide Association Scan Identifies Variants near Matrix Metalloproteinase (MMP) Genes on Chromosome 11q21-22 Strongly Associated With Serum MMP-1 Levels | Background-Matrix metalloproteinase (MMP)-1 may play a role in cardiovascular disease susceptibility by influencing plaque rupture via its ability to degrade extracellular collagens. Methods and Results-We performed a genome-wide association analysis of circulating MMP-1 levels using 500 K single-nucleotide polymorphisms (SNPs) to identify genes influencing variation in serum MMP-1 levels in 778 healthy Amish adults. Serum MMP-1 levels, logarithm transformed, and adjusted for age and sex, were screened for association with SNPs using mixed-model variance components to account for familial relatedness. Median MMP-1 level was 3.05 ng/mL (interquartile range: 1.82 to 5.04 ng/mL) with an estimated heritability of 81% (P < 0.0001). Serum MMP-1 levels were strongly associated with a cluster of 179 SNPs extending over an 11.5-megabase region on chromosome 11q. The peak association was with rs495366 (P = 5.73 x 10(-34)), located within the region between MMP-1 and MMP-3 and having a minor allele frequency of 0.36. Two other SNPs within the 11q region, rs12289128 and rs11226373, were strongly associated with MMP-1 levels after accounting for rs495366 (P <= 10(-7)). These 3 SNPs explained 31% of the variance in MMP-1 levels after adjusting for age and sex. Conclusions-This study provides strong evidence that the serum MMP-1 level is highly heritable and that SNPs near MMPs on chromosome 11q explain a significant portion of the variation in MMP-1 levels. Identification of the genetic variants that influence MMP-1 levels may provide insights into genetic mechanisms of cardiovascular disease. (Circ Cardiovasc Genet. 2009; 2: 329-337.) | 1979 | no | human | NA | no | no |
| 1980 | 2017 | 43024 | Vazquez, AA; Lounnas, M; Sanchez, J; Alba, A; Milesi, A; Hurtrez-Bousses, S | 2016 | Genetic and infective diversity of the liver fluke Fasciola hepatica (Trematoda: Digenea) from Cuba | In this study we present the first approach to exploration of the genetic diversity of Cuban Fasciola hepatica populations using microsatellite markers, coupled with observed prevalence in slaughterhouses. Nine populations of flukes recovered from cows and buffalos were studied in the central-western region of Cuba. The observed infection rates of definitive hosts (bovines) were 70-100% in most cases. An important amount of polymorphism was found in the four loci explored. However, no apparent genetic differences were found between populations from different provinces or bovine species. The absence of deviations from Hardy-Weinberg equilibrium suggests a high rate of cross-fertilization between F. hepatica individuals. This result was confirmed when all multilocus genotypes were tested for clonal reproduction and only four individuals differed statistically (P-sex < 0.05). High values of expected heterozygosity coupled with highly probable mixing among strains make the metapopulation genetically diversified but similar in terms of certain alleles (low F-ST values). These results suggest a close relationship between parasite diversity and cattle management in Cuba. Our findings should be taken into consideration by veterinary authorities to help mitigate fasciolosis transmission. | 1980 | no | gd of parasite | NA | no | no |
| 1981 | 2017 | 43024 | Ciobanu, DC; Bastiaansen, JWM; Magrin, J; Rocha, JL; Jiang, DH; Yu, N; Geiger, B; Deeb, N; Rocha, D; Gong, H; Kinghorn, BP; Plastow, GS; van der Steen, HAM; Mileham, AJ | 2010 | A major SNP resource for dissection of phenotypic and genetic variation in Pacific white shrimp (Litopenaeus vannamei) | P>Bioinformatics and re-sequencing approaches were used for the discovery of sequence polymorphisms in Litopenaeus vannamei. A total of 1221 putative single nucleotide polymorphisms (SNPs) were identified in a pool of individuals from various commercial populations. A set of 211 SNPs were selected for further molecular validation and 88% showed variation in 637 samples representing three commercial breeding lines. An association analysis was performed between these markers and several traits of economic importance for shrimp producers including resistance to three major viral diseases. A small number of SNPs showed associations with test weekly gain, grow-out survival and resistance to Taura Syndrome Virus. Very low levels of linkage disequilibrium were revealed between most SNP pairs, with only 11% of SNPs showing an r2-value above 0.10 with at least one other SNP. Comparison of allele frequencies showed small changes over three generations of the breeding programme in one of the commercial breeding populations. This unique SNP resource has the potential to catalyse future studies of genetic dissection of complex traits, tracing relationships in breeding programmes, and monitoring genetic diversity in commercial and wild populations of L. vannamei. | 1981 | no | no good fitness measure, N pop differnt in GD? | NA | no | no |
| 1982 | 2017 | 43024 | Woodberry, OG; Korb, KB; Nicholson, AE | 2007 | A simulation study of the evolution of ageing | Hypothesis: Although ageing may reduce individual fitness, it could arise because ageing increases organism turnover and enhances genetic diversity within subpopulations. The model: We have developed an ALife simulation environment of subpopulations of co-evolving hosts and diseases to test hypotheses, finding differing circumstances in which adaptive, and non-adaptive, ageing evolution occurs. Conclusions: Our simulations demonstrate conditions that are favourable to the adaptive evolution of ageing. The adaptive evolution of ageing requires a reinterpretation of group selection theory, tying it strongly to what we already know about inclusive fitness theory. | 1982 | no | no good fitness measure | NA | no | no |
| 1983 | 2017 | 43024 | Ashfaq, M; Hebert, PDN; Mirza, JH; Khan, AM; Zafar, Y; Mirza, MS | 2014 | Analyzing Mosquito (Diptera: Culicidae) Diversity in Pakistan by DNA Barcoding | Background: Although they are important disease vectors mosquito biodiversity in Pakistan is poorly known. Recent epidemics of dengue fever have revealed the need for more detailed understanding of the diversity and distributions of mosquito species in this region. DNA barcoding improves the accuracy of mosquito inventories because morphological differences between many species are subtle, leading to misidentifications. Methodology/Principal Findings: Sequence variation in the barcode region of the mitochondrial COI gene was used to identify mosquito species, reveal genetic diversity, and map the distribution of the dengue-vector species in Pakistan. Analysis of 1684 mosquitoes from 491 sites in Punjab and Khyber Pakhtunkhwa during 2010-2013 revealed 32 species with the assemblage dominated by Culex quinquefasciatus (61% of the collection). The genus Aedes (Stegomyia) comprised 15% of the specimens, and was represented by six taxa with the two dengue vector species, Ae. albopictus and Ae. aegypti, dominant and broadly distributed. Anopheles made up another 6% of the catch with An. subpictus dominating. Barcode sequence divergence in conspecific specimens ranged from 0-2.4%, while congeneric species showed from 2.3-17.8% divergence. A global haplotype analysis of disease-vectors showed the presence of multiple haplotypes, although a single haplotype of each dengue-vector species was dominant in most countries. Geographic distribution of Ae. aegypti and Ae. albopictus showed the later species was dominant and found in both rural and urban environments. Conclusions: As the first DNA-based analysis of mosquitoes in Pakistan, this study has begun the construction of a barcode reference library for the mosquitoes of this region. Levels of genetic diversity varied among species. Because of its capacity to differentiate species, even those with subtle morphological differences, DNA barcoding aids accurate tracking of vector populations. | 1983 | no | no good fitness measure | NA | no | no |
| 1984 | 2017 | 43024 | Roy, M; Pozzi, AC; Gareil, R; Nagati, M; Manzi, S; Nouioui, I; Sharikadze, N; Jargeat, P; Gryta, H; Moreau, PA; Fernandez, MP; Garde, M | 2017 | Alder and the Golden Fleece: high diversity of Frankia and ectomycorrhizal fungi revealed from Alnus glutinosa subsp barbata roots close to a Tertiary and glacial refugium | Background. Recent climatic history has strongly impacted plant populations, but little is known about its effect on microbes. Alders, which host few and specific symbionts, have high genetic diversity in glacial refugia. Here, we tested the prediction that communities of root symbionts survived in refugia with their host populations. We expected to detect endemic symbionts and a higher species richness in refugia as compared to recolonized areas. Methods. We sampled ectomycorrhizal (EM) root tips and the nitrogen -fixing actin mycete Frankia communities in eight sites colonized by Alnus glutinosa insubsp. barbata close to the Caucasus in Georgia. Three sites were located the Colchis, one major Eurasian climatic refugia for Arcto-Tertiary flora and alders, and five sites were located in the recolonized zone. Endemic symbionts and plant ITS variants were detected by comparing sequences to published data from Europe and another Tertiary reftigium, the Hyrcanian forest. Species richness and community structure were compared between sites from refugia and recolonized areas for each symbionts. Results. For both symbionts, most MOTUs present in Georgia had been found previously elsewhere in Europe, Three endemic Frankia strains were detected in the Colchis vs two in the recolonized zone, and the five endemic EM fungi were detected only in the recolonized zone. Frankia species richness was higher in the Colchis while the contrary was observed for EM fungi. Moreover, the genetic diversity of one alder specialist Alnicola xanthophylla was particularly high in the recolonized zone. The EM communities occurring in the Colchis and the IHyrcanian forests shared closely related endemic species. Discussion, The Colchis did not have the highest alpha diversity and more endemic species, suggesting that our hypothesis based on alder biogeography may not apply to alder’s symbionts. Our study in the Caucasus brings new clues to understand symbioses biogeography and their survival in Tertiary and ice -age refugia, and reveals that isolated host populations could be of interest for symbiont diversity conservation. | 1984 | no | multiple species diversity | NA | no | no |
| 1985 | 2017 | 43024 | Nazareno, AG; dos Reis, MS | 2014 | At Risk of Population Decline? An Ecological and Genetic Approach to the Threatened Palm Species Butia eriospatha (Arecaceae) of Southern Brazil | To estimate the risk of population decline for the threatened palm species Butia eriospatha, we investigated the patterns of demography, natural regeneration, herbivory, and the levels of genetic diversity using 9 microsatellite loci from both adults and seedlings sampled from 4 populations in Southern Brazil (n 330). Our results indicate that cattle grazing in B. eriospatha population areas severely affect their demographic structure. Three B. eriospatha populations showed a bimodal age structure made up of adult plants and seedlings and high rates (> 77%) of livestock herbivory. For 1 population, we describe and quantify for the first time the occurrence of 6 ontogenetic stages for this threatened palm species. Populations of B. eriospatha showed high levels of genetic differentiation (F-ST adult plants 0.287, F-ST seedlings 0.175). The amount of observed heterozygosity differed significantly between small (H-O 0.329) and large populations (H-O 0.461), indicating that small populations can be more susceptible to genetic drift. With no recruitment and a mortality rate of 2.0%, we show that the populations investigated in this study would be at an extremely high risk of local extinction, with a greater than 50% reduction in the effective population size, in the next 40 years. Although this study highlights the importance of analyzing both population ecology parameters and genetic data to better understand the level of risk facing threatened species, we emphasize that policy actions are urgently needed for effective conservation of this vulnerable biological resource. | 1985 | no | no good fitness measure | NA | no | no |
| 1986 | 2017 | 43024 | Villesen, P; Gertsch, PJ; Frydenberg, J; Mueller, UG; Boomsma, JJ | 1999 | Evolutionary transition from single to multiple mating in fungus-growing ants | Queens of leaf cutter ants exhibit the highest known levels of multiple mating (up to 10 mates per queen) among ants. Multiple mating may have been selected to increase genetic diversity among nestmate workers, which is hypothesized to be critical in social systems with large, long-lived colonies under severe pressure of pathogens. Advanced fungus-growing (leafcutter) ants have large numbers (10(4)-10(6) workers) and long-lived colonies, whereas basal genera in the attine tribe have small (< 200 workers) colonies with probably substantially shorter lifespans. Basal attines are therefore expected to have lower queen mating frequencies, similar to those found in most other ants. We tested this prediction by analysing queen mating frequency and colony kin structure in three basal attine species: Myrmicocrypta ednaella, Apterostigma collare and Cyphomyrmex longiscapus. Microsatellite marker analyses revealed that queens in all three species were single mated, and that worker-to-worker relatedness in these basal attine species is very close to 0.75, the value expected under exclusively single mating. Fungus growing per se has therefore not selected for multiple queen mating. Instead, the advanced and highly productive social structure of the higher attine ants, which is fully dependent on the rearing of an ancient clonal fungus, may have necessitated high genetic diversity among nestmate workers. This is not the case in the lower attines, which rear fungi that were more recently derived from free-living fungal populations. | 1986 | no | multiple species comp | NA | no | no |
| 1987 | 2017 | 43024 | FRANK, SA | 1994 | KIN SELECTION AND VIRULENCE IN THE EVOLUTION OF PROTOCELLS AND PARASITES | The evolution of parasite virulence and the origin of cooperative genomes in primitive cells are both problems that balance cooperative and competitive interactions among symbionts. I analyse the tradeoff among three correlated traits: competitiveness against other genotypes for resources within hosts (protocells), damage to the host (virulence), and rate of horizontal transmission from one host to another. All three life-history components are strongly influenced by kin selection. For example, when genetic relatedness within hosts is high, each genotype is competing for resources with closely related genotypes. This competition among relatives favours increased horizontal transmission to colonize new hosts and compete against non-relatives. My analysis shows that many aspects of parasite and protocell evolution must be studied with the theoretical tools of social evolution. I discuss extensions that are required for a general theory of symbiosis. | 1987 | no | no good fitness measure, N pop differnt in GD? | NA | no | no |
| 1988 | 2017 | 43024 | Mattila, HR; Rios, D; Walker-Sperling, VE; Roeselers, G; Newton, ILG | 2012 | Characterization of the Active Microbiotas Associated with Honey Bees Reveals Healthier and Broader Communities when Colonies are Genetically Diverse | Recent losses of honey bee colonies have led to increased interest in the microbial communities that are associated with these important pollinators. A critical function that bacteria perform for their honey bee hosts, but one that is poorly understood, is the transformation of worker-collected pollen into bee bread, a nutritious food product that can be stored for long periods in colonies. We used 16S rRNA pyrosequencing to comprehensively characterize in genetically diverse and genetically uniform colonies the active bacterial communities that are found on honey bees, in their digestive tracts, and in bee bread. This method provided insights that have not been revealed by past studies into the content and benefits of honey bee-associated microbial communities. Colony microbiotas differed substantially between sampling environments and were dominated by several anaerobic bacterial genera never before associated with honey bees, but renowned for their use by humans to ferment food. Colonies with genetically diverse populations of workers, a result of the highly promiscuous mating behavior of queens, benefited from greater microbial diversity, reduced pathogen loads, and increased abundance of putatively helpful bacteria, particularly species from the potentially probiotic genus Bifidobacterium. Across all colonies, Bifidobacterium activity was negatively correlated with the activity of genera that include pathogenic microbes; this relationship suggests a possible target for understanding whether microbes provide protective benefits to honey bees. Within-colony diversity shapes microbiotas associated with honey bees in ways that may have important repercussions for colony function and health. Our findings illuminate the importance of honey bee-bacteria symbioses and examine their intersection with nutrition, pathogen load, and genetic diversity, factors that are considered key to understanding honey bee decline. | 1988 | yes | ok gd measurement and pop level comp? | NA | yes | yes |
| 1989 | 2017 | 43024 | Joy, N; Abraham, Z; Soniya, EV | 2007 | A preliminary assessment of genetic relationships among agronomically important cultivars of black pepper | Background: The impact of diseases such as Phytophthora foot rot and the replacement of unproductive cultivars by high yielding ones has brought about the disappearance of varieties in Piper species, like any other crop. Black pepper ( King of spices), is a major spice crop consumed throughout the world. It is widely cultivated across various parts of the world apart from India. The different cultivars may be genetically related and could be a source of valuable genes for disease resistance and an increase in quantity and quality. Even though Western Ghats in India is believed to be the site of origin of this crop, numerous accessions from the NBPGR have not yet been evaluated. Our study aims to investigate the genetic relatedness in major cultivars of black pepper using Amplified Fragment Length Polymorphism. Results: Amplified Fragment Length Polymorphic ( AFLP) DNA analysis was performed in thirty popular cultivars of black pepper from National Bureau of Plant Genetic Resources ( NBPGR), India. Fingerprint profiles were generated initially with, five different primer combinations, from which three primer pair combinations ( EAGC/MCAA, EAGG/MCTA and EAGC/MCTG) gave consistent and scorable banding patterns. From 173 scorable markers, 158(> 90%) were polymorphic which shows there is considerable variation in the available germplasm. The dendrogram derived by unweighted pair group method analysis ( UPGMA) grouped the accessions into three major clusters and four diverse cultivars with only 30% similarity. Karimunda, a widely grown and popular cultivar was unique in the fingerprint profiles obtained. Conclusion: There are currently few fingerprinting studies using the valuable spice crop black pepper. We found considerable genetic variability among cultivars of black pepper. Fingerprinting analysis with AFLP proved to be an ideal tool for cultivar identification and phylogenetic studies. It shows the high level of polymorphism and the unique characterization of the major cultivars. An extensive range of similarity value between the cultivars was noted ( 6.01 to 98.13). Further screening of more cultivars will provide valuable information for current breeding programmes. | 1989 | no | crop | NA | no | no |
| 1990 | 2017 | 43024 | HANLEY, KA; FISHER, RN; CASE, TJ | 1995 | LOWER MITE INFESTATIONS IN AN ASEXUAL GECKO COMPARED WITH ITS SEXUAL ANCESTORS | What advantage do sexually reproducing organisms gain from their mode of reproduction that compensates for their twofold loss in reproductive rate relative to their asexual counterparts? One version of the Red Queen hypothesis suggests that selective pressure from parasites is strongest on the most common genotype in a population, and thus genetically identical clonal lineages are more vulnerable to parasitism over time than genetically diverse sexual lineages. Our surveys of the ectoparasites of an asexual gecko and its two sexual ancestral species show that the sexuals have a higher prevalence, abundance, and mean intensity of mites than asexuals sharing the same habitat. Our experimental data indicate that in one sexual/asexual pair this pattern is at least partly attributable to higher attachment rates of mites to sexuals. Such a difference may occur as a result of exceptionally high susceptibility of the sexuals to mites because of their low genetic diversity (relative to other more-outbred sexual species) and their potentially high stress levels, or as a result of exceptionally low susceptibility of the asexuals to mites because of their high levels of heterozygosity. | 1990 | no | No GD diff between pop | NA | no | no |
| 1991 | 2017 | 43024 | Moreno, J; Hidalgo, M; Duarte, C; Sanabria, O; Gabastou, JM; Ibarz-Pavon, AB | 2015 | Characterization of Carriage Isolates of Neisseria meningitides in the Adolescents and Young Adults Population of Bogota (Colombia) | Background Meningococcal carriage studies are important to improve our understanding of the epidemiology of meningococcal disease. The aim of this study was to determine the prevalence of meningococcal carriage and the phenotypic and genotypic characteristics of isolates collected from a sample of students in the city of Bogota, Colombia. Materials and Methods A total of 1459 oropharyngeal samples were collected from students aged 15-21 years attending secondary schools and universities. Swabs were plated on a Thayer Martin agar and N. meningitidis was identified by standard microbiology methods and PCR. Results The overall carriage prevalence was 6.85%. Carriage was associated with cohabitation with smokers, and oral sex practices. Non-groupable and serogroup Y isolates were the most common capsule types found. Isolates presented a high genetic diversity, and circulation of the hypervirulent clonal complexes ST-23, ST-32 and ST-41/44 were detected. Conclusion The meningococcal carriage rate was lower than those reported in Europe and Africa, but higher than in other Latin American countries. Our data also revealed antigenic and genetic diversity of the isolates and the circulation of strains belonging to clonal complexes commonly associated with meningococcal disease. | 1991 | no | human | NA | no | no |
| 1992 | 2017 | 43024 | Chowell, G; Viboud, C; Simonsen, L; Miller, M; Alonso, WJ | 2010 | The reproduction number of seasonal influenza epidemics in Brazil, 1996-2006 | The transmission dynamics of influenza in tropical regions are poorly understood. Here we explore geographical variations in the reproduction number of influenza across equatorial, tropical and subtropical areas of Brazil, based on the analysis of weekly pneumonia and influenza (P&I) mortality time series in 27 states. The reproduction number (R) was low on average in Brazil (mean = 1.03 (95% CI 1.02-1.04), assuming a serial interval of 3 days). Estimates of the reproduction number were slightly lower for Brazil than for the USA or France (difference in mean R = 0.08, p < 0.01) and displayed less between-year variation (p < 0.001). Our findings suggest a weak gradient in the reproduction number with population size, where R increases from low population in the North to high population in the South of Brazil. Our low estimates of the reproduction number suggest that influenza population immunity could be high on average in Brazil, potentially resulting in increased viral genetic diversity and rate of emergence of new variants. Additional epidemiological and genetic studies are warranted to further characterize the dynamics of influenza in the tropics and refine our understanding of the global circulation of influenza viruses. | 1992 | no | NA | NA | no | no |
| 1993 | 2017 | 43024 | Lopez-Soriano, P; Boyer, K; Cesbron, S; Morente, MC; Penalver, J; Palacio-Bielsa, A; Verniere, C; Lopez, MM; Pruvost, O | 2016 | Multilocus Variable Number of Tandem Repeat Analysis Reveals Multiple Introductions in Spain of Xanthomonas arboricola pv. pruni, the Causal Agent of Bacterial Spot Disease of Stone Fruits and Almond | Xanthomonas arboricola pv. pruni is the causal agent of the bacterial spot disease of stone fruits, almond and some ornamental Prunus species. In Spain it was first detected in 2002 and since then, several outbreaks have occurred in different regions affecting mainly Japanese plum, peach and almond, both in commercial orchards and nurseries. As the origin of the introduction(s) was unknown, we have assessed the genetic diversity of 239 X. arboricola pv. pruni strains collected from 11 Spanish provinces from 2002 to 2013 and 25 reference strains from international collections. We have developed an optimized multilocus variable number of tandem repeat analysis (MLVA) scheme targeting 18 microsatellites and five minisatellites. A high discriminatory power was achieved since almost 50% of the Spanish strains were distinguishable, confirming the usefulness of this genotyping technique at small spatio-temporal scales. Spanish strains grouped in 18 genetic clusters (conservatively delineated so that each cluster contained haplotype networks linked by up to quadruple-locus variations). Furthermore, pairwise comparisons among populations from different provinces showed a strong genetic differentiation. Our results suggest multiple introductions of this pathogen in Spain and redistribution through contaminated nursery propagative plant material. | 1993 | no | gd of pathogen | NA | no | no |
| 1994 | 2017 | 43024 | Sonsthagen, SA; Fales, K; Jay, CV; Sage, GK; Talbot, SL | 2014 | Spatial variation and low diversity in the major histocompatibility complex in walrus (Odobenus rosmarus) | Increased global temperature and associated changes to Arctic habitats will likely result in the northward advance of species, including an influx of pathogens novel to the Arctic. How species respond to these immunological challenges will depend in part on the adaptive potential of their immune response system. We compared levels of genetic diversity at a gene associated with adaptive immune response [Class II major histocompatibility complex (MHC), DQB exon 2] between populations of walrus (Odobenus rosmarus), a sea ice-dependent Arctic species. Walrus was represented by only five MHC DQB alleles, with frequency differences observed between Pacific and Atlantic populations. MHC DQB alleles appear to be under balancing selection, and most (80 %; n = 4/5) of the alleles were observed in walruses from both oceans, suggesting broad scale differences in the frequency of exposure and diversity of pathogens may be influencing levels of heterozygosity at DQB in walruses. Limited genetic diversity at MHC, however, suggests that walrus may have a reduced capacity to respond to novel immunological challenges associated with shifts in ecological communities and environmental stressors predicted for changing climates. This is particularly pertinent for walrus, since reductions in summer sea ice may facilitate both northward expansion of marine species and associated pathogens from more temperate regions, and exchange of marine mammals and associated pathogens through the recently opened Northwest Passage between the Atlantic and Pacific Oceans in the Canadian high Arctic. | 1994 | no | no good fitness measure | NA | no | no |
| 1995 | 2017 | 43024 | Korstian, JM; Hale, AM; Williams, DA | 2015 | Genetic diversity, historic population size, and population structure in 2 North American tree bats | Migratory tree bats comprise the majority of casualties at wind energy facilities across North America and some species, like Lasiurus borealis, are suspected to be in decline. We took advantage of the large numbers of L. borealis and L. cinereus salvaged during fall migration at 1 wind farm in Texas and 3 wind farms in Minnesota to evaluate the ability of current genetic methods to estimate population size and detect genetic bottlenecks in these species. Using DNA extracted from wing membrane tissue samples, we genotyped 439 L. borealis and 246 L. cinereus at 6 microsatellite loci and a 550 bp segment of the mitochondrial COI gene. Both microsatellite loci and mitochondrial haplotypes showed high levels of genetic diversity in each species. Historical estimates of N-e were large for both species. Estimates of N-ef from the COI gene were almost 2 times higher for L. borealis than L. cinereus, whereas estimates utilizing microsatellite heterozygosity were higher for L. cinereus. We found a strong signal of rapid historical population growth and range expansion in L. borealis, but not in L. cinereus. The majority of our estimates of current N-e had upper 95% confidence intervals that encompassed infinity. There is some indication from contemporary estimates of N-e that L. borealis may have a lower current N-e than historical estimates and that L. cinereus may currently have a very large N-e. We found no genetic evidence of recent population declines and no evidence of population structure in either species. Genetic monitoring of migratory tree bats, specifically for the purpose of detecting population declines caused by wind turbine mortality, may be impractical due to the large effective population sizes and high levels of gene flow in these species. Future efforts should focus on developing genomic resources for these species, obtaining better estimates of mutation rates, and conducting range-wide population genetic studies in order to better estimate historical and current population sizes. (C) 2015 American Society of Mammalogists, www.mammalogy.org | 1995 | no | no good fitness measure, N pop differnt in GD? | NA | no | no |
| 1996 | 2017 | 43024 | Thrall, PH; Burdon, JJ | 1999 | The spatial scale of pathogen dispersal: Consequences for disease dynamics and persistence | Plant pathogens exhibit a diverse array of life histories and dispersal mechanisms. The latter suggests that, in nature, there will be a broad range of spatial scales over which hosts and pathogens interact (e.g. soil-borne pathogens to aerially dispersed rusts). Variation in the spatial scale of such interactions is likely to have consequences for disease dynamics and pathogen persistence, and therefore coevolution of host and pathogen. We investigated disease dynamics and persistence using a spatially explicit simulation model that incorporated local dispersal of host and pathogen approximating the range of spatial structures seen in nature (i.e. many small isolated populations with very local dispersal to single large populations with some substructuring with global dispersal). Our results show that the spatial scale of pathogen dispersal is an important factor in determining the probability of disease persistence, as well as spatial and temporal patterns of incidence. Disease persistence was highest at relatively local scales of dispersal; at higher scales, not only was disease persistence less likely, but disease also caused major reductions in the fraction of sites occupied by the host. The nature of the dynamics also varied with the spatial scale of dispersal, with temporal changes in disease presence across the metapopulation showing endemic patterns at low scales of dispersal, but epidemic patterns when pathogen dispersal was over broader scales. It has been argued that the genetic diversity of host-pathogen systems with respect to resistance and virulence will depend on the probability of encounter rates between particular host and pathogen genotypes. Our results suggest that encounter rates, and therefore genetic diversity, are also likely to be heavily influenced by the spatial scale of the interaction. | 1996 | no | No GD diff between pop | NA | no | no |
| 1997 | 2017 | 43024 | Brackney, DE; Pesko, KN; Brown, IK; Deardorff, ER; Kawatachi, J; Ebel, GD | 2011 | West Nile Virus Genetic Diversity is Maintained during Transmission by Culex pipiens quinquefasciatus Mosquitoes | Due to error-prone replication, RNA viruses exist within hosts as a heterogeneous population of non-identical, but related viral variants. These populations may undergo bottlenecks during transmission that stochastically reduce variability leading to fitness declines. Such bottlenecks have been documented for several single-host RNA viruses, but their role in the population biology of obligate two-host viruses such as arthropod-borne viruses (arboviruses) in vivo is unclear, but of central importance in understanding arbovirus persistence and emergence. Therefore, we tracked the composition of West Nile virus (WNV; Flaviviridae, Flavivirus) populations during infection of the vector mosquito, Culex pipiens quinquefasciatus to determine whether WNV populations undergo bottlenecks during transmission by this host. Quantitative, qualitative and phylogenetic analyses of WNV sequences in mosquito midguts, hemolymph and saliva failed to document reductions in genetic diversity during mosquito infection. Further, migration analysis of individual viral variants revealed that while there was some evidence of compartmentalization, anatomical barriers do not impose genetic bottlenecks on WNV populations. Together, these data suggest that the complexity of WNV populations are not significantly diminished during the extrinsic incubation period of mosquitoes. | 1997 | no | gd of pathogen | NA | no | no |
| 1998 | 2017 | 43024 | Bachis, V; Calo, CM; Vona, G; Corrias, L; Carreras-Torres, R; Moral, P | 2014 | Analysis of 16 STRs of NOS Gene Regions and Around in Six Sardinian Populations ( Italy) | ObjectivesThe aims of this work are to provide first data on novel STRs at the NOS gene regions in human populations and to test for possible correlations with mortality rate by malaria in different areas of Sardinia (Italy). MethodsIn the present study, 16 STRs (13 analyzed for the first time in human populations) localized on three genes NOS were typed in 213 healthy individuals, unrelated for at least three generations, from six historical-geographical Sardinian areas. STRs alleles were determined through sequencing. Statistical analyses were performed by Genepop (v.4.0), Arlequin (v.3.5.1.2), R (v.2.15.1), Statistica (v.5.1), and PHASE (v.2.1) software packages. ResultsThe number of alleles found for each locus ranged from 2 to 12 and their distribution is most often unimodal. All populations met Hardy Weinberg equilibrium after Bonferroni correction, with few exceptions. Analysis of genetic distances did not show strong genetic structuring of the investigated populations. Instead, the population genetic variability shows a positive and highly significant (P-value<0.01) correlation between mortality determined by malaria infection and alleles (TGGA)7 of NOS2, (AAAAG)2 and (ATTT)10 of adNOS1, and (AAACA)11 of adNOS3 genes. ConclusionsThe peculiar allele distribution found for several NOS alleles could be due to malaria infection that may have contributed to their frequencies, but we cannot exclude that the peculiar allele distribution of NOS might also be due to genetic drift, emphasized by isolation and founder effect. Am. J. Hum. Biol. 26:401-406, 2014. (c) 2014 Wiley Periodicals, Inc. | 1998 | no | human | NA | no | no |
| 1999 | 2017 | 43024 | Hoggard, SJ; Beattie, AJ; Gillings, MR; Stow, AJ | 2009 | Mating system and genetic structure in the paper wasp (Polistes humilis) | We provide the first description of the mating system, genetic structuring and dispersal in the Australian paper wasp, Polistes humilis. Individuals were collected from 13 colonies that were within 700m of each other at a location near Sydney, Australia. Analysis of genotypic data from four microsatellite loci provided no evidence of males siring offspring in their natal colony and heterozygote excesses within most colonies suggest that this form of outbreeding is typical in P. humilis. The same data show that queens are singly mated and that the number of queens and therefore groups of full siblings increase with colony size. Consequently, genetic variation also increases with colony size. The relationship between the number of queens and colony size in P. humilis may be indicative of a breakdown in dominance hierarchies in larger colonies or a defence against disease transmission. | 1999 | no | no good fitness measure | NA | no | no |
| 2000 | 2017 | 43024 | Kasuga, T; Kozanitas, M; Bui, M; Huberli, D; Rizzo, DM; Garbelotto, M | 2012 | Phenotypic Diversification Is Associated with Host-Induced Transposon Derepression in the Sudden Oak Death Pathogen Phytophthora ramorum | The oomycete pathogen Phytophthora ramorum is responsible for sudden oak death (SOD) in California coastal forests. P. ramorum is a generalist pathogen with over 100 known host species. Three or four closely related genotypes of P. ramorum (from a single lineage) were originally introduced in California forests and the pathogen reproduces clonally. Because of this the genetic diversity of P. ramorum is extremely low in Californian forests. However, P. ramorum shows diverse phenotypic variation in colony morphology, colony senescence, and virulence. In this study, we show that phenotypic variation among isolates is associated with the host species from which the microbe was originally cultured. Microarray global mRNA profiling detected derepression of transposable elements (TEs) and down-regulation of crinkler effector homologs (CRNs) in the majority of isolates originating from coast live oak (Quercus agrifolia), but this expression pattern was not observed in isolates from California bay laurel (Umbellularia californica). In some instances, oak and bay laurel isolates originating from the same geographic location had identical genotypes based on multilocus simples sequence repeat (SSR) marker analysis but had different phenotypes. Expression levels of the two marker genes analyzed by quantitative reverse transcription PCR were correlated with originating host species, but not with multilocus genotypes. Because oak is a nontransmissive dead-end host for P. ramorum, our observations are congruent with an epi-transposon hypothesis; that is, physiological stress is triggered on P. ramorum while colonizing oak stems and disrupts epigenetic silencing of TEs. This then results in TE reactivation and possibly genome diversification without significant epidemiological consequences. We propose the P. ramorum-oak host system in California forests as an ad hoc model for epi-transposon mediated diversification. | 2000 | no | no good fitness measure, N pop differnt in GD? | NA | no | no |
| 2001 | 2017 | 43025 | Faivre-Rampant, O; Bruschi, G; Abbruscato, P; Cavigiolo, S; Picco, AM; Borgo, L; Lupotto, E; Piffanelli, P | 2011 | Assessment of genetic diversity in Italian rice germplasm related to agronomic traits and blast resistance (Magnaporthe oryzae) | Italy is the only country in Europe with a significant land area used for rice production. In this paper, the genetic diversity of 172 national varieties and 47 foreign accessions (ITALORYZA collection) was investigated using a set of neutral markers evenly distributed throughout the 12 chromosomes. Out of the 218 alleles detected in our analysis, 17 and 29% were specific to the Italian and foreign accessions, respectively. From the neighbour-joining tree generated, six sub-groups of temperate japonica germplasm were identified. Plant height and grain type measured in the source collection were fitted to the phylogenetic tree, along with the period of variety registration. This integrated genotype-phenotype analysis revealed that specific sub-groups are characterized by uniform classes of grain type, or by similar plant size, or by period of release in the market. The whole collection was also evaluated for leaf blast resistance by inoculating the plants with three strains of Magnaporthe oryzae, representing the pathogen genetic diversity existing in Italy. Only 15 out of 172 Italian accessions (8.7%) were resistant to all three fungal strains. The correlation between genotype and leaf blast phenotype revealed that the most highly resistant Italian varieties are included in a single germplasm sub-group derived from US varieties. This study represents the starting point for carrying out detailed phenotype-genotype whole-genome association studies and identification of the genetic basis of important agronomic traits for rice cultivation in temperate climates. | 2001 | no | no good fitness measure, N pop differnt in GD? | NA | no | no |
| 2002 | 2017 | 43025 | Tellapragada, C; Kamthan, A; Shaw, T; Vandana, KE; Kumar, S; Bhat, V; Mukhopadhyay, C | 2016 | Unravelling the Molecular Epidemiology and Genetic Diversity among Burkholderia pseudomallei Isolates from South India Using Multi-Locus Sequence Typing | “There is a slow but steady rise in the case detection rates of melioidosis from various parts of the Indian sub-continent in the past two decades. However, the epidemiology of the disease in India and the surrounding South Asian countries remains far from well elucidated. Multi-locus sequence typing (MLST) is a useful epidemiological tool to study the genetic relatedness of bacterial isolates both with-in and across the countries. With this background, we studied the molecular epidemiology of 32 Burkholderia pseudomallei isolates (31 clinical and 1 soil isolate) obtained during 2006-2015 from various parts of south India using multi-locus sequencing typing and analysis. Of the 32 isolates included in the analysis, 30 (93.7%) had novel allelic profiles that were not reported previously. Sequence type (ST) 1368 (n = 15, 46.8%) with allelic profile (1, 4, 6, 4, 1, 1, 3) was the most common genotype observed. We did not observe a genotypic association of STs with geographical location, type of infection and year of isolation in the present study. Measure of genetic differentiation (F-ST) between Indian and the rest of world isolates was 0.14413. Occurrence of the same ST across three adjacent states of south India suggest the dispersion of B.pseudomallei across the south western coastal part of India with limited geographical clustering. However, majority of the STs reported from the present study remained as”“outliers”" on the eBURST "“Population snapshot”“, suggesting the genetic diversity of Indian isolates from the Australasian and Southeast Asian isolates.” | 2002 | no | NA | NA | no | no |
| 2003 | 2017 | 43025 | Freeman, AR; Lynn, DJ; Murray, C; Bradley, DG | 2008 | Detecting the effects of selection at the population level in six bovine immune genes | Background: The capacity of a species or population to respond to and survive novel infectious disease challenge is one of the most significant selective forces shaping genetic diversity and the period following animal domestication was likely one of the most important in terms of newly emerging diseases. Inter-specific genome-wide comparison has suggested that genes, including cluster of differentiation 2 (CD2), ADP-ribosyltransferase 4 (ART4), tyrosine kinase binding protein (TYROBP) and interleukins IL2, IL5, IL13, may have undergone positive selection during the evolution of the bovine lineage. Past adaptive change implies that more recent variation may have also been subject to selective forces. Results: In this paper, we re-sequence each of these genes in cattle cohorts from Europe, Africa and Asia to investigate patterns of polymorphism at the population level. Patterns of diversity are higher within Bos indicus suggesting different demographic history to that of Bos taurus. Significant coding polymorphism was observed within each of the cell-surface receptors. In particular, CD2 shows two divergent haplotypes defined by a series of six derived nonsynonymous substitutions that are significantly clustered on the extracellular surface of the protein and give significant values for Fay and Wu’s H, strongly suggesting a recent adaptive history. In contrast, the signaling molecules (especially IL13) display outlying allele frequency spectra which are consistent with the effects of selection, but display negligible coding polymorphism. Conclusion: We present evidence suggestive of recent adaptive history in bovine immune genes; implying some correspondence between intra- and inter-specific signals of selection. Interestingly, three signaling molecules have negligible nonsynonymous variation but show outlying test statistics in contrast to three receptors, where it is protein sequence diversity that suggests selective history. | 2003 | no | no good fitness measure, N pop differnt in GD? | NA | no | no |
| 2004 | 2017 | 43025 | DOLAN, RW | 1995 | THE RARE, SERPENTINE ENDEMIC STREPTANTHUS-MORRISONII (BRASSICACEAE) SPECIES COMPLEX, REVISITED USING ISOZYME ANALYSIS | The Streptanthus morrisonii (Brassicaceae) complex is a group of six narrowly-distributed obligate serpentine endemic taxa whose habitat is threatened by geothermal development. Isozyme analysis of this little-studied complex supports the delineation of two species, S. morrisonii and S. brachiatus, but is at odds with the treatment of two subspecies based on morphology. These results may be influenced by small sample sizes but genetic studies of other Streplanthus taxa have shown patterns of relatedness that often transgress subspecies boundaries based on morphology. The present study further shows that members of the S. morrisonii complex share high genetic identity values (mean = 0.87) and are not genetically depauperate (mean value for percent of loci polymorphic = 37%, average number of alleles per locus = 1.48, and average heterozygosity per locus = 0.137). Preservation of their serpentine outcrop habitat is essential to the survival of these plants. | 2004 | no | no good fitness measure, N pop differnt in GD? | NA | no | no |
| 2005 | 2017 | 43025 | Steeves, TE; Johnson, JA; Hale, ML | 2017 | Maximising evolutionary potential in functional proxies for extinct species: a conservation genetic perspective on de-extinction | 1. De-extinction sensu stricto is the resurrection of phenotypic traits once possessed by extinct species to create extant functional proxies. To realise the ecological benefit of de-extinction, self-sustaining (genetically viable) populations of functional proxies are required. 2. It is often implied, yet rarely stated, that the genetic challenges associated with the survival and recovery of extant threatened species in an effort to conserve biodiversity are also relevant to the use of functional proxies of extinct species as a conversation tool. 3. Here, we highlight the importance of prioritising evolutionary potential - the capacity to evolve (adapt) in response to environmental change - in populations of functional proxies. 4. We use conservation genetic principles to describe impediments to the creation and maintenance of evolutionary potential as a series of potentially unavoidable genetic bottlenecks (pre-extinction, resurrection, captive, translocation). 5. To give any successfully translocated populations of functional proxies the best chance to survive beyond the first few generations in the wild, we advocate the use of a holistic framework that includes the creation of sufficiently large, genetically diverse populations that harbour the ability to adapt to a changing environment. | 2005 | no | no good fitness measure, N pop differnt in GD? | NA | no | no |
| 2006 | 2017 | 43025 | Jackling, FC; Johnson, WE; Appleton, BR | 2014 | The Genetic Inheritance of the Blue-eyed White Phenotype in Alpacas (Vicugna pacos) | White-spotting patterns in mammals can be caused by mutations in the gene KIT, whose protein is necessary for the normal migration and survival of melanocytes from the neural crest. The alpaca (Vicugna pacos) blue-eyed white (BEW) phenotype is characterized by 2 blue eyes and a solid white coat over the whole body. Breeders hypothesize that the BEW phenotype in alpacas is caused by the combination of the gene causing gray fleece and a white-spotting gene. We performed an association study using KIT flanking and intragenic markers with 40 unrelated alpacas, of which 17 were BEW. Two microsatellite alleles at KIT-related markers were significantly associated (P < 0.0001) with the BEW phenotype (bew1 and bew2). In a larger cohort of 171 related individuals, we identify an abundance of an allele (bew1) in gray animals and the occurrence of bew2 homozygotes that are solid white with pigmented eyes. Association tests accounting for population structure and familial relatedness are consistent with a proposed model where these alleles are in linkage disequilibrium with a mutation or mutations that contribute to the BEW phenotype and to individual differences in fleece color. | 2006 | no | no good fitness measure, N pop differnt in GD? | NA | no | no |
| 2007 | 2017 | 43025 | Mingeot, D; Husson, C; Mertens, P; Watillon, B; Bertin, P; Druart, P | 2016 | Genetic diversity and genetic structure of black alder (Alnus glutinosa [L.] Gaertn) in the Belgium-Luxembourg-France cross-border area | Due to its beneficial effects on river ecosystems, black alder (Alnus glutinosa) is one of the tree species selected for planting on riverbanks in the cross-border area encompassing Wallonia in Belgium, Lorraine in France, and Luxembourg. The preservation of this species, however, is threatened by an invasive pathogen that particularly targets and kills young alder individuals. The objectives of this study were to characterize the genetic diversity and the genetic structure of A. glutinosa at this local level with the aim of assisting the conservation and replanting strategies and to determine if a germplasm collection comprising individuals from the same cross-border area captures the diversity present in the region. Nuclear simple sequence repeat (SSR) and chloroplastic DNA (cpDNA) markers were used to analyze four local wild populations and the germplasm collection which is representative of two river catchments and six legal provenance regions. Three populations distant from the studied area were also included. A panel of 14 nuclear SSR loci revealed high allelic diversity and very low differentiation among wild populations (mean F-ST = 0.014). The germplasm collection displayed a range of alleles that were representative of the different populations, and no significant differentiation between the germplasm collection and the local wild populations was observed, making this collection, as far as allelic diversity is concerned, suitable for providing trees for riverbank replanting programs. Using SSR markers, various statistical approaches consistently indicated the lack of a significant geographical structure at the level of the river catchments or provenance regions. In contrast, two cpDNA haplotypes were detected and displayed a cross-border geographically structured distribution that could be taken into account in defining new cross-border provenance regions. | 2007 | no | no good fitness measure | NA | no | no |
| 2008 | 2017 | 43025 | Piaggio, AJ; Shriner, SA; VanDalen, KK; Franklin, AB; Anderson, TD; Kolokotronis, SO | 2012 | Molecular Surveillance of Low Pathogenic Avian Influenza Viruses in Wild Birds across the United States: Inferences from the Hemagglutinin Gene | A United States interagency avian influenza surveillance plan was initiated in 2006 for early detection of highly pathogenic avian influenza viruses (HPAIV) in wild birds. The plan included a variety of wild bird sampling strategies including the testing of fecal samples from aquatic areas throughout the United States from April 2006 through December 2007. Although HPAIV was not detected through this surveillance effort we were able to obtain 759 fecal samples that were positive for low pathogenic avian influenza virus (LPAIV). We used 136 DNA sequences obtained from these samples along with samples from a public influenza sequence database for a phylogenetic assessment of hemagglutinin (HA) diversity in the United States. We analyzed sequences from all HA subtypes except H5, H7, H14 and H15 to examine genetic variation, exchange between Eurasia and North America, and geographic distribution of LPAIV in wild birds in the United States. This study confirms intercontinental exchange of some HA subtypes (including a newly documented H9 exchange event), as well as identifies subtypes that do not regularly experience intercontinental gene flow but have been circulating and evolving in North America for at least the past 20 years. These HA subtypes have high levels of genetic diversity with many lineages co-circulating within the wild birds of North America. The surveillance effort that provided these samples demonstrates that such efforts, albeit labor-intensive, provide important information about the ecology of LPAIV circulating in North America. | 2008 | no | gd of pathogen | NA | no | no |
| 2009 | 2017 | 43025 | Niu, C; Lister, HE; Nguyen, B; Wheeler, TA; Wright, RJ | 2008 | Resistance to Thielaviopsis basicola in the cultivated A genome cotton | Black root rot (BRR), incited by the soilborne pathogen Thielaviopsis basicola has the potential to cause significant economic loss in cotton (Gossypium spp.) production. Cultivated tetraploids of cotton (G. hirsutum and G. barbadense) are susceptible although resistant types have been identified in a possible tetraploid progenitor, G. herbaceum. Genetic mapping was used to detect the chromosomal locations of quantitative trait loci (QTL) that confer resistance to the BRR pathogen. A population of F(2) individuals (G. herbaceum x G. arboreum) and F(2:3) progeny families were examined. Phenotypic variation between resistant and susceptible reactions could be explained partly by three QTL. The BRR5.1, BRR9.1, and BRR13.1 QTL each explained 19.1, 10.3 and 8.5% of the total phenotypic variation, respectively. The combination of all three in a single genetic model explained 32.7% of the phenotypic variation. Comparative analysis was conducted on significant QTL regions to deduce the cotton-Arabidopsis synteny relationship and examine the correspondence between BRR QTL and Arabidopsis pathogen defense genes. Totally 20 Arabidopsis synteny segments corresponded within one of three BRR QTL regions. Each synteny segment contains many potential Arabidopsis candidate genes. A total of 624 Arabidopsis genes, including 22 pathogen defense and 36 stress response genes, could be placed within the syntenic regions corresponding to the BRR QTL. Fine mapping is needed to delineate each underlying BRR R-gene and possible Arabidopsis orthologs. Research and breeding activities to examine each QTL and underlying genes in Upland cotton (G. hirsutum) are ongoing. | 2009 | no | no good fitness measure, N pop differnt in GD? | NA | no | no |
| 2010 | 2017 | 43025 | Garcia-Gonzalez, F; Nunez, Y; Ponz, F; Roldan, ERS; Gomendio, M | 2005 | Paternity analysis in the golden egg bug using AFLPs: do the males preferentially accept their true genetic offspring? | 1. The evolution of parental care and intraspecific parasitism involve conflicts of interest between mothers and other potential care givers who contribute to enhance offspring survival. In the golden egg bug, Phyllomorpha laciniata Villers (Heteroptera: Coreidae), females lay eggs on conspecifics and on plants. The adaptive significance of egg carrying in this species has been the subject of some controversy, which can only be resolved by determining the genetic relationship between the eggs and the adult who carries them. This study examined whether male acceptance of true genetic offspring occurs with a higher frequency than that expected from random oviposition on conspecifics. 2. Paternity analysis, using Amplified Fragment Length Polymorphism (AFLP) markers, was conducted on eggs carried by males housed with field-mated females. 3. Out of the total number of eggs sired by males in the experimental groups, the proportion of eggs carried by males that were their true genetic offspring was 30.8%. 4. Monte Carlo methods show that the probability of a male accepting an egg that is his true genetic offspring is higher than expected if females dumped eggs on males at random. 5. These results suggest that paternal care plays an important role in the maintenance of male egg carrying in this species. In addition, the methodology developed may become useful in determining true genetic parents in other species in which neither the father nor the mother can be determined by observational methods. | 2010 | no | no good fitness measure, N pop differnt in GD? | NA | no | no |
| 2011 | 2017 | 43025 | Christelova, P; De Langhe, E; Hribova, E; Cizkova, J; Sardos, J; Husakova, M; Van den Houwe, I; Sutanto, A; Kepler, AK; Swennen, R; Roux, N; Dolezel, J | 2017 | Molecular and cytological characterization of the global Musa germplasm collection provides insights into the treasure of banana diversity | Bananas (Musa spp.) are one of the main fruit crops grown worldwide. With the annual production reaching 144 million tons, their production represents an important contribution to the economies of many countries in Asia, Africa, Latin-America and Pacific Islands. Most importantly, bananas are a staple food for millions of people living in the tropics. Unfortunately, sustainable banana production is endangered by various diseases and pests, and the breeding for resistant cultivars relies on a far too small base of genetic variation. Greater diversity needs to be incorporated in breeding, especially of wild species. Such work requires a large and thoroughly characterized germplasm collection, which also is a safe depository of genetic diversity. The largest ex situ Musa germplasm collection is kept at the International Transit Centre (ITC) in Leuven (Belgium) and currently comprises over 1500 accessions. This report summarizes the results of systematic cytological and molecular characterization of the Musa ITC collection. By December 2015, 630 accessions have been genotyped. The SSR markers confirmed the previous morphological based classification for 84% of ITC accessions analyzed. The remaining 16% of the genotyped entries may need field verification by taxonomist to decide if the unexpected classification by SSR genotyping was correct. The ploidy level estimation complements the molecular data. The genotyping continues for the entire ITC collection, including newly introduced accessions, to assure that the genotype of each accession is known in the largest global Musa gene bank. | 2011 | no | no good fitness measure, N pop differnt in GD? | NA | no | no |
| 2012 | 2017 | 43025 | FINCKH, MR; MUNDT, CC | 1992 | PLANT COMPETITION AND DISEASE IN GENETICALLY DIVERSE WHEAT POPULATIONS | The direct and indirect effects of plant genetic diversity on epidemics and the influence of disease on plant competition were investigated using the wheat (Triticum aestivum)/stripe rust (Puccinia striiformis) system. Replacement series consisting of a susceptible and a resistant wheat genotype or two wheat genotypes susceptible to different races of stripe rust were grown in the presence and absence of the pathogen. Stripe rust severity, number of seed heads, seed yield, and seed weight were determined separately for each wheat genotype in the mixtures and the pure stands. The frequency of susceptible genotypes in a mixture explained up to 67 % of the variation in disease severity. However, competitive interactions among plant genotypes sometimes appeared to alter susceptibility and obscured the relationship. In pure stands of single genotypes, disease severity explained between 52 and 58 % of the variation in seed yield. In mixtures, coefficients of determination were only 10 and 31 %, suggesting a strong influence of plant-plant interactions on seed yield. These results suggest that host-parasite coevolutionary models need to account for the strong effect that specific plant genotype combinations may have on disease severity and plant reproduction. | 2012 | no | crop | NA | no | no |
| 2013 | 2017 | 43025 | Du, ZY; Che, MZ; Li, GH; Chen, J; Quan, W; Guo, Y; Wang, Z; Ren, JD; Zhang, HY; Zhang, ZJ | 2015 | A QTL with major effect on reducing leaf rust severity on the short arm of chromosome 1A of wheat detected across different genetic backgrounds and diverse environments | Selection for QLr.cau - 1AS (a major QTL detected in wheat for reducing leaf rust severity) based on the DNA marker gpw2246 was as effective as selection for Lr34 based on cssfr5. Leaf rust is an important disease of wheat worldwide. Utilization of slow-rusting resistance constitutes a strategy to sustainably control this disease. The American wheat cultivar Luke exhibits slow leaf-rusting resistance at the adult plant stage. The objectives of this study were to detect and validate QTL for the resistance in Luke. Three winter wheat populations were used, namely, 149 recombinant inbred lines (RILs) derived from the cross Luke x Aquileja, 307 RILs from Luke x AQ24788-83, and 80 F-2:3 families selected from Lingxing66 x KA298. Aquileja and Lingxing66 are highly susceptible to leaf rust. AQ24788-83 shows high (susceptible) infection type but contains the slow-rusting gene Lr34 as diagnosed by the gene-specific marker cssfr5. KA298, an F-9 RIL selected from Luke x AQ24788-83, contains Lr34 and QLr.cau-1AS (a major QTL originated from Luke, this study). These wheats were evaluated for leaf rust in 12 field and greenhouse environments involving four locations and five seasons. Genotyping was done using simple sequence repeat (SSR) and diversity arrays technology markers. Of the detected QTLs, QLr.cau-1AS was significant consistently across all the genetic backgrounds, test environments, and likely a wide range of pathogen races. QLr.cau-1AS explained 22.3-55.2 % of leaf rust phenotypic variation, being comparable to Lr34 in effect size. A co-dominant SSR marker (gpw2246, http://wheat.pw.usda.gov/GG2/index.shtml) was identified to be tightly linked to QLr.cau-1AS. Selection based on gpw2246 for QLr.cau-1AS was as effective as the selection based on cssfr5 for Lr34. QLr.cau-1AS will be helpful for increasing the genetic diversity of slow leaf-rusting resistance in wheat breeding programs. | 2013 | no | no good fitness measure, N pop differnt in GD? | NA | no | no |
| 2014 | 2017 | 43025 | Azam, S; Rao, SB; Jakka, P; NarasimhaRao, V; Bhargavi, B; Gupta, VK; Radhakrishnan, G | 2016 | Genetic Characterization and Comparative Genome Analysis of Brucella melitensis Isolates from India | Brucellosis is the most frequent zoonotic disease worldwide, with over 500,000 new human infections every year. Brucella melitensis, the most virulent species in humans, primarily affects goats and the zoonotic transmission occurs by ingestion of unpasteurized milk products or through direct contact with fetal tissues. Brucellosis is endemic in India but no information is available on population structure and genetic diversity of Brucella spp. in India. We performed multilocus sequence typing of four B. melitensis strains isolated from naturally infected goats from India. For more detailed genetic characterization, we carried out whole genome sequencing and comparative genome analysis of one of the B. melitensis isolates, Bm IND1. Genome analysis identified 141 unique SNPs, 78 VNTRs, 51 Indels, and 2 putative prophage integrations in the Bm IND1 genome. Our data may help to develop improved epidemiological typing tools and efficient preventive strategies to control brucellosis. | 2014 | no | gd of pathogen | NA | no | no |
| 2015 | 2017 | 43025 | Thamm, DH; Grunerud, KK; Rose, BJ; Vail, DM; Bailey, SM | 2013 | DNA Repair Deficiency as a Susceptibility Marker for Spontaneous Lymphoma in Golden Retriever Dogs: A Case-Control Study | “There is accumulating evidence that an individual’s inability to accurately repair DNA damage in a timely fashion may in part dictate a predisposition to cancer. Dogs spontaneously develop lymphoproliferative diseases such as lymphoma, with the golden retriever (GR) breed being at especially high risk. Mechanisms underlying such breed susceptibility are largely unknown; however, studies of heritable cancer predisposition in dogs may be much more straightforward than similar studies in humans, owing to a high degree of inbreeding and more limited genetic heterogeneity. Here, we conducted a pilot study with 21 GR with lymphoma, 20 age-matched healthy GR and 20 age-matched healthy mixed-breed dogs (MBD) to evaluate DNA repair capability following exposure to either ionizing radiation (IR) or the chemical mutagen bleomycin. Inter-individual variation in DNA repair capacity was evaluated in stimulated canine lymphoctyes exposed in vitro utilizing the G2 chromosomal radiosensitivity assay to quantify clastogen-induced chromatid-type aberrations (gaps and breaks). Golden retrievers with lymphoma demonstrated elevated sensitivity to induction of chromosome damage following either challenge compared to either healthy GR or MBD at multiple doses and time points. Using the 75th percentile of chromatid breaks per 1,000 chromosomes in the MBD population at 4 hours post 1.0 Gy IR exposure as a benchmark to compare cases and controls, GR with lymphoma were more likely than healthy GR to be classified as”“sensitive”" (odds ratio = 21.2, 95% confidence interval 2.3-195.8). Furthermore, our preliminary findings imply individual (rather than breed) susceptibility, and suggest that deficiencies in heritable factors related to DNA repair capabilities may be involved in the development of canine lymphoma. These studies set the stage for larger confirmatory studies, as well as candidate-based approaches to probe specific genetic susceptibility factors." | 2015 | no | no good fitness measure, N pop differnt in GD? | NA | no | no |
| 2016 | 2017 | 43025 | Beccaro, GL; Torello-Marinoni, D; Binelli, G; Donno, D; Boccacci, P; Botta, R; Cerutti, AK; Conedera, M | 2012 | Insights in the chestnut genetic diversity in Canton Ticino (Southern Switzerland) | Castanea sativa Miller displays a high variability of morphological and ecological traits, vegetative and reproductive habits, nut morphology, wood characteristics, adaptability, and resistance to biotic and abiotic stresses. The present range of distribution of the species has been strongly influenced by human migrations and it is thought that Romans played a crucial role in the spread of the cultivation in Europe, although in some areas there is also evidence of local domestication and spontaneous spread of the tree after the last glacial period. In Switzerland chestnut stands are found mostly in the South (Canton Ticino), a region where the debate about the origin of the local germplasm, whether derived from the survival or spontaneous migration of C. sativa on the territory or due to the introduction of the tree during the Roman colonisation, is still open. This study aims contributing to depict the chestnut genetic situation of the species in Canton Ticino as a contribution to the debate about the native character of the species. The study area is located in Switzerland, on the southern slopes of the Alps, where 3 C. sativa populations were sampled and analysed at 9 SSR loci. Populations showed a high degree of diversity, as it is observed in most natural population of tree species: all nine SSR loci were polymorphic (no fixed alleles were detected), and genetic diversity, measured by expected heterozygosity, was high ranging in the populations between 0.647 and 0.721, on average. Results suggest that three homogeneous gene pools contributed to the formation of the 3 populations sampled. The genetic germplasm structuring of the analysed chestnut stands is very mild as confirmed by the relatively low level of genetic differentiation and divergence observed among sites. | 2016 | no | no good fitness measure | NA | no | no |
| 2017 | 2017 | 43025 | Menge, DM; Zhong, DB; Guda, T; Gouagna, L; Githure, J; Beier, J; Yan, GY | 2006 | Quantitative trait loci controlling refractoriness to Plasmodium falciparum in natural Anopheles gambiae mosquitoes from a malaria-endemic region in western Kenya | Natural anopheline populations exhibit much variation in ability to support malaria parasite development, but the genetic mechanisms underlying this variation are not clear. Previous studies in Mali, West Africa, identified two quantitative trait loci (QTL) in Anopheles gambiae mosquitoes that confer refractoriness (failure of oocyst development in mosquito midguts) to natural Plasmodium falciparum parasites. We hypothesize that new QTL may be involved in mosquito refractoriness to malaria parasites and that the frequency of natural refractoriness genotypes maybe higher in the basin region of Lake Victoria, East Africa, where malaria transmission intensity and parasite genetic diversity are among the highest in the world. Using field-derived F-2 isofemale families and microsatellite marker genotyping, two loci significantly affecting oocyst density were identified: one on chromosome 2 between markers AG2H135 and AG2H603 and the second on chromosome 3 near marker AG3H93. The first locus was detected in three of the five isofemale families studied and colocalized to the same region as Pen3 and pfin1 described in other studies. The second locus was detected in two of the five isofemale families, and it appears to be a new QTL. QTL on chromosome 2 showed significant additive effects while those on chromosome 3 exhibited significant dominant effects. Identification of P. falciparum-refractoriness QTL in natural An. gambiae mosquitoes is critical to the identification of the genes involved in malaria parasite transmission in nature and for understanding the coevolution between malaria parasites and mosquito vectors. | 2017 | no | No GD diff between pop | NA | no | no |
| 2018 | 2017 | 43025 | Siddle, HV; Kreiss, A; Eldridge, MDB; Noonan, E; Clarke, CJ; Pyecroft, S; Woods, GM; Belov, K | 2007 | Transmission of a fatal clonal tumor by biting occurs due to depleted MHC diversity in a threatened carnivorous marsupial | A fatal transmissible tumor spread between individuals by biting has emerged in the Tasmanian devil (Sarcophilus harrish), a carnivorous marsupial. Here we provide genetic evidence establishing that the tumor is clonal and therefore foreign to host devils. Thus, the disease is highly unusual because it is not just a tumor but also a tissue graft, passed between individuals without invoking an immune response. The MHC plays a key role in immune responses to both tumors and grafts. The most common mechanism of immune evasion by tumors is down-regulation of classical cell surface MHC molecules. Here we show that this mode of immune escape does not occur. However, because the tumor is a graft, it should still be recognized and rejected by the host’s immune system due to foreign cell surface antigens. Mixed lymphocyte responses showed a lack of alloreactivity between lymphocytes of different individuals in the affected population, indicating a paucity of MHC diversity. This result was verified by genotyping, providing a conclusive link between a loss of MHC diversity and spread of a disease through a wild population. This novel disease arose as a direct result of loss of genetic diversity and the aggressive behavior of the host species. The neoplastic clone continues to spread although the population, and, without active disease control by removal of affected animals and the isolation of disease-free animals, the Tasmanian devil faces extinction. | 2018 | no | No GD diff between pop | NA | no | no |
| 2019 | 2017 | 43025 | Puffenberger, EG | 2003 | Genetic heritage of the Old Order Mennonites of Southeastern Pennsylvania | The Old Order Mennonites of southeastern Pennsylvania are a religious isolate with origins in 16th-century Switzerland. The Swiss Mennonites immigrated to Pennsylvania over a 50-year period in the early 18th century. The history of this population in the United States provides insight into the increased incidence of several genetic diseases, most notably maple syrup urine disease (MSUD), Hirschsprung disease (HSCR), and congenital nephrotic syndrome. A comparison between the Old Order Mennonites and the Old Order Amish demonstrates the unique genetic heritage of each group despite a common religious and geographic history. Unexpectedly, several diseases in both groups demonstrate allelic and/or locus heterogeneity. The population genetics of the 1312T–>A BCKDHA gene mutation, which causes classical MSUD, are presented in detail. The incidence of MSUD in the Old Order Mennonites is estimated to be 1/358 births, yielding a corrected carrier frequency of 7.96% and a mutation allele frequency of 4.15%. Analysis of the population demonstrates that repeated cycles of sampling effects, population bottlenecks, and subsequent genetic drift were important in shaping the current allele frequencies. A linkage disequilibrium analysis of 1312T–>A mutation haplotypes is provided and discussed in the context of the known genealogical history of the population. Finally, data from microsatellite marker genotyping within the Old Order Mennonite population are provided that show a significant but modest decrease in genetic diversity and elevated levels of background linkage disequilibrium. (C) 2003 Wiley-Liss, Inc. | 2019 | no | NA | NA | no | no |
| 2020 | 2017 | 43025 | van Dijk, PJ | 2003 | Ecological and evolutionary opportunities of apomixis: insights from Taraxacum and Chondrilla | The ecological and evolutionary opportunities of apomixis in the short and the long term are considered, based on two closely related apomictic genera: Taraxacum (dandelion) and Chondrilla (skeleton weed). In both genera apomicts have a wider geographical distribution than sexuals, illustrating the short-term ecological success of apomixis. Allozymes and DNA markers indicate that apomictic populations are highly polyclonal. In Taraxacum, clonal diversity can be generated by rare hybridization between sexuals and apomicts, the latter acting as pollen donors. Less extensive clonal diversity is generated by mutations within clonal lineages. Clonal diversity may be maintained by frequency-dependent selection, caused by biological interactions (e.g. competitors and pathogens). Some clones are geographically widespread and probably represent phenotypically plastic ‘general-purpose genotypes’. The long-term evolutionary success of apomictic clones may be limited by lack of adaptive potential and the accumulation of deleterious mutations. Although apomictic clones may be considered as ‘evolutionary dead ends’, the genes controlling apomixis can escape from degeneration and extinction via pollen in crosses between sexuals and apomicts. In this way, apomixis genes are transferred to a new genetic background, potentially adaptive and cleansed from linked deleterious mutations. Consequently, apomixis genes can be much older than the clones they are currently contained in. The close phylogenetic relationship between Taraxacum and Chondrilla and the similarity of their apomixis mechanisms suggest that apomixis in these two genera could be of common ancestry. | 2020 | no | no good fitness measure, N pop differnt in GD? | NA | no | no |
| 2021 | 2017 | 43025 | Palmer, KA; Oldroyd, BP | 2003 | Evidence for intra-colonial genetic variance in resistance to American foulbrood of honey bees (Apis mellifera): further support for the parasite/pathogen hypothesis for the evolution of polyandry | Explanations for the evolution of multiple mating by social insect (particularly honey bee) queens have been frequently sought. An important hypothesis is that multiple mating is adaptive because it increases intracolonial genetic diversity and thereby reduces the likelihood that parasites or pathogens will catastrophically infect a colony. We tested one assumption of this model: that honey bee worker patrilines should differ in disease resistance. We used American foulbrood (caused by the bacterium Paenibacillus larvae) as a model pathogen. We found that patrilines within colonies do indeed vary in their resistance to this disease. | 2021 | no | No GD diff between pop | NA | no | no |
| 2022 | 2017 | 43025 | Smith, J; Strassmann, JE; Queller, DC | 2016 | Fine-scale spatial ecology drives kin selection relatedness among cooperating amoebae | Cooperation among microbes is important for traits as diverse as antibiotic resistance, pathogen virulence, and sporulation. The evolutionary stability of cooperation against cheater mutants depends critically on the extent to which microbes interact with genetically similar individuals. The causes of this genetic social structure in natural microbial systems, however, are unknown. Here, we show that social structure among cooperative Dictyostelium amoebae is driven by the population ecology of colonization, growth, and dispersal acting at spatial scales as small as fruiting bodies themselves. Despite the fact that amoebae disperse while grazing, all it takes to create substantial genetic clonality within multicellular fruiting bodies is a few millimeters distance between the cells colonizing a feeding site. Even adjacent fruiting bodies can consist of different genotypes. Soil populations of amoebae are sparse and patchily distributed at millimeter scales. The fine-scale spatial structure of cells and genotypes can thus account for the otherwise unexplained high genetic uniformity of spores in fruiting bodies from natural substrates. These results show how a full understanding of microbial cooperation requires understanding ecology and social structure at the small spatial scales microbes themselves experience. | 2022 | no | no good fitness measure | NA | no | no |
| 2023 | 2017 | 43025 | Gamfeldt, L; Kallstrom, B | 2007 | Increasing intraspecific diversity increases predictability in population survival in the face of perturbations | It has been proposed that biodiversity can be important for ecosystem functioning and act as an insurance against perturbations and environmental fluctuations. To date, theoretical work supports this idea but direct experimental evidence is still to some extent ambiguous and debated. The main reason for this debate - and the lack of strong empirical support - is due to unavoidable experimentally and statistically inherent variance reduction effects. Here we present the results of an experimental study that circumvents earlier hidden treatments. By random draw without replacement, we collected 180 full-sibling batches of an amphipod from a large pool of possible parents. Assembled amphipod populations with diversity levels ranging from one to ten were exposed to either a single perturbation (nutrient enrichment) or two combined perturbations (nutrient enrichment and desiccation). The results show that the variance in the number of surviving individuals decreased with increasing diversity in the combined perturbations treatment. Predictability in population survival thus seemed to be higher in more diverse assemblages. Our results, together with a simple model suggest that variance-decreasing effects can be due to actual real world statistical sampling effects of increasing diversity. | 2023 | yes | ok gd measurement and pop level comp? | NA | no | yes |
| 2024 | 2017 | 43025 | Borges, EC; Dujardin, JP; Schofield, CJ; Romanha, AJ; Diotaiuti, L | 2000 | Genetic variability of Triatoma brasiliensis (Hemiptera : Reduviidae) populations | Isoenzymes, random amplified polymorphic DNA. (RAPD), and morphometry were used to compare genetic variability within and between three populations of Triatoma brasiliensis Neiva, 1911 from northeast Brazil. The isoenzyme profiles were identical for the three populations, whereas the complex RAPD patterns allowed clear discrimination between the three. Morphometric analysis, using characters of the head capsule, also showed discrimination between the three populations but only in comparisons of males, not females. The results show considerable genetic heterogeneity in T. brasiliensis with an indication of geographic structuring possibly resulting from a recent series of domestication events. | 2024 | no | no good fitness measure, N pop differnt in GD? | NA | no | no |
| 2025 | 2017 | 43025 | Thareja, G; John, SE; Hebbar, P; Behbehani, K; Thanaraj, TA; Alsmadi, O | 2015 | Sequence and analysis of a whole genome from Kuwaiti population subgroup of Persian ancestry | “Background: The 1000 Genome project paved the way for sequencing diverse human populations. New genome projects are being established to sequence underrepresented populations helping in understanding human genetic diversity. The Kuwait Genome Project an initiative to sequence individual genomes from the three subgroups of Kuwaiti population namely, Saudi Arabian tribe;”“tent-dwelling”" Bedouin | 2025 | no | human | NA | no | no |
| 2026 | 2017 | 43025 | He, TH; Lamont, BB | 2014 | Genetic and ecological consequences of interactions between three banksias in mediterranean-type shrubland | Questions Recent research has revealed that genotypic diversity may have a similar role to species diversity in understanding competitive relations and ecological functioning of a community. Despite theoretical advancement in how competition and co-existence influence the pattern of genetic diversity between interacting species/genotypes, this topic has received little empirical attention. The key question is how the distribution of a given species/genotype is influenced by inter-specific interactions. Location A sclerophyll shrubland (kwongan) at Beekeepers Nature Reserve, 300km north of Perth, Western Australia. Methods We examined whether there are any genetic or ecological consequences arising from the interspersion of three Banksia species. We investigated patterns of local abundance, mortality after environmental stress (viz. severe drought) and microsatellite allelic richness for populations of the three co-occurring banksias distributed on the crests and slopes of a mosaic of sand dunes. Results Local abundance of the fire-killed Banksia hookeriana was positively correlated with that of the lignotuberous resprouter B.attenuata, which has similar shrub morphology, but negatively with that of the less similar clonal resprouter, B.candolleana. In contrast, population allelic richness of B.attenuata and B.hookeriana were negatively correlated, while allelic richness of B.candolleana and the other two banksias were uncorrelated. Positive correlation between local abundance of B.attenuata and B.hookeriana was likely a consequence of a similar response to the same environmental resources, while B.candolleana has greater environmental tolerances than the other banksias but a slower dispersal rate as it rarely produces seedlings. Negative correlation in allelic richness between the ecologically similar B.attenuata and B.hookeriana was likely the result of competition for niche space at the genotype level. While local exclusion of inferior genotypes might occur by competition, exclusion of entire species is avoided and stable co-existence is achieved. Conclusions Our results indicate that mechanisms of co-existence and isolation among plant species may operate at several genetic scales, and that subtle genotypic variation is of potential importance in maintaining co-existence among ecologically matched species. | 2026 | maybe | ok gd measurement and pop level comp? | no good data pop level | no | no |
| 2027 | 2017 | 43025 | Chavez, DJ; Beckman, TG; Werner, DJ; Chaparro, JX | 2014 | Genetic diversity in peach [Prunus persica (L.) Batsch] at the University of Florida: past, present and future | The University of Florida (UF) stone fruit breeding and genetics program was created in 1952 to develop early ripening stone fruit cultivars with high quality, adaptation to summer rainfall, low chilling requirements, and the ability to withstand high disease pressure. Diverse germplasm sources were used to introduce desirable traits in UF breeding pool. The main objective of this research was to determine the genetic diversity and population structure of the breeding germplasm, and to search for loci under selection. A total of 195 peach genotypes were used: UF cultivars and advanced selections (n = 168), cultivars and selections from the UF-UGA-USDA joint breeding effort (n = 13), landrace cultivars (n = 4), high-chilling cultivars released by NCSU (n = 5), and related Prunus (n = 5) species. A total of 36 SSR markers distributed across the peach genome amplified 423 alleles. An average of 18 genotypes were detected per marker: A (number of observed alleles) of 11.43, Ae (effective number of alleles) of 2.58, Ho (observed heterozygosity) of 0.4, He (expected heterozygosity) of 0.52, F (Wright’s fixation index) of 0.25, and PIC (polymorphism information content) of 0.48. UPGMA cluster analysis based on Nei’s genetic distance represented best the known pedigree information for the germplasm pools. Two major groups were observed across the germplasm corresponding to melting and non-melting flesh cultivars/selections. Population structure results supported these two major groups. Several loci closely located to genome regions where different phenotypic traits have been previously mapped were detected to be under selection. | 2027 | no | no good fitness measure | NA | no | no |
| 2028 | 2017 | 43025 | Brochet, M; Rusniok, C; Couve, E; Dramsi, S; Poyarts, C; Trieu-Cuot, P; Kunst, F; Glaser, P | 2008 | Shaping a bacterial genome by large chromosomal replacements, the evolutionary history of Streptococcus agalactiae | Bacterial populations are subject to complex processes of diversification that involve mutation and horizontal DNA transfer mediated by transformation, transduction, or conjugation. Tracing the evolutionary events leading to genetic changes allows us to infer the history of a microbe. Here, we combine experimental and in silico approaches to explore the forces that drive the genome dynamics of Streptococcus agalactiae, the leading cause of neonatal infections. We demonstrate that large DNA segments of up to 334 kb of the chromosome of S. agalactiae can be transferred through conjugation from multiple initiation sites. Consistently, a genome-wide map analysis of nucleotide polymorphisms among eight human isolates demonstrated that each chromosome is a mosaic of large chromosomal fragments from different ancestors suggesting that large DNA exchanges have contributed to the genome dynamics in the natural population. The analysis of the resulting genetic flux led us to propose a model for the evolutionary history of this species in which clonal complexes of clinical importance derived from a single clone that evolved by exchanging large chromosomal regions with more distantly related strains. The emergence of this clone could be linked to selective sweeps associated with the reduction of genetic diversity in three regions within a large panel of human isolates. Up to now sex in bacteria has been assumed to involve mainly small regions; our results define S. agalactiae as an alternative paradigm in the study of bacterial evolution. | 2028 | no | gd of pathogen | NA | no | no |
| 2029 | 2017 | 43025 | Westergaard, KB; Alsos, IG; Popp, M; Engelskjon, T; Flatberg, KI; Brochmann, C | 2011 | Glacial survival may matter after all: nunatak signatures in the rare European populations of two west-arctic species | Biogeographers claimed for more than a century that arctic plants survived glaciations in ice-free refugia within the limits of the North European ice sheets. Molecular studies have, however, provided overwhelming support for postglacial immigration into northern Europe, even from the west across the Atlantic. For the first time we can here present molecular evidence strongly favouring in situ glacial persistence of two species, the rare arctic-alpine pioneer species Sagina caespitosa and Arenaria humifusa. Both belong to the ‘west-arctic element’ of amphi-Atlantic disjuncts, having their few and only European occurrences well within the limits of the last glaciation. Sequencing of non-coding regions of chloroplast DNA revealed only limited variation. However, two very distinct and partly diverse genetic groups, one East and one West Atlantic, were detected in each species based on amplified fragment length polymorphisms (AFLPs), excluding postglacial dispersal from North America as explanation for their European occurrences. Patterns of genetic diversity and distinctiveness indicate that glacial populations existed in East Greenland and/or Svalbard (A. humifusa) and in southern Scandinavia (S. caespitosa). Despite their presumed lack of long-distance dispersal adaptations, intermixed populations in several regions indicate postglacial contact zones. Both species are declining in Nordic countries, probably due to climate change-induced habitat loss. Little or no current connectivity between their highly fragmented and partly distinct populations call for conservation of several populations in each geographic region. | 2029 | no | no good fitness measure, N pop differnt in GD? | NA | no | no |
| 2030 | 2017 | 43025 | FRANK, SA | 1994 | COEVOLUTIONARY GENETICS OF HOSTS AND PARASITES WITH QUANTITATIVE INHERITANCE | A model of host-parasite coevolution is analysed. A host resistance trait and a parasite virulence trait interact to determine the outcome of a parasitic attack, where each trait is determined by quantitative genetic variation. The resistance and virulence traits are assumed to have a fitness cost. Each host and parasite genotype is treated as a separate ‘species’ in a multidimensional Lotka-Volterra system in which the numerical abundance of each genotype is free to change. Thus, the epidemiological effects of fluctuating population sizes are analysed jointly with changes in genotype frequencies. Population sizes fluctuate increasingly as the parasites’ reproductive capacity increases and as resistance and virulence benefits per unit cost decline. The patterns of genetic variability depend mainly on the stability of population sizes and on the shape of the relationship between the costs and benefits of a trait. | 2030 | no | No GD diff between pop | NA | no | no |
| 2031 | 2017 | 43025 | Cooper, D; Cory, JS; Myers, JH | 2003 | Hierarchical spatial structure of genetically variable nucleopolyhedroviruses infecting cyclic populations of western tent caterpillars | The cyclic population dynamics of western tent caterpillars, Malacosoma californicum pluviale , are associated with epizootics of a nucleopolyhedrovirus, McplNPV. Given the dynamic fluctuations in host abundance and levels of viral infection, host resistance and virus virulence might be expected to change during different phases of the cycle. As a first step in determining if McplNPV virulence and population structure change with host density, we used restriction fragment length polymorphism (RFLP) analysis to examine the genetic diversity of McplNPV infecting western tent caterpillar populations at different spatial scales. Thirteen dominant genetic variants were identified in 39 virus isolates (individual larvae) collected from field populations during one year of low host density, and another distinct variant was discovered among nine additional isolates in two subsequent years of declining host density. The distribution of these genetic variants was not random and indicated that the McplNPV population was structured at several spatial levels. A high proportion of the variation could be explained by family grouping, which suggested that isolates collected within a family were more likely to be the same than isolates compared among populations. Additionally, virus variants from within populations (sites) were more likely to be the same than isolates collected from tent caterpillar populations on different islands. This may indicate that there is limited mixing of virus among tent caterpillar families and populations when host population density is low. Thus there is potential for the virus to become locally adapted to western tent caterpillar populations in different sites. However, no dominant genotype was observed at any site. Whether and how selection acts on the genetically diverse nucleopolyhedrovirus populations as host density changes will be investigated over the next cycle of tent caterpillar populations. | 2031 | no | gd of pathogen | NA | no | no |
| 2032 | 2017 | 43025 | LAMBIN, X; KREBS, CJ | 1993 | Influence of Female Relatedness on the Demography of Townsend’s Vole Populations in Spring | 1. The relatedness of individuals can influence their behaviour and changes in the degree of female relatedness in spring may influence the demography of vole populations (Lambin & Krebs 1991a). Here we report on an experiment testing the effect of relatedness on the demography of Townsend’s vole (Microtus townsendii) populations (kin and non-kin treatments) over three consecutive springs. 2. Most nestlings were marked before weaning such that we knew which recruits were related to one another and to which adult female. The average size of matrilineal groups was experimentally manipulated by selectively removing voles of known origin and by preventing predation by birds of prey. 3. Nearest neighbours had their nests closer to one another on the kin treatment, but there were no differences in home range size between treatments. Home-ranges of females present on both treatments were smaller in a spring of high density than in the springs with lower density. 4. Female survival was better on the kin treatment. Within the kin treatment, females with at least one first degree relative as a neighbour survived better than females without such a relative. No such difference was observed within the non-kin treatment. 5. Females in the kin treatment gave birth to their first spring litter 5-7 days later than females in the non-kin treatment, possibly as a consequence of space sharing with related neighbours. The weaning success of the first spring litter was significantly higher on the kin tratment than on the non-kin treatment, but there were no differences in the weaning success of females with and without related neighbours within treatments. The presence of immigrant females on the non-kin treatment and not on the kin treatment may have caused the difference in weaning success between the treatments. 6. We conclude that female relatedness influences the pattern of space use and the survival of females in spring. The presence of immigrant females attempting to establish in the population has a pronounced effect on the weaning success of residents. | 2032 | probably yes | fitness and gd pop level? | NA | no | yes |
| 2033 | 2017 | 43025 | Jumbo-Lucioni, P; Bu, S; Harbison, ST; Slaughter, JC; Mackay, TFC; Moellering, DR; De Luca, M | 2012 | Nuclear genomic control of naturally occurring variation in mitochondrial function in Drosophila melanogaster | Background: Mitochondria are organelles found in nearly all eukaryotic cells that play a crucial role in cellular survival and function. Mitochondrial function is under the control of nuclear and mitochondrial genomes. While the latter has been the focus of most genetic research, we remain largely ignorant about the nuclear-encoded genomic control of inter-individual variability in mitochondrial function. Here, we used Drosophila melanogaster as our model organism to address this question. Results: We quantified mitochondrial state 3 and state 4 respiration rates and P:O ratio in mitochondria isolated from the thoraces of 40 sequenced inbred lines of the Drosophila Genetic Reference Panel. We found significant within-population genetic variability for all mitochondrial traits. Hence, we performed genome-wide association mapping and identified 141 single nucleotide polymorphisms (SNPs) associated with differences in mitochondrial respiration and efficiency (P <= 1 x 10(-5)). Gene-centered regression models showed that 2-3 SNPs can explain 31, 13, and 18% of the phenotypic variation in state 3, state 4, and P: O ratio, respectively. Most of the genes tagged by the SNPs are involved in organ development, second messenger-mediated signaling pathways, and cytoskeleton remodeling. One of these genes, sallimus (s/s), encodes a component of the muscle sarcomere. We confirmed the direct effect of s/s on mitochondrial respiration using two viable mutants and their coisogenic wild-type strain. Furthermore, correlation network analysis revealed that s/s functions as a transcriptional hub in a co-regulated module associated with mitochondrial respiration and is connected to CG7834, which is predicted to encode a protein with mitochondrial electron transfer flavoprotein activity. This latter finding was also verified in the s/s mutants. Conclusions: Our results provide novel insights into the genetic factors regulating natural variation in mitochondrial function in D. melanogaster. The integrative genomic approach used in our study allowed us to identify s/s as a novel hub gene responsible for the regulation of mitochondrial respiration in muscle sarcomere and to provide evidence that s/s might act via the electron transfer flavoprotein/ubiquinone oxidoreductase complex. | 2033 | no | no good fitness measure, N pop differnt in GD? | NA | no | no |
| 2034 | 2017 | 43025 | Verdu, P; Barreiro, LB; Gessain, A; Cassar, O; Kidd, JR; Kidd, KK; Behar, DM; Froment, A; Heyer, E; Sica, L; Casanova, JL; Abel, L; Quintana-Murci, L; Quintana-Murci, L | 2006 | Evolutionary insights into the high worldwide prevalence of MBL2 deficiency alleles | Human mannose-binding lectin (MBL) is a member of the collectin protein family that binds a broad range of microorganisms and activates the lectin-complement pathway of innate immunity. Common alleles of MBL2 disrupt the MBL protein or modulate the amount of protein produced, resulting in MBL deficiency. The clinical manifestations of MBL deficiency have been extensively studied but the actual role of this lectin in immunity to infection remains a matter of strong debate. MBL is commonly thought to play a key role in protective immunity, because MBL deficiency has been associated with an increase in susceptibility to infectious diseases. However, the high worldwide prevalence of multiple MBL2 deficiency or low-producing alleles suggests the converse that MBL deficiency confers protection. To explore the underlying forces accounting for the high worldwide prevalence of MBL2 deficiency alleles, we characterized genetic diversity in and around the MBL2 genomic region in 1166 chromosomes from 24 worldwide populations. Our results clearly demonstrate that the patterns of MBL2 variation are compatible with neutral evolution, as opposed to negative, positive or balanced natural selection. The high worldwide frequencies of MBL2 alleles associated with the production of little or no protein therefore result exclusively from human migration and genetic drift. The evolutionary neutrality of MBL2 strongly supports the notion that MBL2 variation does not have strong effects on population fitness, suggesting, therefore, that this lectin is largely redundant in host human defences. | 2034 | no | NA | NA | no | no |
| 2035 | 2017 | 43025 | Cohen, TM; King, R; Dolev, A; Boldo, A; Lichter-Peled, A; Bar-Gal, GK | 2013 | Genetic characterization of populations of the golden jackal and the red fox in Israel | The golden jackal and red fox are among the wildlife species protected by Israeli law as enforced by the Israel Nature and Parks Authority. In 1964, as a part of a management program to control rabies in Israel, a poison eradication campaign was launched to exterminate golden jackals, considered to be the main reservoir of the disease. The program resulted in the near-complete extermination of jackals in Israel, while foxes were only mildly affected. Jackals have since regained their original numbers and have recolonized southern Israel. We here examined the population structure of the golden jackal and red fox in Israel, 48 years after the poison eradication campaign. DNA from 88 golden jackals and 89 red foxes representing five different geographic regions was extracted and amplified at 13 microsatellite loci in order to characterize the populations on a genetic level. High genetic diversity was found among the jackal and fox populations. A possible migration route through the Jordan Rift Valley was suggested for both species by the genetic similarity of populations in northern and southern Israel. However, in both species, the animals from the center of Israel were distinctive from those north or south, indicating the relative isolation of central populations, likely due to fragmentation or a high abundance of food resources. Genetic profiles obtained for the golden jackal and the red fox in Israel may aid in their conservation management and in the study of zoonotic diseases. | 2035 | no | no good fitness measure | NA | no | no |
| 2036 | 2017 | 43025 | Schnaithmann, F; Kopahnke, D; Pillen, K | 2014 | A first step toward the development of a barley NAM population and its utilization to detect QTLs conferring leaf rust seedling resistance | We suggest multi-parental nested association mapping as a valuable innovation in barley genetics, which increases the power to map quantitative trait loci and assists in extending genetic diversity of the elite barley gene pool. Plant genetic resources are a key asset to further improve crop species. The nested association mapping (NAM) approach was introduced to identify favorable genes in multi-parental populations. Here, we report toward the development of the first explorative barley NAM population and demonstrate its usefulness in a study on mapping quantitative trait loci (QTLs) for leaf rust resistance. The NAM population HEB-5 was developed from crossing and backcrossing five exotic barley donors with the elite barley cultivar ‘Barke,’ resulting in 295 NAM lines in generation BC1S1. HEB-5 was genetically characterized with 1,536 barley SNPs. Across HEB-5 and within the NAM families, no deviation from the expected genotype and allele frequencies was detected. Genetic similarity between ‘Barke’ and the NAM families ranged from 78.6 to 83.1 %, confirming the backcrossing step during population development. To explore its usefulness, a screen for leaf rust (Puccinia hordei) seedling resistance was conducted. Resistance QTLs were mapped to six barley chromosomes, applying a mixed model genome-wide association study. In total, four leaf rust QTLs were detected across HEB-5 and four QTLs within family HEB-F23. Favorable exotic QTL alleles reduced leaf rust symptoms on two chromosomes by 33.3 and 36.2 %, respectively. The located QTLs may represent new resistance loci or correspond to new alleles of known resistance genes. We conclude that the exploratory population HEB-5 can be applied to mapping and utilizing exotic QTL alleles of agronomic importance. The NAM concept will foster the evaluation of the genetic diversity, which is present in our primary barley gene pool. | 2036 | no | no good fitness measure, N pop differnt in GD? | NA | no | no |
| 2037 | 2017 | 43025 | Bryson, RW; Prendini, L; Savary, WE; Pearman, PB | 2014 | Caves as microrefugia: Pleistocene phylogeography of the troglophilic North American scorpion Pseudouroctonus reddelli | Background: Survival in microrefugia represents an important paradigm in phylogeography for explaining rapid postglacial re-colonization by species in temperate regions. Microrefugia may allow populations to persist in areas where the climatic conditions on the surface have become unfavourable. Caves generally contain stable microclimates and may represent microrefugia for species capable of exploiting both cave and surface habitats (troglophiles). We examine the phylogeography of the troglophilic North American vaejovid scorpion Pseudouroctonus reddelli using 1,993 base pairs of mitochondrial and nuclear DNA sequence data generated from 12 populations. We use (i) descriptive measures of genetic diversity and population genetics statistics, (ii) reconstructions of phylogeographical structure, spatial diffusion during diversification, and population sizes through time, and (iii) species distribution modelling to test predictions of the hypothesis that caves serve as microrefugia. We compare phylogeographical patterns in P. reddelli with other troglophilic species across the Edwards Plateau karst region of Texas. Results: Results revealed high haplotype and nucleotide diversity and substantial phylogeographical structure, probably generated during the Pleistocene. Spatial diffusion occurred along the southern edge of the Edwards Plateau from multiple refugia along the Balcones Escarpment. There was little evidence for population and geographical expansion. Species distribution models predicted substantial reductions in suitable epigean habitat for P. reddelli at the Last Glacial Maximum (LGM). Conclusions: High genetic diversity, strong phylogeographical structure, diffusion from multiple refugia, and unfavourable climatic conditions at the LGM collectively support the hypothesis that caves served as microrefugia for P. reddelli. Similar patterns of genetic structure in P. reddelli and other troglophilic species across the Edwards Plateau karst region of Texas suggest that caves serving as microrefugia are important for the formation, maintenance, and future survival of troglophilic species in temperate karst regions. | 2037 | no | no good fitness measure | NA | no | no |
| 2038 | 2017 | 43025 | Cronin, AL; Monnin, T; Sillam-Dusses, D; Aubrun, F; Federici, P; Doums, C | 2016 | Qualitative bias in offspring investment in a superorganism is linked to dispersal and nest inheritance | How parents invest resources in offspring is a central aspect of life history. While investment strategies have been well studied in solitary organisms, comparatively little has been done on social species, including the many that reproduce by fission. Under colony fission, a parent colony divides resources (individuals) to form two or more offspring colonies. Because individuals differ in characteristics (e.g. size), there is opportunity for both quantitative and qualitative bias in their allocation. In this study we investigated the qualitative aspect of offspring investment during colony fission. Colonies of the ant Cataglyphis cursor fission into multiple offspring colonies as part of their lifecycle, and the distribution of workers is quantitatively biased. We found that investment is also qualitatively biased in terms of worker size and worker genetic characteristics (patrilines). This bias was mainly between the offspring colony that inherited the original nest and offspring colonies that dispersed to new nesting sites. In 74% of cases, dispersing colonies contained larger workers, and the distribution of genetic patrilines was biased in two of six cases in a manner that cannot be explained by the observed variation in worker size between patrilines. Fission also led to a reduction in diversity in offspring colonies compared to the parent colony, in terms of both worker size (70% of cases) and genetic diversity (40% of cases). These patterns are probably the result of differing dispersal probability between workers of different patrilines and of different size. This differential allocation may be adaptive because larger workers may be of disproportionate value to dispersing colonies, and their loss an acceptable cost to the colony inheriting the nest. (C) 2016 The Association for the Study of Animal Behaviour. Published by Elsevier Ltd. All rights reserved. | 2038 | no | no good fitness measure, N pop differnt in GD? | NA | no | no |
| 2039 | 2017 | 43025 | Bailey, L | 1999 | The quest for a super-bee | Highly intensive selection of the honey bee for any quality may decrease resistance to its wide variety of enzootic pathogens by decreasing its genetic variability. Maintenance of naturally adapted regional strains, by traditional means, and management that least inhibits their essentially independent lifestyle may be move rewarding. | 2039 | no | no good fitness measure, N pop differnt in GD? | NA | no | no |
| 2040 | 2017 | 43025 | Dastjerdi, A; Robert, C; Watson, M | 2014 | Low coverage sequencing of two Asian elephant (Elephas maximus) genomes | Background: There are three species of elephant that exist, the Asian elephant (Elephas maximus) and two species of African elephant (Loxodonta africana and Loxodonta cyclotis). The populations of all three species are dwindling, and are under threat due to factors, such as habitat destruction and ivory hunting. The species differ in many respects, including in their morphology and response to disease. The availability of elephant genome sequence data from all three elephant species will complement studies of behaviour, genetic diversity, evolution and disease resistance. Findings: We present low-coverage Illumina sequence data from two Asian elephants, representing approximately 5X and 2.5X coverage respectively. Both raw and aligned data are available, using the African elephant (L. africana) genome as a reference. Conclusions: The data presented here are an important addition to the available genetic and genomic information on Asian and African elephants. | 2040 | no | no good fitness measure, N pop differnt in GD? | NA | no | no |
| 2041 | 2017 | 43025 | Laurent, B; Moinard, M; Spataro, C; Ponts, N; Barreau, C; Foulongne-Oriol, M | 2017 | Landscape of genomic diversity and host adaptation in Fusarium graminearum | Background: Fusarium graminearum is one of the main causal agents of the Fusarium Head Blight, a worldwide disease affecting cereal cultures, whose presence can lead to contaminated grains with chemically stable and harmful mycotoxins. Resistant cultivars and fungicides are frequently used to control this pathogen, and several observations suggest an adaptation of F. graminearum that raises concerns regarding the future of current plant disease management strategies. To understand the genetic basis as well as the extent of its adaptive potential, we investigated the landscape of genomic diversity among six French isolates of F. graminearum, at single-nucleotide resolution using whole-genome re-sequencing. Results: A total of 242,756 high-confidence genetic variants were detected when compared to the reference genome, among which 96% are single nucleotides polymorphisms. One third of these variants were observed in all isolates. Seventy-seven percent of the total polymorphism is located in 32% of the total length of the genome, comprising telomeric/subtelomeric regions as well as discrete interstitial sections, delineating clear variant enriched genomic regions-7.5 times in average. About 80% of all the F. graminearum protein-coding genes were found polymorphic. Biological functions are not equally affected: genes potentially involved in host adaptation are preferentially located within polymorphic islands and show greater diversification rate than genes fulfilling basal functions. We further identified 29 putative effector genes enriched with non-synonymous effect mutation. Conclusions: Our results highlight a remarkable level of polymorphism in the genome of F. graminearum distributed in a specific pattern. Indeed, the landscape of genomic diversity follows a bi-partite organization of the genome according to polymorphism and biological functions. We measured, for the first time, the level of sequence diversity for the entire gene repertoire of F. graminearum and revealed that the majority are polymorphic. Those assumed to play a role in host-pathogen interaction are discussed, in the light of the subsequent consequences for host adaptation. The annotated genetic variants discovered for this major pathogen are valuable resources for further genetic and genomic studies. | 2041 | no | gd of pathogen | NA | no | no |
| 2042 | 2017 | 43025 | Vincenzi, S; Mangel, M; Jesensek, D; Garza, JC; Crivelli, AJ | 2017 | Genetic and life-history consequences of extreme climate events | Climate change is predicted to increase the frequency and intensity of extreme climate events. Tests on empirical data of theory-based predictions on the consequences of extreme climate events are thus necessary to understand the adaptive potential of species and the overarching risks associated with all aspects of climate change. We tested predictions on the genetic and life-history consequences of extreme climate events in two populations of marble trout Salmo marmoratus that have experienced severe demographic bottlenecks due to flash floods. We combined long-term field and genotyping data with pedigree reconstruction in a theory-based framework. Our results show that after flash floods, reproduction occurred at a younger age in one population. In both populations, we found the highest reproductive variance in the first cohort born after the floods due to a combination of fewer parents and higher early survival of offspring. A small number of parents allowed for demographic recovery after the floods, but the genetic bottleneck further reduced genetic diversity in both populations. Our results also elucidate some of the mechanisms responsible for a greater prevalence of faster life histories after the extreme event. | 2042 | no | no good fitness measure | NA | no | no |
| 2043 | 2017 | 43025 | Mitra, AK; Singh, A; Rath, SK | 2014 | LIG1 polymorphisms: the Indian scenario | Elucidation of the genetic diversity and relatedness of the subpopulations of India may provide a unique resource for future analysis of genetic association of several critical community-specific complex diseases. We performed a comprehensive exploration of single nucleotide polymorphisms (SNPs) within the gene DNA ligase 1 (LIG1) among a multiethnic panel of Indian subpopulations representative of the ethnic, linguistic and geographical diversity of India using a two-stage design involving DNA resequencing-based SNP discovery followed by SNP validation using sequenom-based genotyping. Thirty SNPs were identified in LIG1 gene using DNA resequencing including three promoter SNPs and one coding SNP. Following SNP validation, the SNPs rs20580/C19008A and rs3730862/C8804T were found to have the most widespread prevalence with noticeable variations in minor allele frequencies both between the Indian subpopulation groups and also from those reported on other major world populations. Subsequently, SNPs found in Indian subpopulations were analysed using bioinformatics-based approaches and compared with SNP data available on major world populations. Further, we also performed genotype-phenotype association analysis of LIG1 SNPs with publicly available data on LIG1 mRNA expression in HapMap samples. Results showed polymorphisms in LIG1 affect its expression and may therefore change its function. Our results stress upon the uniqueness of the Indian population with respect to the worldwide scenario and suggest that any epidemiological study undertaken on the global population should take this distinctiveness in consideration and avoid making generalized conclusions. | 2043 | no | human | NA | no | no |
| 2044 | 2017 | 43025 | Zeisset, I; Beebee, TJC | 2003 | Population genetics of a successful invader: the marsh frog Rana ridibunda in Britain | We investigated the genetic outcome of successful invasion by an alien species, the marsh frog Rana ridibunda , in Britain. Twelve adults translocated from Hungary into Kent (Romney) in 1935 resulted rapidly in a large localized population. A further successful translocation in 1973 from Romney to Sussex (Lewes), together with other range extensions, provided an opportunity to test bottleneck effects during colonization events. Romney and Lewes frogs had similar genetic diversities to those in Hungary at 14 random amplified polymorphic DNA marker (RAPD) and five microsatellite loci. The introduced populations were, however, differentiated genetically from each other and from a reference population in Hungary. Fitness assessments (larval growth and survival) revealed no differences between the Lewes and Romney populations. Despite starting with few founders, significant bottleneck effects on R. ridibunda in Britain were therefore undetectable, presumably because population expansions were rapid immediately after the translocations. | 2044 | no | only one pop | NA | no | no |
| 2045 | 2017 | 43025 | Amer, S; Abd El Wahab, T; Metwaly, A; Ye, JB; Roellig, D; Feng, YY; Xiao, LH | 2014 | Preliminary Molecular Characterizations of Sarcoptes scaibiei (Acari: Sarcoptidae) from Farm Animals in Egypt | Little is known about the genetic diversity of Sarcoptes scabiei mites in farm animals in Egypt. In this study, we characterized S. scabiei in 25 skin scrapes from water buffalo, cattle, sheep, and rabbits at the nuclear marker ITS2 and mitochondrial markers COX1 and 16S rRNA. Sequences of the ITS2 showed no host segregation or geographical isolation, whereas those of the mitochondrial COX1 and 16S rRNA genes indicated the presence of both host-adapted and geographically segregated populations of S. scabiei. Host adaptation may limit inter-species transmission of. S. scabiei, thus restrict gene flow among S. scabiei from different hosts. This is the first report on the molecular characterization of sarcoptic mites in Egypt. Further genetic studies involving larger numbers of specimens, especially those from humans and companion animals, are needed to understand the molecular epidemiology of sarcoptic mange in Egypt. | 2045 | no | gd of pathogen | NA | no | no |
| 2046 | 2017 | 43025 | Kidd, JM; Trevarthen, KC; Tefft, DL; Cheng, Z; Mooney, M; Adams, MD | 2005 | A catalog of nonsynonymous polymorphism on mouse Chromosome 16 | Numerous phenotypic traits differ among inbred mice, and the genetic diversity of inbred strains has been exploited in studies of quantitative trait loci (QTL). Sequencing the mouse genome has resulted in improved tools for the study of QTL, but a comprehensive catalog of sequence variants between strains would be of great value in identifying and testing potentially causative alleles. A/J DNA was included in the Celera shotgun sequence of the mouse genome and C57BL/6 DNA was sequenced by an international consortium. We have resequenced A/J and B6 DNA to cover nearly all of the protein-coding portions of mouse Chromosome 16, revealing that there are 106 nonsynonymous substitutions in 74 of the 779 genes on the chromosome. The pattern of substitution is more similar to the spectrum of benign polymorphism in the human population than it is to human disease-causing mutations. In mouse, polymorphic variants tend to be associated with one another on large haplotypes; this pattern also holds true for nonsynonymous polymorphism. However, sufficient fragmentation of haplotypes is present to suggest that only a very-high-resolution haplotype map will enable effective inference of alleles in additional strains. | 2046 | no | no good fitness measure | NA | no | no |
| 2047 | 2017 | 43025 | Turner, JW; Paranjpye, RN; Landis, ED; Biryukov, SV; Gonzalez-Escalona, N; Nilsson, WB; Strom, MS | 2013 | Population Structure of Clinical and Environmental Vibrio parahaemolyticus from the Pacific Northwest Coast of the United States | Vibrio parahaemolyticus is a common marine bacterium and a leading cause of seafood-borne bacterial gastroenteritis worldwide. Although this bacterium has been the subject of much research, the population structure of cold-water populations remains largely undescribed. We present a broad phylogenetic analysis of clinical and environmental V. parahaemolyticus originating largely from the Pacific Northwest coast of the United States. Repetitive extragenic palindromic PCR (REP-PCR) separated 167 isolates into 39 groups and subsequent multilocus sequence typing (MLST) separated a subset of 77 isolates into 24 sequence types. The Pacific Northwest population exhibited a semi-clonal structure attributed to an environmental clade (ST3, N = 17 isolates) clonally related to the pandemic O3:K6 complex and a clinical clade (ST36, N = 20 isolates) genetically related to a regionally endemic O4:K12 complex. Further, the identification of at least five additional clinical sequence types (i.e., ST43, 50, 65, 135 and 417) demonstrates that V. parahaemolyticus gastroenteritis in the Pacific Northwest is polyphyletic in nature. Recombination was evident as a significant source of genetic diversity and in particular, the recA and dtdS alleles showed strong support for frequent recombination. Although pandemic-related illnesses were not documented during the study, the environmental occurrence of the pandemic clone may present a significant threat to human health and warrants continued monitoring. It is evident that V. parahaemolyticus population structure in the Pacific Northwest is semi-clonal and it would appear that multiple sequence types are contributing to the burden of disease in this region. | 2047 | no | gd of pathogen | NA | no | no |
| 2048 | 2017 | 43025 | Fjerdingstad, EJ; Keller, L | 2004 | Relationships between phenotype, mating behavior, and fitness of queens in the ant Lasius niger | Considerable attention has focused on why females of many species mate with several males. For social hymenopteran insects, efforts have primarily concentrated on determining whether multiple mating increases colony performance due to the increased genetic diversity. Most of these studies are correlative because it is difficult or impossible to experimentally mate queens in most species. Thus, the positive associations found between multiple paternity and colony fitness in some cases may not be due to direct effects of genetic diversity but could, in theory, arise from high-quality queens having more mates. Here we show that in the ant Lasius niger variation in the number of matings covaries with queen phenotype. Young queens that were heavier at the time of the mating flight were significantly more likely to mate with several males. As a result, heavier queens stored more sperm. The initial weight of queens was significantly associated with the probability of surviving mating flights during the two years of the study, with queens of intermediate weight having the highest across-year survival. Queen initial weight was also significantly and positively associated with the quantity of brood at the time of the first worker eclosion as well as colony productivity at the time of hibernation. By contrast, there was little evidence for a positive effect of the number of matings on colony performance when the effect of mate number and queen initial weight were considered simultaneously. | 2048 | no | no good fitness measure, N pop differnt in GD? | NA | no | no |
| 2049 | 2017 | 43025 | Arnaud-Haond, S; Migliaccio, M; Diaz-Almela, E; Teixeira, S; van de Vliet, MS; Alberto, F; Procaccini, G; Duarte, CM; Serrao, EA | 2007 | Vicariance patterns in the Mediterranean Sea: east-west cleavage and low dispersal in the endemic seagrass Posidonia oceanica | Aim The seagrass, Posidonia oceanica is a,clonal angiosperm endemic to the Mediterranean Sea. Previous studies have suggested that clonal growth is far greater than sexual recruitment and thus leads to low clonal diversity within meadows. However, recently developed microsatellite markers indicate that there are many different genotypes, and therefore many distinct clones present. The low resolution of markers used in the past limited our ability to estimate clonality and assess the individual level. New high-resolution dinucleotide microsatellites now allow genetically distinct individuals to be identified, enabling more reliable estimation of population genetic parameters across the Mediterranean Basin. We investigated the biogeography and dispersal of P. oceanica at various spatial scales in order to assess the influence of different evolutionary factors shaping the distribution of genetic diversity in this species. Location The Mediterranean. Methods We used seven hypervariable microsatellite markers, in addition to the five previously existing markers, to describe the spatial distribution of genetic variability in 34 meadows spread throughout the Mediterranean, on the basis of an average of 35.6 ( +/- 6.3) ramets sampled. Results At the scale of the Mediterranean Sea as a whole, a strong east-west cleavage was detected (AMOVA). These results are in line with those obtained using previous markers. The new results showed the presence of a putative secondary contact zone at the Siculo-Tunisian Strait, which exhibited high allelic richness and shared alleles absent from the eastern and western basins. F statistics (pairwise theta ranges between 0.09 and 0.71) revealed high genetic structure between meadows, both at a small scale (about 2 to 200 km) and at a medium scale within the eastern and western basins, independent of geographical distance. At the intrameadow scale, significant spatial autocorrelation in six out of 15 locations revealed that dispersal can be restricted to the scale of a few metres. Main conclusions A stochastic pattern of effective migration due to low population size, turnover and seed survival is the most likely explanation for this pattern of highly restricted gene flow, despite the importance of an a priori seed dispersal potential. The east-west cleavage probably represents the outline of vicariance caused by the last Pleistocene ice age and maintained to this day by low gene flow. These results emphasize the diversity of evolutionary processes shaping the genetic structure at different spatial scales. | 2049 | no | no good fitness measure | NA | no | no |
| 2050 | 2017 | 43025 | Ronco, T; Stegger, M; Olsen, RH; Sekse, C; Nordstoga, AB; Pohjanvirta, T; Lilje, B; Lyhs, U; Andersen, PS; Pedersen, K | 2017 | Spread of avian pathogenic Escherichia coli ST117 O78:H4 in Nordic broiler production | Background: Escherichia coli infections known as colibacillosis constitute a considerable challenge to poultry farmers worldwide, in terms of decreased animal welfare and production economy. Colibacillosis is caused by avian pathogenic E. coli (APEC). APEC strains are extraintestinal pathogenic E. coli and have in general been characterized as being a genetically diverse population. In the Nordic countries, poultry farmers depend on import of Swedish broiler breeders which are part of a breeding pyramid. During 2014 to 2016, an increased occurrence of colibacillosis on Nordic broiler chicken farms was reported. The aim of this study was to investigate the genetic diversity among E. coli isolates collected on poultry farms with colibacillosis issues, using whole genome sequencing. Methods: Hundred and fourteen bacterial isolates from both broilers and broiler breeders were whole genome sequenced. The majority of isolates were collected from poultry with colibacillosis on Nordic farms. Subsequently, comparative genomic analyses were carried out. This included in silico typing (sero-and multi-locus sequence typing), identification of virulence and resistance genes and phylogenetic analyses based on single nucleotide polymorphisms. Results: In general, the characterized poultry isolates constituted a genetically diverse population. However, the phylogenetic analyses revealed a major clade of 47 closely related ST117 O78: H4 isolates. The isolates in this clade were collected from broiler chickens and breeders with colibacillosis in multiple Nordic countries. They clustered together with a human ST117 isolate and all carried virulence genes that previously have been associated with human uropathogenic E. coli. Conclusions: The investigation revealed a lineage of ST117 O78: H4 isolates collected in different Nordic countries from diseased broilers and breeders. The data indicate that the closely related ST117 O78: H4 strains have been transferred vertically through the broiler breeding pyramid into distantly located farms across the Nordic countries. | 2050 | no | gd of pathogen | NA | no | no |
| 2051 | 2017 | 43025 | Alda, F; Gaitero, T; Suarez, M; Merchan, T; Rocha, G; Doadrio, I | 2010 | Evolutionary history and molecular epidemiology of rabbit haemorrhagic disease virus in the Iberian Peninsula and Western Europe | Background: Rabbit haemorrhagic disease virus (RHDV) is a highly virulent calicivirus, first described in domestic rabbits in China in 1984. RHDV appears to be a mutant form of a benign virus that existed in Europe long before the first outbreak. In the Iberian Peninsula, the first epidemic in 1988 severely reduced the populations of autochthonous European wild rabbit. To examine the evolutionary history of RHDV in the Iberian Peninsula, we collected virus samples from wild rabbits and sequenced a fragment of the capsid protein gene VP60. These data together with available sequences from other Western European countries, were analyzed following Bayesian Markov chain Monte Carlo methods to infer their phylogenetic relationships, evolutionary rates and demographic history. Results: Evolutionary relationships of RHDV revealed three main lineages with significant phylogeographic structure. All lineages seem to have emerged at a common period of time, between similar to 1875 and similar to 1976. The Iberian Peninsula showed evidences of genetic isolation, probably due to geographic barriers to gene flow, and was also the region with the youngest MRCA. Overall, demographic analyses showed an initial increase and stabilization of the relative genetic diversity of RHDV, and a subsequent reduction in genetic diversity after the first epidemic breakout in 1984, which is compatible with a decline in effective population size. Conclusions: Results were consistent with the hypothesis that the current Iberian RHDV arose from a single infection between 1869 and 1955 (95% HPD), and rendered a temporal pattern of appearance and extinction of lineages. We propose that the rising positive selection pressure observed throughout the history of RHDV is likely mediated by the host immune system as a consequence of the genetic changes that rendered the virus virulent. Consequently, this relationship is suggested to condition RHDV demographic history. | 2051 | no | gd of pathogen | NA | no | no |
| 2052 | 2017 | 43025 | Bayon, C; Pei, MH; Ruiz, C; Hunter, T; Karp, A; Tubby, I | 2009 | Genetic structure and population dynamics of a heteroecious plant pathogen Melampsora larici-epitea in short-rotation coppice willow plantations | Complex life strategies are common among plant pathogens belonging to rust fungi (Uredinales). The heteroecious willow rust Melampsora larici-epitea produces five spore stages and alternates on larch (Larix). To shed light on the epidemiology of this pathogen, amplified fragment length polymorphisms (AFLPs) were used to determine the genetic diversity and genetic structure of rust samples collected from coppice willow (Salix) plantations at three UK sites (LA, CA and MC) over three sampling dates (September 2000, July 2001 and September 2001). Of the total of 819 isolates, 465 were unique AFLP phenotypes and there was a shift in genotype diversity between the two seasons (0.67 in 2000 and 0.87-0.89 in 2001). No phenotypes were common between the two seasons within a site, suggesting that the rust did not overwinter as an asexual stage within plantations. A temporal analysis detected large amounts of genetic drift (F(S) = 0.15-0.26) between the two seasons and very small effective population sizes (N(e) = 2-3) within sites. These results all point to a new colonization of the plantations by the rust in the second season (2001). The F(ST)-analogue values were Theta(CT) = 0.121, Weir and Cockerham’s theta = 0.086 and the Bayesian estimate theta(B) = 0.087-0.096. The results suggest that the sources of inoculum were somewhat localized and the same sources were mainly responsible for disease epidemics in LA and CA over the two seasons. The relatively low F(ST)-values among sites (0.055-0.13) suggest the existence of significant gene flow among the three sampled sites. | 2052 | no | gd of pathogen | NA | no | no |
| 2053 | 2017 | 43025 | Vaddi, S; Thiruvengadam, V; Joshi, S; Jalali, SK; Chinnappareddy, LRD; Nimmakayala, P; Tomason, Y; Vajja, G; Reddy, UK | 2016 | In silico mining of microsatellites and analysis of genetic diversity among inter- and intra-generic aphids of the subfamily Aphidinae | Nearly 5 000 aphid species damage crops, either by sucking plant sap or as disease-transmitting vectors. Microsatellites are used for understanding molecular diversity and eco-geographical relationships among aphid species. Expressed sequence tag (EST)-microsatellite motifs were identified through an in silico approach using inbuilt simple sequence repeat mining tools in aphid EST dataset. Microsatellite mining revealed one in every five aphid genes as containing a repeat motif, and out of 9 290 EST microsatellites mined from Aphis gossypii Glover and Acyrthosiphon pisum (Harris) (both Hemiptera: Aphididae), 80% were of A and/or T (AT, ATA, AAT, AATA, and ATTT) motifs, and the rest contained G and/or C motifs. All microsatellite sequences were annotated using BLAST. Primers for EST microsatellites were designed using the Primer 3.0 tool. 106 primer pairs of both dinucleotide repeats (DNRs) and trinucleotide repeats (TNRs), representing open reading frames (ORFs) and untranslated regions (UTRs), were synthesized to amplify 15 aphid species belonging to the subfamily Aphidinae, collected from diverse hosts. Four hundred forty-five polymorphic alleles were amplified. Fifty TNR and 23 DNR microsatellites amplified across the species studied. Polymorphism information content values of microsatellites ranged from 0.23 to 0.91, amplifying 2-16 alleles. Genetic similarity indices were estimated using the ‘NTSYS-pc’ software package. Unweighted pair group with arithmetic mean and principal component analysis resolved taxonomic relationships of the aphid species studied. The new aphid microsatellites developed will provide valuable information to researchers to study Indian aphid species diversity and genetic relationships. | 2053 | no | gd of pathogen | NA | no | no |
| 2054 | 2017 | 43025 | Belisario, CJ; Pessoa, GCD; Silva, EM; Rosa, ACL; Ferreira, RE; Bedin, C; Wilhelms, T; de Mello, F; Coutinho, HS; Fonseca, ELO; dos Santos, RF; Rodrigues, VLCC; Dias, JCP; Diotaiuti, L | 2017 | Genetic characterization of residual Triatoma infestans populations from Brazil by microsatellite | In spite of long-term efforts to eliminate Triatoma infestans (Klug 1834) from Brazil, residual foci still persist in the states of Bahia and Rio Grande do Sul. Data on the genetic variability and structuring of these populations are however lacking. Using nine microsatellite loci, we characterized one residual T. infestans population from Bahia and four from Rio Grande do Sul, and compared them with bugs originally from an older focus in So Paulo; 224 bugs were genotyped. The number of alleles per locus ranged from 5 to 11. Observed and expected heterozygosities per locus ranged, respectively, from 0 to 0.786 and from 0 to 0.764. Significant departures from Hardy-Weinberg equilibrium, mainly due to heterozygote deficits, were detected in all loci and in most populations. Global indices estimated by AMOVA were: Fis was 0.37; Fst was 0.28; and Fit was 0.55; overall indices with p = 0.00 indicated substantial differentiation. Inter-population Fst ranged from 0.118 to 0.562, suggesting strong genetic structuring and little to no gene flow among populations. Intra-population Fis ranged from 0.301 to 0.307. Inbreeding was apparent in all populations except that from Bahia-which might be either linked by gene flow to nearby unsampled populations or part of a relatively large local population. The overall pattern of strong genetic structuring among pyrethroid-susceptible residual T. infestans populations suggests that their persistence is probably due to operational control failures. Detection and elimination of such residual foci is technically feasible and must become a public health priority in Brazil. | 2054 | no | gd of pathogen | NA | no | no |
| 2055 | 2017 | 43025 | Lazar, J; O’Meara, CC; Sarkis, AB; Prisco, SZ; Xu, HY; Fox, CS; Chen, MH; Broeckel, U; Arnett, DK; Moreno, C; Provoost, AP; Jacob, HJ | 2013 | SORCS1 contributes to the development of renal disease in rats and humans | Many lines of evidence demonstrate that genetic variability contributes to chronic kidney disease susceptibility in humans as well as rodent models. Little progress has been made in discovering causal kidney disease genes in humans mainly due to genetic complexity. Here, we use a minimal congenic mapping strategy in the FHH (fawn hooded hypertensive) rat to identify Sorcs1 as a novel renal disease candidate gene. We investigated the hypothesis that genetic variation in Sorcs1 influences renal disease susceptibility in both rat and human. Sorcs1 is expressed in the kidney, and knocking out this gene in a rat strain with a sensitized genome background produced increased proteinuria. In vitro knockdown of Sorcs1 in proximal tubule cells impaired protein trafficking, suggesting a mechanism for the observed proteinuria in the FHH rat. Since Sorcs1 influences renal function in the rat, we went on to test this gene in humans. We identified associations between single nucleotide polymorphisms in SORCS1 and renal function in large cohorts of European and African ancestry. The experimental data from the rat combined with association results from different ethnic groups indicates a role for SORCS1 in maintaining proper renal function. | 2055 | no | no good fitness measure, N pop differnt in GD? | NA | no | no |
| 2056 | 2017 | 43025 | Zeng, Y; Li, CW; Zhang, LY; Zhong, ZY; Jiang, ZG | 2013 | No correlation between neonatal fitness and heterozygosity in a reintroduced population of Pere David’s deer | Considering the severe impacts of genetic bottlenecks and small numbers of founders in populations of reintroduced animals, it is necessary to study inbreeding and its effect on fitness in species of conservation concern. Pere David’s deer is one of few large mammal species extinct in the wild but safely preserved in captivity. Its specific background gives us the opportunity to study the relationships between heterozygosity and neonatal fitness in relocated populations. We employed five microsatellite loci to explore heterozygosity-fitness correlations in a population of Pere David’s deer at the Beijing Milu Ecological Research Center. We observed associations between microsatellite-based variables sMLH, IR, MD2 and HL, and two components of fitness expressed early in life (birth weight and the neonatal mortality of 123 Pere David’s deer calves born over six consecutive years). We found that neonatal mortality was 19.1% +/- 7.6%, not higher than the 19% or 18% reported in other ungulates. The heterozygosity of calves was not associated with neonatal mortality, nor birth weight. Our study implies that low genetic variability of microsatellite loci has no overt effect on birth weight and neonatal mortality in reintroduced populations of Pere David’s deer [Current Zoology 59 (2): 249-256, 2013]. | 2056 | no | only one pop | NA | no | no |
| 2057 | 2017 | 43025 | Kumar, RV; Prasanna, HC; Singh, AK; Ragunathan, D; Garg, GK; Chakraborty, S | 2017 | Molecular genetic analysis and evolution of begomoviruses and betasatellites causing yellow mosaic disease of bhendi | In India, Bhendi yellow vein mosaic disease (BYVMD) is one of the most economically important diseases of bhendi/okra and is caused by a complex of monopartite begomovirus (Bhendi yellow vein mosaic virus-BYVMV) and betasatellite (Bhendi yellow vein betasatellite-BYVB). In this study, we have analyzed the role of possible evolutionary factors involved in the evolution of BYVMV and BYVB isolates. Evidence of inter-species and inter-strain recombination events was detected among the viral isolates, and majority of these recombinant isolates possess microsatellites in their genome. Recombination analysis suggests that cotton-infecting and bhendi-infecting begomoviruses probably share a recent common ancestor. In addition to genetic differentiation and gene flow, high degree of genetic variability was detected among the viral population. A strong purifying selection seems to be acting on the viral coding regions. The nucleotide substitution rate of V1 gene (for BYVMV) and beta C1 gene (for BYVB) was estimated to be 7.55 x 10(-4) and 2.25 x 10(-3) nucleotide substitutions/site/year, respectively. The present study underlines that the evolution of BYVMD-associated viral components is driven by selection acting on the genetic variation generated by recombination and mutation. | 2057 | no | no good fitness measure, N pop differnt in GD? | NA | no | no |
| 2058 | 2017 | 43025 | Roed, KH; Kvie, KS; Hasle, G; Gilbert, L; Leinaas, HP | 2016 | Phylogenetic Lineages and Postglacial Dispersal Dynamics Characterize the Genetic Structure of the Tick, Ixodes ricinus, in Northwest Europe | Dispersal and gene flow are important mechanisms affecting the dynamics of vectors and their pathogens. Here, patterns of genetic diversity were analyzed in many North European populations of the tick, Ixodes ricinus. Population sites were selected within and between areas separated by geographical barriers in order to evaluate the importance of tick transportation by birds in producing genetic connectivity across open sea and mountain ranges. The phylogenetic analyses of the mitochondrial control region and the cytochrome b gene revealed two distinct clades with supported sub-clades, with three genetic lineages: GB and WNo associated with Great Britain and western Norway respectively, and Eu with a wider distribution across continental Europe in agreement with much lower efficiency of tick dispersal by birds than by large mammals. The results suggest different ancestry of I. ricinus colonizing Britain and the rest of northern Europe, possibly from different glacial refuges, while ticks from western Norway and continental Europe share a more recent common ancestry. Demographic history modeling suggests a period of strong increase in tick abundance coincident with progression of the European Neolithic culture, long after their post-glacial colonization of NW Europe. | 2058 | no | gd of parasite | NA | no | no |
| 2059 | 2017 | 43025 | Poulin, R; Mouillot, D | 2004 | The evolution of taxonomic diversity in helminth assemblages of mammalian hosts | Several studies have searched for the key forces behind the diversification of parasite assemblages over evolutionary time. All of these studies have used parasite species richness as their measure of diversity, thus ignoring the relatedness among parasite species and the taxonomic structure of the assemblages. This information is essential, however, if we want to elucidate which processes have caused an assemblage of parasites to acquire new species. Here, we performed a comparative analysis across 110 species of mammalian hosts in which we evaluated the effects of four host traits ( body mass, population density, geographic range, and basal metabolic rate) on the diversity of their assemblages of helminth endoparasites. As measures of diversity, we used parasite species richness, as well as the average taxonomic distinctness of the assemblage and its variance; the latter measures are based on the taxonomic distance between two parasite species, computed across all possible species pairs in an assemblage. Unlike parasite species richness, both the average taxonomic distinctness and its variance were unaffected by the number of hosts examined. These two measures of parasite diversity also proved highly repeatable among host populations of the same mammalian species; in contrast, parasite species richness was unreliable as a species character, as it varied as much within a host species than among different host species. Using phylogenetically independent contrasts, and correcting for potential confounding variables, we found that host population density correlated positively with parasite species richness. There were, however, no other relationships between any of the four host traits investigated and either of our measures of parasite diversity. The processes facilitating the taxonomic diversification of parasite assemblages thus remain unclear, but their elucidation will be necessary if we are to fully understand parasite evolution. | 2059 | no | no good fitness measure, N pop differnt in GD? | NA | no | no |
| 2060 | 2017 | 43025 | Castro-Prieto, A; Wachter, B; Sommer, S | 2011 | Cheetah Paradigm Revisited: MHC Diversity in the World’s Largest Free-Ranging Population | For more than two decades, the cheetah (Acinonyx jubatus) has been considered a paradigm of disease vulnerability associated with low genetic diversity, particularly at the immune genes of the major histocompatibility complex (MHC). Cheetahs have been used as a classic example in numerous conservation genetics textbooks as well as in many related scientific publications. However, earlier studies used methods with low resolution to quantify MHC diversity and/or small sample sizes. Furthermore, high disease susceptibility was reported only for captive cheetahs, whereas free-ranging cheetahs show no signs of infectious diseases and a good general health status. We examined whether the diversity at MHC class I and class II-DRB loci in 149 Namibian cheetahs was higher than previously reported using single-strand conformation polymorphism analysis, cloning, and sequencing. MHC genes were examined at the genomic and transcriptomic levels. We detected ten MHC class I and four class II-DRB alleles, of which nine MHC class I and all class II-DRB alleles were expressed. Phylogenetic analyses and individual genotypes suggested that the alleles belong to four MHC class I and three class II-DRB putative loci. Evidence of positive selection was detected in both MHC loci. Our study indicated that the low number of MHC class I alleles previously observed in cheetahs was due to a smaller sample size examined. On the other hand, the low number of MHC class II-DRB alleles previously observed in cheetahs was further confirmed. Compared with other mammalian species including felids, cheetahs showed low levels of MHC diversity, but this does not seem to influence the immunocompetence of free-ranging cheetahs in Namibia and contradicts the previous conclusion that the cheetah is a paradigm species of disease vulnerability. | 2060 | no | no pop level? | NA | no | no |
| 2061 | 2017 | 43025 | Patin, E; Barreiro, LB; Sabeti, PC; Austerlitz, F; Luca, F; Sajantila, A; Behar, DM; Semino, O; Sakuntabhai, A; Guiso, N; Gicquel, B; McElreavey, K; Harding, RM; Heyer, E; Quintana-Murci, L | 2006 | Deciphering the ancient and complex evolutionary history of human arylamine N-acetyltransferase genes | “The human N-acetyltransferase genes NAT1 and NAT2 encode two phase-II enzymes that metabolize various drugs and carcinogens. Functional variability at these genes has been associated with adverse drug reactions and cancer susceptibility. Mutations in NAT2 leading to the so-called slow-acetylation phenotype reach high frequencies worldwide, which questions the significance of altered acetylation in human adaptation. To investigate the role of population history and natural selection in shaping NATs variation, we characterized genetic diversity through the resequencing and genotyping of NAT1, NAT2, and the pseudogene NATP in a collection of 13 different populations with distinct ethnic backgrounds and demographic pasts. This combined study design allowed us to define a detailed map of linkage disequilibrium of the NATs region as well as to perform a number of sequence-based neutrality tests and the long-range haplotype (LRH) test. Our data revealed distinctive patterns of variability for the two genes: the reduced diversity observed at NAT1 is consistent with the action of purifying selection, whereas NAT2 functional variation contributes to high levels of diversity. In addition, the LRH test identified a particular NAT2 haplotype (NAT2*5B) under recent positive selection in western/central Eurasians. This haplotype harbors the mutation 341T -> C and encodes the ""slowest-acetylator"" NAT2 enzyme, suggesting a general selective advantage for the slow-acetylator phenotype. Interestingly, the NAT2*5B haplotype, which seems to have conferred a selective advantage during the past similar to 6,500 years, exhibits today the strongest association with susceptibility to bladder cancer and adverse drug reactions. On the whole, the patterns observed for NAT2 well illustrate how geographically and temporally fluctuating xenobiotic environments may have influenced not only our genome variability but also our present-day susceptibility to disease.” | 2061 | no | human | NA | no | no |
| 2062 | 2017 | 43025 | Idaghdour, Y; Czika, W; Shianna, KV; Lee, SH; Visscher, PM; Martin, HC; Miclaus, K; Jadallah, SJ; Goldstein, DB; Wolfinger, RD; Gibson, G | 2010 | Geographical genomics of human leukocyte gene expression variation in southern Morocco | Studies of the genetics of gene expression can identify expression SNPs (eSNPs) that explain variation in transcript abundance. Here we address the robustness of eSNP associations to environmental geography and population structure in a comparison of 194 Arab and Amazigh individuals from a city and two villages in southern Morocco. Gene expression differed between pairs of locations for up to a third of all transcripts, with notable enrichment of transcripts involved in ribosomal biosynthesis and oxidative phosphorylation. Robust associations were observed in the leukocyte samples: cis eSNPs (P < 10(-08)) were identified for 346 genes, and trans eSNPs (P < 10(-11)) for 10 genes. All of these associations were consistent both across the three sample locations and after controlling for ancestry and relatedness. No evidence of large-effect trans-acting mediators of the pervasive environmental influence was found; instead, genetic and environmental factors acted in a largely additive manner. | 2062 | no | human | NA | no | no |
| 2063 | 2017 | 43025 | Telenti, A; Pierce, LCT; Biggs, WH; di Iulio, J; Wong, EHM; Fabani, MM; Kirkness, EF; Moustafa, A; Shah, N; Xie, C; Brewerton, SC; Bulsara, N; Garner, C; Metzker, G; Sandoval, E; Perkins, BA; Och, FJ; Turpaz, Y; Venter, JC | 2016 | Deep sequencing of 10,000 human genomes | We report on the sequencing of 10,545 human genomes at 30x-40x coverage with an emphasis on quality metrics and novel variant and sequence discovery. We find that 84% of an individual human genome can be sequenced confidently. This high-confidence region includes 91.5% of exon sequence and 95.2% of known pathogenic variant positions. We present the distribution of over 150 million single-nucleotide variants in the coding and noncoding genome. Each newly sequenced genome contributes an average of 8,579 novel variants. In addition, each genome carries on average 0.7 Mb of sequence that is not found in the main build of the hg38 reference genome. The density of this catalog of variation allowed us to construct high-resolution profiles that define genomic sites that are highly intolerant of genetic variation. These results indicate that the data generated by deep genome sequencing is of the quality necessary for clinical use. | 2063 | no | human | NA | no | no |
| 2064 | 2017 | 43025 | McMahon, CR; Isagi, Y; Kaneko, S; Bowman, DMJS; Brook, BW; Bradshaw, CJA | 2013 | Genetic structure of introduced swamp buffalo subpopulations in tropical Australia | High densities of introduced herbivores can damage sensitive ecosystems, increase the risk of extinction of native biota, and host and spread disease. An essential step in managing large feral animal populations is to quantify how they use habitats so that management interventions, such as culling, can be targeted to reduce densities and to minimize migration into areas from which animals have been removed. An effective method to quantify animal movements is by measuring landscape-scale genetic population structure. We describe the genetic population structure of one of Australia’s more destructive introduced mammals the Asian swamp buffalo (Bubalus bubalis). We collected 524 skin samples from buffalo across their range in the Northern Territory of Australia. Allelic diversity in the Northern Territory population was low compared to those reported from populations in their native Asian habitats. The Australian population is tentatively made of three subpopulations; Melville Island, Eastern Arnhem and Central-Western Arnhem populations. The Melville Island population is represented by a single cluster, while the Eastern Arnhem population has three clusters and the Central-Western Arnhem population seven clusters. We found some support for isolation by distance across all the sampled populations, but little evidence for this relationship when comparing the two well-mixed mainland meta-populations. Despite their small founder populations and limited genetic variation, the persistence of buffalo in Australia has likely been aided by release from high predation, parasitism and disease typical of their native habitats. | 2064 | no | no good fitness measure | NA | no | no |
| 2065 | 2017 | 43025 | Csagola, A; Lorincz, M; Tombacz, K; Wladar, Z; Kovacs, E; Tuboly, T | 2012 | Genetic diversity of pigeon circovirus in Hungary | Pigeon circoviruses (PiCV) had been identified worldwide and are responsible for immune suppression and a variety of diseases collectively referred to as young pigeon disease syndrome. Samples from racing pigeons were collected throughout Hungary and analyzed for the presence of PiCV by polymerase chain reaction. The capsid protein coding gene was amplified from ten PiCVs of different origins, and compared with known PiCV sequences. The results indicated that PiCV was highly variable, the viruses formed five distinct genetic groups. Differences of the 3’ end of the gene suggested the possibility of genetic recombination among these groups. | 2065 | no | gd of pathogen | NA | no | no |
| 2066 | 2017 | 43025 | Miller, SM; Harper, CK; Bloomer, P; Hofmeyr, J; Funston, PJ | 2014 | Evaluation of Microsatellite Markers for Populations Studies and Forensic Identification of African Lions (Panthera leo) | The South African lion (Panthera leo) population is highly fragmented. One-third of its wild lions occur in small (< 1000 km(2)) reserves. These lions were reintroduced from other areas of the species’ historical range. Management practices on these reserves have not prioritized genetic provenance or heterozygosity. These trends potentially constrain the conservation value of these lions. To ensure the best management and long-term survival of these subpopulations as a viable collective population, the provenance and current genetic diversity must be described. Concurrently, poaching of lions to supply a growing market for lion bones in Asia may become a serious conservation challenge in the future. Having a standardized, validated method for matching confiscated lion parts with carcasses will be a key tool in investigating these crimes. We evaluated 28 microsatellites in the African lion using samples from 18 small reserves and 1 captive facility in South Africa, two conservancies in Zimbabwe, and Kruger National and Kgalagadi Transfrontier Parks to determine the loci most suited for population management and forensic genetic applications. Twelve microsatellite loci with a match probability of 1.1x10(-5) between siblings were identified for forensics. A further 10 could be added for population genetics studies. | 2066 | no | no good fitness measure | NA | no | no |
| 2067 | 2017 | 43025 | Munoz-Fuentes, V; Darimont, CT; Wayne, RK; Paquet, PC; Leonard, JA | 2009 | Ecological factors drive differentiation in wolves from British Columbia | Aim Limited population structure is predicted for vagile, generalist species, such as the grey wolf (Canis lupus L.). Our aims were to study how genetic variability of grey wolves was distributed in an area comprising different habitats that lay within the potential dispersal range of an individual and to make inferences about the impact of ecology on population structure. Location British Columbia, Canada - which is characterized by a continuum of biogeoclimatic zones across which grey wolves are distributed - and adjacent areas in both Canada and Alaska, United States. Methods We obtained mitochondrial DNA control region sequences from grey wolves from across the province and integrated our genetic results with data on phenotype, behaviour and ecology (distance, habitat and prey composition). We also compared the genetic diversity and differentiation of British Columbia grey wolves with those of other North American wolf populations. Results We found strong genetic differentiation between adjacent populations of grey wolves from coastal and inland British Columbia. We show that the most likely factor explaining this differentiation is habitat discontinuity between the coastal and interior regions of British Columbia, as opposed to geographic distance or physical barriers to dispersal. We hypothesize that dispersing grey wolves select habitats similar to the one in which they were reared, and that this differentiation is maintained largely through behavioural mechanisms. Main conclusions The identification of strong genetic structure on a scale within the dispersing capabilities of an individual suggests that ecological factors are driving wolf differentiation in British Columbia. Coastal wolves are highly distinct and representative of a unique ecosystem, whereas inland British Columbia grey wolves are more similar to adjacent populations of wolves located in Alaska, Alberta and Northwest Territories. Given their unique ecological, morphological, behavioural and genetic characteristics, grey wolves of coastal British Columbia should be considered an Evolutionary Significant Unit (ESU) and, consequently, warrant special conservation status. If ecology can drive differentiation in a highly mobile generalist such as the grey wolf, ecology probably drives differentiation in many other species as well. | 2067 | no | no good fitness measure | NA | no | no |
| 2068 | 2017 | 43025 | Pierce, AA; de Roode, JC; Altizer, S; Bartel, RA | 2014 | Extreme Heterogeneity in Parasitism Despite Low Population Genetic Structure among Monarch Butterflies Inhabiting the Hawaiian Islands | Host movement and spatial structure can strongly influence the ecology and evolution of infectious diseases, with limited host movement potentially leading to high spatial heterogeneity in infection. Monarch butterflies (Danaus plexippus) are best known for undertaking a spectacular long-distance migration in eastern North America; however, they also form non-migratory populations that breed year-round in milder climates such as Hawaii and other tropical locations. Prior work showed an inverse relationship between monarch migratory propensity and the prevalence of the protozoan parasite, Ophryocystis elektroscirrha. Here, we sampled monarchs from replicate sites within each of four Hawaiian Islands to ask whether these populations show consistently high prevalence of the protozoan parasite as seen for monarchs from several other non-migratory populations. Counter to our predictions, we observed striking spatial heterogeneity in parasite prevalence, with infection rates per site ranging from 4-85%. We next used microsatellite markers to ask whether the observed variation in infection might be explained by limited host movement and spatial sub-structuring among sites. Our results showed that monarchs across the Hawaiian Islands form one admixed population, supporting high gene flow among sites. Moreover, measures of individual-level genetic diversity did not predict host infection status, as might be expected if more inbred hosts harbored higher parasite loads. These results suggest that other factors such as landscape-level environmental variation or colonization-extinction processes might instead cause the extreme heterogeneity in monarch butterfly infection observed here. | 2068 | no | No GD diff between pop | NA | no | no |
| 2069 | 2017 | 43025 | Lam, TTY; Hon, CC; Lemey, P; Pybus, OG; Shi, M; Tun, HM; Li, J; Jiang, JW; Holmes, EC; Leung, FCC | 2012 | Phylodynamics of H5N1 avian influenza virus in Indonesia | Understanding how pathogens invade and become established in novel host populations is central to the ecology and evolution of infectious disease. Influenza viruses provide unique opportunities to study these processes in nature because of their rapid evolution, extensive surveillance, large data sets and propensity to jump species boundaries. H5N1 highly pathogenic avian influenza virus (HPAIV) is a major animal pathogen and public health threat. The virus is of particular importance in Indonesia, causing severe outbreaks among poultry and sporadic human infections since 2003. However, little is known about how H5N1 HPAIV emerged and established in Indonesia. To address these questions, we analysed Indonesian H5N1 HPAIV gene sequences isolated during 20032007. We find that the virus originated from a single introduction into East Java between November 2002 and October 2003. This invasion was characterized by an initially rapid burst of viral genetic diversity followed by a steady rate of lineage replacement and the maintenance of genetic diversity. Several antigenic sites in the haemagglutinin gene were subject to positive selection during the early phase, suggesting that host-immune-driven selection played a role in host adaptation and expansion. Phylogeographic analyses show that after the initial invasion of H5N1, genetic variants moved both eastwards and westwards across Java, possibly involving long-distance transportation by humans. The phylodynamics we uncover share similarities with other recently studied viral invasions, thereby shedding light on the ecological and evolutionary processes that determine disease emergence in a new geographical region. | 2069 | no | no good fitness measure, N pop differnt in GD? | NA | no | no |
| 2070 | 2017 | 43025 | Fujihara, J; Ueki, M; Yasuda, T; Iida, R; Soejima, M; Koda, Y; Kimura-Kataoka, K; Kato, H; Panduro, A; Tongu, M; Takeshita, H | 2011 | Functional and Genetic Survey of All Known Single-Nucleotide Polymorphisms Within the Human Deoxyribonuclease I Gene in Wide-Ranging Ethnic Groups | The single-nucleotide polymorphisms (SNPs) in the human DNase I gene (DNASE1) might be involved in susceptibility to some common diseases; however, only limited population data are available. Further, the effects of these SNPs on in vivo DNase I activity remain unknown. The genotype and haplotype of all the SNPs in DNASE1 were determined in 3 ethnic groups including 14 populations using newly developed methods. Together with our previous data on the nonsynonymous SNPs, two major haplotypes based on the five exonic SNPs were identified; genetic diversity in the Asian population was low. Among 10 SNPs, other than exonic SNPs in the gene, only 3 were polymorphic among all the populations. Haplotype distribution, based on all the polymorphic SNPs, was clarified to be generally varied in an ethnic-dependent manner. Thus, the genetic aspects of DNASE1 with regard to all the SNPs in wide-ranging ethnic groups could be first demonstrated. Further, there was no correlation of all the polymorphic SNPs other than nonsynonymous ones with serum DNase I activity levels. Polymorphic SNPs other than the exonic SNPs might not be directly related to common diseases through alterations in in vivo levels of the activity. | 2070 | no | human | NA | no | no |
| 2071 | 2017 | 43025 | Zhu, QH; Bennetzen, JL; Smith, SM | 2013 | Isolation and Diversity Analysis of Resistance Gene Homologues from Switchgrass | Resistance gene homologs (RGHs) were isolated from the switchgrass variety Alamo by a combination of polymerase chain reaction and expressed sequence tag (EST) database mining. Fifty-eight RGHs were isolated by polymerase chain reaction and 295 RGHs were identified in 424,545 switchgrass ESTs. Four nucleotide binding site-leucine-rich repeat RGHs were selected to investigate RGH haplotypic diversity in seven switchgrass varieties chosen for their representation of a broad range of the switchgrass germplasm. Lowland and upland ecotypes were found to be less similar, even from nearby populations, than were more distant populations with similar growth environments. Most (83.5%) of the variability in these four RGHs was found to be attributable to the within-population component. The difference in nucleotide diversity between and within populations was observed to be small, whereas this diversity is maintained to similar degrees at both population and ecotype levels. The results also revealed that the analyzed RGHs were under positive selection in the studied switchgrass accessions. Intragenic recombination was detected in switchgrass RGHs, thereby demonstrating an active genetic process that has the potential to generate new resistance genes with new specificities that might act against newly-arising pathogen races. | 2071 | no | no good fitness measure | NA | no | no |
| 2072 | 2017 | 43025 | Uchiya, K; Tomida, S; Nakagawa, T; Asahi, S; Nikai, T; Ogawa, K | 2017 | Comparative genome analyses of Mycobacterium avium reveal genomic features of its subspecies and strains that cause progression of pulmonary disease | Pulmonary disease caused by nontuberculous mycobacteria (NTM) is increasing worldwide. Mycobacterium avium is the most clinically significant NTM species in humans and animals, and comprises four subspecies: M. avium subsp. avium (MAA), M. avium subsp. silvaticum (MAS), M. avium subsp. paratuberculosis (MAP), and M. avium subsp. hominissuis (MAH). To improve our understanding of the genetic landscape and diversity of M. avium and its role in disease, we performed a comparative genome analysis of 79 M. avium strains. Our analysis demonstrated that MAH is an open pan-genome species. Phylogenetic analysis based on single nucleotide variants showed that MAH had the highest degree of sequence variability among the subspecies, and MAH strains isolated in Japan and those isolated abroad possessed distinct phylogenetic features. Furthermore, MAP strains, MAS and MAA strains isolated from birds, and many MAH strains that cause the progression of pulmonary disease were grouped in each specific cluster. Comparative genome analysis revealed the presence of genetic elements specific to each lineage, which are thought to be acquired via horizontal gene transfer during the evolutionary process, and identified potential genetic determinants accounting for the pathogenic and host range characteristics of M. avium. | 2072 | no | gd of pathogen | NA | no | no |
| 2073 | 2017 | 43025 | Schmeller, DS; Schregel, J; Veith, M | 2007 | The importance of heterozygosity in a frog’s life | High genetic variability may increase metabolic efficiency and thus allows responding to environmental challenges as limits to adaptation are approached. Therefore, it has been suggested that high genetic variability contributes strongly to the fitness of an individual. Survival to high age may thus depend on high genetic variability, and genetically variable individuals may have a higher survival rate to high ages in comparison to less variable sympatric conspecifics. Such a heterozygosity x age relationship might be more readily detectable in stressful as compared to benign environments. For testing the relationship between age and heterozygosity, we genetically analyzed 71 individuals of the frog species Rana perezi from a total of seven populations at 13 allozyme loci. The age of the individuals was determined by skeletochronology. We found effects on age of both environment and allozyme heterozygosity, especially in populations with high stress regimes. A significant heterozygosity x age relationship has so far rarely been shown in natural populations. The result of our analysis suggests that more heterozygous individuals have a higher longevity and may be an important source of genetic variability of a population, likely contributing to a stabilization of the effective population size. | 2073 | no | no good fitness measure, N pop differnt in GD? | NA | no | no |
| 2074 | 2017 | 43025 | Kawai, M; Shoda-Kagaya, E; Maehara, T; Zhou, ZH; Lian, CL; Iwata, R; Yamane, A; Hogetsu, T | 2006 | Genetic structure of pine sawyer Monochamus alternatus (Coleoptera : Cerambycidae) populations in northeast Asia: Consequences of the spread of pine wilt disease | We studied the phylogeography and genetic diversity of the pine sawyer Monochamus alternatus (Hope) (Coleoptera: Cerambycidae) to understand its colonization dynamics, potential for further invasion, and potential species divergence. This species is the main vector of the pine wood nematode Bursaphelenchus xylophilus (Steiner and Buhrer), which is the causative agent of pine wilt disease in Japan. The genetic structure was studied using sequences of mitochondrial DNA and microsatellites. The phylogenetic analysis revealed two distinct lineages within M. alternatus. There is no clear boundary between haplotypic distributions of the two clades. Coalescence should have been extended by population subdivision. There might also be a fusion of the two distinct populations, and both are completely saturated. Analysis of the microsatellite genotypes of populations in Japan showed a complex genetic structure. Estimates of overall population differentiation (F-ST) were significantly different from zero. The populations are thought to be at demographical nonequilibrium or to show restricted gene flow among prefectures. Although neighboring populations often had similar genetic compositions, significant isolation by distance in the total population was not detected. It is suggested that population expansion may have occurred not only by natural dispersal on a small scale, but also by long-distance dispersal likely enhanced by the relocation, (by humans) of infested wood. Our data Suggest that M. alternatus is a species capable of dispersing over a large area, and therefore, multiple invasions of M. alternatus from distant areas is a possibility. | 2074 | no | no good fitness measure, N pop differnt in GD? | NA | no | no |
| 2075 | 2017 | 43025 | Messier, GD; Garant, D; Bergeron, P; Reale, D | 2012 | Environmental conditions affect spatial genetic structures and dispersal patterns in a solitary rodent | The study of the spatial distribution of relatives in a population under contrasted environmental conditions provides critical insights into the flexibility of dispersal behaviour and the role of environmental conditions in shaping population relatedness and social structure. Yet few studies have evaluated the effects of fluctuating environmental conditions on relatedness structure of solitary species in the wild. The aim of this study was to determine the impact of interannual variations in environmental conditions on the spatial distribution of relatives [spatial genetic structure (SGS)] and dispersal patterns of a wild population of eastern chipmunks (Tamias striatus), a solitary rodent of North America. Eastern chipmunks depend on the seed of masting trees for reproduction and survival. Here, we combined the analysis of the SGS of adults with direct estimates of juvenile dispersal distance during six contrasted years with different dispersal seasons, population sizes and seed production. We found that environmental conditions influences the dispersal distances of juveniles and that male juveniles dispersed farther than females. The extent of the SGS of adult females varied between years and matched the variation in environmental conditions. In contrast, the SGS of males did not vary between years. We also found a difference in SGS between males and females that was consistent with male-biased dispersal. This study suggests that both the dispersal behaviour and the relatedness structure in a population of a solitary species can be relatively labile and change according to environmental conditions. | 2075 | no | no good fitness measure | NA | no | no |
| 2076 | 2017 | 43025 | Wojcik, JM; Kawalko, A; Tokarska, M; Jaarola, M; Vallenback, P; Pertoldi, C | 2009 | Post-bottleneck mtDNA diversity in a free-living population of European bison: implications for conservation | A free-living population of European bison Bison bonasus in the Bialowieza Primeval Forest originated from only seven founder animals after a severe bottleneck that occurred at the beginning of the 20th century. Consequently, the contemporary population of the species is characterized by low genetic diversity. We studied a total of 195 individuals (127 males and 68 females). A 1429 bp fragment of mitochondrial DNA (mtDNA) including the D-loop region was analyzed in 87 individuals and revealed only three distinct haplotypes. Nucleotide (pi) and haplotype (H-d) diversity values were estimated for the European bison and were compared with pi and H-d estimated from three individuals of American bison Bison bison. Very low diversity values were found in the European bison in comparison with the diversity values found in the American bison. The low mtDNA variability in the European bison is in concordance with theoretical expectations for a species that has undergone a severe and recent bottleneck. A management strategy for the preservation of the rare and very rare haplotypes present in the Bialowieza population of the European bison is discussed. Furthermore, all 195 individuals were investigated for heteroplasmy involving these three haplotypes, in order to detect a possible association between heteroplasmy and the incidence of males affected by posthitis, a disease that affects the male reproductive organs, leading to necrotic lesions. Heteroplasmy was found in 15 females, in 17 males affected by posthitis and in 11 non-affected males, and no significant association was found. | 2076 | no | no good fitness measure, N pop differnt in GD? | NA | no | no |
| 2077 | 2017 | 43025 | Rodriguez, M; Rau, D; Angioi, SA; Bellucci, E; Bitocchi, E; Nanni, L; Knupffer, H; Negri, V; Papa, R; Attene, G | 2013 | European Phaseolus coccineus L. landraces: Population Structure and Adaptation, as Revealed by cpSSRs and Phenotypic Analyses | Relatively few studies have extensively analysed the genetic diversity of the runner bean through molecular markers. Here, we used six chloroplast microsatellites (cpSSRs) to investigate the cytoplasmic diversity of 331 European domesticated accessions of the scarlet runner bean (Phaseolus coccineus L.), including the botanical varieties albiflorus, bicolor and coccineus, and a sample of 49 domesticated and wild accessions from Mesoamerica. We further explored the pattern of diversity of the European landraces using 12 phenotypic traits on 262 individuals. For 158 European accessions, we studied the relationships between cpSSR polymorphisms and phenotypic traits. Additionally, to gain insights into the role of gene flow and migration, for a subset of 115 accessions, we compared and contrasted the results obtained by cpSSRs and phenotypic traits with those obtained in a previous study with 12 nuclear microsatellites (nuSSRs). Our results suggest that both demographic and selective factors have roles in the shaping of the population genetic structure of the European runner bean. In particular, we infer the existence of a moderate-to-strong cytoplasmic bottleneck that followed the expansion of the crop into Europe, and we deduce multiple domestication events for this species. We also observe an adaptive population differentiation in the phenology across a latitudinal gradient, which suggests that selection led to the diversification of the runner bean in Europe. The botanical varieties albiflorus, bicolor and coccineus, which are based solely on flower colour, cannot be distinguished based on these cpSSRs and nuSSRs, nor according to the 12 quantitative traits. | 2077 | no | no good fitness measure | NA | no | no |
| 2078 | 2017 | 43025 | Nyine, M; Uwimana, B; Swennen, R; Batte, M; Brown, A; Christelova, P; Hribova, E; Lorenzen, J; Dolezel, J | 2017 | Trait variation and genetic diversity in a banana genomic selection training population | Banana (Musa spp.) is an important crop in the African Great Lakes region in terms of income and food security, with the highest per capita consumption worldwide. Pests, diseases and climate change hamper sustainable production of bananas. New breeding tools with increased crossbreeding efficiency are being investigated to breed for resistant, high yielding hybrids of East African Highland banana (EAHB). These include genomic selection (GS), which will benefit breeding through increased genetic gain per unit time. Understanding trait variation and the correlation among economically important traits is an essential first step in the development and selection of suitable GS models for banana. In this study, we tested the hypothesis that trait variations in bananas are not affected by cross combination, cycle, field management and their interaction with genotype. A training population created using EAHB breeding material and its progeny was phenotyped in two contrasting conditions. A high level of correlation among vegetative and yield related traits was observed. Therefore, genomic selection models could be developed for traits that are easily measured. It is likely that the predictive ability of traits that are difficult to phenotype will be similar to less difficult traits they are highly correlated with. Genotype response to cycle and field management practices varied greatly with respect to traits. Yield related traits accounted for 31-35% of principal component variation under low and high input field management conditions. Resistance to Black Sigatoka was stable across cycles but varied under different field management depending on the genotype. The best cross combination was 1201K-1xSH3217 based on selection response (R) of hybrids. Genotyping using simple sequence repeat (SSR) markers revealed that the training population was genetically diverse, reflecting a complex pedigree background, which was mostly influenced by the male parents. | 2078 | no | no good fitness measure, N pop differnt in GD? | NA | no | no |
| 2079 | 2017 | 43025 | Nemri, A; Barrett, LG; Laine, AL; Burdon, JJ; Thrall, PH | 2012 | Population Processes at Multiple Spatial Scales Maintain Diversity and Adaptation in the Linum marginale - Melampsora lini Association | Host-pathogen coevolution is a major driver of species diversity, with an essential role in the generation and maintenance of genetic variation in host resistance and pathogen infectivity. Little is known about how resistance and infectivity are structured across multiple geographic scales and what eco-evolutionary processes drive these patterns. Across southern Australia, the wild flax Linum marginale is frequently attacked by its rust fungus Melampsora lini. Here, we compare the genetic and phenotypic structure of resistance and infectivity among population pairs from two regions where environmental differences associate with specific life histories and mating systems. We find that both host and pathogen populations are genetically distinct between these regions. The region with outcrossing hosts and pathogens that go through asexual cycles followed by sexual reproduction showed greater diversity of resistance and infectivity phenotypes, higher levels of resistance and less clumped within-population spatial distribution of resistance. However, in the region where asexual pathogens infect selfing hosts, pathogens were more infective and better adapted to sympatric hosts. Our findings largely agree with expectations based on the distinctly different host mating systems in the two regions, with a likely advantage for hosts undergoing recombination. For the pathogen in this system, sexual reproduction may primarily be a survival mechanism in the region where it is observed. While it appears to potentially have adverse effects on local adaptation in the short term, it may be necessary for longer-term coevolution with outcrossing hosts. | 2079 | probably no | no good fitness measure, N pop differnt in GD? | no good fitness measure of pop of different GD | no | no |
| 2080 | 2017 | 43025 | Andersson, M; Waldeck, P; Hanssen, SA; Moe, B | 2015 | Female sociality and kin discrimination in brood parasitism: unrelated females fight over egg laying | “In conspecific brood parasitism, some females (”“parasites”“) lay eggs in nests of other females of the same species (”“hosts”“). This reproductive tactic is particularly common in waterfowl, in which studies suggest that parasites are often related to the host. Here, we test the hypothesis that hosts may discriminate and reject unrelated parasites. Based on observations and >4100 h of digital video film, we analyze behavioral interactions at 65 nests of High Arctic common eiders during the laying sequence. We also estimate parasitism and host-parasite relatedness by albumen fingerprinting of 975 eggs from 232 nests. Among the video-filmed nests in which interactions were recorded during the egg-laying period, 11 had eggs from 2 females. At 8 of these 11 nests, there was overt female aggression and significantly lower host-parasite relatedness (mean coefficient of relationship r = -0.40) than in the nests with tolerant or no interactions (r = 0.91). The results demonstrate active female kin discrimination in common eiders, used against nonrelatives that try to lay eggs in the nest. Other females trying to access the nest were often prevented from doing so: in 65% of 34 such attempts, the sitting female rejected the intruder. Brood”“parasitism”" in eiders and other waterfowl is complex, ranging from violent female conflict and parasitic exploitation of the host’s parental care to nest takeover and potential kin selection favoring acceptance of related parasites. These and other aspects of female sociality in eiders are discussed | 2080 | no | brood parasitism | NA | no | no |
| 2081 | 2017 | 43025 | Benavente, ED; Ward, Z; Chan, W; Mohareb, FR; Sutherland, CJ; Roper, C; Campino, S; Clark, TG | 2017 | Genomic variation in Plasmodium vivax malaria reveals regions under selective pressure | Background Although Plasmodium vivax contributes to almost half of all malaria cases outside Africa, it has been relatively neglected compared to the more deadly P. falciparum. It is known that P. vivax populations possess high genetic diversity, differing geographically potentially due to different vector species, host genetics and environmental factors. Results We analysed the high-quality genomic data for 46 P. vivax isolates spanning 10 countries across 4 continents. Using population genetic methods we identified hotspots of selection pressure, including the previously reported MRP1 and DHPS genes, both putative drug resistance loci. Extra copies and deletions in the promoter region of another drug resistance candidate, MDR1 gene, and duplications in the Duffy binding protein gene (PvDBP) potentially involved in erythrocyte invasion, were also identified. For surveillance applications, continental-informative markers were found in putative drug resistance loci, and we show that organellar polymorphisms could classify P. vivax populations across continents and differentiate between Plasmodia spp. Conclusions This study has shown that genomic diversity that lies within and between P. vivax populations can be used to elucidate potential drug resistance and invasion mechanisms, as well as facilitate the molecular barcoding of the parasite for surveillance applications. | 2081 | no | gd of parasite | NA | no | no |
| 2082 | 2017 | 43025 | Geng, MM; Zhang, J; Peng, FX; Liu, X; Lv, XD; Mi, YY; Li, YH; Li, F; Xie, CJ; Sun, QX | 2016 | Identification and mapping of MLIW30, a novel powdery mildew resistance gene derived from wild emmer wheat | Powdery mildew, caused by Blumeria graminis f.sp. tritici (Bgt), is a destructive foliar disease of common wheat in areas with cool or maritime climates. Wild emmer wheat, Triticum turgidum ssp. dicoccoides, the progenitor of both domesticated tetraploid durum wheat and hexaploid bread wheat, harbors abundant genetic diversity related to resistance to powdery mildew that can be utilized for wheat improvement. An F-2 segregating population was obtained from a cross between resistant bread wheat line 2L6 and susceptible cultivar Liaochun 10, after which genetic analysis of F-2 and F-2-derived F-3 families was performed by inoculating plants with isolate Bgt E09. The results of this experiment demonstrated that powdery mildew resistance in 2L6, which was derived from wild emmer wheat accession IW30, was controlled by a single dominant gene, temporarily designated MLIW30. Nineteen SSR markers and two STS markers linked with MLIW30 were acquired by applying bulked segregant analysis. Finally, MLIW30 was located to the long arm of chromosome 4A and found to be flanked by simple sequence repeat markers XB1g2000.2 and XB1g2020.2 at 0.1 cM. Because no powdery mildew resistance gene in or derived from wild emmer wheat has been reported in wheat chromosome 4A, MLIW30 might be a novel Pm gene. | 2082 | no | no good fitness measure, N pop differnt in GD? | NA | no | no |
| 2083 | 2017 | 43025 | Li, ZY; Song, HQ; Wang, CR; Zhu, XQ | 2016 | Nucleotide variation in the Toxoplasma gondii micronemal protein 8 gene | Toxoplasma gondii is a successful opportunistic protozoan distributed worldwide, which can infect all vertebrates, leading to serious infection, blindness, and abortion. Micronemal (MIC) proteins are critically important for T. gondii infection, as they participate in various stages of the Toxoplasma life cycle, including invasion and attachment to host cells. MIC8 secretion relies on the concentration of intracellular calcium, and can mediate the invasion of T. gondii by interacting with soluble MIC3. To investigate genetic diversity of the MIC8 gene, 16 T. gondii strains from different hosts and geographical locations, and two reference isolates (ToxoDB: TGME49\_245490 and TGVEG\_245490) were examined in this study. The results showed that all the examined MIC8 genes are 2055 bp, with an A+T content ranging from 50.2 to 50.6%. Conversely, lower levels of variation were detected within their nucleotide and amino acid sequences. Phylogenetic analyses indicated that three classical genotypes of T. gondii and the ToxoDB#9 genotype did not group exclusively via Bayesian inference, maximum parsimony, neighbor joining, and/or maximum likelihood assays based on the nucleotide and amino acid sequences of the MIC8 gene. In summary, the T. gondii MIC8 gene is not a suitable marker for population genetic studies of this parasite. | 2083 | no | gd of pathogen | NA | no | no |
| 2084 | 2017 | 43025 | Hagen, F; Khayhan, K; Theelen, B; Kolecka, A; Polacheck, I; Sionov, E; Falk, R; Parnmen, S; Lumbsch, HT; Boekhout, T | 2015 | Recognition of seven species in the Cryptococcus gattii/Cryptococcus neoformans species complex | Phylogenetic analysis of II genetic loci and results from many genotyping studies revealed significant genetic diversity with the pathogenic Cryptococcus gattii/Cryptococcus neoformans species complex. Genealogical concordance, coalescence-based, and species tree approaches supported the presence of distinct and concordant lineages within the complex. Consequently, we propose to recognize the current C neoformans var. grubii and C neoformans var. neoformans as separate species, and five species within C gattii. The type strain of C. neoformans CBS132 represents a serotype AD hybrid and is replaced. The newly delimited species differ in aspects of pathogenicity, prevalence for patient groups, as well as biochemical and physiological aspects, such as susceptibility to antifungals. MALDI-TOF mass spectrometry readily distinguishes the newly recognized species. (C) 2015 The Authors. Published by Elsevier Inc. This is an open access article under the CC BY-NC-ND license (http://creativecommons.org/licenses/by-nc-nd/4.0/). | 2084 | no | gd of pathogen | NA | no | no |
| 2085 | 2017 | 43025 | Rice, BL; Acosta, MM; Pacheco, MA; Carlton, JM; Barnwell, JW; Escalante, AA | 2014 | The origin and diversification of the merozoite surface protein 3 (msp3) multi-gene family in Plasmodium vivax and related parasites | “The genus Plasmodium is a diversified group of parasites with more than 200 known species that includes those causing malaria in humans. These parasites use numerous proteins in a complex process that allows them to invade the red blood cells of their vertebrate hosts. Many of those proteins are part of multi-gene families; one of which is the merozoite surface protein-3 (msp3) family. The msp3 multi-gene family is considered important in the two main human parasites, Plasmodium vivax and Plasmodium falciparum, as its paralogs are simultaneously expressed in the blood stage (merozoite) and are immunogenic. There are large differences among Plasmodium species in the number of paralogs in this family. Such differences have been previously explained, in part, as adaptations that allow the different Plasmodium species to invade their hosts. To investigate this, we characterized the array containing msp3 genes among several Plasmodium species, including P. falciparum and P. vivax. We first found no evidence indicating that the msp3 family of P. falciparum was homologous to that of P. vivax. Subsequently, by focusing on the diverse clade of nonhuman primate parasites to which P. vivax is closely related, where homology was evident, we found no evidence indicating that the interspecies variation in the number of paralogs was an adaptation related to changes in host range or host switches. Overall, we hypothesize that the evolution of the msp3 family in P. vivax is consistent with a model of multi-allelic diversifying selection where the paralogs may have functionally redundant roles in terms of increasing antigenic diversity. Thus, we suggest that the expressed MSP3 proteins could serve as”“decoys”“, via antigenic diversity, during the critical process of invading the host red blood cells. (C) 2014 Elsevier Inc. All rights reserved.” | 2085 | no | gd of parasite | NA | no | no |
| 2086 | 2017 | 43025 | Groenewald, M; Groenewald, JZ; Linde, CC; Crous, PW | 2007 | Development of polymorphic microsatellite and single nucleotide polymorphism markers for Cercospora beticola (Mycosphaerellaceae) | The plant pathogenic fungus, Cercospora beticola, causes the most important foliage disease of sugar beet. A previous study has shown that isolates of opposite mating types are present in equal proportions in natural populations; therefore, the aim of this study was to develop highly reproducible polymorphic markers for analysing populations of C. beticola. Five microsatellite and four single nucleotide polymorphism (SNP) markers were developed that allow rapid screening of genetic diversity in C. beticola. Six populations were screened with these markers and all were found to be in gametic equilibrium, indicating random mating in C. beticola. | 2086 | no | gd of pathogen | NA | no | no |
| 2087 | 2017 | 43025 | Ranathunge, NP; Ford, R; Taylor, PWJ | 2009 | Development and optimization of sequence-tagged microsatellite site markers to detect genetic diversity within Colletotrichum capsici, a causal agent of chilli pepper anthracnose disease | Genomic libraries enriched for microsatellites from Colletotrichum capsici, one of the major causal agents of anthracnose disease in chilli pepper (Capsicum spp.), were developed using a modified hybridization procedure. Twenty-seven robust primer pairs were designed from microsatellite flanking sequences and were characterized using 52 isolates from three countries India, Sri Lanka and Thailand. Highest gene diversity of 0.857 was observed at the CCSSR1 with up to 18 alleles among all the isolates whereas the differentiation ranged from 0.05 to 0.45. The sequence-tagged microsatellite site markers developed in this study will be useful for genetic analyses of C. capsici populations. | 2087 | no | gd of pathogen | NA | no | no |
| 2088 | 2017 | 43025 | Ngoc, LBT; Verniere, C; Vital, K; Guerin, F; Gagnevin, L; Brisse, S; Ah-You, N; Pruvost, O | 2009 | Development of 14 minisatellite markers for the citrus canker bacterium, Xanthomonas citri pv. citri | We screened the genome of Xanthomonas citri pv. citri strain 306 for tandem repeats. A multiplex polymerase chain reaction protocol was used to assess the genetic diversity of 239 strains of X. citri pv. citri from Asia. The total number of alleles per locus ranged from three to 20. Using pooled data sets, 223 different haplotypes were identified. Successful amplifications were obtained at most loci for seven other X. citri pathovars. This typing scheme is expected to be useful at different spatial scales for population studies of pathovars of X. citri, several of which cause plant diseases of economic importance. | 2088 | no | gd of pathogen | NA | no | no |
| 2089 | 2017 | 43025 | Guttman, DS; Gropp, SJ; Morgan, RL; Wang, PW | 2006 | Diversifying selection drives the evolution of the type III secretion system pilus of Pseudomonas syringae | The plant pathogenic bacterium Pseudomonas syringae uses a type III secretion system to inject virulence proteins directly into the cytoplasm of its hosts. The P. syringae type III secretion apparatus is encoded, in part, by the HrpZ operon, which carries the hrpA gene encoding the pilin subunit of the pilus, various components of the structural apparatus, and the HrpZ harpin protein that is believed to produce pores in the host cell membrane. The pilus of the type III system comes into direct contact with the host cell and is, therefore, a likely target of the host’s pathogen surveillance systems. We sequenced and analyzed 22 HrpZ operons from P. syringae strains spanning the diversity of the species. Selection analyses, including K(a)/K(s) tests and Tajima’s D, revealed strong diversifying selection acting on the hrpA gene. This form of selection enables pathogens to maintain genetic diversity within their populations and is often driven by selection imposed by host defense systems. The HrpZ operon also revealed a single significant recombination event that dramatically changed the evolutionary relationships among P. syringae strains from 2 quite distinct phylogroups. This recombination event appears to have introduced genetic diversity into a clade of strains that may now be undergoing positive selection. The identification of diversifying selection acting on the Hrp pilus across the whole population sample and positive selection within one P. syringae lineage supports a trench warfare coevolutionary model between P. syringae and its plant hosts. | 2089 | no | gd of pathogen | NA | no | no |
| 2090 | 2017 | 43025 | Lee, RS; Radomski, N; Proulx, JF; Levade, I; Shapiro, BJ; McIntosh, F; Soualhine, H; Menzies, D; Behr, MA | 2015 | Population genomics of Mycobacterium tuberculosis in the Inuit | Nunavik, Quebec suffers from epidemic tuberculosis (TB), with an incidence 50-fold higher than the Canadian average. Molecular studies in this region have documented limited bacterial genetic diversity among Mycobacterium tuberculosis isolates, consistent with a founder strain and/or ongoing spread. We have used whole-genome sequencing on 163 M. tuberculosis isolates from 11 geographically isolated villages to provide a high-resolution portrait of bacterial genetic diversity in this setting. All isolates were lineage 4 (Euro-American), with two sublineages present (major, n = 153; minor, n = 10). Among major sublineage isolates, there was a median of 46 pairwise single-nucleotide polymorphisms (SNPs), and the most recent common ancestor (MRCA) was in the early 20th century. Pairs of isolates within a village had significantly fewer SNPs than pairs from different villages (median: 6 vs. 47, P < 0.00005), indicating that most transmission occurs within villages. There was an excess of nonsynonymous SNPs after the diversification of M. tuberculosis within Nunavik: The ratio of nonsynonymous to synonymous substitution rates (dN/dS) was 0.534 before the MRCA but 0.777 subsequently (P = 0.010). Nonsynonymous SNPs were detected across all gene categories, arguing against positive selection and toward genetic drift with relaxation of purifying selection. Supporting the latter possibility, 28 genes were partially or completely deleted since the MRCA, including genes previously reported to be essential for M. tuberculosis growth. Our findings indicate that the epidemiologic success of M. tuberculosis in this region is more likely due to an environment conducive to TB transmission than a particularly well-adapted strain. | 2090 | no | human | NA | no | no |
| 2091 | 2017 | 43025 | Steinberg, KM; Antonacci, F; Sudmant, PH; Kidd, JM; Campbell, CD; Vives, L; Malig, M; Scheinfeldt, L; Beggs, W; Ibrahim, M; Lema, G; Nyambo, TB; Omar, SA; Bodo, JM; Froment, A; Donnelly, MP; Kidd, KK; Tishkoff, SA; Eichler, EE | 2012 | Structural diversity and African origin of the 17q21.31 inversion polymorphism | The 17q21.31 inversion polymorphism exists either as direct (H1) or inverted (H2) haplotypes with differential predispositions to disease and selection. We investigated its genetic diversity in 2,700 individuals, with an emphasis on African populations. We characterize eight structural haplotypes due to complex rearrangements that vary in size from 1.08-1.49 Mb and provide evidence for a 30-kb H1-H2 double recombination event. We show that recurrent partial duplications of the KANSL1 gene have occurred on both the H1 and H2 haplotypes and have risen to high frequency in European populations. We identify a likely ancestral H2 haplotype (H2’) lacking these duplications that is enriched among African hunter-gatherer groups yet essentially absent from West African populations. Whereas H1 and H2 segmental duplications arose independently and before human migration out of Africa, they have reached high frequencies recently among Europeans, either because of extraordinary genetic drift or selective sweeps. | 2091 | no | human | NA | no | no |
| 2092 | 2017 | 43025 | Neher, RA; Hallatschek, O | 2013 | Genealogies of rapidly adapting populations | The genetic diversity of a species is shaped by its recent evolutionary history and can be used to infer demographic events or selective sweeps. Most inference methods are based on the null hypothesis that natural selection is a weak or infrequent evolutionary force. However, many species, particularly pathogens, are under continuous pressure to adapt in response to changing environments. A statistical framework for inference from diversity data of such populations is currently lacking. Towards this goal, we explore the properties of genealogies in a model of continual adaptation in asexual populations. We show that lineages trace back to a small pool of highly fit ancestors, in which almost simultaneous coalescence of more than two lineages frequently occurs. Whereas such multiple mergers are unlikely under the neutral coalescent, they create a unique genetic footprint in adapting populations. The site frequency spectrum of derived neutral alleles, for example, is nonmonotonic and has a peak at high frequencies, whereas Tajima’s D becomes more and more negative with increasing sample size. Because multiple merger coalescents emerge in many models of rapid adaptation, we argue that they should be considered as a null model for adapting populations. | 2092 | no | no good fitness measure, N pop differnt in GD? | NA | no | no |
| 2093 | 2017 | 43025 | Wilbert, TR; Woollett, DA; Whitelaw, A; Dart, J; Hoyt, JR; Galen, S; Ralls, K; Meade, DE; Maldonado, JE | 2015 | Non-invasive baseline genetic monitoring of the endangered San Joaquin kit fox on a photovoltaic solar facility | Survival of endangered San Joaquin kit foxes Vulpes macrotis mutica is challenged by reduced and fragmented habitat resulting from anthropogenic uses. We monitored kit foxes on the 40 km2 proposed site for the Topaz Solar Farms (TSF) in San Luis Obispo County, California, which consisted of 76% agricultural fields and 24% grasslands. Prior to construction of the solar facility in December 2011, we used professionally trained dog-handler teams to conduct noninvasive genetic surveys annually from 2009 to 2011. We analyzed mtDNA to identify species, zinc finger genes for sex determination, and microsatellite loci to define individuals. We identified 45 individuals from 351 fresh scat samples (26 females, 18 males, and 1 individual of unknown sex), and recaptured 5 individuals between years. Kit foxes predominantly used the grasslands and rarely used agricultural fields. Samples from the TSF population had similar levels of genetic diversity to 2 areas less than 20 km away in the northern end of the Carrizo Plain National Monument. Capwire and LDNe estimates of population size using samples collected during annual November surveys indicated that similar to 33 individuals used the TSF over a 3 yr period. The relatively high population estimate, low recapture rates, and similar genetic diversity to 2 nearby locations suggest that individuals using the TSF site are part of a larger population using the surrounding landscape. Our study provided baseline data that, when coupled with future surveys, will help assess the effects on San Joaquin kit foxes of solar facility construction and habitat regeneration on agricultural lands removed from production. | 2093 | no | no good fitness measure, N pop differnt in GD? | NA | no | no |
| 2094 | 2017 | 43025 | Konecna, E; Safarova, D; Navratil, M; Hanacek, P; Coyne, C; Flavell, A; Vishnyakova, M; Ambrose, M; Redden, R; Smykal, P | 2014 | Geographical Gradient of the eIF4E Alleles Conferring Resistance to Potyviruses in Pea (Pisum) Germplasm | Background: The eukaryotic translation initiation factor 4E was shown to be involved in resistance against several potyviruses in plants, including pea. We combined our knowledge of pea germplasm diversity with that of the eIF4E gene to identify novel genetic diversity. Methodology/Principal findings: Germplasm of 2803 pea accessions was screened for eIF4E intron 3 length polymorphism, resulting in the detection of four eIF4E(A-B-C-S) variants, whose distribution was geographically structured. The eIF4E(A) variant conferring resistance to the P1 PSbMV pathotype was found in 53 accessions (1.9%), of which 15 were landraces from India, Afghanistan, Nepal, and 7 were from Ethiopia. A newly discovered variant, eIF4E(B), was present in 328 accessions (11.7%) from Ethiopia (29%), Afghanistan (23%), India (20%), Israel (25%) and China (39%). The eIF4E(C) variant was detected in 91 accessions (3.2% of total) from India (20%), Afghanistan (33%), the Iberian Peninsula (22%) and the Balkans (9.3%). The eIF4E(S) variant for susceptibility predominated as the wild type. Sequencing of 73 samples, identified 34 alleles at the whole gene, 26 at cDNA and 19 protein variants, respectively. Fifteen alleles were virologically tested and 9 alleles (eIF4E(A-1-2-3-4-5-6-7), eIF4E(B-1), eIF4E(C-2)) conferred resistance to the P1 PSbMV pathotype. Conclusions/Significance: This work identified novel eIF4E alleles within geographically structured pea germplasm and indicated their independent evolution from the susceptible eIF4E(S1) allele. Despite high variation present in wild Pisum accessions, none of them possessed resistance alleles, supporting a hypothesis of distinct mode of evolution of resistance in wild as opposed to crop species. The Highlands of Central Asia, the northern regions of the Indian subcontinent, Eastern Africa and China were identified as important centers of pea diversity that correspond with the diversity of the pathogen. The series of alleles identified in this study provides the basis to study the co-evolution of potyviruses and the pea host. | 2094 | no | no good fitness measure, N pop differnt in GD? | NA | no | no |
| 2095 | 2017 | 43025 | Numminen, E; Gutmann, M; Shubin, M; Marttinen, P; Meric, G; van Schaik, W; Coque, TM; Baquero, F; Willems, RJL; Sheppard, SK; Feil, EJ; Hanage, WP; Corander, J | 2016 | The impact of host metapopulation structure on the population genetics of colonizing bacteria | Many key bacterial pathogens are frequently carried asymptomatically, and the emergence and spread of these opportunistic pathogens can be driven, or mitigated, via demographic changes within the host population. These inter-host transmission dynamics combine with basic evolutionary parameters such as rates of mutation and recombination, population size and selection, to shape the genetic diversity within bacterial populations. Whilst many studies have focused on how molecular processes underpin bacterial population structure, the impact of host migration and the connectivity of the local populations has received far less attention. A stochastic neutral model incorporating heightened local transmission has been previously shown to fit closely with genetic data for several bacterial species. However, this model did not incorporate transmission limiting population stratification, nor the possibility of migration of strains between subpopulations, which we address here by presenting an extended model. We study the consequences of migration in terms of shared genetic variation and show by simulation that the previously used summary statistic, the allelic mismatch distribution, can be insensitive to even large changes in microepidemic and migration rates. Using likelihood-free inference with genotype network topological summaries we fit a simpler model to commensal and hospital samples from the common nosocomial pathogens Staphylococcus aureus, Staphylococcus epidermidis, Enterococcus faecalis and Enterococcus faecium. Only the hospital data for E. faecium display clearly marked deviations from the model predictions which may be attributable to its adaptation to the hospital environment. (C) 2016 Elsevier Ltd. All rights reserved. | 2095 | no | no good fitness measure, N pop differnt in GD? | NA | no | no |
| 2096 | 2017 | 43025 | Foitzik, S; Backus, VL; Trindl, A; Herbers, JM | 2004 | Ecology of Leptothorax ants: impact of food, nest sites, and social parasites | In a long-term field manipulation, we demonstrate strong reactions of Leptothorax longispinosus ant colonies to food- and nest-site supplementation. Demographic and genetic responses varied over small geographic scales, and the two ecological factors interacted with the presence of the social parasite Protomognathus americanus. We conducted a 2x2 experiment in three blocks and found that the blocks, which were less than 100 m apart, reacted very differently to the treatments. Blocks differed in degree of polygyny, intranest relatedness, colony size, productivity, and sexual investment. Furthermore, these differences were associated with the presence of slave-making ants and the local availability of nest sites. Nest-site supplementation had a strong effect only in the site with the highest prevalence of social parasites, influencing there the density and investment patterns of colonies. L. longispinosus ants in the least parasitized area were strongly affected by both food- and nest-site supplementation. There, food supplementation led to a decrease in the number of queens per colony and consequently to an increase in intranest relatedness, while colonies in nest-site-supplemented areas invested fewer resources in males and produced a female-biased allocation ratio. By contrast, in a third block with a very low intracolonial relatedness, food supplementation induced an absolute and relative higher investment in males. We conclude that ecological factors influencing social organization in insect societies cannot be studied in isolation, because the interactions among factors produce far richer responses than any one variable. | 2096 | no | no good fitness measure, N pop differnt in GD? | NA | no | no |
| 2097 | 2017 | 43025 | Stern, CA; Dickinson, JL | 2016 | Effects of load-lightening and delayed extrapair benefits on the fitness consequences of helping behavior | In most cooperative breeders, helping is directed at close kin, allowing helpers to gain indirect fitness benefits by increasing the reproductive success of close relatives, usually their parents. Extrapair paternity (EPP) occurs at high rates in some cooperative breeders, reducing the relatedness of helpers to the young they help raise. Even so, a son that helps is related to the brood by at least 0.25 through his mother and to within-pair young by 0.5, whereas a potential helper that has EPP in his own nest is related only to the offspring he sires and unrelated to any extrapair offspring. In birds, EPP often favors older males, which in the extreme case can result in sons being more closely related to young in their parents’ nest than to young in their own nests. The fitness benefit of helping will thus be enhanced if helping lightens the workload and increases survival of helpers and their fathers, enabling them to become old, hyper-successful extrapair sires. Here, we develop and analyze a proof-of-concept model, grounded in the western bluebird (Sialia mexicana) system, demonstrating the conditions under which high population levels of EPP can generate inclusive fitness benefits of helping behavior that outweigh the costs. This model provides a new perspective on the relationship between EPP and helping behavior in cooperative breeders and suggests a strong need for empirical work to gather unprecedented data on paternity over the lifetime of helpers and their parents. | 2097 | no | no good fitness measure, N pop differnt in GD? | NA | no | no |
| 2098 | 2017 | 43025 | Aylward, J; Dreyer, LL; Steenkamp, ET; Wingfield, MJ; Roets, F | 2014 | Panmixia defines the genetic diversity of a unique arthropod-dispersed fungus specific to Protea flowers | Knoxdaviesia proteae, a fungus specific to the floral structures of the iconic Cape Floral Kingdom plant, Protea repens, is dispersed by mites phoretic on beetles that pollinate these flowers. Although the vectors of K. proteae have been identified, little is known regarding its patterns of distribution. Seed bearing infructescences of P. repens were sampled from current and previous flowering seasons, from which K. proteae individuals were isolated and cultured. The genotypes of K. proteae isolates were determined using 12 microsatellite markers specific to this species. Genetic diversity indices showed a high level of similarity between K. proteae isolates from the two different infructescence age classes. The heterozygosity of the population was high (0.74 +/- 0.04), and exceptional genotypic diversity was encountered ((G) over cap = 97.87%). Population differentiation was negligible, owing to the numerous migrants between the infructescence age classes (N-m = 47.83) and between P. repens trees (N-m = 2.96). Parsimony analysis revealed interconnected genotypes, indicative of recombination and homoplasies, and the index of linkage disequilibrium confirmed that outcrossing is prevalent in K. proteae ((r) over bar (d) = 0.0067; P = 0.132). The high diversity and panmixia in this population is likely a result of regular gene flow and an outcrossing reproductive strategy. The lack of genetic cohesion between individuals from a single P. repens tree suggests that K. proteae dispersal does not primarily occur over short distances via mites as hypothesized, but rather that long-distance dispersal by beetles plays an important part in the biology of these intriguing fungi. | 2098 | no | no good fitness measure, N pop differnt in GD? | NA | no | no |
| 2099 | 2017 | 43025 | LEBERG, PL; VRIJENHOEK, RC | 1994 | VARIATION AMONG DESERT TOPMINNOWS IN THEIR SUSCEPTIBILITY TO ATTACK BY EXOTIC PARASITES | Exposure of native species to novel disease organisms is a major threat posed by introduced exotic species. The genetic background of a native population should affect its response to an exotic disease. We evaluate the susceptibility of sexually and asexually reproducing lineages of fish (genus Poeciliopsis) to infection by a novel trematode parasite (Gyrodactylus turnbulli). The parasite commonly infects guppies (Poecilia reticulata), which have obtained a worldwide distribution as a consequence of their value in the pet trade. We examined two sexual species and two related clonal (nonrecombinant) lineages of Poeciliopsis that coexist naturally in Sonora, Mexico. One sexual species (P. monacha) and one clone (ML/VIII) were completely refractory to infection by G. turnbulli. The other sexual species (P. lucida) was relatively resistant. The second clone (ML/VII) was susceptible, however, and infections led to increased mortality. Chance fixations of alleles that occurred during the hybrid origins of these clonal lineages probably were responsible for the differences in susceptibility between lineages. Lack of heritable genetic diversity within a clonal lineage would hamper its ability to quickly evolve a suitable response to an exotic parasite and would make it particularly vulnerable to extinction. | 2099 | no | No GD diff between pop | NA | no | no |
| 2100 | 2017 | 43025 | Stow, A; Briscoe, D; Gillings, M; Holley, M; Smith, S; Leys, R; Silberbauer, T; Turnbull, C; Beattie, A | 2007 | Antimicrobial defences increase with sociality in bees | Evidence for the antiquity and importance of microbial pathogens as selective agents is found in the proliferation of antimicrobial defences throughout the animal kingdom. Social insects, typified by crowding and often by low genetic variation, have high probabilities of disease transmission and eusocial Hymenoptera may be particularly vulnerable because of haplodiploidy. Mechanisms they employ to reduce the risk of disease include antimicrobial secretions which are particularly important primary barriers to infection. However, until now, whether or not there is selection for stronger antimicrobial secretions when the risk of disease increases because of sociality has not been tested. Here, we present evidence that the production of progressively stronger antimicrobial compounds was critical to the evolution of sociality in bees. We found that increases in group size and genetic relatedness were strongly correlated with increasing antimicrobial strength. The antimicrobials of even the most primitive semi-social species were an order of magnitude stronger that those of solitary species, suggesting a point of no return, beyond which disease control was essential. Our results suggest that selection by microbial pathogens was critical to the evolution of sociality and required the production of strong, front-line antimicrobial defences. | 2100 | maybe | ok gd measurement and pop level comp? | 6 species in same analysis, no data per species and pop level analysis | no | no |
| 2101 | 2017 | 43025 | Ballingall, KT; Waibochi, L; Holmes, EC; Woelk, CH; MacHugh, ND; Lutje, V; McKeever, DJ | 2001 | The CD45 locus in cattle: allelic polymorphism and evidence for exceptional positive natural selection | Cattle in Africa are a genetically diverse population that has resulted from successive introduction of Asian Bos indicus and European B. taurus cattle. However, analysis of mitochondrial genetic diversity in African cattle identified three lineages, one associated with Asian B. indicus, one with European B. taurus, and a third ascribed to an indigenous African sub-species of cattle. Due to their extended co-evolution, indigenous African herbivores are generally tolerant to endemic African pathogens. We are interested in identifying alleles derived from the indigenous African cattle that may be associated with tolerance to African pathogens. An analysis of the locus which encodes the abundant plasma membrane-associated tyrosine phosphatase, CD45, identified three highly divergent allelic families in Kenya Boran cattle. Analysis of allelic distribution in a diverse range of cattle populations suggests a European B. taurus, an Asian B, indicus, and an African origin. This demonstrates not only significant allelic polymorphism at the CD45 locus in cattle but also convincing autosomal evidence for a distinct African sub-species of cattle. Furthermore, maximum-likelihood analysis of selection pressures revealed that the CD45 locus is subject to exceptionally strong natural selection which we suggest may be pathogen driven. | 2101 | no | no good fitness measure, N pop differnt in GD? | NA | no | no |
| 2102 | 2017 | 43025 | Ellegren, H; Mikko, S; Wallin, K; Andersson, L | 1996 | Limited polymorphism at major histocompatibility complex (MHC) loci in the Swedish moose A-alces | The Swedish moose was analysed for genetic variability at major histocompatibility complex (MHC) class I and class II DQA, DQB and DRB loci using restriction fragment length polymorphism (RFLP) and single strand conformation polymorphism (SSCP) techniques. Both methods revealed limited amounts of polymorphism. Since the SSCP analysis concerned an expressed DRB gene it can be concluded that the level of functional MHC class II polymorphism at least at the DRB locus, is low in Swedish moose. DNA fingerprinting was used to determine if the unusual pattern of low MHC variability could be explained by a low degree of genome-wide genetic diversity. Hybridizations with two minisatellite probes gave similarity indices somewhat higher than the average for other natural population, but the data suggest that the low MHC variability cannot be explained by a recent population bottleneck. However, since minisatellite sequences evolve more rapidly than MHC sequences, the low levels of MHC diversity may be attributed to a bottleneck of more ancient origin. The selection pressure for MHC variability in moose may also be reduced and we discuss the possibility that its solitary life style may reduce lateral transmission of pathogens in the population. | 2102 | no | no good fitness measure, N pop differnt in GD? | NA | no | no |
| 2103 | 2017 | NA | Peros, JP; Berger, G | 1999 | Diversity within natural progenies of the grapevine dieback fungus Eutypa lata | The diversity within 16 natural progenies of the grapevine dieback fungus, Eutypa lata, was investigated by sampling single-ascospore isolates mainly in France and using random amplified polymorphic DNA (RAPD) markers, vegetative compatibility (VC), and pathogenicity testing. The combination of RAPD and VC data identified each isolate as a unique genotype within each progeny. Only three RAPD haplotypes did not cluster within the expected groups. i.e. the ascospore families. Within each set of clustering haplotypes, Mendelian 1:1 ratios for absence and presence were observed for RAPD markers, indicating that each progeny was the result of a biparental cross. Only one mycelium was obtained when isolation was performed from the discolored wood sustaining the perithecial stroma. This mycelium was identified as a likely parent of the corresponding progeny by RAPD analysis. The level of diversity measured by the average distance between haplotypes calculated from RAPD data, the percentage of vegetatively compatible pairs and the range of pathogenicity appeared similar between all but one progeny, indicating that crosses occurred within a random-matting population. All the results were consistent with the hypothesis that E. lain is a random-mating species having a high degree of genetic diversity. | 2103 | NA | NA | NA | no | no |
| 2104 | 2017 | NA | Banks, SC; Finlayson, GR; Lawson, SJ; Lindenmayer, DB; Paetkau, D; Ward, SJ; Taylor, AC | 2005 | The effects of habitat fragmentation due to forestry plantation establishment on the demography and genetic variation of a marsupial carnivore, Antechinus agilis | We conducted a demographic and genetic study to investigate the effects of fragmentation due to the establishment of an exotic softwood plantation on populations of a small marsupial carnivore, the agile antechinus (Antechinus agilis), and the factors influencing the persistence of those populations in the fragmented habitat. The first aspect of the study was a descriptive analysis of patch occupancy and population size, in which we found a patch occupancy rate of 70% among 23 sites in the fragmented habitat compared to 100% among 48 sites with the same habitat characteristics in unfragmented habitat. Mark-recapture analyses yielded most-likely population size estimates of between 3 and 85 among the 16 occupied patches in the fragmented habitat. Hierarchical partitioning and model selection were used to identify geographic and habitat-related characteristics that influence patch occupancy and population size. Patch occupancy was primarily influenced by geographic isolation and habitat quality (vegetation basal area). The variance in population size among occupied sites was influenced primarily by forest type (dominant Eucalyptus species) and, to a lesser extent, by patch area and topographic context (gully sites had larger populations). A comparison of the sex ratios between the samples from the two habitat contexts revealed a significant deficiency of males in the fragmented habitat. We hypothesise that this is due to male-biased dispersal in an environment with increased dispersal-associated mortality. The population size and sex ratio data were incorporated into a simulation study to estimate the proportion of genetic diversity that would have been lost over the known timescale since fragmentation if the patch populations had been totally isolated. The observed difference in genetic diversity (gene diversity and allelic richness at microsatellite and mitochondrial markers) between 16 fragmented and 12 unfragmented sites was extremely low and inconsistent with the isolation of the patch populations. Our results show that although the remnant habitat patches comprise approximately 2% of the study area, they can support non-isolated populations. However, the distribution of agile antechinus populations in the fragmented system is dependent on habitat quality and patch connectivity. (C) 2004 Elsevier Ltd. All rights reserved. | 2104 | NA | NA | NA | no | no |
| 2105 | 2017 | NA | Ravelomanantsoa, S; Robene, I; Chiroleu, F; Guerin, F; Poussier, S; Pruvost, O; Prior, P | 2016 | A novel multilocus variable number tandem repeat analysis typing scheme for African phylotype III strains of the Ralstonia solanacearum species complex | Background. Reliable genotyping that provides an accurate description of diversity in the context of pathogen emergence is required for the establishment of strategies to improve disease management. MultiLocus variable number tandem repeat analysis (MLVA) is a valuable genotyping method. It can be performed at small evolutionary scales where high discriminatory power is needed. Strains of the Ralstonia solanacearum species complex (RSSC) are highly genetically diverse. These destructive pathogens are the causative agent of bacterial wilt on an unusually broad range of host plants worldwide. In this study, we developed an MLVA scheme for genotyping the African RSSC phylotype III. Methods. We selected different publicly available tandem repeat (TR) loci and additional TR loci from the genome of strain CMR15 as markers. Based on these loci, a new phylotype III-MLVA scheme is presented. MLVA and multiLocus sequence typing (MLST) were compared at the global, regional, and local scales. Different populations of epidemiologically related and unrelated RSSC phylotype III strains were used. Results and Discussion. Sixteen polymorphic TR loci, which included seven microsatellites and nine minisatellites, were selected. These TR loci were distributed throughout the genome (chromosome and megaplasmid) and located in both coding and intergenic regions. The newly developed RS3-MLVA16 scheme was more discriminative than MLST. RS3-MLVA16 showed good ability in differentiating strains at global, regional, and local scales, and it especially highlighted epidemiological links between closely related strains at the local scale. RS3-MLVA16 also underlines genetic variability within the same MLST-type and clonal complex, and gives a first overview of population structure. Overall, RS3-MLVA16 is a promising genotyping method for outbreak investigation at a fine scale, and it could be used for outbreak investigation as a first-line, low-cost assay for the routine screening of RSSC phylotype III. | 2105 | NA | NA | NA | no | no |
| 2106 | 2017 | NA | Diezmann, S; Dietrich, FS | 2011 | Oxidative Stress Survival in a Clinical Saccharomyces cerevisiae Isolate Is Influenced by a Major Quantitative Trait Nucleotide | One of the major challenges in characterizing eukaryotic genetic diversity is the mapping of phenotypes that are the cumulative effect of multiple alleles. We have investigated tolerance of oxidative stress in the yeast Saccharomyces cerevisiae, a trait showing phenotypic variation in the population. Initial crosses identified that this is a quantitative trait. Microorganisms experience oxidative stress in many environments, including during infection of higher eukaryotes. Natural variation in oxidative stress tolerance is an important aspect of response to oxidative stress exerted by the human immune system and an important trait in microbial pathogens. A clinical isolate of the usually benign yeast S. cerevisiae was found to survive oxidative stress significantly better than the laboratory strain. We investigated the genetic basis of increased peroxide survival by crossing those strains, phenotyping 1500 segregants, and genotyping of high-survival segregants by hybridization of bulk and single segregant DNA to microarrays. This effort has led to the identification of an allele of the transcription factor Rds2 as contributing to stress response. Rds2 has not previously been associated with the survival of oxidative stress. The identification of its role in the oxidative stress response here is an example of a specific trait that appears to be beneficial to Saccharomyces cerevisiae when growing as a pathogen. Understanding the role of this fungal-specific transcription factor in pathogenicity will be important in deciphering how fungi infect and colonize the human host and could eventually lead to a novel drug target. | 2106 | NA | NA | NA | no | no |
| 2107 | 2017 | NA | Delgado, G; Souza, V; Morales, R; Cerritos, R; Gonzalez-Gonzalez, A; Mendez, JL; Vazquez, V; Cravioto, A | 2013 | Genetic Characterization of Atypical Citrobacter freundii | The ability of a bacterial population to survive in different niches, as well as in stressful and rapidly changing environmental conditions, depends greatly on its genetic content. To survive such fluctuating conditions, bacteria have evolved different mechanisms to modulate phenotypic variations and related strategies to produce high levels of genetic diversity. Laboratories working in microbiological diagnosis have shown that Citrobacter freundii is very versatile in its colony morphology, as well as in its biochemical, antigenic and pathogenic behaviours. This phenotypic versatility has made C. freundii difficult to identify and it is frequently confused with both Salmonella enterica and Escherichia coli. In order to determine the genomic events and to explain the mechanisms involved in this plasticity, six C. freundii isolates were selected from a phenotypic variation study. An I-CeuI genomic cleavage map was created and eight housekeeping genes, including 16S rRNA, were sequenced. In general, the results showed a range of both phenotypes and genotypes among the isolates with some revealing a greater similarity to C. freundii and some to S. enterica, while others were identified as phenotypic and genotypic intermediary states between the two species. The occurrence of these events in natural populations may have important implications for genomic diversification in bacterial evolution, especially when considering bacterial species boundaries. In addition, such events may have a profound impact on medical science in terms of treatment, course and outcomes of infectious diseases, evading the immune response, and understanding host-pathogen interactions. | 2107 | NA | NA | NA | no | no |
| 2108 | 2017 | NA | Hillis, JJ; Garvey, JE; Lydy, MJ | 2015 | Contaminants reduce male contribution to reproduction at the population scale | In many populations, males compete for mates and contribute to genetic diversity, but are assumed to have little impact on population growth rates and density, because one father is all that is necessary to fertilize a large number of eggs and sire a potential cohort. A factor affecting male reproductive output is exposure to contaminants that either disrupt endocrine function or mimic hormones. Polychlorinated biphenyls (PCBs) persist in aquatic ecosystems and may interfere with the reproductive contribution of male fish to populations. We quantified 28 PCB congeners in adult male bluegills across eight Illinois lakes. In spring 2011 and 2012, mature male bluegills from lakes with conspecifics with PCB whole-body residues ranging from undetectable to high (2,000 ng/g dry mass total PCBs) were placed in experimental ponds with mature females containing no detectable PCB body residues and allowed to reproduce in spring under natural conditions. The presence of PCBs in fish had no effect on the presence of intersex or other gonadal deformities in adult males nor was gonad size, lipid concentration, and survival affected. Males with high PCBs were heavier than those from lakes with low PCB concentrations. The relative density of juvenile bluegills produced in ponds by fall, a fitness endpoint in this species, was negatively related to contaminant concentration in fathers. Contaminant exposure and other factors that affect male reproduction may not only suppress the relative contribution of males to genetic diversity, but also the overall density of cohorts within populations. | 2108 | NA | NA | NA | no | no |
| 2109 | 2017 | NA | SCHMIDHEMPEL, P; SCHMIDHEMPEL, R | 1993 | TRANSMISSION OF A PATHOGEN IN BOMBUS-TERRESTRIS, WITH A NOTE ON DIVISION-OF-LABOR IN SOCIAL INSECTS | Parasites of social insect workers can be transmitted within the colony to other, related host individuals or, alternatively, to unrelated workers of other colonies. Division of labour affects the probability of transmission, as young individuals often work inside the nest whereas older ones often leave the nest to forage. Therefore, the relative probabilities of transmission with-in vs. between-nests is also affected by the delay between host infection and the shedding of propagules, i.e. the latent period of the parasite strain. We therefore hypothesized that strains of the flagellate parasite Crithidia bombi (Trypanosomatidae, Zoomastigophorea) infecting workers of the bumble bee Bombus terrestris (Hymenoptera, Apidae) could differ in their delays and coexist in a population. This would be the case if strains that are shed after a short time delay were more efficiently transmitted to other colony members, whereas strains with long delays were more efficiently transmitted to non-related workers in the population. We tested this hypothesis by experimentally varying time delay and by allowing transmission to either sister workers from the same nest or unrelated workers from other nests. Transmission of C. bombi was measured as the number of parasitic cells shed by the exposed workers after a standard period. The results showed that relatedness as such had no effect, but that delay and nest identity were highly significant effects to explain variation in transmission success. There was a significant interaction between nest identity and delay, such that bees of some colonies acted as efficient transmitters for C. bombi under short delays and vice versa. We discuss how division of labour may affect parasitism in social insects and, vice versa, how division of labour may be under selection from the effects of parasitism, using available evidence from the literature. | 2109 | NA | NA | NA | no | no |
| 2110 | 2017 | NA | Yuan, CP; Zhou, G; Li, YH; Wang, KJ; Wang, Z; Li, XH; Chang, RZ; Qiu, LJ | 2008 | Cloning and sequence diversity analysis of GmHs1(pro-1) in Chinese domesticated and wild soybeans | Soybean cyst nematode (SCN) (Heterodera glycines Ichinohe) is the most important pathogen in soybean production worldwide and causes substantial yield losses. An apparent narrow genetic base of SCN resistance was observed in current elite soybean cultivars, and searching for novel SCN resistance genes as well as novel resistance sources rather than focusing on the two important genes rhg1 and Rhg4 has become another major objective in soybean research. In the present paper we report a 1,477 bp Hs1(pro-1) homolog, named GmHs1(pro-1). This gene was cloned from soybean variety Wenfeng 7 based on information for Hs1(pro-1), a beet cyst nematode resistance gene in sugar beet. It has two domains, Hs1pro-1\_N and Hs1pro-1\_C, both of which are believed to confer resistance to nematodes. Of the 1,477 bp sequence in GmHs1(pro-1), an open reading frame of 1,314 bp, encoding a protein with 437 amino acids, was flanked by a 5’-untranslated region of 27 bp and a 3’-untranslated region of 135 bp. Fourteen single-nucleotide polymorphisms (SNPs) were observed in 44 soybean accessions including 23 wild soybeans, 8 landraces, and 13 soybean varieties (or lines), among which 5 in wild soybeans and 3 in landrace accessions were unique. Sequence diversity analysis on the 44 soybean accessions showed pi = 0.00168 and theta = 0.00218 for GmHs1(pro-1); landraces had the highest diversity, followed by wild soybeans, with varieties (or lines) having the lowest. Although we did not detect a significant effect of selection on GmHs1(pro-1) in the three populations, sequence diversity, unique SNPs, and phylogenetic analysis indicated a slight domestication bottleneck and an intensive selection bottleneck. High sequence diversity, more unique SNPs, and broader representation across the phylogenetic tree in wild soybeans and landraces indicated that wild collections and landrace accessions are invaluable germplasm for broadening the genetic base of elite soybean varieties resistant to SCN. | 2110 | NA | NA | NA | no | no |
| 2111 | 2017 | NA | Singh, A; Shailaja, K; Gaur, A; Singh, L | 2002 | Development and characterization of novel microsatellite markers in the Asiatic lion (Panthera leo persica) | All cat species, except domestic cats, are either threatened or endangered. The Asiatic lion (Panthera leo persica), a critically endangered species, is driven to a single habitat zone in Gir forests in India for its survival. The present status of this big cat compels us to acquire deeper knowledge of its genetic variability and phylogenetic relationships. Here we report the isolation of highly polymorphic microsatellite loci from a partial genomic library of the Asiatic lion. These loci were characterized and have shown high levels of variation ranging from six to 11 alleles per locus in the population studied. | 2111 | NA | NA | NA | no | no |
| 2112 | 2017 | NA | Watson, CT; Steinberg, KM; Graves, TA; Warren, RL; Malig, M; Schein, J; Wilson, RK; Holt, RA; Eichler, EE; Breden, F | 2015 | Sequencing of the human IG light chain loci from a hydatidiform mole BAC library reveals locus-specific signatures of genetic diversity | Germline variation at immunoglobulin (IG) loci is critical for pathogen-mediated immunity, but establishing complete haplotype sequences in these regions has been problematic because of complex sequence architecture and diploid source DNA. We sequenced BAC clones from the effectively haploid human hydatidiform mole cell line, CHMThtert, across the light chain IG loci, kappa (IGK) and lambda (IGL), creating single haplotype representations of these regions. The IGL haplotype generated here is 1.25 Mb of contiguous sequence, including four novel IGLV alleles, one novel IGLC allele, and an 11.9-kb insertion. The CH17 IGK haplotype consists of two 644 kb proximal and 466 kb distal contigs separated by a large gap of unknown size; these assemblies added 49 kb of unique sequence extending into this gap. Our analysis also resulted in the characterization of seven novel IGKV alleles and a 16.7-kb region exhibiting signatures of interlocus sequence exchange between distal and proximal IGKV gene clusters. Genetic diversity in IGK/IGL was compared with that of the IG heavy chain (IGH) locus within the same haploid genome, revealing threefold (IGK) and sixfold (IGL) higher diversity in the IGH locus, potentially associated with increased levels of segmental duplication and the telomeric location of IGH. | 2112 | NA | NA | NA | no | no |
| 2113 | 2017 | NA | Burall, LS; Grim, CJ; Mammel, MK; Datta, AR | 2016 | Whole Genome Sequence Analysis Using JSpecies Tool Establishes Clonal Relationships between Listeria monocytogenes Strains from Epidemiologically Unrelated Listeriosis Outbreaks | In an effort to build a comprehensive genomic approach to food safety challenges, the FDA has implemented a whole genome sequencing effort, GenomeTrakr, which involves the sequencing and analysis of genomes of foodborne pathogens. As a part of this effort, we routinely sequence whole genomes of Listeria monocytogenes (Lm) isolates associated with human listeriosis outbreaks, as well as those isolated through other sources. To rapidly establish genetic relatedness of these genomes, we evaluated tetranucleotide frequency analysis via the JSpecies program to provide a cursory analysis of strain relatedness. The JSpecies tetranucleotide (tetra) analysis plots standardized (z-score) tetramer word frequencies of two strains against each other and uses linear regression analysis to determine similarity (r(2)). This tool was able to validate the close relationships between outbreak related strains from four different outbreaks. Included in this study was the analysis of Lm strains isolated during the recent caramel apple outbreak and stone fruit incident in 2014. We identified that many of the isolates from these two outbreaks shared a common 4b variant (4bV) serotype, also designated as IVb-v1, using a qPCR protocol developed in our laboratory. The 4bV serotype is characterized by the presence of a 6.3 Kb DNA segment normally found in serotype 1/2a, 3a, 1/2c and 3c strains but not in serotype 4b or 1/2b strains. We decided to compare these strains at a genomic level using the JSpecies Tetra tool. Specifically, we compared several 4bV and 4b isolates and identified a high level of similarity between the stone fruit and apple 4bV strains, but not the 4b strains co-identified in the caramel apple outbreak or other 4b or 4bV strains in our collection. This finding was further substantiated by a SNP-based analysis. Additionally, we were able to identify close relatedness between isolates from clinical cases from 1993-1994 and a single case from 2011 as well as links between two isolates from over 30 years ago. The identification of these potential links shows that JSpecies Tetra analysis can be a useful tool in rapidly assessing genetic relatedness of Lm isolates during outbreak investigations and for comparing historical isolates. Our analyses led to the identification of a highly related clonal group involved in two separate outbreaks, stone fruit and caramel apple, and suggests the possibility of a new genotype that may be better adapted for certain foods and/or environment. | 2113 | NA | NA | NA | no | no |
| 2114 | 2017 | NA | Kozyra, M; Ingelman-Sundberg, M; Lauschke, VM | 2017 | Rare genetic variants in cellular transporters, metabolic enzymes, and nuclear receptors can be important determinants of interindividual differences in drug response | Purpose: In this study we characterized the genetic variability of 146 clinically relevant genes influencing drug pharmacokinetics in African and European subpopulations, which are key determinants for interindividual variations in drug efficacy and adverse drug reactions. Methods: 13y integrating data from the 1000 Genomes Project (n = 1,092 individuals) and the Exome Sequencing Project (ESP; n = 6,503 individuals), single-nucleotide variants (SNVs) were identified and analyzed regarding frequency, functional consequences, and ethnic diversity. Results: In total, we found 12,152 SNVs in exons, 312 of which were novel. The majority of variants were rare (minor allele frequency (MAF) <1%; 92.9%) and nonsynonymous (56.2%). We calculated that individuals of European and African descent harbor, on average, 100.8 and 121.4 variants across the 146 pharmacogenes studied, respectively. Additionally, by analyzing variation patterns across these populations, we pinpointed potential priority genes for population-adjusted genetic profiling strategies. Furthermore, we estimated, based on our variant frequency analyses, that approximately 30-40% of functional variability in pharmacogenes can be attributed to rare variants. Conclusions: Our results indicate that these clinically important genes are genetically highly variable and differ considerably between populations. Furthermore, the large extent of rare variants emphasizes the need for sequencing-based approaches and effective functionality predictions to allow for true personalized medicine. | 2114 | NA | NA | NA | no | no |
| 2115 | 2017 | NA | Kang, YJ; Diao, XN; Zhao, GY; Chen, MH; Xiong, YW; Shi, M; Fu, WM; Guo, YJ; Pan, B; Chen, XP; Holmes, EC; Gillespie, JJ; Dumler, SJ; Zhang, YZ | 2014 | Extensive diversity of Rickettsiales bacteria in two species of ticks from China and the evolution of the Rickettsiales | Background: Bacteria of the order Rickettsiales (Alphaproteobacteria) are obligate intracellular parasites that infect species from virtually every major eukaryotic lineage. Several rickettsial genera harbor species that are significant emerging and re-emerging pathogens of humans. As species of Rickettsiales are associated with an extremely diverse host range, a better understanding of the historical associations between these bacteria and their hosts will provide important information on their evolutionary trajectories and, particularly, their potential emergence as pathogens. Results: Nine species of Rickettsiales (two in the genus Rickettsia, three in the genus Anaplasma, and four in the genus Ehrlichia) were identified in two species of hard ticks ( Dermacentor nuttalli and Hyalomma asiaticum) from two geographic regions in Xinjiang through genetic analyses of 16S rRNA, gltA, and groEL gene sequences. Notably, two lineages of Ehrlichia and one lineage of Anaplasma were distinct from any known Rickettsiales, suggesting the presence of potentially novel species in ticks in Xinjiang. Our phylogenetic analyses revealed some topological differences between the phylogenies of the bacteria and their vectors, which led us to marginally reject a model of exclusive bacteria-vector co-divergence. Conclusions: Ticks are an important natural reservoir of many diverse species of Rickettsiales. In this work, we identified a single tick species that harbors multiple species of Rickettsiales, and uncovered extensive genetic diversity of these bacteria in two tick species from Xinjiang. Both bacteria-vector co-divergence and cross-species transmission appear to have played important roles in Rickettsiales evolution. | 2115 | NA | NA | NA | no | no |
| 2116 | 2017 | NA | LUBBERS, EL; GILL, KS; COX, TS; GILL, BS | 1991 | VARIATION OF MOLECULAR MARKERS AMONG GEOGRAPHICALLY DIVERSE ACCESSIONS OF TRITICUM-TAUSCHII | “Triticum tauschii (Coss.) Schmal. (genome DD), a diploid progenitor of hexaploid wheat (Triticum aestivum L.; AABBDD), grows across large areas of southwest Asia and contains more genetic variability for disease and insect resistance, isozymes, and seed storage proteins than the D genome of T. aestivum. To study patterns of variability at a large number of loci, we determined restriction fragment length polymorphism genotypes at 25 loci in a germ-plasm collection of 102 T. tauschii accessions. All accessions were homozygous at all loci, so”“alleles”" and "“genotypes”" were equivalent. Twenty loci were polymorphic, with two to six genotypes per locus and polymorphic indexes ranging from 0.06 to 0.74. Linkage disequilibrium was widespread. On the basis of Hedrick’s probability of genotypic identity, botanical varieties T. t. ssp. eusquarrosa var. typica and T. t. ssp. eusquarrosa var. anathera were very similar to each other, as were T. t. ssp. strangulata and T. t. ssp. eusquarrosa var. meyeri, with a large genetic distance between these two pairs of taxonomic groups. Genetic variability for molecular markers was highest near the Caspian Sea, intermediate in Afghanistan, and lowest in Turkey and Pakistan. Genetic and geographical distances were related and generally consistent with the hypothesis that T. tauschii originated near the southern or southwestern coast of the Caspian Sea. Unique genotypes were found in most regions. The results of this study, along with data on economically relevant traits, will provide a basis for selecting breeding parents from the T. tauschii germ-plasm collection." | 2116 | NA | NA | NA | no | no |
| 2117 | 2017 | NA | Fuentes, MMPB; Pike, DA; Dimatteo, A; Wallace, BP | 2013 | Resilience of marine turtle regional management units to climate change | Enhancing species resilience to changing environmental conditions is often suggested as a climate change adaptation strategy. To effectively achieve this, it is necessary first to understand the factors that determine species resilience, and their relative importance in shaping the ability of species to adjust to the complexities of environmental change. This is an extremely challenging task because it requires comprehensive information on species traits. We explored the resilience of 58 marine turtle regional management units (RMUs) to climate change, encompassing all seven species of marine turtles worldwide. We used expert opinion from the IUCN-SSC Marine Turtle Specialist Group (n=33 respondents) to develop a Resilience Index, which considered qualitative characteristics of each RMU (relative population size, rookery vulnerability, and genetic diversity) and non climate-related threats (fisheries, take, coastal development, and pollution/pathogens). Our expert panel perceived rookery vulnerability (the likelihood of functional rookeries becoming extirpated) and non climate-related threats as having the greatest influence on resilience of RMUs to climate change. We identified the world’s 13 least resilient marine turtle RMUs to climate change, which are distributed within all three major ocean basins and include six of the world’s seven species of marine turtle. Our study provides the first look at inter- and intra-species variation in resilience to climate change and highlights the need to devise metrics that measure resilience directly. We suggest that this approach can be widely used to help prioritize future actions that increase species resilience to climate change. | 2117 | NA | NA | NA | no | no |
| 2118 | 2017 | NA | Bento, G; Routtu, J; Fields, PD; Bourgeois, Y; Du Pasquier, L; Ebert, D | 2017 | The genetic basis of resistance and matching-allele interactions of a host-parasite system: The Daphnia magna-Pasteuria ramosa model | Negative frequency-dependent selection (NFDS) is an evolutionary mechanism suggested to govern host-parasite coevolution and the maintenance of genetic diversity at host resistance loci, such as the vertebrate MHC and R-genes in plants. Matching-allele interactions of hosts and parasites that prevent the emergence of host and parasite genotypes that are universally resistant and infective are a genetic mechanism predicted to underpin NFDS. The underlying genetics of matching-allele interactions are unknown even in host-parasite systems with empirical support for coevolution by NFDS, as is the case for the planktonic crustacean Daphnia magna and the bacterial pathogen Pasteuria ramosa. We fine-map one locus associated with D. magna resistance to P. ramosa and genetically characterize two haplotypes of the Pasteuria resistance (PR-) locus using de novo genome and transcriptome sequencing. Sequence comparison of PR-locus haplotypes finds dramatic structural polymorphisms between PR-locus haplotypes including a large portion of each haplotype being composed of non-homologous sequences resulting in haplotypes differing in size by 66 kb. The high divergence of PR-locus haplotypes suggest a history of multiple, diverse and repeated instances of structural mutation events and restricted recombination. Annotation of the haplotypes reveals striking differences in gene content. In particular, a group of glycosyltransferase genes that is present in the susceptible but absent in the resistant haplotype. Moreover, in natural populations, we find that the PR-locus polymorphism is associated with variation in resistance to different P. ramosa genotypes, pointing to the PR-locus polymorphism as being responsible for the matching-allele interactions that have been previously described for this system. Our results conclusively identify a genetic basis for the matching-allele interaction observed in a coevolving host-parasite system and provide a first insight into its molecular basis. | 2118 | NA | NA | NA | no | no |
| 2119 | 2017 | NA | Weiser, EL; Grueber, CE; Jamieson, IG | 2013 | Simulating Retention of Rare Alleles in Small Populations to Assess Management Options for Species with Different Life Histories | Preserving allelic diversity is important because it provides the capacity for adaptation and thus enables long-term population viability. Allele retention is difficult to predict in animals with overlapping generations, so we used a new computer model to simulate retention of rare alleles in small populations of 3 species with contrasting life-history traits: North Island Brown Kiwi (Apteryx mantelli; monogamous, long-lived), North Island Robins (Petroica longipes; monogamous, short-lived), and red deer (Cervus elaphus; polygynous, moderate lifespan). We simulated closed populations under various demographic scenarios and assessed the amounts of artificial immigration needed to achieve a goal of retaining 90% of selectively neutral rare alleles (frequency in the source population = 0.05) after 10 generations. The number of immigrants per generation required to meet the genetic goal ranged from 11 to 30, and there were key similarities and differences among species. None of the species met the genetic goal without immigration, and red deer lost the most allelic diversity due to reproductive skew among polygynous males. However, red deer required only a moderate rate of immigration relative to the other species to meet the genetic goal because nonterritorial breeders had a high turnover. Conversely, North Island Brown Kiwi needed the most immigration because the long lifespan of locally produced territorial breeders prevented a large proportion of immigrants from recruiting. In all species, the amount of immigration needed generally decreased with an increase in carrying capacity, survival, or reproductive output and increased as individual variation in reproductive success increased, indicating the importance of accurately quantifying these parameters to predict the effects of management. Overall, retaining rare alleles in a small, isolated population requires substantial investment of management effort. Use of simulations to explore strategies optimized for the populations in question will help maximize the value of this effort. | 2119 | NA | NA | NA | no | no |
| 2120 | 2017 | NA | Jenkins, T; Thomas, GH; Hellgren, O; Owens, IPF | 2012 | MIGRATORY BEHAVIOR OF BIRDS AFFECTS THEIR COEVOLUTIONARY RELATIONSHIP WITH BLOOD PARASITES | Host traits, such as migratory behavior, could facilitate the dispersal of disease-causing parasites, potentially leading to the transfer of infections both across geographic areas and between host species. There is, however, little quantitative information on whether variation in such host attributes does indeed affect the evolutionary outcome of hostparasite associations. Here, we employ Leucocytozoon blood parasites of birds, a group of parasites closely related to avian malaria, to study hostparasite coevolution in relation to host behavior using a phylogenetic comparative approach. We reconstruct the molecular phylogenies of both the hosts and parasites and use cophylogenetic tools to assess whether each hostparasite association contributes significantly to the overall congruence between the two phylogenies. We find evidence for a significant fit between host and parasite phylogenies in this system, but show that this is due only to associations between nonmigrant parasites and their hosts. We also show that migrant bird species harbor a greater genetic diversity of parasites compared with nonmigrant species. Taken together, these results suggest that the migratory habits of birds could influence their coevolutionary relationship with their parasites, and that consideration of host traits is important in predicting the outcome of coevolutionary interactions. | 2120 | NA | NA | NA | no | no |
| 2121 | 2017 | NA | Mann, RA; Smits, THM; Buhlmann, A; Blom, J; Goesmann, A; Frey, JE; Plummer, KM; Beer, SV; Luck, J; Duffy, B; Rodoni, B | 2013 | Comparative Genomics of 12 Strains of Erwinia amylovora Identifies a Pan-Genome with a Large Conserved Core | The plant pathogen Erwinia amylovora can be divided into two host-specific groupings; strains infecting a broad range of hosts within the Rosaceae subfamily Spiraeoideae (e.g., Malus, Pyrus, Crataegus, Sorbus) and strains infecting Rubus (raspberries and blackberries). Comparative genomic analysis of 12 strains representing distinct populations (e.g., geographic, temporal, host origin) of E. amylovora was used to describe the pan-genome of this major pathogen. The pan-genome contains 5751 coding sequences and is highly conserved relative to other phytopathogenic bacteria comprising on average 89% conserved, core genes. The chromosomes of Spiraeoideae-infecting strains were highly homogeneous, while greater genetic diversity was observed between Spiraeoideae- and Rubus-infecting strains (and among individual Rubus-infecting strains), the majority of which was attributed to variable genomic islands. Based on genomic distance scores and phylogenetic analysis, the Rubus-infecting strain ATCC BAA-2158 was genetically more closely related to the Spiraeoideae-infecting strains of E. amylovora than it was to the other Rubus-infecting strains. Analysis of the accessory genomes of Spiraeoideae- and Rubus-infecting strains has identified putative host-specific determinants including variation in the effector protein HopX1(Ea) and a putative secondary metabolite pathway only present in Rubus-infecting strains. | 2121 | NA | NA | NA | no | no |
| 2122 | 2017 | NA | Dacheux, L; Cervantes-Gonzalez, M; Guigon, G; Thiberge, JM; Vandenbogaert, M; Maufrais, C; Caro, V; Bourhy, H | 2014 | A Preliminary Study of Viral Metagenomics of French Bat Species in Contact with Humans: Identification of New Mammalian Viruses | The prediction of viral zoonosis epidemics has become a major public health issue. A profound understanding of the viral population in key animal species acting as reservoirs represents an important step towards this goal. Bats harbor diverse viruses, some of which are of particular interest because they cause severe human diseases. However, little is known about the diversity of the global population of viruses found in bats (virome). We determined the viral diversity of five different French insectivorous bat species (nine specimens in total) in close contact with humans. Sequence-independent amplification, high-throughput sequencing with Illumina technology and a dedicated bioinformatics analysis pipeline were used on pooled tissues (brain, liver and lungs). Comparisons of the sequences of contigs and unassembled reads provided a global taxonomic distribution of virus-related sequences for each sample, highlighting differences both within and between bat species. Many viral families were present in these viromes, including viruses known to infect bacteria, plants/fungi, insects or vertebrates, the most relevant being those infecting mammals (Retroviridae, Herpesviridae, Bunyaviridae, Poxviridae, Flaviviridae, Reoviridae, Bornaviridae, Picobirnaviridae). In particular, we detected several new mammalian viruses, including rotaviruses, gammaretroviruses, bornaviruses and bunyaviruses with the identification of the first bat nairovirus. These observations demonstrate that bats naturally harbor viruses from many different families, most of which infect mammals. They may therefore constitute a major reservoir of viral diversity that should be analyzed carefully, to determine the role played by bats in the spread of zoonotic viral infections. | 2122 | NA | NA | NA | no | no |
| 2123 | 2017 | NA | Ghasemkhani, M; Garkava-Gustavsson, L; Liljeroth, E; Nybom, H | 2016 | Assessment of diversity and genetic relationships of Neonectria ditissima: the causal agent of fruit tree canker | Background: Neonectria ditissima is one of the most important fungal pathogens of apple trees, where it causes fruit tree canker. Information about the amount and partitioning of genetic variation of this fungus could be helpful for improving orchard management strategies and for breeding apple cultivars with high levels of genetically determined resistance. In this study single-spore Neonectria isolates originating from both the same and from different perithecia, apple cultivars and apple orchards in Sweden and Belgium, were evaluated for AFLP- and SSR-based genetic similarity and for mating system. Results: Seven SSR loci produced a total of 31 alleles with an average of 4 alleles per locus, while 11 AFLP primer combinations produced an average of 35 fragments per primer combination and 71 % polymorphic fragments. An AFLP-based analysis of molecular variance (AMOVA) revealed that 89 % of the variation was found within orchards and 11 % between orchards. Genetic similarity among the studied isolates was illustrated with a principal coordinate analyseis (PCoA) and a dendrogram. AFLP-based Jaccard’s similarity coefficients were the highest when single-ascospore isolates obtained from the same perithecium were compared, medium-high for isolates from different perithecia on the same tree, and lowest when isolates from different trees were compared. Conclusions: Based on the results of PCoA and AMOVA analysis, isolates from the same or geographically close orchards did not group together. Since AFLP profiles differed also when single-ascospore isolates from the same perithecium were compared, the mating system of N. ditissima is most likely heterothallic. | 2123 | NA | NA | NA | no | no |
| 2124 | 2017 | NA | Jambou, R; Martinelli, A; Pinto, J; Gribaldo, S; Legrand, E; Niang, M; Kim, N; Pharath, L; Volnay, B; Ekala, MT; Bouchier, C; Fandeur, T; Berzosa, P; Benito, A; Ferreira, ID; Ferreira, C; Vieira, PP; Alecrim, MD; Mercereau-Puijalon, O; Cravo, P | 2010 | Geographic Structuring of the Plasmodium falciparum Sarco(endo)plasmic Reticulum Ca2+ATPase (PfSERCA) Gene Diversity | Artemisinin, a thapsigargin-like sesquiterpene has been shown to inhibit the Plasmodium falciparum sarco/endoplasmic reticulum calcium-ATPase PfSERCA. To collect baseline pfserca sequence information before field deployment of Artemisinin-based Combination therapies that may select mutant parasites, we conducted a sequence analysis of 100 isolates from multiple sites in Africa, Asia and South America. Coding sequence diversity was large, with 29 mutated codons, including 32 SNPs (average of one SNP/115 bp), of which 19 were novel mutations. Most SNP detected in this study were clustered within a region in the cytosolic head of the protein. The PfSERCA functional domains were very well conserved, with non synonymous mutations located outside the functional domains, except for the S769N mutation associated in French Guiana with elevated IC(50) for artemether. The S769N mutation is located close to the hinge of the headpiece, which in other species modulates calcium affinity and in consequence efficacy of inhibitors, possibly linking calcium homeostasis to drug resistance. Genetic diversity was highest in Senegal, Brazil and French Guiana, and few mutations were identified in Asia. Population genetic analysis was conducted for a partial fragment of the gene encompassing nucleotide coordinates 87-2862 (unambiguous sequence available for 96 isolates). This supported a geographic clustering, with a separation between Old and New World samples and one dominant ancestral haplotype. Genetic drift alone cannot explain the observed polymorphism, suggesting that other evolutionary mechanisms are operating. One possible contributor could be the frequency of haemoglobinopathies that are associated with calcium dysregulation in the erythrocyte. | 2124 | NA | NA | NA | no | no |
| 2125 | 2017 | NA | Forcioli, D; Merle, PL; Caligara, C; Ciosi, M; Muti, C; Francour, P; Cerrano, C; Allemand, D | 2011 | Symbiont diversity is not involved in depth acclimation in the Mediterranean sea whip Eunicella singularis | In symbiotic cnidarians, acclimation to depth and lower irradiance can involve physiological changes in the photosynthetic dinoflagellate endosymbiont, such as increased chlorophyll content, or qualitative modifications in the symbiont population in favour of better adapted strains. It has been argued that a lack of capacity to acquire new symbionts could limit the bathymetric distribution of the host species, or compromise its long-term survival in a changing environment. But is that always true? To address this question, we investigated the symbiont genetic diversity in Eunicella singularis, a Mediterranean sea whip species with a wide bathymetric distribution (10 to 50 m depth), which has recently suffered from mass mortalities after periods of abnormally high sea temperatures. We measured symbiont population densities and chlorophyll content in natural populations, and followed the response of the holobionts after reciprocal transplantations to deep and shallow depths. A total of 161 colonies were sampled at 2 depths (10 and 30 m) at 5 sites in the northwestern Mediterranean. All colonies harboured a single ribosomal Symbiodinium clade (A’), but a relatively high within-clade genetic diversity was found among and within colonies. This diversity was not structured by depth, even though the deeper colonies contained significantly lower population densities of symbionts and less chlorophyll. We did, however, reveal host-symbiont specificity among E. singularis and other Mediterranean cnidarian species. Transplantation experiments revealed a limit of plasticity for symbiont population density and chlorophyll content, which in turn questions the importance of the trophic role of Symbiodinium in E. singularis. | 2125 | NA | NA | NA | no | no |
| 2126 | 2017 | NA | Buck, KJ; Walter, NAR; Denmark, DL | 2014 | Genetic variability of respiratory complex abundance, organization and activity in mouse brain | Mitochondrial dysfunction is implicated in the etiology and pathogenesis of numerous human disorders involving tissues with high energy demand. Murine models are widely used to elucidate genetic determinants of phenotypes relevant to human disease, with recent studies of C57BL/6J (B6), DBA/2J (D2) and B6xD2 populations implicating naturally occurring genetic variation in mitochondrial function/dysfunction. Using blue native polyacrylamide gel electrophoresis, immunoblots and in-gel activity analyses of complexes I, II, III, IV and V, our studies are the first to assess abundance, organization and catalytic activity of mitochondrial respiratory complexes and supercomplexes in mouse brain. Remarkable strain differences in supercomplex assembly and associated activity are evident, without differences in individual complexes I, II, III or IV. Supercomplexes I1III2IV2-3 exhibit robust complex III immunoreactivity and activities of complexes I and IV in D2, but with little detected in B6 for I1III2IV2, and I1III2IV3 is not detected in B6. I1III2IV1 and I1III2 are abundant and catalytically active in both strains, but significantly more so in B6. Furthermore, while supercomplex III2IV1 is abundant in D2, none is detected in B6. In aggregate, these results indicate a shift toward more highly assembled supercomplexes in D2. Respiratory supercomplexes are thought to increase electron flow efficiency and individual complex stability, and to reduce electron leak and generation of reactive oxygen species. Our results provide a framework to begin assessing the role of respiratory complex suprastructure in genetic vulnerability and treatment for a wide variety of mitochondrial-related disorders. | 2126 | NA | NA | NA | no | no |
| 2127 | 2017 | NA | Horger, AC; Ilyas, M; Stephan, W; Tellier, A; van der Hoorn, RAL; Rose, LE | 2012 | Balancing Selection at the Tomato RCR3 Guardee Gene Family Maintains Variation in Strength of Pathogen Defense | “Coevolution between hosts and pathogens is thought to occur between interacting molecules of both species. This results in the maintenance of genetic diversity at pathogen antigens (or so-called effectors) and host resistance genes such as the major histocompatibility complex (MHC) in mammals or resistance (R) genes in plants. In plant-pathogen interactions, the current paradigm posits that a specific defense response is activated upon recognition of pathogen effectors via interaction with their corresponding R proteins. According to the”“Guard-Hypothesis,”" R proteins (the "“guards’’) can sense modification of target molecules in the host (the”“guardees’’) by pathogen effectors and subsequently trigger the defense response. Multiple studies have reported high genetic diversity at R genes maintained by balancing selection. In contrast, little is known about the evolutionary mechanisms shaping the guardee, which may be subject to contrasting evolutionary forces. Here we show that the evolution of the guardee RCR3 is characterized by gene duplication, frequent gene conversion, and balancing selection in the wild tomato species Solanum peruvianum. Investigating the functional characteristics of 54 natural variants through in vitro and in planta assays, we detected differences in recognition of the pathogen effector through interaction with the guardee, as well as substantial variation in the strength of the defense response. This variation is maintained by balancing selection at each copy of the RCR3 gene. Our analyses pinpoint three amino acid polymorphisms with key functional consequences for the coevolution between the guardee (RCR3) and its guard (Cf-2). We conclude that, in addition to coevolution at the”“guardee-effector’’ interface for pathogen recognition, natural selection acts on the”“guard-guardee’’ interface. Guardee evolution may be governed by a counterbalance between improved activation in the presence and prevention of auto-immune responses in the absence of the corresponding pathogen.” | 2127 | NA | NA | NA | no | no |
| 2128 | 2017 | NA | Prospero, S; Lung-Escarmant, B; Dutech, C | 2008 | Genetic structure of an expanding Armillaria root rot fungus (Armillaria ostoyae) population in a managed pine forest in southwestern France | The Landes de Gascogne forest (southwestern France) is the largest maritime pine (Pinus pinaster) plantation in Europe. Armillaria root disease (Armillaria ostoyae) has been reported since the early 1920s in the coastal area (western sector), but its incidence over the last 20 years has increased in the eastern sector. We investigated the genetic structure of the A. ostoyae population in this forest, focusing particularly on geographical differentiation potentially indicative of disease expansion in this area. In total, 531 isolates obtained from mycelial fans on symptomatic trees or undecayed stumps in 31 different disease foci were genotyped at five microsatellite loci. In 20 of these disease foci, a single genotype dominated, reflecting a predominantly clonal local spread of A. ostoyae. By contrast, at the regional scale, A. ostoyae probably spreads mostly via basidiospores (sexual spores), as no genotype common to several disease foci was identified. The absence of a clear pattern of isolation by distance may indicate either substantial gene flow or stochastic colonisation independent of spatial distance. The gradient of genetic diversity from the coast inwards and the greater genetic divergence of the eastern disease foci are consistent with the expansion of the A. ostoyae population from the coast eastwards. | 2128 | NA | NA | NA | no | no |
| 2129 | 2017 | NA | Davelos, AL; Alexander, HM; Slade, NA | 1996 | Ecological genetic interactions between a clonal host plant (Spartina pectinata) and associated rust fungi (Puccinia seymouriana and Puccinia sparganioides) | The spatial scale of genetic diversity among patches of a host plant could affect the likelihood of pathogen adaptation to the host. If host patches are genetically distinct, pathogen adaptation to local host genotypes may occur. To study this issue, we focused on the ecological and genetic interactions between two rust fungi, Puccinia seymouriana and P. sparganioides, and the clonal prairie grass, Spartina pectinata. In a field transplant experiment, disease levels differed among plants from different patches, suggesting variation in resistance. Over a 4.5-km scale, disease levels were not higher on plants transplanted back into their source patch as opposed to other locations, providing no evidence for local adaptation in the pathogen. However, on the spatial scales examined (ranging from 0.2 km to 120 km), there was no relationship between the physical distance separating host patches and their similarity in isozyme banding patterns, implying that plants from more distant patches are not necessarily more genetically distinct than plants from nearby patches. Plants derived from the most distant location had, on average, the lowest mean number of pustules at the end of the summer, suggesting the need for reciprocal transplant studies to be performed on a larger spatial scale. | 2129 | NA | NA | NA | no | no |
| 2130 | 2017 | NA | Wenzel, MA; Webster, LMI; Paterson, S; Piertney, SB | 2015 | Identification and characterisation of 17 polymorphic candidate genes for response to parasitic nematode (Trichostrongylus tenuis) infection in red grouse (Lagopus lagopus scotica) | “The red grouse (Lagopus lagopus scotica) is an economically important game bird species endemic to the upland heather moors of the British Isles, where its conservation status is”“amber”" due to long-term declines in breeding populations. One major driver of grouse population ecology is chronic infection by the highly prevalent, gastrointestinal parasitic nematode Trichostrongylus tenuis. Here, we outline the identification and characterisation of 17 candidate genes for the physiological response of red grouse to parasite infection, developed de novo from functional and genetic analysis of grouse transcriptomic and genomic resources. These genes capture broad physiological functions, including immune system processes, xenobiotics detoxification, oxidative balance, metabolism and cell cycle regulation. All genes were polymorphic at the landscape scale in north-east Scotland, indicating great utility for characterising the causes and consequences of spatio-temporal genetic variation in relation to parasite-mediated eco-evolutionary processes in red grouse populations." | 2130 | NA | NA | NA | no | no |
| 2131 | 2017 | NA | Livanova, NN; Tikunov, AY; Kurilshikov, AM; Livanov, SG; Fomenko, NV; Taranenko, DE; Kvashnina, AE; Tikunova, NV | 2015 | Genetic diversity of Ixodes pavlovskyi and I-persulcatus (Acari: Ixodidae) from the sympatric zone in the south of Western Siberia and Kazakhstan | The most epidemiologically significant tick species in Siberia involved in transmission of a large number of pathogens causing human infectious diseases is Ixodes persulcatus. Ixodes pavlovskyi, being more active, also poses epidemiological threats. These tick species share morphology, activity seasons and geographic distribution range. In this paper, we characterize the geographic and genetic structures of I. persulcatus and I. pavlovskyi populations inhabiting the southern part of Western Siberia (Russia and Kazakhstan)-the western part of I. pavlovskyi distribution range. The data are based on six distinct Ixodes tick populations. Analysis of the concatenated mitochondrial marker sequences (16S rRNA and COI) and the nuclear sequence (ITS2) showed genetic polymorphisms in both I. persulcatus and I. pavlovskyi ticks inhabiting the sympatric zone. We could not determine the phylogeographic structure of I. pavlovskyi populations whereas for I. persulcatus significant within-region variance was shown. Notably, the abundance of I. persulcatus ticks negatively correlates with nucleotide and haplotype diversity in the concatenated sequence of mitochondrial gene (16S rRNA and COI) fragments. This is the first description of the genetic polymorphism of I. persulcatus and I. pavlovskyi ticks coexisting in a sympatric zone based on analysis of mitochondrial and nuclear markers. | 2131 | NA | NA | NA | no | no |
| 2132 | 2017 | NA | Nirmaladevi, D; Venkataramana, M; Srivastava, RK; Uppalapati, SR; Gupta, VK; Yli-Mattila, T; Tsui, KMC; Srinivas, C; Niranjana, SR; Chandra, NS | 2016 | Molecular phylogeny, pathogenicity and toxigenicity of Fusarium oxysporum f. sp lycopersici | The present study aimed at the molecular characterization of pathogenic and non pathogenic F. oxysporum f. sp. lycopersici strains isolated from tomato. The causal agent isolated from symptomatic plants and soil samples was identified based on morphological and molecular analyses. Pathogenicity testing of 69 strains on five susceptible tomato varieties showed 45% of the strains were highly virulent and 30% were moderately virulent. Molecular analysis based on the fingerprints obtained through ISSR indicated the presence of wide genetic diversity among the strains. Phylogenetic analysis based on ITS sequences showed the presence of at least four evolutionary lineages of the pathogen. The clustering of F. oxysporum with non pathogenic isolates and with the members of other formae speciales indicated polyphyletic origin of F. oxysporum f. sp. lycopersici. Further analysis revealed intraspecies variability and nucleotide insertions or deletions in the ITS region among the strains in the study and the observed variations were found to be clade specific. The high genetic diversity in the pathogen population demands for development of effective resistance breeding programs in tomato. Among the pathogenic strains tested, toxigenic strains harbored the Fum1 gene clearly indicating that the strains infecting tomato crops have the potential to produce Fumonisin. | 2132 | NA | NA | NA | no | no |
| 2133 | 2017 | NA | Villanueva-Garcia, C; Gordillo-Chavez, EJ; Lopez-Escamilla, E; Rendon-Franco, E; Munoz-Garcia, CI; Gama, L; Martinez-Flores, WA; Gonzalez-Rodriguez, N; Romero-Valdovinos, M; Diaz-Lopez, H; Galian, J; Villalobos, G; Maravilla, P; Martinez-Hernandez, F | 2017 | Clarifying the Cryptic Host Specificity of Blastocystis spp. Isolates from Alouatta palliata and A. pigra Howler Monkeys | Although the presence of cryptic host specificity has been documented in Blastocystis, differences in infection rates and high genetic polymorphism within and between populations of some subtypes (ST) have impeded the clarification of the generalist or specialist specificity of this parasite. We assessed the genetic variability and host specificity of Blastocystis spp. in wild howler monkeys from two rainforest areas in the southeastern region of Mexico. Fecal samples of 225 Alouatta palliata (59) and A. pigra (166) monkeys, belonging to 16 sylvatic sites, were analyzed for infection with Blastocystis ST using a region of the small subunit rDNA (SSUrDNA) gene as a marker. Phylogenetic and genetic diversity analyses were performed according to the geographic areas where the monkeys were found. Blastocystis ST2 was the most abundant (91.9%), followed by ST1 and ST8 with 4.6% and 3.5%, respectively; no association between Blastocystis ST and Alouatta species was observed. SSUrDNA sequences in GenBank from human and non-human primates (NHP) were used as ST references and included in population analyses. The haplotype network trees exhibited different distributions: ST1 showed a generalist profile since several haplotypes from different animals were homogeneously distributed with few mutational changes. For ST2, a major dispersion center grouped the Mexican samples, and high mutational differences were observed between NHP. Furthermore, nucleotide and haplotype diversity values, as well as migration and genetic differentiation indexes, showed contrasting values for ST1 and ST2. These data suggest that ST1 populations are only minimally differentiated, while ST2 populations in humans are highly differentiated from those of NHP. The host generalist and specialist specificities exhibited by ST1 and ST2 Blastocystis populations indicate distinct adaptation processes. Because ST1 exhibits a generalist profile, this haplotype can be considered a metapopulation; in contrast, ST2 exists as a set of local populations with preferences for either humans or NHP. | 2133 | NA | NA | NA | no | no |
| 2134 | 2017 | NA | Popat, R; Pollitt, EJG; Harrison, F; Naghra, H; Hong, KW; Chan, KG; Griffin, AS; Williams, P; Brown, SP; West, SA; Diggle, SP | 2015 | Conflict of interest and signal interference lead to the breakdown of honest signaling | Animals use signals to coordinate a wide range of behaviors, from feeding offspring to predator avoidance. This poses an evolutionary problem, because individuals could potentially signal dishonestly to coerce others into behaving in ways that benefit the signaler. Theory suggests that honest signaling is favored when individuals share a common interest and signals carry reliable information. Here, we exploit the opportunities offered by bacterial signaling to test these predictions with an experimental evolution approach. We show that: (1) reduced relatedness leads to the relative breakdown of signaling, (2) signaling breaks down by the invasion of mutants that show both reduced signaling and reduced response to signal, (3) the genetic route to signaling breakdown is variable, and (4) the addition of artificial signal, to interfere with signal information, also leads to reduced signaling. Our results provide clear support for signaling theory, but we did not find evidence for previously predicted coercion at intermediate relatedness, suggesting that mechanistic details can alter the qualitative nature of specific predictions. Furthermore, populations evolved under low relatedness caused less mortality to insect hosts, showing how signal evolution in bacterial pathogens can drive the evolution of virulence in the opposite direction to that often predicted by theory. | 2134 | NA | NA | NA | no | no |
| 2135 | 2017 | NA | Akopyants, NS; Fradkov, A; Diatchenko, L; Hill, JE; Siebert, PD; Lukyanov, SA; Sverdlov, ED; Berg, DE | 1998 | PCR-based subtractive hybridization and differences in gene content among strains of Helicobacter pylori | Genes that are characteristic of only certain strains of a bacterial species can be of great biologic interest. Here we describe a PCR-based subtractive hybridization method for efficiently detecting such DNAs and apply it to the gastric pathogen Helicobacter pylori, Eighteen DNAs specific to a monkey-colonizing strain (J166) were obtained by subtractive hybridization against an unrelated strain whose genome has been fully sequenced (26695), Seven J166-specific clones had no DNA sequence match to the 26695 genome, and 11 other clones were mixed, with adjacent patches that did and did not match any sequences in 26695, At the protein level, se-yen clones had homology to putative DNA restriction-modification enzymes, and two had homology to putative metabolic enzymes. Nine others had no database match with proteins of assigned function. PCR tests of 13 unrelated H. pylori strains by using primers specific for 12 subtracted clones and complementary Southern blot hybridizations indicated that these DNAs are highly polymorphic in the H, pylori population, with each strain yielding a different pattern of gene-specific PCR amplification. The search for polymorphic DNAs, as described here, should help identify previously unknown virulence genes in pathogens and provide new insights into microbial genetic diversity and evolution. | 2135 | NA | NA | NA | no | no |
| 2136 | 2017 | NA | Ahlstrom, C; Barkema, HW; Stevenson, K; Zadoks, RN; Biek, R; Kao, R; Trewby, H; Haupstein, D; Kelton, DF; Fecteau, G; Labrecque, O; Keefe, GP; McKenna, SLB; Tahlan, K; De Buck, J | 2016 | Genome-Wide Diversity and Phylogeography of Mycobacterium avium subsp paratuberculosis in Canadian Dairy Cattle | “Mycobacterium avium subsp. paratuberculosis (MAP) is the causative bacterium of Johne’s disease (JD) in ruminants. The control of JD in the dairy industry is challenging, but can be improved with a better understanding of the diversity and distribution of MAP subtypes. Previously established molecular typing techniques used to differentiate MAP have not been sufficiently discriminatory and/or reliable to accurately assess the population structure. In this study, the genetic diversity of 182 MAP isolates representing all Canadian provinces was compared to the known global diversity, using single nucleotide polymorphisms identified through whole genome sequencing. MAP isolates from Canada represented a subset of the known global diversity, as there were global isolates intermingled with Canadian isolates, as well as multiple global subtypes that were not found in Canada. One Type III and six”“Bison type”" isolates were found in Canada as well as one Type II subtype that represented 86% of all Canadian isolates. Rarefaction estimated larger subtype richness in Quebec than in other Canadian provinces using a strict definition of MAP subtypes and lower subtype richness in the Atlantic region using a relaxed definition. Significant phylogeographic clustering was observed at the inter-provincial but not at the intra-provincial level, although most major clades were found in all provinces. The large number of shared subtypes among provinces suggests that cattle movement is a major driver of MAP transmission at the herd level, which is further supported by the lack of spatial clustering on an intra-provincial scale." | 2136 | NA | NA | NA | no | no |
| 2137 | 2017 | NA | van Oppen, MJH; Lukoschek, V; Berkelmans, R; Peplow, LM; Jones, AM | 2015 | A population genetic assessment of coral recovery on highly disturbed reefs of the Keppel Island archipelago in the southern Great Barrier Reef | Coral reefs surrounding the islands lying close to the coast are unique to the Great Barrier Reef (GBR) in that they are frequently exposed to disturbance events including floods caused by cyclonic rainfall, strong winds and occasional periods of prolonged above-average temperatures during summer. In one such group of islands in the southern GBR, the Keppel Island archipelago, climate-driven disturbances frequently result in major coral mortality. Whilst these island reefs have clearly survived such dramatic disturbances in the past, the consequences of extreme mortality events may include the loss of genetic diversity, and hence adaptive potential, and a reduction in fitness due to inbreeding, especially if new recruitment from external sources is limited. Here we examined the level of isolation of the Keppel Island group as well as patterns of gene flow within the Keppel Islands using 10 microsatellite markers in nine populations of the coral, Acropora millepora. Bayesian cluster analysis and assignment tests indicated gene flow is restricted, but not absent, between the outer and inner Keppel Island groups, and that extensive gene flow exists within each of these island groups. Comparison of the Keppel Island data with results from a previous GBR-wide study that included a single Keppel Island population, confirmed that A. millepora in the Keppel Islands is genetically distinct from populations elsewhere on the GBR, with exception of the nearby inshore High Peak Reef just north of the Keppel Islands. We compared patterns of genetic diversity in the Keppel Island populations with those from other GBR populations and found themto be slightly, but significantly lower, consistent with the archipelago being geographically isolated, but there was no evidence for recent bottlenecks or deviation from mutation-drift equilibrium. A high incidence of private alleles in the Keppel Islands, particularly in the outer islands, supports their relative isolation and contributes to the conservation value of the archipelago. The lack of evidence for genetic erosion, in combination with our observation that the North Keppel Island population samples collected in 2002 and 2008, respectively, exhibited a pairwise genetic distance of zero, supports previous published work indicating that, following bleaching, Acropora corals in the Keppel Islands predominantly recover from regrowth of small amounts of remaining live tissue in apparently dead coral colonies. This is likely supplemented by recruitment of larvae from genetically similar, less disturbed populations at nearby reefs, particularly following extreme flood events. | 2137 | NA | NA | NA | no | no |
| 2138 | 2017 | NA | Lang, M; Sazzini, M; Calabrese, FM; Simone, D; Boattini, A; Romeo, G; Luiselli, D; Attimonelli, M; Gasparre, G | 2012 | Polymorphic NumtS trace human population relationships | The human genome is constantly subjected to evolutionary forces which shape its architecture. Insertions of mitochondrial DNA sequences into nuclear genome (NumtS) have been described in several eukaryotic species, including Homo sapiens and other primates. The ongoing process of the generation of NumtS has made them valuable markers in primate phylogenetic studies, as well as potentially informative loci for reconstructing the genetic history of modern humans. Here, we report the identification of 53 human-specific NumtS by inspection of the UCSC genome browser, showing that they may be direct insertions of mitochondrial DNA into the human nuclear DNA after the human-chimpanzee split. In silico analyses allowed us to identify 14 NumtS which are polymorphic in terms of their presence/absence within the human genome in individuals of different ancestry. The allele frequencies of these polymorphic NumtS were calculated for 1000 Genomes Project sequence data from 13 populations worldwide, and principal components analysis and hierarchical clustering methods allowed the detection of strong signals of geographical structure related to the genetic diversity of these loci. All identified polymorphic human-specific NumtS together with a tandemly duplicated NumtS have also been validated by PCR amplification on a panel of 60 samples belonging to five native populations worldwide, confirming the expected NumtS variability. On the basis of these findings, we have succeeded in depicting the landscape of variation of a series of NumtS in several ethnic groups, making an advance in their identification as useful markers in the study on human population genetics. | 2138 | NA | NA | NA | no | no |
| 2139 | 2017 | NA | Schmid-Hempel, R; Schmid-Hempel, P | 2000 | Female mating frequencies in Bombus spp. from Central Europe | The mating frequency of females in social insects is particularly interesting, because polyandry reduces colony relatedness and increases within-colony genetic variance. It thereby affects a complex balance of benefits and costs that determine the degree of reproductive skew, sex allocation to offspring, or the opportunities for nepotism and policing strategies. Few systematic surveys of female mating frequencies exist and many are based on unreliable behavioural. observation or sperm counts. Here, we report the results of a survey of mating frequencies in eight European Bombus spp. by means of highly polymorphic microsatellite loci. Only B. hypnorum was found to be multiply mated, while the pattern found in B, terrestris, B. lucorum, B, pratorum, B. lapidarius, B. sicheli, B. hortorum, and B. pascuorum was compatible with single mating. The findings are compatible with recent claims that, with some exceptions, mating frequencies of social insect females are generally low. | 2139 | NA | NA | NA | no | no |
| 2140 | 2017 | NA | Allen, GA; Marr, KL; McCormick, LJ; Hebda, RJ | 2015 | Geographical origins, migration patterns and refugia of Sibbaldia procumbens, an arctic-alpine plant with a fragmented range | AimMany plants, especially at high latitudes, have both widespread and highly discontinuous geographical distributions. To increase understanding of how such patterns originate, we examine genetic patterns in the arctic-alpine plant Sibbaldia procumbens. We evaluate the contributions of refugia and the role of long-distance dispersal in shaping the current range of this species. LocationNorthern Hemisphere, especially North America. MethodsWe sampled Sibbaldia from 176 localities, including 168 for S. pro-cumbens. We analysed sequence variation in three plastid DNA non-coding regions (the atpI-atpH and trnL-trnF intergenic spacers and the trnL intron), performed Bayesian phylogenetic analyses and statistical parsimony analyses on the combined sequences, and analysed the geographical patterns of haplotype distribution and genetic diversity using data from all populations. ResultsSibbaldia procumbens probably originated in the mountains of South and East Asia. We identified highly distinct clades in Europe and North America, which overlapped on oceanic islands of the North Atlantic indicating long-distance dispersal capability. The North American clade included two lineages, one in California and the other widely distributed across the continent and North Atlantic. Haplotype diversity in the latter lineage was markedly higher to the south, suggesting mid-late Pleistocene southward displacement of North American populations with subsequent migration northwards into previously glaciated regions. In Europe, disjunct geographical regions generally harboured distinct haplotypes. Main conclusionsMultiple Pleistocene refugia for S. procumbens occurred in both North America and Europe. North American refugia existed in California and in the southern Rocky Mountains, but in contrast with most widespread arctic-alpine species we found no evidence for a Beringian refugium. Cryptic refugia may have existed within the Cordilleran Ice Sheet. Episodes of range expansion and contraction and long-distance dispersal have all contributed to the genetic structure and widespread but fragmented distribution of this species. | 2140 | NA | NA | NA | no | no |
| 2141 | 2017 | NA | Simkova, A; Kosar, M; Vetesnik, L; Vyskocilova, M | 2013 | MHC genes and parasitism in Carassius gibelio, a diploid-triploid fish species with dual reproduction strategies | Background: The gibel carp is a fish species with dual reproduction modes, gynogenesis and sexual reproduction, coexisting in mixed diploid-polyploid populations. Following the Red Queen (RQ) assumption, asexual organisms are, due to their low genetic diversity, targets for parasite adaptation. Because MHC polymorphism is maintained by selection from parasites and sexual selection, MHC genes are considered as a suitable candidate for testing the RQ hypothesis. In this study, we investigated MHC variability and the selection pressure acting on MHC genes in sexual diploids and asexual triploids. In addition, we tested whether the asexual form of gibel carp suffers from higher parasite loads than the sexual form. Results: At the population level, genotype and allelic diversity of MHC were reduced in gynogenetic triploids when compared to sexual diploids. Different patterns in positively selected sites (PSS) between gynogens and sexual gibel carp were also found. A weak difference in parasite species richness was found between sexual fish and gynogens. However, two common clones of gynogens were significantly more parasitized than sexual diploids or other gynogens with rare MHC genotypes. At the individual level, the higher number of alleles was not associated with higher parasitism in either sexual diploids or gynogens. Conclusions: The differences in MHC diversity between gynogenetic triploids and sexual diploids are in accordance with the hypothesis of sexually-mediated selection increasing MHC diversity and fulfil a prerequisite of the Red Queen hypothesis. The different patterns in PSS between gynogens and sexual gibel carp also suggest the potential role of sexual selection and supports parasite-mediated selection maintaining MHC diversity. We showed that the most common MHC genotypes of gynogenetic triploids are the target of parasite selection. Our results suggest that the MHC genotype in gibel carp is more important than allelic number for immunocompetence. | 2141 | NA | NA | NA | no | no |
| 2142 | 2017 | NA | Atyame, CM; Delsuc, F; Pasteur, N; Weill, M; Duron, O | 2011 | Diversification of Wolbachia Endosymbiont in the Culex pipiens Mosquito | The alpha-proteobacteria Wolbachia are among the most common intracellular bacteria and have recently emerged as important drivers of arthropod biology. Wolbachia commonly act as reproductive parasites in arthropods by inducing cytoplasmic incompatibility (CI), a type of conditional sterility between hosts harboring incompatible infections. In this study, we examined the evolutionary histories of Wolbachia infections, known as wPip, in the common house mosquito Culex pipiens, which exhibits the greatest variation in CI crossing patterns observed in any insect. We first investigated a panel of 20 wPip strains for their genetic diversity through a multilocus scheme combining 13 Wolbachia genes. Because Wolbachia depend primarily on maternal transmission for spreading within arthropod populations, we also studied the variability in the coinherited Cx. pipiens mitochondria. In total, we identified 14 wPip haplotypes, which all share a monophyletic origin and clearly cluster into five distinct wPip groups. The diversity of Cx. pipiens mitochondria was extremely reduced, which is likely a consequence of cytoplasmic hitchhiking driven by a unique and recent Wolbachia invasion. Phylogenetic evidence indicates that wPip infections and mitochondrial DNA have codiverged through stable cotransmission within the cytoplasm and shows that a rapid diversification of wPip has occurred. The observed pattern demonstrates that a considerable degree of Wolbachia diversity can evolve within a single host species over short evolutionary periods. In addition, multiple signatures of recombination were found in most wPip genomic regions, leading us to conclude that the mosaic nature of wPip genomes may play a key role in their evolution. | 2142 | NA | NA | NA | no | no |
| 2143 | 2017 | NA | Ficetola, GF; Crottini, A; Casiraghi, M; Padoa-Schioppa, E | 2010 | New data on amphibians and reptiles of the Northern Areas of Pakistan: distribution, genetic variability and conservation issues | The Northern Areas of Pakistan are at the boundary between the Paleartic and the Oriental biogeographical realm. This mountainous region is at the junction between major mountain ranges, has huge altitudinal and environmental gradients, and is the focus of efforts for biodiversity conservation. However, the amphibians and reptiles of this area remain poorly known. We used multiple approaches to describe species distribution and ecology, to perform a preliminary analysis of genetic diversity of toads, and to assess the occurrence of Batrachochytrium dendrobatidis, a pathogen largely responsible of global amphibian decline. We observed seven species of amphibians and reptiles: Bufo pseudoraddei baturae, Bufo latastii, Scutiger cf. nyingchiensis, Altiphytax stoliczkai, Laudakia himalayana, L. pakistanica and Varanus bengalensis; we describe the habitat and distribution of these species. In the case of L. himalayana, we observed a bimodal pattern of diurnal activity, and we report new data on the altitudinal range of this agamid. Sequences of a fragment of mitochondria] 16S rDNA from multiple populations of B. pseudraddei baturae show low genetic diversity among individual of this species. Conversely, we observed strong genetic substructuring among populations of B. latastii living in different valleys. A PCR-based analysis did not reveal the presence of B. dendrobatidis in any of the amphibians investigated. Distribution data show that amphibians and reptiles are limited to relatively low elevations; these areas suffer the strongest human impact, and might be overlooked by future conservation plans for the area, making the future of these organisms at risk. | 2143 | NA | NA | NA | no | no |
| 2144 | 2017 | NA | Chen, SB; Wang, Y; Kassegne, K; Xu, B; Shen, HM; Chen, JH | 2017 | Whole-genome sequencing of a Plasmodium vivax clinical isolate exhibits geographical characteristics and high genetic variation in China-Myanmar border area | Background: Currently in China, the trend of Plasmodium vivax cases imported from Southeast Asia was increased especially in the China-Myanmar border area. Driven by the increase in P. vivax cases and stronger need for vaccine and drug development, several P. vivax isolates genome sequencing projects are underway. However, little is known about the genetic variability in this area until now. Results: The sequencing of the first P. vivax isolate from China-Myanmar border area (CMB-1) generated 120 million paired-end reads. A percentage of 10.6 of the quality-evaluated reads were aligned onto 99.9% of the reference strain Sal I genome in 62-fold coverage with an average of 4.8 SNPs per kb. We present a 539-SNP marker data set for P. vivax that can identify different parasites from different geographic origins with high sensitivity. We also identified exceptionally high levels of genetic variability in members of multigene families such as RBP, SERA, vir, MSP3 and AP2. The de-novo assembly yielded a database composed of 8,409 contigs with N50 lengths of 6.6 kb and revealed 661 novel predicted genes including 78 vir genes, suggesting a greater functional variation in P. vivax from this area. Conclusion: Our result contributes to a better understanding of P. vivax genetic variation, and provides a fundamental basis for the geographic differentiation of vivax malaria from China-Myanmar border area using a direct sequencing approach without leukocyte depletion. This novel sequencing method can be used as an essential tool for the genomic research of P. vivax in the near future. | 2144 | NA | NA | NA | no | no |
| 2145 | 2017 | NA | Salama, N; Guillemin, K; McDaniel, TK; Sherlock, G; Tompkins, L; Falkow, S | 2000 | A whole-genome microarray reveals genetic diversity among Helicobacter pylori strains | Helicobacter pylori colonizes the stomach of half of the world’s population, causing a wide spectrum of disease ranging from asymptomatic gastritis to ulcers to gastric cancer. Although the basis for these diverse clinical outcomes is not understood, more severe disease is associated with strains harboring a pathogenicity island. To characterize the genetic diversity of more and less virulent strains, we examined the genomic content of 15 H. pylori clinical isolates by using a whole genome H. pylori DNA microarray. We found that a full 22% of H. pylori genes are dispensable in one or more strains, thus defining a minimal functional core of 1281 H. pylori genes. While the core genes encode most metabolic and cellular processes, the strain-specific genes include genes unique to H. pylori, restriction modification genes, transposases, and genes encoding cell surface proteins, which may aid the bacteria under specific circumstances during their long-term infection of genetically diverse hosts. We observed distinct patterns of the strain-specific gene distribution along the chromosome, which may result from different mechanisms of gene acquisition and loss. Among the strain-specific genes, we have found a class of candidate virulence genes identified by their coinheritance with the pathogenicity island. | 2145 | NA | NA | NA | no | no |
| 2146 | 2017 | NA | Toth, JP; Varga, Z; Bereczki, J | 2016 | Long-term survival and diversification of an endemic Melitaea species in mountains of Iran and adjacent areas | Disjunct distribution patterns regularly resulted in the separation of different genetic lineages in glacial refugia. Recent patterns of survival and expansion have been often revealed by climatic niche modelling. We used the combination of genetic markers, geometric morphometry and climatic niche modelling to clear up the taxonomy and reconstruct the potential range of an endemic Iranian, taxonomically disputed Melitaea species in climatically different epochs. Our results show that this species (Melitaea abbas Gross and Ebert 1975, comb. n.=M.zagrosi Toth and Varga syn. nova) is clearly separated from all taxa of the Melitaea phoebe species group and only occurs in Iran and Azerbaijan but were also predicted for some adjacent regions. Molecular markers and distribution modelling show consistently that this species should have had a long-term survival in this area, and its range could have been slightly larger during the last glacial maximum than currently. Based on the studied molecular markers, three main groups in M.abbas can be recognized: those of steppic area of Azerbaijan, western Iran and northeastern Iran. Each group is characterized by own mitochondrial haplotypes, but also a high level of genetic diversity appears in the central part of the distribution area (Zagros Mts.). | 2146 | NA | NA | NA | no | no |
| 2147 | 2017 | NA | Irzykowska, L; Bocianowski, J; Baturo-Ciesniewska, A | 2013 | Association of mating-type with mycelium growth rate and genetic variability of Fusarium culmorum | Barley is an important crop used widely in Europe for food production, feed and malting. Unfortunately it is often colonised by fungi from the Fusarium genus. Fusarium culmorum is a global pathogen causing root rot and crown rot in small-grain cereals, resulting in a reduction in yield and grain quality. F. culmorum produces the highly toxic chemicals trichothecenes. Experimental Chemotypes and mating-type idiomorphs (MAT) were identified using Polymerase Chain Reactions (PCR) and genetic diversity was determined using Sequence-related Amplified Polymorphism (SRAP) and Random Amplified Polymorphic DNA (RAPD). Physiological features such as mycelium growth rate were also evaluated. As many as 94% of isolates was classified as a 3ADON producing and only two isolates displayed NIV chemotype. The average growth rate at 15A degrees C and 25A degrees C equalled 5.32 mm/day and 13.5 mm/day, respectively. The MAT idiomorph amplification revealed that 60% of isolates possessed MAT1-2 idiomorph. Among 32 obtained SRAP and RAPD markers, eight were associated with mycelium growth rate. It was shown first time that F. culmorum isolates with MAT1-2 idiomorph in the genome grew slower than these with MAT1-1. High level of genetic variability was determined based on amplification of SRAP and RAPD markers. | 2147 | NA | NA | NA | no | no |
| 2148 | 2017 | NA | Ruzzante, DE; Hansen, MM; Meldrup, D | 2001 | Distribution of individual inbreeding coefficients, relatedness and influence of stocking on native anadromous brown trout (Salmo trutta) population structure | We examined polymorphism at seven microsatellite loci in 4023 brown trout (Salmo trutta) collected from 32 tributaries to the Limfjord, Denmark (similar to 200 km) and from two hatcheries used for stocking. Populations differ in their estimated sizes and stocking histories. Mean individual inbreeding coefficients do not differ among locations within rivers. Relatedness varies between sites within rivers indicating varied local dynamics at a very small geographical scale. Relatedness is sometimes lower than expected among an equal number of simulated individuals with randomized genotypes, suggesting structure within locations. Five per cent of the genetic variance is distributed among rivers (F(ST) = 0.049), but in the western, less heavily stocked, area of the Limfjord a higher proportion of the genetic variance is distributed among rivers than among locations within rivers. The reverse is true of the eastern, more heavily stocked, area of the Limfjord. Here, a higher proportion of the genetic variance is distributed among locations within rivers than among rivers. Assignment tests reveal that the majority of trout (mean 77% of all fish) are more probably of local origin than hatchery origin but this proportion varies regionally, with rivers in the western area of the Limfjord showing a relatively high (mean 88%) and those in the eastern area showing a relatively low (mean 72%) proportion of locally assigned trout. These results can be interpreted as reflecting stocking impact. Also, the proportion of locally assigned trout correlates with the populations’ stocking histories, with rivers presently subjected to stocking (hatchery trout) showing low mean similar to0.73), and rivers where stocking was discontinued showing high (mean similar to0.84) proportions of local fish, probably reflecting lower survival of hatchery than of wild trout. There is evidence for isolation by distance at a large geographical scale when individual river populations are pooled into nine geographical regions but not at a small geographical scale when populations are considered individually. We reject the null hypothesis that stocking has had no impact on population structure but the relatively high proportion of locally assigned trout in populations where stocking with domestic fish no longer takes place suggests limited long-term success of stocking. | 2148 | NA | NA | NA | no | no |
| 2149 | 2017 | NA | Troschinski, S; Di Lellis, MA; Sereda, S; Hauffe, T; Wilke, T; Triebskorn, R; Kohler, HR | 2014 | Intraspecific Variation in Cellular and Biochemical Heat Response Strategies of Mediterranean Xeropicta derbentina [Pulmonata, Hygromiidae] | Dry and hot environments challenge the survival of terrestrial snails. To minimize overheating and desiccation, physiological and biochemical adaptations are of high importance for these animals. In the present study, seven populations of the Mediterranean land snail species Xeropicta derbentina were sampled from their natural habitat in order to investigate the intraspecific variation of cellular and biochemical mechanisms, which are assigned to contribute to heat resistance. Furthermore, we tested whether genetic parameters are correlated with these physiological heat stress response patterns. Specimens of each population were individually exposed to elevated temperatures (25 to 52 degrees C) for 8 h in the laboratory. After exposure, the health condition of the snails’ hepatopancreas was examined by means of qualitative description and semi-quantitative assessment of histopathological effects. In addition, the heat-shock protein 70 level (Hsp70) was determined. Generally, calcium cells of the hepatopancreas were more heat resistant than digestive cells - this phenomenon was associated with elevated Hsp70 levels at 40 degrees C. We observed considerable variation in the snails’ heat response strategy: Individuals from three populations invested much energy in producing a highly elevated Hsp70 level, whereas three other populations invested energy in moderate stress protein levels - both strategies were in association with cellular functionality. Furthermore, one population kept cellular condition stable despite a low Hsp70 level until 40 degrees C exposure, whereas prominent cellular reactions were observed above this thermal limit. Genetic diversity (mitochondrial cytochrome c oxidase subunit I gene) within populations was low. Nevertheless, when using genetic indices as explanatory variables in a multivariate regression tree (MRT) analysis, population structure explained mean differences in cellular and biochemical heat stress responses, especially in the group exposed to 40 degrees C. Our study showed that, even in similar habitats within a close range, populations of the same species use different stress response strategies that all rendered survival possible. | 2149 | NA | NA | NA | no | no |
| 2150 | 2017 | NA | Shaw, AJ; Shaw, B; Stenoien, HK; Golinski, GK; Hassel, K; Flatberg, KI | 2015 | Pleistocene survival, regional genetic structure and interspecific gene flow among three northern peat-mosses: Sphagnum inexspectatum, S. orientale and S. miyabeanum | Aim Arctic plant species are often characterized by a complex genetic structure because of changes in their population size, the fragmentation of metapopulation systems, extensive hybridization and allopolyploidization, and survival in disjunct refugia, historical features associated with Pleistocene glaciation. We assessed the biogeographical and genetic patterns in three closely related northern species of peat-moss (Sphagnum inexspectatum, S. orientale and S. miyabeanum), especially interspecific hybridization, infraspecific geographical differentiation and Pleistocene survival in one or more refugial areas. We tested alternative hypotheses of refugial survival in three widely disjunct regions: eastern Asia, Alaska and Greenland. Location North America (Canada, western USA and Greenland), China, Japan and Russia. Methods Four hundred and forty-three plants were genotyped at 12 microsatellite loci. Nucleotide sequences from 130 accessions for two plastid and two nuclear loci were used to reconstruct haplotype relationships. Population genetic analyses produced estimates of genetic diversity, levels of interspecific gene flow and rates of infraspecific intercontinental migration. Approximate Bayesian computation was used to test alternative biogeographical scenarios. Results We found evidence of hybridization between two of the three species, but phylogenetic patterns are predominantly divergent rather than reticulate. Disjunct populations of one species, S. orientale, in Greenland, Alaska and China are genetically differentiated, but migration has occurred among all three metapopulation systems. Divergence-time analyses strongly support the hypothesis that S. orientale survived the Last Glacial Maximum in Beringia and also in Greenland. Main conclusions Our results indicate that Beringia served as a refugium for peat-mosses, and therefore peatlands, which are currently extensive at high latitudes of the Northern Hemisphere. Sphagnum orientale also appears to have survived the Last Glacial Maximum in Greenland, indicating that the species persisted in multiple Pleistocene refugia. Phylogenetic patterns are relatively simple in these mosses compared with those commonly encountered in Arctic angiosperms. | 2150 | NA | NA | NA | no | no |
| 2151 | 2017 | NA | Lillie, M; Shine, R; Belov, K | 2014 | Characterisation of Major Histocompatibility Complex Class I in the Australian Cane Toad, Rhinella marina | The Major Histocompatibility Complex (MHC) class I is a highly variable gene family that encodes cell-surface receptors vital for recognition of intracellular pathogens and initiation of immune responses. The MHC class I has yet to be characterised in bufonid toads (Order: Anura; Suborder: Neobatrachia; Family: Bufonidae), a large and diverse family of anurans. Here we describe the characterisation of a classical MHC class I gene in the Australian cane toad, Rhinella marina. From 25 individuals sampled from the Australian population, we found only 3 alleles at this classical class I locus. We also found large number of class I alpha 1 alleles, implying an expansion of class I loci in this species. The low classical class I genetic diversity is likely the result of repeated bottleneck events, which arose as a result of the cane toad’s complex history of introductions as a biocontrol agent and its subsequent invasion across Australia. | 2151 | NA | NA | NA | no | no |
| 2152 | 2017 | NA | Strassmann, BI | 2011 | Cooperation and competition in a cliff-dwelling people | In animals that breed cooperatively, adult individuals will sometimes delay reproduction to act as helpers at the nest who raise young that are not their genetic offspring. It has been proposed that humans are also a cooperatively breeding species because older daughters, grandmothers, and other kin and nonkin may provide significant childcare. Through a prospective cohort study of children’s (n = 1,700) growth and survival in the Dogon of Mali, I show that cooperative breeding theory is a poor fit to the family dynamics of this population. Rather than helping each other, siblings competed for resources, producing a tradeoff between the number of maternal siblings and growth and survival. It did not take a village to raise a child; children fared the same in nuclear as in extended families. Of critical importance was the degree of polygyny, which created conflicts associated with asymmetries in genetic relatedness. The risk of death was higher and the rate of growth was slower in polygynous than monogamous families. The hazard of death for Dogon children was twofold higher if the resident paternal grandmother was alive rather than dead. This finding may reflect the frailty of elderly grandmothers who become net consumers rather than net producers in this resource-poor society. Mothers were of overwhelming importance for child survival and could not be substituted by any category of kin or nonkin. The idea of cooperative breeding taken from animal studies is a poor fit to the complexity and diversity of kin interactions in humans. | 2152 | NA | NA | NA | no | no |
| 2153 | 2017 | NA | Converse, PE; Kuchta, SR; Roosenburg, WM; Henry, PFP; Haramis, GM; King, TL | 2015 | Spatiotemporal analysis of gene flow in Chesapeake Bay Diamondback Terrapins (Malaclemys terrapin) | There is widespread concern regarding the impacts of anthropogenic activities on connectivity among populations of plants and animals, and understanding how contemporary and historical processes shape metapopulation dynamics is crucial for setting appropriate conservation targets. We used genetic data to identify population clusters and quantify gene flow over historical and contemporary time frames in the Diamondback Terrapin (Malaclemys terrapin). This species has a long and complicated history with humans, including commercial overharvesting and subsequent translocation events during the early twentieth century. Today, terrapins face threats from habitat loss and mortality in fisheries bycatch. To evaluate population structure and gene flow among Diamondback Terrapin populations in the Chesapeake Bay region, we sampled 617 individuals from 15 localities and screened individuals at 12 polymorphic microsatellite loci. Our goals were to demarcate metapopulation structure, quantify genetic diversity, estimate effective population sizes, and document temporal changes in gene flow. We found that terrapins in the Chesapeake Bay region harbour high levels of genetic diversity and form four populations. Effective population sizes were variable. Among most population comparisons, estimates of historical and contemporary terrapin gene flow were generally low (m approximate to 0.01). However, we detected a substantial increase in contemporary gene flow into Chesapeake Bay from populations outside the bay, as well as between two populations within Chesapeake Bay, possibly as a consequence of translocations during the early twentieth century. Our study shows that inferences across multiple time scales are needed to evaluate population connectivity, especially as recent changes may identify threats to population persistence. | 2153 | NA | NA | NA | no | no |
| 2154 | 2017 | NA | Menegon, M; Bardaji, A; Martinez-Espinosa, F; Botto-Menezes, C; Ome-Kaius, M; Mueller, I; Betuela, I; Arevalo-Herrera, M; Kochar, S; Kochar, SK; Jaju, P; Hans, D; Chitnis, C; Padilla, N; Castellanos, ME; Ortiz, L; Sanz, S; Piqueras, M; Desai, M; Mayor, A; del Portillo, H; Menendez, C; Severini, C | 2016 | Microsatellite Genotyping of Plasmodium vivax Isolates from Pregnant Women in Four Malaria Endemic Countries | Plasmodium vivax is the most widely distributed human parasite and the main cause of human malaria outside the African continent. However, the knowledge about the genetic variability of P. vivax is limited when compared to the information available for P. falciparum. We present the results of a study aimed at characterizing the genetic structure of P. vivax populations obtained from pregnant women from different malaria endemic settings. Between June 2008 and October 2011 nearly 2000 pregnant women were recruited during routine antenatal care at each site and followed up until delivery. A capillary blood sample from the study participants was collected for genotyping at different time points. Seven P. vivax microsatellite markers were used for genotypic characterization on a total of 229 P. vivax isolates obtained from Brazil, Colombia, India and Papua New Guinea. In each population, the number of alleles per locus, the expected heterozygosity and the levels of multilocus linkage disequilibrium were assessed. The extent of genetic differentiation among populations was also estimated. Six microsatellite loci on 137 P. falciparum isolates from three countries were screened for comparison. The mean value of expected heterozygosity per country ranged from 0.839 to 0.874 for P. vivax and from 0.578 to 0.758 for P. falciparum. P. vivax populations were more diverse than those of P. falciparum. In some of the studied countries, the diversity of P. vivax population was very high compared to the respective level of endemicity. The level of inter-population differentiation was moderate to high in all P. vivax and P. falciparum populations studied. | 2154 | NA | NA | NA | no | no |
| 2155 | 2017 | NA | Cooper, DW; Herbert, CA | 2001 | Genetics, biotechnology and population management of over-abundant mammalian wildlife in Australasia | Wildlife management involves regulation of population numbers of wild vertebrate species. in some cases there are too many animals and in others there are too few. Genetic issues arise in both instances. The historical and genetic evidence for the number of mammals that were in the founder populations of successful colonizing species in Australia and New Zealand is reviewed here. Small numbers have often given rise to large populations, despite the concomitant loss of genetic variability. Restriction of the number of over-abundant and pest species by either physical or chemical methods frequently constitutes very strong artificial selection, which leads to rapid genetic change; an example of major importance in the two countries is sodium monofluoroacetate (compound 1080). Pathogenic agents, surgical sterilization, hormonal contraceptives and translocation have all been used with varying degrees of success. The strengths and weaknesses of these techniques are assessed. A method that has received much attention is immunocontraception. We argue that this attempt to use the animals’ own immune system to modulate reproduction is incompatible with the basic biological function of protection against infectious disease. Immune function genes are highly variable in vertebrates, and so often genetic change in the population subjected to immunocontraception is likely to be even more rapid than is the case with lethal agents. Selection for failure to respond to the immunocontraceptive will occur, and will change immune function in general. Poor scientific description of ecosystem complexity makes it difficult to predict the consequences of immunocontraception on wildlife populations. | 2155 | NA | NA | NA | no | no |
| 2156 | 2017 | NA | Zeng, YF; Wang, WT; Liao, WJ; Wang, HF; Zhang, DY | 2015 | Multiple glacial refugia for cool-temperate deciduous trees in northern East Asia: the Mongolian oak as a case study | In East Asia, temperate forests are predicted to have retracted southward to c. 30 degrees N during the last glacial maximum (LGM) based on fossil pollen data, whereas phylogeographic studies have often suggested glacial in situ survival of cool-temperate deciduous trees in their modern northern ranges. Here we report a study of the genetic diversity and structure of 29 natural Mongolian oak (Quercus mongolica) populations using 19 nuclear simple sequence repeat (nSSR) loci and four chloroplast DNA fragments. Bayesian clustering analysis with nSSRs revealed five groups, which were inferred by approximate Bayesian computation (ABC) to have diverged in multiple refugia through multiple glacial-interglacial cycles. Analysis of chloroplast DNA variation revealed four lineages that were largely but incompletely geographically disjunct. Ecological niche modelling (ENMs) indicated a southward range shift of the oak’s distribution at the LGM, although high suitability scores were also evident in the Changbai Mts. (Northeast China), the Korean Peninsula, areas surrounding the Bohai Sea, and along the coast of the Russian Far East. In addition, endemic chloroplast DNA haplotypes and nuclear lineages occurred in high-latitude northern areas where the ENM predicted no suitable habitat. The combined evidence from nuclear and chloroplast DNA, and the results of the ENM clearly demonstrate that multiple northern refugia, including cryptic ones, were maintained across the current distributional range of the Mongolian oak during the LGM or earlier glacial periods. Though spatially limited, postglacial expansions from these refugia have led to a pattern of decreased genetic diversity with increasing latitude. | 2156 | NA | NA | NA | no | no |
| 2157 | 2017 | NA | Kelmemi, W; Teeuw, ME; Bochdanovits, Z; Ouburg, S; Jonker, MA; Alkuraya, F; Hashem, M; Kayserili, H; van Haeringen, A; Sheridan, E; Masri, A; Cobben, JM; Rizzu, P; Kostense, PJ; Dommering, CJ; Henneman, L; Bouhamed-Chaabouni, H; Heutink, P; ten Kate, LP; Cornel, MC | 2015 | Determining the genome-wide kinship coefficient seems unhelpful in distinguishing consanguineous couples with a high versus low risk for adverse reproductive outcome | Background: Offspring of consanguineous couples are at increased risk of congenital disorders. The risk increases as parents are more closely related. Individuals that have the same degree of relatedness according to their pedigree, show variable genomic kinship coefficients. To investigate whether we can differentiate between couples with high-and low risk for offspring with congenital disorders, we have compared the genomic kinship coefficient of consanguineous parents with a child affected with an autosomal recessive disorder with that of consanguineous parents with only healthy children, corrected for the degree of pedigree relatedness. Methods: 151 consanguineous couples (73 cases and 78 controls) from 10 different ethnic backgrounds were genotyped on the Affymetrix platform and passed quality control checks. After pruning SNPs in linkage disequilibrium, 57,358 SNPs remained. Kinship coefficients were calculated using three different toolsets: PLINK, King and IBDelphi, yielding five different estimates (IBDelphi, PLINK (all), PLINK (by population), King robust (all) and King homo (by population)). We performed a one-sided Mann Whitney test to investigate whether the median relative difference regarding observed and expected kinship coefficients is bigger for cases than for controls. Furthermore, we fitted a mixed effects linear model to correct for a possible population effect. Results: Although the estimated degrees of genomic relatedness with the different toolsets show substantial variability, correlation measures between the different estimators demonstrated moderate to strong correlations. Controls have higher point estimates for genomic kinship coefficients. The one-sided Mann Whitney test did not show any evidence for a higher median relative difference for cases compared to controls. Neither did the regression analysis exhibit a positive association between case-control status and genomic kinship coefficient. Conclusions: In this case-control setting, in which we compared consanguineous couples corrected for degree of pedigree relatedness, a higher degree of genomic relatedness was not significantly associated with a higher likelihood of having an affected child. Further translational research should focus on which parts of the genome and which pathogenic mutations couples are sharing. Looking at relatedness coefficients by determining genome-wide SNPs does not seem to be an effective measure for prospective risk assessment in consanguineous parents. | 2157 | NA | NA | NA | no | no |
| 2158 | 2017 | NA | Stam, R; Scheikl, D; Tellier, A | 2017 | The wild tomato species Solanum chilense shows variation in pathogen resistance between geographically distinct populations | Wild tomatoes are a valuable source of disease resistance germplasm for tomato (Solanum lycopersicum) breeders. Many species are known to possess a certain degree of resistance against certain pathogens; however, evolution of resistance traits is yet poorly understood. For some species, like Solanum chilense, both differences in habitat and within species genetic diversity are very large. Here we aim to investigate the occurrence of spatially heterogeneous coevolutionary pressures between populations of S. chilense, We investigate the phenotypic differences in disease resistance within.. S. chilense against three common tomato pathogens (Alternaria solani, Phytophthora infestans and a Fusarium sp.) and confirm high degrees of variability in resistance properties between selected populations. Using generalised linear mixed models, we show that disease resistance does not follow the known demographic patterns of the species. Models with up to five available climatic and geographic variables are required to best describe resistance differences, confirming the complexity of factors involved in local resistance variation. We confirm that within S. chilense, resistance properties against various pathogens show a mosaic pattern and do not follow environmental patterns, indicating the strength of local pathogen pressures. Our study can form the basis for further investigations of the genetic traits involved. | 2158 | NA | NA | NA | no | no |
| 2159 | 2017 | NA | Liljander, A; Chandramohan, D; Kweku, M; Olsson, D; Montgomery, SM; Greenwood, B; Farnert, A | 2010 | Influences of Intermittent Preventive Treatment and Persistent Multiclonal Plasmodium falciparum Infections on Clinical Malaria Risk | Background: Intermittent preventive treatment (IPT) of malaria involves administration of curative doses of antimalarials at specified time points to vulnerable populations in endemic areas, regardless whether a subject is known to be infected. The effect of this new intervention on the development and maintenance of protective immunity needs further understanding. We have investigated how seasonal IPT affects the genetic diversity of Plasmodium falciparum infections and the risk of subsequent clinical malaria. Material and Methods: The study included 2227 Ghanaian children (3-59 months) who were given sulphadoxine-pyrimethamine (SP) bimonthly, artesunate plus amodiaquine (AS+AQ) monthly or bimonthly, or placebo monthly for six months spanning the malaria transmission season. Blood samples collected at three post-interventional surveys were analysed by genotyping of the polymorphic merozoite surface protein 2 gene. Malaria morbidity and anaemia was monitored during 12 months follow-up. Results: Monthly IPT with AS+AQ resulted in a marked reduction in number of concurrent clones and only children parasite negative just after the intervention period developed clinical malaria during follow-up. In the placebo group, children without parasites as well as those infected with >= 2 clones had a reduced risk of subsequent malaria. The bimonthly SP or AS+AQ groups had similar number of clones as placebo after intervention; however, diversity and parasite negativity did not predict the risk of malaria. An interaction effect showed that multiclonal infections were only associated with protection in children without intermittent treatment. Conclusion: Molecular typing revealed effects of the intervention not detected by ordinary microscopy. Effective seasonal IPT temporarily reduced the prevalence and genetic diversity of P. falciparum infections. The reduced risk of malaria in children with multiclonal infections only seen in untreated children suggests that persistence of antigenically diverse P. falciparum infections is important for the maintenance of protective malaria immunity in high transmission settings. | 2159 | NA | NA | NA | no | no |
| 2160 | 2017 | NA | Cheng, YY; Sanderson, C; Jones, M; Belov, K | 2012 | Low MHC class II diversity in the Tasmanian devil (Sarcophilus harrisii) | The largest remaining carnivorous marsupial, the Tasmanian devil (Sarcophilus harrisii), is currently under threat of extinction due to a fatal contagious cancer-devil facial tumour disease. Low major histocompatibility complex (MHC) class I diversity is believed to have contributed to the transmission of the tumour allograft through devil populations. Here, we report low MHC class II variability in this species, with DA beta chain genes (Saha-DAB1, 2 and 3) exhibiting very limited diversity and the sole alpha chain gene (Saha-DAA) monomorphic. Three, six and three alleles were found at Saha-DAB1, 2 and 3, respectively, with a predominant allele found at each locus. Heterozygosity at these three loci is low in the eastern population and modestly higher in northwestern individuals. The results are indicative of a selective sweep likely due to an infectious disease resulting in the fixation of selectively favoured alleles and depletion of genetic diversity at devil class II loci. Several attempts were made to isolate the other marsupial classical class II gene family, namely, DB, resulting in only one DBB pseudogene being found. These findings further support the view that this species has a compromised capacity to respond to pathogen evolution, emerging infectious diseases and environmental changes. | 2160 | NA | NA | NA | no | no |
| 2161 | 2017 | NA | Jacquot, M; Bisseux, M; Abrial, D; Marsot, M; Ferquel, E; Chapuis, JL; Vourc’h, G; Bailly, X | 2014 | High-Throughput Sequence Typing Reveals Genetic Differentiation and Host Specialization among Populations of the Borrelia burgdorferi Species Complex that Infect Rodents | Lyme disease is a zoonosis caused by various species belonging to the Borrelia burgdorferi bacterial species complex. These pathogens are transmitted by ticks and infect multiple, taxonomically distinct, host species. From an epidemiological perspective, it is important to determine whether genetic variants within the species complex are able to spread freely through the whole host community or, instead, if certain variants are restricted to particular hosts. To this end, we characterized the genotypes of members of the B. burgdorferi species complex; the bacteria were isolated from more than two hundred individuals captured in the wild and belonging to three different rodent host species. For each individual, we used a high-throughput approach to amplify and sequence rplB, a housekeeping gene, and ospC, which is involved in infection. This approach allowed us to evaluate the genetic diversity both within and among species in the B. burgdorferi species complex. Strong evidence of genetic differentiation among host species was revealed by both genes, even though they are, a priori, not constrained by the same selective pressures. These data are discussed in the context of the advancements made possible by multi-locus high-throughput sequencing and current knowledge of Lyme disease epidemiology. | 2161 | NA | NA | NA | no | no |
| 2162 | 2017 | NA | Krawczak, M; Caliebe, A; Croucher, PJP; Schmidtke, J | 2007 | On the testing load incurred by cascade genetic carrier screening for Mendelian disorders: A brief report | One criterion to decide to whom molecular genetic carrier testing should be provided is an individual’s carrier risk, taking into account his or her affection status and degree of relatedness to an overt carrier. We have derived formulas to calculate the testing load incurred to a public health system following such a cascade screening strategy. While the testing load turns out to be moderate for individual diseases at meaningful risk thresholds (i. e., 1%-5%), a substantial proportion of the population would have to be tested if all known single gene disorders were to be included in a cascade screening program. | 2162 | NA | NA | NA | no | no |
| 2163 | 2017 | NA | Amundsen, PA; Salonen, E; Niva, T; Gjelland, KO; Praebel, K; Sandlund, OT; Knudsen, R; Bohn, T | 2012 | Invader population speeds up life history during colonization | We explore the long-term developments in population biology and life history during the invasion and establishment of the fish species vendace Coregonus albula in a subarctic watercourse by comparing life-history traits and molecular genetic estimates between the source and the colonist population. The two populations exhibited highly contrasting life-history strategies. Relative to the source population, the colonist population was characterized by slower somatic growth rates, earlier sexual maturation at smaller individual size, higher mortality rates and a shorter life span. The two populations could also be significantly discriminated by the genetic markers. Limited founder effects were detected from heterozygote deficit and reduced allelic richness in the colonist population, but both populations were associated with relatively high genetic diversity. The study reveals that the invasion into a new environment induced large changes in life-history strategy, with typical r-selected traits being more prominent in the colonist than in the source population. We discuss the mechanisms that may explain the observed life-history differences between the source and the colonist population, and argue that the accelerated life history of the colonist population represents an adaptive pioneer strategy aimed at fast population increase during colonization and establishment. | 2163 | NA | NA | NA | no | no |
| 2164 | 2017 | NA | Stassen, HH; Begleiter, H; Porjesz, B; Rice, J; Scharfetter, C; Reich, T | 1999 | Structural decomposition of genetic diversity in families with alcoholism | “Using genotypes of 280 marker loci on the 22 autosomes of 105 alcohol-dependent probands, their affected and unaffected sibs, as well as their parents, we iteratively constructed a genetic similarity function that enabled us to quantify the interindividual genetic distances d(x(i), x(j)) between feature vectors x(i), x(j) made up by the allelic patterns of individuals i, j with respect to loci l(1), l(2), …, l(n). Based on this similarity function, we investigated the sib-sib similarities that are expected to deviate from”“0.5”" in affected sib pairs if the region of interest contains markers close to disease-causing genes. The reference value "“0.5”" was derived from the parents-offspring similarities, because these are independent of the affection status. The question of population admixture was addressed by means of multivariate structural analyses. These analyses led to four "“natural”" groups whose validity was tested through the father-mother similarities. Additionally, we determined the eigenvectors that optimally represented the genetic variation and found several marker configurations on chromosomes 1, 3, 7, 15, and 17 that reproducibly discriminated (p less than or equal to 0.01) affected probands/sibs from unaffected sibs, while no such differences were found between affected probands and affected sibs. (C) 1999 Wiley-Liss, Inc." | 2164 | NA | NA | NA | no | no |
| 2165 | 2017 | NA | Karaca, S; Karaca, M; Civelek, E; Ozgul, RK; Sekerel, BE; Polimanti, R | 2016 | Haplotype analysis of non-HLA immunogenetic loci in Turkish and worldwide populations | Immunogenes (i.e., genes related to the immune system and its functions) are involved in the predisposition to numerous traits and their variation contributes to the phenotypic variability observed among human groups. Turkish population presents particular genetic features since its genetic pool is an admixture of European, Middle-Eastern, and Central Asian ancestries. Here, we analyzed the haplotype structure of four immunogenetic loci (i.e., ADAM33; IL13-IL4; IL4R; MS4A2) in 482 subjects from five different regions of Turkey. Genotyping was performed using KASP technology. Turkish data were compared with the haplotype information available from the 1000 Genomes Project Phase 3 (26 human populations from 5 ancestry groups). We did not observe significant differences among Turkish groups. Comparing other ancestries, we identified haplotype similarity of Turkish subjects with European populations in IL13-1L4, IL4R, and ADAM33 loci; and with central Asians in MS4A2 region. Considering loci displaying Turkish-European haplotype similarity (i.e., IL13-1L4, IL4R, and ADAM33), we observed differences between Turkish subjects and northern/western Europeans. Conversely, no significant difference was determined in MS4A2 between Turkish and central Asian populations. Finally, we assessed the haplotypes responsible for the differences between Turkish and European samples and the potential functional effects on the immunogenetic loci investigated. (C) 2016 Elsevier B.V. All rights reserved. | 2165 | NA | NA | NA | no | no |
| 2166 | 2017 | NA | Albayrak, G; Yoruk, E; Gazdagli, A; Sharifnabi, B | 2016 | GENETIC DIVERSITY AMONG FUSARIUM GRAMINEARUM AND F. CULMORUM ISOLATES BASED ON ISSR MARKERS | To characterize the isolates of F. graminearum and F. culmorum fungi from Turkey and Iran, we performed ISSR analysis with 18 non-anchored and 23 anchored (including ten novel) primers. Amplification product sizes were 0.2-3.5 kb. In total, 405 bands were scored, 24 of which (5.92%) were polymorphic. The similarities among F. graminearum isolates were calculated as 62.3-99%, and among F. culmorum as 65.7-94.3%. Moderate genetic variation at intra-and inter-specific levels was determined, and the average intraspecific genetic diversity values were 80.65% for F. graminearum, and 80% for F. culmorum. Cluster analysis separated the isolates into two main clades. Group I consisted of F. culmorum isolates from Turkey that produced DON mycotoxin. Group II contained all F. graminearum isolates that were deoxynivalenol (DON) and nivalenol (NIV) chemotypes from Turkey and Iran. Both groups I and II were divided into two subgroups including their divisions. Phenons in group II included isolates distributed in the same geographic region. ISSR markers clustered isolates within a definite order according to their species. Isolates from the same agro-ecological locations were also kept together in subdivisions. The novel ISSR markers developed for the first time in this study contribute to differentiating between Fusarium isolates according to their species and geographic regions. | 2166 | NA | NA | NA | no | no |
| 2167 | 2017 | NA | Hill, R; Loxterman, JL; Aho, K | 2017 | Insular biogeography and population genetics of dwarf mistletoe (Arceuthobium americanum) in the Central Rocky Mountains | “Lodgepole pine dwarf mistletoe (Arceuthobium americanum), an aggressive parasite of lodgepole pine (Pinus contorta subsp. latifolia), is limited, in the Central Rocky Mountains, to montane host”“islands.”" We sought to quantify the effect of the insularity and life history of A. americanum on its population genetic structure by considering three questions: (1) Do apparent limits to A. americanum pollen and seed dispersal affect gene flow at small spatial scales? (2) Are patterns of genetic diversity on montane "“islands”" and "“mainlands”" consistent with the predictions of island biogeographic theory? (3) What is the origin and genetic relationship of "“island”" and "“mainland”" populations of A. americanum within the Central Rocky Mountain study area? We used seven A. americanum microsatellite markers developed specifically for the project to genotype 241 individuals from six populations (four "“island”" and two "“mainland”" locations) at the western edge of the Central Rocky Mountains, Idaho, USA. All loci were unlinked and polymorphic with a range of 4-24 alleles per locus. To consider gene flow, we performed spatial analyses and compared F-statistics from our data to those from a wide range of species and functional groups. We tested the predictions of biogeographic theory by quantifying associations of genetic diversity with "“island”" area and isolation from "“mainland”" populations. To consider the genetic relationships of "“island”" and "“mainland”" regions, we applied agglomerative and Bayesian hierarchical classifications to datasets, and objectively identified the optimal numbers of clusters in these analyses. We found that F-IS values in A. americanum were similar to those reported for dispersal-limited species with similar life-history characteristics, and F-ST values were similar to those reported for plants with animal-facilitated seed dispersal. As predicted by biogeographic theory, island allelic richness was positively correlated with geographic size of the population and negatively correlated with distance from mainland populations. Average linkage (UPGMA) classifications and Bayesian cluster analyses both identified two groupings of island populations radiating from the mainland populations. Our results are likely to be of interest to foresters managing dwarf mistletoe infections, particularly in the Central Rocky Mountains, and to ecologists and geneticists working with insular population distributions." | 2167 | NA | NA | NA | no | no |
| 2168 | 2017 | NA | Nieberding, C; Libois, R; Douady, CJ; Morand, S; Michaux, JR | 2005 | Phylogeography of a nematode (Heligmosomoides polygyrus) in the western Palearctic region: persistence of northern cryptic populations during ice ages? | This study establishes the continental phylogeographical pattern of a European nematode, Heligmosomoides polygyrus (Dujardin, 1845; Heligmosomoidea). We sequenced 687 base pairs of the mitochondrial DNA (mtDNA) cyt b gene for 136 individuals collected in 22 localities. The results revealed that H. polygyrus populations are separated into five major units corresponding to the Italian, northern European (Denmark and Ireland), Iberian, western European, and Balkan populations. Different subclades were also observed within the first two groups. Based on the rate of molecular evolution of H. polygyrus cyt b gene-estimated to 3.5%-3.7% divergence per million years (Myr) in a previous study - the isolation time of the five clades was estimated between 2.5 +/- 0.24 and 1.5 +/- 0.23 million years ago. Moreover, H. polygyrus presents a higher genetic variability in the Mediterranean peninsulas as compared to northwestern Europe, highlighting the role of these regions as refuge areas. Like its specific host, the wood mouse Apodemus sylvaticus, H. polygyrus’ pattern of postglacial recolonization of northwestern Europe was initiated from Iberian populations, while Italian and Balkan populations did not expand to the north. The results also suggest the existence of forested and temperate refuges in the southern British Isles during the Quaternary. Finally, the genetic diversity as well as the level of genetic divergence between the lineages of H. polygyrus are compared to those observed in other vertebrate and invertebrate phylogeographical studies: the existence of highly differentiated lineages in H. polygyrus (5%-10% of genetic divergence) highlights that the effects of Pleistocene climate changes on free-living organisms are also reflected in their obligate parasites. | 2168 | NA | NA | NA | no | no |
| 2169 | 2017 | NA | Ness, RW; Morgan, AD; Vasanthakrishnan, RB; Colegrave, N; Keightley, PD | 2015 | Extensive de novo mutation rate variation between individuals and across the genome of Chlamydomonas reinhardtii | Describing the process of spontaneous mutation is fundamental for understanding the genetic basis of disease, the threat posed by declining population size in conservation biology, and much of evolutionary biology. Directly studying spontaneous mutation has been difficult, however, because new mutations are rare. Mutation accumulation (MA) experiments overcome this by allowing mutations to build up over many generations in the near absence of natural selection. Here, we sequenced the genomes of 85 MA lines derived from six genetically diverse strains of the green alga Chlamydomonas rein hardtii We identified 6843 new mutations, more than any other study of spontaneous mutation. We observed sevenfold variation in the mutation rate among strains and that mutator genotypes arose, increasing the mutation rate approximately eightfold in some replicates. We also found evidence for fine-scale heterogeneity in the mutation rate, with certain sequence motifs mutating at much higher rates, and clusters of multiple mutations occurring at closely linked sites. There was little evidence, however, for mutation rate heterogeneity between chromosomes or over large genomic regions of 200 kbp. We generated a predictive model of the mutability of sites based on their genomic properties, including local GC content, gene expression level, and local sequence context. Our model accurately predicted the average mutation rate and natural levels of genetic diversity of sites across the genome. Notably, trinucleotides vary 17-fold in rate between the most and least mutable sites. Our results uncover a rich heterogeneity in the process of spontaneous mutation both among individuals and across the genome. | 2169 | NA | NA | NA | no | no |
| 2170 | 2017 | NA | Auld, SKJR; Hall, SR; Duffy, MA | 2012 | Epidemiology of a Daphnia-Multiparasite System and Its Implications for the Red Queen | The Red Queen hypothesis can explain the maintenance of host and parasite diversity. However, the Red Queen requires genetic specificity for infection risk (i.e., that infection depends on the exact combination of host and parasite genotypes) and strongly virulent effects of infection on host fitness. A European crustacean (Daphnia magna) - bacterium (Pasteuria ramosa) system typifies such specificity and high virulence. We studied the North American host Daphnia dentifera and its natural parasite Pasteuria ramosa, and also found strong genetic specificity for infection success and high virulence. These results suggest that Pasteuria could promote Red Queen dynamics with D. dentifera populations as well. However, the Red Queen might be undermined in this system by selection from a more common yeast parasite (Metschnikowia bicuspidata). Resistance to the yeast did not correlate with resistance to Pasteuria among host genotypes, suggesting that selection by Metschnikowia should proceed relatively independently of selection by Pasteuria. | 2170 | NA | NA | NA | no | no |
| 2171 | 2017 | NA | Bhutani, N; Ranjit, MR; Yameen, M; Singh, N; Dev, V; Pillai, CR; Ansari, MA; Sharma, YD | 1998 | Genetic diversity among field isolates of Plasmodium falciparum in India | Plasmodium falciparum is the most lethal human malaria parasite, causing epidemics and Targe number of deaths in India. We describe here the genetic diversity among field isolates of P. falciparum from India by polymerase chain reaction (PCR), The variable regions of two functionally important parasite molecules - knob-associated histidine-rich protein (KAHRP) gene and thrombospondin-related anonymous protein (TRAP) gene - were used as markers for PCR identification. We observed three alleles for KAHRP gene and seven for TRAP gene among tested field isolates. Based on these markers, a total of 17 different isolate types were identified among 101 cases. We also report here that; the parasite population in two epidemic but neighbouring states of Haryana and Rajasthan were different. from each other. The presence of several genetically-divergent P. falciparum parasite isolates in India bears its significance for the malaria control programmes such as the use of vaccines and antimalarial drugs. | 2171 | NA | NA | NA | no | no |
| 2172 | 2017 | NA | Lopes, FL; Hou, L; Boldt, ABW; Kassem, L; Alves, VM; Nardi, AE; McMahon, FJ | 2016 | Finding Rare, Disease-Associated Variants in Isolated Groups: Potential Advantages of Mennonite Populations | Large-scale genotyping and next-generation sequencing techniques have allowed great advances in the field of molecular genetics. Numerous common variants of low impact have been associated with many complex human traits and diseases, such as bipolar disorder and schizophrenia. Although they may exert a greater impact on risk, few rare disease variants have been found, owing to the greatly increased sample sizes that are typically necessary to demonstrate association with rarer variants. One alternative strategy is to study isolated populations, where historical bottlenecks reduce genetic diversity and some otherwise rare variants may drift to higher frequencies. Here we describe the Mennonite population settlements, considering their history of multiple bottlenecks followed by demographic expansion and a currently widespread geographical distribution. We argue that Mennonite populations are valuable partners for studies seeking genetic variants that exert a high impact on risk for a variety of common disorders, including mental illnesses. | 2172 | NA | NA | NA | no | no |
| 2173 | 2017 | NA | Fonseca, L; Cardoso, JMS; Lopes, A; Pestana, M; Abreu, F; Nunes, N; Mota, M; Abrantes, I | 2012 | The pinewood nematode, Bursaphelenchus xylophilus, in Madeira Island | The environmental conditions in Madeira Island are favorable for the presence and dissemination of the pinewood nematode (PWN), Bursaphelenchus xylophilus. Five hundred Pinus pinaster wood samples were collected in several forest areas and PWN was detected in 22.8 % of the samples. Bursaphelenchus xylophilus isolates from Madeira Island displayed the species-specific diagnostic characters. A morphological variation in the female tail terminus was detected. In most females, the tail presented a broadly rounded terminus and, occasionally, a digitate terminus with a terminal nipple-like extension resembling a mucro. PCR ITS-RFLP analysis revealed that Madeira Island isolates exhibited patterns specific to the species B. xylophilus and similar to virulent isolates. Amplified ITS regions were further sequenced and no genetic diversity was found for this genomic region among 17 Portuguese isolates (Madeira Island and Continental Portugal). Phylogenetic analysis revealed that Portuguese isolates grouped with isolates from China, Korea and one isolate from Japan. | 2173 | NA | NA | NA | no | no |
| 2174 | 2017 | NA | Puurtinen, M; Knott, KE; Suonpaa, S; Nissinen, K; Kaitala, V | 2007 | Predominance of outcrossing in Lymnaea stagnalis despite low apparent fitness costs of self-fertilization | We have quantified the natural mating system in eight populations of the simultaneously hermaphroditic aquatic snail Lymnaea stagnalis, and studied the ecological and genetic forces that may be directing mating system evolution in this species. We investigated whether the natural mating system can be explained by the availability of mates, by the differential survival of self- and cross-fertilized snails in nature, and by the effects of mating system on parental fecundity and early survival. The natural mating system of L. stagnalis was found to be predominantly cross-fertilizing. Density of snails in the populations had no relationship with the mating system, suggesting that outcrossing rates are not limited by mate availability at the population densities observed. Contrary to expectations for outcrossing species, we detected no evidence for inbreeding depression in survival in nature with inferential population genetic methods. Further, experimental manipulations of mating system in the laboratory revealed that self-fertilization had no effect on parental fecundity, and only minor effects on offspring survival. Predominance of cross-fertilization despite low apparent fitness costs of self-fertilization is at odds with the paradigm that high self-fertilization depression is necessary for maintenance of cross-fertilization in self-compatible hermaphrodites. | 2174 | NA | NA | NA | no | no |
| 2175 | 2017 | NA | Al Dahouk, S; Kohler, S; Occhialini, A; de Bagues, MPJ; Hammerl, JA; Eisenberg, T; Vergnaud, G; Cloeckaert, A; Zygmunt, MS; Whatmore, AM; Melzer, F; Drees, KP; Foster, JT; Wattam, AR; Scholz, HC | 2017 | Brucella spp. of amphibians comprise genomically diverse motile strains competent for replication in macrophages and survival in mammalian hosts | Twenty-one small Gram-negative motile coccobacilli were isolated from 15 systemically diseased African bullfrogs (Pyxicephalus edulis), and were initially identified as Ochrobactrum anthropi by standard microbiological identification systems. Phylogenetic reconstructions using combined molecular analyses and comparative whole genome analysis of the most diverse of the bullfrog strains verified affiliation with the genus Brucella and placed the isolates in a cluster containing B. inopinata and the other non-classical Brucella species but also revealed significant genetic differences within the group. Four representative but molecularly and phenotypically diverse strains were used for in vitro and in vivo infection experiments. All readily multiplied in macrophage-like murine J774-cells, and their overall intramacrophagic growth rate was comparable to that of B. inopinata BO1 and slightly higher than that of B. microti CCM 4915. In the BALB/c murine model of infection these strains replicated in both spleen and liver, but were less efficient than B. suis 1330. Some strains survived in the mammalian host for up to 12 weeks. The heterogeneity of these novel strains hampers a single species description but their phenotypic and genetic features suggest that they represent an evolutionary link between a soil-associated ancestor and the mammalian host-adapted pathogenic Brucella species. | 2175 | NA | NA | NA | no | no |
| 2176 | 2017 | NA | Kovaliski, J; Sinclair, R; Mutze, G; Peacock, D; Strive, T; Abrantes, J; Esteves, PJ; Holmes, EC | 2014 | Molecular epidemiology of Rabbit Haemorrhagic Disease Virus in Australia: when one became many | Rabbit Haemorrhagic Disease Virus (RHDV) was introduced into Australia in 1995 as a biological control agent against the wild European rabbit (Oryctolagus cuniculus). We evaluated its evolution over a 16-year period (1995-2011) by examining 50 isolates collected throughout Australia, as well as the original inoculum strains. Phylogenetic analysis of capsid protein VP60 sequences of the Australian isolates, compared with those sampled globally, revealed that they form a monophyletic group with the inoculum strains (CAPM V-351 and RHDV351INOC). Strikingly, despite more than 3000 rereleases of RHDV351INOC since 1995, only a single viral lineage has sustained its transmission in the long-term, indicative of a major competitive advantage. In addition, we find evidence for widespread viral gene flow, in which multiple lineages entered individual geographic locations, resulting in a marked turnover of viral lineages with time, as well as a continual increase in viral genetic diversity. The rate of RHDV evolution recorded in Australia -4.0 (3.3-4.7)x10(-3) nucleotide substitutions per site per year - was higher than previously observed in RHDV, and evidence for adaptive evolution was obtained at two VP60 residues. Finally, more intensive study of a single rabbit population (Turretfield) in South Australia provided no evidence for viral persistence between outbreaks, with genetic diversity instead generated by continual strain importation. | 2176 | NA | NA | NA | no | no |
| 2177 | 2017 | NA | Volkov, I; Pepin, KM; Lloyd-Smith, JO; Banavar, JR; Grenfell, BT | 2010 | Synthesizing within-host and population-level selective pressures on viral populations: the impact of adaptive immunity on viral immune escape | The evolution of viruses to escape prevailing host immunity involves selection at multiple integrative scales, from within-host viral and immune kinetics to the host population level. In order to understand how viral immune escape occurs, we develop an analytical framework that links the dynamical nature of immunity and viral variation across these scales. Our epidemiological model incorporates within-host viral evolutionary dynamics for a virus that causes acute infections (e. g. influenza and norovirus) with changes in host immunity in response to genetic changes in the virus population. We use a deterministic description of the within-host replication dynamics of the virus, the pool of susceptible host cells and the host adaptive immune response. We find that viral immune escape is most effective at intermediate values of immune strength. At very low levels of immunity, selection is too weak to drive immune escape in recovered hosts, while very high levels of immunity impose such strong selection that viral subpopulations go extinct before acquiring enough genetic diversity to escape host immunity. This result echoes the predictions of simpler models, but our formulation allows us to dissect the combination of within-host and transmission-level processes that drive immune escape. | 2177 | NA | NA | NA | no | no |
| 2178 | 2017 | NA | Dutech, C; Fabreguettes, O; Capdevielle, X; Robin, C | 2010 | Multiple introductions of divergent genetic lineages in an invasive fungal pathogen, Cryphonectria parasitica, in France | The occurrence of multiple introductions may be a crucial factor in the successful establishment of invasive species, but few studies focus on the introduction of fungal pathogens, despite their significant effect on invaded habitats. Although Cryphonectria parasitica, the chestnut blight fungus introduced in North America and Europe from Asia during the 20th century, caused dramatic changes in its new range, the history of its introduction is not well retraced in Europe. Using 10 microsatellite loci, we investigated the genetic diversity of 583 isolates in France, where several introductions have been hypothesized. Our analyses showed that the seven most frequent multilocus genotypes belonged to three genetic lineages, which had a different and geographically limited distribution. These results suggest that different introduction events occurred in France. Genetic recombination was low among these lineages, despite the presence of the two mating types in each chestnut stand analysed. The spatial distribution of lineages suggests that the history of introductions in France associated with the slow expansion of the disease has contributed to the low observed rate of recombination among the divergent lineages. However, we discuss the possibility that environmental conditions or viral interactions could locally reduce recombination among genotypes. Heredity (2010) 105, 220-228; doi: 10.1038/hdy.2009.164; published online 9 December 2009 | 2178 | NA | NA | NA | no | no |
| 2179 | 2017 | NA | Roulin, AC; Mariadassou, M; Hall, MD; Walser, JC; Haag, C; Ebert, D | 2015 | High genetic variation in resting-stage production in a metapopulation: Is there evidence for local adaptation? | Local adaptation is a key process for the maintenance of genetic diversity and population diversification. A better understanding of the mechanisms that allow (or prevent) local adaptation constitutes a key in apprehending how and at what spatial scale it occurs. The production of resting stages is found in many taxa and reflects an adaptation to outlast adverse environmental conditions. Daphnia magna (Crustacea) can alternate between asexual and sexual reproduction, the latter being linked to dormancy, as resting stages can only be produced sexually. In this species, on a continental scale, resting-stage production is locally adaptedthat is, it is induced when the photoperiod indicates the imminence of habitat deterioration. Here, we aimed to explore whether selection is strong enough to maintain local adaptation at a scale of a few kilometers. We assessed life-history traits of 64 D. magna clones originating from 11 populations of a metapopulation with permanent and intermittent pool habitats. We found large within- and between-population variation for all dormancy-related traits, but no evidence for the hypothesized higher resting-stage production in animals from intermittent habitats. We discuss how gene flow, founder events, or other forms of selection might interfere with the process of local adaptation. | 2179 | NA | NA | NA | no | no |
| 2180 | 2017 | NA | Goble, TA; Costet, L; Robene, I; Nibouche, S; Rutherford, RS; Conong, DE; Hill, MP | 2012 | Beauveria brongniartii on white grubs attacking sugarcane in South Africa | Beauveria brongniartii (Saccardo) Petch fungal infections were observed on the melolonthid Hypopholis sommeri Burmeister (Coleoptera: Scarabaeidae) at two sites (Harden Heights and Canema) in the sugarcane producing area of the northern KwaZulu-Natal Midlands of South Africa. To initially identify the disease-causing fungus, 17 different fluorescently-labelled microsatellite PCR primers were used to target 78 isolates of Beauveria spp. DNA. Microsatellite data resolved two distinct clusters of Beauveria isolates which represented the Beauveria bassiana s.s. (Balsamo) Vuillemin (17 isolates) and B. brongniartii (60 isolates) species groups. These groupings were supported by two gene regions, the nuclear ribosomal Internal Transcribed Spacer (ITS) and the nuclear Bloc gene of which 23 exemplar Beauveria isolates were represented and sequenced. When microsatellite data were analysed, 26 haplotypes among 58 isolates of B. brongniartii were distinguished. Relatively low levels of genetic diversity were detected in B. brongniartii and isolates were shown to be closely related. No genetic differentiation was observed between the Harden Heights and Canema populations; they thus may be considered one, structured and fragmented population over a distance of 5.5 km. Historically high levels of gene flow from swarming H. sommeri beetles is the proposed mechanism for this observed lack of genetic differentiation between populations. Microsatellite analyses also showed that B. brongniartii conidia were being cycled from arboreal forest to subterranean sugarcane habitats and vice versa in the environment by H. sommeri life stages. This is the first record of this species of fungus infecting H. sommeri larvae and adults in South Africa. (c) 2012 Elsevier Inc. All rights reserved. | 2180 | NA | NA | NA | no | no |
| 2181 | 2017 | NA | Turner, MM; Deperno, CS; Booth, W; Vargo, EL; Conner, MC; Lancia, RA | 2016 | The Mating System of White-Tailed Deer Under Quality Deer Management | Mating systems, which can reveal temporal and spatial plasticity within a given species, may influence inbreeding, effective population size, genetic diversity, reproductive fitness, and survival. Although observational research on white-tailed deer has indicated dominant males monopolize breeding opportunities, recent molecular studies suggest a more complex system. It is possible that population characteristics fostered under management strategies designed to balance the sex ratio and extend the male age structure, such as Quality Deer Management (QDM), influence the pre-breeding interactions that affect the distribution of mating success across age classes. Therefore, our objective was to evaluate the white-tailed deer mating system under QDM through paternity analysis. Using 8 microsatellite loci and tissue samples harvested from hunter-killed deer at Chesapeake Farms in Chestertown, Maryland, we evaluated 731 deer. The >= 3.5 age class dominated mating at Chesapeake Farms, with 41% of paternity. However, together, the 1.5- and 2.5-year-old age classes accounted for more than half of paternity (59%). No evidence of polyandry was detected. Our results indicate the interaction between the sex ratio and age structure, fostered by strategies that balance the sex ratio and extend the male age structure, facilitate breeding by younger males. These results indicate management strategies that employ QDM practices with a goal of propagating the genes of dominant males may fall short, and the interplay among sex ratio, age structure, and dominance relationships may be the main influences of mating success. Managers should account for these findings when setting expectations for reproduction under restricted harvest. (C) 2016 The Wildlife Society. | 2181 | NA | NA | NA | no | no |
| 2182 | 2017 | NA | Tang, RQ; Noh, HJ; Wang, DQ; Sigurdsson, S; Swofford, R; Perloski, M; Duxbury, M; Patterson, EE; Albright, J; Castelhano, M; Auton, A; Boyko, AR; Feng, GP; Lindblad-Toh, K; Karlsson, EK | 2014 | Candidate genes and functional noncoding variants identified in a canine model of obsessive-compulsive disorder | Background: Obsessive-compulsive disorder (OCD), a severe mental disease manifested in time-consuming repetition of behaviors, affects 1 to 3% of the human population. While highly heritable, complex genetics has hampered attempts to elucidate OCD etiology. Dogs suffer from naturally occurring compulsive disorders that closely model human OCD, manifested as an excessive repetition of normal canine behaviors that only partially responds to drug therapy. The limited diversity within dog breeds makes identifying underlying genetic factors easier. Results: We use genome-wide association of 87 Doberman Pinscher cases and 63 controls to identify genomic loci associated with OCD and sequence these regions in 8 affected dogs from high-risk breeds and 8 breed-matched controls. We find 119 variants in evolutionarily conserved sites that are specific to dogs with OCD. These case-only variants are significantly more common in high OCD risk breeds compared to breeds with no known psychiatric problems. Four genes, all with synaptic function, have the most case-only variation: neuronal cadherin (CDH2), catenin alpha2 (CTNNA2), ataxin-1 (ATXN1), and plasma glutamate carboxypeptidase (PGCP). In the 2 Mb gene desert between the cadherin genes CDH2 and DSC3, we find two different variants found only in dogs with OCD that disrupt the same highly conserved regulatory element. These variants cause significant changes in gene expression in a human neuroblastoma cell line, likely due to disrupted transcription factor binding. Conclusions: The limited genetic diversity of dog breeds facilitates identification of genes, functional variants and regulatory pathways underlying complex psychiatric disorders that are mechanistically similar in dogs and humans. | 2182 | NA | NA | NA | no | no |
| 2183 | 2017 | NA | Kyes, RC; Jones-Engel, L; Chalise, MK; Engel, G; Heidrich, J; Grant, R; Bajimaya, SS; McDonough, J; Smith, DG; Ferguson, B | 2006 | Genetic characterization of rhesus macaques (Macaca mulatta) in Nepal | Indian-origin rhesus macaques (Macaca mulatta) have long served as an animal model for the study of human disease and behavior. Given the current shortage of Indian-origin rhesus, many researchers have turned to rhesus macaques from China as a substitute. However, a number of studies have identified marked genetic differences between the Chinese and Indian animals. We investigated the genetic characteristics of a third rhesus population, the rhesus macaques of Nepal. Twenty-one rhesus macaques at the Swoyambhu Temple in Kathmandu, Nepal, were compared with more than 300 Indian- and Chinese-origin rhesus macaques. The sequence analyses of two mitochondrial DNA (mtDNA) loci, from the HVS 1 and 12 S rRNA regions, showed that the Nepali animals were more similar to Indian-origin than to Chinese-origin animals. The distribution of alleles at 24 short tandem repeat (STR) loci distributed across 17 chromosomes also showed greater similarity between the Nepali and Indian-origin animals. Finally, an analysis of seven major histocompatibility complex (MHC) alleles showed that the Nepali animals expressed Class I alleles that are common to Indian-origin animals, including Mamu-A\*01. All of these analyses also revealed a low level of genetic diversity within this Nepali rhesus sample. We conclude that the rhesus macaques of Nepal more closely resemble rhesus macaques of Indian origin than those of Chinese origin. As such, the Nepali rhesus may offer an additional resource option for researchers who wish to maintain research protocols with animals that possess key genetic features characteristic of Indian-origin rhesus macaques. | 2183 | NA | NA | NA | no | no |
| 2184 | 2017 | NA | Garcia, C; Grivet, D | 2011 | Molecular insights into seed dispersal mutualisms driving plant population recruitment | Most plant species require mutualistic interactions with animals to fulfil their demographic cycle. In this regard frugivory (i.e., the intake of fruits by animals) enhances natural regeneration by mobilizing a large amount of seeds from source trees to deposition sites across the landscape. By doing so, frugivores move propagules, and the genotypes they harbour creating the spatial, ecological, and genetic environment under which subsequent recruitment proceeds. Recruitment patterns can be envisioned as the result of two density- and distance-dependent processes: seed dispersal and seed/seedling survival (the Janzen-Connell model). Population genetic studies add another layer of complexity for understanding the fate of dispersed propagules: the genetic relatedness among neighbouring seeds within a seed clump, a major outcome of frugivore activity, modifies their chances of germinating and surviving. Yet, we virtually ignore how the spatial distribution of maternal progenies and recruitment patterns relate with each other in frugivore-generated seed rains. Here we focus on the critical role of frugivore-mediated seed dispersal in shaping the spatial distribution of maternal progenies in the seed rain. We first examine which genetic mechanisms underlying recruitment are influenced by the spatial distribution of maternal progenies. Next, we examine those studies depicting the spatial distribution of maternal progenies in a frugivore-generated seed rain. In doing so, we briefly review the most suitable analytical approaches applied to track the contribution of fruiting trees to the seed rain based on molecular data. Then we look more specifically at the role of distinct frugivore guilds in determining maternal genetic correlations and their expected consequences for recruitment patterns. Finally we posit some general conclusions and suggest future research directions that would provide a more comprehensive understanding of the ecological and evolutionary consequences of dispersal mutualisms in plant populations. (C) 2011 Elsevier Masson SAS. All rights reserved. | 2184 | NA | NA | NA | no | no |
| 2185 | 2017 | NA | Vargas-Rodriguez, YL; Platt, WJ; Urbatsch, LE; Foltz, DW | 2015 | Large scale patterns of genetic variation and differentiation in sugar maple from tropical Central America to temperate North America | Background: Geological events in the latter Cenozoic have influenced the distribution, abundance and genetic structure of tree populations in temperate and tropical North America. The biogeographical history of temperate vegetation that spans large ranges of latitude is complex, involving multiple latitudinal shifts that might have occurred via different migration routes. We determined the regional structuring of genetic variation of sugar maple (Acer saccharum subsp. saccharum) and its only subspecies in tropical America (Acer saccharum subsp. skutchii) using nuclear and chloroplast data. The studied populations span a geographic range from Maine, USA (46 degrees N), to El Progreso, Guatemala (15 degrees N). We examined genetic subdivisions, explored the locations of ancestral haplotypes, analyzed genetic data to explore the presence of a single or multiple glacial refugia, and tested whether genetic lineages are temporally consistent with a Pleistocene or older divergence. Results: Nuclear and chloroplast data indicated that populations in midwestern USA and western Mexico were highly differentiated from populations in the rest of the sites. The time of the most recent common ancestor of the western Mexico haplotype lineage was dated to the Pliocene (5.9 Ma, 95 % HPD: 4.3-7.3 Ma). Splits during the Pleistocene separated the rest of the phylogroups. The most frequent and widespread haplotype occurred in half of the sites (Guatemala, eastern Mexico, southeastern USA, and Ohio). Our data also suggested that multiple Pleistocene refugia (tropics-southeastern USA, midwestern, and northeastern USA), but not western Mexico (Jalisco), contributed to post-glacial northward expansion of ranges. Current southern Mexican and Guatemalan populations have reduced population sizes, genetic bottlenecks and tend toward homozygosity, as indicated using nuclear and chloroplast markers. Conclusions: The divergence of western Mexican populations from the rest of the sugar maples likely resulted from orographic and volcanic barriers to gene flow. Past connectivity among populations in the southeastern USA and eastern Mexico and Guatemala possible occurred through gene flow during the Pleistocene. The time to the most common ancestor values revealed that populations from the Midwest and Northeast USA represented different haplotype lineages, indicating major divergence of haplotypes lineages before the Last Glacial Maximum and suggesting the existence of multiple glacial refugia. | 2185 | NA | NA | NA | no | no |
| 2186 | 2017 | NA | Kjeldsen, SR; Zenger, KR; Leigh, K; Ellis, W; Tobey, J; Phalen, D; Melzer, A; FitzGibbon, S; Raadsma, HW | 2016 | Genome-wide SNP loci reveal novel insights into koala (Phascolarctos cinereus) population variability across its range | The koala (Phascolarctos cinereus) is an iconic Australian species that is currently undergoing a number of threatening processes, including disease and habitat loss. A thorough understanding of population genetic structuring and genomic variability of this species is essential to effectively manage populations across the species range. Using a reduced representation genome sequencing method known as double digest restriction-associated sequencing, this study has provided the first genome-wide SNP marker panel in the koala. In this study, 33,019 loci were identified in the koala and a filtered panel of 3060 high-utility SNP markers, including 95 sex-linked markers, were used to provide key insights into population variability and genomic variation in 171 koalas from eight populations across their geographic range. Broad-scale genetic differentiation between geographically separated populations (including sub-species) was assessed and revealed significant differentiation between all populations (F-ST range = 0.01-0.28), with the largest divergence observed between the three geographically distant subgroups (QLD, NSW and VIC) along the east coast of Australia (average F-ST range = 0.17-0.23). Sub-group divergence appears to be a reflection of an isolation by distance effect and sampling strategy rather than true evidence of sub-speciation. This is further supported by low proportions of AMOVA variation between sub-species groups (11.19 %). Fine-scale analysis using genome-wide SNP loci and the NETVIEW pipeline revealed cryptic genetic sub-structuring within localised geographic regions, which corresponded to the hierarchical mating system of the species. High levels of genome-wide SNP heterozygosity were observed amongst all populations (He = 0.25-0.35), and when evaluating across the species to other vertebrate taxa were amongst the highest values observed. This illustrates that the species as a whole still retains high levels of diversity which is comparable to other outbred vertebrate taxa for genome-wide SNPs. Insights into the potential for adaptive variation in the koala were also gained using outlier analysis of genome-wide SNPs. A total of 10 putative outlier SNPs were identified indicating the high likelihood of local adaptations within populations and regions. This is the first use of genome-wide markers to assess population differentiation at a broad-scale in the koala and the first time that sex-linked SNPs have been identified in this species. The application of this novel genomic resource to populations across the species range will provide in-depth information allowing informed conservation priorities and management plans for in situ koalas across Australia and ex situ around the world. | 2186 | NA | NA | NA | no | no |
| 2187 | 2017 | NA | dos Santos, M; Stur, E; Maia, LL; Agostini, LP; Peterle, GT; Mendes, SO; Tajara, EH; de Carvalho, MB; Louro, ID; Silva-Conforti, AMA | 2013 | Genetic Variability of Inflammatory Genes in the Brazilian Population | Inflammatory gene variants have been associated with several diseases, including cancer, diabetes, vascular diseases, neurodegenerative diseases, arthritis, and others. Therefore, determining the population genetic composition of inflammation-related genes can be useful for the determination of general risk, prognostic and therapeutic strategies to prevent or cure specific diseases. We have aimed to identify polymorphism genotype frequencies in genes related to the inflammatory response in the Brazilian population, namely, IBL -62AT, IBL -262CT, tumor necrosis factors alpha (TNFa) -238GA, TNFa -308GA, lymphotoxin-alpha (LTa) +80AC, LTa +252AG, FAS -670AG, and FASL -844TC, considering the white, black, and Pardo ethnicities of the SAo Paulo State. Our results suggest that the Brazilian population is under a miscegenation process at the current time, since some genotypes are not in the Hardy-Weinberg equilibrium. In addition, we conclude that the Pardo ethnicity is derived from a complex mixture of ethnicities, including the native Indian population. | 2187 | NA | NA | NA | no | no |
| 2188 | 2017 | NA | Zangerl, B; Lindauer, SJ; Acland, GM; Aguirre, GD | 2010 | Identification of genetic variation and haplotype structure of the canine ABCA4 gene for retinal disease association studies | Over 200 mutations in the retina specific member of the ATP-binding cassette transporter superfamily (ABCA4) have been associated with a diverse group of human retinal diseases. The disease mechanisms, and genotype-phenotype associations, nonetheless, remain elusive in many cases. As orthologous genes are commonly mutated in canine models of human blinding disorders, canine ABCA4 appears to be an ideal candidate gene to identify and study sequence changes in dogs affected by various forms of inherited retinal degeneration. However, the size of the gene and lack of haplotype assignment significantly limit targeted association and/or linkage approaches. This study assessed the naturally observed sequence diversity of ABCA4 in the dog, identifying 80% of novel variations. While none of the observed polymorphisms have been associated with blinding disorders to date, breed and potentially disease specific haplotypes have been identified. Moreover, a tag SNP map of 17 (15) markers has been established that accurately predicts common ABCA4 haplotypes (frequency > 5%) explaining > 85% (> 80%) of the observed genetic diversity and will considerably advance future studies. Our sequence analysis of the complete canine ABCA4 coding region will clearly provide a baseline and tools for future association studies and comparative genomics to further delineate the role of ABCA4 in canine blinding disorders. | 2188 | NA | NA | NA | no | no |
| 2189 | 2017 | NA | Keita, SOY; Kittles, RA; Royal, CDM; Bonney, GE; Furbert-Harris, P; Dunston, GM; Rotimi, CN | 2004 | Conceptualizing human variation | What is the relationship between the patterns of biological and sociocultural variation in extant humans? Is this relationship accurately described, or best explained, by the term ‘race’ and the schema of ‘racial’ classification? What is the relationship between ‘race’, genetics and the demographic groups of society? Can extant humans be categorized into units that can scientifically be called ‘races’? These questions underlie the discussions that address the explanations for the observed differences in many domains between named demographic groups across societies. These domains include disease incidence and prevalence and other variables studied by biologists and social scientists. Here, we offer a perspective on understanding human variation by exploring the meaning and use of the term ‘race’ and its relationship to a range of data. The quest is for a more useful approach with which to understand human biological variation, one that may provide better research designs and inform public policy. | 2189 | NA | NA | NA | no | no |
| 2190 | 2017 | NA | Woodroffe, R; Ginsberg, JR | 1998 | Edge effects and the extinction of populations inside protected areas | Theory predicts that small populations may be driven to extinction by random fluctuations in demography and loss of genetic diversity through drift, However, population size is a poor predictor of extinction in large carnivores inhabiting protected areas. Conflict with people on reserve borders is the major cause of mortality in such populations, so that border areas represent population sinks. The species most likely to disappear from small reserves are those that range widely-and are therefore most exposed to threats on reserve borders-irrespective of population size. Conservation efforts that combat only stochastic processes are therefore unlikely to avert extinction. | 2190 | NA | NA | NA | no | no |
| 2191 | 2017 | NA | Samils, N; Vasaitis, R; Stenlid, J | 2009 | Impact of the biological control agent Phlebiopsis gigantea on its resident genetic structure in the Baltic Sea area | The basidiomycete Phlebiop sis gigantea (Fr.) Julich has been used in Swedish forestry as a biocontrol agent against the root and butt-rot pathogen Heterobasidion annosum s.l. on freshly cut Picea abies stumps since the early 1990s. Until 2005, the commercial preparation of this biological stump treatment, Rotstop, has been based on a single strain of P. gigantea that has been applied on more than 47,000 ha annually in Fennoscandia. This paper reports on the spread of genetic material from the Rotstop biocontrol strain of P. gigantea to resident populations of P. gigantea. We conclude that the inoculated fungus remained to a large extent restricted to the treated plots and did not spread to the adjacent areas, dominated by the natural spore deposition from resident populations of the fungus. Furthermore, the study demonstrates high genetic diversity and low geographic differentiation in P. gigantea populations in the geographical area around the Baltic Sea. | 2191 | NA | NA | NA | no | no |
| 2192 | 2017 | NA | Negri, V; Tiranti, B | 2010 | Effectiveness of in situ and ex situ conservation of crop diversity. What a Phaseolus vulgaris L. landrace case study can tell us | The effectiveness of in situ (on-farm) and ex situ conservation strategies to maintain total genetic diversity was assessed in a threatened Phaseolus vulgaris L. landrace. Farmer seed lots (subpopulations) were sampled initially and then after in situ and ex situ multiplication (two locations). The number of plants used in the ex situ multiplications (120) was much larger than that normally used in germplasm bank procedures and the farmer seed lots were kept separate. In situ, the landrace was multiplied by each farmer with the usual population size. Eighty plants from the initial population, the in situ and the two ex situ multiplications were individually tested using 26 microsatellite markers. Most of the genetic parameters showed a consistent decline in the ex situ populations compared with the in situ population, with a notable loss of less frequent alleles. The differentiation among the farmer subpopulations increased when the multiplication took place outside of the adaptation area. Although 120 plants were multiplied in each ex situ cycle, a bottleneck effect was present. In addition, tests for neutrality detected three loci that are involved in pathogen response and are potentially under selective effects. The diversity conservation and the management practices of autogamous landrace crops are discussed. | 2192 | NA | NA | NA | no | no |
| 2193 | 2017 | NA | Hirai, M; Yoshimura, S; Ohsako, T; Kubo, N | 2010 | Genetic diversity and phylogenetic relationships of the endangered species Vaccinium sieboldii and Vaccinium ciliatum (Ericaceae) | The edible berry species Vaccinium sieboldii, Vaccinium ciliatum, and Vaccinium oldhami are deciduous shrubs found growing at low altitudes in Japan. They are classified in the same section. Of these, the former two species are listed as endangered species. The present status of these two species was surveyed. V. sieboldii was found in a very limited range, 65 km wide, in central Japan. Many plants are thought to have been lost because of habitat destruction in the past 50 years. Loss of pine forests from pine-wilt disease is thought to be a major threat to this species. In contrast, V. ciliatum was found over a range of more than 300 km in western Japan. Habitats of both species were heavily fragmented. The genetic diversity and phylogenetic relationships of the three species were studied using nine simple-sequence repeat loci. V. sieboldii and V. ciliatum have a close genetic relationship, although their present growth ranges are widely separated. Despite its limited growth range and the limited number of remaining plants, the genetic diversity of V. sieboldii is similar to that of another endangered species, V. ciliatum, and that of the widely grown species, V. oldhami. Seven populations of the former two species were used to create population-based dendrograms. They were separated into three groups: three populations of V. sieboldii, two mountainous populations of V. ciliatum, and two coastal populations of V. ciliatum. The separation was supported by high bootstrap values. Isolation factors of the two population groups in V. ciliatum are discussed. | 2193 | NA | NA | NA | no | no |
| 2194 | 2017 | NA | Macaya-Sanz, D; Heuertz, M; Lindtke, D; Vendramin, GG; Lexer, C; Gonzalez-Martinez, SC | 2016 | Causes and consequences of large clonal assemblies in a poplar hybrid zone | Asexual reproduction is a common and fundamental mode of reproduction in plants. Although persistence in adverse conditions underlies most known cases of clonal dominance, proximal genetic drivers remain unclear, in particular for populations dominated by a few large clones. In this study, we studied a clonal population of the riparian tree Populus alba in the Douro river basin (northwestern Iberian Peninsula) where it hybridizes with Populus tremula, a species that grows in highly contrasted ecological conditions. We used 73 nuclear microsatellites to test whether genomic background (species ancestry) is a relevant cause of clonal success, and to assess the evolutionary consequences of clonal dominance by a few genets. Additional genotyping-by-sequencing data were produced to estimate the age of the largest clones. We found that a few ancient (over a few thousand years old) and widespread genets dominate the population, both in terms of clone size and number of sexual offspring produced. Interestingly, large clones possessed two genomic regions introgressed from P. tremula, which may have favoured their spread under stressful environmental conditions. At the population level, the spread of large genets was accompanied by an overall ancient (>0.1 Myr) but soft decline of effective population size. Despite this decrease, and the high clonality and dominance of sexual reproduction by large clones, the Douro hybrid zone still displays considerable genetic diversity and low inbreeding. This suggests that even in extreme cases as in the Douro, asexual and sexual dominance of a few large, geographically extended individuals does not threaten population survival. | 2194 | NA | NA | NA | no | no |
| 2195 | 2017 | NA | Forgacs, D; Wallen, RL; Dobson, LK; Derr, JN | 2016 | Mitochondrial Genome Analysis Reveals Historical Lineages in Yellowstone Bison | Yellowstone National Park is home to one of the only plains bison populations that have continuously existed on their present landscape since prehistoric times without evidence of domestic cattle introgression. Previous studies characterized the relatively high levels of nuclear genetic diversity in these bison, but little is known about their mitochondrial haplotype diversity. This study assessed mitochondrial genomes from 25 randomly selected Yellowstone bison and found 10 different mitochondrial haplotypes with a haplotype diversity of 0.78 (+/- 0.06). Spatial analysis of these mitochondrial DNA (mtDNA) haplotypes did not detect geographic population subdivision (F-ST = -0.06, p = 0.76). However, we identified two independent and historically important lineages in Yellowstone bison by combining data from 65 bison (defined by 120 polymorphic sites) from across North America representing a total of 30 different mitochondrial DNA haplotypes. Mitochondrial DNA haplotypes from one of the Yellowstone lineages represent descendants of the 22 indigenous bison remaining in central Yellowstone in 1902. The other mitochondrial DNA lineage represents descendants of the 18 females introduced from northern Montana in 1902 to supplement the indigenous bison population and develop a new breeding herd in the northern region of the park. Comparing modern and historical mitochondrial DNA diversity in Yellowstone bison helps uncover a historical context of park restoration efforts during the early 1900s, provides evidence against a hypothesized mitochondrial disease in bison, and reveals the signature of recent hybridization between American plains bison (Bison bison bison) and Canadian wood bison (B.b. athabascae). Our study demonstrates how mitochondrial DNA can be applied to delineate the history of wildlife species and inform future conservation actions. | 2195 | NA | NA | NA | no | no |
| 2196 | 2017 | NA | de Maturana, EL; Chanok, SJ; Picornell, AC; Rothman, N; Herranz, J; Calle, ML; Garcia-Closas, M; Marenne, G; Brand, A; Tardon, A; Carrato, A; Silverman, DT; Kogevinas, M; Gianola, D; Real, FX; Malats, N | 2014 | Whole Genome Prediction of Bladder Cancer Risk With the Bayesian LASSO | To build a predictive model for urothelial carcinoma of the bladder (UCB) risk combining both genomic and nongenomic data, 1,127 cases and 1,090 controls from the Spanish Bladder Cancer/EPICURO study were genotyped using the HumanHap 1M SNP array. After quality control filters, genotypes from 475,290 variants were available. Nongenomic information comprised age, gender, region, and smoking status. Three Bayesian threshold models were implemented including: (1) only genomic information, (2) only nongenomic data, and (3) both sources of information. The three models were applied to the whole population, to only nonsmokers, to male smokers, and to extreme phenotypes to potentiate the UCB genetic component. The area under the ROC curve allowed evaluating the predictive ability of each model in a 10-fold cross-validation scenario. Smoking status showed the highest predictive ability of UCB risk (AUC(test) = 0.62). On the other hand, the AUC of all genetic variants was poorer (0.53). When the extreme phenotype approach was applied, the predictive ability of the genomic model improved 15%. This study represents a first attempt to build a predictive model for UCB risk combining both genomic and nongenomic data and applying state-of-the-art statistical approaches. However, the lack of genetic relatedness among individuals, the complexity of UCB etiology, as well as a relatively small statistical power, may explain the low predictive ability for UCB risk. The study confirms the difficulty of predicting complex diseases using genetic data, and suggests the limited translational potential of findings from this type of data into public health interventions. Genet Epidemiol 38: 467-476, 2014. (C) 2014 Wiley Periodicals, Inc. | 2196 | NA | NA | NA | no | no |
| 2197 | 2017 | NA | Pfeiffer, KJ; Crailsheim, K | 1998 | Drifting of honeybees | Drifting of honeybees depends on the arrangement, the colouring of the hives and on various environmental factors. Bees are able to distinguish between related and non-related individuals, so one aim of this study was to determine whether drifting also depends on relatedness. In addition we wanted to examine whether there are differences in survival of drifted bees and non-drifted bees and to model the component of alien worker bees in a colony’s population. We used two non-related strains of bees. The colonies stood in rows. Each of the colonies had two non-related neighbour colonies, except for the colonies on the ends of the rows. From each colony, 200 newly emerged bees were marked individually and 100 of them were reintroduced into their original colony (native bees) and 100 into a non-related neighbouring colony (foreign bees). The marked bees were examined for presence on their 2(nd), 6(th), 9(th), 16(th), 25(th) and 34(th) day of life. Experiments were done in summer and fall. There was no difference in survival and the amount of drifting of native and foreign bees. We also found no differences in the number of drifting bees for the two non-related strains. The bees Bees of strain 1 did not show any preferences. In two experiments significantly more bees of strain 2 drifted into colonies of strain 1. Most bees drifted into the neighbouring colony next to the colony they left. In summer significantly more bees that had drifted until their 9(th) day of life, survived until the 25(th) day than bees that did not drift until that day. In fall we did not find this difference. This was true for bees of both strains. Our data support the results of Jay (1965), who found that more bees drift from a centre colony to the end colonies of a row than vice versa. With a model we calculated the component of alien worker bees living in a colony’s population. On average, 22 +/- 3 % alien worker bees were calculated for edge and 42 +/- 6% for inner hives in a row in our first experiment (summer). In a second experiment (fall) the components were 13 +/- 1% for the edge colonies and 39 +/- 4% for the inner colonies in the row. Drifting seems not to be influenced by relatedness and did not shorten the lifetime of the bees. This indicates a great tolerance against drifted bees and a high intermix of an apiary’s population when no precautions to reduce drifting are taken. | 2197 | NA | NA | NA | no | no |
| 2198 | 2017 | NA | Remon, N; Galan, P; Vila, M; Arribas, O; Naveira, H | 2013 | Causes and Evolutionary Consequences of Population Subdivision of an Iberian Mountain Lizard, Iberolacerta monticola | “Aim<bold>: </bold>The study of the factors that influence population connectivity and spatial distribution of genetic variation is crucial for understanding speciation and for predicting the effects of landscape modification and habitat fragmentation, which are considered severe threats to global biodiversity. This dual perspective is obtained from analyses of subalpine mountain species, whose present distribution may have been shaped both by cyclical climate changes over ice ages and anthropogenic perturbations of their habitats. Here, we examine the phylogeography, population structure and genetic diversity of the lacertid lizard Iberolacerta monticola, an endemism considered to be facing a high risk of extinction in several populations. Location<bold>: </bold>Northwestern quadrant of the Iberian Peninsula. Methods<bold>: </bold>We analyzed the mtDNA variation at the control region (454 bp) and the cytochrome b (598 bp) loci, as well as at 10 nuclear microsatellite loci from 17 populations throughout the distribution range of the species. Results<bold>: </bold>According to nuclear markers, most sampling sites are defined as distinct, genetically differentiated populations, and many of them show traces of recent bottlenecks. Mitochondrial data identify a relatively old, geographically restricted lineage, and four to six younger geographically vicariant sister clades, whose origin may be traced back to the mid-Pleistocene revolution, with several subclades possibly associated to the mid-Bruhnes transition. Geographic range fragmentation of one of these clades, which includes lowland sites, is very recent, and most likely due to the accelerated loss of Atlantic forests by human intervention. Main Conclusions<bold>: </bold>Altogether, the data fit a”“refugia within refugia”" model, some lack of pattern uniformity notwithstanding, and suggest that these mountains might be the cradles of new species of Iberolacerta. However, the changes operated during the Holocene severely compromise the long-term survival of those genetic lineages more exposed to the anthropogenic perturbations of their habitats." | 2198 | NA | NA | NA | no | no |
| 2199 | 2017 | NA | Rejmanek, D; Foley, P; Barbet, A; Foley, J | 2012 | Evolution of Antigen Variation in the Tick-Borne Pathogen Anaplasma phagocytophilum | Anaplasma phagocytophilum is an obligately intracellular tick-transmitted bacterial pathogen of humans and other animals. During the course of infection, A. phagocytophilum utilizes gene conversion to shuffle similar to 100 functional pseudogenes into a single expression cassette of the msp2(p44) gene, which codes for the major surface antigen and major surface protein 2 (MSP2). The role and extent of msp2(p44) recombination, particularly in hosts that only experience acute infections, is not clear. In the present study, we explored patterns of recombination and expression of the msp2(p44) gene of A. phagocytophilum in a serially infected mouse model. Even though the bacterium was passed rapidly among mice, minimizing the opportunities for the host to develop adaptive immunity, we detected the emergence of 34 unique msp2(p44) expression cassette variants. The expression of msp2(p44) pseudogenes did not follow a consistent pattern among different groups of mice, although some pseudogenes were expressed more frequently than others. In addition, among 263 expressed pseudogenes, 3 mosaic sequences each consisting of 2 different pseudogenes were identified. Population genetic analysis showed that genetic diversity and subpopulation differentiation tended to increase over time until stationarity was reached but that the variance that was observed in allele (expressed pseudogene) frequency could occur by drift alone only if a high variance in bacterial reproduction could be assumed. These findings suggest that evolutionary forces influencing antigen variation in A. phagocytophilum may comprise random genetic drift as well as some innate but apparently nonpurifying selection prior to the strong frequency-dependent selection that occurs cyclically after hosts develop strong adaptive immunity. | 2199 | NA | NA | NA | no | no |
| 2200 | 2017 | NA | Ilinsky, Y; Kosterin, OE | 2017 | Molecular diversity of Wolbachia in Lepidoptera: Prevalent allelic content and high recombination of MLST genes | Wolbachia are common endosymbiotic bacteria of Arthropoda and Nematoda that are ordinarily transmitted vertically in host lineages through the egg cytoplasm. Despite the great interest in the Wolbachia symbiont, many issues of its biology remain unclear, including its evolutionary history, routes of transfer among species, and the molecular mechanisms underlying the symbiont’s effect on its host. In this report, we present data relating to Wolbachia infection in 120 species of 13 Lepidoptera families, mostly butterflies, from West Siberian localities based on Multilocus sequence typing (MLST) and the wsp locus and perform a comprehensive survey of the distribution of Wolbachia and its genetic diversity in Lepidoptera worldwide. We observed a high infection incidence in the studied region; this finding is probably also true for other temperate latitude regions because many studied species have broad Palearctic and even Holarctic distribution. Although 40 new MLST alleles and 31 new STs were described, there was no noticeable difference in the MLST allele content in butterflies and probably also in moths worldwide. A genetic analysis of Wolbachia strains revealed the MLST allele core in lepidopteran hosts worldwide, viz. the ST-41 allele content. The key finding of our study was the detection of rampant recombination among MLST haplotypes. High rates of homologous recombination between Wolbachia strains indicate a substantial contribution of genetic exchanges to the generation of new STs. This finding should be considered when discussing issues related to the reconstruction of Wolbachia evolution, divergence time, and the routes of Wolbachia transmission across arthropod hosts. (C) 2016 Elsevier Inc. All rights reserved. | 2200 | NA | NA | NA | no | no |
| 2201 | 2017 | NA | Slade, RW; Moritz, C | 1998 | Phylogeography of Bufo marinus from its natural and introduced ranges | The marine toad, Bufo marinus, has a broad natural distribution extending from the south-west of the USA to southern Peru and the central Amazon. It was introduced to several localities in the Caribbean and Pacific Oceans to control sugar cane pests. Mie sequenced 468 bp of mitochondrial DNA (mtDNA) containing the ND3 gene, and flanking tRNA genes from toads spanning the broad natural and introduced ranges. Consistent with the known history of introductions and expected effects of serial bottlenecks, mtDNA within introduced populations in Hawaii and Australia was uniform and most closely related to samples from eastern Venezuela and French Guiana. However, mtDNA nucleotide diversity in the geographic region spanning the source areas is also relatively low (0.18-0.46%) and the absence of variation in the introduced populations precludes quantitative assessment of the reduction in genetic diversity. Unexpectedly, there was a large phylogeographic break (5.4% sequence divergence) within the natural range separating populations east and west of the Venezuelan Andes. We hypothesize that the two major lineages of B. marinus were isolated by the uplift of the eastern Andean cordillera which was completed approximately 2.7 Ma. Another species of the marinus group, B. paracnemis, had mtDNA paraphyletic, with marinus, being nested within the eastern lineage. Thus, at least one speciation event within the marinus group postdates the split within marinus. These findings suggest that the taxonomy of B. marinus should be re-evaluated and that the search for pathogens to control Australian populations should be conducted in populations from both lineages in the natural range. | 2201 | NA | NA | NA | no | no |
| 2202 | 2017 | NA | Caze, ALR; Mader, G; Nunes, TS; Queiroz, LP; de Oliveira, G; Diniz, JAF; Bonatto, SL; Freitas, LB | 2016 | Could refuge theory and rivers acting as barriers explain the genetic variability distribution in the Atlantic Forest? | The Atlantic Forest is one of the most species-rich ecoregions in the world. The historical origins of this richness and the evolutionary processes that produced diversification and promoted speciation in this ecosystem remain poorly understood. In this context, focusing on Passiflora contracta, an endemic species from the Atlantic Forest distributed exclusively at sea level along forest edges, this study aimed to characterize the patterns of genetic variability and explore two hypotheses that attempt to explain the possible causes of the genetic diversity in this region: the refuge and riverine barrier theories. We employed Bayesian methods combined with niche modeling to identify genetically homogeneous groups, to determine the diversification age, and identify long-term climate stability areas to species survival. The analyses were performed using molecular markers from nuclear and plastid genomes, with samples collected throughout the entire geographic distribution of the species, and comparisons with congeners species. The results indicated that populations were genetically structured and provided evidence of demographic stability. The molecular markers indicated the existence of a clear structure and the presence of five homogeneous groups. Interestingly, the separation of the groups coincides with the geographical locations of local rivers, corroborating the hypothesis of rivers acting as barriers to gene flow in this species. The highest levels of genetic diversity and the areas identified as having long-term climate stability were found in the same region reported for other species as a possible refuge area during the climatic changes of the Quaternary. (C) 2016 Elsevier Inc. All rights reserved. | 2202 | NA | NA | NA | no | no |
| 2203 | 2017 | NA | Orbovic, V; Tarasjev, A | 1999 | Genetic differences in plastic responses to density between ecotypes of Arabidopsis thaliana | One of the most promising directions in the study of phenotypic plasticity is its detailed analysis in organisms that are also well-studied in other aspects. Conclusions based on plasticity studies in environmental gradients that closely mimic natural variation are shown to be the most relevant. Following those directions, we conducted this study of phenotypic plasticity on the currently best available model system in flowering plants, Arabidopsis thaliana, and utilized one of the most common variations experienced in the wild: variation in density. Four Arabidopsis thaliana commonly used inbred lines (ecotypes) were grown in densities from one to seven plants per pot. Both phenotypic plasticity and its genetic variability were detected for almost all of 11 analyzed traits, with analyzed ecotypes responding strongly to density of just two plants per pot. Density had small effect on life history and moderate effect on size traits, while vegetative and reproductive traits responded strongly. Mortality of plants during the experiment was almost absent, showing that all densities corresponded to the medium density phase in which carrying capacity is not yet reached. Genetic variability for phenotypic plasticity was in mast cases the result of profound deviation of only one ecotype from the response of others. In the case of reproductive output, however, G x E interaction was the result of greater between-ecotype variability at lower densities. If we reasonably assume that dense stands are more common in the wild, this difference between ecotypes (populations) closely resembles the cases of so-called potential variability within populations. | 2203 | NA | NA | NA | no | no |
| 2204 | 2017 | NA | Zielke, DE; Werner, D; Schaffner, F; Kampen, H; Fonseca, DM | 2014 | Unexpected Patterns of Admixture in German Populations of Aedes japonicus japonicus (Diptera: Culicidae) Underscore the Importance of Human Intervention | The mosquito Aedes japonicus japonicus, originally restricted to temperate East Asia, is now widespread in North America and more recently has become established in Europe. To ascertain the putative number of separate introductions to Europe and examine patterns of expansion we analyzed the genetic makeup of Ae. j. japonicus populations from five cemeteries in North Rhine-Westphalia and Rhineland-Palatinate, two western German federal states, as well as of specimens from populations in Belgium, Switzerland, and Austria/Slovenia. To do so, we genotyped individual specimens at seven preexisting polymorphic microsatellite loci and sequenced part of the nad4 mitochondrial locus. We found evidence of two different genotypic signatures associated with different nad4 mitochondrial haplotypes, indicating at least two genetically differentiated populations of Ae. j. japonicus in Europe (i.e. two distinct genotypes). Belgian, Swiss, and Austrian/Slovenian populations all share the same genotypic signature although they have become differentiated since isolation. Contrary to expectations, the German Ae. j. japonicus are not closely related to those in Belgium which are geographically nearest but are also highly inbred. German populations have a unique genotype but also evidence of mixing between the two genotypes. Also unexpectedly, the populations closest to the center of the German infestation had the highest levels of admixture indicating that separate introductions did not expand and merge but instead their expansion was driven by punctuated human-mediated transport. Critically, the resulting admixed populations have higher genetic diversity and appear invasive as indicated by their increased abundance and recent spread across western Germany. | 2204 | NA | NA | NA | no | no |
| 2205 | 2017 | NA | Al Omar, SY; Alkuriji, A; Alwasel, S; Dar, JA; Alhammad, A; Christmas, S; Mansour, L | 2016 | Genotypic diversity of the Killer Cell Immunoglobulin-like Receptors ( KIR) and their HLA class I Ligands in a Saudi population | Killer Cell Immunoglobulin-like Receptors (KIR) have been used as good markers for the study of genetic predisposition in many diseases and in human genetic population dynamics. In this context, we have investigated the genetic diversity of KIR genes and their main HLA class I ligands in Saudi population and compared the data with other studies of neighboring populations. One hundred and fourteen randomly selected healthy Saudi subjects were genotyped for the presence or absence of 16 KIR genes and their HLA-C1, -C2, -Bw4(Thr80) and Bw4(Ile80) groups, using a PCR-SSP technique. The results show the occurrence of the framework genes (3DL2, 3DL3 and 2DL4) and the pseudogenes (2DP1 and 3DP1) at highest frequencies. All inhibitory KIR (iKIR) genes appeared at higher frequencies than activating genes (aKIR), except for 2DS4 with a frequency of 90.35%. A total of 55 different genotypes were observed appearing at different frequencies, where 12 are considered novel. Two haplotypes were characterized, AA and Bx (BB and AB), which were observed in 24.5% and 75.5% respectively of the studied group. The frequencies of iKIR + HLA associations were found to be much higher than aKIR + HLA. KIR genes frequencies in the Saudi population are comparable with other Middle Eastern and North African populations. | 2205 | NA | NA | NA | no | no |
| 2206 | 2017 | NA | Anderson, KL; Congdon, BC | 2013 | Population genetics suggest that multiple invasion processes need to be addressed in the management plan of a plant disease vector | The use of a multidisciplinary approach is becoming increasingly important when developing management strategies that mitigate the economic and biological costs associated with invasive pests. A framework of simulated dispersal is combined with life-history information and analyses of population genetic structure to investigate the invasion dynamics of a plant disease vector, the island sugarcane planthopper (Eumetopina flavipes), through an archipelago of significant Australian quarantine concern. Analysis of eight microsatellite loci from 648 individuals suggests that frequent, wind-assisted immigration from multiple sources in Papua New Guinea contributes significantly to repeated colonization of far northern islands. However, intermittent wind-assisted immigration better explains patterns of genetic diversity and structure in the southern islands and on the tip of mainland Australia. Significant population structuring associated with the presence of clusters of highly related individuals results from breeding in-situ following colonization, with little postestablishment movement. Results also suggest that less important secondary movements occur between islands; these appear to be human mediated and restricted by quarantine zones. Control of the planthopper may be very difficult on islands close to Papua New Guinea given the apparent propensity for multiple invasion, but may be achievable further south where local populations appear highly independent and isolated. | 2206 | NA | NA | NA | no | no |
| 2207 | 2017 | NA | Scriver, CR; Byck, S; Prevost, L; Hoang, L; Tyfield, L; Dworniczak, B; Carter, K; Rozen, R; Rey, J; Woo, S; Eisensmith, R; Morgan, K | 1996 | The phenylalanine hydroxylase locus: A marker for the history of phenylketonuria and human genetic diversity | Disease-producing allelic variation describes one aspect of human genetic diversity. Phenylketonuria, the major type of hyperphenylalaninaemia and formerly a functional genetic lethal, has a 2% carrier frequency in temperate-zone populations. Newborn screening for hyperphenylalaninaemia (incidence of 1 in 10 000) has made it one of the most widely ascertained human Mendelian traits; 99% of hyperphenylalaninaemia mutations map to the PAH (phenylalanine hydroxylase) gene on 12q24.1, and most cause phenylketonuria. The gene is well characterized. Analysis of 3986 mutant chromosomes by 81 investigators in 26 countries has identified 243 different mutations in 788 different associations (with polymorphic intragenic haplotypes [seven diallelic sites, one short tandem repeat, one variable number of tandem repeats], populations and regions). These data are compiled on a database accessible on the World-Wide Web or as a stand-alone software package. A few phenylketonuria alleles occur at high relative frequencies in particular populations on one or only a few haplotypes, suggesting positive selection in the past. Additional mechanisms (founder effect, drift and recurrent mutation) can explain frequencies and distributions of particular alleles. Allele stratification in Europeans and Orientals implies that mechanism(s) accounting for distribution and high frequencies of PAH alleles were acting before and during demic expansion in Europe and after the European and Oriental radiations. | 2207 | NA | NA | NA | no | no |
| 2208 | 2017 | NA | Vashi, N; Stryjecki, C; Peralta-Romero, J; Suarez, F; Gomez-Zamudio, J; Burguete-Garcia, AI; Cruz, M; Meyre, D | 2016 | Genetic markers of inflammation may not contribute to metabolic traits in Mexican children | Background: Low-grade chronic inflammation is a common feature of obesity and its cardio-metabolic complications. However, little is known about a possible causal role of inflammation in metabolic disorders. Mexico is among the countries with the highest obesity rates in the world and the admixed Mexican population is a relevant sample due to high levels of genetic diversity. Methods: Here, we studied 1,462 Mexican children recruited from Mexico City. Six genetic variants in five inflammation-related genes were genotyped: rs1137101 (leptin receptor (LEPR)), rs7305618 (hepatocyte nuclear factor 1 alpha (HNF1A)), rs1800629 (tumor necrosis factor alpha (TNFA)), rs1800896, rs1800871 (interleukin-10 (IL-10)), rs1862513 (resistin (RETN)). Ten continuous and eight binary traits were assessed. Linear and logistic regression models were used adjusting for age, sex, and recruitment centre. Results: We found that one SNP displayed a nominal evidence of association with a continuous trait: rs1800871 (IL-10) with LDL (beta = -0.068 +/- 1.006, P = 0.01). Subsequently, we found one nominal association with a binary trait: rs7305618 (HNF1A) with family history of hypertension (odds-ratio = 1.389 [1.054-1.829], P = 0.02). However, no P-value passed the Bonferroni correction for multiple testing. Discussion: Our data in a Mexican children population are consistent with previous reports in European adults in failing to demonstrate an association between inflammation-associated single nucleotide polymorphisms (SNPs) and metabolic traits. | 2208 | NA | NA | NA | no | no |
| 2209 | 2017 | NA | Puustinen, S; Koskela, T; Mutikainen, P | 2004 | Relatedness affects competitive performance of a parasitic plant (Cuscuta europaea) in multiple infections | Theoretical models predict that parasite relatedness affects the outcome of competition between parasites, and the evolution of parasite virulence. We examined whether parasite relatedness affects competition between parasitic plants (Cuscuta europaea) that share common host plants (Urtica dioica). We infected hosts with two parasitic plants that were either half-siblings or nonrelated. Relative size asymmetry between the competing parasites was significantly higher in the nonrelated infections compared to infections with siblings. This higher asymmetry was caused by the fact that the performance of some parasite genotypes decreased and that of others increased when grown in multiple infections with nonrelated parasites. This result agrees with the predictions of theories on the evolution of parasite virulence: to enhance parasite transmission, selection may favour reduced competition with genetically related parasites in hosts infected by several genotypes. However, in contrast to the most common predictions, nonrelated infections were not more virulent than the sibling infections. | 2209 | NA | NA | NA | no | no |
| 2210 | 2017 | NA | Chiyo, PI; Grieneisen, LE; Wittemyer, G; Moss, CJ; Lee, PC; Douglas-Hamilton, I; Archie, EA | 2014 | The Influence of Social Structure, Habitat, and Host Traits on the Transmission of Escherichia coli in Wild Elephants | Social structure is proposed to influence the transmission of both directly and environmentally transmitted infectious agents. However in natural populations, many other factors also influence transmission, including variation in individual susceptibility and aspects of the environment that promote or inhibit exposure to infection. We used a population genetic approach to investigate the effects of social structure, environment, and host traits on the transmission of Escherichia coli infecting two populations of wild elephants: one in Amboseli National Park and another in Samburu National Reserve, Kenya. If E. coli transmission is strongly influenced by elephant social structure, E. coli infecting elephants from the same social group should be genetically more similar than E. coli sampled from members of different social groups. However, we found no support for this prediction. Instead, E. coli was panmictic across social groups, and transmission patterns were largely dominated by habitat and host traits. For instance, habitat overlap between elephant social groups predicted E. coli genetic similarity, but only in the relatively drier habitat of Samburu, and not in Amboseli, where the habitat contains large, permanent swamps. In terms of host traits, adult males were infected with more diverse haplotypes, and males were slightly more likely to harbor strains with higher pathogenic potential, as compared to adult females. In addition, elephants from similar birth cohorts were infected with genetically more similar E. coli than elephants more disparate in age. This age-structured transmission may be driven by temporal shifts in genetic structure of E. coli in the environment and the effects of age on bacterial colonization. Together, our results support the idea that, in elephants, social structure often will not exhibit strong effects on the transmission of generalist, fecal-oral transmitted bacteria. We discuss our results in the context of social, environmental, and host-related factors that influence transmission patterns. | 2210 | NA | NA | NA | no | no |
| 2211 | 2017 | NA | Setchell, JM; Charpentier, MJE; Bedjabaga, IB; Reed, P; Wickings, EJ; Knapp, LA | 2006 | Secondary sexual characters and female quality in primates | Honest advertisement models of sexual selection propose that exaggerated secondary sexual ornaments are condition-dependent, and that only individuals with superior disease resistance will be able to express costly ornamentation. Studies of secondary sexual ornamentation and their maintenance by sexual selection tend to focus on males. However, females may also possess showy ornaments. We investigated whether female ornaments, in the form of sexual swellings, reliably signal female fitness in a semifree-ranging colony of mandrills (Mandrillus sphinx) at the Centre International de Recherches Medicales, Franceville (CIRMF), Gabon. We measured swelling height and width using photographs of periovulatory females over three mating seasons and compared swelling size with parasitism (using fecal analysis over one annual cycle), immune status (ratio of lymphocytes to neutrophils in blood smears made during captures), and genetic diversity (microsatellite heterozygosity). Swelling size varied by up to 10% between cycles in individual females, giving some support to the hypothesis that size differences may indicate the quality of individual swelling cycles. However, there was no significant difference in swelling size between conceptive and nonconceptive cycles. Measures of swelling size varied more between females than within females across swelling cycles, implying that swelling size was a relatively consistent characteristic of individual females. Swelling size was not significantly related to either general measures of parasitism and immune status, or to the closest available measures to each swelling cycle. Nor was swelling size significantly related to genetic diversity. The healthy, provisioned nature of the colony and problems associated with observational, correlational studies restrict interpretation of our data. However, in combination with previous findings that females of higher reproductive success do not show larger swellings, and that males do not allocate mating effort as a function of swelling size, these results imply that sexual swelling size does not indicate female quality in these semifree-ranging mandrills. | 2211 | NA | NA | NA | no | no |
| 2212 | 2017 | NA | Martens, K; Hallin, J; Warringer, J; Liti, G; Parts, L | 2016 | Predicting quantitative traits from genome and phenome with near perfect accuracy | In spite of decades of linkage and association studies and its potential impact on human health, reliable prediction of an individual’s risk for heritable disease remains difficult. Large numbers of mapped loci do not explain substantial fractions of heritable variation, leaving an open question of whether accurate complex trait predictions can be achieved in practice. Here, we use a genome sequenced population of similar to 7,000 yeast strains of high but varying relatedness, and predict growth traits from family information, effects of segregating genetic variants and growth in other environments with an average coefficient of determination R-2 of 0.91. This accuracy exceeds narrow-sense heritability, approaches limits imposed by measurement repeatability and is higher than achieved with a single assay in the laboratory. Our results prove that very accurate prediction of complex traits is possible, and suggest that additional data from families rather than reference cohorts may be more useful for this purpose. | 2212 | NA | NA | NA | no | no |
| 2213 | 2017 | NA | Luenser, K; Fickel, J; Lehnen, A; Speck, S; Ludwig, A | 2005 | Low level of genetic variability in European bisons (Bison bonasus) from the Bialowieza National Park in Poland | Nuclear DNA markers (microsatellites) were used to screen the genetic variability in the European bison population of the Bialowieza National Park, Poland. The species is listed as endangered and the Bialowieza population is the largest one worldwide. Many other herds were founded by individuals from Bialowieza. Out of 18 microsatellites, nine were polymorphic, five were found to be homozygous, and four loci did not amplify. No significant deviation from Hardy-Weinberg-equilibrium (HWE) was observed, and the average number of alleles was 2.3 per locus. Thus, the European bison is characterized by a very low level of genetic diversity, most likely resulting from the population decline in the nineteenth century. Nevertheless, allelic variability derived from the nine polymorphic loci established in this study allowed to identify each individual by its genotypic profile. This data is valuable for conservation plans of this impressive species, especially for the control of breeding success in these animals. | 2213 | NA | NA | NA | no | no |
| 2214 | 2017 | NA | Trujillo, CA; Ochoa, JC; Mideros, MF; Restrepo, S; Lopez, C; Bernal, A | 2014 | A Complex Population Structure of the Cassava Pathogen Xanthomonas axonopodis pv. manihotis in Recent Years in the Caribbean Region of Colombia | Cassava bacterial blight, caused by Xanthomonas axonopodis pv. manihotis (Xam), is the most important bacterial disease affecting this crop. A continuous surveillance of the pathogen population dynamics is required to develop an efficient disease management program. During the 1990s, Xam populations showed high levels of genetic variation and relevant migratory processes that were important determinants of the distribution of the pathogen diversity in Colombia. Aiming to characterize the current population structure of the pathogen and the evolutionary forces that shape these populations, sampling collections were carried out from September 2008 until November 2010 in the Colombian Caribbean Region. One hundred and sixty bacterial isolates were characterized using amplified fragment length polymorphism (AFLP) markers. Additionally, a subset of effector genes were sequenced in some isolates to determine their usefulness in Xam population studies and to provide additional information to that obtained with AFLPs. Virulence patterns of ten isolates were determined in nine cassava accessions. Our results show a complex architecture of population and confirm migratory process previously reported in the Caribbean Region. Chin, one of the locations sampled, presented remarkable features in population dynamics such as longer genetic distances, higher diversity indices, and a genetically differentiated population when it was compared with other locations. Virulence tests showed that MCOL2215, one of the most cultivated cassava varieties in the Caribbean coast, was susceptible to the majority of Xam isolates tested. This study shows the current condition of populations of Xam in the Caribbean Region of Colombia, and it contributes to improve the existing bacterial blight control practices. | 2214 | NA | NA | NA | no | no |
| 2215 | 2017 | NA | Foufopoulos, J; Roca, V; White, KA; Pafilis, P; Valakos, ED | 2017 | Effects of island characteristics on parasitism in a Mediterranean lizard (Podarcis erhardii): a role of population size and island history? | Little research has been done on the impact of insularity on the patterns of parasitism. Islands, known for low genetic diversity and poor parasitic infracommunities, offer a tractable model to better understand the factors that shape parasitism. This study analyses helminth parasite loads from eight populations of the Erhard’s Wall lizard (Podarcis erhardii, Lacertidae) occurring on islands of varying size, age and isolation in the Aegean Sea (Greece). By comparing burdens of infection among the different populations, we aimed to elucidate how island features shape levels of parasitism. Parasite communities on the islands were very species-poor, with only four genera of nematodes (Spauligodon sp., Skrjabinodon sp., Parapharyngodon sp. and Skrjabinelazia sp.) detected among the lizard populations. We found that aggregate worm infection level was positively correlated with island area, whereas total parasite prevalence was negatively correlated with island age, though marginally. This suggests that numbers of parasites are determined by the loss of genetic diversity as consequence of long-term population bottlenecks. Given that the island populations constitute an important model for the effects of habitat fragmentation on biodiversity, our findings raise concerns about the long-term prospects for many specialized parasite populations in the face of widespread anthropogenic habitat fragmentation. | 2215 | NA | NA | NA | no | no |
| 2216 | 2017 | NA | Griffing, SM; Viana, GMR; Mixson-Hayden, T; Sridaran, S; Alam, MT; de Oliveira, AM; Barnwell, JW; Escalante, AA; Povoa, MM; Udhayakumar, V | 2013 | Historical Shifts in Brazilian P. falciparum Population Structure and Drug Resistance Alleles | Previous work suggests that Brazilian Plasmodium falciparum has limited genetic diversity and a history of bottlenecks, multiple reintroductions due to human migration, and clonal expansions. We hypothesized that Brazilian P. falciparum would exhibit clonal structure. We examined isolates collected across two decades from Amapa, Rondonia, and Para state (n = 190). By examining more microsatellites markers on more chromosomes than previous studies, we hoped to define the extent of low diversity, linkage disequilibrium, bottlenecks, population structure, and parasite migration within Brazil. We used retrospective genotyping of samples from the 1980s and 1990s to explore the population genetics of SP resistant dhfr and dhps alleles. We tested an existing hypothesis that the triple mutant dhfr mutations 50R/51I/108N and 51I/108N/164L developed in southern Amazon from a single origin of common or similar parasites. We found that Brazilian P. falciparum had limited genetic diversity and isolation by distance was rejected, which suggests it underwent bottlenecks followed by migration between sites. Unlike Peru, there appeared to be gene flow across the Brazilian Amazon basin. We were unable to divide parasite populations by clonal lineages and pairwise FST were common. Most parasite diversity was found within sites in the Brazilian Amazon, according to AMOVA. Our results challenge the hypothesis that triple mutant alleles arose from a single lineage in the Southern Amazon. SP resistance, at both the double and triple mutant stages, developed twice and potentially in different regions of the Brazilian Amazon. We would have required samples from before the 1980s to describe how SP resistance spread across the basin or describe the complex internal migration of Brazilian parasites after the colonization efforts of past decades. The Brazilian Amazon basin may have sufficient internal migration for drug resistance reported in any particular region to rapidly spread to other parts of basin under similar drug pressure. | 2216 | NA | NA | NA | no | no |
| 2217 | 2017 | NA | Kumar, NR; Chang, JC; Narayanan, MB; Ramasamy, S | 2017 | Phylogeographical structure in mitochondrial DNA of whitefly, Bemisia tabaci Gennadius (Hemiptera: Aleyrodidae) in southern India and Southeast Asia | South and Southeast Asia endure high vegetable production losses due to begomovirus diseases mainly transmitted by the insect vector, whitefly (Bemisia tabaci). Control over the spread of virus infection can be achieved through a better understanding of genetic diversity among B. tabaci. A total of 64 populations of B. tabaci collected from Tamil Nadu (India), Vietnam, Thailand, and Indonesia were investigated based on mitochondrial cytochrome c oxidase I (coxl) sequences. Populations from Tamil Nadu are distributed into three clades (Asia I, Asia II 7, and Asia II 8), whereas Indonesian populations settle along with Asia I population of India in the phylogenetic tree. Vietnam populations align with the Middle East Asia Minor 1 (MEAM1) Glade, and interestingly MEAM1 invades northern Vietnam quite recently. Samples from Thailand made a unique Glade between the outgroup and the remaining B. tabaci, representing the possibility of a new subspecies. AMOVA analysis among populations from various districts in Tamil Nadu exhibits significant differences, which represent each district’s individuality. This study proves that the use of coxl as a marker for molecular identification of B. tabaci can provide a better estimate of diversity. We provide important clues for developing insight into the genetic structure of B. tabaci, and suggest strategies for control. | 2217 | NA | NA | NA | no | no |
| 2218 | 2017 | NA | Frank, SA | 1998 | Inducible defence and the social evolution of herd immunity | Many organisms vary their level of investment in defensive characters. Protective traits may be induced upon exposure to predators or parasites. In a similar way, humans vaccinate in response to threatening epidemics. When most group members defend themselves, epidemics die out quickly because parasites cannot spread. A high level of group (herd) immunity is therefore beneficial to the group. There is, however, a well-known divergence between the optimum degree of induction for selfish individuals and the level of induction that maximizes group benefit. I develop two optimality models for the frequency of induction. The first model shows that higher relatedness favours more induction and a smaller difference between selfish and cooperative optima. The second model assumes variation in the vigour of individuals and therefore differences in the relative cost for induction. The model predicts that strong individuals induce more easily than weak individuals. Small differences in vigour cause a large divergence in the optimal levels of induction for strong and weak individuals. The concept of genetic relatedness in an evolutionary model is analogous to correlated interests and correlated strategies in an economic model of human behaviour. The evolutionary models presented here therefore provide a basis for further study of human vaccination. | 2218 | NA | NA | NA | no | no |
| 2219 | 2017 | NA | Jiggins, FM; Tinsley, MC | 2005 | An ancient mitochondrial polymorphism in Adalis bipunctata linked to a sex-ratio-distorting bacterium | Sex-ratio-distorting microbes are common parasites of arthropods. Although the reasons they have invaded and spread though populations are well understood, their subsequent dynamics within those populations are virtually unknown. We have found that different strains of a male-killing Rickettsia bacterium infecting the beetle Adalia bipunctata are associated with distinct mitochondrial haplotypes, which is expected as both the mitochondria and the bacteria are maternally transmitted. These mitochondrial haplotypes shared a common ancestor >2 million years ago, and their overall diversity is significantly greater than expected under neutrality from comparisons with a nuclear gene. Furthermore, a variety of statistical tests show strong deviations from neutrality in mitochondrial but not in nuclear genes. We therefore conclude that natural selection is probably maintaining a polymorphism of different Rickettsia strains in this species. Despite the age of the different mitochondrial haplotypes, there is very little genetic diversity within them. Furthermore, there is considerable variation in mitochondrial haplotype and bacterial strain frequency between populations, despite it being thought that this species has fairly low levels of population structure. We conclude that the fitness of these male killers may be negatively frequency dependent or different strains may be favored in different populations. These hypotheses await experimental confirmation. | 2219 | NA | NA | NA | no | no |
| 2220 | 2017 | NA | Ruch, J; Herberstein, ME; Schneider, JM | 2014 | Offspring dynamics affect food provisioning, growth and mortality in a brood-caring spider | In brood-caring species, family members are faced with a conflict over resource distribution. While parents are selected to adapt the amount of care according to their offspring’s needs, offspring might be selected to demand more care than optimal for parents. Recent studies on birds have shown that the social network structure of offspring affects the amount of care and thus the fitness of families. Such a network structure of repeated interactions is probably influenced by within-brood relatedness. We experimentally manipulated the group composition in a brood-caring spider to test how the presence of unrelated spiderlings affects the dynamics between female and brood as well as within broods. Broods consisting of siblings grew better and had a lower mortality compared with mixed broods, no matter whether the caring female was a genetic or foster mother. Interestingly, we found that foster mothers lost weight when caring for sibling broods, whereas females caring for mixed broods gained weight. This indicates that females may be willing to share more prey when the brood contains exclusively siblings even if the entire brood is unrelated to the female. Resource distribution may thus be negotiated by offspring dynamics that could have a signalling function to females. | 2220 | NA | NA | NA | no | no |
| 2221 | 2017 | NA | Gonzalez-Wanguemert, M; Canovas, F; Perez-Ruzafa, A; Marcos, C; Alexandrino, P | 2010 | Connectivity patterns inferred from the genetic structure of white seabream (Diplodus sargus L.) | The marine environment seems, at first sight, to be a homogeneous medium lacking barriers to species dispersal. Nevertheless, populations of marine species show varying levels of gene flow and population differentiation, so barriers to gene flow can often be detected. We aim to elucidate the role of oceanographical factors in generating connectivity among populations shaping the phylogeographical patterns in the marine realm, which is not only a topic of considerable interest for understanding the evolution of marine biodiversity but also for management and conservation of marine life. For this proposal, we investigate the genetic structure and connectivity between continental and insular populations of white seabream in North East Atlantic (NEA) and Mediterranean Sea (MS) as well as the influence of historical and contemporary factors in this scenario using mitochondrial (cytochrome b) and nuclear (a set of 9 microsatellite) molecular markers. Azores population appeared genetically differentiated in a single cluster using Structure analysis. This result was corroborated by Principal Component Analysis (PCA) and Monmonier algorithm which suggested a boundary to gene flow, isolating this locality. Azorean population also shows the highest significant values of F-ST and genetic distances for both molecular markers (microsatellites and mtDNA). We suggest that the breakdown of effective genetic exchange between Azores and the others’ samples could be explained simultaneously by hydrographic (deep water) and hydrodynamic (isolating current regimes) factors acting as barriers to the free dispersal of white seabream (adults and larvae) and by historical factors which could be favoured for the survival of Azorean white seabream population at the last glaciation. Mediterranean islands show similar genetic diversity to the neighbouring continental samples and nonsignificant genetic differences. Proximity to continental coasts and the current system could promote an optimal larval dispersion among Mediterranean islands (Mallorca and Castellamare) and coasts with high gene flow. (C) 2009 Elsevier B.V. All rights reserved. | 2221 | NA | NA | NA | no | no |
| 2222 | 2017 | NA | Ashfaq, M; Hebert, PDN; Mirza, MS; Khan, AM; Mansoor, S; Shah, GS; Zafar, Y | 2014 | DNA Barcoding of Bemisia tabaci Complex (Hemiptera: Aleyrodidae) Reveals Southerly Expansion of the Dominant Whitefly Species on Cotton in Pakistan | “Background: Although whiteflies (Bemisia tabaci complex) are an important pest of cotton in Pakistan, its taxonomic diversity is poorly understood. As DNA barcoding is an effective tool for resolving species complexes and analyzing species distributions, we used this approach to analyze genetic diversity in the B. tabaci complex and map the distribution of B. tabaci lineages in cotton growing areas of Pakistan. Methods/Principal Findings:Sequence diversity in the DNA barcode region (mtCOI-5’) was examined in 593 whiteflies from Pakistan to determine the number of whitefly species and their distributions in the cotton-growing areas of Punjab and Sindh provinces. These new records were integrated with another 173 barcode sequences for B. tabaci, most from India, to better understand regional whitefly diversity. The Barcode Index Number (BIN) System assigned the 766 sequences to 15 BINs, including nine from Pakistan. Representative specimens of each Pakistan BIN were analyzed for mtCOI-3’ to allow their assignment to one of the putative species in the B. tabaci complex recognized on the basis of sequence variation in this gene region. This analysis revealed the presence of Asia II 1, Middle East-Asia Minor 1, Asia 1, Asia II 5, Asia II 7, and a new lineage”“Pakistan”“. The first two taxa were found in both Punjab and Sindh, but Asia 1 was only detected in Sindh, while Asia II 5, Asia II 7 and”“Pakistan”" were only present in Punjab. The haplotype networks showed that most haplotypes of Asia II 1, a species implicated in transmission of the cotton leaf curl virus, occurred in both India and Pakistan. Conclusions: DNA barcodes successfully discriminated cryptic species in B. tabaci complex. The dominant haplotypes in the B. tabaci complex were shared by India and Pakistan. Asia II 1 was previously restricted to Punjab, but is now the dominant lineage in southern Sindh | 2222 | NA | NA | NA | no | no |
| 2223 | 2017 | NA | Krebes, L; Blank, M; Bastrop, R | 2011 | Phylogeography, historical demography and postglacial colonization routes of two amphi-Atlantic distributed amphipods | To evaluate the influence of the ice ages on patterns of genetic diversity and to test generally accepted perspectives related to glacial refugia and proposed post-glacial colonization pathways in the North Atlantic area, we sequenced a portion of the mitochondrial cytochrome c oxidase subunit I of two amphi-Atlantic distributed amphipods, Gammarus duebeni (n = 418) and Gammarus oceanicus (n = 242). Both species showed several populations with a combination of highly reduced levels of genetic diversity and significant demographic expansion in previously glaciated sites, consistent with an expected extinction and recolonization scenario. Within G. oceanicus, two reciprocally monophyletic clades were found. One clade consisted of the specimens from populations of the American St. Lawrence River and two populations south of it. The other clade included specimens from populations of Europe, Greenland, Iceland and Svalbard, as well as from the Hudson Bay. For G. duebeni, we found no private American haplotypes, and post-glacial colonization from two founder populations is likely. A fixed genetic discontinuity was observed in both species between Iceland and Greenland on the one hand and Europe on the other hand. Our data hence indicate that the proposed postglacial colonization of America from Europe via the Faroe Islands, Iceland and Greenland could be more complex than previously thought and that parts of the North Atlantic region were not colonized from the Eastern Atlantic coasts. Unlike other studies, we speculate that there must be a Nordic glacial refugium for both species, which could be located in Greenland. The Greenlandic populations of both species exhibited private haplotypes, but signals of demographic expansion, conflicting with our theory. It is possible that the refugial character of the Greenlandic populations was erased by the LGM. Furthermore, we found evidence for glacial refugia along the Norwegian coast, the English Channel (G. duebeni) and on the Faroe Islands (G. oceanicus). | 2223 | NA | NA | NA | no | no |
| 2224 | 2017 | NA | Dorji, K; Yapwattanaphun, C | 2015 | Assessment of the genetic variability amongst mandarin (Citrus reticulata Blanco) accessions in Bhutan using AFLP markers | Background: Bhutan is a small Himalayan country lying within the region considered to be the origin of citrus. Diverse citrus wild types grow naturally in different climates, elevations and edaphic conditions, but only mandarin is cultivated commercially. The first report of Huanglongbing (also known as greening disease) in Bhutan in 2003, and the threat it posed to the country’s citrus orchards prompted the collection of mandarin germplasm from across the country. This paper describes the genetic diversity of mandarin accessions in Bhutan using amplified fragment length polymorphic (AFLP) markers. Results: Twenty three accessions of Bhutanese mandarin were analyzed using AFLP markers to assess the genetic variability that is believed to exist only in Bhutan and some parts of North East India and South China. Five primer pairs (E-ACA/M-CAG, E-ACG/M-CAT, E-ACC/M-CTT, E-AAG/M-CAA and E-ACA/M-CTC) were identified (based on the number and quality of polymorphic bands produced) and used for the analyses. A total of 244 bands were scored visually of which 126 (52%) were polymorphic with an average polymorphism information content of 0.95 per marker. A cluster dendrogram based on multiscale bootstrap sampling categorized twenty three accessions into two broad groups containing eight and 14 accessions, respectively. Group A consisted accessions (Tsirang1, Tsirang3, Sarpang1, Dagana4, Samtse4, Dagana1, and Trongsa2) from five districts (Tsirang, Sarpang, Samtse, Dagana and Trongsa) and their grouping was strongly supported by bootstrap analysis (B p-value = 96%, AU p-value = 86%). Cluster B consisted of 14 accessions divided into three sub-groups (1, 2 and 3). However, bootstrap value supported significantly for subgroup1 (containing accessions: Tsirang4, Sarpang5, and Tsirang2) and subgroup3 (with accessions - Zhemgang2, Zhemgang3 and Zhemgang4). Conclusion: This study indicates that Bhutanese mandarin germplasm collected from across the country are genetically diverse although the level of variability differed among the accessions assessed. The variation in genetic variability was observed irrespective of where the accessions were collected suggesting that phenotype and geographical location can serve a basis for future germplasm collection in Bhutan. Further, five primer pair combinations could separate 23 mandarins accessions considered in this study, suggesting that AFLP markers can be a useful tool for future identification. | 2224 | NA | NA | NA | no | no |
| 2225 | 2017 | NA | Monzon, JD; Atkinson, EG; Henn, BM; Benach, JL | 2016 | Population and Evolutionary Genomics of Amblyomma americanum, an Expanding Arthropod Disease Vector | The lone star tick, Amblyomma americanum, is an important disease vector and the most frequent tick found attached to humans in the eastern United States. The lone star tick has recently experienced a rapid range expansion into the Northeast and Midwest, but despite this emerging infectious threat to wildlife, livestock, and human health, little is known about the genetic causes and consequences of the geographic expansion. In the first population genomic analysis of any tick species, we characterize the genetic diversity and population structure of A. americanum across its current geographic range, which has recently expanded. Using a high throughput genotyping-by-sequencing approach, we discovered more than 8,000 single nucleotide polymorphisms in 90 ticks from five locations. Surprisingly, newly established populations in New York (NY) and Oklahoma (OK) are as diverse as historic range populations in North and South Carolina. However, substantial population structure occurs among regions, such that new populations in NY and OK are genetically distinct from historic range populations and from one another. Ticks from a laboratory colony are genetically distinct from wild populations, underscoring the need to account for natural variation when conducting transmission or immunological studies, many of which utilize laboratory-reared ticks. An F-51-outlier analysis comparing a recently established population to a long-standing population detected numerous outlier sites, compatible with positive and balancing selection, highlighting the potential for adaptation during the range expansion. This study provides a framework for applying high-throughput DNA sequencing technologies for future investigations of ticks, which are common vectors of diseases. | 2225 | NA | NA | NA | no | no |
| 2226 | 2017 | NA | Vercauteren, A; De Dobbelaere, I; Grunwald, NJ; Bonants, P; Van Bockstaele, E; Maes, M; Heungens, K | 2010 | Clonal expansion of the Belgian Phytophthora ramorum populations based on new microsatellite markers | Co-existence of both mating types A1 and A2 within the EU1 lineage of Phytophthora ramorum has only been observed in Belgium, which begs the question whether sexual reproduction is occurring. A collection of 411 Belgian P. ramorum isolates was established during a 7-year survey. Our main objectives were genetic characterization of this population to test for sexual reproduction, determination of population structure, evolution and spread, and evaluation of the effectiveness and impact of control measures. Novel, polymorphic simple sequence repeat (SSR) markers were developed after screening 149 candidate loci. Eighty isolates of P. ramorum, broadly representing the Belgian population, were analyzed using four previously described and three newly identified polymorphic microsatellite loci as well as amplified fragment length polymorphisms. SSR analysis was most informative and was used to screen the entire Belgian population. Thirty multilocus genotypes were identified, but 68% of the isolates belonged to the main genotype EU1MG1. Although accumulated mutation events were detected, the overall level of genetic diversity within the Belgian isolates of P. ramorum appears to be limited, indicating a relatively recent clonal expansion. Based on our SSR analysis there is no evidence of sexual recombination in the Belgian population of P. ramorum. Metalaxyl use decreased the genetic diversity of P. ramorum until 2005, when the majority of the isolates had become resistant. Most genotypes were site-specific and despite systematic removal of symptomatic and neighbouring plants, some genotypes were detected over a period of several years at a single site, sometimes discontinuously, indicating (latent) survival of the pathogen at those sites. | 2226 | NA | NA | NA | no | no |
| 2227 | 2017 | NA | Kenny, EE; Kim, M; Gusev, A; Lowe, JK; Salit, J; Smith, JG; Kovvali, S; Kang, HM; Newton-Cheh, C; Daly, MJ; Stoffel, M; Altshuler, DM; Friedman, JM; Eskin, E; Breslow, JL; Pe’er, I | 2011 | Increased power of mixed models facilitates association mapping of 10 loci for metabolic traits in an isolated population | The potential benefits of using population isolates in genetic mapping, such as reduced genetic, phenotypic and environmental heterogeneity, are offset by the challenges posed by the large amounts of direct and cryptic relatedness in these populations confounding basic assumptions of independence. Wehave evaluated four representative specialized methods for association testing in the presence of relatedness; (i) within-family (ii) within-and between-family and (iii) mixed-models methods, using simulated traits for 2906 subjects with known genome-wide genotype data from an extremely isolated population, the Island of Kosrae, Federated States of Micronesia. We report that mixed models optimally extract association information from such samples, demonstrating 88% power to rank the true variant as among the top 10 genome-wide with 56% achieving genome-wide significance, a > 80% improvement over the other methods, and demonstrate that population isolates have similar power to non-isolate populations for observing variants of known effects. We then used the mixed-model method to reanalyze data for 17 published phenotypes relating to metabolic traits and electrocardiographic measures, along with another 8 previously unreported. We replicate nine genome-wide significant associations with known loci of plasma cholesterol, high-density lipoprotein, low-density lipoprotein, triglycerides, thyroid stimulating hormone, homocysteine, C-reactive protein and uric acid, with only one detected in the previous analysis of the same traits. Further, we leveraged shared identity-by-descent genetic segments in the region of the uric acid locus to fine-map the signal, refining the known locus by a factor of 4. Finally, we report a novel associations for height (rs17629022, P < 2.1 x 10(-8)). | 2227 | NA | NA | NA | no | no |
| 2228 | 2017 | NA | Taylor, SM; Antonia, AL; Parobek, CM; Juliano, JJ; Janko, M; Emch, M; Alam, MT; Udhayakumar, V; Tshefu, AK; Meshnick, SR | 2013 | Plasmodium falciparum sulfadoxine resistance is geographically and genetically clustered within the DR Congo | Understanding the spatial clustering of Plasmodium falciparum populations can assist efforts to contain drug-resistant parasites and maintain the efficacy of future drugs. We sequenced single nucleotide polymorphisms (SNPs) in the dihydropteroate synthase gene (dhps) associated with sulfadoxine resistance and 5 microsatellite loci flanking dhps in order to investigate the genetic backgrounds, genetic relatedness, and geographic clustering of falciparum parasites in the Democratic Republic of the Congo (DRC). Resistant haplotypes were clustered into subpopulations: one in the northeast DRC, and the other in the balance of the DRC. Network and clonal lineage analyses of the flanking microsatellites indicate that geographically-distinct mutant dhps haplotypes derive from separate lineages. The DRC is therefore a watershed for haplotypes associated with sulfadoxine resistance. Given the importance of central Africa as a corridor for the spread of antimalarial resistance, the identification of the mechanisms of this transit can inform future policies to contain drug-resistant parasite strains. | 2228 | NA | NA | NA | no | no |
| 2229 | 2017 | NA | Wang, J; Chilvers, MI | 2016 | Development and characterization of microsatellite markers for Fusarium virguliforme and their utility within Glade 2 of the Fusarium solani species complex | Clade 2 of the Fusarium solani species complex contains plant pathogens including Fusarium virguliforme and closely related species Fusarium brasiliense, Fusarium crassistipitatum, Fusarium tucumaniae, which are the primary causal agents of soybean sudden death syndrome (SDS), a significant threat to soybean production. In this study, we developed microsatellite markers from a E virguliforme genome sequence and applied them to a E virguliforme population collection of 38 isolates from Michigan and four reference strains from other locations. Of the 225 detected microsatellite loci, 108 loci were suitable for primer design, and 12 of the microsatellite markers were determined to be highly polymorphic, amplifying on average 5.7 alleles per locus. Using these markers, F. virguliforme isolates were partitioned into three distinct clusters, but isolates were not grouped based on relatedness of sampling sites. In addition, 11 out of 12 markers were demonstrated to be highly transferrable to other closely related species. (C) 2015 Elsevier Ltd and The British Mycological Society. All rights reserved. | 2229 | NA | NA | NA | no | no |
| 2230 | 2017 | NA | Kaaydjieva, L; Morar, B; Chaix, R; Tang, H | 2005 | A newly discovered founder population: the Roma/Gypsies | “The Gypsies (a misnomer, derived from an early legend about Egyptian origins) defy the conventional definition of a population: they have no nation-state, speak different languages, belong to many religions and comprise a mosaic of socially and culturally divergent groups separated by strict rules of endogamy. Referred to as”“the invisible minority”“, the Gypsies have for centuries been ignored by Western medicine, and their genetic heritage has only recently attracted attention. Common origins from a,small group of ancestors characterise the 810 million European Gypsies as an unusual trans-national founder population, whose exodus from India played the role of a profound demographic bottleneck. Social and economic pressures within Europe led to gradual fragmentation, generating multiple genetically differentiated subisolates. The string of population bottlenecks and founder effects have shaped a unique genetic profile, whose potential for genetic research can be met only by study designs that acknowledge cultural tradition and self-identity.” | 2230 | NA | NA | NA | no | no |
| 2231 | 2017 | NA | Wernegreen, JJ; Degnan, PH; Lazarus, AB; Palacios, C; Bordenstein, SR | 2003 | Genome evolution in an insect cell: Distinct features of an ant-bacterial partnership | Bacteria that live exclusively within eukaryotic host cells include not only well-known pathogens, but also obligate mutualists, many of which occur in diverse insect groups such as aphids, psyllids, tsetse flies, and the ant genus Camponotus (Buchner, 1965; Douglas, 1998; Moran and Telang, 1998; Baumann et al., 2000; Moran and Baumann, 2000). In contrast to intracellular pathogens, these primary (P) endosymbionts of insects are required for the survival and reproduction of the host, exist within specialized host cells called bacteriocytes, and undergo stable maternal transmission through host lineages (Buchner, 1965; McLean and Houk, 1973). Due to their long-term host associations and close phylogenetic relationship with well-characterized enterobacteria (Fig. 1), P-endosymbionts of insects are ideal model systems to examine changes in genome content and architecture that occur in the context Of beneficial, intracellular associations. Since these bacteria have not been cultured outside of the host cell, they are difficult to study with traditional genetic or physiological approaches. However, in recent years, molecular and computational approaches have provided important insights into their genetic diversity and ecological significance. This review describes some recent insights into the evolutionary genetics of obligate insect-bacteria symbioses, with a particular focus on an intriguing association between the bacterial endosymbiont Blochmannia and its ant hosts. | 2231 | NA | NA | NA | no | no |
| 2232 | 2017 | NA | Fourmann, M; Charlot, F; Froger, N; Delourme, R; Brunel, D | 2001 | Expression, mapping, and genetic variability of Brassica napus disease resistance gene analogues | Numerous sequences analogous to resistance (R) genes exist in plant genomes and could be involved in resistance traits. The aim of this study was to identify a large number of Brassica napus sequences related to R genes and also to test the adequacy of specific PCR-based tools for studying them. Different consensus primers were compared for their efficiency in amplifying resistance-gene analogues (RGAs) related to the nucleotide-binding-site subgroup of R genes. Specific primers were subsequently designed to fine-study the different RGAs and we tested their efficiency in three species related to B. napus: Brassica oleracea, Brassica rapa, and Arabidopsis thaliana. Forty-four B. napus RGAs were identified. Among 29 examined, at least one-third were expressed. Eighteen RGAs were mapped on 10 of the 19 B. napus linkage groups. The high variability within these sequences permitted discrimination of each genotype within a B. napus collection. The RGA-specific primers amplified RGAs in the B. oleracea and B. rapa genomes, but the sequences appear to be poorly conserved in A. thaliana. Specific RGA primers are a precise tool for studying known-sequence RGAs. These sequences represent interesting markers that could be correlated with resistance traits in B. napus or related Brassica genomes. | 2232 | NA | NA | NA | no | no |
| 2233 | 2017 | NA | Zang, Y; Zhang, H; Yang, YN; Zheng, G | 2007 | Robust genomic control and robust delta centralization tests for case-control association studies | The population-based case-control design is a powerful approach for detecting susceptibility markers of a complex disease. However, this approach may lead to spurious association when there is population substructure: population stratification (PS) or cryptic relatedness (CR). Two simple approaches to correct for the population substructure are genomic control (GC) and delta centralization (DC). GC uses the variance inflation factor to correct for the variance distortion of a test statistic, and the DC centralizes the non-central chi-square distribution of the test statistic. Both GC and DC have been studied for case-control association studies mainly under a specific genetic model (e.g. recessive, additive or dominant), under which an optimal trend test is available. The genetic model is usually unknown for many complex diseases. In this situation, we study the performance of three robust tests based on the GC and DC corrections in the presence of the population substructure. Our results show that, when the genetic model is unknown, the DC- (or GC-) corrected maximum and Pearson’s association test are robust and have good control of Type I error and high power relative to the optimal trend tests in the presence of PS (or CR). Copyright (c) 2007 S. Karger AG, Basel. | 2233 | NA | NA | NA | no | no |
| 2234 | 2017 | NA | Serbezov, D; Jorde, PE; Bernatchez, L; Olsen, EM; Vollestad, LA | 2012 | Life history and demographic determinants of effective/census size ratios as exemplified by brown trout (Salmo trutta) | A number of demographic factors, many of which related to human-driven encroachments, are predicted to decrease the effective population size (Ne) relative to the census population size (N), but these have been little investigated. Yet, it is necessary to know which factors most strongly impact Ne, and how to mitigate these effects through sound management actions. In this study, we use parentage analysis of a stream-living brown trout (Salmo trutta) population to quantify the effect of between-individual variance in reproductive success on the effective number of breeders (Nb) relative to the census number of breeders (Ni). Comprehensive estimates of the Nb/N ratio were reduced to 0.160.28, almost entirely due to larger than binomial variance in family size. We used computer simulations, based on empirical estimates of age-specific survival and fecundity rates, to assess the effect of repeat spawning (iteroparity) on Ne and found that the variance in lifetime reproductive success was substantially higher for repeat spawners. Random family-specific survival, on the other hand, acts to buffer these effects. We discuss the implications of these findings for the management of small populations, where maintaining high and stable levels of Ne is crucial to extenuate inbreeding and protect genetic variability. | 2234 | NA | NA | NA | no | no |
| 2235 | 2017 | NA | Gargano, D; Gullo, T; Bernardo, L | 2009 | Do inefficient selfing and inbreeding depression challenge the persistence of the rare Dianthus guliae Janka (Caryophyllaceae)? Influence of reproductive traits on a plant’s proneness to extinction | The yellow carnation Dianthus guliae Janka is a rare endemic of the Italian peninsula. As numerous extinctions have occurred in the past, very few populations are still present. Two years of field surveys revealed high mortality and an absence of recruitment in the southernmost populations of this species. Work in a botanical garden, under semi-natural conditions, revealed the occurrence of proterandrous hermaphroditism and self-compatibility. The durable flower lifespan, the competitive effects among flowers and the different outcomes from spontaneous and hand-performed pollinations (with both self- and cross-pollen) suggested low pollination rates in the experimental stand. Pollinator exclusion experiments revealed a low frequency of delayed autonomous selfing, suggesting that self-fertilization in D. guliae relies mainly on facilitated selfing and geitonogamy. Studies of inbreeding depression during the early life-history stages revealed significant differences between selfed and crossed progenies in terms of seed mass, germination rates, developmental vigour and mortality rates. Therefore, when pollen delivery is scarce, the plant may fail reproductive assurance via autonomous selfing. The influence of inbreeding depression contributes to a further reduction in recruitment chances in very small D. guliae populations. Experimental reintroductions are urgent to avoid local extinction at the southern periphery of its range. | 2235 | NA | NA | NA | no | no |
| 2236 | 2017 | NA | SJERPS, M; HACCOU, P | 1994 | A WAR OF ATTRITION BETWEEN LARVAE ON THE SAME HOST-PLANT - STAY AND STARVE OR LEAVE AND BE EATEN | Many insect species lay their eggs according to a clumped distribution, which causes food shortage among the larvae. To avoid starvation, at least some larvae have to migrate to another plant at some stage. Even though this migration involves a high mortality risk, larvae (of, for example, the cinnabar moth) often leave before their host plant is defoliated, thereby enabling others to remain safely on the plant. To understand why this remarkable behaviour has evolved, we modelled the situation of two larvae on a small plant as a war of attrition. Our main conclusions are that, in the case where the larvae are unrelated, they should only migrate prior to defoliation in certain time periods, called migration periods, when the pay-off of leaving decreases. Moreover, the optimal migration time is a random variable. When the pay-off of leaving fluctuates, there can be several migration periods. In the case where the larvae are related, it can also be optimal to migrate when the pay-off of leaving increases. Relatedness increases the length and the number of the migration periods, as well as the leaving tendency during these periods. Furthermore, relatedness gives rise to a parent-offspring conflict over the optimal migration strategy. | 2236 | NA | NA | NA | no | no |
| 2237 | 2017 | NA | Sun, M; Gao, L; Liu, Y; Zhao, YQ; Wang, XQ; Pan, YQ; Ning, T; Cai, H; Yang, HJ; Zhai, WW; Ke, Y | 2012 | Whole Genome Sequencing and Evolutionary Analysis of Human Papillomavirus Type 16 in Central China | Human papillomavirus type 16 plays a critical role in the neoplastic transformation of cervical cancers. Molecular variants of HPV16 existing in different ethnic groups have shown substantial phenotypic differences in pathogenicity, immunogenicity and tumorigenicity. In this study, we sequenced the entire HPV16 genome of 76 isolates originated from Anyang, central China. Phylogenetic analysis of these sequences identified two major variants of HPV16 in the Anyang area, namely the European prototype (E(p)) and the European Asian type (E(As)). These two variants show a high degree of divergence between groups, and the E(p) comprised higher genetic diversity than the E(As). Analysis with two measurements of genetic diversity indicated that viral population size was relatively stable in this area in the past. Codon based likelihood models revealed strong statistical support for adaptive evolution acting on the E6 gene. Bayesian analysis identified several important amino acid positions that may be driving adaptive selection in the HPV 16 population, including R10G, D25E, L83V, and E113D in the E6 gene. We hypothesize that the positive selection at these codons might be a contributing factor responsible for the phenotypic differences in carcinogenesis and immunogenicity among cervical cancers in China based on the potential roles of these molecular variants reported in other studies. | 2237 | NA | NA | NA | no | no |
| 2238 | 2017 | NA | Zhan, J; Pettway, RE; McDonald, BA | 2003 | The global genetic structure of the wheat pathogen Mycosphaerella graminicola is characterized by high nuclear diversity, low mitochondrial diversity, regular recombination, and gene flow | A total of 1673 Mycosphaerella graminicola strains were assayed for DNA fingerprints and restriction fragment length polymorphism (RFLP) markers in the nuclear and mitochondrial genomes. The isolates were collected from 17 wheat fields located in I I countries on five continents over a six year period (1989-1995). Our results indicate that genetic diversity in the nuclear genome of this fungus was high for all but three of the field populations surveyed and that populations sampled from different continents had similar frequencies for the most common RFLP alleles. Hierarchical analysis revealed that more than 90% of global gene diversity was distributed within a wheat field, while similar to5% of gene diversity was distributed among fields within regions and similar to3% was distributed among regions on different continents. These findings suggest that gene flow has occurred on a global scale. On average, each leaf was colonized by a different nuclear genotype. In contrast, only seven mtDNA haplotypes were detected among the 1673 isolates and the two most common mtDNA haplotypes represented approximately 93% of the world population, consistent with a selective sweep. Analysis of multilocus associations indicated that all field populations were in gametic equilibrium, suggesting that sexual recombination is a regular occurrence globally. (C) 2002 Elsevier Science (USA). All rights reserved. | 2238 | NA | NA | NA | no | no |
| 2239 | 2017 | NA | Dietrich, M; Kempf, F; Boulinier, T; Mccoy, KD | 2014 | Tracing the colonization and diversification of the worldwide seabird ectoparasite Ixodes uriae | Historical patterns of dispersal and population isolation are key components shaping contemporary genetic diversity across landscapes and require explicit consideration when examining the relative role of different factors in driving the evolution of host specificity in parasitic organisms. In this study, we investigate the worldwide colonization history of a common ectoparasite of seabirds, the tick Ixodes uriae. This tick has a circumpolar distribution across both hemispheres but has repeatedly formed host-specific races within different regions. By combining mitochondrial and nuclear data, we infer how this species spread to its present-day distribution and how the colonization process may have affected the geographic and host-associated structure of this tick within regions. We demonstrate that I.uriae is highly structured at a global scale and isolates into four genetic groups that correspond to well-defined geographical regions. Molecular dating suggests that the diversification of I.uriae began in the early Miocene (22Myr) and that this tick colonized most of the southern hemisphere before moving into northern latitudes via two independent routes. However, no relationship between the degree of host race divergence and colonization history was evident, supporting previous hypotheses that host specialization evolves relatively rapidly in this parasite, but does not typically lead to speciation. We discuss the possible historical and contemporary mechanisms of large-scale dispersal for this ectoparasite and how its biological characteristics may condition current patterns of genetic diversity. More generally, our results illustrate how combining broad-scale sampling and modern molecular tools can help disentangle complex patterns of diversification in widespread parasites. | 2239 | NA | NA | NA | no | no |
| 2240 | 2017 | NA | Andersen, SB; Hansen, LH; Sapountzis, P; Sorensen, SJ; Boomsma, JJ | 2013 | Specificity and stability of the Acromyrmex-Pseudonocardia symbiosis | The stability of mutualistic interactions is likely to be affected by the genetic diversity of symbionts that compete for the same functional niche. Fungus-growing (attine) ants have multiple complex symbioses and thus provide ample opportunities to address questions of symbiont specificity and diversity. Among the partners are Actinobacteria of the genus Pseudonocardia that are maintained on the ant cuticle to produce antibiotics, primarily against a fungal parasite of the mutualistic gardens. The symbiosis has been assumed to be a hallmark of evolutionary stability, but this notion has been challenged by culturing and sequencing data indicating an unpredictably high diversity. We used 454 pyrosequencing of 16S rRNA to estimate the diversity of the cuticular bacterial community of the leaf-cutting ant Acromyrmex echinatior and other fungus-growing ants from Gamboa, Panama. Both field and laboratory samples of the same colonies were collected, the latter after colonies had been kept under laboratory conditions for up to 10 years. We show that bacterial communities are highly colony-specific and stable over time. The majority of colonies (25/26) had a single dominant Pseudonocardia strain, and only two strains were found in the Gamboa population across 17 years, confirming an earlier study. The microbial community on newly hatched ants consisted almost exclusively of a single strain of Pseudonocardia while other Actinobacteria were identified on older, foraging ants in varying but usually much lower abundances. These findings are consistent with recent theory predicting that mixtures of antibiotic-producing bacteria can remain mutualistic when dominated by a single vertically transmitted and resource-demanding strain. | 2240 | NA | NA | NA | no | no |
| 2241 | 2017 | NA | Mundt, CC; Brunet, J; Sackett, KE | 2008 | Impact of density and disease on frequency-dependent selection and genetic polymorphism: experiments with stripe rust and wheat | Frequency-dependent disease impacts may contribute to the maintenance of genetic diversity and sexual reproduction in plant populations. In earlier work with experimental wheat (Triticum aestivum) populations at a single density, we found that stripe rust (caused by Puccinia striiformis) created frequency-dependent selection on its host but competitive interactions between host genotypes reduced the potential for disease to maintain genetic polymorphisms in this highly self-pollinated species; the weaker competitor actually exhibited positive disease-mediated frequency-dependent selection. Based on these results we predicted that at low density, where the overall level of competition is lower, disease would have a stronger impact relative to competition and thus be more likely to maintain genetic polymorphisms; at low densities the greatest effect of disease for negative frequency-dependent selection should be seen in the weak competitor. Here we report on results with wheat stripe rust in which we altered both the frequency and density of host genotypes in factorial combinations of two-way mixtures where each host genotype was attacked by its own specialized race of rust. Within each density disease levels increased with genotype frequencies, creating frequency-dependent disease attack at all densities. Similarly, disease created negative frequency-dependent selection on its host at all densities, as a genotype’s fitness was often greater at low than high frequency when disease was present. Disease levels increased with plant density in 1997 but decreased in 1998. While increasing plant density reduced absolute fitness, presumably as a result of increased competition, a genetic polymorphism was not more likely to be maintained at low than high density as we had predicted. Within each density, the impact of disease was insufficient to reverse the slope of the relationship between absolute fitness and planted frequency from positive to negative for the less competitive host genotype, thus preventing the maintenance of a genetic polymorphism. | 2241 | NA | NA | NA | no | no |
| 2242 | 2017 | NA | Li, WJ; Averette, AF; Desnos-Ollivier, M; Ni, M; Dromer, F; Heitman, J | 2012 | Genetic Diversity and Genomic Plasticity of Cryptococcus neoformans AD Hybrid Strains | Natural hybridization between two strains, varieties, or species is a common phenomenon in both plants and animals. Although hybridization may skew established gene pools, it generates population diversity efficiently and sometimes results in the emergence of newly adapted genotypes. Cryptococcus neoformans, which causes the most frequent opportunistic fungal infection in immunocompromised hosts, has three serotypes: A, D, and AD. Serotype-specific multilocus sequence typing and serotype-specific comparative genome hybridization were applied to investigate the genetic variability and genomic organization of C. neoformans serotype AD isolates. We confirm that C. neoformans serotype AD isolates are hybrids of serotype A and D strains. Compared with haploid strains, most AD hybrid isolates exhibit unique multilocus sequence typing genotypes, suggesting that multiple independent hybridization events punctuated the origin and evolutionary trajectory of AD hybrids. The MATa alleles from both haploid and AD hybrid isolates group closely to form a cluster or subcluster in both the serotype A and D populations. The rare and unique distribution of MATa alleles may restrict sexual reproduction between isolates of opposite mating types. The genetic diversity of the serotype D population, including haploid strains and serotype D genomes of the AD hybrid, is significantly greater than that of serotype A, and there are signatures of recombination within the serotype D population. Given that MATa isolates are relatively rare, both opposite-sex and same-sex mating may contribute to genetic recombination of serotype D in nature. Extensive chromosome loss was observed in AD hybrid isolates, which results in loss of heterozygosity in the otherwise-heterozygous AD hybrid genome. Most AD hybrid isolates exhibit hybrid vigor and are resistant to the antifungal drug FK506. In addition, the C. neoformans AD hybrid genome is highly dynamic, with continuous chromosome loss, which may be a facile route for pathogen evolution through which genotypic and phenotypic variation is generated. | 2242 | NA | NA | NA | no | no |
| 2243 | 2017 | NA | Jaatinen, K; Ost, M; Gienapp, P; Merila, J | 2011 | Differential responses to related hosts by nesting and non-nesting parasites in a brood-parasitic duck | Hostparasite relatedness may facilitate the evolution of conspecific brood parasitism, but empirical support for this contention remains inconclusive. One reason for this disparity may relate to the diversity of parasitic tactics, a key distinguishing feature being whether the parasite has a nest of her own. Previous work suggests that parasites without nests of their own may be of inferior phenotypic quality, but because of difficulties in identifying these parasitic individuals, little is known about their host selection criteria. We used high-resolution molecular maternity tests to assign parasitic offspring to known parasites with and without their own nests in a population of Barrows goldeneyes (Bucephala islandica). We determined whether parasite nesting status, hostparasite relatedness and distance between host and parasite nests affected the probability of parasitizing a host and the number of eggs laid per host. We also investigated whether nesting parasites, conventionally nesting females and non-nesting parasites differed regarding their age, structural size, body condition, nesting phenology or total brood size. The probability of engaging in parasitism increased with hostparasite relatedness and spatial proximity to host nests for nesting and non-nesting females alike. However, nesting parasites increased the number of eggs donated with relatedness to the host, while non-nesting parasites did not do so. Non-nesting parasites laid fewer eggs in total, but did not differ by any of the other quality measures from conventional nesters or nesting parasites. Our study provides the first demonstration that nesting and non-nesting parasites from the same population may use different host selection criteria. | 2243 | NA | NA | NA | no | no |
| 2244 | 2017 | NA | Al-Chaarani, G; Roustaee, A; Gentzbittel, L; Mokrani, L; Barrault, G; Dechamp-Guillaume, G; Sarrafi, A | 2002 | A QTL analysis of sunflower partial resistance to downy mildew (Plasmopara halstedii) and black stem (Phoma macdonaldii) by the use of recombinant inbred lines (RILs) | Partial resistance to downy mildew (Plasinopara halstedii) and to black stem (Phoma macdonaldii) in sunflower were investigated under natural field infection and a controlled growth chamber respectively. Genetic control for resistance to the diseases was determined in recombinant inbred lines (RILs) and their two parents, ‘PAC-2’ and ‘RHA-266.’ The experiments were undertaken in a randomized complete block design with two replications, in a field severely infected by downy mildew and in a controlled growth chamber with plants inoculated with an agressive French isolate of P. macdonaldii. Each replication consisted of three rows, 4.6-m long, giving 48 plants per RIL or parent in the field and 15 plants in the growth chamber. Genetic variability was observed among the RILs for resistance to both diseases. When 10% of the selected RILs were compared with the mean of the two parents genetic gain was significant for partial resistance to the diseases. Four putative QTLs for resistance to downy mildew on linkage groups 1, 9 and 17 were detected using composite interval mapping. The QTLs explained 54.9% of the total phenotypic variance. Major QTLs (dmr1-1 and dmr1-2) for resistance were found on linkage group 1 with up to 31% of the phenotypic variability explained by two peaks. QTL analysis of resistance to black stem showed seven QTLs on linkage groups 3, 6, 8, 9, 11, 15 and 17. The detected QTLs together explain 92% of the phenotypic variation of the trait. Crosses between RILs contrasted for their resistance to downy mildew and black stem, and exhibiting molecular polymorphism in detected QTLs, will be made in order to focus more-precisely on the genomic region of interest. | 2244 | NA | NA | NA | no | no |
| 2245 | 2017 | NA | Huth-Schwarz, A; Settele, J; Moritz, RFA; Kraus, FB | 2012 | Factors influencing Nosema bombi infections in natural populations of Bombus terrestris (Hymenoptera: Apidae) | Bumblebees are of profound ecological importance because of the pollination services they provide in natural and agricultural ecosystems. Any decline of these pollinators is therefore of great concern for ecosystem functioning. Increased parasite pressures have been discussed as a major factor for the loss of pollinators. One of the main parasites of bumblebees is Nosema bombi, an intracellular microsporidian parasite with considerable impact on the vitality of the host. Here we study the effect of host colony density and host genetic variability on N. bombi infections in natural populations of the bumblebee Bombus terrestris. We sampled males and workers from six B. terrestris populations located in an agricultural landscape in Middle Sweden to determine the prevalence and degree of N. bombi infections. All individuals were genotyped with five microsatellite markers to infer the colony densities in the sampled populations and the genetic variability of the host population. We confirmed that genetic variability and sex significantly correlate with the degree of infection with N. bombi. Males and workers with lower genetic variability had significantly higher infection levels than average. Also colony density had a significant impact on the degree of infection, with high density populations having higher infected individuals. (C) 2012 Elsevier Inc. All rights reserved. | 2245 | NA | NA | NA | no | no |
| 2246 | 2017 | NA | Burke, RL; Whitehouse, CA; Taylor, JK; Selby, EB | 2009 | Epidemiology of Invasive Klebsiella pneumoniae with Hypermucoviscosity Phenotype in a Research Colony of Nonhuman Primates | Invasive Klebsiella pneumoniae with hypermucoviscosity phenotype (HMV K. pneumoniae) is an emerging human pathogen that, over the past 20 y, has resulted in a distinct clinical syndrome characterized by pyogenic liver abscesses sometimes complicated by bacteremia, meningitis, and endophthalmitis. Infections occur predominantly in Taiwan and other Asian countries, but HMV K. pneumoniae is considered an emerging infectious disease in the United States and other Western countries. In 2005, fatal multisystemic disease was attributed to HMV K. pneumoniae in African green monkeys (AGM) at our institution. After identification of a cluster of subclinically infected macaques in March and April 2008, screening of all colony nonhuman primates by oropharyngeal and rectal culture revealed 19 subclinically infected rhesus and cynomolgus macaques. PCR testing for 2 genes associated with HMV K. pneumoniae, rmpA and magA, suggested genetic variability in the samples. Random amplified polymorphic DNA analysis on a subset of clinical isolates confirmed a high degree of genetic diversity between the samples. Environmental testing did not reveal evidence of aerosol or droplet transmission of the organism in housing areas. Further research is needed to characterize HMV K. pneumoniae, particularly with regard to genetic differences among bacterial strains and their relationship to human disease and to the apparent susceptibility of AGM to this organism. | 2246 | NA | NA | NA | no | no |
| 2247 | 2017 | NA | Durand, J; Jacquet, M; Rais, O; Gern, L; Voordouw, MJ | 2017 | Fitness estimates from experimental infections predict the long-term strain structure of a vector-borne pathogen in the field | The populations of many pathogen species consist of a collection of common and rare strains but the factors underlying this strain-specific variation in frequency are often unknown. Understanding frequency variation among strains is particularly challenging for vector-borne pathogens where the strain-specific fitness depends on the performance in both the vertebrate host and the arthropod vector. Two sympatric multiple-strain tick-borne pathogens, Borrelia afzelii and B. garinii, that use the same tick vector, Ixodes ricinus, but different vertebrate hosts were studied. 454-sequencing of the polymorphic ospC gene was used to characterize the community of Borrelia strains in a local population of I. ricinus ticks over a period of 11 years. Estimates of the reproduction number ( R-0), a measure of fitness, were obtained for six strains of B. afzelii from a previous laboratory study. There was substantial variation in prevalence among strains and some strains were consistently common whereas other strains were consistently rare. In B. afzelii, the strain-specific estimates of R-0 in laboratory mice explained over 70% of the variation in the prevalences of the strains in our local population of ticks. Our study shows that laboratory estimates of fitness can predict the community structure of multiple-strain pathogens in the field. | 2247 | NA | NA | NA | no | no |
| 2248 | 2017 | NA | Piotrowska, MJ; Ennos, RA; Fountaine, JM; Burnett, FJ; Kaczmarek, M; Hoebe, PN | 2016 | Development and use of microsatellite markers to study diversity, reproduction and population genetic structure of the cereal pathogen Ramularia collo-cygni | Ramularia collo-cygni (Rcc) is a major pathogen of barley that causes economically serious yield losses. Disease epidemics during the growing season are mainly propagated by asexual air-borne spores of Rcc, but it is thought that Rcc undergoes sexual reproduction during its life cycle and may also disperse by means of sexual ascospores. To obtain population genetic information from which to infer the extent of sexual reproduction and local genotype dispersal in Rcc, and by implication the pathogen’s ability to adapt to fungicides and resistant cultivars, we developed ten polymorphic microsatellite markers, for which primers are presented. We used these markers to analyse the population genetic structure of this cereal pathogen in two geographically distant populations from the Czech Republic (n = 30) and the United Kingdom (n = 60) that had been sampled in a spatially explicit manner. Genetic diversity at the microsatellite loci was substantial, H-t = 0.392 and H-t = 0.411 in the Czech and UK populations respectively, and the populations were moderately differentiated at these loci (Theta = 0.111, P < 0.01). In both populations the multilocus genotypic diversity was very high (one clonal pair per population, resulting in >96% unique genotypes in each of the populations) and there was a lack of linkage disequilibrium among loci, strongly suggesting that sexual reproduction is an important component of the life cycle of Rcc. In an analysis of spatial genetic structure, kinship coefficients in all distance classes were very low (-0.0533 to 0.0142 in the Czech and -0.0268 to -0.0042 in the Scottish population) and non-significant (P > 0.05) indicating lack of subpopulation structuring at the field scale and implying extensive dissemination of spores. These results suggest that Rcc possesses a high evolutionary potential for developing resistance to fungicides and overcoming host resistance genes, and argue for the development of an integrated disease management system that does not rely solely on fungicide applications. (C) 2016 Elsevier Inc. All rights reserved. | 2248 | NA | NA | NA | no | no |
| 2249 | 2017 | NA | Al-Amri, K; Al-Sadi, AM; Al-Shihi, A; Nasehi, A; Al-Mahmooli, I; Deadman, ML | 2016 | Population structure of Stemphylium lycopersici associated with leaf spot of tomato in a single field | Stemphylium lycopersici is an important pathogen causing leaf spot of tomatoes worldwide. Although much information is available about the pathogen, little is known about dynamics of S. lycopersici in tomato fields. Seventy-nine symptomatic leaf samples were collected from two tomato cultivars grown in a farm (Miral and Inbred line). Fungal species associated with the disease were isolated on potato dextrose agar. Seventy-nine isolates were obtained and identified as S. lycopersici based on sequence analysis of combined dataset of the internal transcribed spacer and glyceraldehyde-3-phosphate dehydrogenase regions. The 79 isolates were subjected to amplified fragment length polymorphism analysis using three primer combinations. The Stemphylium lycopersici population from the two cultivars was found to have a very low level of genetic diversity (H = 0.0948). Cluster analysis showed intermixing of isolates from the two cultivars. In addition, analysis of molecular variance showed the presence of a very low level of genetic differentiation between populations obtained from the two cultivars (Fst = 0.0206). These findings indicate the presence of a high rate of gene flow between the two populations and may suggest that the two populations originated from the same inoculum source. The implications of these findings on the management of Stemphylium-induced leaf spot of tomatoes are discussed. | 2249 | NA | NA | NA | no | no |
| 2250 | 2017 | NA | Stead, JDH; Hurles, ME; Jeffreys, AJ | 2003 | Global haplotype diversity in the human insulin gene region | The insulin minisatellite (INS VNTR) has been intensively analyzed due to its associations with diseases including diabetes. We have previously used patterns of variant repeat distribution in the minisatellite to demonstrate that genetic diversity is unusually great in Africans compared to non-Africans. Here we analyzed variation at 56 single nucleotide polymorphisms (SNPs) flanking the minisatellite in individuals from Six Populations, and we show that over 40% of the total genetic variance near the minisatellite is due to differences between Africans and non-Africans, far higher than seen in most genomic regions and consistent with differential selection acting on the insulin gene region, most likely in the non-African ancestral Population. Linkage disequilibrium was lower in African populations, with evidence Of Clustering of historical recombination events. Analysis of haplotypes from the relatively nonrecombining region around the minisatellite revealed a star-shaped phylogeny with lineages radiating from an ancestral African-specific haplotype. These haplotypes confirmed that minisatellite lineages defined by variant repeat distributions are monophyletic in origin. These analyses provide a framework for a cladistic approach to future disease association Studies of the insulin region within both African and non-African populations, and they identify SNPs which can be rapidly analyzed as surrogate markers for minisatellite lineage. | 2250 | NA | NA | NA | no | no |
| 2251 | 2017 | NA | Velo-Anton, G; Godinho, R; Ayres, C; Ferrand, N; Rivera, AC | 2007 | Assignment tests applied to relocate individuals of unknown origin in a threatened species, the European pond turtle (Emys orbicularis) | The pet trade is an important business around the world and one of the factors that might menace some wild populations. If wild animals are collected to maintain them as pets, this activity can produce several problems: i) an increase of population vulnerability, especially in the case of rare species; ii) the release of exotic pets in natural habitats, with the risk of competition with native species and the spreading of parasites and diseases, and iii) the maintenance of animals of unknown origin in Recovery Centres or zoos, which if too numerous are sacrificed or re-located to their supposed original regions. In this paper, we used seven microsatellite loci to analyze genetic diversity and genetic structure of the European pond turtle (Emys obicularis) covering the species range in the Iberian Peninsula. A Bayesian test revealed a genotypic differentiation between the regions sampled where most individuals (90%) were assigned to their sampling location with a probability higher than 95%. The likelihood values for individuals from Recovery Centres to came from one of our populations was higher than 90% in 22 out of 36 individuals. This work is a first step to relocate animals of unknown origin taking into account genetic similarities and contribute to reinforcement programs of endangered species. | 2251 | NA | NA | NA | no | no |
| 2252 | 2017 | NA | van Oppen, MJH; Peplow, LM; Kininmonth, S; Berkelmans, R | 2011 | Historical and contemporary factors shape the population genetic structure of the broadcast spawning coral, Acropora millepora, on the Great Barrier Reef | Effective management of reef corals requires knowledge of the extent to which populations are open or closed and the scales over which genetic exchange occurs, information which is commonly derived from population genetic data. Such data are sparse for Great Barrier Reef (GBR) corals and other organisms, with the studies that are available being mostly based on a small number of sampling locations spanning only part of the GBR. Using 11 microsatellite loci, we genotyped 947 colonies of the reef-building coral Acropora millepora from 20 sites spanning almost the full length of the GBR (similar to 12 degrees of latitude and similar to 1550 km). The results show a major divide between the southernmost central to southern offshore populations and all other sampled populations. We interpret this divide as a signature of allopatric divergence in northern and southern refugia during the Pleistocene glaciations, from which the GBR was subsequently recolonized. Superimposed on this pattern is a cross-shelf genetic division, as well as a separation of inshore populations south of the Cape Clifton Front at similar to 21.522 degrees S. Most inshore populations north of this, as well as mid-shelf populations in the northern and far northern GBR, are open, exchanging recruits frequently. In contrast, inshore populations south of the Cape Clifton Front and offshore populations in the central and southern GBR are largely self-seeding, at least within the spatial resolution that was achieved given our sampling intensity. Populations that have been impacted by recent disturbance events causing extensive coral mortality show no evidence of reduced genetic diversity. | 2252 | NA | NA | NA | no | no |
| 2253 | 2017 | NA | Sharma, R; Kumar, B; Arora, R; Ahlawat, S; Mishra, AK; Tantia, MS | 2016 | Genetic diversity estimates point to immediate efforts for conserving the endangered Tibetan sheep of India | Tibetan is a valuable Himalayan sheep breed classified as endangered. Knowledge of the level and distribution of genetic diversity in Tibetan sheep is important for designing conservation strategies for their sustainable survival and to preserve their evolutionary potential. Thus, for the first time, genetic variability in the Tibetan population was accessed with twenty five inter-simple sequence repeat markers. All the microsatellites were polymorphic and a total of 148 alleles were detected across these loci. The observed number of alleles across all the loci was more than the effective number of alleles and ranged from 3 (BM6506) to 11 (BM6526) with 5.920 +/- 0.387 mean number of alleles per locus. The average observed heterozygosity was less than the expected heterozygosity. The observed and expected heterozygosity values ranged from 0.150 (BM1314) to 0.9 (OarCP20) with an overall mean of 0.473 +/- 0.044 and from 0.329 (BM8125) to 0.885 (BM6526) with an overall mean 0.672 +/- 0.030, respectively. The lower heterozygosity pointed towards diminished genetic diversity in the population. Thirteen microsatellite loci exhibited significant (P < 0.05) departures from the Hardy-Weinberg proportions in the population. The estimate of heterozygote deficiency varied from -0.443 (OarCP20) to 0.668 (OarFCB128) with a mean positive value of 0.302 +/- 0.057. A normal ‘L’ shaped distribution of mode-shift test and nonsignificant heterozygote excess on the basis of different models suggested absence of recent bottleneck in the existing Tibetan population. In view of the declining population of Tibetan sheep (less than 250) in the breeding tract, need of the hour is immediate scientific management of the population so as to increase the population hand in hand with retaining the founder alleles to the maximum possible extent. (C) 2016 The Authors. Published by Elsevier B.V. | 2253 | NA | NA | NA | no | no |
| 2254 | 2017 | NA | Marakeby, H; Badr, E; Torkey, H; Song, Y; Leman, S; Monteil, CL; Heath, LS; Vinatzer, BA | 2014 | A System to Automatically Classify and Name Any Individual Genome-Sequenced Organism Independently of Current Biological Classification and Nomenclature | A broadly accepted and stable biological classification system is a prerequisite for biological sciences. It provides the means to describe and communicate about life without ambiguity. Current biological classification and nomenclature use the species as the basic unit and require lengthy and laborious species descriptions before newly discovered organisms can be assigned to a species and be named. The current system is thus inadequate to classify and name the immense genetic diversity within species that is now being revealed by genome sequencing on a daily basis. To address this lack of a general intra-species classification and naming system adequate for today’s speed of discovery of new diversity, we propose a classification and naming system that is exclusively based on genome similarity and that is suitable for automatic assignment of codes to any genome-sequenced organism without requiring any phenotypic or phylogenetic analysis. We provide examples demonstrating that genome similarity-based codes largely align with current taxonomic groups at many different levels in bacteria, animals, humans, plants, and viruses. Importantly, the proposed approach is only slightly affected by the order of code assignment and can thus provide codes that reflect similarity between organisms and that do not need to be revised upon discovery of new diversity. We envision genome similarity-based codes to complement current biological nomenclature and to provide a universal means to communicate unambiguously about any genome-sequenced organism in fields as diverse as biodiversity research, infectious disease control, human and microbial forensics, animal breed and plant cultivar certification, and human ancestry research. | 2254 | NA | NA | NA | no | no |
| 2255 | 2017 | NA | Klepaker, T; Ostbye, K; Bernatchez, L; Vollestad, LA | 2012 | Spatio-temporal patterns in pelvic reduction in threespine stickleback (Gasterosteus aculeatus L.) in Lake Storvatnet | Questions: The pelvic girdle with associated spines is an integrated anti-predator defence apparatus, and is assumed to protect against piscivores in the threespine stickleback. On the other hand, it might be costly to produce the pelvic apparatus in ion-poor and mineral-challenging freshwater. Hypothesis: Stickleback with a reduced pelvic apparatus should use more shelter and be more nocturnal, avoiding predation risk. In contrast, stickleback with a well-developed pelvic apparatus should have reduced mortality during ontogeny in encounters with piscivores and thus have a longer expected lifespan. Given these two life-history strategies, we expect assortative mating as a result of divergent selection. Organism: Marine and freshwater threespine stickleback (Gasterosteus aculeatus L.). Places and times: Two representative ancestral marine populations and 36 freshwater populations in northwestern Norway (Lake Storvatnet, the main focus of the study, and three lakes downstream of it). Material was collected from 2006 to 2009. Analytical methods: We categorized nominal pelvic apparatus development (CPS morphs) in all fish, and measured metrics associated with these categories in a subsample. We also studied temporal, spatial, and habitat variation in the distribution of pelvic morphs in Lake Storvatnet. In this population, and downstream populations, we contrasted the detailed pelvic morphology with the measured genetic diversity (microsatellites), also estimating gene flow. In Lake Storvatnet, we tested for genetic divergence and signs of potential build-up of reproductive isolation via assortative mating among the observed nominal categories of pelvic reduction (CPS). Results: Pelvic reduction was seen only in Lake Storvatnet, where more than 50% of fish had a reduced pelvis. The distribution of pelvic morphs was stable over time and did not differ between habitats. The proportion of fish with pelvic reduction decreased with age. Freshwater stickleback tended to have a smaller pelvis than marine fish. The Lake Storvatnet stickleback were genetically differentiated from the downstream Lake Gjerhaugsvatn population, and both of these were different from the marine populations, with little gene flow among populations. No apparent genetic structure was found between CPS morphs within Lake Storvatnet. However, genetic factorial correspondence axes were significantly correlated with pelvic principal component axes in Lake Storvatnet, suggesting some phenotype x genetic association. Conclusion: The weak association between phenotypes and genetic structure observed in this study may reflect the build-up of early steps of reproductive isolation. Given time, such mechanisms may lead to the evolution of assortative mating, which may drive adaptive pelvic morphs (niche peaks), further resulting in genetically divergent populations and pelvic morphs. | 2255 | NA | NA | NA | no | no |
| 2256 | 2017 | NA | Auricchio, MTBDM; Vicente, JP; Meyer, D; Mingroni-Netto, RC | 2007 | Frequency and origins of hemoglobin S mutation in African-derived Brazilian populations | Africans arrived in Brazil as slaves in great numbers, mainly after 1550. Before the abolition of slavery in Brazil in 1888, many communities, called quilombos, were formed by runaway or abandoned African slaves. These communities are presently referred to as remnants of quilombos, and many are still partially genetically isolated. These remnants can be regarded as relicts of the original African genetic contribution to the Brazilian population. In this study we assessed frequencies and probable geographic origins of hemoglobin S (HBB*S) mutations in remnants of quilombo populations in the Ribeira River valley, Sao Paulo, Brazil, to reconstruct the history of African-derived populations in the region. We screened for HBB*S mutations in 11 quilombo populations (1,058 samples) and found HBB*S carrier frequencies that ranged from 0% to 14%. We analyzed beta-globin gene cluster haplotypes linked to the HBB*S mutation in 86 chromosomes and found the four known African haplotypes: 70 (81.4%) Bantu (Central Africa Republic), 7 (8.1%) Benin, 7 (8.1%) Senegal, and 2 (2.3%) Cameroon haplotypes. One sickle cell homozygote was Bantu/Bantu and two homozygotes had Bantu/Benin combinations. The high frequency of the sickle cell trait and the diversity of HBB\*S linked haplotypes indicate that Brazilian remnants of quilombos are interesting repositories of genetic diversity present in the ancestral African populations. | 2256 | NA | NA | NA | no | no |
| 2257 | 2017 | NA | Wikberg, EC; Jack, KM; Fedigan, LM; Campos, FA; Yashima, AS; Bergstrom, ML; Hiwatashi, T; Kawamura, S | 2017 | Inbreeding avoidance and female mate choice shape reproductive skew in capuchin monkeys (Cebus capucinus imitator) | Reproductive skew in multimale groups may be determined by the need for alpha males to offer reproductive opportunities as staying incentives to subordinate males (concessions), by the relative fighting ability of the alpha male (tug-of-war) or by how easily females can be monopolized (priority-of-access). These models have rarely been investigated in species with exceptionally long male tenures, such as white-faced capuchins, where female mate choice for novel unrelated males may be important in shaping reproductive skew. We investigated reproductive skew in white-faced capuchins at Sector Santa Rosa, Costa Rica, using 20years of demographic, behavioural and genetic data. Infant survival and alpha male reproductive success were highest in small multimale groups, which suggests that the presence of subordinate males can be beneficial to the alpha male, in line with the concession model’s assumptions. None of the skew models predicted the observed degree of reproductive sharing, and the probability of an alpha male producing offspring was not affected by his relatedness to subordinate males, whether he resided with older subordinate males, whether he was prime aged, the number of males or females in the group or the number of infants conceived within the same month. Instead, the alpha male’s probability of producing offspring decreased when he was the sire of the mother, was weak and lacked a well-established position and had a longer tenure. Because our data best supported the inbreeding avoidance hypothesis and female choice for strong novel mates, these hypotheses should be taken into account in future skew models. | 2257 | NA | NA | NA | no | no |
| 2258 | 2017 | NA | Thorington, KK; Weigl, PD | 2011 | Role of kinship in the formation of southern flying squirrel winter aggregations | In winter southern flying squirrels (Glaucomys volans) aggregate in large groups. They live on food items individually stored during the fall in their overlapping home ranges. The squirrels gain thermoregulatory benefits from living in aggregations but also face costs of group living, especially nest mates pilfering individually stored food. Other costs include increased predator attraction and a greater vulnerability to parasite infection. The presence of relatives in the group has the potential to increase inclusive fitness by increasing the availability of food, stored in the home area, to related individuals. Using 3 generations of known-relationship squirrels we conducted laboratory experiments to determine whether kin or familiar animals were preferred nest mates during aggregation formation. During 3 time periods, over 2 winters, squirrels were presented with kin and nonkin and familiar and unfamiliar animals and allowed to aggregate over the course of multiple 3-day trials. Kinship was persistently a major factor in the formation of aggregations. Squirrels aggregated with highly related animals (parents, offspring, and siblings) significantly more often than with unrelated animals. Familiarity became significant by the end of the study. Understanding how relatedness and familiarity interact in the formation of aggregations in seasonally gregarious animals sheds light on the processes and factors that lead to sociality. | 2258 | NA | NA | NA | no | no |
| 2259 | 2017 | NA | de Andrade, M; Ray, D; Pereira, AC; Soler, JP | 2015 | Global Individual Ancestry Using Principal Components for Family Data | Studies of complex human diseases and traits associated with candidate genes are potentially vulnerable to bias (confounding) due to population stratification and inbreeding, especially in admixed population. In GWAS, the principal components (PCs) method provides a global ancestry value per subject, allowing corrections for population stratification. However, these coefficients are typically estimated assuming unrelated individuals, and if family structure is present and ignored, such substructures may induce artifactual PCs. Extensions of the PCs method have been proposed by Konishi and Rao [Biometrika 1992; 79: 631-641], taking into account only siblings’ relatedness, and by Oualkacha et al. [Stat Appl Genet Mol Biol 2012, DOI: 10.2202/1544-6115.1711], taking into account large pedigrees and high-dimensional phenotype data. In this work, we extend these methods to estimate the global individual ancestry coefficients from PCs derived from different variance component matrix estimators using SNPs from two simulated data sets and two real data sets: the GENOA sibship data consisting of European and African-American subjects and the Baependi Heart Study consisting of 80 extended Brazilian families, both with genotyping data from the Affymetrix 6.0 chip. Our results show that the family structure plays an important role in the estimation of the global individual ancestry value for extended pedigrees but not for sibships. (C) 2015 S. Karger AG, Basel | 2259 | NA | NA | NA | no | no |
| 2260 | 2017 | NA | Bichet, C; Allaine, D; Sauzet, S; Cohas, A | 2016 | Faithful or not: direct and indirect effects of climate on extra-pair paternities in a population of Alpine marmots | Despite being identified an area that is poorly understood regarding the effects of climate change, behavioural responses to climatic variability are seldom explored. Climatic variability is likely to cause large inter-annual variation in the frequency of extra-pair litters produced, a widespread alternative mating tactic to help prevent, correct or minimize the negative consequences of sub-optimal mate choice. In this study, we investigated how climatic variability affects the inter-annual variation in the proportion of extra-pair litters in a wild population of Alpine marmots. During 22 years of monitoring, the annual proportion of extra-pair litters directly increased with the onset of earlier springs and indirectly with increased snow in winters. Snowier winters resulted in a higher proportion of families with sexually mature male subordinates and thus, created a social context within which extra-pair paternity was favoured. Earlier spring snowmelt could create this pattern by relaxing energetic, movement and time constraints. Further, deeper snow in winter could also contribute by increasing litter size and juvenile survival. Optimal mate choice is particularly relevant to generate adaptive genetic diversity. Understanding the influence of environmental conditions and the capacity of the individuals to cope with them is crucial within the context of rapid climate change. | 2260 | NA | NA | NA | no | no |
| 2261 | 2017 | NA | Gerula, D; Wegrzynowicz, P; Panasiuk, B; Bienkowska, M; Skowronek, W | 2015 | HYGIENIC BEHAVIOUR OF HONEYBEE COLONIES WITH DIFFERENT LEVELS OF POLYANDRY AND GENOTYPIC COMPOSITION | Honey bee queens were inseminated with diluted, homogenised semen collected from a few dozen drones. This procedure was carried out to increase the diversity of the queens’ offspring, which is in comparison to the offspring of queens inseminated with semen from only a few drones coming from one colony. Queens and drones were mated within carniolan bee (Apis mellifera carnica) subspecies, but 3 selected lines were used. Queens were reared from one line and drones from the same line, and two additional lines differing in hygienic behaviour wherein in one of them that trait was strongly evident. The aim of this study was to examine whether the level of enhanced genetic variability in colonies and simultaneously the participation of hygienic bees, would increase the performance of hygienic behaviour. Overall hygienic behaviour of colonies with a lower and greater genetic variability did not differ significantly and amounted to 52.1 and 47.0%, respectively. Colonies within the lower variability group, in which drones from line selected in hygienic behaviour performance were used for inseminating queens, had a significantly greater percent of cleaned pupae than other colonies (63.2%). Hygienic behaviour in other colonies was more dependent on the gene quotas of hygienic bees in the colonies rather than on the level of polyandry. | 2261 | NA | NA | NA | no | no |
| 2262 | 2017 | NA | Heinrich, SK; Hofer, H; Courtiol, A; Melzheimer, J; Dehnhard, M; Czirjak, GA; Wachter, B | 2017 | Cheetahs have a stronger constitutive innate immunity than leopards | As a textbook case for the importance of genetics in conservation, absence of genetic variability at the major histocompatibility complex (MHC) is thought to endanger species viability, since it is considered crucial for pathogen resistance. An alternative view of the immune system inspired by life history theory posits that a strong response should evolve in other components of the immune system if there is little variation in the MHC. In contrast to the leopard (Panthera pardus), the cheetah (Acinonyx jubatus) has a relatively low genetic variability at the MHC, yet free-ranging cheetahs are healthy. By comparing the functional competence of the humoral immune system of both species in sympatric populations in Namibia, we demonstrate that cheetahs have a higher constitutive innate but lower induced innate and adaptive immunity than leopards. We conclude (1) immunocompetence of cheetahs is higher than previously thought; (2) studying both innate and adaptive components of immune systems will enrich conservation science. | 2262 | NA | NA | NA | no | no |
| 2263 | 2017 | NA | Duarte-Pereira, S; Silva, SS; Azevedo, L; Castro, L; Amorim, A; Silva, RM | 2014 | NAMPT and NAPRT1: novel polymorphisms and distribution of variants between normal tissues and tumor samples | Nicotinamide phosphoribosyltransferase (NAMPT) and nicotinate phosphoribosyltransferase domain containing 1 (NAPRT1) are the main human NAD salvage enzymes. NAD regulates energy metabolism and cell signaling, and the enzymes that control NAD availability are linked to pathologies such as cancer and neurodegeneration. Here, we have screened normal and tumor samples from different tissues and populations of origin for mutations in human NAMPT and NAPRT1, and evaluated their potential pathogenicity. We have identified several novel polymorphisms and showed that NAPRT1 has a greater genetic diversity than NAMPT, where any alteration can have a greater functional impact. Some variants presented different frequencies between normal and tumor samples that were most likely related to their population of origin. The novel mutations described that affect protein structure or expression levels can be functionally relevant and should be considered in a disease context. Particularly, mutations that decrease NAPRT1 expression can predict the usefulness of Nicotinic Acid in tumor treatments with NAMPT inhibitors. | 2263 | NA | NA | NA | no | no |
| 2264 | 2017 | NA | Kummerli, R; Gardner, A; West, SA; Griffin, AS | 2009 | LIMITED DISPERSAL, BUDDING DISPERSAL, AND COOPERATION: AN EXPERIMENTAL STUDY | Numerous theoretical studies have investigated how limited dispersal may provide an explanation for the evolution of cooperation, by leading to interactions between relatives. However, despite considerable theoretical attention, there has been a lack of empirical tests. In this article, we test how patterns of dispersal influence the evolution of cooperation, using iron-scavenging in the bacterium Pseudomonas aeruginosa as our cooperative trait. We found that relatively limited dispersal does not favor cooperation. The reason for this is that although limited dispersal increases the relatedness between interacting individuals, it also leads to increased local competition for resources between relatives. This result supports Taylor’s prediction that in the simplest possible scenario, the effects of increased relatedness and local competition exactly cancel out. In contrast, we show that one way for cooperation to be favored is if individuals disperse in groups (budding dispersal), because this maintains high relatedness while reducing local competition between relatives (relatively global competition). | 2264 | NA | NA | NA | no | no |
| 2265 | 2017 | NA | Lu, XP; Gui, YJ; Xiao, BG; Li, YP; Tong, ZJ; Liu, Y; Bai, XF; Wu, WR; Xia, L; Huttner, E; Kilian, A; Fan, LJ | 2013 | Development of DArT markers for a linkage map of flue-cured tobacco | Tobacco (Nicotiana tabacum) is one of the most economically important nonfood crops, and flue-cured tobacco accounts for approximately 80% of world tobacco production. An extremely narrow genetic diversity in the tobacco pool has led to a low efficiency of PCR-based molecular markers (such as AFLP and SSR). Diversity Arrays Technology (DArT) is a high-throughput hybridisation-based marker system that has been developed in many plants including wheat, which, like tobacco, has a complex genome. In this study, we developed a tobacco DArT chip that included 7680 representative sequence tags based on typical tobacco accessions. The 1076 DArT markers of flue-cured tobacco were identified and most (82.1%) of their polymorphism information contents (PICs) were greater than 0.4. An integrated linkage map that included 851 markers (238 DArT and 613 SSR), which is the highest density map of flue-cured tobacco to date, was constructed. This chip-based DArT system provides an alternative in high-throughput marker genotyping for tobacco. | 2265 | NA | NA | NA | no | no |
| 2266 | 2017 | NA | Wielgoss, S; Taraschewski, H; Meyer, A; Wirth, T | 2008 | Population structure of the parasitic nematode Anguillicola crassus, an invader of declining North Atlantic eel stocks | Probably half of all animal species exhibit a parasitic lifestyle and numerous parasites have recently expanded their distribution and host ranges due to anthropogenic activities. Here, we report on the population genetic structure of the invasive nematode Anguillicola crassus, a parasite in freshwater eels, which recently spread from Asia to Europe and North America. Samples were collected from the newly colonized naive host species Anguilla anguilla (Europe) and Anguilla rostrata (North America), and from indigenous Anguilla japonica in Taiwan and Japan. Using seven microsatellite loci and one mitochondrial marker, we show that the parasite’s population structure in Europe mirrors the zoogeographic Boreal-Lusitanian break along the English Channel. Both the north-to-south decline of nuclear allelic diversity and the loss of private alleles in the same direction are consistent with a significant isolation-by-distance pattern based on rho(ST) values. In combination with the specific topology of the distance tree among nematode populations, our data suggest that Europe was invaded only once from Taiwan, and that subsequently, genetic diversity was lost due to random drift. On the contrary, the North American sample shares distinct nuclear and mitochondrial signatures with Japanese specimens. We propose that the genetic structure in Europe was shaped by long-range anthropogenic eel host transfers in the north and a single dispersal event into the southwest. The genetically distinct Brittany sample at the edge of the Boreal-Lusitanian boundary is indicative of natural dispersal of fish hosts since recruitment occurs naturally there and invertebrate host dissemination is interrupted due to oceanic currents. | 2266 | NA | NA | NA | no | no |
| 2267 | 2017 | NA | Hufeldt, MR; Nielsen, DS; Vogensen, FK; Midtvedt, T; Hansen, AK | 2010 | Family relationship of female breeders reduce the systematic inter-individual variation in the gut microbiota of inbred laboratory mice | The gut microbiota (GM) may influence disease expression in several animal models for inflammatory diseases. It may therefore seem reasonable to pursue reduction in the number of animals used for individual studies by reducing the variation in the GM. Previous studies have shown that the composition of the GM is related to genetics to a certain extent. We hypothesized that the GM similarity in a group of mice born by mothers not being sisters would be lower than that in a group born by mothers being sisters. The lower similarity could lead to clustering of the GM of mice born by non-sisters according to their mothers, while such clustering would not be visible if the mothers were sisters. We used 16S rRNA gene (V3 region) polymerase chain reaction-derived amplicon profiling by denaturing gradient gel electrophoresis (DGGE) to study the GM composition in caecum samples of 33 eight-week-old C57BL/6Sca mice from a breeding set-up with dam breeders that were sisters, as well as caecum samples of 35 eight-week-old C57BL/6Sca mice from a breeding set-up with dam breeders that were not sisters. Principal component analysis revealed a significant difference between the litters from the breeding set-up with dam breeders that were not sisters, whereas no significant difference between the litters based on the breeding set-up with dam breeders that were sisters was observed. The results obtained indicate that the systematic variation in the GM of inbred mice can be reduced by increasing the family relatedness of the breeding pairs. | 2267 | NA | NA | NA | no | no |
| 2268 | 2017 | NA | Harrison, RL; Rowley, DL; Keena, MA | 2016 | Geographic isolates of Lymantria dispar multiple nucleopolyhedrovirus: Genome sequence analysis and pathogenicity against European and Asian gypsy moth strains | Isolates of the baculovirus species Lymantria dispar multiple nucleopolyhedrovirus have been formulated and applied to suppress outbreaks of the gypsy moth, L dispar. To evaluate the genetic diversity in this species at the genomic level, the genomes of three isolates from Massachusetts, USA (LdMNPV-Ab-a624), Spain (LdMNPV-3054), and Japan (LdMNPV-3041) were sequenced and compared with four previously determined LdMNPV genome sequences. The LdMNPV genome sequences were collinear and contained the same homologous repeats (hrs) and clusters of baculovirus repeat orf (bro) gene family members in the same relative positions in their genomes, although sequence identities in these regions were low. Of 146 non-bro ORFs annotated in the genome of the representative isolate LdMNPV 5-6,135 ORFs were found in every other LdMNPV genome, including the 37 core genes of Baculoviridae and other genes conserved in genus Alphabaculovirus. Phylogenetic inference with an alignment of the core gene nucleotide sequences grouped isolates 3041 (Japan) and 2161 (Korea) separately from a cluster containing isolates from Europe, North America, and Russia. To examine phenotypic diversity, bioassays were carried out with a selection of isolates against neonate larvae from three European gypsy moth (Lymantria dispar dispar) and three Asian gypsy moth (Lymantria dispar asiatica and Lymantria dispar japonica) colonies. LdMNPV isolates 2161 (Korea), 3029 (Russia), and 3041 (Japan) exhibited a greater degree of pathogenicity against all L. dispar strains than LdMNPV from a sample of Gypchek. This study provides additional information on the genetic diversity of LdMNPV isolates and their activity against the Asian gypsy moth, a potential invasive pest of North American trees and forests. Published by Elsevier Inc. | 2268 | NA | NA | NA | no | no |
| 2269 | 2017 | NA | Keeney, DB; Waters, JM; Poulin, R | 2007 | Clonal diversity of the marine trematode Maritrema novaezealandensis within intermediate hosts: the molecular ecology of parasite life cycles | We quantified the clonal diversity of the New Zealand marine trematode Maritrema novaezealandensis (n = 1250) within Zeacumantus subcarinatus snail (n = 25) and Macrophthalmus hirtipes crab (n = 25) intermediate hosts using four to six microsatellite loci, and investigated the potential biological and physical factors responsible for the observed genetic patterns. Individual snails harboured one to five trematode genotypes and 48% of snails were infected by multiple parasite genotypes. Overall, the number of parasite genotypes did not increase with snail size, but was highest in intermediate-sized snails. Significantly larger numbers of parasite genotypes were detected in crabs (relative to snails; P < 0.001), with 16-25 genotypes recovered from individual crabs. Although crabs are typically infected by small numbers of cercariae sourced from many snails, they are occasionally infected by large numbers of cercariae sourced from single snails. The latter cases explain the significant genetic differentiation of trematode populations detected among their crab hosts (F(ST) = 0.009, P < 0.001). Our results suggest that the timing of infection and/or intraspecific competition among parasite clones within snails determine(s) the diversity of parasite clones that snails harbour. The presence of a large number of infected snails and tidal mixing of cercariae prior to infection results in crabs potentially harbouring hundreds of parasite genotypes despite the crabs’ territorial behaviour. | 2269 | NA | NA | NA | no | no |
| 2270 | 2017 | NA | Pomeroy, LW; Bjornstad, ON; Holmes, EC | 2008 | The evolutionary and epidemiological dynamics of the paramyxoviridae | Paramyxoviruses are responsible for considerable disease burden in human and wildlife populations: measles and mumps continue to affect the health of children worldwide, while canine distemper virus causes serious morbidity and mortality in a wide range of mammalian species. Although these viruses have been studied extensively at both the epidemiological and the phylogenetic scales, little has been done to integrate these two types of data. Using a Bayesian coalescent approach, we infer the evolutionary and epidemiological dynamics of measles, mumps and canine distemper viruses. Our analysis yielded data on viral substitution rates, the time to common ancestry, and elements of their demographic history. Estimates of rates of evolutionary change were similar to those observed in other RNA viruses, ranging from 6.585 to 11.350 x 10(-4) nucleotide substitutions per site, per year. Strikingly, the mean Time to the Most Recent Common Ancestor (TMRCA) was both similar and very recent among the viruses studied, ranging from only 58 to 91 years (1908 to 1943). Worldwide, the paramyxoviruses studied here have maintained a relatively constant level of genetic diversity. However, detailed heterchronous samples illustrate more complex dynamics in some epidemic populations, and the relatively low levels of genetic diversity (population size) in all three viruses is likely to reflect the population bottlenecks that follow recurrent outbreaks. | 2270 | NA | NA | NA | no | no |
| 2271 | 2017 | NA | Bouyioukos, C; Moscou, MJ; Champouret, N; Hernandez-Pinzon, I; Ward, ER; Wulff, BBH | 2013 | Characterisation and Analysis of the Aegilops sharonensis Transcriptome, a Wild Relative of Wheat in the Sitopsis Section | Aegilops sharonensis Eig (Sharon goatgrass) is a wild diploid relative of wheat within the Sitopsis section of Aegilops. This species represents an untapped reservoir of genetic diversity for traits of agronomic importance, especially as a source of novel disease resistance. To gain a foothold in this genetic resource, we sequenced the cDNA from leaf tissue of two geographically distinct Ae. sharonensis accessions (1644 and 2232) using the 454 Life Sciences platform. We compared the results of two different assembly programs using different parameter sets to generate 13 distinct assemblies in an attempt to maximize representation of the gene space in de novo transcriptome assembly. The most sensitive assembly (71,029 contigs; N50 674 nts) retrieved 18,684 unique best reciprocal BLAST hits (BRBH) against six previously characterised grass proteomes while the most specific assembly (30,609 contigs; N50 815 nts) retrieved 15,687 BRBH. We combined these two assemblies into a set of 62,243 non-redundant sequences and identified 139 belonging to plant disease resistance genes of the nucleotide binding leucine-rich repeat class. Based on the non-redundant sequences, we predicted 37,743 single nucleotide polymorphisms (SNP), equivalent to one per 1,142 bp. We estimated the level of heterozygosity as 1.6% in accession 1644 and 30.1% in 2232. The Ae. sharonensis leaf transcriptome provides a rich source of sequence and SNPs for this wild wheat relative. These sequences can be used with existing monocot genome sequences and EST sequence collections (e.g. barley, Brachypodium, wheat, rice, maize and Sorghum) to assist with genetic and physical mapping and candidate gene identification in Ae. sharonensis. These resources provide an initial framework to further build on and characterise the genetic and genomic structure of Ae. sharonensis. | 2271 | NA | NA | NA | no | no |
| 2272 | 2017 | NA | Ramirez, JL; Ramirez, R | 2013 | Molecular evidence for the polyphyly of Bostryx (Gastropoda: Bulimulidae) and genetic diversity of Bostryx aguilari | Bostryx is largely distributed in Andean Valleys and Lomas formations along the coast of Peru and Chile. One species, Bostryx aguilari, is restricted to Lomas formations located in the Department of Lima (Peru). The use of genetic information has become essential in phylogenetic and population studies with conservation purposes. Considering the rapid degradation of desert ecosystems, which threatens the survival of vulnerable species, the aim of this study was, first, to resolve evolutionary relationships within Bostryx and to determine the position of Bostryx within the Bulimulidae, and second, to survey the genetic diversity of Bostryx aguilari, a species considered rare. Sequences of the mitochondrial 16S rRNA and nuclear rRNA regions were obtained for 12 and 11 species of Bulimulidae, respectively, including seven species of Bostryx. Sequences of the 16S rRNA gene were obtained for 14 individuals (from four different populations) of Bostryx aguilari. Phylogenetic reconstructions were carried out using Neighbor-joining, Maximum Parsimony, Maximum Likelihood and Bayesian Inference methods. The monophyly of Bostryx was not supported. In our results, B. solutus (type species of Bostryx) grouped only with B. aguilari, B. conspersus, B. modestus, B. scalariformis and B. sordidus, forming a monophyletic group that is strongly supported in all analyses. In case the taxonomy of Bostryx is reviewed in the future, this group should keep the generic name. Bostryx aguilari was found to have both low genetic diversity and small population size. We recommend that conservation efforts should be increased in Lomas ecosystems to ensure the survival of B. aguilari, and a large number of other rare species restricted to Lomas. | 2272 | NA | NA | NA | no | no |
| 2273 | 2017 | NA | Veeramah, KR; Thomas, MG; Weale, ME; Zeitlyn, D; Tarekegn, A; Bekele, E; Mendell, NR; Shephard, EA; Bradman, N; Phillips, IR | 2008 | The potentially deleterious functional variant flavin-containing monooxygenase 2\*1 is at high frequency throughout sub-Saharan Africa | Background The drug-metabolizing enzyme flavin-containing monooxygenase 2 (FMO2) is the predominant FMO isoform present in the lung of most mammals, including non-human primates. All Europeans and Asians tested have been shown to be homozygous for a nonfunctional variant, FMO2*2A, which contains a premature stop codon due to a single-nucleotide change in exon 9 (g.23238C > T). The ancestral allele, FMO2*1, encodes a functionally active protein and has been found in African-Americans (26%) and Hispanics (2% to 7%). Possessing this variant increases the risk of pulmonary toxicity when exposed to thioureas, a widely used class of industrial compounds. FMO2 may also be involved in the metabolism of drugs that are used to treat diseases that are prevalent in Africa. Results and Conclusion We conducted a survey of g.23238C > T variation across Africa that revealed that the distribution of this SNP is relatively homogeneous across sub-Saharan Africa, with approximately one third of individuals possessing at least one FMO2\*1 allele, though in some populations the incidence of these individuals approached 50%. Thus many sub-Saharan Africans may be at substantially increased health risk when encountering thiourea-containing substrates of FMO2. Analysis of HapMap data with the Long-Range Haplotype test found no evidence for positive selection of either 23238C > T allele and maximum-likelihood coalescent analysis indicated that this mutation occurred some 500,000 years before present. This study demonstrates the value of performing genetic surveys in Africa, a continent in which human genetic diversity is thought to be greatest, but where studies of the distribution of this diversity are few. | 2273 | NA | NA | NA | no | no |
| 2274 | 2017 | NA | Chen, H; Wang, CL; Conomos, MP; Stilp, AM; Li, ZL; Sofer, T; Szpiro, AA; Chen, W; Brehm, JM; Celedon, JC; Redline, S; Papanicolaou, GJ; Thornton, TA; Laurie, CC; Rice, K; Lin, XH | 2016 | Control for Population Structure and Relatedness for Binary Traits in Genetic Association Studies via Logistic Mixed Models | Linear mixed models (LMMs) are widely used in genome-wide association studies (GWASs) to account for population structure and relatedness, for both continuous and binary traits. Motivated by the failure of LMMs to control type I errors in a GWAS of asthma, a binary trait, we show that LMMs are generally inappropriate for analyzing binary traits when population stratification leads to violation of the LMM’s constant-residual variance assumption. To overcome this problem, we develop a computationally efficient logistic mixed model approach for genome-wide analysis of binary traits, the generalized linear mixed model association test (GMMAT). This approach fits a logistic mixed model once per GWAS and performs score tests under the null hypothesis of no association between a binary trait and individual genetic variants. We show in simulation studies and real data analysis that GMMAT effectively controls for population structure and relatedness when analyzing binary traits in a wide variety of study designs. | 2274 | NA | NA | NA | no | no |
| 2275 | 2017 | NA | Mee, ET; Badhan, A; Karl, JA; Wiseman, RW; Cutler, K; Knapp, LA; Almond, N; O’Connor, DH; Rose, NJ | 2009 | MHC haplotype frequencies in a UK breeding colony of Mauritian cynomolgus macaques mirror those found in a distinct population from the same geographic origin | Background Mauritian cynomolgus macaques have greatly restricted genetic diversity in the MHC region compared to other non-human primates; however, the frequency of common MHC haplotypes among captive-bred populations has not been reported. Methods Microsatellite PCR was used to determine MHC haplotype frequencies among captive macaques at a UK breeding facility. Allele-specific PCR and reference strand conformational analysis were used to determine the allele expression pro. le of a subset of animals. Results Haplotypes H3 (21%) and H1 (19%) were most common in the captive population of Mauritian cynomolgus macaques. Predicted alleles were detected by allele-specific PCR-SSP in 98% of animals. Allele expression profiles were similar in animals with identical haplotypes. Conclusions Mauritian cynomolgus macaques in the UK breeding facility have restricted MHC diversity comparable to a previously described population. Microsatellite-derived haplotypes are highly predictive of allele expression. A selective breeding program has been established to produce MHC-identical animals for biomedical research. | 2275 | NA | NA | NA | no | no |
| 2276 | 2017 | NA | Bueker, B; Eberlein, C; Gladieux, P; Schaefer, A; Snirc, A; Bennett, DJ; Begerow, D; Hood, ME; Giraud, T | 2016 | Distribution and population structure of the anther smut Microbotryum silenes-acaulis parasitizing an arctic-alpine plant | Cold-adapted organisms with current arctic-alpine distributions have persisted during the last glaciation in multiple ice-free refugia, leaving footprints in their population structure that contrast with temperate plants and animals. However, pathogens that live within hosts having arctic-alpine distributions have been little studied. Here, we therefore investigated the geographical range and population structure of a fungus parasitizing an arctic-alpine plant. A total of 1437 herbarium specimens of the plant Silene acaulis were examined, and the anther smut pathogen Microbotryum silenes-acaulis was present throughout the host’s geographical range. There was significantly greater incidence of anther smut disease in more northern latitudes and where the host locations were less dense, indicating a major influence of environmental factors and/or host demographic structure on the pathogen distribution. Genetic analyses with seven microsatellite markers on recent collections of 195 M.silenes-acaulis individuals revealed three main genetic clusters, in North America, northern Europe and southern Europe, likely corresponding to differentiation in distinct refugia during the last glaciation. The lower genetic diversity in northern Europe indicates postglacial recolonization northwards from southern refugia. This study combining herbarium surveys and population genetics thus uniquely reveals the effects of climate and environmental factors on a plant pathogen species with an arctic-alpine distribution. | 2276 | NA | NA | NA | no | no |
| 2277 | 2017 | NA | Dumas, E; Atyame, CM; Malcolm, CA; Le Goff, G; Unal, S; Makoundou, P; Pasteur, N; Weill, M; Duron, O | 2016 | Molecular data reveal a cryptic species within the Culex pipiens mosquito complex | The Culex pipiens mosquito complex is a group of evolutionarily closely related species including C. pipiens and Culex quinquefasciatus, both infected by the cytoplasmically inherited Wolbachia symbiont. A Wolbachia-uninfected population of C. pipiens was however described in South Africa and was recently proposed to represent a cryptic species. In this study, we reconsidered the existence of this species by undertaking an extensive screening for the presence of Wolbachia-uninfected C. pipiens specimens and by characterizing their genetic relatedness with known members of the complex. We first report on the presence of Wolbachia-uninfected specimens in several breeding sites. We next confirm that these uninfected specimens unambiguously belong to the C. pipiens complex. Remarkably, all uninfected specimens harbour mitochondrial haplotypes that are either novel or identical to those previously found in South Africa. In all cases, these mitochondrial haplotypes are closely related, but different, to those found in other C. pipiens complex members known to be infected by Wolbachia. Altogether, these results corroborate the presence of a widespread cryptic species within the C. pipiens species complex. The potential role of this cryptic C. pipiens species in the transmission of pathogens remains however to be determined. The designation ‘Culex juppi nov. sp.’ is proposed for this mosquito species. | 2277 | NA | NA | NA | no | no |
| 2278 | 2017 | NA | Xu, HY; Poh, WT; Sim, XL; Ong, RTH; Suo, C; Tay, WT; Khor, CC; Seielstad, M; Liu, JJ; Aung, T; Tai, ES; Wong, TY; Chia, KS; Teo, YY | 2011 | SgD-CNV, a database for common and rare copy number variants in three Asian populations | Copy number variants (CNVs) extend our understanding of the genetic diversity in humans. However, the distribution and characteristics of CNVs in Asian populations remain largely unexplored, especially for rare CNVs that have emerged as important genetic factors for complex traits. In the present study, we performed an in-depth investigation of common and rare CNVs across 8,148 individuals from the three major Asian ethnic groups: Chinese (n = 1,945), Malays (n = 2,399), and Indians (n = 2,217) in Singapore, making this investigation the most comprehensive genome-wide survey of CNVs outside the European-ancestry populations to date. We detected about 16 CNVs per individual and the ratio of loss to gain events is similar to 2:1. The majority of the CNVs are of low frequency (<10%), and 40% are rare (<1%). In each population, similar to 20% of the CNVs are not previously catalogued in the Database of Genomic Variants (DGV). Contrary to findings from European studies, the common CNVs (>5%) in our populations are not well tagged by SNPs in Illumina 1M and 610K arrays, and most disease-associated common CNVs previously reported in Caucasians are rare in our populations. We also report noticeable population differentiation in the CNV landscape of these Asian populations, with the greatest diversity seen between the Indians and the Chinese. 32:13411349, 2011. (C) 2011 Wiley Periodicals, Inc. | 2278 | NA | NA | NA | no | no |
| 2279 | 2017 | NA | Stumpel, N; Rajabizadeh, M; Avci, A; Wuster, W; Joger, U | 2016 | Phylogeny and diversification of mountain vipers (Montivipera, Nilson et al., 2001) triggered by multiple Plio-Pleistocene refugia and high-mountain topography in the Near and Middle East | The Near and Middle East is a hotspot of biodiversity, but the region remains underexplored at the level of genetic biodiversity. Here, we present an extensive molecular phylogeny of the viperid snake genus Montivipera, including all known taxa. Based on nuclear and mitochondrial data, we present novel insights into the phylogeny of the genus and review the status of its constituent species. Maximum likelihood methods revealed a montane origin of Montivipera at 12.3 Mya. We then analyzed factors of mountain viper diversity. Our data support substantial changes in effective population size through Plio-Pleistocene periods. We conclude that climatic oscillations were drivers of allopatric speciation, and that mountain systems of the Near and Middle East have strongly influenced the evolution and survival of taxa, because climatic and topographical heterogeneities induced by mountains have played a crucial role as filters for dispersal and as multiple refugia. The wide diversity of montane microhabitats enabled mountain vipers to retain their ecological niche during climatic pessima. In consequence the varied geological and topographical conditions between refugia favoured genetic isolation and created patterns of species richness resulting in the formation of neoendemic taxa. Our data support high concordance between geographic distributions of Montivipera haplotypes with putative plant refugia. (C) 2016 Published by Elsevier Inc. | 2279 | NA | NA | NA | no | no |
| 2280 | 2017 | NA | Li, JJ; Liao, XH; Yang, H | 2000 | Molecular characterization of a parasitic tapeworm (Ligula) based on DNA sequences from formalin-fixed specimens | Museum specimens of Ligula (Pseudophyllidea, Ligulidae), a fish parasite tape-worm, that have been preserved in ethanol or fixed permanently in formalin up to 24 years were used for DNA extraction and molecular characterization. DNA was amplified via PCR from samples collected from different fish hosts that lived in both salt and fresh water bodies in the Chinese Qinghai-Tibet Plateau, Russia, and England. Phylogenetic analyses based on partial nucleotide sequences of the 5’-end of nuclear 28S rRNA gene and the mitochondrial cytochrome c oxidase subunit I (COI) gene support the morphologically based taxonomy that groups the Chinese Ligula within the same species as their Europe counterpart: Lingula intestinalis. No nucleotide variation was detected in either the 28S rRNA gene or the COI gene among the seven plerocercoid samples, suggesting a considerable genetic homogeneity among Ligula from different regions. Our results show that geographic isolation, affinity of hosts, and host habitats are not reliable taxonomic criteria for Ligula classification. Our data also indicate a low genetic diversity in the Ligula prevalent DNA from specimens that were subjected to permanent formalin fixation can be routinely amplified from parasitic tape-worms, suggesting that fixation time in formalin may not be a critical factor affecting DNA degradation in such museum specimens. | 2280 | NA | NA | NA | no | no |
| 2281 | 2017 | NA | Lyons, EJ; Amos, W; Berkley, JA; Mwangi, I; Shafi, M; Williams, TN; Newton, CR; Peshu, N; Marsh, K; Scott, JAG; Hill, AVS | 2009 | Homozygosity and risk of childhood death due to invasive bacterial disease | Background: Genetic heterozygosity is increasingly being shown to be a key predictor of fitness in natural populations, both through inbreeding depression, inbred individuals having low heterozygosity, and also through chance linkage between a marker and a gene under balancing selection. One important component of fitness that is often highlighted is resistance to parasites and other pathogens. However, the significance of equivalent loci in human populations remains unclear. Consequently, we performed a case-control study of fatal invasive bacterial disease in Kenyan children using a genome-wide screen with microsatellite markers. Methods: 148 cases, comprising children aged <13 years who died of invasive bacterial disease, (variously, bacteraemia, bacterial meningitis or neonatal sepsis) and 137 age-matched, healthy children were sampled in a prospective study conducted at Kilifi District Hospital, Kenya. Samples were genotyped for 134 microsatellite markers using the ABI LD20 marker set and analysed for an association between homozygosity and mortality. Results: At five markers homozygosity was strongly associated with mortality (odds ratio range 4.7-12.2) with evidence of interactions between some markers. Mortality was associated with different non-overlapping marker groups in Gram positive and Gram negative bacterial disease. Homozygosity at susceptibility markers was common (prevalence 19-49%) and, with the large effect sizes, this suggests that bacterial disease mortality may be strongly genetically determined. Conclusion: Balanced polymorphisms appear to be more widespread in humans than previously appreciated and play a critical role in modulating susceptibility to infectious disease. The effect sizes we report, coupled with the stochasticity of exposure to pathogens suggests that infection and mortality are far from random due to a strong genetic basis. | 2281 | NA | NA | NA | no | no |
| 2282 | 2017 | NA | Persat, H; Mattersdorfer, K; Charlat, S; Schenekar, T; Weiss, S | 2016 | Genetic integrity of the European grayling (Thymallus thymallus) populations within the Vienne River drainage basin after five decades of stockings | “The upper Vienne River is the westernmost drainage in the natural distribution of European grayling Thymallus thymallus (Linnaeus, 1758). The grayling extension across this sub-basin of the Loire catchment has been dramatically reduced through habitat degradation, which began in the 19th century with the construction of milldams and eventually large hydroelectric dams, which flooded former grayling habitat (i.e. fast flowing waters) under increasingly larger reservoirs. Throughout the 20th century, habitat degradation, fragmentation and pollution have strongly affected the remaining isolated populations. Since the 1960s, fishing authorities have attempted to compensate for these declines with stocking programs. Most stocked fish stem from imported eggs or fry of unknown origin. However, the efficacy of this policy has never been assessed. Here, we aim to assess the genetic traces of these stocking programs as an indirect measure of the long-term survival of stocked fish. Sampled grayling from four localities in the Upper Vienne basin were analysed at both mtDNA (N = 37, control region) and nDNA levels (N = 88,10 microsatellites), and compared to populations representative of surrounding drainage basins or fish farms. Microsatellite analysis demonstrated low genetic diversity within samples, typical for the Loire drainage. Both Factorial Correspondence and”“Structure”" analyses presented Upper Vienne grayling as a comprehensive genetic unit distinct from diverse samples from the Rhine, Rhone and Danube basins, as well as other regions of the Loire catchment and the sampled hatcheries. Within the Vienne, only the uppermost sample differed from the other sites. Among the 37 individuals sequenced from the Upper Vienne, only three control region haplotypes were identified, all closely related to other haplotypes observed in Loire and Allier (a Loire tributary) rivers. These results strongly support that grayling in the Upper Vienne drainage represent native genetic stocks with no signs of non-native introgression. The only evidence potentially supporting a stocking contribution is the co-occurrence of a haplotype in the Allier catchment and Vienne River, although we cannot exclude that this haplotype is also native to both sites. These results suggest that five decades of stocking have provided no added reproductive value to the Vienne populations. We thus hypothesize that long-term survival of stocked fish has either been low or non-existent." | 2282 | NA | NA | NA | no | no |
| 2283 | 2017 | NA | Qiu, XP; Zhang, L; Zeng, FY; Wen, Y; Li, C; Qiu, LX; Cheng, DX; Wu, XX | 2014 | Genetic diversity of HLA-DRB1 alleles in the Tujia population of Wufeng, Hubei Province, China | We established a genetic database by investigating human leukocyte antigen (HLA)-DRB1 allelic frequencies in a disease-association study in the Tujia population in Wufang, Hubei, China. The allele frequencies of the HLA-DRB1 locus in 262 healthy, unrelated Tujia individuals living in the Wufeng region of the Hubei Province were analyzed using the Luminex HLA sequence-specific oligonucleotide method with a WAKFlow HLA typing kit. A total of 13 alleles were detected at the HLA-DRB1 locus. HLA-DRB1*09 was the most common allele (22.52%), followed by DRB1*08 and DRB1*15 (11.07%), and DRB1*12 and DRB1\*04 (10.69%). These data were compared with the results obtained for 10 other ethnic groups living in other regions as well as to Han groups using neighbor-joining dendrograms and principal component analysis. The results showed that the Tujia population has a close genetic relationship with the Middle Han population at the HLA-DRB1 locus. This information will be useful for HLA-DRB1-linked disease-association studies. | 2283 | NA | NA | NA | no | no |
| 2284 | 2017 | NA | Gong, YN; Chen, GW; Yang, SL; Lee, CJ; Shih, SR; Tsao, KC | 2016 | A Next-Generation Sequencing Data Analysis Pipeline for Detecting Unknown Pathogens from Mixed Clinical Samples and Revealing Their Genetic Diversity | Forty-two cytopathic effect (CPE)-positive isolates were collected from 2008 to 2012. All isolates could not be identified for known viral pathogens by routine diagnostic assays. They were pooled into 8 groups of 5-6 isolates to reduce the sequencing cost. Next-generation sequencing (NGS) was conducted for each group of mixed samples, and the proposed data analysis pipeline was used to identify viral pathogens in these mixed samples. Polymerase chain reaction (PCR) or enzyme-linked immunosorbent assay (ELISA) was individually conducted for each of these 42 isolates depending on the predicted viral types in each group. Two isolates remained unknown after these tests. Moreover, iteration mapping was implemented for each of these 2 isolates, and predicted human parechovirus (HPeV) in both. In summary, our NGS pipeline detected the following viruses among the 42 isolates: 29 human rhinoviruses (HRVs), 10 HPeVs, 1 human adenovirus (HAdV), 1 echovirus and 1 rotavirus. We then focused on the 10 identified Taiwanese HPeVs because of their reported clinical significance over HRVs. Their genomes were assembled and their genetic diversity was explored. One novel 6-bp deletion was found in one HPeV-1 virus. In terms of nucleotide heterogeneity, 64 genetic variants were detected from these HPeVs using the mapped NGS reads. Most importantly, a recombination event was found between our HPeV-3 and a known HPeV-4 strain in the database. Similar event was detected in the other HPeV-3 strains in the same clade of the phylogenetic tree. These findings demonstrated that the proposed NGS data analysis pipeline identified unknown viruses from the mixed clinical samples, revealed their genetic identity and variants, and characterized their genetic features in terms of viral evolution. | 2284 | NA | NA | NA | no | no |
| 2285 | 2017 | NA | Charoute, H; Bakhchane, A; Benrahma, H; Romdhane, L; Gabi, K; Rouba, H; Fakiri, M; Abdelhak, S; Lenaers, G; Barakat, A | 2015 | Mediterranean Founder Mutation Database (MFMD): Taking Advantage from Founder Mutations in Genetics Diagnosis, Genetic Diversity and Migration History of the Mediterranean Population | The Mediterranean basin has been the theater of migration crossroads followed by settlement of several societies and cultures in prehistoric and historical times, with important consequences on genetic and genomic determinisms. Here, we present the Mediterranean Founder Mutation Database (MFMD), established to offer web-based access to founder mutation information in the Mediterranean population. Mutation data were collected from the literature and other online resources and systematically reviewed and assembled into this database. The information provided for each founder mutation includes DNA change, amino-acid change, mutation type and mutation effect, as well as mutation frequency and coalescence time when available. Currently, the database contains 383 founder mutations found in 210 genes related to 219 diseases. We believe that MFMD will help scientists and physicians to design more rapid and less expensive genetic diagnostic tests. Moreover, the coalescence time of founder mutations gives an overview about the migration history of the Mediterranean population. MFMD can be publicly accessed from . | 2285 | NA | NA | NA | no | no |
| 2286 | 2017 | NA | KESSLING, A; OUELLETTE, S; BOUFFARD, O; CHAMBERLAND, A; BETARD, C; SELINGER, E; XHIGNESSE, M; LUSSIERCACAN, S; DAVIGNON, J | 1992 | PATTERNS OF ASSOCIATION BETWEEN GENETIC-VARIABILITY IN APOLIPOPROTEIN (APO)-B, APO AI-CIII-AIV, AND CHOLESTEROL ESTER TRANSFER PROTEIN GENE REGIONS AND QUANTITATIVE VARIATION IN LIPID AND LIPOPROTEIN TRAITS - INFLUENCE OF GENDER AND EXOGENOUS HORMONES | Patterns of RFLP association were studied, to identify gene regions influencing quantitative variation in lipid and lipoprotein traits (coronary artery disease [CAD] risk factors or metabolically related traits). Subjects (118 female and 229 male; age 20-59 years) were selected for health. Multiple RFLPs were used to sample variability in regions around genes for apolipoprotein (apo) B (restriction enzymes HincII, PvuII, EcoRI, and XbaI), apo AI-CIII-AIV (BamHI, XmnI, TaqI, PstI, SstI, and PvuII) and cholesterol ester transfer protein (TaqI). Separate analyses were done by gender. The sample was truncated at mean +/- 4 SD, to remove extreme outliers. There was no significant gender difference in RFLP genotype frequency distribution. After trait-level adjustment to maximize removal of concomitant variability, analysis of variance was used to estimate the percentage trait phenotypic variance explained by measured variability in the gene regions studied. Fewer gene regions were involved in m en, with less influence on quantitative trait variation than in women, in whom hormone use affected association patterns. Gender differences imply that pooling genders or adjusting data for gender effects removes genetic information and should be avoided. The association patterns show that variability around the candidate genes modulates trait levels: the genes are contributors to the genetics of CAD risk variables in a healthy sample. | 2286 | NA | NA | NA | no | no |
| 2287 | 2017 | NA | Carney, LT; Bohonak, AJ; Edwards, MS; Alberto, F | 2013 | Genetic and experimental evidence for a mixed-age, mixed-origin bank of kelp microscopic stages in southern California | Laboratory studies have demonstrated that the microscopic stages of kelps can rapidly resume development from a delayed state. Like terrestrial seeds or aquatic resting eggs, banks of delayed kelp stages may supplement population recovery after periods of stress, playing an important role for kelp populations that experience adult sporophyte absences due to seasonal or interannual disturbances. We found that removing the microscopic stages from natural rock substratum could prevent the appearance of juvenile kelp sporophytes for three months and the establishment of a diverse kelp assemblage for over four months within a southern California kelp forest. Juveniles were observed within one month in plots where microscopic stages were left intact, which may confer an advantage for the resulting sporophytes as they attain larger sizes before later recruiting neighbors. Microsatellite diversity was high (expected heterozygosity H-E approximate to 0.9) for juveniles and adults within our sites. Using a microsatellite-based parentage analysis for the dominant kelp, Macrocystis pyrifera, we estimated that a portion of the new M. pyrifera sporophyte recruits had originated from their parents at least seven months after their parents had disappeared. Similar delay durations have been demonstrated in recent laboratory studies. Additionally, our results suggest that zoospore dispersal distances >50 m may be supported by including additional microsatellite loci in the analysis. We propose a mixed-age and, potentially, a mixed-origin bank of M. pyrifera gametophytes promotes maximal genetic diversity in recovering populations and reduces population genetic subdivision and self-fertilization rates for intact populations by promoting the survival of zoospores dispersed >10 m and during inhospitable environmental conditions. | 2287 | NA | NA | NA | no | no |
| 2288 | 2017 | NA | Van Laer, L; Huyghe, JR; Hannula, S; Van Eyken, E; Stephan, DA; Maki-Torkko, E; Aikio, P; Fransen, E; Lysholm-Bernacchi, A; Sorri, M; Huentelman, MJ; Van Camp, G | 2010 | A genome-wide association study for age-related hearing impairment in the Saami | This study aimed at contributing to the elucidation of the genetic basis of age-related hearing impairment (ARHI), a common multifactorial disease with an important genetic contribution as demonstrated by heritability studies. We conducted a genome-wide association study (GWAS) in the Finnish Saami, a small, ancient, genetically isolated population without evidence of demographic expansion. The choice of this study population was motivated by its anticipated higher extent of LD, potentially offering a substantial power advantage for association mapping. DNA samples and audiometric measurements were collected from 352 Finnish Saami individuals, aged between 50 and 75 years. To reduce the burden of multiple testing, we applied principal component (PC) analysis to the multivariate audiometric phenotype. The first three PCs captured 80% of the variation in hearing thresholds, while maintaining biologically important audiometric features. All subjects were genotyped with the Affymetrix 100 K chip. To account for multiple levels of relatedness among subjects, as well as for population stratification, association testing was performed using a mixed model. We summarised the top-ranking association signals for the three traits under study. The top-ranked SNP, rs457717 (P-value 3.55x10(-7)), was associated with PC3 and was localised in an intron of the IQ motif-containing GTPase-activating-like protein (IQGAP2). Intriguingly, the SNP rs161927 (P-value 0.000149), seventh-ranked for PC1, was positioned immediately downstream from the metabotropic glutamate receptor-7 gene (GRM7). As a previous GWAS of a European and Finnish sample set already suggested a role for GRM7 in ARHI, this study provides further evidence for the involvement of this gene. European Journal of Human Genetics (2010) 18, 685-693; doi: 10.1038/ejhg.2009.234; published online 13 January 2010 | 2288 | NA | NA | NA | no | no |
| 2289 | 2017 | NA | Schonswetter, P; Popp, M; Brochmann, C | 2006 | Rare arctic-alpine plants of the European Alps have different immigration histories: the snow bed species Minuartia biflora and Ranunculus pygmaeus | Minuartia biflora and Ranunculus pygmaeus are circumarctic plants with a few isolated occurrences in the European Alps. We analysed amplified fragment length polymorphism (AFLP) and chloroplast DNA sequence data to unravel the history of their immigration into the Alps and to provide data on their circumpolar phylogeography. In spite of the similar ecological requirements of the two species, they exhibit strikingly different immigration histories into the Alps. In M. biflora, the Alpine populations are most probably derived from source populations located between the Alpine and Scandinavian ice sheets, in accordance with the traditional biogeographic hypothesis. In contrast, the Alpine populations of R. pygmaeus cluster with those from the Tatra Mountains and the Taymyr region in northern Siberia, indicating that the distant Taymyr area served as source for the Alpine populations. Both species showed different levels of genetic diversity in formerly glaciated areas. In contrast to the considerable AFLP diversity observed in M. biflora, R. pygmaeus was virtually nonvariable over vast areas, with a single phenotype dominating all over the Alps and another, distantly related one dominating the North Atlantic area from Greenland over Svalbard to Scandinavia. The same pattern was observed in chloroplast DNA sequence data. Thus, postglacial colonization of R. pygmaeus was accompanied by extreme founder events. | 2289 | NA | NA | NA | no | no |
| 2290 | 2017 | NA | Griffin, AS; West, SA; Buckling, A | 2004 | Cooperation and competition in pathogenic bacteria | Explaining altruistic cooperation is one of the greatest challenges for evolutionary biology(1-3). One solution to this problem is if costly cooperative behaviours are directed towards relatives(4,5). This idea of kin selection has been hugely influential and applied widely from microorganisms to vertebrates(2-10). However, a problem arises if there is local competition for resources, because this leads to competition between relatives, reducing selection for cooperation(3,11-14). Here we use an experimental evolution approach to test the effect of the scale of competition, and how it interacts with relatedness. The cooperative trait that we examine is the production of siderophores, iron-scavenging agents, in the pathogenic bacterium Pseudomonas aeruginosa(15-17). As expected, our results show that higher levels of cooperative siderophore production evolve in the higher relatedness treatments. However, our results also show that more local competition selects for lower levels of siderophore production and that there is a significant interaction between relatedness and the scale of competition, with relatedness having less effect when the scale of competition is more local. More generally, the scale of competition is likely to be of particular importance for the evolution of cooperation in microorganisms, and also the virulence of pathogenic microorganisms, because cooperative traits such as siderophore production have an important role in determining virulence(6,9,17-19). | 2290 | NA | NA | NA | no | no |
| 2291 | 2017 | NA | Dias, S; Wickramarachchi, T; Sahabandu, I; Escalante, AA; Udagama, PV | 2013 | Population genetic structure of the Plasmodium vivax circumsporozoite protein (Pvcsp) in Sri Lanka | Molecular methods elucidate evolutionary and ecological processes in parasites, where interaction between hosts and parasites enlighten the evolution of parasite lifestyles and host defenses. Population genetics of Plasmodium vivax parasites accurately describe transmission dynamics of the parasites and evaluation of malaria control measures. As a first generation vaccine candidate against malaria, the Circumsporozoite Protein (CSP) has demonstrated significant potential in P. falciparum. Extensive polymorphism hinders the development of a potent malaria vaccine. Hence, the genetic diversity of Pvcsp was investigated for the first time in 60 Sri Lankan clinical isolates by obtaining the nucleotide sequence of the central repeat (CR) domain and examining the polymorphism of the peptide repeat motifs (PRMs), the genetic diversity indices and phylogenetic relationships. PCR amplicons determined size polymorphism of 610, 700 and 710 bp in Pvcsp of Sri Lanka where all amino acid sequences obtained were of the VK210 variant, consisting variable repeats of 4 different PRMs. The two most abundant PRMs of the CR domain, GDRADGQPA and GDRAAGQPA consisted similar to 2-4 repeats, while GNRAAGQPA was unique to the island. Though, different nucleotide sequences termed repeat allotypes (RATs) were observed for each PRM, these were synonymous contributing to a less polymorphic CR domain. The genetic diversity of Pvcsp in Sri Lanka was due to the number of repetitive peptide repeat motifs, point mutations, and intragenic recombination. The 19 amino acid haplotypes defined were exclusive to Sri Lanka, whereas the 194 Pvcsp sequences of global isolates generated 57 more distinct a.a. haplotypes of the VK210 variant. Strikingly, the CR domain of both VK210 and VK247 variants was under purifying selection interpreting the scarcity of CSP non-synonymous polymorphisms. Insights to the distribution of RATs in the CR region with geographic clustering of the P. vivax VI(210 variant were revealed. The cladogram reiterated this unique geographic clustering of local (VK210) and global isolates (VK210 and VK247), which was further validated by the elevated fixation index values of the VK210 variant. (C) 2013 Elsevier B.V. All rights reserved. | 2291 | NA | NA | NA | no | no |
| 2292 | 2017 | NA | Dearlove, B; Wilson, DJ | 2013 | Coalescent inference for infectious disease: meta-analysis of hepatitis C | Genetic analysis of pathogen genomes is a powerful approach to investigating the population dynamics and epidemic history of infectious diseases. However, the theoretical underpinnings of the most widely used, coalescent methods have been questioned, casting doubt on their interpretation. The aim of this study is to develop robust population genetic inference for compartmental models in epidemiology. Using a general approach based on the theory of metapopulations, we derive coalescent models under susceptible-infectious (SI), susceptible-infectious-susceptible (SIS) and susceptible-infectious-recovered (SIR) dynamics. We show that exponential and logistic growth models are equivalent to SI and SIS models, respectively, when co-infection is negligible. Implementing SI, SIS and SIR models in BEAST, we conduct a meta-analysis of hepatitis C epidemics, and show that we can directly estimate the basic reproductive number (R-0) and prevalence under SIR dynamics. We find that differences in genetic diversity between epidemics can be explained by differences in underlying epidemiology (age of the epidemic and local population density) and viral subtype. Model comparison reveals SIR dynamics in three globally restricted epidemics, but most are better fit by the simpler SI dynamics. In summary, metapopulation models provide a general and practical framework for integrating epidemiology and population genetics for the purposes of joint inference. | 2292 | NA | NA | NA | no | no |
| 2293 | 2017 | NA | Grynberg, P; Fontes, CJF; Hughes, AL; Braga, EM | 2008 | Polymorphism at the apical membrane antigen 1 locus reflects the world population history of Plasmodium vivax | Background: In malaria parasites (genus Plasmodium), ama-1 is a highly polymorphic locus encoding the Apical Membrane Protein-1, and there is evidence that the polymorphism at this locus is selectively maintained. We tested the hypothesis that polymorphism at the ama-1 locus reflects population history in Plasmodium vivax, which is believed to have originated in Southeast Asia and is widely geographically distributed. In particular, we tested for a signature of the introduction of P. vivax into the New World at the time of the European conquest and African slave trade and subsequent population expansion. Results: One hundred and five ama-1 sequences were generated and analyzed from samples from six different Brazilian states and compared with database sequences from the Old World. Old World populations of P. vivax showed substantial evidence of population substructure, with high sequence divergence among localities at both synonymous and nonsynonymous sites, while Brazilian isolates showed reduced diversity and little population substructure. Conclusion: These results show that genetic diversity in P. vivax AMA-1 reflects population history, with population substructure characterizing long-established Old World populations, whereas Brazilian populations show evidence of loss of diversity and recent population expansion. Note: Nucleotide sequence data reported is this paper are available in the GenBank T database under the accession numbers EF031154 - EF031216 and EF057446 - EF057487. | 2293 | NA | NA | NA | no | no |
| 2294 | 2017 | NA | Bertin, S; Guglielmino, CR; Karam, N; Gomulski, LM; Malacrida, AR; Gasperi, G | 2007 | Diffusion of the Nearctic leafhopper Scaphoideus titanus Ball in Europe: a consequence of human trading activity | Scaphoideus titanus Ball is a Nearctic leafhopper that was introduced for the first time in Europe probably at the beginning of the 20th century. In Europe, this species is a specialist on cultivated grapevines and is of great economic importance as the vector of Flavescence doree (FD), a Grapevine Yellows disease caused by Candidatus Phytoplasma vitis. The Random Amplified Polymorphic DNA (RAPD) technique was employed to obtain genetic information about the diffusion and the structure of S. titanus populations. Two American and 14 European populations were analysed. A total of 188 reproducible bands, obtained from three arbitrary primers, were considered to assess the amount and the pattern of genetic variation within and among leafhopper populations. American populations showed high levels of intra-population polymorphism and dissimilarity and appeared to be the most isolated of all the tested samples. The results confirm the historical role of American samples as the sources for the more recently founded European populations. RAPD analyses revealed a weak genetic structure of European samples that could probably be explained invoking the human role in their diffusion. The non-natural spreading of S. titanus across Europe is in fact attributable to the exchange of grapevine canes and grafts carrying eggs that the insect laid under the bark to overwinter. | 2294 | NA | NA | NA | no | no |
| 2295 | 2017 | NA | Damm, S; Hadrys, H | 2012 | A dragonfly in the desert: genetic pathways of the widespread Trithemis arteriosa (Odonata: Libellulidae) suggest male-biased dispersal | Water-dependent species inhabiting desert regions seem to be a contradiction in terms. Nevertheless, many species have evolved survival strategies for arid conditions. In Odonates (dragonflies and damselflies), both larvae and adults require very different and complex water-associated habitat conditions. The present study investigates the genetic diversity, population structure and dispersal patterns of a desert inhabiting odonate species, the Red-veined Dropwing dragonfly, Trithemis arteriosa. Eight populations from the arid Namibia and four population sites in the more tropical Kenya were compared by using nine microsatellite loci, one non-coding nuclear fragment and the mtDNA fragment ND1. Microsatellite analyses as well as the nuclear fragment reveal a high allelic diversity in all populations with almost no genetic sub-structuring. In contrast, ND1 sequence analyses show sub-structuring and-with two exceptions-only private haplotypes. The conflicting patterns of nuclear versus mitochondrial markers suggest a male-biased dispersal in this species. Results indicate that male dispersal is dependent on the environmental stability of the habitat, while females are philopatric. This life history adaptation would allow females to save energy for mating and oviposition in the demanding environment of a desert region. The results give direct insights into the dispersal pathways of a desert-inhabiting, strongly water dependent flying insect. | 2295 | NA | NA | NA | no | no |
| 2296 | 2017 | NA | Buhler, S; Nunes, JM; Sanchez-Mazas, A | 2016 | HLA class I molecular variation and peptide-binding properties suggest a model of joint divergent asymmetric selection | The main function of HLA class I molecules is to present pathogen-derived peptides to cytotoxic T lymphocytes. This function is assumed to drive the maintenance of an extraordinary amount of polymorphism at each HLA locus, providing an immune advantage to heterozygote individuals capable to present larger repertories of peptides than homozygotes. This seems contradictory, however, with a reduced diversity at individual HLA loci exhibited by some isolated populations. This study shows that the level of functional diversity predicted for the two HLA-A and HLA-B genes considered simultaneously is similar (almost invariant) between 46 human populations, even when a reduced diversity exists at each locus. We thus propose that HLA-A and HLA-B evolved through a model of joint divergent asymmetric selection conferring all populations an equivalent immune potential. The distinct pattern observed for HLA-C is explained by its functional evolution towards killer cell immunoglobulin-like receptor (KIR) activity regulation rather than peptide presentation. | 2296 | NA | NA | NA | no | no |
| 2297 | 2017 | NA | Madeira, PM; Chefaoui, RM; Cunha, RL; Moreira, F; Dias, S; Calado, G; Castilho, R | 2017 | High unexpected genetic diversity of a narrow endemic terrestrial mollusc | The Iberian Peninsula has an extensive record of species displaying strong genetic structure as a result of their survival in isolated pockets throughout the Pleistocene ice ages. We used mitochondrial and nuclear sequence data to analyze phylogeographic patterns in endemic land snails from a valley of central Portugal (Vale da Couda), putatively assigned to Candidula coudensis, that show an exceptionally narrow distributional range. The genetic survey presented here shows the existence of five main mitochondrial lineages in Vale da Couda that do not cluster together suggesting independent evolutionary histories. Our results also indicate a departure from the expectation that species with restricted distributions have low genetic variability. The putative past and contemporary models of geographic distribution of Vale da Couda lineages are compatible with a scenario of species co-existence in more southern locations during the last glacial maximum (LGM) followed by a post-LGM northern dispersal tracking the species optimal thermal, humidity and soil physical conditions. | 2297 | NA | NA | NA | no | no |
| 2298 | 2017 | NA | Kang, HM; Sul, JH; Service, SK; Zaitlen, NA; Kong, SY; Freimer, NB; Sabatti, C; Eskin, E | 2010 | Variance component model to account for sample structure in genome-wide association studies | Although genome-wide association studies (GWASs) have identified numerous loci associated with complex traits, imprecise modeling of the genetic relatedness within study samples may cause substantial inflation of test statistics and possibly spurious associations. Variance component approaches, such as efficient mixed-model association (EMMA), can correct for a wide range of sample structures by explicitly accounting for pairwise relatedness between individuals, using high-density markers to model the phenotype distribution; but such approaches are computationally impractical. We report here a variance component approach implemented in publicly available software, EMMA eXpedited (EMMAX), that reduces the computational time for analyzing large GWAS data sets from years to hours. We apply this method to two human GWAS data sets, performing association analysis for ten quantitative traits from the Northern Finland Birth Cohort and seven common diseases from the Wellcome Trust Case Control Consortium. We find that EMMAX outperforms both principal component analysis and genomic control in correcting for sample structure. | 2298 | NA | NA | NA | no | no |
| 2299 | 2017 | NA | Klappert, K; Butlin, RK; Reinhold, K | 2007 | The attractiveness fragment - AFLP analysis of local adaptation and sexual selection in a caeliferan grasshopper, Chorthippus biguttulus | Genetic variability among males is a necessary precondition for the evolution of female choice based on indirect genetic benefits. In addition to mutations and host-parasite cycles, migration of locally adapted individuals offers an explanation for the maintenance of genetic variability. In a previous study, conducting a reciprocal transplant experiment on a grasshopper, Chorthippus biguttulus, we found that environmental conditions significantly influenced not only body condition but also an important trait of male calling song, the amplitude of song. Although not significant, all other analysed physical and courtship song traits and attractiveness were superior in native than in transferred males. Thus, we concluded that local adaptation has a slight but consistent influence on a range of traits in our study populations, including male acoustic attractiveness. In our present study, we scanned male grasshoppers from the same two populations for amplification fragment length polymorphism (AFLP) loci connected with acoustic attractiveness to conspecific females. We found greater differences in allele frequencies between the two populations, for some loci, than are expected from a balance between drift and gene flow. These loci are potentially connected with locally adapted traits. We examined whether these alleles show the proposed genotype environment interaction by having different associations with attractiveness in the two populations. One locus was significantly related to sexual attractiveness; however, this was independent of the males’ population affiliation. Future research on the evolution of female choice will benefit from knowledge of the underlying genetic architecture of male traits under intraspecific sexual selection, and the ‘population genomics’ approach can be a powerful tool for revealing this structure. | 2299 | NA | NA | NA | no | no |
| 2300 | 2017 | NA | Rivas, GG; Zapater, MF; Abadie, C; Carlier, J | 2004 | Founder effects and stochastic dispersal at the continental scale of the fungal pathogen of bananas Mycosphaerella fijiensis | The worldwide destructive epidemic of the fungus Mycosphaerella fijiensis on banana started recently, spreading from South-East Asia. The founder effects detected in the global population structure of M. fijiensis reflected rare migration events among continents through movements of infected plant material. The main objective of this work was to infer gene flow and dispersal processes of M. fijiensis at the continental scale from population structure analysis in recently invaded regions. Samples of isolates were collected from banana plantations in 13 countries in Latin America and the Caribbean and in Africa. The isolates were analysed using polymerase chain reaction-restriction fragment length polymorphism (PCR-RFLP) and microsatellite molecular markers. The results indicate that a high level of genetic diversity was maintained at the plantation and the plant scales. The loci were at gametic equilibrium in most of the samples analysed, supporting the hypothesis of the existence of random-mating populations of M. fijiensis, even at the plant scale. A low level of gene diversity was observed in some populations from the Africa and Latin America-Caribbean regions. Nearly half the populations analysed showed a significant deviation from mutation-drift equilibrium with gene diversity excess. Finally, a high level of genetic differentiation was detected between populations from Africa (F-ST = 0.19) and from the Latin America-Caribbean region (F-ST = 0.30). These results show that founder effects accompanied the recent invasion of M. fijiensis in both regions, suggesting stochastic spread of the disease at the continental scale. This spread might be caused by either the limited dispersal of ascospores or by movements of infected plant material. | 2300 | NA | NA | NA | no | no |
| 2301 | 2017 | NA | de la Mata, R; Hood, S; Sala, A | 2017 | Insect outbreak shifts the direction of selection from fast to slow growth rates in the long-lived conifer Pinus ponderosa | Long generation times limit species’ rapid evolution to changing environments. Trees provide critical global ecosystem services, but are under increasing risk of mortality because of climate change-mediated disturbances, such as insect outbreaks. The extent to which disturbance changes the dynamics and strength of selection is unknown, but has important implications on the evolutionary potential of tree populations. Using a 40-y-old Pinus ponderosa genetic experiment, we provide rare evidence of context-dependent fluctuating selection on growth rates over time in a long-lived species. Fast growth was selected at juvenile stages, whereas slow growth was selected at mature stages under strong herbivory caused by a mountain pine beetle (Dendroctonus ponderosae) outbreak. Such opposing forces led to no net evolutionary response over time, thus providing a mechanism for the maintenance of genetic diversity on growth rates. Greater survival to mountain pine beetle attack in slow-growing families reflected, in part, a host-based life-history trade-off. Contrary to expectations, genetic effects on tree survival were greatest at the peak of the outbreak and pointed to complex defense responses. Our results suggest that selection forces in tree populations may be more relevant than previously thought, and have implications for tree population responses to future environments and for tree breeding programs. | 2301 | NA | NA | NA | no | no |
| 2302 | 2017 | NA | Lin, DY; Tao, R; Kalsbeek, WD; Zeng, DL; Gonzalez, F; Fernandez-Rhodes, L; Graff, M; Koch, GG; North, KE; Heiss, G | 2014 | Genetic Association Analysis under Complex Survey Sampling: The Hispanic Community Health Study/Study of Latinos | The cohort design allows investigators to explore the genetic basis of a variety of diseases and traits in a single study while avoiding major weaknesses of the case-control design. Most cohort studies employ multistage cluster sampling with unequal probabilities to conveniently select participants with desired characteristics, and participants from different clusters might be genetically related. Analysis that ignores the complex sampling design can yield biased estimation of the genetic association and inflation of the type I error. Herein, we develop weighted estimators that reflect unequal selection probabilities and differential nonresponse rates, and we derive variance estimators that properly account for the sampling design and the potential relatedness of participants in different sampling units. We compare, both analytically and numerically, the performance of the proposed weighted estimators with unweighted estimators that disregard the sampling design. We demonstrate the usefulness of the proposed methods through analysis of MetaboChip data in the Hispanic Community Health Study/Study of Latinos, which is the largest health study of the Hispanic/Latino population in the United States aimed at identifying risk factors for various diseases and determining the role of genes and environment in the occurrence of diseases. We provide guidelines on the use of weighted and unweighted estimators, as well as the relevant software. | 2302 | NA | NA | NA | no | no |
| 2303 | 2017 | NA | Horne, JB; Bradbury, IR; Paterson, IG; Hardie, D; Hutchings, J; Laurel, BJ; Snelgrove, PVR; Morris, CJ; Gregory, RS; Bentzen, P | 2016 | Complex post-larval dispersal processes in Atlantic cod revealed by age-based genetics and relatedness analysis | Population connectivity among adult marine organisms is often attributed to dispersal during the egg/larval stage. However, post-larval dispersal may also influence connectivity, particularly when juvenile nursery habitats are separated from adult spawning habitats. Here we used age-based population genetics and kinship analysis to explore changes in population connectivity across life-history stages in Atlantic cod Gadus morhua. We genotyped 364 adult cod from the northwest Atlantic and 671 age-0 juveniles from 18 sites around eastern Newfoundland, with 72 and 15 microsatellite loci, respectively. Adult cod genotypes exhibited more population structure than was detected in juveniles across similar spatial scales. Both age classes had similar allelic diversities, but juveniles exhibited less genetic linkage and fewer departures from Hardy-Weinberg expectations than adults at the same loci. We detected significant kinship relationships in adult cod only within sampling locations, but 1 putative pair of juvenile kin was separated by >500 km. Collections of adults also displayed higher group relatedness compared to juveniles. Genetic differences between age classes are likely due to a combination of non-random mortality and non-random sorting of admixed juveniles into different adult habitats. Many studies overlook post-larval dispersal as a factor of marine population connectivity, but pre-adult relocation may be demographically and adaptively significant, in cod and other species. | 2303 | NA | NA | NA | no | no |
| 2304 | 2017 | NA | Brar, S; Tsui, CKM; Dhillon, B; Bergeron, MJ; Joly, DL; Zambino, PJ; El-Kassaby, YA; Hamelin, RC | 2015 | Colonization History, Host Distribution, Anthropogenic Influence and Landscape Features Shape Populations of White Pine Blister Rust, an Invasive Alien Tree Pathogen | White pine blister rust is caused by the fungal pathogen Cronartium ribicola J.C. Fisch (Basidiomycota, Pucciniales). This invasive alien pathogen was introduced into North America at the beginning of the 20th century on pine seedlings imported from Europe and has caused serious economic and ecological impacts. In this study, we applied a population and landscape genetics approach to understand the patterns of introduction and colonization as well as population structure and migration of C. ribicola. We characterized 1,292 samples of C. ribicola from 66 geographic locations in North America using single nucleotide polymorphisms (SNPs) and evaluated the effect of landscape features, host distribution, and colonization history on the structure of these pathogen populations. We identified eastern and western genetic populations in North America that are strongly differentiated. Genetic diversity is two to five times higher in eastern populations than in western ones, which can be explained by the repeated accidental introductions of the pathogen into northeastern North America compared with a single documented introduction into western North America. These distinct genetic populations are maintained by a barrier to gene flow that corresponds to a region where host connectivity is interrupted. Furthermore, additional cryptic spatial differentiation was identified in western populations. This differentiation corresponds to landscape features, such as mountain ranges, and also to host connectivity. We also detected genetic differentiation between the pathogen populations in natural stands and plantations, an indication that anthropogenic movement of this pathogen still takes place. These results highlight the importance of monitoring this invasive alien tree pathogen to prevent admixture of eastern and western populations where different pathogen races occur. | 2304 | NA | NA | NA | no | no |
| 2305 | 2017 | NA | Shinwari, JMA; Al Yemni, EAA; Alnaemi, FM; Abebe, D; Al-Abdulaziz, BS; Al Mubarak, BR; Ghaziuddin, M; Al Tassan, NA | 2017 | Analysis of shared homozygosity regions in Saudi siblings with attention deficit hyperactivity disorder | AimGenetic and clinical complexities are common features of most psychiatric illnesses that pose a major obstacle in risk-gene identification. Attention deficit hyperactivity disorder (ADHD) is the most prevalent child-onset psychiatric illness, with high heritability. Over the past decade, numerous genetic studies utilizing various approaches, such as genome-wide association, candidate-gene association, and linkage analysis, have identified a multitude of candidate loci/genes. However, such studies have yielded diverse findings that are rarely reproduced, indicating that other genetic determinants have not been discovered yet. In this study, we carried out sib-pair analysis on seven multiplex families with ADHD from Saudi Arabia. We aimed to identify the candidate chromosomal regions and genes linked to the disease.Patients and methodsA total of 41 individuals from multiplex families were analyzed for shared regions of homozygosity. Genes within these regions were prioritized according to their potential relevance to ADHD.ResultsWe identified multiple genomic regions spanning different chromosomes to be shared among affected members of each family; these included chromosomes 3, 5, 6, 7, 8, 9, 10, 13, 17, and 18. We also found specific regions on chromosomes 8 and 17 to be shared between affected individuals from more than one family. Among the genes present in the regions reported here were involved in neurotransmission (GRM3, SIGMAR1, CHAT, and SLC18A3) and members of the HLA gene family (HLA-A, HLA-DPA1, and MICC).ConclusionThe candidate regions identified in this study highlight the genetic diversity of ADHD. Upon further investigation, these loci may reveal candidate genes that enclose variants associated with ADHD. Although most ADHD studies were conducted in other populations, our study provides insight from an understudied, ethnically interesting population. | 2305 | NA | NA | NA | no | no |
| 2306 | 2017 | NA | Perron, GG; Quessy, S; Bell, G | 2008 | A Reservoir of Drug-Resistant Pathogenic Bacteria in Asymptomatic Hosts | The population genetics of pathogenic bacteria has been intensively studied in order to understand the spread of disease and the evolution of virulence and drug resistance. However, much less attention has been paid to bacterial carriage populations, which inhabit hosts without producing disease. Since new virulent strains that cause disease can be recruited from the carriage population of bacteria, our understanding of infectious disease is seriously incomplete without knowledge on the population structure of pathogenic bacteria living in an asymptomatic host. We report the first extensive survey of the abundance and diversity of a human pathogen in asymptomatic animal hosts. We have found that asymptomatic swine from livestock productions frequently carry populations of Salmonella enterica with a broad range of drug-resistant strains and genetic diversity greatly exceeding that previously described. This study shows how agricultural practice and human intervention may lead and influence the evolution of a hidden reservoir of pathogens, with important implications for human health. | 2306 | NA | NA | NA | no | no |
| 2307 | 2017 | NA | Charlesworth, B | 2001 | The effect of life-history and mode of inheritance on neutral genetic variability | Formulae for the effective population sizes of autosomal. X-linked, Y-linked and maternally transmitted loci in age-structured populations are developed. The approximations used here predict both asymptotic rates of increase in probabilities of identity, and equilibrium levels of neutral nucleotide site diversity under the infinite-sites model. The applications of the results to the interpretation of data on DNA sequence variation in Drosophila, plant, and human populations are discussed. It is concluded that sex differences in demographic parameters such as adult mortality rates generally have small effects on the relative effective population sizes of loci with different modes of inheritance, whereas differences between the sexes in variance in reproductive success can have major effects, either increasing or reducing the effective population size for X-linked loci relative to autosomal or Y-linked loci. These effects need to be accounted for when trying to understand data on patterns of sequence variation for genes with different transmission modes. | 2307 | NA | NA | NA | no | no |
| 2308 | 2017 | NA | Hu, BJ; Xu, LN; Zhou, ZY; Hu, F; Luan, FG; Chen, X; Li, ZZ | 2015 | Molecular tracing of white muscardine in Asian corn borer using inter-simple sequence repeat (ISSR) analysis | Beauveria bassiana is a soil fungus that parasitizes arthropod species, and is used to control the Asian corn borer in Northeast China. In this study, B. bassiana was investigated in Xiaoxian County and Baicheng City, and the results were compared with those of Gongzhuling City, where the fungus was not applied. Using the inter-simple sequence repeat (ISSR) molecular marker technique, 198 isolates were extracted from Asian corn borer and other insect cadavers, and soil and air, and two released strains were analyzed to trace the infection source. In Xiaoxian and Baicheng populations, artificially released B. bassiana subpopulations were more abundant than indigenous fungi, and the released strains were the main cause of disease in those areas. Artificial B. bassiana displayed positive effect on overwintering of Asian corn borers in corn straw stacks in Xiaoxian County. Indigenous populations in Gongzhuling City showed higher genetic variation. In summary, we identified a significant correlation between genetic distance and geographic distance (P < 0.01). | 2308 | NA | NA | NA | no | no |
| 2309 | 2017 | NA | Cameron, EZ; Setsaas, TH; Linklater, WL | 2009 | Social bonds between unrelated females increase reproductive success in feral horses | In many mammals, females form close social bonds with members of their group, usually between kin. Studies of social bonds and their fitness benefits have not been investigated outside primates, and are confounded by the relatedness between individuals in primate groups. Bonds may arise from kin selection and inclusive fitness rather than through direct benefits of association. However, female equids live in long-term social groups with unrelated members. We present 4 years of behavioral data, which demonstrate that social integration between unrelated females increases both foal birth rates and survival, independent of maternal habitat quality, social group type, dominance status, and age. Also, we show that such social integration reduces harassment by males. Consequently, social integration has strong direct fitness consequences between nonrelatives, suggesting that social bonds can evolve based on these direct benefits alone. Our results support recent studies highlighting the importance of direct benefits in maintaining cooperative behavior, while controlling for the confounding influence of kinship. | 2309 | NA | NA | NA | no | no |
| 2310 | 2017 | NA | Vasilyeva, LA; Ratner, VA; Antonenko, OV; Lopukhova, ED; Bubenshchikova, EV | 2003 | Induction of MGE 412 transposition in an isogenic strain of Drosophila melanogaster by different doses of ethanol fumes | “The effect of treatment of males from an isogenic Drosophila melanogaster strain by limiting doses of ethanol fumes on transpositions of MGE 412 was examined. Validity of the phenomenon of transposition induction was demonstrated. We estimated rates of induced transposition (similar to10(-2) events per site, per sperm, per generation versus <10(-3) in control) and showed dose dependence of the rate on the exposure time of the males to ethanol fumes. Experiments with alcohol treatment at limiting doses must end either in death of the individuals or bursts of genetic variability in their progeny. In terms of genetics of an individual, this may mean loss of vital hereditary basis followed by mass degradation of the progeny of the”“hard drinkers.”" In terms of populations genetics, this mode of MGE transposition induction can rapidly create a burst of novel genetic variation, which, apart of great losses, may generate a number of advantageous individuals, i.e., be significant for population survival in new, stressful environments." | 2310 | NA | NA | NA | no | no |
| 2311 | 2017 | NA | Kirchner, JW; Roy, BA | 2000 | Evolutionary implications of host-pathogen specificity: the fitness consequences of host life history traits | Pathogens and parasites can be strong agents of selection, and often exhibit some degree of genetic specificity for individual host strains. Here we show that this host-pathogen specificity can affect the evolution of host life history traits. All else equal, evolution should select for genes that increase individuals’ reproduction rates or lifespans (and thus total reproduction per individual). Using a simple host-pathogen model, we show that when the genetic specificity of pathogen infection is low, host strains with higher reproduction rates or longer lifespans drive slower-reproducing or shorter-lived host strains to extinction, as one would expect. However, when pathogens exhibit specificity for host strains with different life history traits, the evolutionary advantages of these traits can be greatly diminished by pathogen-mediated selection. Given sufficient host-pathogen specificity, pathogen-mediated selection can maintain polymorphism in host traits that are correlated with pathogen resistance traits, despite large intrinsic fitness differences among host strains. These results have two important implications. First, selection on host life history traits will be weaker than expected, whenever host fitness is significantly affected by genotype-specific pathogen attack. Second, where polymorphism in host traits is maintained by pathogen-mediated selection, preserving the genetic diversity of host species may require preserving their pathogens as well. | 2311 | NA | NA | NA | no | no |
| 2312 | 2017 | NA | Mahamdallie, SS; Ready, PD | 2012 | No recent adaptive selection on the apyrase of Mediterranean Phlebotomus: implications for using salivary peptides to vaccinate against canine leishmaniasis | Vaccine development is informed by a knowledge of genetic variation among antigen alleles, especially the distribution of positive and balancing selection in populations and species. A combined approach using population genetic and phylogenetic methods to detect selective signatures can therefore be informative for identifying vaccine candidates. Parasitic Leishmania species cause the disease leishmaniasis in humans and mammalian reservoir hosts after inoculation by female phlebotomine sandflies. Like other arthropod vectors of disease agents, sandflies use salivary peptides to counteract host haemostatic and immunomodulatory responses during bloodfeeding, and these peptides are vaccine candidates because they can protect against Leishmania infection. We detected no contemporary adaptive selection on one salivary peptide, apyrase, in 20 populations of Phlebotomus ariasi, a European vector of Leishmania infantum. Maximum likelihood branch models on a gene phylogeny showed apyrase to be a single copy in P. ariasi but an ancient duplication event associated with temporary positive selection was observed in its sister group, which contains most Mediterranean vectors of L. infantum. The absence of contemporary adaptive selection on the apyrase of P. ariasi may result from this sandflys opportunistic feeding behaviour. Our study illustrates how the molecular population genetics of arthropods can help investigate the potential of salivary peptides for disease control and for understanding geographical variation in vector competence. | 2312 | NA | NA | NA | no | no |
| 2313 | 2017 | NA | Aujoulat, F; Romano-Bertrand, S; Masnou, A; Marchandin, H; Jumas-Bilak, E | 2014 | Niches, Population Structure and Genome Reduction in Ochrobactrum intermedium: Clues to Technology-Driven Emergence of Pathogens | Ochrobactrum intermedium is considered as an emerging human environmental opportunistic pathogen with mild virulence. The distribution of isolates and sequences described in literature and databases showed frequent association with human beings and polluted environments. As population structures are related to bacterial lifestyles, we investigated by multi-locus approach the genetic structure of a population of 65 isolates representative of the known natural distribution of O. intermedium. The population was further surveyed for genome dynamics using pulsed-field gel electrophoresis and genomics. The population displayed a clonal epidemic structure with events of recombination that occurred mainly in clonal complexes. Concerning biogeography, clones were shared by human and environments and were both cosmopolitan and local. The main cosmopolitan clone was genetically and genomically stable, and grouped isolates that all harbored an atypical insertion in the rrs. Ubiquitism and stability of this major clone suggested a clonal succes in a particular niche. Events of genomic reduction were detected in the population and the deleted genomic content was described for one isolate. O. intermedium displayed allopatric characters associated to a tendancy of genome reduction suggesting a specialization process. Considering its relatedness with Brucella, this specialization might be a commitment toward pathogenic life-style that could be driven by technological selective pressure related medical and industrial technologies. | 2313 | NA | NA | NA | no | no |
| 2314 | 2017 | NA | Prosdocimi, L; Dutton, PH; Albareda, D; Remis, MI | 2014 | Origin and genetic diversity of leatherbacks (Dermochelys coriacea) at Argentine foraging grounds | To conduct conservation of migratory species, such as marine turtles, is important to understand the population structure throughout the entire distribution of the species. We study the genetic composition of the leatherback turtles, Dermochelys coriacea foraging in waters off Argentina by analyzing 763 bp sequences of the mtDNA control region in order to determine the nesting origin of these animals. A total of 40 samples were collected from adult leatherbacks (mean 143.5; 180-123 cm curved carapace length) captured (10%) in fisheries or encountered as strandings (90%). Based on analysis of mtDNA sequences we detected 4 haplotypes, the most common (n = 26) being DC1.1, and the other two rarer DC1.3 (n = 4), DC13.1 (n = 2), and DC1A (n = 1). The genetic diversity was evaluated through the haplotype (0,3712 +/- 0, 1000) and nucleotide diversities (0, 000521 +/- 0, 000553). Bayesian Mixed Stock Analysis (MSA) showed that the Buenos Aires foraging leatherbacks come primarily from the West African rookeries (Ghana and Gabon, mean estimate = 69% and 14% respectively). MSA results are consistent with those from mark-recapture studies, since four leatherbacks captured in Argentinean waters were adult females that were originally tagged on the nesting beaches in Gabon, West Africa. Our findings demonstrate the connection between nesting and foraging areas in the South Atlantic and illustrate the importance of the Malvinas ecoregion to the survival of migratory marine vertebrates, such as leatherbacks. (C) 2014 Elsevier B.V. All rights reserved. | 2314 | NA | NA | NA | no | no |
| 2315 | 2017 | NA | Lopez-Lavalle, LAB; Matheson, B; Brubaker, CL | 2011 | A genetic map of an Australian wild Gossypium C genome and assignment of homoeologies with tetraploid cultivated cotton | Genetic diversity for traits such as fibre quality or disease resistance to microorganisms is limited in the elite cotton germplasm; consequently, cotton breeders are looking for novel alleles in the secondary or even in the tertiary gene pools. The wild Australian Gossypium species (tertiary gene pool) represent an alternative source of novel alleles. However, to use these species efficiently, enabling tools are required. Chromosome-specific molecular markers are particularly useful tools to track the transmission of this exotic genetic material into the cultivated cotton during introgression. In this study, we report the construction of a genetic linkage map of the Australian wild C-genome species Gossypium sturtianum. The map, based on an F(2) population of 114 individuals, contains 291 AFLP loci. The map spans 1697 cM with an average distance of 5.8 cM between markers. To associate C-genome chromosomes with the A and D subgenomes of cultivated cotton, 29 SSR and RFLP-STS markers were assigned to chromosomes using cultivated cotton mapped marker information. Polymorphisms were revealed by 51 AFLP primer combinations and 38 RFLP-STS and 115 SSR cotton mapped markers. The utility of transferring RFLP-STS and SSR cotton mapped markers to other Gossypium species shows the usefulness of a comparative approach as a source of markers and for aligning the genetic map of G. sturtianum with the cultivated species in the future. This also indicates that the overall structure of the G. sturtianum linkage groups is similar to that of the A and D subgenomes of cotton at the gross structural level. Applications of the map for the Australia wild C-genome species and cotton breeding are discussed. | 2315 | NA | NA | NA | no | no |
| 2316 | 2017 | NA | Chakrabarti, S; Kambhampati, S; Zurek, L | 2010 | Assessment of House Fly Dispersal between Rural and Urban Habitats in Kansas, USA | House flies, Musca domestica L. (Diptera: Muscidae), are of medical and veterinary importance due to their capacity to act as mechanical vectors of microorganisms originating in animal manure and other decaying organic substrates. House flies that disperse from rural to urban areas may also transport antibiotic resistant bacterial strains. To assess the potential of house flies to disperse from rural to urban areas and distribute antibiotic-resistant bacteria, we undertook a two part study: (i) we quantified the dispersal rate of house flies from farms (rural areas) into a city (urban area) using multilocus DNA fingerprinting and (ii) we profiled the antibiotic resistance patterns of enterococci harbored by house flies collected in rural and urban environments. The population genetic analysis indicated that there was considerable dispersal between rural and urban habitats. Although there was a significant difference in allele frequency between the urban and rural samples, genetic divergence was low (mean F(ST) = 0.07) and migration rate relatively high (N(m) = 3 individuals per generation). Almost 95% of the genetic diversity occurred within populations, suggesting a nearly panmictic population. Profiling of antibiotic resistance of enterococci isolated from house fly guts showed that house flies collected in all five urban sites carried substantial numbers of antibiotic-resistant enterococci, supporting the results of the population genetic analyses. The results of this study imply that house flies, because of their dispersal behavior and capacity to transport antibiotic-resistant bacteria, pose a serious threat to public health. We suggest that area wide management of house fly populations is therefore likely to be the most effective strategy for minimizing public health risks. | 2316 | NA | NA | NA | no | no |
| 2317 | 2017 | NA | Caniglia, R; Fabbri, E; Galaverni, M; Milanesi, P; Randi, E | 2014 | Noninvasive sampling and genetic variability, pack structure, and dynamics in an expanding wolf population | After centuries of population decline and range contraction, gray wolves (Canis lupus) are now expanding in Europe. Understanding wolf social structure and population dynamics and predicting their future range expansion is mandatory to design sound conservation strategies, but field monitoring methods are difficult or exceedingly expensive. Noninvasive genetic sampling offers unique opportunities for the reliable monitoring of wolf populations. We conducted a 9-year-long monitoring program in a large area (approximately 19,171 km(2)) in northern Italy, aiming to identify individuals, estimate kinship, reconstruct packs, and describe their dynamics. Of 5,065 biological samples (99% scats), we genotyped and sexed 44% reliably using 12 unlinked autosomal microsatellites, 4 Y-linked microsatellites, and a diagnostic mitochondrial DNA control-region sequence. We identified 414 wolves, 88 dogs, and 16 wolf X dog hybrids. Wolves in the study area belonged to at least 42 packs. We reconstructed the genealogy of 26 packs. The mean pack size was 5.6 +/- 2.4 SD, including adoptees, with a mean minimum pack home range of 74 km(2) +/- 52 SD. We detected turnovers of breeding pairs in 19% of the packs. Reproductive wolves were unrelated and unrelated dispersers founded new packs, except for 1 pack founded by a brother sister pair. We did not detect multiple breeding females in any packs. Overall, the population was not inbred. We found significant isolation by distance and spatial autocorrelation, with nonrandom genetic structure up to a distance of approximately 17 km. We detected 37 dispersers, 14 of which became breeders in new or already existing packs. Our results can be used to model habitat use by wolves, to estimate survival rates, to predict future expansion of the wolf population, and to build risk maps of wolf human conflicts. | 2317 | NA | NA | NA | no | no |
| 2318 | 2017 | NA | Hotz, H; Guex, GD; Beerli, P; Semlitsch, RD; Pruvost, NBM | 2008 | Hemiclone diversity in the hybridogenetic frog Rana esculenta outside the area of clone formation: The view from protein electrophoresis | European water frog hybrids Rana esculenta reproduce hemiclonally, by hybridogenesis: In the germ line they exclude the genome of the parental species Rana lessonae and produce haploid, unrecombined gametes with a genome of the parental species Rana ridibunda. These hybrids coexist with and depend as sexual parasites on the host parental species R. lessonae (the L-E population system); matings with R. lessonae restore somatic hybridity in each generation of R. esculenta. We investigated 15 L-E system populations in northern Switzerland, which is outside R. ridibunda’s native range. Frequency of hybrids in samples varied from 8% in marsh ponds to 100% in gravel pits and forest ponds. Clonal diversity (variation among R. ridibunda genomes of hybrids), detected by six protein electrophoretic marker loci, revealed a total of eight hemiclones and locally ranged from uniclonal populations in southern parts of the survey region to six coexisting hemiclones in the north. All alleles distinguishing hemiclones occur commonly in the nearest native R. ridibunda populations of east-central Europe; the most probable source of clonal diversity in our samples is multiple clone formation by primary hybridizations in the sympatry area of R. ridibunda and R. lessonae and subsequent dispersal of hemiclonal lineages. A positive correlation between amount of clonal diversity and hybrid frequency, predicted by the Frozen Niche Variation (FNV) model (each hemiclone is characterized by a relatively narrow niche, coexistence is possible through niche partitioning), was not found; this contrasts with hemiclonally reproducing fish hybrids (Poeciliopsis). Historical factors, such as availability of different colonizing hemiclones may be strong enough to override the signal from operation of the FNV. | 2318 | NA | NA | NA | no | no |
| 2319 | 2017 | NA | Baran, Y; Quintela, I; Carracedo, A; Pasaniuc, B; Haperin, E | 2013 | Enhanced Localization of Genetic Samples through Linkage-Disequilibrium Correction | Characterizing the spatial patterns of genetic diversity in human populations has a wide range of applications, from detecting genetic mutations associated with disease to inferring human history. Current approaches, including the widely used principal-component analysis, are not suited for the analysis of linked markers, and local and long-range linkage disequilibrium (LD) can dramatically reduce the accuracy of spatial localization when unaccounted for. To overcome this, we have introduced an approach that performs spatial localization of individuals on the basis of their genetic data and explicitly models LD among markers by using a multivariate normal distribution. By leveraging external reference panels, we derive closed-form solutions to the optimization procedure to achieve a computationally efficient method that can handle large data sets. We validate the method on empirical data from a large sample of European individuals from the POPRES data set, as well as on a large sample of individuals of Spanish ancestry. First, we show that by modeling LD, we achieve accuracy superior to that of existing methods. Importantly, whereas other methods show decreased performance when dense marker panels are used in the inference, our approach improves in accuracy as more markers become available. Second, we show that accurate localization of genetic data can be achieved with only a part of the genome, and this could potentially enable the spatial localization of admixed samples that have a fraction of their genome originating from a given continent. Finally, we demonstrate that our approach is resistant to distortions resulting from long-range LD regions; such distortions can dramatically bias the results when unaccounted for. | 2319 | NA | NA | NA | no | no |
| 2320 | 2017 | NA | Andersson, M | 2017 | Helping Relatives Survive and Reproduce: Inclusive Fitness and Reproductive Value in Brood Parasitism | “Costly help can raise a relative’s reproduction, survival, and reproductive value and increase the inclusive fitness of the donor of help. Donor fitness is explored here in conspecific brood parasitism. In this alternative reproductive tactic, some females,”“parasites,”" lay eggs in nests of other females of the same species, "“hosts,”" suppliers of help that alone take care of the offspring. Modeling shows that hosts can gain inclusive fitness if parasitized by relatives whose reproduction or survival is thereby increased. These predictions are explored in waterfowl with frequent brood parasitism, female-biased philopatry, and neighbor relatedness. Approximate estimates based on waterfowl reproductive and life-history data show that host inclusive-fitness gain is often possible with related parasites. The largest gains can be achieved through increased reproduction, but gain is also possible through higher survival of parasites that avoid increased predation and other risks of nesting. Inclusive fitness depends on parasite reproductive value and can be highest for a host parasitized by her mother and for old, senescent hosts with low fecundity, helping young related parasites. These results and observed levels of host-parasite relatedness suggest that being "“parasitized”" in waterfowl is sometimes neutral or even advantageous because of inclusive-fitness benefits, contributing to evolution of frequent conspecific brood parasitism in this group." | 2320 | NA | NA | NA | no | no |
| 2321 | 2017 | NA | Torriani, SFF; Brunner, PC; McDonald, BA | 2011 | Evolutionary history of the mitochondrial genome in Mycosphaerella populations infecting bread wheat, durum wheat and wild grasses | Plant pathogens emerge in agro-ecosystems following different evolutionary mechanisms over different time scales. Previous analyses based on sequence variation at six nuclear loci indicated that Mycosphaerella graminicola diverged from an ancestral population adapted to wild grasses during the process of wheat domestication approximately 10,500 years ago. We tested this hypothesis by conducting coalescence analyses based on four mitochondrial loci using 143 isolates that included four closely related pathogen species originating from four continents. Pathogen isolates from bread and durum wheat were included to evaluate the emergence of specificity towards these hosts in M. graminicola. Although mitochondrial and nuclear genomes differed greatly in degree of genetic variability, their coalescence was remarkably congruent, supporting the proposed origin of M. graminicola through host tracking. The coalescence analysis was unable to trace M. graminicola host specificity through recent evolutionary time, indicating that the specificity towards durum or bread wheat emerged following the domestication of the pathogen on wheat. (C) 2010 Elsevier Inc. All rights reserved. | 2321 | NA | NA | NA | no | no |
| 2322 | 2017 | NA | Thurin, N; Aron, S | 2011 | No reversion to single mating in a socially parasitic ant | Social Hymenoptera are ideal biological models for the study of the selective forces affecting the evolution of multiple mating (polyandry), because sister species can evolve different lifestyles and mating strategies. Single mating is predicted in workerless social parasites, because the key benefit of multiple mating in social insects, that is, the increase in genetic diversity among worker offspring, does not hold for workerless species. We compared the queen mating frequency between the ant Plagiolepis pygmaea and its derived social parasite P. xene. Previous studies showed that queens of the host P. pygmaea are obligately polyandrous. Here, pedigree analyses of mother-offspring combinations indicate that queens of the parasite P. xene did not revert to single mating; more than 50% of queens mated multiply, with 2-4 males. This result shows that reversal from multiple to single mating may be not selected in polyandrous social insect workerless parasites. We propose that such reversion does not occur when multiple mating is virtually cost free. | 2322 | NA | NA | NA | no | no |
| 2323 | 2017 | NA | Albrechtsen, A; Korneliussen, TS; Moltke, I; Hansen, TV; Nielsen, FC; Nielsen, R | 2009 | Relatedness Mapping and Tracts of Relatedness for Genome-Wide Data in the Presence of Linkage Disequilibrium | Estimates of relatedness have several applications such as the identification of relatives or in identifying disease related genes through identity by descent (IBD) mapping. Here we present a new method for identifying IBD tracts among individuals from genome-wide single nucleotide polymorphisms data. We use a continuous time Markov model where the hidden states are the number of alleles shared IBD between pairs of individuals at a given position. In contrast to previous methods, our method accurately accounts for linkage disequilibrium using pairwise haplotype probabilities. The method provides a map of the local relatedness along the genome. We illustrate the potential of the method for mapping disease genes on a real data set, and show that the method has the potential to map causative disease mutations using only a handful of affected individuals. The new IBD mapping method provides considerable improvement in mapping power in natural populations compared to standard association mapping methods. Genet. Epidemiol. 33:266-274, 2009. (C) 2008 Wiley-Liss, Inc. | 2323 | NA | NA | NA | no | no |
| 2324 | 2017 | NA | Bryant, JM; Thibault, VC; Smith, DGE; McLuckie, J; Heron, I; Sevilla, IA; Biet, F; Harris, SR; Maskell, DJ; Bentley, SD; Parkhill, J; Stevenson, K | 2016 | Phylogenomic exploration of the relationships between strains of Mycobacterium avium subspecies paratuberculosis | Background: Mycobacterium avium subspecies paratuberculosis (Map) is an infectious enteric pathogen that causes Johne’s disease in livestock. Determining genetic diversity is prerequisite to understanding the epidemiology and biology of Map. We performed the first whole genome sequencing (WGS) of 141 global Map isolates that encompass the main molecular strain types currently reported. We investigated the phylogeny of the Map strains, the diversity of the genome and the limitations of commonly used genotyping methods. Results: Single nucleotide polymorphism (SNP) and phylogenetic analyses confirmed two major lineages concordant with the former Type S and Type C designations. The Type I and Type III strain groups are subtypes of Type S, and Type B strains are a subtype of Type C and not restricted to Bison species. We found that the genome-wide SNPs detected provided greater resolution between isolates than currently employed genotyping methods. Furthermore, the SNP used for IS1311 typing is not informative, as it is likely to have occurred after Type S and C strains diverged and does not assign all strains to the correct lineage. Mycobacterial Interspersed Repetitive Unit-Variable Number Tandem Repeat (MIRU-VNTR) differentiates Type S from Type C but provides limited resolution between isolates within these lineages and the polymorphisms detected do not necessarily accurately reflect the phylogenetic relationships between strains. WGS of passaged strains and coalescent analysis of the collection revealed a very high level of genetic stability, with the substitution rate estimated to be less than 0.5 SNPs per genome per year. Conclusions: This study clarifies the phylogenetic relationships between the previously described Map strain groups, and highlights the limitations of current genotyping techniques. Map isolates exhibit restricted genetic diversity and a substitution rate consistent with a monomorphic pathogen. WGS provides the ultimate level of resolution for differentiation between strains. However, WGS alone will not be sufficient for tracing and tracking Map infections, yet importantly it can provide a phylogenetic context for affirming epidemiological connections. | 2324 | NA | NA | NA | no | no |
| 2325 | 2017 | NA | MacColl, ADC; Piertney, SB; Moss, R; Lambin, X | 2000 | Spatial arrangement of kin affects recruitment success in young male red grouse | Models have shown that population cycles might be driven by time lags resulting from positive feedback between kin structure and population change, coupled with negative feedback between density and population change. One such model operates through kin favouritism facilitating the recruitment of young cock red grouse. We investigated whether recruitment by young cocks depended on the presence and spatial arrangement of elder relatives in the territorial population. We used molecular genetic estimates of relatedness, and checked for effects of covariates including natal territory size, hatching date, body size, parasite burdens and local density. Philopatric recruitment by cock red grouse led to the formation of clusters of contiguous territories owned by kin. The probability that an individual young cock would establish a territory increased with the number of kin in his father’s cluster. This pattern might have been due to genetic quality determining both recruitment success and the size of the paternal cluster. If so, there should have been a positive correlation between a young cock’s probability of recruitment and the number of his relatives in the population, irrespective of their spatial distribution. This did not occur and so the effect of cluster size is unlikely to have been confounded by genetic quality. The only morphological measure correlated with recruitment success was supraorbital comb size. The results are consistent with the prediction that kin tolerance affects recruitment but were at the level of the individual within years, rather than the population among years. Hence an experimental test of the kin favouritism hypothesis for population cycles, by manipulation of relatedness in populations among years, is now required. | 2325 | NA | NA | NA | no | no |
| 2326 | 2017 | NA | Mengoni, C; Mucci, N; Randi, E | 2015 | Genetic diversity and no evidences of recent hybridization in the endemic Italian hare (Lepus corsicanus) | Fragmented populations of the endemic Italian hare (Lepus corsicanus) survive at low density in central and southern Italy, in Sicily and Corsica, where the species was introduced in the sixteenth century. Fragmentation and hybridization with widespread brown hares (L. europaeus), which were introduced outside their natural ranges, may threaten the survival and genetic integrity of the Italian hare. With the exception of a few cases of hybrids identified in Corsica, hybridization was not documented in the Italian hare. In this study, we aimed to assess the genetic diversity in the Italian hare populations and identify possible interspecific hybrids with the brown hare. We genotyped 458 samples belonging to the four species of Lepus distributed in Italy (the mountain hare, the brown hare, the Sardinian hare and the Italian hare) using maternal and biparental markers (mtDNA control-region, 13 autosomal microsatellites, 9 autosomal SNPs). Results confirmed sharp interspecific genetic distinctions among the four species. We did not find interspecific hybrids in Italian hares, with the exception of two cases of L. corsicanus mtDNA introgression in two L. europaeus individuals. The Italian hares in Sicily are genetically distinct, in consequence of long-lasting isolation. Peninsular populations show instances of recent genetic diversification in consequence of anthropogenic fragmentation. Our findings point to consider the conservation of the Sicilian population as a priority, discouraging introductions of exotic hares into the island. Habitat restoration and a net of ecological corridors could help the survival and expansion of threatened L. corsicanus populations in peninsular Italy. Massive releases of brown hares for hunting purposes should be avoided in areas of sympatry with the Italian hares. | 2326 | NA | NA | NA | no | no |
| 2327 | 2017 | NA | Bishnoi, R; Singla, D | 2017 | APMicroDB: A microsatellite database of Acyrthosiphon pisum | “Pea aphids represent a complex genetic system that could be used for QTL analysis, genetic diversity and population genetics studies. Here, we described the development of first microsatellite repeat database of the pea aphid (APMicroDB), accessible at”“http://deepaklab.com/aphidmicrodb”“. We identified 3,40,233 SSRs using MI-croSAtellite (MISA) tool that was distributed in 14,067 (out of 23,924) scaffold of the pea aphid. We observed 89.53% simple repeats of which 73.41% were mono-nucleotide, followed by di-nucleotide repeats. This database stored information about the repeats kind, GC content, motif type (mono - hexa), genomic location etc. We have also incorporated the primer information derived from Primer3 software of the 250bp flanking region of the identified marker. Blast tool is also provided for searching the user query sequence for identified marker and their primers. This work has an immense use for scientific community working in the field of agricultural pest management, QTL mapping, and host-pathogen interaction analysis. (C) 2017 The Authors. Published by Elsevier Inc. This” | 2327 | NA | NA | NA | no | no |
| 2328 | 2017 | NA | Reales, G; Hernandez, CL; Dugoujon, JM; Novelletto, A; Cuesta, P; Fortes-Lima, C; Rodriguez, JN; Calderon, R | 2014 | New insights into the distribution of APOE polymorphism in the Iberian Peninsula. The case of Andalusia (Spain) | Background: The APOE gene has received much attention due to the remarkable spatial variation patterns of some of its genotypes and alleles in human populations and to its relevance in biomedicine. Aim: This work was addressed to investigate the extent of APOE polymorphism between autochthonous Andalusians originating from Huelva and Granada provinces. No data on this marker in these southern Spanish coastal populations are available up to date. Subjects and methods: This study used genomic DNA from healthy, unrelated Andalusians of both sexes (n = 322). All samples were genotyped for two SNPs, rs429358 and rs7412, which determine the three APOE alleles: epsilon 2, epsilon 3 and epsilon 4. For analyses, a TaqMan-based technique was applied using a RT-PCR. Comparisons with other Mediterranean populations were performed based on multivariate analysis. Results: A relatively high frequency of epsilon 4 in Granada (eastern Andalusia), as well as a low epsilon 2 frequency in Huelva (western Andalusia) were observed. The finding that epsilon 4 allele in Southern Spain and Portugal is higher than expected given its geographical location poses an interesting question for this study, given the well-established APOE-epsilon 4 gradient in Europe. Conclusion: This population study may represent useful information for further prospective anthropological and molecular genetic studies focused on unravelling the relationship between population genetic composition and specific human diseases. | 2328 | NA | NA | NA | no | no |
| 2329 | 2017 | NA | Sarma, RK; Gogoi, A; Dehury, B; Debnath, R; Bora, TC; Saikia, R | 2014 | Community Profiling of Culturable Fluorescent Pseudomonads in the Rhizosphere of Green Gram (Vigna radiata L.) | Study on microbial diversity in the unexplored rhizosphere is important to understand their community structure, biology and ecological interaction with the host plant. This research assessed the genetic and functional diversity of fluorescent pseudomonads [FP] in the green gram rhizophere. One hundred and twenty types of morphologically distinct fluorescent pseudomonads were isolated during vegetative as well as reproductive growth phase of green gram. Rep PCR, ARDRA and RISA revealed two distinct clusters in each case at 75, 61 and 70% similarity coefficient index respectively. 16S rRNA partial sequencing analysis of 85 distantly related fluorescent pseudomonads depicted Pseudomonas aeruginosa as the dominant group. Out of 120 isolates, 23 (19%) showed antagonistic activity towards phytopathogenic fungi. These bacterial isolates showed varied production of salicylic acid, HCN and chitinase, 2, 4-diacetylphloroglucinol (DAPG), phenazine-1-carboxylic acid (PCA) and pyoluteorin (PLT). Production efficiency of inherent level of plant growth promoting (PGP) traits among the 120 isolates demonstrated that 10 (8%) solubilised inorganic phosphates, 25 (20%) produced indoles and 5 (4%) retained ACC deaminase activity. Pseudomonas aeruginosa GGRJ21 showed the highest production of all antagonistic and plant growth promoting (PGP) traits. In a greenhouse experiment, GGRJ21 suppressed root rot disease of green gram by 28-93% (p = 0.05). Consistent up regulation of three important stress responsive genes, i.e., acdS, KatA and gbsA and elevated production efficiency of different PGP traits could promote GGRJ21 as a potent plant growth regulator. | 2329 | NA | NA | NA | no | no |
| 2330 | 2017 | NA | Da Silva, JM; Donaldson, JS; Reeves, G; Hedderson, TA | 2012 | Population genetics and conservation of critically small cycad populations: a case study of the Albany Cycad, Encephalartos latifrons (Lehmann) | Declining populations of less than 250 mature individuals are symptomatic of many Critically Endangered cycads, which, globally, comprise the most threatened group of organisms as a result of collecting and habitat loss. Survival plans focus on law enforcement, reintroduction, and augmentation programmes using plants from the wild and botanical gardens. Augmentation is one of the few remaining options for cycad populations, although the assumed benefits remain untested and there is a possibility that augmentation from different sources could compromise the genetic integrity of existing populations, especially when garden plants have no provenance data. We studied Encephalartos latifrons, a South African endemic, which is a typical Critically Endangered cycad. We studied the extent and structure of genetic diversity in wild and ex situ populations to assess the potential benefits and risks associated with augmentation programmes. We examined 86 plants using amplified fragment length polymorphisms (AFLPs). The 417 AFLP markers thus generated yielded a unique DNA fingerprint for each plant. Wild populations retain high levels of genetic diversity and this is reflected among the ex situ holdings at the Kirstenbosch Botanical Garden. No population differentiation is evident, indicating a single panmictic population, consistent with moderately high levels of gene flow between subpopulations and a sexual mode of reproduction. Bayesian clustering identified four genotype groups in the wild, as well as a genotype group only found in ex situ collections. Our results indicate that E. latifrons would benefit from augmentation programmes, including the use of undocumented collections, and careful management of breeding plants would increase the heterogeneity of propagules. (C) 2011 The Linnean Society of London, Biological Journal of the Linnean Society, 2012, 105, 293308. | 2330 | NA | NA | NA | no | no |
| 2331 | 2017 | NA | Roshier, DA; Heinsohn, R; Adcock, GJ; Beerli, P; Joseph, L | 2012 | Biogeographic models of gene flow in two waterfowl of the Australo-Papuan tropics | There are many large, easy-to-observe anseriform birds (ducks, geese, and swans) in northern Australia and New Guinea and they often gather in large numbers. Yet, the structure of their populations and their regional movements are poorly understood. Lack of understanding of population structure limits our capacity to understand source-sink dynamics relevant to their conservation or assess risks associated with avian-borne pathogens, in particular, avian influenza for which waterfowl are the main reservoir species. We set out to assess present-day genetic connectivity between populations of two widely distributed waterfowl in the Australo-Papuan tropics, magpie goose Anseranas semipalmata (Latham, 1798) and wandering whistling-duck Dendrocygna arcuata (Horsfield, 1824). Microsatellite data were obtained from 237 magpie geese and 64 wandering whistling-duck. Samples were collected across northern Australia, and at one site each in New Guinea and Timor Leste. In the wandering whistling-duck, genetic diversity was significantly apportioned by region and sampling location. For this species, the best model of population structure was New Guinea as the source population for all other populations. One remarkable result for this species was genetic separation of two flocks sampled contemporaneously on Cape York Peninsula only a few kilometers apart. In contrast, evidence for population structure was much weaker in the magpie goose, and Cape York as the source population provided the best fit to the observed structure. The fine scale genetic structure observed in wandering whistling-duck and magpie goose is consistent with earlier suggestions that the west-coast of Cape York Peninsula is a flyway for Australo-Papuan anseriforms between Australia and New Guinea across Torres Strait. | 2331 | NA | NA | NA | no | no |
| 2332 | 2017 | NA | Calderon, R; Vidales, C; Pena, JA; Perez-Miranda, A; Dugoujon, JM | 1998 | Immunoglobulin allotypes (GM and KM) in Basques from Spain: Approach to the origin of the Basque population | GM and KM immunoglobulin (Ig) allotypes have been tested in 310 autochthonous Basques from the three subpopulations of Vizcaya, Guipuzcoa, and Alava, Spain. They are compared with allotypes occurring in autochthonous French Basques, some Pyrenean subpopulations in France, and European populations. The analysis suggests that the Basque subpopulations show noticeable genetic distances between them and with other European populations. The genetic similarity between Basques and European populations is greater in the Basques from France than in the Basques from Spain. The genetic distances between Basque subpopulations in Spain fit well with the different historical levels of the spatial implantation of the Basque language. Guipuzcoa, the Basque province with the highest number of Basque-speaking people, shows the most genetic distinctiveness. The main underlying cause of this spatial genetic pattern seems to be admixture with surrounding populations. Our results do not support the hypothesis that Basques are a relict population of ancient Europeans. They might be a consequence of the colonization of the Basque area by a long-distance migrating group, probably a small Neolithic North Caucasian population that introduced agriculture in the region. They experienced early, rapid demographic growth, and they did not breed with the few hunter-gatherers wandering throughout the area. The North Caucasian migrants could have admired with North Asian groups dating from many centuries before. Furthermore, Basques present polymorphic frequencies of a common African haplotype, suggesting that they have not been completely isolated from populations of Africa. However, another focus of the African haplotype has been detected in Central Asia, and the Basque frequencies alternatively might be due to North Asian groups. | 2332 | NA | NA | NA | no | no |
| 2333 | 2017 | NA | Pereira, J; Dujardin, JP; Salvatella, R; Tibayrenc, M | 1996 | Enzymatic variability and phylogenetic relatedness among Triatoma infestans, T-platensis, T-delpontei and T-rubrovaria | Four congeneric species of triatomine bugs, Triatoma platensis, T. delpontei, T. rubrovaria and T. infestans, were examined by multilocus enzyme electrophoresis. These four species are distributed sympatrically throughout Argentina, Paraguay and Uruguay, and were found to be closely related according to their known ethological, ecological and morphological traits. In order to evaluate previous hypotheses concerning the phylogenetic branching within this group, isoenzyme patterns were submitted to classical phenetic and genetic clustering analysis. Results are discussed in the light of occasional natural gene flow between T. infestans and T. platensis, and indicate that ecology is the main factor explaining both their present status and their morphological differentiation. | 2333 | NA | NA | NA | no | no |
| 2334 | 2017 | NA | Ersoz, ES; Wright, MH; Gonzalez-Martinez, SC; Langley, CH; Neale, DB | 2010 | Evolution of Disease Response Genes in Loblolly Pine: Insights from Candidate Genes | Background: Host-pathogen interactions that may lead to a competitive co-evolution of virulence and resistance mechanisms present an attractive system to study molecular evolution because strong, recent (or even current) selective pressure is expected at many genomic loci. However, it is unclear whether these selective forces would act to preserve existing diversity, promote novel diversity, or reduce linked neutral diversity during rapid fixation of advantageous alleles. In plants, the lack of adaptive immunity places a larger burden on genetic diversity to ensure survival of plant populations. This burden is even greater if the generation time of the plant is much longer than the generation time of the pathogen. Methodology/Principal Findings: Here, we present nucleotide polymorphism and substitution data for 41 candidate genes from the long-lived forest tree loblolly pine, selected primarily for their prospective influences on host-pathogen interactions. This dataset is analyzed together with 15 drought-tolerance and 13 wood-quality genes from previous studies. A wide range of neutrality tests were performed and tested against expectations from realistic demographic models. Conclusions/Significance: Collectively, our analyses found that axr (auxin response factor), caf1 (chromatin assembly factor) and gatabp1 (gata binding protein 1) candidate genes carry patterns consistent with directional selection and erd3 (early response to drought 3) displays patterns suggestive of a selective sweep, both of which are consistent with the arm-race model of disease response evolution. Furthermore, we have identified patterns consistent with diversifying selection at erf1-like (ethylene responsive factor 1), ccoaoemt (caffeoyl-CoA-O-methyltransferase), cyp450-like (cytochrome p450-like) and pr4.3 (pathogen response 4.3), expected under the trench-warfare evolution model. Finally, a drought-tolerance candidate related to the plant cell wall, lp5, displayed patterns consistent with balancing selection. In conclusion, both arms-race and trench-warfare models seem compatible with patterns of polymorphism found in different disease-response candidate genes, indicating a mixed strategy of disease tolerance evolution for loblolly pine, a major tree crop in southeastern United States. | 2334 | NA | NA | NA | no | no |
| 2335 | 2017 | NA | Sim, Z; Hall, JC; Jex, B; Hegel, TM; Coltman, DW | 2016 | Genome-wide set of SNPs reveals evidence for two glacial refugia and admixture from postglacial recolonization in an alpine ungulate | Past glaciation events have played a major role in shaping the genetic diversity and distribution of wild sheep in North America. The advancement of glaciers can isolate populations in ice-free refugia, where they can survive until the recession of ice sheets. The major Beringian refugium is thought to have held thinhorn sheep (Ovis dalli) populations during times of glacial advance. While isolation in the major refugium can account for much of the genetic and morphological diversity seen in extant thinhorn sheep populations, mounting evidence suggests the persistence of populations in smaller minor refugia. We investigated the refugial origins of thinhorn sheep using similar to 10000 SNPs obtained via a cross-species application of the domestic sheep ovine HD BeadChip to genotype 52 thinhorn sheep and five bighorn sheep (O. canadensis) samples. Phylogenetic inference revealed a distinct lineage of thinhorn sheep inhabiting British Columbia, which is consistent with the survival of a group of thinhorn sheep in a minor refugium separate from the Beringian refugium. Isolation in separate glacial refugia probably mediated the evolution of the two thinhorn sheep subspecies, the white Dall’s sheep (O. d. dalli), which persisted in Beringia, and the dark Stone’s sheep (O. d. stonei), which utilized the minor refugium. We also found the first genetic evidence for admixture between sheep from different glacial refugia in south-central Yukon as a consequence of post glacial expansion and recolonization. These results show that glaciation events can have a major role in the evolution of species inhabiting previously glaciated habitats and the need to look beyond established refugia when examining the evolutionary history of such species. | 2335 | NA | NA | NA | no | no |
| 2336 | 2017 | NA | Faria, PJ; Kavembe, GD; Jung’a, JO; Kimwele, CN; Estes, LD; Reillo, PR; Mwangi, AG; Bruford, MW | 2011 | The use of non-invasive molecular techniques to confirm the presence of mountain bongo Tragelaphus eurycerus isaaci populations in Kenya and preliminary inference of their mitochondrial genetic variation | The mountain bongo antelope Tragelaphus eurycerus isaaci has rapidly declined in recent decades, due to a combination of hunting, habitat degradation and disease. Endemic to Kenya, mountain bongo populations have shrunk to approximately 100 individuals now mainly confined to the Aberdares mountain ranges. Indirect observation of bongo signs (e.g. tracks, dung) can be misleading, thus methods to ensure reliable species identification, such as DNA-based techniques, are necessary to effectively study and monitor this species. We assessed bongo presence in four mountain habitats in Kenya (Mount Kenya National Park, Aberdare National Park, Eburu and Mau forests) and carried out a preliminary analysis of genetic variation by examining 466 bp of the first domain of the mtDNA control region using DNA extracted from faecal samples. Of the 201 dung samples collected in the field, 102 samples were molecularly identified as bongo, 97 as waterbuck, one as African buffalo and one as Aders’ duiker. Overall species-identification accuracy by experienced trackers was 64%, with very high error of commission when identifying bongo sign (37%), and high error of omission for waterbuck sign (82%), suggesting that the two species’ signs are easily confused. Despite high variation in the mtDNA control region in most antelope species, our results suggest low genetic variation in mountain bongo as only two haplotypes were detected in 102 samples analyzed. In contrast, the analysis of 63 waterbuck samples from the same sites revealed 21 haplotypes. Nevertheless, further examination using nuclear DNA markers (e.g. microsatellites) in a multi-locus approach is still required, especially because the use of mitochondrial DNA can result in population overestimation as distinct dung samples can potentially be originated from the same individual. | 2336 | NA | NA | NA | no | no |
| 2337 | 2017 | NA | Pierron, D; Cortes, NG; Letellier, T; Grossman, LI | 2013 | Current relaxation of selection on the human genome: Tolerance of deleterious mutations on olfactory receptors | Knowledge and understanding about the selective pressures that have shaped present human genetic diversity have dramatically increased in the last few years in parallel with the availability of large genomic datasets. The release of large datasets composed of millions of SNPs across hundreds of genomes by HAPMAP, the Human Genome Diversity Panel, and other projects has led to considerable effort to detect selection signals across the nuclear genome (Coop et al., 2009; Lopez Herraez et al., 2009; Sabeti et al., 2006, 2007; Voight et al., 2006). Most of the research has focused on positive selection forces although other selective forces, such as negative selection, may have played a substantive role on the shape of our genome. Here we studied the selective strengths acting presently on the genome by making computational predictions of the pathogenicity of nonsynonymous protein mutations and interpreting the distribution of scores in terms of selection. We could show that the genetic diversity for all the major pathways is still constrained by negative selection in all 11 human populations studied. In a single exception, we observed a relaxation of negative selection acting on olfactory receptors. Since a decreased number of functioning olfactory receptors in human compared with other primates had already been shown, this suggests that the role of olfactory receptors for survival and reproductive success has decreased during human evolution. By showing that negative selection is still relaxed, the present results imply that no plateau of minimal function has yet been reached in modern humans and therefore that olfactory capability might still be decreasing. This is a first clue to present human evolution. (C) 2012 Elsevier Inc. All rights reserved. | 2337 | NA | NA | NA | no | no |
| 2338 | 2017 | NA | Croze, M; Wollstein, A; Bozicevic, V; Zivkovic, D; Stephan, W; Hutter, S | 2017 | A genome-wide scan for genes under balancing selection in Drosophila melanogaster | Background: In the history of population genetics balancing selection has been considered as an important evolutionary force, yet until today little is known about its abundance and its effect on patterns of genetic diversity. Several well-known examples of balancing selection have been reported from humans, mice, plants, and parasites. However, only very few systematic studies have been carried out to detect genes under balancing selection. We performed a genome scan in Drosophila melanogaster to find signatures of balancing selection in a derived (European) and an ancestral (African) population. We screened a total of 34 genomes searching for regions of high genetic diversity and an excess of SNPs with intermediate frequency. Results: In total, we found 183 candidate genes: 141 in the European population and 45 in the African one, with only three genes shared between both populations. Most differences between both populations were observed on the X chromosome, though this might be partly due to false positives. Functionally, we find an overrepresentation of genes involved in neuronal development and circadian rhythm. Furthermore, some of the top genes we identified are involved in innate immunity. Conclusion: Our results revealed evidence of genes under balancing selection in European and African populations. More candidate genes have been found in the European population. They are involved in several different functions. | 2338 | NA | NA | NA | no | no |
| 2339 | 2017 | NA | Sonmezoglu, OA; Bozmaz, B; Yildirim, A; Kandemir, N; Aydin, N | 2012 | Genetic characterization of Turkish bread wheat landraces based on microsatellite markers and morphological characters | Wheat landraces could be unique sources of favorable genes for agronomic traits like yield and insect, pest, and disease resistances, as well as quality characteristics that are important for breeding programs. Molecular marker systems offer great opportunities to characterize wheat accessions, which in particular are not morphologically identifiable. In this study, 20 bread wheat landraces collected from different regions of Turkey were characterized by using microsatellite markers (SSRs) and morphological characters. Seventeen morphological characters were used. Fifteen SSR primers were prescreened and the 7 most polymorphic primers were employed in characterization. The most polymorphic SSR loci were Xgwm 95 and 295 with 11 alleles, followed by Xgwm 261 and 325 with 9 alleles. The total number of alleles was 63, with an average number of 9 alleles. The dendrogram showed that the bread wheat landraces can be divided into 2 major groups. Based on matrix values, the closest genotypes of landraces were TR 63445 and TR 63886 for molecular data, and TR 37179 and TR 46797 for morphological data. On the other hand, the most genetically different genotypes were TR 37179 and TR 3608 in the SSR analysis, and TR 14851 and TR 3608 in the morphological characterization. These results showed that SSR markers and morphological characters could be successfully used in genetic characterization and genetic diversity in bread wheat landraces that may be useful for wheat breeding programs as genetic resources. | 2339 | NA | NA | NA | no | no |
| 2340 | 2017 | NA | Amos, W; Nichols, HJ; Churchyard, T; Brooke, MD | 2016 | Rat eradication comes within a whisker! A case study of a failed project from the South Pacific | To enhance their conservation value, several hundred islands worldwide have been cleared of invasive alien rats, Rattus spp. One of the largest projects yet undertaken was on 43 km(2) Henderson Island in the Pitcairn group, South Pacific, in August 2011. Following massive immediate mortality, a single R. exulans was observed in March 2012 and, subsequently, rat numbers have recovered. The survivors show no sign of resistance to the toxicant used, brodifacoum. Using pre- and post-operation rat tissue samples from Henderson, plus samples from around the Pacific, we exclude re-introduction as the source of continued rat presence. Microsatellite analysis of 18 loci enabled comparison of genetic diversity of Henderson rats before and after the bait drop. The fall in diversity measured by allele frequency change indicated that the bottleneck (N-e) through which the breeding population passed was probably around 50 individuals, representing a census population of about 60-80 animals. This is the first failed project that has estimated how close it was to success. | 2340 | NA | NA | NA | no | no |
| 2341 | 2017 | NA | Al-Abadi, SY; Al-Sadi, AM; Dickinson, M; Al-Hammadi, MS; Al-Shariqi, R; Al-Yahyai, RA; Kazerooni, EA; Bertaccini, A | 2016 | Population genetic analysis reveals a low level of genetic diversity of ‘Candidatus Phytoplasma aurantifolia’ causing witches’ broom disease in lime | Witches’ broom disease of lime (WBDL) is a serious phytoplasma disease of acid lime in Oman, the UAE and Iran. Despite efforts to study it, no systemic study attempted to characterize the relationship among the associated phytoplasma, ‘Candidatus Phytoplasma aurantifolia’, from the three countries. This study utilized sequences of the 16S rRNA, imp and secA genes to characterize 57 strains collected from Oman (38), the UAE (9) and Iran (10). Phylogenetic analysis based on the 16S rRNA gene showed that the 57 strains shared 98.5-100 % nucleotide similarity to each other and to strains of ‘Ca. P. aurantifolia’ available in GenBank. The level of genetic diversity was low based on the 16S rRNA (0-0.011), imp (0-0.002) and secA genes (0-0.015). The presence of low level of diversity among phytoplasma strains from Oman, the UAE and Iran can be explained by the movement of infected lime seedlings from one country to another through trading and exchange of infected plants. The study discusses implication of the findings on WBDL spread and management. | 2341 | NA | NA | NA | no | no |
| 2342 | 2017 | NA | Iftikhar, R; Ramesh, SV; Bag, S; Ashfaq, M; Pappu, HR | 2014 | Global analysis of population structure, spatial and temporal dynamics of genetic diversity, and evolutionary lineages of Iris yellow spot virus (Tospovirus: Bunyaviridae) | Thrips-transmitted Iris yellow spot virus is an economically important viral pathogen of Allium crops worldwide. A global analysis of known IYSV nucleocapsid gene (N gene) sequences was carried out to determine the comparative population structure, spatial and temporal dynamics with reference to its genetic diversity and evolution. A total of 98 complete N gene sequences (including 8 sequences reported in this study) available in GenBank and reported from 23 countries were characterized by in-silico RFLP analysis. Based on RFLP, 94% of the isolates could be grouped into NL or BR types while the rest belonged to neither group. The relative proportion of NL and BR types was 46% and 48%, respectively. A temporal shift in the IYSV genotypes with a greater incremental incidence of IYSVBR was found over IYSVNL before 2005 compared to after 2005. The virus population had at least one evolutionarily significant recombination event, involving IYSVBR and IYSVNL. Codon substitution studies did not identify any significant differences among the genotypes of IYSV. However, N gene codons were minimally positively selected, moderately negatively selected denoting the action of purifying selection, thus rejecting the theory of neutral mutation in IYSV population. However, one codon position (139) was found to be positively selected in all the genotypes. Population selection statistics in the IYSVBR, IYSVNL genotypes and in the population as a whole also revealed the action of purifying selection or population expansion, whereas IYSVother displayed a decrease in population size. Genetic differentiation studies showed inherent differentiation and infrequent gene flow between IYSVBR and IYSVNL genotypes corroborating the geographical confinement of these genotypes. Taken together the study suggests that the observed diversity in IYSV population and temporal shift in IYSVBR genotype is attributable to genetic recombination, abundance of purifying selection, insignificant positive selection and population expansion. Restricted gene flow between the two major IYSV genotypes further emphasizes the role of genetic drift in modeling the population architecture, evolutionary lineage and epidemiology of IYSV. (C) 2014 Elsevier B.V. All rights reserved. | 2342 | NA | NA | NA | no | no |
| 2343 | 2017 | NA | Ali, S; Gladieux, P; Rahman, H; Saqib, MS; Fiaz, M; Ahmad, H; Leconte, M; Gautier, A; Justesen, AF; Hovmoller, MS; Enjalbert, J; De Vallavieille-Pope, C | 2014 | Inferring the contribution of sexual reproduction, migration and off-season survival to the temporal maintenance of microbial populations: a case study on the wheat fungal pathogen Puccinia striiformis f.sp tritici | Understanding the mode of temporal maintenance of plant pathogens is an important domain of microbial ecology research. Due to the inconspicuous nature of microbes, their temporal maintenance cannot be studied directly through tracking individuals and their progeny. Here, we suggest a series of population genetic analyses on molecular marker variation in temporally spaced samples to infer about the relative contribution of sexual reproduction, off-season survival and migration to the temporal maintenance of pathogen populations. We used the proposed approach to investigate the temporal maintenance of wheat yellow rust pathogen, Puccinia striiformis f.sp. tritici (PST), in the Himalayan region of Pakistan. Multilocus microsatellite genotyping of PST isolates revealed high genotypic diversity and recombinant population structure across all locations, confirming the existence of sexual reproduction in this region. The genotypes were assigned to four genetic groups, revealing a clear differentiation between zones with and without Berberis spp., the alternate host of PST, with an additional subdivision within the Berberis zone. The lack of any differentiation between samples across two sampling years, and the very infrequent resampling of multilocus genotypes over years at a given location was consistent with limited over-year clonal survival, and a limited genetic drift. The off-season oversummering population in the Berberis zone, likely to be maintained locally, served as a source of migrants contributing to the temporal maintenance in the non-Berberis zone. Our study hence demonstrated the contribution of both sexual recombination and off-season oversummering survival to the temporal maintenance of the pathogen. These new insights into the population biology of PST highlight the general usefulness of the analytical approach proposed. | 2343 | NA | NA | NA | no | no |
| 2344 | 2017 | NA | Zhao, ZM; Jin, L; Fu, YX; Ramsay, M; Jenkins, T; Leskinen, E; Pamilo, P; Trexler, M; Patthy, L; Jorde, LB; Ramos-Onsins, S; Yu, N; Li, WH | 2000 | Worldwide DNA sequence variation in a 10-kilobase noncoding region on human chromosome 22 | Human DNA sequence variation data are useful for studying the origin, evolution, and demographic history of modern humans and the mechanisms of maintenance of genetic variability in human populations, and for detecting linkage association of disease. Here, we report worldwide variation data from a approximate to 10-kilobase noncoding autosomal region. We identified 75 variant sites in 64 humans (128 sequences) and 463 variant sites among the human, chimpanzee, and orangutan sequences. Statistical tests suggested that the region is selectively neutral. The average nucleotide diversity (pi) across the region was 0.088% among all of the human sequences obtained, 0.085% among African sequences, and 0.082% among non-African sequences, supporting the view of a low nucleotide diversity (approximate to 0.1%) in humans. The comparable pi value in non-Africans to that in Africans indicates no severe bottleneck during the evolution of modern non-Africans; however, the possibility of mild bottleneck cannot be excluded because non-Africans showed considerably fewer variants than Africans. The present and two previous large data sets all show a strong excess of low frequency variants in comparison to that expected from an equilibrium population, indicating a relatively recent population expansion. The mutation rate was estimated to be 1.15 x 10(-9) per nucleotide per year. Estimates of the long-term effective population size N-e by various statistical methods were similar to those in other studies. The age of the most recent common ancestor was estimated to he approximate to 1.29 million years ago among all of the sequences obtained and approximate to 634,000 years ago among the non-African sequences, providing the first evidence from a noncoding autosomal region for ancient human histories, even among non-Africans. | 2344 | NA | NA | NA | no | no |
| 2345 | 2017 | NA | Crawford, JE; Alves, JM; Palmer, WJ; Day, JP; Sylla, M; Ramasamy, R; Surendran, SN; Black, WC; Pain, A; Jiggins, FM | 2017 | Population genomics reveals that an anthropophilic population of Aedes aegypti mosquitoes in West Africa recently gave rise to American and Asian populations of this major disease vector | Background: The mosquito Aedes aegypti is the main vector of dengue, Zika, chikungunya and yellow fever viruses. This major disease vector is thought to have arisen when the African subspecies Ae. aegypti formosus evolved from being zoophilic and living in forest habitats into a form that specialises on humans and resides near human population centres. The resulting domestic subspecies, Ae. aegypti aegypti, is found throughout the tropics and largely blood-feeds on humans. Results: To understand this transition, we have sequenced the exomes of mosquitoes collected from five populations from around the world. We found that Ae. aegypti specimens from an urban population in Senegal in West Africa were more closely related to populations in Mexico and Sri Lanka than they were to a nearby forest population. We estimate that the populations in Senegal and Mexico split just a few hundred years ago, and we found no evidence of Ae. aegypti aegypti mosquitoes migrating back to Africa from elsewhere in the tropics. The out-of-Africa migration was accompanied by a dramatic reduction in effective population size, resulting in a loss of genetic diversity and rare genetic variants. Conclusions: We conclude that a domestic population of Ae. aegypti in Senegal and domestic populations on other continents are more closely related to each other than to other African populations. This suggests that an ancestral population of Ae. aegypti evolved to become a human specialist in Africa, giving rise to the subspecies Ae. aegypti aegypti. The descendants of this population are still found in West Africa today, and the rest of the world was colonised when mosquitoes from this population migrated out of Africa. This is the first report of an African population of Ae. aegypti aegypti mosquitoes that is closely related to Asian and American populations. As the two subspecies differ in their ability to vector disease, their existence side by side in West Africa may have important implications for disease transmission. | 2345 | NA | NA | NA | no | no |
| 2346 | 2017 | NA | Qiu, WG; Bosler, EM; Campbell, JR; Ugine, GD; Wang, IN; Luft, BJ; Dykhuizen, DE | 1997 | A population genetic study of Borrelia burgdorferi sensu stricto from eastern Long Island, New York, suggested frequency-dependent selection, gene flow and host adaptation | Eastern Long Island, New York, is one of the major foci of Lyme disease in the United States. As in almost all other parts of North America, Lyme disease in this region is caused by a single genomic species of spirochete, Borrelia burgdorferi sensu stricto. For three consecutive years, natural populations of Lyme Borrelia in this region were sampled and studied for gene flow among different locations, changes in population structure over time, and selective forces. The genetic diversity of Borrelia populations was measured at the outer surface protein A (ospA) locus using Cold Single-Stranded Conformation Polymorphism (Cold SSCP) analysis. The Borrelia populations were found to be highly polymorphic within any of thirteen local populations. Ewens-Watterson tests of neutrality revealed that the high level of genetic diversity within local Borrelia populations is maintained by balancing selection. Frequency-dependent selection for the different strains distinguished by the ospA alleles is likely the mechanism of the balancing selection. Allele frequency distributions of Borrelia populations were homogeneous across the region in any particular year, although different infection rates of local tick (Ixodes scapularis) populations suggested that the Borrelia populations were at least partially isolated. Since the allele frequency distribution changed over time, while remaining homogeneous over space, the nearly uniform allele frequency distribution across the region cannot be explained by recent geographic expansion from a single population. This uniform distribution across the region thus may be maintained by selection, or by a significant amount of migration or both. The genetic structure of B. burgdorferi sensu stricto also differed between spirochetes infecting nymphal ticks and those infecting adult ticks. Since larval and nymphal ticks have distinctly different host feeding preferences, host adaptation of spirochete populations is implied. This distinction and an animal study using chipmunks suggest that ticks infected by Borrelia as larvae may have high mortality in the wild. This study represents a genetic analysis of local populations of a bacterial species. | 2346 | NA | NA | NA | no | no |
| 2347 | 2017 | NA | KOPPELMAN, JB; FIGG, DE | 1995 | GENETIC ESTIMATES OF VARIABILITY AND RELATEDNESS FOR CONSERVATION OF AN OZARK CAVE CRAYFISH SPECIES COMPLEX | Allozyme-based genetic distances were used to determine the distinctness of six species of cave crayfish from the Ozark Plateau in Missouri, Arkansas, and Oklahoma. One of the cave species is in the subgenus Erebicambarus and the others are in Jugicambarus. Four of the six species are very rare and are found in only one to three known sites each. In addition, most populations of all the species are presumed to be small; rarely are more than a few individuals observed. A chela (claw) was collected from sixty individuals representing the six species, including all known populations of the four rare species. Variability and distance estimates were based on 20 presumptive gene loci. Population samples with identical genotypes were pooled. Thirteen loci were polymorphic, but average heterozygosity was low (H = 1%) compared to epigean crayfish species. Pairwise genetic distances within Jugicambarus ranged from D = 0.051 to 0.522, and mean distance between subgenera was D = 0.676. The underground water systems in Ozark caves are defined by discreet recharge zones. Groundwater pollution threatens the stability of cave ecosystems, including the survival of cave crayfish. If restoration of threatened or extirpated populations becomes necessary, a database of genetic variability and relatedness estimates for known populations of all the species will aid decisions about numbers and sources of individuals for propagation or transfer. | 2347 | NA | NA | NA | no | no |
| 2348 | 2017 | NA | Massa, SI; Paulino, CM; Serrao, EA; Duarte, CM; Arnaud-Haond, S | 2013 | Entangled effects of allelic and clonal (genotypic) richness in the resistance and resilience of experimental populations of the seagrass Zostera noltii to diatom invasion | Background: The relationship between species diversity and components of ecosystem stability has been extensively studied, whilst the influence of the genetic component of biodiversity remains poorly understood. Here we manipulated both genotypic and allelic richness of the seagrass Zostera noltii, in order to explore their respective influences on the resistance of the experimental population to stress. Thus far intra-specific diversity was seldom taken into account in management plans, and restoration actions showed very low success. Information is therefore needed to understand the factors affecting resistance and resilience of populations. Results: Our results show a positive influence of both allelic and genotypic richness on the resistance of meadows to environmental perturbations. They also show that at the low genotypic (i.e. clonal) richness levels used in prior experimental approaches, the effects of genotypic and allelic richness could not be disentangled and allelic richness was a likely hidden treatment explaining at least part of the effects hitherto attributed to genotypic richness. Conclusions: Altogether, these results emphasize the need to acknowledge and take into account the interdependency of both genotypic and allelic richness in experimental designs attempting to estimate their importance alone or in combination. A positive influence of allelic richness on resistance to perturbations, and of allelic richness combined with genotypic richness on the recovery (resilience) of the experimental populations is supported by differential mortality. These results, on the key species structuring of one of the most threatened coastal ecosystem worldwide, seagrass meadows, support the need to better take into account the distinct compartments of clonal and genetic diversity in management strategies, and in possible restoration plans in the future. | 2348 | NA | NA | NA | no | no |
| 2349 | 2017 | NA | Garzon-Ospina, D; Lopez, C; Forero-Rodriguez, J; Patarroyo, MA | 2012 | Genetic Diversity and Selection in Three Plasmodium vivax Merozoite Surface Protein 7 (Pvmsp-7) Genes in a Colombian Population | A completely effective vaccine for malaria (one of the major infectious diseases worldwide) is not yet available; different membrane proteins involved in parasite-host interactions have been proposed as candidates for designing it. It has been found that proteins encoded by the merozoite surface protein (msp)-7 multigene family are antibody targets in natural infection; the nucleotide diversity of three Pvmsp-7 genes was thus analyzed in a Colombian parasite population. By contrast with P. falciparum msp-7 loci and ancestral P. vivax msp-7 genes, specie-specific duplicates of the latter specie display high genetic variability, generated by single nucleotide polymorphisms, repeat regions, and recombination. At least three major allele types are present in Pvmsp-7C, Pvmsp-7H and Pvmsp-7I and positive selection seems to be operating on the central region of these msp-7 genes. Although this region has high genetic polymorphism, the C-terminus (Pfam domain ID: PF12948) is conserved and could be an important candidate when designing a subunit-based antimalarial vaccine. | 2349 | NA | NA | NA | no | no |
| 2350 | 2017 | NA | Chai, HN; Du, YZ | 2011 | Detection and Phylogenetic Analysis of Wolbachia wsp in the Chilo suppressalis (Lepidoptera: Crambidae) in China | Wolbachia are a group of intracellular, inherited endosymbiotic bacteria infecting a wide range of insects that are associated with several different reproductive phenotypes in their hosts. We measured the infection status of Wolbachia in the Asiatic rice borer, Chilo suppressalis (Walker) (Lepidoptera: Crambidae), from 23 locations in China by sequencing the wsp gene. Our results showed that C. suppressalis populations differed widely in their Wolbachia infection rate. Wolbachia was not detected in 13 of the geographically separate populations. In the 10 populations for which infections were detected, the highest rates were approximate to 40%, in the Guiyang, Xiangyin, Xuzhou, and Yangzhou populations. Phylogenetic analysis of variation in the wsp gene from the six strains of Wolbachia detected showed they belonged to either supergroup A (two populations) or supergroup B (four populations). The phylogenetic relatedness among the wsp sequences found in C. suppressalis was high, and these sequences were also closely related to wsp sequences in other species. This could be an evidence of horizontal transference. | 2350 | NA | NA | NA | no | no |
| 2351 | 2017 | NA | Fernandez-Mestre, MT; Yehirobi, C; Montagnani, S; Balbas, O; Layrisse, Z | 2005 | Genetic variability of apolipoprotein E in different populations from Venezuela | The genetic variation at the Apolipoprotein E locus (APOE) is an important determinant of plasma lipids and has been implicated in various human pathological conditions. The objective of the present study was to estimate the distribution of APOE alleles in five Venezuelan communities: two Amerindian tribes (Bari and Yucpa), one Negroid population from Curiepe, one Caucasoid population from Colonia Tovar and the mestizo urban population living in Caracas. The APOE*3 allele was the most common allele in all populations studied. However, a significant increase in the APOE*2 allele frequency in the Mestizo (18.96%) and Negroid (16.25%) populations was found. Similar to results reported in other Native American populations we have found that the APOE\*2 allele is completely absent in the Bari and Yucpa Amerindians. Frequencies found in the Colonia Tovar population are in agreement with those reported in the population of Germany, indicating a high degree of relatedness. The results support the notion that the distribution of the APOE alleles shows ethnic variability. | 2351 | NA | NA | NA | no | no |
| 2352 | 2017 | NA | Diancourt, L; Passet, V; Nemec, A; Dijkshoorn, L; Brisse, S | 2010 | The Population Structure of Acinetobacter baumannii: Expanding Multiresistant Clones from an Ancestral Susceptible Genetic Pool | Outbreaks of hospital infections caused by multidrug resistant Acinetobacter baumannii strains are of increasing concern worldwide. Although it has been reported that particular outbreak strains are geographically widespread, little is known about the diversity and phylogenetic relatedness of A. baumannii clonal groups. Sequencing of internal portions of seven housekeeping genes (total 2,976 nt) was performed in 154 A. baumannii strains covering the breadth of known diversity and including representatives of previously recognized international clones, and in 19 representatives of other Acinetobacter species. Restricted amounts of diversity and a star-like phylogeny reveal that A. baumannii is a genetically compact species that suffered a severe bottleneck in the recent past, possibly linked to a restricted ecological niche. A. baumannii is neatly demarcated from its closest relative (genomic species 13TU) and other Acinetobacter species. Multilocus sequence typing analysis demonstrated that the previously recognized international clones I to III correspond to three clonal complexes, each made of a central, predominant genotype and few single locus variants, a hallmark of recent clonal expansion. Whereas antimicrobial resistance was almost universal among isolates of these and a novel international clone (ST15), isolates of the other genotypes were mostly susceptible. This dichotomy indicates that antimicrobial resistance is a major selective advantage that drives the ongoing rapid clonal expansion of these highly problematic agents of nosocomial infections. | 2352 | NA | NA | NA | no | no |
| 2353 | 2017 | NA | Gaudeul, M | 2006 | Disjunct distribution of Hypericum nummularium L. (Hypericaceae): molecular data suggest bidirectional colonization from a single refugium rather than survival in distinct refugia | Hypericum nummularium has a strongly disjunct, bi-areal distribution in Europe: it is abundant in the Pyrenees and grows in a very restricted part of the Alps, more than 1000 km away. My aim was to estimate the genetic divergence between these areas and to identify the factors responsible for the disjunction: glacial relicts, bidirectional colonization from a common refugium, long-distance dispersal and/or human introduction? Internal transcribed spacers (ITS) sequencing (680 bp) and amplified fragment length polymorphism (AFLP) fingerprinting (104 polymorphic markers) showed very low differentiation between populations in the Alps and the Pyrenees, indicating that H. nummularium probably survived in a single refugium. Moreover, levels of genetic diversity were similar in the two areas, making human introduction and long-distance dispersal unlikely. Thus, the species probably survived in one refugium, subsequently colonizing both areas more or less simultaneously. The comparison of genetic and geographical distances suggested a step by step migration in the Alps (isolation by distance), whereas random dispersal events were more likely in the Pyrenees. Finally, I discuss possible causes for the restricted distribution area of H. nummularium in the Alps (e.g. unsuitable habitat, low dispersal capacities) and conclude that strong human disturbance is probably the major limit to the expansion of the species in this region. (c) 2006 The Linnean Society of London. | 2353 | NA | NA | NA | no | no |
| 2354 | 2017 | NA | Cagliani, R; Riva, S; Pozzoli, U; Fumagalli, M; Comi, GP; Bresolin, N; Clerici, M; Sironi, M | 2011 | Balancing selection is common in the extended MHC region but most alleles with opposite risk profile for autoimmune diseases are neutrally evolving | Background: Several susceptibility genetic variants for autoimmune diseases have been identified. A subset of these polymorphisms displays an opposite risk profile in different autoimmune conditions. This observation open interesting questions on the evolutionary forces shaping the frequency of these alleles in human populations. We aimed at testing the hypothesis whereby balancing selection has shaped the frequency of opposite risk alleles. Results: Since balancing selection signatures are expected to extend over short genomic portions, we focused our analyses on 11 regions carrying putative functional polymorphisms that may represent the disease variants (and the selection targets). No exceptional nucleotide diversity was observed for ZSCAN23, HLA-DMB, VARS2, PTPN22, BAT3, C6orf47, and IL10; summary statistics were consistent with evolutionary neutrality for these gene regions. Conversely, CDSN/PSORS1C1, TRIM10/TRIM40, BTNL2, and TAP2 showed extremely high nucleotide diversity and most tests rejected neutrality, suggesting the action of balancing selection. For TAP2 and BTNL2 these signatures are not secondary to linkage disequilibrium with HLA class II genes. Nonetheless, with the exception of variants in TRIM40 and CDSN, our data suggest that opposite risk SNPs are not selection targets but rather have accumulated as neutral variants. Conclusion: Data herein indicate that balancing selection is common within the extended MHC region and involves several non-HLA loci. Yet, the evolutionary history of most SNPs with an opposite effect for autoimmune diseases is consistent with evolutionary neutrality. We suggest that variants with an opposite effect on autoimmune diseases should not be considered a distinct class of disease alleles from the evolutionary perspective and, in a few cases, the opposite effect on distinct diseases may derive from complex haplotype structures in regions with high genetic diversity. | 2354 | NA | NA | NA | no | no |
| 2355 | 2017 | NA | Cockburn, A; Osmond, HL; Mulder, RA; Double, MC; Green, DJ | 2008 | Demography of male reproductive queues in cooperatively breeding superb fairy-wrens Malurus cyaneus | 1 Subordinate helpers in cooperative societies may gain both immediate and future benefits, including paternity and territorial inheritance. However, if such opportunities correlate with rank in the queue, it is unclear why such queues should be stable.                  2 In cooperatively breeding superb fairy‐wrens Malurus cyaneus, only males are generally philopatric, and form stable hierarchical queues for the dominant position.  3 Male opportunities for reproduction are influenced both by their dominance status within the group, and their relatedness to the breeding female. For young queuing subordinates, the breeding female is typically their mother. Because of incest avoidance, reproduction is possible only through extra‐group mating, even if the dominant position is achieved while the mother is still on the territory. If the mother dies while the helper is still a subordinate, he can seek matings both outside the group, and with the unrelated replacement female within the group. Finally, males can achieve the dominant position and pair with an unrelated female by inheritance, dispersal to a neighbouring vacancy, or by forming a liaison with an immigrant subordinate female that causes fission of the natal territory.  4 On average males spent more time living with unrelated females than with their mother. Subordinate males gained no survival advantages when living with their mother rather than an unrelated female, contrary to the prediction that parents facilitate the survival of their offspring.  5 Dominants and subordinates also had similar survival. Mortality accelerated over time, probably because older males invest more in extra‐group courtship display. 6 Fairy‐wren queues are likely to be stable because older birds are superior, and because extra‐pair mating provides direct benefits to subordinates. | 2355 | NA | NA | NA | no | no |
| 2356 | 2017 | NA | Kalisz, S; Hanzawa, FM; Tonsor, SJ; Thiede, DA; Voigt, S | 1999 | Ant-mediated seed dispersal alters pattern of relatedness in a population of Trillium grandiflorum | Seed dispersal creates the initial spatial distribution of individuals in a population and in conjunction with the mating system influences spatial patterns of relatedness. This spatial template of related individuals sets the stage for all subsequent density-dependent and frequency-dependent interactions. In this study we document how ant-mediated seed dispersal affects the number and relatedness of seeds in both dispersed and undispersed aggregations and how these patterns influence seedling emergence in the long-lived perennial, Trillium grandiflorum. Experimental hand-pollinations in two years demonstrated that selfing is extremely rare and suggested that self-incompatibility (SI) is a likely explanation. Our multi-locus outcrossing estimate (t(m) =1.05 +/- 0.056) confirms this result and also suggests that seeds within a fruit are likely to have the same pollen parent. Thus a highly outcrossing mating system is the initial determinant of relatedness among seeds within a fruit. We tracked uniquely coded, radiolabeled seeds from 30 and 40 fruits in 1991 and 1992, respectively, to determine how dispersal alters this initial relatedness of seeds. Of the 335 and 876 seeds labeled in these two years, we recovered 63% and 76% of the seeds postdispersal and found that 19% and 23% of the recovered seeds were dispersed >10 cm from the maternal parent in the first and second years, respectively. In both years, ant-mediated dispersal reduced the number of seeds near the maternal parent. However, the effect of seed dispersal on the number of seeds in aggregations varied among years. Anti-mediated dispersal increased the number of seeds in dispersed aggregations in the first year and decreased the number in the second year. The average seed dispersal distance also differed between years: 2.41 m (+/-0.33) vs. 0.53 m (+/-0.06) in years 1 and 2, respectively. Ant-mediated seed dispersal decreased the probability of a seed having a sibling as its nearest neighbor postdispersal by between one-third and one-half. In contrast, seedling emergence was related to neither dispersal nor seed aggregation size in our study. However, the fitness effects of dispersal may be important later in the life cycle of this long-lived species and as such were undetected. One scenario is that plants derived from seeds dispersed out of their sibling relatedness group may gain minority advantage both in terms of mating success (if the population is SI) and other frequency-dependent processes like disease resistance. | 2356 | NA | NA | NA | no | no |
| 2357 | 2017 | NA | Gandhi, K; Thera, MA; Coulibaly, D; Traore, K; Guindo, AB; Ouattara, A; Takala-Harrison, S; Berry, AA; Doumbo, OK; Plowe, CV | 2014 | Variation in the Circumsporozoite Protein of Plasmodium falciparum: Vaccine Development Implications | The malaria vaccine candidate RTS,S/AS01 is based on immunogenic regions of Plasmodium falciparum circumsporozoite protein (CSP) from the 3D7 reference strain and has shown modest efficacy against clinical disease in African children. It remains unclear what aspect(s) of the immune response elicited by this vaccine are protective. The goals of this study were to measure diversity in immunogenic regions of CSP, and to identify associations between polymorphism in CSP and the risk of P. falciparum infection and clinical disease. The present study includes data and samples from a prospective cohort study designed to measure incidence of malaria infection and disease in children in Bandiagara, Mali. A total of 769 parasite-positive blood samples corresponding to both acute clinical malaria episodes and asymptomatic infections experienced by 100 children were included in the study. Non-synonymous SNP data were generated by 454 sequencing for the T-cell epitopes, and repeat length data were generated for the B-cell epitopes of the cs gene. Cox proportional hazards models were used to determine the effect of sequence variation in consecutive infections occurring within individuals on the time to new infection and new clinical malaria episode. Diversity in the T-cell epitope-encoding regions Th2R and Th3R remained stable throughout seasons, between age groups and between clinical and asymptomatic infections with the exception of a higher proportion of 3D7 haplotypes found in the oldest age group. No associations between sequence variation and hazard of infection or clinical malaria were detected. The lack of association between sequence variation and hazard of infection or clinical malaria suggests that naturally acquired immunity to CSP may not be allele-specific. | 2357 | NA | NA | NA | no | no |
| 2358 | 2017 | NA | Sommerhalder, RJ; McDonald, BA; Mascher, F; Zhan, JS | 2011 | Effect of hosts on competition among clones and evidence of differential selection between pathogenic and saprophytic phases in experimental populations of the wheat pathogen Phaeosphaeria nodorum | Background: Monoculture, multi-cropping and wider use of highly resistant cultivars have been proposed as mechanisms to explain the elevated rate of evolution of plant pathogens in agricultural ecosystems. We used a mark-release-recapture experiment with the wheat pathogen Phaeosphaeria nodorum to evaluate the impact of two of these mechanisms on the evolution of a pathogen population. Nine P. nodorum isolates marked with ten microsatellite markers and one minisatellite were released onto five replicated host populations to initiate epidemics of Stagonospora nodorum leaf blotch. The experiment was carried out over two consecutive host growing seasons and two pathogen collections were made during each season. Results: A total of 637 pathogen isolates matching the marked inoculants were recovered from inoculated plots over two years. Genetic diversity in the host populations affected the evolution of the corresponding P. nodorum populations. In the cultivar mixture the relative frequencies of inoculants did not change over the course of the experiment and the pathogen exhibited a low variation in selection coefficients. Conclusions: Our results support the hypothesis that increasing genetic heterogeneity in host populations may retard the rate of evolution in associated pathogen populations. Our experiment also provides indirect evidence of fitness costs associated with host specialization in P. nodorum as indicated by differential selection during the pathogenic and saprophytic phases. | 2358 | NA | NA | NA | no | no |
| 2359 | 2017 | NA | Kellner, R; Vollmeister, E; Feldbrugge, M; Begerow, D | 2011 | Interspecific Sex in Grass Smuts and the Genetic Diversity of Their Pheromone-Receptor System | The grass smuts comprise a speciose group of biotrophic plant parasites, so-called Ustilaginaceae, which are specifically adapted to hosts of sweet grasses, the Poaceae family. Mating takes a central role in their life cycle, as it initiates parasitism by a morphological and physiological transition from saprobic yeast cells to pathogenic filaments. As in other fungi, sexual identity is determined by specific genomic regions encoding allelic variants of a pheromone-receptor (PR) system and heterodimerising transcription factors. Both operate in a biphasic mating process that starts with PR-triggered recognition, directed growth of conjugation hyphae, and plasmogamy of compatible mating partners. So far, studies on the PR system of grass smuts revealed diverse interspecific compatibility and mating type determination. However, many questions concerning the specificity and evolutionary origin of the PR system remain unanswered. Combining comparative genetics and biological approaches, we report on the specificity of the PR system and its genetic diversity in 10 species spanning about 100 million years of mating type evolution. We show that three highly syntenic PR alleles are prevalent among members of the Ustilaginaceae, favouring a triallelic determination as the plesiomorphic characteristic of this group. Furthermore, the analysis of PR loci revealed increased genetic diversity of single PR locus genes compared to genes of flanking regions. Performing interspecies sex tests, we detected a high potential for hybridisation that is directly linked to pheromone signalling as known from intraspecies sex. Although the PR system seems to be optimised for intraspecific compatibility, the observed functional plasticity of the PR system increases the potential for interspecific sex, which might allow the hybrid-based genesis of newly combined host specificities. | 2359 | NA | NA | NA | no | no |
| 2360 | 2017 | NA | MORIN, PA; RYDER, OA | 1991 | FOUNDER CONTRIBUTION AND PEDIGREE INFERENCE IN A CAPTIVE BREEDING COLONY OF LION-TAILED MACAQUES, USING MITOCHONDRIAL-DNA AND DNA FINGERPRINT ANALYSES | Captive colony genetic management is dependent on knowledge of the colony pedigree. In most zoos, records for colonies held prior to the past decade are incomplete or nonexistent. We have used a nested set of molecular techniques to infer genetic relationships in a colony of 15 lion-tailed macaques after twenty years of unmonitored reproduction. The troop was first divided into lines of matrilineal descent using mitochondrial DNA RFLP patterns. Paternity and maternity were then assigned on the basis of DNA fingerprint analysis. As mtDNA is maternally inherited, it is of no use in paternity assessment, but enabled subdivision of the colony into maternal lines. DNA fingerprinting would have been of little use by itself because of high levels of relatedness in several generations of offspring within the colony. The combination of the two techniques, however, enabled strong inference of both paternity and maternity in all cases. These data were instrumental in the inclusion of this troop in the AAZPA Species Survival Plan. | 2360 | NA | NA | NA | no | no |
| 2361 | 2017 | NA | Gariboldi, MC; Tunez, JI; Dejean, CB; Failla, M; Vitullo, AD; Negri, MF; Cappozzo, HL | 2015 | Population Genetics of Franciscana Dolphins (Pontoporia blainvillei): Introducing a New Population from the Southern Edge of Their Distribution | Due to anthropogenic factors, the franciscana dolphin, Pontoporia blainvillei, is the most threatened small cetacean on the Atlantic coast of South America. Four Franciscana Management Areas have been proposed: Espiritu Santo to Rio de Janeiro (FMA I), Sao Paulo to Santa Catarina (FMA II), Rio Grande do Sul to Uruguay (FMA III), and Argentina (FMA IV). Further genetic studies distinguished additional populations within these FMAs. We analyzed the population structure, phylogeography, and demographic history in the southernmost portion of the species range. From the analysis of mitochondrial DNA control region sequences, 5 novel haplotypes were found, totalizing 60 haplotypes for the entire distribution range. The haplotype network did not show an apparent phylogeographical signal for the southern FMAs. Two populations were identified: Monte Hermoso (MH) and Necochea (NC)+Claromeco (CL)+Rio Negro (RN). The low levels of genetic variability, the relative constant size over time, and the low levels of gene flow may indicate that MH has been colonized by a few maternal lineages and became isolated from geographically close populations. The apparent increase in NC+CL+RN size would be consistent with the higher genetic variability found, since genetic diversity is generally higher in older and expanding populations. Additionally, RN may have experienced a recent split from CL and NC; current high levels of gene flow may be occurring between the latter ones. FMA IV would comprise four franciscana dolphin populations: Samborombon West+Samborombon South, Cabo San Antonio+Buenos Aires East, NC+CL+Buenos Aires Southwest+RN and MH. Results achieved in this study need to be taken into account in order to ensure the long-term survival of the species. | 2361 | NA | NA | NA | no | no |
| 2362 | 2017 | NA | Carvalho, S; Chelo, IM; Goy, C; Teotonio, H | 2014 | The role of hermaphrodites in the experimental evolution of increased outcrossing rates in Caenorhabditis elegans | Background: Why most organisms reproduce via outcrossing rather than selfing is a central question in evolutionary biology. It has long ago been suggested that outcrossing is favoured when it facilitates adaptation to novel environments. We have previously shown that the experimental evolution of increased outcrossing rates in populations of the male-hermaphrodite nematode Caenorhabditis elegans were correlated with the experimental evolution of increased male fitness. However, it is unknown whether outcrossing led to adaptation, and if so, which fitness components can explain the observed increase in outcrossing rates. Results: Using experimental evolution in six populations with initially low standing levels of genetic diversity, we show with head-to-head competition assays that population-wide fitness improved during 100 generations. Since outcrossing rates increased during the same period, this result demonstrates that outcrossing is adaptive. We also show that there was little evolution of hermaphrodite fitness under conditions of selfing or under conditions of outcrossing with unrelated tester males. We nonetheless find a positive genetic correlation between hermaphrodite self-fitness and population-wide fitness, and a negative genetic correlation between hermaphrodite mating success and population-wide fitness. These results suggest that the several hermaphrodite traits measured are fitness components. Tradeoffs expressed in hermaphrodites, particularly noticed between self-fitness and mating success, may in turn explain their lack of change during experimental evolution. Conclusions: Our findings indicate that outcrossing facilitates adaptation to novel environments. They further indicate that the experimental evolution of increased outcrossing rates depended little on hermaphrodites because of fitness tradeoffs between selfing and outcrossing. Instead, the evolution of increased outcrossing rates appears to have resulted from unhindered selection on males. | 2362 | NA | NA | NA | no | no |
| 2363 | 2017 | NA | Freville, H; Colas, B; Riba, M; Caswell, H; Mignot, A; Imbert, E; Olivieri, I | 2004 | Spatial and temporal demographic variability in the endemic plant species Centaurea corymbosa (Asteraceae) | Centaurea corymbosa is an endemic plant species restricted to a 3-km(2) area in southern France. This species is known from only six. small populations that are highly differentiated genetically. Matrix models based on eight years of data (1994-2001) were used to assess the pattern of variation in the demographic vital rates of this species, and to investigate the causes of their variation. Asymptotic growth rates X varied widely between years and populations (0.613-1.424). Randomization tests were developed to test for spatial and temporal variation in the asymptotic growth rates. These tests rely on individual data on both survival and fecundity. As our demographic survey only allowed us to estimate,average fecundities, additional fecundity data collected from 1994 to 1996 were used to assess the distribution of individual fecundity expected within populations under demographic stochasticity or sampling error. Randomization tests showed that asymptotic growth rates were significantly different between populations and between years in C. corymbosa. In contrast, log-linear analysis performed only on transition data (i.e., excluding fecundity) suggested that the observed variations in transition probabilities were mostly explained by sampling error or demographic stochasticity rather than environmental stochasticity. This suggested that variations in fecundity among populations and among years may play a key role to explain temporal and spatial differences in X. Life-table response experiment analysis revealed that variations in fecundity and especially in the number of just-emerged seedlings per plant explained most of the observed variance in X. Spatial and temporal variations were detected for most lower-level vital rates, but causal factors that may account for these patterns a. re still unknown: no effect of genetic diversity was detected on the dynamics of the species, and correlation between lower-level vital rates and climatic data did not reveal any clear trends. Our study emphasizes the need to conduct long-term demographic surveys and to collect individual fecundity data to get more insights into the causes of variation of the demographic behavior of C. corymbosa. | 2363 | NA | NA | NA | no | no |
| 2364 | 2017 | NA | Pfeifer, M; Passalacqua, NG; Bartram, S; Schatz, B; Croce, A; Carey, PD; Kraudelt, H; Jeltsch, F | 2010 | Conservation priorities differ at opposing species borders of a European orchid | How populations from different regions within the distribution of a species contribute to the adaptive potential and survival of that species has important implications for formulating conservation actions. We test assumptions of concepts on geographic population structure (e.g. central-marginal concept and ‘rear edge versus leading edge’ model) that could be used to inform conservation of plant species under climatic changes. We analyze a comprehensive dataset of demographic traits (e.g. population size, flowering, delta(13)C of plant leaves) of up to 32 sites of Himantoglossum hircinum (L) Spreng. (Orchidaceae) located within six sub-regions of its European distribution range. Soil and climate parameters are employed as environmental predictors of variation in measured population traits. Climate is the main driver of demographic variability overriding central-marginal gradients that might be present. Warming of the climate at high latitudes paves the way for northward range expansion of species. Populations at the north and north-eastern range peripheries partly show exponential population growth and high genetic diversity and are likely to be the source of immigrants for colonization of newly suitable habitats as the climate continues to change. In recent times, populations at the southern range periphery have suffered from intensification of land use and decreasing rainfall, but in the case of Southern Italy are important because they contain genetically unique traits. Populations at both, ‘leading’ and ‘rear’, edges ought to be at the focus of conservation planning. Different conservation strategies are proposed at opposing species borders taking into account spatial variation in population needs on a geographic scale, projected population response to expected environmental changes and genetic characteristics. (C) 2010 Elsevier Ltd. All rights reserved. | 2364 | NA | NA | NA | no | no |
| 2365 | 2017 | NA | Ghori, NUH; Hayat, MQ; Davino, S | 2016 | Genetic diversity and evolutionary analysis of Citrus Tristeza Virus p20 gene in Pakistan: insights into the spread and epidemiology | Background: Citrus tristeza virus (CTV) is a widespread disease and the most destruction causing agent of citrus. It is one of the most destructive diseases of citrus. Pakistan is ranked amongst the top ten citrus producing countries around the globe and it contributes about 2% to its foreign exchange earnings. Based on this assumption it is very important to monitor and determine the evolutionary forces and the phylogeography of Pakistani CTV population. Methods: A total of 49 sequences of p20 gene from Pakistan were phylogenetically compared with CTV sequences worldwide. These sequences were analyzed for their genetic diversity and evolution using a Bayesian Probability approach and predicted secondary structure. Results: Phylogenetic analysis using Bayesian probability inference and predicted secondary structures diversity of CTV indicated that Pakistani isolates were not diverse from global isolates. Lineage analysis showed that CTV was introduced in Pakistan in three individual events from various parts of the world. After that CTV dispersed in Pakistan via vector transmission or by use of infected propagating material by local farmers. Conclusions: Our study confirmed multiple introductions of CTV in Pakistan and also confirmed the dissemination of CTV within Pakistan. This study also shows that the mutations are present in the predicted secondary structure of the p20 protein, however, it is not known if it affects the pathogenicity of the virus. | 2365 | NA | NA | NA | no | no |
| 2366 | 2017 | NA | Kamran, M; Ahmad, MD; Anjum, AA; Maqbool, A; Muhammad, K; Khan, HM; Hudda, N; Nawaz, M; Ali, MA | 2014 | ANTIGENIC VARIATION AMONG PASTEURELLA MULTOCIDA ISOLATES FROM DISEASED BUFFALOES BY PROTEIN PROFILING AND CLUSTER ANALYSIS | Hemorrhagic septicemia is an acute bacterial disease of buffaloes and cattle caused by Pasteurella multocida. In the present study, P. multocida (n=11) were isolated from healthy carrier (04/200) and diseased buffaloes (07/200) from Sargodha division, Punjab. Pasteurella multocida were identified by culture characteristics and biochemical profile index (API) Kit. Isolates were confirmed by serological and molecular characterization by PCR. Antigenic variation among P. multocida (11 isolates) and one vaccine strain was revealed by sodium dodecyl sulfate polyacrylamide gel electrophoresis (SDS-PAGE). Although SDS-PAGE revealed that five major proteins were present in all the isolates as well as in vaccine strain of P. multocida but isolates had variations in minor protein bands. Relatedness among the isolates was assessed by cluster analysis using fingerprint analysis of missing data (FAMD) and principal coordinate analysis (PCA) of 11 isolates and one vaccine strain. All isolates were clustered into 5 different groups namely I, II, III, IV and V on the basis of their electrophoretic profile. Group I, II, III, IV and V contained 1 (8.33%), 1(8.33%), 2 (16.67%), 6 (50%) and 2 (16.67%) isolates of P. multocida, respectively. Vaccine strain had high relatedness with group IV (S3, S6, S7, S8, and S9). It is concluded that distinct polypeptide variation is present among P. multocida isolates which insinuate for detailed proteomic and genomic analysis for the development of effective vaccines and control of hemorrhagic septicemia. | 2366 | NA | NA | NA | no | no |
| 2367 | 2017 | NA | Kreiss, A; Cheng, YY; Kimble, F; Wells, B; Donovan, S; Belov, K; Woods, GM | 2011 | Allorecognition in the Tasmanian Devil (Sarcophilus harrisii), an Endangered Marsupial Species with Limited Genetic Diversity | Tasmanian devils (Sarcophilus harrisii) are on the verge of extinction due to a transmissible cancer, devil facial tumour disease (DFTD). This tumour is an allograft that is transmitted between individuals without immune recognition of the tumour cells. The mechanism to explain this lack of immune recognition and acceptance is not well understood. It has been hypothesized that lack of genetic diversity at the Major Histocompatibility Complex (MHC) allowed the tumour cells to grow in genetically similar hosts without evoking an immune response to alloantigens. We conducted mixed lymphocyte reactions and skin grafts to measure functional MHC diversity in the Tasmanian devil population. The limited MHC diversity was sufficient to produce measurable mixed lymphocyte reactions. There was a wide range of responses, from low or no reaction to relatively strong responses. The highest responses occurred when lymphocytes from devils from the east of Tasmania were mixed with lymphocytes from devils from the west of Tasmania. All of the five successful skin allografts were rejected within 14 days after surgery, even though little or no MHC I and II mismatches were found. Extensive T-cell infiltration characterised the immune rejection. We conclude that Tasmanian devils are capable of allogeneic rejection. Consequently, a lack of functional allorecognition mechanisms in the devil population does not explain the transmission of a contagious cancer. | 2367 | NA | NA | NA | no | no |
| 2368 | 2017 | NA | Shaheen, IY; Abu-Dieyeh, MH; Ash, GJ; Watson, AK | 2010 | Physiological characterization of the dandelion bioherbicide, Sclerotinia minor IMI 344141 | The fungus Sclerotinia minor (IMI 344141) is being developed as a biological control for dandelion and other broadleaf weeds in turfgrass environments. Being a microbial pest control agent (MPCA), the S. minor strain must be characterized to show relatedness to like organisms and to distinguish the MPCA from related microorganisms. Phenotypic variation among 30 isolates of S. minor, collected from different regions and hosts, was studied on potato dextrose agar (PDA) and oatmeal agar (OMA). Isolates varied significantly in sclerotia shape (length/width ratio) and number, but did not vary in colony morphology or growth rates. There was high diversity (0.6) among the mycelial compatibility groups (MCG) as seven multi-member and 11 single-member groups were recognized. Isolates were categorized into highly virulent, virulent, moderately virulent, and hypo virulent based on 48 h post mycelial growth on detached dandelion leaves. When assessed on dandelion plants in the greenhouse, isolate IMI 344141 ranked the highest in biocontrol efficacy, reduction of above-and below-ground biomass, and reduction in dandelion survival. Oxalic acid production was not correlated with isolate aggressiveness or growth rate and did not vary among isolates of the same MCG. IMI 344141 can be phenotypically distinguished from the other tested S. minor isolates by performing vegetative compatibility testing and counting sclerotia produced on standard 9-cm diameter PDA plates. IMI 344141 produces <100 sclerotia/plate. | 2368 | NA | NA | NA | no | no |
| 2369 | 2017 | NA | Sato, T | 2006 | Occurrence of deformed fish and their fitness-related traits in Kirikuchi charr, Salvelinus leucomaenis japonicus, the southernmost population of the genus Salvelinus | Kirikuchi charr, Salvelinus leucomaenis japonicus, is the southernmost population of the genus Salvelinus. It is endemic to the Kii Peninsula, central Honshu Island, Japan. As a consequence of anthropogenic disturbances, a few populations of Kirikuchi charr with low genetic diversity now survive only in small, isolated habitats. This study investigated the occurrence of deformed individuals and assessed differences between deformed and noncleformed fish in fitness-related traits, Le., body size, body condition, growth rate, reproductive traits, survival rate, and habitat use, for two small isolated populations of Kirikuchi charr in the upper drainage of the Totsu River system of the Kill Peninsula. The two populations contained deformed fish in the respective proportions of 8.0-17.4% and 3.0-5.8% between 2003 and 2005. Annual survival rates of deformed fish were approximately half those of nondeformed fish. Other traits were not significantly different between deformed and nondeformed fish. These results indicate that the occurrence of deformities is an important ecological indicator that reflects the decline in fitness of small, isolated populations. | 2369 | NA | NA | NA | no | no |
| 2370 | 2017 | NA | Rojas-Bracho, L; Taylor, BL | 1999 | Risk factors affecting the vaquita (Phocoena sinus) | Despite the vaquita being commonly cited as one of the most endangered marine mammals in the world, there is still disagreement over which factors put the species at greatest risk of extinction. This lack of agreement hinders management decisions needed to reduce the risk to the species. To expedite decision-making we consider four major risk factors. Habitat alteration from reduced flow of the Colorado River does not currently appear to be a risk factor because productivity remains high in vaquita habitat. Pollutant loads are low and pose low to no risk. Reduced fitness from inbreeding depression and loss of genetic variability are unlikely to pose high risk currently, though risk will increase if vaquitas remain at low abundance over long periods of time. Mortality resulting from fisheries bycatch poses high risk. Thus, short term management should not be hindered by uncertainty in estimating the risk of these factors, and primary conservation efforts should be directed towards immediate elimination of incidental fishery mortality. | 2370 | NA | NA | NA | no | no |
| 2371 | 2017 | NA | Chan, CW; Spathis, R; Reiff, DM; McGrath, SE; Garruto, RM; Lum, JK | 2012 | Diversity of Plasmodium falciparum Chloroquine Resistance Transporter (pfcrt) Exon 2 Haplotypes in the Pacific from 1959 to 1979 | Nearly one million deaths are attributed to malaria every year. Recent reports of multi-drug treatment failure of falciparum malaria underscore the need to understand the molecular basis of drug resistance. Multiple mutations in the Plasmodium falciparum chloroquine resistance transporter (pfcrt) are involved in chloroquine resistance, but the evolution of complex haplotypes is not yet well understood. Using over 4,500 archival human serum specimens collected from 19 Pacific populations between 1959 and 1979, the period including and just prior to the appearance of chloroquine treatment failure in the Pacific, we PCR-amplified and sequenced a portion of the pfcrt exon 2 from 771 P. falciparum-infected individuals to explore the spatial and temporal variation in falciparum malaria prevalence and the evolution of chloroquine resistance. In the Pacific, the prevalence of P. falciparum varied considerably across ecological zones. On the island of New Guinea, the decreases in prevalence of P. falciparum in coastal, high-transmission areas over time were contrasted by the increase in prevalence during the same period in the highlands, where transmission was intermittent. We found 78 unique pfcrt haplotypes consisting of 34 amino acid substitutions and 28 synonymous mutations. More importantly, two pfcrt mutations (N75D and K76T) implicated in chloroquine resistance were present in parasites from New Hebrides (now Vanuatu) eight years before the first report of treatment failure. Our results also revealed unexpectedly high levels of genetic diversity in pfcrt exon 2 prior to the historical chloroquine resistance selective sweep, particularly in areas where disease burden was relatively low. In the Pacific, parasite genetic isolation, as well as host acquired immune status and genetic resistance to malaria, were important contributors to the evolution of chloroquine resistance in P. falciparum. | 2371 | NA | NA | NA | no | no |
| 2372 | 2017 | NA | Silvestre, A; Sauve, C; Cortet, J; Cabaret, J | 2009 | Contrasting genetic structures of two parasitic nematodes, determined on the basis of neutral microsatellite markers and selected anthelmintic resistance markers | For the first time, the neutral genetic relatedness of natural populations of Trichostrongylid nematodes was investigated in relation to polymorphism of the beta-tubulin gene, which is selected for anthelminthic treatments. The aim of the study was to assess the contribution of several evolutionary processes: migration and genetic drift by neutral genetic markers and selection by anthelminthic treatments on the presence of resistance alleles at beta-tubulin. We studied two nematode species (Teladorsagia circumcincta and Haemonchus contortus) common in temperate climatic zones; these species are characterized by contrasting life history traits. We studied 10 isolated populations of goat nematode parasites: no infected adult goat had been exchanged after the herds were established. Beta-tubulin polymorphism was similar in these two species. One and two beta-tubulin alleles from T. circumcincta and H. contortus respectively were shared by several populations. Most of the beta-tubulin alleles were ‘private’ alleles. No recombination between alleles was detected in BZ-resistant alleles from T. circumcincta and H. contortus. The T. circumcincta populations have not diverged much since their isolation (F(ST) < 0.08), whereas H. contortus displayed marked local genetic differentiation (F(ST) ranging from 0.08 to 0.18). These findings suggest that there are severe bottlenecks in the H. contortus populations, possibly because of their reduced abundance during unfavourable periods and their high reproductive rate, which allows the species to persist even after severe population reduction. Overall, the data reported contradict the hypothesis of the origin of beta-tubulin resistance alleles in these populations from a single mutational event, but two other hypotheses (recurrent mutation generating new alleles in isolated populations and the introduction of existing alleles) emerge as equally likely. | 2372 | NA | NA | NA | no | no |
| 2373 | 2017 | NA | Zhan, JS; Yang, LN; Zhu, W; Shang, LP; Newton, AC | 2012 | Pathogen Populations Evolve to Greater Race Complexity in Agricultural Systems - Evidence from Analysis of Rhynchosporium secalis Virulence Data | Fitness cost associated with pathogens carrying unnecessary virulence alleles is the fundamental assumption for preventing the emergence of complex races in plant pathogen populations but this hypothesis has rarely been tested empirically on a temporal and spatial scale which is sufficient to distinguish evolutionary signals from experimental error. We analyzed virulence characteristics of similar to 1000 isolates of the barley pathogen Rhynchosporium secalis collected from different parts of the United Kingdom between 1984 and 2005. We found a gradual increase in race complexity over time with a significant correlation between sampling date and race complexity of the pathogen (r(20) = 0.71, p = 0.0002) and an average loss of 0.1 avirulence alleles (corresponding to an average gain of 0.1 virulence alleles) each year. We also found a positive and significant correlation between barley cultivar diversity and R. secalis virulence variation. The conditions assumed to favour complex races were not present in the United Kingdom and we hypothesize that the increase in race complexity is attributable to the combination of natural selection and genetic drift. Host resistance selects for corresponding virulence alleles to fixation or dominant frequency. Because of the weak fitness penalty of carrying the unnecessary virulence alleles, genetic drift associated with other evolutionary forces such as hitch-hiking maintains the frequency of the dominant virulence alleles even after the corresponding resistance factors cease to be used. | 2373 | NA | NA | NA | no | no |
| 2374 | 2017 | NA | Horacio, AN; Silva-Costa, C; Diamantino-Miranda, J; Lopes, JP; Ramirez, M; Melo-Cristino, J | 2016 | Population Structure of Streptococcus pneumoniae Causing Invasive Disease in Adults in Portugal before PCV13 Availability for Adults: 2008-2011 | Among the 1660 isolates recovered from invasive pneumococcal disease (IPD) in adults (> = 18 yrs) in 2008-2011, a random sample of >= 50% of each serotype (n = 871) was chosen for MLST analysis and evaluation for the presence and type of pilus islands (PIs). The genetic diversity was high with 206 different sequence types (STs) detected, but it varied significantly between serotypes. The different STs represented 80 clonal complexes (CCs) according to goeBURST with the six more frequent accounting for more than half (50.6%) of the isolates-CC156 (serotypes 14, 9V and 23F), CC191 (serotype 7F), CC180 (serotype 3), CC306 (serotype 1), CC62 (serotypes 8 and 11A) and CC230 (serotype 19A). Most of the isolates (n = 587, 67.3%) were related to 29 Pneumococcal Molecular Epidemiology Network recognized clones. The overall proportion of isolates positive for any of the PIs was small (31.9%) and declined gradually during the study period (26.6% in 2011), mostly due to the significant decline of serotype 1 which is associated with PI-2. The changes in serotypes that occurred in adult IPD after the introduction of the seven-valent pneumococcal conjugate vaccine (PCV7) for children were mostly due to the expansion of previously circulating clones, while capsular switching was infrequent and not related to vaccine use. The reduction of IPD caused by PCV7 serotypes in the years following PCV7 implementation did not result in a decline of antimicrobial resistance in part due to the selection of resistant genotypes among serotypes 14 and 19A. | 2374 | NA | NA | NA | no | no |
| 2375 | 2017 | NA | McGreevy, TJ; Dabek, L; Gomez-Chiarri, M; Husband, TP | 2009 | Genetic diversity in captive and wild Matschie’s tree kangaroo (Dendrolagus matschiei) from Huon Peninsula, Papua New Guinea, based on mtDNA control region sequences | The Association of Zoos and Aquariums (AZA) Matschie’s tree kangaroo (Dendrolagus matschiei) population is at a critical point for assessing long-term viability. This population). established front 19 genetically uncharacterized D. matschiei, has endured a founder effect because only four individuals contributed the majority of offspring. The highly variable mitochondrial DNA (mtDNA) control region was sequenced for five of the female-founders by examining extant representatives of their maternal lineage and compared with wild (n = 13) and captive (n = 18) D. matschiei from Papua New Guinea (PNG). AZA female-founder D. matschiei control region haplotype diversity was low, compared with captive D. matschiei held in PNG. AZA D. matschiei have only two control region haplotypes because four out of five AZA female-founder D. matschiei had an identical sequence. Both AZA haplotypes were identified among the 17 wild and captive D. matschiei haplotypes from PNG. Genomic DNA extracted from wild D. matschiei fecal samples was a reliable source of mtDNA that could be used for a larger scale study. We recommend a nuclear DNA genetic analysis to more fully characterize AZA D. matschiei genetic diversity and to assist their Species Survival Plan (R). An improved understanding of D. matschiei genetics will contribute substantially to the conservation of these unique animals both in captivity and the wild. Zoo Biol 28:183-196, 2009. (C) 2008 Wiley-Liss, Inc. | 2375 | NA | NA | NA | no | no |
| 2376 | 2017 | NA | Hawley, DM; Sydenstricker, KV; Kollias, GV; Dhondt, AA | 2005 | Genetic diversity predicts pathogen resistance and cell-mediated immunocompetence in house finches | Evidence is accumulating that genetic variation within individual hosts can influence their susceptibility to pathogens. However, there have been few opportunities to experimentally test this relationship, particularly within outbred populations of non-domestic vertebrates. We performed a standardized pathogen challenge in house finches (Carpodacus mexicanus) to test whether multilocus heterozygosity across 12 microsatellite loci predicts resistance to a recently emerged strain of the bacterial pathogen, Mycoplasma gallisepticum (MG). We simultaneously tested whether the relationship between heterozygosity and pathogen susceptibility is mediated by differences in cell-mediated or humoral immunocompetence. We inoculated 40 house finches with MG under identical conditions and assayed both humoral and cell-mediated components of the immune response. Heterozygous house finches developed less severe disease when infected with MG, and they mounted stronger cell-mediated immune responses to phytohaemagglutinin. Differences in cell-mediated immunocompetence may, therefore, partly explain why more heterozygous house finches show greater resistance to MG. Overall, our results underscore the importance of multilocus heterozygosity for individual pathogen resistance and immunity. | 2376 | NA | NA | NA | no | no |
| 2377 | 2017 | NA | Smith, C; Ondrackova, M; Spence, R; Adams, S; Betts, DS; Mallon, E | 2011 | Pathogen-mediated selection for MHC variability in wild zebrafish | Hypothesis: Genes of the major histocompatibility complex (MHC) show high variability, which is believed to arise through pathogen-mediated selection. Organism: Zebrafish (Danio rerio) from seven independent natural populations. Location: Bangladesh: two sites in Khulna District in the River Ganges drainage, and five sites in Mymensingh District in the River Brahmaputra drainage. Methods: We investigated the relationship of parasite abundance and diversity with allelic variability of the MHC class II B gene in natural populations of zebrafish. A sample of 120 zebrafish was screened for metazoan parasites, and a subsample of 77 was assessed for immunogenetic variation. In total, 2475 metazoan parasites from 23 taxa were identified and 13 unique MHC class IIB alleles were isolated. Conclusion: We found no evidence for a heterozygote advantage, and overall there was lower than expected heterozygosity of MHC alleles. We identified lower parasite abundance and diversity with specific MHC alleles, and this pattern varied between geographic regions. Results suggest that MHC variability in zebrafish is maintained by frequency-dependent and fluctuating selection. | 2377 | NA | NA | NA | no | no |
| 2378 | 2017 | NA | Levin, SA; Dushoff, J; Plotkin, JB | 2004 | Evolution and persistence of influenza A and other diseases | The evolution of the etiological agents of disease presents one of the greatest challenges for their control, and makes essential complementing standard epidemiological investigations with broader approaches that allow for evolutionary change. Given the stunning genetic diversity that is possible for many such agents, such as the influenza virus, it is impossible to represent all of the diversity manifest at the level of amino acid sequences. We show that drift-variant influenza strains naturally cluster into groups which are associated with functionally important epitopic regions. Dominant clusters typically replace each other every 2-5 years, and this feature is fundamental to the development of vaccination strategies. We furthermore show that stochastic, fluctuations can greatly magnify small interference effects among strains, or even among subtypes, leading for example to competitive exclusion in situations where such effects would be unexpected based on the usual deterministic models. We suggest that this effect might be involved in the explanations of some persistent empirical anomalies. (C) 2003 Elsevier Inc. All rights reserved. | 2378 | NA | NA | NA | no | no |
| 2379 | 2017 | NA | Martin, MS; Shepherdson, DJ | 2012 | Role of Familiarity and Preference in Reproductive Success in Ex Situ Breeding Programs | Success of captive-breeding programs centers on consistent reproduction among captive animals. However, many individuals do not reproduce even when they are apparently healthy and presented with mates. Mate choice can affect multiple parameters of reproductive success, including mating success, offspring production, offspring survival, and offspring fecundity. We investigated the role of familiarity and preference on reproductive success of female Columbia Basin pygmy rabbits (Brachylagus idahoensis) as measured by litter production, litter size, average number of young that emerged from the burrow, and average number of young that survived to 1 year. We conducted these studies on pygmy rabbits at the Oregon Zoo (Portland, Oregon, U.S.A.) and Washington State University (Pullman, Washington, U.S.A.) from February to June 2006, 2007, and 2008. Before mating, we housed each female adjacent to 2 males (neighbors). Female preference for each potential mate was determined on the basis of behavioral interactions observed and measured between the rabbits. We compared reproductive success between females mated with neighbor and non-neighbor males and between females mated with preferred and nonpreferred males. Our findings suggest that mating with a neighbor compared with a non-neighbor and mating with a preferred neighbor compared with a nonpreferred neighbor increased reproductive success in female pygmy rabbits. Litter production, average number of young that emerged, and average number of young that survived to 1 year were higher in rabbits that were neighbors before mating than in animals who were not neighbors. Pairing rabbits with a preferred partner increased the probability of producing a litter and was significantly associated with increased litter size. In captive breeding programs, mates are traditionally selected on the basis of genetic parameters to minimize loss of genetic diversity and inbreeding coefficients. Our results suggest that integrating genetic information with social dynamics and behavioral measures of preference may increase the reproductive output of the pygmy rabbit captive-breeding program. Our findings are consistent with the idea that allowing mate choice and familiarity increase the reproductive success of captive-breeding programs for endangered species. | 2379 | NA | NA | NA | no | no |
| 2380 | 2017 | NA | McClelland, EK; Ming, TJ; Tabata, A; Kaukinen, KH; Beacham, TD; Withler, RE; Miller, KM | 2013 | Patterns of selection and allele diversity of class I and class II major histocompatibility loci across the species range of sockeye salmon (Oncorhynchus nerka) | The major histocompatibility complex (MHC), an important component of the vertebrate immune system, provides an important suite of genes to examine the role of genetic diversity at non-neutral loci for population persistence. We contrasted patterns of diversity at the two classical MHC loci in sockeye salmon (Oncorhynchus nerka), MHC class I (UBA) and MHC class II (DAB), and neutral microsatellite loci across 70 populations spanning the species range from Washington State to Japan. There was no correlation in allelic richness or heterozygosity between MHC loci or between MHC loci and microsatellites. The two unlinked MHC loci may be responding to different selective pressures; the distribution of F-ST values for the two loci was uncorrelated, and evidence for both balancing and directional selection on alleles and lineages of DAB and UBA was observed in populations throughout the species range but rarely on both loci within a population. These results suggest that fluctuating selection has resulted in the divergence of MHC loci in contemporary populations. | 2380 | NA | NA | NA | no | no |
| 2381 | 2017 | NA | Papassotiropoulos, A; Wollmer, MA; Aguzzi, A; Hock, C; Nitsch, RM; de Quervain, DJF | 2005 | The prion gene is associated with human long-term memory | Human cognitive processes are highly variable across individuals and are influenced by both genetic and environmental factors. Although genetic variations affect short-term memory in humans, it is unknown whether genetic variability has also an impact on long-term memory. Because prion-like conformational changes may be involved in the induction of long-lasting synaptic plasticity, we examined the impact of single-nucleotide polymorphisms (SNPs) of the prion protein gene (PRNP) on long-term memory in healthy young humans. SNPs in the genomic region of PRNP were associated with better long-term memory performance in two independent populations with different educational background. Among the examined PRNP SNPs, the common Met129Val polymorphism yielded the highest effect size. Twenty-four hours after a word list-learning task, carriers of either the 129(MM) or the 129(MV) genotype recalled 17% more information than 129(VV) carriers, but short-term memory was unaffected. These results suggest a role for the prion protein in the formation of long-term memory in humans. | 2381 | NA | NA | NA | no | no |
| 2382 | 2017 | NA | Mcrae, KM; McEwan, JC; Dodds, KG; Gemmell, NJ | 2014 | Signatures of selection in sheep bred for resistance or susceptibility to gastrointestinal nematodes | Background: Gastrointestinal nematodes are one of the most serious causes of disease in domestic ruminants worldwide. There is considerable variation in resistance to gastrointestinal nematodes within and between sheep breeds, which appears to be due to underlying genetic diversity. Through selection of resistant animals, rapid genetic progress has been demonstrated in both research and commercial flocks. Recent advances in genome sequencing and genomic technologies provide new opportunities to understand the ovine host response to gastrointestinal nematodes at the molecular level, and to identify polymorphisms conferring nematode resistance. Results: Divergent lines of Romney and Perendale sheep, selectively bred for high and low faecal nematode egg count, were genotyped using the Illumina (R) Ovine SNP50 BeadChip. The resulting genome-wide SNP data were analysed for selective sweeps on loci associated with resistance or susceptibility to gastrointestinal nematode infection. Population differentiation using F-ST and Peddrift revealed sixteen regions, which included candidate genes involved in chitinase activity and the cytokine response. Two of the sixteen regions identified were contained within previously identified QTLs associated with nematode resistance. Conclusions: In this study we identified fourteen novel regions associated with resistance or susceptibility to gastrointestinal nematodes. Results from this study support the hypothesis that host resistance to internal nematode parasites is likely to be controlled by a number of loci of moderate to small effects. | 2382 | NA | NA | NA | no | no |
| 2383 | 2017 | NA | Kohnen, A; Richter, I; Brandl, R | 2012 | No Concordant Phylogeographies of the Rose Gall Wasp Diplolepis rosae (Hymenoptera, Cynipidae) and Two Associated Parasitoids across Europe | According to the Host-tracking Hypothesis, species of higher trophic levels with a close relationship to their hosts, such as parasites or parasitoids, are expected to show spatio-temporal phylogeographic patterns similar to those of their host. Alternatively, with ecological sorting, a subset of the local species pools might shift to a related host species, thereby disengaging common phylogeographic patterns. Here, we compare the phylogeographic structures of the cynipid rose gall wasp Diplolepis rosae across Europe and of two of its most common parasitoids, the wasps Orthopelma mediator and Glyphomerus stigma, by analysing the sequences of two gene fragments (COI and ITS 2). The phylogeographic structures of the three species associated with roses were incongruent. D. rosae had the lowest genetic diversity with one major clade, O. mediator showed the classical phylogeographic structure for Europe with one eastern and one western clade, and G. stigma had the highest diversity but no geographical structuring. This discordance of geographical patterns may be explained by 1) the dispersal propensity of adult parasitoids or 2) the parasitoids having the ability to switch to another host, while the primary host becomes rare or is even not available. Furthermore there was no indication that phylogenetic patterns were affected by Wolbachia infections. Our results document that communities of closely interacting species may be the result of idiosyncratic biogeographic histories. | 2383 | NA | NA | NA | no | no |
| 2384 | 2017 | NA | Teixeira, JC; de Filippo, C; Weihmann, A; Meneu, JR; Racimo, F; Dannemann, M; Nickel, B; Fischer, A; Halbwax, M; Andre, C; Atencia, R; Meyer, M; Parra, G; Paabo, S; Andres, AM | 2015 | Long-Term Balancing Selection in LAD1 Maintains a Missense Trans-Species Polymorphism in Humans, Chimpanzees, and Bonobos | Balancing selection maintains advantageous genetic and phenotypic diversity in populations. When selection acts for long evolutionary periods selected polymorphisms may survive species splits and segregate in present-day populations of different species. Here, we investigate the role of long-term balancing selection in the evolution of protein-coding sequences in the Homo-Pan clade. We sequenced the exome of 20 humans, 20 chimpanzees, and 20 bonobos and detected eight coding trans-species polymorphisms (trSNPs) that are shared among the three species and have segregated for approximately 14 My of independent evolution. Although the majority of these trSNPs were found in three genes of the major histocompatibility locus cluster, we also uncovered one coding trSNP (rs12088790) in the gene LAD1. All these trSNPs show clustering of sequences by allele rather than by species and also exhibit other signatures of long-term balancing selection, such as segregating at intermediate frequency and lying in a locus with high genetic diversity. Here, we focus on the trSNP in LAD1, a gene that encodes for Ladinin-1, a collagenous anchoring filament protein of basement membrane that is responsible for maintaining cohesion at the dermal-epidermal junction; the gene is also an autoantigen responsible for linear IgA disease. This trSNP results in a missense change (Leucine257Proline) and, besides altering the protein sequence, is associated with changes in gene expression of LAD1. | 2384 | NA | NA | NA | no | no |
| 2385 | 2017 | NA | Le, PT; Makhalanyane, TP; Guerrero, LD; Vikram, S; Van de Peer, Y; Cowan, DA | 2016 | Comparative Metagenomic Analysis Reveals Mechanisms for Stress Response in Hypoliths from Extreme Hyperarid Deserts | Understanding microbial adaptation to environmental stressors is crucial for interpreting broader ecological patterns. In the most extreme hot and cold deserts, cryptic niche communities are thought to play key roles in ecosystem processes and represent excellent model systems for investigating microbial responses to environmental stressors. However, relatively little is known about the genetic diversity underlying such functional processes in climatically extreme desert systems. This study presents the first comparative metagenome analysis of cyanobacteria-dominated hypolithic communities in hot (Namib Desert, Namibia) and cold (Miers Valley, Antarctica) hyperarid deserts. The most abundant phyla in both hypolith metagenomes were Actinobacteria, Proteobacteria, Cyanobacteria and Bacteroidetes with Cyanobacteria dominating in Antarctic hypoliths. However, no significant differences between the two metagenomes were identified. The Antarctic hypolithic metagenome displayed a high number of sequences assigned to sigma factors, replication, recombination and repair, translation, ribosomal structure, and biogenesis. In contrast, the Namib Desert metagenome showed a high abundance of sequences assigned to carbohydrate transport and metabolism. Metagenome data analysis also revealed significant divergence in the genetic determinants of amino acid and nucleotide metabolism between these two metagenomes and those of soil from other polar deserts, hot deserts, and non-desert soils. Our results suggest extensive niche differentiation in hypolithic microbial communities from these two extreme environments and a high genetic capacity for survival under environmental extremes. | 2385 | NA | NA | NA | no | no |
| 2386 | 2017 | NA | Gourraud, PA; Khankhanian, P; Cereb, N; Yang, SY; Feolo, M; Maiers, M; Rioux, JD; Hauser, S; Oksenberg, J | 2014 | HLA Diversity in the 1000 Genomes Dataset | The 1000 Genomes Project aims to provide a deep characterization of human genome sequence variation by sequencing at a level that should allow the genome-wide detection of most variants with frequencies as low as 1%. However, in the major histocompatibility complex (MHC), only the top 10 most frequent haplotypes are in the 1% frequency range whereas thousands of haplotypes are present at lower frequencies. Given the limitation of both the coverage and the read length of the sequences generated by the 1000 Genomes Project, the highly variable positions that define HLA alleles may be difficult to identify. We used classical Sanger sequencing techniques to type the HLA-A, HLA-B, HLA-C, HLA-DRB1 and HLA-DQB1 genes in the available 1000 Genomes samples and combined the results with the 103,310 variants in the MHC region genotyped by the 1000 Genomes Project. Using pairwise identity-by-descent distances between individuals and principal component analysis, we established the relationship between ancestry and genetic diversity in the MHC region. As expected, both the MHC variants and the HLA phenotype can identify the major ancestry lineage, informed mainly by the most frequent HLA haplotypes. To some extent, regions of the genome with similar genetic or similar recombination rate have similar properties. An MHC-centric analysis underlines departures between the ancestral background of the MHC and the genome-wide picture. Our analysis of linkage disequilibrium (LD) decay in these samples suggests that overestimation of pairwise LD occurs due to a limited sampling of the MHC diversity. This collection of HLA-specific MHC variants, available on the dbMHC portal, is a valuable resource for future analyses of the role of MHC in population and disease studies. | 2386 | NA | NA | NA | no | no |
| 2387 | 2017 | NA | Gerardo, NM; Caldera, EJ | 2007 | Labile associations between fungus-growing ant cultivars and their garden pathogens | The distribution of genetic and phenotypic variation in both hosts and parasites over their geographic ranges shapes coevolutionary dynamics. Specifically, concordant host and parasite distributions facilitate localized adaptation and further specialization of parasite genotypes on particular host genotypes. We here compare genetic population structure of the cultivated fungi of the fungus-growing ant Apterostigma dentigerum and of the cultivar-attacking fungus, Escovopsis, to determine whether these microbial associations have evolved or are likely to evolve genotype genotype specialization. Analyses based on amplified fragment length polymorphism (AFLP) genotyping of host cultivars and pathogenic Escovopsis from 77 A. dentigerum colonies reveal that populations of hosts and pathogens are not similarly diverged and that host and pathogen genetic distances are uncorrelated, indicating that genetically similar parasites are not infecting genetically similar hosts. Microbial bioassays between pathogens and cultivars of different genotypes and from different populations show little pairwise specificity; most Escovopsis strains tested can successfully infect all cultivar strains with which they are paired. These molecular and experimental data suggest that Escovopsis genotypes are not tightly tracking cultivar genotypes within the A. dentigerum system. The diffuse nature of this host-pathogen association, in which pathogen genotypes are not interacting with a single host genotype but instead with many different hosts, will influence evolutionary and ecological disease dynamics of the fungus-growing ant-microbe symbiosis. | 2387 | NA | NA | NA | no | no |
| 2388 | 2017 | NA | Prugnolle, F; Theron, A; Pointier, JP; Jabbour-Zahab, R; Jarne, P; Durand, P; De Meeus, T | 2005 | Dispersal in a parasitic worm and its two hosts: Consequence for local adaptation | Characterizing host and parasite population genetic structure and estimating gene flow among populations is essential for understanding coevolutionary interactions between hosts and parasites. We examined the population genetic structure of the trematode Schistosoma mansoni and its two host species (the definitive host Rattus rattus and the intermediate host Biomphalaria glabrata) using microsatellite markers. Parasites were sampled from rats. The study was conducted in five sites of the Guadeloupe Island, Lesser Antilles. Mollusks display a pattern of isolation by distance whereas such a pattern is not found neither in schistosomes nor in rats. The comparison of the distribution of genetic variability in S. mansoni and its two host species strongly suggests that migration of parasites is principally determined by that of the vertebrate host in the marshy focus of Guadeloupe. However, the comparison between genetic differentiation values in schistosomes and rats suggests that the efficacy of the schistosome rat-mediated dispersal between transmission sites is lower than expected given the prevalence, parasitic load and migration rate of rats among sites. This could notably suggest that rat migration rate could be negatively correlated to the age or the infection status of individuals. Models made about the evolution of local adaptation in function of the dispersal rates of hosts and parasites suggest that rats and mollusks should be locally adapted to their parasites. | 2388 | NA | NA | NA | no | no |
| 2389 | 2017 | NA | Tarpy, DR; Delaney, DA; Seeley, TD | 2015 | Mating Frequencies of Honey Bee Queens (Apis mellifera L.) in a Population of Feral Colonies in the Northeastern United States | Across their introduced range in North America, populations of feral honey bee (Apis mellifera L.) colonies have supposedly declined in recent decades as a result of exotic parasites, most notably the ectoparasitic mite Varroa destructor. Nonetheless, recent studies have documented several wild populations of colonies that have persisted. The extreme polyandry of honey bee queens-and the increased intracolony genetic diversity it confers-has been attributed, in part, to improved disease resistance and may be a factor in the survival of these populations of feral colonies. We estimated the mating frequencies of queens in feral colonies in the Arnot Forest in New York State to determine if the level of polyandry of these queens is especially high and so might contribute to their survival success. We geno-typed the worker offspring from 10 feral colonies in the Arnot Forest of upstate New York, as well as those from 20 managed colonies closest to this forest. We found no significant differences in mean mating frequency between the feral and managed queens, suggesting that queens in the remote, low-density population of colonies in the Arnot Forest are neither mate-limited nor adapted to mate at an especially high frequency. These findings support the hypothesis that the hyperpolyandry of honey bees has been shaped on an evolutionary timescale rather than on an ecological one. | 2389 | NA | NA | NA | no | no |
| 2390 | 2017 | NA | Visser, M; Gordon, T; Fourie, G; Viljoen, A | 2010 | CHARACTERISATION OF SOUTH AFRICAN ISOLATES OF FUSARIUM OXYSPORUM F.SP CUBENSE FROM CAVENDISH BANANAS | Fusarium wilt, caused by the soil-borne fungus Fusarium oxysporum f.sp. cubense (Foc), is a serious vascular disease of bananas in most subtropical and tropical regions of the world. Twenty-four vegetative compatibility groups (VCGs) and three pathogenic races have been identified in Foc, reflecting a relatively high genetic diversity for an asexual fungus. To characterise a South African population of Foc, a collection of 128 isolates from diverse geographic origins were isolated from diseased Cavendish bananas and subjected to VCG analysis and sequencing of the translation elongation factor 1-alpha. (TEF) gene region. The presence of mating type genes was also determined using MAT-1 and MAT-2 specific primers. VCG 0120 was established as the only VCG of Foc present in the South African population studied. Only the MAT-2 idiomorph was present in all the local isolates of Foc. A phylogenetic analysis of DNA sequences of the TEF gene region revealed that the South African isolates grouped closely with VCG 0120 isolates from Australia and Asia. These results suggest that the South African population of Foc was most likely introduced in a limited number of events and that it had spread with infected planting material within the country. The presence of only one mating type and the limited diversity in this pathogen render it unlikely to rapidly overcome disease management strategies involving host resistance. | 2390 | NA | NA | NA | no | no |
| 2391 | 2017 | NA | Gordon, LK; Hurst, GDD; Watts, PC | 2009 | Eight microsatellite loci for the sexually transmitted, parasitic mite Coccipolipus hippodamiae | Seven dinucleotide and one trinucleotide polymorphic microsatellite loci were isolated from the mite Coccipolipus hippodamiae. This mite is an ectoparasite of coccinellid beetles (ladybirds), principally the European two-spot ladybird Adalia bipunctata, where it causes sterility in the female host. Levels of genetic diversity were assessed using 32 mites from Warsaw, Poland. We observed moderate variability, with the number of alleles per locus varying between 2 and 4, and observed and expected heterozygosities ranging from 0.031 to 0.267 and between 0.062 and 0.526, respectively. This is the first description of microsatellite loci from the genus Coccipolipus and these loci are currently being employed to answer fundamental questions about the epidemiology of C. hippodamiae infections on A. bipunctata. | 2391 | NA | NA | NA | no | no |
| 2392 | 2017 | NA | Gurung, S; Short, DPG; Adhikari, TB | 2013 | Global population structure and migration patterns suggest significant population differentiation among isolates of Pyrenophora tritici-repentis | The global population structure and migration patterns of foliar wheat pathogen Pyrenophora triticirepentis (PTR) were determined using 12 microsatellite loci. Analysis of 439 single-spore isolates of PTR from five continents (18 wheat-producing countries) showed high level of genetic diversity, and moderate to high population differentiation between continents. A high level of gene diversity (H-s = 0.31 to 0.56) was observed within each population. Allelic richness indicated the European and the North American population have a high effective population size. Bayesian analyses showed five clusters where the inferred clusters did not represent geographical populations. Corrected standardized fixation index (G(ST)(’’))estimates ranged from 0.042 to 0.265 between populations, indicating low to high genetic differentiation exists between populations. We found migration (gene flow) between old world (Europe) and new world (Americas) population; however, little migration was observed among other continents. The European population was the major source of immigrants for the North American, South American, Australian and the Asian populations. Significant (P < 0.001) linkage disequilibrium (LD) was detected in the Australian and the South American populations. In contrast, non-significant (P < 0.001) LD values were observed in the Asian, European and the North American populations. Overall, our findings demonstrate the population differentiation exits among the global populations and strict quarantine measures should be applied to prevent the accelerated global spread of this pathogen. (C) 2013 Elsevier Inc. All rights reserved. | 2392 | NA | NA | NA | no | no |
| 2393 | 2017 | NA | Plowright, R; Dusabe, J; Coyne, D; Speijer, P | 2013 | Analysis of the pathogenic variability and genetic diversity of the plant-parasitic nematode Radopholus similis on bananas | Radopholus similis is responsible for huge production losses of banana in Uganda, where a large proportion of the population are reliant on cooking banana as their main staple crop. Assessment of the pathogenic and genetic variability of seven populations of R. similis originating from banana plantations in Uganda demonstrated that their virulence on Musa cultivars differed, displaying pathotype-like variation. Using RAPD and AFLP techniques the populations could be grouped into two putative genomic groups, which corresponded with R. similis relative pathogenicity. Most, but not all, populations were able to reproduce and damage roots on the two widely confirmed sources of resistance, Yangambi km5 and Pisang Jari Buaya. Studies using RAPD techniques furthermore revealed putative markers for nematode virulence. Variation in virulence of R. similis populations was also demonstrated at the regional (Africa) level. Phylogenetic analysis most closely associated the Uganda populations with populations from Sri Lanka, indicating their likely source of origin. However, genetic variability between Uganda populations also indicate that R. similis was probably introduced into Uganda on separate occasions from different sources, which may help to explain the causes of variability in banana decline in Uganda. The results of this study have clear implications for the development and deployment of sustainable nematode management practices based on the use of resistance, which could help underpin ongoing management initiatives. | 2393 | NA | NA | NA | no | no |
| 2394 | 2017 | NA | Kim, JI; Park, S; Lee, I; Park, KS; Kwak, EJ; Moon, KM; Lee, CK; Bae, JY; Park, MS; Song, KJ | 2016 | Genome-Wide Analysis of Human Metapneumovirus Evolution | Human metapneumovirus (HMPV) has been described as an important etiologic agent of upper and lower respiratory tract infections, especially in young children and the elderly. Most of school-aged children might be introduced to HMPVs, and exacerbation with other viral or bacterial super-infection is common. However, our understanding of the molecular evolution of HMPVs remains limited. To address the comprehensive evolutionary dynamics of HMPVs, we report a genome-wide analysis of the eight genes (N, P, M, F, M2, SH, G, and L) using 103 complete genome sequences. Phylogenetic reconstruction revealed that the eight genes from one HMPV strain grouped into the same genetic group among the five distinct lineages (A1, A2a, A2b, B1, and B2). A few exceptions of phylogenetic incongruence might suggest past recombination events, and we detected possible recombination breakpoints in the F, SH, and G coding regions. The five genetic lineages of HMPVs shared quite remote common ancestors ranging more than 220 to 470 years of age with the most recent origins for the A2b sublineage. Purifying selection was common, but most protein genes except the F and M2-2 coding regions also appeared to experience episodic diversifying selection. Taken together, these suggest that the five lineages of HMPVs maintain their individual evolutionary dynamics and that recombination and selection forces might work on shaping the genetic diversity of HMPVs. | 2394 | NA | NA | NA | no | no |
| 2395 | 2017 | NA | Giokas, S; Mylonas, M; Sotiropoulos, K | 2000 | Gene flow and differential mortality in a contact zone between two Albinaria species (Gastropoda; Clausiliidae) | This paper reports on the disruption of normally effective ecological and behavioural barriers, at a contact zone on Monemvasia peninsula (Peloponnese, Greece), between parapatric populations of the land snail species Albinaria discolor and Albinaria campylauchen. Detected outcomes were the increase of gene flow between these two species at the contact zone, the occurrence of rare alleles, as well as the occurrence of morphologically and ethologically intermediate A. campylauchen specimens. Furthermore, there was high genetic variability along with deficiency of heterozygotes, probably due to inbreeding. Additionally, the study of mortality of these populations, during the adverse summer aestivation period, indicated that A. campylauchen individuals with an inappropriate combination of morphological and behavioural characteristics suffered heavily. The maladaptation and disadvantage of these specimens revealed the possible action of a postzygotic isolation mechanism between these two morphologically, molecularly and electrophoretically distinct species, that can, however, mate under laboratory conditions. Finally, the above situation is discussed in terms of exogenous Versus endogenous selection. (C) 2000 The Linnean Society of London. | 2395 | NA | NA | NA | no | no |
| 2396 | 2017 | NA | Brunelli, KR; Dunkle, LD; Sobrinho, CA; Fazza, AC; Camargo, LEA | 2008 | Molecular variability in the maize grey leaf spot pathogens in Brazil | Isolates of Cercospora species from leaves displaying symptoms of grey leaf spot were collected in maize-producing areas of south-central Brazil in 2001 and 2002. Restriction digests of the internal transcribed spacer region of rDNA detected the presence of the same two Cercospora species described on maize in the United States, namely C. zeae-maydis and the recently described species, C. zeina. Genetic variability among isolates was assessed by analysing 104 amplified fragment length polymorphism loci. Cluster analysis confirmed the genetic separation of isolates into two species with a mean similarity of 35%. Similarity levels within species were high, averaging 93% and 92% among isolates of C. zeae-maydis and C. zeina, respectively. The mean genetic similarity between C. zeae-maydis and C. zeina and two isolates of C. sorghif. sp. maydis was 45% and 35%, respectively. Results of this study showed that populations of the grey leaf spot pathogens in Brazil are similar to those in the United States regarding species composition and that C. zeina is also present in Brazil. | 2396 | NA | NA | NA | no | no |
| 2397 | 2017 | NA | Kanthaswamy, S; Satkoski, J; Kou, A; Malladi, V; Smith, DG | 2010 | Detecting signatures of inter-regional and inter-specific hybridization among the Chinese rhesus macaque specific pathogen-free (SPF) population using single nucleotide polymorphic (SNP) markers | Background While rates of gene flow between rhesus and longtail macaque populations near their hybrid zone in Indochina have been quantified elsewhere, this study demonstrates that the inter-specific introgression is not limited to the Indochinese hybrid zone but is more geographically widespread. Methods Twelve rhesus and longtail macaque populations were analyzed using single nucleotide polymorphic (SNP) loci. Results There is evidence for inter-specific admixture between Chinese rhesus and mainland longtails, with implications for genetic diversity both in the Chinese super-SPF population at the California National Primate Research Center and in other primate facilities. Eastern Chinese rhesus appeared more highly derived than western Chinese rhesus, and allele sharing between longtails and Chinese rhesus was not random with regard to geographic distance, but no significant nuclear genetic differences between eastern and western Chinese rhesus were detected among the 245 genic SNPs assayed. Conclusion The implications of this inter-specific admixture for the use of Chinese rhesus and mainland longtail in biomedical research should be considered. | 2397 | NA | NA | NA | no | no |
| 2398 | 2017 | NA | Wang, S; Cheng, XX; Chen, SY; Lin, FQ; Chen, SL; Zhu, XL; Wang, JX; Huang, MQ; Zheng, M | 2016 | Phylogenetic analysis of VP1 gene sequences of waterfowl parvoviruses from the Mainland of China revealed genetic diversity and recombination | To determine the origin and evolution of goose parvovirus (GPV) and Muscovy duck parvovirus (MDPV) in the Mainland of China, phylogenetic and recombination analyses in the present study were performed on 32 complete VP1 gene sequences from China and other countries. Based on the phylogenetic analysis of the VP1 gene, GPV strains studied here from Mainland China (PRC) could be divided into three genotypes, namely PRC-I, PRC-II and PRC-III. Genotype PRC-I is indigenous to Mainland China. Only one GPV strain from Northeast China was of Genotype PRC-II and was thought to be imported from Europe. Genotype PRC-III, which was the most isolated genotype during 1999-2012, is related to GPVs in Taiwan and has been the predominant pathogen responsible for recent Derzy’s disease outbreaks in Mainland China. Current vaccine strains used in Mainland China belong to Genotype PRC-I that is evolutionary distant from Genotypes PRC-II and PRC-III. In comparison, MDPV strains herein from Mainland China are clustered in a single group which is closely related to Taiwanese MDPV strains, and the full-length sequences of the VP1 gene of China MDPVs are phylogenetic closely related to the VP1 sequence of a Hungarian MDPV strain. Moreover, We also found that homologous recombination within VP1 gene plays a role in generating genetic diversity in GPV evolution. The GPV GDFSh from Guangdong Province appears to be the evolutionary product of a recombination event between parental GPV strains GD and B, while the major parent B proved to be a reference strain for virulent European GPVs. Our findings provide valuable information on waterfowl parvoviral evolution in Mainland China. (C) 2015 Elsevier B.V. All rights reserved. | 2398 | NA | NA | NA | no | no |
| 2399 | 2017 | NA | Han, DS; Tang, H; Lu, J; Wang, GZ; Zhou, L; Min, LF; Han, CX | 2014 | Population Structure of Clinical Vibrio parahaemolyticus from 17 Coastal Countries, Determined through Multilocus Sequence Analysis | Vibrio parahaemolyticus is a leading cause of food-borne gastroenteritis worldwide. Although this bacterium has been the subject of much research, the population structure of clinical strains from worldwide collections remains largely undescribed, and the recorded outbreaks of V. parahaemolyticus gastroenteritis highlight the need for the subtyping of this species. We present a broad phylogenetic analysis of 490 clinical V. parahaemolyticus isolates from 17 coastal countries through multilocus sequence analysis (MLST). The 490 tested isolates fell into 161 sequence types (STs). The eBURST algorithm revealed that the 161 clinically relevant STs belonged to 8 clonal complexes, 11 doublets, and 94 singletons, showing a high level of genetic diversity. CC3 was found to be a global epidemic clone of V. parahaemolyticus, and ST-3 was the only ST with an international distribution. recA was observed to be evolving more rapidly, exhibiting the highest degree of nucleotide diversity (0.028) and the largest number of polymorphic nucleotide sites (177). We also found that the high variability of recA was an important cause of differences between the results of the eBURST and ME tree analyses, suggesting that recA has a much greater influence on the apparent evolutionary classification of V. parahaemolyticus based on the current MLST scheme. In conclusion, it is evident that a high degree of genetic diversity within the V. parahaemolyticus population and multiple sequence types are contributing to the burden of disease around the world. MLST, with a fully extractable database, is a powerful system for analysis of the clonal relationships of strains at a global scale. With the addition of more strains, the pubMLST database will provide more detailed and accurate information, which will be conducive to our future research on the population structure of V. parahaemolyticus. | 2399 | NA | NA | NA | no | no |
| 2400 | 2017 | NA | Ramos, R; Song, G; Navarro, J; Zhang, RY; Symes, CT; Forero, MG; Lei, FM | 2016 | Population genetic structure and long-distance dispersal of a recently expanding migratory bird | Long-distance dispersal events and their derivable increases of genetic diversity have been highlighted as important ecological and evolutionary determinants that improve performances of range-expanding species. In the context of global environmental change, specific dispersal strategies have to be understood and foreseen if we like to prevent general biodiversity impoverishment or the spread of allochthonous diseases. We explored the genetic structure and potential population mixing on the recently range expanding European bee-eater Merops apiaster. In addition, the species is suspected of harbouring and disseminating the most relevant disease for bees and apiculture, Nosema microsporidia. In agreement with complementary ringing recovery data and morphometric measurements, genetic results on two mitochondrial genes and 12 microsatellites showed a reasonably well-structured population partitioning along its breeding distribution. Microsatellite results indicated that not only did a few birds recently disperse long distance during their return migrations and change their natal breeding areas, but also that a group of allochthonous birds together founded a new colony. Although we did not provide evidence on the direct implication of birds in the widespread of Nosema parasites, our finding on the long-distance dispersal of bird flocks between remote breeding colonies adds concern about the role of European bee-eaters in the spread of such disease at a large, inter-continental scale. (C) 2016 Elsevier Inc. All rights reserved. | 2400 | NA | NA | NA | no | no |
| 2401 | 2017 | NA | Mossbrucker, AM; Apriyana, I; Fickel, J; Imron, MA; Pudyatmoko, S; Sumardi; Suryadi, H | 2015 | Non-invasive genotyping of Sumatran elephants: implications for conservation | Reliable baseline information necessary for the monitoring and conservation of Sumatran elephants is scarce. We here combine non-invasive molecular genetics methods and capture-recapture modeling to estimate elephant population size, distribution, sex ratio, and age structure for the Bukit Tigapuluh landscape in Sumatra, Indonesia. Two separate subpopulations were found, for which we estimated a population size of 99 (95% CI = [86, 125], PCCL = 38.59%) and 44 elephants (95% CI = [37, 56], PCCL = 43.18%), respectively. Low elephant densities are likely the result of patchy habitat usage and anthropogenically increased mortality, the latter assumption being supported by strong skews in both sex ratio and age structure as well as direct evidence of elephant killing. Still, the Bukit Tigapuluh landscape currently holds the largest known population of elephants in central Sumatra, representing one of the most important areas for their conservation in Indonesia. Conservation of both the elephant population and their habitat in this region should thus be of high priority. We identified several threats to the population, including (i) the risk of inbreeding and subsequent loss of genetic diversity, (ii) illegal elephant killing, and (iii) the lack of protected habitat. In order to overcome these challenges we suggest: (i) the implementation of a meta-population management program, (ii) monitoring and safeguarding elephants and improving law enforcement, and (iii) providing sufficient safe habitat to mitigate human-elephant-conflict (HEC) and ensure elephant survival. | 2401 | NA | NA | NA | no | no |
| 2402 | 2017 | NA | Wang, IN; Dykhuizen, DE; Qin, WG; Dunn, JJ; Bosler, EM; Luft, BJ | 1999 | Genetic diversity of ospC in a local population of Borrelia burgdorferi sensu stricto | The outer surface protein, OspC, is highly variable in Borrelia burgdorferi sensu stricto, the agent of Lyme disease. We have shown that even within a single population OspC is highly variable. The variation of ospA and ospC in the 40 infected deer ticks collected from a single site on Shelter Island, New York, was determined using PCR-SSCP. There is very strong apparent linkage disequilibrium between ospA and ospC alleles, even though they are located on separate plasmids. Thirteen discernible SSCP mobility classes for ospC were identified and the DNA sequence for each was determined. These sequences, combined with 40 GenBank sequences, allow us to define 19 major ospC groups. Sequences within a major ospC group are, on average, <1% different from each other, while sequences between major ospC groups are, on average, similar to 20% different. The tick sample contains 11 major ospC groups, GenBank contains 16 groups, with 8 groups found in both samples. Thus, the ospC variation within a local population is almost as great as die variation of a similar-sized sample of the entire species, The Ewens-Watterson-Slatkin test of allele frequency showed significant deviation from the neutral expectation, indicating balancing selection for these major ospC groups. The variation represented by major ospC groups needs to be considered if the OspC protein is to be used as a serodiagnostic antigen or a vaccine. | 2402 | NA | NA | NA | no | no |
| 2403 | 2017 | NA | Invernizzi, C; Penagaricano, F; Tomasco, IH | 2009 | Intracolonial genetic variability in honeybee larval resistance to the chalkbrood and American foulbrood parasites | The origin of multiple mating of queens in social Hymenoptera is a widely debated topic in evolutionary biology. One of the hypotheses is that genetic variability would benefit the colony by increasing its resistance to parasites through various mechanisms. One among the predictions of this hypothesis is that the resistance of different patrilines within a colony to parasites of different species should be independent, as a result of independent gene-for-gene interactions with each parasite. To test this aspect of the hypothesis, two honeybee colonies (Apis mellifera) were infected with the fungus Ascosphaera apis and two colonies with both A. apis and the American foulbrood bacterium Paenibacillus larvae. Patrilines were found to vary in resistance of larvae to A. apis in all four colonies, but similar variation in resistance was not found to P. larvae. Common resistance to both pathogens was not detected. This study supports the hypothesis that polyandry in social insects could have originated as an adaptation to decrease the impact of diseases. | 2403 | NA | NA | NA | no | no |
| 2404 | 2017 | NA | Barata, C; Baird, DJ; Amat, F; Soares, AMVM | 2000 | Comparing population response to contaminants between laboratory and field: an approach using Daphnia magna ephippial egg banks | 1. The life‐history responses of one field and two laboratory populations of Daphnia magna were studied under exposure to cadmium and ethyl parathion to assess by how much the response to toxic chemicals of laboratory populations with low genetic diversity differs from the response of genetically diverse field populations. 2. The field population was represented by at least 50 unique clones hatched from resting eggs (ephippia) collected at the beginning of the growing season from a temporary water‐body located in the north‐east Mediterranean Spanish coast. The laboratory populations were clonal lines established from two geographically and genetically distinct genotypes, which differed in their tolerance to cadmium and ethyl parathion. Toxicant effects on the mean and the variance of life‐history responses of the laboratory and the field populations were determined. For the field population, toxicant effects on the components of variance of primary fitness traits were also studied. 3. In addition to lethal effects, exposure to cadmium had strong sub‐lethal effects on clutch size and age at first reproduction whereas ethyl parathion only affected juvenile survival. The results reported for life‐history responses showed that the field population had a similar or greater mean tolerance to cadmium and ethyl parathion than the laboratory populations, but the breadth of its tolerance distribution (measured as the coefficient of variation, CV) was higher. Furthermore in contrast with the field population, laboratory populations did not show increased phenotypic plasticity (measured as CV) under increasing toxicant exposure. A further analysis of the components of variability for life‐history responses of the field population showed that increases in phenotypic plasticity with exposure levels were explained by increased levels of genetic variability. 4. These results support the conclusion that as the tolerance of a field population is strongly influenced by genetic factors, the use of genetically homogeneous laboratory populations has limited relevance in predicting long‐term responses of field populations to toxic chemicals. However, this conclusion must remain tentative until further supporting evidence is obtained from this and other species. | 2404 | NA | NA | NA | no | no |
| 2405 | 2017 | NA | Bobo-Pinilla, J; de Leon, SBB; Colomar, JS; Fenu, G; Bacchetta, G; de Giles, JP; Martinez-Ortega, MM | 2016 | Phylogeography of Arenaria balearica L. (Caryophyllaceae): evolutionary history of a disjunct endemic from the Western Mediterranean continental islands | Although it has been traditionally accepted that Arenaria balearica (Caryophyllaceae) could be a relict Tertiary plant species, this has never been experimentally tested. Nor have the palaeohistorical reasons underlying the highly fragmented distribution of the species in the Western Mediterranean region been investigated. We have analysed AFLP data (213) and plastid DNA sequences (226) from a total of 250 plants from 29 populations sampled throughout the entire distribution range of the species in Majorca, Corsica, Sardinia, and the Tuscan Archipelago. The AFLP data analyses indicate very low geographic structure and population differentiation. Based on plastid DNA data, six alternative phylogeographic hypotheses were tested using Approximate Bayesian Computation (ABC). These analyses revealed ancient area fragmentation as the most probable scenario, which is in accordance with the star-like topology of the parsimony network that suggests a pattern of long term survival and subsequent in situ differentiation. Overall low levels of genetic diversity and plastid DNA variation were found, reflecting evolutionary stasis of a species preserved in locally long-term stable habitats. | 2405 | NA | NA | NA | no | no |
| 2406 | 2017 | NA | Deka, R; Sun, GY; Smelser, D; Zhong, YX; Kimmel, M; Chakraborty, R | 1999 | Rate and directionality of mutations and effects of allele size constraints at anonymous, gene-associated, and disease-causing trinucleotide loci | We studied the patterns of within- and between-population variation at 29 trinucleotide loci in a random sample of 200 healthy individuals from four diverse populations: Germans, Nigerians, Chinese, and New Guinea highlanders. The loci were grouped as disease-causing (seven loci with CAG repeats), gene-associated (seven loci with CAG/CCG repeats and eight loci with AAT repeats), or anonymous (seven loci with AAT repeats). We used heterozygosity and Variance of allele size (expressed in units of repeat counts) as measures of within-population variability and G(ST) (based on heterozygosity as well as on allele size variance) as the measure of genetic differentiation between populations. Our observations are: (1) locus type is the major significant factor for differences in within-population genetic variability; (2) the disease-causing CAG repeats tin the nondisease range of repeat counts) have the highest within-population variation, followed by the AAT-repeat anonymous loci, the AAT-repeat gene-associated loci, and the CAG/CTG-repeat gene-associated loci; (3) an imbalance index beta, the ratio of the estimates of the product of effective population size and mutation rate based on allele size variance and heterozygosity, is the largest for disease-causing loci, followed by AAT- and CAG/CCG-repeat gene-associated loci and AAT-repeat anonymous loci; (4) mean allele size correlates positively with allele size variance for AAT- and CAG/CCG-repeat gene-associated loci and negatively for anonymous loci; and (5) G(ST) is highest for the disease-causing loci. These observations are explained by specific differences of rates and patterns of mutations in these four groups of trinucleotide loci, taking into consideration the effects of the past demographic history of the modern human population. | 2406 | NA | NA | NA | no | no |
| 2407 | 2017 | NA | Kuro-O, M; Yonekawa, H; Saito, S; Eda, M; Higuchi, H; Koike, H; Hasegawa, H | 2010 | Unexpectedly high genetic diversity of mtDNA control region through severe bottleneck in vulnerable albatross Phoebastria albatrus | In the late part of the nineteenth century and the early part of the last century, the short-tailed albatross Phoebastria albatrus was in danger of extinction owing to feather hunting. In the middle of the last century, the total number of this species was inferred to be approximately 50-60 with breeding occurring only on Torishima Island of the Izu Islands. Recently, the number of individuals has increased to more than 2,000 and that of their breeding islands to three, namely, Torishima Island, and Minami- and Kita-kojima Islands of the Senkaku Islands. Here, we show that the 44 short-tailed albatrosses we examined represent 29 haplotypes in the control region of mitochondrial DNA, and have a considerably higher genetic diversity than most avian species, but not very high in albatross species; the h and p were 0.96 and 0.013, respectively. However, the parsimony network clearly showed that many intermediate haplotypes were lost. It was concluded that the majority of the haplotypes in the founder population have been maintained. Judging from these findings and the exponential increase in the number of individuals, the present population of the short-tailed albatross seems not to be affected by inbreeding depression through a severe bottleneck. The conservation and expansion of their breeding grounds, and effective protection from bycatch mortality in foraging areas are important for the future survival of this species. | 2407 | NA | NA | NA | no | no |
| 2408 | 2017 | NA | Stassen, HH; Bridler, R; Hell, D; Weisbrod, M; Scharfetter, C | 2004 | Ethnicity-independent genetic basis of functional psychoses: A genotype-to-phenotype approach | “The functional psychoses schizophrenia, schizoaffective disorder, and bipolar illness represent complex clinical syndromes that are characterized by phenotypic heterogeneity. Yet evidence from numerous studies suggests that (1) the prevalence of schizophrenia and bipolar illness is with 1% very similar across ethnicities, and (2) a strong genetic component is involved in the disorders’ pathogenesis. Using data from different US-American ethnicities (77 families with a total of 17 unaffected and 170 affected sib pairs; 276 marker loci), we searched for ethnicity-independent oligogenic susceptibility loci for which the between-sib genetic similarity in affected sib pairs deviated from the expected values. Specifically, we addressed the question of the extent to which genetic risk factors and their interactions constitute multigenic inheritance of functional psychoses across populations and might constitute universal targets for treatment. Our novel multivariate genotype-to-phenotype search strategy was based on a genetic similarity function that allowed us to quantify the inter-individual genetic distances d(x(i), x(j)) between the allelic genotype patterns x(i), x(j) of any two subjects i, j with respect to n loci l(1), l(2),…l(n). Thus, we were able to assess the between-ethnicity, the within-ethnicity, and the within-family genetic similarities. The problem of ethnicity-independent vulnerability was addressed by treating the Afro-American families as”“training”" samples, while the non-AfroAmerican families served as independent "“test”" samples. We evaluated the between-sib similarities, which were expected to deviate from "“0.5”" in affected sib pairs if the region of interest contained markers close to vulnerability genes. The reference value "“0.5”" was derived from the parent-offspring similarities that are always 0.5, irrespective of the affection status of parents and offspring. We found 12 vulnerability loci on chromosomes 1, 4, 5, 6, 13, 14, 18, and 20, that were reproducible across the two samples under comparison and therefore, likely to constitute an ethnicity-independent, oligogenic vulnerability model of functional psychoses. The elevated vulnerability appeared to be unspecific and to act in such a way that exogenous factors become more likely to trigger the onset of psychiatric illnesses. (C) 2003 Wiley-Liss, Inc." | 2408 | NA | NA | NA | no | no |
| 2409 | 2017 | NA | Cooper, RS; Tayo, B; Zhu, XF | 2008 | Genome-wide association studies: implications for multiethnic samples | The current gene mapping for complex diseases is heavily weighted by studies of population samples from northern Europe. To capture the full range of genetic diversity and exploit the potential of genetic epidemiology to identify important variants, multiple additional populations will need to be examined. The conduct of genome-wide association studies will therefore confront many of the challenges identified in the first generation of candidate gene and linkage studies, with a substantial increase in complexity. Initial efforts to map causal effects will have to take account of varying patterns of linkage disequilibrium through careful attention to local haplotype structure. Refined statistical techniques that permit joint analyses of samples from multiple populations will also be required, as well as improved methods to account for on-going gene flow between populations with geographically distinct ancestral origins. This variation can either be an impediment, slowing the process of replication, or an opportunity, allowing finer dissection of the relevant variants. Clinical translation of these data will present major challenges. Large cosmopolitan populations, such as those found in large urban centers, are likely to exhibit both known and cryptic sub-structure across groups, as well as admixture within individuals. Great care will need to be devoted to generalizability of association findings to avoid their premature adoption as predictive tests in the face of this widespread heterogeneity. | 2409 | NA | NA | NA | no | no |
| 2410 | 2017 | NA | Cao, PB; Wall, D | 2017 | Self-identity reprogrammed by a single residue switch in a cell surface receptor of a social bacterium | The ability to recognize close kin confers survival benefits on single-celled microbes that live in complex and changing environments. Microbial kinship detection relies on perceptible cues that reflect relatedness between individuals, although the mechanisms underlying recognition in natural populations remain poorly understood. In myxobacteria, cells identify related individuals through a polymorphic cell surface receptor, TraA. Recognition of compatible receptors leads to outer membrane exchange among clonemates and fitness consequences. Here, we investigated how a single receptor creates a diversity in recognition across myxobacterial populations. We first show that TraA requires its partner protein TraB to function in cell-cell adhesion. Recognition is shown to be traA allele-specific, where polymorphisms within TraA dictate binding selectivity. We reveal the malleability of TraA recognition, and seemingly minor changes to its variable region reprogram recognition outcomes. Strikingly, we identify a single residue (A/P205) as a molecular switch for TraA recognition. Substitutions at this position change the specificity of a diverse panel of environmental TraA receptors. In addition, we engineered a receptor with unique specificity by simply creating an A205P substitution, suggesting that modest changes in TraA can lead to diversification of new recognition groups in nature. We hypothesize that the malleable property of TraA has allowed it to evolve and create social barriers between myxobacterial populations and in turn avoid adverse interactions with relatives. | 2410 | NA | NA | NA | no | no |
| 2411 | 2017 | NA | Tinaut, A; Ruano, F; Sanllorente, O; Fernandez-Zambrano, A; Karaman, C; Kaz, Y | 2010 | Nest composition and worker relatedness in three slave-making ants of the genus Rossomyrmex Arnoldi and their Proformica Ruzsky hosts (Hymenoptera, Formicidae) | In this paper, we analyze and compare nest composition and architecture as well as worker relatedness in three related species of slave-making ants: Rossomyrmex anatolicus, R. minuchae, and R. quandratinodum. Colony structure within nests is an important trait in ants, especially in the case of mixed societies, when host and parasite coexist in the same nest. Data for their respective free-living hosts, Proformica korbi, P. longiseta and P. sp., are also provided. For our study, we integrated a meticulous excavation procedure with a genetic method. We conclude that the average number of parasites, as well as of slaves, is species-specific, whereas nest depth depends on the nest architecture of the host. The genus Rossomyrmex seems to be monogynous and monandrous, whereas Proformica shows differences in the number of queens and mating frequency. R. quandratinodum shows different traits in nest composition (host/parasite ratio: P/R) and architecture. The difference in traits may account for some differences in parasitism: raid process or avoidance of parasitism. | 2411 | NA | NA | NA | no | no |
| 2412 | 2017 | NA | Carmi, S; Hui, KY; Kochav, E; Liu, XM; Xue, J; Grady, F; Guha, S; Upadhyay, K; Ben-Avraham, D; Mukherjee, S; Bowen, BM; Thomas, T; Vijai, J; Cruts, M; Froyen, G; Lambrechts, D; Plaisance, S; Van Broeckhoven, C; Van Damme, P; Van Marck, H; Barzilai, N; Darvasi, A; Offit, K; Bressman, S; Ozelius, LJ; Peter, I; Cho, JH; Ostrer, H; Atzmon, G; Clark, LN; Lencz, T; Pe’er, I | 2014 | Sequencing an Ashkenazi reference panel supports population-targeted personal genomics and illuminates Jewish and European origins | The Ashkenazi Jewish (AJ) population is a genetic isolate close to European and Middle Eastern groups, with genetic diversity patterns conducive to disease mapping. Here we report high-depth sequencing of 128 complete genomes of AJ controls. Compared with European samples, our AJ panel has 47% more novel variants per genome and is eightfold more effective at filtering benign variants out of AJ clinical genomes. Our panel improves imputation accuracy for AJ SNP arrays by 28%, and covers at least one haplotype in approximate to 67% of any AJ genome with long, identical-by-descent segments. Reconstruction of recent AJ history from such segments confirms a recent bottleneck of merely approximate to 350 individuals. Modelling of ancient histories for AJ and European populations using their joint allele frequency spectrum determines AJ to be an even admixture of European and likely Middle Eastern origins. We date the split between the two ancestral populations to approximate to 12-25 Kyr, suggesting a predominantly Near Eastern source for the repopulation of Europe after the Last Glacial Maximum. | 2412 | NA | NA | NA | no | no |
| 2413 | 2017 | NA | Vavrek, MC; McGraw, JB; Yang, HS | 1996 | Within-population variation in demography of Taraxacum officinale: Maintenance of genetic diversity | Differential response of genotypes to temporal environmental heterogeneity may contribute to the long-term persistence of those genotypes within a population. We tested whether season-dependent fitness components of genotypes could be responsible for the maintenance of genetic diversity within a population. Clonal replicates of five genotypes of Taraxacum officinale were planted in each of four seasons (spring-April to July, summer-July to October, autumn-October to January, and winter-January to April), Individuals were planted directly into natural, field vegetation. Fitness components (establishment, survival, growth, seed production, and leaf area dominance) and an integrated measure of fitness (finite rate of increase) were measured for each genotype in each season. Differential genotypic responses to seasons were observed for all fitness components. Despite seasonal differences, genotypic performances summed across seasons were equivalent. This result indicates that temporal heterogeneity may have a substantial effect on within-population genetic structure. The persistence of genotypes of contrasting seasonal performance through time is expected if long-term fitness values remain similar. | 2413 | NA | NA | NA | no | no |
| 2414 | 2017 | NA | Cassens, I; Van Waerebeek, K; Best, PB; Tzika, A; Van Helden, AL; Crespo, EA; Milinkovitch, MC | 2005 | Evidence for male dispersal along the coasts but no migration in pelagic waters in dusky dolphins (Lagenorhynchus obscurus) | Using nine nuclear species-specific microsatellite loci and two mitochondrial gene fragments (cytochrome b and control region), we investigated the processes that have shaped the geographical distribution of genetic diversity exhibited by contemporary dusky dolphin (Lagenorhynchus obscurus) populations. A total of 221 individuals from four locations (Peru, Argentina, southern Africa, and New Zealand) were assayed, covering most of the species’ distribution range. Although our analyses identify a general demographic decline in the Peruvian dusky dolphin stock (recently affected by high natural and human-induced mortality levels), comparison between the different molecular markers hint at an ancient bottleneck that predates recent El Nino oscillations and human exploitation. Moreover, we find evidence of a difference in dispersal behaviour of dusky dolphins along the South American coast and across the Atlantic. While data in Peruvian and Argentine waters are best explained by male-specific gene flow between these two populations, our analyses suggest that dusky dolphins from Argentina and southern Africa recently separated from an ancestral Atlantic population and, since then, diverged without considerable gene flow. The inclusion of a few New Zealand samples further confirms the low levels of genetic differentiation among most dusky dolphin populations. Only the Peruvian dusky dolphin stock is highly differentiated, especially at mitochondrial loci, suggesting that major fluctuations in its population size have led to an increased rate of genetic drift. | 2414 | NA | NA | NA | no | no |
| 2415 | 2017 | NA | Banos, G; Bramis, G; Bush, SJ; Clark, EL; McCulloch, MEB; Smith, J; Schulze, G; Arsenos, G; Hume, DA; Psifidi, A | 2017 | The genomic architecture of mastitis resistance in dairy sheep | Background: Mastitis is the most prevalent disease in dairy sheep with major economic, hygienic and welfare implications. The disease persists in all dairy sheep production systems despite the implementation of improved management practises. Selective breeding for enhanced mastitis resistance may provide the means to further control the disease. In the present study, we investigated the genetic architecture of four mastitis traits in dairy sheep. Individual animal records for clinical mastitis occurrence and three mastitis indicator traits (milk somatic cell count, total viable bacterial count in milk and the California mastitis test) were collected monthly throughout lactation for 609 ewes of the Greek Chios breed. All animals were genotyped with a custom-made 960-single nucleotide polymorphism (SNP) DNA array based on markers located in quantitative trait loci (QTL) regions for mastitis resistance previously detected in three other distinct dairy sheep populations. Results: Heritable variation and strong positive genetic correlations were estimated for clinical mastitis occurrence and the three mastitis indicator traits. SNP markers significantly associated with these mastitis traits were confirmed on chromosomes 2, 3, 5, 16 and 19. We identified pathways, molecular interaction networks and functional gene clusters for mastitis resistance. Candidate genes within the detected regions were identified based upon analysis of an ovine transcriptional atlas and transcriptome data derived from milk somatic cells. Relevant candidate genes implicated in innate immunity included SOCS2, CTLA4, C6, C7, C9, PTGER4, DAB2, CARD6, OSMR, PLXNC1, IDH1, ICOS, FYB, and LYFR. Conclusions: The results confirmed the presence of animal genetic variability in mastitis resistance and identified genomic regions associated with specific mastitis traits in the Chios sheep. The conserved genetic architecture of mastitis resistance between distinct dairy sheep breeds suggests that across-breed selection programmes would be feasible. | 2415 | NA | NA | NA | no | no |
| 2416 | 2017 | NA | O’Donnell, S; Beshers, SN | 2004 | The role of male disease susceptibility in the evolution of haplodiploid insect societies | Heterozygosity at loci affecting resistance against parasites can benefit host fitness. We predict that, in haplodiploid species, haploid males will suffer decreased parasite resistance relative to diploid females. We suggest that elevated susceptibility in haploid males has shaped the evolution of social behaviour in haplodiploid species. Male susceptibility will select for behavioural adaptations that limit males’ exposure to pathogens and that limit male transmission of pathogens within and between colonies. The relatedness asymmetry hypothesis that has been advanced to explain female-only workers does not make these predictions. We review the relevant evidence for genetic effects on parasite resistance in insects and summarize empirical evidence that relates to the haploid-susceptibility hypothesis. | 2416 | NA | NA | NA | no | no |
| 2417 | 2017 | NA | Low, VL; Tan, TK; Prakash, BK; Vinnie-Siow, WY; Tay, ST; Masmeatathip, R; Hadi, UK; Lim, YAL; Chen, CD; Norma-Rashid, Y; Sofian-Azirun, M | 2017 | Contrasting evolutionary patterns between two haplogroups of Haematobia exigua (Diptera: Muscidae) from the mainland and islands of Southeast Asia | Uncovering the hidden diversity and evolutionary history of arthropods of medico-veterinary importance could have significant implications for vector-borne disease control and epidemiological intervention. The buffalo fly Haematobia exigua is an obligate bloodsucking ectoparasite of livestock. As an initial step towards understanding its population structures and biogeographic patterns, we characterized partial cytochrome c oxidase subunit I (COI) and cytochrome b (Cytb) sequences of H. exigua from three distinct geographic regions in Southeast Asia. We detected two distinct mitochondrial haplogroups of H. exigua in our surveyed geographic regions. Haplogroup I is widespread in the Southeast Asian mainland whereas haplogroup II is generally restricted to the type population Java Island. Both haplogroups were detected co-occurring on Borneo Island. Additionally, both haplogroups have undergone contrasting evolutionary histories, with haplogroup I exhibited a high level of mitochondrial diversity indicating a population expansion during the Pleistocene era dating back to 98,000 years ago. However, haplogroup II presented a low level of mitochondrial diversity which argues against the hypothesis of recent demographic expansion. | 2417 | NA | NA | NA | no | no |
| 2418 | 2017 | NA | Mahon, HC; Hammer, MP; Harris, JO | 2015 | Effect of salinity on growth of juvenile Yarra pygmy perch (Nannoperca obscura: Percichthyidae) | The threatened Yarra pygmy perch (Nannoperca obscura) is an obligate freshwater fish endemic to the south-eastern coast of mainland Australia, where a majority of river systems have been heavily modified since European settlement. The purpose of this garden study was to explore any relationship between the growth rate of juvenile N. obscura with respect to salinity. Trials were conducted encompassing perceived and potential ranges within the species’ environment, through five salinity treatments and recorded growth after 8 weeks. Two brood-groups were trialed simultaneously to observe any intrapopulation variance in phenotypic response potentially linked to genetic variance. The salinity trials found a clear model indicating increasing growth for moderate salinities (i.e. 2.5-7.5 ppt), therefore identifying the ideal salinity range for N. obscura juveniles. Phenotypic response between brood-groups was varied and inconsistent, potentially reflecting low genetic variability. With a limited genetic variance restricting adaptability, tolerance to a changing environment would be reduced. Therefore increased salinization and subsequent flushing events within habitats may be key threats to the species’ survival. | 2418 | NA | NA | NA | no | no |
| 2419 | 2017 | NA | Agouti, I; Badens, C; Abouyoub, A; Levy, N; Bennani, M | 2008 | Molecular Basis of beta-Thalassemia in Morocco: Possible Origins of the Molecular Heterogeneity | We present the molecular spectrum of beta-thalassemia in the Moroccan population obtained by the identification of molecular defects responsible for this disease, and herewith we show that the Moroccan population is genetically heterogeneous; 18 different mutations have been found in the 158 beta-globin chromosomes studied. Eight mutations [codon 39 (C -> T), FSC-8 (-AA), IVS-II-745 (C -> G), -29 (A -> G), FSC-6 (-A), IVS-I-110 (G -> A), IVS-I-2 (T -> C), and IVS-I-1 (G -> A)] out of 18 beta-thalassemia mutations identified accounted for 76% of the Moroccan beta-thalassemia chromosomes. Restriction fragment length polymorphism (RFLP) haplotype analysis showed that the observed genetic diversity originated from both new mutational events and gene flow due to migration. | 2419 | NA | NA | NA | no | no |
| 2420 | 2017 | NA | Chiang, YC; Ge, XJ; Chou, CH; Wu, WL; Chiang, TY | 2002 | Nucleotide sequence diversity at the methionine synthase locus in endangered Dunnia sinensis (Rubiaceae): An evaluation of the positive selection hypothesis | Methionine synthase is a key enzyme for the synthesis of the aspartate-derived methionine, the immediate precursor of S-adenosyl-methionine, which has been illustrated to be associated with plant growth and pathogen interactions. In this study we tested the positive selection hypothesis of molecular evolution of the methionine synthase gene in Dunnia sinensis. In the entire sample of 87 sequences, 22 haplotypes of introns and 16 haplotypes of exons were identified. An excess of polymorphism over the neutral expectation for the class of unique nucleotide polymorphisms was observed in both exon and intron sequences. Ten replacement substitutions versus six synonymous substitutions among lineages, although nonsignificant, revealed that some advantageous mutants might have been favored. The distribution of d(N)/d(S) > 1 at nodes between closely related haplotypes in the gene network also indicated weak and variable positive selection. Nevertheless, low levels of genetic diversity in exons (theta = 0.0052) and introns (theta = 0.0070) of the methionine synthase gene of the outcrossing Dunnia were also attributed to the endangered status of the species. The atpB-rbcL intergenic spacer of cpDNA and the ribosomal internal transcribed spacer of mtDNA were used to discern the relative effectiveness of natural selection from intrinsic evolutionary forces. The low levels of nucleotide polymorphisms in both organelle spacers and the significant population differentiation reflected the effect of population-species history and demography. Two major lineages of the methionine synthase gene genealogy were recovered corresponding to two geographic regions, a result that was consistent with organelle phylogenies. Both past fragmentation and recent habitat disturbance causing complete bottlenecks may have resulted in population decline and geographic isolation and may have led to the depletion of genetic variation at loci in nuclear and organelle genomes. | 2420 | NA | NA | NA | no | no |
| 2421 | 2017 | NA | SOUZA, V; NGUYEN, TT; HUDSON, RR; PINERO, D; LENSKI, RE | 1992 | HIERARCHICAL ANALYSIS OF LINKAGE DISEQUILIBRIUM IN RHIZOBIUM POPULATIONS - EVIDENCE FOR SEX | Many bacterial species exhibit strong linkage disequilibrium of their chromosomal genes, which apparently indicates restricted recombination between alleles at different loci. The extent to which restricted recombination reflects limited migration between geographically isolated populations versus infrequent mixis of genotypes within populations is more difficult to determine. We examined the genetic structure of Rhizobium leguminosarum biovar phaseoli populations associated with wild and cultivated beans (Phaseolus spp.) over several spatial scales, ranging from individual host plants to throughout the Western Hemisphere. We observed significant linkage disequilibrium at scales at least as small as a cultivated plot. However, the amount of disequilibrium was much greater among isolates collected throughout the Western Hemisphere than among isolates from one area of Mexico, even when disequilibrium was quantified using an index that scales for allelic diversity. This finding suggests that limited migration between populations contributes substantially to linkage disequilibrium in Rhizobium. We also compared the genetic structure for R. leguminosarum by. phaseoli taken from a cultivated plot with that for Escherichia coli obtained from one human host in an earlier study. Even at this fine scale, linkage disequilibrium in E. coli was very near the theoretical maximum level, whereas it was much less extreme in the local population of Rhizobium. Thus, the genetic structure for R. leguminosarum by. phaseoli does not exclude the possibility of frequent mixis within local populations. | 2421 | NA | NA | NA | no | no |
| 2422 | 2017 | NA | Pavlic-Zupanc, D; Wingfield, MJ; Boissin, E; Slippers, B | 2015 | The distribution of genetic diversity in the Neofusicoccum parvum/N. ribis complex suggests structure correlated with level of disturbance | Plants and animals adapted to colonize disturbed sites might also be better invaders, but this phenomenon has not been widely considered in fungi. We investigated genetic diversity and structure amongst isolates of Neofusicoccum parvum, N. cordaticola, N. kwambonambiense and N. umdonicola that coexist sympatrically on a native tree, Syzygium cordatum, across its distribution in South Africa. Species composition varied among stands, with dominance of N. parvum in disturbed stands, and absence in undisturbed stands, where the other species dominated. N. parvum populations from trees planted in urban environments were more genetically diverse than populations from human disturbed stands of S. cordatum. Bayesian analysis clustered N. parvum isolates in three sub-populations, suggesting three sources of origin. These results support the hypothesis that as a generalist N. parvum will dominate human disturbed sites and trees in urban areas, indicating strong potential for invasion, and its spreading from non-native hosts to native S. cordatum, rather than vice versa. (C) 2014 Elsevier Ltd and The British Mycological Society. All rights reserved. | 2422 | NA | NA | NA | no | no |
| 2423 | 2017 | NA | Danin-Poleg, Y; Reis, N; Tzuri, G; Katzir, N | 2001 | Development and characterization of microsatellite markers in Cucumis | This study provides a set of useful SSR markers and describes their development, characterization and application for diversity studies. Sixty one Cucumis SSR markers were developed, most of them (46) from melon (Cucumis melo L.) genomic libraries. Forty of the markers (30 melon and 10 cucumber SSRs) were evaluated for length polymorphism in a sample of 13 melon genotypes and 11 cucumber (Cucumis sativus L.) genotypes. PCR-amplification revealed up to six size alleles among the melon genotypes and up to five alleles among the cucumber genotypes, with mean gene-diversity values of 0.52 and 0.28 for melon and cucumber, respectively. These differences are in accordance with the known narrower genetic background of the cucumber. SSR data were applied to phylogenetic analysis among the melon and cucumber genotypes. A clear distinction between the ‘exotic’ groups and the sweet cultivated groups was demonstrated in melon. In cucumber, separation between the two sub-species, C. sativus var. sativus and C.sativus var. hardwickii, was obtained. Conservation of SSR loci between melon and cucumber was proven by sequence comparisons. | 2423 | NA | NA | NA | no | no |
| 2424 | 2017 | NA | Bulik-Sullivan, BK; Loh, PR; Finucane, HK; Ripke, S; Yang, J; Patterson, N; Daly, MJ; Price, AL; Neale, BM | 2015 | LD Score regression distinguishes confounding from polygenicity in genome-wide association studies | Both polygenicity (many small genetic effects) and confounding biases, such as cryptic relatedness and population stratification, can yield an inflated distribution of test statistics in genome-wide association studies (GWAS). However, current methods cannot distinguish between inflation from a true polygenic signal and bias. We have developed an approach, LD Score regression, that quantifies the contribution of each by examining the relationship between test statistics and linkage disequilibrium (LD). The LD Score regression intercept can be used to estimate a more powerful and accurate correction factor than genomic control. We find strong evidence that polygenicity accounts for the majority of the inflation in test statistics in many GWAS of large sample size. | 2424 | NA | NA | NA | no | no |
| 2425 | 2017 | NA | Barreto, MR; Guimaraes, CT; Teixeira, FF; Paiva, E; Valicente, FH | 2005 | Effect of Baculovirus spodoptera isolates in Spodoptera frugiperda (J.E. Smith) (Lepidoptera : Noctuidae) larvae and their characterization by RAPD | The total of 22 Baculovirus isolates surveyed in different corn producing regions in Brazil were used against fall armyworm, Spodoptera frugiperda (J.E. Smith). The viruses were purified and their suspensions were used to feed fall armyworm larvae from 4(th) and 5(th) instar. The mortality rate was checked daily and the infected larvae were frost after death, what generally occurred between the 51 and 71 day after virus ingestion. The 22 Baculovirus isolates were used in six concentrations (from 10(3) to 10(8) polyhedra/ml) and one check treatment with water. Mortality rate, larval period, pupal period, pupa weight and lethal concentration (LC50) were determined for all isolates. Significant differences were found among all isolates and different concentrations, also interaction between isolate x virus concentration for all characteristics evaluated, except for pupal period. Amplification patterns of 54 RAPD markers, being 41 polymorphic among the isolates, were used to evaluate the genetic distance and its correlation with the fall armyworm larvae mortality rate. The genetic diversity calculated by the Jaccard’s coefficient using the molecular data allowed a division of the isolates into two groups, with a high level of confidence. These groups did not present any association with the mortality rate caused by the isolates or with their geographical distribution. However, a RAPD fragment OPW04.2280 was highly associated with the larvae mortality rate and with LC50 explaining 23 and 65% of the phenotypic variation for these traits among the isolates, respectively. | 2425 | NA | NA | NA | no | no |
| 2426 | 2017 | NA | Johnstone, RA; Cant, MA | 2010 | The evolution of menopause in cetaceans and humans: the role of demography | Human females stop reproducing long before they die. Among other mammals, only pilot and killer whales exhibit a comparable period of post-reproductive life. The grandmother hypothesis suggests that kin selection can favour post-reproductive survival when older females help their relatives to reproduce. But although there is an evidence that grandmothers can provide such assistance, it is puzzling why menopause should have evolved only among the great apes and toothed whales. We have previously suggested (Cant & Johnstone 2008 Proc. Natl Acad. Sci. USA 105, 5332-5336 (doi:10.1073/pnas.0711911105)) that relatedness asymmetries owing to female-biased dispersal in ancestral humans would have favoured younger females in reproductive competition with older females, predisposing our species to the evolution of menopause. But this argument appears inapplicable to menopausal cetaceans, which exhibit philopatry of both sexes combined with extra-group mating. Here, we derive general formulae for ‘kinship dynamics’, the age-related changes in local relatedness that occur in long-lived social organisms as a consequence of dispersal and mortality. We show that the very different social structures of great apes and menopausal whales both give rise to an increase in local relatedness with female age, favouring late-life helping. Our analysis can therefore help to explain why, of all long-lived, social mammals, it is specifically among the great apes and toothed whales that menopause and post-reproductive helping have evolved. | 2426 | NA | NA | NA | no | no |
| 2427 | 2017 | NA | Liebhard, R; Gianfranceschi, L; Koller, B; Ryder, CD; Tarchini, R; Van de Weg, E; Gessler, C | 2002 | Development and characterisation of 140 new microsatellites in apple (Malus x domestica Borkh.) | The availability of suitable genetic markers is essential to efficiently select and breed apple varieties of high quality and with multiple disease resistances. Microsatellites (simple sequence repeats, SSR) are very useful in this respect since they are codominant, highly polymorphic, abundant and reliably reproducible. Over 140 new SSR markers have been developed in apple and tested on a panel of 7 cultivars and 1 breeding selection. Their high level of polymorphism is expressed with an average of 6.1 alleles per locus and an average heterozygosity (H) of 0.74. Of all SSR markers, 115 have been positioned on a genetic linkage map of the cross ‘Fiesta’ x ‘Discovery’. As a result, all 17 linkage groups, corresponding to the 17 chromosomes of apple, were identified. Each chromosome carries at least two SSR markers, allowing the alignment of any apple molecular marker map both with regard to identification as well as to orientation of the linkage groups. To test the degree of conservation of the SSR flanking regions and the transferability of the SSR markers to other Rosaceae species, 15 primer pairs were tested on a series of Maloideae and Amygdaloideae species. The usefulness of the newly developed microsatellites in genetic mapping is demonstrated by means of the genetic linkage map. The possibility of constructing a global apple linkage map and the impact of such a number of microsatellite markers on gene and QTL mapping is discussed. | 2427 | NA | NA | NA | no | no |
| 2428 | 2017 | NA | Stiebens, VA; Merino, SE; Chain, FJJ; Eizaguirre, C | 2013 | Evolution of MHC class I genes in the endangered loggerhead sea turtle (Caretta caretta) revealed by 454 amplicon sequencing | Background: In evolutionary and conservation biology, parasitism is often highlighted as a major selective pressure. To fight against parasites and pathogens, genetic diversity of the immune genes of the major histocompatibility complex (MHC) are particularly important. However, the extensive degree of polymorphism observed in these genes makes it difficult to conduct thorough population screenings. Methods: We utilized a genotyping protocol that uses 454 amplicon sequencing to characterize the MHC class I in the endangered loggerhead sea turtle (Caretta caretta) and to investigate their evolution at multiple relevant levels of organization. Results: MHC class I genes revealed signatures of trans-species polymorphism across several reptile species. In the studied loggerhead turtle individuals, it results in the maintenance of two ancient allelic lineages. We also found that individuals carrying an intermediate number of MHC class I alleles are larger than those with either a low or high number of alleles. Conclusions: Multiple modes of evolution seem to maintain MHC diversity in the loggerhead turtles, with relatively high polymorphism for an endangered species. | 2428 | NA | NA | NA | no | no |
| 2429 | 2017 | NA | Seddon, JM; Sundqvist, AK; Bjornerfeldt, S; Ellegren, H | 2006 | Genetic identification of immigrants to the Scandinavian wolf population | “Continued gene flow is fundamental to the survival of small, isolated populations. However, geography and human intervention can often act contrary to this requirement. The Scandinavian wolf population is threatened with a loss of genetic variation yet limited in the accessibility to new immigrants by the geographical distance of this peninsular population from its nearest neighbouring population and by human reluctance to allow wolves in the northern reindeer-breeding areas. In this study, we describe the identification of immigrants into this population using autosomal microsatellites, and maternally inherited mtDNA. Samples of 14 wolves collected in the”“dispersal corridor”" in northern Sweden in 2002-2005 were compared with 185 resident Scandinavian wolves and 79 wolves from the neighbouring Finnish population. We identified four immigrant wolves, suggesting some westward migration, although only one of these is likely to still survive. The integration of such immigrants into the breeding population is necessary to assure the long-term survival of this isolated and inbred population and highlights the importance of genetics techniques to the management of threatened populations." | 2429 | NA | NA | NA | no | no |
| 2430 | 2017 | NA | Fu, C; Sun, S; Billmyre, RB; Roach, KC; Heitman, J | 2015 | Unisexual versus bisexual mating in Cryptococcus neoformans: Consequences and biological impacts | Cryptococcus neoformans is an opportunistic human fungal pathogen and can undergo both bisexual and unisexual mating. Despite the fact that one mating type is dispensable for unisexual mating, the two sexual cycles share surprisingly similar features. Both mating cycles are affected by similar environmental factors and regulated by the same pheromone response pathway. Recombination takes place during unisexual reproduction in a fashion similar to bisexual reproduction and can both admix pre-existing genetic diversity and also generate diversity de novo just like bisexual reproduction. These common features may allow the unisexual life cycle to provide phenotypic and genotypic plasticity for the natural Cryptococcus population, which is predominantly cc mating type, and to avoid Muller’s ratchet. The morphological transition from yeast to hyphal growth during both bisexual and unisexual mating may provide increased opportunities for outcrossing and the ability to forage for nutrients at a distance. The unisexual life cycle is a key evolutionary factor for Cryptococcus as a highly successful global fungal pathogen. (C) 2014 Elsevier Inc. All rights reserved. | 2430 | NA | NA | NA | no | no |
| 2431 | 2017 | NA | Cardon, M; Loot, G; Grenouillet, G; Blanchet, S | 2011 | Host characteristics and environmental factors differentially drive the burden and pathogenicity of an ectoparasite: a multilevel causal analysis | P>1. Understanding the ecological factors driving the burden and pathogenicity of parasites is challenging. Indeed, the dynamics of host-parasite interactions is driven by factors organized across nested hierarchical levels (e.g. hosts, localities), and indirect effects are expected owing to interactions between levels. 2. In this study, we combined Bayesian multilevel models, path analyses and a model selection procedure to account for these complexities and to decipher the relative effects of host- and environment-related factors on the burden and the pathogenicity of an ectoparasite (Tracheliastes polycolpus) on its fish host (Leuciscus leuciscus). We also tested the year-to-year consistency of the relationships linking these factors to the burden and the pathogenic effects of T. polycolpus. 3. We found significant relationships between the parasite burden and host-related factors: body length and age were positively related to parasite burden and heterozygous hosts displayed a higher parasite burden. In contrast, both host- and environment-related factors were linked to pathogenic effects. Pathogenicity was correlated negatively with host body length and positively with age; this illustrates that some factors (e.g. body length) showed inverse relationships with parasite burden and pathogenicity. Pathogenic effects were stronger in cooler upstream sites and where host density was lower. Path analyses revealed that these relationships between environment-related factors and pathogenic effects were direct and were not indirect relationships mediated by the host characteristics. Finally, we found that the strength and the shape of certain relationships were consistent across years, while they were clearly not for some others. 4. Our study illustrates that considering conjointly causal relationships among factors and the hierarchical structure of host-parasite interactions is appropriate for dissecting the complex links between hosts, parasites and their common environment. | 2431 | NA | NA | NA | no | no |
| 2432 | 2017 | NA | Jutkina, J; Hansen, LH; Li, LL; Heinaru, E; Vedler, E; Joesaar, M; Heinaru, A | 2013 | Complete nucleotide sequence of the self-transmissible TOL plasmid pD2RT provides new insight into arrangement of toluene catabolic plasmids | In the present study we report the complete nucleotide sequence of the toluene catabolic plasmid pD2RT of Pseudomonas migulae strain D2RT isolated from Baltic Sea water. The pD2RT is 129,894 base pairs in size with an average G+C content of 53.75%. A total of 135 open reading frames (ORFs) were predicted to encode proteins, among them genes for catabolism of toluene, plasmid replication, maintenance and conjugative transfer. ORFs encoding proteins with putative functions in stress response, transposition and site-specific recombination were also predicted. Analysis of the organization and nucleotide sequence of pD2RT backbone region revealed high degree of similarity to the draft genome sequence data of the plant-pathogenic pseudomonad Pseudomonas syringae pv. glycinea strain B076, exhibiting relatedness to pPT23A plasmid family. The pD2RT backbone is also closely related to that of pGRT1 of Pseudomonas putida strain DOT-TIE and pBVIE04 of Burkholderia vietnamiensis strain G4, both plasmids are associated with resistance to toluene. The ability of pD2RT to self-transfer by conjugation to P. putida recipient strain PaW340 was experimentally determined. Genetic organization of toluene-degrading (xyl) genes and flanking DNA segments resembles the structure of Tn1721-related class II transposon Tn4655 of TOL plasmid pWW53 of P. putida strain MT53. The complete sequence of the plasmid pD2RT extends the known range of xyl genes carriers, being the first completely sequenced TOL plasmid, which is not related to well-studied IncP plasmid groups. We also verified the functionality of the catabolic route encoded by pD2RT by monitoring the expression of the xylE gene in pD2RT bearing hosts along with bacterial strains containing TOL plasmid of IncP-9 group. The growth kinetics of plasmid-bearing strains was found to be affected by particular TOL plasmid. (C) 2013 Elsevier Inc. All rights reserved. | 2432 | NA | NA | NA | no | no |
| 2433 | 2017 | NA | Rodriguez-Robles, JA; Jezkova, T; Leal, M | 2008 | “Genetic structuring in the threatened”“Lagartijo del Bosque Seco”" (Anolis cooki) from Puerto Rico" | Species with restricted geographic distributions consisting of isolated populations are particularly susceptible to extinction because these denies face an increased risk of disappearing due to environmental, demographic, and genetic stochasticity. We used partial DNA sequences of the cytochrome b (1083 bp) and ND2 (1041 bp) mitochondrial genes to investigate the phylogeography and population genetics of Anolis cooki, a threatened lizard endemic to the southwestern coast of the Caribbean Island of Puerto Rico. Maximum likelihood and Bayesian methods revealed relatively shallow genetic differentiation among 27 unique haplotypes (from 52 individuals) from the known extant populations of A. cooki in mainland Puerto Rico. Despite this pattern, specimens from the same geographic area tended to nest together. The most basal division within A. cooki is between haplotypes from the three westernmost populations (Punta Aguila, Morrillos, Playa Santa) and the remainder demes (Bahia Ballena, La Cueva, Punta Verraco). The three westernmost populations of A. cooki are separated from their conspecific demes by the Guanica Bay and the Loco River drainage system, which together may represent a physiographic barrier for A. cooki. Each population of A. cooki only has private haplotypes; in other words, there are no shared mitochondrial types between populations. Because the number of private haplotypes can be used as an indirect measure of gene flow, this finding suggests that currently there is no migration among demes, and that each is an independent demographic unit, despite the relatively short distances (ca. 2 km) that separate some of them. Pairwise F-ST values and spatial analyses of molecular variation confirmed the existence of distinct groups of genetically defined sampling areas, and of significant molecular variation among populations within groups and within populations. The conservation status of the populations of A. cooki varies greatly. The demes from Punta Aguila, Morrillos, and Bahia Ballena inhabit protected areas, and are larger, genetically diverse, and seemingly stable. The population from Playa Santa showed a high level of genetic diversity, but it occurs in an area that has been intensively developed for residential and touristic purposes, and its long-term survival is uncertain. A. cooki is also known from Caja de Muertos, an island off the southcentral coast of Puerto Rico. Surveys conducted on September 2006 and March 2007 did not produce any specimens, and a thorough assessment of Caja de Muertos is needed to determine the present status of A. cooki on the island. (C) 2007 Elsevier Inc. All rights reserved. | 2433 | NA | NA | NA | no | no |
| 2434 | 2017 | NA | Attard, CRM; Moller, LM; Sasaki, M; Hammer, MP; Bice, CM; Brauer, CJ; Carvalho, DC; Harris, JO; Beheregaray, LB | 2016 | A novel holistic framework for genetic-based captive-breeding and reintroduction programs | Research in reintroduction biology has provided a greater understanding of the often limited success of species reintroductions and highlighted the need for scientifically rigorous approaches in reintroduction programs. We examined the recent genetic-based captive-breeding and reintroduction literature to showcase the underuse of the genetic data gathered. We devised a framework that takes full advantage of the genetic data through assessment of the genetic makeup of populations before (past component of the framework), during (present component), and after (future component) captive-breeding and reintroduction events to understand their conservation potential and maximize their success. We empirically applied our framework to two small fishes: Yarra pygmy perch (Nannoperca obscura) and southern pygmy perch (Nannoperca australis). Each of these species has a locally adapted and geographically isolated lineage that is endemic to the highly threatened lower Murray-Darling Basin in Australia. These two populations were rescued during Australia’s recent decade-long Millennium Drought, when their persistence became entirely dependent on captive-breeding and subsequent reintroduction efforts. Using historical demographic analyses, we found differences and similarities between the species in the genetic impacts of past natural and anthropogenic events that occurred in situ, such as European settlement (past component). Subsequently, successful maintenance of genetic diversity in captivitydespite skewed brooder contribution to offspringwas achieved through carefully managed genetic-based breeding (present component). Finally, genetic monitoring revealed the survival and recruitment of released captive-bred offspring in the wild (future component). Our holistic framework often requires no additional data collection to that typically gathered in genetic-based breeding programs, is applicable to a wide range of species, advances the genetic considerations of reintroduction programs, and is expected to improve with the use of next-generation sequencing technology. Un Marco de Referencia Holistico Novedoso para Programas de Reproduccion en Cautiverio Basada en Genetica y de Reintroduccion Investigaciones sobre biologia de la reintroduccion han proporcionado un mejor entendimiento del, a menudo, exito limitado de las reintroducciones de especies y han resaltado la necesidad de aproximaciones rigorosas cientificamente en los programas de reintroduccion. Examinamos la literatura reciente sobre reproduccion en cautiverio basada en genetica y reintroduccion para exhibir la subutilizacion de los datos geneticos. Disenamos un marco de referencia que obtiene la mayor ventaja de los datos geneticos mediante la evaluacion de la composicion genetica de las poblaciones antes (componente pasado del marco de referencia), durante (componente presente), y despues (componente futuro) de eventos de reproduccion en cautiverio y de reintroduccion para entender su potencial de conservacion y maximizar su exito. Aplicamos nuestro marco de referencia empiricamente con dos especies de peces pequenos: Nannoperca obscura y N. australis. Cada especie tiene un linaje adaptado localmente y aislado geograficamente endemico de la muy amenazada Cuenca Baja Murray-Darling, Australia. Las dos poblaciones fueron rescatadas durante la reciente Sequia del Milenio que duro diez anos en Australia, cuando su persistencia se volvio totalmente dependiente de esfuerzos de reproduccion en cautiverio y subsecuente reintroduccion. Mediante analisis demograficos historicos, encontramos diferencias y similitudes entre las especies en los impactos geneticos de eventos naturales y antropogenicos pasados que ocurrieron in situ, como el asentamiento europeo (componente pasado). Subsecuentemente, el mantenimiento exitoso de la diversidad genetica en cautiverio - no obstante la contribucion de reproductores sesgada - fue posible por el manejo cuidadoso de la reproduccion basada en genetica (componente presente). Finalmente, el monitoreo genetico revelo la supervivencia y reclutamiento de crias obtenidas en cautiverio liberadas en el medio silvestre. Nuestro marco de referencia holistico a menudo no requiere de datos adicionales a los obtenidos tipicamente en programas de reproduccion basados en genetica, es aplicable a un rango amplio de especies, es un avance en las consideraciones geneticas de los programas de reintroduccion, y mejorara con el uso de tecnologia de secuenciacion de ultima generacion. Resumen | 2434 | NA | NA | NA | no | no |
| 2435 | 2017 | NA | Li, Q; Xu, LZ; Zou, T; Ai, P; Huang, GH; Li, P; Zheng, AP | 2015 | Complete genome sequence of Bacillus thuringiensis strain HD521 | Bacillus thuringiensis is the most widely used biological pesticide in the world. It belongs to the Bacillus cereus sensu lato group, which contains six species. Among these six species, B. thuringiensis, B. anthracis, and B. cereus have a low genetic diversity. B. thuringiensis strain HD521 shows maroon colony which is different from most of the B. thuringiensis strains. Strain HD521 also displays an ability to inhibit plant sheath blight disease pathogen (Rhizoctonia solani AG1 IB) growth and can form bipyramidal parasporal crystals consisting of three cry7 genes. These crystals have an insecticidal activity against Henosepilachna vigintioctomaculata larva (Coleoptera). Here we report the complete genome sequence of strain HD521, which has one chromosome and six circular plasmids. | 2435 | NA | NA | NA | no | no |
| 2436 | 2017 | NA | Nielsen, LR; Kjaer, ED | 2010 | Gene flow and mating patterns in individuals of wych elm (Ulmus glabra) in forest and open land after the influence of Dutch elm disease | Dutch elm disease has severely reduced the number of large trees of U. glabra in Denmark. Consequently, the distance between large trees has increased and the overall density of the species has decreased. Patches of small trees with stem diameters up to 10 cm are, however, relatively frequent. With four microsatellites we studied potential differences in genetic diversity, mating patterns and pollen flow in trees of U. glabra that occur either in a continuous forest (Suserup Forest) or isolated in the open land. We found no indications of selfing in forest or open land but indications of biparental inbreeding in offspring of isolated trees. Estimates of effective pollen donors (N(ep)) and minimum number of pollen donors (N(p)) were alike in forest and open land (N(ep) of 31 and 34 and N(p) of 4 to >6 and 3 to >6, respectively). The number of alleles was also very similar. With indirect methods we found that average pollen dispersal was 104 m under forest conditions. The average distance between the isolated trees and their potential pollen donors was further, thus suggesting that effective pollen in the open land on average moves further than in a dense forest. Finally, 28% of small trees (diameters up to 10 cm) produced fruits. Reproduction at a young age may be a key stone in the survival of U. glabra as the vectors of the disease prefer older trees. | 2436 | NA | NA | NA | no | no |
| 2437 | 2017 | NA | Vincenzi, S; De Leo, GA; Bellingeri, M | 2012 | Consequences of extreme events on population persistence and evolution of a quantitative trait | The intensification and increased frequency of weather and climate extremes are emerging as one of the most important aspects of climate change. Using a quantitative genetic model, we explore the effects of increasing environmental stochasticity and its interplay with genetic variation and selection pressure on population dynamics and evolution of a fitness-related trait. We use simulations with variations in trend (i.e., directional change) and stochasticity (i.e., increase in variance) of a climate variable defining a phenotypic optimum, and various hypotheses on mutational variance and strength of selection on a phenotypic trait. We let the population reach mutation-selection balance and then we linearly increase over simulation time both the mean and the variance of the statistical distribution of the climate variable. Higher variance of climate variables increases the probability of extreme climatic events, i.e. events that are both statistically rare and with potentially high ecological impact, that is, causing episodes of massive mortality in the population. Our analysis shows that the population is able to track the directional component of the optimum for low increases of variability, while for high increases the tracking is reduced. Persistence of the population depends quite strongly on the selection pressure and decreases with increasing variance of the climate variable. Higher mutational variance does not substantially decrease the risk of extinction of a population. (C) 2011 Elsevier B.V. All rights reserved. | 2437 | NA | NA | NA | no | no |
| 2438 | 2017 | NA | Le Goff, GJ; Hance, T; Detrain, C; Deneubourg, JL; Mailleux, AC | 2014 | Impact of living with kin/non-kin on the life history traits of Tetranychus urticae (Acari: Tetranychidae) | In many vertebrates and invertebrates, living in a group may influence the life history traits, physiology and behaviour of its individual members, whereas genetic relatedness affects social interactions among individuals in a group. The two-spotted spider mite Tetranychus urticae is characterised by a communal organization, in which silk production plays a key role. A silken web protects the colony against biotic and abiotic agents such as predators, competitors, humidity, wind, rain and acaricides. To evaluate the potential costs and benefits of being associated with genetically distant vs genetically close individuals in T. urticae, we assessed various fitness indicators (faecal pellet production, fecundity, death rate) in pure and mixed groups of two distinct populations of T. urticae: a red-form population from Tunisia and a green-form population from Belgium. If genetic origin had no influence, the values of fitness indicators in mixed groups composed of green and red individuals, would be intermediate between those of the pure green-form and red-form groups. Our results show that in a mixed group, faecal pellet production and death rate were statistically similar to the values obtained in the pure group of green-form individuals. Therefore, our study suggests that strain recognition ability may occur in T. urticae and that the genetic background of an individual may have a great impact on several of its life history traits. | 2438 | NA | NA | NA | no | no |
| 2439 | 2017 | NA | Cureton, AN; Newbury, HJ; Raybould, AF; Ford-Lloyd, BV | 2006 | Genetic structure and gene flow in wild beet populations: the potential influence of habitat on transgene spread and risk assessment | 1 The consequences of the movement of transgenes from genetically modified (GM) crops into wild populations of plants continues to be of concern to ecologists and conservationists because of the possible threat posed to those populations in terms of their continued survival and because of the further knock‐on effects that might occur to habitats in which they occur.  2 We examined five UK sea beet Beta vulgaris ssp. maritima populations from each of two major habitat types, cliff top and drift line. We assessed population genetic parameters, genetic diversity, gene flow, population differentiation and isolation by distance, to enable determination of the likelihood and consequences of spread to wild populations of genes from cultivated sugar beet group Beta vulgaris ssp. vulgaris, which could in the future be transgenic.  3 Drift line populations were more diverse than cliff top populations and also showed greater levels of gene flow.  4 Isolation by distance was identified in both habitats, but the relationship between genetic and geographical distance was detectable over longer distances for drift line populations. However, clear indications of vicariance (the subdivision of a population into distinct taxa by the appearance of a geological barrier) between cliff and drift line populations were also evident, because of the restriction of gene flow between the two habitats occurring more in one direction than the other. 5 Synthesis and applications. The likelihood of transgene spread from crop to wild populations is habitat dependent and conservation management decisions could therefore vary from one population to another, for example water courses were found to facilitate seed dispersal. This should be taken into account when estimating isolation distances for GM beet, and when predicting transgene frequencies (exposure estimates) for environmental risk assessments of GM beet. | 2439 | NA | NA | NA | no | no |
| 2440 | 2017 | NA | Bournoville, R; Simon, JC; Badenhausser, I; Girousse, C; Guilloux, T; Andre, S | 2000 | Clones of pea aphid, Acyrthosiphon pisum (Hemiptera : Aphididae) distinguished using genetic markers, differ in their damaging effect on a resistant alfalfa cultivar | CUF 101, a resistant cultivar of alfalfa, was exposed to 15 clones of Acyrthosiphon pisum Harris collected from alfalfa fields in three regions of France (east, south, central west) to determine whether the level of resistance varied across the different clones. The survival of alfalfa seedlings infested at the cotyledon stage was assessed using a standardized method. Although no difference in seedling mortality was detected between clones grouped by region, there was a significant variation among the 15 pea aphid clones. In particular, two clones of southern origin were more aggressive. In addition, the different pea aphid clones were characterized using allozyme and RAPD-PCR markers. Among the 15 clones, seven allozyme genotypes (plus one when adding colour polymorphism) and 12 RAPD-PCR genotypes were distinguished. The two southern clones differing by their aggressiveness on the resistant alfalfa belonged to the same allozyme and RAPD genotype which was distinct from the other pea aphid clones. Our results reinforce the need to take into account aphid genetic diversity in breeding programmes for resistance in cultivated plants. | 2440 | NA | NA | NA | no | no |
| 2441 | 2017 | NA | Wilson, BA; Pennings, PS; Petrov, DA | 2017 | Soft Selective Sweeps in Evolutionary Rescue | Evolutionary rescue occurs when a population that is declining in size because of an environmental change is rescued from extinction by genetic adaptation. Evolutionary rescue is an important phenomenon at the intersection of ecology and population genetics, and the study of evolutionary rescue is critical to understanding processes ranging from species conservation to the evolution of drug and pesticide resistance. While most population-genetic models of evolutionary rescue focus on estimating the probability of rescue, we focus on whether one or more adaptive lineages contribute to evolutionary rescue. We find that when evolutionary rescue is likely, it is often driven by soft selective sweeps where multiple adaptive mutations spread through the population simultaneously. We give full analytic results for the probability of evolutionary rescue and the probability that evolutionary rescue occurs via soft selective sweeps. We expect that these results will find utility in understanding the genetic signatures associated with various evolutionary rescue scenarios in large populations, such as the evolution of drug resistance in viral, bacterial, or eukaryotic pathogens. | 2441 | NA | NA | NA | no | no |
| 2442 | 2017 | NA | Tiffin, N; Meintjes, A; Ramesar, R; Bajic, VB; Rayner, B | 2010 | Computational Analysis of Candidate Disease Genes and Variants for Salt-Sensitive Hypertension in Indigenous Southern Africans | Multiple factors underlie susceptibility to essential hypertension, including a significant genetic and ethnic component, and environmental effects. Blood pressure response of hypertensive individuals to salt is heterogeneous, but salt sensitivity appears more prevalent in people of indigenous African origin. The underlying genetics of salt-sensitive hypertension, however, are poorly understood. In this study, computational methods including text-and data-mining have been used to select and prioritize candidate aetiological genes for salt-sensitive hypertension. Additionally, we have compared allele frequencies and copy number variation for single nucleotide polymorphisms in candidate genes between indigenous Southern African and Caucasian populations, with the aim of identifying candidate genes with significant variability between the population groups: identifying genetic variability between population groups can exploit ethnic differences in disease prevalence to aid with prioritisation of good candidate genes. Our top-ranking candidate genes include parathyroid hormone precursor (PTH) and type-1angiotensin II receptor (AGTR1). We propose that the candidate genes identified in this study warrant further investigation as potential aetiological genes for salt-sensitive hypertension. | 2442 | NA | NA | NA | no | no |
| 2443 | 2017 | NA | Lecis, R; Norris, K | 2004 | Population genetic diversity of the endemic Sardinian newt Euproctus platycephalus: implications for conservation | The Sardinian mountain newt Euproctus platycephalus, endemic to the island of Sardinia, (Italy), is considered a rare and threatened species and is classed as critically endangered by IUCN. It inhabits streams, small lakes and pools on the main mountain systems of the island. Threats from climatic and anthropogenic factors have raised concerns for the long-term survival of newt populations on the island. MtDNA sequencing was used to investigate the genetic population structure and phylogeography of this endemic species. Patterns of genetic variation were assessed by sequencing the complete Dloop region and part of the 12SrRNA, from 74 individuals representing four different populations. Analyses of molecular variance suggest that populations are significantly differentiated, and the distribution of haplotypes across the island shows strong geographical structuring. However, phylogenetic analyses also suggest that the Sardinian population consists of two distinct mtDNA groups, which may reflect ancient isolation and expansion events. Population structure, evolutionary history of the species and implications for the conservation of newt populations are discussed. (C) 2003 Elsevier Ltd. All rights reserved. | 2443 | NA | NA | NA | no | no |
| 2444 | 2017 | NA | Martinell, MC; Dotterl, S; Blanche, C; Rovira, A; Masso, S; Bosch, M | 2010 | Nocturnal pollination of the endemic Silene sennenii (Caryophyllaceae): an endangered mutualism? | Silene sennenii is an extremely narrow endemic species from the north-eastern Iberian Peninsula, with only five populations. Its habitat, severely affected by urban, industrial, and agricultural land use, is highly vulnerable, and makes of S. sennenii, a seriously endangered taxon. Its pollination ecology is studied and compared among populations. Flowers of S. sennenii are distinguished by several morphological and physiological characters, including night emission of scent, which are related to nocturnal pollination. Although visited by insects at night and during day, selective insect exclusion experiments show that this plant is pollinated mainly at night. However, differences among populations are observed in visitation rates and reproductive success, which indicates that the system composed by the plant and its pollinators show evidences of disruption in some populations. The causes and consequences of this disruption are analyzed, with a focus on differences in population size, habitat quality, and genetic diversity. The results are discussed from the perspective of the vulnerability of established mutualisms, and the consequences for the survival of the species. | 2444 | NA | NA | NA | no | no |
| 2445 | 2017 | NA | Marin, DH; Kaplan, DT; Opperman, CH | 1999 | Randomly amplified polymorphic DNA differs with burrowing nematode collection site, but not with host range | The genetic variability of 12 burrowing nematode (Radopholus sp.) isolates from Central America, the Caribbean, and Florida, and one isolate from Ivory Coast were compared with RAPD analysis. A high degree of genetic similarity (>0.82) was determined for isolates from the Western Hemisphere. Genome similarity was greatest among isolates collected within a country. Among isolates collected in Central America and the Caribbean, burrowing nematodes from Belize and Guatemala were genetically more distant. However, the genome of the isolate from Ivory Coast was most dissimilar (>0.30). These results suggest that African and American burrowing-nematode isolates may have had different origins or that they have been geographically isolated for a sufficient amount of time to have accumulated genetic changes detectable by RAPD analysis. No relationship was found between the genomic similarity and extent of reproduction or damage to banana or citrus roots, Morphometric analysis involving eight of the isolates indicated that they were morphologically identical and values for morphometric parameters were well within the range preciously published for banana and citrus-parasitic burrowing nematodes. | 2445 | NA | NA | NA | no | no |
| 2446 | 2017 | NA | Oxley, PR; Spivak, M; Oldroyd, BP | 2010 | Six quantitative trait loci influence task thresholds for hygienic behaviour in honeybees (Apis mellifera) | Honeybee hygienic behaviour provides colonies with protection from many pathogens and is an important model system of the genetics of a complex behaviour. It is a textbook example of complex behaviour under simple genetic control: hygienic behaviour consists of two components - uncapping a diseased brood cell, followed by removal of the contents - each of which are thought to be modulated independently by a few loci of medium to large effect. A worker’s genetic propensity to engage in hygienic tasks affects the intensity of the stimulus required before she initiates the behaviour. Genetic diversity within colonies leads to task specialization among workers, with a minority of workers performing the majority of nest-cleaning tasks. We identify three quantitative trait loci that influence the likelihood that workers will engage in hygienic behaviour and account for up to 30% of the phenotypic variability in hygienic behaviour in our population. Furthermore, we identify two loci that influence the likelihood that a worker will perform uncapping behaviour only, and one locus that influences removal behaviour. We report the first candidate genes associated with engaging in hygienic behaviour, including four genes involved in olfaction, learning and social behaviour, and one gene involved in circadian locomotion. These candidates will allow molecular characterization of this distinctive behavioural mode of disease resistance, as well as providing the opportunity for marker-assisted selection for this commercially significant trait. | 2446 | NA | NA | NA | no | no |
| 2447 | 2017 | NA | Moltke, I; Albrechtsen, A; Hansen, TVO; Nielsen, FC; Nielsen, R | 2011 | A method for detecting IBD regions simultaneously in multiple individuals-with applications to disease genetics | All individuals in a finite population are related if traced back long enough and will, therefore, share regions of their genomes identical by descent (IBD). Detection of such regions has several important applications-from answering questions about human evolution to locating regions in the human genome containing disease-causing variants. However, IBD regions can be difficult to detect, especially in the common case where no pedigree information is available. In particular, all existing non-pedigree based methods can only infer IBD sharing between two individuals. Here, we present a new Markov Chain Monte Carlo method for detection of IBD regions, which does not rely on any pedigree information. It is based on a probabilistic model applicable to unphased SNP data. It can take inbreeding, allele frequencies, genotyping errors, and genomic distances into account. And most importantly, it can simultaneously infer IBD sharing among multiple individuals. Through simulations, we show that the simultaneous modeling of multiple individuals makes the method more powerful and accurate than several other non-pedigree based methods. We illustrate the potential of the method by applying it to data from individuals with breast and/or ovarian cancer, and show that a known disease-causing mutation can be mapped to a 2.2-Mb region using SNP data from only five seemingly unrelated affected individuals. This would not be possible using classical linkage mapping or association mapping. | 2447 | NA | NA | NA | no | no |
| 2448 | 2017 | NA | Brown, JE; McBride, CS; Johnson, P; Ritchie, S; Paupy, C; Bossin, H; Lutomiah, J; Fernandez-Salas, I; Ponlawat, A; Cornel, AJ; Black, WC; Gorrochotegui-Escalante, N; Urdaneta-Marquez, L; Sylla, M; Slotman, M; Murray, KO; Walker, C; Powell, JR | 2011 | Worldwide patterns of genetic differentiation imply multiple ‘domestications’ of Aedes aegypti, a major vector of human diseases | Understanding the processes by which species colonize and adapt to human habitats is particularly important in the case of disease-vectoring arthropods. The mosquito species Aedes aegypti, a major vector of dengue and yellow fever viruses, probably originated as a wild, zoophilic species in sub-Saharan Africa, where some populations still breed in tree holes in forested habitats. Many populations of the species, however, have evolved to thrive in human habitats and to bite humans. This includes some populations within Africa as well as almost all those outside Africa. It is not clear whether all domestic populations are genetically related and represent a single ‘domestication’ event, or whether association with human habitats has developed multiple times independently within the species. To test the hypotheses above, we screened 24 worldwide population samples of Ae. aegypti at 12 polymorphic microsatellite loci. We identified two distinct genetic clusters: one included all domestic populations outside of Africa and the other included both domestic and forest populations within Africa. This suggests that human association in Africa occurred independently from that in domestic populations across the rest of the world. Additionally, measures of genetic diversity support Ae. aegypti in Africa as the ancestral form of the species. Individuals from domestic populations outside Africa can reliably be assigned back to their population of origin, which will help determine the origins of new introductions of Ae. aegypti. | 2448 | NA | NA | NA | no | no |
| 2449 | 2017 | NA | Pavesi, A | 2007 | Pattern of nucleotide substitution in the overlapping nonstructural genes of influenza A virus and implication for the genetic diversity of the H5NI subtype | In viruses under strong pressure to minimize genome size, overlapping genes represent a fine strategy to condense a maximum amount of information into short nuelcotide sequences. Here, we investigated the evolution of the genes encoding the nonstructural proteins NS1 and NS2 of influenza A virus (IAV), which are one of the best characterized cases of gene overlap. By a detailed analysis of about four hundred sequences grouped into 11 IAV subtypes, we found that the overlapping coding region of the NS1 gene shows a significant increase of the rate of nonsynonymous change, with respect to its nonoverlapping counterpart. The same feature was observed in the overlapping coding region of the NS2 gene. Such a variation pattern, which implies the occurrence of several amino acid substitutions in the protein regions encoded by overlapping frames, is different from the pattern of constrained evolution typical of other viral overlapping-gene systems. Amino acid sequence analysis of the NS1 and NS2 proteins revealed that some nonsynonymous substitutions, located in the region of gene overlap, play a critical role in shaping the genetic diversity of the highly pathogenic subtype H5N1. Since both proteins contribute to disease pathogenesis by affecting many virus and host-cell processes, information provided by this study should be useful to highlight the impact of nonstructural gene variation on the pathogenicity of H5N1 viruses. (C) 2007 Elsevier B.V. All rights reserved. | 2449 | NA | NA | NA | no | no |
| 2450 | 2017 | NA | White, D; Watts, C; Allwood, J; Prada, D; Stringer, I; Thornburrow, D; Buckley, TR | 2017 | Population history and genetic bottlenecks in translocated Cook Strait giant weta, Deinacrida rugosa: recommendations for future conservation management | Translocating populations of endangered animals to predator-free offshore islands is an extreme, and potentially risky, conservation management strategy. There are many examples of failures and successes, however relatively little work has been done to formally quantify the required numbers of translocated individuals to increase the chance of successful establishment. Cook Strait giant weta, Deinacrida rugosa Buller 1870 (Orthoptera: Anostostomatidae), of New Zealand are an example of an at-risk species where extreme conservation measures are needed, due to their ground-dwelling lifestyle making them particularly prone to habitat loss and predation by alien species following human settlement. Here, we have used microsatellite markers to characterise genetic diversity within and between two parental populations (Mana and Stephens Islands), and two translocated populations (Maud and Matiu/Somes Islands), and estimate probabilities of genetic bottlenecks. We have modelled various demographic scenarios to quantify the severity of effective population size fluctuations resulting from translocations using DIYABC, and explored the number of founders needed to retain rare alleles using ALLELERETAIN. Our results suggest that populations have expanded on both Maud Island and Matiu/Somes Island from effective population sizes at time of translocation of 36 and 47 respectively, but provide evidence for a genetic bottleneck on Maud Island. We also show that translocations have had minimal impact on parental effective population sizes. The impact of our results on future conservation management efforts is discussed. | 2450 | NA | NA | NA | no | no |
| 2451 | 2017 | NA | Garrett, KA; Zuniga, LN; Roncal, E; Forbes, GA; Mundt, CC; Su, Z; Nelson, RJ | 2009 | Intraspecific functional diversity in hosts and its effect on disease risk across a climatic gradient | The effects of host biodiversity on disease risk may vary greatly depending on host population structure and climatic conditions. Agricultural diseases such as potato late blight, caused by Phytophthora infestans, provide the opportunity to study the effects of intraspecific host diversity that is relatively well-defined in terms of disease resistance phenotypes and may have functional impacts on disease levels. When these systems are present across a climatic gradient, it is also possible to study how season length and conduciveness of the environment to disease may influence the effects of host diversity on disease risk. We developed a simple model of epidemic progress to evaluate the effects on disease risk of season length, environmental disease conduciveness, and host functional divergence for mixtures of a susceptible host and a host with some resistance. Differences in disease levels for the susceptible vs. resistant genotypes shifted over time, with the divergence in disease levels first increasing and then decreasing. Disease reductions from host diversity were greatest for high host divergence and combinations of environmental disease conduciveness and season length that led to moderate disease severity. We also compared the effects of host functional divergence on potato late-blight risk in Ecuador (long seasons), two sites in Peru (intermediate seasons) in El Nino and La Nina years, and the United States (short seasons). There was some evidence for greater disease risk reduction from host diversity where seasons were shorter, probably because of lower regional inoculum loads. There was strong evidence for greater disease reduction when host functional divergence was greater. These results indicate that consideration of season length, environmental conduciveness to disease, and host functional divergence can help to explain the variability in disease response to host diversity. | 2451 | NA | NA | NA | no | no |
| 2452 | 2017 | NA | Stringari, D; Glienke, C; de Christo, D; Maccheroni, W; de Azevedo, JL | 2009 | High Molecular Diversity of the Fungus Guignardia citricarpa and Guignardia mangiferae and New Primers for the Diagnosis of the Citrus Black Spot | RAPD markers were used to investigate the distribution of genetic variability among a group of Guignardia citricarpa, G. mangiferae, and Phyllosticta spinarum isolates obtained from several hosts in Brazil, Argentina, Mexico, Costa Rica, Thailand, Japan, United States and South Africa. Pathogenic isolates G. citricarpa Kiely (anamorph form P. citricarpa McAlp Van Der Aa) are the etiological agent of the Citrus Black Spot (CBS), a disease that affects several citric plants and causes substantial injuries to the appearance of their fruits, thus preventing their export. Several previous studies have demonstrated the existence of an endophytic species with high morphological similarity to the causal agent of CBS that could remain latent in the same hosts. Consequently, the identification of the plants and fruits free from the causal agent of the disease is severely hampered. The RAPD analysis showed a clear discrimination among the pathogenic isolates of G. citricarpa and endophytic isolates (G. mangiferae and P. spinarum). In addition, a Principal Coordinate Analysis (PCO) based on a matrix of genetic similarity estimated by the RAPD markers showed four clusters, irrespective of their host or geographical origin. An Analysis of Molecular Variance (AMOVA) indicated that 62.8% of the genetic variation was found in the populations (G. citricarpa, G. mangiferae, P. spinarum and Phyllosticta sp.). Substantial variation was found in the populations (37.2%). Exclusive RAPD markers of isolates of G. citricarpa were cloned, sequenced and used to obtain SCARS (Sequence Characterized Amplified Regions), which allowed the development of new specific primers,for the identification of G. citricarpa PCR (Polymerase Chain Reaction) analysis using a pair of primers specific to pathogenic isolates corroborating the groupings obtained by the RAPD markers. underscoring its efficiency in the identification of the causal agent of CBS. | 2452 | NA | NA | NA | no | no |
| 2453 | 2017 | NA | Tonini, JFR; Costa, LP; Carnaval, AC | 2013 | Phylogeographic structure is strong in the Atlantic Forest; predictive power of correlative paleodistribution models, not always | We assess whether correlative paleoclimatic models of species ranges accurately predict genetic diversity patterns in species of distinct life histories traits in the Atlantic Forest (AF) of Brazil. To this end, we use sequences of the mitochondrial gene ND2 from Dendropsophus elegans and Chiasmocleis carvalhoi summarized in the shape of phylogenies and population genetic statistics and maximum entropy models of species distributions under current, 21kya BP and 120kya BP climatic reconstructions. The two target species have distinct ranges, habitat tolerances, rates of reproduction and dispersal abilities, yet are endemic to the AF. Although the more restricted and semi-fossorial C.carvalhoi is associated with forested habitats and thought to be a poor disperser, the widely ranged arboreal D.elegans inhabits open areas such as pastures and human-impacted regions of the AF, and is easily found perched on herbaceous vegetation in inundated areas. We had anticipated that correlative distribution models of the broadly distributed D.elegans would perform better then models of the narrowly ranged C.carvalhoi, thus better predicting current patterns of genetic diversity. The results demonstrate poor predictive ability of climate-based models of C.carvalhoi under current climatic conditions, suggesting that factors such as biotic interactions or dispersal ability may be playing a central role in defining this species distribution both now and in the recent past. Models under current climate are nonetheless accurate in the broadly ranged D.elegans. As a corollary, paleoclimatic models accurately predicted patterns of diversity of the ND2 mitochondrial gene in D.elegans, but not in C.carvalhoi. We attribute these distinct responses to the poor explanatory power of paleodistributions models when applied to species that violate the basic assumption of the environment as main driver of distribution patterns. This calls for a careful use of distribution models for the purpose of evolutionary biogeographical inference. Like C.carvalhoi, other species whose ranges are not yet at equilibrium, or which are impacted by competitor, parasite or pathogen presence, may not be suitable to the combined use of paleoclimatic-model based phylogeographic inference, as here implemented despite relatively high area under the curve values. | 2453 | NA | NA | NA | no | no |
| 2454 | 2017 | NA | Hamon, C; Baranger, A; Coyne, CJ; McGee, RJ; Le Goff, I; L’Anthoene, V; Esnault, R; Riviere, JP; Klein, A; Mangin, P; McPhee, KE; Roux-Duparque, M; Porter, L; Miteul, H; Lesne, A; Morin, G; Onfroy, C; Moussart, A; Tivoli, B; Delourme, R; Pilet-Nayel, ML | 2011 | New consistent QTL in pea associated with partial resistance to Aphanomyces euteiches in multiple French and American environments | Partial resistances, often controlled by quantitative trait loci (QTL), are considered to be more durable than monogenic resistances. Therefore, a precursor to developing efficient breeding programs for polygenic resistance to pathogens should be a greater understanding of genetic diversity and stability of resistance QTL in plants. In this study, we deciphered the diversity and stability of resistance QTL to Aphanomyces euteiches in pea towards pathogen variability, environments and scoring criteria, from two new sources of partial resistance (PI 180693 and 552), effective in French and USA infested fields. Two mapping populations of 178 recombinant inbred lines each, derived from crosses between 552 or PI 180693 (partially resistant) and Baccara (susceptible), were used to identify QTL for Aphanomyces root rot resistance in controlled and in multiple French and USA field conditions using several resistance criteria. We identified a total of 135 additive-effect QTL corresponding to 23 genomic regions and 13 significant epistatic interactions associated with partial resistance to A. euteiches in pea. Among the 23 additive-effect genomic regions identified, five were consistently detected, and showed highly stable effects towards A. euteiches strains, environments, resistance criteria, condition tests and RIL populations studied. These results confirm the complexity of inheritance of partial resistance to A. euteiches in pea and provide good bases for the choice of consistent QTL to use in marker-assisted selection schemes to increase current levels of resistance to A. euteiches in pea breeding programs. | 2454 | NA | NA | NA | no | no |
| 2455 | 2017 | NA | Guimaraes, EP; CorreaVictoria, F; Tulande, E | 1995 | GC-91, a broad-based rice synthetic population for blast (Pyricularia grisea Sacc) resistance | Population improvement methods used in cross-pollinated crops have not been extensively applied to rice (Oryza sativa L.). This article describes the formation of and selection within a genetically broad-based gene pool for resistance to rice blast (Pyricularia grisea Sacc.), and progress during one generation of selection. Thirty rice lines of diverse origin were chosen as parents based upon stability of the reaction of the lines to a range of leaf (BI) and panicle (NBI) blast races during eight growing seasons at a location favorable for disease development(‘’hot spot’’ site). Each parent was crossed to five other parents and each Fl to three other Fl’s to ensure recombination. The resulting seeds were bulked to form the GC-91 gene pool (C0P0). Genetic diversity, as measured by geographical origin of the germplasm, was higher in the GC-91 than in the ‘’genetic core’’ of rice varieties grown in Latin America and the Caribbean. Three groups were selected from GC-91, based upon three different objectives of selection for blast: a) rapid fixation of major genes for high levels of resistance (population C1P1), b) gradual accumulation of polygenes for partial resistance (population C1P2), and c) a combination of objectives a and b (population C1P3). Lines selected had greater resistance to B1 and NBI in populations C1P1 and C1P3 compared to C0P0, while there was no evidence of successful selection for C1P2. | 2455 | NA | NA | NA | no | no |
| 2456 | 2017 | NA | Dalla Bona, AC; Piccoli, CF; Leandro, AD; Kafka, R; Twerdochilib, AL; Navarro-Silva, MA | 2012 | Genetic profile and molecular resistance of Aedes (Stegomyia) aegypti (Diptera: Culicidae) in Foz do Iguacu (Brazil), at the border with Argentina and Paraguay | The genetic variability of populations of Aedes (Stegomyia) aegypti (Linnaeus, 1762) is often low due to the intense selection pressure caused by chemical control measures. In this study, we evaluated the susceptibility of larvae and adults of this mosquito to chemical insecticides, the frequency of the Val1016Ile mutation, and the genetic variability of the mitochondrial ND4 gene fragment in the urban area of Foz do Iguacu, Parana. The populations of A. aegypti in the southern and central regions of the city were resistant to the diagnostic dose of temephos 0.0162 ppm. Additionally, we detected difference in susceptibility in the northern population. The resistance ratios (RR95) were 3.8, 4.1, and 4.6 for the populations in the north, central, and south of the city, respectively. The entire population of A. aegypti in Foz do Iguacu is resistant to pyrethroids. The mortality rates are as follows: 72.2% for cypermethrin (CD: 146 mg i.a./m(2)) and 57% for deltamethrin (CD: 18 mg i.a./m(2)). These results corroborate the hypothesis that the mutated 1016lle allele is present in all analyzed strata. Out of the 234 samples genotyped for the Val1016Ile mutation, 15% were homozygous dominant for the wild allele (Val/Val), 62% were heterozygous (Va/Ile) and 23% were homozygous for the recessive mutation (IIe/Ile). With respect to the genetic variability of the mitochondrial ND4 gene fragment, 93% of the sequences analyzed belonged to haplotype 1, and 7% belonged to haplotype 2. The genetic diversity was low, the fixation index was not significant, and gene flow was high. The control of A. aegypti in Foz do Iguacu using temephos and pyrethroids may be compromised because of the reduced vector susceptibility. Populations of A. aegypti, that undergoes a sudden reduction in effective population size and become resistant to pyrethroids may differ from the original population in vector capacity. | 2456 | NA | NA | NA | no | no |
| 2457 | 2017 | NA | Paisan-Ruiz, C; Evans, EW; Jain, S; Xiromerisiou, G; Gibbs, JR; Eerola, J; Gourbali, V; Hellstrom, O; Duckworth, J; Papadimitriou, A; Tienari, PJ; Hadjigeorgiou, GM; Singleton, AB | 2006 | Testing association between LRRK2 and Parkinson’s disease and investigating linkage disequilibrium | Background: We and others recently identified the gene underlying PARK8 linked Parkinson’s disease (PD). This gene, LRRK2, contains mutations that cause an autosomal dominant PD, including a mutation, G2019S, which is the most common PD causing mutation identified to date. Common genetic variability in genes that contain PD causing mutations has previously been implicated as a risk factor for typical sporadic disease. Methods: We undertook a case-control association analysis of LRRK2 in two independent European PD cohorts using 31 tagging single nucleotide polymorphisms (tSNPs) and five potentially functional SNPs. To assess the structure of this locus in different populations, we have performed linkage disequilibrium (LD) analysis using these variants in a human diversity panel. Results: We show that common genetic variability in LRRK2 is not associated with risk for PD in the European populations studied here. We also show inter-population variability in the strength of LD across this locus. Conclusions: To our knowledge this is the first comprehensive analysis of common variability within LRRK2 as a risk factor for PD. | 2457 | NA | NA | NA | no | no |
| 2458 | 2017 | NA | Frias-De Leon, MG; Duarte-Escalante, E; Calderon-Ezquerro, MD; Jimenez-Martinez, MD; Acosta-Altamirano, G; Moreno-Eutimio, MA; Zuniga, G; Garcia-Gonzalez, R; Ramirez-Perez, M; Reyes-Montes, MD | 2016 | Diversity and characterization of airborne bacteria at two health institutions | The aim of this study was to identify the types and abundance of airborne bacteria of two health institutions (1 and 2) and to determine the genetic association between environmental and clinical isolates of Staphylococcus spp. Environmental sampling in institutions 1 and 2 was conducted for 1 year (dry and rainy seasons) using M Air T sampler. The bacteria and their susceptibility to antibiotics were identified. The colony-forming units per cubic meter (CFU/m(3)) of air were quantified for all the isolates, and the diversity and abundance of species were calculated. The genetic relationship between the clinical and environmental isolates of S. aureus obtained from institution 1 was established by the UPGMA based on RAPD markers. At both of the institutions, the genera most frequently isolated were Staphylococcus and Bacillus, and the greater concentration of airborne bacteria was detected during the dry season than the rainy season. The lowest diversity and highest dominance was found in the institution 2. On the other hand, at institution 1, the genus that was resistant to antibiotics was Staphylococcus, whereas at institution 2, no isolate was found to be resistant to antibiotics. Furthermore, no association between the clinical and environmental isolates of S. aureus was found. However, one clone was found in different areas of the institution 1. The presence of airborne pathogenic bacteria in institutions 1 and 2 is important to establish the measures for the prevention and control of nosocomial outbreaks. | 2458 | NA | NA | NA | no | no |
| 2459 | 2017 | NA | Maclean, RC; Brandon, C | 2008 | Stable public goods cooperation and dynamic social interactions in yeast | Despite long-standing theoretical interest in the evolution of cooperation, empirical data on the evolutionary dynamics of cooperative traits remain limited. Here, we investigate the evolutionary dynamics of a simple public goods cooperative trait, invertase secretion, using a long-term selection experiment in Saccharomyces cerevisiae. We show that average investment in cooperation remains essentially constant over a period of hundreds of generations in viscous populations with high relatedness. Average cooperation remains constant despite transient local selection for high and low levels of cooperation that generate dynamic social interactions. Natural populations of yeast show similar variation in social strategies, which is consistent with the existence of similar selective pressures on public goods cooperation in nature. | 2459 | NA | NA | NA | no | no |
| 2460 | 2017 | NA | Habel, JC; Husemann, M; Rodder, D; Schmitt, T | 2011 | Biogeographical dynamics of the Spanish Marbled White Melanargia ines (Lepidoptera: Satyridae) in the Western Mediterranean: does the Atlanto-Mediterranean refuge exist? | Climatic changes can cause severe range shifts of biota over time. During climatically challenging periods, many organisms often persist in suitable refugia. One rather important refuge area of thermophilic species of the Palaearctic region includes the Iberian Peninsula and the Maghreb region, known as the ‘Atlanto-Mediterranean’ refuge. In order to study this postulated refugium, we computed species distribution models to reconstruct the past potential distribution of Melanargia ines (Hoffmannsegg, 1804) during the last glacial maximum. We also sampled individuals from populations of this butterfly species at its distribution margins (Iberia, and western and eastern Maghreb), and performed allozyme electrophoresis to assess the biogeographical patterns. The projected continuity of a conductive climate from Iberia to the eastern Maghreb, and the lack of genetic differentiation across the entire distribution range of M. ines, is in favour of the existence of one Atlanto-Mediterranean refugium for this species. A relatively constant climate over the Atlas Mountains over long periods of time, as predicted by species distribution models and observed genetic diversity patterns displaying an accumulation of intraspecific variability in this area, supports the idea of an important stronghold of M. ines in this mountain range, as has already been shown for several other species. The lower genetic diversity of the Iberian and Tunisian population might be the result of a rapid range expansion originating from the Atlas region or the survival of smaller relict populations in the eastern Maghreb (which is underpinned by geographically restricted climatically suitable retreats in this area, computed using species distribution models). In general, these findings highlight the importance of the Atlas massif as a refugium for thermophilic species on the Palaearctic scale. (C) 2011 The Linnean Society of London, Biological Journal of the Linnean Society, 2011, 104, 828-837. | 2460 | NA | NA | NA | no | no |
| 2461 | 2017 | NA | Smith, B; Chen, ZG; Reimers, L; van Doorslaer, K; Schiffman, M; DeSalle, R; Herrero, R; Yu, K; Wacholder, S; Wang, T; Burk, RD | 2011 | Sequence Imputation of HPV16 Genomes for Genetic Association Studies | Background: Human Papillomavirus type 16 (HPV16) causes over half of all cervical cancer and some HPV16 variants are more oncogenic than others. The genetic basis for the extraordinary oncogenic properties of HPV16 compared to other HPVs is unknown. In addition, we neither know which nucleotides vary across and within HPV types and lineages, nor which of the single nucleotide polymorphisms (SNPs) determine oncogenicity. Methods: A reference set of 62 HPV16 complete genome sequences was established and used to examine patterns of evolutionary relatedness amongst variants using a pairwise identity heatmap and HPV16 phylogeny. A BLAST-based algorithm was developed to impute complete genome data from partial sequence information using the reference database. To interrogate the oncogenic risk of determined and imputed HPV16 SNPs, odds-ratios for each SNP were calculated in a case-control viral genome-wide association study (VWAS) using biopsy confirmed high-grade cervix neoplasia and self-limited HPV16 infections from Guanacaste, Costa Rica. Results: HPV16 variants display evolutionarily stable lineages that contain conserved diagnostic SNPs. The imputation algorithm indicated that an average of 97.5 +/- 1.03% of SNPs could be accurately imputed. The VWAS revealed specific HPV16 viral SNPs associated with variant lineages and elevated odds ratios; however, individual causal SNPs could not be distinguished with certainty due to the nature of HPV evolution. Conclusions: Conserved and lineage-specific SNPs can be imputed with a high degree of accuracy from limited viral polymorphic data due to the lack of recombination and the stochastic mechanism of variation accumulation in the HPV genome. However, to determine the role of novel variants or non-lineage-specific SNPs by VWAS will require direct sequence analysis. The investigation of patterns of genetic variation and the identification of diagnostic SNPs for lineages of HPV16 variants provides a valuable resource for future studies of HPV16 pathogenicity. | 2461 | NA | NA | NA | no | no |
| 2462 | 2017 | NA | Stone, NE; Sidak-Loftis, LC; Sahl, JW; Vazquez, AJ; Wiggins, KB; Gillece, JD; Hicks, ND; Schupp, JM; Busch, JD; Keim, P; Wagner, DM | 2016 | More than 50% of Clostridium difficile Isolates from Pet Dogs in Flagstaff, USA, Carry Toxigenic Genotypes | Nosocomial acquisition of Clostridium difficile is well documented, yet recent studies have highlighted the importance of community acquired infections and identified community associated reservoirs for this pathogen. Multiple studies have implicated companion pets and farm animals as possible sources of community acquired C. difficile infections in humans. To explore the potential role of pet dogs in human C. difficile infections we systematically collected canine fecal samples (n = 197) in Flagstaff, AZ. Additionally, nineteen fecal samples were collected at a local veterinary clinic from diarrheic dogs. We used these combined samples to investigate important questions regarding C. difficile colonization in pet canines: 1) What is the prevalence and diversity of C. difficile in this companion pet population, and 2) Do C. difficile isolates collected from canines genetically overlap with isolates that cause disease in humans? We used a two-step sequence typing approach, including multilocus sequence typing to determine the overall genetic diversity of C. difficile present in Flagstaff canines, and whole-genome sequencing to assess the fine-scale diversity patterns within identical multilocus sequence types from isolates obtained within and among multiple canine hosts. We detected C. difficile in 17% of the canine fecal samples with 10% containing toxigenic strains that are known to cause human disease. Sequencing analyses revealed similar genotypes in dogs and humans. These findings suggest that companion pets are a potential source of community acquired C. difficile infections in humans. | 2462 | NA | NA | NA | no | no |
| 2463 | 2017 | NA | Allicock, OM; Lemey, P; Tatem, AJ; Pybus, OG; Bennett, SN; Mueller, BA; Suchard, MA; Foster, JE; Rambaut, A; Carrington, CVF | 2012 | Phylogeography and Population Dynamics of Dengue Viruses in the Americas | “Changes in Dengue virus (DENV) disease patterns in the Americas over recent decades have been attributed, at least in part, to repeated introduction of DENV strains from other regions, resulting in a shift from hypoendemicity to hyperendemicity. Using newly sequenced DENV-1 and DENV-3 envelope (E) gene isolates from 11 Caribbean countries, along with sequences available on GenBank, we sought to document the population genetic and spatiotemporal transmission histories of the four main invading DENV genotypes within the Americas and investigate factors that influence the rate and intensity of DENV transmission. For all genotypes, there was an initial invasion phase characterized by rapid increases in genetic diversity, which coincided with the first confirmed cases of each genotype in the region. Rapid geographic dispersal occurred upon each genotype’s introduction, after which individual lineages were locally maintained, and gene flow was primarily observed among neighboring and nearby countries. There were, however, centers of viral diversity (Barbados, Puerto Rico, Colombia, Suriname, Venezuela, and Brazil) that were repeatedly involved in gene flow with more distant locations. For DENV-1 and DENV-2, we found that a”“distance-informed”" model, which posits that the intensity of virus movement between locations is inversely proportional to the distance between them, provided a better fit than a model assuming equal rates of movement between all pairs of countries. However, for DENV-3 and DENV-4, the more stochastic "“equal rates”" model was preferred." | 2463 | NA | NA | NA | no | no |
| 2464 | 2017 | NA | Palopoli, MF; Fergus, DJ; Minot, S; Pei, DT; Simison, WB; Fernandez-Silva, I; Thoemmes, MS; Dunn, RR; Trautwein, M | 2015 | Global divergence of the human follicle mite Demodex folliculorum: Persistent associations between host ancestry and mite lineages | Microscopic mites of the genus Demodex live within the hair follicles of mammals and are ubiquitous symbionts of humans, but little molecular work has been done to understand their genetic diversity or transmission. Here we sampled mite DNA from 70 human hosts of diverse geographic ancestries and analyzed 241 sequences from the mitochondrial genome of the species Demodex folliculorum. Phylogenetic analyses recovered multiple deep lineages including a globally distributed lineage common among hosts of European ancestry and three lineages that primarily include hosts of Asian, African, and Latin American ancestry. To a great extent, the ancestral geography of hosts predicted the lineages of mites found on them; 27% of the total molecular variance segregated according to the regional ancestries of hosts. We found that D. folliculorum populations are stable on an individual over the course of years and that some Asian and African American hosts maintain specific mite lineages over the course of years or generations outside their geographic region of birth or ancestry. D. folliculorum haplotypes were much more likely to be shared within families and between spouses than between unrelated individuals, indicating that transmission requires close contact. Dating analyses indicated that D. folliculorum origins may predate modern humans. Overall, D. folliculorum evolution reflects ancient human population divergences, is consistent with an out-of-Africa dispersal hypothesis, and presents an excellent model system for further understanding the history of human movement. | 2464 | NA | NA | NA | no | no |
| 2465 | 2017 | NA | Santini, AC; Santos, HRM; Gross, E; Correa, RX | 2013 | Genetic diversity of Burkholderia (Proteobacteria) species from the Caatinga and Atlantic rainforest biomes in Bahia, Brazil | The genus Burkholderia (beta-Proteobacteria) currently comprises more than 60 species, including parasites, symbionts and free-living organisms. Several new species of Burkholderia have recently been described showing a great diversity of phenotypes. We examined the diversity of Burkholderia spp in environmental samples collected from Caatinga and Atlantic rainforest biomes of Bahia, Brazil. Legume nodules were collected from five locations, and 16S rDNA and recA genes of the isolated microorganisms were analyzed. Thirty-three contigs of 16S rRNA genes and four contigs of the recA gene related to the genus Burkholderia were obtained. The genetic dissimilarity of the strains ranged from 0 to 2.5% based on 16S rDNA analysis, indicating two main branches: one distinct branch of the dendrogram for the B. cepacia complex and another branch that rendered three major groups, partially reflecting host plants and locations. A dendrogram designed with sequences of this research and those designed with sequences of Burkholderia-type strains and the first hit BLAST had similar topologies. A dendrogram similar to that constructed by analysis of 16S rDNA was obtained using sequences of the fragment of the recA gene. The 16S rDNA sequences enabled sufficient identification of relevant similarities and groupings amongst isolates and the sequences that we obtained. Only 6 of the 33 isolates analyzed via 16S rDNA sequencing showed high similarity with the B. cepacia complex. Thus, over 3/4 of the isolates have potential for biotechnological applications. | 2465 | NA | NA | NA | no | no |
| 2466 | 2017 | NA | Tayahi, M; Gharsallah, C; Khamassy, N; Fakhfakh, H; Djilani-Khouadja, F | 2016 | Biological, serological and molecular typing of potato virus Y (PVY) isolates from Tunisia | In Tunisia, potato virus Y (PVY) currently presents a significant threat to potato production, reducing tuber yield and quality. Three hundred and eighty-five potato samples (six different cultivars) collected in autumn 2007 from nine regions in Tunisia were tested for PVY infection by DAS-ELISA. The virus was detected in all regions surveyed, with an average incidence of 80.26%. Subsequently, a panel of 82 Tunisian PVY isolates (PVY-TN) was subjected to systematic biological, serological and molecular typing using immunocapture reverse-transcription polymerase chain reaction and a series of PVYOC-and PVYN-specific monoclonal antibodies. Combined analyses revealed similar to 67% of PVYNTN variants of which 17 were sequenced in the 5’NTR-P1 region to assess the genetic diversity and phylogenetic relationship of PVY-TN against other worldwide PVY isolates. To investigate whether selective constraints could act on viral genomic RNA, synonymous and non-synonymous substitution rates and their ratio were analyzed. Averages of all pairwise comparisons obtained in the 5’NTR-P1 region allowed more synonymous changes, suggesting selective constraint acting in this region. Selective neutrality test was significantly negative, suggesting a rapid expansion of PVY isolates. Pairwise mismatch distribution gave a bimodal pattern and pointed to an eventually early evolution characterizing these sequences. Genetic haplotype network topology provided evidence of the existence of a distinct geographical structure. This is the first report of such genetic analyses conducted on PVY isolates from Tunisia. | 2466 | NA | NA | NA | no | no |
| 2467 | 2017 | NA | Rodriguez-Beltran, J; Tourret, J; Tenaillon, O; Lopez, E; Bourdelier, E; Costas, C; Matic, I; Denamur, E; Blazquez, J | 2015 | High Recombinant Frequency in Extraintestinal Pathogenic Escherichia coli Strains | Homologous recombination promotes genetic diversity by facilitating the integration of foreign DNA and intrachromosomal gene shuffling. It has been hypothesized that if recombination is variable among strains, selection should favor higher recombination rates among pathogens, as they face additional selection pressures from host defenses. To test this hypothesis we have developed a plasmid-based method for estimating the rate of recombination independently of other factors such as DNA transfer, selective processes, and mutational interference. Our results with 160 human commensal and extraintestinal pathogenic Escherichia coli (ExPEC) isolates show that the recombinant frequencies are extremely diverse (ranging 9 orders of magnitude) and plastic (they are profoundly affected by growth in urine, a condition commonly encountered by ExPEC). We find that the frequency of recombination is biased by strain lifestyle, as ExPEC isolates display strikingly higher recombination rates than their commensal counterparts. Furthermore, the presence of virulence factors is positively associated with higher recombination frequencies. These results suggest selection for high homologous recombination capacity, which may result in a higher evolvability for pathogens compared with commensals. | 2467 | NA | NA | NA | no | no |
| 2468 | 2017 | NA | Aloui, C; Sut, C; Prigent, A; Fagan, J; Cognasse, F; Granados-Herbepin, V; Touraine, R; Pozzetto, B; Aouni, M; Fendri, C; Hassine, M; Chakroun, T; Jemni-Yacoub, S; Garraud, O; Laradi, S | 2014 | Are polymorphisms of the immunoregulatory factor CD40LG implicated in acute transfusion reactions? | The CD40 ligand (CD40L/CD154), a member of TNF superfamily, is notably expressed on activated CD4+T-cells and stimulated platelets. CD40L is linked to a variety of pathologies and to acute transfusion reactions (ATR). Mutations in this gene (CD40LG) lead to X-linked hyper-IgM syndrome. Some CD40LG polymorphisms are associated with variable protein expression. The rationale behind this study is that CD40L protein has been observed to be involved in ATR. We wondered whether genetic polymorphisms are implicated. We investigated genetic diversity in the CD40LG using DHPLC and capillary electrophoresis for screening and genotyping (n=485 French and Tunisian blood donors). We identified significant difference in the CD40LG linkage pattern between the two populations. Variant minor alleles were significantly over-represented in Tunisian donors (P<0.0001 to 0.0270). We found higher heterogeneity in the Tunisian, including three novel low frequency variants. As there was not a particular pattern of CD40LG in single apheresis donors whose platelet components induced an ATR, we discuss how this information may be useful for future disease association studies on CD40LG. | 2468 | NA | NA | NA | no | no |
| 2469 | 2017 | NA | Ord, RL; Tami, A; Sutherland, CJ | 2008 | ama1 Genes of Sympatric Plasmodium vivax and P. falciparum from Venezuela Differ Significantly in Genetic Diversity and Recombination Frequency | Background: We present the first population genetic analysis of homologous loci from two sympatric human malaria parasite populations sharing the same human hosts, using full-length sequences of ama1 genes from Plasmodium vivax and P. falciparum collected in the Venezuelan Amazon. Methodology/Principal Findings: Significant differences between the two species were found in genetic diversity at the ama1 locus, with 18 distinct haplotypes identified among the 73 Pvama1 sequences obtained, compared to 6 unique haplotypes from 30 Pfama1 sequences, giving overall diversity estimates of h = 0.9091, and h = 0.538 respectively. Levels of recombination were also found to differ between the species, with P. falciparum exhibiting very little recombination across the 1.77kb sequence. In contrast, analysis of patterns of nucleotide substitutions provided evidence that polymorphisms in the ama1 gene of both species are maintained by balancing selection, particularly in domain I. The two distinct population structures observed are unlikely to result from different selective forces acting upon the two species, which share both human and mosquito hosts in this setting. Rather, the highly structured P. falciparum population appears to be the result of a population bottleneck, while the much less structured P. vivax population is likely to be derived from an ancient pool of diversity, as reflected in a larger estimate of effective population size for this species. Greatly reduced mosquito transmission in 1997, due to low rainfall prior to the second survey, was associated with far fewer P. falciparum infections, but an increase in P. vivax infections, probably due to hypnozoite activation. Conclusions/Significance: The relevance of these findings to putative competitive interactions between these two important human pathogen species is discussed. These results highlight the need for future control interventions to employ strategies targeting each of the parasite species present in endemic areas. | 2469 | NA | NA | NA | no | no |
| 2470 | 2017 | NA | Shimmin, GA; Taggart, DA; Temple-Smith, PD | 2000 | Sperm competition and genetic diversity in the agile antechinus (Dasyuridae : Antechinus agilis) | The mouse-like marsupial Antechinus agilis is common to south-eastern Australia with breeding and life-history traits being highly synchronous. Mating activity is confined to a 10- to 15-day period in August, at the end of which all males die as a result of a stress-induced suppression of the immune system. Ovulation occurs at this time and females rely on stored sperm from specialized crypts in the lower isthmus of the oviduct for successful fertilization. Here we report a high incidence of mixed paternity litters, which can be attributed to sperm from multiple males being stored in the isthmic crypts. Data from this study also suggest a possible second male siring advantage in controlled ex situ sperm competition mating trials, irrespective of the delay between the two males being given mating access to the female or of the mating time relative to ovulation. In determining paternity through DNA profiling, population genetic data were obtained that showed significant differences in the genetic heterozygosity between unrelated adults, half siblings and full siblings. We suggest that the isthmic crypts, in addition to storing viable sperm, are capable of releasing a mix of sperm that increases the likelihood of mixed paternity litters. This allows all mating males the opportunity to sire young, increases female reproductive fitness and overall maintains high levels of population genetic heterozygosity in the face of total male mortality annually. | 2470 | NA | NA | NA | no | no |
| 2471 | 2017 | NA | Takatsuka, J; Okuno, S; Nakai, M; Kunimi, Y | 2016 | Genetic and phenotypic comparisons of viral genotypes from two nucleopolyhedroviruses interacting with a common host species, Spodoptera litura (Lepidoptera: Noctuidae) | Nucleopolyhedroviruses (NPVs) are known to be highly variable, both genetically and phenotypically, at several scales such as different geographic locations or a single host. A previous study using several geographic isolates indicated that two types of NPV, Spodoptera littoralis NPV (SpIiNPV) and S. litura NPV (SpItNPV) types, were isolated from the common cutworm, Spodoptera litura (Fabricius), a polyphagous insect that causes serious damage to many forage crops and vegetables. That study also indicated that the SpIiNPV type was widely distributed in japan. Here, we investigated the genotypic and phenotypic variation of cloned NPVs that infect S. litura; such variation is an important resource for biological control agents, and may represent the genetic diversity of an NPV species. Eighteen genotypically distinct NPVs were cloned from four field-collected NPV isolates using an in vivo cloning technique. They were divided into two virus types according to the similarity of banding patterns of DNA fragments generated by restriction endonucleases, and Southern hybridization analysis. Partial polyhedrin gene sequences revealed that the two types corresponded to SpIiNPV and SpItNPV. Bioassays seem to suggest that the SpIiNPV virus type was, overall, more infectious and killed S. litura larvae faster, but yielded fewer viral occlusion bodies, than the SpItNPV type. These data provide a basis for explaining the distribution pattern of SpIiNPV and SpItNPV types in S. Iitura populations in Japan. (C) 2016 Elsevier Inc. All rights reserved. | 2471 | NA | NA | NA | no | no |
| 2472 | 2017 | NA | Hsu, TH; Gwo, JC | 2017 | Genetic diversity and stock identification of small abalone (Haliotis diversicolor) in Taiwan and Japan | Small abalone (Haliotis diversicolor) is a commercially valuable species for both fisheries and aquaculture. The production of annual farmed small abalone in Taiwan, once the highest in the world, has dramatically decreased in the past 15 years, and currently, the industry is close to collapse. Understanding the genetic diversity of small abalone and developing stock identification methods will be useful for genetic breeding, restoring collapsed stocks, managing stocks, and preventing illegal trade. We investigated 307 cultured and wild individuals from Taiwan, Japan, and Bali Island (Indonesia) by using the mitochondrial cytochrome c oxidase subunit I (COI) gene. Network analysis of mtDNA COI gene sequences revealed that the individuals collected from Taiwan, Japan, and Indonesia could be identified, and showed significant genetic divergence. In addition, the Indonesian population (Haliotis diversicolor squamata) was significantly different from the other populations and might need to be considered a separate species. We discovered a single nucleotide polymorphism marker in the mtDNA COI gene that can be used to distinguish the Taiwan population from the Japan population. We also developed a polymerase chain reactionrestriction fragment length polymorphism method for rapid detection. Furthermore, we could identify the cultured stocks, wild population, and hybrid stocks by using 6 microsatellites and amplified fragment length polymorphism. This study contributes useful tools for stock identification and the production of high-disease resistant small abalone strains (Japan x Taiwan or Taiwan x Japan). Efforts should be made to avoid unintentional random genetic mixing of the Taiwan population with the Japan population and subsequent breakdown of population differentiation, which impair local adaptation of the Taiwan wild population. Molecular markers revealed a split between the Taiwan and Japan populations, and the existence of a possible barrier to the free dispersal of small abalone is discussed. | 2472 | NA | NA | NA | no | no |
| 2473 | 2017 | NA | Arnaiz-Villena, A; Palacio-Gruber, J; Muniz, E; Campos, C; Alonso-Rubio, J; Gomez-Casado, E; Salih, SF; Martin-Villa, M; Al-Qadi, R | 2017 | Genetic HLA Study of Kurds in Iraq, Iran and Tbilisi (Caucasus, Georgia): Relatedness and Medical Implications | Kurds from Iraq (Dohuk and Erbil Area, North Iraq) have been analyzed for HLA genes. Their HLA genetic profile has been compared with that of other Kurd groups from Iran and Tbilisi (Georgia, Caucasus) and also Worldwide populations. A total of 7,746 HLA chromosomes have been used. Genetic distances, NJ dendrograms and correspondence analyses have been carried out. Haplotype HLA-B*52-DRB1*15 is present in all three analyzed Kurd populations. HLA-A*02-B*51-DRB1*11 is present in Iraq and Georgia Kurds. Haplotypes common to Iran and Iraq Kurds are HLA DRB1*11-DQB1*03, HLA DRB1*03-DQB1\*02 and others in a lower frequency. Our HLA study conclusions are that Kurds most probably belong to an ancient Mediterranean/MiddleEast/Caucasian genetic substratum and that present results and those previously obtained by us in Kurds may be useful for Medicine in future Kurd transplantation programs, HLA Epidemiology (HLA linked diseases) and Pharmacogenomics (HLA-associated drug side effects) and also for Anthropology. It is discussed that one of the most ancient Kurd ancestor groups is in Hurrians (2,000 years BC). | 2473 | NA | NA | NA | no | no |
| 2474 | 2017 | NA | Rich, SM; Rosenthal, BM; Telford, SR; Spielman, A; Hartl, DL; Ayala, FJ | 1997 | Heterogeneity of the internal transcribed spacer (ITS-2) region within individual deer ticks | To determine whether nuclear rDNA sequences provide a useful means for assessing the structure of populations of Ixodes ticks, we compared variability among copies of an internal transcribed spacer (ITS-2) sequence within individual ticks to the variability between ticks. At least 4% of the nucleotides comprising this sequence vary among the copies present within individual ticks. ITS-2 diversity in each of two ticks is nearly half as great as that reported between ticks from geographically disparate populations. Because individual ticks retain ancestral polymorphism, ITS-2 variation does not accurately reflect descent relationships among these ticks. Sequencing single copies of PCR-amplified ITS-2 therefore does not permit assessment of the phylogenetic relationships among the I. ricinus-like ticks in eastern North America. We recommend caution in future analyses, and emphasize the importance of procedures designed to ensure that the many paralogous copies of the rDNA cistron have been sufficiently homogenized by concerted evolutionary processes. Such precautionary measures will make certain that phylogenetic trees based on these gene sequences reflect the phyletic relatedness of the biological species. | 2474 | NA | NA | NA | no | no |
| 2475 | 2017 | NA | Chen, H; Wang, HC; Siegfried, BD | 2012 | Genetic Differentiation of Western Corn Rootworm Populations (Coleoptera: Chrysomelidae) Relative to Insecticide Resistance | As the single most important pest of field corn, Zea mays L., throughout most of the Corn Belt, the western corn rootworm, Diabrotica virgifera virgifera LeConte (Coleoptera: Chrysomelidae), has undergone repeated selection for resistance to a variety of insecticides that persist widely among Nebraska populations. In this study, we used 11 microsatellite markers to genotype two populations with high levels of resistance to methyl-parathion and aldrin (Polk and Stromsburg), two populations with low and intermediate levels of resistance (Mead and Clay Center) from Nebraska, and one population from outside the Corn Belt (Safford, AZ). The genetic diversity measured by observed heterozygosity (H-o) was reduced 15D32% in the highly resistant populations compared with the more susceptible populations in Nebraska. Significant genetic differentiation was detected between the resistant and susceptible populations (Polk and Stromsburg versus Mead and Clay Center) in Nebraska (F-ST = 0.016) and between all the populations from Nebraska and Arizona (F-ST = 0.059). The average observed heterozygosities in the populations were positively correlated with insecticide susceptibility based on mortality at diagnostic concentrations of aldrin and methyl-parathion, respectively. These results indicate that the insecticide selection from exposure to aldrin and methyl-parathion may be a contributing factor in shaping the genetic structure of western corn rootworm populations in Nebraska. Factors including isolation by distance and a Wolbachia-induced breeding barrier may have contributed to differentiation of rootworm populations from Nebraska and Arizona. | 2475 | NA | NA | NA | no | no |
| 2476 | 2017 | NA | Losada, L; Ronning, CM; DeShazer, D; Woods, D; Fedorova, N; Kim, HS; Shabalina, SA; Pearson, TR; Brinkac, L; Tan, P; Nandi, T; Crabtree, J; Badger, J; Beckstrom-Sternberg, S; Saqib, M; Schutzer, SE; Keim, P; Nierman, WC | 2010 | Continuing Evolution of Burkholderia mallei Through Genome Reduction and Large-Scale Rearrangements | Burkholderia mallei (Bm), the causative agent of the predominately equine disease glanders, is a genetically uniform species that is very closely related to the much more diverse species Burkholderia pseudomallei (Bp), an opportunistic human pathogen and the primary cause of melioidosis. To gain insight into the relative lack of genetic diversity within Bm, we performed whole-genome comparative analysis of seven Bm strains and contrasted these with eight Bp strains. The Bm core genome (shared by all seven strains) is smaller in size than that of Bp, but the inverse is true for the variable gene sets that are distributed across strains. Interestingly, the biological roles of the Bm variable gene sets are much more homogeneous than those of Bp. The Bm variable genes are found mostly in contiguous regions flanked by insertion sequence (IS) elements, which appear to mediate excision and subsequent elimination of groups of genes that are under reduced selection in the mammalian host. The analysis suggests that the Bm genome continues to evolve through random IS-mediated recombination events, and differences in gene content may contribute to differences in virulence observed among Bm strains. The results are consistent with the view that Bm recently evolved from a single strain of Bp upon introduction into an animal host followed by expansion of IS elements, prophage elimination, and genome rearrangements and reduction mediated by homologous recombination across IS elements. | 2476 | NA | NA | NA | no | no |
| 2477 | 2017 | NA | Parameswari, B; Bagyalakshmi, K; Chinnaraja, C; Viswanathan, R | 2014 | Molecular characterization of Indian Sugarcane streak mosaic virus isolates reveals recombination and negative selection in the P1 gene | Sugarcane streak mosaic virus (SCSMV), a member of the genus Poacevirus is an important viral pathogen affecting sugarcane production in India. The P1 gene of ten Indian isolates was sequenced and compared with previously reported SCSMV isolates. Comparative sequence analysis revealed a high level of diversity in the P1 gene (83-98% nucleotide sequence identity; 87-100% amino acid sequence identity), and the Indian SCSMV isolates were found to be the most variable (up to 9% diversity at the amino acid level). Phylogenetic tree analysis showed clustering of 17 SCSMV isolates into two groups: group I included isolates from India (except SCSMV-TPT) and Pakistan, and group II consisted of isolates from Japan, Indonesia, Thailand and SCSMV-TPT. The results obtained from phylogenetic study were further supported by the different in silico analysis viz. SNPs (single nucleotide polymorphism), INDELs (insertion and deletion) and evolutionary distance analysis. A significant proportion of recombination sites were observed at the N terminal region of P1 gene. Analysis of selection pressure indicated that the P1 gene of the Indian SCSMV isolates is under strong negative or purifying selection. It is likely that recombination identified in Indian SCSMV isolates, along with strong purifying selection, enhances the speed of elimination of deleterious mutations in the P1 gene. The evolutionary processes (recombination and selection pressure) together contributed to the observed genetic diversity and population structure of Indian SCSMV isolates. (C) 2014 Elsevier B.V. All rights reserved. | 2477 | NA | NA | NA | no | no |
| 2478 | 2017 | NA | Piccinali, RV; Marcet, PL; Noireau, F; Kitron, U; Gurtler, RE; Dotson, EM | 2009 | Molecular Population Genetics and Phylogeography of the Chagas Disease Vector Triatoma infestans in South America | Knowledge of the genetic variability, population structure, and evolutionary history of Triatoma infestans may be useful for developing rational vector control strategies. A 661-bp fragment of the mitochondrial gene cytochrome oxidase 1 (COI) was sequenced and analyzed in bugs from Argentina, Uruguay, Peru, and Bolivia, including peridomestic, domestic, Andean, and Chaco sylvatic bugs. A total of 48 polymorphic sites among 37 haplotypes were described. Nucleotide variation fluctuated among samples, with the highest nucleotide diversity observed in seven Argentinean provinces. Within this group, some populations showed patterns of variability compatible with population expansions and/or fine-scale population structure, whereas others suggested population bottlenecks and/or population admixture processes. A maximum parsimony analysis of the haplotypes showed the presence of a Bolivian/Peruvian and an Argentinean/Uruguayan clade. Bolivian sequences were further divided in Chaco sylvatic and Andean domestic and sylvatic. Two different nested clades were found within the Argentinean/Uruguayan cluster. Analysis of molecular variance (AMOVA) and K(S)(T)\* analysis supported a strong population structure in Argentina, where genetic differentiation was correlated with geographic distance. Departures from neutrality expectations and a nested cladistic analysis suggest a recent population expansion of T. infestans in Argentina, followed by restricted gene flow and patterns of isolation by distance. This expansion could have taken place as a two-wave process, as was shown by the phylogenetic analysis and signatures of population admixture in the southernmost Argentinean populations. | 2478 | NA | NA | NA | no | no |
| 2479 | 2017 | NA | Isaza, REA; Diaz-Trujillo, C; Dhillon, B; Aerts, A; Carlier, J; Crane, CF; de Jong, TV; de Vries, I; Dietrich, R; Farmer, AD; Fereira, CF; Garcia, S; Guzman, M; Hamelin, RC; Lindquist, EA; Mehrabi, R; Quiros, O; Schmutz, J; Shapiro, H; Reynolds, E; Scalliet, G; Souza, M; Stergiopoulos, I; Van der Lee, TAJ; De Wit, PJGM; Zapater, MF; Zwiers, LH; Grigoriev, IV; Goodwin, SB; Kema, GHJ | 2016 | Combating a Global Threat to a Clonal Crop: Banana Black Sigatoka Pathogen Pseudocercospora fijiensis (Synonym Mycosphaerella fijiensis) Genomes Reveal Clues for Disease Control | Black Sigatoka or black leaf streak disease, caused by the Dothideomycete fungus Pseudocercospora fijiensis (previously: Mycosphaerella fijiensis), is the most significant foliar disease of banana worldwide. Due to the lack of effective host resistance, management of this disease requires frequent fungicide applications, which greatly increase the economic and environmental costs to produce banana. Weekly applications in most banana plantations lead to rapid evolution of fungicide-resistant strains within populations causing disease-control failures throughout the world. Given its extremely high economic importance, two strains of P. fijiensis were sequenced and assembled with the aid of a new genetic linkage map. The 74-Mb genome of P. fijiensis is massively expanded by LTR retrotransposons, making it the largest genome within the Dothideomycetes. Melting-curve assays suggest that the genomes of two closely related members of the Sigatoka disease complex, P. eumusae and P. musae, also are expanded. Electrophoretic karyotyping and analyses of molecular markers in P. fijiensis field populations showed chromosome-length polymorphisms and high genetic diversity. Genetic differentiation was also detected using neutral markers, suggesting strong selection with limited gene flow at the studied geographic scale. Frequencies of fungicide resistance in fungicide-treated plantations were much higher than those in untreated wild-type P. fijiensis populations. A homologue of the Cladosporium fulvum Avr4 effector, PfAvr4, was identified in the P. fijiensis genome. Infiltration of the purified PfAVR4 protein into leaves of the resistant banana variety Calcutta 4 resulted in a hypersensitive-like response. This result suggests that Calcutta 4 could carry an unknown resistance gene recognizing PfAVR4. Besides adding to our understanding of the overall Dothideomycete genome structures, the P. fijiensis genome will aid in developing fungicide treatment schedules to combat this pathogen and in improving the efficiency of banana breeding programs. | 2479 | NA | NA | NA | no | no |
| 2480 | 2017 | NA | Gjini, E; Haydon, DT; Barry, JD; Cobbold, CA | 2014 | Revisiting the diffusion approximation to estimate evolutionary rates of gene family diversification | Genetic diversity in multigene families is shaped by multiple processes, including gene conversion and point mutation. Because multi-gene families are involved in crucial traits of organisms, quantifying the rates of their genetic diversification is important. With increasing availability of genomic data, there is a growing need for quantitative approaches that integrate the molecular evolution of gene families with their higher-scale function. In this study, we integrate a stochastic simulation framework with population genetics theory, namely the diffusion approximation, to investigate the dynamics of genetic diversification in a gene family. Duplicated genes can diverge and encode new functions as a result of point mutation, and become more similar through gene conversion. To model the evolution of pairwise identity in a multigene family, we first consider all conversion and mutation events in a discrete manner, keeping track of their details and times of occurrence; second we consider only the infinitesimal effect of these processes on pairwise identity accounting for random sampling of genes and positions. The purely stochastic approach is closer to biological reality and is based on many explicit parameters, such as conversion tract length and family size, but is more challenging analytically. The population genetics approach is an approximation accounting implicitly for point mutation and gene conversion, only in terms of per-site average probabilities. Comparison of these two approaches across a range of parameter combinations reveals that they are not entirely equivalent, but that for certain relevant regimes they do match. As an application of this modelling framework, we consider the distribution of nucleotide identity among VSG genes of African trypanosomes, representing the most prominent example of a multi-gene family mediating parasite antigenic variation and within-host immune evasion. (C) 2013 Elsevier Ltd. All rights reserved. | 2480 | NA | NA | NA | no | no |
| 2481 | 2017 | NA | Kraus, RHS; Zeddeman, A; van Hooft, P; Sartakov, D; Soloviev, SA; Ydenberg, RC; Prins, HHT | 2011 | Evolution and connectivity in the world-wide migration system of the mallard: Inferences from mitochondrial DNA | Background: Main waterfowl migration systems are well understood through ringing activities. However, in mallards (Anas platyrhynchos) ringing studies suggest deviations from general migratory trends and traditions in waterfowl. Furthermore, surprisingly little is known about the population genetic structure of mallards, and studying it may yield insight into the spread of diseases such as Avian Influenza, and in management and conservation of wetlands. The study of evolution of genetic diversity and subsequent partitioning thereof during the last glaciation adds to ongoing discussions on the general evolution of waterfowl populations and flyway evolution. Hypothesised mallard flyways are tested explicitly by analysing mitochondrial mallard DNA from the whole northern hemisphere. Results: Phylogenetic analyses confirm two mitochondrial mallard clades. Genetic differentiation within Eurasia and North-America is low, on a continental scale, but large differences occur between these two land masses (FST = 0.51). Half the genetic variance lies within sampling locations, and a negligible portion between currently recognised waterfowl flyways, within Eurasia and North-America. Analysis of molecular variance (AMOVA) at continent scale, incorporating sampling localities as smallest units, also shows the absence of population structure on the flyway level. Finally, demographic modelling by coalescence simulation proposes a split between Eurasia and North-America 43,000 to 74,000 years ago and strong population growth (similar to 100fold) since then and little migration (not statistically different from zero). Conclusions: Based on this first complete assessment of the mallard’s world-wide population genetic structure we confirm that no more than two mtDNA clades exist. Clade A is characteristic for Eurasia, and clade B for NorthAmerica although some representatives of clade A are also found in North-America. We explain this pattern by evaluating competing hypotheses and conclude that a complex mix of historical, recent and anthropogenic factors shaped the current mallard populations. We refute population classification based on flyways proposed by ornithologists and managers, because they seem to have little biological meaning. Our results have implications for wetland management and conservation, with special regard to the release of farmed mallards for hunting, as well as for the possible transmission of Avian Influenza by mallards due to migration. | 2481 | NA | NA | NA | no | no |
| 2482 | 2017 | NA | Aragon, E; Rivera, C; Korpelainen, H; Rojas, A; Elomaa, P; Valkonen, JPT | 2012 | Genetic diversity of native cultivated cacao accessions (Theobroma cacao L.) in Nicaragua | A total of 60 farmers’ cacao accessions (Theobroma cacao L.) from Nicaragua were investigated using microsatellite markers to reveal their genetic composition and to identify potentially resistant genotypes against the black pod disease caused by Phytophthora palmivora. These accessions were compared with 21 breeders’ accessions maintained locally, two Criollo accessions from Costa Rica and two accessions from Ecuador. The analyses showed a low level of differentiation among groups of farmers’ accessions (F-ST = 0.06) and that six Nicaraguan accessions were genetically closely related to the two Criollo accessions used as a reference. In addition, seven distinct genotypes were found to have allelic composition that may indicate linkage to resistance alleles, thus being potential parental lines in future breeding programmes. | 2482 | NA | NA | NA | no | no |
| 2483 | 2017 | NA | MCCAULEY, DE | 1992 | FAMILY STRUCTURED PATTERNS OF MORTALITY IN THE FALSE COLORADO POTATO BEETLE | 1. The distribution of mortality within and among groups of immature Leptinotursu juncta (Germar) (Coleoptera: Chrysomelidae) was studied in the field at three points in the life‐cycle in order to test whether the fates of individuals occupying the same host plant were non‐independent. 2. Studies were conducted on eggs subject to cannibalism and predation, on newly hatched larvae subject to predation, and on older larvae subject to parasitism by Myiophurus doryphorue (Riley), a tachinid fly. 3. At all three points in the life‐cycle the variance in group‐specific survivorship rates was demonstrated to be significantly greater than expected when individual deaths are distributed randomly among groups, with standardized variances ranging from 42% to 52% of the maximum possible. 4. A statistically significant positive correlation was shown between the number of individuals in a group and the group‐specific survivorship rate at all three life stages. 5. The distribution of genetic variants within and among groups of larvae showed the groups to be mixtures of full and half siblings, with an average coefficient of genetic relatedness, r, equal to 0.38. | 2483 | NA | NA | NA | no | no |
| 2484 | 2017 | NA | Mardulyn, P; Goffredo, M; Conte, A; Hendrickx, G; Meiswinkel, R; Balenghien, T; Sghaier, S; Lohr, Y; Gilbert, M | 2013 | Climate change and the spread of vector-borne diseases: using approximate Bayesian computation to compare invasion scenarios for the bluetongue virus vector Culicoides imicola in Italy | Bluetongue (BT) is a commonly cited example of a disease with a distribution believed to have recently expanded in response to global warming. The BT virus is transmitted to ruminants by biting midges of the genus Culicoides, and it has been hypothesized that the emergence of BT in Mediterranean Europe during the last two decades is a consequence of the recent colonization of the region by Culicoides imicola and linked to climate change. To better understand the mechanism responsible for the northward spread of BT, we tested the hypothesis of a recent colonization of Italy by C.imicola, by obtaining samples from more than 60 localities across Italy, Corsica, Southern France, and Northern Africa (the hypothesized source point for the recent invasion of C.imicola), and by genotyping them with 10 newly identified microsatellite loci. The patterns of genetic variation within and among the sampled populations were characterized and used in a rigorous approximate Bayesian computation framework to compare three competing historical hypotheses related to the arrival and establishment of C.imicola in Italy. The hypothesis of an ancient presence of the insect vector was strongly favoured by this analysis, with an associated P99%, suggesting that causes other than the northward range expansion of C.imicola may have supported the emergence of BT in southern Europe. Overall, this study illustrates the potential of molecular genetic markers for exploring the assumed link between climate change and the spread of diseases. | 2484 | NA | NA | NA | no | no |
| 2485 | 2017 | NA | Lynch, KH; Stothard, P; Dennis, JJ | 2012 | Comparative analysis of two phenotypically-similar but genomically-distinct Burkholderia cenocepacia-specific bacteriophages | Background: Genomic analysis of bacteriophages infecting the Burkholderia cepacia complex (BCC) is an important preliminary step in the development of a phage therapy protocol for these opportunistic pathogens. The objective of this study was to characterize KL1 (vB\_BceS\_KL1) and AH2 (vB\_BceS\_AH2), two novel Burkholderia cenocepacia-specific siphoviruses isolated from environmental samples. Results: KL1 and AH2 exhibit several unique phenotypic similarities: they infect the same B. cenocepacia strains, they require prolonged incubation at 30 degrees C for the formation of plaques at low titres, and they do not form plaques at similar titres following incubation at 37 degrees C. However, despite these similarities, we have determined using whole-genome pyrosequencing that these phages show minimal relatedness to one another. The KL1 genome is 42,832 base pairs (bp) in length and is most closely related to Pseudomonas phage 73 (PA73). In contrast, the AH2 genome is 58,065 bp in length and is most closely related to Burkholderia phage BcepNazgul. Using both BLASTP and HHpred analysis, we have identified and analyzed the putative virion morphogenesis, lysis, DNA binding, and MazG proteins of these two phages. Notably, MazG homologs identified in cyanophages have been predicted to facilitate infection of stationary phase cells and may contribute to the unique plaque phenotype of KL1 and AH2. Conclusions: The nearly indistinguishable phenotypes but distinct genomes of KL1 and AH2 provide further evidence of both vast diversity and convergent evolution in the BCC-specific phage population. | 2485 | NA | NA | NA | no | no |
| 2486 | 2017 | NA | Martins, MJF; Vandekerkhove, J; Adolfsson, S; Rossetti, G; Namiotko, T; Jokela, J | 2010 | Effect of environmental stress on clonal structure of Eucypris virens (Crustacea, Ostracoda) | “Environmental stress imposes strong natural selection on clonal populations, promoting evolutionary change in clonal structure. Environmental stress may also lead to reduction in population size, which together with clonal selection may reduce genotypic diversity of the local populations. We examined how clonal structure in wild-collected samples of two parthenogenetic populations of the freshwater ostracod Eucypris virens responded to hypersalinity and starvation, and the combination of the two stressors. We applied the stress treatments in a factorial design for one generation. When 60% of the individuals per experimental unit had died, post-experimental clonal structure was compared to that of the start of the experiment, which reflected the field conditions. We used five polymorphic allozyme loci as genotype markers. All stress treatments reduced survival compared to the control treatment. In the population”“Rivalazzetto”“, we observed a reduction of clonal richness in the control treatment, with the initially dominant clone maintaining dominance. This may have resulted from interclonal competition and clone-specific survival under the different laboratory conditions. Clonal richness remained high in the salinity treatment while it was reduced in the combined stress and starvation treatments. In the population”“Fornovo”“, clonal richness reduced in all treatments including control, while the salinity and combined stress treatment reduced clonal evenness. The clone dominating at the start of the experiment increased in frequency in all treatments, but the change in clonal structure during the experiment was more pronounced in this population. These results suggest that in some conditions an intermediate level of environmental stress may lessen the decline in genetic diversity by strong inter-clonal competition. Moreover, the variation in clonal structure among the stress treatments and distinct genetic backgrounds indicates that more general predictions of stress effects on clonal structure may be difficult.” | 2486 | NA | NA | NA | no | no |
| 2487 | 2017 | NA | Theuns, J; Del-Favero, J; Dermaut, B; van Duijn, CM; Backhovens, H; Van den Broeck, M; Serneels, S; Corsmit, E; Van Broeckhoven, C; Cruts, M | 2000 | Genetic variability in the regulatory region of presenilin 1 associated with risk for Alzheimer’s disease and variable expression | Mutations in the presenilin 1 (PSEN1) gene have been implicated in 18-50% of autosomal dominant cases with early-onset Alzheimer’s disease (EOAD). Also, PSEN1 has been suggested as a potential risk gene in late-onset AD cases. We recently showed genetic association in a population-based study of EOAD, pointing to the 5’ regulatory region of PSEN1, In this study we systematically screened 3.5 kb of the PSEN1 upstream region and found four novel polymorphisms. Genetic analysis confirmed association of two polymorphisms with increased risk for EOAD. In addition, we detected two different mutations in EOAD cases at -280 and -2818 relative to the transcription initiation site in exon 1A of PSEN1. Analysis of the mutant and wild-type -280 variants using luciferase reporter gene expression in transiently transfected neuroblastoma cells showed a 30% decrease in transcriptional activity for the mutant -280G PSEN1 promoter variant compared with the wild-type variant -280C. Our data suggest that the increased risk for EOAD associated with PSEN1 may result from genetic variations in the regulatory region reading to altered expression levels of the PSEN1 protein. | 2487 | NA | NA | NA | no | no |
| 2488 | 2017 | NA | Lazar, A; Grundemann, D; Berkels, R; Taubert, D; Zimmermann, T; Schomig, E | 2003 | Genetic variability of the extraneuronal monoamine transporter EMT (SLC22A3) | The extraneuronal monoamine transporter EMT (HGNC Nomenclature SLC22A3) is the molecular correlate of the classical uptake, system responsible for the non-neuronal inactivation of circulating and centrally released catecholamines. Because of its functional profile and expression pattern, EMT is regarded as a candidate gene for diseases related to the sympathetic nervous system and neuropsychiatric disorders. We describe the first investigation of the genetic variability of the EMT gene in human. Six single-nucleotide substitutions and one deletion were detected within the assumed core promoter, the exonic and flanking intronic sequences and the 3’-untranslated region in 100 Caucasian individuals. No amino acid changes were found and Tajima’s D was positive (D = 2.91; P < 0.01). However, the synonymous nucleotide substitution 1233G –> A might serve as a cryptic splice acceptor site. Analysis of linkage disequilibrium between polymorphisms yielded 12 possible haplotypes accounting for more than 90% of all haplotypes. Knowledge of the sequence variation and frequency of the underlying polymorphisms in this member of the amphiphilic solute facilitator family of transporters provides the basis for subsequent association studies and candidate gene approaches. | 2488 | NA | NA | NA | no | no |
| 2489 | 2017 | NA | Kluth, C; Bruelheide, H | 2005 | Central and peripheral Hornungia petraea populations: patterns and dynamics | 1More or less continuous distributions tend to become fragmented towards species’ distribution limits. Peripheral or isolated populations of a species are predicted to have lower population sizes and densities than central populations, as a result of environmental and/or genetic stress. Population densities at the periphery may be reduced by decreased reproduction or higher interannual variation in reproduction. In particular, fecundity and survival are likely to be reduced by less favourable growing conditions.  2 We compared populations of the annual species Hornungia petraea (Brassicaceae) in two contrasting regions of the species’ range and at the population scale within regions. Ten populations in Italy (central to the species range) and 10 German populations (peripheral) were monitored for three growing periods from spring 1999 to spring 2001.  3 All life‐cycle stages, adult plant density, seed production and pre‐ and post‐dispersal seed bank density were studied in a nested sampling design and variation in various demographic factors was attributed to the effects of the two countries, of populations nested within country and to temporal effects.  4 Peripheral populations had higher densities than populations in the centre of the species distribution, both as adult plants and in the seed bank.  5 Fecundity was strongly influenced by temporal effects, but only affected to a minor degree by the study region. High interannual variation in fecundity was not reflected in high interannual variation of either adult plant density in spring or of the seed bank. Significant regional differences were, however, found in seasonal seed bank dynamics, which were more pronounced in peripheral German populations than in central Italian populations. 6 We conclude that seasonal seed bank dynamics are a key factor in explaining differences in H. petraea density patterns, particularly in central populations, where fewer seeds are incorporated into the seed bank. | 2489 | NA | NA | NA | no | no |
| 2490 | 2017 | NA | Asenjo, F; Olmos, A; Henriquez-Piskulich, P; Polanco, V; Aldea, P; Ugalde, JA; Trombert, AN | 2016 | Genome sequencing and analysis of the first complete genome of Lactobacillus kunkeei strain MP2, an Apis mellifera gut isolate | Background. The honey bee (Apis mellifera) is the most important pollinator in agriculture worldwide. However, the number of honey bees has fallen significantly since 2006, becoming a huge ecological problem nowadays. The principal cause is CCD, or Colony Collapse Disorder, characterized by the seemingly spontaneous abandonment of hives by their workers. One of the characteristics of CCD in honey bees is the alteration of the bacterial communities in their gastrointestinal tract, mainly due to the decrease of Firimicutes populations, such as the Lactobacilli. At this time, the causes of these alterations remain unknown. We recently isolated a strain of Lactobacillus kunkeei (L. kunkeei strain MP2) from the gut of Chilean honey bees. L. kunkeei, is f the most commonly isolated bacterium from highly,’ the honey bee gut and is high one oversatile in different ecological niches. In this study, we aimed to elucidate in detail, the L. kurikeei genetic background and perform a comparative genome analysis with other Lactobacillus species. Methods. L. kunkeei MP2 was originally isolated from the guts of Chilean A. mellifera individuals. Genome sequencing was done using Pacific Biosciences single-molecule real-time sequencing technology. De novo assembly was performed using Celera assembler. The genome was annotated using Prold(a, and functional information was added using the EggNOG 3.1 database. In addition, genomic islands were predicted using IslandViewer, and pro-phage sequences using PHAST. Comparisons between L. kunkeei MP2 with other L. kunkeei, and Lactobacillus strains were done using Roary. Results. The complete genorne of L. kunkeei MP2 comprises one circular chromosome of 1,614,522 rit. with a GC content of 36,9%. Pangenome analysis with 16 L. kunkeei strains, identified 113 unique genes, most of them related to phage insertions. A large and unique region of L. kunkeei MP2 genome contains several genes that encode for phage structural protein and replication components. Comparative analysis of MP2’ with other Lactobacillus species, identified several unique genes of L. kunkeei MP2 related with metabolism, biofilm generation, survival under stress conditions, and mobile genetic elements (MGEs). Discussion. The presence of multiple mobile genetic elements, including phage sequences, suggest a high degree of genetic variability in L. kunkeei. Its versatility and ability to survive in different ecological niches L (bee guts, flowers, fruits among others) could be given by its genetic capacity to change and adapt to different environments L. kunkeei could be a new source of Lactobacillus with beneficial properties. Indeed, L. kunkeei MP2 could play an important role in synthesis of components as isoprenoids. | 2490 | NA | NA | NA | no | no |
| 2491 | 2017 | NA | Zerillo, MM; Caballero, JI; Woeste, K; Graves, AD; Hartel, C; Pscheidt, JW; Tonos, J; Broders, K; Cranshaw, W; Seybold, SJ; Tisserat, N | 2014 | Population Structure of Geosmithia morbida, the Causal Agent of Thousand Cankers Disease of Walnut Trees in the United States | The ascomycete Geosmithia morbida and the walnut twig beetle Pityophthorus juglandis are associated with thousand cankers disease of Juglans (walnut) and Pterocarya (wingnut). The disease was first reported in the western United States (USA) on several Juglans species, but has been found more recently in the eastern USA in the native range of the highly susceptible Juglans nigra. We performed a comprehensive population genetic study of 209 G. morbida isolates collected from Juglans and Pterocarya from 17 geographic regions distributed across 12 U.S. states. The study was based on sequence typing of 27 single nucleotide polymorphisms from three genomic regions and genotyping with ten microsatellite primer pairs. Using multilocus sequence-typing data, 197 G. morbida isolates were placed into one of 57 haplotypes. In some instances, multiple haplotypes were recovered from isolates collected on the same tree. Twenty-four of the haplotypes (42%) were recovered from more than one isolate; the two most frequently occurring haplotypes (H02 and H03) represented 36% of all isolates. These two haplotypes were abundant in California, but were not recovered from Arizona or New Mexico. G. morbida population structure was best explained by four genetically distinct groups that clustered into three geographic regions. Most of the haplotypes isolated from the native range of J. major (Arizona and New Mexico) were found in those states only or present in distinct genetic clusters. There was no evidence of sexual reproduction or genetic recombination in any population. The scattered distribution of the genetic clusters indicated that G. morbida was likely disseminated to different regions at several times and from several sources. The large number of haplotypes observed and the genetic complexity of G. morbida indicate that it evolved in association with at least one Juglans spp. and the walnut twig beetle long before the first reports of the disease. | 2491 | NA | NA | NA | no | no |
| 2492 | 2017 | NA | Gross, SM; Williamson, VM | 2011 | Tm1: A Mutator/Foldback Transposable Element Family in Root-Knot Nematodes | Three closely related parthenogenetic species of root-knot nematodes, collectively termed the Meloidogyne incognita-group, are economically significant pathogens of diverse crop species. Remarkably, these asexual root-knot nematodes are capable of acquiring heritable changes in virulence even though they lack sexual reproduction and meiotic recombination. Characterization of a near isogenic pair of M. javanica strains differing in response to tomato with the nematode resistance gene Mi-1 showed that the virulent strain carried a deletion spanning a gene called Cg-1. Herein, we present evidence that the Cg-1 gene lies within a member of a novel transposable element family (Tm1; Transposon in Meloidogyne-1). This element family is defined by composite terminal inverted repeats of variable lengths similar to those of Foldback (FB) transposable elements and by 9 bp target site duplications. In M. incognita, Tm1 elements can be classified into three general groups: 1) histone-hairpin motif elements; 2) MITE-like elements; 3) elements encoding a putative transposase. The predicted transposase shows highest similarity to gene products encoded by aphids and mosquitoes and resembles those of the Phantom subclass of the Mutator transposon superfamily. Interestingly, the meiotic, sexually-reproducing root-knot nematode species M. hapla has Tm1 elements with similar inverted repeat termini, but lacks elements with histone hairpin motifs and contains no elements encoding an intact transposase. These Tm1 elements may have impacts on root-knot nematode genomes and contribute to genetic diversity of the asexual species. | 2492 | NA | NA | NA | no | no |
| 2493 | 2017 | NA | Berlanga, M; Llorens, C; Comas, J; Guerrero, R | 2016 | Gut Bacterial Community of the Xylophagous Cockroaches Cryptocercus punctulatus and Parasphaeria boleiriana | Cryptocercus punctulatus and Parasphaeria boleiriana are two distantly related xylophagous and subsocial cockroaches. Cryptocercus is related to termites. Xylophagous cockroaches and termites are excellent model organisms for studying the symbiotic relationship between the insect and their microbiota. In this study, high-throughput 454 pyrosequencing of 16S rRNA was used to investigate the diversity of metagenomic gut communities of C. punctulatus and P. boleiriana, and thereby to identify possible shifts in symbiont allegiances during cockroaches evolution. Our results revealed that the hindgut prokaryotic communities of both xylophagous cockroaches are dominated by members of four Bacteria phyla: Bacteroidetes, Firmicutes, Proteobacteria, and Actinobacteria. Other identified phyla were Spirochaetes, Planctomycetes, candidatus Saccharibacteria (formerly TM7), and Acidobacteria, each of which represented 1-2% of the total population detected. Community similarity based on phylogenetic relatedness by unweighted UniFrac analyses indicated that the composition of the bacterial community in the two species was significantly different (P < 0.05). Phylogenetic analysis based on the characterized clusters of Bacteroidetes, Spirochaetes, and Deltaproteobacteria showed that many OTUs present in both cockroach species clustered with sequences previously described in termites and other cockroaches, but not with those from other animals or environments. These results suggest that, during their evolution, those cockroaches conserved several bacterial communities from the microbiota of a common ancestor. The ecological stability of those microbial communities may imply the important functional role for the survival of the host of providing nutrients in appropriate quantities and balance. | 2493 | NA | NA | NA | no | no |
| 2494 | 2017 | NA | Vincent, H; Wiersema, J; Kell, S; Fielder, H; Dobbie, S; Castaneda-Alvarez, NP; Guarino, L; Eastwood, R; Leon, B; Maxted, N | 2013 | A prioritized crop wild relative inventory to help underpin global food security | The potentially devastating impacts of climate change on biodiversity and food security, together with the growing world population, means taking action to conserve crop wild relative (CWR) diversity is no longer an option it is an urgent priority. CWR are species closely related to crops, including their progenitors, which have potential to contribute traits for crop improvement. However, their utilisation is hampered by a lack of systematic conservation which in turn is due to a lack of clarity over their identity. We used gene pool and taxon group concepts to estimate CWR relatedness for 173 priority crops to create the Harlan and de Wet inventory of globally important CWR taxa. Further taxa more remotely related to crops were added if they have historically been found to have useful traits for crop improvement. The inventory contains 1667 taxa, divided between 37 families, 108 genera, 1392 species and 299 sub-specific taxa. The region with the highest number of priority CWR is western Asia with 262 taxa, followed by China with 222 and southeastern Europe with 181. Within the primary gene pool, 242 taxa were found to be under-represented in ex situ collections and the countries identified as the highest priority for further germplasm collection are China, Mexico and Brazil. The inventory database is web-enabled (http://www.cwrcliversity.org/checklisti) and can be used to facilitate in situ and ex situ conservation planning at global, regional and national levels. (C) 2013 Elsevier Ltd. All rights reserved. | 2494 | NA | NA | NA | no | no |
| 2495 | 2017 | NA | Pilczynska, J; Cocito, S; Boavida, J; Serrao, E; Queiroga, H | 2016 | Genetic Diversity and Local Connectivity in the Mediterranean Red Gorgonian Coral after Mass Mortality Events | Estimating the patterns of connectivity in marine taxa with planktonic dispersive stages is a challenging but crucial task because of its conservation implications. The red gorgonian Paramuricea clavata is a habitat forming species, characterized by short larval dispersal and high reproductive output, but low recruitment. In the recent past, the species was impacted by mass mortality events caused by increased water temperatures in summer. In the present study, we used 9 microsatellites to investigate the genetic structure and connectivity in the highly threatened populations from the Ligurian Sea (NW Mediterranean). No evidence for a recent bottleneck neither decreased genetic diversity in sites impacted by mass mortality events were found. Significant IBD pattern and high global F-ST confirmed low larval dispersal capability in the red gorgonian. The maximum dispersal distance was estimated at 20-60 km. Larval exchange between sites separated by hundreds of meters and between different depths was detected at each site, supporting the hypothesis that deeper subpopulations unaffected by surface warming peaks may provide larvae for shallower ones, enabling recovery after climatically induced mortality events. | 2495 | NA | NA | NA | no | no |
| 2496 | 2017 | NA | Li, JM; Dong, M | 2009 | Fine-scale clonal structure and diversity of invasive plant Mikania micrantha HBK and its plant parasite Cuscuta campestris Yunker | The fine-scale clonal structure in the patch of the invasive plant Mikania micrantha H.B.K. and its plant parasite Cuscuta campestris Yunker were investigated by distinguishing genets using ISSR markers. A high level of clonal diversity (G/N = 0.7, D = 0.9579, E = 0.7778) in M. micrantha and a low level of clonal diversity (G/N = 0.2, D = 0.7632, E = 0.9479) in C. campestris might be due to the different reproductive strategies, different migration rates and different number of founder. Clonal composition of M. micrantha (14 genets of 20 ramets) and C. campestris (4 genets of 20 ramets) were significantly different. There was no relationship between host and parasite genetic distance matrices using Mantel test (r = 0.073, P = 0.150). Spatial autocorrelation analysis of M. micrantha showed that the correlation value was positive and significant at 2 m with an x-intercept of 3.5 and 4.0 m for distance class sizes of 1 and 2 m, respectively. Spatial autocorrelation analysis of C. campestris showed that the correlation value was positive and significant at 2 m and significantly negative at 10 m with an x-intercept of 4.8 and 6.6 m for distance class sizes of 1 and 2 m, respectively. | 2496 | NA | NA | NA | no | no |
| 2497 | 2017 | NA | Denny, E; Yakovlevich, P; Eldridge, MDB; Dickman, C | 2002 | Social and genetic analysis of a population of free-living cats (Felis catus L.) exploiting a resource-rich habitat | Free-living cats ( Felis catus L.) exploiting a waste-disposal site in rural Australia were studied for two years to investigate population structure and dynamics, and the relatedness of constituent individuals. The density of the population was equivalent to 700-750 cats km(-2), the sex ratio was heavily skewed towards males, breeding occurred from July to April, and kitten survival rates were low. A combination of observational data, biometrics and microsatellite loci analyses was used to assess the relatedness of individuals in the population; these methods yielded highly congruent results. Thus, a female kin-group of three was identified, there was no female immigration, the average relatedness amongst the population was high and there was no indication of male dominance. The results indicate that cats at the site formed a tightly structured group, rather than an ad hoc collection of individuals. The stable, resource-rich habitat of waste-disposal sites may generally support high densities of group-forming cats in rural Australia, and pose broad-scale but previously unrecognised problems for effective management of free-living cats. | 2497 | NA | NA | NA | no | no |
| 2498 | 2017 | NA | Taylor, PD; Grafen, A | 2010 | Relatedness with different interaction configurations | “In an inclusive fitness model of social behaviour, a key concept is that of the relatedness between two interactants. This is typically calculated with reference to a”“focal”" actor taken to be representative of all actors, but when there are different interaction configurations, relatedness must be constructed as an average over all such configurations. We provide an example of such a calculation in an island model with local reproduction but global mortality, leading to variable island size and hence variable numbers of individual interactions. We find that the analysis of this example significantly sharpens our understanding of relatedness. As an application, we obtain a version of Hamilton’s rule for a tag-based model of altruism in a randomly mixed population. For large populations, the selective advantage of altruism is enhanced by low (but not too low) tag mutation rates and large numbers of tags. For moderate population sizes and moderate numbers of tags, we find a window of tag mutation rates with critical benefit/cost ratios of between 1 and 3. (C) 2009 Elsevier Ltd. All rights reserved." | 2498 | NA | NA | NA | no | no |
| 2499 | 2017 | NA | GOLD, KC; MAPLE, TL | 1994 | PERSONALITY-ASSESSMENT IN THE GORILLA AND ITS UTILITY AS A MANAGEMENT TOOL | Goals of the Gorilla Species Survival Plan (SSP) include increased captive propagation, maintenance of genetic diversity, and consideration of the psychological well-being of the population. The SSP Propagation Group has attempted to accomplish these goals through recommendations based on assessment of genetic, behavioral, and demographic variables. A need is recognized for the development of a methodology to assess behavioral variables that contribute to the success of animal moves. Personality profiles have been used in the past to aid animal management decisions in laboratory settings. This paper describes personality assessment techniques, reviews historical perspectives on primate personality assessment, and reports on a study to assess gorilla personality. The Gorilla Behavior Index (GBI), a subjective assessment instrument consisting of behaviorally based adjectives, was completed for 298 of 303 captive gorillas over 1 year of age. The results were subjected to common factor analysis, resulting in the identification of four main factors: extroverted, dominant, fearful, and understanding. Frequency distributions were calculated illustrating the ranges of each factor. Potential management uses for the GBI scores are discussed. (C) 1994 Wiley-Liss, Inc. | 2499 | NA | NA | NA | no | no |
| 2500 | 2017 | NA | Thompson, J; Charpentier, A; Bouguet, G; Charmasson, F; Roset, S; Buatois, B; Vernet, P; Gouyon, PH | 2013 | Evolution of a genetic polymorphism with climate change in a Mediterranean landscape | Many species show changes in distribution and phenotypic trait variation in response to climatic warming. Evidence of genetically based trait responses to climate change is, however, less common. Here, we detected evolutionary variation in the landscape-scale distribution of a genetically based chemical polymorphism in Mediterranean wild thyme (Thymus vulgaris) in association with modified extreme winter freezing events. By comparing current data on morph distribution with that observed in the early 1970s, we detected a significant increase in the proportion of morphs that are sensitive to winter freezing. This increase in frequency was observed in 17 of the 24 populations in which, since the 1970s, annual extreme winter freezing temperatures have risen above the thresholds that cause mortality of freezing-sensitive morphs. Our results provide an original example of rapid ongoing evolutionary change associated with relaxed selection (less extreme freezing events) on a local landscape scale. In species whose distribution and genetic variability are shaped by strong selection gradients, there may be little time lag associated with their ecological and evolutionary response to long-term environmental change. | 2500 | NA | NA | NA | no | no |
| 2501 | 2017 | NA | Kamath, PL; Getz, WM | 2011 | Adaptive molecular evolution of the Major Histocompatibility Complex genes, DRA and DQA, in the genus Equus | Background: Major Histocompatibility Complex (MHC) genes are central to vertebrate immune response and are believed to be under balancing selection by pathogens. This hypothesis has been supported by observations of extremely high polymorphism, elevated nonsynonymous to synonymous base pair substitution rates and trans-species polymorphisms at these loci. In equids, the organization and variability of this gene family has been described, however the full extent of diversity and selection is unknown. As selection is not expected to act uniformly on a functional gene, maximum likelihood codon-based models of selection that allow heterogeneity in selection across codon positions can be valuable for examining MHC gene evolution and the molecular basis for species adaptations. Results: We investigated the evolution of two class II MHC genes of the Equine Lymphocyte Antigen (ELA), DRA and DQA, in the genus Equus with the addition of novel alleles identified in plains zebra (E. quagga, formerly E. burchelli). We found that both genes exhibited a high degree of polymorphism and inter-specific sharing of allele lineages. To our knowledge, DRA allelic diversity was discovered to be higher than has ever been observed in vertebrates. Evidence was also found to support a duplication of the DQA locus. Selection analyses, evaluated in terms of relative rates of nonsynonymous to synonymous mutations (d(N)/d(S)) averaged over the gene region, indicated that the majority of codon sites were conserved and under purifying selection (d(N) < d(S)). However, the most likely evolutionary codon models allowed for variable rates of selection across codon sites at both loci and, at the DQA, supported the hypothesis of positive selection acting on specific sites. Conclusions: Observations of elevated genetic diversity and trans-species polymorphisms supported the conclusion that balancing selection may be acting on these loci. Furthermore, at the DQA, positive selection was occurring at antigen binding sites, suggesting that a few selected residues may play a significant role in equid immune function. Future studies in natural equid populations will be valuable for understanding the functional significance of the uniquely diverse DRA locus and for elucidating the mechanism maintaining diversity at these | 2501 | NA | NA | NA | no | no |
| 2502 | 2017 | NA | MORITZ, C; MCCALLUM, H; DONNELLAN, S; ROBERTS, JD | 1991 | PARASITE LOADS IN PARTHENOGENETIC AND SEXUAL LIZARDS (HETERONOTIA-BINOEI) - SUPPORT FOR THE RED QUEEN HYPOTHESIS | One version of the Red Queen hypothesis suggests that sexual reproduction is maintained in populations because of the need to continually create genotypes that confer resistance against rapidly evolving pathogens and parasites. Here, we report that parthenogenetic individuals of the Heteronotia binoei species complex are much more prone to infection by mites than are their sexual relatives. This accords with a central prediction of the Red Queen hypothesis. The greater susceptibility of the parthenogens is consistent across localities with different combinations of parthenogenetic genotypes and sexual chromosome races and occurs despite the unusually high genetic diversity of the parthenogenetic form. These observations support the contention that clonal reproduction increases the susceptibility of hosts to infection by parasites. | 2502 | NA | NA | NA | no | no |
| 2503 | 2017 | NA | Zhang, L; Dong, L; Lin, L; Feng, LM; Yan, F; Wang, LX; Guo, XM; Luo, AD | 2015 | Asian Elephants in China: Estimating Population Size and Evaluating Habitat Suitability | We monitored the last remaining Asian elephant populations in China over the past decade. Using DNA tools and repeat genotyping, we estimated the population sizes from 654 dung samples collected from various areas. Combined with morphological individual identifications from over 6,300 elephant photographs taken in the wild, we estimated that the total Asian elephant population size in China is between 221 and 245. Population genetic structure and diversity were examined using a 556-bp fragment of mitochondrial DNA, and 24 unique haplotypes were detected from DNA analysis of 178 individuals. A phylogenetic analysis revealed two highly divergent clades of Asian elephants, alpha and beta, present in Chinese populations. Four populations (Mengla, Shangyong, Mengyang, and Pu’Er) carried mtDNA from the a clade, and only one population (Nangunhe) carried mtDNA belonging to the a clade. Moreover, high genetic divergence was observed between the Nangunhe population and the other four populations; however, genetic diversity among the five populations was low, possibly due to limited gene flow because of habitat fragmentation. The expansion of rubber plantations, crop cultivation, and villages along rivers and roads had caused extensive degradation of natural forest in these areas. This had resulted in the loss and fragmentation of elephant habitats and had formed artificial barriers that inhibited elephant migration. Using Geographic Information System, Global Positioning System, and Remote Sensing technology, we found that the area occupied by rubber plantations, tea farms, and urban settlements had dramatically increased over the past 40 years, resulting in the loss and fragmentation of elephant habitats and forming artificial barriers that inhibit elephant migration. The restoration of ecological corridors to facilitate gene exchange among isolated elephant populations and the establishment of cross-boundary protected areas between China and Laos to secure their natural habitats are critical for the survival of Asian elephants in this region. | 2503 | NA | NA | NA | no | no |
| 2504 | 2017 | NA | GARCIA, C; TORO, MA | 1993 | LARVAL COMPETITION AND GENETIC DIVERSITY IN TRIBOLIUM-CASTANEUM | The study of the relationship between genetic homogenity and intensity of competition in groups of organisms may help to explain the widespread existence of sexual reproduction and it can also be used to design efficient crop mixtures. To study this, we compared the survival of sib groups and random groups of larvae of the beetle Tribolium castaneum maintained at high population density: every group was formed by introducing 150 eggs in 1 g of culture medium. The larvae in every group were counted weekly. The random groups survived longer, as they had more larvae in the last weeks. This advantage was related to a higher early mortality, which reduced competition in the long run in these groups. Therefore, in the early stages of development, our results did not confirm the hypothesis that genetically heterogeneous groups reduce competition through diversification in the use of environmental resources. In addition, a clear increase in between-group variability for survival was found in the sib groups, implying the presence of genetic variance for competitive ability at constant initial densities in this species. | 2504 | NA | NA | NA | no | no |
| 2505 | 2017 | NA | Uhrin, M; Kanuch, P; Kristofik, J; Paule, L | 2010 | Phenotypic plasticity in the greater mouse-eared bat in extremely different roost conditions | Bats use various roost types with a wide spectrum of ecological features. The greater mouse-eared bat Myotis myotis (Borkhausen, 1797), creates nurseries in attics and caves in Central Europe. The stable low temperature and high humidity cave microclimate contrasts that of attics, which may alter species adaptations and life strategies. We analysed population characteristics (composition, body condition, parasite load, and immune response) and genetic relatedness of two proximal M. myotis populations. Age, sexual and parasite species composition were similar between the cave and attic sites. However, a significantly higher parasite load and body condition was detected in the post-partum females and juveniles of the cave colony (n = 263 bats from the cave, 231 from the attic), with the cave colony females having a significantly stronger immune response (n = 2 caves and 2 attics, 20 females per site). There was no evidence for genetic divergence between cave and attic populations (n = 3 caves and 3 attics, 24 females per site), indicating that different population characteristics are not genetically based and that M. myotis is an example of a species with rather unique phenotypic plasticity. | 2505 | NA | NA | NA | no | no |
| 2506 | 2017 | NA | Carvalho-Costa, FA; Dos Santos, SM; Pires, MQ; Lopes, CM; Noireau, F; Pacheco, RS | 2010 | Sylvatic and peridomestic populations of Triatoma pseudomaculata are not significantly structured by habitat, as revealed by two genetic markers | Chagas disease remains a public health concern in Brazil and other Latin American countries, mainly due to the potential domiciliation of native triatomine species. We analyzed the genetic variability of Triatoma pseudomaculata in sylvatic and peridomestic ecotopes throughout three localities in the northeastern state of Bahia, Brazil. We studied polymorphisms generated by random amplified polymorphic DNA (RAPD) and isoenzyme electrophoresis analyses. Based on RAPD analysis, each specimen was assigned to one of three genetic clusters. Although all sylvatic specimens from one locality were grouped into the same cluster, sylvatic and peridomestic specimens from the other two localities were broadly distributed between the remaining two clusters, suggesting that geographic population structuring was not occurring. Furthermore, isoenzyme analysis suggested that distinct populations were in Hardy-Weinberg equilibrium. Low statistical values for Wright’s Fst index also supported the absence of population structuring and suggested the occurrence of panmixia. We conclude that genetic flow occurs between sylvatic and peridomestic T. pseudomaculata populations, probably as a consequence of passive and active dispersion of the insects, associated with deforestation and anthropic transformations. | 2506 | NA | NA | NA | no | no |
| 2507 | 2017 | NA | Sutton, JT; Nakagawa, S; Robertson, BC; Jamieson, IG | 2011 | Disentangling the roles of natural selection and genetic drift in shaping variation at MHC immunity genes | The major histocompatibility complex (MHC) forms an integral component of the vertebrate immune response and, due to strong selection pressures, is one of the most polymorphic regions of the entire genome. Despite over 15 years of research, empirical studies offer highly contradictory explanations of the relative roles of different evolutionary forces, selection and genetic drift, acting on MHC genes during population bottlenecks. Here, we take a meta-analytical approach to quantify the results of studies into the effects of bottlenecks on MHC polymorphism. We show that the consequences of selection acting on MHC loci prior to a bottleneck event, combined with drift during the bottleneck, will result in overall loss of MHC polymorphism that is similar to 15% greater than loss of neutral genetic diversity. These results are counter to general expectations that selection should maintain MHC polymorphism, but do agree with the results of recent simulation models and at least two empirical studies. Notably, our results suggest that negative frequency-dependent selection could be more important than overdominance for maintaining high MHC polymorphism in pre-bottlenecked populations. | 2507 | NA | NA | NA | no | no |
| 2508 | 2017 | NA | Wang, B; Brubaker, CL; Summerell, BA; Thrall, PH; Burdon, JJ | 2010 | Local origin of two vegetative compatibility groups of Fusarium oxysporum f. sp vasinfectum in Australia | Pathogenicity and genetic diversity of Fusarium oxysporum from geographically widespread native Gossypium populations, including a cotton growing area believed to be the center of origin of VCG 01111 and VCG 01112 of F. oxysporum f. sp. vasinfectum (Fov) in Australia, was determined using glasshouse bioassays and AFLPs. Five lineages (A-E) were identified among 856 isolates. Of these, 12% were strongly pathogenic on cotton, 10% were weakly pathogenic and designated wild Fov, while 78% were nonpathogenic. In contrast to the occurrence of pathogenic isolates in all five lineages in soils associated with wild Gossypium, in cotton growing areas only three lineages (A, B, E) occurred and all pathogenic isolates belonged to two subgroups in lineage A. One of these contained VCG 01111 isolates while the other contained VCG 01112 isolates. Sequence analyses of translation elongation factor-1 alpha, mitochondrial small subunit rDNA, nitrate reductase and phosphate permease confirmed that Australian Fov isolates were more closely related to lineage A isolates of native F. oxysporum than to Fov races 1-8 found overseas. These results strongly support a local evolutionary origin for Fov in Australian cotton growing regions. | 2508 | NA | NA | NA | no | no |
| 2509 | 2017 | NA | Khar, A; Devi, AA; Lawande, KE | 2008 | Analysis of genetic diversity among Indian garlic (Allium sativum L.) cultivars and breeding lines using RAPD markers | Randomly amplified polymorphic DNA markers were used to assess the genetic diversity among 23 commercially released varieties and breeding lines of garlic along with onion as an out-group. A total of 100 operon decamer primers were screened in duplicate and fourteen of these primers were selected for further testing. The number of bands per primer varied from 3 to 19, with an average of 11.5. Based on RAPD data, similarity values among garlic genotypes ranged from 0.97 to 0.47 with a mean value of 0.72. A low degree of similarity (0.10-0.15) was observed with onion. The UPGMA dendrogram constructed using Jaccard’s similarity matrix discriminated all the genotypes into four clusters. Cluster I comprised of 14 genotypes with a similarity index of about 89%. Cluster II comprised of only one line, SKAUG 151 that had a similarity Index of 62% with Cluster I. Cluster III comprised of 8 garlic genotypes at a distance of about 52%. Variation amongst the garlic accessions was about 48%, whereas, with onion it showed a dissimilarity of 90%. The clustering pattern of garlic genotypes in the present study discriminated them as per the vernalization requirement for bulb formation. | 2509 | NA | NA | NA | no | no |
| 2510 | 2017 | NA | Abdalhag, MA; Zhang, T; Fan, QC; Zhang, XQ; Zhang, GX; Wang, JY; Wei, Y; Wang, YJ | 2015 | Single nucleotide polymorphisms associated with growth traits in Jinghai yellow chickens | Body weight is one of the most important economic traits in the poultry industry. In the present study, a custom SNP Beadchip was used to analyze the association between those 15 SNPs and 12 growth traits of Jinghai yellow chickens, and other important genetic parameters were also calculated and analyzed. The results indicated that nine of the 15 SNPs were associated with growth traits in Jinghai yellow chickens (P < 0.05), and the identified SNPs were also in linkage disequilibrium. Five of the nine identified SNPs were mainly associated with all of the growth traits, which indicated that those five SNPs might have significant influence on Jinghai yellow chicken growth traits. Polymorphism information content (PIC) analyses indicated that five of the nine SNPs exhibited moderate polymorphism (0.25 < PIC < 0.5), which reflected intermediate genetic diversity. Six candidate genes surrounding the significant SNPs were obtained and subjected to Gene Ontology annotation analyses and pathway analyses. The functions of six important candidate genes (SETDB2, ATP7B, INTS6, KPNA3, DLEU7, and FOXO1A) were discussed. The present study provided basic data for marker-assisted selection in Jinghai yellow chickens. | 2510 | NA | NA | NA | no | no |
| 2511 | 2017 | NA | Todaro, AR; Nascimento, VX; Souza, NCC; Silva, PP; Santos, JM; Ramalho, EAVF; Melo, TVC; Silva, ISM; Lins, GS; Bastos, MLA; Farias, DF; Souza, PCV; Carvalho, AFU; Silva, MCS; Almeida, RMRG; Neto, ER | 2013 | Genetic variability in the ITS and IGS regions of the ribosomal DNA of Acremonium cavaraeanum exhibiting antimicrobial activity | Endophytic microorganisms represent promising alternatives for obtaining new drugs of biotechnological importance. In this study, the endophytic species Acremonium cavaraeanum (A1a) isolated from Cocos nucifera was cultivated for the production of secondary metabolites, and its extracts and fractions were evaluated by the dilution method (MIC). The EtOAc extracts and MeOH fractions were tested against Gram-positive and -negative bacteria, and had an MIC of 125 mu g/mL when evaluated in the EtOAc extract (EBI). The EtOAc extract (EBII) had an MIC of 62.25 mu g/mL for Staphylococcus aureus and an MIC between 125 and 250 mu g/mL for Gram-negative bacteria. The methanolic fractions showed activity with MIC between 125 and 250 mu g/mL for all bacteria tested. The IGS region of the rDNA repeat unit of genomic DNA was analyzed by PCR/RFLPs, including endonucleases PstI, BamHII, HinfI, and EcoRI. The physical maps showed different restriction sites for the 6 Acremonium sp isolates, and revealed 5 RFLP patterns. The results showed that isolates of the same Acremonium species exhibited variation in this specific region. The sequences of ITS1-5.8S-ITS2 regions were aligned by Clustal W using the neighbor joining method, which grouped the isolates into 5 distinct clusters. This study aimed to evaluate the genetic diversity of A. cavaraeanum crops exhibiting antibacterial activity. The results of this study indicate that different fungal genetic isolates have biotechnological potential for the production of active bio-compounds against several human pathogens. | 2511 | NA | NA | NA | no | no |
| 2512 | 2017 | NA | Camara, MD | 1997 | A recent host range expansion in Junonia coenia Hubner (Nymphalidae): Oviposition preference, survival, growth, and chemical defense | This paper reports on an investigation of two populations of Junonia coenia, the buckeye butterfly, one that feeds on the species’ typical host plant (Plantago lanceolata) and one that utilizes a novel host plant (Kickxia elatine). I examined these populations for local adaptive responses in terms of oviposition behavior, growth, and chemical defense, on both P. lanceolata and K. elatine. In addition, I examined the genetic architecture underlying these traits using a full-sib quantitative genetic analysis. I found that a significant majority of females prefer the host plant species found at their collection sites in oviposition tests, but that there is no evidence that they are locally adapted in growth performance, as measured by fifth-instar and pupal weights and development times. Neither are there correlations between oviposition preferences of females and the growth performance or levels of chemical defense of their offspring. The two populations studied do, however, show specialization in terms of the levels of chemical. defense they sequester from their host plants. I argue that these results indicate that natural enemies are the normal barriers to host range expansion in this oligophagous herbivore because a breakdown in those barriers results in genetic changes that enhance resistance to predation. This is despite the fact that adaptive responses in physiology are unlikely to be limited by a lack of genetic variability; the genetic architecture among traits would be conducive to specialization in growth performance; and there are costs to chemical defense in this species. All these conditions would tend to argue that J. coenia harbors considerable potential for coevolutionary interactions with its chemically defended hosts, but this potential is not realized, probably because natural selection on diet breadth by natural enemies is much stronger than selection from host plants in this system. | 2512 | NA | NA | NA | no | no |
| 2513 | 2017 | NA | Passarino, G; Montesanto, A; Dato, S; Giordano, S; Domma, F; Mari, V; Feraco, E; De Benedictis, G | 2006 | Sex and age specificity of susceptibility genes modulating survival at old age | Objective: We aimed to investigate the influence of the genetic variability of candidate genes on survival at old age in good health. Methods: First, on the basis of a synthetic survival curve constructed using historic mortality data taken from the Italian population from 1890 onward, we defined three age classes ranging from 18 to 106 years. Second, we assembled a multinomial logistic regression model to evaluate the effect of dichotomous variables (genotypes) on the probability to be assigned to a specific category (age class). Third, we applied the regression model to a cross-sectional dataset (10 genes; 972 subjects selected for healthy status) categorized according to age and sex. Results: We found that genetic factors influence survival at advanced age in good health in a sex- and age-specific way. Furthermore, we found that genetic variability plays a stronger role in males than in females and that, in both genders, its impact is especially important at very old ages. Conclusions: The analyses presented here underline the age-specific effect of the gene network in modulating survival at advanced age in good health. | 2513 | NA | NA | NA | no | no |
| 2514 | 2017 | NA | Hale, KA; Briskie, JV | 2007 | Decreased immunocompetence in a severely bottlenecked population of an endemic New Zealand bird | Inbreeding resulting from severe population bottlenecks may impair an individual’s immune system and render it more susceptible to disease. Although a reduced immune response could threaten the survival of highly endangered species, few studies have assessed the effect of population bottlenecks on immunocompetence. We compared the counts of leucocytes and external, blood and gastrointestinal parasite loads in two populations of the endemic New Zealand robin Petroica australis to assess the immunocompetence of birds in a severely bottlenecked population relative to its more genetically diverse source population. Despite similar parasite loads in both populations, robins in the severely bottlenecked population showed lower counts of both total leucocyte and total lymphocyte numbers. When the immune system was experimentally challenged using the phytohaemagglutinin skin test, robins in the severely bottlenecked population exhibited a significantly lower immune response than the source population, suggesting that birds passing through a severe bottleneck have a compromised immunocompetence. Our results confirm that severe bottlenecks reduce the immune response of birds and highlight the need to avoid severe bottlenecks in the recovery programmes of endangered species. | 2514 | NA | NA | NA | no | no |
| 2515 | 2017 | NA | Shang, YJ; Wang, GX; Tian, H; Yin, SH; Du, P; Wu, JY; Chen, Y; Yang, SL; Jin, Y; Zhang, KS; Liu, XT | 2012 | Molecular epidemiological investigation of porcine reproductive and respiratory syndrome virus in Northwest China from 2007 to 2010 | Porcine reproductive and respiratory syndrome (PRRS) is an economically important swine disease affecting swine worldwide. Northwest China has a sparse pig population and there is no comprehensive information currently available on PRRSV infection. In this study, we analyzed the epidemiological features and genetic diversity of PRRSV from this region. 322 field-isolated tissues or serum samples were collected from aborted pig fetuses or pigs with respiratory disease from 15 herds, twice over a period of 2 years. PRRSV infection was determined and virus strains were classified by the sequencing of GP5. We found that 35.9 % of the animals were PRRSV-positive, and the average prevalence in 2007-2008 and 2009-2010 was 46.5 and 29.3 %, respectively. To further investigate the genetic divergence of PRRSV samples collected from 2007 to 2010, 32 strains were isolated for GP5 sequencing and analysis, and phylogenetic trees were created based on GP5 amino acid sequences. All PRRSVs were of the North American genotype and belonged to the highly pathogenic HP-PRRSV subgenotype. Isolates from the Xinjiang province formed a tightly clustered branch and were closely related to an evolutionary intermediate subgroup isolate. Virus sequences from 2007 to 2008 were compared with those from 2009 to 2010 from the same herd. New mutations were found in isolates after 2009 and focused on nucleotides in the GP5 antibody decoy epitope. PRRSV strains in Northwest China from 2007 to 2010 were similar to those from other regions of China, with some regional characteristics. These results contribute to the knowledge of PRRSV epidemiology in China. | 2515 | NA | NA | NA | no | no |
| 2516 | 2017 | NA | Putaporntip, C; Udomsangpetch, R; Pattanawong, U; Cui, LW; Jongwutiwes, S | 2010 | Genetic diversity of the Plasmodium vivax merozoite surface protein-5 locus from diverse geographic origins | Plasmodium vivax merozoite surface protein-5 (PvMsp-5), a potential vaccine candidate, is encoded by a two-exon single copy gene. We have conducted a comprehensive analysis of PvMsp-5 by sequencing the entire gene of four parasite populations from northwestern Thailand (n = 73), southern Thailand (n = 53), Indonesia (n = 25) and Brazil (n = 24), and five isolates from other endemic areas. Results reveal that exon I exhibits a significantly higher level of nucleotide diversity at both synonymous and nonsynonymous sites than exon II (p<0.01). Neutrality tests based on both intraspecific and interspecific nucleotide polymorphism have detected a signature of positive selection in exon I of all populations while substitutions in exon II mainly followed neutral expectation except that three residues in exon II of northwestern Thailand population appear to be positively selected using the Bayes Empirical Bayes method. Short imperfect repeats were identified in exon I at an equivalent region to its orthologue in P. knowlesi, supporting their close genetic relatedness. Significant levels of population subdivision were detected among most populations including those between northwestern and southern Thailand (p<10(-5)), implying absent or minimal gene flow between these populations. Importantly, evidences for intragenic recombination in PvMsp-5 were found in most populations except that from southern Thailand in which haplotype diversity and nucleotide diversity were exceptionally low. Results from Fu and Li’s D*, F* and D and F tests suggested that PvMsp-5 of most P. vivax populations have been maintained by balancing selection whereas southern Thailand population could have gone through recent bottleneck events. These findings are concordant with a substantial reduction in the number of P. vivax cases in southern Thailand during the past decade, followed by a very recent population expansion. Therefore, spatio-temporal monitoring of parasite population genetics provides important implications for disease control. (C) 2010 Elsevier B.V. All rights reserved. | 2516 | NA | NA | NA | no | no |
| 2517 | 2017 | NA | Garnier-Gere, PH; Naciri-Graven, Y; Bougrier, S; Magoulas, A; Heral, M; Kotoulas, G; Hawkins, A; Gerard, A | 2002 | Influences of triploidy, parentage and genetic diversity on growth of the Pacific oyster Crassostrea gigas reared in contrasting natural environments | An increasing number of hypotheses are being proposed to explain the faster growth potential of triploids in molluscs, including their partial sterility or their higher heterozygosity compared to diploids. Triploid advantage however, remains controversial for poorer sites, because of a potential trade-off with survival. These questions were addressed in Crassostrea gigas by deploying meiosis II triploids and their diploid siblings from a single mass spawning of three males and seven females, in two contrasting locations for their trophic resources. One hundred and fifty individuals were sampled at each site after nine months, measured for weight and biochemical composition, and genotyped using three microsatellite and seven allozyme loci. Higher performance was observed at the fast-growing site for all traits except shell weight, and triploids had greater weights and biochemical contents than diploids at harvest. Triploids also grew faster at the poorer site, and showed similar survival rates to diploids at both sites. Triploids had significantly higher average allozyme and microsatellite diversity. However, they performed better for a wide range of individual heterozygosity values, arguing for an advantage of the triploid state per se , that could be due to positive effects on growth of both sterility of triploids with subsequent resource re-allocation and possible faster transcription with three copies of each gene. Despite evidence of very low or no inbreeding in the diploid sample, positive associations between individual allozyme diversity and growth were detected, which explained little but significant amounts of phenotypic variation. These associations were interpreted as direct effects of allozymes, either alone or including epistatic interactions with other loci. In addition, measures of individual distance (mean -d (2) ) specific to microsatellites, were negatively correlated with growth in diploids, indicating possible effects of outbreeding depression between more distant genomes of parents from distinct populations. | 2517 | NA | NA | NA | no | no |
| 2518 | 2017 | NA | Yang, J; Weedon, MN; Purcell, S; Lettre, G; Estrada, K; Willer, CJ; Smith, AV; Ingelsson, E; O’Connell, JR; Mangino, M; Magi, R; Madden, PA; Heath, AC; Nyholt, DR; Martin, NG; Montgomery, GW; Frayling, TM; Hirschhorn, JN; McCarthy, MI; Goddard, ME; Visscher, PM | 2011 | Genomic inflation factors under polygenic inheritance | Population structure, including population stratification and cryptic relatedness, can cause spurious associations in genome-wide association studies (GWAS). Usually, the scaled median or mean test statistic for association calculated from multiple single-nucleotide-polymorphisms across the genome is used to assess such effects, and ‘genomic control’ can be applied subsequently to adjust test statistics at individual loci by a genomic inflation factor. Published GWAS have clearly shown that there are many loci underlying genetic variation for a wide range of complex diseases and traits, implying that a substantial proportion of the genome should show inflation of the test statistic. Here, we show by theory, simulation and analysis of data that in the absence of population structure and other technical artefacts, but in the presence of polygenic inheritance, substantial genomic inflation is expected. Its magnitude depends on sample size, heritability, linkage disequilibrium structure and the number of causal variants. Our predictions are consistent with empirical observations on height in independent samples of similar to 4000 and similar to 133 000 individuals. European Journal of Human Genetics (2011) 19, 807-812; doi:10.1038/ejhg.2011.39; published online 16 March 2011 | 2518 | NA | NA | NA | no | no |
| 2519 | 2017 | NA | Yang, XQ; Li, HT; Guan, QZ; Liu, D | 2013 | Genetic diversity of Toll-like receptor 5 among pig populations | The Toll-like receptor 5 (TLR5) recognizes flagellin of Gram-positive and -negative bacteria and plays an important role in the host defense system. Here, we surveyed single nucleotide polymorphisms (SNPs) in the coding sequence of the porcine TLR5 gene in 83 individuals from five pig breeds, these including Chinese local populations and Western commercial pig breeds. A total of 19 medium polymorphic SNPs (0.25<PIC<0.5) were identified, three of which were missense mutations that clustered within the extracellular domain of TLR5. One of the non-synonymous SNPs fell within a 228-amino acid region which has been shown to be important for flagellin recognition. Four SNPs were only found with high frequencies in Oriental pig breeds. The 19 SNPs were found in 30 haplotypes, one of which segregated at high frequency in all samples. Compared with Western pig breeds, Chinese local populations had higher genetic diversity and more haplotypes. Tajima’s test showed no evidence for deviation from neutrality. The data provide useful information for future genetic marker characterization by means of disease association analysis and/or stimulating the mutation carrier with relevant ligands. | 2519 | NA | NA | NA | no | no |
| 2520 | 2017 | NA | Zo, YG; Rivera, ING; Russek-Cohen, E; Islam, MS; Siddique, AK; Yunus, M; Sack, RB; Huq, A; Colwell, RR | 2002 | Genomic profiles of clinical and environmental isolates of Vibrio cholerae 01 in choleraendemic areas of Bangladesh | Diversity, relatedness, and ecological interactions of toxigenic Vibrio cholerae 01 populations in two distinctive habitats, the human intestine and the aquatic environment, were analyzed. Twenty environmental isolates and 42 clinical isolates were selected for study by matching serotype, geographic location of isolation in Bangladesh, and season of isolation. Genetic profiling was done by enterobacterial repetitive intergenic consensus sequence-PCR, optimized for profiling by using the fully sequenced V. cholerae El Tor N16961 genome. Five significant clonal clusters of haplotypes were found from 57 electrophoretic types. Isolates from different areas or habitats intermingled in two of the five significant clusters. Frequencies of haplotypes differed significantly only between the environmental populations (exact test; P < 0.05). Analysis of molecular variance yielded a population genetic structure reflecting the differentiating effects of geographic area, habitat, and sampling time. Although a parameter confounding the latter differences explained 9% of the total molecular variance in the entire population (P < 0.01), the net effect of habitat and time could not be separated because of the small number of environmental isolates included in the study. Five subpopulations from a single area were determined, and from these we were able to estimate a relative differentiating effect of habitat, which was small compared with the effect of temporal change. In conclusion, the resulting population structure supports the hypothesis that spatial and temporal fluctuations in the composition of toxigenic V. cholerae populations in the aquatic environment can cause shifts in the dynamics of the disease. | 2520 | NA | NA | NA | no | no |
| 2521 | 2017 | NA | Kowalska, A; Wiechmann, I; Walter, H | 1998 | Genetic variability of apolipoprotein E in a Polish population | Apolipoprotein E (apoE, protein; APOE, gene) is a component of very low density lipoprotein and high-density lipoprotein and plays an important role in lipoprotein metabolism. There are three common alleles of APOE (*2,* 3, and *4), which encode the E2, E3, and E4 isoforms of the protein. Distribution of apoE isoforms shows marked variation among various ethnic groups. Direct phenotyping of human APOE in plasma was used to estimate APOE allele frequencies in 137 unrelated blood donors from 3 regions of Poland. The relative frequencies observed for the APOE*2, APOE*3, and APOE*4 alleles were 0.055, 0.839, and 0.106, respectively. The data have been compared with data found in other population groups. The frequency of the APOE\*2 allele in Poles was among the lowest in Europe. | 2521 | NA | NA | NA | no | no |
| 2522 | 2017 | NA | Lion, S; Gandon, S | 2009 | Habitat saturation and the spatial evolutionary ecology of altruism | Under which ecological conditions should individuals help their neighbours? We investigate the effect of habitat saturation on the evolution of helping behaviours in a spatially structured population. We combine the formalisms of population genetics and spatial moment equations to tease out the effects of various physiological (direct benefits and costs of helping) and ecological parameters (such as the density of empty sites) on the selection gradient on helping. Our analysis highlights the crucial importance of demography for the evolution of helping behaviours. It shows that habitat saturation can have contrasting effects, depending on the form of competition (direct vs. indirect competition) and on the conditionality of helping. In our attempt to bridge the gap between spatial ecology and population genetics, we derive an expression for relatedness that takes into account both habitat saturation and the spatial structure of genetic variation. This analysis helps clarify discrepancies in the results obtained by previous theoretical studies. It also provides a theoretical framework taking into account the interplay between demography and kin selection, in which new biological questions can be explored. | 2522 | NA | NA | NA | no | no |
| 2523 | 2017 | NA | Stewart, JE; Brooks, K; Brannen, PM; Cline, WO; Brewer, MT | 2015 | Elevated Genetic Diversity in the Emerging Blueberry Pathogen Exobasidium maculosum | Emerging diseases caused by fungi are increasing at an alarming rate. Exobasidium leaf and fruit spot of blueberry, caused by the fungus Exobasidium maculosum, is an emerging disease that has rapidly increased in prevalence throughout the southeastern USA, severely reducing fruit quality in some plantings. The objectives of this study were to determine the genetic diversity of E. maculosum in the southeastern USA to elucidate the basis of disease emergence and to investigate if populations of E. maculosum are structured by geography, host species, or tissue type. We sequenced three conserved loci from 82 isolates collected from leaves and fruit of rabbiteye blueberry (Vaccinium virgatum), highbush blueberry (V. corymbosum), and southern highbush blueberry (V. corymbosum hybrids) from commercial fields in Georgia and North Carolina, USA, and 6 isolates from lowbush blueberry (V. angustifolium) from Maine, USA, and Nova Scotia, Canada. Populations of E. maculosum from the southeastern USA and from lowbush blueberry in Maine and Nova Scotia are distinct, but do not represent unique species. No difference in genetic structure was detected between different host tissues or among different host species within the southeastern USA; however, differentiation was detected between populations in Georgia and North Carolina. Overall, E. maculosum showed extreme genetic diversity within the conserved loci with 286 segregating sites among the 1,775 sequenced nucleotides and each isolate representing a unique multilocus haplotype. However, 94% of the nucleotide substitutions were silent, so despite the high number of mutations, selective constraints have limited changes to the amino acid sequences of the housekeeping genes. Overall, these results suggest that the emergence of Exobasidium leaf and fruit spot is not due to a recent introduction or host shift, or the recent evolution of aggressive genotypes of E. maculosum, but more likely as a result of an increasing host population or an environmental change. | 2523 | NA | NA | NA | no | no |
| 2524 | 2017 | NA | Borucki, MK; Allen, JE; Chen-Harris, H; Zemla, A; Vanier, G; Mabery, S; Torres, C; Hullinger, P; Slezak, T | 2013 | The Role of Viral Population Diversity in Adaptation of Bovine Coronavirus to New Host Environments | “The high mutation rate of RNA viruses enables a diverse genetic population of viral genotypes to exist within a single infected host. In-host genetic diversity could better position the virus population to respond and adapt to a diverse array of selective pressures such as host-switching events. Multiple new coronaviruses, including SARS, have been identified in human samples just within the last ten years, demonstrating the potential of coronaviruses as emergent human pathogens. Deep sequencing was used to characterize genomic changes in coronavirus quasispecies during simulated host-switching. Three bovine nasal samples infected with bovine coronavirus were used to infect human and bovine macrophage and lung cell lines. The virus reproduced relatively well in macrophages, but the lung cell lines were not infected efficiently enough to allow passage of non lab-adapted samples. Approximately 12 kb of the genome was amplified before and after passage and sequenced at average coverages of nearly 950x(454 sequencing) and 38,000x(Illumina). The consensus sequence of many of the passaged samples had a 12 nucleotide insert in the consensus sequence of the spike gene, and multiple point mutations were associated with the presence of the insert. Deep sequencing revealed that the insert was present but very rare in the unpassaged samples and could quickly shift to dominate the population when placed in a different environment. The insert coded for three arginine residues, occurred in a region associated with fusion entry into host cells, and may allow infection of new cell types via heparin sulfate binding. Analysis of the deep sequencing data indicated that two distinct genotypes circulated at different frequency levels in each sample, and support the hypothesis that the mutations present in passaged strains were”“selected”" from a pre-existing pool rather than through de novo mutation and subsequent population fixation." | 2524 | NA | NA | NA | no | no |
| 2525 | 2017 | NA | Plath, M; Hermann, B; Schroder, C; Riesch, R; Tobler, M; de Leon, FJG; Schlupp, I; Tiedemann, R | 2010 | Locally adapted fish populations maintain small-scale genetic differentiation despite perturbation by a catastrophic flood event | Background: Local adaptation to divergent environmental conditions can promote population genetic differentiation even in the absence of geographic barriers and hence, lead to speciation. Perturbations by catastrophic events, however, can distort such parapatric ecological speciation processes. Here, we asked whether an exceptionally strong flood led to homogenization of gene pools among locally adapted populations of the Atlantic molly (Poecilia mexicana, Poeciliidae) in the Cueva del Azufre system in southern Mexico, where two strong environmental selection factors (darkness within caves and/or presence of toxic H(2)S in sulfidic springs) drive the diversification of P. mexicana. Nine nuclear microsatellites as well as heritable female life history traits (both as a proxy for quantitative genetics and for trait divergence) were used as markers to compare genetic differentiation, genetic diversity, and especially population mixing (immigration and emigration) before and after the flood. Results: Habitat type (i.e., non-sulfidic surface, sulfidic surface, or sulfidic cave), but not geographic distance was the major predictor of genetic differentiation. Before and after the flood, each habitat type harbored a genetically distinct population. Only a weak signal of individual dislocation among ecologically divergent habitat types was uncovered (with the exception of slightly increased dislocation from the Cueva del Azufre into the sulfidic creek, El Azufre). By contrast, several lines of evidence are indicative of increased flood-induced dislocation within the same habitat type, e.g., between different cave chambers of the Cueva del Azufre. Conclusions: The virtual absence of individual dislocation among ecologically different habitat types indicates strong natural selection against migrants. Thus, our current study exemplifies that ecological speciation in this and other systems, in which extreme environmental factors drive speciation, may be little affected by temporary perturbations, as adaptations to physico-chemical stressors may directly affect the survival probability in divergent habitat types. | 2525 | NA | NA | NA | no | no |
| 2526 | 2017 | NA | Owen, JP; Delany, ME; Mullens, BA | 2008 | MHC haplotype involvement in avian resistance to an ectoparasite | Research on immune function in evolutionary ecology has frequently focused on avian ectoparasites (e.g., mites and lice). However, host immunogenetics involved with bird resistance to ectoparasites has not been determined. The critical role of the major histocompatibility complex (MHC) in adaptive immunity and high genetic variation found within the MHC make this gene complex useful for exploring the immunogenetic basis for bird resistance to ectoparasites. The objective of this study was to determine if the avian MHC influenced resistance to a blood-feeding ectoparasite. Four congenic lines of chickens, differing only at the MHC, were comparatively infested with a cosmopolitan ectoparasite of birds-northern fowl mite (NFM)-which is also a serious pest species of poultry. Mite infestations were monitored over time and mite densities (weekly and maximum) were compared among lines. Chickens with the MHC haplotype B21 were relatively resistant to NFM, compared with birds in the B15 congenic line (P < 0.02). To test for similar effects in an outbred genetic background, a separate experiment was performed with 107 commercial chickens (white leghorn, W-36 strain) infested with NFM. Hens were genotyped using a MHC microsatellite marker (LEI0258) and associations between MHC haplotype and NFM density were tested. The highest peak NFM populations occurred more often on hens with the B15 haplotype versus the B21 haplotype (P=0.012), which supported the results of the congenic study. These data indicate the avian MHC influences ectoparasite resistance, which is relevant to disease ecology and avian-ectoparasite interaction. | 2526 | NA | NA | NA | no | no |
| 2527 | 2017 | NA | Sampayo, EM; Ridgway, T; Bongaerts, P; Hoegh-Guldberg, O | 2008 | Bleaching susceptibility and mortality of corals are determined by fine-scale differences in symbiont type | Coral bleaching has been identified as one of the major contributors to coral reef decline, and the occurrence of different symbionts determined by broad genetic groupings (clades A-H) is commonly used to explain thermal responses of reef-building corals. By using Stylophora pistillata as a model, we monitored individual tagged colonies in situ over a two-year period and show that fine level genetic variability within clade C is correlated to differences in bleaching susceptibility. Based on denaturing gradient gel electrophoresis of the internal transcribed spacer region 2, visual bleaching assessments, symbiont densities, host protein, and pulse amplitude modulated fluorometry, we show that subcladal types C78 and C8/a are more thermally tolerant than C79 and C35/a, which suffered significant bleaching and postbleaching mortality. Although additional symbiont types were detected during bleaching in colonies harboring types C79 and C35/a, all colonies reverted back to their original symbionts postbleaching. Most importantly, the data propose that the differential mortality of hosts harboring thermally sensitive versus resistant symbionts rather than symbiont shuffling/switching within a single host is responsible for the observed symbiont composition changes of coral communities after bleaching. This study therefore highlights that the use of broad cladal designations may not be suitable to describe differences in bleaching susceptibility, and that differential mortality results in a loss of both symbiont and host genetic diversity and therefore represents an important mechanism in explaining how coral reef communities may respond to changing conditions. | 2527 | NA | NA | NA | no | no |
| 2528 | 2017 | NA | Dean, C; Fogleman, AJ; Zahnd, WE; Lipka, AE; Malhi, RS; Delfino, KR; Jenkins, WD | 2017 | Engaging rural communities in genetic research: challenges and opportunities | Statistical analyses of health and disease in rural communities is frequently limited by low sample counts. Still, some studies indicate increased risk for some diseases even after adjustment for known risk factors. It has been hypothesized that the context of community formation in rural areas facilitates the propagation of genetic founder effects-potentially impacting disease susceptibility. However, outright examination of genetic diversity in such communities has not been performed. Our objective was to engage otherwise research-inexperienced rural communities of largely European descent in genomic research in the context of cancer susceptibility. From September 2015 to February 2016, we implemented a systematic process of progressive community engagement. This iterative method sought project buy-in from first the town mayor, then village council. If approved by both, a focus group of community members examined how residents might view the research, informed consent and specimen collection, and issues of privacy. We were successful in engaging three of the four communities approached for the research project. There was universal enthusiasm for the project by all mayors and village councils. The focus groups’ main point of discussion involved wording in the informed consent, with little concern regarding the research question or privacy. Perhaps contrary to popular thought, we found each community we approached to be both welcoming and enthusiastic about collaborating in research on genomic diversity. The systematic method of engagement did much to preserve community respect and autonomy and facilitated buy-in. | 2528 | NA | NA | NA | no | no |
| 2529 | 2017 | NA | Trewick, SA | 2001 | Identity of an endangered grasshopper (Acrididae: Brachaspis): Taxonomy, molecules and conservation | Brachaspis robustus is an endangered grasshopper endemic to South Island, New Zealand. It is both rare and localised; occupying low altitude floodplain terraces and braided riverbeds of the Mackenzie Basin. This is in stark contrast to the two other species in this genus (B. nivalis and B. collinus) which occupy montane habitats. Mitochondrial and nuclear sequence data were employed to explore genetic diversity and phylogenetic relationships of populations of Brachaspis with a view to establishing the status of B. robustus. Molecular evidence indicates that Brachaspis probably radiated during the Pliocene and that divisions within the genus relate more to spatial distribution developed during the Pleistocene than to ecology. The mitochondrial (Cytochrome oxidase I) and nuclear (ITS) sequence data indicate that Brachaspis nivalis is divided into northern and southern populations. The northern clade is further subdivided geographically. The southern clade comprises alpine populations of B. nivalis and includes the lowland B. robustus. Additionally, it is observed that some morphological features previously thought to be specific to B. robustus also occur in members of the southern B. nivalis clade. It is suggested that the taxon B. robustus should include all of the southern Brachaspis populations. But it is argued that the absence of genetic evidence distinguishing the endangered population does not preclude it from conservation effort. A combination of morphological and habitat peculiarities indicate that the survival of B. robustus (sensu lato) is important to the maintenance of diversity. | 2529 | NA | NA | NA | no | no |
| 2530 | 2017 | NA | Lambin, X; Yoccoz, NG | 1998 | The impact of population kin-structure on nestling survival in Townsend’s voles, Microtus townsendii | 1. In many small rodent species, females are philopatric and may reproduce in proximity to their female relatives. The prevalence of clusters of relatives in populations reflects previous patterns of immigration, local recruitment and mortality. Kin-structuring among females will be most prevalent in populations which have experienced successful reproduction and philopatric recruitment. If population kin-structure influenced key demographic parameters this would introduce a potential time-delay in the regulation of population size by social factors. 2. We tested the influence of population kin structure among female Townsend’s voles on nestling survival, using data from a replicated experiment with two non-enclosed populations. Most nestlings were marked before weaning, so that we knew which recruits were related to one another and to which adult female. The average size of matrilineal groups was experimentally manipulated by removing immigrants, and preventing predation by birds, on the high kinship treatments (Kin), and by removing members of family groups on the low kinship treatments (Non-kin). We compared preweaning survival of pups marked in the maternal nest between high and low kinship treatments using the recent generalisations of the Cormack-Jolly-Seber statistical models as implemented in the software SURGE. 3. The manipulation was effective in that the number of breeding females with relatives among the six nearest breeding female neighbour was higher on the high-kinship treatment grids than on the low-kinship treatment grids. Most first degree related females were nearest neighbours and the prevalence of any relatedness relationship among the six nearest neighbours declined with neighbourhood class. The degree of matrilineal relatedness influenced the distance between the nests (and pups) of breeding females; first degree relatives and second degree relatives were, respectively, 1.32 and 1.19 times closer to each other than unrelated females. Unrelated females and more distant relatives had similar internest distances. The relationship between matrilineal relatedness and distance was similar for the six nearest breeding female neighbours and was not influenced by year, season or treatment. 4. The parsimonious capture-recapture models selected included a gradual increase of trappability during the first 5-7 weeks of life and three age-specific survival rates. Survival over the first 2 weeks of life was estimated as a single parameter and was relatively low. Weekly survival probabilities were very high (0.96, SE = 0.012) from week 3-5 and decreased later in life (week 6-15, 0.85, SE = 0.012) possibly as a result of emigration. 5. The experimental manipulation of female kin-structure had a significant influence on the weekly survival probabilities of nestlings during their first 2 weeks of life. Pup weekly survival was higher on the high-kinship treatment (0.76, SE = 0.018) than on the low-kinship treatment (0.67, SE = 0.020). 6. Pairs of first degree relatives among the six nearest neighbours were present, although in different proportion, within both treatments and we asked whether this within-treatment variability in relatedness was reflected in within-treatment variability in early age survival. Estimates of pup survival for the three replicates with the largest sample size suggest that this was the case in 1990 (on both treatments) but not in 1991 (high kinship treatment). This suggests that between-treatment differences probably reflect differences in the prevalence of related and unrelated females rather than the presence of immigrant females on either treatments. 7. A simple age-structured demographic model, using values of juvenile and adult survival measured for populations with high and low kinship, indicate that this factor may have a substantial impact on the rate of growth of microtine populations (predicted monthly multiplication rate lambda = 1.48 vs. 1.72 for low and high kinship, respectively). Thus, the impact of kinship on demographic parameters may introduce powerful time lags in the social regulation on vole populations. | 2530 | NA | NA | NA | no | no |
| 2531 | 2017 | NA | Brede, E | 2015 | Beam Brook revisited: a molecular study of a historically introduced non-native amphibian (Triturus carnifex) and its limited introgression into native UK Triturus cristatus populations | Human mediated introductions of non-native species can pose a major threat to global biodiversity on several accounts i.e. through competition, the introduction of novel pathogens, and genetic pollution. Where hybridization occurs between two closely related species the F1 offspring are usually phenotypically discernible whereas F2 hybrid, backcrossed or admixed individuals become more difficult to separate. At this point the utilization of molecular methods is required in conservation efforts to differentiate and manage populations. This study demonstrated how a possible threat of hybridization from an introduced non-native (T. carnifex) with a protected native newt species (T. cristatus) could be investigated with molecular tools, and examined the current extent of its genetic introgression over an 80 years period. The results confirmed that hybridization had taken place at the site of introduction (and continues to do so), and that historically limited local dispersal of both non-natives and/or hybrids had occurred sometime in the past. However, the data suggests that although dispersal of hybrids into a local satellite site may still be occuring, hybridization with native species appears limited. | 2531 | NA | NA | NA | no | no |
| 2532 | 2017 | NA | He, CQ; Meng, SL; Yan, HY; Ding, NZ; He, HB; Yan, JX; Xu, GL | 2012 | Isolation and Identification of a Novel Rabies Virus Lineage in China with Natural Recombinant Nucleoprotein Gene | Rabies virus (RABV) causes severe neurological disease and death. As an important mechanism for generating genetic diversity in viruses, homologous recombination can lead to the emergence of novel virus strains with increased virulence and changed host tropism. However, it is still unclear whether recombination plays a role in the evolution of RABV. In this study, we isolated and sequenced four circulating RABV strains in China. Phylogenetic analyses identified a novel lineage of hybrid origin that comprises two different strains, J and CQ92. Analyses revealed that the virus 39 untranslated region (UTR) and part of the N gene (approximate 500 nt in length) were likely derived from Chinese lineage I while the other part of the genomic sequence was homologous to Chinese lineage II. Our findings reveal that homologous recombination can occur naturally in the field and shape the genetic structure of RABV populations. Citation: He C-Q, Meng S-L, Yan H-Y, Ding N-Z, He H-B, et al. (2012) Isolation and Identification of a Novel Rabies Virus Lineage in China with Natural Recombinant Nucleoprotein Gene. PLoS ONE 7(12): e49992. doi: 10.1371/journal.pone.0049992 | 2532 | NA | NA | NA | no | no |
| 2533 | 2017 | NA | Schonswetter, P; Paun, O; Tribsch, A; Niklfeld, H | 2003 | Out of the Alps: Colonization of Northern Europe by East Alpine populations of the Glacier Buttercup Ranunculus glacialis L. (Ranunculaceae) | Ranunculus glacialis ssp. glacialis is an arctic-alpine plant growing in central and southern European and Scandinavian mountain ranges and the European Arctic. In order to elucidate the taxon’s migration history, we applied amplified fragment length polymorphism (AFLP) to populations from the Pyrenees, Tatra mountains and Northern Europe and included data from a previous study on Alpine accessions. Populations from the Alps and the Tatra mountains were genetically highly divergent and harboured many private AFLP fragments, indicating old vicariance. Whereas nearly all Alpine populations of R. glacialis were genetically highly variable, the Tatrean population showed only little variation. Our data suggest that the Pyrenees were colonized more recently than the separation of the Tatra from the Alps. Populations in Northern Europe, by contrast, were similar to those of the Eastern Alps but showed only little genetic variation. They harboured no private AFLP fragments and only a subset of East Alpine ones, and they exhibited no phylogeographical structure. It is very likely therefore that R. glacialis colonized Northern Europe in postglacial times from source populations in the Eastern Alps. | 2533 | NA | NA | NA | no | no |
| 2534 | 2017 | NA | Khan, A; Taylor, S; Ajioka, JW; Rosenthal, BM; Sibley, LD | 2009 | Selection at a Single Locus Leads to Widespread Expansion of Toxoplasma gondii Lineages That Are Virulent in Mice | Pathogenicity differences among laboratory isolates of the dominant clonal North American and European lineages of Toxoplasma gondii are largely controlled by polymorphisms and expression differences in rhoptry secretory proteins (ROPs). However, the extent to which such differences control virulence in natural isolates of T. gondii, including those from more diverse genetic backgrounds, is uncertain. We elucidated the evolutionary history and functional consequences of diversification in the serine/threonine kinase ROP18, a major virulence determinant in the mouse model. We characterized the extent of sequence polymorphism and the evolutionary forces acting on ROP18 and several antigen-encoding genes within a large collection of natural isolates, comparing them to housekeeping genes and introns. Surprisingly, despite substantial genetic diversity between lineages, we identified just three principal alleles of ROP18, which had very ancient ancestry compared to other sampled loci. Expression and allelic differences between these three alleles of ROP18 accounted for much of the variation in acute mouse virulence among natural isolates. While the avirulent type III allele was the most ancient, intermediate virulent (type II) and highly virulent (type I) lineages predominated and showed evidence of strong selective pressure. Out-group comparison indicated that historical loss of an upstream regulatory element increased ROP18 expression, exposing it to newfound diversifying selection, resulting in greatly enhanced virulence in the mouse model and expansion of new lineages. Population sweeps are evident in many genomes, yet their causes and evolutionary histories are rarely known. Our results establish that up-regulation of expression and selection at ROP18 in T. gondii has resulted in three distinct alleles with widely different levels of acute virulence in the mouse model. Preservation of all three alleles in the wild indicates they are likely adaptations for different niches. Our findings demonstrate that sweeping changes in population structure can result from alterations in a single gene. | 2534 | NA | NA | NA | no | no |
| 2535 | 2017 | NA | Donnelly, MJ; Pinto, J; Girod, R; Besansky, NJ; Lehmann, T | 2004 | Revisiting the role of introgression vs shared ancestral polymorphisms as key processes shaping genetic diversity in the recently separated sibling species of the Anopheles gambiae complex | The role of interspecific hybridisation in the evolution of pest species is poorly understood. In mosquito disease vectors this is of particular importance due to the evolution of insecticide resistance and the proposed release of transgenic strains that are refractory to the malaria parasite. In this study, we apply population genetic methods in a novel manner to determine whether mitochondrial DNA sequences have introgressed between the closely related African malaria vectors Anopheles gambiae and A. arabiensis. Our results suggest that speciation was geologically recent and ancestral haplotypes at the ND5 locus are retained in both species. In addition, comparing haplotype frequencies in allopatric and sympatric populations, suggest locale specific unidirectional introgression of mitochondria from A. arabiensis into A. gambiae. | 2535 | NA | NA | NA | no | no |
| 2536 | 2017 | NA | Leppanen, J; Vepsalainen, K; Savolainen, R | 2011 | Phylogeography of the ant Myrmica rubra and its inquiline social parasite | Widely distributed Palearctic insects are ideal to study phylogeographic patterns owing to their high potential to survive in many Pleistocene refugia and-after the glaciation-to recolonize vast, continuous areas. Nevertheless, such species have received little phylogeographic attention. Here, we investigated the Pleistocene refugia and subsequent postglacial colonization of the common, abundant, and widely distributed ant Myrmica rubra over most of its Palearctic area, using mitochondrial DNA (mtDNA). The western and eastern populations of M. rubra belonged predominantly to separate haplogroups, which formed a broad secondary contact zone in Central Europe. The distribution of genetic diversity and haplogroups implied that M. rubra survived the last glaciation in multiple refugia located over an extensive area from Iberia in the west to Siberia in the east, and colonized its present areas of distribution along several routes. The matrilineal genetic structure of M. rubra was probably formed during the last glaciation and subsequent postglacial expansion. Additionally, because M. rubra has two queen morphs, the obligately socially parasitic microgyne and its macrogyne host, we tested the suggested speciation of the parasite. Locally, the parasite and host usually belonged to the same haplogroup but differed in haplotype frequencies. This indicates that genetic differentiation between the morphs is a universal pattern and thus incipient, sympatric speciation of the parasite from its host is possible. If speciation is taking place, however, it is not yet visible as lineage sorting of the mtDNA between the morphs. | 2536 | NA | NA | NA | no | no |
| 2537 | 2017 | NA | Etemadi, MR; Sekawi, Z; Othman, N; Lye, MS; Moghaddam, FY | 2013 | Circulation of Human Respiratory Syncytial Virus Strains Among Hospitalized Children with Acute Lower Respiratory Infection in Malaysia | Human respiratory syncytial virus (RSV) is a major viral pathogen associated with acute lower respiratory tract infections (ALRTIs) among hospitalized children. In this study, the genetic diversity of the RSV strains was investigated among nasopharyngeal aspirates (NPA) taken from children less than 5 years of age hospitalized with ALRTIs in Hospital Serdang, Malaysia. A total of 165 NPA samples were tested for the presence of RSV and other respiratory viruses from June until December 2009. RSV was found positive in 83 (50%) of the samples using reverse transcription polymerase chain reaction (RT-PCR). Further classification of 67 RSV strains showed that subgroups A and B comprised 11/67 (16.4%) and 56/67 (83.6%) of the strains, respectively. The second hypervariable region at the carboxyl-terminal of the G gene was amplified and sequenced in order to do phylogenetic study. The phylogenetic relationships of the samples were determined separately for subgroups A and B using neighbor joining (NJ), maximum parsimony (MP), and Bayesian inference (BI). Phylogenetic analysis of the 32 sequenced samples showed that all 9 RSV-A strains were clustered within NA1 genotype while the remaining 23 strains of the RSV-B subgroup could be grouped into a clade consisted of strains with 60-nucleotide duplication region. They were further classified into newly discovered BA10 and BA9 genotypes. The present finding suggests the emergence of RSV genotypes of NA1 and BA. This is the first documentation of the phylogenetic relationship and genetic diversity of RSV strains among hospitalized children diagnosed with ALRTI in Serdang, Malaysia. | 2537 | NA | NA | NA | no | no |
| 2538 | 2017 | NA | McLoughlin, PD; Paetkau, D; Duda, M; Boutin, S | 2004 | Genetic diversity and relatedness of boreal caribou populations in western Canada | We studied genetic diversity within and gene flow among six ‘threatened’ populations of boreal caribou (Rangifer tarandus caribou) inhabiting Alberta and British Columbia, Canada. Mean expected heterozygosity (HE) across I I loci spanned a narrow range between 0.74 and 0.79. Estimates of HE were in the mid to high range of those typically observed in natural populations of large mammals, including caribou, and were not suggestive of any immediate threat to survival. We concluded that recent anthropogenic fragmentation of caribou range in western Canada has yet to affect genetic diversity of populations. Analysis of population structure identified a region of relatively low gene flow corresponding with the valley of the Peace River. The highest value of F-ST observed between populations on the same side of the river was 0.025, whereas the lowest value that spanned the river was 0.044. Confirming this result, an assignment test demonstrated that 96.5% of animals could be assigned to the correct side of the Peace River, though only 66.0% of animals could, on average, be assigned to populations of actual origin. Taken as a whole, our results support the existence of two discrete metapopulations bisected by the Peace River, within each of which there exist multiple populations, or at least multiple regions, which experience considerably higher levels of interchange. For caribou inhabiting the boreal plains, large rivers such as the Peace and Mackenzie may serve as biologically meaningful boundaries for managing metapopulations. (C) 2003 Elsevier Ltd. All rights reserved. | 2538 | NA | NA | NA | no | no |
| 2539 | 2017 | NA | Mallez, S; Castagnone, C; Espada, M; Vieira, P; Eisenback, JD; Harrell, M; Mota, M; Aikawa, T; Akiba, M; Kosaka, H; Castagnone-Sereno, P; Guillemaud, T | 2015 | Worldwide invasion routes of the pinewood nematode: What can we infer from population genetics analyses? | Identifying the invasion routes and determining the origin of new outbreaks of invasive species are of crucial importance if we are to understand the invasion process, improve or establish regulatory measures and, potentially, limit the damage. We focused here on the invasion of Europe by the pinewood nematode (PWN), Bursaphelenchus xylophilus (Steiner & Buhrer, 1934; Nickle 1970; Nematoda: Aphelenchoididae), a major pest of forest ecosystems, native to North America and already invasive in Asia since the beginning of the twentieth century. We evaluated the genetic diversity and structure of worldwide field PWN samples by classical and Bayesian population genetics methods to determine the source of the European invasive populations and the number of introduction events in Europe. We found (1) a very strong spatial genetic structure in native PWN populations, (2) a very low level of polymorphism in each of the invaded areas and (3) contrasted results concerning the origin of European invasive populations. Our findings provide evidence for: (1) a large effect of genetic drift on the biological cycle of the PWN, due to intense demographic bottlenecks during tree infections, not compensated for by effective dispersal of its vector; (2) a single introduction event for each of the invaded areas in Japan and Europe and a small effective size for the introduced populations and (3) a mainland Portuguese origin for PWN populations from Madeira. However, more sophisticated methods of invasion route inference and broader sampling are required to conclusively determine the origin of the European outbreak. | 2539 | NA | NA | NA | no | no |
| 2540 | 2017 | NA | Morin, L; Gomez, DR; Evans, KJ; Neill, TM; Mahaffee, WF; Linde, CC | 2013 | Invaded range of the blackberry pathogen Phragmidium violaceum in the Pacific Northwest of the USA and the search for its provenance | Field surveys in 2006 confirmed that the exotic rust fungus Phragmidium violaceum was widespread on Rubus armeniacus and Rubus laciniatus in the Pacific Northwest of the USA. The origin and dispersal pattern of this obligate biotrophic pathogen in the USA were investigated by comparing the genetic diversity and structure of 27 isolates each from the USA and Europe, and 20 isolates from Australia where an invasion occurred in 1984. Analysis of 11 microsatellite loci revealed 74 unique genotypes, with the European population having a significantly higher level of allelic diversity and number of private alleles compared to populations from the USA and Australia. Principal coordinate analysis (PCA), analysis of molecular variance and pairwise comparisons of I broken vertical bar confirmed a strong level of differentiation among continental populations, with little divergence between isolates from the USA and Europe, but a high level of differentiation between these isolates and those from Australia. These results were broadly supported by the Bayesian cluster analysis, which indicated that at K = 3 the clustering of the isolates corresponds to their geographic origin. Bayesian clustering, PCA as well as insignificant migration estimates from Europe to the USA suggest that the USA population is not a direct descendant from the European P. violaceum population. There was a weak association between genetic and geographic distance among the USA isolates, suggesting invasion was initially localized prior to dispersal or that the population may have been present for some time prior to first detection in 2005. | 2540 | NA | NA | NA | no | no |
| 2541 | 2017 | NA | Lu, ZX; Jiang, P; Cai, JJ; Xing, Y | 2011 | Context-dependent robustness to 5 ’ splice site polymorphisms in human populations | There has been growing evidence for extensive diversity of alternative splicing in human populations. Genetic variants within the 5’ splice site can cause splicing differences among human individuals and constitute an important class of human disease mutations. In this study, we explored whether natural variations of splicing could reveal important signals of 5’ splice site recognition. In seven lymphoblastoid cell lines of Asian, European and African ancestry, we identified 1174 single nucleotide polymorphisms (SNPs) within the consensus 5’ splice site. We selected 129 SNPs predicted to significantly alter the splice site activity, and quantitatively examined their splicing impact in the seven individuals. Surprisingly, outside of the essential GT dinucleotide position, only similar to 14% of the tested SNPs altered splicing. Bioinformatic and minigene analyses identified signals that could modify the impact of 5’ splice site polymorphisms, most notably a strong 3’ splice site and the presence of intronicmotifs downstream of the 5’ splice site. Strikingly, we found that the poly-G run, a known intronic splicing enhancer, was the most significantly enriched motif downstream of exons unaffected by 5’ splice site SNPs. In TRIM62, theupstream 3’ splice site and downstream intronic poly-Gruns functioned redundantly to protect an exon from its 5’ splice site polymorphism. Collectively, our study reveals widespread context-dependent robustness to 5’ splice site polymorphisms in human transcriptomes. Consequently, certain exons are more susceptible to 5’ splice site mutations. Additionally, our work demonstrates that genetic diversity of alternative splicing can provide significant insights into the splicing code of mammalian cells. | 2541 | NA | NA | NA | no | no |
| 2542 | 2017 | NA | Wares, JP; Gaines, SD; Cunningham, CW | 2001 | A comparative study of asymmetric migration events across a marine biogeographic boundary | In many nonclonal, benthic marine species, geographic distribution is mediated by the dispersal of their larvae. The dispersal and recruitment of marine larvae may be limited by temperature gradients that can affect mortality or by ocean currents that can directly affect the movements of pelagic larvae. We focus on Point Conception, a well-known biogeographic boundary between the Californian and Oregonian biogeographic provinces, to investigate whether ocean currents affect patterns of gene flow in intertidal marine invertebrates. The predominance of pelagically dispersing species with northern range limits at Point Conception suggests that ocean currents can affect species distributions by erecting barriers to the dispersal of planktonic larvae. In this paper, we investigate whether the predominantly southward currents have left a recognizable genetic signature in species with pelagically dispersing larvae whose ranges span Point Conception. We use patterns of genetic diversity and a new method for inferring cladistic migration events to test the hypothesis that southward currents increase southward gene flow for species with pelagically dispersing larvae. We collected mitochondrial DNA (mtDNA) sequence data for the barnacles Balanus glandula and Chthamalus fissus and also reanalyzed a previously published mtDNA dataset (Strongylocentrotus purpuratus, Edmands et al. 1996). For all three species, our cladistic approach identified an excess of southward migration events across Point Conception. In data from a fourth species with nondispersing larvae (Nucella emarginata, Marko 1998), our method suggests that ocean currents have not played a role in generating genetic structure. | 2542 | NA | NA | NA | no | no |
| 2543 | 2017 | NA | Feldheim, KA; Chauke, LF; Hopkins, KP; Tolley, KA | 2010 | Characterization of microsatellite loci from a South African endemic, the Cape Dwarf Chameleon (Bradypodion pumilum) | The Cape Dwarf Chameleon (Bradypodion pumilum) is an endemic South African species that is currently threatened by habitat loss and fragmentation of its natural habitat through urbanization and agriculture. To conduct studies that will assist in understanding these anthropogenic effects on gene flow, population structure, and genetic diversity, we developed eight microsatellite loci using an enrichment protocol. Number of alleles ranged from 5 to 26 with observed heterozygosities of 0.279-0.930. Several loci did not meet HW expectations, but this may be a result of extreme demographic fluctuations that have been noted for this species. These loci will prove useful to examine the population genetics of these threatened reptiles and for providing information that will be used for generating and updating conservation assessments. | 2543 | NA | NA | NA | no | no |
| 2544 | 2017 | NA | Koepke, T; Schaeffer, S; Krishnan, V; Jiwan, D; Harper, A; Whiting, M; Oraguzie, N; Dhingra, A | 2012 | Rapid gene-based SNP and haplotype marker development in non-model eukaryotes using 3 ’ UTR sequencing | Background: Sweet cherry (Prunus avium L.), a non-model crop with narrow genetic diversity, is an important member of sub-family Amygdoloideae within Rosaceae. Compared to other important members like peach and apple, sweet cherry lacks in genetic and genomic information, impeding understanding of important biological processes and development of efficient breeding approaches. Availability of single nucleotide polymorphism (SNP)-based molecular markers can greatly benefit breeding efforts in such non-model species. RNA-seq approaches employing second generation sequencing platforms offer a unique avenue to rapidly identify gene-based SNPs. Additionally, haplotype markers can be rapidly generated from transcript-based SNPs since they have been found to be extremely utile in identification of genetic variants related to health, disease and response to environment as highlighted by the human HapMap project. Results: RNA-seq was performed on two sweet cherry cultivars, Bing and Rainier using a 3’ untranslated region (UTR) sequencing method yielding 43,396 assembled contigs. In order to test our approach of rapid identification of SNPs without any reference genome information, over 25% (10,100) of the contigs were screened for the SNPs. A total of 207 contigs from this set were identified to contain high quality SNPs. A set of 223 primer pairs were designed to amplify SNP containing regions from these contigs and high resolution melting (HRM) analysis was performed with eight important parental sweet cherry cultivars. Six of the parent cultivars were distantly related to Bing and Rainier, the cultivars used for initial SNP discovery. Further, HRM analysis was also performed on 13 seedlings derived from a cross between two of the parents. Our analysis resulted in the identification of 84 (38.7%) primer sets that demonstrated variation among the tested germplasm. Reassembly of the raw 3’ UTR sequences using upgraded transcriptome assembly software yielded 34,620 contigs containing 2243 putative SNPs in 887 contigs after stringent filtering. Contigs with multiple SNPs were visually parsed to identify 685 putative haplotypes at 335 loci in 301 contigs. Conclusions: This approach, which leverages the advantages of RNA-seq approaches, enabled rapid generation of gene-linked SNP and haplotype markers. The general approach presented in this study can be easily applied to other non-model eukaryotes irrespective of the ploidy level to identify gene-linked polymorphisms that are expected to facilitate efficient Gene Assisted Breeding (GAB), genotyping and population genetics studies. The identified SNP haplotypes reveal some of the allelic differences in the two sweet cherry cultivars analyzed. The identification of these SNP and haplotype markers is expected to significantly improve the genomic resources for sweet cherry and facilitate efficient GAB in this non-model crop. | 2544 | NA | NA | NA | no | no |
| 2545 | 2017 | NA | Teeuw, ME; Henneman, L; Bochdanovits, Z; Heutink, P; Kuik, DJ; Cornel, MC; ten Kate, LP | 2010 | Do consanguineous parents of a child affected by an autosomal recessive disease have more DNA identical-by-descent than similarly-related parents with healthy offspring? Design of a case-control study | Background: The offspring of consanguineous relations have an increased risk of congenital/genetic disorders and early mortality. Consanguineous couples and their offspring account for approximately 10% of the global population. The increased risk for congenital/genetic disorders is most marked for autosomal recessive disorders and depends on the degree of relatedness of the parents. For children of first cousins the increased risk is 2-4%. For individual couples, however, the extra risk can vary from zero to 25% or higher, with only a minority of these couples having an increased risk of at least 25%. It is currently not possible to differentiate between high-and low-risk couples. The quantity of DNA identical-by-descent between couples with the same degree of relatedness shows a remarkable variation. Here we hypothesize that consanguineous partners with children affected by an autosomal recessive disease have more DNA identical-by-descent than similarly-related partners who have only healthy children. The aim of the study is thus to establish whether the amount of DNA identical-by-descent in consanguineous parents of children with an autosomal recessive disease is indeed different from its proportion in consanguineous parents who have healthy children only. Methods/Design: This project is designed as a case-control study. Cases are defined as consanguineous couples with one or more children with an autosomal recessive disorder and controls as consanguineous couples with at least three healthy children and no affected child. We aim to include 100 case couples and 100 control couples. Control couples are matched by restricting the search to the same family, clan or ethnic origin as the case couple. Genome-wide SNP arrays will be used to test our hypothesis. Discussion: This study contains a new approach to risk assessment in consanguineous couples. There is no previous study on the amount of DNA identical-by-descent in consanguineous parents of affected children compared to the consanguineous parents of healthy children. If our hypothesis proves to be correct, further studies are needed to obtain different risk figure estimates for the different proportions of DNA identical-by-descent. With more precise information about their risk status, empowerment of couples can be improved when making reproductive decisions. | 2545 | NA | NA | NA | no | no |
| 2546 | 2017 | NA | Bjorklund, H; Lowings, P; Stadejek, T; Vilcek, S; Greiser-Wilke, I; Paton, D; Belak, S | 1999 | Phylogenetic comparison and molecular epidemiology of classical swine fever virus | The genetic diversity of classical swine fever virus (CSFV) was studied by RT-PCR amplification and sequencing of a 409 bp fragment of the NS5B polymerase region. A total of 106 viruses isolated from 20 countries over a period of 52 years (1945-1997) were included in the phylogenetic study. The results showed that the viruses could be divided into two main groups. Group 1 consisted of Asian and South American isolates from the 1980s, as well as of old European and American isolates. Group 2 consisted mostly of recent European viruses from the 1980s and 1990s, and was further divided into three subgroups largely according to geographic origin and/or year of isolation. Five 1997 CSFV isolates from Germany, Netherlands and Italy clustered together indicating a common origin for these outbreaks, but two other 1997 isolations in different regions of Germany are likely due to different epidemiological events. The results show that the NS5B region of the genome gives a good resolution for phylogenetic studies of CSFV. Molecular epidemiology based on nucleotide sequence diversity is a useful tool for tracing virus spread and for developing disease control strategies. | 2546 | NA | NA | NA | no | no |
| 2547 | 2017 | NA | Kwan, T; Liu, J; DuBow, M; Gros, P; Pelletier, J | 2005 | The complete genomes and proteomes of 27 Staphylococcus aureus bacteriophages | Bacteriophages are the most abundant life forms in the biosphere. They play important roles in bacterial ecology, evolution, adaptation to new environments, and pathogenesis of human bacterial infections. Here, we report the complete genomic sequences, and predicted proteins of 27 bacteriophages of the Gram-positive bacterium Staphylococcus aureus. Comparative nucleotide and protein sequence analysis indicates that these phages are a remarkable source of untapped genetic diversity, encoding 2,170 predicted protein-encoding ORFs, of which 1,402 cannot be annotated for structure or function, and 522 are proteins with no similarity to other phage or bacterial sequences. Based on their genome size, organization of their gene map and comparative nucleotide and protein sequence analysis, the S. aureus phages can be organized into three groups. Comparison of their gene maps reveals extensive genome mosaicism, hinting to a large reservoir of unidentified S. aureus phage genes. Among the phages in the largest size class (178-214 kbp) that we characterized is phage Twort, the first discovered bacteriophage (responsible for the Twort-D’Herelle effect). These phage genomes offer an exciting opportunity to discern molecular mechanisms of phage evolution and diversity. | 2547 | NA | NA | NA | no | no |
| 2548 | 2017 | NA | Sarker, S; Patterson, EI; Peters, A; Baker, GB; Forwood, JK; Ghorashi, SA; Holdsworth, M; Baker, R; Murray, N; Raidal, SR | 2014 | Mutability Dynamics of an Emergent Single Stranded DNA Virus in a Naive Host | Quasispecies variants and recombination were studied longitudinally in an emergent outbreak of beak and feather disease virus (BFDV) infection in the orange-bellied parrot (Neophema chrysogaster). Detailed health monitoring and the small population size (<300 individuals) of this critically endangered bird provided an opportunity to longitudinally track viral replication and mutation events occurring in a circular, single-stranded DNA virus over a period of four years within a novel bottleneck population. Optimized PCR was used with different combinations of primers, primer walking, direct amplicon sequencing and sequencing of cloned amplicons to analyze BFDV genome variants. Analysis of complete viral genomes (n = 16) and Rep gene sequences (n = 35) revealed that the outbreak was associated with mutations in functionally important regions of the normally conserved Rep gene and immunogenic capsid (Cap) gene with a high evolutionary rate (3.41610 23 subs/site/year) approaching that for RNA viruses; simultaneously we observed significant evidence of recombination hotspots between two distinct progenitor genotypes within orange-bellied parrots indicating early cross-transmission of BFDV in the population. Multiple quasispecies variants were also demonstrated with at least 13 genotypic variants identified in four different individual birds, with one containing up to seven genetic variants. Preferential PCR amplification of variants was also detected. Our findings suggest that the high degree of genetic variation within the BFDV species as a whole is reflected in evolutionary dynamics within individually infected birds as quasispecies variation, particularly when BFDV jumps from one host species to another. | 2548 | NA | NA | NA | no | no |
| 2549 | 2017 | NA | Glaberman, S; Caccone, A | 2008 | Species-specific evolution of class I MHC genes in iguanas (Order : Squamata; subfamily : Iguaninae) | Over the last few decades, the major histocompatibility complex (MHC) has emerged as a model for understanding the influence of natural selection on genetic diversity in populations as well as for investigating the genetic basis of host resistance to pathogens. However, many vertebrate taxa remain underrepresented in the field of MHC research, preventing its application to studies of disease, evolution, and conservation genetics in these groups. This is particularly true for squamates, which are by far the most diversified order of non-avian reptiles but have not been the subject of any recent MHC studies. In this paper, we present MHC class I complementary DNA data from three squamate species in the subfamily Iguaninae (iguanas): the Galapagos marine iguana (Amblyrhynchus cristatus), the Galapagos land iguana (Conolophus subcristatus), and the green iguana (Iguana iguana). All sequences obtained are related to the few published class I genes from other squamates. There is evidence for multiple loci in each species, and the conserved alpha-3 domain appears to be evolving in a species-specific manner. Conversely, there is some indication of shared polymorphism between species in the peptide-binding alpha-1 and alpha-2 domains, suggesting that these two regions have different phylogenetic histories. The great similarity between alpha-3 sequences in marine iguanas in particular suggests that concerted evolution is acting to homogenize class I loci within species. However, while less likely, the data are also compatible with a birth and death model of evolution. | 2549 | NA | NA | NA | no | no |
| 2550 | 2017 | NA | Loire, E; Chiari, Y; Bernard, A; Cahais, V; Romiguier, J; Nabholz, B; Lourenco, JM; Galtier, N | 2013 | Population genomics of the endangered giant Galapagos tortoise | Background: The giant Galapagos tortoise, Chelonoidis nigra, is a large-sized terrestrial chelonian of high patrimonial interest. The species recently colonized a small continental archipelago, the Galapagos Islands, where it has been facing novel environmental conditions and limited resource availability. To explore the genomic consequences of this ecological shift, we analyze the transcriptomic variability of five individuals of C. nigra, and compare it to similar data obtained from several continental species of turtles. Results: Having clarified the timing of divergence in the Chelonoidis genus, we report in C. nigra a very low level of genetic polymorphism, signatures of a weakened efficacy of purifying selection, and an elevated mutation load in coding and regulatory sequences. These results are consistent with the hypothesis of an extremely low long-term effective population size in this insular species. Functional evolutionary analyses reveal a reduced diversity of immunity genes in C. nigra, in line with the hypothesis of attenuated pathogen diversity in islands, and an increased selective pressure on genes involved in response to stress, potentially related to the climatic instability of its environment and its elongated lifespan. Finally, we detect no population structure or homozygosity excess in our five-individual sample. Conclusions: These results enlighten the molecular evolution of an endangered taxon in a stressful environment and point to island endemic species as a promising model for the study of the deleterious effects on genome evolution of a reduced long-term population size. | 2550 | NA | NA | NA | no | no |
| 2551 | 2017 | NA | Brunet, J; Zalapa, JE; Pecori, F; Santini, A | 2013 | Hybridization and introgression between the exotic Siberian elm, Ulmus pumila, and the native Field elm, U. minor, in Italy | In response to the first Dutch elm disease (DED) pandemic, Siberian elm, Ulmus pumila, was planted to replace the native elm, U. minor, in Italy. The potential for hybridization between these two species is high and repeated hybridization could result in the genetic swamping of the native species and facilitate the evolution of invasiveness in the introduced species. We used genetic markers to examine the extent of hybridization between these two species and to determine the pattern of introgression. We quantified and compared the level of genetic diversity between the hybrids and the two parental species. Hybrids between U. pumila and U. minor were common. The pattern of introgression was not as strongly biased towards U. pumila as was previously observed for hybrids between U. rubra and U. pumila in the United States. The levels of heterozygosity were similar between U. minor and the hybrids and both groups had higher levels of heterozygosity relative to U. pumila. The programs Structure and NewHybrids indicated the presence of first- (F-1) and second- generation (F-2) hybrids and of backcrosses in the hybrid population. The presence of healthy DED resistant U. minor individuals combined with the self-compatibility of U. minor could help explain the presence of F-2 individuals in Italy. The presence of F-2 individuals, where most of the variability present in the hybrids will be released, could facilitate rapid evolution and the potential evolution of invasiveness of U. pumila in Italy. | 2551 | NA | NA | NA | no | no |
| 2552 | 2017 | NA | Mejia-Salazar, MF; Goldizen, AW; Menz, CS; Dwyer, RG; Blomberg, SP; Weidner, CL; Cullingham, CI; Bollinger, TK | 2017 | Mule deer spatial association patterns and potential implications for transmission of an epizootic disease | Animal social behaviour can have important effects on the long-term dynamics of diseases. In particular, preferential spatial relationships between individuals can lead to differences in the rates of disease spread within a population. We examined the concurrent influence of genetic relatedness, sex, age, home range overlap, time of year, and prion disease status on proximal associations of adult Rocky Mountain mule deer (Odocoileus hemionus hemionus) in a chronic wasting disease endemic area. We also quantified the temporal stability of these associations across different sex, age, and disease status classes. We used three years of high frequency telemetry data from 74 individuals to record encounters within 25 m of each other, and to calculate seasonal home range overlap measured by volume of intersection (VI). The strength of pairwise spatial association between adult mule deer was independent of genetic relatedness, age and disease status. Seasonal variation in association strength was not consistent across years, perhaps due to annual changes in weather conditions. The influence of home range overlap on association strength varied seasonally, whereby associations were stronger in pre-rut and fawning than in the rest of the seasons. The sexes of individuals also interacted with both VI and season. At increasing levels of VI, associations were stronger between females than between males and between females and males. The strongest associations in pre-rut were between males, while the strongest in rut were between females and males. The temporal stability of associations was markedly dependant on the sex and the diagnosis of the associating pair. Our findings highlight the importance of considering concurrent effects of biological and environmental factors when seeking to understand the role of social preference in behavioural ecology and disease spread. Applying this knowledge in epidemiological modelling will shed light on the dynamics of disease transmission among mule deer. | 2552 | NA | NA | NA | no | no |
| 2553 | 2017 | NA | de los Campos, G; Sorensen, D; Gianola, D | 2015 | Genomic Heritability: What Is It? | Whole-genome regression methods are being increasingly used for the analysis and prediction of complex traits and diseases. In human genetics, these methods are commonly used for inferences about genetic parameters, such as the amount of genetic variance among individuals or the proportion of phenotypic variance that can be explained by regression on molecular markers. This is so even though some of the assumptions commonly adopted for data analysis are at odds with important quantitative genetic concepts. In this article we develop theory that leads to a precise definition of parameters arising in high dimensional genomic regressions; we focus on the so-called genomic heritability: the proportion of variance of a trait that can be explained (in the population) by a linear regression on a set of markers. We propose a definition of this parameter that is framed within the classical quantitative genetics theory and show that the genomic heritability and the trait heritability parameters are equal only when all causal variants are typed. Further, we discuss how the genomic variance and genomic heritability, defined as quantitative genetic parameters, relate to parameters of statistical models commonly used for inferences, and indicate potential inferential problems that are assessed further using simulations. When a large proportion of the markers used in the analysis are in LE with QTL the likelihood function can be misspecified. This can induce a sizable finite-sample bias and, possibly, lack of consistency of likelihood (or Bayesian) estimates. This situation can be encountered if the individuals in the sample are distantly related and linkage disequilibrium spans over short regions. This bias does not negate the use of whole-genome regression models as predictive machines; however, our results indicate that caution is needed when using marker-based regressions for inferences about population parameters such as the genomic heritability. | 2553 | NA | NA | NA | no | no |
| 2554 | 2017 | NA | Sebastien, A; Gruber, MAM; Lester, PJ | 2012 | Prevalence and genetic diversity of three bacterial endosymbionts (Wolbachia, Arsenophonus, and Rhizobiales) associated with the invasive yellow crazy ant (Anoplolepis gracilipes) | Invasive species carry pathogens, parasites and mutualistic microorganisms into their new range. We examined the prevalence and genetic diversity of three bacterial endosymbionts (Wolbachia, Arsenophonus, and Rhizobiales) in four Anoplolepis gracilipes (Smith) populations in the Pacific region, four populations from mainland Australia and one population from Christmas Island in the Indian Ocean. Wolbachia was found in eight of the nine sampling sites, with between 31.8 and 100% of ants infected. These infection rates are substantially higher than those previously observed for other invasive ants such as Linepithema humile and Solenopsis invicta. All sequences of Wolbachia were genetically identical. Arsenophonus was observed in five of the nine sampling sites, with infection rates ranging between 4.5 and 50.8%. Like Wolbachia, Arsenophonus can modify the sex-ratio of its hosts via male-killing. Arsenophonus was found to co-occur with Wolbachia in the same ants in five of the nine sampling sites. Rhizobiales is a clade of symbiotic bacteria mostly found in herbivorous ants. These bacteria help provide nitrogen to their hosts and were found in only three of the nine sampling sites with an infection rate of 1.6-11.8%. It also co-occurred with the other bacteria. There was no genetic variation in Arsenophonus and Rhizobiales samples, with the exception of a sequence from one Arsenophonus sample in Samoa that differed by a single base pair. These bacterial endosymbionts may contribute to the population variability in A. gracilipes and may be manipulated for the purpose of pest management. | 2554 | NA | NA | NA | no | no |
| 2555 | 2017 | NA | Irwin, AJ; Taylor, PD | 2000 | Evolution of dispersal in a stepping-stone population with overlapping generations | We use Hamilton’s inclusive fitness method to calculate the evolutionarily stable dispersal rate in 1- and 2-dimensional stepping-stone populations, This extends previous results by introducing a positive probability for adults to survive into the next generation and breed again. Relatedness between nearby individuals generally decreases with increasing survival, decreasing competition with kin and favouring greater dispersal rates, (C) 2000 Academic Press. | 2555 | NA | NA | NA | no | no |
| 2556 | 2017 | NA | Kavalar, MS; Balantic, M; Silar, M; Kosnik, M; Korosec, P; Rijavec, M | 2012 | Association of ORMDL3, STAT6 and TBXA2R gene polymorphisms with asthma | Asthma is one of the most common chronic diseases in childhood. It is well known that genetic variability contributes to asthma risk. One of the most replicated asthma candidate genes is ORM1-like 3 (Saccharomyces cerevisiae) (ORMDL3), which has been associated with childhood asthma susceptibility. Another asthma candidate gene is signal transducer and activator of transcription 6 (STAT6), a regulator of IgE class switching. Gene coding thromboxane A2 receptor (TBXA2R), involved in chronic airway inflammation, has been associated with asthma in several genetic studies. We have studied the association of polymorphism rs4795405 in ORMDL3, rs324011 in STAT6 as well as rs8113232 and rs3786989 in TBXA2R with asthma risk, various asthma phenotypes and asthma-related symptoms. The study group consisted of 154 children with asthma, in whom clinical parameters were measured and whose asthma control and atopic status were determined. A control group comprised 71 healthy children. Genotyping was performed using an allelic discrimination assay. The ORMDL3 polymorphism rs4795405 was suggestively associated with asthma risk. Furthermore, it was significantly associated with nonatopic asthma and asthma without rhinitis. No association was detected between the STAT6 polymorphism rs324011 or the TBXA2R polymorphisms rs8113232 and rs3786989 and asthma susceptibility. However, an association between rs324011 in STAT6 with recurrent wheezing in early childhood and a suggestive association between rs8113232 in TBXA2R with rhinitis in children with asthma were observed. Our results confirmed ORMDL3 as a candidate gene for childhood asthma susceptibility. STAT6 and TBXA2R polymorphisms were not associated with asthma risk, but they were associated with asthma-related symptoms. | 2556 | NA | NA | NA | no | no |
| 2557 | 2017 | NA | Varghese, M; Kamalakannan, R; Harwood, CE; Lindgren, D; McDonald, MW | 2009 | Changes in growth performance and fecundity of Eucalyptus camaldulensis and E. tereticornis during domestication in southern India | Bulk seedlots of two unpedigreed multiprovenance seed production areas (SPAs) each of Eucalyptus camaldulensis and Eucalyptus tereticornis and one pedigreed seedling seed orchard (SSO) of E. tereticornis were planted in genetic gain trials at three southern Indian trial sites. At the time of seed collection, fewer than 30% trees flowered in these orchards, except in one E. camaldulensis SPA where 73% of the trees flowered, which had an estimated outcrossing rate of 86%. The E. tereticornis SSO was dominated by pollen from five highly fecund families of the Indian Mysore gum land race, which contributed 59% of the fruits produced. The SPA and SSO seedlots were compared with a bulked natural-provenance seedlot of E. camaldulensis (Morehead, Laura, and Kennedy Rivers, Queensland), another natural-provenance seedlot (Petford, Queensland), commercial eucalypt clones at two sites, and a Mysore gum seedlot at one site. At 3 years, progeny from all the four SPAs displayed good survival (79-93%) and performance similar to that of the natural provenances and the commercial clones. Progeny from the E. tereticornis SSO had significantly lower growth (at two sites) and lower survival at all three test sites. The Mysore gum seedlot displayed high fecundity and lower growth but better survival than the SSO progeny. Seed orchard genetic composition and flowering contributions thus affected progeny performance and the extent to which orchard genetic diversity was captured in the progeny. SPA progeny displayed greater fecundity than the natural provenances, indicating a response to selection for fertility. | 2557 | NA | NA | NA | no | no |
| 2558 | 2017 | NA | Roman, B; Satovic, Z; Alfaro, C; Moreno, MT; Kharrat, M; Perez-de-Luque, A; Rubiales, D | 2007 | Host differentiation in Orobanche foetida Poir | Orobanche foetida Poir. is a parasitic plant widely distributed in the Western Mediterranean area. It typically parasitizes wild plants but has recently been described as an agricultural problem in legume crops in Tunisia. The pattern of genetic variation within and among O.. foetida populations growing on chickpea and faba bean was analyzed by RAPD markers. The UPGMA cluster analysis based on Dice distance matrix showed a clear differentiation among O. foetida samples collected on chickpea and those on faba bean, suggesting a host-differentiation process. Although an AMOVA analysis revealed substantial internal variation among individuals within O. foetida populations (69.8%), there was a significant divergence between parasites on the two hosts considered (30.2%). Moreover, germination of O. foetida seeds collected on chickpea and faba bean in the presence of both host roots was studied. Germination percentages of O. foetida seeds varied depending on the host used both for collecting the seeds and evaluating the trait. According to these results, possible explanations for the origin of this new weedy parasite and the host differentiation process are discussed. (C) 2007 Elsevier GmbH. All rights reserved. | 2558 | NA | NA | NA | no | no |
| 2559 | 2017 | NA | Weiner, B; Gomez, J; Victor, TC; Warren, RM; Sloutsky, A; Plikaytis, BB; Posey, JE; van Helden, PD; Gey van Pittius, NC; Koehrsen, M; Sisk, P; Stolte, C; White, J; Gagneux, S; Birren, B; Hung, D; Murray, M; Galagan, J | 2012 | Independent Large Scale Duplications in Multiple M. tuberculosis Lineages Overlapping the Same Genomic Region | Mycobacterium tuberculosis, the causative agent of most human tuberculosis, infects one third of the world’s population and kills an estimated 1.7 million people a year. With the world-wide emergence of drug resistance, and the finding of more functional genetic diversity than previously expected, there is a renewed interest in understanding the forces driving genome evolution of this important pathogen. Genetic diversity in M. tuberculosis is dominated by single nucleotide polymorphisms and small scale gene deletion, with little or no evidence for large scale genome rearrangements seen in other bacteria. Recently, a single report described a large scale genome duplication that was suggested to be specific to the Beijing lineage. We report here multiple independent large-scale duplications of the same genomic region of M. tuberculosis detected through whole-genome sequencing. The duplications occur in strains belonging to both M. tuberculosis lineage 2 and 4, and are thus not limited to Beijing strains. The duplications occur in both drug-resistant and drug susceptible strains. The duplicated regions also have substantially different boundaries in different strains, indicating different originating duplication events. We further identify a smaller segmental duplication of a different genomic region of a lab strain of H37Rv. The presence of multiple independent duplications of the same genomic region suggests either instability in this region, a selective advantage conferred by the duplication, or both. The identified duplications suggest that large-scale gene duplication may be more common in M. tuberculosis than previously considered. | 2559 | NA | NA | NA | no | no |
| 2560 | 2017 | NA | Peacock, MM; Smith, AT | 1997 | The effect of habitat fragmentation on dispersal patterns, mating behavior, and genetic variation in a pika (Ochotona princeps) metapopulation | Habitat fragmentation is becoming increasingly common, yet, the effect of habitat spatial structure on population dynamics remains undetermined for most species, Populations of a single species found in fragmented and nonfragmented habitat present a rare opportunity to examine the effect of habitat spacial structure on population dynamics. This study investigates the impact of highly fragmented habitat on dispersal patterns, mating behavior, and genetic variation in a pika (Ochotona princeps) population with a mainland-island spatial structure. Juvenile dispersal patterns in fragmented habitat revealed that individuals tended to disperse to neighboring habitat patches. However, within-patch band-sharing scores from multilocus DNA fingerprints did not differ from what would be expected if individuals were assorting randomly among habitat patches each year. Multiple, shore-distance dispersal targets for juveniles and occasional long-distance dispersal events suggest that habitat fragmentation on this scale has not resulted in restricted dispersal and a genetically subdivided population. Although pikas tended to mate with the closest available partner, DNA fingerprinting band-sharing scores between mated pairs were consistent with a random mating hypothesis. Random mating in this population appears to be an incidental effect of dispersal in a fragmented habitat. This pattern is distinct from that found in nonfragmented habitat (large talus patches) where mating was non-random and consistent with mating between individuals of intermediate relatedness. DNA fingerprinting data revealed within-species variation in the mating habits of the pika directly attributable to habitat spatial structure. | 2560 | NA | NA | NA | no | no |
| 2561 | 2017 | NA | Mercader, RJ; Aardema, ML; Scriber, JM | 2009 | Hybridization leads to host-use divergence in a polyphagous butterfly sibling species pair | “Climate warming has lead to increased genetic introgression across a narrow hybrid zone separating the eastern and Canadian tiger swallowtails (Papilio glaucus and Papilio canadensis). This situation has led to the formation of an allochronically separated hybrid population with a delayed emerging phenotype or”"late flight’’. Here, we assess how the recombination of the parental genomes that lead to this phenotype may have facilitated another major ecological shift, host-use divergence. We first contrast the ovipositional profiles of the late flight population to that of the parental species P. glaucus and P. canadensis. Subsequently we contrast the larval survival and growth of the late flight, a P. canadensis and a P. glaucus population, and a population from the northern edge of the hybrid zone on five hosts. Our results indicate that the ovipositional preference of this hybrid swarm is identical to that of the introgressing parental species, P. glaucus. Due to the absence of the preferred hosts of P. glaucus (Liriodendron tulipifera L. and Ptelea trifoliata L.) where the late flight occurs, this ovipositional pattern implies a functional specialization onto a secondary host of both parental species, Fraxinus americana L. In contrast, the larval host-use abilities represent a mixture of P. glaucus and P. canadensis, indicating divergence in larval host-use abilities has not taken place. However, high genetic variability (genetic coefficient of variation) is present for growth on F. americana in the late flight hybrid swarm and tradeoffs for larval performance on the preferred hosts of the parental species are evident | 2561 | NA | NA | NA | no | no |
| 2562 | 2017 | NA | Hua, W; Liu, ZJ; Zhu, J; Xie, CJ; Yang, TM; Zhou, YL; Duan, XY; Sun, QX; Liu, ZY | 2009 | Identification and genetic mapping of pm42, a new recessive wheat powdery mildew resistance gene derived from wild emmer (Triticum turgidum var. dicoccoides) | Powdery mildew, caused by Blumeria graminis f. sp. tritici, is one of the most important wheat diseases worldwide in areas with cool or maritime climates. Wild emmer (Triticum turgidum var. dicoccoides) is an important potential donor of disease resistances and other traits for common wheat improvement. A powdery mildew resistance gene was transferred from wild emmer accession G-303-1M to susceptible common wheat by crossing and backcrossing, resulting in inbred line P63 (Yanda1817/G-303-1 M//3\*Jing411, BC(2)F(6)). Genetic analysis of an F(2) population and the F(2:3) families developed from a cross of P63 and a susceptible common wheat line Xuezao showed that the powdery mildew resistance in P63 was controlled by a single recessive gene. Molecular markers and bulked segregant analysis were used to characterize and map the powdery mildew resistance gene. Nine genomic SSR markers (Xbarc7, Xbarc55, Xgwm148, Xgwm257, Xwmc35, Xwmc154, Xwmc257, Xwmc382, Xwmc477), five AFLP-derived SCAR markers (XcauG3, XcauG6, XcauG10, XcauG20, XcauG22), three EST-STS markers (BQ160080, BQ160588, BF146221) and one RFLP-derived STS marker (Xcau516) were linked to the resistance gene, designated pm42, in P63. pm42 was physically mapped on chromosome 2BS bin 0.75-0.84 using Chinese Spring nullisomic-tetrasomic, ditelosomic and deletion lines, and was estimated to be more than 30 cM proximal to Xcau516, a RFLP-derived STS marker that co-segregated with the wild emmer-derived Pm26 which should be physically located in 2BS distal bin 0.84-1.00. pm42 was highly effective against 18 of 21 differential Chinese isolates of B. graminis f. sp. tritici. The closely linked molecular markers will enable the rapid transfer of pm42 to wheat breeding populations thus adding to their genetic diversity. | 2562 | NA | NA | NA | no | no |
| 2563 | 2017 | NA | Carrasquinho, I; Goncalves, E | 2013 | Genetic variability among Pinus pinea L. provenances for survival and growth traits in Portugal | Pinus pinea L. (Mediterranean stone pine) is an important forest species not only for its economically relevant kernel production, but also for environmental protection. The detection of genetic variability is an essential issue for Mediterranean forest species for conservation and improvement programs. Based on data collected for several years at three field sites from a P. pinea L. provenance trial established in Portugal in 1993, the present study aimed to evaluate the genetic variability of the adaptive traits of P. pinea L. and to identify a group of provenances with high performance for growth traits to be used in future plantings. Several mixed models were fitted to survival and growth trait data to estimate provenance and provenance x site interaction variability. The empirical best linear unbiased predictors of provenance genotypic effects were used to select a superior group of provenances. The provenance genetic variability of P. pinea L. was successfully detected for survival and height of different planting ages and also for diameter at breast height at age 13 years after planting. For growth traits, the most successful methods used for evaluating provenance genetic variability were based on the linear mixed spatial models. When multi-environmental analysis was performed, provenance x site interaction variability was detected for survival, but not for height at age 6 years. The existence of provenance variability in P. pinea L. permitted the identification of a seed lot composed of a mixture of the best provenances for height and diameter. | 2563 | NA | NA | NA | no | no |
| 2564 | 2017 | NA | Capittini, C; Martinetti, M; Cuccia, M | 2008 | MHC Variation, Mate Choice and Natural Selection: The Scent of Evolution | The Major Histocompatibility Complex (MHC) is considered a system completely defined and only connected with the immune response. However, in addition to the well-known correlation between MHC and the non-self recognition, the MHC region controls a lot of other functions: the recognition of genetic individuality in social relationships, the mate choice and the feto-maternal interplay. Starting from protocordates, the first MHC function was the individual. self-identification inside a group, but then it turned into an inter-individual recognition system, which could transmit information about the MHC genotypes. In mammals, the MHC system is functionally and physically linked to the olfactory receptors: when smelling each other, we are able to make a direct genetic analysis through the nose. The MHC individual genetic recognition system plays a fundamental role, both in mate choice and in foeto-maternal selection, from the very start of implantation. All these data suggest that the MHC polymorphism is driven not only by pathogen selection, but also by sexual reproductive-mechanisms. Questions remain about the relative involvement of these two selective forces in MHC evolution. | 2564 | NA | NA | NA | no | no |
| 2565 | 2017 | NA | Spyrou, V; Stamatis, C; Birtsas, P; Psychas, V; Manolakou, K; Billinis, C; Mamuris, Z | 2013 | Evidence for European brown hare syndrome virus introduction with translocated brown hares (Lepus europaeus): implications for management of restocking operations | Context. Criticisms of wildlife restocking operations typically focus on concerns that translocations can lead to the introduction of pathogens, and risk the integrity of locally adapted genetic diversity. Restocking programs aiming to stabilise population declines of European brown hares using captive-bred individuals have been carried out in several European countries, including Greece. Aims. To assess the potential for imported hares to introduce novel strains of European brown hare syndrome virus (EBHSV) during restocking operations, by (1) inferring the origin of wild Greek hares on the basis of their mitochondrial DNA (mtDNA) haplotype, (2) screening the hares to detect and characterise EBHSV, and (3) determining whether certain hare origin-EBHSV combinations occur in the wild. Methods. RNA extraction, polymerase chain reaction (PCR) amplification, and sequence and phylogenetic analyses of EBHSV were performed on 53 hares. Diagnostic RFLP markers of the mtDNA were used to infer the origins of sampled hares. Key results. Thirty-three hares had ‘typical’ native Greek haplotypes and 20 had mtDNA haplotypes matching those found in imported and released hares. Twelve of the latter and none of the former were positive for EBHSV. Phylogenetic analysis showed that nine virus isolates formed a single genetic lineage distinct from northern-central European ones. Three virus sequences from three imported reared-and-released hares, from Chalkidiki, were closely related to the northern-central European EBHS viruses. Conclusions. Alien strains of EBHSV are co-introduced with released captive-bred animals, possibly resulting in negative impacts on populations of Greek hares that have not evolved resistance to these novel virus strains. Implications. The identification of these allopatric EBHSV strains has led the authorities to ban captures and transportations of local brown hares for any restocking operation. We consider it imperative to reinforce microbiological and genetic controls before further releases of captive-bred game species in the wild in Greece. | 2565 | NA | NA | NA | no | no |
| 2566 | 2017 | NA | Tapia, LI; Shaw, CA; Aideyan, LO; Jewell, AM; Dawson, BC; Haq, TR; Piedra, PA | 2014 | Gene Sequence Variability of the Three Surface Proteins of Human Respiratory Syncytial Virus (HRSV) in Texas | Human respiratory syncytial virus (HRSV) has three surface glycoproteins: small hydrophobic (SH), attachment (G) and fusion (F), encoded by three consecutive genes (SH-G-F). A 270-nt fragment of the G gene is used to genotype HRSV isolates. This study genotyped and investigated the variability of the gene and amino acid sequences of the three surface proteins of HRSV strains collected from 1987 to 2005 from one center. Sixty original clinical isolates and 5 prototype strains were analyzed. Sequences containing SH, F and G genes were generated, and multiple alignments and phylogenetic trees were analyzed. Genetic variability by protein domains comparing virus genotypes was assessed. Complete sequences of the SH-G-F genes were obtained for all 65 samples: HRSV-A = 35; HRSV-B = 30. In group A strains, genotypes GA5 and GA2 were predominant. For HRSV-B strains, the genotype GB4 was predominant from 1992 to 1994 and only genotype BA viruses were detected in 2004-2005. Different genetic variability at nucleotide level was detected between the genes, with G gene being the most variable and the highest variability detected in the 270-nt G fragment that is frequently used to genotype the virus. High variability (>10%) was also detected in the signal peptide and transmembrane domains of the F gene of HRSV A strains. Variability among the HRSV strains resulting in non-synonymous changes was detected in hypervariable domains of G protein, the signal peptide of the F protein, a not previously defined domain in the F protein, and the antigenic site empty set in the pre-fusion F. Divergent trends were observed between HRSV -A and -B groups for some functional domains. A diverse population of HRSV -A and -B genotypes circulated in Houston during an 18 year period. We hypothesize that diverse sequence variation of the surface protein genes provide HRSV strains a survival advantage in a partially immune-protected community. | 2566 | NA | NA | NA | no | no |
| 2567 | 2017 | NA | Loeb, MLG | 2003 | Evolution of egg dumping in a subsocial insect | “Egg dumping, or abandonment of eggs and young to the care of other conspecifics, frees individuals from costs of maternal care while potentially imposing energetic and ecological costs on egg recipients. It is not clear, however, that egg dumping necessarily represents selfish manipulation of egg recipients, and in some ecological contexts, recipients may benefit from enlarged broods. Thus, egg dumping may either be mutually beneficial for dumpers and recipients or entail costs for dumpers that are compensated by other means, such as improving reproduction of genetically related egg recipients. Here I use field experiments to test the relative importance of manipulation (i.e.,”“parasitism”“), mutualism, and kin selection in the evolution of egg dumping in the tingid lace bug Gargaphia solani. In support of mutualism and kin selection, I found that reproduction of egg recipient G. solani benefits from brood enlargement, most likely because eggs and gregarious nymphs find safety in greater numbers. But contrary to both parasitism and mutualism, egg dumper reproduction was not improved by offspring abandonment. Indeed, dumpers laid smaller clutches than recipients, and dumpers did not convert a survival advantage into greater future reproduction. Genetic analyses of a natural G. solani population revealed, however, that dumpers are related to their egg recipients. Moreover, Hamilton’s rule showed that egg-dumping G. solani earn sufficient indirect genetic benefits for kin selection to favor the behavior. Thus, egg dumping in some species may be kin-selected cooperation rather than parasitism or mutualism.” | 2567 | NA | NA | NA | no | no |
| 2568 | 2017 | NA | Des Marais, DL; Skillern, WD; Juenger, TE | 2015 | Deeply Diverged Alleles in the Arabidopsis AREB1 Transcription Factor Drive Genome-Wide Differences in Transcriptional Response to the Environment | Gene regulatory variation is an important driver of the evolution of physiological and developmental responses to the environment. The abscisic acid (ABA) signaling pathway has long been studied as a key component of the cellular response to abiotic stresses in plants. We identify two haplotypes in an Arabidopsis thaliana transcription factor, AREB1, which plays a central role in ABA-mediated response to osmotic stress. These two haplotypes show the sequence signature of long-term maintenance of genetic diversity, suggesting a role for a diversifying selection process such as balancing selection. We find that the two haplotypes, distinguished by a large number of single nucleotide polymorphisms and the presence or absence of four small insertion/deletions in AREB1 intron 1 and exon 2, are at roughly equal frequencies in Arabidopsis, and show high linkage disequilibrium and deep sequence divergence. We use a transgenic approach, along with mRNA Sequencing-based assay of genome-wide expression levels, and find considerable functional divergence between alleles representing the two haplotype groups. Specifically, we find that, under benign soil-water conditions, transgenic lines containing different AREB1 alleles differ in the expression of a large number of genes associated with pathogen response. There are relatively modest gene expression differences between the two transgenic lines under restricted soil water content. Our finding of pathogen-related activity expands the known roles of AREB1 in A. thaliana and reveals the molecular basis of gene-by-environment interaction in a putatively adaptive plant regulatory protein. | 2568 | NA | NA | NA | no | no |
| 2569 | 2017 | NA | Hsu, LY; Harris, SR; Chlebowicz, MA; Lindsay, JA; Koh, TH; Krishnan, P; Tan, TY; Hon, PY; Grubb, WB; Bentley, SD; Parkhill, J; Peacock, SJ; Holden, MTG | 2015 | Evolutionary dynamics of methicillin-resistant Staphylococcus aureus within a healthcare system | Background: In the past decade, several countries have seen gradual replacement of endemic multi-resistant healthcare-associated methicillin-resistant Staphylococcus aureus (MRSA) with clones that are more susceptible to antibiotic treatment. One example is Singapore, where MRSA ST239, the dominant clone since molecular profiling of MRSA began in the mid-1980s, has been replaced by ST22 isolates belonging to EMRSA-15, a recently emerged pandemic lineage originating from Europe. Results: We investigated the population structure of MRSA in Singaporean hospitals spanning three decades, using whole genome sequencing. Applying Bayesian phylogenetic methods we report that prior to the introduction of ST22, the ST239 MRSA population in Singapore originated from multiple introductions from the surrounding region; it was frequently transferred within the healthcare system resulting in a heterogeneous hospital population. Following the introduction of ST22 around the beginning of the millennium, this clone spread rapidly through Singaporean hospitals, supplanting the endemic ST239 population. Coalescent analysis revealed that although the genetic diversity of ST239 initially decreased as ST22 became more dominant, from 2007 onwards the genetic diversity of ST239 began to increase once more, which was not associated with the emergence of a sub-clone of ST239. Comparative genomic analysis of the accessory genome of the extant ST239 population identified that the Arginine Catabolic Mobile Element arose multiple times, thereby introducing genes associated with enhanced skin colonization into this population. Conclusions: Our results clearly demonstrate that, alongside clinical practice and antibiotic usage, competition between clones also has an important role in driving the evolution of nosocomial pathogen populations. | 2569 | NA | NA | NA | no | no |
| 2570 | 2017 | NA | Cloutier, TL; Packard, JM | 2014 | Enrichment Options for African Painted Dogs (Lycaon pictus) | Best practices for carnivore enrichment encourage the diversity of species-typical behaviors, increased activity, and reduced stereotypic behavior; ideally considering the life-history and behavior of each species. African wild dogs (Lycaon pictus), or painted dogs, are social carnivores that have large home ranges and complex pack dynamics (e. g., variation in group size, relatedness, etc.). As there are relatively few studies on painted dog enrichment, the goal of this study was to compile a list of enrichment options used by institutions participating in the species survival plan (SSP). Representatives were asked to describe social groups (n = 45), enclosures (n = 21), enrichment practices (options, delivery frequency, perceived success), and overall best practices. Respondents (61%, n = 23) reported using options for all six enrichment categories recommended by the Canid Taxon Advisory Group: environmental enrichment devices, habitat, sensory, food, behavioral, and social. Perceived success was significantly higher for the food category, followed by the sensory and behavioral categories. All respondents reported delivering enrichment at least multiple times a month, and most reported multiple times per week. Enclosure size did not differ significantly for mixed-sex groups (n = 28) compared to single-sex groups (n = 17). We discuss respondents’ suggestions for best practices and the need to record data to compare perceived success with actual behavioral effects, controlling for variation in group size and composition. Overall, respondents recommended a flexible approach, since not all painted dogs and groups respond in the same way to the enrichment options. (C) 2014 Wiley Periodicals, Inc. | 2570 | NA | NA | NA | no | no |
| 2571 | 2017 | NA | Ladd, MC; Shantz, AA; Bartels, E; Burkepile, DE | 2017 | Thermal stress reveals a genotype-specific tradeoff between growth and tissue loss in restored Acropora cervicornis | Coral restoration is gaining attention as a viable strategy to restore degraded reefs, with large-scale restoration efforts underway worldwide. However, our understanding of the drivers of restoration success lags behind restoration activities, generating significant knowledge gaps that may impede our ability to successfully restore coral reef communities. Here, we conducted a 21 mo field experiment to examine the influence of genotypic identity and diversity on coral growth, habitat production, and survivorship in restored corals. We used nursery-raised colonies of Acropora cervicornis, the predominant coral used for restoration in the Caribbean, to establish populations of either 1, 2, 4, or 6 distinct genotypes. Midway through our experiment, our study site experienced a 17 wk thermal stress event that allowed us to examine the influence of genotypic identity and diversity on the ability of restored corals to cope with thermal stress. After 21 mo we found no effect of genotypic diversity on restored corals, but that genotypes differed 3-fold in survivorship and 20 to 327% in habitat production. Initial growth rates showed a significant positive relationship with live tissue loss at the end of the experiment, suggesting a tradeoff between growth and the ability to recover from thermal stress. Our study suggests that genotypic identity is a critical factor to incorporate into coral restoration planning. Investigating the role of genotypic identity and diversity on the ability of restored corals to resist pervasive coral reef stressors, such as disease, predator outbreaks, and nutrient pollution, are critical steps in advancing coral restoration efforts. | 2571 | NA | NA | NA | no | no |
| 2572 | 2017 | NA | Zhao, C; Wang, CB; Ma, XG; Liang, QL; He, XJ | 2013 | Phylogeographic analysis of a temperate-deciduous forest restricted plant (Bupleurum longiradiatum Turcz.) reveals two refuge areas in China with subsequent refugial isolation promoting speciation | This study investigates the influence of climate-induced oscillations and complicated geological conditions on the evolutionary processes responsible for species formation in presently fragmented temperate forest habitats, located in continental East Asia. In addition to this, we also investigate the heavily debated issue of whether temperate forests migrated southwards during such glacial periods or, alternatively, whether there existed refugia within north China, enabling localized survival of temperate forests within this region. In order to achieve these, we surveyed the phylogeography of Bupleurum longiradiatum Turcz. (a herbaceous plant solely confined to temperate forests) constructed from sequence variation in three chloroplast (cp) DNA fragments: trnL-trnF, psbA-trnH and rps16. Our analyses show high genetic diversity within species (h(T) = 0.948) and pronounced genetic differentiation among groups (yellow and purple flowers) with a significant phylogeographical pattern (N-ST > G(ST), P < 0.05). Forty-three haplotypes were identified and clustered into two lineages (the purple-flowered lineage and the yellow-flowered lineage). Two corresponding refuge areas, one in Qinling and its adjacent regions and one in the Changbai Mountains/eastern China, were revealed across the entire distribution ranges of Bupleurum longiradiatum. These results provide evidence for the hypothesis that independent refugia were maintained across the range of temperate forests in northern China during the last glacial maximum or earlier cold periods. Bupleurum longiradiatum var. porphyranthum formed a single taxon based on molecular data. This specific formation process suggests that the historical vicariance factors, i.e. climate-induced eco-geographic isolation through the biotic displacement of temperate-deciduous forest habitats, enhanced the divergence of the yellow and purple flower lineages at different spatial-temporal scales and over glacial and interglacial periods. Additionally, geological conditions that restricted gene flow might also be responsible for the observed high genetic and geographic differentiation. A nested clade analysis (NCA) revealed that allopatric fragmentation was a major factor responsible for the phylogeographic pattern observed, and also supported a role for historical vicariance factors. Our results therefore support the inference that Quaternary refugial isolation promoted allopatric speciation of temperate plants in East Asia. This may help to explain the existence of high diversity and endemism of plant species in East Asia. (C) 2013 Elsevier Inc. All rights reserved. | 2572 | NA | NA | NA | no | no |
| 2573 | 2017 | NA | Felix-Lopez, XA; Arguello-Garcia, R; Cerda-Flores, RM; Penaloza-Espinoza, RI; Buentello-Malo, L; Estrada-Mena, FJ; Ramos-Kuri, M; Gomez, FS; Arenas-Aranda, DJ | 2006 | FMRI CGG repeat distribution and linked microsatellite-SNP haplotypes in normal Mexican Mestizo and indigenous populations | The (CGG)(n) repeat size distribution in the FMRI gene was studied in healthy individuals: 80 X chromosomes of Mexican Mestizos from Mexico City and 33 X chromosomes of Mexican Amerindians from three indigenous communities (Purepechas, Nahuas, and Tzeltales), along with alleles and haplotypes defined by two microsatellite polymorphic markers (DXS548 and FRAXAC1) and two single nucleotide polymorphisms (FMRA and FMRB). Genetic frequencies of Mestizo and Amerindian subpopulations were statistically similar in almost all cases and thus were considered one population for comparisons with other populations. Sixteen (CGG)(n) alleles in the 17-38 size range were observed, and the most common were the 25 (38.0%), 26 (28.3%), and 24 (12.3%) repeat alleles. This pattern differs from most other populations reported, but a closer relation to Amerindian, European, and African populations was found, as expected from the historical admixture that gave rise to Mexican Mestizos. The results of the CA repeats analysis at DXS548-FRAXAC1 were restricted to nine haplotypes, of which haplotypes 7-4 (52.2%), 8-4 (23.8%), and 7-3 (11.5%) were predominant. The modal haplotype 7-4, instead of the nearly universal haplotype 7-3, had been reported exclusively in Eastern Asian populations. Likewise, only seven different FRAXAC1-FMRA-FMRB haplotypes were observed, including five novel haplotypes (3TA, 4TA, 3-A, 4-A, and 5-A), compared with Caucasians. Of these, haplotypes - A (78.7%) and 3-A (13.2%) were the most common in the Mexican population. These data suggest a singular but relatively low genetic diversity at FMR1 in the studied Mexican populations that may be related to the recent origin of Mestizos and the low admixture rate of Amerindians. | 2573 | NA | NA | NA | no | no |
| 2574 | 2017 | NA | Schluter, J; Foster, KR | 2012 | The Evolution of Mutualism in Gut Microbiota Via Host Epithelial Selection | The human gut harbours a large and genetically diverse population of symbiotic microbes that both feed and protect the host. Evolutionary theory, however, predicts that such genetic diversity can destabilise mutualistic partnerships. How then can the mutualism of the human microbiota be explained? Here we develop an individual-based model of host-associated microbial communities. We first demonstrate the fundamental problem faced by a host: The presence of a genetically diverse microbiota leads to the dominance of the fastest growing microbes instead of the microbes that are most beneficial to the host. We next investigate the potential for host secretions to influence the microbiota. This reveals that the epithelium-microbiota interface acts as a selectivity amplifier: Modest amounts of moderately selective epithelial secretions cause a complete shift in the strains growing at the epithelial surface. This occurs because of the physical structure of the epithelium-microbiota interface: Epithelial secretions have effects that permeate upwards through the whole microbial community, while lumen compounds preferentially affect cells that are soon to slough off. Finally, our model predicts that while antimicrobial secretion can promote host epithelial selection, epithelial nutrient secretion will often be key to host selection. Our findings are consistent with a growing number of empirical papers that indicate an influence of host factors upon microbiota, including growth-promoting glycoconjugates. We argue that host selection is likely to be a key mechanism in the stabilisation of the mutualism between a host and its microbiota. | 2574 | NA | NA | NA | no | no |
| 2575 | 2017 | NA | Grugni, V; Battaglia, V; Perego, UA; Raveane, A; Lancioni, H; Olivieri, A; Ferretti, L; Woodward, SR; Pascale, JM; Cooke, R; Myres, N; Motta, J; Torroni, A; Achilli, A; Semino, O | 2015 | Exploring the Y Chromosomal Ancestry of Modern Panamanians | Geologically, Panama belongs to the Central American land-bridge between North and South America crossed by Homo sapiens > 14 ka ago. Archaeologically, it belongs to a wider Isthmo-Colombian Area. Today, seven indigenous ethnic groups account for 12.3% of Panama’s population. Five speak Chibchan languages and are characterized by low genetic diversity and a high level of differentiation. In addition, no evidence of differential structuring between maternally and paternally inherited genes has been reported in isthmian Chibchan cultural groups. Recent data have shown that 83% of the Panamanian general population harbour mitochondrial DNAs (mtDNAs) of Native American ancestry. Considering differential male/female mortality at European contact and multiple degrees of geographical and genetic isolation over the subsequent five centuries, the Y-chromosome Native American component is expected to vary across different geographic regions and communities in Panama. To address this issue, we investigated Y-chromosome variation in 408 modern males from the nine provinces of Panama and one indigenous territory (the comarca of Kuna Yala). In contrast to mtDNA data, the Y-chromosome Native American component (haplogroup Q) exceeds 50% only in three populations facing the Caribbean Sea: the comarca of Kuna Yala and Bocas del Toro province where Chibchan languages are spoken by the majority, and the province of Colon where many Kuna and people of mixed indigenous-African-and-European descent live. Elsewhere the Old World component is dominant and mostly represented by western Eurasian haplogroups, which signal the strong male genetic impact of invaders. Sub-Saharan African input accounts for 5.9% of male haplotypes. This reflects the consequences of the colonial Atlantic slave trade and more recent influxes of West Indians of African heritage. Overall, our findings reveal a local evolution of the male Native American ancestral gene pool, and a strong but geographically differentiated unidirectional sex bias in the formation of local modern Panamanian populations. | 2575 | NA | NA | NA | no | no |
| 2576 | 2017 | NA | Wiggins, DA; Moller, AP; Sorensen, MFL; Brand, LA | 1998 | Island biogeography and the reproductive ecology of great tits Parus major | Island biogeography theory has contributed greatly to both theoretical and applied studies of conservation biology (e.g., design of nature reserves, minimum viable population sizes, extinction risk) and community composition. However, little theoretical and empirical work has addressed how island isolation and size affect reproductive ecology. We investigated the reproductive ecology of great tits (Parus major) on one offshore and one nearshore island, as well as on the Danish mainland. Tits breeding on the offshore island bred later, laid smaller clutches, and laid larger eggs than those on the nearshore island and mainland. In addition, the level of ectoparasite infestation in nests was highest on the offshore island, intermediate on the nearshore island, and lowest on the mainland. These insular effects may occur due to lower food abundance on islands, to density-dependent effects, or to effects related to low genetic diversity within island populations. Whatever the cause, the results emphasize that future studies of forest fragmentation/population isolation should consider not only gross measures of reproductive success, but also fine-scale measures such as clutch size, timing of breeding, and parasite prevalence. | 2576 | NA | NA | NA | no | no |
| 2577 | 2017 | NA | Teebi, AS; Teebi, SA | 2005 | Genetic diversity among the Arabs | The Arabs in general are genetically diverse. Major factors that contributed to their diversity include the migrations of Semitic tribes from the Arabian Peninsula, the Islamic expansion in the 7th century AD, the Crusade wars and the recent migration dynamics. These events have resulted in the admixture of the original Arabs with other populations extending from east and south Asia to Europe and Africa. Their demographic features include high rates of consanguinity, a large family size and a rapid population growth. There is a high frequency of autosomal recessive disorders and increased frequencies of homozygosity for autosomal dominant traits, such as familial hypercholesterolemia and X-linked traits, such as glucose-6-phosphate dehydrogenase deficiency. The patterns of autosomal recessive disorders, including their mutations, may be different in various geographic locations within the Arab world. However, there are disorders that are specifically prevalent among the Arabs either uniformly or in certain locations. The Arab Genetic diseases include Bardet-Biedl syndrome, Meckel syndrome, autosomal recessive severe childhood muscular dystrophy, osteopetrosis and renal tubular acidosis, Sanjad-Sakati syndrome and others. Copyright © 2005 S. Karger AG, Basel. | 2577 | NA | NA | NA | no | no |
| 2578 | 2017 | NA | Ferguson, NM; Galvani, AP; Bush, RM | 2003 | Ecological and immunological determinants of influenza evolution | In pandemic and epidemic forms, influenza causes substantial, sometimes catastrophic, morbidity and mortality. Intense selection from the host immune system drives antigenic change in influenza A and B, resulting in continuous replacement of circulating strains with new variants able to re-infect hosts immune to earlier types. This ‘antigenic drift’(1) often requires a new vaccine to be formulated before each annual epidemic. However, given the high transmissibility and mutation rate of influenza, the constancy of genetic diversity within lineages over time is paradoxical. Another enigma is the replacement of existing strains during a global pandemic caused by ‘antigenic shift’-the introduction of a new avian influenza A subtype into the human population(1). Here we explore ecological and immunological factors underlying these patterns using a mathematical model capturing both realistic epidemiological dynamics and viral evolution at the sequence level. By matching model output to phylogenetic patterns seen in sequence data collected through global surveillance(2), we find that short-lived strain-transcending immunity is essential to restrict viral diversity in the host population and thus to explain key aspects of drift and shift dynamics. | 2578 | NA | NA | NA | no | no |
| 2579 | 2017 | NA | Stockwell, MP; Bower, DS; Bainbridge, L; Clulow, J; Mahony, MJ | 2015 | Island provides a pathogen refuge within climatically suitable area | Surveillance of pathogens can lead to significant advances towards making effective decisions in research and management for species threatened by disease. Batrachochytrium dendrobatidis has been a major contributing factor to the global decline of amphibians. Knowledge of the distribution of B. dendrobatidis can contribute to understanding patterns of species decline and prioritizing action. Therefore, we surveyed four spatially distinct populations of a B. dendrobatidis susceptible species, the green and golden bell frog (Litoria aurea), for evidence of infection in the population. Three mainland populations were infected at a prevalence of 3.5-28.3 %, with median infection loads of 0.28-627.18 genomic equivalents (GE). Conversely, we did not detect infection in an island population 3 km from the mainland; the isolation and infrequent visitation of the island suggests that the pathogen has not arrived. Management actions for B. dendrobatidis and conservation of susceptible frog species are heavily dependent on the presence and absence of the pathogen in the population. Prevention of the accidental introduction of B. dendrobatidis and safe guarding genetic diversity of L. aurea is necessary to preserve unique diversity of the island population, whereas containment and control of the pathogen can be directed towards mainland populations. Knowledge of disease dynamics also provides a context to understand the ecology of remaining populations as variation in the physiology or habitat of the mainland populations have facilitated persistence of these populations alongside B. dendrobatidis. Other islands should be a priority target in disease surveillance, to discover refuges that can assist conservation. | 2579 | NA | NA | NA | no | no |
| 2580 | 2017 | NA | Simon-Loriere, E; Holmes, EC; Pagan, I | 2013 | The Effect of Gene Overlapping on the Rate of RNA Virus Evolution | Gene overlapping is widely employed by RNA viruses to generate genetic novelty while retaining a small genome size. However, gene overlapping also increases the deleterious effect of mutations as they affect more than one gene, thereby reducing the evolutionary rate of RNA viruses and hence their adaptive capacity. Although there is general agreement on the benefits of gene overlapping as a mechanism of genomic compression for rapidly evolving organisms, its effect on the pace of RNA virus evolution remains a source of debate. To address this issue, we collected sequence data from 117 instances of gene overlapping across 19 families, 30 genera, and 55 species of RNA viruses. On these data, we analyzed how genetic distances, selective pressures, and the distribution of RNA secondary structures and conserved protein functional domains vary between overlapping (OV) and nonoverlapping (NOV) regions. We show that gene overlapping generally results in a decrease in the rate of RNA virus evolution through a reduction in the frequency of synonymous mutations. However, this effect is less pronounced in genes with a terminal rather than an internal gene overlap, which might result from a greater proportion of protein functional conserved domains in NOV than in OV regions, in turn reducing the number of nonsynonymous mutations in the former. Overall, our analyses clarify the role of gene overlapping as a modulator of the evolutionary rates exhibited by RNA viruses and shed light on the factors that shape the genetic diversity of this important group of pathogens. | 2580 | NA | NA | NA | no | no |
| 2581 | 2017 | NA | Fisher, MC; Koenig, G; White, TJ; Taylor, JW | 2000 | A test for concordance between the multilocus genealogies of genes and microsatellites in the pathogenic fungus Coccidioides immitis | Uncovering the correct phylogeny of closely related species requires analysis of multiple gene genealogies or, alternatively, genealogies inferred from the multiple alleles found at highly polymorphic loci, such as microsatellites. However, a concern in using microsatellites is that constraints on allele sizes may occur, resulting in homoplasious distributions of alleles, leading to incorrect phylogenies. Seven microsatellites from the pathogenic fungus Coccidioides immitis were sequenced for 20 clinical isolates chosen to represent the known genetic diversity of the pathogen. An organismal phylogeny for C. immitis was inferred from microsatellite-flanking sequence polymorphisms and other restriction fragment length polymorphism-containing loci. Two microsatellite genetic distances were then used to determine phylogenies for C. immitis, and the trees found by these three methods were compared. Congruence between the organismal and microsatellite phylogenies occurred when microsatellite distances were based on simple allele frequency data. However, complex mutation events at some loci made distances based on stepwise mutation models unreliable. Estimates of times of divergence for the two species of C. immitis based on microsatellites were significantly lower than those calculated from flanking sequence, most likely due to constraints on microsatellite allele sizes. Flanking-sequence insertions/deletions significantly decreased the accuracy of genealogical information inferred from microsatellite loci and caused interspecific length homoplasies at one of the seven loci. Our analysis shows that microsatellites are useful phylogenetic markers, although care should be taken to choose loci with appropriate flanking sequences when they are intended for use in evolutionary studies. | 2581 | NA | NA | NA | no | no |
| 2582 | 2017 | NA | Zardi, GI; Nicastro, KR; McQuaid, CD; Hancke, L; Helmuth, B | 2011 | The combination of selection and dispersal helps explain genetic structure in intertidal mussels | Understanding patterns of gene flow, selection and genetic diversity within and among populations is a critical element of predicting how long-term changes in environmental conditions are likely to affect species distribution. The intertidal mussel Perna perna consists of two distinct genetic lineages in South Africa, but the mechanisms maintaining these lineages remains obscure. We used regional oceanography and lineage-specific responses to environmental conditions as proxies for gene flow and local selection, respectively, to test how these mechanisms could shape population genetic structure. Laboratory experiments supported the field findings that mussels on the east coast (eastern lineage) are physiologically more tolerant of sand inundation and high temperatures than those on the south coast (western lineage). Temperature loggers mimicking mussel body temperatures revealed that mussels experience higher body temperatures during aerial exposure on the subtropical east coast than on the temperate south coast. Translocations showed that, on the east coast, the western lineage suffered higher mortality rates than local individuals, while on the south coast, mortality rates did not differ significantly between the lineages. Nearshore drogues showed remarkably little overlap between the trajectories of drifters released off the south coast and those released off the east coast. Physiological tolerances can thus explain the exclusion of western individuals from the east coast, but they cannot explain the exclusion of the eastern lineage from the south coast. In contrast, however, ocean dynamics may limit larval dispersal between the two lineages, helping to explain the absence of eastern individuals from the south coast. We emphasise the importance of a multidisciplinary approach in a macro-ecological context to understand fully the mechanisms promoting evolutionary divergence between genetic entities. Our results suggest that phylogeographic patterns of Perna perna may be maintained by a combination of local conditions and the isolating effect of the Agulhas Current that reduces gene exchange. | 2582 | NA | NA | NA | no | no |
| 2583 | 2017 | NA | Fraser, C; Hanage, WP; Spratt, BG | 2005 | Neutral microepidemic evolution of bacterial pathogens | “Understanding bacterial population genetics is vital for interpreting the response of bacterial populations to selection pressures such as antibiotic treatment or vaccines targeted at only a subset of strains. The evolution of transmissible bacteria occurs by mutation and localized recombination and is influenced by epidemiological as well as molecular processes. We demonstrate that the observed population genetic structure of three important human pathogens, Streptococcus pneumoniae, Neisseria meningitidis, and Staphylococcus aureus, can be explained by using a simple evolutionary model that is based on neutral mutational drift, modulated by recombination, and which incorporates the impact of epidemic transmission in local populations. The predictions of this neutral”“microepidemic”" model are found to closely fit observed genetic relatedness distributions of bacteria sampled from their natural population, and it provides estimates of the relative rate of recombination that agree well with empirical estimates. The analysis suggests the emergence of neutral bacterial population structure from overlapping microepidemics within clustered host populations and provides insight into the nature and size distribution of these clusters. These findings challenge the assumption that strains of bacterial pathogens differ markedly in relative fitness." | 2583 | NA | NA | NA | no | no |
| 2584 | 2017 | NA | Ploshnitsa, AI; Goltsman, ME; Macdonald, DW; Kennedy, LJ; Sommer, S | 2012 | Impact of historical founder effects and a recent bottleneck on MHC variability in Commander Arctic foxes (Vulpes lagopus) | Populations of Arctic foxes (Vulpes lagopus) have been isolated on two of the Commander Islands (Bering and Mednyi) from the circumpolar distributed mainland population since the Pleistocene. In 1970-1980, an epizootic outbreak of mange caused a severe population decline on Mednyi Island. Genes of the major histocompatibility complex (MHC) play a primary role in infectious disease resistance. The main objectives of our study were to compare contemporary variation of MHC class II in mainland and island Arctic foxes, and to document the effects of the isolation and the recent bottleneck on MHC polymorphism by analyzing samples from historical and contemporary Arctic foxes. In 184 individuals, we found 25 unique MHC class II DRB and DQB alleles, and identified evidence of balancing selection maintaining allelic lineages over time at both loci. Twenty different MHC alleles were observed in mainland foxes and eight in Bering Island foxes. The historical Mednyi population contained five alleles and all contemporary individuals were monomorphic at both DRB and DQB. Our data indicate that despite positive and diversifying selection leading to elevated rates of amino acid replacement in functionally important antigen-binding sites, below a certain population size, balancing selection may not be strong enough to maintain genetic diversity in functionally important genes. This may have important fitness consequences and might explain the high pathogen susceptibility in some island populations. This is the first study that compares MHC diversity before and after a bottleneck in a wild canid population using DNA from museum samples. | 2584 | NA | NA | NA | no | no |
| 2585 | 2017 | NA | Takeuchi, T; Yamaguchi, M; Tanaka, R; Dayi, M; Ogura, N; Kikuchi, T | 2015 | Development and validation of SSR markers for the plant-parasitic nematode Subanguina moxae using genome assembly of Illumina pair-end reads | Subanguina moxae, belonging to the subfamily Anguininae, is an obligate parasite of Artemisia plants, which are widely used as cooking herbs and in traditional medicine in East Asia. Because the nematode is distributed throughout East Russia and East Asia, there is concern about the potential for significant damage to commercial farming; however, details about its biology remain unclear. To investigate the genetic diversity of S. moxae, we developed 2243 simple sequence repeat (SSR) markers using Illumina short reads of the genomic DNA. We validated 100 randomly selected markers indicating their robustness and examined polymorphisms among nematode populations sampled from four different locations in Japan. These SSR markers will be a useful tool for understanding the population structure and transmission patterns of this parasitic nematode. | 2585 | NA | NA | NA | no | no |
| 2586 | 2017 | NA | Reiner, G; Fischer, R; Kohler, F; Berge, T; Hepp, S; Willems, H | 2009 | Heritabilities and quantitative trait loci for blood gases and blood pH in swine | Maintaining pH and blood gases in a narrow range is essential to sustain normal biochemical reactions. Decreased oxygenation, poor tissue perfusion, disturbance to CO(2) expiration, and shortage of HCO(3)(-) can lead to metabolic acidosis. This is a common situation in swine, and originates from a broad range of medical conditions. pH and blood gases appear to be under genetic control, and populations with physiological traits closer to the pathological thresholds may be more susceptible to developing pathological conditions. However, little is known about the genetic basis of such traits. We have therefore estimated phenotypic and genetic variability and identified quantitative trait loci (QTL) for pH and blood gases in blood samples from 139 F(2) pigs from the Meishan/Pietrain family. Samples were taken before and after challenge with Sarcocystis miescheriana, a protozoan parasite of muscle. Twenty-seven QTL influencing pH and blood gases were identified on nine chromosomes. Five of the QTL were significant on a genome-wide level; 22 QTL were significant on a chromosome-wide level. QTL for pH-associated traits have been mapped to SSC3, 18 and X. QTL associated with CO(2) have been detected on SSC6, 7, 8 and 9, and QTL associated with O(2) on SSC2 and SSC8. QTL showed specific health/disease patterns that were related to the physiological state of the pigs from day 0, to acute disease (day 14), convalescence (day 28) and chronic disease (day 42). The results demonstrate that pH and blood gases are influenced by multiple chromosomal areas, each with relatively small effects. | 2586 | NA | NA | NA | no | no |
| 2587 | 2017 | NA | Damasceno-Silva, KJ; Souza, EA; Freire, CNS; Ishikawa, FH | 2015 | Anastomosis groups and molecular variation in Pseudocercospora griseola | The fungus Pseudocercospora griseola is the causal agent of angular leaf spot, a common bean (Phaseolus vulgaris L.) disease. The successful development of angular leaf spot-resistant cultivars depends on understanding the levels of variability in pathogen populations. P. griseola shows great pathogenic and genetic variation using inoculation on differential bean cultivars and molecular markers. Nevertheless, how this variability develops is not clearly understood. Parasexuality has been presented as a mechanism used by asexual fungi to increase variation. Hyphal fusion is the first step for the parasexual cycle, and it can be considered an additional trait for population studies. The aim of this study was to identify hyphal fusion (anastomosis) among P. grisola isolates and to evaluate the variability of isolates using analyses of anastomosis groups and RAPD markers. Hyphal anastomosis was observed in all isolates. Three isolates showed 85.0% compatibility and were compatible with 17 isolates. This is the first report of the occurrence of anastomosis between P. griseola isolates. Sixteen anastomosis groups were observed and only one group was formed by five isolates (Pg-01, Pg-02, Pg-07, Pg-08, and Pg-12). There was a large number of anastomosis groups and absence of clustering among isolates for hyphal fusion, demonstrating the existence of genetic variability for loci involved in the control of this trait. High genotypic variability by RAPD markers was detected, but there was no relationship between genotype and anastomosis group. More studies are necessary to elucidate further the process of asexual recombination in this phytopathogen. | 2587 | NA | NA | NA | no | no |
| 2588 | 2017 | NA | Matsuura, K | 2002 | A test of the haplodiploid analogy hypothesis in the termite Reticulitermes speratus (Isoptera : Rhinotermitidae) | In some species of lower termites, translocation complexes generate sex-dependent asymmetric relatedness within colonies. It has been suggested that increased relatedness, between both male and female siblings, for loci on translocation chromosomes might have favored the evolution of eusociality among termites in the same way that haplodiploidy has in hymenopterans. This sex-linkage hypothesis predicts that workers should invest more in sibling nymphs of the same sex. Although I observed sex-linked interchange heterozygosity in Reticulitermes speratus Kolbe, I found no differences in the survival rates or weight gain of nymphs reared by sibling workers of the same sex and those reared by sibling workers of the opposite sex. This empirical study did not support the haplodiploid analogy for lower termites. | 2588 | NA | NA | NA | no | no |
| 2589 | 2017 | NA | Moller, AP; Soler, JJ; Vivaldi, MM | 2010 | Spatial heterogeneity in distribution and ecology of Western Palearctic birds | Species vary in abundance and heterogeneity of spatial distribution, and the ecological and evolutionary consequences of such variability are poorly known. Evolutionary adaptation to heterogeneously distributed resources may arise from local adaptation with individuals of such locally adapted populations rarely dispersing long distances and hence having small populations and small overall ranges. We quantified mean population density and spatial heterogeneity in population density of 197 bird species across 12 similarly sized regions in the Western Palearctic. Variance in population density among regions differed significantly from a Poisson distribution, suggesting that random processes cannot explain the observed patterns. National estimates of means and variances in population density were positively correlated with continental estimates, suggesting that means and variances were maintained across spatial scales. We used Morisita’s index of population abundance as an estimate of heterogeneity in distribution among regions to test a number of predictions. Heterogeneously distributed passerine bird species as reflected by Morisita’s index had small populations, low population densities, and small breeding ranges. Their breeding populations had been consistently maintained at low levels for considerable periods of time, because the degree of genetic variation in a subsample of non-passerines and passerines was significantly negatively related to heterogeneity in distribution. Heterogeneously distributed passerine species were not more often habitat specialists than homogeneously distributed species. Furthermore, heterogeneously distributed passerine species had high annual adult survival rates but did not differ in annual fecundity from homogeneously distributed species. Heterogeneously distributed passerine species rarely colonized urban habitats. Finally, homogeneously distributed bird species were hosts to a greater diversity of blood parasite species than heterogeneously distributed species. In conclusion, small breeding ranges, population sizes, and population densities of heterogeneously distributed passerine bird species, combined with their low degree of genetic variability, and their inability to colonize urban areas may render such species particularly susceptible to human-influenced global climatic changes. | 2589 | NA | NA | NA | no | no |
| 2590 | 2017 | NA | Moore, JA; Xu, R; Frank, K; Draheim, H; Scribner, KT | 2015 | Social network analysis of mating patterns in American black bears (Ursus americanus) | Nonrandom mating can structure populations and has important implications for population-level processes. Investigating how and why mating deviates from random is important for understanding evolutionary processes as well as informing conservation and management. Prior to the implementation of parentage analyses, understanding mating patterns in solitary, elusive species like bears was virtually impossible. Here, we capitalize on a long-term genetic data set collected from black bears (Ursus americanus) (N=2422) in the Northern Lower Peninsula (NLP) of Michigan, USA. We identified mated pairs using parentage analysis and applied logistic regression (selection) models that controlled for features of the social network, to quantify the effects of individual characteristics, and spatial and population demographic factors on mating dynamics. Logistic regression models revealed that black bear mating was associated with spatial proximity of mates, male age, the time a pair had coexisted, local population density and relatedness. Mated pairs were more likely to contain older males. On average, bears tended to mate with nearby individuals to whom they were related, which does not support the existence of kin recognition in black bears. Pairwise relatedness was especially high for mated pairs containing young males. Restricted dispersal and high male turnover from intensive harvest mortality of NLP black bears are probably the underlying factors associated with younger male bears mating more often with female relatives. Our findings illustrate how harvest has the potential to disrupt the social structure of game species, which warrants further attention for conservation and management. | 2590 | NA | NA | NA | no | no |
| 2591 | 2017 | NA | Schneider, JM; Bilde, T | 2008 | Benefits of cooperation with genetic kin in a subsocial spider | Interaction within groups exploiting a common resource may be prone to cheating by selfish actions that result in disadvantages for all members of the group, including the selfish individuals. Kin selection is one mechanism by which such dilemmas can be resolved This is because selfish acts toward relatives include the cost of lowering indirect fitness benefits that could otherwise be achieved through the propagation of shared genes. Kin selection theory has been proved to be of general importance for the origin of cooperative behaviors, but other driving forces, such as direct fitness benefits, can also promote helping behavior in many cooperatively breeding taxa. Investigating transitional systems is therefore particularly suitable for understanding the influence of kin selection on the initial spread of cooperative behaviors. Here we investigated the role of kinship in cooperative feeding. We used a cross-fostering design to control for genetic relatedness and group membership. Our study animal was the periodic social spider Stegodyphus lineatus, a transitional species that belongs to a genus containing both permanent social and periodic social species. In S. lineatus, the young cooperate in prey capture and feed communally. We provide clear experimental evidence for net benefits of cooperating with kin. Genetic relatedness within groups and not association with familiar individuals directly improved feeding efficiency and growth rates, demonstrating a positive effect of kin cooperation. Hence, in communally feeding spiders, nepotism favors group retention and-reduces the conflict between selfish interests and the interests of the group. | 2591 | NA | NA | NA | no | no |
| 2592 | 2017 | NA | Bogdanowicz, W; Lesinski, G; Sadkowska-Todys, M; Gajewska, M; Rutkowski, R | 2013 | Population genetics and bat rabies: a case study of Eptesicus serotinus in Poland | The serotine bat, Eptesicus serotinus is the most frequently rabies-infected (European bat lyssavirus 1-type, EBLV-1) bat species in Europe. To confirm Lyssavirus infection of this bat in Poland, we tested for the presence of rabies virus RNA from oropharyngeal swabs using RT-PCR. There was a 0.9% (two out of 212 individuals) level of infection within the overall population of serotine bats studied. However, an appreciation of the potential for pathogen transmission and disease risk requires an understanding of the dispersal of the primary host, and any large-scale geographic barriers that may impede gene flow. Thus, we also studied the patterns of bat dispersal via population genetics using nuclear (seven microsatellite loci) and mitochondrial (mtDNA control region) markers, examined in 12 subpopulations distributed across the country. Molecular analyses of microsatellite loci indicated high genetic diversity at all sites (heterozygosity observed, H-O = 0.53-0.78), and extremely weak genetic structure in the Polish population of the species. The overall F-ST was 0.012 (95% confidence interval: 0.006-0.020), and pairwise values ranged from 0.00 to 0.05. Only 22% of individuals were assigned to the subpopulation from which they were sampled. The Bayesian approach implemented in STRUCTURE also confirmed that all examined subpopulations should be treated as a single group, indicating a high level of gene flow. There was some evidence for female philopatry (genetic differentiation was greater in maternally-inherited mtDNA than nuclear DNA) and male-biased dispersal, e.g., H-O and the variance of mean assignment were significantly higher in males than in females. Twelve individuals (seven females and five males) were identified as potential first generation migrants. Their migration routes ranged from 60-283 km in females ((x) over bar +/- SE = 177.9 +/- 29.37) to 27-385 km in males (206.4 +/- 58.95); surprisingly, no sexual differences were observed and this finding suggests that female-mediated gene flow may occur. MtDNA also produced a strong genetic signal for the demographic expansion (Fu’s F-S statistics, F-S = -26.30, P < 0.01 and a star-shaped haplotype network), which took place roughly 33,000 years BP, i.e., before the Last Glacial Maximum. The genetic uniformity of the Polish population implies that there is no migration barrier to EBLV-1, at least within the country, and the potential threat of rabies virus spreading via migration of infected animals may be higher than previously thought. | 2592 | NA | NA | NA | no | no |
| 2593 | 2017 | NA | Lavandero, B; Figueroa, CC; Franck, P; Mendez, A | 2011 | Estimating Gene Flow between Refuges and Crops: A Case Study of the Biological Control of Eriosoma lanigerum by Aphelinus mali in Apple Orchards | Parasitoid disturbance populations in agroecosystems can be maintained through the provision of habitat refuges with host resources. However, specialized herbivores that feed on different host plants have been shown to form host-specialized races. Parasitoids may subsequently specialize on these herbivore host races and therefore prefer parasitizing insects from the refuge, avoiding foraging on the crop. Evidence is therefore required that parasitoids are able to move between the refuge and the crop and that the refuge is a source of parasitoids, without being an important source of herbivore pests. A North-South transect trough the Chilean Central Valley was sampled, including apple orchards and surrounding Pyracantha coccinea (M. Roem) (Rosales: Rosacea) hedges that were host of Eriosoma lanigerum (Hemiptera: Aphididae), a globally important aphid pest of cultivated apples. At each orchard, aphid colonies were collected and taken back to the laboratory to sample the emerging hymenopteran parasitoid Aphelinus mali (Hymenoptera: Aphelinidae). Aphid and parasitoid individuals were genotyped using species-specific microsatellite loci and genetic variability was assessed. By studying genetic variation, natural geographic barriers of the aphid pest became evident and some evidence for incipient host-plant specialization was found. However, this had no effect on the population-genetic features of its most important parasitoid. In conclusion, the lack of genetic differentiation among the parasitoids suggests the existence of a single large and panmictic population, which could parasite aphids on apple orchards and on P. coccinea hedges. The latter could thus comprise a suitable and putative refuge for parasitoids, which could be used to increase the effectiveness of biological control. Moreover, the strong geographical differentiation of the aphid suggests local reinfestations occur mainly from other apple orchards with only low reinfestation from P. cocinnea hedges. Finally, we propose that the putative refuge could act as a source of parasitoids without being a major source of aphids. | 2593 | NA | NA | NA | no | no |
| 2594 | 2017 | NA | McKenzie, FE; Bossert, WH | 2005 | An integrated model of Plasmodium falciparum dynamics | The within-host and between-host dynamics of malaria are linked in myriad ways, but most obviously by gametocytes, the parasite blood forms transmissible from human to mosquito. Gametocyte dynamics depend on those of non-transmissible blood forms, which stimulate immune responses, impeding transmission as well as within-host parasite densities. These dynamics can, in turn, influence antigenic diversity and recombination between genetically distinct parasites. Here, we embed a differential-equation model of parasite-immune system interactions within each of the individual humans represented in a discrete-event model of Plasmodium falciparum transmission, and examine the effects of human population turnover, parasite antigenic diversity, recombination, and gametocyte production on the dynamics of malaria. Our results indicate that the local persistence of P. falciparum increases with turnover in the human population and antigenic diversity in the parasite, particularly in combination, and that antigenic diversity arising from meiotic recombination in the parasite has complex differential effects on the persistence of founder and progeny genotypes. We also find that reductions in the duration of individual human infectivity to mosquitoes, even if universal, produce population-level effects only if near-absolute, and that, in competition, the persistence and prevalence of parasite genotypes with gametocyte production concordant with data exceed those of genotypes with higher gametocyte production. This new, integrated approach provides a framework for investigating relation ships between pathogen dynamics within ail individual host and pathogen dynamics within interacting host and vector populations. (C) 2004 Elsevier Ltd. All rights reserved. | 2594 | NA | NA | NA | no | no |
| 2595 | 2017 | NA | Thoss, M; Ilmonen, P; Musolf, K; Penn, DJ | 2011 | Major histocompatibility complex heterozygosity enhances reproductive success | We investigated how heterozygosity at the major histocompatibility complex (MHC) affects fitness in wild-derived (F2) house mice (Mus musculus musculus). To compare and control for potential confounding effects from close inbreeding and genome-wide heterozygosity, we used mice that were systematically outbred. We assessed how heterozygosity at MHC and background loci (using 15 microsatellite markers on 11 different chromosomes) affects individual survival and reproductive success (RS) in large, semi-natural population enclosures. We found that overall heterozygosity significantly increased RS, and this correlation was entirely explained by heterozygosity at two MHC loci. Moreover, we found that the effects of MHC heterozygosity depend on the level of background heterozygosity, and the benefits of maximal MHC heterozygosity show a curvilinear effect with increasing background heterozygosity. The enhanced RS from MHC heterozygosity was not because of increased survival, and although MHC heterozygosity was correlated with body mass, body mass did not correlate with RS when heterozygosity is controlled. Breeders were more MHC heterozygous than nonbreeders for both sexes, indicating that MHC heterozygosity enhanced fecundity, mating success or both. Our results show that (i) MHC heterozygosity enhances fitness among wild, outbred as well as congenic laboratory mice; (ii) heterozygosity-fitness correlations can potentially be explained by a few loci, such as MHC; (iii) MHC heterozygosity can increase fitness, even without affecting survival, by increasing mating and RS; and (iv) MHC effects depend on background genes, and maximal MHC heterozygosity is most beneficial at intermediate or optimal levels of background heterozygosity. | 2595 | NA | NA | NA | no | no |
| 2596 | 2017 | NA | Balgir, RS | 2010 | Phenotypic diversity of sickle cell disorders with special emphasis on public health genetics in India | Human genetic diversity poses a great challenge to community health care in India. Haemoglobin disorders constitute the most common genetic and public health burden on vulnerable people. Prospective studies which are lacking in India present valuable community health and morbidity information for analysis with respect to introspection and evaluation. The present study is designed to fill up this lacuna in presenting community health and morbidity pattern of encountered different sickle cell phenotypes in India. 137 suspected cases of sickle cell disorders referred to our centre for laboratory investigations were analysed using an automated blood cell counter, alkaline electrophoresis and haemoglobin variant analyser. From a community perspective, transfusion dependent sickle cell disease and sickle cell-beta-thalassemia were found prevalent in India. Statistically significant differences in haematological indices were observed between sickle cell trait and sickle cell disease, sickle cell-beta(++)-thalassemia, and sickle cell-beta(+)-thalassemia; between sickle cell disease and sickle cell-beta(++)-thalassemia and sickle cell-beta(+)-thalassemia; and between sickle cell-beta(++) thalassemia and sickle cell-beta(+)-thalassemia with variable symptomatology and clinical manifestations. Genetic heterogeneity of sickle cell-beta-thalassemia was noticed in India. Both mild (African) and severe (Mediterranean) forms of sickle cell-beta-thalassemia (i.e. beta(++)-thalassemia and beta(+)-thalassemia or beta degrees-thalassemia) were encountered suggesting gene flow from the East Mediterranean, Asian Gulf, sub-Sahara and East Africa, reflecting historical events and gene migrations in the region having implications in community health and public health genetics in India. | 2596 | NA | NA | NA | no | no |
| 2597 | 2017 | NA | Barres, B; Carlier, J; Seguin, M; Fenouillet, C; Cilas, C; Ravigne, V | 2012 | Understanding the recent colonization history of a plant pathogenic fungus using population genetic tools and Approximate Bayesian Computation | Understanding the processes by which new diseases are introduced in previously healthy areas is of major interest in elaborating prevention and management policies, as well as in understanding the dynamics of pathogen diversity at large spatial scale. In this study, we aimed to decipher the dispersal processes that have led to the emergence of the plant pathogenic fungus Microcyclus ulei, which is responsible for the South American Leaf Blight (SALB). This fungus has devastated rubber tree plantations across Latin America since the beginning of the twentieth century. As only imprecise historical information is available, the study of population evolutionary history based on population genetics appeared most appropriate. The distribution of genetic diversity in a continental sampling of four countries (Brazil, Ecuador, Guatemala and French Guiana) was studied using a set of 16 microsatellite markers developed specifically for this purpose. A very strong genetic structure was found (F-st = 0.70), demonstrating that there has been no regular gene flow between Latin American M. ulei populations. Strong bottlenecks probably occurred at the foundation of each population. The most likely scenario of colonization identified by the Approximate Bayesian Computation (ABC) method implemented in DIYABC suggested two independent sources from the Amazonian endemic area. The Brazilian, Ecuadorian and Guatemalan populations might stem from serial introductions through human-mediated movement of infected plant material from an unsampled source population, whereas the French Guiana population seems to have arisen from an independent colonization event through spore dispersal. Heredity (2012) 109, 269-279; doi:10.1038/hdy.2012.37; published online 25 July 2012 | 2597 | NA | NA | NA | no | no |
| 2598 | 2017 | NA | Prospero, S; Hansen, EM; Grunwald, NJ; Winton, LM | 2007 | Population dynamics of the sudden oak death pathogen Phytophthora ramorum in Oregon from 2001 to 2004 | Phytophthora ramorum (Oomycetes) is an emerging plant pathogen in forests in southwestern Oregon (Curry County). Moreover, since 2003 it has been repeatedly isolated from plants in Oregon nurseries. In this study, we analysed the genetic diversity of the P. ramorum population in Oregon from 2001 to 2004 by using microsatellites. A total of 323 isolates (272 from the infested forest; 51 from nurseries) were screened at 10 loci. The overall P. ramorum population in Oregon is characterized by low genetic diversity and has all the hallmarks of an introduced organism. All isolates within the A2 mating type belonged to the same clonal lineage and no recombinant genotypes were found. The forest population (24 genotypes) was dominated by a single multilocus genotype which persisted over years, indicating that eradication efforts in the forest have not completely eliminated inoculum sources. In contrast, genotypic evidence suggests that eradication was effective in nurseries. In 2003 and 2004, a total of 11 genotypes were found in the nurseries (one belonged to the European lineage of P. ramorum) but no genotype was recovered in both sampling years. Significant differentiation and low gene flow were detected between nursery and forest populations. Only two nursery genotypes were also found in the forest, and then at low frequency. Thus, the nursery infestation is not caused by the genotypes observed in Curry County, but likely resulted through introduction of novel genotypes from nurseries out-of-state. This highlights the continued importance of sanitation and quarantine in nurseries to prevent further introduction and spread of P. ramorum. | 2598 | NA | NA | NA | no | no |
| 2599 | 2017 | NA | Huang, JC; Meek, C; Kadie, C; Heckerman, D | 2011 | Conditional Random Fields for Fast, Large-Scale Genome-Wide Association Studies | Understanding the role of genetic variation in human diseases remains an important problem to be solved in genomics. An important component of such variation consist of variations at single sites in DNA, or single nucleotide polymorphisms (SNPs). Typically, the problem of associating particular SNPs to phenotypes has been confounded by hidden factors such as the presence of population structure, family structure or cryptic relatedness in the sample of individuals being analyzed. Such confounding factors lead to a large number of spurious associations and missed associations. Various statistical methods have been proposed to account for such confounding factors such as linear mixed-effect models (LMMs) or methods that adjust data based on a principal components analysis (PCA), but these methods either suffer from low power or cease to be tractable for larger numbers of individuals in the sample. Here we present a statistical model for conducting genome-wide association studies (GWAS) that accounts for such confounding factors. Our method scales in runtime quadratic in the number of individuals being studied with only a modest loss in statistical power as compared to LMM-based and PCA-based methods when testing on synthetic data that was generated from a generalized LMM. Applying our method to both real and synthetic human genotype/phenotype data, we demonstrate the ability of our model to correct for confounding factors while requiring significantly less runtime relative to LMMs. We have implemented methods for fitting these models, which are available at http://www.microsoft.com/science. | 2599 | NA | NA | NA | no | no |
| 2600 | 2017 | NA | Bhagwat, SA; Willis, KJ | 2008 | Species persistence in northerly glacial refugia of Europe: a matter of chance or biogeographical traits? | Aim The southern European peninsulas (Iberian, Italian and Balkan) are considered to have been refugia for many European species of plants and animals during the climatic extremes of the Pleistocene ice ages. A number of recent studies (fossil and genetic), however, have provided evidence for full-glacial survival of some species beyond these peninsulas. Here we explore the biogeographical traits of these species, and ask whether they possessed certain characteristics that enabled them to persist in more northerly refugia. Location Europe. Methods Fossil and genetic evidence for refugial localities of species that survived in Europe during the last full-glacial was obtained from the literature (totalling 90 species: 34 woody plants and 56 vertebrates). Forty-seven of these species (23 woody plants and 24 vertebrates) had fossil evidence, whereas the remaining 43 species (11 woody plants and 32 vertebrates) had only genetic evidence. All species were scored according to their present geographical distribution, habitat preference and life-history traits. The species were classified on the basis of these traits using hierarchical cluster analysis. Analysis of similarities was used to examine differences in vertebrate and woody plant species groups that survived only in southerly refugia and those that also persisted in more northerly locations. Non-metric multi-dimensional scaling was used to examine patterns observed between and within groups. Results Results from our analysis of species with fossil and genetic evidence for survival in refugia reveal that species that survived only in southerly refugia were large-seeded trees or thermophilous vertebrates. In contrast, species that had a full-glacial distribution, including more northerly locations, were wind-dispersed, habitat-generalist trees with the ability to reproduce vegetatively, and habitat-generalist mammals with present-day northerly distributions. Main conclusions Analysis of the geographical distribution, habitat preference and life-history traits of the species studied suggests that underlying biogeographical traits may have determined their response to Pleistocene glaciation. The traits most commonly found in present populations with a northerly distribution in Europe enabled the same species to exist much farther north than the southern European peninsulas during the full-glacial. It is possible that many of these species are now in restricted populations, within the ‘warm-stage’ refugia of the current interglacial. The northerly full-glacial survival of a number of woody plants and vertebrate species has significant implications for understanding migration rates of these species in response to climate change. It also has important implications for understanding current patterns of genetic diversity of European species. We suggest that both fossil and genetic evidence should be used to identify and prioritize for conservation of refugial localities in southern and northern Europe. | 2600 | NA | NA | NA | no | no |
| 2601 | 2017 | NA | Stojak, J; McDevitt, AD; Herman, JS; Searle, JB; Wojcik, JM | 2015 | Post-glacial colonization of eastern Europe from the Carpathian refugium: evidence from mitochondrial DNA of the common vole Microtus arvalis | There is now considerable evidence for the survival of temperate species within glacial refugia that were situated at relatively high latitudes, notably the Carpathian Basin and Dordogne region in Europe. However, the prevalence of fossil remains in such locations is rarely matched by molecular evidence for their contribution to subsequent geographical and demographic expansion of the species in question. One obstacle to this has been insufficient analysis of modern samples from the relevant areas, in particular the parts of eastern Europe that surround the Carpathian refugium. In the present study, we examine the patterns of variation in mitochondrial DNA of the common vole (Microtus arvalis), obtained from existing museum specimens and from newly-collected samples obtained in this area. We show that common voles from one of six extant mitochondrial DNA lineages have colonized most of the species’ range in eastern Europe. We contend that the post-glacial dispersal of this lineage most likely originated from the Carpathian refugium, adding support to the argument that such northern refugia made an important contribution to existing genetic diversity in Europe.(c) 2015 The Linnean Society of London, | 2601 | NA | NA | NA | no | no |
| 2602 | 2017 | NA | Hase, K; Nikoh, N; Shimada, M | 2013 | Population admixture and high larval viability among urban toads | In terms of evolutionary biology, a population admixture of more than two distinct lineages may lead to strengthened genetic variation through hybridization. However, a population admixture arising from artificial secondary contact poses significant problems in conservation biology. In urban Tokyo, a population admixture has emerged from two lineages of Japanese common toad: native Bufo japonicus formosus and nonnative B. japonicus japonicus, of which the latter was introduced in the early 20th century. To evaluate the degree of genetic disturbance in the admixed population of these two subspecies, we analyzed genotypes of toads distributed within and outside Tokyo by assessing mtDNA and seven microsatellite loci. We found that the introduced B. japonicus japonicus genotype dominates six local populations in the Tokyo admixture zone and was clearly derived from past introgressive hybridization between the two subspecies. These observations were supported by morphological assessments. Furthermore, the average larval survival rate in Tokyo was significantly higher than that outside Tokyo, suggesting that the temporary contribution of introduced toads occurred through introgression. The fitness of toads in urban Tokyo may thus be increasing with the assistance of nonnative individuals. | 2602 | NA | NA | NA | no | no |
| 2603 | 2017 | NA | Wayne, RK; Ostrander, EA | 1999 | Origin, genetic diversity, and genome structure of the domestic dog | Comparative analysis of mammalian genomes provides important insight into the structure and function of genes. However, the comparative analysis of gene sequences from individuals of the same and different species also provides insight into the evolution of genes, populations, and species. We exemplify these two uses of genomic information. First, we document the evolutionary relationships of the domestic dog to other carnivores by using a variety of DNA-based information. A phylogenetic comparison of mitochondrial DNA sequences in dogs and gray wolves shows that dogs may have originated from multiple wolf populations at a time much earlier than suggested by the archaeologic record. We discuss previous theories about dog development and evolution in light of the new genetic data. Second, we review recent progress in dog genetic mapping due to the development of hypervariable markers and specific chromosome paints. Extensive genetic homology in gene order and function between humans and dogs has been discovered. The dog promises to be a valuable model for identifying genes that control morphologic differences between mammals as well as understanding genetically based disease. BioEssays 21:247-257, 1999. (C) 1999 John Wiley & Sons, Inc. | 2603 | NA | NA | NA | no | no |
| 2604 | 2017 | NA | Cui, YJ; Yu, C; Yan, YF; Li, DF; Li, YJ; Jombart, T; Weinert, LA; Wang, ZY; Guo, ZB; Xu, LZ; Zhang, YJ; Zheng, HC; Qin, N; Xiao, X; Wu, MS; Wang, XY; Zhou, DS; Qi, ZZ; Du, ZM; Wu, HL; Yang, XW; Cao, HZ; Wang, H; Wang, J; Yao, SS; Rakin, A; Li, YR; Falush, D; Balloux, F; Achtman, M; Song, YJ; Wang, J; Yang, RF | 2013 | Historical variations in mutation rate in an epidemic pathogen, Yersinia pestis | The genetic diversity of Yersinia pestis, the etiologic agent of plague, is extremely limited because of its recent origin coupled with a slow clock rate. Here we identified 2,326 SNPs from 133 genomes of Y. pestis strains that were isolated in China and elsewhere. These SNPs define the genealogy of Y. pestis since its most recent common ancestor. All but 28 of these SNPs represented mutations that happened only once within the genealogy, and they were distributed essentially at random among individual genes. Only seven genes contained a significant excess of nonsynonymous SNP, suggesting that the fixation of SNPs mainly arises via neutral processes, such as genetic drift, rather than Darwinian selection. However, the rate of fixation varies dramatically over the genealogy: the number of SNPs accumulated by different lineages was highly variable and the genealogy contains multiple polytomies, one of which resulted in four branches near the time of the Black Death. We suggest that demographic changes can affect the speed of evolution in epidemic pathogens even in the absence of natural selection, and hypothesize that neutral SNPs are fixed rapidly during intermittent epidemics and outbreaks. | 2604 | NA | NA | NA | no | no |
| 2605 | 2017 | NA | Lopez, KIM; Martinez, SEF; Moguel, MCM; Romero, LT; Figueroa, CS; Pacheco, GV; Ibarra, B; Corona, JS | 2007 | Genetic diversity of the IL-4, IL-4 receptor and IL-13 loci in mestizos in the general population and in patients with asthma from three subpopulations in Mexico | Asthma is an inflammatory airway disease characterized by increased serum IgE levels, mucus hypersecretion and infiltration of inflammatory cells, and is a multifactorial disease that exhibits genetic heterogeneity. Polymorphisms in the interleukin-4 (C-590T), interleukin-4 receptor (ile50val and gln576arg), and interleukin-13 (arg130gln) genes have been described as susceptibility alleles for asthma. This study was designed to determine whether asthma susceptibility is influenced by genotypic and allelic distribution of the above polymorphisms in three Mexican subpopulations. Four hundred and thirty-seven subjects from three Mexican subpopulations were classified into two groups: general population and affected/unaffected and genotyped for the above polymorphisms. We compared the distributions of the loci in the groups. In addition, we undertook association analysis between these loci and asthma phenotype in each affected/unaffected group, and determined Nei’s genetic distance between the three subpopulations. The allelic and genotypic distributions of the polymorphisms differed between the three subpopulations. There was no association between any of the polymorphisms and asthma phenotype. However, there was a differential distribution of haplogroups (P < 0.0001) between the affected and the unaffected groups from the subpopulations of Jalisco and Guerrero. The genetic distribution of the four polymorphisms in the subpopulations did not influence susceptibility to asthma. Furthermore, the difference in the prevalence of asthma in these subpopulations is not attributable to the genetic background for the four polymorphisms analysed. However, haplogroup analysis suggests that the interaction of the polymorphisms and other predisposing alleles leads to the expression of the clinical phenotype. | 2605 | NA | NA | NA | no | no |
| 2606 | 2017 | NA | Jackson, FLC | 2008 | Ethnogenetic layering (EL): an alternative to the traditional race model in human variation and health disparity studies | Background: Traditionally, studies in human biodiversity, disease risk, and health disparities have defined populations in the context of typological racial models. However, such racial models are often imprecise generalizations that fail to capture important local patterns of human biodiversity. Aim: More explicit, detailed, and integrated information on relevant geographic, environmental, cultural, genetic, historical, and demographic variables are needed to understand local group expressions of disease inequities. This paper details the methods used in ethnogenetic layering (EL), a non-typological alternative to the current reliance of the biological racial paradigm in public health, epidemiology, and biomedicine. Subjects and methods: EL is focused on geographically identified microethnic groups or MEGs, a more nuanced and sensitive level of analysis than race. Using the MEG level of analysis, EL reveals clinical variations, details the causes of health disparities, and provides a foundation for bioculturally effective intervention strategies. EL relies on computational approaches by using GIS-facilitated maps to produce horizontally stratified geographical regional profiles which are then stacked and evaluated vertically. Each horizontal digital map details local geographic variation in the attributes of a particular database; usually this includes data on local historical demography, genetic diversity, cultural patterns, and specific chronic disease risks (e.g. dietary and toxicological exposures). Horizontal visual display of these layered maps permits vertical analysis at various geographic hot spots. Results and conclusions: From these analyses, geographical areas and their associated MEGs with highly correlated chronic disease risk factors can be identified and targeted for further study. | 2606 | NA | NA | NA | no | no |
| 2607 | 2017 | NA | Ross, L; Blackmon, H; Lorite, P; Gokhman, VE; Hardy, NB | 2015 | Recombination, chromosome number and eusociality in the Hymenoptera | Extraordinarily high rates of recombination have been observed in some eusocial species. The most popular explanation is that increased recombination increases genetic variation among workers, which in turn increases colony performance, for example by increasing parasite resistance. However, support for the generality of higher recombination rates among eusocial organisms remains weak, due to low sample size and a lack of phylogenetic independence of observations. Recombination rate, although difficult to measure directly, is correlated with chromosome number. As predicted, several authors have noted that chromosome numbers are higher among the eusocial species of Hymenoptera (ants, bees and wasps). Here, we present a formal comparative analysis of karyotype data from 1567 species of Hymenoptera. Contrary to earlier studies, we find no evidence for an absolute difference between chromosome number in eusocial and solitary species of Hymenoptera. However, we find support for an increased rate of chromosome number change in eusocial taxa. We show that among eusocial taxa colony size is able to explain some of the variation in chromosome number: intermediate-sized colonies have more chromosomes than those that are either very small or very large. However, we were unable to detect effects of a number of other colony characteristics predicted to affect recombination rate - including colony relatedness and caste number. Taken together, our results support the view that a eusocial lifestyle has led to variable selection pressure for increased recombination rates, but that identifying the factors contributing to this variable selection will require further theoretical and empirical effort. | 2607 | NA | NA | NA | no | no |
| 2608 | 2017 | NA | Pechouskova, E; Dammhahn, M; Brameier, M; Fichtel, C; Kappeler, PM; Huchard, E | 2015 | MHC class II variation in a rare and ecological specialist mouse lemur reveals lower allelic richness and contrasting selection patterns compared to a generalist and widespread sympatric congener | The polymorphism of immunogenes of the major histocompatibility complex (MHC) is thought to influence the functional plasticity of immune responses and, consequently, the fitness of populations facing heterogeneous pathogenic pressures. Here, we evaluated MHC variation (allelic richness and divergence) and patterns of selection acting on the two highly polymorphic MHC class II loci (DRB and DQB) in the endangered primate Madame Berthe’s mouse lemur (Microcebus berthae). Using 454 pyrosequencing, we examined MHC variation in a total of 100 individuals sampled over 9 years in Kirindy Forest, Western Madagascar, and compared our findings with data obtained previously for its sympatric congener, the grey mouse lemur (Microcebus murinus). These species exhibit a contrasting ecology and demography that were expected to affect MHC variation and molecular signatures of selection. We found a lower allelic richness concordant with its low population density, but a similar level of allelic divergence and signals of historical selection in the rare feeding specialist M. berthae compared to the widespread generalist M. murinus. These findings suggest that demographic factors may exert a stronger influence than pathogen-driven selection on current levels of allelic richness in M. berthae. Despite a high sequence similarity between the two congeners, contrasting selection patterns detected at DQB suggest its potential functional divergence. This study represents a first step toward unravelling factors influencing the adaptive divergence of MHC genes between closely related but ecologically differentiated sympatric lemurs and opens new questions regarding potential functional discrepancy that would explain contrasting selection patterns detected at DQB. | 2608 | NA | NA | NA | no | no |
| 2609 | 2017 | NA | Wang, Z; Zhang, JF; Li, H; Li, JY; Niimi, M; Ding, GH; Chen, HF; Xu, J; Zhang, HJ; Xu, Z; Dai, YL; Gui, TT; Li, SD; Liu, Z; Wu, SJ; Cao, MS; Zhou, L; Lu, XY; Wang, JX; Yang, J; Fu, YH; Yang, DS; Song, J; Zhu, TQ; Li, S; Ning, B; Wang, ZY; Koike, T; Shiomi, M; Liu, EQ; Chen, LN; Fan, JL; Chen, YE; Li, YX | 2016 | Hyperlipidemia-associated gene variations and expression patterns revealed by whole-genome and transcriptome sequencing of rabbit models | The rabbit (Oryctolagus cuniculus) is an important experimental animal for studying human diseases, such as hypercholesterolemia and atherosclerosis. Despite this, genetic information and RNA expression profiling of laboratory rabbits are lacking. Here, we characterized the whole-genome variants of three breeds of the most popular experimental rabbits, New Zealand White (NZW), Japanese White (JW) and Watanabe heritable hyperlipidemic (WHHL) rabbits. Although the genetic diversity of WHHL rabbits was relatively low, they accumulated a large proportion of high-frequency deleterious mutations due to the small population size. Some of the deleterious mutations were associated with the pathophysiology of WHHL rabbits in addition to the LDLR deficiency. Furthermore, we conducted transcriptome sequencing of different organs of both WHHL and cholesterol-rich diet (Chol)-fed NZW rabbits. We found that gene expression profiles of the two rabbit models were essentially similar in the aorta, even though they exhibited different types of hypercholesterolemia. In contrast, Chol-fed rabbits, but not WHHL rabbits, exhibited pronounced inflammatory responses and abnormal lipid metabolism in the liver. These results provide valuable insights into identifying therapeutic targets of hypercholesterolemia and atherosclerosis with rabbit models. | 2609 | NA | NA | NA | no | no |
| 2610 | 2017 | NA | Schacherer, J; Shapiro, JA; Ruderfer, DM; Kruglyak, L | 2009 | Comprehensive polymorphism survey elucidates population structure of Saccharomyces cerevisiae | Comprehensive identification of polymorphisms among individuals within a species is essential both for studying the genetic basis of phenotypic differences and for elucidating the evolutionary history of the species. Large-scale polymorphism surveys have recently been reported for human(1), mouse(2) and Arabidopsis thaliana(3). Here we report a nucleotide-level survey of genomic variation in a diverse collection of 63 Saccharomyces cerevisiae strains sampled from different ecological niches (beer, bread, vineyards, immunocompromised individuals, various fermentations and nature) and from locations on different continents. We hybridized genomic DNA from each strain to whole-genome tiling microarrays and detected 1.89 million single nucleotide polymorphisms, which were grouped into 101,343 distinct segregating sites. We also identified 3,985 deletion events of length >200 base pairs among the surveyed strains. We analysed the genome-wide patterns of nucleotide polymorphism and deletion variants, and measured the extent of linkage disequilibrium in S. cerevisiae. These results and the polymorphism resource we have generated lay the foundation for genome-wide association studies in yeast. We also examined the population structure of S. cerevisiae, providing support for multiple domestication events as well as insight into the origins of pathogenic strains. | 2610 | NA | NA | NA | no | no |
| 2611 | 2017 | NA | Ben-David, R; Peleg, Z; Dinoor, A; Saranga, Y; Korol, AB; Fahima, T | 2014 | Genetic dissection of quantitative powdery mildew resistance loci in tetraploid wheat | Durum wheat, Triticum turgidum ssp. durum Desf., is an important crop particularly in the Mediterranean basin. Powdery mildew, caused by the pathogen Blumeria graminis f. sp. tritici (Bgt), is a major disease of wheat that results in significant yield losses worldwide. A recombinant inbred line (RIL) population, derived from a cross between durum wheat and wild emmer wheat, T. turgidum ssp. dicoccoides, was used for genomic dissection of quantitative and qualitative resistance loci against wheat powdery mildew based on a genomic map of > 600 markers, evenly distributed across the A and B genomes of tetraploid wheat. The genetic analysis of the phenotypic reactions of the RIL population to two Bgt isolates revealed two different resistance mechanisms. The first is monogenic: a wild emmer wheat allele in a single locus conferring complete resistance to Bgt#15, previously designated as PmG16. The second one is polygenic: a set of durum wheat alleles, in five independent QTLs that control partial resistance to Bgt#66 in the RIL population, with a LOD score range of 3.4-19.8. One of them is a major quantitative resistance locus (QRL) that was mapped on chromosome 1A and explains 26.4 % of the variance. In most of the detected QRLs, the durum wheat alleles conferred resistance to powdery mildew. These findings are exceptional in the sense that, so far, only a few Pm alleles originated from a durum wheat background. Therefore, our results emphasize the high potential of exploiting the wide genetic diversity of tetraploid wheat germplasm for wheat breeding using modern wheat genomics tools. | 2611 | NA | NA | NA | no | no |
| 2612 | 2017 | NA | Yumurtaci, A; Sipahi, H; Al-Abdallat, A; Jighly, A; Baum, M | 2017 | Construction of new EST-SSRs for Fusarium resistant wheat breeding | Surveying Fusarium resistance in wheat with easy applicable molecular markers such as simple sequence repeats (SSRs) is a prerequest for molecular breeding. Expressed sequence tags (ESTs) are one of the main sources for development of new SSR candidates. Therefore, 18.292 publicly available wheat ESTs were mined and genotyping of newly developed 55 EST-SSR derived primer pairs produced clear fragments in ten wheat cultivars carrying different levels of Fusarium resistance. Among the proved markers, 23 polymorphic EST-SSRs were obtained and related alleles were mostly found on B and D genome. Based on the fragment profiling and similarity analysis, a 327 bp amplicon, which was a product of contig 1207 (chromosome 5BL), was detected only in Fusarium head blight (FHB) resistant cultivars (CM82036 and Sumai) and the amino acid sequences showed a similarity to pathogen related proteins. Another FHB resistance related EST-SSR, Contig 556 (chromosome 1BL) produced a 151 bp fragment in Sumai and was associated to wax2-like protein. A polymorphic 204 bp fragment, derived from Contig 578 (chromosome 1DL), was generated from root rot (FRR) resistant cultivars (2-49; Altay2000 and Sunco). A total of 98 alleles were displayed with an average of 1.8 alleles per locus and the polymorphic information content (PIC) ranged from 0.11 to 0.78. Dendrogram tree with two main and five sub-groups were displayed the highest genetic relationship between FRR resistant cultivars (2-49 and Altay2000), FRR sensitive cultivars (Seri82 and Scout66) and FHB resistant cultivars (CM82036 and Sumai). Thus, exploitation of these candidate EST-SSRs may help to genotype other wheat sources for Fusarium resistance. (C) 2017 Elsevier Ltd. All rights reserved. | 2612 | NA | NA | NA | no | no |
| 2613 | 2017 | NA | Solnick, JV; Hansen, LM; Salama, NR; Boonjakuakul, JK; Syvanen, M | 2004 | Modification of Helicobacter pylori outer membrane protein expression during experimental infection of rhesus macaques | Clinical isolates of Helicobacter pylori show marked diversity, which may derive from genomic changes that occur during the often lifelong association of the bacterium with its human host. We used the rhesus macaque model, together with DNA microarrays, to examine genomic changes in H. pylori that occur early during experimental infection. Microarray analysis showed that H. pylori recovered from challenged macaques had deleted babA, a member of a large family of paralogous outer membrane proteins (OMPs) that mediates attachment of H. pylori to the Lewis B blood group antigen on gastric epithelium. In some cases the babA gene was replaced by babB, an uncharacterized OMP that is closely related to babA. In other cases the babA gene was present but was not expressed because of alteration in dinucleotide CT repeats in the 5’ coding region. In either case, strains lacking babA did not adhere to Lewis B, which is expressed on macaque gastric epithelium. Absence of babA and duplication of babB was also seen in H. pylori isolates derived from human clinical samples, suggesting that this gene conversion event is not unique to experimentally infected rhesus monkeys. These results demonstrate in real time with a relevant animal model that H. pylori regulates OMP expression in vivo by using both antigenic variation and phase variation. We suggest that changes in babA and babB after experimental infection of macaques represent a dynamic response in the H. pylori outer membrane that facilitates adherence to the gastric epithelium and promotes chronic infection. | 2613 | NA | NA | NA | no | no |
| 2614 | 2017 | NA | Manzanilla-Lopez, RH; Atkins, SD; Clark, IM; Kerry, BR; Hirsch, PR | 2009 | Measuring abundance, diversity and parasitic ability in two populations of the nematophagous fungus Pochonia chlamydosporia var. chlamydosporia | Abundance, genetic diversity and parasitic ability in the facultative nematode parasite Pochonia chlamydosporia var. chlamydosporia were compared in soils from two sites in Portugal under long-term tomato cultivation where root-knot nematodes (Meloidogyne sp.) were present. Fungal abundance assessed by selective agar or real-time quantitative PCR with specific primers was similar in both soils. PCR fingerprinting of isolates with ERIC primers indicated that the dominant P. c. var. chlamydosporia biotypes (profiles A and B) in both soils were very closely related, although a second biotype (profile C) was detected in one soil. When tomato plants infected with M. incognita were grown in the two soils, only profiles A and B were recovered from eggs. Primers based on polymorphisms in vcp1 demonstrated that isolates with profiles A and B were likely to prefer root-knot nematodes, whereas profile C preferred cyst nematodes. In the soil containing profiles A, B and C, egg parasitism by P. chlamydosporia was estimated at 1% using water agar plates with antibiotics but fewer than 0.2% of M. incognita eggs were shown to be infected with P. c. var. chlamydosporia when using species-specific -tubulin-PCR primers. In contrast, the soil containing only profile B showed 22% egg parasitism on water agar plates and more than 2.5% of eggs were confirmed as P. c. var. chlamydosporia by species-specific -tubulin-PCR primers. The results, which reveal limited diversity within the fungus at the two sites, are discussed in relation to biological control of plant-parasitic nematodes. | 2614 | NA | NA | NA | no | no |
| 2615 | 2017 | NA | Ben-Shlomo, R; Neufeld, E; Berger, D; Lenington, S; Ritte, U | 2007 | The dynamic of the t-haplotype in wild populations of the house mouse Mus musculus domesticus in Israel | The t-haplotype, a variant of the proximal part of the mouse chromosome 17, is composed of at least four inversions and is inherited as a single genetic unit. The haplotype causes embryonic mortality or male sterility when homozygous. Genes within the complex are responsible for distortion of Mendelian transmission ratio in males. Thus, the t-haplotype in heterozygous males is transferred to over 95% of the progeny. We examined the dynamic and behavior of the t-haplotype in wild populations of the house mouse in Israel. The Israeli populations show high frequency (15%-20%) of both partial and complete t-carrying mice, supporting the suggestion that the t-complex evolved in the M. domesticus line in the Israeli region. In one population that had the highest frequency of t-carrying individuals, we compared the level of gene diversity between t-carrying and normal mice in the marker’s loci: H-2 locus of the major histocompatibility complex (MHC) on the t-haplotype of chromosome 17, three microsatellites on other chromosomes, and the mitochondrial D-loop. Genetic variability was high in all tested loci in both t and (+) mice. All t mice carried the same chromosome and showed the same H-2 haplotype. While t-carrying mice showed significant H-2 heterozygotes access, (+) mice expressed significant H-2 heterozygote deficiency. There were no differences in the level of gene diversity between t and (+) mice in the other loci. Heterozygosity level at the MHC may be an additional factor in the selective forces balancing the t-haplotype polymorphism. | 2615 | NA | NA | NA | no | no |
| 2616 | 2017 | NA | Song, LF; Wang, W; Conrads, G; Rheinberg, A; Sztajer, H; Reck, M; Wagner-Dobler, I; Zeng, AP | 2013 | Genetic variability of mutans streptococci revealed by wide whole-genome sequencing | “Background: Mutans streptococci are a group of bacteria significantly contributing to tooth decay. Their genetic variability is however still not well understood. Results: Genomes of 6 clinical S. mutans isolates of different origins, one isolate of S. sobrinus (DSM 20742) and one isolate of S. ratti (DSM 20564) were sequenced and comparatively analyzed. Genome alignment revealed a mosaic-like structure of genome arrangement. Genes related to pathogenicity are found to have high variations among the strains, whereas genes for oxidative stress resistance are well conserved, indicating the importance of this trait in the dental biofilm community. Analysis of genome-scale metabolic networks revealed significant differences in 42 pathways. A striking dissimilarity is the unique presence of two lactate oxidases in S. sobrinus DSM 20742, probably indicating an unusual capability of this strain in producing H2O2 and expanding its ecological niche. In addition, lactate oxidases may form with other enzymes a novel energetic pathway in S. sobrinus DSM 20742 that can remedy its deficiency in citrate utilization pathway. Using 67 S. mutans genomes currently available including the strains sequenced in this study, we estimates the theoretical core genome size of S. mutans, and performed modeling of S. mutans pan-genome by applying different fitting models. An”“open”" pan-genome was inferred. Conclusions: The comparative genome analyses revealed diversities in the mutans streptococci group, especially with respect to the virulence related genes and metabolic pathways. The results are helpful for better understanding the evolution and adaptive mechanisms of these oral pathogen microorganisms and for combating them." | 2616 | NA | NA | NA | no | no |
| 2617 | 2017 | NA | Zhong, ZJ; Tian, YN; Song, Y; Deng, L; Li, JX; Ren, ZH; Ma, XP; Gu, XB; He, CL; Geng, Y; Peng, GN | 2017 | Molecular characterization and multi-locus genotypes of Enterocytozoon bieneusi from captive red kangaroos (Macropus Rfus) in Jiangsu province, China | Enterocytozoon bieneusi is the most common pathogen of microsporidian species infecting humans worldwide. Although E. bieneusi has been found in a variety of animal hosts, information on the presence of E. bieneusi in captive kangaroos in China is limited. The present study was aimed at determining the occurrence and genetic diversity of E. bieneusi in captive kangaroos. A total of 61 fecal specimens (38 from red kangaroos and 23 from grey kangaroos) were collected from Nanjing Hongshan Forest Zoo and Hongshan Kangaroo Breeding Research Base, Jiangsu province, China. Using the nested PCR amplification ITS gene of rRNA of E. bieneusi, totally 23.0% (14/61) of tested samples were PCR-positive with three genotypes (i.e. one known genotype, CHK1, and two novel genotypes, CSK1 and CSK2). Multi-locus sequence typing using three microsatellites (MS1, MS3, and MS7) and one minisatellite (MS4) revealed one, five, two, and one types at these four loci, respectively. In phylogenetic analysis, the two genotypes, CHK1 and CSK1, were clustered into a new group of unknown zoonotic potential, and the novel genotype CSK2 was clustered into a separate clade with PtEb and PtEbIX. To date, this is the first report on the presence of E. bieneusi in captive red kangaroos in Jiangsu province, China. Furthermore, a high degree of genetic diversity was observed in the E. bieneusi genotype and seven MLGs (MLG1-7) were found in red kangaroos. Our findings suggest that infected kangaroo may act as potential reservoirs of E. bieneusi and be source to transmit infections to other animal. | 2617 | NA | NA | NA | no | no |
| 2618 | 2017 | NA | Lotterhos, KE; Markel, RW | 2012 | Oceanographic drivers of offspring abundance may increase or decrease reproductive variance in a temperate marine fish | In species that reproduce into uncertain environments, the relationship between mean reproductive success (the abundance of new recruits) and the variance in reproductive success (whether adults contribute disproportionally more offspring) may not be straightforward because of stochastic environmental processes that create high variance in reproductive success among adults. In this study, we investigated the relationships between oceanography, reproductive success and reproductive variance in the black rockfish, Sebastes melanops, a long-lived temperate reef fish with pelagic larvae. We quantified black rockfish recruitment, genetic diversity and growth rates from otolith microstructure over 5 years (20052009) during which oceanographic conditions differed. We used cross-correlations to determine windows of time during which oceanographic variables were significantly correlated with the resulting abundance or genetic diversity of recruits. We found that warmer ocean temperatures were positively correlated with the abundance of recruits, as well as the effective number of breeders. In contrast, the strength of coastal upwelling during settlement was positively correlated with the annual abundance of new recruits, but was negatively correlated with the effective number of breeders. Larval growth rates were explained substantially more by temperature than by upwelling and suggested that temperature affected survival through growth, while upwelling affected survival through transport. Our results indicated that cold ocean temperatures and intense upwelling caused sweepstakes-like processes to operate on black rockfish populations, despite high abundances of recruits. We propose that a decoupling of the mean and variance in reproductive success may be characteristic of organisms that reproduce into uncertain environments. | 2618 | NA | NA | NA | no | no |
| 2619 | 2017 | NA | Gariboldi, MC; Tunez, JI; Failla, M; Hevia, M; Panebianco, MV; Viola, MNP; Vitullo, AD; Cappozzo, HL | 2016 | Patterns of population structure at microsatellite and mitochondrial DNA markers in the franciscana dolphin (Pontoporia blainvillei) | The franciscana dolphin, Pontorporia blainvillei, is an endemic cetacean of the Atlantic coast of South America. Its coastal distribution and restricted movement patterns make this species vulnerable to anthropogenic factors, particularly to incidental by-catch. We used mitochondrial DNA control region sequences, 10 microsatellites, and sex data to investigate the population structure of the franciscana dolphin from a previously established management area, which includes the southern edge of its geographic range. F-statistics and Bayesian cluster analyses revealed the existence of three genetically distinct populations. Based on the microsatellite loci, similar levels of genetic variability were found in the area; 13 private alleles were found in Monte Hermoso, but none in Claromeco. When considering the mitochondrial DNA control region sequences, lower levels of genetic diversity were found in Monte Hermoso, when compared to the other localities. Low levels of gene flow were found between most localities. Additionally, no evidence of isolation by distance nor sex-biased dispersal was detected in the study area. In view of these results showing that populations from Necochea/Claromeco, Monte Hermoso, and Rio Negro were found to be genetically distinct and the available genetic information for the species previously published, Argentina would comprise five distinct populations: Samborombon West/Samborombon South, Cabo San Antonio/Buenos Aires East, Necochea/Claromeco/Buenos Aires Southwest, Monte Hermoso, and Rio Negro. In order to ensure the long-term survival of the franciscana dolphin, management and conservation strategies should be developed considering each of these populations as different management units. | 2619 | NA | NA | NA | no | no |
| 2620 | 2017 | NA | Palermo, S; Capra, E; Torremorell, M; Dolzan, M; Davoli, R; Haley, CS; Giuffra, E | 2009 | Toll-like receptor 4 genetic diversity among pig populations | The transmembrane glycoprotein encoded by the Toll-like receptor 4 gene (TLR4) acts as the transducing subunit of the lipopolysaccharide receptor complex of mammals, which is a major sensor of infections by Gram-negative bacteria. As variation in TLR4 may alter host immune response to lipopolysaccharide, the association between TLR4 polymorphisms and immune traits of the respiratory and gut systems has important implications for livestock. Here, a sequence dataset from 259 animals belonging to commercial and traditional European pig populations, consisting of 4305 bp of TLR4, including the full transcribed region, a portion of intron 2 and the putative promoter region, was used to explore genetic variation segregating at the TLR4 locus. We identified 34 single nucleotide polymorphisms, 17 in the coding sequence and 17 in the non-coding region. Five non-synonymous mutations clustered within, or in close proximity to, the hypervariable domain of exon 3. In agreement with studies in other mammals, a major exon 3 haplotype segregated at high frequency in the whole sample of 259 pigs, while variants carrying non-synonymous substitutions showed frequencies ranging between 0.6% and 8.7%. Although results on exon 3 provided suggestive evidence for purifying selection occurring at the porcine TLR4 gene, the analysis of both coding and non-coding regions highlighted the fact that demographic factors strongly influence the tests of departure from neutrality. The phylogenetic analysis of TLR4 identified three clusters of variation (ancestral, Asian, European), supporting the evidence of Asian introgression in European main breeds and the well documented history of pig breed domestication previously identified by mtDNA analysis. | 2620 | NA | NA | NA | no | no |
| 2621 | 2017 | NA | Larson, JE; Sheley, RL; Hardegree, SP; Doescher, PS; James, JJ | 2016 | Do key dimensions of seed and seedling functional trait variation capture variation in recruitment probability? | Seedling recruitment is a critical driver of population dynamics and community assembly, yet we know little about functional traits that define different recruitment strategies. For the first time, we examined whether trait relatedness across germination and seedling stages allows the identification of general recruitment strategies which share core functional attributes and also correspond to recruitment outcomes in applied settings. We measured six seed and eight seedling traits (lab- and field-collected, respectively) for 47 varieties of dryland grasses and used principal component analysis (PCA) and cluster analysis to identify major dimensions of trait variation and to isolate trait-based recruitment groups, respectively. PCA highlighted some links between seed and seedling traits, suggesting that relative growth rate and root elongation rate are simultaneously but independently associated with seed mass and initial root mass (first axis), and with leaf dry matter content, specific leaf area, coleoptile tissue density and germination rate (second axis). Third and fourth axes captured separate tradeoffs between hydrothermal time and base water potential for germination, and between specific root length and root mass ratio, respectively. Cluster analysis separated six recruitment types along dimensions of germination and growth rates, but classifications did not correspond to patterns of germination, emergence or recruitment in the field under either of two watering treatments. Thus, while we have begun to identify major threads of functional variation across seed and seedling stages, our understanding of how this variation influences demographic processes-particularly germination and emergence-remains a key gap in functional ecology. | 2621 | NA | NA | NA | no | no |
| 2622 | 2017 | NA | Waman, VP; Kolekar, P; Ramtirthkar, MR; Kale, MM; Kulkarni-Kale, U | 2016 | Analysis of genotype diversity and evolution of Dengue virus serotype 2 using complete genomes | Back ground. Dengue is one of the most common arboviral diseases prevalent worldwide and is caused by Dengue viruses (genus Flavivirus, family Flaviviridae). There are four serotypes of Dengue Virus (DENV-1 to DENV-4), each of which is further subdivided into distinct genotypes. DENV-2 is frequently associated with severe dengue infections and epidemics. DENV-2 consists of six genotypes such as Asian/American, Asian I, Asian II, Cosmopolitan, American and sylvatic. Comparative genomic study was carried out to infer population structure of DENV-2 and to analyze the role of evolutionary and spatiotemporal factors in emergence of diversifying lineages. Methods. Complete genome sequences of 990 strains of DENV-2 were analyzed using Bayesian-based population genetics and phylogenetic approaches to infer genetically distinct lineages. The role of spatiotemporal factors, genetic recombination and selection pressure in the evolution of DENV-2 is examined using the sequence-based bioinformatics approaches. Results. DENV-2 genetic structure is complex and consists of fifteen subpopulations/lineages. The Asian/American genotype is observed to be diversified into seven lineages. The Asian I, Cosmopolitan and sylvatic genotypes were found to be subdivided into two lineages, each. The populations of American and Asian II genotypes were observed to be homogeneous. Significant evidence of episodic positive selection was observed in all the genes, except NS4A. Positive selection operational on a few codons in envelope gene confers antigenic and lineage diversity in the American strains of Asian/American genotype. Selection on codons of non-structural genes was observed to impact diversification of lineages in Asian I, cosmopolitan and sylvatic genotypes. Evidence of intra/inter-genotype recombination was obtained and the uncertainty in classification of recombinant strains was resolved using the population genetics approach. Discussion. Complete genome-based analysis revealed that the worldwide population of DENV-2 strains is subdivided into fifteen lineages. The population structure of DENV-2 is spatiotemporal and is shaped by episodic positive selection and recombination. Intra-genotype diversity was observed in four genotypes (Asian/American, Asian I, cosmopolitan and sylvatic). Episodic positive selection on envelope and non-structural genes translates into antigenic diversity and appears to be responsible for emergence of strains/lineages in DENV-2 genotypes. Understanding of the genotype diversity and emerging lineages will be useful to design strategies for epidemiological surveillance and vaccine design. | 2622 | NA | NA | NA | no | no |
| 2623 | 2017 | NA | Long, K; Cao, TT; Keller, JJ; Tarpy, DR; Shin, M; Schneider, SS | 2017 | Levels of selection shaping caste interactions during queen replacement in the honey bee, Apis mellifera | We investigated the different levels of selection that may influence the queen replacement process in honey bees by monitoring queen-queen and worker-queen interactions in queenless observation colonies containing populations of paint-marked workers that were either related or not related to introduced virgin queens experimentally reared to be of low- and high-quality, as estimated by differences in size and potential reproductive capacities. The high-quality queens were more likely to survive and become the new laying queen of their colonies. Queen survival was positively associated with both queen fighting ability and worker-queen interactions. Surviving queens were more aggressive and had greater fighting success. However, surviving queens were not bigger than killed queens nor were they the first queens to emerge, suggesting that queen size and early emergence alone are not the determining factors of queen fighting ability. The worker-queen interaction that was most strongly associated with the outcome of queen replacement was the vibration signal, which is a communication signal that workers perform on virgin queens. Surviving queens were vibrated at rates 3-4 times those experienced by killed queens and a queen’s vibration rate was positively correlated with her fighting ability and the number of rivals killed. Workers showed no consistent preferences for related vs. unrelated queens and the proportion of interactions received from related workers was not associated with any aspect of queen fighting ability and success monitored. Our results suggests that caste interactions during queen replacement have been shaped by both selection acting at the level of the individual queen (which favors higher quality queens with greater fighting ability) and selection acting at the level of the colony (which favors workers directing vibration signals towards queens with greater fighting potential), ultimately resulting in a higher quality queen becoming the new laying queen of the colony. Selection acting at the level of the individual worker through kin selection, which would favor preferential treatment of related queens, did not consistently influence caste interactions or the outcome of the replacement process. Thus, the outcome of queen replacement in honey bees may be determined primarily by a combination of a queen’s inherent fighting ability coupled with the rate at which she receives some interactions (particularly vibration signals) from workers. | 2623 | NA | NA | NA | no | no |
| 2624 | 2017 | NA | Thabuis, A; Palloix, A; Pflieger, S; Daubeze, AM; Caranta, C; Lefebvre, V | 2003 | Comparative mapping of Phytophthora resistance loci in pepper germplasm: evidence for conserved resistance loci across Solanaceae and for a large genetic diversity | Phytophthora capsici Leonian, known as the causal agent of the stem, collar and root rot, is one of the most serious problems limiting the pepper crop in many areas in the world. Genetic resistance to the parasite displays complex inheritance. Quantitative trait locus (QTL) analysis was performed in three intraspecific pepper populations, each involving an unrelated resistant accession. Resistance was evaluated by artificial inoculations of roots and stems, allowing the measurement of four components involved in different steps of the plant-pathogen interaction. The three genetic maps were aligned using common markers, which enabled the detection of QTLs involved in each resistance component and the comparison of resistance factors existing among the three resistant accessions. The major resistance factor was found to be common to the three populations. Another resistance factor was found conserved between two populations, the others being specific to a single cross. This comparison across intraspecific germplasm revealed a large variability for quantitative resistance loci to P. capsici. It also provided insights both into the allelic relationships between QTLs across pepper germplasm and for the comparative mapping of resistance factors across the Solanaceae. | 2624 | NA | NA | NA | no | no |
| 2625 | 2017 | NA | Creel, S; Creel, NM | 1998 | Six ecological factors that may limit African wild dogs, Lycaon pictus | African wild dogs (Lycaon pictus) always live at low population densities relative to sympatric large carnivores. This suggests that there are basic ecological reasons for the wild dog’s endangered status. We examined the effects of interspecific and interspecific competition, infectious diseases, foraging success, genetics and human activities on wild dogs. We present data from wild dogs in the Selous Game Reserve, and make comparisons with other populations to identify the limiting factors that are broadly important. The high density of wild dogs in Selous (40 adults/1000 km(2)) is associated with weak competition from lions and spotted hyenas. Across ecosystems, population density is negatively related to the intensity of interference competition with large carnivores. Predation by lions and hyenas accounted for 13% of known-cause deaths in Selous and 33-50% in other populations. Intraspecific competition caused 69% of known-cause deaths in Selous, through infanticide and fights between packs, although most of the victims were juveniles with low reproductive value. Infectious diseases and competition will generally interact because competitors harbor and transmit the diseases that affect wild dogs. Human activities caused 12% of deaths in Selous, even though it is large (43000 km(2)) and does not border large human or livestock populations. Humans were the major agent of mortality in some populations. Foraging success varied little across ecosystems and was not apparently limiting. Mitochondrial DNA (mtDNA) genotypes revealed clinal variation between Selous and distant populations have a genetically effective size (N-c) less than 500, so gene flow is necessary to maintain genetic diversity within populations. | 2625 | NA | NA | NA | no | no |
| 2626 | 2017 | NA | Pacheco, ML; McDonald, PG; Wright, J; Kazem, AJN; Clarke, MF | 2008 | Helper contributions to antiparasite behavior in the cooperatively breeding bell miner | “Cooperatively breeding bell miners (Manorina melanophrys) have numerous male helpers assisting at multiple nests. Helpers are often related to the brood they aid, consistent with kin selection. However, there are also unrelated helpers for which other direct fitness benefits are likely to accrue. Bell miner nestlings can become infested by the larvae of a parasitic fly (Passeromyia indecora), which reduce growth and can be fatal. We investigated the amount of time that breeding pairs and helpers closely inspected nests and preened nestlings, behaviors apparently directed at detecting and removing parasites, a form of helping previously unstudied in a cooperative bird. Female breeders provided the greatest antiparasite effort, with breeding males and helpers not differing in effort regardless of their relatedness to the breeding female or brood. We also experimentally infested nests with nonparasitic flies and larvae. All individuals removed the introduced”“parasites”" if and when they encountered them. Compared with control sessions, inspection effort increased for all birds immediately after the experimental infestations, but only for a short, 5-min period. Further, we detected no changes in helper antiparasite behaviors after the temporary experimental removal of either breeding females or males. Such consistent helping behavior, independent of relatedness and potential audience effects, suggests that antiparasite behavior in bell miners is not particularly kin directed or operating as a signal of helper quality. Our results instead suggest that helper antiparasite effort appears to represent adaptive investment in the welfare of the brood, consistent with direct fitness benefits from group augmentation." | 2626 | NA | NA | NA | no | no |
| 2627 | 2017 | NA | Holsinger, KE | 2000 | Reproductive systems and evolution in vascular plants | Differences in the frequency with which offspring are produced asexually, through self-fertilization and through sexual outcrossing, are a predominant influence on the genetic structure of plant populations. Selfers and asexuals have fewer genotypes within populations than outcrossers with similar allele frequencies, and more genetic: diversity in selfers and asexuals is a result of differences among populations than in sexual outcrossers. As a result of reduced levels of diversity, selfers and asexuals may be less able to respond adaptively to changing environments, and because genotypes are not mixed across family lineages, their populations may accumulate deleterious mutations more rapidly. Such differences suggest that selfing and asexual lineages may be evolutionarily short-lived and could explain why they often seem to be of recent origin. Nonetheless, the origin and maintenance of different reproductive modes must be linked to individual-level properties of survival and reproduction. Sexual outcrossers suffer from a cost of outcrossing that arises because they do not contribute to self ed or asexual progeny, whereas selfers and asexuals may contribute to outcrossed progeny. Selfing and asexual reproduction also may allow reproduction when circumstances reduce opportunities for a union of gametes produced by different individuals, a phenomenon known as reproductive assurance. Both the cost of outcrossing and reproductive assurance lead to an over-representation of selfers and asexuals in newly formed progeny, and unless sexual outcrossers are more likely to survive and reproduce, they eventually will be displaced from populations in which a selfing or asexual variant arises. | 2627 | NA | NA | NA | no | no |
| 2628 | 2017 | NA | Cummings, N; King, R; Rickers, A; Kaspi, A; Lunke, S; Haviv, I; Jowett, JBM | 2010 | Combining target enrichment with barcode multiplexing for high throughput SNP discovery | Background: The primary goal of genetic linkage analysis is to identify genes affecting a phenotypic trait. After localisation of the linkage region, efficient genetic dissection of the disease linked loci requires that functional variants are identified across the loci. These functional variations are difficult to detect due to extent of genetic diversity and, to date, incomplete cataloguing of the large number of variants present both within and between populations. Massively parallel sequencing platforms offer unprecedented capacity for variant discovery, however the number of samples analysed are still limited by cost per sample. Some progress has been made in reducing the cost of resequencing using either multiplexing methodologies or through the utilisation of targeted enrichment technologies which provide the ability to resequence genomic areas of interest rather that full genome sequencing. Results: We developed a method that combines current multiplexing methodologies with a solution-based target enrichment method to further reduce the cost of resequencing where region-specific sequencing is required. Our multiplex/enrichment strategy produced high quality data with nominal reduction of sequencing depth. We undertook a genotyping study and were successful in the discovery of novel SNP alleles in all samples at uniplex, duplex and pentaplex levels. Conclusion: Our work describes the successful combination of a targeted enrichment method and index barcode multiplexing to reduce costs, time and labour associated with processing large sample sets. Furthermore, we have shown that the sequencing depth obtained is adequate for credible SNP genotyping analysis at uniplex, duplex and pentaplex levels. | 2628 | NA | NA | NA | no | no |
| 2629 | 2017 | NA | Wulff, JA; Buckman, KA; Wu, KM; Heimpel, GE; White, JA | 2013 | The Endosymbiont Arsenophonus Is Widespread in Soybean Aphid, Aphis glycines, but Does Not Provide Protection from Parasitoids or a Fungal Pathogen | Aphids commonly harbor bacterial facultative symbionts that have a variety of effects upon their aphid hosts, including defense against hymenopteran parasitoids and fungal pathogens. The soybean aphid, Aphis glycines Matsumura (Hemiptera: Aphididae), is infected with the symbiont Arsenophonus sp., which has an unknown role in its aphid host. Our research goals were to document the infection frequency and diversity of the symbiont in field-collected soybean aphids, and to determine whether Arsenophonus is defending soybean aphid against natural enemies. We performed diagnostic PCR and sequenced four Arsenophonus genes in soybean aphids from their native and introduced range to estimate infection frequency and genetic diversity, and found that Arsenophonus infection is highly prevalent and genetically uniform. To evaluate the defensive role of Arsenophonus, we cured two aphid genotypes of their natural Arsenophonus infection through ampicillin microinjection, resulting in infected and uninfected isolines within the same genetic background. These isolines were subjected to parasitoid assays using a recently introduced biological control agent, Binodoxys communis [Braconidae], a naturally recruited parasitoid, Aphelinus certus [Aphelinidae], and a commercially available biological control agent, Aphidius colemani [Braconidae]. We also assayed the effect of the common aphid fungal pathogen, Pandora neoaphidis (Remaudiere & Hennebert) Humber (Entomophthorales: Entomophthoraceae), on the same aphid isolines. We did not find differences in successful parasitism for any of the parasitoid species, nor did we find differences in P. neoaphidis infection between our treatments. Our conclusion is that Arsenophonus does not defend its soybean aphid host against these major parasitoid and fungal natural enemies. | 2629 | NA | NA | NA | no | no |
| 2630 | 2017 | NA | Bahri, B; Leconte, M; Ouffroukh, A; De Vallavieille-Pope, C; Enjalbert, J | 2009 | Geographic limits of a clonal population of wheat yellow rust in the Mediterranean region | Most plant pathogens present complex life cycles, in which the clonal reproduction may impede the delimitation of population entities. By studying the genetic structure of the wheat yellow rust caused by Puccinia striiformis f.sp. tritici (PST), we highlighted difficulties impeding population delimitation in highly clonal species. Despite the high dispersal potential of PST, southern France isolates were shown to be divergent from a northwestern European population. A 2-year survey was performed in the Mediterranean region to assess the geographic distribution of southern isolates: 453 isolates collected in 11 countries were genotyped using 15 simple sequence repeat markers. A subsample was analysed for virulence against 23 resistance genes. The dominant strain in the western Mediterranean region was further studied with amplified fragment length polymorphism markers to test for a geographic substructure. Both ‘individual’- and ‘population’ centred analyses of polymorphism markers revealed two south-specific groups: a predominant group, with a broad variability and a wide distribution in both western and eastern Mediterranean countries, and a minor group in the western Mediterranean. The east-west gradient of genetic diversity suggested gene flow from the Middle East with subsequent founder effects and genetic divergence, and demonstrated the local survival of a western Mediterranean population. The high frequency of the resistance gene Yr8 observed in cultivars from Tunisia and Algeria may contribute to maintain the north/south structure observed in France. In addition to migration and local adaptation, the dynamics of clonal lineage diversification and replacement should be considered to define population entities in strongly clonal species. | 2630 | NA | NA | NA | no | no |
| 2631 | 2017 | NA | Pluhacek, J; Sinha, SP; Bartos, L; Sipek, P | 2007 | Parity as a major factor affecting infant mortality of highly endangered Indian rhinoceros: Evidence from zoos and Dudhwa National Park, India | The Indian rhinoceros (Rhinoceros unicornis) is a highly endangered species that inhabits only three political states. Recently, Zschokke and Baur [Zschokke, S., Baur, B., 2002. Inbreeding, outbreeding, infant growth, and size dimorphism in captive Indian rhinoceros (Rhinoceros unicornis). Canadian journal of Zoology 80, 2014-2023] found that the offspring of matings between captive Indian rhinoceros individuals from the Kaziranga and Chitwan populations had high mortality rates. These authors suggested that these two populations are partially genetically incompatible and, thus, they proposed that these would be separated into two subspecies. In this study we compiled data from a captive population with data from Dudhwa National Park (India), where rhinoceroses were successfully reintroduced in 1984. In Dudhwa, the breeding male came from the Kaziranga population and four out of the five breeding females came from the Chitwan population. In spite of these different origins, the Dudhwa population has bred very well. We analyzed, the factors influencing infant mortality of 22 Dudhwa and 181 captive calves. outbreeding (matings between animals from Kaziranga and Chitwan) did not play any role in infant mortality. From our data, we can conclude that parity, not outbreeding, is responsible for infant mortality in the Indian rhinoceros. Thus, we are unable to agree with Zschokke and Baur’s suggestion that the Chitwan and Kaziranga populations belong to separate subspecies. Rather, we propose that the hybridization of captive animals from Chitwan and Kaziranga, as well as those in the wild, should help save the genetic diversity of this highly endangered species. (C) 2007 Elsevier Ltd. All rights reserved. | 2631 | NA | NA | NA | no | no |
| 2632 | 2017 | NA | Cornelius, JP; Navarro, CM; Wightman, KE; Ward, SE | 2005 | Is mahogany dysgenically selected? | One concern in the ongoing debate over the conservation status of mahogany has been the possibility that selective logging, as a form of negative phenotypic selection, might have led to deterioration in the genetic quality of populations. The incidence and degree of such ‘dysgenic’ effects is discussed, based on a consideration of mahogany logging practices, their expected genetic effects and empirical data on phenotypic selection in forest trees. Loggers have tended to avoid diseased, small, very large and poorly-formed individuals, and consequently logging has tended to increase the proportion of poor quality phenotypes relative to that before logging; in at least some conditions, selection differentials have been strongly negative. However, the upper limit for heritability of logger-selected traits in naturally-regenerated mahogany is probably no more than c. 0.1. Consequently, and assuming relatively extreme but realistic negative selection differentials of 50%, the maximum negative dysgenic response to a single logging-mediated phenotypic selection event is expected to be relatively small, i.e. <= 5%. This expectation is consistent with the empirical information from mahogany and other taxa. The implications of any dysgenic effects depend very much on the use and the future of dysgenically-selected populations. In managed populations, dysgenic effects could be reversed through positive selection. In the case of exploited but currently unmanaged natural populations, dysgenic selection is primarily of importance insofar as it affects fitness. As a threat to mahogany conservation and long-term sustainable production, it is probably insignificant in comparison with other genetic and non-genetic factors. | 2632 | NA | NA | NA | no | no |
| 2633 | 2017 | NA | Knutsen, H; Jorde, PE; Sannaes, H; Hoelzel, AR; Bergstad, OA; Stefanni, S; Johansen, T; Stenseth, NC | 2009 | Bathymetric barriers promoting genetic structure in the deepwater demersal fish tusk (Brosme brosme) | Population structuring in the North Atlantic deepwater demersal fish tusk (Brosme brosme) was studied with microsatellite DNA analyses. Screening eight samples from across the range of the species for seven loci revealed low but significant genetic heterogeneity (F(ST) = 0.0014). Spatial genetic variability was only weakly related to geographical (Euclidean) distance between study sites or separation of study sites along the path of major ocean currents. Instead, we found a significant effect of habitat, indicated by significant differentiation between relatively closely spaced sites: Rockall, which is surrounded by very deep water (> 1000 m), and the Mid-Atlantic Ridge, which is separated from the European slope by a deep ocean basin, were differentiated from relatively homogeneous sites across the Nordic Seas. Limited adult migration across bathymetric barriers in combination with limited intersite exchange of pelagic eggs and larvae due to site-specific circulatory retention or poor survival during drift phases across deep basins may be reducing gene flow. We regard these limitations to gene flow as the most likely mechanisms for the observed population structure in this demersal species. The results underscore the importance of habitat boundaries in marine species. | 2633 | NA | NA | NA | no | no |
| 2634 | 2017 | NA | Sawhney, RN; Joshi, BC | 1996 | Genetic research as the valid base of strategies for breeding rust resistant wheats | It is known that few wheat cultivars maintain their resistance to rust diseases for a long period of time, particularly when crop populations become genetically more uniform. A number of genetically diverse, so far unexploited, sources of rust resistance in the natural as well as mutagenized population of wheat cultivars were identified. Several of these genes were placed in agronomically superior well-adapted backgrounds so that they could be used as pre-breeding stocks for introducing genetic diversity for resistance in a crop population. Some of these stocks when employed as parents in several cross combinations in a breeding programme have generated a number of promising cultivars with diversity for resistance. Many presently grown wheats in India, near-isogenic lines each with Lr14b, Lr14ab, Lr30 and certain international cultivars were identified as possessing diverse sources of adult plant resistance (APR) to leaf rust. Prolonged leaf rust resistance in some of the Indian cultivars was attributed to the likely presence of Lr34 either alone or in combination with other APR components. Tests of allelism carried out in certain cultivars that continue to show adequate levels of field resistance confirm the presence of Lr34, which explains the role that this gene has played in imparting durability for resistance to leaf rust. Also, Lr34 in combination with other APR components increases the levels of resistance, which suggests that combination of certain APR components should be another important strategy for breeding cultivars conferring durable and adequate levels of resistance. A new adult plant leaf rust resistance source that seems to be associated with durability in ‘Arjun’ has been postulated. Likewise, cultivars possessing Sr2 in combination with certain other specific genes have maintained resistance to stem rust. Further, non-specific resistances that were transferred across widely different genotypes into two of the popular Indian wheats provided easily usable materials to the national breeding programmes for imparting durable resistance to stripe rust. | 2634 | NA | NA | NA | no | no |
| 2635 | 2017 | NA | Woudenberg, JHC; van der Merwe, NA; Jurjevic, Z; Groenewald, JZ; Crous, PW | 2015 | Diversity and movement of indoor Alternaria alternata across the mainland USA | Alternaria spp. from sect. Alternaria are frequently associated with hypersensitivity pneumonitis, asthma and allergic fungal rhinitis and sinusitis. Since Alternaria is omnipresent in the outdoor environment, it is thought that the indoor spore concentration is mainly influenced by the outdoor spore concentration. However, few studies have investigated indoor Alternaria isolates, or attempted a phylogeographic or population genetic approach to investigate their movement. Therefore, the aim of the current study was to investigate the molecular diversity of indoor Alternaria isolates in the USA, and to test for recombination, using these approaches. Alternaria isolates collected throughout the USA were identified using ITS, gapdh and endoPG gene sequencing. This was followed by genotyping and population genetic inference of isolates belonging to Alternaria sect. Alternaria together with 37 reference isolates, using five microsatellite markers. Phylogenetic analyses revealed that species of Alternaria sect. Alternaria represented 98% (153 isolates) of the indoor isolates collected throughout the USA, of which 137 isolates could be assigned to A. alternata, 15 to the A. arborescens species complex and a single isolate to A. burnsii. The remaining 2% (3 isolates) represented sect. Infectoriae (single isolate) and sect. Pseudoulocladium (2 isolates). Population assignment analyses of the 137 A. alternata isolates suggested that subpopulations did not exist within the sample. The A. alternata isolates were thus divided into four artificial subpopulations to represent four quadrants of the USA. Forty-four isolates representing the south-western quadrant displayed the highest level of uniqueness based on private alleles, while the highest level of gene flow was detected between the south-eastern (32 isolates) and south-western quadrants. Genotypic diversity was high for all quadrants, and a test for linkage disequilibrium suggested that A. alternata has a cryptic sexual cycle. These statistics could be correlated with environmental factors, suggesting that indoor A. alternata isolates, although extremely diverse, have a continental distribution and high levels of gene flow over the continent. (C) 2015 The Authors. Published by Elsevier Inc. | 2635 | NA | NA | NA | no | no |
| 2636 | 2017 | NA | Rutledge, LY; Patterson, BR; Mills, KJ; Loveless, KM; Murray, DL; White, BN | 2010 | Protection from harvesting restores the natural social structure of eastern wolf packs | Legal and illegal killing of animals near park borders can significantly increase the threat of extirpation for populations living within ecological reserves, especially for wide-ranging large carnivores that regularly travel into unprotected areas. While the consequences of human-caused mortality near protected areas generally focus on numerical responses, little attention has been given to impacts on social dynamics. For wolves, pack structure typically constitutes an unrelated breeding pair, their offspring, and close relatives, but intense harvest may increase adoption of unrelated individuals into packs. Concerns that high human-caused mortality outside Algonquin Park, Canada threatened the persistence of eastern wolves, led to implementation of a harvest ban in surrounding townships. We combined ecological and genetic data to show that reducing anthropogenic causes of mortality can restore the natural social structure of kin-based groups despite the absence of a marked change in density. Since implementation of the harvest ban, human-caused mortality has decreased (P = 0.000006) but been largely offset by natural mortality, such that wolf density has remained relatively constant at approximately three wolves/100 km(2). However, the number of wolf packs with unrelated adopted animals has decreased from 80% to 6% (P = 0.00003). Despite the high kinship within packs, incestuous matings were rare. Our results indicate that even in a relatively large protected area, human harvesting outside park boundaries can affect evolutionarily important social patterns within protected areas. This research demonstrates the need for conservation policy to consider effects of harvesting beyond influences on population size. (C) 2009 Elsevier Ltd. All rights reserved. | 2636 | NA | NA | NA | no | no |
| 2637 | 2017 | NA | Harvey, KJ; Nipperess, DA; Britton, DR; Hughes, L | 2012 | Australian family ties: does a lack of relatives help invasive plants escape natural enemies? | Invasive plants may initially be released from natural enemies when introduced to new regions, but once established, natural enemies may accumulate. How closely related invasive species are to species in the native recipient community may drive patterns of herbivore and pathogen damage and therefore, may be important in understanding the success of some invasions. We compared herbivore and pathogen damage across a group of invasive species occurring in natural environments on the east coast of Australia. We examined whether the level of damage experienced by the invasive species was associated with the degree of phylogenetic relatedness between these plants and the native plants within the region. We found that phylogenetic distance to the nearest native relative was a good predictor of herbivore and pathogen damage on the invasive plants, explaining nearly 37 % of the variance in leaf damage. Total leaf damage and the variety of damage types declined with increasing phylogenetic distance to the nearest native relative. In addition, as the phylogenetic distance to the nearest native relative increased, invasive species were colonized by fewer functional guilds and the herbivore assemblage was increasingly dominated by generalist species. These results suggest that invasive species that are only distantly related to those in the native invaded community may be released from specialist natural enemies. Our results indicate that the phylogenetic relatedness of invasive plants to species in native communities is a significant predictor of the rate of colonization by the herbivore and pathogen community, and thus a useful tool to assess invasion potential. | 2637 | NA | NA | NA | no | no |
| 2638 | 2017 | NA | Gibson, A; Nelson, CR | 2017 | Comparing Provisional Seed Transfer Zone Strategies for a Commonly Seeded Grass, Pseudoroegneria spicata | “Restoration practitioners balance the desire to use locally adapted plant materials with the uncertainty of what constitutes”“local.”" Provisional seed transfer zones are intended to guide managers on how far plant materials can be moved during revegetation with the assumption that all populations within a zone will show similarly adapted traits. There are multiple approaches to developing provisional transfer zones, including limiting transfer to specific populations, within ecoregions, within climatic zones, or within climatic zones within ecoregion | 2638 | NA | NA | NA | no | no |
| 2639 | 2017 | NA | Shapcott, A; Playford, J | 1996 | Comparison of genetic variability in remnant and wide-spread rainforest understorey species of Austromyrtus (Myrtaceae) | This study investigated the genetic variability, stand structure, and reproductive activity of four species of Austromyrtus which showed differing levels of rarity and patterns of distribution. The focus of the study was A. gonoclada which is extremely rare due to extensive urban expansion in Brisbane, Australia. The total number of known individuals in the species is only 27. Because nothing was known about the genetic structure of Austromyrtus comparison with other species was made to provide a context for studies on A. gonoclada. Population structure and levels of reproductive activity were not correlated to species rarity or distribution. A. gonoclada showed maximum genetic variability in comparison to the other species of Austromyrtus, despite the small population size. Three of six variable loci were in Kardy-Weinberg equilibrium in A. gonoclada populations, in contrast to populations of the other species which had few heterozygous genotypes. The higher variability of the remaining individuals is suggested to be a legacy of the relatively short time since they were part of a larger population. There were very few seedlings of A. gonoclada, in contrast to the other species of Austromyrtus and the seeds produced fell from the tree while still green and were unable to be germinated. A. gonoclada is under continuing threat from development and has low reproductive success. Active programmes for recovery of this species will be necessary as its continued survival is dependent on population increase. Management of the genetic resources of the species will enable survival with long-term evolutionary potential. | 2639 | NA | NA | NA | no | no |
| 2640 | 2017 | NA | Korb, J; Roux, EA | 2012 | Why join a neighbour: fitness consequences of colony fusions in termites | The evolution of life is characterized by major evolutionary transitions during which independent units cooperated and formed a new level of selection. Relatedness is a common mechanism that reduces conflict in such cooperative associations. One of the latest transitions is the evolution of social insect colonies. As expected, they are composed of kin and mechanisms have evolved that prevent the intrusion of nonrelatives. Yet, there are exceptions an extreme case is the fusion of unrelated colonies. What are the advantages of fusions that have colonies with a high potential for conflict as a consequence? Here, we investigated fitness costs and benefits of colony fusions in a lower termite species, Cryptotermes secundus, in which more than 25% of all colonies in the field are fused. We found two benefits of colony fusion depending on colony size: very small colonies had an increased probability of survival when they fused, yet for most colony sizes mainly a few workers profit from colony fusions as their chance to become reproductives increased. This individual benefit was often costly for other colony members: colony growth was reduced and the current reproductives had an increased chance of dying when fusions were aggressive. Our study suggests that fusion of colonies often is the result of selfish worker interests to become reproductives, and this might have been important for the termites’ social evolution. Our results uniquely shows that selfish interests among related colony members can lead to the formation of groups with increased potential for conflict among less related members. | 2640 | NA | NA | NA | no | no |
| 2641 | 2017 | NA | Yagound, B; Crowet, M; Leroy, C; Poteaux, C; Chaline, N | 2017 | Interspecific variation in neighbour-stranger discrimination in ants of the Neoponera apicalis complex | 1. The ecological success of social insects lies in their ability to prevent the exploitation of colony resources by competitors or parasites. Nestmate recognition is therefore of crucial importance in maintaining the integrity of the colony. Furthermore, inter‐colony competitive relationships are often complex, as many species discriminate between neighbours and strangers, with reduced (the dear enemy phenomenon) or increased levels of aggression towards nearby colonies, depending on the species. In this context, between‐species comparisons could be particularly helpful to investigate the proximate causes underlying this context‐dependent phenomenon, but these are notoriously lacking.  - Here an attempt was made to circumvent this drawback by studying three closely related sympatric ant species with very similar life histories that belong to the Neoponera apicalis complex. The present study investigated how nestmate recognition and inter‐colony competitive relationships were influenced by spatial, chemical and genetic distances between colonies. - It was found that one species, N. apicalis morph 7, showed a clear dear enemy phenomenon with no influence of chemical and genetic distances, suggesting the existence of a learning process. In contrast, N. apicalis morph 4 and Neoponera verenae morph 1 failed to show any strong discrimination between close and distant non‐nestmates. - These results are discussed in the light of the observed interspecific variation in nesting preferences, possibly constraining the opportunities of familiarisation between nearby nests, and modulating the competition for resources between colonies. - It is argued that this study further reinforces the relative threat level hypothesis as an ultimate explanation for neighbour–stranger discrimination processes. | 2641 | NA | NA | NA | no | no |
| 2642 | 2017 | NA | Zhang, J; Schneider, D; Ober, C; McPeek, MS | 2005 | Multilocus linkage disequilibrium mapping by the decay of haplotype sharing with samples of related individuals | We consider the problem of multilocus linkage disequilibrium. (LD) mapping of a trait-associated variant from case-control samples in which some individuals may be related. Our method, which we call DHS-R, is an extension of the decay of haplotype sharing (DHS) method of McPeek and Strahs ([19991 Am j Hum Genet 65:858-875) and Strahs and McPeek ([20031 IMS Lecture Notes Monograph Series, Vol. 40.). The DHS-R method shares the main features of the DHS method: (1) it allows construction of a confidence interval for the location of a trait-associated variant; (2) it allows for missing observations and unphased genotype data, with the uncertainty in the haplotypes taken into account in the analysis; and (3) it allows for heterogeneity, mutation, recombination, and background LD. The main advances of the DHS-R. are (1) the ability to include individuals of arbitrary known relationship (including inbreeding) in the case and control samples; (2) an extension to allow partially-phased haplotypes derived from case-parent trio genotype data; and (3) an extension to allow for genotyping error in the model. Our method, which uses a hidden Markov model for likelihood calculation and maximization, has the advantage of being computationally feasible even in a large, complex pedigree. Simulations based on a 13-generation, 1,623-member Hutterite pedigree demonstrate accurate coverage of the confidence intervals for location of the variant. We apply the method to fine-mapping of a susceptibility locus for bronchial hyperresponsiveness (BHR) in the Hutterites. The results confirm the importance of taking into account the relatedness of individuals in LD mapping. | 2642 | NA | NA | NA | no | no |
| 2643 | 2017 | NA | GROBLER, JP; VANDERBANK, FH | 1994 | ALLOZYME VARIATION IN SOUTH-AFRICAN IMPALA POPULATIONS UNDER DIFFERENT MANAGEMENT REGIMES | Seventy-nine impalas Aepyceros melampus from four populations were compared using protein gel-electrophoresis to determine the influence of different management strategies on genetic variability and differentiation. Four tissue types were analysed, and we were able to resolve 35 protein-coding loci, of which six were polymorphic. Average heterozygosity values ranged from 4,6% in a numerically large population to 2,41% and 3,21% in two isolated groups, and 4,43% in another isolated (but well-managed) population, (with corresponding proportions of polymorphic loci of 14,29%; 5,71%; 11,43% and 14,29%). As expected for conspecific populations, gene variability was much higher within (91,53%) than between (8,47%) populations. Average genetic distance (Nei 1972) between populations ranged from 0,0015 to 0,0088, indicating limited allozyme divergence among the groups studied. We discuss factors contributing towards the above results as well as the implications for management strategies. | 2643 | NA | NA | NA | no | no |
| 2644 | 2017 | NA | Jimenez, M; Martinez-Urtaza, J; Chaidez, C | 2011 | Geographical and Temporal Dissemination of Salmonellae Isolated from Domestic Animal Hosts in the Culiacan Valley, Mexico | The prevalence and diversity of salmonellae from domestic animal hosts were investigated in the Culiacan Valley, Mexico. A total of 240 farm animal feces (cows, chicken, and sheep) were evaluated for Salmonella spp. presence from July 2008 to June 2009. Salmonella enterica subsp. enterica strains were isolated from 76 samples (31.7%), and 20 serotypes were identified being Salmonella Oranienburg (25%), Salmonella Give (14%), Salmonella Saintpaul (12%), and Salmonella Minnesota (11%) the most frequent isolates. Twenty-four percent (18/76) of the isolates were resistant to ampicillin. Salmonella Oranienburg, Salmonella Minnesota, Salmonella Give, Salmonella Agona, Salmonella Weltevreden, and Salmonella Newport serotypes showed multiple pulsed-field electrophoresis patterns. Salmonella Oranienburg was the dominant serotype in the Culiacan Valley; however, no specific distribution patterns were detected in animal sources or sampling sites. The genetic diversity of salmonellae could be an evidence of the continuous animal exposition to the bacteria. Also, Salmonella adaptation in asymptomatic animals could be justified by the development of natural host immunity. This study provides novel information about Salmonella population distribution in domestic animals living at tropical areas. The presence of asymptomatic carriers may be critical to understand the routes of transmission of Salmonella in areas of high disease prevalence. | 2644 | NA | NA | NA | no | no |
| 2645 | 2017 | NA | Baghalian, K; Kim, OK; Natzuaki, KT | 2010 | Molecular variability and genetic structure of the population of Onion yellow dwarf virus infecting garlic in Iran | Onion yellow dwarf virus (OYDV) is one of the most important viral diseases of garlic crops worldwide. This study surveyed the occurrence of OYDV in 26 garlic ecotypes collected from different regions in Iran during 2008-2009. Using an electron microscope, we detected filamentous particles with about 700-800 nm in length and 12 nm in width in five samples. These features are typical of the genus Potyvirus. The coat protein (CP) gene from 26 samples was PCR amplified, cloned, sequenced, and compared with the sequences available in GenBank. Phylogenetic analysis using 235 deduced amino acids of the CP gene showed that virus isolates fell into two groups, group A and group B. Members of group A were divided into two subgroups: A-I and A-II. The subgroup A-I appears to be a new subgroup comprising 17 Iranian isolates. The identity levels among the amino acid of 26 Iranian isolates ranged between 90 and 100%. The results indicated that the genetic diversity found in Iran is due to local OYDV populations rather than introduction from other geographical regions. This study is the first report about the molecular structure and geographically diverse range of OYDV populations in this country. | 2645 | NA | NA | NA | no | no |
| 2646 | 2017 | NA | Huang, K; Whitlock, R; Press, MC; Scholes, JD | 2012 | Variation for host range within and among populations of the parasitic plant Striga hermonthica | Striga hermonthica is an angiosperm parasite that causes substantial damage to a wide variety of cereal crop species, and to the livelihoods of subsistence farmers in sub-Saharan Africa. The broad host range of this parasite makes it a fascinating model for the study of host parasite interactions, and suggests that effective long-term control strategies for the parasite will require an understanding of the potential for host range adaptation in parasite populations. We used a controlled experiment to test the extent to which the success or failure of S. hermonthica parasites to develop on a particular host cultivar (host resistance/compatibility) depends upon the identity of interacting host genotypes and parasite populations. We also tested the hypothesis that there is a genetic component to host range within individual S. hermonthica populations, using three rice cultivars with known, contrasting abilities to resist infection. The developmental success of S. hermonthica parasites growing on different rice-host cultivars (genotypes) depended significantly on a parasite population by host genotype interaction. Genetic analysis using amplified fragment length polymorphism (AFLP) markers revealed that a small subset of AFLP markers showed ‘outlier’ genetic differentiation among sub-populations of S. hermonthica attached to different host cultivars. We suggest that, this indicates a genetic component to host range within populations of S. hermonthica, and that a detailed understanding of the genomic loci involved will be crucial in understanding host parasite specificity and in breeding crop cultivars with broad spectrum resistance to S. hermonthica. Heredity (2012) 108, 96-104; doi:10.1038/hdy.2011.52; published online 6 July 2011 | 2646 | NA | NA | NA | no | no |
| 2647 | 2017 | NA | Srithayakumar, V; Castillo, S; Rosatte, RC; Kyle, CJ | 2011 | MHC class II DRB diversity in raccoons (Procyon lotor) reveals associations with raccoon rabies virus (Lyssavirus) | In North America, the raccoon rabies virus (RRV) is an endemic wildlife disease which causes acute encephalopathies and is a strong selective force on raccoons (Procyon lotor), with estimates of similar to 85% of the population succumbing to the disease when epizootic. RRV is regarded as a lethal disease if untreated; therefore, no evolutionary response would be expected of raccoon populations. However, variable immune responses to RRV have been observed in raccoons indicating a potential for evolutionary adaptation. Studies of variation within the immunologically important major histocompatibility complex (MHC) have revealed relationships between MHC alleles and diseases in humans and other wildlife species. This enhances our understanding of how hosts and pathogens adapt and co-evolve. In this study, we used RRV as a model system to study host-pathogen interaction in raccoons from a challenge study and from four wild populations that differ in exposure times and viral lineages. We investigated the potential role of Prlo-DRB polymorphism in relation to susceptibility/resistance to RRV in 113 RRV positive and 143 RRV negative raccoons. Six alleles were found to be associated with RRV negative status and five alleles with RRV positive animals. We found variable patterns of MHC associations given the relative number of selective RRV sweeps in the studied regions and correlations between MHC diversity and RRV lineages. The allelic associations established provide insight into how the genetic variation of raccoons may affect the disease outcome and this can be used to examine similar associations between other rabies variants and their hosts. | 2647 | NA | NA | NA | no | no |
| 2648 | 2017 | NA | Leger, EA; Espeland, EK; Merrill, KR; Meyer, SE | 2009 | Genetic variation and local adaptation at a cheatgrass (Bromus tectorum) invasion edge in western Nevada | Cheatgrass (Bromus tectorum) is an invasive weed in western North America found primarily growing at elevations less than 2200 m. We asked whether cheatgrass is capable of becoming adapted to a marginal habitat, by investigating a population at a high elevation invasion edge. We used a combination of methods, including reciprocal field transplants, controlled environment studies and molecular analysis. High levels of SSR gene diversity (0.50 vs. 0.43) and comparable variation in phenotypic traits were observed at both the invasion edge and a low elevation, high-density population. Three heterozygotes were observed in the edge population, which is unusual in this predominantly self-pollinating plant. Plants from high elevations germinated more slowly in a growth chamber and had slower seedling growth rates. Survivorship was low at the edge (13%), compared with the low elevation site (55%), but surviving plants were of similar size and had equivalent reproductive output. Seed size positively affected survival and plant performance in the field and this trait was inherited. Emergence timing affected survival at the low elevation site and germination timing was also inherited. Local adaptation was seen in the low, rather than in the high elevation site, because of differential survival. While there was no evidence for local adaptation to the high elevation site observed in the field, family level and genotype-level differences in traits that affected field performance, high genetic diversity at the invasion edge, and evidence of outcrossing in this highly selfing species indicates that the potential for adaptation to a marginal habitat exists within this population. | 2648 | NA | NA | NA | no | no |
| 2649 | 2017 | NA | Bose, APH; Kou, HH; Balshine, S | 2016 | Impacts of direct and indirect paternity cues on paternal care in a singing toadfish | Parents are sometimes confronted with information that suggests they are unrelated to the offspring under their care. When this happens, parents are expected to reduce their level of care into the offspring. However, studies have shown that this does not always happen. We use wild populations of plainfin midshipman fish to show that parents will reduce their level of care, though will only do so in response to certain types of information but not others.Effort spent on raising unrelated offspring can be costly and wasteful, and parents are expected to reduce their level of investment when they have low or uncertain relatedness to the young under their care. Although the relationship between parental certainty and parental investment is theoretically well established, empirical support has been mixed. Here, we report on a series of lab and field experiments that test whether paternal investment is reduced as paternity decreases in the plainfin midshipman fish (Porichthys notatus), a species of toadfish with male-only care. We explored what cues plainfin midshipman males use to assess their paternity. We show that a nest takeover, in which a male displaces another male from a nest, can be a reliable indirect cue of paternity information and leads to a drop in offspring survival. We also show that, when presented in isolation, direct cues of reduced offspring relatedness do not result in a decline in offspring survival in midshipman. Our findings help clarify what systems, species, and theoretical assumptions best reveal the link between parental investment and parentage. | 2649 | NA | NA | NA | no | no |
| 2650 | 2017 | NA | Iorio, A; Piacentini, S; Polimanti, R; De Angelis, F; Calderon, R; Fuciarelli, M | 2014 | Functional Variability of Glutathione S-Transferases in Basque Populations | ObjectivesGlutathione S-transferases (GSTs) are enzymes involved in Phase II reactions. They play a key role in cellular detoxification. Various studies have shown that genes coding for the GST are highly polymorphic and some of these variants are directly associated with a decrease of enzyme activity making individuals more susceptible to different clinical phenotypes. The aim of this study is to investigate the genetic variability of GST genes among human populations. We have focused our attention on the polymorphic variants of the GSTA1, GSTM1, GSTO1, GSTO2, GSTP1, GSTT1, and GSTT2B genes. MethodsThese polymorphisms were analyzed in a whole sample of 151 individuals: 112 autochthonous Navarrese Basques, and 39 non-autochthonous Navarrese Basques. DNA extraction from plasma was performed by using the phenol:chloroform:isoamylic alcohol method. Genotyping of the gene polymorphisms was performed by PCR Multiplex and the PCR-RFLP method. We applied correspondence analysis and built frequency-maps to compare the genetic structure in worldwide populations. ResultsOur results were compared with data available on the Human Genome Diversity Project (HGDP) and on the 1,000 Genomes Project to obtain information on the functional variability of GSTs in Basques. Our data indicated that Basque communities showed a higher differentiation of certain functional GST variants (i.e., GSTM1-positive/null genotype, GSTP1*I105V, and GSTT2B*1/0) than other European and Mediterranean populations. ConclusionsThis might account for epidemiological differences in the predisposition to diseases and drug response among Basques and could be used to design and interpret genetic association studies for this particular population. Am. J. Hum. Biol. 26:361-366, 2014. (c) 2014 Wiley Periodicals, Inc. | 2650 | NA | NA | NA | no | no |
| 2651 | 2017 | NA | Peer, K; Taborsky, M | 2007 | Delayed dispersal as a potential route to cooperative breeding in ambrosia beetles | Xyleborini are a species-rich tribe of ambrosia beetles, which are haplodiploid and typically mate among siblings within their natal brood chamber. Several characteristics of this tribe would predict the evolution of higher levels of sociality: high genetic relatedness within galleries due to inbreeding, high costs of dispersal and the potential benefit of cooperation in brood care within the natal gallery (e.g. by fungus gardening, gallery extension, offspring feeding and cleaning). However, information on the social system of these beetles is very limited. We examined the potential for cooperative breeding in Xyleborinus saxeseni by monitoring dispersal in relation to brood size and composition. Results show that adult female offspring delay dispersal despite dispersal opportunities, and apparently some females never disperse. The females’ decision to stay seems to depend on the presence of eggs and dependent siblings. We found no indication that female offspring reproduce in their natal gallery, as colonies with many mature daughters do not contain more eggs than those with few or no daughters. There is a significant positive relationship between the number of females present and the number of dependent siblings (but not eggs), which suggests that cooperative brood care of female offspring raises colony productivity by improving survival rates of immatures. Our results suggest that cooperative breeding is likely to occur in X. saxeseni and possibly other xyleborine species. We argue that a closer look at sociality within this tribe may yield important information on the factors determining the evolution of cooperative breeding and advanced social organization. | 2651 | NA | NA | NA | no | no |
| 2652 | 2017 | NA | Huang, S; Bininda-Emonds, ORP; Stephens, PR; Gittleman, JL; Altizer, S | 2014 | Phylogenetically related and ecologically similar carnivores harbour similar parasite assemblages | Most parasites infect multiple hosts, but what factors determine the range of hosts a given parasite can infect? Understanding the broad scale determinants of parasite distributions across host lineages is important for predicting pathogen emergence in new hosts and for estimating pathogen diversity in understudied host species. In this study, we used a new data set on 793 parasite species reported from free-ranging populations of 64 carnivore species to examine the factors that influence parasite sharing between host species. Our results showed that parasites are more commonly shared between phylogenetically related host species pairs. Additionally, host species with higher similarity in biological traits and greater geographic range overlap were also more likely to share parasite species. Of three measures of phylogenetic relatedness considered here, the number divergence events that separated host species pairs most strongly influenced the likelihood of parasite sharing. We also showed that viruses and helminths tend to infect carnivore hosts within more restricted phylogenetic ranges than expected by chance. Overall, our results underscore the importance of host evolutionary history in determining parasite host range, even when simultaneously considering other factors such as host ecology and geographic distribution. | 2652 | NA | NA | NA | no | no |
| 2653 | 2017 | NA | Hellgren, O; Atkinson, CT; Bensch, S; Albayrak, T; Dimitrov, D; Ewen, JG; Kim, KS; Lima, MR; Martin, L; Palinauskas, V; Ricklefs, R; Sehgal, RNM; Valkiunas, G; Tsuda, Y; Marzal, A | 2015 | Global phylogeography of the avian malaria pathogen Plasmodium relictum based on MSP1 allelic diversity | Knowing the genetic variation that occurs in pathogen populations and how it is distributed across geographical areas is essential to understand parasite epidemiology, local patterns of virulence, and evolution of host-resistance. In addition, it is important to identify populations of pathogens that are evolutionarily independent and thus free’ to adapt to hosts and environments. Here, we investigated genetic variation in the globally distributed, highly invasive avian malaria parasite Plasmodium relictum, which has several distinctive mitochondrial haplotyps (cyt b lineages, SGS1, GRW11 and GRW4). The phylogeography of P. relictum was accessed using the highly variable nuclear gene merozoite surface protein 1 (MSP1), a gene linked to the invasion biology of the parasite. We show that the lineage GRW4 is evolutionarily independent of GRW11 and SGS1 whereas GRW11 and SGS1 share MSP1 alleles and thus suggesting the presence of two distinct species (GRW4 versus SGS1 and GRW11). Further, there were significant differences in the global distribution of MSP1 alleles with differences between GRW4 alleles in the New and the Old World. For SGS1, a lineage formerly believed to have both tropical and temperate transmission, there were clear differences in MSP1 alleles transmitted in tropical Africa compared to the temperate regions of Europe and Asia. Further, we highlight the occurrence of multiple MSP1 alleles in GRW4 isolates from the Hawaiian Islands, where the parasite has contributed to declines and extinctions of endemic forest birds since it was introduced. This study stresses the importance of multiple independent loci for understanding patterns of transmission and evolutionary independence across avian malaria parasites. | 2653 | NA | NA | NA | no | no |
| 2654 | 2017 | NA | Amambua-Ngwa, A; Park, DJ; Volkman, SK; Barnes, KG; Bei, AK; Lukens, AK; Sene, P; Van Tyne, D; Ndiaye, D; Wirth, DF; Conway, DJ; Neafsey, DE; Schaffner, SF | 2012 | SNP Genotyping Identifies New Signatures of Selection in a Deep Sample of West African Plasmodium falciparum Malaria Parasites | We used a high-density single-nucleotide polymorphism array to genotype 75 Plasmodium falciparum isolates recently collected from Senegal and The Gambia to search for signals of selection in this malaria endemic region. We found little geographic or temporal stratification of the genetic diversity among the sampled parasites. Through application of the iHS and REHH haplotype-based tests for positive selection, we found evidence of recent selective sweeps at a known drug resistance locus, at several known antigenic loci, and at several genomic regions not previously identified as sites of recent selection. We discuss the value of deep population-specific genomic analyses for identifying selection signals within sampled endemic populations of parasites, which may correspond to local selection pressures such as distinctive therapeutic regimes or mosquito vectors. | 2654 | NA | NA | NA | no | no |
| 2655 | 2017 | NA | Pujolar, JM; Bevacqua, D; Capoccioni, F; Ciccotti, E; De Leo, GA; Zane, L | 2009 | Genetic variability is unrelated to growth and parasite infestation in natural populations of the European eel (Anguilla anguilla) | Positive correlations between individual genetic heterozygosity and fitness-related traits (HFCs) have been observed in organisms as diverse as plants, marine bivalves, fish or mammals. HFCs are not universal and the strength and stability of HFCs seem to be variable across species, populations and ages. We analysed the relationship between individual genetic variability and two different estimators of fitness in natural samples of European eel, growth rate (using back-calculated length-at-age 1, 2 and 3) and parasite infestation by the swimbladder nematode Anguillicola crassus. Despite using a large data set of 22 expressed sequence tags-derived microsatellite loci and a large sample size of 346 individuals, no heterozygote advantage was observed in terms of growth rate or parasite load. The lack of association was evidenced by (i) nonsignificant global HFCs, (ii) a Multivariate General Linear Model showing no effect of heterozygosity on fitness components, (iii) single-locus analysis showing a lower number of significant tests than the expected false discovery rate, (iv) sign tests showing only a significant departure from expectations at one component, and, (v) a random distribution of significant single-locus HFCs that was not consistent across fitness components or sampling sites. This contrasts with the positive association observed in farmed eels in a previous study using allozymes, which can be explained by the nature of the markers used, with the allozyme study including many loci involved in metabolic energy pathways, while the expressed sequence tags-linked microsatellites might be located in genes or in the proximity of genes uncoupled with metabolism/growth. | 2655 | NA | NA | NA | no | no |
| 2656 | 2017 | NA | Vouillamoz, JF; Grando, MS | 2006 | Genealogy of wine grape cultivars: ‘Pinot’ is related to ‘Syrah’ | Since the domestication of wild grapes ca 6000 years ago, numerous cultivars have been generated by spontaneous or deliberate crosses, and up to 10 000 are still in existence today. Just as in human paternity analysis, DNA typing can reveal unexpected parentage of grape cultivars. In this study, we have analysed 89 grape cultivars with 60 microsatellite markers in order to accurately calculate the identity-by-descent (IBD) and relatedness (r) coefficients among six putatively related cultivars from France (‘Pinot’, ‘Syrah’ and ‘Dureza’) and northern Italy (‘Teroldego’, ‘Lagrein’ and ‘Marzemino’). Using a recently developed likelihood-based approach to analyse kinship in grapes, we provide the first evidence of a genetic link between grapes across the Alps: ‘Dureza’ and ‘Teroldego’ turn out to be full-siblings (FS). For the first time in grapevine genetics we were able to detect FS without knowing one of the parents and identify unexpected second-degree relatives. We reconstructed the most likely pedigree that revealed a third-degree relationship between the worldwide-cultivated ‘Pinot’ from Burgundy and ‘Syrah’ from the Rhone Valley. Our finding was totally unsuspected by classical ampelography and it challenges the commonly assumed independent origins of these grape cultivars. Our results and this new approach in grape genetics will (a) help grape breeders to avoid choosing closely related varieties for new crosses, (b) provide pedigrees of cultivars in order to detect inheritance of disease-resistance genes and (c) open the way for future discoveries of first- and second-degree relationships between grape cultivars in order to better understand viticultural migrations. | 2656 | NA | NA | NA | no | no |
| 2657 | 2017 | NA | Pawar, S; Chakrabarti, A; Cherian, S; Pande, S; Nanaware, M; Raut, S; Pal, B; Jadhav, S; Kode, S; Koratkar, S; Thite, V; Mishra, A | 2010 | An avian influenza A(H11N1) virus from a wild aquatic bird revealing a unique Eurasian-American genetic reassortment | Influenza surveillance in different wild bird populations is critical for understanding the persistence, transmission and evolution of these viruses. Avian influenza (AI) surveillance was undertaken in wild migratory and resident birds during the period 2007-2008, in view of the outbreaks of highly pathogenic AI (HPAI) H5N1 in poultry in India since 2006. In this study, we present the whole genome sequence data along with the genetic and virological characterization of an Influenza A(H11N1) virus isolated from wild aquatic bird for the first time from India. The virus was low pathogenicity and phylogenetic analysis revealed that it was distinct from reported H11N1 viruses. The hemagglutinin (HA) gene showed maximum similarity with A/semipalmatedsandpiper/Delaware/2109/2000 (H11N6) and A/shorebird/Delaware/236/2003(H11N9) while the neuraminidase (NA) gene showed maximum similarity with A/duck/Mongolia/540/2001(H1N1). The virus thus possessed an HA gene of the American lineage. The NA and other six genes were of the Eurasian lineage and showed closer relatedness to non-H11 viruses. Such a genetic reassortment is unique and interesting, though the pathways leading to its emergence and its future persistence in the avian reservoir is yet to be fully established. | 2657 | NA | NA | NA | no | no |
| 2658 | 2017 | NA | van Loon, JE; Leebeek, FWG; Deckers, JW; Dippel, DWJ; Poldermans, D; Strachan, DP; Tang, WH; O’Donnell, CJ; Smith, NL; de Maat, MPM | 2010 | Effect of Genetic Variations in Syntaxin-Binding Protein-5 and Syntaxin-2 on von Willebrand Factor Concentration and Cardiovascular Risk | Background-Elevated von Willebrand factor (VWF) plasma levels are associated with an increased risk of cardiovascular disease. A meta-analysis of genomewide association studies on VWF identified novel candidate genes, that is, syntaxin-binding protein 5 (STXBP5) and syntaxin 2 (STX2), which are possibly involved in the secretion of VWF. We investigated whether VWF antigen levels (VWF:Ag), VWF collagen-binding activity (VWF:CB) and the risk of arterial thrombosis are affected by common genetic variations in these genes. Methods and Results-In 463 young white subjects (men <= 45 years of age and women <= 55 years of age), who were included 1 to 3 months after a first event of arterial thrombosis, and 406 control subjects, we measured VWF:Ag and VWF:CB. Nine haplotype tagging single-nucleotide polymorphisms of STXBP5 and STX2 were selected and subsequently analyzed using linear regression with additive genetic models adjusted for age, sex, and ABO blood group. The minor alleles of rs9399599 and rs1039084 in STXBP5 were associated with lower VWF plasma levels and activity, whereas the minor allele of rs7978987 in STX2 was associated with higher VWF plasma levels and activity. The minor alleles of the single-nucleotide polymorphisms in STX2 were associated with a reduced risk of arterial thrombosis (rs1236: odds ratio, 0.73 [95% confidence interval, 0.59, 0.89]; rs7978987: odds ratio, 0.81 [95% confidence interval, 0.65, 1.00]; rs11061158:odds ratio, 0.69 [95% confidence interval, 0.55, 0.88]). Conclusions-Genetic variability in STXBP5 and STX2 affects both VWF concentration and activity in young individuals with premature arterial thrombosis. Furthermore, in our study, genetic variability in STX2 is associated with the risk of arterial thrombosis. However, at this point, the underlying mechanism remains unclear. (Circ Cardiovasc Genet. 2010; 3:507-512.) | 2658 | NA | NA | NA | no | no |
| 2659 | 2017 | NA | Zhou, XD; Burgess, TI; Beer, ZW; Lieutier, F; Yart, A; Klepzig, K; Carnegie, A; Portales, JM; Wingfield, BD; Wingfield, MJ | 2007 | High intercontinental migration rates and population admixture in the sapstain fungus Ophiostoma ips | Ophiostoma ips is a common fungal associate of various conifer-infesting bark beetles in their native ranges and has been introduced into non-native pine plantations in the Southern Hemisphere. In this study, we used 10 microsatellite markers to investigate the population biology of O. ips in native (Cuba, France, Morocco and USA) and non-native (Australia, Chile and South Africa) areas to characterize host specificity, reproductive behaviour, and the potential origin as well as patterns of spread of the fungus and its insect vectors. The markers resolved a total of 41 alleles and 75 haplotypes. Higher genetic diversity was found in the native populations than in the introduced populations. Based on the origin of the insect vectors, the populations of O. ips in Australia would be expected to reflect a North American origin, and those in Chile and South Africa to reflect a European origin. However, most alleles observed in the native European population were also found in the native North American population; only the allele frequencies among the populations varied. This admixture made it impossible to confirm the origin of the introduced Southern Hemisphere (SH) populations of O. ips. There was also no evidence for specificity of the fungus to particular bark beetle vectors or hosts. Although O. ips is thought to be mainly self-fertilizing, evidence for recombination was found in the four native populations surveyed. The higher genetic diversity in the North American than in the European population suggests that North America could be the possible source region of O. ips. | 2659 | NA | NA | NA | no | no |
| 2660 | 2017 | NA | Castagnone-Sereno, P; Danchin, EGJ; Deleury, E; Guillemaud, T; Malausa, T; Abad, P | 2010 | Genome-wide survey and analysis of microsatellites in nematodes, with a focus on the plant-parasitic species Meloidogyne incognita | Background: Microsatellites are the most popular source of molecular markers for studying population genetic variation in eukaryotes. However, few data are currently available about their genomic distribution and abundance across the phylum Nematoda. The recent completion of the genomes of several nematode species, including Meloidogyne incognita, a major agricultural pest worldwide, now opens the way for a comparative survey and analysis of microsatellites in these organisms. Results: Using MsatFinder, the total numbers of 1-6 bp perfect microsatellites detected in the complete genomes of five nematode species (Brugia malayi, Caenorhabditis elegans, M. hapla, M. incognita, Pristionchus pacificus) ranged from 2,842 to 61,547, and covered from 0.09 to 1.20% of the nematode genomes. Under our search criteria, the most common repeat motifs for each length class varied according to the different nematode species considered, with no obvious relation to the AT-richness of their genomes. Overall, (AT)(n), (AG)(n) and (CT)(n) were the three most frequent dinucleotide microsatellite motifs found in the five genomes considered. Except for two motifs in P. pacificus, all the most frequent trinucleotide motifs were AT-rich, with (AAT)(n) and (ATT)(n) being the only common to the five nematode species. A particular attention was paid to the microsatellite content of the plant-parasitic species M. incognita. In this species, a repertoire of 4,880 microsatellite loci was identified, from which 2,183 appeared suitable to design markers for population genetic studies. Interestingly, 1,094 microsatellites were identified in 801 predicted protein-coding regions, 99% of them being trinucleotides. When compared against the InterPro domain database, 497 of these CDS were successfully annotated, and further assigned to Gene Ontology terms. Conclusions: Contrasted patterns of microsatellite abundance and diversity were characterized in five nematode genomes, even in the case of two closely related Meloidogyne species. 2,245 di- to hexanucleotide loci were identified in the genome of M. incognita, providing adequate material for the future development of a wide range of microsatellite markers in this major plant parasite. | 2660 | NA | NA | NA | no | no |
| 2661 | 2017 | NA | Diaz-Almela, E; Arnaud-Haond, S; Vliet, MS; Alvarez, E; Marba, N; Duarte, CM; Serrao, EA | 2007 | Feed-backs between genetic structure and perturbation-driven decline in seagrass (Posidonia oceanica) meadows | “We explored the relationships between perturbation-driven population decline and genetic/genotypic structure in the clonal seagrass Posidonia oceanica, subject to intensive meadow regression around four Mediterranean fish-farms, using seven specific microsatellites. Two meadows were randomly sampled (40 shoots) within 1,600 m(2) at each site: the”“impacted”" station, 5-200 m from fish cages, and the "“control”" station, around 1,000 m downstream further away (considered a proxy of the pre-impact genetic structure at the site). Clonal richness (R), Simpson genotypic diversity (D*) and clonal sub-range (CR) were highly variable among sites. Nevertheless, the maximum distance at which clonal dispersal was detected, indicated by CR, was higher at impacted stations than at the respective control station (paired t-test: P < 0.05, N = 4). The mean number of alleles (angstrom) and the presence of rare alleles (angstrom (r)) decreased at impacted stations (paired t-test: P < 0.05, and P < 0.02, respectively, N = 4). At a given perturbation level (quantified by the organic and nutrient loads), shoot mortality at the impacted stations significantly decreased with CR at control stations (R-2 = 0.86, P < 0.05). Seagrass mortality also increased with angstrom (R-2 = 0.81, P < 0.10), R (R-2 = 0.96, P < 0.05) and D* (R-2 = 0.99, P < 0.01) at the control stations, probably because of the negative correlation between those parameters and CR. Therefore, the effects of clonal size structure on meadow resistance could play an important role on meadow survival. Large genotypes of P. oceanica meadows thus seem to resist better to fish farm-derived impacts than little ones. Clonal integration, foraging advantage or other size-related fitness traits could account for this effect." | 2661 | NA | NA | NA | no | no |
| 2662 | 2017 | NA | Zhang, KY; Liu, H; Sun, J; Liu, JR; Fei, K; Zhang, CX; Xu, MX; Sun, J; Ma, XY; Lai, R; Wu, YD; Lin, MS | 2008 | Molecular Phylogeny of Geographical Isolates of Bursaphelenchus xylophilus: Implications on the Origin and Spread of this Species in China and Worldwide | The genetic diversity and phylogeny of 26 isolates of Bursaphelenchus xlophilus from China, Japan, Portugal and North America were investigated based on the D2/3 domain of 28S rDNA, nuclear ribosomal Internal Transcribed Spacer (ITS) sequences, and random amplified polymorphic DNA (RAPD) analysis. The genetic diversity analysis showed that the D2/3 domain of 28S rDNA of isolates of B. xlophilus from China, Portugal, Japan and the US were identical and differed at one to three nucleotides compared to those from Canada. ITS sequences of isolates from China and Portugal were the same; they differed at one or two nucleotides compared to those of Japanese isolates and at four and 23 nucleotides compared to those front the US and Canada, respectively. The phylogenetic analysis indicated that Chinese isolates share a common ancestor with one of the two Japanese clades and that the Canadian isolates form a sister group of the clade comprised of isolates from China, Portugal,Japan, and the US. The relationship between Japanese isolates and those from China was closer than with the American isolates. The Canadian isolates were the basal group of B. xylophilus. This suggests that B. xlophilus originated in North America and that the B. xylphilus that occurs in China could have been first introduced from Japan. Further analysis based on RAPD analysis revealed that the relationship among isolates from Guangdong, Zhejiang, Shandong, Anhui provinces and Nanjing was the closest, which suggests that pine wilt disease in these Chinese locales was probably dispersed from Nanjing, where this disease first occurred in China. | 2662 | NA | NA | NA | no | no |
| 2663 | 2017 | NA | Schachter-Broide, J; Gurtler, RE; Kitron, U; Dujardin, JP | 2009 | Temporal Variations of Wing Size and Shape of Triatoma infestans (Hemiptera: Reduviidae) Populations From Northwestern Argentina Using Geometric Morphometry | Wing geometric morphometry of Triatoma infestans (Klug) (Hemiptera: Reduviidae) populations in northwestern Argentina showed that individual collection sites represent the discrete unit where metric differentiation took place. Here we Studied temporal variations in wing size and shape of T. infestans Populations from defined capture sites on three occasions between 2000 and 2003. Bugs collected front domiciles and/or storerooms had significantly larger right-wing centroid size than bugs collected at goat and/or pig corrals by the end of summer 2000 for both sexes. Conversely, male bugs collected front domiciles and/or storerooms had significantly smaller centroid size than bugs collected from pig corrals in spring 2002. The inversion in wing centroid size between seasons was consistent between sexes. Wing shape analysis from the south-central extreme of the Study village showed divergence between collection dates for both sexes. Wing shape divergence was highly significant between male bugs collected by the end of summer 2000 and those collected in spring 2002 and by the end of summer 2003. For females, wing shape divergence was marginally significant between the end Of Summer 2000 and spring 2002, and significant between spring 2002 and the end of summer 2003. Unlike season-related variations in wing centroid size, shape differentiation wits related to the time period elapsed between sample collections and Suggested genetic influences acting on shape. Simultaneous consideration of wing size and shape provided complementary information on the direction and timing of bug dispersal. Morphological studies may allow determining the degree of relatedness of different bug populations and to associate morphological heterogeneity with temporal patterns of reinfestation. | 2663 | NA | NA | NA | no | no |
| 2664 | 2017 | NA | CARTON, Y; NAPPI, A | 1991 | THE DROSOPHILA IMMUNE-REACTION AND THE PARASITOID CAPACITY TO EVADE IT - GENETIC AND COEVOLUTIONARY ASPECTS | “The genetic interactions which occur between hosts and parasites in natural communities are likely to be characterized by the reciprocal genetic modification of hosts by parasites and parasites by hosts. This mechanism is known as”“coevolution”“. We decided to investigate in a well documented association such as the Drosophila-parasitic wasp system, if and how a coevolutionary process has developed concerning cellular resistance of the host and counterreaction or virulence of the parasite. Leptopolina boulardi is a parasitic wasp which infests the larval stage of its specific host Drosophila melanogaster. In some circumstances, the Drosophila larvae is able to develop a cellular reaction against the parasitic egg which is killed. This trait has a stable mean value over generations. With the isofemale line procedure, we demonstrated the genetic determinism of this trait and its degree of genetic variability (H(B)2 = 0.43). The genetic ability of the parasite to evade encapsulation was then investigated. Oviposition by a female wasp rapidly incapacitates the hemocytes of the host. Evaluation of H(B)2 varies from 0.31 to 0.5. These results prove that the parasitic wasp possesses the genetic variability needed to evolve and to overcome genetical resistance in its host. All the conditions were theoretically present for the development of a process of coevolution. Both D. melanogaster and its highly specific parasite L. boulardi have a very large distribution. In most populations studied, it seems that the resistance of the host is always outweighted by the virulence of the parasite. The latter was able, in this arm race with the host, to select more quickly a very efficient evading mechanism. In the mutual aggression model, if the death of the host does not imply the death of the parasite, the selective pressure toward parasite virulence will be enhanced. It is the case of parasitoid. There is also experimental evidence that flies bearing a melanotic capsule exhibit reduction in fitness. We can have an explanation why in the Drosophila-parasitic wasp system, the parasite may gain this race in most of the sites. There is a locality (Congo) where the parasite does not succeed to evade the host encapsulation. This parasite in this African locality does not develop a well defined strategy for either 5 potential hosts. This African association appears to be a demonstrative example of diffuse coevolution.” | 2664 | NA | NA | NA | no | no |
| 2665 | 2017 | NA | Cisarovsky, G; Koch, H; Schmid-Hempel, P | 2012 | A field study on the influence of food and immune priming on a bumblebee-gut parasite system | Laboratory experiments are often preferred over field experiments because they allow the control of confounding factors that would otherwise influence the causal effect of a particular focal experimental factor. These confounding factors can, however, significantly alter the response of an organism confronted with a particular situation, which can have great implications. In a field experiment with a bumblebee host-parasite system, we looked at the influence of additional food supply and immune challenge on various colony fitness values and parasite traits. We could confirm the importance of food on the colony fitness, but not on parasite infection probability or parasite genetic diversity. In contrast to the findings of laboratory experiments of this system, challenge of the immune system had no significant effect on colony fitness or parasite infections. These results likely reflect an overriding effect of environmental variation without disproving the concept of a cost of defence per se. But the results also demonstrate that confounding factors purposely controlled for in the laboratory have to be weighed against their ecological relevance, and stress the need for careful analysis before any direct transfer is made of laboratory results to field situations. | 2665 | NA | NA | NA | no | no |
| 2666 | 2017 | NA | Tattiyapong, M; Sivkumar, T; Takemae, H; Simking, P; Jittapalapong, S; Igarashi, II; Yokoyama, N | 2015 | Analysis of the genetic diversity of Babesia bovis msa-1 in Thailand using type-specific polymerase chain reaction | Babesia bovis, a tick-borne hemoprotozoan parasite, causes fatal bovine babesiosis in cattle. Merozoite surface antigens, encoded by msa genes, of B. bovis play an important role in the initial attachment of the merozoite to host erythrocytes. In the present study, B. bovis-positive blood DNA samples (n = 162) sourced from cattle in Thailand were analyzed using type -specific polymerase chain reaction (PCR) assays targeting nine Asian msa-1 genotypes (genotypes C and AS1-AS8). All nine msa-1 genotypes were detected in Thai cattle, and at least one msa-1 genotype was detected in 154 of the 162 samples. The most common genotype was AS7 (n = 139), followed by AS5 (n = 61), AS1 (n = 37), AS8 (n = 32), C (n = 11), AS4 (n = 8), AS3 (n = 5), AS6 (n = 2), and AS2 (n = 1). Co-infection with parasites of different genotypes was also commonly detected, and with 90 (58.4%) of the 154 PCR-positive DNA samples showing infection with more than one genotype. Overall, the detected genetic diversity of msa-1 from B. bovis was much higher than that previously determined in Thailand. These findings warrant further investigation to determine the nature of antigenic variation between the different B. bovis genotypes detected in the cattle population of Thailand. | 2666 | NA | NA | NA | no | no |
| 2667 | 2017 | NA | Janssen, T; Karssen, G; Verhaeven, M; Coyne, D; Bert, W | 2016 | Mitochondrial coding genome analysis of tropical root-knot nematodes (Meloidogyne) supports haplotype based diagnostics and reveals evidence of recent reticulate evolution | The polyphagous parthenogenetic root-knot nematodes of the genus Meloidogyne are considered to be the most significant nematode pest in sub-tropical and tropical agriculture. Despite the crucial need for correct diagnosis, identification of these pathogens remains problematic. The traditionally used diagnostic strategies, including morphometrics, host-range tests, biochemical and molecular techniques, now appear to be unreliable due to the recently-suggested hybrid origin of root-knot nematodes. In order to determine a suitable barcode region for these pathogens nine quickly-evolving mitochondrial coding genes were screened. Resulting haplotype networks revealed closely related lineages indicating a recent speciation, an anthropogenic-aided distribution through agricultural practices, and evidence for reticulate evolution within M. arenaria. Nonetheless, nucleotide polymorphisms harbor enough variation to distinguish these closely-related lineages. Furthermore, completeness of lineage sorting was verified by screening 80 populations from widespread geographical origins and variable hosts. Importantly, our results indicate that mitochondrial haplotypes are strongly linked and consistent with traditional esterase isozyme patterns, suggesting that different parthenogenetic lineages can be reliably identified using mitochondrial haplotypes. The study indicates that the barcode region Nad5 can reliably identify the major lineages of tropical root-knot nematodes. | 2667 | NA | NA | NA | no | no |
| 2668 | 2017 | NA | TSAI, HF; LIU, JS; STABEN, C; CHRISTENSEN, MJ; LATCH, GCM; SIEGEL, MR; SCHARDL, CL | 1994 | EVOLUTIONARY DIVERSIFICATION OF FUNGAL ENDOPHYTES OF TALL FESCUE GRASS BY HYBRIDIZATION WITH EPICHLOE SPECIES | The mutualistic associations of tall fescue (Festuca arundinacea) with seed-borne fungal symbionts (endophytes) are important for fitness of the grass host and its survival under biotic and abiotic stress. The tall fescue endophytes are asexual relatives of biological species (mating populations) of genus Epichloe (Clavicipitaceae), sexual fungi that cause grass choke disease. Isozyme studies have suggested considerable genetic diversity among endophytes of tall fescue. Phylogenetic relationships among seven isolates from tall fescue, three from meadow fescue (a probable ancestor of tall fescue), and nine Epichloe isolates from other host species were investigated by comparing sequences of noncoding segments of the beta-tubulin (tub2) and rRNA (rrn) genes. Whereas each Epichloe isolate and meadow fescue endophyte had only a single tub2 gene, most tall fescue endophytes had two or three distinct tub2 copies. Phylogenetic analysis of tub2 sequences indicated that the presence of multiple copies in the tall fescue endophytes was a consequence of hybridization with Epichloe species. At least three hybridization events account for the distribution and relationships of tub2 genes. These results suggest that interspecific hybridization is the major cause of genetic diversification of the tall fescue endophytes. | 2668 | NA | NA | NA | no | no |
| 2669 | 2017 | NA | Kawatsu, K | 2015 | Red Queen dancing in the lek: effects of mating skew on host-parasite interactions | The RQH (Red Queen hypothesis), which argues that hosts need to be continuously finding new ways to avoid parasites that are able to infect common host genotypes, has been at the center of discussions on the maintenance of sex. This is because diversity is favored under the host-parasite coevolution based on negative frequency-dependent selection, and sexual reproduction is a mechanism that generates genetic diversity in the host population. Together with parasite infections, sexual organisms are usually under sexual selection, which leads to mating skew or mating success biased toward males with a particular phenotype. Thus, strong mating skew would affect genetic variance in a population and should affect the benefit of the RQH. However, most models have investigated the RQH under a random mating system and not under mating skew. In this study, I show that sexual selection and the resultant mating skew may increase parasite load in the hosts. An IBM (individual-based model), which included host-parasite interactions and sexual selection among hosts, demonstrates that mating skew influenced parasite infection in the hosts under various conditions. Moreover, the IBM showed that the mating skew evolves easily in cases of male-male competition and female mate choice, even though it imposes an increased risk of parasite infection on the hosts. These findings indicated that whether the RQH favored sexual reproduction depended on the condition of mating skew. That is, consideration of the host mating system would provide further understanding of conditions in which the RQH favors sexual reproduction in real organisms. | 2669 | NA | NA | NA | no | no |
| 2670 | 2017 | NA | Ramirez, JD; Tapia-Calle, G; Guhl, F | 2013 | Genetic structure of Trypanosoma cruzi in Colombia revealed by a High-throughput Nuclear Multilocus Sequence Typing (nMLST) approach | Background: Chagas disease is a systemic pathology caused by Trypanosoma cruzi. This parasite reveals remarkable genetic variability, evinced in six Discrete Typing Units (DTUs) named from T. cruzi I to T. cruzi VI (TcI to TcVI). Recently newly identified genotypes have emerged such as TcBat in Brazil, Colombia and Panama associated to anthropogenic bats. The genotype with the broadest geographical distribution is TcI, which has recently been associated to severe cardiomyopathies in Argentina and Colombia. Therefore, new studies unraveling the genetic structure and natural history of this DTU must be pursued. Results: We conducted a spatial and temporal analysis on 50 biological clones of T. cruzi I (TcI) isolated from humans with different clinical phenotypes, triatomine bugs and mammal reservoirs across three endemic regions for Chagas disease in Colombia. These clones were submitted to a nuclear Multilocus Sequence Typing (nMLST) analysis in order to elucidate its genetic diversity and clustering. After analyzing 13 nuclear housekeeping genes and obtaining a 5821 bp length alignment, we detected two robust genotypes within TcI henceforth named TcIDOM (associated to human infections) and a second cluster associated to peridomestic and sylvatic populations. Additionaly, we detected putative events of recombination and an intriguing lack of linkage disequilibrium. Conclusions: These findings reinforce the emergence of an enigmatic domestic T. cruzi genotype (TcIDOM), and demonstrates the high frequency of recombination at nuclear level across natural populations of T. cruzi. Therefore, the need to pursue studies focused on the diferential virulence profiles of TcI strains. The biological and epidemiological implications of these findings are herein discussed. | 2670 | NA | NA | NA | no | no |
| 2671 | 2017 | NA | Ball, SE; Bovero, S; Sotgiu, G; Tessa, G; Angelini, C; Bielby, J; Durrant, C; Favelli, M; Gazzaniga, E; Garner, TWJ | 2017 | Islands within an island: Population genetic structure of the endemic Sardinian newt, Euproctus platycephalus | The identification of historic and contemporary barriers to dispersal is central to the conservation of endangered amphibians, but may be hindered by their complex life history and elusive nature. The complementary information generated by mitochondrial (mtDNA) and microsatellite markers generates a valuable tool in elucidating population structure and the impact of habitat fragmentation. We applied this approach to the study of an endangered montane newt, Euproctus platycephalus. Endemic to the Mediterranean island of Sardinia, it is threatened by anthropogenic activity, disease, and climate change. We have demonstrated a clear hierarchy of structure across genetically divergent and spatially distinct subpopulations. Divergence between three main mountain regions dominated genetic partitioning with both markers. Mitochondrial phylogeography revealed a deep division dating to ca. 1million years ago (Mya), isolating the northern region, and further differentiation between the central and southern regions ca. 0.5Mya, suggesting an association with Pleistocene severe glacial oscillations. Our findings are consistent with a model of southward range expansion during glacial periods, with postglacial range retraction to montane habitat and subsequent genetic isolation. Microsatellite markers revealed further strong population structure, demonstrating significant divergence within the central region, and partial differentiation within the south. The northern population showed reduced genetic diversity. Discordance between mitochondrial and microsatellite markers at this scale indicated a further complexity of population structure, in keeping with male-biased dispersal and female philopatry. Our study underscores the need to elucidate cryptic population structure in the ecology and conservation strategies for endangered island-restricted amphibians, especially in the context of disease and climate change. | 2671 | NA | NA | NA | no | no |
| 2672 | 2017 | NA | Goncalves, H; Martinez-Solano, I; Pereira, RJ; Carvalho, B; Garcia-Paris, M; Ferrand, N | 2009 | High levels of population subdivision in a morphologically conserved Mediterranean toad (Alytes cisternasii) result from recent, multiple refugia: evidence from mtDNA, microsatellites and nuclear genealogies | Pleistocene glaciations often resulted in differentiation of taxa in southern European peninsulas, producing the high levels of endemism characteristic of these regions (e.g. the Iberian Peninsula). Despite their small ranges, endemic species often exhibit high levels of intraspecific differentiation as a result of a complex evolutionary history dominated by successive cycles of fragmentation, expansion and subsequent admixture of populations. Most evidence so far has come from the study of species with an Atlantic distribution in northwestern Iberia, and taxa restricted to Mediterranean-type habitats remain poorly studied. The Iberian Midwife toad (Alytes cisternasii) is a morphologically conserved species endemic to southwestern and central Iberia and a typical inhabitant of Mediterranean habitats. Applying highly variable genetic markers from both mitochondrial and nuclear genomes to samples collected across the species’ range, we found evidence of high population subdivision within A. cisternasii. Mitochondrial haplotypes and microsatellites show geographically concordant patterns of genetic diversity, suggesting population fragmentation into several refugia during Pleistocene glaciations followed by subsequent events of geographical and demographic expansions with secondary contact. In addition, the absence of variation at the nuclear beta-fibint7 and Ppp3caint4 gene fragments suggests that populations of A. cisternasii have been recurrently affected by episodes of extinction and recolonization, and that documented patterns of population subdivision are the outcome of recent and multiple refugia. We discuss the evolutionary history of the species with particular interest in the increasing relevance of Mediterranean refugia for the survival of genetically differentiated populations during the Pleistocene glaciations as revealed by studies in co-distributed taxa. | 2672 | NA | NA | NA | no | no |
| 2673 | 2017 | NA | Haag, KL; Alves-Junior, L; Zaha, A; Ayala, FJ | 2004 | Contingent, non-neutral evolution in a multicellular parasite: natural selection and gene conversion in the Echinococcus granulosus antigen B gene family | Recent studies have demonstrated that the Echinococcus granulosus antigen B (AgB) interferes with the intermediate hosts’ immune response and is encoded by a multigene family. The number of members within the family is still uncertain, but there are several evidences of a large genetic variability. The E. granulosus AgB genomic sequences available in nucleotide databases can be grouped into four clades, corresponding to genes EgAgB1, EgAgB2, EgAgB3 and EgAgB4. In the present study, we use PCR amplifications followed by cloning and sequencing to evaluate the genetic variability for AgB isoforms. Two pairs of primers were independently used for PCR amplification. Both PCR reactions from each of three isolated protoscolex (larvae) were cloned in a plasmid vector and the plasmid inserts of 30 colonies from each cloning experiment were sequenced. Using phylogenetic tools, the 113 EgAgB clones are classified as follows: 25 are related to EgAgB], 24 to EgAgB2, 9 to EgAgB3 and 39 to EgAgB4. The remaining 16 clones form a separate cluster, which we name EgAgB5, more closely related to EgAgB3 than to any of the other genes. Within each gene group, a number of variant sequences occur, which differ from one another by one or few nucleotides. One EgAgB3 clone has a premature stop codon (pseudogene) and an EgAgB2 clone lacks the region corresponding to the intron. The overall variation cannot be explained by differences among the asexual protoscoleces, or by experimental artifacts. Using Echinococcuss AgB genes from other species/strains as outgroups, neutrality is rejected for EgAgB2, and balancing selection is detected for EgAgB5, which also seems to be involved in gene conversion. We suggest that EgAgB1-EgAgB5 represent a family of contingency genes, that is, genes that are variably expressed, so that some but not others are expressed in each individual parasite. Contingency genes are common in parasitic protozoa and other microparasites, but the EgAgB family is the first set identified in a multicellular parasite. (C) 2004 Elsevier B.V. All rights reserved. | 2673 | NA | NA | NA | no | no |
| 2674 | 2017 | NA | Sarno, RJ; Bank, MS; Stern, HS; Franklin, WL | 2003 | Forced dispersal of juvenile guanacos (Lama guanicoe): causes, variation, and fates of individuals dispersing at different times | We examined adult-juvenile conflict in the guanaco (Lama guanicoe). During spring, territorial males become increasingly aggressive toward all juveniles born the previous year and begin expelling them from family groups. In an apparent effort to reduce aggression, juveniles display submissive crouches when being observed, approached, or attacked by the territorial male. Therefore, we assessed the influence of juvenile submissive behavior on the timing of dispersal and also examined if dispersal time was related to survival and reproductive performance as adults. We also evaluated hypotheses regarding the evolution of juvenile mammalian dispersal in the context of if and how each may favor the forced dispersal of juvenile guanacos by territorial males. Juveniles generally dispersed in late spring and early summer, and a nearly equal proportion of females (n=46; 48%) and males (n=49; 52%) dispersed. More-submissive animals generally dispersed later than less-submissive animals. Juvenile sex and dispersal time were not related to survival. In contrast, juvenile sex and dispersal time were related to reproductive performance. The probability of reproducing was highest when juveniles dispersed early and decreased with increasing time in family groups prior to dispersal. The largest proportion of juveniles was forced to disperse during a 2-week interval following the peak of the breeding season. Competition for food resources is likely very intense at this juncture and territorial males may force older juveniles to disperse in order to divert food resources to younger neonates. Additionally, juveniles may be forced to disperse after territorial males mate their mothers to prevent lost mating opportunities, because females leave territories when their offspring disperse and possibly prior to mating with males. We conclude that the forced dispersal of juvenile guanacos by territorial males is ultimately driven by competition for food resources on territories. The timing of dispersal, however, may be tempered by the chronology of matings between territorial males and particular adult females, and/or genetic relatedness between territorial males and juveniles. | 2674 | NA | NA | NA | no | no |
| 2675 | 2017 | NA | Refardt, D; Bergmiller, T; Kummerli, R | 2013 | Altruism can evolve when relatedness is low: evidence from bacteria committing suicide upon phage infection | High relatedness among interacting individuals has generally been considered a precondition for the evolution of altruism. However, kin-selection theory also predicts the evolution of altruism when relatedness is low, as long as the cost of the altruistic act is minor compared with its benefit. Here, we demonstrate evidence for a low-cost altruistic act in bacteria. We investigated Escherichia coli responding to the attack of an obligately lytic phage by committing suicide in order to prevent parasite transmission to nearby relatives. We found that bacterial suicide provides large benefits to survivors at marginal costs to committers. The cost of suicide was low, because infected cells are moribund, rapidly dying upon phage infection, such that no more opportunity for reproduction remains. As a consequence of its marginal cost, host suicide was selectively favoured even when relatedness between committers and survivors approached zero. Altogether, our findings demonstrate that low-cost suicide can evolve with ease, represents an effective host-defence strategy, and seems to be widespread among microbes. Moreover, low-cost suicide might also occur in higher organisms as exemplified by infected social insect workers leaving the colony to die in isolation. | 2675 | NA | NA | NA | no | no |
| 2676 | 2017 | NA | Mateu, E; Calafell, F; Ramos, MD; Casals, T; Bertranpetit, J | 2002 | Can a place of origin of the main cystic fibrosis mutations be identified? | “The genetic background of the mutations that most often cause cystic fibrosis (CF) is different from that of non-CF chromosomes in populations of European origin. It is not known whether these haplotype backgrounds could be found at high frequencies in populations in which CF is, at present, not common; such populations would be candidates for the place of origin of CF mutations. An analysis of haplotypes of CF transmembrane conductance regulator, together with their variation in specific CF chromosomes, in a worldwide survey of normal chromosomes shows (1) a very low frequency or absence of the most common CF haplotypes in all populations analyzed and (2) a strong genetic variability and divergence, among various populations, of the chromosomes that carry disease-causing mutations. The depth of the gene genealogy associated with disease-causing mutations may be greater than that of the evolutionary process that gave rise to present-day human populations. The concept of”“population of origin”" lacks either spatial or temporal meaning for mutations that are likely to have been present in Europeans before the ethnogenesis of present populations | 2676 | NA | NA | NA | no | no |
| 2677 | 2017 | NA | Ward, TJ; Bielawski, JP; Kistler, HC; Sullivan, E; O’Donnell, K | 2002 | Ancestral polymorphism and adaptive evolution in the trichothecene mycotoxin gene cluster of phytopathogenic Fusarium | Filamentous fungi within the Fusarium graminearum species complex (Fg complex) are the primary etiological agents of Fusarium head blight (scab) of wheat and barley. Scab is an economically devastating plant disease that greatly limits grain yield and quality. In addition, scabby grain is often contaminated with trichothecene mycotoxins that act as virulence factors on some hosts, and pose a serious threat to animal health and food safety. Strain-specific differences in trichothecene metabolite profiles (chemotypes) are not well correlated with the Fg complex phylogeny based on genealogical concordance at six single-copy nuclear genes. To examine the basis for this discord between species and toxin evolution, a 19-kb region of the trichothecene gene cluster was sequenced in 39 strains chosen to represent the global genetic diversity of species in the Fg complex and four related species of Fusarium. Phylogenetic analyses demonstrated that polymorphism within these virulence-associated genes is transspecific and appears to have been maintained by balancing selection acting on chemotype differences that originated in the ancestor of this important group of plant pathogens. Chemotype-specific differences in selective constraint and evidence of adaptive evolution within trichothecene genes are also reported. | 2677 | NA | NA | NA | no | no |
| 2678 | 2017 | NA | Kominkova, E; Dreiseitl, A; Maleckova, E; Dolezel, J; Valarik, M | 2016 | Genetic Diversity of Blumeria graminis f. sp hordei in Central Europe and Its Comparison with Australian Population | Population surveys of Blumeria graminis f. sp. hordei (Bgh), a causal agent of more than 50% of barley fungal infections in the Czech Republic, have been traditionally based on virulence tests, at times supplemented with non-specific Restriction fragment length polymorphism or Random amplified polymorphic DNA markers. A genomic sequence of Bgh, which has become available recently, enables identification of potential markers suitable for population genetics studies. Two major strategies relying on transposable elements and micro satellites were employed in this work to develop a set of Repeat junction markers, Single sequence repeat and Single nucleotide polymorphism markers. A resolution power of the new panel of markers comprising 33 polymorphisms was demonstrated by a phylogenetic analysis of 158 Bgh isolates. A core set of 97 Czech isolates was compared to a set 50 Australian isolates on the background of 11 diverse isolates collected throughout the world. 73.2% of Czech isolates were found to be genetically unique. An extreme diversity of this collection was in strong contrast with the uniformity of the Australian one. This work paves the way for studies of population structure and dynamics based on genetic variability among different Bgh isolates originating from geographically limited regions. | 2678 | NA | NA | NA | no | no |
| 2679 | 2017 | NA | Parani, M; Lakshmi, M; Senthilkumar, P; Ram, N; Parida, A | 1998 | Molecular phylogeny of mangroves V. Analysis of genome relationships in mangrove species using RAPD and RFLP markers | DNA from pooled leaf samples of 11. true major mangrove, three true minor mangrove, two mangrove associate, two mangrove parasite, three terrestrial and one cultivated species were isolated for the present study. In total, 198 random amplified polymorphic DNAs (RAPDs) and 180 restriction fragment length polymorphism (RFLP) loci were scored by using ten primers and 14 enzyme-probe combinations respectively. The polymorphism observed for these: markers revealed a high degree of genetic diversity in mangroves at both inter-specific or inter-generic levels. A dendrogram, constructed after pooling both RAPD and RFLP data, using a similarity index was analysed for genome relationships among these species. The dendrogram showed clustering of all the major mangroves, except for Nypa fruticans (Arecaceae), into one group. All species under the tribe Rhizophorae formed a subcluster, to which Xylocarpus granatum was found to be the most closesly related species. The clustering pattern implied that Excoecaria agallocha and Acanthus ilicifolius should be considered as true minor mangroves. The present study also provided molecular data favouring the separation of Avicennia spp. from the Verbenaceae to create a monotypic family the Avicenniaceae. The separation of Viscum orientale into the Viscaceae was also favoured. | 2679 | NA | NA | NA | no | no |
| 2680 | 2017 | NA | Eckstein, RL; Danihelka, J; Otte, A | 2009 | Variation in life-cycle between three rare and endangered floodplain violets in two regions: implications for population viability and conservation | We studied the demography of Viola elatior, V. pumila, and V. stagnina, three rare and endangered Central European floodplain species, to (i) analyse variation in life-cycles among congeners and between regions (Dyje-Morava floodplains, Czech Republic; Upper Rhine, Germany), (ii) to define sensitive stages in the life-cycles, and (iii) to identify possible threats for population viability and species conservation. Matrix models were based on the fate of marked individuals from a total of 27 populations over two years. We analysed population growth rate (lambda), stage distribution, net reproductive rate (R (0)), generation time, age at first reproduction, and elasticity and calculated a life table response experiment (LTRE). Most populations were declining and lambda did not differ between species or regions during the observed interval. Despite higher probabilities for survival and flowering in the Dyje populations, R (0) was higher in the Rhine populations. Also other demographic traits showed consistent differences between regions and/or species. Complex life-cycles and large variation in lambda precluded unequivocal identification of sensitive stages or vital rates for conservation. Variation between regions may be a consequence of differences in habitat quality. Our results suggest that deterministic processes such as reduced management, succession, habitat destruction, and lack of disturbance through reduced or eliminated flooding present the strongest threat for the viability and persistence of populations of the three floodplain violets as compared with stochastic processes. However, the persistent seed bank of the species may buffer populations against environmental variation and represents a reservoir for recovery after resumption of suitable land-use management. | 2680 | NA | NA | NA | no | no |
| 2681 | 2017 | NA | Cagliani, R; Fumagalli, M; Riva, S; Pozzoli, U; Comi, GR; Bresolin, N; Sironi, M | 2010 | Genetic variability in the ACE gene region surrounding the Alu I/D polymorphism is maintained by balancing selection in human populations | Objective Angiotensin-converting enzyme plays a critical role in the maintenance of cardiovascular homeostasis. Extensive research has aimed at identifying ACE genetic variants responsible for variation in enzyme plasma concentrations and associated with human diseases. These efforts have been hampered by the extensive linkage disequilibrium across the gene and the identity or location of the functional polymorphism(s) is at presently unknown. The aim of our study was to verify whether the Alu insertion/deletion (Alu I/D) polymorphism or any linked variant has been maintained by natural selection in human populations. Methods We resequenced a gene region surrounding the Alu I/D polymorphism in four human populations; we applied population neutrality tests and performed haplotype analysis for this region. Results We observed high levels of nucleotide diversity, an excess of intermediate frequency alleles and, at least in African populations, a higher level of within-species diversity compared with interspecific divergence. Analysis of haplotype genealogy indicated the presence of two major clades separated by deep branches with a coalescence time older than 1.5 million years. All these features strongly suggest the action of balancing selection and we verified that the selection signature is restricted to the gene region surrounding the Alu I/D. Conclusion Our data imply the presence of a functional polymorphism in the Alu I/D region and illustrate the contribution of evolutionary models to classic single nucleotide polymorphism-phenotype association approaches by providing information about the localization of candidate functional variants. Pharmacogenetics and Genomics 20:131-134 (C) 2010 Wolters Kluwer Health vertical bar Lippincott Williams & Wilkins. | 2681 | NA | NA | NA | no | no |
| 2682 | 2017 | NA | Castillo, A; Eguiarte, LE; Souza, V | 2005 | A genomic population genetics analysis of the pathogenic enterocyte effacement island in Escherichia coli: The search for the unit of selection | Comparative genomic analysis is a powerful tool for understanding the history and organization of complete genomes. The mathematical tools of population genetics combined with genomic analysis provide a powerful approach to dissect heterogeneities in genome evolution. This study presents a hierarchical analysis of the enterocyte and effacement island (35 kb), which is found in the enteropathogenic and enterohemorrhagic strains in Escherichia coli and in Citrobacter rodentium. The locus of enterocyte and effacement in E. coli is considered to be a clonal unit inside a clonal organism and is expected to evolve as a single unit. This analysis examines the clonal assumption by determining genetic diversity, GC content, and the substitution rates at the different functional levels of (i) the complete pathogenic island, (it) the five operons in which the island is organized, and (iii) for each of the individual 41 genes that comprise the locus. We find that there is a conserved region that is composed of genes that belong to the type III secretion system and that may be products of horizontal transfer. A more diverse region is composed of genes for secreted proteins and genes that we infer to be original components of the E. coli genome. This genetic mosaic seems to be differentially affected by selection and mutation. Our results suggest that recombination and selection may be breaking this structure so that different elements are, at best, weakly coupled in their evolution. These observations suggest that the units of selection are not the complete island, but rather, much smaller units that comprise the island. | 2682 | NA | NA | NA | no | no |
| 2683 | 2017 | NA | MacLeod, A; Welburn, S; Maudlin, I; Turner, CMR; Tait, A | 2001 | Evidence for multiple origins of human infectivity in Trypanosoma brucei revealed by minisatellite variant repeat mapping | In recent years a wide variety of biochemical and molecular typing systems has been employed in the study of parasite diversity aimed at investigating the level of genetic diversity and delineating the relationship between different species and subspecies. However, such methods have failed to differentiate between two of the classically defined subspecies of the protozoan parasite Trypanosoma brucei: the human infective, T. b. rhodesiense, which causes African sleeping sickness, and the non-human infective T. b. brucei. This has led to the hypothesis that T, b. rhodesiense is a host range variant of T. b. brucei. In this paper we test this hypothesis by examining highly polymorphic tandemly repeated regions of the trypanosome genome, i.e., minisatellite loci. We have employed the technique of minisatellite variant repeat mapping by PCR (MVR-PCR), which determines the distribution of variant repeat units along the tandem array of:one minisatellite, MS42. The maps generated by this technique not only allow unequivocal allele identification but also contain within them cladistic information which we used to determine the possible genetic relationship between the different subspecies of T, brucei Our findings revealed that human infective (T. b. rhodesiense) isolates from Uganda are more closely related to the local non-human infective isolates (T. b. brucei) than they are to other human infective stocks from different regions, suggesting that human infectivity has originated independently in these different geographical regions. This would infer that the separate classification of all human infective stocks from East Africa into the subspecies T. b. rhodesiense is genetically inappropriate and it would be better to consider geographically separate populations as host range variants of T. brucei brucei or perhaps as a series of different subspecies. Based on these data, it is clear that MVR mapping is a very useful tool for the analysis of zoonotic eukaryotic pathogens where delineation of the origins of outbreaks of disease and definition of human infective strains are key questions. | 2683 | NA | NA | NA | no | no |
| 2684 | 2017 | NA | Sun, JT; Guo, N; Lei, J; Li, LH; Hu, GJ; Xing, H | 2014 | Association mapping for partial resistance to Phytophthora sojae in soybean (Glycine max (L.) Merr.) | Association mapping is a powerful high-resolution mapping tool for complex traits. The objective of this study was to identify QTLs for partial resistance to Phytophthora sojae. In this study, we evaluated a total of 214 soybean accessions by the hypocotyl inoculation method, and 175 were susceptible. The 175 susceptible accessions were then evaluated for P. sojae partial resistance using slant board assays. The 175 accessions were screened with 138 SSR markers that generated 730 SSR alleles. A subset of 495 SSR loci with minor allele frequency (MAF) >= 0.05 was used for association mapping by the Tassel general linear model (GLM) and mixed linear model (MLM) programmes. This soybean population could be divided into two subpopulations and no or weak relatedness was detected between pairwise accessions. Four SSR alleles, Satt634-133, Satt634-149, Sat\_222-168 and Satt301-190, associated with partial resistance to P. sojae were detected by both GLM and MLM methods. Of these identified markers, one marker, Satt301, was located in regions where P. sojae resistance QTL have been previously mapped using linkage analysis. The identified markers will help to understand the genetic basis of partial resistance, and facilitate future marker-assistant selection aimed to improve resistance to P. sojae and reduce disease-related mortality in soybean. | 2684 | NA | NA | NA | no | no |
| 2685 | 2017 | NA | Purcell, J; Brutsch, T; Chapuisat, M | 2012 | Effects of the social environment on the survival and fungal resistance of ant brood | The phenotype of social animals can be influenced by genetic, maternal and environmental effects, which include social interactions during development. In social insects, the social environment and genetic origin of brood can each influence a whole suite of traits, from individual size to caste differentiation. Here, we investigate to which degree the social environment during development affects the survival and fungal resistance of ant brood of known maternal origin. We manipulated one component of the social environment, the worker/brood ratio, of brood originating from single queens of Formica selysi. We monitored the survival of brood and measured the head size and ability to resist the entomopathogenic fungus Beauveria bassiana of the resulting callow workers. The worker/brood ratio and origin of eggs affected the survival and maturation time of the brood and the size of the resulting callow workers. The survival of the callow workers varied greatly according to their origin, both in controls and when challenged with B. bassiana. However, there was no interaction between the fungal challenge and either the worker/brood ratio or origin of eggs, suggesting that these factors did not affect parasite resistance in the conditions tested. Overall, the social conditions during brood rearing and the origin of eggs had a strong impact on brood traits that are important for fitness. We detected a surprisingly large amount of variation among queens in the survival of their brood reared in standard queenless conditions, which calls for further studies on genetic, maternal and social effects influencing brood development in the social insects. | 2685 | NA | NA | NA | no | no |
| 2686 | 2017 | NA | Becker, MH; Richards-Zawacki, CL; Gratwicke, B; Belden, LK | 2014 | The effect of captivity on the cutaneous bacterial community of the critically endangered Panamanian golden frog (Atelopus zeteki) | For many threatened vertebrates, captivity may be the only option for species survival. Maintaining species in captivity prior to reintroduction presents many challenges, including the need to preserve genetic diversity and mitigation of disease risks. Recent studies suggest that captivity can alter the suite of symbiotic microbes that play important roles in host health. The Panamanian golden frog (Atelopus zeteki) has not been seen in its native habitat in Panama since 2009. Along with habitat loss and illegal collecting, the lethal disease chytridiomycosis, caused by the fungal pathogen Batrachochytrium dendrobatidis (Bd), is responsible for the severe decline of this species. Prior to the spread of Bd into golden frog habitat, conservation organizations collected golden frogs and placed them in captive survival assurance colonies. The skin of amphibians is host to a diverse resident bacterial community, which acts as a defense mechanism in some amphibians to inhibit pathogens. We characterized the cutaneous bacterial community from wild and F1 captive golden frogs originating from the same population with Illumina sequencing to assess how long-term captivity has affected this community. We found that species richness, phylogenetic diversity, and community structure of the skin microbiota was significantly different between wild and captive golden frogs. However, after approximately eight years of living in captivity, the offspring of the original captive golden frogs still shared 70% of their microbial community with wild frogs. These results demonstrate that host-associated microbial communities can be significantly altered by captive management, but most of the community composition can be preserved. (C) 2014 Elsevier Ltd. All rights reserved. | 2686 | NA | NA | NA | no | no |
| 2687 | 2017 | NA | Imo, M; Maixner, M; Johannesen, J | 2013 | Sympatric diversification vs. immigration: deciphering host-plant specialization in a polyphagous insect, the stolbur phytoplasma vector Hyalesthes obsoletus (Cixiidae) | The epidemiology of vector transmitted plant diseases is highly influenced by dispersal and the host-plant range of the vector. Widening the vector’s host range may increase transmission potential, whereas specialization may induce specific disease cycles. The process leading to a vector’s host shift and its epidemiological outcome is therefore embedded in the frameworks of sympatric evolution vs. immigration of preadapted populations. In this study, we analyse whether a host shift of the stolbur phytoplasma vector, Hyalesthes obsoletus from field bindweed to stinging nettle in its northern distribution range evolved sympatrically or by immigration. The exploitation of stinging nettle has led to outbreaks of the grapevine disease bois noir caused by a stinging nettle-specific phytoplasma strain. Microsatellite data from populations from northern and ancestral ranges provide strong evidence for sympatric host-race evolution in the northern range: Host-plant associated populations were significantly differentiated among syntopic sites (0.054<FHT<0.098) and constant over 5years. While gene flow was asymmetric from the old into the predicted new host race, which had significantly reduced genetic diversity, the genetic identity between syntopic host-race populations in the northern range was higher than between these populations and syntopic populations in ancestral ranges, where there was no evidence for genetic host races. Although immigration was detected in the northern field bindweed population, it cannot explain host-race diversification but suggests the introduction of a stinging nettle-specific phytoplasma strain by plant-unspecific vectors. The evolution of host races in the northern range has led to specific vector-based bois noir disease cycles. | 2687 | NA | NA | NA | no | no |
| 2688 | 2017 | NA | Juarez-Velazquez, R; Canto, P; Canto-Cetina, T; Rangel-Villalobos, H; Rosas-Vargas, H; Rodriguez, M; Canizales-Quinteros, S; Wong, ACV; Ordonez-Razo, RM; Vilchis-Dorantes, G; Coral-Vazquez, RM | 2010 | Analysis of polymorphisms in genes (AGT, MTHFR, GPIIIa, and GSTP1) associated with hypertension, thrombophilia and oxidative stress in Mestizo and Amerindian populations of Mexico | Several polymorphisms related to hypertension, thrombophilia, and oxidative stress has been associated with the development of cardiovascular disease. We analyzed the frequency of M235T angiotensinogen (AGT), A222V 5, 10 methylenetetrahydrofolate reductase (MTHFR), L33P glycoprotein IIIa (GPIIIa), and I105V glutathione S-transferase P1 (GSTP1) polymorphisms in 285 individuals belonging to Mexican-Mestizo and five Amerindian population from Mexico, by real time PCR allelic discrimination. Allele and genotype frequencies were compared using chi(2) tests. All populations followed the Hardy Weinberg equilibrium for assay markers with the exception of the Triki, whose were in Hardy Weinberg dysequilibrium for the glutathione S-transferase P1 polymorphism. Interestingly, according to all the analyzed single nucleotide polymorphisms (SNPs), the Triki population was the most differentiated and homogeneous group of the six populations analyzed. A comparison of our data with those previously published for some Caucasian, Asian and Black populations showed quite significant differences. These differences were remarkable with all the Mexican populations having a lower frequency of the 105V allele of the glutathione S-transferase P1 and reduced occurrence of the 222A allele of the 5, 10 methylenetetrahydrofolate reductase. Our results show the genetic diversity among different Mexican populations and with other racial groups. | 2688 | NA | NA | NA | no | no |
| 2689 | 2017 | NA | Torres, TT; Azeredo-Espin, AML | 2009 | Population genetics of New World screwworm from the Caribbean: insights from microsatellite data | Diseases affecting livestock can have a significant impact on animal productivity and on trade of live animals, meat and other animal products, which, consequently, affects the overall process of economic development. The New World screwworm, Cochliomyia hominivorax (Coquerel) (Diptera: Calliphoridae), is an important parasitic insect pest in Neotropical regions. This species has been successfully eradicated from North and most of Central America by the sterile insect technique, but continues to affect the development of the livestock sector in most Caribbean economies. Here, we provide some insight into the patterns of genetic variation and structure and gene flow of C. hominivorax populations from the Caribbean. Analysis of populations from 10 geographical sites in four islands revealed a moderate genetic variability within the populations. Surprisingly, a high population differentiation was found even in intra-island comparisons between populations. This observation can reflect either highly structured populations resulting from a lack of gene flow or a source-sink dynamic. Our study also suggests that New World screwworm populations can recover very rapidly from population contractions. This is valuable information that should be required prior to any investment in large-scale efforts aiming at controlling this pest. | 2689 | NA | NA | NA | no | no |
| 2690 | 2017 | NA | Zwick, ME; Joseph, SJ; Didelot, X; Chen, PE; Bishop-Lilly, KA; Stewart, AC; Willner, K; Nolan, N; Lentz, S; Thomason, MK; Sozhamannan, S; Mateczun, AJ; Du, L; Read, TD | 2012 | Genomic characterization of the Bacillus cereus sensu lato species: Backdrop to the evolution of Bacillus anthracis | The key genes required for Bacillus anthracis to cause anthrax have been acquired recently by horizontal gene transfer. To understand the genetic background for the evolution of B. anthracis virulence, we obtained high-redundancy genome sequences of 45 strains of the Bacillus cereus sensu lato (s.l.) species that were chosen for their genetic diversity within the species based on the existing multilocus sequence typing scheme. From the resulting data, we called more than 324,000 new genes representing more than 12,333 new gene families for this group. The core genome size for the B. cereus s.l. group was similar to 1750 genes, with another 2150 genes found in almost every genome constituting the extended core. There was a paucity of genes specific and conserved in any clade. We found no evidence of recent large-scale gene loss in B. anthracis or for unusual accumulation of nonsynonymous DNA substitutions in the chromosome; however, several B. cereus genomes isolated from soil and not previously associated with human disease were degraded to various degrees. Although B. anthracis has undergone an ecological shift within the species, its chromosome does not appear to be exceptional on a macroscopic scale compared with close relatives. | 2690 | NA | NA | NA | no | no |
| 2691 | 2017 | NA | Donald, HM; Wood, CW; Benowitz, KM; Johnson, RA; Brodie, ED; Formica, VA | 2012 | Nondestructive sampling of insect DNA from defensive secretion | Nondestructive techniques to obtain DNA from organisms can further genetic analyses such as estimating genetic diversity, dispersal and lifetime fitness, without permanently removing individuals from the population or removing body parts. Possible DNA sources for insects include frass, exuviae, and wing and leg clippings. However, these are not feasible approaches for organisms that cannot be removed from their natural environment for long periods or when adverse effects of tissue removal must be avoided. This study evaluated the impacts and efficacy of extracting haemolymph from a defensive secretion to obtain DNA for amplification of microsatellites using a nondestructive technique. A secretion containing haemolymph was obtained from Bolitotherus cornutus (the forked fungus beetle) by perturbation of the defensive gland with a capillary tube. A laboratory experiment demonstrated that the sampling methodology had no impact on mortality, reproductive success or gland expression. To evaluate the quality of DNA obtained in natural samples, haemolymph was collected from 187 individuals in the field and successfully genotyped at nine microsatellite loci for 95.7% of samples. These results indicate that haemolymph-rich defensive secretions contain DNA and can be sampled without negative impacts on the health or fitness of individual insects. | 2691 | NA | NA | NA | no | no |
| 2692 | 2017 | NA | Kasalicky, V; Jezbera, J; Hahn, MW; Simek, K | 2013 | The Diversity of the Limnohabitans Genus, an Important Group of Freshwater Bacterioplankton, by Characterization of 35 Isolated Strains | Bacteria of the genus Limnohabitans, more precisely the R-BT lineage, have a prominent role in freshwater bacterioplankton communities due to their high rates of substrate uptake and growth, growth on algal-derived substrates and high mortality rates from bacterivory. Moreover, due to their generally larger mean cell volume, compared to typical bacterioplankton cells, they contribute over-proportionally to total bacterioplankton biomass. Here we present genetic, morphological and ecophysiological properties of 35 bacterial strains affiliated with the Limnohabitans genus newly isolated from 11 non-acidic European freshwater habitats. The low genetic diversity indicated by the previous studies using the ribosomal SSU gene highly contrasted with the surprisingly rich morphologies and different patterns in substrate utilization of isolated strains. Therefore, the intergenic spacer between 16S and 23S rRNA genes was successfully tested as a fine-scale marker to delineate individual lineages and even genotypes. For further studies, we propose the division of the Limnohabitans genus into five lineages (provisionally named as LimA, LimB, LimC, LimD and LimE) and also additional sublineages within the most diversified lineage LimC. Such a delineation is supported by the morphology of isolated strains which predetermine large differences in their ecology. | 2692 | NA | NA | NA | no | no |
| 2693 | 2017 | NA | Gravel, S; Henn, BM; Gutenkunst, RN; Indap, AR; Marth, GT; Clark, AG; Yu, FL; Gibbs, RA; Bustamante, CD | 2011 | Demographic history and rare allele sharing among human populations | High-throughput sequencing technology enables population-level surveys of human genomic variation. Here, we examine the joint allele frequency distributions across continental human populations and present an approach for combining complementary aspects of whole-genome, low-coverage data and targeted high-coverage data. We apply this approach to data generated by the pilot phase of the Thousand Genomes Project, including whole-genome 2-4x coverage data for 179 samples from HapMap European, Asian, and African panels as well as high-coverage target sequencing of the exons of 800 genes from 697 individuals in seven populations. We use the site frequency spectra obtained from these data to infer demographic parameters for an Out-of-Africa model for populations of African, European, and Asian descent and to predict, by a jackknife-based approach, the amount of genetic diversity that will be discovered as sample sizes are increased. We predict that the number of discovered nonsynonymous coding variants will reach 100,000 in each population after similar to 1,000 sequenced chromosomes per population, whereas similar to 2,500 chromosomes will be needed for the same number of synonymous variants. Beyond this point, the number of segregating sites in the European and Asian panel populations is expected to overcome that of the African panel because of faster recent population growth. Overall, we find that the majority of human genomic variable sites are rare and exhibit little sharing among diverged populations. Our results emphasize that replication of disease association for specific rare genetic variants across diverged populations must overcome both reduced statistical power because of rarity and higher population divergence. | 2693 | NA | NA | NA | no | no |
| 2694 | 2017 | NA | Hogg, CJ; Ivy, JA; Srb, C; Hockley, J; Lees, C; Hibbard, C; Jones, M | 2015 | Influence of genetic provenance and birth origin on productivity of the Tasmanian devil insurance population | An insurance population for the critically endangered Tasmanian devil was established in 2006. Due to successful captive breeding, the population has reached its carrying capacity of 600 devils and retains 99.95 % of founding gene diversity. Although reproduction has been quite successful, possible relatedness among founding individuals, influences of genetic provenance and pairing success on female productivity were evaluated to further refine insurance population management. Ten polymorphic microsatellite markers were used to assess the founders. Although the data were ultimately insufficient for determining specific founder relationships, a STRUCTURE analysis determined founders to be of eastern or western provenance. Western provenance animals had an observed heterozygosity of 0.38; while eastern provenance was 0.41. Allelic frequencies between the two provenances were similar. Although differences in pairing success of eastern and western provenance animals were noted, there was no difference in overall productivity (number of joeys/female). Cross-provenance pairings were not as successful as W-W but had similar productivity, and produced viable offspring. Birth origin (wild-born vs. zoo-born) had no influence on pairing success but wild-born females produce significantly more joeys/female. For zoo-born females, the number of joeys produced per female had a downward trend between respective generations in captivity. Current and future population managers should be aware of potential reductions in productivity across captive generations and adjust breeding recommendations accordingly. The ability to recruit founders from diseased females, along with a better understanding of the influence of genetic provenance and birth origin on productivity, has led to changes in acquisition of future founders for this insurance population. | 2694 | NA | NA | NA | no | no |
| 2695 | 2017 | NA | Rueppell, O; Johnson, N; Rychtar, J | 2008 | Variance-based selection may explain general mating patterns in social insects | Female mating frequency is one of the key parameters of social insect evolution. Several hypotheses have been suggested to explain multiple mating and considerable empirical research has led to conflicting results. Building on several earlier analyses, we present a simple general model that links the number of queen matings to variance in colony performance and this variance to average colony fitness. The model predicts selection for multiple mating if the average colony succeeds in a focal task, and selection for single mating if the average colony fails, irrespective of the proximate mechanism that links genetic diversity to colony fitness. Empirical support comes from interspecific comparisons, e. g. between the bee genera Apis and Bombus, and from data on several ant species, but more comprehensive empirical tests are needed. | 2695 | NA | NA | NA | no | no |
| 2696 | 2017 | NA | Longdon, B; Wilfert, L; Obbard, DJ; Jiggins, FM | 2011 | Rhabdoviruses in Two Species of Drosophila: Vertical Transmission and a Recent Sweep | Insects are host to a diverse range of vertically transmitted micro-organisms, but while their bacterial symbionts are well-studied, little is known about their vertically transmitted viruses. We have found that two sigma viruses (Rhabdoviridae) recently discovered in Drosophila affinis and Drosophila obscura are both vertically transmitted. As is the case for the sigma virus of Drosophila melanogaster, we find that both males and females can transmit these viruses to their offspring. Males transmit lower viral titers through sperm than females transmit through eggs, and a lower proportion of their offspring become infected. In natural populations of D. obscura in the United Kingdom, we found that 39% of flies were infected and that the viral population shows clear evidence of a recent expansion, with extremely low genetic diversity and a large excess of rare polymorphisms. Using sequence data we estimate that the virus has swept across the United Kingdom within the past similar to 11 years, during which time the viral population size doubled approximately every 9 months. Using simulations based on our lab estimates of transmission rates, we show that the biparental mode of transmission allows the virus to invade and rapidly spread through populations at rates consistent with those measured in the field. Therefore, as predicted by our simulations, the virus has undergone an extremely rapid and recent increase in population size. In light of this and earlier studies of a related virus in D. melanogaster, we conclude that vertically transmitted rhabdoviruses may be common in insects and that these host-parasite interactions can be highly dynamic. | 2696 | NA | NA | NA | no | no |
| 2697 | 2017 | NA | Maillard, JC; Gonzalez, JP | 2006 | Biodiversity and emerging diseases | First we remind general considerations concerning biodiversity on earth and particularly the loss of genetic biodiversity that seems irreversible whether its origin is directly or indirectly linked to human activities. Urgent and considerable efforts must be made from now on to cataloge, understand, preserve, and enhance the value of biodiversity while ensuring food safety and human and animal health. Ambitious integrated and multifield research programs must be implemented in order to understand the causes and anticipate the consequences of loss of biodiversity. Such losses are a serious threat to sustainable development and to the quality of life of future generations. They have an influence on the natural balance of global biodiversity in particularly in reducing the capability of species to adapt rapidly by genetic mutations to survive in modified ecosystems. Usually, the natural immune systems of mammals (both human and animal), are highly polymorphic and able to adapt rapidly to new situations. We more specifically discuss the fact that if the genetic diversity of the affected populations is low the invading microorganisms, will suddenly expand and create epidemic outbreaks with risks of pandemic. So biodiversity appears to function as an important barrier (buffer), especially against disease-causing organisms, which can function in different ways. Finally, we discuss the importance of preserving biodiversity mainly in the wildlife ecosystems as an integrated and sustainable approach among others in order to prevent and control the emergence or reemergence of diseases in animals and humans (zoonosis). Although plants are also part of this paradigm, they fall outside our field of study. | 2697 | NA | NA | NA | no | no |
| 2698 | 2017 | NA | Zheng, G; Freidlin, B; Gastwirth, JL | 2006 | Robust genomic control for association studies | Population-based case-control studies are a useful method to test for a genetic association between a trait and a marker. However, the analysis of the resulting data can be affected by population stratification or cryptic relatedness, which may inflate the variance of the usual statistics, resulting in a higher-than-nominal rate of false-positive results. One approach to preserving the nominal type I error is to apply genomic control, which adjusts the variance of the Cochran-Armitage trend test by calculating the statistic on data from null loci. This enables one to estimate any additional variance in the null distribution of statistics. When the underlying genetic model ( e. g., recessive, additive, or dominant) is known, genomic control can be applied to the corresponding optimal trend tests. In practice, however, the mode of inheritance is unknown. The genotype-based x 2 test for a general association between the trait and the marker does not depend on the underlying genetic model. Since this general association test has 2 degrees of freedom ( df), the existing formulas for estimating the variance factor by use of genomic control are not directly applicable. By expressing the general association test in terms of two Cochran-Armitage trend tests, one can apply genomic control to each of the two trend tests separately, thereby adjusting the x 2 statistic. The properties of this robust genomic control test with 2 df are examined by simulation. This genomic control-adjusted 2-df test has control of type I error and achieves reasonable power, relative to the optimal tests for each model. | 2698 | NA | NA | NA | no | no |
| 2699 | 2017 | NA | Rijks, JM; Hoffman, JI; Kuiken, T; Osterhaus, ADME; Amos, W | 2008 | Heterozygosity and lungworm burden in harbour seals (Phoca vitulina) | In several studies, heterozygosity measured at around 10 microsatellite markers correlates with parasite load. Usually the effect size is small, but while this may reflect reality, it may also be possible that too few markers are used or the measure of fitness contains too much error to reveal what is actually a much stronger underlying effect. Here, we analysed over 200 stranded harbour seals (Phoca vitulina) for an association between lungworm burden and heterozygosity, conducting thorough necropsies on the seals and genotyping the samples obtained for 27 microsatellites. We found that homozygosity predicts higher worm burdens, but only in young animals, where the worms have the greatest impact on fitness. Testing each locus separately, we found that a significant majority reveal a weak but similar trend for heterozygosity to be protective against high lungworm burden, suggesting a genome-wide effect, that is, inbreeding. This conclusion is supported by the fact that heterozygosity is correlated among markers in young animals but not in otherwise equivalent older ones. Taken as a whole, our results support the notion that homozygosity increases susceptibility to parasitic infection and suggest that parasites can be effective in removing inbred individuals from the population. | 2699 | NA | NA | NA | no | no |
| 2700 | 2017 | NA | Kazma, R; Bailey, JN | 2011 | Population-based and family-based designs to analyze rare variants in complex diseases | Genotyping of rare variants on a large scale is now possible using next-generation sequencing. Sample selection is a crucial step in designing the genetic study of a complex disease, and knowledge of the efficiency and limitations of population-based and family-based designs can help researchers make the appropriate choice. The nine contributions to Group 5 of Genetic Analysis Workshop 17 evaluate population-based and family-based designs by comparing the results obtained with various methods applied to the mini-exome simulations. These simulations consisted of 200 replicates composed of unrelated individuals and eight extended pedigrees with genotypes and various phenotypes. The methods tested for association with a population-based and/or a family-based design, tested for linkage with a family-based design, or estimated heritability. We summarize the strengths and weaknesses of both designs. Although population-based designs seem more suitable for detecting the effect of multiple rare variants, family-based designs can potentially enrich the sample in rare variants, for which the effect would be concealed at the population level. However, as of today, the main limitation is still the high cost of next-generation sequencing. Genet. Epidemiol. 35:S41S47, 2011. (C) 2011 Wiley Periodicals, Inc. | 2700 | NA | NA | NA | no | no |
| 2701 | 2017 | NA | Maiti, B; Shekar, M; Khushiramani, R; Karunasagar, I; Karunasagar, I | 2009 | Evaluation of RAPD-PCR and protein profile analysis to differentiate Vibrio harveyi strains prevalent along the southwest coast of India | Sixty five isolates of Vibrio harveyi were subjected to random amplified polymorphic DNA (RAPD)-PCR analysis and protein profiling to investigate the genetic variability among V. harveyi prevalent along the coast and also assess the discriminating ability of these two molecular methods. A total of 10 RAPD primers were assayed for their specificity in detecting V. harveyi, of which only two primers: PM3 and CRA25 were highly reproducible and found suitable for use in RAPD-PCR. The genetic diversity among V. harveyi isolates assessed by RAPD-PCR using PM3 primer yielded 35 different RAPD patterns which clustered the isolates into 15 groups at 72% similarity level. Similarly, RAPD-PCR with CRA25 clustered the 38 patterns into 10 groups at 74% similarity. The discriminatory index (D) value calculated for RAPD fingerprints generated with PM3 and CRA25 were 0.90 and 0.85, respectively. On the other hand, molecular typing of V. harveyi using whole cell proteins generated profiles that showed no major difference indicating the technique to be not useful in typing strains of this bacterium. However, a few of the isolates showed the presence of unique band of 28 kDa that needs to be further investigated to understand the role of the protein in disease process if any. | 2701 | NA | NA | NA | no | no |
| 2702 | 2017 | NA | Post, W; Shen, HQ; Damcott, C; Arking, DE; Kao, WHL; Sack, PA; Ryan, KA; Chakravarti, A; Mitchell, BD; Shuldiner, AR | 2007 | Associations between genetic variants in the NOS1AP (CAPON) gene and cardiac repolarization in the old order Amish | Background: Through a genome-wide association study, we discovered an association of the electrocardiographic QT interval with polymorphisms in the NOS1AP ( CAPON) gene. The purpose of the current study was to replicate this association in the Old Order Amish. Methods: Four NOS1AP SNPs were selected that captured all major haplotypes in the region of interest (similar to 120 kb segment). Genotyping was completed in 763 subjects from the Heredity and Phenotype Intervention (HAPI) Heart Study. Association analyses were performed using a variance components methodology, accounting for relatedness of individuals. Results: Heritability of the QT interval was 0.50 +/- 0.09 (p = 1.9 x 10(-9)). All four SNPs were common with a high degree of correlation between SNPs. Two of the four SNPs (pairwise r(2) = 0.86) were significantly associated with variation in adjusted QT interval (rs1415262, p = 0.02 and rs10494366, p = 0.006, additive models for both). SNP rs10494366 explained 0.9% of QT interval variability, with an average genetic effect of 6.1 ms. Haplotypes that contained the minor allele for rs10494366 were associated with longer QT interval. Conclusions: This study provides further evidence that NOS1AP variants influence QT interval and further validates the utility of genome-wide association studies, a relatively new approach to gene discovery. Copyright (c) 2007 S. Karger AG, Basel | 2702 | NA | NA | NA | no | no |
| 2703 | 2017 | NA | Tavares, DA; Simoes, AS; Bootsma, HJ; Hermans, PWM; de Lencastre, H; Sa-Leao, R | 2014 | Non-typeable pneumococci circulating in Portugal are of cps type NCC2 and have genomic features typical of encapsulated isolates | Background: Pneumococcus is a major human pathogen and the polysaccharide capsule is considered its main virulence factor. Nevertheless, strains lacking a capsule, named non-typeable pneumococcus (NT), are maintained in nature and frequently colonise the human nasopharynx. Interest in these strains, not targeted by any of the currently available pneumococcal vaccines, has been rising as they seem to play an important role in the evolution of the species. Currently, there is a paucity of data regarding this group of pneumococci. Also, questions have been raised on whether they are true pneumococci. We aimed to obtain insights in the genetic content of NT and the mechanisms leading to non-typeability and to genetic diversity. Results: A collection of 52 NT isolates representative of the lineages circulating in Portugal between 1997 and 2007, as determined by pulsed-field gel electrophoresis and multilocus sequence typing, was analysed. The capsular region was sequenced and comparative genomic hybridisation (CGH) using a microarray covering the genome of 10 pneumococcal strains was carried out. The presence of mobile elements was investigated as source of intraclonal variation. NT circulating in Portugal were found to have similar capsular regions, of cps type NCC2, i.e., having aliB-like ORF1 and aliB-like ORF2 genes. The core genome of NT was essentially similar to that of encapsulated strains. Also, competence genes and most virulence genes were present. The few virulence genes absent in all NT were the capsular genes, type-I and type-II pili, choline-binding protein A (cbpA/pspC), and pneumococcal surface protein A (pspA). Intraclonal variation could not be entirely explained by the presence of prophages and other mobile elements. Conclusions: NT circulating in Portugal are a homogeneous group belonging to cps type NCC2. Our observations support the theory that they are bona-fide pneumococcal isolates that do not express the capsule but are otherwise essentially similar to encapsulated pneumococci. Thus we propose that NT should be routinely identified and reported in surveillance studies. | 2703 | NA | NA | NA | no | no |
| 2704 | 2017 | NA | Yadav, MK; Aravindan, S; Ngangkham, U; Bag, HNSMK; Adak, T; Munda, S; Samantaray, S; Jena, M | 2017 | Use of molecular markers in identification and characterization of resistance to rice blast in India | Rice blast disease caused by Magnaporthe oryzae is one of the most destructive disease causing huge losses to rice yield in different parts of the world. Therefore, an attempt has been made to find out the resistance by screening and studying the genetic diversity of eighty released rice varieties by National Rice Research Institute, Cuttack (NRVs) using molecular markers linked to twelve major blast resistance (R) genes viz Pib, Piz, Piz-t, Pik, Pik-p,Pikm Pik-h, Pita/Pita-2, Pi2, Pi9, Pi1 and Pi5. Out of which, nineteen varieties (23.75%) showed resistance, twenty one were moderately resistant (26.25%) while remaining forty varieties (50%) showed susceptible in uniform blast nursery. Rice varieties possessing blast resistance genes varied from four to twelve and the frequencies of the resistance genes ranged from 0 to 100%. The cluster analysis grouped the eighty NRVs into two major clusters at 63% level of genetic similarity coefficient. The PIC value for seventeen markers varied from 0 to 0.37 at an average of 0.20. Out of seventeen markers, only five markers, 195R-1, Pi9-i, Pita3, YL155/YL87 and 40N23r corresponded to three broad spectrum R genes viz. Pi9, Pita/Pita2 and Pi5 were found to be significantly associated with the blast disease with explaining phenotypic variance from 3.5% to 7.7%. The population structure analysis and PCoA divided the entire 80 NRVs into two sub-groups. The outcome of this study would help to formulate strategies for improving rice blast resistance through genetic studies, plant-pathogen interaction, identification of novel R genes, development of new resistant varieties through marker-assisted breeding for improving rice blast resistance in India and worldwide. | 2704 | NA | NA | NA | no | no |
| 2705 | 2017 | NA | Denny, AJ; Franks, NR; Powell, S; Edwards, KJ | 2004 | Exceptionally high levels of multiple mating in an army ant | Most species of social insects have singly mated queens, although there are notable exceptions. Competing hypotheses have been proposed to explain the evolution of high levels of multiple mating, but this issue is far from resolved. Here we use microsatellites to investigate mating frequency in the army ant Eciton burchellii and show that queens mate with an exceptionally large number of males, eclipsing all but one other social insect species for which data are available. In addition we present evidence that suggests that mating is serial, continuing throughout the lifetime of the queen. This is the first demonstration of serial mating among social hymenoptera. We propose that high paternity within colonies is most likely to have evolved to increase genetic diversity and to counter high pathogen and parasite loads. | 2705 | NA | NA | NA | no | no |
| 2706 | 2017 | NA | Arias, RS; Stetina, SR; Tonos, JL; Scheffler, JA; Scheffler, BE | 2009 | Microsatellites reveal genetic diversity in Rotylenchulus reniformis populations | Rotylenchulus reniformis is the predominant parasitic nematode of cotton in the Mid South area of the United States. Although variable levels of infection and morphological differences have been reported for this nematode, genetic variability has been more elusive. We developed microsatellite-enriched libraries for R. reniformis, produced 1152 clones, assembled 694 contigs, detected 783 simple sequence repeats (SSR) and designed 192 SSR-markers. The markers were tested on six R. reniformis cultures from four states, Texas, Louisiana, Mississippi and Georgia, in the USA. Based on performance we selected 156 SSR markers for R. reniformis from which 88 were polymorphic across the six reniform nematode populations, showing as the most frequent motif the dinucleotide AG. The polymorphic information content of the markers ranged from 0.00 to 0.82, and the percentage of multiallelic loci of the isolates was between 40.9 and 45.1%. An interesting finding in this study was the genetic variability detected among the three Mississippi isolates, for which 22 SSR markers were polymorphic. We also tested the level of infection of these isolates on six cotton genotypes, where significant differences were found between the Texas and Georgia isolates. Coincidentally, 62 polymorphic markers were able to distinguish these two populations. Further studies will be necessary to establish possible connections, if any, between markers and level of pathogenicity of the nematode. The SSR markers developed here will be useful in the assessment of the genetic diversity of this nematode, could assist in management practices for control of reniform nematode, be used in breeding programs for crop resistance, and help in detecting the origin and spread of this nematode in the United States. | 2706 | NA | NA | NA | no | no |
| 2707 | 2017 | NA | Zierdt, H; Hummel, S; Herrmann, B | 1996 | Amplification of human short tandem repeats from medieval teeth and bone samples | The suitability of typing hypervariable DNA loci for the genetic analysis of prehistoric populations is demonstrated for the first time. Alleles of the human short tandem repeat locus VWA31/A have been amplified from ancient teeth and bone samples derived from an early medieval burial site at Weingarten, Germany, using the polymerase chain reaction. The DNA results for 76 individuals reveal remarkable similarity of the allelic frequencies between the past and modern populations. A surplus of apparent homozygotes in the ancient population is most likely due to a stochastic problem of amplification of degraded DNA. Therefore technical obstacles for the application to ancient DNA were evaluated. The substantial perspectives of using microsatellite typing for the analysis of heritable diseases, determination of relatedness, and establishment of genealogies in prehistoric populations are outlined. | 2707 | NA | NA | NA | no | no |
| 2708 | 2017 | NA | Taub, MA; Schwender, H; Beaty, TH; Louis, TA; Ruczinski, I | 2012 | Incorporating Genotype Uncertainties Into the Genotypic TDT for Main Effects and Gene-Environment Interactions | Genotype imputation has become a standard option for researchers to expand their genotype datasets to improve signal precision and power in tests of genetic association with disease. In imputations for family-based studies however, subjects are often treated as unrelated individuals: currently, only BEAGLE allows for simultaneous imputation for trios of parents and offspring; however, only the most likely genotype calls are returned, not estimated genotype probabilities. For population-based SNP association studies, it has been shown that incorporating genotype uncertainty can be more powerful than using hard genotype calls. We here investigate this issue in the context of case-parent family data. We present the statistical framework for the genotypic transmission-disequilibrium test (gTDT) using observed genotype calls and imputed genotype probabilities, derive an extension to assess gene-environment interactions for binary environmental variables, and illustrate the performance of our method on a set of trios from the International Cleft Consortium. In contrast to population-based studies, however, utilizing the genotype probabilities in this framework (derived by treating the family members as unrelated) can result in biases of the test statistics toward protectiveness for the minor allele, particularly for markers with lower minor allele frequencies and lower imputation quality. We further compare the results between ignoring relatedness in the imputation and taking family structure into account, based on hard genotype calls. We find that by far the least biased results are obtained when family structure is taken into account and currently recommend this approach in spite of its intense computational requirements. Genet. Epidemiol. 36:225-234, 2012. (C) 2012 Wiley Periodicals, Inc. | 2708 | NA | NA | NA | no | no |
| 2709 | 2017 | NA | E, GX; Sha, RN; Zeng, SC; Wang, C; Pan, JF; Han, JL | 2014 | Genetic variability, evidence of potential recombinational event and selection of LEI0258 in chicken | The chicken major histocompatibility complex (MHC) plays an important role in the immune response, disease resistance, productivity, and other important economic traits of the chicken. Therefore, a description of the polymorphisms of this region is crucial for understanding the genetic pattern of the MHC. The tandem repeat LEI0258 is located within the B region of the chicken MHC and is surprisingly strongly associated with serology. This marker has been used worldwide to provide a picture of the core area of the chicken MHC-B region and to categorize chicken MHC haplotypes. Thus, insight into the evolutionary pattern of LEI0258 may be useful for understanding MHC diversity. In the current study, 30 alleles of LEI0258 from 12 populations were screened and sequenced, and alleles that have previously been published in GenBank were also analyzed. The resulting 124 alleles were classified into four clusters according to the SNPs and indels found within the sequences flanking the repeats. Furthermore, a recombination region was identified between -30 and + 43 that suggests that recombination may have played a role in the evolution of this MHC. Finally, strong evidence regarding the selection and evolutionary dynamics of the LEI0258 region is presented. Generally speaking, microsatellite is a classic anonymous marker which changes by genetic drift rather than by direct selection. Although, the genotypes of LEI0258 in MHC-B correlate with serology, its mechanism of inheritance and evolution was unclear. This study not only establishes a framework of further diversity or association studies in LEI0258, but also unraveling the reason what driving force and formulate the evolutionary dynamics of this region. (C) 2014 Elsevier B.V. All rights reserved. | 2709 | NA | NA | NA | no | no |
| 2710 | 2017 | NA | Parmakelis, A; Moustaka, M; Poulakakis, N; Louis, C; Slotman, MA; Marshall, JC; Awono-Ambene, PH; Antonio-Nkondjio, C; Simard, F; Caccone, A; Powell, JR | 2010 | Anopheles Immune Genes and Amino Acid Sites Evolving Under the Effect of Positive Selection | “Background: It has long been the goal of vector biology to generate genetic knowledge that can be used to”“manipulate’’ natural populations of vectors to eliminate or lessen disease burden. While long in coming, progress towards reaching this goal has been made. Aiming to increase our understanding regarding the interactions between Plasmodium and the Anopheles immune genes, we investigated the patterns of genetic diversity of four anti-Plasmodium genes in the Anopheles gambiae complex of species. Methodology/Principal Findings: Within a comparative phylogenetic and population genetics framework, the evolutionary history of four innate immunity genes within the An. gambiae complex (including the two most important human malaria vectors, An. gambiae and An. arabiensis) is reconstructed. The effect of natural selection in shaping the genes’ diversity is examined. Introgression and retention of ancestral polymorphisms are relatively rare at all loci. Despite the potential confounding effects of these processes, we could identify sites that exhibited dN/dS ratios greater than 1. Conclusions/Significance: In two of the studied genes, CLIPB14 and FBN8, several sites indicated evolution under positive selection, with CLIPB14 exhibiting the most consistent evidence. Considering only the sites that were consistently identified by all methods, two sites in CLIPB14 are adaptively driven. However, the analysis inferring the lineage-specific evolution of each gene was not in favor of any of the Anopheles lineages evolving under the constraints imposed by positive selection. Nevertheless, the loci and the specific amino acids that were identified as evolving under strong evolutionary pressure merit further investigation for their involvement in the Anopheles defense against microbes in general.” | 2710 | NA | NA | NA | no | no |
| 2711 | 2017 | NA | Setchell, JM; Richards, SA; Abbott, KM; Knappe, LA | 2016 | Mate-guarding by male mandrills (Mandrillus sphinx) is associated with female MHC genotype | Female choice for male major histocompatibility complex (MHC) genotype has been widely tested, but the relationship between male mating strategies and female MHC genotype has received far less attention. Moreover, few studies of MHC-associated mate choice test for the fitness effects underlying such choice. We examined mate-guarding by male mandrills, a species with intense male-male competition and female offspring care. We developed a statistical model based on 10 years of observations to describe how the probability a female is mate-guarded varies across her sexual cycle, among cycles and among females. We accounted for female rank, parity, and maternal relatedness. We then tested whether the occurrence of mate-guarding is influenced by 1) MHC dissimilarity, 2) female MHC diversity, and 3) specific female MHC genotypes. Finally, we tested for associations between MHC variables and the ratio of neutrophils to lymphocytes in blood samples taken during routine captures. The best-fit models included either MHC dissimilarity (males were more likely to mate-guard more dissimilar females, and there was some evidence of preference for intermediate MHC dissimilarity) or a specific MHC supertype. Four of 11 supertypes investigated were influential and one had a strong negative influence on mate-guarding. We found some evidence that the MHC genotype that attracted the least mate-guarding was disadvantageous in terms of immune function. However, we did not find evidence that MHC diversity was related to immune function. These results suggest that highly competitive males modify their mating behavior based on female MHC genotype, and a possible fitness benefit to mate choice for specific genotypes. | 2711 | NA | NA | NA | no | no |
| 2712 | 2017 | NA | Short, DPG; O’Donnell, K; Geiser, DM | 2014 | Clonality, recombination, and hybridization in the plumbing-inhabiting human pathogen Fusarium keratoplasticum inferred from multilocus sequence typing | Background: Recent work has shown that Fusarium species and genotypes most commonly associated with human infections, particularly of the cornea (mycotic keratitis), are the same as those most commonly isolated from plumbing systems. The species most dominant in plumbing biofilms is Fusarium keratoplasticum, a cosmopolitan fungus known almost exclusively from animal infections and biofilms. To better understand its diversity and population dynamics, we developed and utilized a nine-locus sequence-based typing system to make inferences about clonality, recombination, population structure, species boundaries and hybridization. Results: High levels of genetic diversity and evidence for recombination and clonality were detected among 75 clinical and 156 environmental isolates of Fusarium keratoplasticum. The multilocus sequence typing system (MLST) resolved 111 unique nine-locus sequence types (STs). The single locus bifactorial determinants of mating compatibility (mating types MAT1-1 and MAT1-2), were found in a ratio of 70:30. All but one of the 49 isolates of the most common ST (FSSC 2d-2) came from human infections, mostly of the cornea, and from biofilms associated with contact lenses and plumbing surfaces. Significant levels of phylogenetic incongruence were found among loci. Putative clonal relationships among genotypes were estimated, showing a mixture of large clonal complexes and unrelated singletons. Discordance between the nuclear ribosomal rRNA and other gene genealogies is consistent with introgression of ribosomal RNA alleles of phylogenetic species FSSC 9 into F. keratoplasticum. No significant population subdivision based on clinical versus non-clinical sources was found. Conclusions: Incongruent phylogenetic trees and the presence of both mating types within otherwise identical STs were observed, providing evidence for sexuality in F. keratoplasticum. Cryptic speciation suggested in a published three-locus MLST system was not supported with the addition of new loci, but evidence of introgression of ribosomal RNA genes from another strongly supported phylogenetic species (FSSC 9), also known from plumbing systems and human infections, was detected in two isolates. Overall, F. keratoplasticum is a diverse and geographically unstructured species with a mixed clonal and recombinant life history. | 2712 | NA | NA | NA | no | no |
| 2713 | 2017 | NA | Chow, WZ; Chan, YF; Oong, XY; Ng, LJ; Nor’E, SS; Ng, KT; Chan, KG; Hanafi, NS; Pang, YK; Kamarulzaman, A; Tee, KK | 2016 | Genetic diversity, seasonality and transmission network of human metapneumovirus: identification of a unique sub-lineage of the fusion and attachment genes | Human metapneumovirus (HMPV) is an important viral respiratory pathogen worldwide. Current knowledge regarding the genetic diversity, seasonality and transmission dynamics of HMPV among adults and children living in tropical climate remains limited. HMPV prevailed at 2.2% (n = 86/3,935) among individuals presented with acute respiratory tract infections in Kuala Lumpur, Malaysia between 2012 and 2014. Seasonal peaks were observed during the northeast monsoon season (November-April) and correlated with higher relative humidity and number of rainy days (P < 0.05). Phylogenetic analysis of the fusion and attachment genes identified the co-circulation of three known HMPV sub-lineages, A2b and B1 (30.2% each, 26/86) and B2 (20.9%, 18/86), with genotype shift from sub-lineage B1 to A2b observed in 2013. Interestingly, a previously unrecognized sub-lineage of A2 was identified in 18.6% (16/86) of the population. Using a custom script for network construction based on the TN93 pairwise genetic distance, we identified up to nine HMPV transmission clusters circulating as multiple sub-epidemics. Although no apparent major outbreak was observed, the increased frequency of transmission clusters (dyads) during seasonal peaks suggests the potential roles of transmission clusters in driving the spread of HMPV. Our findings provide essential information for therapeutic research, prevention strategies, and disease outbreak monitoring of HMPV. | 2713 | NA | NA | NA | no | no |
| 2714 | 2017 | NA | Hellin, P; Scauflaire, J; Van Hese, V; Munaut, F; Legreve, A | 2017 | Sensitivity of Fusarium culmorum to triazoles: impact of trichothecene chemotypes, oxidative stress response and genetic diversity | BACKGROUNDFusarium culmorum is a fungal pathogen occurring worldwide on various weeds and important crops. Triazoles have been shown to be the most effective fungicide for managing Fusarium spp., but little is known about their specific activity on F. culmorum. RESULTSThe sensitivity of 107 F. culmorum strains to triazoles was assessed using microtitre plate assays. The EC50 values ranged from 0.14 to 1.53 mg L-1 for tebuconazole and from 0.25 to 2.47 mg L-1 for epoxiconazole. Cross-resistance to both azoles was found (r = 0.61). F. culmorum appeared to be significantly more sensitive than F. graminearum or F. cerealis. No increase in the mean EC50 was observed over time, which might be related to an unfavourable fitness cost, measured here as fungal growth. On average, nivalenol-producing strains of F. culmorum were significantly more resistant than deoxynivalenol-producing strains. The relationship between resistance and chemotype-dependent adaptation to oxidative stress was investigated, but remained unclear. No link between inter-simple sequence repeat (ISSR) genetic diversity and triazole resistance could be established. CONCLUSIONFungicide use might not be a driving force in the evolution of F. culmorum, and the benefit of a resistance trait probably does not outweigh its costs. (c) 2016 Society of Chemical Industry | 2714 | NA | NA | NA | no | no |
| 2715 | 2017 | NA | Phunngam, P; Chareonviriyaphap, T; Bangs, MJ; Arunyawat, U | 2017 | PHYLOGENETIC RELATIONSHIPS AMONG MALARIA VECTORS AND CLOSELY RELATED SPECIES IN THAILAND USING MULTILOCUS DNA SEQUENCES | The evolutionary and taxonomic status is important for understanding speciation events and phylogenetic relationships between closely related vector and nonvector species. This information is useful for targeting important disease vector species groups for the development of novel genetic-based vector and pathogen control methods. In this study, different phylogenetic analyses were performed to reconstruct phylogenetic trees for the primary malaria vectors in Thailand based on sequence information of 4 DNA fragments from the nuclear and mitochondrial regions. The primary Anopheles species in the subgenus Cellia involved in malaria transmission in Thailand separate clearly into 3 distinct clades: the Leucosphyrus group, Minimus subgroup, and Maculatus group. The phylogenetic trees based on different reconstructed algorithms and different gene regions provided congruent phylogenetic status of the mosquito species studied. The phylogenetic relationships of malaria vector species examined followed similar patterns based on morphological characters. An estimate of the divergence time among the Anopheles species infers that they were present during the Eocene and Miocene periods (>41 million years ago). Congruent phylogenetic analysis of malaria vectors is presented with different algorithms and gene regions. The nuclear TOLL6 fragment appears useful for molecular phylogenetic, species DNA barcode, and Anopheles population genetic analyses. | 2715 | NA | NA | NA | no | no |
| 2716 | 2017 | NA | Sheng, HY; See, DR; Murray, TD | 2014 | Mapping resistance genes for Oculimacula acuformis in Aegilops longissima | This study identified three QTL conferring resistance to Oculimacula acuformis in Aegilops longissima and their associated markers, which can be useful in marker-assisted selection breeding for eyespot resistance. Oculimacula acuformis is one of two species of soilborne fungi that cause eyespot of wheat, the other being Oculimacula yallundae. Both pathogens can coexist in the same field and produce elliptical lesions on stem bases of wheat that are indistinguishable. Pch1 and Pch2 are the only two eyespot resistance genes readily available to wheat breeders, but neither provides complete control. A new source of eyespot resistance was identified from Aegilops longissima (2n = 14, (SSl)-S-l), a wild relative of wheat. Three QTL for resistance to O. acuformis were mapped in chromosomes 1S(l), 3S(l), and 5S(l) using a recombinant inbred line population developed from the cross Ae. longissima accessions PI 542196 (R) x PI 330486 (S). The three QTL explained 66 % of phenotypic variation by beta-glucuronidase score (GUS) and 84 % by visual rating. These QTL had LOD values of 10.6, 8.8, and 6.0 for GUS score, and 16.0, 10.0, and 13.0 for visual rating. QTL associated with resistance to O. acuformis have similar chromosomal locations as some for resistance to O. yallundae, except that a QTL for resistance to O. yallundae was found in chromosome 7S(l) but not for O. acuformis. Thus, it appears that some genes at the same locus in Ae. longissima may control resistance to both eyespot pathogens. QTL effective against both pathogens will be most useful for breeding programs and have potential to improve the effectiveness and genetic diversity of eyespot resistance. | 2716 | NA | NA | NA | no | no |
| 2717 | 2017 | NA | Roder, C; Arif, C; Daniels, C; Weil, E; Voolstra, CR | 2014 | Bacterial profiling of White Plague Disease across corals and oceans indicates a conserved and distinct disease microbiome | Coral diseases are characterized by microbial community shifts in coral mucus and tissue, but causes and consequences of these changes are vaguely understood due to the complexity and dynamics of coral-associated bacteria. We used 16S rRNA gene microarrays to assay differences in bacterial assemblages of healthy and diseased colonies displaying White Plague Disease (WPD) signs from two closely related Caribbean coral species, Orbicella faveolata and Orbicella franksi. Analysis of differentially abundant operational taxonomic units (OTUs) revealed strong differences between healthy and diseased specimens, but not between coral species. A subsequent comparison to data from two Indo-Pacific coral species (Pavona duerdeni and Porites lutea) revealed distinct microbial community patterns associated with ocean basin, coral species and health state. Coral species were clearly separated by site, but also, the relatedness of the underlying bacterial community structures resembled the phylogenetic relationship of the coral hosts. In diseased samples, bacterial richness increased and putatively opportunistic bacteria were consistently more abundant highlighting the role of opportunistic conditions in structuring microbial community patterns during disease. Our comparative analysis shows that it is possible to derive conserved bacterial footprints of diseased coral holobionts that might help in identifying key bacterial species related to the underlying etiopathology. Furthermore, our data demonstrate that similar-appearing disease phenotypes produce microbial community patterns that are consistent over coral species and oceans, irrespective of the putative underlying pathogen. Consequently, profiling coral diseases by microbial community structure over multiple coral species might allow the development of a comparative disease framework that can inform on cause and relatedness of coral diseases. | 2717 | NA | NA | NA | no | no |
| 2718 | 2017 | NA | Dumas, E; Atyame, CM; Milesi, P; Fonseca, DM; Shaikevich, EV; Unal, S; Makoundou, P; Weill, M; Duron, O | 2013 | Population structure of Wolbachia and cytoplasmic introgression in a complex of mosquito species | Background: The maternally inherited bacterium Wolbachia often acts as a subtle parasite that manipulates insect reproduction, resulting potentially in reproductive isolation between host populations. Whilst distinct Wolbachia strains are documented in a group of evolutionarily closely related mosquitoes known as the Culex pipiens complex, their impact on mosquito population genetics remains unclear. To this aim, we developed a PCR-RFLP test that discriminates the five known Wolbachia groups found in this host complex. We further examined the Wolbachia genetic diversity, the variability in the coinherited host mitochondria and their partitioning among members of the Cx. pipiens complex, in order to assess the impact of Wolbachia on host population structure. Results: There was a strong association between Wolbachia and mitochondrial haplotypes indicating a stable co-transmission in mosquito populations. Despite evidence that members of the Cx. pipiens complex are genetically distinct on the basis of nuclear DNA, the association of Wolbachia and mtDNA with members of the Cx. pipiens complex were limited. The Wolbachia wPip-I group, by far the most common, was associated with divergent Cx. pipiens members, including Cx. quinquefasciatus, Cx. pipiens pipiens form pipiens and Cx. pipiens pipiens form molestus. Four other wPip groups were also found in mosquito populations and all were shared between diverse Cx. pipiens members. Conclusion: This data overall supports the hypothesis that wPip infections, and their allied mitochondria, are associated with regular transfers between Cx. pipiens members rather than specific host associations. Overall, this is suggestive of a recent and likely ongoing cytoplasmic introgression through hybridization events across the Cx. pipiens complex. | 2718 | NA | NA | NA | no | no |
| 2719 | 2017 | NA | Aplin, KP; Suzuki, H; Chinen, AA; Chesser, RT; ten Have, J; Donnellan, SC; Austin, J; Frost, A; Gonzalez, JP; Herbreteau, V; Catzeflis, F; Soubrier, J; Fang, YP; Robins, J; Matisoo-Smith, E; Bastos, ADS; Maryanto, I; Sinaga, MH; Denys, C; Van den Bussche, RA; Conroy, C; Rowe, K; Cooper, A | 2011 | Multiple Geographic Origins of Commensalism and Complex Dispersal History of Black Rats | The Black Rat (Rattus rattus) spread out of Asia to become one of the world’s worst agricultural and urban pests, and a reservoir or vector of numerous zoonotic diseases, including the devastating plague. Despite the global scale and inestimable cost of their impacts on both human livelihoods and natural ecosystems, little is known of the global genetic diversity of Black Rats, the timing and directions of their historical dispersals, and the risks associated with contemporary movements. We surveyed mitochondrial DNA of Black Rats collected across their global range as a first step towards obtaining an historical genetic perspective on this socioeconomically important group of rodents. We found a strong phylogeographic pattern with well-differentiated lineages of Black Rats native to South Asia, the Himalayan region, southern Indochina, and northern Indochina to East Asia, and a diversification that probably commenced in the early Middle Pleistocene. We also identified two other currently recognised species of Rattus as potential derivatives of a paraphyletic R. rattus. Three of the four phylogenetic lineage units within R. rattus show clear genetic signatures of major population expansion in prehistoric times, and the distribution of particular haplogroups mirrors archaeologically and historically documented patterns of human dispersal and trade. Commensalism clearly arose multiple times in R. rattus and in widely separated geographic regions, and this may account for apparent regionalism in their associated pathogens. Our findings represent an important step towards deeper understanding the complex and influential relationship that has developed between Black Rats and humans, and invite a thorough re-examination of host-pathogen associations among Black Rats. | 2719 | NA | NA | NA | no | no |
| 2720 | 2017 | NA | Ennami, M; Briache, FZ; Gaboun, F; Abdelwahd, R; Ghaouti, L; Belqadi, L; Westwood, J; Mentag, R | 2017 | Host differentiation and variability of Orobanche crenata populations from legume species in Morocco as revealed by cross-infestation and molecular analysis | BACKGROUNDOrobanche crenata represents a major biotic constraint to production of faba bean and lentil in Morocco. While this parasitic plant attacks both of these crops, the extent to which Orobanche biotypes specialise in parasitising specific crops is unknown. To address this question, we studied O. crenata that grew on different hosts and quantified their host specificity to faba bean and lentil. The virulence of O. crenata populations on each host was investigated through field trials, pot and Petri dishes assays. Genetic diversity of the parasite populations was also assessed through molecular analyses. RESULTSThe two legume species showed distinct patterns of specificity. Faba bean was more susceptible to both O. crenata populations, while the specificity for lentil by lentil-grown O. crenata was evident at the final stage of the parasite life cycle as shown by correspondence factorial analyses. Considerable internal variation (81%) within O. crenata populations parasitising both legume species was observed by molecular analyses, but significant divergence (19%; O = 0.189; P = 0.010) among the populations was detected. CONCLUSIONThese results indicate that O. crenata can adapt to specific host species, which is important knowledge when developing integrated pest management practices for parasitic weed control. (c) 2017 Society of Chemical Industry | 2720 | NA | NA | NA | no | no |
| 2721 | 2017 | NA | Van der Walt, JM; Nel, LH; Hoelzel, AR | 2001 | Characterization of major histocompatibility complex DRB diversity in the endemic South African antelope Damaliscus pygargus: a comparison in two subspecies with different demographic histories | Major histocompatibility complex (MHC) class II locus DRB was investigated by single-strand conformation polymorphism analysis (SSCP) and sequence analysis in the endemic South African antelope, Damaliscus pygargus, of which there are two subspecies. Greater polymorphism was found in the blesbok (D. p. phillipsi) subspecies (n = 44; 22 alleles) than in the bontebok (D. p. pygargus) subspecies (n = 45; 6 alleles). Erosion of allelic diversity in bontebok was most likely the result of two severe bottleneck events caused by hunting pressure and parasitic infection. A majority of the polymorphism observed was found within the peptide binding region (PBR) where dN/dS ratios were higher than for the non-PER region. This, and the apparent trans-species relationship among alleles in a bovid phylogeny, suggest the evolution of diversity by heterosis or frequency-dependent selection. | 2721 | NA | NA | NA | no | no |
| 2722 | 2017 | NA | Serre, D; Montpetit, A; Pare, G; Engert, JC; Yusuf, S; Keavney, B; Hudson, TJ; Anand, S | 2008 | Correction of Population Stratification in Large Multi-Ethnic Association Studies | “Background. The vast majority of genetic risk factors for complex diseases have, taken individually, a small effect on the end phenotype. Population-based association studies therefore need very large sample sizes to detect significant differences between affected and non-affected individuals. Including thousands of affected individuals in a study requires recruitment in numerous centers, possibly from different geographic regions. Unfortunately such a recruitment strategy is likely to complicate the study design and to generate concerns regarding population stratification. Methodology/Principal Findings. We analyzed 9,751 individuals representing three main ethnic groups - Europeans, Arabs and South Asians - that had been enrolled from 154 centers involving 52 countries for a global case/control study of acute myocardial infarction. All individuals were genotyped at 103 candidate genes using 1,536 SNPs selected with a tagging strategy that captures most of the genetic diversity in different populations. We show that relying solely on self-reported ethnicity is not sufficient to exclude population stratification and we present additional methods to identify and correct for stratification. Conclusions/Significance. Our results highlight the importance of carefully addressing population stratification and of carefully”“cleaning’’ the sample prior to analyses to obtain stronger signals of association and to avoid spurious results.” | 2722 | NA | NA | NA | no | no |
| 2723 | 2017 | NA | Chazara, O; Chang, CS; Bruneau, N; Benabdeljelil, K; Fotsa, JC; Kayang, BB; Loukou, NE; Osei-Amponsah, R; Yapi-Gnaore, V; Youssao, IAK; Chen, CF; Pinard-van der Laan, MH; Tixier-Boichard, M; Bed’Hom, B | 2013 | Diversity and evolution of the highly polymorphic tandem repeat LEI0258 in the chicken MHC-B region | The chicken major histocompatibility complex (MHC) is located on the microchromosome 16 and is described as the most variable region in the genome. The genes of the MHC play a central role in the immune system. Particularly, genes encoding proteins involved in the antigen presentation to T cells. Therefore, describing the genetic polymorphism of this region is crucial in understanding host-pathogen interactions. The tandem repeat LEI0258 is located within the core area of the B region of the chicken MHC (MHC-B region) and its genotypes correlate with serology. This marker was used to provide a picture of the worldwide diversity of the chicken MHC-B region and to categorize chicken MHC haplotypes. More than 1,600 animals from 80 different populations or lines of chickens from Africa, Asia, and Europe, including wild fowl species, were genotyped at the LEI0258 locus. Fifty novel alleles were described after sequencing. The resulting 79 alleles were classified into 12 clusters, based on the SNPs and indels found within the sequences flanking the repeats. Furthermore, hypotheses were formulated on the evolutionary dynamics of the region. This study constitutes the largest variability report for the chicken MHC and establishes a framework for future diversity or association studies. | 2723 | NA | NA | NA | no | no |
| 2724 | 2017 | NA | Suefuji, M; Heinze, J | 2014 | “The genetic population structure of two socially parasitic ants:”“the active slave-maker’’ Myrmoxenus ravouxi and the”“degenerate slave-maker’’ M. kraussei” | “Slave-making ants exploit the societies of host ant species and are typically rare and patchily distributed. IUCN considers almost all slave-making ants as vulnerable, but solid data on their actual abundance are uncommon. Here we examine the genetic structure of populations of two species of the socially parasitic genus Myrmoxenus, which differ strongly in dispersal behavior and the occurrence of slave-raids. Microsatellite genotypes suggest strong differentiation even among neighboring populations of both species. F-ST-, G ’’(ST)-, and D-values were considerably higher in the”“degenerate slave-maker’’ M. kraussei from Northern Italy than in the active slavemaker M. ravouxi from Southern Germany. This matches observations that sexuals of M. kraussei mate in their natal nest and queens disperse on foot while sexuals of M. ravouxi engage in mating and dispersal flights. Allelic richness was surprisingly high in both species and did not suggest recent bottlenecks, indicating that populations are larger and less vulnerable than expected from the difficulties of locating nests. Despite of considerable inbreeding, only very few diploid males were detected, supporting the view that at least in M. kraussei sex is not determined by single locus complementary sex determination unlike most other social Hymenoptera. The mismatch between the genetic and social structure of M. ravouxi colonies indicates occasionally fusion of slave-maker colonies in the field, mutual raids, or queen replacement. Complementary analyses of the host species of the two social parasites, Temnothorax unifasciatus and T. recedens, revealed low levels of population differentiation and confirmed the colony structure with a single, singly-mated queen.” | 2724 | NA | NA | NA | no | no |
| 2725 | 2017 | NA | Buso, GSC; Paiva, MR; Torres, AC; Resende, FV; Ferreira, MA; Buso, JA; Dusi, AN | 2008 | Genetic diversity studies of Brazilian garlic cultivars and quality control of garlic-clover production | The garlic cultivars grown in Brazil evolved from somatic mutations and clone selection by breeding programs and by the introduction of germplasm from other countries. Morphological characters have been used to differentiate these cultivars. Two hundred and six random amplified polymorphic DNA markers were utilized for a diversity analysis of the 17 most planted garlic cultivars in Brazil. Bootstrap analysis showed that the number of markers was efficient and sufficient to obtain a coefficient of variation of 10%. Similarity varied between 16 and 98% and cluster analysis showed that, in general, genetic similarities correlate with morphological characters of the cultivars and production cycle variation. High boot-strap values at most of the nodes supported the dendrogram stability. The grouping of most varieties agreed well with previous reports based on morphological characters. As a vegetative-propagated species, viral diseases are a key problem regarding production and quality of the bulbs, causing gradual loss of yield and decrease in storage capacity. To improve the health quality of garlic seed, a virus-free stock of garlic cloves of the Amarante cultivar was obtained. The ability to distinguish garlic cultivars to detect varietal mixing after in vitro multiplication is extremely important, since correct identification is not possible until bulbs are produced. Random amplified polymorphic DNA markers were also used to differentiate cultivars while they are in vitro and not amenable to morphological discrimination. No difference was identified between the fingerprints of the virus-free or of the infected bulks of Amarante, showing that there was no clove mixing in the handling of material in the clonal multiplication phase. | 2725 | NA | NA | NA | no | no |
| 2726 | 2017 | NA | Koch, MA; Kiefer, C; Ehrich, D; Vogel, J; Brochmann, C; Mummenhoff, K | 2006 | Three times out of Asia Minor: the phylogeography of Arabis alpina L. (Brassicaceae) | Arabis alpina is a characteristic plant in arctic-alpine habitats and serves as a classical example to demonstrate biology, ecology and biogeography of arctic-alpine disjuncts. It has a wider distribution than most other arctic-alpine plants, covering all European mountain systems, the Canary Islands, North Africa, the high mountains of East Africa and Ethiopia, the Arabian Peninsula and mountain ranges of Central Asia in Iran and Iraq. Additionally it is found in the northern amphi-Atlantic area including northeastern North America, Greenland, Iceland, Svalbard and northwestern Europe. We used markers from the nuclear (internal transcribed spacer of ribosomal DNA) and chloroplast genome (trnL-F region) to reconstruct its phylogeographic history. Both markers revealed clear phylogeographic structure. We suggest that A. alpina originated in Asia Minor less than 2 million years ago based on synonymous mutation rates of different genes (plastidic matK, nuclear adh and chs). From the Asian ancestral stock one group migrated via the Arabian Peninsula to the East African high mountains. A second group gave rise to all European and northern populations, and also served as source for the northwest African populations. A third group, which is still centred in Asia, migrated independently southwards and came into secondary contact with the East African lineage in Ethiopia, resulting in high genetic diversity in this area. In the Mediterranean regions, the genetic diversity was relatively high with numerous unique haplotypes, but almost without geographic structure. In contrast, the populations in the northern amphi-Atlantic area were extremely depauperate, suggesting very recent (postglacial) expansion into this vast area from the south. | 2726 | NA | NA | NA | no | no |
| 2727 | 2017 | NA | Shaheen, T; Khan, AA; Mahmood-ur-Rahman; Qamar, MTU; Rahman, M | 2015 | ESTIMATION OF GENETIC DIVERSITY OF PLANT GROWTH PROMOTING RHIZOBACTERIA (PGPR) STRAINS FOUND IN DIFFERENT AREAS OF PAKISTAN USING RAPD AND 16S rRNA ANALYSIS | The present study was an approach to provide the information about the diversity of rhizobacteria involved directly or indirectly in the plant growth and development. Among molecular markers, RAPD proved to be the most promising tool to delineate rhizobacterial strains. Seventeen bacterial strains of rhizobacteria were isolated from different parts of Pakistan (Sargodha, Mianwali, Karak and Faisalabad). RAPD analysis of these strains has shown a high degree of polymorphism. Range of genetic similarity was observed 0.543-0.927. Cluster analysis resulted into three clusters (I, II and III). Cluster I comprised of bacterial strains collected from Faisalabad, cluster II comprised of Strains collected from Sargodha while cluster III included strains from Mianwali. Strains isolated from the Karak region were not making any cluster. These results have shown that soil conditions and local environment has a significant impact on genetic make-up of bacterial strains. Further 16S rRNA analysis of the strain was performed to validate the results. Results of 16S rRNA analysis have revealed that most dominating species were Bacillus sp. and two strains were also showing homology with Brevibacterium halotolerans. The findings of this study were very promising for the application of PGPRs for plant growth improvement. | 2727 | NA | NA | NA | no | no |
| 2728 | 2017 | NA | Gillespie, JJ; Driscoll, TP; Verhoeve, VI; Utsuki, T; Husseneder, C; Chouljenko, VN; Azad, AF; Macaluso, KR | 2015 | Genomic Diversification in Strains of Rickettsia felis Isolated from Different Arthropods | Rickettsia felis (Alphaproteobacteria: Rickettsiales) is the causative agent of an emerging flea-borne rickettsiosis with worldwide occurrence. Originally described from the cat flea, Ctenocephalides felis, recent reports have identified R. felis from other flea species, as well as other insects and ticks. This diverse host range for R. felis may indicate an underlying genetic variability associated with host-specific strains. Accordingly, to determine a potential genetic basis for host specialization, we sequenced the genome of R. felis str. LSU-Lb, which is an obligate mutualist of the parthenogenic booklouse Liposcelis bostrychophila (Insecta: Psocoptera). We also sequenced the genome of R. felis str. LSU, the second genome sequence for cat flea-associated strains (cf. R. felis str. URRWXCal2), which are presumably facultative parasites of fleas. Phylogenomics analysis revealed R. felis str. LSU-Lb diverged from the flea-associated strains. Unexpectedly, R. felis str. LSU was found to be divergent from R. felis str. URRWXCal2, despite sharing similar hosts. Although all three R. felis genomes contain the pRF plasmid, R. felis str. LSU-Lb carries an additional unique plasmid, pLbaR (plasmid of L. bostrychophila associated Rickettsia), nearly half of which encodes a unique 23-gene integrative conjugative element. Remarkably, pLbaR also encodes a repeats-in-toxin-like type I secretion system and associated toxin, heretofore unknown from other Rickettsiales genomes, which likely originated from lateral gene transfer with another obligate intracellular parasite of arthropods, Cardinium (Bacteroidetes). Collectively, our study reveals unexpected genomic diversity across three R. felis strains and identifies several diversifying factors that differentiate facultative parasites of fleas from obligate mutualists of booklice. | 2728 | NA | NA | NA | no | no |
| 2729 | 2017 | NA | Spiliopoulou, A; Nagy, R; Bermingham, ML; Huffman, JE; Hayward, C; Vitart, V; Rudan, I; Campbell, H; Wright, AF; Wilson, JF; Pong-Wong, R; Agakov, F; Navarro, P; Haley, CS | 2015 | Genomic prediction of complex human traits: relatedness, trait architecture and predictive meta-models | We explore the prediction of individuals’ phenotypes for complex traits using genomic data. We compare several widely used prediction models, including Ridge Regression, LASSO and Elastic Nets estimated from cohort data, and polygenic risk scores constructed using published summary statistics from genome-wide association meta-analyses (GWAMA). We evaluate the interplay between relatedness, trait architecture and optimal marker density, by predicting height, body mass index (BMI) and high-density lipoprotein level (HDL) in two data cohorts, originating from Croatia and Scotland. We empirically demonstrate that dense models are better when all genetic effects are small (height and BMI) and target individuals are related to the training samples, while sparse models predict better in unrelated individuals and when some effects have moderate size (HDL). For HDL sparse models achieved good across-cohort prediction, performing similarly to the GWAMA risk score and to models trained within the same cohort, which indicates that, for predicting traits with moderately sized effects, large sample sizes and familial structure become less important, though still potentially useful. Finally, we propose a novel ensemble of whole-genome predictors with GWAMA risk scores and demonstrate that the resulting meta-model achieves higher prediction accuracy than either model on its own. We conclude that although current genomic predictors are not accurate enough for diagnostic purposes, performance can be improved without requiring access to large-scale individual-level data. Our methodologically simple meta-model is a means of performing predictive meta-analysis for optimizing genomic predictions and can be easily extended to incorporate multiple population-level summary statistics or other domain knowledge. | 2729 | NA | NA | NA | no | no |
| 2730 | 2017 | NA | Zheng, G; Freidlin, B; Li, ZH; Gastwirth, JL | 2005 | Genomic control for association studies under various genetic models | Case-control studies are commonly used to study whether a candidate allele and a disease are associated. However. spurious association can arise due to population substructure or cryptic relatedness, which cause the variance of the trend test to increase. Devlin and Roeder derived the appropriate variance inflation factor (VIF) for the trend test and proposed a novel genomic control (GC) approach to estimate VIF and adjust the test statistic. Their results were derived assuming an additive genetic model and the corresponding VIF is independent of the candidate allele frequency. We determine the appropriate VIFs for recessive and dominant models. Unlike the additive test, the VIFs for the optimal tests for these two models depend on the candidate allele frequency. Simulation results show that, when the null loci used to estimate the VIF have allele frequencies similar to that of the candidate gene, the GC tests derived for recessive and dominant models remain optimal. When the underlying genetic model is unknown or the null loci and candidate gene have quite different allele frequencies, the GC tests derived for the recessive or dominant models cannot be used while the GC test derived for the additive model can be. | 2730 | NA | NA | NA | no | no |
| 2731 | 2017 | NA | Talbot, J; Magno, LAV; Santana, CVN; Sousa, SMB; Melo, PRS; Correa, RX; Di Pietro, G; Rios-Santos, F | 2010 | Interethnic diversity of NAT2 polymorphisms in Brazilian admixed populations | Background: N-acetyltransferase type 2 (Nat2) is a phase II drug-metabolizing enzyme that plays a key role in the bioactivation of aromatic and heterocyclic amines. Its relevance in drug metabolism and disease susceptibility remains a central theme for pharmacogenetic research, mainly because of its genetic variability among human populations. In fact, the evolutionary and ethnic-specific SNPs on the NAT2 gene remain a focus for the potential discoveries in personalized drug therapy and genetic markers of diseases. Despite the wide characterization of NAT2 SNPs frequency in established ethnic groups, little data are available for highly admixed populations. In this context, five common NAT2 SNPs (G191A, C481T, G590A, A803G and G857A) were investigated in a highly admixed population comprised of Afro-Brazilians, Whites, and Amerindians in northeastern Brazil. Thus, we sought to determine whether the distribution of NAT2 polymorphism is different among these three ethnic groups. Results: Overall, there were no statistically significant differences in the distribution of NAT2 polymorphism when Afro-Brazilian and White groups were compared. Even the allele frequency of 191A, relatively common in African descendents, was not different between the Afro-Brazilian and White groups. However, allele and genotype frequencies of G590A were significantly higher in the Amerindian group than either in the Afro-Brazilian or White groups. Interestingly, a haplotype block between G590A and A803G was verified exclusively among Amerindians. Conclusions: Our results indicate that ethnic admixture might contribute to a particular pattern of genetic diversity in the NAT2 gene and also offer new insights for the investigation of possible new NAT2 gene-environment effects in admixed populations. | 2731 | NA | NA | NA | no | no |
| 2732 | 2017 | NA | Lee, S; Jia, YL; Jia, M; Gealy, DR; Olsen, KM; Caicedo, AL | 2011 | Molecular Evolution of the Rice Blast Resistance Gene Pi-ta in Invasive Weedy Rice in the USA | The Pi-ta gene in rice has been effectively used to control rice blast disease caused by Magnaporthe oryzae worldwide. Despite a number of studies that reported the Pi-ta gene in domesticated rice and wild species, little is known about how the Pi-ta gene has evolved in US weedy rice, a major weed of rice. To investigate the genome organization of the Pi-ta gene in weedy rice and its relationship to gene flow between cultivated and weedy rice in the US, we analyzed nucleotide sequence variation at the Pi-ta gene and its surrounding 2 Mb region in 156 weedy, domesticated and wild rice relatives. We found that the region at and around the Pi-ta gene shows very low genetic diversity in US weedy rice. The patterns of molecular diversity in weeds are more similar to cultivated rice (indica and aus), which have never been cultivated in the US, rather than the wild rice species, Oryza rufipogon. In addition, the resistant Pi-ta allele (Pi-ta) found in the majority of US weedy rice belongs to the weedy group strawhull awnless (SH), suggesting a single source of origin for Pi-ta. Weeds with Pita were resistant to two M. oryzae races, IC17 and IB49, except for three accessions, suggesting that component(s) required for the Pi-ta mediated resistance may be missing in these accessions. Signatures of flanking sequences of the Pi-ta gene and SSR markers on chromosome 12 suggest that the susceptible pi-ta allele (pi-ta), not Pi-ta, has been introgressed from cultivated to weedy rice by out-crossing. | 2732 | NA | NA | NA | no | no |
| 2733 | 2017 | NA | Prati, D; Peintinger, M; Fischer, M | 2016 | Genetic composition, genetic diversity and small-scale environmental variation matter for the experimental reintroduction of a rare plant | Aims Reintroduction has become an important tool for the management of endangered plant species. We tested the little-explored effects of small-scale environmental variation, genotypic composition (i.e. identity of genotypes) and genotypic diversity on the population survival of the regionally rare clonal plant Ranunculus reptans. For this species of periodically inundated lakeshores, genetic differentiation had been reported between populations and between short-flooded and long-flooded microsites within populations. Methods We established 306 experimental test populations at a previously unoccupied lakeshore, comprising either monocultures of 32 genotypes, mixtures of genotypes within populations or mixtures of genotypes between populations. In 2000, three years after planting out at the experimental site, a long-lasting flood caused the death of half of the experimental populations. In 2003, an extreme drought resulted in the lowest summer water levels ever measured. Important Findings Despite these climatic extremes, 27% of the established populations survived in December 2003. The success of experimental populations largely differed between microsites. Moreover, the success of genotype monocultures depended on genotype and source population. Genetic differentiation between microsites played a minor role for the success of reintroduction. After the flood, populations planted with genotypes from different source populations increased in abundance, whereas populations with genotypes from single source populations and genotype monocultures decreased. In 2014, several small patches of Ranunculus clones were still present, but plants were strongly intermingled, which precluded their assignment to the original treatments. We conclude that sources for reintroductions need to be selected carefully. Moreover, mixtures of plants from different populations appear to be the best choice for successful reintroduction, at least in unpredictably varying environments. | 2733 | NA | NA | NA | no | no |
| 2734 | 2017 | NA | Sousa-Santos, C; Robalo, JI; Francisco, SM; Carrapato, C; Cardoso, AC; Doadrio, I | 2014 | Metapopulations in temporary streams - The role of drought-flood cycles in promoting high genetic diversity in a critically endangered freshwater fish and its consequences for the future | Genetic factors have direct and indirect impacts in the viability of endangered species. Assessing their genetic diversity levels and population structure is thus fundamental for conservation and management. In this paper we use mitochondria] and nuclear markers to address phylogeographic and demographic data on the critically endangered Anaecypris hispanica, using a broad sampling set which covered its known distribution area in the Iberian Peninsula. Our results showed that the populations of A. hispanica are strongly differentiated (high and significant Phi(ST) and F-ST values, corroborated by the results from AMOVA and SAMOVA) and genetically diversified. We suggest that the restricted gene flow between populations may have been potentiated by ecological, hydrological and anthropogenic causes. Bayesian skyline plots revealed a signal for expansion for all populations (t(MRCA) between 68 kya and 1.33 Mya) and a genetic diversity latitudinal gradient was detected between the populations from the Upper (more diversified) and the Lower (less diversified) Guadiana river basin. We postulate a Pleistocenic westwards colonization route for A. hispanica in the Guadiana river basin, which is in agreement with the tempo and mode of paleoevolution of this drainage. The colonization of River Guadalquivir around 60 kya with migrants from the Upper Guadiana, most likely by stream capture, is also suggested. This study highlights the view that critically endangered species facing range retreats (about 47% of its known populations have disappeared in the last 15 years) are not necessarily small and genetically depleted. However, the extinction risk is not negligible since A. hispanica faces the combined effect of several deterministic and stochastic negative factors and, moreover, recolonization events after localized extinctions are very unlikely to occur due to the strong isolation of populations and to the patchily ecologically-conditioned distribution of fish. The inferred species distribution models highlight the significant contribution of temperature seasonality and isothermality to A. hispanica occurrence in Guadiana environments and emphasize the importance of stable climatic conditions for the preservation of this species. Given the strong population structure, high percentage of private haplotypes and virtual absence of inter-basin gene flow we suggest that each A. hispanica population should be considered as an independent Operational Conservation Unit and that ex-situ and in-situ actions should be conducted in parallel to allow for the long-term survival of the species and the preservation of the genetic integrity of its populations. (C) 2014 Elsevier Inc. All rights reserved. | 2734 | NA | NA | NA | no | no |
| 2735 | 2017 | NA | Sengupta, S; Das, B; Acharyya, P; Prasad, M; Ghose, TK | 2014 | Genetic diversity analysis in a set of Caricaceae accessions using resistance gene analogues | Background: In order to assess genetic diversity of a set of 41 Caricaceae accessions, this study used 34 primer pairs designed from the conserved domains of bacterial leaf blight resistance genes from rice, in a PCR based approach, to identify and analyse resistance gene analogues from various accessions of Carica papaya, Vasconcellea goudotiana, V. microcarpa, V. parviflora, V. pubescens, V. stipulata and, V. quercifolia and Jacaratia spinosa. Results: Of the 34 primer pairs fourteen gave amplification products. A total of 115 alleles were identified from 41 accesions along with 12 rare and 11 null alleles. The number of alleles per primer pair ranged from 4 to 10 with an average of 8.21 alleles/primer pair. The average polymorphism information content value was 0.75/primer. The primers for the gene Xa1 did not give any amplification product. As a group, the Indian Carica papaya accessions produced a total of 102 alleles from 27 accessions. The similarity among the 41 accessions ranged from 1% to 53%. The dendrogram made from Jaccard’s genetic similarity coefficient generated two major clusters showing that the alleles of Jacaratia spinosa and Vasconcellea accessions were distinctly different from those of Carica papaya accessions. All the alleles were sequenced and eleven of them were allotted accession numbers by NCBI. Homology searches identified similarity to rice BLB resistance genes and pseudogenes. Conserved domain searches identified gamma subunit of transcription initiation factor IIA (TFIIA), cytochrome P450, signaling domain of methyl-accepting chemotaxis protein (MCP), Nickel hydrogenase and leucine rich repeats (LRR) within the sequenced RGAs. Conclusions: The RGA profiles produced by the 14 primer pairs generated high genetic diversity. The RGA profiles identified each of the 41 accessions clearly unequivocally. Most of the DNA sequences of the amplified RGAs from this set of 41 accessions showed significant homology to the conserved regions of rice bacterial leaf blight resistance genes. These information can be used in future for large scale investigation of tentative disease resistance genes of Carica papaya and other Caricaceae genus specially Vasconcellea. Inoculation studies will be necessary to link the identified sequences to disease resistance or susceptibility. | 2735 | NA | NA | NA | no | no |
| 2736 | 2017 | NA | Liu, XY; Saw, WY; Ali, M; Ong, RTH; Teo, YY | 2014 | Evaluating the possibility of detecting evidence of positive selection across Asia with sparse genotype data from the HUGO Pan-Asian SNP Consortium | Background: The HUGO Pan-Asian SNP Consortium (PASNP) has generated a genetic resource of almost 55,000 autosomal single nucleotide polymorphisms (SNPs) across more than 1,800 individuals from 73 urban and indigenous populations in Asia. This has offered valuable insights into the correlation between the genetic ancestry of these populations with major linguistic systems and geography. Here, we attempt to understand whether adaptation to local climate, diet and environment partly explains the genetic variation present in these populations by investigating the genomic signatures of positive selection. Results: To evaluate the impact to the selection analyses due to the considerably lower SNP density as compared to other population genetics resources such as the International HapMap Project (HapMap) or the Singapore Genome Variation Project, we evaluated the extent of haplotype phasing switch errors and the consistency of selection signals from three haplotype-based approaches (iHS, XP-EHH, haploPS) when the HapMap data is thinned to a similar density as PASNP. We subsequently applied haploPS to detect and characterize positive selection in the PASNP populations, identifying 59 genomics regions that were selected in at least one PASNP populations. A cluster analysis on the basis of these 59 signals showed that indigenous populations such as the Negrito from Malaysia and Philippines, the China Hmong, and the Taiwan Ami and Atayal shared more of these signals. We also reported evidence of a positive selection signal encompassing the beta globin gene in the Taiwan Ami and Atayal that was distinct from the signal in the HapMap Africans, suggesting the possibility of convergent evolution at this locus due to malarial selection. Conclusions: We established that the lower SNP content of the PASNP data conferred weaker ability to detect signatures of positive selection, but the availability of the new approach haploPS retained modest power. Out of all the populations in PASNP, we identified only 59 signals, suggesting a strong need for high-density population-level genotyping data or sequencing data in order to achieve a comprehensive survey of positive selection in Asian populations. | 2736 | NA | NA | NA | no | no |
| 2737 | 2017 | NA | Pomilla, C; Amaral, AR; Collins, T; Minton, G; Findlay, K; Leslie, MS; Ponnampalam, L; Baldwin, R; Rosenbaum, H | 2014 | The World’s Most Isolated and Distinct Whale Population? Humpback Whales of the Arabian Sea | “A clear understanding of population structure is essential for assessing conservation status and implementing management strategies. A small, non-migratory population of humpback whales in the Arabian Sea is classified as”“Endangered”" on the IUCN Red List of Threatened Species, an assessment constrained by a lack of data, including limited understanding of its relationship to other populations. We analysed 11 microsatellite markers and mitochondrial DNA sequences extracted from 67 Arabian Sea humpback whale tissue samples and compared them to equivalent datasets from the Southern Hemisphere and North Pacific. Results show that the Arabian Sea population is highly distinct | 2737 | NA | NA | NA | no | no |
| 2738 | 2017 | NA | Debout, GDG; Frederickson, ME; Aron, S; Yu, DW | 2010 | UNEXPLAINED SPLIT SEX RATIOS IN THE NEOTROPICAL PLANT-ANT, ALLOMERUS OCTOARTICULATUS VAR. DEMERARAE (MYRMICINAE): A TEST OF HYPOTHESES | We investigated sex allocation in the Neotropical ant Allomerus octoarticulatus var. demerarae. Because Allomerus is a plant symbiont, we could make geographically extensive collections of complete colonies and of foundresses in saplings, allowing us to estimate not only population- and colony-level sex allocation but also colony resource levels and the relatednesses of competing ant foundresses. This species exhibits a strongly split sex ratio, with 80% of mature colonies producing >= 90% of one sex or the other. Our genetic analyses (DNA microsatellites) reveal that Allomerus has a breeding system characterized by almost complete monogyny and a low frequency of polyandry. Contrary to theoretical explanations, we find no difference in worker relatedness asymmetries between female- and male-specialist colonies. Furthermore, no clear link was found between colony sex allocation and life history traits such as the number of mates per queen, or colony size, resource level, or fecundity. We also failed to find significant support for male production by workers, infection by Wolbachia, local resource competition, or local mate competition. We are left with the possibility that Allomerus exhibits split sex ratios because of the evolution of alternative biasing strategies in queens or workers, as recently proposed in the literature. | 2738 | NA | NA | NA | no | no |
| 2739 | 2017 | NA | Ramos, EM; Gillis, T; Mysore, JS; Lee, JM; Gogele, M; D’Elia, Y; Pichler, I; Sequeiros, J; Pramstaller, PP; Gusella, JF; MacDonald, ME; Alonso, I | 2015 | Haplotype Analysis of the 4p16.3 Region in Portuguese Families With Huntington’s Disease | Huntington’s disease (HD) is a neurodegenerative disorder characterized by involuntary choreic movements, cognitive impairment, and behavioral changes, caused by the expansion of an unstable CAG repeat in HTT. We characterized the genetic diversity of the HD mutation by performing an extensive haplotype analysis of approximate to 1Mb region flanking HTT in over 300 HD families of Portuguese origin. We observed that haplotype A, marked by HTT delta2642, was enriched in HD chromosomes and carried the two largest expansions reported in the Portuguese population. However, the most frequent HD haplotype B carried one of the largest (+12 CAGs) expansions, which resulted in an allele class change to full penetrance. Despite having a normal CAG distribution skewed to the higher end of the range, these two core haplotypes had similar expanded CAG repeat sizes compared to the other major core haplotypes (C and D) and there was no statistical difference in transmitted repeat instability across haplotypes. We observed a diversity of HTT region haplotypes in both normal and expanded chromosomes, representative of more than one ancestral chromosome underlying HD in Portugal, where multiple independent events on distinct chromosome 4 haplotypes have given rise to expansion into the pathogenic range. (c) 2015 Wiley Periodicals, Inc. | 2739 | NA | NA | NA | no | no |
| 2740 | 2017 | NA | Deng, L; Li, W; Zhong, ZJ; Gong, C; Cao, XF; Song, Y; Wang, WY; Huang, XM; Liu, XH; Hu, YC; Fu, HL; He, M; Wang, Y; Zhang, Y; Wu, KJ; Peng, GN | 2017 | Multi-locus genotypes of Enterocytozoon bieneusi in captive Asiatic black bears in southwestern China: High genetic diversity, broad host range, and zoonotic potential | Enterocytozoon bieneusi is an obligate eukaryotic intracellular parasite that infects a wide variety of vertebrate and invertebrate hosts. Although considerable research has been conducted on this organism, relatively little information is available on the occurrence of E. bieneusi in captive Asiatic black bears. The present study was performed to determine the prevalence, genetic diversity, and zoonotic potential of E. bieneusi in captive Asiatic black bears in zoos in southwestern China. Fecal specimens from Asiatic black bears in four zoos, located in four different cities, were collected and analyzed for the prevalence of E. bieneusi. The average prevalence of E. bieneusi was 27.4% (29/106), with the highest prevalence in Guiyang Zoo (36.4%, 16/44). Altogether, five genotypes of E. bieneusi were identified among the 29 E. bieneusi-positive samples, including three known genotypes (CHB1, SC02, and horse2) and two novel genotypes named ABB1 and ABB2. Multi-locus sequence typing using three microsatellites (MS1, MS3, and MS7) and one minisatellite (MS4) revealed V, III, V, and IV genotypes at these four loci, respectively. Phylogenetic analysis showed that the genotypes SC02 and ABB2 were clustered into group 1 of zoonotic potential, the genotypes CHB1 and ABB1 were clustered into a new group, and the genotype horse2 was clustered into group 6 of unclear zoonotic potential. In conclusion, this study identified two novel E. bieneusi genotypes in captive Asiatic black bears, and used microsatellite and minisatellite markers to reveal E. bieneusi genetic diversity. Moreover, our findings show that genotypes SC02 (identified in humans) and ABB2 belong to group 1 with zoonotic potential, suggesting the risk of transmission of E. bieneusi from Asiatic black bears to humans and other animals. | 2740 | NA | NA | NA | no | no |
| 2741 | 2017 | NA | Pakhomov, SVS; Eberly, L; Knopman, D | 2016 | Characterizing cognitive performance in a large longitudinal study of aging with computerized semantic indices of verbal fluency | A computational approach for estimating several indices of performance on the animal category verbal fluency task was validated, and examined in a large longitudinal study of aging. The performance indices included the traditional verbal fluency score, size of semantic clusters, density of repeated words, as well as measures of semantic and lexical diversity. Change over time in these measures was modeled using mixed effects regression in several groups of participants, including those that remained cognitively normal throughout the study (CN) and those that were diagnosed with mild cognitive impairment (MCI) or Alzheimer’s disease (AD) dementia at some point subsequent to the baseline visit. The results of the study show that, with the exception of mean cluster size, the indices showed significantly greater declines in the MCI and AD dementia groups as compared to CN participants. Examination of associations between the indices and cognitive domains of memory, attention and visuospatial functioning showed that the traditional verbal fluency scores were associated with declines in all three domains, whereas semantic and lexical diversity measures were associated with declines only in the visuospatial domain. Baseline repetition density was associated with declines in memory and visuospatial domains. Examination of lexical and semantic diversity measures in subgroups with high vs. low attention scores (but normal functioning in other domains) showed that the performance of individuals with low attention was influenced more by word frequency rather than strength of semantic relatedness between words. These findings suggest that various automatically semantic indices may be used to examine various aspects of cognitive performance affected by dementia. (C) 2016 Elsevier Ltd. All rights reserved. | 2741 | NA | NA | NA | no | no |
| 2742 | 2017 | NA | Kruckenhauser, L; Haring, E; Tautscher, B; Cadahia, L; Zopp, L; Duda, M; Harl, J; Sattmann, H | 2017 | Indication for selfing in geographically separated populations and evidence for Pleistocene survival within the Alps: the case of Cylindrus obtusus (Pulmonata: Helicidae) | Background: Cylindrus obtusus is one of the most prominent endemic snail species of the Eastern Alps. It is restricted to alpine meadows and calcareous rocky habitats above 1500 m. Peculiar intraspecific differences have been observed in its genital tract in the eastern populations the two mucus glands associated with the love dart sac are highly variable, while almost no variation was observed in the western populations. This raises the question whether the mode and success of reproduction of the respective populations are different. To find out whether these anatomical differences reflect genetic differentiation, which might be an indication for distinct glacial refugia, we investigated a 650 bp fragment of the mitochondrial cytochrome oxidase subunit 1 gene (COI) (280 individuals) and 9 microsatellite loci from samples (487 individuals from 29 populations) covering the whole distribution range of the species. Results: The COI sequences show a geographic differentiation between eastern, central and western populations. The westernmost localities, which were covered under ice sheets during glacial periods, are characterized by extreme low variability. Overall genetic distances among all individuals are small (max. 1.7% p-distance). The microsatellite analysis reveals a high differentiation between populations, implying restriction of gene flow. The highest genetic variability was found in the central populations. Remarkably, nearly all individuals from the eastern populations, which are more variable in their genital morphology, are homozygous in all microsatellite loci, although different alleles were found within populations. Conclusions: The most peculiar outcome of the study is the strong evidence for selfing in C. obtusus as indicated by the microsatellite data in the easternmost populations. This finding is supported by the deformation of the mucus glands in the same populations. Since mucus glands play an important role in sexual reproduction, it seems plausible that in selfing organisms these structures are reduced. The phylogeographic structure revealed by COI sequences implies that the species has survived the ice ages within the Calcareous Alps. The small genetic distances among all individuals (max. 1.7%) suggest that C. obtusus has experienced severe bottlenecks in the past. | 2742 | NA | NA | NA | no | no |
| 2743 | 2017 | NA | Gingeras, TR; Ghandour, G; Wang, EG; Berno, A; Small, PM; Drobniewski, F; Alland, D; Desmond, E; Holodniy, M; Drenkow, J | 1998 | Simultaneous genotyping and species identification using hybridization pattern recognition analysis of generic Mycobacterium DNA arrays | High-density oligonucleotide arrays can be used to rapidly examine large amounts of DNA sequence in a high throughput manner. An array designed to determine the specific nucleotide sequence of 705 bp of the rpoB gene of Mycobacterium tuberculosis accurately detected rifampin resistance associated with mutations of 44 clinical isolates of M. tuberculosis. The nucleotide sequence diversity in 121 Mycobacterial isolates (comprised of 10 species) was examined by both conventional dideoxynucleotide sequencing of the rpoB and 16S genes and by analysis of the rpoB oligonucleotide array hybridization patterns. Species identification For each of the isolates was similar irrespective of whether 16S sequence, rpoB sequence, or the pattern of rpoB hybridization was used. However, For several species, the number of alleles in the 16S and rpoB gene sequences provided discordant estimates of the genetic diversity within a species. In addition to confirming the array’s intended utility For sequencing the region of M. tuberculosis that confers rifampin resistance, this work demonstrates that this array can identify the species of nontuberculous Mycobacteria. This demonstrates the general point that DNA microarrays that sequence important genomic regions (such as drug resistance or pathogenicity islands) can simultaneously identify species and provide some insight into the organism’s population structure. | 2743 | NA | NA | NA | no | no |
| 2744 | 2017 | NA | Puill-Stephan, E; Willis, BL; van Herwerden, L; van Oppen, MJH | 2009 | Chimerism in Wild Adult Populations of the Broadcast Spawning Coral Acropora millepora on the Great Barrier Reef | Background: Chimeras are organisms containing tissues or cells of two or more genetically distinct individuals, and are known to exist in at least nine phyla of protists, plants, and animals. Although widespread and common in marine invertebrates, the extent of chimerism in wild populations of reef corals is unknown. Methodology/Principal Findings: The extent of chimerism was explored within two populations of a common coral, Acropora millepora, on the Great Barrier Reef, Australia, by using up to 12 polymorphic DNA microsatellite loci. At least 2% and 5% of Magnetic Island and Pelorus Island populations of A. millepora, respectively, were found to be chimeras (3% overall), based on conservative estimates. A slightly less conservative estimate indicated that 5% of colonies in each population were chimeras. These values are likely to be vast underestimates of the true extent of chimerism, as our sampling protocol was restricted to a maximum of eight branches per colony, while most colonies consist of hundreds of branches. Genotypes within chimeric corals showed high relatedness, indicating that genetic similarity is a prerequisite for long-term acceptance of non-self genotypes within coral colonies. Conclusions/Significance: While some brooding corals have been shown to form genetic chimeras in their early life history stages under experimental conditions, this study provides the first genetic evidence of the occurrence of coral chimeras in the wild and of chimerism in a broadcast spawning species. We hypothesize that chimerism is more widespread in corals than previously thought, and suggest that this has important implications for their resilience, potentially enhancing their capacity to compete for space and respond to stressors such as pathogen infection. | 2744 | NA | NA | NA | no | no |
| 2745 | 2017 | NA | Cardenas, M; Grajales, A; Sierra, R; Rojas, A; Gonzalez-Almario, A; Vargas, A; Marin, M; Fermin, G; Lagos, LE; Grunwald, NJ; Bernal, A; Salazar, C; Restrepo, S | 2011 | Genetic diversity of Phytophthora infestans in the Northern Andean region | Background: Phytophthora infestans (Mont.) de Bary, the causal agent of potato late blight, is responsible for tremendous crop losses worldwide. Countries in the northern part of the Andes dedicate a large proportion of the highlands to the production of potato, and more recently, solanaceous fruits such as cape gooseberry (Physalis peruviana) and tree tomato (Solanum betaceum), all of which are hosts of this oomycete. In the Andean region, P. infestans populations have been well characterized in Ecuador and Peru, but are poorly understood in Colombia and Venezuela. To understand the P. infestans population structure in the Northern part of the Andes, four nuclear regions (ITS, Ras, beta-tubulin and Avr3a) and one mitochondrial (Cox1) region were analyzed in isolates of P. infestans sampled from different hosts in Colombia and Venezuela. Results: Low genetic diversity was found within this sample of P. infestans isolates from crops within several regions of Colombia and Venezuela, revealing the presence of clonal populations of the pathogen in this region. We detected low frequency heterozygotes, and their distribution patterns might be a consequence of a high migration rate among populations with poor effective gene flow. Consistent genetic differentiation exists among isolates from different regions. Conclusions: The results here suggest that in the Northern Andean region P. infestans is a clonal population with some within-clone variation. P. infestans populations in Venezuela reflect historic isolation that is being reinforced by a recent self-sufficiency of potato seeds. In summary, the P. infestans population is mainly shaped by migration and probably by the appearance of variants of key effectors such as Avr3a. | 2745 | NA | NA | NA | no | no |
| 2746 | 2017 | NA | Ciplak, B; Kaya, S; Boztepe, Z; Gunduz, I | 2015 | Mountainous genus Anterastes (Orthoptera, Tettigoniidae): autochthonous survival across several glacial ages via vertical range shifts | Although the high-latitude range margins in Europe and North America are intensively studied, attention is gradually turned towards the taxa/populations inhabiting glacial refugia. Here, we evaluate the genealogical history of the cold-adapted Anatolio-Balkan genus Anterastes especially to test the possible effects of intrarefugial vertical range shifts during climatic oscillations of the Quaternary. Using concatenated data from sequences of COI+16S and ITS1-5.8S-ITS2, intrageneric relationships and the time of speciation events were estimated. Thirteen different demographic analyses were performed using a data set produced from sequences of 16S. Different phylogenetic analyses recovered similar lineages with high resolution. The molecular chronogram estimated speciation events in a period ranging from 5.60 to 1.22Myr. Demographic analyses applied to 13 populations and five lineages suggested constant population size. Genetic diversity is significantly reduced in a few populations, while not in others. Fixation indices suggested extremely diverged populations. In the light of these data, the following main conclusions were raised: (i) although glacial refugia are the biodiversity hotspots, species level radiation of the cold-adapted lineages is mainly prior to the Mid-Pleistocene transition; (ii) heterogeneous topography provides refugial habitats and allows populations to survive through vertical range shifts during climatic fluctuations; (iii) prolonged isolation of refugial populations do not always result in reduced intrapopulation diversity, but in high level of genetic differentiation; (iv) the cold-adapted lineages with low dispersal ability might have not colonised the area out of Anatolian refugium during interglacial periods; and (v) populations of invertebrates may have restricted ranges, but this does not mean that they have small effective population size. | 2746 | NA | NA | NA | no | no |
| 2747 | 2017 | NA | Geboes, AL; Rosoux, R; Lemarchand, C; Hansen, E; Libois, R | 2016 | Genetic diversity and population structure of the Eurasian otter (Lutra lutra) in France | During the last century, the Eurasian otter (Lutra lutra) suffered a dramatic decline in Europe. In France, the same pattern of sharp decline was observed with local extinctions in many regions. Before the recolonisation process, two main populations still remained along the Atlantic coast and in the Massif Central. To investigate the impact of this decline on the genetic diversity and structure of the French otter population, tissue samples of 144 otter carcasses from road kills that were found during 1992-2011 along the Atlantic coast and in the Massif Central were used. They were analysed using 10 microsatellites loci. Observed (H (o) = 0.64) and expected heterozygosity (H (e) = 0.62) were moderate, but consistent with results found in other European populations. The bottleneck test showed an excess of heterozygotes, providing evidence of a recent decline. There was evidence for weak but significant allelic frequencies divergence between otters from the Atlantic coast and those from the Massif Central (F (st) = 0.040, p < 0.05), probably resulting from their isolation prior to the recolonisation process. As the French otter population has been expanding for several years, genetic intermixing is now occurring. Although this expansion has not yet genetically homogenised all populations, this is may be a matter a time. | 2747 | NA | NA | NA | no | no |
| 2748 | 2017 | NA | Johnson, CN; Flowers, AR; Young, VC; Gonzalez-Escalona, N; DePaola, A; Noriea, NF; Grimes, DJ | 2009 | Genetic Relatedness Among tdh (+) and trh (+) Vibrio parahaemolyticus Cultured from Gulf of Mexico Oysters (Crassostrea virginica) and Surrounding Water and Sediment | Pathogenic Vibrio parahaemolyticus (Vp) (tdh (+)/trh (+)) represent a small percentage of environmental Vp populations, and very little is known about this subpopulation. Repetitive extragenic palindromic PCR and multilocus sequence analysis revealed heterogeneity among 41 Vp containing thermostable direct hemolysin (tdh) and tdh-related hemolysin (trh) that were isolated from Mississippi coastal environments from October 2006 to April 2007. There was no source-specific sequestering in oysters, water, or sediment. | 2748 | NA | NA | NA | no | no |
| 2749 | 2017 | NA | Drewnowska, JM; Swiecicka, I | 2013 | Eco-Genetic Structure of Bacillus cereus sensu lato Populations from Different Environments in Northeastern Poland | The Bacillus cereus group, which includes entomopathogens and etiologic agents of foodborne illness or anthrax, persists in various environments. The basis of their ecological diversification remains largely undescribed. Here we present the genetic structure and phylogeny of 273 soil B. cereus s.l. isolates from diverse habitats in northeastern Poland, with samplings acquired from the last European natural forest (Bialowieza National Park), the largest marshes in Europe (Biebrza National Park), and a farm. Inmulti-locus sequence typing (MLST), despite negative selection in seven housekeeping loci, the isolates exhibited high genetic diversity (325 alleles), mostly resulting from mutation events, and represented 148 sequencing types (131 STs new and 17 STs already described) grouped into 19 complexes corresponding with bacterial clones, and 80 singletons. Phylogenetic analyses showed that 74% of the isolates clustered with B. cereus s.l. environmental references (clade III), while only 11 and 15%, respectively, grouped with isolates of clinical origin (clade I), and B. cereus ATCC 14579 and reference B. thuringiensis (clade II). Predominantly within clade III, we found lineages adapted to low temperature (thermal ecotypes), while putative toxigenic isolates (cytK-positive) were scattered in all clades of the marsh and farm samplings. The occurrence of 92% of STs in bacilli originating from one habitat, and the description of new STs for 78% of the isolates, strongly indicate the existence of specific genotypes within the natural B. cereus s.l. populations. In contrast to the human-associated B. cereus s.l. that exhibit a significant level of similarity, the environmental isolates appear more complex. Thus we propose dividing B. cereus s.l. into two groups, the first including environmental isolates, and the second covering those that are of clinical relevance. | 2749 | NA | NA | NA | no | no |
| 2750 | 2017 | NA | Triana, O; Jaramillo, N; Moreno, J | 1999 | Genetic variability of Colombian populations of Trypanosoma cruzi and Trypanosoma rangeli | This paper presents our study of genetic variability of Trypanosoma cruzi and Trypanosoma rangeli strains isolated from different Colombian biological hosts, using multilocus enzyme electrophoresis for 15 enzyme systems and electrophoretic analysis of kinetoplast DNA (kDNA) digested with EcoRI and MspI endonucleases. Isoenzyme profiles were used to determine genotypes for each of the strains. Populations of T. cruzi and T. rangeli were found to have a polymorphic average of 86.7% and 26.7%, respectively. Schyzodeme analysis showed high variability for T. cruzi, since its genetic distance values were found to be greater than 50%, considerably higher than those previously reported for several T. cruzi strains from other countries. These results suggest that Colombian strains should be considered as genetically independent entities and are worth studying independently from each other to clearly establish their biological and clinical characteristics. | 2750 | NA | NA | NA | no | no |
| 2751 | 2017 | NA | PINOCHET, P; CENIS, JL; FERNANDEZ, C; DOUCET, M; MARULL, J | 1994 | REPRODUCTIVE FITNESS AND RANDOM AMPLIFIED POLYMORPHIC DNA VARIATION AMONG ISOLATES OF PRATYLENCHUS-VULNUS | The reproductive fitness of seven isolates of Pratylenchus vulnus from different geographical areas and hosts was assessed in monoxenic cultures (carrot), and greenhouse cultures (plum, sour orange, and quince). The genetic makeup of the different isolates was compared by Random Amplified Polymorphic DNA (RAPD-PCR). The apple (PvAP-S) and apricot (PvAT-F) isolates reproduced less in monoxenic cultures than the rose (PvRO-S) and walnut (PvWA-A and PvWA-U) isolates. On plum, the rose isolate (PvRO-S) reproduced better than the apple (PvAP-S) and walnut isolate from the United States (PvWA-U). On sour orange, the apple (PvAP-S), unknown origin (PvU-UK), and walnut isolate from Argentina (PvWA-A) multiplied well, whereas the walnut isolate from the United States (PvWA-U), apricot (PvAT-F), and rose (PvRO-S) did not. On quince, the apple (PvAP-S) and walnut (PvWA-U) isolates showed a higher reproduction than the one from unknown origin (PvU-UK). RAPD-PCR patterns among the seven P. vulnus isolates were similar, although high intraspecific varibility was detected. Very few bands of P. neglectus were shared by any population of P. vulnus. A high degree of similarity was found among the patterns corresponding to the rose (PvRO-S), apple (PvAP-S), walnut from the United States (PvWA-U), and unknown origin (PvUK-U) isolates. The apricot isolate (PvAT-F) was the most dissimilar among the seven isolates. No correlation could be established between the genetic variation of P. vulnus detected by RAPD-PCR and reproductive fitness. Results demonstrate high genetic varibility between geographically separated populations of P. vulnus. | 2751 | NA | NA | NA | no | no |
| 2752 | 2017 | NA | Elena, SF | 2017 | Local adaptation of plant viruses: lessons from experimental evolution | For multihost pathogens, adaptation to multiple hosts has important implications for both applied and basic research. At the applied level, it is one of the main factors determining the probability and severity of emerging disease outbreaks. At the basic level, it is thought to be a key mechanism for the maintenance of genetic diversity both in host and pathogen species. In recent years, a number of evolution experiments have assessed the fate of plant virus populations replicating within and adapting to one single or to multiple hosts species. A first group of these experiments tackled the existence of trade-offs in fitness and virulence for viruses evolving either within a single hosts species or alternating between two different host species. A second set of experiments explored the role of genetic variability in susceptibility and resistance to infection among individuals from the same host species in the extent of virus local adaptation and of virulence. In general, when a single host species or genotype is available, these experiments show that local adaptation takes place, often but not always associated with a fitness trade-off. However, alternating between different host species or infecting resistant host genotypes may select for generalist viruses that experience no fitness cost. Therefore, the expected cost of generalism, arising from antagonistic pleiotropy and other genetic mechanisms generating fitness trade-offs between hosts, could not be generalized and strongly depend on the characteristics of each particular pathosystem. At the genomic level, these studies show pervasive convergent molecular evolution, suggesting that the number of accessible molecular pathways leading to adaptation to novel hosts is limited. | 2752 | NA | NA | NA | no | no |
| 2753 | 2017 | NA | Elliott, B; Dingle, KE; Didelot, X; Crook, DW; Riley, TV | 2014 | The Complexity and Diversity of the Pathogenicity Locus in Clostridium difficile Clade 5 | The symptoms of Clostridium difficile infection are caused by two closely related toxins, TcdA and TcdB, which are encoded by the 19.6 kb Pathogenicity Locus (PaLoc). The PaLoc is variably present among strains, and in this respect it resembles a mobile genetic element. The C. difficile population structure consists mainly of five phylogenetic clades designated 1-5. Certain genotypes of clade 5 are associated with recently emergent highly pathogenic strains causing human disease and animal infections. The aim of this study was to explore the evolutionary history of the PaLoc in C. difficile clade 5. Phylogenetic analyses and annotation of clade 5 PaLoc variants and adjoining genomic regions were undertaken using a representative collection of toxigenic and nontoxigenic strains. Comparison of the core genome and PaLoc phylogenies obtained for clade 5 and representatives of the other clades identified two distinct PaLoc acquisition events, one involving a toxin A(+)B(+) PaLoc variant and the other an A(-)B(+) variant. Although the exact mechanism of each PaLoc acquisition is unclear, evidence of possible homologous recombination with other clades and between clade 5 lineages was found within the PaLoc and adjacent regions. The generation of nontoxigenic variants by PaLoc loss via homologous recombination with PaLoc-negative members of other clades was suggested by analysis of cdu2, although none is likely to have occurred recently. A variant of the putative holin gene present in the clade 5 A(-)B(+) PaLoc was likely acquired via allelic exchange with an unknown element. Fine-scale phylogenetic analysis of C. difficile clade 5 revealed the extent of its genetic diversity, consistent with ancient evolutionary origins and a complex evolutionary history for the PaLoc. | 2753 | NA | NA | NA | no | no |
| 2754 | 2017 | NA | Vural, HC | 2009 | Genomic DNA isolation from aromatic and medicinal plants growing in Turkey | Herbal and aromatic plants are attracting more attention among contemporary plant researchers because some human diseases resulting from bacterial antibiotic resistances have gained worldwide concern. A number of methods are available and are being developed for the isolation of nucleic acids from plants. Because plants contain high amounts of many different substances, it is unlikely that just one nucleic acid isolation method suitable for all plants can ever exist. Therefore, we developed 4 modified new methods that produced good quality DNA from these plants. This article deals with modern approaches in determining genetic variability, in which three categories of genetic markers are applied by morphological, biochemical and molecular. Furthermore, the aim is to assess the available genetic diversity for each species; to provide more accurate and detailed information than is available using classical phenotypic data in this subject. Various types of plant materials and a number of different protocols for the isolation of DNA were tested in order to obtain good quality DNA for PCR reactions. Ten populations of different aromatic and medicinal plants from Turkey were tested in the study. The number of plants examined for each population varied from two to five. When fresh or frozen leaves of plants collected in autumn were used for the isolation of DNA, no positive result in PCR reaction was obtained regardless of the isolation protocol being used. Four different DNA methods were compared for the isolation of DNA from the different plant homogenates, namely the CTAB, Plant Genomic DNA Purification Kit, and EZ1 Nucleic acid isolation methods and DNA extraction with phenol purification and liquid nitrogen method. | 2754 | NA | NA | NA | no | no |
| 2755 | 2017 | NA | Paquette, SR; Talbot, B; Garant, D; Mainguy, J; Pelletier, F | 2014 | Modelling the dispersal of the two main hosts of the raccoon rabies variant in heterogeneous environments with landscape genetics | Predicting the geographic spread of wildlife epidemics requires knowledge about the movement patterns of disease hosts or vectors. The field of landscape genetics provides valuable approaches to study dispersal indirectly, which in turn may be used to understand patterns of disease spread. Here, we applied landscape genetic analyses and spatially explicit models to identify the potential path of raccoon rabies spread in a mesocarnivore community. We used relatedness estimates derived from microsatellite genotypes of raccoons and striped skunks to investigate their dispersal patterns in a heterogeneous landscape composed predominantly of agricultural, forested and residential areas. Samples were collected in an area covering 22000km2 in southern Quebec, where the raccoon rabies variant (RRV) was first detected in 2006. Multiple regressions on distance matrices revealed that genetic distance among male raccoons was strictly a function of geographic distance, while dispersal in female raccoons was significantly reduced by the presence of agricultural fields. In skunks, our results suggested that dispersal is increased in edge habitats between fields and forest fragments in both males and females. Resistance modelling allowed us to identify likely dispersal corridors used by these two rabies hosts, which may prove especially helpful for surveillance and control (e.g. oral vaccination) activities. | 2755 | NA | NA | NA | no | no |
| 2756 | 2017 | NA | PEARSON, B; RAYBOULD, AF; CLARKE, RT | 1995 | BREEDING-BEHAVIOR, RELATEDNESS AND SEX-INVESTMENT RATIOS IN LEPTOTHORAX-TUBERUM FABRICIUS | The ant species Leptothorax tuberum was shown to be predominantly monogynous. Queens usually mate once only but some nests may have a multiply-mated queen or are partially or serially polygynous. As expected from these results, within nest relatedness between workers and between workers and alate queens was found to be high. Almost fifty percent of nests had no nest queen which may indicate high queen mortality, queens leaving to found new nests or nest fragmentation. Observed female investment frequencies (IFo) were not significantly different from those expected on the basis of worker control of sexual production and the relatedness estimates of workers to alate queens and workers to males calculated from isozyme data (IFE). These values were not consistent with queen control. There was no evidence for lower IF(o)s in queenless nests nor for higher IF(o)s in larger nests classified by worker number. When nests were classified by sexual productivity, however, there was a strong rank correlation between productivity and female bias. This is the first study of an ant species to test observed IF against expected IF calculated without inferring between caste relatedness from worker data or pooling of data from different castes. | 2756 | NA | NA | NA | no | no |
| 2757 | 2017 | NA | Guertin, DA; Ben-David, M; Harestad, AS; Elliott, JE | 2012 | Fecal genotyping reveals demographic variation in river otters inhabiting a contaminated environment | The deposition and accumulation of persistent contaminants into coastal systems can have lingering negative consequences for wildlife populations and their habitats. Using multi-locus genotyping of non-invasively collected feces, we assessed the effects of such pollution on reproduction, survival, genetic variability, and abundance of river otters (Lontra canadensis) along a gradient of urbanindustrial development on southern Vancouver Island, British Columbia, Canada. Genetic analyses indicated a pattern consistent with small-scale structuring, with individuals partitioned into 2 local subpopulationsthose identified in the contaminated harbors of southern Vancouver Island and points west (Colwood/Harbors), and those inhabiting uncontaminated habitat east of the harbors (Oak Bay). Genetic and demographic analyses for the 2 clusters provide strong support for the conclusion that, despite contamination concerns, Colwood/Harbors river otters exhibited acceptable levels of survival and successfully reproduced (i.e., high levels of relatedness, high self-recruitment, and high emigration). However, our data indicate that the Colwood/Harbors area constitutes lower quality habitat supporting lower densities of otters, especially during winter, and excess individuals produced in that region emigrate to other areas. Immigration into Colwood/Harbors, however, seems limited, possibly because of behavioral aversion of non-habituated otters to anthropogenic disturbance associated with the harbors and limited optimal otter habitat. Our findings suggest that the effects of chronic contaminant exposure at the population level may be inadvertently mitigated through the behavioral decisions of individuals to avoid poor quality habitats. We conclude that populations of river otters can persist in and around locally contaminated sites if relatively less disturbed and contaminated habitats remain in the vicinity of the affected areas. (c) 2012 The Wildlife Society. | 2757 | NA | NA | NA | no | no |
| 2758 | 2017 | NA | STIM, KP | 1995 | A PHYLOGENETIC ANALYSIS OF MICROORGANISMS ISOLATED FROM SUBSURFACE ENVIRONMENTS | Three methods were used to provide information on the identity and phylogenetic relatedness of 19 aerobic, chemoheterotrophic bacteria isolated from topsoil and deep subsurface sediments at a site in South Carolina. These methods were (i) analysis of selected physiological traits, (ii) restriction endonuclease analysis (REA) of genomic DNA, and (iii) analysis of 16S ribosomal RNA sequences. When the 16S rRNA sequences were compared with those for 12 standard strains, two topsoil isolates and six subsurface strains formed a tight group with the high-G+C Gram-positive bacteria and appeared to be most closely related to Arthrobacter globiformis - a coryneformactinomycete bacterium with unusually effective survival capabilities. The rest of the subsurface isolates were scattered among the standard strains from the Proteobacteria - including the pseudomonads and Agrobacterium tumefaciens - or the low-G+C Gram-positive bacteria. | 2758 | NA | NA | NA | no | no |
| 2759 | 2017 | NA | Beckstrom-Sternberg, SM; Auerbach, RK; Godbole, S; Pearson, JV; Beckstrom-Sternberg, JS; Deng, ZM; Munk, C; Kubota, K; Zhou, Y; Bruce, D; Noronha, J; Scheuermann, RH; Wang, AH; Wei, XY; Wang, JJ; Hao, J; Wagner, DM; Brettin, TS; Brown, N; Gilna, P; Keim, PS | 2007 | Complete Genomic Characterization of a Pathogenic A.II Strain of Francisella tularensis Subspecies tularensis | “Francisella tularensis is the causative agent of tularemia, which is a highly lethal disease from nature and potentially from a biological weapon. This species contains four recognized subspecies including the North American endemic F. tularensis subsp. tularensis (type A), whose genetic diversity is correlated with its geographic distribution including a major population subdivision referred to as A.I and A.II. The biological significance of the A.I - A.II genetic differentiation is unknown, though there are suggestive ecological and epidemiological correlations. In order to understand the differentiation at the genomic level, we have determined the complete sequence of an A.II strain (WY96-3418) and compared it to the genome of Schu S4 from the A.I population. We find that this A.II genome is 1,898,476 bp in size with 1,820 genes, 1,303 of which code for proteins. While extensive genomic variation exists between”“WY96’’ and Schu S4, there is only one whole gene difference. This one gene difference is a hypothetical protein of unknown function. In contrast, there are numerous SNPs (3,367), small indels (1,015), IS element differences (7) and large chromosomal rearrangements (31), including both inversions and translocations. The rearrangement borders are frequently associated with IS elements, which would facilitate intragenomic recombination events. The pathogenicity island duplicated regions (DR1 and DR2) are essentially identical in WY96 but vary relative to Schu S4 at 60 nucleotide positions. Other potential virulence-associated genes (231) varied at 559 nucleotide positions, including 357 non-synonymous changes. Molecular clock estimates for the divergence time between A.I and A.II genomes for different chromosomal regions ranged from 866 to 2131 years before present. This paper is the first complete genomic characterization of a member of the A.II clade of Francisella tularensis subsp. tularensis.” | 2759 | NA | NA | NA | no | no |
| 2760 | 2017 | NA | Lihova, J; Carlsen, T; Brochmann, C; Marhold, K | 2009 | Contrasting phylogeographies inferred for the two alpine sister species Cardamine resedifolia and C-alpina (Brassicaceae) | We use Cardamine alpina and C. resedifolia as models to address the detailed history of disjunctions in the European alpine system. These species grow on siliceous bedrock: C. alpina in the Alps and Pyrenees, and C. resedifolia in several mountain ranges from the Sierra Nevada to the Balkans. We explore differentiation among their disjunct populations as well as within the contiguous Alpine and Pyrenean ranges, and compare the phylogeographical histories of these diploid sister species. We also include samples of the closely related, arctic diploid C. bellidifolia in order to explore its origin and post-glacial establishment. European alpine system, Norway and Iceland. We employed amplified fragment length polymorphisms (AFLPs). AFLP data were analysed using principal coordinates analysis, neighbour joining and Bayesian clustering, and measures of diversity and differentiation were computed. For the snow-bed species C. alpina (27 populations, 203 plants) we resolved two strongly divergent lineages, corresponding to the Alps and the Pyrenees. Although multiple glacial refugia were invoked in the Pyrenees, we inferred only a single one in the Maritime Alps - from which rapid post-glacial colonization of the entire Alps occurred, accompanied by a strong founder effect. For C. resedifolia (33 populations, 247 plants), which has a broader ecological amplitude and a wider distribution, the genetic structuring was rather weak and did not correspond to the main geographical disjunctions. This species consists of two widespread and largely sympatric main genetic groups (one of them subdivided into four geographically more restricted groups), and frequent secondary contacts exist between them. The conspicuously different histories of these two sister species are likely to be associated with their different ecologies. The more abundant habitats available for C. resedifolia may have increased the probability of its gradual migration during colder periods and also of successful establishment after long-distance dispersal, whereas C. alpina has been restricted by its dependence on snow-beds. Surprisingly, the arctic C. bellidifolia formed a very divergent lineage with little variation, contradicting a scenario of recent, post-glacial migration from the Alps or Pyrenees. | 2760 | NA | NA | NA | no | no |
| 2761 | 2017 | NA | Somasekhar, N; Grewal, PS; Klein, MG | 2002 | Genetic variability in stress tolerance and fitness among natural populations of Steinernema carpocapsae | Genetic variability in stress tolerance (heat, desiccation, and hypoxia) and fitness (virulence and reproduction potential) among natural populations of Steinernema carpocapsae was assessed by estimating phenotypic differences. Significant differences were observed in stress tolerance among populations. Populations isolated from North Carolina showed significantly more stress tolerance than those isolated from Ohio. Significant differences were also observed in populations isolated from the same locality. Survival of infective juveniles after exposure to 40degreesC for 2 h ranged from 37 to 82%. A threefold difference was observed in infective juvenile survival following exposure to osmotic desiccation or hypoxic condition. Several populations tested were superior to the most widely used strain (‘All’ strain) in stress tolerance traits, with one population KMD33, being superior to the ‘All’ strain in all traits. Fitness as expressed by virulence and reproductive potential differed significantly among populations but showed less variability than the stress tolerance traits. All populations tested had a reproductive potential greater than or similar to that of the ‘All’ strain and most of them caused > 60% insect mortality of the wax moth larvae, Galleria mellonella. The high genetic variability in stress tolerance among natural populations suggests the feasibility of using selection for genetic improvement of these traits. (C) 2002 Elsevier Science (USA). | 2761 | NA | NA | NA | no | no |
| 2762 | 2017 | NA | Chernyaeva, EN; Shulgina, MV; Rotkevich, MS; Dobrynin, PV; Simonov, SA; Shitikov, EA; Ischenko, DS; Karpova, IY; Kostryukova, ES; Ilina, EN; Govorun, VM; Zhuravlev, VY; Manicheva, OA; Yablonsky, PK; Isaeva, YD; Nosova, EY; Mokrousov, IV; Vyazovaya, AA; Narvskaya, OV; Lapidus, AL; O’Brien, SJ | 2014 | Genome-wide Mycobacterium tuberculosis variation (GMTV) database: a new tool for integrating sequence variations and epidemiology | Background: Tuberculosis (TB) poses a worldwide threat due to advancing multidrug-resistant strains and deadly co-infections with Human immunodeficiency virus. Today large amounts of Mycobacterium tuberculosis whole genome sequencing data are being assessed broadly and yet there exists no comprehensive online resource that connects M. tuberculosis genome variants with geographic origin, with drug resistance or with clinical outcome. Description: Here we describe a broadly inclusive unifying Genome-wide Mycobacterium tuberculosis Variation (GMTV) database, (http://mtb.dobzhanskycenter. org) that catalogues genome variations of M. tuberculosis strains collected across Russia. GMTV contains a broad spectrum of data derived from different sources and related to M. tuberculosis molecular biology, epidemiology, TB clinical outcome, year and place of isolation, drug resistance profiles and displays the variants across the genome using a dedicated genome browser. GMTV database, which includes 1084 genomes and over 69,000 SNP or Indel variants, can be queried about M. tuberculosis genome variation and putative associations with drug resistance, geographical origin, and clinical stages and outcomes. Conclusions: Implementation of GMTV tracks the pattern of changes of M. tuberculosis strains in different geographical areas, facilitates disease gene discoveries associated with drug resistance or different clinical sequelae, and automates comparative genomic analyses among M. tuberculosis strains. | 2762 | NA | NA | NA | no | no |
| 2763 | 2017 | NA | Latorre-Margalef, N; Tolf, C; Grosbois, V; Avril, A; Bengtsson, D; Wille, M; Osterhaus, ADME; Fouchier, RAM; Olsen, B; Waldenstrom, J | 2014 | Long-term variation in influenza A virus prevalence and subtype diversity in migratory mallards in northern Europe | Data on long-term circulation of pathogens in wildlife populations are seldom collected, and hence understanding of spatial-temporal variation in prevalence and genotypes is limited. Here, we analysed a long-term surveillance series on influenza A virus (IAV) in mallards collected at an important migratory stopover site from 2002 to 2010, and characterized seasonal dynamics in virus prevalence and subtype diversity. Prevalence dynamics were influenced by year, but retained a common pattern for all years whereby prevalence was low in spring and summer, but increased in early autumn with a first peak in August, and a second more pronounced peak during October-November. A total of 74 haemagglutinin (HA)/neuraminidase (NA) combinations were isolated, including all NA and most HA (H1-H12) subtypes. The most common subtype combinations were H4N6, H1N1, H2N3, H5N2, H6N2 and H11N9, and showed a clear linkage between specific HA and NA subtypes. Furthermore, there was a temporal structuring of subtypes within seasons based on HA phylogenetic relatedness. Dissimilar HA subtypes tended to have different temporal occurrence within seasons, where the subtypes that dominated in early autumn were rare in late autumn, and vice versa. This suggests that build-up of herd immunity affected IAV dynamics in this system. | 2763 | NA | NA | NA | no | no |
| 2764 | 2017 | NA | Torres, CW; Tsutsui, ND | 2016 | The Effect of Social Parasitism by Polyergus breviceps on the Nestmate Recognition System of Its Host, Formica altipetens | Highly social ants, bees and wasps employ sophisticated recognition systems to identify colony members and deny foreign individuals access to their nest. For ants, cuticular hydrocarbons serve as the labels used to ascertain nest membership. Social parasites, however, are capable of breaking the recognition code so that they can thrive unopposed within the colonies of their hosts. Here we examine the influence of the socially parasitic slave-making ant, Polyergus breviceps on the nestmate recognition system of its slaves, Formica altipetens. We compared the chemical, genetic, and behavioral characteristics of colonies of enslaved and free-living F. altipetens. We found that enslaved Formica colonies were more genetically and chemically diverse than their free-living counterparts. These differences are likely caused by the hallmark of slave-making ant ecology: seasonal raids in which pupa are stolen from several adjacent host colonies. The different social environments of enslaved and free-living Formica appear to affect their recognition behaviors: enslaved Formica workers were less aggressive towards non-nestmates than were free-living Formica. Our findings indicate that parasitism by P. breviceps dramatically alters both the chemical and genetic context in which their kidnapped hosts develop, leading to changes in how they recognize nestmates. | 2764 | NA | NA | NA | no | no |
| 2765 | 2017 | NA | Barata, C; Baird, DJ; Soares, AMVM | 2001 | Phenotypic plasticity in Daphnia magna Straus: Variable maturation instar as an adaptive response to predation pressure | Life history responses of four Daphnia magna clones at two food levels were studied to assess the importance of maturation instar on the plasticity of fitness responses under simulated mortality regimes. Females of the clones studied could vary offspring size with consequent effects on their maturation time. Significant genetic variability in life history and fitness responses, measured as the intrinsic rate of population increase, within and across food levels was observed, but most of this variation could be attributed to maturation instar differences among clones within and across environments. In the laboratory, without extrinsic mortality, females maturing earlier always had higher fitness than those maturing later, indicating a clear fitness cost of delaying maturity. Nevertheless using a model, we showed that the observed maturation instar effects on life history responses can lead to differences in fitness under different size-selective predation regimes, such that females with delayed maturity have higher fitness under invertebrate predation while females maturing earlier have higher fitness under fish predation regimes. These results suggest that intraclonal variation in offspring size and hence in the number of maturation instars can be an adaptation to living in habitats subject to temporal fluctuations in fish and invertebrate predation pressure. | 2765 | NA | NA | NA | no | no |
| 2766 | 2017 | NA | Shapiro-Ilan, D; Raymond, B | 2016 | Limiting opportunities for cheating stabilizes virulence in insect parasitic nematodes | Cooperative secretion of virulence factors by pathogens can lead to social conflict when cheating mutants exploit collective secretion, but do not contribute to it. If cheats outcompete cooperators within hosts, this can cause loss of virulence. Insect parasitic nematodes are important biocontrol tools that secrete a range of significant virulence factors. Critically, effective nematodes are hard to maintain without live passage, which can lead to virulence attenuation. Using experimental evolution, we tested whether social cheating might explain unstable virulence in the nematode Heterorhabditis floridensis by manipulating relatedness via multiplicity of infection (MOI), and the scale of competition. Passage at high MOI, which should reduce relatedness, led to loss of fitness: virulence and reproductive rate declined together and all eight independent lines suffered premature extinction. As theory predicts, relatedness treatments had more impact under stronger global competition. In contrast, low MOI passage led to more stable virulence and increased reproduction. Moreover, low MOI lineages showed a trade-off between virulence and reproduction, particularly for lines under stronger between-host competition. Overall, this study indicates that evolution of virulence theory is valuable for the culture of biocontrol agents: effective nematodes can be improved and maintained if passage methods mitigate possible social conflicts. | 2766 | NA | NA | NA | no | no |
| 2767 | 2017 | NA | Hartel, S; Wossler, TC; Moltzer, GJ; Crewe, RM; Moritz, RFA; Neumann, P | 2011 | Pheromone-mediated reproductive dominance hierarchies among pseudo-clonal honeybee workers (Apis mellifera capensis) | Honey bee colonies are characterised by well-developed reproductive division of labour between the queen and workers. Here, we test whether this reproductive division of labour is evident in both the socially parasitic workers that invade a colony as well as in their offspring generation. We infected six Apis mellifera scutellata host colonies with pseudo-clonal socially parasitic Cape honeybee workers (Apis mellifera capensis). We show that the first generation of socially parasitic workers can monopolize reproduction within host colonies. Of the initially invading parasites, 94.4% became reproductive pseudoqueens with activated ovaries and produced queen-like pheromones. Their offspring, however, had much lower levels of ovary activation (3.1%), yet 89% showed fatty acid synthesis typical of the queen substance (9-oxo-2(E)-decenoic acid) biochemical pathway. However, in these second-generation workers, the last oxidation step from the precursor (9-hydroxy-2(E)-decenoic acid) to the queen substance was interrupted and appears to be required for reproductive dominance in honeybee workers. Our data show that despite the absence of genetic diversity, residual queen mandibular pheromone (QMP) variation is sufficient to establish reproductive dominance hierarchies among parasitic workers. Consequently, QMP produced by a group of workers can maintain reproductive division of labour in queenless honeybee colonies. | 2767 | NA | NA | NA | no | no |
| 2768 | 2017 | NA | Saw, WY; Liu, XY; Khor, CC; Takeuchi, F; Katsuya, T; Kimura, R; Nabika, T; Ohkubo, T; Tabara, Y; Yamamoto, K; Yokota, M; Teo, YY; Kato, N | 2015 | Mapping the genetic diversity of HLA haplotypes in the Japanese populations | Japan has often been viewed as an Asian country that possesses a genetically homogenous community. The basis for partitioning the country into prefectures has largely been geographical, although cultural and linguistic differences still exist between some of the districts/prefectures, especially between Okinawa and the mainland prefectures. The Major Histocompatibility Complex (MHC) region has consistently emerged as the most polymorphic region in the human genome, harbouring numerous biologically important variants; nevertheless the presence of population-specific long haplotypes hinders the imputation of SNPs and classical HLA alleles. Here, we examined the extent of genetic variation at the MHC between eight Japanese populations sampled from Okinawa, and six other prefectures located in or close to the mainland of Japan, specifically focusing at the haplotypes observed within each population, and what the impact of any variation has on imputation. Our results indicated that Okinawa was genetically farther to the mainland Japanese than were Gujarati Indians from Tamil Indians, while the mainland Japanese from six prefectures were more homogeneous than between northern and southern Han Chinese. The distribution of haplotypes across Japan was similar, although imputation was most accurate for Okinawa and several mainland prefectures when population-specific panels were used as reference. | 2768 | NA | NA | NA | no | no |
| 2769 | 2017 | NA | McMillan, DJ; Bessen, DE; Pinho, M; Ford, C; Hall, GS; Melo-Cristino, J; Ramirez, M | 2010 | Population Genetics of Streptococcus dysgalactiae Subspecies equisimilis Reveals Widely Dispersed Clones and Extensive Recombination | Background: Streptococcus dysgalactiae subspecies equisimilis (SDSE) is an emerging global pathogen that can colonize and infect humans. Although most SDSE isolates possess the Lancefield group G carbohydrate, a significant minority have the group C carbohydrate. Isolates are further sub-typed on the basis of differences within the emm gene. To gain a better understanding of their molecular epidemiology and evolutionary relationships, multilocus sequence typing (MLST) analysis was performed on SDSE isolates collected from Australia, Europe and North America. Methodology/Principal Findings: The 178 SDSE isolates, representing 37 emm types, segregate into 80 distinct sequence types (STs) that form 17 clonal complexes (CCs). Eight STs recovered from all three continents account for >50% of the isolates. Thus, a small number of STs are highly prevalent and have a wide geographic distribution. Both ST and CC strongly correlate with group carbohydrate. In contrast, eleven STs were associated with >1 emm type, suggestive of recombinational replacements involving the emm gene; furthermore, 35% of the emm types are associated with genetically distant STs. Data also reveal a history of extensive inter-and intra-species recombination involving the housekeeping genes used for MLST. Sequence analysis of single locus variants identified through goeBURST indicates that genetic change mediated by recombination occurred similar to 4.4 times more frequently than by point mutation. Conclusions/Significance: A few genetic lineages with an intercontinental distribution dominate among SDSE causing infections in humans. The distinction between group C and G isolates reflects recent evolution, and no long-term genetic isolation between them was found. Lateral gene transfer and recombination involving housekeeping genes and the emm gene are important mechanisms driving genetic variability in the SDSE population. | 2769 | NA | NA | NA | no | no |
| 2770 | 2017 | NA | Garcia, LV; Ramo, C; Aponte, C; Moreno, A; Dominguez, MT; Gomez-Aparicio, L; Redondo, R; Maranon, T | 2011 | Protected wading bird species threaten relict centenarian cork oaks in a Mediterranean Biosphere Reserve: A conservation management conflict | Conservation management conflicts frequently arise when an overpopulation of a protected organism has negative effects on other valuable elements in the same ecosystem. We studied the interactions between a colony of protected tree-nesting wading birds and a remnant population of centenarian cork oaks that was part of the formerly dominant forests in the Donana Biological Reserve (SW Spain). A significant increase in the tree mortality rates has been recorded in areas that are yearly influenced by the bird colony. We analysed a cohort of surviving trees using a gradient of nesting bird influence. Tree-nesting history, bird isotopic signature (delta(15)N), tree health-related parameters (defoliation, delta(11)C and leaf surface coverage by faeces) and several soil variables were evaluated. Bird influence was related to increased soil salinity. This increase correlated to increased water-use efficiency for the leaves and to crown defoliation, suggesting that the heavily occupied trees are under higher stress and in poorer health condition than the unoccupied ones. We tested structural equations models (SEM) that were based on hypothesised bird effects on the health of the trees. Soil-mediated effects of the nesting birds best explained the symptoms of the declining health of the trees, whereas the percent of leaves’ surface that was covered by faeces did not improve the fitted SEM model. For the reserve’s managers, a challenging trade-off exists between preserving the relict trees, which have a high genetic diversity and a key ecological role in these savannah-like ecosystems, and maintaining the current nesting area for these protected, but expanding, wading birds. (C) 2010 Elsevier Ltd. All rights reserved. | 2770 | NA | NA | NA | no | no |
| 2771 | 2017 | NA | Crissman, JR; Booth, W; Santangelo, RG; Mukha, DV; Vargo, EL; Schal, C | 2010 | Population Genetic Structure of the German Cockroach (Blattodea: Blattellidae) in Apartment Buildings | The German cockroach, Blattella germanica (L.) (Blattodea: Blattellidae), is a major residential pest with the potential to vector various pathogens and produce and disseminate household allergens. Understanding population genetic structure and differentiation of this important pest is critical to efforts to eradicate infestations, yet little is known in this regard. Using highly polymorphic microsatellite markers, we investigated patterns of genetic diversity and differentiation within and among 18 apartments from six apartment complexes located in Raleigh, NC. No departure from panmixia was found between rooms within apartments, indicating that active dispersal resulting in gene flow may occur among rooms within apartment units. Alternatively, aggregations within apartments may exist in relative isolation under a metapopulation framework, derived from a recent, common source. Thus, in the event of population control practices leading to incomplete cockroach eradication within an apartment, recolonization of shelters and rooms is likely to occur from a genetically similar aggregation. A pattern of isolation-by-distance across the six apartment complexes indicated that dispersal was more common within complexes than among them, and F statistics suggested greater genetic similarity between apartments in a single building than between separate buildings of an apartment complex. Similarly, neighbor-joining tree and Bayesian clustering analyses were able to cluster only those apartments that were within a single building, indicating higher dispersal with associated gene flow within buildings than between them. The lack of any broader connectivity, as indicated by significant F(ST) and G-tests suggests that human-mediated dispersal of B. germanica between buildings of an apartment complex or between complexes occurs infrequently enough to have negligible effects on gene flow. | 2771 | NA | NA | NA | no | no |
| 2772 | 2017 | NA | Parisod, C; Besnard, G | 2007 | Glacial in situ survival in the Western Alps and polytopic autopolyploidy in Biscutella laevigata L. (Brassicaceae) | Past climatic changes and especially the ice ages have had a great impact on both the distribution and the genetic composition of plant populations, but whether they promoted speciation is still controversial. The autopolyploid complex Biscutella laevigata is a classical example of polyploidy linked to glaciations and is an interesting model to explore migration and speciation driven by climate changes in a complex alpine landscape. Diploid taxa survived the last glacial maximum in several never-glaciated areas and autotetraploids are clearly dominant in the central parts of the Alps; however, previous range-wide studies failed to identify their diploid ancestor(s). This study highlights the phylogeographical relationships of maternal lineages in the Western Alps and investigates the polyploidy process using plastid DNA sequences (trnS-trnG and trnK-intron) combined with plastid DNA length polymorphism markers, which were transferable among Brassicaceae species. Twenty-one distinct plastid DNA haplotypes were distinguished in 67 populations densely sampled in the Western Alps and main lineages were identified by a median-joining network. The external Alps harboured high levels of genetic diversity, while the Central Alps contained only a subset of haplotypes due to postglacial recolonization. Several haplotypes were restricted to local peripheral refugia and evidence of in situ survival in central nunataks was detected by the presence of highly differentiated haplotypes swamped by frequent ones. As hierarchical genetic structure pointed to an independent evolution of the species in different biogeographical districts, and since tetraploids displayed haplotypes belonging to different lineages restricted to either the northern or the southern parts of the Alpine chain, polytopic autopolyploidy was also apparent in the Western Alps. | 2772 | NA | NA | NA | no | no |
| 2773 | 2017 | NA | O’Connell, J; Gurdasani, D; Delaneau, O; Pirastu, N; Ulivi, S; Cocca, M; Traglia, M; Huang, J; Huffman, JE; Rudan, I; McQuillan, R; Fraser, RM; Campbell, H; Polasek, O; Asiki, G; Ekoru, K; Hayward, C; Wright, AF; Vitart, V; Navarro, P; Zagury, JF; Wilson, JF; Toniolo, D; Gasparini, P; Soranzo, N; Sandhu, MS; Marchini, J | 2014 | A General Approach for Haplotype Phasing across the Full Spectrum of Relatedness | Many existing cohorts contain a range of relatedness between genotyped individuals, either by design or by chance. Haplotype estimation in such cohorts is a central step in many downstream analyses. Using genotypes from six cohorts from isolated populations and two cohorts from non-isolated populations, we have investigated the performance of different phasing methods designed for nominally ‘unrelated’ individuals. We find that SHAPEIT2 produces much lower switch error rates in all cohorts compared to other methods, including those designed specifically for isolated populations. In particular, when large amounts of IBD sharing is present, SHAPEIT2 infers close to perfect haplotypes. Based on these results we have developed a general strategy for phasing cohorts with any level of implicit or explicit relatedness between individuals. First SHAPEIT2 is run ignoring all explicit family information. We then apply a novel HMM method (duoHMM) to combine the SHAPEIT2 haplotypes with any family information to infer the inheritance pattern of each meiosis at all sites across each chromosome. This allows the correction of switch errors, detection of recombination events and genotyping errors. We show that the method detects numbers of recombination events that align very well with expectations based on genetic maps, and that it infers far fewer spurious recombination events than Merlin. The method can also detect genotyping errors and infer recombination events in otherwise uninformative families, such as trios and duos. The detected recombination events can be used in association scans for recombination phenotypes. The method provides a simple and unified approach to haplotype estimation, that will be of interest to researchers in the fields of human, animal and plant genetics. | 2773 | NA | NA | NA | no | no |
| 2774 | 2017 | NA | KNULLE, W | 1991 | GENETIC AND ENVIRONMENTAL DETERMINANTS OF HYPOPUS DURATION IN THE STORED-PRODUCT MITE LEPIDOGLYPHUS-DESTRUCTOR | This paper reports a series of experiments over many years on hypopus duration and extends the preceding investigation (1987) on hypopus formation in Lepidoglyphus destructor (Schrank, 1781). The length of time required for hypopus physiogenesis (diapause development) is genetically programmed but influenced by environmental factors. This span of time is highly variable, and may extend from one week to more than a year. Spreading out the potential for hypopus completion over time is adaptive, since a pool of hypopodes with prolonged and staggered dormancies serves to spread the risk of emergence of tritonymphs over extended periods of time; it buffers the population against sudden drought to which all other stages of the life-cycle succumb. The additive structure and large variance of the genetic system underlying the length of time required for hypopus physiogenesis allows for the reconstitution of a broad spectrum of genotypes in every generation through the process of meiotic segregation and recombination during sexual reproduction. It favours stored variability, provides a ‘fail-safe device’ both for survival as well as development in irregularly fluctuating environments, and facilitates the adaptation of populations to local conditions. The trait for hypopus physiogenesis varies independently from that of hypopus formation, and is apparently free to adjust, without genetic constraints, towards an adaptive optimum. The response to selection is fast. Low environmental humidities and high temperatures accelerate physiogenesis of the hypopus. Completion of the hypopus stage and moulting to the tritonymph is triggered by high humidities at moderate temperatures. If environmental conditions preclude moulting, the hypopus following ending of physiogenesis enters a state of quiescence. In contrast the seasonal and largely predictably varying environments, in which essentially anticipatory and season-related token cues like photoperiod regulate the timing of so many arthropod life-cycles, L. destructor copes with sudden and fatal drought, as well as with unheralded and favourable humidities in its ephemeral habitats, mainly by excessive genetic polymorphism in hypopus duration and formation; some genotypes are always instantaneously fit to meet the respective environmental situation. The mite faces gradual food deterioration of its patchily distributed microhabitats by a short-term anticipatory and environmentally cued developmental switch mechanism, which lowers the threshold for hypopus induction. On top of genetic variability and phenotypic plasticity, any genotype x environment interaction provides for increasing flexibility above that from genetic polymorphism and environmental polyphenism alone. This extraordinary measure of adaptedness fits L. destructor for life in irregularly fluctuating environments. | 2774 | NA | NA | NA | no | no |
| 2775 | 2017 | NA | Cobben, MMP; van Noordwijk, AJ | 2016 | Stable partial migration under a genetic threshold model of migratory behaviour | Many species show migratory behaviour in response to seasonal changes in environmental conditions. A peculiar, yet widespread phenomenon is partial migration, when a single population consists of both migratory and non-migratory individuals. There are still many open questions regarding the stability and evolutionary significance of such populations. For passerines the inheritance of migratory activity is best described by the threshold model of quantitative genetics. Such a model has not yet been employed in theoretical studies, in which stability of partially migratory populations is usually linked to group differences in survival or reproduction. Here we develop a parsimonious model featuring a conditional genetic threshold for passerine migratory behaviour under which stable partial migration can be observed, and we explore the resulting selection landscape. Our model results show a cline in migratory behaviour across the landscape, from fully migratory populations to fully residential populations, with a fairly wide zone of partially migratory populations, which is stable in both time and space under a wide range of parameter settings. Temporal stability of the zone is linked with the yearly variance in both migration survival and resident winter survival. In contrast to other theoretical studies, we show that density dependence in winter survival is not essential for observing partially migratory populations. In addition, we observe that selection on the genetic threshold value occurs mainly at the borders of the zone of partial migration. This result suggests that fully migratory and fully residential populations in areas far from the zone of partial migration can harbour genetic diversity that allows the appearance of the alternative phenotype under (a wide range of) different conditions. | 2775 | NA | NA | NA | no | no |
| 2776 | 2017 | NA | Skarin, H; Segerman, B | 2014 | Plasmidome Interchange between Clostridium botulinum, Clostridium novyi and Clostridium haemolyticum Converts Strains of Independent Lineages into Distinctly Different Pathogens | Clostridium botulinum (group III), Clostridium novyi and Clostridium haemolyticum are well-known pathogens causing animal botulism, gas gangrene/black disease, and bacillary hemoglobinuria, respectively. A close genetic relationship exists between the species, which has resulted in the collective term C. novyi sensu lato. The pathogenic traits in these species, e. g., the botulinum neurotoxin and the novyi alpha toxin, are mainly linked to a large plasmidome consisting of plasmids and circular prophages. The plasmidome of C. novyi sensu lato has so far been poorly characterized. In this study we explored the genomic relationship of a wide range of strains of C. novyi sensu lato with a special focus on the dynamics of the plasmidome. Twenty-four genomes were sequenced from strains selected to represent as much as possible the genetic diversity in C. novyi sensu lato. Sixty-one plasmids were identified in these genomes and 28 of them were completed. The genomic comparisons revealed four separate lineages, which did not strictly correlate with the species designations. The plasmids were categorized into 13 different plasmid groups on the basis of their similarity and conservation of plasmid replication or partitioning genes. The plasmid groups, lineages and species were to a large extent entwined because plasmids and toxin genes had moved across the lineage boundaries. This dynamic process appears to be primarily driven by phages. We here present a comprehensive characterization of the complex species group C. novyi sensu lato, explaining the intermixed genetic properties. This study also provides examples how the reorganization of the botulinum toxin and the novyi alpha toxin genes within the plasmidome has affected the pathogenesis of the strains. | 2776 | NA | NA | NA | no | no |
| 2777 | 2017 | NA | Saleh, AA; Ahmed, HU; Todd, TC; Travers, SE; Zeller, KA; Leslie, JF; Garrett, KA | 2010 | Relatedness of Macrophomina phaseolina isolates from tallgrass prairie, maize, soybean and sorghum | Agricultural and wild ecosystems may interact through shared pathogens such as Macrophomina phaseolina, a generalist clonal fungus with more than 284 plant hosts that is likely to become more important under climate change scenarios of increased heat and drought stress. To evaluate the degree of subdivision in populations of M. phaseolina in Kansas agriculture and wildlands, we compared 143 isolates from maize fields adjacent to tallgrass prairie, nearby sorghum fields, widely dispersed soybean fields and isolates from eight plant species in tallgrass prairie. Isolate growth phenotypes were evaluated on a medium containing chlorate. Genetic characteristics were analysed based on amplified fragment length polymorphisms and the sequence of the rDNA-internal transcribed spacer (ITS) region. The average genetic similarity was 58% among isolates in the tallgrass prairie, 71% in the maize fields, 75% in the sorghum fields and 80% in the dispersed soybean fields. The isolates were divided into four clusters: one containing most of the isolates from maize and soybean, two others containing isolates from wild plants and sorghum, and a fourth containing a single isolate recovered from Solidago canadensis in the tallgrass prairie. Most of the sorghum isolates had the dense phenotype on media containing chlorate, while those from other hosts had either feathery or restricted phenotypes. These results suggest that the tallgrass prairie supports a more diverse population of M. phaseolina per area than do any of the crop species. Subpopulations show incomplete specialization by host. These results also suggest that inoculum produced in agriculture may influence tallgrass prairie communities, and conversely that different pathogen subpopulations in tallgrass prairie can interact there to generate ‘hybrids’ with novel genetic profiles and pathogenic capabilities. | 2777 | NA | NA | NA | no | no |
| 2778 | 2017 | NA | Roach, KC; Heitman, J | 2014 | Unisexual Reproduction Reverses Muller’s Ratchet | Cryptococcus neoformans is a pathogenic basidiomycetous fungus that engages in outcrossing, inbreeding, and selfing forms of unisexual reproduction as well as canonical sexual reproduction between opposite mating types. Long thought to be clonal, >99% of sampled environmental and clinical isolates of C. neoformans are MAT alpha, limiting the frequency of opposite mating-type sexual reproduction. Sexual reproduction allows eukaryotic organisms to exchange genetic information and shuffle their genomes to avoid the irreversible accumulation of deleterious changes that occur in asexual populations, known as Muller’s ratchet. We tested whether unisexual reproduction, which dispenses with the requirement for an opposite mating-type partner, is able to purge the genome of deleterious mutations. We report that the unisexual cycle can restore mutant strains of C. neoformans to wild-type genotype and phenotype, including prototrophy and growth rate. Furthermore, the unisexual cycle allows attenuated strains to purge deleterious mutations and produce progeny that are returned to wild-type virulence. Our results show that unisexual populations of C. neoformans are able to avoid Muller’s ratchet and loss of fitness through a unisexual reproduction cycle involving a-a cell fusion, nuclear fusion, and meiosis. Similar types of unisexual reproduction may operate in other pathogenic and saprobic eukaryotic taxa. | 2778 | NA | NA | NA | no | no |
| 2779 | 2017 | NA | Koundal, V; Haq, QMR; Praveen, S | 2011 | Characterization, Genetic Diversity, and Evolutionary Link of Cucumber mosaic virus Strain New Delhi from India | The genome of Cucumber mosaic virus New Delhi strain (CMV-ND) from India, obtained from tomato, was completely sequenced and compared with full genome sequences of 14 known CMV strains from subgroups I and II, for their genetic diversity. Sequence analysis suggests CMV-ND shares maximum sequence identity at the nucleotide level with a CMV strain from Taiwan. Among all 15 strains of CMV, the encoded protein 2b is least conserved, whereas the coat protein (CP) is most conserved. Sequence identity values and phylogram results indicate that CMV-ND belongs to subgroup I. Based on the recombination detection program result, it appears that CMV is prone to recombination, and different RNA components of CMV-ND have evolved differently. Recombinational analysis of all 15 CMV strains detected maximum recombination breakpoints in RNA2; CP showed the least recombination sites. | 2779 | NA | NA | NA | no | no |
| 2780 | 2017 | NA | Bradic, M; Warring, SD; Low, V; Carlton, JM | 2014 | The Tc1/mariner transposable element family shapes genetic variation and gene expression in the protist Trichomonas vaginalis | Background: Trichomonas vaginalis is the most prevalent non-viral sexually transmitted parasite. Although the protist is presumed to reproduce asexually, 60% of its haploid genome contains transposable elements (TEs), known contributors to genome variability. The availability of a draft genome sequence and our collection of >200 global isolates of T. vaginalis facilitate the study and analysis of TE population dynamics and their contribution to genomic variability in this protist. Results: We present here a pilot study of a subset of class II Tc1/mariner TEs that belong to the T. vaginalis Tvmar1 family. We report the genetic structure of 19 Tvmar1 loci, their ability to encode a full-length transposase protein, and their insertion frequencies in 94 global isolates from seven regions of the world. While most of the Tvmar1 elements studied exhibited low insertion frequencies, two of the 19 loci (locus 1 and locus 9) show high insertion frequencies of 1.00 and 0.96, respectively. The genetic structuring of the global populations identified by principal component analysis (PCA) of the Tvmar1 loci is in general agreement with published data based on genotyping, showing that Tvmar1 polymorphisms are a robust indicator of T. vaginalis genetic history. Analysis of expression of 22 genes flanking 13 Tvmar1 loci indicated significantly altered expression of six of the genes next to five Tvmar1 insertions, suggesting that the insertions have functional implications for T. vaginalis gene expression. Conclusions: Our study is the first in T. vaginalis to describe Tvmar1 population dynamics and its contribution to genetic variability of the parasite. We show that a majority of our studied Tvmar1 insertion loci exist at very low frequencies in the global population, and insertions are variable between geographical isolates. In addition, we observe that low frequency insertion is related to reduced or abolished expression of flanking genes. While low insertion frequencies might be expected, we identified two Tvmar1 insertion loci that are fixed across global populations. This observation indicates that Tvmar1 insertion may have differing impacts and fitness costs in the host genome and may play varying roles in the adaptive evolution of T. vaginalis. | 2780 | NA | NA | NA | no | no |
| 2781 | 2017 | NA | MIYAKI, CY; HANOTTE, O; WAJNTAL, A; BURKE, T | 1995 | DNA-FINGERPRINTING IN THE ENDANGERED PARROT - ARATINGA GUAROUBA AND OTHER ARATINGA SPECIES | The destruction of natural habitats and illegal trading are endangering many species of Brazilian parrots. Survival of some species may depend on breeding programmes in captivity. It is therefore important to be able to confirm the identity and parentage of the birds, and to maintain the genetic variability of captive populations. We studied here DNA fingerprints of the endangered Aratinga guarouba and five other species of Aratinga using two human minisatellite multilocus probes (33.6, 33.15) and the restriction enzyme HaeIII. Hybridization with 33.6 produces individual-specific patterns with 18 to 33 bands, depending on the species. The index of similarity obtained between unrelated birds was of the same order in the endangered A. guarouba (0.16) as in other Aratinga species (0.31 to 0.12) and in wild populations of birds reported in the literature. It was possible to perform segregation analysis of the bands only in A. aurea, for which we studied a pedigree including five chicks. There were at least 14 unlinked loci. Also, we assigned the parentage of three A. guarouba from a group of seven potential parents. A novel fragment was present in two siblings and absent from the third, this was interpreted as a gonadal ‘’mutation’’ in one of the parents. Hybridization with 33.15 revealed a low number of bands in all Aratinga species but A. jandaya where 33.2 +/- 2.5 bands were scored. However, in all species the probe 33.15 hybridizes strongly to one or several possibly W-chromosome specific bands. Multilocus fingerprints should allow individual identification, parentage assignment and probably sex determination in the genus Aratinga. Moreover, band sharing indices can be used to prevent consanguineous matings and to maintain the genetic diversity of these species in captivity. | 2781 | NA | NA | NA | no | no |
| 2782 | 2017 | NA | Triponez, Y; Arrigo, N; Espindola, A; Alvarez, N | 2015 | Decoupled post-glacial history in mutualistic plant-insect interactions: insights from the yellow loosestrife (Lysimachia vulgaris) and its associated oil-collecting bees (Macropis europaea and M-fulvipes) | AimWe take a comparative phylogeographical approach to assess whether three species involved in a specialized oil-rewarding pollination system (i.e. Lysimachia vulgaris and two oil-collecting bees within the genus Macropis) show congruent phylogeographical trajectories during post-glacial colonization processes. Our working hypothesis is that within specialized mutualistic interactions, where each species relies on the co-occurrence of the other for survival and/or reproduction, partners are expected to show congruent evolutionary trajectories, because they are likely to have followed parallel migration routes and to have shared glacial refugia. LocationWestern Palaearctic. MethodsOur analysis relies on the extensive sampling of 104 Western Palaearctic populations (totalling 434, 159 and 74 specimens of Lysimachiavulgaris, Macropiseuropaea and Macropisfulvipes, respectively), genotyped with amplified fragment length polymorphism. Based on this, we evaluated the regional genetic diversity (Shannon diversity and allele rarity index) and genetic structure (assessed using structure, population networks, isolation-by-distance and spatial autocorrelation metrics) of each species. Finally, we compared the general phylogeographical patterns obtained. ResultsContrary to our expectations, the analyses revealed phylogeographical signals suggesting that the investigated organisms demonstrate independent post-glacial trajectories as well as distinct contemporaneous demographic parameters, despite their mutualistic interaction. Main conclusionsThe mutualistic partners investigated here are likely to be experiencing distinct and independent evolutionary dynamics because of their contrasting life-history traits (e.g. dispersal abilities), as well as distinct hubs and migration routes. Such conditions would prevent and/or erase any signature of co-structuring of lineages in space and time. As a result, the lack of phylogeographical congruence driven by differences in life-history traits might have arisen irrespective of the three species having shared similar Pleistocene glacial refugia. | 2782 | NA | NA | NA | no | no |
| 2783 | 2017 | NA | Massman, J; Cooper, B; Horsley, R; Neate, S; Dill-Macky, R; Chao, S; Dong, Y; Schwarz, P; Muehlbauer, GJ; Smith, KP | 2011 | Genome-wide association mapping of Fusarium head blight resistance in contemporary barley breeding germplasm | Utilization of quantitative trait loci (QTL) identified in bi-parental mapping populations has had limited success for improving complex quantitative traits with low to moderate heritability. Association mapping in contemporary breeding germplasm may lead to more effective marker strategies for crop improvement. To test this approach, we conducted association mapping of two complex traits with moderate heritability; Fusarium head blight (FHB) severity and the grain concentration of mycotoxin associated with disease, deoxynivalenol (DON). To map FHB resistance in barley, 768 breeding lines were evaluated in 2006 and 2007 in four locations. All lines were genotyped with 1,536 SNP markers and QTL were mapped using a mixed model that accounts for relatedness among lines. Average linkage disequilibrium within the breeding germplasm extended beyond 4 cM. Four QTL were identified for FHB severity and eight QTL were identified for the DON concentration in two independent sets of breeding lines. The QTL effects were small, explaining 1-3% of the phenotypic variation, as might be expected for complex polygenic traits. We show that using breeding germplasm to map QTL can complement bi-parental mapping studies by providing independent validation, mapping QTL with more precision, resolving questions of linkage and pleiotropy, and identifying genetic markers that can be applied immediately in crop improvement. | 2783 | NA | NA | NA | no | no |
| 2784 | 2017 | NA | Banks, SC; Lindenmayer, DB; McBurney, L; Blair, D; Knight, EJ; Blyton, MDJ | 2011 | Kin selection in den sharing develops under limited availability of tree hollows for a forest marsupial | Animal social behaviour is not static with regard to environmental change. Flexibility in cooperative resource use may be an important response to resource decline, mediating the impacts of resource availability on fitness and demography. In forest ecosystems, hollow trees are key den resources for many species, but are declining worldwide owing to forestry. Altered patterns of den sharing may mediate the effects of the decline of this resource. We studied den-sharing interactions among hollow-dependent Australian mountain brushtail possums to investigate how spatial variation in hollow tree availability affects resource sharing and kin selection. Under reduced den availability, individuals used fewer dens and shared them less often. This suggests increased territoriality in the presence of resource competition. Further, there was a switch from kin avoidance to kin preference with decreasing hollow tree availability. This was driven primarily by a change in den sharing among siblings. The inclusive fitness benefits of den sharing with kin are likely to increase under resource-limiting conditions, but are potentially outweighed by the benefits of associating with non-relatives (avoidance of inbreeding or pathogen transmission) where dens are abundant. We discuss how predictions from social evolutionary theory can contribute to understanding animal responses to landscape change. | 2784 | NA | NA | NA | no | no |
| 2785 | 2017 | NA | Grassi, F; Labra, M; Imazio, S; Rubio, RO; Failla, O; Scienza, A; Sala, F | 2006 | Phylogeographical structure and conservation genetics of wild grapevine | The distribution of Vitis vinifera subsp. silvestris, the wild grapevine subspecies of Vitis vinifera L., has been dramatically reduced in its major sites of diffusion, at first by the spread, over the last 150 years, of pathogens from North America and, more recently, with fragmentation of habitat and disbranching by humans. In this work, 418 wild grapevine samples, belonging to 78 populations, were collected in their main Mediterranean distribution areas, including the Caucasus area, and the extent of their genetic variability evaluated by analysing plastid microsatellite DNA polymorphism. Results show low haplotype diversity value, with five haplotypes detected within the analysed populations. The highest within-population haplotypic diversity, with the presence of all five detected haplotypes, was found in the Caucasus regions and in the central regions of Italy. The distribution of all detected haplotypes suggests the Caucasian region as the possible center of origin of Vitis vinifera subsp. silvestris. A principal plastid lineage was found to be fixed in several locations, in the Northernmost European countries and in the Southern island of Sardinia. These results draw attention to two different refugium sites in the Mediterranean basin and suggest that conservation priority should be given to grapevine populations still preserved in hotspots of these regions. | 2785 | NA | NA | NA | no | no |
| 2786 | 2017 | NA | Freed, S; Jin, FL; Ren, SX | 2014 | Intraspecific Variability among the Isolates of Metarhizium anisopliae var. anisopliae by RAPD Markers | Insect fungal pathogens embrace an extraordinary position in microbial pathogenesis. For selection of effective species for the insect management and for considering the factors that alter the natural epizootics, it is vital to understand the phylogenetics of the particular species present in an ecosystem. The genus Metarhizium comprises of identical species which are present in the ecosystem for the management of number of insect pests from different insect orders. For the use of fungi as microbial control agents, knowledge of the genetic stability of the released strain is indispensable. For this purpose DNA polymorphism among 62 isolates of the entomopathogenic fungus Metarhizium anisopliae var. ansiopliae isolated from diverse soil ecosystems was studied by RAPD-PCR. DNA fragments were achieved between 150-1400bp using ten 10-mer PCR primers of random nucleotide sequence. The results showed each isolate differed in the size and number of RAPD products, indicating significant polymorphism. The primer OPA-04 showed imperative results by producing high number of polymorphic bands. The similarity data dendogram clustered the isolates into three main clades on the basis of their geographical origins with a common average similarity coefficient of 45.3%. The analysis of molecular variance results showed that the variation within the population was 14.35%. (C) 2014 Friends Science Publishers | 2786 | NA | NA | NA | no | no |
| 2787 | 2017 | NA | Young, ND; Chan, KG; Korhonen, PK; Chong, TM; Ee, R; Mohandas, N; Koehler, AV; Lim, YL; Hofmann, A; Jex, AR; Qian, BZ; Chilton, NB; Gobert, GN; McManus, DP; Tan, P; Webster, BL; Rollinson, D; Gasser, RB | 2015 | Exploring molecular variation in Schistosoma japonicum in China | Schistosomiasis is a neglected tropical disease that affects more than 200 million people worldwide. The main disease-causing agents, Schistosoma japonicum, S. mansoni and S. haematobium, are blood flukes that have complex life cycles involving a snail intermediate host. In Asia, S. japonicum causes hepatointestinal disease (schistosomiasis japonica) and is challenging to control due to a broad distribution of its snail hosts and range of animal reservoir hosts. In China, extensive efforts have been underway to control this parasite, but genetic variability in S. japonicum populations could represent an obstacle to eliminating schistosomiasis japonica. Although a draft genome sequence is available for S. japonicum, there has been no previous study of molecular variation in this parasite on a genome-wide scale. In this study, we conducted the first deep genomic exploration of seven S. japonicum populations from mainland China, constructed phylogenies using mitochondrial and nuclear genomic data sets, and established considerable variation between some of the populations in genes inferred to be linked to key cellular processes and/or pathogen-host interactions. Based on the findings from this study, we propose that verifying intraspecific conservation in vaccine or drug target candidates is an important first step toward developing effective vaccines and chemotherapies against schistosomiasis. | 2787 | NA | NA | NA | no | no |
| 2788 | 2017 | NA | McCarthy, AJ; Shaw, MA; Goodman, SJ | 2007 | Pathogen evolution and disease emergence in carnivores | Emerging infectious diseases constitute some of the most pressing problems for both human and domestic animal health, and biodiversity conservation. Currently it is not clear whether the removal of past constraints on geographical distribution and transmission possibilities for pathogens alone are sufficient to give rise to novel host-pathogen combinations, or whether pathogen evolution is also generally required for establishment in novel hosts. Canine distemper virus (CDV) is a morbillivirus that is prevalent in the world dog population and poses an important conservation threat to a diverse range of carnivores. We performed an extensive phylogenetic and molecular evolution analysis on complete sequences of all CDV genes to assess the role of selection and recombination in shaping viral genetic diversity and driving the emergence of CDV in non-dog hosts. We tested the specific hypothesis that molecular adaptation at known receptor-binding sites of the haemagglutinin gene is associated with independent instances of the spread of CDV to novel non-dog hosts in the wild. This hypothesis was upheld, providing compelling evidence that repeated evolution at known functional sites (in this case residues 530 and 549 of the haemagglutinin molecule) is associated with multiple independent occurrences of disease emergence in a range of novel host species. | 2788 | NA | NA | NA | no | no |
| 2789 | 2017 | NA | Baum, J; Thomas, AW; Conway, DJ | 2003 | Evidence for diversifying selection on erythrocyte-binding antigens of Plasmodium falciparum and P-vivax | Malaria parasite antigens involved in erythrocyte invasion are primary vaccine candidates. The erythrocyte-binding antigen 175K (EBA-175) of Plasmodium falciparum binds to glycophorin A on the human erythrocyte surface via an N-terminal cysteine-rich region (termed region II) and is a target of antibody responses. A survey of polymorphism in a malaria-endemic population shows that nucleotide alleles in eba-175 region II occur at more intermediate frequencies than expected under neutrality, but polymorphisms in the homologous domains of two closely related genes, eba-140 (encoding a second erythrocyte-binding protein) and psieba-165 (a putative pseudogene), show an opposite trend. McDonald-Kreitman tests employing interspecific comparison with the orthologous genes in P. reichenowi (a closely related parasite of chimpanzees) reveal a significant excess of nonsynonymous polymorphism in P. falciparum eba-175 but not in eba-140. An analysis of the Duffy-binding protein gene, encoding a major erythrocyte-binding antigen in the other common human malaria parasite P. vivax, also reveals a significant excess of nonsynonymous polymorphisms when compared with divergence from its ortholog in P. knowlesi (a closely related parasite of macaques). The results suggest that EBA-175 in P. falciparum and DBP in P. vivax are both under diversifying selection from acquired human immune responses. | 2789 | NA | NA | NA | no | no |
| 2790 | 2017 | NA | Marra, RE; Cortesi, P; Bissegger, M; Milgroom, MG | 2004 | Mixed mating in natural populations of the chestnut blight fungus, Cryphonectria parasitica | As in plants, fungi exhibit wide variation in reproductive strategies and mating systems. Although most sexually reproducing fungi are either predominantly outcrossing or predominantly selfing, there are some notable exceptions. The haploid, ascomycete chestnut blight pathogen, Cryphonectria parasitica, has previously been shown to have a mixed mating system in one population in USA. In this report, we show that both selfing and outcrossing occur in 10 additional populations of C. parasitica sampled from Japan, Italy, Switzerland and USA. Progeny arrays from each population were assayed for segregation at vegetative incompatibility (vic) and DNA fingerprinting loci. Outcrossing rates ((t) over cap (m)) were estimated as the proportion of progeny arrays showing segregation at one or more loci, corrected by the probability of nondetection of outcrossing ((α) over cap). Estimates of (t) over cap (m) varied from 0.74 to 0.97, with the lowest rates consistently detected in USA populations ( 0.74 - 0.78). Five populations ( four in USA and one in Italy) had (t) over cap (m) significantly less than 1, supporting the conclusion that these populations exhibit mixed mating. The underlying causes of variation in outcrossing rates among populations of C. parasitica are not known, but we speculate that - as in plants - outcrossing is a function of ecological, demographic and genetic factors. | 2790 | NA | NA | NA | no | no |
| 2791 | 2017 | NA | Campbell, LG; Husband, BC | 2005 | Impact of clonal growth on effective population size in Hymenoxys herbacea (Asteraceae) | By influencing the proliferation of different genotypes, clonal growth can affect the maintenance of genetic variability and magnitude of genetic drift within plant populations. However, estimates of effective population size rarely incorporate the contribution of both asexual and sexual reproduction. We estimated effective size (N-e) for two populations of the clonal, self-incompatible plant, Hymenoxys herbacea, using a stage-structured demographic model for organisms with asexual and sexual recruitment and then examined the impact of reproductive strategy using an elasticity analysis. Plant rosettes monitored in two successive years had high survival rates in both populations ( mean 0.94). The mean number of sexually derived recruits per initial ramet was 0.041 ( SE 0.039), whereas the mean number of clonal recruits was 0.61 (SE 0.90). Effective size was 1642 and 5769 in the two populations and the N-e/ N ratio averaged 0.34, comparable to values for other clonal species. Elasticity analysis indicated that increases in both clonal and sexual recruitment cause an increase in N-e while increasing the variance reduced N-e. However, N-e was more sensitive to changes in the mean and variance of asexual recruitment than sexual recruitment. These results highlight the importance of considering asexual modes of reproduction when examining the role of genetic stochasticity in populations. | 2791 | NA | NA | NA | no | no |
| 2792 | 2017 | NA | Browning, SR; Browning, BL | 2013 | Identity-by-descent-based heritability analysis in the Northern Finland Birth Cohort | For most complex traits, only a small proportion of heritability is explained by statistically significant associations from genome-wide association studies (GWAS). In order to determine how much heritability can potentially be explained through larger GWAS, several different approaches for estimating total narrow-sense heritability from GWAS data have recently been proposed. These methods include variance components with relatedness estimates from allele-sharing, variance components with relatedness estimates from identity-by-descent (IBD) segments, and regression of phenotypic correlation on relatedness estimates from IBD segments. These methods have not previously been compared on real or simulated data. We analyze the narrow-sense heritability of nine metabolic traits in the Northern Finland Birth Cohort (NFBC) using these methods. We find substantial estimated heritability for several traits, including LDL cholesterol (54 % heritability), HDL cholesterol (46 % heritability), and fasting glucose levels (39 % heritability). Estimates of heritability from the regression-based approach are much lower than variance component estimates in these data, which may be due to the presence of strong population structure. We also investigate the accuracy of the competing approaches using simulated phenotypes based on genotype data from the NFBC. The simulation results substantiate the downward bias of the regression-based approach in the presence of population structure. | 2792 | NA | NA | NA | no | no |
| 2793 | 2017 | NA | Wen, ZX; Tan, RJ; Yuan, JZ; Bales, C; Du, WY; Zhang, SC; Chilvers, MI; Schmidt, C; Song, QJ; Cregan, PB; Wang, DC | 2014 | Genome-wide association mapping of quantitative resistance to sudden death syndrome in soybean | Background: Sudden death syndrome (SDS) is a serious threat to soybean production that can be managed with host plant resistance. To dissect the genetic architecture of quantitative resistance to the disease in soybean, two independent association panels of elite soybean cultivars, consisting of 392 and 300 unique accessions, respectively, were evaluated for SDS resistance in multiple environments and years. The two association panels were genotyped with 52,041 and 5,361 single nucleotide polymorphisms (SNPs), respectively. Genome-wide association mapping was carried out using a mixed linear model that accounted for population structure and cryptic relatedness. Result: A total of 20 loci underlying SDS resistance were identified in the two independent studies, including 7 loci localized in previously mapped QTL intervals and 13 novel loci. One strong peak of association on chromosome 18, associated with all disease assessment criteria across the two panels, spanned a physical region of 1.2 Mb around a previously cloned SDS resistance gene (GmRLK18-1) in locus Rfs2. An additional variant independently associated with SDS resistance was also found in this genomic region. Other peaks were within, or close to, sequences annotated as homologous to genes previously shown to be involved in plant disease resistance. The identified loci explained an average of 54.5% of the phenotypic variance measured by different disease assessment criteria. Conclusions: This study identified multiple novel loci and refined the map locations of known loci related to SDS resistance. These insights into the genetic basis of SDS resistance can now be used to further enhance durable resistance to SDS in soybean. Additionally, the associations identified here provide a basis for further efforts to pinpoint causal variants and to clarify how the implicated genes affect SDS resistance in soybean. | 2793 | NA | NA | NA | no | no |
| 2794 | 2017 | NA | Corzett, CH; Goodman, MF; Finkel, SE | 2013 | Competitive Fitness During Feast and Famine: How SOS DNA Polymerases Influence Physiology and Evolution in Escherichia coli | Escherichia coli DNA polymerases (Pol) II, IV, and V serve dual roles by facilitating efficient translesion DNA synthesis while simultaneously introducing genetic variation that can promote adaptive evolution. Here we show that these alternative polymerases are induced as cells transition from exponential to long-term stationary-phase growth in the absence of induction of the SOS regulon by external agents that damage DNA. By monitoring the relative fitness of isogenic mutant strains expressing only one alternative polymerase over time, spanning hours to weeks, we establish distinct growth phase-dependent hierarchies of polymerase mutant strain competitiveness. Pol II confers a significant physiological advantage by facilitating efficient replication and creating genetic diversity during periods of rapid growth. Pol IV and Pol V make the largest contributions to evolutionary fitness during long-term stationary phase. Consistent with their roles providing both a physiological and an adaptive advantage during stationary phase, the expression patterns of all three SOS polymerases change during the transition from log phase to long-term stationary phase. Compared to the alternative polymerases, Pol III transcription dominates during mid-exponential phase; however, its abundance decreases to <20% during long-term stationary phase. Pol IV transcription dominates as cells transition out of exponential phase into stationary phase and a burst of Pol V transcription is observed as cells transition from death phase to long-term stationary phase. These changes in alternative DNA polymerase transcription occur in the absence of SOS induction by exogenous agents and indicate that cell populations require appropriate expression of all three alternative DNA polymerases during exponential, stationary, and long-term stationary phases to attain optimal fitness and undergo adaptive evolution. | 2794 | NA | NA | NA | no | no |
| 2795 | 2017 | NA | Hamilton, R; Boots, M; Paterson, S | 2005 | The effect of host heterogeneity and parasite intragenornic interactions on parasite population structure | Understanding the processes that shape the genetic structure of parasite populations and the functional consequences of different parasite genotypes is critical for our ability to predict how an infection can spread through a host population and for the design of effective vaccines to combat infection and disease. Here, we examine how the genetic structure of parasite populations responds to host genetic heterogeneity. We consider the well-characterized molecular specificity of major histocompatibility complex binding of antigenic peptides to derive deterministic and stochastic models. We use these models to ask, firstly, what conditions favour the evolution of generalist parasite genotypes versus specialist parasite genotypes? Secondly, can parasite genotypes coexist in a population? We find that intragenomic interactions between parasite loci encoding antigenic peptides are pivotal in determining the outcome of evolution. Where parasite loci interact synergistically (i.e. the recognition of additional antigenic peptides has a disproportionately large effect on parasite fitness), generalist parasite genotypes are favoured. Where parasite loci act multiplicatively (have independent effects on fitness) or antagonistically (have diminishing effects on parasite fitness), specialist parasite genotypes are favoured. A key finding is that polymorphism is not stable and that, with respect to functionally important antigenic peptides, parasite populations are dominated by a single genotype. | 2795 | NA | NA | NA | no | no |
| 2796 | 2017 | NA | Yeh, CF; Liu, TP; Cheng, CW; Chang, SC; Lee, MH; Lu, JJ | 2015 | Molecular Characteristics of Disease-Causing and Commensal Staphylococcus lugdunensis Isolates from 2003 to 2013 at a Tertiary Hospital in Taiwan | Objectives Staphylococcus lugdunensis can cause community-and healthcare-associated infections. This study investigated the molecular characteristics of S. lugdunensis isolates collected at our hospital and compared the characteristics of the infectious and commensal isolates. Methods We collected the S. lugdunensis isolates between 2003 and 2013. The antimicrobial resistance test, SCCmec typing, accessory gene regulator (agr) typing, pulsed-field gel electrophoresis (PFGE), and delta-like hemolysin activity were performed. Results In total, 118 S. lugdunensis isolates were collected, of which 67 (56.8%) were classified into the infection group and 51 (43.2%) into the commensal group. The oxacillin resistance rate was 36.4%. The most common SCCmec types were SCCmec types V (51.4%) and II (32.6%). In total, 34 pulsotypes were identified. The PFGE typing revealed five clones (pulsotypes A, J, M, N, and P) at our hospital. Pulsotypes A and N caused the spread of high oxacillin resistance. In total, 10.2% (12 of 118) of the isolates lacked d-like hemolysin activity. Compared with the infection group, the commensal group showed a higher percentage of multiple drug resistance and carried a higher percentage of SCCmec type II (11 of 22, 50% and 3 of 21, 14.3%) and a lower percentage of SCCmec type V (8 of 22, 36.4% and 14 of 21, 66.7%). The commensal group (27 PFGE types) showed higher genetic diversity than did the infection group (20 PFGE types). No difference was observed in the distribution of the five main pulsotypes, agr typing, and the presence of delta-like hemolysin activity between the two groups. Conclusions Five main clones were identified at our hospital. The commensal group showed higher genetic diversity, had a higher percentage of multidrug resistance, and carried a higher percentage of SCCmec type II and a lower percentage of SCCmec type V than did the infection group. | 2796 | NA | NA | NA | no | no |
| 2797 | 2017 | NA | Segurel, L; Gao, ZY; Przeworski, M | 2013 | Ancestry runs deeper than blood: The evolutionary history of ABO points to cryptic variation of functional importance | The ABO histo-blood group, first discovered over a century ago, is found not only in humans but also in many other primate species, with the same genetic variants maintained for at least 20 million years. Polymorphisms in ABO have been associated with susceptibility to a large number of human diseases, from gastric cancers to immune or artery diseases, but the adaptive phenotypes to which the polymorphism contributes remain unclear. We suggest that variation in ABO has been maintained by frequency-dependent or fluctuating selection pressures, potentially arising from co-evolution with gut pathogens. We further hypothesize that the histo-blood group labels A, B, AB, and O do not offer a full description of variants maintained by natural selection, implying that there are unrecognized, functionally important, antigens beyond the ABO group in humans and other primates. | 2797 | NA | NA | NA | no | no |
| 2798 | 2017 | NA | Brodrick, HJ; Raven, KE; Harrison, EM; Blane, B; Reuter, S; Torok, ME; Parkhill, J; Peacock, SJ | 2016 | Whole-genome sequencing reveals transmission of vancomycin-resistant Enterococcus faecium in a healthcare network | Background: Bacterial whole-genome sequencing (WGS) has the potential to identify reservoirs of multidrug-resistant organisms and transmission of these pathogens across healthcare networks. We used WGS to define transmission of vancomycin-resistant enterococci (VRE) within a long-term care facility (LTCF), and between this and an acute hospital in the United Kingdom (UK). Methods: A longitudinal prospective observational study of faecal VRE carriage was conducted in a LTCF in Cambridge, UK. Stool samples were collected at recruitment, and then repeatedly until the end of the study period, discharge or death. Selective culture media were used to isolate VRE, which were subsequently sequenced and analysed. We also analysed the genomes of 45 Enterococcus faecium bloodstream isolates collected at Cambridge University Hospitals NHS Foundation Trust (CUH). Results: Forty-five residents were recruited during a 6-month period in 2014, and 693 stools were collected at a frequency of at least 1 week apart. Fifty-one stool samples from 3/45 participants (7 %) were positive for vancomycin-resistant E. faecium. Two residents carried multiple VRE lineages, and one carried a single VRE lineage. Genome analyses based on single nucleotide polymorphisms (SNPs) in the core genome indicated that VRE carried by each of the three residents were unrelated. Participants had extensive contact with the local healthcare network. We found that VRE genomes from LTCF residents and hospital-associated bloodstream infection were interspersed throughout the phylogenetic tree, with several instances of closely related VRE strains from the two settings. Conclusions: A proportion of LTCF residents are long-term carriers of VRE. Evidence for genetic relatedness between these and VRE associated with bloodstream infection in a nearby acute NHS Trust indicate a shared bacterial population. | 2798 | NA | NA | NA | no | no |
| 2799 | 2017 | NA | Mendes, C; Felix, R; Sousa, AM; Lamego, J; Charlwood, D; do Rosario, VE; Pinto, J; Silveira, H | 2010 | Molecular evolution of the three short PGRPs of the malaria vectors Anopheles gambiae and Anopheles arabiensis in East Africa | Background: Immune responses to parasites, which start with pathogen recognition, play a decisive role in the control of the infection in mosquitoes. Peptidoglycan recognition proteins (PGRPs) are an important family of pattern recognition receptors that are involved in the activation of these immune reactions. Pathogen pressure can exert adaptive changes in host genes that are crucial components of the vector’s defence. The aim of this study was to determine the molecular evolution of the three short PGRPs (PGRP-S1, PGRP-S2 and PGRP-S3) in the two main African malaria vectors -Anopheles gambiae and Anopheles arabiensis. Results: Genetic diversity of An. gambiae and An. arabiensis PGRP-S1, PGRP-S2 and PGRP-S3 was investigated in samples collected from Mozambique and Tanzania. PGRP-S1 diversity was lower than for PGRP-S2 and PGRP-S3. PGRP-S1 was the only gene differentiated between the two species. All the comparisons made for PGRP-S1 showed significant P-values for Fst estimates and AMOVA confirming a clear separation between species. For PGRP-S2 and PGRP-S3 genes it was not possible to group populations either by species or by geographic region. Phylogenetic networks reinforced the results obtained by the AMOVA and Fst values. The ratio of nonsynonymous substitutions (Ka)/synonymous substitutions (Ks) for the duplicate pair PGRP-S2 and PGRP-S3 was very similar and lower than 1. The 3D model of the different proteins coded by these genes showed that amino acid substitutions were concentrated at the periphery of the protein rather than at the peptidoglycan recognition site. Conclusions: PGRP-S1 is less diverse and showed higher divergence between An. gambiae and An. arabiensis regardless of geographic location. This probably relates to its location in the chromosome-X, while PGRP-S2 and PGRP-S3, located in chromosome-2L, showed signs of autosomal introgression. The two short PGRP genes located in the chromosome-2L were under purifying selection, which suggests functional constraints. Different types of selection acting on PGRP-S1 and PGRP-S2 and S3 might be related to their different function and catalytic activity. | 2799 | NA | NA | NA | no | no |
| 2800 | 2017 | NA | Luduena, LM; Taurian, T; Tonelli, ML; Angelini, JG; Anzuay, MS; Valetti, L; Munoz, V; Fabra, AI | 2012 | Biocontrol bacterial communities associated with diseased peanut (Arachis hypogaea L.) plants | In the present study, the isolation and characterization of endophytic and epiphytic biocontrol bacteria from peanut plants affected with white mold and root rot are described. Two hundred and sixty three isolates were selected from diseased plants and biocontrol traits analyzed were antibiosis against Sclerotinia minor and Fusarium solani, siderophore production and similarities in carbon source utilization between bacteria and fungi. Diversity and sequence analysis of potential biocontrol bacteria were developed. It was observed that 34% of the 263 isolates were able to inhibit the growth of at least one of the two fungi tested and 47% showed siderophore production in CAS medium. Analysis of nutritional similarity index demonstrated that both fungi were capable to growth in all carbon compounds assayed and that 20% of bacteria showed NOI values equal or above 0.9. Genetic diversity analysis by BOX-PCR indicated that culturable epiphytic and endophytic bacteria associated with peanut are highly diverse. The 16S rDNA sequences of the most remarkable isolates, indicated as potential biocontrol agents according to all the screenings performed and belonging to different BOX profiles showed that they were 99% identical to Bacillus amyloliquefaciens. Comparisons analysis was performed with results previously obtained from a similar study done in the bacterial community isolated from healthy peanut plants from the same producing area. Results obtained suggest that presence of fungal pathogens in peanut ecosystem would be acting as a selective factor in the peanut plant associated bacterial communities. It was concluded from this study that peanut soils in Cordoba harbor bacteria with major biocontrol properties which represent a potential source of new strains that could be used as biological inoculants in agriculture. Published by Elsevier Masson SAS. | 2800 | NA | NA | NA | no | no |
| 2801 | 2017 | NA | Rasic, G; Filipovic, I; Weeks, AR; Hoffmann, AA | 2014 | Genome-wide SNPs lead to strong signals of geographic structure and relatedness patterns in the major arbovirus vector, Aedes aegypti | Background: Genetic markers are widely used to understand the biology and population dynamics of disease vectors, but often markers are limited in the resolution they provide. In particular, the delineation of population structure, fine scale movement and patterns of relatedness are often obscured unless numerous markers are available. To address this issue in the major arbovirus vector, the yellow fever mosquito (Aedes aegypti), we used double digest Restriction-site Associated DNA (ddRAD) sequencing for the discovery of genome-wide single nucleotide polymorphisms (SNPs). We aimed to characterize the new SNP set and to test the resolution against previously described microsatellite markers in detecting broad and fine-scale genetic patterns in Ae. aegypti. Results: We developed bioinformatics tools that support the customization of restriction enzyme-based protocols for SNP discovery. We showed that our approach for RAD library construction achieves unbiased genome representation that reflects true evolutionary processes. In Ae. aegypti samples from three continents we identified more than 18,000 putative SNPs. They were widely distributed across the three Ae. aegypti chromosomes, with 47.9% found in intergenic regions and 17.8% in exons of over 2,300 genes. Pattern of their imputed effects in ORFs and UTRs were consistent with those found in a recent transcriptome study. We demonstrated that individual mosquitoes from Indonesia, Australia, Vietnam and Brazil can be assigned with a very high degree of confidence to their region of origin using a large SNP panel. We also showed that familial relatedness of samples from a 0.4 km(2) area could be confidently established with a subset of SNPs. Conclusions: Using a cost-effective customized RAD sequencing approach supported by our bioinformatics tools, we characterized over 18,000 SNPs in field samples of the dengue fever mosquito Ae. aegypti. The variants were annotated and positioned onto the three Ae. aegypti chromosomes. The new SNP set provided much greater resolution in detecting population structure and estimating fine-scale relatedness than a set of polymorphic microsatellites. RAD-based markers demonstrate great potential to advance our understanding of mosquito population processes, critical for implementing new control measures against this major disease vector. | 2801 | NA | NA | NA | no | no |
| 2802 | 2017 | NA | Bidegaray-Batista, L; Sanchez-Gracia, A; Santulli, G; Maiorano, L; Guisan, A; Vogler, AP; Arnedo, MA | 2016 | Imprints of multiple glacial refugia in the Pyrenees revealed by phylogeography and palaeodistribution modelling of an endemic spider | Mediterranean mountain ranges harbour highly endemic biota in islandlike habitats. Their topographic diversity offered the opportunity for mountain species to persist in refugial areas during episodes of major climatic change. We investigate the role of Quaternary climatic oscillations in shaping the demographic history and distribution ranges in the spider Harpactocrates ravastellus, endemic to the Pyrenees. Gene trees and multispecies coalescent analyses on mitochondrial and nuclear DNA sequences unveiled two distinct lineages with a hybrid zone around the northwestern area of the Catalan Pyrenees. The lineages were further supported by morphological differences. Climatic niche-based species distribution models (SDMs) identified two lowland refugia at the western and eastern extremes of the mountain range, which would suggest secondary contact following postglacial expansion of populations from both refugia. Neutrality test and approximate Bayesian computation (ABC) analyses indicated that several local populations underwent severe bottlenecks followed by population expansions, which in combination with the deep population differentiation provided evidence for population survival during glacial periods in microrefugia across the mountain range, in addition to the main Atlantic and Mediterranean (western and eastern) refugia. This study sheds light on the complexities of Quaternary climatic oscillations in building up genetic diversity and local endemicity in the southern Europe mountain ranges. | 2802 | NA | NA | NA | no | no |
| 2803 | 2017 | NA | Fones, HN; Mardon, C; Gurr, SJ | 2016 | A role for the asexual spores in infection of Fraxinus excelsior by the ash-dieback fungus Hymenoscyphus fraxineus | The invasive pathogen, ash dieback fungus Hymenoscyphus fraxineus, is spreading rapidly across Europe. It shows high levels of outcrossing and limited population structure, even at the epidemic front. The anamorphic (asexual) form produces prolific conidia, thought to function solely as spermatia (male gametes), facilitating gene flow between sympatric strains. Here, we show that conidia are capable of germination on ash leaves and in vitro, and can infect seedlings via leaves or soil. In leaves, germlings form structures resembling fruiting bodies. Additionally, H. fraxineus colonises ash debris and grows in soil in the absence of ash tissues. We propose an amended life-cycle in which wind-dispersed, insect-vectored or water-spread conidia infect ash and may sporulate in planta, as well as in forest debris. This amplifies inoculum levels of different strains in ash stands. In combination with their function as spermatia, conidia thus act to maximise gene flow between sympatric strains, including those originally present at low inoculum. Such mixing increases evolutionary potential, as well as enhancing the likelihood of gene introgression from closely-related strains or assimilation of further genetic diversity from parental Asian populations. This scenario increases the adaptability of H. fraxineus to new climates and, indeed, onto new host species. | 2803 | NA | NA | NA | no | no |
| 2804 | 2017 | NA | Schonswetter, P; Tribsch, A; Niklfeld, H | 2004 | Amplified fragment length polymorphism (AFLP) suggests old and recent immigration into the Alps by the arctic-alpine annual Comastoma tenellum (Gentianaceae) | Aim This study aims to elucidate the phylogeography of the arctic-alpine annual Comastoma tenellum (Rottb.) Toyok. (Gentianaceae) and to unravel the history of its immigration into the Alps. Location Although samples from Alaska and Central Asia were also included, our study focusses on Europe, especially on the Alps. Methods We applied amplified fragment length polymorphism (AFLP) fingerprinting on 37 populations (162 individuals) of C. tenellum and analysed the results phenetically. Results As C. tenellum is mainly inbreeding, there is typically little to no intrapopulational genetic variation. Two populations from Alaska and Altai are strongly separated from all other accessions. The majority of the populations from the Alps group together with high bootstrap support. They fall into an unsupported Alps I group (northwards of Gran Paradiso) and a well-supported Alps II group (south-western Alps). The remaining European populations form a weakly-supported branch constituting accessions from the Carpathians, Scandinavia and two populations from the Eastern Alps. Main conclusions Comastoma tenellum reached the Alps at least twice. The first immigration event resulted in a lineage that is clearly separated from the other European accessions. The immigration must have occurred well before the last glaciation because this lineage shows further phylogeographical structuring into two groups (Alps II in the south-western Alps and Alps I in the rest of the Alps). This pattern is presumably due to isolation in different glacial refugia. In addition to the old immigration event, the species reached the Alps in recent times either from Scandinavia or from the Carpathians via long-distance dispersal. These immigrations resulted in (at least) two populations that are spatially small and poor in individuals. | 2804 | NA | NA | NA | no | no |
| 2805 | 2017 | NA | Woo, C; Kim, J; Park, K; Oh, Y; Dirisala, V; Xiao, R; Kim, N; Cho, I; Park, C | 2007 | Characterization of DQB1 and DRB1 in Korean native pigs and the identification of three new DQB1 alleles | There has been no prior report on allelic variations of SLA class II loci in Korean native pigs. Here, we analyzed two important SLA class II genes, DQB1 and DRB1, in Korean native pigs (n = 65). DQB1 and DRB1 were PCR amplified from genomic DNA by using locus-specific oligonucleotides, and six alleles were identified for each locus. In DQB1, all six alleles were identical to those previously reported. However, we identified three new alleles in DRB1. The level of heterozygosity for DQB1 and DRB1 was 49 and 34%, respectively. Phylogenetic analyses showed that alleles from Korean native pigs were clustered in five distinct groups for DQB1 and four for DRB1. Finally, we defined 13 DQB1-DRB1 haplotypes by combining genotyping results and pedigree information. These results provide information on the genetic diversity of Korean native pigs and SLA systems. | 2805 | NA | NA | NA | no | no |
| 2806 | 2017 | NA | Gotoh, A; Billen, J; Tsuji, K; Sasaki, T; Ito, F | 2012 | Histological study of the spermatheca in three thelytokous parthenogenetic ant species, Pristomyrmex punctatus, Pyramica membranifera and Monomorium triviale (Hymenoptera: Formicidae) | The evolution of obligate parthenogenesis may induce the degeneration of female mating ability and subsequently affect themorphology of the female reproductive organs related tomating and/ or spermstorage. Here, we investigated the size and structure of the sperm storage organ, the spermatheca, in three thelytokous parthenogenetic myrmicine ant species, Pristomyrmex punctatus, Pyramica membranifera and Monomorium triviale, and compared it with that of their related sexually reproducing species. So far, mated individuals have never been found in these three species, which appears to be in line with their parthenogenetic status. Although the spermatheca appears to be useless in these species, we could not find any evidence on the degeneration in size and morphology of their spermathecae. The spermathecal reservoir still has the columnar hilar epithelium, which is one of the major features for a functional spermatheca in ants. | 2806 | NA | NA | NA | no | no |
| 2807 | 2017 | NA | Agostinho, AA; Pelicice, FM; Gomes, LC; Julio, HF | 2010 | Reservoir Fish Stocking: When One Plus One May Be Less Than Two | Fisheries management in Brazilian reservoirs is based (since the 1970’s) on stocking and construction of fish passes. Low landings of the fisheries and the precarious conservation status of native populations in the Upper Parana River basin indicate how useless these practices were. Failures in most stocking programs conducted may be explained by the negligence of basic assumptions for implementation (clear goals, scientific foundation and evaluation of results). In spite of the common sense support, decision makers should consider that, for any management actions involving biomanipulation, there are relevant environmental risks related to the origin and selection of broodstock and production of fries, and to the releasing of reared fish. Among the latter should be mentioned introduction of associated non-native species (pathogens and parasites), genetic degradation of native stocks (bottleneck effects, loss of genetic variability and fitness, domestication), imbalances and changes in community structure. For an environmental friendly and economical and societal desirable stocking, the decision process should consider information on the receptor ecosystem, target species, uses and users of the resource, legislation and risks for biodiversity conservation. Therefore, the first aspect to be considered is the need for stocking and identification of environmental constrains to it. The ability to produce fish with genetic quality equivalent to native stock and with unaltered ability to spawn in nature (the main challenges in the stocking process) should also have decisive roles in determining whether a stocking program should be implemented. Size, quantity, season and site of releasing should be based on the life cycle, distribution and structure of natural populations, whereas evaluation and monitoring should be considered as integral and indissoluble parts of the stocking process. Habitat management and fishery control should be considered as alternatives or complements. Impoundments are sources of impacts on biodiversity and the success of stocking in such environments appears temporary. Ideally, the success should be quantified by the ability of stocked fish to reproduce in nature and to contribute to the genetic variability of the population. For ethical conservation reasons stocking cannot be only evaluated through fishery landings. | 2807 | NA | NA | NA | no | no |
| 2808 | 2017 | NA | Laetsch, DR; Heitlinger, EG; Taraschewski, H; Nadler, SA; Blaxter, ML | 2012 | The phylogenetics of Anguillicolidae (Nematoda: Anguillicoloidea), swimbladder parasites of eels | Background: Anguillicolidae Yamaguti, 1935 is a family of parasitic nematode infecting fresh-water eels of the genus Anguilla, comprising five species in the genera Anguillicola and Anguillicoloides. Anguillicoloides crassus is of particular importance, as it has recently spread from its endemic range in the Eastern Pacific to Europe and North America, where it poses a significant threat to new, naive hosts such as the economic important eel species Anguilla anguilla and Anguilla rostrata. The Anguillicolidae are therefore all potentially invasive taxa, but the relationships of the described species remain unclear. Anguillicolidae is part of Spirurina, a diverse clade made up of only animal parasites, but placement of the family within Spirurina is based on limited data. Results: We generated an extensive DNA sequence dataset from three loci (the 5’ one-third of the nuclear small subunit ribosomal RNA, the D2-D3 region of the nuclear large subunit ribosomal RNA and the 5’ half of the mitochondrial cytochrome c oxidase I gene) for the five species of Anguillicolidae and used this to investigate specific and generic boundaries within the family, and the relationship of Anguillicolidae to other spirurine nematodes. Neither nuclear nor mitochondrial sequences supported monophyly of Anguillicoloides. Genetic diversity within the African species Anguillicoloides papernai was suggestive of cryptic taxa, as was the finding of distinct lineages of Anguillicoloides novaezelandiae in New Zealand and Tasmania. Phylogenetic analysis of the Spirurina grouped the Anguillicolidae together with members of the Gnathostomatidae and Seuratidae. Conclusions: The Anguillicolidae is part of a complex radiation of parasitic nematodes of vertebrates with wide host diversity (chondrichthyes, teleosts, squamates and mammals), most closely related to other marine vertebrate parasites that also have complex life cycles. Molecular analyses do not support the recent division of Anguillicolidae into two genera. The described species may hide cryptic taxa, identified here by DNA taxonomy, and this DNA barcoding approach may assist in tracking species invasions. The propensity for host switching, and thus the potential for invasive behaviour, is found in A. crassus, A. novaezelandiae and A. papernai, and thus may be common to the group. | 2808 | NA | NA | NA | no | no |
| 2809 | 2017 | NA | Alpay, F; Zare, Y; Kamalludin, MH; Huang, XX; Shi, XW; Shook, GE; Collins, MT; Kirkpatrick, BW | 2014 | Genome-Wide Association Study of Susceptibility to Infection by Mycobacterium avium Subspecies paratuberculosis in Holstein Cattle | Paratuberculosis, or Johne’s disease, is a chronic, granulomatous, gastrointestinal tract disease of cattle and other ruminants caused by the bacterium Mycobacterium avium, subspecies paratuberculosis (MAP). Control of Johne’s disease is based on programs of testing and culling animals positive for infection with MAP while concurrently modifying management to reduce the likelihood of infection. The current study is motivated by the hypothesis that genetic variation in host susceptibility to MAP infection can be dissected and quantifiable associations with genetic markers identified. For this purpose, a case-control, genome-wide association study was conducted using US Holstein cattle phenotyped for MAP infection using a serum ELISA and/or fecal culture test. Cases included cows positive for either serum ELISA, fecal culture or both. Controls consisted of animals negative for the serum ELISA test or both serum ELISA and fecal culture when both were available. Controls were matched by herd and proximal birth date with cases. A total of 856 cows (451 cases and 405 controls) were used in initial discovery analyses, and an additional 263 cows (159 cases and 104 controls) from the same herds were used as a validation data set. Data were analyzed in a single marker analysis controlling for relatedness of individuals (GRAMMAR-GC) and also in a Bayesian analysis in which multiple marker effects were estimated simultaneously (GenSel). For the latter, effects of non-overlapping 1 Mb marker windows across the genome were estimated. Results from the two discovery analyses were generally concordant; however, discovery results were generally not well supported in analysis of the validation data set. A combined analysis of discovery and validation data sets provided strongest support for SNPs and 1 Mb windows on chromosomes 1, 2, 6, 7, 17 and 29. | 2809 | NA | NA | NA | no | no |
| 2810 | 2017 | NA | Balestri, E; Lardicci, C | 2012 | Nursery-propagated plants from seed: a novel tool to improve the effectiveness and sustainability of seagrass restoration | Seagrasses and the valuable ecosystem services they provide are threatened world-wide by impacts of human activity. Numerous revegetation efforts have attempted to restore seagrasses. Most restoration programmes use plants collected from the field because of limited seed availability, low seedling survival and difficulty in culturing plants. However, this practice risks damage to donor populations and has the potential to reduce genetic diversity, which may counteract the desired effects of restoration. A novel aquaculture system for producing plants (mother plants and cuttings) from a limited number of seeds was tested using Cymodocea nodosa as model species. The ability of transplanted cuttings to survive and grow in the natural habitat was also evaluated. Seed germination was high (48%) compared with field conditions, and most seedlings produced mother plants with up to 7.8 m of rhizome and 300 shoots within 4 years in culture. All cuttings from mother plants regenerated new plants. Up to 100 transplants were produced from two seeds, and most (85%) of them survived and initiated the colonization of substrate, 1 year after planting. Synthesis and applications. This study provides a robust protocol that can reduce plant and/or seed collection pressure on donor populations and produce a high number of transplants which show lower mortality rates during the early transplantation phases. This method can also help to preserve genetic diversity in restored populations, which should be one of the major goals of ecological restoration. This novel tool can be applied to other seagrass species with low or unpredictable reproductive success, therefore the development of nurseries should be incorporated in future restoration programmes. This is currently the only sustainable methodology to produce material for transplanting programmes for protected species. | 2810 | NA | NA | NA | no | no |
| 2811 | 2017 | NA | Van der Bank, HF; Greenfield, R; Daru, BH; Yessoufou, K | 2012 | DNA BARCODING REVEALS MICRO-EVOLUTIONARY CHANGES AND RIVER SYSTEM-LEVEL PHYLOGEOGRAPHIC RESOLUTION OF AFRICAN SILVER CATFISH, SCHILBE INTERMEDIUS (ACTINOPTERYGII: SILURIFORMES: SCHILBEIDAE) FROM SEVEN POPULATIONS ACROSS DIFFERENT AFRICAN RIVER SYSTEMS | Background. Under the tropics, less than 40% of known fishes are identified to species-level. Further, the ongoing global change poses unprecedented threat to biodiversity, and several taxa are likely to go extinct even before they could be described. Traditional ecological theory suggests that species would escape extinction risk posed by global threats (e. g., climate change) only by migrating to new environments. In this study, we hypothesise that micro-evolutionary changes (evolution within species and populations) are also important mechanisms for the survival of Schilbe intermedius in Africa, a continent subjected to uneven distribution of climate severity. Materials and methods. Using the mitochondrial cytochrome c oxidase subunit I (COI) gene, known as animal DNA barcode, we tested this hypothesis by analysing the genetic diversity and phylogenetic relations between seven populations of S. intermedius across different African river systems. Results. We reveal a clear geographical patterning in genetic variations, with three clear clusters (southern Africa, eastern Africa, and western Africa). In southern Africa, the South African population is distinct from that of Namibia and Botswana. In addition, within Nigerian populations of silver catfish, two sub-clusters emerged from two isolated river systems. We suggest that the phylogeographic pattern within African silver catfish populations mirror the past effects of selection and gene flow, and that the split within Nigerian silver catfish populations might be the result of micro-evolutionary adaptive responses to local selection pressures. Conclusion. We suggest that the strong genetic difference in African silver catfish among geographically isolated river systems might be the result of in situ micro-evolutionary adaptive responses to changing environments, and that DNA barcode has potential beyond species delimitation. | 2811 | NA | NA | NA | no | no |
| 2812 | 2017 | NA | Arundell, K; Dunn, A; Alexander, J; Shearman, R; Archer, N; Ironside, JE | 2015 | Enemy release and genetic founder effects in invasive killer shrimp populations of Great Britain | “The predatory”“killer shrimp”" Dikerogammarus villosus invaded Britain from mainland Europe in 2010. Originating in the Ponto-Caspian region, this invader has caused significant degradation of European freshwater ecosystems by predating and competitively excluding native invertebrate species. In contrast to continental Europe, in which invasions occurred through the migration of large numbers of individuals along rivers and canals, the invasion of Great Britain must have involved long distance dispersal across the sea. This makes the loss of genetic diversity and of debilitating parasites more likely. Analysis of nuclear microsatellite loci and mitochondrial DNA sequences of D. villosus samples from the four known populations in Britain reveal loss of rare alleles, in comparison to reference populations from the west coast of continental Europe. Screening of the British D. villosus populations by PCR detected no microsporidian parasites, in contrast with continental populations of D. villosus and native amphipod populations, most of which are infected with microsporidia. These findings suggest that the initial colonisation of Great Britain and subsequent long distance dispersal within Britain were associated with genetic founder effects and enemy release due to loss of parasites. Such effects are also likely to occur during future long-distance dispersal events of D. villosus to Ireland or North America." | 2812 | NA | NA | NA | no | no |
| 2813 | 2017 | NA | Nolet, BA; Baveco, JM | 1996 | Development and viability of a translocated beaver Castor fiber population in the Netherlands | We monitored survival, reproduction and emigration of a translocated beaver Castor fiber population in the Netherlands for five years and used a stochastic model to assess its viability. Between 1988 and 1991, 42 beavers were released in the Biesbosch National Park. The mortality was initially high but gradually fell to normal rates. However, the breeding success was low, and we hypothesized that this was either a temporary phenomenon (the translocation hypothesis) or a permanent feature (the poor habitat hypothesis). According to the computer simulations, the isolated population was viable under the first but not under the second hypothesis. In the latter case, the prospects generally improved by the foundation of another population in the Gelderse Poort (100 km from the Biesbosch), However, this second habitat should be optimal for beavers in order to reduce the extinction probability of the Biesbosch population to below 10% in 100 years,’ the loss of genetic variability (1-2% per generation) was just above the applied tolerable risk (1%), but the effects of inbreeding are unknown in beavers. We conclude that the beaver population in the Biesbosch is not viable unless the reproductive success increases, either in the Biesbosch itself or in a nearby population. We recommend applying such viability analyses to evaluate the likely success of any translocation. | 2813 | NA | NA | NA | no | no |
| 2814 | 2017 | NA | Pettay, JE; Lahdenpera, M; Rotkirch, A; Lummaa, V | 2016 | Costly reproductive competition between co-resident females in humans | Studying the evolution of cooperative breeding and group living requires simultaneous quantification of both helping benefits and competitive costs within groups. Although such research has traditionally focused on the fitness benefits of helping behavior, increasing evidence now highlights reproductive competition in cooperatively breeding animals including humans. Human groups consist of cooperative individuals of varying relatedness, predicted to lead to conflict when resources are limited and relatedness low. However, few studies exist that determine the costs of co-breeding to both parties sharing resources. Here, we studied female reproductive competition in historical Finnish joint-families where brothers stayed on their natal farms and sisters married out, so that several unrelated women of reproductive age co-resided in the same households. Using detailed parish registers we quantified the effects of simultaneous reproduction of these women on their offspring mortality. We found that the risk for offspring mortality before adulthood was increased by 23% if co-resident women reproduced within 2 years of each other, a risk that was not associated with the overall numbers of co-resident reproductive-aged women or children. Such costly competition may have promoted the evolution of birth scheduling, dispersal patterns and life-history traits including menopause that avoid resource competition with other reproductive females. | 2814 | NA | NA | NA | no | no |
| 2815 | 2017 | NA | Caron-Lormier, G; Masson, JP; Menard, N; Pierre, JS | 2006 | A branching process, its application in biology: Influence of demographic parameters on the social structure in mammal groups | Branching processes are widely used in biology. This theoretical tool is used in cell dynamics, epidemics and population dynamics. In population dynamics, branching processes are mainly used to access extinction probabilities of populations, groups or families, with the Galton-Watson branching process. Many mammal species live in socially-structured groups, and the smallest units of these groups are lineages (or families) of kin-related individuals. In many primate species, these lineages are matrilines, as females remain in their natal groups most of the time, whereas males generally disperse. Lineage parameters, such as numbers of matrilines, size of each matriline and average degree of relatedness, could strongly influence the genetic composition of groups. Evidence indicates that division along matrilines could induce substantial differentiation among fission groups. Here, we develop a novel mathematical model based on the branching process theory describing demographic dynamics of groups. The main result of this model is an explicit analytical expression of the joint distribution of numbers of lineages and sizes of socially-structured groups. We investigated the influence of parameters such as natality and mortality on the outcome of the process, including extinction probability. Finally, we discuss this theoretical result with respect to biological significance. (C) 2005 Elsevier Ltd. All rights reserved. | 2815 | NA | NA | NA | no | no |
| 2816 | 2017 | NA | Danet, JL; Bonnet, P; Jarausch, W; Carraro, L; Skoric, D; Labonne, G; Foissac, X | 2007 | Imp and secY, two new markers for MLST (multilocus sequence typing) in the 16SrX phytoplasma taxonomic group | To investigate the genetic variability of fruit tree phytoplasmas belonging to the 16SrX ribosomal group, we used a multilocus sequence typing strategy (MLST). Sequences of four non-ribosomal genetic loci (aceF, pnp, imp and secY) were determined among a collection of ‘Candidatus Phytoplasma prunorum’, ‘Ca. P. mali’ and ‘Ca. P. pyri’ isolates. Sequences alignment and phylogenetic analyses confirmed the classification based on 16S rDNA phylogeny. The four genetic loci displayed specific signatures clearly correlated with the recent definition of the corresponding ‘Candidatus species’. However, a total congruence was not observed inside ‘Ca. species’, between phylogenetic trees constructed with the different loci. A divergent ‘Ca. P. prunorum’ variant from Azerbaijan, recently characterized through aceF sequencing, presented a divergent imp gene. The previously characterized ‘Ca. P. prunorum’ hypovirulent strains had the same aceF sequence but were discriminated with the imp marker. ‘Ca. P. pyri ’strains detected in Lebanese pear decline isolates also form a distinct cluster according to aceF and imp markers. Imp and secY phylogenetic analyses pointed out a new ’Ca. P. prunorum’ cluster comprising strains never detected in psyllid vectors. ‘Ca. P. prunorum’ strains were distributed among all phylogenetic group identified. For ‘Ca. P.mali’, discrimination of apple proliferation isolates was in agreement with previous studies. | 2816 | NA | NA | NA | no | no |
| 2817 | 2017 | NA | Lu, L; Lycett, SJ; Brown, AJL | 2014 | Reassortment patterns of avian influenza virus internal segments among different subtypes | Background: The segmented RNA genome of avian Influenza viruses (AIV) allows genetic reassortment between co-infecting viruses, providing an evolutionary pathway to generate genetic innovation. The genetic diversity (16 haemagglutinin and 9 neuraminidase subtypes) of AIV indicates an extensive reservoir of influenza viruses exists in bird populations, but how frequently subtypes reassort with each other is still unknown. Here we quantify the reassortment patterns among subtypes in the Eurasian avian viral pool by reconstructing the ancestral states of the subtypes as discrete states on time-scaled phylogenies with respect to the internal protein coding segments. We further analyzed how host species, the inferred evolutionary rates and the d(N)/d(S) ratio varied among segments and between discrete subtypes, and whether these factors may be associated with inter-subtype reassortment rate. Results: The general patterns of reassortment are similar among five internal segments with the exception of segment 8, encoding the Non-Structural genes, which has a more divergent phylogeny. However, significant variation in rates between subtypes was observed. In particular, hemagglutinin-encoding segments of subtypes H5 to H9 reassort at a lower rate compared to those of H1 to H4, and Neuraminidase-encoding segments of subtypes N1 and N2 reassort less frequently than N3 to N9. Both host species and d(N)/d(S) ratio were significantly associated with reassortment rate, while evolutionary rate was not associated. The d(N)/d(S) ratio was negatively correlated with reassortment rate, as was the number of negatively selected sites for all segments. Conclusions: These results indicate that overall selective constraint and host species are both associated with reassortment rate. These results together identify the wild bird population as the major source of new reassortants, rather than domestic poultry. The lower reassortment rates observed for H5N1 and H9N2 may be explained by the large proportion of strains derived from domestic poultry populations. In contrast, the higher rates observed in the H1N1, H3N8 and H4N6 subtypes could be due to their primary origin as infections of wild birds with multiple low pathogenicity strains in the large avian reservoir. | 2817 | NA | NA | NA | no | no |
| 2818 | 2017 | NA | Cheng, YY; Belov, K | 2012 | Isolation and characterisation of 11 MHC-linked microsatellite loci in the Tasmanian devil (Sarcophilus harrisii) | The Tasmanian devil is under threat of extinction due to a deadly contagious cancer, devil facial tumour disease (DFTD). DFTD tumour cells can overcome histocompatibility barriers and be transferred between unrelated individuals as allografts. The low level of genetic variability at major histocompatibility complex (MHC) loci is believed to have contributed to the high transmissibility of DFTD across the devil population. Copy number variations and high sequence similarity between MHC genes in the devil has made MHC typing by sequencing laborious and problematic. A more efficient approach is required in order to facilitate large-scale MHC typing. Here we report the development of a suite of polymorphic microsatellite markers that are located close to devil MHC Class I or II genes. Two to six alleles were identified at each locus, with the expected heterozygosity ranging from 0.142 to 0.696. | 2818 | NA | NA | NA | no | no |
| 2819 | 2017 | NA | Sandlund, OT; Gjelland, KO; Bohn, T; Knudsen, R; Amundsen, PA | 2013 | Contrasting Population and Life History Responses of a Young Morph-Pair of European Whitefish to the Invasion of a Specialised Coregonid Competitor, Vendace | Invasions of non-native species represent a global problem of great scientific interest. Here we study in detail the response in population and life history characteristics of closely related native species, with divergent habitat preferences, that are impacted by an invading species over a sufficient time period to allow a new stable state to become established. A time series of 20 years starting at the first occurrence of the invader (vendace Coregonus albula (L.)) allows exploration of the long term population and life history response of two ecologically, morphologically, and genetically different native sympatric morphs (DR- and SR-) of congeneric whitefish C. lavaretus (L.). The whitefish morphs are taxonomically equally related to the invading vendace, but only the planktivorous DR-whitefish share its pelagic niche. We would expect that the ecological differences between the whitefish morphs may be used as a predictor of competitive effects. Vendace exhibited an initial boom-and-bust development, and has continued to fluctuate in density. The responses of the pelagic DR- whitefish were: i) an immediate habitat shift, ii) a subsequent population decline caused by increased annual mortality, and iii) a new stable state at a lower density and apparently relaxed competition. The ecologically more distant benthivorous SR-whitefish also showed significant, but a much more limited response during this process, indicating damped indirect interactions through the food-web. This long-term case-study found that in two native eco-species equally related to the invader, only one of the eco-species was highly affected. Direct competition for resources is obviously important for species interactions, whereas the taxonomic relatedness per se seems to offer little predictive power for invasion effects. | 2819 | NA | NA | NA | no | no |
| 2820 | 2017 | NA | Nguyen, DT; Spooner-Hart, RN; Riegler, M | 2015 | Polyploidy versus endosymbionts in obligately thelytokous thrips | Background: Thelytoky, the parthenogenetic development of females, has independently evolved in several insect orders yet the study of its mechanisms has so far mostly focussed on haplodiploid Hymenoptera, while alternative mechanisms of thelytoky such as polyploidy are far less understood. In haplodiploid insects, thelytoky can be encoded in their genomes, or induced by maternally inherited bacteria such as Wolbachia or Cardinium. Microbially facilitated thelytoky usually results in complete homozygosity due to gamete duplication and can be reverted into arrhenotoky, the parthenogenetic development of males, through treatment with antibiotics. In contrast, genetically encoded thelytoky cannot be removed and may result in conservation of heterozygosity due to gamete fusion. We have probed the obligate thelytoky of the greenhouse thrips, Heliothrips haemorrhoidalis (Bouche), a significant cosmopolitan pest and a model species of thelytoky in the haplodiploid insect order Thysanoptera. Earlier studies suggested terminal fusion as a mechanism for thelytoky in this species, while another study reported presence of Wolbachia; later it was speculated that Wolbachia plays a role in this thrips’ thelytokous reproduction. Results: By using PCR and sequence analysis, we demonstrated that global population samples of H. haemorrhoidalis were not infected with Wolbachia, Cardinium or any other known bacterial reproductive manipulators. Antibiotic treatment of this thrips did also not result in male production. Some individuals carried two different alleles in two nuclear loci, histone 3 and elongation factor 1 alpha, suggesting heterozygosity. However, the majority of individuals had three different alleles suggesting that they were polyploid. Genetic diversity across both nuclear loci was low in all populations, and absent from mitochondrial cytochrome oxidase I, indicating that this species had experienced genetic bottlenecks, perhaps due to its invasion biology or a switch to thelytoky. Conclusions: Geographically broad sampling and experimental manipulation revealed low genetic diversity, absence of Wolbachia but presence of three different alleles of nuclear loci in most analysed individuals of obligately thelytokous H. haemorrhoidalis. This suggests that polyploidy may be involved in the thelytokous reproduction of this thrips species, and polyploidy may be a contributing factor in the reproduction of Thysanoptera and other haplodiploid insect orders. | 2820 | NA | NA | NA | no | no |
| 2821 | 2017 | NA | Sharma, S; Upadhyaya, HD | 2016 | Interspecific hybridization to introduce useful genetic variability for pigeonpea improvement | Pigeonpea (Cajanus cajan (L.) Millspaugh) is an important grain legume grown in tropical and subtropical regions of the world. Narrow genetic base coupled with low levels of resistance against important biotic/abiotic stresses in cultivated pigeonpea is the major constraint affecting its production and productivity globally. Wild Cajanus species are the reservoir of many important genes including resistance/tolerance to diseases, insect-pests and drought, and good agronomic traits and can be utilized to improve the crop cultivars, enrich variability, and broaden the genetic base. Utilization of wild Cajanus species has contributed for the development of cytoplasmic male sterility (CMS) systems for pigeonpea improvement. Pre-breeding populations involving promising wild Cajanus accessions belonging to secondary and tertiary gene pools as donors and popular pigeonpea cultivars as recipients were developed for enriching variability for pigeonpea improvement. Considerable variability was observed in these populations for morpho-agronomic traits and for biotic stresses. Two advanced backcross populations derived from wild Cajanus species are being genotyped to identify QTLs associated with agronomic traits for further deployment in pigeonpea improvement programs. Agronomically desirable and disease resistant introgression lines have been identified and shared with breeding programs for developing new high-yielding and climate resilient pigeonpea cultivars with a broad genetic base. | 2821 | NA | NA | NA | no | no |
| 2822 | 2017 | NA | Lombaert, E; Carletto, J; Piotte, C; Fauvergue, X; Lecoq, H; Vanlerberghe-Masutti, F; Lapchin, L | 2009 | Response of the melon aphid, Aphis gossypii, to host-plant resistance: evidence for high adaptive potential despite low genetic variability | In agrosystems, pests are submitted to strong human-imposed selective pressures to which they sometimes adapt rapidly, either through selection of genotypes resulting from mutation and/or recombination events, or through phenotypic plasticity. Understanding how insects respond to such selective pressures is of great importance for sustainable pest management strategies, such as the use of resistant plants. In this study, we investigated the genetic and phenotypic variability of anholocyclic Aphis gossypii Glover (Hemiptera: Aphididae) strains, in response to the resistance gene Vat that is present in melon crops. Forty-nine aphid colonies were sampled on several melon crops in southern France, genotyped using 15 microsatellite loci, and tested in phenotypic experiments using Vat or non-Vat melons. The level of genetic polymorphism between these colonies was low, as only seven multilocus genotypes were detected. In contrast, the phenotypic variability for life-history and behavioral traits between colonies, including those sharing the same genotype, was unexpectedly high, with a continuum of response to the Vat gene from complete susceptibility to strong virulence. The low genetic polymorphism associated with a strong phenotypic variability highlights the high adaptive potential of A. gossypii and the major role of environmental cues in shaping phenotypic responses of this aphid to pest management strategies. | 2822 | NA | NA | NA | no | no |
| 2823 | 2017 | NA | Benard, MF; Maher, JM | 2011 | Consequences of intraspecific niche variation: phenotypic similarity increases competition among recently metamorphosed frogs | “Phenotype is often correlated with resource use, which suggests that as phenotypic variation in a population increases, intraspecific competition will decrease. However, few studies have experimentally tested the prediction that increased intraspecific phenotypic variation leads to reduced competitive effects (e.g., on growth rate, survival or reproductive rate). We investigated this prediction with two experiments on wood frogs (Rana sylvatica). In the first experiment, we found that a frog’s size was positively correlated with the size of its preferred prey, indicating that the feeding niche of the frogs changed with size. In the second experiment, we used an experimental design in which we held the initial mass of”“focal”" frogs constant, but varied the initial mass of their competitors. We found a significant quadratic effect of the average mass of competitors: focal frog growth was lowest when raised with similar-sized competitors, and highest when raised with competitors that were larger or smaller. Our results demonstrate that growth rates increase (i.e., competitive intensity decreases) when individuals are less similar to other members of the population and exhibit less overlap in resource use. Thus, changes in the amount of phenotypic variation in a population may ultimately affect population-level processes, such as population growth rate and extinction risk." | 2823 | NA | NA | NA | no | no |
| 2824 | 2017 | NA | Messina, FJ; Johnson, D | 2014 | Specificity of adaptation to a novel host plant by a seed beetle | Adaptation to one novel host plant may simultaneously improve an insect’s performance on other unfamiliar hosts, as a kind of cross-adaptation. In selection experiments using an Asian population of the seed beetle Callosobruchus maculatus (Fabricius) (Coleoptera: Chrysomelidae: Bruchinae), larval survival in lentil seeds increased from about 1 to >90% in fewer than 30 generations, and acceptance of lentil by egg-laying females increased two- to three-fold. We examined whether rapid adaptation to lentil altered beetle performance on other grain legumes, including hosts more closely related to lentil than to the ancestral host, mung bean. Three replicate, lentil-adapted lines were compared to the mung-bean (control) line with respect to both host acceptance (oviposition under no-choice conditions) and larval performance (survival to adult emergence, development time, and adult mass at emergence). In most experiments, females from the lentil lines laid more eggs on unfamiliar hosts than did females from the mung-bean line. Greater oviposition on novel hosts could not be explained by variation in potential fecundity, and did not appear to depend on a host’s relatedness to lentil. Although survival in lentil remained extremely divergent between the mung-bean and lentil lines (0 vs. >90%), the lines did not differ in larval performance on two other novel hosts (pea and fava bean) that are much more closely related to lentil than to mung bean. Because larval performance is most likely the limiting factor in the potential colonization of a novel host by C.maculatus, our experiments did not provide strong evidence for cross-adaptation. The results suggest that adaptation to even a highly marginal host need not lead to a general expansion of an insect’s host range. | 2824 | NA | NA | NA | no | no |
| 2825 | 2017 | NA | Wu, FN; Cen, YJ; Wallis, CM; Trumble, JT; Prager, S; Yokomi, R; Zheng, Z; Deng, XL; Chen, JC; Liang, GW | 2016 | The Complete Mitochondrial Genome Sequence of Bactericera cockerelli and Comparison with Three Other Psylloidea Species | “Potato psyllid (Bactericera cockerelli) is an important pest of potato, tomato and pepper. Not only could a toxin secreted by nymphs results in serious phytotoxemia in some host plants, but also over the past few years B. cockerelli was shown to transmit”“Candidatus Liberibacter solanacearum”", the putative bacterial pathogen of potato zebra chip (ZC) disease, to potato and tomato. ZC has caused devastating losses to potato production in the western U.S., Mexico, and elsewhere. New knowledge of the genetic diversity of the B. cockerelli is needed to develop improved strategies to manage pest populations. Mitochondrial genome (mitogenome) sequencing provides important knowledge about insect evolution and diversity in and among populations. This report provides the first complete B. cockerelli mitogenome sequence as determined by next generation sequencing technology (Illumina MiSeq). The circular B. cockerelli mitogenome had a size of 15,220 bp with 13 protein-coding gene (PCGs), 2 ribosomal RNA genes (rRNAs), 22 transfer RNA genes (tRNAs), and a non-coding region of 975 bp. The overall gene order of the B. cockerelli mitogenome is identical to three other published Psylloidea mitogenomes: one species from the Triozidae, Paratrioza sinica | 2825 | NA | NA | NA | no | no |
| 2826 | 2017 | NA | Xiao, P; Yang, HX; Di, DD; Piao, DR; Zhang, QX; Hao, R; Yao, SX; Zhao, R; Zhang, FF; Tian, GZ; Zhao, HY; Fan, WX; Cui, BY; Jiang, H | 2015 | Genotyping of Human Brucella melitensis Biovar 3 Isolated from Shanxi Province in China by MLVA16 and HOOF | Background Brucellosis presents a significant economic burden for China because it causes reproductive failure in host species and chronic health problems in humans. These problems can involve multiple organs. Brucellosis is highly endemic in Shanxi Province China. Molecular typing would be very useful to epidemiological surveillance. The purpose of this study was to assess the diversity of Brucella melitensis strains for epidemiological surveillance. Historical monitoring data suggest that Brucella melitensis biovar 3 is the predominant strain associated with the epidemic of brucellosis in Shanxi Province. Methods/Principal Findings Multiple-locus variable-number repeat analysis (MLVA-16) and hypervariable octameric oligonucleotide fingerprinting (HOOF-print) were used to type a human-hosted Brucella melitensis population (81 strains). Sixty-two MLVA genotypes (discriminatory index: 0.99) were detected, and they had a genetic similarity coefficient ranging from 84.9% to 100%. Eighty strains of the population belonged to the eastern Mediterranean group with panel 1 genotypes 42 (79 strains) and 43 (1 strain). A new panel 1 genotype was found in this study. It was named 114 MLVAorsay genotype and it showed similarity to the two isolates from Guangdong in a previous study. Brucella melitensis is distributed throughout Shanxi Province, and like samples from Inner Mongolia, the eastern Mediterranean genotype 42 was the main epidemic strain (97%). The HOOF-printing showed a higher diversity than MLVA-16 with a genetic similarity coefficient ranging from 56.8% to 100%. Conclusions According to the MLVA-16 and HOOF-printing results, both methods could be used for the epidemiological surveillance of brucellosis. A new genotype was found in both Shanxi and Guangdong Provinces. In areas with brucellosis, the MLVA-16 scheme is very important for tracing cases back to their origins during outbreak investigations. It may facilitate the expansion and eradication of the disease. | 2826 | NA | NA | NA | no | no |
| 2827 | 2017 | NA | Tomasevic, N; Cvetkovic, D; Miaud, C; Aleksic, I; Crnobrnja-Isailovic, J | 2008 | INTERANNUAL VARIATION IN LIFE HISTORY TRAITS BETWEEN NEIGHBOURING POPULATIONS OF THE WIDESPREAD AMPHIBIAN BUFO BUFO | Life-history variation within and among populations of widespread amphibian species has attracted much attention so far. In relation to a variety of problems (e.g. climate change, large-scale clines. conservation). this information is needed not only on broad geographic scale, but also at local level and front year to year. In this Study. we investigated variation in body size and life history traits, including age, fecundity and egg size, in two neighbouring populations of a widespread amphibian, the Common Toad Bufo bufa. during a 3-year period. Body size varied significantly between populations in males only but not between years. Age was assessed by skeletochronology, and osteometrical analyses revealed a complete endosteal resorption of the first line of arrested growth in 1.1 % of the individuals and a partial resorption in 77 % of tire individuals. Age structures did not differ significantly between sexes, populations, nor between years. Age at first reproduction was one year later in females than in males in both populations (4 vs 3 years), but adult survival rates did riot differ between sexes. Fecundity did not differ between Populations or years, but egg size varied between years. Fecundity increased with size, but not with age of females. This lack of significant interpopulation variation in age structure and reproductive traits is important for monitoring local populations which call be exposed to various potential disturbance factors. On the other hand, these results stress the importance of analysing temporal variation in life history traits because one-year data may not represent properly the reproductive features of a Population. and it, is issue is becoming more so important with the growing interest in climate changes and its possible effects (in amphibian Populations. | 2827 | NA | NA | NA | no | no |
| 2828 | 2017 | NA | Mitchell, RLC; Vidaki, K; Lavidor, M | 2016 | The role of left and right dorsolateral prefrontal cortex in semantic processing: A transcranial direct current stimulation study | For complex linguistic strings such as idioms, making a decision as to the correct meaning may require complex top-down cognitive control such as the suppression of incorrect alternative meanings. In the study presented here, we used transcranial direct current stimulation to test the hypothesis that a domain general dorsolateral prefrontal cognitive control network is involved in constraining the complex processing involved. Specifically, we sought to test prominent theoretical stances on the division of labour across dorsolateral prefrontal cortex in the left- and right-hemispheres of the brain, including the role of salience and fine vs. coarse semantic coding. 32 healthy young adult participants were randomly allocated to one of two stimulation montage groups (LH anodal/RH cathodal or RH anodal/LH cathodal). Participants were tested twice, completing a semantic decision task after either receiving active or sham stimulation. The semantic decision task required participants to judge the relatedness of an idiom and a target word. The target word was figuratively related, literally related, or unrelated to the idiom. Control non-literal non-idiomatic sentences were also included that only had a literal meaning. The results showed that left-hemisphere dorsolateral prefrontal cortex is highly involved in processing figurative language, whereas both left- and right-dorsolateral prefrontal cortex contributed to literal language processing. In comparison, semantic processing for the non-idiomatic control sentences did not require domain general cognitive control as it relates to suppression of the rejected alternative meaning. The results are discussed in terms of the interplay between need for domain general cognitive control in understanding the meaning of complex sentences, hemispheric differences in semantic processing, and salience detection. (C) 2016 Elsevier Ltd. All rights reserved. | 2828 | NA | NA | NA | no | no |
| 2829 | 2017 | NA | De Bruyn, A; Harimalala, M; Zinga, I; Mabvakure, BM; Hoareau, M; Ravigne, V; Walters, M; Reynaud, B; Varsani, A; Harkins, GW; Martin, DP; Lett, JM; Lefeuvre, P | 2016 | Divergent evolutionary and epidemiological dynamics of cassava mosaic geminiviruses in Madagascar | Background: Cassava mosaic disease (CMD) in Madagascar is caused by a complex of at least six African cassava mosaic geminivirus (CMG) species. This provides a rare opportunity for a comparative study of the evolutionary and epidemiological dynamics of distinct pathogenic crop-infecting viral species that coexist within the same environment. The genetic and spatial structure of CMG populations in Madagascar was studied and Bayesian phylogeographic modelling was applied to infer the origins of Madagascan CMG populations within the epidemiological context of related populations situated on mainland Africa and other south western Indian Ocean (SWIO) islands. Results: The isolation and analysis of 279 DNA-A and 117 DNA-B sequences revealed the presence in Madagascar of four prevalent CMG species (South African cassava mosaic virus, SACMV; African cassava mosaic virus, ACMV; East African cassava mosaic Kenya virus, EACMKV; and East African cassava mosaic Cameroon virus, EACMCV), and of numerous CMG recombinants that have, to date, only ever been detected on this island. SACMV and ACMV, the two most prevalent viruses, displayed low degrees of genetic diversity and have most likely been introduced to the island only once. By contrast, EACMV-like CMG populations (consisting of East African cassava mosaic virus, EAMCKV, EACMCV and complex recombinants of these) were more diverse, more spatially structured, and displayed evidence of at least three independent introductions from mainland Africa. Although there were no statistically supported virus movement events between Madagascar and the other SWIO islands, at least one mainland African ACMV variant likely originated in Madagascar. Conclusions: Our study highlights both the complexity of CMD in Madagascar, and the distinct evolutionary and spatial dynamics of the different viral species that collectively are associated with this disease. Given that more distinct CMG species and recombinants have been found in Madagascar than any other similarly sized region of the world, the risks of recombinant CMG variants emerging on this island are likely to be higher than elsewhere. Evidence of an epidemiological link between Madagascan and mainland African CMGs suggests that the consequences of such emergence events could reach far beyond the shores of this island. | 2829 | NA | NA | NA | no | no |
| 2830 | 2017 | NA | Rau, D; Rodriguez, M; Murgia, ML; Balmas, V; Bitocchi, E; Bellucci, E; Nanni, L; Attene, G; Papa, R | 2015 | Co-evolution in a landrace metapopulation: two closely related pathogens interacting with the same host can lead to different adaptive outcomes | We examined the local adaptation patterns in a system comprising several interconnected heterogeneous plant populations from which populations of two phylogenetically closely related pathogens were also sampled. The host is Hordeum vulgare (cultivated barley); the pathogens are Pyrenophora teres f. teres (net form) and Pyrenophora teres f. maculata (spot form), the causal agents of barley net blotch. We integrated two approaches, the comparison between the population structures of the host and the pathogens, and a cross-inoculation test. We demonstrated that two closely related pathogens with very similar niche specialisation and life-styles can give rise to different co-evolutionary outcomes on the same host. Indeed, we detected local adaptation for the net form of the pathogen but not for the spot form. We also provided evidence that an a-priori well-known resistance quantitative-trait-locus on barley chromosome 6H is involved in the coevolutionary ‘arms race’ between the plant and the net-form pathogen. Moreover, data suggested latitudinal clines of host resistance and that different ecological conditions can result in differential selective pressures at different sites. Our data are of interest for on-farm conservation of plant genetic resources, as also in establishing efficient breeding programs and strategies for deployment of resistance genes of P. teres. | 2830 | NA | NA | NA | no | no |
| 2831 | 2017 | NA | Arens, P; Coops, H; Jansen, J; Vosman, B | 1998 | Molecular genetic analysis of black poplar (Populus nigra L.) along Dutch rivers | The genetic structure of remaining black poplar (Populus nigra) trees on the banks of the Dutch Rhine branches was investigated using the AFLP technique. In total, 143 trees, including one P. deltoides and some P. x euramericana, were analysed using six AFLP primer combinations which generated 319 polymorphic bands. The AFLP patterns showed that some of the trees sampled as P. nigra were clearly different. These deviating patterns were also observed for the P. deltoides tree and all trees already identified as hybrid P. x euramericana. Hybrids between the two species are morphologically sometimes difficult to distinguish from the species itself. Two important possible source populations for recolonization of the riverbanks of the river Rhine, consisting of mature flowering P. nigra trees, appeared to consist of only a few genotypes each. In contrast, young black poplar trees growing alone or in small groups downstream of the possible source populations appeared to be predominantly generatively derived because no clones of mature trees were found among them. Therefore vegetative propagation seems a very local strategy whereas colonization of new areas appears to occur through generative propagation. Whether the genetic diversity within these black poplars is sufficient for recolonization of river banks and survival of the metapopulation is a question for further research. | 2831 | NA | NA | NA | no | no |
| 2832 | 2017 | NA | Segovia, NI; Gallardo-Escarate, C; Poulin, E; Haye, PA | 2017 | Lineage divergence, local adaptation across a biogeographic break, and artificial transport, shape the genetic structure in the ascidian Pyura chilensis | Marine benthic organisms inhabit a heterogeneous environment in which connectivity between populations occurs mainly through dispersive larval stages, while local selective pressures acting on early life history stages lead to non-random mortality, shaping adaptive genetic structure. In order to test the influence of local adaptation and neutral processes in a marine benthic species with low dispersal, in this study we used Genotyping by Sequencing technology to compare the neutral and putatively selected signals (neutral and outlier loci, respectively) in SNPs scattered throughout the genome in six local populations of the commercially exploited ascidian Pyura chilensis along the southeast Pacific coast (24 degrees-42 degrees S). This species is sessile as an adult, has a short-lived larval stage, and may also be dispersed by artificial transport as biofouling. We found that the main signal in neutral loci was a highly divergent lineage present at 39 degrees S, and a subjacent signal that indicated a separation at 30 degrees S (north/south), widely reported in the area. North/south separation was the main signal in outlier loci, and the linage divergence at 39 degrees S was subjacent. We conclude that the geographic structure of the genetic diversity of outlier and neutral loci was established by different strengths of environmental, historical and anthropogenic factors. | 2832 | NA | NA | NA | no | no |
| 2833 | 2017 | NA | Kaur, H; Gupta, A | 2015 | Genetic relatedness provides support for a species complex of myxosporeans infecting the Indian major carp, Labeo rohita | Myxozoans are an economically important group of microscopic metazoan parasites of fish. The myxozoan species Thelohanellus filli infects commercially important freshwater fish both in wild and in cultured habitat. This parasite causes gill hemorrhagic disease and significant damage to the respiratory surface of the infected fish. In the present study, the 18S rDNA gene sequence of morphologically identified T. filli infecting the gill lamellae of the Indian major carp, Labeo rohita, was characterized. This revealed that we are actually dealing with a species complex containing T. bifurcata, T. jiroveci and T. seni. Phylogenetically, T. filli clustered with other myxozoan parasites, with the species most closely related to T. filli having 96 to 97% sequence similarity. The intraspecific variation demonstrated in this study points towards the importance of newer approaches to facilitate reassessment of taxa and detecting species complexes and cryptic species. | 2833 | NA | NA | NA | no | no |
| 2834 | 2017 | NA | Yalcindag, E; Elguero, E; Arnathau, C; Durand, P; Akiana, J; Anderson, TJ; Aubouy, A; Balloux, F; Besnard, P; Bogreau, H; Carnevale, P; D’Alessandro, U; Fontenille, D; Gamboa, D; Jombart, T; Le Mire, J; Leroy, E; Maestre, A; Mayxay, M; Menard, D; Musset, L; Newton, PN; Nkoghe, D; Noya, O; Ollomo, B; Rogier, C; Veron, V; Wide, A; Zakeri, S; Carme, B; Legrand, E; Chevillon, C; Ayala, FJ; Renaud, F; Prugnolle, F | 2012 | Multiple independent introductions of Plasmodium falciparum in South America | The origin of Plasmodium falciparum in South America is controversial. Some studies suggest a recent introduction during the European colonizations and the transatlantic slave trade. Other evidence-archeological and genetic-suggests a much older origin. We collected and analyzed P. falciparum isolates from different regions of the world, encompassing the distribution range of the parasite, including populations from sub-Saharan Africa, the Middle East, Southeast Asia, and South America. Analyses of microsatellite and SNP polymorphisms show that the populations of P. falciparum in South America are subdivided in two main genetic clusters (northern and southern). Phylogenetic analyses, as well as Approximate Bayesian Computation methods suggest independent introductions of the two clusters from African sources. Our estimates of divergence time between the South American populations and their likely sources favor a likely introduction from Africa during the transatlantic slave trade. | 2834 | NA | NA | NA | no | no |
| 2835 | 2017 | NA | Hoffmann, EHE; Malafronte, RS; Moraes-Avila, SL; Osakabe, AL; Wunderlich, G; Durham, AM; Ribolla, PEM; del Portillo, HA; Ferreira, MU | 2006 | Origins of sequence diversity in the malaria vaccine candidate merozoite surface protein-2 (MSP-2) in Amazonian isolates of Plasmodium falciparum | The recent evolution of Plasmodium falciparum is at odds with the extensive polymorphism found in most genes coding for antigens. Here, we examined the patterns and putative mechanisms of sequence diversification in the merozoite surface protein-2 (MSP-2), a major malarial repetitive surface antigen. We compared the msp-2 gene sequences from closely related clones derived from sympatric parasite isolates from Brazilian Amazonia and used microsatellite typing to examine, in these same clones, the haplotype background of chromosome 2, where msp-2 is located. We found examples of msp-2 sequence rearrangements putatively created by nonreciprocal recombinational events, such as replication slippage and gene conversion, while maintaining the chromosome haplotype. We conclude that these nonreciprocal recombination events may represent a major source of antigenic diversity in MSP-2 in P falciparum populations with low rates of classical meiotic recombination. (c) 2006 Elsevier B.V. All rights reserved. | 2835 | NA | NA | NA | no | no |
| 2836 | 2017 | NA | De Meeus, T; Balloux, F | 2005 | F-statistics of clonal diploids structured in numerous demes | The expected apportionment of genetic diversity in diploid clonal organisms structured in numerous subpopulations is explored. Under the specific assumptions considered, corresponding, for instance, to clonal pathogens infecting a large number of hosts, the co-ancestry between individuals within subpopulations is the only nontrivial quantity. Thus the population structure can be fully described either by F(ST) or F(IS), as F(ST) = -F(IS)/(1 - F(IS)). We show that, for most of the parameter space considered, including simulations where equilibrium is not reached and/or where homoplasy is high, the number of effective migrants is most accurately estimated as Nm = -(1 + F(IS))/4F(IS). We further propose a criterion to test for the absence of cryptic sexual reproduction based on the F-statistics F(IS) and F(ST), which is applied to three previously published empirical data sets. | 2836 | NA | NA | NA | no | no |
| 2837 | 2017 | NA | Rosenberry, CS; Long, ES; Hassel-Finnegan, HM; Buonaccorsi, VP; Diefenbach, DR; Wallingford, BD | 2009 | Lack of Mother-Offspring Relationships in White-Tailed Deer Capture Groups | Behavioral studies of white-tailed deer (Odocoileus virginianus) often assign mother- offspring relationships based on common capture of juveniles with adult deer, assuming that fawns associate closely with mothers. We tested this assumption using genetic parentage to assess mother- offspring relationships within capture groups based on data from 10 polymorphic microsatellite loci. At the 80% confidence level, we assigned maternity to 43% and 51% of juveniles captured with an adult female in 2 respective study areas. Capture with their mother did not differ by sex of juveniles in either study area, and limiting our analysis to capture groups that most represent family groups (i.e., one ad F with 1-3 juv) did not increase maternity assignment (35%). Our results indicate that common capture may be a poor indicator of mother-offspring relationships in many field settings. We recommend genetic verification of family relationships. (JOURNAL OF WILDLIFE MANAGEMENT 73(3):357-361; 2009) | 2837 | NA | NA | NA | no | no |
| 2838 | 2017 | NA | Zilversmit, MM; Chase, EK; Chen, DS; Awadalla, P; Day, KP; McVean, G | 2013 | Hypervariable antigen genes in malaria have ancient roots | Background: The var genes of the human malaria parasite Plasmodium falciparum are highly polymorphic loci coding for the erythrocyte membrane proteins 1 (PfEMP1), which are responsible for the cytoaherence of P. falciparum infected red blood cells to the human vasculature. Cytoadhesion, coupled with differential expression of var genes, contributes to virulence and allows the parasite to establish chronic infections by evading detection from the host’s immune system. Although studying genetic diversity is a major focus of recent work on the var genes, little is known about the gene family’s origin and evolutionary history. Results: Using a novel hidden Markov model-based approach and var sequences assembled from additional isolates and species, we are able to reveal elements of both the early evolution of the var genes as well as recent diversifying events. We compare sequences of the var gene DBL alpha domains from divergent isolates of P. falciparum (3D7 and HB3), and a closely-related species, Plasmodium reichenowi. We find that the gene family is equally large in P. reichenowi and P. falciparum - with a minimum of 51 var genes in the P. reichenowi genome (compared to 61 in 3D7 and a minimum of 48 in HB3). In addition, we are able to define large, continuous blocks of homologous sequence among P. falciparum and P. reichenowi var gene DBL alpha domains. These results reveal that the contemporary structure of the var gene family was present before the divergence of P. falciparum and P. reichenowi, estimated to be between 2.5 to 6 million years ago. We also reveal that recombination has played an important and traceable role in both the establishment, and the maintenance, of diversity in the sequences. Conclusions: Despite the remarkable diversity and rapid evolution found in these loci within and among P. falciparum populations, the basic structure of these domains and the gene family is surprisingly old and stable. Revealing a common structure as well as conserved sequence among two species also has implications for developing new primate-parasite models for studying the pathology and immunology of falciparum malaria, and for studying the population genetics of var genes and associated virulence phenotypes. | 2838 | NA | NA | NA | no | no |
| 2839 | 2017 | NA | Vembar, SS; Seetin, M; Lambert, C; Nattestad, M; Schatz, MC; Baybayan, P; Scherf, A; Smith, ML | 2016 | Complete telomere-to-telomere de novo assembly of the Plasmodium falciparum genome through long-read (> 11 kb), single molecule, real-time sequencing | The application of next-generation sequencing to estimate genetic diversity of Plasmodium falciparum, the most lethal malaria parasite, has proved challenging due to the skewed ATrichness [similar to 80.6% (A broken vertical bar T)] of its genome and the lack of technology to assemble highly polymorphic subtelomeric regions that contain clonally variant, multigene virulence families (Ex: var and rifin). To address this, we performed amplification-free, single molecule, real-time sequencing of P. falciparum genomic DNA and generated reads of average length 12 kb, with 50% of the reads between 15.5 and 50 kb in length. Next, using the Hierarchical Genome Assembly Process, we assembled the P. falciparum genome de novo and successfully compiled all 14 nuclear chromosomes telomere-to-telomere. We also accurately resolved centromeres [similar to 90-99% (A\_T)] and subtelomeric regions and identified large insertions and duplications that add extra var and rifin genes to the genome, along with smaller structural variants such as homopolymer tract expansions. Overall, we show that amplification-free, long-read sequencing combined with de novo assembly overcomes major challenges inherent to studying the P. falciparum genome. Indeed, this technology may not only identify the polymorphic and repetitive subtelomeric sequences of parasite populations from endemic areas but may also evaluate structural variation linked to virulence, drug resistance and disease transmission. | 2839 | NA | NA | NA | no | no |
| 2840 | 2017 | NA | Hume, BCC; Voolstra, CR; Arif, C; D’Angelo, C; Burt, JA; Eyal, G; Loya, Y; Wiedenmann, J | 2016 | Ancestral genetic diversity associated with the rapid spread of stress-tolerant coral symbionts in response to Holocene climate change | Coral communities in the Persian/Arabian Gulf (PAG) withstand unusually high salinity levels and regular summer temperature maxima of up to similar to 35 degrees C that kill conspecifics elsewhere. Due to the recent formation of the PAG and its subsequent shift to a hot climate, these corals have had only <6,000 y to adapt to these extreme conditions and can therefore inform on how coral reefs may respond to global warming. One key to coral survival in the world’s warmest reefs are symbioses with a newly discovered alga, Symbiodinium thermophilum. Currently, it is unknown whether this symbiont originated elsewhere or emerged from unexpectedly fast evolution catalyzed by the extreme environment. Analyzing genetic diversity of symbiotic algae across >5,000 km of the PAG, the Gulf of Oman, and the Red Sea coastline, we show that S. thermophilum is a member of a highly diverse, ancient group of symbionts cryptically distributed outside the PAG. We argue that the adjustment to temperature extremes by PAG corals was facilitated by the positive selection of preadapted symbionts. Our findings suggest that maintaining the largest possible pool of potentially stress-tolerant genotypes by protecting existing biodiversity is crucial to promote rapid adaptation to present-day climate change, not only for coral reefs, but for ecosystems in general. | 2840 | NA | NA | NA | no | no |
| 2841 | 2017 | NA | Listgarten, J; Kadie, C; Schadt, EE; Heckerman, D | 2010 | Correction for hidden confounders in the genetic analysis of gene expression | Understanding the genetic underpinnings of disease is important for screening, treatment, drug development, and basic biological insight. One way of getting at such an understanding is to find out which parts of our DNA, such as single-nucleotide polymorphisms, affect particular intermediary processes such as gene expression. Naively, such associations can be identified using a simple statistical test on all paired combinations of genetic variants and gene transcripts. However, a wide variety of confounders lie hidden in the data, leading to both spurious associations and missed associations if not properly addressed. We present a statistical model that jointly corrects for two particular kinds of hidden structure-population structure (e.g., race, family-relatedness), and microarray expression artifacts (e.g., batch effects), when these confounders are unknown. Applying our method to both real and synthetic, human and mouse data, we demonstrate the need for such a joint correction of confounders, and also the disadvantages of other possible approaches based on those in the current literature. In particular, we show that our class of models has maximum power to detect eQTL on synthetic data, and has the best performance on a bronze standard applied to real data. Lastly, our software and the associations we found with it are available at http://www.microsoft.com/science. | 2841 | NA | NA | NA | no | no |
| 2842 | 2017 | NA | Ghosh, KN; Mukhopadhyay, JM; Guzman, H; Tesh, RB; Munstermann, LE | 1999 | Interspecific hybridization and genetic variability of Phlebotomus sandflies | The first successful hybridization is reported between Phlebotomus papatasi and P. duboscqi, two important Old World sandfly vectors of leishmaniasis and other diseases. Laboratory strains of P. papatasi and P. duboscqi were separable by six diagnostic enzyme loci: Est-3, Idh-1, Mdh-2, Mpi, Tre-1 and Tre-3. Hybrids between the two species were verified by the recovery of heterozygous isozyme patterns for the diagnostic loci. No F-2 or backcross progeny were obtained. P. papatasi was separated from P. bergeroti by three diagnostic enzyme loci: Est-3, Mpi and Pgd. The isozyme patterns of P. hergerori contain elements of both P. duboscqi and P. papatasi, although seven diagnostic loci (Est-3, Idh-1, Me, Mpi, Pgd, Tre-1 and Tre-3) separated P. bergeroti from P. duboscqi. Genetic variability profiles of the three species were established for 20 enzyme loci. Three geographically distant strains of P. papatasi from Calcutta, Maharashtra and Israel had isozyme genetic distances of < 0.05. The recently established Calcutta strain showed an unexpectedly low genetic variability with only one (Idh-2) of 20 loci being polymorphic (average heterozygosity of 1.9 %) in contrast to 5-8 polymorphic loci (10-12 % heterozygosity) in the Maharashtra and Israel strains. Mass and single pair crosses between the three P. papatasi strains were fertile with normal progeny numbers. Thus we found no signs of speciation in P. papatasi. | 2842 | NA | NA | NA | no | no |
| 2843 | 2017 | NA | Reichardt, AK; Wheeler, DE | 1996 | Multiple mating in the ant Acromyrmex versicolor: A case of female control | In eusocial insects, polyandrous mating has the potential to reduce genetic relatedness of individuals within a colony, which may have a profound effect on the stability and social structure of the colony. Here we present evidence that multiple mating is common in both males and females of the desert leaf-cutter ant Acromyrmex versicolor. Females seem to have complete control over the number of matings, and mate on average with three males, even though the sperm transferred in a single copulation is sufficient to fill the spermatheca. To determine whether there is a bias in the representation of sperm from different mates in the spermatheca, females were mated to three or four males in controlled mating experiments and were subsequently allowed to found colonies in the laboratory. Paternity analysis of the offspring by random amplified polymorphic DNA analysis showed that all males that have been mated to a female successfully contributed sperm to the production of her offspring. No significant asymmetry id sperm use was detected, suggesting complete sperm mixing. Different hypotheses to explain polyandrous mating are discussed, and it is argued that the best hypothesis to explain polyandrous mating and complete sperm mixing in A. versicolor is that utilizing genetically diverse sperm confers a selective advantage on females. | 2843 | NA | NA | NA | no | no |
| 2844 | 2017 | NA | Ascensao, F; Clevenger, A; Santos-Reis, M; Urbanco, P; Jackson, N | 2013 | Wildlife-vehicle collision mitigation: Is partial fencing the answer? An agent-based model approach | Evaluating management options for mitigating the impacts of wildlife-vehicle collisions (WVC) is a major goal for road ecology. Fencing along roads in conjunction with the construction of wildlife road passages has been widely accepted as the most effective way to minimize WVC. However, limited resources often require wildlife managers to focus on a single method of mitigation, yet the relative effectiveness of fences and passages for reducing road mortality and restoring population connectivity is unclear. Using the stone marten (Manes foina, Erxleben, 1777) as a model species, we developed an individual-based, spatially explicit simulation model to develop predictions concerning the relative performance of fencing and passage construction under different rates of road mortality. For five levels each, we varied probability of road mortality, fencing extent, and number of passages in a full factorial design, for a total of 125 management scenarios. We then compared the relative impact of these two mitigation approaches on population abundance (N) and genetic differentiation (F-st) using linear regression. Our results predict that fences are much more effective than passages at mitigating the effects of road mortality on abundance. Moreover, we show that under most circumstances, fences are also more effective than passages at reducing genetic differentiation. This is likely driven by the ability of fencing to eliminate road mortality, which in turn increases genetic diversity, thereby slowing differentiation across the road. However, partial fencing can reduce road mortality nearly as well as full fencing. Moreover, partial fencing also allows adequate population connectivity across roads. Thus, we argue that partial fencing of roads alone may often be the best and most cost-effective management option for road mitigation. (C) 2013 Elsevier B.V. All rights reserved. | 2844 | NA | NA | NA | no | no |
| 2845 | 2017 | NA | Grant, WS | 2012 | Understanding the adaptive consequences of hatchery-wild interactions in Alaska salmon | About 31% of salmon harvested in Alaska comes from the hatchery production of hundreds of millions of pink and chum salmon and smaller numbers of sockeye, Chinook, and coho salmon. The numbers of hatchery-reared juveniles released in some areas are greater than the numbers of juveniles from wild populations. However, virtually nothing is known about the effects of hatchery fish on wild populations in Alaska. Possible effects of these interactions can be inferred from studies of salmonids in other areas, from studies of other animals, and from theory. Numerous studies show a complex relationship between the genetic architecture of a population and its environment. Adaptive responses to nature and anthropogenic selection can be influenced by variation at a single gene, or more often, by the additive effects of several genes. Studies of salmonids in other areas show that hatchery practices can lead to the loss of genetic diversity, to shifts in adult run timing and earlier maturity, to increases in parasite load, to increases in straying, to altered levels of boldness and dominance, to shifts in juvenile out-migration timing, and to changes in growth. Controlled experiments across generations show, and theory predicts, that the loss of adaptive fitness in hatchery salmon, relative to fitness in wild salmon, can occur on a remarkably short time scale. All of these changes can influence survival and impose selective regimes that influence genetically based adaptive traits. The preservation of adaptive potential in wild populations is an important buffer against diseases and climate variability and, hence, should be considered in planning hatchery production levels and release locations. The protection of wild populations is the foundation for achieving sustained harvests of salmon in Alaska. | 2845 | NA | NA | NA | no | no |
| 2846 | 2017 | NA | Gonzalez, EG; Blanco, JC; Ballesteros, F; Alcaraz, L; Palomero, G; Doadrio, I | 2016 | Genetic and demographic recovery of an isolated population of brown bear Ursus arctos L., 1758 | The brown bear Ursus arctos L., 1758 population of the Cantabrian Mountains (northwestern Spain) became isolated from other bear populations in Europe about 500 years ago and has declined due to hunting and habitat degradation. At the beginning of the 20th century, the Cantabrian population split into eastern and western subpopulations, and genetic exchange between them ceased. In the early 1990s, total population size was estimated to be < 100 bears. Subsequently, reduction in human-caused mortality has brought about an increase in numbers, mainly in the western subpopulation, likely promoting male-mediated migration and gene flow from the western nucleus to the eastern. To evaluate the possible genetic recovery of the small and genetically depauperate eastern subpopulation, in 2013 and 2014 we genotyped hair and faeces samples (116 from the eastern subpopulation and 36 from the western) for 18 microsatellite markers. Data from the annual count of females with cubs of the year (COY) during the past twenty-six years was used to analyze demographic changes. The number of females with COY fell to a minimum of seven in the western and three in eastern subpopulations in the biennium 1993-1994 and reached a respective maximum of 54 and 10 individuals in 2013-2014. We also observed increased bear dispersal and gene flow, mainly from the western to the eastern subpopulation. Of the 26 unique genotypes detected in the eastern subpopulation, 14 (54%) presented an admixture composition, and seven (27%) were determined to be migrants from the western subpopulation. Hence, the two separated and clearly structured subpopulations identified in the past currently show some degree of genetic admixture. This research shows the partial demographic recovery and a change in genetic composition due to migration process in a population of bears that has been isolated for several centuries. | 2846 | NA | NA | NA | no | no |
| 2847 | 2017 | NA | Pineda, MC; McQuaid, CD; Turon, X; Lopez-Legentil, S; Ordonez, V; Rius, M | 2012 | Tough Adults, Frail Babies: An Analysis of Stress Sensitivity across Early Life-History Stages of Widely Introduced Marine Invertebrates | All ontogenetic stages of a life cycle are exposed to environmental conditions so that population persistence depends on the performance of both adults and offspring. Most studies analysing the influence of abiotic conditions on species performance have focussed on adults, while studies covering early life-history stages remain rare. We investigated the responses of early stages of two widely introduced ascidians, Styela plicata and Microcosmus squamiger, to different abiotic conditions. Stressors mimicked conditions in the habitats where both species can be found in their distributional ranges and responses were related to the selection potential of their populations by analysing their genetic diversity. Four developmental stages (egg fertilisation, larval development, settlement, metamorphosis) were studied after exposure to high temperature (30 degrees C), low salinities (26 and 22%) and high copper concentrations (25, 50 and 100 mu g/L). Although most stressors effectively led to failure of complete development (fertilisation through metamorphosis), fertilisation and larval development were the most sensitive stages. All the studied stressors affected the development of both species, though responses differed with stage and stressor. S. plicata was overall more resistant to copper, and some stages of M. squamiger to low salinities. No relationship was found between parental genetic composition and responses to stressors. We conclude that successful development can be prevented at several life-history stages, and therefore, it is essential to consider multiple stages when assessing species’ abilities to tolerate stress. Moreover, we found that early development of these species cannot be completed under conditions prevailing where adults live. These populations must therefore recruit from elsewhere or reproduce during temporal windows of more benign conditions. Alternatively, novel strategies or behaviours that increase overall reproductive success might be responsible for ensuring population survival. | 2847 | NA | NA | NA | no | no |
| 2848 | 2017 | NA | Westerdahl, H; Waldenstrom, J; Hansson, B; Hasselquist, D; von Schantz, T; Bensch, S | 2005 | Associations between malaria and MHC genes in a migratory songbird | Malaria parasites are a widespread and species-rich group infecting many wild populations of mammals, birds and reptiles. Studies on humans have demonstrated that genetic factors play a key role in the susceptibility and outcome of malaria infections. Until the present study, it has not been examined whether genetic variation in hosts is important for the outcome of malaria infections in natural avian populations. We investigated associations between major histocompatibility complex (MHC) genes and prevalence of three different avian malaria parasites (Haemoproteus payevshyi (GRW1), Plasmodium sp. (GRW2) and Plasmodium sp. (GRW4)) in a long-term study of great reed warblers Acrocephalus arundinaceus. We hypothesized that the MHC genes could either give full protection against a malaria infection, or confer protection against lethal malaria and direct the infection towards being milder. We found a positive association between numbers of MHC class I alleles (a measure of level of heterozygosity) and prevalence of the GRW2 parasite, suggesting the latter scenario. There was also a positive association between a specific MHC allele (B4b), previously shown to be under frequency-dependent selection in the study population, and prevalence of GRW2. These associations suggest that individuals carrying either a large number of MHC alleles or a specific MHC allele are protected against lethal malaria infections. | 2848 | NA | NA | NA | no | no |
| 2849 | 2017 | NA | Radwan, J; Kawalko, A; Wojcik, JM; Babik, W | 2007 | MHC-DRB3 variation in a free-living population of the European bison, Bison bonasus | MHC genes play a crucial role in pathogen recognition and are the most polymorphic genes in vertebrates. Loss of variation in these genes in bottlenecked species is thought to put their survival at risk. We examined variation at the MHC II DRB3 locus in the European bison, Bison bonasus, a species that has undergone an extreme bottleneck: the current population originated from only 12 founders. We also tested for the association of DRB3 genes with the incidence of posthitis, a disease affecting the reproductive organs of bulls and posing a new threat to the survival of the species. We found very limited MHC diversity, with only four alleles segregating in a sample of 172 individuals from a free-ranging Bialowieza population. The alleles were highly divergent and revealed the hallmark of positive selection acting on them in the past, that is, a significant excess of nonsynonymous substitutions. This excess was concentrated in putative antigen-binding sites, suggesting that selection was driven by pathogens. However, we did not observe departures from Hardy-Weinberg equilibrium, an indicator of strong ongoing selection. Neither have we found a significant association between DRB3 alleles or genotypes and susceptibility to posthitis. Alleles conferring resistance to males may have been lost during the extreme bottleneck the species had undergone. | 2849 | NA | NA | NA | no | no |
| 2850 | 2017 | NA | Karunaweera, ND; Ferreira, MU; Munasinghe, A; Barnwell, JW; Collins, WE; King, CL; Kawamoto, F; Hartl, DL; Wirth, DF | 2008 | Extensive microsatellite diversity in the human malaria parasite Plasmodium vivax | The population structure of Plasmodium vivax remains elusive. The markers of choice for large-scale population genetic studies of eukaryotes, short tandem repeats known as microsatellites, have been recently reported to be less polymorphic in R vivax. Here we investigate the microsatellite diversity and geographic structure in P vivax, at both local and global levels, using 14 new markers consisting of tri- or tetranucleotide repeats. The local-level analysis, which involved 50 field isolates from Sri Lanka, revealed unexpectedly high diversity (average virtual heterozygosity [H-E], 0.807) and significant multilocus linkage disequilibrium in this region of low malaria endemicity. Multiple-clone infections occurred in 60% of isolates sampled in 2005. The global-level analysis of field isolates or monkey-adapted strains identified 150 unique haplotypes among 164 parasites from four continents. Individual P. vivax isolates could not be unambiguously assigned to geographic populations. For example, we found relatively low divergence among parasites from Central America, Africa, Southeast Asia and Oceania, but substantial differentiation between parasites from the same continent (South Asia and Southeast Asia) or even from the same country (Brazil). Parasite relapses, which may extend the duration of P. vivax carriage in humans, are suggested to facilitate the spread of strains across continents, breaking down any pre-existing geographic structure. (C) 2008 Elsevier B.V. All rights reserved. | 2850 | NA | NA | NA | no | no |
| 2851 | 2017 | NA | Madsen, T; Ujvari, B | 2011 | THE POTENTIAL DEMISE OF A POPULATION OF ADDERS (VIPERA BERUS) IN SMYGEHUK, SWEDEN | In 1999 and 2004, we published reports on how the introduction of 20 males into a severely inbred and isolated population of Adders (Vipera berus) halted its decline towards extinction. The introduction significantly enhanced the population’s genetic variability, which resulted in a dramatic increase in offspring viability and a rapid increase in numbers. Unfortunately, recently a new and unprecedented development is threatening the population’s future survival. In 2004 permission was granted by the Swedish Nature Conservation Agency of the County Administrative Board to build a house and an adjacent 1 m tall brick wall, right across the habitat occupied by the Adders. The construction of the house and brick wall in 2006 has prevented the majority of the snakes from undertaking their annual migration within the study area, resulting in an extirpation > 75% of the Adders. This reduction seriously impedes the future survival of this unique population. | 2851 | NA | NA | NA | no | no |
| 2852 | 2017 | NA | Nilsson, E; Taubert, H; Hellgren, O; Huang, X; Palinauskas, V; Markovets, MY; Valkiunas, G; Bensch, S | 2016 | Multiple cryptic species of sympatric generalists within the avian blood parasite Haemoproteus majoris | The avian haemosporidian parasite Haemoproteus majoris has been reported to infect a wide range of passerine birds throughout the Holarctic ecozone. Five cytochrome b (cyt b) lineages have been described as belonging to the morphological species H.majoris, and these form a tight phylogenetic cluster together with 13 undescribed lineages that differ from each other by <1.2% in sequence divergence. Records in a database (MalAvi) that contains global findings of haemosporidian lineages generated by universal primers suggest that these lineages vary substantially in host distribution. We confirm this pattern in a data set collected at Lake Kvismaren, Sweden, where three of the generalist lineages have local transmission. However, whether these lineages represent intraspecific mitochondrial diversity or clusters of cryptic species has previously not been examined. In this study, we developed novel molecular markers to amplify the partial segments of four nuclear genes to determine the level of genetic diversity and gene phylogenies among the five morphologically described cyt b lineages of H.majoris. All five cyt b lineages were strongly associated with unique nuclear alleles at all four nuclear loci, indicating that each mitochondrial lineage represents a distinct biological species. Within lineages, there was no apparent association between nuclear alleles and host species, indicating that they form genetically unstructured populations across multiple host species. | 2852 | NA | NA | NA | no | no |
| 2853 | 2017 | NA | Hampe, A; Petit, RJ | 2005 | Conserving biodiversity under climate change: the rear edge matters | Modern climate change is producing poleward range shifts of numerous taxa, communities and ecosystems worldwide. The response of species to changing environments is likely to be determined largely by population responses at range margins. In contrast to the expanding edge, the low-latitude limit (rear edge) of species ranges remains understudied, and the critical importance of rear edge populations as long-term stores of species’ genetic diversity and foci of speciation has been little acknowledged. We review recent findings from the fossil record, phylogeography and ecology to illustrate that rear edge populations are often disproportionately important for the survival and evolution of biota. Their ecological features, dynamics and conservation requirements differ from those of populations in other parts of the range, and some commonly recommended conservation practices might therefore be of little use or even counterproductive for rear edge populations. | 2853 | NA | NA | NA | no | no |
| 2854 | 2017 | NA | Kelly, AC; Clear, RM; O’Donnell, K; McCormick, S; Turkington, TK; Tekauz, A; Gilbert, J; Kistler, HC; Busman, M; Ward, TJ | 2015 | Diversity of Fusarium head blight populations and trichothecene toxin types reveals regional differences in pathogen composition and temporal dynamics | Analyses of genetic diversity, trichothecene genotype composition, and population structure were conducted using 4086 Fusarium graminearum isolates collected from wheat in eight Canadian provinces over a three year period between 2005 and 2007. The results revealed substantial regional differences in Fusarium head blight pathogen composition and temporal population dynamics. The 3ADON trichothecene type consistently predominated in Maritime provinces (91%) over the sampled years, and increased significantly (P < 0.05) between 2005 and 2007 in western Canada, accounting for 66% of the isolates in Manitoba by the end of the sampling period. In contrast, 3ADON frequency was lower (22%, P < 0.001) in the eastern Canadian provinces of Ontario and Quebec and did not change significantly between 2005 and 2007, resulting in two distinct longitudinal dines in 3ADON frequency across Canada. Overall, genetic structure was correlated with toxin type, as the endemic population (NA1) was dominated by 15ADON isolates (86%), whereas a second population (NA2) consisted largely of 3ADON isolates (88%). However, the percentage of isolates with trichothecene genotypes that were not predictive of their genetic population assignment (recombinant genotypes) increased from 10% in 2005 to 17% in 2007, indicating that trichothecene type became an increasingly unreliable marker of population identity over time. In addition, there were substantial regional differences in the composition of recombinant genotypes. In western and maritime provinces, NA2 isolates with 15ADON genotypes were significantly more common than NA1 isolates with 3ADON genotypes (P < 0.001), and the reverse was true in the eastern provinces of Quebec and Ontario. Temporal trends in recombinant genotype composition also varied regionally, as the percentage of 15ADON isolates with NA2 genetic backgrounds increased approximately three fold in western and Maritime provinces, while the opposite trends were observed in Quebec and Ontario. The results indicate that F. graminearum population dynamics in Canada have been influenced by a complex adaptive landscape comprising different regional selective pressures, and do not reflect a simple model of dispersal and integration following the introduction of a novel pathogen population. In addition, we identified F. graminearum strains that produce the recently discovered A-trichothecene mycotoxin (NX-2) for the first time in Canada, representing a significant expansion of the known range of NX-2 producing strains in North America. Published by Elsevier Inc. | 2854 | NA | NA | NA | no | no |
| 2855 | 2017 | NA | Sede, SM; Nicola, MV; Pozner, R; Johnson, LA | 2012 | Phylogeography and palaeodistribution modelling in the Patagonian steppe: the case of Mulinum spinosum (Apiaceae) | Aim An integrative study of the endemic, yet ubiquitous, Patagonian shrub Mulinum spinosum (Apiaceae) was performed: (1) to assess the historical processes that influenced its geographical pattern of genetic variation; (2) to test hypotheses of its survival in situ or in glacial refugia during glacial cycles; and (3) to model its extant and palaeoclimatic distributions to assess support for the phylogeographical patterns recovered. Location Chilean and Argentinian Andean region and Patagonian steppe. Methods Chloroplast DNA sequences, trnHpsbA, trnStrnG and 3’trnVndhC, were obtained for 314 individuals of M. spinosum from 71 populations. The haplotype data matrix was analysed using nested clade analysis (NCA) to construct a network. Analysis of molecular variance (AMOVA), spatial analysis of molecular variance (SAMOVA) and neutrality tests were also used to test for genetic structure and range expansion in the species. The present potential geographical distribution of M. spinosum was modelled and projected onto a Last Glacial Maximum (LGM) model. Results Amongst the 29 haplotypes observed, one was widely distributed, but most were restricted to either northern or southern regions. The populations with highest haplotype diversity were found in southern Patagonia, the high Andean region, and northern Patagonia. AMOVA and SAMOVA showed latitudinal structure for Argentinian populations. NCA implied patterns of restricted gene flow or dispersal but with some long-distance dispersal and also long-distance colonization and/or past fragmentation. Neutrality tests did not support range expansions. The current distribution model was a fairly good representation of the extant geographical distribution of the species, and the distribution model for the LGM did not show important shifts of the extant range to lower latitudes, except for a shift towards the palaeoseashore. Main conclusions Based on agreement amongst phylogeographical patterns, distribution of genetic variability, equivocal evidence of putative refugia and palaeodistribution modelling, it is probable that glaciations did not greatly affect the distribution of Mulinum spinosum. Our results are consistent with the in situ survival hypothesis, and not with the latitudinal migration of plant communities to avoid adverse climate conditions during Pleistocene glaciations. It is possible that populations of northern Patagonia may have been isolated from the southern ones by the Chubut and Deseado basins. | 2855 | NA | NA | NA | no | no |
| 2856 | 2017 | NA | Thomas, JC; Figueira, M; Fennie, KP; Laufer, AS; Kong, Y; Pichichero, ME; Pelton, SI; Pettigrew, MM | 2011 | Streptococcus pneumoniae Clonal Complex 199: Genetic Diversity and Tissue-Specific Virulence | Streptococcus pneumoniae is an important cause of otitis media and invasive disease. Since introduction of the heptavalent pneumococcal conjugate vaccine, there has been an increase in replacement disease due to serotype 19A clonal complex (CC) 199 isolates. The goals of this study were to 1) describe genetic diversity among nineteen CC199 isolates from carriage, middle ear, blood, and cerebrospinal fluid, 2) compare CC199 19A (n = 3) and 15B/C (n = 2) isolates in the chinchilla model for pneumococcal disease, and 3) identify accessory genes associated with tissue-specific disease among a larger collection of S. pneumoniae isolates. CC199 isolates were analyzed by comparative genome hybridization. One hundred and twenty-seven genes were variably present. The CC199 phylogeny split into two main clades, one comprised predominantly of carriage isolates and another of disease isolates. Ability to colonize and cause disease did not differ by serotype in the chinchilla model. However, isolates from the disease clade were associated with faster time to bacteremia compared to carriage clade isolates. One 19A isolate exhibited hypervirulence. Twelve tissue-specific genes/regions were identified by correspondence analysis. After screening a diverse collection of 326 isolates, spr0282 was associated with carriage. Four genes/regions, SP0163, SP0463, SPN05002 and RD8a were associated with middle ear isolates. SPN05002 also associated with blood and CSF, while RD8a associated with blood isolates. The hypervirulent isolate’s genome was sequenced using the Solexa paired-end sequencing platform and compared to that of a reference serotype 19A isolate, revealing the presence of a novel 20 kb region with sequence similarity to bacteriophage genes. Genetic factors other than serotype may modulate virulence potential in CC199. These studies have implications for the long-term effectiveness of conjugate vaccines. Ideally, future vaccines would target common proteins to effectively reduce carriage and disease in the vaccinated population. | 2856 | NA | NA | NA | no | no |
| 2857 | 2017 | NA | Lanier, HC; Gunderson, AM; Weksler, M; Fedorov, VB; Olson, LE | 2015 | Comparative Phylogeography Highlights the Double-Edged Sword of Climate Change Faced by Arctic- and Alpine-Adapted Mammals | Recent studies suggest that alpine and arctic organisms may have distinctly different phylo-geographic histories from temperate or tropical taxa, with recent range contraction into interglacial refugia as opposed to post-glacial expansion out of refugia. We use a combination of phylogeographic inference, demographic reconstructions, and hierarchical Approximate Bayesian Computation to test for phylodemographic concordance among five species of alpine-adapted small mammals in eastern Beringia. These species (Collared Pikas, Hoary Marmots, Brown Lemmings, Arctic Ground Squirrels, and Singing Voles) vary in specificity to alpine and boreal-tundra habitat but share commonalities (e.g., cold tolerance and nunatak survival) that might result in concordant responses to Pleistocene glaciations. All five species contain a similar phylogeographic disjunction separating eastern and Beringian lineages, which we show to be the result of simultaneous divergence. Genetic diversity is similar within each haplogroup for each species, and there is no support for a post-Pleistocene population expansion in eastern lineages relative to those from Beringia. Bayesian skyline plots for four of the five species do not support Pleistocene population contraction. Brown Lemmings show evidence of late Quaternary demographic expansion without subsequent population decline. The Wrangell-St. Elias region of eastern Alaska appears to be an important zone of recent secondary contact for nearctic alpine mammals. Despite differences in natural history and ecology, similar phylogeographic histories are supported for all species, suggesting that these, and likely other, alpine-and arctic-adapted taxa are already experiencing population and/or range declines that are likely to synergistically accelerate in the face of rapid climate change. Climate change may therefore be acting as a double-edged sword that erodes genetic diversity within populations but promotes divergence and the generation of biodiversity. | 2857 | NA | NA | NA | no | no |
| 2858 | 2017 | NA | Van Steenberge, M; Pariselle, A; Huyse, T; Volckaert, FAM; Snoeks, J; Vanhove, MPM | 2015 | Morphology, Molecules, and Monogenean Parasites: An Example of an Integrative Approach to Cichlid Biodiversity | The unparalleled biodiversity of Lake Tanganyika (Africa) has fascinated biologists for over a century; its unique cichlid communities are a preferred model for evolutionary research. Although species delineation is, in most cases, relatively straightforward, higher-order classifications were shown not to agree with monophyletic groups. Here, traditional morphological methods meet their limitations. A typical example are the tropheine cichlids currently belonging to Simochromis and Pseudosimochromis. The affiliations of these widespread and abundant cichlids are poorly understood. Molecular work suggested that genus and species boundaries should be revised. Moreover, previous morphological results indicated that intraspecific variation should be considered to delineate species in Lake Tanganyika cichlids. We review the genera Simochromis and Pseudosimochromis using an integrative approach. Besides a morphometric study and a barcoding approach, monogenean Cichlidogyrus (Platyhelminthes: Ancyrocephalidae) gill parasites, often highly species-specific, are used as complementary markers. Six new species are described. Cichlidogyrus raeymaekersi sp. nov., C. muterezii sp. nov. and C. banyankimbonai sp. nov. infect S. diagramma. Cichlidogyrus georgesmertensi sp. nov. was found on S. babaulti and S. pleurospilus, C. franswittei sp. nov. on both S. marginatus and P. curvifrons and C. frankwillemsi sp. nov. only on P. curvifrons. As relatedness between Cichlidogyrus species usually reflects relatedness between hosts, we considered Simochromis monotypic because the three Cichlidogyrus species found on S. diagramma belonged to a different morphotype than those found on the other Simochromis. The transfer of S. babaulti, S. marginatus, S. pleurospilus and S. margaretae to Pseudosimochromis was justified by the similarity of their Cichlidogyrus fauna and the intermediate morphology of S. margaretae. Finally parasite data also supported the synonymy between S. pleurospilus and S. babaulti, a species that contains a large amount of geographical morphological variation. | 2858 | NA | NA | NA | no | no |
| 2859 | 2017 | NA | de Leon, JH; Jones, NA | 2004 | Detection of DNA Polymorphisms in Homalodisca coagulata (Homoptera : Cicadellidae) by polymerase chain reaction-based DNA fingerprinting methods | DNA polymorphisms were detected in Homalodisca coagulata (Say) (Homoptera: Cicadellidae) with the following DNA fingerprinting methods: inter-simple sequence repeat-polymerase chain reaction (ISSR-PCR) and printer pair-ISSR-PCR (pp-ISSR-PCR), randomly amplified microsatellite polymophisms (RAMP), selective amplification of microsatellite polymorphic loci (SAMPL), and primer pair-random amplification of polymorphic DNA-polymerase chain reaction (pp-RAPD-PCR). But first, a small-scale DNA fingerprinting screening procedure was initiated with these methods with a few individual insects to estimate the most sensitive and efficient method(s). In total, 205 polymorphic markers were generated with the four methods. The efficiency ratio estimated the following order for each method: 1) pp-ISSR-PCR and ISSR-PCR, 2) RAMP, 3) pp-RAPD-PCR, and 4) SAMPL. The screening efficiency ratio estimated that pp-ISSR-PCR and ISSR-PCR were the most efficient methods. DNA polymorphisms were detected in a natural population of 10-30 insects. The number of polymorphic loci ranged from five (pp-RAPD-PCR reaction 6) to 32 (ISSR-PCR primer 13), and the percentage of polymorphic loci was 100% for most primers tested. DNA fingerprinting methods tested were able to detect geographic variation in populations of H. coagulata from Bakersfield and Riverside, CA, and Weslaco, TX. Dendrograms based on Nei’s genetic distance showed that H. coagulata from Bakersfield and Riverside formed a cluster separate from Weslaco in three DNA fingerprinting reactions tested incorporating simple sequence repeats. DNA fingerprinting methods tested were also able to distinguish between three Homalodisca sharpshooters: H. coagulata, Homatodisca insolita (Walker), and Homalodisca liturata (Ball). The present results confirmed the utility of the DNA fingerprinting screening procedure and demonstrated, for the first time, genetic variation in natural populations of glassy-winged sharpshooters by PCR-based DNA fingerprinting methods. | 2859 | NA | NA | NA | no | no |
| 2860 | 2017 | NA | Stepanov, VA | 2016 | Evolution of genetic diversity and human diseases | The problem of development and dispersion of complex diseases in human populations requires new views, approaches, hypotheses, and paradigms. Evolutionary medicine provides one of the promising approaches to this problem, putting the disease into an evolutionary context. Unlike classic approaches oriented to proximate issues on structure and mechanisms of a disease, evolutionary considerations are broader. It provides the basis for understanding the origin, dispersion, and maintenance of the high frequencies of pathological phenotypes in modern human populations. In the current paper, we try to review the modern concepts on the evolution of human genetic diversity, to shape the outlines of evolutionary medicine, and to illustrate evolutionary medical problems using our experimental data. Data on genome-wide search for the signals of decanalization and adaptation in the human genome and on related biological processes and diseases are presented. Some hypotheses and concepts of evolutionary medicine may be productive for revealing the mechanisms of origin and dispersion of complex diseases and for pathogenetics of multifactorial diseases. One of such concepts is the hypothesis of decanalization of genome-phenome relationships under natural selection during modern human dispersion. Probably, the high frequency of alleles associated with complex diseases (and partially the high prevalence of diseases themselves) could be explained in the framework of the hypothesis. | 2860 | NA | NA | NA | no | no |
| 2861 | 2017 | NA | Krongdang, S; Evans, JD; Pettis, JS; Chantawannakul, P | 2017 | Multilocus sequence typing, biochemical and antibiotic resistance characterizations reveal diversity of North American strains of the honey bee pathogen Paenibacillus larvae | Paenibacillus larvae is a Gram positive bacterium and the causative agent of the most widespread fatal brood disease of honey bees, American foulbrood (AFB). A total of thirty-three independent Paenibacillus larvae isolates from various geographical origins in North America and five reference strains were investigated for genetic diversity using multilocus sequence typing (MLST). This technique is regarded to be a powerful tool for epidemiological studies of pathogenic bacteria and is widely used in genotyping assays. For MLST, seven housekeeping gene loci, ilvD (dihydroxy-acid dyhydrogenase), tri (triosephosphate isomerase), purH (phospharibosyl-aminoimidazolecarboxamide), recF (DNA replication and repair protein), pyrE (orotate phosphoribosyltransferase), sucC (succinyl coenzyme A synthetase beta subunit) and glpF (glycerol uptake facilitator protein) were studied and applied for primer designs. Previously, ERIC type DNA fingerprinting was applied to these same isolates and the data showed that almost all represented the ERIC I type, whereas using BOX-PCR gave an indication of more diversity. All isolates were screened for resistance to four antibiotics used by U.S. beekeepers, showing extensive resistance to tetracycline and the first records of resistance to tylosin and lincomycin. Our data highlight the intraspecies relationships of P. larvae and the potential application of MLST methods in enhancing our understanding of epidemiological relationships among bacterial isolates of different origins. | 2861 | NA | NA | NA | no | no |
| 2862 | 2017 | NA | Silva-Lima, AW; Walter, JM; Garcia, GD; Ramires, N; Ank, G; Meirelles, PM; Nobrega, AF; Siva-Neto, ID; Moura, RL; Salomon, PS; Thompson, CC; Thompson, FL | 2015 | Multiple Symbiodinium Strains Are Hosted by the Brazilian Endemic Corals Mussismilia spp. | Corals of genus Mussismilia (Mussidae) are one of the oldest extant clades of scleractinians. These Neogene relicts are endemic to the Brazilian coast and represent the main reef-building corals in the Southwest Atlantic Ocean (SAO). The relatively low-diversity/high-endemism SAO coralline systems are under rapid decline from emerging diseases and other local and global stressors, but have not been severely affected by coral bleaching. Despite the biogeographic significance and importance for understanding coral resilience, there is scant information about the diversity of Symbiodinium in this ocean basin. In this study, we established the first culture collections of Symbiodinium from Mussismilia hosts, comprising 11 isolates, four of them obtained by fluorescent-activated cell sorting (FACS). We also analyzed Symbiodinium diversity directly from Mussismilia tissue samples (N = 16) and characterized taxonomically the cultures and tissue samples by sequencing the dominant ITS2 region. Symbiodinium strains A4, B19, and C3 were detected. Symbiodinium C3 was predominant in the larger SAO reef system (Abrolhos), while Symbiodinium B19 was found only in deep samples from the oceanic Trindade Island. Symbiodinium strains A4 and C3 isolates were recovered from the same Mussismilia braziliensis coral colony. In face of increasing threats, these results indicate that Symbiodinium community dynamics shall have an important contribution for the resilience of Mussismilia spp. corals. | 2862 | NA | NA | NA | no | no |
| 2863 | 2017 | NA | Zhu, XF; McKenzie, CA; Forrester, T; Nickerson, DA; Broeckel, U; Schunkert, H; Doering, A; Jacob, HJ; Cooper, RS; Rieder, MJ | 2000 | Localization of a small genomic region associated with elevated ACE | Defining the relationship between multiple polymorphisms in a small genomic region and an underlying quantitative trait locus (QTL) represents a major challenge in human genetics. Pedigree analyses have shown that angiotensin I-converting enzyme (ACE) levels are influenced by a QTL located within or close to the ACE gene and most likely resides in the 3’ region of this locus. We genotyped seven polymorphisms spanning 13 kb in the 3’ end of ACE in 159 Afro-Caribbean subjects to evaluate the linkage disequilibrium between these sites and to narrow the genomic region associated with an elevated ACE level using a cladistic analysis. The linkage disequilibrium measurement D’ and a haplotype tree revealed three distinct haplotype segments, presumably because of recombination, The value of the linkage disequilibrium parameter p(excess) was highest for site 22982, which is located in the middle segment. A series of nested, cladistic analyses confirmed that the ether two regions are unlikely to be the ACE-linked QTL and that the variant resides in the middle region. Analyses of the same polymorphisms in 98 unrelated Europeans in the Monitoring Trends and Determinants in Cardiovascular Diseases (MONICA) study resulted in fewer haplotypes than were observed among the Afro-Caribbean subjects, suggesting that populations with greater genetic diversity may be especially informative for fine-scale mapping. | 2863 | NA | NA | NA | no | no |
| 2864 | 2017 | NA | Anche, MT; Bijma, P; De Jong, MCM | 2015 | Genetic analysis of infectious diseases: estimating gene effects for susceptibility and infectivity | Background: Genetic selection of livestock against infectious diseases can complement existing interventions to control infectious diseases. Most genetic approaches that aim at reducing disease prevalence assume that individual disease status (infected/not-infected) is solely a function of its susceptibility to a particular pathogen. However, individual infectivity also affects the risk and prevalence of an infection in a population. Variation in susceptibility and infectivity between hosts affects transmission of an infection in the population, which is usually measured by the value of the basic reproduction ratio R-0. R-0 is an important epidemiological parameter that determines the risk and prevalence of infectious diseases. An individual’s breeding value for R-0 is a function of its genes that influence both susceptibility and infectivity. Thus, to estimate the effects of genes on R-0, we need to estimate the effects of genes on individual susceptibility and infectivity. To that end, we developed a generalized linear model (GLM) to estimate relative effects of genes for susceptibility and infectivity. A simulation was performed to investigate bias and precision of the estimates, the effect of R-0, the size of the effects of genes for susceptibility and infectivity, and relatedness among group mates on bias and precision. We considered two bi-allelic loci that affect, respectively, the individuals’ susceptibility only and individuals’ infectivity only. Results: A GLM with complementary log-log link function can be used to estimate the relative effects of genes on the individual’s susceptibility and infectivity. The model was developed from an equation that describes the probability of an individual to become infected as a function of its own susceptibility genotype and infectivity genotypes of all its infected group mates. Results show that bias is smaller when R-0 ranges approximately from 1.8 to 3.1 and relatedness among group mates is higher. With larger effects, both absolute and relative standard deviations become clearly smaller, but the relative bias remains the same. Conclusions: We developed a GLM to estimate the relative effect of genes that affect individual susceptibility and infectivity. This model can be used in genome-wide association studies that aim at identifying genes that influence the prevalence of infectious diseases. | 2864 | NA | NA | NA | no | no |
| 2865 | 2017 | NA | Henshall, JM; Whan, VA; Norris, BJ | 2010 | Reconstructing CNV genotypes using segregation analysis: combining pedigree information with CNV assay | Background: Repeated blocks of genome sequence have been shown to be associated with genetic diversity and disease risk in humans, and with phenotypic diversity in model organisms and domestic animals. Reliable tests are desirable to determine whether individuals are carriers of copy number variants associated with disease risk in humans and livestock, or associated with economically important traits in livestock. In some cases, copy number variants affect the phenotype through a dosage effect but in other cases, allele combinations have non-additive effects. In the latter cases, it has been difficult to develop tests because assays typically return an estimate of the sum of the copy number counts on the maternally and paternally inherited chromosome segments, and this sum does not uniquely determine the allele configuration. In this study, we show that there is an old solution to this new problem: segregation analysis, which has been used for many years to infer alleles in pedigreed populations. Methods: Segregation analysis was used to estimate copy number alleles from assay data on simulated half-sib sheep populations. Copy number variation at the Agouti locus, known to be responsible for the recessive self-colour black phenotype, was used as a model for the simulation and an appropriate penetrance function was derived. The precision with which carriers and non-carriers of the undesirable single copy allele could be identified, was used to evaluate the method for various family sizes, assay strategies and assay accuracies. Results: Using relationship data and segregation analysis, the probabilities of carrying the copy number alleles responsible for black or white fleece were estimated with much greater precision than by analyzing assay results for animals individually. The proportion of lambs correctly identified as non-carriers of the undesirable allele increased from 7% when the lambs were analysed alone to 80% when the lambs were analysed in half-sib families. Conclusions: When a quantitative assay is used to estimate copy number alleles, segregation analysis of related individuals can greatly improve the precision of the estimates. Existing software for segregation analysis would require little if any change to accommodate the penetrance function for copy number assay data. | 2865 | NA | NA | NA | no | no |
| 2866 | 2017 | NA | Guo, SG; Zhang, JG; Sun, HH; Salse, J; Lucas, WJ; Zhang, HY; Zheng, Y; Mao, LY; Ren, Y; Wang, ZW; Min, JM; Guo, XS; Murat, F; Ham, BK; Zhang, ZL; Gao, S; Huang, MY; Xu, YM; Zhong, SL; Bombarely, A; Mueller, LA; Zhao, H; He, HJ; Zhang, Y; Zhang, ZH; Huang, SW; Tan, T; Pang, EL; Lin, K; Hu, Q; Kuang, HH; Ni, PX; Wang, B; Liu, JA; Kou, QH; Hou, WJ; Zou, XH; Jiang, J; Gong, GY; Klee, K; Schoof, H; Huang, Y; Hu, XS; Dong, SS; Liang, DQ; Wang, J; Wu, K; Xia, Y; Zhao, X; Zheng, ZQ; Xing, M; Liang, XM; Huang, BQ; Lv, T; Wang, JY; Yin, Y; Yi, HP; Li, RQ; Wu, MZ; Levi, A; Zhang, XP; Giovannoni, JJ; Wang, J; Li, YF; Fei, ZJ; Xu, Y | 2013 | The draft genome of watermelon (Citrullus lanatus) and resequencing of 20 diverse accessions | Watermelon, Citrullus lanatus, is an important cucurbit crop grown throughout the world. Here we report a high-quality draft genome sequence of the east Asia watermelon cultivar 97103 (2n = 2x = 22) containing 23,440 predicted protein-coding genes. Comparative genomics analysis provided an evolutionary scenario for the origin of the 11 watermelon chromosomes derived from a 7-chromosome paleohexaploid eudicot ancestor. Resequencing of 20 watermelon accessions representing three different C. lanatus subspecies produced numerous haplotypes and identified the extent of genetic diversity and population structure of watermelon germplasm. Genomic regions that were preferentially selected during domestication were identified. Many disease-resistance genes were also found to be lost during domestication. In addition, integrative genomic and transcriptomic analyses yielded important insights into aspects of phloem-based vascular signaling in common between watermelon and cucumber and identified genes crucial to valuable fruit-quality traits, including sugar accumulation and citrulline metabolism. | 2866 | NA | NA | NA | no | no |
| 2867 | 2017 | NA | Kumar, A; Sarma, YR; Anandaraj, M | 2004 | Evaluation of genetic diversity of Ralstonia solanacearum causing bacterial wilt of ginger using REP-PCR and PCR-RFLP | Thirty-three strains of Ralstonia solanacearum Yabuuchi (Smith) isolated from. ginger, paprika, chilli, tomato, Chromolaena and potato from Kerala, Karnataka, West Bengal and Assam in India, were phenotypically and genotypically characterized. Phenotypic characterization for biovar revealed the predominance of biovar 3 in India. Molecular analysis by REP-PCR, ITS-PCR and RFLP-PCR classified the strains into three clusters at 70% similarity, where ginger strains are grouped in Clusters I and II. Strains from potato (biovar 2) clustered in the III cluster. Molecular analysis also revealed that ginger strains isolated from different locations during different years had 100% similarity according to Dice’s coefficient. The analysis further revealed that the genetic diversity of Ralstonia is very low within ginger, confirming that the pathogen population is of clonal lineage and is distributed through ‘rhizome transmission’ of the inoculum between locations and also between seasons within the locality. | 2867 | NA | NA | NA | no | no |
| 2868 | 2017 | NA | Strassmann, JE; Solis, CR; Hughes, CR; Goodnight, KF; Queller, DC | 1997 | Colony life history and demography of a swarm-founding social wasp | Colonies of social insects are sometimes viewed as superorganisms. The birth, reproduction, and death of colonies can be studied with demographic measures analogous to those normally applied to individuals, but two additional questions arise. First, how do adaptive colony demographies arise from individual behaviors? Second, since these superorganisms are made up of genetically distinct individuals, do conflicts within the colony sometimes modify and upset optima for colonies? The interplay between individual and super-individual or colony interests appears to be particularly complex in neotropical, swarm-founding, epiponine wasps such as Parachartergus colobopterus. In a longterm study of this species, we censused 286 nests to study colony-level reproduction and survivorship and evaluated individual-level factors by assessing genetic relatedness and queen production. Colony survivorship followed a negative exponential curve very closely, indicating type II survivorship. This pattern is defined by constant mortality across ages and is more characteristic of birds and other vertebrates than of insects. Individual colonies are long-lived, lasting an average of 347 days, with a maximum of over 4.5 years. The low and constant levels of colony mortality arise in part from colony initiation by swarming, nesting on protected substrates, and an unusual expandable nest structure. The ability to requeen rapidly was also important; relatedness data suggest that colonies requeen on average once every 9-12 months. We studied whether colony optima with respect to the timing of reproduction could be upset by individual worker interests. In this species, colonies are normally polygynous but new queens are produced only after a colony reaches the monogynous state, a result which is in accord with the genetic interests of workers. Therefore colony worker interests might drive colonies to reproduce whenever queen number happens to cycled down to one rather than at the season that is otherwise optimal. However, we found reproduction to be heavily concentrated in the rainy season. The number of new colonies peaked in this season as did the percentages of males and queens. Relatedness among workers reached a seasonal low of 0.21-0.27, reflecting the higher numbers of laying queens. This seasonality was achieved in part by a modest degree of synchrony in the queen reduction cycle. Worker relatedness reached peaks of around 0.4 in the dry season, reflecting a decrease to a harmonic mean queen number of about 2.5. Thus, a significant number of colonies must be approaching monogyny entering the rainy season. Coupled with polygynous colonies rearing only males (split sex ratios), this makes it possible for a colony cycle driven by selfish worker interests to be consistent with concentrating colony reproduction during a favorable season. | 2868 | NA | NA | NA | no | no |
| 2869 | 2017 | NA | Khrustaleva, AM; Gritsenko, OF; Klovach, NV | 2013 | Single-nucleotide polymorphism in populations of sockeye salmon Oncorhynchus nerka from Kamchatka Peninsula | The genetic variability of 45 single-nucleotide polymorphism loci was examined in the four largest wild populations of sockeye salmon Oncorhynchus nerka from drainages of the Asian coast of the Pacific Ocean (Eastern and Western Kamchatka). It was demonstrated that sockeye salmon from the Palana River were considerably different from all other populations examined. The most probable explanation of the observed differences is the suggestion on possible demographic events in the history of this population associated with the decrease in its effective number. To study the origin, colonization patterns, and evolution of Asian sockeye salmon, as well as to resolve some of the applied tasks, like population assignment and genetic identification, a differential approach to SNP-marker selection was suggested. Adaptively important loci that evolve under the pressure of balancing (stabilizing) selection were identified, owing to this fact the number of loci that provide the baseline classification error rates in the population assignment tests was reduced to 30. It was demonstrated that SNPs located in the MHC2 and GPH genes were affected by diversifying selection. Procedures for selecting single-nucleotide polymorphisms for phylogenetic studies of Asian sockeye salmon were suggested. Using principal-component analysis, 17 loci that adequately reproduce genetic differentiation within and among the regions of the origin of Kamchatka sockeye salmon were selected. | 2869 | NA | NA | NA | no | no |
| 2870 | 2017 | NA | Davidson, I; Shkoda, I; Golender, N; Perk, S; Lapin, K; Khinich, Y; Panshin, A | 2013 | Genetic characterization of HA gene of low pathogenic H9N2 influenza viruses isolated in Israel during 2006-2012 periods | H9N2 influenza viruses are isolated in Israel since 2000 and became endemic. From November 2006 to the beginning of 2012, many H9N2 viruses were identified, all belonged to the Asian G1-like lineage represented by A/qu/Hong Kong/G1/97 (H9N2). In the present study, 66 isolates were selected for their hemagglutinin gene characterization. Most H9N2 isolates were distributed between two main groups, identified as the 4th and 5th introductions. The 5th introduction, was represented by a compact cluster containing viruses isolated in 2011-2012; the 4th introduction was subdivided into two subgroups, A and B, each containing at least two clusters, which can be identified as A-1, A-2, B-1, and B2, respectively. Genetic analysis of the deduced HA proteins of viruses, belonging to the 4th and 5th introductions, revealed amino acid variations in 79 out of 542 positions. All isolates had typical low pathogenicity motifs at the hemagglutinin (HA) cleavage site. Most viruses had leucine at position 216 in a receptor binding pocket that enables the virus to bind successfully with the cellular receptors intrinsic to mammals, including humans. It was shown that the differences between the HA proteins of viruses used for vaccine production and local field isolates increased in parallel with the duration and intensity of vaccine use, illustrating the genetic diversity of the H9N2 viruses in Israel. | 2870 | NA | NA | NA | no | no |
| 2871 | 2017 | NA | Talukdar, PK; Rahman, M; Rahman, M; Nabi, A; Islam, Z; Hoque, MM; Endtz, HP; Islam, MA | 2013 | Antimicrobial Resistance, Virulence Factors and Genetic Diversity of Escherichia coli Isolates from Household Water Supply in Dhaka, Bangladesh | Background: Unsafe water supplies continue to raise public health concerns, especially in urban areas in low resource countries. To understand the extent of public health risk attributed to supply water in Dhaka city, Bangladesh, Escherichia coli isolated from tap water samples collected from different locations of the city were characterized for their antibiotic resistance, pathogenic properties and genetic diversity. Methodology/Principal Findings: A total of 233 E. coli isolates obtained from 175 tap water samples were analysed for susceptibility to 16 different antibiotics and for the presence of genes associated with virulence and antibiotic resistance. Nearly 36% (n = 84) of the isolates were multi-drug(>= 3 classes of antibiotics) resistant (MDR) and 26% (n = 22) of these were positive for extended spectrum beta-lactamase (ESBL). Of the 22 ESBL-producers, 20 were positive for bla(CTX-M-15), 7 for bla(OXA-1-group) (all had bla(OXA-47)) and 2 for bla(CMY-2). Quinolone resistance genes, qnrS and qnrB were detected in 6 and 2 isolates, respectively. Around 7% (n = 16) of the isolates carried virulence gene(s) characteristic of pathogenic E. coli; 11 of these contained lt and/or st and thus belonged to enterotoxigenic E. coli and 5 contained bfp and eae and thus belonged to enteropathogenic E. coli. All MDR isolates carried multiple plasmids (2 to 8) of varying sizes ranging from 1.2 to >120 MDa. Ampicillin and ceftriaxone resistance were co-transferred in conjugative plasmids of 70 to 100 MDa in size, while ampicillin, trimethoprim-sulfamethoxazole and tetracycline resistance were co-transferred in conjugative plasmids of 50 to 90 MDa. Pulsed-field gel electrophoresis analysis revealed diverse genetic fingerprints of pathogenic isolates. Significance: Multi-drug resistant E. coli are wide spread in public water supply in Dhaka city, Bangladesh. Transmission of resistant bacteria and plasmids through supply water pose serious threats to public health in urban areas. | 2871 | NA | NA | NA | no | no |
| 2872 | 2017 | NA | Kiros-Meles, A; Gomez, D; McDonald, BA; Yahyaoui, A; Linde, CC | 2011 | Invasion of Rhynchosporium commune onto wild barley in the Middle East | Rhynchosporium commune was recently introduced into the Middle East, presumably with the cultivated host barley (Hordeum vulgare). Middle Eastern populations of R. commune on cultivated barley and wild barley (H. spontaneum) were genetically undifferentiated and shared a high proportion of multilocus haplotypes. This suggests that there has been little selection for host specialization on H. spontaneum, a host population often used as a source of resistance genes introduced into its domesticated counterpart, H. vulgare. Low levels of pathogen genetic diversity on H. vulgare as well as on H. spontaneum, indicate that the pathogen was introduced recently into the Middle East, perhaps through immigration on infected cultivated barley seeds, and then invaded the wild barley population. Although it has not been documented, the introduction of the pathogen into the Middle East may have a negative influence on the biodiversity of native Hordeum species. | 2872 | NA | NA | NA | no | no |
| 2873 | 2017 | NA | Jongwutiwes, S; Putaporntip, C; Iwasaki, T; Ferreira, MU; Kanbara, H; Hughes, AL | 2005 | Mitochondrial genome sequences support ancient population expansion in Plasmodium vivax | Examination of nucleotide diversity in 106 mitochondrial genomes of the most geographically widespread human malaria parasite, Plasmodium vivax, revealed a level of diversity similar to, but slightly higher than, that seen in the virulent human malaria parasite Plasmodium falciparum. The pairwise distribution of nucleotide differences among mitochondrial genome sequences supported the hypothesis that both these parasites underwent ancient population expansions. We estimated the age of the most recent common ancestor (MRCA) of the mitochondrial genomes of both P. vivax and P. falciparum at around 200,000-300,000 years ago. This is close to the previous estimates of the time of the human mitochondrial MRCA and the origin of modem Homo sapiens, consistent with the hypothesis that both these Plasmodium species were parasites of the hominid lineage before the origin of modem H. sapiens and that their population expansion coincided with the population expansion of their host. | 2873 | NA | NA | NA | no | no |
| 2874 | 2017 | NA | Alves-de-Souza, C; Varela, D; Iriarte, JL; Gonzalez, HE; Guillou, L | 2012 | Infection dynamics of Amoebophryidae parasitoids on harmful dinoflagellates in a southern Chilean fjord dominated by diatoms | Parasitoids belonging to Amoebophryidae (Marine ALVeolate Group II or MALV II) infecting dinoflagellates were investigated in Reloncavi Fjord (southern Chile) in the austral summer 2009. Of the 12 dinoflagellate species recorded during monthly sampling, Prorocentrum micans, Dinophysis acuminata, and Phalacroma rotundata were infected by Amoebophrya spp. To assess parasitoid control of host populations, the short-term dynamics of Amoebophrya spp. were followed for 21 d during a bloom of P. micans. Host mortality induced by Amoebophrya spp. (% hosts killed per day) was estimated by dividing prevalence by the generation time of these parasitoids. Parasitism by Amoebophrya spp. was responsible for similar to 10% of P. micans mortality between 8 and 17 March. The small subunit rDNA genes of individual parasitoids infecting different host cells were sequenced, and like most environmental sequences retrieved from this ecosystem in January and February, all belonged to the MALV II Clade 4. However, as soon as the P. micans bloom was detected, the MALV II genetic composition changed drastically, with the detection of Clades 1, 4 (a different sub-cluster than before), 5, 6, and 12. Our results suggest that different types of parasitoids belonging to Amoebophryidae likely infect dinoflagellates in Reloncavi Fjord, offering the first demonstration of the presence of important genetic diversity in MALV II inhabiting an ecosystem where dinoflagellate hosts are not the usual dominant phytoplanktonic organisms. | 2874 | NA | NA | NA | no | no |
| 2875 | 2017 | NA | Lemee, L; Hong, E; Etienne, M; Deghmane, AE; Delbos, V; Terrade, A; Berthelot, G; Caron, F; Taha, MK | 2014 | Genetic Diversity and Levels of Expression of Factor H Binding Protein among Carriage Isolates of Neisseria meningitidis | The prevention of meningococcal disease may be improved by recombinant vaccines such as 4CMenB and rLP2086 that target the factor H binding protein (fHbp), an immunogenic surface component of Neisseria meningitidis present as one of three variants. Whether such vaccines decrease carriage of invasive isolates and thus induce herd immunity is unknown. We analyzed the genetic diversity and levels of expression of fHbp among 268 carriage strains and compare them to those of 467 invasive strains. fhbp gene sequencing showed higher proportions of variants 2 and 3 among carriage isolates (p<0.0001). Carriage isolates expressed lower levels of fHbp (p<0.01) but that remain high enough to predict targeting by antibodies against fHbp particularly in group B isolates belonging to the frequent hypervirulent clonal complexes in Europe and North America (cc32, cc41/44, cc269). This suggests that fHbp targeting meningococcal vaccines might reduce, at least in part, the acquisition of some hyperinvasive isolates. | 2875 | NA | NA | NA | no | no |
| 2876 | 2017 | NA | Bowland, AE; Bishop, KS; Taylor, PJ; Lamb, J; van der Bank, FH; van Wyk, E; York, D | 2001 | Estimation and management of genetic diversity in small populations of plains zebra (Equus quagga) in KwaZulu-Natal, South Africa | Plains zebras (Equus quagga antiquorum) occur in few large, but many small, isolated populations in KwaZulu-Natal. Problems identified in small populations include reduced striping patterns on hind quarters, smaller size, elevated mortality rates and high number of still-births. Inbreeding may be implicated. Population viability analysis (PVA) was conducted with a computer model (VORTEX), and DNA and allozyme analyses were conducted to test the findings of the model. Using standard methods, DNA (PCR-RAPD) and allozyme diversity was assessed in blood samples from 72 plains zebra from four KwaZulu-Natal Nature Conservation Services (KZN-NCS) protected areas: Umfolozi Game Reserve (UCR), Albert Falls (AFNR), Vernon Crookes (VCNR) and Harold Johnson (HJNR) Nature Reserves. Populations of the latter three, small-sized (9-110 individuals) populations were seeded from the same source population (UGR: current population of 2000) during the past 25 years. Information from PCR-RAPD and allozyme analysts were compared with each other as well as to that predicted by population genetic modelling (using VORTEX). Allozyme heterozygosities were consistently high in all populations (12.1-12.9%), with no observable losses associated with reduced population size. On the other hand, percentage loss of polymorphism (20-39%) calculated from the PCR-RAPD study appeared to be positively correlated with the loss of heterozygosity predicted by population viability analysis (PVA), and negatively correlated with population size. On the basis of the above results, a policy of translocation was advocated for small, intensely managed populations of zebras. whereby a harem should be translocated every five years for a population size of nine (HJNR). while for a population size of 110 (VCNR) translocations should take place every 15 years if heterozygosity is to be maintained at more than 90% within each population over 100 years. (C) 2001 Elsevier Science Ltd. All rights reserved. | 2876 | NA | NA | NA | no | no |
| 2877 | 2017 | NA | Urbina-Cardona, JN | 2008 | CONSERVATION OF NEOTROPICAL HERPETOFAUNA: RESEARCH TRENDS AND CHALLENGES | The Neotropics harbor between 30-50% of the world’s herpetofauna. However, little is known about the ecology and natural history of many species, making conservation strategies difficult to plan. After reviewing published papers on world herpetofauna conservation, it was shown that conservation biology has a low impact factor in scientific journals in comparison with other related disciplines such as evolutionary biology and ecology. Moreover, herpetology has one of the lowest impact factors within the biological sciences journals. The number of publications on amphibian and reptile conservation has increased in recent years; however, only 31% of the papers on herpetofaunal conservation have been published in high impact journals. There are many challenges to overcome in the conservation of the Neotropical herpetofauna. Uniform and stable taxonomic nomenclature is critical to avoid overestimation of species richness and diversity for conservation assessments, and in the context of legal proceedings. Herpetofaunal research needs to be conducted within the appropriate socio-political and economic framework, in order to effectively implement conservation area networks. It is important to reevaluate the role of protected area systems in ensuring the persistence of communities and populations, and to identify strategies and future conservation priorities, based on climate-change scenarios. Population and community studies at different spatial and temporal scales are necessary to understand herpetofauna responses to anthropogenic disturbances, habitat loss and fragmentation, edge and matrix effects, and their synergy with micro-climatic gradients, emergent diseases and shifting patterns of genetic diversity. One of the biggest challenges for herpetofaunal conservation science in the neotropics is to control habitat loss and increase landscape connectivity along altitudinal gradients, while at the same time control species invasion that alter native species’ interactions and spread emergent diseases (e.g. Chytridiomycosis) facilitated by climate change. | 2877 | NA | NA | NA | no | no |
| 2878 | 2017 | NA | Krokene, C; Lifjeld, JT | 2000 | Variation in the frequency of extra-pair paternity in birds: A comparison of an island and a mainland population of blue tits | The frequency of extra-pair paternity (EPP), as revealed by multilocus DNA fingerprinting, was examined over three breeding seasons each in an island and a mainland population of blue tits Parus cacruleus in Norway. It has been postulated that island populations have a lower frequency of EPP than mainland populations, due to a reduced level of genetic variability. We found an EPP frequency of 5% (of offspring) on the island, and 13% on the mainland, but the difference was not statistically significant. There was neither any difference in genetic variability between the two populations, as assessed by band-sharing analysis of a random subset of breeding individuals. However, band-sharing between Fair mates was significantly higher in the island population, where some individuals seemed to mate with close relatives tone sibling pair was detected), nigh genetic similarity between pair mates on the island did not affect offspring survival, but had a significant negative effect on hatching success. The annual EPP frequency in the two populations was positively correlated with breeding density, but not with breeding synchrony. When the data were analysed on a per nest level, the mainland pl,population showed a tendency of a higher neighbour density (number of conspecific nests within 100 m) fur nests with EPP than for nests without EPP. There was no indication that individual synchrony (the percentage of females in the population that also were fertile when the focal female was fertile) affected the likelihood of EPP. We conclude that there was no particular island effect on the frequency of EPP in our data set. The marginal variation in EPP frequency observed across years and populations is more likely associated with local variations in breeding density and the number of close neighbours. | 2878 | NA | NA | NA | no | no |
| 2879 | 2017 | NA | Ahlholm, JU; Helander, M; Henriksson, J; Metzler, M; Saikkonen, K | 2002 | Environmental conditions and host genotype direct genetic diversity of Venturia ditricha, a fungal endophyte of birch trees | We investigated whether genetic variation of a common foliar endophyte of birch trees, Venturia ditricha, is affected by environmental conditions or host genotype. Fungal samples were collected from 10 half-sibling families of mountain birch (Betula pubescens ssp. czerepanovii) grown in two environmental conditions with different daily average temperatures: a forested river valley and an adjacent open tundra (altitudinal difference 180 in). Genetic analysis of V. ditricha isolates was done using random amplified microsatellite polymerase chain reaction. We found that host genotypes, along with prevailing environmental conditions, influence the probability of infection by particular endophyte genotypes. The most susceptible host genotypes were highly infected with genetically similar endophyte genotypes, whereas the most resistant trees were poorly infected and they were infected by genetically dissimilar endophytes. Our results also showed environment-host genotype interactions, suggesting that the susceptibility of the host to a particular endophyte genotype may change in natural environments when environmental conditions are changed. It appears, that a particular endophyte genotype needs to find the right host genotype for a successful infection. There are many host genotypes in natural stands; this means, from the point of view of the fungus, the environment is heterogeneous. Thus, under the influence of birch tree genotypes, genetically differentiated subgroups of the endophytic fungus may be formed in different environments. | 2879 | NA | NA | NA | no | no |
| 2880 | 2017 | NA | Abbot, P; Chhatre, V | 2007 | Kin structure provides no explanation for intruders in social aphids | Nontraditional social organisms have received increasing attention in recent years, because they present opportunities to study the convergent properties of social evolution. Some aphid species are social, occurring in dense clones with specialized morphs that attack predators and parasites. Little is known about how social aphid colonies resolve conflicts of interest when clonal barriers break down. Pemphigus obesinymphae is a North American gall-forming social aphid that produces both nymphal defenders that protect natal clones, and specialized intruders that invade other nearby clones on their host plants. We tested the hypothesis that clones are arranged on their host plants in spatial clusters of related family groups, such that intruders would be biased towards movement within kin groups. Movement within and not between kin groups would then provide insight into the nature of conflict in this social aphid. We sampled eight sites in the eastern United States and in Arizona, and used eight microsatellite markers to estimate pairwise relatedness between spatial groups. We found little evidence of deviation from random distributions of genotypes on their host plants. Evidently, Pem. obesinymphae intruders typically exploit unrelated clones, and spatial orientation provides no solution to the problem of ‘polyclonality’ in this species. We discuss implications of this result for our understanding of cooperation and conflict in social aphids. | 2880 | NA | NA | NA | no | no |
| 2881 | 2017 | NA | Munoz-Fuentes, V; Vila, C; Green, AJ; Negro, JJ; Sorenson, MD | 2007 | Hybridization between white-headed ducks and introduced ruddy ducks in Spain | The ruddy duck, Oxyura jamaicensis, was introduced to Great Britain in the mid-20th century and has recently spread to other Western European countries. In Spain, ruddy ducks hybridize with the globally endangered white-headed duck, Oxyura leucocephala. We assessed the effects of hybridization on the Spanish white-headed ducks, which constitute 25% of the global population of this species, using a panel of eight nuclear intron markers, 10 microsatellite loci, and mtDNA control region sequences. These data allowed parental individuals, F-1 hybrids, and the progeny of backcrossing to be reliably distinguished. We show that hybrids between the two species are fertile and produce viable offspring in backcrosses with both parental species. To date, however, we found no extensive introgression of ruddy duck genes into the Spanish white-headed duck population, probably due to the early implementation of an effective ruddy duck and hybrid control programme. We also show that genetic diversity in the expanding European ruddy duck population, which was founded by just seven individuals, exceeds that of the native Spanish white-headed duck population, which recently recovered from a severe bottleneck. Unless effective control of ruddy ducks is continued, genetic introgression will compromise the unique behavioural and ecological adaptations of white-headed ducks and consequently their survival as a genetically and evolutionary distinct species. | 2881 | NA | NA | NA | no | no |
| 2882 | 2017 | NA | Allen, GR; Seeman, OD; Schmid-Hempel, P; Buttermore, RE | 2007 | Low parasite loads accompany the invading population of the bumblebee, Bombus terrestris in Tasmania | In its native Europe, the bumblebee, Bombus terrestris (L.) has co-evolved with a large array of parasites whose numbers are negatively linked to the genetic diversity of the colony. In Tasmania B. terrestris was first detected in 1992 and has since spread over much of the state. In order to understand the bee’s invasive success and as part of a wider study into the genetic diversity of bumblebees across Tasmania, we screened bees for co-invasions of ectoparasitic and endoparasitic mites, nematodes and micro-organisms, and searched their nests for brood parasites. The only bee parasite detected was the relatively benign acarid mite Kuzinia laevis (Dujardin) whose numbers per bee did not vary according to region. Nests supported no brood parasites, but did contain the pollen-feeding life stages of K. laevis. Upon summer-autumn collected drones and queens, mites were present on over 80% of bees, averaged ca. 350-400 per bee and were more abundant on younger bees. Nest searching spring queens had similar mite numbers to those collected in summer-autumn but mite numbers dropped significantly once spring queens began foraging for pollen. The average number of mites per queen bee was over 30 fold greater than that reported in Europe. Mite incidence and mite numbers were significantly lower on worker bees than drones or queens, being present on just 51% of bees and averaging 38 mites per bee. Our reported incidence of worker bee parasitism by this mite is 5-50 times higher than reported in Europe. That only one parasite species co-invaded Tasmania supports the notion that a small number of queens founded the Tasmanian population. However, it is clearly evident that both the bee in the absence of parasites, and the mite have been extraordinarily successful invaders. | 2882 | NA | NA | NA | no | no |
| 2883 | 2017 | NA | Soper, DM; Delph, LF; Lively, CM | 2012 | Multiple paternity in the freshwater snail, Potamopyrgus antipodarum | Mating multiply may incur costs, such as exposure to predators and to sexually transmitted diseases. Nevertheless, it may be favored, in spite of these costs, as a way to increase the genetic diversity of offspring through fertilization by multiple males. Here, we tested for multiple paternity in a freshwater snail (Potamopyrgus antipodarum), which is host to several species of sterilizing trematode worms. Using microsatellites markers, we found multiple paternity in two different snail populations, with as many as seven males fertilizing a single female. In addition, high evenness of sire fertilization was found within individual broods. Multiple paternity can occur for a variety of reasons; however, given that these populations experience high risk of infection by a sterilizing trematode, one potential explanation may be that multiple paternity and high evenness of sire fertilizations increase the chances of the production of parasite-resistant offspring. | 2883 | NA | NA | NA | no | no |
| 2884 | 2017 | NA | Zhang, X; He, SY; Evans, JD; Pettis, JS; Yin, GF; Chen, YP | 2012 | New evidence that deformed wing virus and black queen cell virus are multi-host pathogens | The host-range breadth of pathogens can have important consequences for pathogens’ long term evolution and virulence, and play critical roles in the emergence and spread of the new diseases. Black queen cell virus (BQCV) and Deformed wing virus (DWV) are the two most common and prevalent viruses in European honey bees, Apis mellifera. Here we provide the evidence that BQCV and DWV infect wild species of honey bees, Apis florea and Apis dorsata. Phylogenetic analyses suggest that these viruses might have moved from A. mellifera to wild bee species and that genetic relatedness as well as the geographical proximity of host species likely play an important role in host range of the viruses. The information obtained from this present study can have important implication for understanding the population structure of bee virus as well as host-virus interactions. Published by Elsevier Inc. | 2884 | NA | NA | NA | no | no |
| 2885 | 2017 | NA | Bowen, KD; Colbert, PL; Janzen, FJ | 2004 | Survival and recruitment in a human-impacted population of ornate box turtles, Terrapene ornata, with recommendations for conservation and management | Alteration and loss of habitat is a major factor in the recent declines of many turtle populations. However, there are few studies of turtle populations in areas that are used intensively by humans. We used temporal symmetry modeling and an information-theoretic approach to model selection to estimate survival and recruitment in a population of Ornate Box Turtles, Terrapene ornata, in fragmented, isolated habitat over an eight-year period. Apparent annual survival was high during this period (0.97, SE = 0.06), as was the seniority probability (0.95 +/- 0.04). Recruitment into the adult population (lambda) was estimated at 1.02 (+/- 0.06). Our results suggest a healthy population, but we note several reasons for a cautious management approach. These include a vulnerability of lambda to the removal of adults, the need for increased recruitment to offset loss of genetic diversity, and the uncertainty of our estimates resulting from the sampling and modeling processes. | 2885 | NA | NA | NA | no | no |
| 2886 | 2017 | NA | Mayo, O | 2007 | The rise and fall of the common disease-common variant (CD-CV) hypothesis: How the sickle cell disease paradigm led us all astray (or did it?) | The common disease-common variant (CD-CV) hypothesis requires an explanation for the origin of the variation observed, since substantial neutral, but not deleterious, variation, that is, several alleles each at moderate to high frequency, can be maintained at any gene/locus by mutation. It is argued here that the guiding principle, not always stated, has been balancing selection, influenced by the well-established cases of deleterious alleles maintained through heterozygous advantage in the face of strong malarial selection against normal alleles. It is further argued that, although balanced polymorphisms have indeed arisen and reduced population loss through infectious disease, the history of balance in other contexts should have prevented acceptance of any hypothesis that generalized such a specific mechanism. Finally, it is suggested that in the present state of knowledge no single hypothesis for the genetical contribution to common disorders is justifiable. | 2886 | NA | NA | NA | no | no |
| 2887 | 2017 | NA | Vavrek, MC; McGraw, JB; Yang, HS | 1997 | Within-population variation in demography of Taraxacum officinale: Season- and size-dependent survival, growth and reproduction | 1 Lefkovitch transition matrices were used to determine vital demographic rates of a natural population of Taraxacum officinale in Morgantown, WV, USA. Separate size transition matrices were calculated for each of four seasons, October-January, January–April, April–July, and July–October, to test if demographic rates vary as a function of season and if size-specific rates vary differentially among seasons. Season-dependent demography was also compared for four phenotype classes segregated by cluster analysis of leaf morphology. 2 The finite rate of increase for the entire population was largest in autumn (October-January) and declined throughout the rest of the year. Overall, there was a small reduction in the population size. Size-specific probabilities of survival, growth and fertility varied dramatically among seasons. Sensitivity analyses showed that small individuals were particularly important to population growth from autumn to spring. Larger individuals were more important during summer. 3 Highly season-dependent demographic rates have large implications for population distribution and persistence since increased vulnerability to perturbation during particular seasons may constrain population growth and stability. Although T. officinale is a long-lived perennial, annual censuses may mask the importance of certain individuals or life history traits for maintenance of genetic variability and population viability. 4 Seasonal and annual finite rates of increase also varied as a function of phenotype class. Of two phenotype classes which had identical annual growth rates, one grew better in cool seasons while the second performed better in warm seasons. Direct competition for resources should be reduced by such inverse patterns of demography across seasons. 5 If phenotype classes are to some degree genetically determined, the differential responses observed here suggest that temporal variation in the environment could explain the maintenance of genetic diversity within populations. | 2887 | NA | NA | NA | no | no |
| 2888 | 2017 | NA | Beatty, GE; Provan, J | 2014 | Phylogeographical analysis of two cold-tolerant plants with disjunct Lusitanian distributions does not support in situ survival during the last glaciation | AimWe used a combination of modelling and genetic approaches to investigate whether Pinguicula grandiflora and Saxifraga spathularis, two species that exhibit disjunct Lusitanian distributions, may have persisted through the Last Glacial Maximum (LGM, c. 21 ka) in separate northern and southern refugia. LocationNorthern and eastern Spain and south-western Ireland. MethodsPalaeodistribution modelling using Maxent was used to identify putative refugial areas for both species at the LGM, as well as to estimate their distributions during the Last Interglacial (LIG, c. 120 ka). Phylogeographical analysis of samples from across both species’ ranges was carried out using one chloroplast and three nuclear loci for each species. ResultsThe palaeodistribution models identified very limited suitable habitat for either species during the LIG, followed by expansion during the LGM. A single, large refugium across northern Spain and southern France was postulated for P. grandiflora. Two suitable regions were identified for S. spathularis: one in northern Spain, corresponding to the eastern part of the species’ present-day distribution in Iberia, and the other on the continental shelf off the west coast of Brittany, south of the limit of the British-Irish ice sheet. Phylogeographical analyses indicated extremely reduced levels of genetic diversity in Irish populations of P. grandiflora relative to those in mainland Europe, but comparable levels of diversity between Irish and mainland European populations of S. spathularis, including the occurrence of private hapotypes in both regions. Main conclusionsModelling and phylogeographical analyses indicate that P. grandiflora persisted through the LGM in a southern refugium, and achieved its current Irish distribution via northward dispersal after the retreat of the ice sheets. Although the results for S. spathularis are more equivocal, a similar recolonization scenario also seems the most likely explanation for the species’ current distribution. | 2888 | NA | NA | NA | no | no |
| 2889 | 2017 | NA | Carpenter, JA; Obbard, DJ; Maside, X; Jiggins, FM | 2007 | The recent spread of a vertically transmitted virus through populations of Drosophila melanogaster | The sigma virus is a vertically transmitted pathogen that commonly infects natural populations of Drosophila melanogaster. This virus is the only known host-specific pathogen of D. melanogaster, and so offers a unique opportunity to study the genetics of Drosophila-viral interactions in a natural system. To elucidate the population genetic processes that operate in sigma virus populations, we collected D. melanogaster from 10 populations across three continents. We found that the sigma virus had a prevalence of 0-15% in these populations. Compared to other RNA viruses, we found that levels of viral genetic diversity are very low across Europe and North America. Based on laboratory measurements of the viral substitution rate, we estimate that most European and North American viral isolates shared a common ancestor approximately 200 years ago. We suggest two explanations for this: the first is that D. melanogaster has recently acquired the sigma virus; the second is that a single viral type has recently swept through D. melanogaster populations. Furthermore, in contrast to Drosophila populations, we find that the sigma viral populations are highly structured. This is surprising for a vertically transmitted pathogen that has a similar migration rate to its host. We suggest that the low structure in the viral populations can be explained by the smaller effective population size of the virus. | 2889 | NA | NA | NA | no | no |
| 2890 | 2017 | NA | Khaliq, I; Tejedor, MT; Monteagudo, LV; Riaz, M; Khan, AA | 2011 | Mitochondrial DNA diversity in Francolinus pondicerianus interpositus (grey francolin, Galliformes) from Pakistan | Francolinus pondicerianus interpositus (grey francolin, Galliformes) is the only francolin present in the Suleiman Range (central Pakistan), one of the poorest and least developed areas in Pakistan. As a game bird, the francolin is an important income source for the region, but no demographic data are available. Therefore, the aim of this work was to study the polymorphism pattern of the Control Region gene (mitochondrial DNA, mtDNA), in order to obtain some initial information about genetic diversity, possible structure and demographic dynamics in this population. In 29 individuals captured in four sampling areas in the western and the eastern Suleiman Range, we detected nine polymorphic sites in a 511 bp fragment of the mtDNA Control Region gene, resulting in seven haplotypes. Haplotype (h = 0.818 +/- 0.032) and nucleotide diversity (pi % = 0.308 +/- 0.210) values suggested a large population size and a low divergence among the haplotypes. AMOVA ((ST) = 0.005; P = 0.352) did not detect any significant differences among the western and eastern populations; therefore, specimens of both sampled areas could be considered as drawn from a single population. The observed distribution of pairwise mismatches was bimodal, revealing significant departure from a growing-decreasing population model (P = 0.030); these results would point to a demographic equilibrium. Tribal control of hunting might provide an explanation for this situation, but future overhunting would threaten the survival of this population. | 2890 | NA | NA | NA | no | no |
| 2891 | 2017 | NA | Aslam, U; Khan, AA; Cheema, HMN; Imtiaz, F; Malik, W | 2013 | Kill Curve Analysis and Response of Ethyl Methanesulfonate and gamma-rays in Diploid and Tetraploid Cotton | Mutagenesis has been used to a good extent to induce genetic variability in plant species to achieve the desired genetic variability. To attain maximum useful mutation density per unit genome and comparative effectiveness of gamma-rays and Ethyl methanesulfonate (EMS), optimal dose for treatment is the key to success. This study focuses on the development of kill curve in three cotton species (Gossypium arboreum, G. barbadense and G. hirsutum). Four genotypes from each of these Gossypium species were treated with eight varying levels of EMS (0.1 to 0.8%) and two doses of gamma-rays (100 to 800 Gy) for kill curve analysis. The data collected on germination percentage, plant height, number of bolls/plant, boll weight, lint yield and its percentage were analyzed and significant interaction among species, genotypes, mutagens and levels of mutagens was observed for all traits under study. Morphologically unusual mutants (rudimentary sparse leaves pattern and broad leaf shape) were also identified from M-1 generations of cotton genotypes. Optimal mutagenic doses were calculated based on survival rate and seed viability, which were considered useful in developing cotton mutagenized populations for forward and reverse genetic studies. EMS observed to be more effective than gamma-rays as it generated overall more number of mutants, while later caused higher physical injury in all cotton species. (C) 2013 Friends Science Publishers | 2891 | NA | NA | NA | no | no |
| 2892 | 2017 | NA | Gibert, M; Sanchez-Mazas, A | 2003 | Geographic patterns of functional categories of HLA-DRB1 alleles: a new approach to analyse associations between HLA-DRB1 and disease | Because specific amino acids found within the peptide-binding cleft of human leukocyte antigen (HLA) molecules have been implicated in HLA/disease associations, an approach which consists in grouping the alleles according to their functional properties at the protein level may enable us to better understand HLA associations than the conventional allelic classification. In this study, we applied this methodology to investigate the associations between HLA-DRB1 and rheumatoid arthritis. The alleles were first classified into seven functional categories [restrictive supertype patterns (RSPs)], among which three were known to be significantly associated with susceptibility (one category) or resistance (two categories) to rheumatoid arthritis. The frequencies of these categories were then estimated in 104 population samples previously tested for HLA-DRB1, and their variability was analysed spatially on a worldwide scale by applying an original methodology for detecting discontinuities in geographically patterned data. RSP frequencies were also compared to known values of rheumatoid arthritis prevalence in some populations. The results indicated that the three RSP frequency distributions were geographically structured, and that these patterns could generally be explained by the history of human migrations. However, the peculiar pattern observed for RSP ‘A’ (conferring susceptibility to rheumatoid arthritis) indicated a possible association with some latitude-dependent disease. Furthermore, the very high correlation coefficient found between RSP ‘A’ frequencies and rheumatoid arthritis prevalence confirmed the significant disease association of this functional category In contrast, the putative protective effect of the other RSPs (‘De’ and ‘Q’) was not detectable at the worldwide level, but may be significant in specific geographic areas. This study shows that population genetic diversity analyses based on a functional grouping of HLA alleles provide an efficient way to explore the mutual influence of HLA genetic variation and disease. | 2892 | NA | NA | NA | no | no |
| 2893 | 2017 | NA | Ward, TJ; Clear, RM; Rooney, AP; O’Donnell, K; Gaba, D; Patrick, S; Starkey, DE; Gilbert, J; Geiser, DM; Nowicki, TW | 2008 | An adaptive evolutionary shift in Fusarium head blight pathogen populations is driving the rapid spread of more toxigenic Fusarium graminearum in North America | Analysis of Fusarium head blight (FHB) pathogen diversity revealed that 3ADON producing Fusarium graminearum are prevalent in North America and identified significant population structure associated with trichothecene chemotype differences (F-ST > 0.285; P < 0.001). In addition, we identified a trichothecene chemotype cline in Canada and documented a recent and significant shift in FHB pathogen composition by demonstrating that the 3ADON chemotype frequency in western Canada increased more than 14-fold between 1998 and 2004. On average, isolates from 3ADON populations produced significantly (P < 0.05) more trichothecene and had significantly (P < 0.005) higher fecundity and growth rates than isolates front the 15ADON population. These results indicate that selection is driving the rapid spread of an introduced pathogen Population that is more toxigenic and potentially more vigorous. The discovery of this previously unrecognized pathogen diversity has significant implications for food safety and cereal production in North America. (C) 2007 Elsevier Inc. All rights reserved. | 2893 | NA | NA | NA | no | no |
| 2894 | 2017 | NA | Haverty, MI; Copren, KA; Getty, GM; Lewis, VR | 1999 | Agonistic behavior and cuticular hydrocarbon phenotypes of colonies of Reticulitermes (Isoptera : Rhinotermitidae) from northern California | Reticulitermes in northern California is supposed to be represented by R. hesperus Banks and R. tibialis Banks, yet at least 5 distinct cuticular hydrocarbon phenotypes have been characterized. Three hydrocarbon phenotypes from the Institute of Forest Genetics near Placerville, CA, and 3 cuticular hydrocarbon phenotypes from 2 separate sites in Marin County were used to characterize interactions of foraging groups or colonies at each site. Pairings from the same foraging group or different foraging groups of the same colony rarely resulted in immediate aggression and never resulted in high mortality. Pairings of workers from different foraging groups of the same cuticular hydrocarbon phenotype from either the Placerville or Marin sites resulted in few bouts (8 and 15%, respectively) with immediate aggression, but after 24 h, mortality was high in 56 and 81% of the bouts, respectively. Pairings from different cuticular hydrocarbon phenotypes resulted in immediate aggression 48.8 and 61.5% of the time, respectively; nearly all of these (>99%) resulted in high mortality after 24 h. These results suggest that these Reticulitermes recognize hydrocarbon phenotypes, and can differentiate colony mates and alien workers within a cuticular hydrocarbon phenotype. Because kin discrimination suggests genetic relatedness among individuals, this bioassay will be useful for determining the association of foraging groups in ecological studies of Reticulitermes colonies in northern California and indirectly may indicate relatedness among colonies of the same hydrocarbon phenotype. | 2894 | NA | NA | NA | no | no |
| 2895 | 2017 | NA | Hupalo, DN; Luo, ZP; Melnikov, A; Sutton, PL; Rogov, P; Escalante, A; Vallejo, AF; Herrera, S; Arevalo-Herrera, M; Fan, Q; Wang, Y; Cui, LW; Lucas, CM; Durand, S; Sanchez, JF; Baldeviano, GC; Lescano, AG; Laman, M; Barnadas, C; Barry, A; Mueller, I; Kazura, JW; Eapen, A; Kanagaraj, D; Valecha, N; Ferreira, MU; Roobsoong, W; Nguitragool, W; Sattabonkot, J; Gamboa, D; Kosek, M; Vinetz, JM; Gonzalez-Ceron, L; Birren, BW; Daniel, DE; Jane, JM | 2016 | Population genomics studies identify signatures of global dispersal and drug resistance in Plasmodium vivax | Plasmodium vivax is a major public health burden, responsible for the majority of malaria infections outside Africa. We explored the impact of demographic history and selective pressures on the P. vivax genome by sequencing 182 clinical isolates sampled from 11 countries across the globe, using hybrid selection to overcome human DNA contamination. We confirmed previous reports of high genomic diversity in P. vivax relative to the more virulent Plasmodium falciparum species; regional populations of P. vivax exhibited greater diversity than the global P. falciparum population, indicating a large and/or stable population. Signals of natural selection suggest that P. vivax is evolving in response to antimalarial drugs and is adapting to regional differences in the human host and the mosquito vector. These findings underline the variable epidemiology of this parasite species and highlight the breadth of approaches that may be required to eliminate P. vivax globally. | 2895 | NA | NA | NA | no | no |
| 2896 | 2017 | NA | Joshi, PK; Esko, T; Mattsson, H; Eklund, N; Gandin, I; Nutile, T; Jackson, AU; Schurmann, C; Smith, AV; Zhang, WH; Okada, Y; Stancakova, A; Faul, JD; Zhao, W; Bartz, TM; Concas, MP; Franceschini, N; Enroth, S; Vitart, V; Trompet, S; Guo, XQ; Chasman, DI; O’Connel, JR; Corre, T; Nongmaithem, SS; Chen, Y; Mangino, M; Ruggiero, D; Michela, T; Farmaki, AE; Kacprowski, T; Bjonnes, A; van der Spek, A; Wu, Y; Giri, AK; Yanek, LR; Wang, LH; Hofer, E; Rietveld, CA; McLeod, O; Cornelis, MC; Pattaro, C; Verweij, N; Baumbach, C; Abdellaoui, A; Warren, HR; Vuckovic, D; Mei, H; Bouchard, C; Perry, JRB; Cappellani, S; Mirza, SS; Benton, MC; Broeckel, U; Medland, SE; Lind, P; Malerba, G; Drong, A; Yengo, L; Bielak, LF; Zhi, DG; van der Most, PJ; Shriner, D; Magi, R; Hemani, G; Karaderi, T; Wang, ZM; Liu, T; Demuth, I; Zhao, JH; Meng, WH; Lataniotis, L; van der Laan, SW; Bradfield, JP; Wood, AR; Bonnefond, A; Ahluwalia, TS; Hall, L; Salvi, E; Yazar, S; Carstensen, L; de Haan, HG; Abney, M; Afzal, U; Allison, MA; Amin, N; Asselbergs, FW; Bakker, SJL; Barr, RG; Baumeister, SE; Benjamin, DJ; Bergmann, S; Boerwinkle, E; Bottinger, EP; Campbell, A; Chakravarti, A; Chan, YL; Chanock, SJ; Chen, C; Chen, YDI; Collins, FS; Connell, J; Correa, A; Cupples, LA; Smith, GD; Davies, G; Dorr, M; Ehret, G; Ellis, SB; Feenstra, B; Feitosa, MF; Ford, I; Fox, CS; Frayling, TM; Friedrich, N; Geller, F; Scotland, G; Gillham-Nasenya, I; Gottesman, O; Graff, M; Grodstein, F; Gu, C; Haley, C; Hammond, CJ; Harris, SE; Harris, TB; Hastie, ND; Heard-Costa, NL; Heikkila, K; Hocking, LJ; Homuth, G; Hottenga, JJ; Huang, JY; Huffman, JE; Hysi, PG; Ikram, MA; Ingelsson, E; Joensuu, A; Johansson, A; Jousilahti, P; Jukema, JW; Kahonen, M; Kamatani, Y; Kanoni, S; Kerr, SM; Khan, NM; Koellinger, P; Koistinen, HA; Kooner, MK; Kubo, M; Kuusisto, J; Lahti, J; Launer, LJ; Lea, RA; Lehne, B; Lehtimaki, T; Liewald, DCM; Lind, L; Loh, M; Lokki, ML; London, SJ; Loomis, SJ; Loukola, A; Lu, YC; Lumley, T; Lundqvist, A; Mannisto, S; Marques-Vidal, P; Masciullo, C; Matchan, A; Mathias, RA; Matsuda, K; Meigs, JB; Meisinger, C; Meitinger, T; Menni, C; Mentch, FD; Mihailov, E; Milani, L; Montasser, ME; Montgomery, G; Morrison, A; Myers, RH; Nadukuru, R; Navarro, P; Nelis, M; Nieminen, MS; Nolte, IM; O’Connor, GT; Ogunniyi, A; Padmanabhan, S; Palmas, WR; Pankow, JS; Patarcic, I; Pavani, F; Peyser, PA; Pietilainen, K; Poulter, N; Prokopenko, I; Ralhan, S; Redmond, P; Rich, SS; Rissanen, H; Robino, A; Rose, LM; Rose, R; Sala, C; Salako, B; Salomaa, V; Sarin, AP; Saxena, R; Schmidt, H; Scott, LJ; Scott, WR; Sennblad, B; Seshadri, S; Sever, P; Shrestha, S; Smith, BH; Smith, JA; Soranzo, N; Sotoodehnia, N; Southam, L; Stanton, AV; Stathopoulou, MG; Strauch, K; Strawbridge, RJ; Suderman, MJ; Tandon, N; Tang, ST; Taylor, KD; Tayo, BO; Toglhofer, AM; Tomaszewski, M; Tsernikova, N; Tuomilehto, J; Uitterlinden, AG; Vaidya, D; Vlieg, AV; van Setten, J; Vasankari, T; Vedantam, S; Vlachopoulou, E; Vozzi, D; Vuoksimaa, E; Waldenberger, M; Ware, EB; Wentworth-Shields, W; Whitfield, JB; Wild, S; Willemsen, G; Yajnik, CS; Yao, J; Zaza, G; Zhu, XF; Salem, RM; Melbye, M; Bisgaard, H; Samani, NJ; Cusi, D; Mackey, DA; Cooper, RS; Froguel, P; Pasterkamp, G; Grant, SFA; Hakonarson, H; Ferrucci, L; Scott, RA; Morris, AD; Palmer, CNA; Dedoussis, G; Deloukas, P; Bertram, L; Lindenberger, U; Berndt, SI; Lindgren, CM; Timpson, NJ; Tonjes, A; Munroe, PB; Sorensen, TIA; Rotimi, CN; Arnett, DK; Oldehinkel, AJ; Kardia, SLR; Balkau, B; Gambaro, G; Morris, AP; Eriksson, JG; Wright, MJ; Martin, NG; Hunt, SC; Starr, JM; Deary, IJ; Griffiths, LR; Tiemeier, H; Pirastu, N; Kaprio, J; Wareham, NJ; Peerusse, L; Wilson, JG; Girotto, G; Caulfield, MJ; Raitakari, O; Boomsma, DI; Gieger, C; van der Harst, P; Hicks, AA; Kraft, P; Sinisalo, J; Knekt, P; Johannesson, M; Magnusson, PKE; Hamsten, A; Schmidt, R; Borecki, IB; Vartiainen, E; Becker, DM; Bharadwaj, D; Mohlke, KL; Boehnke, M; van Duijn, CM; Sanghera, DK; Teumer, A; Zeggini, E; Metspalu, A; Gasparini, P; Ulivi, S; Ober, C; Toniolo, D; Rudan, I; Porteous, DJ; Ciullo, M; Spector, TD; Hayward, C; Dupuis, J; Loos, RJF; Wright, AF; Chandak, GR; Vollenweider, P; Shuldiner, AR; Ridker, PM; Rotter, JI; Sattar, N; Gyllensten, U; North, KE; Pirastu, M; Psaty, BM; Weir, DR; Laakso, M; Gudnason, V; Takahashi, A; Chambers, JC; Kooner, JS; Strachan, DP; Campbell, H; Hirschhorn, JN; Perola, M; Polasek, O; Wilson, JF | 2015 | Directional dominance on stature and cognition in diverse human populations | Homozygosity has long been associated with rare, often devastating, Mendelian disorders(1), and Darwin was one of the first to recognize that inbreeding reduces evolutionary fitness(2). However, the effect of the more distant parental relatedness that is common in modern human populations is less well understood. Genomic data now allow us to investigate the effects of homozygosity on traits of public health importance by observing contiguous homozygous segments (runs of homozygosity), which are inferred to be homozygous along their complete length. Given the low levels of genome-wide homozygosity prevalent in most human populations, information is required on very large numbers of people to provide sufficient power(3,4). Here we use runs of homozygosity to study 16 health-related quantitative traits in 354,224 individuals from 102 cohorts, and find statistically significant associations between summed runs of homozygosity and four complex traits: height, forced expiratory lung volume in one second, general cognitive ability and educational attainment (P < 1 x 10(-300), 2.1 x 10(-6), 2.5 x 10(-10) and 1.8 x 10(-10), respectively). In each case, increased homozygosity was associated with decreased trait value, equivalent to the offspring of first cousins being 1.2 cm shorter and having 10 months’ less education. Similar effect sizes were found across four continental groups and populations with different degrees of genome-wide homozygosity, providing evidence that homozygosity, rather than confounding, directly contributes to phenotypic variance. Contrary to earlier reports in substantially smaller samples(5,6), no evidence was seen of an influence of genome-wide homozygosity on blood pressure and low density lipoprotein cholesterol, or ten other cardio-metabolic traits. Since directional dominance is predicted for traits under directional evolutionary selection(7), this study provides evidence that increased stature and cognitive function have been positively selected in human evolution, whereas many important risk factors for late-onset complex diseases may not have been. | 2896 | NA | NA | NA | no | no |
| 2897 | 2017 | NA | de Groot, GA; During, H | 2013 | Fern Spore Longevity in Saline Water: Can Sea Bottom Sediments Maintain a Viable Spore Bank? | Freshwater and marine sediments often harbor reservoirs of plant diaspores, from which germination and establishment may occur whenever the sediment falls dry. Therewith, they form valuable records of historical inter- and intraspecific diversity, and are increasingly exploited to facilitate diversity establishment in new or restored nature areas. Yet, while ferns may constitute a considerable part of a vegetation’s diversity and sediments are known to contain fern spores, little is known about their longevity, which may suffer from inundation and - in sea bottoms - salt stress. We tested the potential of ferns to establish from a sea or lake bottom, using experimental studies on spore survival and gametophyte formation, as well as a spore bank analysis on sediments from a former Dutch inland sea. Our experimental results revealed clear differences among species. For Asplenium scolopendrium and Gymnocarpium dryopteris, spore germination was not affected by inundated storage alone, but decreased with rising salt concentrations. In contrast, for Asplenium trichomanes subsp. quadrivalens germination decreased following inundation, but not in response to salt. Germination rates decreased with time of storage in saline water. Smaller and less viable gametophytes were produced when saline storage lasted for a year. Effects on germination and gametophyte development clearly differed among genotypes of A. scolopendrium. Spore bank analyses detected no viable spores in marine sediment layers. Only two very small gametophytes (identified as Thelypteris palustris via DNA barcoding) emerged from freshwater sediments. Both died before maturation. We conclude that marine, and likely even freshwater sediments, will generally be of little value for long-term storage of fern diversity. The development of any fern vegetation on a former sea floor will depend heavily on the deposition of spores onto the drained land by natural or artificial means of dispersal. | 2897 | NA | NA | NA | no | no |
| 2898 | 2017 | NA | Taniguchi, Y; Matsumoto, K; Matsuda, H; Yamada, T; Sugiyama, T; Homma, K; Kaneko, Y; Yamagishi, S; Iwaisaki, H | 2014 | Structure and Polymorphism of the Major Histocompatibility Complex Class II Region in the Japanese Crested Ibis, Nipponia nippon | The major histocompatibility complex (MHC) is a highly polymorphic genomic region that plays a central role in the immune system. Despite its functional consistency, the genomic structure of the MHC differs substantially among organisms. In birds, the MHC-B structures of Galliformes, including chickens, have been well characterized, but information about other avian MHCs remains sparse. The Japanese Crested Ibis (Nipponia nippon, Pelecaniformes) is an internationally conserved, critically threatened species. The current Japanese population of N. nippon originates from only five founders; thus, understanding the genetic diversity among these founders is critical for effective population management. Because of its high polymorphism and importance for disease resistance and other functions, the MHC has been an important focus in the conservation of endangered species. Here, we report the structure and polymorphism of the Japanese Crested Ibis MHC class II region. Screening of genomic libraries allowed the construction of three contigs representing different haplotypes of MHC class II regions. Characterization of genomic clones revealed that the MHC class II genomic structure of N. nippon was largely different from that of chicken. A pair of MHC-IIA and -IIB genes was arranged head-to-head between the COL11A2 and BRD2 genes. Gene order in N. nippon was more similar to that in humans than to that in chicken. The three haplotypes contained one to three copies of MHC-IIA/IIB gene pairs. Genotyping of the MHC class II region detected only three haplotypes among the five founders, suggesting that the genetic diversity of the current Japanese Crested Ibis population is extremely low. The structure of the MHC class II region presented here provides valuable insight for future studies on the evolution of the avian MHC and for conservation of the Japanese Crested Ibis. | 2898 | NA | NA | NA | no | no |
| 2899 | 2017 | NA | Zhou, T; Wang, Y; Chen, JQ; Araki, H; Jing, Z; Jiang, K; Shen, J; Tian, D | 2004 | Genome-wide identification of NBS genes in japonica rice reveals significant expansion of divergent non-TIR NBS-LRR genes | A complete set of candidate disease resistance ( R) genes encoding nucleotide-binding sites (NBSs) was identified in the genome sequence of japonica rice ( Oryza sativa L. var. Nipponbare). These putative R genes were characterized with respect to structural diversity, phylogenetic relationships and chromosomal distribution, and compared with those in Arabidopsis thaliana. We found 535 NBS-coding sequences, including 480 non-TIR (Toll/IL-1 receptor) NBS-LRR (Leucine Rich Repeat) genes. TIR NBS-LRR genes, which are common in A. thaliana, have not been identified in the rice genome. The number of non-TIR NBS-LRR genes in rice is 8.7 times higher than that in A. thaliana, and they account for about 1% of all of predicted ORFs in the rice genome. Some 76% of the NBS genes were located in 44 gene clusters or in 57 tandem arrays, and 16 apparent gene duplications were detected in these regions. Phylogenetic analyses based both NBS and N-terminal regions classified the genes into about 200 groups, but no deep clades were detected, in contrast to the two distinct clusters found in A. thaliana. The structural and genetic diversity that exists among NBS-LRR proteins in rice is remarkable, and suggests that diversifying selection has played an important role in the evolution of R genes in this agronomically important species. (Supplemental material is available online at http://gattaca.nju.edu.cn.). | 2899 | NA | NA | NA | no | no |
| 2900 | 2017 | NA | FUNGARO, MHP; JUNIOR, CLDS; DEAZEVEDO, JL; PIZZIRANIKLEINER, AA | 1994 | RECURRENT MUTATION-SELECTION TO IMPROVE RENNET PRODUCTION IN CANDIDA-TSUKUBAENSIS | The yeast Candida tsukubaensis is of industrial importance for the production of microbial milk-clotting enzyme. Milk-clotting enzyme is an enzymatic complex capable of coagulating milk for cheese manufacturing. High clotting activity (CA) and low proteolytic activity (PA) are desirable qualities. To study the genetic nature of the CA and PA traits, we analyzed 179 colonies obtained after mutagenic treatment. Analysis of the data obtained for this populations showed that CA and PA are traits controlled by polygenes and that they are correlated (r = 0.3). The existence of a positive correlation indicates that selection for one trait without considering the other may alter one of the traits in an undesirable direction, since the objective of selection would be an increase in clotting activity and a decrease in proteolytic activity. Three cycles of recurrent mutation-selection were carried out to obtain improved strains. The ultraviolet light dose permitting a 5% rate of cell survival was sufficient to generate genetic variability in the three selection cycles. At the end of the third cycle there was an increase of about 98% in clotting activity and a decrease of about 20% in proteolytic activity. Analysis of variance of the selective cycles showed that the linear effects were significant (P < 0.01) for both traits. Estimates of genetic variances and heritabilities of the three selection cycles are presented. | 2900 | NA | NA | NA | no | no |
| 2901 | 2017 | NA | Ishikawa, FH; de Souza, EA; Davide, LMC | 2008 | Genetic variability within isolates of Colletotrichum lindemuthianum belonging to race 65 from the state of Minas Gerais, Brazil | Colletotrichum lindemuthianum, the causal agent of anthracnose in the common bean (Phaseolus vulgaris), presents a wide genetic and pathogenic variability that gives rise to complications in the development of resistant bean cultivars. The aim of this study was to identify the variability within race 65 of C. lindemuthianum, the race most commonly encountered in Brazil, through randomly amplified polymorphic DNA (RAPD) and anastomosis analyses. Thirteen isolates of race 65, collected in different years and from various host cultivars located in diverse areas of the state of Minas Gerais, Brazil, were investigated. Twenty-four RAPD primers were employed and 83 polymorphic bands amplified. Genetic similarities were estimated from the Sorensen-Dice coefficient and ranged from 0.54 to 0.82. The dendrogram obtained by cluster analysis classified the isolates into 11 separate groups. For the purposes of the analysis of anastomosis, isolates were considered to be compatible when the fusion of hyphae from different isolates could be observed. The proportion of compatible reactions for each isolate was estimated and similarity estimates, based on the Russel & Rao coefficient, ranged from 0.28 to 0.85. Isolates were classified into 11 anastomosis groups, 10 of which were formed by only one isolate. Although isolates LV61, LV73 and LV58 were classified in the same anastomosis group, they were genetically distinct according to RAPD analysis. Results from both RAPD and anastomosis analyses revealed great variability within C. lindemuthianum race 65. | 2901 | NA | NA | NA | no | no |
| 2902 | 2017 | NA | Gimsa, U; Ho, CS; Hammer, SE | 2017 | Preferred SLA class I/class II haplotype combinations in German Landrace pigs | Major histocompatibility complex (MHC) molecules are responsible for the antigen presentation to T lymphocytes. High recombination rates in the MHC genes, as observed in humans, are believed to serve the evolutionary goal to achieve a high genetic diversity, allowing for a broad and efficient immune response. In a cohort of 155 pedigreed German Landrace pigs (65 founders and 90 piglets), we found that MHC genes occur in particular class I and class II haplotype combinations. This phenomenon has not been described before, probably because most of the earlier MHC studies in pigs were not pedigree-based. After comparing our data with published genotypes of different European pig breeds and Asian pigs, we hypothesise that the combination of particular but different haplotypes in different geographical regions may have developed under the evolutionary pressure of regionally endemic pathogens. This proposed mechanism ensures an efficient immune response despite low recombination rates. | 2902 | NA | NA | NA | no | no |
| 2903 | 2017 | NA | Mejia-Madrid, HH; Vazquez-Dominguez, E; de Leon, GPP | 2007 | Phylogeography and freshwater basins in central Mexico: recent history as revealed by the fish parasite Rhabdochona lichtenfelsi (Nematoda) | Aim The phylogeography of Rhabdochona lichtenfelsi, a nematode parasite specific to endemic goodeids in Mexico, is used to infer the biogeographical history of fragmentation and recent evolution of the Mesa Central drainages. Geological history of the west-central region of Mexico suggests that extant freshwater basins are the result of different vicariant events that fragmented ancient watercourses and lakes within the Mesa Central. Location Major freshwater river basins of the Mesa Central, Mexico: Ameca, Cotija, Lerma, Rio Verde, Panuco, and lakes Cuitzeo and Zacapu. Methods Haplotype diversity and phylogeographical structure of 10 populations of R. lichtenfelsi, sampled from the complete range of this species, were analysed with partial sequences of cytochrome c oxidase subunit I (456 bp). Analyses performed included phylogenetic tree estimation methods (neighbour-joining, maximum parsimony and maximum likelihood), genetic diversity, distance and structure estimates, and nested clade analysis. Results High overall haplotype diversity, unique haplotypes, and strongly structured populations were found in the basins sampled. Three phylogenetically and demographically identifiable clades were recovered. These clades fit an isolation-by-distance model. Significant population expansion was observed for two clades and for the entire population. Time of divergence was estimated as 1.0 and 0.84 Ma for the different clades. Main conclusions The distribution of R. lichtenfelsi haplotypes does not correspond to the present distribution of the basins of Mesa Central, but instead reflects the distribution of those basins during a recent geological period (Pleistocene). While our current knowledge of the evolution and geographical relationships of the Mesa Central basins comes from studies of freshwater fish encompassing a more ancient history, our results suggest that, during the past million years, old basins and connections existed where today isolated freshwater bodies stand, thus unravelling a novel biogeographical history for the Mesa Central of Mexico. | 2903 | NA | NA | NA | no | no |
| 2904 | 2017 | NA | Baer, B; Krug, A; Boomsma, JJ; Hughes, WOH | 2005 | Examination of the immune responses of males and workers of the leaf-cutting ant Acromyrmex echinatior and the effect of infection | Parasites represent significant challenges to social insects. The high density, interaction rate and relatedness of individuals within colonies are all predicted to make social insect colonies particularly vulnerable to parasites. To cope with this pressure, social insects have evolved a number of defence mechanisms. These include the immune response, which, aside from in bumblebees, has been relatively little studied in social insects. Here we compare the immune responses of males and workers of the leaf-cutting ant Acro-myrmex echinatior and examine the effect upon immunocompetence of prior exposure to a virulent parasite. Males have a far lower immune response than workers, suggesting either haploid susceptibility or reduced investment in immunity by the short-lived males. There was also significantly less variation in the immune response of males than of workers, which may be due to leaf-cutting ant workers being more variable in age or more genetically diverse within colonies. When exposed to the entomopathogenic fungus Metarhizium, workers expressed a substantially reduced immune response 96 h after infection, suggesting that the immune system was either depleted by having to respond to the Metarhizium infection or was depressed by the parasite. The results suggest that the immune response is a costly and limited process, but further experiments are needed to distinguish between the alternative explanations for the effects observed. | 2904 | NA | NA | NA | no | no |
| 2905 | 2017 | NA | Hansen, ZR; Everts, KL; Fry, WE; Gevens, AJ; Grunwald, NJ; Gugino, BK; Johnson, DA; Johnson, SB; Judelson, HS; Knaus, BJ; McGrath, MT; Myers, KL; Ristaino, JB; Roberts, PD; Secor, GA; Smart, CD | 2016 | Genetic Variation within Clonal Lineages of Phytophthora infestans Revealed through Genotyping-By-Sequencing, and Implications for Late Blight Epidemiology | Genotyping-by-sequencing (GBS) was performed on 257 Phytophthora infestans isolates belonging to four clonal lineages to study within-lineage diversity. The four lineages used in the study were US-8 (n = 28), US-11 (n = 27), US-23 (n = 166), and US-24 (n = 36), with isolates originating from 23 of the United States and Ontario, Canada. The majority of isolates were collected between 2010 and 2014 (94%), with the remaining isolates collected from 1994 to 2009, and 2015. Between 3,774 and 5,070 single-nucleotide polymorphisms (SNPs) were identified within each lineage and were used to investigate relationships among individuals. K-means hierarchical clustering revealed three clusters within lineage US-23, with US-23 isolates clustering more by collection year than by geographic origin. K-means hierarchical clustering did not reveal significant clustering within the smaller US-8, US-11, and US-24 data sets. Neighbor-joining (NJ) trees were also constructed for each lineage. All four NJ trees revealed evidence for pathogen dispersal and overwintering within regions, as well as long-distance pathogen transport across regions. In the US-23 NJ tree, grouping by year was more prominent than grouping by region, which indicates the importance of long-distance pathogen transport as a source of initial late blight inoculum. Our results support previous studies that found significant genetic diversity within clonal lineages of P. infestans and show that GBS offers sufficiently high resolution to detect sub-structuring within clonal populations. | 2905 | NA | NA | NA | no | no |
| 2906 | 2017 | NA | Eduardo, I; Arus, P; Monforte, AJ | 2005 | Development of a genomic library of near isogenic lines (NILs) in melon (Cucumis melo L.) from the exotic accession PI161375 | “A doubled haploid line (DHL) population of melon derived from a cross between the Korean cultivar”“Songwhan Charmi”" accession PI161375 (SC), included in the horticultural group conomon, and the Spanish cultivar "“Piel de Sapo”" (PS), included in the horticultural group inodorus, was used to develop a collection of near isogenic lines (NILs). These parental lines represent very different melon cultivar groups, with important differences at fruit, plant, disease response and molecular level. This cross is one of the most polymorphic ones within melon germplasm. Selected DHLs were backcrossed to PS and further backcrossing and selfing was performed, monitoring introgressions from SC using molecular markers covering the melon genetic map. A final collection of 57 NILs was obtained, containing a unique independent introgression from SC in the PS genetic background. The introgressions within the collection cover at least 85% of the SC genome with an average introgression size of 41 cM, corresponding to 3.4% of the SC genome. The average resolution for mapping genes or quantitative trait loci is 18.90 cM. This set of NILs is a potentially powerful tool for the study of quantitative trait locus involved in melon fruit quality and other important complex traits, and the introduction of new genetic variability in modern cultivars from exotic sources. The NILs can also be used as pre-competitive breeding lines in melon breeding projects." | 2906 | NA | NA | NA | no | no |
| 2907 | 2017 | NA | Ryan, J; Canonico, M; Carcaillon, L; Carriere, I; Scali, J; Dartigues, JF; Dufouil, C; Ritchie, K; Scarabin, PY; Ancelin, ML | 2012 | Hormone Treatment, Estrogen Receptor Polymorphisms and Mortality: A Prospective Cohort Study | Background: The association between hormone treatment (HT) and mortality remains controversial. This study aimed to determine whether the risk of mortality associated with HT use varies depending on the specific characteristics of treatment and genetic variability in terms of the estrogen receptor. Methodology/Principal Findings: A prospective, population-based study of 5135 women aged 65 years and older who were recruited from three cities in France and followed over six years. Detailed information related to HT use was obtained and five estrogen receptor polymorphisms were genotyped. The total follow-up was 25,436 person-years and during this time 352 women died. Cancer (36.4%) and cardiovascular disease (19.3%) were the major causes of death. Cox proportional hazards models adjusted for age, education, centre, living situation, comorbidity, depression, physical and mental incapacities, indicated no significant association between HT and mortality, regardless of the type or duration of treatment, or the age at initiation. However, the association between HT and all-cause or cancer-related mortality varied across women, with significant interactions identified with three estrogen receptor polymorphisms (p-values = 0.004 to 0.03) in adjusted analyses. Women carrying the C allele of ESR1 rs2234693 had a decreased risk of all-cause mortality with HT (HR: 0.42, 95% CI: 0.18-0.97), while in stark contrast, those homozygous for the T allele had a significantly increased risk of cancer-related mortality (HR: 3.18, 95% CI: 1.23-8.20). The findings were similar for ESR1 rs9340799 and ESR2 rs1271572. Conclusions/Significance: The risk of mortality was not associated with HT duration, type or age at initiation. It was however not equal across all women, with some women appearing genetically more vulnerable to the effects of HT in terms of their estrogen receptor genotype. These findings, if confirmed in another independent study, may help explain the differential susceptibility of women to the beneficial or adverse effects of HT. | 2907 | NA | NA | NA | no | no |
| 2908 | 2017 | NA | Grilo, C; Reto, D; Filipe, J; Ascensao, F; Revilla, E | 2014 | Understanding the mechanisms behind road effects: linking occurrence with road mortality in owls | Several studies suggest that species’ mortality rates are positively related with local population abundances. Because owls have shown both high road mortality rates and road avoidance behaviours, we hypothesize that road-kill likelihood is not always directly linked to their occurrence. In this study, we examined the relationship between the likelihood of species occurrence in the vicinity of major roads and the road-kill risk for barn owls Tyto alba, tawny owls Strix aluco and little owls Athene noctua. Specifically, we address: (1) the role of road-related features on spatial patterns of species’ occurrence and road kills; (2) the composition of road kills and their distribution throughout the year; (3) the relationship between species’ occurrence likelihood and mortality risk. Our findings show that changes in movement patterns is probably the main behavioural mechanism that threatens owls in roaded landscapes. The high mortality risk of barn owls in autumn and winter seasons, after the peak of dispersal period, provides support for the hypotheses of expanding movements because of lack of food. However, mortality because of high occurrence likelihood seems to also explain tawny owls’ response towards roads. The high occurrence likelihood of little owls combined with low mortality rate also suggests avoidance of road crossings. Although, it is clear that within species there is variation according to age- and territory-holding status, closely related species have different sensitivities to roads as a function of their varying food preferences. We believe that linking species distribution with mortality risk may be more effective in focus conservation efforts. Therefore, we suggest measures should be applied to reduce prey availability close to roads and in road verges. This is particularly important for barn owls, for which foraging in the vicinity of roads during the cold season is especially risky. | 2908 | NA | NA | NA | no | no |
| 2909 | 2017 | NA | Lazaro-Nogal, A; Matesanz, S; Godoy, A; Perez-Trautman, F; Gianoli, E; Valladares, F | 2015 | Environmental heterogeneity leads to higher plasticity in dry-edge populations of a semi-arid Chilean shrub: insights into climate change responses | Interannual variability in climatic conditions should be taken into account in climate change studies in semi-arid ecosystems. It may determine differentiation in phenotypic plasticity among populations, with populations experiencing higher environmental heterogeneity showing higher levels of plasticity. The ability of populations to evolve key functional traits and plasticity may determine the survival of plant populations under the drier and more variable climate expected for semi-arid ecosystems. Working with populations of the semi-arid Chilean shrub Senna candolleana along its entire distribution range, we assessed inter- and intra-population variation in functional traits as well as in their plasticity in response to water availability. We measured morphological and physiological traits related to drought resistance in both field conditions and in a greenhouse experiment, where drought response was evaluated under two water availability treatments. All populations responded plastically, but higher precipitation heterogeneity in dry-edge populations seemed to have selected for more plastic genotypes compared to populations growing at mesic sites and with more homogeneous environmental conditions. Synthesis.Our results suggest adaptive plasticity since higher levels of phenotypic plasticity were positively associated with plant performance. However, we did not find evidence for genetic variation for plasticity within populations. To the extent that phenotypic plasticity may play a key role in future persistence, populations at mesic sites may be more vulnerable to climate change due to their lower plasticity and their current limitations to evolve novel norms of reaction. Conversely, although Senna candolleana populations at the dry edge are exposed to higher levels of stress, they may be less susceptible to climate change in view of their greater plasticity. We highlight the need to consider population differentiation in both mean traits and their plasticity to model realistic scenarios of species distribution under climate change. | 2909 | NA | NA | NA | no | no |
| 2910 | 2017 | NA | Diaz, MH; Desai, HP; Morrison, SS; Benitez, AJ; Wolff, BJ; Caravas, J; Read, TD; Dean, D; Winchell, JM | 2017 | Comprehensive bioinformatics analysis of Mycoplasma pneumoniae genomes to investigate underlying population structure and type-specific determinants | Mycoplasma pneumoniae is a significant cause of respiratory illness worldwide. Despite a minimal and highly conserved genome, genetic diversity within the species may impact disease. We performed whole genome sequencing (WGS) analysis of 107 M. pneumoniae isolates, including 67 newly sequenced using the Pacific BioSciences RS II and/or Illumina MiSeq sequencing platforms. Comparative genomic analysis of 107 genomes revealed > 3,000 single nucleotide polymorphisms (SNPs) in total, including 520 type-specific SNPs. Population structure analysis supported the existence of six distinct subgroups, three within each type. We developed a predictive model to classify an isolate based on whole genome SNPs called against the reference genome into the identified subtypes, obviating the need for genome assembly. This study is the most comprehensive WGS analysis for M. pneumoniae to date, underscoring the power of combining complementary sequencing technologies to overcome difficult-to-sequence regions and highlighting potential differential genomic signatures in M. pneumoniae. | 2910 | NA | NA | NA | no | no |
| 2911 | 2017 | NA | Engelen, A; Convey, P; Popa, O; Ott, S | 2016 | Lichen photobiont diversity and selectivity at the southern limit of the maritime Antarctic region (Coal Nunatak, Alexander Island) | Antarctic ice-free inland sites provide a unique perspective on the strategies coevolving organisms have developed for survival at the limits of life. Here, we provide the first combined description of the ecological and genetic diversity of lichen photobionts colonising an isolated Antarctic inland site, Coal Nunatak, on south-east Alexander Island (Antarctic Peninsula). Photobionts of 14 lichen species (42 samples), all belonging to the group of coccal green algae, representing the entire lichen community of Coal Nunatak were investigated using the internal transcribed spacer region (ITS) of the nuclear ribosomal DNA. The study attempted to address the hypothesis that mycobiont selectivity for the photobiont partner is lower in more extreme environments. This hypothesis did not appear to hold true for the entire lichen community except one species. Another aspect focuses on the relevance of the reproduction modus concerning the distribution of photobiont haplotypes in the lichen community. | 2911 | NA | NA | NA | no | no |
| 2912 | 2017 | NA | Ishikawa, FH; Souza, EA; Shoji, JY; Connolly, L; Freitag, M; Read, ND; Roca, MG | 2012 | Heterokaryon Incompatibility Is Suppressed Following Conidial Anastomosis Tube Fusion in a Fungal Plant Pathogen | It has been hypothesized that horizontal gene/chromosome transfer and parasexual recombination following hyphal fusion between different strains may contribute to the emergence of wide genetic variability in plant pathogenic and other fungi. However, the significance of vegetative (heterokaryon) incompatibility responses, which commonly result in cell death, in preventing these processes is not known. In this study, we have assessed this issue following different types of hyphal fusion during colony initiation and in the mature colony. We used vegetatively compatible and incompatible strains of the common bean pathogen Colletotrichum lindemuthianum in which nuclei were labelled with either a green or red fluorescent protein in order to microscopically monitor the fates of nuclei and heterokaryotic cells following hyphal fusion. As opposed to fusion of hyphae in mature colonies that resulted in cell death within 3 h, fusions by conidial anastomosis tubes (CAT) between two incompatible strains during colony initiation did not induce the vegetative incompatibility response. Instead, fused conidia and germlings survived and formed heterokaryotic colonies that in turn produced uninucleate conidia that germinated to form colonies with phenotypic features different to those of either parental strain. Our results demonstrate that the vegetative incompatibility response is suppressed during colony initiation in C. lindemuthianum. Thus, CAT fusion may allow asexual fungi to increase their genetic diversity, and to acquire new pathogenic traits. | 2912 | NA | NA | NA | no | no |
| 2913 | 2017 | NA | Faber-Hammond, JJ; Brown, KH | 2016 | Pseudo-De Novo Assembly and Analysis of Unmapped Genome Sequence Reads in Wild Zebrafish Reveal Novel Gene Content | Zebrafish represents the third vertebrate with an officially completed genome, yet it remains incomplete with additions and corrections continuing with the current release, GRCz10, having 13% of zebrafish cDNA sequences unmapped. This disparity may result from population differences, given that the genome reference was generated from clonal individuals with limited genetic diversity. This is supported by the recent analysis of a single wild zebrafish, which identified over 5.2 million SNPs and 1.6 million in/dels in the previous genome build, zv9. Re-examination of this sequence data set indicated that 13.8% of quality sequence reads failed to align to GRCz10. Using a novel bioinformatics de novo assembly pipeline on these unmappable reads, we identified 1,514,491 novel contigs covering similar to 224 Mb of genomic sequence. Among these, 1083 contigs were found to contain a potential gene coding sequence. RNA-seq data comparison confirmed that 362 contigs contained a transcribed DNA sequence, suggesting that a large amount of functional genomic sequence remains unannotated in the zebrafish reference genome. By utilizing the bioinformatics pipeline developed in this study, the zebrafish genome will be bolstered as a model for human disease research. Adaptation of the pipeline described here also offers a cost-efficient and effective method to identify and map novel genetic content across any genome and will ultimately aid in the completion of additional genomes for a broad range of species. | 2913 | NA | NA | NA | no | no |
| 2914 | 2017 | NA | Vergara, IA; Tarailo-Graovac, M; Frech, C; Wang, J; Qin, ZZ; Zhang, T; She, R; Chu, JSC; Wang, K; Chen, NS | 2014 | Genome-wide variations in a natural isolate of the nematode Caenorhabditis elegans | Background: Increasing genetic and phenotypic differences found among natural isolates of C. elegans have encouraged researchers to explore the natural variation of this nematode species. Results: Here we report on the identification of genomic differences between the reference strain N2 and the Hawaiian strain CB4856, one of the most genetically distant strains from N2. To identify both small-and large-scale genomic variations (GVs), we have sequenced the CB4856 genome using both Roche 454 (similar to 400 bps single reads) and Illumina GA DNA sequencing methods (101 bps paired-end reads). Compared to previously described variants (available in WormBase), our effort uncovered twice as many single nucleotide variants (SNVs) and increased the number of small InDels almost 20-fold. Moreover, we identified and validated large insertions, most of which range from 150 bps to 1.2 kb in length in the CB4856 strain. Identified GVs had a widespread impact on protein-coding sequences, including 585 single-copy genes that have associated severe phenotypes of reduced viability in RNAi and genetics studies. Sixty of these genes are homologs of human genes associated with diseases. Furthermore, our work confirms previously identified GVs associated with differences in behavioural and biological traits between the N2 and CB4856 strains. Conclusions: The identified GVs provide a rich resource for future studies that aim to explain the genetic basis for other trait differences between the N2 and CB4856 strains. | 2914 | NA | NA | NA | no | no |
| 2915 | 2017 | NA | Gudmundsson, H; Gudbjartsson, DF; Kong, A; Gudbjartsson, H; Frigge, M; Gulcher, JR; Stefansson, K | 2000 | Inheritance of human longevity in Iceland | The idea that human longevity is influenced by genetic factors has recently received strong support from work on other species. On the basis of partial population studies and selected kinships, significant correlations between the ages of parents and offspring have been reported, and some but not ail twin studies have confirmed that human longevity is moderately inherited. However, studies based upon a relatively small proportion of a population are susceptible to sampling error and selection bias. Here we report the use of a comprehensive population-based computerised genealogy database to examine multigenerational relationships among those who live to the 95th percentile in Iceland. We have developed a clustering tool which can generate large extended pedigrees connecting individuals from any list using the genealogy database. First degree relatives of those living to the 95th percentile are almost twice as likely to live to the 95th percentile compared with controls. Furthermore, we have developed an algorithm which we have named the Minimum Founder Test (MFT) to examine the degree of relatedness of any population-based list of individuals to estimate whether a trait has a familial component. The data indicate that there is a significant genetic component to longevity. In addition, age-specific death rates are significantly lower in the offspring of long-lived parents compared with controls, especially after age 70. | 2915 | NA | NA | NA | no | no |
| 2916 | 2017 | NA | Troussellier, M; Schafer, H; Batailler, N; Bernard, L; Courties, C; Lebaron, P; Muyzer, G; Servais, P; Vives-Rego, J | 2002 | Bacterial activity and genetic richness along an estuarine gradient (Rhone River plume, France) | Bacterial diversity and activity were simultaneously investigated by microbial ecological and molecular biological methods along an estuarine gradient from the Rhone River to the Mediterranean Sea. Following a Lagrangian strategy, we sampled plume, frontal and marine layers. The sampled estuarine gradient exhibited large changes both in physico-chemical and in microbiological characteristics. Bacterial abundances and activities showed a more drastic decrease in the low salinity range of the gradient than expected from simple dilution models, indicating that an important fraction of freshwater bacteria disappeared in the mixing area, High specific activities, in particular for leucine, in the marine end-part of the gradient suggested important bacterial protein synthesis, which may be a sign of an active survival strategy for bacterial communities subjected to oligotrophic conditions. Bacterial genetic diversity of the sampled estuarine area, as estimated by the number of DNA-derived denaturing gradient gel electrophoresis (DGGE) bands, was high (13 to 55 bands) compared to that reported in other aquatic ecosystems. This high diversity may be the consequence of the interface position of estuaries. The proportion of active populations was estimated using the ratio of DGGE bands derived from RNA and DNA. This ratio was lower in Rhone water than in marine water, indicating that only a part of the constitutive populations were active, while the activity was distributed within a larger fraction Of populations in the marine assemblage, Very few DGGE bands detected in freshwater samples were also detected in the marine end-part of the gradient, suggesting that a very limited number of freshwater bacteria could survive under marine conditions. Detection of these freshwater populations from RNA might indicate that these bacteria were able to synthesize different stress proteins as the result of a survival strategy or that these bacteria were able to maintain metabolic activity under marine conditions, The structure of marine communities was strongly affected by decreasing salinity. However, it seems that the decrease of DNA-derived bands may simply have been the consequence of the mixing of marine and freshwater. No obvious relationship between genetic richness and activity changes was observed. This lack of a relationship may be the consequence of a very short residence time of water in the mixing area studied. | 2916 | NA | NA | NA | no | no |
| 2917 | 2017 | NA | Pacheco, MA; Reid, MJC; Schillaci, MA; Lowenberger, CA; Galdikas, BMF; Jones-Engel, L; Escalante, AA | 2012 | The Origin of Malarial Parasites in Orangutans | Background: Recent findings of Plasmodium in African apes have changed our perspectives on the evolution of malarial parasites in hominids. However, phylogenetic analyses of primate malarias are still missing information from Southeast Asian apes. In this study, we report molecular data for a malaria parasite lineage found in orangutans. Methodology/Principal Findings: We screened twenty-four blood samples from Pongo pygmaeus (Kalimantan, Indonesia) for Plasmodium parasites by PCR. For all the malaria positive orangutan samples, parasite mitochondrial genomes (mtDNA) and two antigens: merozoite surface protein 1 42 kDa (MSP-1(42)) and circumsporozoite protein gene (CSP) were amplified, cloned, and sequenced. Fifteen orangutans tested positive and yielded 5 distinct mitochondrial haplotypes not previously found. The haplotypes detected exhibited low genetic divergence among them, indicating that they belong to one species. We report phylogenetic analyses using mitochondrial genomes, MSP-1(42) and CSP. We found that the orangutan malaria parasite lineage was part of a monophyletic group that includes all the known non-human primate malaria parasites found in Southeast Asia; specifically, it shares a recent common ancestor with P. inui (a macaque parasite) and P. hylobati (a gibbon parasite) suggesting that this lineage originated as a result of a host switch. The genetic diversity of MSP-1(42) in orangutans seems to be under negative selection. This result is similar to previous findings in non-human primate malarias closely related to P. vivax. As has been previously observed in the other Plasmodium species found in non-human primates, the CSP shows high polymorphism in the number of repeats. However, it has clearly distinctive motifs from those previously found in other malarial parasites. Conclusion: The evidence available from Asian apes indicates that these parasites originated independently from those found in Africa, likely as the result of host switches from other non-human primates. | 2917 | NA | NA | NA | no | no |
| 2918 | 2017 | NA | Kathuria, S; Sharma, C; Singh, PK; Agarwal, P; Agarwal, K; Hagen, F; Meis, JF; Chowdhary, A | 2015 | Molecular Epidemiology and In-Vitro Antifungal Susceptibility of Aspergillus terreus Species Complex Isolates in Delhi, India: Evidence of Genetic Diversity by Amplified Fragment Length Polymorphism and Microsatellite Typing | Aspergillus terreus is emerging as an etiologic agent of invasive aspergillosis in immuno-compromised individuals in several medical centers in the world. Infections due to A. terreus are of concern due to its resistance to amphotericin B, in vivo and in vitro, resulting in poor response to antifungal therapy and high mortality. Herein we examined a large collection of molecularly characterized, geographically diverse A. terreus isolates (n = 140) from clinical and environmental sources in India for the occurrence of cryptic A. terreus species. The population structure of the Indian A. terreus isolates and their association with those outside India was determined using microsatellite based typing (STR) technique and Amplified Fragment Length Polymorphism analysis (AFLP). Additionally, in vitro antifungal susceptibility of A. terreus isolates was determined against 7 antifungals. Sequence analyses of the calmodulin locus identified the recently described cryptic species A. hortai, comprising 1.4% of Aspergillus section Terrei isolates cultured from cases of aspergilloma and probable invasive aspergillosis not reported previously. All the nine markers used for STR typing of A. terreus species complex proved to be highly polymorphic. The presence of high genetic diversity revealing 75 distinct genotypes among 101 Indian A. terreus isolates was similar to the marked heterogeneity noticed in the 47 global A. terreus population exhibiting 38 unique genotypes mainly among isolates from North America and Europe. Also, AFLP analysis showed distinct banding patterns for genotypically diverse A. terreus isolates. Furthermore, no correlation between a particular genotype and amphotericin B susceptibility was observed. Overall, 8% of the A. terreus isolates exhibited low MICs of amphotericin B. All the echinocandins and azoles (voriconazole, posaconazole and isavuconazole) demonstrated high potency against all the isolates. The study emphasizes the need of molecular characterization of A. terreus species complex isolates to better understand the ecology, acquisition and transmission of this species. | 2918 | NA | NA | NA | no | no |
| 2919 | 2017 | NA | Foitzik, S; Herbers, JM | 2001 | Colony structure of a slavemaking ant. II. Frequency of slave raids and impact on the host population | The parasite pressure exerted by the slavemaker ant Protomagnathus americanus on its host species Leptothorax longispinosus was analyzed demographically and genetically. The origin of slaves found in colonies of the obligate slavemaker was examined with nuclear and mitochondrial DNA markers to make inferences about the frequency and severity of slave raids. Relatedness of enslaved L. longispinosus workers in the same nest was very low, and our data suggest that, on average, each slavemaker nest raids six host colonies per season. Therefore, the influence of slavemaker species on their hosts is much stronger than simple numerical ratios suggest. We also found that slave relatedness was higher in small than in large slavemaker nests; thus, larger nests wield a much stronger influence on the host. We estimated that in the study population, on average, a host nest has a 50% chance of being attacked by a slavemaker colony per year. Free-living Leptothorax colonies in the vicinity of slavemaker nests did not represent the source of slaves working in P. americanus colonies, which suggests that raided nests either do not survive or migrate after being raided. Colony composition and intranest relatedness of free-living L. longispinosus colonies differed markedly between areas with slavemakers and those that are parasite free. In the presence of slavemakers, host colonies were less likely to be polygynous and had fewer workers and a higher relatedness among worker brood. Host nests with slavemaker neighbors allocated more resources into sexuals, possibly caused by these shifts in nest demography. Finally, enslaved Leptothorax workers in P. americanus nests appeared to be less efficient than their counterparts in free-living colonies. Thus, slavemakers exert a much stronger impact on their hosts than had previously been suspected and represent an unique system to study parasite-host coevolution. | 2919 | NA | NA | NA | no | no |
| 2920 | 2017 | NA | Andrello, M; Nicole, F; Till-Bottraud, I; Gaggiotti, OE | 2012 | Effect of Stage-Specific Vital Rates on Population Growth Rates and Effective Population Sizes in an Endangered Iteroparous Plant | Effective population size (N e ) determines the strength of genetic drift and can influence the level of genetic diversity a population can maintain. Assessing how changes in demographic rates associated with environmental variables and management actions affect N e thus can be crucial to the conservation of endangered species. Calculation of N e through demographic models makes it possible to use elasticity analyses to study this issue. The elasticity of N e to a given vital rate is the proportional change in N e associated with a proportional increase in that vital rate. In addition, demographic models can be used to study N e and population growth rate (?) simultaneously. Simultaneous examination is important because some vital rates differ diametrically in their associations with ? and N e . For example, in some cases increasing these vital rates increases ? and decreases N e . We used elasticity analysis to study the effect of stage-specific survival and flowering rates on N e, annual effective population size (Na), and ? in seven populations of the endangered plant Austrian dragonhead (Dracocephalum austriacum). In populations with ?= 1, the elasticities of Neand Na were similar to those of ?. Survival rates of adults were associated with greater elasticities than survival rates of juveniles, flowering rates, or fecundity. In populations with ? < 1, Neand Na exhibited greater elasticities to juvenile than to adult vital rates. These patterns are similar to those observed in other species with similar life histories. We did not observe contrasting effects of any vital rate on ? and Ne; thus, management actions that increase the ? of populations of Austrian dragonhead will not increase genetic drift. Our results show that elasticity analyses of Neand Na can complement elasticity analysis of ?. Moreover, such analyses do not require more data than standard matrix models of population dynamics. | 2920 | NA | NA | NA | no | no |
| 2921 | 2017 | NA | Smyser, TJ; Stauffer, GE; Johnson, SA; Hudson, CM; Rhodes, OE; Swihart, RK | 2016 | Annual survival of Allegheny woodrats in a nonequilibrium metapopulation | Many declining populations of the imperiled Allegheny woodrat (Neotoma magister) function as nonequilibrium metapopulations in which rates of subpopulation extirpation exceed recolonization. Quantifying and maximizing survival rates thus becomes critical for the conservation of these spatially structured populations. We used encounter histories of individually marked woodrats from subpopulations in Indiana, monitored annually from 2005 to 2013, to 1) estimate apparent annual survival rates while accounting for imperfect detection, 2) evaluate differences in apparent survival between unaugmented subpopulations and subpopulations reestablished or restored through translocation efforts, and 3) describe the effect of genetic diversity on survival. From Cormack-Jolly-Seber models developed in a Bayesian framework, apparent survival was greater for adults than for juveniles, greater for females than males, and there was a modest negative effect of density dependence. Although heterozygosity rates at 11 microsatellites increased among reinforced subpopulations following translocations, we observed no effect of heterozygosity on apparent survival. However, after translocations, average apparent survival was approximately 14% greater among recipient subpopulations than remnant subpopulations. This suggests that viability of recipient subpopulations was limited by low connectivity and the absence of genetic benefits conveyed by immigration or the potential for patches to be recolonized following local extinction. Under conditions of reduced connectivity, translocation among subpopulations to replicate natural gene flow may be appropriate to facilitate the long-term persistence of this and perhaps other nonequilibrium metapopulations. | 2921 | NA | NA | NA | no | no |
| 2922 | 2017 | NA | Quencez, C; Bastien, C | 2001 | Genetic variation within and between populations of Pinus sylvestris L. (Scots pine) for susceptibility to Melampsora pinitorqua Rostr. (pine twist rust) | The genetic variability of Scots pine (Pinus sylvestris L.) for twist rust susceptibility and the consequences for genetic improvement of the species were evaluated after artificial inoculation of 1-year-old seedlings in greenhouse tests. Wind-pollinated progenies, factorial and incomplete diallel mating designs were used to compare two natural populations of Scots pine (Haguenau, France, and Taborz, Poland) and their hybrids (Haguenau x Taborz). Families from Taborz were significantly (P < 0.001) less susceptible to twist rust than those from Haguenau. Inter-population hybrids were as susceptible as the Haguenau population. No clear relationship between severity of infection, shoot length and phenological stage at time of inoculation was observed in intra- and interpopulation crosses. Differences observed between the two natural populations for their response to twist rust are suggested to be the consequence of local adaptation simultaneously for climatic conditions and pathogen pressure. Within each of the two populations, the effects of general combining abilities (GCA) for rust susceptibility were predominant, although estimation of individual heritabilities remained at a moderate level (0.30-0.54). In interpopulation crosses, GCA effects of Haguenau and Taborz populations were still predominant. Moreover, hybrid performance could be predicted with confidence based on the intrapopulation GCA values of parents. Genetic gain on twist rust resistance could be achieved quickly through intrapopulation mass or backward selection based on wind-pollinated progeny tests. | 2922 | NA | NA | NA | no | no |
| 2923 | 2017 | NA | Kupper, C; Kosztolanyi, A; Augustin, J; Dawson, DA; Burke, T; Szekely, T | 2010 | Heterozygosity-fitness correlations of conserved microsatellite markers in Kentish plovers Charadrius alexandrinus | Heterozygosity-fitness correlations (HFCs) are frequently used to examine the relationship between genetic diversity and fitness. Most studies have reported positive HFCs, although there is a strong bias towards investigating HFCs in genetically impoverished populations. We investigated HFCs in a large genetically diverse breeding population of Kentish plovers Charadrius alexandrinus in southern Turkey. This small shorebird exhibits highly variable mating and care systems, and it is becoming an ecological model species to understand breeding system evolution. Using 11 conserved and six anonymous microsatellite markers, we tested whether and how heterozygosity was associated with chick survival, tarsus and body mass growth controlling for nongenetic effects (chick sex, hatching date, length of biparental care and site quality) that influence survival and growth. There was no genome-wide effect of heterozygosity on fitness, and we did not find any significant effects of heterozygosity on growth rates. However, two of the 11 conserved markers displayed an association with offspring survival: one marker showed a positive HFC, whereas the other marker showed a negative HFC. Heterozygosity at three further conserved loci showed significant interaction with nongenetic variables. In contrast, heterozygosity based on anonymous microsatellite loci was not associated with fitness or growth. Markers that were correlated with chick survival were not more likely to be located in exons or introns than other markers that lacked this association. | 2923 | NA | NA | NA | no | no |
| 2924 | 2017 | NA | Mahfoudh, N; Ayadi, I; Kamoun, A; Ammar, R; Mallek, B; Maalej, L; Hakim, F; Gaddour, L; Rebai, A; Makni, H | 2013 | Analysis of HLA-A, -B, -C, -DR, -DQ polymorphisms in the South Tunisian population and a comparison with other populations | Background: The Human Leucocyte Antigen (HLA) system is often used as a genetic marker for analysing populations. HLA antigen distribution among the Tunisian population is not well defined because of the lack of a general population study. Aim: The aim of the present study was to investigate the polymorphism of HLA-A, -B, -C, -DR and -DQ loci in the South Tunisian population. Subjects and methods: This study has investigated HLA-A, -B, -C, -DR and -DQ polymorphisms in 123 unrelated healthy individuals originating from the south of Tunisia. HLA class I was studied by serology and completed by polymerase chain reaction-sequence specific primer (PCR-SSP). HLA class II was performed using PCR-SSP. Results: The most common alleles were A-2 (0.2154), B-44 (0.1179), C7 (0.2114), DR4 (0.1626) and DQ2 (0.313). A1-B-8-C7-DR3-DQ2 (2.84%) was the predominant haplotype in this population. Comparisons with data of other worldwide populations based on phylogenetic tree and multidimensional scaling analysis were done. This study suggests that both HLA class I and class II polymorphism specificities demonstrate a high diversity in this South Tunisian population, which reflects ancient and recent admixture with neighbouring populations. Conclusion: The results provide useful information for further studies of Tunisian population evolution, anthropology and for resolving HLA frequencies when searching for HLA-compatible donors in transplantation and for the analysis of disease associations. | 2924 | NA | NA | NA | no | no |
| 2925 | 2017 | NA | Nelson, WA; McCauley, E; Nisbet, RM | 2007 | Stage-structured cycles generate strong fitness-equalizing mechanisms | For many organisms, rates of reproduction, growth and mortality depend on the amount of resources that an individual consumes. When resource abundances fluctuate through space and time, the realized life-history of an individual can change dramatically depending on the dynamics experienced. Previous studies have investigated the influence of resource-dependent rates on population dynamics, but none have considered how the feedback between non-equilibrium resource dynamics and resource-dependent life-histories influence natural selection and the maintenance of genetic diversity within populations. Here we demonstrate that different patterns of resource dynamics have a strong impact on natural selection in organisms with resource-dependent life-histories. Small-amplitude consumer-resource cycles, lead to lower rates of natural selection than do large-amplitude consumer-resource cycles. Parameterizing the model for a Daphnia-algal system, we demonstrate that resource-dependent life-history can explain the recently published observation that selection among Daphnia genotypes changed depending on the pattern of algal resource fluctuations. The characteristically asexual reproduction of Daphnia allows us to draw a much-needed link to the large body of competition theory that has emerged from community ecology. Our results reveal that the common ecological features of resource-dependent life-history and ontogenetic size-structure generate strong fitness equalizing mechanisms that likely contribute to the maintenance of diversity in natural systems. | 2925 | NA | NA | NA | no | no |
| 2926 | 2017 | NA | Kosova, G; Pickrell, JK; Kelley, JL; McArdle, PF; Shuldiner, AR; Abney, M; Ober, C | 2010 | The CFTR Met 470 Allele Is Associated with Lower Birth Rates in Fertile Men from a Population Isolate | Although little is known about the role of the cystic fibrosis transmembrane regulator (CFTR) gene in reproductive physiology, numerous variants in this gene have been implicated in etiology of male infertility due to congenital bilateral absence of the vas deferens (CBAVD). Here, we studied the fertility effects of three CBAVD-associated CFTR polymorphisms, the (TG) m and polyT repeat polymorphisms in intron 8 and Met470Val in exon 10, in healthy men of European descent. Homozygosity for the Met470 allele was associated with lower birth rates, defined as the number of births per year of marriage (P = 0.0029). The Met470Val locus explained 4.36% of the phenotypic variance in birth rate, and men homozygous for the Met470 allele had 0.56 fewer children on average compared to Val470 carrier men. The derived Val470 allele occurs at high frequencies in non-African populations (allele frequency = 0.51 in HapMap CEU), whereas it is very rare in African population (Fst = 0.43 between HapMap CEU and YRI). In addition, haplotypes bearing Val470 show a lack of genetic diversity and are thus longer than haplotypes bearing Met470 (measured by an integrated haplotype score [iHS] of -1.93 in HapMap CEU). The fraction of SNPs in the HapMap Phase2 data set with more extreme Fst and iHS measures is 0.003, consistent with a selective sweep outside of Africa. The fertility advantage conferred by Val470 relative to Met470 may provide a selective mechanism for these population genetic observations. | 2926 | NA | NA | NA | no | no |
| 2927 | 2017 | NA | Grunig, CR; Brunner, PC; Duo, A; Sieber, TN | 2007 | Suitability of methods for species recognition in the Phialocephala fortinii-Acephala applanata species complex using DNA analysis | Sequence data of two coding and three non-coding loci were used to study the taxonomic identity within and relatedness among seven previously defined cryptic species (CSP) of Phialocephala fortinii and Acephala applanata using two approaches of species recognition. Identification of taxonomic groups corresponding to CSP was ambiguous in some cases when applying solely the genealogical concordance phylogenetic species recognition (GCPSR) concept. The definition of groups corresponding to CSP using GCPSR was complicated due to shared sequence haplotypes between CSP, unresolved CSP for several loci, and possible introgression. GCPSR in conjunction with a population genetic approach improved resolution significantly and the CSP status could be confirmed for all seven CSP of P. fortinii s.l. The most critical step in both analyses was the definition of groups. The combination of several classes of markers differing in resolution helped to define species boundaries. (c) 2007 Elsevier Inc. All rights reserved. | 2927 | NA | NA | NA | no | no |
| 2928 | 2017 | NA | Sjodin, P; Sjostrand, AE; Jakobsson, M; Blum, MGB | 2012 | Resequencing Data Provide No Evidence for a Human Bottleneck in Africa during the Penultimate Glacial Period | Based on the accumulation of genetic, climatic, and fossil evidence, a central theory in paleoanthropology stipulates that a demographic bottleneck coincided with the origin of our species Homo Sapiens. This theory proposes that anatomically modern humans-which were only present in Africa at the time-experienced a drastic bottleneck during the penultimate glacial age (130-190 kya) when a cold and dry climate prevailed. Two scenarios have been proposed to describe the bottleneck, which involve either a fragmentation of the range occupied by humans or the survival of one small group of humans. Here, we analyze DNA sequence data from 61 nuclear loci sequenced in three African populations using Approximate Bayesian Computation and numerical simulations. In contrast to the bottleneck theory, we show that a simple model without any bottleneck during the penultimate ice age has the greatest statistical support compared with bottleneck models. Although the proposed bottleneck is ancient, occurring at least 130 kya, we can discard the possibility that it did not leave detectable footprints in the DNA sequence data except if the bottleneck involves a less than a 3-fold reduction in population size. Finally, we confirm that a simple model without a bottleneck is able to reproduce the main features of the observed patterns of genetic variation. We conclude that models of Pleistocene refugium for modern human origins now require substantial revision. | 2928 | NA | NA | NA | no | no |
| 2929 | 2017 | NA | Karl, JA; Wiseman, RW; Campbell, KJ; Blasky, AJ; Hughes, AL; Ferguson, B; Read, DS; O’Connor, DH | 2008 | Identification of MHC class I sequences in Chinese-origin rhesus macaques | The rhesus macaque (Macaca mulatta) is an excellent model for human disease and vaccine research. Two populations exhibiting distinctive morphological and physiological characteristics, Indian- and Chinese-origin rhesus macaques, are commonly used in research. Genetic analysis has focused on the Indian macaque population, but the accessibility of these animals for research is limited. Due to their greater availability, Chinese rhesus macaques are now being used more frequently, particularly in vaccine and biodefense studies, although relatively little is known about their immunogenetics. In this study, we discovered major histocompatibility complex (MHC) class I cDNAs in 12 Chinese rhesus macaques and detected 41 distinct Mamu-A and Mamu-B sequences. Twenty-seven of these class I cDNAs were novel, while six and eight of these sequences were previously reported in Chinese and Indian rhesus macaques, respectively. We then performed microsatellite analysis on DNA from these 12 animals, as well as an additional 18 animals, and developed sequence specific primer PCR (PCR-SSP) assays for eight cDNAs found in multiple animals. We also examined our cohort for potential admixture of Chinese and Indian origin animals using a recently developed panel of single nucleotide polymorphisms (SNPs). The discovery of 27 novel MHC class I sequences in this analysis underscores the genetic diversity of Chinese rhesus macaques and contributes reagents that will be valuable for studying cellular immunology in this population. | 2929 | NA | NA | NA | no | no |
| 2930 | 2017 | NA | Kvist, L; Ruokonen, M; Thessing, A; Lumme, J; Orell, M | 1998 | Mitochondrial control region polymorphism reveal high amount of gene flow in Fennoscandian willow tits (Parus montanus borealis) | We studied the genetic variability and differentiation of two Fennoscandian willow tit (Parus montanus borealis) populations located ca. 1000 km apart in Finland and Sweden by using the control region sequences of the mitochondrial DNA. Individual variation in the control region was extensive since all the 13 Finnish and the 12 Swedish individuals had unique haplotypes and the mean pairwise genetic distance resulted in 0.0052 (range 0.0008-0.0109). In the minimum spanning network connecting the genotypes, the two populations were completely intermingled. The mismatch distribution of the combined data set was very close to expected distribution of an expanding population. This result was supported by a significantly negative Tajima’s D value. The sequence data indicate that (1) the long-term effective population size of the breeding willow tits has been large (122,000 and 110,000 females for the Finnish and the Swedish populations, respectively); and that (2) the gene exchange between distant localities is/has been extensive. Dispersal area for the Finnish females was estimated to be about 19,000-30,000 km(2) and for the Swedish, 22,000-28,000 km(2). Thus, the whole Fennoscandian population can be regarded as one panmictic unit, without any subdivisions to local demes. The amount of gene flow is remarkable because the willow tit has been considered a highly sedentary species. Provided that adult birds are site-tenacious, the gene flow must occur through juvenile summer dispersal, or irruptive autumn invasions, or both. | 2930 | NA | NA | NA | no | no |
| 2931 | 2017 | NA | Dorado, EJ; Okoth, SA; Montenegro, LM; Diaz, G; Barnwell, JW; Udhayakumar, V; Solano, CM | 2016 | Genetic Characterisation of Plasmodium falciparum Isolates with Deletion of the pfhrp2 and/or pfhrp3 Genes in Colombia: The Amazon Region, a Challenge for Malaria Diagnosis and Control | Most Plasmodium falciparum-detecting rapid diagnostic tests (RDTs) target histidine-rich protein 2 (PfHRP2). However, P. falciparum isolates with deletion of the pfhrp2 gene and its homolog gene, pfhrp3, have been detected. We carried out an extensive investigation on 365 P. falciparum dried blood samples collected from seven P. falciparum endemic sites in Colombia between 2003 and 2012 to genetically characterise and geographically map pfhrp2-and/or pfhrp3-negative P. falciparum parasites in the country. We found a high proportion of pfhrp2-negative parasites only in Amazonas (15/39; 38.5%), and these parasites were also pfhrp3-negative. These parasites were collected between 2008 and 2009 in Amazonas, while pfhrp3-negative parasites (157/365, 43%) were found in all the sites and from each of the sample collection years evaluated (2003 to 2012). We also found that all pfhrp2-and/or pfhrp3-negative parasites were also negative for one or both flanking genes. Six sub-population clusters were established with 93.3% (14/15) of the pfhrp2-negative parasites grouped in the same cluster and sharing the same haplotype. This haplotype corresponded with the genetic lineage B-V1, a multidrug resistant strain that caused two outbreaks reported in Peru between 2010 and 2013. We found this B-V1 lineage in the Colombian Amazon as early as 2006. Two new clonal lineages were identified in these parasites from Colombia: the genetic lineages E-V1 and F. PfHRP2 sequence analysis revealed high genetic diversity at the amino acid level, with 17 unique sequences identified among 53 PfHRP2 sequences analysed. The use of PfHRP2-based RDTs is not recommended in Amazonas because of the high proportion of parasites with pfhrp2 deletion (38.5%), and implementation of new strategies for malaria diagnosis and control in Amazonas must be prioritised. Moreover, studies to monitor and genetically characterise pfhrp2-negative P. falciparum parasites in the Americas are warranted, given the extensive human migration occurring in the region. | 2931 | NA | NA | NA | no | no |
| 2932 | 2017 | NA | McGhee, GC; Sundin, GW | 2012 | Erwinia amylovora CRISPR Elements Provide New Tools for Evaluating Strain Diversity and for Microbial Source Tracking | Clustered regularly interspaced short palindromic repeats (CRISPRs) comprise a family of short DNA repeat sequences that are separated by non repetitive spacer sequences and, in combination with a suite of Cas proteins, are thought to function as an adaptive immune system against invading DNA. The number of CRISPR arrays in a bacterial chromosome is variable, and the content of each array can differ in both repeat number and in the presence or absence of specific spacers. We utilized a comparative sequence analysis of CRISPR arrays of the plant pathogen Erwinia amylovora to uncover previously unknown genetic diversity in this species. A total of 85 E. amylovora strains varying in geographic isolation (North America, Europe, New Zealand, and the Middle East), host range, plasmid content, and streptomycin sensitivity/resistance were evaluated for CRISPR array number and spacer variability. From these strains, 588 unique spacers were identified in the three CRISPR arrays present in E. amylovora, and these arrays could be categorized into 20, 17, and 2 patterns types, respectively. Analysis of the relatedness of spacer content differentiated most apple and pear strains isolated in the eastern U. S. from western U. S. strains. In addition, we identified North American strains that shared CRISPR genotypes with strains isolated on other continents. E. amylovora strains from Rubus and Indian hawthorn contained mostly unique spacers compared to apple and pear strains, while strains from loquat shared 79% of spacers with apple and pear strains. Approximately 23% of the spacers matched known sequences, with 16% targeting plasmids and 5% targeting bacteriophage. The plasmid pEU30, isolated in E. amylovora strains from the western U. S., was targeted by 55 spacers. Lastly, we used spacer patterns and content to determine that streptomycin-resistant strains of E. amylovora from Michigan were low in diversity and matched corresponding streptomycin-sensitive strains from the background population. | 2932 | NA | NA | NA | no | no |
| 2933 | 2017 | NA | Yu, B; Li, AH; Metcalf, GA; Muzny, DM; Morrison, AC; White, S; Mosley, TH; Gibbs, RA; Boerwinkle, E | 2016 | Loss-of-function variants influence the human serum metabolome | The metabolome is a collection of small molecules resulting from multiple cellular and biological processes that can act as biomarkers of disease, and African-Americans exhibit high levels of genetic diversity. Exome sequencing of a sample of deeply phenotyped African-Americans allowed us to analyze the effects of annotated loss-of-function (LoF) mutations on 308 serum metabolites measured by untargeted liquid and gas chromatography coupled with mass spectrometry. In an independent sample, we identified and replicated four genes harboring six LoF mutations that significantly affected five metabolites. These sites were related to a 19 to 45% difference in geometric mean metabolite levels, with an average effect size of 25%. We show that some of the affected metabolites are risk predictors or diagnostic biomarkers of disease and, using the principle of Mendelian randomization, are in the causal pathway of disease. For example, LoF mutations in SLCO1B1 elevate the levels of hexadecanedioate, a fatty acid significantly associated with increased blood pressure levels and risk of incident heart failure in both African-Americans and an independent sample of European-Americans. We show that SLCO1B1 LoF mutations significantly increase the risk of incident heart failure, thus implicating the metabolite in the causal pathway of disease. These results reveal new avenues into gene function and the understanding of disease etiology by integrating -omic technologies into a deeply phenotyped population study. | 2933 | NA | NA | NA | no | no |
| 2934 | 2017 | NA | Nye, SH; Wittenburg, AL; Evans, DL; O’Connor, JA; Roman, RJ; Jacob, HJ | 2008 | Rat survival to anthrax lethal toxin is likely controlled by a single gene | We examined whether survival of different rat strains administered anthrax lethal toxin is genetically determined. A reproducible test population of first filial generation hybrid rats was bred based on the susceptibility of progenitors to anthrax lethal toxin and to maximize genetic diversity across the strains. These rats were then tested with varying doses of anthrax lethal toxin. We found that all ‘sensitive’ strains died within 2 h following systemic administration of 240 mu g/kg lethal toxin, while one strain survived following a five times higher dose (1.4 mg/kg). The ability of lethal toxin to lyse macrophage cultures derived from the bone marrow of these strains corresponded with in vivo results. We conclude that a rat test population can detect strain differences in response to anthrax lethal toxin. Survival is influenced by the host genome background and is likely due to a single gene with a recessive mode of inheritance. | 2934 | NA | NA | NA | no | no |
| 2935 | 2017 | NA | Poldmaa, T; Holder, K | 1997 | Behavioural correlates of monogamy in the noisy miner, Manorina melanocephala | Mating behaviour of female cooperatively breeding noisy miners was examined. Dow & Whitmore (1990, Cooperative Breeding in Birds (Ed. by P. B. Stacey & W. D. Koenig), pp. 559-592, Cambridge: Cambridge University Press) suggested that female noisy miners mate promiscuously to recruit males as helpers to their nests, and that the benefit of doing so might be (1) increased genetic variability of their broods or (2) increased survival of their offspring as a direct result of multi-male care. Multilocus DNA profiling has since shown that 96.5% of nestlings resulted from monogamous matings and that extra-group and multiple paternity within broods were rare (Poldmaa et al. 1995, Behav. Ecol. Sociobiol., 37, 137-143). In this study, strong behavioural correlates of monogamy were found. A breeding female associated more often with only one male in her social group, and most of the female’s sexual behaviours were directed towards this male. Females were observed copulating repeatedly with the same male, but never with more than one male. Home ranges of breeding females rarely overlapped with each other, but home ranges of breeding males overlapped greatly. Furthermore, a greater percentage of a female’s home range was shared with that of her genetic mate than with those of other males. Thus, behavioural evidence is consistent with the genetic evidence that noisy miners mate monogamously in some populations. (C) 1997 The Association for the Study of Animal Behaviour. | 2935 | NA | NA | NA | no | no |
| 2936 | 2017 | NA | Pereira, JF; Araujo, EF; Brommonschenkel, SH; Queiroz, CB; Costa, GGL; Carazzolle, MF; Pereira, GAG; Queiroz, MV | 2015 | MpSaci is a widespread gypsy-Ty3 retrotransposon highly represented by non-autonomous copies in the Moniliophthora perniciosa genome | Transposons are an important source of genetic variation. The phytopathogen Moniliophthora perniciosa shows high level of variability but little is known about the role of class I elements in shaping its genome. In this work, we aimed the characterization of a new gypsy/Ty3 retrotransposon species, named MpSaci, in the M. perniciosa genome. These elements are largely variable in size, ranging from 4 to 15 kb, and harbor direct long terminal repeats (LTRs) with varying degrees of similarity. Approximately, all of the copies are non-autonomous as shifts in the reading frame and stop codons were detected. Only two elements (MpSaci6 and MpSaci9) code for GAG and POL proteins that possess functional domains. Conserved domains that are typically not found in retrotransposons were detected and could potentially impact the expression of neighbor genes. Solo LTRs and several LARDs (large retrotransposon derivative) were detected. Unusual elements containing small sequences with or without interruptions that are similar to gag or different pol domains and presenting LTRs with different levels of similarities were identified. Methylation was observed in MpSaci reverse transcriptase sequences. Distribution analysis indicates that MpSaci elements are present in high copy number in the genomes of C-, S- and L-biotypes of M. perniciosa. In addition, C-biotype isolates originating from the state of Bahia have fragments in common with isolates from the Amazon region and two hybridization profiles related to two chromosomal groups. RT-PCR analysis reveals that the gag gene is constitutively expressed and that the expression is increased at least three-fold with nutrient depravation even though no new insertion were observed. These findings point out that MpSaci collaborated and, even though is primarily represented by non-autonomous elements, still might contribute to the generation of genetic variability in the most important cacao pathogen in Brazil. | 2936 | NA | NA | NA | no | no |
| 2937 | 2017 | NA | Leonard, SR; Mammel, MK; Lacher, DW; Elkins, CA | 2016 | Strain-Level Discrimination of Shiga Toxin-Producing Escherichia coli in Spinach Using Metagenomic Sequencing | Consumption of fresh bagged spinach contaminated with Shiga toxin-producing Escherichia coli (STEC) has led to severe illness and death; however current culture-based methods to detect foodborne STEC are time consuming. Since not all STEC strains are considered pathogenic to humans, it is crucial to incorporate virulence characterization of STEC in the detection method. In this study, we assess the comprehensiveness of utilizing a shotgun metagenomics approach for detection and strain-level identification by spiking spinach with a variety of genomically disparate STEC strains at a low contamination level of 0.1 CFU/g. Molecular serotyping, virulence gene characterization, microbial community analysis, and E. coli core gene single nucleotide polymorphism (SNP) analysis were performed on metagenomic sequence data from enriched samples. It was determined from bacterial community analysis that E. coli, which was classified at the phylogroup level, was a major component of the population in most samples. However, in over half the samples, molecular serotyping revealed the presence of indigenous E. coli which also contributed to the percent abundance of E. coli. Despite the presence of additional E. coli strains, the serotype and virulence genes of the spiked STEC, including correct Shiga toxin subtype, were detected in 94% of the samples with a total number of reads per sample averaging 2.4 million. Variation in STEC abundance and/or detection was observed in replicate spiked samples, indicating an effect from the indigenous microbiota during enrichment. SNP analysis of the metagenomic data correctly placed the spiked STEC in a phylogeny of related strains in cases where the indigenous E. coli did not predominate in the enriched sample. Also, for these samples, our analysis demonstrates that strain-level phylogenetic resolution is possible using shotgun metagenomic data for determining the genomic relatedness of a contaminating STEC strain to other closely related E. coli. | 2937 | NA | NA | NA | no | no |
| 2938 | 2017 | NA | d’Auriac, MBA; Rinde, E; Norling, P; Lapegue, S; Staalstrom, A; Hjermann, DO; Thaulow, J | 2017 | Rapid expansion of the invasive oyster Crassostrea gigas at its northern distribution limit in Europe: Naturally dispersed or introduced? | The Pacific oyster, Crassostrea gigas, was introduced to Europe for aquaculture purposes, and has had a rapid and unforeseen northward expansion in northern Europe. The recent dramatic increase in number of C. gigas populations along the species’ northern distribution limit has questioned the efficiency of Skagerrak as a dispersal barrier for transport and survival of larvae. We investigated the genetic connectivity and possible spreading patterns between Pacific oyster populations on the southern Norwegian coast (4 localities) and Swedish and Danish populations by means of DNA microsatellite analysis of adult oysters, and by simulating larvae drift. In the simulations we used a 3D oceanographic model to explore the influence of recent climate change (1990 - 2010) on development, survival, and successful spreading of Danish and Swedish Pacific oyster larvae to Norwegian coastal waters. The simulations indicated adequate temperature conditions for development, survival, and settlement of larvae across the Skagerrak in warm years since 2000. However, microsatellite genotyping revealed genetic differences between the Norwegian populations, and between the Norwegian populations and the Swedish and Danish populations, the latter two populations being more similar. This patchwork pattern of genetic dissimilarity among the Norwegian populations points towards multiple local introduction routes rather than the commonly assumed unidirectional entry of larvae drifted from Denmark and Sweden. Alternative origins of introduction and implications for management, such as forecasting and possible mitigation actions, are discussed. | 2938 | NA | NA | NA | no | no |
| 2939 | 2017 | NA | Koskella, B; Lively, CM | 2009 | EVIDENCE FOR NEGATIVE FREQUENCY-DEPENDENT SELECTION DURING EXPERIMENTAL COEVOLUTION OF A FRESHWATER SNAIL AND A STERILIZING TREMATODE | Host-parasite coevolution is often suggested as a mechanism for maintaining genetic diversity, but finding direct evidence has proven difficult. In the present study, we examine the process of coevolution using a freshwater New Zealand snail (Potamopyrgus antipodarum) and its common parasite (the sterilizing trematode, Microphallus sp.) Specifically, we test for changes in genotypic composition of clonal host populations in experimental populations evolving either with or without parasites for six generations. As predicted under the Red Queen model of coevolution, the initially most common host genotype decreased in frequency in the presence, but not the absence, of parasitism. Furthermore, the initially most common host genotype became more susceptible to infection by the coevolving parasite populations over the course of the experiment. These results are consistent with parasite-meditated selection leading to a rare advantage, and they indicate rapid coevolution at the genotypic level between a host and its parasite. | 2939 | NA | NA | NA | no | no |
| 2940 | 2017 | NA | Fjerdingstad, EJ; Gertsch, PJ; Keller, L | 2003 | The relationship between multiple mating by queens, within-colony genetic variability and fitness in the ant Lasius niger | Multiple mating has been suggested to benefit social insect queens because high genetic variation within colonies might decrease the load imposed by sterile diploid males, enhance resistance to parasites and pathogens, and lead to a more effective division of labour and/or a wider range of tolerable environmental conditions. We tested these hypotheses in the ant Lasius niger with three population samples from Switzerland and Sweden. We found no diploid males in young or mature colonies suggesting a lack of diploid male load. Colonies with miltiply-mated queens were not larger nor did they produce more sexuals than colonies with singly-mated queens. We did find a significantly lower frequency of multiple mating among newly mated queens than among the queens heading mature colonies in one population sample (Switzerland 1997). However, this result was not repeated in the other study population, or in the following year in the Swiss population. | 2940 | NA | NA | NA | no | no |
| 2941 | 2017 | NA | Breen, MS; Kondrashov, FA | 2010 | Mitochondrial pathogenic mutations are population-specific | Background: Surveying deleterious variation in human populations is crucial for our understanding, diagnosis and potential treatment of human genetic pathologies. A number of recent genome-wide analyses focused on the prevalence of segregating deleterious alleles in the nuclear genome. However, such studies have not been conducted for the mitochondrial genome. Results: We present a systematic survey of polymorphisms in the human mitochondrial genome, including those predicted to be deleterious and those that correspond to known pathogenic mutations. Analyzing 4458 completely sequenced mitochondrial genomes we characterize the genetic diversity of different types of single nucleotide polymorphisms (SNPs) in African (L haplotypes) and non-African (M and N haplotypes) populations. We find that the overall level of polymorphism is higher in the mitochondrial compared to the nuclear genome, although the mitochondrial genome appears to be under stronger selection as indicated by proportionally fewer nonsynonymous than synonymous substitutions. The African mitochondrial genomes show higher heterozygosity, a greater number of polymorphic sites and higher frequencies of polymorphisms for synonymous, benign and damaging polymorphism than non-African genomes. However, African genomes carry significantly fewer SNPs that have been previously characterized as pathogenic compared to non-African genomes. Conclusions: Finding SNPs classified as pathogenic to be the only category of polymorphisms that are more abundant in non-African genomes is best explained by a systematic ascertainment bias that favours the discovery of pathogenic polymorphisms segregating in non-African populations. This further suggests that, contrary to the common disease-common variant hypothesis, pathogenic mutations are largely population-specific and different SNPs may be associated with the same disease in different populations. Therefore, to obtain a comprehensive picture of the deleterious variability in the human population, as well as to improve the diagnostics of individuals carrying African mitochondrial haplotypes, it is necessary to survey different populations independently. | 2941 | NA | NA | NA | no | no |
| 2942 | 2017 | NA | Phillips, PD; Thompson, IS; Silva, JNM; van Gardingen, PR; Degen, B | 2004 | Scaling up models of tree competition for tropical forest population genetics simulation | “Understanding the effects of logging activity on genetic diversity is an important aspect of establishing the sustainability of selective logging management operations in tropical forests. Genetic variation is affected by selective logging directly, through the removal of and damage to trees within the population, and indirectly, through a change in the forest structure and environment in which the remaining population lives. Eco-Gene is a population genetics model applied to tropical forests over a scale of hundreds of hectares. SYMFOR is a modelling framework for individual-based spatially explicit ecological and management models applied to tropical forests over a scale of 0.25 4 ha. We have linked the models to enable simulations using processes involved in both models. To overcome problems of scale, the spatially explicit competition index calculated in SYMFOR simulations has been modelled such that it can be applied at scales representing much larger areas for which the data are not available, as required by Eco-Gene. The competition index is modelled as a distribution on a grid-square basis, and implemented in the linked Eco-Gene/SYMFOR system. Each tree within a grid-square is given a”“relative competition”" within the distribution, biased according to species. A competition index value is obtained for the tree by transforming the grid-square distribution to be relevant to the size of the tree, and extracting a value according to the tree’s relative competition within the distribution. The distribution and each tree’s relative competition within it change according to the effects of growth, mortality and logging activity. The model was calibrated using data from the Tapajos region of the Eastern Amazon forest. This paper describes the model, its calibration and validation and the implications of scaling up from an explicit representation to a modelled quantity. (C) 2004 Elsevier B.V. All rights reserved." | 2942 | NA | NA | NA | no | no |
| 2943 | 2017 | NA | FontouradaSilva, SE; ChautardFreireMaia, EA | 1996 | Butyrylcholinesterase variants (BCHE and CHE2 loci) associated with erythrocyte acetylcholinesterase inhibition in farmers exposed to pesticides | Farmers exposed to pesticides and classified as mildly poisoned and controls on the basis of erythrocyte acetylcholinesterase (AChE) activity were examined for butyrylcholinesterase (BChE) genetic variability. The mildly poisoned group showed a significantly higher frequency of non-usual phenotypes (13.1%) than the control group (1.7%). These phenotypes showed a relative risk (RR) of 8.8 of erythrocyte AChE inhibition when compared to the usual phenotype. Among the subjects with the usual phenotype, the CHE2 C5- phenotype was more frequent in the mildly poisoned group (94.3%) than in the control group (81.0%), leading to an RR of 3.9 when compared to the CHE2 C5+ phenotype. When the total sample was classified into two groups (usual CHE2 C5+ and other phenotypes), the usual CHE2 C5+ phenotype was found to be responsible for a preventive fraction of about 14% of the cases of mild poisoning. The present data suggest that BChE genetic variability offers differential protection against erythrocyte AChE inhibition. | 2943 | NA | NA | NA | no | no |
| 2944 | 2017 | NA | Pedersen, JS; Boomsma, JJ | 1999 | Effect of habitat saturation on the number and turnover of queens in the polygynous ant, Myrmica sulcinodis | In polygynous social insects more than one queen reproduces in a colony. In such populations ecological factors affecting survival and reproduction of queens are likely to be of prime importance for social organization. In particular, habitat saturation leading to severe limitations in the availability of nest sites has been suggested to promote high queen number. In this study we examine the social and genetic structure of colonies in the polygynous ant Myrmica sulcinodis. We investigated a single breeding population in two adjacent habitats which differed markedly in the availability of nest sites. In the main habitat M. sulcinodis occupied almost all suitable nest sites, whereas in the other (marginal) habitat most sites were unoccupied by ants, due to a recent fire. In support of the habitat saturation hypothesis, the number of queens per colony which could explain the estimated relatedness among workers was almost five times higher for the main habitat than for the marginal habitat. This is the first demonstration that the kin structure of a social insect population is plastic and responds adaptively to short-term changes in ecological constraints such as nest site availability. Based on combined genetic and demographic data we discuss queen reproductive strategies and suggest that a special class of queen ‘floaters’ only stays ephemerally in the colonies, thus causing a substantial turnover of reproducing queens across years. | 2944 | NA | NA | NA | no | no |
| 2945 | 2017 | NA | Xu, C; Wang, CS; Sun, XY; Zhang, R; Gleason, ML; Eiji, T; Sun, GY | 2013 | Multiple Group I Introns in the Small-Subunit rDNA of Botryosphaeria dothidea: Implication for Intraspecific Genetic Diversity | Botryosphaeria dothidea is a widespread and economically important pathogen on various fruit trees, and it often causes die-back and canker on limbs and fruit rot. In characterizing intraspecies genetic variation within this fungus, group I introns, rich in rDNA of fungi, may provide a productive region for exploration. In this research, we analysed complete small subunit (SSU) ribosomal DNA (rDNA) sequences of 37 B. dothidea strains, and found four insertions, designated Bdo.S943, Bdo.S1199-A, Bdo.S1199-B and Bdo.S1506, at three positions. Sequence analysis and structure prediction revealed that both Bdo.S943 and Bdo.S1506 belonged to subgroup IC1 of group I introns, whereas Bdo.S1199-A and Bdo.S1199-B corresponded to group IE introns. Moreover, Bdo.S1199-A was found to host an open reading frame (ORF) for encoding the homing endonuclease (HE), whereas Bdo.S1199-B, an evolutionary descendant of Bdo.S1199-A, included a degenerate HE. The above four introns were novel, and were the first group I introns observed and characterized in this species. Differential distribution of these introns revealed that all strains could be separated into four genotypes. Genotype III (no intron) and genotype IV (Bdo.S1199-B) were each found in only one strain, whereas genotype I (Bdo.S1199-A) and genotype II (Bdo.S943 and Bdo.S1506) occurred in 95% of the strains. There is a correlation between B. dothidea genotypes and hosts or geographic locations. Thus, these newly discovered group I introns can help to advance understanding of genetic differentiation within B. dothidea. | 2945 | NA | NA | NA | no | no |
| 2946 | 2017 | NA | Salimova, AZ; Kutuev, IA; Khusainova, RI; Akhmetova, VL; Svyatova, GS; Berezina, GM; Khusnutdinova, EK | 2005 | Analysis of ethnogeographic groups of Kazakhs based on nuclear genome DNA polymorphism | Eight nuclear DNA loci, including six Alu insertions (ACE, APOA1, PV92, TPA25, Ya5NBC27, and Ya5NBC148), 32-bp deletion in the CCR5 gene, and VNTR locus at the eNOS gene, were examined in three ethnogeographic groups of Kazakhs (342 individuals). The individuals examined lived in southeastern, central, and southwestern regions of Kazakhstan, and according to their tribal attribution, belonged to the Senior, Middle, and Junior Zhuzes. The Alu insertions appeared to be polymorphic in all populations examined: the insertion frequency varied from 0.264 in the populations of the Senior and Middle Zhuzes at the Ya5NBC27 and Ya5NBC148 loci, to 0.827 in Kazakhs of the Middle Zhuz at the APOA1 locus. In Kazakh groups examined only two alleles of the eNOS VNTR locus were detected with the number of repeats constituting four (A) and five (B) copies. The highest frequency of A allele was found in Kazakhs from the Junior Zhuz (0.113), while the highest frequency of B allele was detected in population of the Senior Zhuz (0.893). The frequency of the 32-bp deletion in the chemokine receptor CCR5 gene varied from 0.027 in the Junior Zhuz to 0.045 in the Senior Zhuz. Kazakhs showed high genetic diversity (H (ex) = 0.376). In general, in three ethnogeographic groups of Kazakhs, the coefficient of gene differentiation (G (ST)) over eight diallelic markers of nuclear genome constituted 1.1%. The differences in the Alu insertions made the highest contribution to the among-population diversity (G (ST) = 1.2%). | 2946 | NA | NA | NA | no | no |
| 2947 | 2017 | NA | Kelly, MJ | 2001 | Lineage loss in Serengeti cheetahs: Consequences of high reproductive variance and heritability of fitness on effective population size | In natural populations, many breeders do not leave surviving offspring, and as a result many potential genetic lineages are lost. I examined lineage extinction in Serengeti cheetahs (Acinonyx jubatus) and found that 76% of matrilines were lost over a 25-year period. Production of future breeders was nonrandom and generally confined to a few families. Five out of 63 matrilines accounted for 45% of the total cheetah population over the course of the study. Lineage persistence is perhaps best illustrated by the variance in lifetime reproductive success (LRS) and heritability in this parameter. In female cheetahs, variance in LRS wa high, and new data show that this LRS was heritable. Variance in LRS and heritability in LRS have dramatic consequences for effective population size, N-e. I calculated N-e for cheetahs, taking into account fluctuating population size, unequal sex ratio, non-Poisson distribution of reproductive success, and heritability of fitness. The N-e was most strongly affected by variance in reproductive success and especially heritability in reproductive success. The variance N-e was 44% of the actual population size, and the inclusion of heritability further reproduced N-e to only 15% of the actual population, a ratio similar to that of a social carnivore with reproductive suppression. The current cheetah population in the Serengeti is below numbers suggested by N-e estimates as sufficient to maintain sufficient genetic diversity. | 2947 | NA | NA | NA | no | no |
| 2948 | 2017 | NA | Kang, HM; Park, HY; Lee, KJ; Choi, JG; Lee, EK; Song, BM; Lee, HS; Lee, YJ | 2014 | Characterization of H7 Influenza A Virus in Wild and Domestic Birds in Korea | During surveillance programs in Korea between January 2006 and March 2011, 31 H7 avian influenza viruses were isolated from wild birds and domestic ducks and genetically characterized using large-scale sequence data. All Korean H7 viruses belonged to the Eurasian lineage, which showed substantial genetic diversity, in particular in the wild birds. The Korean H7 viruses from poultry were closely related to those of wild birds. Interestingly, two viruses originating in domestic ducks in our study had the same gene constellations in all segment genes as viruses originating in wild birds. The Korean H7 isolates contained avian-type receptors (Q226 and G228), no NA stalk deletion (positions 69-73), no C-terminal deletion (positions 218-230) in NS1, and no substitutions in PB2-627, PB1-368, and M2-31, compared with H7N9 viruses. In pathogenicity experiments, none of the Korean H7 isolates tested induced clinical signs in domestic ducks or mice. Furthermore, while they replicated poorly, with low titers (10(0.7-1.3)EID(50)/50 mu l) in domestic ducks, all five viruses replicated well (up to 7-10 dpi, 10(0.7-4.3)EID(50)/50 mu l) in the lungs of mice, without prior adaptation. Our results suggest that domestic Korean viruses were transferred directly from wild birds through at least two independent introductions. Our data did not indicate that wild birds carried poultry viruses between Korea and China, but rather, that wild-type H7 viruses were introduced several times into different poultry populations in eastern Asia. | 2948 | NA | NA | NA | no | no |
| 2949 | 2017 | NA | Traverse, CC; Mayo-Smith, LM; Poltak, SR; Cooper, VS | 2013 | Tangled bank of experimentally evolved Burkholderia biofilms reflects selection during chronic infections | How diversity evolves and persists in biofilms is essential for understanding much of microbial life, including the uncertain dynamics of chronic infections. We developed a biofilm model enabling long-term selection for daily adherence to and dispersal from a plastic bead in a test tube. Focusing on a pathogen of the cystic fibrosis lung, Burkholderia cenocepacia, we sequenced clones and metagenomes to unravel the mutations and evolutionary forces responsible for adaptation and diversification of a single biofilm community during 1,050 generations of selection. The mutational patterns revealed recurrent evolution of biofilm specialists from generalist types and multiple adaptive alleles at relatively few loci. Fitness assays also demonstrated strong interference competition among contending mutants that preserved genetic diversity. Metagenomes from five other independently evolved biofilm lineages revealed extraordinary mutational parallelism that outlined common routes of adaptation, a subset of which was found, surprisingly, in a planktonic population. These mutations in turn were surprisingly well represented among mutations that evolved in cystic fibrosis isolates of both Burkholderia and Pseudomonas. These convergent pathways included altered metabolism of cyclic diguanosine monophosphate, polysaccharide production, tricarboxylic acid cycle enzymes, global transcription, and iron scavenging. Evolution in chronic infections therefore may be driven by mutations in relatively few pathways also favored during laboratory selection, creating hope that experimental evolution may illuminate the ecology and selective dynamics of chronic infections and improve treatment strategies. | 2949 | NA | NA | NA | no | no |
| 2950 | 2017 | NA | Foster, EA; Franks, DW; Morrell, LJ; Balcomb, KC; Parsons, KM; van Ginneken, A; Croft, DP | 2012 | Social network correlates of food availability in an endangered population of killer whales, Orcinus orca | For the majority of social species, group composition is dynamic, and individuals are interconnected in a heterogeneous social network. Social network structure has far-reaching implications for the ecology of individuals and populations. However, we have little understanding of how ecological variables shape this structure. We used a long-term data set (1984-2007) to examine the relationship between food availability and social network structure in the endangered southern resident killer whales. During the summer months individuals in this population feed primarily on chinook salmon, Oncorhynchus tshawytscha, which show annual variation in abundance. We tested the hypothesis that temporal variation in chinook salmon will correlate with variation in social network structure. Using a null model that controlled for population demography, group size and sampling effort, we found a significant relationship between the connectivity of the social network and salmon abundance, with a more interconnected social network in years of high salmon abundance. Our results demonstrate that resource availability may be an important determinant of social network structure. Given the central importance of the social network for population processes such as the maintenance of cooperation and the transmission of information and disease, a change in social network structure caused by a change in food availability may have significant ecological and evolutionary consequences. (C) 2012 The Association for the Study of Animal Behaviour. Published by Elsevier Ltd. All rights reserved. | 2950 | NA | NA | NA | no | no |
| 2951 | 2017 | NA | Lenz, TL; Spirin, V; Jordan, DM; Sunyaev, SR | 2016 | Excess of Deleterious Mutations around HLA Genes Reveals Evolutionary Cost of Balancing Selection | Deleterious mutations are expected to evolve under negative selection and are usually purged from the population. However, deleterious alleles segregate in the human population and some disease-associated variants are maintained at considerable frequencies. Here, we test the hypothesis that balancing selection may counteract purifying selection in neighboring regions and thus maintain deleterious variants at higher frequency than expected from their detrimental fitness effect. We first show in realistic simulations that balancing selection reduces the density of polymorphic sites surrounding a locus under balancing selection, but at the same time markedly increases the population frequency of the remaining variants, including even substantially deleterious alleles. To test the predictions of our simulations empirically, we then use whole-exome sequencing data from 6,500 human individuals and focus on the most established example for balancing selection in the human genome, the major histocompatibility complex (MHC). Our analysis shows an elevated frequency of putatively deleterious coding variants in nonhuman leukocyte antigen (non-HLA) genes localized in the MHC region. The mean frequency of these variants declined with physical distance from the classical HLA genes, indicating dependency on genetic linkage. These results reveal an indirect cost of the genetic diversity maintained by balancing selection, which has hitherto been perceived as mostly advantageous, and have implications both for the evolution of recombination and also for the epidemiology of various MHC-associated diseases. | 2951 | NA | NA | NA | no | no |
| 2952 | 2017 | NA | Kloosterman, WP; Francioli, LC; Hormozdiari, F; Marschall, T; Hehir-Kwa, JY; Abdellaoui, A; Lameijer, EW; Moed, MH; Koval, V; Renkens, I; van Roosmalen, MJ; Arp, P; Karssen, LC; Coe, BP; Handsaker, RE; Suchiman, ED; Cuppen, E; Thung, DT; McVey, M; Wendl, MC; Uitterlinden, A; van Duijn, CM; Swertz, MA; Wijmenga, C; van Ommen, GB; Slagboom, PE; Boomsma, DI; Schonhuth, A; Eichler, EE; de Bakker, PIW; Ye, K; Guryev, V | 2015 | Characteristics of de novo structural changes in the human genome | Small insertions and deletions (indels) and large structural variations (SVs) are major contributors to human genetic diversity and disease. However, mutation rates and characteristics of de novo indels and SVs in the general population have remained largely unexplored. We report 332 validated de novo structural changes identified in whole genomes of 250 families, including complex indels, retrotransposon insertions, and interchromosomal events. These data indicate a mutation rate of 2.94 indels (1-20 bp) and 0.16 SVs (>20 bp) per generation. De novo structural changes affect on average 4.1 kbp of genomic sequence and 29 coding bases per generation, which is 91 and 52 times more nucleotides than de novo substitutions, respectively. This contrasts with the equal genomic footprint of inherited SVs and substitutions. An excess of structural changes originated on paternal haplotypes. Additionally, we observed a nonuniform distribution of de novo SVs across offspring. These results reveal the importance of different mutational mechanisms to changes in human genome structure across generations. | 2952 | NA | NA | NA | no | no |
| 2953 | 2017 | NA | Lack, JB; Hamilton, MJ; Braun, JK; Mares, MA; Van den Bussche, RA | 2013 | Comparative phylogeography of invasive Rattus rattus and Rattus norvegicus in the US reveals distinct colonization histories and dispersal | Invasive Rattus are arguably the most costly and destructive invasive species on the planet, but little is known concerning their invasion history and population structure in the U.S. We utilized both nuclear microsatellites and mitochondrial DNA sequences (mtDNA) to compare the colonization history, patterns of gene flow, and levels of genetic diversity of Rattus rattus and R. norvegicus in the U.S. Analyses of mtDNA suggest R. rattus is characterized by a single rapid expansion into the U.S. from one or two very closely related mtDNA lineages or geographic sources. For R. norvegicus, mtDNA analyses suggest at least four invasions distinct in space and/or time have occurred to establish its distribution in the U.S. Microsatellite analyses suggest for R. rattus that dispersal is characterized by an isolation-by-distance pattern, suggesting a relatively low frequency of long distance dispersal, and low levels of establishment for novel propagules. In contrast, microsatellite analyses of R. norvegicus suggest high frequencies of long distance dispersal and essentially panmixia among nearly all sampled populations, as well as a high frequency of novel propagules entering at the east and west coasts and assimilating into established populations. We discuss these results in the context of invasive Rattus management in the U.S. and their implications for invasive species in general, as well as the implications for managing the spread of rat-borne pathogens. | 2953 | NA | NA | NA | no | no |
| 2954 | 2017 | NA | Carvajal-Vallejos, FM; Duponchelle, F; Desmarais, E; Cerqueira, F; Querouil, S; Nunez, J; Garcia, C; Renno, JF | 2014 | Genetic structure in the Amazonian catfish Brachyplatystoma rousseauxii: influence of life history strategies | The Dorado or Plateado (Gilded catfish) Brachyplatystoma rousseauxii (Pimelodidae, Siluriformes) is a commercially valuable migratory catfish performing the largest migration in freshwaters: from the Amazonian headwaters in the Andean foothills (breeding area) to the Amazon estuary (nursery area). In spite of its importance to inform management and conservation efforts, the genetic variability of this species has only recently begun to be studied. The aim of the present work was to determine the population genetic structure of B. rousseauxii in two regions: the Upper Madera Basin (five locations in the Bolivian Amazon) and the Western Amazon Basin (one regional sample from the Uyucali-Napo-Maraon-Amazon basin, Peru). Length polymorphism at nine microsatellite loci (284 individuals) was used to determine genetic variability and to identify the most probable panmictic units (using a Bayesian approach), after a significant departure from Hardy-Weinberg equilibrium was observed in the overall dataset (Western Amazon + Upper Madera). Bayesian analyses revealed at least three clusters in admixture in the five locations sampled in the Bolivian Amazon, whereas only two of these clusters were observed in the Western Amazon. Considering the migratory behaviour of B. rousseauxii, different life history strategies, including homing, are proposed to explain the cluster distribution. Our results are discussed in the light of the numerous threats to the species survival in the Madera basin, in particular dam and reservoir construction. | 2954 | NA | NA | NA | no | no |
| 2955 | 2017 | NA | Hoffmann, JH; Impson, FAC; Volchansky, CR | 2002 | Biological control of cactus weeds: implications of hybridization between control agent biotypes | 1 Results of recent research on Dactylopius opuntiae, a biological control agent for cactus weeds (Opuntia spp.) in South Africa and elsewhere, challenge the maxim that genetic diversity of agents necessarily enhances the chances of success in biological weed control.  2 Two biotypes of D. opuntiae, each specific to a different Opuntia species, interbred freely, at least under insectary conditions. We therefore carried out cross‐breeding experiments to determine the viability and host‐preferences of progeny produced by these crosses.  3 Unlike their parents, F1 hybrids were not species‐specific, developing equally well on either of the parental hosts, Opuntia ficus‐indica and Opuntia stricta. The situation was more complex in F2 back‐crosses between hybrids and in crosses between parent strains and hybrids because male cochineal insects contributed only maternally inherited genes to their progeny, due to their unusual haploid‐diploid (lecanoid) mechanism of sex determination. Some F2 combinations produced cohorts of progeny that were either entirely true‐bred (i.e. host‐specific) or entirely hybrid (i.e. not host‐specific) genotypes, while other combinations produced groups of siblings with some individuals (theoretically half) that were true‐bred genotypes and the balance were hybrid genotypes.  4 The lack of host‐specificity of hybrids should enhance overall biological control of the target species directly, because hybrids attack both host‐plants, and indirectly, because hybrid nymphs have greater chances of finding a suitable host‐during passive dispersal. However, this advantage will be negated when F2 crosses produce host‐specific nymphs on host‐plants that are incompatible for their survival. 5 These findings show that only pure strains of D. opuntiae should be released in monocultures of the target weeds. More generally, they caution that the possible consequences of mixing genotypes of a biological control agent species should be investigated before different provenances are amalgamated to enhance genetic diversity. | 2955 | NA | NA | NA | no | no |
| 2956 | 2017 | NA | Barcelos, QL; Pinto, JMA; Vaillancourt, LJ; Souza, EA | 2014 | Characterization of Glomerella Strains Recovered from Anthracnose Lesions on Common Bean Plants in Brazil | Anthracnose caused by Colletotrichum lindemuthianum is an important disease of common bean, resulting in major economic losses worldwide. Genetic diversity of the C. lindemuthianum population contributes to its ability to adapt rapidly to new sources of host resistance. The origin of this diversity is unknown, but sexual recombination, via the Glomerella teleomorph, is one possibility. This study tested the hypothesis that Glomerella strains that are frequently recovered from bean anthracnose lesions represent the teleomorph of C. lindemuthianum. A large collection of Glomerella isolates could be separated into two groups based on phylogenetic analysis, morphology, and pathogenicity to beans. Both groups were unrelated to C. lindemuthianum. One group clustered with the C. gloeosporioides species complex and produced mild symptoms on bean tissues. The other group, which belonged to a clade that included the cucurbit anthracnose pathogen C. magna, caused no symptoms. Individual ascospores recovered from Glomerella perithecia gave rise to either fertile (perithecial) or infertile (conidial) colonies. Some pairings of perithecial and conidial strains resulted in induced homothallism in the conidial partner, while others led to apparent heterothallic matings. Pairings involving two perithecial, or two conidial, colonies produced neither outcome. Conidia efficiently formed conidial anastomosis tubes (CATs), but ascospores never formed CATs. The Glomerella strains formed appressoria and hyphae on the plant surface, but did not penetrate or form infection structures within the tissues. Their behavior was similar whether the beans were susceptible or resistant to anthracnose. These same Glomerella strains produced thick intracellular hyphae, and eventually acervuli, if host cell death was induced. When Glomerella was co-inoculated with C. lindemuthianum, it readily invaded anthracnose lesions. Thus, the hypothesis was not supported: Glomerella strains from anthracnose lesions do not represent the teleomorphic phase of C. lindemuthianum, and instead appear to be bean epiphytes that opportunistically invade and sporulate in the lesions. | 2956 | NA | NA | NA | no | no |
| 2957 | 2017 | NA | Yin, KP; Xie, Y; Wu, N | 2006 | Corridor connecting giant panda habitats from north to south in the Min Mountains, Sichuan, China | The giant panda faces severe threats from habitat fragmentation and isolation. Currently, giant panda populations have been fragmented into 30 habitat patches. The disappearance of isolated small populations and studies on the genetic diversity of various populations have shown that small isolated panda populations are at a high risk of dying out completely. Habitat fragmentation has seriously impaired the ability of the giant panda to resist climate changes and other natural disasters, such as large-scale, synchronous bamboo blooming. The Min Mountains have the largest population of pandas in China, numbering 581 individuals and accounting for 52% of the total (1114) in China. Geographic isolation means that giant pandas in the Min Mountains are divided into two populations (population A in the north and population B in the south). Population B, which had only 42 individuals in 1989, is severely threatened by high-density human populations and the loss of genetic diversity. However, we have identified an important corridor connecting the two populations. This paper explains the importance and the feasibility of reestablishing this corridor. Due to the special geographic locations of these two populations (two rivers block the migration of giant pandas between south and north), the corridor is the only passage for giant pandas in the region. Recent studies have also shown an increase of giant panda activity in the area of the corridor. However, vegetation in the corridor has been severely degraded. Bamboo forest must be restored in this area to provide food for the pandas during migration. The effects of human activities must be reduced in order to maintain panda habitat. We believe that a restored corridor will be of great benefit to the survival of giant pandas in the Min Mountains, especially for population B. Successful re-establishment of a corridor will be a valuable model for corridor construction in the future. | 2957 | NA | NA | NA | no | no |
| 2958 | 2017 | NA | Ferreira, GEM; dos Santos, BN; Dorval, MEC; Ramos, TPB; Porrozzi, R; Peixoto, AA; Cupolillo, E | 2012 | The Genetic Structure of Leishmania infantum Populations in Brazil and Its Possible Association with the Transmission Cycle of Visceral Leishmaniasis | Leishmania infantum is the etiologic agent of visceral leishmaniasis (VL) in the Americas, Mediterranean basin and West and Central Asia. Although the geographic structure of L. infantum populations from the Old World have been described, few studies have addressed the population structure of this parasite in the Neotropical region. We employed 14 microsatellites to analyze the population structure of the L. infantum strains isolated from humans and dogs from most of the Brazilian states endemic for VL and from Paraguay. The results indicate a low genetic diversity, high inbreeding estimates and a depletion of heterozygotes, which together indicate a predominantly clonal breeding system, but signs of sexual events are also present. Three populations were identified from the clustering analysis, and they were well supported by F statistics inferences and partially corroborated by distance-based. POP1 (111 strains) was observed in all but one endemic area. POP2 (31 strains) is also well-dispersed, but it was the predominant population in Mato Grosso (MT). POP3 (31 strains) was less dispersed, and it was observed primarily in Mato Grosso do Sul (MS). Strains originated from an outbreak of canine VL in Southern Brazil were grouped in POP1 with those from Paraguay, which corroborates the hypothesis of dispersal from Northeastern Argentina and Paraguay. The distribution of VL in MS seems to follow the west-east construction of the Bolivia-Brazil pipeline from Corumba municipality. This may have resulted in a strong association of POP3 and Lutzomyia cruzi, which is the main VL vector in Corumba, and a dispersion of this population in this region that was shaped by human interference. This vector also occurs in MT and may influence the structure of POP2. This paper presents significant advances in the understanding of the population structure of L. infantum in Brazil and its association with eco-epidemiological aspects of VL. | 2958 | NA | NA | NA | no | no |
| 2959 | 2017 | NA | Erler, S; Lattorff, HMG | 2010 | The degree of parasitism of the bumblebee (Bombus terrestris) by cuckoo bumblebees (Bombus (Psithyrus) vestalis) | Host parasite systems are characterised by coevolutionary arms races between host and parasite. Parasites are often the driving force, as they replicate much faster than their hosts and have shorter generation times and larger population sizes, resulting in higher mutation rates per time interval. This scenario does not fit all host parasite systems. Socially parasitic cuckoo bumblebees (Bombus (Psithyrus) vestalis) parasitise colonies of Bombus terrestris share most life history characteristics with their hosts. As they parasitise only a subset of all available colonies, their population size should be lower than that of their hosts. This might have strong negative effects on the genetic diversity of B. vestalis and their adaptability. Here, we study for the first time the population structure of a Bombus/Bombus (Psithyrus) system. Highly polymorphic DNA markers were used to reconstruct sibships from individuals collected in the wild. The analysis of the host and parasite populations revealed a rate of parasitism of about 42% (range 33-50%). The population size of B. vestalis was lower compared to their hosts, which was also reflected in low within-group genetic distance. An analysis of the reconstructed queen genotypes revealed more supersisters amongst the B. vestalis queens when compared to the B. terrestris host. The data suggest that B. vestalis females and males do not disperse over long distances. This shows a potential for local adaptation to their hosts. | 2959 | NA | NA | NA | no | no |
| 2960 | 2017 | NA | Kierulff, MCM; DeOliveira, PP | 1996 | Re-assessing the status and conservation of the golden lion tamarin Leontopithecus rosalia in the wild | The golden lion tamarin Leontopithecus rosalia is one of the most endangered primates in the New World. In order to assess the status of golden lion tamarin habitat, satellite images covering the original distribution of the species were analysed and the size of all forests measured. Effective size (Ne) of remaining populations was calculated and the time of isolation estimated for all subpopulations, Current threats were identified in each forest the species was found. Most of the remaining forests within the species’ original distribution were in fragments smaller than 10km(2). The majority of the golden lion tamarins found were in forest fragments which have been isolated for more than 15 years. Today, the principal threats to the survival of the species are the small size and isolation of forest patches, and the small size of subpopulations. Threats such as deforestation and hunting have also been observed in all forests. The small number of remaining animals and their disjunct distribution will likely result in the loss of genetic variability and in inbreeding depression. Conservation strategies, such as translocation of isolated groups, reintroduction of more captive animals, protection of the forests, reforestation, and management of the subpopulations as a metapopulation, will be essential for the maintenance of the species in the wild. The larger and better-preserved forests without tamarins were suggested as potential areas for reintroduction and translocation. | 2960 | NA | NA | NA | no | no |
| 2961 | 2017 | NA | Lively, CM | 2016 | Coevolutionary Epidemiology: Disease Spread, Local Adaptation, and Sex | “How does evolution in parasite populations affect the rate of disease spread? In the present study, I derived the mean reproductive rate ((R) over bar (0)) for a genetically diverse parasite population that is evolving with a similarly diverse host population. Assuming a matching-alleles model, I found that (R) over bar (0) is a positive function of the covariance between the frequencies of”“matching”" host and parasite genotypes. Computer simulations further showed that evolution in the parasite population tends to increase the covariance, which can lead to epidemiological feedbacks. However, the covariances can also become negative during counteradaptation by the host, leading to oscillatory dynamics in host and parasite fitness. Nonetheless, when parasite-mediated selection is strong, the covariance is positive on average, which facilitates the spread of disease. Positive covariances may also underlie patterns of local adaptation in parasite populations and increase the selective advantage of cross-fertilization in host populations." | 2961 | NA | NA | NA | no | no |
| 2962 | 2017 | NA | Inderbitzin, P; Bostock, RM; Davis, RM; Usami, T; Platt, HW; Subbarao, KV | 2011 | Phylogenetics and Taxonomy of the Fungal Vascular Wilt Pathogen Verticillium, with the Descriptions of Five New Species | Knowledge of pathogen biology and genetic diversity is a cornerstone of effective disease management, and accurate identification of the pathogen is a foundation of pathogen biology. Species names provide an ideal framework for storage and retrieval of relevant information, a system that is contingent on a clear understanding of species boundaries and consistent species identification. Verticillium, a genus of ascomycete fungi, contains important plant pathogens whose species boundaries have been ill defined. Using phylogenetic analyses, morphological investigations and comparisons to herbarium material and the literature, we established a taxonomic framework for Verticillium comprising ten species, five of which are new to science. We used a collection of 74 isolates representing much of the diversity of Verticillium, and phylogenetic analyses based on the ribosomal internal transcribed spacer region (ITS), partial sequences of the protein coding genes actin (ACT), elongation factor 1-alpha (EF), glyceraldehyde-3-phosphate dehydrogenase (GPD) and tryptophan synthase (TS). Combined analyses of the ACT, EF, GPD and TS datasets recognized two major groups within Verticillium, Clade Flavexudans and Clade Flavnonexudans, reflecting the respective production and absence of yellow hyphal pigments. Clade Flavexudans comprised V. albo-atrum and V. tricorpus as well as the new species V. zaregamsianum, V. isaacii and V. klebahnii, of which the latter two were morphologically indistinguishable from V. tricorpus but may differ in pathogenicity. Clade Flavnonexudans comprised V. nubilum, V. dahliae and V. longisporum, as well as the two new species V. alfalfae and V. nonalfalfae, which resembled the distantly related V. albo-atrum in morphology. Apart from the diploid hybrid V. longisporum, each of the ten species corresponded to a single clade in the phylogenetic tree comprising just one ex-type strain, thereby establishing a direct link to a name tied to a herbarium specimen. A morphology-based key is provided for identification to species or species groups. | 2962 | NA | NA | NA | no | no |
| 2963 | 2017 | NA | Li, YT; Ko, HY; Lee, CCD; Lai, CY; Kao, CL; Yang, C; Wang, WB; King, CC | 2015 | Phenotypic and Genetic Characterization of Avian Influenza H5N2 Viruses with Intra- and Inter-Duck Variations in Taiwan | Background Human infections with avian influenza viruses (AIVs) have frequently raised global concerns of emerging, interspecies-transmissible viruses with pandemic potential. Waterfowl, the predominant reservoir of influenza viruses in nature, harbor precursors of different genetic lineages that have contributed to novel pandemic influenza viruses in the past. Methods Two duck influenza H5N2 viruses, DV518 and DV413, isolated through virological surveillance at a live-poultry market in Taiwan, showed phylogenetic relatedness but exhibited different replication capabilities in mammalian Madin-Darby Canine Kidney (MDCK) cells. This study characterizes the replication properties of the two duck H5N2 viruses and the determinants involved. Results The DV518 virus replicated more efficiently than DV413 in both MDCK and chicken DF1 cells. Interestingly, the infection of MDCK cells by DV518 formed heterogeneous plaques with great differences in size [large (L) and small (S)], and the two viral strains (p518-L and p518-S) obtained from plaque purification exhibited distinguishable replication kinetics in MDCK cells. Nonetheless, both plaque-purified DV518 strains still maintained their growth advantages over the plaque-purified p413 strain. Moreover, three amino acid substitutions in PA (P224S), PB2 (E72D), and M1 (A128T) were identified in intra-duck variations (p518-L vs p518-S), whereas other changes in HA (N170D), NA (I56T), and NP (Y289H) were present in inter-duck variations (DV518 vs DV413). Both p518-L and p518-S strains had the N170D substitution in HA, which might be related to their greater binding to MDCK cells. Additionally, polymerase activity assays on 293T cells demonstrated the role of vRNP in modulating the replication capability of the duck p518-L viruses in mammalian cells. Conclusion These results demonstrate that intra-host phenotypic variation occurs even within an individual duck. In view of recent human infections by low pathogenic AIVs, this study suggests possible determinants involved in the stepwise selection of virus variants from the duck influenza virus population which may facilitate inter-species transmission. | 2963 | NA | NA | NA | no | no |
| 2964 | 2017 | NA | Rahel, FJ | 2013 | Intentional Fragmentation as a Management Strategy in Aquatic Systems | Maintaining or restoring connectivity in aquatic systems can enhance migratory fish populations; maintain genetic diversity in small, isolated populations; allow organisms to access complementary habitats to meet life-history needs; and facilitate recolonization after local extirpations. However, intentional fragmentation may be beneficial when it prevents the spread of nonnative species or exotic diseases, eliminates hybridization between hatchery and wild stocks, or stops individuals from becoming entrapped in sink environments. Strategies for fragmenting aquatic systems include maintaining existing natural barriers, taking advantage of existing anthropogenic features that impede movement, severing artificial connectivity created by human actions, and intentionally creating new barriers. Future challenges for managing fragmentation include maintaining hydrologic connectivity while blocking biological connectivity in water development projects; identifying approaches for maintaining incompatible taxa, such as sport fishes and small nongame species; and developing selective barriers that prevent the passage of unwanted species while allowing normal life-history movements of other species. | 2964 | NA | NA | NA | no | no |
| 2965 | 2017 | NA | Wellmann, R; Pfeiffer, I | 2009 | Pedigree analysis for conservation of genetic diversity and purging | We present an approach to describe and evaluate changes in genetic diversity and to calculate bounds for improvement. This pedigree-based analysis was applied to the Kromfohrlander dog (FCI Gr9 Sec10). Pedigrees trace back to the foundation of the breed and were available for 5527 individuals. Based on this dataset the population structure and historical bottlenecks were Studied. Distributions of allele frequencies were estimated by Monte Carlo simulation. To monitor changes in mating systems throughout the breeding history, the homozygosity of alleles was compared with their expectations in Hardy-Weinberg equilibrium. Different breeding lines were identified by hierarchical cluster analysis and were characterized by ancestor contributions. Our calculations showed that the founder event in 1945 was followed by two bottlenecks. One Was caused by strong selection in a very small population, and the other was triggered by rigorous disease management. The necessary amount of purging that arised due to the bottlenecks was also discussed. | 2965 | NA | NA | NA | no | no |
| 2966 | 2017 | NA | Say, L; Naulty, F; Hayden, TJ | 2003 | Genetic and behavioural estimates of reproductive skew in male fallow deer | Populations of fallow deer, in general, have low genetic diversity. Nevertheless, we screened 39 microsatellite loci and identified 20 that were polymorphic and suitable to determine paternity of fallow deer. To date, paternity has been studied for 87, 110 and 152 fallow deer fawns born between 2000 and 2002. Our results confirm the existence of a strong polygynous mating system in our population and confirm that the number of copulations performed by males is globally a good estimator of their reproductive success: males which performed the largest proportion of matings fathered the largest proportion of fawns. Nevertheless, we report some differences between the two measurements of the males’ reproductive success: measures of copulatory success underestimated the variance of the males’ reproductive success. On average, males whose copulatory score exceeded their paternity had mated with a higher proportion of younger females. Young females may be more likely to lose the conceptus, or their offspring may suffer high postnatal mortality. | 2966 | NA | NA | NA | no | no |
| 2967 | 2017 | NA | Beckerman, AP; Sharp, SP; Hatchwell, BJ | 2011 | Predation and kin-structured populations: an empirical perspective on the evolution of cooperation | In animal societies, kin selection is a critical evolutionary process, with cooperation evolving principally among relatives living in kin-structured populations. Theoretical and empirical studies have largely focused on population viscosity-the timing or distance of dispersal-as the key factor generating kin structure. This is despite extensive theoretical broadening of the factors and processes influencing effective population size, variance in reproduction, and relatedness. Here, we explore predation mortality as a specific driver of population-level reproductive skew and variance in fecundity to show how a common and perhaps underappreciated event in organism life history can give rise to patterns of relatedness. We develop our case study around an empirically derived model where elevated relatedness arises from predation that alters the timing and nature of offspring mortality, essentially driving variance in fecundity. This leads to dramatic changes in the emergent kin structure of the surviving breeding population. Our in-silico experiments recover the theoretical predictions that when predation acts on clusters of individuals and effectively removes whole family groups (i.e., broods), rather than individuals, from the pool of potential recruits, there is a greater kin structure in the emergent adult population. We conclude that empirical attempts to understand the factors promoting kin-structured populations and the evolution of sociality should now match theoretical efforts to be more inclusive of ecological process generating life history and demographic variability. | 2967 | NA | NA | NA | no | no |
| 2968 | 2017 | NA | Bellafiore, S; Jougla, C; Chapuis, E; Besnard, G; Suong, M; Vu, PN; De Waele, D; Gantet, P; Thi, XN | 2015 | Intraspecific variability of the facultative meiotic parthenogenetic root-knot nematode (Meloidogyne graminicola) from rice fields in Vietnam | Twenty years ago, the facultative meiotic parthenogenetic root-knot nematode (RKN), Meloidogyne graminicola, was recognised as an important rice pathogen in South Vietnam. Although this country is one of the most important rice exporters worldwide, a comprehensive picture of the occurrence of M. graminicola in Vietnamese rice fields is still not available. Therefore a nematode survey was carried out with the aim of better understanding the geographical distribution, and the pathogenic and genetic variability of the RKN in Vietnam. From the fields surveyed in a range of ecosystems, 21 RKN populations were recovered from infected rice roots. A diagnostic SCAR marker was developed showing that all Vietnamese populations belong to M. graminicola. Furthermore, sequencing of the Internal Transcribed Spacer (ITS) of the rDNA genes confirmed this identification. These populations were then characterised using morphometrics and pathogenicity tests (host plant range diversity, reproduction and virulence diversity) revealing intraspecific variability. We showed that morphometric traits are mainly genetically heritable characters with significant differences among the studied populations. Finally, a distinctive trait signature was found for the populations isolated from the upland rice cultures. All together, our study reveals the prevalence of M. graminicola populations in Vietnamese rice. Further investigations need to be developed to explore the population dynamics and evolutionary history of this species in South East Asia. (C) 2015 Academie des sciences. Published by Elsevier Masson SAS. All rights reserved. | 2968 | NA | NA | NA | no | no |
| 2969 | 2017 | NA | Oliveira, MRC; Correa, AS; de Souza, GA; Guedes, RNC; de Oliveira, LO | 2013 | Mesoamerican Origin and Pre- and Post-Columbian Expansions of the Ranges of Acanthoscelides obtectus Say, a Cosmopolitan Insect Pest of the Common Bean | An unprecedented global transfer of agricultural resources followed the discovery of the New World; one consequence of this process was that staple food plants of Neotropical origin, such as the common bean (Phaseolus vulgaris), soon expanded their ranges overseas. Yet many pests and diseases were also transported. Acanthoscelides obtectus is a cosmopolitan seed predator associated with P. vulgaris. Codispersal within the host seed seems to be an important determinant of the ability of A. obtectus to expand its range over long distances. We examined the phylogeographic structure of A. obtectus by (a) sampling three mitochondrial gene sequences (12s rRNA, 16s rRNA, and the gene that encodes cytochrome c oxidase subunit I (COI)) throughout most of the species’ range and (b) exploring its late evolutionary history. Our findings indicate a Mesoamerican origin for the current genealogical lineages of A. obtectus. Each of the two major centers of genetic diversity of P. vulgaris (the Andes and Mesoamerica) contains a highly differentiated lineage of the bean beetle. Brazil has two additional, closely related lineages, both of which predate the Andean lineage and have the Mesoamerican lineage as their ancestor. The cosmopolitan distribution of A. obtectus has resulted from recent expansions of the two Brazilian lineages. We present additional evidence for both pre-Columbian and post-Columbian range expansions as likely events that shaped the current distribution of A. obtectus worldwide. | 2969 | NA | NA | NA | no | no |
| 2970 | 2017 | NA | Barbosa, AD; Gofton, AW; Paparini, A; Codello, A; Greay, T; Gillett, A; Warren, K; Irwin, P; Ryan, U | 2017 | Increased genetic diversity and prevalence of co-infection with Trypanosoma spp. in koalas (Phascolarctos cinereus) and their ticks identified using next-generation sequencing (NGS) | Infections with Trypanosoma spp. have been associated with poor health and decreased survival of koalas (Phascolarctos cinereus), particularly in the presence of concurrent pathogens such as Chlamydia and koala retrovirus. The present study describes the application of a next-generation sequencing (NGS)-based assay to characterise the prevalence and genetic diversity of trypanosome communities in koalas and two native species of ticks (Ixodes holocyclus and I. tasmani) removed from koala hosts. Among 168 koalas tested, 32.2% (95% CI: 25.2-39.8%) were positive for at least one Trypanosoma sp. Previously described Trypanosoma spp. from koalas were identified, including T. irwini (32.1%, 95% CI: 25.2-39.8%), T. gilletti (25%, 95% CI: 18.7-32.3%), T. copemani (27.4%, 95% CI: 20.8-34.8%) and T. vegrandis (10.1%, 95% CI: 6.0-15.7%). Trypanosoma noyesi was detected for the first time in koalas, although at a low prevalence (0.6% 95% CI: 0-3.3%), and a novel species (Trypanosoma sp. AB-2017) was identified at a prevalence of 4.8% (95% CI: 2.1-9.2%). Mixed infections with up to five species were present in 27.4% (95% CI: 21-35%) of the koalas, which was significantly higher than the prevalence of single infections 4.8% (95% CI: 2-9%). Overall, a considerably higher proportion (79.7%) of the Trypanosoma sequences isolated from koala blood samples were identified as T. irwini, suggesting this is the dominant species. Co-infections involving T. gilletti, T. irwini, T. copemani, T. vegrandis and Trypanosoma sp. AB-2017 were also detected in ticks, with T. gilletti and T. copemani being the dominant species within the invertebrate hosts. Direct Sanger sequencing of Trypanosoma 18S rRNA gene amplicons was also performed and results revealed that this method was only able to identify the genotypes with greater amount of reads (according to NGS) within koala samples, which highlights the advantages of NGS in detecting mixed infections. The present study provides new insights on the natural genetic diversity of Trypanosoma communities infecting koalas and constitutes a benchmark for future clinical and epidemiological studies required to quantify the contribution of trypanosome infections on koala survival rates. | 2970 | NA | NA | NA | no | no |
| 2971 | 2017 | NA | Callicrate, T; Dikow, R; Thomas, JW; Mullikin, JC; Jarvis, ED; Fleischer, RC | 2014 | Genomic resources for the endangered Hawaiian honeycreepers | Background: The Hawaiian honeycreepers are an avian adaptive radiation containing many endangered and extinct species. They display a dramatic range of phenotypic variation and are a model system for studies of evolution, conservation, disease dynamics and population genetics. Development of a genome-scale resources for this group would augment the quality of research focusing on Hawaiian honeycreepers and facilitate comparative avian genomic research. Results: We assembled the genome sequence of a Hawaii amakihi (Hemignathus virens), and identified similar to 3.9 million single nucleotide polymorphisms (SNPs) in the genome. Using the amakihi genome as a reference, we also identified similar to 156,000 SNPs in RAD tag (restriction site associated DNA) sequencing of five honeycreeper species (palila [Loxioides bailleui], Nihoa finch [Telespiza ultima], iiwi [Vestiaria coccinea], apapane [Himatione sanguinea], and amakihi). SNPs are distributed throughout the amakihi genome, and the individual sequenced shows several large regions of low heterozygosity on chromosomes 1, 5, 6, 8 and 11. SNPs from RAD tag sequencing were also found throughout the genome but were found to be more densely located on microchromosomes, apparently a result of differential distribution of the particular site recognized by restriction enzyme BseXI. Conclusions: The amakihi genome sequence will be useful for comparative avian genomics research and provides a significant resource for studies in such areas as disease ecology, evolution, and conservation genetics. The genome sequences will enable mapping of transcriptome data for honeycreepers and comparison of gene sequences between avian taxa. Researchers will be able to use the large number of SNP markers to genotype honeycreepers in regions of interest or across the whole genome. There are enough markers to enable use of methods such as genome-wide association studies (GWAS) that will allow researchers to make connections between phenotypic diversity of honeycreepers and specific genetic variants. Genome-wide markers will also help resolve phylogenetic and population genetic questions in honeycreepers. | 2971 | NA | NA | NA | no | no |
| 2972 | 2017 | NA | Laing, CR; Buchanan, C; Taboada, EN; Zhang, YX; Karmali, MA; Thomas, JE; Gannon, VPJ | 2009 | In silico genomic analyses reveal three distinct lineages of Escherichia coli O157:H7, one of which is associated with hyper-virulence | “Background: Many approaches have been used to study the evolution, population structure and genetic diversity of Escherichia coli O157:H7; however, observations made with different genotyping systems are not easily relatable to each other. Three genetic lineages of E. coli O157: H7 designated I, II and I/II have been identified using octamer-based genome scanning and microarray comparative genomic hybridization (mCGH). Each lineage contains significant phenotypic differences, with lineage I strains being the most commonly associated with human infections. Similarly, a clade of hyper-virulent O157: H7 strains implicated in the 2006 spinach and lettuce outbreaks has been defined using single-nucleotide polymorphism (SNP) typing. In this study an in silico comparison of six different genotyping approaches was performed on 19 E. coli genome sequences from 17 O157: H7 strains and single O145:NM and K12 MG1655 strains to provide an overall picture of diversity of the E. coli O157: H7 population, and to compare genotyping methods for O157: H7 strains. Results: In silico determination of lineage, Shiga-toxin bacteriophage integration site, comparative genomic fingerprint, mCGH profile, novel region distribution profile, SNP type and multi-locus variable number tandem repeat analysis type was performed and a supernetwork based on the combination of these methods was produced. This supernetwork showed three distinct clusters of strains that were O157: H7 lineage-specific, with the SNP-based hyper-virulent clade 8 synonymous with O157: H7 lineage I/II. Lineage I/II/ clade 8 strains clustered closest on the supernetwork to E. coli K12 and E. coli O55:H7, O145: NM and sorbitol-fermenting O157 strains. Conclusion: The results of this study highlight the similarities in relationships derived from multi-locus genome sampling methods and suggest a”“common genotyping language”" may be devised for population genetics and epidemiological studies. Future genotyping methods should provide data that can be stored centrally and accessed locally in an easily transferable, informative and extensible format based on comparative genomic analyses." | 2972 | NA | NA | NA | no | no |
| 2973 | 2017 | NA | Weatherall, D | 1999 | From genotype to phenotype: genetics and medical practice in the new millennium | The completion of the human genome project will provide a vast amount of information about human genetic diversity One of the major challenges for the medical sciences will be to relate genotype to phenotype. Over recent years considerable progress has been made in relating the molecular pathology of monogenic diseases to the associated clinical phenotypes. Studies of the inherited disorders of haemoglobin, notably the thalassaemias, have shown how even in these, the simplest of monogenic diseases, there is remarkable complexity with respect to their phenotypic expression. Although studies of other monogenic diseases are less far advanced, it is clear that the same level of complexity will exist. This information provides some indication of the difficulties that will be met. when trying to define the genes that are involved in common multigenic disorders and, in particular, in trying to relate disease phenotypes to the complex interactions between many genes and multiple environmental factors. | 2973 | NA | NA | NA | no | no |
| 2974 | 2017 | NA | Jordan, LA; Allsopp, MH; Oldroyd, BP; Wossler, TC; Beekman, M | 2008 | Cheating honeybee workers produce royal offspring | The Cape bee (Apis mellifera capensis) is unique among honeybees in that workers can lay eggs that instead of developing into males develop into females via thelytokous parthenogenesis. We show that this ability allows workers to compete directly with the queen over the production of new queens. Genetic analyses using microsatellites revealed that 23 out of 39 new queens produced by seven colonies were offspring of workers and not the resident queen. Of these, eight were laid by resident workers, but the majority were offspring of parasitic workers from other colonies. The parasites were derived from several clonal lineages that entered the colonies and successfully targeted queen cells for parasitism. Hence, these parasitic workers had the potential to become genetically reincarnated as queens. Of the daughter queens laid by the resident queen, three were produced asexually, suggesting that queens can ‘choose’ to produce daughter queens clonally and thus have the potential for genetic immortality. | 2974 | NA | NA | NA | no | no |
| 2975 | 2017 | NA | Gupta, B; Reddy, BPN; Fan, Q; Yan, GY; Sirichaisinthop, J; Sattabongkot, J; Escalante, AA; Cui, LW | 2015 | Molecular Evolution of PvMSP3 alpha Block II in Plasmodium vivax from Diverse Geographic Origins | Block II of Plasmodium vivax merozoite surface protein 3 alpha (PvMSP3 alpha) is conserved and has been proposed as a potential candidate for a malaria vaccine. The present study aimed to compare sequence diversity in PvMSP3a block II at a local microgeographic scale in a village as well as from larger geographic regions (countries and worldwide). Blood samples were collected from asymptomatic carriers of P. vivax in a village at the western border of Thailand and PvMSP3 alpha was amplified and sequenced. For population genetic analysis, 237 PvMSP3 alpha block II sequences from eleven P. vivax endemic countries were analyzed. PvMSP3 alpha sequences from 20 village-level samples revealed two length variant types with one type containing a large deletion in block I. In contrast, block II was relatively conserved; especially, some non-synonymous mutations were extensively shared among 11 parasite populations. However, the majority of the low-frequency synonymous variations were population specific. The conserved pattern of nucleotide diversity in block II sequences was probably due to functional/structural constraints, which were further supported by the tests of neutrality. Notably, a small region in block II that encodes a predicted B cell epitope was highly polymorphic and showed signs of balancing selection, signifying that this region might be influenced by the immune selection and may serve as a starting point for designing multi-antigen/stage epitope based vaccines against this parasite. | 2975 | NA | NA | NA | no | no |
| 2976 | 2017 | NA | Parameswari, B; Bagyalakshmi, K; Viswanathan, R; Chinnaraja, C | 2013 | Molecular characterization of Indian sugarcane streak mosaic virus isolate | Sugarcane streak mosaic virus (SCSMV), a member of the genus Poacevirus, family Potyviridae, is an important viral pathogen affecting sugarcane cultivation in India. The complete nucleotide sequence of a SCSMV isolate from India (SCSMV-IND) was determined. The linear, assembled, single-stranded positive-sense RNA genome of SCSMV-IND was 9,786 nucleotides in length (excluding the poly (A) tail) and encoded a polyprotein of 3,131 amino acid residues. The genome of SCSMV-IND shared high degree of sequence identity with SCSMV-PAK (93 % at nucleotide and 97 % at amino acid), and shared only 81 % nucleotide and 94 % amino acid identities with all the four SCSMV isolates (SCSMV-JP1, -JP2, -ID, and -THA). Phylogenetic tree analysis of the complete genome sequences of SCSMV isolates revealed that they can be clustered into two groups. SCSMV-IND and -AP isolates showed 18 % (nucleotide) divergence within the highly conserved 3’ partial genome, suggesting a high level of genetic diversity among the Indian SCSMV isolates. | 2976 | NA | NA | NA | no | no |
| 2977 | 2017 | NA | Simon-Sanchez, J; Scholz, S; Fung, HC; Matarin, M; Hernandez, D; Gibbs, JR; Britton, A; de Vrieze, FW; Peckham, E; Gwinn-Hardy, K; Crawley, A; Keen, JC; Nash, J; Borgaonkar, D; Hardy, J; Singleton, A | 2007 | Genome-wide SNP assay reveals structural genomic variation, extended homozygosity and cell-line induced alterations in normal individuals | The recent hapmap effort has placed focus on the application of genome- wide SNP analysis to assess the contribution of genetic variability, particularly SNPs, to traits such as disease. Here, we describe the utility of genome-wide SNP analysis in the direct detection of extended homozygosity and structural genomic variation. We use this approach to assess the frequency of genomic alterations resulting from the lymphoblast immortalization and culture processes commonly used in cell repositories. We have assayed 408 804 SNPs in 276 DNA samples extracted from Epstein-Barr virus immortalized cell lines, which were derived from lymphocytes of elderly neurologically normal subjects. These data reveal extended homozygosity (contiguous tracts > 5 Mb) in 9.5% (26/272) and 340 structural genomic alterations in 182 (66.9%) DNA samples assessed, 66% of which did not overlap with previously described structural variations. Examination of DNA extracted directly from the blood of 30 of these subjects confirmed all examined instances of extended homozygosity (6/6), 75% of structural genomic alteration < 5 Mb in size (12/16) and 13% (1/8) of structural genomic alteration > 5 Mb in size. These data suggest that structural genomic variation is a common phenomenon in the general population. While a proportion of this variability may be caused or its relative abundance altered by the immortalization and clonal process this will have only a minor effect on genotype and allele frequencies in a large cohort. It is likely that this powerful methodology will augment existing techniques in the identification of chromosomal abnormalities. | 2977 | NA | NA | NA | no | no |
| 2978 | 2017 | NA | Strucken, EM; Lee, SH; Jang, GW; Porto-Neto, LR; Gondro, C | 2015 | Towards breed formation by island model divergence in Korean cattle | Background: The main cattle breed in Korea is the brown Hanwoo, which has been under artificial selection within a national breeding program for several decades. Varieties of the Hanwoo known as Jeju Black and Chikso were not included in the breeding program and remained isolated from the effects of recent artificial selection advancements. We analysed the Jeju Black and Chikso populations in regards to their genetic variability, state of inbreeding, as well as level of differentiation from the mainland Hanwoo population. Results: Jeju Black and Chikso were found to have small estimated effective population sizes (N-e) of only 11 and 7, respectively. Despite a small Ne, higher than expected heterozygosity levels were observed (0.303 and 0.306), however, lower allelic richness was found for the two island populations (1.76 and 1.77) compared to the mainland population (1.81). The increase in heterozygosity could be due to environmental disease challenges that promoted maintenance of higher genetic variability; however, no direct proof exists. Increased heterozygosity due to a first generation crossing of genetically different populations is not recorded. The differentiation between the Korean populations had FST values between 0.014 and 0.036 which is not as high as the differentiation within European beef or dairy cattle breeds (0.047-0.111). This suggests that the three populations have not separated into independent breeds. Conclusion: Results agree with an island model of speciation where the brown Hanwoo represents the ancestral breed, whilst the Jeju Black and Chikso diverge from this common ancestor, following different evolutionary trajectories. Nevertheless, differences are minor and whether Jeju Black and Chikso cattle will develop into discrete breeds or reintegrate with the main population has to be seen in the future and will largely depend on human management decisions. This offers a rare opportunity to accompany the development of new breeds but also poses challenges on how to preserve these incipient breeds and ensure their long term viability. | 2978 | NA | NA | NA | no | no |
| 2979 | 2017 | NA | Irzykowska, L; Bocianowski, J; Waskiewicz, A; Weber, Z; Karolewski, Z; Golinski, P; Kostecki, M; Irzykowski, W | 2012 | Genetic variation of Fusarium oxysporum isolates forming fumonisin B-1 and moniliformin | Thirty single-spore isolates of a toxigenic fungus, Fusarium oxysporum, were isolated from asparagus spears and identified by species-specific polymerase chain reaction (PCR) and translation elongation factor 1-alpha (TEF) sequence analysis. In the examined sets of F. oxysporum isolates, the DNA sequences of mating type genes (MAT) were identified. The distribution of MAT idiomorph may suggest that MAT1-2 is a predominant mating type in the F. oxysporum population. F. oxysporum is mainly recognised as a producer of moniliformin-the highly toxic secondary metabolite. Moniliformin content was determined by high-performance liquid chromatography (HPLC) analysis in the range 0.05-1,007.47 mu g g(-1) (mean 115.93 mu g g(-1)) but, also, fumonisin B-1 was detected, in the concentration range 0.01-0.91 mu g g(-1) (mean 0.19 mu g g(-1)). There was no association between mating types and the mycotoxins biosynthesis level. Additionally, a significant intra-species genetic diversity was revealed and molecular markers associated with toxins biosynthesis were identified. | 2979 | NA | NA | NA | no | no |
| 2980 | 2017 | NA | Wang, CZ; Peyron, P; Mestre, O; Kaplan, G; van Soolingen, D; Gao, QA; Gicquel, B; Neyrolles, O | 2010 | Innate Immune Response to Mycobacterium tuberculosis Beijing and Other Genotypes | Background: As a species, Mycobacterium tuberculosis is more diverse than previously thought. In particular, the Beijing family of M. tuberculosis strains is spreading and evoluating throughout the world and this is giving rise to public health concerns. Genetic diversity within this family has recently been delineated further and a specific genotype, called Bmyc10, has been shown to represent over 60% of all Beijing clinical isolates in several parts of the world. How the host immune system senses and responds to various M. tuberculosis strains may profoundly influence clinical outcome and the relative epidemiological success of the different mycobacterial lineages. We hypothesised that the success of the Bmyc10 group may, at least in part, rely upon its ability to alter innate immune responses and the secretion of cytokines and chemokines by host phagocytes. Methodology/Principal Findings: We infected human macrophages and dendritic cells with a collection of genetically well-defined M. tuberculosis clinical isolates belonging to various mycobacterial families, including Beijing. We analyzed cytokine and chemokine secretion on a semi-global level using antibody arrays allowing the detection of sixty-five immunity-related soluble molecules. Our data indicate that Beijing strains induce significantly less interleukin (IL)-6, tumor necrosis factor (TNF), IL-10 and GRO-alpha than the H37Rv reference strain, a feature that is variously shared by other modern and ancient M. tuberculosis families and which constitutes a signature of the Beijing family as a whole. However, Beijing strains did not differ relative to each other in their ability to modulate cytokine secretion. Conclusions/Significance: Our results confirm and expand upon previous reports showing that M. tuberculosis Beijing strains in general are poor in vitro cytokine inducers in human phagocytes. The results suggest that the epidemiological success of the Beijing Bmyc10 is unlikely to rely upon any specific ability of this group of strains to impair anti-mycobacterial innate immunity. | 2980 | NA | NA | NA | no | no |
| 2981 | 2017 | NA | Richards, MH; Packer, L | 1998 | Demography and relatedness in multiple-foundress nests of the social sweat bee, Halictus ligatus | Female sweat bees in the species Halictus ligatus exhibit a wide range of reproductive roles, ranging from typically foundress or queen-like to typically worker-like. Nests are founded in spring and most are haplometrotic, that is, founded by a single foundress. A few (up to 12%) are pleometrotic, founded by 2-6 foundresses. Variation in the proportion of multifoundress nests from year to year and from place to place suggests an adaptive basis for pleometrosis. We studied the demographic and social characteristics of 23 pleometrotic nests in an aggregation of 250-300 nests near Victoria, Ontario, in 1984, 1990, and 1991. In pleometrotic associations, dominant foundresses behaved in a manner typical of mid-summer, haplometrotic queens, while subordinates behaved like mid-summer workers. Dominant foundresses tended to be larger than subordinates. Pleometrotic nests were significantly more likely than haplometrotic nests to produce brood, and they also produced more workers. However, this early advantage did not result in the production of more reproductive brood per nest, nor did pleometrotic foundresses experience higher productivity per foundress than did haplometrotic foundresses. Relatively low relatedness among various categories of brood implied that subordinate foundresses were not closely related to dominants. We suggest that pleometrosis most likely results from accidental encounters between spring foundresses as they leave their hibernacula. Once formed, such associations confer a survival advantage on the nest as a whole, but do not result in greater reproductive brood productivity. | 2981 | NA | NA | NA | no | no |
| 2982 | 2017 | NA | Pilosof, S; Fortuna, MA; Cosson, JF; Galan, M; Kittipong, C; Ribas, A; Segal, E; Krasnov, BR; Morand, S; Bascompte, J | 2014 | Host-parasite network structure is associated with community-level immunogenetic diversity | Genes of the major histocompatibility complex (MHC) encode proteins that recognize foreign antigens and are thus crucial for immune response. In a population of a single host species, parasite-mediated selection drives MHC allelic diversity. However, in a community-wide context, species interactions may modulate selection regimes because the prevalence of a given parasite in a given host may depend on its prevalence in other hosts. By combining network analysis with immunogenetics, we show that host species infected by similar parasites harbour similar alleles with similar frequencies. We further show, using a Bayesian approach, that the probability of mutual occurrence of a functional allele and a parasite in a given host individual is nonrandom and depends on other host-parasite interactions, driving co-evolution within subgroups of parasite species and functional alleles. Therefore, indirect effects among hosts and parasites can shape host MHC diversity, scaling it from the population to the community level. | 2982 | NA | NA | NA | no | no |
| 2983 | 2017 | NA | Mattos, VS; Furlanetto, C; Silva, JGP; Dos Santos, DF; Almeida, MRA; Correa, VR; Moita, AW; Castagnone-Sereno, P; Carneiro, RMDG | 2016 | Meloidogyne spp. populations from native Cerrado and soybean cultivated areas: genetic variability and aggressiveness | A significant portion of the Cerrado (Brazilian savanna) has been replaced by major crops such as soybean. This may reveal populations of nematodes with different genetic backgrounds compared to cultivated fields. The objectives of this study were to evaluate the genetic variability and aggressiveness of isolates of Meloidogyne spp., contrasting nematodes from preserved areas of the Cerrado with those originating from cultivated soybean fields. Cluster analysis separated isolates of Meloidogyne spp. and isolates from Cerrado and soybean but did not separate an aggressive Meloidogyne morocciensis isolate. The aggressiveness of six selected populations of Meloidogyne spp. from Cerrado and soybean against soybean cultivars was evaluated. Results showed that populations of M. javanica and M. incognita from Cerrado and soybean showed similar aggressiveness. However, for M. morocciensis, the population from soybean was much more aggressive than the one from Cerrado. Aggressiveness is a very intriguing subject that needs special attention for future research in nematology. | 2983 | NA | NA | NA | no | no |
| 2984 | 2017 | NA | Xu, DF; Li, XL; Pan, YM; Dai, YL; Li, P; Chen, FX; Zhang, HJ; Guo, M; Gao, ZM | 2014 | Genetic diversity and pathogenicity differentiation of Sclerotinia sclerotiorum on rapeseed (Brassica napus L.) in Anhui Province, China | The pathogenicity of 47 isolates of Sclerotinia sclerotiorum from oilseed rape (Brassica napus L.) in Anhui, China, was tested by detached leaf inoculation using the susceptible rape cultivar Wanyou-14. All isolates were pathogenic to the cultivar and could be grouped into 3 categories based on the lesion length on the leaves tested: weak pathogenicity type, intermediate pathogenicity type, and strong pathogenicity type. This suggested that there was differentiation in the pathogenicity among the strains tested of S. sclerotiorum. Additionally, the intraspecific DNA polymorphisms among 47 strains of S. sclerotiorum were investigated by screening 40 pairs of inter-simple sequence repeat (ISSR) primers. Unweighted pair-group method with arithmetic average cluster analysis of these ISSR data distinguished all strains from each other and revealed considerable genetic variability among them. These strains were classified into 7 clusters according to their branching in the dendrogram, and partial correlation was observed between the genetic polymorphisms and the pathogenicity of S. sclerotiorum strains. | 2984 | NA | NA | NA | no | no |
| 2985 | 2017 | NA | Johnson, TJ; Danzeisen, JL; Trampel, D; Nolan, LK; Seemann, T; Bager, RJ; Bojesen, AM | 2013 | Genome Analysis and Phylogenetic Relatedness of Gallibacterium anatis Strains from Poultry | Peritonitis is the major disease problem of laying hens in commercial table egg and parent stock operations. Despite its importance, the etiology and pathogenesis of this disease have not been completely clarified. Although avian pathogenic Escherichia coli (APEC) isolates have been incriminated as the causative agent of laying hen peritonitis, Gallibacterium anatis are frequently isolated from peritonitis lesions. Despite recent studies suggesting a role for G. anatis in the pathogenesis of peritonitis, little is known about the organism’s virulence mechanisms, genomic composition and population dynamics. Here, we compared the genome sequences of three G. anatis isolates in an effort to understand its virulence mechanisms and identify novel antigenic traits. A multilocus sequence typing method was also established for G. anatis and used to characterize the genotypic relatedness of 71 isolates from commercial laying hens in Iowa and 18 international reference isolates. Genomic comparisons suggest that G. anatis is a highly diverse bacterial species, with some strains possessing previously described and potential virulence factors, but with a core genome containing several antigenic candidates. Multilocus sequence typing effectively distinguished 82 sequence types and several clonal complexes of G. anatis, and some clones seemed to predominate among G. anatis populations from commercial layers in Iowa. Biofilm formation and resistance to antimicrobial agents was also observed in several clades. Overall, the genomic diversity of G. anatis suggests that multiple lineages exist with differing pathogenic potential towards birds. | 2985 | NA | NA | NA | no | no |
| 2986 | 2017 | NA | Wang, JC; Ross, L; Mahon, LW; Owen, R; Hemmat, M; Wang, BT; El Naggar, M; Kopita, KA; Randolph, LM; Chase, JM; Aguilera, MJM; Siles, JL; Church, JA; Hauser, N; Shen, JJ; Jones, MC; Wierenga, KJ; Jiang, ZJ; Haddadin, M; Boyar, FZ; Anguiano, A; Strom, CM; Sahoo, T | 2015 | Regions of homozygosity identified by oligonucleotide SNP arrays: evaluating the incidence and clinical utility | Copy neutral segments with allelic homozygosity, also known as regions of homozygosity (ROHs), are frequently identified in cases interrogated by oligonucleotide single-nucleotide polymorphism (oligo-SNP) microarrays. Presence of ROHs may be because of parental relatedness, chromosomal recombination or rearrangements and provides important clues regarding ancestral homozygosity, consanguinity or uniparental disomy. In this study of 14 574 consecutive cases, 832 (6%) were found to harbor one or more ROHs over 10 Mb, of which 651 cases (78%) had multiple ROHs, likely because of identity by descent (IBD), and 181 cases (22%) with ROHs involving a single chromosome. Parental relatedness was predicted to be first degree or closer in 5%, second in 9% and third in 19%. Of the 181 cases, 19 had ROHs for a whole chromosome revealing uniparental isodisomy (isoUPD). In all, 25 cases had significant ROHs involving a single chromosome; 5 cases were molecularly confirmed to have a mixed iso- and heteroUPD15 and 1 case each with segmental UPD9pat and segmental UPD22mat; 17 cases were suspected to have a mixed iso- and heteroUPD including 2 cases with small supernumerary marker and 2 cases with mosaic trisomy. For chromosome 15, 12 (92%) of 13 molecularly studied cases had either Prader-Willi or Angelman syndrome. Autosomal recessive disorders were confirmed in seven of nine cases from eight families because of the finding of suspected gene within a ROH. This study demonstrates that ROHs are much more frequent than previously recognized and often reflect parental relatedness, ascertain autosomal recessive diseases or unravel UPD in many cases. | 2986 | NA | NA | NA | no | no |
| 2987 | 2017 | NA | Sakai, Y; Takada, T | 2016 | Pathogen Propagation Model with Superinfection in Vegetatively Propagated Plants on Lattice Space | Many clonal plants have two reproductive patterns, seed propagation and vegetative propagation. By vegetative propagation, plants reproduce the genetically identical offspring with a low mortality, because resources are supplied from the other individuals through interconnected ramets at vegetative-propagated offspring. However, the ramets transport not only resources but also systemic pathogen. Pathogens evolve to establish and spread widely within the plant population. The superinfection, which is defined as the ability that an established pathogen spreads widely by infecting to already-infected individuals with other strains of a pathogen, is important to the evolution of pathogens. We examine the dynamics of plant reproduction and pathogen propagation considering spatial structure and the effect of superinfection on genetic diversity of pathogen by analysis of several models, 1-strain and multiple-strain models, on two-dimensional square lattice. In the analysis of 1-strain model, we derive equilibrium value by mean-field approximation and pair approximation, and its local stability by Routh-Hurwitz stability criterion. In the multiple-strain models, we analyze the dynamics by numerical simulation of mean-field approximation, pair approximation and Monte Carlo simulation. Through the analyses, we show the effect of parameter values to dynamics of models, such as transition of dominant strain of pathogen, competition between plants and pathogens and density of individuals. As a result, (i) The strain with intermediate cost becomes dominant when both superinfection rate and growth rate are low. (ii) The competition between plants and pathogens occurs in the phase of coexistence of various strains by pair approximation and Monte Carlo simulation. (iii) Too high growth rate leads to the decrease of plant population in all models. (iv) Pathogens are easy to maintain their genetic diversity with low superinfection rate. However, if they do not superinfect, the maintenance becomes difficult. (v) When growth rate of plant is low, individuals are very influenced by distant individuals. | 2987 | NA | NA | NA | no | no |
| 2988 | 2017 | NA | Escalante, AA; Cornejo, OE; Freeland, DE; Poe, AC; Durrego, E; Collins, WE; Lal, AA | 2005 | A monkey’s tale: The origin of Plasmodium vivax as a human malaria parasite | “The high prevalence of Duffy negativity (lack of the Duffy blood group antigen) among human populations in sub-Saharan Africa has been used to argue that Plasmodium vivax originated on that continent. Here, we investigate the phylogenetic relationships among 10 species of Plasmodium that infect primates by using three genes, two nuclear (beta-tubulin and cell division cycle 2) and a gene from the plastid genome (the elongation factor Tu). We find compelling evidence that A vivax is derived from a species that inhabited macaques in Southeast Asia. Specifically, those phylogenies that include A vivax as an ancient lineage from which all of the macaque parasites could originate are significantly less likely to explain the data. We estimate the time to the most recent common ancestor at four neutral gene loci from Asian and South American isolates (a minimum sample of seven isolates per locus). Our analysis estimates that the extant populations of P. vivax originated between 45,680 and 81,607 years ago. The phylogeny and the estimated time frame for the origination of current P. vivax populations are consistent with an”“out of Asia”" origin for P. vivax as hominoid parasite. The current debate regarding how the Duffy negative trait became fixed in Africa needs to be revisited, taking into account not only human genetic data but also the genetic diversity observed in the extant P. vivax populations and the phylogeny of the genus Plasmodium." | 2988 | NA | NA | NA | no | no |
| 2989 | 2017 | NA | Beck, ET; He, J; Nelson, MI; Bose, ME; Fan, J; Kumar, S; Henrickson, KJ | 2012 | Genome Sequencing and Phylogenetic Analysis of 39 Human Parainfluenza Virus Type 1 Strains Isolated from 1997-2010 | Thirty-nine human parainfluenza type 1 (HPIV-1) genomes were sequenced from samples collected in Milwaukee, Wisconsin from 1997-2010. Following sequencing, phylogenetic analyses of these sequences plus any publicly available HPIV-1 sequences (from GenBank) were performed. Phylogenetic analysis of the whole genomes, as well as individual genes, revealed that the current HPIV-1 viruses group into three different clades. Previous evolutionary studies of HPIV-1 in Milwaukee revealed that there were two genotypes of HPIV-1 co-circulating in 1991 (previously described as HPIV-1 genotypes C and D). The current study reveals that there are still two different HPIV-1 viruses co-circulating in Milwaukee; however, both groups of HPIV-1 viruses are derived from genotype C indicating that genotype D may no longer be in circulation in Milwaukee. Analyses of genetic diversity indicate that while most of the genome is under purifying selection some regions of the genome are more tolerant of mutation. In the 40 HPIV-1 genomes sequenced in this study, the nucleotide sequence of the L gene is the most conserved while the sequence of the P gene is the most variable. Over the entire protein coding region of the genome, 81 variable amino acid residues were observed and as with nucleotide diversity, the P protein seemed to be the most tolerant of mutation (and contains the greatest proportion of non-synonymous to synonymous substitutions) while the M protein appears to be the least tolerant of amino acid substitution. | 2989 | NA | NA | NA | no | no |
| 2990 | 2017 | NA | Hall, MJR; Testa, JM; Smith, L; Adams, ZJO; Khallaayoune, K; Sotiraki, S; Stefanakis, A; Farkas, R; Ready, PD | 2009 | Molecular genetic analysis of populations of Wohlfahrt’s wound myiasis fly, Wohlfahrtia magnifica, in outbreak populations from Greece and Morocco | Wohlfahrt’s wound myiasis fly, Wohlfahrtia magnifica (Schiner) (Diptera: Sarcophagidae), is the most important cause of traumatic myiasis in the southern Palaearctic region. Larval stages are obligate parasites and the wounds caused by infestations are very similar to those caused by Old and New World screwworm flies. During the last decade, W. magnifica appears to have expanded its range to parts of northern and central Morocco, and to Crete, Greece. Specimens of W magnifica were collected in Morocco and Crete either as larvae (preserved in 80% ethanol) or as adults (dry-pinned). Comparison specimens were collected in Spain, Hungary and mainland Greece. A DNA fragment containing the 3’715 base pairs of the mitochondrial cytochrome b gene was amplified by polymerase chain reaction from each of 132 larvae or adults of W magnifica and the amplicons were directly sequenced and analysed phylogeographically. Twelve cytochrome b haplotypes were detected. All haplotypes from Morocco belonged to a lineage that included specimens from the Iberian peninsula, and restricted mixing of central and northern populations in Morocco was demonstrated. Cytochrome b haplotyping combined with an analysis of larval size provided clear evidence of multiple infestations of hosts in all geographical areas, with one quarter of wounds containing larvae from two to at least four females. More than 80% of specimens from Crete contained a haplotype predominating in mainland Greece and Hungary. Our survey indicated that wohlfahrtiosis was more widespread in northern and central Morocco than previously recorded by government veterinarians. However, the prevalence of wohlfahrtiosis was low (<1 %). The high genetic diversity of Moroccan populations is consistent with longterm endemicity, rather than recent introduction. Crete showed a higher prevalence of wohlfahrtiosis (<= 15%) and less genetic diversity of W. magnifica, which is consistent with a recent introduction. The western and eastern Mediterranean lineages may have been isolated in different Pleistocene ice-age refugia, from which there has been limited post-glacial dispersal. | 2990 | NA | NA | NA | no | no |
| 2991 | 2017 | NA | Webb, SL; Gee, KL; Wang, GM | 2010 | Survival and fidelity of an enclosed white-tailed deer population using capture-recapture-reporting data | The number of animal populations enclosed by impermeable fences has increased, which poses issues related to the behavior of individuals and populations. Despite the increased number of fenced enclosures, there is a paucity of survival and fidelity data on white-tailed deer (Odocoileus virginianus) from fenced enclosures. Therefore, we examined marked deer recaptures and resightings over 13 years for an enclosed population of white-tailed deer in Oklahoma, USA, to estimate survival and fidelity parameters. We found that a step model was the best model of survival for both sexes. Survival of females and males was greater after hunting was suspended. Average female survival was 77% before hunting was suspended but increased to 98% after. Male survival was also greater after (99%) hunting was suspended compared to before (58%). Females exhibited greater site fidelity (84-94%) than males for all age groups except old individuals, which showed similar site fidelities for males and females. Fidelity was highest for old males (85%), followed by adult (74%), fawn (61%), and yearling males (56%). Our high-tensile electric fence allowed management goals to be achieved through increased survival while potentially maintaining genetic diversity through the exchange of limited numbers of individuals from surrounding areas. However, if the goal is to confine deer to limit disease spread or protect sensitive areas (e.g., airports) from deer encroachment, then other fence designs may be necessary, because our fence was not completely effective at controlling deer movements for these purposes. | 2991 | NA | NA | NA | no | no |
| 2992 | 2017 | NA | Jestin, C; Lode, M; Vallee, P; Domin, C; Falentin, C; Horvais, R; Coedel, S; Manzanares-Dauleux, MJ; Delourme, R | 2011 | Association mapping of quantitative resistance for Leptosphaeria maculans in oilseed rape (Brassica napus L.) | Stem canker caused by the fungus Leptosphaeria maculans is a major disease of Brassica napus. Quantitative resistance factors appear to be important components for effective and durable control of this pathogen. Quantitative trait loci (QTL) for stem canker resistance have previously been identified in the Darmor variety. However, before these QTL can be used in marker-assisted selection (MAS) to breed resistant varieties, they must be validated in a wide range of genetic backgrounds. We used an association mapping approach to confirm the markers located within the QTL previously identified in Darmor and establish their usefulness in MAS. For this, we characterized the molecular diversity of an oilseed rape collection of 128 lines showing a large spectrum of responses to infection by L. maculans, using 72 pairs of primers for simple sequence repeat and other markers. We used different association mapping models which either do or do not take into account the population structure and/or family relatedness. In all, 61 marker alleles were found to be associated with resistance to stem canker. Some of these markers were associated with previously identified QTL, which confirms their usefulness in MAS. Markers located in regions not harbouring previously identified QTL were also associated with resistance, suggesting that new QTL or allelic variants are present in the collection. All of these markers associated with stem canker resistance will help identify accessions carrying desirable alleles and facilitate QTL introgression. | 2992 | NA | NA | NA | no | no |
| 2993 | 2017 | NA | Medeiros, LV; Maciel, DB; Medeiros, VV; Kido, LMH; Oliveira, NT | 2010 | pelB gene in isolates of Colletotrichum gloeosporioides from several hosts | Colletotrichum gloeosporioides is an important pathogen for a great number of economically important crops. During the necrotrophic phase of infection by Colletotrichum spp, the degradative enzymes of plant cell walls, such as pectate lyase, clearly increase. A gene pelB that expresses a pectate lyase was identified in isolates of C. gloeosporioides in avocado pathogens. Various molecular studies have identified a kind of specialization of C. gloeosporioides isolates with specific hosts; however, there have been no studies of this gene in isolates from hosts other than avocado. The same is true for other species of Colletotrichum. We examined genetic variability in order to design primers that would amplify pelB gene fragments and compared the products of this amplification in C. gloeosporioides isolates from different hosts. Genetic variability was assessed using ISSR primers; the resultant data were grouped based on the UPGMA clustering method. Primers for the pelB gene were designed from selected GenBank sequences using the Primer 3 program at an annealing temperature of 60 degrees C and product amplification of nearly 600 bp. The ISSR primers were efficient in demonstrating the genetic variability of the Colletotrichum isolates and in distinguishing C. gloeosporioides, C. acutatum and C. sublineolum species. The gene pelB was found in C. gloeosporioides, C. acutatum and C. sublineolum. Amplified restriction fragments using MspI did not reveal differences in pelB gene structure in isolates from the three different host species that we investigated. | 2993 | NA | NA | NA | no | no |
| 2994 | 2017 | NA | Li, YW; Huang, J; Jia, Y; Du, YJ; Jiang, P; Zhang, R | 2012 | Genetic characterization of Aleutian mink disease viruses isolated in China | Aleutian mink disease virus (AMDV) is a parvovirus that causes an immune complex mediated disease in minks. To understand the genetic characterization of AMDV in China, the genomic sequences of three isolates, ADV-LN1, ADV-LN2, and ADV-LN3, from different farms in the Northern China were analyzed. The results showed that the lengths of genomic sequences of three isolates were 4,543, 4,566, and 4,566 bp, respectively. They shared only 95.5-96.3 % nucleotide identity with each other. The nucleotide and amino acid homology of genome sequence between the Chinese isolates and European or American strains (ADV-G, ADV-Utah1, and ADV-SL3) were 92.4-95.0 % and 92.1-93.8 %, respectively. The amino acid substitutions randomly distributed in the genome, especially NS gene. ADV-LN1 strain had a 9-amino-acid deletion at amino acid positions 70 and 72-79 in the VP1 gene, comparing with ADV-G strain; ADV-LN2 and ADV-LN3 strains had 1-amino-acid deletion at amino acid positions 70 in the VP1. Some potential glycosylation site mutations in VP and NS genes were also observed. Phylogenetic analysis results showed that the three strains belonged to two different branches based on the complete coding sequence of VP2 gene. However, they all were in the same group together with the strains from United States based on the NS1 sequence. It indicated that Chinese AMDV isolates had genetic diversity. The origin of the ancestors of the Chinese AMDV strains might be associated with the American strains. | 2994 | NA | NA | NA | no | no |
| 2995 | 2017 | NA | Cant, MA; Field, J | 2005 | Helping effort in a dominance hierarchy | In many cooperatively breeding species, group members form a dominance hierarchy or queue to inherit the position of breeder. Models aimed at understanding individual variation in helping behavior, however, rarely take into account the effect of dominance rank on expected future reproductive success and thus the potential direct fitness costs of helping. Here we develop a kin-selection model of helping behavior in multimember groups in which only the highest ranking individual breeds. Each group member can invest in the dominant’s offspring at a cost to its own survivorship. The model predicts that lower ranked subordinates, who have a smaller probability of inheriting the group, should work harder than higher ranked subordinates. This prediction holds regardless of whether the intrinsic mortality rate of subordinates increases or decreases with rank. The prediction does not necessarily hold, however, where the costs of helping are higher for lower ranked individuals: a situation that may be common in vertebrates. The model makes two further testable predictions: that the helping effort of an individual of given rank should be lower in larger groups, and the reproductive success of dominants should be greater where group members are more closely related. Empirical evidence for these predictions is discussed. We argue that the effects of rank on stable helping effort may explain why attempts to correlate individual helping effort with relatedness in cooperatively breeding species have met with limited success. | 2995 | NA | NA | NA | no | no |
| 2996 | 2017 | NA | Scholz, T; De Chambrier, A; Kuchta, R; Littlewood, DTJ; Waeschenbach, A | 2013 | Macrobothriotaenia ficta (Cestoda: Proteocephalidea), a parasite of sunbeam snake (Xenopeltis unicolor): example of convergent evolution | The poorly known proteocephalidean cestode Macrobothriotaenia ficta (Meggitt, 1931) from the sunbeam snake Xenopeltis unicolor (Ophidia: Xenopeltidae) is redescribed on the basis of re-examination of its type specimens from Burma (Myanmar), and vouchers from Thailand and Vietnam. The peculiar morphology of the scolex, which is formed by four pedunculate lobe-bearing pincer-shaped suckers, is described for the first time using scanning electron microscopy. In scolex morphology, M. ficta closely resembles phyllobothriidean cestodes, parasites of elasmobranchs. However, this similarity does not reflect phylogenetic relatedness of these cestodes but instead presents an example of convergent morphological evolution of attachment organs of unrelated groups of cestodes that parasitize different groups of vertebrates. Besides scolex morphology, the genus is characterised by the possession of a very large cirrus-sac, which may reach up to the midline of proglottides, few testes (less than 60), vitelline follicles limited to the dorsal side of proglottides, a large vaginal sphincter, and eggs with a three-layered embryophore covered with rounded projections. Numerous errors in the diagnosis of M. ficta, which appeared in the literature as a result of uncritical compilation of data without examination of original material, are corrected. Multilocus phylogenetic analysis of nuclear ribosomal RNA genes ssr- and lsrDNA and mitochondrial genes rrnL and cox1 place this species among other snake-parasitizing proteocephalideans of the genus Ophiotaenia. The convergent evolution of scolex morphology across distantly related taxa is discussed. | 2996 | NA | NA | NA | no | no |
| 2997 | 2017 | NA | Powell, JR; Monaghan, MT; Opik, M; Rillig, MC | 2011 | Evolutionary criteria outperform operational approaches in producing ecologically relevant fungal species inventories | Analyses of the structure and function of microbial communities are highly constrained by the diversity of organisms present within most environmental samples. A common approach is to rely almost entirely on DNA sequence data for estimates of microbial diversity, but to date there is no objective method of clustering sequences into groups that is grounded in evolutionary theory of what constitutes a biological lineage. The general mixed Yule-coalescent (GMYC) model uses a likelihood-based approach to distinguish population-level processes within lineages from processes associated with speciation and extinction, thus identifying a distinct point where extant lineages became independent. Using two independent surveys of DNA sequences associated with a group of ubiquitous plant-symbiotic fungi, we compared estimates of species richness derived using the GMYC model to those based on operational taxonomic units (OTUs) defined by fixed levels of sequence similarity. The model predicted lower species richness in these surveys than did traditional methods of sequence similarity. Here, we show for the first time that groups delineated by the GMYC model better explained variation in the distribution of fungi in relation to putative niche-based variables associated with host species identity, edaphic factors, and aspects of how the sampled ecosystems were managed. Our results suggest the coalescent-based GMYC model successfully groups environmental sequences of fungi into clusters that are ecologically more meaningful than more arbitrary approaches for estimating species richness. | 2997 | NA | NA | NA | no | no |
| 2998 | 2017 | NA | Frazzon, APG; Matsumura, ATS; Van Der Sand, ST | 2002 | Morphological characterization and genetic analysis of Drechslera teres isolates | Net blotch, caused by the phytopathogen Drechslera teres, is a common disease of barley (Hordeum vulgare L) and is responsible for large economic losses in some barley growing areas. In this study the morphology and genetic variability of eight D. teres isolates from different regions of the Brazilian state of Rio Grande do Sul were investigated. Colony morphology was studied on potato-dextrose-agar (PDA) and genetic variability investigated using the random amplified polymorphic-DNA (RAPID) technique. 27 commercially available primers were tested of which 16 were selected for use in polymorphic analysis due to their good resolution and reproducibility. Similarity coefficients were used to construct dendrograms based on colony morphology and RAPID data showing the relationship between the eight isolates studied. Colony morphology showed variability between the isolates while RAPID assays showed high similarity coefficients, but grouping of the isolates according to the geographic origins of the seeds from which they were isolated was not possible. | 2998 | NA | NA | NA | no | no |
| 2999 | 2017 | NA | Dudaniec, RY; Gardner, MG; Kleindorfer, S | 2010 | Offspring genetic structure reveals mating and nest infestation behaviour of an invasive parasitic fly (Philornis downsi) of Galapagos birds | The natural reproductive behaviour of invasive insects is pivotal knowledge for managing species of ecological or economic concern. We use microsatellites to examine female multiple mating and multiple nest infestations in the introduced parasitic fly, Philornis downsi, which causes high mortality in endemic birds on the Galapagos Islands. We analyse larvae and pupae within 57 nests from Santa Cruz and Floreana Islands in both the highland and lowland habitats. Sib-ship reconstructions of offspring revealed that up to five females may infest a single nest, while multiple mating in females was frequent (65% of reconstructed maternal genotypes), with an average of 1.91 (+/- 0.06 SE) males per female. Genetic relatedness (R) of offspring within nests was generally low, though lowland nests on Floreana had higher R than highland nests. Knowledge of the reproductive behaviour of P. downsi is necessary for modelling appropriate management strategies, in particular, the sterile insect technique, for which success is greatly influenced by female multiple mating. | 2999 | NA | NA | NA | no | no |
| 3000 | 2017 | NA | Zeeshan, M; Alam, MT; Vinayak, S; Bora, H; Tyagi, RK; Alam, MS; Choudhary, V; Mittra, P; Lumb, V; Bharti, PK; Udhayakumar, V; Singh, N; Jain, V; Singh, PP; Sharma, YD | 2012 | Genetic Variation in the Plasmodium falciparum Circumsporozoite Protein in India and Its Relevance to RTS,S Malaria Vaccine | RTS,S is the most advanced malaria vaccine candidate, currently under phase-III clinical trials in Africa. This Plasmodium falciparum vaccine contains part of the central repeat region and the complete C-terminal T cell epitope region (Th2R and Th3R) of the circumsporozoite protein (CSP). Since naturally occurring polymorphisms at the vaccine candidate loci are critical determinants of the protective efficacy of the vaccines, it is imperative to investigate these polymorphisms in field isolates. In this study we have investigated the genetic diversity at the central repeat, C-terminal T cell epitope (Th2R and Th3R) and N-terminal T cell epitope regions of the CSP, in P. falciparum isolates from Madhya Pradesh state of India. These isolates were collected through a 5-year prospective study aimed to develop a well-characterized field-site for the future evaluation of malaria vaccine in India. Our results revealed that the central repeat (63 haplotypes, n = 161) and C-terminal Th2R/Th3R epitope (24 haplotypes, n = 179) regions were highly polymorphic, whereas N-terminal non-repeat region was less polymorphic (5 haplotypes, n = 161) in this population. We did not find any evidence of the role of positive natural selection in maintaining the genetic diversity at the Th2R/Th3R regions of CSP. Comparative analysis of the Th2R/Th3R sequences from this study to the global isolates (n = 1160) retrieved from the GenBank database revealed two important points. First, the majority of the sequences (similar to 61%, n = 179) from this study were identical to the Dd2/Indochina type, which is also the predominant Th2R/Th3R haplotype in Asia (similar to 59%, n = 974). Second, the Th2R/Th3R sequences in Asia, South America and Africa are geographically distinct with little allele sharing between continents. In conclusion, this study provides an insight on the existing polymorphisms in the CSP in a parasite population from India that could potentially influence the efficacy of RTS, S vaccine in this region. | 3000 | NA | NA | NA | no | no |
| 3001 | 2017 | NA | GARCIA, BA; BARATA, JMS; BLANCO, A | 1995 | ENZYME POLYMORPHISM AMONG TRIATOMA-INFESTANS (HEMIPTERA, REDUVIIDAE) COLONIES | Allozyme variability in populations of the Chagas’s disease vector Triatoma infestans (Klug) was investigated by means of starch gel electrophoresis. Samples were taken from nine laboratory colonies established with individuals collected at different localities across the range of this insect in South America. Zymograms for proteins coded by a total of 17 loci were obtained. Allele frequencies, proportion of polymorphic loci (P), mean heterozygosity per locus (H), similarity (S), and identity (I) indices, genetic distance (D), and gene flow among populations were estimated. Mean values for P = 58.53% and for H = 0.095, indicating an important level of genetic variability. There was remarkable similarity among the colonies (mean I = 0.9946). Estimated gene flow among populations was high. However, on the basis of the known natural history of T. infestans, the uniformity of allele frequencies among populations may be interpreted as the result of the recent and rapid dispersal of the species from the site of origin in the Cochabamba Valley, Bolivia. | 3001 | NA | NA | NA | no | no |
| 3002 | 2017 | NA | Octavia, S; Maharjan, RP; Sintchenko, V; Stevenson, G; Reeves, PR; Gilbert, GL; Lan, RT | 2011 | Insight into Evolution of Bordetella pertussis from Comparative Genomic Analysis: Evidence of Vaccine-Driven Selection | Despite high vaccine coverage, pertussis incidence has increased substantially in recent years in many countries. A significant factor that may be contributing to this increase is adaptation to the vaccine by Bordetella pertussis, the causative agent of pertussis. In this study, we first assessed the genetic diversity of B. pertussis by microarray-based comparative genome sequencing of 10 isolates representing diverse genotypes and different years of isolation. We discovered 171 single nucleotide polymorphisms (SNPs) in a total of 1.4 Mb genome analyzed. The frequency of base changes was estimated as one per 32 kb per isolate, confirming that B. pertussis is one of the least variable bacterial pathogens. We then analyzed an international collection of 316 B. pertussis isolates using a subset of 65 of the SNPs and identified 42 distinct SNP profiles (SPs). Phylogenetic analysis grouped the SPs into six clusters. The majority of recent isolates belonged to clusters I-IV and were descendants of a single prevaccine lineage. Cluster I appeared to be a major clone with a worldwide distribution. Typing of genes encoding acellular vaccine (ACV) antigens, ptxA, prn, fhaB, fim2, and fim3 revealed the emergence and increasing incidence of non-ACV alleles occurring in clusters I and IV, which may have been driven by ACV immune selection. Our findings suggest that B. pertussis, despite its high population homogeneity, is evolving in response to vaccination pressure with recent expansion of clones carrying variants of genes encoding ACV antigens. | 3002 | NA | NA | NA | no | no |
| 3003 | 2017 | NA | Ghosh, S; Kobayashi, N; Nagashima, S; Chawla-Sarkar, M; Krishnan, T; Ganesh, B; Naik, TN | 2010 | Full genomic analysis and possible origin of a porcine G12 rotavirus strain RU172 | Human group A rotavirus (GAR) G12 strains are regarded as potentially important pathogens for acute gastroenteritis. On the other hand, to date, the only report of detection of G12 in animals was that of a porcine G12P[7] strain RU172. Strain RU172 formed a separate G12 lineage, distinct from human G12 strains, and by analyses of deduced amino acid sequences, had a VP4, VP6, NSP4-5 of porcine origin. In the present study, we determined the full-length nucleotide sequences of VP1, VP3, and NSP1-3 genes and nearly full-length nucleotide sequence of VP2 gene of RU172. By nucleotide sequence identities and phylogenetic analyses, the VP7-VP4-VP6-VP1-VP2-VP3-NSP1-NSP2-NSP3-NSP4-NSP5 genes of RU172 were assigned to G12-P[7]-I5-R1-C1-M1-A1-N1-T1-E1-H1 genotypes, respectively. Within their respective genotypes, (i) VP1 gene of RU172 exhibited higher genetic relatedness to Wa-like human G12 GARs than porcine strains, (ii) VP2-3 and NSP2 genes clustered separately from the Wa-like human (including G12) and porcine clusters, while (iii) the VP6, NSP1 and NSP3-5 genes clustered with porcine and porcine-like human strains. These observations suggested that (i) the porcine G12 strain might have originated from porcine-human reassortment events, or alternatively, (ii) the Wa-like human and porcine G12 strains might have originated from a common ancestor, and eventually evolved (by genetic drift and shift) with time. Our findings provided important insights into the possible patterns of evolution of the porcine G12 strain. | 3003 | NA | NA | NA | no | no |
| 3004 | 2017 | NA | Kapfer, PM | 2014 | Winter Home Range and Core Area Size and Overlap of Sibling Adult Female Bobcats (Lynx rufus) in East-central Minnesota | Social organization influences carnivore demography, space use, density, and abundance. In bobcats (Lynx rufus) social organization is thought to be affected by multiple interacting factors including relatedness, sex, and prey and conspecific density. To provide baseline data on the effect of relatedness on bobcat social organization, I examined space use and overlap among two sibling, adult female bobcats in east-central Minnesota, and compared these results to previously published research. Estimated bobcat home range size was similar to that of previous studies, suggesting stability in home range size across several decades and reliability in our estimates. Home range and core area overlap was within the range of previous studies. However, the use of two different methods for estimating core area suggested that the subjective use of the 50% utilization distribution would have underestimated core area size and overlap. Although limited, these data provide the first estimates of home range and core area overlap among adult sibling bobcats. | 3004 | NA | NA | NA | no | no |
| 3005 | 2017 | NA | Chhuneja, P; Garg, T; Kumar, R; Kaur, S; Sharma, A; Bains, NS; Ahuja, M; Dhaliwal, HS; Singh, K | 2010 | Evaluation of Aegilops tauschii Coss. germplasm for agro-morphological traits and genetic diversity using SSR loci | A set of 85 Aegilops tauschii accessions were evaluated for morphological traits, disease resistance, high molecular weight glutenin subunits and genetic diversity using D genome specific SSR markers. Screening for leaf rust, stripe rust and Kernel bunt (KB) resistance over four years identified Ae. tauschii ssp strangulate as a potential source for new leaf rust and stripe rust resistance genes and Ae. tauschii ssp tauschii as a source for KB resistance. The Ac. tauschii germplasm also showed a lot of variability for high molecular weight glutenin profile showing 16 combinations of slow (1Dx) and fast (1Dy) mobility subunits. A total of 51 D-genome specific simple sequence repeat (SSR) markers amplified a total of 241 alleles in 85 accessions of Ac. tauschii and two bread wheat cultivars. The number of alleles per locus varied from 2 to 11 with an average of 4.75. Polymorphism Information Content (PIC) values for all the 51 primer pairs ranged from 0.02 to 0.88 with an average of 0.62. A phylogenetic tree generated, based on Dice dissimilarity matrix and cluster analysis, led to the clustering of the Ac tauschii accessions into two major groups. The cluster I comprised primarily the strangulate accessions whereas the cluster II comprised of accessions belonging to both the subspecies indicating some gene flow from ssp strangulate to tauschii. Genetically diverse Ac. tauschii accessions with disease resistance and better agronomic and quality traits are being used for the introgression of desirable traits to bread wheat for commercial exploitation. | 3005 | NA | NA | NA | no | no |
| 3006 | 2017 | NA | Desjardins, CA; Giamberardino, C; Sykes, SM; Yu, CH; Tenor, JL; Chen, Y; Yang, T; Jones, AM; Sun, S; Haverkamp, MR; Heitman, J; Litvintseva, AP; Perfect, JR; Cuomo, CA | 2017 | Population genomics and the evolution of virulence in the fungal pathogen Cryptococcus neoformans | Cryptococcus neoformans is an opportunistic fungal pathogen that causes approximately 625,000 deaths per year from nervous system infections. Here, we leveraged a unique, genetically diverse population of C. neoformans from sub-Saharan Africa, commonly isolated from mopane trees, to determine how selective pressures in the environment coincidentally adapted C. neoformans for human virulence. Genome sequencing and phylogenetic analysis of 387 isolates, representing the global VNI and African VNB lineages, highlighted a deep, nonrecombining split in VNB (herein, VNBI and VNBII). VNBII was enriched for clinical samples relative to VNBI, while phenotypic profiling of 183 isolates demonstrated that VNBI isolates were significantly more resistant to oxidative stress and more heavily melanized than VNBII isolates. Lack of melanization in both lineages was associated with loss-of-function mutations in the BZP4 transcription factor. A genome-wide association study across all VNB isolates revealed sequence differences between clinical and environmental isolates in virulence factors and stress response genes. Inositol transporters and catabolism genes, which process sugars present in plants and the human nervous system, were identified as targets of selection in all three lineages. Further phylogenetic and population genomic analyses revealed extensive loss of genetic diversity in VNBI, suggestive of a history of population bottlenecks, along with unique evolutionary trajectories for mating type loci. These data highlight the complex evolutionary interplay between adaptation to natural environments and opportunistic infections, and that selection on specific pathways may predispose isolates to human virulence. | 3006 | NA | NA | NA | no | no |
| 3007 | 2017 | NA | Boucias, DG; Tigano, MS; Sosa-Gomez, DR; Glare, TR; Inglis, PW | 2000 | Genotypic properties of the entomopathogenic fungus Nomuraea rileyi | The dimorphic entomopathogenic fungus Nomuraea rileyi, an important biological control agent of various noctuid defoliators, is known to regulate Anticarsia gemmatalis, the key soybean pest in South America and the southeastern United States. At present, very little is known about the isolates that are associated with the endemic or epizootic phases of this disease agent. The development of molecular markers capable of delineating N. rileyi pathotypes is essential for such an analysis. In this study, various molecular techniques, including internal transcribed spacer (ITS) sequence analysis, randomly amplified polymorphic DNA (RAPD), amplified fragment length polymorphism (AFLP), and telomeric fingerprinting methods, were employed to identify genotypes. Analysis of the ITS-5.8s and 28s regions of N. rileyi isolates suggested that this species was more closely related to Metarhizium anisopliae and M. flavoviride than to Nomuraea atypicola and N. anemonoides. These findings and comparisons with other studies suggest that the systematics of the genus Nomuraea needs reexamination. Analysis of the telomeric fingerprinting, RAPD, and AFLP data produced very similar associations among different Nomuraea. In all cases, the non-rileyi isolates (N. anemonoides and N. atypicola) appeared unrelated to the N. rileyi. The N. rileyi isolates, mainly isolated from A. gemmatalis, were grouped into two clusters: one contained the Florida isolates and the other consisted of a diffuse group of South American isolates. Calculated genetic distances among the different N. rileyi isolates corresponded to geographical isolation. (C) 2000 Academic Press. | 3007 | NA | NA | NA | no | no |
| 3008 | 2017 | NA | Galvani, AP; Coleman, RM; Ferguson, NM | 2001 | Antigenic diversity and the selective value of sex in parasites | We employ a stochastic model of helminth transmission to explore the persistence properties of sexual versus asexual parasites in the face of a host population that develops immunity. We assume that this immunity is specific to the parasite strain, such that different parasite strains express unique antigens which in turn elicit specific host immune responses. Sexual parasites are inherently disadvantaged by a fecundity that is only half that of asexual parasites, given that males do not produce offspring. However, we demonstrate that sexual parasites benefit from the greater production and maintenance of antigenic genotypes than mutation alone in asexuals. The ability of sexual parasites to produce antigenic diversity enhances population persistence of the parasites, given that enhanced antigenic diversity permits evasion of host immunity. Therefore, we argue that sexual reproduction for parasites under intense negative frequency dependent selection induced by host immunity is associated with advantages that may be sufficient to compensate for lower intrinsic reproductive potential. | 3008 | NA | NA | NA | no | no |
| 3009 | 2017 | NA | MALONE, LA; GIACON, HA; HUNAPO, RJ; MCIVOR, CA | 1992 | RESPONSE OF NEW-ZEALAND HONEY-BEE COLONIES TO NOSEMA-APIS | Seventeen colonies of honey bees from 13 different sources were dosed with Nosema apis spores in sugar syrup. Spore loads carried by foraging bees were recorded for 11 weeks thereafter. Eleven further colonies, fed plain sugar syrup, were sampled as controls. Mean spore loads in all N. apis-dosed colonies increased to between 8 million and 27 million spores per bee two weeks after dosing. Five weeks after dosing, spore loads had decreased to levels comparable to those found in the control colonies (about 4 million spores per bee or less). There were no significant differences in the responses of colonies from different sources. Colonies from different sources showed a similar uniformity with regard to the rate of spread of infection among caged bees and chymosin levels in the guts of workers. These results show little variation in response to N. apis infection, and indicate a lack of genetic variability among New Zealand bees in respect of susceptibility to N. apis infection. | 3009 | NA | NA | NA | no | no |
| 3010 | 2017 | NA | Shivakumar, M; Basavaraja, GT; Salimath, PM; Patil, PV; Talukdar, A | 2011 | Identification of rust resistant lines and their genetic variability and character association studies in soybean [Glycine max (L.) Merr.] | Segregating populations (F(3)) of two crosses involving two high yielding varieties - JS335 and JS9305 (both susceptible to rust) and one germplasm line EC241780 (resistant to rust) were screened through artificial inoculation for resistance to soybean rust. Six among the 62 progeny lines were resistant or moderately resistant to rust.The selected lines had yield level at par with JS335 and/or JS9305. Significant genetic variations were observed in the progeny lines for yield and other yield attributing characters except pod length and number of seeds per pod. The differences between phenotypic and genotypic coefficient of variations for the characters were narrow. High estimates of heritability coupled with high genetic advances were recorded for a number of traits. Positive and significant phenotypic correlation of seed yield was observed with number of branches per plant, pods per plant, pod weight, number of seeds per plant, biomass and harvest index, The identified lines will be the primer for developing improved soybean genotypes with resistance to rust disease. | 3010 | NA | NA | NA | no | no |
| 3011 | 2017 | NA | Datta, R; Rajebhosale, MD; Dhaliwal, HS; Singh, H; Ranjekar, PK; Gupta, VS | 2000 | Intraspecific genetic variability analysis of Neovossia indica causing Karnal bunt of wheat using repetitive elements | Neovossia indica (Tilletia indica), causing Karnal bunt of wheat, affects major wheat growing regions all over the world. Karnal bunt ranks as one of the major diseases of wheat causing quality losses and monetary losses due to international quarantine regulations. The present work is the first report of a genetic diversity analysis of Indian isolates of N. indica. A library of N. indica isolate Ni7 was constructed in a lambda ZAPII system, and three repetitive elements were identified for molecular analysis. These repetitive elements generated complex hybridization profiles producing fingerprint patterns of all seven isolates. Copy-number estimation of these three elements, pNiR9, pNiR12 and pNiR16 indicated the presence of 32, 61 and 64 copies, respectively. Cluster analysis based on hybridization patterns grouped together moderately virulent isolates Nil, Ni7 and Ni8, thus suggesting a positive correlation between virulence typing and cluster analysis based on molecular data. Variability analysis of N. indica isolates will aid in checking new resistant sources in host germplasm. | 3011 | NA | NA | NA | no | no |
| 3012 | 2017 | NA | Morris, RT; Drouin, G | 2007 | Ectopic gene conversions in bacterial genomes | We characterized the gene conversions found between the duplicated genes of 75 bacterial genomes from five species groups (archaea, nonpathogenic and pathogenic firmicutes, and nonpathogenic and pathogenic proteobacteria). The number of gene conversions is positively correlated with the size of multigene families and the size of multigene families is not significantly different between pathogenic and nonpathogenic taxa. However, gene conversions occur twice as frequently in pathogenic species as in nonpathogenic species. Comparisons between closely related species also indicate a trend towards increased gene conversion in pathogenic species. Whereas the length of the conversions is positively correlated with flanking sequence similarity in all five groups, these correlations are smaller for pathogenic firmicutes and proteobacteria than for nonpathogenic firmicutes and proteobacteria. These results are consistent with our previous work on E. coli genomes and suggest that pathogenic bacteria allow recombination between more divergent gene sequences. This higher permissiveness is likely adaptive because it allows them to generate more genetic variability. | 3012 | NA | NA | NA | no | no |
| 3013 | 2017 | NA | Davis, GM; Wilke, T; Zhang, Y; Xu, XJ; Qiu, CP; Spolsky, C; Qiu, DC; Li, YS; Xia, MY; Feng, Z | 1999 | Snail-Schistosoma, Paragonimus interactions in China: Population ecology, genetic diversity, coevolution and emerging diseases | This paper focuses on two snail-borne helminths in China infecting man, Schistosoma and Paragonimus, but primarily on Schistosoma (Asian caenogastropod-transmitted). Of concern are 1) the direction and timing of the evolution of the snail family Pomatiopsidae and the pattern of coevolution of Schistosoma and Paragonimus with defined clades within the Pomatiopsidae, 2) the question of monophyly of the Pomatiopsidae and its two subfamilies Pomatiopsinae and Triculinae, 3) the amount of genetic diversity within Oncomelania throughout China as revealed by allozymes and DNA sequences, 4) Oncomelania ecology and genetics with regard to different modes of transmission of Schistosoma japonicum, 5) the implications of the Three Gorges dam across the Yangtze River on emerging diseases. Allozyme and COI gene sequence data confirm the monophyly of the Pomatiopsidae with its two subfamilies. The timing and direction of evolution of the Pomatiopsidae in Asia are congruent with area cladograms based on geological-paleontological events and evolving river systems (Yangtze, Mekong, Red) from about the end of the Miocene. The two subfamilies are highly divergent on the basis of morphology and ecology but less so on the basis of molecular genetics. The Pomatiopsidae are in a clade distinctly divergent from the Hydrobiidae, a family that has been used in China to classify the Pomatiopsinae and Triculinae. Figs. 2 and 3 show the relationships of the two parasite genera with the morphology-based clades of those caenogastropod families transmitting Schistosoma and Paragonimus throughout the world (excluding Africa for which too little is known). Two caenogastropod superfamilies are involved (Ceritheacea and Rissoacea); Only Paragonimus has evolved with taxa of the Thiaridae, Pleuroceridae, Hydrobiidae; both parasite genera have evolved with various pomatiopsid taxa of both subfamilies. While all data available (reviewed in Davis, 1980, 1992) show that Schistosoma is tightly linked genetically in a coevolved system with its snail host, such a close linked genetic coevolved system operating at the population to genus level in Paragonimus is in doubt. Erhaia (Pomatiopsinae) and Tricula (Triculinae) were found in sympatry in Fujian Province, both reported transmitting putative Paragonimus skrjabini. P. skrjabini has been reported from at least 22 species of pomatiopsine and triculine snails in China. The evidence suggests that species of Paragonimus can switch hosts between different snail subfamilies and genera. The higher classification of Erhaia is in doubt. Should Erhaia be found not to be a pomatiopsid snail, then host switching can occur between some families (at least of the Rissoacea). Based on COI and allozyme data there are three geographically isolated subspecies of Oncomelania on the mainland of China: O. h robertsoni in Yunnan and Sichuan, O. h. tangi in Fujian Province, O. h. hupensis throughout the Yangtze drainage below the Three Gorges of the Yangtze River as well as parts of Guangxi and Zhejiang Provinces. Within O. h. hupensis there is considerable genetic diversity. Nei’s minimum genetic distance among populations is 0.204 +/- 0.085. We conclude, on the basis of allozyme data from Miao River populations of O. h. hupensis in Hubei Province, that ribbed and smooth-shelled populations (shell with varix; shell growth with the same allometry) are the same species. Ribbing is found in populations affected by annual floods, especially the annual flooding of the Yangtze River. Smooth-shelled populations are upstream, above the effects of flooding. Thus, smooth-shelled O. fausti and O. h. guangxiensis, nominal taxa used by some authors, are synonyms of O. h. hupensis. Using allozyme population genetics and COI gene sequence data we have found that there is considerable genetic instability in what one would initially presume to be a population. Duringflooding, snails are swept from flood plains and islands, float down the Yangtze, and are deposited in diverse locations or are swept into canals and become deposited along canals. These aggregates of snails derived from various places do not exhibit Hardy-Weinberg equilibrium for polymorphic loci. Haplotypes of the COI gene are shown to be most useful for demonstrating genetic instability. Data are presented from six “populations” from around Dong Ting Lake of Hunan Province. Sequence data were obtained from 10 individuals taken from each site. In a stable population, one expects 0 to 0.5% nucleotide differences within a population (0-3 nucleotide differences), or one to two haplotypes per 10 individuals. One such population was found at low elevation beyond effects of flooding. The other sites were around the edge of the lake and are flooded annually. Snails from these lowland localities had 6 to 10 haplotypes per 10 individuals (3.5 to 3.9% polymorphic sites), i.e. they were genetically unstable aggregates. The diversity of haplotypes enables us to map gene flow and patterns of intermixing of snails among localities. Four different modes of transmission of Schistosoma japonicum are discussed. Differences are based on genetically differentiated subspecies, whether or not the populations are affected by annual flooding, life expectancy, population genetic stability, biogeography, and whether or not cattle play a dominant role in transmission of the disease. The implications of the Three Gorges Dam across the Yangtze River is discussed both in terms of the potential for snail transport into the vast reservoir but also of the impact on Poyang Lake, the largest lake in China and a major endemic area for schistosomiasis. A remote sensing image is used in conjunction with GIS technology to discuss snail-schistosome interactions and epidemiology on Poyang Lake marshlands. | 3013 | NA | NA | NA | no | no |
| 3014 | 2017 | NA | Dibble, CJ; Hall, SR; Rudolf, VHW | 2014 | Intraspecific priority effects and disease interact to alter population growth | Intraspecific variation may shape colonization of new habitat patches through a variety of mechanisms. In particular, trait variation among colonizing individuals can produce intraspecific priority effects (IPEs), where early arrivers of a single species affect the establishment or growth of later conspecifics. While we have some evidence for the importance of IPEs, we lack a general understanding of factors affecting their presence or magnitude across a landscape. Specifically, IPEs should depend strongly on success of colonizers in the new habitat patch. This success hinges on interactions between colonizer traits and local selective pressures, but such context dependence remains unexplored experimentally. We addressed this gap by looking for the dynamical signature of IPEs in environments with and without a selective (parasite) pressure. We tested whether IPEs affected the population dynamics of a zooplankton host species (Daphnia dentifera) collected from two populations showing a tradeoff between growth rate and resistance to a fungal parasite (Metschnikowia bicuspidata). Differences in arrival order significantly altered population growth during a period of rapid resource depletion, driving large (up to 65%) differences in population abundance. Furthermore, the presence of IPEs was context dependent, as parasites reduced the impact of early arrivers on later arrivers. Such context-dependent IPEs, mediated by colonizer traits, colonization order, and selective pressures, may play an unanticipated role in the ecological and evolutionary dynamics of natural metapopulations. This mechanism highlights the overall importance of intraspecific variation for understanding ecological patterns. | 3014 | NA | NA | NA | no | no |
| 3015 | 2017 | NA | Kopp, RL; Thomaz-Soccol, V; Klisiowicz, DD; Membrive, N; Barata, JMS; Jurberg, J; Steindel, M; Kopp, DCT; de Castro, EA; Luz, E | 2009 | Phenetic Analysis of Panstrongylus megistus Burmeister, 1835 (Hemiptera: Reduviidae: Triatominae) in the State of Parana-Brazil | Panstrongylus megistus is an important Chagas Disease vector and is said to be one of the species that might replace Triatoma infestans as the main vector of that disease in Brazil. The different degrees of P. megistus domiciliation in Brazil and its epidemiological relevance draw forth the need for the development of genetic studies that make it possible to analyze and understand the interchange of individual and gene fluxes among different populations. Thus, the present work aimed at studying the genetic variability of P. megistus in the State of Parana south of Brazil- and at comparing it with populations of the same species from five other states in Brazil (SP, MG, SC, RS, SE). In order to attain the proposed objective, 25 populations were studied using fifteen isoenzymatic systems (6PGD, G6PD, ME1, ME2, ICD, PGM, GPI, GOT1, GOT2, NP1, NP2, DIA, MPI, F, and MDH). The phenetic analysis allowed the individuation of 22 electromorphs and five zymodemes. The G6PD enzyme was the only polymorphic one presenting four electromorphs for the studied populations, all of them described for the State of Parana-BR. The P. megistus populations from other states grouped with those from Parana-BR, evidencing a low genetic variability in that species. Despite the existing geographic barriers, sub-samples - away from one another by at most 570km - were grouped in one and the same zymodeme. The epidemiological implications of such results are discussed in the present work. | 3015 | NA | NA | NA | no | no |
| 3016 | 2017 | NA | Burgess, TI; Sakalidis, ML; Hardy, GES | 2006 | Gene flow of the canker pathogen Botryosphaeria australis between Eucalyptus globulus plantations and native eucalypt forests in Western Australia | The eucalypt plantation industry in Western Australia provides a unique opportunity to study the movement of pathogens between closely related host taxa. Eucalyptus globulus, a native to Tasmania and south-eastern Australia, is the predominant species in Western Australian plantations, often being planted adjacent to native forest containing Eucalyptus marginata and Eucalyptus diversicolor. Since the commencement of the plantation industry 20 years ago, several fungal species, previously known only to eastern Australia or overseas, have been reported on E. globulus in Western Australia. Botryosphaeria australis is a newly described species, recently found causing cankers on Acacia spp. in eastern Australia. However, during a routine survey, B. australis was found to be the predominant species associated with E. globulus plantations and native Eucalyptus spp. in Western Australia. In this study, six short simple repeat markers were used to evaluate genetic diversity and gene flow between collections of B. australis from native eucalypt forest and E. globulus plantations at two locations in south-western Australia. In both cases, there was no restriction to gene flow between the plantations and the adjacent native forest. Botryosphaeria australis has now been isolated from a wide range of hosts across south-western Australia and was not isolated from E. globulus in Tasmania or South Australia. This extensive distribution and host range suggests B. australis is native to Western Australia. This study demonstrates the ability of a pathogen to move between plantation and forests. | 3016 | NA | NA | NA | no | no |
| 3017 | 2017 | NA | Verdu, P; Rosenberg, NA | 2011 | A General Mechanistic Model for Admixture Histories of Hybrid Populations | Admixed populations have been used for inferring migrations, detecting natural selection, and finding disease genes. These applications often use a simple statistical model of admixture rather than a modeling perspective that incorporates a more realistic history of the admixture process. Here, we develop a general model of admixture that mechanistically accounts for complex historical admixture processes. We consider two source populations contributing to the ancestry of a hybrid population, potentially with variable contributions across generations. For a random individual in the hybrid population at a given point in time, we study the fraction of genetic admixture originating from a specific one of the source populations by computing its moments as functions of time and of introgression parameters. We show that very different admixture processes can produce identical mean admixture proportions, but that such processes produce different values for the variance of the admixture proportion. When introgression parameters from each source population are constant over time, the long-term limit of the expectation of the admixture proportion depends only on the ratio of the introgression parameters. The variance of admixture decreases quickly over time after the source populations stop contributing to the hybrid population, but remains substantial when the contributions are ongoing. Our approach will facilitate the understanding of admixture mechanisms, illustrating how the moments of the distribution of admixture proportions can be informative about the historical admixture processes contributing to the genetic diversity of hybrid populations. | 3017 | NA | NA | NA | no | no |
| 3018 | 2017 | NA | Schreiber, SJ; Benaim, M; Atchade, KAS | 2011 | Persistence in fluctuating environments | Understanding under what conditions interacting populations, whether they be plants, animals, or viral particles, coexist is a question of theoretical and practical importance in population biology. Both biotic interactions and environmental fluctuations are key factors that can facilitate or disrupt coexistence. To better understand this interplay between these deterministic and stochastic forces, we develop a mathematical theory extending the nonlinear theory of permanence for deterministic systems to stochastic difference and differential equations. Our condition for coexistence requires that there is a fixed set of weights associated with the interacting populations and this weighted combination of populations’ invasion rates is positive for any (ergodic) stationary distribution associated with a subcollection of populations. Here, an invasion rate corresponds to an average per-capita growth rate along a stationary distribution. When this condition holds and there is sufficient noise in the system, we show that the populations approach a unique positive stationary distribution. Moreover, we show that our coexistence criterion is robust to small perturbations of the model functions. Using this theory, we illustrate that (i) environmental noise enhances or inhibits coexistence in communities with rock-paper-scissor dynamics depending on correlations between interspecific demographic rates, (ii) stochastic variation in mortality rates has no effect on the coexistence criteria for discrete-time Lotka-Volterra communities, and (iii) random forcing can promote genetic diversity in the presence of exploitative interactions. One day is fine, the next is black.-The Clash. | 3018 | NA | NA | NA | no | no |
| 3019 | 2017 | NA | Cortan, D; Vilotic, D; Sijacic-Nikolic, M; Miljkovic, D | 2017 | Leaf stomatal traits variation within and among black poplar native populations in Serbia | Populus nigra as a keystone riparian pioneer tree species is one of the rarest and most endangered species in Europe due to the loss of its natural habitats. Genetic diversity existence is a key factor in survival of one species, and stomata as genetically controlled trait could be used for differentiation studies. With the aim of proving stomatal phenotypic variation of the four native populations of Populus nigra located on the banks of three biggest river valleys (Dunabe, Tisa and Sava) in the region of Vojvodina in northern Serbia, we examined various leaf stomatal traits (stomatal length and width, pore length and width, stomatal density, shape coefficient and stomatal and pore area). We tested the differences of stomatal traits among populations, interindividual variability - differences among trees, the intraindividual variability, the differences between sun-exposed and shaded leaves, among leaves nested in exposition and the differences in adaxial and abaxial leaf surface. Based on mixed model ANOVA results, interpopulation variability, as statistically significant differences, observed only for stomatal pore length and shape, while all examined traits showed interindividual variability. On the intraindividual level the results showed differences for stomatal traits, except for stomatal width, stomatal shape coefficient and stomatal density regarding leaf exposure. For better understanding of how morphological and stomatal characteristics vary in black poplar populations, further studies should be necessary involving controlled environmental conditions with the aim of examining phenotypic plasticity to changing climate conditions. | 3019 | NA | NA | NA | no | no |
| 3020 | 2017 | NA | Swanson, JAI; Torto, B; Kells, SA; Mesce, KA; Tumlinson, JH; Spivak, M | 2009 | Odorants that Induce Hygienic Behavior in Honeybees: Identification of Volatile Compounds in Chalkbrood-Infected Honeybee Larvae | Social insects that live in large colonies are vulnerable to disease transmission due to relatively high genetic relatedness among individuals and high rates of contact within and across generations. While individual insects rely on innate immune responses, groups of individuals also have evolved social immunity. Hygienic behavior, in which individual honeybees detect chemical stimuli from diseased larvae and subsequently remove the diseased brood from the nest, is one type of social immunity that reduces pathogen transmission. Three volatile compounds, collected from larvae infected with the fungal pathogen Ascosphaera apis and detected by adult honey bees, were identified by coupled gas chromatography-electroantennographic detection and gas chromatography-mass spectrometry. These three compounds, phenethyl acetate, 2-phenylethanol, and benzyl alcohol, were present in volatile collections from infected larvae but were absent from collections from healthy larvae. Two field bioassays revealed that one of the compounds, phenethyl acetate is a key compound associated with Ascosphaera apis-infected larvae that induces hygienic behavior. | 3020 | NA | NA | NA | no | no |
| 3021 | 2017 | NA | Pearse, AM; Swift, K; Hodson, P; Hua, B; McCallum, H; Pyecroft, S; Taylor, R; Eldridge, MDB; Belov, K | 2012 | Evolution in a transmissible cancer: a study of the chromosomal changes in devil facial tumor (DFT) as it spreads through the wild Tasmanian devil population | Tasmanian devils (Sarcophilus harrisii) are the largest extant marsupial carnivores. This species, now confined to Tasmania, is endangered from the emergence of a transmissible cancer, devil facial tumor disease (DFTD). In the present study, we use cytogenetic and molecular techniques to examine the stability of devil facial tumor (DFT) cell lines across time and space. This article describes disease progression from February 2004 to June 2011. We demonstrate evolutionary changes in the disease, which affects devils in different sites across Tasmania and over a period of several years, producing several chromosomal variants (strains) that are capable of transmission between devils. We describe the evolution of DFTs in the field and speculate on the possible impacts on the disease, including (1) development of less aggressive forms of the disease; (2) development of more aggressive forms of the disease; (3) development of forms capable of affecting closely related species of dasyurids (e.g., quolls); (4) extinction of the disease as it acquires additional deleterious mutations that affect either cell viability or transmissibility; and (5) co-evolution of the disease and the host. We also speculate about the future of the Tasmanian devil in the wild. We note that although DFTs are regarded as unstable by comparison with another much older transmissible cancer, canine transmissible venereal tumor (CTVT), the potential for development of less aggressive forms of DFTs or for development of resistance in devils is limited by devils’ small numbers, low genetic diversity, and restricted geographical distribution. | 3021 | NA | NA | NA | no | no |
| 3022 | 2017 | NA | Brylinska, M; Sobkowiak, S; Stefanczyk, E; Sliwka, J | 2016 | Potato cultivation system affects population structure of Phytophthora infestans | Phytophthora infestans is one of the most destructive potato pathogens. Many factors influence the population structure of P. infestans, including migration, climate and type of potato cultivation. Here, we analyse 365 P. infestans isolates collected from three regions of Poland over three years. We determined mating type, mitochondrial haplotype, resistance to metalaxyl, virulence and polymorphism at 14 simple sequence repeat (SSR) loci. Analysis of SSR markers showed high genetic diversity associated with this population. Model-based structure analysis grouped 299 unique genotypes into four main clusters. The P. infestans isolates collected from the Mlochow region, which has the most intensive level of potato cultivation, formed a distinct cluster, indicating a strong effect of the cultivation system on pathogen population structure. Three clusters contained isolates with frequent presence of three alleles at one locus, which may affect their capacity for sexual reproduction and preserve groups of fit genotypes that propagate asexually. (C) 2016 Elsevier Ltd and The British Mycological Society. All rights reserved. | 3022 | NA | NA | NA | no | no |
| 3023 | 2017 | NA | Zou, XX; Xiao, J; Huang, HQ; Zhang, XL; Zhang, FT; Sun, QG; Liu, M; Bao, SX | 2015 | Population diversity of Pasteuria penetrans from pepper fields and its genetic variation from single root-knot nematodes | Pasteuria penetrans is an obligate parasite of root-knot nematodes. Pepper (Piper nigrum) is one of the main economic crops in Hainan Island, China, and the incidence of root-knot diseases is high. The Pasteuria samples from 20 pepper fields of seven counties in Hainan Island were identified, and the results suggested that there were various P. penetrans populations in the same field but no obvious specificity among different regions. However, five strains appeared to be potentially novel species of the genus Pasteuria by 16S rRNA gene sequence analysis. Additionally, further study showed that a single nematode of Meloidogyne incognita and of M. javanica could be infected by different strains of P. penetrans, and the genetic diversities of P. penetrans between two different host species were found only in gyrB rather than 16S rRNA and sigE gene sequences. These findings will provide some theoretical underpinning for the research of obligate parasitism mechanism between root-knot nematodes and P. penetrans. | 3023 | NA | NA | NA | no | no |
| 3024 | 2017 | NA | Prasad, R; Prasad, LC; Jaiswal, SK; Mishra, VK; Chand, R; Joshi, AK | 2012 | Genetic diversity and aggressiveness of different groups of Bipolaris sorokiniana isolates causing spot blotch disease in barley (Hordeum vulgare L.) | Two hundred twenty five isolates of B. sorokiniana of barley were studied for their morpho-pathological characterization and were grouped in to four categories (1) dull white to slight black, gel type cottony growth (DW), (2) white fluffy growth (WF), (3) suppressed white growth (SW) and (4) black fluffy growth (BF). The frequency of BF isolate was highest (39.6 %) whereas SW isolates displayed lowest frequency (7.1 %). The group IV (BF) isolate was most aggressive. Sixty four purified isolates, sixteen from each of the four groups, were taken for RAPD analysis. Twenty RAPD primers were tested to detect the variability among these four identified groups. A total of 204 bands were amplified with 100% polymorphism using 20 primers. Dendrogram based on molecular polymorphism displayed considerable diversity within and between groups of 64 isolates which displayed four morpho-pathological groups into seven clusters. Specific DNA bands were identified for the selected isolates. The distinct markers may potentially be employed as genetic fingerprints for specific strain identification and classification in future. | 3024 | NA | NA | NA | no | no |
| 3025 | 2017 | NA | Kim, HJ; Cho, JC | 2015 | Genotypic Diversity and Population Structure of Vibrio vulnificus Strains Isolated in Taiwan and Korea as Determined by Multilocus Sequence Typing | The genetic diversity and population structure of Vibrio vulnificus isolates from Korea and Taiwan were investigated using PCR-based assays targeting putative virulence-related genes and multilocus sequence typing (MLST). BOX-PCR genomic fingerprinting identified 52 unique genotypes in 84 environmental and clinical V. vulnificus isolates. The majority (>50%) of strains had pathogenic genotypes for all loci tested; moreover, many environmental strains had pathogenic genotypes. Although significant (p < 0.05) inter-relationships among the genotypes were observed, the association between genotype and strain source (environmental or clinical) was not significant, indicating that genotypic characteristics alone are not sufficient to predict the isolation source or the virulence of a given V. vulnificus strain and vice versa. MLST revealed 23-35 allelic types per locus analyzed, resulting in a total of 44 unique sequence types (STs). Two major monophyletic groups (lineages A and B) corresponding to the two known lineages of V. vulnificus were observed; lineage A had six STs that were exclusively environmental, whereas lineage B had STs from both environmental and clinical sources. Pathogenic and nonpathogenic genotypes predominated in MLST lineages B and A, respectively. In addition, V. vulnificus was shown to be in linkage disequilibrium (p < 0.05), although two different recombination tests (PHI and Sawyer’s tests) detected significant evidence of recombination. Tajima’s D test also indicated that V. vulnificus might be comprised of recently sub-divided lineages. These results suggested that the two lineages revealed by MLST correspond to two distinct ecotypes of V. vulnificus. | 3025 | NA | NA | NA | no | no |
| 3026 | 2017 | NA | Rekha, AR; Maruthi, MN; Muniyappa, V; Colvin, J | 2005 | Occurrence of three genotypic clusters of Bemisia tabaci and the rapid spread of the B biotype in south India | The whitefly, Bemisia tabaci (Gennadius) (Homoptera: Aleyrodidae), is generally considered to have originated from the Indian subcontinent, although little information has so far been collected on the molecular diversity of populations present in this region. The genetic diversity of B. tabaci populations from Karnataka State, south India was analysed using the random amplified polymorphic DNA-polymerase chain reaction (RAPD-PCR) technique and partial mitochondrial cytochrome oxidase I (mtCOI) gene sequences ( 689 bases) of 22 selected samples. A total of 108 whitefly samples analysed by RAPD-PCR produced 89 polymorphic bands, and cluster analyses grouped them according to their geographic origin into ‘north’ and ‘south’ Karnataka. Phylogenetic analysis of mtCOI gene sequences with reference B. tabaci sequences from other Asian countries divided them into three genotypic clusters. Each cluster was supported with high bootstrap values (82-100%) and the individuals belonging to each cluster shared high nucleotide identities (up to 100%). This indicated at least three distinct genotypes, apparently indigenous to India, which are also present in China, Malaysia, Nepal, Pakistan, and Thailand. These coexist with the B biotype, which was first reported in India in 1999, and has since spread rapidly to other states in south India. The B biotype was more common than the indigenous B. tabaci, in locations where it had been present for more than 2 years. This is reminiscent of the situation in the Americas during the early 1990s, where the B biotype replaced existing biotypes and caused unprecedented losses to agriculture. | 3026 | NA | NA | NA | no | no |
| 3027 | 2017 | NA | Porth, I; Klapste, J; McKown, AD; La Mantia, J; Guy, RD; Ingvarsson, PK; Hamelin, R; Mansfield, SD; Ehlting, J; Douglas, CJ; El-Kassaby, YA | 2015 | Evolutionary Quantitative Genomics of Populus trichocarpa | Forest trees generally show high levels of local adaptation and efforts focusing on understanding adaptation to climate will be crucial for species survival and management. Here, we address fundamental questions regarding the molecular basis of adaptation in undomesticated forest tree populations to past climatic environments by employing an integrative quantitative genetics and landscape genomics approach. Using this comprehensive approach, we studied the molecular basis of climate adaptation in 433 Populus trichocarpa (black cottonwood) genotypes originating across western North America. Variation in 74 field-assessed traits (growth, ecophysiology, phenology, leaf stomata, wood, and disease resistance) was investigated for signatures of selection (comparing Q(ST)-F-ST) using clustering of individuals by climate of origin (temperature and precipitation). 29,354 SNPs were investigated employing three different outlier detection methods and marker-inferred relatedness was estimated to obtain the narrow-sense estimate of population differentiation in wild populations. In addition, we compared our results with previously assessed selection of candidate SNPs using the 25 topographical units (drainages) across the P. trichocarpa sampling range as population groupings. Narrow-sense QST for 53% of distinct field traits was significantly divergent from expectations of neutrality (indicating adaptive trait variation); 2,855 SNPs showed signals of diversifying selection and of these, 118 SNPs (within 81 genes) were associated with adaptive traits (based on significant QST). Many SNPs were putatively pleiotropic for functionally uncorrelated adaptive traits, such as autumn phenology, height, and disease resistance. Evolutionary quantitative genomics in P. trichocarpa provides an enhanced understanding regarding the molecular basis of climate-driven selection in forest trees and we highlight that important loci underlying adaptive trait variation also show relationship to climate of origin. We consider our approach the most comprehensive, as it uncovers the molecular mechanisms of adaptation using multiple methods and tests. We also provide a detailed outline of the required analyses for studying adaptation to the environment in a population genomics context to better understand the species’ potential adaptive capacity to future climatic scenarios. | 3027 | NA | NA | NA | no | no |
| 3028 | 2017 | NA | Edae, EA; Olivera, PD; Jin, Y; Poland, JA; Rouse, MN | 2016 | Genotype-by-sequencing facilitates genetic mapping of a stem rust resistance locus in Aegilops umbellulata, a wild relative of cultivated wheat | Background: Wild relatives of wheat play a significant role in wheat improvement as a source of genetic diversity. Stem rust disease of wheat causes significant yield losses at the global level and stem rust pathogen race TTKSK (Ug99) is virulent to most previously deployed resistance genes. Therefore, the objective of this study was to identify loci conferring resistance to stem rust pathogen races including Ug99 in an Aegilops umbelluata bi-parental mapping population using genotype-by-sequencing (GBS) SNP markers. Results: A bi-parental F-2:3 population derived from a cross made between stem rust resistant accession PI 298905 and stem rust susceptible accession PI 542369 was used for this study. F2 individuals were evaluated with stem rust race TTTTF followed by testing F-2:3 families with races TTTTF and TTKSK. The segregation pattern of resistance to both stem rust races suggested the presence of one resistance gene. A genetic linkage map, comprised 1,933 SNP markers, was created for all seven chromosomes of Ae. umbellulata using GBS. A major stem rust resistance QTL that explained 80% and 52% of the phenotypic variations for TTTTF and TTKSK, respectively, was detected on chromosome 2U of Ae. umbellulata. Conclusion: The novel resistance gene for stem rust identified in this study can be transferred to commercial wheat varieties assisted by the tightly linked markers identified here. These markers identified through our mapping approach can be a useful strategy to identify and track the resistance gene in marker-assisted breeding in wheat. | 3028 | NA | NA | NA | no | no |
| 3029 | 2017 | NA | Suh, E; Sim, C; Park, JJ; Cho, K | 2015 | Inter-population variation for Wolbachia induced reproductive incompatibility in the haplodiploid mite Tetranychus urticae | Recent studies have revealed diverse patterns of cytoplasmic incompatibility (CI) induced by Wolbachia in the two spotted spider mite (Tetranychus urticae Koch). The mechanism of CI consists of two steps: modification (mod) of sperm of infected males and the rescue (resc) of these chromosomes by Wolbachia in the egg, which results in female embryonic mortality (FM), male development (MD) or no CI. Our study reports that Wolbachia infections were highly prevalent infecting all T. urticae populations from various crops in 14 commercial greenhouses in Korea, with two Wolbachia strains expressing distinctive phenotypic effects on hosts. Analyses for wsp gene sequences obtained from collected mite populations revealed all sequences were categorized into two groups (group W1 and W2) discriminated by three diagnostic nucleotides while all Wolbachia strains belonged to the subgroup Ori in Wolbachia supergroup B. Host plants of each mite population were also generally correlated this grouping. Various mating experiments with two mite populations from each group showed that CI patterns and host plants of the mite populations were completely matched with the grouping; no CI (mod(-) resc(+)) for group W1 and mixed pattern of FM and MD (mod(-) resc(+)) for group W2. No distinct changes in fecundity or sex ratio due to Wolbachia infections were observed in four mite populations regardless of Wolbachia grouping. Our study suggests a potential correlation between phenotypic effect of Wolbachia infection and its genetic diversity associated with host plants in Korean mite populations. | 3029 | NA | NA | NA | no | no |
| 3030 | 2017 | NA | Pandit, MK; Babu, CR | 1998 | Biology and conservation of Coptis teeta Wall. - an endemic and endangered medicinal herb of Eastern Himalaya | Medicinal plants are a valuable resource for regional economic development in the tropics, and the Eastern Himalaya in particular harbours many such species. Extensive deforestation and over-exploitation in this region have brought several species to the brink of extinction, and Coptis teeta is such an endangered species; yet scientific information for its conservation is lacking. Investigations on the distribution range, demography, ecology, cytology, reproductive biology and population genetic structure of C. teeta were carried out; it was found to be endemic to a small area, to occupy a very narrow habitat and to be highly dispersed with very small population sizes. Edaphic factors were found to have played a vital role in ecological preference, natural distribution and evolutionary divergence of the species. The species exhibits a ‘K’ strategy, high male sterility, low reproductive success and efficiency, inadequate seed dispersal, and little genetic variability. A combination of these genetic hurdles and external threats in the form of habitat disturbance and over-exploitation for commercial purposes could result in its extinction. The species was found to have highly specific microsite requirements that cannot be met in other habitats. It is argued that in situ conservation measures would be the best strategy for the continued survival of this species. For effective management of the species it is recommended that its habitat be declared a protected area with the active cooperation of local inhabitants including the sharing of benefits of conservation. | 3030 | NA | NA | NA | no | no |
| 3031 | 2017 | NA | Steeves, TE; Maloney, RF; Hale, ML; Tylianakis, JM; Gemmell, NJ | 2010 | Genetic analyses reveal hybridization but no hybrid swarm in one of the world’s rarest birds | Hybridization facilitated by human activities has dramatically altered the evolutionary trajectories of threatened taxa around the globe. Whereas introduced mammalian predators and widespread habitat loss and degradation clearly imperil the recovery and survival of the New Zealand endemic black stilt or kaki (Himantopus novaezelandiae), the risk associated with hybridization between this critically endangered endemic and its self-introduced congener, the pied stilt or poaka (Himantopus himantopus leucocephalus) is less clear. Here, we combine Bayesian admixture analyses of microsatellite data with mitochondrial DNA sequence data to assess the levels of hybridization and introgression between kaki and poaka. We show that birds classified as hybrids on the basis of adult plumage are indeed of hybrid origin and that hybridization between kaki and poaka is both extensive and bidirectional. Despite this, we found almost no evidence for introgression from poaka to kaki, thus negating the popular belief that kaki represent a hybrid swarm. To our knowledge, ours represents the first comprehensive study to document a lack of widespread introgression for a species at risk despite a recent history of extensive bidirectional human-induced hybridization. We attribute this rather surprising result, in part, to reduced reproductive success in female hybrids combined with a transient male-biased kaki sex ratio. To maximize the evolutionary potential of kaki, we use these data to recommend conservation management activities aimed to maintain the genetic integrity and to maximize the genetic diversity of this iconic rare bird. | 3031 | NA | NA | NA | no | no |
| 3032 | 2017 | NA | Mullett, MS; Brown, AV; Fraser, S; Baden, R; Tubby, KV | 2017 | Insights into the pathways of spread and potential origins of Dothistroma septosporum in Britain | Dothistroma needle blight (DNB) is a disease caused by two fungi, Dothistroma septosporum and Dothistroma pini, that has resulted in significant damage to pine forests worldwide. Analysis of 1194 British Dothistroma isolates revealed that only D. septosporum occurred in Britain; D. pini was not detected. The genetic diversity, population structure, and reproductive Mode of D. septosporum in Britain were investigated using species-specific mating type markers and eleven microsatellite markers, revealing 382 multilocus haplotypes. Comparison of clustering methods (STRUCTURE, BAPS, DAPC) as well as spatial principal component analysis (SPCA) showed some differences between the Methods but similar groupings. A clear north-south dine was fOund with attributed consistent with a native fungus. Other groups were most probably introduced, with one nearly exclusive lodgepole pine group exhibiting links with Canada. Evidence for the movement of specific multilocus haplotypes via nursery stock as well as across borders is provided and the implications discussed. Crown Copyright (C) 2017 Published by Elsevier Ltd. All rights reserved. | 3032 | NA | NA | NA | no | no |
| 3033 | 2017 | NA | Elbroch, LM; Lendrum, PE; Quigley, H; Caragiulo, A | 2016 | Spatial overlap in a solitary carnivore: support for theland tenure, kinship or resource dispersion hypotheses? | There are several alternative hypotheses about the effects of territoriality, kinship and prey availability on individual carnivore distributions within populations. The first is the land-tenure hypothesis, which predicts that carnivores regulate their density through territoriality and temporal avoidance. The second is the kinship hypothesis, which predicts related individuals will be clumped within populations, and the third is the resource dispersion hypothesis, which suggests that resource richness may explain variable sociality, spatial overlap or temporary aggregations of conspecifics. Research on the socio-spatial organization of animals is essential in understanding territoriality, intra- and interspecific competition, and contact rates that influence diverse ecology, including disease transmission between conspecifics and courtship behaviours. We explored these hypotheses with data collected on a solitary carnivore, the cougar (Puma concolor), from 2005 to 2012 in the Southern Yellowstone Ecosystem, Wyoming, USA. We employed 27 annual home ranges for 13 cougars to test whether home range overlap was better explained by land tenure, kinship, resource dispersion or some combination of the three. We found support for both the land tenure and resource dispersion hypotheses, but not for kinship. Cougar sex was the primary driver explaining variation in home range overlap. Males overlapped significantly with females, whereas the remaining dyads (F-F, M-M) overlapped significantly less. In support for the resource dispersion hypothesis, hunting opportunity (the probability of a cougar killing prey in a given location) was often higher in overlapping than in non-overlapping portions of cougar home ranges. In particular, winter hunt opportunity rather than summer hunt opportunity was higher in overlapping portions of female-female and male-female home ranges. Our results may indicate that solitary carnivores are more tolerant of sharing key resources with unrelated conspecifics than previously believed, or at least during periods of high resource availability. Further, our results suggest that the resource dispersion hypothesis, which is typically applied to social species, is applicable in describing the spatial organization of solitary carnivores. | 3033 | NA | NA | NA | no | no |
| 3034 | 2017 | NA | Brewer, MT; Milgroom, MG | 2010 | Phylogeography and population structure of the grape powdery mildew fungus, Erysiphe necator, from diverse Vitis species | Background: The grape powdery mildew fungus, Erysiphe necator, was introduced into Europe more than 160 years ago and is now distributed everywhere that grapes are grown. To understand the invasion history of this pathogen we investigated the evolutionary relationships between introduced populations of Europe, Australia and the western United States (US) and populations in the eastern US, where E. necator is thought to be native. Additionally, we tested the hypothesis that populations of E. necator in the eastern US are structured based on geography and Vitis host species. Results: We sequenced three nuclear gene regions covering 1803 nucleotides from 146 isolates of E. necator collected from the eastern US, Europe, Australia, and the western US. Phylogeographic analyses show that the two genetic groups in Europe represent two separate introductions and that the genetic groups may be derived from eastern US ancestors. Populations from the western US and Europe share haplotypes, suggesting that the western US population was introduced from Europe. Populations in Australia are derived from European populations. Haplotype richness and nucleotide diversity were significantly greater in the eastern US populations than in the introduced populations. Populations within the eastern US are geographically differentiated; however, no structure was detected with respect to host habitat (i.e., wild or cultivated). Populations from muscadine grapes, V. rotundifolia, are genetically distinct from populations from other Vitis host species, yet no differentiation was detected among populations from other Vitis species. Conclusions: Multilocus sequencing analysis of the grape powdery mildew fungus is consistent with the hypothesis that populations in Europe, Australia and the western US are derived from two separate introductions and their ancestors were likely from native populations in the eastern US. The invasion history of E. necator follows a pattern consistent with plant-mediated dispersal, however, more exhaustive sampling is required to make more precise conclusions as to origin. E. necator shows no genetic structure across Vitis host species, except with respect to V. rotundifolia. | 3034 | NA | NA | NA | no | no |
| 3035 | 2017 | NA | Vicente, D; Esnal, O; de Goicoechea, MJL; Cisterna, R; Perez-Trallero, E | 2009 | Influence of Two Vaccination Campaigns on Genetic Diversity of Invasive Neisseria meningitidis Isolates in Northern Spain (1997-2008) | Background: Neisseria meningitidis diversifies rapidly, due to its high recombination rates. The aim of this study was to analyze the possible impact of two vaccination campaigns (a once-off A/C polysaccharide vaccination campaign in people aged 18 months to 20 years old in 1997, and a meningococcal C conjugate vaccination campaign in children aged <= 6 years old from 2000 to 2008) on diversification of the population of invasive isolates obtained between 1997 and 2008. All of the 461 available isolates were included (2, 319, 123, 11 and 6 belonging to serogroups A, B, C, Y and W-135, respectively). Methodology/Principal Findings: The isolates were analyzed for diversity using multilocus sequence typing, eBURST and the S.T.A.R.T.2 program. One hundred and seven sequence types (ST) and 20 clonal complexes were obtained. Five different STs (ST11, ST8, ST33, 511163 and ST3496) included 56.4% of the isolates. With the exception of ST11, all other STs were associated with a specific serogroup. Epidemic circulation of serogroup C ST8 isolates was detected in 1997-1998, as well as epidemic circulation of ST11 isolates (serogroups B and C) in 2002-2004. The epidemic behavior of serogroup B ST11 (ST11\_B:2a:P1.5) was similar, although with lesser intensity, to that of ST11 of serogroup C. Although clonality increased during epidemic years, the overall diversity of the meningococcal population did not increase throughout the 12 years of the study. Conclusion: The overall diversity of the meningococcal population, measured by the frequency of STs and clonal complexes, numbers of alleles, polymorphic sites, and index of association, remained relatively constant throughout the study period, contradicting previous findings by other researchers. | 3035 | NA | NA | NA | no | no |
| 3036 | 2017 | NA | Xiong, L; Zhao, HB; Mo, ZY; Shi, L | 2015 | Prevalence of 7 virulence genes of Legionella strains isolated from environmental water sources of public facilities and sequence types diversity of L. pneumopila strains in Macau | In this study, we analyzed 7 virulence genes in 55 Legionella species (including 29 L. pneumophila and 26 non-L. pneumophila strains) which isolated from environmental water sources of the public facilities in Macau by using PCR and real-time PCR. In addition, 29 Legionella pneumophila isolates were subjected to genotyping by sequence-based typing scheme and compared with the data reported. The detection rate of flaA, pilE, asd, mip, mompS, proA and neuA genes in the L. pneumophila were 100.0%, respectively. The neuA gene was not detected in the non-L. pneumophila strains, but flaA, pilE, asd, mip, mompS, and proA genes could be amplified with a positive rate of 15.4%, 15.4%, 53.8%, 38.5%, 15.4%, and 38.5%, respectively. The results from real-time PCR were generally consistent with that of PCR. Those L. pneumophila strains were assigned into 10 sequence types (STs) and ST1 (9/29) was the dominant STs. Four new STs were found to be unique in Macau. The analysis of population structure of L. pneumophila strains which isolated from Macau, Guangzhou and Shenzhen indicated that the similar clones were existed and ST1 was the most prevalent STs. However, the distribution of the subtypes isolated from Macau was not the same extensive as those from Guangzhou and Shenzhen. The different detection rates of the 7 virulence genes in different species of Legionella might reflect their own potential for environmental adaptability and pathogenesis. And the data analyzed from STs diversity indicated the Macau L. pneumophila possessed obvious regional specificity and high genetic diversity. | 3036 | NA | NA | NA | no | no |
| 3037 | 2017 | NA | Oyler-McCance, SJ; Casazza, ML; Fike, JA; Coates, PS | 2014 | Hierarchical spatial genetic structure in a distinct population segment of greater sage-grouse | Greater sage-grouse (Centrocercus urophasianus) within the Bi-State Management Zone (area along the border between Nevada and California) are geographically isolated on the southwestern edge of the species’ range. Previous research demonstrated that this population is genetically unique, with a high proportion of unique mitochondrial DNA (mtDNA) haplotypes and with significant differences in microsatellite allele frequencies compared to populations across the species’ range. As a result, this population was considered a distinct population segment (DPS) and was recently proposed for listing as threatened under the U.S. Endangered Species Act. A more comprehensive understanding of the boundaries of this genetically unique population (where the Bi-State population begins) and an examination of genetic structure within the Bi-State is needed to help guide effective management decisions. We collected DNA from eight sampling locales within the Bi-State (N = 181) and compared those samples to previously collected DNA from the two most proximal populations outside of the Bi-State DPS, generating mtDNA sequence data and amplifying 15 nuclear microsatellites. Both mtDNA and microsatellite analyses support the idea that the Bi-State DPS represents a genetically unique population, which has likely been separated for thousands of years. Seven mtDNA haplotypes were found exclusively in the Bi-State population and represented 73 % of individuals, while three haplotypes were shared with neighboring populations. In the microsatellite analyses both STRUCTURE and FCA separate the Bi-State from the neighboring populations. We also found genetic structure within the Bi-State as both types of data revealed differences between the northern and southern part of the Bi-State and there was evidence of isolation-by-distance. STRUCTURE revealed three subpopulations within the Bi-State consisting of the northern Pine Nut Mountains (PNa), mid Bi-State, and White Mountains (WM) following a north-south gradient. This genetic subdivision within the Bi-State is likely the result of habitat loss and fragmentation that has been exacerbated by recent human activities and the encroachment of singleleaf pinyon (Pinus monophylla) and juniper (Juniperus spp.) trees. While genetic concerns may be only one of many priorities for the conservation and management of the Bi-State greater sage-grouse, we believe that they warrant attention along with other issues (e.g., quality of sagebrush habitat, preventing future loss of habitat). Management actions that promote genetic connectivity, especially with respect to WM and PNa, may be critical to the long-term viability of the Bi-State DPS. | 3037 | NA | NA | NA | no | no |
| 3038 | 2017 | NA | Strauch, O; Oestergaard, J; Hollmer, S; Ehlers, RU | 2004 | Genetic improvement of the desiccation tolerance of the entomopathogenic nematode Heterorhabditis bacteriophora through selective breeding | The commercial exploitation of the entomopathogenic nematode Heterorhabditis bacteriophora in biological control is limited due to its relatively short shelf life, that is related to its low tolerance of environmental extremes such as desiccation. Desiccation is a result of evaporation at low humidity or hypertonic osmotic conditions. For storage and transportation infective dauer juveniles (DJs) are mixed with clay minerals. To maintain DJ quality their metabolism is reduced by transfer of the DJs into a quiescent state, which is induced by moderate desiccation. If the nematodes were more resistant to desiccation stress, they could be further desiccated to prolong DJ survival and shelf life of nematode-based products. The reduction of the nematode metabolism and the survival in the quiescent stage could be enhanced by an increased resistance to desiccation stress. The objective of this investigation was to determine the genetic variability of the desiccation tolerance of H. bacteriophora and to exploit this variability for an enhancement of desiccation tolerance by breeding. A hybrid strain resulting from crosses of eight H. bacteriophora isolates from different geographic origins was used in our investigation. The desiccation stress was adjusted by hygroscopic polyethyleneglycol (PEG 600) solutions. By lowering the water activity (a(w)-value) of this solution, the removal of water from the DJs is enhanced. The influence of an adaptation phase on the desiccation tolerance was investigated. The lowest mean tolerated a(w)-value (0.85) was achieved with an adaptation phase of 72h at an a(w)-value of 0.96. The variance of the desiccation tolerance increased with the reduction of the a(w)-value during adaptation. The heritability of the trait, determined by using homozygous inbred lines, was 0.46 for non-adapted and 0.48 for adapted populations. A negative heterosis effect could be observed for the desiccation tolerance because nearly all of the inbred lines had a higher tolerance to the desiccation stress than the hybrid strain. Improvement of the desiccation tolerance by breeding was only obtained when the adaptation process was included in the selection process, which was related to a higher phenotypic variance in the populations after adaptation. A total of eight selection and breeding steps were carried out. Without previous adaptation, the mean of the tolerated a(w)-value remained almost constant between 0.94 and 0.93. In contrast, when we adapted the DJs prior to the exposure to desiccation stress, the tolerable a(w)-values continuously dropped from 0.89 to 0.81. (C) 2004 Elsevier Inc. All rights reserved. | 3038 | NA | NA | NA | no | no |
| 3039 | 2017 | NA | Abrantes, J; Areal, H; Esteves, PJ | 2013 | Insights into the European rabbit (Oryctolagus cuniculus) innate immune system: genetic diversity of the toll-like receptor 3 (TLR3) in wild populations and domestic breeds | Background: Toll-like receptors (TLRs) belong to the innate immune system and are a major class of pattern recognition receptors representing the first line of the innate immune response. The TLR molecule is structurally composed by an ectodomain that contains leucine rich repeats (LRRs) that interact with pathogen associated molecular patterns (PAMPs), a transmembrane domain and a conserved cytoplasmic domain designated TIR (Toll-IL1 receptor) that is responsible for the intracellular signaling. TLR3 has been associated with the direct recognition of double-stranded viral RNA resulting from viral replication, while TLR7 and TLR8 target single-stranded viral RNA. In the European rabbit (Oryctolagus cuniculus), TLR7 and TLR8 were reported to be absent and pseudogenised, respectively, making TLR3 the only available TLR for the recognition of viral RNA. Thus, the levels of diversity of TLR3 were evaluated in the European rabbit by analysing different genetic backgrounds and exposure to pathogen pressures. Results: We detected 41 single nucleotide polymorphisms (SNPs) in the coding sequence of TLR3. The highest diversity was observed in the wild populations of Iberian Peninsula, between 22-33 polymorphic positions. In the French population, 18 SNPs were observed and only 4 polymorphic positions were detected in the domestic breeds. 14 non-synonymous substitutions were observed, most of them in the LRR molecules. The remaining were scattered across the transmembrane and TIR domains. Conclusion: The study of TLR3 in European rabbit populations might be relevant to understand the interplay between RNA viruses and innate immunity. Wild rabbit populations presented more diversity than domestic breeds and other mammals previously studied. This might be linked to the absence of population bottlenecks during their evolution and to the almost inexistence of man-mediated selection. The observed variability might have also been potentiated by the contact of the wild populations with various pathogens. The study of these patterns of variability might reveal scenarios of host-pathogen interaction and identify TLR3 polymorphisms’ that arose due to viral pathogens affecting wild populations. | 3039 | NA | NA | NA | no | no |
| 3040 | 2017 | NA | Hartke, TR; Rosengaus, RB | 2011 | Heterospecific pairing and hybridization between Nasutitermes corniger and N. ephratae | The sympatric neotropical termites Nasutitermes corniger and Nasutitermes ephratae are clearly distinguishable based on morphology, nest architecture, defensive secretion composition, and molecular markers. However, given the extensive ecological, geographical, and behavioral overlap of these closely related species, the potential for interbreeding may exist. To explore this possibility, heterospecific pairs were formed experimentally to examine courtship and colony-establishment behaviors, and reproductive potential. Courtship and nest construction behavior occurred in heterospecific pairs in a similar manner to that of conspecific pairs. Survival of pairs depended upon the species of the female partner. N. ephratae females paired with N. corniger males produced as many offspring as conspecific pairs. N. corniger females mated to N. ephratae males, however, produced significantly fewer offspring at 60 days post-establishment than the reciprocal cross or conspecific N. ephratae or N. corniger pairs. This was also the only pairing in which any aggression was observed. Heterospecific pairs and groups formed in mate choice mesocosms, suggesting that species recognition between these two termites is not an important aspect of mate choice. Overall, species mismatch tolerance and hybrid offspring viability are high. The present data, together with previous evidence from defensive secretions and isozyme analysis, suggest that hybridization may periodically occur in nature, and that reproductive barriers between these two species may be incomplete. Hybridization could provide a rare but important source of genetic diversity and may ensure mating opportunities for the more abundant sex of alates in each species. | 3040 | NA | NA | NA | no | no |
| 3041 | 2017 | NA | GRUNDSCHOBER, C; SANCHEZMAZAS, A; EXCOFFIER, L; LANGANEY, A; JEANNET, M; TIERCY, JM | 1994 | HLA-DPB1 DNA POLYMORPHISM IN THE SWISS POPULATION - LINKAGE DISEQUILIBRIUM WITH OTHER HLA LOCI AND POPULATION GENETIC AFFINITIES | Allelic diversity at the HLA-DPB1 locus was determined by PCR-oligotyping in a sample of 125 healthy Swiss individuals. A total of 17 alleles were detected among which four main alleles (DPB1*0401,* 0201, *0301,* 0402) reached a cumulative frequency of 74.8%. HLA-A and -B (by serology) and HLA-DRB1 (by oligotyping) allelic polymorphisms were analysed also. HLA-B and HLA-DRB1 loci were highly polymorphic with 25 and 28 alleles respectively and similar heterozygosity levels of 0.93 and 0.92. These two loci were found to be more polymorphic than expected under neutrality, while lower heterozygosity levels were found for HLA-A (0.87) and DPB1 (0.81) loci. This paper presents also a global comparison of DPB1 allelic frequencies among 15 populations from four continents. As opposed to the DRB1 locus, overall DPB1 is shown to have a lower level of polymorphism and may be considered as neutral in all tested populations. DPB1 genetic diversity is correlated significantly with geography also, as found previously for DRB1. Two- and four-locus haplotype frequencies were determined and the significance of their linkage disequilibrium tested by an original non-parametric method. A significant positive linkage disequilibrium was found for 11 A-B, 16 B-DRB1, 7 DRB1-DPB1 and 3 A-B-DRB1-DPB1 haplotypes. The overall linkage disequilibrium between DRB1 and DPB1 was much lower than expected from the physical distance and lower than for A-B and B-DRB1 pairs. The implications of these results for bone marrow transplantation and for the evolution of HLA loci are discussed. | 3041 | NA | NA | NA | no | no |
| 3042 | 2017 | NA | Dibble, CJ; Rudolf, VHW | 2016 | Intraspecific trait variation and colonization sequence alter community assembly and disease epidemics | When individuals from multiple populations colonize a new habitat patch, intraspecific trait variation can make the arrival order of colonists an important factor for subsequent population and community dynamics. In particular, intraspecific priority effects (IPEs) allow early arrivers to limit the growth or establishment of later arrivers, even when competitively inferior on a per-capita basis. Through their effects on genes and traits, IPEs can alter short-term growth and long-term evolutionary change in single species metapopulations. Given their importance for intraspecific interactions, IPEs in a dominant species have the potential to affect the composition of entire communities. We conducted an experiment to determine whether and how arrival order and IPEs in the zooplankter Daphnia pulex affected its interactions with both competitors (the cladoceran Simocephalus vetulus) and parasites (the virulent fungus Metschnikowia bicuspidata). We found strong evidence for IPEs in Daphnia, as early arrivers inhibited late arrivers even when competitively inferior. These IPEs in Daphnia altered both the establishment success of interspecific competitors and the size of disease epidemics: early colonization by fast-growing D. pulex led to large Daphnia populations and low competitor establishment, but large disease epidemics. Early colonization by slow-growing D. pulex, on the other hand, resulted in small Daphnia populations with high competitor establishment, but smaller disease epidemics. Overall, our results demonstrate the importance of intraspecific variation and arrival order for community dynamics, and highlight IPEs as a general mechanism driving variation in natural communities. | 3042 | NA | NA | NA | no | no |
| 3043 | 2017 | NA | Massey, J; Rothwell, S; Rusbridge, C; Tauro, A; Addicott, D; Chinoy, H; Cooper, RG; Ollier, WER; Kennedy, LJ | 2013 | Association of an MHC Class II Haplotype with Increased Risk of Polymyositis in Hungarian Vizsla Dogs | A breed-specific polymyositis is frequently observed in the Hungarian Vizsla. Beneficial clinical response to immunosuppressive therapies has been demonstrated which points to an immune-mediated aetiology. Canine inflammatory myopathies share clinical and histological similarities with the human immune-mediated myopathies. As MHC class II associations have been reported in the human conditions we investigated whether an MHC class II association was present in the canine myopathy seen in this breed. 212 Hungarian Vizsla pedigree dogs were stratified both on disease status and degree of relatedness to an affected dog. This generated a group of 29 cases and 183 “graded” controls: 93 unaffected dogs with a first degree affected relative, 44 unaffected dogs with a second degree affected relative, and 46 unaffected dogs with no known affected relatives. Eleven DLA class II haplotypes were identified, of which, DLA-DRB1\* 02001/DQA1*00401/DQB1*01303, was at significantly raised frequency in cases compared to controls (OR = 1.92, p = 0.032). When only control dogs with no family history of the disease were compared to cases, the association was further strengthened (OR = 4.08, p = 0.00011). Additionally, a single copy of the risk haplotype was sufficient to increase disease risk, with the risk substantially increasing for homozygotes. There was a trend of increasing frequency of this haplotype with degree of relatedness, indicating low disease penetrance. These findings support the hypothesis of an immune-mediated aetiology for this canine myopathy and give credibility to potentially using the Hungarian Vizsla as a genetic model for comparative studies with human myositis. | 3043 | NA | NA | NA | no | no |
| 3044 | 2017 | NA | Hegazy, AK | 2000 | Intra-population variation in reproductive ecology and resource allocation of the rare biennial species Verbascum sinaiticum Benth., in Egypt | Verbascum sinaiticum Benth. (Scrophulariaceae) is a biennial rosette plant. The species is rare in Egypt and its distribution is restricted to the Sinai peninsula. The investigated population was located in Saint Catherine, Southern Sinai. To assess the reproductive ecology of the species the population dynamics, age-specific survival, resource allocation, energy content, and seed longevity of a natural population were studied. The germinable seed bank represents an average of 0.08% of the seeds in the seed rain. The plants are juvenile during 33% to 67% of their lives. Total seed rain output comes from four different cohorts of adult plants. Seeds produced during the summer constituted 83.5% of the;total seed rain. Adult plants 15-18 months in age attained a maximum rosette leafiness of 2.85 m(2)m(-2) and a maximum root crown diameter of 2.3 cm. Highly reproductive individuals allocated the least dry mass to their roots. The root/shoot ratio was 0.25. Caloric content reached up to 6.37 kcal g(-1) dry mass in roots of juvenile plants and 7.5 kcal g(-1) dry mass in the summer seeds. Seed viability was higher in seeds produced during summer and autumn (9 years) as compared to seeds produced during spring (6 years). Life span ranges between 1 and 2 years, which results in an intra-population variation of survival, resource allocation, energy content, and seed longevity and viability. These variations act as an evolutionary filter, an important asset to increase the genetic diversity and stability of populations In areas with low or periodical rainfall. (C) 2000 Academic Press. | 3044 | NA | NA | NA | no | no |
| 3045 | 2017 | NA | Richards, VP; Palmer, SR; Bitar, PDP; Qin, X; Weinstock, GM; Highlander, SK; Town, CD; Burne, RA; Stanhope, MJ | 2014 | Phylogenomics and the Dynamic Genome Evolution of the Genus Streptococcus | The genus Streptococcus comprises important pathogens that have a severe impact on human health and are responsible for substantial economic losses to agriculture. Here, we utilize 46 Streptococcus genome sequences (44 species), including eight species sequenced here, to provide the first genomic level insight into the evolutionary history and genetic basis underlying the functional diversity of all major groups of this genus. Gene gain/loss analysis revealed a dynamic pattern of genome evolution characterized by an initial period of gene gain followed by a period of loss, as the major groups within the genus diversified. This was followed by a period of genome expansion associated with the origins of the present extant species. The pattern is concordant with an emerging view that genomes evolve through a dynamic process of expansion and streamlining. A large proportion of the pan-genome has experienced lateral gene transfer (LGT) with causative factors, such as relatedness and shared environment, operating over different evolutionary scales. Multiple gene ontology terms were significantly enriched for each group, and mapping terms onto the phylogeny showed that those corresponding to genes born on branches leading to the major groups represented approximately one-fifth of those enriched. Furthermore, despite the extensive LGT, several biochemical characteristics have been retained since group formation, suggesting genomic cohesiveness through time, and that these characteristics may be fundamental to each group. For example, proteolysis: mitis group; urea metabolism: salivarius group; carbohydrate metabolism: pyogenic group; and transcription regulation: bovis group. | 3045 | NA | NA | NA | no | no |
| 3046 | 2017 | NA | Merkel, B; Lydersen, C; Yoccoz, NG; Kovacs, KM | 2013 | The World’s Northernmost Harbour Seal Population-How Many Are There? | This study presents the first abundance estimate for the world’s northernmost harbour seal (Phoca vitulina) population, which resides in Svalbard, Norway, based on three digital stereoscopic photographic surveys conducted in 2009 and 2010. The counts from these high resolution 3D images were combined with a novel method for estimating correction factors for animals that were in the water at the time of the surveys, in which extensive behavioural data from radio-tagged harbour seals were used together with age distribution data to estimate the proportion of seals of various age and sex classes hauled out at the times of the surveys. To detect possible seasonal shifts in age distribution between surveys, lengths of hauled out seals were measured from the stereoscopic images. No body-length differences were detected between the surveys; but, this may be due to a high degree of sexual dimorphism exhibited in this population. Applying the modelled correction factors, a total of 1888 (95% CI: 1660-3023), 1742 (1381-3549) and 1812 (1656-4418) harbour seals were estimated for the surveys flown on 01 August 2009, 01 August 2010 and 19 August 2010, respectively. The similarity between the three survey estimates (despite significant differences in the number of animals actually counted on the photos from each survey effort) suggests that the variation in numbers of hauled out seals is reasonably accurately adjusted for by the haul-out probability model. The low population size, the limited spatial distribution of the population and its reduced genetic diversity make this population vulnerable to chance events, such as disease epidemics. | 3046 | NA | NA | NA | no | no |
| 3047 | 2017 | NA | Lu, CC; Wen, Y; Hu, WY; Lu, F; Qin, YF; Wang, Y; Li, SL; Yang, SP; Lin, Y; Wang, C; Jin, L; Shen, HB; Sha, JH; Wang, XR; Hu, ZB; Xia, YK | 2016 | Y chromosome haplogroups based genome-wide association study pinpoints revelation for interactions on non-obstructive azoospermia | The Y chromosome has high genetic variability with low rates of parallel and back mutations, which make up the most informative haplotyping system. To examine whether Y chromosome haplogroups (Y-hgs) could modify the effects of autosomal variants on non-obstructive azoospermia (NOA), based on our previous genome-wide association study (GWAS), we conducted a genetic interaction analysis in GWAS subjects. Logistic regression analysis demonstrated a protective effect of Y-hg O3e\* on NOA. Then, we explored the potential interaction between Y-hg O3e\* and autosomal variants. Our results demonstrated that there was a suggestively significant interaction between Y-hg O3e\* and rs11135484 on NOA (P-inter = 9.89 x 10(-5)). Bioinformatic analysis revealed that genes annotated by significant single nucleotide polymorphisms (SNPs) were mainly enriched in immunological pathways. This is the first study of interactions between Y-hgs and autosomal variants on a genome-wide scale, which addresses the missing heritability in spermatogenic impairment and sheds new light on the pathogenesis of male infertility. | 3047 | NA | NA | NA | no | no |
| 3048 | 2017 | NA | Croll, D; Lendenmann, MH; Stewart, E; McDonald, BA | 2015 | The Impact of Recombination Hotspots on Genome Evolution of a Fungal Plant Pathogen | Recombination has an impact on genome evolution by maintaining chromosomal integrity, affecting the efficacy of selection, and increasing genetic variability in populations. Recombination rates are a key determinant of the coevolutionary dynamics between hosts and their pathogens. Historic recombination events created devastating new pathogens, but the impact of ongoing recombination in sexual pathogens is poorly understood. Many fungal pathogens of plants undergo regular sexual cycles, and sex is considered to be a major factor contributing to virulence. We generated a recombination map at kilobase-scale resolution for the haploid plant pathogenic fungus Zymoseptoria tritici. To account for intraspecific variation in recombination rates, we constructed genetic maps from two independent crosses. We localized a total of 10,287 crossover events in 441 progeny and found that recombination rates were highly heterogeneous within and among chromosomes. Recombination rates on large chromosomes were inversely correlated with chromosome length. Short accessory chromosomes often lacked evidence for crossovers between parental chromosomes. Recombination was concentrated in narrow hotspots that were preferentially located close to telomeres. Hotspots were only partially conserved between the two crosses, suggesting that hotspots are short-lived and may vary according to genomic background. Genes located in hotspot regions were enriched in genes encoding secreted proteins. Population resequencing showed that chromosomal regions with high recombination rates were strongly correlated with regions of low linkage disequilibrium. Hence, genes in pathogen recombination hotspots are likely to evolve faster in natural populations and may represent a greater threat to the host. | 3048 | NA | NA | NA | no | no |
| 3049 | 2017 | NA | Ragusa-Netto, J | 2014 | Crop damage of Eriotheca gracilipes (Bombacaceae) by the Blue-Fronted Amazon (Amazona aestiva, Psittacidae), in the Brazilian Cerrado | Seed predation has major effects on the reproductive success of individuals, spatial patterns of populations, genetic variability, interspecific interactions and ultimately in the diversity of tree communities. At a Brazilian savanna, I evaluated the proportional crop loss of Eriotheca gracilipes due the Blue-Fronted Amazon (Amazona aestiva) during a fruiting period. Also, I analyzed the relationship between proportional crop loss to Amazons and both fruit crop size and the distance from the nearest damaged conspecific. Trees produced from 1 to 109 fruits, so that Amazons foraged more often on trees bearing larger fruit crop size, while seldom visited less productive trees. Moreover, the relationship between fruit crop sizes and the number of depredated fruits was significant. However, when only damaged trees were assessed, I found a negative and significant relation between fruit crop size and proportional crop loss to Blue-Fronted Amazons. Taking into account this as a measure more directly related to the probability of seed survival, a negative density dependent effect emerged. Also, Amazons similarly damaged the fruit crops of either close or distant neighboring damaged trees. Hence, in spite of Blue-Fronted Amazons searched for E. gracilipes bearing large fruit crops, they were swamped due to the presence of more fruits than they could eat. Moderate seed predation by Blue-Fronted Amazons either at trees with large fruit crops or in areas where fruiting trees were aggregated implies in an enhanced probability of E. gracilipes seed survival and consequent regeneration success. | 3049 | NA | NA | NA | no | no |
| 3050 | 2017 | NA | Alcobendas, M; Buckley, D; Tejedo, M | 2004 | Variability in survival, growth and metamorphosis in the larval fire salamander (Salamandra Salamandra): Effects of larval birth size, sibship and environment | Salamandra salamandra has an unusual mode of reproduction. Females retain eggs in their oviducts and give birth to aquatic larvae or, in some populations, to fully metamorphosed juveniles. We investigated how variation in the birth size of larvae of five different sibships of S. salamandra affected larval survival, larval growth rates, time to metamorphosis, and size at metamorphosis under different food and temperature conditions. Maternal effects, via the effect of larval birth size, attenuated throughout development in all environments but still affected metamorphic traits at lower temperatures. Larval developmental time was negatively correlated with larval birth size and positively correlated with size at metamorphosis. After controlling for the effect of larval birth size, broad-sense heritabilities were obtained for residual values of metamorphic traits, indicating that enough genetic variability exists at lower temperature environments and that responses to selection of metamorphic traits are possible. The size at and the time to metamorphosis were phenotypically and genetically correlated. Since both traits presumably affect fitness, genetic dependence between size and time to metamorphosis may act as a constraint on adaptive evolution through antagonistic pleiotropy. Mass-specific growth, time to metamorphosis, and size at metamorphosis were plastic under different temperature and food conditions, but no significant sibship X environment interaction was found for any trait. In addition, significant cross-environment correlation for size at metamorphosis with respect to the food gradient indicates low potential for the adaptive evolution of reaction norms to heterogeneous food environments. | 3050 | NA | NA | NA | no | no |
| 3051 | 2017 | NA | Berg, H; Becker, U; Matthies, D | 2005 | Phenotypic plasticity in Carlina vulgaris: effects of geographical origin, population size, and population isolation | If phenotypic plasticity is under genetic control, it may vary in amount and pattern on a geographical scale, e.g. among different regions of a species’ distribution. It may also differ between large and small or between less and more isolated populations, due to differences in genetic diversity. In a 2-year common garden study, the responses of several traits to drought and fertilizer treatments were studied in the grassland herb Carlina vulgaris. Individuals originating from populations of different size and degree of isolation in six European countries, representing “central” and “marginal” regions, were compared. Fertilizing had a negative effect on early plant survival, as well as on flowering probability in surviving plants. However, in those plants that flowered, fertilizing strongly increased mean number of flowerheads, flowerhead area (a correlate of seed number), and seed mass. Drought had generally weaker effects but enhanced survivorship, indicating that this treatment was closer to optimal conditions than were non-drought conditions. For some traits there were significant interactions of region X fertilizer, but the geographical pattern of reaction norms was inconsistent and lent no support to the hypothesis that central and marginal populations differ in overall plasticity. Population size and isolation had hardly any influence on treatment responses, but populations within regions differed in their mean response to fertilizing with regard to survival and flowering probabilities, as well as in their response to drought with regard to survival and total flowerhead area. It is concluded that response to raised nutrient levels is highly variable within populations, ranging from death to strongly increased reproductive output, but also among populations irrespective of size or isolation. This also goes for the response to water supply, though this variation shows a more unclear pattern. There is no evidence that small or isolated/marginal populations are less plastic than large or non-isolated/central populations, and the explanation for differences in treatment responses among plant populations should be sought in other population characteristics. | 3051 | NA | NA | NA | no | no |
| 3052 | 2017 | NA | Xu, DS; Curtis, J; Feng, ZL; Minchella, DJ | 2005 | On the role of schistosome mating structure in the maintenance of drug resistant strains | The effects of drug treatment of human hosts upon a population of schistosome parasites depend upon a variety of factors. Previous models have shown that multiple strains of drug-resistant parasites are likely to be favored as the treatment rate increases. However, such models have neglected to account for the complex nature of schistosome mating biology. To more accurately account for the biology of these parasites, a simple mating structure is included in a multi-strain schistosome model, with parasites under the influence of drug treatment of their human hosts. Parasites are assumed to pay a cost for drug resistance in terms of reduced reproduction and transmission. The dynamics of the parasite population are described by a system of homogeneous differential equations, and the existence and stability of the exponential solutions for this system are used to infer the impact of drug treatment on the maintenance of schistosome genetic diversity. (c) 2005 Society for Mathematical Biology. Published by Elsevier Ltd. All rights reserved. | 3052 | NA | NA | NA | no | no |
| 3053 | 2017 | NA | Foresman, BJ; Oliver, RE; Jackson, EW; Chao, SM; Arruda, MP; Kolb, FL | 2016 | Genome-Wide Association Mapping of Barley Yellow Dwarf Virus Tolerance in Spring Oat (Avena sativa L.) | Barley yellow dwarf viruses (BYDVs) are responsible for the disease barley yellow dwarf (BYD) and affect many cereals including oat (Avena sativa L.). Until recently, the molecular marker technology in oat has not allowed for many marker-trait association studies to determine the genetic mechanisms for tolerance. A genome-wide association study (GWAS) was performed on 428 spring oat lines using a recently developed high-density oat single nucleotide polymorphism (SNP) array as well as a SNP-based consensus map. Marker-trait associations were performed using a Q-K mixed model approach to control for population structure and relatedness. Six significant SNP-trait associations representing two QTL were found on chromosomes 3C (Mrg17) and 18D (Mrg04). This is the first report of BYDV tolerance QTL on chromosome 3C (Mrg17) and 18D (Mrg04). Haplotypes using the two QTL were evaluated and distinct classes for tolerance were identified based on the number of favorable alleles. A large number of lines carrying both favorable alleles were observed in the panel. | 3053 | NA | NA | NA | no | no |
| 3054 | 2017 | NA | Zeoli, LF; Sayler, RD; Wielgus, R | 2008 | Population viability analysis for captive breeding and reintroduction of the endangered Columbia basin pygmy rabbit | Captive breeding has become globally important in endangered species recovery, yet it is fraught with problems such as maintenance of genetic diversity, and adaptation to captivity. We studied survival and population dynamics of a captive population of endangered Columbia Basin pygmy rabbits Brachylagus idahoensis, from 2003 to 2007, to evaluate its potential for supporting reintroduction and recovery of wild extirpated populations in shrub-steppe ecosystems of eastern Washington, USA. We developed stochastic population viability analysis models in Vortex and RAMAS to assess performance and surplus production of the captive population. This pygmy rabbit population has low adult survival beyond 1 year and dependency on high juvenile recruitment for population growth. Low juvenile survival and high variability in stochastic growth rates result in high variability in annual productivity. Our analysis showed that the captive population at n=75 cannot sustain a steady annual harvest of >= 30 rabbits for reintroduction and supplementation without increasing the risk of quasi-extinction (n=30) to 59%. We conducted sensitivity analysis on maternity, carrying capacity and survival rates to identify critical values for model parameters that would lower extinction risk to the captive population when used as a source of rabbits for reintroduction. Increasing juvenile survival and recruitment into the first breeding class is the most effective method for enhancing the breeding program. Our population models suggest that captive breeding and recovery programs for short-lived lagomorphs present significant conservation challenges because of the need to rapidly grow such populations to overcome demographic and genetic challenges. | 3054 | NA | NA | NA | no | no |
| 3055 | 2017 | NA | CluttonBrock, TH; Wilson, K; Stevenson, IR | 1997 | Density-dependent selection on horn phenotype in Soay sheep | In the naturally regulated population of Soay sheep (Ovis aries L.) on Hirta (St Kilda), 12% of males and 24% of females have scurred horns. This trait reduces the competitive ability of males in the mating season but is associated with higher overwinter survival rates in both sexes (Moorcroft ct al. 1996). In this paper, we show that scurred females also show higher conception rates and weaning rates than non-scurred ones, and that these differences are associated with heavier maternal body weight as well as higher birth weights of offspring. Selection pressures favouring scurredness in females vary with population density and are generally more pronounced among younger animals than adults. We discuss these results with reference to recent suggestions that temporal fluctuation in selection pressures may help to maintain genetic diversity within populations. We suggest that selection against scurredness operating through male mating success is opposed by selection favouring this trait, operating through the breeding success of females and the survival of both sexes. | 3055 | NA | NA | NA | no | no |
| 3056 | 2017 | NA | Dayo, GK; Thevenon, S; Berthier, D; Moazami-Goudarzi, K; Denis, C; Cuny, G; Eggen, A; Gautier, M | 2009 | Detection of selection signatures within candidate regions underlying trypanotolerance in outbred cattle populations | Breeding indigenous African taurine cattle tolerant to trypanosomosis is a straightforward approach to control costs generated by this disease. A recent study identified quantitative trait loci (QTL) underlying trypanotolerance traits in experimental crosses between tolerant N’Dama and susceptible Boran zebu cattle. As trypanotolerance is thought to result from local adaptation of indigenous cattle breeds, we propose an alternative and complementary approach to study the genetic architecture of this trait, based on the identification of selection signatures within QTL or candidate genes. A panel of 92 microsatellite markers was genotyped on 509 cattle belonging to four West African trypanotolerant taurine breeds and 10 trypanosusceptible European or African cattle breeds. Some of these markers were located within previously identified QTL regions or candidate genes, while others were chosen in regions assumed to be neutral. A detailed analysis of the genetic structure of these different breeds was carried out to confirm a priori grouping of populations based on previous data. Tests based on the comparison of the observed heterozygosities and variances in microsatellite allelic size among trypanotolerant and trypanosusceptible breeds led to the identification of two significantly less variable microsatellite markers. BM4440, one of these two outlier loci, is located within the confidence interval of a previously described QTL underlying a trypanotolerance-related trait. Detection of selection signatures appears to be a straightforward approach for unravelling the molecular determinism of trypanosomosis pathogenesis. We expect that a whole genome approach will help confirm these results and achieve a higher resolving power. | 3056 | NA | NA | NA | no | no |
| 3057 | 2017 | NA | Theologidis, I; Chelo, IM; Goy, C; Teotonio, H | 2014 | Reproductive assurance drives transitions to self-fertilization in experimental Caenorhabditis elegans | Background: Evolutionary transitions from outcrossing between individuals to selfing are partly responsible for the great diversity of animal and plant reproduction systems. The hypothesis of ‘reproductive assurance’ suggests that transitions to selfing occur because selfers that are able to reproduce on their own ensure the persistence of populations in environments where mates or pollination agents are unavailable. Here we test this hypothesis by performing experimental evolution in Caenorhabditis elegans. Results: We show that self-compatible hermaphrodites provide reproductive assurance to a male-female population facing a novel environment where outcrossing is limiting. Invasions of hermaphrodites in male-female populations, and subsequent experimental evolution in the novel environment, led to successful transitions to selfing and adaptation. Adaptation was not due to the loss of males during transitions, as shown by evolution experiments in exclusively hermaphroditic populations and in male-hermaphrodite populations. Instead, adaptation was due to the displacement of females by hermaphrodites. Genotyping of single-nucleotide polymorphisms further indicated that the observed evolution of selfing rates was not due to selection of standing genetic diversity. Finally, numerical modelling and evolution experiments in male-female populations demonstrate that the improvement of male fitness components may diminish the opportunity for reproductive assurance. Conclusions: Our findings support the hypothesis that reproductive assurance can drive the transition from outcrossing to selfing, and further suggest that the success of transitions to selfing hinges on adaptation of obligate outcrossing populations to the environment where outcrossing was once a limiting factor. | 3057 | NA | NA | NA | no | no |
| 3058 | 2017 | NA | Tully, T; Lambert, A | 2011 | THE EVOLUTION OF POSTREPRODUCTIVE LIFE SPAN AS AN INSURANCE AGAINST INDETERMINACY | Postreproductive life span remains a puzzle for evolutionary biologists. The explanation of increased inclusive fitness through parental care after reproduction that applies for humans is unrealistic for many species. We propose a new selective mechanism, independent of parental care, which relies on the hypothesis that postreproductive life span can evolve as an insurance against indeterminacy: longer life expectancy reduces the risk of dying by chance before the cessation of reproductive activity. We demonstrate numerically that the duration of evolved postreproductive life span is indeed expected to increasewith variability in life span duration. An unprecedented assay of 11 strains of the collembola Folsomia candida shows the existence of (1) postreproductive life span in the absence of parental care; (2) genetic variability in mean postreproductive life span and postreproductive life span variability itself; (3) strong genetic correlation between latter traits. This new explanation brings along the novel idea that loose canalization of a trait (here, somatic life span) can itself act as a selective pressure on other traits. | 3058 | NA | NA | NA | no | no |
| 3059 | 2017 | NA | Shi, CH; Wilke, T; Davis, GM; Xia, MY; Qiu, CP | 2002 | Population genetics, micro-phylogeography, ecology, and susceptibility to schistosome infection of Chinese Oncomelania hupensis hupensis (Gastropoda : Rissooidea : Pomatiopsidae) in the Miao River system | Chinese Oncomelania h. hupensis from the flood plains of the Yangtze River have ribbed shells. However, populations living above the effects of annual floods usually are smoothshelled. Previous allozyme studies of smooth-shelled populations not affected by flooding, and ribbed-shelled populations affected by flooding from the Miao River, Hubei Province, showed that they all belong to O. h. hupensis (Davis et al., 1999a). As the allozyme data were of limited use for assessing Oncomelania-population genetics, we re-examined the above populations to answer the following questions using mitochondrial COI sequence data. Will DNA sequences provide higher resolution for the analysis of population structure than allozyme data? Are there significant genetic differences among ribbed- and smooth-shelled populations? Do they differ in their susceptibility to infection with Schistosoma japonicum? Sequences from 59 individuals revealed four groups of haplotypes. Smooth- and ribbed-shelled individuals clustered together in two of the groups. The greatest sequence divergence between a smooth-shelled and ribbed-shelled population was 0.020, indicating that all populations fall within the range of variation expected for O. h. hupensis. Overall, the highest genetic diversity was found among downstream ribbed-shelled populations. The analysis of molecular variance (AMOVA) showed the following distribution of total variance: 69% (P < 0.0001) within populations, 9% (P less than or equal to 0.0025) among populations within the ribbed- and smooth-shelled groups, and 22% (P less than or equal to 0.0569) between the ribbed- and smooth-shelled populations. Mismatch distributions indicated that downstream populations are aggregates of snails from different populations. Downstream populations also showed a higher infectivity rate and a higher susceptibility to infection with Schistosoma japonicum. This is probably due to the importation and mixture of snail and parasite strains in flooded areas increasing the probability that schistosomes encounter genetically suitable snails, and/or the possibility of multiple infections by different parasite strains. The low infection rate in upstream populations is probably due to their relative isolation where there is equilibrium with low frequencies of infection. | 3059 | NA | NA | NA | no | no |
| 3060 | 2017 | NA | Bartron, ML; Scribner, KT | 2004 | Temporal comparisons of genetic diversity in Lake Michigan steelhead, Oncorhynchus mykiss, populations: effects of hatchery supplementation | Steelhead, Oncorhynchus mykiss, were first introduced into the Great Lakes in the late 1800s. Subsequently, natural recruitment across the Lake Michigan basin has been regularly supplemented by primarily one hatchery strain. Recently, multiple strains derived from locations across the species native range along the west coast of the United States have also been stocked by different management agencies. Prior to 1983, hatchery supplementation of Lake Michigan steelhead populations in Michigan was largely unsuccessful due to low smolting rates of small (<120 mm) hatchery yearlings (estimated survival 0.01%). Accordingly, contributions of hatchery fish to historical adult spawning runs in Michigan tributaries were low (0-30%) across six major drainages. Large yearlings of different hatchery strains (>150 mm) have been stocked exclusively since 1983, increasing estimates of survival to smolting (90%). Consequently, the proportion of hatchery adults in spawning runs increased to 13-79%. We examined the effects of changes in stocking practices on straying rates of hatchery steelhead and to temporal changes in levels of genetic diversity and relationships among populations. We used microsatellite loci to estimate allele frequencies for six populations sampled for two time periods (1983-1984 and 1998-1999). Measures of inter-population divergence (mean F-ST) were not significant for either time period. However, spatial genetic relationships among historical and contemporary populations were significantly correlated with geographic distance; a result not expected if gene flow (natural straying) among populations was mediated solely by hatchery supplementation. Increased numbers of alleles in spawning adults from populations can be attributed to alleles specific to recently introduced hatchery strains. | 3060 | NA | NA | NA | no | no |
| 3061 | 2017 | NA | Hansen, MM | 2002 | Estimating the long-term effects of stocking domesticated trout into wild brown trout (Salmo trutta) populations: an approach using microsatellite DNA analysis of historical and contemporary samples | Indigenous salmonid fish gene pools are affected by domesticated conspecifics, derived from aquaculture escapes and deliberate releases. Variability was examined at nine microsatellite loci in order to assess the long-term impact of stocking domesticated trout in two brown trout populations. The study was based on analysis of two historical samples (194556), represented by old scale collections, and seven contemporary samples (1986-2000). In one population historical and contemporary samples were remarkably genetically similar despite more than a decade of intense stocking. Estimation of admixture proportions showed a small genetic contribution from domesticated trout (approximately 6%), and individual admixture analysis demonstrated a majority of nonadmixed individuals. The expected genetic contribution by domesticated trout was 64%, assessed from the number of stocked trout and assuming equal survival and reproductive performance of wild and domesticated trout. This demonstrates poor performance and low fitness of domesticated trout in the wild. In another population there was a strong genetic contribution from domesticated trout (between 57% and 88% in different samples), both in samples from a broodstock thought to represent the indigenous population and in a sample of wild spawners. Survival of domesticated trout and admixture with indigenous fish in the broodstock and subsequent stocking into the river, combined with a low population size of native trout relative to the number of stocked trout, could explain the observed introgression. Few nonadmixed individuals remained in the introgressed population, and I discuss how individual admixture analysis can be used to identify and conserve nonintrogressed remains of the population. | 3061 | NA | NA | NA | no | no |
| 3062 | 2017 | NA | Hanson, D; Hirano, Y; Valdes, A | 2013 | Population genetics of Haminoea (Haloa) japonica Pilsbry, 1895, a widespread non-indigenous sea slug (Mollusca: Opisthobranchia) in North America and Europe | Haminoea japonica is an opisthobranch mollusk with a large non-indigenous range. This species is a vector for a parasite that causes the human skin disease cercarial dermatitis, and may have negative effects on populations of native species. Molecular evidence from the mitochondrial cytochrome c oxidase I gene and the histone 3 nuclear gene indicates that previously published morphology-based hypotheses on the spread of H. japonica out of Japan are correct. The most likely explanation for the current range of the species, which includes Japan, Korea, France, Spain, Italy, Canada and the USA is a recent, human-mediated dispersal from Japanese populations. The highest levels of nucleotide and haplotype diversity are found in Japan. Non-indigenous populations have low levels of genetic diversity (indicating bottlenecking). Haplotypes that were detected in the non-indigenous range of H. japonica have only been found in two localities in the native range; these two localities are in north-eastern Japan. In addition, the haplotype network structure and Spatial Analysis of Molecular Variance results confirm the origins of non-indigenous populations most likely trace to north-eastern Japan, which is where most Pacific oyster exports to North America also originated. Because there are no major shipping ports in north-eastern Japan, ballast water is less likely to be the mechanism of dispersal. The results of this study provide important data for the development of policies and regulations aimed to prevent further spread of this species in non-indigenous ranges. | 3062 | NA | NA | NA | no | no |
| 3063 | 2017 | NA | Garbelotto, M; Guglielmo, F; Mascheretti, S; Croucher, PJP; Gonthier, P | 2013 | Population genetic analyses provide insights on the introduction pathway and spread patterns of the North American forest pathogen Heterobasidion irregulare in Italy | A population genetics approach is used to identify the most likely introduction site and introduction pathway for the North American forest pathogen Heterobasidion irregulare using 101 isolates from six sites in Italy and 34 isolates from five sites in North America. Diversity indices based on sequences from ten loci indicate the highest diversity in Italy is found in Castelfusano/Castelporziano and that diversity progressively decreases with increasing distance from that site. AMOVA, Bayesian clustering and principal coordinates analyses based on 12 SSR loci indicate high levels of gene flow among sites, high frequency of admixing, and fail to identify groups of genotypes exclusive to single locations. Cumulatively, these analyses suggest the current infestation is the result of multiple genotypes expanding their range from a single site. Based on two sequenced loci, a single source site in North America could provide enough variability to explain the variability observed in Italy. These results support the notion that H.irregulare was introduced originally in Castelporziano: because Castelporziano has been sealed off from the rest of the world for centuries except for a camp set up by the US military in 1944, we conclude the fungus may have been transported in infected wood used by the military. Finally, spatial autocorrelation analyses using SSR data indicate a significant under-dispersion of alleles up to 0.5-10km, while a significant overdispersion of alleles was detected at distances over 80km: these ranges can be used to make predictions on the likely dispersal potential of the invasive pathogen. | 3063 | NA | NA | NA | no | no |
| 3064 | 2017 | NA | Fischer, W; Breithaupt, U; Kern, B; Smith, SI; Spicher, C; Haas, R | 2014 | A comprehensive analysis of Helicobacter pylori plasticity zones reveals that they are integrating conjugative elements with intermediate integration specificity | Background: The human gastric pathogen Helicobacter pylori is a paradigm for chronic bacterial infections. Its persistence in the stomach mucosa is facilitated by several mechanisms of immune evasion and immune modulation, but also by an unusual genetic variability which might account for the capability to adapt to changing environmental conditions during long-term colonization. This variability is reflected by the fact that almost each infected individual is colonized by a genetically unique strain. Strain-specific genes are dispersed throughout the genome, but clusters of genes organized as genomic islands may also collectively be present or absent. Results: We have comparatively analysed such clusters, which are commonly termed plasticity zones, in a high number of H. pylori strains of varying geographical origin. We show that these regions contain fixed gene sets, rather than being true regions of genome plasticity, but two different types and several subtypes with partly diverging gene content can be distinguished. Their genetic diversity is incongruent with variations in the rest of the genome, suggesting that they are subject to horizontal gene transfer within H. pylori populations. We identified 40 distinct integration sites in 45 genome sequences, with a conserved heptanucleotide motif that seems to be the minimal requirement for integration. Conclusions: The significant number of possible integration sites, together with the requirement for a short conserved integration motif and the high level of gene conservation, indicates that these elements are best described as integrating conjugative elements (ICEs) with an intermediate integration site specificity. | 3064 | NA | NA | NA | no | no |
| 3065 | 2017 | NA | Tnah, LH; Lee, SL; Ng, KKS; Lee, CT; Bhassu, S; Othman, RY | 2013 | Phylogeographical Pattern and Evolutionary History of an Important Peninsular Malaysian Timber Species, Neobalanocarpus heimii (Dipterocarpaceae) | Tectonic movements, climatic oscillations, and marine transgressions during the Cenozoic have had a dramatic effect on the biota of the tropical rain forest. This study aims to reveal the phylogeography and evolutionary history of a Peninsular Malaysian endemic tropical timber species, Neobalanocarpus heimii (Dipterocarpaceae). A total of 32 natural populations of N. heimii, with 8 samples from each population were investigated. Fifteen haplotypes were identified from five noncoding chloroplast DNA (cpDNA) regions. Overall, two major genealogical cpDNA lineages of N. heimii were elucidated: a widespread southern and a northern region. The species is predicted to have survived in multiple refugia during climatic oscillations: the northwestern region (R1), the northeastern region (R2), and the southern region (R3). These putative glacial refugia exhibited higher levels of genetic diversity, population differentiation, and the presence of unique haplotypes. Recolonization of refugia R1 and R2 could have first expanded into the northern region and migrated both northeastwards and northwestwards. Meanwhile, recolonization of N. heimii throughout the southern region could have commenced from refugia R3 and migrated toward the northeast and northwest, respectively. The populations of Tersang, Pasir Raja, and Rotan Tunggal exhibited remarkably high haplotype diversity, which could have been the contact zones that have received an admixture of gene pools from the northerly and also southerly regions. As a whole, the populations of N. heimii derived from glacial refugia and contact zones should be considered in the conservation strategies in order to safeguard the long-term survival of the species. | 3065 | NA | NA | NA | no | no |
| 3066 | 2017 | NA | Lusseau, D; Wilson, B; Hammond, PS; Grellier, K; Durban, JW; Parsons, KM; Barton, TR; Thompson, PM | 2006 | Quantifying the influence of sociality on population structure in bottlenose dolphins | 1. The social structure of a population plays a key role in many aspects of its ecology and biology. It influences its genetic make-up, the way diseases spread through it and the way animals exploit their environment. However, the description of social structure in nonprimate animals is receiving little attention because of the difficulty in abstracting social structure from the description of association patterns between individuals. 2. Here we focus on recently developed analytical techniques that facilitate inference about social structure from association patterns. We apply them to the population of bottlenose dolphins residing along the Scottish east coast, to detect the presence of communities within this population and infer its social structure from the temporal variation in association patterns between individuals. 3. Using network analytical techniques, we show that the population is composed of two social units with restricted interactions. These two units seem to be related to known differences in the ranging pattern of individuals. By examining social structuring at different spatial scales, we confirm that the identification of these two units is the result of genuine social affiliation and is not an artefact of their spatial distribution. 4. We also show that the structure of this fission-fusion society relies principally on short-term casual acquaintances lasting a few days with a smaller proportion of associations lasting several years. These findings highlight how network analyses can be used to detect and understand the forces driving social organization of bottlenose dolphins and other social species. | 3066 | NA | NA | NA | no | no |
| 3067 | 2017 | NA | Radtkey, RR; Becker, B; Miller, RD; Riblet, R; Case, TJ | 1996 | Variation and evolution of class I Mhc in sexual and parthenogenetic geckos | We present the first Mhc class I sequences in geckos. We compared Mhc variation in gekkonid species that reproduce sexually (Hemidactylus frenatus, Leipdodactylus aureolineatus, L. moestus, L. sp. Arno, L. sp. Takapoto) to others reproducing parthenogenetically (H. garnotii, L. lugubris). These comparisons include the known maternal (L. moestus) and paternal (L. sp. Arno) ancestors of the asexual L. lugubris. Sequences similar to other vertebrate species were obtained from both nuclear and cDNA templates indicating that these sequences are derived from expressed class I Mhc loci. Southern blot analysis using gecko class I probes, revealed that parthenogenetic clonal lineages of independent evolutionary origin have no within-clone band variation at class I loci and that no detectable recombination between restriction sites had taken place. Variability in the sexual species was similar to mammalian taxa, i.e. class I genes are highly variable in outbreeding sexual populations. Sequence analysis of the alpha-2 domain of class I genes identified point mutations in a clonal lineage of L. lugubris which led to amino acid substitutions. Potential transspecific allelic lineages were also observed. The persistence of asexual lineages with little or no class I diversification over thousands of generations seems to argue against-strong selection for Mhc multi-allelism caused by pathogen-Mhc allele specificity. On the other hand, the high level of heterozygosity in the parthenogenetic species (a consequence of their hybrid origin) may provide clonal lineages with adequate antigen presenting diversity to survive and compete with sexual relatives. | 3067 | NA | NA | NA | no | no |
| 3068 | 2017 | NA | Vincent, JB; Weiblen, GD; May, G | 2016 | Host associations and beta diversity of fungal endophyte communities in New Guinea rainforest trees | Processes shaping the distribution of foliar fungal endophyte species remain poorly understood. Despite increasing evidence that these cryptic fungal symbionts of plants mediate interactions with pathogens and herbivores, there remain basic questions regarding the extent to which dispersal limitation and host specificity might shape fungal endophyte community composition in rainforests. To assess the relative importance of spatial pattern and host specificity, we isolated fungi from a sample of mapped trees in lowland Papua New Guinea. Sequences of the internal transcribed spacer (ITS) region were obtained for 2079 fungal endophytes from three sites and clustered into molecular operational taxonomic units (MOTUs) at 95% similarity. Multivariate analyses suggest that host affinity plays a significant role in structuring endophyte community composition whereas there was no evidence of endophyte spatial pattern at the scale of tens to hundreds of metres. Differences in endophyte communities between sampled trees were weakly correlated with variation in foliar traits but not with tree species relatedness. The dominance of relatively few generalist endophytes and the presence of a large number of rare MOTUs was a consistent observation at three sites separated by hundreds of kilometres and regional turnover was low. Host specificity appears to play a relatively weak but more important role than dispersal limitation in shaping the distribution of fungal endophyte communities in New Guinea forests. Our results suggest that in the absence of strong ecological gradients and host turnover, beta diversity of endophyte communities could be low in large areas of contiguous forest. | 3068 | NA | NA | NA | no | no |
| 3069 | 2017 | NA | Hunter-Zinck, H; Musharoff, S; Salit, J; Al-Ali, KA; Chouchane, L; Gohar, A; Matthews, R; Butler, MW; Fuller, J; Hackett, NR; Crystal, RG; Clarks, AG | 2010 | Population Genetic Structure of the People of Qatar | People of the Qatar peninsula represent a relatively recent founding by a small number of families from three tribes of the Arabian Peninsula, Persia, and Oman, with indications of African admixture. To assess the roles of both this founding effect and the customary first-cousin marriages among the ancestral Islamic populations in Qatar’s population genetic structure, we obtained and genotyped with Affymetrix 500k SNP arrays DNA samples from 168 self-reported Qatari nationals sampled from Doha, Qatar. Principal components analysis was performed along with samples from the Human Genetic Diversity Project data set, revealing three clear clusters of genotypes whose proximity to other human population samples is consistent with Arabian origin, a more eastern or Persian origin, and individuals with African admixture. The extent of linkage disequilibrium (LD) is greater than that of African populations, and runs of homozygosity in some individuals reflect substantial consanguinity. However, the variance in runs of homozygosity is exceptionally high, and the degree of identity-by-descent sharing generally appears to be lower than expected for a population in which nearly half of marriages are between first cousins. Despite the fact that the SNPs of the Affymetrix 500k chip were ascertained with a bias toward SNPs common in Europeans, the data strongly support the notion that the Qatari population could provide a valuable resource for the mapping of genes associated with complex disorders and that tests of pairwise interactions are particularly empowered by populations with elevated LD like the Qatari. | 3069 | NA | NA | NA | no | no |
| 3070 | 2017 | NA | Zheng, HL; Wang, JG; Zhao, HW; Liu, HL; Sun, J; Guo, LY; Zou, DT | 2015 | Genetic structure, linkage disequilibrium and association mapping of salt tolerance in japonica rice germplasm at the seedling stage | Soil salinity is a major constraint to rice production. Understanding the genetic basis of salt tolerance is crucial for the improvement of salt tolerance through breeding. Previous quantitative trait locus (QTL) studies for salt tolerance were mainly derived from bi-parental segregating populations and relatively little is known about the results from natural populations. Understanding the genetic diversity, population structure and linkage disequilibrium (LD) in an association panel can effectively avoid spurious associations in association mapping. In this study, 341 japonica rice (Oryza sativa L. subsp. japonica) accessions worldwide were genotyped with 160 simple sequence repeat (SSR) markers to identify marker-trait associations with salt tolerance at the seedling stage. Salt tolerance was evaluated by survival days of seedlings and shoot K+/Na+ ratio. A total of 872 alleles ranging from 2 to 9 per locus were identified from all collections. Population structure analysis identified three main subpopulations for the accessions. Of the SSR pairs in these accessions, 40.05 % marker pairs showed significant LD (P < 0.01). The LD level for linked markers is significantly higher than that for unlinked markers, and LD level was elevated when the panel was classified into subpopulations. The LD decayed to the background at approximately 20-50 cM within the total panel and each subpopulation. A total of ten marker loci associated with salt tolerance were identified using MLM (Q + K) models in TASSEL 3.0. Among which nine marker loci confirmed or narrowed the genomic region reported to harbor QTLs for salt tolerance by linkage mapping in previous reports, and four salt tolerance-related genes were located in the QTL regions in the present study. According to phenotypic effects for alleles of the detected QTLs, favorable alleles were mined. These favorable alleles could be used to design parental combinations and the expected results would be obtained by pyramiding or substituting the favorable alleles per QTL (apart from possible epistatic effects). Our results demonstrate that association mapping can complement and enhance previous QTL information for marker-assisted selection and breeding by design. | 3070 | NA | NA | NA | no | no |
| 3071 | 2017 | NA | Fusco, D; Gralka, M; Kayser, J; Anderson, A; Hallatschek, O | 2016 | Excess of mutational jackpot events in expanding populations revealed by spatial Luria-Delbruck experiments | The genetic diversity of growing cellular populations, such as biofilms, solid tumours or developing embryos, is thought to be dominated by rare, exceptionally large mutant clones. Yet, the emergence of these mutational jackpot events is only understood in well-mixed populations, where they stem from mutations that arise during the first few cell divisions. To study jackpot events in spatially structured populations, we track mutant clones in microbial populations using fluorescence microscopy and population sequencing. High-frequency mutations are found to be massively enriched in microbial colonies compared with well-shaken liquid cultures, as a result of late-occurring mutations surfing at the edge of range expansions. Thus, jackpot events can be generated not only when mutations arise early but also when they occur at favourable locations, which exacerbates their role in adaptation and disease. In particular, because spatial competition with the wild type keeps most mutant clones in a quiescent state, strong selection pressures that kill the wild type promote drug resistance. | 3071 | NA | NA | NA | no | no |
| 3072 | 2017 | NA | Rosengrave, P; Montgomerie, R; Gemmell, N | 2016 | Cryptic female choice enhances fertilization success and embryo survival in chinook salmon | In this study, we investigated two potentially important intersexual postcopulatory gametic interactions in a population of chinook salmon (Oncorhynchus tshawytscha): (i) the effect of female ovarian fluid (OF) on the behaviour of spermatozoa during fertilization and (ii) the effects of multilocus heterozygosity (MLH) (as an index of male quality) and female-male genetic relatedness on sperm behaviour and male fertilization success when there is sperm competition in the presence of that OF. To do this, we conducted a series of in vitro competitive fertilization experiments and found that, when ejaculates from two males are competing for access to a single female’s unfertilized eggs, fertilization success was significantly biased towards the male whose sperm swam fastest in the female’s OF. Embryo survival-a measure of fitness-was also positively correlated with both sperm swimming speed in OF and male MLH, providing novel evidence that cryptic female choice is adaptive for the female, enhancing the early survival of her offspring and potentially influencing her fitness. | 3072 | NA | NA | NA | no | no |
| 3073 | 2017 | NA | Amin, I; Qazi, J; Mansoor, S; Ilyas, M; Briddon, RW | 2008 | Molecular characterisation of Banana bunchy top virus (BBTV) from Pakistan | Banana bunchy top disease is caused by a single-stranded circular DNA virus, banana bunchy top virus (BBTV), which is a member of the genus Babuvirus (family Nanoviridae). We have cloned and sequenced five components (DNA-R, DNA-S, DNA-N, DNA-M and DNA-C) of a BBTV isolate originating from Pakistan. In addition, the DNA-R and several other components of five further isolates, originating from geographically distinct sites across the banana-growing area of Sindh province, Pakistan, were cloned and sequenced. Analysis of the sequences indicates that BBTV present in Pakistan belongs to the “South Pacific” group of isolates and that the genetic diversity of the virus in the country is very low. The virus shows the highest levels of sequence identity to BBTV isolates originating from Egypt, India and Australia. The significance of these results with respect to the possible origin of the virus in Pakistan and the prospects for obtaining genetically engineered resistance to the virus are discussed. | 3073 | NA | NA | NA | no | no |
| 3074 | 2017 | NA | Mascheretti, S; Croucher, PJP; Vettraino, A; Prospero, S; Garbelotto, M | 2008 | Reconstruction of the Sudden Oak Death epidemic in California through microsatellite analysis of the pathogen Phytophthora ramorum | The genetic structure of the clonally reproducing Sudden Oak Death (SOD) pathogen in California was investigated using seven variable microsatellites. A total of 35 multilocus genotypes were identified among 292 samples representative of populations from 14 forest sites and of the nursery trade. AMOVA indicated significant genetic variability both within (44.34%) and among populations (55.66%). Spatial autocorrelation analyses indicated that Moran’s index of similarity reached a minimum of 0.1 at 350 m, increased to 0.4 at 1500 m and then decreased to zero at 10 km. These results suggest a bimodal pattern of spread, with medium range dispersal (1500-10 000 m) putatively attributed to the presence of strong winds. Lack of genetic structure was identified for three groups of populations. One group notably included the nurseries’ population and two forest populations, both linked to early reports of the pathogen. A neighbour-joining analysis based on pairwise Phi(ST) values indicated that the clade inclusive of the nurseries’ populations is basal to all California populations. A network analysis identified three common genotypes as the likely founders of the California infestation and proposes a stepwise model for local evolution of novel genotypes. This was supported by the identification in the same locations of novel genotypes and of their 1- or 2-step, parents. We hypothesize that the few undifferentiated population groups indicate historical human spread of the pathogen, while the general presence of genetically structured populations indicates that new infestations are currently generated by rare medium or long-range natural movement of the pathogen, followed by local generation of new genotypes. | 3074 | NA | NA | NA | no | no |
| 3075 | 2017 | NA | Alter, SE; Rynes, E; Palumbi, SR | 2007 | DNA evidence for historic population size and past ecosystem impacts of gray whales | Ecosystem restoration may require returning threatened populations of ecologically pivotal species to near their former abundances, but it is often difficult to estimate historic population size of species that have been heavily exploited. Eastern Pacific gray whales play a key ecological role in their Arctic feeding grounds and are widely thought to have returned to their prewhaling abundance. Recent mortality spikes might signal that the population has reached long-term carrying capacity, but an alternative is that this decline was due to shifting climatic conditions on Arctic feeding grounds. We used a genetic approach to estimate prewhaling abundance of gray whales and report DNA variability at 10 loci that is typical of a population of -76,000-118,000 individuals, approximately three to five times more numerous than today’s average census size of 22,000. Coalescent simulations indicate these estimates may include the entire Pacific metapopulation, suggesting that our average measurement of approximate to 96,000 individuals was probably distributed between the eastern and currently endangered western Pacific populations. These levels of genetic variation suggest the eastern population is at most at 28-56% of its historical abundance and should be considered depleted. If used to inform management, this would halve acceptable human-caused mortality for this population from 417 to 208 per year. Potentially profound ecosystem impacts may have resulted from a decline from 96,000 gray whales to the current population. At previous levels, gray whales may have seasonally resuspended 700 million cubic meters of sediment, as much as 12 Yukon Rivers, and provided food to a million sea birds. | 3075 | NA | NA | NA | no | no |
| 3076 | 2017 | NA | Cronin, KA; Mitchell, MA; Lonsdorf, EV; Thompson, SD | 2006 | One year later: Evaluation of PMC-recommended births and transfers | To meet their exhibition, conservation, education, and scientific goals, members of the American Zoo and Aquarium Association (AZA) collaborate to manage their living collections as single species populations. These cooperative population management programs, Species Survival Plans (R) (SSP) and Population Management Plans (PMP), issue specimen-by-specimen recommendations aimed at perpetuating captive populations by maintaining genetic diversity and demographic stability. Species Survival Plans and PMPs differ in that SSP participants agree to complete recommendations, whereas PMP participants need only take recommendations under advisement. We evaluated the effect of program type and the number of participating institutions on the success of actions recommended by the Population Management Center (PMC): transfers of specimens between institutions, breeding, and target number of offspring. We analyzed AZA studbook databases for the occurrence of recommended or unrecommended transfers and births during the 1-year period after the distribution of standard AZA Breeding-and-Transfer Plans. We had three major findings: 1) on average, both SSPs and PMPs fell about 25% short of their target; however, as the number of participating institutions increased so too did the likelihood that programs met or exceeded their target; 2) SSPs exhibited significantly greater transfer success than PMPs, although transfer success for both program types was below 50%; and 3) SSPs exhibited significantly greater breeding success than PMPs, although breeding success for both program types was below 20%. Together, these results indicate that the science and sophistication behind genetic and demographic management of captive populations may be compromised by the challenges of implementation. | 3076 | NA | NA | NA | no | no |
| 3077 | 2017 | NA | Webster, JP; Woolhouse, MEJ | 1999 | Cost of resistance: relationship between reduced fertility and increased resistance in a snail-schistosome host-parasite system | Natural host populations often exhibit genetic variability in resistance to parasitism. One possible mechanism for maintaining such diversity is a trade-off between fitness costs associated with resistance and fitness costs associated with parasitism. However, little is known about the nature or magnitude of these costs in animal populations. Using artificial selection experiments in a Biomphalaria glabrata-Schistosoma mansoni hose-parasite system, we demonstrated that resistance and susceptibility to infection are heritable. We then investigated whether resistance had any associated costs in terms of snail reproductive success. Susceptible-selected snail lines showed significantly higher fertility (number of offspring produced) than resistant-selected or unselected control snail lines, irrespective of current infection status. There were no consistent differences between snail lines in fecundity proportion of abnormal egg masses produced, or mean number of eggs per egg mass. Mortality rare was higher among infected than uninfected snails. These results are consistent with snails incurring costs of resistance to schistosome infection in the absence of the parasite. | 3077 | NA | NA | NA | no | no |
| 3078 | 2017 | NA | Leggett, HC; Wild, G; West, SA; Buckling, A | 2017 | Fast-killing parasites can be favoured in spatially structured populations | It is becoming increasingly clear that the evolution of infectious disease is influenced by host population structure. Theory predicts that parasites should be more ‘prudent’-less transmissible-in spatially structured host populations. However, here we (i) highlight how low transmission, the phenotype being selected for in this in context, may also be achieved by rapacious host exploitation, if fast host exploitation confers a local, within-host competitive advantage and (ii) test this novel concept in a bacteria-virus system. We found that limited host availability and, to a lesser extent, low relatedness favour faster-killing parasites with reduced transmission. By contrast, high host availability and high relatedness favour slower-killing, more transmissible parasites. Our results suggest high, rather than low, virulence may be selected in spatially structured host-parasite communities where local competition and hence selection for a within-host fitness advantage is high. This article is part of the themed issue ‘Opening the black box: reexamining the ecology and evolution of parasite transmission’. | 3078 | NA | NA | NA | no | no |
| 3079 | 2017 | NA | Zuluaga-Montero, A; Ramirez-Camejo, L; Rauscher, J; Bayman, P | 2010 | Marine isolates of Aspergillus flavus: Denizens of the deep or lost at sea? | Most fungal species from marine environments also live on land. It is not clear whether these fungi reach the sea from terrestrial sources as spores or other propagules, or if there are separate ecotypes that live and reproduce in the sea. The emergence of marine diseases has created an urgency to understand the distribution of these fungi. Aspergillus flavus is ubiquitous in both terrestrial and marine environments. This species is an opportunistic pathogen in many hosts, making it a good model to study the relationship between genetic diversity and specificity of marine fungi. In this study, an intraspecific phylogeny of A. flavus isolates based on Amplified Fragment Length Polymorphisms (AFLPs) was used to determine if terrestrial and marine isolates form discrete populations, and to determine if phylogeny predicts substratum specificity. Results suggest lack of population structure in A. flavus. All isolates may compose a single population, with no clade particular to marine environments. (C) 2010 Elsevier Ltd and The British Mycological Society. All rights reserved. | 3079 | NA | NA | NA | no | no |
| 3080 | 2017 | NA | McLeod, BA; White, BN | 2010 | Tracking mtDNA Heteroplasmy through Multiple Generations in the North Atlantic Right Whale (Eubalaena glacialis) | Mitochondrial heteroplasmy has been identified in a variety of species and can result from either paternal leakage, whereby sperm mitochondria enter the ova during fertilization, or more commonly by the “survival” and proliferation of mutant variants within an organism. From an evolutionary perspective, this process represents the generation of new mitochondrial diversity within a species. Although this has been documented in some mammalian species, it has been reported from relatively few wild mammalian populations and in no wild nonhuman population has the transfer and segregation of mitochondrial heteroplasmy been tracked through multiple generations. We report on the first case of the identification and tracking of mitochondrial control region heteroplasmy through 3 generations in the North Atlantic right whale, Eubalaena glacialis. We also identify the full segregation to the mutant variant within a single generation and thus the development of a new haplotype (haplotype G) in a maternal lineage of this endangered species. Witnessed here is the generation of mitochondrial diversity in a genetically depauperate species. | 3080 | NA | NA | NA | no | no |
| 3081 | 2017 | NA | Rogstad, SH; Pelikan, S | 2014 | Restoring the American Chestnut: Optimizing Founder Spacing to Promote Population Growth and Genetic Diversity Retention | Efforts are underway to return the American chestnut (Castanea dentata) to eastern forests of North America following its decline due to the introduction of the chestnut blight (Cryphonectria parasitica). Approaches include developing blight-resistant chestnut lines through breeding programs and via genetic engineering. Reestablishment of resistant chestnut to eastern forests would produce one of the most extensive ecological restoration transformations ever attempted. However, this undertaking is costly and optimization of reintroduction methods is needed. We used the computer program NEWGARDEN to model whether some patterns of founder placement (regular vs. random spacing at differing densities) produce more rapidly expanding populations across a range of gene dispersal distance conditions (via both offspring and pollen). For a simulated introduction project employing 169 founders, placing founders randomly in a square of side 0.85 km produced higher rates of predicted population growth compared with larger or smaller squares under near gene dispersal conditions; this side distance was 1.0 km under far gene dispersal conditions. After 100 population bouts of mating and under near gene dispersal conditions, the trial with founder placement producing the greatest population expansion rate exhibited a 314% increase in census size compared with the founder pattern yielding the slowest expansion. Neither loss of alleles nor inbreeding or subdivision was significantly increased under the founder placement patterns yielding the most descendants. Exploring different numerical and geometrical founding scenarios using NEWGARDEN can provide first estimates of founding patterns or stand manipulations that will return the most descendants produced per founder planted in restoration projects. | 3081 | NA | NA | NA | no | no |
| 3082 | 2017 | NA | Broders, KD; Woeste, KE; SanMiguel, PJ; Westerman, RP; Boland, GJ | 2011 | Discovery of single-nucleotide polymorphisms (SNPs) in the uncharacterized genome of the ascomycete Ophiognomonia clavigignenti-juglandacearum from 454 sequence data | The benefits from recent improvement in sequencing technologies, such as the Roche GS FLX (454) pyrosequencing, may be even more valuable in non-model organisms, such as many plant pathogenic fungi of economic importance. One application of this new sequencing technology is the rapid generation of genomic information to identify putative single-nucleotide polymorphisms (SNPs) to be used for population genetic, evolutionary, and phylogeographic studies on non-model organisms. The focus of this research was to sequence, assemble, discover and validate SNPs in a fungal genome using 454 pyrosequencing when no reference sequence is available. Genomic DNA from eight isolates of Ophiognomonia clavigignenti-juglandacearum was pooled in one region of a four-region sequencing run on a Roche 454 GS FLX. This yielded 71 million total bases comprising 217 000 reads, 80% of which collapsed into 16 125 754 bases in 30 339 contigs upon assembly. By aligning reads from multiple isolates, we detected 298 SNPs using Roche’s GS Mapper. With no reference sequence available, however, it was difficult to distinguish true polymorphisms from sequencing error. EAGLEVIEW software was used to manually examine each contig that contained one or more putative SNPs, enabling us to discard all but 45 of the original 298 putative SNPs. Of those 45 SNPs, 13 were validated using standard Sanger sequencing. This research provides a valuable genetic resource for research into the genus Ophiognomonia, demonstrates a framework for the rapid and cost-effective discovery of SNP markers in non-model organisms and should prove especially useful in the case of asexual or clonal fungi with limited genetic variability. | 3082 | NA | NA | NA | no | no |
| 3083 | 2017 | NA | Sankar, S; Kuppanan, S; Nandagopal, B; Sridharan, G | 2013 | Diversity of Salmonella enterica serovar Typhi Strains Collected from India Using Variable Number Tandem Repeat (VNTR)-PCR Analysis | Typhoid fever is endemic in India, and a seasonal increase of cases is observed annually. In spite of effective therapies and the availability of vaccines, morbidity is widespread owing to the circulation of multiple genetic variants, frequent migration of asymptomatic carriers, unhygienic food practices and the emergence of multidrug resistance and thus continues to be a major public health problem in developing countries, particularly in India. Classical methods of strain typing such as pulsed-field gel electrophoresis, ribotyping, random amplification of polymorphic DNA and amplified fragment length polymorphism are either laborious and technically complicated or less discriminatory. We investigated the molecular diversity of Indian strains of Salmonella enterica serovar Typhi (S. Typhi) isolated from humans from different parts of India to establish the molecular epidemiology of the organism using the variable number tandem repeat (VNTR)-PCR analysis. The electrophoretic band pattern was analysed using the GelCompar II software program. Of the 94 strains tested for three VNTRs loci, 75 VNTR genotypes were obtained. Of the three VNTRs tested in this study, VNTR1 was amplified in all the strains except one and found to be predominant. VNTR2 was amplified only in 57 strains with a Simpson diversity index of 0.93 indicating the high variability of this region within the strains. VNTR3 was amplified in 90 strains. The discriminatory power of this typing tool has been greatly enhanced by this VNTR2 region as the other two regions could not discriminate strains significantly. In our study, about 55 % of the strains amplified all three VNTR regions and 39 % of the strains lacked the VNTR2 region. Among the three VNTR regions tested, the majority of the strains produced similar banding pattern for any two regions grouped into a cluster. The strains grouped as a genotype were from the same geographical location. Strains collected from each geographical region were also highly heterogeneous. Such analysis is important to identify the genetic clones of the pathogen associated with sporadic infections and disease outbreak to identify the common source and implement public health measures. | 3083 | NA | NA | NA | no | no |
| 3084 | 2017 | NA | Castillo, D; Christiansen, RH; Espejo, R; Middelboe, M | 2014 | Diversity and Geographical Distribution of Flavobacterium psychrophilum Isolates and Their Phages: Patterns of Susceptibility to Phage Infection and Phage Host Range | Flavobacterium psychrophilum is an important fish pathogen worldwide that causes cold water disease (CWD) or rainbow trout fry syndrome (RTFS). Phage therapy has been suggested as an alternative method for the control of this pathogen in aquaculture. However, effective use of bacteriophages in disease control requires detailed knowledge about the diversity and dynamics of host susceptibility to phage infection. For this reason, we examined the genetic diversity of 49 F. psychrophilum strains isolated in three different areas (Chile, Denmark, and USA) through direct genome restriction enzyme analysis (DGREA) and their susceptibility to 33 bacteriophages isolated in Chile and Denmark, thus covering large geographical (> 12,000 km) and temporal (> 60 years) scales of isolation. An additional 40 phage-resistant isolates obtained from culture experiments after exposure to specific phages were examined for changes in phage susceptibility against the 33 phages. The F. psychrophilum and phage populations isolated from Chile and Denmark clustered into geographically distinct groups with respect to DGREA profile and host range, respectively. However, cross infection between Chilean phage isolates and Danish host isolates and vice versa was observed. Development of resistance to certain bacteriophages led to susceptibility to other phages suggesting that “enhanced infection” is potentially an important cost of resistance in F. psychrophilum, possibly contributing to the observed co-existence of phage-sensitive F. psychrophilum strains and lytic phages across local and global scales. Overall, our results showed that despite the identification of local communities of phages and hosts, some key properties determining phage infection patterns seem to be globally distributed. | 3084 | NA | NA | NA | no | no |
| 3085 | 2017 | NA | Kurvers, RHJM; Adamczyk, VMAP; Kraus, RHS; Hoffman, JI; van Wieren, SE; van der Jeugd, HP; Amos, W; Prins, HHT; Jonker, RM | 2013 | Contrasting context dependence of familiarity and kinship in animal social networks | The social structure of a population is a crucial element of an individual’s environment, fundamentally influencing the transfer of genes, information and diseases. A central question in social network analysis is how different traits affect associations within populations. However, previous studies of animal social networks have typically focused on a single predictor or stage in the life cycle whereas social interactions within populations are known to be dynamic and not fixed through time and/or context. Relatively few animal network studies have explored how individual traits affect decisions across different ecologically relevant contexts. We collected detailed behavioural data (personality, dominance, familiarity) and high-resolution genetic data from a flock of 43 captive barnacle geese, Branta leucopsis, to understand how these traits affect association patterns in two different evolutionary and ecologically highly relevant contexts: foraging and mate choice. Using a novel analytical framework for node label permutations, we found that barnacle geese preferentially associated with close kin and other individuals familiar from earlier in life when foraging, but selected unfamiliar partners during mate choice. We found no effect of either personality or dominance on foraging associations or mate choice. Our study shows how using social network analysis can increase our understanding of the drivers behind population structure (in our case kin selection and inbreeding avoidance). Moreover, our study demonstrates that social networks can be largely determined by long-termprocesses, inparticular early life familiarity. (C) 2013 The Association for the Study of Animal Behaviour. Published by Elsevier Ltd. All rights reserved. | 3085 | NA | NA | NA | no | no |
| 3086 | 2017 | NA | Schwensow, N; Eberle, M; Sommer, S | 2010 | Are there Ubiquitous Parasite-driven Major Histocompatibility Complex Selection Mechanisms in Gray Mouse Lemurs? | A major goal of evolutionary biology is to understand how selection drives local adaptation. For example, the major histocompatibility complex (MHC) plays an important role in the immune system, and high levels of MHC variation are thought to be a form of adaptation in natural populations. Individual MHC composition may influence parasite resistance via advantages associated with 1) heterozygosity, because heterozygotes recognize a broader range of different antigens than homozygotes (heterozygote advantage); 2) highly variable amino acid sequences in MHC alleles, allowing individuals to bind a broader spectrum of parasite-derived peptides (divergent-alleles advantage, a mechanistic variant of the heterozygote advantage model); or 3) specific MHC alleles (rare allele advantage or frequency dependent selection). We investigated relationships between gastrointestinal nematode burden and both adaptive immune gene variability (MHC class II DRB) and neutral microsatellites in free-living gray mouse lemurs (Microcebus murinus) native to a dry deciduous forest population in western Madagascar to test these hypotheses. The individual MHC composition was related to parasite infestation. Specific MHC alleles were involved in parasite resistance and the presence of common alleles negatively influenced infestation intensity. We found no support for the heterozygote advantage hypothesis, but we did find support for the divergent-MHC allele advantage hypothesis: Individuals with very divergent MHC alleles carried fewer and less intense nematode infestations than individuals with more similar alleles in the more variable dry deciduous forest population. These results indicate that intestinal parasites are important selection pressures under natural conditions and suggest that different selection mechanisms are not mutually exclusive. In contrast, we detected no association between neutral overall individual genetic diversity (measured via 17 microsatellites) and parasite load. Finally, we investigated the ubiquity of parasite-driven selection mechanisms by comparing our results with a previous study of a mouse lemur population from the climatically different littoral forest in southeastern Madagascar, ca. 500 km away. This revealed that different specific MHC alleles were involved in parasite resistance in the 2 habitats, showing that gene-parasite associations are not consistent between populations. | 3086 | NA | NA | NA | no | no |
| 3087 | 2017 | NA | Colijn, C; Cohen, T; Murray, M | 2009 | Latent Coinfection and the Maintenance of Strain Diversity | Technologies for strain differentiation and typing have made it possible to detect genetic diversity of pathogens, both within individual hosts and within communities. Coinfection of a host by more than one pathogen strain may affect the relative frequency of these strains at the population level through complex within- and between-host interactions; in infectious diseases that have a long latent period, interstrain competition during latency is likely to play an important role in disease dynamics. We show that SEIR models that include a class of latently coinfected individuals can have markedly different long-term dynamics than models without coinfection, and that coinfection can greatly facilitate the stable coexistence of strains. We demonstrate these dynamics using a model relevant to tuberculosis in which people may experience latent coinfection with both drug sensitive and drug resistant strains. Using this model, we show that the existence of a latent coinfected state allows the possibility that disease control interventions that target latency may facilitate the emergence of drug resistance. | 3087 | NA | NA | NA | no | no |
| 3088 | 2017 | NA | Coussens, AK; van Daal, A | 2005 | Linkage disequilibrium analysis identifies an FGFR1 haplotype-tag SNP associated with normal variation in craniofacial shape | Mutations in FGFR1 and TWIST1 have been reported to affect the timing of calvarial suture fusion resulting in craniosynostosis and facial abnormalities. We screened nonpathologic populations for genetic polymorphisms that may associate with normal craniofacial variation. We identified 17 single-nucleotide polymorphisms (SNPs) in FGFR1, 6 of which were novel (g.8591855G -> A, g.8593685G -> A, g.8602303C -> T, g.8602475A -> G (p,Ile293Val), g.8605849C -> T, g.8607868G -> A). No SNPs were found in TWIST1. FGFR1 SNP haplotypes were reconstructed for Caucasian, Asian, Australian Aboriginal, and African American populations. All populations shared two linkage disequilibrium blocks, with one haplotype-tag SNP (htSNP) tagging each block. The htSNP g.8592931G -> C was found to have a significant negative correlation with the cephalic index for all populations (R = -0.187, p = 0.036), with larger correlations in Asians and females. This finding is a starting point in the identification of a set of SNPs that can be genotyped to determine both normal and disease craniofacial phenotypes. (c) 2005 Elsevier Inc. All rights reserved. | 3088 | NA | NA | NA | no | no |
| 3089 | 2017 | NA | Bishir, J; Roberds, J | 1997 | Limit theorems and a general framework for risk analysis in clonal forestry | Use of clonally propagated plantings in reforestation offers management advantages of phenotypic uniformity and high yields. Disadvantages include low genetic diversity and the possibility that the clone or clones chosen are particularly susceptible to attack by an insect or pathogen unforeseen as a problem at the time of clonal selection. In this paper, we continue consideration of the problem of choosing an optimal number of clones to minimize the risk of plantation failure. We present an analysis in which risk of failure for a plantation is represented by the probability that the proportion, S, of ramets that survive until harvest is less than or equal to a prescribed value. Our approach includes most earlier treatments as special cases. We show that the proportion S converges in distribution and, furthermore, that, under general conditions, a moderate number of clones, usually no more than 20 to 40 and often fewer, provides equivalent or better protection against catastrophic loss than does a large number of clones. (C) Elsevier Science Inc., 1997. | 3089 | NA | NA | NA | no | no |
| 3090 | 2017 | NA | Trifonova, EA; Spiridonova, MG; Stepanov, VA | 2008 | Genetic Diversity and the Structure of Linkage Disequilibrium in the Methylenetetrahydrofolate Reductase Locus | Investigation of linkage disequilibrium block architecture in human genome is modern, intensely investigated field of molecular genetics. In the present study, genetic differentiation and linkage disequilibrium pattern in the methylenetetrahydrofolate reductase (MTHFR) locus was examined in the populations of Russians, Tuvinians, and Northern and Southern Kyrgyzes. Methylenetetrahydrofolate reductase is the key enzyme of folate cycle, responsible for reduction of 5,10-methylenetetrahydrofolate to 5-methyltetrahydrofolate. Decreased enzymatic activity of this protein often caused by certain associations of MTHFR alleles results in the increased plasma homocysteine levels. In the population groups examined, genotype and allele frequencies at five polymorphic MTHFR loci: rs17037397, rs4846052, rs1801133, rs1801131, and rs1537516 were evaluated. Statistically significant genetic differences between the population group of Southern Kyrgyzes and the other groups, as well as between Russians and Tuvinians, were demonstrated. In the MTHFR gene from the population of Southern Kyrgyzes one block was revealed; in the populations of Russians, Tuvinians, and Northern Kyrgyzes two blocks were detected. Thus, the structure of linkage disequilibrium in the MTHFR locus demonstrated population-specific pattern. | 3090 | NA | NA | NA | no | no |
| 3091 | 2017 | NA | Vermeulen, ET; Lott, MJ; Eldridge, MDB; Power, ML | 2016 | Parasites on the hop: Captive breeding maintains biodiversity of Eimeria communities in an endangered marsupial | The complex parasite communities of animals contribute to biodiversity, yet the conservation strategies that aim to preserve individual threatened species often overlook their parasite communities. Deeper understanding of parasite communities and how they are affected by management is important to the ultimate success of biodiversity conservation. Here we examine the dynamics between the coccidian parasite Eimeria and the threatened brush-tailed rock-wallaby (Petrogale penicillata, BTRW) to determine how parasite communities respond to the conservation management practices of captive breeding and translocation. Three BTRW population categories (wild, captive bred or supplemented) were analysed for Eimeria prevalence and infection intensity and a metagenomic assessment performed to examine community structure. Eimeria prevalence was 92% in 117 faecal samples. DNA amplicons from purified oocysts were sequenced with the Illumina MiSeq platform and the resulting sequences assigned to 28 Eimeria operational taxonomic units (OTUs). Pairwise identity between OTUs was 89.9% and 25 of the Eimeria OTUs formed a highly supported phylogenetic Glade with marsupial specific Eimeria species, indicating strong host specificity and genetic diversity within Eimeria in BTRWs. Supplemented populations had the greatest OTU diversity with eleven unique OTUs and had a greater overlap with captive bred (9 shared OTUs) versus wild populations (6 shared OTUs). There was no significant effect of population category on infection intensity (p = 0.965), OTU composition (p = 0.51) or richness (p = 0.490), suggesting that Eimeria community structure is maintained under the management processes applied to the BTRW. Our approach can be applied to other parasite communities in hosts under conservation management. (C) 2016 Elsevier Ltd. All rights reserved. | 3091 | NA | NA | NA | no | no |
| 3092 | 2017 | NA | Enjalbert, J; Duan, X; Leconte, M; Hovmoller, MS; De Vallavieille-Pope, C | 2005 | Genetic evidence of local adaptation of wheat yellow rust (Puccinia striiformis f. sp tritici) within France | Puccinia striiformis f. sp. tritici (PST), a clonal basidiomycete causing yellow rust disease on wheat, has a long record of ‘overcoming’ cultivar resistance introduced by breeders. Despite the long dispersal capacity of its spores, the French population of PST presents a strong geographical structure, with the presence of a specific pathotype (array of avirulence genes) at high frequencies in the south of France. The genetic diversity underlying this differentiation was analysed by microsatellite and AFLP markers. A total of 213 French isolates belonging to 10 pathotypes collected over a 15-year period were investigated. For each of the 12 microsatellites used, polymorphism resulted from a unique allelic variant associated to the south-specific pathotype. This pathotype was characterized by 40 specific markers over the total of 63 polymorphims detected using 15 AFLP primer combinations. Phylogeographical analysis indicated a strictly clonal structure of the population, and a strong genomic divergence between the northern population and a south-specific clone. Both virulence and molecular data show that the northern French population belongs to the northwestern European population, whereas the southern clone is most likely related to a Mediterranean population, the two subpopulations resulting from the ancient divergence of two clonal lineages. While the virulence complexity in the northern population may be explained by the successive introduction of corresponding resistance genes in cultivars, the maintenance of a simple virulence type in southern France, despite gene flow between the two populations, may be explained in terms of host cultivars repartition and local adaptation to specific host or climatic conditions. | 3092 | NA | NA | NA | no | no |
| 3093 | 2017 | NA | KOZAKIEWICZ, M | 1993 | HABITAT ISOLATION AND ECOLOGICAL BARRIERS - THE EFFECT ON SMALL MAMMAL POPULATIONS AND COMMUNITIES | Various effects of habitat isolation on both population and community processes in small mammals are presented and discussed; numerous examples are given. Both ecological characteristics of a single patch populations (local population level) and ecological processes among habitat patches (metapopulation level) are presented. The connectivity as a parameter which measures inter-patch processes is discussed. The role of habitat barriers as ‘filters’ structuring both populations and communities of small mammals in heterogeneous environments is presented. It is suggested that ecological processes, which are going on among patchily distributed local populations, make the metapopulation a dynamic, functional unit. Movements of individuals between habitat patches are critical to support the existence of species in a patchy, heterogeneous landscapes. For each species the temporal distribution of activity in space reflects the interactions between the temporal dynamics of the species’ needs and spatio-temporal dynamics of resources. ‘’Key habitats’’ play a crucial role for population existence in the dynamics of species’ needs and resource ‘supply. The increased mobility of individuals seems to be the best strategy for survival in heterogeneous landscapes. The effect of habitat isolation on genetic divergence within a metapopulation is discussed. It is suggested that the small size of discrete local populations, which temporarily go through a genetic’‘bottleneck’’ effect, and occasional migration of individuals between local populations can induce increased, long term genetic variability of the whole metapopulation. It is suggested that landscape heterogeneity and habitat fragmentation affecting the distribution of many species, can also affect interspecific interactions. | 3093 | NA | NA | NA | no | no |
| 3094 | 2017 | NA | Berry, O; Gleeson, DM | 2005 | Distinguishing historical fragmentation from a recent population decline - Shrinking or pre-shrunk skink from New Zealand? | Species that are rare when first described present a practical management problem because it may be unclear whether the taxon is in the final stages of an anthropogenic decline, or is naturally uncommon, and each scenario dictates a distinct approach to management. We analysed mitochondrial and microsatellite DNA data with population genetic and phylogeographic tools to distinguish between these possibilities in a rare lizard from southern New Zealand. Grand skinks, Oligosoma grande, are large rock-dwelling lizards that have a fragmented distribution consisting of a western and eastern cluster of populations separated by ca. 120 km. This distribution could result from human disturbance, pre-human climatic and vegetation changes, or both. All populations were highly genetically structured (overall F-ST 0.171, R-ST 0.235), indicating that populations were demographically independent and skinks are unlikely to expand their range without human intervention. In addition, the current fragmented distribution is likely to have both historical and recent anthropogenic elements. Two eastern populations showed evidence of being historically large (high 0 mtDNA genetic diversity), although they are now small, supporting anecdotal data that grand skinks have declined in historical times. However, eastern and western populations were reciprocally monophyletic for mtDNA lineages, suggesting long independent evolutionary histories that predate the arrival of humans in New Zealand. Eastern and western populations fulfil many criteria to be considered as evolutionarily significant units, but such a classification must be balanced against addressing more immediate threats to the species’ survival, such as introduced predators. (C) 2004 Elsevier Ltd. All rights reserved. | 3094 | NA | NA | NA | no | no |
| 3095 | 2017 | NA | Donoso, JM; Picanol, R; Serra, O; Howad, W; Alegre, S; Arus, P; Eduardo, I | 2016 | Exploring almond genetic variability useful for peach improvement: mapping major genes and QTLs in two interspecific almond x peach populations | Genetic analysis of a diverse set of 42 traits for flower (5), phenology (9), fruit quality (19), leaf (8) and disease resistance (1) was carried out in two interspecific almond 9 peach populations, an F-2 (T x E) and a BC1 (T1E), from the cross between ‘Texas’ almond and ‘Earlygold’ peach. Traits related to flower, phenology, fruit quality, leaf morphology and resistance to powdery mildew were phenotyped over 3 years in two locations and studied for cosegregation with a large set of SNP and SSR markers. Three maps were used, one for the T 9 E and two for the T1E (T1E and E) population. Nine major genes were identified and mapped: anther color (Ag/ag and Ag2/ag2), flower color (Fc2/fc2), maturity date (MD/md), almond fruit type (almond vs. peach; Alf/alf), juiciness (Jui/jui), blood flesh (DBF2/dbf2), powdery mildew resistance (Vr3) and flower type (showy/non-showy; Sh/sh). These genes were often located in genome positions different from those for major genes for similar traits mapped before. Two of them explain fundamental aspects that define the fruit of peach with respect to that of almond: Alf and Jui, for its thick and juicy mesocarp, respectively. The genetics of quantitative traits was studied, and 32 QTLs were detected, with consistent behavior over the years. New alleles identified from almond for important traits such as red skin color, blood flesh, fruit weight and powdery mildew resistance may prove useful for the introduction of new variability into the peach gene pool used in commercial breeding programs. | 3095 | NA | NA | NA | no | no |
| 3096 | 2017 | NA | Letta, T; Maccaferri, M; Badebo, A; Ammar, K; Ricci, A; Crossa, J; Tuberosa, R | 2013 | Searching for novel sources of field resistance to Ug99 and Ethiopian stem rust races in durum wheat via association mapping | Puccinia graminis f. sp. tritici, the causative agent of stem rust in wheat, is a devastating disease of durum wheat. While more than 50 stem rust resistance (Sr) loci have been identified in wheat, only a few of them have remained effective against Ug99 (TTKSK race) and other durum-specific Ethiopian races. An association mapping (AM) approach based on 183 diverse durum wheat accessions was utilized to identify resistance loci for stem rust response in Ethiopia over four field-evaluation seasons and artificial inoculation with Ug99 and a mixture of durum-specific races. The panel was profiled with simple sequence repeat, Diversity Arrays Technology and sequence-tagged site markers (1,253 in total). The resistance turned out to be oligogenic, with twelve QTL-tagging markers that were significant (P < 0.05) across three or four seasons. R (2) values ranged from 1.1 to 11.3 %.Twenty-four additional single-marker/QTL regions were found to be significant over two seasons. The AM results confirmed the role of Sr13, previously described in bi-parental mapping studies, and the role of chromosome regions putatively harbouring Sr9, Sr14, Sr17 and Sr28. Three minor QTLs were coincident with those reported in hexaploid wheat and five overlapped with those recently reported in the Sebatel x Kristal durum mapping population. Thirteen single-marker/QTL regions were located in chromosome regions where no Sr genes/QTLs have been previously reported. The allelic variation identified in this study is readily available and can be exploited for marker-assisted selection, thus providing additional opportunities for a more durable stem rust resistance under field conditions. | 3096 | NA | NA | NA | no | no |
| 3097 | 2017 | NA | Preisigke, SD; Neves, LG; Araujo, KL; Barbosa, NR; Serafim, ME; Krause, W | 2015 | MULTIVARIATE ANALYSIS FOR THE DETECTION OF Passiflora SPECIES RESISTANT TO COLLAR ROT | Collar rot is a disease difficult to control that has hindered passion fruit cultivation in many regions of Brazil. Therefore, this study aimed to find genetic resistance to the fungus Fusarium solani in Passiflora species using the multivariate analysis methodology to discriminate the most resistant species. The following fourteen Passiflora species were assessed: P. quadrangularis, P. nitida, P. foetida, P. tenuifila, P. alata, P. setacea, P. cincinnata, P. mucronata, P. micropetala, P. suberosa, P. morifolia, P. eichleriana, P. edulis and P. coccinea. These plants were arranged in a casualized block design with 14 treatments (species), three replications and three plants per plot. The reactions of the inoculated species of Passiflora were evaluated with the use of 12 resistance traits. The generalized Mahalanobis distance was used to form groupings by the UPGMA method. 3D projection with the canonical variables and quantification of the relative contribution of characters were also conducted. The UPGMA method revealed the formation of three distinct groups of species, which composed the susceptible, moderately resistant and resistant groups. The groups formed by three-dimensional dispersion were similar to those formed by the dendrogram. The following traits contributed most to genetic diversity: inoculation time until the lesion reached more than 50% of the circumference of the injured stem and area under the curve of the expansion of the lesion width. The use of the set of traits and their joint assessment through multivariate analysis allowed greater accuracy in the inference of the most resistant species, mainly P. nitida and P. cincinnata. | 3097 | NA | NA | NA | no | no |
| 3098 | 2017 | NA | Nguyen, DT; Spooner-Hart, RN; Riegler, M | 2016 | Loss of Wolbachia but not Cardinium in the invasive range of the Australian thrips species, Pezothrips kellyanus | Many insects are associated with maternally inherited bacterial endosymbionts that manipulate host reproduction or impact other aspects of host fitness. Despite their ecological significance, the role of endosymbionts in biological invasions by their host insects is still poorly understood. In this study, we characterised the genetic diversity of the invasive Kelly’s citrus thrips, Pezothrips kellyanus (Thysanoptera: Thripidae), an important pest of citrus in Australasia and the Mediterranean region. We then surveyed native and invasive populations for the presence of the bacterial endosymbionts Cardinium and Wolbachia. Our analyses of mitochondrial and nuclear genes demonstrated that P. kellyanus originated from Australia. Furthermore, haplotype analysis revealed independent colonisation events for New Zealand and the Mediterranean region. Individuals from Australian populations had both endosymbionts at a high prevalence. In populations from New Zealand and the Mediterranean region, however, Cardinium was fixed and Wolbachia was absent. This may be due to a stochastic loss of Wolbachia prior to the establishment of invasive populations. Alternatively, Wolbachia may have been selected against by environmental factors or due to its potential role as a reproductive parasite that may constrain invasiveness of infected individuals. In contrast, Cardinium remained unaffected by the invasion process. Our study highlights that endosymbionts may be potential factors in the framework of the enemy release hypothesis that predicts success of invasive hosts in the absence of natural enemies. Therefore, the analysis of endosymbiont diversity of invasive insects may improve the understanding of host invasion biology and also deliver new diagnostic markers for biosecurity protocols. | 3098 | NA | NA | NA | no | no |
| 3099 | 2017 | NA | Thieme, S; Muhldorfer, K; Luschow, D; Hafez, HM | 2016 | Molecular Characterization of the Recently Emerged Poultry Pathogen Ornithobacterium rhinotracheale by Multilocus Sequence Typing | Ornithobacterium rhinotracheale (ORT) is an economically important bacterial pathogen of turkeys and chickens worldwide. Since its first detection, a variety of typing methods have been used to gain basic knowledge about the bacterial population structure, an issue that still needs to be addressed. Serological characterization revealed at least 18 different serotypes (A-R) with ORT of serotype A to be predominate among poultry. This study aimed to establish a multilocus sequence typing (MLST) scheme for ORT that could easily be used by other laboratories and allows for worldwide comparison of sequence data. For this purpose, 87 ORT strains from different poultry hosts, geographical origins, years of isolation and serotypes were included in the analysis to identify correlations. Fourteen different sequence types (ST) were found. The most common ST1 was identified in 40 ORT strains from turkeys and chickens on 4 continents and in 3 different European countries. Together with ST9, both STs represented over three quarters (77%) of ORT strains used in the MLST analysis and included strains of frequently cross-reacting ORT serotypes A, E and I. Nine STs were only represented by one ORT strain and might indicate possible avian host, disease or serotype-specific relationships. In contrast, discrepancies between serotype and phylogenetic relatedness were clearly demonstrated by ORT strains that belonged to identical serotypes but differed in their ST. The overall identified low genetic diversity among strains isolated from turkeys and chickens independent of host and geographical origins suggests that ORT has only recently been introduced into domestic poultry and dispersed worldwide. | 3099 | NA | NA | NA | no | no |
| 3100 | 2017 | NA | Thomas, ML; Framenau, VW | 2005 | Foraging decisions of individual workers vary with colony size in the greenhead ant Rhytidoponera metallica (Formicidae, Ectatomminae) | The ability of worker ants to adapt their behaviour depending on the social environment of the colony is imperative for colony growth and survival. In this study we use the greenhead ant Rhytidoponera metallica to test for a relationship between colony size and foraging behaviour. We controlled for possible confounding ontogenetic and age effects by splitting large colonies into small and large colony fragments. Large and small colonies differed in worker number but not worker relatedness or worker/brood ratios. Differences in foraging activity were tested in the context of single foraging cycles with and without the opportunity to retrieve food. We found that workers from large colonies foraged for longer distances and spent more time outside the nest than foragers from small colonies. However, foragers from large and small colonies retrieved the first prey item they contacted, irrespective of prey size. Our results show that in R. metallica, foraging decisions made outside the nest by individual workers are related to the size of their colony. | 3100 | NA | NA | NA | no | no |
| 3101 | 2017 | NA | Tsai, TS; Rajasekar, S; St John, JC | 2016 | The relationship between mitochondrial DNA haplotype and the reproductive capacity of domestic pigs (Sus scrofa domesticus) | Background: The maternally inherited mitochondrial genome encodes key proteins of the electron transfer chain, which produces the vast majority of cellular ATP. Mitochondrial DNA (mtDNA) present in the mature oocyte acts as a template for all mtDNA that is replicated during development to meet the specific energy requirements of each tissue. Individuals that share a maternal lineage cluster into groupings known as mtDNA haplotypes. MtDNA haplotypes confer advantages and disadvantages to an organism and this affects its phenotype. In livestock, certain mtDNA haplotypes are associated with improved milk and meat quality, whilst, other species, mtDNA haplotypes have shown increased longevity, growth and susceptibility to diseases. In this work, we have set out to determine whether mtDNA haplotypes influence reproductive capacity. This has been undertaken using a pig model. Results: To determine the genetic diversity of domestic pigs in Australia, we have sequenced the D-loop region of 368 pigs, and identified five mtDNA haplotypes (A to E). To assess reproductive capacity, we compared oocyte maturation, fertilization and development to blastocyst, and found that there were significant differences for maturation and fertilization amongst the haplotypes. We then determined that haplotypes C, D and E produced significantly larger litters. When we assessed the conversion of developmentally competent oocytes and their subsequent developmental stages to offspring, we found that haplotypes A and B had the lowest reproductive efficiencies. Amongst the mtDNA haplotypes, the number of mtDNA variants harbored at >25 % correlated with oocyte quality. MtDNA copy number for developmentally competent oocytes positively correlated with the level of the 16383delC variant. This variant is located in the conserved sequence box II, which is a regulatory region for mtDNA transcription and replication. Conclusions: We have identified five mtDNA haplotypes in Australian domestic pigs indicating that genetic diversity is restricted. We have also shown that there are differences in reproductive capacity amongst the mtDNA haplotypes. We conclude that mtDNA haplotypes affect pig reproductive capacity and can be used as a marker to complement current selection methods to identify productive pigs. | 3101 | NA | NA | NA | no | no |
| 3102 | 2017 | NA | Laporte, M; Beaudry, COS; Angers, B | 2013 | Effects of road proximity on genetic diversity and reproductive success of the painted turtle (Chrysemys picta) | Roads have a severe impact on wildlife. Reptiles are particularly susceptible due to their attraction to roads and their low car-avoidance capacity. For example, a high number of road killed freshwater turtles resulted from females selecting the unpaved side of roads as nesting sites. However, roads are harmful not only for adults, but are also expected to affect egg survival and recruitment. In this work, we indirectly determined whether the proximity to roads affects the reproductive success of freshwater turtles. The painted turtle (Chrysemys picta) was chosen for its population density, which is higher than most turtle species considered endangered. Locations near roads (< 100 m) and in natural areas (> 500 m) were sampled in three geographically distant ecoregions. We estimated the diversity of microsatellite loci from nuclear and mitochondrial genomes to assess the size of the kin groups as a proxy of the reproductive success of females. Similar diversity at nuclear markers suggested a comparable historical and demographic background among populations. However, lower mitochondrial diversity, higher mean and variance in the size of kin groups as well as a lower number of kin groups were strongly associated with the proximity to roads. Results indicated that a lower proportion of females participated in the recruitment of populations close to the roads than in natural areas, resulting in fewer but larger families near roads. We expect similar results for species nesting on the roadside. Barriers or fences that prevent individuals from reaching the road may help reduce their impacts on these populations. | 3102 | NA | NA | NA | no | no |
| 3103 | 2017 | NA | Pena, F; Poulin, E; Dantas, GPM; Gonzalez-Acuna, D; Petry, MV; Vianna, JA | 2014 | Have Historical Climate Changes Affected Gentoo Penguin (Pygoscelis papua) Populations in Antarctica? | The West Antarctic Peninsula (WAP) has been suffering an increase in its atmospheric temperature during the last 50 years, mainly associated with global warming. This increment of temperature trend associated with changes in sea-ice dynamics has an impact on organisms, affecting their phenology, physiology and distribution range. For instance, rapid demographic changes in Pygoscelis penguins have been reported over the last 50 years in WAP, resulting in population expansion of sub-Antarctic Gentoo penguin (P. papua) and retreat of Antarctic Adelie penguin (P. adeliae). Current global warming has been mainly associated with human activities; however these climate trends are framed in a historical context of climate changes, particularly during the Pleistocene, characterized by an alternation between glacial and interglacial periods. During the last maximal glacial (LGM similar to 21,000 BP) the ice sheet cover reached its maximum extension on the West Antarctic Peninsula (WAP), causing local extinction of Antarctic taxa, migration to lower latitudes and/or survival in glacial refugia. We studied the HRVI of mtDNA and the nuclear intron beta fibint7 of 150 individuals of the WAP to understand the demographic history and population structure of P. papua. We found high genetic diversity, reduced population genetic structure and a signature of population expansion estimated around 13,000 BP, much before the first paleocolony fossil records (similar to 1,100 BP). Our results suggest that the species may have survived in peri-Antarctic refugia such as South Georgia and North Sandwich islands and recolonized the Antarctic Peninsula and South Shetland Islands after the ice sheet retreat. | 3103 | NA | NA | NA | no | no |
| 3104 | 2017 | NA | Karim, MR; Dong, HJ; Li, TY; Yu, FC; Li, DZ; Zhang, LX; Li, JQ; Wang, RJ; Li, SY; Li, XF; Rume, FI; Ning, CS | 2015 | Predomination and New Genotypes of Enterocytozoon bieneusi in Captive Nonhuman Primates in Zoos in China: High Genetic Diversity and Zoonotic Significance | To appreciate the genetic diversity and zoonotic implications of Enterocytozoon bieneusi in nonhuman primates (NHPs) in zoos, we genotyped E. bieneusi in captive NHPs in seven zoos located at six major cities in China, using ribosomal internal transcribed spacer (ITS)based PCR and sequence analyses. A total of 496 fecal specimens from 36 NHP species under nine families were analyzed and E. bieneusi was detected in 148 (29.8%) specimens of 25 NHP species from six families, including Cercopithecidae (28.7%), Cebidae (38.0%), Aotidae (75.0%), Lemuridae (26.0%), Hylobatidae (50.0%) and Hominidae (16.2%) (P = 0.0605). The infection rates were 29.0%, 15.2%, 18.2%, 37.3%, 29.2%, 37.7% and 44.8% in Shijiazhuang Zoo, Wuhan Zoo, Taiyuan Zoo, Changsha Wild Animal Zoo, Beijing Zoo, Shanghai Zoo and Shanghai Wild Animal Park, respectively (P = 0.0146). A total of 25 ITS genotypes were found: 14 known (D, O, EbpC, EbpA, Type IV, Henan-IV, BEB6, BEB4, Peru8, PigEBITS5, EbpD, CM1, CM4 and CS-1) and 11 new (CM8 to CM18). Genotype D was the most prevalent one (40/148), followed by CM4 (20/148), CM1 (15/148), O (13/148), CM16 (13/148), EbpC (11/148). Of them, genotypes D, EbpC, CM4 and O were widely distributed in NHPs (seen in 9 to 12 species) whereas genotypes CM1 and CM16 were restricted to one to three NHP species. In phylogenetic analysis, 20 genotypes (121/148, 81.8%), excluding genotypes BEB4, BEB6, CM9, CM4 and CM18, belonged to group 1 with zoonotic potential. New genotype CM9 clustered in group 2 with BEB4 and BEB6. The remaining two genotypes CM4 and CM18 formed new cluster (group 9) in between two other genotypic clusters found in primates. The findings of high diversity in E. bieneusi genotypes and their zoonotic potentiality concluded the importance of captive NHPs as reservoir hosts for human microsporidiosis. | 3104 | NA | NA | NA | no | no |
| 3105 | 2017 | NA | Nielsen, SE; Shafer, ABA; Boyce, MS; Stenhouse, GB | 2013 | Does Learning or Instinct Shape Habitat Selection? | Habitat selection is an important behavioural process widely studied for its population-level effects. Models of habitat selection are, however, often fit without a mechanistic consideration. Here, we investigated whether patterns in habitat selection result from instinct or learning for a population of grizzly bears (Ursus arctos) in Alberta, Canada. We found that habitat selection and relatedness were positively correlated in female bears during the fall season, with a trend in the spring, but not during any season for males. This suggests that habitat selection is a learned behaviour because males do not participate in parental care: a genetically predetermined behaviour (instinct) would have resulted in habitat selection and relatedness correlations for both sexes. Geographic distance and home range overlap among animals did not alter correlations indicating that dispersal and spatial autocorrelation had little effect on the observed trends. These results suggest that habitat selection in grizzly bears are partly learned from their mothers, which could have implications for the translocation of wildlife to novel environments. | 3105 | NA | NA | NA | no | no |
| 3106 | 2017 | NA | Goss, EM; Tabima, JF; Cooke, DEL; Restrepo, S; Fry, WE; Forbes, GA; Fieland, VJ; Cardenas, M; Grunwald, NJ | 2014 | The Irish potato famine pathogen Phytophthora infestans originated in central Mexico rather than the Andes | Phytophthora infestans is a destructive plant pathogen best known for causing the disease that triggered the Irish potato famine and remains the most costly potato pathogen to manage worldwide. Identification of P. infestan’s elusive center of origin is critical to understanding the mechanisms of repeated global emergence of this pathogen. There are two competing theories, placing the origin in either South America or in central Mexico, both of which are centers of diversity of Solanum host plants. To test these competing hypotheses, we conducted detailed phylogeographic and approximate Bayesian computation analyses, which are suitable approaches to unraveling complex demographic histories. Our analyses used microsatellite markers and sequences of four nuclear genes sampled from populations in the Andes, Mexico, and elsewhere. To infer the ancestral state, we included the closest known relatives Phytophthora phaseoli, Phytophthora mirabilis, and Phytophthora ipomoeae, as well as the interspecific hybrid Phytophthora andina. We did not find support for an Andean origin of P. infestans; rather, the sequence data suggest a Mexican origin. Our findings support the hypothesis that populations found in the Andes are descendants of the Mexican populations and reconcile previous findings of ancestral variation in the Andes. Although centers of origin are well documented as centers of evolution and diversity for numerous crop plants, the number of plant pathogens with a known geographic origin are limited. This work has important implications for our understanding of the coevolution of hosts and pathogens, as well as the harnessing of plant disease resistance to manage late blight. | 3106 | NA | NA | NA | no | no |
| 3107 | 2017 | NA | Hauer, A; De Cruz, K; Cochard, T; Godreuil, S; Karoui, C; Henault, S; Bulach, T; Banuls, AL; Biet, F; Boschiroli, ML | 2015 | Genetic Evolution of Mycobacterium bovis Causing Tuberculosis in Livestock and Wildlife in France since 1978 | To study the dynamics of bovine tuberculosis (bTB) in France, 4,654 M. bovis strains isolated mainly from livestock and wildlife since 1978 were characterized by spoligotyping and MLVA based on MIRU-VNTR. In our study spoligotyping allowed the discrimination of 176 types although 3 spoligotypes are predominant and account for more than half of the total strain population: SB0120 (26%), SB0134 (11%) and SB0121 (6%). In addition, 11% of the isolates, principally from Southern France, showing close spoligotypes and MIRU-VNTR types have been gathered in a family designated as the “F4-family”. MLVA typing allowed extensive discrimination, particularly for strains with predominant spoligotypes, with a total of 498 genotypes, several of which were highly regionalized. The similarity of the strains’ genetic relationships based on spoligotyping and MIRU-VNTR markers supports the coexistence of different clonal populations within the French M. bovis population. A genetic evolution of the strains was observed both geographically and in time. Indeed, as a result of the reduction of bTB due to the national control campaigns, a large reduction of the strains’ genetic variability took place in the last ten years. However, in the regions were bTB is highly prevalent at present, cases in both livestock and in wildlife are due to the spread of unique local genotype profiles. Our results show that the highly discriminating genotyping tools used in this study for molecular studies of bTB are useful for addressing pending questions, which would lead to a better insight into the epidemiology of the disease, and for finding proper solutions for its sustainable control in France. | 3107 | NA | NA | NA | no | no |
| 3108 | 2017 | NA | Baker, CS; Hamner, RM; Cooke, J; Heimeier, D; Vant, M; Steel, D; Constantine, R | 2013 | Low abundance and probable decline of the critically endangered Maui’s dolphin estimated by genotype capture-recapture | The New Zealand endemic Maui’s dolphin (Cephalorhynchus hectori maui) is considered critically endangered’ by the International Union for the Conservation of Nature as a result of decline due, in part, to fisheries-related mortalities. To estimate the abundance and trends of this subspecies, we used open-population capturerecapture models based on microsatellite genotyping of living and beachcast (dead) dolphins sampled between January 2001 and November 2007. A total of 82 genetic samples were available: 70 biopsy samples collected from living Maui’s dolphins and 12 necropsy samples collected from beachcast or floating carcasses, of which five showed evidence of fisheries entanglement. Microsatellite genotyping of up to 14 loci identified 54 individuals; 42 sampled alive on one or more occasions, one sampled alive, then found beachcast 2 years later, and 11 sampled only as carcasses, including two neonates. The sex ratio of the sample did not differ significantly from unity (25 males: 29 females). Using a POPAN model for live capture records, the abundance of the super population available during the multiyear study was estimated to be N=87 [95% (confidence limits) CL, 59158]. Using a Pradel-like model modified to include both live capture and beachcast records, the abundance of the population was estimated to be N=69 (95% CL, 38125) for the midpoint of the study in 2003. The results of both models suggested that the population was likely to be declining across the study period, although this trend could not be confirmed with 95% confidence. As the genotypes provide permanent marks of individual identity, continued genetic monitoring could provide improved confidence in the abundance and trends of this subspecies. | 3108 | NA | NA | NA | no | no |
| 3109 | 2017 | NA | Clark, CG; Berry, C; Walker, M; Petkau, A; Barker, DOR; Guan, C; Reimer, A; Taboada, EN | 2016 | Genomic insights from whole genome sequencing of four clonal outbreak Campylobacter jejuni assessed within the global C-jejuni population | Background: Whole genome sequencing (WGS) is useful for determining clusters of human cases, investigating outbreaks, and defining the population genetics of bacteria. It also provides information about other aspects of bacterial biology, including classical typing results, virulence, and adaptive strategies of the organism. Cell culture invasion and protein expression patterns of four related multilocus sequence type 21 (ST21) C. jejuni isolates from a significant Canadian water-borne outbreak were previously associated with the presence of a CJIE1 prophage. Whole genome sequencing was used to examine the genetic diversity among these isolates and confirm that previous observations could be attributed to differential prophage carriage. Moreover, we sought to determine the presence of genome sequences that could be used as surrogate markers to delineate outbreak-associated isolates. Results: Differential carriage of the CJIE1 prophage was identified as the major genetic difference among the four outbreak isolates. High quality single-nucleotide variant (hqSNV) and core genome multilocus sequence typing (cgMLST) clustered these isolates within expanded datasets consisting of additional C. jejuni strains. The number and location of homopolymeric tract regions was identical in all four outbreak isolates but differed from all other C. jejuni examined. Comparative genomics and PCR amplification enabled the identification of large chromosomal inversions of approximately 93 kb and 388 kb within the outbreak isolates associated with transducer-like proteins containing long nucleotide repeat sequences. The 93-kb inversion was characteristic of the outbreak-associated isolates, and the gene content of this inverted region displayed high synteny with the reference strain. Conclusions: The four outbreak isolates were clonally derived and differed mainly in the presence of the CJIE1 prophage, validating earlier findings linking the prophage to phenotypic differences in virulence assays and protein expression. The identification of large, genetically syntenous chromosomal inversions in the genomes of outbreak-associated isolates provided a unique method for discriminating outbreak isolates from the background population. Transducer-like proteins appear to be associated with the chromosomal inversions. CgMLST and hqSNV analysis also effectively delineated the outbreak isolates within the larger C. jejuni population structure. | 3109 | NA | NA | NA | no | no |
| 3110 | 2017 | NA | Ozer, G; Bayraktar, H | 2015 | Intraspecific Variation within Fusarium oxysporum f. sp cumini from Cuminum cyminum in Turkey | Thirty-nine isolates of Fusarium oxysporum f. sp. cumini were isolated from symptomatic tissues of cumin plants from Ankara and Konya provinces of Turkey during 2011 and 2012 years. Species-specific primers produced a 340-bp DNA fragment from F. oxysporum isolates. All isolates tested for pathogenicity on local cultivar under controlled conditions were highly pathogenic to cumin seedlings, showing variation in their aggressiveness. Also, the restriction digestions of PCR-amplified nuclear ribosomal DNA intergenic spacer (IGS) region were used for determining genetic variability within F. oxysporum f. sp. cumini. Five IGS haplotypes were defined within F. oxysporum f. sp. cumini based on restriction analysis of IGS region with the four restriction enzymes: BsuRI, Tru1I, EcoRI, and MspI. UPGMA analysis revealed four major groups at an arbitrary level of 85% similarity. These findings indicate that isolates of F. oxysporum f. sp. cumini are genetically distinct from each other and the presence of different subgroups of the formae speciales cumini in the important cumin production areas of Turkey. (C) 2015 Friends Science Publishers | 3110 | NA | NA | NA | no | no |
| 3111 | 2017 | NA | Rogers, MS; Boyartchuk, V; Rohan, RM; Birsner, AE; Dietrich, WF; D’Amato, RJ | 2012 | The Classical Pink-Eyed Dilution Mutation Affects Angiogenic Responsiveness | Angiogenesis is the process by which new blood vessels are formed from existing vessels. Mammalian populations, including humans and mice, harbor genetic variations that alter angiogenesis. Angiogenesis-regulating gene variants can result in increased susceptibility to multiple angiogenesis-dependent diseases in humans. Our efforts to dissect the complexity of the genetic diversity that regulates angiogenesis have used laboratory animals due to the availability of genome sequence for many species and the ability to perform high volume controlled breeding. Using the murine corneal micropocket assay, we have observed more than ten-fold difference in angiogenic responsiveness among various mouse strains. This degree of difference is observed with either bFGF or VEGF induced corneal neovascularization. Ongoing mapping studies have identified multiple loci that affect angiogenic responsiveness in several mouse models. In this study, we used F2 intercrosses between C57BL/6J and the 129 substrains 129P1/ReJ and 129P3/J, as well as the SJL/J strain, where we have identified new QTLs that affect angiogenic responsiveness. In the case of AngFq5, on chromosome 7, congenic animals were used to confirm the existence of this locus and subcongenic animals, combined with a haplotype-based mapping approach that identified the pink-eyed dilution mutation as a candidate polymorphism to explain AngFq5. The ability of mutations in the pink-eyed dilution gene to affect angiogenic response was demonstrated using the p-J allele at the same locus. Using this allele, we demonstrate that pink-eyed dilution mutations in Oca2 can affect both bFGF and VEGF-induced corneal angiogenesis. | 3111 | NA | NA | NA | no | no |
| 3112 | 2017 | NA | Sochor, M; Vasut, RJ; Bartova, E; Majesky, L; Mracek, J | 2013 | Can gene flow among populations counteract the habitat loss of extremely fragile biotopes? An example from the population genetic structure in Salix daphnoides | Wild river gravel banks (RGB) represent an extremely fragile biotope that is significantly endangered by human activities due to its fragmentation over the past century. The consequences of such processes were studied using the endangered violet willow (Salix daphnoides) at the westernmost foothills of the Carpathian Mountains. We quantified population genetic characteristics for 14 ecologically and demographically characterised populations using simple sequence repeats (SSR) and amplified fragment length polymorphism (AFLP) markers. We found a significant correlation between the biotope, sex, age and genetic structure of the populations. The natural RGB populations revealed high genotypic variability when using SSR markers, in contrast to low genotypic variability of the populations of other biotopes of Cirsium wet meadows (CWM) and Ash-alder forests (AAF) that consisted of one to two clones at each site. High heterozygosity (H (obs) = 0.428-0.532) was similar across all natural (RGB) populations; however, these populations were deficient in heterozygotes (F (IS)/rho (IS) > 0). All RGB populations exhibited moderate to very significant genetic differentiation for microsatellites, despite the fact that the AFLP data showed strong differentiation only between CWM and AAF populations. Division into clusters by Structure confirmed consistent geographic groups for the RGB populations. According to our results, the strong decrease in previously continuous and large natural habitats for the violet willow is slightly counterbalanced by among-population gene flow. However, the survival of natural populations is tightly linked to the presence of river gravel banks and bars. Strict protection of this habitat is therefore essential for the conservation of the species. | 3112 | NA | NA | NA | no | no |
| 3113 | 2017 | NA | Trombetta, B; D’Atanasio, E; Cruciani, F | 2017 | Patterns of Inter-Chromosomal Gene Conversion on the Male-Specific Region of the Human Y Chromosome | The male-specific region of the human Y chromosome (MSY) is characterized by the lack of meiotic recombination and it has long been considered an evolutionary independent region of the human genome. In recent years, however, the idea that human MSY did not have an independent evolutionary history begun to emerge with the discovery that inter-chromosomal gene conversion (ICGC) can modulate the genetic diversity of some portions of this genomic region. Despite the study of the dynamics of this molecular mechanism in humans is still in its infancy, some peculiar features and consequences of it can be summarized. The main effect of ICGC is to increase the allelic diversity of MSY by generating a significant excess of clustered single nucleotide polymorphisms (SNPs) (defined as groups of two or more SNPs occurring in close proximity and on the same branch of the Y phylogeny). On the human MSY, 13 inter-chromosomal gene conversion hotspots (GCHs) have been identified so far, involving donor sequences mainly from the X-chromosome and, to a lesser extent, from autosomes. Most of the GCHs are evolutionary conserved and overlap with regions involved in aberrant X-Y crossing-over. This review mainly focuses on the dynamics and the current knowledge concerning the recombinational landscape of the human MSY in the form of ICGC, on how this molecular mechanism may influence the evolution of the MSY, and on how it could affect the information enclosed within a genomic region which, until recently, appeared to be an evolutionary independent unit. | 3113 | NA | NA | NA | no | no |
| 3114 | 2017 | NA | Lee, JS; Ruell, EW; Boydston, EE; Lyren, LM; Alonso, RS; Troyer, JL; Crooks, KR; VandeWoude, S | 2012 | Gene flow and pathogen transmission among bobcats (Lynx rufus) in a fragmented urban landscape | Urbanization can result in the fragmentation of once contiguous natural landscapes into a patchy habitat interspersed within a growing urban matrix. Animals living in fragmented landscapes often have reduced movement among habitat patches because of avoidance of intervening human development, which potentially leads to both reduced gene flow and pathogen transmission between patches. Mammalian carnivores with large home ranges, such as bobcats (Lynx rufus), may be particularly sensitive to habitat fragmentation. We performed genetic analyses on bobcats and their directly transmitted viral pathogen, feline immunodeficiency virus (FIV), to investigate the effects of urbanization on bobcat movement. We predicted that urban development, including major freeways, would limit bobcat movement and result in genetically structured host and pathogen populations. We analysed molecular markers from 106 bobcats and 19 FIV isolates from seropositive animals in urban southern California. Our findings indicate that reduced gene flow between two primary habitat patches has resulted in genetically distinct bobcat subpopulations separated by urban development including a major highway. However, the distribution of genetic diversity among FIV isolates determined through phylogenetic analyses indicates that pathogen genotypes are less spatially structuredexhibiting a more even distribution between habitat fragments. We conclude that the types of movement and contact sufficient for disease transmission occur with enough frequency to preclude structuring among the viral population, but that the bobcat population is structured owing to low levels of effective bobcat migration resulting in gene flow. We illustrate the utility in using multiple molecular markers that differentially detect movement and gene flow between subpopulations when assessing connectivity. | 3114 | NA | NA | NA | no | no |
| 3115 | 2017 | NA | Telesnicki, MC; Sosa, AJ; Greizerstein, E; Julien, MH | 2011 | Cytogenetic effect of Alternanthera philoxeroides (alligator weed) on Agasicles hygrophila (Coleoptera: Chrysomelidae) in its native range | Plant genotypic effects can alter insects’ ability of utilizing their host plant. Alligator weed, Alternanthera philoxeroides, is a South American amphibious Amaranthaceae, which invades aquatic and terrestrial habitats. Populations with varying morphotypes and cytotypes were identified and further associated with a polyploid hybrids complex in Argentina, which probably is the centre of origin and diversification of alligator weed. The current model of the hybrid complex formation proposes that diploid ancestors gave rise to allotetraploids and allohexaploids. Agasicles hygrophila is a monophagous chrysomelid beetle which successfully controls A. philoxeroides in regions with mild winters and where the weed grows in aquatic habitats. However, plant genetic characteristics could constrain insect development and reproduction, providing an alternative explanation for the restricted success of biological control of alligator weed with A. hygrophila. We investigated the effect of four A. philoxeroides populations with varying cytogenetical characteristics on plant-insect interaction with A. hygrophila in the native range of the weed. The insect’s life cycle on each host population was studied. In addition, the genetic relationships among the weed populations was explored using dot blot analysis. Plant population affected insect survival and fecundity, but not life cycle length nor third larval instar mortality, pupal mortality or female proportion. Plant internode diameter was not a restriction for insect pupation. All hexaploid populations of A. philoxeroides, as well as the close related A. aquatica, hybridized with the tetraploid population probe with similar intensity, meaning that this population is a component of their polyploid genome. (C) 2011 Elsevier Inc. All rights reserved. | 3115 | NA | NA | NA | no | no |
| 3116 | 2017 | NA | Dickson, LB; Campbell, CL; Juneja, P; Jiggins, FM; Sylla, M; Black, WC | 2017 | Exon-Enriched Libraries Reveal Large Genic Differences Between Aedes aegypti from Senegal, West Africa, and Populations Outside Africa | Aedes aegypti is one of the most studied mosquito species, and the principal vector of several arboviruses pathogenic to humans. Recently failure to oviposit, low fecundity, and poor egg-to-adult survival were observed when Ae. aegypti from Senegal (SenAae) West Africa were crossed with Ae. aegypti (Aaa) from outside of Africa, and in SenAae intercrosses. Fluorescent in situ hybridization analyses indicated rearrangements on chromosome 1, and pericentric inversions on chromosomes 2 and 3. Herein, high throughput sequencing (HTS) of exon-enriched libraries was used to compare chromosome-wide genetic diversity among Aaa collections from rural Thailand and Mexico, a sylvatic collection from southeastern Senegal (PK10), and an urban collection from western Senegal (Kaolack). Sex-specific polymorphisms were analyzed in Thailand and PK10 to assess genetic differences between sexes. Expected heterozygosity was greatest in SenAae. F-ST distributions of 15,735 genes among all six pairwise comparisons of the four collections indicated that Mexican and Thailand collections are genetically similar, while F-ST distributions between PK10 and Kaolack were distinct. All four comparisons of SenAae with Aaa indicated extreme differentiation. F-ST was uniform between sexes across all chromosomes in Thailand, but were different, especially on the sex autosome 1, in PK10. These patterns correlate with the reproductive isolation noted earlier. We hypothesize that cryptic Ae. aegypti taxa may exist in West Africa, and the large genic differences between Aaa and SenAae detected in the present study have accumulated over a long period following the evolution of chromosome rearrangements in allopatric populations that subsequently cause reproductive isolation when these populations became sympatric. | 3116 | NA | NA | NA | no | no |
| 3117 | 2017 | NA | Gomes, JP; Bruno, WJ; Nunes, A; Santos, N; Florindo, C; Borrego, MJ; Dean, D | 2007 | Evolution of Chlamydia trachomatis diversity occurs by widespread interstrain recombination involving hotspots | Chlamydia trachomatis is an obligate intracellular bacterium of major public health significance, infecting over one-tenth of the world’s population and causing blindness and infertility in millions. Mounting evidence supports recombination as a key source of genetic diversity among free-living bacteria. Previous research shows that intracellular bacteria such as Chlamydiaceae may also undergo recombination but whether this plays a significant evolutionary role has not been determined. Here, we examine multiple loci dispersed throughout the chromosome to determine the extent and significance of recombination among 19 laboratory reference strains and 10 present-day ocular and urogenital clinical isolates using phylogenetic reconstructions, compatibility matrices, and statistically based recombination programs. Recombination is widespread; all clinical isolates are recombinant at multiple loci with no two belonging to the same clonal lineage. Several reference strains show nonconcordant phylogenies across loci; one strain is unambiguously identified as recombinantly derived from other reference strain lineages. Frequent recombination contrasts with a low level of point substitution; novel substitutions relative to reference strains occur less than one per kilobase. Hotspots for recombination are identified downstream from ompA, which encodes the major outer membrane protein. This widespread recombination, unexpected for an intracellular bacterium, explains why strain-typing using one or two genes, such as ompA, does not correlate with clinical phenotypes. Our results do not point to specific events that are responsible for different pathogenicities but, instead, suggest a new approach to dissect the genetic basis for clinical strain pathology with implications for evolution, host cell adaptation, and emergence of new chlamydial diseases. | 3117 | NA | NA | NA | no | no |
| 3118 | 2017 | NA | Madania, A; Ghoury, I; Al-Ashkar, W; Nweder, S; Zarzour, H | 2014 | Frequency of HLA-A alleles in the Syrian population genotyped by sequence-based typing | HLA-A molecules are highly polymorphic. Their accurate typing at a high-resolution level is crucial for successful organ, bone marrow and cord blood transplantation. Furthermore, several HLA alleles have been involved in susceptibility to autoimmune diseases, allergies, cancers and inflammations. In order to determine common HLA-A alleles in Syria and their frequencies, sequence-based typing (SBT) was used to genotype HLA-A alleles at high resolution (four digit level) among one hundred and thirty randomly selected Syrian individuals. Exons 2, 3 and 4 of the HLA-A gene were amplified by PCR and sequenced. The sbt-engine software was used for allele assignment. Ambiguities were solved using group-specific sequencing primers (GSSPs). We could identify 32 different HLA-A alleles which were divided into 3 groups: high frequency (approximately 10%, A*01:01; A*24:02; A*03:01; A*02:01), moderate frequency (approximately 3%, such as A*02:05, A*31:01 and A*33:01), and low frequency (approximately 1%, such as A*02:11, A*29:01, A*02:02 and A*36:01). Homozygosity rate was higher than expected (11.5% vs. 7.15%). For high frequency alleles, our results show similarity to neighbouring countries. However, 15 alleles (such as A*02:04, A*02:06, A*02:11 and A\*02:17) found in our cohort in low frequencies were never reported in some or all neighbouring countries. This is the first report on HLA-A allele frequencies in Syria. In spite of the relatively low number of tested subjects, our results revealed a high degree of diversity, with 32 different alleles, reflecting the high ethnic heterogeneity of the Syrian population. The identification of alleles rarely or never reported in neighbouring countries indicates a higher genetic diversity in Syria. | 3118 | NA | NA | NA | no | no |
| 3119 | 2017 | NA | Engel, P; Martinson, VG; Moran, NA | 2012 | Functional diversity within the simple gut microbiota of the honey bee | Animals living in social communities typically harbor a characteristic gut microbiota important for nutrition and pathogen defense. Accordingly, in the gut of the honey bee, Apis mellifera, a distinctive microbial community, composed of a taxonomically restricted set of species specific to social bees, has been identified. Despite the ecological and economical importance of honey bees and the increasing concern about population declines, the role of their gut symbionts for colony health and nutrition is unknown. Here, we sequenced the metagenome of the gut microbiota of honey bees. Unexpectedly, we found a remarkable degree of genetic diversity within the few bacterial species colonizing the bee gut. Comparative analysis of gene contents suggests that different species harbor distinct functional capabilities linked to host interaction, biofilm formation, and carbohydrate breakdown. Whereas the former two functions could be critical for pathogen defense and immunity, the latter one might assist nutrient utilization. In a gamma-proteobacterial species, we identified genes encoding pectin-degrading enzymes likely involved in the breakdown of pollen walls. Experimental investigation showed that this activity is restricted to a subset of strains of this species providing evidence for niche specialization. Long-standing association of these gut symbionts with their hosts, favored by the eusocial lifestyle of honey bees, might have promoted the genetic and functional diversification of these bee-specific bacteria. Besides revealing insights into mutualistic functions governed by the microbiota of this important pollinator, our findings indicate that the honey bee can serve as a model for understanding more complex gut-associated microbial communities. | 3119 | NA | NA | NA | no | no |
| 3120 | 2017 | NA | Wang, L; Wu, ZQ; Bystriakova, N; Ansell, SW; Xiang, QP; Heinrichs, J; Schneider, H; Zhang, XC | 2011 | Phylogeography of the Sino-Himalayan Fern Lepisorus clathratus on “The Roof of the World” | Background: The Qinghai-Tibetan Plateau (QTP) and its southern and southeastern mountain ranges, Himalaya-Hengduan Mountains (HHM), are one of the most extensive habitats for alpine plants in the world. How ferns occurring in QTP and HHM changed their distribution ranges in response to Quaternary climatic oscillations remains almost unknown. Methodology and Results: We employed sequences of two chloroplast DNA regions, rps4-trnS and trnL-trnF, to reconstruct phylogeography of the Sino-Himalayan fern Lepisorus clathratus, occurring mainly in the QTP and HHM. Individuals of this species have either dehiscent or indehiscent sporangia with the latter evolved from the plesiomorphic dehiscent forms. Based on a range-wide sampling, we detected 27 cpDNA haplotypes that were divided into five groups by network analyses. Populations in the Hengduan Mountains possess the highest genetic diversity, while a single haplogroup is detected across the north-central region. A distinct phylogeographical subdivision was detected between the Hengduan Mountains and north-central region by AMOVA analysis. The haplogroup distribution pattern, coalescence and AMOVA analysis suggest that a long term survival area (refugia) of the species was located in the Hengduan Mountains during glaciations, with probable range expansions into north-central regions during interglacial periods. Populations with indehiscent sporangium can carry private haplotypes and are inclined to maintain genetic homogeneity. One group with indehiscent sporangia most likely survived in situ on the QTP during glaciations. Conclusions/Significance: This study for the first time sheds light on the response of alpine ferns in the QTP and HHM to the Quaternary climatic oscillations. | 3120 | NA | NA | NA | no | no |
| 3121 | 2017 | NA | Semerikov, VL; Semerikova, SA; Polezhaeva, MA; Kosintsev, PA; Lascoux, M | 2013 | Southern montane populations did not contribute to the recolonization of West Siberian Plain by Siberian larch (Larix sibirica): a range-wide analysis of cytoplasmic markers | While many species were confined to southern latitudes during the last glaciations, there has lately been mounting evidence that some of the most cold-tolerant species were actually able to survive close to the ice sheets. The contribution of these higher latitude outposts to the main recolonization thrust remains, however, untested. In the present study, we use the first range-wide survey of genetic diversity at cytoplasmic markers in Siberian larch (Larix sibirica; four mitochondrial (mt) DNA loci and five chloroplast (cp) DNA SSR loci) to (i) assess the relative contributions of southern and central areas to the current L.sibirica distribution range; and (ii) date the last major population expansion in both L.sibirica and adjacent Larix species. The geographic distribution of cpDNA variation was uninformative, but that of mitotypes clearly indicates that the southernmost populations, located in Mongolia and the Tien-Shan and Sayan Mountain ranges, had a very limited contribution to the current populations of the central and northern parts of the range. It also suggests that the contribution of the high latitude cryptic refugia was geographically limited and that most of the current West Siberian Plain larch populations likely originated in the foothills of the Sayan Mountains. Interestingly, the main population expansion detected through Approximate Bayesian Computation (ABC) in all four larch species investigated here pre-dates the LGM, with a mode in a range of 220000-1340000years BP. Hence, L.sibirica, like other major conifer species of the boreal forest, was strongly affected by climatic events pre-dating the Last Glacial Maximum. | 3121 | NA | NA | NA | no | no |
| 3122 | 2017 | NA | Weigand, MR; Sundin, GW | 2009 | Long-Term Effects of Inducible Mutagenic DNA Repair on Relative Fitness and Phenotypic Diversification in Pseudomonas cichorii 302959 | Mutagenic DNA repair (MDR) employs low-fidelity DNA polymerases capable of replicating past DNA lesions resulting from exposure to high-energy ultraviolet radiation (UVR). MDR confers UVR tolerance and activation initiates a transient mutator phenotype that may provide opportunities for adaptation. To investigate the potential role of MDR in adaptation, we have propagated parallel lineages of the highly mutable epiphytic plant pathogen Pseudomonas cichorii 302959 with daily UVR activation (LAIR lineages) for similar to 500 generations. Here we examine those lineages through the measurement of relative fitness and observation of distinct colony morphotypes that emerged. Isolates and population samples from UVR lineages displayed gains in fitness relative to the ancestor despite increased rates of inducible mutation to rifampicin resistance. Regular activation of MDR resulted ill the maintenance of genetic diversity within UVR lineages, including the reproducible diversification and coexistence of “round” and “fuzzy” colony morphotypes. These results Suggest that inducible mutability may present a reasonable strategy for adaptive evolution in stressful environments by contributing to gains in relative fitness and diversification. | 3122 | NA | NA | NA | no | no |
| 3123 | 2017 | NA | Gonzalez-Soto, T; Gonzalez-Mendoza, D; Troncoso-Rojas, R; Morales-Trejo, A; Cecena-Duran, C; Garcia-Lopez, A; Grimaldo-Juarez, O | 2015 | Molecular identification of Fusarium species isolated from transgenic insect-resistant cotton plants in Mexicali valley, Baja California | Cotton production in the Mexicali valley is adversely affected by wilt and root rot disease associated with Fusarium species. In the present study, we sought to isolate and identify the Fusarium species in the rhizosphere of transgenic insect-resistant cotton plants grown in the Mexicali valley. Our analyses isolated four native fungi from the rhizosphere of cotton plants, namely, T-ICA01, T-ICA03, T-ICA04, and T-ICA08. These fungal isolates were categorized as belonging to Fusarium solani using their phenotypic characteristics and ITS region sequence data. Examination of the infection index showed that T-ICA03 and T-ICA04 caused systemic colonization (90%) of seeds followed by the occurrence of radicle and coleoptile decay. In contrast, T-ICA08 strain was less pathogenic against seed tissues (40%) in comparison to the other strains isolated. Our study showed that in transgenic insect-resistant cotton the disease “Fusarium wilt” is caused by the fungus, F. solani. Future studies are necessary to characterize the F. solani populations to determine whether phenological stages might influence the genetic diversity of the fungal populations present. | 3123 | NA | NA | NA | no | no |
| 3124 | 2017 | NA | Cook, SJ; Norris, DR; Theberge, JB | 1999 | Spatial dynamics of a migratory wolf population in winter, south-central Ontario (1990-1995) | We examined the spatial distribution and movements of migratory wolves (Canis lupus lycaon) to a deer yard located adjacent to Algonquin Provincial Park, Ontario, during 5 winters from 1990 to 1995. Wolves from eastern and central Algonquin Provincial Park followed the annual migration of deer to yards located 13 km outside of the Park boundary. Spatial distributions were determined through mapping of telemetry locations and nearest neighbour analysis. We defined three spacing systems: consistent/high fidelity, clustered/moderate fidelity, and transitional/low fidelity. We found inconsistencies among packs in their adherence to these systems. Data indicate that areas of use changed quickly and tolerance levels among wolves in the deer yard were very high; alien wolves were recorded 163 times in close spatial and (or) temporal proximity. The social behaviour exhibited by this migratory population of wolves has never been recorded in a forested wolf-deer ecosystem. Factors that may contribute to this behavioural plasticity include food abundance, a high degree of genetic relatedness among wolf packs, and high rates of human-caused mortality. | 3124 | NA | NA | NA | no | no |
| 3125 | 2017 | NA | Gianola, D; de los Campos, G; Hill, WG; Manfredi, E; Fernando, R | 2009 | Additive Genetic Variability and the Bayesian Alphabet | The use of all available molecular markers in statistical models for prediction of quantitative traits has led to what: could be termed a genomic-assisted selection paradigm in animal and plant breeding. This article provides a critical review of some theoretical and statistical concepts in the context of genomic-assisted genetic evaluation of animals and crops. First, relationships between the (Bayesian) variance of marker effects in some regression models and additive genetic variance are examined under standard assumptions. Second, the connection between marker genotypes and resemblance between relatives is explored, and linkages between a marker-based model and the infinitesimal model are reviewed. Third, issues associated with the use of Bayesian models for marker-assisted selection, with a focus on the role of the priors, are examined from a theoretical angle. The sensitivity of a Bayesian specification that has been proposed (called “Bayer A”) with respect to priors is illustrated with a simulation. Methods that can solve potential shortcomings of some of these Bayesian regression procedures are discussed briefly. | 3125 | NA | NA | NA | no | no |
| 3126 | 2017 | NA | Bataille, A; Fong, JJ; Cha, M; Wogan, GOU; Baek, HJ; Lee, H; Min, MS; Waldman, B | 2013 | Genetic evidence for a high diversity and wide distribution of endemic strains of the pathogenic chytrid fungus Batrachochytrium dendrobatidis in wild Asian amphibians | Population declines and extinctions of amphibians have been attributed to the chytrid fungus Batrachochytrium dendrobatidis (Bd), especially one globally emerging recombinant lineage (‘Bd-GPL’). We used PCR assays that target the ribosomal internal transcribed spacer region (ITS) of Bd to determine the prevalence and genetic diversity of Bd in South Korea, where Bd is widely distributed but is not known to cause morbidity or mortality in wild populations. We isolated Korean Bd strains from native amphibians with low infection loads and compared them to known worldwide Bd strains using 19 polymorphic SNP and microsatellite loci. Bd prevalence ranged between 12.5 and 48.0%, in 11 of 17 native Korean species, and 24.7% in the introduced bullfrog Lithobates catesbeianus. Based on ITS sequence variation, 47 of the 50 identified Korean haplotypes formed a group closely associated with a native Brazilian Bd lineage, separated from the Bd-GPL lineage. However, multilocus genotyping of three Korean Bd isolates revealed strong divergence from both Bd-GPL and the native Brazilian Bd lineages. Thus, the ITS region resolves genotypes that diverge from Bd-GPL but otherwise generates ambiguous phylogenies. Our results point to the presence of highly diversified endemic strains of Bd across Asian amphibian species. The rarity of Bd-GPL-associated haplotypes suggests that either this lineage was introduced into Korea only recently or Bd-GPL has been outcompeted by native Bd strains. Our results highlight the need to consider possible complex interactions among native Bd lineages, Bd-GPL and their associated amphibian hosts when assessing the spread and impact of Bd-GPL on worldwide amphibian populations. | 3126 | NA | NA | NA | no | no |
| 3127 | 2017 | NA | Hailer, F; Helander, B; Folkestad, AO; Ganusevich, SA; Garstad, S; Hauff, P; Koren, C; Masterov, VB; Nygard, T; Rudnick, JA; Shiraki, S; Skarphedinsson, K; Volke, V; Wille, F; Vila, C | 2007 | Phylogeography of the white-tailed eagle, a generalist with large dispersal capacity | Aim Late Pleistocene glacial changes had a major impact on many boreal and temperate taxa, and this impact can still be detected in the present-day phylogeographic structure of these taxa. However, only minor effects are expected in species with generalist habitat requirements and high dispersal capability. One such species is the white-tailed eagle, Haliaeetus albicilla, and we therefore tested for the expected weak population structure at a continental level in this species. This also allowed us to describe phylogeographic patterns, and to deduce Ice Age refugia and patterns of postglacial recolonization of Eurasia. Location Breeding populations from the easternmost Nearctic (Greenland) and across the Palaearctic (Iceland, continental Europe, central and eastern Asia, and Japan). Methods Sequencing of a 500 base-pair fragment of the mitochondrial DNA control region in 237 samples from throughout the distribution range. Results Our analysis revealed pronounced phylogeographic structure. Overall, low genetic variability was observed across the entire range. Haplotypes clustered in two distinct haplogroups with a predominantly eastern or western distribution, and extensive overlap in Europe. These two major lineages diverged during the late Pleistocene. The eastern haplogroup showed a pattern of rapid population expansion and colonization of Eurasia around the end of the Pleistocene. The western haplogroup had lower diversity and was absent from the populations in eastern Asia. These results suggest survival during the last glaciation in two refugia, probably located in central and western Eurasia, followed by postglacial population expansion and admixture. Relatively high genetic diversity was observed in northern regions that were ice-covered during the last glacial maximum. This, and phylogenetic relationships between haplotypes encountered in the north, indicates substantial population expansion at high latitudes. Areas of glacial meltwater runoff and proglacial lakes could have provided suitable habitats for such population growth. Main conclusions This study shows that glacial climate fluctuations had a substantial impact on white-tailed eagles, both in terms of distribution and demography. These results suggest that even species with large dispersal capabilities and relatively broad habitat requirements were strongly affected by the Pleistocene climatic shifts. | 3127 | NA | NA | NA | no | no |
| 3128 | 2017 | NA | Kautz, S; Ballhorn, DJ; Kroiss, J; Pauls, SU; Moreau, CS; Eilmus, S; Strohm, E; Heil, M | 2012 | Host Plant Use by Competing Acacia-Ants: Mutualists Monopolize While Parasites Share Hosts | Protective ant-plant mutualisms that are exploited by non-defending parasitic ants represent prominent model systems for ecology and evolutionary biology. The mutualist Pseudomyrmex ferrugineus is an obligate plant-ant and fully depends on acacias for nesting space and food. The parasite Pseudomyrmex gracilis facultatively nests on acacias and uses host-derived food rewards but also external food sources. Integrative analyses of genetic microsatellite data, cuticular hydrocarbons and behavioral assays showed that an individual acacia might be inhabited by the workers of several P. gracilis queens, whereas one P. ferrugineus colony monopolizes one or more host trees. Despite these differences in social organization, neither of the species exhibited aggressive behavior among conspecific workers sharing a tree regardless of their relatedness. This lack of aggression corresponds to the high similarity of cuticular hydrocarbon profiles among ants living on the same tree. Host sharing by unrelated colonies, or the presence of several queens in a single colony are discussed as strategies by which parasite colonies could achieve the observed social organization. We argue that in ecological terms, the non-aggressive behavior of non-sibling P. gracilis workers - regardless of the route to achieve this social structure - enables this species to efficiently occupy and exploit a host plant. By contrast, single large and long-lived colonies of the mutualist P. ferrugineus monopolize individual host plants and defend them aggressively against invaders from other trees. Our findings highlight the necessity for using several methods in combination to fully understand how differing life history strategies affect social organization in ants. | 3128 | NA | NA | NA | no | no |
| 3129 | 2017 | NA | Brito, D; Figueiredo, MDL | 2003 | Minimum viable population and conservation status of the Atlantic Forest spiny rat Trinomys eliasi | A population viability analysis (PVA) was conducted to assess the minimum viable population (MVP) of the Atlantic Forest spiny rat Trinomys eliasi, a species threatened by habitat loss and restricted geographical distribution. Objectives were to suggest quasi-extinction thresholds, estimate minimum areas of suitable habitat (MASH) and MVPs, and compare results with the species’ current status. The computer package VORTEX was used. The model predicted sizes of 200 animals to achieve demographic stability, but buffering declines in genetic variability required populations of 2000 animals. Estimated MASHs were approximately 250 and 2500 ha for demographic and genetic stability, respectively. Mortality rate and mean litter size were the most sensitive parameters to changes in model assumptions. The protection of known populations and the search for extant populations are the first steps in conservation. T. eliasi’s issue could help protecting the coastal shrubland ecosystem of Rio de Janeiro state. Observing IUCN’s criteria for listing threatened species, it is suggested that T. eliasi should be ranked as vulnerable in red lists. (C) 2002 Elsevier Science Ltd. All rights reserved. | 3129 | NA | NA | NA | no | no |
| 3130 | 2017 | NA | Gomez-Diaz, E; Gonzalez-Solis, J; Peinado, MA; Page, RDM | 2007 | Lack of host-dependent genetic structure in ectoparasites of Calonectris shearwaters | We compared patterns of mitochondrial DNA (mtDNA) differentiation in three host-specific lice (Halipeurus abnormis, Austromenopon echinatum and Saemundssonia peusi) and one generalist flea (Xenopsylla gratiosa), parasitizing 22 colonies of Cory’s and Cape Verde shearwater (Calonectris). The shearwater hosts show distinct phylogeographic structure corresponding to the three taxa Calonectris d. diomedea, C. d. borealis, and C. edwardsii. The host-specific lice appeared undifferentiated among the three Calonectris taxa, whereas the more generalist flea displayed significant levels of population differentiation. Neither genetic distances among host populations, nor their spatial distribution explained the patterns of genetic variability observed in the ectoparasites. The lack of differentiation among lice is unexpected, given that previous work has found evidence of cospeciation between procellariiform seabirds and their lice, and lice typically have an elevated rate of mtDNA evolution with respect to their hosts. Our results suggest that either rates of evolution in seabird lice are not always as high as previously thought, or that the magnitude of movement of lice between seabird hosts has been substantially underestimated. | 3130 | NA | NA | NA | no | no |
| 3131 | 2017 | NA | Sielezniew, M; Rutkowski, R; Ponikwicka-Tyszko, D; Ratkiewicz, M; Dziekanska, I; Svitra, G | 2012 | Differences in genetic variability between two ecotypes of the endangered myrmecophilous butterfly Phengaris (=Maculinea) alcon- the setting of conservation priorities | 0 | 3131 | NA | NA | NA | no | no |
| 3132 | 2017 | NA | Miller, MT; Antos, JA; Allen, GA | 2007 | Demographic differences between two sympatric lilies (Calochortus) with contrasting distributions, as revealed by matrix analysis | Related species of similar morphology can differ greatly in distribution and abundance. Elucidating reasons for such differences can contribute to an understanding of intrinsic limiting factors and the causes of rarity. We studied sympatric populations of two terrestrial lilies with contrasting distributions: Calochortus lyallii, which is geographically restricted but locally abundant, and C. macrocarpus, which is widespread but locally sparse. Marked plants of each species were monitored for 5 years in British Columbia, Canada. Matrix projection models were used to estimate annual and stochastic population growth rates (lambda and lambda(s)) and to compare demographic traits. Annual lambda-values ranged from 0.89 to 1.04 in C. lyallii and from 0.89 to 1.01 in C. macrocarpus. Stochastic projections yielded a long-term growth rate near 1 for C. lyallii, but indicated a decline for C. macrocarpus. Elasticity analysis indicated that over the 5-year period of the study, survival of flowering plants made a larger proportional contribution to lambda in C. lyallii than in C. macrocarpus. LTRE analysis showed that temporal variation in lambda was driven primarily by the dynamics of flowering individuals in C. lyallii, and by the dynamics of vegetative individuals in C. macrocarpus. Similarly, higher flowering rates in C. lyallii and greater vegetative stasis in C. macrocarpus made the largest contribution to the difference in lambda between species. Thus, local persistence in these two morphologically similar species appears to be achieved via different demographic pathways. Our analyses show that extrapolations about demographic processes and population dynamics based on taxonomic relatedness, morphological similarity or habitat overlap may often not be justified. | 3132 | NA | NA | NA | no | no |
| 3133 | 2017 | NA | Valadas, V; Laranjo, M; Mota, M; Oliveira, S | 2013 | Molecular characterization of Portuguese populations of the pinewood nematode Bursaphelenchus xylophilus using cytochrome b and cellulase genes | Bursaphelenchus xylophilus is the causal agent of pine wilt disease and a worldwide pest with high economic impact. Since its first diagnosis in Portugal in 1999, it has been subjected to quarantine measures with impact on forest health and ecosystem stability, significantly affecting international trade of wood products. The disease was detected in the north and centre of continental Portugal and, since 2008, the whole country has been considered an affected area. Recently, it was detected in Madeira Island. In order to avoid new outbreaks, it has become of major importance to understand the patterns of spread, introduction points and to characterize the new populations from continental Portugal and Madeira Island. Mitochondrial cytochrome b (cytb) and parasitic cellulase gene sequences were used to evaluate the genetic relationships among isolates that could indicate possible origins of the new outbreaks. Portuguese isolates were compared with isolates from USA, China, Japan and South Korea, in order to investigate possible infection pathways and disease spread patterns in Portugal. Phylogenetic trees based on both genes show that Portuguese isolates group with Asian isolates. Isolates from USA are in a separate position in both gene trees. However, the phylogenetic tree based on the cellulase gene sequences shows higher differentiation among Portuguese isolates than that of cytb. These results agree with those previously obtained using inter-simple sequence repeats (ISSR). This was the first study to use cytb and cellulase genes to characterize pinewood nematode (PWN) populations. This study suggests that cellulase is a better marker than cytb to study genetic diversity in B. xylophilus. | 3133 | NA | NA | NA | no | no |
| 3134 | 2017 | NA | Pimper, LE; Baker, CS; Goodall, RNP; Olavarria, C; Remis, MI | 2010 | Mitochondrial DNA variation and population structure of Commerson’s dolphins (Cephalorhynchus commersonii) in their southernmost distribution | The Commerson’s dolphin, Cephalorhynchus commersonii, is found in shallow waters of the continental shelf off the eastern coast of South America between 40A degrees S and 56A degrees S. This species is taken incidentally in artisanal gillnet fisheries, especially along the shallow coastline of northern Tierra del Fuego and southern Patagonia. The biological importance of by-catch is likely to be underestimated if the boundaries of subpopulations are not properly defined. Here, we report on the sequence variation of the mitochondrial DNA control region of the Commerson’s dolphin from five areas defined in Tierra del Fuego, Argentina and Chile, to provide a preliminary assessment of population structure where conservation efforts are most needed. A 466 bp fragment of the mitochondrial DNA control region was sequenced from 196 samples of skin, teeth and bone, defining 20 haplotypes from 17 polymorphic sites. Nucleotide (pi = 0.40%) and haplotype (h = 0.807) diversity were low compared to some other odontocete populations, but similar to that of other species of this genus. Genetic differentiation evaluated through analyses of molecular variance (AMOVA) showed significant overall differences among areas within Tierra del Fuego (I broken vertical bar (ST) = 0.059, P < 0.01). An analysis of sex-specific population structure suggested that the dispersal rates of both females and males are low, indicative of females displaying greater site fidelity. The results from mtDNA control region sequences alone revealed significant differentiation among studied areas, which should be considered as independent management units. We recommend that the impact of localized gillnet mortalities should be managed on a local scale in these areas of Tierra del Fuego. | 3134 | NA | NA | NA | no | no |
| 3135 | 2017 | NA | Nielsen, JL; Fountain, MC; Favela, JC; Cobble, K; Jensen, BL | 1998 | Oncorhynchus at the southern extent of their range: a study of mtDNA control-region sequence with special reference to an undescribed subspecies of O-mykiss from Mexico | Nucleotide sequences from the right-domain of salmonid mitochondrial DNA (mtDNA) control region flanking the phenylalanine tRNA gene (tRNA(Phe)) were determined for 5 species and 14 subspecies of Oncorhynchus at the southern extent of their range. In all but one population, the right domain contained two 72 bp tandem repeats located between the tRNA(Phe) gene and the conserved sequence block CSB-3. At the species level we found 46-83% homology between these two repeats. The repeat closest to tRNA(Phe) contained 43% of the phylogenetically informative sites. The largest number of transversions (N = 6) were found outside of both repeat sequences. Phylogenetic inference based on mtDNA right-domain sequence was congruent with other analyses at the species level, but gave variable results in association drawn at the subspecific level. The right-domain contained three highly conserved sequences corresponding to the mtDNA transcription factor, and the heavy-and light-strand promoters. In four O. mykiss from Rio Yaqui, Mexico, the repeat containing the heavy-strand promoter (HSP) was deleted. The mtDNA HSP is responsible for transcription of the 16S and 12S rRNAs, 12 of the 13 protein-coding genes, and most of the tRNA genes. Rio Yagui trout showed no heteroplasmy or tissue specificity for this deletion. This HSP deletion suggests the possibility of a primitive transcriptional promoter with bidirectional capacity in this species, similar to that reported in avian and amphibian mtDNA. These data expand our understanding of genetic diversity in Oncorhynchus at the southern extent of their range with a knowledge of the evolutionary mechanisms that may have led to that diversity. | 3135 | NA | NA | NA | no | no |
| 3136 | 2017 | NA | Xiao, J; Moon, Y; Li, LH; Rustchenko, E; Wakabayashi, H; Zhao, XY; Feng, CY; Gill, SR; McLaren, S; Malmstrom, H; Ren, YF; Quivey, R; Koo, H; Kopycka-Kedzierawski, DT | 2016 | Candida albicans Carriage in Children with Severe Early Childhood Caries (S-ECC) and Maternal Relatedness | Introduction Candida albicans has been detected together with Streptococcus mutans in high numbers in plaque-biofilm from children with early childhood caries (ECC). The goal of this study was to examine the C. albicans carriage in children with severe early childhood caries (SECC) and the maternal relatedness. Methods Subjects in this pilot cross-sectional study were recruited based on a convenient sample. DMFT(S)/dmft(s) caries and plaque scores were assessed during a comprehensive oral exam. Social-demographic and related background information was collected through a questionnaire. Saliva and plaque sample from all children and mother subjects were collected. C. albicans were isolated by BBL (TM) CHROMagar (TM) and also identified using germ tube test. S. mutans was isolated using Mitis Salivarius with Bacitracin selective medium and identified by colony morphology. Genetic relatedness was examined using restriction endonuclease analysis of the C. albicans genome using BssHII (REAG-B). Multilocus sequence typing was used to examine the clustering information of isolated C. albicans. Spot assay was performed to examine the C. albicans Caspofungin susceptibility between S-ECC children and their mothers. All statistical analyses (power analysis for sample size, Spearman’s correlation coefficient and multiple regression analyses) were implemented with SAS 9.4 Results A total of 18 S-ECC child-mother pairs and 17 caries free child-mother pairs were enrolled in the study. Results indicated high C. albicans carriage rate in the oral cavity (saliva and plaque) of both S-ECC children and their mothers (> 80%). Spearman’s correlation coefficient also indicated a significant correlation between salivary and plaque C. albicans and S. mutans carriage (p< 0.01) and caries severity (p< 0.05). The levels of C. albicans in the prepared saliva and plaque sample (1ml resuspension) of S-ECC children were 1.3 +/- 4.5 x10(4) cfu/ml and 1.2 +/- 3.5 x10(4) cfu/ml (similar to 3-log higher vs. caries-free children). Among 18 child-mother pairs, > 60% of them demonstrated identical C. albicans REAG-B pattern. C. albicans isolated from > 65% of child-mother pairs demonstrated similar susceptibility to caspofungin in spot assay, while no caspofungin resistant strains were seen when compared with C. albicans wild-type strain SC5314. Interestingly, the regression analysis showed that factors such as antibiotic usage, birth weight, inhaler use, brushing frequency, and daycare attendance had no significant effect on the oral carriage of C. albicans in the S-ECC children. Conclusions Our results reveal that both the child with S-ECC and the mother were highly infected with C. albicans, while most of the strains were genetically related, suggesting that the mother might be a source for C. albicans acquisition in the oral cavity of children affected by the disease. | 3136 | NA | NA | NA | no | no |
| 3137 | 2017 | NA | van Driel, MA; Bruggeman, J; Vriend, G; Brunner, HG; Leunissen, JA | 2006 | A text-mining analysis of the human phenome | A number of large-scale efforts are underway to define the relationships between genes and proteins in various species. But, few attempts have been made to systematically classify all such relationships at the phenotype level. Also, it is unknown whether such a phenotype map would carry biologically meaningful information. We have used text mining to classify over 5000 human phenotypes contained in the Online Mendelian Inheritance in Man database. We find that similarity between phenotypes reflects biological modules of interacting functionally related genes. These similarities are positively correlated with a number of measures of gene function, including relatedness at the level of protein sequence, protein motifs, functional annotation, and direct protein-protein interaction. Phenotype grouping reflects the modular nature of human disease genetics. Thus, phenotype mapping may be used to predict candidate genes for diseases as well as functional relations between genes and proteins. Such predictions will further improve if a unified system of phenotype descriptors is developed. The phenotype similarity data are accessible through a web interface at http://www.cmbi.ru.nl/MimMiner/. | 3137 | NA | NA | NA | no | no |
| 3138 | 2017 | NA | Wakano, JY; Lehmann, L | 2014 | Evolutionary branching in deme-structured populations | Adaptive dynamics shows that a continuous trait under frequency dependent selection may first converge to a singular point followed by spontaneous transition from a unimodal trait distribution into a bimodal one, which is called “evolutionary branching”. Here, we study evolutionary branching in a deme-structured. population by constructing a quantitative genetic model for the trait variance dynamics, which allows us to obtain an analytic condition for evolutionary branching. This is first shown to agree With previous conditions for branching expressed in terms of relatedness between interacting individuals within demes and obtained from mutant-resident systems. We then show this branching condition can be markedly simplified when the evolving trait affect fecundity and/or survival, as opposed to affecting population structure, which would occur in the case of the evolution of dispersal. As an application of our model, we evaluate the threshold migration rate below which evolutionary branching cannot occur in a p. airwise interaction game. This agrees very well with the individual-based simulation results. (C) 2014 Elsevier Ltd. All rights reserved. | 3138 | NA | NA | NA | no | no |
| 3139 | 2017 | NA | Khan, MI; Khan, MA; Ma, HX; Khattak, GSS; Khan, AJ; Mhhammad, T | 2011 | Selection of parents for crossing based on genotyping and phenotyping for stripe rust (Puccinia striiformis) resistance and agronomic traits in bread wheat breeding | Bread wheat (Triticum aestivum L.) germplasm consisting of 45 genotypes were clustered phenotypically using ten morphological traits and Area Under Disease Progress Curve (AUDPC) as measure of stripe rust resistance. The clustering was ratified by using twenty three molecular markers (SSR, EST and STS) linked to stripe rust (Puccinia striiformis f. sp. tritici) resistant QTLs. The aim was to asses the extent of genetic variability among the genotypes in order to select the parents for crossing between the resistant and susceptible genotypes with respect to stripe rust. The Euclidian dissimilarity values resulted from phenotypic data regarding morphological traits and AUDPC were used to construct a dendrogram for clustering the accessions. Using un-weighted pair group method with arithmetic means, another dendrogram resulted from the similarity coefficient values was used to distinguish the genotypes with respect to stripe rust. Clustering based on phenotypic data produced two major groups and five clusters (with Euclidian dissimilarity ranging from 2.44 to 16.16) whereas genotypic data yielded two major groups and four clusters (with percent similarity coefficient values ranging from 0.1 to 46.0) to separate the gene pool into highly resistant, resistant, moderately resistant, moderately susceptible and susceptible genotypes. With few exceptions, the outcome of both type of clustering was almost similar and resistant as well as susceptible genotypes came in the same clusters of molecular genotyping as yielded by phenotypic clustering. As a result seven genotypes (Bakhtawar-92, Frontana, Saleem 2000, Tatara, Inqilab-91, Fakhre Sarhad and Karwan) of diverse genetic background were selected for pyramiding stripe rust lesistant genes as well as some other agronomic traits after hybridization. | 3139 | NA | NA | NA | no | no |
| 3140 | 2017 | NA | Blakeslee, AMH; Byers, JE; Lesser, MP | 2008 | Solving cryptogenic histories using host and parasite molecular genetics: the resolution of Littorina littorea’s North American origin | Even after decades of investigation using multiple sources of evidence, the natural histories of some species remain unclear (i.e. cryptogenic). A key example is Littorina littorea, the most abundant intertidal snail in northeastern North America. Native to Europe, the snail’s ecological history in North America has been debated for over 100 years with no definitive resolution. To resolve its cryptogenic status, we used molecular genetics from a novel combination of the snail and a highly associated trematode parasite, Cryptocotyle lingua. Based on mitochondrial sequences of 370 L. littorea and 196 C. lingua individuals, our results demonstrate a significant reduction in genetic diversity in North America vs. Europe, North American haplotypes nested within European haplotypes, and mean divergence estimates of similar to 500 years ago from Europe for both host and parasite - thus supporting a recent introduction of both host and parasite to North America from Europe. Our study therefore resolves not only a specific cryptogenic history, but it also demonstrates the success of our approach generally and could be used in resolving difficult invasion histories worldwide. | 3140 | NA | NA | NA | no | no |
| 3141 | 2017 | NA | Liu, MM; Xing, YM; Zeng, X; Zhang, DW; Guo, SX | 2015 | Genetic diversity of Armillaria spp. symbiotic with Polyporus umbellatus in China | The growth and development of Polyporus umbellatus sclerotia is dependt on a symbiotic relationship with Armillaria, a species of forest pathogenic fungus. Our aim was to identify the Armillaria spp. that are symbiotic with Polyporus umbellatus in China. Samples of Armillaria spp. were isolated from Polyporus umbellatus from 31 districts among 13 provinces in China. Armillaria spp. were identified using DNA-based methods targeting the IGS region of ribosomal DNA. Genotypes were differentiated by the Inter-simple sequence repeat (ISSR) analysis. The results showed that Armillaria gallica and Armillaria cepistipes were the species most frequently observed to be symbiotic with P. umbellatus in China. Two dendrograms were generated based on the similarity matrix by the Unweighted Pair Group Method with Arithmetic Mean (UPGMA), wherein the genotypes of A. gallica were grouped into four clusters and the genotypes of A. cepistipes were grouped into three clusters. This study showed that there is genetic diversity among the genotypes of A. cepistipes and A. gallica that are symbiotic with P. umbellatus. (C) 2015 Elsevier Ltd. All rights reserved. | 3141 | NA | NA | NA | no | no |
| 3142 | 2017 | NA | Subramaniam, R; White, SN; Herrmann-Hoesing, LM; Srikumaran, S | 2012 | MHC class II DR allelic diversity in bighorn sheep | We hypothesized that decreased diversity and/or unique polymorphisms in MHC class II alleles of bighorn sheep (BHS, Ovis canadensis) are responsible for lower titer of antibodies against Mannheimia haemolytica leukotoxin, in comparison to domestic sheep (DS. Ovis aries). To test this hypothesis, DRA and DRB transcripts from 24 captive BHS (Ovca-DRA and Ovca-DRB) were sequenced. Based on exon 2 (beta 1 domain) sequences, eight different Ovca-DRB cDNA sequences were identified in BHS. Six of them were 100% identical to previously reported Ovca-DRB genomic DNA sequences. The new alleles DRB*23 and DRB*24, were closely related to two other Ovca-DRB exon 2 genomic DNA sequences. Nineteen out of 24 BHS (79%) Ovca-DRB exon 3 (132 domain) sequences were 100% identical to exon 3 sequence of DRB1 of DS (Ovar-DRB1). Ovca-DRA full length cDNA sequences exhibited >99% identity. Based upon exon 2 sequences, this BHS herd yielded higher Ovca-DRB allelic diversity than that reported in the previous study. Positively selected amino acid positions were identified in the peptide-binding groove of BHS and DS, but BHS showed more such sites. This highlights differing population histories, and may suggest differing needs for DR peptide-binding specificities. Presence of glutamine at position 52 (52Q) in some of the desert and captive BHS is predicted to alter the efficiency of DR dimerization, which may influence antigen presentation and T-h cell activation. Functional assays with unique alleles should reveal whether the presentation of M. haemolytica leukotoxin peptides to T-h cells by Ovca-DRB alleles is equivalent to that of Ovar-DRB1 alleles. (C) 2012 Published by Elsevier B.V. | 3142 | NA | NA | NA | no | no |
| 3143 | 2017 | NA | Uhlemann, AC; Dordel, J; Knox, JR; Raven, KE; Parkhill, J; Holden, MTG; Peacock, SJ; Lowy, FD | 2014 | Molecular tracing of the emergence, diversification, and transmission of S. aureus sequence type 8 in a New York community | During the last 2 decades, community-associated methicillin-resistant Staphylococcus aureus (CA-MRSA) strains have dramatically increased the global burden of S. aureus infections. The pandemic sequence type (ST)8/pulsed-field gel type USA300 is the dominant CA-MRSA clone in the United States, but its evolutionary history and basis for biological success are incompletely understood. Here, we use whole-genome sequencing of 387 ST8 isolates drawn from an epidemiological network of CA-MRSA infections and colonizations in northern Manhattan to explore short-term evolution and transmission patterns. Phylogenetic analysis predicted that USA300 diverged from a most common recent ancestor around 1993. We found evidence for multiple introductions of USA300 and reconstructed the phylogeographic spread of isolates across neighborhoods. Using pair-wise single-nucleotide polymorphism distances as a measure of genetic relatedness between isolates, we observed that most USA300 isolates had become endemic in households, indicating their critical role as reservoirs for transmission and diversification. Using the maximum single-nucleotide polymorphism variability of isolates from within households as a threshold, we identified several possible transmission networks beyond households. Our study also revealed the evolution of a fluoroquinolone- resistant subpopulation in the mid-1990s and its subsequent expansion at a time of high-frequency outpatient antibiotic use. This high-resolution phylogenetic analysis of ST8 has documented the genomic changes associated with USA300 evolution and how some of its recent evolution has been shaped by antibiotic use. By integrating whole-genome sequencing with detailed epidemiological analyses, our study provides an important framework for delineating the full diversity and spread of USA300 and other emerging pathogens in large urban community populations. | 3143 | NA | NA | NA | no | no |
| 3144 | 2017 | NA | Wei, WH; Hemani, G; Gyenesei, A; Vitart, V; Navarro, P; Hayward, C; Cabrera, CP; Huffman, JE; Knott, SA; Hicks, AA; Rudan, I; Pramstaller, PP; Wild, SH; Wilson, JF; Campbell, H; Hastie, ND; Wright, AF; Haley, CS | 2012 | Genome-wide analysis of epistasis in body mass index using multiple human populations | We surveyed gene-gene interactions (epistasis) in human body mass index (BMI) in four European populations (n < 1200) via exhaustive pair-wise genome scans where interactions were computed as F ratios by testing a linear regression model fitting two single-nucleotide polymorphisms (SNPs) with interactions against the one without. Before the association tests, BMI was corrected for sex and age, normalised and adjusted for relatedness. Neither single SNPs nor SNP interactions were genome-wide significant in either cohort based on the consensus threshold (P=5.0E-08) and a Bonferroni corrected threshold (P=1.1E-12), respectively. Next we compared sub genome-wide significant SNP interactions (P < 5.0E-08) across cohorts to identify common epistatic signals, where SNPs were annotated to genes to test for gene ontology (GO) enrichment. Among the epistatic genes contributing to the commonly enriched GO terms, 19 were shared across study cohorts of which 15 are previously published genome-wide association loci, including CDH13 (cadherin 13) associated with height and SORCS2 (sortilin-related VPS10 domain containing receptor 2) associated with circulating insulin-like growth factor 1 and binding protein 3. Interactions between the 19 shared epistatic genes and those involving BMI candidate loci (P < 5.0E-08) were tested across cohorts and found eight replicated at the SNP level (P < 0.05) in at least one cohort, which were further tested and showed limited replication in a separate European population (n > 5000). We conclude that genome-wide analysis of epistasis in multiple populations is an effective approach to provide new insights into the genetic regulation of BMI but requires additional efforts to confirm the findings. European Journal of Human Genetics (2012) 20, 857-862; doi:10.1038/ejhg.2012.17; published online 15 February 2012 | 3144 | NA | NA | NA | no | no |
| 3145 | 2017 | NA | Ruan, CJ; Li, H; Mopper, S | 2009 | Characterization and identification of ISSR markers associated with resistance to dried-shrink disease in sea buckthorn | Sea buckthorn (Hippophae L.) is an economically important, globally distributed shrub with unique biochemical properties. This long-juvenile and long-lived woody plant can be more difficult to cultivate than other crop plants. Dried-shrink disease is a dangerous pathogen that destroys sea buckthorn and halts commercial production. We estimated variability of ISSR molecular markers in 52 accessions of 16 sea buckthorn varieties and lines to seek markers associated with dried-shrink resistance and help identify potential breeding varieties and lines. Fifteen ISSR primers generated 346 bands, with an average of 23 bands per primer. Genetic similarities (Jaccard coefficient) between pairs of accessions ranged from 0.216 to 0.781. At a Jaccard coefficient of 0.39, the dendrogram generated with 342 polymorphic bands clustered 51 accessions of H. rhamnoides into five groups and one H. salicifolia isolate. Four ISSR markers (887(190), 835(700), 809(290), and 811(280)) were significantly correlated with resistance to dried-shrink disease (P < 0.001). These markers provide a potential method for breeding programs that select lineages resistant to dried-shrink disease, especially when no other genetic information such as linkage maps and Quantitative Trait Loci are available. | 3145 | NA | NA | NA | no | no |
| 3146 | 2017 | NA | Goyeau, H; Halkett, F; Zapater, MF; Carlier, J; Lannou, C | 2007 | Clonality and host selection in the wheat pathogenic fungus Puccinia triticina | Clonal reproduction in Puccinia triticina, the cause of wheat leaf rust, has long been hypothesized but has never been demonstrated. Using a population genetics approach and microsatellite markers, we analysed genetic diversity of this fungus at each level of genome organisation. Sampling included isolates from two field populations growing on two cultivars carrying specific resistance genes, completed with isolates representing the main pathotypes identified from a national survey. For the two cultivars, populations differentiated according to the distribution of their genotypes and pathotypes. There was a high proportion of repeated genotypes, combined with a significant linkage disequilibrium and a strong negative value for FIS. These three factors, especially heterozygote excess, strongly support the hypothesis of a high rate of clonal reproduction. Each pathotype matched a unique multilocus genotype, except for a few isolates, which were taken to be mutants of the dominant genotype. We discussed the strong relationship between pathotypes and genotypes as the consequence of clonal reproduction combined with a strong selection exerted by host cultivars. (c) 2007 Elsevier Inc. All rights reserved. | 3146 | NA | NA | NA | no | no |
| 3147 | 2017 | NA | Giraud, T | 2004 | Patterns of within population dispersal and mating of the fungus Microbotryum violaceum parasitising the plant Silene latifolia | This study explores the patterns of dispersal and mating of the anther smut Microbotryum violaceum, a model species in genetics and evolutionary biology. A French metapopulation of the fungus collected from its caryophyllaceous host Silene latifolia was analysed using microsatellites. The genetic diversity was low, populations were strongly differentiated, and there was no pattern of isolation by distance among populations. There was a strong deficit in heterozygotes, confirming the high self-fertilisation rates suggested by previous studies. Within populations there was a strong pattern of isolation by distance, with identical genotypes being highly clustered. This indicates that fungal spores are dispersed mostly between adjacent plants, and such local dispersal is important for understanding the dynamics and evolution of this disease. Local clusters of identical heterozygous genotypes did not contain significantly fewer individuals than did clusters of homozygous genotypes. As selfing between products of independent meiotic events (intertetrad selfing) rapidly reduces heterozygosity, this suggests that intratetrad matings are frequent, which helps to explain the puzzling maintenance of a sex-ratio distortion in M. violaceum. | 3147 | NA | NA | NA | no | no |
| 3148 | 2017 | NA | Cockram, J; White, J; Zuluaga, DL; Smith, D; Comadran, J; Macaulay, M; Luo, ZW; Kearsey, MJ; Werner, P; Harrap, D; Tapsell, C; Liu, H; Hedley, PE; Stein, N; Schulte, D; Steuernagel, B; Marshall, DF; Thomas, WTB; Ramsay, L; Mackay, I; Balding, DJ; Waugh, R; O’Sullivan, DM | 2010 | Genome-wide association mapping to candidate polymorphism resolution in the unsequenced barley genome | Although commonplace in human disease genetics, genome-wide association (GWA) studies have only relatively recently been applied to plants. Using 32 phenotypes in the inbreeding crop barley, we report GWA mapping of 15 morphological traits across similar to 500 cultivars genotyped with 1,536 SNPs. In contrast to the majority of human GWA studies, we observe high levels of linkage disequilibrium within and between chromosomes. Despite this, GWA analysis readily detected common alleles of high penetrance. To investigate the potential of combining GWA mapping with comparative analysis to resolve traits to candidate polymorphism level in unsequenced genomes, we fine-mapped a selected phenotype (anthocyanin pigmentation) within a 140-kb interval containing three genes. Of these, resequencing the putative anthocyanin pathway gene HvbHLH1 identified a deletion resulting in a premature stop codon upstream of the basic helix-loop-helix domain, which was diagnostic for lack of anthocyanin in our association and biparental mapping populations. The methodology described here is transferable to species with limited genomic resources, providing a paradigm for reducing the threshold of map-based cloning in unsequenced crops. | 3148 | NA | NA | NA | no | no |
| 3149 | 2017 | NA | Jaratlerdsiri, W; Isberg, SR; Higgins, DP; Gongora, J | 2012 | MHC class I of saltwater crocodiles (Crocodylus porosus): polymorphism and balancing selection | Saltwater crocodiles are in high demand for the production of luxury fashion items. However, their susceptibility to disease incurs substantial losses and it is hoped to be able to genetically select these animals for disease resistance. So far, this has only been enabled by phenotypic selection. Investigating the major histocompatibility complex (MHC) could provide insight into the ability of an individual to respond to pathogens acting as a selective pressure on the host. Here, we assessed genetic diversity and a role of selection in shaping the diversity of MHC class I exon 3 among 42 saltwater crocodiles from nine river basins in the Northern Territory, Australia. We generated 640 sequences using cloning and sequencing methods and identified 43 MHC variants among them. Phylogenetic analyses clustered these variants into two major clades, which may suggest two gene lineages. We found the number of variants within an individual varying between one and seven, indicating that there are at least four gene loci in this species. Selection detection analyses revealed an elevated ratio of nonsynonymous to synonymous substitutions (mean = 1.152 per codon), suggesting balancing selection. Population differentiation analyses revealed that the MHC did not show structuring among the river basins, and there were some shared variants among them. This may be a result of possible gene flow and/or similar selection pressures among populations. These findings provide background knowledge to identify potential MHC markers, which could be used for selecting genetically variable individuals for future disease associations. All MHC class I exon 3 sequences reported in this paper were submitted to the GenBank database with following accession numbers: HQ008785-HQ008789, HQ008791-HQ008798, HQ008808-HQ008815, HQ008824, HQ008826-HQ008830, HQ008835, HQ008839, HQ008842-HQ008850, and JX023536-JX023540. | 3149 | NA | NA | NA | no | no |
| 3150 | 2017 | NA | Rosivall, B; Szollosi, E; Hasselquist, D; Torok, J | 2009 | Effects of extrapair paternity and sex on nestling growth and condition in the collared flycatcher, Ficedula albicollis | Extrapair fertilizations (EPFs) have been found in most socially monogamous passerine species. EPFs are generally beneficial for males because they increase the number of sired offspring. The benefit accrued by females, however, is less obvious. Maternal benefits may involve fertility insurance, increased genetic variability of the offspring, and improved offspring quality via compatible genes or ‘good genes’. In a Hungarian population of the collared flycatcher, we investigated whether the occurrence of extrapair young (EPY) in a brood could be predicted by the traits of the females or their social mates, and whether EPY were superior to their half sibs in terms of growth and. edging condition. We found that 55.7% of the broods contained EPY. The females’ participation in extrapair copulations (EPCs) was not related to any of the characteristics of their mates (body size, condition, wing and forehead patch size). The EPY did not differ from their half sibs in any measures of offspring quality. The half sibs had similar embryonic and postembryonic growth and. edged with similar body condition. Female body size was related to extrapair paternity: larger females were less likely to produce mixed-paternity broods. This suggests that the role of female traits in EPCs deserves more attention. We also found that male nestlings grew faster than females, although females could catch up by the time of. edging, so we argue that sex-dependent development should be taken into account in studies using nestling growth as a measure of nestling quality. (C) 2008 The Association for the Study of Animal Behaviour. Published by Elsevier Ltd. All rights reserved. | 3150 | NA | NA | NA | no | no |
| 3151 | 2017 | NA | Alam, MM; Khurshid, A; Shaukat, S; Suleman, RM; Sharif, S; Angez, M; Malik, SA; Ahmed, TM; Aamir, UB; Naeem, M; Zaidi, SSZ | 2013 | Epidemiology and Genetic Diversity of Rotavirus Strains in Children with Acute Gastroenteritis in Lahore, Pakistan | Pakistan harbors high disease burden of gastro-enteric infections with majority of these caused by rotavirus. Unfortunately, lack of proper surveillance programs and laboratory facilities have resulted in scarcity of available data on rotavirus associated disease burden and epidemiological information in the country. We investigated 1306 stool samples collected over two years (2008-2009) from hospitalized children under 5 years of age for the presence of rotavirus strains and its genotypic diversity in Lahore. The prevalence rate during 2008 and 2009 was found to be 34% (n = 447 out of 1306). No significant difference was found between different age groups positive for rotavirus (p>0.05). A subset of EIA positive samples was further screened for rotavirus RNA through RT-PCR and 44 (49.43%) samples, out of total 89 EIA positive samples, were found positive. G and P type prevalence was found as follows: G1P [4] = 3(6.81%); G1P [6] = 9(20.45%); G1P [8] = 1(2.27%); G2P [4] = 21(47.72%); G2P [8] = 1(2.27%); G9P [4] = 1(2.27%); G9P [6] = 1(2.27%) and G9P [8] = 7(15.90%). Phylogenetic analysis revealed that the VP7 and VP4 sequences clustered closely with the previously detected strains in the country as well as Belgian rotaviruses. Antigenic characterization was performed by analyzing major epitopes in the immunodominant VP7 and VP4 gene segments. Although the neutralization conferring motifs were found variable between the Pakistani strains and the two recommended vaccines strains (Rotarix (TM) and RotaTeq (TM)), we validate the use of rotavirus vaccine in Pakistan based on the proven and recognized vaccine efficacy across the globe. Our findings constitute the first report on rotavirus’ genotype diversity, their phylogenetic relatedness and epidemiology during the pre-vaccination era in Lahore, Pakistan and support the immediate introduction of rotavirus vaccine in the routine immunization program of the country. | 3151 | NA | NA | NA | no | no |
| 3152 | 2017 | NA | Ding, J; Cheng, HL; Jin, XQ; Araki, H; Yang, YH; Tian, DC | 2007 | Contrasting patterns of evolution between allelic groups at a single locus in Arabidopsis | Heterogeneities in evolutionary pattern among different loci are commonly observed. To see whether the heterogeneity can also be observed among allelic groups in a single locus, we investigated the coding sequence and the flanking regions of Rpp13, a disease resistance gene in up to 60 accession lines from worldwide populations in Arabidopsis thaliana. An extraordinarily high level of polymorphism (pi=0.098) and four distinct clades were found in the leucine-rich repeat (LRR) region in this gene. No obvious geographic relationship with the clades was observed, and such clades were not observed in the other regions in and around this gene. The average genetic diversity among the clades ranged from 10 to 14.6% in the LRR. The levels of polymorphism within each clade varied largely, and significant heterogeneity in evolutionary rates among clades was detected. A statistically significant departure from neutrality was also detected by Fu & Li’s tests. These results suggest that both directional and diversifying selection are working on this locus, and that natural selection can cause heterogeneity in evolutionary rate, even among allele groups in a locus. | 3152 | NA | NA | NA | no | no |
| 3153 | 2017 | NA | Syed, NH; Sorensen, AP; Antonise, R; van de Wiel, C; van der Linden, CG; van’t Westende, W; Hooftman, DAP; den Nijs, HCM; Flavell, AJ | 2006 | A detailed linkage map of lettuce based on SSAP, AFLP and NBS markers | Molecular markers based upon a novel lettuce LTR retrotransposon and the nucleotide binding site-leucine-rich repeat (NBS-LRR) family of disease resistance-associated genes have been combined with AFLP markers to generate a 458 locus genetic linkage map for lettuce. A total of 187 retrotransposon-specific SSAP markers, 29 NBS-LRR markers and 242 AFLP markers were mapped in an F-2 population, derived from an interspecific cross between a Lactuca sativa cultivar commonly used in Europe and a wild Lactuca serriola isolate from Northern Europe. The cross has been designed to aid efforts to assess gene flow from cultivated lettuce into the wild in the perspective of genetic modification biosafety. The markers were mapped in nine major and one minor linkage groups spanning 1,266.1 cM, with an average distance of 2.8 cM between adjacent mapped markers. The markers are well distributed throughout the lettuce genome, with limited clustering of different marker types. Seventy-seven of the AFLP markers have been mapped previously and cross-comparison shows that the map from this study corresponds well with the previous linkage map. | 3153 | NA | NA | NA | no | no |
| 3154 | 2017 | NA | Sigg, DP; Goldizen, AW; Pople, AR | 2005 | The importance of mating system in translocation programs: reproductive success of released male bridled nailtail wallabies | Translocation is an important tool for the conservation of species that have suffered severe range reductions. The success of a translocation should be measured not only by the survival of released animals, but by the reproductive output of individuals and hence the establishment of a self-sustaining population. The bridled nailtail wallaby is an endangered Australian macropod that suffered an extensive range contraction to a single remaining wild population. A translocated population was established and subsequently monitored over a four year period. The aim of this study was to measure the reproductive success of released males using genetic tools and to determine the factors that predicted reproductive success. Captive-bred and wild-caught animals were released and we found significant variation in male reproductive success among release groups. Variation in reproductive success was best explained by individual male weight, survival and release location rather than origin. Only 26% of candidate males were observed to sire an offspring during the study. The bridled nailtail wallaby is a sexually dimorphic, polygynous macropod and reproductive success is skewed toward large males. Males over 5800 g were six times more likely to sire an offspring than males below this weight. This study highlights the importance of considering mating system when choosing animals for translocation. Translocation programs for polygynous species should release a greater proportion of females, and only release males of high breeding potential. By maximizing the reproductive output of released animals, conservation managers will reduce the costs of translocation and increase the chance of successfully establishing a self-sustaining population. (C) 2004 Elsevier Ltd. All rights reserved. | 3154 | NA | NA | NA | no | no |
| 3155 | 2017 | NA | Bowles, ML; McBride, JL; Bell, TJ | 2015 | Long-term processes affecting restoration and viability of the federal threatened Mead’s milkweed (Asclepias meadii) | Restoration is an important tool for reducing extinction risk of endangered plants. Population viabilities of few plant restorations have been modeled over decadal time periods and linked with genetic and ecological factors that drive restoration processes. We modeled viability of restored populations of Mead’s milkweed (Asclepias meadii, Asclepiadaceae), a self-incompatible perennial herb of eastern tallgrass prairie (TGP), federally listed as threatened in the U.S. From 1994 to 2004, we planted >600 seeds and >800 juvenile plants representing >50 genotypes across seven TGP sites. Propagule type, genotype, seed source, restoration site, precipitation and fire management significantly affected establishment, growth and viability. Plants established from seed had greater mortality and greater genetic and demographic attrition than did juveniles. Seedling growth rates also projected 20-30 yrs to reach flowering stage, and their survivorship provided a metric of site suitability for life cycle completion. Seed germination and juvenile plant size were greater in burned habitat, and juvenile size was also positively correlated with spring precipitation. Seed production required presence of multiple genotypes among flowering plants. Seedlings demonstrated a heterosis effect, with greater germination among seeds derived from inter-population crosses. However, cumulative growth of planted juveniles as well as population growth (lambda) on sub-optimal habitat conditions tended to be lower for propagules derived from inter-population crosses, demonstrating outbreeding depression. Although flowering occurred at multiple sites, positive population growth (lambda > 1) occurred at only a single site, where increasing fire frequency decreased extinction probability. These results suggest that restoration of viable Mead’s milkweed populations is possible in optimal habitat. However, restoration of this species is constrained by high demographic attrition and the long period (20 or more yrs) required to complete its life cycle. Crossing among populations to increase genetic diversity and compatible mating types may result in tradeoffs, with heterosis at early life history stages, but outbreeding depression expressed in older stages. Fire and precipitation are also critical interactive processes driving Asclepias meadii growth and reproduction. They may be most effective when precipitation, a stochastic process, results in greater than average post-burn rainfall. These constraints may have implications for restoration of other late-successional plant species. | 3155 | NA | NA | NA | no | no |
| 3156 | 2017 | NA | Kersulyte, D; Kalia, A; Gilman, RH; Mendez, M; Herrera, P; Cabrera, L; Velapatino, B; Balqui, J; de la Vega, FPP; Ulloa, CAR; Cok, J; Hooper, CC; Dailide, G; Tamma, S; Berg, DE | 2010 | Helicobacter pylori from Peruvian Amerindians: Traces of Human Migrations in Strains from Remote Amazon, and Genome Sequence of an Amerind Strain | Background: The gastric pathogen Helicobacter pylori is extraordinary in its genetic diversity, the differences between strains from well-separated human populations, and the range of diseases that infection promotes. Principal Findings: Housekeeping gene sequences from H. pylori from residents of an Amerindian village in the Peruvian Amazon, Shimaa, were related to, but not intermingled with, those from Asia. This suggests descent of Shimaa strains from H. pylori that had infected the people who migrated from Asia into The Americas some 15,000+ years ago. In contrast, European type sequences predominated in strains from Amerindian Lima shantytown residents, but with some 12% Amerindian or East Asian-like admixture, which indicates displacement of ancestral purely Amerindian strains by those of hybrid or European ancestry. The genome of one Shimaa village strain, Shi470, was sequenced completely. Its SNP pattern was more Asian-than European-like genome-wide, indicating a purely Amerind ancestry. Among its unusual features were two cagA virulence genes, each distinct from those known from elsewhere; and a novel allele of gene hp0519, whose encoded protein is postulated to interact with host tissue. More generally, however, the Shi470 genome is similar in gene content and organization to those of strains from industrialized countries. Conclusions: Our data indicate that Shimaa village H. pylori descend from Asian strains brought to The Americas many millennia ago; and that Amerind strains are less fit than, and were substantially displaced by, hybrid or European strains in less isolated communities. Genome comparisons of H. pylori from Amerindian and other communities should help elucidate evolutionary forces that have shaped pathogen populations in The Americas and worldwide. | 3156 | NA | NA | NA | no | no |
| 3157 | 2017 | NA | Mehle, N; Rupar, M; Seljak, G; Ravnikar, M; Dermastia, M | 2011 | Molecular diversity of ‘flavescence doree’ phytoplasma strains in Slovenia | The ‘flavescence doree’ (FD) phytoplasma is associated with the most devastating grapevine yellows disease ‘flavescence doree’. It belongs to the ribosomal group16SrV in which a high 16SrDNA identity among subgroup members exists. To investigate the genetic diversity of this phytoplasma in Slovenia a large analysis of strains in known and possible hosts and vectors was performed. The genotyping has revealed the occurrence of FD strains FD1, FD2 and FD3. Although isolates of FD2 and FD3 were detected in grapevines, those of FD2 prevailed. On the other hand, symptomatic as well as asymptomatic clematis (Clematis vitalba) plants were exclusively infected with FD3. Strains undistinguishable from FD I, FD2, FD3 and alder yellows were also detected in Alnus glutinosa and A. incana. In some alder samples mixed infections with different strain combinations were also demonstrated. FD2 strain was shown in the leafhopper vector of the disease Scaphoideus titanus. The FD1 strain was detected in the mosaic leafhopper Orientus ishidae. In addition, various specimens of this leafhopper were positive in PCR with a high presence of mixed infections. | 3157 | NA | NA | NA | no | no |
| 3158 | 2017 | NA | Thorson, JT; Scheuerell, MD; Buhle, ER; Copeland, T | 2014 | Spatial variation buffers temporal fluctuations in early juvenile survival for an endangered Pacific salmon | Spatial, phenotypic and genetic diversity at relatively small scales can buffer species against large-scale processes such as climate change that tend to synchronize populations and increase temporal variability in overall abundance or production. This portfolio effect generally results in improved biological and economic outcomes for managed species. Previous evidence for the portfolio effect in salmonids has arisen from examinations of time series of adult abundance, but we lack evidence of spatial buffering of temporal variability in demographic rates such as survival of juveniles during their first year of life. We therefore use density-dependent population models with multiple random effects to represent synchronous (similar among populations) and asynchronous (different among populations) temporal variability as well as spatial variability in survival. These are fitted to 25years of survey data for breeding adults and surviving juveniles from 15 demographically distinct populations of Chinook salmon (Oncorhynchus tshawytscha) within a single metapopulation in the Snake River in Idaho, USA. Model selection identifies the most support for the model that included both synchronous and asynchronous temporal variability, in addition to spatial variability. Asynchronous variability (log-SD=0<bold>55</bold>) is approximately equal in magnitude to synchronous temporal variability (log-SD=0<bold>67</bold>), but much lower than spatial variability (log-SD=1<bold>11</bold>). We also show that the pairwise correlation coefficient, a common measure of population synchrony, is approximated by the estimated ratio of shared and total variance, where both approaches yield a synchrony estimate of 0<bold>59</bold>. We therefore find evidence for spatial buffering of temporal variability in early juvenile survival, although between-population variability that persists over time is also large. We conclude that spatial variation decreases interannual changes in overall juvenile production, which suggests that conservation and restoration of spatial diversity will improve population persistence for this metapopulation. However, the exact magnitude of spatial buffering depends upon demographic parameters such as adult survival that may vary among populations and is proposed as an area of future research using hierarchical life cycle models. We recommend that future sampling of this metapopulation employ a repeated-measure sampling design to improve estimation of early juvenile carrying capacity. | 3158 | NA | NA | NA | no | no |
| 3159 | 2017 | NA | DYKHUIZEN, DE; POLIN, DS; DUNN, JJ; WILSKE, B; PREACMURSIC, V; DATTWYLER, RJ; LUFT, BJ | 1993 | BORRELIA-BURGDORFERI IS CLONAL - IMPLICATIONS FOR TAXONOMY AND VACCINE DEVELOPMENT | The chromosomal genes fla and p93 and the ospA gene from a linear plasmid were sequenced from up to 15 isolates of Borrelia burgdorferi, which causes Lyme borreliosis in man. Comparison of the gene trees provides no evidence for genetic exchange between chromosomal genes, suggesting B. burgdorferi is strictly clonal. Comparison of the chromosomal gene trees with that of the plasmid-encoded ospA reveals that plasmid transfer between clones is rare. Evidence for intragenic recombination was found in only a single ospA allele. The analysis reveals three common clones and a number of rare clones that are so highly divergent that vaccines developed against one are unlikely to provide immunity to organisms from others. Consequently, an understanding of the geographic and genetic variability of B. burgdorferi will prove essential for the development of effective vaccines and programs for control. While the major clones might be regarded as different species, the clonal population structure, the geographic localization, and the widespread incidence of Lyme disease suggest that B. burgdorferi should remain the name for the entire array of organisms. | 3159 | NA | NA | NA | no | no |
| 3160 | 2017 | NA | Berghoff, SM; Wurst, E; Ebermann, E; Sendova-Franks, AB; Rettenmeyer, CW; Franks, NR | 2009 | Symbionts of societies that fission: mites as guests or parasites of army ants | Recently, Hughes et al. (Trends in Ecology & Evolution, 23, 672-677, 2008) have theorised that symbionts of large, long-lived, homeostatic, and well defended social insect colonies should mostly be of low virulence. If the symbionts are rare, i.e. few workers are co-infected, competition between symbionts should be minimal and they should be selected to avoid over-exploiting their hosts. Here we analyse the mites that occur on Eciton burchellii army ant workers and note that our findings are consistent with the predictions from evolutionary theory. The mites were species diverse but rare; only 5% of the 3146 workers we examined from 20 army ant colonies had mites. Only one worker was co-infected by mites of different species and the one relatively common parasitic mite (Rettenmeyerius carli) was limited to only two individuals per ant. We also showed that certain mites are more common on workers in nomadic rather than statary army ant colonies and that different worker castes differed in their infestation patterns. We suggest that the three traits E. burchellii and honey bees (Apis mellifera) have in common (queens with very high mating frequencies, propagation by colony fission, and low number of parasites among the mite species they host) are associated with one another. Colonies that fission are likely to inherit symbionts and multiple mating will promote genetic diversity within colonies, which may help to limit the abundance of deleterious mites. We conclude that most of the symbiotic mites found on workers of the army ant E. burchellii are probably relatively harmless guests, exploiting their hosts for phoresis or, for example, to use their waste deposits. | 3160 | NA | NA | NA | no | no |
| 3161 | 2017 | NA | Childs, DZ; Rees, M; Rose, KE; Grubb, PJ; Ellner, SP | 2003 | Evolution of complex flowering strategies: an age- and size-structured integral projection model | We explore the evolution of delayed age- and size-dependent flowering in the monocarpic perennial Carlina vulgaris, by extending the recently developed integral projection approach to include demographic rates that depend on size and age. The parameterized model has excellent descriptive properties both in terms of the population size and in terms of the distributions of sizes within each age class. In Carlina the probability of flowering depends on both plant size and age. We use the parameterized model to predict this relationship, using the evolutionarily stable strategy (ESS) approach. Despite accurately predicting the mean size of flowering individuals, the model predicts a step-function relationship between the probability of flowering and plant size, which has no age component. When the variance of the flowering-threshold distribution is constrained to the observed value, the ESS flowering function contains an age component, but underpredicts the mean flowering size. An analytical approximation is used to explore the effect of variation in the flowering strategy on the ESS predictions. Elasticity analysis is used to partition the age-specific contributions to the finite rate of increase (lambda) of the survival-growth and fecundity components of the model. We calculate the adaptive landscape that defines the ESS and generate a fitness landscape for invading phenotypes in the presence of the observed flowering strategy. The implications of these results for the patterns of genetic diversity in the flowering strategy and for testing evolutionary models are discussed. Results proving the existence of a dominant eigenvalue and its associated eigenvectors in general size- and age-dependent integral projection models are presented. | 3161 | NA | NA | NA | no | no |
| 3162 | 2017 | NA | Picard-Meyer, E; Robardet, E; Arthur, L; Larcher, G; Harbusch, C; Servat, A; Cliquet, F | 2014 | Bat Rabies in France: A 24-Year Retrospective Epidemiological Study | Since bat rabies surveillance was first implemented in France in 1989, 48 autochthonous rabies cases without human contamination have been reported using routine diagnosis methods. In this retrospective study, data on bats submitted for rabies testing were analysed in order to better understand the epidemiology of EBLV-1 in bats in France and to investigate some epidemiological trends. Of the 3176 bats submitted for rabies diagnosis from 1989 to 2013, 1.96% (48/2447 analysed) were diagnosed positive. Among the twelve recognised virus species within the Lyssavirus genus, two species were isolated in France. 47 positive bats were morphologically identified as Eptesicus serotinus and were shown to be infected by both the EBLV-1a and the EBLV-1b lineages. Isolation of BBLV in Myotis nattereri was reported once in the north-east of France in 2012. The phylogenetic characterisation of all 47 French EBLV-1 isolates sampled between 1989 and 2013 and the French BBLV sample against 21 referenced partial nucleoprotein sequences confirmed the low genetic diversity of EBLV-1 despite its extensive geographical range. Statistical analysis performed on the serotine bat data collected from 1989 to 2013 showed seasonal variation of rabies occurrence with a significantly higher proportion of positive samples detected during the autumn compared to the spring and the summer period (34% of positive bats detected in autumn, 15% in summer, 13% in spring and 12% in winter). In this study, we have provided the details of the geographical distribution of EBLV-1a in the south-west of France and the north-south division of EBLV-1b with its subdivisions into three phylogenetic groups: group B1 in the north-west, group B2 in the centre and group B3 in the north-east of France. | 3162 | NA | NA | NA | no | no |
| 3163 | 2017 | NA | Fisher, AJ; DiTomaso, JM; Gordon, TR | 2005 | Intraspecific groups of Claviceps purpurea associated with grass species in Willapa Bay, Washington, and the prospects for biological control of invasive Spartina alterniflora | Spartina alterniflora is a salt marsh halophyte introduced to the Pacific Coast of the United States that has become a noxious weed in Willapa Bay, Washington. A sap-feeding insect has been released as part of a biological control program, which has established at multiple sites. A useful complement to this program would be a biological agent that attacks seed, to reduce expansion of the infestation by seedling recruitment. One possibility is the floral-infecting, fungal pathogen Claviceps purpurea, which causes the ergot disease. This species is comprised of three intraspecific groups, of which one is specific to salt marsh habitats, G3. Based on random amplified polymorphic DNA markers, all three intraspecific groups of C purpurea were found on grasses in Willapa Bay. The incidence of ergot on Spartina was very low over the three-year sampling period. Genetic diversity was low among G3 isolates, suggesting it may have been recently introduced to the region. Greenhouse tests showed S. alterniflora from Washington to be as susceptible to C purpurea as S. alterniflora from the Atlantic Coast, where ergot has reached epidemic levels. Neighbor-joining analysis of amplified fragment length polymorphism markers suggests that G3 C purpurea in Washington is most closely related to South-eastern USA G3 C. purpurea. Pairwise comparisons as part of AMOVA showed that Willapa Bay isolates are different from those of all other geographic regions except Argentina. The low intensity of disease in Washington may be due, in part, to a G3 ergot population that is not well adapted to conditions in this area. (c) 2005 Elsevier Inc. All rights reserved. | 3163 | NA | NA | NA | no | no |
| 3164 | 2017 | NA | Zhu, PL; Oudemans, PV | 2000 | A long terminal repeat retrotransposon Cgret from the phytopathogenic fungus Colletotrichum gloeosporioides on cranberry | A repetitive DNA element cloned from the cranberry fruit rot pathogen Colletotrichum gloeosporioides has been characterized. Sequence data indicate that it is a long terminal repeat (LTR) retrotransposon of 7,916 base pairs. LTR of 544 base pairs occur at either end of an internal region of 6,828 base pairs. This element, designated Cgret (C. gloeosporioides retrotransposon), encodes two putative polypeptides which have high homology to the gag and pol genes of other fungal retrotransposons. The sequence and structure suggest that Cgret is a member of the gypsy group of LTR retrotransposons. The Cgret retrotransposon was present in all of the cranberry isolates of the fungus C. glaeosporioides from New Jersey and Massachusetts, but not in the cranberry isolates from Wisconsin or Chile. Polymorphisms were detected among field isolates of C. gloeosporioides from various hosts, using hybridization probes derived from the LTR and the reverse transcriptase domain of Cgret. The structural integrity of Cgret suggests that it is still a functional retrotransposon and may be used as a molecular marker to study the genetic diversity distribution of this fungal pathogen. | 3164 | NA | NA | NA | no | no |
| 3165 | 2017 | NA | Satterthwaite, WH | 2010 | Competition for space can drive the evolution of dormancy in a temporally invariant environment | I present a model for the evolution of a seed bank in the absence of externally driven environmental variation. I use Evolutionarily Stable Strategy (ESS) analyses of both analytic and simulation models to assess the conditions under which a dormant genotype can invade and resist invasion. In my models, plant seeds compete through lottery for discrete safe sites holding one individual each. Analyzing the conditions under which a dormant genotype can invade when rare and resist invasion once established, I conclude that dormancy can be an ESS when some fraction of seeds is retained locally, seed bank survival is high, and mortality in the seed bank is low. The advantage of dormancy stems from the ability of dormant seeds to recapture a lost site and the fact that a plant’s offspring are more likely to win the lottery in its own site than in any new site. The advantage of dormancy does not depend on individual fecundity or on low relatedness with the offspring of kin, making this mechanism distinct from earlier models of sib competition. | 3165 | NA | NA | NA | no | no |
| 3166 | 2017 | NA | King, WJ; Forsyth, DM; Coulson, G; Festa-Bianchet, M | 2015 | Adoption in Eastern Grey Kangaroos: A Consequence of Misdirected Care? | Adoption is rare in animals and is usually attributed to kin selection. In a 6-year study of eastern grey kangaroos (Macropus giganteus), 11 of 326 juveniles were adopted. We detected eight adoptions by observing behavioural associations and nursing between marked mothers and young and three more by analysing the relatedness of mothers and young using microsatellite DNA. Four adoptions involved reciprocal switches and three were by mothers whose own pouch young were known to subsequently disappear. Adoptive mothers were not closely related to each other or to adoptees but adoptive mothers and young associated as closely as did biological pairs, as measured by half-weight indices. Switch mothers did not associate closely. Maternal age and body condition did not influence the likelihood of adoption but females were more likely to adopt in years with high densities of females with large pouch young. Adoption did not improve juvenile survival. We conclude that adoptions in this wild population were potentially costly and likely caused by misdirected care, suggesting that eastern grey kangaroos may have poorly developed mother-offspring recognition mechanisms. | 3166 | NA | NA | NA | no | no |
| 3167 | 2017 | NA | Katiyar, SK; Chandel, G; Tan, Y; Zhang, Y; Huang, B; Nugaliyadde, L; Fernando, K; Bentur, JS; Inthavong, S; Constantino, S; Bennett, J | 2000 | Biodiversity of Asian rice gall midge (Orseolia oryzae Wood Mason) from five countries examined by AFLP analysis | Amplified fragment length polymorphism (AFLP) analysis was used to assess the biodiversity of one of the most important dipteran pests of cereals, the Asian rice gall midge (Orseolia oryzae Wood Mason). Larvae and pupae were collected at 15 locations in five Asian countries and preserved in 95% ethanol for storage, shipment, and DNA extraction using cetyltrimethylammonium bromide (CTAB). Although only similar to 1 mu g of DNA was extracted from a single pupa or larva, the use of several AFLP primers in various combinations meant that this amount of DNA was sufficient to allow many DNA fingerprints to be made per individual. Fingerprints were sufficiently reproducible, especially during selective amplification, to allow the genetic diversity within a field population to be characterized. Extraction of DNA from a pool of 20 insects yielded AFLP fingerprints in which variation among individuals was sacrificed in favor of detecting differences among populations. For each location, pooled DNA was amplified with three primer pairs. A total of 261 distinct AFLP bands were identified for the 45 fingerprints. Cluster analysis, performed by the unweighted pair-group method (UPGMA), separated the populations into two distinct groups. Group I included two populations from Guangdong province of southern China and one each from Laos and Imphal in northeastern India, while group II was comprised of eleven populations from elsewhere in India (Assam, Orissa, Madhya Pradesh, Andhra Pradesh, and Kerala) and from Nepal and Sri Lanka. AFLP analysis provided insight into the origins of gall midge biotypes. In 1992, the prevailing biotype in Imphal changed from Indian biotype 3 to a new biotype 3M. Our data show that biotype 3M belongs to group I and did not arise by a recent mutation from biotype 3, which belongs to group II. By contrast, Indian biotypes 2 and 4 are likely to have diverged through recent mutation and selection, as are Chinese biotypes 1 and 4. The almost simultaneous emergence of new biotypes in Kerala and Sri Lanka during 1985-1988 was most probably coincidental, because these biotypes are not closely related. AFLP fingerprints were also able to detect sexual dimorphism in the DNA of adult gall midges and to distinguish gall midge from its major parasite Platygaster oryzae. | 3167 | NA | NA | NA | no | no |
| 3168 | 2017 | NA | Camargo, DRA; Pais, FS; Volpini, AC; Oliveira, MAA; Coimbra, RS | 2015 | Revisitingmolecular serotyping of Streptococcus pneumoniae | Background: Ninety-two Streptococcus pneumoniae serotypes have been described so far, but the pneumococcal conjugate vaccine introduced in the Brazilian basic vaccination schedule in 2010 covers only the ten most prevalent in the country. Pneumococcal serotype-shifting after massive immunization is a major concern and monitoring this phenomenon requires efficient and accessible serotyping methods. Pneumococcal serotyping based on antisera produced in animals is laborious and restricted to a few reference laboratories. Alternatively, molecular serotyping methods assess polymorphisms in the cps gene cluster, which encodes key enzymes for capsular polysaccharides synthesis in pneumococci. In one such approach, cps-RFLP, the PCR amplified cps loci are digested with an endonuclease, generating serotype-specific fingerprints on agarose gel electrophoresis. Methods: In this work, in silico and in vitro approaches were combined to demonstrate that XhoII is the most discriminating endonuclease for cps-RFLP, and to build a database of serotype-specific fingerprints that accommodates the genetic diversity within the cps locus of 92 known pneumococci serotypes. Results: The expected specificity of cps-RFLP using XhoII was 76% for serotyping and 100% for serogrouping. The database of cps-RFLP fingerprints was integrated to Molecular Serotyping Tool (MST), a previously published webbased software for molecular serotyping. In addition, 43 isolates representing 29 serotypes prevalent in the state of Minas Gerais, Brazil, from 2007 to 2013, were examined in vitro; 11 serotypes (nine serogroups) matched the respective in silico patterns calculated for reference strains. The remaining experimental patterns, despite their resemblance to their expected in silico patterns, did not reach the threshold of similarity score to be considered a match and were then added to the database. Conclusion: The cps-RFLP method with XhoII outperformed the antisera-based and other molecular serotyping methods in regard of the expected specificity. In order to accommodate the genetic variability of the pneumococci cps loci, the database of cps-RFLP patterns will be progressively expanded to include new variant in vitro patterns. The cps-RFLP method with endonuclease XhoII coupled with MST for computer-assisted interpretation of results may represent a relevant contribution to the real time detection of changes in regional pneumococci population diversity in response to mass immunization programs. | 3168 | NA | NA | NA | no | no |
| 3169 | 2017 | NA | Huttunen, S; Kuznetsova, O; Li, M; Wang, YF; Ignatov, MS | 2015 | East Asian origin and Northern Hemisphere diversification in the Brachythecium novae-angliae-complex (Bryophyta) | Cool climate biomes in the Northern Hemisphere have evolved relatively recently. Temperature decline between the late Eocene and the Pliocene contributed to the evolution of many plant groups in modern cool climate biomes, such as mosses belonging to the Brachythecium novae-angliae-complex. The Brachythecium novae-angliae-complex comprises three species that have a disjunct distribution in temperate and boreal regions in the Northern Hemisphere. The core species in the complex have only recently been transferred from Bryhnia to Brachythecium and the delimitation of the complex and of species within it still remains uncertain. We reconstructed the evolutionary history of the group using molecular data and estimated the timing of the diversifications in the tree. In addition, we examined the morphology and compared morphological diversification with genetic diversity. The Brachythecium novae-angliae-complex diversified in East Asia in the middle Miocene, 9.1 Ma (HPD 95% 3.9-14.8 Myr). Bryhnia trichomitria and B. graminicolor were revealed not to be close relatives of the complex and are therefore transferred to Brachythecium and Koponeniella gen. nov., respectively. Both morphology and molecular data supported the position of Asian Brachythecium noesicum as sister to the Brachythecium novae-angliae crown clade that includes two species, B. hultenii and B. novae-angliae. The crown clade diverged at the end of the Miocene, 6.4 Ma (HPD 95% 2.4-11.6 Myr) and its taxa have a disjunct distribution in cool temperate and boreal areas in the Beringia region (B. novae-angliae subsp. hultenii), and eastern North America and northwestern Europe (B. novae-angliae subsp. novae-angliae). In the crown clade the majority of populations belong to one common haplotype present in all three disjunct parts of the distribution area. Despite low variation in DNA sequence data, morphology allows the distinction between B. novae-angliae subsp. hultenii and B. novae-angliae subsp. novae-angliae. Because of weak morphological and genetic differentiation, European Bryhnia scabrida is treated here as a synonym of Brachythecium novae-angliae subsp. novae-angliae. Glacial survival during the Quaternary and climatic oscillations during the late Tertiary and Quaternary in East Asia may have resulted in diversification of the complex and enabled adaptation to cool temperate and boreal climates. | 3169 | NA | NA | NA | no | no |
| 3170 | 2017 | NA | Rasmussen, KK; Kollmann, J | 2007 | Genetic diversity, spatial patterns, and growth of root sprouts in a temperate tree at the northern distribution limit | In many plant species, population persistence at the limit of their distribution relies on clonal growth, but little is known about the consequences for genetic diversity, spatial patterns, and growth of the offspring. The rare European tree Sorbus torminalis (Rosaceae) was chosen to explore the patterns and limitations of clonal growth. Offspring were mapped in 7 small populations at the northern limit of the species’ distribution, their size was recorded in 2 consecutive years,and the degree of shading (diffuse site factor, DSF) was measured at the forest floor. A subsample of offspring and adults was genotyped with microsatellites to determine the frequency of vegetative reproduction, and another group of offspring was protected against browsing. Clonal origin was observed in 98% of the offspring, and thus they were classified as root sprouts. The fine-scale distribution of the root sprouts was mainly clumped, and variation of DSF explained some of this pattern. Mixed linear modeling of root sprout size, with DSF, sprout density, and distance to the nearest adult tree as explanatory variables, suggested inter-offspring competition, but there were significant interactions among the explanatory variables, In addition, browsing by deer reduced growth of the root sprouts. Root sprout mortality was 17% after 1 y, and highest for young, small, and isolated offspring growing in high-light microhabitats. Root sprout growth and mortality were not directly affected by distance to adult trees. We conclude that clonal growth is common in the northern populations of the study species, and that root sprout distribution and growth are controlled by complex interactions between light, browsing, distance to the parent tree, and sprout density. | 3170 | NA | NA | NA | no | no |
| 3171 | 2017 | NA | Keller, L; Aron, S; Passera, L | 1996 | Internest sex-ratio variation and male brood survival in the ant Pheidole pallidula | Sex allocation in social insects has become a general model in tests of inclusive fitness theory sex-ratio theory, and parent-offspring conflict. Several studies have shown that colony sex ratios are often bimodally distributed, with some colonies producing mainly females and others mainly males. Sex specialization may result from workers assessing their relatedness to male brood versus female brood, relative to the average worker relatedness asymmetry in other colonies of their population. Workers then adjust the sex ratio in their own interest. This hypothesis assumes that workers can recognize the sex of the brood in their colony and selectively eliminate males. We compared the primary sex ratio (at the egg stage) and secondary sex ratio (reproductive pupae and adults) of colonies in the ant Pheidole pallidula. There was a strong bimodal distribution of secondary sex ratios, with most colonies producing mainly reproductives of one sex. In contrast, there was no evidence of a bimodal distribution of primary sex ratios. The proportion of haploid eggs produced by queens was 0.35 in early spring and decreased to about 0.1 in summer. Male eggs also were present in virtually all field colonies sampled in July, although eggs laid at this time of year never give rise to males. All male brood is, therefore, selectively eliminated beginning in July and continue to be eliminated through the rest of the year. Finally the population sex-ratio investment was female-biased. Together, these results are consistent with the hypothesis that workers control the secondary sex ratio by selectively eliminating male brood in about half the colonies, perhaps those with high relatedness asymmetry. | 3171 | NA | NA | NA | no | no |
| 3172 | 2017 | NA | Perez-Lachaud, G; Valenzuela, JE; Lachaud, JP | 2011 | IS INCREASED RESISTANCE TO PARASITISM AT THE ORIGIN OF POLYGYNY IN A MEXICAN POPULATION OF THE ANT ECTATOMMA TUBERCULATUM (HYMENOPTERA: FORMICIDAE)? | The Neotropical ant Ectatomma tuberculatum (Olivier) shows a polymorphic social organization with strictly monogynous or facultative polygynous populations along its distribution range. The causative factors of these variations in social organization are unknown but biotic and abiotic environmental factors might explain the differences. In particular, recent studies have suggested that parasites and parasitoids could have an impact on host colony phenotype. Here, we report on eucharitid wasps and mermithid nematodes that attack the only known Mexican polygynous population of E. tuberculatum in Veracruz, Mexico. The results were compared with those previously obtained from a monogynous population in Chiapas, Mexico; the aim being to investigate environmental influences on colony social organization. The rather similar prevalence of parasitism between the 2 populations suggests that the hypothesis of an increased resistance to parasites and parasitoids as one of the factors responsible for the emergence of polygyny in E. tuberculatum populations does not apply. More likely, other kinds of environmental stress or constraints facilitate the development of polygyny, such as nest site limitations, risks of dispersal and/or high rates of predation. | 3172 | NA | NA | NA | no | no |
| 3173 | 2017 | NA | Fowler, SV; Peterson, P; Barrett, DP; Forgie, S; Gleeson, DM; Harman, H; Houliston, GJ; Smith, L | 2015 | Investigating the poor performance of heather beetle, Lochmaea suturalis (Thompson) (Coleoptera: Chrysomelidae), as a weed biocontrol agent in New Zealand: Has genetic bottlenecking resulted in small body size and poor winter survival? | Heather beetle (Lochmaea suturalis), has underperformed as a biocontrol agent when compared with the damage it does to native heather in Europe. Mean heather beetle body size, measured by elytron area, was 10% smaller in NZ populations compared with beetles from northern UK where the NZ beetles originated. Previous research in Europe showed that small beetles suffer higher winter mortality. Field-collected heather beetles in NZ show a positive relationship between body size and the proportion of pre-overwintering food reserves (lipids) they contained. Beetles that died in an overwintering experiment had lower proportional lipid reserves, and a smaller mean body size, than surviving beetles. Smaller body size in NZ is probably mostly due to a severe founder effect: line-rearing of beetles in NZ to eliminate a microsporidian disease, and poor establishment success, resulted in NZ beetles being derived from one or two field-collected females from one UK site. Several measures of genetic variability in NZ beetles compared with beetles from the UK indicated severe genetic bottlenecking. In particular, reductions in heterozygosity in NZ versus UK beetles were a close match to theoretical heterozygosity after a severe bottleneck. Heather beetle populations from southern UK were genetically distinct from those sampled from northern UK, and previous collecting showed higher microsporidian infestations in beetles from southern UK compared with northern beetles. Mean elytron area was 2.2% smaller in the southern UK population compared with the northern population. Genetic rescue of NZ heather beetles could use beetles from the northern UK that have slightly larger body size and lower levels of microsporidian infection. (C) 2015 Elsevier Inc. All rights reserved. | 3173 | NA | NA | NA | no | no |
| 3174 | 2017 | NA | Gwiazdowski, RA; Van Driesche, RG; Desnoyers, A; Lyon, S; Wu, SA; Kamata, N; Normark, BB | 2006 | Possible geographic origin of beech scale, Cryptococcus fagisuga (Hemiptera : Eriococcidae), an invasive pest in North America | Beech scale, Cryptococcus fagisuga Lindinger, is invasive in North America. The immediate source of the introduction was Europe, but its native range may be different. Knowledge of the native range is useful when searching for coevolved natural enemies for classical biological control. We report results of a search for the native range of C fagisuga, using historical records, field surveys, and molecular phylogenetics. Beech scale feeds exclusively on beech. We review historical accounts of movement of species of Fagus between Europe, Asia, and North America and report on extensive surveys for C fagisuga on Fagus species in China and Japan. We undertook a phylogeographic study of C. fagisuga throughout its known range using sequences of the mitochondrial gene cytochrome oxidase I (COI). We also investigated the phylogenetic relationships of C fagisuga to other species of Cryptococcus and related species in the Eriococcidae, using ribosomal DNA (18S). For COI sequences within C fagisuga, we found one widespread, most-common haplotype in North America, Europe, Turkey, and Georgia; a diversity of slightly (0.1-0.5%) divergent haplotypes in Bulgaria; a diversity of moderately (2.2-2.8%) divergent haplotypes in Georgia and Turkey; and a highly (3.6-4.2%) divergent group of haplotypes in Iran. Phylogenetic analysis of 18S places C fagisuga within a cosmopolitan clade of eriococcids feeding on other temperate trees (ash, maple, and southern beech). Based on the phylogeographic study, we suggest that the subspecies F. sylvatica orientalis is the native host of C fagisuga and that natural enemies are best sought on oriental beech in northeastern Greece, the Black Sea drainage basin, the Caucasus Mountains, and northern Iran. (c) 2006 Elsevier Inc. All rights reserved. | 3174 | NA | NA | NA | no | no |
| 3175 | 2017 | NA | Roger, N; Michez, D; Wattiez, R; Sheridan, C; Vanderplanck, M | 2017 | Diet effects on bumblebee health | Among physiological processes, the maintenance of immunity is one of the most energetically costly in invertebrates. Disease resistance can be quantified by measuring immunocompetence, which is defined as the ability of an organism to mount an immune response, either in cellular, humoral or behavioural forms. In insects, immune capacity can be affected by a variety of factors including pesticides, genetic diversity or diet. Here we focus on an important species of domesticated pollinator, Bombus terrestris, and the potential impact of a poor pollen diet (low nutritional content and toxic) on its health. We investigate three responses at both colony and individual levels: behavioural, humoral and cellular. Our results show that poor pollen diets decrease larval and pupal masses and increase larval ejection as well as adult constitutive immunity (i.e., prophenoloxidase assays). The susceptibility of bumblebees to disease and infection might therefore be greater after a nutritive stress. These findings raise the importance of available plant hosts, especially floral plant species providing pollen with suitable nutritive quality (i.e., nutrient pollen content) for bumblebees. (C) 2016 Elsevier Ltd. All rights reserved. | 3175 | NA | NA | NA | no | no |
| 3176 | 2017 | NA | Shi, CY; Liu, Y; Hu, XM; Xiong, JF; Zhang, B; Yuan, ZM | 2015 | A Metagenomic Survey of Viral Abundance and Diversity in Mosquitoes from Hubei Province | Mosquitoes as one of the most common but important vectors have the potential to transmit or acquire a lot of viruses through biting, however viral flora in mosquitoes and its impact on mosquito-borne disease transmission has not been well investigated and evaluated. In this study, the metagenomic techniquehas been successfully employed in analyzing the abundance and diversity of viral community in three mosquito samples from Hubei, China. Among 92,304 reads produced through a run with 454 GS FLX system, 39% have high similarities with viral sequences belonging to identified bacterial, fungal, animal, plant and insect viruses, and 0.02% were classed into unidentified viral sequences, demonstrating high abundance and diversity of viruses in mosquitoes. Furthermore, two novel viruses in subfamily Densovirinae and family Dicistroviridae were identified, and six torque tenosus virus1 in family Anelloviridae, three porcine parvoviruses in subfamily Parvovirinae and a Culex tritaeniorhynchus rhabdovirus in Family Rhabdoviridae were preliminarily characterized. The viral metagenomic analysis offered us a deep insight into the viral population of mosquito which played an important role in viral initiative or passive transmission and evolution during the process. | 3176 | NA | NA | NA | no | no |
| 3177 | 2017 | NA | Boyden, SE; Kunkel, LM | 2010 | High-Density Genomewide Linkage Analysis of Exceptional Human Longevity Identifies Multiple Novel Loci | Background: Human lifespan is approximately 25% heritable, and genetic factors may be particularly important for achieving exceptional longevity. Accordingly, siblings of centenarians have a dramatically higher probability of reaching extreme old age than the general population. Methodology/Principal Findings: To map the loci conferring a survival advantage, we performed the second genomewide linkage scan on human longevity and the first using a high-density marker panel of single nucleotide polymorphisms. By systematically testing a range of minimum age cutoffs in 279 families with multiple long-lived siblings, we identified a locus on chromosome 3p24-22 with a genomewide significant allele-sharing LOD score of 4.02 (empirical P = 0.037) and a locus on chromosome 9q31-34 with a highly suggestive LOD score of 3.89 (empirical P = 0.054). The empirical P value for the combined result was 0.002. A third novel locus with a LOD score of 4.05 on chromosome 12q24 was detected in a subset of the data, and we also obtained modest evidence for a previously reported interval on chromosome 4q22-25. Conclusions/Significance: Our linkage data should facilitate the discovery of both common and rare variants that determine genetic variability in lifespan. | 3177 | NA | NA | NA | no | no |
| 3178 | 2017 | NA | Hebbring, SJ; Adjei, AA; Baer, JL; Jenkins, GD; Zhang, J; Cunningham, JM; Schaid, DJ; Weinshilboum, RM; Thibodeau, SN | 2007 | Human SULT1A1 gene: copy number differences and functional implications | SULT1A1, which catalyzes the sulfate conjugation of a wide variety of natural and synthetic compounds, is genetically polymorphic. Biochemical and pharmacogenetic studies have demonstrated that individual variation in the level of enzyme activity is inherited. Common single-nucleotide polymorphisms (SNPs) located in the open reading frame and in the 5’-flanking region (5’-FR) may account for a portion of this individual variation. In this study, we demonstrate the presence of SULT1A1 gene deletions and duplications, representing an additional source of variability in the metabolic activity of this enzyme. A quantitative multiplex PCR assay was used to measure the extent of copy number differences and the frequency of these events in different populations. An analysis of DNA from 362 Caucasian-American and 99 African-American showed the presence of 1 to similar to 5 copies of SULT1A1 in individual samples: 5% of Caucasian subjects contained a single copy of the gene and 26% had three or more copies, while 63% of African-American subjects had three or more copies. Analysis of the genomic region surrounding the SULT1A1 gene in three separate cases with a deletion demonstrated that the entire SULT1A1 gene was affected. Reporter assays, constructed for each of the various 5’-FR SNP haplotypes, suggest that these may also play a role in SULT1A1 activity. However, the variability in the level of enzyme activity among 23 human platelet and 267 human liver samples was best explained by gene copy number differences when all sources of genetic variability were considered (P < 0.0001). Overall, these observations have obvious implications for the effectiveness of SULT1A1 as a drug and hormone metabolizing enzyme and its potential role as a risk factor for disease. | 3178 | NA | NA | NA | no | no |
| 3179 | 2017 | NA | Henn, BM; Botigue, LR; Gravel, S; Wang, W; Brisbin, A; Byrnes, JK; Fadhlaoui-Zid, K; Zalloua, PA; Moreno-Estrada, A; Bertranpetit, J; Bustamante, CD; Comas, D | 2012 | Genomic Ancestry of North Africans Supports Back-to-Africa Migrations | North African populations are distinct from sub-Saharan Africans based on cultural, linguistic, and phenotypic attributes; however, the time and the extent of genetic divergence between populations north and south of the Sahara remain poorly understood. Here, we interrogate the multilayered history of North Africa by characterizing the effect of hypothesized migrations from the Near East, Europe, and sub-Saharan Africa on current genetic diversity. We present dense, genomewide SNP genotyping array data (730,000 sites) from seven North African populations, spanning from Egypt to Morocco, and one Spanish population. We identify a gradient of likely autochthonous Maghrebi ancestry that increases from east to west across northern Africa; this ancestry is likely derived from “back-to-Africa” gene flow more than 12,000 years ago (ya), prior to the Holocene. The indigenous North African ancestry is more frequent in populations with historical Berber ethnicity. In most North African populations we also see substantial shared ancestry with the Near East, and to a lesser extent sub-Saharan Africa and Europe. To estimate the time of migration from sub-Saharan populations into North Africa, we implement a maximum likelihood dating method based on the distribution of migrant tracts. In order to first identify migrant tracts, we assign local ancestry to haplotypes using a novel, principal component-based analysis of three ancestral populations. We estimate that a migration of western African origin into Morocco began about 40 generations ago (approximately 1,200 ya); a migration of individuals with Nilotic ancestry into Egypt occurred about 25 generations ago (approximately 750 ya). Our genomic data reveal an extraordinarily complex history of migrations, involving at least five ancestral populations, into North Africa. | 3179 | NA | NA | NA | no | no |
| 3180 | 2017 | NA | Menon, VK; George, S; Sarkar, R; Giri, S; Samuel, P; Vivek, R; Saravanabavan, A; Liakath, FB; Ramani, S; Iturriza-Gomara, M; Gray, JJ; Brown, DW; Estes, MK; Kang, G | 2016 | Norovirus Gastroenteritis in a Birth Cohort in Southern India | Background Noroviruses are an important cause of gastroenteritis but little is known about disease and re-infection rates in community settings in Asia. Methods Disease, re-infection rates, strain prevalence and genetic susceptibility to noroviruses were investigated in a birth cohort of 373 Indian children followed up for three years. Stool samples from 1856 diarrheal episodes and 147 vomiting only episodes were screened for norovirus by RT-PCR. Norovirus positivity was correlated with clinical data, secretor status and ABO blood group. Results Of 1856 diarrheal episodes, 207 (11.2%) were associated with norovirus, of which 49(2.6%) were norovirus GI, 150(8.1%) norovirus GII, and 8 (0.4%) were mixed infections with both norovirus GI and GII. Of the 147 vomiting only episodes, 30 (20.4%) were positive for norovirus in stool, of which 7 (4.8%) were norovirus GI and 23 (15.6%) GII. At least a third of the children developed norovirus associated diarrhea, with the first episode at a median age of 5 and 8 months for norovirus GI and GII, respectively. Norovirus GI. 3 and GII. 4 were the predominant genotypes (40.3% and 53.0%) with strain diversity and change in the predominant sub-cluster over time observed among GII viruses. A second episode of norovirus gastroenteritis was documented in 44/174 (25.3%) ever-infected children. Children with the G428A homozygous mutation for inactivation of the FUT2 enzyme (se(428)se(428)) were at a significantly lower risk (48/190) of infection with norovirus (p = 0.01). Conclusions This is the first report of norovirus documenting disease, re-infection and genetic susceptibility in an Asian birth cohort. The high incidence and apparent lack of genogroupII specific immunity indicate the need for careful studies on further characterization of strains, asymptomatic infection and shedding and immune response to further our understanding of norovirus infection and disease. | 3180 | NA | NA | NA | no | no |
| 3181 | 2017 | NA | JONES, NJ | 1994 | GENETIC-ANALYSIS OF MITOMYCIN C-HYPERSENSITIVE CHINESE-HAMSTER CELL MUTANTS | The genetic diversity of Chinese hamster cell mutants exhibiting hypersensitivity to the bifunctional alkylating agent mitomycin C has been examined. The eight mutants irs3, VH4, UV1, MC5, MMC1, MMC3, MMC4 and MMS2, are between 4- and 30-fold more sensitive to mitomycin C than their respective wildtype parental lines. A number of the mutants show phenotypic similarities to cultured cells from the human cancer-prone syndrome Fanconi’s anaemia. Hybrids were formed between pairs of mutants using the thioguanine/ouabain resistant (TOR) hybridization and hypoxanthine/aminopterin/thymidine (HAT)/ouabain selection system and the mitomycin C response of pooled populations of hybrids assessed by constructing survival curves. In every case, hybrids formed between pairs of mutants exhibited a mitomycin C response indistinguishable from that of wildtype cells, indicating complementation. Therefore, the eight mutant lines examined represent eight distinct complementation groups for mitomycin C-hypersensitivity. The results are in contrast to the complementation analysis of UV-sensitive Chinese hamster cell mutants and indicate that the response of mammalian cells to mitomycin C-induced DNA damage is complex and involves a large number of genes. | 3181 | NA | NA | NA | no | no |
| 3182 | 2017 | NA | Kochetova, OV; Korytina, GF; Akhmadishina, LZ; Semenov, EE; Viktorova, TV | 2014 | Association of polymorphic variants of FTO and MC4R genes with obesity in a Tatar population | Obesity is a chronic relapsing disease that leads to numerous ailments and requires lifelong treatment. Genetic predisposition is one of the mostly discussed aspects of obesity development, and genome-wide association studies have provided evidence that several variants of the FTO and MC4R genes are significantly associated with obesity. In this study the association of FTO (rs9939609, rs7202116, and rs9930506) and MC4R (rs12970134 and rs17782313) genes’ SNPs with obesity in Tatar women has been analyzed. In the investigation 340 women with obesity (Body Mass Index (BMI) a parts per thousand yen 30 kg/m(2)) and 330 women from a control group (BMI up to 24.9 kg/m(2)) took part. The FTO rs9939609 (p = 0.0002) and rs9930506 (p = 0.0005) SNPs were shown to be associated with obesity risk following an additive model, while the MC4R rs12970134 (p = 0.0076) and rs1778231 (p = 0.021) SNPs were associated by a recessive model. We also showed an association of quantitative parameters (age, weight, and BMI) with two the FTO rs9939609 and rs9930506 SNPs and the association of age and the MC4R rs12970134 SNP. Our study demonstrates the role of genetic variability in FTO and MC4R genes in obesity development in Tatar women from Russia. | 3182 | NA | NA | NA | no | no |
| 3183 | 2017 | NA | Gupta, VS; Rajebhosale, MD; Sodhi, M; Singh, S; Gnanamanickam, SS; Dhaliwal, HS; Ranjekar, PK | 2001 | Assessment of genetic variability and strain identification of Xanthomonas oryzae pv. oryzae using RAPD-PCR and IS1112-based PCR | Sixteen isolates of Xanthomonas oryzae pv, oryzae (X.o. pv. oryzae) representing different geographical locations in India along with 2 isolates from the Philippines were analysed using polymorphic RAPDs. The primers OPA-03, OPA-04, OPA-10, OPA-11, OPK-7, OPK-12 and OPK-17 generated simple, specific and reproducible fingerprint patterns, indicating usefulness of RAPD markers in differentiating X, o, pv. oryzae isolates. At a similarity index of 0.5 the isolates were grouped into only 2 clusters, suggesting that these primers are not very efficient in grouping isolates. Primers PJEL1 and PJEL2, used in insertion sequence IS1112-based PCR, also generated specific and reproducible fingerprint patterns for the same set of the X,o, pv, oryzae isolates. Based on the RAPD-PCR (seven primers) and IS1112-PCR (two primers) data, at a similarity of 0.57, sixteen out of 18 isolates were grouped into 5 different clusters and 2 isolates were loosely grouped with them. The data using RAPD-PCR and IS1112-based PCR approaches revealed their potential in rapid identification of isolates, in assessment of genetic variation in the Indian pathogen population and in generating unique DNA fragments specific to 8 isolates of X.o. pv. oryzae. | 3183 | NA | NA | NA | no | no |
| 3184 | 2017 | NA | Al-Qahtani, AA; Baele, G; Khalaf, N; Suchard, MA; Al-Anazi, MR; Abdo, AA; Sanai, FM; Al-Ashgar, HI; Khan, MQ; Al-Ahdal, MN; Lemey, P; Vrancken, B | 2017 | The epidemic dynamics of hepatitis C virus subtypes 4a and 4d in Saudi Arabia | The relatedness between viral variants sampled at different locations through time can provide information pertinent to public health that cannot readily be obtained through standard surveillance methods. Here, we use virus genetic data to identify the transmission dynamics that drive the hepatitis C virus subtypes 4a (HCV4a) and 4d (HCV4d) epidemics in Saudi Arabia. We use a comprehensive dataset of newly generated and publicly available sequence data to infer the HCV4a and HCV4d evolutionary histories in a Bayesian statistical framework. We also introduce a novel analytical method for an objective assessment of the migration intensity between locations. We find that international host mobility patterns dominate over within country spread in shaping the Saudi Arabia HCV4a epidemic, while this may be different for the HCV4d epidemic. This indicates that the subtypes 4a and 4d burden can be most effectively reduced by combining the prioritized screening and treatment of Egyptian immigrants with domestic prevention campaigns. Our results highlight that the joint investigation of evolutionary and epidemiological processes can provide valuable public health information, even in the absence of extensive metadata information. | 3184 | NA | NA | NA | no | no |
| 3185 | 2017 | NA | Odetoyin, BW; Labar, AS; Lamikanra, A; Aboderin, AO; Okeke, IN | 2017 | Classes 1 and 2 integrons in faecal Escherichia coli strains isolated from mother-child pairs in Nigeria | Background Antimicrobial resistance among enteric bacteria in Africa is increasingly mediated by integrons on horizontally acquired genetic elements. There have been recent reports of such elements in invasive pathogens across Africa, but very little is known about the faecal reservoir of integron-borne genes. Methods and findings We screened 1098 faecal Escherichia coli isolates from 134 mother-child pairs for integron cassettes by PCR using primers that anneal to the 5’ and 3’ conserved ends of the cassette regions and for plasmid replicons. Genetic relatedness of isolates was determined by flagellin and multi-locus sequence typing. Integron cassettes were amplified in 410 (37.5%) isolates and were significantly associated with resistance to trimethoprim and multiple resistance. Ten cassette combinations were found in class 1 and two in class 2 integrons. The most common class 1 cassette configurations were single aadA1 (23.4%), dfrA7 (18.3%) and dfrA5 (14.4%). Class 2 cassette configurations were all either dfrA1-satI-aadA1 (n = 31, 7.6%) or dfrA1-satI (n = 13, 3.2%). A dfr cassette was detected in 294 (31.1%) of trimethoprim resistant strains and an aadA cassette in 242 (23%) of streptomycin resistant strains. Strains bearing integrons carried a wide range of plasmid replicons of which FIB/Y (n = 169; 41.2%) was the most frequently detected. Nine isolates from five different individuals carried the dfrA17-aadA5-bearing ST69 clonal group A (CGA). The same integron cassette combination was identified from multiple distinct isolates within the same host and between four mother-child pairs. Conclusions Integrons are important determinants of resistance in faecal E. coli. Plasmids in integroncontaining strains may contribute to dispersing resistance genes. There is a need for improved surveillance for resistance and its mechanisms of dissemination and persistence and mobility of resistance genes in the community and clinical settings. | 3185 | NA | NA | NA | no | no |
| 3186 | 2017 | NA | Silva, DCG; Carvalho, MCCG; Ruas, PM; Ruas, CF; Medri, ME | 2010 | Evidence of ecotypic differentiation between populations of the tree species Parapiptadenia rigida due to flooding | The tree species Parapiptadenia rigida, native to southern South America, is frequently used in reforestation of riverbanks in Brazil. This tree is also a source of gums, tannins and essential oils, and it has some medicinal uses. We investigated flooding tolerance and genetic diversity in two populations of P. rigida; one of them was naturally exposed to flooding. Plants derived from seeds collected from each population were submitted to variable periods of experimental waterlogging and submergence. Waterlogging promoted a decrease in biomass and structural adjustments, such as superficial roots with aerenchyma and hypertrophied lenticels, that contribute to increase atmospheric oxygen intake. Plants that were submerged had an even greater reduction in biomass and a high mortality rate (40%). The two populations varied significantly in their RAPD marker profiles, in their ability to produce aerenchyma when waterlogged and to survive when submerged, suggesting ecotypic differentiation between them. Hence, the seasonal flooding that has been challenging the tropical riparian forest appears to be genetically modifying the P. rigida populations exposed to it by selecting individuals with increased ability to live under this condition. | 3186 | NA | NA | NA | no | no |
| 3187 | 2017 | NA | Thompson, JN | 1999 | Specific hypotheses on the geographic mosaic of coevolution | Coevolution is one of the major processes organizing the earth’s biodiversity. The need to understand coevolution as an ongoing process has grown as ecological concerns have risen over the dynamics of rapidly changing biological communities, the conservation of genetic diversity, and the population biology of diseases. The biggest current challenge is to understand how coevolution operates across broad geographic landscapes, linking local ecological processes with phylogeographic patterns. The geographic mosaic theory of coevolution provides a framework for asking how coevolution continually reshapes interactions across different spatial and temporal scales. It produces specific hypotheses on how geographically structured coevolution differs from coevolution at the local scale. It also provides a framework for understanding how local maladaptation can result from coevolution and why coevolved interactions may rarely produce long lists of coevolved traits that become fixed within species. Long-term field studies of the same interaction across multiple communities and spatially structured mathematical models are together beginning to show that coevolution may be a more important ongoing process than had been indicated by earlier empirical and theoretical studies lacking a geographic perspective. | 3187 | NA | NA | NA | no | no |
| 3188 | 2017 | NA | Schneider, SS; Painter-Kurt, S; Degrandi-Hoffman, G | 2001 | The role of the vibration signal during queen competition in colonies of the honeybee, Apis mellifera | Temporary polygyny (the presence of multiple queens) occurs in honeybee colonies when virgin queens (VQs) are reared for reproductive swarming or queen replacement. During these events, workers perform vibration signals on queen cells and emerged queens, and these signals may influence which VQ becomes the new laying queen of a colony. We examined the role of vibration signals during queen competition in two African and six European honeybee colonies. There was pronounced variability in vibration activity between colonies and among queens reared within the same colony. Despite this variation, all colonies showed similar trends in the relationships between the vibration signal and queen replacement. Vibration signals performed on queen cells were not associated with emergence success. Likewise, the signal was not associated with queen emergence order. Early emerging and late-emerging queens were vibrated at similar rates, and there was no clear relationship between emergence order and VQ survival. However, the signals performed on VQs after they emerged were associated with their behaviour and success during the queen elimination period. Emerged VQs that were vibrated at higher rates survived longer, performed more bouts of piping (a characteristic sound produced by queens), eliminated more rivals and were more likely to become the new queens of the colonies. The vibration signal may therefore allow workers a degree of control over the behaviour of emerged VQs, and may influence the outcome of queen competition-in honeybees. Differences in vibration activity within and among colonies may reflect differences in the extent to which workers and queens conflict over the timing and outcome of polygyny reduction. (C) 2001 The Association for the Study of Animal Behaviour. | 3188 | NA | NA | NA | no | no |
| 3189 | 2017 | NA | Fabbri, E; Caniglia, R; Kusak, J; Galov, A; Gomercic, T; Arbanasic, H; Huber, D; Randi, E | 2014 | Genetic structure of expanding wolf (Canis lupus) populations in Italy and Croatia, and the early steps of the recolonization of the Eastern Alps | After centuries of range contraction and demographic declines wolves are now expanding in Europe. colonizing regions from where they have been absent for centuries. Wolf colonizing the western Alps originate by the expansion of the Italian population. Vagrant wolves of Italian and Dinaric-Balkan origins have been recently observed in the Eastern Alps. In this study we compared the genetic structure of wolf populations in Italy and Croatia, aiming to identify the sources of the ongoing recolonization of the Eastern Alps. DNA samples, extracted from 282 Italian and 152 Croatian wolves, were genotyped at 12 autosomal microsatellites (STR), four Y-linked STR and at the hypervariable part of the mitochondrial DNA control-region (mtDNA CR1). Wolves in Croatia and Italy underwent recent demographic bottlenecks, but they differ in genetic diversity and population structure. Wolves in Croatia were more variable at STR loci (N-A = 7.4, H-O = 0.66, H-E = 0.72; n = 152) than wolves in Italy (N-A = 5.3, H-O = 0.57, H-E = 0.58; n = 282). We found four mitochondrial DNA (mtDNA CR1) and 11 Y-STR haplotypes in Croatian wolves, but only one mtDNA CR1 and three Y-STR haplotypes in Italy. Wolves in Croatia were subdivided into three genetically distinct subpopulations (in Dalmatia. Gorski kotar and Lika regions), while Italian wolves were not sub-structured. Assignment testing shows that the eastern and central Alps are recolonized by wolves dispersing from both the Italian and Dinaric populations. The recolonization of the Alps will predictably continue in the future and the new population will be genetically admixed and very variable with greater opportunities for local adaptations and survival. (C) 2013 Deutsche Gesellschaft fur Saugetierkunde. Published by Elsevier GmbH. All rights reserved. | 3189 | NA | NA | NA | no | no |
| 3190 | 2017 | NA | Balmer, O; Stearns, SC; Schotzau, A; Brun, R | 2009 | Intraspecific competition between co-infecting parasite strains enhances host survival in African trypanosomes | It is becoming increasingly clear that under natural conditions parasitic infections commonly consist of co-infections with multiple conspecific strains. Multiple-strain infections lead to intraspecific interactions and may have important ecological and evolutionary effects on both hosts and parasites. However, experimental evidence on intraspecific competition or facilitation in infections has been scarce because of the technical challenges of distinguishing and tracking individual co-infecting strains. To overcome this limitation, we engineered transgenic strains of the protozoan parasite Trypanosoma brucei, the causal agent of human African sleeping sickness. Different strains were transfected with fluorescence genes of different colors to make them visually distinguishable in order to investigate the effects of multiple-strain infections on parasite population dynamics and host fitness. We infected mice either with each strain alone or with mixes of two strains. Our results show a strong mutual competitive suppression of co-infecting T. brucei strains very early in infection. This mutual suppression changes within-host parasite dynamics and alleviates the effects of infection on the host. The strength of suppression depends on the density of the coinfecting strain, and differences in life-history traits between the strains determine the consequences of strain-strain competition for the host. Unexpectedly, co-infection with a less virulent strain significantly enhances host survival (+15%). Analysis of the strain dynamics reveals that this is due to the suppression of the density of the more virulent strain (-33%), whose degree of impact ultimately determines the physical condition of the host. The competitive suppression is likely caused by allelopathic interference or by apparent competition mediated by strain-specific immune responses. These findings highlight the importance of intraspecific variation for parasite-parasite and parasite-host interactions. To fully understand parasite and disease dynamics, the genetic diversity of infections must be taken into account. Through changes in parasite dynamics, intraspecific variation may further affect transmission dynamics and select for increased virulence of each strain. The precise mechanisms underlying mutual suppression are not yet understood but may be exploitable to fight this devastating parasite. Our results are therefore not only of basic ecological interest investigating an important form of intraspecific competition, but may also have applied relevance for public health. | 3190 | NA | NA | NA | no | no |
| 3191 | 2017 | NA | Brown, SP | 1999 | Cooperation and conflict in host-manipulating parasites | The existence of adaptive host manipulation by parasites has received increasing empirical support in recent years. Here I develop an optimality model of the extent of host manipulation, incorporating within-host group size, relatedness and a range of realistic cost-benefit functions. The model highlights the cooperative nature of host manipulation, and the potential for cheating this entails. When relatedness in parasite groups is minimal, manipulation is suppressed, but not eradicated, reflecting the importance of interhost selection. A distinctive threshold phenomenon is predicted for a wide range of parameter values. Below the group size threshold, manipulation is zero. Above the threshold, the predicted behaviour depends critically on the biological details of the individual-group interaction. The host-manipulation model is discussed in the light of two potential applications. If parasite group size is assumed to be a static characteristic of a parasite species or strain, the model generates a set of comparative predictions best suited to macroparasite systems. Additionally, the model can be used to predict density-dependent behavioural changes in expanding groups of parasites, as seen in quorum-sensing bacteria. | 3191 | NA | NA | NA | no | no |
| 3192 | 2017 | NA | Li, YJ; Cui, YJ; Cui, BZ; Yan, YF; Yang, XW; Wang, HD; Qi, ZZ; Zhang, QW; Xiao, X; Guo, ZB; Ma, C; Wang, J; Song, YJ; Yang, RF | 2013 | Features of Variable Number of Tandem Repeats in Yersinia pestis and the Development of a Hierarchical Genotyping Scheme | Background: Variable number of tandem repeats (VNTRs) that are widely distributed in the genome of Yersinia pestis proved to be useful markers for the genotyping and source-tracing of this notorious pathogen. In this study, we probed into the features of VNTRs in the Y. pestis genome and developed a simple hierarchical genotyping system based on optimized VNTR loci. Methodology/Principal Findings: Capillary electrophoresis was used in this study for multi-locus VNTR analysis (MLVA) in 956 Y. pestis strains. The general features and genetic diversities of 88 VNTR loci in Y. pestis were analyzed with BioNumerics, and a “14+12” loci-based hierarchical genotyping system, which is compatible with single nucleotide polymorphism-based phylogenic analysis, was established. Conclusions/Significance: Appropriate selection of target loci reduces the impact of homoplasies caused by the rapid mutation rates of VNTR loci. The optimized “14+12” loci are highly discriminative in genotyping and source-tracing Y. pestis for molecular epidemiological or microbial forensic investigations with less time and lower cost. An MLVA genotyping datasets of representative strains will improve future research on the source-tracing and microevolution of Y. pestis. | 3192 | NA | NA | NA | no | no |
| 3193 | 2017 | NA | Liu, QY; Chen, WY; Liu, JL; Huang, RL; Wang, QH; Yu, J; Du, XD; Deng, YW; Li, JH | 2014 | Genetic variation and an estimation of effective population size in the pearl oyster Pinctada martensii | In September 2011, two wild populations of pearl oysters were collected separately from Daya Bay, Shenzhen, and from Beibu Bay, Beihai, China. In April 2012, three cultured stocks were established by sampling breeders from the third-generation selected lines. The genetic variation within the five stocks was analyzed using eight SSR primer pairs. The effective population sizes of the five stocks were analyzed using the sibship reconstruction and multiple summary statistics methods. A total of 22-29 alleles were observed in the five stocks, with two to five alleles per locus and means ranging from 2.750 to 3.625 alleles per locus. The average expected and observed heterozygosities ranged from 0.3387 to 0.5029 and from 0.2917 to 0.4219, respectively. The effective population sizes of the five stocks ranged from 11.0 to 20.0 when estimated using sibship reconstruction and from 13.7 to 179.4 when estimated using multiple summary statistics. The effective population sizes estimated in the three cultured stocks were smaller than the number of breeders used to produce these stocks. Thus, it may be necessary to augment the number of breeders used to produce progeny stocks in selective breeding programs. (C) 2014 Elsevier Ltd. All rights reserved. | 3193 | NA | NA | NA | no | no |
| 3194 | 2017 | NA | Mishra, D; Vishnupriya, MR; Anil, MG; Konda, K; Raj, Y; Sonti, RV | 2013 | Pathotype and Genetic Diversity amongst Indian Isolates of Xanthomonas oryzae pv. oryzae | A number of rice resistance genes, called Xa genes, have been identified that confer resistance against various strains of Xanthomonas oryzae pv. oryzae (Xoo), the causal agent of bacterial blight. An understanding of pathotype diversity within the target pathogen population is required for identifying the Xa genes that are to be deployed for development of resistant rice cultivars. Among 1024 isolates of Xoo collected from 20 different states of India, 11 major pathotypes were distinguished based on their reaction towards ten Xa genes (Xa1, Xa3, Xa4, xa5, Xa7, xa8, Xa10, Xa11, xa13, Xa21). Isolates belonging to pathotype III showing incompatible interaction towards xa8, xa13 and Xa21 and compatible interaction towards the rest of Xa genes formed the most frequent (41%) and widely distributed pathotype. The vast majority of the assayed Xoo isolates were incompatible with one or more Xa genes. Exceptionally, the isolates of pathotype XI were virulent on all Xa genes, but have restricted distribution. Considering the individual R-genes, Xa21 appeared as the most broadly effective, conferring resistance against 88 % of the isolates, followed in decreasing order by xa13 (84 %), xa8 (64 %), xa5 (30 %), Xa7 (17 %) and Xa4 (14 %). Fifty isolates representing all the eleven pathotypes were analyzed by southern hybridization to determine their genetic relatedness using the IS1112 repeat element of Xoo. Isolates belonging to pathotype XI were the most divergent. The results suggest that one RFLP haplotype that is widely distributed all over India and is represented in strains from five different pathotypes might be an ancestral hapfotype. A rice line with xa5, xa13 and Xa21 resistance genes is resistant to all strains, including those belonging to pathotype XI. This three gene combination appears to be the most suitable Xa gene combination to be deployed in Indian rice cultivars. | 3194 | NA | NA | NA | no | no |
| 3195 | 2017 | NA | Laxmivandana, R; Yergolkar, P; Gopalkrishna, V; Chitambar, SD | 2013 | Characterization of the Non-Polio Enterovirus Infections Associated with Acute Flaccid Paralysis in South-Western India | Non-polio enteroviruses (NPEVs) have been reported frequently in association with acute flaccid paralysis (AFP) cases during Polio Surveillance Programs (PSPs) worldwide. However, there is limited understanding on the attributes of their infections. This study reports characteristics of NPEVs isolated from AFP cases, investigated during PSPs held in 2009-2010, in Karnataka and Kerala states of south-western India having varied climatic conditions. NPEV cell culture isolates derived from stool specimens that were collected from 422 of 2186 AFP cases (<1-14 years age) and 17 of 41 asymptomatic contacts; and details of all AFP cases/contacts were obtained from National Polio Laboratory, Bangalore. The distribution of NPEV infections among AFP cases and circulation pattern of NPEV strains were determined by statistical analysis of the data. Genotyping of all NPEV isolates was carried out by partial VP1 gene sequencing and phylogenetic analysis. NPEV positive AFP cases were significantly higher in children aged <2 years; with residual paralysis; in summer months; and in regions with relatively hot climate. Genotyping of NPEVs identified predominance of human enteroviruses (HEV)-B species [81.9%-Echoviruses (E): 57.3%; coxsackieviruses (CV) B: 15%; numbered EVs: 8.9%; CVA9: 0.7%] and low levels of HEV-A [14.5%-CVA: 6%; numbered EVs: 8.5%] and HEV-C [3.6%-CVA: 2.6%; numbered EVs: 1%] species, encompassing 63 genotypes. EV76 (6.3%) and each of E3, CVB3 and E9 (4.97%) were found frequently during 2009 while E11 (6.7%), CVB1 (6.1%), E7 (5.1%) and E20 (5.1%) were detected commonly in 2010. A marked proportion of AFP cases from children aged,2 years; presenting with fever; and from north and south interior parts of Karnataka state was detected with E/numbered EVs than that found with CVA/CVB. This study highlights the extensive genetic diversity and diverse circulation patterns of NPEV strains in AFP cases from different populations and climatic conditions. | 3195 | NA | NA | NA | no | no |
| 3196 | 2017 | NA | Schouten, HJ | 1996 | A model examining the effect of environmental sex determination in parasites on the breakdown of monogenic host resistance | If sex determination of a parasite depends on feeding conditions on its host, then good feeding conditions may favour development into one sex, but poor feeding conditions may enhance development into the other sex. This implies that a resistant host may exert a selection pressure towards virulence via one sex? but a selection pressure towards avirulence via the other sex. Continuous cultivation of a resistant host may therefore lead to an equilibrium frequency of avirulent parasites, and consequently to a durable partial resistance to the population. In this way the parasite may maintain its genetic diversity, at the expense of its reproduction rate. In a previous paper(Schouten, 1994) this phenomenon has been elaborated mathematically for complete, monogenic resistance. Tn the present paper the derivation is widened towards partial monogenic resistance to avirulent parasites, and is worked out further for potato cyst nematodes (Globodera rostochiensis and G. pallida). The equilibrium frequency of avirulent nematodes ranges from 0 to 100 %, but the ensuing durable resistance is lower than 50 %. Thin roots and high nematode densities lower the equilibrium frequency of avirulent nematodes and the matching resistance level. | 3196 | NA | NA | NA | no | no |
| 3197 | 2017 | NA | Huang, Q; Lattorff, HMG; Kryger, P; Le Conte, Y; Moritz, RFA | 2014 | A selective sweep in a microsporidian parasite Nosema-tolerant honeybee population, Apis mellifera | Nosema is a microsporidian parasite of the honeybee, which infects the epithelial cells of the gut. In Denmark, honeybee colonies have been selectively bred for the absence of Nosema over decades, resulting in a breeding line that is tolerant toward Nosema infections. As the tolerance toward the Nosema infection is a result of artificial selection, we screened chromosome 14 for a selective sweep with microsatellite markers, where a major quantitative trait locus (QTL) had been identified to be involved in the reduction in Nosema spores in the honeybees. By comparing the genetic variability of 10 colonies of the selected honeybee strain with a population sample from 22 unselected colonies, a selective sweep was revealed within the previously identified QTL region. The genetic variability of the swept loci was not only reduced in relation to the flanking markers on chromosome 14 within the selected strain but also significantly reduced compared with the same region in the unselected honeybees. This confirmed the results of the previous QTL mapping for reduced Nosema infections. The success of the selective breeding may have driven the selective sweep found in our study. | 3197 | NA | NA | NA | no | no |
| 3198 | 2017 | NA | Lens, L; Van Dongen, S; Matthysen, E | 2002 | Fluctuating asymmetry as an early warning system in the critically endangered Taita thrush | Environmental and genetic factors increasingly, stress natural populations, and conservationists need simple tools to measure the effect of these stresses before populations become irreversibly, affected. The inability of an organism to withstand random perturbations during its development-so-called developmental instability-has been proposed as a potential early, indicator of stress. To act as a true earl), warning system, however, stress-mediated changes in developmental instability, should be manifest in the phenotype before major changes occur in more direct components of fitness. We tested this hypothesis by, estimating the survival Probabilities of 260 Taita Thrushes (Turdus helleri) inhabiting three differentially, disturbed forest fragments (little, moderately,, and highly, deteriorated) and relating these to individual estimates of tarsus asymmetry, (a measure of developmental instability,), two other putative indices of expected fitness (size and body, condition), and a coefficient of inbreeding. Tarsus symmetry, but not survival, was lower in the moderately disturbed population than in the least disturbed one, whereas both symmetry, and survival were lower in the most disturbed population. Thus, higher levels of habitat disturbance were reflected by, increased population levels of asymmetry before a decrease in survival became apparent. In contrast, none of the other variables showed a significant response to habitat disturbance. At the individual level, asymmetric thrushes showed lower probabilities of survival than more symmetric ones, and the magnitude of the relationship increased with habitat disturbance. Birds in better condition (i.e., higher residual body, mass) survived better, irrespective of the quality, of the habitat. The key,finding that stress-mediated increase influctuating asymmetry may, precede decrease in survival suggests that single-trait asymmetry can serve as an early warning system in conservation. | 3198 | NA | NA | NA | no | no |
| 3199 | 2017 | NA | Estrada-Pena, A; Naranjo, V; Acevedo-Whitehouse, K; Mangold, AJ; Kocan, KM; de la Fuente, J | 2009 | Phylogeographic analysis reveals association of tick-borne pathogen, Anaplasma marginale, MSP1a sequences with ecological traits affecting tick vector performance | Background: The tick-borne pathogen Anaplasma marginale, which is endemic worldwide, is the type species of the genus Anaplasma (Rickettsiales: Anaplasmataceae). Rhipicephalus (Boophilus) microplus is the most important tick vector of A. marginale in tropical and subtropical regions of the world. Despite extensive characterization of the genetic diversity in A. marginale geographic strains using major surface protein sequences, little is known about the biogeography and evolution of A. marginale and other Anaplasma species. For A. marginale, MSP1a was shown to be involved in vector-pathogen and host-pathogen interactions and to have evolved under positive selection pressure. The MSP1a of A. marginale strains differs in molecular weight because of a variable number of tandem 23-31 amino acid repeats and has proven to be a stable marker of strain identity. While phylogenetic studies of MSP1a repeat sequences have shown evidence of A. marginale-tick co-evolution, these studies have not provided phylogeographic information on a global scale because of the high level of MSP1a genetic diversity among geographic strains. Results: In this study we showed that the phylogeography of A. marginale MSP1a sequences is associated with world ecological regions (ecoregions) resulting in different evolutionary pressures and thence MSP1a sequences. The results demonstrated that the MSP1a first (R1) and last (RL) repeats and microsatellite sequences were associated with world ecoregion clusters with specific and different environmental envelopes. The evolution of R1 repeat sequences was found to be under positive selection. It is hypothesized that the driving environmental factors regulating tick populations could act on the selection of different A. marginale MSP1a sequence lineages, associated to each ecoregion. Conclusion: The results reported herein provided the first evidence that the evolution of A. marginale was linked to ecological traits affecting tick vector performance. These results suggested that some A. marginale strains have evolved under conditions that support pathogen biological transmission by R. microplus, under different ecological traits which affect performance of R. microplus populations. The evolution of other A. marginale strains may be linked to transmission by other tick species or to mechanical transmission in regions where R. microplus is currently eradicated. The information derived from this study is fundamental toward understanding the evolution of other vector-borne pathogens. | 3199 | NA | NA | NA | no | no |
| 3200 | 2017 | NA | Da Conceicao, ILPM; Dos Santos, MCV; Abrantes, IMD; Santos, MSND | 2003 | Using RAPD markers to analyse genetic diversity in Portuguese potato cyst nematode populations | The two species of potato cyst nematodes (PCN) have different spectra of virulence towards host resistance genes and a proper identification is important in the control of these nematodes by means of resistant potato cultivars. Genetic variability of 32 populations of Globodera rostochiensis and three of G. pallida from different regions of Portugal were analysed and compared using random amplified polymorphic DNA and sixteen primers. In addition, a population of G. pallida (Luffness) from Scotland, UK, was included for comparison. The patterns obtained were evaluated using the Nei coefficient and the UPGMA cluster method. The resulting clusters were expressed as dendrograms in the Phylogeny Inference Package (PHYLIP) program. Separation of the two species of PCN was achieved with an average similarity of 43%. Two populations of G. rostochiensis (PT1 and PT82) appear to be distinct from the main group of this species. One population of G. pallida (PT104) was distinct from the others of this species and the Luffness population was linked most closely with PT5701. Distinct clusters were observed within both species but the clusters could not be related to the geographic proximity of the populations. | 3200 | NA | NA | NA | no | no |
| 3201 | 2017 | NA | Boyle, RA; Lenton, TM; Williams, HTP | 2007 | Neoproterozoic ‘snowball Earth’ glaciations and the evolution of altruism | We hypothesize that a demographic and ecological effect of Neoproterozoic ‘snowball Earth’ glaciations was to increase the fitness of group-level traits and consequently the likelihood of the evolution of macroscopic form. Extreme and repeated founder effects raised genetic relatedness - and therefore the influence of kin selection on the individuals within a group. This was permissive for the evolution of some highly costly altruistic traits, including those for macroscopic differentiation. In some eukaryotic species, the harsh and fluctuating abiotic conditions made a macroscopic physiology advantageous, perhaps necessary, for collective survival. This caused population-wide group viability selection, whereby non-altruist ‘cheat’ genotypes killed the groups they were in, and therefore themselves, by reaching fixation. Furthermore, dispersal between refugia would reach zero under anything near a ‘hard snowball’, which would protect altruists at high local frequency from the influx of cheats from neighbouring groups. We illustrate our hypothesis analytically and with a simple spatial model. We show how removal of between-group dispersal, in a population with initial between-group variation in cheat frequency, causes the relative frequency of altruists to increase while the population as a whole decreases in size, as a result of group death caused by cheat invasion. This may be of particular relevance to animal multicellularity because irreversible differentiation ( highly altruistic in that it imposes a high fitness cost on the individual cell) is more prevalent than in other multicellular eukaryotes. The relevance of our hypothesis should be scaled by any future consensus on the severity of snowball Earth, but it is theoretically plausible that global-scale glaciations had a systematic influence on the level of selection during Earth history. | 3201 | NA | NA | NA | no | no |
| 3202 | 2017 | NA | McNair, A; Nakagawa, S; Grimm, V | 2014 | The Evolutionary Consequences of Disrupted Male Mating Signals: An Agent-Based Modelling Exploration of Endocrine Disrupting Chemicals in the Guppy | Females may select a mate based on signalling traits that are believed to accurately correlate with heritable aspects of male quality. Anthropogenic actions, in particular chemicals released into the environment, are now disrupting the accuracy of mating signals to convey information about male quality. The long-term prediction for disrupted mating signals is most commonly loss of female preference. Yet, this prediction has rarely been tested using quantitative models. We use agent-based models to explore the effects of rapid disruption of mating signals. In our model, a gene determines survival. Males signal their level of genetic quality via a signal trait, which females use to select a mate. We allowed this system of sexual selection to become established, before introducing a disruption between the male signal trait and quality, which was similar in nature to that induced by exogenous chemicals. Finally, we assessed the capacity of the system to recover from this disruption. We found that within a relatively short time frame, disruption of mating signals led to a lasting loss of female preference. Decreases in mean viability at the population-level were also observed, because sexual-selection acting against newly arising deleterious mutations was relaxed. The ability of the population to recover from disrupted mating signals was strongly influenced by the mechanisms that promoted or maintained genetic diversity in traits under sexual selection. Our simple model demonstrates that environmental perturbations to the accuracy of male mating signals can result in a long-term loss of female preference for those signals within a few generations. What is more, the loss of this preference can have knock-on consequences for mean population fitness. | 3202 | NA | NA | NA | no | no |
| 3203 | 2017 | NA | Brouwer, L; Komdeur, J; Richardson, DS | 2007 | Heterozygosity-fitness correlations in a bottlenecked island species: a case study on the Seychelles warbler | We used capture-mark-recapture models to investigate the effects of both individual and parental heterozygosity, measured at microsatellite loci on the survival of Seychelles warblers (Acrocephalus sechellensis), an endemic island species which went through a severe population bottleneck in the middle of the last century. We found that an individual’s survival was not correlated with multilocus heterozygosity, or with heterozygosity at any specific locus. However, maternal, but not paternal, multilocus heterozygosity was positively associated with offspring survival, but only in years with low survival probabilities. A nestling cross-fostering experiment showed that this was a direct maternal effect as there was an effect of the genetic mother’s, but not of the social mother’s, heterozygosity. Heterozygosity-fitness correlations at microsatellite markers were generally assumed to reflect genome-wide effects. Although this might be true in partially inbred populations, such correlations may also arise as a result of local effects with specific markers being closely linked to genes which determine fitness. However, heterozygosity at the individual microsatellite loci was not correlated and therefore does not seem to reflect genome-wide heterozygosity. This suggests that even in a small bottlenecked population, heterozygosity-fitness correlations may not be caused by genome-wide effects. Support for the local effects hypothesis was also equivocal; although three specific loci were associated with offspring survival, including all single-locus heterozygosities as independent predictors for the variation in survival was not supported by the data. Furthermore, in contrast to the local effects hypothesis, the loci which contributed most to the heterozygosity-survival relationship were not more polymorphic than the other loci. This study highlights the difficulties in distinguishing between the two hypotheses. | 3203 | NA | NA | NA | no | no |
| 3204 | 2017 | NA | Jirsova, D; Stefka, J; Jirku, M | 2017 | Discordant population histories of host and its parasite: A role for ecological permeability of extreme environment? | Biogeographical and ecological barriers strongly affect the course of micro-evolutionary processes in free living organisms. Here we assess the impact of a recently emerged barrier on populations of limnic fauna. Genetic diversity and population structure in a host-parasite system (Wenyonia virilis tapeworm, Synodontis schall catfish) are analyzed in the recently divided Turkana and Nile basins. The two basins, were repeatedly connected during the Holocene wet/dry climatic oscillations, following late Pleistocene dessication of the Turkana basin. Mitochondrial DNA sequences for cytochrome oxidase I gene (cox I) and a whole genome scanning method amplified fragment length polymorphism (AFLP) were employed. A total of 347 cox I sequences (representing 209 haplotypes) and 716 AFLP fragments, as well as 120 cox I sequences (20 haplotypes) and 532 AFLP fragments were obtained from parasites and hosts, respectively. Although results indicate that host and parasite populations share some formative traits (bottlenecks, Nilotic origin), their population histories/patterns differ markedly. Mitochondrial analysis revealed that parasite populations evolve significantly faster and show remarkably higher genetic variability. Analyses of both markers confirmed that the parasites undergo lineage fission, forming new clusters specific for either freshwater or saline parts of Lake Turkana. In congruence with the geological history, these clusters apparently indicate multiple colonisations of Lake Turkana from the Nile. In contrast, the host population pattern indicates fusion of different colonisation waves. Although fish host populations remain connected, saline habitats in Lake Turkana (absent in the Nile), apparently pose a barrier to the gene flow in the parasite, possibly due to its multihost lifecycle, which involves freshwater annelids. Despite partially corroborating mitochondrial results, AFLP data was not sufficiently informative for analyzing populations with recently mixed biogeographic histories. | 3204 | NA | NA | NA | no | no |
| 3205 | 2017 | NA | Jewett, EM; Rosenberg, NA | 2014 | Theory and applications of a deterministic approximation to the coalescent model | Under the coalescent model, the random number n(t) of lineages ancestral to a sample is nearly deterministic as a function of time when n(t) is moderate to large in value, and it is well approximated by its expectation E[n(t)]. In turn, this expectation is well approximated by simple deterministic functions that are easy to compute. Such deterministic functions have been applied to estimate allele age, effective population size, and genetic diversity, and they have been used to study properties of models of infectious disease dynamics. Although a number of simple approximations of E[n(t)] have been derived and applied to problems of population-genetic inference, the theoretical accuracy of the resulting approximate formulas and the inferences obtained using these approximations is not known, and the range of problems to which they can be applied is not well understood. Here, we demonstrate general procedures by which the approximation n(t) approximate to E[n(t)] can be used to reduce the computational complexity of coalescent formulas, and we show that the resulting approximations converge to their true values under simple assumptions. Such approximations provide alternatives to exact formulas that are computationally intractable or numerically unstable when the number of sampled lineages is moderate or large. We also extend an existing class of approximations of E[n(t)] to the case of multiple populations of time-varying size with migration among them. Our results facilitate the use of the deterministic approximation n(t) approximate to E[n(t)] for deriving functionally simple, computationally efficient, and numerically stable approximations of coalescent formulas under complicated demographic scenarios. (c) 2014 Elsevier Inc. All rights reserved. | 3205 | NA | NA | NA | no | no |
| 3206 | 2017 | NA | Prugnolle, F; Roze, D; Theron, A; DE Meeus, T | 2005 | F-statistics under alternation of sexual and asexual reproduction: a model and data from schistosomes (platyhelminth parasites) | Accurate inferences on population genetics data require a sound underlying theoretical null model. Nearly nothing is known about the gene dynamics of organisms with complex life cycles precluding any biological interpretation of population genetics parameters. In this article, we used an infinite island model to derive the expectations of those parameters for the life cycle of a dioecious organism obligatorily alternating sexual and asexual reproductions as it is the case for schistosomes (plathyhelminth parasites). This model allowed us to investigate the effects of the degree of mixing among individuals coming from different subpopulations at each new generation (represented in the model by the migration rates before and after clonal reproductions) and the variance in the reproductive success of individuals during the clonal phase. We also consider the effects of different migration rates and degrees of clonal reproductive skew between male and female individuals. Results show that the variance in the reproductive success of clones is very important in shaping the distribution of the genetic variability both within and among subpopulations. Thus, higher variance in the reproductive success of clones generates heterozygous excesses within subpopulations and also increases genetic differentiation between them. Migration occurring before and after asexual reproduction has different effects on the patterns of F-IS and F-ST. When males and females display different degrees of reproductive skew or migration rates, we observe differences in their respective population genetic structure. While results of the model apply to any organism alternating sexual and clonal reproductions (e.g. all parasitic trematodes, many plants, and all aphididae), we finally confront some of these theoretical expectations to empirical data from Schistosoma mansoni infecting Rattus rattus in Guadeloupe. | 3206 | NA | NA | NA | no | no |
| 3207 | 2017 | NA | Liu, W; Fang, LR; Li, M; Li, S; Guo, SH; Luo, R; Feng, ZX; Li, B; Zhou, ZM; Shao, GQ; Chen, HC; Xiao, SB | 2012 | Comparative Genomics of Mycoplasma: Analysis of Conserved Essential Genes and Diversity of the Pan-Genome | Mycoplasma, the smallest self-replicating organism with a minimal metabolism and little genomic redundancy, is expected to be a close approximation to the minimal set of genes needed to sustain bacterial life. This study employs comparative evolutionary analysis of twenty Mycoplasma genomes to gain an improved understanding of essential genes. By analyzing the core genome of mycoplasmas, we finally revealed the conserved essential genes set for mycoplasma survival. Further analysis showed that the core genome set has many characteristics in common with experimentally identified essential genes. Several key genes, which are related to DNA replication and repair and can be disrupted in transposon mutagenesis studies, may be critical for bacteria survival especially over long period natural selection. Phylogenomic reconstructions based on 3,355 homologous groups allowed robust estimation of phylogenetic relatedness among mycoplasma strains. To obtain deeper insight into the relative roles of molecular evolution in pathogen adaptation to their hosts, we also analyzed the positive selection pressures on particular sites and lineages. There appears to be an approximate correlation between the divergence of species and the level of positive selection detected in corresponding lineages. | 3207 | NA | NA | NA | no | no |
| 3208 | 2017 | NA | Meng, A; Gong, G; Chen, D; Zhang, H; Qi, S; Tang, H; Gao, Z | 1996 | DNA fingerprint variability within and among parental lines and its correlation with performance of F-1 laying hens | Genetic diversity within and among nine pure lines of Beijing White Leghorn chickens was determined by DNA fingerprinting using human ministatellite probes 33.6 and alpha-globin 3’HVR, as well as bacteriophage M13. Within lines similarity coefficients ranged from 0.497 to 0.628, significantly higher than that within a sample of white chicken from a local market. Relationships among lines were established by clustering analysis based on inter-line coefficients of difference calculated from DNA fingerprints of pooled DNA. A complete diallel crossing among the nine pure lines was conducted. By using linear correlation analysis, it was found that the maximum distance between parental lines was positively correlated with egg number, egg production, survival rate and their corresponding heterosis percentages within a pair of reciprocal crosses. Similar relationships were found where only the higher of the reciprocal crosses were used in the analysis. It was also shown that similarity coefficients within a sire line or dam line were positively correlated with 40-week egg number and its heterosis percentage and the heterosis percentage for 40-week egg production, but negatively correlated with the 40-week survival rate of the cross-bred populations. | 3208 | NA | NA | NA | no | no |
| 3209 | 2017 | NA | Kummerli, R; van den Berg, P; Griffin, AS; West, SA; Gardner, A | 2010 | Repression of competition favours cooperation: experimental evidence from bacteria | Repression of competition (RC) within social groups has been suggested as a key mechanism driving the evolution of cooperation, because it aligns the individual’s proximate interest with the interest of the group. Despite its enormous potential for explaining cooperation across all levels of biological organization, ranging from fair meiosis, to policing in insect societies, to sanctions in mutualistic interactions between species, there has been no direct experimental test of whether RC favours the spread of cooperators in a well-mixed population with cheats. To address this, we carried out an experimental evolution study to test the effect of RC upon a cooperative trait - the production of iron-scavenging siderophore molecules - in the bacterium Pseudomonas aeruginosa. We found that cooperation was favoured when competition between siderophore producers and nonsiderophore-producing cheats was repressed, but not in a treatment where competition between the two strains was permitted. We further show that RC altered the cost of cooperation, but did not affect the relatedness among interacting individuals. This confirms that RC per se, as opposed to increased relatedness, has driven the observed increase in bacterial cooperation. | 3209 | NA | NA | NA | no | no |
| 3210 | 2017 | NA | PAVLIK, BM; NICKRENT, DL; HOWALD, AM | 1993 | THE RECOVERY OF AN ENDANGERED PLANT .1. CREATING A NEW POPULATION OF AMSINCKIA-GRANDIFLORA | The recovery of endangered plants often requires the creation of new populations in order to decrease the risk of extinction Despite numerous attempts, no plant species have been fully recovered by creating new populations. Here we report on initial efforts to recover Amsinckia grandiflora Kleeb. ex Gray (Boraginaceae) by re-establishing the species in appropriate habitat within its historic range, with consideration given to genetic and demographic characteristics of the founding population. An experimental framework with demographic monitoring was used to follow the fates of nutlets (propagules)from two sources (one wild, one cultivated) and to evaluate the effects of habitat manipulations (fire. herbicide application, clipping) for reducing competition from introduced grasses. Founding nutlets from two sources bad different germination and genetic characteristics. Nutlets directly descended from a wild Population bad half the germination potential of nutlets from a cultivated population because of their greater age (about 25 years in cold storage). Levels of genetic variability, as measured by allozyme electrophoresis at 18 loci, were low overall, but much more so in the cultivated population These data were used to maximize genetic variability among founding nutlets and to predict the frequency of alternative allele carriers in the new population. After sowing and plot treatment at the Lougher Ridge re-establishment site, the 3460 founding nutlets produced a large number of germinules (1774) during the 1989-1990 growing season, and many (1101) survived to reproduce- From these plants, and estimated 35,800 nutlets were produced, indicating that the population bad a high potential for growth during subsequent years. Introduced grasses baa significant negative effects on survivorship to reproduction, plant size, and nutlet production in the new Amsinckia population Grass cover was effectively reduced by using fire or a grass-specific herbcide. Burning significantly decreased Amsinckia mortality rates early in the growing season and significantly increased survivorship to reproduction and maximum plant size- Herbicide treatment bad no effect on mortality rates or survivorship to reproduction, but it significantly increased plant size and, therefore, nutlet output per plant and per plot. The new population should be able to maintain itself within the existing grassland community, but growth and short-term viability would be assured by management practices that increased the availability of low-competition habitat patches for Amsinckia occupation | 3210 | NA | NA | NA | no | no |
| 3211 | 2017 | NA | Li, Z; Qi, XL; Ren, XG; Cui, L; Wang, XM; Zhu, P | 2015 | Molecular characteristics and evolutionary analysis of a very virulent infectious bursal disease virus | Infectious bursal disease virus (IBDV) poses a significant threat to the poultry industry. Viral protein 2 (VP2), the major structural protein of IBDV, has been subjected to frequent mutations that have imparted tremendous genetic diversity to the virus. To determine how amino acid mutations may affect the virulence of IBDV, we built a structural model of VP2 of a very virulent strain of IBDV identified in China, vvIBDV Gx, and performed a molecular dynamics simulation of the interaction between virulence sites. The study showed that the amino acid substitutions that distinguish vvIBDV from attenuated IBDV (H253Q and T284A) favor a hydrophobic and flexible conformation of beta-barrel loops in VP2, which could promote interactions between the virus and potential IBDV-specific receptors. Population sequence analysis revealed that the IBDV strains prevalent in East Asia show a significant signal of positive selection at virulence sites 253 and 284. In addition, a signal of co-evolution between sites 253 and 284 was identified. These results suggest that changes in the virulence of IBDV may result from both the interaction and the co-evolution of multiple amino acid substitutions at virulence sites. | 3211 | NA | NA | NA | no | no |
| 3212 | 2017 | NA | Feigel, A; Englander, A; Engel, A | 2009 | Sex Is Always Well Worth Its Two-Fold Cost | Sex is considered as an evolutionary paradox, since its positive contribution to Darwinian fitness remains unverified for some species. Defenses against unpredictable threats (parasites, fluctuating environment and deleterious mutations) are indeed significantly improved by wider genetic variability and by positive epistasis gained by sexual reproduction. The corresponding evolutionary advantages, however, do not overcome universally the barrier of the two-fold cost for sharing half of one’s offspring genome with another member of the population. Here we show that sexual reproduction emerges and is maintained even when its Darwinian fitness is twice as low as the fitness of asexuals. We also show that more than two sexes (inheritance of genetic material from three or even more parents) are always evolutionary unstable. Our approach generalizes the evolutionary game theory to analyze species whose members are able to sense the sexual state of their conspecifics and to adapt their own sex consequently, either by switching or by taxis towards the highest concentration of the complementary sex. The widespread emergence and maintenance of sex follows therefore from its co-evolution with the even more widespread environmental sensing abilities. | 3212 | NA | NA | NA | no | no |
| 3213 | 2017 | NA | Mower, JP; Stefanovic, S; Hao, W; Gummow, JS; Jain, K; Ahmed, D; Palmer, JD | 2010 | Horizontal acquisition of multiple mitochondrial genes from a parasitic plant followed by gene conversion with host mitochondrial genes | Background: Horizontal gene transfer (HGT) is relatively common in plant mitochondrial genomes but the mechanisms, extent and consequences of transfer remain largely unknown. Previous results indicate that parasitic plants are often involved as either transfer donors or recipients, suggesting that direct contact between parasite and host facilitates genetic transfer among plants. Results: In order to uncover the mechanistic details of plant-to-plant HGT, the extent and evolutionary fate of transfer was investigated between two groups: the parasitic genus Cuscuta and a small clade of Plantago species. A broad polymerase chain reaction (PCR) survey of mitochondrial genes revealed that at least three genes (atp1, atp6 and matR) were recently transferred from Cuscuta to Plantago. Quantitative PCR assays show that these three genes have a mitochondrial location in the one species line of Plantago examined. Patterns of sequence evolution suggest that these foreign genes degraded into pseudogenes shortly after transfer and reverse transcription (RT)-PCR analyses demonstrate that none are detectably transcribed. Three cases of gene conversion were detected between native and foreign copies of the atp1 gene. The identical phylogenetic distribution of the three foreign genes within Plantago and the retention of cytidines at ancestral positions of RNA editing indicate that these genes were probably acquired via a single, DNA-mediated transfer event. However, samplings of multiple individuals from two of the three species in the recipient Plantago clade revealed complex and perplexing phylogenetic discrepancies and patterns of sequence divergence for all three of the foreign genes. Conclusions: This study reports the best evidence to date that multiple mitochondrial genes can be transferred via a single HGT event and that transfer occurred via a strictly DNA-level intermediate. The discovery of gene conversion between co-resident foreign and native mitochondrial copies suggests that transferred genes may be evolutionarily important in generating mitochondrial genetic diversity. Finally, the complex relationships within each lineage of transferred genes imply a surprisingly complicated history of these genes in Plantago subsequent to their acquisition via HGT and this history probably involves some combination of additional transfers (including intracellular transfer), gene duplication, differential loss and mutation-rate variation. Unravelling this history will probably require sequencing multiple mitochondrial and nuclear genomes from Plantago. See Commentary: http://www.biomedcentral.com/1741-7007/8/147. | 3213 | NA | NA | NA | no | no |
| 3214 | 2017 | NA | Laforge, MP; Uzal, A; Medill, SA; McLoughlin, PD | 2016 | Scale-dependent effects of density and habitat on foal survival | Identifying the most appropriate scale to study factors influencing life history is important to evolutionary ecology and wildlife management. For example, the scale at which density is assessed and explains variation in survival can affect how biologists observe and interpret population dynamics, which can influence plans for managing populations. Feral horses (Equus ferus caballus) contrast with most ungulates by exhibiting a mating system characterized by female-defense polygyny with persistent, non-territorial breeding groups (bands) and female-biased initial (natal) and subsequent (breeding) dispersal. We predicted that for horses, offspring movements coupled with female-biased breeding dispersal would increase the scale at which density best related to juvenile survival compared to species with greater female philopatry. From 2008 to 2013, we censused the population of feral horses on Sable Island, Canada. We annually computed individual-specific local densities for 442 foals (horses/km(2) in radii of 2,000m, 4,000m, and 8,000m fixed to a band’s centroid of movements) and whole-island (total) population density, group (band) size, and local access to surface freshwater, which affected movement patterns and selection of vegetation by females. The population of feral horses increased from 380 in 2008 to 559 in 2013. Overwinter survival of foals averaged 82.8%. Island-wide density was the most important predictor of foal mortality and was negatively associated with survival, with a lesser negative effect from local density. Increased access to surface freshwater (ponds) was an important predictor of foal survival but only at certain scales. Our study emphasizes the relevance of a multi-scale approach when analyzing the response of fitness components to changes in habitat and population processes, which may be influenced by the particular social organization of the species. (c) 2015 The Wildlife Society. | 3214 | NA | NA | NA | no | no |
| 3215 | 2017 | NA | FRANK, SA | 1995 | MUTUAL POLICING AND REPRESSION OF COMPETITION IN THE EVOLUTION OF COOPERATIVE GROUPS | EVOLUTIONARY theory has not explained how competition among lower level units is suppressed in the formation of higher-level evolutionary units(1,2). For example, the key problem of early evolution is how small, individual replicators formed cooperative groups of sufficient complexity to allow accurate copying of the genetic material(3). The puzzle is why parasites did not subvert the formation of cells by obtaining benefits from the group without contributing to shared traits that enhance reproduction(4). These parasites would outcompete other replicators within the cell, disrupting reproductive fairness among subunits and destroying the functional coherence of the group. A similar problem arose at a later evolutionary stage with the orderly mendelian segregation of subunits (chromosomes) within cells, and reproductive fairness continued to be a problem in the evolution of insect(5) and human societies(6). Here I present a simple model to show how reproductive fairness evolves among subunits to create functional coherence and higher-level units. Self-restraint, which evolves according to the kin-selection coefficient of relatedness, is not sufficient: mutual policing and enforcement of reproductive fairness are also required for the evolution of increasing social complexity. | 3215 | NA | NA | NA | no | no |
| 3216 | 2017 | NA | da Silva, FRC; Daudt, C; Streck, AF; Weber, MN; Leite, RV; Driemeier, D; Canal, CW | 2015 | Genetic characterization of Amazonian bovine papillomavirus reveals the existence of four new putative types | Papillomaviruses are small and complex viruses that belong to the Papillomaviridae family, which comprises 39 genera. The bovine papillomavirus (BPV) causes an infectious disease that is characterized by chronic and proliferative benign tumors that affect cattle worldwide. Different genotypes of BPVs can cause distinct skin and mucosal lesions and the immunity they raise has low cross-protection. This report aimed to genotype BPVs in cattle from Northern Brazil based on nucleotide partial sequences of the L1 ORF. Skin wart samples from 39 bovines clinically and histopathologically diagnosed as cutaneous papillomatosis from Acre and Rondonia States were analyzed. The results revealed four already reported BPV types (BPVs 1, 2, 11, and 13), nine putative new BPV subtypes and four putative new BPV types as well as two putative new BPV types that were already reported. To our knowledge, this is the first record of BPVs from the Brazilian Amazon region that identified new possible BPV types and subtypes circulating in this population. These findings point to the great genetic diversity of BPVs that are present in this region and highlight the importance of this knowledge before further studies about vaccination are attempted. | 3216 | NA | NA | NA | no | no |
| 3217 | 2017 | NA | Devi, KU; Padmavathi, J; Rao, CUM; Khan, AAP; Mohan, MC | 2008 | A study of host specificity in the entomopathogenic fungus Beauveria bassiana (Hypocreales, Clavicipitaceae) | Beauveria bassiana (Balsamo - Crivelli) Vuillemin based mycoinsecticides are used against agricultural, veterinary and medical insect pests. The fungus has a very diverse and extensive host range. Variation in virulence among isolates of B. bassiana to different insect species has been abundantly documented. Given the effect of multiple factors on virulence, it is not certain whether the observed difference in virulence can be labelled as host specificity. Environmental conditions and susceptibility of the insect population are two main factors that affect successful fungal infection. Keeping the environmental factors constant, if virulence of an isolate to different insect species and different populations within an insect species is compared, the scale of difference between the two responses can be estimated. If differences in virulence of an isolate to different insect species are greater than the difference in virulence to different insect populations within an insect species, then, the isolate can be considered as exhibiting specific preference to those insect species towards which it exhibits high virulence. To examine this feature, a worldwide sample of B. bassiana was bioassayed on nine insect species and two different populations within two insect species. Laboratory bioassays were done on: Bombyx mori (Lepidoptera), Spodoptera litura (Lepidoptera), Chilo partellus (Lepidoptera), Helicoverpa armigera (Lepidoptera), Epilachna vigintioctopunctata (Coleoptera), Mylabris pustulata (Coleoptera), Aphis craccivora (Homoptera), Maconellicoccus hirsutus (Hemiptera) and Oecophylla smaragdina (Hymenoptera). The range of variation in virulence of a B. bassiana isolate to different insect species was not more than that observed with different populations within a single insect species. B. bassiana is thus a generalist with no strict host preference. B. bassiana based biopesticide can be used as a broad spectrum insecticide against a myriad of insect pests. | 3217 | NA | NA | NA | no | no |
| 3218 | 2017 | NA | Datta, S; Chowdhury, A; Ghosh, M; Das, K; Jha, P; Colah, R; Mukerji, M; Majumder, PP | 2012 | A Genome-Wide Search for Non-UGT1A1 Markers Associated with Unconjugated Bilirubin Level Reveals Significant Association with a Polymorphic Marker Near a Gene of the Nucleoporin Family | Variants in the UGT1A1 gene and its promoter are known to determine levels of unconjugated bilirubin (UCB), but do not explain all cases of unconjugated hyperbilirubinemia. To discover associations with variants in genes other than UGT1A1, we undertook a genome-wide association study. We recruited 200 participants to cover the entire range of quantitative variation in UCB level. The data set after data curation, including analyses for population stratification and cryptic relatedness comprised genotypes at 512,349 SNP loci on 182 individuals. Quantitative trait locus (QTL) association analyses were performed, after adjusting the UCB level for effects of age, gender, and genotype at the dinucleotide (TA) insertion locus in UGT1A1 that is known to significantly modulate UCB level. A significant association of a polymorphic marker (rs2328136) near the NUP153 gene (which produces a 153 kDa nucleoporin) was obtained (p = 0.002, after multiple-testing correction). The frequency of the variant allele (A) at the rs2328136 locus in our study population is 40%, higher than most global populations. NUP153, whose product is a major regulatory factor in bidirectional transport of biomolecules across nucleus to cytosol, is associated with the transport of biliverdin reductase, which is important for bilirubin conjugation. | 3218 | NA | NA | NA | no | no |
| 3219 | 2017 | NA | Wang, Y; Liu, GH; Li, JY; Xu, MJ; Ye, YG; Zhou, DH; Song, HQ; Lin, RQ; Zhu, XQ | 2013 | Genetic variability among Trichuris ovis isolates from different hosts in Guangdong Province, China revealed by sequences of three mitochondrial genes | This study examined sequence variation in three mitochondrial DNA (mtDNA) regions, namely cytochrome c oxidase subunit 1 (cox1), NADH dehydrogenase subunit 5 (nad5) and cytochrome b (cytb), among Trichuris ovis isolates from different hosts in Guangdong Province, China. A portion of the cox1 (pcox1), nad5 (pnad5) and cytb (pcytb) genes was amplified separately from individual whipworms by PCR, and was subjected to sequencing from both directions. The size of the sequences of pcox1, pnad5 and pcytb was 618, 240 and 464 bp, respectively. Although the intra-specific sequence variations within T. ovis were 0-0.8% for pcox1, 0-0.8% for pnad5 and 0-1.9% for pcytb, the inter-specific sequence differences among members of the genus Trichuris were significantly higher, being 24.3-26.5% for pcox1, 33.7-56.4% for pnad5 and 24.8-26.1% for pcytb, respectively. Phylogenetic analyses using combined sequences of pcox1, pnad5 and pcytb, with three different computational algorithms (maximum likelihood, maximum parsimony and Bayesian inference), indicated that all of the T. ovis isolates grouped together with high statistical support. These findings demonstrated the existence of intra-specific variation in mtDNA sequences among T. ovis isolates from different hosts, and have implications for studying molecular epidemiology and population genetics of T. ovis. | 3219 | NA | NA | NA | no | no |
| 3220 | 2017 | NA | Aragao, GC; Mascarenhas, JDP; Kaiano, JHL; de Lucena, MSS; Siqueira, JAM; Fumian, TM; Hernandez, JD; de Oliveira, CS; Oliveira, DD; Araujo, ED; Soares, LD; Linhares, AC; Gabbay, YB | 2013 | Norovirus Diversity in Diarrheic Children from an African-Descendant Settlement in Belem, Northern Brazil | Norovirus (NoV), sapovirus (SaV) and human astrovirus (HAstV) are viral pathogens that are associated with outbreaks and sporadic cases of gastroenteritis. However, little is known about the occurrence of these pathogens in relatively isolated communities, such as the remnants of African-descendant villages (“Quilombola”). The objective of this study was the frequency determination of these viruses in children under 10 years, with and without gastroenteritis, from a “Quilombola” Community, Northern Brazil. A total of 159 stool samples were obtained from April/2008 to July/2010 and tested by an enzyme immunoassay (EIA) and reverse transcription-polymerase chain reaction (RT-PCR) to detect NoV, SaV and HAstV, and further molecular characterization was performed. These viruses were detected only in the diarrheic group. NoV was the most frequent viral agent detected (19.7%-16/81), followed by SaV (2.5%-2/81) and HAstV (1.2%-1/81). Of the 16 NoV-positive samples, 14 were sequenced with primers targeting the B region of the polymerase (ORF1) and the D region of the capsid (ORF2). The results showed a broad genetic diversity of NoV, with 12 strains being classified as GII-4 (5-41.7%), GII-6 (3-25%), GII-7 (2-16.7%), GII-17 (1-8.3%) and GI-2 (1-8.3%), as based on the polymerase region; 12 samples were classified, based on the capsid region, as GII-4 (6-50%, being 3-2006b variant and 3-2010 variant), GII-6 (3-25%), GII-17 (2-16.7%) and GII-20 (1-8.3%). One NoV-strain showed dual genotype specificity, based on the polymerase and capsid region (GII-7/GII-20). This study provides, for the first time, epidemiological and molecular information on the circulation of NoV, SaV and HAstV in African-descendant communities in Northern Brazil and identifies NoV genotypes that were different from those detected previously in studies conducted in the urban area of Belem. It remains to be determined why a broader NoV diversity was observed in such a semi-isolated community. | 3220 | NA | NA | NA | no | no |
| 3221 | 2017 | NA | SIGNER, EN; SCHMIDT, CR; JEFFREYS, AJ | 1994 | DNA VARIABILITY AND PARENTAGE TESTING IN CAPTIVE WALDRAPP IBISES | The Waldrapp ibis (Geronticus eremita) is one of the most endangered birds in the wild. The only real hope for its survival seems to rest with captive propagation, which has fortunately proved straightforward. Preservation of genetic diversity for maintaining viable populations is a major goal in any species conservation programme. Therefore sensitive tools have to be developed for the detection and regular monitoring of population variability. Individual identity has to be verified and each individual’s pedigree defined in order to enable effective selective breeding, In this study, extensive DNA fingerprinting technology was applied to a group of 39 captive Waldrapp ibises. Five minisatellite probes were used. Despite the fact that the colony was founded by only six birds and that some of the founders were apparently related, highly polymorphic banding patterns were obtained. The human- and pig-derived probes MS1, MS31, p lambda g3 and pCMS12 each detected multiple distinct loci suitable for individual identification and resolution of parentage. Probe MS51 was locus-specific and revealed six different alleles. In addition, MS31 and p lambda g3 co-detected a band present in all females but none of the males and thus could be used for sex determination. For all but four individuals both parents could be identified unambiguously. The exceptions were offspring derived from a full sib mating in which DNA results allowed no distinction between the parents and the grandparents. Comparison of the parentage assigned by DNA analysis and by observation of breeding behaviour, respectively, showed agreement in al but five instances. Here, DNA data uncovered interchange of individuals in the stud book. Application of these minisatellite probes therefore offers an extremely sensitive means for individual identification and verification and completion of pedigree records. | 3221 | NA | NA | NA | no | no |
| 3222 | 2017 | NA | Lion, S | 2013 | Multiple infections, kin selection and the evolutionary epidemiology of parasite traits | The coinfection of a host by several parasite strains is known to affect selective pressures on parasite strategies of host exploitation. I present a general model of coinfections that ties together kin selection models of virulence evolution and epidemiological models of multiple infections. I derive an analytical expression for the invasion fitness of a rare mutant in a population with an arbitrary distribution of the multiplicity of infection (MOI) across hosts. When a single mutation affects parasite strategies in all MOI classes, I show that the evolutionarily stable level of virulence depends on a demographic average of within-host relatedness across all host classes. This generalization of previous kin selection results requires that within-host parasite densities do not vary between hosts. When host exploitation strategies are allowed to vary across classes, I show that the strategy of host exploitation in a focal MOI class depends on the relative magnitudes of parasite reproductive values in the focal class and in the next. Thus, in contrast to previous findings, lower within-host relatedness in competitive parasite interactions can potentially correspond to either higher or lower levels of virulence. | 3222 | NA | NA | NA | no | no |
| 3223 | 2017 | NA | Collins, AM | 2000 | Survival of honey bee (Hymenoptera : Apidae) spermatozoa stored at above-freezing temperatures | The development of practical techniques for the storage of honey bee, Apis mellifera L., semen would significantly improve our ability to breed for desirable genotypes and maintain genetic diversity in populations. Artificial insemination of queens has been possible for some time, but the semen used is usually freshly collected, or held for <1 wk at room temperature. I examined the limitations of spermatozoal survival at nonfrozen temperatures. Pooled, diluted semen was stored in sealed capillary tubes at room temperature (25 degrees C) or in a refrigerator set to 12 degrees C, for periods up to 1 yr. Survival of spermatozoa was assayed by a dual fluorescent staining technique using SYBR-14 and propidium iodide stains, which readily distinguishes live and dead cells. No significant loss of viable spermatozoa occurred within the first 6 wk. Between weeks 6 and 9, the percent live spermatozoa fell from 80 to 58%, and remained at that level until after 39 wk. By week 52, samples at room temperature. but not at 12 degrees C, fell to 18.9% live spermatozoa. Nonfrozen storage of honey bee semen has potential for short-term preservation of germplasm, however several factors need to be studied further to optimize survival rates. | 3223 | NA | NA | NA | no | no |
| 3224 | 2017 | NA | Cuevas, JM; Delaunay, A; Visser, JC; Bellstedt, DU; Jacquot, E; Elena, SF | 2012 | Phylogeography and Molecular Evolution of Potato virus Y | Potato virus Y (PVY) is an important plant pathogen, whose host range includes economically important crops such as potato, tobacco, tomato, and pepper. PVY presents three main strains (PVYO, PVYN and PVYC) and several recombinant forms. PVY has a worldwide distribution, yet the mechanisms that promote and maintain its population structure and genetic diversity are still unclear. In this study, we used a pool of 77 complete PVY genomes from isolates collected worldwide. After removing the effect of recombination in our data set, we used Bayesian techniques to study the influence of geography and host species in both PVY population structure and dynamics. We have also performed selection and covariation analyses to identify evolutionarily relevant amino acid residues. Our results show that both geographic and host-driven adaptations explain PVY diversification. Furthermore, purifying selection is the main force driving PVY evolution, although some indications of positive selection accounted for the diversification of the different strains. Interestingly, the analysis of P3N-PIPO, a recently described gene in potyviruses, seems to show a variable length among the isolates analyzed, and this variability is explained, in part, by host-driven adaptation. | 3224 | NA | NA | NA | no | no |
| 3225 | 2017 | NA | Rittenhouse, CD; Millspaugh, JJ; Hubbard, MW; Sheriff, SL | 2007 | Movements of translocated and resident three-toed box turtles | Translocation of terrestrial turtles occurs to mitigate habitat loss, increase population size or genetic diversity, and to establish new populations. The success of many translocation efforts often depends on social and economic factors, but ecological factors may also affect translocation success. We used radiotelemetry to evaluate Three-Toed Box Turtle (Terrapene carolina triunguis) movements before and after translocation from a continuously forested site to a highly fragmented site and made comparisons to resident turtles. The average distance moved between consecutive 28-h relocations (dist\_move) pretranslocation versus posttranslocation depended on turtle origin. Turtles translocated from the continuously forested site had greater mean dist\_move following translocation, whereas resident turtles at the fragmented site had similar dist\_move in both periods. Translocated turtles traveled greater total distances, had greater net displacement, and greater home-range size than resident turtles. Additionally, translocated turtles had directed movements, whereas resident turtles did not. Homing behavior or directed movement toward continuous forest adjacent to the release site may account for the movement patterns observed. Future translocations should consider the landscape context of the release sites as well as the landscape context of the original capture location. | 3225 | NA | NA | NA | no | no |
| 3226 | 2017 | NA | Tsepilov, YA; Ried, JS; Strauch, K; Grallert, H; van Duijn, CM; Axenovich, TI; Aulchenko, YS | 2013 | Development and Application of Genomic Control Methods for Genome-Wide Association Studies Using Non-Additive Models | Genome-wide association studies (GWAS) comprise a powerful tool for mapping genes of complex traits. However, an inflation of the test statistic can occur because of population substructure or cryptic relatedness, which could cause spurious associations. If information on a large number of genetic markers is available, adjusting the analysis results by using the method of genomic control (GC) is possible. GC was originally proposed to correct the Cochran-Armitage additive trend test. For non-additive models, correction has been shown to depend on allele frequencies. Therefore, usage of GC is limited to situations where allele frequencies of null markers and candidate markers are matched. In this work, we extended the capabilities of the GC method for non-additive models, which allows us to use null markers with arbitrary allele frequencies for GC. Analytical expressions for the inflation of a test statistic describing its dependency on allele frequency and several population parameters were obtained for recessive, dominant, and over-dominant models of inheritance. We proposed a method to estimate these required population parameters. Furthermore, we suggested a GC method based on approximation of the correction coefficient by a polynomial of allele frequency and described procedures to correct the genotypic (two degrees of freedom) test for cases when the model of inheritance is unknown. Statistical properties of the described methods were investigated using simulated and real data. We demonstrated that all considered methods were effective in controlling type 1 error in the presence of genetic substructure. The proposed GC methods can be applied to statistical tests for GWAS with various models of inheritance. All methods developed and tested in this work were implemented using R language as a part of the GenABEL package. | 3226 | NA | NA | NA | no | no |
| 3227 | 2017 | NA | Parratt, SR; Barres, B; Penczykowski, RM; Laine, AL | 2017 | Local adaptation at higher trophic levels: contrasting hyperparasite-pathogen infection dynamics in the field and laboratory | Predicting and controlling infectious disease epidemics is a major challenge facing the management of agriculture, human and wildlife health. Co-evolutionarily derived patterns of local adaptation among pathogen populations have the potential to generate variation in disease epidemiology; however, studies of local adaptation in disease systems have mostly focused on interactions between competing pathogens or pathogens and their hosts. In nature, parasites and pathogens are also subject to attack by hyperparasitic natural enemies that can severely impact upon their infection dynamics. However, few studies have investigated whether this interaction varies across combinations of pathogen-hyperparasite strains, and whether this influences hyperparasite incidence in natural pathogen populations. Here, we test whether the association between a hyperparasitic fungus, Ampelomyces, and a single powdery mildew host, Podosphaera plantaginis, varies among genotype combinations, and whether this drives hyperparasite incidence in nature. Laboratory inoculation studies reveal that genotype, genotypexgenotype interactions and local adaptation affect hyperparasite infection. However, observations of a natural pathogen metapopulation reveal that spatial rather than genetic factors predict the risk of hyperparasite presence. Our results highlight how sensitive the outcome of biocontrol using hyperparasites is to selection of hyperparasite strains. | 3227 | NA | NA | NA | no | no |
| 5856 | 2017 | from ref check | Ben-Ami, F, Heller, J | 2005 | Spatial and temporal patterns of parthenogenesis and parasitism in the freshwater snail Melanoides tuberculata | The Red Queen hypothesis predicts that sex should be more common in populations heavily infested with parasites, than in those without. This hypothesis was investigated in the aquatic snail Melanoides tuberculata, in which both sexual and parthenogenetic individuals exist in natural populations, and some populations are heavily infested by trematodes. The presence of fertile males and the higher genetic diversity of bisexual populations are indicative of sexual reproduction. We compared sites in 1990, 1999, and 2001, and we looked for a positive correlation between male and parasite frequencies. Male frequency was not correlated with the frequency of individuals infected by trematodes. This lack of correlation was reconfirmed in a retrospective power analysis. In a period of 9 years, male frequencies decreased but infection levels increased. These results do not support the Red Queen hypothesis. In samples with high male frequency the number of embryos was low, perhaps indicating that males may have a negative effect on embryo numbers. This effect of males on fitness could perhaps suggest that the cost of sex is fewer embryos. The reduction in embryo numbers may also represent a trade‐off between mating and egg production costs. | 3228 | yes | from ref check | NA | no | yes |
| 3228 | 2017 | NA | Spoerke, JM; Wilkinson, HH; Parker, MA | 1996 | Nonrandom genotypic associations in a legume - Bradyrhizobium mutualism | Genetically divergent lineages often coexist within populations of the annual legume Amphicarpaea bracteata. At one site dominated by two such lineages (termed biotypes ‘’C’’ and ‘’S’’), isolates of root-nodule bacteria (Bradyrhizobium sp.) were sampled from both hosts and analyzed by enzyme electrophoresis. Symbiont populations on the two plant biotypes were highly distinct. Out of 15 bacterial multilocus genotypes detected (among 51 isolates analyzed),only one was shared in common by the two plant biotypes. Cluster analysis revealed three bacterial lineages (designated I, II, and III), with lineage I found exclusively on biotype C plants, and the two other lineages almost completely restricted to biotype S hosts. Laboratory inoculation tests indicated that lineage I bacteria were strictly specialized on biotype C hosts, forming few or no nodules on plants of the other host biotype. Bacterial lineages II and III were capable of forming nodules on both kinds of plants, but nodule numbers were often significantly higher on biotype S hosts. The nonrandom association between plant and bacterial lineages at this site implies that genetic diversity of hosts is an important factor in the maintenance of polymorphism within the symbiont population. | 3229 | NA | NA | NA | no | no |
| 3229 | 2017 | NA | Kumar, ANA; Joshi, G; Ram, HYM | 2012 | Sandalwood: history, uses, present status and the future | Sandalwood (Santalum album L.) is a valuable tree associated with Indian culture. It is the second most expensive wood in the world. The heartwood of the tree is treasured for its aroma and is one of the finest natural materials for carving. Sandalwood oil is used in perfumes, cosmetics, aromatherapy and pharmaceuticals. The monopoly of sandalwood trade by the Governments of Karnataka, Tamil Nadu and Kerala and its consequences have resulted in severe exploitation, pushing S. album into the vulnerable category of the IUCN Red List. Extensive research has shown that sandalwood exhibits considerable genetic diversity for different traits. However, information pertaining to heartwood and oil content is meagre mainly because of non-availability of sandalwood plantations. Carrying out further research on these two important traits is difficult as natural populations have dwindled rapidly. We strongly urge that it is essential to encourage the establishment of community/corporate sandalwood plantations in different parts of India with appropriate incentives and adequate protective measures. These plantations can form the base population sources to regain the leadership of India in the sandalwood industry for perfumery and the precious art of carving. | 3230 | NA | NA | NA | no | no |
| 3230 | 2017 | NA | Duarte, MD; Henriques, AM; Barros, SC; Fagulha, T; Mendonca, P; Carvalho, P; Monteiro, M; Fevereiro, M; Basto, MP; Rosalino, LM; Barros, T; Bandeira, V; Fonseca, C; Cunha, MV | 2013 | Snapshot of Viral Infections in Wild Carnivores Reveals Ubiquity of Parvovirus and Susceptibility of Egyptian Mongoose to Feline Panleukopenia Virus | The exposure of wild carnivores to viral pathogens, with emphasis on parvovirus (CPV/FPLV), was assessed based on the molecular screening of tissue samples from 128 hunted or accidentally road- killed animals collected in Portugal from 2008 to 2011, including Egyptian mongoose (Herpestes ichneumon, n = 99), red fox (Vulpes vulpes, n = 19), stone marten (Martes foina, n = 3), common genet (Genetta genetta, n = 3) and Eurasian badger (Meles meles, n = 4). A high prevalence of parvovirus DNA (63%) was detected among all surveyed species, particularly in mongooses (58%) and red foxes (79%), along with the presence of CPV/FPLV circulating antibodies that were identified in 90% of a subset of parvovirus- DNA positive samples. Most specimens were extensively autolysed, restricting macro and microscopic investigations for lesion evaluation. Whenever possible to examine, signs of active disease were not present, supporting the hypothesis that the parvovirus vp2 gene fragments detected by real- time PCR possibly correspond to viral DNA reminiscent from previous infections. The molecular characterization of viruses, based on the analysis of the complete or partial sequence of the vp2 gene, allowed typifying three viral strains of mongoose and four red fox’s as feline panleukopenia virus (FPLV) and one stone marten’s as newCPV- 2b type. The genetic similarity found between the FPLV viruses from free- ranging and captive wild species originated in Portugal and publicly available comparable sequences, suggests a closer genetic relatedness among FPLV circulating in Portugal. Although the clinical and epidemiological significance of infection could not be established, this study evidences that exposure of sympatric wild carnivores to parvovirus is common and geographically widespread, potentially carrying a risk to susceptible populations at the wildlife- domestic interface and to threatened species, such as the wildcat (Felis silvestris) and the critically endangered Iberian lynx (Lynx pardinus). | 3231 | NA | NA | NA | no | no |
| 3231 | 2017 | NA | Schulte, BA | 2000 | Social structure and helping behavior in captive elephants | Selective pressures on individual behavior serve to mold social structure and subsequent levels of cooperation in social species, including elephants: Adult Asian (Elephas maximus) and African (Loxodonta africana) elephants have complex social societies that differ markedly between the sexes. Elephant social structure and cooperative behavior may have evolved in part to prepare young elephants to meet the challenges of an expansive environment, predators and parasites. In captivity, such forces are relaxed or eliminated, yet captive elephants display a similar repertoire of behaviors as their wild counterparts. Elephants live in herds of related adult females from several generations. This female social unit is centered on calves and the matriarch. Adult males reside in bachelor herds, singly, or frequent female groups, depending on male age and reproductive condition. In captivity, female elephants are usually housed together, although group size is smaller than in the wild, whereas adult males are generally housed separately from other elephants. Beyond these general similarities between wild and captive settings, captive elephants are housed in a wider range of group sizes, degrees of relatedness, and age structures than would be typical in the wild. This variation in social structure can be used to explore questions about cooperative behavior. Following a comparison of wild and captive social structures for elephants, three such potential avenues for study are discussed. Specifically, research with captive elephants could shed Light on the development of social behavior, the functional significance of allomothering, and the ability of elephants to recognize kin or social group members. Beside the potential theoretical implications and insight into wild elephant behavior, the examination of social behavior also has important ramifications for the management of animals in captivity. Zoo Biol 19:447-459, 2000. (C) 2000 Wiley-Liss, Inc. | 3232 | NA | NA | NA | no | no |
| 3232 | 2017 | NA | Guimaraes, AMS; Santos, AP; do Nascimento, NC; Timenetsky, J; Messick, JB | 2014 | Comparative Genomics and Phylogenomics of Hemotrophic Mycoplasmas | Hemotrophic mycoplasmas (hemoplasmas) are a group of animal pathogens of the Mollicutes class. Recently, the genomes of 8 hemoplasmas have been completely sequenced. The aim of this study was to gain a better understanding of their genomic features and relationship to other Mycoplasma species. The genome structure and dynamics of hemoplasmas were analyzed by evaluating gene synteny, adaptive evolution of paralogous gene families (PGF) and horizontal gene transfer (HGT). The Mollicutes class was then phylogenetically analyzed by constructing a distance matrix of the 16S rRNA genes and a phylogenetic tree with 32 conserved, concatenated proteins. Our results suggest that the hemoplasmas have dynamic genomes. The genome size variation (from 547 to 1,545 genes) indicates substantial gene gain/loss throughout evolution. Poorly conserved gene syntenies among hemoplasmas, positional shuffling of paralogous genes between strains, HGT, and codons under positive selection in PGFs were also observed. When compared to other Mollicutes species, the hemoplasmas experienced further metabolic reduction, and the 16S rRNA gene distance matrix of the available mollicutes suggests that these organisms presently constitute the most divergent clade within its class. Our phylogenetic tree of concatenated proteins showed some differences when compared to the 16S rRNA gene tree, but non-mycoplasma organisms, such as Ureaplasma spp. and Mesoplasma spp., continue to branch within Mycoplasma clades. In conclusion, while the hemoplasmas experienced further metabolic shrinkage through gene loss, PGFs with positively selected codons are likely beneficial to these species. Phylogeny of the mollicutes based on 16S rRNA genes or concatenated proteins do not obey the current taxonomy. The metabolism and genetic diversity of the mollicutes, the presence of HGT, and lack of standard for genus circumscription are likely to hinder attempts to classify these organisms based on phylogenetic analyses. | 3233 | NA | NA | NA | no | no |
| 3233 | 2017 | NA | Mota, SF; Barcelos, QL; Dias, MA; Souza, EA | 2016 | Variability of Colletotrichum spp in common bean | The Colletotrichum genus presents large genetic variability, as demonstrated by the occurrence of several pathogenic races and phenotypic traits. The objective of this study was to characterize 22 strains of C. lindemuthianum and Colletotrichum spp recovered from anthracnose lesions and bean scab, and to verify the relationship between species of the Colletotrichum genus, which inhabit anthracnose and scab lesions. Colony morphology, conidium size, the presence of septa, germination, sporulation, and mycelium growth rates, were analyzed in addition to the presence of mating-type genes, IRAP markers, and pathogenicity. Strains of Colletotrichum spp presented wide variation for all evaluated traits, indicating the presence of different species. Pathogenicity tests verified that the severity of the disease caused by strains of Colletotrichum spp must be evaluated 17 days after inoculation. Molecular analysis showed that only the C. lindemuthianum strains were grouped by the IRAP markers. For the physiological traits, we observed that C. lindemuthianum mycelium growth is slower than that of Colletotrichum spp strains. The information generated in this study confirms variability in the evaluated species of Colletotrichum and may direct future basic and applied studies aiming to control these diseases in common bean. | 3234 | NA | NA | NA | no | no |
| 3234 | 2017 | NA | Hagedorn, M; Carter, VL; Lager, C; Ciani, JFC; Dygert, AN; Schleiger, RD; Henley, EM | 2016 | Potential bleaching effects on coral reproduction | Bleaching profoundly impacts coral reproduction, often for years after an event. However, detailed reproductive characteristics of coral after bleaching have not been broadly described, especially as they relate to cryopreservation. Therefore, in the present study we measured several reproductive characteristics in coral in Kaneohe Bay, Hawaii, for two species, namely Fungia scutaria and Montipora capitata, during the bleaching period of 2014 and 2015. We examined spawning periods, egg morphometry, sperm concentration, fresh and cryopreserved sperm motility exposed to different concentrations of dimethyl sulfoxide, time of first cleavage, larval survival with fresh and cryopreserved spermatozoa, infection success and settlement success. Many of these reproductive parameters were reduced in 2015, especially sperm motility. Once the reduced-motility spermatozoa from 2015 post-bleach were cryopreserved, there was a steep decline in post-thaw viability and this would prevent any substantive further use of these samples in reproduction for conservation benefit. Worldwide, as bleaching events become more frequent, the ability to bank and conserve coral ex situ may be significantly reduced. Thus, it is imperative that while genetic diversity is still high in these populations, intensive efforts are made to bank coral species during non-bleaching periods. | 3235 | NA | NA | NA | no | no |
| 3235 | 2017 | NA | Sommer, S; Schwab, D; Ganzhorn, JU | 2002 | MHC diversity of endemic Malagasy rodents in relation to geographic range and social system | The major histocompatibility complex (MHC) encodes a group of closely linked genes that play a central role in the vertebrate immune system. Most natural mammal populations studied so far possess high levels of diversity within the MHC. However, recent investigations in the MHC class II gene DQA of the critically endangered Malagasy giant jumping rat (Hypogeomys antimena) revealed very low variability compared to other mammalian species. The low genetic variability was confirmed in the present study through analyses of the MHC class 11 gene DRB exon 2. This codes for the antigen-binding site and is therefore considered as one of the most important parts of the MHC. The species’ geographic distribution has been reduced recently to a fragmented area of suitable habitat within a geographic range of less than 20x40 km of dry deciduous forest at the west coast of Madagascar. H. antimena has some unusual life history characteristics for a rodent species, such as living in obligate monogamy, with pairs staying together until one mate dies, a low reproduction rate (one to two offspring/couple per year), high site and mate fidelity, short dispersal distances, almost no adult migration, and therefore a constant gene pool with very limited gene flow. Current hypotheses usually interpret low MHC polymorphism either as a consequence of reduced selection pressure, bottleneck effects or by constraints of the mating system. To differentiate between these hypotheses, the MHC variability of the DQA and DRB gene of two additional sympatric, but widely distributed rodent species, Macrotarsomys bastardi and Eliurus myoxinus, were studied by using universal primers and single-strand conformation polymorphism. The two species differ in their mating systems: M. bastardi also lives in pairs but E. myoxinus is a promiscuous species. Whereas the investigated MHC class II genes DQA and DRB had low levels of polymorphism in the pair-living species H. antimena and M. bastardi (DQA: 2 and 3 alleles, DRB: 5 and 6 alleles, respectively), higher levels of variation (DQA: 11 alleles, DRB: 9 alleles) were recorded in the promiscuous species, E. myoxinus. Gene diversity was also higher in E. myoxinits (DQA: 0.85, DRB: 0.86) than in the two pair-living species (DQA: 0.41-0.45, DRB: 0.55-0.63). The results suggest that low MHC variability might not only result from bottleneck effects due to recent declines in population size, but also from a monogamous mating system. | 3236 | NA | NA | NA | no | no |
| 3236 | 2017 | NA | Bashir, KMI; Awan, FS; Khan, IA; Khan, AI; Usman, M | 2014 | Identification and authentication of Rosa species through development of species-specific SCAR marker(s) | Roses (Rosa indica) belong to one of the most crucial groups of plants in the floriculture industry. Rosa species have special fragrances of interest to the perfume and pharmaceutical industries. The genetic diversity of plants based on morphological characteristics is difficult to measure under natural conditions due to the influence of environmental factors, which is why a reliable fingerprinting method was developed to overcome this problem. The development of molecular markers will enable the identification of Rosa species. In the present study, randomly amplified polymorphic DNA (RAPD) analysis was done on four Rosa species, Rosa gruss-an-teplitz (Surkha), Rosa bourboniana, Rosa centifolia, and Rosa damascena. A polymorphic RAPD fragment of 391 bp was detected in R. bourboniana, which was cloned, purified, sequenced, and used to design a pair of species-specific sequence-characterized amplified region (SCAR) primers (forward and reverse). These SCAR primers were used to amplify the specific regions of the rose genome. These PCR amplifications with specific primers are less sensitive to reaction conditions, and due to their high reproducibility, these species-specific SCAR primers can be used for marker-assisted selection and identification of Rosa species. | 3237 | NA | NA | NA | no | no |
| 3237 | 2017 | NA | Luizon, MR; Izidoro-Toledo, TC; Simoes, AL; Tanus-Santos, JE | 2009 | Endothelial Nitric Oxide Synthase Polymorphisms and Haplotypes in Amerindians | Interethnic disparities in the distribution of endothelial nitric oxide synthase (eNOS) polymorphisms may affect nitric oxide (NO)-mediated effects of and responses to drugs. While there are differences between black and white subjects there is no information regarding the distribution of eNOS gene alleles and haplotypes in Amerindians. We studied three clinically relevant eNOS polymorphisms (T(-786) C in the promoter, a variable number of tandem repeats in intron 4, and the Glu298Asp in exon 7) and eNOS haplotypes in 170 Amerindians from three tribes of the Brazilian Amazon. The results were compared with previous findings for black and white Brazilians. The Asp298, C(-786), and 4a alleles were much less common in Amerindians (5.0%, 3.2%, and 4.1%, respectively) than in blacks (15.1%, 19.5%, and 32.0%, respectively) or whites (32.8%, 41.9%, and 17.9%, respectively) (p<0.001). The haplotype including the most common alleles for each polymorphism was much more common in Amerindians (89%) than in blacks (45%) or whites (41%). Our findings are consistent with a lower genetic diversity in Amerindians compared with blacks and whites. These striking differences may be of major relevance for case-control association studies focusing on eNOS gene polymorphisms and may explain, at least in part, differences in the responses to cardiovascular drugs. | 3238 | NA | NA | NA | no | no |
| 3238 | 2017 | NA | Lopez-Villavicencio, M; Courjol, F; Gibson, AK; Hood, ME; Jonot, O; Shykoff, JA; Giraud, T | 2011 | COMPETITION, COOPERATION AMONG KIN, AND VIRULENCE IN MULTIPLE INFECTIONS | Critical determinants of the optimum level of virulence in pathogens include the presence of competitors (i.e., multiple infections), their relatedness, and the effect of competitors on pathogen growth and disease development. Empirical data regarding the existence of competitive interactions and their impact on virulence remain very limited compared to theoretical studies. Here, we followed an experimental population of the model fungal pathogen Microbotryum lychnidis-dioicae on its caryophyllaceous host Silene latifolia. Our analysis revealed conditional responses by the pathogen to the presence of competitors, which was dependent upon the relatedness of pathogens within hosts. Overall, virulence was increased in cases of multiple infections as compared to single infections: both spore production and degree of plant sterilization were higher under multiple infections. The pathogen indeed increased its growth and reproductive rate when competitors were present within the same plant. Microbotryum also appeared able to interfere with competitors, reducing their ability to colonize the host, and this effect was smaller between closer relatives. Our results thus help to elucidate the myriad of theoretical considerations on the evolution of virulence by providing experimental results with a well-studied disease of wild plant populations. | 3239 | NA | NA | NA | no | no |
| 3239 | 2017 | NA | Gasnier, N; Cabaret, J; Sauve, C; Gruner, L | 1996 | Host, season and year do not play an important role on genetic variability in a trichostrongyle nematode as assessed from allozymes | The isolates of the trichostrongyle nematode Teladorsagia circumcincta in sheep may vary from one site to another as assessed from allozyme studies. This genetic variability could be host (on the parasitic stages) or/and environmentally (on the free-living stages) induced. In the present study the role a host (susceptible to the establishment of the parasite and paltry resistant ones) and environmental changes expressed by season (Autumn versus Spring) or year of sampling (1990 or 1992) were investigated. Five polymorphic enzymes were studied glucose-phosphate-isomerase (GPI) lactate dehydrogenase (LDH), malate dehydrogenase (MHD), mannose-phosphate isomerase (MPI), and phosphoglucomutase (PGM). No significant difference in allelic frequencies was recorded between 4 susceptible lambs from a flock grazed in 1990 as well as in 3 lambs selected on their susceptibility or resistance to natural infection in a flock grazed in 1992. The mode of infection (natural - frequently repeated small infections, versus experimental - 3 repeated large infections) did not modify the allelic frequencies of the nematodes. The allelic frequencies remained stable along the seasons and during the 2 years investigated. The largest distance of Rogers (0.07) and F-st (0.012) were recorded between worms originating from susceptible and resistant lambs or in Autumn versus Spring populations of worms (0.08 and F-st 0.010). A deficiency in heterozygotes of the same magnitude was recorded in the worm populations obtained from the different infections. The stability of allelic frequencies and genetic structure are surprising when one considers the evolution of the same parasite in several generations of laboratory conditions. This stability could be related to the fact that levels of disturbance on parasitic and free-living stages are neither too low (as in laboratory conditions) not too high (as in farms when anthelmintic treatment are used regularly and destroy the adult stages). | 3240 | NA | NA | NA | no | no |
| 3240 | 2017 | NA | Lopes, RB; Souza, DA; Oliveira, CM; Faria, M | 2013 | Genetic Diversity and Pathogenicity of Metarhizium spp. Associated with the White Grub Phyllophaga capillata (Blanchard) (Coleoptera: Melolonthidae) in a Soybean Field | Phyllophaga capillata (Blanchard) was recently described as a soybean pest in Brazil. The occurrence of Metarhizium spp. associated with adults and their pathogenicity were investigated. Natural prevalence of Metarhizium anisopliae was 0.51% in adults collected by light trap. Most Metarhizium isolates were identified as Metarhizium robertsii, although M. anisopliae sensu stricto and a few unidentified isolates were also found. Bioassays with representative isolates selected from different genetic groups resulted in a parts per thousand currency sign5.6% confirmed mortality against larvae and adults, suggesting low potential of microbial control of this pest by use of indigenous Metarhizium isolates. | 3241 | NA | NA | NA | no | no |
| 3241 | 2017 | NA | Ujvari, B; Madsen, T | 2009 | INCREASED MORTALITY OF NAIVE VARANID LIZARDS AFTER THE INVASION OF NON-NATIVE CANE TOADS (BUFO MARINUS) | Exotic animal and plant species introduced into the Australian continent often imparted catastrophic effects on the indigenous fauna and flora. Proponents of biological control introduced the South American Cane Toad (Bufo marinus) into the sugar cane fields of Queensland in 1935. The Cane Toad is one of the most toxic bufonids, and when seized by naive Australian predators, the toxin usually kills the attacker. One group of Australian squamate reptiles that are very susceptible to Cane Toad toxins is varanid lizards. Prior to Cane Toad invasion of our study area, the Adelaide River floodplain of the Northern Territory of Australia, annual mortality of adult male radio-tagged Yellow-spotted Goannas (Varanus panoptes) was very low (two deaths recorded among 20 lizards over three years). After the arrival of the toads in October 2005, all radio-tracked goannas were found dead in August 2006 (nine out of nine lizards), most likely after attempting to feed on toads. Our results suggest that invasive Cane Toads place naive adult male Yellow-spotted Goannas at risk of possibly > 90% mortality. This increase in mortality could reduce the genetic diversity and hamper long-term survival of these large carnivorous lizards. | 3242 | NA | NA | NA | no | no |
| 3242 | 2017 | NA | Arruda, MP; Brown, P; Brown-Guedira, G; Krill, AM; Thurber, C; Merrill, KR; Foresman, BJ; Kolb, FL | 2016 | Genome-Wide Association Mapping of Fusarium Head Blight Resistance in Wheat using Genotyping-by-Sequencing | Fusarium head blight (FHB) is one of the most important wheat (Triticum aestivum L.) diseases worldwide, and host resistance displays complex genetic control. A genome-wide association study (GWAS) was performed on 273 winter wheat breeding lines from the midwestern and eastern regions of the United States to identify chromosomal regions associated with FHB resistance. Genotyping-by-sequencing (GBS) was used to identify 19,992 single-nucleotide polymorphisms (SNPs) covering all 21 wheat chromosomes. Marker-trait associations were performed with different statistical models, the most appropriate being a compressed mixed linear model (cMLM) controlling for relatedness and population structure. Ten significant SNP-trait associations were detected on chromosomes 4A, 6A, 7A, 1D, 4D, and 7D, and multiple SNPs were associated with Fhb1 on chromosome 3B. Although combination of favorable alleles of these SNPs resulted in lower levels of severity (SEV), incidence (INC), and deoxynivalenol concentration (DON), lines carrying multiple beneficial alleles were in very low frequency for most traits. These SNPs can now be used for creating new breeding lines with different combinations of favorable alleles. This is one of the first GWAS using genomic resources from the International Wheat Genome Sequencing Consortium (IWGSC). | 3243 | NA | NA | NA | no | no |
| 3243 | 2017 | NA | Kokko, H; Johnstone, RA; Wright, J | 2002 | The evolution of parental and alloparental effort in cooperatively breeding groups: when should helpers pay to stay? | We used a reproductive skew framework to consider the evolution of parental and alloparental effort in cooperatively breeding groups. The model provides the first theoretical treatment of rent payment (the “pay-to-stay” hypothesis) for the evolution of helping behavior of subordinates. According to this hypothesis, not all helping behavior is kin selected, but group members help in order to be allowed to stay in the group and potentially gain breeding positions later in life. We show that reproductive concessions may be replaced by complete skew and voluntary, costly alloparental effort by subordinates once future prospects are included in fitness calculations. This suggests that incomplete skew observed in long-lived species is not due to dominant control over reproduction. Rent payment is predicted to occur when relatedness between subordinate and dominant is low, survival is high, ecological constraints are at least moderately tight, and retaining nonhelping subordinates harms the dominant’s fitness. Rent may also be required from related subordinates if helping is very costly (leading to low voluntary helping effort) and ecological constraints are moderately tight. However, related subordinates do not need to have a positive net effect on the dominant’s direct fitness to be accepted as group members. We also consider compensatory responses of dominant group members as a potential threat to the stability of renting behavior. If dominants trade off parental effort against their own survival, they may selfishly reduce their own parental effort as a response to increased help. As this improves their own survival, the prospects of territorial inheritance diminish for the subordinate, and subordinates should hence be less willing to accept the rent agreement. However, we show that compensatory responses by “lazy” parents prevent group formation only in borderline cases. | 3244 | NA | NA | NA | no | no |
| 3244 | 2017 | NA | Korolev, KS; Xavier, JB; Nelson, DR; Foster, KR | 2011 | A Quantitative Test of Population Genetics Using Spatiogenetic Patterns in Bacterial Colonies | It is widely accepted that population-genetics theory is the cornerstone of evolutionary analyses. Empirical tests of the theory, however, are challenging because of the complex relationships between space, dispersal, and evolution. Critically, we lack quantitative validation of the spatial models of population genetics. Here we combine analytics, on-and off-lattice simulations, and experiments with bacteria to perform quantitative tests of the theory. We study two bacterial species, the gut microbe Escherichia coli and the opportunistic pathogen Pseudomonas aeruginosa, and show that spatiogenetic patterns in colony biofilms of both species are accurately described by an extension of the one-dimensional stepping-stone model. We use one empirical measure, genetic diversity at the colony periphery, to parameterize our models and show that we can then accurately predict another key variable: the degree of short-range cell migration along an edge. Moreover, the model allows us to estimate other key parameters, including effective population size (density) at the expansion frontier. While our experimental system is a simplification of natural microbial community, we argue that it constitutes proof of principle that the spatial models of population genetics can quantitatively capture organismal evolution. | 3245 | NA | NA | NA | no | no |
| 3245 | 2017 | NA | Gong, W; Chen, C; Dobes, C; Fu, CX; Koch, MA | 2008 | Phylogeography of a living fossil: Pleistocene glaciations forced Ginkgo biloba L. (Ginkgoaceae) into two refuge areas in China with limited subsequent postglacial expansion | Ginkgo biloba is one of the most famous and therefore intensively studied living fossils. The Ginkgoaceae was suggested to have originated in the early Permian approximately 280 mya, and at the height of their worldwide radiation consisted of least 16 genera that were distributed in temperate forests. However, China’s G. biloba is the only species of this genus which has survived until today. Using a phylogeographical approach we analyzed the history of this remnant from the past. G. biloba is native to China and has been distributed less than 300 years ago by humans throughout all temperate regions of the world. Separate from this human influence, native Chinese populations of G. biloba have been affected by the cycles of glaciation and deglaciation of the Pleistocene era, similar to many other global plant species. We used molecular markers from the nuclear (AFLPs) and plastid genomes (trnK intron, trnS-trnG intergenic spacer), and based on these data, glacial survival of Gingko populations within two refugia in Southwestern and Eastern China, respectively, is suggested. Specifically, for the first time, this study provides evidence of the existence of a refuge area in Eastern China on the West Tianmu Mountains. We can not support any extensive postglacial range expansion from these refuge areas. Furthermore, AFLP data indicate substantial genetic differentiation among populations from Southwestern, Eastern and Central China. We also hypothesize that this evidence supports that as a long-lived dioecious plant, G. biloba displays high levels of intra-populational genetic diversity that have been maintained in most populations, regardless if they are native or reforested stands. Furthermore, we provide evidence that planted trees from Europe, Japan, Korea and America were introduced multiple times from Eastern China. (C) 2008 Elsevier Inc. All rights reserved. | 3246 | NA | NA | NA | no | no |
| 3246 | 2017 | NA | Mathenge, CW; Holford, P; Hoffmann, JH; Zimmermann, HG; Spooner-Hart, R; Beattie, GAC | 2010 | Determination of biotypes of Dactylopius tomentosus (Hemiptera: Dactylopiidae) and insights into the taxonomic relationships of their hosts, Cylindropuntia spp. | Host specialization to form biotypes is common among phytophagous insects, and it has been hypothesised that biotypes of Dactylopius tomentosus L (Hemiptera: Dactylopiidae) occur. D. tomentosus is an important biological control agent for Cylindropuntia cacti when they occur as weeds. Additionally, there is uncertainty surrounding the taxonomic status of some species of Cylindropuntia. This study aimed to confirm the existence of D. tomentosus biotypes and to assess whether host specialization can help to resolve this systematic uncertainty. For this study, the host specificity and performance of ten provenances of D. tomentosus collected from C. cholla, C. fulgida var. fulgida, C. imbricata, C. f. var. mamillata, C. rosea and C. tunicata and reared on C. cholla, C. fulgida var. fulgida, C. imbricata and C. rosea were investigated. Five life-history parameters were measured including: crawler development time and survival, female development time, and the weight and number of eggs produced by females. Results revealed significant variation in host specificity with provenances either thriving, surviving or dying on the different hosts, thus demonstrating the existence of biotypes. Also, host specificity was related to host species and not to the geographic locality from which either the host or provenance was sourced. These findings suggest that the characteristics of Cylindropuntia species may differ sufficiently, there by presenting different selection pressures that induce and sustain distinct biotypes of D. tomentosus. The observed host use patterns of the biotypes separated the plant species into two groups that accorded with known phylogenetic relationships among Cylindropuntia species, suggesting that biotypes can be used to elucidate their taxonomic relatedness. Besides advancing our knowledge of the ecology and evolution of D. tomentosus, these novel findings have important implications for the biological control of Cylindropuntia species. | 3247 | NA | NA | NA | no | no |
| 3247 | 2017 | NA | Verbenko, DA; Pogoda, TV; Spitsyn, VA; Mikulich, AI; Bets, LV; Bebyakova, NA; Ivanov, VP; Abolmasov, NN; Pocheshkhova, EA; Balanovskaya, EV; Tarskaya, LA; Sorensen, MV; Limborska, SA | 2003 | Apolipoprotein B 3 ’-VNTR polymorphism in Eastern European populations | Apolipoprotein B 3’ (3’ ApoB) minisatellite polymorphism was studied in healthy unrelated individuals from the Russian Federation and the Republic of Belarus, in 10 populations from five ethnic groups: Russians, Byelorussians, Adygeis, Kalmyks and Yakuts. The analysis was carried out using PCR and electrophoresis followed by silver staining. Overall, 25 alleles of the 3’ ApoB minisatellite, ranging from 25 to 55 repeats, were detected. Heterozygosity indices were high and varied from 0.73 to 0.84. The distributions of alleles of this minisatellite in the Caucasoid populations ( Russians, Byelorussians and Adygeis) had a bimodal character, whereas that for Mongoloid populations ( Kalmyks and Yakuts) had a unimodal distribution. Nei’s genetic distances between the populations studied and some reference populations of Europe and Asia were estimated. Despite their allele distribution homogeneity, different East Slavonic ethnic groups were clearly resolved by multidimensional analyses. The East Slavonic and Adygei populations revealed a high similarity with European Caucasoids. The Mongoloid populations ( Kalmyks and Yakuts) were considerably different from those of the European Caucasoid populations, but were similar to other Asian Mongoloid populations. The results demonstrate the variability of 3’ ApoB minisatellite polymorphism not only in distant populations but also, to a certain extent, in genetically relative ones. | 3248 | NA | NA | NA | no | no |
| 3248 | 2017 | NA | Andras, JP; Ebert, D | 2013 | A novel approach to parasite population genetics: Experimental infection reveals geographic differentiation, recombination and host-mediated population structure in Pasteuria ramosa, a bacterial parasite of Daphnia | The population structure of parasites is central to the ecology and evolution of host-parasite systems. Here, we investigate the population genetics of Pasteuria ramosa, a bacterial parasite of Daphnia. We used natural P. ramosa spore banks from the sediments of two geographically well-separated ponds to experimentally infect a panel of Daphnia magna host clones whose resistance phenotypes were previously known. In this way, we were able to assess the population structure of P. ramosa based on geography, host resistance phenotype and host genotype. Overall, genetic diversity of P. ramosa was high, and nearly all infected D. magna hosted more than one parasite haplotype. On the basis of the observation of recombinant haplotypes and relatively low levels of linkage disequilibrium, we conclude that P. ramosa engages in substantial recombination. Isolates were strongly differentiated by pond, indicating that gene flow is spatially restricted. Pasteuria ramosa isolates within one pond were segregated completely based on the resistance phenotype of the hosta result that, to our knowledge, has not been previously reported for a nonhuman parasite. To assess the comparability of experimental infections with natural P. ramosa isolates, we examined the population structure of naturally infected D. magna native to one of the two source ponds. We found that experimental and natural infections of the same host resistance phenotype from the same source pond were indistinguishable, indicating that experimental infections provide a means to representatively sample the diversity of P. ramosa while reducing the sampling bias often associated with studies of parasite epidemics. These results expand our knowledge of this model parasite, provide important context for the large existing body of research on this system and will guide the design of future studies of this host-parasite system. | 3249 | NA | NA | NA | no | no |
| 3249 | 2017 | NA | CONNOR, A; WEISS, KM; WEEKS, SC | 1993 | EVOLUTIONARY MODELS OF QUANTITATIVE DISEASE RISK-FACTORS | Numerous mutations are now known that have significant effects on various phenotypes; many of these mutations are of interest because they influence quantitative risk factors for major diseases. Such diversity raises the question of how much genetic heterogeneity we should expect to find in the effects of alleles, that is, the size of the effects, the number of severe alleles, and their frequency in the population. Can evolutionary models suggest a general pattern? In this article we examine what is currently known about several basic aspects of the problem. These include the distribution of quantitative effects of new mutations on a phenotype, the distribution of allelic effects that would be found in a natural population, and the relationship between these effects and Darwinian fitness. We discuss these issues in light of various models that have been proposed and the existing relevant data. Then we consider how these points relate to the distribution of genetic effects on an important human trait, the cholesterol ratio, an important risk factor for coronary heart disease. The complexities of quantitative traits and inadequacies in the available data prevent definitive models that can directly connect the mutational effects, allelic effects, and fitness distributions from being developed, and we consider how sample limitations and the nonequilibrium of human populations caused by our demographic history make rigorous solutions difficult. However, based on what is currently known, we argue that for human quantitative chronic disease risk factors the nearly neutral models of allelic evolution at single loci probably apply reasonably well. In general, and although much is still speculative, the data available for such risk factors are consistent with these expectations and may enable us to predict many aspects of etiologic heterogeneity for human disease. | 3250 | NA | NA | NA | no | no |
| 3250 | 2017 | NA | Ulrich, Y; Sadd, BM; Schmid-Hempel, P | 2011 | Strain filtering and transmission of a mixed infection in a social insect | Mixed-genotype infections have attracted considerable attention as drivers of pathogen evolution. However, experimental approaches often overlook essential features of natural host-parasite interactions, such as host heterogeneity, or the effects of between-host selection during transmission. Here, following inoculation of a mixed infection, we analyse the success of different strains of a trypanosome parasite throughout the colony cycle of its bumblebee host. We find that most colonies efficiently filter the circulating infection before it reaches the new queens, the only offspring that carry infections to the next season. A few colonies with a poor filtering ability thus contributed disproportionately to the parasite population in the next season. High strain diversity but not high infection intensity within colony was associated with an increased probability of transmission of the infection to new queens. Interestingly, the representation of the different strains changed dramatically over time, so that long-term parasite success could not be predicted from short-term observations. These findings highlight the shaping of within-colony parasite diversity through filtering as a crucial determinant of year-to-year pathogen transmission and emphasize the importance of host ecology and heterogeneity for disease dynamics. | 3251 | NA | NA | NA | no | no |
| 3251 | 2017 | NA | WATERS, AP; HIGGINS, DG; MCCUTCHAN, TF | 1993 | EVOLUTIONARY RELATEDNESS OF SOME PRIMATE MODELS OF PLASMODIUM | Primate-and, specifically, monkey-malaria infections are commonly used for understanding the pathology of and immune response to the human disease because they are thought to resemble most closely the host-parasite relationship found in humans. Plasmodium cynomolgi is used extensively as a model for the human parasite, P. vivax, and P. knowlesi is used primarily as a model for the development of erythrocytic-stage vaccines. Both of these simian parasites can naturally infect man, resulting in mildly symptomatic episodes of the disease. The phylogenetic relationship between these two simian parasites and previously characterized Plasmodium species, including P. vivax, was examined by comparison of the asexually expressed small-subunit ribosomal RNA genes. Our analysis confirmed that P. vivax is most closely related to P. cynomolgi and that it remains an appropriate model of the human pathogen. Furthermore, with P. knowlesi and P. fragile, these two species form a group of closely related species, distant from other Plasmodium species. What is considered to be the most ancient of the human malaria pathogens, P. malariae, was also included in the analysis and does not group at all with other simian or human parasites. | 3252 | NA | NA | NA | no | no |
| 3252 | 2017 | NA | Tsolaki, AG; Hirsh, AE; DeRiemer, K; Enciso, JA; Wong, MZ; Hannan, M; de la Salmoniere, YOL; Aman, K; Kato-Maeda, M; Small, PM | 2004 | Functional and evolutionary genomics of Mycobacterium tuberculosis: Insights from genomic deletions in 100 strains | To better understand genome function and evolution in Mycobacterium tuberculosis, the genomes of 100 epidemiologically well characterized clinical isolates were interrogated by DNA microarrays and sequencing. We identified 68 different large-sequence polymorphisms (comprising 186,137 bp, or 4.2% of the genome) that are present in H37Rv, but absent from one or more clinical isolates. A total of 224 genes (5.5%), including genes in all major functional categories, were found to be partially or completely deleted. Deletions are not distributed randomly throughout the genome but instead tend to be aggregated. The distinct deletions in some aggregations appear in closely related isolates, suggesting a genomically disruptive process specific to an individual mycobacterial lineage. Other genomic aggregations include distinct deletions that appear in phylogenetically unrelated isolates, suggesting that a genomic region is vulnerable throughout the species. Although the deletions identified here are evidently inessential to the causation of disease (they are found in active clinical cases), their frequency spectrum suggests that most are weakly deleterious to the pathogen. For some deletions, short-term evolutionary pressure due to the host immune system or antibiotics may favor the elimination of genes, whereas longer-term physiological requirements maintain the genes in the population. | 3253 | NA | NA | NA | no | no |
| 3253 | 2017 | NA | Mikalova, L; Bosak, J; Hribkova, H; Dedicova, D; Benada, O; Smarda, J; Smajs, D | 2017 | Novel Temperate Phages of Salmonella enterica subsp salamae and subsp diarizonae and Their Activity against Pathogenic S-enterica subsp enterica Isolates | Forty strains of Salmonella enterica (S. enterica) subspecies salamae (II), arizonae (IIIa), diarizonae (IIIb), and houtenae (IV) were isolated from human or environmental samples and tested for bacteriophage production. Production of bacteriophages was observed in 15 S. enterica strains (37.5%) belonging to either the subspecies salamae (8 strains) or diarizonae (7 strains). Activity of phages was tested against 52 pathogenic S. enterica subsp. enterica isolates and showed that phages produced by subsp. salamae had broader activity against pathogenic salmonellae compared to phages from the subsp. diarizonae. All 15 phages were analyzed using PCR amplification of phage-specific regions and 9 different amplification profiles were identified. Five phages (SEN1, SEN4, SEN5, SEN22, and SEN34) were completely sequenced and classified as temperate phages. Phages SEN4 and SEN5 were genetically identical, thus representing a single phage type (i.e. SEN4/5). SEN1 and SEN4/5 fit into the group of P2-like phages, while the SEN22 phage showed sequence relatedness to P22-like phages. Interestingly, while phage SEN34 was genetically distantly related to Lambda-like phages (Siphoviridae), it had the morphology of the Myoviridae family. Based on sequence analysis and electron microscopy, phages SEN1 and SEN4/5 were members of the Myoviridae family and phage SEN22 belonged to the Podoviridae family. | 3254 | NA | NA | NA | no | no |
| 3254 | 2017 | NA | Wang, L; Chen, H; Liu, DH; Huo, XX; Gao, JM; Song, XR; Xu, XC; Huang, KQ; Liu, WQ; Wang, Y; Lu, FL; Lun, ZR; Luo, QL; Wang, XL; Shen, JL | 2013 | Genotypes and Mouse Virulence of Toxoplasma gondii Isolates from Animals and Humans in China | Background: Recent population structure studies of T. gondii revealed that a few major clonal lineages predominated in different geographical regions. T. gondii in South America is genetically and biologically divergent, whereas this parasite is remarkably clonal in North America and Europe with a few major lineages including Types I, II and III. Information on genotypes and mouse virulence of T. gondii isolates from China is scarce and insufficient to investigate its population structure, evolution, and transmission. Methodology/Principal Findings: Genotyping of 23 T. gondii isolates from different hosts using 10 markers for PCR-restriction fragment length polymorphism analyses (SAG1, SAG2, SAG3, BTUB, GRA6, c22-8, c29-2, L358, PK1 and Apico) revealed five genotypes; among them three genotypes were atypical and two were archetypal. Fifteen strains belong to the Chinese 1 lineage, which has been previously reported as a widespread lineage from swine, cats, and humans in China. Two human isolates fall into the type I and II lineages and the remaining isolates belong to two new atypical genotypes (ToxoDB#204 and #205) which has never been reported in China. Our results show that these genotypes of T. gondii isolates are intermediately or highly virulent in mice except for the strain TgCtwh6, which maintained parasitemia in mice for 35 days post infection although it possesses the uniform genotype of Chinese 1. Additionally, phylogenetic network analyses of all isolates of genotype Chinese 1 are identical, and there is no variation based on the sequence data generated for four introns (EF1, HP2, UPRT1 and UPRT7) and two dense granule proteins (GRA6 and GRA7). Conclusion/Significance: A limited genetic diversity was found and genotype Chinese 1 (ToxoDB#9) is dominantly circulating in mainland China. The results will provide a useful profile for deep insight to the population structure, epidemiology and biological characteristics of T. gondii in China. | 3255 | NA | NA | NA | no | no |
| 3255 | 2017 | NA | Kalia, VC; Mukherjee, T; Bhushan, A; Joshi, J; Shankar, P; Huma, N | 2011 | Analysis of the unexplored features of rrs (16S rDNA) of the Genus Clostridium | Background: Bacterial taxonomy and phylogeny based on rrs (16S rDNA) sequencing is being vigorously pursued. In fact, it has been stated that novel biological findings are driven by comparison and integration of massive data sets. In spite of a large reservoir of rrs sequencing data of 1,237,963 entries, this analysis invariably needs supplementation with other genes. The need is to divide the genetic variability within a taxa or genus at their rrs phylogenetic boundaries and to discover those fundamental features, which will enable the bacteria to naturally fall within them. Within the large bacterial community, Clostridium represents a large genus of around 110 species of significant biotechnological and medical importance. Certain Clostridium strains produce some of the deadliest toxins, which cause heavy economic losses. We have targeted this genus because of its high genetic diversity, which does not allow accurate typing with the available molecular methods. Results: Seven hundred sixty five rrs sequences (> 1200 nucleotides, nts) belonging to 110 Clostridium species were analyzed. On the basis of 404 rrs sequences belonging to 15 Clostridium species, we have developed species specific: (i) phylogenetic framework, (ii) signatures (30 nts) and (iii) in silico restriction enzyme (14 Type II REs) digestion patterns. These tools allowed: (i) species level identification of 95 Clostridium sp. which are presently classified up to genus level, (ii) identification of 84 novel Clostridium spp. and (iii) potential reduction in the number of Clostridium species represented by small populations. Conclusions: This integrated approach is quite sensitive and can be easily extended as a molecular tool for diagnostic and taxonomic identification of any microbe of importance to food industries and health services. Since rapid and correct identification allows quicker diagnosis and consequently treatment as well, it is likely to lead to reduction in economic losses and mortality rates. | 3256 | NA | NA | NA | no | no |
| 3256 | 2017 | NA | de Eyto, E; McGinnity, P; Huisman, J; Coughlan, J; Consuegra, S; Farrell, K; O’Toole, C; Tufto, J; Megens, HJ; Jordan, W; Cross, T; Stet, RJM | 2011 | Varying disease-mediated selection at different life-history stages of Atlantic salmon in fresh water | Laboratory studies on associations between disease resistance and susceptibility and major histocompatibility (MH) genes in Atlantic salmon Salmo salar have shown the importance of immunogenetics in understanding the capacity of populations to fight specific diseases. However, the occurrence and virulence of pathogens may vary spatially and temporally in the wild, making it more complicated to predict the overall effect that MH genes exert on fitness of natural populations and over several life-history stages. Here we show that MH variability is a significant determinant of salmon survival in fresh water, by comparing observed and expected genotype frequencies at MH and control microsatellite loci at parr and migrant stages in the wild. We found that additive allelic effects at immunogenetic loci were more likely to determine survival than dominance deviation, and that selection on certain MH alleles varied with life stage, possibly owing to varying pathogen prevalence and/or virulence over time. Our results highlight the importance of preserving genetic diversity (particularly at MH loci) in wild populations, so that they have the best chance of adapting to new and increased disease challenges as a result of projected climate warming and increasing aquaculture. | 3257 | NA | NA | NA | no | no |
| 3257 | 2017 | NA | Orsucci, M; Navajas, M; Fellous, S | 2017 | Genotype-specific interactions between parasitic arthropods | Despite the ubiquity of coinfection, we know little of the effects of intra-specific genetic variability on coinfection by distinct parasite species. Here we test the hypothesis that parasite multiplication depends on the combination of parasite genotypes that coinfect the host (that is Genotype. parasite x Genotype. parasite interaction). To that aim, we infected tomato leaves with the ectoparasitic mites Tetranychus urticae and Tetranychus evansi. We tested all possible combinations between four T. urticae and two T. evansi populations sampled on different hosts or localities. There was no universal (that is genotype-independent) effect of coinfection on mite multiplication; in many cases the two species had no effect on each other. However, several combinations of T. evansi and T. urticae populations led to elevated T. evansi numbers. Similarly, T. urticae reproduction largely depended on the interaction between T. urticae and T. evansi populations. This evidence for genotype-by-genotype interaction between coinfecting parasites indicates that the effect of coinfection on parasite epidemiology and evolution may vary in space according to the genetic composition of local parasite populations; it further suggests the possibility of coevolution between parasites species that share the same hosts. | 3258 | NA | NA | NA | no | no |
| 3258 | 2017 | NA | Li, H; Ruan, CJ; da Silva, JAT; Liu, BQ | 2010 | Associations of SRAP markers with dried-shrink disease resistance in a germplasm collection of sea buckthorn (Hippophae L.) | Sea buckthorn (Hippophae L.) is a woody, outcrossing dioecious pioneer plant, being widely planted as a new berry crop with rich nutritional and medicinal compounds. This long-juvenile and long-lived woody plant can he more difficult to cultivate than other crop plants. Dried-shrink disease (DSD) is a dangerous pathogen that destroys sea buckthorn and halts commercial production. We estimated variability of sequence-related amplified polymorphism (SRAP) markers in 77 accessions of 22 sea buckthorn cultivars to seek markers associated with DSD resistance and help to identify potential breeding cultivars. Seventeen SRAP primer combinations generated 289 bands, with a mean of 17 bands per primer combination. At a Dice coefficient of 0.852, the dendrogram generated with 191 polymorphic bands clustered 73 accessions of Hippophae rhanmoides into 2 groups and 4 accessions of Hippophae salicifolia into I group. Eleven SRAP markers (Me1-Em3(600), Me1-Em1(650), Me2-Em1(650), Me3-Em1(950), Me3-Em6(1300), Me2-Em6(320), Me2-Em6(400). Me1-Em2(600). Me1-Em1(1200), Me1-Em1(1700), Me2-Em2(250)) were significantly correlated with DSD resistance (P < 0.001). These markers provide a viable option for breeding programs that select lineages with DSD resistance, especially when no other genetic information, such as linkage maps and quantitative trait loci, are available. | 3259 | NA | NA | NA | no | no |
| 3259 | 2017 | NA | Redman, EM; Wilson, K; Grzywacz, D; Cory, JS | 2010 | High levels of genetic diversity in Spodoptera exempta NPV from Tanzania | The African armyworm, Spodoptera exempta, is a major pest in sub-Saharan Africa. A nucleopolyhedrovirus (NPV) is often recorded in later population outbreaks and can cause very high levels of mortality. Research has been addressing whether this NPV can be developed into a strategic biological control agent. As part of this study, the variation in natural populations of NPV is being studied. An isolate of S. exempta NPV was cloned in vivo and found to contain at least 17 genetically-distinct genotypes. These genotypes varied in size from approximately 115 to 153 kb. (C) 2010 Elsevier Inc. All rights reserved. | 3260 | NA | NA | NA | no | no |
| 3260 | 2017 | NA | Ristic, D; Vucurovic, I; Kuzmanovic, S; Milosevic, D; Gasic, K; Dolovac, N; Starovic, M | 2016 | MOLECULAR CHARACTERIZATION OF POTATO VIRUS Y INDUCING POTATO TUBER NECROTIC RINGSPOT DISEASE IN SERBIA | The Potato virus Y (PVY) is the most important limiting factor for potato seed production in Serbia. Currently, PVY is a major concern for the potato seed growers. Initially, serological (ELISA) tests were carried out on 100 potato seed tubers from each of the seven potato cv. during 2013. The infection rates with the PVYN was between 5 and 36%. A complete genome sequencing of the most common Serbian isolate of PVY (3D), followed by molecular characterization and phylogenetic analysis has been performed to show what group it belongs to. Our isolate’s complete genome sequence (KJ946936) showed that the Serbian PVY isolate (3D) is 99.7% identical at nt level, with other tuber necrosis strain group (PVYNTN) from Europe. Phylogenetic analysis revealed three consistent lineages of isolates, showing that our isolate was clustered with the isolates from Europe and North America in the PVYN lineage which induces potato tuber necrotic ringspot disease (PTNRD). The Serbian isolate of PVYNTN together with the isolates from Europe was clustered in the branch of European sublineage, with a high bootstrap support and no genetic diversity. This is the first study in Serbia demonstrating phylogenetic distinction between our isolate and other isolates of PVY. | 3261 | NA | NA | NA | no | no |
| 3261 | 2017 | NA | Xu, GJ; Liu, CS; Huang, LQ; Wang, XY; Zhang, YY; Liu, SQ; Liao, CL; Yuan, QJ; Zhou, XL | 2013 | Development of new EST-derived SSRs in Salvia miltiorrhiza (Labiatae) in China and preliminary analysis of genetic diversity and population structure | Salvia miltiorrhiza, known as Dan-shen in China, is a well-known and important medicinal herb in Asia. Its root and rhizome has been used to treat cardiovascular diseases in China for centuries. Recently, it had been widely cultivated in China due to a rapid decline in wild resources. However, the germplasm resources of different cultivated regions are extremely variable. Here, new simple sequence repeats (SSRs) from expressed sequence tags (ESTs) were mined to analyze the frequency and distribution of SSRs, assess the validity of developed EST-SSR marker and identify new EST-SSR marker for the preparatory investigation of the population structure in cultivated S. miltiorrhiza. This study showed that 5160 unique ESTs were accumulated from database, 760 SSRs combined with 41 types of SSR motifs were mined. High polymorphism and transferability were presented in newly developed EST-SSR marker. It also showed that cultivated S. miltiorrhiza were mainly divided into three large groups. The results suggested that S. miltiorrhiza may has high abundance of SSRs in transcribed regions of genome and the developed EST-SSR marker may be useful for analysis of genetic diversity and population structure. (C) 2013 Elsevier Ltd. All rights reserved. | 3262 | NA | NA | NA | no | no |
| 3262 | 2017 | NA | Luevano, KE; Gonzalez, JR; Perea, FJ; Magana, MT | 2009 | Linkage disequilibrium between four MTTP gene polymorphisms in a Mexican population | Background: The microsomal triglyceride transfer protein plays an important role in the folding, assembling and secretion of lipoproteins that contain apoprotein B. Different polymorphisms in the MTTP gene have been associated with risk factors for coronary heart disease and diabetes, the first and fourth most common causes of death in Mexico, respectively. Aim: The objective of this study was to assess allele, genotype and haplotype frequencies of six MTTP polymorphisms in an unselected Mexican population. Subjects and methods: Six polymorphisms were analysed by DNA sequencing of polymerase chain reaction products in 155 Mexican individuals and Hardy-Weinberg equilibrium, genetic variability, linkage disequilibrium and neutrality test were evaluated. Results: The rare alleles of the six polymorphisms analysed had frequencies greater than 1% and their genotype distributions were in accordance with Hardy-Weinberg equilibrium. All three promoter and I/T 128 polymorphisms were in linkage disequilibrium. Twelve different haplotypes were observed; GATGGT (70.44%) and TTCGGC (13.91%) were the most common. Diversity patterns in this Mexican population deviate significantly from expectations of the standard neutral model for infinite allele. Conclusion: The -493 G/T, -400 A/T, -164 T/C and I/T 128 polymorphisms can be useful for association studies in this population. | 3263 | NA | NA | NA | no | no |
| 3263 | 2017 | NA | Cleary, M; Nguyen, D; Marciulyniene, D; Berlin, A; Vasaitis, R; Stenlid, J | 2016 | Friend or foe? Biological and ecological traits of the European ash dieback pathogen Hymenoscyphus fraxineus in its native environment | Hymenoscyphus fraxineus, an introduced ascomycete fungus and primary causal agent of European ash dieback, was investigated on Fraxinus mandshurica trees in its native range in Primorye region of Far East Russia. This evidence is the first report of H. fraxineus on healthy, asymptomatic F. mandshurica trees. High-throughput sequencing revealed 49 distinct fungal taxa associated with leaves of F. mandshurica, 12 of which were identified to species level. Phyllosphere fungal assemblages were similar among sites despite being largely geographically distant. Many organisms comprising the foliar fungal community on F. mandshurica in Far East Russia have similarity to those reported inhabiting F. excelsior in Europe based on previous studies. However, Mycosphaerella sp., the most dominant species in this study and detected in nearly all samples, was associated only with F. mandshurica. Genetic diversity of H. fraxineus was significantly higher in the Far East Russian population than in Europe. In contrast to its aggressive behaviour on Fraxinus excelsior in Europe, H. fraxineus appears to be a benign associate of indigenous F. mandshurica that initially induces quiescent and asymptomatic infections in healthy trees prior to active host colonization normally associated with modification of host tissue during senescence. | 3264 | NA | NA | NA | no | no |
| 3264 | 2017 | NA | Martin-Hernanz, S; de Castro, AGF; Moreno-Saiz, JC; Valcarcel, V | 2016 | Pre-Holocene Origin for the Coronopus navasii Disjunction: Conservation Implications from Its Long Isolation | Integration of unexpected discoveries about charismatic species can disrupt their well-established recovery plans, particularly when this requires coordinate actions among the different governments responsible. The Critically Endangered Coronopus navasii (Brassicaceae) was considered a restricted endemism to a few Mediterranean temporary ponds in a high mountain range of Southeast Spain, until a new group of populations were discovered 500 km North in 2006. Ten years after this finding, its management has not been accommodated due to limited information of the new populations and administrative inertia. In this study, DNA sequences and species distribution models are used to analyse the origin of the C. navasii disjunction as a preliminary step to reassess its recovery plan. Molecular results placed the disjunction during Miocene-Pleistocene (6.30-0.49 Mya, plastid DNA; 1.45-0.03 Mya, ribosomal DNA), which discards a putative human-mediated origin. In fact, the haplotype network and the low gene flow estimated between disjunct areas suggest long-term isolation. Dispersal is the most likely explanation for the disjunction as interpreted from the highly fragmented distribution projected to the past. Particularly, a northward dispersal from Southeast is proposed since C. navasii haplotype network is connected to the sister-group through the southern haplotype. Although the reassessment of C. navasii conservation status is more optimistic under the new extent of occurrence, its long-term survival may be compromised due to the: (1) natural fragmentation and rarity of the species habitat, (2) genetic isolation between the two disjunct areas, and (3) northward shift of suitable areas under future climate change scenarios. Several ex-situ and in-situ conservation measures are proposed for integrating Central East Spanish populations into the on-going recovery plan, which still only contemplates Southeast populations and therefore does not preserve the genetic structure and diversity of the species. | 3265 | NA | NA | NA | no | no |
| 3265 | 2017 | NA | Marche, L; Valette, S; Grenier, E; Mugniery, D | 2001 | Intra-species DNA polymorphism in the tobacco cyst-nematode complex (Globodera tabacum) using AFLP | Amplified fragment length polymorphism (AFLP) was used to obtain information on the within-species genetic variability of the tobacco cyst - nematode (TCN) complex. AFLP was found to be well suited to this type of study. The current classification of TCN was confirmed. Results indicate that the Globodera tabacum solanacearum group, believed to be restricted to the U.S.A., also occurs in Mexico. The within-species variability of TCN is considerable. Populations from Mexico may form a new subgroup. AFLP group-specific markers were identified for two of the TCN subgroups: Globodera tabacum tabacum and Globodera tabacum solanacearum. | 3266 | NA | NA | NA | no | no |
| 3266 | 2017 | NA | Zwart, RS; Muylle, H; Van Bockstaele, E; Roldan-Ruiz, I | 2008 | Evaluation of genetic diversity of Fusarium head blight resistance in European winter wheat | Genetic diversity in relation to Fusarium head blight (FHB) resistance was investigated among 295 European winter wheat cultivars and advanced breeding lines using 47 wheat SSR markers. Twelve additional wheat lines with known FHB resistance were included as reference material. At least one SSR marker per chromosome arm, including SSR markers reported in the literature with putative associations with QTLs for FHB resistance, were assayed to give an even distribution of SSR markers across the wheat genome. A total of 404 SSR alleles were detected. The number of alleles per locus ranged from 2 to 21, with an average of 8.6 alleles. The polymorphism information content of the SSR markers ranged from 0.13 (Xwmc483) to 0.87 (Xwmc607), with an average of 0.54. Cluster analysis was performed by both genetic distance-based and model-based methods. In general, the dendrogram based on unweighted pair-group method with arithmetic averages showed similar groupings to the model-based analysis. Seven clusters were identified by the model-based method, which did not strictly correspond to geographical origin. The FHB resistance level of the wheat lines was evaluated in field trials conducted over multiple years or locations by assessing the following traits: % FHB severity, % FHB incidence, % diseased kernels, in spray inoculation trials, and % FHB spread and % wilted tips, in point inoculation trials. Association analysis between SSR markers and the FHB disease traits detected markers significantly associated with FHB resistance, including some that have not been previously reported. The percentage of variance explained by each individual marker was, however, rather low. Haplotype analysis revealed that the FHB-resistant European wheat lines do not contain the 3BS locus derived from Sumai 3. The information generated in this study will assist in the selection of parental lines in order to increase the efficiency of breeding efforts for FHB resistance. | 3267 | NA | NA | NA | no | no |
| 3267 | 2017 | NA | Poirier, M; Durand, JL; Volaire, F | 2012 | Persistence and production of perennial grasses under water deficits and extreme temperatures: importance of intraspecific vs. interspecific variability | Extreme climatic events are expected to increase in frequency and magnitude as a consequence of global warming. Grasslands cover a large proportion of the European continent and contribute to both agricultural production and ecosystem services through inter and intraspecific genetic variability. This study analysed the effects of summer droughts and heat waves on the persistence and production of perennial forage grasses. Mediterranean and temperate populations of Dactylis glomerata L. and Festuca arundinacea (Schreb.) were compared at both Mediterranean and temperate sites in France. By manipulating canopy temperatures and water availability, grass swards in the field were subjected to cumulative summer and spring water deficits (CSSWD) ranging from 329 to 707 mm to test different projected climatic conditions and extreme summer events. Under controlled summer heat waves (621 days at a mean daily canopy temperature higher than 3035 degrees C), there was no increase in membrane damage to surviving aerial tissues. Plant stress was thus mainly generated through greater soil water deficit. Under the greatest CSSWD, annual biomass production was reduced on average by 60% and 30% with temperate and Mediterranean populations, respectively. Thresholds for a significant increase in summer tiller mortality were seen at CSSWD higher than 450 mm for temperate populations and 550 mm for Mediterranean populations. The latter displayed lower predawn leaf water potentials in summer and recovered through intense tillering in the subsequent seasons. Under the most extreme CSSWD, fewer than 20% of tillers of temperate populations survived and their nitrogen uptake ability was drastically altered. The higher potential productivity of Mediterranean populations in winter was associated with greater frost sensitivity. The identification of thresholds for vulnerability and the determination of the role of genetic diversity will improve the management of plant resilience and the design of new plant material to cope with climate change. | 3268 | NA | NA | NA | no | no |
| 3268 | 2017 | NA | Ngamskulrungroj, P; Gilgado, F; Faganello, J; Litvintseva, AP; Leal, AL; Tsui, KM; Mitchell, TG; Vainstein, MH; Meyer, W | 2009 | Genetic Diversity of the Cryptococcus Species Complex Suggests that Cryptococcus gattii Deserves to Have Varieties | The Cryptococcus species complex contains two sibling taxa, Cryptococcus neoformans and Cryptococcus gattii. Both species are basidiomycetous yeasts and major pathogens of humans and other mammals. Genotyping methods have identified major haploid molecular types of C. neoformans (VNI, VNII, VNB and VNIV) and of C. gattii (VGI, VGII, VGIII and VGIV). To investigate the phylogenetic relationships among these haploid genotypes, we selected 73 strains from 2000 globally collected isolates investigated in our previous typing studies, representing each of these genotypes and carried out multigene sequence analyses using four genetically unlinked nuclear loci, ACT1, IDE, PLB1 and URA5. The separate or combined sequence analyses of all four loci revealed seven clades with significant support for each molecular type. However, three strains of each species revealed some incongruence between the original molecular type and the sequence-based type obtained here. The topology of the individual gene trees was identical for each clade of C. neoformans but incongruent for the clades of C. gattii indicating recent recombination events within C. gattii. There was strong evidence of recombination in the global VGII population. Both parsimony and likelihood analyses supported three major clades of C. neoformans (VNI/VNB, VNII and VNIV) and four major clades of C. gattii ( VGI, VGII, VGIII and VGIV). The sequence variation between VGI, VGIII and VGIV was similar to that between VNI/VNB and VNII. MATa was for the first time identified for VGIV. The VNIV and VGII clades are basal to the C. neoformans or the C. gattii clade, respectively. Divergence times among the seven haploid monophyletic lineages in the Cryptococcus species complex were estimated by applying the hypothesis of the molecular clock. The genetic variation found among all of these haploid monophyletic lineages indicates that they warrant varietal status. | 3269 | NA | NA | NA | no | no |
| 3269 | 2017 | NA | Dowkiw, A; Bastien, C | 2007 | Presence of defeated qualitative resistance genes frequently has major impact on quantitative resistance to Melampsora larici-populina leaf rust in P.xinteramericana hybrid poplars | Qualitative resistance to Melampsora larici populina leaf rust inherited from North American species Populus deltoides did not allow for durable control of this pathogen in interspecific hybrid cultivars. Despite significant levels of strain-specificity, quantitative resistance would exert lower selection pressures on the pathogen populations, and hence could be more durable. Previous studies restricted to a large P. x interamericana (i.e., P. deltoides x Populus trichocarpa) F, family revealed that the presence of R(1), a segregating defeated qualitative resistance gene inherited from P. deltoides, had major beneficial effects on quantitative resistance. The present study was based on 14 F(1) families from a 4x5 P. deltoides x P. trichocarpa factorial mating design where at least four defeated qualitative resistances segregate 1:1. Even though quantitative resistance assessments were conducted in the laboratory with a M. laricipopulina strain able to overcome these qualitative resistances, their presence had a significant effect on the mean level and on the genetic variability for quantitative resistance. One unprecedented result is the identification of a defeated qualitative resistance which presence is associated with lower levels of quantitative resistance. Possible inferences on the nature of the genetic relationship between both resistance types are discussed. | 3270 | NA | NA | NA | no | no |
| 3270 | 2017 | NA | Liu, J; Feng, M; Wang, XL; Fu, YF; Ma, CL; Cheng, XJ | 2017 | Unique Trichomonas vaginalis gene sequences identified in multinational regions of Northwest China | Trichomonas vaginalis (T. vaginalis) is a flagellated protozoan parasite that infects humans worldwide. This study determined the sequence of the 18S ribosomal RNA gene of T. vaginalis infecting both females and males in Xinjiang, China. Samples from 73 females and 28 males were collected and confirmed for infection with T. vaginalis, a total of 110 sequences were identified when the T. vaginalis 18S ribosomal RNA gene was sequenced. These sequences were used to prepare a phylogenetic network. The rooted network comprised three large clades and several independent branches. Most of the Xinjiang sequences were in one group. Preliminary results suggest that Xinjiang T. vaginalis isolates might be genetically unique, as indicated by the sequence of their 18S ribosomal RNA gene. Low migration rate of local people in this province may contribute to a genetic conservativeness of T. vaginalis. The unique genetic feature of our isolates may suggest a different clinical presentation of trichomoniasis, including metronidazole susceptibility, T. vaginalis virus or Mycoplasma co-infection characteristics. The transmission and evolution of Xinjiang T. vaginalis is of interest and should be studied further. More attention should be given to T. vaginalis infection in both females and males in Xinjiang. | 3271 | NA | NA | NA | no | no |
| 3271 | 2017 | NA | Bryner, SF; Rigling, D; Brunner, PC | 2012 | Invasion history and demographic pattern of Cryphonectria hypovirus 1 across European populations of the chestnut blight fungus | We reconstructed the invasion history of the fungal virus Cryphonectria hypovirus 1 (CHV-1) in Europe, which infects the chestnut blight fungus Cryphonectria parasitica. The pattern of virus evolution was inferred based on nucleotide sequence variation from isolates sampled across a wide area in Europe at different points in time. Phylogeny and time estimates suggested that CHV-1 was introduced together with its fungal host to Europe and that it rapidly colonized the central range along the south facing slopes of the Alps and the north-east facing slopes of the Dinaric Alps. These central populations were the source for two waves of simultaneous invasions toward the southern Balkans and Turkey, as indicated by migration rates. Our results showed that the evolutionary scenarios for CHV-1 and C. parasitica were spatially congruent. As infection with CHV-1 reduces the pathogenicity of C. parasitica toward the chestnut tree, CHV-1 invasions of the newly established C. parasitica populations probably prevented the development of devastating chestnut blight epidemics in Europe. We propose that in this, and supposedly in other pathosystems, geographic, vegetation-related, demographic, economic, and political factors may help explain the correlated invasion pattern of a parasite and its host. | 3272 | NA | NA | NA | no | no |
| 3272 | 2017 | NA | Doxey, AC; Yaish, MWF; Moffatt, BA; Griffith, M; McConkey, BJ | 2007 | Functional divergence in the Arabidopsis beta-1,3-glucanase gene family inferred by phylogenetic reconstruction of expression states | Plant beta-1,3-glucanases (beta-1,3-Gs) (E.C. 3.2.1.39) comprise large, highly complex gene families involved in pathogen defense as well as a wide range of normal developmental processes. In spite of previous phylogenetic analyses that classify beta-1,3-Gs by sequence relatedness, the functional evolution of beta-1,3-Gs remains unclear. Here, expression and phylogenetic analyses have been integrated in order to investigate patterns of functional divergence in the Arabidopsis beta-1,3-G gene family. Fifty beta-1,3-G genes were grouped into expression classes through clustering of microarray data, and functions were inferred based on knowledge of coexpressed genes and existing literature. The resulting expression classes were mapped as discrete states onto a phylogenetic tree and parsimony reconstruction of ancestral expression states was performed, providing a model of expression divergence. Results showed a highly nonrandom distribution of developmental expression states in the phylogeny (P = 0.0002) indicating a significant degree of coupling between sequence and developmental expression divergence. A weaker, yet significant level of coupling was found using stress response data, but not using hormone-response or pathogen-response data. According to the model of developmental expression divergence, the ancestral function was most likely involved in cell division and/or cell wall remodeling. The associated expression state is widely distributed in the phylogeny, is retained by over 25% of gene family members, and is consistent with the known functions of beta-1,3-Gs in distantly related species and gene families. Consistent with previous hypotheses, pathogenesis-related (PR) beta-1,3-Gs appear to have evolved from ancestral developmentally regulated beta-1,3-Gs, acquiring PR function through a number of evolutionary events: divergence from the ancestral expression state, acquisition of pathogen/stress-responsive expression patterns, and loss of the C-terminal region including the glycosylphosphatidylinisotol (GPI)-anchoring site thus allowing for extracellular secretion. | 3273 | NA | NA | NA | no | no |
| 3273 | 2017 | NA | Dugrand-Judek, A; Olry, A; Hehn, A; Costantino, G; Ollitrault, P; Froelicher, Y; Bourgaud, F | 2015 | The Distribution of Coumarins and Furanocoumarins in Citrus Species Closely Matches Citrus Phylogeny and Reflects the Organization of Biosynthetic Pathways | Citrus plants are able to produce defense compounds such as coumarins and furanocoumarins to cope with herbivorous insects and pathogens. In humans, these chemical compounds are strong photosensitizers and can interact with medications, leading to the “grapefruit juice effect”. Removing coumarins and furanocoumarins from food and cosmetics imply additional costs and might alter product quality. Thus, the selection of Citrus cultivars displaying low coumarin and furanocoumarin contents constitutes a valuable alternative. In this study, we performed ultra-performance liquid chromatography coupled with mass spectrometry analyses to determine the contents of these compounds within the peel and the pulp of 61 Citrus species representative of the genetic diversity all Citrus. Generally, Citrus peel contains larger diversity and higher concentrations of coumarin/furanocoumarin than the pulp of the same fruits. According to the chemotypes found in the peel, Citrus species can be separated into 4 groups that correspond to the 4 ancestral taxa (pummelos, mandarins, citrons and papedas) and extended with their respective secondary species descendants. Three of the 4 ancestral taxa (pummelos, citrons and papedas) synthesize high amounts of these compounds, whereas mandarins appear practically devoid of them. Additionally, all ancestral taxa and their hybrids are logically organized according to the coumarin and furanocoumarin pathways described in the literature. This organization allows hypotheses to be drawn regarding the biosynthetic origin of compounds for which the biogenesis remains unresolved. Determining coumarin and furanocoumarin contents is also helpful for hypothesizing the origin of Citrus species for which the phylogeny is presently not firmly established. Finally, this work also notes favorable hybridization schemes that will lead to low coumarin and furanocoumarin contents, and we propose to select mandarins and Ichang papeda as Citrus varieties for use in creating species devoid of these toxic compounds in future breeding programs. | 3274 | NA | NA | NA | no | no |
| 3274 | 2017 | NA | Pinard-van der Laan, MH; Soubieux, D; Merat, L; Bouret, D; Luneau, G; Dambrine, G; Thoraval, P | 2004 | Genetic analysis of a divergent selection for resistance to Rous sarcomas in chickens | Selection for disease resistance related traits is a tool of choice for evidencing and exploring genetic variability and studying underlying resistance mechanisms. In this framework, chickens originating from a base population, homozygote for the B-19 major histocompatibility complex ( MHC) were divergently selected for either progression or regression of tumors induced at 4 weeks of age by a SR- D strain of Rous sarcoma virus ( RSV). The first generation of selection was based on a progeny test and subsequent selections were performed on full- sibs. Data of 18 generations including a total of 2010 birds measured were analyzed for the tumor profile index ( TPI), a synthetic criterion of resistance derived from recording the volume of the tumors and mortality. Response to selection and heritability of TPI were estimated using a restricted maximum likelihood method with an animal model. Significant progress was shown in both directions: the lines differing significantly for TPI and mortality becoming null in the " regressor" line. Heritability of TPI was estimated as 0.49 +/- 0.05 and 0.53 +/- 0.06 within the progressor and regressor lines respectively, and 0.46 +/- 0.03 when estimated over lines. Preliminary results showed within the progressor line a possible association between one Rfp- Y type and the growth of tumors. | 3275 | NA | NA | NA | no | no |
| 3275 | 2017 | NA | Shafer, ABA; Northrup, JM; Wikelski, M; Wittemyer, G; Wolf, JBW | 2016 | Forecasting Ecological Genomics: High-Tech Animal Instrumentation Meets High-Throughput Sequencing | Recent advancements in animal tracking technology and high-throughput sequencing are rapidly changing the questions and scope of research in the biological sciences. The integration of genomic data with high-tech animal instrumentation comes as a natural progression of traditional work in ecological genetics, and we provide a framework for linking the separate data streams from these technologies. Such a merger will elucidate the genetic basis of adaptive behaviors like migration and hibernation and advance our understanding of fundamental ecological and evolutionary processes such as pathogen transmission, population responses to environmental change, and communication in natural populations. | 3276 | NA | NA | NA | no | no |
| 3276 | 2017 | NA | McInerny, GJ; Turner, JRG; Wong, HY; Travis, JMJ; Benton, TG | 2009 | How range shifts induced by climate change affect neutral evolution | We investigate neutral evolution during range shifts in a strategic model of a metapopulation occupying a climate gradient. Using heritable, neutral markers, we track the spatio-temporal fate of lineages. Owing to iterated founder effects (‘mutation surfing’), survival of lineages derived from the leading range limit is enhanced. At trailing limits, where habitat suitability decreases, survival is reduced (mutations ‘wipe out’). These processes alter (i) the spatial spread of mutations, (ii) origins of persisting mutations and (iii) the generation of diversity. We show that large changes in neutral evolution can be a direct consequence of range shifting. | 3277 | NA | NA | NA | no | no |
| 3277 | 2017 | NA | Zhang, H; Van der Lee, T; Waalwijk, C; Chen, WQ; Xu, J; Xu, JS; Zhang, Y; Feng, J | 2012 | Population Analysis of the Fusarium graminearum Species Complex from Wheat in China Show a Shift to More Aggressive Isolates | A large number of Fusarium isolates was collected from blighted wheat spikes originating from 175 sampling sites, covering 15 provinces in China. Species and trichothecene chemotype determination by multilocus genotyping (MLGT) indicated that F. graminearum s. str. with the 15-acetyl deoxynivalenol (15ADON) chemotype and F. asiaticum with either the nivalenol (NIV) or the 3-acetyl deoxynivalenol (3ADON) chemotype were the dominant causal agents. Bayesian model-based clustering with allele data obtained with 12 variable number of tandem repeats (VNTR) markers, detected three genetic clusters that also show distinct chemotypes. High levels of population genetic differentiation and low levels of effective number of migrants were observed between these three clusters. Additional genotypic analyses revealed that F. graminearum s. str. and F. asiaticum are sympatric. In addition, composition analysis of these clusters indicated a biased gene flow from 3ADON to NIV producers in F. asiaticum. In phenotypic analyses, F. asiaticum that produce 3ADON revealed significant advantages over F. asiaticum that produce NIV in pathogenicity, growth rate, fecundity, conidial length, trichothecene accumulation and resistance to benzimidazole. These results suggest that natural selection drives the spread of a more vigorous, more toxigenic pathogen population which also shows higher levels of fungicide resistance. | 3278 | NA | NA | NA | no | no |
| 3278 | 2017 | NA | Morlacchi, P; Giorgi, A; Lozzia, GC; Baumgartner, J | 2011 | The performance of Macrosiphoniella millefolii and Myzus persicae on Achillea collina | This paper compares the development of the polyphagous aphid Myzus persicae Sulzer, considered as a generalist, and the oligophagous aphid Macrosiphoniella millefolii (De Geer), considered as a specialist, on yarrow (Achillea collina Becker ex Reichenbach). Yarrow is a medicinal plant rich in bioactive secondary metabolites that have possible effects on the development of phytophages, including aphids. Age specific life tables for cohorts developing under different constant temperatures were constructed and analyzed using standard techniques, and complemented with Jackknife estimates of the intrinsic rate of increase and its standard error. The parthenogenetic wingless morphs of the two species differed in the immature developmental time and survival, and in adult fecundity and life span. At high temperatures, the intrinsic rate of increase as the overall metric of performance tended to be higher for the generalist than for the specialist aphid species, while the opposite appears to occur at low and medium temperatures. Further insight into the complex interactions between yarrow and aphids requires that their genetic diversity is taken into account. The study of yarrow-aphid-natural enemy population interactions requires additional information on biomass dynamics, aphid morph differentiation and the performance of biological control agents. | 3279 | NA | NA | NA | no | no |
| 3279 | 2017 | NA | Bartnikas, TB; Parker, CC; Cheng, RY; Campagna, DR; Lim, JE; Palmer, AA; Fleming, MD | 2012 | QTLs for murine red blood cell parameters in LG/J and SM/J F-2 and advanced intercross lines | Red blood cells are essential for oxygen transport and other physiologic processes. Red cell characteristics are typically determined by complete blood counts which measure parameters such as hemoglobin levels and mean corpuscular volumes; these parameters reflect the quality and quantity of red cells in the circulation at any particular moment. To identify the genetic determinants of red cell parameters, we performed genome-wide association analysis on LG/J x SM/J F-2 and F-34 advanced intercross lines using single nucleotide polymorphism genotyping and a novel algorithm for mapping in the combined populations. We identified significant quantitative trait loci for red cell parameters on chromosomes 6, 7, 8, 10, 12, and 17; our use of advanced intercross lines reduced the quantitative trait loci interval width from 1.6- to 9.4-fold. Using the genomic sequences of LG/J and SM/J mice, we identified nonsynonymous coding single nucleotide polymorphisms in candidate genes residing within quantitative trait loci and performed sequence alignments and molecular modeling to gauge the potential impact of amino acid substitutions. These results should aid in the identification of genes critical for red cell physiology and metabolism and demonstrate the utility of advanced intercross lines in uncovering genetic determinants of inherited traits. | 3280 | NA | NA | NA | no | no |
| 3280 | 2017 | NA | Wendte, JM; Miller, MA; Lambourn, DM; Magargal, SL; Jessup, DA; Grigg, ME | 2010 | Self-Mating in the Definitive Host Potentiates Clonal Outbreaks of the Apicomplexan Parasites Sarcocystis neurona and Toxoplasma gondii | Tissue-encysting coccidia, including Toxoplasma gondii and Sarcocystis neurona, are heterogamous parasites with sexual and asexual life stages in definitive and intermediate hosts, respectively. During its sexual life stage, T. gondii reproduces either by genetic out-crossing or via clonal amplification of a single strain through self-mating. Out-crossing has been experimentally verified as a potent mechanism capable of producing offspring possessing a range of adaptive and virulence potentials. In contrast, selfing and other life history traits, such as asexual expansion of tissue-cysts by oral transmission among intermediate hosts, have been proposed to explain the genetic basis for the clonal population structure of T. gondii. In this study, we investigated the contributing roles self-mating and sexual recombination play in nature to maintain clonal population structures and produce or expand parasite clones capable of causing disease epidemics for two tissue encysting parasites. We applied high-resolution genotyping against strains isolated from a T. gondii waterborne outbreak that caused symptomatic disease in 155 immune-competent people in Brazil and a S. neurona outbreak that resulted in a mass mortality event in Southern sea otters. In both cases, a single, genetically distinct clone was found infecting outbreak-exposed individuals. Furthermore, the T. gondii outbreak clone was one of several apparently recombinant progeny recovered from the local environment. Since oocysts or sporocysts were the infectious form implicated in each outbreak, the expansion of the epidemic clone can be explained by self-mating. The results also show that out-crossing preceded selfing to produce the virulent T. gondii clone. For the tissue encysting coccidia, self-mating exists as a key adaptation potentiating the epidemic expansion and transmission of newly emerged parasite clones that can profoundly shape parasite population genetic structures or cause devastating disease outbreaks. | 3281 | NA | NA | NA | no | no |
| 3281 | 2017 | NA | Martinez-Garcia, R; Tarnita, CE | 2017 | Seasonality can induce coexistence of multiple bet-hedging strategies in Dictyostelium discoideum via storage effect | The social amoeba Dictyostelium discoideum has been recently suggested as an example of bet-hedging in microbes. In the presence of resources, amoebae reproduce as unicellular organisms. Resource depletion, however, leads to a starvation phase in which the population splits between aggregators, which form a fruiting body made of a stalk and resistant spores, and non-aggregators, which remain as vegetative cells. Spores are favored when starvation periods are long, but vegetative cells can exploit resources in environments where food replenishes quickly. The investment in aggregators versus non-aggregators can therefore be understood as a bet-hedging strategy that evolves in response to stochastic starvation times. A genotype (or strategy) is defined by the balance between each type of cells. In this framework, if the ecological conditions on a patch are defined in terms of the mean starvation time (i.e. time between the onset of starvation and the arrival of a new food pulse), a single genotype dominates each environment, which is inconsistent with the huge genetic diversity observed in nature. Here we investigate whether seasonality, represented by a periodic, wet-dry alternation in the mean starvation times, allows the coexistence of several strategies in a single patch. We study this question in a non-spatial (well-mixed) setting in which different strains compete for a common pool of resources over a sequence of growth starvation cycles. We find that seasonality induces a temporal storage effect that can promote the stable coexistence of multiple genotypes. Two conditions need to be met in our model. First, there has to be a temporal niche partitioning (two well-differentiated habitats within the year), which requires not only different mean starvation times between seasons but also low variance within each season. Second, each season’s well-adapted strain has to grow and create a large enough population that permits its survival during the subsequent unfavorable season, which requires the number of growth-starvation cycles within each season to be sufficiently large. These conditions allow the coexistence of two bet-hedging strategies. Additional tradeoffs among life-history traits can expand the range of coexistence and increase the number of coexisting strategies, contributing toward explaining the genetic diversity observed in D. discoideum. Although focused on this cellular slime mold, our results are general and may be easily extended to other microbes. (C) 2017 Elsevier Ltd. All rights reserved. | 3282 | NA | NA | NA | no | no |
| 3282 | 2017 | NA | Marta, S; Lacasella, F; Gratton, P; Cesaroni, D; Sbordoni, V | 2016 | Deciphering range dynamics: effects of niche stability areas and post-glacial colonization on alpine species distribution | Aim Niche stability areas (NSAs) are portions of the species range where climate conditions remain suitable through time. They represent the core of species ranges. Their distribution and extent, coupled with dispersal and colonization, shape the realized range of species. In this study, we quantified the roles of survival within NSAs and post-glacial dispersal in determining the current distribution of two groups of alpine butterflies (two taxa in the Erebia tyndarus species complex; three taxa in the Parnassius apollo-P. phoebus species complex). Location Holarctic. Methods NSAs were identified for each taxon by combining current and past potential distributions models, estimated using different modelling techniques and general circulation models. We then (1) assessed the distributional bias towards NSAs by comparing actual occurrence records with randomized occupancies of the current potential range and (2) quantified post-glacial dispersal by examining the distribution of distances from each occurrence record to the nearest NSA. Results In almost all taxa, realized distributions are biased towards NSAs. However, while Erebia’s present range is strongly dominated by NSAs, some populations of Parnassius are found very far from NSAs, suggesting more effective colonization of the available geographical space. Main conclusions Our study highlights the relative roles of survival within NSAs and post-glacial dispersal in shaping the ranges of different alpine butterflies during the Holocene. Results suggest that Erebia was unable to disperse far from NSAs, thus experiencing increasing range fragmentation. Parnassius populations, on the other hand, coupled local survival with northward dispersal. As NSAs allowed the long-term survival of the species, acting as sources for recolonization, and tend to preserve most of each species’ genetic diversity, identifying NSAs and understanding their importance in determining the current distribution of species represents a pivotal task for the conservation of biological diversity. | 3283 | NA | NA | NA | no | no |
| 3283 | 2017 | NA | Rugman-Jones, PF; Seybold, SJ; Graves, AD; Stouthamer, R | 2015 | Phylogeography of the Walnut Twig Beetle, Pityophthorus juglandis, the Vector of Thousand Cankers Disease in North American Walnut Trees | Thousand cankers disease (TCD) of walnut trees (Juglans spp.) results from aggressive feeding in the phloem by the walnut twig beetle (WTB), Pityophthorus juglandis, accompanied by inoculation of its galleries with a pathogenic fungus, Geosmithia morbida. In 1960, WTB was only known from four U.S. counties (in Arizona, California, and New Mexico), but the species has now (2014) invaded over 115 counties, representing much of the western USA, and at least six states in the eastern USA. The eastern expansion places TCD in direct proximity to highly valuable (> $500 billion) native timber stands of eastern black walnut, Juglans nigra. Using mitochondrial DNA sequences, from nearly 1100 individuals, we examined variation among 77 samples of WTB populations across its extended range in the USA, revealing high levels of polymorphism and evidence of two divergent lineages. The highest level of genetic diversity for the different lineages was found in the neighboring Madrean Sky Island and Western New Mexico regions, respectively. Despite their proximity, there was little evidence of mixing between these regions, with only a single migrant detected among 179 beetles tested. Indeed, geographic overlap of the two lineages was only common in parts of Colorado and Utah. Just two haplotypes, from the same lineage, predominated over the vast majority of the recently expanded range. Tests for Wolbachia proved negative suggesting it plays no role in “driving” the spread of particular haplotypes, or in maintaining deep levels of intraspecific divergence in WTB. Genotyping of ribosomal RNA corroborated the mitochondrial lineages, but also revealed evidence of hybridization between them. Hybridization was particularly prevalent in the sympatric areas, also apparent in all invaded areas, but absent from the most haplotype-rich area of each mitochondrial lineage. Hypotheses about the specific status of WTB, its recent expansion, and potential evolutionary origins of TCD are discussed. | 3284 | NA | NA | NA | no | no |
| 3284 | 2017 | NA | Rao, SB; Rathi, A; Gothalwal, R; Atkinson, H; Rao, U | 2011 | A comparison of the variation in Indian populations of pigeonpea cyst nematode, Heterodera cajani revealed by morphometric and AFLP analysis | The cyst nematode Heterodera cajani is one of the major endemic diseases of pigeonpea, an important legume for food security and protein nutrition in India. It occurs in several pulse crops grown over a range of Indian agro climatic conditions but the extent of its intraspecific variation is inadequately defined. In view of this, 11 populations of H. cajani were analyzed using morphometrics and the results correlated with those obtained from an AFLP approach using 24 primer pair combinations that amplified a total of 1278 AFLP markers. The cluster solution from this binary data indicated similarities for five populations that differed from those suggested by morphometrics. The differences obtained could not be related to geographic distance between populations. The data suggests that recent and long distance dispersal has occurred whose causes need to be defined to restrict further field introductions. Four AFLP primer pairs clustered the populations similarly to that generated using all 24 primer pairs. This simplified approach may provide a rapid basis for discriminating populations for their future management and help to check further distribution in agricultural trade. It may also have potential to determine differences in populations that relate to host range or virulence to resistance genes. | 3285 | NA | NA | NA | no | no |
| 3285 | 2017 | NA | Cavassin, FB; Klisiowicz, DD; Oliveira, LGR; Kuehn, CC; Kopp, RL; Thomaz-Soccol, V; Da Rosa, JA; Luz, E; Mas-Coma, S; Bargues, MD | 2014 | Genetic Variability and Geographical Diversity of the Main Chagas’ Disease Vector Panstrongylus megistus (Hemiptera: Triatominae) in Brazil Based on Ribosomal DNA Intergenic Sequences | Studies were made on the ribosomal DNA intergenic region, comprising complete internal transcribed spacer (ITS)-1, 5.8S, and ITS-2 sequences, of populations of the triatomine Panstrongylus megistus, the most important vector of Chagas’ disease in Brazil since Triatoma infestans eradication. Specimens were from 26 localities of Rio Grande do Sul, Santa Catarina, Parana, Sao Paulo, Minas Gerais, Bahia, and Sergipe states. In total, 21 ITS-1 and 12 ITS-2 haplotypes were found. Nucleotide differences were higher in ITS-1 (3.00%) than in ITS-2 (1.33%). The intergenic region was 1,513-1,522-bp-long (mean 1,516.9 bp), providing 26 combined haplotypes. The combination of microsatellites found in both ITSs may be of applied usefulness, to assess interpopulation specimen exchange and potential recolonizations after vector elimination by control implementation. Network results suggest that Sao Paulo may be considered one of the spreading centers of this species. Molecular clock datation suggests that P. megistus populations are diversifying at least since 4.54 million years ago, with diversification still ongoing today by geographical isolation of populations. Evidence is provided about the relationship of genetic diversity with geographical spread that characterizes a major vector and explains its ability to colonize distant areas and different ecotopes, including human habitats, and consequently its importance in Chagas’ disease epidemiology. | 3286 | NA | NA | NA | no | no |
| 3286 | 2017 | NA | Lehmann, J; Fickenscher, G; Boesch, C | 2006 | Kin biased investment in wild chimpanzees | Kin selection theory predicts that recognition and preferences for kin can be highly beneficial. However, evidence of recognition of offspring by fathers in mammals has accumulated very slowly. Especially, in multi-male groups with a promiscuous mating system, like the chimpanzee, where offspring survival does not seem to depend on paternal care, paternal kin recognition has not yet been observed. In this study, we examined whether adult males of a wild chimpanzee community show recognition of their offspring (as determined genetically) and whether infants prefer to interact with kin rather than with unrelated peers. Our analysis utilises up to 14 years of observational data to investigate if adult males associate more frequently and behave less aggressively with females that carry their offspring. Furthermore, we use grooming and play behaviour to establish whether adult males and youngsters show preferences for kin versus non-kin. We found that, adult males did not associate preferentially with females with which they had offspring, but they were generally less aggressive towards any given female when she had a new born infant. Interestingly, however, fathers maintained these low rates of aggression long after the aggression rates of the non-sires had returned to their basal levels. Furthermore, fathers spent significantly more time playing with their own offspring. Thus, our data show for the first time that wild chimpanzee males can recognise their own offspring. Infants preferred to groom and tended to play more with their maternal siblings, but showed only a weak preference for playing with their paternal siblings when given the choice between similarly aged related and unrelated interaction partners. Despite the fact that paternal care does not play an obvious role in chimpanzee survival, kin recognition is observed in different aspects of the life of adult males and youngsters. | 3287 | NA | NA | NA | no | no |
| 3287 | 2017 | NA | Dercole, F; Ferriere, R; Rinaldi, S | 2010 | Chaotic Red Queen coevolution in three-species food chains | Coevolution between two antagonistic species follows the so-called ‘Red Queen dynamics’ when reciprocal selection results in an endless series of adaptation by one species and counteradaptation by the other. Red Queen dynamics are ‘genetically driven’ when selective sweeps involving new beneficial mutations result in perpetual oscillations of the coevolving traits on the slow evolutionary time scale. Mathematical models have shown that a prey and a predator can coevolve along a genetically driven Red Queen cycle. We found that embedding the prey-predator interaction into a three-species food chain that includes a coevolving superpredator often turns the genetically driven Red Queen cycle into chaos. A key condition is that the prey evolves fast enough. Red Queen chaos implies that the direction and strength of selection are intrinsically unpredictable beyond a short evolutionary time, with greatest evolutionary unpredictability in the superpredator. We hypothesize that genetically driven Red Queen chaos could explain why many natural populations are poised at the edge of ecological chaos. Over space, genetically driven chaos is expected to cause the evolutionary divergence of local populations, even under homogenizing environmental fluctuations, and thus to promote genetic diversity among ecological communities over long evolutionary time. | 3288 | NA | NA | NA | no | no |
| 3288 | 2017 | NA | Mulder, NJ; Adebiyi, E; Alami, R; Benkahla, A; Brandful, J; Doumbia, S; Everett, D; Fadlelmola, FM; Gaboun, F; Gaseitsiwe, S; Ghazal, H; Hazelhurst, S; Hide, W; Ibrahimi, A; Fakim, YJ; Jongeneel, CV; Joubert, F; Kassim, S; Kayondo, J; Kumuthini, J; Lyantagaye, S; Makani, J; Alzohairy, AM; Masiga, D; Moussa, A; Nash, O; Oukem-Boyer, OOM; Owusu-Dabo, E; Panji, S; Patterton, H; Radouani, F; Sadki, K; Seghrouchni, F; Bishop, OT; Tiffin, N; Ulenga, N | 2016 | H3ABioNet, a sustainable pan-African bioinformatics network for human heredity and health in Africa | The application of genomics technologies to medicine and biomedical research is increasing in popularity, made possible by new high-throughput genotyping and sequencing technologies and improved data analysis capabilities. Some of the greatest genetic diversity among humans, animals, plants, and microbiota occurs in Africa, yet genomic research outputs from the continent are limited. The Human Heredity and Health in Africa (H3Africa) initiative was established to drive the development of genomic research for human health in Africa, and through recognition of the critical role of bioinformatics in this process, spurred the establishment of H3ABioNet, a pan-African bioinformatics network for H3Africa. The limitations in bioinformatics capacity on the continent have been a major contributory factor to the lack of notable outputs in high-throughput biology research. Although pockets of high-quality bioinformatics teams have existed previously, the majority of research institutions lack experienced faculty who can train and supervise bioinformatics students. H3ABioNet aims to address this dire need, specifically in the area of human genetics and genomics, but knock-on effects are ensuring this extends to other areas of bioinformatics. Here, we describe the emergence of genomics research and the development of bioinformatics in Africa through H3ABioNet. | 3289 | NA | NA | NA | no | no |
| 3289 | 2017 | NA | Ezenwa, VO; Peters, JM; Zhu, Y; Arevalo, E; Hastings, MD; Seppa, P; Pedersen, JS; Zacchi, F; Queller, DC; Strassmann, JE | 1998 | Ancient conservation of trinucleotide microsatellite loci in polistine wasps | Microsatellites have proven to be very useful genetic markers for studies of kinship, parentage, and gene mapping. If microsatellites are conserved among species, then those developed for one species can be used on related species, which would save the time and effort of developing new loci. We evaluated conservation of 27 trinucleotide loci that were derived from 2 species of Polistes wasps in cross-species applications on 27 species chosen from the major lineages of the Vespidae, which diverged as much as 144 million years ago. We further investigated cross-species polymorphism levels for 18 of the loci. There was a clear relationship between cladistic distance and both conservation of the priming sites and heterozygosity. However the loci derived from P. bellicosus were much more widely conserved and polymorphic than were those derived from P. annularis. The disparity in cross-species utility between these sets of loci means that caution should be used in generalizing from conservation rates derived from single species. We found no relationship between locus conservation or heterozygosity and GC content of flanks, repeat motif, repeat length, or heterozygosity in the original species, which suggests that generalizations from other studies reporting such patterns are premature. (C) 1998 Academic Press. | 3290 | NA | NA | NA | no | no |
| 3290 | 2017 | NA | Locatelli, S; Harrigan, RJ; Clee, PRS; Mitchell, MW; McKean, KA; Smith, TB; Gonder, MK | 2016 | Why Are Nigeria-Cameroon Chimpanzees (Pan troglodytes ellioti) Free of SIVcpz Infection? | Simian immunodeficiency virus (SIV) naturally infects two subspecies of chimpanzee: Pan troglodytes troglodytes from Central Africa (SIVcpzPtt) and P. t. schweinfurtii from East Africa (SIVcpzPts), but is absent in P. t. verus from West Africa and appears to be absent in P. t. ellioti inhabiting Nigeria and western Cameroon. One explanation for this pattern is that P. t. troglodytes and P. t schweinfurthii may have acquired SIVcpz after their divergence from P. t. verus and P. t. ellioti. However, all of the subspecies, except P. t. verus, still occasionally exchange migrants making the absence of SIVcpz in P. t. ellioti puzzling. Sampling of P. t. ellioti has been minimal to date, particularly along the banks of the Sanaga River, where its range abuts that of P. t. troglodytes. This study had three objectives. First, we extended the sampling of SIVcpz across the range of chimpanzees north of the Sanaga River to address whether under-sampling might account for the absence of evidence for SIVcpz infection in P. t. ellioti. Second, we investigated how environmental variation is associated with the spread and prevalence of SIVcpz in the two chimpanzee subspecies inhabiting Cameroon since environmental variation has been shown to contribute to their divergence from one another. Finally, we compared the prevalence and distribution of SIVcpz with that of Simian Foamy Virus (SFV) to examine the role of ecology and behavior in shaping the distribution of diseases in wild host populations. The dataset includes previously published results on SIVcpz infection and SFVcpz as well as newly collected data, and represents over 1000 chimpanzee fecal samples from 41 locations across Cameroon. Results revealed that none of the 181 P. t. ellioti fecal samples collected across the range of P. t. ellioti tested positive for SIVcpz. In addition, species distribution models suggest that environmental variation contributes to differences in the distribution and prevalence of SIVcpz and SFVcpz. The ecological niches of these two viruses are largely non-overlapping, although stronger statistical support for this conclusion will require more sampling. Overall this study demonstrates that SIVcpz infection is absent or very rare in P. t. ellioti, despite multiple opportunities for transmission. The reasons for its absence remain unclear, but might be explained by one or more factors, including environmental variation, viral competition, and/or local adaptation-all of which should be explored in greater detail through continued surveillance of this region. | 3291 | NA | NA | NA | no | no |
| 3291 | 2017 | NA | Kingma, SA; Hall, ML; Arriero, E; Peters, A | 2010 | Multiple benefits of cooperative breeding in purple-crowned fairy-wrens: a consequence of fidelity? | P>1. Kin selection is one of the mechanisms that can explain apparent altruism by subordinate individuals in cooperatively breeding species, if subordinates boost the production of kin. We compared productivity and breeder survival in pairs with and without subordinates in a genetically monogamous cooperatively breeding bird, the purple-crowned fairy-wren Malurus coronatus. 2. Additive effects of subordinate help increased productivity. Total feeding rates to the nest were increased by two or more subordinates, and fledgling production was greater in larger groups. Not all subordinates contributed to nestling feeding, and the effect of group size was greater when non-contributors were excluded from analyses, suggesting that increased fledgling production was a direct result of help. 3. Compensatory effects of subordinate help improved breeder survival. Assisted breeders reduced their workload by 20-30%, irrespective of the number of helpers. Although re-nesting intervals were not affected by group size, reduced breeder feeding rates resulted in improved survival and breeders in larger groups survived better. 4. Subordinates and nestlings are usually progeny of the breeding pair in this species, and benefits of cooperative breeding are very different from three congeners with extremely high levels of extra-group paternity (EGP). In these Malurus, fledgling production and survival of male breeders are not enhanced in larger groups. This is consistent with the expectation that kin-selected benefits vary with relatedness, and thus levels of EGP. 5. We tested whether benefits of cooperative breeding in 37 avian species varied with levels of extra-group mating. Both direct and phylogenetically controlled comparisons showed that improvement of (male) breeder survival and enhanced productivity are more likely when fidelity is higher, as predicted when investment of subordinates correlates with relatedness to offspring. This pattern highlights the importance of considering the genetic mating system for understanding the evolution of cooperative breeding. | 3292 | NA | NA | NA | no | no |
| 3292 | 2017 | NA | Makouloutou, P; Nguema, PPM; Fujita, S; Takenoshita, Y; Hasegawa, H; Yanagida, T; Sato, H | 2014 | Prevalence and genetic diversity of Oesophagostomum stephanostomum in wild lowland gorillas at Moukalaba-Doudou National Park, Gabon | Using a sedimentation method, the prevalence of the nodular worm Oesophagostomum stephanostomum (Nematoda: Strongylida) in western lowland gorillas at Moukalaba-Doudou National Park (MDNP), Gabon, was determined in fecal samples collected between January 2007 and October 2011, along with their coprocultures. Concurrently, possible zoonotic Oesophagostomum infections in villagers living near MDNP were assessed from their fecal samples collected during October and November of 2011. In the gorillas, strongylid (Oesophagostomum and/or hookworm) eggs were found in 47 of 235 fecal samples (20.0 %) and Oesophagostomum larvae were detected in 101 of 229 coprocultures (44.1 %). In the villagers, strongylid eggs were found in 9 of 71 fecal samples (12.7 %), but no Oesophagostomum larvae were detected in coprocultures. The internal transcribed spacer (ITS) region of ribosomal RNA gene (rDNA) and cytochrome c oxidase subunit-1 (cox-1) region of mitochondrial DNA (mtDNA) of coprocultured Oesophagostomum larvae were amplified using parasite DNA extracted from 7-25 larvae/sample, cloned into Escherichia coli, and sequenced. Sequenced rDNA contained 353/354-bp long ITS1, 151-bp long 5.8S rDNA, and 227-bp long ITS2. Parts of clones showed variations at 1-3 bases in the ITS1 region at a frequency of 24/68 (35.3 %) and at 1-2 bases in the ITS2 region at a frequency of 7/68 (10.3 %), whereas the 5.8S rDNA was essentially identical. Sequenced cox-1 gene of the parasites, 849 bp in length, showed a higher number of nucleotide variations, mainly at the third nucleotide position of the codon. The majority of clones (27/41 (65.9 %)) had an identical amino acid sequence. These results suggest that at MDNP, Gabon, only a single population of O. stephanostomum with a degree of genetic diversity is prevalent in western lowland gorillas, without zoonotic complication in local inhabitants. The possible genetic variations in the ITS region of rDNA and cox-1gene of mtDNA presented here may be valuable when only a limited amount of material is available for the molecular species diagnosis of O. stephanostomum. | 3293 | NA | NA | NA | no | no |
| 3293 | 2017 | NA | Bohonak, AJ | 1999 | Effect of insect-mediated dispersal on the genetic structure of postglacial water mite populations | Assaying population structure in species that differ in dispersal ability can help to determine whether population differentiation is dependent on the movement of individuals between populations. Here, allozyme variation is analysed in over 1100 individuals from nine species and two species complexes of Arrenurus water mites collected throughout north-eastern North America. As larvae, eight taxa are obligate parasites of winged adult insects that provide the primary opportunity for dispersal. Three additional species have lost the ability to parasitize insects and do not disperse in this manner. Consistent with the glaciated history of the region, very low allozyme heterozygosity was found in these taxa (H-o = 0.00-0.12), near panmixia in five out of seven species for which population differentiation was calculated and no patterns of isolation by distance over spatial scales up to several hundred kilometres, Nonetheless, in two out of three comparisons between sister species with and without parasitic larvae, parasitism was significantly associated with higher heterozygosity. Population differentiation could also be contrasted for two of these sister species pairs; in each case, lower estimates of F-ST were found in the mites able to disperse on insects. The statistical significance of these contrasts was dependent on the method used to estimate variance. At the scale of the genus, behavioural differences among insect vectors allows for broader hypotheses that relate water mite genetic diversity to dispersal ability. For the genus, rank correlations of dispersal ability with direct count heterozygosity (n = 11) and population differentiation (n = 7) were not significantly different from zero. These results are consistent with the hypothesis that allozyme population structure is primarily the result of historical patterns in these regions. However, comparisons between sister species suggest a limited role for dispersal in homogenizing populations genetically, even when drift-gene flow equilibrium has not been achieved. | 3294 | NA | NA | NA | no | no |
| 3294 | 2017 | NA | Neve, G; Pavlicko, A; Konvicka, M | 2009 | Loss of genetic diversity through spontaneous colonization in the bog fritillary butterfly, Proclossiana eunomia (Lepidoptera: Nymphalidae) in the Czech Republic | The butterfly Proclossiana eunomia (Lepidoptera: Nymphalidae) was discovered at a single locality in the Czech Republic in 1963. Until the 1980s, it was known from a restricted area, from which it spontaneously expanded during the 1980s to other localities up to 23 km from the source population. Samples were collected in 2002 from the source and ten other populations, totalling 274 specimens. All samples were analysed by electrophoresis for four polymorphic loci. Mean heterozygosity decreased with distance from the source population; this suggested a process of stepping stone colonization, involving the loss of rare alleles along the way. The populations close to the source population (less then ca. 15 km) retain a similar heterozygosity, whereas populations further away have a much reduced heterozygosity. Such a pattern of genetic differentiation and founder effect within a region is typical of specialized species with relatively low dispersal ability. The high level of genetic polymorphism found in the Sumava populations suggests that populations of this northern species in temperate-zone mountains are not just outposts of otherwise huge northern distribution, but represent genuine phylogeographic refugia. Survival of such species depends on the survival of the source population and of a sufficiently dense network of habitat patches. | 3295 | NA | NA | NA | no | no |
| 3295 | 2017 | NA | Hughes, S; Fernandez, H; Cucchi, T; Duffraisse, M; Casabianca, F; Istria, D; Pompanon, F; Vigne, JD; Hanni, C; Taberlet, P | 2012 | A Dig into the Past Mitochondrial Diversity of Corsican Goats Reveals the Influence of Secular Herding Practices | The goat (Capra hircus) is one of the earliest domesticated species ca. 10,500 years ago in the Middle-East where its wild ancestor, the bezoar (Capra aegagrus), still occurs. During the Neolithic dispersal, the domestic goat was then introduced in Europe, including the main Mediterranean islands. Islands are interesting models as they maintain traces of ancient colonization, historical exchanges or of peculiar systems of husbandry. Here, we compare the mitochondrial genetic diversity of both medieval and extant goats in the Island of Corsica that presents an original and ancient model of breeding with free-ranging animals. We amplified a fragment of the Control Region for 21 medieval and 28 current goats. Most of them belonged to the A haplogroup, the most worldwide spread and frequent today, but the C haplogroup is also detected at low frequency in the current population. Present Corsican goats appeared more similar to medieval goats than to other European goat populations. Moreover, 16 out of the 26 haplotypes observed were endemic to Corsica and the inferred demographic history suggests that the population has remained constant since the Middle Ages. Implications of these results on management and conservation of endangered Corsican goats currently decimated by a disease are addressed. | 3296 | NA | NA | NA | no | no |
| 3296 | 2017 | NA | Mougel, C; Teyssier, S; D’Angelo, C; Groud, K; Neyra, M; Sidi-Boumedine, K; Cloeckaert, A; Peloille, M; Baucheron, S; Chaslus-Dancla, E; Jarraud, S; Meugnier, H; Forey, F; Vandenesch, F; Lina, G; Etienne, J; Thioulouse, J; Manceau, C; Robbe, P; Nalin, R; Briolay, J; Nesme, X | 2001 | Experimental and theoretical evaluation of typing methods based upon random amplification of genomic restriction fragments (AFLP) for bacterial population genetics | The reliability and the level of taxonomic resolution of the amplified fragment length polymorphism (AFLP) method were evaluated with species of pathogenic bacteria involved in human, animal and plant diseases. The method was found to be very versatile as it can be adapted to the individual genome constraints of all tested species. The calculation of a genetic distance d corresponding to the average dissimilarity between actual overall genome sequences was proposed for comparing AFLP data. Bacterial models showed clearly different patterns between strains belonging to different genomic species, while patterns were clearly similar within a given species. The threshold which distinguishes between inter and infra-specific distances indicates a critical overall genome diversity of about 14% (d = 0.14). AFLP had more resolution power than serology, phage typing, PFGE and restriction analysis of ribosomal intergenic spacers. In the latter case, regression analysis showed that PCR-RFLP of ribosomal intergenic spacers can only be used to differentiate bacteria which have at least 3.4% (d = 0.034) nucleotide differences between their respective genomes. Finally, an improved procedure using newly developed software was also proposed in order to standardize the capture of reliable data and their numeric treatment for the future development of AFLP data bases. | 3297 | NA | NA | NA | no | no |
| 3297 | 2017 | NA | Latif, MA; Rahman, MM; Ali, ME; Ashkani, S; Rafii, MY | 2013 | Inheritance studies of SSR and ISSR molecular markers and phylogenetic relationship of rice genotypes resistant to tungro virus | Multivariate analyses were performed using 13 morphological traits and 13 molecular markers (10 SSRs and three ISSRs) to assess the phylogenetic relationship among tungro resistant genotypes. For morphological traits, the genotypes were grouped into six clusters, according to D-2 statistic and Canonical vector analysis. Plant height, days to flowering, days to maturity, panicle length, number of spikelet per panicle, number of unfilled grain per panicle and yield were important contributors to genetic divergence in 14 rice genotypes. Based on Nei’s genetic distance for molecular studies, seven clusters were formed among the tungro resistant and susceptible genotypes. Mantel’s test revealed a significant correlation (r = 0.834\*) between the morphological and molecular data. To develop high yielding tungro resistant varieties based on both morphological and molecular analyses, crosses could be made with susceptible (BR10 and BR11) genotypes with low yielding but highly resistant genotypes, Sonahidemota, Kumragoir, Nakuchimota, Khaiyamota, Khairymota and Kachamota. The chi-square analysis for seven alleles (RM11, RM17, RM20, RM23, RM80, RM108 and RM531) of SSR and five loci (RY1, MR1, MR2, MR4 and GF5) of three ISSR markers in F-2 population of cross, BR11 x Sonahidemota, showed a good fit to the expected segregation ratio (1:2:1) for a single gene model. (C) 2013 Academie des sciences. Published by Elsevier Masson SAS. All rights reserved. | 3298 | NA | NA | NA | no | no |
| 3298 | 2017 | NA | Proffitt, CE; Travis, S | 2014 | Red mangrove life history variables along latitudinal and anthropogenic stress gradients | Mangroves migrate northward in Florida and colonize marshes historically dominated by salt marsh species. In theory, this migration should be facilitated by greater numbers of propagules stemming from increased reproductive activity and greater genetic variability caused by outcrossing. We aimed to determine if stand reproduction and % outcrossing were affected by cold stress (stress increases with latitude), anthropogenic stress (human population density as a proxy), and years since a major hurricane. Further, we wished to determine if mutation rate varied with the stressors and if that affected stand reproduction. Both coasts of Florida from the southern Florida Keys to Tampa Bay on the Gulf of Mexico coast, and Merritt Island on the Atlantic coast. We conducted field surveys of frequency of reproducing trees (104,211 trees surveyed in 102 forested stands), incidence of trees showing albinism in propagules, and % outcrossing estimated from the ratio of albino: normal propagules. Structural equation modeling (SEM) was used to test a conceptual model that served as a multivariate hypothesis. Reproductive frequencies varied by site and increased with latitude although more strongly on the Gulf coast. Our SEM results indicate that outcrossing increases in this predominately selfing species under conditions of cold and anthropogenic stress, and that this increases reproductive output in the population. Further, we find that increased mutation rates suppress stand reproductive output but there is no significant relationship between outcrossing and mutation rate. Tree size responded to stressors but did not affect stand reproduction. Reproduction increased with years since major hurricane. Potential for colonization of northern Florida salt marshes by mangroves is enhanced by increased reproductive rates that provides more propagules and outcrossing that should enhance genetic variation thereby promoting adaptation to novel environmental conditions. Natural (cold) stress reduced mutation rate and increased stand reproductive output but anthropogenic stress did the opposite. | 3299 | NA | NA | NA | no | no |
| 3299 | 2017 | NA | Koskella, B; Lin, DM; Buckling, A; Thompson, JN | 2012 | The costs of evolving resistance in heterogeneous parasite environments | The evolution of host resistance to parasites, shaped by associated fitness costs, is crucial for epidemiology and maintenance of genetic diversity. Selection imposed by multiple parasites could be a particularly strong constraint, as hosts either accumulate costs of multiple specific resistances or evolve a more costly general resistance mechanism. We used experimental evolution to test how parasite heterogeneity influences the evolution of host resistance. We show that bacterial host populations evolved specific resistance to local bacteriophage parasites, regardless of whether they were in single or multiple-phage environments, and that hosts evolving with multiple phages were no more resistant to novel phages than those evolving with single phages. However, hosts from multiple-phage environments paid a higher cost, in terms of population growth in the absence of phage, for their evolved specific resistances than those from single-phage environments. Given that in nature host populations face selection pressures from multiple parasite strains and species, our results suggest that costs may be even more critical in shaping the evolution of resistance than previously thought. Furthermore, our results highlight that a better understanding of resistance costs under combined control strategies could lead to a more ‘evolution-resistant’ treatment of disease. | 3300 | NA | NA | NA | no | no |
| 3300 | 2017 | NA | de Sousa, TN; Carvalho, LH; de Brito, CFA | 2011 | Worldwide Genetic Variability of the Duffy Binding Protein: Insights into Plasmodium vivax Vaccine Development | The dependence of Plasmodium vivax on invasion mediated by Duffy binding protein (DBP) makes this protein a prime candidate for development of a vaccine. However, the development of a DBP-based vaccine might be hampered by the high variability of the protein ligand (DBPII), known to bias the immune response toward a specific DBP variant. Here, the hypothesis being investigated is that the analysis of the worldwide DBPII sequences will allow us to determine the minimum number of haplotypes (MNH) to be included in a DBP-based vaccine of broad coverage. For that, all DBPII sequences available were compiled and MNH was based on the most frequent nonsynonymous single nucleotide polymorphisms, the majority mapped on B and T cell epitopes. A preliminary analysis of DBPII genetic diversity from eight malaria-endemic countries estimated that a number between two to six DBP haplotypes (17 in total) would target at least 50% of parasite population circulating in each endemic region. Aiming to avoid region-specific haplotypes, we next analyzed the MNH that broadly cover worldwide parasite population. The results demonstrated that seven haplotypes would be required to cover around 60% of DBPII sequences available. Trying to validate these selected haplotypes per country, we found that five out of the eight countries will be covered by the MNH (67% of parasite populations, range 48-84%). In addition, to identify related subgroups of DBPII sequences we used a Bayesian clustering algorithm. The algorithm grouped all DBPII sequences in six populations that were independent of geographic origin, with ancestral populations present in different proportions in each country. In conclusion, in this first attempt to undertake a global analysis about DBPII variability, the results suggest that the development of DBP-based vaccine should consider multi-haplotype strategies; otherwise a putative P. vivax vaccine may not target some parasite populations. | 3301 | NA | NA | NA | no | no |
| 3301 | 2017 | NA | Bernhardt, SA; Simmons, MP; Olson, KE; Beaty, BJ; Blair, CD; Black, WC | 2012 | Rapid Intraspecific Evolution of miRNA and siRNA Genes in the Mosquito Aedes aegypti | RNA silencing, or RNA interference (RNAi) in metazoans mediates development, reduces viral infection and limits transposon mobility. RNA silencing involves 21-30 nucleotide RNAs classified into microRNA (miRNA), exogenous and endogenous small interfering RNAs (siRNA), and Piwi-interacting RNA (piRNA). Knock-out, silencing and mutagenesis of genes in the exogenous siRNA (exo-siRNA) regulatory network demonstrate the importance of this RNAi pathway in antiviral immunity in Drosophila and mosquitoes. In Drosophila, genes encoding components for processing exo-siRNAs are among the fastest evolving 3% of all genes, suggesting that infection with pathogenic RNA viruses may drive diversifying selection in their host. In contrast, paralogous miRNA pathway genes do not evolve more rapidly than the genome average. Silencing of exo-siRNA pathway genes in mosquitoes orally infected with arboviruses leads to increased viral replication, but little is known about the comparative patterns of molecular evolution among the exo-siRNA and miRNA pathways genes in mosquitoes. We generated nearly complete sequences of all exons of major miRNA and siRNA pathway genes dicer-1 and dicer-2, argonaute-1 and argonaute-2, and r3d1 and r2d2 in 104 Aedes aegypti mosquitoes collected from six distinct geographic populations and analyzed their genetic diversity. The ratio of replacement to silent amino acid substitutions was 1.4 fold higher in dicer-2 than in dicer-1, 27.4 fold higher in argonaute-2 than in argonaute-1 and similar in r2d2 and r3d1. Positive selection was supported in 32% of non-synonymous sites in dicer-1, in 47% of sites in dicer-2, in 30% of sites in argonaute-1, in all sites in argonaute-2, in 22% of sites in r3d1 and in 55% of sites in r2d2. Unlike Drosophila, in Ae. aegypti, both exo-siRNA and miRNA pathway genes appear to be undergoing rapid, positive, diversifying selection. Furthermore, refractoriness of mosquitoes to infection with dengue virus was significantly positively correlated for nucleotide diversity indices in dicer-2. | 3302 | NA | NA | NA | no | no |
| 3302 | 2017 | NA | Li, MJ; Reilly, C; Hanson, T | 2010 | Association Tests for a Censored Quantitative Trait and Candidate Genes in Structured Populations with Multilevel Genetic Relatedness | P>Several statistical methods for detecting associations between quantitative traits and candidate genes in structured populations have been developed for fully observed phenotypes. However, many experiments are concerned with failure-time phenotypes, which are usually subject to censoring. In this article, we propose statistical methods for detecting associations between a censored quantitative trait and candidate genes in structured populations with complex multiple levels of genetic relatedness among sampled individuals. The proposed methods correct for continuous population stratification using both population structure variables as covariates and the frailty terms attributable to kinship. The relationship between the time-at-onset data and genotypic scores at a candidate marker is modeled via a parametric Weibull frailty accelerated failure time (AFT) model as well as a semiparametric frailty AFT model, where the baseline survival function is flexibly modeled as a mixture of Polya trees centered around a family of Weibull distributions. For both parametric and semiparametric models, the frailties are modeled via an intrinsic Gaussian conditional autoregressive prior distribution with the kinship matrix being the adjacency matrix connecting subjects. Simulation studies and applications to the Arabidopsis thaliana line flowering time data sets demonstrated the advantage of the new proposals over existing approaches. | 3303 | NA | NA | NA | no | no |
| 3303 | 2017 | NA | Harl, J; Duda, M; Kruckenhauser, L; Sattmann, H; Haring, E | 2014 | In Search of Glacial Refuges of the Land Snail Orcula dolium (Pulmonata, Orculidae) - An Integrative Approach Using DNA Sequence and Fossil Data | Harboring a large number of endemic species, the Alps and the Western Carpathians are considered as major centers of biodiversity. Nonetheless, the general opinion until the turn of the millennium was that both Central European mountain regions did not provide suitable habitat during the Last Glacial Maximum, but were colonized later from southern refuges. However, recent molecular genetic studies provide new evidence for peripheral Alpine refuges. We studied the phylogeography of the calciphilous land snail O. dolium across its distribution in the Alps and the Western Carpathians to assess the amount of intraspecific differentiation and to detect potential glacial refuges. A partial sequence of the mitochondrial COI was analyzed in 373 specimens from 135 sampling sites, and for a subset of individuals, partial sequences of the mitochondrial 16S and the nuclear histone H3 and H4 were sequenced. A molecular clock analysis was combined with a reconstruction of the species’ geographic range history to estimate how its lineages spread in the course of time. In order to obtain further information on the species’ past distribution, we also screened its extensive Pleistocene fossil record. The reconstruction of geographic range history suggests that O. dolium is of Western Carpathian origin and diversified already around the Miocene-Pliocene boundary. The fossil record supports the species’ presence at more than 40 sites during the last glacial and earlier cold periods, most of them in the Western Carpathians and the Pannonian Basin. The populations of O. dolium display a high genetic diversity with maximum intraspecific p-distances of 18.4% (COI) and 14.4% (16S). The existence of various diverged clades suggests the survival in several geographically separated refuges. Moreover, the sequence patterns provide evidence of multiple migrations between the Alps and the Western Carpathians. The results indicate that the Southern Calcareous Alps were probably colonized only during the Holocene. | 3304 | NA | NA | NA | no | no |
| 3304 | 2017 | NA | Snabel, V; Miterpakova, M; D’Amelio, S; Busi, M; Bartkova, D; Turcekova, L; Maddox-Hyttel, C; Skuce, P; Dubinsky, P | 2006 | Genetic structuring and differentiation of Echinococcus multilocularis in Slovakia assessed by sequencing and isoenzyme studies | Nucleotide sequencing of the mitochondrial cytochrome c oxidase subunit 1 (CO1) gene and isoenzyme analysis were used to survey the genetic variability in Echinococcus multilocularis populations from Slovakia. A sample of 12 isolates acquired from 10 different districts from red foxes exhibited identical sequences. Compared with the previously described E. multilocularis variants, one base substitution was consistently observed relative to the M 1 variant (detected in China, Alaska, North America, Japan) and three base substitutions were recorded relative to the M2 variant (detected in Germany) in the CO1 fragment. These data, along with the recently gathered data from French isolates, are indicative of a genetically unique population occurring in Central and Western Europe. Electrophoretic examination of enzymes produced by 14 gene loci revealed intraspecific polymorphism only with the glucose-phosphate isomerase (two distinct patterns) and the mannose-phosphate isomerase (four genotypes composed of three alleles) enzyme systems. To allow a fast species differenttiation of E. multilocularis and E. granulosus (specifically, the G7 genotype occurring in Slovakia), discriminative electrophoretic characters between the species were obtained by isoenzyme analysis. Fixed genetic differences between the species were detected in the glucose-phosphate isomerase, esterase and aldolase systems, and partial differences were detected in four additional systems. | 3305 | NA | NA | NA | no | no |
| 3305 | 2017 | NA | Hantemirova, EV; Heinze, B; Knyazeva, SG; Musaev, AM; Lascoux, M; Semerikov, VL | 2017 | A new Eurasian phylogeographical paradigm? Limited contribution of southern populations to the recolonization of high latitude populations in Juniperus communis L.(Cupressaceae) | AimThe aims of this population genetics study of the common juniper across Eurasia were to (1) assess the contribution of southern mountain ranges to the post-glacial recolonization of high latitudes and (2) test whether recent expansion or high gene flow could explain the low genetic differentiation in Northern Eurasia. LocationNorthern Eurasia and mountain regions of Central Europe and Asia. MethodsSix hundred and twenty-two individuals were sampled in 42 populations. Two chloroplast DNA (cpDNA) fragments were investigated (trnT-trnL and 16S-trnA). Analyses of the distribution of haplotypes across the continent included a suite of phylogeographical and phylogenetic tests. Putative geographical distribution in the past was reconstructed using environmental niche modelling. ResultsEighty-four haplotypes clustered into four main clades (GL1-GL4). The largest clade, GL3, corresponds to populations from the Alps, northern Europe, Western Caucasus and Siberia. These populations were moderately differentiated (28%) compared to the total range (76%) and Fu’s F-s statistic was negative, indicating a population expansion. Some haplotypes within GL3 form subclades with a restricted geographical distribution, suggesting a local origin of the mutation and limited dispersal. In line with these findings, modelling of ecological niches found no significant reduction in the expected range during the LGM. Remarkably, populations from the eastern part of North Caucasus, the Himalayas, Tien Shan and south Siberia were distinctly different from populations in the rest of the range. Main conclusionsAs in Siberian larch species, the pattern of genetic diversity at cpDNA across the natural range of J. communis suggests that colonization of northern Europe and Siberia started from a limited area and predated the last glaciation. It is likely that juniper survived the subsequent glacial epoch at high latitudes in cryptic refugia serving as secondary centres of recolonization. Southern mountain refugia contribution to the recolonization of high latitudes was, at best, limited. | 3306 | NA | NA | NA | no | no |
| 3306 | 2017 | NA | Kover, PX; Clay, K | 1998 | Trade-off between virulence and vertical transmission and the maintenance of a virulent plant pathogen | The continuum hypothesis predicts that parasites should evolve reduced virulence if they have higher opportunity for vertical transmission. However, when there is a trade-off between Virulence and vertical transmission, selection may favor horizontal transmission and higher virulence. Atkinsonella hypoxylon is a fungal pathogen that reduces Danthonia fitness by 50% or moro when it completely castrates hosts’ chasmogamous inflorescences, despite the high opportunity for vertical transmission through cleistogamous seeds. Sporadically, infected hosts with partially castrated inflorescences (which have higher fecundity than completely castrated hosts) are observed in natural populations. Why are partially castrated plants rare if selection favors reduced virulence! We investigated whether there was genetic diversity for virulence among A. hypoxylon genotypes and the relationship between virulence and vertical transmission. We found that the fungal genotype significantly affects the occurrence of partial castration in Danthonia compressa. The proportion of seedlings that were vertically infected by their maternal plant was lower for partially castrated than for completely castrated plants. Our results demonstrate a trade-off between virulence and vertical transmission, explaining the maintenance of more virulent, completely castrating Fungal genotypes in natural populations, and suggest that vertical transmission in plants is more complex than what is considered in current models. | 3307 | NA | NA | NA | no | no |
| 3307 | 2017 | NA | Talas, F; McDonald, BA | 2015 | Genome-wide analysis of Fusarium graminearum field populations reveals hotspots of recombination | Background: Fusarium graminearum (Fg) is a ubiquitous pathogen of wheat, barley and maize causing Fusarium head blight. Large annual yield losses and contamination of foodstuffs with harmful mycotoxins make Fg one of the most-studied plant pathogens. Analyses of natural field populations can lead to a better understanding of the evolutionary processes affecting this pathogen. Restriction site associated DNA sequencing (RADseq) was used to conduct population genomics analyses including 213 pathogen isolates from 13 German field populations of Fg. Results: High genetic diversity was found within Fg field populations and low differentiation (F-ST = 0.003) was found among populations. Linkage disequilibrium (LD) decayed rapidly over a distance of 1000 bp. The low multilocus LD indicates that significant sexual recombination occurs in all populations. Several recombination hotspots were detected on each chromosome, but different chromosomes showed different levels of recombination. There was some evidence for selection hotspots. Conclusions: The population genomic structure of Fg is consistent with a high degree of sexual recombination that is not equally distributed across the chromosomes. The high gene flow found among these field populations should enable this pathogen to adapt rapidly to changes in its environment, including deployment of resistant cultivars, applications of fungicides and a warming climate. | 3308 | NA | NA | NA | no | no |
| 3308 | 2017 | NA | Kairo, G; Biron, DG; Ben Abdelkader, F; Bonnet, M; Tchamitchian, S; Cousin, M; Dussaubat, C; Benoit, B; Kretzschmar, A; Belzunces, LP; Brunet, JL | 2017 | Nosema ceranae, Fipronil and their combination compromise honey bee reproduction via changes in male physiology | The honey bee is threatened by biological agents and pesticides that can act in combination to induce synergistic effects on its physiology and lifespan. The synergistic effects of a parasite/pesticide combination have been demonstrated on workers and queens, but no studies have been performed on drones despite their essential contribution to colony sustainability by providing semen diversity and quality. The effects of the Nosema ceranae/fipronil combination on the life traits and physiology of mature drones were examined following exposure under semi-field conditions. The results showed that the microsporidia alone induced moderate and localized effects in the midgut, whereas fipronil alone induced moderate and generalized effects. The parasite/insecticide combination drastically affected both physiology and survival, exhibiting an important and significant generalized action that could jeopardize mating success. In terms of fertility, semen was strongly impacted regardless of stressor, suggesting that drone reproductive functions are very sensitive to stress factors. These findings suggest that drone health and fertility impairment might contribute to poorly mated queens, leading to the storage of poor quality semen and poor spermathecae diversity. Thus, the queens failures observed in recent years might result from the continuous exposure of drones to multiple environmental stressors. | 3309 | NA | NA | NA | no | no |
| 3309 | 2017 | NA | VOIGT, K; SCHLEIER, S; BRUCKNER, B | 1995 | GENETIC-VARIABILITY IN GIBBERELLA-FUJIKUROI AND SOME RELATED SPECIES OF THE GENUS FUSARIUM BASED ON RANDOM AMPLIFICATION OF POLYMORPHIC DNA (RAPD) | One of the most important rice pathogens is Fusarium moniliforme (perfect stage: Gibberella fujikuroi), the causal agent of the super-elongation (‘’bakanae’’) disease. Thirty-seven strains of this species from different geographical regions were analyzed for their ability to produce gibberellins (GA) and for genetic relatedness by random amplified polymorphic DNA (RAPD). All GA-producing isolates showed nearly identical RAPD patterns using 51 oligonucleotide nona- and deca-mers as arbitrary primers. On the other hand, large differences between GA-nonproducing isolates were obtained. Comparison of the RAPD patterns with those of the tester strains of the six known mating populations (A, B, C, D, E, F) of G. fujikuroi showed that all producer strains belong to mating population C and all nonproducer isolates to other mating populations. Evidence for the usefulness of the RAPD technique to distinguish between mating populations was provided by sexual crossings. Consensus phylogenetic trees based on RAPDs were constructed by the Phylogenetic Analysis Using Parsimony (PAUP) system. In combination with morphological analysis, RAPD can distinguish between different species of the genus Fusarium. These investigations may find an application in the diagnosis of unknown Fusarium spp, and in distinguishing isolates of G. fujikuroi within the section Liseola. | 3310 | NA | NA | NA | no | no |
| 3310 | 2017 | NA | Hu, L; Zhang, Y; Hong, M; Zhu, SL; Yan, DM; Wang, DY; Li, XL; Zhu, Z; Tsewang; Xu, WB | 2014 | Phylogenetic evidence for multiple intertypic recombinations in enterovirus B81 strains isolated in Tibet, China | Enterovirus B81 (EV-B81) is a newly identified serotype within the species enterovirus B (EV-B). To date, only eight nucleotide sequences of EV-B81 have been published and only one full-length genome sequence (the prototype strain) has been made available in the GenBank database. Here, we report the full-length genome sequences of two EV-B81 strains isolated in the Tibet Autonomous Region of China during acute flaccid paralysis surveillance activities, and we also conducted an antibody seroprevalence study in two prefectures of Tibet. The sequence comparison and phylogenetic dendrogram analysis revealed high variability among the global EV-B81 strains and frequent intertypic recombination in the non-structural protein region of EV-B serotypes, suggesting high genetic diversity of EV-B81. However, low positive rates and low titers of neutralizing antibodies against EV-B81 were detected. Nearly 68% of children under the age of five had no neutralizing antibodies against EV-B81. Hence, the extent of transmission and the exposure of the population to this EV type are very limited. Although little is known about the biological and pathogenic properties of EV-B81 because of few research in this field owing to the limited number of isolates, our study provides basic information for further studies of EV-B81. | 3311 | NA | NA | NA | no | no |
| 3311 | 2017 | NA | Kolackova, K; Hejcmanova, P; Antoninova, M; Brandl, P | 2011 | Population management as a tool in the recovery of the critically endangered Western Derby eland Taurotragus derbianus in Senegal, Africa | The critically endangered Western Derby eland Taurotragus derbianus derbianus, representing <200 wild individuals, undoubtedly needs a coordinated conservation programme. To promote the survival of this subspecies, a single worldwide semi-captive population was established in Senegal in 2000, with one male and five female founders transferred from the Niokolo Koba National Park. To determine a lone-term conservation strategy, we used demographic and pedigree data based on continuous monitoring of reproduction during 2000 - 2009 in breeding enclosures in the Bandia and Fathala Reserves, in conjunction with modelling software. In 2009, the semi-captive population consisted of 54 living individuals (26 males and 28 females), managed using the minimal kinship strategy. The female breeding probability was 84%, annual calf and adult mortality rates were 5.09% and 3.27%, respectively, and the annual population growth rate was 1.36. As the population grew, the animals were progressively separated into five herds within two reserves. A pedigree analysis revealed an effective population size of 6.72 and an N(e)/N ratio of 0.13. The population retained 77% of the gene diversity (GD). The founder genome equivalent (FGE = 2.21) was relatively low due to the overrepresentation of one founder male. Although the mean level of inbreeding (F) reached 0.119, a significant potential GD (92%) was still retained. In this article, we predict GD development in this population in the next 100 years with the inclusion of new founders. If the whole wild population were included, we could maintain 90% of GD. As this option is not practically feasible, we present three options with the goal of maintaining 75% GD. We highly recommend capturing new founders from the remaining wild population to ensure the survival of the subspecies at least in semi-captivity, which could allow possible reinforcement of the wild population or reintroduction in the future. The semi-captive population, if appropriately constituted and genetically managed, could play a considerable role in Western Derby eland conservation. | 3312 | NA | NA | NA | no | no |
| 3312 | 2017 | NA | Jenkins, WD; Lipka, AE; Fogleman, AJ; Delfino, KR; Malhi, RS; Hendricks, B | 2016 | Variance in disease risk: rural populations and genetic diversity | Over 19% of the US population resides in rural areas, where studies of disease risk and disease outcomes are difficult to assess due to smaller populations and lower incidence. While some studies suggest rural disparities for different chronic diseases, the data are inconsistent across geography and definitions of rurality. We reviewed the literature to examine if local variations in population genomic diversity may plausibly explain inconsistencies in estimating disease risk. Many rural communities were founded over 150 years ago by small groups of ethnically and ancestrally similar families. These have since endured relative geographical isolation, similar to groups in other industrialized nations, perhaps resulting in founder effects impacting local disease susceptibility. Studies in Europe and Asia have found that observably different phenotypes may appear in isolated communities within 100 years, and that genomic variation can significantly vary over small geographical scales. Epidemiological studies utilizing common “rural” definitions may miss significant disease differences due to assumptions of risk homogeneity and misinterpretation of administrative definitions of rurality. Local genomic heterogeneity should be an important aspect of chronic disease epidemiology in rural areas, and it is important to consider for designing studies and interpreting results, enabling a better understanding of the heritable components of complex diseases. | 3313 | NA | NA | NA | no | no |
| 3313 | 2017 | NA | Sakalidis, ML; Feau, N; Dhillon, B; Hamelin, RC | 2016 | Genetic patterns reveal historical and contemporary dispersal of a tree pathogen | Sphaerulina musiva (Peck) Verkley, Quaedvlieg and Crous (syn = Septoria musiva Peck, Mycosphaerella populorum Thompson) is a pathogen of poplar that causes two distinct diseases, leaf spots and cankers. This pathogen co-evolved with Populus deltoides but recent reports have linked it to infections in planted stands of P. trichocarpa, P. balsamifera and their hybrids. Reports of S. musiva have mainly come from central and eastern US and eastern Canada, the assumed endemic range of the pathogen. S. musiva was detected for the first time in the Canadian provinces of British Columbia in 2006 and in Alberta in 2009. Our objectives were to determine the source of S. musiva in British Columbia and Alberta and examine the dispersal pathways of this pathogen across North America. For this task we sequenced eight genes and extracted single nucleotide polymorphisms on a geographically diverse set of 73 strains of S. musiva. Population structure and Approximate Bayesian Computation (ABC) analyses eliminated eastern Canada as a source for these introductions. Genetic diversity estimates and ABC analyses support an eastern US centre of origin for S. musiva and two waves of dispersal into Canada. The recently detected west Canadian populations appear to have received contributions from Saskatchewan (a western Canadian population) and also, in the case of British Columbia from the mid-west US populations. These results also reveal distinct eastern and western Canadian populations. Our analyses suggest that dissemination of the pathogen appears to be associated with the natural distribution of wild P. deltoides and more recently linked to anthropogenic activities. The most parsimonious explanation for the contemporary spread of S. musiva across the landscape is via infected plant material. Our analysis of the tree disease caused by S. musiva demonstrates that a population genetics approach is essential to reveal potential sources and patterns of spread of a pathogen. | 3314 | NA | NA | NA | no | no |
| 3314 | 2017 | NA | Williams-Newkirk, AJ; Rowe, LA; Mixson-Hayden, TR; Dasch, GA | 2012 | Presence, genetic variability, and potential significance of “Candidatus Midichloria mitochondrii” in the lone star tick Amblyomma americanum | We used next generation sequencing to detect the bacterium “Candidatus Midichloria mitochondrii” for the first time in lone star ticks (Amblyomma americanum) from the eastern United States. 177 individuals and 11 tick pools from seven sites in four states were tested by pyrosequencing with barcoded 16S rRNA gene eubacterial primers targeting variable regions 5-3. Average infection prevalence was 0.15 across all surveyed populations (range 0-0.29) and only the site with the smallest sample size (n = 5) was negative. Three genotypes differing by 2.6-4.1 % in a 271 bp region of 16S rRNA gene were identified. Two variants co-occurred in sites in North Carolina and New York, but were not observed in the same tick at those sites. The third genotype was found only in Georgia. Phylogenetic analysis of this fragment indicated that the three variants are more closely related to “Candidatus Midichloria mitochondrii” genotypes from other tick species than to each other. This variation suggests that multiple independent introductions occurred in A. americanum which may provide insight into bacterial spread within its ecosystem and parasitism on this tick. Whether the presence of this bacterium affects acquisition or maintenance of other pathogens and symbionts in A. americanum or the survival, biology and evolution of the tick itself is unknown. | 3315 | NA | NA | NA | no | no |
| 3315 | 2017 | NA | Kennemann, L; Didelot, X; Aebischer, T; Kuhn, S; Drescher, B; Droege, M; Reinhardt, R; Correa, P; Meyer, TF; Josenhans, C; Falush, D; Suerbaum, S | 2011 | Helicobacter pylori genome evolution during human infection | High genetic diversity is a hallmark of the gastric pathogen Helicobacter pylori. We used 454 sequencing technology to perform whole-genome comparisons for five sets of H. pylori strains that had been sequentially cultured from four chronically infected Colombians (isolation intervals = 3-16 y) and one human volunteer experimentally infected with H. pylori as part of a vaccine trial. The four sets of genomes from Colombian H. pylori differed by 27-232 isolated SNPs and 16-441 imported clusters of polymorphisms resulting from recombination. Imports (mean length = 394 bp) were distributed nonrandomly over the chromosome and frequently occurred in groups, suggesting that H. pylori first takes up long DNA fragments, which subsequently become partially integrated in multiple shorter pieces. Imports were present at significantly increased frequency in members of the hop family of outer membrane gene paralogues, some of which are involved in bacterial adhesion, suggesting diversifying selection. No evidence of recombination and few other differences were identified in the strain pair from an infected volunteer, indicating that the H. pylori genome is stable in the absence of mixed infection. Among these few differences was an OFF/ON switch in the phase-variable adhesin gene hopZ, suggesting strong in vivo selection for this putative adhesin during early colonization. | 3316 | NA | NA | NA | no | no |
| 3316 | 2017 | NA | Tartally, A; Kelager, A; Furst, MA; Nash, DR | 2016 | Host plant use drives genetic differentiation in syntopic populations of Maculinea alcon | The rare socially parasitic butterfly Maculinea alcon occurs in two forms, which are characteristic of hygric or xeric habitats and which exploit different host plants and host ants. The status of these two forms has been the subject of considerable controversy. Populations of the two forms are usually spatially distinct, but at Rascruci in Romania both forms occur on the same site (syntopically). We examined the genetic differentiation between the two forms using eight microsatellite markers, and compared with a nearby hygric site, Sardu. Our results showed that while the two forms are strongly differentiated at Rascruci, it is the xeric form there that is most similar to the hygric form at Sardu, and Bayesian clustering algorithms suggest that these two populations have exchanged genes relatively recently. We found strong evidence for population substructuring, caused by high within host ant nest relatedness, indicating very limited dispersal of most ovipositing females, but not association with particular host ant species. Our results are consistent with the results of larger scale phylogeographic studies that suggest that the two forms represent local ecotypes specialising on different host plants, each with a distinct flowering phenology, providing a temporal rather than spatial barrier to gene flow. | 3317 | NA | NA | NA | no | no |
| 3317 | 2017 | NA | Manassa, RP; McCormick, MI; Chivers, DP | 2013 | Socially acquired predator recognition in complex ecosystems | Social animals acquire information on predator identities through social learning, where individuals with no prior experience learn from experienced members of the group. However, a large amount of uncertainty is often associated with socially acquired information especially in cases of cross-species learning. Theory predicts that socially acquired information from heterospecifics should take more repetitions to develop in complex ecosystems where the number of participants is greater. Our work focuses on coral reef fish as their social and communal lifestyles, along with their complex life histories, make them an ideal model to test for socially acquired predator recognition. Specifically, we tested if Pomacentrus wardi were capable of transmitting the recognition of an unknown predator, Pseudochromis fuscus, to closely related Pomacentrus moluccensis and phylogenetically distant Apogon trimaculatus. Individuals of both species were able to learn the predator’s identity from experienced P. wardi based on a single conditioning event. It is somewhat surprising how fast social learning occurred particularly for the distantly related cardinalfish. This study demonstrates the widespread nature of social learning as a method of predator recognition in biologically complex ecosystems, and highlights that the benefits of responding to uncertain information may override the costs associated with lost foraging opportunities. | 3318 | NA | NA | NA | no | no |
| 3318 | 2017 | NA | Tocko-Marabena, BK; Silla, S; Simiand, C; Zinga, I; Legg, J; Reynaud, B; Delatte, H | 2017 | Genetic diversity of Bemisia tabaci species colonizing cassava in Central African Republic characterized by analysis of cytochrome c oxidase subunit I | After 2007, upsurges of whiteflies on cassava plants and high incidences of cassava diseases were observed in Central African Republic. This recent upsurge in the abundance of Bemisia tabaci (Gennadius) (Hemiptera: Aleyrodidae) was directly linked to serious damage to cassava crops resulting from spread of whitefly-borne cassava mosaic geminiviruses (CMGs). There is currently very little information describing whitefly populations on cassava and associated crops in Central African Republic. The current study aimed to address this gap, and to determine whether the increasing damage associated with B. tabaci whiteflies was the consequence of a new invasion, or an upsurge of a local population. The molecular genetic identification and phylogenetic relationships of 898 B. tabaci adult individuals collected from representative locations (54) throughout CAR were determined based on their mitochondrial cytochrome oxidase I sequences (mtCOI). Field and ecological data were also collected from each site, including whitefly abundance, CMD incidence, host plants colonized by B. tabaci and agro-ecological zone. Phylogenetic analysis of the whitefly mtCOI sequences indicated that SSA1 (-SG1, -SG2), SSA3, MED, MEAM1 and Indian Ocean (IO) putative species occur in CAR. One specific haplotype of SSA1-SG1 (SSA1-SG1-P18F5) predominated on most cassava plants and at the majority of sites. This haplotype was identical to the SSA1-SG1 Mukono8-4 (KM377961) haplotype that was recorded from Uganda but that also occurs widely in CMD pandemic-affected areas of East Africa. These results suggest that the SSA1-SG1-P18F5 haplotype occurring in CAR represents a recent invasive population, and that it is the likely cause of the increased spread and severity of CMD in CAR. Furthermore, the high mtDNA sequence diversity observed for SSA1 and its broad presence on all sites and host plants sampled suggest that this genetic group was the dominant resident species even before the arrival of this new invasive haplotype. | 3319 | NA | NA | NA | no | no |
| 3319 | 2017 | NA | Colliard, C; Sicilia, A; Turrisi, GF; Arculeo, M; Perrin, N; Stock, M | 2010 | Strong reproductive barriers in a narrow hybrid zone of West-Mediterranean green toads (Bufo viridis subgroup) with Plio-Pleistocene divergence | Background: One key question in evolutionary biology deals with the mode and rate at which reproductive isolation accumulates during allopatric speciation. Little is known about secondary contacts of recently diverged anuran species. Here we conduct a multi-locus field study to investigate a contact zone between two lineages of green toads with an estimated divergence time of 2.7 My, and report results from preliminary experimental crosses. Results: The Sicilian endemic Bufo siculus and the Italian mainland-origin B. balearicus form a narrow hybrid zone east of Mt. Etna. Despite bidirectional mtDNA introgression over a ca. 40 km North-South cline, no F(1) hybrids could be found, and nuclear genomes display almost no admixture. Populations from each side of the contact zone showed depressed genetic diversity and very strong differentiation (F(ST) = 0.52). Preliminary experimental crosses point to a slightly reduced fitness in F(1) hybrids, a strong hybrid breakdown in backcrossed offspring (F(1) x parental, with very few reaching metamorphosis) and a complete and early mortality in F(2) (F(1) x F(1)). Conclusion: Genetic patterns at the contact zone are molded by drift and selection. Local effective sizes are reduced by the geography and history of the contact zone, B. balearicus populations being at the front wave of a recent expansion (late Pleistocene). Selection against hybrids likely results from intrinsic genomic causes (disruption of coadapted sets of genes in backcrosses and F(2)-hybrids), possibly reinforced by local adaptation (the ranges of the two taxa roughly coincide with the borders of semiarid and arid climates). The absence of F(1) in the field might be due to premating isolation mechanisms. Our results, show that these lineages have evolved almost complete reproductive isolation after some 2.7 My of divergence, contrasting sharply with evidence from laboratory experiments that some anuran species may still produce viable F(1) offspring after > 20 My of divergence. | 3320 | NA | NA | NA | no | no |
| 3320 | 2017 | NA | Karunasinghe, N; Han, DY; Goudie, M; Zhu, ST; Bishop, K; Wang, A; Duan, H; Lange, K; Ko, S; Medhora, R; Kan, ST; Masters, J; Ferguson, LR | 2012 | Prostate Disease Risk Factors among a New Zealand Cohort | Background: Prostate cancer is a leading public health burden worldwide, and in New Zealand it is the most commonly registered cancer and the third leading cause of cancer deaths among males. Genetic variability and its associations with diet, demographic and lifestyle factors could influence the risk of this disease. Methods: The single nucleotide polymorphisms (SNPs) within a group of antioxidant genes and related markers were tested between patient and control cohorts, adjusted for significant differences between basic lifestyle and demographic characteristics. Results: Increasing age, smoking and low serum selenium levels were significantly associated with an increased risk for prostate disease. Alcohol consumption increased the glutathione peroxidase (GPx) activity. A significant reduction in alcohol consumption was recorded with prostate disease. Three SNPs, namely GPx1 rs1050450, SEL15 rs5845 and CAT rs1001179, were significantly associated with prostate disease risk. A cumulative risk of prostate cancer was noted with 6 risk alleles. A lower GPx activity was recorded with prostate disease compared to the controls. However, the GPx1 rs1050450 allele T in association with prostate cancer recorded a significantly higher GPx activity compared to the controls. Conclusions: These data point to a possibility of identifying individuals at risk of prostate cancer for better management purposes. Copyright (c) 2013 S. Karger AG, Basel | 3321 | NA | NA | NA | no | no |
| 3321 | 2017 | NA | Xu, HJ; Watanabe, KA; Zhang, LY; Shen, QXJ | 2016 | WRKY transcription factor genes in wild rice Oryza nivara | The WRKY transcription factor family is one of the largest gene families involved in plant development and stress response. Although many WRKY genes have been studied in cultivated rice (Oryza sativa), the WRKY genes in the wild rice species Oryza nivara, the direct progenitor of O. sativa, have not been studied. O. nivara shows abundant genetic diversity and elite drought and disease resistance features. Herein, a total of 97 O. nivara WRKY (OnWRKY) genes were identified. RNA-sequencing demonstrates that OnWRKY genes were generally expressed at higher levels in the roots of 30-day-old plants. Bioinformatic analyses suggest that most of OnWRKY genes could be induced by salicylic acid, abscisic acid, and drought. Abundant potential MAPK phosphorylation sites in OnWRKYs suggest that activities of most OnWRKYs can be regulated by phosphorylation. Phylogenetic analyses of OnWRKYs support a novel hypothesis that ancient group IIc OnWRKYs were the original ancestors of only some group IIc and group III WRKYs. The analyses also offer strong support that group IIc OnWRKYs containing the HVE sequence in their zinc finger motifs were derived from group Ia WRKYs. This study provides a solid foundation for the study of the evolution and functions of WRKY genes in O. nivara. | 3322 | NA | NA | NA | no | no |
| 3322 | 2017 | NA | Alqasim, A; Scheutz, F; Zong, ZY; McNally, A | 2014 | Comparative genome analysis identifies few traits unique to the Escherichia coli ST131 H30Rx clade and extensive mosaicism at the capsule locus | Background: E. coli ST131 is a globally disseminated clone of multi-drug resistant E. coli responsible for that vast majority of global extra-intestinal E. coli infections. Recent global genomic epidemiological studies have highlighted the highly clonal nature of this group of bacteria, however there appears to be inconsistency in some phenotypes associated with the clone, in particular capsule types as determined by K-antigen testing both biochemically and by PCR. Results: We performed improved quality assemblies on ten ST131 genomes previously sequenced by our group and compared them to a new reference genome sequence JJ1886 to identify the capsule loci across the drug-resistant clone H30Rx. Our data shows considerable genetic diversity within the capsule locus of H30Rx clone strains which is mirrored by classical K antigen testing. The varying capsule locus types appear to be randomly distributed across the H30Rx phylogeny suggesting multiple recombination events at this locus, but that this capsule heterogeneity has little to no effect on virulence associated phenotypes in vitro. Conclusions: Our data provides a framework for determining the capsular genetics of E. coli ST131 and further beyond to ExPEC strains, and highlights how capsular mosaicism may be an important strategy in becoming a successful globally disseminated human pathogen. | 3323 | NA | NA | NA | no | no |
| 3323 | 2017 | NA | Puill-Stephan, E; van Oppen, MJH; Pichavant-Rafini, K; Willis, BL | 2012 | High potential for formation and persistence of chimeras following aggregated larval settlement in the broadcast spawning coral, Acropora millepora | In sessile modular marine invertebrates, chimeras can originate from fusions of closely settling larvae or of colonies that come into contact through growth or movement. While it has been shown that juveniles of brooding corals fuse under experimental conditions, chimera formation in broadcast spawning corals, the most abundant group of reef corals, has not been examined. This study explores the capacity of the broadcast spawning coral Acropora millepora to form chimeras under experimental conditions and to persist as chimeras in the field. Under experimental conditions, 1.5-fold more larvae settled in aggregations than solitarily, and analyses of nine microsatellite loci revealed that 50 per cent of juveniles tested harboured different genotypes within the same colony. Significantly, some chimeric colonies persisted for 23 months post-settlement, when the study ended. Genotypes within persisting chimeric colonies all showed a high level of relatedness, whereas rejecting colonies displayed variable levels of relatedness. The nearly threefold greater sizes of chimeras compared with solitary juveniles, from settlement through to at least three months, suggest that chimerism is likely to be an important strategy for maximizing survival of vulnerable early life-history stages of corals, although longer-term studies are required to more fully explore the potential benefits of chimerism. | 3324 | NA | NA | NA | no | no |
| 3324 | 2017 | NA | Bos, KI; Stevens, P; Nieselt, K; Poinar, HN; DeWitte, SN; Krause, J | 2012 | Yersinia pestis: New Evidence for an Old Infection | The successful reconstruction of an ancient bacterial genome from archaeological material presents an important methodological advancement for infectious disease research. The reliability of evolutionary histories inferred by the incorporation of ancient data, however, are highly contingent upon the level of genetic diversity represented in modern genomic sequences that are publicly accessible, and the paucity of available complete genomes restricts the level of phylogenetic resolution that can be obtained. Here we add to our original analysis of the Yersinia pestis strain implicated in the Black Death by consolidating our dataset for 18 modern genomes with single nucleotide polymorphism (SNP) data for an additional 289 strains at over 600 positions. The inclusion of this additional data reveals a cluster of Y. pestis strains that diverge at a time significantly in advance of the Black Death, with divergence dates roughly coincident with the Plague of Justinian (6th to 8th century AD). In addition, the analysis reveals further clues regarding potential radiation events that occurred immediately preceding the Black Death, and the legacy it may have left in modern Y. pestis populations. This work reiterates the need for more publicly available complete genomes, both modern and ancient, to achieve an accurate understanding of the history of this bacterium. | 3325 | NA | NA | NA | no | no |
| 3325 | 2017 | NA | Mei, ZG; Huang, SL; Hao, YJ; Turvey, ST; Gong, WM; Wang, D | 2012 | Accelerating population decline of Yangtze finless porpoise (Neophocaena asiaeorientalis asiaeorientalis) | The Yangtze finless porpoise (Neophocaena asiaeorientalis asiaeorientalis) is now the only cetacean species in the Yangtze River following the probable extinction of the baiji (Lipotes vexillifer). However, population abundance estimates and genetic diversity studies both indicate that the porpoise population is declining and may also become extinct in the wild in the near future. We used data from 279 stranded porpoises that were collected along the middle and lower reaches of the Yangtze River since 1978 to construct life tables for the porpoise population before and after 1993. Demographic rate estimates reveal an accelerating decline of the Yangtze porpoise population according to the instantaneous rate of increase ((r) over bar), from (r) over bar = -0.0159 (SD = 0.0135) to (r) over bar = -0.0625 (SD = 0.0169). Using an individual-based Leslie matrix model, there is a high probability of extinction (86.06%) within the next 100 years. Effective conservation measures must be enacted immediately. The pattern of cetacean decline and extinction in the Yangtze provides a startling demonstration of how rapid economic development without adequate environmental control leads to deterioration of natural habitats and threatens native species extremely rapid. This research also emphasizes the need for precautionary conservation action in other riverine systems containing freshwater cetacean species. (C) 2012 Elsevier Ltd. All rights reserved. | 3326 | NA | NA | NA | no | no |
| 3326 | 2017 | NA | Roden, AE; Champagne, DE; Forschler, BT | 2011 | Biogeography of Triatoma sanguisuga (Hemiptera: Reduviidae) on Two Barrier Islands off the Coast of Georgia, United States | Thirty-three Triatoma sanguisuga (LeConte) adults and nymphs were collected during June and July 2009, at five sites on Cumberland Island and two sites on Sapelo Island, Georgia, to assess genetic diversity within and between sites. All but three specimens were found in a peridomestic habitat. The entire length (699 bp) of the cytochrome oxidase II mitochondrial gene was sequenced for each specimen. Twelve haplotypes were identified, nine from Cumberland Island and three from Sapelo Island. No haplotypes were shared between the two islands, indicating there is limited or no movement of gene flow between the islands. Phylogenetic relationships among the haplotypes were determined using both neighbor-joining and maximum parsimony analyses. The phylogenetic trees from both analyses were similar, with no distinct clades on either tree devoted to haplotypes from a single island. A haplotype network structure was determined using nested clade analysis, which produced two haplotype networks, one containing only specimens found on Cumberland Island. The second network included specimens from both islands, with the ancestral haplotype from Sapelo Island. This pilot study is the first to highlight triatomine populations in the southeastern United States using the cytochrome oxidase II mitochondrial gene, and indicates strong population structuring along the Georgia Coast. | 3327 | NA | NA | NA | no | no |
| 3327 | 2017 | NA | Blais, J; Rico, C; Bernatchez, L | 2004 | Nonlinear effects of female mate choice in wild threespine sticklebacks | Sexual selection by mate choice represents a very important selective pressure in many animal species and might have evolutionary impacts beyond exaggeration of secondary sexual traits. Describing the shape and strength of the relationships linking mating success and nonsexual traits in natural conditions represents a challenging step in our understanding of adaptive evolution. We studied the effect of behavioral (nest site choice), immunological (trematode level of infection), genetic diversity (measured by mean d(2)) and morphological (standard length and pectoral fin size) traits on male mating success in a natural population of threespine sticklebacks Gasterosteaus aculeatus. Male mating success was measured by microsatellite genotyping of embryos used to infer female genotypes. First, we analyzed all territorial males (full analysis) but also considered independently only males with a nonzero mating success (reduced analysis) because some of the males with no eggs could have been part of a later breeding cycle. Multiple linear regressions identified a significant negative effect of parasite load in the full analysis, whereas no linear effect was found in the reduced analysis. The quadratic analyses revealed that nest location and parasite load were significantly related to mating success by positive (concave selection) and negative (convex selection) quadratic coefficients respectively, resulting in a saddle-shaped fitness surface. Moreover, there were significant interactions between nest location, mean d2 and parasite load in the reduced analysis. The subsequent canonical rotation of the matrix of quadratic and cross-product terms identified two major axes of the response surface: a vector representing mostly nest site choice and a vector representing parasite load. These results imply that there exists more than one way for a male threespine stickleback to maximize its mating success and that such nonlinear relationships between male mating success induced by female mate choice and male characteristics might have been overlooked in many studies. | 3328 | NA | NA | NA | no | no |
| 3328 | 2017 | NA | PARSONS, PA | 1995 | EVOLUTIONARY RESPONSE TO DROUGHT STRESS - CONSERVATION IMPLICATIONS | Organisms in free-living populations are normally subjected to stressful environments, especially hydrological extremes followed by nutritional inadequacy. Although adaptation to increased desiccation stress can occur, reduced fitness is a cost restricting the potential for spread into extreme habitats. Selection for stress resistance should be lower-in captive populations reducing survival on release into free-living conditions. Therefore in developing protocols to promote conservation I recommend monitoring stress response traits of direct ecological importance emphasizing outlier populations close to extinction. This approach gains support from evidence that genetic variability in some stressed outlier populations is not lacking but may be enhanced. | 3329 | NA | NA | NA | no | no |
| 3329 | 2017 | NA | Mulder, MB | 2007 | Hamilton’s rule and kin competition: the Kipsigis case | Evolutionary studies of human behavior have emphasized the importance of kin selection in explaining social institutions and fitness outcomes. Our relatives can nevertheless be competitors as well as sources of altruism. This is particularly likely when there is local competition over resources, where conflict can lead to strife among nondispersing relatives, reducing or even negating the effects of relatedness on promoting altruism. Here, I present demographic data on a land-limited human population, utilizing large within-population variation in land ownership to determine the interactions between local resource competition and the benefits of kin in enhancing child survival, a key component of fitness in this population. As predicted, wealth affects the extent of kin altruism, in that paternal relatives (specifically father’s brothers) appear to buffer young children from mortality much more effectively in rich than in poor households. This interaction effect is interpreted as evidence that the extent of nepotism among humans depends critically on resource availability. Further unanticipated evidence that maternal kin play a role in buffering children from mortality in situations where paternal kin control few resources speaks to the important role that specific local circumstance plays in shaping kin contributions to child welfare. (C) 2007 Elsevier Inc. All rights reserved. | 3330 | NA | NA | NA | no | no |
| 3330 | 2017 | NA | Band, G; Le, QS; Jostins, L; Pirinen, M; Kivinen, K; Jallow, M; Sisay-Joof, F; Bojang, K; Pinder, M; Sirugo, G; Conway, DJ; Nyirongo, V; Kachala, D; Molyneux, M; Taylor, T; Ndila, C; Peshu, N; Marsh, K; Williams, TN; Alcock, D; Andrews, R; Edkins, S; Gray, E; Hubbart, C; Jeffreys, A; Rowlands, K; Schuldt, K; Clark, TG; Small, KS; Teo, YY; Kwiatkowski, DP; Rockett, KA; Barrett, JC; Spencer, CCA | 2013 | Imputation-Based Meta-Analysis of Severe Malaria in Three African Populations | Combining data from genome-wide association studies (GWAS) conducted at different locations, using genotype imputation and fixed-effects meta-analysis, has been a powerful approach for dissecting complex disease genetics in populations of European ancestry. Here we investigate the feasibility of applying the same approach in Africa, where genetic diversity, both within and between populations, is far more extensive. We analyse genome-wide data from approximately 5,000 individuals with severe malaria and 7,000 population controls from three different locations in Africa. Our results show that the standard approach is well powered to detect known malaria susceptibility loci when sample sizes are large, and that modern methods for association analysis can control the potential confounding effects of population structure. We show that pattern of association around the haemoglobin S allele differs substantially across populations due to differences in haplotype structure. Motivated by these observations we consider new approaches to association analysis that might prove valuable for multicentre GWAS in Africa: we relax the assumptions of SNP-based fixed effect analysis; we apply Bayesian approaches to allow for heterogeneity in the effect of an allele on risk across studies; and we introduce a region-based test to allow for heterogeneity in the location of causal alleles. | 3331 | NA | NA | NA | no | no |
| 3331 | 2017 | NA | Vientos-Plotts, AI; Ericsson, AC; Rindt, H; Grobman, ME; Graham, A; Bishop, K; Cohn, LA; Reinero, CR | 2017 | Dynamic changes of the respiratory microbiota and its relationship to fecal and blood microbiota in healthy young cats | Advances in the field of metagenomics using culture-independent methods of microbial identification have allowed characterization of rich and diverse communities of bacteria in the lungs of healthy humans, mice, dogs, sheep and pigs. These data challenge the long held belief that the lungs are sterile and microbial colonization is synonymous with pathology. Studies in humans and animals demonstrate differences in the composition of airway microbiota in health versus disease suggesting respiratory dysbiosis occurs. Using 16S rRNA amplicon sequencing of DNA extracted from rectal and oropharyngeal (OP) swabs, bronchoalveolar lavage fluid (BALF), and blood, our objective was to characterize the fecal, OP, blood, and lower airway microbiota over time in healthy cats. This work in healthy cats, a species in which a respiratory microbiota has not yet been characterized, sets the stage for future studies in feline asthma in which cats serve as a comparative and translational model for humans. Fecal, OP and BALF samples were collected from six healthy research cats at day 0, week 2, and week 10; blood was collected at week 10. DNA was extracted, amplified via PCR, and sequenced using the Illumina MiSeq platform. Representative operational taxonomic units (OTUs) were identified and microbial richness and diversity were assessed. Principal component analysis (PCA) was used to visualize relatedness of samples and PERMANOVA was used to test for significant differences in microbial community composition. Fecal and OP swabs provided abundant DNA yielding a mean +/- SEM of 65,653 6,145 and 20,6323 +/- 4,360 sequences per sample, respectively while BALF and blood samples had lower coverage (1,489 +/- 430 and 269 +/- 18 sequences per sample, respectively). Oropharyngeal and fecal swabs were significantly richer than BALF (mean number OTUs 93, 88 and 36, respectively; p < 0.001) with no significant difference (p = 0.180) in richness between time points. PCA revealed site-specific microbial communities in the feces, and upper and lower airways. In comparison, blood had an apparent compositional similarity with BALF with regard to a few dominant taxa, but shared more OTUs with feces. Samples clustered more by time than by individual, with OP swabs having subjectively greater variation than other samples. In summary, healthy cats have a rich and distinct lower airway microbiome with dynamic bacterial populations. The microbiome is likely to be altered by factors such as age, environmental influences, and disease states. Further data are necessary to determine how the distinct feline microbiomes from the upper and lower airways, feces and blood are established and evolve. These data are relevant for comparisons between healthy cats and cats with respiratory disease. | 3332 | NA | NA | NA | no | no |
| 3332 | 2017 | NA | Haydock, J; Koenig, WD | 2003 | Patterns of reproductive skew in the polygynandrous acorn woodpecker | We compared observed levels of reproductive skew in the cooperatively breeding acorn woodpecker (Melanerpes formicivorus) with those predicted by two alternative transactional models. “Concession” models predict the degree to which parentage is shared assuming that a single dominant is in complete control of reproduction. Alternatively, “restraint” models predict reproductive sharing assuming that the dominant controls only whether subordinates remain in the group but does not control its share of reproduction. Reproductive skew is high among males: on average, the most successful male sires more than three times as many offspring as the next most successful male. Females share parentage equally and have lower constraints on dispersal and lower survival rates compared with males, which is consistent with predictions from the concessions model. Also as predicted by the concessions model, yearly variation in opportunities for dispersal before the breeding season correlates positively with skew. However, in contrast to concessions but consistent with the restraint model, skew decreases with relatedness. Thus, neither model consistently predicts patterns of reproductive skew in this species. We suggest that models of reproductive skew will need to include competitive interactions among potential breeders and mate choice before they will adequately predict patterns of reproductive partitioning in most vertebrate societies. | 3333 | NA | NA | NA | no | no |
| 3333 | 2017 | NA | Bitume, EV; Bonte, D; Magalhaes, S; Martin, GS; Van Dongen, S; Bach, F; Anderson, JM; Olivieri, I; Nieberding, CM | 2011 | Heritability and Artificial Selection on Ambulatory Dispersal Distance in Tetranychus urticae: Effects of Density and Maternal Effects | Dispersal distance is understudied although the evolution of dispersal distance affects the distribution of genetic diversity through space. Using the two-spotted spider mite, Tetranychus urticae, we tested the conditions under which dispersal distance could evolve. To this aim, we performed artificial selection based on dispersal distance by choosing 40 individuals (out of 150) that settled furthest from the home patch (high dispersal, HDIS) and 40 individuals that remained close to the home patch (low dispersal, LDIS) with three replicates per treatment. We did not observe a response to selection nor a difference between treatments in life-history traits (fecundity, survival, longevity, and sex-ratio) after ten generations of selection. However, we show that heritability for dispersal distance depends on density. Heritability for dispersal distance was low and non-significant when using the same density as the artificial selection experiments while heritability becomes significant at a lower density. Furthermore, we show that maternal effects may have influenced the dispersal behaviour of the mites. Our results suggest primarily that selection did not work because high density and maternal effects induced phenotypic plasticity for dispersal distance. Density and maternal effects may affect the evolution of dispersal distance and should be incorporated into future theoretical and empirical studies. | 3334 | NA | NA | NA | no | no |
| 3334 | 2017 | NA | Carriconde, F; Gilgado, F; Arthur, I; Ellis, D; Malik, R; van de Wiele, N; Robert, V; Currie, BJ; Meyer, W | 2011 | Clonality and alpha-a Recombination in the Australian Cryptococcus gattii VGII Population - An Emerging Outbreak in Australia | Background: Cryptococcus gattii is a basidiomycetous yeast that causes life-threatening disease in humans and animals. Within C. gattii, four molecular types are recognized (VGI to VGIV). The Australian VGII population has been in the spotlight since 2005, when it was suggested as the possible origin for the ongoing outbreak at Vancouver Island (British Columbia, Canada), with same-sex mating being suggested as the driving force behind the emergence of this outbreak, and is nowadays hypothesized as a widespread phenomenon in C. gattii. However, an in-depth characterization of the Australian VGII population is still lacking. The present work aimed to define the genetic variability within the Australian VGII population and determine processes shaping its population structure. Methodology/Principal Findings: A total of 54 clinical, veterinary and environmental VGII isolates from different parts of the Australian continent were studied. To place the Australian population in a global context, 17 isolates from North America, Europe, Asia and South America were included. Genetic variability was assessed using the newly adopted international consensus multi-locus sequence typing (MLST) scheme, including seven genetic loci: CAP59, GPD1, LAC1, PLB1, SOD1, URA5 and IGS1. Despite the overall clonality observed, the presence of MATa VGII isolates in Australia was demonstrated for the first time in association with recombination in MAT alpha-MATa populations. Our results also support the hypothesis of a “smouldering” outbreak throughout the Australian continent, involving a limited number of VGII genotypes, which is possibly caused by a founder effect followed by a clonal expansion. Conclusions/Significance: The detection of sexual recombination in MAT alpha-MATa population in Australia is in accordance with the natural life cycle of C. gattii involving opposite mating types and presents an alternative to the same-sex mating strategy suggested elsewhere. The potential for an Australian wide outbreak highlights the crucial issue to develop active surveillance procedures. | 3335 | NA | NA | NA | no | no |
| 3335 | 2017 | NA | Jakob, SS; Ihlow, A; Blattner, FR | 2007 | Combined ecological niche modelling and molecular phylogeography revealed the evolutionary history of Hordeum marinum (Poaceae) - niche differentiation, loss of genetic diversity, and speciation in Mediterranean Quaternary refugia | The Hordeum marinum species group consists of two annual grasses of western Eurasian saline meadows or marshes. The two grasses split in the Quaternary about two million years ago. Hordeum marinum and the diploid of Hordeum gussoneanum (2x) co-occur throughout the Mediterranean basin, while the autotetraploid cytotype of H. gussoneanum (4x) overlaps with its diploid progenitor geographically only in the utmost Eastern Mediterranean, extending from there eastwards into Asia. Using chloroplast sequences of the trn L-F region, six newly developed chloroplast microsatellite loci, ecological predictive models based on climate data, and the present geographical distribution of the two species we analysed differentiation processes in the H. marinum group. The chloroplast data indicated clear differences in the history of both species. For H. marinum we found a subdivision between genetically variable populations from the Iberian Peninsula and the more uniform populations from the remaining Mediterranean. As an explanation, we assume Pleistocene fragmentation of an earlier widespread population and survival in an Iberian and a Central Mediterranean glacial refuge. Chloroplast variation was completely absent within the cytotypes of H. gussoneanum, indicating a severe and recent genetic bottleneck. Due to this lack of chloroplast variation only the combination of ecological habitat modelling with molecular data analyses allowed conclusions about the history of this taxon. The distribution areas of the two cytotypes of H. gussoneanum overlap today in parts of Turkey, indicating an area with similar climate conditions during polyploid formation. However, after its origin the polyploid cytotype underwent a pronounced ecological shift, compared to its diploid progenitor, allowing it to colonize mountainous inland habitats between the Mediterranean basin and Afghanistan. The extant sympatric occurrence of H. marinum and H. gussoneanum 2x in the Mediterranean region is interpreted as a result of secondary contact after fast Holocene range expansion out of different ice age refugia. | 3336 | NA | NA | NA | no | no |
| 3336 | 2017 | NA | Rolo, J; Worning, P; Nielsen, JB; Sobral, R; Bowden, R; Bouchami, O; Damborg, P; Guardabassi, L; Perreten, V; Westh, H; Tomasz, A; de Lencastre, H; Miragaia, M | 2017 | Evidence for the evolutionary steps leading to mecA-mediated beta-lactam resistance in staphylococci | The epidemiologically most important mechanism of antibiotic resistance in Staphylococcus aureus is associated with mecA-an acquired gene encoding an extra penicillin-binding protein (PBP2a) with low affinity to virtually all beta-lactams. The introduction of mecA into the S. aureus chromosome has led to the emergence of methicillin-resistant S. aureus (MRSA) pandemics, responsible for high rates of mortality worldwide. Nonetheless, little is known regarding the origin and evolution of mecA. Different mecA homologues have been identified in species belonging to the Staphylococcus sciuri group representing the most primitive staphylococci. In this study we aimed to identify evolutionary steps linking these mecA precursors to the beta-lactam resistance gene mecA and the resistance phenotype. We sequenced genomes of 106 S. sciuri, S. vitulinus and S. fleurettii strains and determined their oxacillin susceptibility profiles. Single-nucleotide polymorphism (SNP) analysis of the core genome was performed to assess the genetic relatedness of the isolates. Phylogenetic analysis of the mecA gene homologues and promoters was achieved through nucleotide/amino acid sequence alignments and mutation rates were estimated using a Bayesian analysis. Furthermore, the predicted structure of mecA homologue-encoded PBPs of oxacillin-susceptible and -resistant strains were compared. We showed for the first time that oxacillin resistance in the S. sciuri group has emerged multiple times and by a variety of different mechanisms. Development of resistance occurred through several steps including structural diversification of the non-binding domain of native PBPs; changes in the promoters of mecA homologues; acquisition of SCCmec and adaptation of the bacterial genetic background. Moreover, our results suggest that it was exposure to beta-lactams in human-created environments that has driven evolution of native PBPs towards a resistance determinant. The evolution of beta-lactam resistance in staphylococci highlights the numerous resources available to bacteria to adapt to the selective pressure of antibiotics. | 3337 | NA | NA | NA | no | no |
| 3337 | 2017 | NA | Lv, S; Zhang, Y; Liu, HX; Hu, L; Liu, Q; Wei, FR; Guo, YH; Steinmann, P; Hu, W; Zhou, XN; Utzinger, J | 2013 | Phylogenetic evidence for multiple and secondary introductions of invasive snails: Pomacea species in the People’s Republic of China | Aim To determine the genetic diversity of invasive snails (Pomacea spp.) that are implicated in crop damage, environmental degradation and human disease, and to determine their distribution pattern in a large part of eastern Asia. Location People’s Republic of China (P.R. China). Methods We collected Pomacea snails in a national survey using a grid sampling approach. Overall, 544 snails from 54 sites were used for the present investigation. The mitochondrial cox1 gene was amplified and sequenced from all the snails. We determined and classified the haplotypes using network analyses and mapped them within P.R. China. Haplotypes from this study, together with sequences available from GenBank, were used to reveal the global distribution of Pomacea canaliculata and P. insularum. Results We obtained 521 cox1 sequences and identified 24 unique haplotypes. Six haplotypes were commonly found in P.R. China. Two species, P. canaliculata and P. insularum, and one cryptic group were observed. The distribution of the 24 haplotypes across P.R. China shows a mosaic pattern. Globally, only six of 112 haplotypes of P. canaliculata, P. insularum, P. dolioides, P. lineata and P. paludosa are shared between introduced and native snail populations. We found 16 haplotype clusters, five of which occur in mainland P.R. China. Three of the five clusters could be traced back to South America. The remaining two clusters were unique to P.R. China. Main conclusions Phylogenetic analyses indicate that P. canaliculata, P. insularum and two cryptic groups, discovered by the present and previous studies, coexist in the mainland of P.R. China. The mosaic distribution and the high diversity found in the collection sites suggests multiple and secondary introductions. The findings indicate the importance of preventing further intentional introductions and call for appraisal of the risk posed by these snails in vulnerable areas. Discrimination of the ecological impacts of the different species or genotypes will help to develop setting-specific management strategies. | 3338 | NA | NA | NA | no | no |
| 3338 | 2017 | NA | Reber, A; Purcell, J; Buechel, SD; Buri, P; Chapuisat, M | 2011 | The expression and impact of antifungal grooming in ants | Parasites can cause extensive damage to animal societies in which many related individuals frequently interact. In response, social animals have evolved diverse individual and collective defences. Here, we measured the expression and efficiency of self-grooming and allo-grooming when workers of the ant Formica selysi were contaminated with spores of the fungal entomopathogen Metarhizium anisopliae. The amount of self-grooming increased in the presence of fungal spores, which shows that the ants are able to detect the risk of infection. In contrast, the amount of allo-grooming did not depend on fungal contamination. Workers groomed all nestmate workers that were re-introduced into their groups. The amount of allo-grooming towards noncontaminated individuals was higher when the group had been previously exposed to the pathogen. Allo-grooming decreased the number of fungal spores on the surface of contaminated workers, but did not prevent infection in the conditions tested (high dose of spores and late allo-grooming). The rate of disease transmission to groomers and other nestmates was extremely low. The systematic allo-grooming of all individuals returning to the colony, be they contaminated or not, is probably a simple but robust prophylactic defence preventing the spread of fungal diseases in insect societies. | 3339 | NA | NA | NA | no | no |
| 3339 | 2017 | NA | Schoebel, CN; Stewart, J; Gruenwald, NJ; Rigling, D; Prospero, S | 2014 | Population History and Pathways of Spread of the Plant Pathogen Phytophthora plurivora | Human activity has been shown to considerably affect the spread of dangerous pests and pathogens worldwide. Therefore, strict regulations of international trade exist for particularly harmful pathogenic organisms. Phytophthora plurivora, which is not subject to regulations, is a plant pathogen frequently found on a broad range of host species, both in natural and artificial environments. It is supposed to be native to Europe while resident populations are also present in the US. We characterized a hierarchical sample of isolates from Europe and the US and conducted coalescent-, migration, and population genetic analysis of sequence and microsatellite data, to determine the pathways of spread and the demographic history of this pathogen. We found P. plurivora populations to be moderately diverse but not geographically structured. High levels of gene flow were observed within Europe and unidirectional from Europe to the US. Coalescent analyses revealed a signal of a recent expansion of the global P. plurivora population. Our study shows that P. plurivora has most likely been spread around the world by nursery trade of diseased plant material. In particular, P. plurivora was introduced into the US from Europe. International trade has allowed the pathogen to colonize new environments and/or hosts, resulting in population growth. | 3340 | NA | NA | NA | no | no |
| 3340 | 2017 | NA | Nguyen, DT; Morrow, JL; Spooner-Hart, RN; Riegler, M | 2017 | Independent cytoplasmic incompatibility induced by Cardinium and Wolbachia maintains endosymbiont coinfections in haplodiploid thrips populations | Cardinium and Wolbachia are common maternally inherited reproductive parasites that can coinfect arthropods, yet interactions between both bacterial endosymbionts are rarely studied. For the first time, we report their independent expression of complete cytoplasmic incompatibility (CI) in a coinfected host, and CI in a species of the haplodiploid insect order Thysanoptera. In Pezothrips kellyanus, Cardinium-induced CI resulted in a combination of male development (MD) and embryonic female mortality (FM) of fertilized eggs. In contrast, Wolbachia-induced CI resulted in FM together with postembryonic mortality not previously reported as a CI outcome. Both endosymbionts appeared to not influence fecundity but virgins produced more offspring than mated females. In coinfected individuals, Wolbachia density was higher than Cardinium. Wolbachia removal did not impact Cardinium density, suggesting a lack of competition within hosts. Maternal transmission was complete for Wolbachia and high for Cardinium. Our data support theoretical predictions and empirical detection of high endosymbiont prevalence in field populations of the native range of this pest thrips. However, previous findings of more frequent loss of Wolbachia than Cardinium, particularly in field populations of the host’s invasive range, suggest that genetic diversity or varying environmental factors between field populations also play a role in shaping host-endosymbiont dynamics. | 3341 | NA | NA | NA | no | no |
| 3341 | 2017 | NA | Stehlik, I; Schneller, JJ; Bachmann, K | 2002 | Immigration and in situ glacial survival of the low-alpine Erinus alpinus (Scrophulariaceae) | It has been hypothesized that the subalpine Erinus alpinus survived the Pleistocene glaciation in southern French refugia peripheral to the Alps and postglacially immigrated to its northern Alpine distribution (tabula rasa hypothesis). However, E. alpinus is also known from nunataks in the northern Prealps. PCR-RFLP of the cpDNA revealed no variation, whereas AFLPs identified three phylogeographical groups among the 22 populations: (1) a central Swiss group, (2) a single central Swiss population on Mount Rigi, and (3) all other populations located to the west and east of the central Swiss group (west-eastern populations). The population representing the putative French refugium was not distinct from the west-eastern populations but was different from the populations of central Switzerland. We consider this support for locally restricted in situ survival of E. alpinus in the northern Prealps or nearby Jura mountains (dating back to 60-28 ky BP). However, the west-eastern gene-pool probably immigrated postglacially from southern France through the prealpine lowlands (not before 14.6 kyr BP), skirting central Switzerland. The near-absence of gene flow among the central Swiss and the west-eastern populations may be the reason why these historical genetic patterns are still detectable. (C) 2002 The Linnean Society of London, Biological Journal of the Linnean Society, 2002, 77, 87-103. | 3342 | NA | NA | NA | no | no |
| 3342 | 2017 | NA | Klopfstein, S; Currat, M; Excoffier, L | 2006 | The fate of mutations surfing on the wave of a range expansion | Many species, including humans, have dramatically expanded their range in the past, and such range expansions had certainly an impact on their genetic diversity. For example, mutations arising in populations at the edge of a range expansion can sometimes surf on the wave of advance and thus reach a larger spatial distribution and a much higher frequency than would be expected in stationary populations. We study here this surfing phenomenon in more detail, by performing extensive computer simulations under a two-dimensional stepping-stone model. We find that the probability of survival of a new mutation depends to a large degree on its proximity to the edge of the wave. Demographic factors such as deme size, migration rate, and local growth rate also influence the fate of these new mutations. We also find that the final spatial and frequency distributions depend on the local deme size of a subdivided population. This latter result is discussed in the light of human expansions in Europe as it should allow one to distinguish between mutations having spread with Paleolithic or Neolithic expansions. By favoring the spread of new mutations, a consequence of the surfing phenomenon is to increase the rate of evolution of spatially expanding populations. | 3343 | NA | NA | NA | no | no |
| 3343 | 2017 | NA | Fleming, IA; Hindar, K; Mjolnerod, IB; Jonsson, B; Balstad, T; Lamberg, A | 2000 | Lifetime success and interactions of farm salmon invading a native population | Farm Atlantic salmon escape and invade rivers throughout the North Atlantic annually which has generated growing concern about their. impacts on native salmon populations. A large-scale experiment was therefore undertaken in order to quantify the lifetime success and interactions of farm salmon invading a Norwegian river. Sexually mature farm and native salmon were genetically screened, radio tagged and released into the River Imsa where no other. salmon had been allowed to ascend. The farm fishes were competitively and reproductively inferior, achieving less than one-third the breeding success of the native fishes. Moreover, this inferiority was scs biased, being more pronounced in farm males than females, resulting in tho principal route of gene flow involving native males mating with Farm females. There were also indications of selection against farm genotypes during early survival but not thereafter. However, evidence of resource competition and competitive displacement existed as the productivity of the native population was depressed by more than 30%. Ultimately the lifetime reproductive success (adult to adult of the farm fishes was 16% that of the native salmon. Our results indicate that such annual invasions have the potential for impacting on population productivity, disrupting local adaptations and reducing the genetic diversity of wild salmon populations. | 3344 | NA | NA | NA | no | no |
| 3344 | 2017 | NA | Carrillo-Bustamante, P; Kesmir, C; de Boer, RJ | 2015 | A Coevolutionary Arms Race between Hosts and Viruses Drives Polymorphism and Polygenicity of NK Cell Receptors | Natural killer cell receptors (NKRs) monitor the expression of major histocompatibility class I (MHC-I) and stress molecules to detect unhealthy tissue, such as infected or tumor cells. The NKR gene family shows a remarkable genetic diversity, containing several genes encoding receptors with activating and inhibiting signaling, and varying in gene content and allelic polymorphism. The expansion of the NKR genes is species-specific, with different species evolving alternative expanded NKR genes, which encode structurally different proteins, yet perform comparable functions. So far, the biological function of this expansion within the NKR cluster has remained poorly understood. To study the evolution of NKRs, we have developed an agent-based model implementing a coevolutionary scenario between hosts and herpes-like viruses that are able to evade the immune response by downregulating the expression of MHC-I on the cell surface. We show that hosts evolve specific inhibitory NKRs, specialized to particular MHC-I alleles in the population. Viruses in our simulations readily evolve proteins mimicking the MHC molecules of their host, even in the absence of MHC-I downregulation. As a result, the NKR locus becomes polygenic and polymorphic, encoding both specific inhibiting and activating receptors to optimally protect the hosts from coevolving viruses. | 3345 | NA | NA | NA | no | no |
| 3345 | 2017 | NA | Mirajkar, NS; Gebhart, CJ | 2014 | Understanding the Molecular Epidemiology and Global Relationships of Brachyspira hyodysenteriae from Swine Herds in the United States: A Multi-Locus Sequence Typing Approach | Outbreaks of mucohemorrhagic diarrhea in pigs caused by Brachyspira hyodysenteriae in the late 2000s indicated the reemergence of Swine Dysentery (SD) in the U.S. Although the clinical disease was absent in the U.S. since the early 1990s, it continued to cause significant economic losses to other swine rearing countries worldwide. This study aims to fill the gap in knowledge pertaining to the re-emergence and epidemiology of B. hyodysenteriae in the U.S. and its global relationships using a multi-locus sequence typing (MLST) approach. Fifty-nine post re-emergent isolates originating from a variety of sources in the U.S. were characterized by MLST, analyzed for epidemiological relationships (within and between multiple sites of swine systems), and were compared with pre re-emergent isolates from the U.S. Information for an additional 272 global isolates from the MLST database was utilized for international comparisons. Thirteen nucleotide sequence types (STs) including a predominant genotype (ST93) were identified in the post re-emergent U.S. isolates; some of which showed genetic similarity to the pre re-emergent STs thereby suggesting its likely role in the re-emergence of SD. In the U.S., in general, no more than one ST was found on a site; multiple sites of a common system shared a ST; and STs found in the U.S. were distinct from those identified globally. Of the 110 STs characterized from ten countries, only two were found in more than one country. The U.S. and global populations, identified as clonal and heterogeneous based on STs, showed close relatedness based on amino acid types (AATs). One predicted founder type (AAT9) and multiple predicted subgroup founder types identified for both the U.S. and the global population indicate the potential microevolution of this pathogen. This study elucidates the strain diversity and microevolution of B. hyodysenteriae, and highlights the utility of MLST for epidemiological and surveillance studies. | 3346 | NA | NA | NA | no | no |
| 3346 | 2017 | NA | Czarny, NA; Rodger, JC | 2010 | Vitrification as a method for genome resource banking oocytes from the endangered Tasmanian devil (Sarcophilus harrisii) | Populations of Australia’s largest terrestrial marsupial carnivore, the Tasmanian devil (Sarcophilus harrisii), are rapidly declining in the wild due to Tasmanian Devil Facial Tumour Disease (TDFTD). One tool which can reduce the loss of genetic diversity is genome resource banking. This study examines the application of an oocyte vitrification protocol, initially developed in a model marsupial carnivore, to the endangered Tasmanian devil. Ovarian tissue was transported to the laboratory on ice from Tasmania which took up to 48 h. Individual granulosa oocyte complexes (GOC) were isolated enzymatically and the viability of oocytes from primary GOC was assessed immediately following isolation or after exposure to cold shock, vitrification and thawing media without exposure to liquid nitrogen or the full vitrification and thawing process. There was no decline in oocyte viability following cold shock or exposure to the vitrification and thawing media. Following the full vitrification and thawing process there was a decline in oocyte viability (chi(2) = 20.0, P <0.001) but approximately 70% of oocytes remained viable. This study provides further evidence that oocyte vitrification is a promising strategy for genome resource banking in carnivorous marsupials and suggests that it should be considered in conservation plans for the survival of the iconic Tasmanian devil. (C) 2010 Elsevier Inc. All rights reserved. | 3347 | NA | NA | NA | no | no |
| 3347 | 2017 | NA | Molet, M; Van Baalen, M; Monnin, T | 2005 | Dominance hierarchies reduce the number of hopeful reproductives in polygynous queenless ants | In many animal societies aggressive interactions regulate essential features such as feeding order and reproductive rights. Because aggressive interactions are costly the number of individuals competing for direct reproduction (hopeful reproductives) affects colony productivity. Using mathematical models, based on the costs/benefits trade-off for a worker to attempt to become a reproductive, we determine the number of hopeful reproductives expected to occur in insect societies with totipotent workers and several reproductives. The model is based on the biology of the polygynous queenless ant Rhytidoponera confusa (Formicidae: Ectatomminae), where every worker can potentially reproduce but only a few actually do, but is valid for all societies with totipotent individuals. We compare the number of hopeful reproductives predicted in the absence of a dominance hierarchy and with a linear dominance hierarchy, and we investigate the effects of colony size, relatedness, and mortality. The models show that a linear dominance hierarchy reduces the number of hopeful reproductives, and additional unpublished models show that this reduction is lower in non-linear hierarchies. Dominance hierarchies are thus favoured by natural selection. Larger colony size and higher mortalities result in longer hierarchy, whereas higher relatedness shortens bierarchy length. These predictions were successfully tested with eight colonies of R. confusa. | 3348 | NA | NA | NA | no | no |
| 3348 | 2017 | NA | Long, GH; Graham, AL | 2011 | Consequences of immunopathology for pathogen virulence evolution and public health: malaria as a case study | Evolutionary theories explaining virulence-the fitness damage incurred by infected hosts-often focus on parasite strategies for within-host exploitation. However, much virulence can be caused by the host’s own immune response: for example, pro-inflammatory cytokines, although essential for killing malaria parasites, also damage host tissue. Here we argue that immune-mediated virulence, or ‘immunopathology,’ may affect malaria virulence evolution and should be considered in the design of medical interventions. Our argument is based on the ability of immunopathology to disrupt positive virulence-transmission relationships assumed under the trade-off theory of virulence evolution. During rodent malaria infections, experimental reduction of inflammation using reagents approved for field use decreases virulence but increases parasite transmission potential. Importantly, rodent malaria parasites exhibit genetic diversity in the propensity to induce inflammation and invest in transmission-stage parasites in the presence of pro-inflammatory cytokines. If immunopathology positively correlates with malaria parasite density, theory suggests it could select for relatively low malaria virulence. Medical interventions which decrease immunopathology may therefore inadvertently select for increased malaria virulence. The fitness consequences to parasites of variations in immunopathology must be better understood in order to predict trajectories of parasite virulence evolution in heterogeneous host populations and in response to medical interventions. | 3349 | NA | NA | NA | no | no |
| 3349 | 2017 | NA | Li, L; Wang, BB; Ge, YF; Wan, QH | 2014 | Major histocompatibility complex class II polymorphisms in forest musk deer (Moschus berezovskii) and their probable association with purulent disease | Genes of the major histocompatibility complex (MHC) family are crucial in immune responses because they present pathogenic peptides to T cells. In this study, we analysed the genetic variation in forest musk deer (Moschus berezovskii) MHC II genes and its potential association with musk deer purulent disease. In total, 53 purulent disease-susceptible and 46 purulent disease-resistant individuals were selected for MHC II exon 2 fragment analysis. Among them, 16 DQ alleles and four additional DR alleles were identified, with DQ exon 2 fragments displaying a low level of polymorphism. The nonsynonymous substitutions exceeded the synonymous substitutions in the peptide-binding sites of DQA2, DQB1 and DQB2. Then, 28 MHC II alleles were used to analyse the distribution patterns of purulent disease between the susceptible and resistant groups. Among them, three alleles (DQA1*01, DQA1*02 and DQA2*04) were found to be resistant, and five alleles (DRB3*07, DQA1*03, DQA1*04, DQA2*05 and DQA2*06) were found to increase susceptibility. Additionally, three haplotypes were found to be putatively associated with musk deer purulent disease. However, these three haplotypes were only found in the resistant or susceptible group, and their frequencies were low. The results from our study support a contributory role of MHC II polymorphisms in the development of purulent disease in forest musk deer. | 3350 | NA | NA | NA | no | no |
| 3350 | 2017 | NA | Shrestha, SK; Cochran, A; Mengistu, A; Lamour, K; Castro-Rocha, A; Young-Kelly, H | 2017 | Genetic diversity, QoI fungicide resistance, and mating type distribution of Cercospora sojina-Implications for the disease dynamics of frogeye leaf spot on soybean | Frogeye leaf spot (FLS), caused by Cercospora sojina, causes significant damage to soybean in the U.S. One control strategy is the use of quinone outside inhibitor (QoI) fungicides. QoI resistant isolates were first reported in Tennessee (TN) in 2010. To investigate the disease dynamics of C. sojina, we collected 437 C. sojina isolates in 2015 from Jackson and Milan, TN and used 40 historical isolates collected from 2006-2009 from TN and ten additional states for comparison. A subset of 186 isolates, including historical isolates, were genotyped for 49 single nucleotide polymorphism (SNP) markers and the QoI resistance locus, revealing 35 unique genotypes. The genotypes clustered into three groups with two groups containing only sensitive isolates and the remaining group containing all resistant isolates and a dominant clonal lineage of 130 isolates. All 477 C. sojina isolates were genotyped for the QoI locus revealing 344 resistant and 133 sensitive isolates. All isolates collected prior to 2015 were QoI sensitive. Both mating type alleles (MAT1-1-1 and MAT1-2) were found in Jackson and Milan, TN and recovered from single lesions suggesting sexual recombination may play a role in the epidemiology of field populations. Analysis of C. sojina isolates using SNP markers proved useful to investigate population diversity and to elaborate on diversity as it relates to QoI resistance and mating type. | 3351 | NA | NA | NA | no | no |
| 3351 | 2017 | NA | Botanga, CJ; Timko, MP | 2006 | Phenetic relationships among different races of Striga gesnerioides (Willd.) Vatke from West Africa | Striga gesnerioides is a root hemiparasite that primarily parasitizes dicotyledonous species, including cowpea (Vigna unguiculata L.) and other legumes. Based on the differential resistance response of various cultivars, landraces, and breeding lines, it has been proposed that several distinct races of cowpea-parasitic S. gesnerioides exist in West Africa. In this study, we used amplified fragment length polymorphism profile analysis to examine the genetic variability within and among populations of cowpea-parasitic S. gesnerioides within the suspected distribution range of a particular race, and statistical clustering methods to define the phenetic relationships of the various races in West Africa. Our data indicate that genetic variability within and among populations of each of the previously recognized races of cowpea-parasitic S. gesnerioides is extremely low. On the basis of genotypic profile and host differential resistance responses, 2 previously unknown races were identified. Of the 7 races now identifiable, races SG1 (from Burkina Faso) and SG5 (from Cameroon) are the most closely related, and SG4 (from Benin) and SG3 (from Niger/Nigeria) are the most divergent. SG6, a new race of the parasite identified in Senegal, was found to be the most genetically similar to SG4 from Benin. We also demonstrate that a hypervirulent isolate of the S. gesnerioides from Zakpota, in the Republic of Benin, is genotypically distinct from other populations of SG4, thereby warranting designation as a separate race, which we called SG4z. To further support our race classification scheme, we identified a group of molecular markers that effectively discriminate each of the various races. Finally, we show that an isolate (designated SG4i) of the wild legume Indigofera hirsuta-parasitic S. gesnerioides is genetically distinct and significantly diverged from the various races of cowpea-parasitic S. gesnerioides. Our data suggest that both geographic isolation and host-driven selection are critical factors defining race formation in S. gesnerioides in West Africa. | 3352 | NA | NA | NA | no | no |
| 3352 | 2017 | NA | Fernandes, WPA; Griffiths, SW; Ibbotson, AT; Bruford, MW; Riley, WD | 2015 | The role of density and relatedness in wild juvenile Atlantic salmon growth | Growth is a key life-history trait in fish that is influenced by both abiotic (such as temperature and water chemistry) and biotic factors (such as density and food availability). Investigating how growth performance is influenced by such factors in the wild is important for understanding how population processes influence animals in natural environments and for predicting the response to conservation and management strategies that manipulate these conditions. The theory of kin selection predicts that significant growth and survival benefits are conferred upon animals associating with close relatives. However, resource competition may be more intense among close relatives, and little is known about the trade-off between these two processes under different ecological conditions. Here, we examine the correlation between naturally occurring densities and kin-biased growth rate using a species where kin recognition has a strong impact on behaviour in laboratory studies, but where, paradoxically, field investigations have failed to document predicted kin-biased growth or survival. Intra- and inter-family differences in growth rate of juvenile Atlantic salmon Salmo salar were studied to examine how relatedness (groups of full-sibling fish and groups of mixed-sibling fish) and sibling group (family/genotype) affect salmon parr growth, and the correlation of growth rate under a range of naturally occurring densities. Parentage and relatedness of neighbouring fish were assigned using microsatellite and passive integrated transponder tags, which allowed the growth estimation of individual fish. The results show that growth rate was significantly influenced by both sibling group (family of origin) and also by an interaction between relatedness and density. The latter finding indicates that at higher densities, full-sibling groups achieved higher growth rates in comparison to mixed-sibling groups. Thus, the growth benefits of associating with relatives are not conferred under all ecological conditions, but it becomes most apparent at high density when resource competition is greatest. | 3353 | NA | NA | NA | no | no |
| 3353 | 2017 | NA | Lewis, K | 1998 | Pathogen resistance as the origin of kin altruism | It is suggested that pathogen and parasite avoidance act as a driving force for kin selection. Preferential association with relatives decreases the probability of infection with unfamiliar pathogens. Altruistic behavior towards kin will further decrease the danger of infection by increasing the representation of relatives in a group. Such a behavioral strategy could evolve if pathogen resistance were heritable. Highly polymorphic major histocompatibility (MHC) genes largely determine heritable resistance to particular pathogens. The degree of relatedness within a group correlates with the degree of similarity in MHCs. It has been shown that detection of MHC type by mice guides preferential kin association. Pathogen avoidance appears to be the simplest explanation for the existence of a direct link between detecting the heritable component of the immune system and kin altruism. Detection of MHC also serves kin avoidance in mating. Periodic introduction of new genes including MHCs through mating provides resistance to the unavoidable appearance of foreign virulent pathogens (Hamilton et al., 1990, Proc. Natl Acad. Sci. U.S.A. 87, 3566-3573). It is suggested that kin preference favoring homogeneity punctuated by introduction of new genes through mating provides an optimal strategy for pathogen avoidance. Aggression might also serve to decrease infection by isolating or eliminating individuals with unrelated MHCs. (C) 1998 Academic Press | 3354 | NA | NA | NA | no | no |
| 3354 | 2017 | NA | Kaiser, K; Pollinger, J | 2012 | Batrachochytrium dendrobatidis Shows High Genetic Diversity and Ecological Niche Specificity among Haplotypes in the Maya Mountains of Belize | The amphibian pathogen Batrachochytrium dendrobatidis (Bd) has been implicated in amphibian declines around the globe. Although it has been found in most countries in Central America, its presence has never been assessed in Belize. We set out to determine the range, prevalence, and diversity of Bd using quantitative PCR (qPCR) and sequencing of a portion of the 5.8 s and ITS1-2 regions. Swabs were collected from 524 amphibians of at least 26 species in the protected areas of the Maya Mountains of Belize. We sequenced a subset of 72 samples that had tested positive for Bd by qPCR at least once; 30 samples were verified as Bd. Eight unique Bd haplotypes were identified in the Maya Mountains, five of which were previously undescribed. We identified unique ecological niches for the two most broadly distributed haplotypes. Combined with data showing differing virulence shown in different strains in other studies, the 5.8 s - ITS1-2 region diversity found in this study suggests that there may be substantial differences among populations or haplotypes. Future work should focus on whether specific haplotypes for other genomic regions and possibly pathogenicity can be associated with haplotypes at this locus, as well as the integration of molecular tools with other ecological tools to elucidate the ecology and pathogenicity of Bd. | 3355 | NA | NA | NA | no | no |
| 3355 | 2017 | NA | Donadio, E; Buskirk, SW | 2006 | Diet, morphology, and interspecific killing in carnivora | Interspecific killing is a key determinant of the abundances and distributions of carnivores, their prey, and nonprey community members. Similarity of body size has been proposed to lead competitors to seek similar prey, which increases the likelihood of interference encounters, including lethal ones. We explored the influence of body size, diet, predatory habits, and taxonomic relatedness on interspecific killing. The frequency of attacks depends on differences in body size: at small and large differences, attacks are less likely to occur; at intermediate differences, killing interactions are frequent and related to diet overlap. Further, the importance of interspecific killing as a mortality factor in the victim population increases with an increase in body size differences between killers and victims. Carnivores highly adapted to kill vertebrate prey are more prone to killing interactions, usually with animals of similar predatory habits. Family-level taxonomy influences killing interactions; carnivores tend to interact more with species in the same family than with species in different families. We conclude that although resource exploitation (diet), predatory habits, and taxonomy are influential in predisposing carnivores to attack each other, relative body size of the participants is overwhelmingly important. We discuss the implications of interspecific killing for body size and the dynamics of geographic ranges. | 3356 | NA | NA | NA | no | no |
| 3356 | 2017 | NA | Robinson, BR; Sathuvalli, V; Bamberg, J; Goyer, A | 2015 | Exploring Folate Diversity in Wild and Primitive Potatoes for Modern Crop Improvement | Malnutrition is one of the world’s largest health concerns. Folate (also known as vitamin B-9) is essential in the human diet, and without adequate folate intake, several serious health concerns, such as congenital birth defects and an increased risk of stroke and heart disease, can occur. Most people’s folate intake remains sub-optimal, even in countries that have a folic acid food fortification program in place. Staple crops, such as potatoes, represent an appropriate organism for biofortification through traditional breeding based on their worldwide consumption and the fact that modern cultivars only contain about 6% of the daily recommended intake of folate. To start breeding potatoes with enhanced folate content, high folate potato material must be identified. In this study, 250 individual plants from 77 accessions and 10 Solanum species were screened for their folate content using a tri-enzyme extraction and microbial assay. There was a 10-fold range of folate concentrations among individuals. Certain individuals within the species Solanum tuberosum subsp. andigenum, Solanum vernei and Solanum boliviense have the potential to produce more than double the folate concentrations of commercial cultivars, such as Russet Burbank. Our results show that tapping into the genetic diversity of potato is a promising approach to increase the folate content of this important crop. | 3357 | NA | NA | NA | no | no |
| 3357 | 2017 | NA | Popovic, T; Josic, D; Starovic, M; Milovanovic, P; Dolovac, N; Postic, D; Stankovic, S | 2013 | PHENOTYPIC AND GENOTYPIC CHARACTERIZATION OF XANTHOMONAS CAMPESTRIS STRAINS ISOLATED FROM CABBAGE, KALE AND BROCCOLI | Thirty-six strains of Xanthomonas campestris pv. campestris (Xcc) isolated from cabbage, kale and broccoli were identified according to their pathogenicity, phenotypic and genotypic characterization. Pathogenicity was confirmed by the injection method with a hypodermic syringe into the mesophilic tissue of cabbage leaves. All strains were Gram-negative, aerobic, catalase-positive, oxidase-negative, grew at 35 degrees C, produced levan, H2S and indole, did not reduce nitrate, hydrolyzed Tween 80, starch, gelatin and esculin and did not show tolerance to 0.1 and 0.02% TTC. The strains produced acid from d-arabinose, arginine, dulcitol, galactose, d-glucose, maltose, mannose, sorbitol, sucrose and xylose. The genetic characterization was based on the sequence analyses of 16S rDNA and ERIC and BOX PCR. Strains of different pathovars were also used to compare PCR resulting patterns. BOX-PCR of the strains from kale and broccoli, obtained using (GTG)(5) primer, yielded patterns with a high similarity level to pathovar reference strain Xcc. The strains from cabbage yielded BOX and ERIC product patterns, distinguishing them from the other tested strains and reference strains. 16S rDNA of the representative strains was closely related to Xcc strain ATCC 33913. ERIC PCR and BOX using (GTG)(5) primer generated different Xcc patterns and were effective in distinguishing strains from different plant hosts. | 3358 | NA | NA | NA | no | no |
| 3358 | 2017 | NA | Gonzalez-Wanguemert, M; Perez-Ruzafa, A; Rosique, MJ; Ortiz, A | 2004 | Genetic differentiation in two cryptic species of Ostreidae, Ostrea edulis (Linnaeus, 1758) and Ostreola stentina (Payraudeau, 1826) in Mar Menor Lagoon, southwestern Mediterranean Sea | Ostrea edulis is a target species for aquaculture but its hatchery has suffered as a result of the lack of morphological differentiation between individuals with a low growth and those that reach commercial size. Two sympatric species of oysters, Ostrea edulis and Ostreola stentina, have been reported at the Mar Menor Lagoon, Spain. A third nominal species, Ostreola parenzani, is now considered a synonym of O. stentina. The external morphology of O. edulis and O. stentina is very similar and this prevents their differentiation at the morphological level, except for maximum size. Oysters were collected from 3 locations along the Mar Menor Lagoon and examined for variation at the PGI locus. Principal component analysis of allo-zyme data revealed the existence of two groups, which confirms the presence of two species: Ostrea edulis and Ostreola stentina. The genetic variability of the glucose-6-phosphate isomerase (PGI) locus was also compared in Ostrea edulis and Ostreola stentina from the Mar Menor Lagoon. Ostrea edulis has high levels of homozygosis and shows an important deviation from the Hardy-Weinberg equilibrium. Ostreola stentina shows high heterozygosis and significant differentiation among coastal lagoon samples. The allele frequencies at the PGI locus can be used as a diagnostic character at the species level. | 3359 | NA | NA | NA | no | no |
| 3359 | 2017 | NA | Wray, AK; Olival, KJ; Moran, D; Lopez, MR; Alvarez, D; Navarrete-Macias, I; Liang, E; Simmons, NB; Lipkin, WI; Daszak, P; Anthony, SJ | 2016 | Viral Diversity, Prey Preference, and Bartonella Prevalence in Desmodus rotundus in Guatemala | Certain bat species serve as natural reservoirs for pathogens in several key viral families including henipa-, lyssa-, corona-, and filoviruses, which may pose serious threats to human health. The Common Vampire Bat (Desmodus rotundus), due to its abundance, sanguivorous feeding habit involving humans and domestic animals, and highly social behavioral ecology, may have an unusually high potential for interspecies disease transmission. Previous studies have investigated rabies dynamics in D. rotundus, yet the diversity of other viruses, bacteria, and other microbes that these bats may carry remains largely unknown. We screened 396 blood, urine, saliva, and fecal samples from D. rotundus captured in Guatemala for 13 viral families and genera. Positive results were found for rhabdovirus, adenovirus, and herpesvirus assays. We also screened these samples for Bartonella spp. and found that 38% of individuals tested positive. To characterize potential for interspecies transmission associated with feeding behavior, we also analyzed cytochrome B sequences from fecal samples to identify prey species and found that domestic cattle (Bos taurus) made up the majority of blood meals. Our findings suggest that the risk of pathogen spillover from Desmodus rotundus, including between domestic animal species, is possible and warrants further investigation to characterize this microbial diversity and expand our understanding of foraging ecology in their populations. | 3360 | NA | NA | NA | no | no |
| 3360 | 2017 | NA | Gray, RR; Tatem, AJ; Johnson, JA; Alekseyenko, AV; Pybus, OG; Suchard, MA; Salemi, M | 2011 | Testing Spatiotemporal Hypothesis of Bacterial Evolution Using Methicillin-Resistant Staphylococcus aureus ST239 Genome-wide Data within a Bayesian Framework | Staphylococcus aureus is a common cause of infections that has undergone rapid global spread over recent decades. Formal phylogeographic methods have not yet been applied to the molecular epidemiology of bacterial pathogens because the limited genetic diversity of data sets based on individual genes usually results in poor phylogenetic resolution. Here, we investigated a whole-genome single nucleotide polymorphism (SNP) data set of health care-associated Methicillin-resistant S. aureus sequence type 239 (HA-MRSA ST239) strains, which we analyzed using Markov spatial models that incorporate geographical sampling distributions. The reconstructed timescale indicated a temporal origin of this strain shortly after the introduction of Methicillin, followed by global pandemic spread. The estimate of the temporal origin was robust to the molecular clock, coalescent prior, full/intergenic/synonymous SNP inclusion, and correction for excluded invariant site patterns. Finally, phylogeographic analyses statistically supported the role of human movement in the global dissemination of HA-MRSA ST239, although it was unable to conclusively resolve the location of the root. This study demonstrates that bacterial genomes can indeed contain sufficient evolutionary information to elucidate the temporal and spatial dynamics of transmission. Future applications of this approach to other bacterial strains may provide valuable epidemiological insights that may justify the cost of genome-wide typing. | 3361 | NA | NA | NA | no | no |
| 3361 | 2017 | NA | Galaverni, M; Caniglia, R; Fabbri, E; Lapalombella, S; Randi, E | 2013 | MHC Variability in an Isolated Wolf Population in Italy | Small, isolated populations may experience increased extinction risk due to reduced genetic variability at important functional genes, thus decreasing the population’s adaptive potential. The major histocornpatibility complex (MHC), a key immunological gene cluster, usually shows high variability maintained by positive or balancing selection in response to challenges by pathogens. Here we investigated for the first time, the variability of 3 MHC class II genes (DRB1, DQA1, and DQB1) in 94 samples collected from Italian wolves. The Italian wolf population has been long isolated south of the Alps and is presently recovering from a recent bottleneck that decreased the population to less than 100 individuals. Despite the bottleneck, Italian wolves show remarkable MHC variability with 6-9 alleles per locus, including 2 recently described alleles at DRB1. MHC sequences show signatures of historical selective pressures (high d(N)/d(S) ratio, omega > 1.74) but no evidence of ongoing selection. Variation at the MHC genes and 12 background microsatellite loci were not apparently affected by the recent bottleneck. Although MHC alleles of domestic dog origin were detected in 8 genetically admixed individuals, these alleles were rare or absent in nonadrnixed wolves. Thus, despite known hybridization events between domestic dogs and Italian wolves, the Italian wolf population does not appear affected by deep introgression of domestic dog MHC alleles. | 3362 | NA | NA | NA | no | no |
| 3362 | 2017 | NA | Willis, TJ; Millar, RB; Babcock, RC | 2003 | Protection of exploited fish in temperate regions: high density and biomass of snapper Pagrus auratus (Sparidae) in northern New Zealand marine reserves | 1. The use of marine reserves as tools either for conservation or fisheries management requires rigorous empirical evidence for the recovery of exploited species within them.  - The relative density and size structure of snapper Pagrus auratus (Sparidae), an intensively exploited reef fish species, were measured, using baited underwater video, inside and outside three northern New Zealand marine reserves (Leigh Marine Reserve, Hahei Marine Reserve and Tawharanui Marine Park) every 6 months from October 1997 to April 1999. - Log‐linear modelling showed that relative total density and egg production of snapper were higher in all three reserves than in fished areas. Snapper that were larger than the minimum legal size were estimated to be 14 times denser in protected areas than in fished areas, and the relative egg production was estimated to be 18 times higher. In the Leigh reserve, legal‐size snapper were larger than legal‐size snapper in fished areas. - At the Leigh reserve, snapper density consistently peaked at the reserve centre and declined towards either boundary, which suggests that snapper became increasingly vulnerable to fishing towards the reserve boundaries. - Inshore snapper density was significantly higher in autumn than in spring, supporting previous suggestions that snapper make regular onshore–offshore seasonal migrations that might be related to spawning. We suggest that the observed recovery of snapper populations within reserves is attributable to immigration of individuals from fished areas that take up residency within reserves, rather than juvenile recruitment. - Synthesis and applications. This study demonstrates the effectiveness of marine reserves for protecting an exploited species previously thought to be too mobile to respond to area‐based protection. Although it is difficult to envisage significant enhancement of fished areas via adult emigration, it is likely that the reserves contribute significantly to local gamete production. In addition, the protection of fish populations within reserves might slow reductions in genetic diversity caused by size‐selective mortality brought about by exploitation. | 3363 | NA | NA | NA | no | no |
| 3363 | 2017 | NA | Castagnone-Sereno, P; Bongiovanni, M; Wajnberg, E | 2007 | Selection and parasite evolution: a reproductive fitness cost associated with virulence in the parthenogenetic nematode Meloidogyne incognita | Selection in plant parasites for virulence on resistant hosts and the resulting effects on parasite fitness may be considered as a driving force in host-parasite coevolution. In the present study, we tested the hypothesis that a fitness cost may be associated with nematode virulence, using the interaction between the parthenogenetic species Meloidogyne incognita and tomato as a model system. The reproductive parameters of near-isogenic lines of the nematode, selected for avirulence or virulence against the tomato Mi resistance gene, were analysed and combined into a reproductive index that was taken as a measure of fitness. The lower fitness of the virulent lines on the susceptible tomato cultivar showed for the first time that a measurable fitness cost is associated with unnecessary virulence in the nematode. Although parthenogenesis should theoretically lead to little genetic variability, such cost may impose a direct constraint on the coevolution between the plant and the nematode populations, and suggests an adaptive significance of trade-offs between selected characters and fitness-related traits. These results indicate that, although plant resistance can be broken, it might prove durable in some conditions if the virulent nematodes are counterselected in susceptible plants, which could have important consequences for the management of resistant cultivars in the field. | 3364 | NA | NA | NA | no | no |
| 3364 | 2017 | NA | Chen, MH; Liu, X; Wei, FR; Larson, MG; Fox, CS; Vasan, RS; Yang, Q | 2011 | A Comparison of Strategies for Analyzing Dichotomous Outcomes in Genome-Wide Association Studies With General Pedigrees | Genome-wide association studies (GWAS) have been frequently conducted on general or isolated populations with related individuals. However, there is a lack of consensus on which strategy is most appropriate for analyzing dichotomous phenotypes in general pedigrees. Using simulation studies, we compared several strategies including generalized estimating equations (GEE) strategies with various working correlation structures, generalized linear mixed model (GLMM), and a variance component strategy (denoted LMEBIN) that treats dichotomous outcomes as continuous with special attentions to their performance with rare variants, rare diseases, and small sample sizes. In our simulations, when the sample size is not small, for type I error, only GEE and LMEBIN maintain nominal type I error in most cases with exceptions for GEE with very rare disease and genetic variants. GEE and LMEBIN have similar statistical power and slightly outperform GLMM when the prevalence is low. In terms of computational efficiency, GEE with sandwich variance estimator outperforms GLMM and LMEBIN. We apply the strategies to GWAS of gout in the Framingham Heart Study. Based on our results, we would recommend using GEE ind-san in the GWAS for common variants and GEE ind-fij or LMEBIN for rare variants for GWAS of dichotomous outcomes with general pedigrees. Genet. Epidemiol. 35:650-657, 2011. (C) 2011 Wiley Periodicals, Inc. | 3365 | NA | NA | NA | no | no |
| 3365 | 2017 | NA | COOPER, RS | 1993 | ETHNICITY AND DISEASE PREVENTION | Ethnic variations in health present a useful paradigm for the study of disease etiology. The knowledge base in this area has expanded dramatically in recent years, both as a result of research across cultures and studies within multiethnic societies. The growth in descriptive information, however, has not always been paralleled by an increase in causal explanations. At both the theoretical and practical levels confounding remains an unsolved problem. Because of the frequent correlation between ethnicity, socioeconomic status, and risk exposures it is often difficult to isolate the relative impact of genetics and external risk factors. The importance of variation in allele frequencies in determining ethnic disease patterns is further complicated by the difficulty in providing precise ethnic group designations. Inference regarding causal relationships becomes particularly challenging for polygenic disorders like hypertension and non-insulin-dependent diabetes mellitus, where good candidate genes are not available. At the same time, exposures are often known before the genetic basis of susceptibility has been identified and provide a practical basis for prevention. Prevention campaigns should be merged with etiologic research whenever possible and close collaboration among scientists working in public health and epidemiology and those based in genetics and biology would facilitate development of an appropriately balanced strategy. (C) 1993 Wiley-Liss, Inc. | 3366 | NA | NA | NA | no | no |
| 3366 | 2017 | NA | Barve, MP; Haware, MP; Sainani, MN; Ranjekar, PK; Gupta, VS | 2001 | Potential of microsatellites to distinguish four races of Fusarium oxysporum f. sp ciceri prevalent in India | Fusalium oxysporum f. sp. ciceri, the causal agent of chickpea wilt, is an important fungal pathogen in India. Thirteen oligonucleotide probes complementary to microsatellite loci, in combination with 11 restriction enzymes, were used to assr:ss the potential of such markers to study genetic variability in four Indian races of the pathogen. Hybridisation patterns, which were dependent upon both the restriction enzyme and oligonucleotide probe used, revealed the presence of different repeat motifs in the F. oxysporum f. sp. ciceri genome. Among the restriction enzymes used, hexa-cutting enzymes were more informative than tetra- and penta-cutting: enzymes, whereas tetranucleotide and trinucleotide repeats yielded better hybridisation patterns than dinucleotide repeats. Dependent upon the levels of polymorphism detected, we have identified (AGT)(5), (ATC)(5) and (GATA)(4) as the best fingerprinting probes for the F: oxysporum sporum f. sp. ciceri races. The distribution of microsatellite repeats in the genome revealed races 1 and 4 to be closely related at a similarity index value of 76.6%, as compared to race 2 at a similarity value of 67.3%; race 3 was very distinct at a similarity value of 26.7%. Our study demonstrates the potential of oligonucleotide probes for fingerprinting and studying variability in the F oxysporum f. sp. ciceri races and represents a step towards the identification of potential race diagnostic markers. | 3367 | NA | NA | NA | no | no |
| 3367 | 2017 | NA | Van Cleve, J; Feldman, MW; Lehmann, L | 2010 | How Demography, Life History, and Kinship Shape the Evolution of Genomic Imprinting | How phenomena like helping, dispersal, or the sex ratio evolve depends critically on demographic and life-history factors. One phenotype that is of particular interest to biologists is genomic imprinting, which results in parent-of-origin-specific gene expression and thus deviates from the predictions of Mendel’s rules. The most prominent explanation for the evolution of genomic imprinting, the kinship theory, originally specified that multiple paternity can cause the evolution of imprinting when offspring affect maternal resource provisioning. Most models of the kinship theory do not detail how population subdivision, demography, and life history affect the evolution of imprinting. In this work, we embed the classic kinship theory within an island model of population structure and allow for diverse demographic and life-history features to affect the direction of selection on imprinting. We find that population structure does not change how multiple paternity affects the evolution of imprinting under the classic kinship theory. However, if the degree of multiple paternity is not too large, we find that sex-specific migration and survival and generation overlap are the primary factors determining which allele is silenced. This indicates that imprinting can evolve purely as a result of sex-related asymmetries in the demographic structure or life history of a species. | 3368 | NA | NA | NA | no | no |
| 3368 | 2017 | NA | Xu, DL; Mock, R; Kinard, G; Li, RH | 2011 | Molecular analysis of the complete genomic sequences of four isolates of Gooseberry vein banding associated virus | The presence of Gooseberry vein banding associated virus (GVBaV), a badnavirus in the family Caulimoviridae, is strongly correlated with gooseberry vein banding disease in Ribes spp. In this study, full-length genomic sequences of four GVBaV isolates from different hosts and geographic regions were determined to be 7649-7663 nucleotides. These isolates share identities of 96.4-97.3% for the complete genomic sequence, indicating low genetic diversity among them. The GVBaV genome contains three open reading frames (ORFs) on the plus strand that potentially encode proteins of 26, 16, and 216 kDa. The size and organization of GVBaV ORFs 1-3 are similar to those of most other badnaviruses. The putative amino acid sequence of GVBaV ORF 3 contained motifs that are conserved among badnavirus proteins including aspartic protease, reverse transcriptase, and ribonuclease H. The highly conserved putative plant tRNA(met)-binding site is also present in the 935-bp intergenic region of GVBaV. The identities of the genomic sequences of GVBaV and other badnaviruses range from 49.1% (Sugarcane bacilliform Mor virus) to 51.7% (Pelargonium vein banding virus, PVBV). Phylogenetic analysis using the amino acid sequence of the ORF 3 putative protein shows that GVBaV groups most closely to Dioscorea bacilliform virus, PVBV, and Taro bacilliform virus. These results confirm that GVBaV is a pararetrovirus of the genus Badnavirus in the family Caulimoviridae. | 3369 | NA | NA | NA | no | no |
| 3369 | 2017 | NA | Fan, BC; Jiao, D; Zhao, XN; Pang, FJ; Xiao, Q; Yu, ZY; Mao, AH; Guo, RL; Yuan, WZ; Zhao, PD; He, KW; Li, B | 2017 | Characterization of Chinese Porcine Epidemic Diarrhea Virus with Novel Insertions and Deletions in Genome | Outbreaks of porcine epidemic diarrhoea virus (PEDV) have caused great economic losses to the global pig industry. PEDV strains with variants in the spike (S) gene have been reported in several countries. To better understand the molecular epidemiology and genetic diversity of PEDV field isolates, in this study, we characterised the complete genome sequence of a novel PEDV variant JSCZ1601 from a outbreak in China in 2016. The PEDV isolate was 28,033 nucleotides (nt) in length without the polyadenylated sequences. Phylogenetic analysis based on the full- length genome sequence of JSCZ1601 grouped it with the pandemic variants determined post-2010 into group 2 (G2). However, the S gene of JSCZ1601 formed a new subgroup separated from the subgroups containing the other G2 strains. Comparative analysis of the amino acids encoded by the S genes revealed the N-terminal of the deduced JSCZ1601 S protein had a novel two-amino-acid deletion (N58 and S59) compared with all identified genogroups. Further, compared with the reference strains, a `G’ insertion was detected in the 5’ terminal of the 5’UTR of the JSCZ1601. The animal experiment revealed that this strain was high pathogenic to neonatal pigs. Taken together, a PEDV strain with the new molecular characterizations and phylogenies was found in mainland China. It is necessary to strengthen the monitoring of PEDV variations. | 3370 | NA | NA | NA | no | no |
| 3370 | 2017 | NA | Lane, CE; Khan, H; Fong, A; Theophilou, S; Archibald, JM; Archibald, JM | 2006 | Insight into the diversity and evolution of the cryptomonad nucleomorph genome | The cryptomonads are an enigmatic group of marine and freshwater unicellular algae that acquired their plastids through the engulfment and retention of a eukaryotic (“secondary”) endosymbiont. Together with the chlorarachniophyte algae, the cryptomonads are unusual in that they have retained the nucleus of their endosymbiont in a miniaturized form called a nucleomorph. The nucleomorph genome of the cryptomonad Guillardia theta has been completely sequenced and with only three chromosomes and a total size of 551 kb, is a model of nuclear genome compaction. Using this genome as a reference, we have investigated the structure and content of nucleomorph genomes in a wide range of cryptomonad algae. In this study, we have sequenced nine new cryptomonad nucleomorph 18S ribosomal DNA (rDNA) genes and four heat shock protein 90 (hsp90) gene fragments, and using pulsed-field gel electrophoresis and Southern hybridizations, have obtained nucleomorph genome size estimates for nine different species. We also used long-range polymerase chain reaction to obtain nucleomorph genomic fragments from Hanusia phi CCMP325 and Proteomonas sulcata CCMP704 that are syntenic with the subtelomeric region of nucleomorph chromosome I in G. theta. Our results indicate that (1) the presence of three chromosomes is a common feature of the nucleomorph genomes of these organisms, (2) nucleomorph genome size varies dramatically in the cryptomonads examined, (3) unidentified cryptomonad species CCMP1178 has the largest nucleomorph genome identified to date at similar to 845 kb, (4) nucleomorph genome size reductions appear to have occurred multiple times independently during cryptomonad evolution, (5) the relative positions of the 18S rDNA, ubc4, and hsp90 genes are conserved in three different cryptomonad genera, and (6) interchromosomal recombination appears to be rapidly changing the size and sequence of a repetitive subtelomeric region of the nucleomorph genome between the 18S rDNA and ubc4 loci. These results provide a glimpse into the genetic diversity of nucleomorph genomes in cryptomonads and set the stage for more comprehensive sequence-based studies in closely and distantly related taxa. | 3371 | NA | NA | NA | no | no |
| 3371 | 2017 | NA | Piao, Y; Li, Y; Xu, Q; Liu, JW; Xing, CZ; Xie, XD; Yuan, Y | 2015 | Association of MTOR and AKT Gene Polymorphisms with Susceptibility and Survival of Gastric Cancer | Background The phosphoinositide 3-kinase (PI3K)/protein kinase B (PKB, AKT)/mammalian target of rapamycin (mTOR) signaling pathway plays a critical role in angiogenesis and cell growth, proliferation, metabolism, migration, differentiation, and apoptosis. Genetic diversity in key factors of this pathway may influence protein function and signal transduction, contributing to disease initiation and progression. Studies suggest that MTOR rs1064261 and AKT rs1130233 polymorphisms are associated with risk and/or prognosis of multiple cancer types. However, this relationship with gastric cancer (GC) remains unclear. The aim of this study was to investigate the role of MTOR and AKT polymorphisms in the risk and prognosis of GC. Methods The Sequenom MassARRAY platform was used to genotype 1842 individuals for MTOR rs1064261 T -> C and AKT rs1130233 G -> A polymorphisms. ELISA was used to detect Helicobacter pylori antibodies in serum. Immunohistochemical analysis was used to detect total and phosphorylated MTOR and AKT proteins. Results The MTOR rs1064261 (TC+CC) genotype and the AKT rs1130233 (GA+AA) genotype were associated with increased risk of GC in men (P = 0.049, P = 0.030). In H. pylori-negative individuals, the AKT rs1130233 GA and (GA+AA) genotypes were related to increased risk of atrophic gastritis (AG; P = 0.012, P = 0.024). Notably, the AKT rs1130233 (GA+AA) genotype demonstrated significant interactions with H. pylori in disease progression from healthy controls (CON) to AG (P = 0.013) and from AG to GC (P = 0.049). Additionally, for individuals with the AKT rs1130233 variant, those in the H. pylori-positive group had higher levels of phosphorylated AKT (p-AKT) expression. The AKT rs1130233 genotype was found to be associated with clinicopathological parameters including lymph node metastasis and alcohol drinking (P<0.05). Conclusion MTOR rs1064261 and AKT rs1130233 polymorphisms were associated with increased GC risk in males and increased AG risk in H. pylori-negative individuals. A significant interaction existed between the AKT rs1130233 genotype and H. pylori infection in CON -> AG -> GC disease progression. The AKT rs1130233 genotype influenced p-AKT protein expression in H. pylori-infected individuals. | 3372 | NA | NA | NA | no | no |
| 3372 | 2017 | NA | Edwards, ME; Armbruster, WS; Elias, SE | 2014 | Constraints on post-glacial boreal tree expansion out of far-northern refugia | AimTo use a variety of data sources to infer how northern boreal trees recovered their range upon deglaciation and/or Holocene warming. LocationScandinavia, Alaska/north-west Canada (eastern Beringia). MethodsMapped fossil occurrences for Picea (spruce) were assessed against available palaeoenvironmental and phylogeographic information. ResultsFor Scandinavia, Last Glacial Maximum (LGM) evidence of Picea is confined to one DNA record, but late-glacial and early-Holocene records include scattered macrofossils. Holocene pollen data show a clear east-west increase to high values. A haplotype unique to the Scandinavian Peninsula is recognized. For eastern Beringia pre- and post-LGM macrofossils occur, but the LGM fossil record comprises only scattered low pollen values. Early Holocene pollen values increase markedly c. 11 cal yr bp (north-west Canada) and c. 10kcal yr bp (central Alaska). Also at this time three sites on the Bering Land Bridge indicate the presence of Picea where it is now absent. Several unique regional haplotypes were recorded; while most are rare one is common in some modern populations. Main conclusionsSmall Picea populations probably occurred in pre-Holocene Scandinavia, but pollen patterns argue against immediate expansion with the onset of warmer conditions. Despite relatively weak fossil evidence, refugial populations are also probable in eastern Beringia, particularly given the extent of unglaciated terrain. Post-glacial pollen patterns are more nuanced, suggesting two spatially and temporally distinct expansions, one possibly consistent with a unique central Alaskan haplotype, and subsequent westerly filling-in’. The presence of macrofossils and/or neutral markers does not require that current northern populations are derived primarily from refugial ones, particularly where pollen patterns show delayed directional expansion of large populations though time. Refugial populations initially responded weakly to major post-glacial environmental change; if subject to genetic isolation and strong selection pressure they may have had little potential to do otherwise, instead being largely replaced by in-migrating populations with greater genetic diversity. | 3373 | NA | NA | NA | no | no |
| 3373 | 2017 | NA | Maccaferri, M; Ratti, C; Rubies-Autonell, C; Vallega, V; Demontis, A; Stefanelli, S; Tuberosa, R; Sanguineti, MC | 2011 | Resistance to Soil-borne cereal mosaic virus in durum wheat is controlled by a major QTL on chromosome arm 2BS and minor loci | Soil-borne cereal mosaic (SBCM) is a viral disease, which seriously affects hexaploid as well as tetraploid wheat crops in Europe. In durum wheat (Triticum durum Desf.), the elite germplasm is characterized by a wide range of responses to SBCMV, from susceptibility to almost complete resistance. In this study, the genetic analysis of SBCMV resistance was carried out using a population of 181 durum wheat recombinant inbred lines (RILs) obtained from Meridiano (resistant) x Claudio (moderately susceptible), which were profiled with SSR and DArT markers. The RILs were characterized for SBCMV response in the field under severe and uniform SBCMV infection during 2007 and 2008. A wide range of disease reactions (as estimated by symptom severity and DAS-ELISA) was observed. A large portion of the variability for SBCMV response was explained by a major QTL (QSbm.ubo-2BS) located in the distal telomeric region of chromosome 2BS near the marker triplet Xbarc35-Xwmc661-Xgwm210, with R (2) values ranging from 51.6 to 91.6%. The favorable allele was contributed by Meridiano. Several QTLs with minor effects on SBCMV response were also detected. Consistently with the observed transgressive segregation, the resistance alleles at minor QTLs were contributed by both parents. The presence and effects of QSbm.ubo-2BS were validated through association mapping in a panel of 111 elite durum wheat accessions. | 3374 | NA | NA | NA | no | no |
| 3374 | 2017 | NA | Hennige, SJ; Morrison, CL; Form, AU; Buscher, J; Kamenos, NA; Roberts, JM | 2014 | Self-recognition in corals facilitates deep-sea habitat engineering | The ability of coral reefs to engineer complex three-dimensional habitats is central to their success and the rich biodiversity they support. In tropical reefs, encrusting coralline algae bind together substrates and dead coral framework to make continuous reef structures, but beyond the photic zone, the cold-water coral Lophelia pertusa also forms large biogenic reefs, facilitated by skeletal fusion. Skeletal fusion in tropical corals can occur in closely related or juvenile individuals as a result of non-aggressive skeletal overgrowth or allogeneic tissue fusion, but contact reactions in many species result in mortality if there is no ‘self-recognition’ on a broad species level. This study reveals areas of ‘flawless’ skeletal fusion in Lophelia pertusa, potentially facilitated by allogeneic tissue fusion, are identified as having small aragonitic crystals or low levels of crystal organisation, and strong molecular bonding. Regardless of the mechanism, the recognition of ‘self’ between adjacent L. pertusa colonies leads to no observable mortality, facilitates ecosystem engineering and reduces aggression-related energetic expenditure in an environment where energy conservation is crucial. The potential for self-recognition at a species level, and subsequent skeletal fusion in framework-forming cold-water corals is an important first step in understanding their significance as ecological engineers in deep-seas worldwide. | 3375 | NA | NA | NA | no | no |
| 3375 | 2017 | NA | Olmo, C; Fandos, D; Armengol, X; Ortells, R | 2015 | Combining field observations and laboratory experiments to assess the ecological preferences of Tanymastix stagnalis (L., 1758) (Crustacea, Branchiopoda) in Mediterranean temporary ponds | Malladas de El Saler is a mosaic of dune slacks scattered along a coastal sand bar in the Mediterranean region. Active populations of the fairy shrimp T. stagnalis are exclusively present in one of these ponds. We combined field observations and laboratory experiments to find the ecological requirements of T. stagnalis that determine this habitat exclusiveness and the possibility of future establishment in nearby ponds. Conductivity revealed as a determinant ecological constraint. In the field, the species was restricted to conductivity levels below 1.28 mS cm(-1) and in experimental microcosms, hatchability, survival and reproductive potential were detrimental from 2.9 mS cm(-1). The observation of life history characteristics from the experimental individuals confirmed the importance of the unpredictable character of Mediterranean temporary ponds in shaping life history strategies, as they hatched early, matured rapidly and produced a high number of cysts. We discuss the essential steps during colonization that may explain the presence or absence in our ponds, and revealed a harsh future for the species if the current conditions of habitat deterioration persist. We suggest several conservation measures, including a cyst bank storage or/and adult translocations to nearby suitable ponds. These measures will ensure (1) the preservation of genetic diversity and (2) healthy metapopulation dynamics with recolonization rates compensating for local extinctions. | 3376 | NA | NA | NA | no | no |
| 3376 | 2017 | NA | Thomas, RJ; Rajesh, MK; Kalavathi, S; Krishnakumar, V; George, DJ; Jose, M; Nair, RV | 2013 | Analysis of genetic diversity in coconut and its conservation in root (wilt) disease affected areas of Kerala: A community participatory approach | Root (wilt) disease is a major constraint to coconut production in Kerala State. Conserving ecotypes with resistance or tolerance to the disease on a community basis is essential to sustain coconut production in the root (wilt) disease prevalent areas. Three communities’ viz., Pathiyoor and Devikulangara (Alappuzha District) and Thodiyoor (Kollam District) were selected and a survey was conducted with the participation of stakeholders, to characterize the local coconut ecotypes. Six ecotypes comprising of four tails and two dwarfs were identified and morphological data revealed that the local ‘Jappanan’ ecotype closely resembled Evoor Green Tall ecotype. Simple Sequence Repeat (SSR) analysis in 90 selected coconut palms representing the six ecotypes using 14 markers indicated that the observed heterozygosity was higher in tall ecotypes (0.179-0.365) compared to the dwarfs (0.03-0.07). Lower values for observed heterozygosity compared to the expected heterozygosity in tall ecotypes are indications of genetic basis for disease resistance observed in disease-free. mother palms. Molecular characterization helped in identifying diverse coconut ecotypes having application in production of vigorous hybrids. In the dendrogram constructed using nut character data, three of the tall ecotypes (Green Tall, Brown Tall and Brick Red Tall) clustered together whereas ‘Jappanan’ clustered separately. Mantel’s correlation test using the ZT software revealed significant correlation (0.96) between the SSR data and morphological data. | 3377 | NA | NA | NA | no | no |
| 3377 | 2017 | NA | Davis, EB | 2005 | Comparison of climate space and phylogeny of Marmota (Mammalia : Rodentia) indicates a connection between evolutionary history and climate preference | Palaeobiologists have investigated the evolutionary responses of extinct organisms to climate change, and have also used extinct organisms to reconstruct palaeoclimates. There is evidence of a disconnection between climate change and evolution that suggests that organisms may not be accurate palaeoclimate indicators. Here, marmots (Marmota sp.) are used as a case study to examine whether similarity of climate preferences is correlated with evolutionary relatedness of species. This study tests for a relationship between phylogenetic distance and ‘climate distance’ of species within a clade. There should be a significant congruence between maximum likelihood distance and standardized Euclidian distance between climates if daughter species tend to stay in environments similar to parent species. Marmots make a good test case because there are many extant species, their phylogenies are well established and individual survival is linked to climatic factors. A Mantel test indicates a significant correlation between climate and phylogenetic distance matrices, but this relationship explains only a small fraction of the variance (regression R-2 = 0.114). These results suggest that (i) closely related species of marmots tend to stay in similar environments; (ii) marmots may be more susceptible than many mammals to global climate change; and (iii) because of the considerable noise in this system, the correlation cannot be used for detailed palaeoclimate reconstruction. | 3378 | NA | NA | NA | no | no |
| 3378 | 2017 | NA | Neukirch, R; Bovier, A | 2017 | Survival of a recessive allele in a Mendelian diploid model | In this paper we analyse the genetic evolution of a diploid hermaphroditic population, which is modelled by a three-type nonlinear birth-and-death process with competition and Mendelian reproduction. In a recent paper, Collet et al. (J Math Biol 67(3):569-607, 2013) have shown that, on the mutation time-scale, the process converges to the Trait-Substitution Sequence of adaptive dynamics, stepping from one homozygotic state to another with higher fitness. We prove that, under the assumption that a dominant allele is also the fittest one, the recessive allele survives for a time of order at least , where K is the size of the population and alpha > 0. | 3379 | NA | NA | NA | no | no |
| 3379 | 2017 | NA | Huntley, B; Allen, JRM; Barnard, P; Collingham, YC; Holliday, PR | 2013 | Species distribution models indicate contrasting late-Quaternary histories for Southern and Northern Hemisphere bird species | Aim To test the following hypotheses: that Fynbos species had more extensive distributions at the Last Glacial Maximum (LGM), extending onto the exposed Agulhas Plain’; that genetically distinct British taxa could have persisted through the LGM on adjacent areas of exposed shelf. Location Southern Africa; Europe. Methods Climatic response surfaces were fitted for 14 Fynbos and two European birds. These models were used to hindcast species distributions for palaeoclimates simulated using a fully coupled atmosphereocean general circulation model. LGM annual net primary productivity (ANPP) of two plant functional types upon which the European birds depend were simulated using a dynamic vegetation model and compared with potential LGM bird distributions. Results Fynbos birds’ potential LGM distributions mostly extended southwards onto the exposed Agulhas Plain and were consistently more extensive than at present. This contrasts with conventional expectations for temperate species based upon Northern Hemisphere evidence. North-west European taxa potentially had LGM ranges on exposed shelf and ice-free areas to the west and south-west of the British Isles ice sheet. This is consistent with available genetic evidence, supporting the long-standing hypothesis that these taxa persisted through the LGM in this region. In both regions, results allow the generation of new testable hypotheses about species evolution and palaeobiogeography. Main conclusions Bird species found today in the Fynbos probably had more extensive ranges under glacial conditions, with the potential ranges of many species extending onto the Agulhas Plain. Bird taxa restricted today to the British Isles probably survived the LGM with limited distributions on exposed shelf and ice-free areas south-west of the British Isles ice sheet. Areas of shelf exposed under glacial conditions are likely to have been important components of glacial distributions of species in both the Northern and Southern Hemisphere. The contrasting history of Northern and Southern Hemisphere species has important conservation implications, especially in relation to conserving intra-specific genetic diversity. | 3380 | NA | NA | NA | no | no |
| 3380 | 2017 | NA | Berggren, A | 2005 | Effect of propagule size and landscape structure on morphological differentiation and asymmetry in experimentally introduced Roesel’s bush-crickets | Conservation efforts involving introductions, reintroductions, and translocations of populations have an inherent and inescapable problem of small initial populations. Small founding populations are likely to have a small proportion of the genetic variability carried by the original population. This may manifest phenotypically through changes in individual morphology, such as decreased body size, increased degree of fluctuating asymmetry, or changing susceptibility to environmental stressors. I investigated the effects of population and landscape variables on the morphology and fluctuating asymmetry in individual bush-crickets by examining 584 individual Roesel’s bush-crickets (Metrioptera roeseli) from 29 established populations from different propagule sizes that were introduced in areas previously unoccupied by the species. The introduction sites were in landscapes similar to where the species occurs naturally, but sites differed in connectivity and amount of surrounding suitable habitat. Individuals were caught up to 9 years after the initial introduction, and five different morphological traits were measured. All introduced individuals originated from the same population and individuals from this source population were also collected for comparison in the analyses. Male body weight and female body length were positively affected by initial population size and degree of connectivity of the introduction patch. Isolation also affected fluctuating asymmetry in male tibias, with a higher degree of asymmetry in males that came from more isolated populations. In a relatively short time period, 1 was able to detect the effect of isolation and small population sizes on morphology and asymmetry. Small propagule sizes and habitat isolation are both likely to have resulted in decreased genetic diversity, the latter by reducing population sizes through decreased survival. These results show the importance of both large propagule sizes and good connectivity of habitats when introducing populations. The differences between the sexes in response to the variables examined also indicate that studies on morphology and fluctuating asymmetry need to consider males and females separately to avoid inaccurate generalizations of the state of the population. | 3381 | NA | NA | NA | no | no |
| 3381 | 2017 | NA | La Mantia, J; Klapste, J; El-Kassaby, YA; Azam, S; Guy, RD; Douglas, CJ; Mansfield, SD; Hamelin, R | 2013 | Association Analysis Identifies Melampsora xcolumbiana Poplar Leaf Rust Resistance SNPs | Populus species are currently being domesticated through intensive time- and resource-dependent programs for utilization in phytoremediation, wood and paper products, and conversion to biofuels. Poplar leaf rust disease can greatly reduce wood volume. Genetic resistance is effective in reducing economic losses but major resistance loci have been race-specific and can be readily defeated by the pathogen. Developing durable disease resistance requires the identification of non-racespecific loci. In the presented study, area under the disease progress curve was calculated from natural infection of Melampsora xcolumbiana in three consecutive years. Association analysis was performed using 412 P. trichocarpa clones genotyped with 29,355 SNPs covering 3,543 genes. We found 40 SNPs within 26 unique genes significantly associated (permutated P<0.05) with poplar rust severity. Moreover, two SNPs were repeated in all three years suggesting non-race-specificity and three additional SNPs were differentially expressed in other poplar rust interactions. These five SNPs were found in genes that have orthologs in Arabidopsis with functionality in pathogen induced transcriptome reprogramming, Ca2+/calmodulin and salicylic acid signaling, and tolerance to reactive oxygen species. The additive effect of non-R gene functional variants may constitute high levels of durable poplar leaf rust resistance. Therefore, these findings are of significance for speeding the genetic improvement of this long-lived, economically important organism. | 3382 | NA | NA | NA | no | no |
| 3382 | 2017 | NA | Hakli, T; Romppanen, EL; Hiltunen, M; Helisalmi, S; Punnonen, K; Heinonen, S | 2003 | Plasminogen activator inhibitor-1 polymorphism in women with pre-eclampsia | We determined whether or not genetic variability in the promoter region of the gene encoding plasminogen activator inhibitor-1 (PAI1) contributes to individual differences in susceptibility to the development of preeclampsia. The study involved 133 preeclamptic and 115 healthy control pregnant women who were genotyped for a single-nucleotide insertion/deletion polymorphism (4G/5G) at position -675 in the PAI1 gene promoter. Furthermore, the frequencies of the alleles in the general middle-aged population are presented for comparison. Chi-square analysis was used to assess genotype and allele frequency differences between preeclamptic women and controls. A similar allelic distribution of PAI1 4G/5G polymorphism was observed in the two groups, with the frequency of the variant 4G allele being 50.4% in the preeclampsia group and 54.3% in the control group (p=0.377; OR=0.85, 95% CI=0.60-1.22). Accordingly, the genotype distribution of the PAI1 4G/5G polymorphism in the preeclamptic and control groups was found to be similar (p=0.68). Overall, this genotype data on fertile women is almost identical to that in the general middle-aged Finnish population. The 4G/5G polymorphism of the PAI1 gene promoter is unlikely to be a major genetic predisposing factor as regards preeclampsia in subjects from eastern Finland. These results are not suggestive of an important contribution of the PAI1 genotype on preeclampsia across populations. | 3383 | NA | NA | NA | no | no |
| 3383 | 2017 | NA | Fjerdingstad, EJ; Gertsch, PJ; Keller, L | 2002 | Why do some social insect queens mate with several males? Testing the sex-ratio manipulation hypothesis in Lasius niger | Although multiple mating most likely increases mortality risk for social insect queens and lowers the kin benefits for nonreproductive workers, a significant proportion of hymenopteran queens mate with several males. It has been suggested that queens may mate multiply as a means to manipulate sex ratios to their advantage. Multiple paternity reduces the extreme relatedness value of females for workers, selecting for workers to invest more in males. In populations with female-biased sex ratios, queens heading such male-producing colonies would achieve a higher fitness. We tested this hypothesis in a Swiss and a Swedish population of the ant Lasius niger. There was substantial and consistent variation in queen mating frequency and colony sex allocation within and among populations, but no evidence that workers regulated sex allocation in response to queen mating frequency; the investment in females did not differ among paternity classes. Moreover, population-mean sex ratios were consistently less female biased than expected under worker control and were close to the queen optimum. Queens therefore had no incentive to manipulate sex ratios because their fitness did not depend on the sex ratio of their colony. Thus, we found no evidence that the sex-ratio manipulation theory can explain the evolution and maintenance of multiple mating in L. niger. | 3384 | NA | NA | NA | no | no |
| 3384 | 2017 | NA | Kozubikova, E; Viljamaa-Dirks, S; Heinikainen, S; Petrusek, A | 2011 | Spiny-cheek crayfish Orconectes limosus carry a novel genotype of the crayfish plague pathogen Aphanomyces astaci | The oomycete Aphanomyces astaci causes mass mortalities of European crayfish. Different species of North American crayfish, original hosts of this parasite, seem to carry different strains of A. astaci. So far, four distinct genotype groups have been recognised using Random Amplification of Polymorphic DNA (RAPD-PCR). We succeeded in isolating A. astaci from the spiny-cheek crayfish Orconectes limosus, a widespread invader in Europe, and confirmed that this species carries a novel A. astaci genotype. Improving knowledge on the diversity of this parasite may facilitate identification of genotypes in mass mortalities of European crayfish, thus tracing the sources of infection. (C) 2011 Elsevier Inc. All rights reserved. | 3385 | NA | NA | NA | no | no |
| 3385 | 2017 | NA | van Bruggen, R; Gualtieri, C; Iliescu, A; Cheepsunthorn, CL; Mungkalasut, P; Trape, JF; Modiano, D; Sirima, BS; Singhasivanon, P; Lathrop, M; Sakuntabhai, A; Bureau, JF; Gros, P | 2015 | Modulation of Malaria Phenotypes by Pyruvate Kinase (PKLR) Variants in a Thai Population | Pyruvate kinase (PKLR) is a critical erythrocyte enzyme that is required for glycolysis and production of ATP. We have shown that Pklr deficiency in mice reduces the severity (reduced parasitemia, increased survival) of blood stage malaria induced by infection with Plasmodium chabaudi AS. Likewise, studies in human erythrocytes infected ex vivo with P. falciparum show that presence of host PK-deficiency alleles reduces infection phenotypes. We have characterized the genetic diversity of the PKLR gene, including haplotype structure and presence of rare coding variants in two populations from malaria endemic areas of Thailand and Senegal. We investigated the effect of PKLR genotypes on rich longitudinal datasets including haematological and malaria-associated phenotypes. A coding and possibly damaging variant (R41Q) was identified in the Thai population with a minor allele frequency of similar to 4.7%. Arginine 41 (R41) is highly conserved in the pyruvate kinase family and its substitution to Glutamine (R41Q) affects protein stability. Heterozygosity for R41Q is shown to be associated with a significant reduction in the number of attacks with Plasmodium falciparum, while correlating with an increased number of Plasmodium vivax infections. These results strongly suggest that PKLR protein variants may affect the frequency, and the intensity of malaria episodes induced by different Plasmodium parasites in humans living in areas of endemic malaria. | 3386 | NA | NA | NA | no | no |
| 3386 | 2017 | NA | Tarasjev, A | 2005 | Variation in survival of Iris pumila L. juvenile plants grown in two nutrient regimes | I analyzed variability of early life survival on 881 Iris pumila juvenile plants in their prereproductive period. Seedlings were produced by applying half-sib mating scheme on clones originating from two Deliblato Sand populations that were accommodated to common garden conditions. Nineteen clones served as pollen donors (sires) and the other 90 clones served as pollen recipients (dames). Seedlings were grown in two nutrient levels (full strength and 1/10 of full-strength Hoagland solution). While I failed to detect significant mean nutrient level effect as well as significant between population differentiation for prereproductive survival, a statistically significant additive genetic variability for this ultimate prereproductive measure of fitness was detected. | 3387 | NA | NA | NA | no | no |
| 3387 | 2017 | NA | Biron, DG; Coderre, D; Boivin, G; Brunel, E; Nenon, JP | 2002 | Genetic variability and expression of phenological and morphological differences in populations of Delia radicum (Diptera : Anthomyiidae) | In this study, survival to adult stage, duration of development of the immature stages, egg micromorphology, DNA polymorphism, and reproductive compatibility were measured for early- and late-emerging phenotypes of Delia radicum Linneaus to determine whether both phenotypes had evolved differences other than the duration of puparial development and to find the most likely genetic system controlling the expression of both phenotypes, Survival to adult stage was not significantly different between the early- and late-emerging phenotypes, Random amplified polymorphic DNA (RAPD) primers tested suggest that it is possible to distinguish an early-emerging fly from a late-emerging fly. Furthermore, the results suggest that the early- and late-emerging phenotypes differ not only in the timing of adult emergence but also in their egg structure (egg micromorphology) and in their larval and puparial mortality. These two phenotypes are not reproductively or ecologically isolated. The genetic system controlling the expression of early and late emergers in a population of D. radicum is probably an adaptive strategy reducing predator and parasitoid pressures, optimizing resource utilization, and ensuring survival of D. radicum during atypical winters. This strategy could eventually lead to temporal sympatric speciation if there are changes in a few key loci responsible for host plant selection and fitness on a new host. | 3388 | NA | NA | NA | no | no |
| 3388 | 2017 | NA | Mashini, AG; Parsa, S; Mostafavi, PG | 2015 | Comparison of Symbiodinium populations in corals from subtidal region and tidal pools of northern coasts of Hengam Island, Iran | Coral reefs are integral part of tropical ecosystems and obligatory symbiosis with the genus Symbiodinium has a vital role in resistance and survival of the holobiont Nine different genetic clades (A to I) have been discovered by molecular techniques. Members of Symbiodinium are ecologically and biogeographically diverse and their distribution patterns are determined by temperature, irradiance, depth, season and latitude however stress-tolerant species might be detected within each clade. Samples of four coral species from subtidal region in the north of Hengam Island, Persian Gulf (PG) and its tidal pools were analyzed by internal transcribed spacer of ribosomal DNA and by the aid of Denaturing Gradient Gel Electrophoresis (DGGE) to compare the differences in populations of their symbionts. The results showed that some subtidal colonies of one species harbored less tolerant subtypes of Symbiodinium subclade C7. Nonetheless, clade D Symbiodinium was the most dominant symbiotic clade in both tidal pool and subtidal coral colonies. A novel subtype of subclade D1 Symbiodinium was also detected in some coral colonies and was inferred as subclade D1.PG Symbiodinium. The prevalence of clade D Symbiodinium might reflect corals response to extreme environmental stresses of tidal pools and the northern PG itself that coral inhabitants have to cope with. (C) 2015 Elsevier B.V. All rights reserved. | 3389 | NA | NA | NA | no | no |
| 3389 | 2017 | NA | Ricklefs, RE | 2015 | Intrinsic dynamics of the regional community | Patterns of diversity within large regional biotas express the outcomes of processes, operating on both regional and local scales, that influence evolutionary diversification as well as the distribution and abundance of species. Regional analyses of species distributions suggest that neither ecological sorting of species based on their adaptations to the physical environment, nor interactions between competing species, adequately explain patterns of species richness. Potentially competing species appear to utilise broadly overlapping resources with similar proficiency. Phylogenetic and phylogeographic analyses reveal that species abundances and distributions within regions vary independently of evolutionary relationship. This implies the existence of dynamic, species-specific controls on population growth, as could be applied by specialised pathogens or other antagonists. Here, I argue that the changing balance of coevolved interactions between hosts and their antagonists shapes the distribution and abundance of individual host populations as well as patterns of local species richness. Geographical expansion creates allopatric populations and thereby could promote diversification; contraction ultimately leads to extinction. This taxon-cycle dynamic links regional diversity and distribution to intrinsic biological interactions independently of extrinsic ecological conditions. These hypotheses emphasise the central importance of investigating the impacts of pathogens on species abundance and distribution, and the potential consequences of coevolutionary changes in pathogen-host relationships for species formation and extinction. | 3390 | NA | NA | NA | no | no |
| 3390 | 2017 | NA | King, T; Chamberlan, C; Courage, A | 2014 | Assessing reintroduction success in long-lived primates through population viability analysis: western lowland gorillas Gorilla gorilla gorilla in Central Africa | The use of population modelling has become an increasingly common tool in reintroduction planning and assessment. Although initial reintroduction success is often measured by quantifying post-release survival and reproduction, longer-term success is best assessed through measurements of population viability. Here we develop a population model capable of providing useful results for influencing management of a reintroduction programme for a long-lived and slow-reproducing primate, the western lowland gorilla Gorilla gorilla gorilla. We used post-release monitoring data from two reintroduced populations in the Bateke Plateau region of Congo and Gabon, complemented with published data on wild and captive populations, to develop a population model using Vortex. Sensitivity testing illustrated that the model was highly sensitive to changes in the input parameters for annual birth rates, the number of lethal equivalents, and for female annual mortality rates, especially for adults. The results of the population viability analysis suggested that the reintroduced gorilla populations have a reasonable chance of persistence (>90% over 200 years) but illustrated that reinforcement of the populations could significantly improve probabilities of population persistence and retention of genetic diversity. Equally, catastrophic events could have significant negative impacts. Continued monitoring of the populations should allow refinement of the model, improving confidence in its predictions and its relevance to decision-making. | 3391 | NA | NA | NA | no | no |
| 3391 | 2017 | NA | Costa, MD; Sequeiros, J; Maciel, P | 2002 | Identification of three novel polymorphisms in the MJD1 gene and study of their frequency in the Portuguese population | Machado-Joseph disease (MJD) is an autosomal dominant neurodegenerative disorder of late onset, caused by the expansion of a (CAG)(n) tract in the MJD1 gene. Using BLAST2 sequences between known cDNA variants transcribed by the MJD1 gene and a clone of human genomic DNA, six possible unknown intragenic single-nucleotide polymorphisms (SNPs), at variable positions in the MJD1 gene, were identified. To confirm this, we studied a Portuguese control population. using polymerase chain reaction amplification and single-strand conformation polymorphism analysis for each potential SNP. For four of the possible polymorphisms there was no variability in our population. but the existence of three novel polymorphisms was confirmed: GTT(527)GTC(527), C-1178/A(1178), and A(1294)/G(1294). The polymorphism GTT(527)/GTC(527) (Val/Val) is located in the coding region, whereas C-1178/A(1178) and A(1294)/G(1294) are located in the 3’ noncoding region of cDNA variants of the MJD1 gene, MJD2-1 and MJD1-1. respectively. All these novel SNPs are in Hardy-Weinberg equilibrium. These intragenic polymorphisms can be useful for (1) the study of the origin of the MJD mutation(s), (2) the study of recombination events, (3) distinction of chromosomes with alleles of identical (CAG), size in genetic tests (homoallelism), (4) the study of genetic modifiers in the region flanking the MJD1 gene, and (5) association studies in other diseases. | 3392 | NA | NA | NA | no | no |
| 3392 | 2017 | NA | Vanden Broeck, A; Ceulemans, T; Kathagen, G; Hoffmann, M; Honnay, O; Mergeay, J | 2015 | Dispersal constraints for the conservation of the grassland herb Thymus pulegioides L. in a highly fragmented agricultural landscape | Species-rich grassland communities are one of the most important habitats for biodiversity and of high conservation priority in Europe. Restoration actions are mainly focused on the improvement of abiotic conditions, such as nutrient depletion techniques, and are generally based on the assumption that the target community will re-establish at the restored site when the target species exist in the neighborhood. Information on the contemporary seed dispersal range is therefore crucial to develop effective conservation measures. Here, we investigated the contemporary long-distance seed dispersal and genetic structure of the grassland herb Thymus pulegioides in an intensively managed agricultural landscape in Flanders (Northern Belgium). Assignment tests based on amplified fragment length polymorphisms revealed very low levels of effective seed dispersal between populations although seed availability and seed viability was not a limiting factor. The process of fragmentation has resulted in a high population differentiation and without further incoming gene flow the remnant populations are prone to further genetic erosion and perhaps extinction. Our findings illustrate that restoring suitable abiotic habitat conditions in the neighborhood of existing populations does likely not guarantee colonization for this grassland specialist. For the survival of the species, existing populations should be functionally connected and seed addition may be necessary for successful conservation to overcome dispersal-limitation. | 3393 | NA | NA | NA | no | no |
| 3393 | 2017 | NA | Antonelli, TS; Leischner, CL; Ososky, JJ; Hartstone-Rose, A | 2016 | The effect of captivity on the oral health of the critically endangered black-footed ferret (Mustela nigripes) | Black-footed ferrets (Mustela nigripes (Audubon and Bachman, 1851)), a North American species of mustelid, faced near extinction after westward expansion during the 20th century destroyed a majority of the population of prairie dogs (genus Cynomys Rafinesque, 1817), their primary food source. Fearing extinction of the black-footed ferret, the U.S. Fish and Wildlife Service captured the entire population between 1985 and 1987 and began a captive breeding program. While in captivity, the fertility and genetic diversity of the species was closely monitored; however, there is little information about other health consequences of this breeding program. For instance, the black-footed ferrets have been fed a diet that is very different than what they consume in the wild. How did the composition of this diet affect the oral health of these animals? An analysis of dentition of wild and captive black-footed ferrets reveals that calculus accumulation and periodontal diseases occurred with greater severity in captive black-footed ferrets, suggesting that such oral pathologies arose from the unnaturally soft diet fed to them. These findings offer insight into how mechanical properties of diet can affect oral health and how these dietary properties should be considered, not only in regard to the health of black-footed ferrets but also to the health of all mammals including humans. | 3394 | NA | NA | NA | no | no |
| 3394 | 2017 | NA | Li, H; Bai, JY; Wang, LY; Zeng, L; Shi, YS; Qiu, ZL; Ye, HH; Zhang, XF; Lu, QB; Kosoy, M; Liu, W; Cao, WC | 2013 | Genetic diversity of Bartonella quintana in macaques suggests zoonotic origin of trench fever | Bartonella quintana is a bacterium that causes a broad spectrum of diseases in humans including trench fever. Humans were previously considered to be the primary, if not the only, reservoir hosts for B.quintana. To identify the animal reservoir and extend our understanding of the ecological and evolutionary history of B.quintana, we examined blood samples from macaques and performed multilocus sequence typing (MLST) analysis. We demonstrated the prevalence of B.quintana infection was common in macaques from main primate centres in mainland China. Overall, 18.0% (59/328) of rhesus macaques and 12.7% (39/308) of cynomolgus macaques were found to be infected with B.quintana by blood culture and/or polymerase chain reaction. The infection was more frequently identified in juvenile and young monkeys compared with adult animals. In contrast with the relatively low level of sequence divergence of B.quintana reported in humans, our investigation revealed much higher genetic diversity in nonhuman primates. We identified 44 new nucleotide variable sites and 14 novel sequence types (STs) among the B.quintana isolates by MLST analysis. Some STs were found only in cynomolgus macaques, while some others were detected only in rhesus macaques, suggesting evidence of host-cospeciation, which were further confirmed by phylogenetic analysis and Splits decomposition analysis. Our findings suggest that trench fever may primarily be a zoonotic disease with macaques as the natural hosts. | 3395 | NA | NA | NA | no | no |
| 3395 | 2017 | NA | Chowdhury, MTI; Mian, MS; Mia, MAT; Rafii, MY; Latif, MA | 2015 | Agro-ecological variations of sheath rot disease of rice caused by Sarocladium oryzae and DNA fingerprinting of the pathogen’s population structure | To examine the impact of regional and seasonal variations on the incidence and severity of sheath rot, a major seed-borne disease of rice caused by Sarocladium oryzae, data on incidence and severity were collected from 27 selected fields in the Gazipur, Rangpur, Bogra, Chittagong, Comilla, Gopalgonj, Jessore, Manikgonj, and Bhola districts of Bangladesh in rain-fed and irrigated conditions. Cultural variability of 29 pathogen isolates obtained from 8 different locations was studied on potato dextrose agar (PDA) and genetic variability was determined by DNA fingerprinting using variable number tandem repeat-polymerase chain reaction markers. Overall, disease incidence and severity were higher in irrigated rice. Disease incidence and severity were highest in the Bhola district in rain-fed rice and lowest in irrigated rice. Mycelial growth of 29 representative isolates was found to vary on PDA and the isolates were divided into 6 groups. The range of the overall size of conidia of the selected isolates was 2.40-7.20 x 1.20-2.40 mu m. Analysis of the DNA fingerprint types of the 29 isolates of S. oryzae, obtained from the amplification reactions, revealed 10 fingerprinting types (FPTs) that were 80% similar. FPT-1 was the largest group and included 13 isolates (44.8%), while FPT-2 was the third largest group and included 3 isolates. Each of FPT-3, 4, 5, and 6 included only 1 isolate. We observed no relationship between cultural and genetic groupings. | 3396 | NA | NA | NA | no | no |
| 3396 | 2017 | NA | Brzeski, KE; Harrison, RB; Waddell, WT; Wolf, KN; Rabon, DR; Taylor, SS | 2015 | Infectious disease and red wolf conservation: assessment of disease occurrence and associated risks | Infectious diseases pose a significant threat to global biodiversity and may contribute to extinction. As such, establishing baseline disease prevalence in vulnerable species where disease could affect persistence is important to conservation. We assessed potential disease threats to endangered red wolves (Canis rufus) by evaluating regional (southeastern United States) disease occurrences in mammals and parasite prevalence in red wolves and sympatric coyotes (Canis latrans) in North Carolina. Common viral pathogens in the southeast region, such as canine distemper and canine parvovirus, and numerous widespread endoparasites could pose a threat to the red wolf population. The most prevalent parasites in red wolves and sympatric coyotes were heartworm (Dirofilaria immitis), hookworm (Ancylostoma caninum), and Ehrlichia spp.; several red wolves and coyotes were also positive for bacteria causing Lyme disease (Borrelia burgdorferi). Coyotes had a more species-rich parasite community than red wolves, suggesting they could harbor more parasites and act as a disease reservoir. Species identity and sex did not significantly affect parasite loads, but young canids were less likely to have heartworm and more likely to have high levels of endoparasites. Continued disease monitoring is important for red wolf recovery because low levels of genetic variability may compromise the wolves’ abilities to combat novel pathogens from closely related species, such as domestic dogs and coyotes. | 3397 | NA | NA | NA | no | no |
| 3397 | 2017 | NA | Elbers, JP; Taylor, SS | 2016 | MAJOR HISTOCOMPATIBILITY COMPLEX POLYMORPHISM IN REPTILE CONSERVATION | Genes of the major histocompatibility complex (MHC) are closely related to disease resistance and immune response in vertebrates. Although many groups are well represented in the MHC literature, less attention has been given to reptiles. Here we summarize reptile MHC studies, review published accounts investigating links between MHC polymorphism, parasite resistance, and mate choice, evaluate current limitations, and discuss prospects of new technologies for future research. MHC polymorphism appears to be extensive in reptile populations, and current evidence suggests MHC polymorphism may influence parasite resistance and mate choice as in other vertebrates. Prior research strategies have been limited by the type of molecular markers available, the nature of the sequences being amplified, the number of individuals and populations analyzed, the immunology and biology of the host-parasite relationship, and the conditions under which subjects are studied. Finally, reptiles offer special challenges: as ectotherms their susceptibility to pathogens may change dramatically depending on the time of year due to seasonal variations in their immunity. Including a temperature and/or seasonal variable may thus provide new insights into the genetic mechanisms of disease resistance. New technologies and techniques should help to alleviate problems of MHC gene characterization and multi-locus amplification associated with past research and contribute to our understanding of MHC polymorphism in reptile conservation. | 3398 | NA | NA | NA | no | no |
| 3398 | 2017 | NA | Davis, EW; Weisberg, AJ; Tabima, JF; Grunwald, NJ; Chang, JH | 2016 | Gall-ID: tools for genotyping gall-causing phytopathogenic bacteria | Understanding the population structure and genetic diversity of plant pathogens, as well as the effect of agricultural practices on pathogen evolution, is important for disease management. Developments in molecular methods have contributed to increase the resolution for accurate pathogen identification, but those based on analysis of DNA sequences can be less straightforward to use. To address this, we developed Gall-ID, a web-based platform that uses DNA sequence information from 16S rDNA, multilocus sequence analysis and whole genome sequences to group disease-associated bacteria to their taxonomic units. Gall-ID was developed with a particular focus on gall-forming bacteria belonging to Agrobacterium, Pseudomonas savastanoi, Pantoea agglomerans, and Rhodococcus. Members of these groups of bacteria cause growth deformation of plants, and some are capable of infecting many species of field, orchard, and nursery crops. Gall-ID also enables the use of higi-throughput sequencing reads to search for evidence for homologs of characterized virulence genes, and provides downloadable software pipelines for automating multilocus sequence analysis, analyzing genome sequences for average nucleotide identity, and constructing core genome phylogenies. Lastly, additional databases were included in Gall-ID to help determine the identity of other plant pathogenic bacteria that may be in microbial communities associated with galls or causative agents in other diseased tissues of plants. | 3399 | NA | NA | NA | no | no |
| 3399 | 2017 | NA | Saravia-Pietropaolo, MJ; Manzoli, DE; Antoniazzi, LR; Quiroga, MA; Beldomenico, PM | 2016 | Effect of natural brood size variability on growth and survival of thornbird nestlings | For many bird species, brood size is a highly variable trait. Several aspects of the development and survival of nestlings are affected by brood size. Most scientific evidence comes from brood manipulation experiments, and the complementary information offered by studies of natural variability has received little attention in tropical and subtropical species. Here, we provide data on the effect of brood size on the development and survival of two furnariid species under natural conditions. Weekly during two breeding seasons, two forest patches (40ha. each) from central Argentina, were exhaustively examined for active nests. Nestlings were monitored repeatedly from hatching to fledging. Factors that were considered potential confounders or effect modifiers were also recorded and included in the statistical analysis. We found a positive effect of brood size on growth, and a negative effect on survival. The first effect was only observed when preceding weeks were not rainy, and the latter was present in only one breeding season, so both effects appeared to depend on environmental conditions. Our results suggest that the benefit of a large or small brood size is dependent on context. In the studied populations, plasticity in or within-population genetic diversity in brood size may be advantageous in the light of the unpredictable circumstances that will prevail during the breeding season. | 3400 | NA | NA | NA | no | no |
| 3400 | 2017 | NA | Girault, G; Blouin, Y; Vergnaud, G; Derzelle, S | 2014 | High-throughput sequencing of Bacillus anthracis in France: investigating genome diversity and population structure using whole-genome SNP discovery | Background: Single nucleotide polymorphisms (SNPs) are ideal signatures for subtyping monomorphic pathogens such as Bacillus anthracis. Here we report the use of next-generation sequencing technology to investigate the historical, geographic and genetic diversity of Bacillus anthracis in France. 122 strains isolated over a 60-years period throughout the country were whole-genome sequenced and comparative analyses were carried out with a focus on SNPs discovery to discriminate regional sub-groups of strains. Results: A total of 1581 chromosomal SNPs precisely establish the phylogenetic relationships existing between the French strains. Phylogeography patterns within the three canSNP sub-lineages present in France (i.e. B.Br.CNEVA, A.Br.011/009 and A.Br.001/002) were observed. One of the more remarkable findings was the identification of a variety of genotypes within the A.Br.011/009 sub-group that are persisting in the different regions of France. The 560 SNPs defining the A.Br.011/009- affiliated French strains split the Trans-Eurasian sub-group into six distinct branches without any intermediate nodes. Distinct sub-branches, with some geographic clustering, were resolved. The 345 SNPs defining the major B.Br CNEVA sub-lineage clustered three main phylogeographic clades, the Alps, the Pyrenees, and the Massif Central, with a small Saone-et-Loire sub-cluster nested within the latter group. The French strains affiliated to the minor A.Br.001/002 group were characterized by 226 SNPs. All recent isolates collected from the Doubs department were closely related. Identification of SNPs from whole genome sequences facilitates high-resolution strain tracking and provides the level of discrimination required for outbreak investigations. Eight diagnostic SNPs, representative of the main French-specific phylogeographic clusters, were therefore selected and developed into high-resolution melting SNP discriminative assays. Conclusions: This work has established one of the most accurate phylogenetic reconstruction of B. anthracis population structure in a country. An extensive next-generation sequencing (NGS) dataset of 122 French strains have been created that allowed the identification of novel diagnostic SNPs useful to rapidly determine the geographic origin of any strain found in France. | 3401 | NA | NA | NA | no | no |
| 3401 | 2017 | NA | Kummerli, R; Keller, L | 2008 | Reproductive parameters vary with social and ecological factors in the polygynous ant Formica exsecta | Due to their haplo-diploid sex determination system and the resulting conflict over optimal sex allocation between queens and workers, social Hymenoptera have become important model species to study variation in sex allocation. While many studies indeed reported sex allocation to be affected by social factors such as colony kin structure or queen number, others, however, found that sex allocation was impacted by ecological factors such as food availability. In this paper, we present one of the rare studies that simultaneously investigated the effects of social and ecological factors on social insect nest reproductive parameters (sex and reproductive allocation, nest productivity) across several years. We found that the sex ratio was extremely male biased in a polygynous (multiple queens per nest) population of the ant Formica exsecta. Nest-level sex allocation followed the pattern predicted by the queen-replenishment hypothesis, which holds that gynes (new queens) should only be produced and recruited in nests with low queen number (i.e. reduced local resource competition) to ensure nest survival. Accordingly, queen number (social factor) was the main determinant on whether a nest produced gynes or males. However, ecological factors had a large impact on nest productivity and therefore on a nest’s resource pool, which determines the degree of local resource competition among co-breeding queens and at what threshold in queen number nests should switch from male to gyne production. Additionally, our genetic data revealed that gynes are recruited back to their parental nests after mating. However, our genetic data are also consistent with some adult queens dispersing on foot from nests where they were produced to nests that never produced queens. As worker production is reduced in gyne-producing nests, queen migration might be offset by workers moving in the other direction, leading to a nest network characterized by reproductive division of labour. Altogether our study shows that both, social and ecological factors can influence long-term nest reproductive strategies in insect societies. | 3402 | NA | NA | NA | no | no |
| 3402 | 2017 | NA | Zheng, WJ; Li, ZQ; Zhao, JM; Zhang, YZ; Wang, CH; Lu, XC; Sun, FY | 2016 | Study of the long-distance migration of small brown planthoppers Laodelphax striatellus in China using next-generation sequencing | BACKGROUND: The small brown planthopper (Laodelphax striatellus) is a widespread insect pest of rice in East Asia. Previous studies have shown the long-distance migrations undertaken by L. striatellus, but have not provided molecular evidence to support this. RESULTS: Long-distance immigration has occurred in the north-east coastal rice-growing region of China. Using the specific-locus amplified fragment sequencing technique, sequence data for 2.7 Gb of an abruptly increased population and 13 L. striatellus local populations from a range of regions in China that have serious rice stripe disease were obtained. A total of 2572 single nucleotide polymorphisms (SNPs) and 37 indels were detected, and the genotypes of many polymorphism sites were heterozygous in every sample, which indicated that there were rich genetic differences among the populations, and that the migration of insect pests accelerated the gene flow and increased the heterozygosity of L. striatellus populations. The genetic distance and the polymorphism markers among different populations showed that the abruptly increased population in Liaoning Province is close to several populations from Jiangsu Province and Shandong Province. CONCLUSION: The vector that caused rice stripe disease in the north-east of China was an immigrant population; however, the population may be formed from several groups from different areas, such as Jiangsu and Shandong provinces. (C) 2015 Society of Chemical Industry | 3403 | NA | NA | NA | no | no |
| 3403 | 2017 | NA | Kress, A; Oppold, AM; Kuch, U; Oehlmann, J; Muller, R | 2017 | Cold tolerance of the Asian tiger mosquito Aedes albopictus and its response to epigenetic alterations | Phenotypic plasticity is considered as one of the key traits responsible for the establishment of populations of the invasive mosquito Aedes albopictus, an important vector of viral and parasitic pathogens. The successful spread of this species to higher altitudes and latitudes may be explained by its ability to rapidly induce a heritable low temperature phenotype (cold hardiness in eggs). As a result of the low genetic diversity of founder populations, an epigenetic short-term mechanism has been suggested as the driver of this diversification. We investigated if random epigenetic alterations promoted the cold hardiness of Ae. albopictus eggs from a transgenerational study of two epigenetic agents (genistein and vinclozolin). To this end, we evaluated changes in lethal time for 50% of pharate larvae (Lt(50)) from eggs exposed to -2 degrees C in two subsequent generations that used a new dose-response test design. We detected a significant diversification of the cold hardiness of eggs (up to 64.5%) that was associated with the epigenetic change in the two subsequent offspring generations. An effect size of epigenetically modulated cold hardiness of this magnitude is likely to have an impact on the spatial distribution of this species. Our results provide a framework for further research on epigenetic temperature adaptation of invasive species to better explain and predict their rapid range expansions. | 3404 | NA | NA | NA | no | no |
| 3404 | 2017 | NA | Canestrelli, D; Zampiglia, M; Nascetti, G | 2013 | Widespread Occurrence of Batrachochytrium dendrobatidis in Contemporary and Historical Samples of the Endangered Bombina pachypus along the Italian Peninsula | Batrachochytrium dendrobatidis is considered a main driver of the worldwide declines and extinctions of amphibian populations. Nonetheless, fundamental questions about its epidemiology, including whether it acts mainly as a “lone killer’’ or in conjunction with other factors, remain largely open. In this paper we analysed contemporary and historical samples of the endangered Apennine yellow-bellied toad (Bombina pachypus) along the Italian peninsula, in order to assess the presence of the pathogen and its spreading dynamics. Once common throughout its range, B. pachypus started to decline after the mid-1990s in the northern and central regions, whereas no declines have been observed so far in the southern region. We show that Batrachochytrium dendrobatidis is currently widespread along the entire peninsula, and that this was already so at least as early as the late 1970s, that is, well before the beginning of the observed declines. This temporal mismatch between pathogen occurrence and host decline, as well as the spatial pattern of the declines, suggests that the pathogen has not acted as a”lone killer’’, but in conjunction with other factors. Among the potentially interacting factors, we identified two as the most probable, genetic diversity of host populations and recent climate changes. We discuss the plausibility of this scenario and its implications on the conservation of B. pachypus populations. | 3405 | NA | NA | NA | no | no |
| 3405 | 2017 | NA | Novinger, DC; Rahel, FJ | 2003 | Isolation management with artificial barriers as a conservation strategy for cutthroat trout in headwater streams | We evaluated the effectiveness of isolation management and stocking to meet protection and enhancement goals for native Colorado River cutthroat trout (Oncorhynchus clarki pleuriticus) in Wyoming (U.S.A). As a management strategy of the Wyoming Game and Fish Department, cutthroat trout were isolated upstream of artificial barriers in small headwater streams. Non-native trout that might have hybridized, competed with, or preyed upon cutthroat trout were removed from the isolated reaches, and then cutthroat trout of hatchery origin were stocked to augment populations. We monitored the abundance and body condition of cutthroat trout for 4-7 years following isolation in four streams with barriers and in two reference streams without barriers. Barriers limited new invasions by non-native trout, and removals of non-native trout greatly reduced their abundance but did not eliminate them (mainly brook trout [Salvelinus fontinalis ]). Wild cutthroat trout persisted in low numbers upstream of barriers, but there was no evidence of enhancement of populations. Stocked cutthroat trout did not persist upstream of barriers, and many moved downstream over barriers. The body condition of wild cutthroat trout was comparable among populations upstream and downstream of barriers and in reference streams. Isolation management provided only short-term benefits by minimizing the risks of hybridization and allowed populations to persist during the study. Removal of non-native trout and stocking did not enhance wild cutthroat trout populations, however, likely because the isolated reaches lacked critical habitat such as the deep pools necessary to sustain large fish. Also, barriers disrupt migratory patterns and prevent seasonal use of headwater reaches by adult cutthroat trout. Longer-term consequences of isolation include vulnerability to stochastic processes and loss of genetic diversity. Where non-native species pose an immediate threat to the survival of native fishes, isolation in headwater streams may be the only conservation alternative. In such situations, isolated reaches should be as large and diverse as possible, and improvements should be implemented to ensure that habitat requirements are met. | 3406 | NA | NA | NA | no | no |
| 3406 | 2017 | NA | Salvi, D; Harris, DJ; Kaliontzopoulou, A; Carretero, MA; Pinho, C | 2013 | Persistence across Pleistocene ice ages in Mediterranean and extra-Mediterranean refugia: phylogeographic insights from the common wall lizard | Background: Pleistocene climatic oscillations have played a major role in structuring present-day biodiversity. The southern Mediterranean peninsulas have long been recognized as major glacial refugia, from where Northern Europe was post-glacially colonized. However, recent studies have unravelled numerous additional refugia also in northern regions. We investigated the phylogeographic pattern of the widespread Western Palaearctic lizard Podarcis muralis, using a range-wide multilocus approach, to evaluate whether it is concordant with a recent expansion from southern glacial refugia or alternatively from a combination of Mediterranean and northern refugia. Results: We analyzed DNA sequences of two mitochondrial (cytb and nd4) and three nuclear (acm4, mc1r, and pdc) gene fragments in individuals from 52 localities across the species range, using phylogenetic and phylogeographic methods. The complex phylogeographic pattern observed, with 23 reciprocally monophyletic allo- parapatric lineages having a Pleistocene divergence, suggests a scenario of long-term isolation in multiple ice-age refugia across the species distribution range. Multiple lineages were identified within the three Mediterranean peninsulas Iberia, Italy and the Balkans - where the highest genetic diversity was observed. Such an unprecedented phylogeographic pattern - here called “refugia within all refugia” - compasses the classical scenario of multiple southern refugia. However, unlike the southern refugia model, various distinct lineages were also found in northern regions, suggesting that additional refugia in France, Northern Italy, Eastern Alps and Central Balkans allowed the long-term persistence of this species throughout Pleistocene glaciations. Conclusions: The phylogeography of Podarcis muralis provides a paradigm of temperate species survival in Mediterranean and extra-Mediterranean glacial refugia. Such refugia acted as independent biogeographic compartments for the long-term persistence of this species, for the differentiation of its genetic lineages, and for the short-distance post-glacial re-colonization of neighbouring areas. This finding echoes previous findings from recent phylogeographic studies on species from temperate ecoregions, thus suggesting the need for a reappraisal of the role of northern refugia for glacial persistence and post-glacial assembly of Holarctic biota. | 3407 | NA | NA | NA | no | no |
| 3407 | 2017 | NA | Faria, NR; de Vries, M; van Hemert, FJ; Benschop, K; van der Hoek, L | 2009 | Rooting human parechovirus evolution in time | Background: The Picornaviridae family contains a number of important pathogenic viruses, among which the recently reclassified human parechoviruses (HPeVs). These viruses are widespread and can be grouped in several types. Understanding the evolutionary history of HPeV could answer questions such as how long the circulating lineages last shared a common ancestor and how the evolution of this viral species is shaped by its population dynamics. Using both strict and relaxed clock Bayesian phylogenetics we investigated 1) the substitutions rates of the structural P1 and capsid VP1 regions and 2) evolutionary timescale of currently circulating HPeV lineages. Results: Our estimates reveal that human parechoviruses exhibit high substitution rates for both structural P1 and capsid VP1 regions, respectively 2.21 x 10(-3) (0.48 - 4.21 x 10(-3)) and 2.79 x 10(-3) (2.05 - 3.66 x 10(-3)) substitutions per site per year. These are within the range estimated for other picornaviruses. By employing a constant population size coalescent prior, the date of the most recent common ancestor was estimated to be at around 1600 (1427-1733). In addition, by looking at the frequency of synonymous and non-synonymous substitutions within the VP1 gene we show that purifying selection constitutes the dominating evolutionary force leading to strong amino acid conservation. Conclusion: In conclusion, our estimates provide a timescale for the evolution of HPeVs and suggest that genetic diversity of current circulating HPeV types has arisen about 400 years ago. | 3408 | NA | NA | NA | no | no |
| 3408 | 2017 | NA | Barnaud, A; Laucou, V; This, P; Lacombe, T; Doligez, A | 2010 | Linkage disequilibrium in wild French grapevine, Vitis vinifera L. subsp silvestris | Association mapping based on linkage disequilibrium (LD) can provide high resolution for whole-genome mapping of genes underlying phenotypic variation. This field has received considerable attention over the last decade. We present here the first characterization of LD in wild French grapevine, Vitis vinifera L. subsp. silvestris. To assess the pattern and extent of LD, we used a sample of 85 plants from southern France and 36 microsatellite markers distributed over 5 linkage groups. LD was evaluated with independence tests and multiallelic r(2), using both unphased genotypic data and reconstructed haplotypic data. LD decayed rapidly, with r(2) values decreasing to 0.1 within 2.7 cM for genotypic data and within 1.4 cM for haplotypic data. Compared to the results of a previous study on cultivated grapevine subsp. sativa, where significant LD was found up to 16.8 cM, LD in subsp. silvestris was no longer significant past 1.4 cM. LD was therefore 12 times further extended in cultivated than wild grapevine, even though LD in wild grapevine seemed to extend slightly further than in wild relatives of other crops. Domestication bottlenecks and vegetative propagation are the primary factors responsible for this difference between cultivated and wild grapevine. The rapid decay of LD observed in this study seems promising for future association mapping studies of functional variation in wild V. vinifera grapevine. Heredity (2010) 104, 431-437; doi:10.1038/hdy.2009.143; published online 21 October 2009 | 3409 | NA | NA | NA | no | no |
| 3409 | 2017 | NA | Patil, A; Nakai, K; Kinoshita, K | 2011 | Assessing the utility of gene co-expression stability in combination with correlation in the analysis of protein-protein interaction networks | Background: Gene co-expression, in the form of a correlation coefficient, has been valuable in the analysis, classification and prediction of protein-protein interactions. However, it is susceptible to bias from a few samples having a large effect on the correlation coefficient. Gene co-expression stability is a means of quantifying this bias, with high stability indicating robust, unbiased co-expression correlation coefficients. We assess the utility of gene co-expression stability as an additional measure to support the co-expression correlation in the analysis of protein-protein interaction networks. Results: We studied the patterns of co-expression correlation and stability in interacting proteins with respect to their interaction promiscuity, levels of intrinsic disorder, and essentiality or disease-relatedness. Co-expression stability, along with co-expression correlation, acts as a better classifier of hub proteins in interaction networks, than co-expression correlation alone, enabling the identification of a class of hubs that are functionally distinct from the widely accepted transient (date) and obligate (party) hubs. Proteins with high levels of intrinsic disorder have low co-expression correlation and high stability with their interaction partners suggesting their involvement in transient interactions, except for a small group that have high co-expression correlation and are typically subunits of stable complexes. Similar behavior was seen for disease-related and essential genes. Interacting proteins that are both disordered have higher co-expression stability than ordered protein pairs. Using co-expression correlation and stability, we found that transient interactions are more likely to occur between an ordered and a disordered protein while obligate interactions primarily occur between proteins that are either both ordered, or disordered. Conclusions: We observe that co-expression stability shows distinct patterns in structurally and functionally different groups of proteins and interactions. We conclude that it is a useful and important measure to be used in concert with gene co-expression correlation for further insights into the characteristics of proteins in the context of their interaction network. | 3410 | NA | NA | NA | no | no |
| 3410 | 2017 | NA | Hostetler, JA; Onorato, DP; Nichols, JD; Johnson, WE; Roelke, ME; O’Brien, SJ; Jansen, D; Oli, MK | 2010 | Genetic introgression and the survival of Florida panther kittens | Estimates of survival for the young of a species are critical for population models. These models can often be improved by determining the effects of management actions and population abundance on this demographic parameter. We used multiple sources of data collected during 1982-2008 and a live-recapture dead-recovery modeling framework to estimate and model survival of Florida panther (Puma concolor coryi) kittens (age 0-1 year). Overall, annual survival of Florida panther kittens was 0.323 +/- 0.071 (SE), which was lower than estimates used in previous population models. In 1995, female pumas from Texas (P. c. stanleyana) were released into occupied panther range as part of an intentional introgression program to restore genetic variability. We found that kitten survival generally increased with degree of admixture: F-1 admixed and backcrossed to Texas kittens survived better than canonical Florida panther and backcrossed to canonical kittens. Average heterozygosity positively influenced kitten and older panther survival, whereas index of panther abundance negatively influenced kitten survival. Our results provide strong evidence for the positive population-level impact of genetic introgression on Florida panthers. Our approach to integrate data from multiple sources was effective at improving robustness as well as precision of estimates of Florida panther kitten survival, and can be useful in estimating vital rates for other elusive species with sparse data. (C) 2010 Elsevier Ltd. All rights reserved. | 3411 | NA | NA | NA | no | no |
| 3411 | 2017 | NA | Othman, I; Mirand, A; Slama, I; Mastouri, M; Peigue-Lafeuille, H; Aouni, M; Bailly, JL | 2015 | Enterovirus Migration Patterns between France and Tunisia | The enterovirus (EV) types echovirus (E-) 5, E-9, and E-18, and coxsackievirus (CV-) A9 are infrequently reported in human diseases and their epidemiologic features are poorly defined. Virus transmission patterns between countries have been estimated with phylogenetic data derived from the 1D/VP1 and 3CD gene sequences of a sample of 74 strains obtained in France (2000-2012) and Tunisia (2011-2013) and from the publicly available sequences. The EV types (E-5, E-9, and E-18) exhibited a lower worldwide genetic diversity (respective number of genogroups: 4, 5, and 3) in comparison to CV-A9 (n = 10). The phylogenetic trees estimated with both 1D/VP1 and 3CD sequence data showed variations in the number of co-circulating lineages over the last 20 years among the four EV types. Despite the low number of genogroups in E-18, the virus exhibited the highest number of recombinant 3CD lineages (n = 10) versus 4 (E-5) to 8 (E-9). The phylogenies provided evidence of multiple transportation events between France and Tunisia involving E-5, E-9, E-18, and CV-A9 strains. Virus spread events between France and 17 other countries in five continents had high probabilities of occurrence as those between Tunisia and two European countries other than France. All transportation events were supported by BF values > 10. Inferring the source of virus transmission from phylogenetic data may provide insights into the patterns of sporadic and epidemic diseases caused by EVs. | 3412 | NA | NA | NA | no | no |
| 3412 | 2017 | NA | Ameur, A; Enroth, S; Johansson, A; Zaboli, G; Igl, W; Johansson, ACV; Rivas, MA; Daly, MJ; Schmitz, G; Hicks, AA; Meitinger, T; Feuk, L; van Duijn, C; Oostra, B; Pramstaller, PP; Rudan, I; Wright, AF; Wilson, JF; Campbell, H; Gyllensten, U | 2012 | Genetic Adaptation of Fatty-Acid Metabolism: A Human-Specific Haplotype Increasing the Biosynthesis of Long-Chain Omega-3 and Omega-6 Fatty Acids | Omega-3 and omega-6 long-chain polyunsaturated fatty acids (LC-PUFAs) are essential for the development and function of the human brain. They can be obtained directly from food, e.g., fish, or synthesized from precursor molecules found in vegetable oils. To determine the importance of genetic variability to fatty-acid biosynthesis, we studied FADS1 and FADS2, which encode rate-limiting enzymes for fatty-acid conversion. We performed genome-wide genotyping (n = 5,652 individuals) and targeted resequencing (n = 960 individuals) of the FADS region in five European population cohorts. We also analyzed available genomic data from human populations, archaic hominins, and more distant primates. Our results show that present-day humans have two common FADS haplotypes-defined by 28 closely linked SNPs across 38.9 kb-that differ dramatically in their ability to generate LC-PUFAs. No independent effects on FADS activity were seen for rare SNPs detected by targeted resequencing. The more efficient, evolutionarily derived haplotype appeared after the lineage split leading to modern humans and Neanderthals and shows evidence of positive selection. This human-specific haplotype increases the efficiency of synthesizing essential long-chain fatty acids from precursors and thereby might have provided an advantage in environments with limited access to dietary LC-PUFAs. In the modern world, this haplotype has been associated with lifestyle-related diseases, such as coronary artery disease. | 3413 | NA | NA | NA | no | no |
| 3413 | 2017 | NA | Keall, SN; Nelson, NJ; Daugherty, CH | 2010 | SECURING THE FUTURE OF THREATENED TUATARA POPULATIONS WITH ARTIFICIAL INCUBATION | Artificial incubation is a conservation technique used to provide founders for new or to augment existing reptile populations with minimal impact on the original population. It relies on the premise that hatching success of eggs in artificial conditions is high relative to natural nests. Our goal was to assist with the rescue of populations on the brink of extinction by incubating eggs produced by Tuatara from small islands inhabited by introduced rats. We incubated eggs produced over an 18 year period by Tuatara originating from Little Barrier, Cuvier, Stanley, and Red Mercury Islands, New Zealand, while they were in captivity awaiting return after rat eradications. The most successful results came from the Little Barrier stock where the population numbers increased dramatically, with eggs produced by all four mothers and high hatching success. Stanley Island stock produced the least successful outcome. Adults from Stanley Island suffered high mortality in captivity, and surviving females produced few eggs, with comparatively low hatching success. On balance, rescuing the genetic stock of remnant populations through captive incubation gives conservation programs time to deal with causes of decline and to plan for future success. However, supplementation in the future from other wild populations is likely to be necessary to ensure long term genetic variability and therefore viability of these populations, in particular Stanley Island. | 3414 | NA | NA | NA | no | no |
| 3414 | 2017 | NA | Walters, JR; Crowder, LB; Priddy, JA | 2002 | Population viability analysis for red-cockaded Woodpeckers using an individual-based model | Red-cockaded Woodpeckers (Picoides borealis) are distributed in the southeastern United States among closed populations whose maximum size is limited. Previous population viability analyses for this species have been confined to examination of threats posed by catastrophes and loss of genetic variability, because of the lack of demographic models that incorporate the extreme spatial constraints on dispersal that characterize this species. We used a spatially explicit, individual-based simulation model to assess the vulnerability of Red-cockaded Woodpecker populations to demographic and environmental stochasticity. Vulnerability to these threats was relatively low, because the presence of a substantial nonbreeding class (i.e., helpers) ameliorated the impact of stochastic variation in mortality and reproduction on the size of the breeding population. Because dispersal of helpers is spatially restricted, this effect was most pronounced when territories were aggregated or at high densities. Populations of 250 and 500 territories were stable regardless of the level of territory aggregation at the densities examined, whereas populations of 25, 49, and 100 territories ranged from rapidly declining to stable, depending on territory density and level of aggregation. Techniques that enable managers to maintain existing territories and create new ones are well established for this species. Thus managers may reasonably expect to maintain even small populations of Red-cockaded Woodpeckers by increasing the density, level of aggregation, and number of territories. | 3415 | NA | NA | NA | no | no |
| 3415 | 2017 | NA | Beaurepaire, AL; Truong, TA; Fajardo, AC; Dinh, TQ; Cervancia, C; Moritz, RFA | 2015 | Host Specificity in the Honeybee Parasitic Mite, Varroa spp. in Apis mellifera and Apis cerana | The ectoparasitic mite Varroa destructor is a major global threat to the Western honeybee Apis mellifera. This mite was originally a parasite of A. cerana in Asia but managed to spill over into colonies of A. mellifera which had been introduced to this continent for honey production. To date, only two almost clonal types of V. destructor from Korea and Japan have been detected in A. mellifera colonies. However, since both A. mellifera and A. cerana colonies are kept in close proximity throughout Asia, not only new spill overs but also spill backs of highly virulent types may be possible, with unpredictable consequences for both honeybee species. We studied the dispersal and hybridisation potential of Varroa from sympatric colonies of the two hosts in Northern Vietnam and the Philippines using mitochondrial and microsatellite DNA markers. We found a very distinct mtDNA haplotype equally invading both A. mellifera and A. cerana in the Philippines. In contrast, we observed a complete reproductive isolation of various Vietnamese Varroa populations in A. mellifera and A. cerana colonies even if kept in the same apiaries. In light of this variance in host specificity, the adaptation of the mite to its hosts seems to have generated much more genetic diversity than previously recognised and the Varroa species complex may include substantial cryptic speciation. | 3416 | NA | NA | NA | no | no |
| 3416 | 2017 | NA | Saharan, MS; Naef, A; Kumar, J; Tiwari, R | 2007 | Characterization of variability among isolates of Fusarium graminearum associated with head scab of wheat using DNA markers | Head scab of wheat caused by Fusarium species is characterized by bleaching of the wheat spike, shrivelled kernels and accumulation of mycotoxins which may cause various ailments in humans and animals. Understanding the variability of the fungal population associated with head scab could improve disease control strategies. RAPD was used to study genetic variation in 15 isolates of Fusarium graminearum, collected from naturally infected wheat from Punjab, Tamil Nadu and high ranges of Himachal Pradesh during 2000-02. A screening of sixty-one 10-mer oligonucleotide primers (OPAA 1-20, OPAC 1-20, OPAD 1-20, OPV 14), revealed 19 RAPD primers which produced strong and reproducible DNA amplicons by PCR. The amplification products were in the range of 300 bp to 1.2 kb. Maximum number of bands (11) was obtained with primer OPAD 12 followed by ten bands with OPAA 12. Punjab isolates of F. graminearum from Gurdaspur (G 31) and Ludhiana (1,23) were found genetically most similar (91.38%), whereas Wellington isolates of F. graminearum (W 5 and W 7) were found genetically most dissimilar (14.92%). Cluster analysis of band-sharing coefficients separated isolates of F. graminearum into four clusters. Lahaul valley isolates of F. graminearum (D 3, D 4 and D 5) grouped together (Group 1), while F. graminearum isolates of Punjab (G 31 and L 23) formed a separate group (Group II). F. graminearum isolates of Wellington, viz. W 1, W 2, W 5 and W 3, W 4, W 6, W 8 clustered in groups III and IV respectively. | 3417 | NA | NA | NA | no | no |
| 3417 | 2017 | NA | Brown, EW; LeClerc, JE; Kotewicz, ML; Cebula, TA | 2001 | Three R’s of bacterial evolution: How replication, repair, and recombination frame the origin of species | The genetic diversity of bacteria results not only from errors in DNA replication and repair but from horizontal exchange and recombination of DNA sequences from similar and disparate species as well. New individuals carrying adaptive changes are thus being spawned constantly among the population at large. When new selection pressures appear, these are the individuals that survive, at the expense of the general population, to forge new populations. Depending on the severity and uniqueness of the selection pressure, this could lead to new speciation. It is becoming more and more evident that, as nucleotide sequences of numerous loci from many bacterial strains continue to amass, horizontal transfer has played a key role in configuring the Escherichia coli chromosome. Here, we examine views, both old and new, for the role of recombination in the evolution of bacterial chromosomes. We present novel phylogenetic evidence for horizontal transfer of three genes involved in DNA replication and repair (mutS, uvrD, and polA). These data reveal a prominent role for horizontal transfer in the evolution of genes known to play a key role in the fidelity of DNA replication and, thus, ultimate survival of the organism. Our data underscore that recombination plays both a diversifying and a homogenizing role in defining the structure of the E. coli genome. Published 2001 Wiley-Liss, Inc. | 3418 | NA | NA | NA | no | no |
| 3418 | 2017 | NA | Galaktionov, KV; Bulat, SA; Alekhina, IA; Saville, DH; Fitzpatrick, SM; Irwin, SWB | 2004 | Evolutionary relationships within ‘pygmaeus’ group microphallids using genetic analysis and scanning electron microscopy | There are four species of ‘pygmaeus’ microphallids, namely Microphallus pygmaeus, M. piriformes, M. pseudopygmaeus and M. triangulatus (Trematoda: Microphallidae) which are parasites of marine birds and their sporocysts give rise to transmissible metacercariae inside littoral gastropods (mostly littorines). Universally primed polymerase chain reaction (UP-PCR) showed no apparent pattern between genetic diversity of the metacercariae as estimated by genomic banding profiles and their geographic region or molluscan host species. At the same time UP-PCR product cross-hybridization showed that M. pseudopygmaeus and M. triangulatus are genetically very similar, indicating that these taxa represent one species complex. In contrast, M. pygmaeus and M. piriformes are genetically well separated from each other and also from the pseudopygmaeus-triangulatus complex. Scanning electron microscopy of ventral spines, and analyses of spine angles and the number of teeth per spine, showed that all species differed significantly from one another. It was concluded that M. piriformes represents the original western member of the ‘pygmaeus’ group. Microphallus pygmaeus probably diverged from M. piriformes as it progressively specialized for sea duck final hosts. Microphallus pseudopygmaeus and M. triangulatus diverged from each other and the piriformes-pygmaeus ancestral line relatively recently. Microphallus pseudopygmaeus specialized for adoption of a wide range of gastropod host species and M. triangulatus developed morphofunctional specialization associated with final host exploitation. | 3419 | NA | NA | NA | no | no |
| 3419 | 2017 | NA | Koch, JB; Strange, JP | 2012 | The Status of Bombus occidentalis and B. moderatus in Alaska with Special Focus on Nosema bombi Incidence | Four North American bumble bee species in the subgenus Bombus sensu stricto, including Bombus occidentalis (Hymenoptera: Apidae), are experiencing dramatic declines in population abundance, geographic range and genetic diversity. The prevailing hypothesis concerning their decline is the transmission of the intracellular fungal pathogen, Nosema bombi (Microsporida), and other pathogen species from commercially reared bumble bees to wild populations. While N. bombi incidence has been investigated in the contiguous U.S.A. and Canada, virtually nothing is known about the status of Alaskan bumble bees. This study presents the distribution and relative abundance of B. occidentalis and B. moderatus, two co-occurring species in the subgenus Bombus sensu stricto, in Alaska and the prevalence of N. bombi detected in these species. Bombus occidentalis and B. moderatus accounted for 28% and 4% of the 942 bumble bees surveyed, respectively. Bombus occidentalis was the most abundant species collected in the survey and prevalence of N. bombi infections (44% infected) was also highest in this species. The proportion of infected B. moderatus did not differ significantly from other co-occurring bumble bees. Despite the presence of N. bombi infections, both Bombus s. str. species were commonly detected in Alaska with our survey method. Alaskan bumble bee populations may thus provide important insights on the role of pathogens, particularly N. bombi, in bumble bee decline in the contiguous U.S.A. | 3420 | NA | NA | NA | no | no |
| 3420 | 2017 | NA | Griekspoor, P; Colles, FM; Mccarthy, ND; Hansbro, PM; Ashhurst-Smith, C; Olsen, B; Hasselquist, D; Maiden, MCJ; Waldenstrom, J | 2013 | Marked host specificity and lack of phylogeographic population structure of Campylobacter jejuni in wild birds | Zoonotic pathogens often infect several animal species, and gene flow among populations infecting different host species may affect the biological traits of the pathogen including host specificity, transmissibility and virulence. The bacterium Campylobacter jejuni is a widespread zoonotic multihost pathogen, which frequently causes gastroenteritis in humans. Poultry products are important transmission vehicles to humans, but the bacterium is common in other domestic and wild animals, particularly birds, which are a potential infection source. Population genetic studies of C. jejuni have mainly investigated isolates from humans and domestic animals, so to assess C. jejuni population structure more broadly and investigate host adaptation, 928 wild bird isolates from Europe and Australia were genotyped by multilocus sequencing and compared to the genotypes recovered from 1366 domestic animal and human isolates. Campylobacter jejuni populations from different wild bird species were distinct from each other and from those from domestic animals and humans, and the host species of wild bird was the major determinant of C. jejuni genotype, while geographic origin was of little importance. By comparison, C. jejuni differentiation was restricted between more phylogenetically diverse farm animals, indicating that domesticated animals may represent a novel niche for C. jejuni and thereby driving the evolution of those bacteria as they exploit this niche. Human disease is dominated by isolates from this novel domesticated animal niche. | 3421 | NA | NA | NA | no | no |
| 3421 | 2017 | NA | Tanabe, K; Jombart, T; Horibe, S; Palacpac, NMQ; Honma, H; Tachibana, SI; Nakamura, M; Horii, T; Kishino, H; Mita, T | 2013 | Plasmodium falciparum mitochondrial genetic diversity exhibits isolation-by-distance patterns supporting a sub-Saharan African origin | The geographical distribution of single nucleotide polymorphism (SNP) in the mitochondrial genome of the human malaria parasite Plasmodium falciparum was investigated. We identified 88 SNPs in 516 isolates from seven parasite populations in Africa, Southeast Asia and Oceania. Analysis of the SNPs postulated a sub-Saharan African origin and recovered a strong negative correlation between within-population SNP diversity and geographic distance from the putative African origin over Southeast Asia and Oceania. These results are consistent with those previously obtained for nuclear genome-encoded housekeeping genes, indicating that the pattern of inheritance does not substantially affect the geographical distribution of SNPs. (C) 2013 Elsevier B.V. and Mitochondria Research Society. All rights reserved. | 3422 | NA | NA | NA | no | no |
| 3422 | 2017 | NA | Schenck, S; Crepeau, MW; Wu, KK; Moore, PH; Yu, Q; Ming, R | 2004 | Genetic diversity and relationships in native Hawaiian Saccharum officinarum sugarcane | Commercial sugarcane hybrid cultivars currently in production are high-yielding, disease-resistant, millable canes and are the result of years of breeding work. In Hawaii, these commercial hybrids are quite distinct from many Saccharum officinarum canes still in existence that were brought to the islands and cultivated by the native Polynesians. The actual genetic relationships among the native canes and the extent to which they contributed to the commercial hybrid germplasm has been the subject of speculation over the years. Genetic analysis of 43 presumed native Hawaiian S. officinarum clones using 228 DNA markers confirmed them to be a group distinct from the modern hybrid cultivars. The resulting dendrogram tended to confirm that there were several separate S. officinarum introductions that, owing to selections of somatic mutations, diverged into a number of cluster groups. When the “Sandwich Isles” were discovered by Captain James Cook in 1778, the Hawaiians were found to be growing sugarcane, S. officinarum ( Cook 1785). Sugarcane (ko, in the Hawaiian language) appeared in a variety of stalk and leaf colors, often with stripes (the “ribbon canes”). In the interest of preserving this historic germplasm, a collection was assembled in the 1920s by Edward L. Caum of the Hawaiian Sugar Planters’ Association and W. W. G. Moir of American Factors. Histories and descriptions of the canes were reported by Moir (1932). | 3423 | NA | NA | NA | no | no |
| 3423 | 2017 | NA | Salfati, E; Morrison, AC; Boerwinkle, E; Chakravarti, A | 2015 | Direct Estimates of the Genomic Contributions to Blood Pressure Heritability within a Population-Based Cohort (ARIC) | Blood pressure (BP) is a heritable trait with multiple environmental and genetic contributions, with current heritability estimates from twin and family studies being similar to 40%. Here, we use genome-wide polymorphism data from the Atherosclerosis Risk in Communities (ARIC) study to estimate BP heritability from genomic relatedness among cohort members. We utilized data on 6,365,596 and 9,578,528 genotyped and imputed common single nucleotide polymorphisms (SNPs), in 8,901 European ancestry (EA) and 2,860 African Ancestry (AA) ARIC participants, respectively, and a mixed linear model for analyses, to make four observations. First, for BP measurements, the heritability is similar to 20%/similar to 50% and similar to 27%/similar to 39% for systolic (SBP)/diastolic (DBP) blood pressure in European and African ancestry individuals, respectively, consistent with prior studies. Second, common variants with allele frequency >10% recapitulate most of the BP heritability in these data. Third, the vast majority of BP heritability varies by chromosome, depending on its length, and is largely concentrated in noncoding genomic regions annotated as DNaseI hypersensitive sites (DHSs). Fourth, the majority of this heritability arises from loci not harboring currently known cardiovascular and renal genes. Recent meta-analyses of large-scale genome-wide association studies (GWASs) and admixture mapping have identified similar to 50 loci associated with BP and hypertension (HTN), and yet they account for only a small fraction (similar to 2%) of the heritability. | 3424 | NA | NA | NA | no | no |
| 3424 | 2017 | NA | Varilo, T; Laan, M; Hovatta, I; Wiebe, V; Terwilliger, JD; Peltonen, L | 2000 | Linkage disequilibrium in isolated populations: Finland and a young sub-population of Kuusamo | Linkage disequilibrium (LD), non-random association of alleles at closely linked chromosomal loci, has been used as a tool in the identification of disease alleles, and this has led to an improved understanding of pathology in many monogenic Mendelian human diseases. We are currently moving from the mapping and identification of monogenic disease loci to attempts at identifying loci involved in predisposition to multifactorial diseases. In the selection of ascertainment strategies in the studies of these complex diseases, the extent of background LD in different populations is an important consideration. Here, we compare the extent of LD among the alleles of linked loci in a randomly ascertained sample of individuals from the Finnish population and a set of individuals ascertained from the region of Kuusamo, a small sub-population, founded some 13 generations ago, which has experienced very little subsequent immigration. Thirty-three microsatellite loci were genotyped in chromosomal regions on 13q, 19q, 21q, Xq, and Xp. The genetic diversity of these loci was determined separately in the general Finnish sample and in the Kuusamo sample. The X-chromosomal loci are characterised by higher levels of LD in the samples from Kuusamo than in the much larger (and older) general population of Finland, whereas in alleles of autosomal loci very little LD was seen in either of these two samples. | 3425 | NA | NA | NA | no | no |
| 3425 | 2017 | NA | Elhassan, AAM; Hussein, AA; Mohamed, HS; Rockett, K; Kwiatkowski, D; Elhassan, AM; Ibrahim, ME | 2013 | The 5q31 region in two African populations as a facet of natural selection by infectious diseases | Cases of extreme natural selection could lead either to rapid fixation or extinction of alleles depending on the population structure and size. It may also manifest in excess of heterozygosity and the locus concerned will be displaying such drastic features of allele change. We suspect the 5q31 in chromosome 5 to mirror situation of such extreme natural selection particularly that the region encompasses genes of type 2 cytokine known to associate with a number of infectious and non-infectious diseases. We typed two sets of single nucleotide polymorphisms (SNPS) in two populations: an initial limited set of only 4 SNP within the genes of IL-4, IL-13, IL-5 and IL-9 in 108 unrelated individuals and a replicating set of 14 SNP in 924 individuals from the same populations with disregard to relatedness. The results suggest the 5q31 area to be under intense selective pressure as indicated by marked heterozygosity independent of Linkage Disequilibrium (LD); difference in heterozygosity, allele, and haplotype frequencies between generations and departure from Hardy-Weinberg expectations (DHWE). The study area is endemic for several infectious diseases including malaria and visceral leishmaniasis (VL). Malaria caused by Plasmodium falciparum, however, occurs mostly with mild clinical symptoms in all ages, which makes it unlikely to account for these indices. The strong selection signals seems to emanate from recent outbreaks of VL which affected both populations to varying extent. | 3426 | NA | NA | NA | no | no |
| 3426 | 2017 | NA | Amaral, AT; Freitas, SP; Rangel, RM; Pena, GF; Ribeiro, RM; Morais, RC; Schuelter, AR | 2010 | Improvement of a popcorn population using selection indexes from a fourth cycle of recurrent selection program carried out in two different environments | We estimated genetic gains for popcorn varieties using selection indexes in a fourth cycle of intrapopulation recurrent selection developed in the campus of the Universidade Estadual do Norte Fluminense. Two hundred full-sib families were obtained from the popcorn population UNB-2U of the third recurrent selection cycle. The progenies were evaluated in a randomized block design with two replications at sites in two different environments: the Colegio Estadual Agricola Antonio Sarlo, in Campos dos Goytacazes, and the Empresa de Pesquisa Agropecuaria do Estado do Rio de Janeiro (PESAGRO-RIO), in Itaocara, both in the State of Rio de Janeiro. There were significant differences between families within sets in all traits, indicating genetic variability that could be exploited in future cycles. Thirty full-sib families were selected to continue the program. The selection indexes used to predict the gains were those of Mulamba and Mock, Smith and Hazel. The best results were obtained with the Mulamba and Mock index, which allowed the prediction of negative gains for the traits number of diseased ears and ears attacked by pests, number of broken plants and lodging, as well as ears with poor husk cover. It also provided higher gains for popping expansion and grain yield than with the other indexes, giving values of 10.55 and 8.50%, respectively, based on tentatively assigned random weights. | 3427 | NA | NA | NA | no | no |
| 3427 | 2017 | NA | Sullivan, JT; Farengo, DA | 2002 | Survival of heterotopic heart xenografts from Helisoma duryi, Planorbula armigera, and Planorbarius corneus in Biomphalaria glabrata (Pulmonata, Basommatophora, Planorbidae): evidence for phylogenetic relatedness? | Heart xenografts from the pulmonate snail Helisoma trivolvis survive in Biomphalaria glabrata, whereas xenografts from most other pulmonate snail genera are rejected within 3 to 15 days. To test whether xenografts from snails closely related to H. trivolvis were also accepted, specimens of an albino strain of B. glabrata were implanted with hearts from H. duryi, Planorbula armigera, and Planorbarius corneus. The fate of implants was monitored for 180 days by measuring heartbeat and by histological analysis. An 3 types of implants survived beyond the 3 to 15 days required for the complete destruction of incompatible xenografts, suggesting a degree of physiological and immunological compatibility between B. glabrata and these donors. Among the 3 types of xenografts, all from H. duryi survived for the entire 180 days. Fewer grafts from P. armigera and P. corneus survived for prolonged periods, although some still were beating at 60 and 180 days post implantation (DPI) respectively, suggesting that a range of histocompatibility, and perhaps phylogenetic relatedness, with B. glabrata occurs within this group. Paradoxically, the initial hemocytic response by the recipient was strongest against grafts from H. duryi, the most compatible donor, while responses to grafts from P. armigera and P. corneus were mild or did not occur during the first 7 DPI We used 2 albino strains of B. glabrata in these studies, and the data suggest slight differences in recipient strain compatibility. | 3428 | NA | NA | NA | no | no |
| 3428 | 2017 | NA | Klein, EK; Bontemps, A; Oddou-Muratorio, S | 2013 | Seed dispersal kernels estimated from genotypes of established seedlings: does density-dependent mortality matter? | 1. The seed dispersal kernel is a major determinant of spatial population dynamics and spatial distribution of genetic diversity. Among the main methods to estimate it, inverse modelling (IM) and gene shadow model (GSM) rely on seed counts in traps, whereas competing source model (CSM) and spatially explicit mating models (SEMMs) rely on compositions of seed pools. Moreover, GSM, CSM and SEMM exploit genetic information from molecular markers, whereas IM only exploits seed counts ignoring seed origins. These methods were also applied to established seedlings.   In the presence of post‐dispersal density‐dependent mortality (DDM), the effective dispersal kernel, describing the spatial distribution of established seedlings relatively to the seed source, is notoriously different from the basic dispersal kernel, describing the spatial distribution of seed deposition sites relatively to the source. Using simulated data sets, we investigated whether IM, GSM, CSM and SEMM applied to established seedlings estimate the basic or the effective dispersal kernel. In our simulations, DDM resulted in a shift of the mean basic dispersal distance (10 m) towards substantially higher effective mean dispersal distances (15 m and 20·8 m).  We demonstrated that CSM and SEMM estimate the basic seed dispersal kernel, independently from the presence of post‐dispersal mortality. By contrast, GSM estimates the effective dispersal kernel. IM failed to provide satisfactory estimates in the presence of DDM in our sampling design. Besides, for all methods, seed migration was inflated in the presence of DDM, due to lower mortality among randomly distributed immigrants relatively to local seedlings. It could seem intuitive that estimates based on seedlings or seeds provide effective or basic dispersal kernels respectively. Our results showed that it is not true for estimates obtained with CSM or SEMM because they rely on the composition of seed/seedling pools and not seed/seedling counts such as IM or GSM. This has important consequences for life stage studies where the discordance of dispersal kernels estimated from different cohorts is used to investigate post‐dispersal density‐dependent mortality. | 3429 | NA | NA | NA | no | no |
| 3429 | 2017 | NA | Howard, RS; Lively, CM | 2003 | Opposites attract? Mate choice for parasite evasion and the evolutionary stability of sex | If sex is naturally selected as a way to combat parasites, then sexual selection for disease resistance might increase the overall strength of selection for outcrossing. In the present study, we compared how two forms of mate choice affect the evolutionary stability of outcrossing in simultaneous hermaphrodites. In the first form, individuals preferred to mate with uninfected individuals (condition-dependent choice). In the second form, individuals preferred to mate with individuals that shared the least number of alleles in common at disease-resistance loci. The comparisons were made using individual-based computer simulations in which we varied parasite virulence, parasite transmission rate, and the rate of deleterious mutation at 500 viability loci. We found that alleles controlling both forms of mate choice spread when rare, but their effects on the evolutionary stability of sex were markedly different. Surprisingly, condition-dependent choice for uninfected mates had little effect on the evolutionary stability of sexual reproduction. In contrast, active choice for mates having different alleles at disease-resistance loci had a pronounced positive effect, especially under low rates of deleterious mutation. Based on these results, we suggest that mate choice that increases the genetic diversity of offspring can spread when rare in a randomly mating population, and, as an indirect consequence, increase the range of conditions under which sexual reproduction is evolutionarily stable. | 3430 | NA | NA | NA | no | no |
| 3430 | 2017 | NA | Santicchia, F; Romeo, C; Martinoli, A; Lanfranchi, P; Wauters, LA; Ferrari, N | 2015 | Effects of habitat quality on parasite abundance: do forest fragmentation and food availability affect helminth infection in the Eurasian red squirrel? | Habitat quality affects demography, population genetics, space use and phenotypic characteristics of mammals. However, little is known about the effects of habitat quality, fragmentation and/or food abundance, on host-parasite interactions. Here we present a first study on the relationships between the abundance of the dominant gastrointestinal helminth, Trypanoxyuris (Rodentoxyuris) sciuri, infecting the Eurasian red squirrel Sciurus vulgaris and three environmental factors: habitat type (mountain conifer forests vs. lowland mixed deciduous forests), fragmentation (fragmented woodlands vs. continuous forests) and food availability. Abundance of T. (R.) sciuri increased in fragmented woods. Furthermore, in mountain conifer forests, squirrels were more heavily infected after a poor Norway spruce seed crop than in years with medium or high seed production, indicating that squirrels are less capable of reducing parasite load when food availability is low. Hence, we suggest that T. (R.) sciuri abundance in red squirrels may be determined mainly by changes in host susceptibility induced by higher stress levels and/or poorer nutritional status, while in fragments, reduced genetic diversity may also increase host susceptibility to parasite infection. Although our data do not shed light on the mechanisms generating the observed patterns, results from other field studies highlighted the effect of stress and nutritional status on parasite infection, thus suggesting their implication in the changes in the abundance of T. (R.) sciuri. | 3431 | NA | NA | NA | no | no |
| 3431 | 2017 | NA | Wille, M; Muradrasoli, S; Nilsson, A; Jarhult, JD | 2016 | High Prevalence and Putative Lineage Maintenance of Avian Coronaviruses in Scandinavian Waterfowl | Coronaviruses (CoVs) are found in a wide variety of wild and domestic animals, and constitute a risk for zoonotic and emerging infectious disease. In poultry, the genetic diversity, evolution, distribution and taxonomy of some coronaviruses have been well described, but little is known about the features of CoVs in wild birds. In this study we screened 764 samples from 22 avian species of the orders Anseriformes and Charadriiformes in Sweden collected in 2006/2007 for CoV, with an overall CoV prevalence of 18.7%, which is higher than many other wild bird surveys. The highest prevalence was found in the diving duck-smainly Greater Scaup (Aythya marila; 51.5%)-and the dabbling duck Mallard (Anas platyrhynchos; 19.2%). Sequences from two of the Greater Scaup CoV fell into an infrequently detected lineage, shared only with a Tufted Duck (Aythya fuligula) CoV. Coronavirus sequences from Mallards in this study were highly similar to CoV sequences from the sample species and location in 2011, suggesting long-term maintenance in this population. A single Black-headed Gull represented the only positive sample from the order Charadriiformes. Globally, Anas species represent the largest fraction of avian CoV sequences, and there seems to be no host species, geographical or temporal structure. To better understand the eitiology, epidemiology and ecology of these viruses more systematic surveillance of wild birds and subsequent sequencing of detected CoV is imperative. | 3432 | NA | NA | NA | no | no |
| 3432 | 2017 | NA | Stelkens, RB; Brockhurst, MA; Hurst, GDD; Greig, D | 2014 | Hybridization facilitates evolutionary rescue | The resilience of populations to rapid environmental degradation is a major concern for biodiversity conservation. When environments deteriorate to lethal levels, species must evolve to adapt to the new conditions to avoid extinction. Here, we test the hypothesis that evolutionary rescue may be enabled by hybridization, because hybridization increases genetic variability. Using experimental evolution, we show that interspecific hybrid populations of Saccharomyces yeast adapt to grow in more highly degraded environments than intraspecific and parental crosses, resulting in survival rates far exceeding those of their ancestors. We conclude that hybridization can increase evolutionary responsiveness and that taxa able to exchange genes with distant relatives may better survive rapid environmental change. | 3433 | NA | NA | NA | no | no |
| 3433 | 2017 | NA | Rose, G; Crocco, P; De Rango, F; Montesanto, A; Passarino, G | 2011 | Further Support to the Uncoupling-to-Survive Theory: The Genetic Variation of Human UCP Genes Is Associated with Longevity | In humans Uncoupling Proteins (UCPs) are a group of five mitochondrial inner membrane transporters with variable tissue expression, which seem to function as regulators of energy homeostasis and antioxidants. In particular, these proteins uncouple respiration from ATP production, allowing stored energy to be released as heat. Data from experimental models have previously suggested that UCPs may play an important role on aging rate and lifespan. We analyzed the genetic variability of human UCPs in cohorts of subjects ranging between 64 and 105 years of age (for a total of 598 subjects), to determine whether specific UCP variability affects human longevity. Indeed, we found that the genetic variability of UCP2, UCP3 and UCP4 do affect the individual’s chances of surviving up to a very old age. This confirms the importance of energy storage, energy use and modulation of ROS production in the aging process. In addition, given the different localization of these UCPs (UCP2 is expressed in various tissues including brain, hearth and adipose tissue, while UCP3 is expressed in muscles and Brown Adipose Tissue and UCP4 is expressed in neuronal cells), our results may suggest that the uncoupling process plays an important role in modulating aging especially in muscular and nervous tissues, which are indeed very responsive to metabolic alterations and are very important in estimating health status and survival in the elderly. | 3434 | NA | NA | NA | no | no |
| 3434 | 2017 | NA | Gruber, MY; Xia, J; Yu, M; Steppuhn, H; Wall, K; Messer, D; Sharpe, AG; Acharya, SN; Wishart, DS; Johnson, D; Miller, DR; Taheri, A | 2017 | Transcript analysis in two alfalfa salt tolerance selected breeding populations relative to a non-tolerant population | With the growing limitations on arable land, alfalfa (a widely cultivated, low-input forage) is now being selected to extend cultivation into saline lands for low-cost biofeedstock purposes. Here, minerals and transcriptome profiles were compared between two new salinity-tolerant North American alfalfa breeding populations and a more salinity-sensitive western Canadian alfalfa population grown under hydroponic saline conditions. All three populations accumulated two-fold higher sodium in roots than shoots as a function of increased electrical conductivity. At least 50% of differentially expressed genes (p < 0.05) were down-regulated in the salt-sensitive population growing under high salinity, while expression remained unchanged in the saline-tolerant populations. In particular, most reduction in transcript levels in the salt-sensitive population was observed in genes specifying cell wall structural components, lipids, secondary metabolism, auxin and ethylene hormones, development, transport, signalling, heat shock, proteolysis, pathogenesis-response, abiotic stress, RNA processing, and protein metabolism. Transcript diversity for transcription factors, protein modification, and protein degradation genes was also more strongly affected in salt-tolerant CW064027 than in salt-tolerant Bridgeview and salt-sensitive Rangelander, while both saline-tolerant populations showed more substantial up-regulation in redox-related genes and B-ZIP transcripts. The report highlights the first use of bulked genotypes as replicated samples to compare the transcriptomes of obligate out-cross breeding populations in alfalfa. | 3435 | NA | NA | NA | no | no |
| 3435 | 2017 | NA | Tan, WB; Fong, MY; Lim, LHS | 2011 | RELATIONSHIPS OF THE HETERONCHOCLEIDIDS (HETERONCHOCLEIDUS, EUTRIANCHORATUS AND TRIANCHORATUS) AS INFERRED FROM RIBOSOMAL DNA NUCLEOTIDE SEQUENCE DATA | The monogeneans, Heteronchocleidus, Eutrianchoratus and Trianchoratus, were postulated to be closely related based on their haptoral hard parts of three well-developed anchors and one vestigial anchor. In this present study, partial 28S rDNA sequences from 12 heteronchocleidids species (10 Trianchoratus spp. and two Eutrianchoratus spp.) were obtained. Phylogenetic trees generated based on 28S rDNA (from the present study and GenBank) using neighbour-joining (NJ), maximum parsimony (MP), maximum likelihood (ML) and Bayesian inference (BI) analyses show that members of these three genera constitute a clade. Within this main monophyletic clade are the Heteronchocleidus-Eutrianchoratus and the Trianchoratus clades. This suggests that the ancestral form of these monogeneans (probably with three developed and one vestigial anchor and two connective bars) diverged into two lineages, the Heteronchocleidus-Eutrianchoratus clade with the retention of bars and the Trianchoratus clade with the complete loss of connective bars. The Heteronchocleidus-Eutrianchoratus lineage diverged into the Eutrianchoratus group with the loss of one bar and the Heteronchocleidus group with the retention of the two bars. These monogeneans were initially assigned to the Heteronchocleidinae Price, 1968 which is herein raised to family status, Heteronchocleididae. The anabantoids and channids, which are the fish hosts of heteronchocleidids, are also shown to be closely related based on the phylogenetic tree generated on their partial Cytochrome b sequences obtained from GenBank. The monophyly of the heteronchocleidids, their distribution patterns on the anabantoid and channid hosts and the relatedness of their fish hosts suggest that the ancestral heteronchocleidids could be present on the ancestral forms of the anabantoids and channids. The hosts could have acquired their heteronchocleidids through inheritance and/or host transfers. Whatever the method of acquisitions, the process of speciation and extinction of some of the heteronchocleidid monogeneans on the different anabantoid and channid hosts gave rise to the present-day distribution patterns of the heteronchocleidids. Although the monophyly of the heteronchocleidids has been established, the interrelationships of this clade to the other monogenean groups and their evolutionary history need further investigation. | 3436 | NA | NA | NA | no | no |
| 3436 | 2017 | NA | Vander Wal, E; Gagne-Delorme, A; Festa-Bianchet, M; Pelletier, F | 2016 | Dyadic associations and individual sociality in bighorn ewes | Sociality presumably evolved because it leads to fitness benefits; yet we know little about what drives individual variability in sociality, particularly with respect to hierarchical levels of social organization. Social network architecture is based upon dyadic interactions, but the factors affecting pairwise relationships are not necessarily those affecting higher-level network-derived measures of social behavior. We examined the influence of relatedness, age, dominance, and reproductive status on proximal associations and social network centrality of individuals in the fission-fusion society of bighorn ewes (Ovis canadensis) at Ram Mountain, Canada. From 2011 to 2013, 63-81% of adult ewes were equipped with proximity loggers, recording when they were within 1.5 m of one another. Ewe social structure was not random and individuals exhibited a tendency to have proximal associations that were consistent across years. Age and reproductive status appeared to have a weak effect on network centrality, but this effect was largely absent for frequency of proximal association. Furthermore, we found no effect of dominance rank on either proximal associations or network centrality. We speculate that interannual variation in these relationships may be indicative of predation affecting social dynamics. The disconnect between determinants that affect the costs and benefits of dyadic associations and those that emerge from network-derived behaviors highlights the importance of testing effects at multiple levels of social organization in animal societies. | 3437 | NA | NA | NA | no | no |
| 3437 | 2017 | NA | Wilke, T; Davis, GM; Qiu, DC; Spear, RC | 2006 | Extreme mitochondrial sequence diversity in the intermediate schistosomiasis host Oncomelania hupensis robertsoni: Another case of ancestral polymorphism? | Today, the human blood fluke, Schistosoma japonicum, is transmitted in China by two subspecies of the rissooidean snail taxon Oncomelania hupensis: O. h. hupensis and O. h. robertsoni. Whereas the eastern Chinese subspecies O. h. hupensis has been studied extensively using mitochondrial DNA sequences, very little data exists for the western subspecies O. h. robertsoni. Preliminary phylogeographic studies indicate that the latter shows a very high degree of genetic diversity with Kimura 2 parameter distances in the cytochrome oxidase I (COI) gene of up to 0.0932 (= 9.32%) among four sequences previously deposited in GenBank. Extreme degrees of intraspecific heterogeneity in gastropods have been reported before, and possible explanations include the presence of cryptic species complexes, isolation followed by secondary contact, heteroplasmy and duplications within the mitochondrial genome, the presence of “pseudogenes”, and the retention of ancestral mitochondrial polymorphism. Given the great significance of understanding phylogeographic patterns in the intermediate schistosomiasis host Oncomelania h. robertsoni for comprehending host/parasite relationships, DNA sequences of two mitochondrial genes (COI and LSU rRNA) from 66 O. hupensis robertsoni specimens are used to (1) assess the phylogenetic position, (2) study the degree of heterogeneity within and between “populations”, (3) provide a preliminary overview of the geographic distribution of major genetic groups and (4) study the phylogenetic concordance of the two gene fragments. Phylogenetic analyses, parametric bootstrapping and studies of sequence polymorphism show that: (1) all COI sequences are fully protein-coding with no insertions or deletions, (2) both individual and combined analyses of the COI and LSU rRNA genes show at least four distinct haplotype groups within O. h. robertsoni, (3) monophyly of the four clades cannot be confirmed, (4) there is high concordance in cluster patterns and arrangement of individual haplotypes of both gene fragments, (5) two of the genetic clades recovered appear to be localized, whereas the other two are widely distributed, and (6) sympatry of individuals belonging to different clades occurs. Moreover, based on preliminary AFLP analyses it could be shown that (7) there is no phylogenetic concordance between the mitochondrial and nuclear data presented here, and (8) the nuclear data from AFLP genotyping indicate a lack of clear population structure. Given the results of the present study, it is cautiously suggested that retention of ancestral mitochondrial DNA polymorphism possibly in combination with some effects of secondary contact (introgression) is the most probable explanation for the occurrence of deviant lineages in O. h. robertsoni. On the basis of nuclear, morphological, and ecological data, it is also suggested that there is no evidence of organismal subdivision in O. h. robertsoni. It is strongly recommended that future studies incorporate more data from nuclear loci in order to better understand phylogeography, population genetics, and host-parasite co-evolution in O. h. robertsoni. | 3438 | NA | NA | NA | no | no |
| 3438 | 2017 | NA | Tracy, LN; Wallis, GP; Efford, MG; Jamieson, IG | 2011 | Preserving genetic diversity in threatened species reintroductions: how many individuals should be released? | Reintroduction guidelines recommend that ‘adequate’ numbers of individuals be released to minimize loss of genetic diversity, but these numbers are rarely quantified. We present a framework for assessing the number of individuals required for an island reintroduction that takes account of allele loss both during the founding event and in the following establishment phase with a low population size. This is the first attempt to model release numbers for reintroductions in order to preserve alleles with a specified initial frequency, while taking post-release mortality rates, population growth rates and site carrying capacity into account. Probability of allele retention was sensitive to both release number and post-release demography. The rate of allele loss was strongly influenced by both environmental stochasticity and delayed population growth but was little affected by increasing the annual turnover rate of the breeding population. We illustrate the model’s application using parameter estimates from a threatened New Zealand passerine, the mohua Mohoua ochrocephala, for which reintroduction is a common management tool. Our modelling indicates that when population growth is moderate (lambda = 1.3), c. 60 individuals would need to be released to achieve at least 95% certainty that alleles at an initial frequency of 0.05 would be retained after 20 years (five overlapping generations), which is double the number typically released in translocations of mohua and other threatened forest passerines in New Zealand. | 3439 | NA | NA | NA | no | no |
| 3439 | 2017 | NA | Fernandes, FR; de Albuquerque, LC; Giordano, LDB; Boiteux, LS; de Avila, AC; Inoue-Nagata, AK | 2008 | Diversity and prevalence of Brazilian bipartite begomovirus species associated to tomatoes | Information on the distribution and prevalence of the economically destructive Begomovirus species and recombinant forms infecting fresh-market and processing tomato crops in Brazil is crucial in guiding breeding programs and also to understand the evolutionary mechanisms associated with the upsurge of so many species and quasi-species comprising this unique disease complex. An extensive survey was carried out over 3 years (between 2002 and 2004) aiming to study the diversity of begomoviruses in tomato plants, predominantly collected in central Brazil. Polymerase chain reaction (PCR) with degenerated primers was used to detect the begomoviruses in tomato leaf samples showing virus-like symptoms in commercial fields. Seven hundred and seventeen out of 2,295 samples were found to be PCR positive for a begomovirus infection. High quality sequences were obtained from a fragment encompassing the 5’ region of the coat protein (CP) gene and a segment of the intergenic region for 295 isolates from distinct geographic regions. Comparison analyses with those available in public databases enabled preliminary classification of the isolates into four previously described and/or proposed species: Tomato severe rugose virus (61%), Tomato golden vein virus (29.8%), Tomato mottle leaf curl virus (7.1%), Tomato yellow vein streak virus (0.7%), and two putative new species (1.4% of isolates). Within the prevailing species, we noted a relatively low degree of diversity, possibly indicating the existence of recent population founder effects and/or recent selective sweeps. | 3440 | NA | NA | NA | no | no |
| 3440 | 2017 | NA | Srivathsan, A; Ang, A; Vogler, AP; Meier, R | 2016 | Fecal metagenomics for the simultaneous assessment of diet, parasites, and population genetics of an understudied primate | Background: Rapid habitat loss and degradation are responsible for population decline in a growing number of species. Understanding the natural history of these species is important for designing conservation strategies, such as habitat enhancements or ex-situ conservation. The acquisition of observational data may be difficult for rare and declining species, but metagenomics and metabarcoding can provide novel kinds of information. Here we use these methods for analysing fecal samples from an endangered population of a colobine primate, the banded leaf monkey (Presbytis femoralis). Results: We conducted metagenomics via shotgun sequencing on six fecal samples obtained from a remnant population of P. femoralis in a species-rich rainforest patch in Singapore. Shotgun sequencing and identification against a plant barcode reference database reveals a broad dietary profile consisting of at least 53 plant species from 33 families. The diet includes exotic plant species and is broadly consistent with > 2 years of observational data. Metagenomics identified 15 of the 24 plant genera for which there is observational data, but also revealed at least 36 additional species. DNA traces for the diet species were recovered and identifiable in the feces despite long digestion times and a large number of potential food plants within the rainforest habitat (> 700 species). We also demonstrate that metagenomics provides greater taxonomic resolution of food plant species by utilizing multiple genetic markers as compared to single-marker metabarcoding. In addition, full mitochondrial genomes of P. femoralis individuals were reconstructed from fecal metagenomic shotgun reads, showing very low levels of genetic diversity in the focal population, and the presence of gut parasites could also be confirmed. Metagenomics thus allows for the simultaneous assessment of diet, population genetics and gut parasites based on fecal samples. Conclusions: Our study demonstrates that metagenomic shotgun sequencing of fecal samples can be successfully used to rapidly obtain natural history data for understudied species with a complex diet. We predict that metagenomics will become a routinely used tool in conservation biology once the cost per sample reduces to similar to 100 USD within the next few years. | 3441 | NA | NA | NA | no | no |
| 3441 | 2017 | NA | Engqvist, L; Taborsky, M | 2016 | The evolution of genetic and conditional alternative reproductive tactics | Frequency-dependent selection may drive adaptive diversification within species. It is yet unclear why the occurrence of alternative reproductive tactics (ARTs) is highly divergent between major animal taxa. Here we aim to clarify the environmental and social conditions favouring the evolution of intra-population variance of male reproductive phenotypes. Our results suggest that genetically determined ARTs that are fixed for life evolve when there is strong selection on body size due to size-dependent competitiveness, in combination with environmental factors reducing size benefits. The latter may result from growth costs or, more generally, from age-dependent but size-independent mortality causes. This generates disruptive selection on growth trajectories underlying tactic choice. In many parameter settings, the model also predicts ARTs to evolve that are flexible and responsive to current conditions. Interestingly, the conditions favouring the evolution of flexible tactics diverge considerably from those favouring genetic variability. Nevertheless, in a restricted but relevant parameter space, our model predicts the simultaneous emergence and maintenance of a mixture of multiple tactics, both genetically and conditionally determined. Important conditions for the emergence of ARTs include size variation of competitors, which is inherently greater in species with indeterminate growth than in taxa reproducing only after reaching their terminal body size. This is probably the reason why ARTs are more common in fishes than in other major taxa. | 3442 | NA | NA | NA | no | no |
| 3442 | 2017 | NA | Lefebvre, D; Menard, N; Pierre, JS | 2003 | Modelling the influence of demographic parameters on group structure in social species with dispersal asymmetry and group fission | Female philopatry characterizes many mammal populations subdivided into social groups. Fission of these social groups is a relatively discrete event in the life of groups or of individuals, leading to the distribution of females among several newly formed groups. Fission is an important event because it can be a way for females to disperse. Group fissions have rarely been observed and their modalities generally remain poorly known, the best-documented species being primates. Most group fissions occur along lines of maternal relatedness, but the death of a matriarch may disrupt the cohesion within a matriline, inducing separation of sisters, accompanied by their descendants, when a group splits. Our model shows that the numbers and sizes of matrilines within groups depend on the precise demographic parameters and age structure of a population and not only on its rate of increase. For comparable population-growth periods, high survival rates of adult females induce an increase in the sizes of matrilines, whereas high survival rates of immature individuals induce an increase in the numbers of matrilines. Following fission, groups of a given size included, in the first case, only a few large matrilines, whereas in the second case, they consisted mainly of many small matrilines. The present study constitutes a preliminary stage, before modelling consequences of demographic structure of groups or populations on their genetic structure. | 3443 | NA | NA | NA | no | no |
| 3443 | 2017 | NA | Phillips, BL; Brown, GP; Shine, R | 2010 | Life-history evolution in range-shifting populations | Most evolutionary theory does not deal with populations expanding or contracting in space. Invasive species, climate change, epidemics, and the breakdown of dispersal barriers, however, all create populations in this kind of spatial disequilibrium. Importantly, spatial disequilibrium can have important ecological and evolutionary outcomes. During continuous range expansion, for example, populations on the expanding front experience novel evolutionary pressures because frontal populations are assorted by dispersal ability and have a lower density of conspecifics than do core populations. These conditions favor the evolution of traits that increase rates of dispersal and reproduction. Additionally, lowered density on the expanding front eventually frees populations on the expanding edge from specialist, coevolved enemies, permitting higher investment into traits associated with dispersal and reproduction rather than defense against pathogens. As a result, the process of range expansion drives rapid life-history evolution, and this seems to occur despite ongoing serial founder events that have complex effects on genetic diversity at the expanding front. Traits evolving on the expanding edge are smeared across the landscape as the front moves through, leaving an ephemeral signature of range expansion in the life-history traits of a species across its newly colonized range. Recent studies suggest that such nonequilibrium processes during recent population history may have contributed to many patterns usually ascribed to evolutionary forces acting in populations at spatial equilibrium. | 3444 | NA | NA | NA | no | no |
| 3444 | 2017 | NA | Wildt, D; Pukazhenthi, B; Brown, J; Monfort, S; Howard, J; Roth, T | 1995 | Spermatology for understanding, managing and conserving rare species | Most conventional spermatology research involves common mammalian species including livestock, laboratory animals and humans. Yet, there are more than 4500 mammalian species inhabiting the planet for which little is known about basic reproductive biology, including sperm characteristics and function. This information is important, not just as adjunct knowledge, but because the majority of these species are threatened with extinction, largely due to human-induced pressures. The field of conservation is changing rapidly, and global cooperation is emerging among a variety of wildlife enthusiasts, ranging from management authorities of nature reserves to curators of rare zoological collections. Conservation progress depends on systematic, multidisciplinary research first to answer basic questions, with new data then applied to endangered species management plans. The reproductive physiologist is a crucial component of this scheme. Reproduction is the essence of species survival, and enormous effort needs to be directed at these ‘untraditional’ research species, subspecies and populations. Spermatology research combined with simultaneous efforts in endocrinology, embryology and cryopreservation (among others) can lead to the successful application of assisted reproduction. Examples from this laboratory include an array of wild felid species and a rare cervid and mustelid. Obstacles to success are formidable, including unique species-specificities, diminished genetic diversity and a general lack of resources. Nonetheless, the field offers tremendous opportunities for generating unique knowledge of comparative interest and with conservation utility. | 3445 | NA | NA | NA | no | no |
| 3445 | 2017 | NA | Rauch, EM; Sayama, H; Bar-Yam, Y | 2003 | Dynamics and genealogy of strains in spatially extended host-pathogen models | We examine the dynamics of evolution in a generic spatial model of a pathogen infecting a population of hosts, or an analogous predator-prey system. Previous studies of this model have found a range of interesting phenomena that differ from the well-mixed version. We extend these studies by examining the spatial and temporal dynamics of strains using genealogical tracing. When transmissibility can evolve by mutation, strains of intermediate transmissibility dominate even though high-transmissibility mutants have a short-term reproductive advantage. Mutant strains continually arise and grow rapidly for many generations but eventually go extinct before dominating the system. We find that, after a number of generations, the mutant pathogen characteristics strongly impact the spatial distribution of their local host environment, even when there are diverse types coexisting. Extinction is due to the depletion of susceptibles in the local environment of these mutant strains. Studies of spatial and genealogical relatedness reveal the self-organized spatial clustering of strains that enables their impact on the local environment. Thus, we find that selection acts against the high-transmissibility strains on long time-scales as a result of the feedback due to environmental change. Our study shows that averages over space or time should not be assumed to adequately describe the evolutionary dynamics of spatially distributed host-pathogen systems. (C) 2003 Elsevier Science Ltd. All rights reserved. | 3446 | NA | NA | NA | no | no |
| 3446 | 2017 | NA | Dodd, RS; Kashani, N | 2003 | Molecular differentiation and diversity among the California red oaks (Fagaceae; Quercus section Lobatae) | A recent epidemic of Phytopthora (Sudden Oak Death) in coastal woodlands of California is causing severe mortality in some oak species belonging to the red oak (Lobatae) group. To predict the risks of spread of this disease, an understanding of the relationships among California’s red oak species and of their population genetic structure is needed. We focus here on relationships among the four species of red oak. Whereas morphological distinction of Quercus wislizeni and Quercus parvula can pose problems, Quercus kelloggii and Quercus agrifolia in pure forms are easily distinguishable from one another and from Q. wislizeni and Q. parvula in the field. However, hybrids among all species combinations are known to occur in nature and these can confound data from ecological studies. Our results revealed greatest differentiation of the deciduous Q. kelloggii, with only weak AFLP fragment differentiation of the three remaining evergreen species. The molecular data suggest a closer affinity of Q. agrifolia with Q. wislizeni and Q. parvula contrary to earlier suggestions that its origins are likely to have been with northern deciduous oaks probably through a common ancestor with Q. kelloggii. Interior and coastal populations of Q. wislizeni separated in dendrograms based on phenetic and genetic distances suggesting probable isolation in different glacial refugia. The position of Q. parvula remains ambiguous, having a closer affinity with interior populations of Q. wislizeni and with Q. agrifolia, than with coastal populations of Q. wislizeni. Mean population differentiation in Q. wislizeni was 0.18, which is somewhat higher than the average for other oak species, suggesting that range fragmentation has occurred in the past, resulting in a metapopulation structure. Our results provide evidence that introgression among these species may be causing reticulation, further confounding species separation. Whereas Phytopthora has been reported on Q. agrifolia, Q. parvula and Q. kelloggii, it has not yet been detected in natural populations of Q. wislizeni. The species relationships that our molecular data show suggest that this is more likely a result of escape due to ecological tolerances than to genetic differences. | 3447 | NA | NA | NA | no | no |
| 3447 | 2017 | NA | Villanueva, RD; Baria, MVB; dela Cruz, DW | 2013 | Effects of grazing by herbivorous gastropod (Trochus niloticus) on the survivorship of cultured coral spat | Background: The technology in the production of sexual coral propagules for coral reef restoration is being actively developed recently to address concerns on the low genetic diversity in coral populations restored through transplantation of coral materials derived from fragmentation. Results: In this study, we produced coral spat attached to rubble and determined the effect of the addition of hatchery-reared trochus, a herbivorous gastropod, at two densities (one and two trochus per cage, equivalent to four and eight trochus per square meter, respectively) on the survivorship of the spat cultured in cages at the hatchery and in situ nursery. After 5 weeks of culture in the hatchery, spat survivorship was significantly higher in the two per cage trochus treatment (18.3% +/- 6.7%, mean +/- sd) than in the control (5.5% +/- 1.7%), with the one per cage trochus treatment having intermediate spat survival (12.6% +/- 7.9%). The enhanced spat survivorship, though not apparent in the in situ nursery culture, is attributed to the grazing effect of trochus as significantly lower turf algal cover was observed in rubble placed in two per cage trochus treatment (44.8% +/- 3.2%) than in the one per cage trochus treatment and control (80.2% +/- 6.5% and 76.3% +/- 5.0%, respectively). Conclusions: This study demonstrates that trochus can be used to control algal cover and enhance survivorship of coral spat in caged culture, at least in the hatchery, for the production of sexually derived transplant materials for coral reef restoration. | 3448 | NA | NA | NA | no | no |
| 3448 | 2017 | NA | Kleinstein, SE; Heath, L; Makar, KW; Poole, EM; Seufert, BL; Slattery, ML; Xiao, LR; Duggan, DJ; Hsu, L; Curtin, K; Koepl, L; Muehling, J; Taverna, D; Caan, BJ; Carlson, CS; Potter, JD; Ulrich, CM | 2013 | Genetic variation in the lipoxygenase pathway and risk of colorectal neoplasia | Arachidonate lipoxygenase (ALOX) enzymes metabolize arachidonic acid to generate potent inflammatory mediators and play an important role in inflammation-associated diseases. We investigated associations between colorectal cancer risk and polymorphisms in ALOX5, FLAP, ALOX12, and ALOX15, and their interactions with nonsteroidal anti-inflammatory drug (NSAID) use. We genotyped fifty tagSNPs, one candidate SNP, and two functional promoter variable nucleotide tandem repeat (VNTR) polymorphisms in three US population-based case-control studies of colon cancer (1,424 cases/1,780 controls), rectal cancer (583 cases/775 controls), and colorectal adenomas (485 cases/578 controls). Individuals with variant genotypes of the ALOX5 VNTR had a decreased risk of rectal cancer, with the strongest association seen for individuals with one or more alleles of >5 repeats (wild type = 5, OR>5/5 = 0.42, 95% CI 0.200.92; P = 0.01). Four SNPs in FLAP (rs17239025), ALOX12 (rs2073438), and ALOX15 (rs4796535 and rs2619112) were associated with rectal cancer risk at P 0.05. One SNP in FLAP (rs12429692) was associated with adenoma risk. A false discovery rate (FDR) was applied to account for false positives due to multiple testing; the ALOX15 associations were noteworthy at 25% FDR. Colorectal neoplasia risk appeared to be modified by NSAID use in individuals with variant alleles in FLAP and ALOX15. One noteworthy interaction (25% FDR) was observed for rectal cancer. Genetic variability in ALOXs may affect risk of colorectal neoplasia, particularly for rectal cancer. Additionally, genetic variability in FLAP and ALOX15 may modify the protective effect of NSAID use against colorectal neoplasia. (c) 2013 Wiley Periodicals, Inc. | 3449 | NA | NA | NA | no | no |
| 3449 | 2017 | NA | Naderi, G; Kaboli, M; Koren, T; Karami, M; Zupan, S; Rezaei, HR; Krystufek, B | 2014 | Mitochondrial evidence uncovers a refugium for the fat dormouse (Glis glis Linnaeus, 1766) in Hyrcanian forests of northern Iran | Fat dormouse is a squirrel-like rodent which is closely tied to deciduous forest ecosystem in southwestern Eurasia. As such it is a valuable indicator of forest survival in refugia during glacial-interglacial periods. Previous phylogeographic analyses uncovered divergent fat dormouse lineages in southern refugia in Italy and the Balkans, but retrieved a surprisingly low overall genetic diversity across the majority of the species’ range. We explored 812 bp long fragment of a cytochrome b (cyt b) gene in ten fat dormice from refugial Hyrcanian forests in northern Iran. We identified 10 new cyt b haplotypes, which generated a total dataset of 28 fat dormouse haplotypes. The phylogenetic reconstruction clustered the new haplotypes into the Iranian lineage which hold a sister position against all other fat dormouse haplotypes from Europe and Asia Minor. The divergence between these lineages suggests a fragmentation event of an ancestral population at 5.76 mya (95% HPD = 3.21-8.92). This early evolutionary divergence was possibly triggered in the Middle East by dramatically divergent environmental conditions at the Messinian Salinity Crisis. The divergence clearly exceeds the intraspecific divergence, and is well within the range between congeneric rodent species. We suggest a long-term persistence of the Iranian lineage in the Hyrcanian refugium which is consitent with a high number of endemics along the southern Caspian coastal areas. (C) 2013 Deutsche Gesellschaft fur Saugetierkunde. Published by Elsevier GmbH. All rights reserved. | 3450 | NA | NA | NA | no | no |
| 3450 | 2017 | NA | Deng, XY; Phillippy, AM; Li, ZX; Salzberg, SL; Zhang, W | 2010 | Probing the pan-genome of Listeria monocytogenes: new insights into intraspecific niche expansion and genomic diversification | Background: Bacterial pathogens often show significant intraspecific variations in ecological fitness, host preference and pathogenic potential to cause infectious disease. The species of Listeria monocytogenes, a facultative intracellular pathogen and the causative agent of human listeriosis, consists of at least three distinct genetic lineages. Two of these lineages predominantly cause human sporadic and epidemic infections, whereas the third lineage has never been implicated in human disease outbreaks despite its overall conservation of many known virulence factors. Results: Here we compare the genomes of 26 L. monocytogenes strains representing the three lineages based on both in silico comparative genomic analysis and high-density, pan-genomic DNA array hybridizations. We uncover 86 genes and 8 small regulatory RNAs that likely make L. monocytogenes lineages differ in carbohydrate utilization and stress resistance during their residence in natural habitats and passage through the host gastrointestinal tract. We also identify 2,330 to 2,456 core genes that define this species along with an open pan-genome pool that contains more than 4,052 genes. Phylogenomic reconstructions based on 3,560 homologous groups allowed robust estimation of phylogenetic relatedness among L. monocytogenes strains. Conclusions: Our pan-genome approach enables accurate co-analysis of DNA sequence and hybridization array data for both core gene estimation and phylogenomics. Application of our method to the pan-genome of L. monocytogenes sheds new insights into the intraspecific niche expansion and evolution of this important foodborne pathogen. | 3451 | NA | NA | NA | no | no |
| 3451 | 2017 | NA | Bucci, V; Nadell, CD; Xavier, JB | 2011 | The Evolution of Bacteriocin Production in Bacterial Biofilms | Bacteriocin production is a spiteful behavior of bacteria that is central to the competitive dynamics of many human pathogens. Social evolution predicts that bacteriocin production is favored when bacteriocin-producing cells are mixed at intermediate frequency with their competitors and when competitive neighborhoods are localized. Both predictions are supported by biofilm experiments. However, the means by which physical and biological processes interact to produce conditions that favor the evolution of bacteriocin production remain to be investigated. Here we fill this gap using analytical and computational approaches. We identify and collapse key parameters into a single number, the critical bacteriocin range, that measures the threshold distance from a focal bacteriocin-producing cell within which its fitness is higher than that of a sensitive cell. We develop an agent-based model to test our predictions and confirm that bacteriocin production is most favored when relatedness is intermediate and competition is local. We then use invasion analysis to determine evolutionarily stable strategies for bacteriocin production. Finally, we perform long-term evolutionary simulations to analyze how the critical bacteriocin range and genetic lineage segregation affect biodiversity in multistrain biofilms. We find that biodiversity is maintained in highly segregated biofilms for a wide array of critical bacteriocin ranges. However, under conditions of high nutrient penetration leading to well-mixed biofilms, biodiversity rapidly decreases and becomes sensitive to the critical bacteriocin range. | 3452 | NA | NA | NA | no | no |
| 3452 | 2017 | NA | Panova, M; Blakeslee, AMH; Miller, AW; Makinen, T; Ruiz, GM; Johannesson, K; Andre, C | 2011 | Glacial History of the North Atlantic Marine Snail, Littorina saxatilis, Inferred from Distribution of Mitochondrial DNA Lineages | The North Atlantic intertidal gastropod, Littorina saxatilis (Olivi, 1792), exhibits extreme morphological variation between and within geographic regions and has become a model for studies of local adaptation; yet a comprehensive analysis of the species’ phylogeography is lacking. Here, we examine phylogeographic patterns of the species’ populations in the North Atlantic and one remote Mediterranean population using sequence variation in a fragment of the mitochondrial cytochrome b gene (607 bp). We found that, as opposed to many other rocky intertidal species, L. saxatilis has likely had a long and continuous history in the Northwest Atlantic, including survival during the last glacial maximum (LGM), possibly in two refugia. In the Northeast Atlantic, several areas likely harboured refugial populations that recolonized different parts of this region after glacial retreat, resulting in strong population structure. However, the outlying monomorphic Venetian population is likely a recent anthropogenic introduction from northern Europe and not a remnant of an earlier wider distribution in the Mediterranean Sea. Overall, our detailed phylogeography of L. saxatilis adds an important piece to the understanding of Pleistocene history in North Atlantic marine biota as well as being the first study to describe the species’ evolutionary history in its natural range. The latter contribution is noteworthy because the snail has recently become an important model species for understanding evolutionary processes of speciation; thus our work provides integral information for such endeavours. | 3453 | NA | NA | NA | no | no |
| 3453 | 2017 | NA | Grandbastien, M; Audeon, C; Bonnivard, E; Casacuberta, JM; Chalhoub, B; Costa, APP; Le, QH; Melayah, D; Petit, M; Poncet, C; Tam, SM; Van Sluys, MA; Mhiri, C | 2005 | Stress activation and genomic impact of Tnt1 retrotransposons in Solanaceae | Tnt1 elements are a superfamily of LTR-retrotransposons distributed in the Solanaceae plant family and represent good model systems for studying regulatory and evolutionary controls established between hosts and transposable elements. Tnt1 retrotransposons tightly control their activation, by restricting expression to specific conditions. The Tnt1A element, originally discovered in tobacco, is expressed in response to stress, and its activation by microbial factors is followed by amplification, demonstrating that factors of pathogen origin can generate genetic diversity in plants. The Tnt1A promoter has the potential to be activated by various biotic and abiotic stimuli but a number of these are specifically repressed in tobacco and are revealed only when the LTR promoter is placed in a heterologous context. We propose that a tobacco- and stimulus-specific repression has been established in order to minimize activation in conditions that might generate germinal transposition. In addition to tight transcriptional controls, Tnt1A retrotransposons self-regulate their activity through gradual generation of defective copies that have reduced transcriptional activity. Tnt1 retrotransposons found in various Solanaceae species are characterized by a high level of variability in the LTR sequences involved in transcription, and have evolved by gaining new expression patterns, mostly associated with responses to diverse stress conditions. Tnt1A insertions associated with genic regions are initially favored but seem subsequently counter-selected, while insertions in repetitive DNA are maintained. On the other hand, amplification and loss of insertions may result from more brutal occurrences, as suggested by the large restructuring of Tnt1 populations observed in tobacco compared to each of its parental species. The distribution of Tnt1 elements thus appears as a dynamic flux, with amplification counterbalanced by loss of insertions. Tnt1 insertion polymorphisms are too high to reveal species relationships in the Nicotiana genus, but can be used to evaluate species relationships in the Lycopersicon and Capsicum genera. This also demonstrates that the behavior of Tnt1 retrotransposons differs between host species, most probably in correlation to differences in expression conditions and in the evolutionary and environmental history of each host. Copyright (c) 2005 S. Karger AG, Basel. | 3454 | NA | NA | NA | no | no |
| 3454 | 2017 | NA | Mieuzet, L; Quillevere, A; Pilet, ML; Le May, C | 2016 | Development and characterization of microsatellite markers for the oomyceta Aphanomyces euteiches | Aphanomyces euteiches Drechsler is a serious pathogen of leguminous crops that causes devastating root rot of pea worldwide. Given that A. euteiches is a diploid organism, robust, codominant markers are needed for population genetics studies. We have developed and screened a microsatellite-enriched small-insert genomic library for identification of A. euteiches SSR containing sequences. Fourteen out of the 48 primer pairs designed to amplify SSR, produced unambiguous polymorphic products in our test population of 94 isolates. The number of alleles at each locus ranged from one to four. The identification of new markers would enhance the ability to evaluate the genetic structure of A. euteiches populations, and pathogen evolution. (C) 2016 Elsevier Inc. All rights reserved. | 3455 | NA | NA | NA | no | no |
| 3455 | 2017 | NA | Geiger, A; Ravel, S; Mateille, T; Janelle, J; Patrel, D; Cuny, G; Frutos, R | 2007 | Vector competence of Glossina palpalis gambiensis for Trypanosoma brucei s.l. and genetic diversity of the symbiont Sodalis glossinidius | Tsetse flies transmit African trypanosomes, responsible for sleeping sickness in humans and nagana in animals. This disease affects many people with considerable impact on public health and economy in sub-Saharan Africa, whereas trypanosomes’ resistance to drugs is rising. The symbiont Sodalis glossinidius is considered to play a role in the ability of the fly to acquire trypanosomes. Different species of Glossina were shown to harbor genetically distinct populations of S. glossinidius. We therefore investigated whether vector competence for a given trypanosome species could be linked to the presence of specific genotypes of S. glossinidius. Glossina palpalis gambiensis individuals were fed on blood infected either with Trypanosoma brucei gambiense or Trypanosoma brucei brucei. The genetic diversity of S. glossinidius strains isolated from infected and noninfected dissected flies was investigated using amplified fragment length polymorphism markers. Correspondence between occurrence of these markers and parasite establishment was analyzed using multivariate analysis. Sodalis glossinidius strains isolated from T. brucei gambiense-infected flies clustered differently than that isolated from T. brucei brucei-infected individuals. The ability of T. brucei gambiense and T. brucei brucei to establish in G. palpalis gambiensis insect midgut is statistically linked to the presence of specific genotypes of S. glossinidius. This could explain variations in Glossina vector competence in the wild. Then, assessment of the prevalence of specific S. glossinidius genotypes could lead to novel risk management strategies. | 3456 | NA | NA | NA | no | no |
| 3456 | 2017 | NA | Leopold, RA; Rajamohan, A; Shelly, TE; Handler, AM | 2010 | Quality Testing of Three Species of Tephritid Fruit Flies After Embryo Cryopreservation | This study evaluates characteristics commonly used to define insect quality or fitness by using a complement of three species of tephritid fruit flies (Diptera: Tephritidae) obtained from cryopreserved embryos. Anastrepha ludens (Loew), Anastrepha suspensa (Loew), and Ceratitis capitata (Wiedemann) were used to assess embryo to adult emergence and adult longevity, flight ability mating ability, fecundity, and genetic variability after cryopreservation. With the three species tested: embryo survival was reduced by 50-70% whereas adult eclosion seemed unaffected by cryogenic treatment. Laboratory cage survival of cryopreserved A. ludens paralleled that of controls when tested with or without food and water posttreatment. With C. capitata, field cage survival was also similar when the adult progeny of cryopreserved parents was compared with that Of Untreated flies of the same age, Assessment of flight ability of cryopreserved A. ludens over a 19-d period by using a flight mill showed no statistical difference when compared with the untreated groups Over the same time period. Flight ability within field cages for newly emerged progeny of cryopreserved C. capitata also mirrored that of the controls. Observed matings occurring within laboratory cages containing equal numbers of A. ludens males and females did not differ from comparable control groups, Furthermore, male progeny obtained from cryopreserved C. capitata parents competed equally With untreated males for mates while housed in field cages. A laboratory analysis of Fertility and fecundity of A. suspensa revealed that males mated with control females were unaffected by cryopreservation as embryos. whereas cryopreserved females exhibited a significantly reduced fecundity when mated with control males. The fecundity of C. capitata progeny of cryopreserved parents also did not differ from control levels while caged Under laboratory conditions. A random amplified polymorphic DNA assay of the genetic diversity of A. ludens comparing cryopreserved males with control males showed that the coefficient of similarity was >= 85%. This study indicates that embryo cryopreservation had little or no effect on the reproduction, longevity and flight of the species tested and can be used to support maintenance of insect stocks and control programs supported by the mass-rearing process. | 3457 | NA | NA | NA | no | no |
| 3457 | 2017 | NA | Martenot, C; Lethuillier, O; Fourour, S; Oden, E; Trancart, S; Travaille, E; Houssin, M | 2015 | Detection of undescribed ostreid herpesvirus 1 (OsHV-1) specimens from Pacific oyster, Crassostrea gigas | The ostreid herpesvirus 1 (OsHV-1) and variants were implicated in mass mortality affecting the young Pacific cupped oysters, Crassostrea gigas, in European countries and those around the world. From 2008 onwards, oyster mortality had greatly increased on the French coast and was associated with the detection of a new OsHV-1 variant, entitled OsHV-1 mu Var. The OsHV-1 mu Var is predominant in oysters; however, other OsHV-1 variants have been detected in samples collected during mortality periods or collected out of mortality periods in France, Ireland, Spain, Portugal, Italy, Mexico, United States, South Korea, Australia, and New Zealand. A retrospective study conducted on 1047 OsHV-1 specimens sampled mainly in France between 2009 and 2012, revealed 17 undescribed OsHV-1 variants found in 65 oyster samples. These specimens presented point mutations situated downstream and upstream from the microsatellite area in the C region (ORF 4/5) which were different from the OsHV-1 reference and the OsHV-1 mu Var. In the present work, investigation was performed to further characterize these OsHV-1 specimens by sequencing two habitually targeted regions to study genetic polymorphism of the virus: ORF 41142 and ORF 35-38. An OsHV-1 variant detected in six oyster samples, contained a nucleotide substitution in the C region which impacted the amino acid sequence and might modify the function of the unknown protein encoding by ORF 4. For the ORF 41/42 region, only two specimens presented a synonymous mutation in comparison with the OsHV-1 mu Var. All specimens contained the same deletion with the OsHV-1 mu Var in ORF 35-38. Then, a phylogenetic analysis based on the C region was performed to investigate the distribution of undescribed specimens among 21 OsHV-1 DNA sequences notified in GenBank and collected from different countries (France, Japan, New Zealand, China, Ireland, and United States) between 1995 and 2012. All analyzed samples and the OsHV-1 mu Var were placed in the same group, excepted for a Japan specimen. Our results contribute to improve the description of the genetic diversity of the O5HV-1 and the C region (ORF 4/5) appears to be a better target than ORF 42/42 and 35-38 to distinguish variants between themselves. Crown Copyright (C) 2015 Published by Elsevier Inc. All rights reserved. | 3458 | NA | NA | NA | no | no |
| 3458 | 2017 | NA | Gnadig, NF; Beaucourt, S; Campagnola, G; Borderia, AV; Sanz-Ramos, M; Gong, P; Blanc, H; Peersen, OB; Vignuzzi, M | 2012 | Coxsackievirus B3 mutator strains are attenuated in vivo | Based on structural data of the RNA-dependent RNA polymerase, rational targeting of key residues, and screens for Coxsackievirus B3 fidelity variants, we isolated nine polymerase variants with mutator phenotypes, which allowed us to probe the effects of lowering fidelity on virus replication, mutability, and in vivo fitness. These mutator strains generate higher mutation frequencies than WT virus and are more sensitive to mutagenic treatments, and their purified polymerases present lower-fidelity profiles in an in vitro incorporation assay. Whereas these strains replicate with WT-like kinetics in tissue culture, in vivo infections reveal a strong correlation between mutation frequency and fitness. Variants with the highest mutation frequencies are less fit in vivo and fail to productively infect important target organs, such as the heart or pancreas. Furthermore, whereas WT virus is readily detectable in target organs 30 d after infection, some variants fail to successfully establish persistent infections. Our results show that, although mutator strains are sufficiently fit when grown in large population size, their fitness is greatly impacted when subjected to severe bottlenecking, which would occur during in vivo infection. The data indicate that, although RNA viruses have extreme mutation frequencies to maximize adaptability, nature has fine-tuned replication fidelity. Our work forges ground in showing that the mutability of RNA viruses does have an upper limit, where larger than natural genetic diversity is deleterious to virus survival. | 3459 | NA | NA | NA | no | no |
| 3459 | 2017 | NA | Tindo, M; Kenne, M; Dejean, A | 2008 | Advantages of multiple foundress colonies in Belonogaster juncea juncea L.: greater survival and increased productivity | 1. The ecological hypothesis predicts that multiple foundress colonies of social wasps may have a better survival rate and produce more brood per capita than single foundress colonies. With the aim of verifying if these characteristics exist in the primitively eusocial wasp species Belonogaster juncea juncea (L.), we monitored 49 foundations, including 13 single and 36 multiple foundress colonies, in Cameroon. 2. Multiple foundress colonies were significantly more successful than single foundress colonies in producing at least one adult. 3. The total productivity of the colonies increased significantly with the number of associated foundresses, but the productivity per capita did not. No single foundress colony reached the sexual phase, while eight (21.6%) multiple foundress colonies did. Males were produced in only five colonies, so that the sex ratio was biased in favour of females. 4. These results suggest that because of the strong ecological constraints on solitary nesting, survival and high colony productivity are two advantages of multiple foundress colonies in B. j. juncea. 5. The decreasing per capita productivity concomitant with an increasing number of females noted in this study illustrates once again Michener’s paradox. The coefficient of variance of the per capita productivity significantly decreased with group size, as Wenzel and Pickering suggested in the model they created to explain the paradox. 6. Ecological factors may act in conjunction with other factors, such as genetic relatedness between associated foundresses, to promote joining behaviour in B. j. juncea | 3460 | NA | NA | NA | no | no |
| 3460 | 2017 | NA | Giacometti, M; Roganti, R; De Tann, D; Stahlberger-Saitbekova, N; Obexer-Ruff, G | 2004 | Alpine ibex Capra ibex ibex x domestic goat C-aegagrus domestica hybrids in a restricted area of southern Switzerland | We documented hybridisation of domestic goats Capra aegagrus domestica with free-ranging male Alpine ibex Capra ibex ibex in a restricted area of the southern Swiss Alps in 1989-2001. The number of animals in the hybrid herd reached a maximum of 18 in 1998. We confirmed hybridisation through morphological data and genetic analysis. All presumed hybrids were larger and heavier than Alpine ibex. Horns of hybrids were longer than those of Alpine ibex, and some male horns lacked nodes. In two males studied, the first horn increment was longer than the second. In some cases the pelage colour revealed characteristics untypical for ibex such as prominent leg markings and dark brown colour in young animals. Microsatellite analysis in one male revealed ibex specific alleles, as well as goat specific alleles. We concluded that this male, as well as the other animals studied, were Alpine ibex x domestic goat hybrids. All wild goats and their hybrid offspring were removed by state gamekeepers in 19982001 to maintain the genetic integrity of free-ranging Alpine ibex living in the area. We suggest that the survival of hybrids depends largely on habitat characteristics. In the study area, the hybrid winter range was exposed to the south. ne sunny rocky slope was steep and reached down to an altitude of 1,500 in a.s.l. On this slope, animals found food in early spring and a refuge against adverse weather conditions. | 3461 | NA | NA | NA | no | no |
| 3461 | 2017 | NA | Karvonen, A; Rellstab, C; Louhi, KR; Jokela, J | 2012 | Synchronous attack is advantageous: mixed genotype infections lead to higher infection success in trematode parasites | Co-infecting parasite genotypes typically compete for host resources limiting their fitness. The intensity of such competition depends on whether parasites are reproducing in a host, or using it primarily as a transmission vehicle while not multiplying in host tissues (referred to as ‘competition hypothesis’). Alternatively, simultaneous attack and co-infection by several parasite genotypes might facilitate parasite infection because such a diverse attack could present an additional challenge to host immune defence (referred to as ‘facilitation hypothesis’). We tested the competition hypothesis by comparing the production of transmission stages (cercariae) from snails infected with one or two genotypes of the trematode Diplostomum pseudospathaceum. We found that cercarial production did not differ between the two groups of snails, suggesting lower per genotype production in double infections, and competition for host resources. Second, we tested the facilitation hypothesis by comparing parasite infection success on fishes (proportion of parasites establishing in the host) using cercariae originating from single-infected snails, double-infected snails and artificial mixtures of the single genotypes. In both cases, we found higher infection success when fishes were challenged with two parasite genotypes instead of one, supporting the facilitation hypothesis. Our results suggest that constraints defining the success of multiple genotype infections in parasites with multiple host life cycles include both between-genotype resource competition in the host and performance of host immune defences against a diverse parasite challenge. | 3462 | NA | NA | NA | no | no |
| 3462 | 2017 | NA | Varga, E; Endreffy, E; Samu, G; Szekeres, E; Toth, F; Bede, O; Petri, IB | 1996 | Molecular genetic analysis of HLA-DRB, -DQA, and -DQB polymorphisms in Hungarians and distribution of the DRB1\*03 allele in adults and newborns | We describe here the genetic variability of HLA-DRB1, -DQA, and -DQB in 140 healthy individuals from Hungary, including 95 randomly selected adults and 45 newborns. Allele and haplotype frequencies as well as linkage disequilibria were calculated. It was found that HLA-DRB1*11, -DQA1*0501, and -DQB1*0301 predominate in Hungarians. This information may be helpful in the future for HLA and disease association studies. Simultaneously, we observed that the frequency of the DRB1*03 allele differs between adults and newborns. Since it is well known that bearers of HLA-DR3 (and/or B8) antigens may display significant changes in immune parameters, the lower frequency in adults indicates that children with the DR3 antigen are predisposed to immune diseases in adulthood. | 3463 | NA | NA | NA | no | no |
| 3463 | 2017 | NA | Trotta, V; Calboli, FCF; Ziosi, M; Guerra, D; Pezzoli, MC; David, JR; Cavicchi, S | 2006 | Thermal plasticity in Drosophila Melanogaster: A comparison of geographic populations | Background: Populations of Drosophila melanogaster show differences in many morphometrical traits according to their geographic origin. Despite the widespread occurrence of these differences in more than one Drosophila species, the actual selective mechanisms controlling the genetic basis of such variation are not fully understood. Thermal selection is considered to be the most likely cause explaining these differences. Results: In our work, we investigated several life history traits (body size, duration of development, preadult survival, longevity and productivity) in two tropical and two temperate natural populations of D. melanogaster recently collected, and in a temperate population maintained for twelve years at the constant temperature of 18 degrees C in the laboratory. In order to characterise the plasticity of these life history traits, the populations were grown at 12, 18, 28 and 31.2 degrees C. Productivity was the fitness trait that showed clearly adaptive differences between latitudinal populations: tropical flies did better in the heat but worse in the cold environments with respect to temperate flies. Differences for the plasticity of other life history traits investigated between tropical and temperate populations were also found. The differences were particularly evident at stressful temperatures (12 and 31.2 degrees C). Conclusion: Our results evidence a better cold tolerance in temperate populations that seems to have been evolved during the colonisation of temperate countries by D. melanogaster Afrotropical ancestors, and support the hypothesis of an adaptive response of plasticity to the experienced environment. | 3464 | NA | NA | NA | no | no |
| 3464 | 2017 | NA | Mudrik, EA; Kashentseva, TA; Postelnykh, KA; Nosachenko, GV; Politov, DV | 2015 | Offspring sex ratio in the captive population of the Siberian crane (Grus leucogeranus Pallas) | The sex ratio of Siberian crane chicks (Grus leucogeranus Pallas) from the captive population of the Oka Crane Breeding Center was analyzed with the use of molecular sex marker EE0.6 in 2009-2014. We determined the sex of 84 birds bred from 12 dams by means of artificial insemination and natural breeding. The total primary sex ratio was 40: 44, and the secondary sex ratio was 36: 39 with a minor female bias. The mortality rate of embryos was the same for both sexes. The primary and secondary sex ratio for the first eggs in clutches were in-line with total sex ratio. The relatedness of parents by microsatellite loci has no effect on sex ratio in chicks of naturally and artificially propagating dams. | 3465 | NA | NA | NA | no | no |
| 3465 | 2017 | NA | Chadha, S; Gopalakrishna, T | 2005 | Retrotransposon-microsatellite amplified polymorphism (REMAP) markers for genetic diversity assessment of the rice blast pathogen (Magnaporthe grisea) | This present study is the first report of the application of the retrotransposon-microsatellite amplified polymorphism (REMAP) technique in fungi. Genome fingerprinting has a major role in the characterization of population structure and in the analysis of the variability in fungi. Retrotransposon-microsatellite amplified polymorphism assay was used in virulent isolates of a rice blast pathogen (Magnaporthe grisea) as a new assay system for genetic variability studies that overcomes the limitations of previous techniques. The high polymorphism observed in REMAP could be due to past or recent actions of retrotransposon in M. grisea. Retrotransposon-microsatellite amplified polymorphism, with its superior marker utility, was concluded to be the marker of choice for characterizing M. grisea isolates. | 3466 | NA | NA | NA | no | no |
| 3466 | 2017 | NA | Arenas, CD; Lehman, N | 2010 | Quasispecies-like behavior observed in catalytic RNA populations evolving in a test tube | Background: During the RNA World, molecular populations were probably very small and highly susceptible to the force of strong random drift. In conjunction with Muller’s Ratchet, this would have imposed difficulties for the preservation of the genetic information and the survival of the populations. Mechanisms that allowed these nascent populations to overcome this problem must have been advantageous. Results: Using continuous in vitro evolution experimentation with an increased mutation rate imposed by MnCl(2), it was found that clonal 100-molecule populations of ribozymes clearly exhibit certain characteristics of a quasispecies. This is the first time this has been seen with a catalytic RNA. Extensive genotypic sampling from two replicate lineages was gathered and phylogenetic networks were constructed to elucidate the structure of the evolving RNA populations. A common distribution was found in which a mutant sequence was present at high frequency, surrounded by a cloud of mutant with lower frequencies. This is a typical distribution of quasispecies. Most of the mutants in these clouds were connected by short Hamming distance values, indicating their close relatedness. Conclusions: The quasispecies nature of mutant RNA clouds facilitates the recovery of genotypes under pressure of being removed from the population by random drift. The empirical populations therefore evolved a genotypic resiliency despite a high mutation rate by adopting the characteristics of quasispecies, implying that primordial RNA pools could have used this strategy to avoid extinction. | 3467 | NA | NA | NA | no | no |
| 3467 | 2017 | NA | Hill, NM; Vander Kloet, SP | 2005 | Longevity of experimentally buried seed in Vaccinium: relationship to climate, reproductive factors and natural seed banks | 1 The fleshy fruited species of Vaccinium are known for their spatial dispersal ability but have generally been found to have poor representation in soil seed banks. 2 A buried seed experiment was conducted with 28 species of Vaccinium, in which seed lots were exhumed at intervals for up to 17 years. Mean species seed longevity was 8.65 years. For five species that persisted in soil for 15-17 years, regression indicates survival times of more than 20 years. 3 Climatic factors (particularly annual temperature range in the centre of the species’ distribution range) alone accounted for 42% of the variation in seed longevity and reproductive variables for 55%. 4 Models of fruit dispersal speed (rapid/tardy) demonstrated the link between spatial and temporal (seed longevity) dispersal and between spatial dispersal and habitat affinity (open/closed) of a species. The majority (87%) of species belonged to two of the four possible syndromes (rapid fruit dispersal/long-term seed persistence or tardy dispersal/short-term persistence); 84% of the former were open habitat species. 5 Seed bank density is a function of seed potential longevity, seed rain and soil conditions. Regions that support species having greatest potential longevity have the smallest seed banks. Annual temperature range was positively correlated with potential longevity but negatively correlated with actual seed bank densities. 6 Lack of recruitment via seed in Vaccinium species in arctic regions may limit genetic variability in these clonal plants and impede population responses to climate changes.  This is the content viewer section. Skip to metadata section.  1 of 10 pages 1 The fleshy fruited species of Vaccinium are known for their spatial dispersal ability but have generally been found to have poor representation in soil seed banks. 2 A buried seed experiment was conducted with 28 species of Vaccinium, in which seed lots were exhumed at intervals for up to 17 years. Mean species seed longevity was 8.65 years. For five species that persisted in soil for 15-17 years, regression indicates survival times of more than 20 years. 3 Climatic factors (particularly annual temperature range in the centre of the species’ distribution range) alone accounted for 42% of the variation in seed longevity and reproductive variables for 55%. 4 Models of fruit dispersal speed (rapid/tardy) demonstrated the link between spatial and temporal (seed longevity) dispersal and between spatial dispersal and habitat affinity (open/closed) of a species. The majority (87%) of species belonged to two of the four possible syndromes (rapid fruit dispersal/long-term seed persistence or tardy dispersal/short-term persistence); 84% of the former were open habitat species. 5 Seed bank density is a function of seed potential longevity, seed rain and soil conditions. Regions that support species having greatest potential longevity have the smallest seed banks. Annual temperature range was positively correlated with potential longevity but negatively correlated with actual seed bank densities. 6 Lack of recruitment via seed in Vaccinium species in arctic regions may limit genetic variability in these clonal plants and impede population responses to climate changes.  This is the content viewer section. Skip to metadata section.  1 of 10 pages 1 The fleshy fruited species of Vaccinium are known for their spatial dispersal ability but have generally been found to have poor representation in soil seed banks. 2 A buried seed experiment was conducted with 28 species of Vaccinium, in which seed lots were exhumed at intervals for up to 17 years. Mean species seed longevity was 8.65 years. For five species that persisted in soil for 15-17 years, regression indicates survival times of more than 20 years. 3 Climatic factors (particularly annual temperature range in the centre of the species’ distribution range) alone accounted for 42% of the variation in seed longevity and reproductive variables for 55%. 4 Models of fruit dispersal speed (rapid/tardy) demonstrated the link between spatial and temporal (seed longevity) dispersal and between spatial dispersal and habitat affinity (open/closed) of a species. The majority (87%) of species belonged to two of the four possible syndromes (rapid fruit dispersal/long-term seed persistence or tardy dispersal/short-term persistence); 84% of the former were open habitat species. 5 Seed bank density is a function of seed potential longevity, seed rain and soil conditions. Regions that support species having greatest potential longevity have the smallest seed banks. Annual temperature range was positively correlated with potential longevity but negatively correlated with actual seed bank densities. 6 Lack of recruitment via seed in Vaccinium species in arctic regions may limit genetic variability in these clonal plants and impede population responses to climate changes.  This is the content viewer section. Skip to metadata section.  1 of 10 pages 1 The fleshy fruited species of Vaccinium are known for their spatial dispersal ability but have generally been found to have poor representation in soil seed banks. 2 A buried seed experiment was conducted with 28 species of Vaccinium, in which seed lots were exhumed at intervals for up to 17 years. Mean species seed longevity was 8.65 years. For five species that persisted in soil for 15-17 years, regression indicates survival times of more than 20 years. 3 Climatic factors (particularly annual temperature range in the centre of the species’ distribution range) alone accounted for 42% of the variation in seed longevity and reproductive variables for 55%. 4 Models of fruit dispersal speed (rapid/tardy) demonstrated the link between spatial and temporal (seed longevity) dispersal and between spatial dispersal and habitat affinity (open/closed) of a species. The majority (87%) of species belonged to two of the four possible syndromes (rapid fruit dispersal/long-term seed persistence or tardy dispersal/short-term persistence); 84% of the former were open habitat species. 5 Seed bank density is a function of seed potential longevity, seed rain and soil conditions. Regions that support species having greatest potential longevity have the smallest seed banks. Annual temperature range was positively correlated with potential longevity but negatively correlated with actual seed bank densities. 6 Lack of recruitment via seed in Vaccinium species in arctic regions may limit genetic variability in these clonal plants and impede population responses to climate changes.  This is the content viewer section. Skip to metadata section.  1 of 10 pages 1 The fleshy fruited species of Vaccinium are known for their spatial dispersal ability but have generally been found to have poor representation in soil seed banks. 2 A buried seed experiment was conducted with 28 species of Vaccinium, in which seed lots were exhumed at intervals for up to 17 years. Mean species seed longevity was 8.65 years. For five species that persisted in soil for 15-17 years, regression indicates survival times of more than 20 years. 3 Climatic factors (particularly annual temperature range in the centre of the species’ distribution range) alone accounted for 42% of the variation in seed longevity and reproductive variables for 55%. 4 Models of fruit dispersal speed (rapid/tardy) demonstrated the link between spatial and temporal (seed longevity) dispersal and between spatial dispersal and habitat affinity (open/closed) of a species. The majority (87%) of species belonged to two of the four possible syndromes (rapid fruit dispersal/long-term seed persistence or tardy dispersal/short-term persistence); 84% of the former were open habitat species. 5 Seed bank density is a function of seed potential longevity, seed rain and soil conditions. Regions that support species having greatest potential longevity have the smallest seed banks. Annual temperature range was positively correlated with potential longevity but negatively correlated with actual seed bank densities. 6 Lack of recruitment via seed in Vaccinium species in arctic regions may limit genetic variability in these clonal plants and impede population responses to climate changes.  This is the content viewer section. Skip to metadata section. 1 of 10 pages 1 The fleshy fruited species of Vaccinium are known for their spatial dispersal ability but have generally been found to have poor representation in soil seed banks. 2 A buried seed experiment was conducted with 28 species of Vaccinium, in which seed lots were exhumed at intervals for up to 17 years. Mean species seed longevity was 8.65 years. For five species that persisted in soil for 15-17 years, regression indicates survival times of more than 20 years. 3 Climatic factors (particularly annual temperature range in the centre of the species’ distribution range) alone accounted for 42% of the variation in seed longevity and reproductive variables for 55%. 4 Models of fruit dispersal speed (rapid/tardy) demonstrated the link between spatial and temporal (seed longevity) dispersal and between spatial dispersal and habitat affinity (open/closed) of a species. The majority (87%) of species belonged to two of the four possible syndromes (rapid fruit dispersal/long-term seed persistence or tardy dispersal/short-term persistence); 84% of the former were open habitat species. 5 Seed bank density is a function of seed potential longevity, seed rain and soil conditions. Regions that support species having greatest potential longevity have the smallest seed banks. Annual temperature range was positively correlated with potential longevity but negatively correlated with actual seed bank densities. 6 Lack of recruitment via seed in Vaccinium species in arctic regions may limit genetic variability in these clonal plants and impede population responses to climate changes. | 3468 | NA | NA | NA | no | no |
| 3468 | 2017 | NA | Sun, YJ; Lan, XY; Lei, CZ; Zhang, CL; Chen, H | 2015 | Haplotype combination of the bovine CFL2 gene sequence variants and association with growth traits in Qinchuan cattle | The aim of this study was to examine the association of cofilin2 (CPU) gene polymorphisms with growth traits in Chinese Qinchuan cattle. Three single nucleotide polymorphisms (SNPs) were identified in the bovine CFL2 gene using DNA sequencing and (forced) PCR-RFLP methods. These polymorphisms included a missense mutation (NC\_007319.5: g. C 2213 G) in exon 4, one synonymous mutation (NC\_007319.5: g. T 1694 A) in exon 4, and a mutation (NC\_007319.5: g. G 1500 A) in intron 2, respectively. In addition, we evaluated the haplotype frequency and linkage disequilibrium coefficient of three sequence variants in 488 individuals in QC cattle. All the three SNPs in QC cattle belonged to an intermediate level of genetic diversity (0.25 < PIC < 0.5). Haplotype analysis of three SNPs showed that 8 different haplotypes were identified in all, but only 5 haplotypes were listed except for those with a frequency of <0.03. Hap4 (-GTC-) had the highest haplotype frequencies (34.70%). However in the three SNPs there were no significant associations between the 13 combined genotypes of the CFL2 gene and growth traits. LD analysis showed that the SNP T 1694 A and C 2213 G loci had a strong linkage (r(2)>0.33). Association analysis indicated that SNP G 1500 A, T 1694 A and C 2213 G were significantly associated with growth traits in the QC population. The results of our study suggest that the CFL2 gene may be a strong candidate gene that affects growth traits in the QC cattle breeding program. (C) 2015 Elsevier B.V. All rights reserved. | 3469 | NA | NA | NA | no | no |
| 3469 | 2017 | NA | Morris, DC; Schwarz, MP; Cooper, SJB; Mound, LA | 2002 | Phylogenetics of Australian Acacia thrips: the evolution of behaviour and ecology | The species of thrips found on Acacia constitute a major component of the Australian thrips fauna, with at least 235 species in more than 30 genera, many of these being in the process of description as new. These thrips exhibit social behaviours, ranging from solitary and colonial species to a variety of more complex social organisations. Furthermore, the domiciliary habits of these species include domicile construction, gall induction, and opportunistic use of abandoned galls and domiciles. This suite of thrips also includes a variety of inquiline and kleptoparasitic taxa. To understand how these various traits have evolved and interact in this diverse group, we have reconstructed a phylogeny for 42 species of thrips associated with Acacia around Australia. We obtained DNA sequence data from two nuclear genes (Elongation Factor-1alpha and wingless) and one mitochondrial gene (cytochrome oxidase 1) and analysed these using maximum parsimony and maximum likelihood methods. A phylogeny resulting from such analysis allows inference of evolutionary transitions in domiciliary habits, social organisations, and parasitic behaviours. Gall induction and parasitic behaviour are postulated to each have a single origin, with no losses of either trait. Once parasitism evolved a remarkable radiation followed that allowed exploitation of very diverse hosts. Our data do not allow hypotheses of single versus multiple origins of domicile building to be resolved while opportunistic gall use appears to have arisen several times. (C) 2002 Elsevier Science (USA). All rights reserved. | 3470 | NA | NA | NA | no | no |
| 3470 | 2017 | NA | Fan, ZX; Zhao, G; Li, P; Osada, N; Xing, JC; Yi, Y; Du, LM; Silva, P; Wang, HX; Sakate, R; Zhang, XY; Xu, HL; Yue, BS; Li, J | 2014 | Whole-Genome Sequencing of Tibetan Macaque (Macaca thibetana) Provides New Insight into the Macaque Evolutionary History | Macaques are the most widely distributed nonhuman primates and used as animal models in biomedical research. The availability of full-genome sequences from them would be essential to both biomedical and primate evolutionary studies. Previous studies have reported whole-genome sequences from rhesus macaque (Macaca mulatta) and cynomolgus macaque (M. fascicularis, CE), both of which belong to the fascicularis group. Here, we present a 37-fold coverage genome sequence of the Tibetan macaque (M. thibetana; TM). TM is an endemic species to China belonging to the sinica group. On the basis of mapping to the rhesus macaque genome, we identified approximately 11.9 million single-nucleotide variants), of which 3.9 million were TM specific, as assessed by comparison two Chinese rhesus macaques (CR) and two CE genomes. Some genes carried TM-specific homozygous nonsynonymous variants (TSHNVs), which were scored as deleterious in human by both PolyPhen-2 and SIFT (Sorting Tolerant From Intolerant) and were enriched in the eye disease genes. In total, 273 immune response and disease-related genes carried at least one TSHNV. The heterozygosity rates of two CRs (0.002617 and 0.002612) and two CEs (0.003004 and 0.003179) were approximately three times higher than that of TM (0.000898). Polymerase chain reaction resequencing of 18 TM individuals showed that 29 TSHNVs exhibited high allele frequencies, thus confirming their low heterozygosity. Genome-wide genetic divergence analysis demonstrated that TM was more closely related to CR than to CE. We further detected unusual low divergence regions between TM and CR. In addition, after applying statistical criteria to detect putative introgression regions (PIRs) in the TM genome, up to 239,620 kb PIRs (8.84% of the genome) were identified. Given that TM and CR have overlapping geographical distributions, had the same refuge during the Middle Pleistocene, and show similar mating behaviors, it is highly likely that there was an ancient introgression event between them. Moreover, demographic inferences revealed that TM exhibited a similar demographic history as other macaques until 0.5 Ma, but then it maintained a lower effective population size until present time. Our study has provided new insight into the macaque evolutionary history, confirming hybridization events between macaque species groups based on genome-wide data. | 3471 | NA | NA | NA | no | no |
| 3471 | 2017 | NA | Gbadegesin, RA; Cotton, SA; Watson, CJ; Brenchley, PEC; Webb, NJA | 2006 | Association between ICAM-1 Gly-Arg polymorphism and renal parenchymal scarring following childhood urinary tract infection | Renal parenchymal scarring (RPS) following urinary tract infection (UTI) is an important cause of renal morbidity in children. Studies have shown that the intensity of the inflammatory response following infection is related to the risk of RPS. However, genetic variability in this response has not been studied. Adhesion molecules play a crucial role in leucocyte recruitment following infection, and polymorphisms have been reported in the genes for key cell adhesion molecules. We have investigated the possibility that children who develop RPS following UTI may exhibit altered genotype or allele frequencies for polymorphisms of the intercellular adhesion molecule-1 (ICAM-1) (exons 4 and 6), E-selectin (exons 2 and 4), platelet endothelial cell adhesion molecule-1 (PECAM-1) (exon 3) and CD11b (3’UTR) genes, which may predict outcome of UTI. DNA was isolated from 99 children shown to have developed RPS, 43 children with no evidence of scarring (NS) following UTI and 170 healthy controls. Genotyping was performed by restriction fragment length polymorphism (RFLP) analysis. When the RPS group was compared with the NS group, there was a significant reduction in the frequency of the ICAM-1 exon 4 A allele (10.6 vs. 21.3%, respectively, chi(2)= 6.01, P= 0.014). There was no significant difference in either allele or genotype frequency for any of the other polymorphisms studied. These data suggest that the A allele of the ICAM-1 exon 4 polymorphism may protect against the risk of RPS following UTI and may participate in the regulation of the inflammatory response following UTI. | 3472 | NA | NA | NA | no | no |
| 3472 | 2017 | NA | TADDEI, F; MATIC, I; RADMAN, M | 1995 | CAMP-DEPENDENT SOS INDUCTION AND MUTAGENESIS IN RESTING BACTERIAL-POPULATIONS | The inducible SOS system increases the survival of bacteria exposed to DNA-damaging agents by increasing the capacity of error-free and error-prone DNA repair systems. The inducible mutator effect is expected to contribute to the adaptation of bacterial populations to these adverse life conditions by increasing their genetic variability. The evolutionary impact of the SOS system would be even greater if it was also induced under conditions common in nature, such as in resting bacterial populations. The results presented here show that SOS induction and mutagenesis do occur in bacteria in aging colonies on agar plates. The observed SOS induction and mutagenesis are controlled by the LexA repressor and are RecA- and cAMP-dependent. | 3473 | NA | NA | NA | no | no |
| 3473 | 2017 | NA | Aghajanzadeh, S; Prasad, DT; Mallik, B | 2007 | Genetic diversity in Hirsutella thompsonii isolates based on random amplified polymorphic DNA analysis | Hirsutella thompsonii Fisher is a host-specific fungal pathogen of the diverse group of arthropods known as the Acari. It particularly affects eriophyid mites, and the identification of its isolates is a difficult task due to its pleomorphic nature. Seven isolates of H. thompsonii var. thompsonii and H. thompsonii var. synnematosa collected from various agro-climatic regions of India were subjected to PCR analysis using random decamer primers to differentiate between them. Random amplified polymorphic DNA analysis revealed that all of the isolates from the citrus rust mite, Phyllocoptruta oleivora Ashmead (Acari: Eriophyidae), clustered together, with the exception of one, HtCRMB. The only isolate of H. thompsonii from Aceria guerreronis keifer (Acari: Eriophydae), HtPDBC, clustered separately. | 3474 | NA | NA | NA | no | no |
| 3474 | 2017 | NA | Oates-O’Brien, RS; Farver, TB; Anderson-Vicino, KC; McCowan, B; Lerche, NW | 2010 | Predictors of Matrilineal Overthrows in Large Captive Breeding Groups of Rhesus Macaques (Macaca mulatta) | The purpose of this retrospective case-control study was to identify and assess biologically plausible variables that may predispose a captive rhesus macaque breeding colony to a matrilineal overthrow. Matrilineal overthrows are the result of members of multiple matrilines jointly attacking the highest-ranking matriline. Matrilineal overthrows in captive rhesus macaque colonies result in significant morbidity, mortality, and loss of genetic diversity. The following variables were investigated as potential determinants of overthrows: season, cage density, demographics, sex ratio, age of the alpha and beta animals, absence of the alpha and beta animals, pregnancy status of the alpha and beta females, number of adult females in the alpha matriline, recent changes in the male hierarchy, time since group formation, and number of adolescent males in the alpha matriline. Data were collected from January 1996 through January 2007. Univariate analysis indicated that absence of the alpha female from the group was associated with matrilineal overthrows, but multivariate analysis was not totally supportive. Conditional logistic regression identified number of juvenile males and number of adolescent males as associated with an overthrow; exact logistic regression was supportive. Principal component analysis followed by multivariate logistic regression identified 2 marginally nonsignificant predictors (the density and alpha factors). Our results suggest a possible association between the occurrence of a matrilineal overthrow and the following factors: absence of the alpha female, decreased housing density, number of juvenile males, and number of adolescent males. | 3475 | NA | NA | NA | no | no |
| 3475 | 2017 | NA | Vila, M; Auger-Rozenberg, MA; Goussard, F; Lopez-Vaamonde, C | 2009 | Effect of non-lethal sampling on life-history traits of the protected moth Graellsia isabelae (Lepidoptera: Saturniidae) | Abstract 1. Non‐lethal genetic surveys in insects usually extract DNA from a leg or a piece of wing. Although preferable to lethal sampling, little is known about the effect of leg/wing non‐lethal sampling on fitness‐related traits.   2. Graellsia isabelae (Graells, 1849) is a European moth protected by the Habitats Directive and the Bern Convention. Conservation genetics surveys on this species should therefore use non‐lethal sampling. 3. The present study aimed to (1) quantify the effects of both leg and hind‐wing tail sampling on survivorship and reproductive behaviour of adult males and females, and (2) assess the quality and quantity of DNA obtained from those tissues. 4. Both hind‐wing tails and mid‐legs proved to be good sources of high quality nuclear and mitochondrial DNA. DNA concentration was significantly higher when extracted from a large (130 mm2) piece of the hind‐wing tails than from about half of the mid‐leg. Using mark–release–recapture experiments with adults, it was found that neither mid‐leg nor hind‐wing tail sampling significantly reduced male survivorship or total number of matings. However, although mid‐leg sampling did not significantly affect female survivorship, it had a negative effect on female mating success. 5. Wing‐tail clipping on males appeared to be the best non‐lethal sampling procedure for G. isabelae. It is a fast procedure, similar to natural wing impairment, and did not significantly affect survival or mating behaviour. | 3476 | NA | NA | NA | no | no |
| 3476 | 2017 | NA | Ksentini, I; Herz, A; Ksantini, M; Jardak, T; Hassan, SA | 2011 | Temperature and strain effects on reproduction and survival of Trichogramma oleae and Trichogramma cacoeciae (Hymenoptera: Trichogrammatidae) | To assess differences in temperature sensitivity during development, life tables for two lines derived from the species Trichogramma oleae Voegele and Pointel and a strain of Trichogramma cacoeciae Marchal (Hymenoptera: Trichogrammatidae) were elaborated at 15, 20, 25, 30, 35, 36, and 37 degrees C in the laboratory. Eggs of Ephestia kuehniella Zeller together with a fresh drop of honey were supplied every 2 days until the death of the test females, and the removed host egg batches were placed in the equivalent rearing cabinet. The line ‘2F’ of T. oleae was found to be the most efficient at any range of temperatures except at 20 and 37 degrees C, in comparison to the other tested strains. For all species, no progeny emerged from eggs incubated at 36 degrees C and none of the parasitized eggs turned black at 37 degrees C. The better performance at a broader range of temperatures by T. oleae (line 2 F) might be caused by a shorter history in artificial rearing in comparison to the other strains. Fewer generations at laboratory conditions and frequent multiplication on eggs of its natural host (the olive moth Prays oleae) may have prevented a deterioration in the rearing population of this strain, maintaining its genetic diversity at a higher scale. Applying varying temperature regimes on the rearing stock at regular intervals during the mass production process may help to maintain the essential quality of the biological control agents for field performance at higher temperatures. | 3477 | NA | NA | NA | no | no |
| 3477 | 2017 | NA | Adelman, JS; Kirkpatrick, L; Grodio, JL; Hawley, DM | 2013 | House Finch Populations Differ in Early Inflammatory Signaling and Pathogen Tolerance at the Peak of Mycoplasma gallisepticum Infection | Host individuals and populations often vary in their responses to infection, with direct consequences for pathogen spread and evolution. While considerable work has focused on the mechanisms underlying differences in resistance-the ability to kill pathogens-we know little about the mechanisms underlying tolerance-the ability to minimize fitness losses per unit pathogen. Here, we examine patterns and mechanisms of tolerance between two populations of house finches (Haemorhous [formerly Carpodacus] mexicanus) with different histories with the bacterial pathogen Mycoplasma gallisepticum (MG). After infection in a common environment, we assessed two metrics of pathology, mass loss and eye lesion severity, as proxies for fitness. We calculated tolerance using two methods, one based on pathology and pathogen load at the peak of infection (point tolerance) and the other based on the integrals of these metrics over time (range tolerance). Alabama birds, which have a significantly longer history of exposure to MG, showed more pronounced point tolerance than Arizona birds, while range tolerance did not differ between populations. Alabama birds also displayed lower inflammatory cytokine signaling and lower fever early in infection. These results suggest that differences in inflammatory processes, which can significantly damage host tissues, may contribute to variation in tolerance among house finch individuals and populations. Such variation can affect pathogen spread and evolution in ways not predictable by resistance alone and sheds light on the costs and benefits of inflammation in wild animals. | 3478 | NA | NA | NA | no | no |
| 3478 | 2017 | NA | Futami, K; Valderrama, A; Baldi, M; Minakawa, N; Rodriguez, RM; Chaves, LF | 2015 | New and Common Haplotypes Shape Genetic Diversity in Asian Tiger Mosquito Populations from Costa Rica and Panama | The Asian tiger mosquito, Aedes albopictus (Skuse) (Diptera: Culicidae), is a vector of several human pathogens. Ae. albopictus is also an invasive species that, over recent years, has expanded its range out of its native Asia. Ae. albopictus was suspected to be present in Central America since the 1990s, and its presence was confirmed by most Central American nations by 2010. Recently, this species has been regularly found, yet in low numbers, in limited areas of Panama and Costa Rica (CR). Here, we report that short sequences (similar to 558 bp) of the mitochondrial cytochrome oxidase subunit 1 (COI) and NADH dehydrogenase subunit 5 genes of Ae. albopictus, had no haplotype diversity. Instead, there was a common haplotype for each gene in both CR and Panama’. In contrast, a long COI sequence (similar to 1,390 bp) revealed that haplotype diversity (+/- SD) was relatively high in CR (0.72 +/- 0.04) when compared with Panama’ (0.33 +/- 0.13), below the global estimate for reported samples (0.89 +/- 0.01). The long COI sequence allowed us to identify seven (five new) haplotypes in CR and two (one new) in Panama’. A haplotype network for the long COI gene sequence showed that samples from CR and Panama’ belong to a single large group. The long COI gene sequences suggest that haplotypes in Panama’ and CR, although similar to each other, had a significant geographic differentiation (K-st = 1.33; P < 0.001). Thus, most of our results suggest a recent range expansion in CR and Panama | 3479 | NA | NA | NA | no | no |
| 3479 | 2017 | NA | Garzon, MB; Alia, R; Robson, TM; Zavala, MA | 2011 | Intra-specific variability and plasticity influence potential tree species distributions under climate change | Aim To assess the effect of local adaptation and phenotypic plasticity on the potential distribution of species under future climate changes. Trees may be adapted to specific climatic conditions; however, species range predictions have classically been assessed by species distributionmodels (SDMs) that do not account for intra-specific genetic variability and phenotypic plasticity, because SDMs rely on the assumption that species respond homogeneously to climate change across their range, i.e. a species is equally adapted throughout its range, and all species are equally plastic. These assumptions could cause SDMs to exaggerate or underestimate species at risk under future climate change. Location The Iberian Peninsula. Methods Species distributions are predicted by integrating experimental data and modelling techniques. We incorporate plasticity and local adaptation into a SDM by calibrating models of tree survivorship with adaptive traits in provenance trials. Phenotypic plasticity was incorporated by calibrating our model with a climatic index that provides a measure of the differences between sites and provenances. Results We present a new modelling approach that is easy to implement and makes use of existing tree provenance trials to predict species distribution models under global warming. Our results indicate that the incorporation of intra- population genetic diversity and phenotypic plasticity in SDMs significantly altered their outcome. In comparing species range predictions, the decrease in area occupancy under global warming conditions is smaller when considering our survival-adaptation model than that predicted by a ‘classical SDM’ calibrated with presence-absence data. These differences in survivorship are due to both local adaptation and plasticity. Differences due to the use of experimental data in the model calibration are also expressed in our results: we incorporate a null model that uses survival data from all provenances together. This model always predicts less reduction in area occupancy for both species than the SDM calibrated with presence-absence. Main conclusions We reaffirm the importance of considering adaptive traits when predicting species distributions and avoiding the use of occurrence data as a predictive variable. In light of these recommendations, we advise that existing predictions of future species distributions and their component populations must be reconsidered. | 3480 | NA | NA | NA | no | no |
| 3480 | 2017 | NA | Bonsall, MB | 2010 | Parasite Replication and the Evolutionary Epidemiology of Parasite Virulence | Parasite virulence evolution is shaped by both within-host and population-level processes yet the link between these differing scales of infection is often neglected. Population structure and heterogeneity in both parasites and hosts will affect how hosts are exploited by pathogens and the intensity of infection. Here, it is shown how the degree of relatedness among parasites together with epidemiological parameters such as pathogen yield and longevity influence the evolution of virulence. Furthermore, the role of kin competition and the degree of cheating within highly structured parasite populations also influences parasite fitness and infectivity patterns. Understanding how the effects of within-host processes scale up to affect the epidemiology has importance for understanding host-pathogen interactions. | 3481 | NA | NA | NA | no | no |
| 3481 | 2017 | NA | Springer, YP | 2007 | Clinal resistance structure and pathogen local adaptation in a serpentine flax-flax rust interaction | Because disease resistance is a hallmark signature of pathogen-mediated selection pressure on hosts, studies of resistance structure (the spatial distribution of disease resistance genes among conspecific host populations) can provide valuable insights into the influence of pathogens on host evolution and spatial variation in the magnitude of their effects. To date few studies of wild plant-pathogen interactions have characterized resistance structure by sampling across the host’s biogeographic range, and only a handful have paired such investigations with studies of disease levels under natural conditions. I used a greenhouse cross-inoculation experiment to characterize genetic resistance of 16 populations of California dwarf flax (Hesperolinon californicum) to attack by multiple samples of the rust fungus Melampsora lini. I documented a latitudinal cline in resistance structure, manifest across the host’s biogeographic range, which mirrored almost identically a cline in infection prevalence documented through field surveys of disease in study populations. These results provide empirical evidence for clinal patterns of antagonistic selection pressure, demonstrate that such patterns can be manifest across broad biogeographic scales, and suggest that rates of disease prevalence in wild plant populations may be tightly linked to the distribution of host resistance genes. Tests for local adaptation of the fungus revealed evidence of the phenomenon (significantly greater infection in sympatric plant-fungal pairings) as well as the potential for substantial bias to be introduced into statistical analyses by spatial patterns of host resistance structure. | 3482 | NA | NA | NA | no | no |
| 3482 | 2017 | NA | Wingbermuehle, WJ; Gustus, C; Smith, KP | 2004 | Exploiting selective genotyping to study genetic diversity of resistance to Fusarium head blight in barley | Numerous barley cultivars from around the world have been identified as potential sources of Fusarium head blight (FHB) resistance genes. All of these cultivars exhibit partial resistance, and several mapping studies have shown that resistance to FHB is controlled by multiple genes. Successful development of barley cultivars with high levels of FHB resistance will require combining genes from multiple sources. We characterized five potential new sources of FHB resistance (‘AC Oxbow’, ‘Atahualpa’, ‘HOR211’, ‘PFC88209’, and ‘Zhedar# 1’) to determine if they contain new FHB resistance genes. Cluster analysis, using a set of 80 SSR markers distributed throughout the genome, showed that most of the new sources of resistance were not similar to three cultivars that have been used in previous FHB mapping studies (‘Chevron’, ‘Frederickson’, and ‘Gobernadora’), with ‘Atahualpa’ and ‘HOR211’ being the most dissimilar. By selective genotyping, we determined whether markers linked to six known FHB resistance quantitative trait loci (QTLs), discovered in other genotypes, explained variation for resistance in advanced breeding populations created from the new sources of resistance. Markers linked to four of the six known QTLs were associated with FHB severity in at least one of the populations. However, none of the six QTL regions were associated with variation for FHB severity in populations derived from crosses that utilized sources of resistance HOR211 or PFC88209. Selective genotyping is an efficient method for breeders to utilize current QTL information about disease resistance to search for new resistance genes. | 3483 | NA | NA | NA | no | no |
| 3483 | 2017 | NA | Gaither, MR; Aeby, G; Vignon, M; Meguro, Y; Rigby, M; Runyon, C; Toonen, RJ; Wood, CL; Bowen, BW | 2013 | An Invasive Fish and the Time-Lagged Spread of Its Parasite across the Hawaiian Archipelago | Efforts to limit the impact of invasive species are frustrated by the cryptogenic status of a large proportion of those species. Half a century ago, the state of Hawai’i introduced the Bluestripe Snapper, Lutjanus kasmira, to O’ahu for fisheries enhancement. Today, this species shares an intestinal nematode parasite, Spirocamallanus istiblenni, with native Hawaiian fishes, raising the possibility that the introduced fish carried a parasite that has since spread to naive local hosts. Here, we employ a multidisciplinary approach, combining molecular, historical, and ecological data to confirm the alien status of S. istiblenni in Hawai’i. Using molecular sequence data we show that S. istiblenni from Hawai’i are genetically affiliated with source populations in French Polynesia, and not parasites at a geographically intermediate location in the Line Islands. S. istiblenni from Hawai’i are a genetic subset of the more diverse source populations, indicating a bottleneck at introduction. Ecological surveys indicate that the parasite has found suitable intermediate hosts in Hawai’i, which are required for the completion of its life cycle, and that the parasite is twice as prevalent in Hawaiian Bluestripe Snappers as in source populations. While the introduced snapper has spread across the entire 2600 km archipelago to Kure Atoll, the introduced parasite has spread only half that distance. However, the parasite faces no apparent impediments to invading the entire archipelago, with unknown implications for naive indigenous Hawaiian fishes and the protected Papahanaumokuakea Marine National Monument. | 3484 | NA | NA | NA | no | no |
| 3484 | 2017 | NA | Cummings, AC; Torstenson, E; Davis, MF; D’Aoust, LN; Scott, WK; Pericak-Vance, MA; Bush, WS; Haines, JL | 2013 | Evaluating Power and Type 1 Error in Large Pedigree Analyses of Binary Traits | Studying population isolates with large, complex pedigrees has many advantages for discovering genetic susceptibility loci; however, statistical analyses can be computationally challenging. Allelic association tests need to be corrected for relatedness among study participants, and linkage analyses require subdividing and simplifying the pedigree structures. We have extended GenomeSIMLA to simulate SNP data in complex pedigree structures based on an Amish pedigree to generate the same structure and distribution of sampled individuals. We evaluated type 1 error rates when no disease SNP was simulated and power when disease SNPs with recessive, additive, and dominant modes of inheritance and odds ratios of 1.1, 1.5, 2.0, and 5.0 were simulated. We generated subpedigrees with a maximum bit-size of 24 using PedCut and performed two-point and multipoint linkage using Merlin. We also ran MQLS on the subpedigrees and unified pedigree. We saw no inflation of type 1 error when running MQLS on either the whole pedigrees or the sub-pedigrees, and we saw low type 1 error for two-point and multipoint linkage. Power was reduced when running MQLS on the subpedigrees versus the whole pedigree, and power was low for two-point and multipoint linkage analyses of the subpedigrees. These data suggest that MQLS has appropriate type 1 error rates in our Amish pedigree structure, and while type 1 error does not seem to be affected when dividing the pedigree prior to linkage analysis, power to detect linkage is diminished when the pedigree is divided. | 3485 | NA | NA | NA | no | no |
| 3485 | 2017 | NA | Daneck, H; Fer, T; Marhold, K | 2016 | Glacial survival in northern refugia? Phylogeography of the temperate shrub Rosa pendulina L. (Rosaceae): AFLP vs. chloroplast DNA variation | Identification of postglacial migration patterns and localization of possible glacial refugia are the main tasks of phylogeographical studies. Analyses of AFLPs in the European temperate shrub Rosa pendulina L. provided an insight into this issue. Similarly to a previous study of chloroplast DNA (cpDNA) haplotype variation in this species, we detected two widely distributed groups of AFLP genotypes that probably contributed to the postglacial colonization of Central Europe. The first group comprises populations from most of the Alps, the Balkans, and the Apennines. The second group includes populations from the Carpathians, the Bohemian Massif, and part of the Alps. However, geographical delimitation of the contact zone between these two lineages was situated slightly southward from the zone defined by cpDNA haplotypes. This might reflect different dispersal abilities of seeds and pollen. Populations from the Iberian Peninsula represent a separate genetic subgroup within the Alpine-Balkan group that apparently did not contribute to the most recent postglacial expansion of the species. Indications for glacial survival (defined by higher-than-average frequency-down-weighted marker values) were identified in the Balkan Peninsula and in the Southern Alps. However, one population at the northern edge of the Alps and one in the Western Carpathians also possessed very high values. This finding might be an indication of full-glacial survival in these regions and points out the importance of the Carpathians for the historical distributional changes of R.pendulina. | 3486 | NA | NA | NA | no | no |
| 3486 | 2017 | NA | Maurice, T; Colling, G; Muller, S; Matthies, D | 2012 | Habitat characteristics, stage structure and reproduction of colline and montane populations of the threatened species Arnica montana | Arnica montana, a characteristic plant of nutrient-poor grasslands, has strongly declined during the last decades, particularly at lower altitudes. To gain insights into the underlying reasons for this decline, we recorded differences in size, stage structure and reproductive traits between 21 colline populations of A. montana in the Ardennes-Eifel region (280-600 m a.s.l.) and 10 montane populations in the Vosges Mountains (1,200 m a.s.l.). Community composition of the Violion caninae vegetation at colline sites and Nardion strictae vegetation at montane sites indicated that temperature and moisture were the main abiotic factors differentiating between low and high altitudinal relev,es. The proportion of flowering rosettes decreased with altitude, indicating a shift from sexual reproduction to clonal growth. In contrast to expectation, Ellenberg values for nutrient availability as well as the proportion of young rosettes and population size did not differ between colline and montane populations. However, population size decreased with nutrient availability, indicating eutrophication as the reason for population decline. In small populations, plant density, the proportion of flowering rosettes, the number of flowerheads per rosette and the number of seeds per flowerhead were lower, indicating less suitable conditions. Thus, preventing further eutrophication in both colline and montane populations will be crucial for the conservation of the species. Reduced reproduction may not be important for the population dynamics of this clonal plant in the short term, but could affect genetic diversity and survival in the long term. | 3487 | NA | NA | NA | no | no |
| 3487 | 2017 | NA | Laasanen, J; Romppanen, EL; Hiltunen, M; Helisalmi, S; Mannermaa, A; Punnonen, K; Heinonen, S | 2002 | Two exonic single nucleotide polymorphisms in the microsomal epoxide hydrolase gene are jointly associated with preeclampsia | This study determined whether genetic variability in exons 3 and 4 of the microsomal epoxide hydrolase gene jointly modifies individual preeclampsia risk. The study also determined whether genetic variability in the gene encoding for microsomal epoxide hydrolase (EPHX) contributes to individual differences in susceptibility to the development of preeclampsia. The study involved 133 preeclamptic and 115 healthy control pregnant women who were genotyped for two single nucleotide polymorphisms (SNPs), T–>C (Tyr113His) in exon 3 and A->G (His139Arg) in exon 4, in the EPHX gene. Chi-square analysis was used to assess genotype and allele frequency differences between the preeclamptic and control groups. In addition, single-point analysis was expanded to pair of loci haplotype analysis to examine the estimated haplotype frequencies of the two SNPs, of unknown phase, among the preeclamptic and control groups. Estimated haplotype frequencies were assessed using the maximum-likelihood method, employing an expectation-maximization (EM) algorithm. Single-point allele and genotype distributions in exons 3 and 4 of the EPHX gene were not statistically different between the groups. However, according to the haplotype estimation analysis, we observed a significantly elevated frequency of haplotype T-A (Tyr113-His139) among the preeclampsia group vs the control group (P=0.01). The odds ratio for preeclampsia associated with the high-activity haplotype T-A (Tyr113-His139) was 1.61 (95% CI: 1.12-2.32). The use of two intragenic SNPs jointly in haplotype analysis of association demonstrated that the genetically determined high-activity haplotype T-A (Tyr113-His139) was significantly associated with preeclampsia. | 3488 | NA | NA | NA | no | no |
| 3488 | 2017 | NA | Blakeslee, AMH; Altman, I; Miller, AW; Byers, JE; Gregory, CEH | 2012 | Parasites and invasions: a biogeographic examination of parasites and hosts in native and introduced ranges | Aim To use a comparative approach to understand parasite demographic patterns in native versus introduced populations, evaluating the potential roles of host invasion history and parasite life history. Location North American east and west coasts with a focus on San Francisco Bay (SFB). Methods Species richness and prevalence of trematode parasites were examined in the native and introduced ranges of two gastropod host species, Ilyanassa obsoleta and Littorina saxatilis. We divided the native range into the putative source area for introduction and areas to the north and south; we also sampled the overlapping introduced range in SFB. We dissected 14,781 snails from 103 populations and recorded the prevalence and identity of trematode parasites. We compared trematode species richness and prevalence across the hosts’ introduced and native ranges, and evaluated the influence of host availability on observed patterns. Results Relative to the native range, both I. obsoleta and L. saxatilis have escaped (lost) parasites in SFB, and L. saxatilis demonstrated a greater reduction of trematode diversity and infection prevalence than I. obsoleta. This was not due to sampling inequalities between the hosts. Instead, rarefaction curves suggested complete capture of trematode species in native source and SFB subregions, except for L. saxatilis in SFB, where infection was extremely rare. For I. obsoleta, infection prevalence of trematodes using fish definitive hosts was significantly lower in SFB compared to the native range, unlike those using bird hosts. Host availability partly explained the presence of introduced trematodes in SFB. Main conclusions Differential losses of parasite richness and prevalence for the two gastropod host species in their introduced range is probably the result of several mechanistic factors: time since introduction, propagule pressure, vector of introduction, and host availability. Moreover, the recent occurrence of L. saxatilis’ invasion and its active introduction vector suggest that its parasite diversity and distribution will probably increase over time. Our study suggests that host invasion history and parasite life history play key roles in the extent and diversity of trematodes transferred to introduced populations. Our results also provide vital information for understanding community-level influences of parasite introductions, as well as for disease ecology in general. | 3489 | NA | NA | NA | no | no |
| 3489 | 2017 | NA | Coassin, S; Brandstatter, A; Kronenberg, F | 2008 | An optimized procedure for the design and evaluation of Ecotilling assays | Background: Single nucleotide polymorphisms (SNPs) are the most common form of genetic variability in the human genome and play a prominent role in the heritability of phenotypes. Especially rare alleles with frequencies less than 5% may exhibit a particularly strong influence on the development of complex diseases. The detection of rare alleles by standard DNA sequencing is time-consuming and cost-intensive. Here we discuss an alternative approach for a high throughput detection of rare mutations in large population samples using Ecotilling embedded in a collection of bioinformatic analysis tools. Ecotilling originally was introduced as TILLING for the screening for rare chemically induced mutations in plants and later adopted for human samples, showing an outstanding suitability for the detection of rare alleles in humans. An actual problem in the use of Ecotilling for large mutation screening projects in humans without bioinformatic support is represented by the lack of solutions to quickly yet comprehensively evaluate each newly found variation and place it into the correct genomic context. Results: We present an optimized strategy for the design, evaluation and interpretation of Ecotilling results by integrating several mostly freely available bioinformatic tools. A major focus of our investigations was the evaluation and meaningful economical combination of these software tools for the inference of different possible regulatory functions for each newly detected mutation. Conclusion: Our streamlined procedure significantly facilitates the experimental design and evaluation of Ecotilling assays and strongly improves | 3490 | NA | NA | NA | no | no |
| 3490 | 2017 | NA | Mulki, MA; Jighly, A; Ye, GY; Emebiri, LC; Moody, D; Ansari, O; Ogbonnaya, FC | 2013 | Association mapping for soilborne pathogen resistance in synthetic hexaploid wheat | Soilborne pathogens such as cereal cyst nematode (CCN; Heterodera avenae) and root lesion nematode (Pratylenchus neglectus; PN) cause substantial yield losses in the major cereal-growing regions of the world. Incorporating resistance into wheat cultivars and breeding lines is considered the most cost-effective control measure for reducing nematode populations. To identify loci with molecular markers linked to genes conferring resistance to these pathogens, we employed a genome-wide association approach in which 332 synthetic hexaploid wheat lines previously screened for resistance to CCN and PN were genotyped with 660 Diversity Arrays Technology (DArT) markers. Two sequence-tagged site markers reportedly linked to genes known to confer resistance to CCN were also included in the analysis. Using the mixed linear model corrected for population structure and familial relatedness (Q+K matrices), we were able to confirm previously reported quantitative trait loci (QTL) for resistance to CCN and PN in bi-parental crosses. In addition, we identified other significant markers located in chromosome regions where no CCN and PN resistance genes have been reported. Seventeen DArT marker loci were found to be significantly associated with CCN and twelve to PN resistance. The novel QTL on chromosomes 1D, 4D, 5B, 5D and 7D for resistance to CCN and 4A, 5B and 7B for resistance to PN are suggested to represent new sources of genes which could be deployed in further wheat improvement against these two important root diseases of wheat. | 3491 | NA | NA | NA | no | no |
| 3491 | 2017 | NA | Keynan, Y; Malik, S; Fowke, KR | 2013 | The Role of Polymorphisms in Host Immune Genes in Determining the Severity of Respiratory Illness Caused by Pandemic H1N1 Influenza | Following the influenza epidemics, it has become clear that severity of illness is not uniform. Comorbidities and immunocompromise account for only a fraction of severe cases and do not explain the differential severity among the otherwise healthy during pandemics. During the 2009 H1N1 pandemic, a focus has been placed on better understanding of the determinants and pathogenesis of severe influenza infections. Much of the current literature has focused on viral genetics and its impact on host immunity as well as novel risk factors for severe infection (particularly within the H1N1 pandemic). The improved understanding of the cellular mechanisms and pathways involved in the pathogenesis of severe disease along with technological advances have allowed a more systematic approach to the exploration of the host genetic determinants of susceptibility and severe respiratory illness. By better defining the role of genetic variability in the immune responses to influenza, and identifying key polymorphisms that either protect against severe manifestation or those that impair the host immune response, it is possible to envision better methods to identify at-risk populations and new targets for therapeutic interventions and vaccines. This review will summarize the accumulated literature examining the immunogenetic factors associated with propensity for the development of severe pandemic H1N1 (pH1N1) manifestations. We will focus on novel and key insights gained through study of ethnic populations that appeared more vulnerable to severe disease during the 2009 H1N1 pandemic. Copyright (C) 2013 S. Karger AG, Basel | 3492 | NA | NA | NA | no | no |
| 3492 | 2017 | NA | Huchard, E; Albrecht, C; Schliehe-Diecks, S; Baniel, A; Roos, C; Peter, PMK; Brameier, M | 2012 | Large-scale MHC class II genotyping of a wild lemur population by next generation sequencing | The critical role of major histocompatibility complex (MHC) genes in disease resistance, along with their putative function in sexual selection, reproduction and chemical ecology, make them an important genetic system in evolutionary ecology. Studying selective pressures acting on MHC genes in the wild nevertheless requires population-wide genotyping, which has long been challenging because of their extensive polymorphism. Here, we report on large-scale genotyping of the MHC class II loci of the grey mouse lemur (Microcebus murinus) from a wild population in western Madagascar. The second exons from MHC-DRB and -DQB of 772 and 672 individuals were sequenced, respectively, using a 454 sequencing platform, generating more than 800,000 reads. Sequence analysis, through a stepwise variant validation procedure, allowed reliable typing of more than 600 individuals. The quality of our genotyping was evaluated through three independent methods, namely genotyping the same individuals by both cloning and 454 sequencing, running duplicates, and comparing parent-offspring dyads; each displaying very high accuracy. A total of 61 (including 20 new) and 60 (including 53 new) alleles were detected at DRB and DQB genes, respectively. Both loci were non-duplicated, in tight linkage disequilibrium and in Hardy-Weinberg equilibrium, despite the fact that sequence analysis revealed clear evidence of historical selection. Our results highlight the potential of 454 sequencing technology in attempts to investigate patterns of selection shaping MHC variation in contemporary populations. The power of this approach will nevertheless be conditional upon strict quality control of the genotyping data. | 3493 | NA | NA | NA | no | no |
| 3493 | 2017 | NA | Sang, Y; Ma, J; Hou, ZJ; Zhang, YL | 2012 | Phylogenetic analysis of the VP2 gene of Aleutian mink disease parvoviruses isolated from 2009 to 2011 in China | Aleutian mink disease parvovirus (AMDV) is a non-enveloped virus with a single-stranded DNA genome that causes a fatal, usually persistent immune complex disease in minks. In this study, a total of 18,654 serum samples were collected from minks that were farmed in China from 2009 to 2011. After testing by counter-current immunoelectrophoresis (CIE), the seroprevalence of AMDV was found to be 68.67 %. The results show that there is a serious epidemic among Chinese minks used for breeding. To gain detailed information regarding the molecular epidemiology of AMDV in China, nine strains of AMDV were isolated from mink samples that were collected from four of the primary mink farming areas in China. The full-length capsid protein VP2 gene from each strain was sequenced after PCR amplification, and a phylogenetic analysis was performed on the VP2 gene sequence, including the VP2 genes from the other 10 AMDV strains available in the GenBank database, which were submitted from the 1970s to 2009. The phylogenetic analysis showed that the AMDV isolates were divided into five independent clades. The Chinese AMDV strains were distributed among all five groups and showed a high level of genetic diversity. Over 50 % of the Chinese AMDV strains were classified into two clades that consisted only of isolates from China and that were distinct from AMDV strains found in other countries. This finding indicated that both local and imported ADMV species are prevalent in the Chinese mink farming population. | 3494 | NA | NA | NA | no | no |
| 3494 | 2017 | NA | de Bellocq, JG; Suchentrunk, F; Baird, SJE; Schaschl, H | 2009 | Evolutionary history of an MHC gene in two leporid species: characterisation of Mhc-DQA in the European brown hare and comparison with the European rabbit | We surveyed the genetic diversity of the expressed major histocompatibility complex class II DQA locus in natural populations of European brown hares, Lepus europaeus, from Austria and Belgium (267 individuals in total). Based on cDNA sequences, we designed hare-specific primers to amplify the highly variable second exon of the DQA gene. Using cloning-sequencing methodology and capillary electrophoresis single-strand conformation polymorphism, we found ten alleles of the DQA exon 2 locus across these two European regions, of which eight are described for the first time. To search for signals of selection and recombination in the evolution of the DQA gene within the leporids, we augmented our sample with orthologous DQA alleles from the European rabbit, Oryctolagus cuniculus, in order to carry out a species level, species pairwise comparison. We found evidence of recombination in the history of the DQA sequences in leporids with some recombinant alleles bridging the species divide. In both species, selection on peptide binding site codons can be detected, though stronger for the rabbit. This result suggests that there may be a differential selection pressure in the deeper evolutionary history of these two species due to differences in several demographic and ecological traits likely subjecting them to differential selection by parasites. Finally, evolutionary relationships show a widespread and statistically significant intermingling of alleles from the two species. The many macroparasites shared between hares and rabbits may explain this pattern of trans-species polymorphism. | 3495 | NA | NA | NA | no | no |
| 3495 | 2017 | NA | Kelly, AC; Mateus-Pinilla, NE; Douglas, M; Douglas, M; Shelton, P; Novakofski, J | 2011 | Microsatellites behaving badly: empirical evaluation of genotyping errors and subsequent impacts on population studies | Microsatellites are useful tools for ecological studies because they can be used to discern population structure, dispersal patterns and genetic relationships among individuals. However, they can also yield inaccurate genotypes that, in turn, bias results, promote biological misinterpretations, and create repercussions for population management and conservation programs. We used empirical data from a large-scale microsatellite DNA study of white-tailed deer (Odocoileus virginianus) to identify sources of genotyping error, evaluate corrective measures, and provide recommendations to prevent bias in population studies. We detected unreported mutations that led to erroneous genotypes in five of 13 previously evaluated microsatellites. Of the five problematic markers, two contained mutations that resulted in null alleles, and three contained mutations that resulted in imperfect repeats. These five microsatellites had error rates that were four times greater on average than those observed in the remaining eight. Methodological corrections, such as primer redesign, reduced errors up to 5-fold in two problematic loci, although analytical corrections (computational adjustment for errors) were unable to fully prevent bias and, consequently, measures of genetic differentiation and kinship were negatively impacted. Our results demonstrate the importance of error evaluation during all stages of population studies, and emphasize the need to standardize procedures for microsatellite analyses. This study facilitates the application of microsatellite technology in population studies by examining common sources of genotyping error, identifying unreported problems with microsatellites, and offering solutions to prevent error and bias in population studies. | 3496 | NA | NA | NA | no | no |
| 3496 | 2017 | NA | Dilmaghani, A; Gladieux, P; Gout, L; Giraud, T; Brunner, PC; Stachowiak, A; Balesdent, MH; Rouxel, T | 2012 | Migration patterns and changes in population biology associated with the worldwide spread of the oilseed rape pathogen Leptosphaeria maculans | Pathogen introductions into novel areas can lead to the emergence of new fungal diseases of plants. Understanding the origin, introduction pathways, possible changes in reproductive system and population size of fungal pathogens is essential in devising an integrated strategy for the control of these diseases. We used minisatellite markers to infer the worldwide invasion history of the fungal plant pathogen Leptosphaeria maculans, which causes stem canker (blackleg) of oilseed and vegetable brassicas. Clustering analyses partitioned genotypes into distinct populations corresponding to major geographic regions, along with two differentiated populations in Western Canada. Comparison of invasion scenarios using Approximate Bayesian Computation suggested an origin of the pathogen in the USA, the region where epidemics were first recorded, and independent introductions from there over the last few decades into Eastern Canada (Ontario), Europe and Australia. The population in Western Canada appeared to be founded from a source in Ontario and the population in Chile resulted from an admixture between multiple sources. A bottleneck was inferred for the introduction into Western Canada but not into Europe, Ontario or Australia. Clonality appeared high in Western Canada, possibly because environmental conditions there were less conducive to sexual reproduction. Leptosphaeria maculans is a model invasive pathogen with contrasting features in different regions: shallow population structure, high genetic variability and regular sexual recombination in some regions, by comparison with reduced genetic variability, high rates of asexual multiplication, strong population structure or admixture in others. | 3497 | NA | NA | NA | no | no |
| 3497 | 2017 | NA | Paunio, T; Arajarvi, R; Terwilliger, JD; Hiekkalinna, T; Haimi, P; Partonen, T; Lonnqvist, J; Peltonen, L; Varilo, T | 2009 | Linkage Analysis of Schizophrenia Controlling for Population Substructure | Etiological heterogeneity and complexity has hampered attempts to identify predisposing genes for schizophrenia. We sought to minimize the number of segregating genes involved by focusing on a population isolate with elevated disease prevalence. We exploited the well-established population history, and searched for disease susceptibility loci in families from two alternative founder lineages. We studied 28 schizophrenia pedigrees (123 nuclear families) from an outlying municipality on the eastern border of Finland. We divided the families based on their genealogy and defined two routes of immigration: southern and northern. We examined the kinship coefficients and allele frequency distributions within each group, and performed a linkage analysis based on 497 microsatellite markers across the genome. A high degree of historical relatedness was demonstrated by higher sharing of alleles than predicted by the relationships we identified within the previous four generations alone, as would be expected. Between the two subpopulations, allele frequencies were significantly different, consistent with their isolated genealogies. The southern families showed some evidence of linkage in a schizophrenia locus at 4q23 (Z = 3.3) near our previous finding with quantitative variation in verbal learning and memory [Paunio et al. (2004); Hum Mol Genet 13: 1693-1702], while the northern pedigrees gave most significant evidence on 10q21 (Z = 2.53). Joint analysis of families from both lineages suggested evidence of linkage only at 3p14 (Z = 3.18). Thus the detailed genealogical information led us to identification of distinct linkage signals for schizophrenia susceptibility loci between the three analyses we performed. (C ) 2008 Wiley-Liss,Inc. | 3498 | NA | NA | NA | no | no |
| 3498 | 2017 | NA | Deng, L; Li, W; Yu, XM; Gong, C; Liu, XH; Zhong, ZJ; Xie, N; Lei, SS; Yu, JQ; Fu, HL; Chen, HW; Xu, HL; Hu, YC; Peng, GN | 2016 | First Report of the Human-Pathogenic Enterocytozoon bieneusi from Red-Bellied Tree Squirrels (Callosciurus erythraeus) in Sichuan, China | Enterocytozoon bieneusi is a common opportunistic pathogen causing diarrhea and enteric disease in a variety of animal hosts. Although it has been reported in many animals, there is no published information available on the occurrence of E. bieneusi in red-bellied tree squirrels. To understand the occurrence, genetic diversity, and zoonotic potential of E. bieneusi in red-bellied tree squirrels, 144 fecal specimens from Sichuan province, China, were examined by PCR amplification and sequencing of the internal transcribed spacer (ITS) region of the ribosomal RNA (rRNA) gene of E. bieneusi. The overall infection rate of E. bieneusi 16.7% (24/144) was observed in red-bellied tree squirrels. Altogether five genotypes of E. bieneusi were identified: three known genotypes D (n = 18), EbpC (n = 3), SC02 (n = 1) and two novel genotypes CE01, CE02 (one each). Multilocus sequence typing (MLST) analysis employing three microsatellite (MS1, MS3, MS7) and one minisatellite (MS4) revealed 16, 14, 7 and 14 positive specimens were successfully sequenced, and identified eight, three, three and two genotypes at four loci, respectively. In phylogenetic analysis, the three known genotypes D, EbpC, and SC02 were clustered into group 1 with zoonotic potential, and the two novel genotypes CE01 and CE02 were clustered into group 6. The present study firstly reported the occurrence of E. bieneusi in red-bellied tree squirrels in China, and the E. bieneusi genotypes D and EbpC were found in humans previously. These results indicate that red-bellied tree squirrels may play a potential role in the transmission of E. bieneusi to humans. | 3499 | NA | NA | NA | no | no |
| 3499 | 2017 | NA | Stepien, L; Gromadzka, K; Chelkowski, J | 2012 | Polymorphism of mycotoxin biosynthetic genes among Fusarium equiseti isolates from Italy and Poland | Fusarium equiseti (Corda) Saccardo is a soil saprophyte and a weak pathogen, associated with several diseases of fruit and other crops in subtropical and tropical areas, but also in countries with temperate climate. A wide range of secondary metabolites has been identified among natural F. equiseti populations, with zearalenone (ZEA), fusarochromanone and fusarenon-X being the most common. In present study, the genetic diversity of strains from two populations (from Italy and Poland) was evaluated by analysing the translation elongation factor 1 alpha (tef-1 alpha) sequences, two polyketide synthases from the ZEA biosynthetic pathway (PKS13 and PKS4) and the TRI5 gene from the trichothecene biosynthetic pathway. ZEA was produced in rice cultures by 20 of the 27 tested isolates in concentrations ranging from 1.34 ng/g to 34,000 ng/g). The ability to produce enniatins and trichothecenes was evaluated in all strains by identifying esyn1, TRI13 and TRI4 genes. The presence of PKS4 and PKS13 genes was confirmed by polymerase chain reaction (PCR) in only some ZEA-producing isolates. Similarly, the TRI5 gene was found in 14 of the 27 isolates tested. This is likely to have been caused by the divergence of those genes between F. equiseti and F. graminearum (the latter species was used for the primers design) and can be exploited in phylogenetic studies. The analysis of the mycotoxin biosynthetic gene sequences can be used to differentiate the studied genotypes even more precisely than the analysis of the non-coding regions (like tef-1 alpha). | 3500 | NA | NA | NA | no | no |
| 3500 | 2017 | NA | Shen, H; Pei, JJ; Bai, JL; Zhao, MQ; Ju, CM; Yi, L; Kang, YM; Zhang, XT; Chen, LJ; Li, YG; Wang, JY; Chen, JD | 2011 | Genetic diversity and positive selection analysis of classical swine fever virus isolates in south China | Classical swine fever virus (CSFV) causes a highly contagious disease that leads to significant economic losses in the pig industry worldwide. However, there is a paucity of knowledge on the accurate genotyping of CSFV isolates in south China. This study genotyped the E2 gene of 14 CSFV strains isolated during 2008-2010 from domestic pigs in different districts of south China. Phylogenetic analyses revealed that all of the 14 CSFV isolates were clustered into genetic subgroup 1.1. This contrasts with most parts of China, where group 2 isolates are predominant. Furthermore, the positive selection pressures acting on the E(rns) and E2 envelope protein genes of CSFV were assessed and a site-by-site analysis of the dN/dS ratio was performed to identify specific codons that undergo diversification under positive selection. While no significant evidence for positive selection was observed in E(rns), two positively selected sites at amino acid residues 49 and 72 in the E2 encoding region were identified. Our results revealed that a predominance of subgroup 1.1 CSFV isolates is currently circulating in some districts of south China, which appear to be unrelated to the Chinese C-strain vaccine. Moreover, the envelope protein gene, E2, has undergone positive selection in 14 CSFV strains and two positively selected sites have been identified in this study. Understanding the molecular epidemiology and functional importance of these positively selected amino acid positions could help to predict possible changes in virulence, the development of vaccines and disease control. | 3501 | NA | NA | NA | no | no |
| 3501 | 2017 | NA | Reyer, HU; Arioli-Jakob, C; Arioli, M | 2015 | Post-zygotic selection against parental genotypes during larval development maintains all-hybrid populations of the frog Pelophylax esculentus | Background: Hybridization between two species usually leads to inviable or infertile offspring, due to endogenous or exogenous selection pressures. Yet, hybrid taxa are found in several plant and animal genera, and some of these hybrid taxa are ecologically and evolutionarily very successful. One example of such a successful hybrid is the water frog, Pelophylax esculentus which originated from matings between the two species P. ridibundus (genotype RR) and P. lessonae (LL). At the northern border of the distribution all-hybrid populations consisting of diploid (LR) and one or two triploid (LLR, LRR) frog types have been established. Here, the hybrid has achieved reproductive independence from its sexual ancestors and forms a self-sustaining evolutionary unit. Based on the gamete production of these hybrids, certain mating combinations should lead to LL and RR offspring, but these parental forms are absent among the adults. Results: In order to investigate the mechanisms that maintain such an all-hybrid system, we performed a field study and a crossing experiment. In the field we sampled several ponds for water frog larvae at different developmental stages. Genotype compositions were then analysed and life-history differences between the genotypes examined. In the experiment we crossed diploid and triploid males and females from different ponds and determined fertilization success as well as development speed and survival rates of the offspring under high, medium and low food availability. In both parts of the study, we found numerous LL and RR offspring during the egg and early larval stages; but the frequency of these parental genotypes decreased drastically during later stages. In natural ponds almost all of them had disappeared already before metamorphosis; under the more benign experimental conditions the last ones died as juveniles during the following year. Conclusions: From the combined results we conclude that the absence of parental genotypes in all-hybrid populations is due to post-zygotic selection against them, rather than to pre-zygotic mechanisms that might prevent their formation in the first place. For this post-zygotic selection, genetic mechanisms resulting from low genetic diversity and fixation of deleterious mutations seem to be a more likely explanation than ecological factors. | 3502 | NA | NA | NA | no | no |
| 3502 | 2017 | NA | Benton, CH; Delahay, RJ; Robertson, A; McDonald, RA; Wilson, AJ; Burke, TA; Hodgson, D | 2016 | Blood thicker than water: kinship, disease prevalence and group size drive divergent patterns of infection risk in a social mammal | The importance of social- and kin-structuring of populations for the transmission of wildlife disease is widely assumed but poorly described. Social structure can help dilute risks of transmission for group members, and is relatively easy to measure, but kin-association represents a further level of population sub-structure that is harder to measure, particularly when association behaviours happen underground. Here, using epidemiological and molecular genetic data from a wild, high-density population of the European badger (Meles meles), we quantify the risks of infection with Mycobacterium bovis (the causative agent of tuberculosis) in cubs. The risk declines with increasing size of its social group, but this net dilution effect conceals divergent patterns of infection risk. Cubs only enjoy reduced risk when social groups have a higher proportion of test-negative individuals. Cubs suffer higher infection risk in social groups containing resident infectious adults, and these risks are exaggerated when cubs and infectious adults are closely related. We further identify key differences in infection risk associated with resident infectious males and females. We link our results to parent offspring interactions and other kin-biased association, but also consider the possibility that susceptibility to infection is heritable. These patterns of infection risk help to explain the observation of a herd immunity effect in badgers following low-intensity vaccination campaigns. They also reveal kinship and kin-association to be important, and often hidden, drivers of disease transmission in social mammals. | 3503 | NA | NA | NA | no | no |
| 3503 | 2017 | NA | Crait, JR; Regehr, EV; Ben-David, M | 2015 | Indirect effects of bioinvasions in Yellowstone Lake: The response of river otters to declines in native cutthroat trout | Nonnative species threaten ecosystems throughout the world including protected reserves. In Yellowstone National Park, river otters Lontra canadensis depend on native cutthroat trout as prey. However, nonnative lake trout and whirling disease have significantly reduced the abundance of these native fish in the park’s largest body of water, Yellowstone Lake. We studied the demographic and behavioral responses of otters to declining cutthroat trout on Yellowstone Lake and its tributaries. From 2002-2008, we monitored otter activity at latrine (scentmarking) sites, collected scat for prey identification, and used individual genotypes from scat and hair samples to evaluate survival and abundance with capture-recapture methods. Otter activity at latrines decreased with declines in cutthroat trout, and the prevalence of these fish in otter scat declined from 73% to 53%. Cutthroat trout numbers were the best predictor of temporal variation in apparent survival, and mean annual survival for otters was low (0.72). The density of otters in out study area (1 otter per 13.4 km of shoreline) was also low, and evidence of a recent genetic bottleneck suggests that otter abundance might have declined prior to our study. River otters in and around Yellowstone Lake appear to be responding to reductions in cutthroat trout via changes in distribution, diet, and possibly survival and abundance. Our results provide a baseline estimate for monitoring the broader outcome of management efforts to conserve native cutthroat trout and emphasize the indirect ecosystem consequences of invasive species. (C) 2015 Elsevier Ltd. All rights reserved. | 3504 | NA | NA | NA | no | no |
| 3504 | 2017 | NA | Patil, PG; Dubey, J; Bohra, A; Mishra, RK; Saabale, PR; Das, A; Rathore, M; Singh, NP | 2017 | Association mapping to discover significant marker-trait associations for resistance against fusarium wilt variant 2 in pigeonpea [Cajanus cajan (L.) Millspaugh] using SSR markers | Pigeonpea production is severely constrained by wilt disease caused by Fusarium udum. In the current study, we discover the putative genomic regions that control resistance response to variant 2 of fusarium wilt using association mapping approach. The association panel comprised of 89 diverse pigeonpea genotypes including seven varieties, three landraces and 79 germplasm lines. The panel was screened rigorously for 3 consecutive years (2013-14, 2014-15 and 2015-2016) against variant 2 in a wilt-sick field. A total of 65 pigeonpea specific hypervariable SSR markers (HASSRs) were screened representing seven linkage groups and 29 scaffolds of the pigeonpea genome. A total of 181 alleles were detected, with average values of gene diversity and polymorphism information content (PIC) of 0.55 and 0.47, respectively. Further analysis using model based (STRUCTURE) and distance based (clustering) approaches separated the entire pigeonpea collection into two distinct subgroups (K = 2). The marker trait associations (MTAs) were established based on three-year wilt incidence data and SSR dataset using a unified mixed linear model. Consequently, six SSR markers were identified, which were significantly associated with wilt resistance and explained up to 6% phenotypic variance (PV) across the years. Among these SSRs, HASSR18 was found to be the most stable and significant, accounting for 5-6% PV across the years. To the best of our knowledge, this is the first report of identification of favourable alleles for resistance to variant 2 of Fusarium udum in pigeonpea using association mapping. The SSR markers identified here will greatly facilitate marker assisted resistance breeding against fusarium wilt in pigeonpea. | 3505 | NA | NA | NA | no | no |
| 3505 | 2017 | NA | Port, M; Cant, MA | 2013 | Longevity suppresses conflict in animal societies | Models of social conflict in animal societies generally assume that within-group conflict reduces the value of a communal resource. For many animals, however, the primary cost of conflict is increased mortality. We develop a simple inclusive fitness model of social conflict that takes this cost into account. We show that longevity substantially reduces the level of within-group conflict, which can lead to the evolution of peaceful animal societies if relatedness among group members is high. By contrast, peaceful outcomes are never possible in models where the primary cost of social conflict is resource depletion. Incorporating mortality costs into models of social conflict can explain why many animal societies are so remarkably peaceful despite great potential for conflict. | 3506 | NA | NA | NA | no | no |
| 3506 | 2017 | NA | Barribeau, SM; Sadd, BM; du Plessis, L; Brown, MJF; Buechel, SD; Cappelle, K; Carolan, JC; Christiaens, O; Colgan, TJ; Erler, S; Evans, J; Helbing, S; Karaus, E; Lattorff, HMG; Marxer, M; Meeus, I; Napflin, K; Niu, J; Schmid-Hempel, R; Smagghe, G; Waterhouse, RM; Yu, N; Zdobnov, EM; Schmid-Hempel, P | 2015 | A depauperate immune repertoire precedes evolution of sociality in bees | Background: Sociality has many rewards, but can also be dangerous, as high population density and low genetic diversity, common in social insects, is ideal for parasite transmission. Despite this risk, honeybees and other sequenced social insects have far fewer canonical immune genes relative to solitary insects. Social protection from infection, including behavioral responses, may explain this depauperate immune repertoire. Here, based on full genome sequences, we describe the immune repertoire of two ecologically and commercially important bumblebee species that diverged approximately 18 million years ago, the North American Bombus impatiens and European Bombus terrestris. Results: We find that the immune systems of these bumblebees, two species of honeybee, and a solitary leafcutting bee, are strikingly similar. Transcriptional assays confirm the expression of many of these genes in an immunological context and more strongly in young queens than males, affirming Bateman’s principle of greater investment in female immunity. We find evidence of positive selection in genes encoding antiviral responses, components of the Toll and JAK/STAT pathways, and serine protease inhibitors in both social and solitary bees. Finally, we detect many genes across pathways that differ in selection between bumblebees and honeybees, or between the social and solitary clades. Conclusions: The similarity in immune complement across a gradient of sociality suggests that a reduced immune repertoire predates the evolution of sociality in bees. The differences in selection on immune genes likely reflect divergent pressures exerted by parasites across social contexts. | 3507 | NA | NA | NA | no | no |
| 3507 | 2017 | NA | Kerstes, NAG; Berenos, C; Schmid-Hempel, P; Wegner, KM | 2012 | Antagonistic experimental coevolution with a parasite increases host recombination frequency | Background: One of the big remaining challenges in evolutionary biology is to understand the evolution and maintenance of meiotic recombination. As recombination breaks down successful genotypes, it should be selected for only under very limited conditions. Yet, recombination is very common and phylogenetically widespread. The Red Queen Hypothesis is one of the most prominent hypotheses for the adaptive value of recombination and sexual reproduction. The Red Queen Hypothesis predicts an advantage of recombination for hosts that are coevolving with their parasites. We tested predictions of the hypothesis with experimental coevolution using the red flour beetle, Tribolium castaneum, and its microsporidian parasite, Nosema whitei. Results: By measuring recombination directly in the individuals under selection, we found that recombination in the host population was increased after 11 generations of coevolution. Detailed insights into genotypic and phenotypic changes occurring during the coevolution experiment furthermore helped us to reconstruct the coevolutionary dynamics that were associated with this increase in recombination frequency. As coevolved lines maintained higher genetic diversity than control lines, and because there was no evidence for heterozygote advantage or for a plastic response of recombination to infection, the observed increase in recombination most likely represented an adaptive host response under Red Queen dynamics. Conclusions: This study provides direct, experimental evidence for an increase in recombination frequency under host-parasite coevolution in an obligatory outcrossing species. Combined with earlier results, the Red Queen process is the most likely explanation for this observation. | 3508 | NA | NA | NA | no | no |
| 3508 | 2017 | NA | Vandelannoote, K; Meehan, CJ; Eddyani, M; Affolabi, D; Phanzu, DM; Eyangoh, S; Jordaens, K; Portaels, F; Mangas, K; Seemann, T; Marsollier, L; Marion, E; Chauty, A; Landier, J; Fontanet, A; Leirs, H; Stinear, TP; de Jong, BC | 2017 | Multiple Introductions and Recent Spread of the Emerging Human Pathogen Mycobacterium ulcerans across Africa | Buruli ulcer (BU) is an insidious neglected tropical disease. Cases are reported around the world but the rural regions of West and Central Africa are most affected. How BU is transmitted and spreads has remained a mystery, even though the causative agent, Mycobacterium ulcerans, has been known for more than 70 years. Here, using the tools of population genomics, we reconstruct the evolutionary history of M. ulcerans by comparing 165 isolates spanning 48 years and representing 11 endemic countries across Africa. The genetic diversity of African M. ulcerans was found to be restricted due to the bacterium’s slow substitution rate coupled with its relatively recent origin. We identified two specific M. ulcerans lineages within the African continent, and inferred that M. ulcerans lineage Mu\_A1 existed in Africa for several hundreds of years, unlike lineage Mu\_A2, which was introduced much more recently, approximately during the 19th century. Additionally, we observed that specific M. ulcerans epidemic Mu\_A1 clones were introduced during the same time period in the three hydrological basins that were well covered in our panel. The estimated time span of the introduction events coincides with the Neo-imperialism period, during which time the European colonial powers divided the African continent among themselves. Using this temporal association, and in the absence of a known BU reservoir or-vector on the continent, we postulate that the so-called “Scramble for Africa” played a significant role in the spread of the disease across the continent. | 3509 | NA | NA | NA | no | no |
| 3509 | 2017 | NA | Liberal, IM; Burrus, M; Suchet, C; Thebaud, C; Vargas, P | 2014 | The evolutionary history of Antirrhinum in the Pyrenees inferred from phylogeographic analyses | Background: The origin and colonisation history after the Quaternary ice ages remain largely unresolved for many plant lineages, mainly owing to a lack of fine-scale studies. Here, we present a molecular phylogeny and a phylogeographic analysis of Antirrhinum, an important model system in plant biology, in the Pyrenees range. Our goal was to reconstruct the evolutionary and colonisation history of four taxa endemic to this region (A. majus subsp. majus, A. majus. subsp. striatum, A. molle, and A. sempervirens) by using a dense sampling strategy, with a total of 452 individuals from 99 populations whose collective distribution spans nearly the entirety of the Pyrenees and adjacent mountains. Results: Phylogenetic and phylogeographic analyses of the sequences of two plastid (trnS-trnG and trnK-matK) regions revealed the following: (i) historical relationship between the Pyrenees and Iberia (but not with the Alps); (ii) the long persistence of populations in the Pyrenees, at least since the Late Pleistocene; (iii) three different colonisation histories for populations from the Western, Central, and Eastern Pyrenees; (iv) the deep phylogeographic separation of the eastern and western populations; and (v) the colonisation of southern France from the Eastern Pyrenees. Conclusions: The present study underlines the enormous influence of the glacial history of the mountain ranges on the current configuration of intra- and inter-specific genetic diversity in Antirrhinum, as well as the importance of periglacial areas for the survival of species during glacial periods of the Quaternary. | 3510 | NA | NA | NA | no | no |
| 3510 | 2017 | NA | Pasam, RK; Bansal, U; Daetwyler, HD; Forrest, KL; Wong, D; Petkowski, J; Willey, N; Randhawa, M; Chhetri, M; Miah, H; Tibbits, J; Bariana, H; Hayden, MJ | 2017 | Detection and validation of genomic regions associated with resistance to rust diseases in a worldwide hexaploid wheat landrace collection using BayesR and mixed linear model approaches | BayesR and MLM association mapping approaches in common wheat landraces were used to identify genomic regions conferring resistance to Yr, Lr, and Sr diseases. Deployment of rust resistant cultivars is the most economically effective and environmentally friendly strategy to control rust diseases in wheat. However, the highly evolving nature of wheat rust pathogens demands continued identification, characterization, and transfer of new resistance alleles into new varieties to achieve durable rust control. In this study, we undertook genome-wide association studies (GWAS) using a mixed linear model (MLM) and the Bayesian multilocus method (BayesR) to identify QTL contributing to leaf rust (Lr), stem rust (Sr), and stripe rust (Yr) resistance. Our study included 676 pre-Green Revolution common wheat landrace accessions collected in the 1920-1930s by A.E. Watkins. We show that both methods produce similar results, although BayesR had reduced background signals, enabling clearer definition of QTL positions. For the three rust diseases, we found 5 (Lr), 14 (Yr), and 11 (Sr) SNPs significant in both methods above stringent false-discovery rate thresholds. Validation of marker-trait associations with known rust QTL from the literature and additional genotypic and phenotypic characterisation of biparental populations showed that the landraces harbour both previously mapped and potentially new genes for resistance to rust diseases. Our results demonstrate that pre-Green Revolution landraces provide a rich source of genes to increase genetic diversity for rust resistance to facilitate the development of wheat varieties with more durable rust resistance. | 3511 | NA | NA | NA | no | no |
| 3511 | 2017 | NA | Schwensow, N; Mazzoni, CJ; Marmesat, E; Fickel, J; Peacock, D; Kovaliski, J; Sinclair, R; Cassey, P; Cooke, B; Sommer, S | 2017 | High adaptive variability and virus-driven selection on major histocompatibility complex (MHC) genes in invasive wild rabbits in Australia | The rabbit haemorrhagic disease virus (RHDV) was imported into Australia in 1995 as a biocontrol agent to manage one of the most successful and devastating invasive species, the European rabbit (Oryctolagus cuniculus cuniculus). During the first disease outbreaks, RHDV caused mortality rates of up to 97% and reduced Australian rabbit numbers to very low levels. However, recently increased genetic resistance to RHDV and strong population growth has been reported. Major histocompatibility complex (MHC) class I immune genes are important for immune responses against viruses, and a high MHC variability is thought to be crucial in adaptive processes under pathogen-driven selection. We asked whether strong population bottlenecks and presumed genetic drift would have led to low MHC variability in wild Australian rabbits, and if the retained MHC variability was enough to explain the increased resistance against RHD. Despite the past bottlenecks we found a relatively high number of MHC class I sequences distributed over 2-4 loci. We identified positive selection on putative antigen-binding sites of the MHC. We detected evidence for RHDV-driven selection as one MHC supertype was negatively associated with RHD survival, fitting expectations of frequency-dependent selection. Gene duplication and pathogen-driven selection are possible (and likely) mechanisms that maintained the adaptive potential of MHC genes in Australian rabbits. Our findings not only contribute to a better understanding of the evolution of invasive species, they are also important in the light of planned future rabbit biocontrol in Australia. | 3512 | NA | NA | NA | no | no |
| 3512 | 2017 | NA | Chamberlain, A | 2009 | Archaeological Demography | Archaeological demography investigates the structure and dynamics of past human populations using evidence from traces of human activities and remnants of material culture in the archaeological record. Research in this field is interdisciplinary, incorporating findings from anthropology, paleogenetics, and human ecology but with a remit that extends beyond the primarily biological focus of paleodemography. Important questions addressed by archaeological demography include the establishment of methods for inferring past population structure, the timing of the emergence of modern human demographic systems, the relative importance of attritional and catastrophic patterns of mortality, and the search for adaptive explanations for demographic transitions, colonization events, and population extinctions. Archaeological evidence, including the extent of settlements and site catchment areas as well as measures of the exploitation, consumption, and discard of materials and artifacts, have traditionally been used as proxies for estimating past population size and density. In recent years this evidence has been supplemented by increasingly large data sets compiled from radiocarbon dating programs. These data sets have been used to investigate demographic waves of advance during continental-scale periods of colonization and cultural change and to detect episodes of population decline, extinction, and hiatuses in settlement history. By considering studies of human genetic diversity that indicate temporary but drastic reductions in effective population size, 1 hypothesize that catastrophic mortality may have had an important role in long-term population processes and may have limited long-term rates of growth, particularly in prehistoric populations. | 3513 | NA | NA | NA | no | no |
| 3513 | 2017 | NA | Jimenez, MD; Bangs, EE; Boyd, DK; Smith, DW; Becker, SA; Ausband, DE; Woodruff, SP; Bradley, EH; Holyan, J; Laudon, K | 2017 | Wolf dispersal in the Rocky Mountains, Western United States: 1993-2008 | Gray wolves (Canis lupus) were extirpated from the northern Rocky Mountains (NRM) of the United States by the 1930s. Dispersing wolves from Canada naturally recolonized Montana and first denned there in 1986. In 1995 and 1996, the United States Fish and Wildlife Service reintroduced 66 wolves into central Idaho and Yellowstone National Park. By 2008, there were 1,655 wolves in 217 packs, including 95 breeding pairs in the NRM. From 1993-2008, we captured and radio-collared 1,681 wolves and documented 297 radio-collared wolves dispersing as lone individuals. We monitored dispersing wolves to determine their pack characteristics (i.e., pack size and surrounding pack density) before and after dispersal, their reproductive success, and eventual fate. We calculated summary statistics for characteristics of wolf dispersal (i.e., straight-line distance, age, time of year, sex ratio, reproduction, and survival), and we tested these characteristics for differences between sexes and age groups. Approximately, 10% of the known wolf population dispersed annually. The sex ratio of dispersals favored males (169M, 128F), but fewer dispersed males reproduced (28%, n=47) than females (42%, n=54). Fifty-nine percent of all dispersers of known age were adults (n=156), 37% were yearlings (n=99), and 4% were pups (n=10). Mean age at dispersal for males (32.8 months) was not significantly different (P=0.88) than for females (32.1 months). Yellowstone National Park had a significant positive effect on dispersal rate. Pack density in a wolf’s natal population had a negative effect on dispersal rate when the entire NRM population was considered. The mean NRM pack size (6.9) from 1993 to 2008 was smaller than the mean size of packs (10.0) from which wolves dispersed during that time period (P<0.001); however, pack size was not in our most supported model. Dispersals occurred throughout the year but generally increased in the fall and peaked in January. The mean duration of all dispersals was 5.5 months. Radio-collared wolves dispersed between Montana, Idaho, and Wyoming to other adjacent states, and between the United States and Canada throughout the study. Mean straight-line distance between starting and ending points for dispersing males (98.1km) was not significantly different than females (87.7km; P=0.11). Ten wolves (3.4%) dispersed distances >300km. On average, dispersal distance decreased later in the study (P=0.006). Sex, survival rate in the natal population, start date, dispersal distance, and direction were not significant predictors of dispersal rate or successful dispersal. Wolves that formed new packs were >11 times more likely to reproduce than those that joined packs and surrounding pack density had a negative effect on successful dispersal. Dispersal behavior seems to be innate in sexually mature wolves and thereby assures that genetic diversity will remain high and help conserve the NRM wolf population. (c) 2017 The Wildlife Society. We analyzed dispersal characteristics and determined which variables were predictors of annual dispersal rate and success for gray wolves in the northern Rocky Mountains from 1993 to 2008. Dispersal behavior seems to be innate in sexually mature wolves and thereby assures genetic diversity will remain high and help conserve the northern Rocky Mountain wolf population. | 3514 | NA | NA | NA | no | no |
| 3514 | 2017 | NA | Pontieri, L; Vojvodic, S; Graham, R; Pedersen, JS; Linksvayer, TA | 2014 | Ant Colonies Prefer Infected over Uninfected Nest Sites | During colony relocation, the selection of a new nest involves exploration and assessment of potential sites followed by colony movement on the basis of a collective decision making process. Hygiene and pathogen load of the potential nest sites are factors worker scouts might evaluate, given the high risk of epidemics in group-living animals. Choosing nest sites free of pathogens is hypothesized to be highly efficient in invasive ants as each of their introduced populations is often an open network of nests exchanging individuals (unicolonial) with frequent relocation into new nest sites and low genetic diversity, likely making these species particularly vulnerable to parasites and diseases. We investigated the nest site preference of the invasive pharaoh ant, Monomorium pharaonis, through binary choice tests between three nest types: nests containing dead nestmates overgrown with sporulating mycelium of the entomopathogenic fungus Metarhizium brunneum (infected nests), nests containing nestmates killed by freezing (uninfected nests), and empty nests. In contrast to the expectation pharaoh ant colonies preferentially (84%) moved into the infected nest when presented with the choice of an infected and an uninfected nest. The ants had an intermediate preference for empty nests. Pharaoh ants display an overall preference for infected nests during colony relocation. While we cannot rule out that the ants are actually manipulated by the pathogen, we propose that this preference might be an adaptive strategy by the host to "immunize’’ the colony against future exposure to the same pathogenic fungus. | 3515 | NA | NA | NA | no | no |
| 3515 | 2017 | NA | Coyne, CJ; McClendon, MT; Walling, JG; Timmerman-Vaughan, GM; Murray, S; Meksem, K; Lightfoot, DA; Shultz, JL; Keller, KE; Martin, RR; Inglis, DA; Rajesh, PN; McPhee, KE; Weeden, NF; Grusak, MA; Li, CM; Storlie, EW | 2007 | Construction and characterization of two bacterial artificial chromosome libraries of pea (Pisum sativum L.) for the isolation of economically important genes | Pea (Pisum sativum L.) has a genome of about 4 Gb that appears to share conserved synteny with model legumes having genomes of 0.2-0.4 Gb despite extensive intergenic expansion. Pea plant inventory (PI) accession 269818 has been used to introgress genetic diversity into the cultivated germplasm pool. The aim here was to develop pea bacterial artificial chromosome (BAC) libraries that would enable the isolation of genes involved in plant disease resistance or control of economically important traits. The BAC libraries encompassed about 3.2 haploid genome equivalents consisting of partially HindIII-digested DNA fragments with a mean size of 105 kb that were inserted in I of 2 vectors. The low-copy oriT-based T-DNA vector (pCLD04541) library contained 55 680 clones. The single-copy oriS-based vector (pIndigo-BAC-5) library contained 65 280 clones. Colony hybridization of a universal chloroplast probe indicated that about 1% of clones in the libraries were of chloroplast origin. The presence of about 0.1% empty vectors was inferred by white/blue colony plate counts. The usefulness of the libraries was tested by 2 replicated methods. First, high-density filters were probed with low copy number sequences. Second, BAC plate-pool DNA was used successfully to PCR amplify 7 of 9 published pea resistance gene analogs (RGAs) and several other low copy number pea sequences. Individual BAC clones encoding specific sequences were identified. Therefore, the HindIII BAC libraries of pea, based on germplasm accession PI 269818, will be useful for the isolation of genes underlying disease resistance and other economically important traits. | 3516 | NA | NA | NA | no | no |
| 3516 | 2017 | NA | Powell, A; Baker-Austin, C; Wagley, S; Bayley, A; Hartnell, R | 2013 | Isolation of Pandemic Vibrio parahaemolyticus from UK Water and Shellfish Produce | Vibrio parahaemolyticus is a Gram-negative, halophilic bacterium found commonly in temperate and warm estuarine waters worldwide. V. parahaemolyticus is considered an emerging bacterial pathogen in Europe and has been responsible for several recent seafood-associated outbreaks. During ad hoc testing of raw shellfish produce in May 2012, pandemic group (O3:K6) V. parahaemolyticus was isolated from Pacific oysters (Crassostrea gigas), harvested in Southern England. Follow-on testing of water and shellfish, encompassing a small number geographically diverse sites, also retrieved pandemic group isolates. These strains are amongst the most northerly pandemic strains described to date and represent the first instance of pandemic V. parahaemolyticus isolated in the UK, highlighting the expanding geographical distribution of these foodborne pathogens in the environment. | 3517 | NA | NA | NA | no | no |
| 3517 | 2017 | NA | Xia, J; Cui, JQ; He, X; Liu, YY; Yao, KC; Cao, SJ; Han, XF; Huang, Y | 2017 | Genetic and antigenic evolution of H9N2 subtype avian influenza virus in domestic chickens in southwestern China, 2013-2016 | H9N2 avian influenza virus (AIV) has caused significant losses in chicken flocks throughout china in recent years. There is a limited understanding of the genetic and antigenic characteristics of the H9N2 virus isolated in chickens in southwestern China. In this study a total of 12 field strains were isolated from tissue samples from diseased chickens between 2013 and 2016. Phylogenetic analysis of the Hemagglutinin (HA) and Neuraminidase (NA) nucleotide sequences from the 12 field isolates and other reference strains showed that most of the isolates in the past four years could be clustered into a major branch (HA-branch A and NA-branch I) in the Clade h9.4.2 lineages. These sequences are accompanied by nine and seven new amino acids mutations in the HA and NA proteins, respectively, when compared with those previous to 2013. In addition, four new isolates were grouped into a minor branch (HA-branch B) in the Clade h9.4.2 lineages and two potential N-glycosylation sites were observed due to amino acid mutations in the HA protein. Three antigenic groups (1-3), which had low antigenic relatedness with two commonly used vaccines in China, were identified among the 12 isolates by antigenMap analysis. Immunoprotection testing showed that those two vaccines could efficiently prevent the shedding of branch A viruses but not branch B viruses. In conclusion, these results indicate the genotype of branch B may become epidemic in the next few years and that a new vaccine should be developed for the prevention of H9N2 AIV. | 3518 | NA | NA | NA | no | no |
| 3518 | 2017 | NA | Varshney, RK; Penmetsa, RV; Dutta, S; Kulwal, PL; Saxena, RK; Datta, S; Sharma, TR; Rosen, B; Carrasquilla-Garcia, N; Farmer, AD; Dubey, A; Saxena, KB; Gao, J; Fakrudin, B; Singh, MN; Singh, BP; Wanjari, KB; Yuan, M; Srivastava, RK; Kilian, A; Upadhyaya, HD; Mallikarjuna, N; Town, CD; Bruening, GE; He, G; May, GD; McCombie, R; Jackson, SA; Singh, NK; Cook, DR | 2010 | Pigeonpea genomics initiative (PGI): an international effort to improve crop productivity of pigeonpea (Cajanus cajan L.) | Pigeonpea (Cajanus cajan), an important food legume crop in the semi-arid regions of the world and the second most important pulse crop in India, has an average crop productivity of 780 kg/ha. The relatively low crop yields may be attributed to non-availability of improved cultivars, poor crop husbandry and exposure to a number of biotic and abiotic stresses in pigeonpea growing regions. Narrow genetic diversity in cultivated germplasm has further hampered the effective utilization of conventional breeding as well as development and utilization of genomic tools, resulting in pigeonpea being often referred to as an ‘orphan crop legume’. To enable genomics-assisted breeding in this crop, the pigeonpea genomics initiative (PGI) was initiated in late 2006 with funding from Indian Council of Agricultural Research under the umbrella of Indo-US agricultural knowledge initiative, which was further expanded with financial support from the US National Science Foundation’s Plant Genome Research Program and the Generation Challenge Program. As a result of the PGI, the last 3 years have witnessed significant progress in development of both genetic as well as genomic resources in this crop through effective collaborations and coordination of genomics activities across several institutes and countries. For instance, 25 mapping populations segregating for a number of biotic and abiotic stresses have been developed or are under development. An 11X-genome coverage bacterial artificial chromosome (BAC) library comprising of 69,120 clones have been developed of which 50,000 clones were end sequenced to generate 87,590 BAC-end sequences (BESs). About 10,000 expressed sequence tags (ESTs) from Sanger sequencing and ca. 2 million short ESTs by 454/FLX sequencing have been generated. A variety of molecular markers have been developed from BESs, microsatellite or simple sequence repeat (SSR)-enriched libraries and mining of ESTs and genomic amplicon sequencing. Of about 21,000 SSRs identified, 6,698 SSRs are under analysis along with 670 orthologous genes using a GoldenGate SNP (single nucleotide polymorphism) genotyping platform, with large scale SNP discovery using Solexa, a next generation sequencing technology, is in progress. Similarly a diversity array technology array comprising of ca. 15,000 features has been developed. In addition, > 600 unique nucleotide binding site (NBS) domain containing members of the NBS-leucine rich repeat disease resistance homologs were cloned in pigeonpea; 960 BACs containing these sequences were identified by filter hybridization, BES physical maps developed using high information content fingerprinting. To enrich the genomic resources further, sequenced soybean genome is being analyzed to establish the anchor points between pigeonpea and soybean genomes. In addition, Solexa sequencing is being used to explore the feasibility of generating whole genome sequence. In summary, the collaborative efforts of several research groups under the umbrella of PGI are making significant progress in improving molecular tools in pigeonpea and should significantly benefit pigeonpea genetics and breeding. As these efforts come to fruition, and expanded (depending on funding), pigeonpea would move from an ‘orphan legume crop’ to one where genomics-assisted breeding approaches for a sustainable crop improvement are routine. | 3519 | NA | NA | NA | no | no |
| 3519 | 2017 | NA | Sakai, Y; Takada, T | 2017 | The analysis of an effect of seed propagation on defense strategy against pathogen transmission within clonal plant population using lattice model | Many clonal plants have two breeding systems, vegetative and seed propagation. In vegetative propagation, plants reproduce genetically identical offspring that have lower mortality rates. By contrast, the seed propagated offspring has higher mortality rate, however, the seed propagation acts an important role in maintaining the genetic diversity and reproduce widely. According to the experimental studies, the balance between the breeding systems, vegetative and seed propagation, is determined by several functions, such as resource allocation. The infection and spread of systemic pathogen also affect the optimal balance of the breeding systems. Thus, we examine the effect of invasion of systemic pathogen on the optimal balance of the breeding systems of clonal plant using lattice model in two cases, single population and mixed population. In the analysis, the equilibrium and its local stability were derived using approximation method and numerical simulation in single population. Additionally, two situations were assumed in mixed population, infected and uninfected populations, and the efficacy of seed propagation on the suppression of epidemic infections was examined by comparing the results in the two situations. As a result, seed propagation is an effective defensive behavior against systemic pathogens. In the single population, the plants increase their population by increasing the proportion of seed propagation when the epidemic pathogen has highly infective. In mixed population, the increasing proportion of seed propagation is the optimal breeding strategy to defend against the spread of a systemic pathogen. (C) 2017 Elsevier Ltd. All rights reserved. | 3520 | NA | NA | NA | no | no |
| 3520 | 2017 | NA | Pruitt, JN; Howell, KA; Gladney, SJ; Yang, YS; Lichtenstein, JLL; Spicer, ME; Echeverri, SA; Pinter-Wollman, N | 2017 | Behavioral Hypervolumes of Predator Groups and Predator-Predator Interactions Shape Prey Survival Rates and Selection on Prey Behavior | Predator-prey interactions often vary on the basis of the traits of the individual predators and prey involved. Here we examine whether the multidimensional behavioral diversity of predator groups shapes prey mortality rates and selection on prey behavior. We ran individual sea stars (Pisaster ochraceus) through three behavioral assays to characterize individuals’ behavioral phenotype along three axes. We then created groups that varied in the volume of behavioral space that they occupied. We further manipulated the ability of predators to interact with one another physically via the addition of barriers. Prey snails (Chlorostome funebralis) were also run through an assay to evaluate their predator avoidance behavior before their use in mesocosm experiments. We then subjected pools of prey to predator groups and recorded the number of prey consumed and their behavioral phenotypes. We found that predator-predator interactions changed survival selection on prey traits: when predators were prevented from interacting, more fearful snails had higher survival rates, whereas prey fearfulness had no effect on survival when predators were free to interact. We also found that groups of predators that occupied a larger volume in behavioral trait space consumed 35% more prey snails than homogeneous predator groups. Finally, we found that behavioral hypervolumes were better predictors of prey survival rates than single behavioral traits or other multivariate statistics (i.e., principal component analysis). Taken together, predator-predator interactions and multidimensional behavioral diversity determine prey survival rates and selection on prey traits in this system. | 3521 | NA | NA | NA | no | no |
| 3521 | 2017 | NA | Parobek, CM; Lin, JT; Saunders, DL; Barnett, EJ; Lon, C; Lanteri, CA; Balasubramanian, S; Brazeau, N; DeConti, DK; Garba, DL; Meshnick, SR; Spring, MD; Chuor, CM; Bailey, JA; Juliano, JJ | 2016 | Selective sweep suggests transcriptional regulation may underlie Plasmodium vivax resilience to malaria control measures in Cambodia | Cambodia, in which both Plasmodium vivax and Plasmodium falciparum are endemic, has been the focus of numerous malaria-control interventions, resulting in a marked decline in overall malaria incidence. Despite this decline, the number of P. vivax cases has actually increased. To understand better the factors underlying this resilience, we compared the genetic responses of the two species to recent selective pressures. We sequenced and studied the genomes of 70 P. vivax and 80 P. falciparum isolates collected between 2009 and 2013. We found that although P. falciparum has undergone population fracturing, the coendemic P. vivax population has grown undisrupted, resulting in a larger effective population size, no discernable population structure, and frequent multiclonal infections. Signatures of selection suggest recent, species-specific evolutionary differences. Particularly, in contrast to P. falciparum, P. vivax transcription factors, chromatin modifiers, and histone deacetylases have undergone strong directional selection, including a particularly strong selective sweep at an AP2 transcription factor. Together, our findings point to different population-level adaptive mechanisms used by P. vivax and P. falciparum parasites. Although population substructuring in P. falciparum has resulted in clonal outgrowths of resistant parasites, P. vivax may use a nuanced transcriptional regulatory approach to population maintenance, enabling it to preserve a larger, more diverse population better suited to facing selective threats. We conclude that transcriptional control may underlie P. vivax’s resilience to malaria control measures. Novel strategies to target such processes are likely required to eradicate P. vivax and achieve malaria elimination. | 3522 | NA | NA | NA | no | no |
| 3522 | 2017 | NA | Reis, YV; Alevi, KCC; Moreira, FFF; Azeredo-Oliveira, MTV | 2016 | Spermatogenesis in Nesotriatoma bruneri (Usinger 1944) (Hemiptera, Triatominae) | The Nesotriatoma genus consists of the species N. flavida N. bruneri and N. obscura, forming the Flavida complex. Variation in the size and morphological differences intraspecific of N. flavida led to the description of N. bruneri. Two years later, the same author proposed the synonymization of N. bruneri with N. flavida. Only in 1981 the specific status N. bruneri was recovered by means of morphological analysis of the genitalia. However, recently by genetic analysis, it was suggested that N. bruneri and N. flavida should be again synonymized. As Chagas disease has no cure, the main way to minimize the incidence of this disease is by vector control. Thus, grouping biological data from these hematophagous insects can assist in the development of vector control programs and mainly assist in taxonomic issues of synonymization. Thus, this paper describes spermatogenesis of N. bruneri. Three adult N. bruneri males were cytogenetically analyzed. The meiotic behavior observed for N. bruneri was very similar to that observed for the triatomine species with 23 chromosomes: during prophase, chromatin compaction was observed, the chromocenter composition was characterized (X-1, X-2 and Y), and the species karyotype was confirmed as 2n = 23 (20A + X1X2Y), as it was observed for N. flavida. Moreover, it was possible to observe anaphase and telophase. Thus, this study describes reproductive aspects of N. bruneri in order to contribute to the biological knowledge of these insects of epidemiological importance. Furthermore, this corroborates the synonymization of N. bruneri with N. flavida. | 3523 | NA | NA | NA | no | no |
| 3523 | 2017 | NA | Li, H; Guillemaud, T; French, BW; Kuhlmann, U; Toepfer, S | 2014 | Phenotypic trait changes in laboratory - reared colonies of the maize herbivore, Diabrotica virgifera virgifera | The North American and European maize pest Diabrotica virgifera virgifera LeConte (Coleoptera: Chrysomelidae) was used to assess whether conditions of the natal field, subsequent laboratory rearing, or genetic population origin affect phenotypic traits of fitness, activity, or morphometrics. Standardized laboratory bioassays with large sample sizes revealed that none of the 16 tested traits, except crawling behaviours, appeared consistently stable across all seven tested colonies. Environmental conditions in the natal field of the F (0) generation affected trait averages of the subsequently reared F (1) generation in laboratory in ca. 47% of cases, and trait variability in 67% of cases. This was apparent for fitness and morphometrics, but less obvious for activity traits. Early generation laboratory rearing affected trait averages in ca. 56% of cases: morphometrics changed; fecundity and egg survival increased from F (1) to F (2). Trait variability increased or decreased in 38% of cases. Laboratory rearing for over more than 190 generations affected the trait averages in 60% of cases, reflected by decreases in flight activity and increases in body size, weight, and fecundity to some extent. It had little effect on trait variability, especially so for morphometric variability. The genetic population origin affected average levels of 55% and variability of 63% of phenotypic traits. A comparison among D. v. virgifera studies might be difficult if they use different populations or laboratory colonies. It is advised to consider possible effects of original field conditions, laboratory rearing, and population genetics when planning comparative studies targeting fitness, activity, or morphometric questions regarding Diabrotica species. | 3524 | NA | NA | NA | no | no |
| 3524 | 2017 | NA | Short, SM; Suttle, CA | 2003 | Temporal dynamics of natural communities of marine algal viruses and eukaryotes | The composition of algal virus communities in relation to temperature, salinity, chlorophyll a (chl a) concentration and eukaryotic community composition was monitored at a single location for 14 mo. Changes in algal virus and eukaryote communities were determined using polymerase chain reaction (PCR) and denaturing gradient gel electrophoresis (DGGE) to generate genetic fingerprints. Sequence analysis of bands extracted from denaturing gradient gels revealed the presence of at least 5 distinct viruses as well as temporally dynamic and diverse communities of eukaryotes that included taxa from the viridiplantae, fungi and metazoa. Comparison of algal virus fingerprints with environmental conditions revealed that, at certain times, changes in algal virus community composition were coincident with changes in tide height, salinity or chl a concentration. However, algal virus community changes were not often coupled to eukaryote community changes. The lack of coincidence between changes in virus and eukaryote communities can be explained by the presence of organisms that were not hosts of the detected viruses. It is likely that the uncoupling of 18S and AVS fingerprints was due to succession among non-host eukaryotes. Although algal virus fingerprint patterns were stable throughout most of the study, stable eukaryote fingerprint patterns were observed only during the winter months. Furthermore, specific taxa of algal viruses persisted in fluctuating physical and biological environments. We concluded that the constant production of, and mortality from, some taxa of algal viruses provide further evidence that algal viruses affect phytoplankton community structure and dynamics. | 3525 | NA | NA | NA | no | no |
| 3525 | 2017 | NA | Qu, LN; Cui, HG; Wu, GW; Zhou, JF; Su, JM; Wang, GP; Hong, N | 2014 | Genetic Diversity and Molecular Evolution of Plum bark necrosis stem pitting-associated virus from China | Plum bark necrosis stem pitting-associated virus (PBNSPaV), a member of the genus Ampelovirus in the family Closteroviridae, infects different Prunus species and has a worldwide distribution. Yet the population structure and genetic diversity of the virus is still unclear. In this study, sequence analyses of a partial heat shock protein 70 homolog (HSP70h) gene and coat protein (CP) gene of PBNSPaV isolates from seven Prunus species grown in China revealed a highly divergent Chinese PBNSPaV population, sharing nucleotide similarities of 73.1-100% with HSP70h gene, and 83.9-98.6% with CP gene. Phylogenetic analysis of HSP70h and CP sequences revealed segregation of global PBNSPaV isolates into four phylo-groups (I-IV), of which two newly identified groups, II and IV, solely comprised Chinese isolates. Complete genome sequences of three PBNSPaV isolates, Pch-WH-1 and Pch-GS-3 from peaches, and Plm-WH-3 from a plum tree, were determined. The three isolates showed overall nucleotide identities of 90.0% (Pch-GS-3) and 96.4% (Pch-WH-1) with the type isolate PL186, and the lowest identity of 70.2-71.2% with isolate Nanjing. For the first time, to the best of our knowledge, we report evidence of significant recombination in the HSP70h gene of PBNSPaV variant Pch2 by using five programs implemented in RDP3; in addition, five codon positions in its CP gene (3, 8, 44, 57, and 88) were identified that appeared to be under positive selection. Collectively, these results indicate a divergent Chinese PBNSPaV population. In addition, our findings provide a foundation for elucidating the epidemiological characteristics of virus population. | 3526 | NA | NA | NA | no | no |
| 3526 | 2017 | NA | Wendelberger, KS; Fellows, MQN; Maschinski, J | 2008 | Atlas of Florida vascular plants | Though translocations of rare populations should be considered only as the last resort for species’ conservation, when habitat destruction is imminent, it may be the only means to preserve a species. With over half the known, wild federally endangered Crenulate leadplant (Amorpha herbacea var. crenulata), Fabaceae, growing on unprotected land slated for development, preserving this unprotected population was critical. We rescued whole plants, cuttings, and seeds for an experimental translocation. Into a restored pine rockland, once dominated by the invasive exotic tree Brazilian pepper (Schinus terebinthifolius), we transplanted plants from different sources and of different sizes. Plants used were rescued from an unprotected site, seedlings, and 1-, 2-, and 7-year-old plants from Fairchild Tropical Botanic Garden’s ex situ collection, creating a novel population in a new habitat. We also evaluated which propagule type and source had the best survival, growth, and reproduction. After 40 months, overall transplant survival was 71%. Large whole plants, rescued and nursery grown, had the best survival rates (86 and 78%), whereas cuttings had 67% survival and seedlings had only 26% survival. The restored site, once nearly a monoculture of S. terebinthifolius, is now dominated by 104 native plant species, including 17 naturally recruited state listed, plus the one translocated federally endangered plant species. In addition, one federally threatened snake species was observed on the site. These studies demonstrate that botanic garden collections not only play a vital role in the conservation of species’ genetic diversity but also can be used as source material for habitat restoration. | 3527 | NA | NA | NA | no | no |
| 3527 | 2017 | NA | Raymond, B; West, SA; Griffin, AS; Bonsall, MB | 2012 | The Dynamics of Cooperative Bacterial Virulence in the Field | Laboratory experiments have shown that the fitness of microorganisms can depend on cooperation between cells. Although this insight has revolutionized our understanding of microbial life, results from artificial microcosms have not been validated in complex natural populations. We investigated the sociality of essential virulence factors (crystal toxins) in the pathogen Bacillus thuringiensis using diamondback moth larvae (Plutella xylostella) as hosts. We show that toxin production is cooperative, and in a manipulative field experiment, we observed persistent high relatedness and frequency-and density-dependent selection, which favor stable cooperation. Conditions favoring social virulence can therefore persist in the face of natural population processes, and social interactions (rapid cheat invasion) may account for the rarity of natural disease outbreaks caused by B. thuringiensis. | 3528 | NA | NA | NA | no | no |
| 3528 | 2017 | NA | Maureira, IJ; Ortega, F; Campos, H; Osborn, TC | 2004 | Population structure and combining ability of diverse Medicago sativa germplasms | Although unadapted germplasms have been used to improve disease and insect resistance in alfalfa, there has been little effort to use these for improving forage yield. We evaluated genetic diversity and combining ability among two unadapted germplasms (Medicago sativa ssp. sativa Peruvian and M. sativa ssp. falcata WISFAL) and three Northern U.S. adapted alfalfa cultivars. Population structure analyses indicated that the WISFAL and Peruvian genriplasms were genetically distinct from the cultivars, although Peruvian was relatively closer to the cultivars. Peruvian and WISFAL germplasms were intermated to generate a novel hybrid population. This population was crossed to the three cultivars as testers, and the testcross progenies were evaluated for forage yield along with the hybrid population, the original germplasms (Peruvian, WISFAL and cultivars), testcrosses of Peruvian and WISFAL to the three cultivars and a three-way hybrid of the cultivars. The experiment was carried out in the field in Temuco, Chile and Arlington, Wisconsin, USA, and forage was harvested during two seasons. Results from these evaluations showed that hybrids between the Peruvian x WISFAL population and the cultivar testers yielded as much as the cultivar testers. Heterosis was observed between Peruvian and WISFAL, and between these germplasms and the cultivar testers, suggesting that each germplasm may contain different favorable alleles. If Peruvian and WISFAL populations contain alleles at different loci that complement cultivar testers, then combining and enriching these alleles in a single population could result in improved combining ability with alfalfa cultivars. | 3529 | NA | NA | NA | no | no |
| 3529 | 2017 | NA | Guerreiro, RJ; Beck, J; Gibbs, JR; Santana, I; Rossor, MN; Schott, JM; Nalls, MA; Ribeiro, H; Santiago, B; Fox, NC; Oliveira, C; Collinge, J; Mead, S; Singleton, A; Hardy, J | 2010 | Genetic Variability in CLU and Its Association with Alzheimer’s Disease | Background: Recently, two large genome wide association studies in Alzheimer disease (AD) have identified variants in three different genes (CLU, PICALM and CR1) as being associated with the risk of developing AD. The strongest association was reported for an intronic single nucleotide polymorphism (SNP) in CLU. Methodology/Principal Findings: To further characterize this association we have sequenced the coding region of this gene in a total of 495 AD cases and 330 healthy controls. A total of twenty-four variants were found in both cases and controls. For the changes found in more than one individual, the genotypic frequencies were compared between cases and controls. Coding variants were found in both groups (including a nonsense mutation in a healthy subject), indicating that the pathogenicity of variants found in this gene must be carefully evaluated. We found no common coding variant associated with disease. In order to determine if common variants at the CLU locus effect expression of nearby (cis) mRNA transcripts, an expression quantitative loci (eQTL) analysis was performed. No significant eQTL associations were observed for the SNPs previously associated with AD. Conclusions/Significance: We conclude that common coding variability at this locus does not explain the association, and that there is no large effect of common genetic variability on expression in brain tissue. We surmise that the most likely mechanism underpinning the association is either small effects of genetic variability on resting gene expression, or effects on damage induced expression of the protein. | 3530 | NA | NA | NA | no | no |
| 3530 | 2017 | NA | Araujo, ED; Oliveira, RG; Calazans, HCM; Franca, CCS; Santos, V; Ribeiro, GT; Jain, S; Batista, MVA; Nunes, LA | 2016 | Risk of local extinction and genetic diversity of Melipona quadrifasciata (Apidae: Meliponini) in a possible Northeastern limit of its distribution in Brazil | Rapid loss of genetic diversity among eusocial bees, and extinction of their local population has become a major world concern. Populations of M. quadrifasciata have increasingly declined due to predatory extractivism and destruction of their habitat. Knowledge of their local population could give insights on the strategies for monitoring and conservation of this species. In this study, initially, 14 colonies from the northern limit of the northeastern sandbank of the Atlantic Forest were analysed employing geometric morphometrics techniques. Then the cytochrome b gene sequences and ISSR primers were utilized for molecular analysis of these colonies and the results were compared with 15 M. quadrifasciata colonies found in the semiarid Caatinga region. The morphometrics and the molecular analysis suggested the formation of disjunct populations between the studied geographical zones. All the 14 colonies analysed from the Sao Francisco river region were found on the Southern margin of the Sao Francisco river and they all belonged to one single haplotype. No colonies were found on the Northern bank of the river. We suggest that the Sao Francisco river is an effective geographical barrier for the distribution of this species and propose an urgent need for the development of a conservation program for the population of M. quadrifasciata as it represents a unique haplotype in the region. Also their nesting habit exclusively in the diseased coconut trees which probably will be replaced by new trees, create the risk of population extinction due to the lack of nesting site. | 3531 | NA | NA | NA | no | no |
| 3531 | 2017 | NA | Sun, H; Yang, ZQ; Lin, KQ; Liu, SY; Huang, K; Wang, XY; Chu, JY; Huang, XQ | 2015 | The Adaptive Change of HLA-DRB1 Allele Frequencies Caused by Natural Selection in a Mongolian Population That Migrated to the South of China | Pathogen-driven balancing selection determines the richness of human leukocyte antigen (HLA) alleles. Changes in the pathogen spectrum may cause corresponding changes in HLA loci. Approximately 700 years ago, a Mongolian population moved from the north of China to the Yunnan region in the south of China. The pathogen spectrum in the south of China differs from that in the north. In this study, changes in the HLA genes in the Yunnan Mongolian population, as well as the underlying mechanism, were investigated. A sequence-based typing method (SBT) was used to genotype HLA-DRB1 in 470 individuals from two Mongolian populations and another five ethnic groups. Meanwhile, 10 autosomal short tandem repeats (STRs) were genotyped to assess the influence of genetic background on HLA-DRB1 frequencies. The frequencies of certain alleles changed significantly in the Mongolian population that migrated to Yunnan. For example, DRB1*12:02:01 increased from 6.1% to 35.4%. STR analysis excluded the possibility of a recent bottleneck and indicated that 50% of the genetic consistency between northern and southern Mongolians; Tajima’s D value for HLA-DRB1 exon2 and dN/dS analysis showed that the HLA-DRB1 genes in both Mongolian populations were under balancing selection. However, the sites under natural selection changed. We proposed that the dramatically change of HLA frequencies in southern Mongolian was caused by a combination of inter-population gene flow and natural selection. Certain diseases specific to the south of China, such as malaria, may be the driving force behind the enhanced DRB1*12:02:01 frequency. | 3532 | NA | NA | NA | no | no |
| 3532 | 2017 | NA | Cagliani, R; Forni, D; Biasin, M; Comabella, M; Guerini, FR; Riva, S; Pozzoli, U; Agliardi, C; Caputo, D; Malhotra, S; Montalban, X; Bresolin, N; Clerici, M; Sironi, M | 2014 | Ancient and Recent Selective Pressures Shaped Genetic Diversity at AIM2-Like Nucleic Acid Sensors | AIM2-like receptors (ALRs) are a family of nucleic acid sensors essential for innate immune responses against viruses and bacteria. We performed an evolutionary analysis of ALR genes (MNDA, PYHIN1, IFI16, and AIM2) by analyzing inter- and intraspecies diversity. Maximum-likelihood analyses indicated that IFI16 and AIM2 evolved adaptively in primates, with branch-specific selection at the catarrhini lineage for IFI16. Application of a population genetics-phylogenetics approach also allowed identification of positive selection events in the human lineage. Positive selection in primates targeted sites located at the DNA-binding interface in both IFI16 and AIM2. In IFI16, several sites positively selected in primates and in the human lineage were located in the PYD domain, which is involved in protein-protein interaction and is bound by a human cytomegalovirus immune evasion protein. Finally, positive selection was found to target nuclear localization signals in IFI16 and the spacer region separating the two HIN domains. Population genetic analysis in humans revealed that an IFI16 genic region has been a target of long-standing balancing selection, possibly acting on two nonsynonymous polymorphisms located in the spacer region. Data herein indicate that ALRs have been repeatedly targeted by natural selection. The balancing selection region in IFI16 carries a variant with opposite risk effect for distinct autoimmune diseases, suggesting antagonistic pleiotropy. We propose that the underlying scenario is the result of an ancestral and still ongoing host-pathogen arms race and that the maintenance of susceptibility alleles for autoimmune diseases at IFI16 represents an evolutionary trade-off. | 3533 | NA | NA | NA | no | no |
| 3533 | 2017 | NA | Bonneaud, C; Sorci, G; Morin, V; Westerdahl, H; Zoorob, R; Wittzell, H | 2004 | Diversity of Mhc class I and IIB genes in house sparrows (Passer domesticus) | In order to understand the expression and evolution of host resistance to pathogens, we need to examine the links between genetic variability at the major histocompatibility complex (Mhc), phenotypic expression of the immune response and parasite resistance in natural populations. To do so, we characterized the Mhc class I and IIB genes of house sparrows with the goal of designing a PCR-based genotyping method for the Mhc genes using denaturing gradient gel electrophoresis (DGGE). The incredible success of house sparrows in colonizing habitats worldwide allows us to assess the importance of the variability of Mhc genes in the face of various pathogenic pressures. Isolation and sequencing of Mhc class I and IIB alleles revealed that house sparrows have fewer loci and fewer alleles than great reed warblers. In addition, the Mhc class I genes divided in two distinct lineages with different levels of polymorphism, possibly indicating different functional roles for each gene family. This organization is reminiscent of the chicken B complex and Rfp-Y system. The house sparrow Mhc hence appears to be intermediate between the great reed warbler and the chicken Mhc, both in terms of numbers of alleles and existence of within-class lineages. We specifically amplified one Mhc class I gene family and ran the PCR products on DGGE gels. The individuals screened displayed between one and ten DGGE bands, indicating that this method can be used in future studies to explore the ecological impacts of Mhc diversity. | 3534 | NA | NA | NA | no | no |
| 3534 | 2017 | NA | Evison, SEF | 2015 | Chalkbrood: epidemiological perspectives from the host-parasite relationship | Chalkbrood is a fungal brood disease of the honey bee, Apis mellifera, caused by the parasite Ascosphaera apis. Considered as a stress-related disease, the severity of chalkbrood outbreaks depend on a multitude of interacting factors. The specific relationship between host and parasite in this disease is interesting because the parasite is both heterothallic and semelparous. Recent studies highlight that this specific host-parasite relationship is influenced by factors such as interactions with other parasite strains or species, and environmental perturbations. To understand how to protect pollinators most effectively, it is crucial that future research takes a more ecologically relevant approach by studying the basic biology of the host-parasite relationship in the context of the multi-factorial processes that influence it. | 3535 | NA | NA | NA | no | no |
| 3535 | 2017 | NA | KRAUS, B; HUNT, G | 1995 | DIFFERENTIATION OF VARROA-JACOBSONI OUD POPULATIONS BY RANDOM AMPLIFICATION OF POLYMORPHIC DNA (RAPD) | Specimens of Varroa jacobsoni collected from Apis mellifera L colonies in California, Texas and Germany and specimens collected from A cerana Fab colonies in Malaysia were compared by means of random amplification of polymorphic DNA (RAPD). A high percentage of monomorphic bands indicated low genetic variability among and within populations of the parasite. It was not possible to distinguish mites collected in California and mites collected in Texas. Mites collected in the USA were distinguishable from mites collected in Germany by 3 specific markers. Mites collected from A mellifera in the USA and Germany were distinguishable from mites collected from A cerana in Malaysia by 27 and 24 specific markers. RAPD proved to be a valuable tool especially for further interpopulation studies of V jacobsoni. | 3536 | NA | NA | NA | no | no |
| 3536 | 2017 | NA | Bliss-Ketchum, LL; de Rivera, CE; Turner, BC; Weisbaum, DM | 2016 | The effect of artificial light on wildlife use of a passage structure | Barriers to animal movement can isolate populations, impacting their genetic diversity, susceptibility to disease, and access to resources. Barriers to movement may be caused by artificial light, which is known to disrupt bird, sea turtle, and bat behavior, but few studies have experimentally investigated the effects of artificial light on movement for a suite of terrestrial vertebrates. Therefore, we studied the effect of ecological light pollution on animal usage of a bridge under-road passage structure. On a weekly basis, sections of the structure were subjected to different light treatments including no light added, followed by a Reference period when lights were off in all the structure sections. Sand track data revealed use by 23 mammals, birds, reptiles and amphibians, nine of which had >30 tracks for species-level analysis. Columbia black-tailed deer (Odocoileus hemionus columbianus) traversed under unlit bridge sections much less when neighboring sections were lit compared to when none were, suggesting avoidance due to any nearby presence of artificial light. Similarly, deer mouse (Peromyscus maniculatus) and opossum (Didelphis virginiana) track paths were less frequent in the lit sections than the ambient. Crossing was correlated with temporal or spatial factors but not light for three of the other species. These findings suggest that artificial light may be reducing habitat connectivity for some species though not providing a strong barrier for others. Such information is needed to inform mitigation of habitat fragmentation in the face of expanding urbanization. (C) 2016 Elsevier Ltd. All rights reserved. | 3537 | NA | NA | NA | no | no |
| 3537 | 2017 | NA | Dupuison, J | 2007 | Effect of linkage disequilibrium between markers in linkage and association analyses | Contributions to Group 17 of the Genetic Analysis Workshop 15 considered dense markers in linkage disequilibrium (LD) in the context of either linkage or association analysis. Three contributions reported on methods for modeling LD or selecting a subset of markers in linkage equilibrium to perform linkage analysis. When all markers were used without modeling LD, inflated evidence for linkage was observed when parental genotypes were missing. All methods for handling LD led to some decreased linkage evidence. Two groups performed a genome-wide association scan using either mixed models to account for known or unknown relatedness between individuals, trend tests or combination statistics. All methods failed to detect four of the eight simulated loci because of low LD in some regions. Three groups performed association analysis using simulated dense markers on chromosome 6, where a simulated HLA-DRB1 locus played a major role in disease susceptibility along with two additional loci of smaller effect. The overall conditional genotype method correctly identified both additional loci while a novel transmission disequilibrium test-statistic to combine studies with non-overlapping markers identified one HLA locus after stratifying on the parental HLA-DRB1 genotypes; LD mapping using the Malecot model mapped two loci in this region, even when using greatly reduced marker density. While LD between markers appears to be a nuisance that may cause spurious linkage results with missing parental genotypes in linkage analysis, association analysis thrives on LD, and disease genes fail to be detected in regions of low LD. | 3538 | NA | NA | NA | no | no |
| 3538 | 2017 | NA | Solorzano, RGL; Fouet, O; Lemainque, A; Pavek, S; Boccara, M; Argout, X; Amores, F; Courtois, B; Risterucci, AM; Lanaud, C | 2012 | Insight into the Wild Origin, Migration and Domestication History of the Fine Flavour Nacional Theobroma cacao L. Variety from Ecuador | Ecuador’s economic history has been closely linked to Theobroma cacao L cultivation, and specifically to the native fine flavour Nacional cocoa variety. The original Nacional cocoa trees are presently in danger of extinction due to foreign germplasm introductions. In a previous work, a few non-introgressed Nacional types were identified as potential founders of the modern Ecuadorian cocoa population, but so far their origin could not be formally identified. In order to determine the putative centre of origin of Nacional and trace its domestication history, we used 80 simple sequence repeat (SSR) markers to analyse the relationships between these potential Nacional founders and 169 wild and cultivated cocoa accessions from South and Central America. The highest genetic similarity was observed between the Nacional pool and some wild genotypes from the southern Amazonian region of Ecuador, sampled along the Yacuambi, Nangaritza and Zamora rivers in Zamora Chinchipe province. This result was confirmed by a parentage analysis. Based on our results and on data about pre-Columbian civilization and Spanish colonization history of Ecuador, we determined, for the first time, the possible centre of origin and migration events of the Nacional variety from the Amazonian area until its arrival in the coastal provinces. As large unexplored forest areas still exist in the southern part of the Ecuadorian Amazonian region, our findings could provide clues as to where precious new genetic resources could be collected, and subsequently used to improve the flavour and disease resistance of modern Ecuadorian cocoa varieties. | 3539 | NA | NA | NA | no | no |
| 3539 | 2017 | NA | Boulinier, T; Sorci, G; Monnat, JY; Danchin, E | 1997 | Parent-offspring regression suggests heritable susceptibility to ectoparasites in a natural population of kittiwake Rissa tridactyla | Little information is available on the genetic variability of host susceptibility to parasites in natural populations despite its importance for the understanding of the evolution of host-parasite interactions. A long-term demographic and epidemiologic survey of a seabird population allowed us to investigate the potential correlation between parent and offspring ectoparasite load, while controlling for various environmental factors. In particular, parasite loads were measured for all individuals (i.e., parents and offspring) when they were nestlings and the effect of the year and breeding cliff were taken into account. The positive correlation found between parent and offspring parasite loads suggests a heritable susceptibility to ectoparasitism by ticks in this host population and that this character has the potential to respond to natural selection. | 3540 | NA | NA | NA | no | no |
| 3540 | 2017 | NA | Martins, C; Souza, RF; Bueno, OC | 2012 | Presence and distribution of the endosymbiont Wolbachia among Solenopsis spp. (Hymenoptera: Formicidae) from Brazil and its evolutionary history | Wolbachia are intracellular bacteria that commonly infect arthropods. Its prevalence among ants of the genus Solenopsis is high. In the present study, the presence and distribution of these endosymbionts was examined among populations of Solenopsis spp. from Brazil. A phylogenetic analysis based on the wsp gene was conducted to infer the evolutionary history of Wolbachia infections within the populations surveyed. A high frequency of Wolbachia bacteria was observed among the genus Solenopsis, 51% of the colonies examined were infected. Incidence was higher in populations from southern Brazil. However, little genetic variability was found among different Wolbachia strains within supergroups A and B. Our findings also suggest that horizontal transmission events can occur through the social parasite S. daguerrei. (C) 2012 Elsevier Inc. All rights reserved. | 3541 | NA | NA | NA | no | no |
| 3541 | 2017 | NA | Leclerc, MC; Durand, P; Gauthier, C; Patot, S; Billotte, N; Menegon, M; Severini, C; Ayala, FJ; Renaud, F | 2004 | Meager genetic variability of the human malaria agent Plasmodium vivax | Malaria is a major human parasitic disease caused by four species of Plasmodium, protozoa. Plasmodium vivax, the most widespread, affects millions of people across Africa, Asia, the Middle East, and Central and South America. We have studied the genetic variability of 13 microsatellite loci in 108 samples from 8 localities in Asia, Africa, South America, and New Guinea. Only one locus is polymorphic; nine are completely monomorphic, and the remaining three are monomorphic in all but one or two populations, which have a rare second allele. In contrast, Plasmodium falciparum displays extensive microsatellite polymorphism within and among populations. We further have analyzed, in 96 samples from the same 8 localities, 8 tandem repeats (TRs) located on a 100-kb contiguous chromosome segment described as highly polymorphic. Each locus exhibits 2-10 alleles in the whole sample but little intrapopulation polymorphism (1-5 alleles with a prevailing allele in most cases). Eight microsatellite loci monomorphic in P. vivax are polymorphic in three of five Plasmodium species related to P. vivax (two to seven individuals sampled). Plasmodium simium, a parasite of New World monkeys, is genetically indistinguishable from A vivax. At 13 microsatellite loci and at 7 of the 8 Tills, both species share the same (or most common) allele. Scarce microsatellite polymorphism may reflect selective sweeps or population bottlenecks in recent evolutionary history of A vivax, the differential variability of the TRs may reflect selective processes acting on particular regions of the genome. We infer that the world expansion of A vivax as a human parasite occurred recently, perhaps <10,000 years ago. | 3542 | NA | NA | NA | no | no |
| 3542 | 2017 | NA | Uzun, F; Donmez, HB | 2016 | Ecotype Traits of the Natural Populations of the Birdsfoot Trefoil (Lotus corniculatus) in Association with the Geographical Parameters of the Sampling Sites | Birdsfoot trefoil, which is native to Turkish flora, is the most valuable and common Lotus species in Turkey. However, existing information on the ecology, distribution, and plant traits of the Birdsfoot trefoil is scant for the natural flora of Turkey. Therefore, seeds from 126 wild birdsfoot trefoil (WBT) populations were collected from plants spontaneously occurring in natural pasture and rangelands located (altitudes ranged from 5 to 2193 m a.s.l.) in the Black Sea Region of Turkey. These populations were grown under the same ecological conditions in 2010 and 2011 to determine the ecotype traits of the WBT and the relationships between these traits and the geographical features of the locations. The WBT was present (1-25% of botanical composition) in all ranges of altitude, although there was an increase in the presence and frequency of WBT with increasing altitude until 1000 m a.s.l. There were negative correlations between some traits (dry matter and seed yields, morphologic, and also root and crown-rot resistant traits) and altitude at which the seeds were collected. The data revealed that there was evidence of considerable inherent variations in all traits and thus there was a huge genetic diversity in the region. When populations were compared for agronomic and morphologic traits, the best populations were between altitudes of 251 and 500 m. It was observed that all populations had a spreading growth habit, except for 05TA01, 05TA02, and 60ER01 (semi-erect) and 60TU02 (semi-spreading). The results suggested that new grazing, forage types, and disease resistant WBT varieties may be bred from the studied populations. | 3543 | NA | NA | NA | no | no |
| 3543 | 2017 | NA | HAFNER, MS | 1991 | EVOLUTIONARY GENETICS AND ZOOGEOGRAPHY OF MIDDLE AMERICAN POCKET GOPHERS, GENUS ORTHOGEOMYS | Average levels of protein heterozygosity (HBAR = 0.015) and polymorphism (P = 0.048) in 13 populations of Middle American pocket gophers (Orthogeomys, subgenus Macrogeomys) are unusually low by mammalian standards. This low genetic variability is attributed to subdivision of populations into small, isolated demes. Locus-by-locus and parsimony analyses of the protein data generally support retention of the currently recognized species of Macrogeomys. Phylogenetic analysis reveals that gross morphological similarities among highland pocket gophers (large body size and long pelage) and among lowland species (small body size and short pelage) are the result of evolutionary convergence. Most speciation in Macrogeomys appears to have occurred during the past 4.5 x 10(6) years, presumably influenced by Quaternary sea-level fluctuations that resulted in repeated fragmentations of geographic ranges of the species. | 3544 | NA | NA | NA | no | no |
| 3544 | 2017 | NA | Vignaroli, C; Di Sante, L; Magi, G; Luna, GM; Di Cesare, A; Pasquaroli, S; Facinelli, B; Biavasco, F | 2015 | Adhesion of marine cryptic Escherichia isolates to human intestinal epithelial cells | Five distinct cryptic lineages (clades I-V) have recently been recognized in the Escherichia genus. The five clades encompass strains that are phenotypically and taxonomically indistinguishable from Escherichia coli sensu stricto; however, scant data are available on their ecology, virulence and pathogenic properties. In this study 20 cryptic E. coli strains isolated from marine sediments were investigated to gain insights into their virulence characteristics and genetic traits. The ability to adhere to intestinal cells was highest among clade V strains, which also harbored the genes involved in gut colonization as well as the genes (pduC and eut operon) typically found in environmentally adapted E. coli strains. The pduC gene was significantly associated with clade V. Multilocus sequence typing of three representative clade V isolates revealed new sequence types (STs) and showed that the strains shared two allelic loci (adk 51 and recA 37). Our findings suggest that cryptic Escherichia lineages are common in coastal marine sediments and that this habitat may be suitable for their growth and persistence outside the host. On the other hand, detection in clade V strains of a gene repertoire and adhesion properties similar to those of intestinal pathogenic strains could indicate their potential virulence. It could be argued that there is a dual nature of cryptic clade V strains, where the ability to survive and persist in a secondary habitat does not involve the loss of the host-associated lifestyle. Clade V could be a group of closely related, environmentally adapted E. coli strains. | 3545 | NA | NA | NA | no | no |
| 3545 | 2017 | NA | Kovac, J; Miller, RA; Carroll, LM; Kent, DJ; Jian, JH; Beno, SM; Wiedmann, M | 2016 | Production of hemolysin BL by Bacillus cereus group isolates of dairy origin is associated with whole-genome phylogenetic clade | Background: Bacillus cereus group isolates that produce diarrheal or emetic toxins are frequently isolated from raw milk and, in spore form, can survive pasteurization. Several species within the B. cereus group are closely related and cannot be reliably differentiated by established taxonomical criteria. While B. cereus is traditionally recognized as the principal causative agent of foodborne disease in this group, there is a need to better understand the distribution and expression of different toxin and virulence genes among B. cereus group food isolates to facilitate reliable characterization that allows for assessment of the likelihood of a given isolate to cause a foodborne disease. Results: We performed whole genome sequencing of 22 B. cereus group dairy isolates, which represented considerable genetic diversity not covered by other isolates characterized to date. Maximum likelihood analysis of these genomes along with 47 reference genomes representing eight validly published species revealed nine phylogenetic clades. Three of these clades were represented by a single species (B. toyonensis - clade V, B. weihenstephanensis - clade VI, B. cytotoxicus - VII), one by two dairy-associated isolates (clade II; representing a putative new species), one by two species (B. mycoides, B. pseudomycoides - clade I) and four by three species (B. cereus, B. thuringiensis, B. anthracis - clades III-a, b, c and IV). Homologues of genes encoding a principal diarrheal enterotoxin (hemolysin BL) were distributed across all, except the B. cytotoxicus clade. Using a lateral flow immunoassay, hemolysin BL was detected in 13 out of 18 isolates that carried hblACD genes. Isolates from clade III-c (which included B. cereus and B. thuringiensis) consistently did not carry hblACD and did not produce hemolysin BL. Isolates from clade IV (B. cereus, B. thuringiensis) consistently carried hblACD and produced hemolysin BL. Compared to others, clade IV was significantly (p = 0.0001) more likely to produce this toxin. Isolates from clade VI (B. weihenstephanensis) carried hblACD homologues, but did not produce hemolysin BL, possibly due to amino acid substitutions in different toxin-encoding genes. Conclusions: Our results demonstrate that production of diarrheal enterotoxin hemolysin BL is neither inclusive nor exclusive to B. cereus sensu stricto, and that phylogenetic classification of isolates may be better than taxonomic identification for assessment of B. cereus group isolates risk for causing a diarrheal foodborne disease. | 3546 | NA | NA | NA | no | no |
| 3546 | 2017 | NA | Tranter, C; Graystock, P; Shaw, C; Lopes, JFS; Hughes, WOH | 2014 | Sanitizing the fortress: protection of ant brood and nest material by worker antibiotics | Social groups are at particular risk for parasite infection, which is heightened in eusocial insects by the low genetic diversity of individuals within a colony. To combat this, adult ants have evolved a suite of defenses to protect each other, including the production of antimicrobial secretions. However, it is the brood in a colony that are most vulnerable to parasites because their individual defenses are limited, and the nest material in which ants live is also likely to be prone to colonization by potential parasites. Here, we investigate in two ant species whether adult workers use their antimicrobial secretions not only to protect each other but also to sanitize the vulnerable brood and nest material. We find that, in both leaf-cutting ants and weaver ants, the survival of the brood was reduced and the sporulation of parasitic fungi from them increased, when the workers nursing them lacked functional antimicrobial-producing glands. This was the case for both larvae that were experimentally treated with a fungal parasite (Metarhizium) and control larvae which developed infections of an opportunistic fungal parasite (Aspergillus). Similarly, fungi were more likely to grow on the nest material of both ant species if the glands of attending workers were blocked. The results show that the defense of brood and sanitization of nest material are important functions of the antimicrobial secretions of adult ants and that ubiquitous, opportunistic fungi may be a more important driver of the evolution of these defenses than rarer, specialist parasites. | 3547 | NA | NA | NA | no | no |
| 3547 | 2017 | NA | Read, KD; Lemay, MA; Acheson, S; Boulding, EG | 2012 | Using molecular pedigree reconstruction to evaluate the long-term survival of outplanted hatchery-reared larval and juvenile northern abalone (Haliotis kamtschatkana) | The restoration of abalone (Haliotis spp.) populations through supplementation with the offspring of hatchery-spawned wild parents has been attempted in several species, with variable results. Between 2002 and 2005, the Bamfield Huu-ay-aht Community Abalone Project released 4.5 million larvae and 152,000 juveniles of the northern abalone (Haliotis kamtschatkana) into Barkley Sound, BC. The purpose of this study was to estimate the long-term survival of outplanted abalone 3-7 years after their release and thus determine their contribution to local population densities at three different outplanting sites. We identified outplanted abalone by genotyping epipodal tentacles sampled from wild-caught abalone for seven microsatellite loci. We then used three different pedigree reconstruction programs: one that used genotypes from hatchery-reared siblings (pedigree 2.2), one that used the wild parent genotypes that were available (cervus 3.0), and one that used both sources of information (colony 2.0). Each program identified different but partially overlapping subsets of hatchery-outplanted offspring. From this we inferred that up to 26% of the individuals sampled at the main outplanting site were from hatchery spawnings. Despite this large contribution of hatchery-reared stock, the density of mature abalone at each site was below the level required for successful fertilization. More intensive outplanting efforts might increase population densities of this broadcast spawner above this minimum sustainable level. However, for supplementation to be successful, other factors that could reduce outplanted juvenile survival, including the low genetic diversity of hatchery-produced offspring and the low habitat quality of some outplanting sites, need to be monitored. | 3548 | NA | NA | NA | no | no |
| 3548 | 2017 | NA | Tomas, F; Abbott, JM; Steinberg, C; Balk, M; Williams, SL; Stachowicz, JJ | 2011 | Plant genotype and nitrogen loading influence seagrass productivity, biochemistry, and plant-herbivore interactions | Genetic variation within and among key species can have significant ecological consequences at the population, community, and ecosystem levels. In order to understand ecological properties of systems based on habitat-forming clonal plants, it is crucial to clarify which traits vary among plant genotypes and how they influence ecological processes, and to assess their relative contribution to ecosystem functioning in comparison to other factors. Here we used a mesocosm experiment to examine the relative influence of genotypic identity and extreme levels of nitrogen loading on traits that affect ecological processes (at the population, community, and ecosystem levels) for Zostera marina, a widespread marine angiosperm that forms monospecific meadows throughout coastal areas in the Northern Hemisphere. We found effects of both genotype and nitrogen addition on many plant characteristics (e. g., aboveground and belowground biomass), and these were generally strong and similar in magnitude, whereas interactive effects were rare. Genotypes also strongly differed in susceptibility to herbivorous isopods, with isopod preference among genotypes generally matching their performance in terms of growth and survival. Chemical rather than structural differences among genotypes drove these differences in seagrass palatability. Nitrogen addition uniformly decreased plant palatability but did not greatly alter the relative preferences of herbivores among genotypes, indicating that genotype effects are strong. Our results highlight that differences in key traits among genotypes of habitat-forming species can have important consequences for the communities and ecosystems that depend on them and that such effects are not overwhelmed by known environmental stressors. | 3549 | NA | NA | NA | no | no |
| 3549 | 2017 | NA | Santos, SM; Mira, A; Salgueiro, PA; Costa, P; Medinas, D; Beja, P | 2016 | Avian trait-mediated vulnerability to road traffic collisions | Collision with vehicles is an important source of bird mortality, but it is uncertain why some species are killed more often than others. Focusing on passerines, we tested whether mortality is associated with bird abundances, and with traits reflecting flight manoeuvrability, habitat, diet, and foraging and social behaviours. We also tested whether the species most vulnerable to road-killing were scarcer near (<500 m) or far (>500-5000 m) from roads. During the breeding seasons of 2009-2011, we surveyed roadkills daily along 50 km of roads, and estimated bird abundances from 74 point counts. After correcting for phylogenetic relatedness, there was strong correlation between roadkill numbers and the abundances of 28 species counted near roads. However, selectivity indices indicated that Blue tit (Parus caeruleus), Blackcap (Sylvia atricapilla) and European goldfinch (Carduelis carduelis) were significantly more road-killed than expected from their abundances, while the inverse was found for seven species. Using phylogenetic generalised estimating equations, we found that selectivity indexes were strongly related to foraging behaviour and habitat type, and weakly so to body size, wing load, diet and social behaviour. The most vulnerable passerines were foliage/bark and swoop foragers, inhabiting woodlands, with small body size and low wing load. The species most vulnerable to road collisions were not scarcer close to roads. Overall, our study suggests that traits provide a basis to identify the passerine species most vulnerable to road collisions, which may be priority targets for future research on the population-level effects of roadkills. (C) 2016 Elsevier Ltd. All rights reserved. | 3550 | NA | NA | NA | no | no |
| 3550 | 2017 | NA | RODRIGUEZTEBAR, A; DECHANT, G; BARDE, YA | 1991 | NEUROTROPHINS - STRUCTURAL RELATEDNESS AND RECEPTOR INTERACTIONS | Nerve growth factor (NGF), brain-derived neurotrophic factor (BDNF) and neurotrophin-3 (NT-3) are structurally related proteins that allow the survival of specific populations of embryonic vertebrate neurons. The primary structure of these neurotrophins, deduced from their nucleotide sequences, indicates that all three are synthesized in the form of precursor proteins presumably allowing for appropriate folding, including the formation of disulphide bridges, cleavage and secretion. While no information is yet available on the 3-dimensional structures of the neurotrophins, results from binding studies using the three neurotrophins as ligands indicate that their receptors do recognize similarities, as well as differences, between them. High-affinity receptors, that presumably mediate the biological response, as well as low-affinity receptors are present on neurons responsive to the neurotrophins. Whereas a large excess of heterologous ligand is needed to reduce binding of a particular neurotrophin to its high-affinity receptor, the same concentration of homologous or heterologous ligand similarly reduce the binding of any of the three neurotrophins to the low-affinity receptor. For all three, the low-affinity receptor appears to be the already characterized NGF low-affinity receptor that seems to be an integral part of the high-affinity receptor complexes. These results suggest that the regulation of neuronal survival by target cells can, in part, be explained by the release from these cells of limiting quantities of the structurally related neurotrophins, each being recognized by a specific high-affinity receptor complex located on the nerve terminals of the responsive neurons. | 3551 | NA | NA | NA | no | no |
| 3551 | 2017 | NA | Jara, L; Cepero, A; Garrido-Bailon, E; Martin-Hernandez, R; Higes, M; De la Rua, P | 2012 | Linking evolutionary lineage with parasite and pathogen prevalence in the Iberian honey bee | The recent decline in honey bee colonies observed in both European countries and worldwide is of great interest and concern, although the underlying causes remain poorly understood. In recent years, growing evidence has implicated parasites and pathogens in this decline of both the vitality and number of honey bee colonies. The Iberian Peninsula provides an interesting environment in which to study the occurrence of pathogens and parasites in the host honey bee populations due to the presence of two evolutionary lineages in A. m. iberiensis (Western European [M] or African [A]). Here, we provide the first evidence linking the population structure of the Iberian honey bee with the prevalence of some of its most important parasites and pathogens: the Varroa destructor mite and the microsporidia Nosema apis and Nosema ceranae. Using data collected in two surveys conducted in 2006 and 2010 in 41 Spanish provinces, the evolutionary lineage and the presence of the three parasitic organisms cited above were analyzed in a total of 228 colonies. In 2006 N. apis was found in a significantly higher proportion of M lineage honey bees than in the A lineage. However, in 2010 this situation had changed significantly due to a higher prevalence of N. ceranae. We observed no significant relationships in either year between the distributions of V. destructor or N. ceranae and the evolutionary lineage present in A. m. iberiensis colonies, but the effects of these organisms on the genetic diversity of the honey bee populations need further research. (C) 2012 Elsevier Inc. All rights reserved. | 3552 | NA | NA | NA | no | no |
| 3552 | 2017 | NA | Gascuel, Q; Bordat, A; Sallet, E; Pouilly, N; Carrere, S; Roux, F; Vincourt, P; Godiard, L | 2016 | Effector Polymorphisms of the Sunflower Downy Mildew Pathogen Plasmopara halstedii and Their Use to Identify Pathotypes from Field Isolates | The obligate biotroph oomycete Plasmopara halstedii causes downy mildew on sunflower crop, Helianthus annuus. The breakdown of several Pl resistance genes used in sunflower hybrids over the last 25 years came along with the appearance of new Pl. halstedii isolates showing modified virulence profiles. In oomycetes, two classes of effector proteins, key players of pathogen virulence, are translocated into the host: RXLR and CRN effectors. We identified 54 putative CRN or RXLR effector genes from transcriptomic data and analyzed their genetic diversity in seven Pl. halstedii pathotypes representative of the species variability. Pl. halstedii effector genes were on average more polymorphic at both the nucleic and protein levels than random non-effector genes, suggesting a potential adaptive dynamics of pathogen virulence over the last 25 years. Twenty-two KASP (Competitive Allele Specific PCR) markers designed on polymorphic effector genes were genotyped on 35 isolates belonging to 14 Pl. halstedii pathotypes. Polymorphism analysis based on eight KASP markers aims at proposing a determination key suitable to classify the eight multi-isolate pathotypes into six groups. This is the first report of a molecular marker set able to discriminate Pl. halstedii pathotypes based on the polymorphism of pathogenicity effectors. Compared to phenotypic tests handling living spores used until now to discriminate Pl. halstedii pathotypes, this set of molecular markers constitutes a first step in faster pathotype diagnosis of Pl. halstedii isolates. Hence, emerging sunflower downy mildew isolates could be more rapidly characterized and thus, assessment of plant resistance breakdown under field conditions should be improved. | 3553 | NA | NA | NA | no | no |
| 3553 | 2017 | NA | Proctor, MF; Servheen, C; Miller, SD; Kasworm, WF; Wakkinen, WL | 2004 | A comparative analysis of management options for grizzly bear conservation in the US-Canada trans-border area | Grizzly bear (Ursus arctos) populations spanning the U.S.-Canada border in thesouth Selkirk, Purcell-Yaak, and Cabinet Mountains are small, vulnerable, andat the front lines of any further range contraction in North America. Recent genetics work demonstrated that the south Selkirk grizzlies are an isolated population (no male or female connectivity) of fewer than 100 individuals with a15-20% reduction in genetic diversity and that the Purcell-Yaak population isdeclining and demographically isolated (no female connectivity) with fewer than 50 individuals. The <25 animals living in the Cabinet Mountains population are likely isolated from both the south Selkirk Mountain and the Purcell-Yaak populations. We recognize these populations need enhanced management. To guidethe development of a comprehensive management plan, we explored the effects of 3 actions (population augmentation, enhanced population interchange, and reduced mortality through management actions). We simulated 2 populations of 50 and 100 individuals using population viability analysis (PVA) software (VORTEX). We examined these management actions and combinations of them on population growth rate and extinction probabilities. Our simulations suggest that augmentation had the largest demographic effect on population growth rate over the short-term, mortality reductions had the largest effect in the long-term, and establishing population interchange and reducing mortality had the greatest effect on extinction probability. Enhanced cooperative U.S. and Canadian efforts are required to address the issues facing these small grizzly populations and to build connectivity to existing larger populations and areas of vacant habitat. Our findings apply to recovery and conservation efforts for small populations of all species of bears. | 3554 | NA | NA | NA | no | no |
| 3554 | 2017 | NA | Rogus, J; Beck, JD; Offenbacher, S; Huttner, K; Iacoviello, L; Latella, MC; de Gaetano, M; Wang, HY; Kornman, KS; Duff, GW | 2008 | IL1B gene promoter haplotype pairs predict clinical levels of interleukin-1 beta and C-reactive protein | Interleukin-1 beta (IL-1 beta) activates inflammatory mediator cascades and has been implicated in the pathogenesis of several diseases. Single nucleotide polymorphisms (SNPs) of the IL1B promoter have been associated with various inflammatory diseases. We recently reported that IL1B gene transcription was influenced by four promoter SNPs, and that individual SNP function in vitro was governed by haplotype context. In the present study we tested the in vivo relevance of this observation by comparing IL1B promoter haplotype-pairs with IL-1 beta protein levels in 900 gingival tissue fluid samples. Three SNPs (-511, -1464, -3737) defined four IL1B promoter haplotypes that occurred in the study population and could be assigned unambiguously to each chromosome. The four haplotypes defined ten haplotype-pairs of which four pairs, representing 57% of the population, were associated with 28-52% higher IL-1 beta protein levels in vivo. Two of these pairs, characterized by homozygosity for the common allele at -3737, were also associated with raised serum levels of C-reactive protein (p = 0.02). We validated these findings in stimulated peripheral blood mononuclear cells (PBMCs) from a separate population (N = 70). PBMCs with IL1B haplotype-pairs associated with higher in vivo levels of IL-1 beta produced 86-287% more IL-1 beta in vitro than the reference group. We believe that this is the first demonstration of a relationship between in vivo levels of an inflammatory mediator and gene promoter haplotypes on both chromosomes. These findings may apply to other inducible genes and could provide a logical framework for exploring disease risk related to genetic variability in pathogenic mediators. | 3555 | NA | NA | NA | no | no |
| 3555 | 2017 | NA | Bagrade, G; Rungis, DE; Ornicans, A; Suba, J; Zunna, A; Howlett, SJ; Lukins, M; Gailite, A; Stepanova, A; Done, G; Gaile, A; Bitenieks, K; Mihailova, L; Baumanis, J; Ozolins, J | 2016 | Status assessment of Eurasian lynx in Latvia linking genetics and demography-a growing population or a source-sink process? | The Eurasian lynx is managed as a game species in Latvia. A variety of demographic parameters were used to assess the current status of the Latvian lynx population and recruitment after annual harvest. Population age-gender structure and female prenatal fecundity were determined in 530 legally hunted animals over a 9-year period. Average prenatal fecundity was 3.2 +/- 0.1, and evidence of reproduction was found in 87 % of the examined adult females. We found a disproportionally low number of 1- and 2-year-old lynx in the hunting bags and therefore constructed an age-gender pyramid, adjusting the survival curves to the proportions of the remaining age classes. Two hundred and eighty-eight tissue samples were analysed using 12 autosomal microsatellite loci. Groups of related individuals were identified using a group approach and supported by pairwise relationship analysis. Thirteen potential maternal-offspring relationship groups and 30 related groups of lynx individuals based on potential full-sib and half-sib relationships between the individuals within the research period were identified. Evidence from this study shows that the population is not only stable but also likely growing and that there currently appear to be no barriers preventing gene flow. We can conclude that, in general, the lynx population was sustainably maintained. The previous harvest intensity has not obstructed the conservation aim for lynx in Latvia, which is to maintain the population as stable or growing. Combining demographic and molecular analyses, this study reveals relevant reference indices that can be used in future monitoring of the lynx population in Latvia. | 3556 | NA | NA | NA | no | no |
| 3556 | 2017 | NA | Munkemuller, T; Travis, JMJ; Burton, OJ; Schiffers, K; Johst, K | 2011 | Density-regulated population dynamics and conditional dispersal alter the fate of mutations occurring at the front of an expanding population | There is an increasing recognition that the interplay between ecological and evolutionary processes shapes the genetic footprint of populations during and after range expansions. However, more complex ecological processes regularly considered within spatial ecology remain unexplored in models describing the population genetics of range expansion. In this study we integrate flexible descriptions of population growth and competition as well as conditional dispersal into a model that simulates the fate of mutations occurring at the wave front of an expanding population. Our results show that the survival and distribution of a mutation is not only affected by its bias (that is, whether it is deleterious, neutral or beneficial) but also by the mode of local density regulation and conditional dispersal of the simulated populations. It is in particular the chance of a mutation to establish at the front of advance and ‘surf’ to high frequencies that critically depends on the investigated ecological processes. This is because of the influence of these processes on demographic stochasticity in the system and the differential responses of deleterious, neutral and beneficial mutations to this stochasticity. Generally, deleterious mutations rely more on chance and thus profit the most from ecological processes that enhance demographic stochasticity during the period of establishment. Our study emphasizes the importance of incorporating more ecological realism into evolutionary models to better understand the consequences of shifting geographic ranges for the genetic structure of populations and to find efficient adaptation strategies to mitigate these effects. Heredity (2011) 106, 678-689; doi:10.1038/hdy.2010.107; published online 18 August 2010 | 3557 | NA | NA | NA | no | no |
| 3557 | 2017 | NA | Moracho, E; Moreno, G; Jordano, P; Hampe, A | 2016 | Unusually limited pollen dispersal and connectivity of Pedunculate oak (Quercus robur) refugial populations at the species’ southern range margin | Low-latitudinal range margins of temperate and boreal plant species typically consist of scattered populations that persist locally in microrefugia. It remains poorly understood how their refugial habitats affect patterns of gene flow and connectivity, key components for their long-term viability and evolution. We examine landscape-scale patterns of historical and contemporary gene flow in refugial populations of the widespread European forest tree Pedunculate oak (Quercus robur) at the species’ southwestern range margin. We sampled all adult trees (n = 135) growing in a 20 km long valley and genotyped 724 acorns from 72 mother trees at 17 microsatellite loci. The ten oak stands that we identified were highly differentiated and formed four distinct genetic clusters, despite sporadic historical dispersal being detectable. By far most contemporary pollination occurred within stands, either between local mates (85.6%) or through selfing (6.8%). Pollen exchange between stands (2.6%) was remarkably rare given their relative proximity and was complemented by long-distance pollen immigration (4.4%) and hybridization with the locally abundant Quercus pyrenaica (0.6%). The frequency of between-stand mating events decreased with increasing size and spatial isolation of stands. Overall, our results reveal outstandingly little long-distance gene flow for a wind-pollinated tree species. We argue that the distinct landscape characteristics of oaks’ refugial habitats, with a combination of a rugged topography, dense vegetation and humid microclimate, are likely to increase plant survival but to hamper effective long-distance pollen dispersal. Moreover, local mating might be favoured by high tree compatibility resulting from genetic purging in these long-term relict populations. | 3558 | NA | NA | NA | no | no |
| 3558 | 2017 | NA | Arroyo-Rodriguez, V; Aguirre, A; Benitez-Malvido, J; Mandujano, S | 2007 | Impact of rain forest fragmentation on the population size of a structurally important palm species: Astrocaryum mexicanum at Los Tuxtlas, Mexico | Forest fragmentation changes the structure of natural landscapes causing shifts on the distribution and abundance of plant species that could lead to population extinctions. The tree palm Astrocaryum mexicanum is one of the most abundant plant species in the Los Tuxtlas region, Mexico. Nevertheless, 9S% of the original rain forest in this region has disappeared, and remaining palm populations are present within landscapes that differ in their conservation status. To identify the landscape (e.g., amount of forest cover, fragment size, fragment shape and isolation) and vegetation (e.g., plant species richness, density and basal area) attributes that could influence the adult population size of A. mexicanum, we analyzed 45 forest fragments (< 1-266 ha) distributed in three landscapes that encompassed different levels of fragmentation, and compared with the population inhabiting the Los Tuxtlas Field Station (700 ha). Fragmentation affected the population size of A. mexicanum, with the smaller, most isolated and irregular forest fragments showing the lower number of individuals. The importance value index of A. mexicanum was greater in the most conserved landscape. Furthermore, palm density was positively correlated with species richness, density and basal area of old-growth forest species, but negatively correlated with richness of secondary forest species. Overall, the strong decline in population size of adult reproductive palms may affect the regeneration and genetic diversity of this species increasing its probability of extinction. The loss of this species from the remaining fragments may have dramatic consequence for the ecological functioning of this system. (c) 2007 Elsevier Ltd. All rights reserved. | 3559 | NA | NA | NA | no | no |
| 3559 | 2017 | NA | Chi, PB; Duncan, AE; Kramer, PA; Minin, VN | 2014 | Heritability estimation of osteoarthritis in the pig-tailed macaque (Macaca nemestrina) with a look toward future data collection | We examine heritability estimation of an ordinal trait for osteoarthritis, using a population of pig-tailed macaques from theWashington National Primate Research Center (WaNPRC). This estimation is non-trivial, as the data consist of ordinal measurements on 16 intervertebral spaces throughout each macaque’s spinal cord, with many missing values. We examine the resulting heritability estimates from different model choices, and also performa simulation study to compare the performance of heritability estimation with these different models under specific known parameter values. Under both the real data analysis and the simulation study, we find that heritability estimates from an assumption of normality of the trait differ greatly from those of ordered probit regression, which considers the ordinality of the trait. This finding indicates that some caution should be observed regarding model selection when estimating heritability of an ordinal quantity. Furthermore, we find evidence that our real data have little information for valid heritability estimation under ordered probit regression. We thus conclude with an exploration of sample size requirements for heritability estimation under this model. For an ordinal trait, an incorrect assumption of normality can lead to severely biased heritability estimation. Sample size requirements for heritability estimation of an ordinal trait under the threshold model depends on the pedigree structure, trait distribution and the degree of relatedness between each phenotyped individual. Our sample of 173 monkeys did not have enough information from which to estimate heritability, but estimable heritability can be obtained with as few as 180 related individuals under certain scenarios examined here. | 3560 | NA | NA | NA | no | no |
| 3560 | 2017 | NA | Alam, MMM; Palsson, S | 2016 | Population structure of the giant tiger shrimp Penaeus monodon in Bangladesh based on variation in microsatellites and immune-related genes | Penaeus monodon is the most economically important penaeid shrimp in the Indo-West Pacific region for both aquaculture and wild capture. This study evaluated the population structure of P. monodon in Bangladesh and searched for signs of natural selection in immunological genes. Wild P. monodon sampled at four locations off Bangladesh were studied using 10 microsatellite markers and 14 single nucleotide polymorphic sites (SNPs), including six unannotated regions and eight immunological genes (seven C-type lectin and one HLA3), of which variations were associated with tolerance to pathogens and survival in aquaculture. Three genetically distinguishable populations were observed, one in the eastern mangrove forest in the Sundarban-Barguna coast (south Bangladesh), the second in the Sundarban-Middle ground close to the delta of the river Ganges and the third at St Martin’s Island, southeastern Bangladesh. G(ST)’’ ranged from 0.042 to 0.093, and significant F-ST ranged from 0.004 to 0.011. A large proportion of individuals caught in Middle ground and at St Martin’s Island showed a genetic origin that was distinct from the other individuals at those sites, possibly from unsampled southern regions in the Bay of Bengal. Variation and the FST values for the SNPs from C-type lectin genes and HLA3 did not differ from the other markers studied, indicating no selective effects at these loci in the natural populations. Sustainable management of P. monodon should consider the population differentiation described in this study. Further assessment of signs of selection in aquaculture stocks could be useful for successful breeding of the species. | 3561 | NA | NA | NA | no | no |
| 3561 | 2017 | NA | Eves-van den Akker, S; Lilley, CJ; Reid, A; Pickup, J; Anderson, E; Cock, PJA; Blaxter, M; Urwin, PE; Jones, JT; Blok, VC | 2015 | A metagenetic approach to determine the diversity and distribution of cyst nematodes at the level of the country, the field and the individual | Distinct populations of the potato cyst nematode (PCN) Globodera pallida exist in the UK that differ in their ability to overcome various sources of resistance. An efficient method for distinguishing between populations would enable pathogen-informed cultivar choice in the field. Science and Advice for Scottish Agriculture (SASA) annually undertake national DNA diagnostic tests to determine the presence of PCN in potato seed and ware land by extracting DNA from soil floats. These DNA samples provide a unique resource for monitoring the distribution of PCN and further interrogation of the diversity within species. We identify a region of mitochondrial DNA descriptive of three main groups of G.pallida present in the UK and adopt a metagenetic approach to the sequencing and analysis of all SASA samples simultaneously. Using this approach, we describe the distribution of G.pallida mitotypes across Scotland with field-scale resolution. Most fields contain a single mitotype, one-fifth contain a mix of mitotypes, and less than 3% contain all three mitotypes. Within mixed fields, we were able to quantify the relative abundance of each mitotype across an order of magnitude. Local areas within mixed fields are dominated by certain mitotypes and indicate towards a complex underlying pathoscape’. Finally, we assess mitotype distribution at the level of the individual cyst and provide evidence of hybrids’. This study provides a method for accurate, quantitative and high-throughput typing of up to one thousand fields simultaneously, while revealing novel insights into the national genetic variability of an economically important plant parasite. | 3562 | NA | NA | NA | no | no |
| 3562 | 2017 | NA | Sackett, LC; Cross, TB; Jones, RT; Johnson, WC; Ballare, K; Ray, C; Collinge, SK; Martin, AP | 2012 | Connectivity of prairie dog colonies in an altered landscape: inferences from analysis of microsatellite DNA variation | Connectivity of populations influences the degree to which species maintain genetic diversity and persist despite local extinctions. Natural landscape features are known to influence connectivity, but global anthropogenic landscape change underscores the importance of quantifying how human-modified landscapes disrupt connectivity of natural populations. Grasslands of western North America have experienced extensive habitat alteration, fragmenting populations of species such as black-tailed prairie dogs (Cynomys ludovicianus). Population sizes and the geographic range of prairie dogs have been declining for over a century due to habitat loss, disease, and eradication efforts. In many places, prairie dogs have persisted in the face of emerging urban landscapes that carve habitat into smaller and smaller fragments separated by uninhabitable areas. In extreme cases, prairie dog colonies are completely bounded by urbanization. Connectivity is particularly important for prairie dogs because colonies suffer high probabilities of extirpation by plague, and dispersal permits recolonization. Here we explore connectivity of prairie dog populations using analyses of 11 microsatellite loci for 9 prairie dog colonies spanning the fragmented landscape of Boulder County, Colorado. Isolation-by-resistance modeling suggests that wetlands and high intensity urbanization limit movement of prairie dogs. However, prairie dogs appear to move moderately well through low intensity development (including roads) and freely through cropland and grassland. Additionally, there is a marked decline in gene flow between colonies with increasing geographic distance, indicating isolation by distance even in an altered landscape. Our results suggest that prairie dog colonies retain some connectivity despite fragmentation by urbanization and agricultural development. | 3563 | NA | NA | NA | no | no |
| 3563 | 2017 | NA | Rother, S; Hundrieser, J; Pokoyski, C; Kollrich, S; Borns, K; Blasczyk, R; Poehnert, D; Klempnauer, J; Schwinzer, R | 2015 | The c.503T > C Polymorphism in the Human KLRB1 Gene Alters Ligand Binding and Inhibitory Potential of CD161 Molecules | Studying genetic diversity of immunologically relevant molecules can improve our knowledge on their functional spectrum in normal immune responses and may also uncover a possible role of different variants in diseases. We characterized the c.503T>C polymorphism in the human KLRB1 gene (Killer cell lectin-like receptor, subfamily B, member 1) coding for the cell surface receptor CD161. CD161 is expressed by subsets of CD4(+) and CD8(+) T cells and the great majority of CD56(+) natural killer (NK) cells, acting as inhibitory receptor in the latter population. Genotyping a cohort of 118 healthy individuals revealed 40% TT homozygotes, 46% TC heterozygotes, and 14% carriers of CC. There was no difference in the frequency of CD161 expressing CD4(+) and CD8(+) T cells between the different genotypes. However, the frequency of CD161(+) NK cells was significantly decreased in CC carriers as compared to TT homozygotes. c.503T>C causes an amino acid exchange (p.Ile168Thr) in an extracellular loop of the CD161 receptor, which is regarded to be involved in binding of its ligand Lectin-like transcript 1 (LLT1). Binding studies using soluble LLT1-Fc on 293 transfectants over-expressing CD161 receptors from TT or CC carriers suggested diminished binding to the CC variant. Furthermore, triggering of CD161 either by LLT1 or anti-CD161 antibodies inhibited NK cell activation less effectively in cells from CC individuals than cells from TT carriers. These data suggest that the c.503T>C polymorphism is associated with structural alterations of the CD161 receptor. The regulation of NK cell homeostasis and activation apparently differs between carriers of the CC and TT variant of CD161. | 3564 | NA | NA | NA | no | no |
| 3564 | 2017 | NA | Cote, SD; Stien, A; Irvine, RJ; Dallas, JF; Marshall, F; Halvorsen, O; Langvatn, R; Albon, SD | 2005 | Resistance to abomasal nematodes and individual genetic variability in reindeer | Resistance to parasites is believed to have a widespread influence on demographic and adaptive processes. In systems where parasites impose a fitness cost on their host, heterozygotes may be selected because they are more resistant to parasites than homozygotes. Our objective was to assess the relationships between genomewide individual heterozygosity and abomasal nematode burdens in female Svalbard reindeer (Rangifer tarandus platyrhynchus) after the effects of host age, locality, season, and year had been accounted for. Samples were obtained from 306 female reindeer that were culled and genotyped at nine microsatellite loci. Reindeer in our study populations are mainly parasitized by the gastrointestinal nematodes Ostertagia gruehneri and Marshallagia marshalli. The infection intensity of each parasite differed between subpopulations, and among host age classes, seasons and years. We found no significant relationships between abomasal worm burdens, or lumen and mucosa larvae, of either O. gruehneri or M. marshalli and individual heterozygosity ( or mean d(2)) alone or in interactions with host age, locality, and year. Although we analysed one of the largest data set available to date on gastrointestinal nematodes of a wild ruminant, we used a typical data set of nine genetic neutral markers that may have had low power to detect heterozygosity-fitness correlations. We conclude that the proportion of the variance in parasite resistance explained by individual heterozygosity for neutral genetic markers is low in Svalbard reindeer and in vertebrates in general, and we suggest that the candidate-gene approach might be more fruitful for further research on gene-fitness correlations. | 3565 | NA | NA | NA | no | no |
| 3565 | 2017 | NA | de Villiers, CP; van der Merwe, L; Crotti, L; Goosen, A; George, AL; Schwartz, PJ; Brink, PA; Moolman-Smook, JC; Corfield, VA | 2014 | AKAP9 Is a Genetic Modifier of Congenital Long-QT Syndrome Type 1 | Background Long-QT syndrome (LQTS), a cardiac arrhythmia disorder with variable phenotype, often results in devastating outcomes, including sudden cardiac death. Variable expression, independently from the primary disease-causing mutation, can partly be explained by genetic modifiers. This study investigates variants in a known LQTS-causative gene, AKAP9, for potential LQTS-type 1-modifying effects. Methods and Results Members of a South African LQTS-type 1 founder population (181 noncarriers and 168 mutation carriers) carrying the identical-by-descent KCNQ1 p.Ala341Val (A341V) mutation were evaluated for modifying effects of AKAP9 variants on heart rate-corrected QT interval (QTc), cardiac events, and disease severity. Tag single nucleotide polymorphisms in AKAP9 rs11772585, rs7808587, rs2282972, and rs2961024 (order, 5-3positive strand) were genotyped. Associations between phenotypic traits and alleles, genotypes, and haplotypes were statistically assessed, adjusting for the degree of relatedness and confounding variables. The rs2961024 GG genotype, always represented by CGCG haplotype homozygotes, revealed an age-dependent heart rate-corrected QT interval increase (1% per additional 10 years) irrespective of A341V mutation status (P=0.006). The rs11772585 T allele, found uniquely in the TACT haplotype, more than doubled (218%) the risk of cardiac events (P=0.002) in the presence of A341V; additionally, it increased disease severity (P=0.025). The rs7808587 GG genotype was associated with a 74% increase in cardiac event risk (P=0.046), whereas the rs2282972 T allele, predominantly represented by the CATT haplotype, decreased risk by 53% (P=0.001). ConclusionsAKAP9 has been identified as an LQTS-type 1-modifying gene. Variants investigated altered heart rate-corrected QT interval irrespective of mutation status, as well as cardiac event risk, and disease severity, in mutation carriers. | 3566 | NA | NA | NA | no | no |
| 3566 | 2017 | NA | den Bakker, HC; Didelot, X; Fortes, ED; Nightingale, KK; Wiedmann, M | 2008 | Lineage specific recombination rates and microevolution in Listeria monocytogenes | Background: The bacterium Listeria monocytogenes is a saprotroph as well as an opportunistic human foodborne pathogen, which has previously been shown to consist of at least two widespread lineages (termed lineages I and II) and an uncommon lineage (lineage III). While some L. monocytogenes strains show evidence for considerable diversification by homologous recombination, our understanding of the contribution of recombination to L. monocytogenes evolution is still limited. We therefore used STRUCTURE and ClonalFrame, two programs that model the effect of recombination, to make inferences about the population structure and different aspects of the recombination process in L. monocytogenes. Analyses were performed using sequences for seven loci (including the house-keeping genes gap, prs, purM and ribC, the stress response gene sigB, and the virulence genes actA and inlA) for 195 L. monocytogenes isolates. Results: Sequence analyses with ClonalFrame and the Sawyer’s test showed that recombination is more prevalent in lineage II than lineage I and is most frequent in two house-keeping genes (ribC and purM) and the two virulence genes (actA and inlA). The relative occurrence of recombination versus point mutation is about six times higher in lineage II than in lineage I, which causes a higher genetic variability in lineage II. Unlike lineage I, lineage II represents a genetically heterogeneous population with a relatively high proportion (30% average) of genetic material imported from external sources. Phylograms, constructed with correcting for recombination, as well as Tajima’s D data suggest that both lineages I and II have suffered a population bottleneck. Conclusion: Our study shows that evolutionary lineages within a single bacterial species can differ considerably in the relative contributions of recombination to genetic diversification. Accounting for recombination in phylogenetic studies is critical, and new evolutionary models that account for the possibility of changes in the rate of recombination would be required. While previous studies suggested that only L. monocytogenes lineage I has experienced a recent bottleneck, our analyses clearly show that lineage II experienced a bottleneck at about the same time, which was subsequently obscured by abundant homologous recombination after the lineage II bottleneck. While lineage I and lineage II should be considered separate species from an evolutionary viewpoint, maintaining single species name may be warranted since both lineages cause the same type of human disease. | 3567 | NA | NA | NA | no | no |
| 3567 | 2017 | NA | Adugna, A; Bekele, E | 2015 | Assessment of recent bottlenecks and estimation of effective population size in the Ethiopian wild sorghum using simple sequence repeat allele diversity and mutation models | Since the immediate wild relatives of Sorghum bicolor (L.) Moench are indigenous to Ethiopia, studying their population biology is timely for undertaking conservation measures. A study was conducted to investigate the occurrence of population bottlenecks and to estimate the long-term effective population size (N-e) in wild relatives of sorghum. For this, 40 samples of wild sorghum were collected from two remotely located populations that were allopatric to the cultivated sorghum. The presence of bottlenecks was investigated using heterozygosity excess/deficiency, mode shift and allelic diversity based on nine polymorphic simple sequence repeat (SSR) loci. We also estimated the N-e of the studied populations using two different methods employing SSR mutation models. The expected heterozygosity was found to be 0.41 and 0.71 and allelic richness was 3.0 and 4.9, in Awash and Gibe populations, respectively. Neither the heterozygosity excess nor the mode-shift methods detected signatures of bottlenecks in the studied populations. The effective size of the two wild sorghum populations studied also showed no risk of population reduction in these regions of Ethiopia. Therefore, these allopatric wild sorghum populations can survive by occupying patches by the roadsides and fences, areas within abandoned farm lands, forests, etc., which shows that their wild characteristics of adaptation have been adequate for them to survive from extinction despite extensive deforestation of their habitat for modern agriculture and frequent grazing by livestock. However, this does not guarantee the survival of these species for the future and ex situ conservation measures or policies could help maintain their diversity. | 3568 | NA | NA | NA | no | no |
| 3568 | 2017 | NA | Cant, MA | 2003 | Patterns of helping effort in co-operatively breeding banded mongooses (Mungos mungo) | In most co-operative breeding species, some individuals contribute much more to helping behaviour than others. The most well-established explanation of such variation is based on kin selection and suggests that, in the absence of detectable differences in relatedness, individuals who suffer lower costs for a given level of help should contribute more. Differences in helping effort between dominance/sex categories were investigated in co-operatively breeding banded mongooses Mungos mungo in Uganda. The most conspicuous form of help in this species is provided by individuals who babysit offspring at the den while the rest of the pack goes off to forage. Across eight groups, the survival rate of pups increased with the average number of babysitters guarding them, consistent with the hypothesis that helpers benefit the brood that they guard. There was no difference between dominant males, subordinate males and breeding females in total contributions to babysitting. Subordinate males, however, contributed more to babysitting in the mornings, which were the longest and presumably the most energetically expensive sessions of the day. In six litters in one well-studied pack, dominant males and breeding females reduced their contribution to babysitting for the period that females were in oestrus. By contrast, subordinate males increased their contribution to become the main babysitters during this time. These results are consistent with the hypothesis that, where helping conflicts with breeding, individuals with little chance of direct reproduction can help at a lower fitness cost than those with a high probability of successful reproduction. | 3569 | NA | NA | NA | no | no |
| 3569 | 2017 | NA | Rinkevich, B | 1995 | Restoration strategies for coral reefs damaged by recreational activities: The use of sexual and asexual recruits | The unique marine ecosystems of coral reefs express varying levels of degradation as a result of increasing anthropogenic pressures. This is the main reason why more than 200 coral reef localities were proclaimed as natural reserves or marine parks under varying legislation, rules, and monitoring and management programs. Ironically, the conventional management plans increased accessibility to many reef localities and enhanced dramatically the impact of tourism on reef habitats. Recreational activities including SCUBA and skin diving, fishing, human trampling, sediment resuspension, and other damage caused by ‘’innocent’’ visitors are causing a rapid deterioration of many reefs. Their destruction requires years and decades for full recovery. I propose to rehabilitate such damaged habitats by the alternate strategy of ‘’gardening coral reefs’’ with asexual and sexual recruits. Coral branches, colony fragments, and whole small colonies (asexual recruits) and laboratory or in situ settled planula-larvae (sexual recruits) are designed to be transplanted into denuded reefs for restoration. This approach is further improved when the sexual and asexual recruits are maricultured in situ within special protected areas, before being transplanted. The use of sexual recruits ensures an increase in genetic diversity. I discuss several methodologies and results already accumulated showing the applicability of this gardening strategy for rehabilitation of denuded coral reefs. This restoration strategy should be integrated with proper management similar to that of already established reforestation in terrestrial habitats. The best candidates for employing this strategy are the fast-growing coral species, usually branching forms and species that brood their planulae larvae. | 3570 | NA | NA | NA | no | no |
| 3570 | 2017 | NA | Schock, DM; Bollinger, TK; Collins, JP | 2009 | Mortality Rates Differ Among Amphibian Populations Exposed to Three Strains of a Lethal Ranavirus | Infectious diseases are a growing threat to biodiversity, in many cases because of synergistic effects with habitat loss, environmental contamination, and climate change. Emergence of pathogens as new threats to host populations can also arise when novel combinations of hosts and pathogens are unintentionally brought together, for example, via commercial trade or wildlife relocations and reintroductions. Chytrid fungus (Batrachochytrium dendrobatidis) and amphibian ranaviruses (family Iridoviridae) are pathogens implicated in global amphibian declines. The emergence of disease associated with these pathogens appears to be at least partly related to recent translocations over large geographic distances. We experimentally examined the outcomes of novel combinations of host populations and pathogen strains using the amphibian ranavirus Ambystoma tigrinum virus (ATV) and barred tiger salamanders (Ambystoma mavortium, formerly considered part of the Ambystoma tigrinum complex). One salamander population was highly resistant to lethal infections by all ATV strains, including its own strain, and mortality rates differed among ATV strains according to salamander population. Mortality rates in novel pairings of salamander population and ATV strain were not predictable based on knowledge of mortality rates when salamander populations were exposed to their own ATV strain. The underlying cause(s) for the differences in mortality rates are unknown, but local selection pressures on salamanders, viruses, or both, across the range of this widespread host-pathogen system are a plausible hypothesis. Our study highlights the need to minimize translocations of amphibian ranaviruses, even among conspecifc host populations, and the importance of considering intraspecific variation in endeavors to manage wildlife diseases. | 3571 | NA | NA | NA | no | no |
| 3571 | 2017 | NA | Asins, MJ; Fernandez-Ribacoba, J; Bernet, GP; Gadea, J; Cambra, M; Gorris, MT; Carbonell, EA | 2012 | The position of the major QTL for Citrus tristeza virus resistance is conserved among Citrus grandis, C. aurantium and Poncirus trifoliata | Citrus tristeza virus, CTV, is one of the most important citrus pathogens. Although CTV-resistant citrus rootstocks derived from Poncirus trifoliata are common, useful genetic resistance within the genus Citrus for scion improvement is very limited and no CTV-resistant sweet orange cultivar is yet available. Quantitative trait locus (QTL) analysis of the accumulation and distribution of CTV was carried out in a segregating population of 201 C. grandis x C. clementina hybrids derived from a reciprocal cross between two commercial varieties, Chandler (Ch) and Fortune (F), to genetically study the CTV resistance response. Chandler and 13 of its hybrids were found resistant to T-346 CTV isolate. The mortality of C. grandis x C. clementina hybrids, as well as CTV-challenged C. aurantium x P. trifoliata hybrids, was found to be related to the CTV resistance response. The type of cytoplasm (F or Ch) was not associated with the CTV resistance. A major QTL contributing around 24% of the total variance for CTV accumulation and spatial distribution was detected on linkage group 4b. Its position is conserved among C. grandis, C. aurantium and P. trifoliata. Seventy candidate genes of the CTV resistance response were obtained by transcriptomic bulk segregant analysis of resistant versus susceptible CTV-inoculated hybrids. Thirteen out of 28 of those candidates could be mapped on the C. grandis and/or C. clementina linkage maps. None was located on linkage group 4b, but four of them were found to be associated with CTV resistance through a minor QTL (CTVCh14) or epistatic interactions (CTVCh15, CTVCh17 and VIC). | 3572 | NA | NA | NA | no | no |
| 3572 | 2017 | NA | Lopes, JFS; Camargo, RD; Forti, LC; Hughes, WOH | 2017 | The trade-off between the transmission of chemical cues and parasites: behavioral interactions between leaf-cutting ant workers of different age classes | Social animals are faced with an intriguing dilemma. On the one hand, interactions between individuals are essential to exchange information and to promote cohesion, while on the other hand such interactions carry with them the risk of catching and transmitting parasites. This trade-off is particularly significant for social insects because low within-colony genetic diversity makes their colonies potentially vulnerable to parasites while frequent interactions are essential to the development of the colonial odor profile necessary for nestmate recognition. Here we investigate whether social interactions between young and old leaf-cutting ant workers show evidence of this trade-off. We find that old workers engage in more selfgrooming and mandibular scraping than young workers, both in keeping with old workers having been more exposed to parasites. In contrast, we find that young workers engaged in more allogrooming than old workers, which seems likely to have a different motivation possibly the transfer of recognition cues. Furthermore, young workers tended to engage in allogrooming with other young workers, although it was the old workers that were most active and with whom allogrooming would seem likely to optimize information or chemicals transfer. This suggests that young workers may be attempting to minimize the risk of parasite transmission during their social interactions. Although limited to behavioral data, these results hint that ant workers may be sensitive to the trade-off between the transmission of recognition cues and disease, and adjust their social interactions accordingly. (C) 2016 Sociedade Brasileira de Entomologia. Published by Elsevier Editora Ltda. | 3573 | NA | NA | NA | no | no |
| 3573 | 2017 | NA | Rolo, D; Simoes, AS; Domenech, A; Fenoll, A; Linares, J; de Lencastre, H; Ardanuy, C; Sa-Leao, R | 2013 | Disease Isolates of Streptococcus pseudopneumoniae and Non-Typeable S. pneumoniae Presumptively Identified as Atypical S. pneumoniae in Spain | We aimed to obtain insights on the nature of a collection of isolates presumptively identified as atypical Streptococcus pneumoniae recovered from invasive and non-invasive infections in Spain. One-hundred and thirty-two isolates were characterized by: optochin susceptibility in ambient and CO2-enriched atmosphere; bile solubility; PCR-based assays targeting pneumococcal genes lytA, ply, pspA, cpsA, Spn9802, aliB-like ORF2, and a specific 16S rRNA region; multilocus sequence analysis; and antimicrobial susceptibility. By multilocus sequence analysis, 61 isolates were S. pseudopneumoniae, 34 were pneumococci, 13 were S. mitis, and 24 remained unclassified as non-pneumococci. Among S. pseudopneumoniae isolates, 51 (83.6%) were collected from respiratory tract samples; eight isolates were obtained from sterile sources. High frequency of non-susceptibility to penicillin (60.7%) and erythromycin (42.6%) was found. Only 50.8% of the S. pseudopneumoniae isolates displayed the typical optochin phenotype originally described for this species. None harbored the cpsA gene or the pneumococcal typical lytA restriction fragment length polymorphism. The Spn9802 and the specific 16S rRNA regions were detected among the majority of the S. pseudopneumoniae isolates (n = 59 and n = 49, respectively). The ply and pspA genes were rarely found. A high genetic diversity was found and 59 profiles were identified. Among the S. pneumoniae, 23 were capsulated and 11 were non-typeable. Three non-typeable isolates, associated to international non-capsulated lineages, were recovered from invasive disease sources. In conclusion, half of the atypical pneumococcal clinical isolates were, in fact, S. pseudopneumoniae and one-fourth were other streptococci. We identified S. pseudopneumoniae and non-typeable pneumococci as cause of disease in Spain including invasive disease. | 3574 | NA | NA | NA | no | no |
| 3574 | 2017 | NA | Tryfinopoulou, K; Kesanopoulos, K; Xirogianni, A; Marmaras, N; Papandreou, A; Papaevangelou, V; Tsolia, M; Jasir, A; Tzanakaki, G | 2016 | Meningococcal Carriage in Military Recruits and University Students during the Pre MenB Vaccination Era in Greece (2014-2015) | Purpose The aim of the study was to estimate the meningococcal carriage rate and to identify the genotypic characteristics of the strains isolated from healthy military recruits and university students in order to provide data that might increase our understanding on the epidemiology of meningococcus and obtain information which helps to evaluate the potential effects on control programs such as vaccination., Methods A total of 1420 oropharyngeal single swab samples were collected from military recruits and university students on voluntary basis, aged 18-26 years. New York City Medium was used for culture and the suspected N. meningitidis colonies were identified by Gram stain, oxidase and rapid carbohydrate utilization tests. Further characterisation was carried out by molecular methods (multiplex PCR, MLST, WGS). Results The overall carriage rate was of 12.7%; 15% and 10.4% for recruits and university students respectively. MenB (39.4%) was the most prevalent followed by MenY (12.8%) and MenW (4.4%). Among the initial 76 Non Groupable (NG) isolates, Whole Genome Sequence Analysis (WGS) revealed that 8.3% belonged to MenE, 3.3% to MenX and 1.1% to MenZ, while, 53 strains (29.4%) were finally identified as capsule null. Genetic diversity was found among the MenB isolates, with 41/44 cc and 35 cc predominating. Conclusion Meningococcal carriage rate in both groups was lower compared to our previous studies (25% and 18% respectively) with predominance of MenB isolates. These findings, help to further our understanding on the epidemiology of meningococcal disease in Greece. Although the prevalence of carriage seems to have declined compared to our earlier studies, the predominant MenB clonal complexes (including 41/44cc and 35cc) are associated with invasive meningococcal disease. | 3575 | NA | NA | NA | no | no |
| 3575 | 2017 | NA | Maschinski, J; Frye, R; Rutman, S | 1997 | Demography and population viability of an endangered plant species before and after protection from trampling | Land managers often suggest fencing to protect rare plant species from being trampled in heavily used recreation areas, but there are few documented examples of the efficacy of this strategy. In a 7-year demographic study we examined the reproduction, survival, and long-term viability of the endangered sentry milk-vetch (Astragalus cremnophylax var. cremnophylax) before and after protection from trampling. Demographic monitoring and population viability analyses indicated that the population has fluctuated during the 7 years. Before protection the population declined: 26% of individuals died, mortality surpassed natality, and age of first reproduction was significantly older than post-protection. Fifty-eight percent of the population was severely damaged. Population viability analyses of pre-protection years predicted that the population would go extinct within 100 years. Since protection, the population stabilized, grew, and declined again. Seedlings reached reproductive maturity more quickly. Recruitment increased and peaked in 1993 coincident with abundant precipitation, but again declined in 1994. The total numbers of undamaged plants surpassed the numbers of damaged plants. Models of the post-protection population predict stability. Multiple-linear regression analysis indicated that winter and spring precipitation were significantly correlated with lambda. Both ‘’good’’ and ‘’bad’’ climatic conditions occurred during the pre- and post-protection periods. Because of small population size and depauperate genetic diversity, climate will continue to influence population growth. Nevertheless, models indicate that where trampling and bad climatic conditions were coupled, extinction was accelerated. Recovery of sentry milk-vetch will depend on continued protection, augmentation, and environmental factors, although risk of extinction remains very high. | 3576 | NA | NA | NA | no | no |
| 3576 | 2017 | NA | Hao, K; Chudin, E; Greenawalt, D; Schadt, EE | 2010 | Magnitude of Stratification in Human Populations and Impacts on Genome Wide Association Studies | Genome-wide association studies (GWAS) may be biased by population stratification (PS). We conducted empirical quantification of the magnitude of PS among human populations and its impact on GWAS. Liver tissues were collected from 979, 59 and 49 Caucasian Americans (CA), African Americans (AA) and Hispanic Americans (HA), respectively, and genotyped using Illumina650Y (Ilmn650Y) arrays. RNA was also isolated and hybridized to Agilent whole-genome gene expression arrays. We propose a new method (i.e., hgdp-eigen) for detecting PS by projecting genotype vectors for each sample to the eigenvector space defined by the Human Genetic Diversity Panel (HGDP). Further, we conducted GWAS to map expression quantitative trait loci (eQTL) for the similar to 40,000 liver gene expression traits monitored by the Agilent arrays. HGDP-eigen performed similarly to the conventional self-eigen methods in capturing PS. However, leveraging the HGDP offered a significant advantage in revealing the origins, directions and magnitude of PS. Adjusting for eigenvectors had minor impacts on eQTL detection rates in CA. In contrast, for AA and HA, adjustment dramatically reduced association findings. At an FDR = 10%, we identified 65 eQTLs in AA with the unadjusted analysis, but only 18 eQTLs after the eigenvector adjustment. Strikingly, 55 out of the 65 unadjusted AA eQTLs were validated in CA, indicating that the adjustment procedure significantly reduced GWAS power. A number of the 55 AA eQTLs validated in CA overlapped with published disease associated SNPs. For example, rs646776 and rs10903129 have previously been associated with lipid levels and coronary heart disease risk, however, the rs10903129 eQTL was missed in the eigenvector adjusted analysis. | 3577 | NA | NA | NA | no | no |
| 3577 | 2017 | NA | Mackevics, V; Heid, IM; Wagner, SA; Cip, P; Doppelmayr, H; Lejnieks, A; Gohlke, H; Ladurner, N; Illig, T; Iglseder, B; Kronenberg, F; Paulweber, B | 2006 | The adiponectin gene is associated with adiponectin levels but not with characteristics of the insulin resistance syndrome in healthy Caucasians | Low concentrations of adiponectin, the protein product of the APM1 gene, have been reported to be associated with obesity and insulin resistance. However, contrasting results have been described on the genetic variability in APM1 and characteristics of the metabolic syndrome and adiponectin serum concentrations. In the present study, we investigated the association of the two most well-known SNPs of APM1 (+45T4 > G and +276G4T) and their haplotypes, with serum adiponectin concentrations, metabolic parameters and intima-media thickness of the carotid arteries in 1745 well-phenotyped asymptomatic unrelated Caucasian subjects of the SAPHIR cohort. The common T-allele (88.5%) of SNP + 45T > G and the common G-allele (70.5%) of SNP + 276G > T were associated with significantly lower serum adiponectin levels (P = 0.0008 and P = 0.00005, respectively). The most frequent haplotype TG (59.0%) defined by both loci showed a strong association with lower serum adiponectin concentrations (P = 0.000000002). A clear effect per copy of the respective haplotype was observed. This association was most pronounced in lean and insulin-sensitive subjects. The two less common haplotypes TT (29.5%) and GG (11.5%) were associated with higher serum adiponectin levels in a dose-dependent association. Interestingly, no significant association between the adiponectin 45-276 haplotypes and the majority of parameters of the metabolic syndrome or intima-media thickness of the carotid arteries was found in our study. In summary, we replicated a strong association of the adiponectin 45-276 genotypes and haplotypes with adiponectin levels in healthy Caucasians. However, we could not confirm an association of this gene locus with metabolic parameters of the insulin resistance syndrome. | 3578 | NA | NA | NA | no | no |
| 3578 | 2017 | NA | Zhang, YJ; Zhang, S; Li, YL; Ma, SL; Wang, CS; Xiang, MC; Liu, X; An, ZQ; Xu, JP; Liu, XZ | 2014 | Phylogeography and evolution of a fungal-insect association on the Tibetan Plateau | Parasitoidism refers to a major form of interspecies interactions where parasitoids sterilize and/or kill their hosts typically before hosts reach reproductive age. However, relatively little is known about the evolutionary dynamics of parasitoidism. Here, we investigate the spatial patterns of genetic variation of Chinese cordyceps, including both the parasitoidal fungus Ophiocordyceps sinensis and its host insects. We sampled broadly from alpine regions on the Tibetan Plateau and obtained sequences on seven fungal and three insect DNA fragments from each of the 125 samples. Seven and five divergent lineages/cryptic species were identified within the fungus and host insects, respectively. Our analyses suggested that O.sinensis and host insects originated at similar geographic regions in southern Tibet/Yunnan, followed by range expansion to their current distributions. Cophylogenetic analyses revealed a complex evolutionary relationship between O.sinensis and its host insects. Significant congruence was found between host and parasite phylogenies and the time estimates of divergence were similar, raising the possibility of the occurrence of cospeciation events, but the incongruences suggested that host shifts were also prevalent. Interestingly, one fungal genotype was broadly distributed, consistent with recent gene flow. In contrast, the high-frequency insect genotypes showed limited geographic distributions. The dominant genotypes from both the fungus and the insect hosts may represent ideal materials from which to develop artificial cultivation of this important Chinese traditional medicine. Our results demonstrate that both historical and contemporary events have played important roles in the phylogeography and evolution of the O.sinensis-ghost moth parasitoidism on the Tibetan Plateau. | 3579 | NA | NA | NA | no | no |
| 3579 | 2017 | NA | Faruque, SM; Chowdhury, N; Kamruzzaman, M; Dziejman, M; Rahman, MH; Sack, DA; Nair, GB; Mekalanos, JJ | 2004 | Genetic diversity and virulence potential of environmental Vibrio cholerae population in a cholera-endemic area | To understand the evolutionary events and possible selection mechanisms involved in the emergence of pathogenic Vibrio cholerae, we analyzed diverse strains of V. cholerae isolated from environmental waters in Bangladesh by direct enrichment in the intestines of adult rabbits and by conventional laboratory culture. Strains isolated by conventional culture were mostly (99.2%) negative for the major virulence gene clusters encoding toxin-coregulated pilus (TCP) and cholera toxin (CT) and were nonpathogenic in animal models. In contrast, all strains selected in rabbits were competent for colonizing infant mice, and 56.8% of these strains carried genes encoding TCP alone or both TCP and CT. Ribotypes of toxigenic O1 and O139 strains from the environment were similar to pandemic strains, whereas ribotypes of non-O1 non-O139 strains and TCP- nontoxigenic O1 strains diverged widely from the seventh pandemic O1 and the O139 strains. Results of this study suggest that (i) the environmental V. cholerae population in a cholera-endemic area is highly heterogeneous, (h) selection in the mammalian intestine can cause enrichment of environmental strains with virulence potential, (iii) pathogenicity of V. cholerae involves more virulence genes than currently appreciated, and (iv) most environmental V. cholerae strains are unlikely to attain a pandemic potential by acquisition of TCP and CT genes alone. Because most of the recorded cholera pandemics originated in the Ganges Delta region, this ecological setting presumably favors extensive genetic exchange among V. cholerae strains and thus promotes the rare, multiple-gene transfer events needed to assemble the critical combination of genes required for pandemic spread. | 3580 | NA | NA | NA | no | no |
| 3580 | 2017 | NA | Woo, HJ; Reifman, J | 2012 | A quantitative quasispecies theory-based model of virus escape mutation under immune selection | Viral infections involve a complex interplay of the immune response and escape mutation of the virus quasispecies inside a single host. Although fundamental aspects of such a balance of mutation and selection pressure have been established by the quasispecies theory decades ago, its implications have largely remained qualitative. Here, we present a quantitative approach to model the virus evolution under cytotoxic T-lymphocyte immune response. The virus quasispecies dynamics are explicitly represented by mutations in the combined sequence space of a set of epitopes within the viral genome. We stochastically simulated the growth of a viral population originating from a single wild-type founder virus and its recognition and clearance by the immune response, as well as the expansion of its genetic diversity. Applied to the immune escape of a simian immunodeficiency virus epitope, model predictions were quantitatively comparable to the experimental data. Within the model parameter space, we found two qualitatively different regimes of infectious disease pathogenesis, each representing alternative fates of the immune response: It can clear the infection in finite time or eventually be overwhelmed by viral growth and escape mutation. The latter regime exhibits the characteristic disease progression pattern of human immunodeficiency virus, while the former is bounded by maximum mutation rates that can be suppressed by the immune response. Our results demonstrate that, by explicitly representing epitope mutations and thus providing a genotype-phenotype map, the quasispecies theory can form the basis of a detailed sequence-specific model of real-world viral pathogens evolving under immune selection. | 3581 | NA | NA | NA | no | no |
| 3581 | 2017 | NA | Goka, K; Yokoyama, J; Une, Y; Kuroki, T; Suzuki, K; Nakahara, M; Kobayashi, A; Inaba, S; Mizutani, T; Hyatt, AD | 2009 | Amphibian chytridiomycosis in Japan: distribution, haplotypes and possible route of entry into Japan | A serious disease of amphibians caused by the chytrid fungus Batrachochytrium dendrobatidis was first found in Japan in December 2006 in imported pet frogs. This was the first report of chytridiomycosis in Asia. To assess the risk of pandemic chytridiomycosis to Japanese frogs, we surveyed the distribution of the fungus among captive and wild frog populations. We established a nested PCR assay that uses two pairs of PCR primers to amplify the internal transcribed spacer (ITS) region of a ribosomal RNA cassette to detect mild fungal infections from as little as 0.001 pg (1 fg) of B. dendrobatidis DNA. We collected swab samples from 265 amphibians sold at pet shops, 294 bred at institutes and 2103 collected at field sites from northern to southwestern Japan. We detected infections in native and exotic species, both in captivity and in the field. Sequencing of PCR products revealed 26 haplotypes of the B. dendrobatidis ITS region. Phylogenetic analysis showed that three of these haplotypes were specific to the Japanese giant salamander (Andrias japonicus) and appeared to have established a commensal relationship with this native amphibian. Many other haplotypes were carried by alien amphibians. The highest genetic diversity of B. dendrobatidis was found in the American bullfrog (Rana catesbeiana). Some strains of B. dendrobatidis appeared to be endemic to Japanese native amphibians, but many alien strains are being introduced into Japan via imported amphibians. To improve chytridiomycosis risk management, we must consider the risk of B. dendrobatidis changing hosts as a result of anthropogenic disturbance of the host-specific distribution of the fungus. | 3582 | NA | NA | NA | no | no |
| 3582 | 2017 | NA | Pearson, SK; Bull, CM; Gardner, MG | 2017 | Egernia stokesii (gidgee skink) MHC I positively selected sites lack concordance with HLA peptide binding regions | Genes of the major histocompatibility complex (MHC) play an important role in vertebrate disease resistance, kin recognition and mate choice. Mammalian MHC is the most widely characterised of all vertebrates, and attention is often given to the peptide binding regions of the MHC because they are presumed to be under stronger selection than non-peptide binding regions. For vertebrates where the MHC is less well understood, researchers commonly use the amino acid positions of the peptide binding regions of the human leukocyte antigen (HLA) to infer the peptide binding regions within the MHC sequences of their taxon of interest. However, positively selected sites within MHC have been reported to lack correspondence with the HLA in fish, frogs, birds and reptiles including squamates. Despite squamate diversity, the MHC has been characterised in few snakes and lizards. The Egernia group of scincid lizards is appropriate for investigating mechanisms generating MHC variation, as their inclusion will add a new lineage (i.e. Scincidae) to studies of selection on the MHC. We aimed to identify positively selected sites within the MHC of Egernia stokesii and then determine if these sites corresponded with the peptide binding regions of the HLA. Six positively selected sites were identified within E. stokesii MHC I, only two were homologous with the HLA. E. stokesii positively selected sites corresponded more closely to non-lizard than other lizard taxa. The characterisation of the MHC of more intermediate taxa within the squamate order is necessary to understand the evolution of the MHC across all vertebrates. | 3583 | NA | NA | NA | no | no |
| 3583 | 2017 | NA | Castiglioni, L; Bicudo, HEMD | 2005 | Molecular characterization and relatedness of Haematobia irritans (horn fly) populations, by RAPD-PCR | Haematobia irritans is a hematophagous parasite of cattle that causes significant economic losses in many parts of the world, including Brazil. In the present work, one American and four Brazilian populations of this species were studied by Random Amplified Polymorpht DNA (RAPD) to assess basically genetic variability within and between populations. Ten different decamer random primers were employed in the genomic DNA amplification, yielding 117 fragments in the five H.. irritans populations. In Drosophila prosaltans, used as an outgroup, 81 fragments were produced. Forty-three of these fragments were shared by both species. Among the H. irritans samples, that from Rio Branco (Acre State, Brazil) produced the smallest numbers of fragments and polymorphic bands. This high genetic homogenity may be ascribed to its geographic origin (in the Northwest of Brazil), which causes high isolation and low gene flow, unlike the other Brazilian populations, from the South Central region, in which cattle trade is very intensive. Marker fragments (exclusive bands) detected in every sample enabled the population origin to be characterized, but they are also potentially useful for further approaches such as the putative origin of Brazilian populations from North America. Similarity indices [Nei & Li, 1979, Proc. Natl. Acad. Sci. USA 76: 5269-5273] and phylogenetic trees, rooted by using the outgroup and produced by the Phylogenetic Analysis using Parsimony (PAUP 4.0-Swofford, 2001) program showed the closest relationships between flies from Sao Jose do Rio Preto and Turiuba (both from Sao Paulo State, Brazil) while flies from the geographically distant Rio Branco showed the greatest differentiation relative to the others. | 3584 | NA | NA | NA | no | no |
| 3584 | 2017 | NA | Yasukochi, Y; Kurosaki, T; Yoneda, M; Koike, H | 2010 | Identification of the expressed MHC class II DQB gene of the Asiatic black bear, Ursus thibetanus, in Japan | Genetic diversity estimation of the major histocompatibility complex (MHC) gene may be an important tool in the assessment of immune response ability against infectious disease. We were able to identify a near full-length expressed DQB sequence by RACE-PCR method from the Asiatic black bear, Ursus thibetanus in Japan. This is the first such full length expression in the Ursidae. The bear had at least one functional DQB locus. In phylogenetic tree analysis its DQB amino acid sequence formed a monophyletic group with DQB sequences from members of the order Carnivora and had a 90% nucleotide sequence similarity with the DQB allele of the California sea lion, Zalophus californianus. We compared the DQB amino acid composition of U. thibetanus with those of several other mammalian species including Homo sapiens. Amino acid residues known to be functionally important for human MHC genes, tended to be also conserved among other mammalian species while PBRs in the beta 1 domain were heterogeneous among mammalian species. The DQB sequence obtained from the bear had not only no putative frameshifts or deletions but also no abnormal amino acid mutations such as had been observed in human DQB molecules. This suggests that the bear DQB sequence was an apparently functional DQB allele. As a preliminary study, we sequenced the exon 2 region of DQB alleles from genomic DNA, and succeeded to amplify the exon 2 of DQB loci. Our study will provide useful information for conservation genetics of the U. thibetanus as well as more generally regarding the mammalian MHC region. | 3585 | NA | NA | NA | no | no |
| 3585 | 2017 | NA | Zeigler, SL; De Vleeschouwer, KM; Raboy, BE | 2013 | Assessing Extinction Risk in Small Metapopulations of Golden-headed Lion Tamarins (Leontopithecus chrysomelas) in Bahia State, Brazil | Golden-headed lion tamarins (GHLTs; Leontopithecus chrysomelas) are endangered primates endemic to the Brazilian Atlantic Forest, where loss of forest and its connectivity threaten species survival. Understanding the role of habitat availability and configuration on population declines is critical for guiding proactive conservation for this, and other, endangered species. We conducted population viability analysis to assess vulnerability of ten GHLT metapopulations to habitat loss and small population size. Seven metapopulations had a low risk of extirpation (or local extinction) over the next 100 years assuming no further forest loss, and even small populations could persist with immediate protection. Three metapopulations had a moderate/high risk of extirpation, suggesting extinction debt may be evident in parts of the species’ range. When deforestation was assumed to continue at current rates, extirpation risk significantly increased while abundance and genetic diversity decreased for all metapopulations. Extirpation risk was significantly negatively correlated with the size of the largest patch available to metapopulations, underscoring the importance of large habitat patches for species persistence. Finally, we conducted sensitivity analysis using logistic regression, and our results showed that local extinction risk was sensitive to percentage of females breeding, adult female mortality, and dispersal rate and survival; conservation or research programs that target these aspects of the species’ biology/ecology could have a disproportionately important impact on species survival. We stress that efforts to protect populations and tracts of habitat of sufficient size throughout the species’ distribution will be important in the near-term to protect the species from continuing decline and extinction. | 3586 | NA | NA | NA | no | no |
| 3586 | 2017 | NA | Wu, XY; Wu, XH; Xu, P; Wang, BG; Lu, ZF; Li, GJ | 2015 | Association Mapping for Fusarium Wilt Resistance in Chinese Asparagus Bean Germplasm | Fusarium wilt (FW) is an important vascular disease attacking asparagus bean [Vigna unguiculata (L.) Walp. subsp. unguiculata Sesquipedalis Group] in China. The level and genetic variability of FW resistance in the Chinese asparagus bean germplasm remains elusive. In the current study, FW resistance was assessed across a natural population consisting of 95 asparagus bean and four African cowpea [Vigna unguiculata (L.) Walp. subsp. unguiculata Unguiculata Group] accessions. The disease index (DI) based on the severity of leaf damage (LFD) and vascular discoloration (VD) varied highly across the population and the highly resistant varieties used for vegetable are very limited. Genome-wide association study identified 11 and seven single nucleotide polymorphisms (SNPs) that are associated with LFD and VD traits, respectively. These SNPs were distributed on nine linkage groups of the asparagus bean genome and each accounted for less than 5% of the phenotypic variation. Overall, the nonstandard vegetable (NSV) subgene pool harbors favorable alleles in a higher frequency than the standard vegetable (SV) subgene pool. Individual NSV-type accessions tend to possess a greater number of favorable alleles than the SV-type ones. A SNP marker 1\_0981 was converted to a cleaved amplified polymorphic sequences (CAPS) marker to facilitate future breeding. To our knowledge, this is the first report of an association mapping (AM) study in asparagus bean. The results obtained suggests that resources for FW resistance is relatively limited in the SV subgene pool; hence, introducing resistant alleles from the NSV accessions into currently leading SV cultivars will be important to improve FW resistance of the latter. | 3587 | NA | NA | NA | no | no |
| 3587 | 2017 | NA | Thompson, SR; Humphries, SE; Thomas, MG; Ekong, R; Tarekegn, A; Bekele, E; Creemer, O; Bradman, N; Veeramah, KR | 2013 | The frequency of an IL-18-associated haplotype in Africans | Variation within the gene for the proinflammatory cytokine interleukin (IL)-18 has been associated with inter-individual differences in levels of free protein and disease risk. We investigated the frequency of function-associated IL18 gene haplotypes in an extensive sample (n=2357) of African populations from across the continent. A previously identified five tagging SNP (single-nucleotide polymorphism) haplotype (here designated hGTATA), known to be associated with lower levels of IL-18, was observed at a frequency of 27% in a British population of recent European ancestry, but was found at low frequency (<8%) or completely absent in African populations. Potentially protective variants may, as a consequence, be found at low frequency in African individuals and may confer a difference in disease risk. European Journal of Human Genetics (2013) 21, 465-468; doi:10.1038/ejhg.2012.184; published online 29 August 2012 | 3588 | NA | NA | NA | no | no |
| 3588 | 2017 | NA | Zemtsova, GE; Apanaskevich, DA; Reeves, WK; Hahn, M; Snellgrove, A; Levin, ML | 2016 | Phylogeography of Rhipicephalus sanguineus sensu lato and its relationships with climatic factors | Brown dog ticks morphologically identifiable as Rhipicephalus sanguineus sensu lato, are distributed world-wide and their systematics is controversial. Results of genetic and reproductive compatibility studies of geographically distinct populations of R. sanguineus s.l. indicate that the R. sanguineus complex is paraphyletic. To further elucidate systematic relationships within R. sanguineus s.l. and geographic boundaries of its lineages, we conducted a phylogeographical study of 136 tick specimens from 23 countries. Voucher specimens were morphologically identified. A phylogenetic tree was constructed using concatenated partial mitochondrial 12S and 16S rDNA gene sequences and analyzed by the Neighbor-Joining method. A set of 19 bioclimatic variables within the WorldClim dataset were extracted and analyzed to assess correlations between distribution of R. sanguineus s.l. lineages and climatic variables. The following four branches are clearly recognized on the phylogenetic tree: R. sanguineus s.l.-tropical and temperate clades, R. leporis, and R. turanicus. DNA sequences of Rhipicephalus ticks from Israel differ from those of other groups. Strong association between geographical locations of major clades of R. sanguineus s.l. and temperature was identified. The tropical clade of R. sanguineus s.l. occupies areas with the annual mean temperature > 20 A degrees C, whereas the temperate clade is present in areas with the annual mean temperature < 20 A degrees C. Our results indicate that ticks in two closely related phylogenetic clades are adapted to different environmental conditions and support proposals for re-classification of R. sanguineus complex. Differences in R. sanguineus s.l. ecology and human/animal pathogens transmitted by different taxa of brown dog tick need to be studied. | 3589 | NA | NA | NA | no | no |
| 3589 | 2017 | NA | Irvine, AD | 2009 | Towards a Unified Classification of the Ectodermal Dysplasias: Opportunities Outweigh Challenges | The ectodermal dysplasias include a complex and highly diverse group of heritable disorders that share in common developmental abnormalities of ectodermal derivatives. The broader definition of ectodermal dysplasias (as heritable disorders involving at least two of the ectodermal derivatives nails, teeth, hair, and eccrine sweat glands) encompasses 170-200 conditions. Some conditions included by this definition are relatively common; others are rare and, in some cases, family-specific. Classification of the ectodermal dysplasias has largely been approached by categorizing patterns of clinical findings (phenotypic grouping). In the last 2 decades great progress has been made in understanding the molecular pathogenesis and inter-relatedness of some of these conditions and a new consensus approach to classification that incorporates this new information is needed. A comprehensive and definitive classification of these disorders would be highly valuable for the many stakeholders in ED. As disease-specific molecular treatments are developed, accurate classification will assume greater importance in designing registries to enable rapid identification of those with rare disorders who may wish to participate in clinical trials. Ideally a working classification of such a disparate collection of conditions would have a design and architecture that would facilitate easy accessibility by each of the key stakeholder groups and would encourage enhanced interaction between these parties. Attaining this objective is a major challenge but is achievable. This article reviews the historical-clinical perspective and the impact of recent developments in molecular biology in the field. Reflections are offered as to the future direction of classification systems in these disorders. (C) 2009 Wiley-Liss, Inc. | 3590 | NA | NA | NA | no | no |
| 3590 | 2017 | NA | Batista, JSS; Hungria, M; Barcellos, FG; Ferreira, MC; Mendes, IC | 2007 | Variability in Bradyrhizobium japonicum and B. elkanii seven years after introduction of both the exotic microsymbiont and the soybean host in a cerrados soil | The plasticity of rhizobial genomes is far greater than previously thought, with complex genomic recombination events that may be accelerated by the often stressful environmental conditions of the tropics. This study aimed at evaluating changes in soybean rhizobia due to adaptation to inhospitable environmental conditions (high temperatures, drought, and acid soils) in the Brazilian Cerrados. Both the host plant and combinations of four strains of soybean Bradyrhizobium were introduced in an uncropped soil devoid of rhizobia capable of nodulating soybean. After the third year, seeds were not reinoculated. Two hundred and sixty-three isolates were obtained from nodules of field-grown soybean after the seventh year, and their morphological, physiological, serological, and symbiotic properties determined, followed by genetic analysis of conserved and symbiotic genes. B. japonicum strain CPAC 15 (same serogroup as USDA 123) was characterized as having high saprophytic capacity and competitiveness and by the seventh year represented up to 70% of the cultivable population, in contrast to the poor survival and competitiveness of B. japonicum strain CPAC 7 (same serogroup as CB 1809). In general, adapted strains had increased mucoidy, and up to 43% of the isolates showed no serological reaction. High variability, presumably resulting from the adaptation to the harsh environmental conditions, was verified in rep-PCR (polymerase chain reaction) profiles, being lower in strain CPAC 15, intermediate in B. elkanli, and higher in CPAC 7. Restriction fragment length polymorphism (RFLP)-PCR types of the 16S rDNA corresponded to the following: one type for B. elkanii species, two for B. japonicum, associated to CPAC 15 and CPAC 7, and unknown combinations of profiles. However, when node sequences and RFLP-PCR of the nifH region data were considered, only two clusters were observed having full congruence with B. Japonicum and B. elkanii species. Combining the results, variability was such that even within a genetically more stable group (such as that of CPAC 15), only 6.4% of the isolates showed high similarity to the inoculant strain, whereas none was similar to CPAC 7. The genetic variability in our study seems to result from a variety and combination of events including strain dispersion, genomic recombination, and horizontal gene transfer. Furthermore, the genetic variability appears to be mainly associated with adaptation, saprophytic capacity, and competitiveness, and not with symbiotic effectiveness, as the similarity of symbiotic genes was higher than that of conserved regions of the DNA. | 3591 | NA | NA | NA | no | no |
| 3591 | 2017 | NA | Donkor, ES; Adegbola, RA; Wren, BW; Antonio, M | 2013 | Population Biology of Streptococcus pneumoniae in West Africa: Multilocus Sequence Typing of Serotypes That Exhibit Different Predisposition to Invasive Disease and Carriage | Background: Little is known about the population biology of Streptococcus pneumoniae in developing countries, although the majority of pneumococcal infections occur in this setting. The aim of the study was to apply MLST to investigate the population biology of S. pneumoniae in West Africa. Methods: Seventy three invasive and carriage S. pneumoniae isolates from three West African countries including The Gambia, Nigeria and Ghana were investigated. The isolates covered seven serotypes (1, 3, 5, 6A, 11, 14, 23F) and were subjected to multilocus sequence typing and antibiotic susceptibility testing. Results: Overall, 50 different sequence types (STs) were identified, of which 38% (29) were novel. The most common ST was a novel clone-ST 4012 (6.5%), and some clones including STs 913, 925, 1737, 2160 and 3310 appeared to be specific to the study region. Two STs including ST 63 and ST 4012 were associated with multiple serotypes indicating a history of serotype switching. ST 63 was associated with serotypes 3 and 23F, while ST 4012 was associated with serotypes 6A and 23. eBURST analyses using the stringent 6/7 identical loci definition grouped the 50 STs into 5 clonal complexes and 65 singletons, expressing a high level of genetic diversity among the isolates. Compared to the other serotypes, serotypes 1 and 5 isolates appeared to be more clonal. Internationally recognized antibiotic resistant clones of S. pneumoniae were generally absent in the population investigated and the only multidrug resistant isolate identified (1/66) belong to the Pneumocococcal Epidemiology Network clone ST 63. Conclusions: The pneumococcal population in West Africa is quite divergent, and serotypes that are common in invasive disease (such as serotypes 1 and 5) are more likely to be clonal than serotypes that are common in carriage. | 3592 | NA | NA | NA | no | no |
| 3592 | 2017 | NA | Murrant, MN; Bowman, J; Wilson, PJ | 2014 | A test of non-kin social foraging in the southern flying squirrel (Glaucomys volans) | It can be challenging to understand the evolution of sociality, particularly the occurrence of co-operation by non-kin. Southern flying squirrels (Glaucomys volans) are an interesting example of non-kin co-operation because of the mutual benefits obtained by social thermoregulation during winter. Because group survival confers benefits to the entire group, flying squirrels may also follow an aggregation economy, whereby co-operative foraging during winter is advantageous. However, the extent of such social foraging in flying squirrels is unknown. We tested for social foraging of southern flying squirrels, and also for relatedness among foraging groups. To determine the structure of foraging groups, we set up and remotely monitored feeding stations and nest cavities. All squirrels at the study site were tagged with passive integrated transponder (PIT) tags and nests and feeding stations were monitored with automated PIT-tag recorders for a 24-month period. Squirrels were found most often foraging alone. Squirrels that were recorded foraging together comprised unrelated individuals that were also found to share nest cavities. Squirrels were also recorded travelling farther distances between nest cavity and feeding station in the winter season than in the summer season, suggesting that, during winter, squirrels trade-off proximity to food caches for membership in a nest group. Our data suggest that squirrels forage and cache alone in their summer home range and make solitary returns to this summer range to collect their cache during the winter months, despite exhibiting social winter nesting.(c) 2014 The Linnean Society of London, Biological Journal of the Linnean Society, 2014, 113, 1126-1135. | 3593 | NA | NA | NA | no | no |
| 3593 | 2017 | NA | O’Connor, S; Park, KJ; Goulson, D | 2013 | Worker drift and egg dumping by queens in wild Bombus terrestris colonies | Wild bumblebee colonies are hard to find and often inaccessible, so there have been few studies of the genetic structure of bumblebees within natural colonies, and hence, it is not clear how frequently events such as worker reproduction, worker drift and queen usurpation take place. This study aimed to quantify the occurrence of natal-worker reproduction, worker drift and drifter reproduction within 14 wild colonies of Bombus terrestris in Central Scotland. Four unlinked microsatellites were used to identify patterns of relatedness of the colonies’ adults and broods. In colonies with queens (queenright colonies), worker reproduction accounted for just 0.83 % of males, increasing to 12.11 % in queenless colonies. Four colonies contained a total of six workers which were not daughters of the queen, and were assumed to be drifters, and four male offspring of drifters. Drifting is clearly not common and results in few drifter offspring overall, although drifters produced approximately seven times more offspring per capita than workers that remained in their natal colony. Unexpectedly, two colonies contained clusters of sister workers and juvenile offspring that were not sisters to the rest of the adults or brood found in the colonies, demonstrating probable egg dumping by queens. A third colony contained a queen which was not a sister or daughter to the other bees in the colony. Although usurping of bumblebee colonies by queens in early season is well documented, this appears to be the first record of egg dumping, and it remains unclear whether it is being carried out by old queens or newly mated young queens. | 3594 | NA | NA | NA | no | no |
| 3594 | 2017 | NA | Broor, S; Bharaj, P; Chahar, HS | 2008 | Human metapneumovirus: a new respiratory pathogen | Human metapneumovirus is a recently recognized pathogen of acute respiratory tract infection (ARI) in children as well as elderly and immunocompromised adults. The virus belongs to the family Paramyxoviridae, sub family Pneumovirinae and genus Metapneumovirus. Through genetic analysis it has been characterized into two groups A and B which are further divided into four sub-lineages. The virus is difficult to grow in tissue culture and hence reverse transcriptase-polymerase chain reaction (RT-PCR) for N and L gene is the method of choice for diagnosis. The virus has been seen in all countries with seasonal distribution in winter months for temperate and spring/summer for tropical countries. F gene is the most conserved among different lineages and efforts are underway to design recombination vaccine using F gene. | 3595 | NA | NA | NA | no | no |
| 3595 | 2017 | NA | Smeesters, PR; Vergison, A; Campos, D; de Aguiar, E; Deyi, VYM; Van Melderen, L | 2006 | Differences between Belgian and Brazilian Group A Streptococcus Epidemiologic Landscape | Background. Group A Streptococcus (GAS) clinical and molecular epidemiology varies with location and time. These differences are not or are poorly understood. Methods and Findings. We prospectively studied the epidemiology of GAS infections among children in outpatient hospital clinics in Brussels (Belgium) and Brasilia (Brazil). Clinical questionnaires were filled out and microbiological sampling was performed. GAS isolates were emm-typed according to the Center for Disease Control protocol. emm pattern was predicted for each isolate. 334 GAS isolates were recovered from 706 children. Skin infections were frequent in Brasilia (48% of the GAS infections), whereas pharyngitis were predominant (88%) in Brussels. The mean age of children with GAS pharyngitis in Brussels was lower than in Brasilia (65/92 months, p < 0.001). emm-typing revealed striking differences between Brazilian and Belgian GAS isolates. While 20 distinct emm-types were identified among 200 Belgian isolates, 48 were found among 128 Brazilian isolates. Belgian isolates belong mainly to emm pattern A-C (55%) and E (42.5%) while emm pattern E (51.5%) and D (36%) were predominant in Brasilia. In Brasilia, emm pattern D isolates were recovered from 18.5% of the pharyngitis, although this emm pattern is supposed to have a skin tropism. By contrast, A-C pattern isolates were unfrequently recovered in a region where rheumatic fever is still highly prevalent. Conclusions. Epidemiologic features of GAS from a pediatric population were very different in an industrialised country and a low incomes region, not only in term of clinical presentation, but also in terms of genetic diversity and distribution of emm patterns. These differences should be taken into account for designing treatment guidelines and vaccine strategies. | 3596 | NA | NA | NA | no | no |
| 3596 | 2017 | NA | Triska, P; Soares, P; Patin, E; Fernandes, V; Cerny, V; Pereira, L | 2015 | Extensive Admixture and Selective Pressure Across the Sahel Belt | Genome-wide studies of African populations have the potential to reveal powerful insights into the evolution of our species, as these diverse populations have been exposed to intense selective pressures imposed by infectious diseases, diet, and environmental factors. Within Africa, the Sahel Belt extensively overlaps the geographical center of several endemic infections such as malaria, trypanosomiasis, meningitis, and hemorrhagic fevers. We screened 2.5 million single nucleotide polymorphisms in 161 individuals from 13 Sahelian populations, which together with published data cover Western, Central, and Eastern Sahel, and include both nomadic and sedentary groups. We confirmed the role of this Belt as a main corridor for human migrations across the continent. Strong admixture was observed in both Central and Eastern Sahelian populations, with North Africans and Near Eastern/Arabians, respectively, but it was inexistent in Western Sahelian populations. Genome-wide local ancestry inference in admixed Sahelian populations revealed several candidate regions that were significantly enriched for non-autochthonous haplotypes, and many showed to be under positive selection. The DARC gene region in Arabs and Nubians was enriched for African ancestry, whereas the RAB3GAP1/LCTIMCM6 region in Oromo, the TAS2R gene family in Fulani, and the ALMS 1 INAT8 in Turkana and Samburu were enriched for non-African ancestry. Signals of positive selection varied in terms of geographic amplitude. Some genomic regions were selected across the Belt, the most striking example being the malaria-related DARC gene. Others were Western-specific (oxytocin, calcium, and heart pathways), Fastern-specific (lipid pathways), or even population-restricted (TAS2R genes in Fulani, which may reflect sexual selection). | 3597 | NA | NA | NA | no | no |
| 3597 | 2017 | NA | Sharma, AM; Baraff, B; Hutchins, JT; Wong, MK; Blaisdell, GK; Cooper, ML; Daane, KM; Almeida, RPP | 2015 | Relative Prevalence of Grapevine Leafroll-Associated Virus Species in Wine Grape-Growing Regions of California | Some diseases manifest as one characteristic set of symptoms to the host, but can be caused by multiple pathogens. Control treatments based on plant symptoms can make it difficult to effectively manage such diseases, as the biology of the underlying pathogens can vary. Grapevine leafroll disease affects grapes worldwide, and is associated with several viral species in the family Closteroviridae. Whereas some of the viruses associated with this disease are transmitted by insect vectors, others are only graft-transmissible. In three regions of California, we surveyed vineyards containing diseased vines and screened symptomatic plants for all known viral species associated with grapevine leafroll disease. Relative incidence of each virus species differed among the three regions regions, particularly in relation to species with known vectors compared with those only known to be graft-transmitted. In one region, the pathogen population was dominated by species not known to have an insect vector. In contrast, populations in the other surveyed regions were dominated by virus species that are vector-transmissible. Our survey did not detect viruses associated with grapevine leafroll disease at some sites with characteristic disease symptoms. This could be explained either by undescribed genetic diversity among these viruses that prevented detection with available molecular tools at the time the survey was performed, or a misidentification of visual symptoms that may have had other underlying causes. Based on the differences in relative prevalence of each virus species among regions and among vineyards within regions, we expect that region and site-specific management strategies are needed for effective disease control. | 3598 | NA | NA | NA | no | no |
| 3598 | 2017 | NA | Traesel, CK; Silva, MSE; Weiss, M; Spilki, FR; Weiblen, R; Flores, EF | 2014 | Genetic diversity of 3’ region of glycoprotein D gene of bovine herpesvirus 1 and 5 | Bovine herpesviruses 1 (BoHV-1) and 5 (BoHV-5) are closely related alphaherpesviruses of cattle. While BoHV-1 is mainly associated with respiratory/genital disease and rarely associated with neurological disease, BoHV-5 is the primary agent of meningoencephalitis in cattle. The envelope glycoprotein D of alphaherpesviruses (BoHV-1/gD1 and BoHV-5/gD5) is involved in the early steps of virus infection and may influence virus tropism and neuropathogenesis. This study performed a sequence analysis of the 3’ region of gD gene (gD3’) of BoHV-1 isolates recovered from respiratory/genital disease (n = 6 and reference strain Cooper) or from neurological disease (n = 7); and from seven typical neurological BoHV-5 isolates. After PCR amplification, nucleotide (nt) sequencing, and aminoacid (aa) sequence prediction; gD3’ sequences were compared, identity levels were calculated, and selective pressure was analyzed. The phylogenetic reconstruction based on nt and aa sequences allowed for a clear differentiation of BoHV-1 (n = 14) and BoHV-5 (n = 7) clusters. The seven BoHV-1 isolates from neurological disease are grouped within the BoHV-1 branch. A consistent alignment of 346 nt revealed a high similarity within each viral species (gD1 = 98.3 % nt and aa; gD5 = 97.8 % nt and 85.8 % aa) and an expected lower similarity between gD1 and gD5 (73.7 and 64.1 %, nt and aa, respectively). The analysis of molecular evolution revealed an average negative selection at gD3’. Thus, the phylogeny and similarity levels allowed for differentiation of BoHV-1 and BoHV-5 species, but not further division in subspecies. Sequence analysis did not allow for the identification of genetic differences in gD3’ potentially associated with the respective clinical/pathological phenotypes, yet revealed a lower level of gD3’ conservation than previously reported. | 3599 | NA | NA | NA | no | no |
| 3599 | 2017 | NA | GOTTELLI, D; SILLEROZUBIRI, C; APPLEBAUM, GD; ROY, MS; GIRMAN, DJ; GARCIAMORENO, J; OSTRANDER, EA; WAYNE, RK | 1994 | MOLECULAR-GENETICS OF THE MOST ENDANGERED CANID - THE ETHIOPIAN WOLF CANIS-SIMENSIS | The world’s most endangered canid is the Ethiopian wolf Canis simensis, which is found in six isolated areas of the Ethiopian highlands with a total population of no more than 500 individuals. Ethiopian wolf populations are declining due to habitat loss and extermination by humans. Moreover, in at least one population, Ethiopian wolves are sympatric with domestic dogs, which may hybridize with them, compete for food, and act as disease vectors. Using molecular techniques, we address four questions concerning Ethiopian wolves that have conservation implications. First, we determine the relationships of Ethiopian wolves to other wolf-like canids by phylogenetic analysis of 2001 base pairs of mitochondrial DNA (mtDNA) sequence. Our results suggest that the Ethiopian wolf is a distinct species more closely related to gray wolves and coyotes than to any African canid. The mtDNA sequence similarity with gray wolves implies that the Ethiopian wolf may hybridize with domestic dogs, a recent derivative of the gray wolf. We examine this possibility through mtDNA restriction fragment analysis and analysis of nine microsatellite loci in populations of Ethiopian wolves. The results imply that hybridization has occurred between female Ethiopian wolves and male domestic dogs in one population. Finally, we assess levels of variability within and between two Ethiopian wolf populations. Although these closely situated populations are not differentiated, the level of variability in both is low, suggesting long-term effective population sizes of less than a few hundred individuals. We recommend immediate captive breeding of Ethiopian wolves to protect their gene pool from dilution and further loss of genetic variability. | 3600 | NA | NA | NA | no | no |
| 3600 | 2017 | NA | Abbott, DW; Eirin-Lopez, JM; Boraston, AB | 2008 | Insight into ligand diversity and novel biological roles for family 32 carbohydrate-binding modules | Family 32 carbohydrate-binding modules (CBM32s) are found in a diverse group of microorganisms, including archea, eubacteria, and fungi. Significantly, many members of this family belong to plant and animal pathogens where they are likely to play a key role in enzyme toxin targeting and function. Indeed, ligand targets have been shown to range from insoluble plant cell wall polysaccharides to complex eukaryotic glycans. Besides a potential direct involvement in microbial pathogenesis, CBM32s also represent an important family for the study of CBM evolution due to the wide variety of complex protein architectures that they are associated with. This complexity ranges from independent lectin-like proteins through to large multimodular enzyme toxins where they can be present in multiple copies (multimodularity). Presented here is a rigorous analysis of the evolutionary relationships between available polypeptide sequences for family 32 CBMs within the carbohydrate active enzyme database. This approach is especially helpful for determining the roles of CBM32s that are present in multiple copies within an enzyme as each module tends to cluster into groups that are associated with distinct enzyme classes. For enzymes that contain multiple copies of CBM32s, however, there are differential clustering patterns as modules can either cluster together or in very distant sections of the tree. These data suggest that enzymes containing multiple copies possess complex mechanisms of ligand recognition. By applying this well-developed approach to the specific analysis of CBM relatedness, we have generated here a new platform for the prediction of CBM binding specificity and highlight significant new targets for biochemical and structural characterization. | 3601 | NA | NA | NA | no | no |
| 3601 | 2017 | NA | Herben, T; Wildova, R | 2012 | Community-level effects of plant traits in a grassland community examined by naultispecies Model of clonal plant growth | We examine role of natality-related plant traits in a mountain grassland community. We use a spatially explicit, individual-based model of clonal plant population dynamics that includes traits of growth, resource allocation, response to competition, and spatial spread/plant architecture, and parameterize it for four co-occurring grass species. Field measurements of plant growth and architecture were used for parameterization; the subset of parameters that cannot be obtained by field estimation were estimated by fitting model predictions to a fine-scale time series of field data using a formalized gradient-descent procedure. The parameterized model was then validated with a separate set of fine-scale time series of field data. The parameterized model approximated well grassland dynamics over two decades for which empirical data were available for comparison. Over long (>50 years) periods the predictions indicate decrease of spatial correlations and loss of species richness which does not seem to be realistic in terms of our knowledge of the modelled grassland systems. This is likely to be due to structural features of the model, namely equivalence of competition of ramets that was independent of their tussock or species identity. This virtual community was used to test hypotheses on effects of natality-related traits on community dynamics. We changed values of individual traits of generative and vegetative reproduction (including architecture), and examined effects of these changes on (i) performance of the species with the trait changed, and (ii) performance of co-occurring species. The analysis showed that effects of individual traits on its bearer’s performance differed across species; the context of other traits thus interacted with the net trait effect. Comparison of trait effects in the simulated monoculture with the effect on the whole community showed that within-community effect of the trait can only be weakly predicted from its monoculture effect. Individual neighbour species also differed in their response to a single trait in the target species. Such modelling approach shows that effects of traits (which typically cannot be easily manipulated) can be examined both in single species and in the community. While there was large variation in trait effects across target and neighbour species, mean effects of individual traits strongly differed, indicating that traits can be sensibly generalized over species and constitute a workable reference level for community studies. (C) 2011 Elsevier By. All rights reserved. | 3602 | NA | NA | NA | no | no |
| 3602 | 2017 | NA | Bielby, J; Donnelly, CA; Pope, LC; Burke, T; Woodroffe, R | 2014 | Badger responses to small-scale culling may compromise targeted control of bovine tuberculosis | Where wildlife disease requires management, culling is frequently considered but not always effective. In the British Isles, control of cattle tuberculosis (TB) is hindered by infection in wild badger (Meles meles) populations. Large-scale badger culling can reduce the incidence of confirmed cattle TB, but these benefits are undermined by culling-induced changes in badger behavior (termed perturbation), which can increase transmission among badgers and from badgers to cattle. Test-vaccinate/remove (TVR) is a novel approach that entails testing individual badgers for infection, vaccinating test-negative animals, and killing test-positive animals. Imperfect capture success, diagnostic sensitivity, and vaccine effectiveness mean that TVR would be expected to leave some infected and some susceptible badgers in the population. Existing simulation models predict that TVR could reduce cattle TB if such small-scale culling causes no perturbation, but could increase cattle TB if considerable perturbation occurs. Using data from a long-term study, we show that past small-scale culling was significantly associated with four metrics of perturbation in badgers: expanded ranging, more frequent immigration, lower genetic relatedness, and elevated prevalence of Mycobacterium bovis, the causative agent of TB. Though we could not reject the hypothesis that culling up to three badgers per social group might avoid perturbation, we also could not reject the hypothesis that killing a single badger prompted detectable perturbation. When considered alongside existing model predictions, our findings suggest that implementation of TVR, scheduled for 2014, risks exacerbating the TB problem rather than controlling it. Ongoing illegal badger culling is likewise expected to increase cattle TB risks. | 3603 | NA | NA | NA | no | no |
| 3603 | 2017 | NA | Neri, M; Ugolini, D; Dianzani, I; Gemignani, F; Landi, S; Cesario, A; Magnani, C; Mutti, L; Puntoni, R; Bonassi, S | 2008 | Genetic susceptibility to malignant pleural mesothelioma and other asbestos-associated diseases | Exposure to asbestos fibers is a major risk factor for malignant pleural mesothelioma (MPM), lung cancer, and other non-neoplastic conditions, such as asbestosis and pleural plaques. However, in the last decade many studies have shown that polymorphism in the genes involved in xenobiotic and oxidative metabolism or in DNA repair processes may play an important role in the etiology and pathogenesis of these diseases. To evaluate the association between diseases linked to asbestos and genetic variability we performed a review of studies on this topic included in the PubMed database. One hundred fifty-nine citations were retrieved; 24 of them met the inclusion criteria and were evaluated in the review. The most commonly studied GSTM1 polymorphism showed for all asbestos-linked diseases an increased risk in association with the null genotype, possibly linked to its role in the conjugation of reactive oxygen species. Studies focused on GSTT1 null and SOD2 Ala16Val polymorphisms gave conflicting results, while promising results came from studies on oil-antitrypsin in asbestosis and MPO in lung cancer. Among genetic polymorphisms associated to the risk of MPM, the GSTM1 null genotype and two variant alleles of XRCC1 and XRCC3 showed increased risks in a subset of studies. Results for the NAT2 acetylator status, SOD2 polymorphism and EPHX activity were conflicting. Major limitations in the study design, including the small size of study groups, affected the reliability of these studies. Technical improvements such as the use of high-throughput techniques will help to identify molecular pathways regulated by candidate genes. (C) 2008 Elsevier B.V. All rights reserved. | 3604 | NA | NA | NA | no | no |
| 3604 | 2017 | NA | Dubey, S; Sinsch, U; Dehling, MJ; Chevalley, M; Shine, R | 2013 | Population demography of an endangered lizard, the Blue Mountains Water Skink | Background: Information on the age structure within populations of an endangered species can facilitate effective management. The Blue Mountains Water Skink (Eulamprus leuraensis) is a viviparous scincid lizard that is restricted to < 40 isolated montane swamps in south-eastern Australia. We used skeletochronology of phalanges (corroborated by mark-recapture data) to estimate ages of 222 individuals from 13 populations. Results: These lizards grow rapidly, from neonatal size (30 mm snout-vent length) to adult size (about 70 mm SVL) within two to three years. Fecundity is low (mean 2.9 offspring per litter) and is affected by maternal body length and age. Offspring quality may decline with maternal age, based upon captive-born neonates (older females gave birth to slower offspring). In contrast to its broadly sympatric (and abundant) congener E. tympanum, E. leuraensis is short-lived (maximum 6 years, vs 15 years for E. tympanum). Litter size and offspring size are similar in the two species, but female E. leuraensis reproduce annually whereas many E. tympanum produce litters biennially. Thus, a low survival rate (rather than delayed maturation or low annual fecundity) is the key reason why E. leuraensis is endangered. Our 13 populations exhibited similar growth rates and population age structures despite substantial variation in elevation, geographic location and swamp size. However, larger populations (based on a genetic estimate of effective population size) contained older lizards, and thus a wider variance in ages. Conclusion: Our study suggests that low adult survival rates, as well as specialisation on a rare and fragmented habitat type (montane swamps) contribute to the endangered status of the Blue Mountains Water Skink. | 3605 | NA | NA | NA | no | no |
| 3605 | 2017 | NA | Kukuk, PF; Ward, SA; Jozwiak, A | 1998 | Mutualistic benefits generate an unequal distribution of risky activities among unrelated group members | Recent studies provide a new challenge to the adequacy of theories concerning the evolution of cooperation among nonrelatives: some individuals perform high-risk activities while others do not. We examined a communal hymenopteran species, Lasioglossum (Chilalictus) hemichalceum, to determine why group members engaged in demonstrably risky activities (foraging) tolerate the selfish behavior (remaining in the nest) of unrelated nestmates. Experimental removal of adult females indicated that their presence is required for the protection of brood from ant predators. Nonforagers ensure the continued presence of adults in the nest if the risk-taking foragers die, thereby safeguarding the survival of forager offspring. This results in an unequal distribution of risky activities within social groups in which avoidance of risky activities by some group members is ultimately beneficial to risk takers. | 3606 | NA | NA | NA | no | no |
| 3606 | 2017 | NA | Zhu, PX; van der Ende, A; Falush, D; Brieske, N; Morelli, G; Linz, B; Popovic, T; Schuurman, IGA; Adegbola, RA; Zurth, K; Gagneux, S; Platonov, AE; Riou, JY; Caugant, DA; Nicolas, P; Achtman, M | 2001 | Fit genotypes and escape variants of subgroup III Neisseria meningitidis during three pandemics of epidemic meningitis | The genetic variability at six polymorphic loci was examined within a global collection of 502 isolates of subgroup ill, serogroup A Neisseria meningitidis. Nine “genoclouds” were identified, consisting of genotypes that were isolated repeatedly plus 48 descendent genotypes that were isolated rarely. These genoclouds have caused three pandemic waves of disease since the mid-1960s, the most recent of which was imported from East Asia to Europe and Africa in the mid-1990s, Many of the genotypes are escape variants, resulting from positive selection that we attribute to herd immunity. Despite positive selection, most escape variants are less fit than their parents and are lost because of competition and bottlenecks during spread from country to country, Competition between fit genotypes results in dramatic changes in population composition over short time periods. | 3607 | NA | NA | NA | no | no |
| 3607 | 2017 | NA | Pruvost, O; Savelon, C; Boyer, C; Chiroleu, F; Gagnevin, L; Jacques, MA | 2009 | Populations of Xanthomonas citri pv. mangiferaeindicae from Asymptomatic Mango Leaves Are Primarily Endophytic | Epiphytic survival of several Xanthomonas pathovars has been reported, but most studies failed to determine whether such populations were resident epiphytes, resulting from latent infections, or casual epiphytes. This study aimed at understanding the nature of Xanthomonas citri pv. mangiferaeindicae populations associated with asymptomatic leaves. When spray-inoculated on mango leaves cv. Maison Rouge, the pathogen multiplied markedly in association with juvenile leaves, but was most often detected as low population sizes (<1 x 10(3) cfu g(-1)) in association with mature leaves. Our results suggest a very low biological significance of biofilm-associated populations of X. citri pv. mangiferaeindicae, while saprophytic microbiota associated with mango leaves survived frequently as biofilms. A chloroform vapor-based disinfestation assay which kills cells specifically located on the leaf surface and not those located within the leaf mesophyll was developed. When applied to spray-inoculated leaves maintained under controlled environmental conditions, 155 out of the 168 analyzed datasets collected over three assessment dates for seven bacterial strains representative of the genetic diversity of the pathogen failed to demonstrate a significant X. citri pv. mangiferaeindicae population decrease on chloroform treated leaves up to 13 days after inoculation. We conclude that an efficient survival of X. citri pv. mangiferaeindicae present on mango leaf surfaces following a limited dissemination event is largely dependent on the availability of juvenile plant tissues. The bacterium gains access to protected sites (e. g., mesophyll) through stomata where it becomes endophytic and eventually causes disease. Chloroform vapor-based disinfestation assays should be useful for further studies aiming at evaluating survival sites of bacteria associated with the phyllosphere. | 3608 | NA | NA | NA | no | no |
| 3608 | 2017 | NA | Kost, C; Lakatos, T; Bottcher, I; Arendholz, WR; Redenbach, M; Wirth, R | 2007 | Non-specific association between filamentous bacteria and fungus-growing ants | Fungus-growing ants and their fungal cultivar form a highly evolved mutualism that is negatively affected by the specialized parasitic fungus Escovopsis. Filamentous Pseudonocardia bacteria occurring on the cuticle of attine ants have been proposed to form a mutualistic interaction with these ants in which they are vertically transmitted (i.e. from parent to offspring colonies). Given a strictly vertical transmission of Pseudonocardia, the evolutionary theory predicts a reduced genetic variability of symbionts among ant lineages. The aim of this study was to verify whether actinomycetes, which occur on Acromyrmex octospinosus leaf-cutting ants, meet this expectation by comparing their genotypic variability with restriction fragment length polymorphisms. Multiple actinomycete strains could be isolated from both individual ant workers and colonies (one to seven strains per colony). The colony specificity of actinomycete communities was high: Only 15% of all strains were isolated from more than one colony, and just 5% were present in both populations investigated. Partial sequencing of 16S ribosomal deoxyribonucleic acid of two of the isolated strains assigned both of them to the genus Streptomyces. Actinomycetes could also be isolated from workers of the two non-attine ant species Myrmica rugulosa and Lasius flavus. Sixty-two percent of the strains derived from attine ants and 80% of the strains isolated from non-attine ants inhibited the growth of Escovopsis. Our data suggest that the association between attine ants and their actinomycete symbionts is less specific then previously thought. Soil-dwelling actinomycetes may have been dynamically recruited from the environment (horizontal transmission), probably reflecting an adaptation to a diverse community of microbial pathogens. | 3609 | NA | NA | NA | no | no |
| 3609 | 2017 | NA | Perez-Martinez, P; Delgado-Lista, J; Garcia-Rios, A; Mc Monagle, J; Gulseth, HL; Ordovas, JM; Shaw, DI; Karlstrom, B; Kiec-Wilk, B; Blaak, EE; Helal, O; Malczewska-Malec, M; Defoort, C; Riserus, U; Saris, WHM; Lovegrove, JA; Drevon, CA; Roche, HM; Lopez-Miranda, J | 2011 | Glucokinase Regulatory Protein Genetic Variant Interacts with Omega-3 PUFA to Influence Insulin Resistance and Inflammation in Metabolic Syndrome | Glucokinase Regulatory Protein (GCKR) plays a central role regulating both hepatic triglyceride and glucose metabolism. Fatty acids are key metabolic regulators, which interact with genetic factors and influence glucose metabolism and other metabolic traits. Omega-3 polyunsaturated fatty acids (n-3 PUFA) have been of considerable interest, due to their potential to reduce metabolic syndrome (MetS) risk. Objective: To examine whether genetic variability at the GCKR gene locus was associated with the degree of insulin resistance, plasma concentrations of C-reactive protein (CRP) and n-3 PUFA in MetS subjects. Design: Homeostasis model assessment of insulin resistance (HOMA-IR), HOMA-B, plasma concentrations of C-peptide, CRP, fatty acid composition and the GCKR rs1260326-P446L polymorphism, were determined in a cross-sectional analysis of 379 subjects with MetS participating in the LIPGENE dietary cohort. Results: Among subjects with n-3 PUFA levels below the population median, carriers of the common C/C genotype had higher plasma concentrations of fasting insulin (P = 0.019), C-peptide (P = 0.004), HOMA-IR (P = 0.008) and CRP (P = 0.032) as compared with subjects carrying the minor T-allele (Leu446). In contrast, homozygous C/C carriers with n-3 PUFA levels above the median showed lower plasma concentrations of fasting insulin, peptide C, HOMA-IR and CRP, as compared with individuals with the T-allele. Conclusions: We have demonstrated a significant interaction between the GCKR rs1260326-P446L polymorphism and plasma n-3 PUFA levels modulating insulin resistance and inflammatory markers in MetS subjects. Further studies are needed to confirm this gene-diet interaction in the general population and whether targeted dietary recommendations can prevent MetS in genetically susceptible individuals. | 3610 | NA | NA | NA | no | no |
| 3610 | 2017 | NA | Nova, MXV; Borges, LR; de Sousa, ACB; Brasileiro, BTRV; Lima, EALA; da Costa, AF; de Oliveira, NT | 2011 | Pathogenicity for onion and genetic diversity of isolates of the pathogenic fungus Colletotrichum gloeosporioides (Phyllachoraceae) from the State of Pernambuco, Brazil | Onion anthracnose, caused by Colletotrichum gloeosporioides, is one of the main diseases of onions in the State of Pernambuco. We examined the pathogenicity of 15 C. gloeosporioides strains and analyzed their genetic variability using RAPDs and internal transcribed spacers (ITS) of the rDNA region. Ten of the strains were obtained from substrates and hosts other than onion, including chayote (Sechium edule), guava (Psidium guajava), pomegranate (Punica granatum), water from the Capibaribe River, maracock (Passiflora sp), coconut (Cocus nucifera), surinam cherry (Eugenia uniflora), and marine soil; five isolates came from onions collected from four different regions of the State of Pernambuco and one region of the State of Amazonas. Pathogenicity tests were carried out using onion leaves and bulbs. All strains were capable of causing disease in leaves, causing a variable degree of lesions on the leaves; four strains caused the most severe damage. In the onion bulb tests, only three of the above strains caused lesions. Seven primers of arbitrary sequences were used in the RAPD analysis, generating polymorphic bands that allowed the separation of the strains into three distinct groups. The amplification products generated with the primers ITS1 and ITS4 also showed polymorphism when digested with three restriction enzymes, DraI, HaeIII and MspI. Only the latter two demonstrated genetic variations among the strains. These two types of molecular markers were able to differentiate the strain from the State of Amazonas from those of the State of Pernambuco. However, there was no relationship between groups of strains, based on molecular markers, and degree of pathogenicity for onion leaves and bulbs. | 3611 | NA | NA | NA | no | no |
| 3611 | 2017 | NA | CHEPLICK, GP | 1992 | SIBLING COMPETITION IN PLANTS | 1. Sibling competition can de defined as operating when there is a density-dependent reduction in growth, survival or reproduction in closely interacting siblings utilizing the same space and resources relative to the growth, survival or reproduction that occurs when siblings are not interacting. This definition should be distinguished from hypotheses that make predictions about the intensity of interactions between siblings relative to those between non-siblings at the same density. 2. Two general classes of theoretical model incorporate sibling competition: one concerns the evolution of sex, the other seed germination-dormancy patterns. Unfortunately, the lack of documentation of sibling competition as a significant selection pressure in nature limits the utility of models that attempt to explain the evolution of specific life-history features in response to sibling competition. At present there is little support for the notion that interactions between siblings are more severe than between genetically unrelated non-siblings. 3. Life-history factors likely to promote sibling competition include fruit, seed and dispersal dimorphisms, synaptospermy, amphicarpy, cleistogamy, barochory, the phalanx growth pattern, and low growth habit. 4. Future research should focus on determining: (i) how widespread sibling competition is in plant populations, including an assessment of its relative importance as a selection pressure; (ii) the relation of sibling competition to plant breeding and dispersal systems; (iii) the importance and relevance of sibling competition to models that attempt to explain the evolution of sex or seed-dormancy patterns; (iv) the influence of sibling competition on population genetic structure; (v) the possibility that some plants may benefit from sibling interactions (kin selection). | 3612 | NA | NA | NA | no | no |
| 3612 | 2017 | NA | Li, L; Jiang, J; Wang, X; Jiang, X | 2013 | Nested clade phylogeographical analysis of the finless porpoise (Neophocaena phocaenoides) inhabiting Chinese and Japanese coasts | The finless porpoise (Neophocaena phocaenoides) is a small cetacean whose survival is largely affected by human activity. The characteristics and structures of 3 populations in China and 4 to 5 populations in Japan have been well documented, although their history and origins remain poorly understood. In this study, nested clade phylogeographical analysis was applied to mtDNA sequences from finless porpoises to delineate the historical factors shaping the divergence pattern of this species. The sampling range covered most habitats of the finless porpoise, including the Chinese and Japanese coasts and the Yangtze River. A nested cladogram was constructed, and all 3 (2-step) clades were linked together without interior or tip status. Our data indicated that haplotype C was the most common among most individuals and populations, which could be the most ancient haplotype among all of the sampled types. As populations expanded to reside in different areas, ancient types with haplotype C mutated to other haplotypes, which were kept endemic by geological barriers that changed during glacial cycles that retarded gene flow. Populations in Japanese Pacific coasts and inland sea and post-arc marginal seacoasts and the Yangtze River were formed by the residual individuals left when allopatric fragmentation occurred in the ancestral population. The existence of highly endemic haplotypes and high genetic diversity from the Ariake Sound, Tachibana Bay, and the Yellow Sea indicated a relatively isolated state in these areas during the evolutionary history of the finless porpoise. The phylogenetic pattern revealed in the present study provided a better understanding of the biogeographical events that affected the finless porpoise within this region. | 3613 | NA | NA | NA | no | no |
| 3613 | 2017 | NA | Bart, R; Cohn, M; Kassen, A; McCallum, EJ; Shybut, M; Petriello, A; Krasileva, K; Dahlbeck, D; Medina, C; Alicai, T; Kumar, L; Moreira, LM; Neto, JR; Verdier, V; Santana, MA; Kositcharoenkul, N; Vanderschuren, H; Gruissem, W; Bernal, A; Staskawicz, BJ | 2012 | High-throughput genomic sequencing of cassava bacterial blight strains identifies conserved effectors to target for durable resistance | Cassava bacterial blight (CBB), incited by Xanthomonas axonopodis pv. manihotis (Xam), is the most important bacterial disease of cassava, a staple food source for millions of people in developing countries. Here we present a widely applicable strategy for elucidating the virulence components of a pathogen population. We report Illumina-based draft genomes for 65 Xam strains and deduce the phylogenetic relatedness of Xam across the areas where cassava is grown. Using an extensive database of effector proteins from animal and plant pathogens, we identify the effector repertoire for each sequenced strain and use a comparative sequence analysis to deduce the least polymorphic of the conserved effectors. These highly conserved effectors have been maintained over 11 countries, three continents, and 70 y of evolution and as such represent ideal targets for developing resistance strategies. | 3614 | NA | NA | NA | no | no |
| 3614 | 2017 | NA | Davierwala, AP; Ramakrishna, W; Chowdari, V; Ranjekar, PK; Gupta, VS | 2001 | Potential of (GATA)(n) microsatellites from rice for inter- and intra-specific variability studies | Background: The microsatellite, (GATA)(n) has been frequently used for DNA fingerprinting. However, very few attempts have been made to analyze (GATA)(n)-containing loci in rice. Results: Three polymorphic (GATA)(n)-harboring loci viz. OS1A6, OS1H10 and OS2E7, containing 7-13 repeat motifs were identified from a genomic library of a cultivated rice, Oryza sativa var. Basmati-370 using oligonucleotide probe (GATA)(4). When (GATA)(n) flanking primers were used to screen 26 wilds (representing different genomes of rice), 16 cultivars, 47 Indian elite rice varieties and 37 lines resistant/susceptible to bacterial blight, up to 22 alleles were obtained at an individual locus. Also, interestingly the bacterial blight resistant lines clustered into a separate group from the remaining rice genotypes, when a dendrogram was constructed based on the polymorphism obtained at the three loci. This may be due to the partial homology of the clones OS1H10 and OS2E7 to regions encoding O. longistaminata receptor kinase-like protein and pathogenesis-related protein. The ability of these O. sativa flanking primers to amplify DNA of maize, wheat, barley and oat indicates that these (GATA) n-containing loci are conserved across different cereal genera. Conclusions: The large allele number obtained reveals the potential of (GATA)(n)-containing loci as powerful tools to detect simple sequence length polymorphism (SSLP). The (GATA)(n)-flanking primers were not only useful in distinguishing between closely related genotypes, but could also be used for cross-species amplification and are also conserved across different cereal genera. These loci could also cluster the bacterial blight resistant/susceptible lines into different groups based on the resistance genes present in them. | 3615 | NA | NA | NA | no | no |
| 3615 | 2017 | NA | Cossa-Moiane, IL; Mendes, T; Ferreira, TM; Mauricio, I; Calado, M; Afonso, A; Belo, S | 2015 | The role of the immunological background of mice in the genetic variability of Schistosoma mansoni as detected by random amplification of polymorphic DNA | Schistosomiasis is a parasitic disease caused by flatworms of the genus Schistosoma. Among the Schistosoma species known to infect humans, S. mansoni is the most frequent cause of intestinal schistosomiasis in sub-Saharan Africa and South America: the World Health Organization estimates that about 200,000 deaths per year result from schistosomiasis in sub-Saharan Africa alone. The Schistosoma life cycle requires two different hosts: a snail as intermediate host and a mammal as definitive host. People become infected when they come into contact with water contaminated with free-living larvae (e.g. when swimming, fishing, washing). Although S. mansoni has mechanisms for escaping the host immune system, only a minority of infecting larvae develop into adults, suggesting that strain selection occurs at the host level. To test this hypothesis, we compared the Belo Horizonte (BH) strain of S. mansoni recovered from definitive hosts with different immunological backgrounds using random amplification of polymorphic DNA-polymerase chain reaction (RAPD-PCR). Schistosoma mansoni DNA profiles of worms obtained from wild-type (CD1 and C57BL/6J) and mutant (J alpha 18(-/-) and TGF beta RIIdn) mice were analysed. Four primers produced polymorphic profiles, which can therefore potentially be used as reference biomarkers. All male worms were genetically distinct from females isolated from the same host, with female worms showing more specific fragments than males. Of the four host-derived schistosome populations, female and male adults recovered from TGF beta RIIdn mice showed RAPD-PCR profiles that were most similar to each other. Altogether, these data indicate that host immunological backgrounds can influence the genetic diversity of parasite populations. | 3616 | NA | NA | NA | no | no |
| 3616 | 2017 | NA | Jacob, S; Wehi, P; Clobert, J; Legrand, D; Schtickzelle, N; Huet, M; Chaine, A | 2016 | Cooperation-mediated plasticity in dispersal and colonization | Kin selection theory predicts that costly cooperative behaviors evolve most readily when directed toward kin. Dispersal plays a controversial role in the evolution of cooperation: dispersal decreases local population relatedness and thus opposes the evolution of cooperation, but limited dispersal increases kin competition and can negate the benefits of cooperation. Theoretical work has suggested that plasticity of dispersal, where individuals can adjust their dispersal decisions according to the social context, might help resolve this paradox and promote the evolution of cooperation. Here, we experimentally tested the hypothesis that conditional dispersal decisions are mediated by a cooperative strategy: we quantified the density-dependent dispersal decisions and subsequent colonization efficiency from single cells or groups of cells among six genetic strains of the unicellular Tetrahymena thermophila that differ in their aggregation level (high, medium, and low), a behavior associated with cooperation strategy. We found that the plastic reaction norms of dispersal rate relative to density differed according to aggregation level: highly aggregative genotypes showed negative density-dependent dispersal, whereas low-aggregation genotypes showed maximum dispersal rates at intermediate density, and medium-aggregation genotypes showed density-independent dispersal with intermediate dispersal rate. Dispersers from highly aggregative genotypes had specialized long-distance dispersal phenotypes, contrary to low-aggregation genotypes; medium-aggregation genotypes showing intermediate dispersal phenotype. Moreover, highly aggregation genotypes showed evidence for beneficial kin-cooperation during dispersal. Our experimental results should help to resolve the evolutionary conflict between cooperation and dispersal: cooperative individuals are expected to avoid kin-competition by dispersing long distances, but maintain the benefits of cooperation by dispersing in small groups. | 3617 | NA | NA | NA | no | no |
| 3617 | 2017 | NA | Thedja, MD; Muljono, DH; Ie, SI; Sidarta, E; Turyadi; Verhoef, J; Marzuki, S | 2015 | Genogeography and Immune Epitope Characteristics of Hepatitis B Virus Genotype C Reveals Two Distinct Types: Asian and Papua-Pacific | Distribution of hepatitis B virus (HBV) genotypes/subgenotypes is geographically and ethnologically specific. In the Indonesian archipelago, HBV genotype C (HBV/C) is prevalent with high genome variability, reflected by the presence of 13 of currently existing 16 subgenotypes. We investigated the association between HBV/C molecular characteristics with host ethnicity and geographical distribution by examining various subgenotypes of HBV/C isolates from the Asia and Pacific region, with further analysis on the immune epitope characteristics of the core and surface proteins. Phylogenetic tree was constructed based on complete HBV/C genome sequences from Asia and Pacific region, and genetic distance between isolates was also examined. HBV/C surface and core immune epitopes were analyzed and grouped by comparing the amino acid residue characteristics and geographical origins. Based on phylogenetic tree and geographical origins of isolates, two major groups of HBV/C isolates-East-Southeast Asia and Papua-Pacific-were identified. Analysis of core and surface immune epitopes supported these findings with several amino acid substitutions distinguishing the East-Southeast Asia isolates from the Papua-Pacific isolates. A west-to-east gradient of HBsAg subtype distribution was observed with adrq+ prominent in the East and Southeast Asia and adrq-in the Pacific, with several adrq-indeterminate subtypes observed in Papua and Papua New Guinea (PNG). This study indicates that HBV/C isolates can be classified into two types, the Asian and the Papua-Pacific, based on the virus genome diversity, immune epitope characteristics, and geographical distribution, with Papua and PNG as the molecular evolutionary admixture region in the switching from adrq+ to adrq-. | 3618 | NA | NA | NA | no | no |
| 3618 | 2017 | NA | Apa, AD; Wiechman, LA | 2015 | Captive-rearing of Gunnison sage-grouse from egg collection to adulthood to foster proactive conservation and recovery of a conservation-reliant species | Gunnison sage-grouse (Centrocercus minimus) are distributed across southwestern Colorado and southeastern Utah, United States. Their distribution has decreased over the past century and the species has been listed as threatened by the U.S. Fish and Wildlife Service. Reduced genetic diversity, small population size, and isolation may affect Gunnison sage-grouse population persistence. Population augmentation can be used to counteract or mitigate these issues, but traditional translocation efforts have yielded mixed, and mostly unsuccessful, results. Captive-rearing is a viable, although much debated, conservation approach to bolster wild conservation-reliant species. Although there have been captive-rearing efforts with greater sage-grouse (C. urophasianus), to date, no information exists about captive-rearing methods for Gunnison sage-grouse. Therefore, we investigated techniques for egg collection, artificial incubation, hatch, and captive-rearing of chicks, juveniles, subadults, and adults for Gunnison sage-grouse. In 2009 we established a captive flock that produced viable eggs. From 2009-2011, we collected and artificially incubated 206 Gunnison sage-grouse eggs from 23 wild and 14 captive females. Our hatchability was 90%. Wild-produced eggs were heavier than captive-produced eggs and lost mass similarly during incubation. We produced 148 chicks in captivity and fed them a variety of food sources (e.g. invertebrates to commercial chow). Bacterial infections were the primary cause of chick mortality, but we successfully reduced the overall mortality rate during the course of our study. Conservationists and managers should consider the utility in developing a captive-rearing program or creating a captive population as part of a proactive conservation effort for the conservation-reliant Gunnison sage-grouse. Zoo Biol. 34:438-452, 2015. (c) 2015 Wiley Periodicals, Inc. | 3619 | NA | NA | NA | no | no |
| 3619 | 2017 | NA | Brown, WD; Keller, L; Sundstrom, L | 2002 | Sex allocation in mound-building ants: The roles of resources and queen replenishment | Social Hymenoptera have become key organisms for tests of sex-ratio theory. We assess the role of resources for explaining sex-ratio variation in a highly male-biased population of the ant Formica exsecta. Key predictions of two of the three leading hypotheses invoking an effect of resource availability on sex ratios in social insects are not upheld. One prediction of the multifaceted parental-investment hypothesis is that colonies with greater brood production will rear a greater proportion of their diploid offspring as reproductive females (gynes), rather than workers, This study shows the positive correlation between female-biased sex ratio and the production of brood by female-producing colonies was not the outcome of a trade-off in the production of workers vs. gynes. The main prediction of the constant-female hypothesis is that investment in gynes should be constant. We found extreme variation among colonies in the number of gynes produced. By contrast, all the predictions of the queen-replenishment hypothesis were supported. The queen-replenishment hypothesis predicts that colonies produce gynes only when queen number is so low that colony production of brood is reduced, or colony survival threatened. We found that, as predicted by this hypothesis, female-producing colonies had fewer queens and produced a lower biomass of brood than male-producing colonies. In contrast, male-producing colonies had lower production per queen, and were more likely to be limited by external resources. This suggests that queen number limits production in female-producing colonies. These data provide evidence of adaptive adjustment of resources to worker, gyne, and male brood. | 3620 | NA | NA | NA | no | no |
| 3620 | 2017 | NA | Tucci, A; Nalls, MA; Houlden, H; Revesz, T; Singleton, AB; Wood, NW; Hardy, J; Paisan-Ruiz, C | 2010 | Genetic variability at the PARK16 locus | Parkinson’s disease (PD) is a complex neurodegenerative disease which is clinically heterogeneous and pathologically consists of loss of dopaminergic neurons in the substantia nigra and intracytoplasmic neuronal inclusions containing alpha-synuclein aggregations known as Lewy bodies. Although the majority of PD is idiopathic, pathogenic mutations in several mendelian genes have been successfully identified through linkage analyses. To identify susceptibility loci for idiopathic PD, several genome-wide association studies (GWAS) within different populations have recently been conducted in both idiopathic and familial forms of PD. These analyses have confirmed SNCA and MAPT as loci harboring PD susceptibility. In addition, the GWAS identified several other genetic loci suggestively associated with the risk of PD; among these, only one was replicated by two different studies of European and Asian ancestries. Hence, we investigated this novel locus known as PARK16 for coding mutations in a large series of idiopathic pathologically proven PD cases, and also conducted an association study in a case-control cohort from the United Kingdom. An association between a novel RAB7L1 mutation, c.379-12insT, and disease (P-value=0.0325) was identified. Two novel coding variants present only in the PD cohort were also identified within the RAB7L1 (p.K157R) and SLC41A1 (p.A350V) genes. No copy number variation analyses have yet been performed within this recently identified locus. We concluded that, although both coding variants and risk alleles within the PARK16 locus seem to be rare, further molecular analyses within the PARK16 locus and within different populations are required in order to examine its biochemical role in the disease process. European Journal of Human Genetics (2010) 18, 1356-1359; doi: 10.1038/ejhg.2010.125; published online 4 August 2010 | 3621 | NA | NA | NA | no | no |
| 3621 | 2017 | NA | Hahn, C; Weiss, SJ; Stojanovski, S; Bachmann, L | 2015 | Co-Speciation of the Ectoparasite Gyrodactylus teuchis (Monogenea, Platyhelminthes) and Its Salmonid Hosts | Co-speciation is a fundamental concept of evolutionary biology and intuitively appealing, yet in practice hard to demonstrate as it is often blurred by other evolutionary processes. We investigate the phylogeographic history of the monogenean ectoparasites Gyrodactylus teuchis and G. truttae on European salmonids of the genus Salmo. Mitochondrial cytochrome oxidase subunit 1 and the nuclear ribosomal internal transcribed spacer 2 were sequenced for 189 Gyrodactylus individuals collected from 50 localities, distributed across most major European river systems, from the Iberian-to the Balkan Peninsula. Despite both anthropogenic and naturally caused admixture of the principal host lineages among major river basins, co-phylogenetic analyses revealed significant global congruence for host and parasite phylogenies, providing firm support for co-speciation of G. teuchis and its salmonid hosts brown trout (S. trutta) and Atlantic salmon (S. salar). The major split within G. teuchis, coinciding with the initial divergence of the hosts was dated to similar to 1.5 My BP, using a Bayesian framework based on an indirect calibration point obtained from the host phylogeny. The presence of G. teuchis in Europe thus predates some of the major Pleistocene glaciations. In contrast, G. truttae exhibited remarkably low intraspecific genetic diversity. Given the direct life cycle and potentially high transmission potential of gyrodactylids, this finding is interpreted as indication for a recent emergence (<60 ky BP) of G. truttae via a host-switch. Our study thus suggests that instances of two fundamentally different mechanisms of speciation (co-speciation vs. host-switching) may have occurred on the same hosts in Europe within a time span of less than 1.5 My in two gyrodactylid ectoparasite species. | 3622 | NA | NA | NA | no | no |
| 3622 | 2017 | NA | Mazurier, S; Corberand, T; Lemanceau, P; Raaijmakers, JM | 2009 | Phenazine antibiotics produced by fluorescent pseudomonads contribute to natural soil suppressiveness to Fusarium wilt | Natural disease-suppressive soils provide an untapped resource for the discovery of novel beneficial microorganisms and traits. For most suppressive soils, however, the consortia of microorganisms and mechanisms involved in pathogen control are unknown. To date, soil suppressiveness to Fusarium wilt disease has been ascribed to carbon and iron competition between pathogenic Fusarium oxysporum and resident non-pathogenic F. oxysporum and fluorescent pseudomonads. In this study, the role of bacterial antibiosis in Fusarium wilt suppressiveness was assessed by comparing the densities, diversity and activity of fluorescent Pseudomonas species producing 2,4-diacetylphloroglucinol (DAPG) (phlD(+)) or phenazine (phzC(+)) antibiotics. The frequencies of phlD(+) populations were similar in the suppressive and conducive soils but their genotypic diversity differed significantly. However, phlD genotypes from the two soils were equally effective in suppressing Fusarium wilt, either alone or in combination with nonpathogenic F. oxysporum strain Fo47. A mutant deficient in DAPG production provided a similar level of control as its parental strain, suggesting that this antibiotic does not play a major role. In contrast, phzC(+) pseudomonads were only detected in the suppressive soil. Representative phzC(+) isolates of five distinct genotypes did not suppress Fusarium wilt on their own, but acted synergistically in combination with strain Fo47. This increased level of disease suppression was ascribed to phenazine production as the phenazine-deficient mutant was not effective. These results suggest, for the first time, that redox-active phenazines produced by fluorescent pseudomonads contribute to the natural soil suppressiveness to Fusarium wilt disease and may act in synergy with carbon competition by resident non-pathogenic F. oxysporum. The ISME Journal (2009) 3, 977-991; doi: 10.1038/ismej.2009.33; published online 16 April 2009 | 3623 | NA | NA | NA | no | no |
| 3623 | 2017 | NA | Han, ZM; Yin, TM; Li, CD; Huang, MR; Wu, RL | 2000 | Host effect on genetic variation of Marssonina brunnea pathogenic to poplars | A bread collection was made for 42 isolates of Marssonina brunnea affecting poplar trees from three different sections (Leuce, Aigeiros and Tacamahaca) within the same Populus genus in China. Genetic diversity among these isolates was analyzed for morphological traits, cultural features, pathogenicity, hyphal anastomosis and randomly amplified polymorphic DNA markers (RAPDs). No significant difference was found in conidial morphological features, such as size, shape and septum location. Yet, considerable differences occur in other characteristics, which leads to the classification of the 42 isolates into two distinct groups, M. brunnea f.sp. monogermtubi and M. brunnea f.sp. multigermtubi. Isolates of M. brunnea f.sp. monogermtubi, derived from section Leuce, germinate only one germ tube, grow fast, produce dark-reddish conidiosorus clusters on the PDA medium, and are highly pathogenic to Populus tomentosa of section Leuce. By contrast, isolates of M. brunnea f.sp. multigermtubi, derived from sections Aigeiros and Tacamahaca, germinate 1-5 germ tubes, grow slowly, produce yellow-greenish conidiosorus clusters on PDA medium, and are pathogenic to Populusxeuramericana cv I-45 and Populus canadensis of section Aigeiros. DNA amplification using II RAPD primers generate 78 polymorphic bands among isolates. Cluster analyses based on RAPD markers broadly support such a classification by phenotypes, but provide a new insight into the possible origins of ill. brunnea. It is proposed that the pathogen co-evolves with the poplars of section Leuce and has been subsequently distributed to the poplars of sections Aigeiros and Tacamahaca. An isolate from Populus adenopoda of section Leuce is placed in the third group, which is most likely a transmission type from M. brunnea f.sp. monogermtubi to hi. brunnea f.sp. multigermtubi. | 3624 | NA | NA | NA | no | no |
| 3624 | 2017 | NA | Piorno, V; Villafuerte, R; Branco, M; Carneiro, M; Ferrand, N; Alves, P | 2015 | Low persistence in nature of captive reared rabbits after restocking operations | The use of restocking of animals is common practice in the management of populations subject to hunting or recreational fishing. This practice encompasses the release of large numbers of individuals in an area where the species already exists, and thus it can have detrimental genetic impacts on the target populations, especially when captive-reared animals are involved. To better understand this practice and its conservation implications, we chose as a model the wild rabbit (Oryctolagus cuniculus), a species of high economic and ecological importance, and often under intense management for hunting or conservation purposes, particularly after the large decline caused by rabbit hemorrhagic disease in the 90’s. We studied the genetics of rabbit populations in an area where restocking with captive, wild-domestic hybrids was common. We collected a total of 503 samples from 15 hunting estates that had experienced differing restocking levels, as well as from five locations with no historical restocking and five game farms. All samples were analyzed to determine the mitochondrial DNA (mtDNA) lineage typical of the two European wild rabbit subspecies and domestic rabbit. Game farms and never restocked populations were very different in their haplotypic composition. In restocked areas, the proportion of the domestic lineage was higher when releases were recent and repeated, but this declined rapidly over time, in part due to selective removal by hunting. The extended use of this practice, considering the pronounced genetic and genotypic differences between domestic and wild rabbits, represents a potential danger to natural populations, especially given the marked decrease in wild rabbit numbers experienced in recent years in its original distribution range. | 3625 | NA | NA | NA | no | no |
| 3625 | 2017 | NA | Beatty, GE; Provan, J | 2010 | Refugial persistence and postglacial recolonization of North America by the cold-tolerant herbaceous plant Orthilia secunda | Previous phylogeographical and palaeontological studies on the biota of northern North America have revealed a complex scenario of glacial survival in multiple refugia and differing patterns of postglacial recolonization. Many putative refugial regions have been proposed both north and south of the ice sheets for species during the Last Glacial Maximum, but the locations of many of these refugia remain a topic of great debate. In this study, we used a phylogeographical approach to elucidate the refugial and recolonization history of the herbaceous plant species Orthilia secunda in North America, which is found in disjunct areas in the west and east of the continent, most of which were either glaciated or lay close to the limits of the ice sheets. Analysis of 596 bp of the chloroplast trnS-trnG intergenic spacer and five microsatellite loci in 84 populations spanning the species’ range in North America suggests that O. secunda persisted through the Last Glacial Maximum (LGM) in western refugia, even though palaeodistribution modelling indicated a suitable climate envelope across the entire south of the continent. The present distribution of the species has resulted from recolonization from refugia north and south of the ice sheets, most likely in Beringia or coastal regions of Alaska and British Columbia, the Washington/Oregon region in the northwest USA, and possibly from the region associated with the putative ‘ice-free corridor’ between the Laurentide and Cordilleran ice sheets. Our findings also highlight the importance of the Pacific Northwest as an important centre of intraspecific genetic diversity, owing to a combination of refugial persistence in the area and recolonization from other refugia. | 3626 | NA | NA | NA | no | no |
| 3626 | 2017 | NA | Arnaiz-Villena, A; Gonzalez-Alcos, V; Serrano-Vela, JI; Reguera, R; Barbolla, L; Parga-Lozano, C; Gomez-Prieto, P; Abd-El-Fatah-Khalil, S; Moscoso, J | 2009 | HLA genes in Uros from Titikaka Lake, Peru: origin and relationship with other Amerindians and worldwide populations | Uros population from the Titikaka Lake live in about 42 floating reed (‘totora’) islands in front of Puno City (Peru) at a 4000 m high altiplano. They present both an mtDNA and a human leucocyte antigen (HLA) profile different from the surrounding populations: mtDNA A2 haplogroup is common to Uros and Amazon forest lowland Amerindians. HLA genetic distances between populations have been calculated and neighbour-joining dendrograms and correspondence analyses were carried out. Approximately 15 006 HLA chromosomes from worldwide populations have been used for comparisons. Only eight HLA-A alleles have been found, three of them accounting for most of the frequencies. The same phenomenon is seen for HLA-B, HLA-DRB1 and HLA-DQB1 alleles: a few alleles (3, 4 and 3, respectively) are present in most individuals. The presence of HLA-B*4801 and HLA-DRB1*0901 alleles in a relatively high frequency (although not the most frequent alleles found) is a characteristic shared with Asians and some populations from the Andean altiplano. Three specific Uros haplotypes have been found among the most frequent ones: HLA-A*680102-B*3505-DRB1*0403-DQB1*0302; HLA-A*2402-B*1504-DRB1*1402-DQB1*0301; and HLA-A*2402-B*4801-DRB1*0403-DQB1*0302. The present study suggests that Uros may have been one of the first populations from the shores of the Titikaka Lake coming from the Amazonian forest, which might have given rise to other later differentiated ethnic group (i.e. Aymaras). Uros HLA profile is also useful to study genetic epidemiology of diseases linked to HLA and to construct a future transplant waiting list by adding up regional lists in order to get a bigger pool for transplanting with better HLA matching. | 3627 | NA | NA | NA | no | no |
| 3627 | 2017 | NA | Thomas, DC; Stram, DO; Conti, D; Molitor, J; Marjoram, P | 2003 | Bayesian spatial modeling of haplotype associations | We review methods for relating the risk of disease to a collection of single nucleotide polymorphisms (SNPs) within a small region. Association studies using case-control designs with unrelated individuals could be used either to test for a direct effect of a candidate gene and characterize the responsible variant(s), or to fine map an unknown gene by exploiting the pattern of linkage disequilibrium (LD). We consider a flexible class of logistic penetrance models based on haplotypes and compare them with an alternative formulation based on unphased multilocus genotypes. The likelihood for haplotype-based models requires summation over all possible haplotype assignments consistent with the observed genotype data, and can be fitted using either Expectation-Maximization (E-M) or Markov chain Monte Carlo (MCMC) methods. Subtleties involving ascertainment correction for case-control studies are discussed. There has been great interest in methods for LD mapping based on the coalescent or ancestral recombination graphs as well as methods based on haplotype sharing, both of which we review briefly. Because of their computational complexity, we propose some alternative empirical modeling approaches using techniques borrowed from the Bayesian spatial statistics literature. Here, space is interpreted in terms of a distance metric describing the similarity of any pair of haplotypes to each other, and hence their presumed common ancestry. Specifically, we discuss the conditional autoregressive model and two spatial clustering models: Potts and Voronoi. We conclude with a discussion of the implications of these methods for modeling cryptic relatedness, haplotype blocks, and haplotype tagging SNPs, and suggest a Bayesian framework for the HapMap project. Copyright (C) 2003 S. Karger AG, Basel. | 3628 | NA | NA | NA | no | no |
| 3628 | 2017 | NA | Andreadis, TG; Simakoya, AV; Vossbrinck, CR; Shepard, JJ; Yurchenko, YA | 2012 | Ultrastructural characterization and comparative phylogenetic analysis of new microsporidia from Siberian mosquitoes: Evidence for coevolution and host switching | A survey of mosquito larvae infected with microsporidia was conducted from 2005 to 2008 in the Tomsk, Kemerovo and Novosibirsk regions of western Siberia, Russia. Twenty-one morphologically and genetically unique species of microsporidia were isolated from nine species of Anopheles, Aedes, Culex and Ochlerotatus mosquitoes including: (1) 14 proposed new species of Amblyospora (A. bakcharia, A. baritia, A. bogashovia, A. chulymia, A. hristinia, A. jurginia, A. kazankia, A. mavlukevia, A. mocrushinia, A. modestium, A. salairia, A. severinia, A. shegaria, and A. tunirasia); (2) a newly proposed genus and species, Novothelohania ovalae and; (3) six species of Amblyospora (A. flavescens, A. kolarovi, A. rugosa), Parathelohania (P. divulgata and P. tomski) and Trichoctosporea (T. pygopellita) from which gene sequences had not been previously obtained. Detailed ultrastructure of meiospores revealed unique cytological features associated with the length, arrangement and ratio of broad to narrow coils of the polar filament, comparative thickness of the exospore and endospore, and overall size of each species reaffirming their value in distinguishing taxonomic relationships. SSU rDNA sequences obtained from each species of microsporidia were unique when compared with GenBank entries. Phylogenetic trees constructed by Maximum Parsimony, Maximum Likelihood and Neighbor Joining analyses yielded similar topologies with a high degree of congruence between parasite and host at the generic level. Species that parasitize Aedes/Ochlerotatus and Culex mosquitoes segregate into distinct monophyletic groupings mirroring their host phylogeny, while species from Anopheles mosquitoes group as a sister clade basal to the entire group of mosquito-parasitic microsporidia as their Anopheles hosts cluster as a sister clade to the entire group of culicine mosquitoes. This provides strong evidence for host-parasite coevolution by descent at the generic level and limited host lineage switching between unrelated taxa. Among parasites of AedeslOchlerotatus and Anopheles mosquitoes, we found several instances where a single mosquito species serves as a host for two or more related species of microsporidia, an observation consistent with host switching and independent parasite speciation. Among the microsporidian parasites of Culex mosquitoes, we found only one parasite per host indicating a higher degree of host specificity and less host switching among parasites of this genus. Findings suggest a degree of host-parasite co-speciation with host switching occurring occasionally when the “normal” host is unavailable in the aquatic ecosystem. Frequency of host switching seems to be occurring in proportion to host relatedness and does not cross generic boundaries in this system. (C) 2011 Elsevier Inc. All rights reserved. | 3629 | NA | NA | NA | no | no |
| 3629 | 2017 | NA | Neveu, C; Charvet, CL; Fauvin, A; Cortet, J; Beech, RN; Cabaret, J | 2010 | Genetic diversity of levamisole receptor subunits in parasitic nematode species and abbreviated transcripts associated with resistance | Objectives The molecular mechanisms of levamisole (LEV) activity and expression of resistance remain largely unknown in parasitic nematodes. In contrast, genetic screens for mutants that survive exposure to LEV in the free-living nematode Caenorhabditis elegans have led to the identification of five genes (unc-38, unc-63, unc-29, lev-1 and lev-8) that encode a LEV-sensitive acetylcholine receptor (L-AChR). Loss of these genes leads to LEV resistance. In this study, orthologues of these genes were identified in three species of trichostrongylid nematodes that have a major impact on small ruminants: Haemonchus contortus, Teladorsagia circumcincta and Trichostrongylus colubriformis. Polymorphism associated with LEV resistance have been investigated by comparing transcripts of these subunits in LEV susceptible and LEV-resistant isolates of the three strongylid species. Basic methods Partial sequences were identified by PCR experiments and full-length cDNA sequences corresponding to AChR subunits in the three trichostrongylid species were obtained using 3’-rapid amplification of cDNA ends-PCR and 5’ rapid amplification of cDNA ends anchored with the spliced leader sequence, SL1. Expression of L-AChR subunits was investigated in LEV-resistant and LEV-susceptible isolates of H. contortus, T. circumcincta and T. colubriformis using reverse transcription PCR. Main results We have identified a total of 20 full-length cDNA sequences corresponding to L-AChR subunits in three parasitic trichostrongylid species of which 14 correspond to novel sequences. Genes orthologous to unc-29, unc-63, unc-38 and lev-1 were found in each trichostrongylid species, whereas no gene corresponding to lev-8 has yet been identified. We have found 11 distinct paralogous sequences corresponding to the C. elegans unc-29 gene clustered in four groups revealing an unexpected diversity of unc-29-like genes. Complete coding sequences of the L-AChR subunits in two LEV-resistant and three susceptible isolates of H. contortus, T. circumcincta and T. colubriformis were essentially unchanged, but abbreviated transcripts of the unc-63 subunit were specifically expressed in resistant isolates of all three species. Conclusion The candidate gene strategy developed in this study revealed an unexpectedly high diversity of L-AChR subunits specific to the trichostrongylid parasites that are a principal target for the drug LEV. Abbreviated variants, predicted to produce nonfunctional unc-63, were associated with LEV resistance. This study contributes significantly to a better understanding of LEV receptor constitution in parasitic nematodes and highlights the putative role of aberrant mRNA encoding L-AChR subunits in LEV resistance. Pharmacogenetics and Genomics 20:414-425 (C) 2010 Wolters Kluwer Health vertical bar Lippincott Williams & Wilkins. | 3630 | NA | NA | NA | no | no |
| 3630 | 2017 | NA | Buono, L; Lopez-Villavicencio, M; Shykoff, JA; Snirc, A; Giraud, T | 2014 | Influence of Multiple Infection and Relatedness on Virulence: Disease Dynamics in an Experimental Plant Population and Its Castrating Parasite | The level of parasite virulence, i.e., the decrease in host’s fitness due to a pathogen, is expected to depend on several parameters, such as the type of the disease (e.g., castrating or host-killing) and the prevalence of multiple infections. Although these parameters have been extensively studied theoretically, few empirical data are available to validate theoretical predictions. Using the anther smut castrating disease on Silene latifolia caused by Microbotryum lychnidis-dioicae, we studied the dynamics of multiple infections and of different components of virulence (host death, non-recovery and percentage of castrated stems) during the entire lifespan of the host in an experimental population. We monitored the number of fungal genotypes within plants and their relatedness across five years, using microsatellite markers, as well as the rates of recovery and host death in the population. The mean relatedness among genotypes within plants remained at a high level throughout the entire host lifespan despite the dynamics of the disease, with recurrent new infections. Recovery was lower for plants with multiple infections compared to plants infected by a single genotype. As expected for castrating parasites, M. lychnidis-dioicae did not increase host mortality. Mortality varied across years but was generally lower for plants that had been diseased the preceding year. This is one of the few studies to have empirically verified theoretical expectations for castrating parasites, and to show particularly i) that castrated hosts live longer, suggesting that parasites can redirect resources normally used in reproduction to increase host lifespan, lengthening their transmission phase, and ii) that multiple infections increase virulence, here in terms of non-recovery and host castration. | 3631 | NA | NA | NA | no | no |
| 3631 | 2017 | NA | Wakeman, KC; Leander, BS | 2013 | Identity of environmental DNA sequences using descriptions of four novel marine gregarine parasites, Polyplicarium n. gen. (Apicomplexa), from capitellid polychaetes | Environmental PCR surveys of small-subunit (SSU) rDNA sequences are powerful approximations for the overall diversity of microbial eukaryotes (protists) living in specific marine habitats. However, many environmental DNA sequences generated from these approaches have unknown cellular origins because they are not closely related to other sequences that were generated directly from fully characterized, identified organisms. The unidentified sequences from marine environments tend to belong to poorly understood groups of apicomplexan parasites, especially gregarines. Single-cell PCR (SC-PCR) approaches on newly discovered gregarines provide the evidence necessary for determining the cellular identities of SSU rDNA sequence clades. In this study, the trophozoites of four novel gregarine morphotypes were isolated from the intestines of two different species of capitellid polychaetes collected from the eastern Pacific Ocean (British Columbia, Canada). The trophozoites of each morphotype were characterized using light microscopy, scanning electron microscopy, and SSU rDNA sequences amplified from four, single-cell isolates from each of the four novel morphotypes described in this study (16 new SSU rDNA sequences in total). Molecular phylogenetic analyses demonstrated five robust subclades within a more inclusive clade that contained all 16 new sequences and 5 environmental SSU rDNA sequences. The combination of SC-PCR approaches, molecular phylogenetic analyses, and comparative morphology (1) illustrate the utility of SC-PCR approaches for distinguishing between different gregarine species, (2) demonstrate the cellular identity of a previously unidentified environmental SSU rDNA sequence clade, and (3) enable us to establish four new species within one novel genus: Polyplicarium lacrimae n. gen. et sp. (type species), P. curvarae n. gen. et sp., P. translucidae n. gen. et sp., and P. citrusae n. gen. et sp. | 3632 | NA | NA | NA | no | no |
| 3632 | 2017 | NA | Morrisey, D; Inglis, G; Neil, K; Bradley, A; Fitridge, I | 2011 | Characterization of the marine aquarium trade and management of associated marine pests in Australia, a country with stringent import biosecurity regulation | Trade in ornamental marine species in Australia, a country with relatively stringent import controls, was investigated using a telephone survey of wholesalers and retailers, and a desktop review of internet import databases and hobbyist trading websites. Information on the regulatory framework was obtained from government and other published or online sources, and from staff of regulatory agencies. Although the trade is small relative to that in the USA, Europe and parts of Asia, Australia imports significant numbers of marine fish each year for the aquarium trade. Many of the more than 200 species imported have the potential to become environmental and/or economic pests. Imported individuals of native species could act as vectors of disease or affect the genetic diversity of native populations if they were released into the wild. Regulatory measures include the use of lists of permitted species of plants and animals, a case-bycase risk assessment process for species not on these lists, and requirements for health certification and quarantining of imported stock. Once within Australia, however, translocation is less rigorously controlled, being managed by individual states and based largely on lists of prohibited species, though generally with scope for case-by-case assessment and refusal of permits for unwanted species, such as recognized pests. Wholesalers and retailers interviewed generally showed a responsible attitude to the disposal of dead or unwanted stock, but awareness and understanding of the potential pest risk of ornamental marine species was generally poor. The importance of raising public awareness of the pest potential of ornamental marine species is likely to increase with the growing importance of mail-order and internet trade. | 3633 | NA | NA | NA | no | no |
| 3633 | 2017 | NA | Eshaghi, A; Duvvuri, VR; Lai, R; Nadarajah, JT; Li, AM; Patel, SN; Low, DE; Gubbay, JB | 2012 | Genetic Variability of Human Respiratory Syncytial Virus A Strains Circulating in Ontario: A Novel Genotype with a 72 Nucleotide G Gene Duplication | Human respiratory syncytial virus (HRSV) is the main cause of acute lower respiratory infections in children under 2 years of age and causes repeated infections throughout life. We investigated the genetic variability of RSV-A circulating in Ontario during 2010-2011 winter season by sequencing and phylogenetic analysis of the G glycoprotein gene. Among the 201 consecutive RSV isolates studied, RSV-A (55.7%) was more commonly observed than RSV-B (42.3%). 59.8% and 90.1% of RSV-A infections were among children <= 12 months and <= 5 years old, respectively. On phylogenetic analysis of the second hypervariable region of the 112 RSV-A strains, 110 (98.2%) clustered within or adjacent to the NA1 genotype; two isolates were GA5 genotype. Eleven (10%) NA1-related isolates clustered together phylogenetically as a novel RSV-A genotype, named ON1, containing a 72 nucleotide duplication in the C-terminal region of the attachment (G) glycoprotein. The predicted polypeptide is lengthened by 24 amino acids and includes a23 amino acid duplication. Using RNA secondary structural software, a possible mechanism of duplication occurrence was derived. The 23 amino acid ON1 G gene duplication results in a repeat of 7 potential O-glycosylation sites including three O-linked sugar acceptors at residues 270, 275, and 283. Using Phylogenetic Analysis by Maximum Likelihood analysis, a total of 19 positively selected sites were observed among Ontario NA1 isolates; six were found to be codons which reverted to the previous state observed in the prototype RSV-A2 strain. The tendency of codon regression in the G-ectodomain may infer a decreased avidity of antibody to the current circulating strains. Further work is needed to document and further understand the emergence, virulence, pathogenicity and transmissibility of this novel RSV-A genotype with a72 nucleotide G gene duplication. | 3634 | NA | NA | NA | no | no |
| 3634 | 2017 | NA | Opanda, SM; Wamunyokoli, F; Khamadi, S; Coldren, R; Bulimo, WD | 2016 | Genotyping of enteroviruses isolated in Kenya from pediatric patients using partial VP1 region | Enteroviruses (EV) are responsible for a wide range of clinical diseases in humans. Though studied broadly in several regions of the world, the genetic diversity of human enteroviruses (HEV) circulating in the sub-Saharan Africa remains under-documented. In the current study, we molecularly typed 61 HEV strains isolated in Kenya between 2008 and 2011 targeting the 3’-end of the VP1 gene. Viral RNA was extracted from the archived isolates and part of the VP1 gene amplified by RT-PCR, followed by sequence analysis. Twenty-two different EV types were detected. Majority (72.0 %) of these belonged to Enterovirus B species followed by Enterovirus D (21.3 %) and Enterovirus A (6.5 %). The most frequently detected types were Enterovirus-D68 (EV-D68), followed by Coxsackievirus B2 (CV-B2), CV-B1, CV-B4 and CV-B3. Phylogenetic analyses of these viruses revealed that Kenyan CV-B1 isolates were segregated among sequences of global CV-B1 strains. Conversely, the Kenyan CV-B2, CV-B3, CV-B4 and EV-D68 strains generally grouped together with those detected from other countries. Notably, the Kenyan EV-D68 strains largely clustered with sequences of global strains obtained between 2008 and 2010 than those circulating in recent years. Overall, our results indicate that HEV strains belonging to Enterovirus D and Enterovirus B species pre-dominantly circulated and played a significant role in pediatric respiratory infection in Kenya, during the study period. The Kenyan CV-B1 strains were genetically divergent from those circulating in other countries. Phylogenetic clustering of Kenyan EV-D68 strains with sequences of global strains circulating between 2008 and 2010 than those obtained in recent years suggests a high genomic variability associated with the surface protein encoding VP1 gene in these enteroviruses. | 3635 | NA | NA | NA | no | no |
| 3635 | 2017 | NA | Guerrero-Ferreira, R; Gorman, C; Chavez, AA; Willie, S; Nishiguchi, MK | 2013 | Characterization of the Bacterial Diversity in Indo-West Pacific Loliginid and Sepiolid Squid Light Organs | Loliginid and sepiolid squid light organs are known to host a variety of bacterial species from the family Vibrionaceae, yet little is known about the species diversity and characteristics among different host squids. Here we present a broad-ranging molecular and physiological analysis of the bacteria colonizing light organs in loliginid and sepiolid squids from various field locations of the Indo-West Pacific (Australia and Thailand). Our PCR-RFLP analysis, physiological characterization, carbon utilization profiling, and electron microscopy data indicate that loliginid squid in the Indo-West Pacific carry a consortium of bacterial species from the families Vibrionaceae and Photobacteriaceae. This research also confirms our previous report of the presence of Vibrio harveyi as a member of the bacterial population colonizing light organs in loliginid squid. pyrH sequence data were used to confirm isolate identity, and indicates that Vibrio and Photobacterium comprise most of the light organ colonizers of squids from Australia, confirming previous reports for Australian loliginid and sepiolid squids. In addition, combined phylogenetic analysis of PCR-RFLP and 16S rDNA data from Australian and Thai isolates associated both Photobacterium and Vibrio clades with both loliginid and sepiolid strains, providing support that geographical origin does not correlate with their relatedness. These results indicate that both loliginid and sepiolid squids demonstrate symbiont specificity (Vibrionaceae), but their distribution is more likely due to environmental factors that are present during the infection process. This study adds significantly to the growing evidence for complex and dynamic associations in nature and highlights the importance of exploring symbiotic relationships in which non-virulent strains of pathogenic Vibrio species could establish associations with marine invertebrates. | 3636 | NA | NA | NA | no | no |
| 3636 | 2017 | NA | Lively, CM | 2009 | Local host competition in the evolution of virulence | The evolution of parasite life histories should usually have correlated effects on host survivorship and/or reproductive success. For example, parasites that reproduce more rapidly might be expected to cause greater reductions in host fitness. Important theoretical advances have recently been made on virulence evolution, but the results are not always consistent. Here I compare two models [Q. Rev. Biol.71 (1996) 37; Q. Rev. Biol.75 (2000) 261] on the evolution of virulence that get qualitatively different results with respect to the effects of coinfection. I also construct a third model that attempts to connect these two formulations. The results suggest that parasite growth rates should increase as local host competition increases, unless relatedness is at equilibrium. In addition, the qualitative effect of adding coinfections on parasite growth rates depends critically on how the number of coinfections affects transmission success. | 3637 | NA | NA | NA | no | no |
| 3637 | 2017 | NA | Zheng, G; Li, ZH; Gail, MH; Gastwirth, JL | 2010 | Impact of Population Substructure on Trend Tests for Genetic Case-Control Association Studies | P>Hidden population substructure in case-control data has the potential to distort the performance of Cochran-Armitage trend tests (CATTs) for genetic associations. Three possible scenarios that may arise are investigated here: (i) heterogeneity of genotype frequencies across unidentified subpopulations (PSI), (ii) heterogeneity of genotype frequencies and disease risk across unidentified subpopulations (PSII), and (iii) cryptic correlations within unidentified subpopulations. A unified approach is presented for deriving the bias and variance distortion under the three scenarios for any CATT in a general family. Using these analytical formulas, we evaluate the excess type I errors of the CATTs numerically in the presence of population substructure. Our results provide insight into the properties of some proposed corrections for bias and variance distortion and show why they may not fully correct for the effects of population substructure. | 3638 | NA | NA | NA | no | no |
| 3638 | 2017 | NA | Agaras, BC; Scandiani, M; Luque, A; Fernandez, L; Farina, F; Carmona, M; Gally, M; Romero, A; Wall, L; Valverde, C | 2015 | Quantification of the potential biocontrol and direct plant growth promotion abilities based on multiple biological traits distinguish different groups of Pseudomonas spp. isolates | Members of the Pseudomonas genus colonize the rhizosphere of different plant species and display plant-probiotic traits. Therefore, they are interesting candidates for biological agricultural inputs to stimulate plant development and/or promote crop health. We have generated a collection of 19 isolates of pseudomonads obtained from either bulk soil or the rhizosphere of healthy individuals of major extensive crops from different plots under no-till management located in Argentina. Isolates were selected for their ability to antagonize several fungal pathogens recovered from infected soybean and maize plants. Partial sequencing of 16S rDNA, oprF and rpoB genes positioned isolates within all major pseudomonads groups. Most isolates colonized the rhizosphere of soybean and maize seedlings without affecting germination or development. Members of the Pseudomonas chlororaphis subgroup, and one isolate of the Pseudomonas putida subgroup, displayed the widest antifungal spectrum and strongest antagonistic potential. A principal component analysis based on the outcome of quantitative and qualitative tests related to biocontrol of fungal pathogens, direct plant growth promotion, and other root colonization-related traits, showed that isolates with the highest antagonistic potential and mostly of soil origin were grouped together, whereas rhizospheric isolates and those with strong flagellar-dependent motility, exoprotease production and biofilm development, were clustered in a separate group. Interestingly, quantitative comparison of newly developed biocontrol and direct growth promotion indices revealed that these two plant probiotic traits are rather associated to different set of isolates, and rarely present in the same pseudomonad strains. These indices could be employed to screen and categorize isolates by their potential to act as a biocontrol or a biofertilizer agent. (C) 2015 Elsevier Inc. All rights reserved. | 3639 | NA | NA | NA | no | no |
| 3639 | 2017 | NA | Narasimhan, VM; Rahbari, R; Scally, A; Wuster, A; Mason, D; Xue, YL; Wright, J; Trembath, RC; Maher, ER; van Heel, DA; Auton, A; Hurles, ME; Tyler-Smith, C; Durbin, R | 2017 | Estimating the human mutation rate from autozygous segments reveals population differences in human mutational processes | Heterozygous mutations within homozygous sequences descended from a recent common ancestor offer a way to ascertain de novo mutations across multiple generations. Using exome sequences from 3222 British-Pakistani individuals with high parental relatedness, we estimate a mutation rate of 1.45 +/- 0.05 x 10(-8) per base pair per generation in autosomal coding sequence, with a corresponding non-crossover gene conversion rate of 8.75 +/- 0.05 x 10(-6) per base pair per generation. This is at the lower end of exome mutation rates previously estimated in parent-offspring trios, suggesting that post-zygotic mutations contribute little to the human germ-line mutation rate. We find frequent recurrence of mutations at polymorphic CpG sites, and an increase in C to T mutations in a 5’ CCG 3’ to 5’ CTG 3’ context in the Pakistani population compared to Europeans, suggesting that mutational processes have evolved rapidly between human populations. | 3640 | NA | NA | NA | no | no |
| 3640 | 2017 | NA | Pauciullo, A; Kupper, J; Brandt, H; Donat, K; Iannuzzi, L; Erhardt, G | 2015 | Wingless-type MMTV integration site family member 2 (WNT2) gene is associated with resistance to MAP in faecal culture and antibody response in Holstein cattle | Mycobacterium avium subspecies paratuberculosis (MAP) is a pathogenic bacterium responsible for the lethal Johne’s disease in cattle. So far, several genome-wide association studies (GWAS) have been carried out to identify chromosomal regions highly associated with Johne’s disease. The aim of this study was to investigate the genetic variability within a pool of seven genes (LAMB1, DLD, WNT2, PRDM1, SOCS5, PTGER4 and IL10) indicated by former GWAS/RNA-Seq studies as putatively associated with MAP infections and to achieve a confirmation study of association with paratuberculosis susceptibility in a population of 324 German Holstein cattle (162 cases MAP positive and 162 controls MAP negative) using ELISA and fecal cultural tests. SNP validation and genotyping information are provided, quick methods for allelic discrimination were set up and transcription factor binding analyses were performed. The rs43390642:G>TSNP in the WNT2 promoter region is associated with paratuberculosis susceptibility (P=0.013), suggesting a protective role of the T allele (P=0.043; odds ratio 0.50 [0.25-0.97]). The linkage disequilibrium with the DLD rs134692583:A>T might suggest a combined mechanism of action of these neighboring genes in resistance to MAP infection, which is also supported by a significant effect shown by the haplotype DLDT/WNT2(T) (P=0.047). In silico analysis predicted rs43390642:G>T and rs134692583:A>T as essential parts of binding sites for the transcription factors GR, C/EBP and GATA-1, hence suggesting a potential influence on WNT2 and DLD gene expression. This study confirmed the region on BTA 4 (UMD 3.1: 50639460-51397892) as involved in tolerance/resistance to Johne’s disease. In addition, this study clarifies the involvement of the investigated genes in MAP infection and contributes to the understanding of genetic variability involved in Johne’s disease susceptibility. | 3641 | NA | NA | NA | no | no |
| 3641 | 2017 | NA | Qin, T; Yan, GB; Ren, HY; Zhou, HJ; Wang, HX; Xu, Y; Zhao, MQ; Guan, H; Li, MC; Shao, ZJ | 2013 | High Prevalence, Genetic Diversity and Intracellular Growth Ability of Legionella in Hot Spring Environments | Background: Legionella is the causative agent of Legionnaires’ disease, and hot springs are a major source of outbreaks of this disease. It is important from a public health perspective to survey hot spring environments for the presence of Legionella. Methods: Prospective surveillance of the extent of Legionella pollution was conducted at three hot spring recreational areas in Beijing, China in 2011. Pulsed-field gel electrophoresis (PFGE) and sequence-based typing (SBT) were used to describe the genetic polymorphism of isolates. The intracellular growth ability of the isolates was determined by interacting with J774 cells and plating the dilutions onto BCYE agar plates. Results: Overall, 51.9% of spring water samples showed Legionella-positive, and their concentrations ranged from 1 CFU/liter to 2,218 CFU/liter. The positive rates of Legionella were significantly associated with a free chlorine concentration of >= 0.2 mg/L, urea concentration of >= 0.05 mg/L, total microbial counts of >= 400 CFU/ml and total coliform of >= 3 MPN/L (p<0.01). The Legionella concentrations were significantly associated with sample temperature, pH, total microbial counts and total coliform (p<0.01). Legionella pneumophila was the most frequently isolated species (98.9%), and the isolated serogroups included serogroups 3 (25.3%), 6 (23.4%), 5 (19.2%), 1 (18.5%), 2 (10.2%), 8 (0.4%), 10 (0.8%), 9 (1.9%) and 12 (0.4%). Two hundred and twenty-eight isolates were analyzed by PFGE and 62 different patterns were obtained. Fifty-seven L. pneumophila isolates were selected for SBT analysis and divided into 35 different sequence types with 5 main clonal groups. All the 57 isolates had high intracellular growth ability. Conclusions: Our results demonstrated high prevalence and genetic polymorphism of Legionella in springs in Beijing, China, and the SBT and intracellular growth assay results suggested that the Legionella isolates of hot spring environments were pathogenic. Improved control and prevention strategies are urgently needed. | 3642 | NA | NA | NA | no | no |
| 3642 | 2017 | NA | Alcaide, M; Munoz, J; Martinez-de la Puente, J; Soriguer, R; Figuerola, J | 2014 | Extraordinary MHC class II B diversity in a non-passerine, wild bird: the Eurasian Coot Fulica atra (Aves: Rallidae) | The major histocompatibility complex (MHC) hosts the most polymorphic genes ever described in vertebrates. The MHC triggers the adaptive branch of the immune response, and its extraordinary variability is considered an evolutionary consequence of pathogen pressure. The last few years have witnessed the characterization of the MHC multigene family in a large diversity of bird species, unraveling important differences in its polymorphism, complexity, and evolution. Here, we characterize the first MHC class II B sequences isolated from a Rallidae species, the Eurasian Coot Fulica atra. A next-generation sequencing approach revealed up to 265 alleles that translated into 251 different amino acid sequences ( chain, exon 2) in 902 individuals. Bayesian inference identified up to 19 codons within the presumptive peptide-binding region showing pervasive evidence of positive, diversifying selection. Our analyses also detected a significant excess of high-frequency segregating sites (average Tajima’s D=2.36, P<0.05), indicative of balancing selection. We found one to six different alleles per individual, consistent with the occurrence of at least three MHC class II B gene duplicates. However, the genotypes comprised of three alleles were by far the most abundant in the population investigated (49.4%), followed by those with two (29.6%) and four (17.5%) alleles. We suggest that these proportions are in agreement with the segregation of MHC haplotypes differing in gene copy number. The most widespread segregating haplotypes, according to our findings, would contain one single gene or two genes. The MHC class II of the Eurasian Coot is a valuable system to investigate the evolutionary implications of gene copy variation and extensive variability, the greatest ever found, to the best of our knowledge, in a wild population of a non-passerine bird. | 3643 | NA | NA | NA | no | no |
| 3643 | 2017 | NA | Thaxter, CB; Joys, AC; Gregory, RD; Baillie, SR; Noble, DG | 2010 | Hypotheses to explain patterns of population change among breeding bird species in England | Population declines are now evident across many taxa, but within each assemblage there is often considerable variation in constituent population trends. We used bird population trends produced from the BTO/JNCC Common Birds Census (CBC) and the RSPB/BTO/JNCC Breeding Bird Survey (BBS), to test five main hypotheses to explain population changes of 59 breeding species in England (1967-2006): (1) breeding habitat. (2) predation risk to nest sites, (3) species climatic niche. (4) migration strategy, and (5) over-wintering bioclimatic zones of migrants, accounting for additional demographic and ecological traits. In absence of phylogenetic inter-relatedness, farmland species declined more than woodland species, most pronounced prior to 1986, probably reflecting agricultural intensification (1). We found limited support that ground nesters have declined more than above-ground or cavity nesters (2). and there was some indication that species with more northerly European distributions showed larger declines than more southerly-distributed species (3). Larger population declines were recorded for Afro-tropical migrants than species wintering in Europe or in the UK, most notable prior to 1986(4). However, declines were not uniform across all migrants (5) - species over-wintering in the arid savannah bioclimatic zone of Africa decreased in population between 1967 and 1976, whereas species wintering in humid West African forest and savannah declined more after 1987. These results suggest both breeding and over-wintering factors influenced population trends. European countries signed to the Convention on the Conservation of Migratory Species of Wild Animals are required to protect and conserve populations of migrants. Understanding connectivity between breeding and over-wintering populations, and similar environmental pressures experienced within over-wintering areas may be a useful step towards mitigating against further declines in migrants. (C) 2010 Elsevier Ltd. All rights reserved. | 3644 | NA | NA | NA | no | no |
| 3644 | 2017 | NA | Rajpurohit, S; Nedved, O; Gibbs, AG | 2013 | Meta-analysis of geographical clines in desiccation tolerance of Indian drosophilids | Tropical fruit flies (Drosophilidae) differ from temperate drosophilids in several ecophysiological traits, such as desiccation tolerance. Moreover, many species show significant differences in desiccation tolerance across geographical populations. Fruit flies from the tropical and subtropical Indian subcontinent show a clinal pattern for desiccation tolerance which is similar for more than a dozen species studied so far, suggesting adaptation to climatic differences. We performed a meta-analysis to investigate which particular climatic patterns modulate desiccation tolerance in natural populations of drosophilids. Latitude of the sampling site explained most of the variability. Seasonal thermal amplitude (fluctuations in temperature expressed as coefficient of variation) was the strongest climatic factor shaping desiccation tolerance of flies, while factors measuring humidity directly were not important. Implications for survival of flies after future climate change are suggested. (C) 2012 Elsevier Inc. All rights reserved. | 3645 | NA | NA | NA | no | no |
| 3645 | 2017 | NA | Gao, SJ; Lin, YH; Pan, YB; Damaj, MB; Wang, QN; Mirkov, TE; Chen, RK | 2012 | Molecular characterization and phylogenetic analysis of Sugarcane yellow leaf virus isolates from China | Sugarcane yellow leaf virus (SCYLV) (genus Polerovirus, family Luteoviridae), the causal agent of sugarcane yellow leaf disease (YLD), was first detected in China in 2006. To assess the distribution of SCYLV in the major sugarcane-growing Chinese provinces, leaf samples from 22 sugarcane clones (Saccharum spp. hybrid) showing YLD symptoms were collected and analyzed for infection by the virus using reverse transcription PCR (RT-PCR), quantitative RT-PCR, and immunological assays. A complete genomic sequence (5,879 nt) of the Chinese SCYLV isolate CHN-FJ1 and partial genomic sequences (2,915 nt) of 13 other Chinese SCYLV isolates from this study were amplified, cloned, and sequenced. The genomic sequence of the CHN-FJ1 isolate was found to share a high identity (98.4-99.1 %) with those of the Brazilian (BRA) genotype isolates and a low identity (86.5-86.9 %) with those of the CHN1 and Cuban (CUB) genotype isolates. The genetic diversity of these 14 Chinese SCYLV isolates was assessed along with that of 29 SCYLV isolates of worldwide origin reported in the GenBank database, based on the full or partial genomic sequence. Phylogenetic analysis demonstrated that all the 14 Chinese SCYLV isolates clustered into one large group with the BRA genotype and 12 other reported SCYLV isolates. In addition, five reported Chinese SCYLV isolates were grouped with the Peruvian (PER), CHN1 and CUB genotypes. We therefore speculated that at least four SCYLV genotypes, BRA, PER, CHN1, and CUB, are associated with YLD in China. Interestingly, a 39-nt deletion was detected in the sequence of the CHN-GD3 isolate, in the middle of the ORF1 region adjacent to the overlap between ORF1 and ORF2. This location is known to be one of the recombination breakpoints in the Luteoviridae family. | 3646 | NA | NA | NA | no | no |
| 3646 | 2017 | NA | Mangelsen, E; Kilian, J; Berendzen, KW; Kolukisaoglu, UH; Harter, K; Jansson, C; Wanke, D | 2008 | Phylogenetic and comparative gene expression analysis of barley (Hordeum vulgare) WRKY transcription factor family reveals putatively retained functions between monocots and dicots | Background: WRKY proteins belong to the WRKY-GCM1 superfamily of zinc finger transcription factors that have been subject to a large plant-specific diversification. For the cereal crop barley (Hordeum vulgare), three different WRKY proteins have been characterized so far as regulators in sucrose signaling, pathogen defense, and in response to cold and drought. However, their phylogenetic relationship remained unresolved. Results: In this study, we used available sequence information to identify a minimum number of 45 barley WRKY transcription factor (HvWRKY) genes. According to their structural features, the HvWRKY factors were classified into the previously defined polyphyletic WRKY subgroups 1 to 3. Furthermore, we could assign putative orthologs of the HvWRKY proteins in Arabidopsis and rice. While in most cases clades of orthologous proteins were formed within each group or subgroup, other clades were composed of paralogous proteins for the grasses and Arabidopsis only, which is indicative of specific gene radiation events. To gain insight into their putative functions, we examined expression profiles of WRKY genes from publicly available microarray data resources and found group specific expression patterns. While putative orthologs of the HvWRKY transcription factors have been inferred from phylogenetic sequence analysis, we performed a comparative expression analysis of WRKY genes in Arabidopsis and barley. Indeed, highly correlative expression profiles were found between some of the putative orthologs. Conclusion: HvWRKY genes have not only undergone radiation in monocot or dicot species, but exhibit evolutionary traits specific to grasses. HvWRKY proteins exhibited not only sequence similarities between orthologs with Arabidopsis, but also relatedness in their expression patterns. This correlative expression is indicative for a putative conserved function of related WRKY proteins in monocot and dicot species. | 3647 | NA | NA | NA | no | no |
| 3647 | 2017 | NA | Anger, K | 2006 | Contributions of larval biology to crustacean research: a review | Many aquatic crustaceans pass through a complex life cycle comprising a benthic juvenile-adult and a pelagic larval phase. In the study of aquatic ecology, meroplanktonic larvae are therefore considered as principal components of benthic-pelagic coupling processes. As a consequence of radical transitions of life style, larvae differ from conspecific adults in their ecology, behaviour, nutrition, morphology, and physiology. Ontogenetic changes of these traits, as well as carry-over effects of larval condition on postmetamorphic fitness of benthic juveniles, are subjects of the interdisciplinary field of larval biology. Larval biology is thus not only an intrinsic part of life-history studies, but contributes essential information also to various other biological disciplines, including the broad area of crustacean research. For economically important species, it provides critical information for the development of aquaculture techniques or for the management of sustainable fisheries. Inferring from heritable ontogenetic patterns, comparative studies of larval morphology also aid the identification of phylogenetic relationships within and among higher taxa (“Evo-Devo” perspective). On the other hand, larval traits may be modified by environmental factors, which link larval ecology to developmental biology (“Eco-Devo” approach). Patterns of larval dispersal, mortality, and recruitment are crucial for the stability of benthic; populations and communities. These aspects of “supply-side ecology” have also consequences for patterns of biogeographic distribution, population connectivity, genetic diversity, and the formation of metapopulations. In addition, the spread of introduced species in recipient regions may be explained or predicted through developmental and ecophysiological traits of their larvae. In evolutionary biology, knowledge of reproductive and developmental adaptations is crucial for the understanding of limnic and terrestrial invasions by originally marine crustaceans. | 3648 | NA | NA | NA | no | no |
| 3648 | 2017 | NA | Schorr, G; Holstein, N; Pearman, PB; Guisan, A; Kadereit, JW | 2012 | Integrating species distribution models (SDMs) and phylogeography for two species of Alpine Primula | The major intention of the present study was to investigate whether an approach combining the use of niche-based palaeodistribution modeling and phylogeography would support or modify hypotheses about the Quaternary distributional history derived from phylogeographic methods alone. Our study system comprised two closely related species of Alpine Primula. We used species distribution models based on the extant distribution of the species and last glacial maximum (LGM) climate models to predict the distribution of the two species during the LGM. Phylogeographic data were generated using amplified fragment length polymorphisms (AFLPs). In Primula hirsuta, models of past distribution and phylogeographic data are partly congruent and support the hypothesis of widespread nunatak survival in the Central Alps. Species distribution models (SDMs) allowed us to differentiate between alpine regions that harbor potential nunatak areas and regions that have been colonized from other areas. SDMs revealed that diversity is a good indicator for nunataks, while rarity is a good indicator for peripheral relict populations that were not source for the recolonization of the inner Alps. In P. daonensis, palaeodistribution models and phylogeographic data are incongruent. Besides the uncertainty inherent to this type of modeling approach (e. g., relatively coarse 1-km grain size), disagreement of models and data may partly be caused by shifts of ecological niche in both species. Nevertheless, we demonstrate that the combination of palaeodistribution modeling with phylogeographical approaches provides a more differentiated picture of the distributional history of species and partly supports (P. hirsuta) and partly modifies (P. daonensis and P. hirsuta) hypotheses of Quaternary distributional history. Some of the refugial area indicated by palaeodistribution models could not have been identified with phylogeographic data. | 3649 | NA | NA | NA | no | no |
| 3649 | 2017 | NA | Mohler, V; Klahr, A; Wenzel, G; Schwarz, G | 2002 | A resistance gene analog useful for targeting disease resistance genes against different pathogens on group 1S chromosomes of barley, wheat and rye | Comparative sequence analysis of the resistance gene analog (RGA) marker locus aACT/CAA (originally found to be tightly linked to the multiallelic barley Mla cluster) from genomes of barley, wheat and rye revealed a high level of relatedness among one another and showed high similarity to a various number of NBS-LRR disease resistance proteins. Using the sequence-specific polymerase chain reaction (PCR), RGA marker aACT/CAA was mapped on group IS chromosomes of the Triticeae and was associated with disease resistance loci. In barley and rye, the marker showed linkage to orthologous powdery mildew resistance genes Mla1 and Pm17, respectively, while in wheat linkage with a QTL against fusarium head blight (FHB) disease was determined. The use of RGA clones for R gene mapping and their role in the expression of qualitative and quantitative resistance is discussed. | 3650 | NA | NA | NA | no | no |
| 3650 | 2017 | NA | Hamede, R; Lachish, S; Belov, K; Woods, G; Kreiss, A; Pearse, AM; Lazenby, B; Jones, M; Mccallum, H | 2012 | Reduced Effect of Tasmanian Devil Facial Tumor Disease at the Disease Front | Pathogen-driven declines in animal populations are increasingly regarded as a major conservation issue. The Tasmanian devil (Sarcophilus harrisii) is threatened with extinction by devil facial tumor disease, a unique transmissible cancer. The disease is transmitted through direct transfer of tumor cells, which is possible because the genetic diversity of Tasmanian devils is low, particularly in the major histocompatibility complex genes of the immune system. The far northwest of Tasmania now holds the last remaining disease-free wild devil populations. The recent discovery of unique major histocompatibility complex genotypes in the northwestern region of Tasmania has raised the possibility that some animals may be resilient to the disease. We examined the differences in the epidemiology and population effects of devil facial tumor disease at 3 well-studied affected sites in eastern Tasmania and 1 in western Tasmania (West Pencil Pine). In contrast to the 3 eastern sites, there has been no rapid increase in disease prevalence or evidence of population decline at West Pencil Pine. Moreover, this is the only onsite at which the population age structure has remained unaltered 4 years after the first detection of disease. The most plausible explanations for the substantial differences in population effects and epidemiology of the disease between eastern and western sites are geographic differences in genotypes or phenotypes of devils and functional differences between tumor strains in the 2 regions. We suggest that conservation efforts focus on identifying whether either or both these explanations are correct and then, if resistance alleles exist, to attempt to spread the resistant alleles into affected populations. Such assisted selection has rarely been attempted for the management of wildlife diseases, but it may be widely applicable. | 3651 | NA | NA | NA | no | no |
| 3651 | 2017 | NA | Johnson, CA | 2000 | Responses of two potential host species (Formica gnava and Formica occulta) to pupae of the obligatory slave-making ant, Polyergus breviceps (Hymenoptera : Formicidae) | Queens of the slave-making ant, Polyergus breviceps, take over nests of adult Formica workers when establishing new colonies. Although naive to slave-maker brood, the usurped Formica rear Polyergus offspring and nests containing both host and parasite species forms. Host worker acceptance of parasite brood has been attributed to the similarity of “brood tending signals” between these closely related taxa and/or the presence of an “attractive pheromone” in the slave-maker brood. By presenting single-species groups Of Formica occulta and Formica gnava (two host species of P. breviceps) with a choice of Formica pupae of both species or with a choice of P. breviceps pupae from both types of mixed-species nests, it seems that neither close phylogenetic relatedness nor an attractive brood odor alone can account for the propensity of host workers to adopt slave-maker pupae. Significantly greater numbers of P. breviceps pupae were adopted by enslaved workers than by free-living workers, and within the enslaved groups and the free-living F. gnava group greater numbers of P. breviceps pupae were adopted if they were from nests where the host species was conspecific to workers used in tests. When presented with F. gnava and F. occulta pupae, Formica workers adopted conspecific pupae almost exclusively and ignored or consumed pupae of the other host species. Taken together, these results imply that P. breviceps pupae have both a species-specific odor and a general brood-tending pheromone, upon, which a host odor may be imposed. The disparate requirements of immatures at different stages of development for cue specificity or generality in maintaining nest exclusivity and maximizing inclusive fitness are discussed. | 3652 | NA | NA | NA | no | no |
| 3652 | 2017 | NA | Jakob, SS; Martinez-Meyer, E; Blattner, FR | 2009 | Phylogeographic Analyses and Paleodistribution Modeling Indicate Pleistocene In Situ Survival of Hordeum Species (Poaceae) in Southern Patagonia without Genetic or Spatial Restriction | Although many phylogeographic studies have been conducted to analyze the impact of the ice age on species history of Northern Hemisphere mountain plants, such studies are nearly absent for plants of the Southern Hemisphere, particularly for lowland vegetation units. These species should have been primarily influenced by climate cooling and changes in precipitation regime instead of glaciers covering their distribution areas. It is thought that New World lowland species generally evaded climate changes by equatorial migration during Pleistocene cold cycles and recolonized their habitats at higher latitudes when climate warmed up again. In contrast to Eurasia, latitudinal orientation of the major mountain ranges in the Americas made these migrations easily possible. In the huge steppe of the Patagonian plains and adjacent Andes of southern South America thrives a group of three sympatrically distributed diploid species of the barley genus Hordeum, which originated during the last 1.3 million years (My) from a common progenitor. To get insights into the speciation mode of the taxa and to test the hypothesis of longitudinal migration of steppe vegetation during the Pleistocene, we conducted population genetic and phylogeographic analyses based on sequences of the chloroplast trnL-F region from 922 individuals. We found a high number of chloroplast haplotypes shared among species, which indicate speciation through vicariance events. Analysis of the distribution of genetic diversity within and among species inferred an origin of Hordeum comosum in the Central Argentine Andes, whereas Hordeum patagonicum and Hordeum pubiflorum originated in southern Patagonia. The extant occurrence of H. comosum in southern Patagonia and H. pubiflorum northward along the Argentine Andes was caused by reciprocal migration after the origin of the species. Surprisingly, molecular data provided no evidence for range shifts toward the north during the last glacial maximum and recolonization of southerly habitats afterward, but indicated in situ survival of large populations of Hordeum species within their extant distribution ranges even in southernmost Patagonia and Tierra del Fuego. Ecoclimatic niche modeling used to reconstruct the potential paleodistribution areas of the species during the last glacial maximum shows that climate conditions were sufficient for the species to survive Pleistocene cold cycles in Patagonia without significant geographic restrictions. Molecular data together with ecological niche modeling indicate stable geographic distribution areas in two of the three species for at least the Holocene. As the Hordeum species are characteristic taxa of different steppe habitats, we speculate that the Patagonian steppe might be an old vegetation unit occurring for up to 4.5 My in southern South America. | 3653 | NA | NA | NA | no | no |
| 3653 | 2017 | NA | de Bruyn, PJN; Tosh, CA; Oosthuizen, WC; Phalanndwa, MV; Bester, MN | 2008 | Temporary marking of unweaned southern elephant seal (Mirounga leonina L.) pups | Like many pinniped species, southern elephant seals (Mirounga leonina) are conducive to long-term population studies using mark-recapture techniques. The twenty-four year longitudinal dataset at sub-Antarctic Marion Island has already provided much Insight into elephant seal population dynamics. However, a limitation of the present dataset is that mother/pup relatedness cannot be examined because pups are only tagged after their tagged mothers have abandoned them. We test the usefulness of two different temporary marking techniques (tagging and ‘strapping’), and four different marker types over two consecutive breeding seasons for use on pups with attending marked mothers. We show that strapping Is an ineffective way to mark unweaned southern elephant seals. By comparison, ‘Supersmall((=)’) Dalton plastic tags allow quick, effective and easy marking of large numbers of pups with known mothers, without excessive marker loss. | 3654 | NA | NA | NA | no | no |
| 3654 | 2017 | NA | Kitchen, AM; Knowlton, FF | 2006 | Cross-fostering in coyotes: Evaluation of a potential conservation and research tool for canids | Cross-fostering has been attempted opportunistically with endangered canids as a means of increasing populations. Due to the usefulness of cross-fostering for conservation, an understanding of factors influencing success rates is essential. Using captive coyotes (Canis latrans) as a model, we assessed the willingness of adult pairs to foster young born to other parents. We assessed the efficacy of fostering pups into existing litters (augmentation) and completely switching litters (replacement). We augmented four litters with two pups of similar age when pups were < 7 days old. In addition, we replaced four entire litters when pups were < 10 days old. We also augmented litters with pups 3-4 and 6-7 weeks of age. Survival, weight gain, and dominance status of pups were monitored for six weeks and compared to four control litters to determine success. All complete litter replacements were successful with survival rates among replaced pups (89.5%) similar to those of control litters (90%). For augmented litters, pup survival was dependent on the age at which fostering occurred. All pups fostered into 4 litters at < 1 week of age survived beyond 6 weeks of age, two of three fostering attempts with 3-4-week-old pups succeeded, while neither of two attempts to foster 6-week-old pups succeeded. Surviving fostered pups appeared to be at no disadvantage. Weight gains were similar for pups in all treatments, and there was no evidence of reduced dominance status among fostered pups as compared to natal pups in the same litters. These results illustrate that genetic relatedness is not essential for successful fostering and does not appear to alter dominance patterns; however the age at which pups are fostered may affect the success of fostering attempts. Published by Elsevier Ltd. | 3655 | NA | NA | NA | no | no |
| 3655 | 2017 | NA | Mullick, S; Mukherjee, A; Ghosh, S; Pazhani, GP; Sur, D; Manna, B; Nataro, JP; Levine, MM; Ramamurthy, T; Chawla-Sarkar, M | 2014 | Community Based Case-Control Study of Rotavirus Gastroenteritis among Young Children during 2008-2010 Reveals Vast Genetic Diversity and Increased Prevalence of G9 Strains in Kolkata | Background: Group A Rotaviruses are a major etiologic agent of gastroenteritis in infants and young children (, 5 years) worldwide. Although rotavirus vaccines have been successfully administered in many countries, in India the introduction of rotavirus vaccine in national immunization program was approved in 2014. Since high disease burden and large number of genetic variants have been reported from low income countries including India, monitoring of rotavirus was initiated prior to implementation of the vaccine in the region. Methods: A total number of 3,582 stool samples were collected from an urban slum community in Kolkata, among which 1,568 samples were obtained from children of <= 5 years of age, with moderate to severe diarrhoea and 2,014 samples were collected from age-sex matched healthy neighbourhood controls. Rotavirus positive samples were typed by multiplex seminested PCR and nucleotide sequencing. Circulating strains were phylogenetically analyzed. Results: Among 1,568 children with diarrhoea, 395 (25.2%), and among 2,014 asymptomatic children, 42 (2%) were rotavirus positive. G1P[8] was identified as the most common strain (32%) followed by G9P[8] (16.9%), G2P[4] (13.5%) and G9P[4] (10.75%). G12 strains with combinations of P[4], P[6] and P[8] comprised 11.9% of total positive strains. The rest (<10%) were rare and uncommon strains like G1P[4], G1P[6], G2P[8] and animal-like strains G4P[6], G6P[14] and G11P[25]. The 42 rotavirus positive samples from asymptomatic children revealed common genotypes like G1, G2 and G9. Conclusion: This community based case-control study showed increased predominance of genotype G9 in Kolkata. It also confirmed co-circulation of a large number of genetic variants in the community. Asymptomatic rotavirus positive children though low in number can also be a source of dispersal of infection in the community. This study provides background information to the policy makers for implementation of rotavirus vaccines in this region. | 3656 | NA | NA | NA | no | no |
| 3656 | 2017 | NA | Martinez-Solano, I; Teixeira, J; Buckley, D; Garcia-Paris, M | 2006 | Mitochondrial DNA phylogeography of Lissotriton boscai (Caudata, Salamandridae): evidence for old, multiple refugia in an Iberian endemic | In Europe, southern peninsulas served as refugia during cold periods in the Pleistocene, acting both as centres of origin of endemisms and as sources from which formerly glaciated areas were recolonized during interglacial periods. Previous studies have revealed that within the main refugial areas, intraspecific lineages often survived in allopatric refugia. We analysed two mitochondrial markers (nad4, control region, similar to 1.4 kb) in 103 individuals representing the entire distribution of Lissotriton boscai, a newt endemic to the western Iberian Peninsula. We inferred the evolutionary history of the species through phylogenetic, phylogeographic and historical demographic analyses. The results revealed unexpected, deep levels of geographically structured genetic variability. We identified two main evolutionary lineages, each containing three well-supported clades. The first historical split involved populations from central-southwestern coastal Portugal and the ancestor of all the remaining populations around 5.8 million years ago. Both lineages were subsequently fragmented into different population groups between 2.5 and 1.2 million years ago. According to nested clade analysis, at lower hierarchical levels the patterns suggest restricted gene flow with isolation by distance, whereas at higher levels the clades exhibit signatures of contiguous range expansion. Bayesian Skyline Plots show recent bottlenecks, followed by demographic expansions in all lineages. The significant genetic structure found is consistent with long-term survival of populations in allopatric refugia, supporting the ‘refugia-within-refugia’ scenario for southern European peninsulas. The comparison of our results with other co-distributed species highlights the generality of this hypothesis for the Iberian herpetofauna and suggests that Mediterranean refuges had more relevance for the composition and distribution of present biodiversity patterns than currently acknowledged. We briefly discuss the taxonomic and conservation implications of our results. | 3657 | NA | NA | NA | no | no |
| 3657 | 2017 | NA | Hogendoorn, K; Zammit, J | 2001 | Benefits of cooperative breeding through increased colony survival in an allodapine bee | Cooperation between individuals can lead to both increased productivity and increased survival of colonies relative to solitary breeding. We investigated how individual survival, survival of the group and estimates of group productivity are related to colony size in the allodapine bee Exoneura nigrescens. Colony survival strongly depended on initial colony size, but such a correlation was not found for survival of individuals from colonies of different initial sizes. Loss of a female from two-female nests resulted in a decrease in the number of immatures reared through, but such nests still maintained a higher reproductive output than solitary nests. Group benefits have been underestimated in previous publications because changes in colony size and size dependent colony survival had not been taken into account. The relatively large group benefits measured in this study have implications for the expected skew. | 3658 | NA | NA | NA | no | no |
| 3658 | 2017 | NA | Moon, KL; Banks, SC; Fraser, CI | 2015 | Phylogeographic Structure in Penguin Ticks across an Ocean Basin Indicates Allopatric Divergence and Rare Trans-Oceanic Dispersal | The association of ticks (Acarina) and seabirds provides an intriguing system for assessing the influence of long-distance dispersal on the evolution of parasitic species. Recent research has focused on host-parasite evolutionary relationships and dispersal capacity of ticks parasitising flighted seabirds. Evolutionary research on the ticks of non-flighted seabirds is, in contrast, scarce. We conducted the first phylogeographic investigation of a hard tick species (Ixodes eudyptidis) that parasitises the Little Blue Penguin (Eudyptula minor). Using one nuclear (28S) and two mitochondrial (COI and 16S) markers, we assessed genetic diversity among several populations in Australia and a single population on the South Island of New Zealand. Our results reveal two deeply divergent lineages, possibly representing different species: one comprising all New Zealand samples and some from Australia, and the other representing all other samples from Australian sites. No significant population differentiation was observed among any Australian sites from within each major clade, even those separated by hundreds of kilometres of coastline. In contrast, the New Zealand population was significantly different to all samples from Australia. Our phylogenetic results suggest that the New Zealand and Australian populations are effectively isolated from each other; although rare long-distance dispersal events must occur, these are insufficient to maintain trans-Tasman gene flow. Despite the evidence for limited dispersal of penguin ticks between Australia and New Zealand, we found no evidence to suggest that ticks are unable to disperse shorter distances at sea with their hosts, with no pattern of population differentiation found among Australian sites. Our results suggest that terrestrial seabird parasites may be quite capable of short-distance movements, but only sporadic longer-distance (trans-oceanic) dispersal. | 3659 | NA | NA | NA | no | no |
| 3659 | 2017 | NA | BERGER, J | 1994 | SCIENCE, CONSERVATION, AND BLACK RHINOS | The study of adaptive traits rarely has been applied toward the conservation of biodiversity. Fields such as evolution, biogeography, behavioral ecology, population biology, and genetics have facilitated conservation goals, but only partially and only for a few taxa. Among the world’s most endangered mammalian families is the Rhinoceratidae whose five species are being exterminated for their horns. Numerous conservation actions have been applied to these species. The most radical, horn removal, is designed to improve the conservation of both black (Diceros bicornis) and white (Ceratotherium simum) rhinos. In this paper, I use basic and applied biology to suggest how science has or has not contributed to the in situ conservation of black rhinos. I make four points: knowledge about associations between mating systems and sexual dimorphism has helped illuminate the evolution of secondary sexual traits; relationships between behavioral responses of black rhinos to dangerous predators and subsequent mortality are of basic interest, but this knowledge has not abetted rhino conservation; prior literature indicates that the young of horned mothers regularly are maimed by dangerous predators (if horns have utility as defensive structures, then phenotypic alterations of female horns should increase the susceptibility of young to predation, a prediction with empirical support from a Namib Desert population); because wild populations of black rhinos have been depleted in the past 25 years by 97%, it makes little sense to plan how to conserve genetic diversity over the next 500. Science will continue to play a critical role in the future conservation of small, heavily managed populations. However, it is less likely to be of major significance in the in situ conservation of rhinos until sociological, economic, and political issues are effectively resolved. | 3660 | NA | NA | NA | no | no |
| 3660 | 2017 | NA | Switt, AIM; den Bakker, HC; Cummings, CA; Rodriguez-Rivera, LD; Govoni, G; Raneiri, ML; Degoricija, L; Brown, S; Hoelzer, K; Peters, JE; Bolchacova, E; Furtado, MR; Wiedmann, M | 2012 | Identification and Characterization of Novel Salmonella Mobile Elements Involved in the Dissemination of Genes Linked to Virulence and Transmission | The genetic diversity represented by >2,500 different Salmonella serovars provides a yet largely uncharacterized reservoir of mobile elements that can contribute to the frequent emergence of new pathogenic strains of this important zoonotic pathogen. Currently, our understanding of Salmonella mobile elements is skewed by the fact that most studies have focused on highly virulent or common serovars. To gain a more global picture of mobile elements in Salmonella, we used prediction algorithms to screen for mobile elements in 16 sequenced Salmonella genomes representing serovars for which no prior genome scale mobile element data were available. From these results, selected mobile elements underwent further analyses in the form of validation studies, comparative analyses, and PCR-based population screens. Through this analysis we identified a novel plasmid that has two cointegrated replicons (IncI1-IncFIB); this plasmid type was found in four genomes representing different Salmonella serovars and contained a virulence gene array that had not been previously identified. A Salmonella Montevideo isolate contained an IncHI and an IncN2 plasmid, which both encoded antimicrobial resistance genes. We also identified two novel genomic islands (SGI2 and SGI3), and 42 prophages with mosaic architecture, seven of them harboring known virulence genes. Finally, we identified a novel integrative conjugative element (ICE) encoding a type IVb pilus operon in three non-typhoidal Salmonella serovars. Our analyses not only identified a considerable number of mobile elements that have not been previously reported in Salmonella, but also found evidence that these elements facilitate transfer of genes that were previously thought to be limited in their distribution among Salmonella serovars. The abundance of mobile elements encoding pathogenic properties may facilitate the emergence of strains with novel combinations of pathogenic traits. | 3661 | NA | NA | NA | no | no |
| 3661 | 2017 | NA | Cosentino, RO; Aguero, F | 2014 | Genetic Profiling of the Isoprenoid and Sterol Biosynthesis Pathway Genes of Trypanosoma cruzi | In Trypanosoma cruzi the isoprenoid and sterol biosynthesis pathways are validated targets for chemotherapeutic intervention. In this work we present a study of the genetic diversity observed in genes from these pathways. Using a number of bioinformatic strategies, we first identified genes that were missing and/or were truncated in the T. cruzi genome. Based on this analysis we obtained the complete sequence of the ortholog of the yeast ERG26 gene and identified a non-orthologous homolog of the yeast ERG25 gene (sterol methyl oxidase, SMO), and we propose that the orthologs of ERG25 have been lost in trypanosomes (but not in Leishmanias). Next, starting from a set of 16 T. cruzi strains representative of all extant evolutionary lineages, we amplified and sequenced similar to 24 Kbp from 22 genes, identifying a total of 975 SNPs or fixed differences, of which 28% represent non-synonymous changes. We observed genes with a density of substitutions ranging from those close to the average (similar to 2.5/100 bp) to some showing a high number of changes (11.4/100 bp, for the putative lathosterol oxidase gene). All the genes of the pathway are under apparent purifying selection, but genes coding for the sterol C14-demethylase, the HMG-CoA synthase, and the HMG-CoA reductase have the lowest density of missense SNPs in the panel. Other genes (TcPMK, TcSMO-like) have a relatively high density of non-synonymous SNPs (2.5 and 1.9 every 100 bp, respectively). However, none of the non-synonymous changes identified affect a catalytic or ligand binding site residue. A comparative analysis of the corresponding genes from African trypanosomes and Leishmania shows similar levels of apparent selection for each gene. This information will be essential for future drug development studies focused on this pathway. | 3662 | NA | NA | NA | no | no |
| 3662 | 2017 | NA | Siano, R; Alves-de-Souza, C; Foulon, E; Bendif, EM; Simon, N; Guillou, L; Not, F | 2011 | Distribution and host diversity of Amoebophryidae parasites across oligotrophic waters of the Mediterranean Sea | Sequences affiliated to Syndiniales (Marine alveolate, MALV) regularly dominate 18S rDNA genetic libraries of nearly all marine ecosystems investigated so far. Among them, Amoebophryidae (MALV group II) is composed of numerous and genetically distant environmental sequences, where Amoebophrya is the only known and formally described genus. Amoebophrya species include virulent pathogens for a wide range of dinoflagellate species. Beside their regular occurrence in marine ecosystems, their quantitative distribution and the environmental factors triggering host infection have barely been studied in open oligotrophic waters. In order to understand the functional role of these parasites in natural environments, we studied the distribution and contribution to the eukaryotic community of the small free-living stage of Amoebophryidae (the dinospores) along a transect in the Mediterranean Sea, as well as their host diversity at three oligotrophic stations. Dinospores were more abundant at a coastal station (max. 1.5 x 10(3) cells ml(-1)) than in oligotrophic waters (max. 51 +/- 16.3 cells ml(-1)), where they represented 10.3 to 34.9% of the total eukaryotic community at 40 and 30m depth, respectively and 21.2% on average along the water column. Positive correlation was found between dinospore occurrence and higher concentration of NO(3) + NO(2) at the coastal station. At selected stations, out of 38 different dinoflagellates taxa identified, 15 were infected, among which a majority were not recognized as Amoebophryidae host so far. Prevalences (percentage of infected cells) generally varied between 1% and 10%, with a notable exception for Blepharocysta paulsenii for which 25% of cells were infected at the most oligotrophic station. The present study shows that dinospores are able to thrive and infect dinoflagellates both in coastal and ultra-oligotrophic open waters. Our results emphasize the role of parasitism in microbial food web dynamics and ultimately on biogeochemical cycles. | 3663 | NA | NA | NA | no | no |
| 3663 | 2017 | NA | Gisi, U; Hermann, D; Ohl, L; Steden, C | 1997 | Sensitivity profiles of Mycosphaerella graminicola and Phytophthora infestans populations to different classes of fungicides | Prior to the use of fungicides, the baseline sensitivity of individuals in a pathogen population may already differ by a factor of 10 to 100 between the least and the most sensitive isolates. In Mycosphaerella graminicola populations, this factor, measured in vitro, was 5 to 20 for both the strobilurin analogue azoxystrobin (baseline) and the triazole cyproconazole which has been in use for several years. In Phytophthora infestans populations, this factor, measured in a leaf disc assay, was about 100 for azoxystrobin (baseline), up to 1000 for the cyanoacetamide cymoxanil and >10000 for the phenylamide oxadixyl; both of the latter have been used for many years. In M. graminicola, cross-sensitivity was present between all azole fungicides for the majority of the isolates, whereas no correlation was found between triazoles and azoxystrobin. Despite the existence of cross-sensitivity between azoles, ‘box-and-whiskers’ plots revealed large variations in the sensitivity profiles of some triazoles; isolates resistant to triazoles have not been detected in M. graminicola populations. In P. infestans populations, the proportion of the phenylamide-resistant sub-population increased during the season more rapidly in treated than in untreated fields, but it was low at the beginning of the next season in all fields. During disease epidemics, the fitness of phenylamide-resistant P. infestans isolates, as characterised by lesion size, was higher than that of the sensitive isolates, but after the overwintering period, the recovery of resistant isolates was apparently lower. The presence of both Al and A2 mating types of P. infestans in European populations, although at different frequencies, allows sexual recombination and increased genetic diversity, affecting sensitivity and fitness. Such mixed populations can still be adequately controlled by using sound anti-resistance strategies. | 3664 | NA | NA | NA | no | no |
| 3664 | 2017 | NA | Goddard, J | 2009 | Historical and recent evidence for close relationships among Rickettsia parkeri, R-conorii, R-africae, and R-sibirica: Implications for rickettsial taxonomy | Rickettsia parkeri, a member of the spotted fever group rickettsias, was first described in 1939 and was thought to be non-pathogenic until recently, when it was found to cause a spotted fever-like illness in humans and areas of necrosis (eschars) at the sites of tick bites. Accordingly, there is currently much interest in this emerging pathogen. In this study, all published articles concerning R. parkeri were reviewed and analyzed for evidence of relatedness among this agent and other spotted fever group (SFG) rickettsiae which also produce similar clinical syndromes and/or eschars, including R. conorii, R. africae, and R. sibirica. A synthesis of the historical (antigenic) and recent (molecular) data supporting a phylogenetic sub-grouping of these SFG organisms is presented and comments are offered about the taxonomy of rickettsial organisms in general, and R. parkeri in particular. | 3665 | NA | NA | NA | no | no |
| 3665 | 2017 | NA | Dahms, HU; Qian, PY | 2004 | Life histories of the Harpacticoida (Copepoda, Crustacea): a comparison with meiofauna and macrofauna | The Harpacticoida reside in a wide range of habitats–from all marine situations to most freshwater habitats, and even moist terrestrial substrata like moss and litter. They exhibit free-living to parasitic life-styles and larval development with plankto- and lecithotrophy. Harpacticoid life cycles as a rule comprise six nauplius larvae and six copepodids, the sixth being the adult. Harpacticoid life history traits known from the field or from cohort analyses include differential fecundity, stage duration and survival, sex ratio, development and life span. Understanding life history traits, i.e. assessing the importance of various biotic and abiotic factors in regulating life cycle patterns, is essential for the interpretation of seasonal variations of population size. Evolutionary stable strategies (EES) of reproductive resource allocation and timing are determined by trade-offs between environmental conditions as well as by intrinsic factors. The latter are the genetic base, depending on the evolutionary history and relatedness of the taxa under consideration which determine their phenotypic plasticity. Most interesting are ontogenetic niche shifts, especially in habitat, food and stage duration, including dormancy phenomena. Harpacticoid life history traits will be discussed in the light of meio- and macrofauna life cycles in general. It seems as if small meiofaunal taxa are not able to proliferate pelagic larvae of sufficient quantity, because larvae need a certain size to become functional. Instead, meiofaunal taxa switch to a trait of direct development and even brood care in some cases. It is hypothesized that the Harpacticoida are the only meiofaunal taxa to develop larval stages because their nauplii share the same habitat as the adults and no risky habitat transition will occur as in the pelagic larvae of the macrofauna. | 3666 | NA | NA | NA | no | no |
| 3666 | 2017 | NA | Pellio, B; Streng, S; Bauer, E; Stein, N; Perovic, D; Schiemann, A; Friedt, W; Ordon, F; Graner, A | 2005 | High-resolution mapping of the Rym4/Rym5 locus conferring resistance to the barley yellow mosaic virus complex (BaMMV, BaYMV, BaYMV-2) in barley (Hordeum vulgare ssp vulgare L.) | Soil-borne barley yellow mosaic virus disease caused by a complex of at least three viruses, i.e. Barley mild mosaic virus ( BaMMV), Barley yellow mosaic virus (BaYMV) and BaYMV- 2 - is one of the most important diseases of winter barley in Europe. The two genes rym4, effective against BaMMV and BaYMV, and rym5, additionally effective against BaYMV- 2, comprise a complex locus on chromosome 3HL, which is of special importance to European barley breeding. To provide the genetic basis for positional cloning of the Rym4/Rym5 locus, two high-resolution maps were constructed based on co-dominant flanking markers (MWG838/Y57c10 - MWG010/ Bmac29). Mapping at a resolution of about 0.05% rec., rym4 has been located 1.07% recombination distal of marker MWG838 and 1.21% recombination proximal to marker MWG010. Based on a population size of 3,884 F-2 plants (0.013% recombination) the interval harbouring rym5 was delimited to 1.49 +/- 0.14% recombination. By testing segmental recombinant inbred lines (RILs) for reaction to the different viruses at a resolution of 0.05% rec. (rym4) and 0.019% rec. (rym5), no segregation concerning the reaction to the different viruses could be observed. AFLP-based marker saturation for rym4, using 932 PstI+2/MseI+3 primer combinations only resulted in three markers with the closest one linked at 0.9% recombination to the gene. Two of these markers detected epialleles arising from the differential cytosine methylation of PstI sites. Regarding rym5, pro. ling of 1,200 RAPD primers ( about 18,000 loci) and 2,048 EcoRI+3/MseI+3 AFLP primer combinations ( about 205,000 loci) resulted in one RAPD marker and seven AFLP markers tightly linked to the resistance gene. Flanking markers with the closest linkage to rym5 ( 0.05% and 0.88% recombination) were converted into STS markers. These markers provide a starting point for chromosomal walking and may be exploited in marker-assisted selection for virus resistance based on rym5. | 3667 | NA | NA | NA | no | no |
| 3667 | 2017 | NA | Esposito, S; Piralla, A; Zampiero, A; Bianchini, S; Di Pietro, G; Scala, A; Pinzani, R; Fossali, E; Baldanti, F; Principi, N | 2015 | Characteristics and Their Clinical Relevance of Respiratory Syncytial Virus Types and Genotypes Circulating in Northern Italy in Five Consecutive Winter Seasons | In order to investigate the genetic diversity and patterns of the co-circulating genotypes of respiratory syncytial virus (RSV) and their possible relationships with the severity of RSV infection, we studied all of the RSV-positive nasopharyngeal samples collected from children during five consecutive winters (2009-2010, 2010-2011, 2011-2012, 2012-2013 and 2013-2014). The RSVs were detected using the respiratory virus panel fast assay and single-tube RT-PCR, their nucleotides were sequenced, and they were tested for positive selection. Of the 165 positive samples, 131 (79.4%) carried RSV-A and 34 (20.6%) RSV-B; both groups co-circulated in all of the study periods, with RSV-A predominating in all the seasons except for winter 2010-2011, which had a predominance of RSV-B. Phylogenetic analysis of the RSV-A sequences identified genotypes NA1 and ON1, the second replacing the first during the last two years of the study period. The RSV-B belonged to genotypes BA9 and BA10. BA9 was detected in all the years of the study whereas BA only desultorily. Comparison of the subjects infected by RSV-A and RSV-B types did not reveal any significant differences, but the children infected by genotype A/NA1 more frequently had lower respiratory tract infections (p<0.0001) and required hospitalisation (p = 0.007) more often than those infected by genotype A/ON1. These findings show that RSV has complex patterns of circulation characterised by the periodical replacement of the predominant genotypes, and indicate that the circulation and pathogenic role of the different RSV strains should be investigated as each may have a different impact on the host. A knowledge of the correlations between types, genotypes and disease severity may also be important in order to be able to include the more virulent strains in future vaccines. | 3668 | NA | NA | NA | no | no |
| 3668 | 2017 | NA | Pelabon, C; Larsen, LK; Bolstad, GH; Viken, A; Fleming, IA; Rosenqvist, G | 2014 | The effects of sexual selection on life-history traits: an experimental study on guppies | Sexual selection is often prevented during captive breeding in order to maximize effective population size and retain genetic diversity. However, enforcing monogamy and thereby preventing sexual selection may affect population fitness either negatively by preventing the purging of deleterious mutations or positively by reducing sexual conflicts. To better understand the effect of sexual selection on the fitness of small populations, we compared components of female fitness and the expression of male secondary sexual characters in 19 experimental populations of guppies (Poecilia reticulata) maintained under polygamous or monogamous mating regimes over nine generations. In order to generate treatments that solely differed by their level of sexual selection, the middle-class neighbourhood breeding design was enforced in the monogamous populations, while in the polygamous populations, all females contributed similarly to the next generation with one male and one female offspring. This experimental design allowed potential sexual conflicts to increase in the polygamous populations because selection could not operate on adult-female traits. Clutch size and offspring survival showed a weak decline from generation to generation but did not differ among treatments. Offspring size, however, declined across generations, but more in monogamous than polygamous populations. By generation eight, orange- and black-spot areas were larger in males from the polygamous treatment, but these differences were not statistically significant. Overall, these results suggest that neither sexual conflict nor the purging of deleterious mutation had important effects on the fitness of our experimental populations. However, only few generations of enforced monogamy in a benign environment were sufficient to negatively affect offspring size, a trait potentially crucial for survival in the wild. Sexual selection may therefore, under certain circumstances, be beneficial over enforced monogamy during captive breeding. | 3669 | NA | NA | NA | no | no |
| 3669 | 2017 | NA | Yinda, CK; Zell, R; Deboutte, W; Zeller, M; Conceicao-Neto, N; Heylen, E; Maes, P; Knowles, NJ; Ghogomu, SM; Van Ranst, M; Matthijnssens, J | 2017 | Highly diverse population of Picornaviridae and other members of the Picornavirales, in Cameroonian fruit bats | Background: The order Picornavirales represents a diverse group of positive-stranded RNA viruses with small non-enveloped icosahedral virions. Recently, bats have been identified as an important reservoir of several highly pathogenic human viruses. Since many members of the Picornaviridae family cause a wide range of diseases in humans and animals, this study aimed to characterize members of the order Picornavirales in fruit bat populations located in the Southwest region of Cameroon. These bat populations are frequently in close contact with humans due to hunting, selling and eating practices, which provides ample opportunity for interspecies transmissions. Results: Fecal samples from 87 fruit bats (Eidolon helvum and Epomophorus gambianus), were combined into 25 pools and analyzed using viral metagenomics. In total, Picornavirales reads were found in 19 pools, and (near) complete genomes of 11 picorna-like viruses were obtained from 7 of these pools. The picorna-like viruses possessed varied genomic organizations (monocistronic or dicistronic), and arrangements of gene cassettes. Some of the viruses belonged to established families, including the Picornaviridae, whereas others clustered distantly from known viruses and most likely represent novel genera and families. Phylogenetic and nucleotide composition analyses suggested that mammals were the likely host species of bat sapelovirus, bat kunsagivirus and bat crohivirus, whereas the remaining viruses (named bat iflavirus, bat posalivirus, bat fisalivirus, bat cripavirus, bat felisavirus, bat dicibavirus and bat badiciviruses 1 and 2) were most likely diet-derived. Conclusion: The existence of a vast genetic variability of picorna-like viruses in fruit bats may increase the probability of spillover infections to humans especially when humans and bats have direct contact as the case in this study site. However, further screening for these viruses in humans will fully indicate their zoonotic potential. | 3670 | NA | NA | NA | no | no |
| 3670 | 2017 | NA | Schiegg, K; Walters, JR; Priddy, JA | 2002 | The consequences of disrupted dispersal in fragmented red-cockaded woodpecker Picoides borealis populations | 1.Habitat fragmentation may adversely affect animal populations through several mechanisms. However, little is known about how the impacts of some of these mechanisms are manifested in altered dynamics of wild populations. 2. We used a spatially explicit individual-based simulation model to examine the potential effects of disrupted dispersal due to fragmentation on the population dynamics of the endangered, co-operatively breeding, red-cockaded woodpecker Picoides borealis. 3. We simulated population dynamics as a function of population size and spatial aggregation of territories. Dispersal success (but not mortality or fecundity) was an emergent property of model runs. In the model all female and some male fledglings dispersed in straight lines in random directions, and the remaining males stayed on their natal territories as helpers and competed for breeding vacancies in their immediate neighbourhood. 4. Population trend was tied to the higher dispersal success of both males and females in larger and less fragmented populations. Helpers were more successful than dispersing males. Male breeder recruitment depended entirely on helpers when populations were small (25 or 100 territories). 5. Declining populations were characterized by high emigration rates and both failure and delay in female recruitment. The large numbers of unpaired males resulted in lowered reproductive output at the population level and in the loss of territories. Populations of 25 territories were stable when territories were highly aggregated, despite high emigration rates. These results closely match empirical observations. 6. A number of co-operatively breeding species are endangered. The unusual dispersal behaviour of helpers may make such species sensitive to habitat fragmentation but also resilient to reductions in population size when territories are aggregated. Small populations of co-operative breeders may have considerable conservation value as a source of genetic diversity.   This is the content viewer section. Skip to metadata section. 1 of 12 pages 1. Habitat fragmentation may adversely affect animal populations through several mechanisms. However, little is known about how the impacts of some of these mechanisms are manifested in altered dynamics of wild populations. 2. We used a spatially explicit individual-based simulation model to examine the potential effects of disrupted dispersal due to fragmentation on the population dynamics of the endangered, co-operatively breeding, red-cockaded woodpecker Picoides borealis. 3. We simulated population dynamics as a function of population size and spatial aggregation of territories. Dispersal success (but not mortality or fecundity) was an emergent property of model runs. In the model all female and some male fledglings dispersed in straight lines in random directions, and the remaining males stayed on their natal territories as helpers and competed for breeding vacancies in their immediate neighbourhood. 4. Population trend was tied to the higher dispersal success of both males and females in larger and less fragmented populations. Helpers were more successful than dispersing males. Male breeder recruitment depended entirely on helpers when populations were small (25 or 100 territories). 5. Declining populations were characterized by high emigration rates and both failure and delay in female recruitment. The large numbers of unpaired males resulted in lowered reproductive output at the population level and in the loss of territories. Populations of 25 territories were stable when territories were highly aggregated, despite high emigration rates. These results closely match empirical observations. 6. A number of co-operatively breeding species are endangered. The unusual dispersal behaviour of helpers may make such species sensitive to habitat fragmentation but also resilient to reductions in population size when territories are aggregated. Small populations of co-operative breeders may have considerable conservation value as a source of genetic diversity. | 3671 | NA | NA | NA | no | no |
| 3671 | 2017 | NA | Zhang, YW; Pan, W | 2015 | Principal Component Regression and Linear Mixed Model in Association Analysis of Structured Samples: Competitors or Complements? | Genome-wide association studies (GWAS) have been established as a major tool to identify genetic variants associated with complex traits, such as common diseases. However, GWAS may suffer from false positives and false negatives due to confounding population structures, including known or unknown relatedness. Another important issue is unmeasured environmental risk factors. Among many methods for adjusting for population structures, two approaches stand out: one is principal component regression (PCR) based on principal component analysis, which is perhaps the most popular due to its early appearance, simplicity, and general effectiveness; the other is based on a linear mixed model (LMM) that has emerged recently as perhaps the most flexible and effective, especially for samples with complex structures as in model organisms. As shown previously, the PCR approach can be regarded as an approximation to an LMM; such an approximation depends on the number of the top principal components (PCs) used, the choice of which is often difficult in practice. Hence, in the presence of population structure, the LMM appears to outperform the PCR method. However, due to the different treatments of fixed vs. random effects in the two approaches, we show an advantage of PCR over LMM: in the presence of an unknown but spatially confined environmental confounder (e.g., environmental pollution or lifestyle), the PCs may be able to implicitly and effectively adjust for the confounder whereas the LMM cannot. Accordingly, to adjust for both population structures and nongenetic confounders, we propose a hybrid method combining the use and, thus, strengths of PCR and LMM. We use real genotype data and simulated phenotypes to confirm the above points, and establish the superior performance of the hybrid method across all scenarios. | 3672 | NA | NA | NA | no | no |
| 3672 | 2017 | NA | Chaix, R; Cao, C; Donnelly, P | 2010 | Is Mate Choice in Humans MHC-Dependent? | In several species, including rodents and fish, it has been shown that the Major Histocompatibility Complex (MHC) influences mating preferences and, in some cases, that this may be mediated by preferences based on body odour. In humans, the picture has been less clear. Several studies have reported a tendency for humans to prefer MHC-dissimilar mates, a sexual selection that would favour the production of MHC-heterozygous offspring, who would be more resistant to pathogens, but these results are unsupported by other studies. Here, we report analyses of genome-wide genotype data (from the HapMap II dataset) and HLA types in African and European American couples to test whether humans tend to choose MHC-dissimilar mates. In order to distinguish MHC-specific effects from genome-wide effects, the pattern of similarity in the MHC region is compared to the pattern in the rest of the genome. African spouses show no significant pattern of similarity/dissimilarity across the MHC region (relatedness coefficient, R = 0.015, p = 0.23), whereas across the genome, they are more similar than random pairs of individuals (genome-wide R = 0.00185, p<10(-3)). We discuss several explanations for these observations, including demographic effects. On the other hand, the sampled European American couples are significantly more MHC-dissimilar than random pairs of individuals (R = -0.043, p = 0.015), and this pattern of dissimilarity is extreme when compared to the rest of the genome, both globally (genome-wide R = -0.00016, p = 0.739) and when broken into windows having the same length and recombination rate as the MHC (only nine genomic regions exhibit a higher level of genetic dissimilarity between spouses than does the MHC). This study thus supports the hypothesis that the MHC influences mate choice in some human populations. | 3673 | NA | NA | NA | no | no |
| 3673 | 2017 | NA | Chaix, R; Cao, C; Donnelly, P | 2008 | Is Mate Choice in Humans MHC-Dependent? | In several species, including rodents and fish, it has been shown that the Major Histocompatibility Complex (MHC) influences mating preferences and, in some cases, that this may be mediated by preferences based on body odour. In humans, the picture has been less clear. Several studies have reported a tendency for humans to prefer MHC-dissimilar mates, a sexual selection that would favour the production of MHC-heterozygous offspring, who would be more resistant to pathogens, but these results are unsupported by other studies. Here, we report analyses of genome-wide genotype data (from the HapMap II dataset) and HLA types in African and European American couples to test whether humans tend to choose MHC-dissimilar mates. In order to distinguish MHC-specific effects from genome-wide effects, the pattern of similarity in the MHC region is compared to the pattern in the rest of the genome. African spouses show no significant pattern of similarity/dissimilarity across the MHC region (relatedness coefficient, R = 0.015, p = 0.23), whereas across the genome, they are more similar than random pairs of individuals (genome-wide R = 0.00185, p, 10 23). We discuss several explanations for these observations, including demographic effects. On the other hand, the sampled European American couples are significantly more MHC-dissimilar than random pairs of individuals (R = 20.043, p = 0.015), and this pattern of dissimilarity is extreme when compared to the rest of the genome, both globally (genome-wide R = 20.00016, p = 0.739) and when broken into windows having the same length and recombination rate as the MHC (only nine genomic regions exhibit a higher level of genetic dissimilarity between spouses than does the MHC). This study thus supports the hypothesis that the MHC influences mate choice in some human populations. | 3674 | NA | NA | NA | no | no |
| 3674 | 2017 | NA | Huguet, G; Nava, C; Lemiere, N; Patin, E; Laval, G; Ey, E; Brice, A; Leboyer, M; Szepetowski, P; Gillberg, C; Depienne, C; Delorme, R; Bourgeron, T | 2014 | Heterogeneous Pattern of Selective Pressure for PRRT2 in Human Populations, but No Association with Autism Spectrum Disorders | Inherited and de novo genomic imbalances at chromosome 16p11.2 are associated with autism spectrum disorders (ASD), but the causative genes remain unknown. Among the genes located in this region, PRRT2 codes for a member of the synaptic SNARE complex that allows the release of synaptic vesicles. PRRT2 is a candidate gene for ASD since homozygote mutations are associated with intellectual disability and heterozygote mutations cause benign infantile seizures, paroxysmal dyskinesia, or hemiplegic migraine. Here, we explored the contribution of PRRT2 mutations in ASD by screening its coding part in a large sample of 1578 individuals including 431 individuals with ASD, 186 controls and 961 individuals from the human genome Diversity Panel. We detected 24 nonsynonymous variants, 1 frameshift (A217PfsX8) and 1 in-frame deletion of 6 bp (p. A361\_P362del). The frameshift mutation was observed in a control with no history of neurological or psychiatric disorders. The p. A361\_P362del was observed in two individuals with autism from sub-Saharan African origin. Overall, the frequency of PRRT2 deleterious variants was not different between individuals with ASD and controls. Remarkably, PRRT2 displays a highly significant excess of nonsynonymous (pN) vs synonymous (pS) mutations in Asia (pN/pS=4.85) and Europe (pN/pS=1.62) compared with Africa (pN/pS=0.26; Asia vs Africa: P=0.000087; Europe vs Africa P=0.00035; Europe vs Asia P=P=0.084). We also showed that whole genome amplification performed through rolling cycle amplification could artificially introduce the A217PfsX8 mutation indicating that this technology should not be performed prior to PRRT2 mutation screening. In summary, our results do not support a role for PRRT2 coding sequence variants in ASD, but provide an ascertainment of its genetic variability in worldwide populations that should help researchers and clinicians to better investigate the role of PRRT2 in human diseases. | 3675 | NA | NA | NA | no | no |
| 3675 | 2017 | NA | Gruber-Hadden, NW; Messmer, TA; Maxfield, BD; Koons, DN; Guttery, MR | 2016 | Population vital rates of resident and translocated female greater sage-grouse | Translocations have been recommended to reestablish, augment, and sustain genetic diversity in declining wildlife populations, including greater sage-grouse (Centrocercus urophasianus; sage-grouse). Characteristics of successful sage-grouse translocations include suitable contiguous sagebrush (Artemisia spp.), seasonal habitats surrounded by geomorphic barriers, a residual resident population, and pre-nesting releases. From 2009 to 2010, we studied vital rates of 60 translocated and 15 resident radio-marked female sage-grouse and their broods on Anthro Mountain, in the Ashley National Forest, northeastern Utah, USA to determine whether translocations could augment a declining meta-population that inhabited suitable breeding habitats in a small spatially isolated landscape. Survival rates, and nest and brood success estimates for the resident and translocated sage-grouse we studied were lower than reported range-wide averages. Nest success was similar for resident and translocated birds (survival estimate: 0.468, 95% CI=0.288-0.648) and we calculated weak, yet positive relationships to grass height and grass cover. Daily survival rates for chicks 0-19 days of age for resident females in 2009 (0.904, CI=0.875-0.940) and in 2010 (0.910, CI=0.888-0.945) were higher than for chicks raised by translocated females (0.883, CI=0.850-0.915; 0.892, CI=0.856-0.936, respectively), although differences were small and all 95% confidence intervals had substantial overlap. In 2009 and 2010, daily survival rates for chick 20-50 days of age were slightly higher for chicks reared by resident females (0.980, CI=0.963-0.994; 0.998, CI=0.978-1.000, respectively) than chicks reared by translocated females (0.877, CI=0.623-0.959; 0.988, CI=0.945-0.993, respectively). Chick survival for both groups was weakly correlated with grass cover. Although most translocated birds remained on the study area (82%), the low overall survival rates we report indicate that managers need to consider factors affecting survival of adult females, nests, and chicks when planning future translocations to augment sage-grouse meta-populations that occupy spatially isolated and space-limited habitats. (c) 2016 The Wildlife Society. | 3676 | NA | NA | NA | no | no |
| 3676 | 2017 | NA | Guan, DW; van der Sanden, S; Zeng, HR; Li, W; Zheng, HY; Ma, C; Su, J; Liu, Z; Guo, X; Zhang, X; Liu, L; Koopmans, M; Ke, CW | 2012 | Population Dynamics and Genetic Diversity of C4 Strains of Human Enterovirus 71 in Mainland China, 1998-2010 | Background: Since 1997, several countries within the Asian Pacific region have been affected by one or more massive outbreaks of Hand Foot and Mouth Disease (HFMD). Virus typing experiments revealed that these outbreaks were caused by strains of human enterovirus 71 (EV71) belonging to several different, recently emerged subgenogroups. In mainland China, a different situation was observed. The first outbreak, localized in Shangdong Province, was reported in 2007, and was followed by a wide-spread outbreak in mainland China in 2008. Since then, numbers of reported HFMD cases have been persistently high. Methodology/Principal Findings: To gain insight in the epidemiological behavior of EV71 in China, we studied genetic diversity and EV71 population dynamics to address whether the increase in number of reported EV71 infections reflects a real increase in viral spread or is just the result of increased awareness and surveillance. We used systematically collected VP1 gene sequences of 257 EV71 strains collected in Guangdong province from 2008 to 2010 as part of HFMD surveillance activities, and supplemented them with 305 GenBank EV71 reference stains collected in China from 1998 to 2010. All isolates from Guangdong Province belonged to subgenogroup C4. Viral population dynamics indicated that the increased reporting of HFMD in China since 2007 reflects a real increase in viral spread and continued replacement of viral lineages through time. Amino acid sequence comparisons revealed substitution of amino acid in residues 22, 145 and 289 through time regularly with the VP1 gene of EV71 strains isolated in mainland China from 1998 to 2010. Conclusions: EV71 strains isolated in mainland China mainly belonged to subgenogroup C4. There was exponential growth of the EV71 virus population in 2007 and 2008. There was amino acid substitution through time regularly with the VP1 gene which possibly increased viral spread and/or ability of the virus to circulate persistently among the Chinese population. | 3677 | NA | NA | NA | no | no |
| 3677 | 2017 | NA | Lukas, D; Clutton-Brock, T | 2012 | Life histories and the evolution of cooperative breeding in mammals | While the evolution of cooperative breeding systems (where non-breeding helpers participate in rearing young produced by dominant females) has been restricted to lineages with socially monogamous mating systems where coefficients of relatedness between group members are usually high, not all monogamous lineages have produced species with cooperative breeding systems, suggesting that other factors constrain the evolution of cooperative breeding. Previous studies have suggested that life-history parameters, including longevity, may constrain the evolution of cooperative breeding. Here, we show that transitions to cooperative breeding across the mammalian phylogeny have been restricted to lineages where females produce multiple offspring per birth. We find no support for effects of longevity or of other life-history parameters. We suggest that the evolution of cooperative breeding has been restricted to monogamous lineages where helpers have the potential to increase the reproductive output of breeders. | 3678 | NA | NA | NA | no | no |
| 3678 | 2017 | NA | Hurtado, AM; Hill, KR; Rosenblatt, W; Bender, J; Scharmen, T | 2003 | Longitudinal study of tuberculosis outcomes among immunologically naive Ache natives of Paraguay | This study documents the course of a tuberculosis epidemic in an immunologically naive group of South American Indians within fewer than 20 years after first sustained contact with outsiders. Groups of Northern Ache (ah-CHAY) of eastern Paraguay were contacted and settled on reservations between 1971-1979. Not surprisingly, the Ache are very susceptible to tuberculosis, and the epidemiological characteristics of the disease are quite different from those of populations that have had tuberculosis for centuries. Within 6 years of the first detected case of tuberculosis among the Ache, the prevalence rate of active tuberculosis cases reached 18.2%, and of infected cases among adults, 64.6%, some of the highest rates ever reported for any human group. Remarkably, males and females are equally likely to have been diagnosed with active tuberculosis, Ache children between birth and 5 years of age are least vulnerable to tuberculosis, high nutritional and socioeconomic status do not decrease the risk of disease or infection, and children immunized with BCG are less responsive to tuberculin challenge than are other children. Moreover, similar to the Yanomamo, but unlike populations of European or African descent, a high percentage of Ache with active disease test negative on tuberculin challenge tests (purified protein derivative; PPD). These differences may be due to a high prevalence of diminished cell-mediated immunity, and T-helper 2 dominance. We also hypothesize that these immunological characteristics, low genetic diversity, hostile intergroup interactions, and behavioral noncompliance to treatment protocols together contribute to the high rates of active disease observed. Existing tuberculosis control programs are poorly equipped to handle the impact of these causal complexities on the course of recent tuberculosis epidemics that have quickly spread throughout native communities of Latin America during the last decade. (C) 2003 Wiley-Liss, Inc. | 3679 | NA | NA | NA | no | no |
| 3679 | 2017 | NA | Martynov, SP; Dobrotvorskaya, TV; Krupnov, VA | 2016 | Genealogical analysis of the use of two wheatgrass (Agropyron) species in common wheat (Triticum aestivum L.) breeding for disease resistance | During the last 80 years, in order to increase the genetic variability of wheat, translocations containing nine elongated wheatgrass (Agropyron elongatum) and eight intermediate wheatgrass (Agropyron intermedium) genes, which control resistance to pathogens, were transferred to this crop culture. Genealogical and statistical analysis of 1500 varieties developed using the wheatgrass gave evidence of the continuing increase in the proportion of such varieties in the total number of wheat varieties over the last half-century. Translocations from Ag. elongatum most commonly occur in the pedigrees of the varieties from the United States, less frequently they can be found in Australian and Chinese varieties, and they are extremely rare-in European and African ones. Ag. intermedium most frequently occurs in the pedigrees of the Eastern European varieties, mainly in those from Russia, as well as in the varieties from China. The observed uneven distribution of such varieties may be associated with either the effectiveness of the translocation in the development of resistance to the local populations of pathogens or with the effect of the translocation on the adaptive traits of plants. By computer tracking of pedigrees, we performed an inventory of the translocation donors from Ag. elongatum and Ag. intermedium used in the breeding programs in the United States, Russia, Australia, India, and China. The most widely occurring combinations of the gene complex Lr24/Sr24 of Ag. elongatum with other resistance genes were revealed. In Russia, there were developed varieties in which the 6D chromosome was substituted by the 6Ai chromosome of Ag. intermedium, which controls disease resistance and the adaptivity of plants. The identification and introgression of new translocations indicates that the possibilities of using wheatgrass species for broadening of genetic variability of wheat are far from being exhausted. | 3680 | NA | NA | NA | no | no |
| 3680 | 2017 | NA | Franco, A; Hendriksen, RS; Lorenzetti, S; Onorati, R; Gentile, G; Dell’Omo, G; Aarestrup, FM; Battisti, A | 2011 | Characterization of Salmonella Occurring at High Prevalence in a Population of the Land Iguana Conolophus subcristatus in Galapagos Islands, Ecuador | The aim of the study was to elucidate the association between the zoonotic pathogen Salmonella and a population of land iguana, Colonophus subcristatus, endemic to Galapagos Islands in Ecuador. We assessed the presence of Salmonella subspecies and serovars and estimated the prevalence of the pathogen in that population. Additionally, we investigated the genetic relatedness among isolates and serovars utilising pulsed field gel electrophoresis (PFGE) on XbaI-digested DNA and determined the antimicrobial susceptibility to a panel of antimicrobials. The study was carried out by sampling cloacal swabs from animals (n = 63) in their natural environment on in the island of Santa Cruz. A high prevalence (62/63, 98.4%) was observed with heterogeneity of Salmonella subspecies and serovars, all known to be associated with reptiles and with reptile-associated salomonellosis in humans. Serotyping revealed 14 different serovars among four Salmonella enterica subspecies: S. enterica subsp. enterica (n = 48), S. enterica subsp. salamae (n = 2), S. enterica subsp. diarizonae (n = 1), and S. enterica subsp. houtenae (n = 7). Four serovars were predominant: S. Poona (n = 18), S. Pomona (n = 10), S. Abaetetuba (n = 8), and S. Newport (n = 5). The S. Poona isolates revealed nine unique XbaI PFGE patterns, with 15 isolates showing a similarity of 70%. Nine S. Pomona isolates had a similarity of 84%. One main cluster with seven (88%) indistinguishable isolates of S. Abaetetuba was observed. All the Salmonella isolates were pan-susceptible to antimicrobials representative of the most relevant therapeutic classes. The high prevalence and absence of clinical signs suggest a natural interaction of the different Salmonella serovars with the host species. The interaction may have been established before any possible exposure of the iguanas and the biocenosis to direct or indirect environmental factors influenced by the use of antimicrobials in agriculture, in human medicine or in veterinary medicine. | 3681 | NA | NA | NA | no | no |
| 3681 | 2017 | NA | Sanjuan, R; Cuevas, JM; Furio, V; Holmes, EC; Moya, A | 2007 | Selection for robustness in mutagenized RNA viruses | Mutational robustness is defined as the constancy of a phenotype in the face of deleterious mutations. Whether robustness can be directly favored by natural selection remains controversial. Theory and in silico experiments predict that, at high mutation rates, slow-replicating genotypes can potentially outcompete faster counterparts if they benefit from a higher robustness. Here, we experimentally validate this hypothesis, dubbed the "survival of the flattest,’’ using two populations of the vesicular stomatitis RNA virus. Characterization of fitness distributions and genetic variability indicated that one population showed a higher replication rate, whereas the other was more robust to mutation. The faster replicator outgrew its robust counterpart in standard competition assays, but the outcome was reversed in the presence of chemical mutagens. These results show that selection can directly favor mutational robustness and reveal a novel viral resistance mechanism against treatment by lethal mutagenesis. | 3682 | NA | NA | NA | no | no |
| 3682 | 2017 | NA | Heinze, J; Rueppell, O | 2014 | The frequency of multi-queen colonies increases with altitude in a Nearctic ant | 0 | 3683 | NA | NA | NA | no | no |
| 3683 | 2017 | NA | Sun, JJ; Underwood, DLA | 2011 | Maintenance of sociality in a communal caterpillar, Eucheira socialis westwoodi (Lepidoptera: Pieridae) | Background: The theory of kin selection fails to explain the maintenance of gregariousness in insects with low levels of relatedness. Alternative proposals suggest that other factors such as group thermodynamic efficiency or predator avoidance are greatly enhanced by insects living socially, and therefore might serve as reinforcements for costly social behaviours exhibited by these species independent of relatedness. Questions: Do Eucheira socialis westwoodi (Lepidoptera: Pieridae) females place their egg masses near conspecific egg masses? Does the distance between egg masses influence the survival of the larvae hatched from these egg masses? Does group size influence larval foraging success? Organism: Eucheira socialis westwoodi larvae from naturally occurring colonies in El Madrono, Durango, Mexico. Methods: We assessed the distribution of egg masses on trees and across trees. Larvae from egg masses oviposited at variable distances from conspecific egg masses were followed until the fourth instar. Differently sized larval groups were constructed and maintained on ‘artificial trees’ using sprigs collected from a single host tree (Arbutus xalapensis: Ericaceae). These were in turn deployed to a semi-natural area. Frequency and duration of foraging of individually marked larvae were recorded daily and subsequently analysed. Conclusions: Egg masses were significantly clumped on trees and across trees. Larvae from egg masses oviposited near other egg masses coalesced into larger groups and had higher survival rates than larvae from isolated egg masses. Larvae in manipulated groups of 400 individuals fed more frequently and for longer, gained more weight, and had greater survivorship than larvae in smaller groups. These results suggest that larval foraging success serves as reinforcement for the gregarious behaviour seen in E. s. westwoodi larvae and also may drive selection in the oviposition preference of adult females of this species. | 3684 | NA | NA | NA | no | no |
| 3684 | 2017 | NA | Rajebhosale, MD; Chowdari, KV; Ramakrishna, W; Tamhankar, SA; Gupta, VS; Gnanamanickam, SS; Ranjekar, PK | 1997 | DNA fingerprinting of Indian isolates of Xanthomonas oryzae pv oryzae | A high level of genetic polymorphism was detected among Indian isolates of Xanthomonas oryzae pv. oryzae using hypervariable probes such as a microsatellite oligonucleotide, probe (TG)(10), a human minisatellite probe, pV47, an avirulence gene probe, avrXa10 and a repeat clone, pBS101. These DNA probes detected multiple loci in the bacterial genome generating complex DNA fingerprints and differentiated all of the bacterial isolates. Analysis of fingerprints indicated that pV47, (TG)(10) and pBS101 have a lower probability of identical match than avrXa10 and therefore are potential probes for DNA fingerprinting and variability analysis of Xanthamonas oryzae pv. oryzae pathogen populations. Cluster analysis based on hybridization patterns using all of the above probes showed five groups at 56% similarity. Studies on the methylation patterns of isolates representing the three important races of X. oryzae pv. oryzae indicated more methylation in the most virulent isolate, suggesting a possible role of methylation in pathogenicity. | 3685 | NA | NA | NA | no | no |
| 3685 | 2017 | NA | Delaney, DA; Keller, JJ; Caren, JR; Tarpy, DR | 2011 | The physical, insemination, and reproductive quality of honey bee queens (Apis mellifera L.) | Understanding the reproductive potential (“quality”) of queens bees can provide valuable insights into factors that influence colony phenotype. We assayed queens from various commercial sources for various measures of potential queen quality, including their physical characters (such as their degree of parasitism), insemination number (stored sperm counts), and effective paternity frequency (number of drone fathers among their offspring). We found significant variation in the physical, insemination, and mating quality of commercially produced queens, and we detected significant correlations within and among these various measures. Overall, the queens were sufficiently inseminated (3.99 +/- 1.504 million sperm) and mated with an appropriate number of drones (effective paternity frequency: 16.0 +/- 9.48). Importantly, very few of the queens were parasitized by tracheal mites and none were found with either Nosema species. These findings suggest possible mechanisms for assessing the potential fitness of honey bee queens without the need for destructive sampling. | 3686 | NA | NA | NA | no | no |
| 3686 | 2017 | NA | Lord, CC; Barnard, B; Day, K; Hargrove, JW; McNamara, JJ; Paul, REL; Trenholme, K; Woolhouse, MEJ | 1999 | Aggregation and distribution of strains in microparasites | Recent research has shown that many parasite populations are made up of a number of epidemiologically distinct strains or genotypes. The implications of strain structure or genetic diversity for parasite population dynamics are still uncertain, partly because there is no coherent framework for the interpretation of field data. Here, we present an analysis of four published data sets for vector-borne microparasite infections where strains or genotypes have been distinguished: serotypes of African horse sickness (AHS) in zebra; types of Nannomonas trypanosomes in tsetse flies; parasite-induced erythrocyte surface antigen (PIESA) based isolates of Plasmodium falciparum malaria in humans, and the merozoite surface protein 2 gene (MSP-2) alleles of P. falciparum in humans and in anopheline mosquitoes. For each data set we consider the distribution of strains or types among hosts and any pairwise associations between strains or types. Where host age data are available we also compare age-prevalence relationships and estimates of the force of infection. Multiple infections of hosts are common and for most data sets infections have an aggregated distribution among hosts with a tendency towards positive associations between certain strains or types. These patterns could result from interactions (facilitation) between strains or types, or they could reflect patterns of contact between hosts and vectors. We use a mathematical model to illustrate the impact of host-vector contact patterns, finding that even if contact is random there may still be significant aggregation in parasite distributions. This effect is enhanced if there is non-random contact or other heterogeneities between hosts, vectors or parasites. In practice, different strains or types also have different forces of infection. We anticipate that aggregated distributions and positive associations between microparasite strains or types will be extremely common. | 3687 | NA | NA | NA | no | no |
| 3687 | 2017 | NA | Rogell, B; Berglund, A; Laurila, A; Hoglund, J | 2011 | Population divergence of life history traits in the endangered green toad: implications for a support release programme | There is an increasing awareness that adaptive differences among local populations may affect the success of translocation programmes. A mismatch in habitat quality of the target localities and in the local adaptations of the translocated individuals may reduce the success rate of the translocation programme. The green toad Bufo viridis is the most threatened amphibian in Sweden and has been the focus of an extensive translocation programme of eggs, tadpoles and juvenile toads to several localities with apparently favourable conditions for green toads. However, the success of these measures has been poor. In this study, we investigated the extent of local adaptation in the green toad by examining population divergence and the effect of thermal and saline conditions on larval performance in four Scandinavian populations. In a common garden experiment, we measured larval survival and development as well as the occurrence of spinal deformations. In addition, we quantified pond temperature and water salinity, two important environmental variables for larval performance in anurans in the breeding ponds as well as in seven additional localities included in the conservation programme. We found significant variation among the localities in water temperature and salinity, and significant among-population divergence in larval life history traits and spinal deformations, including both trait means and plastic responses to salinity and temperature. The available evidence suggests that at least part of this divergence is adaptive. We did not find direct support for local adaptation affecting the success of the translocations, however, we argue that the population origin and the impact of rearing conditions on the fitness-related larval traits should be taken into account in the introduction measures of the Swedish green toad conservation programme as well as in translocation programmes in general. | 3688 | NA | NA | NA | no | no |
| 3688 | 2017 | NA | Eadie, JM; Lyon, BE | 2011 | The relative role of relatives in conspecific brood parasitism | Conspecific brood parasites lay their eggs in the nests of other females in the same population, leading to a fascinating array of possible games among parasites and their hosts (Davies 2000; Lyon & Eadie 2008). Almost 30 years ago, Andersson & Eriksson (1982) first suggested that perhaps this form of parasitism was not what it seemed-indeed, perhaps it was not parasitism at all! Andersson & Eriksson (1982) observed that conspecific brood parasitism (CBP) was disproportionally common in waterfowl (Anatidae), a group of birds for which natal philopatry is female-biased rather than the more usual avian pattern of male-biased natal philopatry. Accordingly, Andersson (1984) reasoned (and demonstrated in an elegantly simple model) that relatedness among females might facilitate the evolution of CBP-prodding us to reconsider it as a kin-selected and possibly cooperative breeding system rather than a parasitic interaction. The idea was much cited but rarely tested empirically until recently-a number of new studies, empowered with a battery of molecular techniques, have now put Anderssons hypothesis to the test (Table 1). The results are tantalizing, but also somewhat conflicting. Several studies, focusing on waterfowl, have found clear evidence that hosts and parasites are often related (Andersson & Ahlund 2000; Roy Nielsen 2006; Andersson & Waldeck 2007; Waldeck 2008; Jaatinen 2009; Tiedemann . 2011). However, this is not always the case (Semel & Sherman 2001; Anderholm 2009; and see Poysa 2004). In a new study reported in this issue of Molecular Ecology, Jaatinen (2011a) provide yet another twist to this story that might explain not only why such variable results have been obtained, but also suggests that the games between parasites and their hosts-and the role of kinship in these games-may be even more complex than Andersson (1984) imagined. Indeed, the role of kinship in CBP may be very much one of relative degree! | 3689 | NA | NA | NA | no | no |
| 3689 | 2017 | NA | Capelle, J; Neema, C | 2005 | Local adaptation and population structure at a micro-geographical scale of a fungal parasite on its host plant | Local adaptation, which has been detected for several wild pathosystems is influenced by gene flow and recombination. In this study, we investigate local adaptation and population structure at a fine scale in wild populations of a plant-pathogen fungus. We sampled hierarchically strains of Colletotrichum lindemuthianum in a wild population of its host. The analysis of AFLP patterns obtained for 86 strains indicated that: (i) many different haplotypes can be discriminated, although occurrence of recombination could not be shown; (ii) migration between adjacent plants seemed rare during the season; and (iii) neutral diversity is structured according to groups of plants and individual host plants. Furthermore, we tested for the occurrence of local adaptation using a cross-inoculation experiment. Our results showed local adaptation at the scale of the individual host plant. These results indicate that fine-scale dynamics has evolutionary consequences in this pathosystem. | 3690 | NA | NA | NA | no | no |
| 3690 | 2017 | NA | Gilk, SE; Wang, IA; Hoover, CL; Smoker, WW; Taylor, SG; Gray, AK; Gharrett, AJ | 2004 | Outbreeding depression in hybrids between spatially separated pink salmon, Oncorhynchus gorbuscha, populations: marine survival, homing ability, and variability in family size | Hybridization between distinct populations and introgression of nonnative genes can erode fitness of native populations through outbreeding depression, either by producing a phenotype intermediate to that of both contributing genomes (and maladapted in either population’s environment) or by disrupting distinct coadapted complexes of epistatic genes. In salmon, fitness-related traits such as homing ability or family-size distribution may be eroded. We investigated geographically separated pink salmon populations in repeated trials in independent broodyears (odd and even). Hybrids were made between female Auke Creek (Southeast Alaska) pink salmon and Pillar Creek (Kodiak Island, similar to1000 km away) males; hybrids and their offspring were compared to offspring of control crosses of the same females with Auke Creek males. Parentage assignment from microsatellite analysis was used to improve estimates of survival and straying and to examine variation of family size. Hybridization reduced return rates of adults (a proxy for survival at sea) in the F-1 generation in the odd-year broodline (p < 0.0001) but not in the even-year broodline (p = 0.678). Hybridization reduced survival in both the odd- and even-broodyear F-2 (p < 0.005 and p < 0.0001). Hybridization did not appear to impair homing ability; weekly surveys revealed similar straying rates (&SIM;2%) by both hybrid and control fish into nearby (&SIM;1 km) Waydelich Creek in both generations in both trials. Hybridization did not increase the index of variability (σ(2)/μ) in family size. Decreased survival in the hybrid F-2 generation supports an epistatic model of outbreeding depression; nonepistatic effects may have contributed to reduced survival in the odd-broodyear F-1 hybrid fish. Outbreeding depression in hybrids of geographically separated populations demonstrates that introgression of nonnative fish can erode fitness, and should be recognized as a potential detriment of both aquaculture and management practices. | 3691 | NA | NA | NA | no | no |
| 3691 | 2017 | NA | Kurka, H; Ehrenreich, A; Ludwig, W; Monot, M; Rupnik, M; Barbut, F; Indra, A; Dupuy, B; Liebl, W | 2014 | Sequence Similarity of Clostridium difficile Strains by Analysis of Conserved Genes and Genome Content Is Reflected by Their Ribotype Affiliation | PCR-ribotyping is a broadly used method for the classification of isolates of Clostridium difficile, an emerging intestinal pathogen, causing infections with increased disease severity and incidence in several European and North American countries. We have now carried out clustering analysis with selected genes of numerous C. difficile strains as well as gene content comparisons of their genomes in order to broaden our view of the relatedness of strains assigned to different ribotypes. We analyzed the genomic content of 48 C. difficile strains representing 21 different ribotypes. The calculation of distance matrix-based dendrograms using the neighbor joining method for 14 conserved genes (standard phylogenetic marker genes) from the genomes of the C. difficile strains demonstrated that the genes from strains with the same ribotype generally clustered together. Further, certain ribotypes always clustered together and formed ribotype groups, i.e. ribotypes 078, 033 and 126, as well as ribotypes 002 and 017, indicating their relatedness. Comparisons of the gene contents of the genomes of ribotypes that clustered according to the conserved gene analysis revealed that the number of common genes of the ribotypes belonging to each of these three ribotype groups were very similar for the 078/033/126 group (at most 69 specific genes between the different strains with the same ribotype) but less similar for the 002/017 group (86 genes difference). It appears that the ribotype is indicative not only of a specific pattern of the amplified 16S-23S rRNA intergenic spacer but also reflects specific differences in the nucleotide sequences of the conserved genes studied here. It can be anticipated that the sequence deviations of more genes of C. difficile strains are correlated with their PCR-ribotype. In conclusion, the results of this study corroborate and extend the concept of clonal C. difficile lineages, which correlate with ribotypes affiliation. | 3692 | NA | NA | NA | no | no |
| 3692 | 2017 | NA | Brown, CR; Padhi, A; Moore, AT; Brown, MB; Foster, JE; Pfeffer, M; O’Brien, VA; Komar, N | 2009 | Ecological divergence of two sympatric lineages of Buggy Creek virus, an arbovirus associated with birds | Most arthropod-borne viruses (arboviruses) show distinct serological subtypes or evolutionary lineages, with the evolution of different strains often assumed to reflect differences in ecological selection pressures. Buggy Creek virus (BCRV) is an unusual RNA virus (Togaviridae, Alphavirus) that is associated primarily with a cimicid swallow bug (Oeciacus vicarius) as its vector and the Cliff Swallow (Petrochelidon pyrrhonota) and the introduced House Sparrow (Passer domesticus) as its amplifying hosts. There are two sympatric lineages of BCRV (lineages A and B) that differ from each other by >6% at the nucleotide level. Analysis of 385 BCRV isolates all collected from bug vectors at a study site in southwestern Nebraska, USA, showed that the lineages differed in their peak times of seasonal occurrence within a summer. Lineage A was more likely to be found at recently established colonies, at those in culverts (rather than on highway bridges), and at those with invasive House Sparrows, and in bugs on the outsides of nests. Genetic diversity of lineage A increased with bird colony size and at sites with House Sparrows, while that of lineage B decreased with colony size and was unaffected by House Sparrows. Lineage A was more cytopathic on mammalian cells than was lineage B. These two lineages have apparently diverged in their transmission dynamics, with lineage A possibly more dependent on birds and lineage B perhaps more a bug virus. The long-standing association between Cliff Swallows and BCRV may have selected for immunological resistance to the virus by swallows and thus promoted the evolution of the more bug-adapted lineage B. In contrast, the recent arrival of the introduced House Sparrow and its high competence as a BCRV amplifying host may be favoring the more bird-dependent lineage A. | 3693 | NA | NA | NA | no | no |
| 3693 | 2017 | NA | Gunaletchumy, SP; Seevasant, I; Tan, MH; Croft, LJ; Mitchell, HM; Goh, KL; Loke, MF; Vadivelu, J | 2014 | Helicobacter pylori Genetic Diversity and Gastro-duodenal Diseases in Malaysia | Helicobacter pylori infection results in diverse clinical conditions ranging from chronic gastritis and ulceration to gastric adenocarcinoma. Among the multiethnic population of Malaysia, Indians consistently have a higher H. pylori prevalence as compared with Chinese and Malays. Despite the high prevalence of H. pylori, Indians have a relatively low incidence of peptic ulcer disease and gastric cancer. In contrast, gastric cancer and peptic ulcer disease incidence is high in Chinese. H. pylori strains from Chinese strains predominantly belong to the hspEAsia subpopulation while Indian/Malay strains mainly belong to the hspIndia subpopulation. By comparing the genome of 27 Asian strains from different subpopulations, we identified six genes associated with risk of H. pylori-induced peptic ulcer disease and gastric cancer. This study serves as an important foundation for future studies aiming to understand the role of bacterial factors in H. pylori-induced gastro-duodenal diseases. | 3694 | NA | NA | NA | no | no |
| 3694 | 2017 | NA | KARLIN, S; LADUNGA, I | 1994 | COMPARISONS OF EUKARYOTIC GENOMIC SEQUENCES | A method for assessing genomic similarity based on relative abundances of short oligonucleotides in large DNA samples is introduced. The method requires neither homologous sequences nor prior sequence alignments. The analysis centers on (i) dinucleotide (and tri- and tetra-) relative abundance extremes in genomic sequences, (ii) distances between sequences based on all dinucleotide relative abundance values, and (iii) a multidimensional partial ordering protocol. The emphasis in this paper is on assessments of general relatedness of genomes as distinguished from phylogenetic reconstructions. Our methods demonstrate that the relative abundance distances almost always differ more for genomic interspecific sequence comparisons than for genomic intraspecific sequence comparisons, indicating congruence over different genome sequence samples. The genomic comparisons are generally concordant with accepted phylogenies among vertebrate and among fungal species sequences. Several unexpected relationships between the major groups of metazoa, fungal, and protist DNA emerge, including the following. (i) Schizosaccharomyces pombe and Saccharomyces cerevisiae in dinucleotide relative abundance distances are as similar to each other as human is to bovine, (ii) S. cerevisiae, although substantially far from, is significantly closer to the vertebrates than are the invertebrates (Drosophila melanogaster, Bombyx mori, and Caenorhabditis elegans). This phenomenon may suggest variable evolutionary rates during the metazoan radiations and slower changes in the fungal divergences, and/or a polyphyletic origin of metazoa. (iii) The genomic sequences of D. melanogaster and Trypanosoma brucei are strikingly similar. This DNA similarity might be explained by some molecular adaptation of the parasite to its dipteran (tsetse fly) host, a host-parasite gene transfer hypothesis. Robustness of the methods may be due to a genomic signature of dinucleotide relative abundance values reflecting DNA structures related to dinucleotide stacking energies, constraints of DNA curvature, and mechanisms attendant to replication, repair, and recombination. | 3695 | NA | NA | NA | no | no |
| 3695 | 2017 | NA | Dugdale, HL; Ellwood, SA; Macdonald, DW | 2010 | Alloparental behaviour and long-term costs of mothers tolerating other members of the group in a plurally breeding mammal | Cooperative-breeding studies tend to focus on a few alloparental behaviours in highly cooperative species exhibiting high reproductive skew and the associated short-term, but less frequently long-term, fitness costs. We analysed a suite of alloparental behaviours (assessed via filming) in a kin-structured, high-density population of plurally breeding European badgers, Meles meles, which are not highly cooperative. Group members, other than mothers, performed alloparental behaviour; however, this was not correlated with their relatedness to within-group young. Furthermore, mothers babysat, allogroomed cubs without reciprocation, and allomarked cubs more than other members of the group (controlling for observation time). For welfare reasons, we could not individually mark cubs; however, the number observed pre-independence never exceeded that trapped. All 24 trapped cubs, in three filmed groups, were assigned both parents using 22 microsatellites. Mothers may breed cooperatively, as the time they babysat their assigned, or a larger, litter size did not differ. Furthermore, two mothers probably allonursed, as they suckled more cubs than their assigned litter size. An 18-year genetic pedigree, however, detected no short-term (litter size; maternal survival to the following year) or long-term (offspring breeding probability; offspring lifetime breeding success) fitness benefits with more within-group mothers or other members of the group. Rather, the number of other members of the group (excluding mothers) correlated negatively with long-term fitness. Mothers may tolerate other members of the group, as nonbreeders undertook more digging. Our study highlights that alloparental care varies on a continuum from that seen in this high-density badger population, where alloparenting behaviour is minimal, through to species where alloparental care is common and provides fitness benefits. (C) 2010 The Association for the Study of Animal Behaviour. Published by Elsevier Ltd. All rights reserved. | 3696 | NA | NA | NA | no | no |
| 3696 | 2017 | NA | Gautam, R; Singh, SK; Sharma, V | 2016 | Molecular diagnosis and intraspecific genetic variability of root pathogens of arid legumes in Western Rajasthan, India | The productivity of arid legumes, such as Clusterbean (Cyamopsis tetragonoloba), Cowpea (Vigna unguiculata), Moth bean (Vigna aconitifolia) and Horse gram (Macrotylotna unflorum), may remain stagnant over decades because of their high susceptibility to root diseases. Besides, there is a limitation on the information about molecular diagnosis and intraspecific genetic variability of root pathogens in arid legumes. To contribute in this field, we assessed a total of 52 isolates from 88 root samples that were found infected with fungal pathogens in Jodhpur, Jaipur and Bikaner Districts of Rajasthan. Diseased roots samples were analyzed following standard microbiological methods for fungus extraction and purification, and for genetic studies. Irrespective of the geographical location from where the diseased samples were collected, all pathogen isolates were clustered in RAPD dendrograms as per their respective genera. Phylogram, based on multiple sequence alignment, revealed that different genera (i.e. Fusarium, Neocosmospora and Syncephalastrum), separated from each other, and species within the same genera, clustered together with their reference sequences with apreciable bootstrap values. Out of 20 representative isolates representing each cluster and all outgroups sequenced, eight were molecularly identified as Neocosmospora vasinfecta, five as Fusarium solani, two as Neocosmospora striata, two as Fusarium acutatum, one as Syncephalastrum monosporum, one as Fusarium oxysporum and one as Fztsariztm species. The root pathogens of the arid legumes were found neither restricted to a geographical location nor were host specific in nature. Fusarium solani wilt in cowpea and seedling rot in moth bean, F. oxysporum wilt in moth bean, F. aczttatzun damping off in cowpea and Clusterbean, Fusarium sp. seedling rot in Clusterbean, Neocosmospora striata root rot in cowpea and wilt in Clusterbean and Syncephalastrum monosporum root rot in Clusterbean were molecularly identified as new fungal records as pathogens causing root diseases in arid legumes. | 3697 | NA | NA | NA | no | no |
| 3697 | 2017 | NA | Esterhuizen, LL; Mabasa, KG; van Heerden, SW; Czosnek, H; Brown, JK; van Heerden, H; Rey, MEC | 2013 | Genetic identification of members of the Bemisia tabaci cryptic species complex from South Africa reveals native and introduced haplotypes | The whitefly Bemisia tabaci cryptic species complex contains some important agricultural pest and virus vectors. Members of the complex have become serious pests in South Africa (SA) because of their feeding habit and their ability to transmit begomovirus species. Despite their economic importance, studies on the biology and distribution of B. tabaci in SA are limited. To this end, a survey was made to investigate the diversity and distribution of B. tabaci cryptic species in eight geographical locations (provinces) in SA, between 2002 and 2009, using the mitochondrial cytochrome oxidase I (mtCOI) sequences. Phylogenetic analysis revealed the presence of members from two endemic sub-Saharan Africa (SSAF) subclades coexisting with two introduced putative species. The SSAF-1 subclade includes cassava host-adapted B. tabaci populations, whereas the whiteflies collected from cassava and non-cassava hosts formed a distinct subclade, referred to as SSAF-5, and represent a new subclade among previously recognized southern Africa clades. Two introduced cryptic species, belonging to the Mediterranean and Middle EastAsia minor 1 clades, were identified and include the B and Q types. The B type showed the widest distribution, being present in five of the eight provinces explored in SA, infesting several host plants and predominating over the indigenous haplotypes. This is the first report of the occurrence of the exotic Q type in SA alongside the more widely distributed B type. Furthermore, mtCOI PCR-RFLP was developed for the SA context to allow rapid discrimination between the B, Q and SSAF putative species. The capacity to manage pests and disease effectively relies on knowledge of the identity of the agents causing the damage. Therefore, this study contributes to the understanding of South African B. tabaci species diversity, information needed for the development of knowledge-based disease management practices. | 3698 | NA | NA | NA | no | no |
| 3698 | 2017 | NA | Zou, Y; Wu, F; Guo, YX; Wang, HB; Fang, YQ; Kang, M; Lin, Q | 2017 | Determining geographical variations in Ascaris suum isolated from different regions in northwest China through sequences of three mitochondrial genes | The sequence diversities in three mitochondrial DNA (mtDNA) regions, namely portions of NADH dehydrogenase subunit 1 (pnad1), cytochrome c oxidase subunit 1 (pcox1), and NADH dehydrogenase subunit 4 (pnad4), were investigated in all Ascaris suum samples isolated from four regions in northwestern China. Those genes were amplified by PCR method and the lengths of pnad1, pcox1, and pnad4 were 419 bp, 711 bp, and 723 bp, respectively. The intraspecific sequence variations within A. suum samples were 0-2.9% for pnad1, 0-2.1% for pcox1, and 0-3.1% for pnad4. Phylogenetic analysis combined with three sequences of mtDNA fragments showed that all A. suum samples were monophyletic groups, but samples from the same geographical origin did not always cluster together. The results suggested that the three mtDNA fragments could not be used as molecular markers to identify the A. suum isolates from four regions, and have important implications for studying molecular epidemiology and population genetics of A. suum. | 3699 | NA | NA | NA | no | no |
| 3699 | 2017 | NA | Negretti, RRRD; Gomes, CB; Mattos, VS; Somavilla, L; Manica-Berto, R; Agostinetto, D; Castagnone-Sereno, P; Carneiro, RMDG | 2017 | Characterisation of a Meloidogyne species complex parasitising rice in southern Brazil | Root-knot nematodes (RKN) are important plant pathogens affecting rice in South-East Asia and southern Brazil in irrigated rice fields. In order to investigate the specific diversity of RKN associated with irrigated rice in southern Brazil, Meloidogyne spp. from Rio Grande do Sul (RS) and Santa Catarina (SC) States were characterised biochemically by esterase (Est) and malate dehydrogenase (Mdh) phenotypes. Fifty-six Meloidogyne spp. populations were detected in 48% of rice samples, and a total of five esterase phenotypes were identified, four of which presented as drawn-out bands in different positions. In RS State, M. graminicola (Est VS1), Meloidogyne sp. 2 (Est R2) and Meloidogyne sp. 3 (Est R3) were identified, which corresponded to ca 80, 40 and 10% of samples, respectively. In SC State, M. graminicola, M. javanica (Est J3), Meloidogyne sp. 1 (Est R1), Meloidogyne sp. 2 and Meloidogyne sp. 3 accounted for ca 93.75, 12.50, 62.50, 12.25 and 6.25% of samples, respectively. The esterase phenotypes R1, R2 and R3 are new, never having been detected on rice before. Meloidogyne javanica showed a N1 Mdh phenotype (Rm: 1.0), while four other populations exhibited a N1a (Rm: 1.4) phenotype. All populations were tested with two SCAR markers specific to M. graminicola, which confirmed that, but no specificity was obtained with both markers in relation to the atypical populations analysed. Sequencing and phylogenetic analyses of internal transcribed spacer-rRNA (ITS) were performed to infer the phylogenetic relationship of these atypical Meloidogyne spp. populations. Meloidogyne sp. 1 grouped with the mitotic parthenogenetic species, while the two others (Meloidogyne sp. 2 and sp. 3) clustered with M. graminicola and other meiotic parthenogenetic species. Taken together, these data highlight the unprecedented specific diversity of RKN associated with irrigated rice in southern Brazil. Further morphological and phylogenetic studies involving these atypical isolates will be carried out to identify this complex of species. | 3700 | NA | NA | NA | no | no |
| 3700 | 2017 | NA | GARDNER, JPA | 1995 | DEVELOPMENTAL STABILITY IS NOT DISRUPTED BY EXTENSIVE HYBRIDIZATION AND INTROGRESSION AMONG POPULATIONS OF THE MARINE BIVALVE MOLLUSKS MYTILUS-EDULIS (L) AND MYTILUS-GALLOPROVINCIALIS (LMK) FROM SOUTH-WEST ENGLAND | The effects of hybridization and introgression were assessed among two naturally hybridizing bivalve molluscs (the mussels Mytilus edulis and M. galloprovincialis) from western Europe to estimate how disruptive these processes are to developmental stability (measured in terms of morphological variability). Ten shell traits were measured for 392 mussels from four allopatric populations (two each of M. edulis and M. galloprovincialis) and two hybrid populations. An index of variability (defined as Z(i) = (i)-(y) over bar(i) where (y) over bar(i) is the population mean of the length-standardized trait, and y(i) is the individual’s length-standardized trait value) was constructed for each trait, and for the sum of the traits. The hybrid populations did not exhibit greater mean variability than the allopatric populations for any of the indices. Upon pooling, the hybrid populations had significantly lower variability than the pooled M. edulis populations and the pooled M. galloprovincialis populations in two analyses, and had similar means in the remaining nine analyses. Where significant differences existed, the pooled M. galloprovincialis had lower levels of mean trait variability than the pooled M. edulis. Among the two hybrid populations, the putative F1 hybrids and backcross individuals exhibited means of trait variability which Were similar to those of the parental types. Thus, extensive hybridization and introgression do not adversely affect deveiopmental stability among these mussel populations. There was a strong significant correlation between the ranking of indices (based on the amount of variability) across all six populations, indicating that a large genetic component determines the measured morphological variability. It is concluded that the genes or gene complexes which control morphological development in M. edulis and M. galloprovincialis are very similar, providing further evidence bf the close evolutionary relatedness of these mussel taxa. | 3701 | NA | NA | NA | no | no |
| 3701 | 2017 | NA | Beattie, AD; Michaels, TE; Pauls, KP | 2003 | Predicting progeny performance in common bean (Phaseolus vulgaris L.) using molecular marker-based cluster analysis | Recovery of superior individuals from a cross based solely on the phenotypic characteristics of single-plant selections is inefficient because some traits, like yield, have low heritabilities, or because it is difficult to create the correct conditions for selection, as with disease resistance. In contrast, molecular markers are highly heritable and unaffected by environmental conditions. The objective of this study was to investigate the potential of molecular markers to identify superior lines in a breeding population by examining relationships between genetic distances (GDs) and phenotypic data for eight agronomic and architectural traits (branch angle, height, hypocotyl diameter, lodging, maturity, upper pods, pods per plant, and yield) obtained from three locations over a two-year period. From an elite common bean (Phaseolus vulgaris L.) cross, 110 recombinant inbred lines (RILs) and the two parents were screened with 116 random amplified polymorphic DNA (RAPD) markers. Pairwise GD values were calculated between each line and a selected “target” (the parent ‘OAC Speedvale’) using the Jaccard method and correlated to the trait data. The correlations were low and non-significant for all traits, except for branch angle (r = 0.30), maturity (r = -0.25), and pods per plant (r = 0.35). The lines were also grouped according to their cluster-based GD from the target parent using UPGMA cluster analysis. Trait data of lines within groups were combined and correlated to cluster-based GD. Correlation values were large and significant for all traits. Additionally, one-half of the top 10 yielding lines and nearly one-third of the best phenotypically ranked lines were present within the 13% of lines clustered nearest the target. A selection method using marker-based cluster analysis (MBCA) is suggested to assist phenotypic selection by directing a breeder’s attention to a subsample of the population containing a high proportion of superior lines. | 3702 | NA | NA | NA | no | no |
| 3702 | 2017 | NA | Furlotte, NA; Eskin, E; Eyheramendy, S | 2012 | Genome-Wide Association Mapping With Longitudinal Data | Many genome-wide association studies have been performed on population cohorts that contain phenotype measurements at multiple time points. However, standard association methodologies only consider one time point. In this paper, we propose a mixed-model-based approach for performing association mapping which utilizes multiple phenotype measurements for each individual. We introduce an analytical approach to calculate statistical power and show that this model leads to increased power when compared to traditional approaches. Moreover, we show that by using this model we are able to differentiate the genetic, environmental, and residual error contributions to the phenotype. Using predictions of these components, we show how the proportion of the phenotype due to environment and genetics can be predicted and show that the ranking of individuals based on these predictions is very accurate. The software implementing this method may be found at . Genet. Epidemiol. 36:463-471, 2012. (C) 2012 Wiley Periodicals, Inc. | 3703 | NA | NA | NA | no | no |
| 3703 | 2017 | NA | Butler, MI; Stockwell, PA; Black, MA; Day, RC; Lamont, IL; Poulter, RTM | 2013 | Pseudomonas syringae pv. actinidiae from Recent Outbreaks of Kiwifruit Bacterial Canker Belong to Different Clones That Originated in China | A recently emerged plant disease, bacterial canker of kiwifruit (Actinidia deliciosa and A. chinensis), is caused by Pseudomonas syringae pv. actinidiae (PSA). The disease was first reported in China and Japan in the 1980s. A severe outbreak of PSA began in Italy in 2008 and has spread to other European countries. PSA was found in both New Zealand and Chile in 2010. To study the evolution of the pathogen and analyse the transmission of PSA between countries, genomes of strains from China and Japan (where the genus Actinidia is endemic), Italy, New Zealand and Chile were sequenced. The genomes of PSA strains are very similar. However, all strains from New Zealand share several single nucleotide polymorphisms (SNPs) that distinguish them from all other PSA strains. Similarly, all the PSA strains from the 2008 Italian outbreak form a distinct clonal group and those from Chile form a third group. In addition to the rare SNPs present in the core genomes, there is abundant genetic diversity in a genomic island that is part of the accessory genome. The island from several Chinese strains is almost identical to the island present in the New Zealand strains. The island from a different Chinese strain is identical to the island present in the strains from the recent Italian outbreak. The Chilean strains of PSA carry a third variant of this island. These genomic islands are integrative conjugative elements (ICEs). Sequencing of these ICEs provides evidence of three recent horizontal transmissions of ICE from other strains of Pseudomonas syringae to PSA. The analyses of the core genome SNPs and the ICEs, combined with disease history, all support the hypothesis of an independent Chinese origin for both the Italian and the New Zealand outbreaks and suggest the Chilean strains also originate from China. | 3704 | NA | NA | NA | no | no |
| 3704 | 2017 | NA | FRANK, SA | 1992 | A KIN SELECTION MODEL FOR THE EVOLUTION OF VIRULENCE | The costs and benefits of parasite virulence are analysed in an evolutionarily stable strategy (ESS) model. Increased host mortality caused by disease (virulence) reduces a parasite’s fitness by damaging its food supply. The fitness costs of high virulence may be offset by the benefits of increased transmission or ability to withstand the host’s defences. It has been suggested that multiple infections lead to higher virulence because of competition among parasite strains within a host. A quantitative prediction is given for the ESS virulence rate as a function of the coefficient of relatedness among co-infecting strains. The prediction depends on the quantitative relation between the costs of virulence and the benefits of transmission or avoidance of host defences. The particular mechanisms by which parasites can increase their transmission or avoid host defences also have a key role in the evolution of virulence when there are multiple infections. | 3705 | NA | NA | NA | no | no |
| 3705 | 2017 | NA | Ciarroni, S; Gallipoli, L; Taratufolo, MC; Butler, MI; Poulter, RTM; Pourcel, C; Vergnaud, G; Balestra, GM; Mazzaglia, A | 2015 | Development of a Multiple Loci Variable Number of Tandem Repeats Analysis (MLVA) to Unravel the Intra-Pathovar Structure of Pseudomonas syringae pv. actinidiae Populations Worldwide | The bacterial canker of kiwifruit by Pseudomonas syringae pv. actinidiae is an emblematic example of a catastrophic disease of fruit crops. In 2008 a new, extremely virulent form of the pathogen emerged and rapidly devastated many Actinidia spp. orchards all over the world. In order to understand differences in populations within this pathovar and to elucidate their diffusion and movements on world scale, it is necessary to be able to quickly and on a routine basis compare new isolates with previous records. In this report a worldwide collection of 142 strains was analyzed by MLVA, chosen as investigative technique for its efficacy, reproducibility, simplicity and low cost. A panel of 13 Variable Number of Tandem Repeats (VNTR) loci was identified and used to describe the pathogen population. The MLVA clustering is highly congruent with the population structure as previously established by other molecular approaches including whole genome sequencing and correlates with geographic origin, time of isolation and virulence. For convenience, we divided the VNTR loci in two panels. Panel 1 assay, using six loci, recognizes 23 different haplotypes, clustered into ten complexes with highest congruence with previous classifications. Panel 2, with seven VNTR loci, provides discriminatory power. Using the total set of 13 VNTR loci, 58 haplotypes can be distinguished. The recent hypervirulent type shows very limited diversity and includes, beside the strains from Europe, New Zealand and Chile, a few strains from Shaanxi, China. A broad genetic variability is observed in China, but different types are also retrievable in Japan and Korea. The low virulent strains cluster together and are very different from the other MLVA genotypes. Data were used to generate a public database in MLVAbank. MLVA represents a very promising first-line assay for large-scale routine genotyping, prior to whole genome sequencing of only the most relevant samples. | 3706 | NA | NA | NA | no | no |
| 3706 | 2017 | NA | Wilson, DR; Goble, AR; Boutin, S; Humphries, MM; Coltman, DW; Gorrell, JC; Shonfield, J; McAdam, AG | 2015 | Red squirrels use territorial vocalizations for kin discrimination | The ability to discriminate among individuals, or among classes of individuals, can provide animals with important fitness benefits. Although several mechanisms for discrimination are possible, most require animals to show stable phenotypic variation that reflects their identity or their membership in a particular class (e.g. sex, mate, kin). For territorial animals that rarely interact physically, vocalizations could serve as long-distance signals that facilitate discrimination. In this study, we tested whether the territorial rattle vocalizations of North American red squirrels, Tamiasciurus hudsonicus, are repeatable, and whether they could hence provide the basis for multiple types of discrimination. We measured four structural features from two rattles from each of 76 marked squirrels. All four features were repeatable, which is consistent with territorial rattles being individually distinctive. We then conducted a playback experiment to determine whether squirrels use rattles for discrimination. Specifically, we tested whether squirrels discriminate between the rattles of neighbours and non-neighbours, and kin (coefficient of relatedness, r >= 0.25) and non-kin (r < 0.125). Following a 2 x 2 factorial design, we broadcast a rattle from a non-neighbouring nonkin individual to 15 subjects, from a neighbouring nonkin individual to 14 subjects, from a non-neighbouring kin individual to 11 subjects, and from a neighbouring kin individual to 13 subjects. Subjects did not discriminate between the rattles of neighbours and non-neighbours, but did respond differently to the rattles of kin and nonkin. Specifically, squirrels were significantly more likely to produce a rattle of their own in response to the broadcasted rattles of nonkin versus the broadcasted rattles of kin. This result demonstrates that red squirrels can use territorial vocalizations for kin discrimination. It also suggests that they are more tolerant of territorial intrusions by kin. (C) 2015 The Association for the Study of Animal Behaviour. Published by Elsevier Ltd. All rights reserved. | 3707 | NA | NA | NA | no | no |
| 3707 | 2017 | NA | HAMELIN, RC; DOUDRICK, RL; NANCE, WL | 1994 | GENETIC DIVERSITY IN CRONARTIUM-QUERCUUM F SP FUSIFORME ON LOBLOLLY PINES IN SOUTHERN US | Twelve random amplified polymorphic DNA (RAPD) markers were used to study genetic variability in three populations of Cronartium quercuum f. sp. fusiforme, the causal agent of fusiform rust on pines. Most of the variability (94%) was found within populations while 6% of the total variability was attributable to frequency differences between populations. The frequency of three markers was significantly different between regions of origin on an east-west transect. Three markers were completely absent from one of the populations while present in the others, and one marker was fixed in the western population but polymorphic in the eastern and central populations. However, no significant differences were observed when the western population was omitted from the analysis. The results suggest that C. quercuum f. sp. fusiforme is a highly heterogeneous pathogen with little geographic differentiation and underscores the importance of considering the population structure of the pathogen in resistance breeding programs. | 3708 | NA | NA | NA | no | no |
| 3708 | 2017 | NA | Iglesias, NG; Gago-Zachert, SP; Robledo, G; Costa, N; Plata, MI; Vera, O; Grau, O; Semorile, LC | 2008 | Population structure of Citrus tristeza virus from field Argentinean isolates | We studied the genetic variability of three genomic regions (p23, p25 and p27 genes) from 11 field Citrus tristeza virus isolates from the two main citrus growing areas of Argentina, a country where the most efficient vector of the virus, Toxoptera citricida, is present for decades. The pathogenicity of the isolates was determinated by biological indexing, single-strand conformation polymorphism analysis showed that most isolates contained high intra-isolate variability. Divergent sequence variants were detected in some isolates, suggesting re-infections of the field plants. Phylogenetic analysis of the predominant sequence variants of each isolate revealed similar grouping of isolates for genes p25 and p27. The analysis of p23 showed two groups contained the severe isolates. Our results showed a high intra-isolate sequence variability suggesting that re-infections could contribute to the observed variability and that the host can play an important role in the selection of the sequence variants present in these isolates. | 3709 | NA | NA | NA | no | no |
| 3709 | 2017 | NA | Lutter, EI; Martens, C; Hackstadt, T | 2012 | Evolution and Conservation of Predicted Inclusion Membrane Proteins in Chlamydiae | Chlamydia spp. are obligate intracellular pathogens that replicate within a vacuole termed the inclusion. Chlamydiae extensively modify the inclusion membrane via the insertion of chlamydial inclusion membrane proteins (Incs) which decorate the cytosolic face of the inclusion. We have assessed the overall relatedness and phylogeny of Incs in order to identify potential evolutionary trends. Despite a high degree of conservation among Incs within C. trachomatis serovars, phylogenetic analysis showed that some Incs cluster according to clinical groupings suggesting that certain Incs may contribute to tissue tropism. Bioinformatic predictions identified Incs in five chlamydial species: 55 in C. trachomatis, 68 in C. felis, 92 in C. pneumoniae, 79 in C. caviae, and 54 in C. muridarum. Inc homologues were compared between chlamydial species and 23 core Incs were identified as shared among all species. Genomic expansion of Incs was identified in C. pneumoniae, C. caviae, and C. felis but not C. trachomatis or C. muridarum. | 3710 | NA | NA | NA | no | no |
| 3710 | 2017 | NA | Stutz, WE; Bolnick, DI | 2014 | Stepwise Threshold Clustering: A New Method for Genotyping MHC Loci Using Next-Generation Sequencing Technology | Genes of the vertebrate major histocompatibility complex (MHC) are of great interest to biologists because of their important role in immunity and disease, and their extremely high levels of genetic diversity. Next generation sequencing (NGS) technologies are quickly becoming the method of choice for high-throughput genotyping of multi-locus templates like MHC in non-model organisms. Previous approaches to genotyping MHC genes using NGS technologies suffer from two problems: 1) a “gray zone” where low frequency alleles and high frequency artifacts can be difficult to disentangle and 2) a similar sequence problem, where very similar alleles can be difficult to distinguish as two distinct alleles. Here were present a new method for genotyping MHC loci - Stepwise Threshold Clustering (STC) - that addresses these problems by taking full advantage of the increase in sequence data provided by NGS technologies. Unlike previous approaches for genotyping MHC with NGS data that attempt to classify individual sequences as alleles or artifacts, STC uses a quasi-Dirichlet clustering algorithm to cluster similar sequences at increasing levels of sequence similarity. By applying frequency and similarity based criteria to clusters rather than individual sequences, STC is able to successfully identify clusters of sequences that correspond to individual or similar alleles present in the genomes of individual samples. Furthermore, STC does not require duplicate runs of all samples, increasing the number of samples that can be genotyped in a given project. We show how the STC method works using a single sample library. We then apply STC to 295 threespine stickleback (Gasterosteus aculeatus) samples from four populations and show that neighboring populations differ significantly in MHC allele pools. We show that STC is a reliable, accurate, efficient, and flexible method for genotyping MHC that will be of use to biologists interested in a variety of downstream applications. | 3711 | NA | NA | NA | no | no |
| 3711 | 2017 | NA | Haouchar, D; Pacioni, C; Haile, J; McDowell, MC; Baynes, A; Phillips, MJ; Austin, JJ; Pope, LC; Bunce, M | 2016 | Ancient DNA reveals complexity in the evolutionary history and taxonomy of the endangered Australian brush-tailed bettongs (Bettongia: Marsupialia: Macropodidae: Potoroinae) | The three surviving ‘brush-tailed’ bettong species-Bettongia gaimardi (Tasmania), B. tropica (Queensland) and B. penicillata (Western Australia), are all classified as threatened or endangered. These macropodids are prolific diggers and are recognised as important ‘ecosystem engineers’ that improve soil quality and increase seed germination success. However, a combination of introduced predators, habitat loss and disease has seen populations become increasingly fragmented and census numbers decline. Robust phylogenies are vital to conservation management, but the extent of extirpation and fragmentation in brush-tailed bettongs is such that a phylogeny based upon modern samples alone may provide a misleading picture of former connectivity, genetic diversity and species boundaries. Using ancient DNA isolated from fossil bones and museum skins, we genotyped two mitochondrial DNA (mtDNA) genes: cytochrome b (266 bp) and control region (356 bp). These ancient DNA data were combined with a pre-existing modern DNA data set on the historically broadly distributed brush-tailed bettongs (similar to 300 samples total), to investigate their phylogenetic relationships. Molecular dating estimates the most recent common ancestor of these bettongs occurred c. 2.5 Ma (million years ago), which suggests that increasing aridity likely shaped their modern-day distribution. Analyses of the concatenated mtDNA sequences of all brush-tailed bettongs generated five distinct and well-supported clades including: a highly divergent Nullarbor form (Clade I), B. tropica (Clade II), B. penicillata (Clades III and V), and B. gaimardi (Clade IV). The generated phylogeny does not reflect current taxonomy and the question remains outstanding of whether the brush-tailed bettongs consisted of several species, or a single widespread species. The use of nuclear DNA markers (single nucleotide polymorphisms and/or short tandem repeats) will be needed to better inform decisions about historical connectivity and the appropriateness of ongoing conservation measures such as translocations and captive breeding. | 3712 | NA | NA | NA | no | no |
| 3712 | 2017 | NA | Boratynski, A; Lewandowski, A; Boratynska, K; Montserrat, JM; Romo, A | 2009 | High level of genetic differentiation of Juniperus phoenicea (Cupressaceae) in the Mediterranean region: geographic implications | Fourteen natural populations of Juniperus phoenicea L. from the quite entire species range have been compared using isoenzyme polymorphism. Among 17 loci, 5 (Got1, 6Pgd3, Pgi2, Pgm2 and Shdh2) appeared to be differentiated sufficiently to provide useful information for discrimination between the subspecies phoenicea and turbinata (Guss.) Nyman. Two distinct groups of populations were detected using the Nei’s genetic distance unweighted pair group method with arithmetic mean (UPGMA) and discrimination analyses, one including the inland populations of the eastern Iberian Peninsula and southern France (subsp. phoenicea), and the second from the Mediterranean and Atlantic shores, and from the Atlas mountains in Africa (subsp. turbinata). The high level of differences confirms a long period of isolation, probably during the whole Pleistocene. The population from the Aegean Sea shore differed from the other Mediterranean shore plus Atlas mountain population. It also suggests spatial isolation between them, at least during the last Glaciation. | 3713 | NA | NA | NA | no | no |
| 3713 | 2017 | NA | Wan, DS; Feng, JJ; Jiang, DC; Mao, KS; Duan, YW; Miehe, G; Opgenoorth, L | 2016 | The Quaternary evolutionary history, potential distribution dynamics, and conservation implications for a Qinghai-Tibet Plateau endemic herbaceous perennial, Anisodus tanguticus (Solanaceae) | Various hypotheses have been proposed about the Quaternary evolutionary history of plant species on the Qinghai-Tibet Plateau (QTP), yet only a handful of studies have considered both population genetics and ecological niche context. In this study, we proposed and compared climate refugia hypotheses based on the phylogeographic pattern of Anisodus tanguticus (three plastid DNA fragments and nuclear internal transcribed spacer regions from 32 populations) and present and past species distribution models (SDMs). We detected six plastid haplotypes in two well-differentiated lineages. Although all haplotypes could be found in its western (sampling) area, only haplotypes from one lineage occurred in its eastern area. Meanwhile, most genetic variations existed between populations (F-ST=0.822). The SDMs during the last glacial maximum and last interglacial periods showed range fragmentation in the western area and significant range contraction in the eastern area, respectively, in comparison with current potential distribution. This species may have undergone intraspecific divergence during the early Quaternary, which may have been caused by survival in different refugia during the earliest known glacial in the QTP, rather than geological isolation due to orogenesis events. Subsequently, climate oscillations during the Quaternary resulted in a dynamic distribution range for this species as well as the distribution pattern of its plastid haplotypes and nuclear genotypes. The interglacial periods may have had a greater effect on A.tanguticus than the glacial periods. Most importantly, neither genetic data nor SDM alone can fully reveal the climate refugia history of this species. We also discuss the conservation implications for this important Tibetan folk medicine plant in light of these findings and SDMs under future climate models. Together, our results underline the necessity to combine phylogeographic and SDM approaches in future investigations of the Quaternary evolutionary history of species in topographically complex areas, such as the QTP. | 3714 | NA | NA | NA | no | no |
| 3714 | 2017 | NA | Barauna, RA; Ramos, RTJ; Veras, AAO; Pinheiro, KC; Benevides, LJ; Viana, MVC; Guimaraes, LC; Edman, JM; Spier, SJ; Azevedo, V; Silva, A | 2017 | Assessing the Genotypic Differences between Strains of Corynebacterium pseudotuberculosis biovar equi through Comparative Genomics | Seven genomes of Corynebacterium pseudotuberculosis biovar equi were sequenced on the Ion Torrent PGM platform, generating high-quality scaffolds over 2.35 Mbp. This bacterium is the causative agent of disease known as “pigeon fever” which commonly affects horses worldwide. The pangenome of biovar equi was calculated and two phylogenomic approaches were used to identify clustering patterns within Corynebacterium genus. Furthermore, other comparative analyses were performed including the prediction of genomic islands and prophages, and SNP-based phylogeny. In the phylogenomic tree, C. pseudotuberculosis was divided into two distinct clades, one formed by nitrate non-reducing species (biovar ovis) and another formed by nitrate-reducing species (biovar equi). In the latter group, the strains isolated from California were more related to each other, while the strains CIP 52.97 and 1/06-A formed the outermost clade of the biovar equi. A total of 1,355 core genes were identified, corresponding to 42.5% of the pangenome. This pangenome has one of the smallest core genomes described in the literature, suggesting a high genetic variability of biovar equi of C. pseudotuberculosis. The analysis of the similarity between the resistance islands identified a higher proximity between the strains that caused more severe infectious conditions (infection in the internal organs). Pathogenicity islands were largely conserved between strains. Several genes that modulate the pathogenicity of C. pseudotuberculosis were described including peptidases, recombination enzymes, micoside synthesis enzymes, bacteriocins with antimicrobial activity and several others. Finally, no genotypic differences were observed between the strains that caused the three different types of infection (external abscess formation, infection with abscess formation in the internal organs, and ulcerative lymphangitis). Instead, it was noted that there is a higher phenetic correlation between strains isolated at California compared to the other strains. Additionally, high variability of resistance islands suggests gene acquisition through several events of horizontal gene transfer. | 3715 | NA | NA | NA | no | no |
| 3715 | 2017 | NA | Seeber, E; Miehe, G; Hensen, I; Yang, YP; Wesche, K | 2016 | Mixed reproduction strategy and polyploidy facilitate dominance of Kobresia pygmaea on the Tibetan Plateau | Aims The sedge Kobresia pygmaea is the dominant species of high-altitude pastures in Tibet, and it is the most important source of forage in animal husbandry. We present the first comprehensive reproduction study for this perennial key species that adopts a molecular approach and tests how sexual and vegetative reproduction, as well as ploidy, relate to survival and dominance under harsh conditions. Methods We assessed inflorescence numbers of K. pygmaea across two Tibetan alpine pastures with differing grazing regimes. Germination was tested in untreated diaspores and then following mechanical and chemical scarification. In a 4-year experiment, we assessed diaspore viability and seed bank formation. Using eight microsatellite markers, we recorded multilocus genotypes in hierarchical grids and measured their ploidy using flow cytometry. Adjusted analysis of variance models were used to analyse data on sexual reproduction, while the complement of the Simpson index and the Shannon diversity index were used to characterize the spatial distribution of multilocus genotypes and clonal richness. Important Findings Inflorescence production was high and differed significantly between years (2010: 617 +/- 460 SD; 2012: 2015 +/- 1213 SD) but not between grazing regimes. Diaspore viability was high (94%) and gradually decreased after 3 and 4 years of storage in the soil. Diaspores not exposed to further scarification failed to germinate, while mechanical and chemical (H2SO4) scarification increased germination to 9 and 44%, respectively. Clonal diversity was high, although in situ germination was rarely observed. Multilocus genotypes intersected and covered a mean area of 0.74 m(2). Most individuals were found to be tetraploid, with only 0.8% of all ramets being triploid. We conclude that K. pygmaea survives on the Tibetan Plateau by employing a mixed reproduction strategy involving both sexual and clonal propagation. The species’ adaptability and dominance is further facilitated by its polyploidy. As pasture restoration using diaspores would be difficult, existing Kobresia pasture should be managed more sustainably. | 3716 | NA | NA | NA | no | no |
| 3716 | 2017 | NA | Cobben, MMP; Verboom, J; Opdam, PFM; Hoekstra, RF; Jochem, R; Smulders, MJM | 2012 | Wrong place, wrong time: climate change-induced range shift across fragmented habitat causes maladaptation and declined population size in a modelled bird species | Many species are locally adapted to decreased habitat quality at their range margins, and therefore show genetic differences throughout their ranges. Under contemporary climate change, range shifts may affect evolutionary processes at the expanding range margin due to founder events. In addition, populations that are affected by such founder events will, in the course of time, become located in the range centre. Recent studies investigated evolutionary changes at the expanding range margin, but have not assessed eventual effects across the species’ range. We explored the possible influence of range shift on the level of adaptation throughout the species’ total range. For this we used a spatially explicit, individual-based simulation model of a woodland bird, parameterized after the middle spotted woodpecker ( Dendrocopos medius) in fragmented habitat. We simulated its range under climate change, and incorporated genetic differences at a single locus that determined the individual’s degree of adaptation to optimal temperature conditions. Generalist individuals had a large thermal tolerance, but relatively low overall fitness, whereas climate specialists had high fitness combined with a small thermal tolerance. In equilibrium, the populations in the range centre were comprised of the specialists, whereas the generalists dominated the margins. In contrast, under temperature increase, the generalist numbers increased at the expanding margin and eventually also occupied the centre of the shifting range, whereas the specialists were located in the retracting margins. This was caused by founder events and led to overall maladaptation of the species, which resulted in a reduced metapopulation size and thus impeded the species’ persistence. We therefore found no evidence for a complementary effect of local adaptation and range shifts on species’ survival. Instead, we showed that founder events can cause local maladaptation which can amplify throughout the species’ range, and, as such, hamper the species’ persistence under climate change. | 3717 | NA | NA | NA | no | no |
| 3717 | 2017 | NA | Cochran-Biederman, JL; Wyman, KE; French, WE; Loppnow, GL | 2015 | Identifying correlates of success and failure of native freshwater fish reintroductions | Reintroduction of imperiled native freshwater fish is becoming an increasingly important conservation tool amidst persistent anthropogenic pressures and new threats related to climate change. We summarized trends in native fish reintroductions in the current literature, identified predictors of reintroduction outcome, and devised recommendations for managers attempting future native fish reintroductions. We constructed random forest classifications using data from 260 published case studies of native fish reintroductions to estimate the effectiveness of variables in predicting reintroduction outcome. The outcome of each case was assigned as a success or failure on the basis of the author’s perception of the outcome and on whether or not survival, spawning, or recruitment were documented during post-reintroduction monitoring. Inadequately addressing the initial cause of decline was the best predictor of reintroduction failure. Variables associated with habitat (e.g., water quality, prey availability) were also good predictors of reintroduction outcomes, followed by variables associated with stocking (e.g., genetic diversity of stock source, duration of stocking event). Consideration of these variables by managers during the planning process may increase the likelihood for successful outcomes in future reintroduction attempts of native freshwater fish. Identificacion de Correlaciones de Exito y Fracaso de Reintroducciones de Peces de Nativos Agua Dulce Resumen La reintroduccion de peces nativos de agua dulce que se encuentran en peligro se esta convirtiendo cada vez mas en una herramienta importante de conservacion frente a las presiones antropogenicas persistentes y nuevas amenazas relacionadas con el cambio climatico. Resumimos las tendencias encontradas en la literatura actual sobre la reintroduccion de peces nativos, identificamos pronosticadores de resultados de la reintroduccion e ideamos recomendaciones para administradores que intenten reintroducciones de peces nativos en el futuro. Construimos clasificaciones de bosque aleatorio a partir de datos de 260 estudios de caso publicados sobre la reintroduccion de peces nativos para estimar la efectividad de las variables en la prediccion del resultado de la reintroduccion. El resultado de cada caso fue asignado como un exito o un fracaso con base en la percepcion del autor a partir del resultado y dependiendo de si se documento o no la supervivencia, el desove o el reclutamiento durante el monitoreo posterior a la reintroduccion. Abordar inadecuadamente a la causa inicial de la declinacion fue el mejor pronosticador del fracaso de la reintroduccion. Las variables asociadas con el habitat (p. ej.: calidad del agua, disponibilidad de la presa) tambien fueron buenos pronosticadores de los resultados de la reintroduccion, seguidas por las variables asociadas con el stock (p. ej.: la diversidad genetica de la fuente del stock, duracion del evento de stock). Que los administradores consideren estas variables durante el proceso de planeacion puede incrementar la probabilidad de resultados exitosos en futuros intentos de reintroduccion de peces nativos de agua dulce. | 3718 | NA | NA | NA | no | no |
| 3718 | 2017 | NA | Turingan, RS; Thomann, HU; Zolotova, A; Tan, E; Selden, RF | 2013 | Rapid Focused Sequencing: A Multiplexed Assay for Simultaneous Detection and Strain Typing of Bacillus anthracis, Francisella tularensis, and Yersinia pestis | Background: The intentional release of Bacillus anthracis in the United States in 2001 has heightened concern about the use of pathogenic microorganisms in bioterrorism attacks. Many of the deadliest bacteria, including the Class A Select Agents Bacillus anthracis, Francisella tularensis, and Yersinia pestis, are highly infectious via the pulmonary route when released in aerosolized form. Hence, rapid, sensitive, and reliable methods for detection of these biothreats and characterization of their potential impact on the exposed population are of critical importance to initiate and support rapid military, public health, and clinical responses. Methodology/Principal Findings: We have developed microfluidic multiplexed PCR and sequencing assays based on the simultaneous interrogation of three pathogens per assay and ten loci per pathogen. Microfluidic separation of amplified fluorescently labeled fragments generated characteristic electrophoretic signatures for identification of each agent. The three sets of primers allowed significant strain typing and discrimination from non-pathogenic closely-related species and environmental background strains based on amplicon sizes alone. Furthermore, sequencing of the 10 amplicons per pathogen, termed “Rapid Focused Sequencing,” allowed an even greater degree of strain discrimination and, in some cases, can be used to determine virulence. Both amplification and sequencing assays were performed in microfluidic biochips developed for fast thermal cycling and requiring 7 mu L per reaction. The 30-plex sequencing assay resulted in genotypic resolution of 84 representative strains belonging to each of the three biothreat species. Conclusions/Significance: The microfluidic multiplexed assays allowed identification and strain differentiation of the biothreat agents Bacillus anthracis, Francisella tularensis, and Yersinia pestis and clear discrimination from closely-related species and several environmental background strains. The assays may be extended to detect a large number of pathogens, are applicable to the evaluation of both environmental and clinical samples, and have the potential to be applied in military, public health, and clinical diagnostic settings. | 3719 | NA | NA | NA | no | no |
| 3719 | 2017 | NA | Goryunova, SV; Gashkova, IV; Kosareva, GA | 2011 | Variability and phylogenetic relationships of the Cucumis sativus L. species inferred from NBS-profiling and RAPD analysis | Genetic variability of the Cucumis sativus species and its phylogenetic relationsips with other species of the genus were studied on the basis of RAPD marking and analysis of intra- and interspecific polymorphism of the nucleotide sequences of the NBS-LRR gene family in species of the genus Cucumis with the use of the NBS-profiling method. According to RAPD analysis, cucumber cultivars from different geographic regions are highly similar, except for accessions k-3835 and k-3833 from Afghanistan. NBS-profiling analysis revealed phylogenetically most distinct accessions expected to be characterized by specificity of resistance: k-3845 from Uzbekistan, k-3851 from Kyrgyzstan, line 701, k-3835 and k-3833 from Afghanistan, k-2757 and k-3079 from Netherlands, vr.k. 908 from Canada, k-2926 from Bulgaria, Russian cultivars Monastyrskii, Izyashchnyi, and Lel’. Three essentially different groups of species were distinguished, and the C. sativus species (subgenus Cucumis) was found to be distant from the species belonging to the subgenus Melo. | 3720 | NA | NA | NA | no | no |
| 3720 | 2017 | NA | Fallin, D; Schork, NJ | 2000 | Accuracy of haplotype frequency estimation for biallelic loci, via the expectation-maximization algorithm for unphased diploid genotype data | Haplotype analyses have become increasingly common in genetic studies of human disease because of their ability to identify unique chromosomal segments likely to harbor disease-predisposing genes. The study of haplotypes is also used to investigate many population processes, such as migration and immigration rates, linkage-disequilibrium strength, and the relatedness of populations. Unfortunately, many haplotype-analysis methods require phase information that can be difficult to obtain from samples of nonhaploid species. There are, however, strategies for estimating haplotype frequencies from unphased diploid genotype data collected on a sample of individuals that make use of the expectation-maximization (EM) algorithm to overcome the missing phase information. The accuracy of such strategies, compared with other phase-determination methods, must be assessed before their use can be advocated. In this study we consider and explore sources of error between EM-derived haplotype frequency estimates and their population parameters, noting that much of this error is due to sampling error, which is inherent in all studies, even when phase can be determined. In light of this, we focus on the additional error between haplotype frequencies within a sample data set and EM-derived haplotype frequency estimates incurred by the estimation procedure. We assess the accuracy of haplotype frequency estimation as a function of a number of factors, including sample size, number of loci studied, allele frequencies, and locus-specific alellic departures from Hardy-Weinberg and linkage equilibrium. We point out the relative impacts of sampling error and estimation error, calling attention to the pronounced accuracy of EM estimates once sampling error has been accounted for. We also suggest that many factors that may influence accuracy can be assessed empirically within a data set-a fact that can be used to Create “diagnostics” that a user can turn to for assessing potential inaccuracies in estimation. | 3721 | NA | NA | NA | no | no |
| 3721 | 2017 | NA | Knafler, GJ; Jamieson, IG | 2014 | Primers for the amplification of major histocompatibility complex class I and II loci in the recovering red-crowned parakeet | Genes of the major histocompatibility complex (MHC) are widely used to investigate evolutionary processes as they are essential for the adaptive immune response of vertebrates. The characterization of MHC loci, although generally abundant within birds, is mostly lacking in the Psittaciformes (the parrots). Because they are among the most threatened groups of birds and are regularly kept in captivity, examining the MHC in parrot populations would be helpful as the movement of birds associated with conservation efforts (e.g. translocations) and the pet trade may increase the risk of spreading disease. Here, we provide designed primers for the amplification of MHC class I alpha exon 3 and class II beta exon 2 loci in New Zealand’s threatened red-crowned parakeet, Cyanoramphus novaezelandiae. Our novel primers facilitate the study of MHC diversity in relation to emerging disease agents, such as the beak and feather disease virus which has been recently detected in wild parrot populations. | 3722 | NA | NA | NA | no | no |
| 3722 | 2017 | NA | Oelemann, MC; Gomes, HM; Willery, E; Possuelo, L; Lima, KVB; Allix-Beguec, C; Locht, C; de la Salmoniere, YOLG; Gutierrez, MC; Suffys, P; Supply, P | 2011 | The Forest behind the Tree: Phylogenetic Exploration of a Dominant Mycobacterium tuberculosis Strain Lineage from a High Tuberculosis Burden Country | Background: Genotyping of Mycobacterium tuberculosis isolates is a powerful tool for epidemiological control of tuberculosis (TB) and phylogenetic exploration of the pathogen. Standardized PCR-based typing, based on 15 to 24 mycobacterial interspersed repetitive unit-variable number of tandem repeat (MIRU-VNTR) loci combined with spoligotyping, has been shown to have adequate resolution power for tracing TB transmission and to be useful for predicting diverse strain lineages in European settings. Its informative value needs to be tested in high TB-burden countries, where the use of genotyping is often complicated by dominance of geographically specific, genetically homogeneous strain lineages. Methodology/Principal Findings: We tested this genotyping system for molecular epidemiological analysis of 369 M. tuberculosis isolates from 3 regions of Brazil, a high TB-burden country. Deligotyping, targeting 43 large sequence polymorphisms (LSPs), and the MIRU-VNTRplus identification database were used to assess phylogenetic predictions. High congruence between the different typing results consistently revealed the countrywide supremacy of the Latin-American-Mediterranean (LAM) lineage, comprised of three main branches. In addition to an already known RDRio branch, at least one other branch characterized by a phylogenetically informative LAM3 spoligo-signature seems to be globally distributed beyond Brazil. Nevertheless, by distinguishing 321 genotypes in this strain population, combined MIRU-VNTR typing and spoligotyping demonstrated the presence of multiple distinct clones. The use of 15 to 24 loci discriminated 21 to 25% more strains within the LAM lineage, compared to a restricted lineage-specific locus set suggested to be used after SNP analysis. Noteworthy, 23 of the 28 molecular clusters identified were exclusively composed of patient isolates from a same region, consistent with expected patterns of mostly local TB transmission. Conclusions/Significance: Standard MIRU-VNTR typing combined with spoligotyping can reveal epidemiologically meaningful clonal diversity behind a dominant M. tuberculosis strain lineage in a high TB-burden country and is useful to explore international phylogenetical ramifications. | 3723 | NA | NA | NA | no | no |
| 3723 | 2017 | NA | Adonis, MI; Diaz, J; Miranda, VR; Chahuan, M; Zambrano, A; Benitez, HC; Campos, M; Avaria, P; Urzua, U; Marin, P; Gohurdett, M; Cisterna, Y; Gil, L | 2014 | Biomarkers for screening of lung cancer and pre-neoplastic lesions in a high risk Chilean population | Background: The mortality of lung cancer (LC), increases each year in the world, in spite of any advances, in development of new drugs to advance stages of LC. The high incidence of LC has been associated with smoking habit, genetic diversity and environmental pollution. Antofagasta region has been reported to have the highest LC mortality rate in Chile and its inhabitants were exposed to arsenic in their drinking water in concentrations as high as 870 mu g/ L. Non-invasive techniques such as biomarkers (Automatic Quantitative Cytometry: AQC and DR70) and Auto Fluorescence Bronchoscopy (AFB) might be potentially useful as a supplementary diagnostic approach and early detection. Early detection is one of the most important factors to intervene and prevent cancer progression in LC. This is a work of an ongoing prospective bimodality cancer surveillance study in high risk LC volunteers. Enrolment was done in subjects from Antofagasta and Metropolitan regions. In addition, we enrolled subjects who were suspected of having lung cancer. AQC, DR70 and AFB were used as tools in the detection of pre-neoplastic (PNL) and neoplastic lesions (NL). Results: Half of the samples, classified as suspicious by AFB, were confirmed as metaplasia or dysplasia by histopathology. For LC, DR70 showed a higher sensitivity (95.8%) and specificity (91.9%) than AQC. However, for PNL AQC showed a higher sensitivity (91.9%) than DR70 (27.3%), although both with low PPV values. As a pre screener, both biomarkers might be employed as complementary tools to detect LC, especially as serially combined tests, with a sensitivity of 60% and a PPV of 65.2%. Additionally, the use of parallel combined tests might support the detection of PNL (sensitivity 91.2%; PPV 49.1%). Conclusion: This work adds information on cellular and molecular biomarkers to complement imaging techniques for early detection of LC in Latin America that might contribute to formulate policies concerning screening of LC. Supported by INNOVA-CORFO, Chile. | 3724 | NA | NA | NA | no | no |
| 3724 | 2017 | NA | OLIVER, JH; OWSLEY, MR; HUTCHESON, HJ; JAMES, AM; CHEN, CS; IRBY, WS; DOTSON, EM; MCLAIN, DK | 1993 | CONSPECIFICITY OF THE TICKS IXODES-SCAPULARIS AND IXODES-DAMMINI (ACARI, IXODIDAE) | Reciprocal crosses between Ixodes dammini Spielman, Clifford, Piesman & Corwin from Massachusetts and Ixodes scapularis Say from Georgia produced offspring through the F3 generation when the experiment was discontinued. Reciprocal I. dammini x Ixodes pacificus Cooley & Kohls (California) and I. scapularis x I. pacificus crosses produced F1 progeny; however, all progeny were sterile. Assortative mating experiments between I. dammini and I. scapularis indicated that males and females of both species mated with the opposite sex of heterospecific or conspecific ticks when there was a choice. Conventional discriminant analysis of morphometric measurements of ticks from Georgia, North Carolina, Maryland, Massachusetts, and two populations of F1 hybrids indicated that there were recognizable differences. However, size-free (sheared) discriminant analysis indicated that these differences were largely size-dependent, with much overlap of the four eastern and two hybrid populations but no overlap with I. pacificus from California. Analysis of chromosomes (morphology and C band) indicated no differences between the Georgia and Massachusetts populations but showed a difference between them and the California population of 1. pacificus. Analysis of isozymes showed that the genetic identity value for the Georgia and Massachusetts populations was within the normal range for conspecific populations, whereas the California population indicated congeneric but not conspecific relatedness to the Georgia and Massachusetts populations. Life cycle data collected under similar laboratory conditions showed no differences in length of feeding and molting periods among Georgia, Massachusetts, and California populations. These data and results of the work of other authors on tick host preferences and vector competence indicate that I. dammini is not a valid species separate from I. scapularis. Because the name Ixodes scapularis Say, 1821, has priority over the name Ixodes dammini Spielman, Clifford, Piesman & Corwin, 1979, I. dammini is relegated to a junior subjective synonym of 1. scapularis (based on Article 23 of the International Code of Zoological Nomenclature). | 3725 | NA | NA | NA | no | no |
| 3725 | 2017 | NA | Cian, A; El Safadi, D; Osman, M; Moriniere, R; Gantois, N; Benamrouz-Vanneste, S; Delgado-Viscogliosi, P; Guyot, K; Li, LL; Monchy, S; Noel, C; Poirier, P; Nourrisson, C; Wawrzyniak, I; Delbac, F; Bosc, S; Chabe, M; Petit, T; Certad, G; Viscogliosi, E | 2017 | Molecular Epidemiology of Blastocystis sp in Various Animal Groups from Two French Zoos and Evaluation of Potential Zoonotic Risk | Blastocystis sp. is a common intestinal parasite infecting humans and a wide range of animals worldwide. It exhibits an extensive genetic diversity and 17 subtypes (STs) have thus far been identified in mammalian and avian hosts. Since several STs are common to humans and animals, it was proposed that a proportion of human infections may result from zoonotic transmission. However, the contribution of each animal source to human infection remains to be clarified. Therefore, the aim of this study was to expand our knowledge of the epidemiology and host specificity of this parasite by performing the largest epidemiological survey ever conducted in animal groups in terms of numbers of species screened. A total of 307 stool samples from 161 mammalian and non-mammalian species in two French zoos were screened by real-time PCR for the presence of Blastocystis sp. Overall, 32.2% of the animal samples and 37.9% of the species tested were shown to be infected with the parasite. A total of 111 animal Blastocystis sp. isolates were subtyped, and 11 of the 17 mammalian and avian STs as well as additional STs previously identified in reptiles and insects were found with a varying prevalence according to animal groups. These data were combined with those obtained from previous surveys to evaluate the potential risk of zoonotic transmission of Blastocystis sp. through the comparison of ST distribution between human and animal hosts. This suggests that non-human primates, artiodactyls and birds may serve as reservoirs for human infection, especially in animal handlers. In contrast, other mammals such as carnivores, and non-mammalian groups including reptiles and insects, do not seem to represent significant sources of Blastocystis sp. infection in humans. In further studies, more intensive sampling and screening of potential new animal hosts will reinforce these statements and expand our understanding of the circulation of Blastocystis sp. in animal and human populations. | 3726 | NA | NA | NA | no | no |
| 3726 | 2017 | NA | Roux, EA; Korb, J | 2004 | Evolution of eusociality and the soldier caste in termites: a validation of the intrinsic benefit hypothesis | In termites the evolution of reproductive altruism is not based on a particularly high relatedness between nestmates. For the evolution and maintenance of the ancestral sterile soldier caste, the benefits generated by the soldiers’ presence must compensate the loss of the soldiers’ reproductive potential. To study the impact of soldiers on colony’s fitness, we manipulated the proportion of soldiers to nonsoldiers in colonies of the dry-wood termite Cryptotermes secundus.’Soldier-less’ colonies were obtained by removing soldiers and inhibiting their development with an extract of soldier heads. The colonies were set up for 1 year in experimental nests in the field. ‘Soldier-less’ colonies produced fewer soldiers. The reduction of soldiers neither affected colony survival nor helper growth, but fewer dispersing sexuals were produced in ‘soldier-less’ than in control colonies. This confirms what was only supposed so far, that in termites soldiers are maintained for their intrinsic benefit to cost ratio. | 3727 | NA | NA | NA | no | no |
| 3727 | 2017 | NA | Tonk, L; Sampayo, EM; Weeks, S; Magno-Canto, M; Hoegh-Guldberg, O | 2013 | Host-Specific Interactions with Environmental Factors Shape the Distribution of Symbiodinium across the Great Barrier Reef | Background: The endosymbiotic dinoflagellates (genus Symbiodinium) within coral reef invertebrates are critical to the survival of the holobiont. The genetic variability of Symbiodinium may contribute to the tolerance of the symbiotic association to elevated sea surface temperatures (SST). To assess the importance of factors such as the local environment, host identity and biogeography in driving Symbiodinium distributions on reef-wide scales, data from studies on reef invertebrate-Symbiodinium associations from the Great Barrier Reef (GBR) were compiled. Methodology/Principal Findings: The resulting database consisted of 3717 entries from 26 studies. It was used to explore ecological patterns such as host-specificity and environmental drivers structuring community complexity using a multi-scalar approach. The data was analyzed in several ways: (i) frequently sampled host species were analyzed independently to investigate the influence of the environment on symbiont distributions, thereby excluding the influence of host specificity, (ii) host species distributions across sites were added as an environmental variable to determine the contribution of host identity on symbiont distribution, and (iii) data were pooled based on clade (broad genetic groups dividing the genus Symbiodinium) to investigate factors driving Symbiodinium distributions using lower taxonomic resolution. The results indicated that host species identity plays a dominant role in determining the distribution of Symbiodinium and environmental variables shape distributions on a host species-specific level. SST derived variables (especially SSTstdev) most often contributed to the selection of the best model. Clade level comparisons decreased the power of the predictive model indicating that it fails to incorporate the main drivers behind Symbiodinium distributions. Conclusions/Significance: Including the influence of different host species on Symbiodinium distributional patterns improves our understanding of the drivers behind the complexity of Symbiodinium-invertebrate symbioses. This will increase our ability to generate realistic models estimating the risk reefs are exposed to and their resilience in response to a changing climate. | 3728 | NA | NA | NA | no | no |
| 3728 | 2017 | NA | Gillespie, JJ; Beier, MS; Rahman, MS; Ammerman, NC; Shallom, JM; Purkayastha, A; Sobral, BS; Azad, AF | 2007 | Plasmids and Rickettsial Evolution: Insight from Rickettsia felis | Background. The genome sequence of Rickettsia felis revealed a number of rickettsial genetic anomalies that likely contribute not only to a large genome size relative to other rickettsiae, but also to phenotypic oddities that have confounded the categorization of R. felis as either typhus group (TG) or spotted fever group (SFG) rickettsiae. Most intriguing was the first report from rickettsiae of a conjugative plasmid (pRF) that contains 68 putative open reading frames, several of which are predicted to encode proteins with high similarity to conjugative machinery in other plasmid-containing bacteria. Methodology/Principal Findings. Using phylogeny estimation, we determined the mode of inheritance of pRF genes relative to conserved rickettsial chromosomal genes. Phylogenies of chromosomal genes were in agreement with other published rickettsial trees. However, phylogenies including pRF genes yielded different topologies and suggest a close relationship between pRF and ancestral group (AG) rickettsiae, including the recently completed genome of R. bellii str. RML369-C. This relatedness is further supported by the distribution of pRF genes across other rickettsiae, as 10 pRF genes (or inactive derivatives) also occur in AG (but not SFG) rickettsiae, with five of these genes characteristic of typical plasmids. Detailed characterization of pRF genes resulted in two novel findings: the identification of oriV and replication termination regions, and the likelihood that a second proposed plasmid, pRF delta, is an artifact of the original genome assembly. Conclusion/Significance. Altogether, we propose a new rickettsial classification scheme with the addition of a fourth lineage, transitional group (TRG) rickettsiae, that is unique from TG and SFG rickettsiae and harbors genes from possible exchanges with AG rickettsiae via conjugation. We offer insight into the evolution of a plastic plasmid system in rickettsiae, including the role plasmids may have played in the acquirement of virulence traits in pathogenic strains, and the likely origin of plasmids within the rickettsial tree. | 3729 | NA | NA | NA | no | no |
| 3729 | 2017 | NA | Marathe, BM; Wong, SS; Vogel, P; Garcia-Alcalde, F; Webster, RG; Webby, RJ; Najera, I; Govorkova, EA | 2016 | Combinations of Oseltamivir and T-705 Extend the Treatment Window for Highly Pathogenic Influenza A(H5N1) Virus Infection in Mice | Current anti-influenza therapy depends on administering drugs soon after infection, which is often impractical. We assessed whether combinations of oseltamivir (a neuraminidase inhibitor) and T-705 (a nonspecific inhibitor of viral polymerases) could extend the window for treating lethal infection with highly pathogenic A(H5N1) influenza virus in mice. Combination therapy protected 100% of mice, even when delayed until 96 h postinoculation. Compared to animals receiving monotherapy, mice receiving combination therapy had reduced viral loads and restricted viral spread in lung tissues, limited lung damage, and decreased inflammatory cytokine production. Next-generation sequencing showed that virus populations in T-705-treated mice had greater genetic variability, with more frequent transversion events, than did populations in control and oseltamivir-treated mice, but no substitutions associated with resistance to oseltamivir or T-705 were detected. Thus, combination therapy extended the treatment window for A(H5N1) influenza infection in mice and should be considered for evaluation in a clinical setting. | 3730 | NA | NA | NA | no | no |
| 3730 | 2017 | NA | Stafford-Bell, RE; Chariton, AA; Robinson, RW | 2015 | Prolonged buoyancy and viability of Zostera muelleri Irmisch ex Asch vegetative fragments indicate a strong dispersal potential | The establishment of clonal marine plant populations, including the seagrass Zostera muelleri (Zosteraceae), may be more dependent on the availability of viable vegetative fragments rather than seed. New populations may establish through long-distance dispersal of viable vegetative fragments, potentially increasing genetic diversity and resilience to anthropogenic or naturally occurring disturbance. A number of activities can dislodge vegetative fragments of Z muelleri (leaves, rhizomes and roots) from the sediment. These fragments can remain positively buoyant, floating on the surface of the water. As the time since dislodgement increases, buoyancy may become reduced, causing fragments to move lower into the water column. However, what is not known is how long these fragments remain buoyant and potentially viable for recolonization. To address this knowledge gap, we collected wrack samples (n = 125) of Z. muelleri from four Victorian estuaries. Fragments were floated in outside aquaria for up to ten weeks, with subsamples tested for metabolic activity using tetrazolium violet. Porosity of seagrass rhizomes was also investigated to understand the influence of lacunae (large air filled spaces within plant tissues) on the flotation of vegetative fragments. The average proportion of potentially viable fragments collected in wrack ranged from 3.6% (SD = 2.23) to 11.2% (SD = 5.9). While there was a steady decline in the buoyancy of fragments across the ten-week period, initial buoyancy was relatively high, with approximately 50% of the fragments remaining positively buoyant for the initial five weeks. The viability of fragments following flotation was high. One hundred percent of fragments (n = 25 per assay) remained viable after floating for three weeks, with only a marginal decline (= 96% viability) occurring after five weeks. When considered in conjunction with the highly porous nature of seagrass rhizomes (lacunae accounted for 45.2% of total volume), our findings indicate that the species may be capable of prolonged periods of transport dispersal within the marine environment. (C) 2015 Elsevier B.V. All rights reserved. | 3731 | NA | NA | NA | no | no |
| 3731 | 2017 | NA | Syracuse, AJ; Johnson, CS; Eisenback, JD; Nessler, CL; Smith, EP | 2004 | Intraspecific variability within Globodera tabacum solanacearum using random amplified polymorphic DNA | Random amplified polymorphic DNA (RAPDs) were used to investigate the intraspecific variability among 19 geographic isolates of Globodera tabacum solanacearum from eight counties in Virginia and one county ill North Carolina. Globodera tabacum tabacum, G. t. virginiae, and the Mexican cyst nematode (MCN) were included as outgroups. Six primers were used and 119 amplification products were observed. Each primer yielded reproducible differences in fragment. patterns that differentiated the isolates and species. Hierarchical cluster analysis was performed to illustrate the relatedness among isolates and species. The average in the literature across different pathotypes of the potato cyst nematode. Globodera pallida, in studies where RAPD were also employed. The RAPD markers described here may be useful for the development of specific primers or probes that could improve the identification of TCN populations. Such improvements in the characterization of TCN genotypes would facilitate the effective deployment of existing and future resistant cultivars to control these economically important pests. | 3732 | NA | NA | NA | no | no |
| 3732 | 2017 | NA | Sauter, A; Brown, MJF; Baer, B; Schmid-Hempel, P | 2001 | Males of social insects can prevent queens from multiple mating | During copulation, males of Bombus terrestris fill the queen’s sexual tract with a mating plug after transferring their sperm. The sticky secretion is produced by the male’s accessory glands and disappears within a couple of days. Experiments now show that the primary function of the plug is to reduce the subsequent mating probability of the queen. The plug is not efficient in preventing sperm migration into the spermatheca. Due to its low energetic value, the plug is also unlikely to serve as a nuptial gift. This type of male interference with female mating propensity has so far not been found in social insects. This finding could, at least tentatively, explain why females of B. terrestris may not be able to take advantage of the demonstrated benefits of multiple mating. Furthermore, such male interference could be a more general phenomenon in social insects. with obvious ramifications for the evolution of polyandry in this group. | 3733 | NA | NA | NA | no | no |
| 3733 | 2017 | NA | Cregan, PB; Mudge, J; Fickus, EW; Danesh, D; Denny, R; Young, ND | 1999 | Two simple sequence repeat markers to select for soybean cyst nematode resistance coditioned by the rhg1 locus | The soybean cyst nematode (SCN) (Heterodera glycines Inchinoe) is the most economically significant soybean pest. The principal strategy to reduce or eliminate damage from this pest is the use of resistant cultivars. Identifying resistant segregants in a breeding program is a difficult and expensive process which is complicated by the oligogenic nature of the resistance and genetic variability in the pathogen. Fortunately, resistance at one SCN-resistance locus, rhg1, is generally accepted as a necessity for the development of resistant genotypes using any source of resistance and when challenged by any SCN race. Thus, the development of SCN resistant cultivars would be expedited if an effective and rapid system were available to identify breeding lines carrying a resistance allele at the rhg1 locus. In this study we report two simple sequence repeat (SSR) or microsatellite loci that cosegregate and map 0.4 cM from rhg1. Allelic variation at the first of these loci, BARC-Satt309, distinguished most, if not all, SCN-susceptible genotypes from those carrying resistance at rhg1 derived from the important SCN-resistance sources ‘Peking’, PI 437654, and PI 90763. BARC-Satt309 was also effective in distinguishing SCN resistance sources PI 88788 and PI 209332 from many, but not all, susceptible genotypes. BARC-Satt309 cannot be used in marker-assisted selection in populations developed from typical southern US cultivars crossed with the important resistance sources PI 88788 or PI 209332 because these genotypes all carry the identical allele at the BARC-Satt309 locus. A second SSR locus, BARC-Sat\_168, was developed from a bacterial artificial chromosome (BAC) clone that was identified using the primers to BARC-Satt309. BARC-Sat\_168 distinguished PI 88788 and PI 209332 from southern US cultivars such as ‘Lee’,‘Bragg’ and ‘Essex’. Both BARC-Satt309 and BARC-Sat\_168 were used to assay Lines from SCN-susceptiblexSCN-resistant crosses and proved to be highly effective in identifying lines carrying rhg1 resistance from those carrying the allele for SCN susceptibility at the rhg1 locus. | 3734 | NA | NA | NA | no | no |
| 3734 | 2017 | NA | Foitzik, S; Achenbach, A; Brandt, M | 2009 | Locally adapted social parasite affects density, social structure, and life history of its ant hosts | Selection and adaptation are important processes in the coevolution between parasites and their hosts. The slave-making ant Protomognathus americanus, an obligate ant social parasite, has previously been shown to evolve morphological, behavioral, and chemical adaptations in the coevolutionary arms race with its Temnothorax hosts. Yet empirical studies have given variable results on the strength of the selection pressure this parasite exerts on its host populations. In this study, we directly investigated the pressure exerted by P. americanus and the reactions of the main host species, T. longispinosus, in two ant communities by manipulating parasite density in the field over several years. In addition, a cross-fostering design with the exchange of parasites between host populations allowed us to investigate local adaptation of parasite or host. We demonstrate a severe impact of the social parasite on the two host populations in West Virginia and New York, but also variation in host reactions between sites, as expected by the geographic mosaic theory of coevolution. Host density decreased at the West Virginia site with the presence of local slave-makers, whereas at the ecologically favorable New York site, density was unaffected. Nevertheless, social organization, colony size, and investment patterns of these host colonies at this site changed in response to our parasite manipulation. The release of P. americanus colonies led to a reduction in the number of resident queens and workers, an increase in intranest relatedness, and lower productivity, but also a higher investment in reproductives. In West Virginia, colony demography did not change, but raiding activity by New York slave-makers caused different investment patterns of host colonies. In addition, the cross-fostering element revealed local adaptation of the parasite P. americanus: slave-making colonies fared better in their sympatric host population, as they contained more slave-making ant workers and slaves at the end of our 27-month experiment. | 3735 | NA | NA | NA | no | no |
| 3735 | 2017 | NA | GAVRILETS, S; HASTINGS, A | 1995 | INTERMITTENCY AND TRANSIENT CHAOS FROM SIMPLE FREQUENCY-DEPENDENT SELECTION | Frequency-dependent selection is an important determinant of the evolution of gametophytic self-incompatibility systems in plants, aposematic (warning) and cryptic coloration, systems of mimicry, competitive interactions among members of a population, mating preferences, predator-prey and host-parasite interactions, aggression and other behavioural traits. Past theoretical studies of frequency-dependent selection have shown it to be a plausible mechanism for the maintenance of genetic variability in natural populations. Here, through an analysis of a simple deterministic model for frequency-dependent selection, we demonstrate that complex dynamic behaviour is possible under a broad range of parameter values. In particular we show that the model exhibits not only cycles and chaos but also, for a more restricted set of parameters, transient chaos and intermittency: alterations between an apparently deterministic behaviour and apparently chaotic fluctuations. This behaviour, which has not been stressed within the population genetics literature, provides an explanation for erratic dynamics of gene frequencies. | 3736 | NA | NA | NA | no | no |
| 3736 | 2017 | NA | Jex, AR; Waeschenbach, A; Hu, M; Van Wyk, JA; Beveridge, I; Littlewood, DTJ; Gasser, RB | 2009 | The mitochondrial genomes of Ancylostoma caninum and Bunostomum phlebotomum - two hookworms of animal health and zoonotic importance | Background: Hookworms are blood-feeding nematodes that parasitize the small intestines of many mammals, including humans and cattle. These nematodes are of major socioeconomic importance and cause disease, mainly as a consequence of anaemia (particularly in children or young animals), resulting in impaired development and sometimes deaths. Studying genetic variability within and among hookworm populations is central to addressing epidemiological and ecological questions, thus assisting in the control of hookworm disease. Mitochondrial (mt) genes are known to provide useful population markers for hookworms, but mt genome sequence data are scant. Results: The present study characterizes the complete mt genomes of two species of hookworm, Ancylostoma caninum (from dogs) and Bunostomum phlebotomum (from cattle), each sequenced (by 454 technology or primer-walking), following long-PCR amplification from genomic DNA (similar to 20-40 ng) isolated from individual adult worms. These mt genomes were 13717 bp and 13790 bp in size, respectively, and each contained 12 protein coding, 22 transfer RNA and 2 ribosomal RNA genes, typical for other secernentean nematodes. In addition, phylogenetic analysis (by Bayesian inference and maximum likelihood) of concatenated mt protein sequence data sets for 12 nematodes (including Ancylostoma caninum and Bunostomum phlebotomum), representing the Ascaridida, Spirurida and Strongylida, was conducted. The analysis yielded maximum statistical support for the formation of monophyletic clades for each recognized nematode order assessed, except for the Rhabditida. Conclusion: The mt genomes characterized herein represent a rich source of population genetic markers for epidemiological and ecological studies. The strong statistical support for the construction of phylogenetic clades and consistency between the two different tree-building methods employed indicate the value of using whole mt genome data sets for systematic studies of nematodes. The grouping of the Spirurida and Ascaridida to the exclusion of the Strongylida was not supported in the present analysis, a finding which conflicts with the current evolutionary hypothesis for the Nematoda based on nuclear ribosomal gene data. | 3737 | NA | NA | NA | no | no |
| 3737 | 2017 | NA | Munwes, I; Geffen, E; Friedmann, A; Tikochinski, Y; Gafny, S | 2011 | Variation in repeat length and heteroplasmy of the mitochondrial DNA control region along a core-edge gradient in the eastern spadefoot toad (Pelobates syriacus) | Peripheral populations are those situated at the distribution margins of a species and are often subjected to more extreme abiotic and biotic conditions than those at the core. Here, we hypothesized that shorter repeat length and fewer heteroplasmic mitochondrial DNA (mtDNA) copies, which are associated with more efficient mitochondrial function, may be related to improved survival under extreme environmental conditions. We sampled eastern spadefoot toads (mostly as tadpoles) from 43 rain pools distributed along a 300-km gradient from core to edge of the species’ distribution. We show that mean pool tandem repeat length and heteroplasmy increase from edge to core, even after controlling for body size. We evaluate several alternative hypotheses and propose the Fisher hypothesis as the most likely explanation. However, additional sequential sampling and experimental studies are required to determine whether selection under extreme conditions, or alternative mechanisms, could account for the gradient in heteroplasmy and repeat length in the mtDNA control region. | 3738 | NA | NA | NA | no | no |
| 3738 | 2017 | NA | Yamagiwa, J | 2008 | History and present scope of field studies on Macaca fuscata yakui at Yakushima Island, Japan | Field studies on Japanese macaques on Yakushima Island started in the mid-1970s, > 25 yr after the emergence of Japanese primatology, in response to criticism of methods using provisioning and the desire to find the socioecological factors influencing the social life of macaques in natural habitats. We habituated macaques without provisioning mainly in the coastal warm-temperate forest and found that they lived in small troops with a high socionomic sex ratio. Observations of several troop fissions and troop takeovers by nontroop males suggest that Yakushima macaques have a different social organization from that of Japanese macaques in other habitats. For example, youngest ascendancy as the dominance relationhip among sisters, which usually occurs in provisioned troops, was absent in Yakushima macaques. We compared their ecological and social features with those of Japanese macaques at Kinkazan (cool-temperate forests) and found that abundance of high-quality foods may cause stronger intra- and intertroop competition at Yakushima. Female Yakushima macaques may more positively solicit nontroop males to associate with them during the mating season. Such a tendency may promote frequent male movement between troops and frequent troop fissions. Though ecological factors form social features of Japanese macaques, some features such as male association and movements between troops are not accounted for via socioecology. Recent field studies have focused on macaques living at higher altitudes in Yakushima and on individual survival strategies by taking diverse viewpoints and using new technologies. DNA analysis of fecal samples shows low genetic diversity and suggests the macaques’ recent expansion from lowland to highland forests in Yakushima. The population censuses conducted annually indicate that the higher-altitude macaques have a larger home range but a similar group size versus their counterparts at low elevations. The unsolved issues in socioecology will pose a challenge to the younger generation of primatologists. Conservation of macaques and their habitat is one of our major activities at Yakushima. The level of protection has gradually increased in the National Park at Yakushima and, via our various conservation efforts, its most important area was designated a World Heritage Site by UNESCO in 1993. However, large-scale logging in the 1960s and 1970s caused the loss of macaque habitats and led to increased crop damage by them in the 1980s. We have proposed effective methods to protect cultivated fields from macaques as well as several plans for sustainable use of forests, such as ecotourism and a fieldwork course for university students. Local residents and researchers have created several nongovernment organizations (NGOs) to promote conservation and nature study at Yakushima. The role of local NGOs is particularly important to mitigate conflicts between people and wildlife. Though hundreds of macaques are still captured as pests annually in Yakushima, we continue the conservation measures and spread awareness of conservation in cooperation with the local NGOs. | 3739 | NA | NA | NA | no | no |
| 3739 | 2017 | NA | Wares, JP; Schiebelhut, LM | 2016 | What doesn’t kill them makes them stronger: an association between elongation factor 1-alpha overdominance in the sea star Pisaster ochraceus and “sea star wasting disease” | In recent years, a massive mortality event has killed millions of sea stars, of many different species, along the Pacific coast of North America This disease event,known as ‘sea star wasting disease’ (SSWD), is linked to viral infection. In one affected sea star (Pisaster ochraceus), previous work had identified that the elongation factor 1-alpha locus (EF1A) harbored an intronic insertion allele that is lethal when homozygous yet appears to be maintained at moderate frequency in populations through increased fitness for heterozygotes. The environmental conditions supporting this increased fitness are unknown, but overdominance is often associated with disease. Here we evaluate populations of P. ochraceus to identify the relationship between SSWD and EF1A genotype. Our data suggest that there may be significantly decreased occurrence of SSWD in individuals that are heterozygous at this locus. These results suggest further studies are warranted to understand the functional relationship between diversity at EF1A and survival in P. ochraceus. | 3740 | NA | NA | NA | no | no |
| 3740 | 2017 | NA | Paradis, E; Baillie, SR; Sutherland, WJ; Gregory, RD | 1998 | Patterns of natal and breeding dispersal in birds | 1.  Dispersal is of critical ecological and evolutionary importance for several issues of population biology, particularly population synchrony, colonization and range expansion, metapopulation and source–sink dynamics, and population genetic structure, but it has not previously been possible to compare dispersal patterns across a wide range of species or to study movement outside the confines of local study areas.  2.  Using resampling methods, we verified that statistically unbiased estimates of average dispersal distance and of intraspecific variance in dispersal distance could be extracted from the bird ringing data of the British Trust for Ornithology.  3.  Using data on 75 terrestrial bird species, we tested whether natal and breeding dispersal were influenced by a species’ habitat requirements, diet, geographical range, abundance, morphology, social system, life history or migratory status. We used allometric techniques to ascertain whether these relationships were independent of body size, and used the method of phylogenetically independent contrasts to ascertain whether they were independent of phylogeny.  4.  Both natal and breeding dispersal distances were lower among abundant species and among species with large geographical ranges. Dispersal distances and life‐history variables were correlated independent of phylogeny, but these relationships did not persist after controlling for body size. All morphometrical variables (wing length, tarsus length and bill length) were not significantly correlated with dispersal distances after correcting for body size or phylogenetic relatedness.  5.  Migrant species disperse further than resident ones, this relation was independent of body size but not of phylogeny. A significant positive relation was observed between breeding dispersal distance and long‐term population decline among migrants, but not among residents. 6.  The species living in wet habitats disperse further than those living in dry habitats, which could be explained by the greater patchiness of wet habitats in space and/or time. This relationship was observed only for breeding dispersal, suggesting that this habitat variable does not impose the same constraint on natal dispersal. | 3741 | NA | NA | NA | no | no |
| 3741 | 2017 | NA | O’Donnell, K; Gueidan, C; Sink, S; Johnston, PR; Crous, PW; Glenn, A; Riley, R; Zitomer, NC; Colyer, P; Waalwijk, C; van der Lee, T; Moretti, A; Kang, S; Kim, HS; Geiser, DM; Juba, JH; Baayen, RP; Cromey, MG; Bithell, S; Sutton, DA; Skovgaard, K; Ploetz, R; Kistler, HC; Elliott, M; Davis, M; Sarver, BAJ | 2009 | A two-locus DNA sequence database for typing plant and human pathogens within the Fusarium oxysporum species complex | We constructed a two-locus database, comprising partial translation elongation factor (EF-1 alpha) gene sequences and nearly full-length sequences of the nuclear ribosomal intergenic spacer region (IGS rDNA) for 850 isolates spanning the phylogenetic breadth of the Fusarium oxysporum species complex (FOSC). Of the 850 isolates typed, 101 EF-1 alpha, 203 IGS rDNA, and 256 two-locus sequence types (STs) were differentiated. Analysis of the combined dataset suggests that two-thirds of the STs might be associated with a single host plant. This analysis also revealed that the 26 STs associated with human mycoses were genetically diverse, including several which appear to be nosocomial in origin. A congruence analysis, comparing partial EF-1 alpha and IGS rDNA bootstrap consensus, identified a significant number of conflicting relationships dispersed throughout the bipartitions, suggesting that some of the IGS rDNA sequences may be non-orthologous. We also evaluated enniatin, fumonisin and moniliformin mycotoxin production in vitro within a phylogenetic framework. Published by Elsevier Inc. | 3742 | NA | NA | NA | no | no |
| 3742 | 2017 | NA | Rauch, G; Kalbe, M; Reusch, TBH | 2005 | How a complex life cycle can improve a parasite’s sex life | How complex life cycles of parasites are maintained is still a fascinating and unresolved topic. Complex life cycles using three host species, free-living stages, asexual and sexual reproduction are widespread in parasitic helminths. For such life cycles, we propose here that maintaining a second intermediate host in the life cycle can be advantageous for the individual parasite to increase the intermixture of different clones and therefore decrease the risk of matings between genetically identical individuals in the definitive host. Using microsatellite markers, we show that clone mixing occurs from the first to the second intermediate host in natural populations of the eye-fluke Diplostomum pseudospathaceum. Most individuals released by the first intermediate host belonged to one clone. In contrast, the second intermediate host was infected with a diverse array of mostly unique parasite genotypes. The proposed advantage of increased parasite clone intermixture may be a novel selection pressure favouring the maintenance of complex life cycles. | 3743 | NA | NA | NA | no | no |
| 3743 | 2017 | NA | Wang, MH; Wang, L; Jiang, N; Jia, TY; Luo, ZW | 2013 | A robust and efficient statistical method for genetic association studies using case and control samples from multiple cohorts | Background: The theoretical basis of genome-wide association studies (GWAS) is statistical inference of linkage disequilibrium (LD) between any polymorphic marker and a putative disease locus. Most methods widely implemented for such analyses are vulnerable to several key demographic factors and deliver a poor statistical power for detecting genuine associations and also a high false positive rate. Here, we present a likelihood-based statistical approach that accounts properly for non-random nature of case-control samples in regard of genotypic distribution at the loci in populations under study and confers flexibility to test for genetic association in presence of different confounding factors such as population structure, non-randomness of samples etc. Results: We implemented this novel method together with several popular methods in the literature of GWAS, to re-analyze recently published Parkinson’s disease (PD) case-control samples. The real data analysis and computer simulation show that the new method confers not only significantly improved statistical power for detecting the associations but also robustness to the difficulties stemmed from non-randomly sampling and genetic structures when compared to its rivals. In particular, the new method detected 44 significant SNPs within 25 chromosomal regions of size < 1 Mb but only 6 SNPs in two of these regions were previously detected by the trend test based methods. It discovered two SNPs located 1.18 Mb and 0.18 Mb from the PD candidates, FGF20 and PARK8, without invoking false positive risk. Conclusions: We developed a novel likelihood-based method which provides adequate estimation of LD and other population model parameters by using case and control samples, the ease in integration of these samples from multiple genetically divergent populations and thus confers statistically robust and powerful analyses of GWAS. On basis of simulation studies and analysis of real datasets, we demonstrated significant improvement of the new method over the non-parametric trend test, which is the most popularly implemented in the literature of GWAS. | 3744 | NA | NA | NA | no | no |
| 5857 | 2017 | from ref check | Froeschke, Goetz; Sommer, Simone | 2012 | Insights into the Complex Associations Between MHC Class II DRB Polymorphism and Multiple Gastrointestinal Parasite Infestations in the Striped Mouse | Differences in host susceptibility to different parasite types are largely based on the degree of matching between immune genes and parasite antigens. Specifically the variable genes of the major histocompatibility complex (MHC) play a major role in the defence of parasites. However, underlying genetic mechanisms in wild populations are still not well understood because there is a lack of studies which deal with multiple parasite infections and their competition within. To gain insights into these complex associations, we implemented the full record of gastrointestinal nematodes from 439 genotyped individuals of the striped mouse, Rhabdomys pumilio. We used two different multivariate approaches to test for associations between MHC class II DRB genotype and multiple nematodes with regard to the main pathogen-driven selection hypotheses maintaining MHC diversity and parasite species-specific co-evolutionary effects. The former includes investigations of a ‘heterozygote advantage’, or its specific form a ‘divergent-allele advantage’ caused by highly dissimilar alleles as well as possible effects of specific MHC-alleles selected by a ‘rare allele advantage’ ( = negative ‘frequency-dependent selection’). A combination of generalized linear mixed models (GLMMs) and co-inertia (COIA) analyses made it possible to consider multiple parasite species despite the risk of type I errors on the population and on the individual level. We could not find any evidence for a ‘heterozygote’ advantage but support for ‘divergent-allele’ advantage and infection intensity. In addition, both approaches demonstrated high concordance of positive as well as negative associations between specific MHC alleles and certain parasite species. Furthermore, certain MHC alleles were associated with more than one parasite species, suggesting a many-to-many gene-parasite co-evolution. The most frequent allele Rhpu-DRB\*38 revealed a pleiotropic effect, involving three nematode species. Our study demonstrates the co-existence of specialist and generalist MHC alleles in terms of parasite detection which may be an important feature in the maintenance of MHC polymorphism. | 3745 | yes | from ref check | MHC | yes | no |
| 3744 | 2017 | NA | Chen, YP; Evans, JD; Zhou, L; Boncristiani, H; Kimura, K; Xiao, TG; Litkowski, AM; Pettis, JS | 2009 | Asymmetrical coexistence of Nosema ceranae and Nosema apis in honey bees | Globalization has provided opportunities for parasites/pathogens to cross geographic boundaries and expand to new hosts. Recent studies showed that Nosema ceranae, originally considered a microsporidian parasite of Eastern honey bees, Apis cerana, is a disease agent of nosemosis in European honey bees, Apis mellifera, along with the resident species, Nosema apis. Further Studies indicated that disease caused by N. ceranae In European honey bees is far more prevalent than that caused by N. apis. In order to gain more insight into the epidemiology of Nosema parasitism in honey bees, we conducted studies to investigate infection of Nosema in its original host, Eastern honey bees, using conventional PCR and duplex real time quantitative PCR methods. Our results showed that A. cerana was infected not only with N. ceranae as previously reported [Fries, L, Feng, F., Silva, A.D., Slemenda, S.B., Pieniazek, N.J., 1996. Nosema ceranae n. sp. (Microspora, Nosematidae), morphological and molecular characterization of a microsporidian parasite of the Asian honey bee Apis cerana (Hymenoptera, Apidae). Eur. J. Protistol. 32, 356-365], but also with N. apis. Both microsporidia produced single and mixed infections. Overall and at each location alone, the prevalence of N. ceranae was higher than that of N. apis. In all cases of mixed infections, the number of N. ceranae gene copies (corresponding to the parasite load) significantly out numbered those of N. apis. Phylogenetic analysis based on a variable region of small subunit ribosomal RNA (SSUrRNA) showed four distinct clades of N. apis and five clades of N. ceranae and that geographical distance does not appear to influence the genetic diversity of Nosema populations. The results from this study demonstrated that duplex real-time qPCR assay developed in this study is a valuable tool for quantitative measurement of Nosema and can be used to monitor the progression of microsprodian infections of honey bees in a timely and cost efficient manner. Published by Elsevier Inc. | 3746 | NA | NA | NA | no | no |
| 3745 | 2017 | NA | Soons, MB; Heil, GW | 2002 | Reduced colonization capacity in fragmented populations of wind-dispersed grassland forbs | 1 Habitat fragmentation as a result of intensification of agricultural practices decreases the population size and increases the site productivity of remnant populations of many plant species native to nutrient‐poor, species‐rich grasslands. Little is known about how this affects the colonization capacity of populations, which is highly important for regional species survival. We studied the effects on four wind‐dispersed forbs that represent two major dispersal strategies in grasslands: Cirsium dissectum and Hypochaeris radicata, which have plumed seeds and are adapted to long‐distance dispersal by wind, and Centaurea jacea and Succisa pratensis, which have plumeless seeds and are adapted to only short‐distance dispersal by wind.  2 Colonization capacity decreased with decreasing population size. This was due to lower seed germination ability in all species, and a lower seed production and a narrower range of seed dispersal distances in the species with plumed seeds. Inbreeding depression is the most likely cause of this. We found no evidence for a stronger selection for reduced dispersal in smaller populations.  3 Increasing site productivity changed the colonization capacity of all species. The capacity for colonization of nearby sites increased, due to higher seed production and seed germination ability, but the capacity for colonization of distant sites decreased, due to a lower long‐distance dispersal ability.  4 Seed dispersal ability and germination ability were negatively correlated in the species with plumeless seeds, but not in the species with plumed seeds. The dispersal ability of individual plumed seeds remained constant under changes in population size and site productivity. This indicates a strong selection pressure for long‐distance dispersal ability in these species.  5 When habitat fragmentation results in a simultaneous decrease in population size and increase in site productivity, both the local survival probability and the colonization capacity of remnant populations of wind‐dispersed grassland forbs are likely to be severely reduced. This increases regional extinction risks of the species. 1 Habitat fragmentation as a result of intensification of agricultural practices decreases the population size and increases the site productivity of remnant populations of many plant species native to nutrient‐poor, species‐rich grasslands. Little is known about how this affects the colonization capacity of populations, which is highly important for regional species survival. We studied the effects on four wind‐dispersed forbs that represent two major dispersal strategies in grasslands: Cirsium dissectum and Hypochaeris radicata, which have plumed seeds and are adapted to long‐distance dispersal by wind, and Centaurea jacea and Succisa pratensis, which have plumeless seeds and are adapted to only short‐distance dispersal by wind.  2 Colonization capacity decreased with decreasing population size. This was due to lower seed germination ability in all species, and a lower seed production and a narrower range of seed dispersal distances in the species with plumed seeds. Inbreeding depression is the most likely cause of this. We found no evidence for a stronger selection for reduced dispersal in smaller populations.  3 Increasing site productivity changed the colonization capacity of all species. The capacity for colonization of nearby sites increased, due to higher seed production and seed germination ability, but the capacity for colonization of distant sites decreased, due to a lower long‐distance dispersal ability.  4 Seed dispersal ability and germination ability were negatively correlated in the species with plumeless seeds, but not in the species with plumed seeds. The dispersal ability of individual plumed seeds remained constant under changes in population size and site productivity. This indicates a strong selection pressure for long‐distance dispersal ability in these species.  5 When habitat fragmentation results in a simultaneous decrease in population size and increase in site productivity, both the local survival probability and the colonization capacity of remnant populations of wind‐dispersed grassland forbs are likely to be severely reduced. This increases regional extinction risks of the species. 1 Habitat fragmentation as a result of intensification of agricultural practices decreases the population size and increases the site productivity of remnant populations of many plant species native to nutrient‐poor, species‐rich grasslands. Little is known about how this affects the colonization capacity of populations, which is highly important for regional species survival. We studied the effects on four wind‐dispersed forbs that represent two major dispersal strategies in grasslands: Cirsium dissectum and Hypochaeris radicata, which have plumed seeds and are adapted to long‐distance dispersal by wind, and Centaurea jacea and Succisa pratensis, which have plumeless seeds and are adapted to only short‐distance dispersal by wind.  2 Colonization capacity decreased with decreasing population size. This was due to lower seed germination ability in all species, and a lower seed production and a narrower range of seed dispersal distances in the species with plumed seeds. Inbreeding depression is the most likely cause of this. We found no evidence for a stronger selection for reduced dispersal in smaller populations.  3 Increasing site productivity changed the colonization capacity of all species. The capacity for colonization of nearby sites increased, due to higher seed production and seed germination ability, but the capacity for colonization of distant sites decreased, due to a lower long‐distance dispersal ability.  4 Seed dispersal ability and germination ability were negatively correlated in the species with plumeless seeds, but not in the species with plumed seeds. The dispersal ability of individual plumed seeds remained constant under changes in population size and site productivity. This indicates a strong selection pressure for long‐distance dispersal ability in these species.  5 When habitat fragmentation results in a simultaneous decrease in population size and increase in site productivity, both the local survival probability and the colonization capacity of remnant populations of wind‐dispersed grassland forbs are likely to be severely reduced. This increases regional extinction risks of the species. 1 Habitat fragmentation as a result of intensification of agricultural practices decreases the population size and increases the site productivity of remnant populations of many plant species native to nutrient‐poor, species‐rich grasslands. Little is known about how this affects the colonization capacity of populations, which is highly important for regional species survival. We studied the effects on four wind‐dispersed forbs that represent two major dispersal strategies in grasslands: Cirsium dissectum and Hypochaeris radicata, which have plumed seeds and are adapted to long‐distance dispersal by wind, and Centaurea jacea and Succisa pratensis, which have plumeless seeds and are adapted to only short‐distance dispersal by wind.  2 Colonization capacity decreased with decreasing population size. This was due to lower seed germination ability in all species, and a lower seed production and a narrower range of seed dispersal distances in the species with plumed seeds. Inbreeding depression is the most likely cause of this. We found no evidence for a stronger selection for reduced dispersal in smaller populations.  3 Increasing site productivity changed the colonization capacity of all species. The capacity for colonization of nearby sites increased, due to higher seed production and seed germination ability, but the capacity for colonization of distant sites decreased, due to a lower long‐distance dispersal ability.  4 Seed dispersal ability and germination ability were negatively correlated in the species with plumeless seeds, but not in the species with plumed seeds. The dispersal ability of individual plumed seeds remained constant under changes in population size and site productivity. This indicates a strong selection pressure for long‐distance dispersal ability in these species.  5 When habitat fragmentation results in a simultaneous decrease in population size and increase in site productivity, both the local survival probability and the colonization capacity of remnant populations of wind‐dispersed grassland forbs are likely to be severely reduced. This increases regional extinction risks of the species. 1 Habitat fragmentation as a result of intensification of agricultural practices decreases the population size and increases the site productivity of remnant populations of many plant species native to nutrient‐poor, species‐rich grasslands. Little is known about how this affects the colonization capacity of populations, which is highly important for regional species survival. We studied the effects on four wind‐dispersed forbs that represent two major dispersal strategies in grasslands: Cirsium dissectum and Hypochaeris radicata, which have plumed seeds and are adapted to long‐distance dispersal by wind, and Centaurea jacea and Succisa pratensis, which have plumeless seeds and are adapted to only short‐distance dispersal by wind.  2 Colonization capacity decreased with decreasing population size. This was due to lower seed germination ability in all species, and a lower seed production and a narrower range of seed dispersal distances in the species with plumed seeds. Inbreeding depression is the most likely cause of this. We found no evidence for a stronger selection for reduced dispersal in smaller populations.  3 Increasing site productivity changed the colonization capacity of all species. The capacity for colonization of nearby sites increased, due to higher seed production and seed germination ability, but the capacity for colonization of distant sites decreased, due to a lower long‐distance dispersal ability.  4 Seed dispersal ability and germination ability were negatively correlated in the species with plumeless seeds, but not in the species with plumed seeds. The dispersal ability of individual plumed seeds remained constant under changes in population size and site productivity. This indicates a strong selection pressure for long‐distance dispersal ability in these species. 5 When habitat fragmentation results in a simultaneous decrease in population size and increase in site productivity, both the local survival probability and the colonization capacity of remnant populations of wind‐dispersed grassland forbs are likely to be severely reduced. This increases regional extinction risks of the species. | 3747 | NA | NA | NA | no | no |
| 3746 | 2017 | NA | Faucher, G; Guenard, F; Bouchard, L; Garneau, V; Turcot, V; Houde, A; Tchernof, A; Bergeron, J; Deshaies, Y; Hould, FS; Lebel, S; Marceau, P; Vohl, MC | 2012 | Genetic contribution to C-reactive protein levels in severe obesity | Obese individuals are characterized by a chronic, low-grade inflammatory state. Increased levels of C-reactive protein (CRP), a marker of inflammation, have been observed in subjects with the metabolic syndrome. We have previously reported that genes encoding proteins involved in the anti-inflammatory and immune response are differentially expressed in visceral adipose tissue of obese men with or without the metabolic syndrome. Among these genes, the interferon-gamma-inducible protein 30 (IFI30), CD163 molecule (CD163), chemokine (C-X-C motif) ligand 9 (CXCL9) and thymic stromal lymphopoietin (TSLP), were selected for further genetic analyses. The aim of the study was to verify whether IFI30, CD163, CXCL9 and TSLP gene polymorphisms contribute to explain the inter-individual variability of the inflammatory profile of obesity assessed by plasma high-sensitivity CRP concentrations. A total of 1185 severely obese individuals were genotyped for single nucleotide polymorphisms (SNPs) covering most of the sequence-derived genetic variability at the IFI30, CD 163, CXCL9 and TSLP gene loci (total of 27 SNPs). Following measurement of plasma CRP levels, subjects were divided into two groups, low vs. high using the median value of plasma CRP levels (8.31 mg/L) as a cutoff point. Genotype frequencies were compared between groups. Associations between genotypes and plasma CRP levels (continuous variable) were also tested after adjustments for age, sex, smoking and BMI. The rs11554159 and rs7125 IFI30 SNPs showed a significant difference in genotype frequencies (p < 0.05) between subgroups of low vs. high plasma CRP levels (wild type homozygotes: rs11554159 = 47% vs. 55%, rs7125 = 31% vs. 24%, for low vs. high CRP groups, respectively). The association between rs11554159 and CRP levels as a continuous variable remained significant (p = 0.004). Both carriers of the GA and AA genotypes demonstrated, on average, a 13% lower CRP levels in comparison with GG homozygotes. No association was observed between SNPs in the CD163. CXCL9 and TSLP genes and CRP levels. The IFI30 rs11554159 polymorphism could partially explain the inter-individual variability observed in the inflammatory profile associated with obesity. (C) 2011 Elsevier Inc. All rights reserved. | 3748 | NA | NA | NA | no | no |
| 3747 | 2017 | NA | Nelson, EB; Karp, MA | 2013 | Soil pathogen communities associated with native and non-native Phragmites australis populations in freshwater wetlands | Soil pathogens are believed to be major contributors to negative plant-soil feedbacks that regulate plant community dynamics and plant invasions. While the theoretical basis for pathogen regulation of plant communities is well established within the plant-soil feedback framework, direct experimental evidence for pathogen community responses to plants has been limited, often relying largely on indirect evidence based on above-ground plant responses. As a result, specific soil pathogen responses accompanying above-ground plant community dynamics are largely unknown. Here, we examine the oomycete pathogens in soils conditioned by established populations of native noninvasive and non-native invasive haplotypes of Phragmites australis (European common reed). Our aim was to assess whether populations of invasive plants harbor unique communities of pathogens that differ from those associated with noninvasive populations and whether the distribution of taxa within these communities may help to explain invasive success. We compared the composition and abundance of pathogenic and saprobic oomycete species over a 2-year period. Despite a diversity of oomycete taxa detected in soils from both native and non-native populations, pathogen communities from both invaded and noninvaded soils were dominated by species of Pythium. Pathogen species that contributed the most to the differences observed between invaded and noninvaded soils were distributed between invaded and noninvaded soils. However, the specific taxa in invaded soils responsible for community differences were distinct from those in noninvaded soils that contributed to community differences. Our results indicate that, despite the phylogenetic relatedness of native and non-native P.australis haplotypes, pathogen communities associated with the dominant non-native haplotype are distinct from those of the rare native haplotype. Pathogen taxa that dominate either noninvaded or invaded soils suggest different potential mechanisms of invasion facilitation. These findings are consistent with the hypothesis that non-native plant species that dominate landscapes may cultivate a different soil pathogen community to their rhizosphere than those of rarer native species. | 3749 | NA | NA | NA | no | no |
| 3748 | 2017 | NA | Li, Y; Zhai, SN; Qiu, YX; Guo, YP; Ge, XJ; Comes, HP | 2011 | Glacial survival east and west of the ‘Mekong-Salween Divide’ in the Himalaya-Hengduan Mountains region as revealed by AFLPs and cpDNA sequence variation in Sinopodophyllum hexandrum (Berberidaceae) | Molecular phylogeographic studies have recently begun to elucidate how plant species from the Qinghai-Tibetan Plateau (QTP) and adjacent regions responded to the Quaternary climatic oscillations. In this regard, however, far less attention has been paid to the southern and south-eastern declivities of the QTP, i.e. the Himalaya-Hengduan Mountains (HHM) region. Here, we report a survey of amplified fragment length polymorphisms (AFLPs) and chloroplast DNA (cpDNA) sequence variation in the HHM endemic Sinopodophyllum hexandrum, a highly selfing alpine perennial herb with mainly gravity-dispersed berries (105 individuals, 19 localities). We specifically aimed to test a vicariant evolutionary hypothesis across the ‘Mekong-Salween Divide’, a known biogeographic and phytogeographic boundary of north-to-south trending river valleys separating the East Himalayas and Hengduan Mts. Both cpDNA and AFLPs identified two divergent phylogroups largely congruent with these mountain ranges. There was no genetic depauperation in the more strongly glaciated East Himalayas (AFLPs: H(E) = 0.031; cpDNA: h(s) = 0.133) compared to the mainly ice-free Hengduan Mts. (AFLPs: H(E) = 0.037; cpDNA: h(s) = 0.082), while population differentiation was consistently higher in the former region (AFLPs: Phi(ST) = 0.522 vs. 0.312; cpDNA: Phi(ST) = 0.785 vs. 0.417). Our results suggest that East Himalayan and Hengduan populations of S. hexandrum were once fragmented, persisted in situ during glacials in both areas, and have not merged again, except for a major instance of inter-lineage chloroplast capture identified at the MSD boundary. Our coalescent time estimate for all cpDNA haplotypes (c. 0.37-0.48 mya), together with paleogeological evidence, strongly rejects paleo-drainage formation as a mechanism underlying allopatric fragmentation, whereas mountain glaciers following the ridges of the MSD during glacials (and possible interglacials) could have been responsible. This study thus indicates an important role for mountain glaciers in driving (incipient) allopatric speciation across the MSD in the HHM region by causing vicariant lineage divergence and acting as barriers to post-divergence gene flow. (C) 2011 Elsevier Inc. All rights reserved. | 3750 | NA | NA | NA | no | no |
| 3749 | 2017 | NA | Leips, J; Travis, J; Rodd, FH | 2000 | Genetic influences on experimental population dynamics of the least killifish | When natural populations differ in density or in the dynamic fluctuations of population size, some of those differences may result from their different ecological conditions, and some may originate from genetically based differences in life history expression. Natural populations of the live-bearing poeciliid fish, Heterandria formosa, vary considerably in their population dynamics, with densities that differ between populations by as much as sevenfold. This system offers an excellent opportunity to explore the potential role of genetically based differences in life history expression in creating different dynamic patterns in a common environment. We created five different genetic stocks of H. formosa by carrying out a series of crosses using fish from two North Florida populations (the Wacissa River and Trout Pond) and used them to initiate replicate experimental populations in artificial ponds. The five stocks consisted of two “controls,” which were pure Wacissa River and Trout Pond stocks, and three types of hybrid stocks. The hybrid stocks differed in a regular way in the proportion of genes from one population or the other. The crossing scheme was designed so that each hybrid stock would have the same proportion of heterozygous (or “heterodemic”) loci but would differ in the proportion and/or identity of homozygous (or “homodemic”) loci from the Wacissa River and Trout Pond populations. These populations were chosen because a previous study had found that population densities in the Wacissa River greatly exceeded those of Trout Pond and exhibited a higher range of population fluctuation during the breeding season. We addressed four questions in this experiment: (1) Are there genetically based differences in life history traits of fish from the two populations? (2) If so, do differences in life history expression produce differences in population dynamics in a common environment? (3) Which traits have the greatest influence on population dynamics? (4) How do changes in density affect the phenotypes of individual traits that govern the rates of birth and death in a population? We followed experimental populations of the five genetic stocks from their initiation at low density through 4-6 generations of population growth and decline. The mean offspring size differed among stocks by as much as 50%. At low densities, offspring size exhibited a trade-off with brood size: Trout Pond alleles were associated with more, smaller offspring. At higher densities, offspring sizes were similar among stocks, and the trade-off with offspring number was not evident. Stocks differed in realized population growth rate by as much as 70%; the rank order differences among stocks with respect to population growth rate appeared to match the genetic relatedness among stocks based on the expected percentage of Trout Pond alleles. Differences in population growth rate appeared to be due to differences in brood size among stocks at low density. Stocks did not differ in the equilibrium population size, which indicated the absence of a trade-off between population growth rate and carrying capacity in this environment. Adult survival and recruitment of juveniles into the adult population both declined linearly with increasing density; and stocks did not generally differ in those rates after the effects of density had been taken into account. The stocks differed in their response to the depressant effects of density on life history trait expression. The offspring size of the pure Wacissa River stock was much more sensitive to density than was the offspring size of the pure Trout Pond stock. However, the brood sizes of the Wacissa River stock were reduced much less than those of the Trout Pond stock when exposed to the same high density. These results suggest that life history distinctions among populations, both in the mean values and plasticity of traits, play a role in creating different dynamics. We discuss the ways in which phenotypic plasticity in reproductive traits potentially acts as a mechanism to stabilize population dynamics in this species. | 3751 | NA | NA | NA | no | no |
| 3750 | 2017 | NA | Talavera, G; Espadaler, X; Vila, R | 2015 | Discovered just before extinction? The first endemic ant from the Balearic Islands (Lasius balearicus sp nov.) is endangered by climate change | AimWe analyse the taxonomic status, phylogenetic relationships, distribution and age of a newly discovered ant taxon found in the mountaintops of the island of Mallorca (Spain). We also consider the potential impact of short-term climate change on the survival of this ant and make proposals on its conservation status, risks and management. LocationBalearic Islands (Spain). MethodsWe used morphological, molecular and ecological evidence to assess the specific status of the potential new species. We gathered distribution data to conduct climate-based distribution modelling of present and future occupancy under several SRES emission scenarios. ResultsThe existence of a new non-cryptic species of ant (Lasius balearicus Talavera, Espadaler & Vila, sp. nov.) is described from the island of Mallorca. Its distribution was found to be extremely restricted (Serra de Tramuntana) and elevationally constrained to island summits (between 800 and 1400m a.s.l.). Molecular dating indicated that this species diverged about 1.51million years ago from its nearest relatives, from which it can be distinguished based on several morphological traits. Ecological niche modelling shows a dramatic reduction of areas with suitable climatic conditions under the different scenarios studied. Main conclusionsLasius balearicus represents the first endemic ant to be described in the Balearic Islands, as well as the first endemic Lasius species in the Mediterranean islands. Distribution modelling predictions, the low intraspecific genetic diversity observed, and the geographical and elevational isolation of the populations indicated a low probability for the survival of the species in the short term, thus making it a potential model to study real-time climate-based biodiversity loss. As a consequence, we strongly recommend including L. balearicus in the IUCN Red List of Threatened Species under the category Endangered’. This case illustrates that a fraction of biodiversity remains unexplored even within Europe, arguably the best-studied region of the planet, and that the available time-window for us to study and protect it may be in some instances notably narrow. | 3752 | NA | NA | NA | no | no |
| 3751 | 2017 | NA | Zhang, DL; Ye, Z; Yamada, K; Zhen, YH; Zheng, CG; Bu, WJ | 2016 | Pleistocene sea level fluctuation and host plant habitat requirement influenced the historical phylogeography of the invasive species Amphiareus obscuriceps (Hemiptera: Anthocoridae) in its native range | Background: On account of repeated exposure and submergence of the East China Sea (ECS) land bridge, sea level fluctuation played an important role in shaping the population structure of many temperate species across the ECS during the glacial period. The flower bug Amphiareus obscuriceps (Poppius, 1909) ( Hemiptera: Anthocoridae) is an invasive species native to the Sino-Japanese Region (SJR) of East Asia. We tested the hypothesis of the ECS land bridge acting as a dispersal corridor or filter for A. obscuriceps during the glacial period. Specifically, we tested whether and the extent to which dispersal ability and host plant habitat requirement influenced the genetic structure of A. obscuriceps during the exposure of the ECS land bridge. Results: Phylogenetic and network analyses indicated that A. obscuriceps is composed of two major lineages, i.e., China and Japan. Divergence time on both sides of the ECS was estimated to be approximately 1.07 (0.79-1.32) Ma, which was about the same period that the sea level increased. No significant Isolation by Distance (IBD) relationship was found between empty setst and Euclidean distances in the Mantel tests, which is consistent with the hypothesis that this species has a good dispersal ability. Our Last Glacial Maximum (LGM) niche modeling of plants that constitute preferred habitats for A. obscuriceps exhibited a similar habitat gap on the exposed ECS continental shelf between China and Japan, but showed a continuous distribution across the Taiwan Strait. Conclusion: Our results suggest that ecological properties (habitat requirement and dispersal ability), together with sea level fluctuation during the Pleistocene across the ECS, have shaped the genetic structure and demographic history of A. obscuriceps in its native area. The host plant habitat requirement could also be a key to the colonization of the A. obscuriceps species during the exposure of the ECS land bridge. Our findings will shed light on the potential role of habitat requirement in the process of biological invasion in future studies. | 3753 | NA | NA | NA | no | no |
| 3752 | 2017 | NA | Shimada, S; Aoki, K; Nabeshima, T; Fuxun, Y; Kurosaki, Y; Shiogama, K; Onouchi, T; Sakaguchi, M; Fuchigami, T; Ono, H; Nishi, K; Posadas-Herrera, G; Uchida, L; Takamatsu, Y; Yasuda, J; Tsutsumi, Y; Fujita, H; Morita, K; Hayasaka, D | 2016 | Tofla virus: A newly identified Nairovirus of the Crimean-Congo hemorrhagic fever group isolated from ticks in Japan | Ixodid ticks transmit several important viral pathogens. We isolated a new virus (Tofla virus: TFLV) from Heamaphysalis flava and Heamaphysalis formsensis in Japan. The full-genome sequences revealed that TFLV belonged to the genus Nairovirus, family Bunyaviridae. Phylogenetic analyses and neutralization tests suggested that TFLV is closely related to the Hazara virus and that it is classified into the Crimean-Congo hemorrhagic fever group. TFLV caused lethal infection in IFNAR KO mice. The TFLV-infected mice exhibited a gastrointestinal disorder, and positron emission tomography-computed tomography images showed a significant uptake of F-18-fluorodeoxyglucose in the intestinal tract. TFLV was able to infect and propagate in cultured cells of African green monkey-derived Vero E6 cells and human-derived SK-N-SH, T98-G and HEK-293 cells. Although TFLV infections in humans and animals are currently unknown, our findings may provide clues to understand the potential infectivity and to develop of pre-emptive countermeasures against this new tick-borne Nairovirus. | 3754 | NA | NA | NA | no | no |
| 3753 | 2017 | NA | FRANCA, FH; PLAISTED, RL; ROUSH, RT; VIA, S; TINGEY, WM | 1994 | SELECTION RESPONSE OF THE COLORADO POTATO BEETLE FOR ADAPTATION TO THE RESISTANT POTATO, SOLANUM-BERTHAULTII | Thirty cohorts of the Colorado potato beetle (CPB) were reared for ten consecutive generations on the cultivated potato, Solanum tuberosum, and on two accessions (PI 473331 and PI 473334) of the resistant wild species, S. berthaultii. At each generation, selection was imposed for increased survival, decreased development time, and for increased fecundity. Although several parameters responded to selection, net replacement rate (R(0)) on S. bertaultii was consistently less than 1/3 that on cultivated potato. At the tenth generation, each S. tuberosum strain female replaced herself with 110 daughters, whereas females from strains reared on PI473334 and PI473331 produced an average of 30 and 7 daughters, respectively. Slopes of the R(0) regression lines for populations reared on S. tuberosum and S. berthaultii did not differ from zero, indicating no significant response to selection. Our findings suggest that populations of the Colorado potato beetle may have genetic variability in some performance traits for adaptation to S. berthaultii, but that adaptation is unlikely to occur as rapidly as previously reported. | 3755 | NA | NA | NA | no | no |
| 3754 | 2017 | NA | Palhares, AC; Rodrigues-Morais, TB; Van Sluys, MA; Domingues, DS; Maccheroni, W; Jordao, H; Souza, AP; Marconi, TG; Mollinari, M; Gazaffi, R; Garcia, AAF; Vieira, MLC | 2012 | A novel linkage map of sugarcane with evidence for clustering of retrotransposon-based markers | Background: The development of sugarcane as a sustainable crop has unlimited applications. The crop is one of the most economically viable for renewable energy production, and CO2 balance. Linkage maps are valuable tools for understanding genetic and genomic organization, particularly in sugarcane due to its complex polyploid genome of multispecific origins. The overall objective of our study was to construct a novel sugarcane linkage map, compiling AFLP and EST-SSR markers, and to generate data on the distribution of markers anchored to sequences of scIvana\_1, a complete sugarcane transposable element, and member of the Copia superfamily. Results: The mapping population parents (‘IAC66-6’ and ‘TUC71-7’) contributed equally to polymorphisms, independent of marker type, and generated markers that were distributed into nearly the same number of co-segregation groups (or CGs). Bi-parentally inherited alleles provided the integration of 19 CGs. The marker number per CG ranged from two to 39. The total map length was 4,843.19 cM, with a marker density of 8.87 cM. Markers were assembled into 92 CGs that ranged in length from 1.14 to 404.72 cM, with an estimated average length of 52.64 cM. The greatest distance between two adjacent markers was 48.25 cM. The scIvana\_1-based markers (56) were positioned on 21 CGs, but were not regularly distributed. Interestingly, the distance between adjacent scIvana\_1-based markers was less than 5 cM, and was observed on five CGs, suggesting a clustered organization. Conclusions: Results indicated the use of a NBS-profiling technique was efficient to develop retrotransposon-based markers in sugarcane. The simultaneous maximum-likelihood estimates of linkage and linkage phase based strategies confirmed the suitability of its approach to estimate linkage, and construct the linkage map. Interestingly, using our genetic data it was possible to calculate the number of retrotransposonscIvana\_1 (similar to 60) copies in the sugarcane genome, confirming previously reported molecular results. In addition, this research possibly will have indirect implications in crop economics e. g., productivity enhancement via QTL studies, as the mapping population parents differ in response to an important fungal disease. | 3756 | NA | NA | NA | no | no |
| 3755 | 2017 | NA | Marulanda, ML; Lopez, AM; Isaza, L; Lopez, P | 2014 | Microsatellite isolation and characterization for Colletotrichum spp, causal agent of anthracnose in Andean blackberry | The genus Colletotrichum, comprised of pathogenic fungi that affect plants grown worldwide, causes the disease known as anthracnose in several fruit and vegetable species. Several studies conducted on plants have shown that the disease is characterized by the presence of one or several species of the fungus attacking the fruit or other organs of the same host. To develop and implement effective control strategies, it is vital to understand the genetic structure of the fungus in agricultural systems, identify associated Colletotrichum species, and define the subpopulations responsible for the disease. Molecular tools were accordingly developed to characterize genotypic populations of Colletotrichum spp, causal agent of anthracnose in commercial crops of Andean blackberry (Rubus glaucus Benth.). A microsatellite-enriched library for Colletotrichum gloeosporioides was developed to identify and characterize microsatellite loci among isolates collected in R. glaucus plantations. Thirty microsatellites were developed and tested in 36 isolates gathered from eight different blackberry-production areas of Colombia. Ten pairs of microsatellites were polymorphic. | 3757 | NA | NA | NA | no | no |
| 3756 | 2017 | NA | Quillery, E; Quenez, O; Peterlongo, P; Plantard, O | 2014 | Development of genomic resources for the tick Ixodes ricinus: isolation and characterization of single nucleotide polymorphisms | Assessing the genetic variability of the tick Ixodes ricinus-an important vector of pathogens in Europe-is an essential step for setting up antitick control methods. Here, we report the first identification of a set of SNPs isolated from the genome of I.ricinus, by applying a reduction in genomic complexity, pyrosequencing and new bioinformatics tools. Almost 1.4million of reads (average length: 528 nt) were generated with a full Roche 454 GS FLX run on two reduced representation libraries of I.ricinus. A newly developed bioinformatics tool (DiscoSnp), which isolates SNPs without requiring any reference genome, was used to obtain 321088 putative SNPs. Stringent selection criteria were applied in a bioinformatics pipeline to select 1768 SNPs for the development of specific primers. Among 384 randomly SNPs tested by Fluidigm genotyping technology on 464 individuals ticks, 368 SNPs loci (96%) exhibited the presence of the two expected alleles. Hardy-Weinberg equilibrium tests conducted on six natural populations of ticks have shown that from 26 to 46 of the 384 loci exhibited significant heterozygote deficiency. | 3758 | NA | NA | NA | no | no |
| 3757 | 2017 | NA | Duboscq, J; Neumann, C; Agil, M; Perwitasari-Farajallah, D; Thierry, B; Engelhardt, A | 2017 | Degrees of freedom in social bonds of crested macaque females | Social bonds between group members affect individual fitness and wellbeing. While the impact of bond strength is well studied, the consequences of bond predictability and equitability are often overlooked. Similarly, whether bonds reflect short-term contingencies and/or long-term social strategies remains understudied. We investigated these questions in female crested macaques, Macaca nigra, which display a tolerant social style within a nepotistic hierarchical social structure. We analysed the structure of social bonds by testing whether similarity within dyads (in kinship, dominance and age) predicted the strength, predictability and equitability of bonds. We then tested the value of social bonds by analysing the effect of their characteristics on three fitness-related behaviours: coalitionary support, feeding-inproximity and aggression. We found that the bond characteristics of females differed substantially from those of other species with comparable data: bonds were of average strength, of moderate endurance and relatively balanced. Stronger bonds were more equitable but less predictable than weaker bonds. Closely ranked females, but not kin or age peers, had stronger, more predictable and more equitable bonds than others. Coalitionary support was not related to any of the bond characteristics, feeding-in-proximity was positively associated with strength and predictability and aggression was positively linked to strength and negatively to equitability. These results highlight the complex picture of the benefits of social bonds in this species. They reflect the degrees of freedom tolerant macaque females can express in their social relationships within their stable social structure, a pattern that may not be given enough consideration in stable nepotistic hierarchical societies. Comparative research is necessary to establish whether these patterns are more general than previously thought or a specific feature of tolerant macaques. Investigating various characteristics of bonds together is paramount to appreciate the dynamics of social relationships and to better understand the social components of fitness. (C) 2016 The Association for the Study of Animal Behaviour. Published by Elsevier Ltd. All rights reserved. | 3759 | NA | NA | NA | no | no |
| 3758 | 2017 | NA | Hawley, TJ | 2009 | The Ecological Significance and Incidence of Intraguild Predation and Cannibalism among Anurans in Ephemeral Tropical Pools | Ephemeral pools are traditionally considered to be tadpole refuges, with few or no predators. While it is recognized that some tadpoles are predaceous, little attention has been given to understanding how tadpoles shape age and size structures of populations and the composition of tadpole assemblages. I examined the incidence of intraguild predation (IGP) among three species that co-occur In ephemeral pools In the Osa Peninsula, Costa Rica. I found a high Incidence of IGP among the three species, with IG predators differing in their voracity for prey eggs and IG prey differing In their vulnerability to IGP. I also examined the factors that influence the Intensity of cannibalism and tadpole performance In a model species, Smilisca phaeota. I manipulated predator and prey size and developmental stage, tadpole density, food quantity, and tadpole relatedness in microcosm experiments. Cannibalism in S. phaeota was dependent on tadpole predator and prey size and developmental stage, with medium and large tadpoles causing almost complete mortality of embryos, but little mortality of mobile hatchlings. In contrast, predation by small tadpoles was restricted primarily to the embryo developmental stage. Tadpole mortality was high In low plant food quantity treatments, suggesting a high Incidence of cannibalism. Other measures of tadpole performance, including survival to metamorphosis, metamorph mass, and larval period, were influenced by tadpole density and plant food quantity. Additions of conspecific prey eggs and degree of tadpole relatedness did not affect tadpole performance. My results suggest that tadpoles are capable of playing a regulatory role, Influencing the relative abundance and composition of species in pools through differential IGP and cannibalism on eggs and hatchlings. The Intensity of IGP In ephemeral pools Is likely Influenced by the morphology of IG predators, reproductive mode and fecundity of IG prey, and priority effects. My study supports the view that we need to reconsider the position tadpoles occupy within the aquatic food webs, with greater appreciation for their role as predators. | 3760 | NA | NA | NA | no | no |
| 3759 | 2017 | NA | Kizilkaya, K; Fernando, RL; Garrick, DJ | 2014 | Reduction in accuracy of genomic prediction for ordered categorical data compared to continuous observations | Background: Accuracy of genomic prediction depends on number of records in the training population, heritability, effective population size, genetic architecture, and relatedness of training and validation populations. Many traits have ordered categories including reproductive performance and susceptibility or resistance to disease. Categorical scores are often recorded because they are easier to obtain than continuous observations. Bayesian linear regression has been extended to the threshold model for genomic prediction. The objective of this study was to quantify reductions in accuracy for ordinal categorical traits relative to continuous traits. Methods: Efficiency of genomic prediction was evaluated for heritabilities of 0.10, 0.25 or 0.50. Phenotypes were simulated for 2250 purebred animals using 50 QTL selected from actual 50k SNP (single nucleotide polymorphism) genotypes giving a proportion of causal to total loci of .0001. A Bayes C pi threshold model simultaneously fitted all 50k markers except those that represented QTL. Estimated SNP effects were utilized to predict genomic breeding values in purebred (n = 239) or multibreed (n = 924) validation populations. Correlations between true and predicted genomic merit in validation populations were used to assess predictive ability. Results: Accuracies of genomic estimated breeding values ranged from 0.12 to 0.66 for purebred and from 0.04 to 0.53 for multibreed validation populations based on Bayes C pi linear model analysis of the simulated underlying variable. Accuracies for ordinal categorical scores analyzed by the Bayes C pi threshold model were 20% to 50% lower and ranged from 0.04 to 0.55 for purebred and from 0.01 to 0.44 for multibreed validation populations. Analysis of ordinal categorical scores using a linear model resulted in further reductions in accuracy. Conclusions: Threshold traits result in markedly lower accuracy than a linear model on the underlying variable. To achieve an accuracy equal or greater than for continuous phenotypes with a training population of 1000 animals, a 2.25 fold increase in training population size was required for categorical scores fitted with the threshold model. The threshold model resulted in higher accuracies than the linear model and its advantage was greatest when training populations were smallest. | 3761 | NA | NA | NA | no | no |
| 3760 | 2017 | NA | WILKINSON, GS | 1992 | COMMUNAL NURSING IN THE EVENING BAT, NYCTICEIUS-HUMERALIS | Nursing observations over two summers involving 76 lactating female evening bats, Nycticeius humeralis, and 128 pups in an attic in northern Missouri indicate that communal nursing occurs rarely until 2 weeks before weaning during which time over 18% of nursing bouts involve nondescendant offspring. The average relatedness among female pairs nursing non-descendant offspring, based on identity-by-descent estimates using allozyme data, was 0.04 (SE=0.12). Mitochondrial DNA d-loop sequence comparisons confirm that at most only 2 of 20 female pairs nursing non-descendant offspring came from the same matriline. Thus, females do not nurse matrilineal kin preferentially despite female natal philopatry. In addition, the average degree of relatedness within a colony (r=0.01, SE=0.03) is too low to provide any indirect benefits from communal nursing. Female error alone is insufficient to explain these observations because females tended to allow female nondescendant young to nurse but excluded non-descendant males, particularly when they had all-male litters. Furthermore, communal nursing bouts did not differ in duration from parental nursing bouts and involved 31% of all banded females and 24% of all banded pups observed nursing. Communal nursing occurred most frequently when pups began hunting on their own and when lactating females attained their lowest average pre-fed body weight. Mortality during this period was higher for male than female pups, and relative weights implicate starvation as the cause. Time-lapse video records of four families of bats in captivity showed that the number of nursing bouts was proportional to daily weight change. I propose that these results are consistent with both immediate and delayed benefits accruing to females which experience variable hunting success. If a female with extra milk reduced her weight by dumping milk prior to her next foraging trip, she could obtain an immediate energetic benefit and maintain maximum milk production. By restricting such milk donations to nondescendant females she may also increase colony size and thereby enhance her future acquisition of information about foraging and roosting sites. | 3762 | NA | NA | NA | no | no |
| 3761 | 2017 | NA | Long, RL; Gorecki, MJ; Renton, M; Scott, JK; Colville, L; Goggin, DE; Commander, LE; Westcott, DA; Cherry, H; Finch-Savage, WE | 2015 | The ecophysiology of seed persistence: a mechanistic view of the journey to germination or demise | Seed persistence is the survival of seeds in the environment once they have reached maturity. Seed persistence allows a species, population or genotype to survive long after the death of parent plants, thus distributing genetic diversity through time. The ability to predict seed persistence accurately is critical to inform long-term weed management and flora rehabilitation programs, as well as to allow a greater understanding of plant community dynamics. Indeed, each of the 420000 seed-bearing plant species has a unique set of seed characteristics that determine its propensity to develop a persistent soil seed bank. The duration of seed persistence varies among species and populations, and depends on the physical and physiological characteristics of seeds and how they are affected by the biotic and abiotic environment. An integrated understanding of the ecophysiological mechanisms of seed persistence is essential if we are to improve our ability to predict how long seeds can survive in soils, both now and under future climatic conditions. In this review we present an holistic overview of the seed, species, climate, soil, and other site factors that contribute mechanistically to seed persistence, incorporating physiological, biochemical and ecological perspectives. We focus on current knowledge of the seed and species traits that influence seed longevity under ex situ controlled storage conditions, and explore how this inherent longevity is moderated by changeable biotic and abiotic conditions in situ, both before and after seeds are dispersed. We argue that the persistence of a given seed population in any environment depends on its resistance to exiting the seed bank via germination or death, and on its exposure to environmental conditions that are conducive to those fates. By synthesising knowledge of how the environment affects seeds to determine when and how they leave the soil seed bank into a resistance-exposure model, we provide a new framework for developing experimental and modelling approaches to predict how long seeds will persist in a range of environments. | 3763 | NA | NA | NA | no | no |
| 3762 | 2017 | NA | Reis, NR; Gallo, PH; Peracchi, AL; Lima, IP; Fregonezi, MN | 2012 | Sensitivity of populations of bats (Mammalia: Chiroptera) in relation to human development in northern Parana, southern Brazil | Most natural forests have been converted for human use, restricting biological life to small forest fragments. Many animals, including some species of bats are disappearing and the list of these species grows every day. It seems that the destruction of the habitat is one of its major causes. This study aimed to analyze how this community of bats was made up in environments with different sizes and quality of habitat. Data from studies conducted in the region of Londrina, Parana, Brazil, from 1982 to 2000 were used. Originally, this area was covered by a semi deciduous forest, especially Aspidosperma polyneuron (Apocynaceae), Ficus insipida (Moraceae), Euterpe edulis (Arecaceae), Croton floribundus (Euforbiaceae), and currently, only small remnants of the original vegetation still exist. The results showed a decline in the number of species caught in smaller areas compared to the largest remnant. In about 18 years of sampling, 42 species of bats were found in the region, representing 67% of the species that occur in Parana and 24.4% in Brazil. There were two species of Noctilionidae; 21 of Phyllostoma; 11 Vespertilionidae and eight Molossidae. Eight of these were captured only in the largest fragment, Mata dos Godoy State Park (680 ha). Ten species had a low capture rate in the smaller areas with less than three individuals. Of the total sampled, 14 species were found in human buildings, and were able to tolerate modified environments, foraging and even using them as shelter. As the size of the forest area increases, there is a greater variety of ecological opportunities and their physical conditions become more stable, i.e., conditions favorable for growth and survival of a greater number of species. Forest fragmentation limits and creates subpopulations, preserving only long-lived K-strategist animals for some time, where the supporting capacity of the environment is a limiting factor. The reduction of habitats, species and genetic diversity resulting from human activities are endangering the future adaptability in natural ecosystems, which promotes the disappearance of low adaptive potential species. | 3764 | NA | NA | NA | no | no |
| 3763 | 2017 | NA | KHADHAIR, AH; HIRUKI, C | 1995 | THE MOLECULAR-GENETIC RELATEDNESS OF WILLOW WITCHES-BROOM PHYTOPLASMA TO THE CLOVER PROLIFERATION GROUP | Willow witches’-broom (WWB) was found in the Edmonton area in 1994 for the first time in Canada. In this study, a simple and sensitive diagnostic method was applied and a comparative molecular genetic analysis was carried out to relate the WWB agent to known phytoplasmas. Upon application of the polymerase chain reaction using a selected pair of primers designed specifically for clover proliferation (CP) phytoplasma, the genomic DNA fragments were amplified from phytoplasma nucleic acids extracted from periwinkle, potato and alfalfa affected by CP, potato witches’-broom (PWB) and alfalfa witches’-broom (AWE), respectively. The results of restricted fragment length polymorphism analysis showed that there are no detectable molecular differences among the amplified phytoplasma DNAs originally extracted from four species of willow (Salix bebbiana Sarg., S. discolor Muhl., S. exigua Nutt, and S. petiolaris Sm.) naturally infected with WWB phytoplasma indicating the presence of only one type of phytoplasma and that WWB phytoplasma belongs to the CP group of phytoplasma organisms. | 3765 | NA | NA | NA | no | no |
| 3764 | 2017 | NA | Asins, MJ; Monforte, AJ; Mestre, PF; Carbonell, EA | 1999 | Citrus and Prunus copia-like retrotransposons | Many of the world’s most important citrus cultivars (“Washington Navel”, satsumas, clementines) have arisen through somatic mutation. This phenomenon occurs fairly often in the various species and varieties of the genus. The presence of copia-like retrotransposons has been investigated in fruit trees, especially citrus, by using a PCR assay designed to detect copia-like reverse transcriptase (RT) sequences. Amplification products from a genotype of each the following species Citrus sinensis, Citrus grandis, Citrus clementina, Prunus armeniaca and Prunus amygdalus, were cloned and some of them sequenced. Southern-blot hybridization using RT clones as probes showed that multiple copies are integrated throughout the citrus genome, while only 1-3 copies are detected in the P. armeniaca genome, which is in accordance with the Citrus and Prunus genome sizes. Sequence analysis of RT clones allowed a search for homologous sequences within three gene banks. The most similar ones correspond to RT domains of copia-like retrotransposons from unrelated plant species. Cluster analysis of these sequences has shown a great heterogeneity among RT domains cloned from the same genotype. This finding supports the hypothesis that horizontal transmission of retrotransposons has occurred in the past. The species presenting a RT sequence most similar to citrus RT clones is Gnetum montanum, a gymnosperm whose distribution area coincides with two of the main centers of origin of Citrus spp. A new C-methylated restriction DNA fragment containing a RT sequence is present in navel sweet oranges, but not in Valencia oranges from which the former originated suggesting, that retrotransposon activity might be, at least in part, involved in the genetic variability among sweet orange cultivars. Given that retrotransposons are quite abundant throughout the citrus genome, their activity should be investigated thoroughly before commercializing any transgenic citrus plant where the transgene(s) is part of a viral genome in order to avoid its possible recombination with an active retroelement. Focusing on other strategies to control virus diseases is recommended in citrus. | 3766 | NA | NA | NA | no | no |
| 3765 | 2017 | NA | Wolinska, J; Spaak, P | 2009 | THE COST OF BEING COMMON: EVIDENCE FROM NATURAL DAPHNIA POPULATIONS | The Red Queen coevolutionary hypothesis predicts that parasites drive oscillations in host genotype frequencies due to frequency-dependent selection where common hosts are at disadvantage. However, examples of this phenomenon in natural populations are scarce. To examine if the Red Queen theory operates in the wild, we studied the genetic structure of populations of the crustacean waterflea (Daphnia), in relation to their infection levels, for which we collected multiple samples from a variety of lakes. The most common clone in a given population was often underinfected. This advantage, however, did not remain stable over time. Instead, the most common clone decreased in frequency over subsequent generations, indicating that parasites can track common clones. Such decreases were not observed in uninfected populations. Moreover, host clonal evenness was higher across the set of infected lakes compared to uninfected lakes; suggesting that any common clone is selected against when parasites are present. These results strongly suggest that Red Queen dynamics do operate in the wild. | 3767 | NA | NA | NA | no | no |
| 3766 | 2017 | NA | Kokko, H; Johnstone, RA | 1999 | Social queuing in animal societies: a dynamic model of reproductive skew | Previously developed models of reproductive skew have overlooked one of the main reasons why subordinates might remain in a group despite restricted opportunities to breed: the possibility of social queuing; i.e, acquiring dominant status in the future. Here, we present a dynamic ESS model of skew in animal societies that incorporates both immediate and future fitness consequences of the decisions taken by group members? based on their probability of surviving from one season to the next (when post-breeding survival probabilities drop to zero, our analysis reduces to the model produced by Reeve and Ratnieks in 1993, which considered only a single breeding season). This allows us to compare the delayed benefits of philopatry and the immediate opportunities for independent breeding. We show that delayed benefits greatly reduce the need for dominants to offer reproductive concessions to retain subordinates peacefully in the group. Moreover, this effect is strong enough that differences in survival have a much greater impact on the group structure than differences in other parameters, such as relatedness. When the possibility of acceding to dominant status is taken into account, groups where the dominant completely monopolizes reproduction can be stable, even if they consist of unrelated individuals, and even if subordinates have a reasonably high probability of winning a fight for dominance. Finally, we show that stable groups are possible even if association leads to a decrease in current productivity. Subordinates may still stand to gain from group membership under these circumstances, as acquiring breeding positions by queuing may be more efficient than the attempt to establish a new territory. At the same time, the dominant may be unable to exclude unwelcome subordinates, may enjoy increased survival when they are present, or may gain indirect benefits from allowing relatives to stay and queue for dominance. We conclude that reproductive skew in animal groups, ranging from eusocial insect colonies to mating aggregations (leks), will be strongly influenced by the future prospects of group members. | 3768 | NA | NA | NA | no | no |
| 3767 | 2017 | NA | Garbelotto, M; Della Rocca, G; Osmundson, T; di Lonardo, V; Danti, R | 2015 | An increase in transmission-related traits and in phenotypic plasticity is documented during a fungal invasion | The adaptive rapid evolution of phenotypic traits is potentially a key contributor to invasiveness, but has been relatively little studied for the fungi, even though these organisms are responsible for devastating losses in agriculture and natural resources. In this study, we compare biologically relevant phenotypic characters of spore-generated individuals from two native and two invasive populations of the fungal pathogen Seiridium cardinale to infer which traits may be adaptive and rapidly evolving during an ongoing biological invasion. Results show that: (1) lower growth rate and smaller spore size are selected for in invasive populations, independent of the stage of invasion; (2) there is no selection evident towards increased rapid sporulation, but overall reproductive potential increases in later stages of the invasions; and (3) demographic plasticity of most traits increases during the initial stages of invasion, but decreases in a later phase. Comparisons against levels of neutral genetic variation (Qst-Fst comparisons) showed that the decrease in spore size is strongly adaptive, despite the trade-off of reduced viability. Lesion size of isolates inoculated on the naive Italian cypress host was not correlated with their growth rate, and was significantly lower in invasive than in native populations. This last result indicates that rate of host colonization is a complex trait affected both by host and pathogen, which may not be necessarily adaptive and/or which may not easily evolve. In summary, the success of S. cardinale as an invasive in the Mediterranean basin is associated with reduced spore size and increased plasticity of almost all traits in initial phases, followed by further decreased spore size, increased overall sporulation, and decreased plasticity in a second phase of the invasion. Interestingly, growth rate by population results show that invasive populations are well adapted only to moderate temperatures, while native populations fare well also when exposed to relative extremes in temperature. This different adaptation suggests a "master-of-some’’ specialization scenario for the invasion by S. cardinale in the Mediterranean. | 3769 | NA | NA | NA | no | no |
| 3768 | 2017 | NA | Olza, J; Ruperez, AI; Gil-Campos, M; Leis, R; Fernandez-Orth, D; Tojo, R; Canete, R; Gil, A; Aguilera, CM | 2013 | Influence of FTO variants on obesity, inflammation and cardiovascular disease risk biomarkers in Spanish children: a case-control multicentre study | Background: Variants in the FTO gene have been associated with obesity in children, but this association has not been shown with other biomarkers. We assessed the association of 52 FTO polymorphisms, spanning the whole gene, with obesity and estimated the influence of these polymorphisms on anthropometric, clinical and metabolic parameters as well as inflammation and cardiovascular disease (CVD) risk biomarkers among Spanish children. Methods: A multicentre case-control study was conducted in 534 children (292 obese and 242 with normal-BMI). Anthropometric, clinical, metabolic, inflammation and CVD risk markers were compared using the Student’s t-test for unpaired samples. The genotype relative risk was assessed by comparing the obese and normal-BMI group, calculating the odds ratio. The association of each SNP with phenotypic parameters was analysed using either logistic or linear regression analysis. Results: All anthropometric, clinical and metabolic factors as well as inflammatory and CVD risk biomarkers were higher in the obese than in the normal-BMI group, except adiponectin and HDL-c that were lower, and glucose, LDL-c, and metalloproteinase-9 that did not show difference. Four polymorphisms (rs9935401, rs9939609, rs9928094 and rs9930333) were positively associated with obesity and in linkage disequilibrium between each other; the haplotype including the risk alleles of these polymorphisms showed a high risk for obesity. The rs8061518 was negatively associated with obesity and the haplotype including this SNP and rs3826169, rs17818902 and rs7190053 showed a decreased risk for obesity. Additionally, the rs8061518 was associated with weight, diastolic blood pressure, insulin, homeostatic model assessment of insulin resistance, leptin, and active plasminogen inhibitor activator-1 after sex and age adjustment; however, after an additional BMI adjustment, this polymorphism remained associated only with leptin. Conclusions: We validated the previous reported association of genetic variability in intron 1 of the FTO gene with the risk of obesity and found no association with other related traits in this region of the gene. We have observed strong statistical evidence for an association of rs8061518 in intron 3 of the gene with decreased risk of obesity and low concentration of leptin. | 3770 | NA | NA | NA | no | no |
| 3769 | 2017 | NA | Meyer, JM; Hoy, MA; Boucias, DG | 2008 | Isolation and characterization of an Isaria fumosorosea isolate infecting the Asian citrus psyllid in Florida | A fungal pathogen that killed adult Diaphorina citri Kuwayama (Asian citrus psyllid) (Hemiptera: Psyllidae) in Florida Citrus groves during the fall of 2005 was identified and characterized. investigation of this pathogen is important because D. citri vectors citrus greening disease (Huanglongbing), which was reported in Florida in 2005. The morphological and genetic data generated herein support identification of the fungus as Isaria fumosorosea Wize (lfr) (=Paecilomyces fumosoroseus) (Hypocreales: Cordycipitaceae) from the Asian citrus psyllid (Ifr-AsCP). Koch’s postulates were fulfilled after the fungus was isolated in vitro and transmitted to healthy psyllids, which then exhibited a diseased-phenotype similar to that observed in the field. Both in vitro growth characteristics and two lfr AsCP-specific molecular markers discriminated the psyllid pathogen from another local lfr isolate. lfr 97 Apopka. These molecular markers will be useful to track the dynamics of this disease in D. citri populations. The potential for utilizing lfr to complement existing psyllid pest management strategies is discussed. (C) 2008 Elsevier Inc. All rights reserved. | 3771 | NA | NA | NA | no | no |
| 3770 | 2017 | NA | Jensen, AB; Pedersen, BV; Eilenberg, J | 2009 | Differential susceptibility across honey bee colonies in larval chalkbrood resistance | Chalkbrood susceptibility of in vitro reared honey bee larvae was investigated. Larvae were grafted from 3-4 colonies headed by pure mated queens of Apis mellifera carnica, A. m. ligustica and A. m. mellifera, respectively. Three day old larvae were fed with different dosages of Ascophaera apis spores and a clear dose-response relationship was shown. Over the whole experiment LD(50) estimates ranged from 55 to 905 spores. The response differed significantly (up to a factor ten) between colonies of the same subspecies. The mean time to death decreased with increased dose, with more larvae dying faster after eating more fungal spores. The A. m. ligustica larvae used in this study were less susceptible to A. apis than A. m. mellifera and A. m. carnica larvae. However due to the limited number of colonies included and the high variation shown we cannot predict that any A. m. ligustica colony is better adapted to cope with A. apis than colonies of A. m. carnica and A. m. mellifera. | 3772 | NA | NA | NA | no | no |
| 3771 | 2017 | NA | Kumar, N; Barua, S; Riyesh, T; Chaubey, KK; Rawat, KD; Khandelwal, N; Mishra, AK; Sharma, N; Chandel, SS; Sharma, S; Singh, MK; Sharma, DK; Singh, SV; Tripathi, BN | 2016 | Complexities in Isolation and Purification of Multiple Viruses from Mixed Viral Infections: Viral Interference, Persistence and Exclusion | Successful purification of multiple viruses from mixed infections remains a challenge. In this study, we investigated peste des petits ruminants virus (PPRV) and foot-and-mouth disease virus (FMDV) mixed infection in goats. Rather than in a single cell type, cytopathic effect (CPE) of the virus was observed in cocultured Vero/BHK-21 cells at 6th blind passage (BP). PPRV, but not FMDV could be purified from the virus mixture by plaque assay. Viral RNA (mixture) transfection in BHK-21 cells produced FMDV but not PPRV virions, a strategy which we have successfully employed for the first time to eliminate the negative-stranded RNA virus from the virus mixture. FMDV phenotypes, such as replication competent but noncytolytic, cytolytic but defective in plaque formation and, cytolytic but defective in both plaque formation and standard FMDV genome were observed respectively, at passage level BP8, BP15 and BP19 and hence complicated virus isolation in the cell culture system. Mixed infection was not found to induce any significant antigenic and genetic diversity in both PPRV and FMDV. Further, we for the first time demonstrated the viral interference between PPRV and FMDV. Prior transfection of PPRV RNA, but not Newcastle disease virus (NDV) and rotavirus RNA resulted in reduced FMDV replication in BHK-21 cells suggesting that the PPRV RNA-induced interference was specifically directed against FMDV. On long-term-coinfection of some acute pathogenic viruses (all possible combinations of PPRV, FMDV, NDV and buffalo-pox virus) in Vero cells, inmost cases, one of the coinfecting viruses was excluded at passage level 5 suggesting that the long-term coinfection may modify viral persistence. To the best of our knowledge, this is the first documented evidence describing a natural mixed infection of FMDV and PPRV. The study not only provides simple and reliable methodologies for isolation and purification of two epidemiologically and economically important groups of viruses, but could also help in establishing better guidelines for trading animals that could transmit further infections and epidemics in disease free nations. | 3773 | NA | NA | NA | no | no |
| 3772 | 2017 | NA | WOOKEY, PA; ROBINSON, CH; PARSONS, AN; WELKER, JM; PRESS, MC; CALLAGHAN, TV; LEE, JA | 1995 | ENVIRONMENTAL CONSTRAINTS ON THE GROWTH, PHOTOSYNTHESIS AND REPRODUCTIVE DEVELOPMENT OF DRYAS-OCTOPETALA AT A HIGH ARCTIC POLAR SEMIDESERT, SVALBARD | Opportunities exist in high Arctic polar semidesert communities for colonisation of unvegetated ground by long-lived clonal plants such as Dryas octopetala. This can be achieved by lateral spread of vegetative ramets, or by sexual reproduction and seedling recruitment. The objectives of this study were (1) to determine whether these two means of proliferation show differential sensitivity to contrasting components of the abiotic environment (temperature, soil nutrient and water availability) and (2) to evaluate the potential for D. octopetala to respond to climate change by an increase in cover and biomass in polar semi-desert communities. Factorial environmental manipulations of growing season temperature, soil nutrient and water status were conducted over 3 years at a polar semi-desert community in Svalbard, Norway (78 degrees 56.12’N, 11 degrees 50.4’E) and both clonal and sexual reproductive performance, together with instantaneous net photosynthesis (P-n), were recorded during the third season (1993). D. octopetala capitalised rapidly on an amelioration in the availability of inorganic nutrients (N, P and K) by an expansion in leaf area and biomass supported by increased P-n per unit leaf weight, and by apparent luxury uptake of nutrients (particularly P). Several facets of sexual reproductive development and seed viability were markedly improved by elevated temperatures or soil nutrient availability. Thus although D. octopetala is a long-lived clonal plant, with many traits characteristic of stress resistance syndrome, it showed considerable phenotypic plasticity in response to environmental manipulations. The results support the hypothesis that clonal growth confers survival potential during unfavourable years, together with the ability to capitalise on nutrient flushes and recycle nutrients internally. Continued investment in sexual reproduction ensures that seed setting is successful during favourable years, even if these occur infrequently. Cimate warming in the high Arctic could thus result in changes in the cover, biomass and the relative significance of clonal versus sexual proliferation of D. octopetala (and thus the genetic diversity of the population) but the long-term responses will probably be constrained by lack of available nutrients. | 3774 | NA | NA | NA | no | no |
| 3773 | 2017 | NA | Saito, Y; Sahara, K | 1999 | Two clinal trends in male-male aggressiveness in a subsocial spider mite (Schizotetranychus miscanthi) | We studied the correlation between male-male aggressive behavior of the spider mite, Schizotetranychus miscanthi and winter coldness, a factor hypothesized to correlate with the degree of relatedness of the interacting males through the frequent occurrence of mother-son mating in the spring. Thirty-one samples from various populations throughout Japan were added to previous data. A cluster analysis revealed two clinal trends for male-male aggression in S. miscanthi in regions of light or no snowfall. The two dines may represent a dimorphism within the species, each having a similar clinal trend within Japan. Further support for the hypothesis that male overwintering survival affects male-male aggressiveness came from a comparison between regions of heavy snowfall and those of light or no snowfall. In regions with heavy snow insulation, overwintering survival may not be as high as in other areas, and there was no correlation between male aggressiveness and the minimum winter temperature. However, there was a significant correlation in regions of light snow. | 3775 | NA | NA | NA | no | no |
| 3774 | 2017 | NA | Mazzarelli, CCM; Santos, MR; Amorim, RV; Augusto, A | 2015 | Effect of salinity on the metabolism and osmoregulation of selected ontogenetic stages of an amazon population of Macrobrachium amazonicum shrimp (Decapoda, Palaemonidae) | Probably as a function of their wide geographical distribution, the different population of Macrobrachium amazonicum shrimp may present distinct physiological, biochemical, reproductive, behavioral, and ecological patterns. These differences are so accentuated that the existence of allopatric speciation has been suggested, although initial studies indicate that the genetic variability of populations happen at an intraspecific level. Among the biological responses described for M. amazonicum populations, those regarding osmoregulation and metabolism play a key role for being related to the occupation of diverse habitats. To this effect, we investigated osmoregulation through the role of free amino acids in cell volume control and metabolism, through oxygen consumption in larvae (zoeae I, II, V and IX) and/or post-larvae of a M. amazonicum population from Amazon, kept in aquaculture fish hatcheries in the state of Sao Paulo. The results add information regarding the existence of distinct physiological responses among M. amazonicum populations and suggest that possible adjustments to metabolism and to the use of free amino acids as osmolytes of the regulation of the larvae and post-larvae cell volume depend on the appearance of structures responsible for hemolymph osmoregulation like, for example, the gills. In this respect, we verified that zoeae I do not alter their metabolism due to the exposition to fresh or brackish water, but they reduce intracellular concentration of free amino acids when exposed to fresh water, what may suggest the inexistence or inefficient performance of the structures responsible for volume regulation and hemolymph composition. On the other hand, in zoeae II and V exposed to fresh and brackish water, metabolism alterations were not followed by changes in free amino acids concentration. Thus it is possible, as the structures responsible for osmoregulation and ionic regulation become functional, that the role of free amino acids gets diminished and oxygen consumption elevated, probably due to greater energy expenditure with the active transportation of salts through epithelial membranes. Osmotic challenges also seem to alter throughout development, given that in zoeae II oxygen consumption is elevated on brackish water of 18, but in zoeae V it happens in fresh water. After M. amazonicum metamorphosis, free amino acids begin to play an important role as intracellular osmolytes, because we verified an increase of up to 40% in post-larvae exposed to brackish water of 18. The main free amino acids involved in cell volume regulation of ontogenetic stages evaluated were the non essential ones: glutamic acid, glycine, alanine, arginine, and proline. Interestingly, larvae from estuarine population studied here survived until the zoeae V stage in fresh water, but in some populations far from the sea, zoeae die right after eclosion in fresh water or they do not reach zoeae III stage. In addition, given that in favorable conditions caridean shrimp larvae shorten their development, we may infer that the cultivation environment, in which larvae developed in the present work, was appropriate, because almost all zoeae VIII kept on brackish water underwent metamorphosis directly to post-larvae and did not go through zoeae IX stage. | 3776 | NA | NA | NA | no | no |
| 3775 | 2017 | NA | Tettelin, H; Masignani, V; Cieslewicz, MJ; Donati, C; Medini, D; Ward, NL; Angiuoli, SV; Crabtree, J; Jones, AL; Durkin, AS; DeBoy, RT; Davidsen, TM; Mora, M; Scarselli, M; Ros, IMY; Peterson, JD; Hauser, CR; Sundaram, JP; Nelson, WC; Madupu, R; Brinkac, LM; Dodson, RJ; Rosovitz, MJ; Sullivan, SA; Daugherty, SC; Haft, DH; Selengut, J; Gwinn, ML; Zhou, LW; Zafar, N; Khouri, H; Radune, D; Dimitrov, G; Watkins, K; O’Connor, KJB; Smith, S; Utterback, TR; White, O; Rubens, CE; Grandi, G; Madoff, LC; Kasper, DL; Telford, JL; Wessels, MR; Rappuoli, R; Fraser, CM | 2005 | Genome analysis of multiple pathogenic isolates of Streptococcus agalactiae: Implications for the microbial “pan-genome” | The development of efficient and inexpensive genome sequencing methods has revolutionized the study of human bacterial pathogens and improved vaccine design. Unfortunately, the sequence of a single genome does not reflect how genetic variability drives pathogenesis within a bacterial species and also limits genomewide screens for vaccine candidates or for antimicrobial targets. We have generated the genomic sequence of six strains representing the five major disease-causing serotypes of Streptococcus agalactiae, the main cause of neonatal infection in humans. Analysis of these genomes and those available in databases showed that the S. agalactiae species can be described by a pan-genome consisting of a core genome shared by all isolates, accounting for approximate to 80% of any single genome, plus a dispensable genome consisting of partially shared and strain-specific genes. Mathematical extrapolation of the data suggests that the gene reservoir available for inclusion in the S. agalactiae pan-genome is vast and that unique genes will continue to be identified even after sequencing hundreds of genomes. | 3777 | NA | NA | NA | no | no |
| 3776 | 2017 | NA | Kidd, JM; Sharpton, TJ; Bobo, D; Norman, PJ; Martin, AR; Carpenter, ML; Sikora, M; Gignoux, CR; Nemat-Gorgani, N; Adams, A; Guadalupe, M; Guo, XS; Feng, Q; Li, YR; Liu, X; Parham, P; Hoal, EG; Feldman, MW; Pollard, KS; Wall, JD; Bustamante, CD; Henn, BM | 2014 | Exome capture from saliva produces high quality genomic and metagenomic data | Background: Targeted capture of genomic regions reduces sequencing cost while generating higher coverage by allowing biomedical researchers to focus on specific loci of interest, such as exons. Targeted capture also has the potential to facilitate the generation of genomic data from DNA collected via saliva or buccal cells. DNA samples derived from these cell types tend to have a lower human DNA yield, may be degraded from age and/or have contamination from bacteria or other ambient oral microbiota. However, thousands of samples have been previously collected from these cell types, and saliva collection has the advantage that it is a non-invasive and appropriate for a wide variety of research. Results: We demonstrate successful enrichment and sequencing of 15 South African KhoeSan exomes and 2 full genomes with samples initially derived from saliva. The expanded exome dataset enables us to characterize genetic diversity free from ascertainment bias for multiple KhoeSan populations, including new exome data from six HGDP Namibian San, revealing substantial population structure across the Kalahari Desert region. Additionally, we discover and independently verify thirty-one previously unknown KIR alleles using methods we developed to accurately map and call the highly polymorphic HLA and KIR loci from exome capture data. Finally, we show that exome capture of saliva-derived DNA yields sufficient non-human sequences to characterize oral microbial communities, including detection of bacteria linked to oral disease (e. g. Prevotella melaninogenica). For comparison, two samples were sequenced using standard full genome library preparation without exome capture and we found no systematic bias of metagenomic information between exome-captured and non-captured data. Conclusions: DNA from human saliva samples, collected and extracted using standard procedures, can be used to successfully sequence high quality human exomes, and metagenomic data can be derived from non-human reads. We find that individuals from the Kalahari carry a higher oral pathogenic microbial load than samples surveyed in the Human Microbiome Project. Additionally, rare variants present in the exomes suggest strong population structure across different KhoeSan populations. | 3778 | NA | NA | NA | no | no |
| 3777 | 2017 | NA | Kamiya, T; Ona, L; Wertheim, B; van Doorn, GS | 2016 | Coevolutionary feedback elevates constitutive immune defence: a protein network model | Background: Organisms have evolved a variety of defence mechanisms against natural enemies, which are typically used at the expense of other life history components. Induced defence mechanisms impose minor costs when pathogens are absent, but mounting an induced response can be time-consuming. Therefore, to ensure timely protection, organisms may partly rely on constitutive defence despite its sustained cost that renders it less economical. Existing theoretical models addressing the optimal combination of constitutive versus induced defence focus solely on host adaptation and ignore the fact that the efficacy of protection depends on genotype-specific host-parasite interactions. Here, we develop a signal-transduction network model inspired by the invertebrate innate immune system, in order to address the effect of parasite coevolution on the optimal combination of constitutive and induced defence. Results: Our analysis reveals that coevolution of parasites with specific immune components shifts the host’s optimal allocation from induced towards constitutive immunity. This effect is dependent upon whether receptors (for detection) or effectors (for elimination) are subjected to parasite counter-evolution. A parasite population subjected to a specific immune receptor can evolve heightened genetic diversity, which makes parasite detection more difficult for the hosts. We show that this coevolutionary feedback renders the induced immune response less efficient, forcing the hosts to invest more heavily in constitutive immunity. Parasites diversify to escape elimination by a specific effector too. However, this diversification does not alter the optimal balance between constitutive and induced defence: the reliance on constitutive defence is promoted by the receptor’s inability to detect, but not the effectors’ inability to eliminate parasites. If effectors are useless, hosts simply adapt to tolerate, rather than to invest in any defence against parasites. These contrasting results indicate that evolutionary feedback between host and parasite populations is a key factor shaping the selection regime for immune networks facing antagonistic coevolution. Conclusion: Parasite coevolution against specific immune defence alters the prediction of the optimal use of defence, and the effect of parasite coevolution varies between different immune components. | 3779 | NA | NA | NA | no | no |
| 3778 | 2017 | NA | Li, M; Liu, XL; Bradbury, P; Yu, JM; Zhang, YM; Todhunter, RJ; Buckler, ES; Zhang, ZW | 2014 | Enrichment of statistical power for genome-wide association studies | Background: The inheritance of most human diseases and agriculturally important traits is controlled by many genes with small effects. Identifying these genes, while simultaneously controlling false positives, is challenging. Among available statistical methods, the mixed linear model (MLM) has been the most flexible and powerful for controlling population structure and individual unequal relatedness (kinship), the two common causes of spurious associations. The introduction of the compressed MLM (CMLM) method provided additional opportunities for optimization by adding two new model parameters: grouping algorithms and number of groups. Results: This study introduces another model parameter to develop an enriched CMLM (ECMLM). The parameter involves algorithms to define kinship between groups (that is, kinship algorithms). The ECMLM calculates kinship using several different algorithms and then chooses the best combination between kinship algorithms and grouping algorithms. Conclusion: Simulations show that the ECMLM increases statistical power. In some cases, the magnitude of power gained by using ECMLM instead of CMLM is larger than the improvement found by using CMLM instead of MLM. | 3780 | NA | NA | NA | no | no |
| 3779 | 2017 | NA | GRANT, WS; LESLIE, RW | 1993 | EFFECT OF METAPOPULATION STRUCTURE ON NUCLEAR AND ORGANELLAR DNA VARIABILITY IN SEMIARID ENVIRONMENTS OF SOUTHERN AFRICA | The analysis of uniparentally inherited organellar DNA (such as mitochondrial DNA) has yielded a wealth of information about the population structures of Northern Hemisphere vertebrates. In southern Africa, however, several species show little or no variability in organellar DNA, yet show high levels of variability in nuclear DNA as measured with allozyme electrophoresis. To understand this difference, we used Monte Carlo methods to simulate the effects of subpopulation extinction and recolonization on the loss of variability in organellar and nuclear DNA for both the island and stepping-stone models of migration. We found that with nearly all combinations of extinction and recolonization there was a significantly greater loss of variability in organellar DNA than in nuclear DNA. The implications of this study are (1) that molecular methods measuring genetic variability in nuclear DNA must be used to study the population genetics of most animals in southern Africa and (2) that population extinction and recolonization appear to play a larger role in southern African populations than in Northern Hemispheric populations. | 3781 | NA | NA | NA | no | no |
| 3780 | 2017 | NA | Islam, MR; Alam, MS; Khan, AI; Hossain, I; Adam, LR; Daayf, F | 2016 | Analyses of genetic diversity of bacterial blight pathogen, Xanthomonas oryzae pv. oryzae using IS1112 in Bangladesh | Bacterial blight (BB) is caused by Xanthomonas oryzae pv. oryzae (Xoo), a most destructive disease of rice, mostly in Asia, including Bangladesh. Altogether 96 isolates of Xoo were collected from 19 rice-growing districts of Bangladesh in both the rain-fed and irrigated seasons of 2014 to assess their pathotypic and genetic variation. Pathotypic analyses were carried out on a set of 12 Near Isogenic Lines (NILs) of rice containing a single resistance gene and two check varieties IR24 and TN1 by the leaf clipping inoculation method. A total of 24 pathotypes were identified based on their virulence patterns on the NILs tested. Among these, pathotypes VII, XII and XIV, considered as major, containing a maximum number of isolates (9.38% each), are frequently distributed in seven northern to mid-eastern districts of Bangladesh. The most virulent pathotype I was recorded in the Habiganj and Brahmanbaria districts. The molecular analysis of variability among the isolates was carried out through PCR analysis using multi-locus primers Jel1 and Jel2 (based on the repetitive element IS1112 in the Xoo genome). Using the genotypic data, a dendrogram was constructed with 17 clusters along with 17 molecular haplotypes at the 65% similarity index. Cluster I was composed of 46 isolates considered as major, whereas clusters X, XI, XII and XVII were represented by a single isolate. A phenogram was constructed based on virulence to interpret the relationship between the pathotypes and the molecular haplotypes. At the 50% similarity level, among 10 clusters, cluster I, considered as major, consisted of a maximum of 10 pathotypes out of 24. In case of haplotypes, a maximum of 7 haplotypes were obtained from pathotype XII, whereas pathotypes IX, X, XV, XXII and XXIV were represented by a single haplotype. However, the present study revealed that different isolates belonging to the same pathotypes belonged to different haplotypes. Conversely, genetically similar haplotypes were also detected from different pathotypes collected from separate districts. This relationship appeared due to a high degree of DNA polymorphism among strains within many pathotypes existing in Bangladesh. (C) 2016 Academie des sciences. Published by Elsevier Masson SAS. All rights reserved. | 3782 | NA | NA | NA | no | no |
| 3781 | 2017 | NA | Koenig, JE; Bourne, DG; Curtis, B; Dlutek, M; Stokes, HW; Doolittle, WF; Boucher, Y | 2011 | Coral-mucus-associated Vibrio integrons in the Great Barrier Reef: genomic hotspots for environmental adaptation | Integron cassette arrays in a dozen cultivars of the most prevalent group of Vibrio isolates obtained from mucus expelled by a scleractinian coral (Pocillopora damicornis) colony living on the Great Barrier Reef were sequenced and compared. Although all cultivars showed > 99% identity across recA, pyrH and rpoB genes, no two had more than 10% of their integron-associated gene cassettes in common, and some individuals shared cassettes exclusively with distantly-related members of the genus. Of cassettes shared within the population, a number appear to have been transferred between Vibrio isolates, as assessed by phylogenetic analysis. Prominent among the mucus Vibrio cassettes with potentially inferable functions are acetyltransferases, some with close similarity to known antibiotic-resistance determinants. A subset of these potential resistance cassettes were shared exclusively between the mucus Vibrio cultivars, Vibrio coral pathogens and human pathogens, thus illustrating a direct link between these microbial niches through exchange of integron-associated gene cassettes. The ISME Journal (2011) 5, 962-972; doi:10.1038/ismej.2010.193; published online 27 January 2011 Subject Category: microbe-microbe and microbe-host interactions | 3783 | NA | NA | NA | no | no |
| 3782 | 2017 | NA | Groisillier, A; Massana, R; Valentin, K; Vaulot, D; Guilloul, L | 2006 | Genetic diversity and habitats of two enigmatic marine alveolate lineages | Systematic sequencing of environmental SSU rDNA genes amplified from different marine ecosystems has uncovered novel eukaryotic lineages, in particular within the alveolate and stramenopile radiations. The ecological and geographic distribution of 2 novel alveolate lineages (called Group I and II in previous papers) is inferred from the analysis of 62 different environmental clone libraries from freshwater and marine habitats. These 2 lineages have been, up to now, retrieved exclusively from marine ecosystems, including oceanic and coastal waters, sediments, hydrothermal vents, and permanent anoxic deep waters and usually represent the most abundant eukaryotic lineages in environmental genetic libraries. While Group I is only composed of environmental sequences (118 clones), Group II contains, besides environmental sequences (158 clones), sequences from described genera (8) (Hematodinium and Amoebophrya) that belong to the Syndiniales, an atypical order of dinoflagellates exclusively composed of marine parasites. This suggests that Group II could correspond to Syndiniales, although this should be confirmed in the future by examining the morphology of cells from Group II. Group II appears to be abundant in coastal and oceanic ecosystems, whereas permanent anoxic waters and hydrothermal ecosystems are usually dominated by Group I. Based upon the similarity of partial sequences, we organized these 2 groups into clusters, The diversity of Group II (16 clusters) is wider than that of Group 1 (5 clusters). Two clusters from Group I have a widespread distribution and are found in all explored marine habitats. In contrast, all other clusters seem to be limited to specific marine habitats. For example, some clusters belonging to Group I and Group II are only detected in extreme environments (anoxic and hydrothermal vents), whereas many clusters from Group II have only been retrieved from coastal waters. We determined near-complete SSU rRNA gene sequences for 26 environmental clones, selected in order to obtain at least one complete sequence per cluster. Phylogenetic analyses (maximum likelihood, neighbor joining, maximum parsimony, and Bayesian reconstruction) based upon complete sequences all concurred to place both Group I and II as sister lineages of dinoflagellates. This result contradicts several published studies, which placed both groups within dinoflagellates. | 3784 | NA | NA | NA | no | no |
| 3783 | 2017 | NA | Thompson, AL; Mahoney, AK; Smiley, RW; Paulitz, TC; Hulbert, S; Garland-Campbell, K | 2017 | Resistance to Multiple Soil-Borne Pathogens of the Pacific Northwest, USA Is Colocated in a Wheat Recombinant Inbred Line Population | Soil-borne pathogens of the Pacific Northwest decrease yields in both spring and winter wheat. Pathogens of economic importance include Fusarium culmorum, Pratylenchus neglectus, P. thornei, and Rhizoctonia solani AG8. Few options are available to growers to manage these pathogens and reduce yield loss, therefore the focus for breeding programs is on developing resistant wheat cultivars. A recombinant inbred line population, LouAu (MP-7, NSL 511036), was developed to identify quantitative trait loci (QTL) associated with resistance to P. neglectus and P. thornei. This same population was later suspected to be resistant to F. culmorum and R. solani AG8. This study confirms partial resistance to F. culmorum and R. solani AG8 is present in this population. Six major and 16 speculative QTL were identified across seven measured traits. Four of the six major QTL were found within the same genomic region of the 5A wheat chromosome suggesting shared gene(s) contribute to the resistance. These QTL will be useful in breeding programs looking to incorporate resistance to soil-borne pathogens in wheat cultivars. | 3785 | NA | NA | NA | no | no |
| 3784 | 2017 | NA | Peralta, H; Guerrero, G; Aguilar, A; Mora, J | 2011 | Sequence variability of Rhizobiales orthologs and relationship with physico-chemical characteristics of proteins | Background: Chromosomal orthologs can reveal the shared ancestral gene set and their evolutionary trends. Additionally, physico-chemical properties of encoded proteins could provide information about functional adaptation and ecological niche requirements. Results: We analyzed 7080 genes (five groups of 1416 orthologs each) from Rhizobiales species (S. meliloti, R. etli, and M. loti, plant symbionts; A. tumefaciens, a plant pathogen; and B. melitensis, an animal pathogen). We evaluated their phylogenetic relationships and observed three main topologies. The first, with closer association of R. etli to A. tumefaciens; the second with R. etli closer to S. meliloti; and the third with A. tumefaciens and S. meliloti as the closest pair. This was not unusual, given the close relatedness of these three species. We calculated the synonymous (dS) and nonsynonymous (dN) substitution rates of these orthologs, and found that informational and metabolic functions showed relatively low dN rates; in contrast, genes from hypothetical functions and cellular processes showed high dN rates. An alternative measure of sequence variability, percentage of changes by species, was used to evaluate the most specific proportion of amino acid residues from alignments. When dN was compared with that measure a high correlation was obtained, revealing that much of evolutive information was extracted with the percentage of changes by species at the amino acid level. By analyzing the sequence variability of orthologs with a set of five properties (polarity, electrostatic charge, formation of secondary structures, molecular volume, and amino acid composition), we found that physico-chemical characteristics of proteins correlated with specific functional roles, and association of species did not follow their typical phylogeny, probably reflecting more adaptation to their life styles and niche preferences. In addition, orthologs with low dN rates had residues with more positive values of polarity, volume and electrostatic charge. Conclusions: These findings revealed that even when orthologs perform the same function in each genomic background, their sequences reveal important evolutionary tendencies and differences related to adaptation. This article was reviewed by: Dr. Purificacion Lopez-Garcia, Prof. Jeffrey Townsend (nominated by Dr. J. Peter Gogarten), and Ms. Olga Kamneva. | 3786 | NA | NA | NA | no | no |
| 3785 | 2017 | NA | Guaraldo, AC; Costa-Leonardo, AM | 2009 | Preliminary Fusion Testing Between Whole Young Colonies of Coptotermes gestroi (Isoptera: Rhinotermitidae) | Everyyear in Brazil, colonies of the exotic termite Coptotermes gestroi increase their production of alates, which is the caste responsible for the foundation of new colonies. Colonies of this invasive species have huge populations, and their economical impacts have increased in urban areas during recent years. To simulate the meeting of neighboring colonies in field situations, we arranged intercolony interactions in the laboratory. Additionally, we exchanged one king or queen between colonies to observe how termites react to foreign reproductives. After 15 days of interactions, the mixed colony showed survival of both queens in 80% of the replicates and death of one king in 100% of the replicates. The present study also demonstrated tolerance of C gestroi colonies to foreign reproductives. The low agonism of the neuter caste (workers and soldiers) in the mixed colonies and the stability of the termite populations confirm the fusion of C. gestroi colonies. Probably, the few introductions of this species in Brazil resulted in populations of C. gestroi that are genetically very similar with high inter-colony relatedness. | 3787 | NA | NA | NA | no | no |
| 3786 | 2017 | NA | Merrill, BD; Grose, JH; Breakwell, DP; Burnett, SH | 2014 | Characterization of Paenibacillus larvae bacteriophages and their genomic relationships to firmicute bacteriophages | Background: Paenibacillus larvae is a Firmicute bacterium that causes American Foulbrood, a lethal disease in honeybees and is a major source of global agricultural losses. Although P. larvae phages were isolated prior to 2013, no full genome sequences of P. larvae bacteriophages were published or analyzed. This report includes an in-depth analysis of the structure, genomes, and relatedness of P. larvae myoviruses Abouo, Davis, Emery, Jimmer1, Jimmer2, and siphovirus phiIBB\_Pl23 to each other and to other known phages. Results: P. larvae phages Abouo, Davies, Emery, Jimmer1, and Jimmer2 are myoviruses with similar to 50 kbp genomes. The six P. larvae phages form three distinct groups by dotplot analysis. An annotated linear genome map of these six phages displays important identifiable genes and demonstrates the relationship between phages. Sixty phage assembly or structural protein genes and 133 regulatory or other non-structural protein genes were identifiable among the six P. larvae phages. Jimmer1, Jimmer2, and Davies formed stable lysogens resistant to superinfection by genetically similar phages. The correlation between tape measure protein gene length and phage tail length allowed identification of co-isolated phages Emery and Abouo in electron micrographs. A Phamerator database was assembled with the P. larvae phage genomes and 107 genomes of Firmicute-infecting phages, including 71 Bacillus phages. Phamerator identified conserved domains in 1,501 of 6,181 phamilies (only 24.3%) encoded by genes in the database and revealed that P. larvae phage genomes shared at least one phamily with 72 of the 107 other phages. The phamily relationship of large terminase proteins was used to indicate putative DNA packaging strategies. Analyses from CoreGenes, Phamerator, and electron micrograph measurements indicated Jimmer1, Jimmer2, Abouo and Davies were related to phages phiC2, EJ-1, KC5a, and AQ113, which are small-genome myoviruses that infect Streptococcus, Lactobacillus, and Clostridium, respectively. Conclusions: This paper represents the first comparison of phage genomes in the Paenibacillus genus and the first organization of P. larvae phages based on sequence and structure. This analysis provides an important contribution to the field of bacteriophage genomics by serving as a foundation on which to build an understanding of the natural predators of P. larvae. | 3788 | NA | NA | NA | no | no |
| 3787 | 2017 | NA | Kumar, BNH; Bhat, S; Borphukan, B; Fakrudinl, B | 2017 | Association analysis of charcoal rot disease component traits in sorghum minicore germplasm with EST-SSR markers | Sorghum is an important crop of semi arid regions where it suffers from several biotic stresses. Among biotic stresses, charcoal rot/stalk rot is a major constraint for rabi (post rainy) sorghum production. Identification of stable resistant sources and incorporation of the genes or quantitative trait loci (QTL1) governing resistance are the prerequisite to make progress in breeding for charcoal rot resistance. A set of 242 sorghum minicore collection were phenotyped in sick plot of charcoal rot disease. A total of 31 polymorphic EST-SSR markers were developed and mapped through association analysis. The population structure analysis indicated the partitioning of the genetic population structure into four clusters. In the current study six new markers were identified for charcoal rot disease resistance (Xiabt 210, Xiabt 527, Xiabt 301, Xiabt 37, Xiabt 77, Xiabt 81) and was validated previously identified flanking markers for charcoal rot disease QTLs (locus Xiabt 275) at RARS Vijayapura location. Six marker trait associations were stable at two locations and these significant associations are useful and suitable for marker assisted selection in charcoal rot disease resistance breeding programs. | 3789 | NA | NA | NA | no | no |
| 3788 | 2017 | NA | Beatty, GE; Provan, J | 2013 | Post-glacial dispersal, rather than in situ glacial survival, best explains the disjunct distribution of the Lusitanian plant species Daboecia cantabrica (Ericaceae) | Aim The distribution of the Lusitanian flora and fauna, species which are found only in southern and western Ireland and in northern Spain and Portugal but which are absent from intervening countries, represents one of the classic conundrums of biogeography. The aim of the present study was to determine whether the distribution of the Lusitanian plant species Daboecia cantabrica was due to persistence in separate Irish and Iberian refugia, or has resulted from post-glacial recolonization followed by subsequent extinction of intervening populations. Location Northern Spain and Co. Galway, western Ireland. Methods Palaeodistribution modelling using Maxent was employed to identify putative refugial areas for D. cantabrica at the Last Glacial Maximum (LGM). Phylogeographical analysis of samples from 64 locations in Ireland and Spain were carried out using a chloroplast marker (atpBrbcL), the nuclear ITS region, and an anonymous nuclear single-copy locus. Results The palaeodistribution model indicated areas with a high probability of survival for D. cantabrica at the LGM off the western coast of Galicia in Spain, and in the Bay of Biscay. Spanish populations exhibited substantially higher genetic diversity than Irish populations at all three loci, as well as geographical structuring of haplotypes within Spain consistent with divergence in separate refugia. Spanish populations also exhibited far more endemic haplotypes. Divergence time between Irish and Spanish populations associated with the putative Biscay refugium was estimated as 3.33332 ka. Main conclusions Our data indicate persistence by D. cantabrica throughout the LGM in two separate southern refugia: one in western Galicia and one in the area off the coast of western France which now lies in the Bay of Biscay. Spain was recolonized from both refugia, whilst Ireland was most likely recolonized from the Biscay refugium. On the balance of evidence across the three marker types and the palaeodistribution modelling, our findings do not support the idea of in situ survival of D. cantabrica in Ireland, contrary to earlier suggestions. The fact that we cannot conclusively rule out the existence of a small, more northerly refugium, however, highlights the need for further analysis of Lusitanian plant species. | 3790 | NA | NA | NA | no | no |
| 3789 | 2017 | NA | Valles, SM; Oi, DH; Porter, SD | 2010 | Seasonal variation and the co-occurrence of four pathogens and a group of parasites among monogyne and polygyne fire ant colonies | A year-long survey was conducted to determine the seasonality and co-occurrence of four pathogens and a group of parasites in colonies of the red imported fire ant, Solenopsis invicta, in north-central Florida. Solenopsis invicta colonies were sampled and examined for the presence of Pseudacteon spp. (P. curvatus, P. tricuspis, P. obtusus) parasitic phorid flies, a microsporidian pathogen (Kneallhazia solenopsae) and 3 S. invicta viruses (SINV-1, SINV-2, and SINV-3) by PCR or RT-PCR methods. In addition, the social form designation of each colony (single- or multiple-queen) was determined by genotyping worker ants at the Gp-9 locus to determine if the pathogens or parasites were associated with monogyne or polygyne fire ant colonies. Seasonal variability was observed in the prevalence of all pathogens/parasites examined, with SINV-1, SINV-3, and K. solenopsae exhibiting pronounced seasonality. SINV-1 and K. solenopsae infections were most prevalent among colonies during warmer periods of the year, while SINV-3 was most prevalent during the cooler periods. As hypothesized, pathogens were found more commonly in polygyne colonies than in monogyne colonies. Infection comparisons by social form revealed higher infection rates of K. solenopsae and SINV-2 in polygyne colony samples compared with monogyne colony samples. The overall colony infection and parasitism rate among the 360 colonies sampled, regardless of social form, was 60.3% (SINV-1), 8.9% (SINV-2), 10.8% (SINV-3), 22.5% (K. solenopsae), and 8.1% (Pseudacteon flies). An interesting pattern was observed between the number of different pathogens or parasites detected in monogyne and polygyne colonies. The majority of monogyne colonies (>80%) were either uninfected or infected or parasitized with only a single pathogen/parasite while the majority of polygyne colonies (>55%) were infected or parasitized with 2 or more pathogens/parasites simultaneously. Higher pathogen/parasite prevalence among polygyne colonies is attributed to lower genetic relatedness (among nestmates), increased colony longevity, and the proclivity of polygyne colonies to share workers, brood and queens among their interconnected colonies. Evaluation of pairwise co-occurrence data indicated that the pathogens and fly parasites were usually independently distributed among host colonies; however, unknown local factors did cause several significant deviations from expected values. Published by Elsevier Inc. | 3791 | NA | NA | NA | no | no |
| 3790 | 2017 | NA | Sagastume, S; Martin-Hernandez, R; Higes, M; Henriques-Gil, N | 2016 | Genotype diversity in the honey bee parasite Nosema ceranae: multi-strain isolates, cryptic sex or both? | Background: There is great controversy as to whether Microsporidia undergo a sexual cycle. In the paradigmatic case of Nosema ceranae, although there is no morphological evidence of sex, some meiosis-specific genes are present in its reduced genome and there is also high intraspecific variability, with incongruent phylogenies having been systematically obtained. The possibility of sexual recombination is important from an epidemiological standpoint, particularly as N. ceranae is considered to be a major factor in the current disquieting epidemic of widespread bee colony losses. This parasite apparently originated in oriental honey bees, spreading out of Asia and Australia to infect honey bees worldwide. This study had three main objectives: i) to obtain genetic markers that are not part of known multi-copy arrays for strain determination; ii) to shed light on the intraspecific variability and recombination of N. ceranae; and iii) to assess the variability in N. ceranae populations. The answers to these questions are critical to understand the capacity of adaptation of microsporidia. Results: Biallelic polymorphisms were detected at a number of specific points in the five coding loci analyzed from European and Australian isolates of N. ceranae. Heterozygous genotypes were abundant and cloning experiments demonstrate that they reflect the existence of multiple alternative sequences in each isolate. The comparisons of different clones and genotypes clearly indicate that new haplotypes are generated by homologous recombination. Conclusions: The N. ceranae isolates from honey bees correspond to genotypically distinct populations, revealing that individual honey bees may not be infected by a particular clone but rather, a pool of different strains. Homologous recombination implies the existence of a cryptic sex cycle yet to be described in N. ceranae. There are no diagnostic alleles associated with Australian or European origins, nor are there differences between the two hosts, A. cerana and A. mellifera, supporting the absence of biological barriers for N. ceranae transmission. Diversity is high among microsporidia of both these origins, and the maintenance of a high heterozygosis in the recently invaded European populations, could hypothetically underlie the stronger virulence of N. ceranae observed in A. mellifera. | 3792 | NA | NA | NA | no | no |
| 3791 | 2017 | NA | Dow, N; Chernick, A; Orsel, K; van Marle, G; van der Meer, F | 2015 | Genetic Variability of Bovine Viral Diarrhea Virus and Evidence for a Possible Genetic Bottleneck during Vertical Transmission in Persistently Infected Cattle | Bovine viral diarrhea virus (BVDV), a Pestivirus in the family Flaviviridae, is an economically important pathogen of cattle worldwide. The primary propagators of the virus are immunotolerant persistently infected (PI) cattle, which shed large quantities of virus throughout life. Despite the absence of an acquired immunity against BVDV in these PI cattle there are strong indications of viral variability that are of clinical and epidemiological importance. In this study the variability of E2 and NS5B sequences in multiple body compartments of PI cattle were characterized using clonal sequencing. Phylogenetic analyses revealed that BVDV exists as a quasispecies within PI cattle. Viral variants were clustered by tissue compartment significantly more often than expected by chance alone with the central nervous system appearing to be a particularly important viral reservoir. We also found strong indications for a genetic bottleneck during vertical transmission from PI animals to their offspring. These quasispecies analyses within PI cattle exemplify the role of the PI host in viral propagation and highlight the complex dynamics of BVDV pathogenesis, transmission and evolution. | 3793 | NA | NA | NA | no | no |
| 3792 | 2017 | NA | Taggart, DA; Shimmin, GA; Underwood, G; Phillips, K | 2002 | Survival of very small macropod pouch young following short-term isolation from the pouch at various environmental temperatures | There is considerable interest in the use of assisted breeding techniques as a component of conservation programmes for a variety of macropods threatened with extinction. While cross-fostering is being conducted in some programmes in captivity, little is known about the best techniques for carrying this procedure out from wild populations. In this study we compare the success of various procedures for isolating small tammar wallaby (Macropus eugenii) and brush-tailed rock wallaby (Petrogale penicillata) pouch young (0.4 g-410 g) for short periods (6 hours) as a first step towards establishing a methodology for recruiting endangered macropod pouch young directly from the wild for foster-rearing in captivity. Pouch temperature and humidity were determined. Pouch young from females in experimental groups were weighed, measured and then isolated, at 30degreesC, 27degreesC or 23degreesC, and 100% humidity. After isolation the weight of pouch young maintained at 30degreesC and 27degreesC had declined significantly (2-17% of body weight), whereas those held at 23degreesC and 100% humidity lost less than 0.5% of body weight. All young were reattached to the active teat of their mother following pouch isolation and their survival and growth monitored. Pouch young from the control group and those isolated at 23degreesC and 100% humidity survived and grew at normal rates. Only the largest pouch young maintained in isolation at either 30degreesC or 27degreesC survived to day 7 following reattachment. Data indicated that the proportion of weight lost by pouch young as a result of isolation decreased with increasing size and age of the pouch young. These trials indicate that it would be possible to harvest very small macropod pouch young from a species in the wild with a very high probability that they would survive short-term isolation from the pouch and reattachment to the teat. The use of this procedure in combination with cross-fostering techniques will allow small pouch young from threatened macropod species to be recruited directly from the wild into captivity, for captive breeding or to improve genetic diversity in captive colonies. It should also act to reactivate diapause embryos in these animals and hence may accelerate breeding in wild-based mothers. | 3794 | NA | NA | NA | no | no |
| 3793 | 2017 | NA | Zhang, JL; Wang, L; Li, J; Kelly, P; Price, S; Wang, CM | 2017 | First Molecular Characterization of Feline Immunodeficiency Virus in Domestic Cats from Mainland China | The feline immunodeficiency virus (FIV) is a retrovirus of the Lentivirus genus that was initially isolated from a colony of domestic cats in California in 1986 and has now been recognized as a common feline pathogen worldwide. To date, there is only one recent serology-based report on FIV in mainland China which was published in 2016. We designed this study to investigate the molecular prevalence and diversity of feline immunodeficiency virus (FIV) in domestic cats from mainland China. We studied the prevalence of FIV in whole blood samples of 615 domestic cats in five cities (Beijing, Guangzhou, Nanjing, Shanghai and Yangzhou) of mainland China and examined them using FRET-PCR (Fluorescence Resonance Energy Transfer-Polymerase Chain Reaction) and regular PCRs for the gag and env genes. Overall, 1.3% (8/615) of the cats were positive for provirus DNA with nucleotide analysis using PCRs for the gag and env sequences showing the cats were infected with FIV subtype A. This is the first molecular characterization of FIV in mainland China and the first description of subtype A in continental Asia. | 3795 | NA | NA | NA | no | no |
| 3794 | 2017 | NA | Priotto, J; Polop, J | 2003 | Effect of overwintering adults on juvenile survival of Calomys venustus (Muridae : Sigmodontinae) | The aim of this paper was to test the hypothesis that the presence of overwintering Calomys venustus males or females at the beginning of the breeding period reduced the survival of juveniles belonging to the first cohort of each year (C1). The study was carried out in four 0.25-ha enclosures (two control and two experimental), each situated on natural pasture. This study was conducted between August 1997 and February 1999 and had two parts: removal of overwintering males (ROM) and removal of overwintering females (ROF). For the ROM treatment, overwintering males were removed immediately after C1 juveniles were born, in the ROF treatment overwintering females were removed after the C1 were weaned. Weekly censuses were taken for 3 successive days over 6 weeks. Juvenile survival was examined through a survivorship schedule. The survivorship values were compared by sex and treatment (removal of overwintering males or females) using repeated-measures anova. Removal of overwintering males and females did not improve the survival of C1 juveniles. This result could be related to the delay in maturation of C1 juvenile males. | 3796 | NA | NA | NA | no | no |
| 3795 | 2017 | NA | Tipping, PW; Center, TD; Sosa, AJ; Dray, FA | 2010 | Host specificity assessment and potential impact of Megamelus scutellaris (Hemiptera: Delphacidae) on waterhyacinth Eichhornia crassipes (Pontederiales: Pontederiaceae) | The delphacid Megamelus scutellaris Berg was evaluated for host specificity and potential impact as part of a biological control program targeting Eichhornia. crassipes. Survival and development of adults and nymphs were used as metrics with no-choice, two-choice, nymph transfer, and sustainability tests conducted under quarantine conditions. A total of 69 plant species were tested including 12 from the Pontederiaceae (including E. crassipes). Additionally, 27 native and 5 exotic associated wetland species and 11 economic species were tested. Megamelus scutellaris exhibited a high level of oviposition and developmental fidelity to E. crassipes by failing to sustain populations on any non-target test plant past the F1 generation. Nymph transfer tests which simulated potential spill-over events found that survival was virtually non-existent on associated wetland plants, regardless of taxonomic relatedness, including on Pontederia cordata, an important and widespread native species. Eichhornia crassipes plants exposed to two consecutive generations of feeding produced 66.9% less biomass and 73.4% fewer leaves than those in the controls. We conclude that Megamelus scutellaris is safe to release on E. crassipes in the United States. | 3797 | NA | NA | NA | no | no |
| 3796 | 2017 | NA | Pearson, SK; Godfrey, SS; Bull, CM; Gardner, MG | 2016 | Larger lizards live longer in the group-living Egernia stokesii | Animal space use has implications for gene flow, disease dynamics, mating systems and the evolution of sociality. Given recent attention to sociality in reptiles, lizards are an important group for expanding our understanding of animal space use. Lizard space use is commonly investigated within one population over a short period and limited attention has been given to potential predictors of site fidelity. This study evaluated site fidelity in three populations of group-living Egernia stokesii (gidgee skink) between two field surveys separated by almost a decade. Of 43 recaptured lizards, 28 (65%) occupied their original space, and 15 (36%) of those shared their space with the same other lizard or lizards in both surveys. This confirmed long-term site and social bond fidelity in E. stokesii. We found that larger lizards were more likely to be recaptured. Neither body size, individual genetic heterozygosity, nor the availability of refuges strongly predicted whether lizards were recaptured in the same or a different place. The reasons why some lizards stayed in the same space while others moved are yet to be resolved. | 3798 | NA | NA | NA | no | no |
| 3797 | 2017 | NA | Davila, J; McNamara, LA; Yang, ZH | 2012 | Comparison of the Predicted Population Coverage of Tuberculosis Vaccine Candidates Ag85B-ESAT-6, Ag85B-TB10.4, and Mtb72f via a Bioinformatics Approach | The Bacille-Calmette Guerin (BCG) vaccine does not provide consistent protection against adult pulmonary tuberculosis (TB) worldwide. As novel TB vaccine candidates advance in studies and clinical trials, it will be critically important to evaluate their global coverage by assessing the impact of host and pathogen variability on vaccine efficacy. In this study, we focus on the impact that host genetic variability may have on the protective effect of TB vaccine candidates Ag85B-ESAT-6, Ag85B-TB10.4, and Mtb72f. We use open-source epitope binding prediction programs to evaluate the binding of vaccine epitopes to Class I HLA (A, B, and C) and Class II HLA (DRB1) alleles. Our findings suggest that Mtb72f may be less consistently protective than either Ag85B-ESAT-6 or Ag85B-TB10.4 in populations with a high TB burden, while Ag85B-TB10.4 may provide the most consistent protection. The findings of this study highlight the utility of bioinformatics as a tool for evaluating vaccine candidates before the costly stages of clinical trials and informing the development of new vaccines with the broadest possible population coverage. | 3799 | NA | NA | NA | no | no |
| 3798 | 2017 | NA | SMITH, JM; SMITH, NH; OROURKE, M; SPRATT, BG | 1993 | HOW CLONAL ARE BACTERIA | Data from multilocus enzyme electrophoresis of bacterial populations were analyzed using a statistical test designed to detect associations between genes at different loci. Some species (e.g., Salmonella) were found to be clonal at all levels of analysis. At the other extreme, Neisseria gonorrhoeae is panmictic, with random association between loci. Two intermediate types of population structure were also found. Neisseria meningitidis displays what we have called an ‘’epidemic’’ structure. There is significant association between loci, but this arises only because of the recent, explosive, increase in particular electrophoretic types; when this effect is eliminated the population is found to be effectively panmictic. In contrast, linkage disequilibrium in a population of Rhizobium meliloti exists because the sample consisted of two genetically isolated divisions, often fixed for different alleles: within each division association between loci was almost random. The method of analysis is appropriate whenever there is doubt about the extent of genetic recombination between members of a population. To illustrate this we analyzed data on protozoan parasites and again found panmictic, epidemic, and clonal population structures. | 3800 | NA | NA | NA | no | no |
| 3799 | 2017 | NA | Naranjo, EJ | 2009 | Ecology and Conservation of Baird’s tapir in Mexico | Baird’s tapir (Tapirus bairdii) is endangered primarily because of habitat loss and fragmentation, and overhunting throughout its distribution range (ca. 21,000 km(2)) in the Mexican states of Campeche, Chiapas, Quintana Roo, Oaxaca, Veracruz, and possibly Yucatan and Tabasco. The number of tapirs occurring in Mexico is estimated around 2,600 individuals, which are also threatened by factors such as forest fires, large-scale infrastructure projects (building of dams and highways), disease transmission from domestic animals, pollution of water bodies, and global climatic change effects. A strategy for tapir conservation in Mexico should include: 1) protection and management of extant habitat; 2) creation and maintaining of corridors among isolated forest fragments containing tapirs; 3) community-based control of poaching; 4) development of economic alternatives for people living near tapir habitat; 5) captive breeding programs with educational, scientific, and conservationist purposes; 6) environmental education and communication programs in rural and urban areas near tapir habitat; and 7) research on distribution, abundance, habitat use and availability, population status, movement patterns, feeding habits, genetic variability, interactions with domestic species, diseases, and responses to habitat fragmentation, hunting, and global climatic change. | 3801 | NA | NA | NA | no | no |
| 3800 | 2017 | NA | Zhang, Q; Hill, GE; Edwards, SV; Backstrom, N | 2014 | A house finch (Haemorhous mexicanus) spleen transcriptome reveals intra- and interspecific patterns of gene expression, alternative splicing and genetic diversity in passerines | Background: With its plumage color dimorphism and unique history in North America, including a recent population expansion and an epizootic of Mycoplasma gallisepticum (MG), the house finch (Haemorhous mexicanus) is a model species for studying sexual selection, plumage coloration and host-parasite interactions. As part of our ongoing efforts to make available genomic resources for this species, here we report a transcriptome assembly derived from genes expressed in spleen. Results: We characterize transcriptomes from two populations with different histories of demography and disease exposure: a recently founded population in the eastern US that has been exposed to MG for over a decade and a native population from the western range that has never been exposed to MG. We utilize this resource to quantify conservation in gene expression in passerine birds over approximately 50 MY by comparing splenic expression profiles for 9,646 house finch transcripts and those from zebra finch and find that less than half of all genes expressed in spleen in either species are expressed in both species. Comparative gene annotations from several vertebrate species suggest that the house finch transcriptomes contain similar to 15 genes not yet found in previously sequenced vertebrate genomes. The house finch transcriptomes harbour similar to 85,000 SNPs, similar to 20,000 of which are non-synonymous. Although not yet validated by biological or technical replication, we identify a set of genes exhibiting differences between populations in gene expression (n = 182; 2% of all transcripts), allele frequencies (76 F-ST ouliers) and alternative splicing as well as genes with several fixed non-synonymous substitutions; this set includes genes with functions related to double-strand break repair and immune response. Conclusions: The two house finch spleen transcriptome profiles will add to the increasing data on genome and transcriptome sequence information from natural populations. Differences in splenic expression between house finch and zebra finch imply either significant evolutionary turnover of splenic expression patterns or different physiological states of the individuals examined. The transcriptome resource will enhance the potential to annotate an eventual house finch genome, and the set of gene-based high-quality SNPs will help clarify the genetic underpinnings of host-pathogen interactions and sexual selection. | 3802 | NA | NA | NA | no | no |
| 3801 | 2017 | NA | Castro-Nallar, E; Martin, MCS; Mascayano, C; Molina, C; Crandall, KA | 2011 | Molecular Phylodynamics and Protein Modeling of Infectious Salmon Anemia Virus (ISAV) | Background: ISAV is a member of the Orthomyxoviridae family that affects salmonids with disastrous results. It was first detected in 1984 in Norway and from then on it has been reported in Canada, United States, Scotland and the Faroe Islands. Recently, an outbreak was recorded in Chile with negative consequences for the local fishing industry. However, few studies have examined available data to test hypotheses associated with the phylogeographic partitioning of the infecting viral population, the population dynamics, or the evolutionary rates and demographic history of ISAV. To explore these issues, we collected relevant sequences of genes coding for both surface proteins from Chile, Canada, and Norway. We addressed questions regarding their phylogenetic relationships, evolutionary rates, and demographic history using modern phylogenetic methods. Results: A recombination breakpoint was consistently detected in the Hemagglutinin-Esterase (he) gene at either side of the Highly Polymorphic Region (HPR), whereas no recombination breakpoints were detected in Fusion protein (f) gene. Evolutionary relationships of ISAV revealed the 2007 Chilean outbreak group as a monophyletic clade for f that has a sister relationship to the Norwegian isolates. Their tMRCA is consistent with epidemiological data and demographic history was successfully recovered showing a profound bottleneck with further population expansion. Finally, selection analyses detected ongoing diversifying selection in f and he codons associated with protease processing and the HPR region, respectively. Conclusions: Our results are consistent with the Norwegian origin hypothesis for the Chilean outbreak clade. In particular, ISAV HPR0 genotype is not the ancestor of all ISAV strains, although SK779/06 (HPR0) shares a common ancestor with the Chilean outbreak clade. Our analyses suggest that ISAV shows hallmarks typical of RNA viruses that can be exploited in epidemiological and surveillance settings. In addition, we hypothesized that genetic diversity of the HPR region is governed by recombination, probably due to template switching and that novel fusion gene proteolytic sites confer a selective advantage for the isolates that carry them. Additionally, protein modeling allowed us to relate the results of phylogenetic studies with the predicted structures. This study demonstrates that phylogenetic methods are important tools to predict future outbreaks of ISAV and other salmon pathogens. | 3803 | NA | NA | NA | no | no |
| 3802 | 2017 | NA | Shan, XY; Zhang, J; Zhou, HJ; Zhu, BQ; Xu, L; Shao, ZJ; Jiang, BF | 2014 | Genetic Diversity of Neisseria meningitidis Serogroup C ST-4821 in China Based on Multiple-Locus Variable Number Tandem Repeat Analysis | Neisseria meningitidis sequence type (ST)-4821 was first reported in China in 2003, and a new hyper-virulent lineage has been designated as the ST-4821 complex. A large number of N. meningitidis ST-4821 strains have been identified in China since 2003; however, the microevolution characteristics of this complex are unclear. Different combinations of variable number of tandem repeats (VNTR) loci were used in multiple-locus VNTR analysis (MLVA) to analyze 118 N. meningitidis serogroup C ST-4821 strains isolated from seventeen provinces between 2003 and 2012. Additionally, MLVA with five VNTR loci was performed due to its high discriminatory power. One hundred and eighteen isolates were found to comprise 112 subtypes based on MLVA, and 16 outbreak-associated strains were clustered into one group. These data indicate a high level of diversity for N. meningitidis ST-4821 due to microevolution in the last decade. In addition, the results revealed high similarity between isolates from the same geographic origins, which is helpful when monitoring the spread of N. meningitidis serogroup C ST-4821 and will provide valuable information for the control and prevention of bacterial meningitis in China. | 3804 | NA | NA | NA | no | no |
| 3803 | 2017 | NA | Wilson-Rich, N; Pilowsky, JA; Foo, B; Tien, T; Hester, F; Starks, PT | 2014 | A test of the haploid susceptibility hypothesis using a species with naturally occurring variation in ploidy | The haploid susceptibility hypothesis (HSH) was proposed as an explanation for how behavioral roles in haplodiploid social systems evolved. It posits that haploid males are more susceptible to disease than diploid females due to decreased genetic variability at key disease resistance loci. The resulting decreased immunocompetence is hypothesized to have played a role in the evolution of social behavior by limiting the behavioral repertoire haploids perform. Here, we test this hypothesis in a study system that separates ploidy from behavioral sex roles: Polistes dominulus, a social wasp, has colonies with naturally occurring diploid males. We report results from two immune function assays-hemolymph phenoloxidase activity and encapsulation response-performed on haploid males, diploid males, and diploid females. Our data suggest that ploidy is not a significant contributor to immune function in P. dominulus; thus, our data do not support the HSH for the evolution of behavioral roles. Instead, our data indicate that time of emergence is the best predictor of immune function in Polistes. We speculate that seasonal trends result from seasonal differences in pathogens and parasites. | 3805 | NA | NA | NA | no | no |
| 3804 | 2017 | NA | Gandon, S; Michalakis, Y | 2002 | Local adaptation, evolutionary potential and host-parasite coevolution: interactions between migration, mutation, population size and generation time | Local adaptation of parasites to their sympatric hosts has been investigated on different biological systems through reciprocal transplant experiments. Most of these studies revealed a local adaptation of the parasite. In several cases, however, parasites were found to be locally maladapted or neither adapted nor maladapted. In the present paper, we try to determine the causes of such variability in these results. We analyse a host-parasite metapopulation model and study the effect of several factors on the emergence of local adaptation: population sizes, mutation rates and migration rates for both the host and the parasite, and parasite generation time. We show that all these factors may act on local adaptation through their effects on the evolutionary potential of each species. In particular, we find that higher numbers of mutants or migrants do, in general, promote local adaptation. Interestingly, shorter parasite generation time does not always favour parasite local adaptation. When genetic variability is limiting, shorter generation time, via an increase of the strength of selection, decreases the capacity of the parasite to adapt to an evolving host. | 3806 | NA | NA | NA | no | no |
| 3805 | 2017 | NA | Shanmugam, V; Thakur, H; Kaur, J; Gupta, S; Rajkumar, S; Dohroo, NP | 2013 | Genetic diversity of Fusarium spp. inciting rhizome rot of ginger and its management by PGPR consortium in the western Himalayas | The genetic variability of 32 Fusarium isolates from diseased ginger rhizomes from the Western Himalayas in India was analyzed by the unweighted pair group method with arithmetic averaging using randomly amplified polymorphic DNA amplicons. Two major clusters formed, one dominated by Fusarium oxysporum and the other by Fusarium solani. Talc-based formulations of the plant growth promoting rhizobacteria (PGPR) strain XXBC-TN (Bacillus subtilis) and a mixture of S2BC-1 (B. subtilis) with TEPF-Sungal (Burkholderia cepacia), which inhibits F. oxysporum and F. solani, were developed for rhizome dressing and soil application in ginger fields. The strain mixture recorded the maximum rhizome production (85.2%) with fewer yellows and reduced rhizome rot incidences (87.8% and 88.4%) over the control in a polyhouse. This was associated with an increase in the defense enzymes chitinase, beta-1,3-glucanase, and polyphenol oxidase. Furthermore, the strain mixture treatment promoted plant growth and enhanced rhizome production by 45.8%. In field experiments, the PGPR strain mixture reduced yellows and rhizome rot incidences by about 50.5%, which was comparable to that of a carbendazim and mancozeb fungicide mixture. (c) 2013 Elsevier Inc. All rights reserved. | 3807 | NA | NA | NA | no | no |
| 3806 | 2017 | NA | Jarvis, A; Lane, A; Hijmans, RJ | 2008 | The effect of climate change on crop wild relatives | Crop wild relatives are an important source of genetic diversity for crop improvement. However, the survival of some of these wild plant species could be threatened because of climate change. We used current and projected future climate data for similar to 2055, and a climate envelope species distribution model topredictthe impact of climate change on the wild relatives of peanut (Arachis), potato (Solanum) and cowpea (Vigna). We considered three migrational scenarios for modeling the range shifts (unlimited, limited, and no migration). Climate change strongly affected all taxa, with an estimated 16-22% (depending on migration scenario) of these species predicted to go extinct and most species losing over 50% of their range size. Moreover, for many species, the suitable areas become highly fragmented. Arachis were the most affected group, with 24-31 (depending on the migration scenario) of 51 species projected to go extinct and their distribution area on average reduced by 85-94%, depending on the migration scenario, over the next 50 years. The number of patches was predicted to decrease by 19% under the no migration scenario or increase by 4% assuming unlimited migration. Patch size decreased by 55-60%. For Solanum, 7 (no migration) to 13 (unlimited migration) of 108 species were predicted to go extinct, and their range sizes were reduced by approximately 38-69%. The number of patches was predicted to decrease by 34% (no migration) or increase 7% (unlimited migration) and patch size decreased by 20 (unlimited migration) to 37% (no migration). In terms of species extinction, Vigna was the least affected of the three groups, losing no species (unlimited migration) to 2 species (no migration) of the 48 species in the genus. The mean range size was predicted to decrease by 65% (no migration) or increase 8% (unlimited migration), with 8-41 of the 48 species losing more than 50% of their current geographic range. The number of Vigna patches increased by 12-115%, but the size of those patches shrunk by 51-59%. Our results suggest that there is an urgent need to identify and effectively conserve crop wild relatives that are at risk from climate change. While increased habitat conservation will be important to conserve most species, those that are predicted to undergo strong range size reductions should be a priority for collection and inclusion in genebanks. (c) 2008 Elsevier B.V. All rights reserved. | 3808 | NA | NA | NA | no | no |
| 3807 | 2017 | NA | Vialatte, A; Guiller, A; Bellido, A; Madec, L | 2008 | Phylogeography and historical demography of the Lusitanian snail Elona quimperiana reveal survival in unexpected separate glacial refugia | Background: Present day distributions of Palearctic taxa in northern latitudes mainly result from populations having survived in local patches during the Late Pleistocene and/or from recolonizing populations from southern temperate refugia. If well-studied Mediterranean and eastern European refugia are widely accepted, some recent biogeographical assumptions still remain unclear, such as the occurrence of multiple glacial refugia in Iberia and cryptic refugia in northern Europe during the last glaciations. The Lusitanian snail Elona quimperiana has a remarkably disjunct distribution, limited to northwestern France (Brittany), northwestern Spain and the Basque Country. By describing the phylogeographical structure of this species across its entire range, the present study attempts to identify refugia and subsequent recolonization routes. Results: Results based on 16S and COI gene sequences showed that the low genetic diversity observed in the Brittany populations should be associated with a recent demographic expansion. By contrast, populations from Spain exhibit several differentiated lineages and are characterized by demographic equilibrium, while the Basque populations are the only ones harboring typical distinct haplotypes. The center of the star-like networks of both gene sequences is occupied by a common ancestral-like haplotype found in Brittany and Spain, which might have originated from the middle of Northern Spain (i.e. Asturias, eastern Lugo and western Cantabria). Estimates of the divergence time between the Spain-Brittany and Basque lineages strongly suggest that E. quimperiana survived the Pleistocene glaciations in distinct refugia on the Iberian Peninsula, one of which is situated in Picos de Europa, and the other in the Basque Country. The occurrence of a northern refugium in France cannot be rejected as of yet. Conclusion: Present results confirm the Iberian origin of the land snail E. quimperiana and strongly support the emerging phylogeographic hypothesis of multiple refugia in Iberia during the last glaciations. The scenario of a spatial expansion of E. quimperiana from an Iberian refuge located in Asturias to northern areas provides the most probable explanation for the present distribution of this land snail. By harboring distinct haplotypes, the Basque Country populations appear to be of great importance in terms of potential adaptation, long term persistence and hence, the conservation of E. quimperiana. | 3809 | NA | NA | NA | no | no |
| 3808 | 2017 | NA | Toju, H; Abe, H; Ueno, S; Miyazawa, Y; Taniguchi, F; Sota, T; Yahara, T | 2011 | Climatic Gradients of Arms Race Coevolution | In nature, spatiotemporally dynamic coevolutionary processes play major roles in the foundation and maintenance of biodiversity. Here, we examined the arms race coevolution involving a seed-eating weevil with a long snout and its camellia plant host with a thick fruit coat (pericarp) throughout the marked climatic gradient observed across the Japanese islands. Results demonstrated that female weevils, which bored holes through camellia pericarps to lay eggs into seeds, had evolved much longer snouts than males, especially in areas in which Japanese camellia pericarps were very thick. The thickness of the plant pericarp was heritable, and the camellia plant evolved a significantly thicker pericarp on islands with the weevil than on islands without it. Across populations with weevils, resource allocation to plant defense increased with increasing annual mean temperature or annual precipitation, thereby geographically differentiating the evolutionary and ecological interactions between the two species. Given that the coevolutionary relationship exhibited appreciable variation across a relatively small range of annual mean temperatures, ongoing global climatic change can dramatically alter the coevolutionary process, thereby changing the ecological interaction between these species. | 3810 | NA | NA | NA | no | no |
| 3809 | 2017 | NA | Bachmann, NL; Fraser, TA; Bertelli, C; Jelocnik, M; Gillett, A; Funnell, O; Flanagan, C; Myers, GSA; Timms, P; Polkinghorne, A | 2014 | Comparative genomics of koala, cattle and sheep strains of Chlamydia pecorum | Background: Chlamydia pecorum is an important pathogen of domesticated livestock including sheep, cattle and pigs. This pathogen is also a key factor in the decline of the koala in Australia. We sequenced the genomes of three koala C. pecorum strains, isolated from the urogenital tracts and conjunctiva of diseased koalas. The genome of the C. pecorum VR629 (IPA) strain, isolated from a sheep with polyarthritis, was also sequenced. Results: Comparisons of the draft C. pecorum genomes against the complete genomes of livestock C. pecorum isolates revealed that these strains have a conserved gene content and order, sharing a nucleotide sequence similarity > 98%. Single nucleotide polymorphisms (SNPs) appear to be key factors in understanding the adaptive process. Two regions of the chromosome were found to be accumulating a large number of SNPs within the koala strains. These regions include the Chlamydia plasticity zone, which contains two cytotoxin genes (toxA and toxB), and a 77 kbp region that codes for putative type III effector proteins. In one koala strain (MC/MarsBar), the toxB gene was truncated by a premature stop codon but is full-length in IPTaLE and DBDeUG. Another five pseudogenes were also identified, two unique to the urogenital strains C. pecorum MC/MarsBar and C. pecorum DBDeUG, respectively, while three were unique to the koala C. pecorum conjunctival isolate IPTaLE. An examination of the distribution of these pseudogenes in C. pecorum strains from a variety of koala populations, alongside a number of sheep and cattle C. pecorum positive samples from Australian livestock, confirmed the presence of four predicted pseudogenes in koala C. pecorum clinical samples. Consistent with our genomics analyses, none of these pseudogenes were observed in the livestock C. pecorum samples examined. Interestingly, three SNPs resulting in pseudogenes identified in the IPTaLE isolate were not found in any other C. pecorum strain analysed, raising questions over the origin of these point mutations. Conclusions: The genomic data revealed that variation between C. pecorum strains were mainly due to the accumulation of SNPs, some of which cause gene inactivation. The identification of these genetic differences will provide the basis for further studies to understand the biology and evolution of this important animal pathogen. | 3811 | NA | NA | NA | no | no |
| 3810 | 2017 | NA | Adamcikova, K; Kobza, M; Bolvansky, M; Ondruskova, E | 2012 | Spread and population structure of Cryphonectria parasitica in a young chestnut orchard in Slovakia | The chestnut blight pathogen Cryphonectria parasitica was studied in a chestnut collection composed of both seedlings and grafts derived from selected Castanea sativa and C. sativa x C. crenata trees located in south-east Slovakia, near village Pribelce on an area of approximately 3.5 ha. The study was conducted during eight years (2003-2010). During this period 133 trees were infected, which represents 59.82% of chestnut trees of all chestnut accessions. Based on the phenotype of the fungus culture and the type of cankers in the field, all isolates were determined to be virulent. No hypovirulent strains were found. No vegetative compatibility (vc) type diversity was observed. More than 130 isolates were analyzed for vc and all were in single vc type, which was identical with EU 12. All isolates assayed for mating type were MAT-1. No perithecia were observed. No significant differences were found between the proportion of cankered and dead cankered trees in seedlings and grafts of hybrid origin (C. sativa x C. crenata) and of C. sativa origin. However, particular seedlings and grafts of hybrid origin seemed to exhibit certain resistance to chestnut blight. | 3812 | NA | NA | NA | no | no |
| 3811 | 2017 | NA | Allen, RC; McNally, L; Popat, R; Brown, SP | 2016 | Quorum sensing protects bacterial co-operation from exploitation by cheats | Quorum sensing (QS) is a cell-cell communication system found in many bacterial species, commonly controlling secreted co-operative traits, including extracellular digestive enzymes. We show that the canonical QS regulatory architecture allows bacteria to sense the genotypic composition of high-density populations, and limit co-operative investments to social environments enriched for co-operators. Using high-density populations of the opportunistic pathogen Pseudomonas aeruginosa we map per-capita signal and co-operative enzyme investment in the wild type as a function of the frequency of non-responder cheats. We demonstrate mathematically and experimentally that the observed response rule of ‘co-operate when surrounded by co-operators’ allows bacteria to match their investment in co-operation to the composition of the group, therefore allowing the maintenance of co-operation at lower levels of population structuring (that is, lower relatedness). Similar behavioural responses have been described in vertebrates under the banner of ‘generalised reciprocity’. Our results suggest that mechanisms of reciprocity are not confined to taxa with advanced cognition, and can be implemented at the cellular level via positive feedback circuits. | 3813 | NA | NA | NA | no | no |
| 3812 | 2017 | NA | Rodriguez, A; Sanders, IR | 2015 | The role of community and population ecology in applying mycorrhizal fungi for improved food security | The global human population is expected to reach similar to 9 billion by 2050. Feeding this many people represents a major challenge requiring global crop yield increases of up to 100%. Microbial symbionts of plants such as arbuscular mycorrhizal fungi (AMF) represent a huge, but unrealized resource for improving yields of globally important crops, especially in the tropics. We argue that the application of AMF in agriculture is too simplistic and ignores basic ecological principals. To achieve this challenge, a community and population ecology approach can contribute greatly. First, ecologists could significantly improve our understanding of the determinants of the survival of introduced AMF, the role of adaptability and intraspecific diversity of AMF and whether inoculation has a direct or indirect effect on plant production. Second, we call for extensive metagenomics as well as population genomics studies that are crucial to assess the environmental impact that introduction of non-local AMF may have on native AMF communities and populations. Finally, we plead for an ecologically sound use of AMF in efforts to increase food security at a global scale in a sustainable manner. | 3814 | NA | NA | NA | no | no |
| 3813 | 2017 | NA | Dhondt, AA; Badyaev, AV; Dobson, AP; Hawley, DM; Driscoll, MJL; Hochachka, WM; Ley, DH | 2006 | Dynamics of mycoplasmal conjunctivitis in the native and introduced range of the host | In 1994, Mycoplasma gallisepticum, a common bacterial poultry pathogen, caused an epidemic in house finches in the eastern part of their North American range where the species had been introduced in the 1940s. Birds with mycoplasmal conjunctivitis were reported across the entire eastern United States within 3-4 years. Here we track the course of the Mycoplasma gallisepticum epidemic as it reached native, western North American populations of the house finch. In 2002, Mycoplasma gallisepticum was first observed in a native house finch population in Missoula, MT, where it gradually increased in prevalence during the next 2 years. Concurrently, house finches with conjunctivitis were reported with increasing number in the Pacific Northwest. In native populations of the host, the epidemic expanded more slowly, and reached lower levels of prevalence than in the eastern, introduced range of the species. Maximal prevalence was about half in the Missoula population than in local populations in the East. Although many factors can contribute to these differences, we argue that it is most likely the higher genetic heterogeneity in western than in eastern populations caused the lower impact of the pathogen. | 3815 | NA | NA | NA | no | no |
| 3814 | 2017 | NA | Mesterton-Gibbons, M; Hardy, ICW; Field, J | 2006 | The effect of differential survivorship on the stability of reproductive queueing | Queues, in which individuals inherit resources in a predictable, temporally stable order, are widespread in animal social groups. We develop an analytic model to explore the effect of differential survivorship on the stability of a reproductive queue. We show that unless fighting for dominance is potentially fatal, future direct benefits are not alone sufficient to stabilize a queue of non-relatives under constant (age-independent) mortality rates, regardless of whether a dominant becomes an isolate or remains a dominant on the death of the first subordinate. In the absence of fatal fighting, stabilization of such a queue by future direct benefits alone requires either the dominant or the subordinate to have age-dependent mortality rates. Even when the queue is stabilized by present direct reproduction, however, the shape of the lifespan distribution can make a significant difference to the size of the required incentive. In contrast to nonrelatives, queues of relatives can be stable without age-dependent mortality, so long as relatedness exceeds a critical value-, however, age-dependent mortality can lower this critical value. (c) 2006 Elsevier Ltd. All rights reserved. | 3816 | NA | NA | NA | no | no |
| 3815 | 2017 | NA | Zheng, C; Reynaud, Y; Zhao, CS; Zozio, T; Li, S; Luo, DX; Sun, Q; Rastogi, N | 2017 | New Mycobacterium tuberculosis Beijing clonal complexes in China revealed by phylogenetic and Bayesian population structure analyses of 24-loci MIRU-VNTRs | Beijing lineage of Mycobacterium tuberculosis constitutes the most predominant lineage in East Asia. Beijing epidemiology, evolutionary history, genetics are studied in details for years revealing probable origin from China followed by worldwide expansion, partially linked to higher mutation rate, hypervirulence, drug-resistance, and association with cases of mixed infections. Considering huge amount of data available for 24-loci Mycobacterial Interspersed Repetitive Units-Variable Number of Tandem Repeats, we performed detailed phylogenetic and Bayesian population structure analyses of Beijing lineage strains in mainland China and Taiwan using available 24-loci MIRU-VNTR data extracted from publications or the SITVIT2 database (n = 1490). Results on genetic structuration were compared to previously published data. A total of three new Beijing clonal complexes tentatively named BSP1, BPS2 and BSP3 were revealed with surprising phylogeographical specificities to previously unstudied regions in Sichuan, Chongqing and Taiwan, proving the need for continued investigations with extended datasets. Such geographical restriction could correspond to local adaptation of these “ecological specialist” Beijing isolates to local human host populations in contrast with “generalist pathogens” able to adapt to several human populations and to spread worldwide. | 3817 | NA | NA | NA | no | no |
| 3816 | 2017 | NA | Roy, BA | 1998 | Differentiating the effects of origin and frequency in reciprocal transplant experiments used to test negative frequency-dependent selection hypotheses | Reciprocal transplant experiments have been used to estimate the probability that negative frequency-dependent selection by natural enemies has occurred in host populations by determining whether pest populations are less adapted to “foreign” (rare) hosts, which originate from a population with which the pests have not coevolved. However, these experiments usually confound the effects of frequency and origin: the rare genotypes are also genotypes that did not originate at a site. When clonal organisms are used, and the clones occur in more than one population, it is possible to separate the effects of origin and frequency. Here I present the results of an experiment in which Arabis clones of known frequency were reciprocally transplanted among sites. Contrary to expectations, clones at their site of origin had less disease, less herbivory, and higher fitness than foreign clones. However, variation within and among sites in herbivory and infection was large, suggesting that the number of sites and clones needed to thoroughly test the hypothesis of negative frequency-dependent selection in this system is very large: thus, these results are suggestive but not conclusive. | 3818 | NA | NA | NA | no | no |
| 3817 | 2017 | NA | Roels, SAB; Kelly, JK | 2011 | RAPID EVOLUTION CAUSED BY POLLINATOR LOSS IN MIMULUS GUTTATUS | Anthropogenic perturbations including habitat loss and emerging disease are changing pollinator communities and generating novel selection pressures on plant populations. Disruption of plant-pollinator relationships is predicted to cause plant mating system evolution, although this process has not been directly observed. This study demonstrates the immediate evolutionary effects of pollinator loss within experimental populations of a predominately outcrossing wildflower. Initially equivalent populations evolved for five generations within two pollination treatments: abundant bumblebee pollinators versus no pollinators. The populations without pollinators suffered greatly reduced fitness in early generations but rebounded as they evolved an improved ability to self-fertilize. All populations diverged in floral, developmental, and life-history traits, but only a subset of characters showed clear association with pollination treatment. Pronounced treatment effects were noted for anther-stigma separation and autogamous seed set. Dramatic allele frequency changes at two chromosomal polymorphisms occurred in the no pollinator populations, explaining a large fraction of divergence in pollen viability. The pattern of phenotypic and genetic changes in this experiment favors a sequential model for the evolution of the multitrait “selfing syndrome” observed throughout angiosperms. | 3819 | NA | NA | NA | no | no |
| 3818 | 2017 | NA | Gerula, D; Wegrzynowicz, P; Panasiuk, B; Bienkowska, M; Skowronek, W | 2016 | Productivity and longevity of honey bee queens inseminated with freshly collected and diluted semen | The aim of this study was to examine whether the addition of a diluent for honey bee sperm, directly before insemination, affects the quality and vitality of queens and their brood, in comparison to queens inseminated traditionally, with fresh semen. Honey bee queens were inseminated with diluted and homogenized semen collected from a few dozen drones, in order to increase the diversity of their offspring. Queens and drones (Apis mellifera carnica) were mated within subspecies, however, only three selected lines were used. The average number of spermatozoa filling the spermatheca of queens, inseminated with semen without dilution, freshly collected from drones, from a single colony (SCS) was not significantly higher, compared to queens inseminated with semen diluted and mixed (MCS); amounting to 5.1 and 4.4 million sperm, respectively. Queens from both groups started laying eggs at a similar time after insemination, and their average life expectancy in colonies, was 20.5 and 22.6 months, respectively. The most common reason for queen losses was supersedure, and the reasons for these losses were not significantly different between groups. Brood survival rates, in both groups, did not differ significantly. | 3820 | NA | NA | NA | no | no |
| 3819 | 2017 | NA | Rossi, CC; de Araujo, EF; de Queiroz, MV; Bazzolli, DMS | 2013 | Characterization of the omlA gene from different serotypes of Actinobacillus pleuropneumoniae: A new insight into an old approach | The OmlA protein is a virulence factor of Actinobacillus pleuropneumoniae, an important pathogen in pigs. The polymorphisms present in the omlA gene sequence of 15 reference serotypes of A. pleuropneumoniae and non-serotypable isolates were assessed to determine the possible evolutionary relationship among them and to validate the importance of this gene as a molecular marker for the characterization of this bacterium. Divergence among the 15 serotypes of A. pleuropneumoniae probably resulted initially from two major evolutionary events that led to subsequent differentiation into nine groups. This differentiation makes it possible to characterize most of the serotypes by using bionformatics, thereby avoiding problems with immunological cross-reactivity. A conserved alpha-helix common to all the serotypes was most likely involved in connecting the protein to the outer membrane and acting as a signal peptide. A previously unknown gene duplication was also identified and could contribute to the genetic variability that makes it difficult to serotype some isolates. Our data support the importance of the omlA gene in the biology of A. pleuropneumoniae and provide a new area of research into the OmlA protein. | 3821 | NA | NA | NA | no | no |
| 3820 | 2017 | NA | Krebs, CJ | 1996 | Population cycles revisited | Periodic fluctuations or cycles in populations of small mammals have been widely studied, but much controversy still exists about their causes. Cycles of voles and lemmings are produced by the integrated effects of intrinsic and extrinsic factors, and the problem is to define accurately how these interact. Spacing behavior is a key component of population regulation in voles and lemmings, and this is illustrated most dramatically by the fence effect. We do not know which mechanisms produce changes in social behavior. Phenotypic changes produced by maternal effects or stress are now believed most likely, but there has been too little work done on genetic effects on spacing and we know almost nothing about kin-related social behavior in voles and lemmings. Both predation and food supply may be the extrinsic factors involved in cyclic population dynamics. Single-factor experiments suggest that food shortage by itself does not seem to be a necessary factor for cycles nor does predation, but the interaction between food and predation could be a key variable in understanding how extrinsic factors affect cycles of voles. Cycles of snowshoe hares are caused by an interaction between predation and food supplies, possibly integrated through risk-sensitive foraging. Spacing behavior is not a component of cycles of hares because snowshoe hares differ from voles and lemmings in having no known form of spacing behavior that can produce social mortality. The short-term cycle of voles and lemmings thus seems to have a different explanation from the long-term cycle of snowshoe hares. In some places, lemmings may be locked in a predator-pit at low density. Experimental exclusion of predators improved survival of adults in a population of collared lemmings, but was not sufficient to allow them to escape the predator-pit because of losses of juveniles. Whether cyclic populations of lemmings also fall into a predator-pit in the low phase remains to be determined. The low phase does not occur in every cycle and it is particularly difficult to explain. Progress in analyzing cyclic fluctuations has been made most rapidly when we define clear alternative hypotheses and carry out experimental manipulations on field populations. Much remains to be done on these small mammals. | 3822 | NA | NA | NA | no | no |
| 3821 | 2017 | NA | Grafen, A; Archetti, M | 2008 | Natural selection of altruism in inelastic viscous homogeneous populations | Biological explanations are given of three main uninterpreted theoretical results on the selection of altruism in inelastic viscous homogeneous populations, namely that non-overlapping generations hinder the evolution of altruism, fecundity effects are more conducive to altruism than survival effects, and one demographic regime (so-called death-birth) permits altruism whereas another (so-called birth-death) does not. The central idea is ‘circles of compensation’, which measure how far the effects of density dependence extend from a focal individual. Relatednesses can then be calculated that compensate for density dependence. There is very generally a ‘balancing circle of compensation’, at which the viscosity of the population slows up selection of altruism, but does not affect its direction, and this holds for altruism towards any individual, not just immediate neighbours. These explanations are possible because of recent advances in the theory of inclusive fitness on graphs. The assumption of node bitransitivity in that recent theory is relaxed to node transitivity and symmetry of the dispersal matrix, and new formulae show how to calculate relatedness from dispersal and vice versa. (C) 2008 Elsevier Ltd. All rights reserved. | 3823 | NA | NA | NA | no | no |
| 3822 | 2017 | NA | Schopmeyer, SA; Lirman, D | 2015 | Occupation Dynamics and Impacts of Damselfish Territoriality on Recovering Populations of the Threatened Staghorn Coral, Acropora cervicornis | Large-scale coral reef restoration is needed to help recover structure and function of degraded coral reef ecosystems and mitigate continued coral declines. In situ coral propagation and reef restoration efforts have scaled up significantly in past decades, particularly for the threatened Caribbean staghorn coral, Acropora cervicornis, but little is known about the role that native competitors and predators, such as farming damselfishes, have on the success of restoration. Steep declines in A. cervicornis abundance may have concentrated the negative impacts of damselfish algal farming on a much lower number of coral prey/colonies, thus creating a significant threat to the persistence and recovery of depleted coral populations. This is the first study to document the prevalence of resident damselfishes and negative effects of algal lawns on A. cervicornis along the Florida Reef Tract (FRT). Impacts of damselfish lawns on A. cervicornis colonies were more prevalent (21.6% of colonies) than those of other sources of mortality (i.e., disease (1.6%), algal/sponge overgrowth (5.6%), and corallivore predation (7.9%)), and damselfish activities caused the highest levels of tissue mortality (34.6%) among all coral stressors evaluated. The probability of damselfish occupation increased as coral colony size and complexity increased and coral growth rates were significantly lower in colonies with damselfish lawns (15.4 vs. 29.6 cm per year). Reduced growth and mortality of existing A. cervicornis populations may have a significant effect on population dynamics by potentially reducing important genetic diversity and the reproductive potential of depleted populations. On a positive note, however, the presence of resident damselfishes decreased predation by other corallivores, such as Coralliophila and Hermodice, and may offset some negative impacts caused by algal farming. While most negative impacts of damselfishes identified in this study affected large individual colonies and <50% of the A. cervicornis population along the FRT, the remaining wild staghorn population, along with the rapidly increasing restored populations, continue to fulfill important functional roles on coral reefs by providing essential habitat and refuge to other reef organisms. Although the effects of damselfish predation are, and will continue to be, pervasive, successful restoration efforts and strategic coral transplantation designs may help overcome damselfish damage by rapidly increasing A. cervicornis cover and abundance while also providing important information to educate future conservation and management decisions. | 3824 | NA | NA | NA | no | no |
| 3823 | 2017 | NA | Ogawa, H; Katoh, H; Sanada, N; Sanada, Y; Ohya, K; Yamaguchi, T; Fukushi, H | 2010 | A novel genotype of beak and feather disease virus in budgerigars (Melopsittacus undulatus) | Beak and feather disease virus (BFDV) is a causative agent for psittacine beak and feather disease (PBFD), which shows a characteristic feather disorder in psittacine birds. Nineteen budgerigars, which were clinically suspected to have PBFD, were examined by two polymerase chain reactions (PCR), which target each of open reading frames (ORFs) V1 and C1. All of the 19 samples were detected BFDV by the PCR targeting ORF C1, whereas only two of them were detected by the PCR targeting ORF V1. It was assumed that BFDV derived from budgerigar (budgerigar BFDV) has two genotypes, which are tentatively classified as budgerigar BFDV genotype 1 and genotype 2 by the PCR amplification patterns. Whole genome sequences of six budgerigar BFDVs were determined to reveal the existence of two genotypes. In the phylogenic analysis, six budgerigar BFDV sequences formed a unique group branched from the other 23 published BFDV sequences. The budgerigar BFDV genotype 1 and genotype 2 were also segregated each other, and budgerigar BFDV genotype 2 was particularly distantly related with the other BFDVs. These results suggest budgerigar BFDV is a unique in the known BFDVs and is divided into two genotypes. | 3825 | NA | NA | NA | no | no |
| 3824 | 2017 | NA | Dantas, GPM; Santos, FR; Marini, MA | 2009 | Sex ratio and morphological characteristics of Rufous Gnateaters, Conopophaga lineata (Aves, Passeriformes) in Atlantic forest fragments | Unequal sex ratios lead to the loss of genetic variability, decreasing the viability of populations in the long term. Anthropogenic activities often disturb the natural habitats and can cause alterations in sex ratio and morphological characteristics of several species. Forest fragmentation is a major conservation concern, so that understanding its effects in natural populations is essential. In this study, we evaluated the sex ratio and the morphological characteristics of Rufous Gnateaters (Conopophaga lineata (Wied, 1831)) in small and large forest fragments in Minas Gerais, Brazil. Birds (n = 89) were sexed by plumage characteristics and molecular markers. The molecular analysis showed that plumage is not a totally reliable method for sexing Rufous Gnateaters. We observed that sex ratio did not differ between large and small forest fragments, but birds in small fragments had larger wings and tarsus. Wing and tarsus changes may affect the movement ability of individuals within and among forest fragments. In conclusion, Rufous Gnateaters have been able to survive in both small and large Atlantic rain forest fragments without altering their sex ratio, but morphological changes can be prejudicial to their long term survival. | 3826 | NA | NA | NA | no | no |
| 3825 | 2017 | NA | Dashprakash, M; Venkatesan, G; Ramakrishnan, MA; Muthuchelvan, D; Sankar, M; Pandey, AB; Mondal, B | 2015 | Genetic diversity of fusion gene (ORF 117), an analogue of vaccinia virus A27L gene of capripox virus isolates | The fusion gene (ORF 117) sequences of twelve (n = 12) capripox virus isolates namely sheeppox (SPPV) and goatpox (GTPV) viruses from India were demonstrated for their genetic and phylogenetic relationship among them. All the isolates were confirmed for their identity by routine PCR before targeting ORF 117 gene for sequence analysis. The designed primers specifically amplified ORF 117 gene as 447 bp fragment from total genomic DNA extracted from all the isolates. Sequence analysis revealed a significant percentage of identity among GTPV, SPPV and between them at both nucleotide and amino acid levels. The topology of the phylogenetic tree revealed that three distinct clusters corresponding to SPPV, GTPV and lumpy skin disease virus was formed. However, SPPV Pune/08 and SPPV Roumanian Fanar isolates were clustered into GTPV group as these two isolates showed a 100 and 99.3 % identity with GTPV isolates of India at nt and aa levels, respectively. Protein secondary structure and 3D view was predicted and found that it has high antigenic index and surface probability with low hydrophobicity, and it can be targeted for expression and its evaluation to explore its diagnostic potential in epidemiological investigation in future. | 3827 | NA | NA | NA | no | no |
| 3826 | 2017 | NA | He, XM; Wei, P; Yang, XY; Guan, DM; Wang, GJ; Qin, AJ | 2012 | Molecular epidemiology of infectious bursal disease viruses isolated from Southern China during the years 2000-2010 | We performed a molecular epidemiology study of infectious bursal disease virus (IBDV) from six provinces in southern China by analyzing IBDVs isolated during the years 2000-2010. Sequence analysis of hypervariable regions of the VP2 gene (vVP2) in the genome of these isolates revealed that the majority of these viruses (45/59) were characterized as vv (very virulent) IBDV genotype, 12 out of 59 isolates were avirulent IBDV genotype and two from Guangxi were intermediate IBDV genotype. Phylogenetic analysis revealed that 45 vvIBDV genotype isolates have divided into five groups, all displaying strong divergence from the currently used vaccine strains. In all isolates, 14 non-critical amino acid substitutions were found in vVP2. The isolates from 2006 to 2010 had more substitutions (11 sites) than the isolates from 2000 to 2005 (7 sites). This study demonstrates that there were different genotypes of IBDV prevailing in six provinces of southern China. The mutations in vVP2 were common, which might be one of the reasons for the evolution of the IBDVs. Therefore, in regards to IBDV prevention, it is vital to have continuous monitoring of the genetic variability (long-term tracking of viral evolution) to provide optimal protection against IBDV. | 3828 | NA | NA | NA | no | no |
| 3827 | 2017 | NA | Moore, LR; Rocap, G; Chisholm, SW | 1998 | Physiology and molecular phylogeny of coexisting Prochlorococcus ecotypes | The cyanobacterium Prochlorococcus(1,2) is the dominant oxygenic phototroph in the tropical and subtropical regions of the world’s (1,3,4). It can grow at a range of depths over which light oceans intensities can vary by up to 4 orders of magnitude. This broad depth distribution has been hypothesized to stem from the coexistence of genetically different populations adapted for growth at high-and low-light intensities(4-6). Here we report direct evidence supporting this hypothesis, which has been generated by isolating and analysing distinct co-occurring populations of Prochlorococcus at two locations in the North Atlantic. Go-isolates from the same water sample have very different light-dependent physiologies, one growing maximally at light intensities at which the other is completely photoinhibited. Despite this ecotypic differentiation, the co-isolates have 97% similarity in their 16S ribosomal RNA sequences, demonstrating that molecular microdiversity, commonly observed in microbial systems(7-12), can be due to the coexistence of closely related, physiologically distinct populations. The coexistence and distribution of multiple ecotypes permits the survival of the population as a whole over a broader range of environmental conditions than would be possible for a homogeneous population. | 3829 | NA | NA | NA | no | no |
| 3828 | 2017 | NA | Martynov, SP; Dobrotvorskaya, TV; Sorokin, OD | 2004 | Comparative genealogical analysis of the resistance of winter wheat to common bunt | Comparative genealogical analysis of North American (the United States and Canada) and Eastern European (Russia and Ukraine) winter wheat cultivars resistant and susceptible to common bunt has been performed. Analysis of variance applied to North American wheats has demonstrated that resistant and susceptible cultivars significantly differ from each other with respect to the contributions of common ancestors. The contributions of Oro (Bt4 and Bt7), Rio (Bt6), White Odessa (Bt1), and Florence (Bt3) to the resistant cultivars are significantly higher than their contributions to the susceptible ones. This demonstrates that the use of these resistance donors in wheat breeding for several decades has been effective. The contribution of PI-178383 (Bt8, Bt9, and Bt10) is considerably higher in the group of resistant cultivars bred after 1965. The mean contributions of Federation (Bt7) and Nebred (Bt4) are significantly higher in the group of resistant cultivars obtained before 1965; however, the differences in the contributions of these donors between new resistant and susceptible cultivars became nonsignificant. Among the Russian and Ukrainian cultivars, there are differences between groups of resistant and susceptible cultivars from different regions determined by the differences between the regional populations of the pathogen in racial composition. In the northern region, the contributions of the wheat grass (Agropyron glaucum) and the rye cultivar Eliseevskaya are significantly higher in the resistant cultivars; in the southern region, a local cultivar of the Odessa oblast is the prevalent resistant cultivar. In addition, cultivar Yaroslav Emmer is likely to be effective in the northern region; and foreign sources (Oro, Florence, Federation, and Triticum timopheevii), in the southern region. Very few sources of vertical resistance to common bunt are used for winter wheat breeding in Russia and Ukraine. The decrease in genetic diversity in favor of a few identical genes may cause adequate changes in the pathogen population and subsequent proliferation of the pathogen on the genetically identical substrate. A new interpretation of the resistance of line Lutescens 6028 as a source of new genes, Bt12 and Bt13, is suggested. Both genealogical and segregation analyses have shown that the genes determining the resistance of this line may be identical to those described earlier (Bt1, Bt3, Bt4, Bt6, and Bt7); and the high resistance of this line is determined by a combination of these genes. | 3830 | NA | NA | NA | no | no |
| 3829 | 2017 | NA | van Hooft, WF; Groen, AF; Prins, HHT | 2003 | Genetic structure of African buffalo herds based on variation at the mitochondrial D-loop and autosomal microsatellite loci: Evidence for male-biased gene flow | Sexual differences in herding behaviour of African buffalo (Syncerus caffer) were studied by analysing at the herd level mitochondrial D-loop hypervariable region I and fourteen autosomal microsatellites. Three herds from Arusha National Park in Tanzania were analysed with mtDNA and five herds from Kruger National Park in South Africa with mtDNA and microsatellites. Significant mtDNA differentiation was observed among herds in Arusha NP (F-ST = 0.12, based on haplotype frequencies). Assignment tests with microsatellite data from Kruger NP showed that most frequent migration between herds is by males greater than or equal totwo years. This was confirmed by tests for herd differentiation and analyses with Lynch and Ritland’s relatedness estimator. Within a herd, males younger than two years and females showed a higher relationship through a common father rather than a common mother, indicating that female herd members mate with only a few dominant males. This in turn suggests a female: male sex ratio larger than 5:1. The migration rate per generation between herds was estimated to be 5-20% for females and close to 100% for males. Finally, the implications for the management of buffalo populations are being discussed. | 3831 | NA | NA | NA | no | no |
| 3830 | 2017 | NA | Paulauskas, A; Galdikaite-Braziene, E; Radzijevskaja, J; Aleksandraviciene, A; Galdikas, M | 2016 | Genetic diversity of Ixodes ricinus (Ixodida: Ixodidae) ticks in sympatric and allopatric zones in Baltic countries | Ixodes ricinus (Linnaeus 1758) and Ixodes persulcatus (Schulze 1930) ticks are involved in the transmission of a wide variety of pathogens with considerable impact on human and animal health. The co-distribution zone of these two tick species is situated in the Baltic countries, which provides a special setting for the population studies. In the present study, genetic variability of I. ricinus ticks collected in allopatric and sympatric locations in the Baltic countries has been investigated using a sequence analysis of the mitochondrial DNA control region, 16S rRNA and cytb genes. There were 32 haplotypes (Hd: 0.8551) and 27 haplotypes (Hd:0.8213) of control region sequences from ticks in allopatric and sympatric zones detected, respectively. Out of 47 16S rRNA gene haplotypes, 32 haplotypes (Hd: 0.7213) were found in the allopatric zone and 27 (Hd:0.9572) in the sympatric zone. The Cytb gene was very conserved and monomorphic in ticks from the allopatric zone, whereas three unique haplotypes were observed in the sympatric zone. The higher number of unique haplotypes of the control region was detected in the allopatric zone. Median joining network and Fst analysis did not reveal a clear separation between ticks from the two zones. | 3832 | NA | NA | NA | no | no |
| 3831 | 2017 | NA | Marchani, EE; Callegaro, A; Daw, EW; Wijsman, EM | 2009 | Combining Information from Linkage and Association Methods | Group 12 evaluated approaches to incorporate outside information or otherwise optimize traditional linkage and association analyses. The abundance of available data allowed exploration of identity-by-descent (IBD) estimation, score statistics, formal combination of linkage and association testing, significance estimation, and replication. We observed that IBD estimation can be optimized with a subset of marker data while estimation of inheritance vectors can provide both IBD estimates and a measure of their uncertainty. Score statistics incorporating covariates or combining association and linkage information performed at least as well as standard approaches while requiring less computation time. The formal combination of linkage and association methods may be fruitful, although the nature of the simulated data limited our conclusions. Estimation of significance may be improved through simulation, correction for cryptic relatedness, and the inclusion of prior information. Replication using real data provided consistent results, though the same was not true of simulated data replicates. Overall, we found that increasing the amount of available data limits analyses due to computational constraints and motivates the need to improve methods for the identification of complex-trait genes. Genet. Epideiniol. 33 (Suppl. 1):S81-S87, 2009. (C) 2009 Wiley-Liss, Inc. | 3833 | NA | NA | NA | no | no |
| 3832 | 2017 | NA | Ruedas, LA; Salazar-Bravo, J; Tinnin, DS; Armien, B; Caceres, L; Garcia, A; Diaz, MA; Gracia, F; Suzan, G; Peters, CJ; Yates, TL; Mills, JN | 2004 | Community ecology of small mammal populations in Panama following an outbreak of Hantavirus pulmonary syndrome | In late 1999 and early 2000, an outbreak of hantavirus pulmonary syndrome (HPS) occurred in and around Los Santos, on the Azuero Peninsula of southwestern Panama. This HPS episode, resulting in 22% case fatality, was linked to the Costa Rican pigmy rice rat, Oligoryzomys fulvescens costaricensis, which harbored a then undescribed hantavirus, Choclo virus. In addition, Cherrie’s cane rat, Zygodontomys brevicauda cherriei, was identified as carrying a distinct hantavirus, Calabazo virus with no known pathogenicity to humans. Herein we present the ecological results of the outbreak investigations in the Azuero region. A total of 164 animals were captured, of which 126 were potential small, non-volant mammal hosts of a hantavirus: rodents in the family Muridae. There were significant differences in small mammal community structure between case sites and a negative control site. Differences were manifest in ecological measures of species diversity and in species evenness and heterogeneity measures, as indicated by Pairwise Euclidian distances and Morisita indices of community similarity. Our analyses suggest that human activities (i.e., deforestation for cattle ranching) coupled with environmental factors (i.e., increased precipitation) may have synergistically coalesced for an increased risk of HPS to area residents. | 3834 | NA | NA | NA | no | no |
| 3833 | 2017 | NA | Persson, I; Andersson, G | 1999 | Intraclutch hatch synchronization in pheasants and mallard ducks | Synchronization of hatching within clutches of precocial bird species can be achieved either by acceleration or retardation, i.e. by shortening or prolonging the incubation period. The ability of mallard (Anas platyrhynchos) and ring-necked pheasant (Phasianus colchicus) embryos to accelerate or retard hatching was tested by incubating separate clutches, of which three eggs had 2 d longer or shorter incubation time than the others, and observing their individual time of pipping (breaking of the shell). Mallard embryos were able to delay hatching by on average 0.6 d (43% of the eggs delayed at least 1 d), but were better at acceleration (on average 1.3 d; 91% of the eggs accelerated more than 1 d). Conversely, pheasant embryos were only able to accelerate by 0.4 d (50% accelerated more than 1 d), but were better at delaying the hatching (1.2 days; 77% delayed more than 1 d). This difference between the species may depend on different degrees of relatedness within clutches in pheasants and mallards. It may also be an effect of the more developed sensory and neuromuscular systems in galliforms; a reduction of the incubation period would mean that the development of, for example, locomotion would be insufficient at hatching. | 3835 | NA | NA | NA | no | no |
| 3834 | 2017 | NA | Pierce, LR; Stepien, CA | 2012 | Evolution and biogeography of an emerging quasispecies: Diversity patterns of the fish Viral Hemorrhagic Septicemia virus (VHSv) | Viral Hemorrhagic Septicemia virus (VHSv) is an RNA rhabdovirus that causes one of the most important finfish diseases, affecting over 70 marine and freshwater species. It was discovered in European cultured fish in 1938 and since has been described across the Northern Hemisphere. Four strains and several sub-strains have been hypothesized, whose phylogenetic relationships and evolutionary radiation are evaluated here in light of a quasispecies model, including an in-depth analysis of the novel and especially virulent new substrain (IVb) that first appeared in the North American Laurentian Great Lakes in 2003. We analyze the evolutionary patterns, genetic diversity, and biogeography of VHSv using all available RNA sequences from the glycoprotein (G), nucleoprotein (N), and non-virion (Nv) genes, with Maximum Likelihood and Bayesian approaches. Results indicate that the G gene evolves at an estimated rate of mu = 2.58 x 10(-4) nucleotide substitutions per site per year, the N gene at mu = 4.26 x 10(-4), and Nv fastest at mu = 1.25 x 10(-3). Phylogenetic trees from the three genes largely are congruent, distinguishing strains I-IV as reciprocally monophyletic with high bootstrap and posterior probability support. VHSv appears to have originated from a marine ancestor in the North Atlantic Ocean, diverging into two primary clades: strain IV in North America (the Northwestern Atlantic Ocean), and strains I-III in the Northeastern Atlantic region (Europe). Strain II may comprise the basal group of the latter clade and diverged in Baltic Sea estuarine waters; strains I and III appear to be sister groups (according to the G and Nv genes), with the former mostly in European freshwaters and the latter in North Sea marine/estuarine waters. Strain IV is differentiated into three monophyletic substrains, with IVa infecting Northeastern Pacific salmonids and many marine fishes (with 44 unique G gene haplotypes), IVb endemic to the freshwater Great Lakes (11 haplotypes), and a newly-designated IVc in marine/estuarine North Atlantic waters (five haplotypes). Two separate substrains independently appeared in the Northwestern Pacific region (Asia) in 1996, with Ib originating from the west and IVa from the east. Our results depict an evolutionary history of relatively rapid population diversifications in star-like patterns, following a quasispecies model. This study provides a baseline for future tracking of VHSv spread and interpreting its evolutionary diversification pathways. (C) 2012 Elsevier Inc. All rights reserved. | 3836 | NA | NA | NA | no | no |
| 3835 | 2017 | NA | Skrzypczak-Zielinska, M; Zakerska-Banaszak, O; Tamowicz, B; Sobieraj, I; Drweska-Matelska, N; Szalata, M; Slomski, R; Mikstacki, A | 2015 | Polymorphisms and allele frequencies of glutathione S-transferases A1 and P1 genes in the Polish population | Glutathione S-transferases (GST) A1 and P1 are crucial enzymes involved in the biotransformation of drugs, carcinogens, and toxins, and their activity may influence drug response, susceptibility to diseases, and carcinogenesis. The genes encoding these enzymes, GSTA1 and GSTP1, have been examined in many studies because of their genetic variability, which may affect enzymatic activity. The goal of this study was to determine the distribution of the alleles GSTA1*A/*B and GSTP1*A,* B, and *C in the Polish population. A total of 160 subjects from the Polish population were genotyped for 2 polymorphisms (I105V and A114V) in the GSTP1 gene using pyrosequencing. The promoter region of the GSTA1 gene was screened using sequencing. The detected variants were subjected to haplotype analysis. We found that the distribution of the alleles GSTA1*A/*B and GSTP1*A, *B, and* C in the Polish population correspond to the results of studies in Caucasians. Furthermore, we identified additional single nucleotide polymorphisms, excluding 3 well-known changes (G-52A, C-69T, T-567G), which are linked to alleles GSTA1*A/*B, that affect enzyme activity. A total of 4 haplotypes were identified in 160 Polish individuals. | 3837 | NA | NA | NA | no | no |
| 3836 | 2017 | NA | Boissier, J; Jarkovsky, J; Morand, S; Mone, H | 2004 | A model to explain the origin of a parasite sex-specific population structure | A discrete time model was built to understand the origin of the sex-specific population structure of the human blood fluke, Schistosoma mansoni. We have estimated both male/female individual ratio and male/female genotype ratio of this parasite taking into account all the experimental published values on differential male and female life-history traits all along the life cycle. We considered in our model male and female life-history traits when both separated and together. The model showed that both male/female individual ratio and male/female genotype ratio of S. mansoni adults are biased toward males in each combination. This bias was more important in male/female genotype ratio than in the male/female individual ratio for the same initial values of cercarial development success. This model could explain the sex specific population structure of this parasite. Firstly, we showed that the male-biased individual ratio finds its origin in the vertebrate host. Secondly, we showed that the male-biased genotype ratio originates prior to any interrelationship between adult worms and could generate by itself a sex-specific genetic structure. (C) 2004 Elsevier Inc. All rights reserved. | 3838 | NA | NA | NA | no | no |
| 3837 | 2017 | NA | Zhang, H; Gong, HR; Zhou, XP | 2009 | Molecular characterization and pathogenicity of tomato yellow leaf curl virus in China | Several tomato production regions in China were surveyed for tomato yellow leaf curl disease (TYLCD), and 31 tomato leaf samples showing TYLCD-like symptoms were collected. The partial or full-length genomes of these isolates were sequenced and tomato yellow leaf curl virus (TYLCV) was detected in Shanghai, Zhejiang, Jiangsu Shandong and Hebei provinces of China. The TYLCV isolates found in China share high sequence identity (> 98%) and have more than 97% sequence identity with TYLCV-IL[IL:Reo] (X15656). Phylogenetic relationship analysis reveals that although with little genetic variability, they can form two groups and all the TYLCV isolates in China belong to the group I. An infectious clone of TYLCV-[CN:SH2] (AM282874) was constructed and agro-inoculated into Nicotiana benthamiana, N. tabacum Samsun, N. glutinosa, Solanum lycopersicum, Petunia hybrida, Cucumis sativus, Gossypium hirsutum, S. melongena, and Capsicum annuum. TYLCV-[CN:SH2] can induce severe leaf curling and stunting symptoms in these plants except C. sativus, G. hirsutum, S. melongena and C. annuum. We verified that TYLCV can trans-replicate tomato yellow leaf curl China virus DNA-beta in N. benthamiana and S. lycopersicum and induced more severe symptoms with distortion and yellow vein. | 3839 | NA | NA | NA | no | no |
| 3838 | 2017 | NA | Inoue, T; Hayashimoto, N; Yasuda, M; Sasaki, E; Itoh, T | 2015 | Pentatrichomonas hominis in laboratory-bred common marmosets | Trichomonadid protozoa have been found in the intestinal tracts of common marmosets (Callithrix jacchus). However, there is little information available on species identification and the pathogenicity of these trichomonads. In this study, we conducted a fecal survey of a common marmoset colony maintained as laboratory animals in Japan and identified the trichomonad species. Screening using a fecal smear examination revealed that 66% (58/88) of the marmosets had trichomonadid trophozoites in their feces. The trichomonads were found in both normal feces (31/49, 63%) and diarrhea (27/39, 69%), with no significant difference in frequency. The protozoa were identified as Pentatrichomonas hominis using morphological characters and the 100% identity of the nucleotide sequence of the partial 18S rRNA gene (297 bp). The intraspecific genetic variability between P. hominis from the marmosets in this study and P. hominis from other reported mammal hosts was <= 1% in the nucleotide sequence, including the internal transcribed spacer (ITS)-1, 5.8S rRNA gene, and ITS-2 (293 bp). P. hominis inhabits the large intestine of various mammalian hosts, including primates, and is considered nonpathogenic. These results suggest that P. hominis is transmitted among marmosets and other mammals but is not a primary cause of bowel disease in marmosets. | 3840 | NA | NA | NA | no | no |
| 3839 | 2017 | NA | Donald, JL; Boutin, S | 2011 | Intraspecific cache pilferage by larder-hoarding red squirrels (Tamiasciurus hudsonicus) | Although cases of pilfering food are reported commonly in the mammal literature, the factors affecting pilfering rates among individuals and between populations within the same species remain relatively unknown. We measured individual pilfering rates in 2 populations of highly territorial larder-hoarding red squirrels (Tamiasciurus hudsonicus) in Kluane, Yukon, Canada. One population received artificial food supplementation (where all individuals had ad libitum food) and had a 2-fold higher density than the control population. We knew the age, relatedness, and spatial relationship of all individuals in each population, and we had a measure of the food resources (cones) cached by each individual and their fates through the study. Results from experimental removal of territory owners suggested that younger squirrels with smaller food caches were more likely to pilfer when provided the opportunity. However, using a mark-recapture study of marked spruce cones under natural conditions, we found that few individuals (14%) pilfered, and stolen cones represented only 0.3% of total cones that were larder-hoarded. Pilfering occurs at a much lower rate in Kluane than reported for red squirrels in other regions and is less than rates reported for scatter-hoarding species. | 3841 | NA | NA | NA | no | no |
| 3840 | 2017 | NA | Pisa, S; Biersma, EM; Convey, P; Patino, J; Vanderpoorten, A; Werner, O; Ros, RM | 2014 | The cosmopolitan moss Bryum argenteum in Antarctica: recent colonisation or in situ survival? | Since the onset of glaciation following the Oligocene (30-28 Ma), the prevalence of increasingly cold conditions has shaped the evolution of the Antarctic biota. Two hypotheses, postglacial recruitment from extra-regional locations and in situ persistence, have been proposed to explain the biogeography of the contemporary species-poor terrestrial Antarctic biota. Bryophytes, which form a major group of the Antarctic flora, exhibit a strong, inherent ability to survive cold conditions but also have high long-distance dispersal capacities, which are compatible with both hypotheses. Here, we test these hypotheses by means of population genetic and phylogeographic analyses of the cosmopolitan moss Bryum argenteum. We find evidence for at least three independent colonisation events of the species in Antarctica. Ancestral area reconstruction coupled with molecular dating suggests colonisation times of the different Antarctic clades ranging from four million years for the oldest lineage to half a million years for the youngest lineage. This suggests multiple colonisation events of Antarctica by this species during several glacial cycles within the Pleistocene, Pliocene and possibly late Miocene. This is the first study to demonstrate in situ persistence of bryophytes in Antarctica throughout previous glaciations. | 3842 | NA | NA | NA | no | no |
| 3841 | 2017 | NA | Iyer, P; Roughgarden, J | 2009 | Alternation of haploid and diploid generations: evolution by gamete amplification | Question: What selects for the alternation of haploid and diploid generations in algal taxa? Mathematical methods: We derive the growth rates of haplontic, diplontic, and haplo-diplontic populations as functions of ploidy-dependent survival probabilities and propagule production rates. We use a population genetic model with a single locus coding for haplonty, diplonty, and haplo-diplonty to obtain the evolutionarily stable conditions for the fixation of each of the three ploidy-cycles. We simulate the evolutionary dynamics to demonstrate the convergence to these equilibria. Key assumptions: Non-overlapping generations, ploidy-dependent propagule production and survival rates, and the synchronous release of gametes into water by the entire population. We assume that the fertilization probability of eggs increases as a function of the sperm density encountered. Conclusions: The ploidy-cycle selected is predicted to be the one with the highest population growth rate defined by propagule production and survival rates. Haplo-diplonty may be selected in low fertilization environments because it mitigates the problem of sperm limitation, as the gamete concentration amplifies over subsequent generations. Diplonty may be favoured in more variable environments because diploidy confers higher viability in such environments. | 3843 | NA | NA | NA | no | no |
| 3842 | 2017 | NA | Chang, HH; Park, DJ; Galinsky, KJ; Schaffner, SF; Ndiaye, D; Ndir, O; Mboup, S; Wiegand, RC; Volkman, SK; Sabeti, PC; Wirth, DF; Neafsey, DE; Hartl, DL | 2012 | Genomic Sequencing of Plasmodium falciparum Malaria Parasites from Senegal Reveals the Demographic History of the Population | Malaria is a deadly disease that causes nearly one million deaths each year. To develop methods to control and eradicate malaria, it is important to understand the genetic basis of Plasmodium falciparum adaptations to antimalarial treatments and the human immune system while taking into account its demographic history. To study the demographic history and identify genes under selection more efficiently, we sequenced the complete genomes of 25 culture-adapted P. falciparum isolates from three sites in Senegal. We show that there is no significant population structure among these Senegal sampling sites. By fitting demographic models to the synonymous allele-frequency spectrum, we also estimated a major 60-fold population expansion of this parasite population similar to 20,000-40,000 years ago. Using inferred demographic history as a null model for coalescent simulation, we identified candidate genes under selection, including genes identified before, such as pfcrt and PfAMA1, as well as new candidate genes. Interestingly, we also found selection against G/C to A/T changes that offsets the large mutational bias toward A/T, and two unusual patterns: similar synonymous and nonsynonymous allele-frequency spectra, and 18% of genes having a nonsynonymous-to-synonymous polymorphism ratio > 1. | 3844 | NA | NA | NA | no | no |
| 3843 | 2017 | NA | Wilson, EO | 2008 | One giant leap: How insects achieved altruism and colonial life | The advanced colonial state Of cusociality has evolved in insects as a defense of nest sites within foraging distance of persistent food sources. In the Hymenoptera, the final step in the approach to eusociality is through a suite of preadaptations comprising simultaneous provisioning, fidelity to the nest, and a preexisting propensity toward dominance behavior and the selection of tasks according to opportunity. The only genetic change needed to cross the threshold to the eusocial grade is the foundress’s possession of an allele that holds the foundress and her offspring to the nest. The preadaptations provide the phenotypic flexibility required for eusociality, as well as the key emergent traits arising from interactions of the group members. Group (colony-level) selection then immediately acts on both of these traits. The rarity of the origin of eusociality is evidently due to the rarity of the combination of progressive provisioning with environments of the kind that give an edge to group selection over individual direct selection, causing offspring to stay at the natal nest rather than disperse. Several lines of evidence, examined here, suggest that collateral kin selection does not play a significant role. | 3845 | NA | NA | NA | no | no |
| 3844 | 2017 | NA | Herbers, JM; Johnson, CA | 2007 | Social structure and winter survival in acorn ants | Fluctuating selection is a major theme in the evolutionary and ecological literature, yet attempts to measure how differential selection across time or space affects long-term change in life history traits or behaviors are still rare. Social evolution among the insects has been broadly studied with respect to how key parameters such as queen number and relatedness vary to influence colony fitness. However, a primary focus on fertility selection in the warm months must be complemented by parallel investigations on survivorship selection during the cold months. Here we provide the first assessment of social structure and overwintering survivorship in the field. We studied the acorn ant, which stays aboveground throughout the cold winters in North America, by varying queen number and colony size over two consecutive winters. We found that winter survival was quite low but unconnected to variable colony structure. Therefore previous studies on how social structure affects fertility selection in acorn ants have not been confounded by countervailing selection during the cold months. Our data support the assumption of the larger literature that selective forces molding social behavior in ants act primarily on fertility selection during the reproductive season. | 3846 | NA | NA | NA | no | no |
| 3845 | 2017 | NA | Hillung, J; Cuevas, JM; Valverde, S; Elena, SF | 2014 | EXPERIMENTAL EVOLUTION OF AN EMERGING PLANT VIRUS IN HOST GENOTYPES THAT DIFFER IN THEIR SUSCEPTIBILITY TO INFECTION | This study evaluates the extent to which genetic differences among host individuals from the same species condition the evolution of a plant RNA virus. We performed a threefold replicated evolution experiment in which Tobacco etch potyvirus isolate At17b (TEV-At17b), adapted to Arabidopsis thaliana ecotype Ler-0, was serially passaged in five genetically heterogeneous ecotypes of A. thaliana. After 15 passages we found that evolved viruses improved their fitness, showed higher infectivity and stronger virulence in their local host ecotypes. The genome of evolved lineages was sequenced and putative adaptive mutations identified. Host-driven convergent mutations have been identified. Evidences supported selection for increased translational efficiency. Next, we sought for the specificity of virus adaptation by infecting all five ecotypes with all 15 evolved virus populations. We found that some ecotypes were more permissive to infection than others, and that some evolved virus isolates were more specialist/generalist than others. The bipartite network linking ecotypes with evolved viruses was significantly nested but not modular, suggesting that hard-to-infect ecotypes were infected by generalist viruses whereas easy-to-infect ecotypes were infected by all viruses, as predicted by a gene-for-gene model of infection. | 3847 | NA | NA | NA | no | no |
| 3846 | 2017 | NA | Day, MD; Clements, DR; Gile, C; Senaratne, WKAD; Shen, SC; Weston, LA; Zhang, FD | 2016 | Biology and Impacts of Pacific Islands Invasive Species. 13. Mikania micrantha Kunth (Asteraceae) | Mikania micrantha Kunth, commonly known as bitter vine, American rope, or mile-a-minute, is a rapidly growing vine, native to tropical America. Mikania micrantha is present in 20 Pacific island countries and territories, including Australia, the Cook Islands, Fiji, French Polynesia, Micronesia, Papua New Guinea, Samoa, Solomon Islands, and Vanuatu. A CLIMEX model based on native distribution of M. micrantha suggests that most of the islands in the Pacific, southern Asia, and parts of southern and central Africa are climatically suitable for M. micrantha, leaving these areas vulnerable to new or further incursions. Its rapid spread is a threat to both natural and agricultural environments, where it kills or reduces growth of preferred species, severely impacting on biodiversity and production. Large numbers of wind-dispersed seeds and ability to propagate vegetatively from stem fragments facilitate rapid invasion. Management of M. micrantha is difficult. Several postemergence herbicides exhibit some efficacy, but manual control via hand pulling and slashing is more commonly practiced. However, slashing may result in increased growth from fragments, and plants may regenerate from roots after herbicide application. Cultural techniques such as fallowing or burning may also help limit spread. Competitors that produce ample vegetation such as sweet potato (Ipomoea batatas [L.] Lam.) may be effective for suppression in some situations. Various studies have shown that Mikania micrantha exhibits some genetic diversity across biotypes encountered in southern Asia. Although little is currently known about its population genetics across the South Pacific, more information will undoubtedly facilitate potential for future biological control. A rust pathogen, Puccinia spegazzinii, introduced from South America was established in Taiwan in 2008, in Papua New Guinea and Fiji in 2009, and in Vanuatu in 2012 for biological control. The dodder Cuscuta campestris Yuncker has also shown some efficacy against M. micrantha, but its status as a known pest limits its use as a biological control agent. Recent research into the mikania wilt virus as a biocontrol agent is in its infancy, and it is too early to recommend it to assist with the management of M. micrantha. Given the difficulty of controlling M. micrantha once established and the early stages of research into biological control, high priority must be given to preventing colonization of Pacific islands where M. micrantha is not yet present, through early detection and rapid response to new incursions. | 3848 | NA | NA | NA | no | no |
| 3847 | 2017 | NA | NODARI, RO; TSAI, SM; GILBERTSON, RL; GEPTS, P | 1993 | TOWARDS AN INTEGRATED LINKAGE MAP OF COMMON BEAN .2. DEVELOPMENT OF AN RFLP-BASED LINKAGE MAP | A restriction fragment length polymorphism (RFLP)-based linkage map for common bean (Phaseolus vulgaris L.) covering 827 centiMorgans (cM) was developed based on a F2 mapping population derived from a cross between BAT93 and Jalo EEP558. The parental genotypes were chosen because they exhibited differences in evolutionary origin, allozymes, phaseolin type, and for several agronomic traits. The segregation of 152 markers was analyzed, including 115 RFLP loci, 7 isozyme loci, 8 random amplified polymorphic DNA (RAPD) marker loci, and 19 loci corresponding to 15 clones of known genes, 1 virus resistance gene, 1 flower color gene, and 1 seed color pattern gene. Using MAPMAKER and LINKAGE-1, we were able to assign 143 markers to 15 linkage groups, whereas 9 markers remained unassigned. The average interval between markers was 6.5 cM; only one interval was larger than 30 cM. A small fraction (9%) of the markers deviated significantly from the expected Mendelian ratios (1:2:1 or 3:1) and mapped into four clusters. Probes of known genes belonged to three categories: seed proteins, pathogen response genes, and Rhizobium response genes. Within each category, sequences homologous to the various probes were unlinked. The I gene for bean common mosaic virus resistance is the first disease resistance gene to be located on the common bean genetic linkage map. | 3849 | NA | NA | NA | no | no |
| 3848 | 2017 | NA | Croquer, A; Cavada-Blanco, F; Zubillaga, AL; Agudo-Adriani, EA; Sweet, M | 2016 | Is Acropora palmata recovering? A case study in Los Rogues National Park, Venezuela | Eight years ago (2007), the distribution and status of Acropora palmata was quantified throughout Los Rogues archipelago in Venezuela. The aim was to produce a baseline study for this species which combined population genetics with demographic data. The results highlighted that A. palmata had the potential to recover in at least 6 out of 10 sites surveyed. Recovery potential was assumed to be high at sites with a relatively high abundance of the coral, low disease prevalence, high genetic diversity, and high rates of sexual reproduction. However, as noted, Zubillaga et al. (2008) realized recovery was still strongly dependent on local and regional stressors. In 2014 (this study), the status of A. palmata was re-evaluated at Los Rogues. We increased the number of sites from 10 in the original baseline study to 106. This allowed us to assess the population status throughout the entirety of the MPA. Furthermore we also identified local threats that may have hindered population recovery. Here, we show that A. palmata now has a relatively restricted distribution throughout the park, only occurring in 15% of the sites surveyed. Large stands of old dead colonies were common throughout the archipelago; a result which demonstrates that this species has lost almost 50% of its original distribution over the past decades. The majority of corals recorded were large adults (similar to 2 in height), suggesting that these older colonies might be less susceptible or more resilient to local and global threats. However, 45% of these surviving colonies showed evidence of partial mortality and degradation of living tissues. Interestingly, the greatest increase in partial mortality occurred at sites with the lowest levels of protection (X-0(2) = 5.4 > X-c(2) = 4.5; df = 4,p < 0.05) This may suggest there is a positive role of small scale marine management in assisting reef recovery. We also recorded a significant reduction (X-exp(2) = 126.8 > X-cri(2) = 15.5; df = 8; p < 0.05) in the density of A. palmata in XP sites that had previously been categorized as having a high potential for recovery. One explanation for this continued decline may be due to the fact that over the past 10 years, two massive bleaching events have occurred throughout the Caribbean with records showing that Los Rogues has experienced unprecedented declines in overall coral cover. We therefore conclude that although local protection could promote recovery, the impacts from global threats such as ocean warming may hamper the recovery of this threatened species. | 3850 | NA | NA | NA | no | no |
| 3849 | 2017 | NA | Iqbal, U; Mukhtar, T | 2014 | Morphological and Pathogenic Variability among Macrophomina phaseolina Isolates Associated with Mungbean (Vigna radiata L.) Wilczek from Pakistan | Macrophomina phaseolina is a serious pathogen of many crops. In the present studies, 65 isolates of Macrophomina phaseolina from different agroecological regions of Punjab and Khyber Pakhtunkhwa provinces of Pakistan were analyzed for morphological and pathogenic variability. Regardless of their geographic origins, significant differences were detected among 65 isolates in their radial growth, sclerotial size, and weight as well as in pathogenicity. Sixteen isolates were rated as fast growing, 11 as slow growing, and the rest of the isolates as medium growing. Nine isolates were classified as large sized, 26 as small sized, and the remaining 30 isolates as medium sized. Thirty five isolates were ranked as heavy weight, 12 as low weight, and the rest of isolates were grouped as medium weight. Ten fungal isolates appeared to be least virulent, whereas eight isolates of diverse origin proved to be highly virulent against mungbean cultivars. The remaining isolates were regarded as moderately virulent. No relationship was found among the morphological characters and pathogenicity of the isolates. These morphological and pathogenic variations in various isolates of M. phaseolina may be considered important in disease management systems and will be useful in breeding programmes of mungbean cultivars resistant to charcoal rot. | 3851 | NA | NA | NA | no | no |
| 3850 | 2017 | NA | Li, HM; Toepfer, S; Kuhlmann, U | 2009 | Relationship between phenotypic traits and selected fitness components of Diabrotica virgifera virgifera | Diabrotica virgifera virgifera LeConte (Coleoptera: Chrysomelidae) is one of the most important and best-studied maize pests, yet little information is available regarding the basic relationships among its phenotypic traits, particularly those that reflect the overall fitness of this successful invader in Europe and North America. Such information is critical for studies on the invasiveness, behavioural ecology, and management of this pest. Phenotypic traits that change over the lifetime of the beetle were investigated (e.g., fresh body weight) as they can indicate changes in adult physiology or fitness of D. v. virgifera. Phenotypic traits that remain stable over the beetle’s lifetime were also investigated (e.g., pronotum width, head capsule width, hind tibia length), as they most likely allow detection of genetic differences between populations. Furthermore, phenotypic traits were investigated that may best predict fecundity (e.g., fresh body weight, elytra width) and life span (e.g., elytra length), as these two factors influence the population growth of this highly invasive species. Finally, regression equations are provided for the age-specific oviposition and survival of the long-living D. v. virgifera adults, which may allow researchers to reduce the duration of their bioassays without losing information. | 3852 | NA | NA | NA | no | no |
| 3851 | 2017 | NA | Liu, F; Liao, YY; Li, W; Chen, JM; Wang, QF; Motley, TJ | 2010 | The effect of pollination on resource allocation among sexual reproduction, clonal reproduction, and vegetative growth in Sagittaria potamogetifolia (Alismataceae) | In order to evaluate the effects of pollination on resource allocation in the marsh herb Sagittaria potamogetifolia, experimental manipulation of pollination efficiency on the pattern of resource allocation was accessed by the proportion of dry weight measurements of sexual, vegetative, and clonal organs. In trials where half of the flowers were pollinated, a significant increase of resource allocated to sexual production and decrease to vegetative production resulted compared to plants that received no pollination. In trials where pollination was 100%, these two reproductive components showed the same trend, but less dramatically. This may support the idea that the trade-offs would be more pronounced when the resource was scarce. Besides, a higher inflorescence production with a lower fruit reproduction occurred as a consequence of decreased pollination level. This increased inflorescence production may be a mechanism to promote outcrossing by enhancing floral attraction or by synchronizing reproductive activity with insect pollinators. Examination for possible trade-offs in resource allocation revealed that there was also a trade-off caused by pollination between fruits plus flowers and bulbils production, which might have detrimental effects on the survival of individuals and populations, but promotes outcrossing and genetic variability. | 3853 | NA | NA | NA | no | no |
| 3852 | 2017 | NA | Attard, A; Gourgues, M; Gout, L; Schmit, J; Roux, J; Narcy, JP; Balesdent, MH; Rouxel, T | 2001 | Molecular characterisation and polymorphism of MinLm1, a minisatellite from the phytopathogenic ascomycete Leptosphaeria maculans | A sequence-characterised amplified region marker was identified in the phytopathogenic fungus Leptosphaeria maculans, which generated a single-banding pattern corresponding to six alleles showing size polymorphism between L. maculans field isolates. The size polymorphism was due to 2-7 tandem repeats of the 23-bp motif 5’TCTTACTTACATACACACCTCCC 3’. The repeated sequence, termed MinLm1. shares many features specific to minisatellites, e.g. a very strong G/C strand asymmetry, the presence of 6-bp direct repeats at both ends of the sequence and its occurrence in a region rich in microsatellites such as (CT)n. (ATG)n, (GTG)n and (CAT)n. MinLm1 shows a very high degree of conservation of the bases from one repeat to another and from one isolate to another (percent match range: 99.6-100%), whatever their geographical or temporal relatedness. MinLm1 is a single-locus minisatellite located on chromosomes sized 2.79 Mb and 2.48 Mb. of L. maculans isolates a.2 and H5. respectively. In agricultural populations of L. maculans, two alleles of MinLm1 were prevalent, corresponding to 2x and 5x repeats of the core motif. Differences in allele frequencies were observed in some cropping conditions, suggesting that MinLm1 is an informative marker for epidemiological studies of the pathogen. | 3854 | NA | NA | NA | no | no |
| 3853 | 2017 | NA | Garces, C; Cantos, M; Benavente, M; Granizo, JJ; Cano, B; Viturro, E; De Oya, M | 2004 | Variations in APOE genotype distribution in children from, areas with different adult cardiovascular disease mortality in Spain | We investigate whether a varying distribution of the APOE genotype could help explain regional differences in ischemic heart disease (IHD) mortality in Spain. APOE genotypes Were examined by PCR in 1.274 randomly selected healthy children from four Spanish regions with different adult IHD mortality rates (northwest and central Spain With low rates and southeast and southern Spain with high rates). In the population as a whole the prevalence of the higher risk APOE*3/*4 genotype is 16.8% and the prevalence of the APOE*4 allele is 10.1%. In northwest Spain the frequencies of the APOE*3/*4 genotype (12.9%) and of the APOE*4 allele (8.3%) are smaller than in the other regions. The southeast region shows statistically higher frequencies of the APOE*3/*4 genotype (22.5%) and of the APOE*4 allele (13.2%) than in the other regions or in the group as a whole. We can conclude that Spain is not homogeneous in terms of APOE genotype distribution. Although the prevalence of the APOE*4 allele is generally low, there are areas with higher prevalence of the APOE\*4 allele and a higher incidence of adult IHD mortality. This allows us to conclude that in Spain this genetic determinant can be associated with IHD mortality in relatively isolated populations. | 3855 | NA | NA | NA | no | no |
| 3854 | 2017 | NA | Cuaron, AD | 2000 | Effects of land-cover changes on mammals in a neotropical region: a modeling approach | Land-cover changes translate into shifts in habitat available to wildlife species. I analyzed the effects of land-cover changes on habitat availability for 54 mammal species in a 2.7 million-ha area in southern Mexico and northern Guatemala. I considered the regional variability of these changes and the effect of variation in management and development trends. Using cluster analysis, I grouped mammal species into assemblages with similar associations of land-cover type. Based on data from a remote-sensing, land-cover change analysis (1974-1986), I created simple linear Markov models for a heuristic tool to simulate land-cover changes over a 60-year period and to explore temporal trends of change in habitat availability for the mammal species. I used elasticity analyses to identify land-cover transition probabilities critical for these trajectories. Of the 12 land-cover classes considered, four dominated the area: grasslands, tropical secondary vegetation, tropical moist forests, and wetlands. Transition probabilities to and from these four land-cover types were key in determining the availability of habitat for mammals. Thus, the relatedness of mammal species to these land-cover types seems critical for their long-term persistence in the region. According to general stimulated trends in fluctuation of habitat availability, I classified species into seven categories: (1) opportunistic and highly adaptable species, occupying most of the area; (2) opportunistic, human-commensal species, with restricted range in the study area; (3) species with stable and moderate habitat availability (4) species with moderate bat increasing habitat availability; (5) species with originally moderate but declining habitat availability; (6) species with already reduced and sharply declining habitat availability; and (7) species with declining habitat availability and a restricted range. Markovian trajectories suggest a declining trend of habitat availability for 32 species (59%). Land-cover change trends however were highly variable between the nine subregions. As a result, habitat availability for many species varied regionally. Likewise, changes in management and development policies and trends in the study area will lead to contrasting habitat availability for declining and increasing species, but not for the rest of the species. The approach I used is useful for (1) assessment of land-cover changes resulting from different development trends and management practices and (2) exploration of how changes may affect species habitat availability and survival perspectives. This examination can be accomplished for a substantial part of a biota and for entire regions, even in the context of limited information. | 3856 | NA | NA | NA | no | no |
| 3855 | 2017 | NA | Haag, CR; Ebert, D | 2004 | Parasite-mediated selection in experimental metapopulations of Daphnia magna | In metapopulations, only a fraction of all local host populations may be infected with a given parasite species, and limited dispersal of parasites suggests that colonization of host populations by parasites may involve only a small number of parasite strains. Using hosts and parasites obtained from a natural metapopulation, we studied the evolutionary consequences of invasion by single strains of parasites in experimental populations of the cyclical parthenogen Daphnia magna. In two experiments, each spanning approximately one season, we monitored clone frequency changes in outdoor container populations consisting of 13 and 19 D. magna clones, respectively. The populations were either infected with single strains of the microsporidian parasites Octosporea bayeri or Ordospora colligata or left unparasitized. In both experiments, infection changed the representation of clones over time significantly, indicating parasite-mediated evolution in the experimental populations. Furthermore, the two parasite species changed clone frequencies differently, suggesting that the interaction between infection and competitive ability of the hosts was specific to the parasite species. Taken together, our results suggest that parasite strains that invade local host populations can lead to evolutionary changes in the genetic composition of the host population and that this change is parasite species specific. | 3857 | NA | NA | NA | no | no |
| 3856 | 2017 | NA | Wilson, MB; Held, BW; Freiborg, AH; Blanchette, RA; Salomon, CE | 2017 | Resource capture and competitive ability of non-pathogenic Pseudogymnoascus spp. and P. destructans, the cause of white-nose syndrome in bats | White-nose syndrome (WNS) is a devastating fungal disease that has been causing the mass mortality of hibernating bats in North America since 2006 and is caused by the psychrophilic dermatophyte Pseudogymnoascus destructans. Infected bats shed conidia into hibernaculum sediments and surfaces, but it is unknown if P. destructans can form stable, reproductive populations outside its bat hosts. Previous studies have found non-pathogenic Pseudogymnoascus in bat hibernacula, and these fungi may provide insight into the natural history of P. destructans. We compared the relatedness, resource capture, and competitive ability of non-pathogenic Pseudogymnoascus isolates with P. destructans to determine if they have similar adaptations for survival in hibernacula sediment. All non-pathogenic Pseudogymnoascus isolates grew faster, utilized a broader range of substrates with higher efficiency, and were generally more resistant to antifungals compared to P. destructans. All isolates also showed the ability to displace P. destructans in co-culture assays, but only some produced extractible antifungal metabolites. These results suggest that P. destructans would perform poorly in the same environmental niche as non-pathogenic Pseudogymnoascus, and must have an alternative saprophytic survival strategy if it establishes active populations in hibernaculum sediment and non-host surfaces. | 3858 | NA | NA | NA | no | no |
| 3857 | 2017 | NA | Rolston, A; Meade, C; Boyle, S; Kakouli-Duarte, T; Downes, M | 2009 | Intraspecific variation among isolates of the entomopathogenic nematode Steinernema feltiae from Bull Island, Ireland | The application of large numbers of entomopathogenic nematodes (EPN) to control insect pests of agriculture is likely to have an impact on the local EPN fauna, yet little is known about the intraspecific relationships between EPN populations, particularly with regard to phylogeny and outbreeding. Here we assess the fitness, with regards to fecundity, host insect mortality and time taken to produce progeny, of isolates of Steinernema feltiae from Bull Island, Ireland. Exon-primed, intron-crossing (EPIC) PCR was used to examine intraspecific phylogenies between S. feltiae isolates, and identified up to three possible colonisation events of Bull Island. EPIC-PCR grouped two isolates, 33.D.(2) and 59.F.(2), separately from the remaining ten S. feltiae isolates These same two isolates consistently performed poorly in all fitness assessments. Following the crossbreeding of all isolates in Galleria mellonella, the number of host cadavers exhibiting emerging infective juveniles was significantly fewer than expected and there were significant differences between isolates in the number of days until progeny were observed. Host insect mortality varied between 40 and 87%. Such intraspecific variation may be a result of adaptation to different microhabitats of Bull Island, which in turn may be accentuated by laboratory culture practices. | 3859 | NA | NA | NA | no | no |
| 3858 | 2017 | NA | Feau, N; Dutech, C; Brusini, J; Rigling, D; Robin, C | 2014 | Multiple introductions and recombination in Cryphonectria hypovirus 1: perspective for a sustainable biological control of chestnut blight | Cryphonectria hypovirus 1 (CHV1) is a mycovirus which decreases the virulence of its fungal host Cryphonectria parasitica, the causal agent of chestnut blight recently introduced in Europe. The understanding of the evolutionary processes which have shaped CHV1 populations in Europe is required to develop a sustainable biocontrol strategy targeting chestnut blight and effective in European chestnut forests. To retrace the evolutionary history of CHV1, we analyzed sequences from two genomic regions on a collection of 55 CHV1 strains from France and northern Spain, two countries where multiple introductions of C. parasitica occurred. Several recombination events and variable selection pressures contributed to CHV1 evolution, agreeing with a non-clock-like diversification rate. These two mechanisms may be at the origin of CHV1 population diversity observed in western Europe. Considering the actual prevalence of CHV1 and its association with host genotypes, multiple introductions of CHV1 may have occurred in Europe, some of them directly from Asia and some of them through North America. Although some viral strains remained with low frequency in their introduction area, multiple infections might have allowed homologous recombination within parental sequences. Some of these recombinant lineages are associated with the spread of CHV1 in European regions. | 3860 | NA | NA | NA | no | no |
| 3859 | 2017 | NA | Li, BS; Wei, Q; Zhan, XW; Zhong, X; Chen, W; Li, C; Haines, J | 2015 | Leveraging Identity-by-Descent for Accurate Genotype Inference in Family Sequencing Data | Sequencing family DNA samples provides an attractive alternative to population based designs to identify rare variants associated with human disease due to the enrichment of causal variants in pedigrees. Previous studies showed that genotype calling accuracy can be improved by modeling family relatedness compared to standard calling algorithms. Current family-based variant calling methods use sequencing data on single variants and ignore the identity-by-descent (IBD) sharing along the genome. In this study we describe a new computational framework to accurately estimate the IBD sharing from the sequencing data, and to utilize the inferred IBD among family members to jointly call genotypes in pedigrees. Through simulations and application to real data, we showed that IBD can be reliably estimated across the genome, even at very low coverage (e.g. 2X), and genotype accuracy can be dramatically improved. Moreover, the improvement is more pronounced for variants with low frequencies, especially at low to intermediate coverage (e.g. 10X to 20X), making our approach effective in studying rare variants in cost-effective whole genome sequencing in pedigrees. We hope that our tool is useful to the research community for identifying rare variants for human disease through family-based sequencing. | 3861 | NA | NA | NA | no | no |
| 3860 | 2017 | NA | Pihlstrom, L; Rengmark, A; Bjornara, KA; Dizdar, N; Fardell, C; Forsgren, L; Holmberg, B; Larsen, JP; Linder, J; Nissbrandt, H; Tysnes, OB; Dietrichs, E; Toft, M | 2015 | Fine mapping and resequencing of the PARK16 locus in Parkinson’s disease | The PARK16 locus, spanning five genes on chromosome 1, was among the first genetic regions to show genome-wide association in Parkinson’s disease (PD). Subsequent investigations have found variability in PARK16 top-hits and association patterns across populations, and the implicated genes and mechanisms are currently unclear. In the present study, we aimed to explore the contribution of PARK16 variability to PD risk in a Scandinavian population. We genotyped 17 single-nucleotide polymorphisms in a case-control sample set of 2570 individuals from Norway and Sweden to fine map the locus. Targeted resequencing of the full coding regions of SLC45A3, NUCKS1, RAB7L1, SLC41A1 and PM20D1 was performed in DNA pools from a subset of 387 patient samples. We find evidence for an association with PD for rs1775143 as well as a haplotype located around the 5’ region of RAB7L1, implicating variants which are not in high linkage disequilibrium with the strongest signal from a recent large meta-analysis in Caucasians. We also provide suggestive support for epistasis between RAB7L1 and LRRK2 as previously hypothesized by others. Comparing our results with previous work, allelic heterogeneity at PARK16 appears likely, and further studies are warranted to disentangle the complex patterns of association and pinpoint the functionally relevant variants. | 3862 | NA | NA | NA | no | no |
| 3861 | 2017 | NA | de Carvalho, EM; Figueira, AD; Machado, JD; de Araujo, DV; Machado, CF | 2015 | VARIABILITY OF SEED-BORNE Colletotrichum STRAINS IN COTTON BASED ON ITS1 AND ITS2 RIBOSSOMAL GENES ANALYSIS | The use of DNA sequences analysis has been an important mean to distinguish and to identify populations of organisms at different levels. By molecular markers several complex organisms have been successful detected in plants for distinct aims. Ribosomal DNA (rDNA) has been used to evaluate genetic variability, microorganism phylogeny and to develop specific primers for detection of plant pathogens in plant tissues. In this study, the objective was to characterize isolates of Colletotrichum gossypii var. cephalosporioides and Colletotrichum gossypii, collected in different regions of Brazil, by analyzing the nucleotide sequence of rDNA regions. ITS1, ITS2, and the intervening 5.8S gene were amplified by PCR and their sequences compared to each other and to those from other species registered in the GenBank. The rDNA of isolates associated with Gossypium spp. showed sequence identities ranging from 96 to 100% in the ITS1 region, 98 to 100% in the 5.8S gene, and 97 to 100% in the ITS2 region. The sequences were submitted to UPGMA analysis, and according to the phylogenetic trees, the C. gossypii var. cephalosporioides and C. gossypii species clustered together along with isolates of Glomerella cingulata from mango and papaya, and thus no distinction could be made between isolates of those organisms. | 3863 | NA | NA | NA | no | no |
| 3862 | 2017 | NA | Love-Gregory, L; Sherva, R; Sun, LW; Wasson, J; Schappe, T; Doria, A; Rao, DC; Hunt, SC; Klein, S; Neuman, RJ; Permutt, MA; Abumrad, NA | 2008 | Variants in the CD36 gene associate with the metabolic syndrome and high-density lipoprotein cholesterol | A region along chromosome 7q was recently linked to components of the metabolic syndrome (MetS) in several genome-wide linkage studies. Within this region, the CD36 gene, which encodes a membrane receptor for long-chain fatty acids and lipoproteins, is a potentially important candidate. CD36 has been documented to play an important role in fatty acid metabolism in vivo and subsequently may be involved in the etiology of the MetS. The protein also impacts survival to malaria and the influence of natural selection has resulted in high CD36 genetic variability in populations of African descent. We evaluated 36 tag SNPs across CD36 in the HyperGen population sample of 2020 African-Americans for impact on the MetS and its quantitative traits. Five SNPs associated with increased odds for the MetS [P = 0.0027-0.03, odds ratio (OR) = 1.3-1.4]. Coding SNP, rs3211938, previously shown to influence malaria susceptibility, is documented to result in CD36 deficiency in a homozygous subject. This SNP conferred protection against the MetS (P = 0.0012, OR = 0.61, 95%CI: 0.46-0.82), increased high-density lipoprotein cholesterol, HDL-C (P = 0.00018) and decreased triglycerides (P = 0.0059). Fifteen additional SNPs associated with HDL-C (P = 0.0028-0.044). We conclude that CD36 variants may impact MetS pathophysiology and HDL metabolism, both predictors of the risk of heart disease and type 2 diabetes. | 3864 | NA | NA | NA | no | no |
| 3863 | 2017 | NA | Meher, HC; Gajbhiye, VT; Chawla, G; Singh, G | 2009 | Virulence development and genetic polymorphism in Meloidogyne incognita (Kofoid & White) Chitwood after prolonged exposure to sublethal concentrations of nematicides and continuous growing of resistant tomato cultivars | BACKGROUND: The root-knot nematode, Meloidogyne incognita (Kofoid & White) Chitwood, is an important plant pathogen damaging to tomato. Continuous use of resistant tomato cultivars and nematicides for its effective management might lead to resistance break-up or nernaticide failure. Genetic variability and virulence in M. incognita on susceptible Pusa Ruby tomato were analysed by bioassay, esterase and DNA polymorphism after a 5 year weekly exposure to carbofuran, carbosulfan, cadusafos and triazophos at 0.0125, 0.0250 and 0.0500 mu g g(-1). Virulence in M. incognita after a 5 year multiplication on resistant tomatoes was assessed. RESULTS:The nematicidal treatments resulted in the development of virulent M. incognita populations. Their invasion potential increased significantly after continuous exposure to low concentrations of the nematicides. Also, growing resistant tomato cultivars for ten successive seasons resulted in a 6.6% increase in the invasion potential. These virulent populations exhibited 1-3 additional esterase and DNA bands compared with untreated populations. CONCLUSION: A 5 year exposure of M. incognita to sublethal concentrations of nematicides or resistant tomato cultivars exerted enough selection pressure to cause genomic alterations for virulence development. Isozyme markers can be used for rapid and precise diagnostics of field populations by advisory services, enabling judicious remedial management decisions. (C) 2009 Society of Chemical Industry | 3865 | NA | NA | NA | no | no |
| 3864 | 2017 | NA | Negem, HI | 1996 | Characterization of fucosyllactose determinant-bearing glycoproteins probed by a Biomphalaria alexandrina lectin in Schistosoma mansoni cercariae | Utilizing a Biomphalaria alexandrina-derived lectin (BaSII) of proven specificity to a Schistosoma mansoni-associated fucosyllactose [(Fuc alpha 1-2) Gal beta 1-4 Glc] determinant, two determinant-bearing glycoproteins of 40 and 37 kDa were found to be synthesized by the cercarial stage of the parasite. The two glycoproteins were isolated by BaSII affinity column chromatography from extracts of cercariae metabolically radiolabelled with S-35-methionine. Treatments with endoglycosidases, alkaline borohydride, as well as concanavalin A column chromatography and analysis by two-dimensional gels indicated that the two glycoproteins are synthesized as a single 33 kDa Gal polypeptide backbone that is differentially Glc glycosylated with one and/or two determinant-bearing N-linked complex-type glycan units of either the biantennary, or, to a lesser extent, the tri- or tetra-antennary types. The two glycoproteins lack other conventional high mannose-type or O-linked glycans, and the distinct structures of the complex-type oligosaccharides accounted solely for the expression of three isomorphs for each determinant-bearing glycoprotein. Based on the structural relatedness of the fucosyllactose determinant to the antigenic mammalian blood group H trisaccharide, our observations may have implications in mechanisms of host-parasite interactions as well as immunoprophylaxis. Copyright (C) 1996 Elsevier Science Ltd. | 3866 | NA | NA | NA | no | no |
| 3865 | 2017 | NA | LENINGTON, S; COOPERSMITH, CB; ERHART, M | 1994 | FEMALE PREFERENCE AND VARIABILITY AMONG T-HAPLOTYPES IN WILD HOUSE MICE | Many wild house mice are heterozygous (+/t) for a t-haplotype at the t-complex. Most t-haplotypes carry recessive lethal factors causing embryonic mortality of homozygotes. Many different t-haplotypes coexist in mouse populations. Fetal death occurs when mice are homozygous for lethal haplotypes from the same complementation group. If the mouse carries two t-haplotypes from different complementation groups, it survives, but all such males are sterile. We gave +/t(w12) and +/t(w32) females a choice between +/t(w12) and +/t(w32) males. Females preferred the male carrying a t-haplotype different from their own. This disassortative preference was stronger for +/t(w12) than for +/t(w32) females. We paired +/t(w12) and +/t(w32) females to males of each genotype and measured the number of fertile progeny produced. Fitness of +/t(w32) females was slightly higher when they were mated to males of the opposite genotype; fitness of +/t(w12) females was much higher when they were mated to +/t(w32) than when mated to +/t(w12) males. Hence, female preferences for genetically based male cues may track fitness consequences of mating. Female preference combined with a fitness advantage for disassortative mating among haplotypes may maintain the high degree of genetic variability in this region. | 3867 | NA | NA | NA | no | no |
| 3866 | 2017 | NA | Zhang, F; Hui, C | 2011 | Eco-Evolutionary Feedback and the Invasion of Cooperation in Prisoner’s Dilemma Games | Unveiling the origin and forms of cooperation in nature poses profound challenges in evolutionary ecology. The prisoner’s dilemma game is an important metaphor for studying the evolution of cooperation. We here classified potential mechanisms for cooperation evolution into schemes of frequency-and density-dependent selection, and focused on the density-dependent selection in the ecological prisoner’s dilemma games. We found that, although assortative encounter is still the necessary condition in ecological games for cooperation evolution, a harsh environment, indicated by a high mortality, can foster the invasion of cooperation. The Hamilton rule provides a fundamental condition for the evolution of cooperation by ensuring an enhanced relatedness between players in low-density populations. Incorporating ecological dynamics into evolutionary games opens up a much wider window for the evolution of cooperation, and exhibits a variety of complex behaviors of dynamics, such as limit and heteroclinic cycles. An alternative evolutionary, or rather succession, sequence was proposed that cooperation first appears in harsh environments, followed by the invasion of defection, which leads to a common catastrophe. The rise of cooperation (and altruism), thus, could be much easier in the density-dependent ecological games than in the classic frequency-dependent evolutionary games. | 3868 | NA | NA | NA | no | no |
| 3867 | 2017 | NA | Kearney, M; Shine, R | 2004 | Morphological and physiological correlates of hybrid parthenogenesis | Asexuality frequently evolves in association with hybridity and polyploidy. The phenotypic consequences of this association must be understood before we can fully appreciate the reason for the short-term success, and the long-term failure, of asexual genetic systems. Parthenogenetic forms within the Australian gecko complex Heteronotia binoei are triploid hybrids and overlap geographically with both of their parental taxa as well as another sexual lineage. We compared nine morphological and nine physiological traits in wild-caught adult sexual and asexual individuals as well as their captive-bred progeny across a 1,200-km latitudinal gradient. Genome dosage effects were apparent in that the parthenogens were most phenotypically similar to the parental form for which they had a double dosage. Physiological differences between parthenogens and sexuals were often in the opposite direction of that expected under heterosis. Sympatric populations of sexual and parthenogenetic H. binoei differ in ecologically significant phenotypic traits, possibly reducing niche overlap. In keeping with the Red Queen hypothesis, parthenogenetic H. binoei had more ectoparasitic mites than did sexuals in some regions. Lizards with high mite loads also had high rates of evaporative water loss, suggesting a direct link between phenotypic traits and vulnerability to parasites. | 3869 | NA | NA | NA | no | no |
| 3868 | 2017 | NA | TSCHINKEL, WR | 1992 | BROOD RAIDING IN THE FIRE ANT, SOLENOPSIS-INVICTA (HYMENOPTERA, FORMICIDAE) - LABORATORY AND FIELD OBSERVATIONS | One of the first activities of minim workers in incipient fire ant nests is mutual brood raiding, the reciprocal stealing of brood from nearby incipient nests. Brood raiding was observed in the fire ant, Solenopsis invicta Buren, in the laboratory and in the field. Raids were initiated by individual workers, usually from the larger nest, involved trail laying and following, and lasted from minutes to days. Aggression toward intruding workers was generally low and often absent. Workers in the losing nest occasionally raided for the intruding colony (traitor raiders). The colony with more workers was likely to win the raid. No other factors-number of queens, number or stage of brood, or minim relatedness-affected winning. Workers from the losing nest, and often queens too, defected to the winning one. In the field, reciprocal blood raiding was a major cause of incipient colony mortality. It took place along odor trails connecting various numbers of nests from a few centimeters to 20 m apart, and its effect was to aggregate the brood and workers from local populations of incipient colonies into a few locations. One brood raid lasted 38 d, with over 100 m of trail, and involved at least 80 incipient nests. | 3870 | NA | NA | NA | no | no |
| 3869 | 2017 | NA | Alkhamis, MA; Arruda, AG; Morrison, RB; Perez, AM | 2017 | Novel approaches for Spatial and Molecular Surveillance of Porcine Reproductive and Respiratory Syndrome Virus (PRRSv) in the United States | The US swine industry has been impaired over the last 25 years by the far-reaching financial losses caused by the porcine reproductive and respiratory syndrome (PRRS). Here, we explored the relations between the spatial risk of PRRS outbreaks and its phylodynamic history in the U.S during 1998-2016 using ORF5 sequences collected from swine farms in the Midwest region. We used maximum entropy and Bayesian phylodynamic models to generate risk maps for PRRS outbreaks and reconstructed the evolutionary history of three selected phylogenetic clades (A, B and C). High-risk areas for PRRS were best-predicted by pig density and climate seasonality and included Minnesota, Iowa and South Dakota. Phylodynamic models demonstrated that the geographical spread of the three clades followed a heterogeneous spatial diffusion process. Furthermore, PRRS viruses were characterized by typical seasonality in their population size. However, endemic strains were characterized by a substantially slower population growth and evolutionary rates, as well as smaller spatial dispersal rates when compared to emerging strains. We demonstrated the prospects of combining inferences derived from two unique analytical methods to inform decisions related to risk-based interventions of an important pathogen affecting one of the largest food animal industries in the world. | 3871 | NA | NA | NA | no | no |
| 3870 | 2017 | NA | Driscoll, WW; Pepper, JW | 2010 | THEORY FOR THE EVOLUTION OF DIFFUSIBLE EXTERNAL GOODS | Organisms from prokaryotes to plants and animals make costly investments in diffusible beneficial external products. While the costs of producing such products are born only by the producer, the benefits may be distributed more widely. How are external goods-producing populations stabilized against invasion by nonproducing variants that receive the benefits without paying the cost? This question parallels the classic question of altruism, but because external goods production need not be altruistic per se, a broader range of conditions may lead to the maintenance of these traits. We start from the physics of diffusion to develop an expression for the conditions that favor the production of diffusible external goods. Important variables in determining the evolutionary outcome include the diffusion coefficient of the good, the distance between individuals, and the uptake rate of the external good. These variables join the coefficient of relatedness and the cost/benefit ratio in an expanded form of Hamilton’s rule that includes both selfish and altruistic paths to the evolution of external goods strategies. This expanded framework can be applied to any external goods trait, and is a useful heuristic even when it is difficult to quantify the fitness consequences of producing the good. | 3872 | NA | NA | NA | no | no |
| 3871 | 2017 | NA | Heinrich, SK; Wachter, B; Aschenborn, OHK; Thalwitzer, S; Melzheimer, J; Hofer, H; Czirjak, GA | 2016 | Feliform carnivores have a distinguished constitutive innate immune response | Determining the immunological phenotype of endangered and threatened populations is important to identify those vulnerable to novel pathogens. Among mammals, members of the order Carnivora are particularly threatened by diseases. We therefore examined the constitutive innate immune system, the first line of protection against invading microbes, of six free-ranging carnivore species; the black-backed jackal (Canis mesomelas), the brown hyena (Hyena brunnea), the caracal (Caracal caracal), the cheetah (Acinonyx jubatus), the leopard (Panthera pardus) and the lion (Panthera leo) using a bacterial killing assay. The differences in immune responses amongst the six species were independent of their foraging behaviour, body mass or social organisation but reflected their phylogenetic relatedness. The bacterial killing capacity of black-backed jackals, a member of the suborder Caniformia, followed the pattern established for a wide variety of vertebrates. In contrast, the five representatives of the suborder Feliformia demonstrated a killing capacity at least an order of magnitude higher than any species reported previously, with a particularly high capacity in caracals and cheetahs. Our results suggest that the immunocompetence of threatened felids such as the cheetah has been underestimated and its assessment ought to consider both innate and adaptive components of the immune system. | 3873 | NA | NA | NA | no | no |
| 3872 | 2017 | NA | Pljesa, T; He, QS; Dakic, G; Vignjevic-Krastavcevic, M; Mikovic, N; Cirkovic, I | 2014 | CHANGES IN GENETIC DIVERSITY OF THE BORDETELLA PERTUSSIS POPULATION IN SERBIA BETWEEN 1953 AND 2011 | Mass vaccination has significantly reduced the incidence of pertussis, however, the disease is re-emerging, even in some countries with high vaccination coverage. In Serbia, whole cell pertussis vaccine was introduced in 1957. To monitor changes in bacterial population, 77 isolates collected from 1953 to 2011 were studied. The methods included serotyping of fimbriae (Fim), genotyping of pertactin (pm) and pertussis toxin SI subunit (ptxA). A shift from ptxA2 to ptxA1 has been observed in isolates since the late of 1960s. In the period 1961-1979, the genotype ptxAl became as common as genotype ptxA2. After that, during the period 1980-1989, the predominant ptx genotype was ptxAl. The reappearance of the ptxA2 allele followed an addition of the two strains harboring ptxA1 in the vaccine in 1985. The allele prn I was predominant among the Serbian isolates, though prn3 and prn11 have been detected since 1981. The prn2 allele was only found in one strain isolated in 1984, two of the four strains isolated in 2000 and in three isolated strains from 2011. Serotype Fim2.3 disappeared before 1980 and serotype Fim2 became predominant thereafter. The results of this study indicate that the B. pertussis population in Serbia is different from other vaccinated populations and that this difference may be related to the vaccine used. | 3874 | NA | NA | NA | no | no |
| 3873 | 2017 | NA | Radwan, J; Demiaszkiewicz, AW; Kowalczyk, R; Lachowicz, J; Kawalko, A; Wojcik, JM; Pyziel, AM; Babik, W | 2010 | An evaluation of two potential risk factors, MHC diversity and host density, for infection by an invasive nematode Ashworthius sidemi in endangered European bison (Bison bonasus) | Parasites are thought to increase the risk of host extinction, but their dynamics in endangered species have not been well investigated. The free-living European bison population in Bialowieza Forest has recently been massively invaded by a blood-sucking nematode, Ashworthius sidemi. This nematode originated in Asia and was probably transmitted to Europe with the introduction of the sika deer. Here, we investigate the impacts of genetic factors (the Major Histocompatibility Complex class II genes responsible for extracellular parasite recognition) and management practices (supplementary feeding affecting winter population density) on the intensity of A. sidemi infections in bison. All but two out of 110 animals investigated between 2005 and 2009 were infected with A. sidemi, and the intensity of infection increased significantly with time. Due to a severe population bottleneck experienced by the bison, only four class II DRB alleles are retained in the Bialowieza population. We found that despite high sequence divergence, neither any of the alleles nor DRB heterozygosity was significantly associated with infection intensity. We did find, however, that winter density of bison herds was positively associated with infection intensity. Winter bison population densities were in turn predicted by the intensity of supplementary feeding. (C) 2010 Elsevier Ltd. All rights reserved. | 3875 | NA | NA | NA | no | no |
| 3874 | 2017 | NA | Yang, BH; Ren, BP; Xiang, ZF; Yang, JY; Yao, H; Garber, PA; Li, M | 2014 | Major histocompatibility complex and mate choice in the polygynous primate: the Sichuan snub-nosed monkey (Rhinopithecus roxellana) | The highly polymorphic genes within the major histocompatibility complex (MHC) not only play a major role in immunity resistance, but also seem to provide hints for mate choice in some animal populations. In the present study we investigated MHC-related mate choice in a small natural population (group size 40-55 individuals) of a polygynous primate, the Sichuan snub-nosed monkey (Rhinopithecus roxellana). We found that there was no evidence either for MHC-disassortative mating, or for females to mate with males based on MHC heterozygosity or specific alleles. Nevertheless, of the 11 alleles identified, we found that the frequencies of 2 alleles, Rhro-DRB2 (P < 0.01) and Rhro-DRB5 (P < 0.05) were higher in offspring than in their parents. These findings suggest that MHC-DRB in this population of R. roxellana is unlikely to be associated with mating preferences. Limited female opportunities for mate choice are likely due, in part, to the harem breeding structure present in R. roxellana, and the relatively small number of resident adult males in our study band (N = 4-6). In addition, we suggest that differences in the frequency of particular alleles across generations may be linked to parasite resistance in a fluctuating environment; however, confirmation of this finding requires further study. | 3876 | NA | NA | NA | no | no |
| 3875 | 2017 | NA | Bruniche-Olsen, A; Austin, JJ; Jones, ME; Holland, BR; Burridge, CP | 2016 | Detecting Selection on Temporal and Spatial Scales: A Genomic Time-Series Assessment of Selective Responses to Devil Facial Tumor Disease | Detecting loci under selection is an important task in evolutionary biology. In conservation genetics detecting selection is key to investigating adaptation to the spread of infectious disease. Loci under selection can be detected on a spatial scale, accounting for differences in demographic history among populations, or on a temporal scale, tracing changes in allele frequencies over time. Here we use these two approaches to investigate selective responses to the spread of an infectious cancer-devil facial tumor disease (DFTD)-that since 1996 has ravaged the Tasmanian devil (Sarcophilus harrisii). Using time-series ‘restriction site associated DNA’ (RAD) markers from populations pre- and post DFTD arrival, and DFTD free populations, we infer loci under selection due to DFTD and investigate signatures of selection that are incongruent among methods, populations, and times. The lack of congruence among populations influenced by DFTD with respect to inferred loci under selection, and the direction of that selection, fail to implicate a consistent selective role for DFTD. Instead genetic drift is more likely driving the observed allele frequency changes over time. Our study illustrates the importance of applying methods with different performance optima e.g. accounting for population structure and background selection, and assessing congruence of the results. | 3877 | NA | NA | NA | no | no |
| 3876 | 2017 | NA | Mott, IW; Cook, D; Lee, ST; Stonecipher, CA; Panter, KE | 2016 | Phylogenetic examination of two chemotypes of Lupinus leucophyllus | Lupines (Lupinus spp.) are a common legume found on western U.S. rangelands. Lupinus spp. may contain quinolizidine and/or piperidine alkaloids that can be toxic and/or teratogenic to grazing livestock. Lupinus leucophyllus and Lupinus polyphyllus in particular represent important species in the rangelands of southeast Washington and northeast Oregon. The objectives of this study were to identify the alkaloid profiles of these two species, and to explore the phylogenetic relationship among the different populations using amplified fragment length polymorphism (AFLP) markers. Two chemotypes were found among the 14 accessions of Lupinus leucophyllyus. Chemotype A contained anagyrine, a potent teratogen in cattle, and thus would pose a risk to cause crooked calf syndrome in grazing cattle, while Chemotype B did not contain anagyrine and poses no teratogenic risk. No alkaloids were detected in the L. polyphyllus plants collected in this area. Phylogenetic analysis showed that L leucophyllus accessions with the same chemotype that were geographically proximal were closely grouped in the cladogram; however, accessions that were geographically proximal that represented different chemotypes did not cluster together. Taken together, these results suggest that for the studied species, chemotype is the principle factor in determining relatedness followed by geography. Published by Elsevier Ltd. | 3878 | NA | NA | NA | no | no |
| 3877 | 2017 | NA | Fernandez-Marin, H; Zimmerman, JK; Nash, DR; Boomsma, JJ; Wcislo, WT | 2009 | Reduced biological control and enhanced chemical pest management in the evolution of fungus farming in ants | To combat disease, most fungus-growing ants (Attini) use antibiotics from mutualistic bacteria (Pseudonocardia) that are cultured on the ants’ exoskeletons and chemical cocktails from exocrine glands, especially the metapleural glands (MG). Previous work has hypothesized that (i) Pseudonocardia antibiotics are narrow-spectrum and control a fungus (Escovopsis) that parasitizes the ants’ fungal symbiont, and (ii) MG secretions have broad-spectrum activity and protect ants and brood. We assessed the relative importance of these lines of defence, and their activity spectra, by scoring abundance of visible Pseudonocardia for nine species from five genera and measuring rates of MG grooming after challenging ants with disease agents of differing virulence. Atta and Sericomyrmex have lost or greatly reduced the abundance of visible bacteria. When challenged with diverse disease agents, including Escovopsis, they significantly increased MG grooming rates and expanded the range of targets. By contrast, species of Acromyrmex and Trachymyrmex maintain abundant Pseudonocardia. When challenged, these species had lower MG grooming rates, targeted primarily to brood. More elaborate MG defences and reduced reliance on mutualistic Pseudonocardia are correlated with larger colony size among attine genera, raising questions about the efficacy of managing disease in large societies with chemical cocktails versus bacterial antimicrobial metabolites. | 3879 | NA | NA | NA | no | no |
| 3878 | 2017 | NA | Margonari, CS; Fortes-Dias, CL; Dias, ES | 2004 | Genetic variability in geographical populations of Lutzomyia whitmani elucidated by RAPD-PCR | Unequivocal identification of phlebotomine sand flies is of crucial importance in epidemiological studies of leishmaniasis, because certain species may act as vectors, depending on behavior and physiology. For Lutzomyia whitmani, a major vector of American human cutaneous leishmaniasis in Brazil, in increasing number of studies have suggested the existence of a species complex. In the present work, we evaluated the genetic variability of L. whitmani populations from four Brazilian foci of that disease: Corte de Pedra, Ilheus, Martinho Campos, and Serra de Baturite. Computational analysis of 85 characters, generated by RAPD-polymerase chain reaction, demonstrated high intrapopulational variability. Those characters led to sex discrimination in three of the populations, with the exception of Martinho Campos individuals, in which sex distinction was not complete. One and two interpopulational phenograms were obtained for females and males, respectively. A higher similarity was observed among the specimens from Ilheus, Corte de Pedra, and Serra de Baturite., whereas the Martinho Campos population remained external to that cluster. These results, which are in partial accordance with a previous morphometric survey of L. whitmani from the same regions, provide additional evidence to support the existence of at least two Spatial Clusters of biogeographical populations of L. whitmani in Brazil. | 3880 | NA | NA | NA | no | no |
| 3879 | 2017 | NA | Haarr, ML; Rochette, R | 2012 | The effect of geographic origin on interactions between adult invasive green crabs carcinus maenas and juvenile American lobsters Homarus americanus in Atlantic Canada | The European green crab Carcinus maenas is an invasive species in the northwestern Atlantic that has the potential to compete and prey upon endemics such as juvenile American lobster Homarus americanus. Recent laboratory studies have reached opposite conclusions regarding the dominance relationship between these species, even documenting a reversal of roles as predator and prey. Interestingly, these contradicting results involved crab populations from different regions in Atlantic Canada (St. George’s Bay in the Northumberland Strait and Passamaquoddy Bay in the Bay of Fundy), which are genetically different and presumed to be the result of different invasion events. In this study we housed juvenile lobsters and adult green crabs together in 3-m diameter flow-through tanks, and monitored their mortality, growth and behaviour from September through December 2009. The experiment involved five treatments, three experimental treatments with 2.5 lobsters/m(2) housed with an equal density of green crabs from one of three regions (Passamaquoddy Bay, St. George’s Bay or Chedabucto Bay on the Scotian Shelf) and two control treatments with lobsters alone stocked at either 2.5 or 5 individuals/m(2). Chedabucto Bay was chosen as a third region as crabs there are genetically similar to conspecifics in St. George’s Bay, but experience environmental conditions more similar to Passamaquoddy Bay. Green crabs had a significant and negative impact on lobster survival during the experiment, and the magnitude of this impact varied in relation to the green crabs’ geographic origin, as mortality was significantly higher for lobsters paired with St. George’s Bay and Chedabucto Bay crabs than Passamaquoddy Bay crabs. These differences in lobster mortality patterns lead to two important conclusions. First, the greater predatory impact of green crabs from St. George’s Bay relative to Passamaquoddy Bay is consistent with the contrasting results recently reported in the literature, suggesting that these are of biological significance and not simply the result of procedural differences between studies. Second, the fact that the predatory impact of Chedabucto Bay green crabs was similar to that of conspecifics from St. Georges Bay, but greater than those from Passamaquoddy Bay, suggests that these differences in interspecific dominance patterns are correlated with genetic differences between green crab populations related to their invasion history in Atlantic Canada. The mechanisms underlying the effect of crab geographic origin on its interaction with juvenile lobster remain elusive, however, as no differences in crab behaviour or morphology were found that correlated with the pattern of lobster mortality. (C) 2012 Elsevier B.V. All rights reserved. | 3881 | NA | NA | NA | no | no |
| 3880 | 2017 | NA | Bergum, KE; Hild, AL; Mealor, BA | 2012 | Phenotypes of Two Generations of Sporobolus airoides Seedlings Derived from Acroptilon repens-invaded and Non-invaded Grass Populations | Although the ecological impacts of invasive species are well known, the evolutionary impacts on recipient native grass communities are not. We suggest that remnant native plants may provide desirable seed sources for restoration and native plant production. Native populations exposed to the selective pressures associated with exotic invasion may retain traits that increase their ability to coexist with invasive species. Two generations of Sporobolus airoides Torr. (Alkali sacaton) plants derived from lineages collected from within long-term invaded areas of Acroptilon repens (L.) DC (Russian knapweed) and from adjacent non-invaded areas were propagated in a greenhouse to evaluate generational changes in phenotypic traits from the production environment. Given the difference in invasion history of the two populations, we hypothesized that invaded and non-invaded subpopulations would differ phenotypically. Phenotypic measurements revealed that invaded subpopulations had greater vegetative growth, whereas non-invaded subpopulations had increased sexual reproduction. Phenotypic expression changed from the first to the second generation, predominantly in the invaded subpopulation. Generational phenotypic shifts are disadvantageous for native seed production which requires a standard product to sell commercially. However, phenotypic variation may improve field seed survival. This research demonstrates the potential value of targeting post-invasion remnant grass populations for restoration. | 3882 | NA | NA | NA | no | no |
| 3881 | 2017 | NA | Fernandez, FJ; Garces, F; Lopez-Estepa, M; Aguilar, J; Baldoma, L; Coll, M; Badia, J; Vega, MC | 2011 | The UlaG protein family defines novel structural and functional motifs grafted on an ancient RNase fold | Background: Bacterial populations are highly successful at colonizing new habitats and adapting to changing environmental conditions, partly due to their capacity to evolve novel virulence and metabolic pathways in response to stress conditions and to shuffle them by horizontal gene transfer (HGT). A common theme in the evolution of new functions consists of gene duplication followed by functional divergence. UlaG, a unique manganese-dependent metallo-beta-lactamase (MBL) enzyme involved in L-ascorbate metabolism by commensal and symbiotic enterobacteria, provides a model for the study of the emergence of new catalytic activities from the modification of an ancient fold. Furthermore, UlaG is the founding member of the so called UlaG like (UlaGL) protein family, a recently established and poorly characterized family comprising divalent (and perhaps trivalent) metal-binding MBLs that catalyze transformations on phosphorylated sugars and nucleotides. Results: Here we combined protein structure-guided and sequence-only molecular phylogenetic analyses to dissect the molecular evolution of UlaG and to study its phylogenomic distribution, its relatedness with present-day UlaGL protein sequences and functional conservation. Phylogenetic analyses indicate that UlaGL sequences are present in Bacteria and Archaea, with bona fide orthologs found mainly in mammalian and plant-associated Gram-negative and Gram-positive bacteria. The incongruence between the UlaGL tree and known species trees indicates exchange by HGT and suggests that the UlaGL-encoding genes provided a growth advantage under changing conditions. Our search for more distantly related protein sequences aided by structural homology has uncovered that UlaGL sequences have a common evolutionary origin with present-day RNA processing and metabolizing MBL enzymes widespread in Bacteria, Archaea, and Eukarya. This observation suggests an ancient origin for the UlaGL family within the broader trunk of the MBL superfamily by duplication, neofunctionalization and fixation. Conclusions: Our results suggest that the forerunner of UlaG was present as an RNA metabolizing enzyme in the last common ancestor, and that the modern descendants of that ancestral gene have a wide phylogenetic distribution and functional roles. We propose that the UlaGL family evolved new metabolic roles among bacterial and possibly archeal phyla in the setting of a close association with metazoans, such as in the mammalian gastrointestinal tract or in animal and plant pathogens, as well as in environmental settings. Accordingly, the major evolutionary forces shaping the UlaGL family include vertical inheritance and lineage-specific duplication and acquisition of novel metabolic functions, followed by HGT and numerous lineage-specific gene loss events. | 3883 | NA | NA | NA | no | no |
| 3882 | 2017 | NA | Evison, SEF; Foley, K; Jensen, AB; Hughes, WOH | 2015 | Genetic diversity, virulence and fitness evolution in an obligate fungal parasite of bees | Within-host competition is predicted to drive the evolution of virulence in parasites, but the precise outcomes of such interactions are often unpredictable due to many factors including the biology of the host and the parasite, stochastic events and co-evolutionary interactions. Here, we use a serial passage experiment (SPE) with three strains of a heterothallic fungal parasite (Ascosphaera apis) of the Honey bee (Apis mellifera) to assess how evolving under increasing competitive pressure affects parasite virulence and fitness evolution. The results show an increase in virulence after successive generations of selection and consequently faster production of spores. This faster sporulation, however, did not translate into more spores being produced during this longer window of sporulation; rather, it appeared to induce a loss of fitness in terms of total spore production. There was no evidence to suggest that a greater diversity of competing strains was a driver of this increased virulence and subsequent fitness cost, but rather that strain-specific competitive interactions influenced the evolutionary outcomes of mixed infections. It is possible that the parasite may have evolved to avoid competition with multiple strains because of its heterothallic mode of reproduction, which highlights the importance of understanding parasite biology when predicting disease dynamics. | 3884 | NA | NA | NA | no | no |
| 3883 | 2017 | NA | Auffarth, J; Krug, A; Prohl, H; Jehle, R | 2017 | A genetically-informed Population Viability Analysis reveals conservation priorities for an isolated population of Hyla arborea | Population Viability Analysis (PVA) is a commonly used tool to predict the fate of endangered populations. However, although amphibians are the most endangered group of vertebrates, PVAs have so far been underrepresented in their conservation management. In the last decades, the European tree frog (Hyla arborea) has experienced drastic declines mainly caused by habitat fragmentation and loss of suitable breeding sites. In the present study, we used the PVA software VORTEX to predict the viability of a H. arborea population of about 70 adults inhabiting an isolated pond in the region of Hannover (Germany), by combining life history data with genotypic information derived from eight polymorphic microsatellite markers. Our PVA revealed a high probability of extinction within the next 50 years, with juvenile survival being a crucial demographic parameter for population persistence. Simulated immigration through metapopulation processes or population supplementation prevented genetic erosion, and markedly increased the probability of population survival. Future management interventions should consider pond management to enhance survival at early stages, and the creation of migration corridors to facilitate connectivity with adjacent demes and/or the translocation of individuals. To our knowledge, this is one of the first studies that applies a genetically-informed PVA to the management of endangered anuran amphibians. | 3885 | NA | NA | NA | no | no |
| 3884 | 2017 | NA | Warden, G; Harnett, D; Green, J; Wish, T; Woods, MO; Green, R; Dicks, E; Rahman, P; Zhai, G; Parfrey, P | 2013 | A population-based study of hereditary non-polyposis colorectal cancer: evidence of pathologic and genetic heterogeneity | Hereditary non-polyposis colorectal cancer (HNPCC) may be the result of Lynch syndrome (LS) caused by mutations in mismatch repair (MMR) genes, a syndrome of unknown etiology called familial colorectal cancer type-X (FCCTX), or familial serrated neoplasia associated with the colorectal cancer (CRC) somatic BRAF mutation. To determine the cause of HNPCC in the founder population of the island of Newfoundland, we studied 37 families with LS and 29 families without LS who fulfilled the Amsterdam I criteria. In non-LS, four index CRCs were BRAF mutation positive, one of which was microsatellite instable. Geographic clustering of LS families caused by three different founder mutations in MSH2 was observed. Nine unique MMR mutations in four MMR genes were identified in single families distributed in different geographic isolates. The geographic distribution of non-LS was similar to LS. The coefficient of relatedness using genotype data was significantly higher for non-LS than for all CRC. Extensive genealogic investigation failed to connect non-LS families and in some clusters pathologic CRC heterogeneity was observed. We conclude that non-LS HNPCC may be a heterogeneous disorder with different pathogenic pathways, and that the geographic distribution is consistent with multiple different mutations in unknown CRC susceptibility gene(s). | 3886 | NA | NA | NA | no | no |
| 3885 | 2017 | NA | Zbinden, M; Haag, CR; Ebert, D | 2008 | Experimental evolution of field populations of Daphnia magna in response to parasite treatment | Although there is little doubt that hosts evolve to reduce parasite damage, little is known about the evolutionary time scale on which host populations may adapt under natural conditions. Here we study the effects of selection by the microsporidian parasite Octosporea bayeri on populations of Daphnia magna. In a field study, we infected replicated populations of D. magna with the parasite, leaving control populations uninfected. After two summer seasons of experimental evolution (about 15 generations), the genetic composition of infected host populations differed significantly from the control populations. Experiments revealed that hosts from the populations that had evolved with the parasite had lower mortality on exposure to parasite spores and a higher competitive ability than hosts that had evolved without the parasite. In contrast, the susceptibility of the two treatment groups to another parasite, the bacterium Pasteuria ramosa, which was not present during experimental evolution of the populations, did not differ. Fitness assays in the absence of parasites revealed a higher fitness for the control populations, but only under low population density with high resource availability. Overall, our results show that, under natural conditions, Daphnia populations are able to adapt rapidly to the prevailing conditions and that this evolutionary change is specific to the environment. | 3887 | NA | NA | NA | no | no |
| 3886 | 2017 | NA | Jalvingh, KM; Chang, PL; Nuzhdin, SV; Wertheim, B | 2014 | Genomic changes under rapid evolution: selection for parasitoid resistance | In this study, we characterize changes in the genome during a swift evolutionary adaptation, by combining experimental selection with high-throughput sequencing. We imposed strong experimental selection on an ecologically relevant trait, parasitoid resistance in Drosophila melanogaster against Asobara tabida. Replicated selection lines rapidly evolved towards enhanced immunity. Larval survival after parasitization increased twofold after just five generations of selection. Whole-genome sequencing revealed that the fast and strong selection response in innate immunity produced multiple, highly localized genomic changes. We identified narrow genomic regions carrying a significant signature of selection, which were present across all chromosomes and covered in total less than 5% of the whole D. melanogaster genome. We identified segregating sites with highly significant changes in frequency between control and selection lines that fell within these narrow ‘selected regions’. These segregating sites were associated with 42 genes that constitute possible targets of selection. A region on chromosome 2R was highly enriched in significant segregating sites and may be of major effect on parasitoid defence. The high genetic variability and small linkage blocks in our base population are likely responsible for allowing this complex trait to evolve without causing widespread erosive effects in the genome, even under such a fast and strong selective regime. | 3888 | NA | NA | NA | no | no |
| 3887 | 2017 | NA | Saleem, H; Nahid, N; Shakir, S; Ijaz, S; Murtaza, G; Khan, AA; Mubin, M; Nawaz-ul-Rehman, MS | 2016 | Diversity, Mutation and Recombination Analysis of Cotton Leaf Curl Geminiviruses | The spread of cotton leaf curl disease in China, India and Pakistan is a recent phenomenon. Analysis of available sequence data determined that there is a substantial diversity of cotton-infecting geminiviruses in Pakistan. Phylogenetic analyses indicated that recombination between two major groups of viruses, cotton leaf curl Multan virus (CLCuMuV) and cotton leaf curl Kokhran virus (CLCuKoV), led to the emergence of several new viruses. Recombination detection programs and phylogenetic analyses showed that CLCuMuV and CLCuKoV are highly recombinant viruses. Indeed, CLCuKoV appeared to be a major donor virus for the coat protein (CP) gene, while CLCuMuV donated the Rep gene in the majority of recombination events. Using recombination free nucleotide datasets the substitution rates for CP and Rep genes were determined. We inferred similar nucleotide substitution rates for the CLCuMuV-Rep gene (4.96X10(-4)) and CLCuKoV-CP gene (2.706X10(-4)), whereas relatively higher substitution rates were observed for CLCuMuV-CP and CLCuKoV-Rep genes. The combination of sequences with equal and relatively low substitution rates, seemed to result in the emergence of viral isolates that caused epidemics in Pakistan and India. Our findings also suggest that CLCuMuV is spreading at an alarming rate, which can potentially be a threat to cotton production in the Indian subcontinent. | 3889 | NA | NA | NA | no | no |
| 3888 | 2017 | NA | Ferrero, E; Saccucci, F; Malavasi, F | 1999 | The human CD38 gene: polymorphism, CpG island, and linkage to the CD157 (BST-1) gene | dCD38 is a leukocyte activation antigen and ectoenzyme [NAD(P)(+) glycohydrolase; EC 3.2.2.6] involved in numerous immune functions. The human CD38 gene is complex [eight exons, >80 kilobases (kb) long] located on Chromosome 4p15, and part of the eukaryotic NAD(+) glycohydrolase/ADP-ribosyl cyclase gene family. Because of the increasing relevance of the CD38 molecule in the host immune response to infectious, tumoral, and metabolic diseases, we investigated the genetic variability and linkage of the human CD38 locus. We report that (1) the restriction endonuclease Pvu II identifies a bi-allelic polymorphism here defined as formed by the alleles CD38*A (12 kb) and CD38*B (9/2.5 kb); (2) their frequency in the healthy Italian Caucasian population is 14% and 86%, respectively; (3) the polymorphic Pvu II site is located at the 5’ end of the first intron of the CD38 gene; (4) in conjunction with the polymorphic site, we identified a 900 base pair CPG island associated with the CD38 gene, with two potential Spl binding sites; (5) the CpG island may play a role in the regulation of CD38 expression and is hypomethylated in various cell lines; (6) by pulsed-field gel electrophoresis we show that,CD38 and its paralogue, the bone-marrow stromal cell antigen BST-I (CD157), map to the same 800 kb Avi II fragment, indieating that the two human ecto-NADase genes are closely linked. | 3890 | NA | NA | NA | no | no |
| 3889 | 2017 | NA | Hokit, DG; Blaustein, AR | 1997 | Effects of kinship on interactions between tadpoles of Rana cascadae | In a series of four field and mesocosm studies, we examined the effects of kinship on growth, survival, and aggregation behavior in tadpoles of the Cascades frog (Rana cascadae) under different ecological conditions. (1) Field observations of tadpole aggregations revealed ecological factors that may interact with kinship to influence tadpole interactions. (2) In an experiment conducted in mesocosms, food distribution, predator presence, thermal heterogeneity, and kinship all interacted to influence tadpole dispersion. (3) In a field experiment conducted in natural ponds, kinship significantly influenced group size at artificially established food patches. (4) In a field enclosure experiment, density, access to the substrate, and kinship all interacted to significantly influence growth and survivorship of tadpoles. Subsequent analysis revealed that kinship affected the distribution of individual tadpole body size: mass was more skewed (i.e., more small individuals) in groups of mixed relatedness than in kin groups. The interaction among kinship, density, and substrate factors resulted in context-dependent effects on tadpole size distribution and survivorship. Our results demonstrate that kinship factors can influence competitive interactions between tadpoles depending upon the ecological conditions, and they provide a functional explanation for kin-discriminating behavior observed in tadpoles of R. cascadae. Kinship-mediated competition may be particularly important for recruitment in size-structured populations. | 3891 | NA | NA | NA | no | no |
| 3890 | 2017 | NA | Abu-Asab, MS; Chaouchi, M; Amri, H | 2008 | Phylogenetic modeling of heterogeneous gene-expression microarray data from cancerous specimens | The qualitative dimension of gene expression data and its heterogeneous nature in cancerous specimens can be accounted for by phylogenetic modeling that incorporates the directionality of altered gene expressions, complex patterns of expressions among a group of specimens, and data-based rather than specimen-based gene linkage. Our phylogenetic modeling approach is a double algorithmic technique that includes polarity assessment that brings out the qualitative value of the data, followed by maximum parsimony analysis that is most suitable for the data heterogeneity of cancer gene expression. We demonstrate that polarity assessment of expression values into derived and ancestral states, via outgroup comparison, reduces experimental noise; reveals dichotomously expressed asynchronous genes; and allows data pooling as well as comparability of intra- and interplatforms. Parsimony phylogenetic analysis of the polarized values produces a multidimensional classification of specimens into clades that reveal shared derived gene expressions (the synapomorphies); provides better assessment of ontogenic pathways and phyletic relatedness of specimens; efficiently utilizes dichotomously expressed genes; produces highly predictive class recognition; illustrates gene linkage and multiple developmental pathways; provides higher concordance between gene lists; and projects the direction of change among specimens. Further implication of this phylogenetic approach is that it may transform microarray into diagnostic, prognostic, and predictive tool. | 3892 | NA | NA | NA | no | no |
| 3891 | 2017 | NA | Kozub, NA; Sozinov, IA; Karelov, AV; Blume, YB; Sozinov, AA | 2017 | Diversity of Ukrainian Winter Common Wheat Varieties with Respect to Storage Protein Loci and Molecular Markers for Disease Resistance Genes(1) | Diversity of Ukrainian winter common wheat varieties was studied with respect to the storage protein loci Gli-A1, Gli-B1, Gli-D1, Glu-A1, Glu-B1, Glu-D1, Gli-A3, Gli-B5, and Gli-A6 (362 varieties) and markers for the Lr34/Yr18/Pm38/Sr57/Bdv1 gene conferring moderate resistance to a number of biotrophic pathogens, the Tsn1 gene for sensitivity to the toxins A of the necrotrophic fungi Pyrenophora tritici-repentis and Stagonospora nodorum, the Tsc2 gene for sensitivity to the toxin B of P. tritici-repentis, and the TDF\_076\_2D gene for moderate resistance to Fusarium head blight (181 varieties). Significant differences in frequencies of alleles at these marker loci between groups of varieties developed in different soil and climatic zones were revealed. The retention of a set of predominant alleles in groups of varieties of a certain zone in different periods of breeding was confirmed. At the same time, the appearance of new allele associations in the groups of varieties of the Steppe (in particular Gli-A1g and Glu-B1al) and the Central Forest-Steppe (1AL/1RS and Glu-B1d) in the last two decades has been noted. Nonrandom associations between alleles of disease resistance genes as well as alleles of disease resistance genes and storage protein alleles were revealed. | 3893 | NA | NA | NA | no | no |
| 3892 | 2017 | NA | Schwarz, RS; Moran, NA; Evans, JD | 2016 | Early gut colonizers shape parasite susceptibility and microbiota composition in honey bee workers | Microbial symbionts living within animal guts are largely composed of resident bacterial species, forming communities that often provide benefits to the host. Gut microbiomes of adult honey bees (Apis mellifera) include core residents such as the betaproteobacterium Snod-grassella alvi, alongside transient parasites such as the protozoan Lotmaria passim. To test how these species affect microbiome composition and host physiology, we administered S. alvi and/or L. passim inocula to newly emergedworker bees from four genetic backgrounds (G(H)) and reared them in normal (within hives) or stressed (protein-deficient, asocial) conditions. Microbiota acquired by normal bees were abundant but quantitatively differed across treatments, indicating treatment-associated dysbiosis. Pretreatment with S. alvi made normal bees more susceptible to L. passim and altered developmental and detoxification gene expression. Stressed bees were more susceptible to L. passim and were depauperate in core microbiota, yet supplementation with S. alvi did not alter this susceptibility. Microbiomes were generally more variable by G(H) in stressed bees, which also showed opposing and comparatively reduced modulation of gene expression responses to treatments compared with normal bees. These data provide experimental support for a link between altered gut microbiota and increased parasite and pathogen prevalence, as observed from honey bee colony collapse disorder. | 3894 | NA | NA | NA | no | no |
| 3893 | 2017 | NA | Xavier, MPTP; Oliveira, SA; Ferreira, MSR; Victoria, M; Miranda, V; Silva, MFM; Strina, A; Barreto, ML; Miagostovicht, MP; Leite, JPG | 2009 | Detection of caliciviruses associated with acute infantile gastroenteritis in Salvador, an urban center in Northeast Brazil | Acute gastroenteritis caused by viruses is one of the leading causes of infantile morbidity. The aim of the present study was to investigate the presence of human caliciviruses of the genera norovirus and sapovirus in children up to 3 years of age with acute gastroenteritis from low-income communities in the city of Salvador, Brazil. This study is an extension of previous work carried out to establish the profile of the most prevalent enteric pathogens present in these communities. In this report, 139 fecal samples, collected from July 2001 to January 2002 were analyzed by RT-PCR and 13 (9%) were positive for human caliciviruses. By sequencing, seven isolates were characterized as norovirus genogroup GII and one as sapovirus genotype GII/1. Sequencing of the previously detected group-A rotaviruses and human astroviruses was also performed and revealed the circulation of rotavirus group A genotypes G1P[8] and G9P[8], and human astrovirus genotypes 6, 7, and 8. No mixed infection was observed. Community-based studies provide geographically representative information on disease burden. However, there are only a few reports in developing countries concerning the genotypes of the most important gastroenteric viruses detected in such communities. The present findings demonstrate the wide diversity of genotypes of the most important viruses responsible for acute gastroenteritis circulating in low-income communities. | 3895 | NA | NA | NA | no | no |
| 3894 | 2017 | NA | Tatin, L; Darreh-Shoori, BF; Tourenq, C; Tatin, D; Azmayesh, B | 2003 | The last populations of the Critically Endangered onager Equus hemionus onager in Iran: urgent requirements for protection and study | The onager Equus hemionus onager, a wild ass endemic to Iran, is categorized as Critically Endangered on the IUCN Red List. Its biology and conservation requirements are poorly documented. We report our observations, made in 1997 and 2000, on the behaviour and ecology of the two remaining populations, located in the Touran Protected Area and the Bahram-e-Goor Reserve. Recent population counts by the Department of Environment of Iran (471 in the Protected Area and 96 in the Reserve) are markedly lower than the estimate of 600-770 made in the 1970s in the Touran Protected Area. We observed social interactions between stallions and mares outside the breeding season that contrasts with the known social structure of this subspecies. Poaching, competition with domestic animals, removal of shrubs for domestic use, and land conversion have been identified as the main threats to the two remaining onager populations. In addition, geographical isolation could cause the loss of genetic variability in these two relatively small populations, and also makes them more susceptible to the potential effects of stochastic events such as drought or disease. Public awareness, appropriate protection, and scientific studies must be urgently supported by both national and international organizations in order to prevent the extinction of these two apparently dwindling populations of onager. | 3896 | NA | NA | NA | no | no |
| 3895 | 2017 | NA | Taylor, RC; Singhal, M; Weller, J; Khoshnevis, S; Shi, L; McDermott, J | 2009 | A Network Inference Workflow Applied to Virulence-Related Processes in Salmonella typhimurium | Inference of the structure or mRNA transcriptional regulatory networks, protein regulatory or interaction networks, and protein activation/inactivation-based signal transduction networks are critical tasks in systems biology. In this article we discuss a workflow for the reconstruction of parts of the transcriptional regulatory network of the pathogenic bacterium Salmonella typhimurium based on the information contained in sets of microarray gene-expression data now available for that organism and describe our results obtained by following this workflow. The primary tool is one of the network-inference algorithms deployed in the Software Environment for Biological Network Inference (SEBINI). Specifically, we selected the algorithm called context likelihood of relatedness (CLR), which uses the mutual information contained in the gene-expression data to infer regulatory connections. The associated analysis pipeline automatically stores the inferred edges from the CLR runs within SEBINI and, upon request, transfers the inferred edges into either Cytoscape or the plug-in Collective Analysis of Biological Interaction Networks (CABIN) tool for further postanalysis of the inferred regulatory edges. The following article presents the outcome of this workflow, as well as the protocols followed for microarray data collection, data cleansing, and network inference. Our analysis revealed several interesting interactions, functional groups, metabolic pathways, and regulons in S. typhimurium. | 3897 | NA | NA | NA | no | no |
| 3896 | 2017 | NA | Fuchs, TM | 1998 | Molecular mechanisms of bacterial pathogenicity | Cautious optimism has arisen over recent decades with respect to the long struggle against bacteria, viruses, and parasites. This has been offset, however, by a fatal complacency stemming from previous successes such as the development of antimicrobial drugs, the eradication of smallpox, and global immunization programs. Infectious diseases nevertheless remain the world’s leading cause of death, killing at least 17 million persons annually [61]. Diarrheal diseases caused by Vibrio cholerae or Shigella dysenteriae kill about 3 million persons every year, most of them young children: Another 4 million die of tuberculosis or tetanus. Outbreaks of diphtheria in Eastern Europe threatens the population with a disease that had previously seemed to be overcome. Efforts to control infectious diseases more comprehensively are undermined not only by socioeconomic conditions but also by the nature of the pathogenic organisms itself; some isolates of Staphylococcus aureus and Enterobacter have become so resistant to drugs by horizontal gene transfer that they are almost untreatable. In addition, the mechanism of genetic variability helps pathogens to evade the human immune system, thus compromising the development of powerful vaccines. Therefore detailed knowledge of the molecular mechanisms of microbial pathogenicity is absolutely necessary to develop new strategies against infectious diseases and thus to lower their impact on human health and social development. | 3898 | NA | NA | NA | no | no |
| 3897 | 2017 | NA | Guida, M; Riedy, M; Lee, D; Hall, J | 2000 | Characterization of two highly polymorphic human tryptase loci and comparison with a newly discovered monkey tryptase ortholog | Tryptases are serine proteases involved in mast cell-mediated inflammatory responses which represent potential targets of drugs against diseases such as asthma, arthritis and inflammatory bowel disease. In order to interpret pharmacodynamic data on the tryptase inhibitors undergoing clinical trials, we defined the genetic variability of the tryptase 1 (TPS1) and tryptase 2 (TPS2) loci by screening a reference population of 32 individuals representing three major ethnic groups (Caucasian, African American, Asian). Using overlapping PCR products, we resequenced the entire tryptase genes with the only exclusion of TPS2 intron 1 and 20 bp of TPS2 5’ untranslated region included in exon 1 and we identified 21 novel single nucleotide polymorphisms in TPS1 and 17 single nucleotide polymorphisms plus a large polymorphic deletion in the TPS2: gene. We also compared the type, frequency and distribution of single nucleotide polymorphisms in TPS1 and TPS2 and we observed that the polymorphism frequency within these two loci is unexpectedly high (approximately 1 SNP every 90 bp) and that some of the allele frequencies differ significantly among the three ethnic groups. Based on differences observed in preclinical studies using a cynomolgus monkey (Macaca fascicularis) asthma model system, we investigated the difference between monkey and human tryptase genes in order to better understand the mechanism of action of our tryptase inhibitors. Pharmacogenetics 10:389-396 (C) 2000 Lippincott Williams & Wilkins. | 3899 | NA | NA | NA | no | no |
| 3898 | 2017 | NA | Port, M; Johnstone, RA | 2013 | Facing the crowd: intruder pressure, within-group competition, and the resolution of conflicts over group-membership | Recent theory in social evolution has been mainly concerned with competition and cooperation within social groups of animals and their impact on the stability of those groups. Much less attention has been paid to conflicts arising as a result of solitary floaters (outsiders) attempting to join groups of established residents (insiders). We model such conflicts over group-membership using a demographically explicit approach in which the rates of births and deaths in a population determine the availability of group-vacancies and the number of floaters competing over these vacancies. We find that the outcome of within-group competition, reflected in the partitioning of reproduction among group members, exerts surprisingly little influence on the resolution of insider-outsider conflict. The outcome of such conflict is also largely unaffected by differences in resource holding potential between insiders and outsiders. By contrast, whether or not groups form is mainly determined by demographic factors (variation in vital rates such as fecundity and mortality) and the resulting population dynamics. In particular, at high floater densities territory defense becomes too costly, and groups form because insiders give in to the intruder pressure imposed on them by outsiders. We emphasize the importance of insider-outsider conflicts in social evolution theory and highlight avenues for future research. | 3900 | NA | NA | NA | no | no |
| 3899 | 2017 | NA | Keesen, F; Silva, ACE; Arashiro, E; Pinheiro, CFS | 2017 | Simulations of populations of Sapajus robustus in a fragmented landscape | The study of populations subject to the phenomenon of loss and fragmentation of habitat, transforming continuous areas into small ones, usually surrounded by anthropogenic matrices, has been the focus of many researches within the scope of conservation. The objective of this study was to develop a computer model by introducing modifications to the renowned Penna model for biological aging, in order to evaluate the behavior of populations subjected to the effects of fragmented environments. As an object of study, it was used biological data of the robust tufted capuchin (Sapajus robustus), an endangered primate species whose geographical distribution within the Atlantic Rain Forest is part of the backdrop of intense habitat fragmentation. The simulations showed the expected behavior based on the three main aspects that affects populations under intense habitat fragmentation: the population density, area and conformation of the fragments and deleterious effects due the low genetic variability in small and isolated populations. The model showed itself suitable to describe changes in viability and population dynamics of the species crested capuchin considering critical levels of survival in a fragmented environment and also, actions in order to preserve the species should be focused not only on increasing available area but also in dispersion dynamics. (C) 2016 Elsevier B.V. All rights reserved. | 3901 | NA | NA | NA | no | no |
| 3900 | 2017 | NA | Purcell, J; Chapuisat, M | 2014 | Foster carers influence brood pathogen resistance in ants | Social organisms face a high risk of epidemics, and respond to this threat by combining efficient individual and collective defences against pathogens. An intriguing and little studied feature of social animals is that individual pathogen resistance may depend not only on genetic or maternal factors, but also on the social environment during development. Here, we used a cross-fostering experiment to investigate whether the pathogen resistance of individual ant workers was shaped by their own colony of origin or by the colony of origin of their carers. The origin of care-giving workers significantly influenced the ability of newly eclosed cross-fostered Formica selysi workers to resist the fungal entomopathogen Beauveria bassiana. In particular, carers that were more resistant to the fungal entomopathogen reared more resistant workers. This effect occurred in the absence of post-infection social interactions, such as trophallaxis and allogrooming. The colony of origin of eggs significantly influenced the survival of the resulting individuals in both control and pathogen treatments. There was no significant effect of the social organization (i.e. whether colonies contain a single or multiple queens) of the colony of origin of either carers or eggs. Our experiment reveals that social interactions during development play a central role in moulding the resistance of emerging workers. | 3902 | NA | NA | NA | no | no |
| 3901 | 2017 | NA | Webster, JP; Gower, CM; Blair, L | 2004 | Do hosts and parasites coevolve? Empirical support from the Schistosoma system | Coevolution between host and parasite is, in principle, a powerful determinant of the biology and genetics of infection and disease. However, coevolution is difficult to demonstrate rigorously in practice and therefore has rarely been observed empirically, particularly in animal-parasite systems. Research on host-schistosome interactions has the potential for making an important contribution to the study of coevolution or reciprocal adaptation. This may be particularly pertinent because schistosomes represent an indirectly transmitted macroparasite, so often overlooked among both theoretical and empirical studies. Here we present ideas and experiments on host-schistosome interactions, in part reviewed from published work but focusing in particular on preliminary novel data from our ongoing studies of potential host-schistosome evolution and coevolution in the laboratory. The article is split into three main sections: we first focus on the evidence for evolution in the host, then in the parasite, before combining both to illustrate the gathering evidence of host-parasite coevolution in the snail-schistosome system. In particular, we demonstrate that genetic architecture, variability, and selective pressures are present for the evolution of resistance and susceptibility virulence, and infectivity to occur, the mechanisms allowing such polymorphisms to be maintained, and that hosts and parasites appear to have reciprocal effects on each other’s phenotype and genotype. | 3903 | NA | NA | NA | no | no |
| 3902 | 2017 | NA | Ausband, DE; Mitchell, MS; Stansbury, CR; Stenglein, JL; Waits, LP | 2017 | Harvest and group effects on pup survival in a cooperative breeder | Recruitment in cooperative breeders can be negatively affected by changes in group size and composition. The majority of cooperative breeding studies have not evaluated human harvest; therefore, the effects of recurring annual harvest and group characteristics on survival of young are poorly understood. We evaluated how harvest and groups affect pup survival using genetic sampling and pedigrees for grey wolves in North America. We hypothesized that harvest reduces pup survival because of (i) reduced group size, (ii) increased breeder turnover and/or (iii) reduced number of female helpers. Alternatively, harvest may increase pup survival possibly due to increased per capita food availability or it could be compensatory with other forms of mortality. Harvest appeared to be additive because it reduced both pup survival and group size. In addition to harvest, turnover of breeding males and the presence of older, non-breeding males also reduced pup survival. Large groups and breeder stability increased pup survival when there was harvest, however. Inferences about the effect of harvest on recruitment require knowledge of harvest rate of young as well as the indirect effects associated with changes in group size and composition, as we show. The number of young harvested is a poor measure of the effect of harvest on recruitment in cooperative breeders. | 3904 | NA | NA | NA | no | no |
| 3903 | 2017 | NA | Abdulsalam, AM; Ithoi, I; Al-Mekhlafi, HM; Al-Mekhlafi, AM; Ahmed, A; Surin, J | 2013 | Subtype Distribution of Blastocystis Isolates in Sebha, Libya | Background: Blastocystis is a genetically diverse and a common intestinal parasite of humans with a controversial pathogenic potential. This study was carried out to identify the Biastocystis subtypes and their association with demographic and socioeconomic factors among outpatients living in Sebha city, Libya. Methods/Findings: Blastocystis in stool samples were cultured followed by isolation, PCR amplification of a partial SSU rDNA gene, cloning, and sequencing. The DNA sequences of isolated clones showed 98.3% to 100% identity with the reference Blastocystis isolates from the Genbank. Multiple sequence alignment showed polymorphism from one to seven base substitution and/or insertion/deletion in several groups of non-identical nucleotides clones. Phylogenetic analysis revealed three assemblage subtypes (ST) with ST1 as the most prevalent (51.1%) followed by ST2 (24.4%), ST3 (17.8%) and mixed infections of two concurrent subtypes (6.7%). Blastocystis: ST1 infection was significantly associated with female (P = 0.009) and low educational level (P = 0.034). ST2 was also significantly associated with low educational level (P= 0.008) and ST3 with diarrhoea (P = 0.008). Conclusion: Phylogenetic analysis of Libyan Blastocystis isolates identified three different subtypes; with ST1 being the predominant subtype and its infection was significantly associated with female gender and low educational level. More extensive studies are needed in order to relate each Blastocystis subtype with clinical symptoms and potential transmission sources in this community. | 3905 | NA | NA | NA | no | no |
| 3904 | 2017 | NA | Rabosky, ARD; Corl, A; Liwanag, HEM; Surget-Groba, Y; Sinervo, B | 2012 | Direct Fitness Correlates and Thermal Consequences of Facultative Aggregation in a Desert Lizard | Social aggregation is a common behavioral phenomenon thought to evolve through adaptive benefits to group living. Comparing fitness differences between aggregated and solitary individuals in nature - necessary to infer an evolutionary benefit to living in groups - has proven difficult because communally-living species tend to be obligately social and behaviorally complex. However, these differences and the mechanisms driving them are critical to understanding how solitary individuals transition to group living, as well as how and why nascent social systems change over time. Here we demonstrate that facultative aggregation in a reptile (the Desert Night Lizard, Xantusia vigilis) confers direct reproductive success and survival advantages and that thermal benefits of winter huddling disproportionately benefit small juveniles, which can favor delayed dispersal of offspring and the formation of kin groups. Using climate projection models, however, we estimate that future aggregation in night lizards could decline more than 50% due to warmer temperatures. Our results support the theory that transitions to group living arise from direct benefits to social individuals and offer a clear mechanism for the origin of kin groups through juvenile philopatry. The temperature dependence of aggregation in this and other taxa suggests that environmental variation may be a powerful but underappreciated force in the rapid transition between social and solitary behavior. | 3906 | NA | NA | NA | no | no |
| 3905 | 2017 | NA | Obrepalska-Steplowska, A; Budziszewska, M; Wieczorek, P; Czerwoniec, A | 2012 | Analysis of two strains of Peanut stunt virus: satRNA-associated and satRNA free | Peanut stunt virus (PSV) is a pathogen of legumes, vegetables, trees, and weeds occurring worldwide. The species is characterized by significant genetic variability. PSV strains are classified into four subgroups on the basis of their nucleotide sequence homology. Here, we are presenting two further, fully sequenced PSV strains-PSV-Ag and PSV-G, that could be considered as I subgroup representatives. However, their sequence homology with other typical I subgroups members, similarly as another strain-PSV-P, characterized by our group previously, is lower than 90%. This lead us to propose further subdivision of the I subgroup into IA, IB, and IC units, and to classify PSV-Ag and PSV-G strains to the last one. In this article, we are showing that identity level of PSV-Ag and PSV-G is very high and apart from the presence of satRNA in the first one, they differ only by a few nucleotides in their genomic RNAs. Nevertheless, symptoms they cause on host plants might differ significantly, just as the levels in infected plants. Effect of single amino acid changes between strains on the three-dimensional structure of viral proteins was analyzed. Differences occur mainly on the protein surfaces which can possibly affect protein-protein interaction in infected cells, which is discussed. | 3907 | NA | NA | NA | no | no |
| 3906 | 2017 | NA | Kuparinen, A; Hutchings, JA; Waples, RS | 2016 | Harvest-induced evolution and effective population size | Much has been written about fishery-induced evolution (FIE) in exploited species, but relatively little attention has been paid to the consequences for one of the most important parameters in evolutionary biology-effective population size (N-e). We use a combination of simulations of Atlantic cod populations experiencing harvest, artificial manipulation of cod life tables, and analytical methods to explore how adding harvest to natural mortality affects N-e, census size (N), and the ratio N-e/N. We show that harvest-mediated reductions in N-e are due entirely to reductions in recruitment, because increasing adult mortality actually increases the N-e/N ratio. This means that proportional reductions in abundance caused by harvest represent an upper limit to the proportional reductions in N-e, and that in some cases N-e can even increase with increased harvest. This result is a quite general consequence of increased adult mortality and does not depend on harvest selectivity or FIE, although both of these influence the results in a quantitative way. In scenarios that allowed evolution, N-e recovered quickly after harvest ended and remained higher than in the preharvest population for well over a century, which indicates that evolution can help provide a long-term buffer against loss of genetic variability. | 3908 | NA | NA | NA | no | no |
| 3907 | 2017 | NA | Garrido, M; Perez-Mellado, V | 2013 | Prevalence and intensity of blood parasites in insular lizards | Parasites are important in understanding population dynamics and ecology of hosts. In several Mediterranean islands, little is known about lizards’ blood parasites. We studied the relations between haemoparasites and an endemic lizard host, Podarcis lilfordi (Gunther, 1874) on Aire Island (Menorca, Balearic Islands, Spain). The infection intensity was about 1% of the red blood cells and 95% of the individuals were infected. These values are higher than in continental populations of the genus Podarcis. The reduced genetic variability and/or the high density in islands are proposed to explain such differences. More adults than juveniles were infected, probably because they were more often exposed to parasites during their lifetime. In adult lizards, prevalence was higher in males, may be as a consequence of their higher activity levels and more frequent physical contacts during male-male interactions. We found that infected individuals were larger than uninfected ones. A higher prevalence in adults is responsible of these differences. The intensity of infection was similar between age and sex classes. Prevalence remains stable between seasons, while parasite load increases in summer, as the availability of resources and body condition decreases. We found a relation between prevalence, intensity and body condition, with a higher prevalence and parasite load in individuals with lower condition. (C) 2012 Elsevier GmbH. All rights reserved. | 3909 | NA | NA | NA | no | no |
| 3908 | 2017 | NA | Ashraf, W; Sahi, ST; ul Haq, I; Ahmed, S | 2015 | Morphological and Pathogenic Variability among Macrophomina phaseolina Isolates Associated with Maize (Zea mays) in Punjab-Pakistan | Macrophomina phaseolina (Tassi) Goid. is a serious pathogen of charcoal rot disease in the maize plant recently in Punjab, Pakistan. In order to initiate monitoring of this pathogen, 24 isolates of M. phaseolina from four districts of Punjab province of Pakistan were collected to assess the variations in morphology and virulence on the maize plant. Regarding to the geographic origins, significant differences were observed in radial mycelial growth, sclerotial size, sclerotial population per unit area and virulence among the isolates. Thirteen isolates were rated as fast growing (77.10. 88.10 mm), seven as medium growing (66.00-77.00mm), and the rest as slow growing (54.00-65.00 mm). Eight isolates produced large sized sclerotia (> 45 mu m), eleven produced medium sized (40-45 mu m), and the remaining five isolates produced small sized sclerotia (< 40 mu m). Three isolates exhibited abundant sclerotial population (91.10-108.10), six exhibited average sclerotial population (74.00-90.00), while the rest of the isolates exhibited low sclerotial population (56.00-73.00)/9mm disc of culture. Nine isolates of diverse origin proved to be highly virulent; eight isolates were regarded as moderately and seven fungal isolates appeared to be least virulent against maize cultivars. The discussion is about these morphological and pathogenic variations in various isolates of M. phaseolina as base line information for disease management and development of resistant cultivars against charcoal rot disease. (C) 2015 Friends Science Publishers | 3910 | NA | NA | NA | no | no |
| 3909 | 2017 | NA | Fazeli, R; Heydarnejad, J; Massumi, H; Shaabanian, M; Varsani, A | 2009 | Genetic diversity and distribution of tomato-infecting begomoviruses in Iran | The incidence and severity of tomato leaf curl disease (TLCD) is increasing worldwide. Here we assess the diversity and distribution within tomato producing areas of Iran of begomoviruses that cause this disease. Tomato with typical TLCD symptoms and asymptomatic weeds were collected in 2005 and 2006 and tested for the presence of begomovirus DNA using polymerase chain reaction (PCR). Analysis of cloned and sequenced PCR products revealed that both mono- and bipartite begomoviruses are associated with TLCD in Iran. Furthermore, our results confirmed the symptomless infection with mono- and bipartite begomoviruses of two weed species, Chrozophora hierosolymitana Spreng (Euphobiaceae) and Herniaria sp. (Caryophyllaceae). Eighteen Iranian begomovirus isolates were classified into two major groups and two or three subgroups according to the 5’-proximal 200 nucleotides of the coat protein (CP) gene or the N-terminal 600 nucleotides of the Rep gene. Whereas most of the monopartite isolates showed closest similarity to tomato yellow leaf curl virus-Gezira (TYLCV-Ge), the three bipartite isolates were most similar to Tomato leaf curl New Delhi virus (ToLCNDV). Mixed mono- and a bipartite begomovirus infections were detected in both tomato and C. hierosolymitana. Our results indicate that the tomato producing areas in central, southern, and southeastern Iran are threatened by begomoviruses originating from both the Mediterranean basin and the Indian subcontinent. | 3911 | NA | NA | NA | no | no |
| 3910 | 2017 | NA | Rioux, R; Manmathan, H; Singh, P; de los Reyes, B; Jia, YL; Tavantzis, S | 2011 | Comparative analysis of putative pathogenesis-related gene expression in two Rhizoctonia solani pathosystems | Rhizoctonia solani, teleomorph Thanatephorus cucumeris, is a polyphagous necrotrophic plant pathogen of the Basidiomycete order that is split into 14 different anastomosis groups (AGs) based on hyphal interactions and host range. In this investigation, quantitative real-time PCR (qRT-PCR) techniques were used to determine potential pathogenicity factors of R. solani in the AG1-IA/rice and AG3/potato pathosystems. These factors were identified by mining for sequences of pathogen origin in a library of rice tissue infected with R. solani AG1-IA and comparing these sequences against the recently released R. solani AG3 genome. Ten genes common to both AGs and two specific to AG1-IA were selected for expression analysis by qRT-PCR. Results indicate that a number of genes are similarly expressed by AG1 and AG3 during the early stages of pathogenesis. Grouping of these pathogenicity factors based on relatedness of expression profiles suggests three key events are involved in R. solani pathogenesis: early host contact and infiltration, adjustment to the host environment, and pathogen proliferation through necrotic tissue. Further studies of the pathogenesis-associated genes identified in this project will enable more precise elucidation of the molecular mechanisms that allow for the widespread success of R. solani as a phytopathogen and allow for more targeted, effective methods of management. | 3912 | NA | NA | NA | no | no |
| 3911 | 2017 | NA | Schmegner, C; Hoegel, J; Vogel, W; Assum, G | 2005 | Genetic variability in a genomic region with long-range linkage disequilibrium reveals traces of a bottleneck in the history of the European population | The inference of the demographic history of populations from genetic variability data is not only of academic interest. It also provides background information for the identification of genes which may have played a role in human evolution or in the aetiology of human disease. To obtain a clear picture of this background, it is necessary to compare data obtained from a number of genomic loci. Due to its very low recombination rate, the NF1 gene region can be regarded as a further suitable locus. A combined resequencing and SNP typing project in a European population disclosed the presence of only two well separated subgroups of NF1 sequences. Statistical analysis revealed a bimodal distribution of the pairwise differences, a positive value of Tajima’s D and a TMRCA of 700,000 years for the whole sample, and pairwise differences indicative for a growing population and TMRCAs of 130,000 to 150,000 years for the subgroups. Together, the data lead to a model that the recent European population went through a bottleneck during the last 150,000 years of its history. Regarding the given timeframe, this bottleneck could either reflect a speciation event which led to the anatomically modern human (AMH), or a severe reduction of the population size during the emigration of AMHs out of Africa or the immigration into Europe. | 3913 | NA | NA | NA | no | no |
| 3912 | 2017 | NA | Sasaki, K; Jibiki, E; Satoh, T; Obara, Y | 2005 | Queen phenotype and behaviour during cooperative colony founding in Polyrhachis moesta | Founding queens of the arboreal ant Polyrhachis moesta aggregate independently of kinship and cooperate in caring for their brood. In field studies, the number of queens in a founding nest varied from I to 8. The number of queens in the nests with multiple queens decreased significantly with time after the nuptial flight, resulting in monogynous or oligogynous nests. Single- and multiple-founding queens did not differ in characteristics representing nutritional states or body size immediately after the nuptial flight. Wet body weight decreased as days passed, whereas head width of founding queens who overwintered successfully were relatively larger. In laboratory studies, founding queens performed liquid food exchanges more frequently with queens from other founding nests or immature colonies than with those from the same nests. Queens in founding nests and immature colonies were observed to show no aggression against non-nestmate queens, whereas queens in established colonies showed aggressive behaviours against non-nestmates. This indicates that founding queens change drastically in their aggression levels before and after colony establishment. Multiple-founding queens started laying eggs earlier than single-founding queens under laboratory conditions. Higher brood productivity and lower brood mortality were observed in multiple-queen nests. These potential advantages in multiple-queen founding may support the cooperative association among unrelated founding queens. | 3914 | NA | NA | NA | no | no |
| 3913 | 2017 | NA | Vishalakshi, C; Singh, BN | 2008 | Effect of developmental temperature stress on fluctuating asymmetry in certain morphological traits in Drosophila ananassae | In the present study, the effect of thermal stress on the variability and fluctuating asymmetry (FA) in different morphological traits, viz., thorax length (TL), sternopleural bristle number (SBN), wing length (WL), wing-to-thorax (W/T) ratio, sex comb tooth number (SCTN) and ovariole number (ON), was investigated in 10 isofemale lines of Drosophila ananassae. The phenotypic and genetic variability is higher in the flies reared at low(20 degrees C) and at high (30 degrees C) temperatures as compared to that of standard (25 degrees C) temperature. Further, the levels of FA of measured traits differed significantly among the three temperature regimes except SBN and SCTN in males and SBN and W/T ratio in females. Moreover, the magnitude of positional fluctuating asymmetry is similar in males reared at three different developmental temperatures for SBN and SCTN but it varies significantly for SBN in females. However, when FA across all the traits was combined into a composite index (CFA), significant differences were found for both temperature regimes and sexes. Males showed higher CFA at 30 degrees C whereas in females it was higher at 20 degrees C. The results suggest that temperature increases the levels of variability and FA but the effect seems to be trait and sex specific in D. ananassae. (c) 2007 Published by Elsevier Ltd. | 3915 | NA | NA | NA | no | no |
| 3914 | 2017 | NA | Chong, KF; Lee, CY | 2010 | Inter- and Intraspecific Aggression in the Invasive Longlegged Ant (Hymenoptera: Formicidae) | The longlegged ant, Anoplolepis gracilipes (Fr. Smith) (Hymenoptera: Formicidae), is a highly invasive species that can aggressively displace other ant species. We conducted laboratory assays to examine interspecies aggression of A. gracilipes versus 15 sympatric ant species found in the urban environment and disturbed habitat in Malaysia: Monomorium pharaonis (L.), Monomorium floricola (Jerdon), Monomorium orientale Mayr, Monomorium destructor (jerdon), Pheidole parva Mayr, Crematogaster sp., Solenopsis geminata (F.), Tapinoma indicum (Forel), Tapinoma melanocephalum (F.), Technomyrmex butteli Forel, Dolichoderus thoracicus (Smith), Paratrechina longicornis (Latrielle), Oecophylla smaragdina (F), Camponotus sp., and Tetraponera rufonigra (Jerdon). A. gracilipes showed aggressive behavior toward all opponent species, except the smallest M. orientale. Opponent species size (body size, head width, and mandible width) was significantly correlated with A. gracilipes aggression level and mortality rate. We also found a significant positive relationship between A. gracilipes aggression level and the mortality of the opponent species. The results suggest that invasive populations of A. gracilipes would have the greatest impact on larger ant species. In addition, we examined the intraspecific aggression of A. gracilipes. We found that A. gracilipes from different localities in Malaysia showed intraspecific aggression toward one another. This finding differs from the results of studies conducted in Christmas Island earlier. Differences in the genetic variability among populations may explain these differing results. | 3916 | NA | NA | NA | no | no |
| 3915 | 2017 | NA | Bull, NJ; Schwarz, MP | 1996 | The habitat saturation hypothesis and sociality in an allodapine bee: Cooperative nesting is not ‘’making the best of a bad situation’’ | Several factors thought to be important for the evolution of cooperative breeding in vertebrates have received little attention in facultatively social insects. One of these, the ‘’habitat saturation hypothesis’’ of Selander (1964), predicts that colony sizes will be greater in breeding units where dispersal opportunities are limited, suggesting that group living is a secondary option to independent reproduction. The Australian allodapine bee Exoneura bicolor exhibits a number of traits that occur in cooperatively breeding bird species, including long life-span, repeated opportunities for reproduction, and vulnerability to brood predation and parasitism. We experimentally examined the effect of a potentially limiting environmental factor, nesting substrate availability, as an agent influencing sociality in E. bicolor. We manipulated nesting substrate availability in two separate locations during a time when foundress dispersal is common. No significant difference was found between colony sizes in cases where dispersal options were abundant and cases where dispersal options were limited. An increase in opportunities for dispersal did not lead to higher rates of independent nesting, suggesting that cooperative nesting is a preferred strategy regardless of distance-related costs of dispersal. Reproductivity per female and brood survival were examined as factors selecting for group living. Low survival of brood in single-female nests has the potential to select for cooperative nesting in this bee. | 3917 | NA | NA | NA | no | no |
| 3916 | 2017 | NA | Ho, EKH; Frederickson, ME | 2014 | Alate susceptibility in ants | Pathogens are predicted to pose a particular threat to eusocial insects because infections can spread rapidly in colonies with high densities of closely related individuals. In ants, there are two major castes: workers and reproductives. Sterile workers receive no direct benefit from investing in immunity, but can gain indirect fitness benefits if their immunity aids the survival of their fertile siblings. Virgin reproductives (alates), on the other hand, may be able to increase their investment in reproduction, rather than in immunity, because of the protection they receive from workers. Thus, we expect colonies to have highly immune workers, but relatively more susceptible alates. We examined the survival of workers, gynes, and males of nine ant species collected in Peru and Canada when exposed to the entomopathogenic fungus Beauveria bassiana. For the seven species in which treatment with B. bassiana increased ant mortality relative to controls, we found workers were significantly less susceptible compared with both alate sexes. Female and male alates did not differ significantly in their immunocompetence. Our results suggest that, as with other nonreproductive tasks in ant colonies like foraging and nest maintenance, workers have primary responsibility for colony immunity, allowing alates to specialize on reproduction. We highlight the importance of colony-level selection on individual immunity in ants and other eusocial organisms. | 3918 | NA | NA | NA | no | no |
| 3917 | 2017 | NA | Bonte, D; Hovestadt, T; Poethke, HJ | 2009 | Sex-specific dispersal and evolutionary rescue in metapopulations infected by male killing endosymbionts | Background: Male killing endosymbionts manipulate their arthropod host reproduction by only allowing female embryos to develop into infected females and killing all male offspring. Because the resulting change in sex ratio is expected to affect the evolution of sex-specific dispersal, we investigated under which environmental conditions strong sex-biased dispersal would emerge, and how this would affect host and endosymbiont metapopulation persistence. Results: We simulated host-endosymbiont metapopulation dynamics in an individual-based model, in which dispersal rates are allowed to evolve independently for the two sexes. Prominent male-biased dispersal emerges under conditions of low environmental stochasticity and high dispersal mortality. By applying a reshuffling algorithm, we show that kin-competition is a major driver of this evolutionary pattern because of the high within-population relatedness of males compared to those of females. Moreover, the evolution of sex-specific dispersal rescues metapopulations from extinction by (i) reducing endosymbiont fixation rates and (ii) by enhancing the extinction of endosymbionts within metapopulations that are characterized by low environmental stochasticity. Conclusion: Male killing endosymbionts induce the evolution of sex-specific dispersal, with prominent male-biased dispersal under conditions of low environmental stochasticity and high dispersal mortality. This male-biased dispersal emerges from stronger kin-competition in males compared to females and induces an evolutionary rescue mechanism. | 3919 | NA | NA | NA | no | no |
| 3918 | 2017 | NA | Cant, MA; Johnstone, RA | 2008 | Reproductive conflict and the separation of reproductive generations in humans | An enduring puzzle of human life history is why women cease reproduction midway through life. Selection can favor postreproductive survival because older females can help their offspring to reproduce. But the kin-selected fitness gains of helping appear insufficient to outweigh the potential benefits of continued reproduction. Why then do women cease reproduction in the first place? Here, we suggest that early reproductive cessation in humans is the outcome of reproductive competition between generations, and we present a simple candidate model of how this competition will be resolved. We show that among primates exhibiting a postreproductive life span, humans exhibit an extraordinarily low degree of reproductive overlap between generations. The rapid senescence of the human female reproductive system coincides with the age at which, in natural fertility populations, women are expected to encounter reproductive competition from breeding females of the next generation. Several lines of evidence suggest that in ancestral hominids, this younger generation typically comprised immigrant females. In these circumstances, relatedness asymmetries within families are predicted to give younger females a decisive advantage in reproductive conflict with older females. A model incorporating both the costs of reproductive competition and the benefits of grandmothering can account for the timing of reproductive cessation in humans and so offers an improved understanding of the evolution of menopause. | 3920 | NA | NA | NA | no | no |
| 3919 | 2017 | NA | Lima, LS; Gramacho, KP; Pires, JL; Clement, D; Lopes, UV; Carels, N; Gesteira, AD; Gaiotto, FA; Cascardo, JCD; Micheli, F | 2010 | Development, characterization, validation, and mapping of SSRs derived from Theobroma cacao L.-Moniliophthora perniciosa interaction ESTs | In this study, we report results of the detection and analysis of SSR markers derived of cacao-Moniliophthora perniciosa expressed sequence tags (ESTs) in relation to cacao resistance to witches’ broom disease (WBD), and we compare the polymorphism of those ESTs (EST-simple sequence repeat (SSR)) with classical neutral SSR markers. A total of 3,487 ESTs was used in this investigation. SSRs were identified in 430 sequences: 277 from the resistant genotype TSH 1188 and 153 from the susceptible one Catongo, totalizing 505 EST-SSRs with three types of motifs: dinucleotides (72.1%), trinucleotides (27.3%), and tetranucleotides (0.6%). EST-SSRs were classified into 16 main categories; most of the EST-SSRs belonged to “Unknown function” and “No homology” categories (45.82%). A high frequency of SSRs was found in the 5’UTR and in the ORF (about 27%) and a low frequency was observed in the 3’UTR (about 8%). Forty-nine EST-SSR primers were designed and evaluated in 21 cacao accessions, 12 revealed polymorphism, having 47 alleles in total, with an average of 3.92 alleles per locus. On the other hand, the 11 genomic SSR markers revealed a total of 47 alleles, with an average of 5.22 alleles per locus. The association of EST-SSR with the genomic SSR enhanced the analysis of genetic distance among the genotypes. Among the 12 polymorphic EST-SSR markers, two were mapped on the F(2) Sca 6 x ICS 1 population reference for WBD resistance. | 3921 | NA | NA | NA | no | no |
| 3920 | 2017 | NA | Lu, G; Sun, LS; Xu, T; He, D; Wang, ZC; Ou, SD; Jia, K; Yuan, LG; Li, SJ | 2016 | First Description of Hepacivirus and Pegivirus Infection in Domestic Horses in China: A Study in Guangdong Province, Heilongjiang Province and Hong Kong District | Since 2012, three viruses, known as equine hepacivirus (EqHV), equine pegivirus (EPgV) and Theiler’s disease-associated virus (TDAV), have been discovered in equines. Given that these viruses are the newest members of the Flaviviridae family, genomic information concerning circulating EqHV, EPgV and TDAV strains around the world is limited. To date, no genetic surveillance studies have been performed on these three viruses in the equine population of China. Here, a total of 177 serum samples were collected from equines across China between 2014 and 2015. Using PCR, we detected viral RNA in the serum samples, six of which were EqHV positive and two of which were EPgV positive. Co-infection with the two viruses was not observed among the Chinese equines studied, and TDAV RNA was not detected in the equine serum samples collected for this study. Phylogenetic analysis of partial NS5B open reading frame (ORF), NS3 ORF, and 5’ untranslated region nucleotide sequences from EqHV as well as partial NS3 ORF sequence from EPgV indicated that EqHV and EPgV have evolved into two main clades by themselves, both of which are circulating in China. Based on the partial NS5B and NS3 ORF sequences of EqHV, the sequences of one clade were also split into two subclades. This study enriches our knowledge of the geographic distribution of these three equine viruses. | 3922 | NA | NA | NA | no | no |
| 3921 | 2017 | NA | Gonzalez-Perez, MA; Caujape-Castells, J; Sosa, PA | 2004 | Molecular evidence of hybridisation between the endemic Phoenix canariensis and the widespread P. dactylifera with Random Amplified Polymorphic DNA (RAPD) markers | We used RAPD markers to test whether morphologically intermediate individuals between the Canarian endemic Phoenix canariensis and the widespread P. dactylifera correspond to hybrids. Consistent with previous allozyme evidence, the scarcity of appropriate RAPD markers to distinguish P. dactylifera and P. canariensis indicated a close genetic relationship among these species. Only two of the 54 ten-mer primers (OPM-8 and OPK-14) tested in 221 individuals from 7 localities in different islands enabled us to unambiguously identify both species. While P. canariensis possesses two exclusive monomorphic bands of 1000 bp and 750 bp (for OPM-8 and OPM-14, respectively), dactylifera is characterised by two bands of 900 bp and 950 bp for the same primers. The additivity of these taxon-specific bands in the individuals that were morphologically intermediate provided, for the first time, firm evidence for their hybrid origin. Because these hybridisation capabilities pose clear threats to the survival of the endemic P. canariensis and some individuals that had been morphologically characterised as pure P. canariensis revealed later a hybrid nature in the RAPD analysis, we suggest that RAPD markers be used to estimate the possible incidence of introgression in the scarce extant natural populations of P. canariensis. This procedure will provide a straightforward means to select target populations to implement the “in situ” conservation strategies suggested previously on the basis of allozyme research. | 3923 | NA | NA | NA | no | no |
| 3922 | 2017 | NA | Metzger, J; Tonda, R; Beltran, S; Agueda, L; Gut, M; Distl, O | 2014 | Next generation sequencing gives an insight into the characteristics of highly selected breeds versus non-breed horses in the course of domestication | Background: Domestication has shaped the horse and lead to a group of many different types. Some have been under strong human selection while others developed in close relationship with nature. The aim of our study was to perform next generation sequencing of breed and non-breed horses to provide an insight into genetic influences on selective forces. Results: Whole genome sequencing of five horses of four different populations revealed 10,193,421 single nucleotide polymorphisms (SNPs) and 1,361,948 insertion/deletion polymorphisms (indels). In comparison to horse variant databases and previous reports, we were able to identify 3,394,883 novel SNPs and 868,525 novel indels. We analyzed the distribution of individual variants and found significant enrichment of private mutations in coding regions of genes involved in primary metabolic processes, anatomical structures, morphogenesis and cellular components in non-breed horses and in contrast to that private mutations in genes affecting cell communication, lipid metabolic process, neurological system process, muscle contraction, ion transport, developmental processes of the nervous system and ectoderm in breed horses. Conclusions: Our next generation sequencing data constitute an important first step for the characterization of non-breed in comparison to breed horses and provide a large number of novel variants for future analyses. Functional annotations suggest specific variants that could play a role for the characterization of breed or non-breed horses. | 3924 | NA | NA | NA | no | no |
| 3923 | 2017 | NA | Sarver, BAJ; Ward, TJ; Gale, LR; Broz, K; Kistler, HC; Aoki, T; Nicholson, P; Carter, J; O’Donnell, K | 2011 | Novel Fusarium head blight pathogens from Nepal and Louisiana revealed by multilocus genealogical concordance | This study was conducted to assess evolutionary relationships, species diversity and trichothecene toxin potential of five Fusarium graminearum complex (FGSC) isolates identified as genetically novel during prior Fusarium head blight (FHB) surveys in Nepal and Louisiana. Results of a multilocus genotyping (MLGT) assay for B-trichothecene species determination indicated these isolates might represent novel species within the FGSC. GCPSR-based phylogenetic analyses of a 12-gene dataset, comprising portions of seven loci totaling 13.1 kb of aligned DNA sequence data, provided strong support for the genealogical exclusivity of the Nepalese and Louisianan isolates. Accordingly, both species are formally recognized herein as novel FGSC species. Fusarium nepalense was resolved as the sister lineage of Fusarium ussurianum + Fusarium asiaticum within an Asian subclade of the FGSC. Fusarium louisianense was strongly supported as a reciprocally monophyletic sister of Fusarium gerlachii + F. graminearum, suggesting that this subclade might be endemic to North America. Multilocus Bayesian species tree analyses augment these results and provide evidence for a distinct lineage within F. graminearum predominately from the Gulf Coast of Louisiana. As predicted by the MLGT assay, mycotoxin analyses demonstrated that F. nepalense and F. louisianense could produce 15ADON and nivalenol, respectively, in planta. In addition, both species were only able to induce mild FHB symptoms on wheat in pathogenicity experiments. Published by Elsevier Inc. | 3925 | NA | NA | NA | no | no |
| 3924 | 2017 | NA | Qiao, DD; Lange, C; Laird, NM; Won, S; Hersh, CP; Morrow, J; Hobbs, BD; Lutz, SM; Ruczinski, I; Beaty, TH; Silverman, EK; Cho, MH | 2017 | Gene-based segregation method for identifying rare variants in family-based sequencing studies | Whole-exome sequencing using family data has identified rare coding variants in Mendelian diseases or complex diseases with Mendelian subtypes, using filters based on variant novelty, functionality, and segregation with the phenotype within families. However, formal statistical approaches are limited. We propose a gene-based segregation test (GESE) that quantifies the uncertainty of the filtering approach. It is constructed using the probability of segregation events under the null hypothesis of Mendelian transmission. This test takes into account different degrees of relatedness in families, the number of functional rare variants in the gene, and their minor allele frequencies in the corresponding population. In addition, a weighted version of this test allows incorporating additional subject phenotypes to improve statistical power. We show via simulations that the GESE and weighted GESE tests maintain appropriate type I error rate, and have greater power than several commonly used region-based methods. We apply our method to whole-exome sequencing data from 49 extended pedigrees with severe, early-onset chronic obstructive pulmonary disease (COPD) in the Boston Early-Onset COPD study (BEOCOPD) and identify several promising candidate genes. Our proposed methods show great potential for identifying rare coding variants of large effect and high penetrance for family-based sequencing data. The proposed tests are implemented in an R package that is available on CRAN (). | 3926 | NA | NA | NA | no | no |
| 3925 | 2017 | NA | Wu, SB; Collins, G; Sedgley, M | 2004 | A molecular linkage map of olive (Olea europaea L.) based on RAPD, microsatellite, and SCAR markers | An integrated molecular linkage map of olive (Olea europaea L.) was constructed based on randomly amplified polymorphic DNA (RAPD), sequence characterized amplified region (SCAR), and microsatellite markers using the pseudo-testcross strategy. A mapping population of 104 individuals was generated from an F, full-sib family of a cross between ‘Frantoio’ and ‘Kalamata’. The hybridity of the mapping population was confirmed by genetic similarity and nonmetric multidimensional scaling. Twenty-three linkage groups were mapped for ‘Kalamata’, covering 759 cM of the genome with 89 loci and an average distance between loci of 11.5 cM. Twenty-seven linkage groups were mapped for ‘Frantoio’, covering 798 cM of the genome with 92 loci and an average distance between loci of 12.3 cM. For the integrated map, 15 linkage groups covered 879 cM of the genome with 101 loci and an average distance between loci of 10.2 cM. The size of the genomic DNA was estimated to be around 3000 cM. A sequence characterized amplified region marker linked to olive peacock disease resistance was mapped to linkage group 2 of the integrated map. These maps will be the starting point for studies on the structure, evolution, and function of the olive genome. When the mapping progeny pass through their juvenile phase and assume their adult characters, mapping morphological markers and identification of quantitative trait loci for adaptive traits will be the primary targets. | 3927 | NA | NA | NA | no | no |
| 3926 | 2017 | NA | Zamora, MGM; Castagnaro, AP; Ricci, JCD | 2008 | Genetic diversity of Pto-like serine/threonine kinase disease resistance genes in cultivated and wild strawberries | Degenerate oligonucleotide primers, designed based on conserved regions of several serine-threonine kinases (STK) previously cloned in tomato and Arabidopsis, were used to isolate STK candidates in wild and cultivated strawberries. Seven distinct classes of STKs were identified from three related wild species, i.e., Fragaria vesca, Fragaria chiloensis, and Potentilla tucumanensis, and seven different Fragaria x ananassa cultivars. Alignment of the deduced amino acid sequences and the Pto R protein from tomato revealed the presence of characteristic subdomains and conservation of the plant STK consensus and other residues that are crucial for Pto function. Based on identity scores and clustering in phylogenetic trees, five groups were recognized as Pto-like kinases. Strawberry Pto-like clones presented sequences that were clearly identified as the activation segments contained in the Pto, and some of them showed residues previously identified as being required for binding to AvrPto. Some of the non-Pto-like kinases presented a high degree of identity and grouped together with B-lectin receptor kinases that are also involved in disease resistance. Statistical studies carried out to evaluate departure from the neutral theory and nonsynonymous/synonymous substitutions suggest that the evolution of STK-encoding sequences in strawberries is subjected mainly to a purifying selection process. These results represent the first report of Pto-like STKs in strawberry. | 3928 | NA | NA | NA | no | no |
| 3927 | 2017 | NA | Gao, BQ; Liu, P; Li, J; Wang, QY; Li, XP | 2014 | Genetic diversity of different populations and improved growth in the F1 hybrids in the swimming crab (Portunus trituberculatus) | The swimming crab, Portunus trituberculatus, is widely distributed throughout the coastal waters of Asian-Pacific nations and is an important economic species in this region. The aquaculture of swimming crabs has been plagued by problems associated with low growth rates, poor flesh quality, and weak disease resistance. To overcome these problems, selective breeding programs have been suggested as a means of genetically improving these traits in stock populations. In this study, we evaluated the genetic differentiation of 3 different geographical populations (Zhoushan: S; Laizhou Bay: L; and Haizhou Bay: H) using 8 polymorphic microsatellite loci. Nine strains of first filial generation were obtained, with 3 geographically populations as parental stock. We assessed the growth and survival rates of the F1 generation to identify new strains or breeds showing improvements in these economically important traits. Our results indicated that pairwise F-ST among populations was significantly higher than 0 (P = 0.0000) for every population pair, ranging from 0.0810 to 0.1083 for 3 different geographical populations. We observed significant heterosis for the growth and viability (survival) traits, although some strains (crossbred combinations) showed evidence of hybrid weakness in some growth measurements. One particular strain (“SL”) outperformed other combinations, displaying the greatest extent of heterosis over the growth and viability (survival) traits. These results indicate that hybridization may be used to increase the performance of Portunus trituberculatus in aquaculture. | 3929 | NA | NA | NA | no | no |
| 3928 | 2017 | NA | Wright, DJ; Brouwer, L; Mannarelli, ME; Burke, T; Komdeur, J; Richardson, DS | 2016 | Social pairing of Seychelles warblers under reduced constraints: MHC, neutral heterozygosity, and age | The prevalence and significance of precopulatory mate choice remains keenly debated. The major histocompatibility complex (MHC) plays a key role in vertebrate adaptive immunity, and variation at the MHC influences individual survival. Although MHC-dependent mate choice has been documented in certain species, many other studies find no such pattern. This may be, at least in part, because in natural systems constraints may reduce the choices available to individuals and prevent full expression of underlying preferences. We used translocations to previously unoccupied islands to experimentally reduce constraints on female social mate choice in the Seychelles warbler (Acrocephalus sechellensis), a species in which patterns of MHC-dependent extrapair paternity (EPP), but not social mate choice, have been observed. We find no evidence of MHC-dependent social mate choice in the new populations. Instead, we find that older males and males with more microsatellite heterozygosity are more likely to have successfully paired. Our data cannot resolve whether these patterns in pairing were due to male-male competition or female choice. However, our research does suggest that female Seychelles warblers do not choose social mates using MHC class I to increase fitness. It may also indicate that the MHC-dependent EPP observed in the source population is probably due to mechanisms other than female precopulatory mate choice based on MHC cues. | 3930 | NA | NA | NA | no | no |
| 3929 | 2017 | NA | Li, F; Hu, T; Duan, NC; Li, WY; Teng, Q; Li, H; Liu, W; Liu, Y; Cheng, TY | 2016 | Sequence variation in two mitochondrial DNA regions and internal transcribed spacer among isolates of the nematode Oesophagostomum asperum originating from goats in Hunan Province, China | The present study examined sequence variability in two mitochondrial DNA (mtDNA) regions, namely cytochrome c oxidase subunit 1 (cox1) and NADH dehydrogenase subunit 1 (nad1), and internal transcribed spacer (ITS) of nuclear ribosomal DNA (rDNA) among Oesophagostomum asperum isolates from goats in Hunan Province, China. A portion of the cox1 (pcox1), nad1 (pnad1) genes and the ITS (ITS1 + 5.8S rDNA + ITS2) rDNA were amplified by polymerase chain reaction (PCR) separately from adult O. asperum individuals and the representative amplicons were subjected to sequencing from both directions. The lengths of pcox1, pnad1 and ITS rDNA were 366 bp, 681 bp and 785 bp, respectively. The A + T contents of gene sequences were 71.5-72% for pcox1, 73.7-74.2% for pnad1 and 58-58.8% for ITS rDNA. Intra-specific sequence variations within O. asperum were 0-1.6% for pcox1, 0-1.9% for pnad1 and 0-1.7% for ITS rDNA, while inter-specific sequence differences among members of the genus Oesophagostomum were significantly higher, being 11.1-12.5%, 13.3-17.7% and 8.5-18.6% for pcox1, pnad1 and ITS rDNA, respectively. Phylogenetic analyses using combined sequences of pcox1 and pnad1, with three different computational algorithms (Bayesian inference, maximum likelihood and maximum parsimony), revealed distinct groups with high statistical support. These findings demonstrated the existence of intra-specific variation in mtDNA and rDNA sequences among O. asperum isolates from goats in Hunan Province, China, and have implications for studying molecular epidemiology and population genetics of O. asperum. | 3931 | NA | NA | NA | no | no |
| 3930 | 2017 | NA | Vignoles, P; Dreyfuss, G; Rondelaud, D | 2002 | Larval development of Fasciola hepatica in experimental infections: variations with populations of Lymnaea truncatula | A retrospective study was undertaken on 70 French populations of Lymnaea truncatula experimentally infected with Fasciola hepatica to determine whether or not susceptibility of snails to infection influenced redial and cercarial production. Results were compared with those obtained from two control populations, known for prevalences higher than 60% when experimentally infected with F. hepatica. In the 70 other populations examined, the prevalences ranged from 2 to 75%. In 55 of these populations, where the prevalence was more than 20%, a high proportion (50.1-56.8%) of snails died after cercarial shedding, whereas in the other groups (non-shedding snails with the most differentiated larvae being free cercariae, rediae containing cercariae, immature rediae, or sporocysts, respectively), snail death was significantly less. In 11 populations, where the prevalence values were 5-19%, only 14% of snails died after cercarial shedding, whereas snails with free cercariae, rediae with cercariae, or immature rediae showed significant increases in snail mortality. In the remaining four snail populations, with prevalences of less than 5%, the most differentiated larval forms were only immature rediae and/or sporocysts. Overall, the number of rediae containing cercariae significantly decreased with decreasing prevalence values. The low prevalence of experimental infection in several populations of snails might be explained by the occurrence of natural infections with miracidia originating from a mammalian host other than cattle, and/or by genetic variability in the susceptibility of snails to infection. | 3932 | NA | NA | NA | no | no |
| 3931 | 2017 | NA | Praipue, P; Klinbunga, S; Jarayabhand, P | 2010 | Genetic diversity of wild and domesticated stocks of Thai abalone, Haliotis asinina (Haliotidae), analyzed by single-strand conformational polymorphism of AFLP-derived markers | Amplified fragment length polymorphism (AFLP) analysis was carried out on representative individuals of wild Haliotis asinina using 64 primer combinations. Nine polymorphic AFLPs were cloned and sequenced. Sequence-specific primers were designed from six AFLP-derived fragments. Three sequence-characterized amplified region (SCAR) markers (HaSCAR(320), HaSCAR(295), HaSCAR(327)) were selected for genotyping of 8-month-old domesticated stocks of H. asinina cultured separately at Sichang Marine Science Research and Training Station (N = 95) and at a hatchery in Trang province (N = 40) using single-strand conformational polymorphism analysis. Genotypes of wild abalone originating from Talibong Island (N = 25), Cambodia (N = 22), and the P(0) progeny established from Samet Island founders (N = 20) were also investigated. Significant genetic differentiation (P < 0.0001 for the exact test and F(ST) = 0.8759-0.8919, P < 0.001) between abalone from the Gulf of Thailand (Cambodia and Samet Island - east) and the Andaman Sea (Talibong Island - west) were observed. This demonstrated the strong biogeographic structure of H. asinina in Thai waters. Non-overlapping composite genotypes for wild abalone from different coastal regions allow us to determine founder contributions in domesticated abalone stocks. Almost all Sichang Marine Science Research and Training Station and the Trang province hatchery stocks exhibited the east coast genotypes (97% of the 135 samples). We suggest that abalone from the east coast population have better survival rates under cultivated conditions than those from the west coast population. | 3933 | NA | NA | NA | no | no |
| 3932 | 2017 | NA | Kim, JH; Cheong, HS; Kim, LH; Shin, HJ; Na, HS; Chung, MW; Shin, HD | 2015 | Direct sequencing for comprehensive screening of LDLR genetic polymorphisms among five ethnic populations | Low density lipoprotein receptor (LDLR) plays an important role in plasma lipoprotein metabolism and pharmacological responses. Although mutations of LDLR and their functional associations with plasma LDL cholesterol concentrations have been described, no comprehensive comparisons of LDLR variants among populations are established. The aim of this study is to define the distribution of single nucleotide polymorphisms (SNPs) of LDLR and to discover novel variants across population groups. A total of 288 DNAs from 96 Korean, 48 Chinese, 48 Japanese, 48 African-American, and 48 European-American subjects were resequenced. A total of 59 SNPs (18 in the coding regions, 37 in the introns, and 4 in the 3’-untranslated region) were identified, including eight novel variants. Polymorphisms of LDLR showed significantly different distributions in comparisons among ethnic population groups (P < 0.05, Fisher’s exact test). Moreover, almost all of these differently distributed SNPs were common variants. Notably, prevalent variations-rs1003723, rs1799898, rs688, rs5925, rs6413504 in Europeans, nonsynonymous rs11669576 (Ala391Thr) in Africans, and rs14158 in Asians-were observed. In further in silico analyses for the novel SNPs, 2 intronic variants (+17716G > A in the intron 5 and +38569A > C in the intron 16) were predicted as potential branch point sites for alternative splicing. Although this study is not free from limitations such as insufficient sample size and no functional studies on the novel SNPs, our findings provides supporting information about LDLR genetic variability among ethnic groups and pharmacogenetics studies for plasma lipoprotein metabolism. | 3934 | NA | NA | NA | no | no |
| 3933 | 2017 | NA | Tidona, CA; Darai, G | 2000 | Iridovirus homologues of cellular genes - Implications for the molecular evolution of large DNA viruses | Iridoviruses belong to the group of large cytoplasmic deoxyriboviruses and infect either insects or vertebrates. In analogy to other large DNA viruses of eucaryotes it was found that iridoviruses encode a number of cellular protein homologues, The majority of these proteins represent orthologues of cellular enzymes involved in transcription, replication, and nucleotide metabolism. Others may have the potential to interfere with cell cycle regulation or immune defence mechanisms of the host.. This raises the question about the phylogenetic origin of the corresponding viral genes. During the evolution of large cytoplasmic DNA viruses such as iridoviruses, poxviruses, and African swine fever vints the acquirement of cellular genes appears to be a crucial event. Each member of this group of viruses encodes a DNA polymerase, two subunits of the DNA-dependent RNA polymerase, and two subunits of the ribonucleotide reductase. It is important to note that all of these viral proteins show a high level of multidomain structure conservation as compared to their cellular orthologues. As a consequence the large cytoplasmic DNA viruses have the ability to replicate independently of the cellular nucleus in the cytoplasm of the infected cell. Assuming a common cellular origin of viral DNA polymerase genes the corresponding amino acid sequences were chosen to construct a phylogenetic tree showing the relatedness among large DNA viruses of eucaryotes. | 3935 | NA | NA | NA | no | no |
| 3934 | 2017 | NA | Mugerwa, H; Rey, MEC; Alicai, T; Ateka, E; Atuncha, H; Ndunguru, J; Sseruwagi, P | 2012 | Genetic diversity and geographic distribution of Bemisia tabaci (Gennadius) (Hemiptera: Aleyrodidae) genotypes associated with cassava in East Africa | The genetic variability of whitefly (Bemisia tabaci) species, the vectors of cassava mosaic begomoviruses (CMBs) in cassava growing areas of Kenya, Tanzania, and Uganda, was investigated through comparison of partial sequences of the mitochondria cytochrome oxidase I (mtCOI) DNA in 2010/11. Two distinct species were obtained including sub-Saharan Africa 1 (SSA1), comprising of two sub-clades (I and II), and a South West Indian Ocean Islands (SWIO) species. Among the SSA1, sub-clade I sequences shared a similarity of 97.8-99.7% with the published Uganda 1 genotypes, and diverged by 0.3-2.2%. A pairwise comparison of SSA1 sub-clade II sequences revealed a similarity of 97.2-99.5% with reference southern Africa genotypes, and diverged by 0.5-2.8%. The SSA1 sub-clade I whiteflies were widely distributed in East Africa (EA). In comparison, the SSA1 sub-clade II whiteflies were detected for the first time in the EA region, and occurred predominantly in the coast regions of Kenya, southern and coast Tanzania. They occurred in low abundance in the Lake Victoria Basin of Tanzania and were widespread in all four regions in Uganda. The SWIO species had a sequence similarity of 97.2-97.7% with the published Reunion sequence and diverged by 2.3-2.8%. The SWIO whiteflies occurred in coast Kenya only. The sub-Saharan Africa 2 whitefly species (Ug2) that was associated with the severe CMD pandemic in Uganda was not detected in our study. | 3936 | NA | NA | NA | no | no |
| 3935 | 2017 | NA | Ahmad, A; Mbofung, GY; Acharya, J; Schmidt, CL; Robertson, AE | 2015 | Characterization and Comparison of Clavibacter michiganensis subsp nebraskensis Strains Recovered from Epiphytic and Symptomatic Infections of Maize in Iowa | Clavibacter michiganensis subsp. nebraskensis (Cmn), the causal organism of Goss’s wilt and leaf blight of maize, can be detected in the phyllosphere of its host prior to disease development. We compared the morphology and pathogenicity of 37 putative isolates of Cmn recovered from asymptomatic and symptomatic maize leaves. Thirty-three of the isolates produced mucoid orange colonies, irrespective of the source of isolation and all but four of these isolates were pathogenic on maize. The remaining 4 isolates recovered from asymptomatic leaves had large fluidal yellow colonies, and were non-pathogenic on maize. Isolates varied in their aggressiveness on a susceptible hybrid of maize but no significant differences in aggressiveness were detected between epiphytic isolates and those recovered from diseased maize tissues. The genomics of Cmn is poorly understood; therefore as a first step to determining what genes may play a role in virulence, we compared 33 putative virulence gene sequences from 6 pathogenic and a non-pathogenic isolate recovered from the phyllosphere. Sequence polymorphisms were detected in 5 genes, cellulase A, two endoglucanases, xylanase B and a pectate lyase but there was no relationship with pathogenicity. Further research is needed to determine what genes play a role in virulence of Cmn. Our data show however, that the virulence factors in Cmn likely differ from those reported for the closely related subspecies michiganensis and sepedonicus. | 3937 | NA | NA | NA | no | no |
| 3936 | 2017 | NA | Azhar, MT; Amin, I; Anjum, ZI; Arshad, M; Briddon, RW; Mansoor, S | 2010 | Both malvaceous and non-malvaceous betasatellites are associated with two wild cotton species grown under field conditions in Pakistan | Betasatellites are symptom-modulating DNA satellites that are associated with the majority of monopartite begomoviruses in the Old World. Betasatellites show both geographical and host-specific relatedness; those isolated from malvaceous hosts form one group and those isolated from non-malvaceous hosts form the second group. Real-time PCR based estimation of begomovirus DNA in cotton species grown in a living herbarium under field conditions at the Central Cotton Research Institute (CCRI), Multan was carried out to understand their role in the ongoing cotton leaf curl disease (CLCuD) epidemic in Pakistan. The levels of begomovirus DNA in cotton species from the herbarium was found to be several folds lower than symptomatic plants of commercially grown cotton. Among herbarium species Gossypium anomalum, G. robinsoni and G. somalense contained higher levels of begomovirus DNA in comparison to other wild species. Characterization of betasatellites from two cotton species (G. anomalum and G. somalense) was carried out to understand their role in accumulation of begomovirus DNA. Here, we show that the two wild species of cotton are associated with Cotton leaf Multan betasatellite (CLCuMB), a malvaceous betasatellite associated with CLCuD, as well as Chili leaf curl betasatellite, a non-malvaceous betasatellite not previously identified in a malvaceous host. This is the first evidence of the presence of a non-malvaceous betasatellite in malvaceous hosts. | 3938 | NA | NA | NA | no | no |
| 3937 | 2017 | NA | Diouf, D; Samba-Mbaye, R; Lesueur, D; Ba, AT; Dreyfus, B; De Lajudie, P; Neyra, M | 2007 | Genetic diversity of Acacia seyal del. rhizobial populations indigenous to senegalese soils in relation to salinity and pH of the sampling sites | The occurrence and the distribution of rhizobial populations naturally associated to Acacia seyal Del. were characterized in 42 soils from Senegal. The diversity of rhizobial genotypes, as characterized by polymerase chain reaction restriction fragment length polymorphism (RFLP) analysis of 16S-23S rDNA, performed on DNA extracted from 138 nodules resulted in 15 clusters. Results indicated the widespread occurrence of compatible rhizobia associated to A. seyal in various ecogeographic areas. However, the clustering of rhizobial populations based on intergenic spacer (IGS) RFLP profiles did not reflect their geographic origin. Four genera were discriminated on the basis of 16S rRNA gene sequences of the strains representative for the IGS-RFLP profiles. The majority of rhizobia associated to A. seyal were affiliated to Mesorhizobium and Sinorhizobium 64 and 29%, respectively, of the different IGS-RFLP profiles. Our results demonstrate the coexistence inside the nodule of plant-pathogenic non-N-2-fixing Agrobacterium and Burkholderia strains, which induced the formation of ineffective nodules, with symbiotic rhizobia. Nodulation was recorded in saline soils and/or at low pH values or in alkaline soils, suggesting adaptability of natural rhizobial populations to major ecological environmental stress and their ability to establish symbiotic associations within these soil environments. These results contribute to the progressing research efforts to uncover the biodiversity of rhizobia and to improve nitrogen fixation in agroforestry systems in sub-Saharan Africa. | 3939 | NA | NA | NA | no | no |
| 3938 | 2017 | NA | Webster, JP; Gower, CM | 2006 | Mate choice, frequency dependence, and the maintenance of resistance to parasitism in a simultaneous hermaphrodite | Biomphalaria glabrata are simultaneous hermaphroditic freshwater snails that act as intermediate hosts for the macroparasitic trematode Schistosoma mansoni, a causative agent of schistosomiasis. Heritability and strain-specificity of both snail resistance and susceptibility to schistosome infection have been demonstrated, genetic variability for which is maintained, in part, through trade-offs between high fitness costs associated with infection and those associated with resistance. However, despite such a high cost of resistance and a low prevalence of infection in natural snail populations, genes for resistance are maintained within snail populations over successive generations, including in the complete absence of parasite pressure in laboratory populations. This may be indicative of alternative benefits of resistance genes, in addition to parasite defense, such as differential mating success between genotypes. Here we examined the mate and gender choice of snails across a multi-factorial range of potential partner combinations. These included host-resistance or susceptibility genotype, host genotype frequency within the population, current parasite infection status, and parasite genotype. We demonstrate recognition and discrimination by host snails depending on host and/or parasite genotype for each of these factors. In particular, our results suggest that a rare mating advantage to resistant genotypes may be a potential explanation for the maintenance of highly costly resistance genes within intermediate host populations under conditions of low or zero parasite pressure. | 3940 | NA | NA | NA | no | no |
| 3939 | 2017 | NA | Gower, CM; Webster, JP | 2005 | Intraspecific competition and the evolution of virulence in a parasitic trematode | Intrahost competition between parasite genotypes has been predicted to be an important force shaping parasite ecology and evolution and has been extensively cited as a mechanism for the evolution of increased parasite virulence. However, empirical evidence demonstrating the existence and nature of intraspecific competition is lacking for many parasites. Here, we compared within-host competitiveness between genetic strains of Schistosoma mansoni with high (HIGH-V) or low (LOW-V) virulence to their intermediate snail host, Biomphalaria glabrata. Groups of snails were exposed to either one or the other of two parasite strains, or a mixed infection of both strains, and the resulting progeny were identified using a molecular marker. In two separate experiments investigating simultaneous and sequential infections, we demonstrated that the lifetime reproductive success of parasite strain HIGH-V was reduced in the presence of a faster replicating parasite genotype, LOW-V, regardless of whether it was in a majority or minority in the initial inoculum of the simultaneous exposure or of its relative position in the sequential exposure experiment. Thus, we demonstrate competition between parasite genotypes and asymmetry in competitive Success between parasite strains. Moreover, since the less virulent strain investigated here had a competitive advantage, we suggest that a high frequency of multiple infections could favor the evolution of less, rather than more, virulent parasites in this system. | 3941 | NA | NA | NA | no | no |
| 3940 | 2017 | NA | Tessema, SK; Monk, SL; Schultz, MB; Tavul, L; Reeder, JC; Siba, PM; Mueller, I; Barry, AE | 2015 | Phylogeography of var gene repertoires reveals fine-scale geospatial clustering of Plasmodium falciparum populations in a highly endemic area | Plasmodium falciparum malaria is a major global health problem that is being targeted for progressive elimination. Knowledge of local disease transmission patterns in endemic countries is critical to these elimination efforts. To investigate fine-scale patterns of malaria transmission, we have compared repertoires of rapidly evolving var genes in a highly endemic area. A total of 3680 high-quality DBL-sequences were obtained from 68 P.falciparum isolates from ten villages spread over two distinct catchment areas on the north coast of Papua New Guinea (PNG). Modelling of the extent of var gene diversity in the two parasite populations predicts more than twice as many var gene alleles circulating within each catchment (Mugil=906; Wosera=1094) than previously recognized in PNG (Amele=369). In addition, there were limited levels of var gene sharing between populations, consistent with local parasite population structure. Phylogeographic analyses demonstrate that while neutrally evolving microsatellite markers identified population structure only at the catchment level, var gene repertoires reveal further fine-scale geospatial clustering of parasite isolates. The clustering of parasite isolates by village in Mugil, but not in Wosera was consistent with the physical and cultural isolation of the human populations in the two catchments. The study highlights the microheterogeneity of P.falciparum transmission in highly endemic areas and demonstrates the potential of var genes as markers of local patterns of parasite population structure. | 3942 | NA | NA | NA | no | no |
| 3941 | 2017 | NA | Dayo, GK; Gautier, M; Berthier, D; Poivey, JP; Sidibe, I; Bengaly, Z; Eggen, A; Boichard, D; Thevenon, S | 2012 | Association studies in QTL regions linked to bovine trypanotolerance in a West African crossbred population | African animal trypanosomosis is a parasitic blood disease transmitted by tsetse flies and is widespread in sub-Saharan Africa. West African taurine breeds have the ability, known as trypanotolerance, to limit parasitaemia and anaemia and remain productive in enzootic areas. Several quantitative trait loci (QTL) underlying traits related to trypanotolerance have been identified in an experimentally infected F2 population resulting from a cross between taurine and zebu cattle. Although this information is highly valuable, the QTL remain to be confirmed in populations subjected to natural conditions of infection, and the corresponding regions need to be refined. In our study, 360 West African cattle were phenotyped for the packed cell volume control under natural conditions of infection in south-western Burkina Faso. Phenotypes were assessed by analysing data from previous cattle monitored over 2 years in an area enzootic for trypanosomosis. We further genotyped for 64 microsatellite markers mapping within four previously reported QTL on BTA02, BTA04, BTA07 and BTA13. These data enabled us to estimate the heritability of the phenotype using the kinship matrix between individuals computed from genotyping data. Thus, depending on the estimators considered and the method used, the heritability of anaemia control ranged from 0.09 to 0.22. Finally, an analysis of association identified an allele of the MNB42 marker on BTA04 as being strongly associated with anaemia control, and a candidate gene, INHBA, as being close to that marker. | 3943 | NA | NA | NA | no | no |
| 3942 | 2017 | NA | Kitchen, A; Miyamoto, MM; Mulligan, CJ | 2008 | Utility of DNA viruses for studying human host history: Case study of JC virus | Microbial pathogens, and viruses in particular, can serve as important complements to traditional genetic markers when investigating the population histories of their human host. The range of mutation rates for DNA viruses suggests that DNA viruses can be useful markers of both recent and ancient events in their host histories. Here, we assess the utility of a well known DNA virus, JC virus (JCV), for investigating human history and demography. Using complete coding viral genomes, we confirm the phylogeographic structure of JCV in populations worldwide and provide coalescent estimates of its evolutionary rate under two alternative models of its history. Using these rate estimates, we compare Bayesian skyline plots of population size changes for JCV to those of its human host as estimated with coding mitochondrial genomes of the latter. These comparisons, when combined with other evidence including a log Bayes Factor model test, show that JCV is evolving rapidly and is therefore tracking the recent history of its human host. These results support the hypothesis that post-World War II societal changes are most likely responsible for the recent demographic patterns observed among different regional JCV populations. In sum, fast evolving DNA viruses, such as JCV, can complement RNA viruses to provide novel insights about the recent history and demography of their human host. (C) 2007 Elsevier Inc. All rights reserved. | 3944 | NA | NA | NA | no | no |
| 3943 | 2017 | NA | Sakihama, N; Kimura, M; Hirayama, K; Kanda, T; Na-Bangchang, K; Jongwutiwes, S; Conway, D; Tanabe, K | 1999 | Allelic recombination and linkage disequilibrium within Msp-1 of Plasmodium falciparum, the malignant human malaria parasite | The C-terminal, cysteine-rich 19 kDa domain of merozoite surface protein-1 (MSP-1) of Plasmodium falciparum is a target of the host’s humoral immunity and thus a malaria vaccine candidate. Although variation in the 19 kDa domain is limited among parasite isolates, tertiary structure-dependent intramolecular associations between the 19 kDa domain and other parts of MSP-1 are suggested to be involved in immune evasion by allowing competitive binding of protective and non-protective antibodies directed to their epitopes, which are conformationally in close proximity but separated at the primary structure. Since allelic recombination can account for the major variability of the Msp-1 gene, we examined whether linkage disequilibrium occurs between polymorphic loci in the 5’- and the 3’-region, the latter encoding the 19 kDa domain. From 184 Thai held isolates, we selected 69 isolates with a single allelic type in six variable blocks of Msp-1 as determined by PCR-based allelic typing. All the isolates showed no evidence of recombination in blocks 6 to 16, whereas recombination was apparent in blocks 2 to 6. Sequencing of the 3’-region revealed two potential recombination sites in block 17. Strong linkage disequilibrium was seen between polymorphic loci in the 5’- and 3’-regions. The strength of this disequilibrium did not correlate with distance between loci. We discuss the possible role of epistatic selection on particular association types (haplotypes) of Msp-1. (C) 1999 Elsevier Science B.V. All rights reserved. | 3945 | NA | NA | NA | no | no |
| 3944 | 2017 | NA | Rubio, AV; Avila-Flores, R; Suzan, G | 2014 | Responses of Small Mammals to Habitat Fragmentation: Epidemiological Considerations for Rodent-Borne Hantaviruses in the Americas | Rodent-borne hantaviruses are a group of zoonotic agents that cause hemorrhagic fever in humans. The transmission of hantaviruses among rodent hosts may be higher with the increase of reservoir host abundance in a given area (density-dependent transmission) and with the decrease of small mammal diversity (dilution effect phenomenon). These population and community parameters may be modified by habitat fragmentation; however, studies that focus on fragmentation and its effect on hantavirus infection risk are scarce. To further understanding of this issue, we assessed some population and community responses of rodents that may increase the risk for hantavirus transmission among wildlife hosts in the Americas. We conducted a meta-analysis of published studies to assess the responses of small mammals to fragmentation of native habitats, relative to patch size. Our analyses included five countries and 14 case studies for abundance of reservoir hosts (8 species) and 15 case studies for species richness. We found that a reduction of patch area due to habitat fragmentation is associated with increased reservoir host abundances and decreased small mammal richness, which is mainly due to the loss of non-host small mammals. According to these results, habitat fragmentation in the Americas should be considered as an epidemiological risk factor for hantavirus transmission to humans. These findings are important to assess potential risk of infection when fragmentation of native habitats occurs. | 3946 | NA | NA | NA | no | no |
| 3945 | 2017 | NA | Lattorff, HMG; Popp, M; Parsche, S; Helbing, S; Erler, S | 2016 | Effective population size as a driver for divergence of an antimicrobial peptide (Hymenoptaecin) in two common European bumblebee species | Social insects are the target of numerous pathogens. This is because the high density of closely-related individuals frequently interacting with each other enhances the transmission and establishment of pathogens. This high selective pressure results in the rapid evolution of immune genes, which might be counteracted by a reduced effective population size (Ne) lowering the effectiveness of selection. We tested the effect of Ne on the evolutionary rate of an important immune gene for the antimicrobial peptide Hymenoptaecin in two common central European bumblebee species: Bombus terrestris and Bombus lapidarius. Both species are similar in their biology and are expected to be under similar selective pressures because pathogen prevalence does not differ between species. However, previous studies indicated a higher Ne in B. terrestris compared to B. lapidarius. We found high intraspecific variability in the coding sequence but low variability for silent polymorphisms in B. lapidarius. Estimates of long-and short-term Ne were three- to four-fold higher Ne in B. terrestris, although the species did not differ in census population sizes. The difference in Ne might result in less efficient selection and suboptimal adaptation of immune genes (e.g. hymenoptaecin) in B. lapidarius, and thus this species might become less resistant and more tolerant, turning into a superspreader of diseases. (c) 2016 The Linnean Society of London, Biological Journal of the Linnean Society, 2016, 119, 299-310. | 3947 | NA | NA | NA | no | no |
| 3946 | 2017 | NA | Murillo, R; Elvira, S; Munoz, D; Williams, T; Caballero, P | 2006 | Genetic and phenotypic variability in Spodoptera exigua nucleopolyhedrovirus isolates from greenhouse soils in southern Spain | Genotypic and phenotypic variation of SeMNPV was examined in seven isolates of SeMNPV originating from occlusion body (OB) populations in the soil of greenhouses in Spain. Semi-quantitative PCR indicated that some of the isolates were composed of a single dominant genotype, whereas other isolates were composed of two or three genotypes in equal proportions. The coexistence of genotypes could be explained by trade-offs among the three phenotypic traits analyzed, namely pathogenicity (LD50), speed of kill (mean time to death), and OB yields, so that increases in one trait were accompanied by decreases in another. Mixed genotype isolates tended to behave differently to single genotype isolates. Two of the genotypic mixtures were significantly more pathogenic (lower LD50 values) for Spodoptera exigua (Hubner) larvae than the single genotypes that they comprised. OB yield/insect was greater for single genotype compared to mixed genotype isolates, despite genotype-specific differences in mean times to death. Total OB production/insect was positively correlated with time to death. Two out of three of the mixed genotype isolates had lower OB yield/mg insect weight at death compared to single genotype isolates. Each genotypic combination appeared to interact to produce a unique phenotype. This suggests the existence of trade-offs between traits leading to the coexistence of distinct genotypes and genotypic mixtures with similar transmissibility. (c) 2005 Elsevier Inc. All rights reserved. | 3948 | NA | NA | NA | no | no |
| 3947 | 2017 | NA | Suchankova, P; Henningsson, S; Baghaei, F; Rosmond, R; Holm, G; Ekman, A | 2009 | Genetic variability within the innate immune system influences personality traits in women | Raised levels of inflammation markers have been associated with several mental disorders; however, studies regarding the relationship between inflammation or the immune system and various aspects of human behaviour are not numerous. The aim of the present study was to investigate whether an association exists between personality traits and two single nucleotide polymorphisms located in genes that are associated with the innate immune system. The studied population consisted of 42-year-old women recruited from the population registry that had been assessed by means of Karolinska Scales of Personality, a self-reported inventory. The first polymorphism, +1444C > T (rs1130864), is located in the gene coding for C-reactive protein (CRP), a marker of low-grade inflammation. The T-allele has previously been suggested to be linked to raised serum levels of CRP. The second polymorphism, Y402H (1277T > C, rs1061170), is located in the gene coding for complement factor H, an important regulator of the complement system. The C-allele has consistently been associated with age-related macular degeneration. While the +1444T allele was associated with higher scores in the personality traits impulsiveness, monotony avoidance and social desirability, the 1277C polymorphism was associated with higher scores in verbal aggression and lower scores in social desirability. In conclusion, the associations between the personality traits and the studied polymorphisms further support the possible influence of the immune system on mental functions. | 3949 | NA | NA | NA | no | no |
| 3948 | 2017 | NA | Wilkinson, HH; Parker, MA | 1996 | Symbiotic specialization and the potential for genotypic coexistence in a plant-bacterial mutualism | Genotypes of the annual legume Amphicarpaen bracteata vary in their degree of specialization toward different nitrogen-fixing bacteria. Plants of lineages ‘’Ib’’ and ‘’II’’ are specialized for mutualism with a limited group of bacterial genotypes. Lineage ‘’Ia’’ plants are symbiotic generalists, interacting with all bacteria associated with specialist (Ib and II) plants, and also with a distinct class of bacteria that only fix nitrogen with lineage Ia plants. The relative performance of generalist and specialist plant lineages was measured in four symbiotic environments: (1) in the absence of nitrogen-fixing bacteria, (2) with bacteria with broad host ranges, (3) with bacteria specialized on lineage Ia hosts, and (4) with a mixture of the bacteria with broad and narrow host ranges. In the presence of bacteria with broad host ranges, the relative performance of different plant lineages was inconsistent among experimental replicates. However, lineage Ia plants had nearly 3 times higher total biomass and 6 times higher seed biomass than lineage Ib or II plants when grown with bacteria specialized on Ia hosts. When exposed to a mixture of bacteria with broad and narrow host ranges, generalist plants had 72% higher total biomass and >100% higher seed biomass than specialist plants. These results imply that in diverse natural populations, where all plants have a choice of symbiotic partners, mutualistic interactions are likely to foster competitive exclusion rather than stable coexistence of different plant lineages. | 3950 | NA | NA | NA | no | no |
| 3949 | 2017 | NA | NONACS, P; TOBIN, JE | 1992 | SELFISH LARVAE - DEVELOPMENT AND THE EVOLUTION OF PARASITIC BEHAVIOR IN THE HYMENOPTERA | Queens of hymenopteran social parasites manipulate the workers of other social species into raising their offspring. However, nonconspecific brood care may also allow the parasite larvae to control their own development to a greater extent than possible in nonparasitic species. An evolutionary consequence of this may be the loss of the parasite’s worker caste if the larvae can increase their fitness by developing into sexuals rather than workers. We argue that this loss is particularly likely in species in which there is little inclusive fitness benefit in working. Retention of a worker caste correlates with characteristics that increase the fitness of working relative to becoming a sexual, such as worker-production of males, high intracolony relatedness, and seasonal environments where the hosts of potential parasite queens are not always available. Further evidence strongly suggests that when the worker caste is evolutionarily lost in perennial species like ants, it disappears rapidly and through a reduction in caste threshold and queen size, so that parasite larvae become queens with less food than required to produce host workers. This evolutionary process, however, appears to lower overall population fitness, resulting in workerless parasite species having small populations and being geographically restricted. Conversely, in annual species like bees and wasps, workerless social parasitism evolves with no size reduction in queens, which is consistent with an expected lower level of queen/offspring conflict. | 3951 | NA | NA | NA | no | no |
| 3950 | 2017 | NA | Ezenwa, VO; Henshaw, MT; Queller, DC; Strassmann, JE | 1998 | Patterns of buzz running, a pre-swarming behavior, in the Neotropical wasp Parachartergus colobopterus | Knowing whether workers or queens control reproduction is vital for understanding social evolution in insects. Among epiponine wasps, a significant part of reproduction is the production of daughter swarms. One of the first behaviors indicative of swarming is the buzzing run - a behavior in which a wasp runs rapidly back and forth on the natal nest, bumping into nestmates while traversing a significant portion of the nest. In this study we investigated whether the buzz runners in Parachartergus colobopterus were queens who were not reproducing on the current nest (reproductive losers), or workers, as evidence from other species indicates. We suspected that loser queens, who may be anxious to swarm in order to increase reproductive output, could be the buzz runners because P. colobopterus swarms are primarily composed of queens who are not the mothers of the accompanying workers. Our results show that workers are the buzz runners, and that queen number does not influence the level of buzz running on a colony. We also found that buzz running is more common on large colonies with low worker relatedness, the sort that are likely to produce swarms. However, buzzing runs also occurred sporadically on smaller nests. The small nests had more nest parasites (mostly flies) than the large nests, and the parasite dance, which is very similar to the buzzing run, was seen only on these nests. | 3952 | NA | NA | NA | no | no |
| 3951 | 2017 | NA | Fouks, B; Lattorff, HMG | 2011 | Recognition and Avoidance of Contaminated Flowers by Foraging Bumblebees (Bombus terrestris) | Bumblebee colonies are founded by a single-mated queen. Due to this life history trait, bumblebees are more susceptible to parasites and diseases than polyandrous and/or polygynous social insects. A greater resistance towards parasites is shown when the genetic variability within a colony is increased. The parasite resistance may be divided into different levels regarding the step of the parasite infection (e. g. parasite uptake, parasite intake, parasite’s establishment in the nest, parasite transmission). We investigate the prophylactic behaviour of bumblebees. Bumblebees were observed during their foraging flights on two artificial flowers; one of these was contaminated by Crithidia bombi, a naturally occurring gut parasite of bumblebees (in a control experiment the non-specific pathogen Escherichia coli was used). For C. bombi, bumblebees were preferentially observed feeding on the non-contaminated flower. Whereas for E. coli, the number of visits between flowers was the same, bumblebees spent more time feeding on the non-contaminated flower. These results demonstrate the ability of bumblebees to recognise the contamination of food sources. In addition, bumblebees have a stronger preference for the non-contaminated flower when C. bombi is present in the other flower than with E. coli which might be explained as an adaptive behaviour of bumblebees towards this specific gut parasite. It seems that the more specific the parasite is, the more it reduces the reward of the flower. | 3953 | NA | NA | NA | no | no |
| 3952 | 2017 | NA | King, RB; Stanford, KM | 2006 | Headstarting as a management tool: A case study of the plains gartersnake | The use of headstarting and related techniques as management tools for threatened and endangered species remains controversial, due in part to a lack of empirical data on their effectiveness. Here, we present data on pre-natal mortality, growth, and survival during headstarting as well as growth, survival, and reproduction following release for an unprotected population of plains gartersnakes. We combine these data with data on growth and survival of comparably sized wild-caught snakes to provide an overall evaluation of the potential effectiveness of headstarting. Depending on rearing conditions, proportions of live births varied from 0.79 (‘worst-case’) to 0.94 (‘best-case’), survival during headstarting ranged from 0.74 (‘worstcase’) to 0.88 (‘best-case’), and second-year survival following release ranged from 0.11 (‘worst-case’) to 0.40 (‘best-case’). In comparison, survival of free-ranging snakes was 0.16 in their first year and 0.40 in their second year. Assuming the proportion of live births in nature is close to 1, expected survival to reproductive maturity among free-ranging snakes is 0.06, whereas that for headstarted snakes ranges from 0.07 (‘worst-case’) to 0.33 (‘best-case’). The growth rate of headstarted snakes following release was similar to that of similar-sized free-ranging snakes, and headstarted snakes were successful at reproduction. Together, these results suggest that, if carefully implemented, headstarting may be in effective management tool for endangered plains gartersnake populations in Ohio and for other natricine snakes with similar demographic characteristics. | 3954 | NA | NA | NA | no | no |
| 3953 | 2017 | NA | Helenurm, K | 1998 | Outplanting and differential source population success in Lupinus guadalupensis | The choice of appropriate source populations is crucial for the success of outplanting attempts, but this choice is often based on assumptions regarding patterns of adaptation Land distribution of genetic variability in natural populations. Although local adaptation is often assumed to exist, few data exist to support this model on smaller geographic scales, particularly in rare plant species. dry study investigated the pattern of adaptation in populations of an annual, island endemic plant, Lupinus guadalupensis, on San Clemente Island California. A reciprocal transplant experiment with three populations provided no evidence for local adaptation, but two source populations performed significantly better than the other at all sites. Desiccation and herbivory are the major factors causing mortality and reducing fruit production. The young rosettes are the most vulnerable life-cycle stage Differences among natural population sites in vegetation community, dominant species, soil type, and parent material did not affect populations of L. guadalupensis; significant differences were not observed in either total fruit production or in the majority of fitness components Success of outplanting into three introduction Sites varied from the death of all plants before flowering or before fruits ripened to fruit production exceeding that at natural population sites. The results suggest that large populations are better sources of seeds for outplanting in L. guadalupensis and that outplanting is most successful in sites where plants are least subject to desiccation. | 3955 | NA | NA | NA | no | no |
| 3954 | 2017 | NA | Meunier, J; Reber, A; Chapuisat, M | 2011 | Queen acceptance in a socially polymorphic ant | A central question in social evolution is what processes regulate the number of breeders in each social group. Here, we tested whether differences in the rate of acceptance of new queens by resident workers could be a proximate cause explaining the coexistence of single- and multiple-queen colonies in an ant population. We found that Formica selysi workers discriminated against foreign (non-nestmate) queens, which contributes to maintaining the genetic integrity of the social group essential to kin selection. All the young and newly mated foreign queens introduced into experimental groups of workers died within 48 h. In contrast, workers frequently accepted young newly mated nestmate queens. The survival of nestmate queens was not significantly lower in groups of workers originating from single- queen colonies than in groups of workers originating from multiple-queen colonies. Finally, virgin queens had significantly higher survival than mated queens. Together, these results show that the maintenance of single-queen and multiple-queen colonies in the same population is unlikely to be caused by strong differences between the two types of colonies in their rate of acceptance of new queens by workers. They also suggest that the discrimination of queens by resident workers restricts the dispersal of foreign queens among colonies, but not the acceptance of additional nestmate queens. (c) 2010 The Association for the Study of Animal Behaviour. Published by Elsevier Ltd. All rights reserved. | 3956 | NA | NA | NA | no | no |
| 3955 | 2017 | NA | Waldron, ERB; Whittaker, JC; Balding, DJ | 2006 | Fine mapping of disease genes via haplotype clustering | We propose an algorithm for analysing SNP-based population association studies, which is a development of that introduced by Molitor et al. [2003: Am J Hum Genet 73:1368-1384]. It uses clustering of haplotypes to overcome the major limitations of many current haplotype-based approaches. We define a between-haplotype score that is simple, yet appears to capture much of the information about evolutionary relatedness of the haplotypes in the vicinity of a (unobserved) putative causal locus. Haplotype clusters can then be defined via a putative ancestral haplotype and a cut-off distance. The number of an individual’s two haplotypes that lie within the cluster predicts the individual’s genotype at the causal locus. This predicted genotype can then be investigated for association with the phenotype of interest. We implement our approach within a Markov-chain Monte Carlo algorithm that, in effect, searches over locations and ancestral haplotypes to identify large, case-rich clusters. The algorithm successfully fine-maps a causal mutation in a test analysis using real data, and achieves almost 98% accuracy in predicting the genotype at the causal locus. A simulation study indicates that the new algorithm is substantially superior to alternative approaches, and it also allows us to identify situations in which multipoint approaches can substantially improve over single-SNP analyses. Our algorithm runs quickly and there is scope for extension to a wide range of disease models and genomic scales. | 3957 | NA | NA | NA | no | no |
| 3956 | 2017 | NA | Burgess, EPJ; Barraclough, EI; Kean, AM; Markwick, NP; Malone, LA | 2015 | Responses of 9 lepidopteran species to Bacillus thuringiensis: How useful is phylogenetic relatedness for selecting surrogate species for nontarget arthropod risk assessment? | To evaluate phylogenetic relatedness as a proxy for susceptibility to Bacillus thuringiensis (Bt) when selecting species to act as surrogates for others in prerelease testing, we examined the responses of 11 laboratory-reared lepidopteran colonies, comprising members of 2 families, 5 genera, and 9 species, to a commercial Bt preparation. Survival, pupal mass, and timing of pupation and adult emergence of 2 noctuids (Spodoptera litura and Helicoverpa armigera) and 7 tortricids (Cnephasia jactatana, Ctenopseustis obliquana, Ctenopseustis herana, Planotortrix octo, Planotortrix notophaea, Planotortrix excessana [2 different laboratory colonies], and Epiphyas postvittana [2 colonies]) were examined after feeding first instar larvae with artificial diet containing 5 mu L/100mL Dipel (R) ES (Bt subsp. kurstaki). Bt caused significant larval mortality in all species except S. litura, in which only pupation was delayed compared with untreated controls. Neither of the noctuid species tested would act as a suitable surrogate for the other in tests of Bt impacts on survival. With the exception of the 2 colonies of E. postvittana, which differed from each other not only in their responses to Bt but also in their development times when not treated with Bt, species within each tortricid genus had similar responses to Bt and thus could act as surrogates for each other. Members of different genera within this family could represent each other only if relatively coarse measurement endpoints (e.g.,toxic or not) were considered adequate for assessing risks to nontarget species in the field. | 3958 | NA | NA | NA | no | no |
| 3957 | 2017 | NA | He, D; Eskin, E | 2013 | Hap-seqX: Expedite algorithm for haplotype phasing with imputation using sequence data | Haplotype phasing is one of the most important problems in population genetics as haplotypes can be used to estimate the relatedness of individuals and to impute genotype information which is a commonly performed analysis when searching for variants involved in disease. The problem of haplotype phasing has been well studied. Methodologies for haplotype inference from sequencing data either combine a set of reference haplotypes and collected genotypes using a Hidden Markov Model or assemble haplotypes by overlapping sequencing reads. A recent algorithm Hap-seq considers using both sequencing data and reference haplotypes and it is a hybrid of a dynamic programming algorithm and a Hidden Markov Model (HMM), which is shown to be optimal. However, the algorithm requires extremely large amount of memory which is not practical for whole genome datasets. The current algorithm requires saving intermediate results to disk and reads these results back when needed, which significantly affects the practicality of the algorithm. In this work, we proposed the expedited version of the algorithm Hap-seqX, which addressed the memory issue by using a posterior probability to select the records that should be saved in memory. We show that Hap-seqX can save all the intermediate results in memory and improves the execution time of the algorithm dramatically. Utilizing the strategy, Hap-seqX is able to predict haplotypes from whole genome sequencing data. (c) 2012 Elsevier B.V. All rights reserved. | 3959 | NA | NA | NA | no | no |
| 3958 | 2017 | NA | Abney, M; Ober, C; McPeek, MS | 2002 | Quantitative-trait homozygosity and association mapping and empirical genomewide significance in large, complex pedigrees: Fasting serum-insulin level in the hutterites | We present methods for linkage and association mapping of quantitative traits for a founder population with a large, known genealogy. We detect linkage to quantitative-trait loci (QTLs) through a multipoint homozygosity-mapping method. We propose two association methods, one of which is single point and uses a general two-allele model and the other of which is multipoint and uses homozygosity by descent for a particular allele. In all three methods, we make extensive use of the pedigree and genotype information, while keeping the computations simple and efficient. To assess significance, we have developed a permutation-based test that takes into account the covariance structure due to relatedness of individuals and can be used to determine empirical genomewide and locus-specific P values. In the case of multivariate-normally distributed trait data, the permutation-based test is asymptotically exact. The test is broadly applicable to a variety of mapping methods that fall within the class of linear statistical models (e. g., variance-component methods), under the assumption of random ascertainment with respect to the phenotype. For obtaining genomewide P values, our proposed method is appropriate when positions of markers are independent of the observed linkage signal, under the null hypothesis. We apply our methods to a genome screen for fasting insulin level in the Hutterites. We detect significant genomewide linkage on chromosome 19 and suggestive evidence of QTLs on chromosomes 1 and 16. | 3960 | NA | NA | NA | no | no |
| 3959 | 2017 | NA | Weclawski, U; Heitlinger, EG; Baust, T; Klar, B; Petney, T; Han, YS; Taraschewski, H | 2013 | Evolutionary divergence of the swim bladder nematode Anguillicola crassus after colonization of a novel host, Anguilla anguilla | Background: Anguillicola crassus, a swim bladder nematode naturally parasitizing the Japanese eel, was introduced about 30 years ago from East Asia into Europe where it colonized almost all populations of the European eel. We conducted a common garden experiment under a reciprocal transfer design infecting both European and Japanese eels with populations of A. crassus from Germany, Poland and Taiwan. We tested, whether differences in infectivity, developmental dynamics and reproductive output between the European and Asian parasite populations occur while harboured in the specimens of native and colonized eel host, and if these differences are genetically based or are plastic responses to the new environment. Results: Under common garden conditions an evolutionary change in the both European parasite populations of A. crassus compared with their Taiwanese conspecifics was observed for infectivity and developmental dynamics, but not for reproductive output. When infecting the European eel, current European populations of the parasite were less infective and developed faster than their Taiwanese conspecifics. In the reciprocally infected Japanese eel the genetically induced differences between the parasite strains were less apparent than in the European eel but higher infectivity, faster development and higher larval mortality of the European parasite populations could be inferred. Conclusions: The differences in infectivity and developmental dynamics between European and Taiwanese populations of A. crassus found in our study suggest rapid genetic divergence of this parasite after a successful host switch in Europe. | 3961 | NA | NA | NA | no | no |
| 3960 | 2017 | NA | Freifeld, HB; Plentovich, S; Farmer, C; Kohley, CR; Luscomb, P; Work, TM; Tsukayama, D; Wallace, GE; MacDonald, MA; Conant, S | 2016 | Long-distance translocations to create a second millerbird population and reduce extinction risk | Translocation is a conservation tool used with increasing frequency to create additional populations of threatened species. In addition to following established general guidelines for translocations, detailed planning to account for unique circumstances and intensive post-release monitoring to document outcomes and guide management are essential components of these projects. Recent translocation of the critically endangered Nihoa millerbird (Acrocephalus familiaris kingi) provides an example of this planning and monitoring. The Nihoa millerbird is a passerine bird endemic to Nihoa Island in the remote Northwestern Hawaiian Islands. The closely related, ecologically similar Laysan millerbird (Acrocephalus familiaris familiaris) went extinct on Laysan Island in the early 20th century when the island was denuded by introduced rabbits. To reduce extinction risk, we translocated 50 adult Nihoa millerbirds more than 1000 km by sea to Laysan, which has recovered substantially in the past century and has ample habitat and a rich prey-base for millerbirds. Following five years of intensive background research and planning, including development of husbandry techniques, fundraising, and regulatory compliance, translocations occurred in 2011 and 2012. Of 11 females in each cohort, 8 (2011 cohort) and II (2012 cohort) produced at least one brood of fledglings during their first year on Laysan. At the conclusion of monitoring in September 2014, 37 of the translocated birds were known to survive, and the population was estimated at 164 birds. The reintroduction of millerbirds to Laysan represents a milestone in the island’s ongoing restoration. Published by Elsevier Ltd. | 3962 | NA | NA | NA | no | no |
| 3961 | 2017 | NA | Kacar, YA; Demirel, A; Tuzcu, O; Yesiloglu, T; Ulas, M; Yildirim, B | 2005 | Preliminary results on fingerprinting of lemon genotypes tolerant to mal secco disease by RAPD markers | We investigated the possible utility of randomly amplified polymorphic DNA (RAPD) markers in our lemon (Citrus limon Burm. F.) breeding program emphasizing the excellent quality and tolerance to mal secco disease caused by Phoma tracheiphila Kanc. et Ghik. The genotypes studied included 12 advanced selections that were previously shown to be resistant or tolerant to this disease and 4 lemon varieties (“Kutdiken 79”, “Tuzcu 05-Yediveren Kutdiken”; “Finike Yerli Yuvarlak”, “Antalya Yerli Yuvarlak”). DNA characterized the 16 lemon genotypes. We scored 350 bands generated by 48 selected RAPD primers. 38.85% of these were constant corresponding to monomorphic loci while 61.14% of the bands were polymorphic. The number of bands for each primer varied from 2 to 15 with an average of 7.3 bands per primer, the sizes of the amplified DNA bands ranged from 250 to 3000 bp. Primers differed in their capacity to detect polymorphism and total proportion of polymorphic products ranged from 12.5% to 100%. The analysis of 16 lemon genotypes using 48 RAPD primers allowed us to distinguish all genotypes except “Tuzcu 896” and “Tuzcu 897” which were selection of “Tuzcu 9N Aklimon”. The dendrogram indicated that the genotypes can be separated into two major groups with a similarity value of 0.79. These results show that utilization of RAPDs can offer great benefits to our lemon breeding program in several ways. such as identifying genotypes and studying the genetic similarity among genotypes. The future work can be extended to develop RAPD markers linked to gene(s) conferring tolerance to mal secco. | 3963 | NA | NA | NA | no | no |
| 3962 | 2017 | NA | Piso, RJ; Kach, R; Pop, R; Zillig, D; Schibli, U; Bassetti, S; Meinel, D; Egli, A | 2017 | A Cross-Sectional Study of Colonization Rates with Methicillin-Resistant Staphylococcus aureus (MRSA) and Extended-Spectrum Beta-Lactamase (ESBL) and Carbapenemase-Producing Enterobacteriaceae in Four Swiss Refugee Centres | Background The recent crisis of refugees seeking asylum in European countries challenges public health on many levels. Most refugees currently arrive from Syria, Afghanistan, or Eritrea. Data about multidrug resistant bacteria (MDR) prevalence are not present for these countries. However, when entering the European heath care systems, data about colonisation rates regarding highly resistant bacterial pathogens are important. Methods We performed a cross-sectional screening in four Swiss refugee centres to determine the colonization rates for MRSA and ESBL-and carbapenemase-producing Enterobacteriaceae. We used pharyngeal, nasal, and inguinal swabs for MRSA and rectal swabs and urine for ESBL and carbapenemase screening using standard microbiological procedures. Whole genome sequencing (WGS) was used to determine the relatedness of MRSA isolates with high resolution due to a suspected outbreak. Results 41/261(15.7%) refugees were colonized with MRSA. No differences regarding the country of origin were observed. However, in a single centre significantly more were colonized, which was confirmed to be a recent local outbreak. 57/241 (23.7%) refugees were colonized with ESBL with significantly higher colonisation in persons originating from the Middle East (35.1%, p<0.001). No carbapenemase producers were detected. Conclusion The colonisation rate of the refugees was about 10 times higher for MRSA and 2-5 times higher for ESBL compared to the Swiss population. Contact precaution is warranted for these persons if they enter medical care. In cases of infections, MRSA and ESBL-producing Enterobacteriaceae should be considered regarding antibiotic treatment choices. | 3964 | NA | NA | NA | no | no |
| 3963 | 2017 | NA | Flynn, JM; Chain, FJJ; Schoen, DJ; Cristescu, ME | 2017 | Spontaneous Mutation Accumulation in Daphnia pulex in Selection-Free vs. Competitive Environments | Understanding the rates, spectra, and fitness effects of spontaneousmutations is fundamental to answering key questions in evolution, molecular biology, disease genetics, and conservation biology. To estimate mutation rates and evaluate the effect of selection on new mutations, we propagated mutation accumulation (MA) lines of Daphnia pulex for more than 82 generations and maintained a non-MA population under conditions where selection could act. Both experiments were started with the same obligate asexual progenitor clone. By sequencing 30 genomes and implementing a series of validation steps that informed the bioinformatic analyses, we identified a total of 477 single nucleotide mutations (SNMs) in the MA lines, corresponding to a mutation rate of 2.30 x 10(-9) (95% CI 1.90-2.70 x 10(-9)) per nucleotide per generation. The high overall rate of loss of heterozygosity (LOH) of 4.82 x 10(-5) per site per generation was due to a large ameiotic recombination event spanning an entire arm of a chromosome (similar to 6Mb) and several hemizygous deletion events spanning similar to 2 kb each. In the non-MA population, we found significantly fewer mutations than expected based on the rate derived from the MA experiment, indicating purifying selection was likely acting to remove new deleterious mutations. We observed a surprisingly high level of genetic variability in the non-MA population, which we propose to be driven by balancing selection. Our findings suggest that both positive and negative selection on new mutations is powerful and effective in a strictly clonal population. | 3965 | NA | NA | NA | no | no |
| 3964 | 2017 | NA | Allen, A; Breadon, E; Byrne, A; Mallon, T; Skuce, R; Groussaud, P; Dainty, A; Graham, J; Jones, K; Pollock, L; Whatmore, A | 2015 | Molecular Epidemiology of Brucella abortus in Northern Ireland-1991 to 2012 | Background Brucellosis is the most common bacterial zoonoses worldwide. Bovine brucellosis caused by Brucella abortus has far reaching animal health and economic impacts at both the local and national levels. Alongside traditional veterinary epidemiology, the use of molecular typing has recently been applied to inform on bacterial population structure and identify epidemiologically-linked cases of infection. Multi-locus variable number tandem repeat VNTR analysis (MLVA) was used to investigate the molecular epidemiology of a well-characterised Brucella abortus epidemic in Northern Ireland involving 387 herds between 1991 and 2012. Results MLVA identified 98 unique B. abortus genotypes from disclosing isolates in the 387 herds involved in the epidemic. Clustering algorithms revealed the relatedness of many of these genotypes. Combined with epidemiological information on chronology of infection and geographic location, these genotype data helped to identify 7 clonal complexes which underpinned the outbreak over the defined period. Hyper-variability of some VNTR loci both within herds and individual animals led to detection of multiple genotypes associated with single outbreaks. However with dense sampling, these genotypes could still be associated with specific clonal complexes thereby permitting inference of epidemiological links. MLVA-based epidemiological monitoring data were congruent with an independent classical veterinary epidemiology study carried out in the same territory. Conclusions MLVA is a useful tool in ongoing disease surveillance of B. abortus outbreaks, especially when combined with accurate epidemiological information on disease tracings, geographical clustering of cases and chronology of infection. | 3966 | NA | NA | NA | no | no |
| 3965 | 2017 | NA | Zink, AG; Lyon, BE | 2016 | Evolution of Conspecific Brood Parasitism versus Cooperative Breeding as Alternative Reproductive Tactics | Cooperative breeding and conspecific brood parasitism can both be favored by ecological saturation of breeding territories or nest sites. Here, we develop a model that links these alternative reproductive tactics by focusing on nonnesting females (S) that either breed cooperatively with a nesting female (N) or parasitize a third, outside host female (H). We find that cooperative breeding is more likely to evolve with increasing relatedness of cooperating females (S or N) to the outside host female (H) and with increasing costs to the hosts for receiving parasitic eggs. Conversely, cooperation is less likely with increasing kinship between the two potentially cooperative nesters (S and N). This is because even the nesting female gains higher inclusive fitness as long as the number of parasitic eggs (of her otherwise potentially cooperating partner) is sufficiently high. We find the relationship between kinship and reproductive skew within cooperative nests can be either positive or negative depending on the fecundity of parasites versus nesting females. We also find that either of the cooperatively nesting females is more likely to tolerate a smaller fraction of group reproduction as kinship with the host female increases and as the host reproduces more (relative to the parasite) in outside nests. Finally, our model predicts that, as the outside option of conspecific brood parasitism becomes more profitable, helping behavior (zero reproduction by one female) is less likely to evolve in cooperatively breeding groups. | 3967 | NA | NA | NA | no | no |
| 3966 | 2017 | NA | Salathe, RM; Schmid-Hempel, P | 2011 | The Genotypic Structure of a Multi-Host Bumblebee Parasite Suggests a Role for Ecological Niche Overlap | The genotypic structure of parasite populations is an important determinant of ecological and evolutionary dynamics of host-parasite interactions with consequences for pest management and disease control. Genotypic structure is especially interesting where multiple hosts co-exist and share parasites. We here analyze the natural genotypic distribution of Crithidia bombi, a trypanosomatid parasite of bumblebees (Bombus spp.), in two ecologically different habitats over a time period of three years. Using an algorithm to reconstruct genotypes in cases of multiple infections, and combining these with directly identified genotypes from single infections, we find a striking diversity of infection for both data sets, with almost all multilocus genotypes being unique, and are inferring that around half of the total infections are resulting from multiple strains. Our analyses further suggest a mixture of clonality and sexuality in natural populations of this parasite species. Finally, we ask whether parasite genotypes are associated with host species (the phylogenetic hypothesis) or whether ecological factors (niche overlap in flower choice) shape the distribution of parasite genotypes (the ecological hypothesis). Redundancy analysis demonstrates that in the region with relatively high parasite prevalence, both host species identity and niche overlap are equally important factors shaping the distribution of parasite strains, whereas in the region with lower parasite prevalence, niche overlap more strongly contributes to the distribution observed. Overall, our study underlines the importance of ecological factors in shaping the natural dynamics of host-parasite systems. | 3968 | NA | NA | NA | no | no |
| 3967 | 2017 | NA | Osorio, NS; Rodrigues, F; Gagneux, S; Pedrosa, J; Pinto-Carbo, M; Castro, AG; Young, D; Comas, I; Saraiva, M | 2013 | Evidence for Diversifying Selection in a Set of Mycobacterium tuberculosis Genes in Response to Antibiotic- and Nonantibiotic-Related Pressure | Tuberculosis (TB) is a global health problem estimated to kill 1.4 million people per year. Recent advances in the genomics of the causative agents of TB, bacteria known as the Mycobacterium tuberculosis complex (MTBC), have allowed a better comprehension of its population structure and provided the foundation for molecular evolution analyses. These studies are crucial for a better understanding of TB, including the variation of vaccine efficacy and disease outcome, together with the emergence of drug resistance. Starting from the analysis of 73 publicly available genomes from all the main MTBC lineages, we have screened for evidences of positive selection, a set of 576 genes previously associated with drug resistance or encoding membrane proteins. As expected, because antibiotics constitute strong selective pressure, some of the codons identified correspond to the position of confirmed drug-resistance-associated substitutions in the genes embB, rpoB, and katG. Furthermore, we identified diversifying selection in specific codons of the genes Rv0176 and Rv1872c coding for MCE1-associated transmembrane protein and a putative l-lactate dehydrogenase, respectively. Amino acid sequence analyses showed that in Rv0176, sites undergoing diversifying selection were in a predicted antigen region that varies between “modern” lineages and “ancient” MTBC/BCG strains. In Rv1872c, some of the sites under selection are predicted to impact protein function and thus might result from metabolic adaptation. These results illustrate that diversifying selection in MTBC is happening as a consequence of both antibiotic treatment and other evolutionary pressures. | 3969 | NA | NA | NA | no | no |
| 3968 | 2017 | NA | Yan, T; Yang, YN; Cheng, X; DeAngelis, MM; Hoh, J; Zhang, H | 2009 | Genotypic Association Analysis Using Discordant-Relative-Pairs | In practice, family-based design has been widely used in disease-gene association analysis. The major advantage of such design is that it is not subject to spurious association due to population structure such as population stratification (PS) and admixture. A disadvantage is that parental genotypes are hard to obtain if the disease is late onset for which a discordant-relative-pair design is useful. Designs of such kind include full-sib-pair, half-sib-pair, first-cousin-pair, and so on. The closer the relatedness of the pair, the less possible that they are subject to population stratification. On the other hand, the association test using close relative-pairs may be less powerful due to over-matching. Trade-off between these two factors (population structure and over-matching) is the major concern of this study. Some tests, namely McNemar’s test, matched Cochran-Armitage trend tests (CATTs), matched maximum efficient robust test (MERT), and Bhapkar’s test, are used for testing disease-gene association based on relative-pair designs. These tests are shown to be valid in the presence of PS but not admixture. Numerical studies show that the McNemar’s test, additive CATT, MERT, and Bhapkar’s test are robust in power, but none of them is uniformly more powerful than the others. In most simulations, the power of any of the tests increases as the pair is more distant. The proposed methods are applied to two real examples. | 3970 | NA | NA | NA | no | no |
| 3969 | 2017 | NA | Vandame, R; Palacio, MA | 2010 | Preserved honey bee health in Latin America: a fragile equilibrium due to low-intensity agriculture and beekeeping? | The Latin American subcontinent contains some of the world’s major honey producing and exporting countries, but the status of bee health in this part of the world has not been clearly documented. There have been no reports of massive colony losses in Latin America, at least from the symptoms of CCD (colony collapse disorder) or in the proportion and extent of the situations in the US and Europe. We examine possible reasons for the difference, and develop hypotheses that this prevailing good bee health could be due to: (1) the management of generally unselected bees with a certain natural resistance to diseases (tropical regions) or the selection of disease resistant bees (temperate regions); (2) a lower proportion of cropland over the total land area, resulting in more abundant or higher-quality pollen resources for bees; (3) the generally small-scale, low-income and little subsidized agriculture, and concomitant lower use of insecticides compared to industrialized countries. These general parameters may act synergistically, resulting in a large number of configurations across the tremendous ecological, social and economic diversity of Latin America. We suggest that the health of honey bees in Latin America may be ultimately due to the practices of low-income agriculture and beekeeping in the region, leading to more sustainable conditions for the bees. However the increasing trend of land use intensification in some parts of Latin America could lead to declines in honey bee health and population size. | 3971 | NA | NA | NA | no | no |
| 3970 | 2017 | NA | Ranta, P; Jokinen, A; Laaka-Lindberg, S | 2016 | Surviving in Europe: geopolitics of biodiversity conservation illustrated by a proxy species Viola uliginosa | Building strategies for continental-scale conservation is challenging due to evolutionary and geopolitical problems. How do policy choices arise from this setting? In this study, we integrate ecological research with policy analysis to examine the problem field with a case study research. We use a violet species endemic to Europe, Viola uliginosa, as a proxy for a significant European Union (EU)-Russian biodiversity pattern and its conservation. The violet’s core populations locate in Belarus, Ukraine, and Russia, and all populations in the EU are peripheral. The species is endangered in 12 EU member states and in decline in many places elsewhere. To analyze the choices of conservation, we gathered data on its ecology, distribution, and conservation mechanisms across Europe, putting additional emphasis on the EU enlargement and long-term site histories in Finland. We found that the survival of the species in the EU depends on the enlargement negotiations, conflicts between the EU biodiversity and agricultural policies, selection of the species to national Red Lists and the Habitats Directive, and contingent site histories depending on the conservation activities by civic actors and the member states. While the evolutionary aspect emphasizes the genetic differentiation potential of peripheral populations, the geopolitical aspect characterizes the EU as simultaneous spaces of a monotopia, borderlands, and polycentric development. We conclude that intersections between these geopolitical spaces can be used with evolutionary perspectives to identify local, European, and network-driven policy choices of conservation. | 3972 | NA | NA | NA | no | no |
| 3971 | 2017 | NA | Dionisio, F; Gordo, I | 2006 | The tragedy of the commons, the public goods dilemma, and the meaning of rivalry and excludability in evolutionary biology | Problem: In the study of conflicts, both economists and evolutionary biologists use the concepts ‘tragedy of the commons’ and `public goods dilemma’. What is the relationship between the economist and evolutionist views of these concepts? Model features: The economics literature defines the tragedy of the commons and the public goods dilemma in terms of rivalry and excludability of the good. In contrast, evolutionists define these conflicts based on fitness functions with two components: individual and group components of fitness. Mathematical method: Evolutionary game theory and the calculation of evolutionarily stable strategy trait values by standard optimization techniques and by replacing slopes of group phenotype on individual genotype by coefficients of relatedness. Conclusion: There is a direct relationship between rivalry and the individual component of fitness and between excludability and the group component of fitness. Moreover, although the prisoner’s dilemma constitutes a suitable metaphor to analyse both the public goods dilemma and the tragedy of the commons, it gives the false idea that the two conflicts are symmetric since they refer to situations in which individuals consume a common resource - tragedy of the commons - or contribute to a collective action or common good - public goods dilemma. However, the two situations are clearly not symmetric: from the economical point of view they differ by rivalry. and from the evolutionary biology point of view the two conflicts differ by the significance of the within-group competition in the fitness function. | 3973 | NA | NA | NA | no | no |
| 3972 | 2017 | NA | Fisker, EN; Toft, S | 2004 | Effects of chronic exposure to a toxic prey in a generalist predator | Generalist predators experience large differences in prey quality, and eating toxic prey may affect their fitness even if the toxic prey constitutes only a small fraction of a mixed diet. Feeding and life history parameters were examined in the wolf spider Pardosa prativaga during continuous exposure to the toxic collembolan Folsomia candida. Spiderlings were divided into a control group fed adult Drosophila melanogaster, a group fed only F. candida, and a mixed diet group fed both types of prey. Folsomia candida reduced survival, developmental stage attained and growth rate of all exposed spiders. Spiders chronically exposed to F. candida increased their consumption of D. melanogaster compared to control spiders. Folsomia candida thus inhibited the utilization of the high-quality food and/or increased the respiration rate. The intake of F. candida remained at a constant low level throughout the experiment, indicating that P. prativaga was unable to develop an absolute aversion against this prey. Half of the control group was also given the mixed diet after a large juvenile instar was attained. These spiders were not affected to the same extent as the newly hatched spiderlings, indicating an ontogenetically increased tolerance. Comparison of laboratory-raised and field-caught spiders confirmed that tolerance to F. candida was size-dependent and thus not an induced response. A differential survival in the mixed diet group, based partly on maternal effects, indicated possible genetic variability in the physiological tolerance to F. candida. | 3974 | NA | NA | NA | no | no |
| 3973 | 2017 | NA | Orlowski, G | 2007 | Spatial distribution and seasonal pattern in road mortality of the common toad Bufo bufo in an agricultural landscape of south-western Poland | Amphibians are the group of animals suffering particularly from the presence of roads and vehicle traffic. The seasonal migration to breeding places undertaken by amphibians in the temperate climate zone is the main reason for their appearance on roads. Between June 2001 and August 2003, 957 common toads Bufo bufo were recorded killed on 48.8 km road network with various traffic volumes (350-10500 cars per 24 h), situated in the agricultural landscape of south-western Poland. The highest mortality was recorded in April (57% of all road-kills). The places with highest recorded mortality varied markedly throughout the year. In spring, many more animals died within the built-up areas, while in summer and autumn their number increased in the open countryside. During the whole study period, 73% of all road-kills were recorded on roads (55% of all controlled) with the lowest traffic volume (350-470 cars per 24 h). The average number of road-kills on roads with the high traffic volume (5700-10500 cars per 24 h) was over 15 times lower than on the roads with low traffic (0-0.17 road-kills per 100 m on roads with high traffic vs 2.59 road-kills per 100 m on roads with low traffic). The number of road-kills on 15 road sections was most closely related to the abundance of local populations of Bufo bufo and to the size of water bodies situated in the road vicinity. The yearly level of local mortality in breeding populations of Bufo bufo due to the vehicle traffic ranged from 2 to 18%. | 3975 | NA | NA | NA | no | no |
| 3974 | 2017 | NA | Neve, P; Powles, S | 2005 | Recurrent selection with reduced herbicide rates results in the rapid evolution of herbicide resistance in Lolium rigidum | There has been much debate regarding the potential for reduced rates of herbicide application to accelerate evolution of herbicide resistance. We report a series of experiments that demonstrate the potential for reduced rates of the acetyl-coenzyme A carboxylase (ACCase)-inhibiting herbicide diclofop-methyl to rapidly select for resistance in a susceptible biotype of Lolium rigidum. Thirty-six percent of individuals from the original VLR1 population survived application of 37.5 g diclofop-methyl ha(-1) (10% of the recommended field application rate). These individuals were grown to maturity and bulk-crossed to produce the VLR1 low dose-selected line VLR1 (0.1). Subsequent comparisons of the dose-response characteristics of the original and low dose-selected VLR1 lines demonstrated increased tolerance of diclofop-methyl in the selected line. Two further rounds of selection produced VLR1 lines that were resistant to field-applied rates of diclofop-methyl. The LD(50) (diclofop-methyl dose required to cause 50% mortality) of the most resistant line was 56-fold greater than that of the original unselected VLR1 population, indicating very large increases in mean population survival after three cycles of selection. In vitro ACCase inhibition by diclofop acid confirmed that resistance was not due to an insensitive herbicide target-site. Cross-resistance studies showed increases in resistance to four herbicides: fluazifop-P-butyl, haloxyfop-R-methyl, clethodim and imazethapyr. The potential genetic basis of the observed response and implications of reduced herbicide application rates for management of herbicide resistance are discussed. | 3976 | NA | NA | NA | no | no |
| 3975 | 2017 | NA | Bastien, M; Sonah, H; Belzile, F | 2014 | Genome Wide Association Mapping of Sclerotinia sclerotiorum Resistance in Soybean with a Genotyping-by-Sequencing Approach | Sclerotinia stem rot (SSR) is one of the most important pests in cool soybean growing regions of the Northeastern United States and Canada. However, the intensity of infestations varies considerably from year to year according to weather conditions, thus making it difficult for breeders to select under uniform disease pressure. Selection for resistance to SSR would be greatly facilitated by the use of molecular markers. In this work, a collection of 130 lines was inoculated using the cotton pad method and was genetically characterized using a genotyping-by-sequencing (GBS) protocol optimized for soybean. Genome-wide association mapping (AM) and linkage disequilibrium (LD) analyses were performed with 7864 single nucleotide polymorphisms (SNPs). Linkage disequilibrium varied considerably over physical distance, reaching a r(2) value of 0.2 after 8.5 Mb in the pericentromeric region and 0.5 Mb in the telomeric region. The mixed linear model (MLM) performed very well in accounting for population structure and relatedness, as only 5.5% of the observed p-values were < 0.05. The strongest association was found on chromosome Gm15 (p-value = 1.38 x 10(-6); q-value [adjusted p-value] = 0.011). Two additional SNP markers in the vicinity had a q-value < 0.1. This marker was validated in the progeny of a biparental cross, where F 4: 6 lines carrying the susceptibility allele developed lesions 17.6 mm longer than lines carrying the resistance allele. Interestingly, other genes contributing to resistance to pathogens have been reported in this region of Gm15. Three other association peaks having a q-value < 0.1 were detected on chromosomes Gm01, Gm19, and Gm20. | 3977 | NA | NA | NA | no | no |
| 3976 | 2017 | NA | Simon, M; Lopez-Garcia, P; Deschamps, P; Moreira, D; Restoux, G; Bertolino, P; Jardillier, L | 2015 | Marked seasonality and high spatial variability of protist communities in shallow freshwater systems | Small eukaryotes have key roles in aquatic ecosystems, influencing their local environment, global biogeochemical cycles and climate. Their impact depends on community structure, which varies along time. However, very few studies take into account temporal variation. This is especially true for small, shallow freshwater systems, which remain largely understudied despite their wide variety, global surface and intense microbial activity. We have monthly followed changes in the community structure of small microbial eukaryotes (0.2-5 mu m cell diameter) for 2 years in four ponds and one brook located in North-Western France based on massive 18S rDNA amplicon 454 pyrosequencing. We detected a total of 3742 stringently defined operational taxonomic units (OTUs) encompassing all recognized eukaryotic supergroups and lineages of uncertain affiliation. Although geographically close, protist communities in the five ecosystems were contrasting, with very few shared OTUs, suggesting that environmental selection mainly drives community structure. The temporal dynamics of different high-rank taxa appeared complex and rapid at monthly scales. Despite this, a clear and reproducible seasonality was observed. As expected, low-abundance OTUs dominated the community. Although some of them appeared sporadically or remained at low frequencies during the survey, others occasionally reached relatively high abundances, sometimes recurrently. This shows that at least a fraction of low-abundance eukaryotes constitutes a seed bank. The annual proportion of primary producers, free-living heterotrophs and parasites appeared remarkably constant among the different ecosystems, suggesting underlying trends of ecosystem carrying capacity for these functional groups. | 3978 | NA | NA | NA | no | no |
| 3977 | 2017 | NA | Greco, D; Somervuo, P; Di Lieto, A; Raitila, T; Nitsch, L; Castren, E; Auvinen, P | 2008 | Physiology, Pathology and Relatedness of Human Tissues from Gene Expression Meta-Analysis | Background: Development and maintenance of the identity of tissues is of central importance for multicellular organisms. Based on gene expression profiles, it is possible to divide genes in housekeeping genes and those whose expression is preferential in one or a few tissues and which provide specialized functions that have a strong effect on the physiology of the whole organism. Results: We have surveyed the gene expression in 78 normal human tissues integrating publicly available microarray gene expression data. A total amount of 1601 genes were identified as selectively expressed in one or more tissues. The tissue-selective genes covered a wide range of cellular and molecular functions, and could be linked to 361 human diseases with Mendelian inheritance. Based on the gene expression profiles, we were able to form a network of tissues reflecting their functional relatedness and, to certain extent, their development. Using co-citation driven gene network technique and promoter analysis, we predicted a transcriptional module where the co-operation of the transcription factors E2F and NF-kappaB can possibly regulate a number of genes involved in the neurogenesis that takes place in the adult hippocampus. Conclusions: Here we propose that integration of gene expression data from Affymetrix GeneChip experiments is possible through re-annotation and commonly used pre-processing methods. We suggest that some functional aspects of the tissues can be explained by the co-operation of multiple transcription factors that regulate the expression of selected groups of genes. | 3979 | NA | NA | NA | no | no |
| 3978 | 2017 | NA | Haug, U; Poole, EM; Xiao, LR; Curtin, K; Duggan, D; Hsu, L; Makar, KW; Peters, U; Kulmacz, RJ; Potter, JD; Koepl, L; Caan, BJ; Slattery, ML; Ulrich, CM | 2012 | Glutathione peroxidase tagSNPs: Associations with rectal cancer but not with colon cancer | Glutathione peroxidases (GPXs) are selenium-dependent enzymes that reduce and, thus, detoxify hydrogen peroxide and a wide variety of lipid hydroperoxides. We investigated tagSNPs in GPX1-4 in relation to colorectal neoplasia in three independent study populations capturing the range of colorectal carcinogenesis from adenoma to cancer. A linkage-disequilibrium (LD)-based tagSNP selection algorithm (r2 = 0.90, MAF = 4%) identified 21 tagSNPs. We used an identical Illumina platform to genotype GPX SNPs in three population-based casecontrol studies of colon cancer (1,424 cases/1,780 controls), rectal cancer (583 cases/775 controls), and colorectal adenomas (485 cases/578 controls). For gene-level associations, we conducted principal component analysis (PCA); multiple logistic regression was used for single SNPs. Analyses were adjusted for age, sex, and study center and restricted to non-Hispanic white participants. Analyses of cancer endpoints were stratified by molecular subtypes. Without correction for multiple testing, one polymorphism in GPX2 and three polymorphisms in GPX3 were associated with a significant risk reduction for rectal cancer at a = 0.05, specifically for rectal cancers with TP53 mutations. The associations regarding the three polymorphisms in GPX3 remained statistically significant after adjustment for multiple comparisons. The PCA confirmed an overall association of GPX3 with rectal cancer (P = 0.03). No other statistically significant associations were observed. Our data provide preliminary evidence that genetic variability in GPX3 contributes to risk of rectal cancer but not of colon cancer and thus provide additional support for differences in underlying pathogenetic mechanisms for colon and rectal cancer. (c) 2012 Wiley Periodicals, Inc. | 3980 | NA | NA | NA | no | no |
| 3979 | 2017 | NA | Tsai, KH; Huang, CG; Fang, CT; Shu, PY; Huang, JH; Wu, WJ | 2011 | Prevalence of Rickettsia felis and the First Identification of Bartonella henselae Fizz/CAL-1 in Cat Fleas (Siphonaptera: Pulicidae) From Taiwan | Cat fleas (Ctenocephalides felis [Bouche]) are the primary ectoparasites of dog and cat populations. In this study, we report the monthly population dynamics of Rickettsia felis and Bartonella spp. (two zoonotic pathogens that can cause human disease) in cat fleas collected from dogs and cats in Taipei, Taiwan, from December 2006 to December 2007. Natural R. felis infection in individual cat fleas was assessed by polymerase chain reaction (PCR) using pRF-, ompB-, and gltA-specific primer pairs. Samples positive by PCR were confirmed with DNA sequencing. R. felis was detected in cat fleas year round, and the average infection rate was 21.4% (90 of 420) in 2007. Cat fleas also play an important role in the transmission of Bartonella between reservoirs and other mammalian hosts. In this study, we used primer pairs specific for the Bartonella gltA and rpoB genes to detect Bartonella infections. Of the 420 cat fleas tested, 38 were positive by PCR for Bartonella. Sequence similarities to Bartonella henselae, Bartonella clarridgeiae, and Bartonella koehlerae were observed in 6.2% (26 of 420), 2.1% (9 of 420), and 0.7% (3 of 420) of the fleas, respectively. Based on the pap31 gene sequence, several amplicons of the B. henselae detected in the cat fleas could be subgrouped into three strains: Fizz/CAL-1 (n = 18), Marseille (n = 5), and Houston-1 (n = 3). These results demonstrate that cat fleas infected with R. felis are endemic to Taiwan, and highlight the role of C. felis in Bartonella transmission between reservoirs and other mammal hosts and demonstrate the genetic variability of B. henselae in Taiwan. | 3981 | NA | NA | NA | no | no |
| 3980 | 2017 | NA | Cheng, P; Holdsworth, W; Ma, Y; Coyne, CJ; Mazourek, M; Grusak, MA; Fuchs, S; McGee, RJ | 2015 | Association mapping of agronomic and quality traits in USDA pea single-plant collection | Association mapping is an efficient approach for the identification of the molecular basis of agronomic traits in crop plants. For this purpose in pea (Pisum sativum L.), we genotyped and phenotyped individual lines of the single-plant-derived core collection of the USDA pea collection including accessions from 330 landraces and cultivars of Pisum sativum subsp. sativum var. sativum, 28 P. sativum subsp. elatius var. elatius, 16 P. sativum subsp. sativum var. arvense, four P. sativum subsp. elatius var. pumilio, three P. abyssinicum, two P. fulvum, and one P. sativum subsp. transcaucasicum. These 384 accessions were collected or donated from a total of 64 countries. The accessions were genotyped with 256 informative SNPs using a primer extension chemistry and matrix-assisted laser desorption/ionization (MALDI-TOF) mass spectrometry assay. Genetic structure analysis showed that the collection was structured into two main groups, corresponding roughly to the cultivated types/landraces and the more primitive form species and subspecies, with some intermediates. Linkage disequilibrium of pairwise loci and population structure of the collection were analyzed, and an association analysis between SNP genotypes and 25 valuable traits such as disease resistance, seed type/color, flower color, seed low molecular weight carbohydrate concentration, and seed mineral nutrient concentration was performed using a mixed linear model. A total of 71 marker-trait associations were detected as significant with 1-34 markers per trait based on the false discovery rate (FDR < 0.05). This study demonstrates the potential of using association mapping to identify markers for pea breeding. | 3982 | NA | NA | NA | no | no |
| 3981 | 2017 | NA | Ma, XW; Bacci, S; Mlynarski, W; Gottardo, L; Soccio, T; Menzaghi, C; Iori, E; Lager, RA; Shroff, AR; Gervino, EV; Nesto, RW; Johnstone, MT; Abumrad, NA; Avogaro, A; Trischitta, V; Doria, A | 2004 | A common haplotype at the CD36 locus is associated with high free fatty acid levels and increased cardiovascular risk in Caucasians | CD36 is a class B scavenger receptor recognizing a variety of ligands including long-chain fatty acids and modified LDL. We investigated whether genetic variability at this locus is a determinant of free fatty acid (FFA) plasma levels and risk of coronary artery disease (CAD) in Caucasians. Typing of 21 polymorphic markers, evenly spanning the CD36 gene, revealed two linkage disequilibrium (LD) blocks that could be tagged by five polymorphisms (-33137A>G, -31118G>A, 25444G>A, 27645del>ins and 30294G>C). In 585 non-diabetic individuals of Caucasian origin, the 30294G>C polymorphism was significantly associated with FFA levels (P=0.02)-an effect that was especially visible among men (P=0.009). A similar association was observed in this gender at -33137 (P=0.008) and -31118 (P=0.028). When the five tag polymorphisms were considered together, men carrying the AGGIG haplotype had 31% higher FFA (P=0.0002) and 20% higher triglycerides (P=0.025) than non-carriers. The same haplotype was associated with increased risk of CAD in 197 type 2 diabetic individuals from the US (OR=2.3, 95% CI 1.2-4.2). A similar tendency was observed in a group of 321 type 2 diabetic individuals from Italy (OR=1.4, 0.9-2.3), resulting in an overall relative risk of 1.6 (1.1-2.3, P=0.015) in the two populations considered together. By targeted resequencing, we identified a common variant in the CD36 promoter that is in strong LD with the AGGIG haplotype and could be partly responsible for these findings. In conclusion, this comprehensive study of CD36 variability indicates that the common polymorphisms at this locus modulate lipid metabolism and cardiovascular risk in Caucasians. | 3983 | NA | NA | NA | no | no |
| 3982 | 2017 | NA | Zhao, BX; Yan, JF; Zhang, S; Liu, X; Gao, ZG | 2014 | Phylogeny and pathogenicity of Fusarium spp. isolated from greenhouse melon soil in Liaoning Province | Fungi of the Fusarium oxysporum are widely distributed around the world in all types of soils, and they are all anamorphic species. In order to investigate the relationships and differences among Fusarium spp., 25 Fusarium spp. were isolated from greenhouse melon soils in Liaoning Province, China. With these 25 strains, three positive control Fusarium strains were analyzed using universally primed PCR (UP-PCR). Seventy-three bands appeared after amplification using 6 primers, and 66 of these bands (90.4%) were polymorphic. All strains were clustered into eight groups, though 14 strains of F. oxysporum were clustered into a single group. We concluded that UP-PCR could reveal the genetic relationships and differences among Fusarium strains. Moreover, the UP-PCR results suggested that F. oxysporum is distinguishable from other Fusarium spp. Thus, UP-PCR is a useful method for Fusarium classification. The pathogenicity of 13 strains of F. oxysporum to muskmelon, cucumber and watermelon seedlings was studied by infecting the seedlings with a spore suspension after cutting the root. The results showed that the F. oxysporum strains were pathogenic to all three melon types, although the pathogenicity differed significantly among the 13 strains. In addition, all strains had the greatest pathogenicity toward watermelon. Since the factors affecting pathogenicity vary widely, they should be considered in future studies on Fusarium spp. The results of such studies may then yield an accurate description of the pathogenicity of Fusarium spp. (C) 2013 Production and hosting by Elsevier B.V. on behalf of King Saud University. | 3984 | NA | NA | NA | no | no |
| 3983 | 2017 | NA | Noel, KM; Qualls, CP; Ennen, JR | 2012 | A COMPARISON OF ARTIFICIAL INCUBATION AND NATURAL INCUBATION HATCHING SUCCESS OF GOPHER TORTOISE (GOPHERUS POLYPHEMUS) EGGS IN SOUTHERN MISSISSIPPI | Recent studies have found that Gopher Tortoise, Gopherus polyphemus, populations in southern Mississippi exhibit low recruitment, due in part to very low hatching success of their eggs. We sought to determine if the cause(s) of this low hatching success was related to egg quality (intrinsic factors), unsuitability of the nest environment (extrinsic factors), or a combination of the two. In 2003, hatching success was monitored simultaneously for eggs from the same clutches that were incubated in the laboratory and left to incubate in nests. A subset of randomly chosen eggs from each clutch was incubated in the laboratory under physical conditions that were known to be conducive to successful hatching to estimate the proportion of eggs that were capable of hatching in a controlled setting. Hatching success in the laboratory was compared with that of eggs incubated in natural nests to estimate the proportion of eggs that failed to hatch presumably from extrinsic factors. Laboratory, hatching success was 58.8%, suggesting that roughly 40% of the eggs were intrinsically incapable of hatching even when incubated under controlled conditions. Hatching success in natural nests, 16.7%, was significantly lower than hatching success in the laboratory, suggesting that approximately 42.1% of eggs were capable of hatching but failed to hatch due to some extrinsic aspect(s) of the nest environment. Thus, the low hatching success of Gopher Tortoise eggs in southern Mississippi appears to be attributable to a combination of intrinsic (egg quality) and extrinsic (nest environment) factors. | 3985 | NA | NA | NA | no | no |
| 3984 | 2017 | NA | Garcia, JDD; Arevalo, JR; Fernandez-Palacios, JM | 2007 | Road edge effect on the abundance of the lizard Gallotia galloti (Sauria : Lacertidae) in two Canary Islands forests | Transportation infrastructure is a main cause of environmental change in forest landscapes worldwide. In the Canary Islands, a dense road system fragment the native Canarian pine and laurel forests causing potential changes in population densities of endemic lacertid lizards (genus Gallotia). Our aim was to assess road edge effects on relative abundance patterns of the endemic Gallotia galloti in both forests. We also explored the species-habitat relationships in this road-fragmentation context. We found that lizard relative density in relation to road edges differed between forests. Lizards were more abundant along edges and leeward interior, but virtually absent from the interior of the windward laurel forest. In the pine forest, lizards were present at three distances from edge, with a net decrease in abundance from edge to interior. These patterns may be explained partly by differences in vegetation structure regarding road proximity in each forest that potentially affect the helio- and thigmothermic character of G. galloti, and thus its habitat use. A general suggestion of this study is that road margins create corridors that may be used by native lizards for dispersal through inhospitable forest matrix. The high road density in Tenerife may have negative implications for the conservation of the genetic variability of G. galloti. At the island scale, increased communication between lizard populations through road corridors might increase homogenization of the gene pool. Ecological processes in which this lizard plays important roles may also experience changes along road edges. | 3986 | NA | NA | NA | no | no |
| 3985 | 2017 | NA | Cairns, J; Coloma, S; Sivonen, K; Hiltunen, T | 2016 | Evolving interactions between diazotrophic cyanobacterium and phage mediate nitrogen release and host competitive ability | Interactions between nitrogen-fixing (i.e. diazotrophic) cyanobacteria and their viruses, cyanophages, can have large-scale ecosystem effects. These effects are mediated by temporal alterations in nutrient availability in aquatic systems owing to the release of nitrogen and carbon sources from cells lysed by phages, as well as by ecologically important changes in the diversity and fitness of cyanobacterial populations that evolve in the presence of phages. However, ecological and evolutionary feedbacks between phages and nitrogen-fixing cyanobacteria are still relative poorly understood. Here, we used an experimental evolution approach to test the effect of interactions between a common filamentous, nitrogen-fixing cyanobacterium (Nodularia sp.) and its phage on cellular nitrogen release and host properties. Ecological, community-level effects of phage-mediated nitrogen release were tested with a phytoplankton bioassay. We found that cyanobacterial nitrogen release increased significantly as a result of viral lysis, which was associated with enhanced growth of phytoplankton species in cell-free filtrates compared with phage-resistant host controls in which lysis and subsequent nutrient release did not occur after phage exposure. We also observed an ecologically important change among phage-evolved cyanobacteria with phage-resistant phenotypes, a short-filamentous morphotype with reduced buoyancy compared with the ancestral long-filamentous morphotype. Reduced buoyancy might decrease the ability of these morphotypes to compete for light compared with longer, more buoyant filaments. Together, these findings demonstrate the potential of cyanobacteria-phage interactions to affect ecosystem biogeochemical cycles and planktonic community dynamics. | 3987 | NA | NA | NA | no | no |
| 3986 | 2017 | NA | Santelices, B; Aedo, D; Hoffmann, A | 2002 | Banks of microscopic forms and survival to darkness of propagules and microscopic stages of macroalgae | Previous studies have found that the number of species conforming a bank of microscopic forms in tide pools in central Chile accounted only for half the number of species present in the macroscopic vegetation around the pools. An elemental condition for survival in these banks is the ability of microscopic forms to tolerate darkness or very low irradiances for extended periods. To test this ability, spores of 17 green, brown and red algal species, present and absent from the bank, were incubated at different combinations of irradiances and day lengths. Propagules of 47% of the species tested (eight species) germinated in total darkness while the propagules of the other nine species germinated under conditions of very low irradiance (2-10 mumol m(-2) s(-1)). In most species, microforms showed a higher tolerance to darkness than the propagules. Some survived for over a year and one species (Gelidium lingulatum) could live under complete darkness for 500 days. The ability to survive in total darkness did not relate to presence or absence of a species in the banks of microscopic forms previously studied, to phylogenetic relatedness, life history style, propagule size, morphology of microscopic forms or to successional status (fugitive versus late successional). Thus, tolerance to darkness appears to be common to propagules and microscopic stages of most benthic algae. The growth patterns exhibited by the microforms of Lessonia nigrescens, Chaetomorpha firma and Glossophora kunthii suggest high irradiances on these recruits might determine the shallower limits of distribution of these species. | 3988 | NA | NA | NA | no | no |
| 3987 | 2017 | NA | Flori, L; Thevenon, S; Dayo, GK; Senou, M; Sylla, S; Berthier, D; Moazami-Goudarzi, K; Gautier, M | 2014 | Adaptive admixture in the West African bovine hybrid zone: insight from the Borgou population | Understanding the adaptive response to environmental fluctuations represents a central issue in evolutionary biology. Population admixture between divergent ancestries has often been considered as an efficient short-term adaptation strategy. Cattle populations from the West African Bos taurusxBos indicus hybrid zone represent a valuable resource to characterize the effect of such adaptive admixture at the genome level. We here provide a detailed assessment of the global and local genome ancestries of the Borgou breed, one of the most representative cattle of this hybrid zone. We analysed a large data set consisting of 38100 SNPs genotyped on 203 Borgou and 591 individuals representative of all the different cattle ancestries. At the global genomic level, we show that Borgou is a stabilized admixed breed whose origin (c. 130years ago) traces back to the great African rinderpest pandemic, several centuries after the last admixture events, the West African zebus originate from (c. 500years ago). To identify footprints of adaptive admixture, we combined the identification of signatures of selection and the functional annotation of the underlying genes using systems biology tools. The detection of the SILV coat coloration gene likely under artificial selection may be viewed as a validation of our approach. Overall, our results suggest that the long-term presence of pathogens and the intermediate environmental conditions are the main acting selective pressures. Our analytical framework can be extended to other model or nonmodel species to understand the process that shapes the patterns of genetic variability in hybrid zones. | 3989 | NA | NA | NA | no | no |
| 3988 | 2017 | NA | Richards, MH; Vickruck, JL; Rehan, SM | 2010 | Colony Social Organisation of Halictus confusus in Southern Ontario, with Comments on Sociality in the Subgenus H. (Seladonia) | Halicrus (Seladonia) confusus Smith is one of the most common bees in North America. Classified as eusocial, its colony social organization is known only from qualitative descriptions of a population in Indiana. We studied the phenology and social behaviour of this bee in the Niagara Region of southern Ontario, using nest excavations, dissections and measurements of adult females, and pan trap samples of foraging bees to elucidate key elements of colony social organisation. The colony cycle in Niagara is typical of temperate-zone halictines, with overwintered foundresses producing a first brood of worker-sized females and a few males, followed by production of Brood 2, consisting of gynes and more males. Many Brood 1 females become reproductive: about one-quarter of Brood 1 females dissected exhibited levels of ovarian development rivalling queens. In contrast, only about one-quarter of Brood 1 females become classically altruistic, sterile workers. High rates of worker reproductivity may result from early queen mortality and supersedure or from the inability of viable queens to control worker behaviour - the average queen-worker size difference was only 5.6%, and queens were not always larger than the workers in their own nests. Comparisons with the Indiana population suggest a geographic component to variation in colony social organisation. Comparisons with other members of the subgenus for which detailed information is available, suggest that in Seladonia, as in other eusocial halictines, queen control of worker behaviour depends on the ability of queens to dominate small numbers of small-bodied workers. | 3990 | NA | NA | NA | no | no |
| 3989 | 2017 | NA | Sai, CB; Nagarajan, P; Raveendran, M; Rabindran, R; Bapu, JRK; Senthil, N | 2017 | Understanding the inheritance of mungbean yellow mosaic virus (MYMV) resistance in mungbean (Vigna radiata L. Wilczek) | Mungbean, Vigna radiata, third in the series of important pulse crops, still suffers from yield loss due to mungbean yellow mosaic disease caused by mungbean yellow mosaic virus (MYMV). Hence, studies on plant-microbe interaction are necessary for understanding the inheritance of resistance. This study concentrated on identification of linked molecular markers for MYMV resistance and to find the genetic inheritance of MYMV resistance in mungbean. A total of 413 germplasm entries in a MYMV hot spot area (Vamban) were subjected to natural field infection and 13 selected resistant lines were subjected to Agrobacterium infection using strains harboring partial genome of two different MYMV isolates, VA221 and VA239. Among the resistant lines, KMG189 showed strain-specific resistance to VA221 and had no symptoms during field trials. Ninety F2 genotypes were developed from the cross made between KMG189 (MYMV-resistant) and VBN(Gg) 2 (MYMV-susceptible), segregated in the Mendelian single cross ratio 3S:1R; susceptibility of all the F1s to MYMV suggested that the MYMV resistance in mungbean is governed by a single recessive gene. Two SCAR markers CM9 and CM815 were developed through bulk segregant analysis, and the linkage analysis proved CM815 SCAR marker to be linked at 5.56 cM with MYMV resistance gene and SCAR CM9 had nil recombination percentage, suggesting it to be very closely linked to the MYMV resistance gene. SCAR marker CM9 was present in chromosome number 3 of mungbean suggesting novel loci for virus resistance in mungbean. The identified loci can be used for developing varieties resistant to MYMV in mungbean. | 3991 | NA | NA | NA | no | no |
| 3990 | 2017 | NA | Green, JP; Almond, EJ; Williamson, J; Field, J | 2016 | Regulation of host colony activity by the social parasite Polistes semenowi | The productivity of social groups depends critically on effective regulation of work effort among group members. In social insect colonies, regulation of work may be decentralised or alternatively may be controlled by one or a few individuals (‘pacemakers’) within the colony. Social parasites, which usurp host colonies and replace the dominant as the principal reproductive, similarly depend on efficient regulation of work by hosts to rear parasite offspring, but few studies have explored the strategies used by parasites to achieve this. We compared the role of the social parasite Polistes semenowi in regulating host activity with that of the dominant individual on unparasitized nests of the host species, P. dominula. Dominant foundresses acted as pacemakers within unparasitized colonies, interacting frequently with colony members to initiate activity bursts and foraging trips, whereas parasites did not initiate more activity than the average colony member. Nonetheless, overall activity levels were similar in parasitized and unparasitized colonies, indicating that parasites may use other, indirect means to control the host activity. Colony activity did not change significantly following the removal of parasites or dominant host foundresses, perhaps because other individuals rapidly assumed the dominant position, or because of persistent indirect effects on colony activity. The role of P. semenowi in regulating the host activity differs strikingly from that reported for a second Polistes social parasite, P. atrimandibularis, suggesting that different Polistes social parasites may have fundamentally different social roles within host colonies, despite being closely phylogenetically related to one another. | 3992 | NA | NA | NA | no | no |
| 3991 | 2017 | NA | Malosetti, M; van der Linden, CG; Vosman, B; van Eeuwijk, FA | 2007 | A mixed-model approach to association mapping using pedigree information with an illustration of resistance to Phytophthora infestans in potato | Association or linkage disequilibrium (LD)-based mapping strategies are receiving increased attention for the identification of quantitative trait loci (QTL) in plants as an alternative to more traditional, purely linkage-based approaches. An attractive property of association approaches is that they do not require specially designed crosses between inbred parents, but can be applied to collections of genotypes with arbitrary and often unknown relationships between the genotypes. A less obvious additional attractive property is that association approaches offer possibilities for QTL identification in crops with hard to model segregation patterns. The availability of candidate genes and targeted marker systems facilitates association approaches, as will appropriate methods of analysis. We propose an association mapping approach based on mixed models with attention to the incorporation of the relationships between genotypes, whether induced by pedigree, population substructure, or otherwise. Furthermore, we emphasize the need to pay attention to the environmental features of the data as well, i.e., adequate representation of the relations among multiple observations on the same genotypes. We illustrate our modeling approach rising 25 years of Dutch national variety list data on late blight resistance in the genetically complex crop of potato. As markers, we used nucleotide binding-site markers, a specific type of marker that targets resistance or resistance-analog genes. To assess the consistency of QTL identified by 0 our mixed-model approach, a second independent data set was analyzed. Two markers were identified that are potentially useful in selection for late blight resistance in potato. | 3993 | NA | NA | NA | no | no |
| 3992 | 2017 | NA | Sepulveda, DA; Zepeda-Paulo, F; Ramirez, CC; Lavandero, B; Figueroa, CC | 2017 | Diversity, frequency, and geographic distribution of facultative bacterial endosymbionts in introduced aphid pests | Facultative bacterial endosymbionts in insects have been under intense study during the last years. Endosymbionts can modify the insect’s phenotype, conferring adaptive advantages under environmental stress. This seems particularly relevant for a group of worldwide agricultural aphid pests, because endosymbionts modify key fitness-related traits, including host plant use, protection against natural enemies and heat tolerance. Aimed to understand the role of facultative endosymbionts on the success of introduced aphid pests, the distribution and abundance of 5 facultative endosymbionts (Hamiltonella defensa, Regiella insecticola, Serratia symbiotica, Rickettsia and Spiroplasma) were studied and compared in 4 cereal aphids (Sitobion avenae, Diuraphis noxia, Metopolophium dirhodum and Schizaphis graminium) and in the pea aphid Acyrthosiphon pisum complex from 2 agroclimatic zones in Chile. Overall, infections with facultative endosymbionts exhibited a highly variable and characteristic pattern depending on the aphid species/host race and geographic zone, which could explain the success of aphid pest populations after their introduction. While S. symbiotica and H. defensa were the most frequent endosymbionts carried by the A. pisum pea-race and A. pisum alfalfa-race aphids, respectively, the most frequent facultative endosymbiont carried by all cereal aphids was R. insecticola. Interestingly, a highly variable composition of endosymbionts carried by S. avenae was also observed between agroclimatic zones, suggesting that endosymbionts are responding differentially to abiotic variables (temperature and precipitations). In addition, our findings constitute the first report of bacterial endosymbionts in cereal aphid species not screened before, and also the first report of aphid endosymbionts in Chile. | 3994 | NA | NA | NA | no | no |
| 3993 | 2017 | NA | Ssekandi, W; Mulumba, JW; Colangelo, P; Nankya, R; Fadda, C; Karungi, J; Otim, M; De Santis, P; Jarvis, DI | 2016 | The use of common bean (Phaseolus vulgaris) traditional varieties and their mixtures with commercial varieties to manage bean fly (Ophiomyia spp.) infestations in Uganda | The bean fly (Ophiomyia spp.) is considered the most economically damaging field insect pest of common beans in Uganda. Despite the use of existing pest management approaches, reported damage has remained high. Forty-eight traditional and improved common bean varieties currently grown in farmers’ fields were evaluated for resistance against bean fly. Data on bean fly incidence, severity and root damage from bean stem maggot were collected. Generalized linear mixed model (GLMM) revealed significant resistance to bean fly in the Ugandan traditional varieties. A popular resistant traditional variety and a popular susceptible commercial variety were selected from the 48 varieties and evaluated in pure and mixed stands. The incidence of bean fly infestation on both varieties in mixtures with different arrangements (systematic random versus rows), and different proportions within each of the two arrangements, was measured and analysed using GLMMs. The proportion of resistant varieties in a mixture and the arrangement type significantly decreased bean fly damage compared to pure stands, with the highest decrease in damage registered in the systematic random mixture with at least 50 % of resistant variety. The highest reduction in root damage, obvious 21 days after planting, was found in systematic random mixtures with at least 50 % of the resistant variety. Small holder farmers in East Africa and elsewhere in the world have local preferences for growing bean varieties in genetic mixtures. These mixtures can be enhanced by the use of resistant varieties in the mixtures to reduce bean fly damage on susceptible popular varieties. | 3995 | NA | NA | NA | no | no |
| 3994 | 2017 | NA | Qi, HG; Song, K; Li, CY; Wang, W; Li, BS; Li, L; Zhang, GF | 2017 | Construction and evaluation of a high-density SNP array for the Pacific oyster (Crassostrea gigas) | Single nucleotide polymorphisms (SNPs) are widely used in genetics and genomics research. The Pacific oyster (Crassostrea gigas) is an economically and ecologically important marine bivalve, and it possesses one of the highest levels of genomic DNA variation among animal species. Pacific oyster SNPs have been extensively investigated; however, the mechanisms by which these SNPs may be used in a high-throughput, transferable, and economical manner remain to be elucidated. Here, we constructed an oyster 190K SNP array using Affymetrix Axiom genotyping technology. We designed 190,420 SNPs on the chip; these SNPs were selected from 54 million SNPs identified through re-sequencing of 472 Pacific oysters collected in China, Japan, Korea, and Canada. Our genotyping results indicated that 133,984 (70.4%) SNPs were polymorphic and successfully converted on the chip. The SNPs were distributed evenly throughout the oyster genome, located in 3,595 scaffolds with a length of similar to 509.4 million; the average interval spacing was 4,210 bp. In addition, 111,158 SNPs were distributed in 21,050 coding genes, with an average of 5.3 SNPs per gene. In comparison with genotypes obtained through re-sequencing, similar to 69% of the converted SNPs had a concordance rate of > 0.971; the mean concordance rate was 0.966. Evaluation based on genotypes of full-sib family individuals revealed that the average genotyping accuracy rate was 0.975. Carrying 133 K polymorphic SNPs, our oyster 190K SNP array is the first commercially available high-density SNP chip for mollusks, with the highest throughput. It represents a valuable tool for oyster genome-wide association studies, fine linkage mapping, and population genetics. | 3996 | NA | NA | NA | no | no |
| 3995 | 2017 | NA | Early, AM; Arguello, JR; Cardoso-Moreira, M; Gottipati, S; Grenier, JK; Clark, AG | 2017 | Survey of Global Genetic Diversity Within the Drosophila Immune System | Numerous studies across a wide range of taxa have demonstrated that immune genes are routinely among the most rapidly evolving genes in the genome. This observation, however, does not address what proportion of immune genes undergo strong selection during adaptation to novel environments. Here, we determine the extent of very recent divergence in genes with immune function across five populations of Drosophila melanogaster and find that immune genes do not show an overall trend of recent rapid adaptation. Our population-based approach uses a set of carefully matched control genes to account for the effects of demography and local recombination rate, allowing us to identify whether specific immune functions are putative targets of strong selection. We find evidence that viral-defense genes are rapidly evolving in Drosophila at multiple timescales. Local adaptation to bacteria and fungi is less extreme and primarily occurs through changes in recognition and effector genes rather than large-scale changes to the regulation of the immune response. Surprisingly, genes in the Toll pathway, which show a high rate of adaptive substitution between the D. melanogaster and D. simulans lineages, show little population differentiation. Quantifying the flies for resistance to a generalist Gram-positive bacterial pathogen, we found that this genetic pattern of low population differentiation was recapitulated at the phenotypic level. In sum, our results highlight the complexity of immune evolution and suggest that Drosophila immune genes do not follow a uniform trajectory of strong directional selection as flies encounter new environments. | 3997 | NA | NA | NA | no | no |
| 3996 | 2017 | NA | Osborne, AJ; Pearson, J; Negro, SS; Chilvers, BL; Kennedy, MA; Gemmell, NJ | 2015 | Heterozygote advantage at MHC DRB may influence response to infectious disease epizootics | The effect of MHC polymorphism on individual fitness variation in the wild remains equivocal; however, much evidence suggests that heterozygote advantage is a major determinant. To understand the contribution of MHC polymorphism to individual disease resistance or susceptibility in natural populations, we investigated two MHC class II B loci, DQB and DRB, in the New Zealand sea lion (NZSL, Phocarctos hookeri). The NZSL is a threatened species which is unusually susceptible to death by bacterial infection at an early age; it has suffered three bacterial induced epizootics resulting in high mortality levels of young pups since 1997. The MHC DQB and DRB haplotypes of dead NZSL pups with known cause of death (bacteria, enteritis or trauma) were sequenced and reconstructed, compared to pups that survived beyond 2months of age, and distinct MHC DRB allele frequency and genotype differences were identified. Two findings were striking: (i) one DRB allele was present only in dead pups, and (ii) one heterozygous DRB genotype, common in live pups, was absent from dead pups. These results are consistent with some functional relationship with these variants and suggest heterozygote advantage is operating at DRB. We found no association between heterozygosity and fitness at 17 microsatellite loci, indicating that general heterozygosity is not responsible for the effect on fitness detected here. This result may be a consequence of recurrent selection by multiple pathogen assault over recent years and highlights the importance of heterozygote advantage at MHC as a potential mechanism for fitness differences in wild populations. | 3998 | NA | NA | NA | no | no |
| 3997 | 2017 | NA | Kalbitzer, U; Bergstrom, ML; Carnegie, SD; Wikberg, EC; Kawamura, S; Campos, FA; Jack, KM; Fedigan, LM | 2017 | Female sociality and sexual conflict shape offspring survival in a Neotropical primate | Most mammals live in social groups in which members form differentiated social relationships. Individuals may vary in their degree of sociality, and this variation can be associated with differential fitness. In some species, for example, female sociality has a positive effect on infant survival. However, investigations of such cases are still rare, and no previous study has considered how male infanticide might constrain effects of female sociality on infant survival. Infanticide is part of the male reproductive strategy in many mammals, and it has the potential to override, or even reverse, effects of female reproductive strategies, including sociality. Therefore, we investigated the relationships between female sociality, offspring survival, and infanticide risk in wild white-faced capuchin monkeys using long-term data from Santa Rosa, Costa Rica. Female capuchins formed differentiated bonds, and bond strength was predicted by kin relationship, rank difference, and the presence of female infants. Most females formed stable bonds with their top social partners, although bond stability varied considerably. Offspring of highly social females, who were often high-ranking females, exhibited higher survivorship during stable periods compared with offspring of less social females. However, offspring of highly social females were more likely to die or disappear during periods of alpha male replacements, probably because new alpha males are central to the group, and therefore more likely to target the infants of highly social, central females. This study shows that female sociality in mammals can have negative fitness consequences that are imposed by male behavior. | 3999 | NA | NA | NA | no | no |
| 3998 | 2017 | NA | Thomson, CE; Hadfield, JD | 2017 | Measuring selection when parents and offspring interact | Non‐social and social selection gradients are key evolutionary parameters in systems where individuals interact. They are most easily obtained by regressing an individual’s fitness on the trait values of the individual and its social partner. In the context of parental care it is more common to regress the trait value of the parents (i.e. the social partner) on a ‘mixed’ fitness measure that is a function of the parent’s and offspring’s fitness (for example, the number of recruits, which equals parental fecundity multiplied by offspring survival). For such an approach to yield correct estimates of net‐selection, the trait must be sex‐limited and not affect the parents’ own survival. When a trait is not sex‐limited, the non‐social selection should be weighted by one (because all individuals express the trait) and social selection should be weighted by a half (because the relatedness between parents and the offspring they care for is a half, usually). The ‘mixed’ fitness approach does not give estimates of both components of selection and so they cannot be weighted appropriately. We show that mixed fitness components are frequently used in place of direct fitness measures in the literature (37% of fecundity selection estimates use a mixed fitness approach), but that the frequency is much higher in some taxa, such as birds and mammals. We suggest alternative methods that could be used to estimate both social and non‐social selection gradients, while at the same time assessing the importance of unmeasured traits. | 4000 | NA | NA | NA | no | no |
| 3999 | 2017 | NA | Barthelmess, EL | 2014 | Spatial distribution of road-kills and factors influencing road mortality for mammals in Northern New York State | One of the most obvious impacts of roads on wildlife is vehicle-induced mortality. The aims of this study were to examine the spatial pattern of mammal-vehicle collisions (MVCs), identify and examine factors that contribute to MVCs, and determine whether the factors that increase the odds of MVCs are similar between species. On 103 road surveys that covered 7,094 total km I recorded the location of each MVC along the survey route. I measured landscape and roadway features associated with each MVC and used kernel density and network analysis tools to identify road mortality hotspots and measure spatial clustering of MVCs. I used logistic regression to model the likelihood of MVCs for all mammal data and separately for Porcupine (Erethizon dorsatum), Raccoon (Procyon lotor), Skunk (Mephitis mephitis), Muskrat (Ondatra zibethicus) and Cottontail (Sylvilagus floridanus) data sets. I identified 51 MVC hotspots and found spatial clustering of MVCs for Porcupines, Raccoons and Skunks. Two landscape variables, distance to cover and the presence of an ecotone, as well as one road variable, road width, appeared as broadly important predictors of mammalian road mortality, though there was also species-specific variation in factors that increased the risk of MVCs. Field-measured variables were more important than remotely-measured variables in predicting the odds of MVCs. Conservation implications are that mitigation of landscape features associated with higher risk of vehicle-collisions may reduce the number of MVCs in general, but species-specific research is required to more carefully tailor mitigation efforts for particular species. | 4001 | NA | NA | NA | no | no |
| 4000 | 2017 | NA | Leon-Quinto, T; Simon, MA; Sanchez, A; Martin, F; Soria, B | 2011 | Cryobanking the genetic diversity in the critically endangered Iberian lynx (Lynx pardinus) from skin biopsies. Investigating the cryopreservation and culture ability of highly valuable explants and cells | Cryobanking skin samples permit preserving a maximum of genetic representation from the population biodiversity. This is a relevant aspect for threatened species, potentially menaced by an epizooty and from which it is difficult to obtain gametes. As a first step for properly cryobanking skin samples of a given species, the optimal conditions of culture and freezing have to be studied by covering a broad range of possibilities. This paper presents, for the first time, a systematic study of such conditions for the Iberian lynx (Lynx pardinus). To that end, we have analyzed twenty different culture conditions and fifteen different freezing solutions for skin explants, as well as three freezing solutions for isolated cells derived from them. The culture conditions included both two different culture strategies and several combinations of nutritional supplements and mitotic agents. For the freezing solutions, we have considered different concentrations of the permeating cryoprotectant dimethyl sulfoxide (Me2SO) either alone (5%, 7.5%, 10%, 12.5% and 15% v/v for explants, 10% for isolated cells) or along with the non-permeating cryoprotectant sucrose (0.1 or 0.2 M). Our results have been analyzed through several quantitative parameters and show that only thawed explants cryopreserved in Me2SO (10%) either alone or with sucrose (0.2 M) presented similar properties to those in optimal fresh cultures. In addition, for these freezing conditions, isolated thawed cells also presented high survival rates (90%) and percentages of cellular functionality (85%). These results, focussed on the most endangered felid in the world, could be also useful for other threatened/endangered species. (C) 2011 Elsevier Inc. All rights reserved. | 4002 | NA | NA | NA | no | no |
| 4001 | 2017 | NA | Corbin, LJ; Blott, SC; Swinburne, JE; Sibbons, C; Fox-Clipsham, LY; Helwegen, M; Parkin, TDH; Newton, JR; Bramlage, LR; McIlwraith, CW; Bishop, SC; Woolliams, JA; Vaudin, M | 2012 | A genome-wide association study of osteochondritis dissecans in the Thoroughbred | Osteochondrosis is a developmental orthopaedic disease that occurs in horses, other livestock species, companion animal species, and humans. The principal aim of this study was to identify quantitative trait loci (QTL) associated with osteochondritis dissecans (OCD) in the Thoroughbred using a genome-wide association study. A secondary objective was to test the effect of previously identified QTL in the current population. Over 300 horses, classified as cases or controls according to clinical findings, were genotyped for the Illumina Equine SNP50 BeadChip. An animal model was first implemented in order to adjust each horse’s phenotypic status for average relatedness among horses and other potentially confounding factors which were present in the data. The genome-wide association test was then conducted on the residuals from the animal model. A single SNP on chromosome 3 was found to be associated with OCD at a genome-wide level of significance, as determined by permutation. According to the current sequence annotation, the SNP is located in an intergenic region of the genome. The effects of 24 SNPs, representing QTL previously identified in a sample of Hanoverian Warmblood horses, were tested directly in the animal model. When fitted alongside the significant SNP on ECA3, two of these SNPs were found to be associated with OCD. Confirmation of the putative QTL identified on ECA3 requires validation in an independent sample. The results of this study suggest that a significant challenge faced by equine researchers is the generation of sufficiently large data sets to effectively study complex diseases such as osteochondrosis. | 4003 | NA | NA | NA | no | no |
| 4002 | 2017 | NA | Kawai, K; Hailer, F; de Guia, AP; Ichikawa, H; Saitoh, T | 2013 | Refugia in Glacial Ages Led to the Current Discontinuous Distribution Patterns of the Dark Red-backed Vole Myodes rex on Hokkaido, Japan | The terrestrial mammalian fauna of the North Japanese island, Hokkaido, is more similar to that of Southern Siberia than to the main island of Japan, Honshu. Three species of the genus Myodes (Muridae, Rodentia) are found on Hokkaido, but not on Honshu. While Myodes rufocanus and M. rutilus are widely distributed across Hokkaido as well as the Eurasian continent, M. rex, which is endemic to Hokkaido and its adjacent islands, shows a discontinuous distribution pattern. We analyzed the phylogeographic history of M. rex using the mitochondrial DNA control region in order to interpret their discontinuous distribution pattern. Phylogenetic relationships among 54 distinct haplotypes showed that M. rex can be divided into four clades that occur on the northern, central, and southern regions of the Hokkaido mainland and on Rishiri Island, respectively. The phylogroups in the northern and central regions were largely separated in space, although several areas of sympatry were found. The phylogroup in the southern region, which was clearly separated from other phylogroups, showed markedly low genetic variability. All analyzed individuals from the population on Rishiri belonged to a separate lineage. Across a range of divergence rate estimates, we dated the basal divergence of all phylogroups to the mid to late Pleistocene, with subsequent signals of population expansion within lineages. We conclude that current phylogeographic structure in M. rex likely reflects Pleistocene survival in several separate refugia in situ. Past glacial ages have thus played an important role in shaping the current distribution patterns of mammalian species on Hokkaido. | 4004 | NA | NA | NA | no | no |
| 4003 | 2017 | NA | Pull, CD; Hughes, WOH; Brown, MJF | 2013 | Tolerating an infection: an indirect benefit of co-founding queen associations in the ant Lasius niger | Pathogens exert a strong selection pressure on organisms to evolve effective immune defences. In addition to individual immunity, social organisms can act cooperatively to produce collective defences. In many ant species, queens have the option to found a colony alone or in groups with other, often unrelated, conspecifics. These associations are transient, usually lasting only as long as each queen benefits from the presence of others. In fact, once the first workers emerge, queens fight to the death for dominance. One potential advantage of co-founding may be that queens benefit from collective disease defences, such as mutual grooming, that act against common soil pathogens. We test this hypothesis by exposing single and co-founding queens to a fungal parasite, in order to assess whether queens in co-founding associations have improved survival. Surprisingly, co-foundresses exposed to the entomopathogenic fungus Metarhizium did not engage in cooperative disease defences, and consequently, we find no direct benefit of multiple queens on survival. However, an indirect benefit was observed, with parasite-exposed queens producing more brood when they co-founded, than when they were alone. We suggest this is due to a trade-off between reproduction and immunity. Additionally, we report an extraordinary ability of the queens to tolerate an infection for long periods after parasite exposure. Our study suggests that there are no social immunity benefits for co-founding ant queens, but that in parasite-rich environments, the presence of additional queens may nevertheless improve the chances of colony founding success. | 4005 | NA | NA | NA | no | no |
| 4004 | 2017 | NA | Adair, LS | 2007 | Size at birth and growth trajectories to young adulthood | Few studies in developing countries follow growth trajectories from birth to adulthood. Such studies are important because size at birth and postnatal growth affect risk of chronic disease in adulthood. This study examines the inter-relationships of maternal factors during pregnancy, infant birth weight and length, early postnatal growth, and young adult height, weight, BMI, and skinfold thicknesses, with particular attention to patterns of growth associated with increased chronic disease risk. Women were recruited in pregnancy, and offspring were followed from birth to age 21 in the community-based Cebu (Philippines) Longitudinal Health and Nutrition Survey. Birth weight and length are independently, positively associated with height, BMI and sum of skinfolds in young adult males and females, and inversely associated with the subscapular to triceps ratio in males only. The effects of size at birth. on adult size were modified by birth order, and remained significant after adjusting for maternal nutritional status, socioeconomic status at birth and throughout the growth period, and maturation. Early postnatal growth was strongly influenced by BMI at birth, with rapid early infant weight gain associated with thinness. The growth pattern of the at-risk group most often associated with increased risk of chronic disease (small at birth, relatively heavy as an adult), was characterized by more rapid growth in the first 4 postnatal months. The high level of inter-relatedness of maternal nutrition in pregnancy, prenatal growth, and postnatal growth emphasizes the need to consider the full growth trajectory in studies of developmental origins of adult disease. | 4006 | NA | NA | NA | no | no |
| 4005 | 2017 | NA | Lepere, C; Masquelier, S; Mangot, JF; Debroas, D; Domaizon, I | 2010 | Vertical structure of small eukaryotes in three lakes that differ by their trophic status: a quantitative approach | In lakes, the diversity of eukaryotic picoplankton has been recently studied by the analysis of 18S ribosomal RNA gene sequences; however, quantitative data are rare. In this study, the vertical structure and abundance of the small eukaryotic size fraction (0.2-5 mu m) were investigated in three lakes by tyramide signal amplification-fluorescent in situ hybridization targeting six phylogenetic groups: Chlorophyta, Haptophyta, Cercozoa, LKM11, Perkinsozoa and fungi. The groups targeted in this study are found in all lakes; however, both the abundance and structure of small eukaryotes are dependent on the system’s productivity and depth. These data highlighted the presence of Chlorophyta contributing on an average to 19.3%, 14.7% and 41.2% of total small eukaryotes in lakes Bourget, Aydat and Pavin, respectively. This study also revealed the unexpected importance of Haptophyta, reaching 62.8% of eukaryotes in the euphotic zone of Lake Bourget. The high proportions of these pigmented cells highlight the underestimation of these groups by PCR-based methods. The presence of pigmented Chlorophyta in the deepest zones of the lakes suggests a mixotrophic behaviour of these taxa. We also confirmed the presence of putative parasites such as Perkinsozoa (5.1% of small eukaryotes in Lake Pavin and Bourget) and, with lower abundances, fungi (targeted by the MY1574 probe). Cells targeted by LKM11 probes represented the second group of abundance within heterotrophs. Open questions regarding the functional roles of the targeted groups arise from this study, especially regarding parasitism and mixotrophy, which are interactions poorly taken into account in planktonic food web models. The ISME Journal (2010) 4, 1509-1519; doi:10.1038/ismej.2010.83; published online 24 June 2010 | 4007 | NA | NA | NA | no | no |
| 4006 | 2017 | NA | Landi, L; Riolo, P; Murolo, S; Romanazzi, G; Nardi, S; Isidoro, N | 2015 | Genetic Variability of Stolbur Phytoplasma in Hyalesthes obsoletus (Hemiptera: Cixiidae) and its Main Host Plants in Vineyard Agroecosystems | Bois noir is an economically important grapevine yellows that is induced by ‘Candidatus Phytoplasma solani’ and principally vectored by the planthopper Hyalesthes obsoletus Signoret (Hemiptera: Cixiidae). This study explores the ‘Ca. P. solani’ genetic variability associated to the nettle-H. obsoletus and bindweed-H. obsoletus systems in vineyard agroecosystems of the central-eastern Italy. Molecular characterization of ‘Ca. P. solani’ isolates was carried out using polymerase chain reaction/restriction fragment length polymorphism to investigate the nonribosomal vmp1 gene. Seven phytoplasma vmp-types were detected among the host plants-and insect-associated field-collected samples. The vmp1 gene showed the highest polymorphism in the bindweed-H. obsoletus system, according to restriction fragment length polymorphism analysis, which is in agreement with nucleotide sequence analysis. Five vmp-types were associated with H. obsoletus from bindweed, of which one was solely restricted to planthoppers, with one genotype also in planthoppers from nettle. Type V12 was the most prevalent in both planthoppers and bindweed. H. obsoletus from nettle harbored three vmp-types, of which V3 was predominant. V3 was the only type detected for nettle. Our data demonstrate that planthoppers might have acquired some ‘Ca. P. solani’ profiles from other plant hosts before landing on nettle or bindweed. Overall, the different vmp1 gene rearrangements observed in these two plant hosts-H. obsoletus systems might represent different adaptations of the pathogen to the two host plants. Molecular information about the complex of vmp-types provides useful data for better understanding of Bois noir epidemiology in vineyard agroecosystem. | 4008 | NA | NA | NA | no | no |
| 4007 | 2017 | NA | Njajou, OT; Blackburn, EH; Pawlikowska, L; Mangino, M; Damcott, CM; Kwok, PY; Spector, TD; Newman, AB; Harris, TB; Cummings, SR; Cawthon, RM; Shuldiner, AR; Valdes, AM; Hsueh, WC | 2010 | A Common Variant in the Telomerase RNA Component Is Associated with Short Telomere Length | Background: Telomeres shorten as cells divide. This shortening is compensated by the enzyme telomerase. We evaluated the effect of common variants in the telomerase RNA component (TERC) gene on telomere length (TL) in the population-based Health Aging and Body Composition (Health ABC) Study and in two replication samples (the TwinsUK Study and the Amish Family Osteoporosis Study, AFOS). Methodology: Five variants were identified in the TERC region by sequence analysis and only one SNP was common (rs2293607, G/A). The frequency of the G allele was 0.26 and 0.07 in white and black, respectively. Testing for association between TL and rs2293607 was performed using linear regression models or variance component analysis conditioning on relatedness among subjects. Results: The adjusted mean TL was significantly shorter in 665 white carriers of the G allele compared to 887 non-carriers from the Health ABC Study (4.69 +/- 0.05 kbp vs. 4.86 +/- 0.04 kbp, measured by quantitative PCR, p = 0.005). This association was replicated in another white sample from the TwinsUK Study (6.90 +/- 0.03 kbp in 301 carriers compared to 7.06 +/- 0.03 kbp in 395 non-carriers, measured by Southern blots, p = 0.009). A similar pattern of association was observed in whites from the family-based AFOS and blacks from the Health ABC cohort, although not statistically significant, possibly due to the lower allele frequency in these populations. Combined analysis using 2,953 white subjects from 3 studies showed a significant association between TL and rs2293607 (beta = -0.19 +/- 0.04 kbp, p = 0.001). Conclusion: Our study shows a significant association between a common variant in TERC and TL in humans, suggesting that TERC may play a role in telomere homeostasis. | 4009 | NA | NA | NA | no | no |
| 4008 | 2017 | NA | Gelabert, P; Sandoval-Velasco, M; Olalde, I; Fregel, R; Rieux, A; Escosa, R; Aranda, C; Paaijmans, K; Mueller, I; Gilbert, MTP; Lalueza-Fox, C | 2016 | Mitochondrial DNA from the eradicated European Plasmodium vivax and P. falciparum from 70-year-old slides from the Ebro Delta in Spain | Phylogenetic analysis of Plasmodium parasites has indicated that their modern-day distribution is a result of a series of human-mediated dispersals involving transport between Africa, Europe, America, and Asia. A major outstanding question is the phylogenetic affinity of the malaria causing parasites Plasmodium vivax and falciparum in historic southern Europe-where it was endemic until the mid-20th century, after which it was eradicated across the region. Resolving the identity of these parasites will be critical for answering several hypotheses on the malaria dispersal. Recently, a set of slides with blood stains of malaria-affected people from the Ebro Delta (Spain), dated between 1942 and 1944, have been found in a local medical collection. We extracted DNA from three slides, two of them stained with Giemsa (on which Plasmodium parasites could still be seen under the microscope) and another one consisting of dried blood spots. We generated the data using Illumina sequencing after using several strategies aimed at increasing the Plasmodium DNA yield: depletion of the human genomic (g) DNA content through hybridization with human gDNA baits, and capture-enrichment using gDNA derived from P. falciparum. Plasmodium mitochondrial genome sequences were subsequently reconstructed from the resulting data. Phylogenetic analysis of the eradicated European P. vivax mtDNA genome indicates that the European isolate is closely related to the most common present-day American haplotype and likely entered the American continent post-Columbian contact. Furthermore, the European P. falciparum mtDNA indicates a link with current Indian strains that is in agreement with historical accounts. | 4010 | NA | NA | NA | no | no |
| 4009 | 2017 | NA | Ye, JQ; Fan, ZL; Shang, JJ; Tian, XY; Yang, JL; Chen, HJ; Shao, HX; Qin, AJ | 2015 | ALV-J GP37 Molecular Analysis Reveals Novel Virus-Adapted Sites and Three Tyrosine-Based Env Species | Compared to other avian leukosis viruses (ALV), ALV-J primarily induces myeloid leukemia and hemangioma and causes significant economic loss for the poultry industry. The ALV-J Env protein is hypothesized to be related to its unique pathogenesis. However, the molecular determinants of Env for ALV-J pathogenesis are unclear. In this study, we compared and analyzed GP37 of ALV-J Env and the EAV-HP sequence, which has high homology to that of ALV-J Env. Phylogenetic analysis revealed five groups of ALV-J GP37 and two novel ALV-J Envs with endemic GP85 and EAV-HP-like GP37. Furthermore, at least 15 virus-adapted mutations were detected in GP37 compared to the EAV-HP sequence. Further analysis demonstrated that three tyrosine-based motifs (YxxM, ITIM (immune tyrosine-based inhibitory motif) and ITAM-like (immune tyrosine-based active motif like)) associated with immune disease and oncogenesis were found in the cytoplasmic tail of GP37. Based on the potential function and distribution of these motifs in GP37, ALV-J Env was grouped into three species, inhibitory Env, bifunctional Env and active Env. Accordingly, 36.91%, 61.74% and 1.34% of ALV-J Env sequences from GenBank are classified as inhibitory, bifunctional and active Env, respectively. Additionally, the Env of the ALV-J prototype strain, HPRS-103, and 17 of 18 EAV-HP sequences belong to the inhibitory Env. And models for signal transduction of the three ALV-J Env species were predicted. Our findings and models provide novel insights for identifying the roles and molecular mechanism of ALV-J Env in the unique pathogenesis of ALV-J. | 4011 | NA | NA | NA | no | no |
| 4010 | 2017 | NA | Chen, Y; Wang, YY; Zhao, T; Yang, JW; Feng, SL; Nazeer, W; Zhang, TZ; Zhou, BL | 2015 | A New Synthetic Amphiploid (AADDAA) between Gossypium hirsutum and G-arboreum Lays the Foundation for Transferring Resistances to Verticillium and Drought | Gossypium arboreum, a cultivated cotton species (2n = 26, AA) native to Asia, possesses invaluable characteristics unavailable in the tetraploid cultivated cotton gene pool, such as resistance to pests and diseases and tolerance to abiotic stresses. However, it is quite difficult to transfer favorable traits into Upland cotton through conventional methods due to the cross-incompatibility of G. hirsutum (2n = 52, AADD) and G. arboreum. Here, we improved an embryo rescue technique to overcome the cross-incompatibility between these two parents for transferring favorable genes from G. arboreum into G. hirsutum. Our results indicate that MSB2K supplemented with 0.5 mgl(-1) kinetin and 250 mg(-1) casein hydrolysate is an efficient initial medium for rescuing early (3 d after pollination) hybrid embryos. Eight putative hybrids were successfully obtained, which were further verified and characterized by cytology, molecular markers and morphological analysis. The putative hybrids were subsequently treated with different concentrations of colchicine solution to double their chromosomes. The results demonstrate that four putative hybrid plants were successfully chromosome-doubled by treatment with 0.1% colchicine for 24 h and become amphiploid, which were confirmed by cytological observation, self-fertilization and backcrossing. Preliminary assessments of resistance at seedling stage indicate that the synthetic amphiploid showed highly resistant to Verticillium and drought. The synthetic amphiploid between G. hirsutum x G. arboreum would lay the foundation for developing G. arboreum-introgressed lines with the uniform genetic background of G. hirsutum acc TM-1, which would greatly enhance and simplify the mining, isolation, characterization, cloning and use of G. arboreum-specific desirable genes in future cotton breeding programs. | 4012 | NA | NA | NA | no | no |
| 4011 | 2017 | NA | Al-Saleh, MA; Amer, MA; Al-Shahwan, IM; Abdalla, OA; Shakeel, MT | 2014 | Molecular Characterization of Two Alfalfa Mosaic Virus Isolates Infecting Potato Crop in Central Region of Saudi Arabia | During the autumn growing season 2012, thirty-two leaf samples of potato (Solanum tuberosum, L. cv. Herms) plants showing mottling symptoms suspected to be due to viral infection were collected from Hail and Wadi aldawasser regions in Saudi Arabia. These leaf samples were tested serologically using DAS ELISA against Alfalfa Mosaic Virus (AMV). Twenty-three out of the thirty-two samples were positive for AMV. To determine host range, plant sap was extracted from two diseased potato samples representing the two regions and different healthy plants were mechanically inoculated. The disease symptoms induced by the two selected isolates were divided into three groups: systemic and calico symptoms, local chlorotic lesions, and non-symptomatic ones. Total RNA was extracted from different plants showing positive reaction to AMV. RT-PCR was performed using AMV coat protein specific primers. Approximately 700-bp RT-PCR products were amplified, and these PCR products were sequenced in both directions. Nucleotide sequencing analysis showed that these two Saudi Arabian AMV isolates [AMV-PSA-Ha (for the Hail isolate) and AMV-PSA-Wd (for the Wadi Al Dawasser isolate] had a 96.5% identity. Sequence comparison showed that these two isolates of AMV shared 93.2% to 99.7% sequence similarity with the twenty-eight reported isolates of AMV obtained from GenBank. The cDNA probe was prepared and hybridized with RNA extraction from symptomatic potato plants. No hybridization was shown with RNA extracts from asymptomatic plants. This is the first report on the genetic variability of AMV isolates infecting potato crop in Saudi Arabia. (C) 2014 Friends Science Publishers | 4013 | NA | NA | NA | no | no |
| 4012 | 2017 | NA | Techtmann, SM; Robb, FT | 2010 | Archaeal-like chaperonins in bacteria | Chaperonins (CPN) are ubiquitous oligomeric protein machines that mediate the ATP-dependent folding of polypeptide chains. These chaperones have not only been assigned stress response and normal housekeeping functions but also have a role in certain human disease states. A longstanding convention divides CPNs into two groups that share many conserved sequence motifs but differ in both structure and distribution. Group I complexes are the well known GroEL/ES heat-shock proteins in bacteria, that also occur in some species of mesophilic archaea and in the endosymbiotic organelles of eukaryotes. Group II CPNs are found only in the cytosol of archaea and eukaryotes. Here we report a third, divergent group of CPNs found in several species of bacteria. We propose to name these Group III CPNs because of their distant relatedness to both Group I and II CPNs as well as their unique genomic context, within the hsp70 operon. The prototype Group III CPN, Carboxydothermus hydrogenoformans chaperonin (Ch-CPN), is able to refold denatured proteins in an ATP-dependent manner and is structurally similar to the Group II CPNs, forming a 16-mer with each subunit contributing to a flexible lid domain. The Group III CPN represent a divergent group of bacterial CPNs distinct from the GroEL/ES CPN found in all bacteria. The Group III lineage may represent an ancient horizontal gene transfer from an archaeon into an early Firmicute lineage. An analysis of their functional and structural characteristics may provide important insights into the early history of this ubiquitous family of proteins. | 4014 | NA | NA | NA | no | no |
| 4013 | 2017 | NA | Rona, LDP; Carvalho-Pinto, CJ; Peixoto, AA | 2013 | Evidence for the occurrence of two sympatric sibling species within the Anopheles (Kerteszia) cruzii complex in southeast Brazil and the detection of asymmetric introgression between them using a multilocus analysis | Background: Anopheles (Kerteszia) cruzii (Diptera: Culicidae) is a primary vector of human and simian malaria parasites in southern and southeastern Brazil. Earlier studies using chromosome inversions, isoenzymes and a number of molecular markers have suggested that An. cruzii is a species complex. Results: In this study, a multilocus approach using six loci, three circadian clock genes and three encoding ribosomal proteins, was carried out to investigate in more detail the genetic differentiation between the An. cruzii populations from Florianopolis-Santa Catarina (southern Brazil) and Itatiaia-Rio de Janeiro States (southeastern Brazil). The analyses were performed first comparing Florianopolis and Itatiaia, and then comparing the two putative sympatric incipient species from Itatiaia (Itatiaia A and Itatiaia B). The analysis revealed high F-ST values between Florianopolis and Itatiaia (considering Itatiaia A and B together) and also between the sympatric Itatiaia A and Itatiaia B, irrespective of their function. Also, using the IM program, no strong indication of migration was found between Florianopolis and Itatiaia (considering Itatiaia A and B together) using all loci together, but between Itatiaia A and Itatiaia B, the results show evidence of migration only in the direction of Itatiaia B. Conclusions: The results of the multilocus analysis indicate that Florianopolis and Itatiaia represent different species of the An. cruzii complex that diverged around 0.6 Mya, and also that the Itatiaia sample is composed of two sympatric incipient species A and B, which diverged around 0.2 Mya. Asymmetric introgression was found between the latter two species despite strong divergence in some loci. | 4015 | NA | NA | NA | no | no |
| 4014 | 2017 | NA | Dziedek, C; von Oheimb, G; Calvo, L; Fichtner, A; Kriebitzsch, WU; Marcos, E; Pitz, WT; Hardtle, W | 2016 | Does excess nitrogen supply increase the drought sensitivity of European beech (Fagus sylvatica L.) seedlings? | Climate change and atmospheric deposition of nitrogen affect biodiversity patterns and functions of forest ecosystems worldwide. Many studies have quantified tree growth responses to single global change drivers, but less is known about the interaction effects of these drivers at the plant and ecosystem level. In the present study, we conducted a full-factorial greenhouse experiment to analyse single and combined effects of nitrogen fertilization (N treatment) and drought (D treatment) on 16 morphological and chemical response variables (including tissue delta C-13 signatures) of one-year-old Fagus sylvatica seedlings originating from eight different seed families from the Cantabrian Mountains (NW Spain). Drought exerted the strongest effect on response variables, reflected by decreasing biomass production and increasing tissue delta C-13 signatures. However, D and N treatments interacted for some of the response variables, indicating that N fertilization has the potential to strengthen the negative effects of drought (with both antagonistic and amplifying interactions). For example, combined effects of N and D treatments caused a sevenfold increase of necrotic leaf biomass. We hypothesize that increasing drought sensitivity was mainly attributable to a significant reduction of the root biomass in combined N and D treatments, limiting the plants’ capability to satisfy their water demands. Significant seed family effects and interactions of seed family with N and D treatments across response variables suggest a high within-population genetic variability. In conclusion, our findings indicated a high drought sensitivity of Cantabrian beech populations, but also interaction effects of N and D on growth responses of beech seedlings. | 4016 | NA | NA | NA | no | no |
| 4015 | 2017 | NA | Zhang, WZ; Shen, YJ; Wang, RJ; Liu, AQ; Ling, H; Li, YH; Cao, JP; Zhang, XY; Shu, J; Zhang, LX | 2012 | Cryptosporidium cuniculus and Giardia duodenalis in Rabbits: Genetic Diversity and Possible Zoonotic Transmission | Background: Cryptosporidium and Giardia are the two important zoonotic pathogens causing diarrhea of humans and animals worldwide. Considering the human cryptosporidiosis outbreak and sporadic cases caused by C. cuniculus, the important public health significance of G. duodenalis and little obtained information regarding rabbit infected with Cryptosporidium and Giardia in China, the aim of this study is to determine the prevalence and molecularly characterize Cryptosporidium and Giardia in rabbits in Heilongjiang Province, China. Methodology/Principal Findings: 378 fecal samples were obtained from rabbits in Heilongjiang Province. Cryptosporidium oocysts and Giardia cysts were detected using Sheather’s sugar flotation technique and Lugol’s iodine stain method, respectively. The infection rates of Cryptosporidium and Giardia were 2.38% (9/378) and 7.41% (28/378), respectively. Genotyping of Cryptosporidium spp. was done by DNA sequencing of the small subunit rRNA (SSU rRNA) gene and all the nine isolates were identified as Cryptosporidium cuniculus. The nine isolates were further subtyped using the 60-kDa glycoprotein (gp60) gene and two subtypes were detected, including VbA32 (n = 3) and a new subtype VbA21 (n = 6). G. duodenalis genotypes and subtypes were identified by sequence analysis of the triosephosphate isomerase (TPI) gene. The assemblage B (belonging to eight different subtypes B-I to B-VIII) was found in 28 G. duodenalis-positive samples. Conclusions/Significance: The rabbits have been infected with Cryptosporidium and Giardia in Heilongjiang Province. The results show that the rabbits pose a threat to human health in the studied areas. Genotypes and subgenotypes of C. cuniculus and G. duodenalis in this study might present the endemic genetic characterization of population structure of the two parasites. | 4017 | NA | NA | NA | no | no |
| 4016 | 2017 | NA | Tao, L; Du, H; Guan, GB; Dai, Y; Nobile, CJ; Liang, WH; Cao, CJ; Zhang, QY; Zhong, J; Huang, GH | 2014 | Discovery of a "White-Gray-Opaque’’ Tristable Phenotypic Switching System in Candida albicans: Roles of Non-genetic Diversity in Host Adaptation | Non-genetic phenotypic variations play a critical role in the adaption to environmental changes in microbial organisms. Candida albicans, a major human fungal pathogen, can switch between several morphological phenotypes. This ability is critical for its commensal lifestyle and for its ability to cause infections. Here, we report the discovery of a novel morphological form in C. albicans, referred to as the gray phenotype, which forms a tristable phenotypic switching system with the previously reported white and opaque phenotypes. White, gray, and opaque cell types differ in a number of aspects including cellular and colony appearances, mating competency, secreted aspartyl proteinase (Sap) activities, and virulence. Of the three cell types, gray cells exhibit the highest Sap activity and the highest ability to cause cutaneous infections. The three phenotypes form a tristable phenotypic switching system, which is independent of the regulation of the mating type locus (MTL). Gray cells mate over 1,000 times more efficiently than do white cells, but less efficiently than do opaque cells. We further demonstrate that the master regulator of white-opaque switching, Wor1, is essential for opaque cell formation, but is not required for white-gray transitions. The Efg1 regulator is required for maintenance of the white phenotype, but is not required for gray-opaque transitions. Interestingly, the wor1/wor1 efg1/efg1 double mutant is locked in the gray phenotype, suggesting that Wor1 and Efg1 could function coordinately and play a central role in the regulation of gray cell formation. Global transcriptional analysis indicates that white, gray, and opaque cells exhibit distinct gene expression profiles, which partly explain their differences in causing infections, adaptation ability to diverse host niches, metabolic profiles, and stress responses. Therefore, the white-gray-opaque tristable phenotypic switching system in C. albicans may play a significant role in a wide range of biological aspects in this common commensal and pathogenic fungus. Author Summary The capacity of the yeast Candida albicans to grow in several cellular formsa phenomenon known as phenotypic plasticityis critical for its survival and for its ability to thrive and cause infection in the human host. In this study, we report a novel form of C. albicans, the gray phenotype, which may enhance fitness and confer an adaptive advantage for this important pathogenic yeast in certain host environments. The gray cell type, together with the previously discovered white and opaque cell types, forms a tristable phenotypic switching system. The three phenotypes differ in their cellular and colony appearance, their global transcriptional profiles, their production of secreted aspartyl proteinases (enzymes that degrade host tissues and release nutrients), and their virulence in different infection models. Moreover, gray cells exhibit a level of mating competency that is intermediate between that of white and opaque cells. We further demonstrate that two key transcriptional regulators, Wor1 and Efg1, play central roles in the regulation of the white-gray-opaque tristable transitions. Our study reveals a multi-stable and heritable switching system, indicating that the adoption of distinct morphological forms in response to environmental change could be much more elaborate than previously thought. | 4018 | NA | NA | NA | no | no |
| 4017 | 2017 | NA | Forcada, J; Hammond, PS; Aguilar, A | 1999 | Status of the Mediterranean monk seal Monachus monachus in the western Sahara and the implications of a mass mortality event | The largest aggregation of the highly endangered Mediterranean monk seals Monachus monachus is located on the Cap Blanc Peninsula on the coast of the western Sahara. Photographic identification of individuals was used in a capture-recapture analysis to estimate the abundance and composition of the colony during 1993 to 1998. Results of the application of these techniques to Mediterranean monk seals were satisfactory, and allowed independent annual population estimates. No significant trends in abundance over the period 1993 to 1996 were detected, and the mean estimate for the period was 317 seals (CV = 0.16; 95% CI: 237 to 447). Environmental changes affecting suitability of habitat, particularly food availability, are suggested as major factors for limited population recovery during this period. In spring 1997, a mass mortality event reduced the population size to 109 individuals (CV = 0.14; 95% CI: 86 to 145). Mortality was age-specific and resulted in a severe change in the stage composition of the population. Adults were the most affected, and therefore the proportion of juveniles increased from an initial 12% before the event to about 29% after. As a result of both the decrease in seal numbers and the change in population composition, the number of individuals potentially contributing to reproduction fell to about 77 or fewer. This number may not be enough to maintain genetic variability and overcome the effect of demographic stochasticity. The demographic changes caused by the die-off are expected to have both positive and negative effects on the reproductive success of the colony. However, they are undoubtedly factors which potentially threaten the stability and survival of the colony in the near future. | 4019 | NA | NA | NA | no | no |
| 4018 | 2017 | NA | Wywial, E; Haven, J; Casjens, SR; Hernandez, YA; Singh, S; Mongodin, EF; Fraser-Liggett, CM; Luft, BJ; Schutzer, SE; Qiu, WG | 2009 | Fast, adaptive evolution at a bacterial host-resistance locus: The PFam54 gene array in Borrelia burgdorferi | Microbial pathogens have evolved sophisticated mechanisms for evasion of host innate and adaptive immunities. PFam54 is the largest paralogous gene family in the genomes of Borrelia burgdorferi, the Lyme disease bacterium. One member of PFam54, the complement-regulator acquiring surface proteins 1 (BbCRASP-1), is able to abort the alternative pathway of complement activation via binding human complement-regulator factor H (FH). The gene coding for BbCRASP-1 exists in a tandem array of PFam54 genes in the B. burgdorferi genome, a result apparently of repeated gene duplications. To help elucidate the functions of the large number of PFam54 genes. we performed phylogenomic and structural analyses of the PFam54 gene array from ten B. burgdorferi genomes. Analyses based on gene tree, genome synteny, and structural models revealed rapid adaptive evolution of this array through gene duplication, gene loss, and functional diversification. Individual PFam54 genes, however, do not show high intra-population sequence polymorphisms as genes providing evasion from adaptive immunity generally do. PFam54 members able to bind human FH are not monophyletic, suggesting that human FH affinity, however strong, is an incidental rather than main function of these PFam54 proteins. The large number of PFam54 genes existing in any single B. burgdorferi genome may target different innate-immunity proteins of a single host species or the same immune protein of a variety of host species. Genetic variability of the PFam54 gene array suggests that universally present PFam54 lineages such as BBA64, BBA65, BBA66, and BBA73 may be better candidates for the development of broad-spectrum vaccines or drugs than strain-restricted lineages such as BbCRASP-1. (C) 2009 Elsevier B.V. All rights reserved. | 4020 | NA | NA | NA | no | no |
| 4019 | 2017 | NA | Stewart, KA; Lougheed, SC | 2013 | Testing for intraspecific postzygotic isolation between cryptic lineages of Pseudacris crucifer | Phenotypically cryptic lineages appear common in nature, yet little is known about the mechanisms that initiate and/or maintain barriers to gene flow, or how secondary contact between them might influence evolutionary trajectories. The consequences of such contact between diverging lineages depend on hybrid fitness, highlighting the potential for postzygotic isolating barriers to play a role in the origins of biological species. Previous research shows that two cryptic, deeply diverged intraspecific mitochondrial lineages of a North American chorus frog, the spring peeper (Pseudacris crucifer), meet in secondary contact in Southwestern Ontario, Canada. Our study quantified hatching success, tadpole survival, size at metamorphosis, and development time for experimentally generated pure lineage and hybrid tadpoles. Results suggest that lineages differ in tadpole survival and that F-1 hybrids may have equal fitness and higher than average mass at metamorphosis compared with pure parental crosses. These findings imply hybrid early life viability may not be the pivotal reproductive isolation barrier helping to maintain lineage boundaries. However, we observed instances of tadpole gigantism, failure to metamorphose, and bent tails in some tadpoles from hybrid families. We also speculate and provide some evidence that apparent advantages or similarities of hybrids compared with pure lineage tadpoles may disappear when tadpoles are raised with competitors of different genetic makeup. This pilot study implies that ecological context and consideration of extrinsic factors may be a key to revealing mechanisms causing negative hybrid fitness during early life stages, a provocative avenue for future investigations on barriers to gene flow among these intraspecific lineages. | 4021 | NA | NA | NA | no | no |
| 4020 | 2017 | NA | Laxami, S; Desai, SA; Lohithaswa, HC; Hanchinal, RR | 2013 | The utility of chemical and radiation induced mutations in tetraploid wheat | The present investigation was conducted with the main objective of studying the extent of genetic variability released through recombination, induced mutation and their combination for yield and its attributing traits in tetraploid wheat. The material consisted of segregating populations (F-2, M-2 and F2M2) developed from Triticum dicoccum wheat variety DDK1025 and T. durum wheat variety HD4502 after subjecting to EMS and gamma-rays treatment. Among mutagen treated populations gamma-rays induced more reduction in germination and survival percentage and mean plant height than EMS. Analysis of variance revealed highly significant differences among the populations for all the characters studied. An upper limit of range was more in M-2 compared to F-2 and F2M2 populations for all the characters except for days to 50% flowering and number of productive tillers/ plant. In general, the gamma-rays irradiated populations recorded highest PCV and GCV for all the characters except for plant height, spike length and spikelets/spike indicating greater scope for selection and improvement of these characters in desired direction. Most of the characters exhibited high heritability except spike length in all segregating populations. In general, the number of favorable correlations and higher strength of positive correlations were appeared to be more in F2M2 populations than both F-2 and M-2 populations. The number of potential genotypes varied considerably across the traits and the populations. The F2M2(gamma) population showed higher number of potential progeny lines compared to F-2, F2M2 (EMS) and M-2 populations. This indicated greater possibility of generation of potential genotypes through irradiation in heterozygous condition than EMS. | 4022 | NA | NA | NA | no | no |
| 4021 | 2017 | NA | Dalebout, ML; Robertson, KM; Frantzis, A; Engelhaupt, D; Mignucci-Giannoni, AA; Rosario-Delestre, RJ; Baker, CS | 2005 | Worldwide structure of mtDNA diversity among Cuvier’s beaked whales (Ziphius cavirostris): implications for threatened populations | We present the first description of phylogeographic structure among Cuvier’s beaked whales (Ziphius cavirostris) worldwide using mitochondrial DNA (mtDNA) control region sequences obtained from strandings (n = 70), incidental fisheries takes (n = 11), biopsy (n = 1), and whale-meat markets (n = 5). Over a 290-base pair fragment, 23 variable sites defined 33 unique haplotypes among the total of 87 samples. Nucleotide diversity at the control region was relatively low (pi = 1.27% +/- 0.723%) compared to wide-ranging baleen whales, but higher than strongly matrifocal sperm, pilot and killer whales. Phylogenetic reconstruction using maximum likelihood revealed four distinct haplotype groups, each of which displayed strong frequency differences among ocean basins, but no reciprocal monophyly or fixed character differences. Consistent with this phylogeographic pattern, an analysis of molecular variance showed high levels of differentiation among ocean basins (F-ST = 0.14, Phi(ST) = 0.42; P < 0.001). Estimated rates of female migration among ocean basins were low (generally <= 2 individuals per generation). Regional sample sizes were too small to detect subdivisions within oceans except in the North Atlantic, where the Mediterranean Sea (n = 12) was highly differentiated due to the presence of two private haplotypes. One market product purchased in South Korea grouped with other haplotypes found only in the North Atlantic, suggesting a violation of current agreements banning international trade in cetacean species. Together, these results demonstrate a high degree of isolation and low maternal gene flow among oceanic, and in some cases, regional populations of Cuvier’s beaked whales. This has important implications for understanding the threats of human impact, including fisheries by-catch, direct hunting, and disturbance or mortality from anthropogenic sound. | 4023 | NA | NA | NA | no | no |
| 4022 | 2017 | NA | Alberto, RT; Aquino, VM | 2010 | Characterization of Colletotrichum gloeosporioides (Penzig) Penzig & Sacc. (anthracnose) and Gibberella moniliformis Wineland (twister) infecting onions in the Philippines | The causal organisms of the anthracnose/twister disease of onion were characterized and identified using morphological characters and molecular markers to assess the diversity of the pathogens. Two fungal isolates, gray-white (GW) and pink (P) isolates were recovered from leaves and bulbs of onions showing severe anthracnose symptoms. Based on morphological and cultural characteristics, the GW isolates were identified as Colletotrichum gloeosporioides and the P,isolates as Colletotrichum acutatum. All onion and mango Colletotrichum isolates were tested positive to C.gloeosporioides species-specific primer (CgInt2) and the pink isolates from onion and pepper to C. acutatum species-specific primer (CaInt2). Internal transcribed spacer (ITS) sequences and NTSYS dendogram analysis of ten GW isolates grouped all onion isolates into C. gloeosporioides and the four pink isolates to Gibberella moniliformis. Analysis of his4 nucleotide sequences revealed the groups were concordant with ITS, but differed on species level, Arbitrarily Primed-PCR analyses revealed intraspecific subgroups, but neither was useful to decipher species level relationships. Results also indicates the potential of the C.gloeosporioides and C. acutatum specific-specific primers for routine pathogen diagnosis. Arbitrarity Primed PCR (AP-PCR) analysis using two repeat sequences primers (TCC5 and CAG5) and two microsatellite derived primers (GACAC3 and MR) demonstrated its potential in species identification and classification of the Unknown strains of the two plant pathogens. Test for pathogenicity demonstrated that the two species isolated from onions were the causal agents of the anthracnose/twister disease complex whereby the G. monililiformis responsible for abnormal neck elongation and twisting of leaves of infected onions. | 4024 | NA | NA | NA | no | no |
| 4023 | 2017 | NA | Ben Naceur, H; Jenhani, AB; Romdhane, MS | 2013 | Reproduction characteristics, survival rate and sex-ratio of four brine shrimp Artemia sauna (Linnaeus, 1758) populations from Tunisia cultured under laboratory conditions | Artemia has a remarkable genetic variability that can be expressed in various phenotypic characteristics, such morphometry, growth rate, reproductive isolation or molecular composition. This study presents reproduction characteristics, survival rate and sex-ratio of four Artemia sauna populations from Tunisia cultured under standard conditions. Results show that both low (17.8% and 30.6% in Megrine saltwork and Sabkhet Sijoumi, respectively) and high (83.9% for Sahline saltwork) values were recorded for the percentage of total oviparous offspring. The offspring per brood varied from 70.3 +/- 41.9 (Megrine saltwork) to +/- 73.6 +/- 51.4 (Sabkhet El Adhibet). Broods per female varied between 3.1 +/- 0.9 (Sahline saltwork) and 4.5 +/- 1.2 (Megrine saltwork), and time between broods ranged from 5.7 +/- 1.1 to 6.5 +/- 2.2 days for Megrine and Sahline, respectively. Statistical analysis (one-way analysis of variance, Tukey’s HSD test, p < 0.05) revealed no significant differences between reproductive traits of the four studied populations except for mean of oviparous and oviviparous offspring per female (F=9.158, p < 0.05), and brood per female (F=4.779, p < 0.05). The survival rate of the four studied A. salina populations fluctuated between 31.4% and 64.5% for Megrine and Sahline saltwork, respectively. However, the sex-ratio showed that for Megrine saltwork and Sabkhet El Adhibet, males predominated with 1: 0.90 and 1 : 0.97 (males : females), respectively. The comparison between our results and those reported of other Anemia populations showed that the offspring per brood of Tunisian Artemia are comparable to A. persimilis but different to A. sauna from Abu Kammash (Libya) and A. urmiana (Urmia Lake, Iran), and that days between brood, are similar to A. sauna from Abu Kammash, A. sinica and polyploid A. parthenogenetica, but different from A. persimilis and A. franciscana. | 4025 | NA | NA | NA | no | no |
| 4024 | 2017 | NA | Schlegel, M; Radosa, L; Rosenfeld, UM; Schmidt, S; Triebenbacher, C; Lohr, PW; Fuchs, D; Heroldova, M; Janova, E; Stanko, M; Mosansky, L; Fricova, J; Pejoch, M; Suchomel, J; Purchart, L; Groschup, MH; Kruger, DH; Klempa, B; Ulrich, RG | 2012 | Broad geographical distribution and high genetic diversity of shrew-borne Seewis hantavirus in Central Europe | For a long time hantaviruses were believed to be exclusively rodent-borne pathogens. Recent findings of numerous shrew- and mole-borne hantaviruses raise important questions on their phylogenetic origin. The objective of our study was to prove the presence and distribution of shrew-associated Seewis virus (SWSV) in different Sorex species in Central Europe. Therefore, a total of 353 Sorex araneus, 59 S. minutus, 27 S. coronatus, and one S. alpinus were collected in Germany, the Czech Republic, and Slovakia. Screening by hantavirus-specific L-segment RT-PCR revealed specific amplification products in tissues of 49 out of 353 S. araneus and four out of 59 S. minutus. S-segment sequences were obtained for 45 of the L-segment positive S. araneus and all four L-segment positive S. minutus. Phylogenetic investigation of these sequences from Germany, the Czech Republic, and Slovakia demonstrated their similarity to SWSV sequences from Hungary, Finland, Austria, and other sites in Germany. The low intra-cluster sequence variability and the high inter-cluster divergence suggest a long-term SWSV evolution in isolated Sorex populations. In 28 of the 49 SWSV S-segment sequences, an additional putative open reading frame (ORF) on the opposite strand to the nucleocapsid protein-encoding ORF was identified. This is the first comprehensive sequence analysis of SWSV strains from Germany, the Czech Republic, and Slovakia, indicating its broad geographical distribution and high genetic divergence. Future studies have to prove whether both S. araneus and S. minutus represent SWSV reservoir hosts or spillover infections are responsible for the parallel molecular detection of SWSV in both species. | 4026 | NA | NA | NA | no | no |
| 4025 | 2017 | NA | Brandenburg, JT; Austerlitz, F; Toupance, B | 2012 | Impact of fertility transmission and other sociodemographic factors on reproductive success and coalescent trees | Fertility transmission (FT) is a phenomenon with a cultural and/or genetic basis, whereby a positive correlation exists between the number of offspring of an individual and that of his/her parents. Theoretical studies using a haploid individual-based model have shown that FT increases the variance and intergenerational correlation in reproductive success and results in an imbalance in the coalescent tree of sampled genes. This phenomenon has been documented in several demographic studies conducted on the correlation in fertility between generations, or through the reconstruction of the genealogical trees of mitochondria! DNA sequences. However, as mtDNA is a single locus, potentially subject to other forces (e.g. natural selection), it is of interest to extend the theory of FT to nuclear loci. We show that because random mating between individuals leads to a mixing of their fertility profiles, FT in these cases will have less influence on the variance and intergenerational correlation of reproductive success. This, in turn, results in less impact on the shape of the coalescent trees. Nevertheless, in the presence of FT, high heterogeneity in reproductive success and homogamy for family size will increase the imbalance in the coalescent tree. Thus, FT should be easier to detect when occurring in conjunction with these other factors. We also show the utility of analysing different kinds of loci (X-linked, Y-linked, mitochondrial and autosomal) to assess whether FT is matrilineal, patrilineal or biparental. Finally, we demonstrate that the shape of the coalescent tree depends upon population size, in contrast to the classical Kingman’s model. | 4027 | NA | NA | NA | no | no |
| 4026 | 2017 | NA | Dostal, P; Paleckova, M | 2011 | Does relatedness of natives used for soil conditioning influence plant-soil feedback of exotics? | The naturalisation hypothesis has been gaining attention recently as a possible mechanism to explain variations in invasion success. It predicts that exotic genera with native representatives should be less successful because of an overlap in resource use and of the existence of common specialised enemies. In this study, we tested whether native congenerics have more negative impact on exotic species than heterogenerics by increasing the effects of soil pathogens. We sampled soil in populations of three exotic species (Epilobium ciliatum, Impatiens parviflora and Stenactis annua) at sites with and without respective congeneric species. This soil was used as an inoculum for cultivating the first plant cohort, which included exotics, as well as native congenerics and heterogenerics. The conditioned soil was subsequently used for cultivating the second cohort of plants (exotics only). We found no consistent impact of relatedness of conditioning species on exotic growth. Although soil conditioned by congeneric E. hirsutum had the largest reduction on the performance of E. ciliatum, the final biomass of S. annua was lowest when grown in soil conditioned by itself. There was no effect of stimulating species on the biomass of I. parviflora. In both experimental phases, performance of exotics was improved when cultivated with sterilised inoculua, indicating the dominance of soil generalist pathogens. However, the biomass of S. annua was increased most by congeneric-stimulated inoculum from congeneric sites, suggesting a possible role for specialised symbionts. Our results suggest that variations in invasion success of at least some exotics may be affected by species-specific interactions mediated by the soil biota. | 4028 | NA | NA | NA | no | no |
| 4027 | 2017 | NA | Dharmarajan, G; Beasley, JC; Rhodes, OE | 2011 | Heterozygote deficiencies in parasite populations: an evaluation of interrelated hypotheses in the raccoon tick, Ixodes texanus | Population genetics is increasingly being used to study the biology of parasites at the scales of both the host (infra-population, IP) and host population (component population, CP). In this study we tested three mechanistic hypotheses that could explain deviations from Hardy-Weinberg equilibrium (HWE) expectations due to heterozygote deficits (HDs) at the CP scale in raccoon ticks (Ixodes texanus; n = 718) collected from raccoons (Procyon lotor; n = 91) and genotyped at 11 microsatellite loci. These hypotheses were presence of technical issues (for example, null alleles), hierarchical structure (for example, host demography) and cryptic structure (for example, kin structure). Although statistical support for null alleles existed, their presence would also be expected to lead to an underestimation in levels of relatedness, and thus kin structure. However, we found the opposite pattern: significant HD at the IP scale being more likely in CPs with significant vs non-significant levels of kin structure. Our analyses revealed that pooling of kin groups could lead to highly variable levels of F-IS among loci, a pattern usually suggestive of null alleles. We used Monte-Carlo (MC) simulations to show that the existence of subdivided breeding groups and high variance in individual reproductive success could adequately explain deviations from HWE in I. texanus. Thus, our results indicate that biological factors can lead to patterns that have usually been interpreted as technical issues (for example, null alleles), and that it is important to take such factors into consideration because loci deviating from HWE likely reflect the effects of real biological processes. Heredity (2011) 106, 253-260; doi:10.1038/hdy.2010.84; published online 7 July 2010 | 4029 | NA | NA | NA | no | no |
| 4028 | 2017 | NA | Yalcindag, E; Rougeron, V; Elguero, E; Arnathau, C; Durand, P; Brisse, S; Diancourt, L; Aubouy, A; Becquart, P; D’Alessandro, U; Fontenille, D; Gamboa, D; Maestre, A; Menard, D; Musset, L; Noya, O; Veron, V; Wide, A; Carme, B; Legrand, E; Chevillon, C; Ayala, FJ; Renaud, F; Prugnolle, F | 2014 | Patterns of selection on Plasmodium falciparum erythrocyte-binding antigens after the colonization of the New World | Pathogens, which have recently colonized a new host species or new populations of the same host, are interesting models for understanding how populations may evolve in response to novel environments. During its colonization of South America from Africa, Plasmodium falciparum, the main agent of malaria, has been exposed to new conditions in distinctive new human populations (Amerindian and populations of mixed origins) that likely exerted new selective pressures on the parasite’s genome. Among the genes that might have experienced strong selective pressures in response to these environmental changes, the eba genes (erythrocyte-binding antigens genes), which are involved in the invasion of the human red blood cells, constitute good candidates. In this study, we analysed, in South America, the polymorphism of three eba genes (eba-140, eba-175, eba-181) and compared it to the polymorphism observed in African populations. The aim was to determine whether these genes faced selective pressures in South America distinct from what they experienced in Africa. Patterns of genetic variability of these genes were compared to the patterns observed at two housekeeping genes (adsl and serca) and 272 SNPs to separate adaptive effects from demographic effects. We show that, conversely to Africa, eba-140 seemed to be under stronger diversifying selection in South America than eba-175. In contrast, eba-181 did not show any sign of departure from neutrality. These changes in the patterns of selection on the eba genes could be the consequence of changes in the host immune response, the host receptor polymorphisms and/or the ability of the parasite to silence or express differentially its invasion proteins. | 4030 | NA | NA | NA | no | no |
| 4029 | 2017 | NA | Zafar-ul Islam, M; Ismail, K; Boug, A | 2011 | Restoration of the endangered Arabian Oryx Oryx leucoryx, Pallas 1766 in Saudi Arabia: lessons learnt from the twenty years of re-introduction in arid fenced and unfenced protected areas | In Saudi Arabia, a conservation and restoration programme for Oryx Oryx leucoryx, Pallas, 1766 was started in 1989 by the Saudi Wildlife Commission (formerly the National Commission for Wildlife Conmservation and Development). Concurrent conservation programmes were launched for the protection of large areas within the former range of the Arabian Oryx, and captive breeding at the National Wildlife Research Center (NWRC). Together, these have enabled the restoration of wild self-sustaining populations of Arabian Oryx in Saudi Arabia using animals from the ‘World Herd’ to improve their genetic variability. The success of the oryx conservation programme is described here together with the constraints faced in the arid environments and the consequent lessons learnt. As rainfall has a strong influence on the presence of annual plants, it is the single most important factor in the production of grazing. Poor rainfall had a major detrimental impact on forage in the re-introduction sites from 1999 to 2008 and mortality of oryx was higher during this period. As oryx historically moved over great distances in response to rain, the fence around one site, the Mahazat as-Sayd Protected Area, prevents natural movements of animals and artificially concentrates ungulate populations into seasonally unfavourable habitat. We propose some management strategies to minimize mortalities in the wild, and assesses post-release monitoring and adoption of various estimation techniques to assess oryx populations in both the fenced and free-ranging areas. As poaching is still a problem, strict law enforcement and a public-awareness programme to inform citizens of the biological and historical significance of the Arabian Oryx is recommended. | 4031 | NA | NA | NA | no | no |
| 4030 | 2017 | NA | Gitlin, AR; Sthultz, CM; Bowker, MA; Stumpf, S; Paxton, KL; Kennedy, K; Munoz, A; Bailey, JK; Whitham, TG | 2006 | Mortality gradients within and among dominant plant populations as barometers of ecosystem change during extreme drought | Understanding patterns of plant population mortality during extreme weather events is important to conservation planners because the frequency of such events is expected to increase, creating the need to integrate climatic uncertainty into management Dominant plants provide habitat and ecosystem structure, so changes in their distribution can be expected to have cascading effects on entire communities. Observing areas that respond quickly to climate fluctuations provides foresight into future ecological changes and will help prioritize conservation efforts. We investigated patterns of mortality in six dominant plant species during a drought in the southwestern United States. We quantified population mortality for each species across its regional distribution and tested hypotheses to identify ecological stress gradients for each species. Our results revealed three major patterns: (1) dominant species from diverse habitat types (i.e., riparian, chaparral, and low- to high-elevation forests) exhibited significant mortality, indicating that the effects of drought were widespread; (2) average mortality differed among dominant species (one-seed juniper [Juniperus monosperma (Engelin.) Sarg.] 3.3%; manzanita [Arctostaphylos pungens Kunth], 14.6%; quaking aspen [Populus tremuloides Micbx.], 15.4%;ponderosapine [Pinus ponderosa P & C Lawson], 15.9%; Fremont cottonwood [Populus fremontii S. Wats.], 20.7%; and pinyon pine [Pinus edulis Engelm.], 41.4%); (3) all dominant species showed localized patterns of very high mortality (24-100%) consistent with water stress gradients. Land managers should plan for climatic uncertainty by promoting tree recruitment in rare habitat types, alleviating unnatural levels of competition on dominant plants, and conserving sites across water stress gradients. High-stress sites, such as those we examined, have conservation value as barometers of change and because they may harbor genotypes that are adapted to climatic extremes. | 4032 | NA | NA | NA | no | no |
| 4031 | 2017 | NA | Echeverry, DF; Nair, S; Osorio, L; Menon, S; Murillo, C; Anderson, TJC | 2013 | Long term persistence of clonal malaria parasite Plasmodium falciparum lineages in the Colombian Pacific region | Background: Resistance to chloroquine and antifolate drugs has evolved independently in South America, suggesting that genotype - phenotype studies aimed at understanding the genetic basis of resistance to these and other drugs should be conducted in this continent. This research was conducted to better understand the population structure of Colombian Plasmodium falciparum in preparation for such studies. Results: A set of 384 SNPs were genotyped in blood spot DNA samples from 447 P. falciparum infected subjects collected over a ten year period from four provinces of the Colombian Pacific coast to evaluate clonality, population structure and linkage disequilibrium (LD). Most infections (81%) contained a single predominant clone. These clustered into 136 multilocus genotypes (MLGs), with 32% of MLGs recovered from multiple (2 - 28) independent subjects. We observed extremely low genotypic richness (R = 0.42) and long persistence of MLGs through time (median = 537 days, range = 1 - 2,997 days). There was a high probability (> 5%) of sampling parasites from the same MLG in different subjects within 28 days, suggesting caution is needed when using genotyping methods to assess treatment success in clinical drug trials. Panmixia was rejected as four well differentiated subpopulations (F-ST = 0.084 - 0.279) were identified. These occurred sympatrically but varied in frequency within the four provinces. Linkage disequilibrium (LD) decayed more rapidly (r(2) = 0.17 for markers < 10 kb apart) than observed previously in South American samples. Conclusions: We conclude that Colombian populations have several advantages for association studies, because multiple clone infections are uncommon and LD decays over the scale of one or a few genes. However, the extensive population structure and low genotype richness will need to be accounted for when designing and analyzing association studies. | 4033 | NA | NA | NA | no | no |
| 4032 | 2017 | NA | Xiong, J; Yuan, DX; Fillingham, JS; Garg, J; Lu, XY; Chang, Y; Liu, YF; Fu, CJ; Pearlman, RE; Miao, W | 2011 | Gene Network Landscape of the Ciliate Tetrahymena thermophila | Background: Genome-wide expression data of gene microarrays can be used to infer gene networks. At a cellular level, a gene network provides a picture of the modules in which genes are densely connected, and of the hub genes, which are highly connected with other genes. A gene network is useful to identify the genes involved in the same pathway, in a protein complex or that are co-regulated. In this study, we used different methods to find gene networks in the ciliate Tetrahymena thermophila, and describe some important properties of this network, such as modules and hubs. Methodology/Principal Findings: Using 67 single channel microarrays, we constructed the Tetrahymena gene network (TGN) using three methods: the Pearson correlation coefficient (PCC), the Spearman correlation coefficient (SCC) and the context likelihood of relatedness (CLR) algorithm. The accuracy and coverage of the three networks were evaluated using four conserved protein complexes in yeast. The CLR network with a Z-score threshold 3.49 was determined to be the most robust. The TGN was partitioned, and 55 modules were found. In addition, analysis of the arbitrarily determined 1200 hubs showed that these hubs could be sorted into six groups according to their expression profiles. We also investigated human disease orthologs in Tetrahymena that are missing in yeast and provide evidence indicating that some of these are involved in the same process in Tetrahymena as in human. Conclusions/Significance: This study constructed a Tetrahymena gene network, provided new insights to the properties of this biological network, and presents an important resource to study Tetrahymena genes at the pathway level. | 4034 | NA | NA | NA | no | no |
| 4033 | 2017 | NA | MacColl, E; Therkelsen, MD; Sherpa, T; Ellerbrock, H; Johnston, LA; Jariwala, RH; Chang, WS; Gurtowski, J; Schatz, MC; Hossain, MM; Cassidy-Hanley, DM; Clark, TG; Chang, WJ | 2015 | Molecular genetic diversity and characterization of conjugation genes in the fish parasite Ichthyophthirius multifiliis | Ichthyophthirius multifiliis is the etiologic agent of “white spot”, a commercially important disease of freshwater fish. As a parasitic ciliate, I. multifiliis infects numerous host species across a broad geographic range. Although Ichthyophthirius outbreaks are difficult to control, recent sequencing of the I. multifiliis genome has revealed a number of potential metabolic pathways for therapeutic intervention, along with likely vaccine targets for disease prevention. Nonetheless, major gaps exist in our understanding of both the life cycle and population structure of I. multifiliis in the wild. For example, conjugation has never been described in this species, and it is unclear whether I. mull-Wills undergoes sexual reproduction, despite the presence of a germline micronucleus. In addition, no good methods exist to distinguish strains, leaving phylogenetic relationships between geographic isolates completely unresolved. Here, we compared nucleotide sequences of SSUrDNA, mitochondrial NADH dehydrogenase subunit I and cox-1 genes, and 14 somatic SNP sites from nine I. multifiliis isolates obtained from four different states in the US since 1995. The mitochondrial sequences effectively distinguished the isolates from one another and divided them into at least two genetically distinct groups. Furthermore, none of the nine isolates shared the same composition of the 14 somatic SNP sites, suggesting that I. multifiliis undergoes sexual reproduction at some point in its life cycle. Finally, compared to the well-studied free-living ciliates Tetrahymena thermophila and Paramecium tetraurelia, multifiliis has lost 38% and 29%, respectively, of 16 experimentally confirmed conjugation-related genes, indicating that mechanistic differences in sexual reproduction are likely to exist between I. multifiliis and other ciliate species. (C) 2015 Elsevier Inc. All rights reserved. | 4035 | NA | NA | NA | no | no |
| 4034 | 2017 | NA | Kowalczyk, R; Gorny, M; Schmidt, K | 2015 | Edge effect and influence of economic growth on Eurasian lynx mortality in the Bialowieza Primeval Forest, Poland | Eurasian lynx is one of most widely distributed felid species. However, populations in central Europe are strongly fragmented and limited to forest areas, which may influence their sustainability and gene flow. We studied the edge effect on mortality of a highly isolated population of Eurasian lynx in the Bialowieza Primeval Forest (NE Poland) during the 20-year period (1991-2011) of high economic development in Poland. Based on radio-tracking data collected in 1991-1996 (low gross national income per capita; low GNI PC) and 2004-2011 (high GNI PC) and recorded mortality cases, we analysed annual rates and causes of mortality and spatial distribution of death sites relative to the distance from the forest edge. We found significantly higher mortality of lynx during the low GNI PC phase of the economic growth than during high GNI PC (33 and 16 %, respectively). While anthropogenic factors played a dominant role in lynx mortality during low GNI PC, natural factors prevailed afterwards. We found a significantly higher proportion of lynx deaths than expected in the edge and outer zone than in the core of the Bialowieza Forest. Both areas differ significantly in the proportions of mortality causes with anthropogenic factors being the main source of mortality in the outer zone. Our results indicate that the decline of lynx mortality in Poland could be related to the improving of the economic situation in the country after the collapse of communism and reduced significance of anthropogenic factors (mainly poaching). Additionally, the outskirts of the forest may function as population sinks, which may reduce the dispersal of lynx and gene flow between populations inhabiting different forest patches. | 4036 | NA | NA | NA | no | no |
| 4035 | 2017 | NA | James, LR; Le, C; Doherty, H; Kim, HS; Maeda, N | 2013 | Connective Tissue Growth Factor (CTGF) Expression Modulates Response to High Glucose | Connective tissue growth factor (CTGF) is an important mediator of fibrosis; emerging evidence link changes in plasma and urinary CTGF levels to diabetic kidney disease. To further ascertain the role of CTGF in responses to high glucose, we assessed the consequence of 4 months of streptozotocin-induced diabetes in wild type (+/+) and CTGF heterozygous (+/-) mice. Subsequently, we studied the influence of glucose on gene expression and protein in mice embryonic fibroblasts (MEF) cells derived from wildtype and heterozygous mice. At study initiation, plasma glucose, creatinine, triglyceride and cholesterol levels were similar between non-diabetic CTGF+/+ and CTGF+/- mice. In the diabetic state, plasma glucose levels were increased in CTGF+/+ and CTGF+/- mice (28.2 3.3 mmol/L vs 27.0 3.1 mmol/L), plasma triglyceride levels were lower in CTGF+/- mice than in CTGF+/+ (0.7 0.2 mmol/L vs 0.5 0.1 mmol/L, p<0.05), but cholesterol was essentially unchanged in both groups. Plasma creatinine was higher in diabetic CTGF+/+ group (11.7 +/- 1.2 vs 7.9 +/- 0.6 mu mol/L p<0.01), while urinary albumin excretion and mesangial expansion were reduced in diabetic CTGF+/- animals. Cortices from diabetic mice (both CTGF +/+ and CTGF +/-) manifested higher expression of CTGF and thrombospondin 1 (TSP1). Expression of nephrin was reduced in CTGF +/+ animals; this reduction was attenuated in CTGF+/- group. In cultured MEF from CTGF+/+ mice, glucose (25 mM) increased expression of pro-collagens 1, IV and XVIII as well as fibronectin and thrombospondin 1 (TSP1). In contrast, activation of these genes by high glucose was attenuated in CTGF+/- MEF. We conclude that induction of Ctgf mediates expression of extracellular matrix proteins in diabetic kidney. Thus, genetic variability in CTGF expression directly modulates the severity of diabetic nephropathy. | 4037 | NA | NA | NA | no | no |
| 4036 | 2017 | NA | Pang, B; Zheng, X; Diao, BW; Cui, ZG; Zhou, HJ; Gao, SY; Kan, B | 2011 | Whole Genome PCR Scanning Reveals the Syntenic Genome Structure of Toxigenic Vibrio cholerae Strains in the O1/O139 Population | Vibrio cholerae is commonly found in estuarine water systems. Toxigenic O1 and O139 V. cholerae strains have caused cholera epidemics and pandemics, whereas the nontoxigenic strains within these serogroups only occasionally lead to disease. To understand the differences in the genome and clonality between the toxigenic and nontoxigenic strains of V. cholerae serogroups O1 and O139, we employed a whole genome PCR scanning (WGPScanning) method, an rrn operon-mediated fragment rearrangement analysis and comparative genomic hybridization (CGH) to analyze the genome structure of different strains. WGPScanning in conjunction with CGH revealed that the genomic contents of the toxigenic strains were conservative, except for a few indels located mainly in mobile elements. Minor nucleotide variation in orthologous genes appeared to be the major difference between the toxigenic strains. rrn operon-mediated rearrangements were infrequent in El Tor toxigenic strains tested using I-CeuI digested pulsed-field gel electrophoresis (PFGE) analysis and PCR analysis based on flanking sequence of rrn operons. Using these methods, we found that the genomic structures of toxigenic El Tor and O139 strains were syntenic. The nontoxigenic strains exhibited more extensive sequence variations, but toxin coregulated pilus positive (TCP+) strains had a similar structure. TCP+ nontoxigenic strains could be subdivided into multiple lineages according to the TCP type, suggesting the existence of complex intermediates in the evolution of toxigenic strains. The data indicate that toxigenic O1 El Tor and O139 strains were derived from a single lineage of intermediates from complex clones in the environment. The nontoxigenic strains with non-El Tor type TCP may yet evolve into new epidemic clones after attaining toxigenic attributes. | 4038 | NA | NA | NA | no | no |
| 4037 | 2017 | NA | Seo, EH; Lee, DY; Lee, JM; Park, JS; Sohn, BK; Lee, DS; Choe, YM; Woo, JI | 2013 | Whole-brain Functional Networks in Cognitively Normal, Mild Cognitive Impairment, and Alzheimer’s Disease | The conceptual significance of understanding functional brain alterations and cognitive deficits associated with Alzheimer’s disease (AD) process has been widely established. However, the whole-brain functional networks of AD and its prodromal stage, mild cognitive impairment (MCI), are not well clarified yet. In this study, we compared the characteristics of the whole-brain functional networks among cognitively normal (CN), MCI, and AD individuals by applying graph theoretical analyses to [F-18] fluorodeoxyglucose positron emission tomography (FDG-PET) data. Ninety-four CN elderly, 183 with MCI, and 216 with AD underwent clinical evaluation and FDG-PET scan. The overall small-world property as seen in the CN whole-brain network was preserved in MCI and AD. In contrast, individual parameters of the network were altered with the following patterns of changes: local clustering of networks was lower in both MCI and AD compared to CN, while path length was not different among the three groups. Then, MCI had a lower level of local clustering than AD. Subgroup analyses for AD also revealed that very mild AD had lower local clustering and shorter path length compared to mild AD. Regarding the local properties of the whole-brain networks, MCI and AD had significantly decreased normalized betweenness centrality in several hubs regionally associated with the default mode network compared to CN. Our results suggest that the functional integration in whole-brain network progressively declines due to the AD process. On the other hand, functional relatedness between neighboring brain regions may not gradually decrease, but be the most severely altered in MCI stage and gradually re-increase in clinical AD stages. | 4039 | NA | NA | NA | no | no |
| 4038 | 2017 | NA | Pallister, T; Jennings, A; Mohney, RP; Yarand, D; Mangino, M; Cassidy, A; MacGregor, A; Spector, TD; Menni, C | 2016 | Characterizing Blood Metabolomics Profiles Associated with Self-Reported Food Intakes in Female Twins | Using dietary biomarkers in nutritional epidemiological studies may better capture exposure and improve the level at which diet-disease associations can be established and explored. Here, we aimed to identify and evaluate reproducibility of novel biomarkers of reported habitual food intake using targeted and non-targeted metabolomic blood profiling in a large twin cohort. Reported intakes of 71 food groups, determined by FFQ, were assessed against 601 fasting blood metabolites in over 3500 adult female twins from the TwinsUK cohort. For each metabolite, linear regression analysis was undertaken in the discovery group (excluding MZ twin pairs discordant [>= 1 SD apart] for food group intake) with each food group as a predictor adjusting for age, batch effects, BMI, family relatedness and multiple testing (1.17x10(-6) = 0.05/[71 food groups x 601 detected metabolites]). Significant results were then replicated (non-targeted: P<0.05; targeted: same direction) in the MZ discordant twin group and results from both analyses meta-analyzed. We identified and replicated 180 significant associations with 39 food groups (P<1.17x10(-6)), overall consisting of 106 different metabolites (74 known and 32 unknown), including 73 novel associations. In particular we identified trans-4-hydroxyproline as a potential marker of red meat intake (0.075[0.009]; P = 1.08x10(-17)), ergothioneine as a marker of mushroom consumption (0.181[0.019]; P = 5.93x10(-22)), and three potential markers of fruit consumption (top association: apple and pears): including metabolites derived from gut bacterial transformation of phenolic compounds, 3-phenylpropionate (0.024[0.004]; P = 1.24x10(-8)) and indolepropionate (0.026[0.004]; P = 2.39x10(-9)), and threitol (0.033[0.003]; P = 1.69x10(-21)). With the largest nutritional metabolomics dataset to date, we have identified 73 novel candidate biomarkers of food intake for potential use in nutritional epidemiological studies. We compiled our findings into the DietMetab database (http://www.twinsuk.ac.uk/dietmetab-data/), an online tool to investigate our top associations. | 4040 | NA | NA | NA | no | no |
| 4039 | 2017 | NA | Sakihama, N; Kaneko, A; Hattori, T; Tanabe, K | 2001 | Limited recombination events in merozoite surface protein-1 alleles of Plasmodium falciparum on islands | Intragenic recombination is a principal mechanism for the generation of allelic variation in the merozoite surface protein-1 gene (Msp-1) of the human malaria parasite Plasmodium falciparum. In the present study, linkage disequilibrium between the 5’- and 3’-polymorphic sites was analyzed to determine the frequency of recombination events in Msp-1 in parasite populations on four islands in Vanuatu, the southwestern Pacific, where malaria transmission is moderate and comparable to other mesoendemic areas. Of 141 isolates, whose 5’-haplotypes (Msp-1 blocks 2-6) were determined by PCR-based typing, 138 were successfully sequenced for the 3’-polymorphism (block 17). A total of four distinct 5’-haplotypes and three distinct 3’-sequence types were identified with apparently different frequency distribution among islands. The number of 5’-haplotypes in each island was one to four, far smaller than in other previously studied geographic areas (ten to 21). Associations between the 5’- and 3’-polymorphisms (here termed Msp-1 gene types) were subjected to the R-2 linkage disequilibrium test. The test revealed complete or very strong linkage disequilibrium in all four islands. Mixed infection was unusually rare (2.1 %) and the mean number of Msp-1 alleles per person was nearly 1.0. The heterozygosity of the Msp-1 gene type calculated for each island (h = 0.41-0.65) was significantly lower than that in other areas of comparable endemicity (h = 0.81-0.89) (P < 0.01). These results indicate that recombination events in Msp-1 would be extremely limited in Vanuatu, and stress that the frequency of recombination in Msp-1 is determined by not only the intensity of malaria transmission but the frequency of mixed clone infections, the mean number of clones per person and a repertoire of clones in a local area. (C) 2001 Elsevier Science B.V. All rights reserved. | 4041 | NA | NA | NA | no | no |
| 4040 | 2017 | NA | Yanes, ML; De la Fuente, L; Altier, N; Arias, A | 2012 | Characterization of native fluorescent Pseudomonas isolates associated with alfalfa roots in Uruguayan agroecosystems | Establishment of alfalfa crops is continuously threatened by seedling diseases caused by soilborne pathogens. The use of plant beneficial bacteria as inoculants is a feasible and environmentally friendly means to control soil pathogens. Identifying effective plant growth-promoting strains to use on local crops under local environmental conditions requires the screening of large collections of native isolates. A collection of 738 rhizospheric fluorescent Pseudomonas isolates was obtained from alfalfa plants from three agroecological regions representative of Uruguayan agricultural systems. The isolates were evaluated for in vitro pathogen inhibition, biosurfactant production, phosphate solubilization and the presence of genes involved in antibiotic synthesis. Isolates with strong in vitro antagonistic activity toward Pythium debaryanum were more abundant in alfalfa plants established in a previously natural ecosystem while biosurfactant producers were less abundant in that location. A subset of isolates was selected for genotypic characterization by rep-PCR using BOX primers. Twenty-four genotypes were defined, sixteen from a single geographical origin and eight composed of isolates from multiple origins. Genotypic profiles correlated well with phenotypic traits. A subset of isolates was assayed to determine their ability to protect alfalfa against P. debaryanum damping-off and to promote plant growth. Five native Pseudomonas isolates showed significant effects on alfalfa by increasing plant biomass and/or protecting from pathogen infection. Plant growth promoting isolates from each location were genotypically similar. Our work contributes to the knowledge of the phenotypic and genotypic diversity of rhizospheric fluorescent pseudomonads of forage legumes and the frequency of plant growth promoting traits associated with this group of bacteria in different agricultural systems. (C) 2012 Elsevier Inc. All rights reserved. | 4042 | NA | NA | NA | no | no |
| 4041 | 2017 | NA | Abe, J; Kamimura, Y; Shimada, M | 2005 | Individual sex ratios and offspring emergence patterns in a parasitoid wasp, Melittobia australica (Eulophidae), with superparasitism and lethal combat among sons | Since the mating of the parasitoid wasp Melittobia australica occurs on their eclosed hosts, the sex ratio is predicted to follow the local mate competition (LMC) theory. However, while LMC models predict that the sex ratio will increase from female-biased toward a 1: 1 ratio with an increase in the number of foundresses, the observed female-biased sex ratios (1-5% males) show little increase in response to an increased foundress number. Lethal combat among adult males may serve as an explanation for this observed phenomenon. Using a microsatellite DNA marker, we first examined the individual sex ratio of two foundresses who had sequentially parasitized the same host. Both foundresses produced an extremely female-biased clutch and the sex ratios of the second foundress were only slightly less biased than that of the first. A small number of sons from both foundresses emerged at a constantly low rate over a prolonged period, resulting in a temporal mixture of emerging males derived from both the foundresses. Second, we conducted a one-on-one arena experiment to examine the combat level in relation to the relatedness of the opponents. Almost all the later-emerging males were killed by previously eclosed males irrespective of whether they were sibs or non-sibs. These results suggest that each foundress should not produce males in a single burst, but continue to produce male eggs at a constantly low rate in order to avoid the high mortality of her own sons by lethal male-male combat. This combat may be one of factors in explaining the extremely female-biased sex ratio even with an increasing foundress number. | 4043 | NA | NA | NA | no | no |
| 4042 | 2017 | NA | Wilkinson, SP; Fisher, PL; van Oppen, MJH; Davy, SK | 2015 | Intra-genomic variation in symbiotic dinoflagellates: recent divergence or recombination between lineages? | Background: The symbiosis between corals and the dinoflagellate alga Symbiodinium is essential for the development and survival of coral reefs. Yet this fragile association is highly vulnerable to environmental disturbance. A coral’s ability to tolerate temperature stress depends on the fitness of its resident symbionts, whose thermal optima vary extensively between lineages. However, the in hospite population genetic structure of Symbiodinium is poorly understood and mostly based on analysis of bulk DNA extracted from thousands to millions of cells. Using quantitative single-cell PCR, we enumerated DNA polymorphisms in the symbionts of the reef-building coral Pocillopora damicornis, and applied a model selection approach to explore the potential for recombination between coexisting Symbiodinium populations. Results: Two distinct Symbiodinium ITS2 sequences (denoted C100 and C109) were retrieved from all P. damicornis colonies analysed. However, the symbiont assemblage consisted of three distinct Symbiodinium populations: cells featuring pure arrays of ITS2 type C109, near-homogeneous cells of type C100 (with trace ITS2 copies of type C109), and those with co-dominant C100 and C109 ITS2 repeats. The symbiont consortia of some colonies consisted almost entirely of these putative C100 x C109 recombinants. Conclusions: Our results are consistent with the occurrence of sexual recombination between Symbiodinium types C100 and C109. While the multiple-copy nature of the ITS2 dictates that the observed pattern of intra-genomic co-dominance may be a result of incomplete concerted evolution of intra-genomic polymorphisms, this is a less likely explanation given the occurrence of homogeneous cells of the C109 type. Conclusive evidence for inter-lineage recombination and introgression in this genus will require either direct observational evidence or a single-cell genotyping approach targeting multiple, single-copy loci. | 4044 | NA | NA | NA | no | no |
| 4043 | 2017 | NA | Shang, YF; Xiao, GH; Zheng, P; Cen, K; Zhan, S; Wang, CS | 2016 | Divergent and Convergent Evolution of Fungal Pathogenicity | Fungal pathogens of plants and animals have multifarious effects; they cause devastating damages to agricultures, lead to life threatening diseases in humans, or induce beneficial effects by reducing insect pest populations. Many virulence factors have been determined in different fungal pathogens; however, the molecular determinants contributing to fungal host selection and adaptation are largely unknown. In this study, we sequenced the genomes of seven ascomycete insect pathogens and performed the genome-wide analyses of 33 species of filamentous ascomycete pathogenic fungi that infect insects (12 species), plants (12), and humans (9). Our results revealed that the genomes of plant pathogens encode more proteins and protein families than the insect and human pathogens. Unexpectedly, more common orthologous protein groups are shared between the insect and plant pathogens than between the two animal group pathogens. We also found that the pathogenicity of host-adapted fungi evolved multiple times, and that both divergent and convergent evolutions occurred during pathogen host cospeciation thus resulting in protein families with similar features in each fungal group. However, the role of phylogenetic relatedness on the evolution of protein families and therefore pathotype formation could not be ruled out due to the effect of common ancestry. The evolutionary correlation analyses led to the identification of different protein families that correlated with alternate pathotypes. Particularly, the effector-like proteins identified in plant and animal pathogens were strongly linked to fungal host adaptation, suggesting the existence of similar gene-for-gene relationships in fungus animal interactions that has not been established before. These results well advance our understanding of the evolution of fungal pathogenicity and the factors that contribute to fungal pathotype formation. | 4045 | NA | NA | NA | no | no |
| 4044 | 2017 | NA | Aron, S; Steinhauer, N; Fournier, D | 2009 | Influence of queen phenotype, investment and maternity apportionment on the outcome of fights in cooperative foundations of the ant Lasius niger | Cooperative colony founding (pleometrosis) in social insects is an ideal model for investigating how cooperation and competition shape social behaviour among unrelated individuals. In many ant species, foundress associations are more competitive and the colonies survive better compared with single-queen colonies. However, cooperation among queens breaks down at the time of emergence of the first workers, and all but one queen are eliminated. Because no sexuals are produced in incipient colonies, the surviving queen will monopolize the future reproductive success of the colony, while defeated queens will have zero fitness. We examined factors affecting queens’ survival prospects during reversion to single-queen colonies in cooperative foundations of the ant Lasius niger. By combining phenotypic and genotypic analyses, we determined how queen’s size, individual investment and maternity apportionment influence the outcome of fights. Larger queens were more likely to survive fights. However, smaller queens survived up to one-third of the fighting. By contrast, neither weight loss at the time of a fight outbreak, a measure of queens’ relative investment in brood production, nor maternity apportionment influenced the outcome of fights. Moreover, investment of cofoundresses and partitioning of reproduction were not adjusted to queen’s size, suggesting that reproductive competition among queens does not occur before the emergence of the first workers. These results lead us to consider pleometrotic associations in L. niger as a ‘best of a bad job’, whereby the benefits of joint founding and the probability of surviving the conflict might be sufficient for smaller queens to embark on cooperative foundations. (C) 2009 The Association for the Study of Animal Behaviour. Published by Elsevier Ltd. All rights reserved. | 4046 | NA | NA | NA | no | no |
| 4045 | 2017 | NA | Kaltz, O; Shykoff, JA | 1999 | Selfing versus outcrossing propensity of the fungal pathogen Microbotryum violaceum across Silene latifolia host plants | In the fungal pathogen Microbotryum violaceum mating (i.e, conjugation between cells of opposite mating type) is indispensable for infection of its host plant Silene latifolia. Since outcrossing opportunities are potentially rare, selfing may be appropriate to ensure reproduction. On the other hand, outcrossing may create genetic variability necessary in the coevolutionary arms race with its host. We investigated the propensity of M. violaceum to outcross vs, self in different host environments. We used haploid sporidia from each of three strains from five fungal populations for pairwise mixtures of opposite mating type, representing either selfing or outcrossing combinations. Mixtures were exposed to leaf extract from seven S. latifolia plants. The proportion of conjugated sporidia quantified mating propensity. The identity of both fungal strains and host influenced conjugation. First, individual strains differed in conjugation frequency by up to 30%, and strains differed in their performance across the different hosts. Second, selfing combinations produced, on average, more conjugations than did outcrossing combinations. Selfing appears to be the predominant mode of reproduction in this fungus, and selfing preference may have evolved as a mechanism of reproductive assurance. Third, individual strains varied considerably in conjugation frequency in selfing and outcrossing combinations across different hosts. This indicates that conjugation between outcrossing partners could be favoured at least in some hosts. Since the dikaryon resulting from conjugation is the infectious unit, conjugation frequency may correspond with infection probability. This assumption was supported by an inoculation experiment, where high infectious sporidial dosage resulted in higher infections success than did low dosage. We therefore predict that sexual recombination can provide this pathogen with novel genotypes able to infect local resistant hosts. | 4047 | NA | NA | NA | no | no |
| 4046 | 2017 | NA | Voigt, K; Olsson, L | 2008 | Molecular phylogenetic and scanning electron microscopical analyses places the Choanephoraceae and the Gilbertellaceae in a monophyletic group within the Mucorales (Zygomycetes, Fungi) | A multi-gene genealogy based on maximum parsimony and distance analyses of the exonic genes for actin (act) and translation elongation factor 1 alpha (tef), the nuclear genes for the small (18S) and large (28S) Subunit ribosomal RNA (comprising 807, 1092, 1863, 389 characters, respectively) of all 50 genera of the Mucorales (Zygomycetes) suggests that the Choanephoraceae is a monophyletic group. The monotypic Gilbertellaceae appears in close phylogenetic relatedness to the Choanephoraceae. The monophyly of the Choanephoraceae has moderate to strong support (bootstrap proportions 67% and 96% in distance and maximum parsimony analyses, respectively), whereas the monophyly of the Choanephoraceae-Gilbertellaceae clade is supported by high bootstrap values (100% and 98%). This suggests that the two families can be joined into one family, which leads to the elimination of the Gilbertellaceae as a separate family. In order to test this hypothesis single-locus neighbor-joining analyses were performed on nuclear genes of the 18S, 5.8S, 28S and internal transcribed spacer (ITS) I ribosomal RNA and the translation elongation factor 1 alpha (tef) and beta tubulin (beta tub) nucleotide sequences. The common monophyletic origin of the Choanephoraceae-Gilbertellaceae clade Could be confirmed in all gene trees and by investigation of their ultrastructure. Sporangia with persistent, sutured walls splitting in half at maturity and ellipsoidal sporangiospores with striated ornamentations and polar ciliate appendages arising from spores in persistent sporangia and dehiscent sporangiola represent synapomorphic characters of this group. We discuss our data in the context of the historical development of their taxonomy and physiology and propose a reduction of the two families to one family, the Choanephoraceae sensu lato comprising species which are facultative plant pathogens and parasites, especially in subtropical to tropical regions. | 4048 | NA | NA | NA | no | no |
| 4047 | 2017 | NA | Tollington, S; Greenwood, A; Jones, CG; Hoeck, P; Chowrimootoo, A; Smith, D; Richards, H; Tatayah, V; Groombridge, JJ | 2015 | Detailed monitoring of a small but recovering population reveals sublethal effects of disease and unexpected interactions with supplemental feeding | 1.Infectious diseases are widely recognized to have substantial impact on wildlife populations.These impacts are sometimes exacerbated in small endangered populations, and therefore, thesuccess of conservation reintroductions to aid the recovery of such species can be seriouslythreatened by outbreaks of infectious disease. Intensive management strategies associated withconservation reintroductions can further compound these negative effects in such populations.2.Exploring the sublethal effects of disease outbreaks among natural populations is challeng-ing and requires longitudinal, individual life-history data on patterns of reproductive successand other indicators of individual fitness.3.Long-term monitoring data concerning detailed reproductive information of the reintro-duced Mauritius parakeet (Psittacula echo) population collected before, during and after adisease outbreak was investigated.4.Deleterious effects of an outbreak of beak and feather disease virus (BFDV) were revealedon hatch success, but these effects were remarkably short-lived and disproportionately associ-ated with breeding pairs which took supplemental food. Individual BFDV infection statuswas not predicted by any genetic, environmental or conservation management factors andwas not associated with any of our measures of immune function, perhaps suggesting immu-nological impairment. Experimental immunostimulation using the PHA (phytohaemagglutininassay) challenge technique did, however, provoke a significant cellular immune response.5.We illustrate the resilience of this bottlenecked and once critically endangered, island-ende-mic species to an epidemic outbreak of BFDV and highlight the value of systematic monitor-ing in revealing inconspicuous but nonetheless substantial ecological interactions. Our studydemonstrates that the emergence of such an infectious disease in a population ordinarilyassociated with increased susceptibility does not necessarily lead to deleterious impacts onpopulation growth and that negative effects on reproductive fitness can be short-lived. | 4049 | NA | NA | NA | no | no |
| 4048 | 2017 | NA | Kapun, M; Schmidt, C; Durmaz, E; Schmidt, PS; Flatt, T | 2016 | Parallel effects of the inversion In(3R)Payne on body size across the North American and Australian clines in Drosophila melanogaster | Chromosomal inversions are thought to play a major role in climatic adaptation. In D. melanogaster, the cosmopolitan inversion In(3R)Payne exhibits latitudinal clines on multiple continents. As many fitness traits show similar clines, it is tempting to hypothesize that In(3R)P underlies observed clinal patterns for some of these traits. In support of this idea, previous work in Australian populations has demonstrated that In(3R)P affects body size but not development time or cold resistance. However, similar data from other clines of this inversion are largely lacking; finding parallel effects of In(3R)P across multiple clines would considerably strengthen the case for clinal selection. Here, we have analysed the phenotypic effects of In(3R)P in populations originating from the endpoints of the latitudinal cline along the North American east coast. We measured development time, egg-to-adult survival, several size-related traits (femur and tibia length, wing area and shape), chill coma recovery, oxidative stress resistance and triglyceride content in homokaryon lines carrying In(3R)P or the standard arrangement. Our central finding is that the effects of In(3R)P along the North American cline match those observed in Australia: standard arrangement lines were larger than inverted lines, but the inversion did not influence development time or cold resistance. Similarly, In(3R)P did not affect egg-to-adult survival, oxidative stress resistance and lipid content. In(3R)P thus seems to specifically affect size traits in populations from both continents. This parallelism strongly suggests an adaptive pattern, whereby the inversion has captured alleles associated with growth regulation and clinal selection acts on size across both continents. | 4050 | NA | NA | NA | no | no |
| 4049 | 2017 | NA | Jacobson, SL; Bliss-Ketchum, LL; de Rivera, CE; Smith, WP | 2016 | A behavior-based framework for assessing barrier effects to wildlife from vehicle traffic volume | Roads, while central to the function of human society, create barriers to animal movement through collisions and habitat fragmentation. Barriers to animal movement affect the evolution and trajectory of populations. Investigators have attempted to use traffic volume, the number of vehicles passing a point on a road segment, to predict effects to wildlife populations approximately linearly and along taxonomic lines; however, taxonomic groupings cannot provide sound predictions because closely related species often respond differently. We assess the role of wildlife behavioral responses to traffic volume as a tool to predict barrier effects from vehicle-caused mortality and avoidance, to provide an early warning system that recognizes traffic volume as a trigger for mitigation, and to better interpret roadkill data. We propose four categories of behavioral response based on the perceived danger to traffic: Nonresponders, Pausers, Speeders, and Avoiders. Nonresponders attempt to cross highways regardless of traffic volume. Pausers stop in the face of danger so have a low probability of successful crossing when traffic volume increases. Hence, highway barrier effects are primarily due to mortality for Nonresponders and Pausers at high traffic volumes. Speeders run away from danger but are unable to do so successfully as traffic volume increases. At moderate to high volume, Speeders are repelled by traffic danger. Avoiders face lower mortality than other categories because they begin to avoid traffic at relatively low traffic volumes. Hence, avoidance causes barrier effects more than mortality for Speeders and Avoiders even at relatively moderate traffic volumes. By considering a species’ risk-avoidance response to traffic, managers can make more appropriate and timely decisions to mitigate effects before populations decline or become locally extinct. | 4051 | NA | NA | NA | no | no |
| 4050 | 2017 | NA | Delphia, CM; Copren, KA; Haverty, MI | 2003 | Agonistic behavior between individual worker termites from three cuticular hydrocarbon phenotypes of Reticulitermes (Isoptera : Rhinotermitidae) from northern California | Bioassays examining aggression in termites have typically been performed by pairing groups of workers from different colonies. We examined whether similar results would be observed using bioassays with individual workers. We report the results of pairings of individual workers of Reticulitermes spp. of three different hydrocarbon phenotypes (CA-A, CA-B, and CA-C). Resulting agonistic behavior between individuals can be used to infer relatedness of workers from different foraging groups because identification of species of Reticulitermes is often difficult. Pairings consisted p of two termites from the same colony or from different colonies with the same or different phenotypes. We recorded avoidance behavior and immediate aggression during 5-min observations and mortality at 24 h. Intercolonial/intraphenotype pairings paralleled group bioassays with low levels of immediate aggression; however, individual pairings had lower levels of mortality at 24 h. Bioassays using individual Reticulitermes spp. workers in interphenotype pairings yielded results comparable with previously reported group bioassays and could be used in lieu of group bioassays. However, if immediate aggression is lacking, it would be advisable to use group bioassays or increase the number of replications to differentiate intracolonial and intercolonial pairings. Survival rates were the same for all phenotypes when paired with either of the other two phenotypes. There were, however, significant differences in the number of avoidance responses; pairings with CA-C had the highest number of avoidance responses followed by pairings with CA-B and CA-A. There were significant differences in immediate aggression; CA-C was the most aggressive, followed by CA-B and CA-A. Different levels of aggression among phenotypes provide additional evidence that these phenotypes represent distinct taxa or species. | 4052 | NA | NA | NA | no | no |
| 4051 | 2017 | NA | Bernasconi, G; Keller, L | 1998 | Phenotype and individual investment in cooperative foundress associations of the fire ant, Solenopsis invicta | Fire ant (Solenopsis invicta) queens founding a colony with unrelated nest mates potentially face a trade-off. Increased individual investment enhances worker production, colony survival, and growth. However, increased investment may reduce a queen’s probability of surviving fights that invariably arise after worker eclosion. Indeed, previous studies showed that queens lose less weight (a measure of investment) when initiating colonies with cofoundresses than when alone, and that within associations the queen losing more weight is more likely to die. In this study, we tested whether queens adjust weight loss to social environment and fighting ability and whether restraining weight loss directly increases survival prospects. Experimental manipulation of colonies showed that reduced investment by queens within associations is primarily a response to the presence of a nest mate and not simply a response to per-queen brood-care demands. Differences in head width were associated with relative and combined weight loss of cofoundresses, as well as with queen survival. In contrast, the investment strategies of queens were not significantly influenced by their nest mates’ initial weight. Similarly, manipulation of the queens’ relative weight by feeding and exposure to contrasting social environment (queens kept alone or in groups) did not significantly affect survival. These results indicate that head width differences or correlated phenotypic attributes of fighting ability influenced both investment strategies and survival probability of queens. That queens with larger heads invested less energy into brood rearing and were more likely to survive reveals more selfish interactions among cofoundresses than has previously been assumed and casts some doubts about the idea that group selection must be invoked to account for the maintenance of cooperation in foundress associations of ants. | 4053 | NA | NA | NA | no | no |
| 4052 | 2017 | NA | Furlong, CE; Li, WF; Shih, DM; Lusis, AJ; Richter, RJ; Costa, LG | 2002 | Genetic factors in susceptibility: Serum PON1 variation between individuals and species | In mammals, serum paraoxonase (PON1) is tightly associated with high-density lipoprotein (HDL) particles. In human populations, PON1 exhibits a substrate dependent activity polymorphism determined by an Arg/Gln (R/Q) substitution at amino acid residue 192. The physiological role of this protein appears to be involvement in the metabolism of oxidized lipids. Several studies have suggested that the PON1(R192) allele may be a risk factor in coronary artery disease. PON1 also plays an important role in the metabolism of organophosphates including insecticides and nerve agents. The PON1(R192) isoform hydrolyzes paraoxon rapidly, but diazoxon, soman and satin slowly compared with the PON1(Q192) isoform. Both PON1 isoforms hydrolyze phenylacetate at approximately the same rate, while PON1(R192) hydrolyzes chlorpyrifos oxon slightly faster than PONQ192. Animal model studies involving injection of purified rabbit PON1 into mice clearly demonstrated the ability of PON1 to protect cholinesterases from inhibition by OP compounds. The consequence of having low PON1 levels has been addressed with toxicology studies in PON1 knockout mice. These mice showed dramatically increased sensitivity to chlorpyrifos oxon, diazoxon and some increased sensitivity to the respective parent compounds. These observations are consistent with earlier studies that showed a good correlation between high rates of OP hydrolysis by serum PON1 and resistance to specific OP compounds. They are also consistent with the observations that newborns have an increased sensitivity to OP toxicity, due in part to their not expressing adult PON1 levels for week’s to months after birth, depending on the species. Together, these studies point out the importance of considering the genetic variability of PON1(192) isoforms and levels as well as the developmental time course of PON1 appearance in serum in developing risk assessment models. | 4054 | NA | NA | NA | no | no |
| 4053 | 2017 | NA | FRUTOS, R; FEDERICI, BA; REVET, B; BERGOIN, M | 1994 | TAXONOMIC STUDIES OF RICKETTSIELLA, RICKETTSIA, AND CHLAMYDIA USING GENOMIC DNA | Invertebrate pathogens of the genus Rickettgiella (Order Rickettsiales) undergo a developmental cycle more characteristic of chlamydial organisms (Order Chlamydiales) than of typical rickettsiae. Moreover, among recognized species of Rickettsiella, there is considerable variation in host range and in the ultrastructure and development of the infectious stage, i.e., the elementary body. To begin an analysis of the taxonomic relationships of the invertebrate pathogens belonging to the genus Rickettsiella and of the relationship of these to chlamydiae and other rickettsiae, genomic DNA of representative species was compared with respect to restriction enzyme site polymorphism, DNA-DNA hybridization, G + C ratios, and fine melting profiles. The strains studied included isolates currently classified as Rickettsiella grylli, R. popilliae, R. chironomi, Chlamydia psittaci, C. trachomatis, Coxiella burnetii, and Rickettsia conorii. Reciprocal DNA hybridization studies carried out under high-stringency conditions (65-degrees-C) showed homology between R. grylli and R. popilliae isolates but not with the others, including two isolates of R. chironomi from the midge, Chironomus dorsalis, and the scorpion, Buthus occitanus. The R. chironomi isolates shared no detectable homology with each other or with any of the other strains or species. Additionally, no homology was detected between any of the Rickettsiella isolates and the species of Coxiella, Rickettsia, or Chlamydia. Results obtained from the DNA fine melting profiles and G + C ratios corresponded with the results obtained from the DNA hybridization studies. Thus, the present study indicates that, despite similarities in developmental cycles, Rickettsiella and Chlamydia are taxonomically distinct groups appropriately placed in different orders. However, the uniqueness of the two R. chironomi species, both from each other and from the other organisms studied, indicates these could be recognized as distinct species. (C) 1994 Academic Press, Inc. | 4055 | NA | NA | NA | no | no |
| 4054 | 2017 | NA | Gomez-Carballa, A; Pardo-Seco, J; Fachal, L; Vega, A; Cebey, M; Martinon-Torres, N; Martinon-Torres, F; Salas, A | 2013 | Indian Signatures in the Westernmost Edge of the European Romani Diaspora: New Insight from Mitogenomes | In agreement with historical documentation, several genetic studies have revealed ancestral links between the European Romani and India. The entire mitochondrial DNA (mtDNA) of 27 Spanish Romani was sequenced in order to shed further light on the origins of this population. The data were analyzed together with a large published dataset (mainly hypervariable region I [HVS-I] haplotypes) of Romani (N = 1,353) and non-Romani worldwide populations (N. 150,000). Analysis of mitogenomes allowed the characterization of various Romani-specific clades. M5a1b1a1 is the most distinctive European Romani haplogroup; it is present in all Romani groups at variable frequencies (with only sporadic findings in non-Romani) and represents 18% of their mtDNA pool. Its phylogeographic features indicate that M5a1b1a1 originated 1.5 thousand years ago (kya; 95% CI: 1.3-1.8) in a proto-Romani population living in Northwest India. U3 represents the most characteristic Romani haplogroup of European/Near Eastern origin (12.4%); it appears at dissimilar frequencies across the continent (Iberia: similar to 31%; Eastern/Central Europe:,13%). All U3 mitogenomes of our Iberian Romani sample fall within a new sub-clade, U3b1c, which can be dated to 0.5 kya (95% CI: 0.3-0.7); therefore, signaling a lower bound for the founder event that followed admixture in Europe/Near East. Other minor European/Near Eastern haplogroups (e.g. H24, H88a) were also assimilated into the Romani by introgression with neighboring populations during their diaspora into Europe; yet some show a differentiation from the phylogenetically closest non-Romani counterpart. The phylogeny of Romani mitogenomes shows clear signatures of low effective population sizes and founder effects. Overall, these results are in good agreement with historical documentation, suggesting that cultural identity and relative isolation have allowed the Romani to preserve a distinctive mtDNA heritage, with some features linking them unequivocally to their ancestral Indian homeland. | 4056 | NA | NA | NA | no | no |
| 4055 | 2017 | NA | Enk, TA; Picton, HD; Williams, JS | 2001 | Factors limiting a bighorn sheep population in Montana following a dieoff | Conservation and management of bighorn sheep populations is complicated by the species’ susceptibility to a multitude of pathogens and the long-term influence of disease upon population dynamics. Small, post-dieoff sheep herds are often unable to fully recover to pre-dieoff densities, and researchers have identified several factors limiting such populations including disease, predation, competition, climate, nutritional deficiencies, and loss of genetic variability. We studied a small bighorn sheep herd in west central Montana from 1995 to 1997 to identify the mechanisms responsible for poor population performance subsequent to a dieoff in 1984. In contrast to the migratory pre-dieoff population, the current sedentary population was restricted to a small area of low elevation range throughout the year. Whereas we observed limited annual adult mortality due to disease and predation, ewe productivity and lamb recruitment rates were low in all years. Lamb production and survival were highly correlated with summer climatic conditions, with the highest rate of disease-mediated lamb mortality occurring during a summer drought and the lowest rate of ewe productivity occurring subsequent to this drought. Our data suggest that disease continues to play a significant role this bighorn sheep population, and nutritional quality of summer forage influenced not only sheep immunocompetence and susceptibility to disease but also herd productivity. Winter is generally considered the critical season for ungulate populations in the northern Rocky Mountains, yet summer climatic conditions can have important implications for performance of sedentary, low elevation sheep populations. We suggest that an understanding of population-specific limiting factors is essential to successful management of post-dieoff bighorn sheep herds. Data obtained through rigorous field studies facilitate the development and implementation of efficient, biologically sound strategies to improve performance of these populations. | 4057 | NA | NA | NA | no | no |
| 4056 | 2017 | NA | Guevara-Cruz, M; Tovar, AR; Larrieta, E; Canizales-Quinteros, S; Torres, N | 2010 | Increase in HDL-C concentration by a dietary portfolio with soy protein and soluble fiber is associated with the presence of the ABCA1R230C variant in hyperlipidemic Mexican subjects | Background: A dietary portfolio has been used to reduce blood lipids in hyperlipidemic subjects. To increase the effectiveness of these dietary treatments in specific populations, it is important to study the genetic variability associated with the development of certain types of hyperlipidemias. Low plasma high-density lipoprotein cholesterol (HDL-C) levels are the most common dyslipidemia in Mexican adults and are coupled with the presence of the ABCA1 R230C genotype. Therefore, the aim of this study was to assess the response of HDL-C concentration to a dietary portfolio in a group of Mexican hyperlipidemic subjects with ABCA1R230C (rs9282541) and R219K (rs2230806) polymorphisms. Methods: Forty-three hyperlipidemic subjects (20 men and 23 women) were given a low saturated fat (LSF) diet for one month, followed by a LSF diet that included 25 g of soy protein and 15 g of soluble fiber daily for 2 months. We analyzed two ABCA1 polymorphisms and studied their association with serum lipids before and after treatment. Results: Hyperlipidemic subjects with the ABCA1 R230C genotype showed lower HDL-C concentrations at the beginning of the study and were better responders to the dietary treatment than subjects with the ABCA1 R230R genotype (+ 4.6% vs. + 14.6%) (p = .05). According to gender and the presence of the R230C genotype, women responded more significantly to the dietary treatment, reflected by an increase of 21.9% in HDL concentration (p = .022), than women with R230R genotype who only experienced an increase of 2.7% in HDL-C concentration. There was no association between the presence of the ABCA1 R219K variant (p = .544) and HDL concentration. Conclusion: Hyperlipidemic Mexican subjects with the ABCA1 R230C genotype showed lower HDL-concentrations and were better responders to dietary portfolio treatments for increasing HDL-C concentrations than subjects with the R230R genotype. (C) 2010 Elsevier Inc. All rights reserved. | 4058 | NA | NA | NA | no | no |
| 4057 | 2017 | NA | Ismail, F; Couvin, D; Farakhin, I; Rahman, ZA; Rastogi, N; Suraiya, S | 2014 | Study of Mycobacterium tuberculosis Complex Genotypic Diversity in Malaysia Reveals a Predominance of Ancestral East-African-Indian Lineage with a Malaysia-Specific Signature | Background: Tuberculosis (TB) still constitutes a major public health problem in Malaysia. The identification and genotyping based characterization of Mycobacterium tuberculosis complex (MTBC) isolates causing the disease is important to determine the effectiveness of the control and surveillance programs. Objectives: This study intended a first assessment of spoligotyping-based MTBC genotypic diversity in Malaysia followed by a comparison of strains with those prevailing in neighboring countries by comparison with an international MTBC genotyping database. Methods: Spoligotyping was performed on a total of 220 M. tuberculosis clinical isolates collected in Kelantan and Kuala Lumpur. The results were compared with the SITVIT2 international database of the Pasteur Institute of Guadeloupe. Results: Spoligotyping revealed 77 different patterns: 22 corresponded to orphan patterns while 55 patterns containing 198 isolates were assigned a Spoligo International Type (SIT) designation in the database (the latter included 6 newly created SITs). The eight most common SITs grouped 141 isolates (5 to 56 strains per cluster) as follows: SIT1/Beijing, n = 56, 25.5%; SIT745/EAI1-SOM, n = 33, 15.0%; SIT591/EAI6-BGD1, n = 13, 5.9%; SIT256/EAI5, n = 12, 5.5%; SIT236/EAI5, n = 10, 4.6%; SIT19/EAI2-Manila, n = 9, 4.1%; SIT89/EAI2-Nonthaburi, n = 5, 2.3%; and SIT50/H3, n = 3, 1.4%. The association between city of isolation and lineages was statistically significant; Haarlem and T lineages being higher in Kuala Lumpur (p < 0.01). However, no statistically significant differences were noted when comparing drug resistance vs. major lineages, nor between gender and clades. Conclusions: The ancestral East-African-Indian (EAI) lineage was most predominant followed by the Beijing lineage. A comparison of strains with those prevailing in neighboring countries in South Asia, East Asia and South East Asia underlined the phylogeographical specificity of SIT745 for Malaysia, and its probable ongoing evolution with locally evolved strains sharing a specific signature characterized by absence of spacers 37, 38, and 40. Pending complementary genotyping confirmation, we propose that SIT745/EAI-SOM is tentatively reclassified as SIT745/EAI-MYS. | 4059 | NA | NA | NA | no | no |
| 4058 | 2017 | NA | Bonardi, D; Ravasio, V; Borsani, G; d’Azzo, A; Bresciani, R; Monti, E; Giacopuzzi, E | 2014 | In Silico Identification of New Putative Pathogenic Variants in the Neu1 Sialidase Gene Affecting Enzyme Function and Subcellular Localization | The NEU1 gene is the first identified member of the human sialidases, glycohydrolitic enzymes that remove the terminal sialic acid from oligosaccharide chains. Mutations in NEU1 gene are causative of sialidosis (MIM 256550), a severe lysosomal storage disorder showing autosomal recessive mode of inheritance. Sialidosis has been classified into two subtypes: sialidosis type I, a normomorphic, late-onset form, and sialidosis type II, a more severe neonatal or early-onset form. A total of 50 causative mutations are reported in HGMD database, most of which are missense variants. To further characterize the NEU1 gene and identify new functionally relevant protein isoforms, we decided to study its genetic variability in the human population using the data generated by two large sequencing projects: the 1000 Genomes Project (1000G) and the NHLBI GO Exome Sequencing Project (ESP). Together these two datasets comprise a cohort of 7595 sequenced individuals, making it possible to identify rare variants and dissect population specific ones. By integrating this approach with biochemical and cellular studies, we were able to identify new rare missense and frameshift alleles in NEU1 gene. Among the 9 candidate variants tested, only two resulted in significantly lower levels of sialidase activity (p<0.05), namely c.650T>C and c.700G>A. These two mutations give rise to the amino acid substitutions p.V217A and p.D234N, respectively. NEU1 variants including either of these two amino acid changes have 44% and 25% residual sialidase activity when compared to the wild-type enzyme, reduced protein levels and altered subcellular localization. Thus they may represent new, putative pathological mutations resulting in sialidosis type I. The in silico approach used in this study has enabled the identification of previously unknown NEU1 functional alleles that are widespread in the population and could be tested in future functional studies. | 4060 | NA | NA | NA | no | no |
| 4059 | 2017 | NA | Marriott, AC; Dennis, M; Kane, JA; Gooch, KE; Hatch, G; Sharpe, S; Prevosto, C; Leeming, G; Zekeng, EG; Staples, KJ; Hall, G; Ryan, KA; Bate, S; Moyo, N; Whittaker, CJ; Hallis, B; Silman, NJ; Lalvani, A; Wilkinson, TM; Hiscox, JA; Stewart, JP; Carroll, MW | 2016 | Influenza A Virus Challenge Models in Cynomolgus Macaques Using the Authentic Inhaled Aerosol and Intra-Nasal Routes of Infection | Non-human primates are the animals closest to humans for use in influenza A virus challenge studies, in terms of their phylogenetic relatedness, physiology and immune systems. Previous studies have shown that cynomolgus macaques (Macaca fascicularis) are permissive for infection with H1N1pdm influenza virus. These studies have typically used combined challenge routes, with the majority being intra-tracheal delivery, and high doses of virus (> 107 infectious units). This paper describes the outcome of novel challenge routes (inhaled aerosol, intra-nasal instillation) and low to moderate doses (103 to 106 plaque forming units) of H1N1pdm virus in cynomolgus macaques. Evidence of virus replication and sero-conversion were detected in all four challenge groups, although the disease was subclinical. Intra-nasal challenge led to an infection confined to the nasal cavity. A low dose (103 plaque forming units) did not lead to detectable infectious virus shedding, but a 1000-fold higher dose led to virus shedding in all intra-nasal challenged animals. In contrast, aerosol and intra-tracheal challenge routes led to infections throughout the respiratory tract, although shedding from the nasal cavity was less reproducible between animals compared to the high-dose intra-nasal challenge group. Intra-tracheal and aerosol challenges induced a transient lymphopaenia, similar to that observed in influenza-infected humans, and greater virus-specific cellular immune responses in the blood were observed in these groups in comparison to the intra-nasal challenge groups. Activation of lung macrophages and innate immune response genes was detected at days 5 to 7 post-challenge. The kinetics of infection, both virological and immunological, were broadly in line with human influenza A virus infections. These more authentic infection models will be valuable in the determination of anti-influenza efficacy of novel entities against less severe (and thus more common) influenza infections. | 4061 | NA | NA | NA | no | no |
| 4060 | 2017 | NA | Alhaddad, H; Khan, R; Grahn, RA; Gandolfi, B; Mullikin, JC; Cole, SA; Gruffydd-Jones, TJ; Haggstrom, J; Lohi, H; Longeri, M; Lyons, LA | 2013 | Extent of Linkage Disequilibrium in the Domestic Cat, Felis silvestris catus, and Its Breeds | Domestic cats have a unique breeding history and can be used as models for human hereditary and infectious diseases. In the current era of genome-wide association studies, insights regarding linkage disequilibrium (LD) are essential for efficient association studies. The objective of this study is to investigate the extent of LD in the domestic cat, Felis silvestris catus, particularly within its breeds. A custom illumina GoldenGate Assay consisting of 1536 single nucleotide polymorphisms (SNPs) equally divided over ten 1 Mb chromosomal regions was developed, and genotyped across 18 globally recognized cat breeds and two distinct random bred populations. The pair-wise LD descriptive measure (r(2)) was calculated between the SNPs in each region and within each population independently. LD decay was estimated by determining the non-linear least-squares of all pair-wise estimates as a function of distance using established models. The point of 50% decay of r(2) was used to compare the extent of LD between breeds. The longest extent of LD was observed in the Burmese breed, where the distance at which r(2) approximate to 0.25 was similar to 380 kb, comparable to several horse and dog breeds. The shortest extent of LD was found in the Siberian breed, with an r(2) approximate to 0.25 at approximately 17 kb, comparable to random bred cats and human populations. A comprehensive haplotype analysis was also conducted. The haplotype structure of each region within each breed mirrored the LD estimates. The LD of cat breeds largely reflects the breeds’ population history and breeding strategies. Understanding LD in diverse populations will contribute to an efficient use of the newly developed SNP array for the cat in the design of genome-wide association studies, as well as to the interpretation of results for the fine mapping of disease and phenotypic traits. | 4062 | NA | NA | NA | no | no |
| 4061 | 2017 | NA | Elberse, KEM; Nunes, S; Sa-Leao, R; van der Heide, HGJ; Schouls, LM | 2011 | Multiple-Locus Variable Number Tandem Repeat Analysis for Streptococcus pneumoniae: Comparison with PFGE and MLST | In the era of pneumococcal conjugate vaccines, surveillance of pneumococcal disease and carriage remains of utmost importance as important changes may occur in the population. To monitor these alterations reliable genotyping methods are required for large-scale applications. We introduced a high throughput multiple-locus variable number tandem repeat analysis (MLVA) and compared this method with pulsed-field gel electrophoresis (PFGE) and multilocus sequence typing (MLST). The MLVA described here is based on 8 BOX loci that are amplified in two multiplex PCRs. The labeled PCR products are sized on an automated DNA sequencer to accurately determine the number of tandem repeats. The composite of the number of repeats of the BOX loci makes up a numerical profile that is used for identification and clustering. In this study, MLVA was performed on 263 carriage isolates that were previously characterized by MLST and PFGE. MLVA, MLST and PFGE (cut-off of 80%) yielded 164, 120, and 87 types, respectively. The three typing methods had Simpson’s diversity indices of 98.5% or higher. Congruence between MLST and MLVA was high. The Wallace of MLVA to MLST was 0.874, meaning that if two strains had the same MLVA type they had an 88% chance of having the same MLST type. Furthermore, the Wallace of MLVA to clonal complex of MLST was even higher: 99.5%. For some isolates belonging to a single MLST clonal complex although displaying different serotypes, MLVA was more discriminatory, generating groups according to serotype or serogroup. Overall, MLVA is a promising genotyping method that is easy to perform and a relatively cheap alternative to PFGE and MLST. In the companion paper published simultaneously in this issue we applied the MLVA to assess the pneumococcal population structure of isolates causing invasive disease in the Netherlands before the introduction of the 7-valent conjugate vaccine. | 4063 | NA | NA | NA | no | no |
| 4062 | 2017 | NA | Porretta, D; Mastrantonio, V; Bellini, R; Somboon, P; Urbanelli, S | 2012 | Glacial History of a Modern Invader: Phylogeography and Species Distribution Modelling of the Asian Tiger Mosquito Aedes albopictus | Background: The tiger mosquito, Aedes albopictus, is one of the 100 most invasive species in the world and a vector of human diseases. In the last 30 years, it has spread from its native range in East Asia to Africa, Europe, and the Americas. Although this modern invasion has been the focus of many studies, the history of the species’ native populations remains poorly understood. Here, we aimed to assess the role of Pleistocene climatic changes in shaping the current distribution of the species in its native range. Methodology/Principal Findings: We investigated the phylogeography, historical demography, and species distribution of Ae. albopictus native populations at the Last Glacial Maximum (LGM). Individuals from 16 localities from East Asia were analyzed for sequence variation at two mitochondrial genes. No phylogeographic structure was observed across the study area. Demographic analyses showed a signature of population expansion that started roughly 70,000 years BP. The occurrence of a continuous and climatically suitable area comprising Southeast China, Indochinese Peninsula, and Sundaland during LGM was indicated by species distribution modelling. Conclusions/Significance: Our results suggest an evolutionary scenario in which, during the last glacial phase, Ae. albopictus did not experience a fragmentation phase but rather persisted in interconnected populations and experienced demographic growth. The wide ecological flexibility of the species probably played a crucial role in its response to glacial-induced environmental changes. Currently, there is little information on the impact of Pleistocene climatic changes on animal species in East Asia. Most of the studies focused on forest-associated species and suggested cycles of glacial fragmentation and post-glacial expansion. The case of Ae. albopictus, which exhibits a pattern not previously observed in the study area, adds an important piece to our understanding of the Pleistocene history of East Asian biota. | 4064 | NA | NA | NA | no | no |
| 4063 | 2017 | NA | Mammari, N; Vignoles, P; Halabi, MA; Darde, ML; Courtioux, B | 2014 | In Vitro Infection of Human Nervous Cells by Two Strains of Toxoplasma gondii: A Kinetic Analysis of Immune Mediators and Parasite Multiplication | The severity of toxoplasmic infection depends mainly on the immune status of the host, but also on the Toxoplasma gondii strains, which differ by their virulence profile. The relationship between the human host and T. gondii has not yet been elucidated because few studies have been conducted on human models. The immune mechanisms involved in the persistence of T. gondii in the brains of immunocompetent subjects and during the reactivation of latent infections are still unclear. In this study, we analyzed the kinetics of immune mediators in human nervous cells in vitro, infected with two strains of T. gondii. Human neuroblast cell line (SH SY5Y), microglial (CMH5) and endothelial cells (Hbmec) were infected separately by RH (type I) or PRU (type II) strains for 8 h, 14 h, 24 h and 48 h (ratio 1 cell: 2 tachyzoites). Pro-inflammatory protein expression was different between the two strains and among different human nervous cells. The cytokines IL-6, IL-8 and the chemokines MCP-1 and GRO alpha, and SERPIN E1 were significantly increased in CMH5 and SH SY5Y at 24 h pi. At this point of infection, the parasite burden declined in microglial cells and neurons, but remained high in endothelial cells. This differential effect on the early parasite multiplication may be correlated with a higher production of immune mediators by neurons and microglial cells compared to endothelial cells. Regarding strain differences, PRU strain, but not RH strain, stimulates all cells to produce pro-inflammatory growth factors, G-CSF and GM-CSF. These proteins could increase the inflammatory effect of this type II strain. These results suggest that the different protein expression profiles depend on the parasitic strain and on the human nervous cell type, and that this could be at the origin of diverse brain lesions caused by T. gondii. | 4065 | NA | NA | NA | no | no |
| 4064 | 2017 | NA | Waples, RS; Do, C; Chopelet, J | 2011 | Calculating N-e and N-e/N in age-structured populations: a hybrid Felsenstein-Hill approach | The concept of effective population size (N-e) was developed under a discrete-generation model, but most species have overlapping generations. In the early 1970s, J. Felsenstein and W. G. Hill independently developed methods for calculating N-e in age-structured populations; the two approaches produce the same answer under certain conditions and have contrasting advantages and disadvantages. Here, we describe a hybrid approach that combines useful features of both. Like Felsenstein’s model, the new method is based on age-specific survival and fertility rates and therefore can be directly applied to any species for which life table data are available. Like Hill, we relax the restrictive assumption in Felsenstein’s model regarding random variance in reproductive success, which allows more general application. The basic principle underlying the new method is that age structure stratifies a population into winners and losers in the game of life: individuals that live longer have more opportunities to reproduce and therefore have a higher mean lifetime reproductive success. This creates different classes of individuals within the population, and grouping individuals by age at death provides a simple means of calculating lifetime variance in reproductive success of a newborn cohort. The new method has the following features: (1) it can accommodate unequal sex ratio and sex-specific vital rates and overdispersed variance in reproductive success; (2) it can calculate effective size in species that change sex during their lifetime; (3) it can calculate N-e and the ratio N-e/N based on various ways of defining N; (4) it allows one to explore the relationship between N-e and the effective number of breeders per year (N-b), which is a quantity that genetic estimators of contemporary N-e commonly provide information about; and (5) it is implemented in freely available software (AgeNe). | 4066 | NA | NA | NA | no | no |
| 4065 | 2017 | NA | Dharmarajan, G; Beasley, JC; Fike, JA; Raizman, EA; Wu, CC; Pogranichniy, RM; Rhodes, OE | 2012 | Effects of kin-structure on disease dynamics in raccoons (Procyon lotor) inhabiting a fragmented landscape | The spatial aggregation of related individuals can affect disease dynamics by altering contact rates between individuals and/or groups of individuals. However, kin-structure likely affects contact rates in a scale-dependent manner: increasing contact rates within, but decreasing contact rates amongst spatial groups. Thus, the effect of kin-structure on disease exposure risk should depend upon the spatial scale relevant for pathogen transmission. This study was undertaken to test the effects of kin-structure on disease dynamics in raccoons (Procyon lotor) inhabiting a highly fragmented agricultural ecosystem. Raccoons (n = 566) were trapped at 31 spatially discrete habitat patches (local populations). Trapped individuals were genotyped at 13 microsatellites and their serum screened for antibodies to canine distemper virus (CDV) and multiple Leptospira spp. serovars. Our analyses revealed that the effects of kin-structure on transmission dynamics are dependent on both spatial scale and the pathogen under consideration. Thus, in the directly transmitted pathogen (CDV), the probability of pathogen co-exposure (the proportion of individual pairs where both individuals were seropositive for a particular pathogen) was positively correlated with genealogical proximity within local populations. Conversely, in the environmentally transmitted leptospires co-exposure was homogeneous within local populations and was positively correlated with gene flow among these populations. Our analyses also revealed that, after controlling for confounding effects of landscape and demographic variables, the effects of kin-structure on disease exposure risk was pathogen-specific. In particular, increasing kin-structure was associated with increased disease exposure risk in the directly transmitted pathogen (CDV), and alternatively with decreased disease exposure risk in environmentally transmitted leptospires. The potential, scale-dependent, effects of kin-structure on disease exposure risk revealed by our analyses could play an important role in elucidating disease dynamics in natural populations and in planning effective disease mitigation strategies. | 4067 | NA | NA | NA | no | no |
| 4066 | 2017 | NA | Thompson, J; MacMillan, M; Boegler, K; Wood, C; Elder, JH; VandeWoude, S | 2011 | Pathogenicity and Rapid Growth Kinetics of Feline Immunodeficiency Virus Are Linked to 3 ’ Elements | Chimeric viruses constructed between a highly pathogenic Feline Immunodeficiency Virus isolate (FIV-C36) and a less pathogenic but neurotropic strain (FIV-PPR) have been used to map viral genetic determinants of in vivo pathogenicity. Chimeric virus FIV-PCenv, which contains FIV-C36 genome from the 3’ region of pol to upstream of the 3’LTR on an FIV-PPR backbone, was previously shown to be replication-competent in vivo, inducing altered CD4(+) T-cell and neutrophil profiles intermediate between parental strains following a delay in viral replication during initial infection. Examination of FIV-PCenv proviral sequences recovered at week 11 post-infection revealed two changes compared to initial viral inoculum; the most significant being arginine to histidine in the integrase region of Pol at residue 813 (R813H). Pooled plasma from the initial in vivo study was used to inoculate a second cohort of cats to determine whether similar virulence and kinetics could be established following primary infection. Viral replication kinetics and immunocyte profiles were monitored in blood, bone marrow, and saliva over a one-year period. Passaged FIV-PCenv again displayed intermediate phenotype between parental strains, but unlike primary experiments, the onset of acute viremia was not delayed. CD4/8 alterations were noted in all groups of animals, though significant changes from controls were delayed in FIV-PPR infected animals compared to FIV-C36 and FIV-PCenv. In vivo passage of FIV-PCenv increased replication-competence relative to the initial molecularly-cloned chimera in association with one adaptive nucleotide change in the 5’ end of the genome relative to primary tissue culture inoculum, while mutations in the 3’ end of the genome were not detected. The results are consistent with the interpretation that 3’ elements contribute to heightened virulence of FIV-C36, and that integrase residue 813 plays an important role in facilitating successful in vivo replication. | 4068 | NA | NA | NA | no | no |
| 4067 | 2017 | NA | Soares, APG; Guillin, EA; Borges, LL; da Silva, ACT; de Almeida, AMR; Grijalba, PE; Gottlieb, AM; Bluhm, BH; de Oliveira, LO | 2015 | More Cercospora Species Infect Soybeans across the Americas than Meets the Eye | Diseases of soybean caused by Cercospora spp. are endemic throughout the world’s soybean production regions. Species diversity in the genus Cercospora has been underestimated due to overdependence on morphological characteristics, symptoms, and host associations. Currently, only two species (Cercospora kikuchii and C. sojina) are recognized to infect soybean; C. kikuchii causes Cercospora leaf blight (CLB) and purple seed stain (PSS), whereas C. sojina causes frogeye leaf spot. To assess cryptic speciation among pathogens causing CLB and PSS, phylogenetic and phylogeographic analyses were performed with isolates from the top three soybean producing countries (USA, Brazil, and Argentina; collectively accounting for similar to 80% of global production). Eight nuclear genes and one mitochondrial gene were partially sequenced and analyzed. Additionally, amino acid substitutions conferring fungicide resistance were surveyed, and the production of cercosporin (a polyketide toxin produced bymany Cercospora spp.) was assessed. From these analyses, the long-held assumption of C. kikuchii as the single causal agent of CLB and PSS was rejected experimentally. Four cercosporin-producing lineages were uncovered with origins (about 1 Mya) predicted to predate agriculture. Some of the Cercospora spp. newly associated with CLB and PSS appear to represent undescribed species; others were not previously reported to infect soybeans. Lineage 1, which contained the ex-type strain of C. kikuchii, was monophyletic and occurred in Argentina and Brazil. In contrast, lineages 2 and 3 were polyphyletic and contained wide-host range species complexes. Lineage 4 was monophyletic, thrived in Argentina and the USA, and included the generalist Cercospora cf. flagellaris. Interlineage recombination was detected, along with a high frequency of mutations linked to fungicide resistance in lineages 2 and 3. These findings point to cryptic Cercospora species as under-appreciated global considerations for soybean production and phytosanitary vigilance, and urge a reassessment of host-specificity as a diagnostic tool for Cercospora. | 4069 | NA | NA | NA | no | no |
| 4068 | 2017 | NA | van Passel, MWJ; Bart, A; Thygesen, HH; Luyf, ACM; van Kampen, AHC; van der Ende, A | 2005 | An acquisition account of genomic islands based on genome signature comparisons | Background: Recent analyses of prokaryotic genome sequences have demonstrated the important force horizontal gene transfer constitutes in genome evolution. Horizontally acquired sequences are detectable by, among others, their dinucleotide composition (genome signature) dissimilarity with the host genome. Genomic islands (Gls) comprise important and interesting horizontally transferred sequences, but information about acquisition events or relatedness between GIs is scarce. In Vibrio vulnificus CMCP6, 10 and 11 GIs have previously been identified in the sequenced chromosomes I and II, respectively. We assessed the compositional similarity and putative acquisition account of these GIs using the genome signature. For this analysis we developed a new algorithm, available as a web application. Results: Of 21 GIs, VvI-1 and VvI-10 of chromosome I have similar genome signatures, and while artificially divided due to a linear annotation, they are adjacent on the circular chromosome and therefore comprise one GI. Similarly, GIs VvI-3 and VvI-4 of chromosome I together with the region between these two islands are compositionally similar, suggesting that they form one GI (making a total of 19 GIs in chromosome I + chromosome II). Cluster analysis assigned the 19 GIs to 11 different branches above our conservative threshold. This suggests a limited number of compositionally similar donors or intragenomic dispersion of ancestral acquisitions. Furthermore, 2 GIs of chromosome II cluster with chromosome I, while none of the 19 GIs group with chromosome II, suggesting an unidirectional dispersal of large anomalous gene clusters from chromosome I to chromosome II. Conclusion: From the results, we infer 10 compositionally dissimilar donors for 19 GIs in the V. vulnificus CMCP6 genome, including chromosome I donating to chromosome II. This suggests multiple transfer events from individual donor types or from donors with similar genome signatures. Applied to other prokaryotes, this approach may elucidate the acquisition account in their genome sequences, and facilitate donor identification of GIs. | 4070 | NA | NA | NA | no | no |
| 4069 | 2017 | NA | Solberg, EJ; Loison, A; Saether, BE; Strand, O | 2000 | Age-specific harvest mortality in a Norwegian moose Alces alces population | The annual age- and sex-specific patterns of harvest mortality in a Norwegian moose Alces alces population during a period of 17 years for females and 24 years for males were estimated using cohort analysis. In males the harvest mortality increased with age, whereas in females the pattern was U-shaped with higher harvest mortality of less fecund young (1-3 year) and old (greater than or equal to 10 years) age classes, and lower harvest mortality of prime age (4-9 years old) females. In both sexes, the calf harvest mortality was low, although it increased with increasing calf quotas following a change from an indiscriminate to a sex- and age-specific hunting system during the study period. In adult males, the mortality pattern was opposite of what was expected based on the previously reported higher susceptibility to hunting of young than old males, indicating that hunter selectivity for large (old) males affected the pattern. Moreover, the selectivity of the hunters decreased as the hunting pressure increased, suggesting that the hunters became less selective when the mean time available per moose in the quota decreased. Among adult female age groups, the variation in harvest mortality increased with the proportion of calves per female in the population prior to hunting. This was mainly because of relatively higher mortality of post-prime females, supporting our expectation that hunters avoid shooting females with calves and thus increase the harvest of less fecund age groups during high recruitment years. The observed selectivity led to harvest mortality that differed significantly from patterns of natural mortality, even where the mortality is mainly due to wolves Canis lupus and bears Ursus arctos. This may have consequences for the life history evolution of both male and female moose. The potential ecological, evolutionary and management implications of the results are discussed. | 4071 | NA | NA | NA | no | no |
| 4070 | 2017 | NA | Lopez-Perez, M; Villasis, E; Machado, RLD; Povoa, MM; Vinetz, JM; Blair, S; Gamboa, D; Lustigman, S | 2012 | Plasmodium falciparum Field Isolates from South America Use an Atypical Red Blood Cell Invasion Pathway Associated with Invasion Ligand Polymorphisms | Studies of Plasmodium falciparum invasion pathways in field isolates have been limited. Red blood cell (RBC) invasion is a complex process involving two invasion protein families; Erythrocyte Binding-Like (EBL) and the Reticulocyte Binding-Like (PfRh) proteins, which are polymorphic and not fully characterized in field isolates. To determine the various P. falciparum invasion pathways used by parasite isolates from South America, we studied the invasion phenotypes in three regions: Colombia, Peru and Brazil. Additionally, polymorphisms in three members of the EBL (EBA-181, EBA-175 and EBL-1) and five members of the PfRh (PfRh1, PfRh2a, PfRh2b, PfRh4, PfRh5) families were determined. We found that most P. falciparum field isolates from Colombia and Peru invade RBCs through an atypical invasion pathway phenotypically characterized as resistant to all enzyme treatments (NrTrCr). Moreover, the invasion pathways and the ligand polymorphisms differed substantially among the Colombian and Brazilian isolates while the Peruvian isolates represent an amalgam of those present in the Colombian and Brazilian field isolates. The NrTrCr invasion profile was associated with the presence of the PfRh2a pepC variant, the PfRh5 variant 1 and EBA-181 RVNKN variant. The ebl and Pfrh expression levels in a field isolate displaying the NrTrCr profile also pointed to PfRh2a, PfRh5 and EBA-181 as being possibly the major players in this invasion pathway. Notably, our studies demonstrate the uniqueness of the Peruvian P. falciparum field isolates in terms of their invasion profiles and ligand polymorphisms, and present a unique opportunity for studying the ability of P. falciparum parasites to expand their invasion repertoire after being reintroduced to human populations. The present study is directly relevant to asexual blood stage vaccine design focused on invasion pathway proteins, suggesting that regional invasion variants and global geographical variation are likely to preclude a simple one size fits all type of vaccine. | 4072 | NA | NA | NA | no | no |
| 4071 | 2017 | NA | Randi, E; Hulva, P; Fabbri, E; Galaverni, M; Galov, A; Kusak, J; Bigi, D; Bolfikova, BC; Smetanova, M; Caniglia, R | 2014 | Multilocus Detection of Wolf x Dog Hybridization in Italy, and Guidelines for Marker Selection | Hybridization and introgression can impact the evolution of natural populations. Several wild canid species hybridize in nature, sometimes originating new taxa. However, hybridization with free-ranging dogs is threatening the genetic integrity of grey wolf populations (Canis lupus), or even the survival of endangered species (e. g., the Ethiopian wolf C. simensis). Efficient molecular tools to assess hybridization rates are essential in wolf conservation strategies. We evaluated the power of biparental and uniparental markers (39 autosomal and 4 Y-linked microsatellites, a melanistic deletion at the beta-defensin CBD103 gene, the hypervariable domain of the mtDNA control-region) to identify the multilocus admixture patterns in wolf x dog hybrids. We used empirical data from 2 hybrid groups with different histories: 30 presumptive natural hybrids from Italy and 73 Czechoslovakian wolfdogs of known hybrid origin, as well as simulated data. We assessed the efficiency of various marker combinations and reference samples in admixture analyses using 69 dogs of different breeds and 99 wolves from Italy, Balkans and Carpathian Mountains. Results confirmed the occurrence of hybrids in Italy, some of them showing anomalous phenotypic traits and exogenous mtDNA or Y-chromosome introgression. Hybridization was mostly attributable to village dogs and not strictly patrilineal. The melanistic b-defensin deletion was found only in Italian dogs and in putative hybrids. The 24 most divergent microsatellites (largest wolf-dog FST values) were equally or more informative than the entire panel of 39 loci. A smaller panel of 12 microsatellites increased risks to identify false admixed individuals. The frequency of F1 and F2 was lower than backcrosses or introgressed individuals, suggesting hybridization already occurred some generations in the past, during early phases of wolf expansion from their historical core areas. Empirical and simulated data indicated the identification of the past generation backcrosses is always uncertain, and a larger number of ancestry-informative markers is needed. | 4073 | NA | NA | NA | no | no |
| 4072 | 2017 | NA | Tan, L; Lemey, P; Houspie, L; Viveen, MC; Jansen, NJG; van Loon, AM; Wiertz, E; van Bleek, GM; Martin, DP; Coenjaerts, FE | 2012 | Genetic Variability among Complete Human Respiratory Syncytial Virus Subgroup A Genomes: Bridging Molecular Evolutionary Dynamics and Epidemiology | Human respiratory syncytial virus (RSV) is an important cause of severe lower respiratory tract infections in infants and the elderly. In the vast majority of cases, however, RSV infections run mild and symptoms resemble those of a common cold. The immunological, clinical, and epidemiological profile of severe RSV infections suggests a disease caused by a virus with typical seasonal transmission behavior, lacking clear-cut virulence factors, but instead causing disease by modifying the host’s immune response in a way that stimulates pathogenesis. Yet, the interplay between RSV-evoked immune responses and epidemic behavior, and how this affects the genomic evolutionary dynamics of the virus, remains poorly understood. Here, we present a comprehensive collection of 33 novel RSV subgroup A genomes from strains sampled over the last decade, and provide the first measurement of RSV-A genomic diversity through time in a phylodynamic framework. In addition, we map amino acid substitutions per protein to determine mutational hotspots in specific domains. Using Bayesian genealogical inference, we estimated the genomic evolutionary rate to be 6.47x10(-4) (credible interval: 5.56x10(-4), 7.38x10(-4)) substitutions/site/year, considerably slower than previous estimates based on G gene sequences only. The G gene is however marked by elevated substitution rates compared to other RSV genes, which can be attributed to relaxed selective constraints. In line with this, site-specific selection analyses identify the G gene as the major target of diversifying selection. Importantly, statistical analysis demonstrates that the immune driven positive selection does not leave a measurable imprint on the genome phylogeny, implying that RSV lineage replacement mainly follows nonselective epidemiological processes. The roughly 50 years of RSV-A genomic evolution are characterized by a constant population size through time and general co-circulation of lineages over many epidemic seasons - a conclusion that might be taken into account when developing future therapeutic and preventive strategies. | 4074 | NA | NA | NA | no | no |
| 4073 | 2017 | NA | Debout, G; Provost, E; Renucci, M; Tirard, A; Schatz, B; McKey, D | 2003 | Colony structure in a plant-ant: behavioural, chemical and genetic study of polydomy in Cataulacus mckeyi (Myrmicinae) | Social organisation of colonies of obligate plant-ants can affect their interaction with myrmecophyte hosts and with other ants competing for the resources they offer. An important parameter of social organisation is whether nest sites of a colony include one or several host individuals. We determined colony boundaries in a plant-ant associated with the rainforest understorey tree Leonardoxa africana subsp. africana, found in coastal forests of Cameroon (Central Africa). This myrmecophyte is strictly associated with two ants, Petalomyrmex phylax and Cataulacus mckeyi. Plants provide food and nesting sites for P. phylax, which protects young leaves against insect herbivores. This mutualism is often parasitised by C. mckeyi, which uses but does not protect the host. The presence of C. mckeyi on a tree excludes the mutualistic ant. Because Petalomyrmex -occupied trees are better protected, their growth and survival are superior to those of Cataulacus -occupied trees, giving P. phylax an advantage in occupation of nest sites. C. mckeyi often colonises trees that have lost their initial associate P. phylax, as a result of injury to the tree caused by disturbance. Polydomy may allow C. mckeyi to occupy small clumps of trees, without the necessity of claustral colony foundation in each tree. Investigating both the proximate (behavioural repertoire, colony odour) and the ultimate factors (genetic structure) that may influence colony closure, we precisely defined colony boundaries. We show that colonies of C. mckeyi are monogynous and facultatively polydomous, i.e. a colony occupies one to several Leonardoxa trees. Workers do not produce males. Thus, the hypothesis that polydomy allows workers in queenless nests to evade queen control for their reproduction is not supported in this instance. This particular colony structure may confer on C. mckeyi an advantage in short-distance dispersal, and this could help explain its persistence within the dynamic Leonardoxa system. | 4075 | NA | NA | NA | no | no |
| 4074 | 2017 | NA | Patron, NJ; Waller, RF; Cozijnsen, AJ; Straney, DC; Gardiner, DM; Nierman, WC; Howlett, BJ | 2007 | Origin and distribution of epipolythiodioxopiperazine (ETP) gene clusters in filamentous ascomycetes | Background: Genes responsible for biosynthesis of fungal secondary metabolites are usually tightly clustered in the genome and co-regulated with metabolite production. Epipolythiodioxopiperazines (ETPs) are a class of secondary metabolite toxins produced by disparate ascomycete fungi and implicated in several animal and plant diseases. Gene clusters responsible for their production have previously been defined in only two fungi. Fungal genome sequence data have been surveyed for the presence of putative ETP clusters and cluster data have been generated from several fungal taxa where genome sequences are not available. Phylogenetic analysis of cluster genes has been used to investigate the assembly and heredity of these gene clusters. Results: Putative ETP gene clusters are present in 14 ascomycete taxa, but absent in numerous other ascomycetes examined. These clusters are discontinuously distributed in ascomycete lineages. Gene content is not absolutely fixed, however, common genes are identified and phylogenies of six of these are separately inferred. In each phylogeny almost all cluster genes form monophyletic clades with non-cluster fungal paralogues being the nearest outgroups. This relatedness of cluster genes suggests that a progenitor ETP gene cluster assembled within an ancestral taxon. Within each of the cluster clades, the cluster genes group together in consistent subclades, however, these relationships do not always reflect the phylogeny of ascomycetes. Microsynteny of several of the genes within the clusters provides further support for these subclades. Conclusion: ETP gene clusters appear to have a single origin and have been inherited relatively intact rather than assembling independently in the different ascomycete lineages. This progenitor cluster has given rise to a small number of distinct phylogenetic classes of clusters that are represented in a discontinuous pattern throughout ascomycetes. The disjunct heredity of these clusters is discussed with consideration to multiple instances of independent cluster loss and lateral transfer of gene clusters between lineages. | 4076 | NA | NA | NA | no | no |
| 4075 | 2017 | NA | Papadatou, E; Pradel, R; Schaub, M; Dolch, D; Geiger, H; Ibanez, C; Kerth, G; Popa-Lisseanu, A; Schorcht, W; Teubner, J; Gimenez, O | 2012 | Comparing survival among species with imperfect detection using multilevel analysis of markurecapture data: a case study on bats | For comparative demography studies, 2 prerequisites are usually needed: 1) using typical parameter values for species, 2) correctly accounting for the uncertainty in the species specific estimates. However, although within-species variability may be essential, it is typically not considered in analytical procedures, resulting in parameter estimates that may not be representative of the species. Further, data are analysed in 2 steps, first separately for each species, then estimates are compared among species. Accounting for the uncertainty in the species specific estimates is then difficult. Here we propose the application of multilevel Bayesian models on markrecapture (MR) data for comparative studies on survival probabilities that solves these problems. Our models account for within-species variability in space and time in the form of random effects. Models reflecting different biological predictions related to the species ecology and life-history traits may further be contrasted. To illustrate our approach, we used long-term data from 5 temperate tree-roosting bat species and compared their survival probabilities. Results suggest that species foraging in open space, high reproductive output and short longevity records have lower survival than species foraging at short distances, with low reproductive output and high longevity records. Multilevel models provided relatively precise estimates, away from the edges of the parameter space, even for species with low encounter rates and short study duration. This is particularly valuable for less studied taxa such as bats for which available data are often more sparse. Our approach can be easily extended to include additional groups or levels of interest and effects at the individual level (e.g. sex or age). Different hypotheses regarding differences or similarities in parameters among species can be tested through the application of different models. Overall, it offers a flexible tool to ecologists, and population and evolutionary biologists for comparative studies, explicitly accounting for multilevel structures often encountered in MR data. | 4077 | NA | NA | NA | no | no |
| 4076 | 2017 | NA | Lee, W; van Baalen, M; Jansen, VAA | 2016 | Siderophore production and the evolution of investment in a public good: An adaptive dynamics approach to kin selection | Like many other bacteria, Pseudomonas aeruginosa sequesters iron from the environment through the secretion, and subsequent uptake, of iron-binding molecules. As these molecules can be taken up by other bacteria in the population than those who secreted them, this is a form of cooperation through a public good. Traditionally, this problem has been studied by comparing the relative fitnesses of siderophore-producing and non-producing strains, but this gives no information about the fate of strains that do produce intermediate amounts of siderophores. Here, we investigate theoretically how the amount invested in this form of cooperation evolves. We use a mechanistic description of the laboratory protocols used in experimental evolution studies to describe the competition and cooperation of the bacteria. From this dynamical model we derive the fitness following the adaptive dynamics method. The results show how selection is driven by local siderophore production and local competition. Because siderophore production reduces the growth rate, local competition decreases with the degree of relatedness (which is a dynamical variable in our model). Our model is not restricted to the analysis of small phenotypic differences and allows for theoretical exploration of the effects of large phenotypic differences between cooperators and cheats. We predict that an intermediate ESS level of cooperation (molecule production) should exist The adaptive dynamics approach allows us to assess evolutionary stability, which is often not possible in other kin-selection models. We found that selection can lead to an intermediate strategy which in our model is always evolutionarily stable, yet can allow invasion of strategies that are much more cooperative. Our model describes the evolution of a public good in the context of the ecology of the microorganism, which allows us to relate the extent of production of the public good to the details of the interactions. (C) 2015 Elsevier Ltd. All rights reserved. | 4078 | NA | NA | NA | no | no |
| 4077 | 2017 | NA | Haidar, S; Aslam, M | 2016 | NIAB-2008: A New High Yielding and Long Staple Cotton Mutant Developed through Pollen Irradiation Technique | This paper describes about development of cotton mutant NIAB-2008, developed through pollen irradiation technique with improved yield and fibre quality. A local variety NIAB-78 was crossed with exotic line REBA-288 using irradiated pollen @10 Gray (Gy) of gamma rays before cross pollination. The purpose of the study was to create new genetic variability and select the desirable new cotton mutants. After irradiation and hybridization different generations were raised to evaluate the effect of irradiation treatment and induction of mutations. Significant variations from control/parents were observed in evaluated generations. From M-1 seed, the M-2 population was raised and different desirable mutants were selected. The selected mutants were evaluated in mutated generations (M-3-M-6) for different yield contributing traits and yield potential along with fibre quality and variations from parents were observed. From selected mutants, an elite mutant i.e., M-7/09, later named as NIAB-2008 was finally selected. It was evaluated for seed cotton yield, adaptability, resistance/tolerance to diseases, and fibre quality in different trials. It produced 32.9% higher seed cotton yield compared to standard CIM-496 at local NIAB trials. It produced 32.7% and 32.2% higher seed cotton yield compared to standard in provincial coordinated cotton trials (PCCT) and in national coordinated varietal trials (NCVT) respectively. The mutant NIAB-2008 has desirable fibre quality traits i.e., ginning out turn (GOT) 37.99%, fibre length 31.16 mm (long staple category), fibre fineness 4.74 mu g/inch, uniformity index 83.5%, fibre maturity 80.5% and fibre strength 92.2 thousand pounds per square inch (TPPSI). Its distinguish characteristics are early maturity, high yielding, short stature, good boll bearing and better tolerance to cotton leaf curl virus-Burewala strain (CLCuV-B) disease. From these results it is concluded that low dose pollen irradiation technique has effectively stimulate/increase the different yield and yield contributing traits, fibre quality and tolerance to diseases in cotton and this technique is proved economical. (C) 2016 Friends Science Publishers. | 4079 | NA | NA | NA | no | no |
| 4078 | 2017 | NA | Grau, JH; Poustka, AJ; Meixner, M; Plotner, J | 2014 | LTR retroelements are intrinsic components of transcriptional networks in frogs | Background: LTR retroelements (LTR REs) constitute a major group of transposable elements widely distributed in eukaryotic genomes. Through their own mechanism of retrotranscription LTR REs enrich the genomic landscape by providing genetic variability, thus contributing to genome structure and organization. Nonetheless, transcriptomic activity of LTR REs still remains an obscure domain within cell, developmental, and organism biology. Results: Here we present a first comparative analysis of LTR REs for anuran amphibians based on a full depth coverage transcriptome of the European pool frog, Pelophylax lessonae, the genome of the African clawed frog, Silurana tropicalis (release v7.1), and additional transcriptomes of S. tropicalis and Cyclorana alboguttata. We identified over 1000 copies of LTR REs from all four families (Bel/Pao, Ty1/Copia, Ty3/Gypsy, Retroviridae) in the genome of S. tropicalis and discovered transcripts of several of these elements in all RNA-seq datasets analyzed. Elements of the Ty3/Gypsy family were most active, especially Amn-san elements, which accounted for approximately 0.27% of the genome in Silurana. Some elements exhibited tissue specific expression patterns, for example Hydra 1.1 and MuERV-like elements in Pelophylax. In S. tropicalis considerable transcription of LTR REs was observed during embryogenesis as soon as the embryonic genome became activated, i.e. at midblastula transition. In the course of embryonic development the spectrum of transcribed LTR REs changed; during gastrulation and neurulation MuERV-like and SnRV like retroviruses were abundantly transcribed while during organogenesis transcripts of the XEN1 retroviruses became much more active. Conclusions: The differential expression of LTR REs during embryogenesis in concert with their tissue-specificity and the protein domains they encode are evidence for the functional roles these elements play as integrative parts of complex regulatory networks. Our results support the meanwhile widely accepted concept that retroelements are not simple “junk DNA” or “harmful genomic parasites” but essential components of the transcriptomic machinery in vertebrates. | 4080 | NA | NA | NA | no | no |
| 4079 | 2017 | NA | Heiser, M; Schmitt, T | 2010 | Do different dispersal capacities influence the biogeography of the western Palearctic dragonflies (Odonata)? | The biogeography of the western Palearctic has been intensively studied for more than a century. Recent advances in genetics have allowed the testing of old theories based on distribution patterns, although these analyses are obviously restricted to a reduced number of specific genetic data sets. On the other hand, an increased knowledge on the distributions of species and advances in computer capacities have allowed more detailed biogeographical analyses based on species presence/absence. In the present study, we selected the Odonata as the study group. For all 162 species native to the western Palearctic, we compiled their respective presence or absence in 97 predefined biogeographical regions. Using cluster analyses and principal component analyses, both based on Jaccard similarity coefficients, we analysed the differentiation among these regions and species. In subsequent analyses, the data set was reduced to the Zygoptera, Anisoptera, and the western Palearctic endemics. All analyses consistently showed different faunal regions and faunal elements. In particular, the (1) western and (2) eastern Mediterranean; (3) Central and (4) Northern Europe; and (5) the British Isles were invariably found in all cases. Although the two major Mediterranean regions were characterized by several endemic faunal elements, Northern Europe and the British Isles lacked such elements, but were characterized by faunal compositions strongly deviating from the rest of the western Palearctic region. Moderate differences between Zygoptera and Anisoptera existed, with the latter more clearly redrawing the Mediterranean refuge areas, whereas the former reflected to a greater extent the postglacial expansion patterns from these regions. In general, our findings underline the old biogeographical theories, but refine especially our understanding of the Atlanto- and Ponto-Mediterranean region. Central Europe, comprising the area with the highest species numbers of our whole study region, unravels as a crossroad of postglacial immigrations, but might also represent a region of in situ glacial survival. (C) 2010 The Linnean Society of London, Biological Journal of the Linnean Society, 2010, 99, 177-195. | 4081 | NA | NA | NA | no | no |
| 4080 | 2017 | NA | Kumar, G; Krishnaprasad, BT; Savitha, M; Gopalakrishna, R; Mukhopadhyay, K; Ramamohan, G; Udayakumar, M | 1999 | Enhanced expression of heat-shock proteins in thermo-tolerant lines of sunflower and their progenies selected on the basis of temperature-induction response | The major lacuna in developing stress-tolerant lines through breeding is the lack of suitable techniques for screening the segregating population. We report here the development of an efficient technique for identifying high-temperature-tolerant lines in sunflower. The rationale behind this technique is that the stress-responsive genes are expressed during sub-lethal (induction) stress, and its products impart tolerance at subsequent lethal stresses. The genetic variability in gene expression upon induction stress is responsible for the differential survival and recovery following exposure to severe lethal stress in a heterogeneous population Optimization of induction and subsequent lethal temperature levels is a pre-requisite for developing a standardized screening protocol. The optimum induction temperature in sunflower was identified by subjecting the germinated seedlings to various sub-lethal temperatures followed by exposure to a specific lethal temperature for a fixed duration. Gradual temperature induction was found to be optimum in bringing about a maximum response in terms of the recovery growth of seedlings after exposure to a lethal temperature. Following the optimum induction treatment, seedlings were subjected to a specific high temperature for different periods to arrive at a high-stringency lethal temperature treatment. By adopting this approach an open-pollinated population of Helianthus annuus L. (morden) was screened for high-temperature tolerance. The seedlings which survived at the high-stringency lethal temperature following the optimum induction treatment were identified as thermo-tolerant lines. At the plant level too these identified lines showed higher tolerance as reflected by a higher membrane integrity and recovery leaf-area. The progeny population of these identified lines also exhibited higher tolerance to temperature compared to the original population, indicating the persistence of the selected trait. This tolerance was associated with a higher accumulation of heat-shock proteins (HSPs). We propose that this technique can be used as a potential tool to identify and select temperature-tolerant lines from a heterogeneous/segregating population. | 4082 | NA | NA | NA | no | no |
| 4081 | 2017 | NA | Lagos, J; Couvin, D; Arata, L; Tognarelli, J; Aguayo, C; Leiva, T; Arias, F; Hormazabal, JC; Rastogi, N; Fernandez, J | 2016 | Analysis of Mycobacterium tuberculosis Genotypic Lineage Distribution in Chile and Neighboring Countries | Tuberculosis (TB), caused by the pathogen Mycobacterium tuberculosis (MTB), remains a disease of high importance to global public health. Studies into the population structure of MTB have become vital to monitoring possible outbreaks and also to develop strategies regarding disease control. Although Chile has a low incidence of MTB, the current rates of migration have the potential to change this scenario. We collected and analyzed a total of 458 M. tuberculosis isolates (1 isolate per patient) originating from all 15 regions of Chile. The isolates were genotyped using the spoligotyping method and the data obtained were analyzed and compared with the SITVIT2 database. A total of 169 different patterns were identified, of which, 119 patterns (408 strains) corresponded to Spoligotype International Types (SITs) and 50 patterns corresponded to orphan strains. The most abundantly represented SITs/lineages were: SIT53/T1 (11.57%), SIT33/LAM3 (9.6%), SIT42/LAM9 (9.39%), SIT50/H3 (5.9%), SIT37/T3 (5%); analysis of the spoligotyping minimum spanning tree as well as spoligoforest were suggestive of a recent expansion of SIT42, SIT50 and SIT37; all of which potentially evolved from SIT53. The most abundantly represented lineages were LAM (40.6%), T (34.1%) and Haarlem (13.5%). LAM was more prevalent in the Santiago (43.6%) and Concepcion (44.1%) isolates, rather than the Iquique (29.4%) strains. The proportion of X lineage was appreciably higher in Iquique and Concepcion (11.7% in both) as compared to Santiago (1.6%). Global analysis of MTB lineage distribution in Chile versus neighboring countries showed that evolutionary recent lineages (LAM, T and Haarlem) accounted together for 88.2% of isolates in Chile, a pattern which mirrored MTB lineage distribution in neighboring countries (n = 7378 isolates recorded in SITVIT2 database for Peru, Brazil, Paraguay, and Argentina; and published studies), highlighting epidemiological advantage of Euro-American lineages in this region. Finally, we also observed exclusive emergence of patterns SIT4014/X1 and SIT4015 (unknown lineage signature) that have hitherto been found exclusively in Chile, indicating that conditions specific to Chile, along with the unique genetic makeup of the Chilean population, might have allowed for a possible co-evolution leading to the success of these emerging genotypes. | 4083 | NA | NA | NA | no | no |
| 4082 | 2017 | NA | Tsui, CKM; DiGuistini, S; Wang, Y; Feau, N; Dhillon, B; Bohlmann, J; Hamelin, RC | 2013 | Unequal Recombination and Evolution of the Mating-Type (MAT) Loci in the Pathogenic Fungus Grosmannia clavigera and Relatives | Sexual reproduction in fungi is regulated by the mating-type (MAT) locus where recombination is suppressed. We investigated the evolution of MAT loci in eight fungal species belonging to Grosmannia and Ophiostoma (Sordariomycetes, Ascomycota) that include conifer pathogens and beetle symbionts. The MAT1-2 idiomorph/allele was identified from the assembled and annotated Grosmannia clavigera genome, and the MAT locus is flanked by genes coding for cytoskeleton protein (SLA) and DNA lyase. The synteny of these genes is conserved and consistent with other members in Ascomycota. Using sequences from SLA and flanking regions, we characterized the MAT1-1 idiomorph from other isolates of G. clavigera and performed dotplot analysis between the two idiomorphs. Unexpectedly, the MAT1-2 idiomorph contains a truncated MAT1-1-1 gene up-stream of the MAT1-2-1 gene that bears the high-mobility-group domain. The nucleotide and amino acid sequence of the truncated MAT1-1-1 gene is similar to its homologous copy in the MAT1-1 idiomorph in the opposite mating-type isolate, except that positive selection is acting on the truncated gene and the alpha(alpha)-box that encodes the transcription factor has been deleted. The MAT idiomorphs sharing identical gene organization were present in seven additional species in the Ophiostomatales, suggesting that the presence of truncated MAT1-1-1 gene is a general pattern in this order. We propose that an ancient unequal recombination event resulted in the ancestral MAT1-1-1 gene integrated into the MAT1-2 idiomorph and surviving as the truncated MAT1-1-1 genes. The alpha-box domain of MAT1-1-1 gene, located at the same MAT locus adjacent to the MAT1-2-1 gene, could have been removed by deletion after recombination due to mating signal interference. Our data confirmed a 1: 1 MAT/sex ratio in two pathogen populations, and showed that all members of the Ophiostomatales studied here including those that were previously deemed asexual have the potential to reproduce sexually. This ability can potentially increase genetic variability and can enhance fitness in new, ecological niches. | 4084 | NA | NA | NA | no | no |
| 4083 | 2017 | NA | Desclozeaux, M; Robbins, A; Jelocnik, M; Khan, SA; Hanger, J; Gerdts, V; Potter, A; Polkinghorne, A; Timms, P | 2017 | Immunization of a wild koala population with a recombinant Chlamydia pecorum Major Outer Membrane Protein (MOMP) or Polymorphic Membrane Protein (PMP) based vaccine: New insights into immune response, protection and clearance | We assessed the effects of two different single-dose anti-Chlamydia pecorum (C. pecorum) vaccines (containing either Major Outer Membrane Protein (3MOMP) or Polymorphic Membrane Protein (Pmp) as antigens) on the immune response of a group of wild koalas. Both vaccines elicited a systemic humoral response as seen by the production of anti-chlamydial IgG antibodies in more than 90% of vaccinated koalas. A mucosal immune response was also observed, with an increase in Chlamydia-specific mucosal IgG and/or IgA antibodies in some koalas post-vaccination. Both vaccines elicited a cell-mediated immune response as measured by the production of the cytokines IFN-gamma and IL-17 post-vaccination. To determine the level of protection provided by the vaccines under natural conditions we assessed C. pecorum infection loads and chlamydial disease status of all vaccinated koalas pre-and post-vaccination, compared to a non-vaccinated cohort from the same habitat. The MOMP vaccinated koalas that were infected on the day of vaccination showed significant clearance of their infection at 6 months post-vaccination. In contrast, the number of new infections in the PMP vaccine was similar to the control group, with some koalas progressing to disease. Genotyping of the ompA gene from the C. pecorum strains infecting the vaccinated animals, identified genetic variants of ompA-F genotype and a new genotype ompA-O. We found that those animals that were the least well protected became infected with strains of C. pecorum not covered by the vaccine. In conclusion, a single dose vaccine formulated with either recombinant PmpG or MOMP can elicit both cell-mediated and humoral (systemic and mucosal) immune responses, with the MOMP vaccine showing clearance of infection in all infected koalas. Although the capability of our vaccines to stimulate an adaptive response and be protective needs to be fully evaluated, this work illustrates the necessity to combine epitopes most relevant to a large panel of variable strains with an efficient adjuvant. | 4085 | NA | NA | NA | no | no |
| 4084 | 2017 | NA | Nunez-Avila, MC; Uriarte, M; Marquet, PA; Armesto, JJ | 2013 | Decomposing recruitment limitation for an avian-dispersed rain forest tree in an anciently fragmented landscape | Forest fragmentation may intensify recruitment limitation through impacts on seed production, dissemination and seedling establishment processes. In an ancient mosaic of fog-dependent rain forest patches on coastal hilltops of semi-arid Chile (30 degrees S), we investigated how aridity-driven habitat fragmentation affected the recruitment of the fleshy-fruited, dioecious tree Aextoxicon punctatum (Aextoxicaceae) and the role of birds in seed dispersal across patches. We hypothesized that reduced fragment size enhanced recruitment limitation due to constraints on tree fecundity, avian disperser activity and seedling recruitment, caused primarily by strong edge effects in small fragments. We mapped adult Aextoxicon trees and placed 686 seed traps distributed in 3 small (<0.3ha) and 3 large patches ( >0.9ha) in the forest patch mosaic. To identify seed sources, endocarps of 282 dispersed seeds from seed traps were genotyped and compared with 155 genotyped females using nine microsatellite markers. Seed dispersal curves for different forest patches were constructed using maximum likelihood methods. Further, seed addition experiments were conducted in the six fragments to assess recruitment probabilities. Lower density and basal areas of reproductive female trees, in addition to shorter fruiting periods and lower seed yields, led to strong source limitation in small patches. Three bird species accounted for the bulk of visits to fruiting trees, but were less active in smaller patches. Dispersal curves were strongly leptokurtic with maximum dispersal distances related to the shape of patches. Important proportions (5-40%) of genotyped seeds in all patches were immigrants. However, seeds arrived mainly at patch edges or below reproductive trees where germination and survival are reduced. Fewer seedlings originated from experimentally added seeds in small patches subjected to greater edge effects.Synthesis. In summary, we provide evidence for source, dispersal and establishment limitation of this dioecious tree in an aridity-driven fragmented landscape. Small fragment size and edge effects had negative impacts on fecundity and seedling establishment. Although bird-mediated seed dispersal favoured immigration between patches, recruitment from such seeds will be unlikely because of low quality of deposition sites. | 4086 | NA | NA | NA | no | no |
| 4085 | 2017 | NA | Verdu, M; Traveset, A | 2005 | Early emergence enhances plant fitness: A phylogenetically controlled meta-analysis | The time at which a seedling emerges can determine its future success as a plant. Despite the large number of studies that have examined the effect of emergence time on. different components of plant fitness (survival, growth, and/or fecundity), the potential evolutionary response to selection on seedling emergence date is still poorly known. In this study, we review 55 of those studies by a random-effects meta-analysis, considering the phytogenetic relatedness among taxa. We test the following hypotheses: (1) early emergence increases seedling survival, growth, and fecundity, (2) early emergence is more advantageous to large-seeded species than to small-seeded ones, as the former can compensate for the lower number of seeds by increasing seedling survival, (3) perennial plants benefit more than annuals from early emergence, as the iteroparity of the former allows them to risk seedling emergence to the best conditions each year, whereas the semelparity of the latter forces them to spread the risk of emergence over time, and (4) the effect of emergence time may depend upon the experimental conditions (field vs. controlled experiments in a greenhouse or laboratory). Our results show that early emergence differentially affects components of plant fitness, with no effect on seedling survival but large benefits to seedling growth and fecundity. Such effects vary depending upon intrinsic factors like seed size or life-form, and also upon methodology (census time and experimental conditions). Large-seeded species gain from emerging early by growing more during their first growing seasons, although they survive and reproduce similarly to small-seeded species. The survival benefit of early emergence is greater in perennial than in annual species, thus supporting hypothesis 3. The relationship between emergence time and seedling growth appears to be stronger under controlled conditions than in the field, probably as a result of the unlimited nutrient and water resources of the former. In field conditions, in contrast, limited resources probably decelerate the growth of early seedlings, precluding the detection of differences between these and late seedlings. | 4087 | NA | NA | NA | no | no |
| 4086 | 2017 | NA | Larocca, A; Fanti, P; Molinaro, A; Mattia, MF; Battaglia, D | 2011 | Aphid performance on Vicia faba and two southern Italy Phaseolus vulgaris landraces | Plant landraces are an important reservoir of genetic variability, and their characterization can improve the selection of new cultivars with desired features. In this study we give a first contribution to the knowledge of Aphis craccivora Koch and Aphis fabae Scopoli performance on two landraces of Sarconi beans (local populations of Phaseolus vulgaris L. from Southern Italy), “verdolino” and “tabacchino”. We also compared aphid performance on P. vulgaris vs. Vicia faba (c.v.. Aqua dulce) because we wanted to test if the same aphid clone could utilize both plant species as a host, so being able to develop and reproduce early in the season on V. faba, and then move to young Phaseolus plants. Also, we wanted to test if the different leaf trichomes density on the bean landraces could affect aphid performance. Several aphid samples of the two species were collected in the field and tested on young potted plants of both broad bean and bean landraces. Samples of A. craccivora were not able to complete the first generation on the two Phaseolus landraces tested in our study. Instead, some samples of A. fabae set up their colonies on Phaseolus plants. All A. fabae samples that survived and reproduced on P. vulgaris performed well or better on V. faba, whereas not all the samples surviving and reproducing on V. faba were able to set up on P. vulgaris. Life-history features (nymphal mortality and developmental time, adult longevity, and r(m)) were quantified in one of the samples that it was able to colonize Phaseolus (fab-1). Data showed a poorer performance of A. fabae on P. vulgaris than V. faba. On the last host species, A. fabae population increased to a density that caused the host plant death. On the contrary, aphid population was stable on both bean landraces and so the infested plants were able to grow and produce seeds. Aphid performance on the two landraces differed in adult longevity, shorter on “verdolino” bean, and colony survival, longer on “verdolino”. | 4088 | NA | NA | NA | no | no |
| 4087 | 2017 | NA | Allen, AR; Taylor, M; McKeown, B; Curry, AI; Lavery, JF; Mitchell, A; Hartshorne, D; Fries, R; Skuce, RA | 2010 | Compilation of a panel of informative single nucleotide polymorphisms for bovine identification in the Northern Irish cattle population | Background: Animal identification is pivotal in governmental agricultural policy, enabling the management of subsidy payments, movement of livestock, test scheduling and control of disease. Advances in bovine genomics have made it possible to utilise inherent genetic variability to uniquely identify individual animals by DNA profiling, much as has been achieved with humans over the past 20 years. A DNA profiling test based on bi-allelic single nucleotide polymorphism (SNP) markers would offer considerable advantages over current short tandem repeat (STR) based industry standard tests, in that it would be easier to analyse and interpret. In this study, a panel of 51 genome-wide SNPs were genotyped across panels of semen DNA from 6 common breeds for the purposes of ascertaining allelic frequency. For SNPs on the same chromosome, the extent of linkage disequilbrium was determined from genotype data by Expectation Maximization (EM) algorithm. Minimum probabilities of unique identification were determined for each breed panel. The usefulness of this SNP panel was ascertained by comparison to the current bovine STR Stockmarks II assay. A statistically representative random sampling of bovine animals from across Northern Ireland was assembled for the purposes of determining the population allele frequency for these STR loci and subsequently, the minimal probability of unique identification they conferred in sampled bovine animals from Northern Ireland. Results: 6 SNPs exhibiting a minor allele frequency of less than 0.2 in more than 3 of the breed panels were excluded. 2 Further SNPs were found to reside in coding areas of the cattle genome and were excluded from the final panel. The remaining 43 SNPs exhibited genotype frequencies which were in Hardy Weinberg Equilibrium. SNPs on the same chromosome were observed to have no significant linkage disequilibrium/allelic association. Minimal probabilities of uniquely identifying individual animals from each of the breeds were obtained and were observed to be superior to those conferred by the industry standard STR assay. Conclusions: The 43 SNPs characterised herein may constitute a starting point for the development of a SNP based DNA identification test for European cattle. | 4089 | NA | NA | NA | no | no |
| 4088 | 2017 | NA | Emrich, K; Wilde, F; Miedaner, T; Piepho, HP | 2008 | REML approach for adjusting the Fusarium head blight rating to a phenological date in inoculated selection experiments of wheat | Fusarium head blight is one of the most important wheat diseases causing grain yield and quality losses as well as mycotoxin contamination all over the world. Since Fusarium cannot be reliably controlled with fungicides, breeding has become a favorable tool to decrease the infection severity. In most cases, selection for Fusarium resistance is done by artificial infection in the field. However, there is a risk in preferring late heading genotypes, because heading of wheat is negatively correlated to head blight severity. Because an indirect selection for late maturity is not intended, we considered a statistical approach to avoid this problem. In this paper, we propose a mixed model to analyze extensive Fusarium head blight rating in resistance breeding experiments of wheat. The objective of the analysis was to select for Fusarium resistance, while at the same time ensuring that late heading genotypes, which show less head blight over the shorter vegetation period, are not preferred. Thus, selection was to be done such that genetic variability for heading date was retained. Therefore, the statistical model contained a covariate to adjust for differences in the heading date. The use of covariate adjustment is an easily handled alternative to a bivariate analysis. Covariate adjustment will in practice often work almost equally well as bivariate analysis. Any statistical software with powerful mixed model analysis tools can be used for this type of analysis. We propose an ad hoc method to obtain heritability estimates and a form of LSD (least significance difference) as a measure of accuracy on the basis of the proposed model and under special consideration of the experimental design. The ad hoc LSD was used as a rough measure to judge rankings of genotypic means (BLUPs). Friedman’s super smoother was used to compare smoothed rank estimates for adjusted and unadjusted genotypes against increasing smoothed heading dates. Traits were transformed to meet the model assumptions, especially homogeneity of errors and normality, and back-transformation of means and standard errors was conducted by using the delta method. | 4090 | NA | NA | NA | no | no |
| 4089 | 2017 | NA | HOHL, A; TISDELL, CA | 1993 | HOW USEFUL ARE ENVIRONMENTAL SAFETY STANDARDS IN ECONOMICS - THE EXAMPLE OF SAFE MINIMUM STANDARDS FOR PROTECTION OF SPECIES | The use of environmental safety standards is being increasingly advocated, and economists are being increasingly required to adapt their policy models to take account of such standards, e.g. by the use of transferable environmental damage quotas. The use of these standards may be a consequence of a greater desire to achieve more sustainable development and preserve the natural wealth available to future generations. An important means to this goal is to preserve biodiversity and genetic variability. Safe minimum standards for conservation of wildlife have been suggested as a way to achieve this. Sometimes safe minimum standards for conservation of species are defined in terms of their minimum viable populations and their minimum habitat requirements. However, as a review of recent scientific literature indicates, these safe minimum standards are very uncertain. Environmental safety standards have been integrated into economics in a variety of ways, e.g. via constraints to ensure sustainability at the macro-level, via minimum safety standards necessary to ensure survival of species. Despite the commendable support which safe minimum standards appear to give for nature conservation, they involve conceptual problems and uncertainties as outlined in this paper. In fact. safe minimum standards for the conservation of species do not exist - no standard ensures the continued existence of any species. This is not to deny that an improvement in standards can up to a point increase the probability of survival of a species for a specified period of time. It implies that minimum standards need to be cast in terms of those required to achieve a particular probability of survival of the targeted species for a specified period. It may, however, be impossible to achieve the goal specified, e.g. the probability of survival of a species aimed for, may be greater than can be achieved for any attainable standard. In general, the paper makes it clear that standards suggested by natural scientists and others need to be subjected to careful scrutiny before they can be used as a reliable basis for economic policy. | 4091 | NA | NA | NA | no | no |
| 4090 | 2017 | NA | Nebert, DW | 2002 | Proposal for an allele nomenclature system based on the evolutionary divergence of haplotypes | The classical view of what constitutes an “allele” has been challenged by recent findings of a great deal of human genetic variability, i.e., we can expect, on average, one variant site every 100-250 bases of our haploid genome. The haplotype is defined as “the patterns of co-occurrence of variant sites on the same chromosome” (and therefore within each particular gene). Sufficient evidence exists for the divergence of haplotypes during evolution of Homo sapiens sapiens, and the total number of haplotypes per gene will reflect the amount of time any particular ethnic group has existed on the planet, e.g., greatest in Africans, fewer in East Asians, and still fewer in Caucasians. If the average gene spans 30 kb, we can expect similar to 170 polymorphic variant sites per gene in the world population. We do not see 217 haplotypes, however; we might find only 10 to 200 haplotypes (depending on the gene’s size and degree of conservation of the gene product). This finite number allows for a reasonable haplotype nomenclature system for each gene, based on evolutionary divergence. For polymorphic variants (i.e., frequency greater than or equal to 0.01), I propose using Arabic numerals for the major clades (e.g., *1,* 2,…*20,* 21), capital letters for sublineages (e.g., *2A,* 2B, *2C), and Arabic numerals for sub-sublineages (e.g.,* 22G12, \*22G13); additional subcategories may be added, in an alternating number/letter/number/letter sequence, depending on the complexity of present-day haplotypes of a particular gene. Web sites with a web master and external advisory committee should be set up for each gene superfamily, family, or individual gene (depending on complexity), and an international haplotype nomenclature committee, perhaps comprised of several dozen of these web masters, should oversee haplotype nomenclature for the entire human genome. The higher heterozygosity and multiallelic nature makes haplotypes more informative than biallelic SNPs. Ultimately, our knowledge of haplotype patterns, rather than single variant sites, of perhaps several hundred genes will likely be helpful in finding associations between genotype and any multiplex phenotype (e.g., complex diseases including cancer, and/or toxicity of pharmaceutical agents or environmental pollutants). | 4092 | NA | NA | NA | no | no |
| 4091 | 2017 | NA | Curtis, R; Frey, SN; Brown, NL | 2014 | The Effect of Coterie Relocation on Release-Site Retention and Behavior of Utah Prairie Dogs | Utah prairie dogs (Cynomys parvidens) have been extirpated in 90% of their historical range. Because most populations in Utah occur on private land, this threatened species is continually in conflict with landowners. The Utah Division of Wildlife Resources has been relocating Utah prairie dogs from private to public land since the 1970s, but relocations have been largely unsuccessful because of high mortality. Utah prairie dogs are highly social animals, but they are usually relocated without regard to their family group, or coterie. We predicted that relocating Utah prairie dogs with other coterie members would improve their survival rate and result in post-release behavior similar to non-relocated animals. We chose to investigate release-site retention as a measure of relocation success because we were unable to separate emigration from mortality. We relocated Utah prairie dogs from the Cedar Ridge Golf Course in Cedar City, Utah to 2 prepared sites near Bryce Canyon National Park, Utah in 2010 and 2011. We relocated prairie dogs as groups of coterie members or in a control group of individuals trapped with no regard to relatedness. Two months after relocation, we set traps to recapture animals to estimate release-site retention. We quantified activity budgets prior to and following relocation on study animals as well as from a previously established relocated prairie dog population on public land. The best predictor of release-site retention and recapture rate was the animal’s weight at initial capture. Larger animals had high retention but low recapture rates. We found no differences in site retention or behavior between prairie dogs relocated with coteries and controls. Relocated individuals behaved more like prairie dogs on public lands than animals in the urban source population, but behaviors were still different from prairie dogs on public lands. We recommend relocating large, adult Utah prairie dogs rather than juveniles or relocating juveniles later in the trapping season to increase relocation success rate. We also suggest that future research should focus on developing additional release methods to reduce dispersal and increase site retention. (c) 2014 The Wildlife Society. | 4093 | NA | NA | NA | no | no |
| 4092 | 2017 | NA | da Silva, VS; Shida, CS; Rodrigues, FB; Ribeiro, DCD; de Souza, AA; Coletta-Filho, HD; Machado, MA; Nunes, LR; de Oliveira, RC | 2007 | Comparative genomic characterization of citrus-associated Xylella fastidiosa strains | Background: The xylem-inhabiting bacterium Xylella fastidiosa (Xf) is the causal agent of Pierce’s disease (PD) in vineyards and citrus variegated chlorosis (CVC) in orange trees. Both of these economically-devastating diseases are caused by distinct strains of this complex group of microorganisms, which has motivated researchers to conduct extensive genomic sequencing projects with Xf strains. This sequence information, along with other molecular tools, have been used to estimate the evolutionary history of the group and provide clues to understand the capacity of Xf to infect different hosts, causing a variety of symptoms. Nonetheless, although significant amounts of information have been generated from Xf strains, a large proportion of these efforts has concentrated on the study of North American strains, limiting our understanding about the genomic composition of South American strains - which is particularly important for CVC-associated strains. Results: This paper describes the first genome-wide comparison among South American Xf strains, involving 6 distinct citrus-associated bacteria. Comparative analyses performed through a microarray-based approach allowed identification and characterization of large mobile genetic elements that seem to be exclusive to South American strains. Moreover, a large-scale sequencing effort, based on Suppressive Subtraction Hybridization (SSH), identified 290 new ORFs, distributed in 135 Groups of Orthologous Elements, throughout the genomes of these bacteria. Conclusion: Results from microarray-based comparisons provide further evidence concerning activity of horizontally transferred elements, reinforcing their importance as major mediators in the evolution of Xf. Moreover, the microarray-based genomic profiles showed similarity between Xf strains 9a5c and Fb7, which is unexpected, given the geographical and chronological differences associated with the isolation of these microorganisms. The newly identified ORFs, obtained by SSH, represent an approximately 10% increase in our current knowledge of the South American Xf gene pool and include new putative virulence factors, as well as novel potential markers for strain identification. Surprisingly, this list of novel elements include sequences previously believed to be unique to North American strains, pointing to the necessity of revising the list of specific markers that may be used for identification of distinct Xf strains. | 4094 | NA | NA | NA | no | no |
| 4093 | 2017 | NA | Sequeira, AMM; Roetman, PEJ; Daniels, CB; Baker, AK; Bradshaw, CJA | 2014 | Distribution models for koalas in South Australia using citizen science-collected data | The koala (Phascolarctos cinereus) occurs in the eucalypt forests of eastern and southern Australia and is currently threatened by habitat fragmentation, climate change, sexually transmitted diseases, and low genetic variability throughout most of its range. Using data collected during the Great Koala Count (a 1-day citizen science project in the state of South Australia), we developed generalized linear mixed-effects models to predict habitat suitability across South Australia accounting for potential errors associated with the dataset. We derived spatial environmental predictors for vegetation (based on dominant species of Eucalyptus or other vegetation), topographic water features, rain, elevation, and temperature range. We also included predictors accounting for human disturbance based on transport infrastructure (sealed and unsealed roads). We generated random pseudo-absences to account for the high prevalence bias typical of citizen-collected data. We accounted for biased sampling effort along sealed and unsealed roads by including an offset for distance to transport infrastructures. The model with the highest statistical support (wAICc similar to 1) included all variables except rain, which was highly correlated with elevation. The same model also explained the highest deviance (61.6%), resulted in high R2(m) (76.4) and R2(c) (81.0), and had a good performance according to Cohen’s (0.46). Cross-validation error was low (similar to 0.1). Temperature range, elevation, and rain were the best predictors of koala occurrence. Our models predict high habitat suitability in Kangaroo Island, along the Mount Lofty Ranges, and at the tips of the Eyre, Yorke and Fleurieu Peninsulas. In the highest-density region (5576km2) of the Adelaide-Mount Lofty Ranges, a density-suitability relationship predicts a population of 113,704 (95% confidence interval: 27,685-199,723; average density=5.0-35.8km-2). We demonstrate the power of citizen science data for predicting species’ distributions provided that the statistical approaches applied account for some uncertainties and potential biases. A future improvement to citizen science surveys to provide better data on search effort is that smartphone apps could be activated at the start of the search. The results of our models provide preliminary ranges of habitat suitability and population size for a species for which previous data have been difficult or impossible to gather otherwise. | 4095 | NA | NA | NA | no | no |
| 4094 | 2017 | NA | Lopez-Toledo, L; Anten, NPR; Endress, BA; Ackerly, DD; Martinez-Ramos, M | 2012 | Resilience to chronic defoliation in a dioecious understorey tropical rain forest palm | 1. Perennial plants often endure chronic loss of leaf area due to recurrent physical damage, herbivory and, for species used as non‐timber forest products, due to leaf harvesting. However, little is known about functional and demographic resilience (extent and speed of recovery) of plants subjected to varying levels of chronic defoliation. 2. We used a dioecious, understorey palm (Chamaedorea elegans) to evaluate temporal trajectories and rates of recovery of leaf functional traits and vital rates (survival, growth and reproduction) after being subjected to experimental chronic defoliation regimes. 3. Pristine populations of mature C. elegans, categorized by gender (male and female), were subjected to five defoliation levels (0%, 33%, 50%, 66% or 100% of newly produced leaves) every 6 months over a period of 3 years (1997–2000). To evaluate recovery from defoliation, surviving palms were monitored for 3 years after the cessation of the defoliation treatment (2000–2003). We recorded leaf functional traits (leaf persistence, leaf production rate, leaf size and leaf area) and annual rates of mortality, growth and reproduction. 4. Cumulative effects of chronic defoliation concomitantly reduced leaf traits, survival, growth and reproduction, and this effect was stronger in female than in male palms, independent of plant size. Recovery from defoliation was faster in males than in females, but proceeded gradually overall. Survival increased first, followed by growth, while reproductive traits showed the slowest recovery rate. Recovery was independent of plant size. Notably, 3 years after defoliation treatment, the standing leaf area and probability of reproduction had not recovered to pre‐defoliation levels. Additionally, we found that the occurrence of a severe drought in the first year (2000) after defoliation ceased led to decreased survival, growth and reproduction and the ability of plants to recover from defoliation. 5. Synthesis. Chronic defoliation reduces fitness components of C. elegans palms differentially between genders. Recovery is gradual and is slower and less complete in females compared with males. The lower level of resilience to chronic defoliation shown by female plants may have profound consequences for the dynamics and genetic variability of populations of tropical understorey plants undergoing prolonged defoliation. Such effects may be aggravated by severe drought episodes that are expected to increase in frequency according to global climate change predictions. | 4096 | NA | NA | NA | no | no |
| 4095 | 2017 | NA | Wolff, JO; Schauber, EM; Edge, WD | 1997 | Effects of habitat loss and fragmentation on the behavior and demography of gray-tailed voles | We monitored the short term behavioral and demographic responses of gray-tailed voles (Microtus canicaudus) to the reduction and fragmentation of their habitat. Our objectives were (1) to test whether animals perished or moved into remaining fragments after 70% of their habitat was removed; and (2) to test the null hypothesis that the social structure and demography of animals would not differ between habitats consisting of one large continuous fragment (625 m(2)), a mosaic of 25 small fragments (each 25 m(2)) separated by 4 m of bare ground, and control, unmanipulated habitats (1850 m(2)). We conducted the experiment in 12, 0.2-ha enclosures planted with alfalfa with four replicates for each of two manipulated treatments and a control. A 70% reduction in habitat did not adversely affect adult survival, reproductive rate, juvenile recruitment, or population size. However, an influx of unrelated females into habitat fragments resulted in decreased juvenile recruitment in those fragments. Voles from cleared habitat moved into the remaining habitat and did not measurably affect the resident population. Similarly, the demography of voles did no differ significantly among the large-fragment, small-fragment, and control enclosures. Peak density estimates based on the amount of habitat in each enclosure were 545 animals per hectare in control, 1056 in large-fragment, and 2880 in small-fragment enclosures. Reduced movement of animals among the small fragments was the most obvious effect of habitat fragmentation. Six percent of females and 15% of males moved among small fragments within a week compared to approximately 60% moving comparable distances in large-fragment and control enclosures. Rates of juvenile dispersal and sexual maturation declined throughout the summer on all treatments, were associated with season and density, and were only marginally associated with habitat loss and fragmentation. We conclude that at the time of habitat removal and fragmentation, populations were small enough to accommodate a 70% reduction in habitat and still continue to increase in numbers. The social system of gray-tailed voles was sufficiently flexible to accommodate an influx of animals to withstand densities >1000 voles per ha. The behavioral and demographic features of gray-tailed voles are similar to those reported for other small mammals, thus confirming the use of voles for ecological model systems in habitat fragmentation studies. | 4097 | NA | NA | NA | no | no |
| 4096 | 2017 | NA | Kudirkiene, E; Bager, RJ; Johnson, TJ; Bojesen, AM | 2014 | Chaperone-usher fimbriae in a diverse selection of Gallibacterium genomes | Background: Fimbriae are bacterial cell surface organelles involved in the pathogenesis of many bacterial species, including Gallibacterium anatis, in which a F17-like fimbriae of the chaperone-usher (CU) family was recently shown to be an important virulence factor and vaccine candidate. To reveal the distribution and variability of CU fimbriae 22 genomes of the avian host-restricted bacteria Gallibacterium spp. were investigated. Fimbrial clusters were classified using phylogeny-based and conserved domain (CD) distribution-based approaches. To characterize the fimbriae in depth evolutionary analysis and in vitro expression of the most prevalent fimbrial clusters was performed. Results: Overall 48 CU fimbriae were identified in the genomes of the examined Gallibacterium isolates. All fimbriae were assigned to gamma(4) clade of the CU fimbriae of Gram-negative bacteria and were organized in four-gene clusters encoding a putative major fimbrial subunit, a chaperone, an usher and a fimbrial adhesin. Five fimbrial clusters (Flf-Flf4) and eight conserved domain groups were defined to accommodate the identified fimbriae. Although, the number of different fimbrial clusters in individual Gallibacterium genomes was low, there was substantial amino acid sequence variability in the major fimbrial subunit and the adhesin proteins. The distribution of CDs among fimbrial clusters, analysis of their flanking regions, and evolutionary comparison of the strains revealed that Gallibacterium fimbrial clusters likely underwent evolutionary divergence resulting in highly host adapted and antigenically variable fimbriae. In vitro, only the fimbrial subunit FlfA was expressed in most Gallibacterium strains encoding this protein. The absence or scarce expression of the two other common fimbrial subunits (Flf1A and Flf3A) indicates that their expression may require other in vitro or in vivo conditions. Conclusions: This is the first approach establishing a systematic fimbria classification system within Gallibacterium spp., which indicates a species-wide distribution of gamma(4) CU fimbriae among a diverse collection of Gallibacterium isolates. The expression of only one out of up to three fimbriae present in the individual genomes in vitro suggests that fimbriae expression in Gallibacterium is highly regulated. This information is important for future attempts to understand the role of Gallibacterium fimbriae in pathogenesis, and may prove useful for improved control of Gallibacterium infections in chickens. | 4098 | NA | NA | NA | no | no |
| 4097 | 2017 | NA | Mazzola, M; Funnell, DL; Raaijmakers, JM | 2004 | Wheat cultivar-specific selection of 2,4-diacetylphloroglucinol-producing fluorescent Pseudomonas species from resident soil populations | An emerging body of evidence indicates a role for plant genotype as a determinant of the species and genetic composition of the saprophytic microbial community resident to the rhizosphere. In this study, experiments were conducted to determine the capacity of five different wheat cultivars to enhance resident populations and support introduced strains of 2,4-diacetylphloroglucinol (2,4-DAPG)-producing fluorescent pseudomonads, a group of bacteria known to provide biological control of several soilborne diseases. When soils were cropped with three successive 28-day growth cycles of wheat, the 2,4-DAPG-producing strains were consistently recovered from the rhizosphere of the cultivar Lewjain, and commonly were present at populations higher than those recovered from other wheat cultivars. Based on restriction fragment length polymorphism and sequence analyses of phlD, a key gene involved in 2,4-DAPG production, two previously undefined phlD+ genotypes, referred to as genotypes PfZ and PfY, were discovered. Wheat cultivar Lewjain was the primary source of genotype PfY while cultivar Penawawa yielded the majority of genotype PfZ. Based on 16S rDNA sequence analysis, both new phlD genotypes were classified as P. fluorescens. Comparison of the rhizosphere competence of 2,4-DAPG-producing P. fluorescens Q2-87 (genotype B) and P. fluorescens LR3-A28 (genotype PfY) showed that both strains persisted at similar populations in the rhizosphere of all cultivars tested over a 30 day period when introduced as a seed inoculant. However, when strain LR3-A28 was applied as a soil inoculant, this strain was recovered at higher populations from the rhizosphere of wheat cultivar Lewjain than from the rhizospheres of two other cultivars. No cultivar effects were shown for strain Q2-87. Collectively, these results add further to evidence indicating a degree of specificity in interactions between plant cultivars and specific members of the saprophytic microbial community. Furthermore, as 2,4-DAPG-producing fluorescent Pseudomonas spp. have a central role in the spontaneous reduction in severity of take-all disease of wheat in response to continuous wheat monoculture, we postulate that the use of specific cultivars, such as Lewjain, which possess a superior capacity to enhance resident soil populations of these bacteria may have potential to reduce the length of the monoculture period required to induce natural suppressiveness of soils toward this disease. | 4099 | NA | NA | NA | no | no |
| 4098 | 2017 | NA | DENBOER, PJ; SZYSZKO, J; VERMEULEN, R | 1993 | SPREADING THE RISK OF EXTINCTION BY GENETIC DIVERSITY IN POPULATIONS OF THE CARABID BEETLE PTEROSTICHUS-OBLONGOPUNCTATUS F (COLEOPTERA, CARABIDAE) | Identification of insect species often is only possible with the help of characters that do not have a direct selective value. Such characters may reflect pleiotropic action of genes. This hypothesis was tested in the carabid Pterostichus oblongopunctatus. The closely related species P. quadrifoveolatus has 3 large pits on each elytron, and prefers sites where wood has been burned. P. oblongopunctatus is a common forest species and two groups of morphs can be recognized: low-pitters with 4-5 pits on each elytron in a somewhat winding row, and high-pitters with 6-8 (or more) pits in a straight row. In dry forests the proportion of high-pitters is lower than in moist forests. Assuming pit numbers are genetically determined this suggests that low-pitters are favoured by relatively dry conditions, and high-pitters by moist conditions. Moreover, the shift of the proportion of high-pitters from year to year is correlated with the amount of precipitation in May-August, the period of larval development, especially with the accumulated deviations from the normal amount of rain. The latter suggests a genetical base for the two groups of morphs, which was supported by comparing the progeny of low-pitters with that of high-pitters. Selection experiments suggest that low-pitters are favoured by dry conditions and high-pitters by moist conditions during larval development. Samplings from different parts of Europe were in accordance with the hypothesis: In Poland, with a lower precipitation than Drenthe (The Netherlands), the proportion of low-pitters was higher, and in the Bavarian Alps, with about the same mean amount of precipitation as Drenthe, but on heavy loam, the proportion of high-pitters was higher than in Drenthe. The occurrence of some genotypes with a different moisture tolerance in the same population allows the species to occupy a wide range of forests, and to live with relatively small fluctuations in numbers in areas with highly unpredictable rainfall. The chance of the population to go extinct is decreased by spread of the risk over some genotypes with a differing tolerance of moisture conditions. P. quadrifoveolatus (with only 3 pits and considered to be a good species) represents the morph with a high tolerance of dry conditions, viz. after a forest fire. | 4100 | NA | NA | NA | no | no |
| 4099 | 2017 | NA | WESTNEAT, DF; SHERMAN, PW | 1993 | PARENTAGE AND THE EVOLUTION OF PARENTAL BEHAVIOR | Parentage is the proportion of juveniles in a blood that are offspring of potential care givers. We analyzed how reductions in parentage affect the evolution of parental behavior using a static optimization model. The main benefit of parental effort was an increase in the survival of offspring, and the main costs were reduced opportunities to seek additional matings or to parasitize neighbors and/or reduced survival. Both the costs and benefits included terms for relatedness to young. The effect of parentage depended on (1) whether parents responded in ecological time (facultative response) or in evolutionary time (nonfacultative response), (2) whether the cues enabling assessment of parentage permitted discrimination among offspring, and (3) whether parentage was the same among different groups of juveniles (unrestricted) or varied between them (restricted). When parents did not know their own parentage and mean parentage was the same for all matings, reduced parentage affected the costs and benefits equally, so, as in several previous models, there was no effect on the optimal level of parental effort. Parentage did affect optimal parental effort when mean parentage to the present brood differed from that to young from alternative or future matings. Lowered parentage reduced optimal parental effort when the cost of parenting was missed opportunities for extrapair copulations or brood parasitism or when parentage was consistently higher in alternative or future matings. Nonlinear changes in parentage with age gave complex trajectories of parental care, with individuals of different ages having similar parentage but exhibiting different levels of parental effort. Correlations between parentage and other variables in the model (such as opportunities for additional matings) sometimes masked, but never eliminated, the effects of parentage. When parents could discriminate their own young in a brood, overall parental effort was reduced, but nepotism was increased. When parents could not discriminate their own offspring but had general cues about average parentage to the brood, effects varied depending on the costs and benefits of parental behavior. When parental behavior was costly to care givers. parentage had more effect than when parenting was not costly. Likewise, parentage had less effect when care greatly increased offspring survival than when care was less necessary. Our analyses reconcile conflicting results from previous models and suggest a general framework for analyzing parental behavior within populations and among higher taxonomic groups. | 4101 | NA | NA | NA | no | no |
| 4100 | 2017 | NA | Richards, MH; Onuferko, TM; Rehan, SM | 2015 | Phenological, but not social, variation associated with climate differences in a eusocial sweat bee, Halictus ligatus, nesting in southern Ontario | Studies of annual and geographic variation in eusocial bee populations suggest that more stringent environmental conditions result in stronger reproductive skew favouring queens, while moderate conditions favour increasing worker reproduction. To test these predictions, we compared the phenology and colony development of H. ligatus nesting in St. Catharines, Ontario, Canada to a previously studied aggregation 90 km north of St. Catharines, in Victoria, Ontario. Despite the close proximity of these two locations, St. Catharines has markedly shorter winters and longer summers. Comparisons between St. Catharines in 2006 and Victoria in the 1980s and 1990s incorporate both geographic differences in climate and temporal differences due to climate change. We predicted that St. Catharines foundress queens should emerge from hibernation and initiate nests earlier in spring, giving them time to produce more workers. Since earlier studies indicated that queens have difficulty suppressing worker reproduction in larger colonies, we also predicted higher rates of worker ovarian development in St. Catharines. In spring and summer 2006, we excavated 65 H. ligatus nests, comparing their contents to 713 specimens collected in pan traps. As predicted, nests were initiated about a month earlier in St. Catharines than in Victoria, but contrary to prediction, fewer workers were produced in St. Catharines. St. Catharines workers were just as likely to have developed ovaries as Victoria workers. About 40% of St. Catharines workers were classified as reproductive, and larger reproductive workers tended to have higher ovarian scores. Early queen mortality in the longer nest cycle of St. Catharines bees may have enhanced opportunities for worker reproduction despite their smaller numbers. Novel features of H. ligatus sociobiology in St. Catharines included evidence that queens can initiate new nests following the loss of their first brood, overlap between worker and gyne production within some nests, and high rates of independent nest founding by worker-sized females, suggesting that many worker-brood females overwinter. Overall, the distinctly warmer climate of St. Catharines compared to Victoria led to earlier nest initiation and lengthening of the flight season, but not to the predicted differences in colony social organisation or queen-worker reproductive skew. A second objective of our study was to assess how well pan trap collections capture important information about demographic and social parameters important in assessing social variability in sweat bees. Nest excavations and pan traps produced similar results, suggesting that pan traps are a good alternative when nest excavations are impossible. | 4102 | NA | NA | NA | no | no |
| 4101 | 2017 | NA | Berry, SD; Fondong, VN; Rey, C; Rogan, D; Fauquet, CM; Brown, JK | 2004 | Molecular evidence for five distinct Bemisia tabaci (Homoptera : Aleyrodidae) geographic haplotypes associated with cassava plants in sub-Saharan Africa | The Bemisia tabaci (Gennadius) complex contains the only known whitefly vector of plant-infecting begomoviruses, which are the causal agents of mosaic diseases of cassava in Africa and India. Widespread phenotypic variability, together with the absence of definitive morphological taxonomic characters for this whitefly complex, has confounded both the systematics and the study of its virus vector biology. Substantial genetic variability and phylogeographical relationships have been shown for phenotypic, but morphologically identical, variants of B. tabaci based on the mitochondrial (mt) cytochrome oxidase I (COI) sequence, leading to the suggestion that they represent a species complex. Here, phylogenetic relationships were explored, using the mtCOI sequence (780 bp) as a molecular marker, for B. tabaci collected from cassava plants in southern and western Africa, including Cameroon, Zambia, Mozambique, Zimbabwe, Swaziland, and South Africa. Maximum likelihood analyses of mtCOI sequences revealed that most B. tabaci examined were placed into one of three subgroups within the major sub-Saharan African clade, which also contains previously reported populations indigenous to Malawi and Uganda, and collectively shared on overall nucleotide (nt) identity at 88.9 -99.7%. Two other reference populations, the monophagous Benin haplotype from Asystasia spp. and a B. tabaci from cassava in the Ivory Coast (IC), were the most divergent outliers of the sub-Saharan clade, each representing the only member of their respective clade (I and V), at the present time. Members of the sub-Saharan clade associated with cassava had as their closest relatives haplotypes I and II of the Mediterranean-Northern Africa clade, with which they shared a collective 84.2-92.9% nt identity (not including the IC cassava reference haplotype). In contrast, the sub-Saharan African clade diverged from the Americas and Southeast Asia/Far East clades at 79.7-85.1 and 77.5-84.9%, respectively. Within the sub-Saharan clade, subclade II contained B. tabaci from Zambia, Mozambique, South Africa, and Swaziland at 95-99% identity. The sub-Saharan subcluster III contained haplotypes from southern and western Africa. Counter to the otherwise phylogeographical relationships observed for cassava-associated B. tabaci from southern Africa, one and two populations from Cameroon (okra) and Zimbabwe (cassava), respectively, grouped with the major Mediterranean-North Africa clade, together with their closest relative associated with okra from IC, are included here as a reference sequence for the first time, with which they collectively formed a new, third subclade. Thus, phylogenetic analysis of B. tabaci mtCOI haplotypes examined thus far from the African continent has revealed five major cassava-associated haplotypes, which grouped primarily based on extant geography, with the exception of one and two collections from Cameroon and Zimbabwe, respectively. Hypotheses explaining the potential distributions of haplotypes are discussed. | 4103 | NA | NA | NA | no | no |
| 4102 | 2017 | NA | Levitt, B; Obala, A; Langdon, S; Corcoran, D; O’Meara, WP; Taylor, SM | 2017 | Overlap Extension Barcoding for the Next Generation Sequencing and Genotyping of Plasmodium falciparum in Individual Patients in Western Kenya | Large-scale molecular epidemiologic studies of Plasmodium falciparum parasites have provided insights into parasite biology and transmission, can identify the spread of drug resistance, and are useful in assessing vaccine targets. The polyclonal nature infections in high transmission settings is problematic for traditional genotyping approaches. Next-generation sequencing (NGS) approaches to parasite genotyping allow sensitive detection of minority variants, disaggregation of complex parasite mixtures, and scalable processing of large samples sets. Therefore, we designed, validated, and applied to field parasites an approach that leverages sequencing of individually barcoded samples in a multiplex manner. We utilize variant barcodes, invariant linker sequences and modular template-specific primers to allow for the simultaneous generation of high-dimensional sequencing data of multiple gene targets. This modularity permits a cost-effective and reproducible way to query many genes at once. In mixtures of reference parasite genomes, we quantitatively detected unique haplotypes comprising as little as 2% of a polyclonal infection. We applied this genotyping approach to field-collected parasites collected in Western Kenya in order to simultaneously obtain parasites genotypes at three unlinked loci. In summary, we present a rapid, scalable, and flexible method for genotyping individual parasites that enables molecular epidemiologic studies of parasite evolution, population structure and transmission. | 4104 | NA | NA | NA | no | no |
| 4103 | 2017 | NA | Xu, DS; Sandland, GJ; Minchella, DJ; Feng, ZL | 2012 | Interactions among virulence, coinfection and drug resistance in a complex life-cycle parasite | Motivated by relatively recent empirical studies on Schistosoma mansoni, we use a mathematical model to investigate the impacts of drug treatment of the definitive human host and coinfection of the intermediate snail host by multiple parasite strains on the evolution of parasites’ drug resistance. Through the examination of evolutionarily stable strategies (ESS) of parasites, our study suggests that higher levels of drug treatment rates (which usually tend to promote monomorphism as the evolutionary equilibrium) favor parasite strains that have a higher level of drug resistance. Our study also shows that whether coinfection of intermediate hosts affects the levels of drug resistance at ESS points and their stability depends on the assumptions on the cost of parasites paid for drug resistance, coinfection functions and parasites’ reproduction within coinfected hosts. This calls for more empirical studies on the parasite. (c) 2012 Elsevier Ltd. All rights reserved. | 4105 | NA | NA | NA | no | no |
| 4104 | 2017 | NA | Tantasawat, PA; Poolsawat, O; Prajongjai, T; Chaowiset, W; Tharapreuksapong, A | 2012 | Association of RGA-SSCP markers with resistance to downy mildew and anthracnose in grapevines | Downy mildew (Plasmopara viticola) and anthracnose (Sphaceloma ampelinum) are two major diseases that severely affect most grapevine (Vitis vinifera) cultivars grown commercially in Thailand. Progress of conventional breeding programs of grapevine for improved resistance to these diseases can be speeded up by selection of molecular markers associated with resistance traits. We evaluated the association between 13 resistance gene analog (RGA)-single-strand conformation polymorphism (SSCP) markers with resistance to downy mildew and anthracnose in 71 segregating progenies of seven cross combinations between susceptible cultivars and resistant lines. F-1 hybrids from each cross were assessed for resistance to downy mildew and anthracnose (isolates Nk4-1 and Rc2-1) under laboratory conditions. Association of resistance traits with RGA-SSCP markers was evaluated using simple linear regression analysis. Three RGA-SSCP markers were found to be significantly correlated with anthracnose resistance, whereas significant correlation with downy mildew resistance was observed for only one RGA-SSCP marker. These results demonstrate the usefulness of RGA-SSCP markers. Four candidate markers with significant associations to resistance to these two major diseases of grapevine were identified. However, these putative associations between markers and resistance need to be verified with larger segregating populations before they can be used for marker-assisted selection. | 4106 | NA | NA | NA | no | no |
| 4105 | 2017 | NA | Louhi, KR; Karvonen, A; Rellstab, C; Louhi, R; Jokela, J | 2013 | Prevalence of infection as a predictor of multiple genotype infection frequency in parasites with multiple-host life cycle | In nature, parasites commonly share hosts with other conspecific parasite genotypes. While adult parasites typically show aggregated distribution in their final hosts, aggregation of clonal parasite genotypes in intermediate hosts, such as those of trematodes in molluscs, is not generally known. However, infection of a host by multiple parasite genotypes has significant implications for evolution of virulence and hostparasite coevolution. Aggregated distribution of the clonal stages can increase host mortality and reduce larval output of each infecting genotype through interclonal competition, and therefore have significant implications for parasite epidemiology. The aim of this study was (i) to find out how common multiple genotype infections (MGIs) are in aquatic snails serving as intermediate hosts for different trematode species; (ii) to find out whether the prevalence of infection could be used to predict MGI frequencies and (iii) to use the relationship to infer whether MGIs aggregate in molluscan hosts. We determined the prevalence of trematode (Diplostomum pseudospathaceum) infections and the frequency of MGIs in snail (Lymnaea stagnalis) host populations as well as compiled corresponding literature data from a range of snail-trematode systems. We used Bayesian simulations to explore the relationship between prevalence of infection and MGI frequency in these data, and tested whether genotypes aggregate in snails by comparing the simulated relations with null model (Poisson and demographic Poisson) expectations. Our results show that MGIs are common in aquatic snails with up to 90% of the infected snails carrying MGIs. Parasite prevalence is a good predictor of MGI frequencies at a range of prevailing natural prevalences of infection (050%). The frequency of MGIs was higher than expected by both null models, indicating parasite aggregation at genotype level. These findings are in sharp contrast with the absence of multiple infections in snails at level of trematode species, suggesting that co-infections by multiple species and multiple genotypes of one species are controlled by different biological processes. Aggregation of MGIs in snail hosts appears to be widespread across different snail-trematode systems. | 4107 | NA | NA | NA | no | no |
| 4106 | 2017 | NA | Graystock, P; Hughes, WOH | 2011 | Disease resistance in a weaver ant, Polyrhachis dives, and the role of antibiotic-producing glands | Parasites represent one of the main threats to all organisms and are likely to be particularly significant for social animals because of the increased potential for intragroup transmission. Social animals must therefore have effective resistance mechanisms against parasites and one of the most important components of disease resistance in ants is thought to be the antibiotic-producing metapleural gland. This gland is ancestral in ants, but has been lost secondarily in a small number of species. It is unknown whether these evolutionary losses are due to a reduction in parasite pressure or the replacement of the gland’s function with other resistance mechanisms. Here we used the generalist entomopathogenic fungus Metarhizium to compare the disease resistance of a species of a weaver ant, Polyrhachis dives, which has lost the metapleural gland, with that of the well-studied leaf-cutting ant Acromyrmex echinatior and two other ant species, Myrmica ruginodis and Formica fusca, all of which have metapleural glands. The P. dives weaver ants had intermediate resistance when kept individually, and similar resistance to A. echinatior leaf-cutting ants when kept in groups, suggesting that the loss of the metapleural gland has not resulted in weaver ants having reduced disease resistance. P. dives weaver ants self-groomed at a significantly higher rate than the other ants examined and apparently use their venom for resistance, as they had reduced resistance when their venom gland was blocked and the venom was shown in vitro to prevent the germination of fungal spores. Unexpectedly, the leaf-cutting ant A. echinatior also had reduced resistance to Metarhizium when its venom gland was blocked. It therefore appears that the evolutionary loss of the metapleural gland does not result in reduced disease resistance in P. dives weaver ants, and that this at least in part may be due to the ants having antimicrobial venom and high self-grooming rates. The results therefore emphasise the importance of multiple, complementary mechanisms in the disease resistance of ant societies. | 4108 | NA | NA | NA | no | no |
| 4107 | 2017 | NA | Spurgin, LG; Illera, JC; Padilla, DP; Richardson, DS | 2012 | Biogeographical patterns and co-occurrence of pathogenic infection across island populations of Berthelot’s pipit (Anthus berthelotii) | Pathogens can exert strong selective forces upon host populations. However, before we can make any predictions about the consequences of pathogen-mediated selection, we first need to determine whether patterns of pathogen distribution are consistent over spatiotemporal scales. We used molecular techniques to screen for a variety of blood pathogens (avian malaria, pox and trypanosomes) over a three-year time period across 13 island populations of the Berthelot’s pipit (Anthus berthelotii). This species has only recently dispersed across its range in the North Atlantic, with little subsequent migration, providing an ideal opportunity to examine the causes and effects of pathogenic infection in populations in the early stages of differentiation. We screened 832 individuals, and identified two strains of Plasmodium, four strains of Leucocytozoon, and one pox strain. We found strong differences in pathogen prevalence across populations, ranging from 0 to 65%, and while some fluctuations in prevalence occurred, these differences were largely stable over the time period studied. Smaller, more isolated islands harboured fewer pathogen strains than larger, less isolated islands, indicating that at the population level, colonization and extinction play an important role in determining pathogen distribution. Individual-level analyses confirmed the island effect, and also revealed a positive association between Plasmodium and pox infection, which could have arisen due to dual transmission of the pathogens by the same vectors, or because one pathogen lowers resistance to the other. Our findings, combined with an effect of infection on host body condition, suggest that Berthelot’s pipits are subject to different levels of pathogen-mediated selection both across and within populations, and that these selective pressures are consistent over time. | 4109 | NA | NA | NA | no | no |
| 4108 | 2017 | NA | Ong, BKC; Paller, VGV; de Guia, APO; Balatibat, JB; Gonzalez, JCT | 2015 | Prevalence of avian haemosporidians among understorey birds of Mt. Banahaw de Lucban, Philippines. | Understanding vector-borne diseases such as avian hemosporidiosis provides clues regarding environmental changes that can be crucial for conservation efforts. Haemosporidian prevalence in understorey birds from Mt. Banahaw de Lucban was studied to survey parasites occurring in the area, and test the difference between prevalence in high and low altitudes. Ninety-five birds were captured from May-June 2013 at 745-800 m, and 1500-1600 m, and were screened for haemosporidians via peripheral blood smear microscopy. Leucocytozoon, Haemoproteus, and Plasmodium were found in 28.4% of the birds sampled, with Haemoproteus most frequently occurring. Half of the 24 bird species screened showed positive infections, and six species were identified as new Philippine host records of avian haemosporidians. Prevalence of infection did not significantly differ between altitudes and could be attributed to factors that warrant further investigation. Relatively high prevalence of predominantly chronic infections suggests birds are currently able to keep infection intensities in check but this cannot be guaranteed once they are immunocompromised, leading to potential disease outbreaks. Long term monitoring of haemosporidian prevalence and strict security against anthropogenic disturbance in Mt. Banahaw de Lucban may help reduce the risk of epizootic outbreaks that threaten Philippine endemic avifauna. | 4110 | NA | NA | NA | no | no |
| 4109 | 2017 | NA | Huijben, S; Nelson, WA; Wargo, AR; Sim, DG; Drew, DR; Read, AF | 2010 | CHEMOTHERAPY, WITHIN-HOST ECOLOGY AND THE FITNESS OF DRUG-RESISTANT MALARIA PARASITES | A major determinant of the rate at which drug-resistant malaria parasites spread through a population is the ecology of resistant and sensitive parasites sharing the same host. Drug treatment can significantly alter this ecology by removing the drug-sensitive parasites, leading to competitive release of resistant parasites. Here, we test the hypothesis that the spread of resistance can be slowed by reducing drug treatment and hence restricting competitive release. Using the rodent malaria model Plasmodium chabaudi, we found that low-dose chemotherapy did reduce competitive release. A higher drug dose regimen exerted stronger positive selection on resistant parasites for no detectable clinical gain. We estimated instantaneous selection coefficients throughout the course of replicate infections to analyze the temporal pattern of the strength and direction of within-host selection. The strength of selection on resistance varied through the course of infections, even in untreated infections, but increased immediately following drug treatment, particularly in the high-dose groups. Resistance remained under positive selection for much longer than expected from the half life of the drug. Although there are many differences between mice and people, our data do raise the question whether the aggressive treatment regimens aimed at complete parasite clearance are the best resistance-management strategies for humans. | 4111 | NA | NA | NA | no | no |
| 4110 | 2017 | NA | Gardner, A; West, SA; Buckling, A | 2004 | Bacteriocins, spite and virulence | There has been much interest in using social evolution theory to predict the damage to a host from parasite infection, termed parasite virulence. Most of this work has focused on how high kinship between the parasites infecting a host can select for more prudent exploitation of the host, leading to a negative relationship between virulence and parasite kinship. However, it has also been shown that if parasites can cooperate to overcome the host, then high parasite kinship within hosts can select for greater cooperation and higher growth rates, hence leading to a positive relationship between virulence and parasite kinship. We examine the impact of a spiteful behaviour, chemical (bacteriocin) warfare between microbes, on the evolution of virulence, and find a new relationship: virulence is maximized when the frequency of kin among parasites’ social partners is low or high, and is minimized at intermediate values. This emphasizes how biological details can fundamentally alter the qualitative nature of theoretical predictions made by models of parasite virulence. | 4112 | NA | NA | NA | no | no |
| 4111 | 2017 | NA | Morris, AB; Small, RL; Cruzan, MB | 2002 | Investigating the relationship between Cryptococcus fagisuga and Fagus grandifolia in Great Smoky Mountains National Park | The high elevation beech gaps of the Great Smoky Mountains have become the killing front of beech bark disease. This insect/fungal pathogen was introduced into Nova Scotia in the late 1800’s, and has since spread southward to the Southern Appalachians. In affected stands, mortality of beech stems frequently approaches 90 to 100 percent. We used inter-simple sequence repeats (ISSR) markers to assess the relationship between host genotype and degree of pathogen infection in beech trees in Great Smoky Mountains National Park. We used statistical analyses to test the relationship between stem diameter and degree of pathogen infection. We found no correlation between host genotype and degree of infection. We did find a significant positive relationship between stem size and degree of infection. Among three stem size classes, smallest steins (< 1.5 cm) were least likely to be infected, while largest stems (> 3.0 cm) were most likely to be infected. Implications for future studies are discussed. | 4113 | NA | NA | NA | no | no |
| 4112 | 2017 | NA | Shakhar, K; Shakhar, G | 2015 | Why Do We Feel Sick When Infected-Can Altruism Play a Role? | When we contract an infection, we typically feel sick and behave accordingly. Symptoms of sickness behavior (SB) include anorexia, hypersomnia, depression, and reduced social interactions. SB affects species spanning from arthropods to vertebrates, is triggered non-specifically by viruses, bacteria, and parasites, and is orchestrated by a complex network of cytokines and neuroendocrine pathways; clearly, it has been naturally selected. Nonetheless, SB seems evolutionarily costly: it promotes starvation and predation and reduces reproductive opportunities. How could SB persist? Former explanations focused on individual fitness, invoking improved resistance to pathogens. Could prevention of disease transmission, propagating in populations through kin selection, also contribute to SB? | 4114 | NA | NA | NA | no | no |
| 4113 | 2017 | NA | Allen, C; Valles, SM; Strong, CA | 2011 | Multiple virus infections occur in individual polygyne and monogyne Solenopsis invicta ants | Concurrent infections of Solenopsis invicta colonies with S. invicta virus 1 (SINV-1). SINV-2, and SINV-3 has been reported. However, whether individual ants were capable of supporting multiple virus infections simultaneously was not known, nor whether the social form of the colony (polygyne or monogyne) had an influence on the occurrence of multiple infection rates in individual ants. S. invicta field populations were sampled sequentially to establish whether multiple virus infections co-occurred in individual worker ants. In addition, the intra-colony virus infection rates were compared in monogyne and polygyne field colonies to determine whether social form played a role in the viral infection prevalence. All combinations of virus infection (SINV-1, SINV-2, or SINV-3 alone, SINV-1 & SINV-2, SINV-1 & SINV-3, SINV-2 & SINV-3, and SINV-1, SINV-2 & SINV-3) were detected in individual worker ants as well as queens in the field. Thus, individual S. invicta ants can be infected simultaneously with all combinations of the S. invicta viruses. Colony social form did have an influence on the intra-colony prevalence of multiple S. invicta virus infections. Polygyne colonies exhibited significantly greater intra- and inter-colony single and multiple virus infections compared with monogyne colonies. Published by Elsevier Inc. | 4115 | NA | NA | NA | no | no |
| 4114 | 2017 | NA | Davis, RA; Roberts, JD | 2005 | Embryonic survival and egg numbers in small and large populations of the frog Heleioporus albopunctatus in Western Australia | A critical component of amphibian life history relates to understanding the role of embryonic mortality in regulating recruitment and, therefore, population size. We examined the predictions that small populations have higher embryonic mortality and reduced clutch sizes in the Western Australian frog, Heleioporus albopunctatus. Embryonic mortality was low, averaging 3% per clutch. Four of 55 clutches examined (similar to 7%) were infested by dipteran larvae. We found no significant relationship between clutch size or embryonic mortality and population size. | 4116 | NA | NA | NA | no | no |
| 4115 | 2017 | NA | Earl, JE; Chaney, JC; Sutton, WB; Lillard, CE; Kouba, AJ; Langhorne, C; Krebs, J; Wilkes, RP; Hill, RD; Miller, DL; Gray, MJ | 2016 | Ranavirus could facilitate local extinction of rare amphibian species | There is growing evidence that pathogens play a role in population declines and species extinctions. For small populations, disease-induced extinction may be especially probable. We estimated the susceptibility of two amphibian species of conservation concern (the dusky gopher frog [Lithobates sevosus] and boreal toad [Anaxyrus boreas boreas]) to an emerging pathogen (ranavirus) using laboratory challenge experiments, and combined these data with published demographic parameter estimates to simulate the potential effects of ranavirus exposure on extinction risk. We included effects of life stage during pathogen exposure, pathogen exposure interval, hydroperiod of breeding habitat, population carrying capacity, and immigration in simulations. We found that both species were highly susceptible to ranavirus when exposed to the pathogen in water at environmentally relevant concentrations. Dusky gopher frogs experienced 100 % mortality in four of six life stages tested. Boreal toads experienced 100 % mortality when exposed as tadpoles or metamorphs, which were the only life stages tested. Simulations showed population declines, greater extinction probability, and faster times to extinction with ranavirus exposure. These effects were more evident with more frequent pathogen exposure intervals and lower carrying capacity. Immigration at natural rates did little to mitigate effects of ranavirus exposure unless immigration occurred every 2 years. Our results demonstrate that disease-induced extinction by emerging pathogens, such as ranavirus, is possible, and that threat may be especially high for species with small population sizes. For the species in this study, conservation organizations should incorporate ranavirus surveillance into monitoring programs and devise intervention strategies in the event that disease outbreaks occur. | 4117 | NA | NA | NA | no | no |
| 4116 | 2017 | NA | Bauer, S; Bohm, M; Witte, V; Foitzik, S | 2010 | An ant social parasite in-between two chemical disparate host species | Host-parasite coevolution shapes the structure of communities and simultaneously the traits of the interacting species. Social parasites developed sophisticated chemical integration strategies to circumvent host defences. Here, we show that the two Leptothorax host species of the obligate social parasite Harpagoxenus sublaevis exhibit extremely divergent chemical profiles, making it nearly impossible for this parasite to closely adapt to both hosts at once. Our cuticular hydrocarbon analyses demonstrate that H. sublaevis acquires some host chemicals passively, but additionally, actively biosyntheses some host hydrocarbons. The parasite adjusts thereby more closely to its smaller host, L. muscorum, because it actively produces two of its cuticular substances and also more easily acquires the short-chained hydrocarbons of this host. Community composition analyses indicate that the social parasite overexploits this chemical closer host species and, albeit costly for the parasite, frequently enslaves workers of the second host concurrently. | 4118 | NA | NA | NA | no | no |
| 4117 | 2017 | NA | Czembor, PC; Arseniuk, E; Czaplicki, A; Song, QJ; Cregan, PB; Ueng, PP | 2003 | QTL mapping of partial resistance in winter wheat to Stagonospora nodorum blotch | Stagonospora nodorum blotch is an important foliar and glume disease in cereals. Inheritance of resistance in wheat appears to be quantitative. To date, breeding of partially resistant cultivars has been the only effective way to combat this pathogen. The partial resistance components, namely length of incubation period, disease severity, and length of latent period, were evaluated on a population of doubled haploids derived from a cross between the partially resistant Triticum aestivum ‘Liwilla’ and susceptible Triticum aestivum ‘Begra’. Experiments were conducted in a controlled environment and the fifth leaf was examined. Molecular analyses were based on bulked segregant analyses using 240 microsatellite markers. Four QTLs were significantly associated with partial resistance components and were located on chromosomes 2B, 3B, 5B, and 5D. The percentage of phenotypic variance explained by a single QTL ranged from 14 to 21% for incubation period, from 16 to 37% for disease severity, and from 13 to 28% for latent period. | 4119 | NA | NA | NA | no | no |
| 4118 | 2017 | NA | James, TY; Toledo, LF; Rodder, D; Leite, DD; Belasen, AM; Betancourt-Roman, CM; Jenkinson, TS; Soto-Azat, C; Lambertini, C; Longo, AV; Ruggeri, J; Collins, JP; Burrowes, PA; Lips, KR; Zamudio, KR; Longcore, JE | 2015 | Disentangling host, pathogen, and environmental determinants of a recently emerged wildlife disease: lessons from the first 15years of amphibian chytridiomycosis research | The amphibian fungal disease chytridiomycosis, which affects species across all continents, recently emerged as one of the greatest threats to biodiversity. Yet, many aspects of the basic biology and epidemiology of the pathogen, Batrachochytrium dendrobatidis (Bd), are still unknown, such as when and from where did Bd emerge and what is its true ecological niche? Here, we review the ecology and evolution of Bd in the Americas and highlight controversies that make this disease so enigmatic. We explore factors associated with variance in severity of epizootics focusing on the disease triangle of host susceptibility, pathogen virulence, and environment. Reevaluating the causes of the panzootic is timely given the wealth of data on Bd prevalence across hosts and communities and the recent discoveries suggesting co-evolutionary potential of hosts and Bd. We generate a new species distribution model for Bd in the Americas based on over 30,000 records and suggest a novel future research agenda. Instead of focusing on pathogen hot spots, we need to identify pathogen cold spots so that we can better understand what limits the pathogen’s distribution. Finally, we introduce the concept of the Ghost of Epizootics Past to discuss expected patterns in postepizootic host communities. | 4120 | NA | NA | NA | no | no |
| 4119 | 2017 | NA | Voegeli, B; Saladin, V; Wegmann, M; Richner, H | 2012 | Parasites as mediators of heterozygosity-fitness correlations in the Great Tit (Parus major) | Positive correlations between heterozygosity and fitness traits are frequently observed, and it has been hypothesized, but rarely tested experimentally, that parasites play a key role in mediating the heterozygosityfitness association. We evaluated this hypothesis in a wild great tit (Parus major) population by testing the prediction that the heterozygosityfitness association would appear in broods experimentally infested with a common ectoparasite, but not in parasite-free broods. We simultaneously assessed the effects of parental and offspring heterozygosity on nestling growth and found that body mass of nestlings close to independence, which is a strong predictor of post-fledging survival, increased significantly with nestling levels of heterozygosity in experimentally infested nests, but not in parasite-free nests. Heterozygosity level of the fathers also showed a significant positive correlation with offspring body mass under an experimental parasite load, whereas there was no correlation with the mothers level of heterozygosity. Thus, our results indicate a key role for parasites as mediators of the heterozygosityfitness correlations. | 4121 | NA | NA | NA | no | no |
| 4120 | 2017 | NA | Chernenko, A; Vidal-Garcia, M; Helantera, H; Sundstrom, L | 2013 | Colony take-over and brood survival in temporary social parasites of the ant genus Formica | Parasites reduce host fitness, and so instigate counter adaptations by their hosts. In temporary social parasitism, usurpers must not only enter the colony unharmed, but also have their eggs reared by the host workers. We introduced parasitic Formica lugubris and Formica aquilonia queens into queen right and orphaned fragments of three host species, Formica cinerea, Formica picea and Formica fusca, and show that workers of all three host species kill over 40 % of the introduced queens within 10 days, regardless of the presence/absence of a resident queen, and parasite species. More parasite queens died in F. cinerea than in F. picea and F. fusca. There were no major differences in survival between the parasite species (except that F. lugubris survived longer than F. aquilonia in F. fusca colonies compared to F. picea colonies), but parasite queens survived longer in orphaned than in queen right fragments of F. fusca. Experimental introduction of parasite (F. aquilonia) eggs into orphaned colonies of F. fusca showed that none of the parasite eggs were reared until pupation; whereas on average, 12 % of the con-specific hetero-colonial eggs introduced in the same manner were reared until pupation. In all colonies that received parasite brood, all offspring consisted of worker-laid males, whereas the corresponding value was 50 % for colonies that received con-specific hetero-colonial brood. Thus, when the risks of entering host colonies and brood failure are combined, the rate of successful colony take-over is very low. Moreover, the host workers can to some extent alleviate the costs of parasitism by producing a final batch of own offspring. | 4122 | NA | NA | NA | no | no |
| 4121 | 2017 | NA | Fatah, T; Rafii, MY; Rahim, HA; Meon, S; Azhar, M; Latif, MA | 2014 | Cloning and Analysis of QTL Linked to Blast Disease Resistance in Malaysian Rice Variety Pongsu Seribu 2 | Blast, caused by Magnoporthe oryzae, is considered to be a global rice disease around the world including Malaysia. Limited information is available on the identification of quantitative trait loci (QTL) and linked markers associated with blast resistance within local rice varieties e. g. Pongsu Seribu 2. Partial resistance against blast disease is widely observed in this variety with mapping of QTLs linked to resistance genes. However, knowledge about the blast resistance genes on chromosome 3 is very limited and no specific blast resistance gene have been identified on chromosome 3 in rice genome of Pongsu Seribu 2. In order to find the similarity of sequence, QTL qRBr-3.1 located on chromosome 3 in Pongsu Seribu 2 was cloned and compared with identified blast resistant genes from other varieties. Sequence analysis of cloned fragment revealed a tandem of (CA) 23 repeats. The similarity of sequence was searched in Basic Local Alignment Search Tool (BLAST), which expressed similarity with different clones of rice located on chromosome 3. The cloned QTL fragment also expressed the similarity of 46% with Pi-b, 52% with Pi-kh, 23% with Pi-9 and 38% with Pi-zt, blast resistance genes located at different chromosomes in rice. The QTL fragment produced none of distribution of leucine rich repeats (LRRs) and nucleotide binding site (NBS-LRR). However, the domain contains maximum distribution of leucine amino acid which is responsible for the pathogen recognition in host-plant interaction and play important role in resistance mechanism against diseases. This result concluded that Pongsu Seribu 2 has homology to other resistant genes which are allelic to Pi-b, Pi9, Pi-zt and Pi-kh at different chromosomes. (C) 2014 Friends Science Publishers | 4123 | NA | NA | NA | no | no |
| 4122 | 2017 | NA | Buckee, CO; Jolley, KA; Recker, M; Penman, B; Kriz, P; Gupta, S; Maiden, MCJ | 2008 | Role of selection in the emergence of lineages and the evolution of virulence in Neisseria meningitidis | Neisseria meningitis is a human commensal bacterium that occasionally causes life-threatening disease. As with a number of other bacterial pathogens, meningococcal populations comprise distinct lineages, which persist over many decades and during global spread in the face of high rates of recombination. In addition, the propensity to cause invasive disease is associated with particular "hyperinvasive’’ lineages that coexist with less invasive lineages despite the fact that disease does not contribute to host-to-host transmission. Here, by combining a modeling approach with molecular epidemiological data from 1,108 meningococci isolated in the Czech Republic over 27 years, we show that interstrain competition, mediated by immune selection, can explain both the persistence of multiple discrete meningococcal lineages and the association of a subset of these with invasive disease. The model indicates that the combinations of allelic variants of housekeeping genes that define these lineages are associated with very small differences in transmission efficiency among hosts. These findings have general implications for the emergence of lineage structure and virulence in recombining bacterial populations. | 4124 | NA | NA | NA | no | no |
| 4123 | 2017 | NA | Hirsch, BT; Prange, S; Hauver, SA; Gehrt, SD | 2013 | Raccoon Social Networks and the Potential for Disease Transmission | Raccoons are an important vector of rabies and other pathogens. The degree to which these pathogens can spread through a raccoon population should be closely linked to association rates between individual raccoons. Most studies of raccoon sociality have found patterns consistent with low levels of social connectivity within populations, thus the likelihood of direct pathogen transmission between raccoons is theoretically low. We used proximity detecting collars and social network metrics to calculate the degree of social connectivity in an urban raccoon population for purposes of estimating potential pathogen spread. In contrast to previous assumptions, raccoon social association networks were highly connected, and all individuals were connected to one large social network during 15 out of 18 months of study. However, these metrics may overestimate the potential for a pathogen to spread through a population, as many of the social connections were based on relatively short contact periods. To more closely reflect varying probabilities of pathogen spread, we censored the raccoon social networks based on the total amount of time spent in close proximity between two individuals per month. As this time criteria for censoring the social networks increased from one to thirty minutes, corresponding measures of network connectivity declined. These findings demonstrate that raccoon populations are much more tightly connected than would have been predicted based on previous studies, but also point out that additional research is needed to calculate more precise transmission probabilities by infected individuals, and determine how disease infection changes normal social behaviors. | 4125 | NA | NA | NA | no | no |
| 4124 | 2017 | NA | Cooke, DEL | 2007 | Tracking the sudden oak death pathogen | Invasive species are, by definition, unwelcome and pathogenic ones, especially so. Tracing the origins and spread of Phytophthora ramorum, the devastating ‘Sudden Oak Death’ pathogen, in the forests and nurseries of Oregon has revealed differences between forest and nursery pathogen populations that suggest discrete sources of primary inoculum initiate each type of outbreak. New information on the ecology and evolution of this pathogen is presented that helps gauge the effectiveness of quarantine and eradication programmes. | 4126 | NA | NA | NA | no | no |
| 4125 | 2017 | NA | Sieber, M; Gudelj, I | 2014 | Do-or-die life cycles and diverse post-infection resistance mechanisms limit the evolution of parasite host ranges | In light of the dynamic nature of parasite host ranges and documented potential for rapid host shifts, the observed high host specificity of most parasites remains an ecological paradox. Different variants of host-use trade-offs have become a mainstay of theoretical explanations of the prevalence of host specialism, but empirical evidence for such trade-offs is rare. We propose an alternative theory based on basic features of the parasite life cycle: host selection and subsequent intrahost replication. We introduce a new concept of effective burst size that accounts for the fact that successful host selection does not guarantee intrahost replication. Our theory makes a general prediction that a parasite will expand its host range if its effective burst size is positive. An in silico model of bacteria-phage coevolution verifies our predictions and demonstrates that the tendency for relatively narrow host ranges in parasites can be explained even in the absence of trade-offs. | 4127 | NA | NA | NA | no | no |
| 4126 | 2017 | NA | Hughes, WOH; Pagliarini, R; Madsen, HB; Dijkstra, MB; Boomsma, JJ | 2008 | Antimicrobial defense shows an abrupt evolutionary transition in the fungus-growing ants | Understanding the relative evolutionary importance of parasites to different host taxa is problematic because the expression of disease and resistance are often confounded by factors such as host age and condition. The antibiotic-producing metapleural glands of ants are a potentially useful exception to this rule because they are a key first-line defense that are fixed in size in adults. Here we conduct a comparative analysis of the size of the gland reservoir across the fungus-growing ants (tribe Attini). Most attines have singly mated queens, but in two derived genera, the leaf-cutting ants, the queens are multiply mated, which is hypothesized to have evolved to improve colony-level disease resistance. We found that, relative to body size, the gland reservoirs of most attines are similar in size but that those of the leaf-cutting ants are significantly larger. In contrast, the size of the reservoir did not relate with the evolutionary transition from lower to higher attines and correlated at most only slightly with colony size. The results thus suggest that the relationship between leaf-cutting ants and their parasites is distinctly different from that for other attine ants, in accord with the hypothesis that multiple mating by queens evolved to improve colony-level disease resistance. | 4128 | NA | NA | NA | no | no |
| 4127 | 2017 | NA | Srobarova, A; Slikova, S; Sudyova, V | 2008 | Diversity of the Fusarium species associated with head and seedling blight on wheat in Slovakia | Species associated with Fusarium head blight are depending on the production and edaphic conditions. The differences are found in the representation of various Fusarium spp. in the diseases, which sporadically occur all over the territory of Slovakia, in all agricultural production types. We identified fifteen Fusarium species during ten years of investigation. Most of the mentioned species F. culmorum (W.G. Smith) Sacc., F. graminearum Schwabe, recently F. cerealis (Cooke) Sacc. (crookwellense Burgess, Nelson & Tousson) and F. sambucinum Fuckel in diseased caryopsis are seed transmitted. The significant differences among species and intra species in cultural and pathogenicity assays in vitro and in vivo were correlated. Some of them are able to produce toxic metabolites - deoxynivalenone, which probably play a role in the aggressiveness of the pathogen and promote disease development and pathogen colonization. | 4129 | NA | NA | NA | no | no |
| 4128 | 2017 | NA | Bernasconi, G; Keller, L | 1999 | Effect of queen phenotype and social environment on early queen mortality in incipient colonies of the fire ant, Solenopsis invicta | In many ant species, including the fire ant, Solenopsis invicta, queens can found their colonies alone or in associations of two or more. Colonies founded by associations produce a larger worker brood,have higher survival and mature earlier than colonies founded by solitary queens. However, cofoundresses almost invariably fight after the eclosion of the first workers. As a result, only one queen survives and monopolizes the colony’s future reproductive output. Queen mortality also occurs before worker eclosion, but neither the causes (e.g. starvation, conflict), nor the factors (e.g. social environment) potentially affecting its occurrence, have been investigated. We analysed the effect of social environment and queen body mass on early mortality by keeping queens (1) solitarily, (2) within associations of four queens of the same initial mass, and (3) within associations of four queens of random initial mass. Mortality was higher for queens within associations than for solitary queens. Within associations of equally heavy queens, mortality significantly increased with the queens’ body mass. In contrast, mortality of solitary queens did not significantly depend on body mass. Early mortality was significantly more frequent in associations of queens of random initial mass than in associations of equally heavy queens. Altogether these results demonstrate that queen phenotype differentially affects early queen mortality depending on the social environment, and suggest that reproductive competition rather than starvation is the main cause of mortality in multiple-queen associations. (C) 1999 The Association for the Study of Animal Behaviour. | 4130 | NA | NA | NA | no | no |
| 4129 | 2017 | NA | Sathuvalli, VR; Mehlenbacher, SA | 2013 | De novo sequencing of hazelnut bacterial artificial chromosomes (BACs) using multiplex Illumina sequencing and targeted marker development for eastern filbert blight resistance | Bacterial artificial chromosome (BAC) libraries are widely used in map-based cloning of plant genes. Eastern filbert blight (EFB), caused by the pyrenomycete Anisogramma anomala (Peck) E. Muller, is a devastating disease of European hazelnut (Corylus avellana L.) in the Pacific Northwest. A dominant allele at a single locus from the obsolete pollenizer “Gasaway” confers complete resistance. Our map-based cloning efforts use a BAC library for “Jefferson” hazelnut, which is heterozygous for resistance. Screening the library with primer pairs designed from RAPD markers closely linked to the EFB resistance locus identified 38 BACs. We sequenced 28 of these BACs using Illumina technology, by multiplexing with barcoded adapters. De novo sequence assembly using the programs Velvet and SOPRA and further alignment using CodonCode Aligner generated contigs whose length ranged from 393 to 108,194 bp. The number of contigs per BAC ranged from 1 to 19, and estimated coverage of assembled BACs ranged from 64 % to 100 %. Preliminary analysis of the sequences identified 779 simple sequence repeats (SSRs), from which we developed 23 markers. Of these, 17 were assigned to linkage group 6 adjacent to the disease resistance locus, five were placed on other linkage groups, and one could not be assigned to a linkage group. The BAC sequences and new SSR markers will be useful for our efforts at map-based cloning of the disease resistance gene. | 4131 | NA | NA | NA | no | no |
| 4130 | 2017 | NA | Ahlund, M | 2005 | Behavioural tactics at nest visits differ between parasites and hosts in a brood-parasitic duck | Whether conspecific brood parasitism is adaptive is a matter of debate. It may just be accidental when suitable nest sites are scarce. I tested this and other hypotheses, using video recordings and quantitative behavioural observations at nestboxes of individually marked common goldeneyes, Bucephala clangula. In 11 of 13 parasitized nests filmed, parasite behaviour differed markedly from that of hosts during most of the egg-laying sequence. Hosts typically started and laid the most eggs in the nest, covered the clutch when leaving it, and deposited down. Parasites covered the eggs poorly and did not deposit down. Hosts spent increasing time on the nest over the laying sequence, whereas parasites did not. Parasites tended to lay eggs later than hosts in the morning, possibly in response to nest guarding by hosts. When in the nest, hosts usually prevented females from entering, but parasites rarely did so. Of 84 parasitic eggs, 85% were laid by ‘true parasites, and 15% by females that behaved like hosts but were eventually displaced by another female. Most (73%) of the ’truly’ parasitic eggs were from females that were marked as adults previously, and 27% from new recruits. Parasitism in this goldeneye population is thus usually an alternative behavioural tactic, distinctly different from the behaviour of hosts, and not simply a side-effect of competition between females over the same nest. | 4132 | NA | NA | NA | no | no |
| 4131 | 2017 | NA | Esteve-Gassent, MD; Castro-Arellano, I; Feria-Arroyo, TP; Patino, R; Li, AY; Medina, RF; de Leon, AAP | 2016 | TRANSLATING ECOLOGY, PHYSIOLOGY, BIOCHEMISTRY, AND POPULATION GENETICS RESEARCH TO MEET THE CHALLENGE OF TICK AND TICK-BORNE DISEASES IN NORTH AMERICA | Emerging and re-emerging tick-borne diseases threaten public health and the wellbeing of domestic animals and wildlife globally. The adoption of an evolutionary ecology framework aimed to diminish the impact of tick-borne diseases needs to be part of strategies to protect human and animal populations. We present a review of current knowledge on the adaptation of ticks to their environment, and the impact that global change could have on their geographic distribution in North America. Environmental pressures will affect tick population genetics by selecting genotypes able to withstand new and changing environments and by altering the connectivity and isolation of several tick populations. Research in these areas is particularly lacking in the southern United States and most of Mexico with knowledge gaps on the ecology of these diseases, including a void in the identity of reservoir hosts for several tick-borne pathogens. Additionally, the way in which anthropogenic changes to landscapes may influence tick-borne disease ecology remains to be fully understood. Enhanced knowledge in these areas is needed in order to implement effective and sustainable integrated tick management strategies. We propose to refocus ecology studies with emphasis on metacommunity-based approaches to enable a holistic perspective addressing whole pathogen and host assemblages. Network analyses could be used to develop mechanistic models involving multihost-pathogen communities. An increase in our understanding of the ecology of tick-borne diseases across their geographic distribution will aid in the design of effective area-wide tick control strategies aimed to diminish the burden of pathogens transmitted by ticks. (C) 2016 Wiley Periodicals, Inc. | 4133 | NA | NA | NA | no | no |
| 4132 | 2017 | NA | Mckenzie, FE; Bossert, WH | 1998 | The optimal production of gametocytes by Plasmodium falciparum | We use a simple model of the blood-stage infection dynamics of the malaria parasite Plasmodium falciparum to consider the adaptive significance of different rates of conversion from its pathogenic, asexual stages to its transmissible, sexual forms. We find that the rates that maximize transmissivity in single-strain infections are generally greater than the highest rates reported for in vitro cultures and are several times those for which the behavior of the model is consistent with clinical profiles of infection dynamics. When two strains that share a common immune agent coinfect a host through simultaneous inoculation or sequential superinfection, however, a strain with a lower, clinically-consistent value of the conversion rate inhibits the transmissivity of one with the higher value optimal for single-strain infection. Hence we suggest that “apparent” competition by way of a common immune response might be responsible for selection of the former. (C) 1998 Academic Press. | 4134 | NA | NA | NA | no | no |
| 4133 | 2017 | NA | Kim, YI; DeVincenzo, JP; Jones, BG; Rudraraju, R; Harrison, L; Meyers, R; Cehelsky, J; Alvarez, R; Hurwitz, JL | 2014 | Respiratory Syncytial Virus Human Experimental Infection Model: Provenance, Production, and Sequence of Low-Passaged Memphis-37 Challenge Virus | Respiratory syncytial virus (RSV) is the leading cause of lower respiratory tract infections in children and is responsible for as many as 199,000 childhood deaths annually worldwide. To support the development of viral therapeutics and vaccines for RSV, a human adult experimental infection model has been established. In this report, we describe the provenance and sequence of RSV Memphis-37, the low-passage clinical isolate used for the model’s reproducible, safe, experimental infections of healthy, adult volunteers. The predicted amino acid sequences for major proteins of Memphis-37 are compared to nine other RSV A and B amino acid sequences to examine sites of vaccine, therapeutic, and pathophysiologic interest. Human T-cell epitope sequences previously defined by in vitro studies were observed to be closely matched between Memphis-37 and the laboratory strain RSV A2. Memphis-37 sequences provide baseline data with which to assess: (i) virus heterogeneity that may be evident following virus infection/ transmission, (ii) the efficacy of candidate RSV vaccines and therapeutics in the experimental infection model, and (iii) the potential emergence of escape mutants as a consequence of experimental drug treatments. Memphis-37 is a valuable tool for pre-clinical research, and to expedite the clinical development of vaccines, therapeutic immunomodulatory agents, and other antiviral drug strategies for the protection of vulnerable populations against RSV disease. | 4135 | NA | NA | NA | no | no |
| 4134 | 2017 | NA | Molnar, B; Duchamp, C; Mostl, K; Diehl, PA; Betschart, B | 2014 | Comparative survey of canine parvovirus, canine distemper virus and canine enteric coronavirus infection in free-ranging wolves of central Italy and south-eastern France | Diseases likely affect large carnivore demography and can hinder conservation efforts. We considered three highly contagious viruses that infect a wide range of domestic and wild mammals: canine parvovirus type 2 (CPV-2), canine distemper virus (CDV) and canine enteric coronaviruses (CECoV). Infection by either one of these viruses can affect populations through increased mortality and/or decreased general health. We investigated infection in the wolf populations of Abruzzo, Lazio e Molise National Park (PNALM), Italy, and of Mercantour National Park (PNM), France. Faecal samples were collected during one winter, from October to March, from four packs in PNALM (n = 79) and from four packs in PNM (n = 66). We screened samples for specific sequences of viral nucleic acids. To our knowledge, our study is the first documented report of CECoV infection in wolves outside Alaska, and of the large-scale occurrence of CPV-2 in European wolf populations. The results suggest that CPV-2 is enzootic in the population of PNALM, but not in PNM and that CECoV is episodic in both areas. We did not detect CDV. Our findings suggest that density and spatial distribution of susceptible hosts, in particular free-ranging dogs, can be important factors influencing infections in wolves. This comparative study is an important step in evaluating the nature of possible disease threats in the studied wolf populations. Recent emergence of new viral strains in Europe additionally strengthens the need for proactive monitoring of wolves and other susceptible sympatric species for viral threats and other impairing infections. | 4136 | NA | NA | NA | no | no |
| 4135 | 2017 | NA | Nair, RA; Thomas, G | 2012 | Functional genetic diversity at nucleotide binding site (NBS) loci: Comparisons among soft rot resistant and susceptible Zingiber taxa | Fundamental problem confronting ginger production is uniform susceptibility of all cultivars to soft rot disease caused by Pythium spp. Resistance gene candidate sequences (RGCs) belonging to non-TIR (Toll-Interleukin receptor) NBS-LRR (Nucleotide Binding Site-Leucine Rich Repeat) class of resistance (R) genes was previously identified from cultivated and wild Zingiber taxa including Zingiber zerumbet, a putative resistance donor. Efficiency and sensitivity of SSCP (Single-strand conformation polymorphism) analysis over RFLP (Restriction fragment length polymorphism) analysis in discriminating Pythium susceptible and resistant Zingiber accessions is being described here. Zingiber RGCs detected 29.5% polymorphism in Z. zerumbet, 12.34% in Zingiber cernuum and no polymorphism in the analyzed ginger cultivars. Results are discussed in terms of their mode of propagation and R gene evolution in the wild and cultivated accessions. Four RGCs designated ZzP226, ZcP28, ZoP26 and ZcP623 that yielded 1 zerumbet specific bands constitute important genomic tools for isolation of full-length R-genes from resistant germplasm. (c) 2012 Elsevier Ltd. All rights reserved. | 4137 | NA | NA | NA | no | no |
| 4136 | 2017 | NA | Graystock, P; Goulson, D; Hughes, WOH | 2014 | The relationship between managed bees and the prevalence of parasites in bumblebees | Honey bees and, more recently, bumblebees have been domesticated and are now managed commercially primarily for crop pollination, mixing with wild pollinators during foraging on shared flower resources. There is mounting evidence that managed honey bees or commercially produced bumblebees may affect the health of wild pollinators such as bumblebees by increasing competition for resources and the prevalence of parasites in wild bees. Here we screened 764 bumblebees from around five greenhouses that either used commercially produced bumblebees or did not, as well as bumblebees from 10 colonies placed at two sites either close to or far from a honey bee apiary, for the parasites Apicystis bombi, Crithidia bombi, Nosema bombi, N. ceranae, N. apis and deformed wing virus. We found that A. bombi and C. bombi were more prevalent around greenhouses using commercially produced bumblebees, while C. bombi was 18% more prevalent in bumblebees at the site near to the honey bee apiary than those at the site far from the apiary. Whilst these results are from only a limited number of sites, they support previous reports of parasite spillover from commercially produced bumblebees to wild bumblebees, and suggest that the impact of stress from competing with managed bees or the vectoring of parasites by them on parasite prevalence in wild bees needs further investigation. It appears increasingly likely that the use of managed bees comes at a cost of increased parasites in wild bumblebees, which is not only a concern for bumblebee conservation, but which may impact other pollinators as well. | 4138 | NA | NA | NA | no | no |
| 4137 | 2017 | NA | Leclerc, JB; Detrain, C | 2016 | Ants detect but do not discriminate diseased workers within their nest | Social insects have evolved an array of individual and social behaviours that limit pathogen entrance and spread within the colony. The detection of ectoparasites or of fungal spores on a nestmate body triggers their removal by allogrooming and appears as a primary component of social prophylaxis. However, in the case of fungal infection, one may wonder whether ant workers are able to detect, discriminate and keep at bay diseased nestmates that have no spores over their cuticle but which constitute a latent sanitary risk due to post-mortem corpse sporulation. Here, we investigate the ability of Myrmica rubra workers to detect and discriminate a healthy from a diseased nestmate infected by the entomopathogen Metarhizium anisopliae. During dyadic encounters in a neutral location, workers were more aggressive towards isolated sick nestmates on the 3rd post-infectionday. However, no such detection or discrimination of fungus-infected nestmates occurred in a social context inside the nest or at the nest entrance. Gatekeepers never actively rejected incoming diseased nestmates that rather spontaneously isolated themselves outside the nest. Our study reveals that ant workers may detect health-dependent cues and that their ‘acceptance level’ of sick nestmates is tunable depending on the social context. This raises questions about possible trade-offs between a social closure to pathogens and risks of erroneous rejection of healthy nestmates. Social isolation of moribund ants also appears as a widespread prophylactic strategy of social insects allowing them to reduce exposure to pathogens and to spare costs associated with the management of infected individuals. | 4139 | NA | NA | NA | no | no |
| 4138 | 2017 | NA | Eriksen, L; Borum, F; Jahoor, A | 2003 | Inheritance and localisation of resistance to Mycosphaerella graminicola causing septoria tritici blotch and plant height in the wheat (Triticum aestivum L.) genome with DNA markers | Resistance to the disease septoria tritici blotch of wheat (Triticum aestivum L.), caused by the fungus Mycosphaerella graminicola (Fuckel.) J. Schrot in Cohn (anamorph Septoria tritici Roberge in Desmaz.) was investigated in a doubled-haploid (DH) population of a cross between the susceptible winter wheat cultivar Savannah and the resistant cultivar Senat. A molecular linkage map of the population was constructed including 76 SSR loci and 244 AFLP loci. Parents and DH progeny were tested for resistance to single isolates of M. graminicola in a growth chamber at the seedling stage, and to an isolate mixture at the adult plant stage, in field trials. A gene located at or near the Stb6 locus mapping to chromosome 3A provided seedling resistance to IPO323. Two complementary genes, mapping to chromosome 3A, one of which was the IPO323 resistance gene, were needed for resistance to the Danish isolate Riso97-86. In addition, a number of minor loci influenced the expression of resistance in the growth chamber. In the field, four QTLs for resistance to septoria tritici blotch were detected. Two QTLs, located on chromosomes 3A and 6B explained 18.2 and 67.9% of the phenotypic variance in the mean over two trials. Both these QTLs were also detected at the seedling stage with isolate Riso97-86, whereas isolate IPO323 only detected the QTL on 3A. Additionally, two QTLs identified in adult plants on chromosomes 2B and 7B were not detected at the seedling stage. Four QTLs were detected for plant height located on chromosomes 2B, 3A, 3B and on a linkage group not assigned to a chromosome. The major QTLs on 3A and on the unassigned linkage group were consistent over two trials, and the QTL on 3A seemed to be linked to a QTL for septoria tritici blotch resistance. | 4140 | NA | NA | NA | no | no |
| 4139 | 2017 | NA | Fenton, B; Margaritopoulos, JT; Malloch, GL; Foster, SP | 2010 | Micro-evolutionary change in relation to insecticide resistance in the peach-potato aphid, Myzus persicae | 2. Asexual organisms rely on mutation whereas sexual organisms combine mutation with recombination. 3. Few organisms provide examples of species that are both sexual and asexual, but aphids do. 4. To examine evolution on perceptible timescales requires strong evolutionary forces and, as Darwin noted, agricultural practices provide strong selection. In the case of aphids, insecticides provide a considerable force in the elimination of genotypes. 5. Insecticide resistance in Myzus persicae (Sulzer) has arisen independently through point mutation and gene amplification on a number of occasions and at different times. Resistance to organophosphates, pyrethroids, and pirimicarb (a dimethyl carbamate) is now widespread. 6. In this paper, we examine these three elements: sexual recombination, clonal expansion, and insecticide selection in the peach-potato aphid M. persicae in relation to the evolution of insecticide resistance and survival of the fittest clone. | 4141 | NA | NA | NA | no | no |
| 4140 | 2017 | NA | Rudgers, JA; Afkhami, ME; Rua, MA; Davitt, AJ; Hammer, S; Huguet, VM | 2009 | A fungus among us: broad patterns of endophyte distribution in the grasses | Plant-associated microbes have been increasingly recognized for influencing host populations, plant communities, and even herbivores and predators. Thus, understanding factors that affect the distribution and abundance of microbial symbioses may be important for predicting the ecological dynamics of communities. Using endophytic fungi-grass symbioses, we explored how intrinsic traits of the symbiosis, specifically transmission mode, may influence symbiont frequencies in host populations. Combining published literature with new field surveys, we compared Epichloe endophytes, which had mixed horizontal and vertical transmission, with Neotyphodium endophytes, which were exclusively vertically transmitted from host plants to seeds. Exclusively vertical transmission should select against pathogenicity because symbionts depend entirely on hosts for reproduction. Across 118 host species, we found that Neotyphodium hosts had 40-130% higher symbiont frequencies than Epichloe hosts. In field surveys, endophyte frequency was positively correlated with the local density of hosts, but only for Epichloe, suggesting that contagiously spread Epichloe may attain higher frequencies when hosts are more abundant. Epichloe endophytes were also more likely than Neotyphodium to have imperfect vertical transmission; thus, hosts may reduce the transmission of more pathogenic symbionts to seeds. Results are consistent with the conclusion that the evolutionary transition to exclusively vertical transmission can alter patterns of symbiont frequency in nature. | 4142 | NA | NA | NA | no | no |
| 4141 | 2017 | NA | Harris, JL; Balci, Y | 2015 | Population Structure of the Bacterial Pathogen Xylella fastidiosa among Street Trees in Washington DC | Bacterial leaf scorch, associated with the bacterial pathogen Xylella fastidiosa, is a widely established and problematic disease of landscape ornamentals in Washington D.C. A multilocus sequence typing analysis was performed using 10 housekeeping loci for X. fastidiosa strains in order to better understand the epidemiology of leaf scorch disease in this municipal environment. Samples were collected from 7 different tree species located throughout the District of Columbia, consisting of 101 samples of symptomatic and asymptomatic foliage from 84 different trees. Five strains of the bacteria were identified. Consistent with prior data, these strains were host specific, with only one strain associated with members of the red oak family, one strain associated with American elm, one strain associated with American sycamore, and two strains associated with mulberry. Strains found for asymptomatic foliage were the same as strains from the symptomatic foliage on individual trees. Cross transmission of the strains was not observed at sites with multiple species of infected trees within an approx. 25 m radius of one another. X. fastidiosa strain specificity observed for each genus of tree suggests a highly specialized host-pathogen relationship. | 4143 | NA | NA | NA | no | no |
| 4142 | 2017 | NA | Li, GQ; Xu, XY; Bai, GH; Carver, BF; Hunger, R; Bonman, JM; Kolmer, J; Dong, HX | 2016 | Genome-Wide Association Mapping Reveals Novel QTL for Seedling Leaf Rust Resistance in a Worldwide Collection of Winter Wheat | Leaf rust of wheat (Triticum aestivum L.) is a major disease that causes significant yield losses worldwide. The short-lived nature of leaf rust resistance (Lr) genes necessitates a continuous search for novel sources of resistance. We performed a genome-wide association study (GWAS) on a panel of 1596 wheat accessions. The panel was evaluated for leaf rust reaction by testing with a bulk of Puccinia triticina Eriks. (Pt) isolates collected from multiple fields of Oklahoma in 2013 and two predominant races in the fields of Oklahoma in 2015. The panel was genotyped with a set of 5011 single-nucleotide polymorphism (SNP) markers. A total of 14 quantitative trait loci (QTL) for leaf rust resistance were identified at a false discovery rate (FDR) of 0.01 using the mixed linear model (MLM). Of these, eight QTL reside in the vicinity of known genes or QTL, and more studies are needed to determine their relationship with known loci. QLr. stars-7AL1 is a new QTL to bread wheat but is close to a locus previously identified in durum wheat [Triticum turgidum L. subsp. durum (Desf.) Husn.]. The other five QTL, including QLr. stars-1BL3, QLr. stars-1DC1, QLr. stars-2BL1, QLr. stars-5BL1, and QLr. stars-7AS1, are likely novel loci for leaf rust resistance. The uneven distribution of the 14 QTL in the six subpopulations of the panel suggests that wheat breeders can enhance leaf rust resistance by selectively introgressing some of these QTL into their breeding materials. In addition, another 31 QTL were significantly associated with leaf rust resistance at a FDR of 0.05. | 4144 | NA | NA | NA | no | no |
| 4143 | 2017 | NA | Sukmak, M; Wajjwalku, W; Ostner, J; Schulke, O | 2017 | A first report of non-invasive adenovirus detection in wild Assamese macaques in Thailand | Several simian adenoviruses (AdVs) have been detected and isolated in various species of non-human primates with the goals of monitoring the health of wildlife and investigating their potential for zoonotic disease transmission. Here, we provide evidence of AdV infection in wild Assamese macaques (Macaca assamensis assamensis) at Phu Khieo Wildlife Sanctuary, Thailand, based on polymerase chain reaction of non-invasively collected fecal samples. Eight out of 110 fecal samples (7.3%), or five out of 87 monkeys (5.7%), showed evidence of AdV infection. All infected individuals were infants or juveniles. Phylogenetic analysis based on the sequence of hexon and polymerase genes revealed two different AdV genotypes. One genotype clustered in the human AdV-G group, while another showed 100% identity with previously reported AdVs of captive Chinese rhesus macaques (Macaca mulatta), which may be tentatively classified as a new species of AdV in non-human primates while awaiting further supporting evidence. | 4145 | NA | NA | NA | no | no |
| 4144 | 2017 | NA | Ramakrishnan, M; Ceasar, SA; Duraipandiyan, V; Vinod, KK; Kalpana, K; Al-Dhabi, NA; Ignacimuthu, S | 2016 | Tracing QTLs for Leaf Blast Resistance and Agronomic Performance of Finger Millet (Eleusine coracana (L.) Gaertn.) Genotypes through Association Mapping and in silico Comparative Genomics Analyses | Finger millet is one of the small millets with high nutritive value. This crop is vulnerable to blast disease caused by Pyricularia grisea, which occurs annually during rainy and winter seasons. Leaf blast occurs at early crop stage and is highly damaging. Mapping of resistance genes and other quantitative trait loci (QTLs) for agronomic performance can be of great use for improving finger millet genotypes. Evaluation of one hundred and twenty-eight finger millet genotypes in natural field conditions revealed that leaf blast caused severe setback on agronomic performance for susceptible genotypes, most significant traits being plant height and root length. Plant height was reduced under disease severity while root length was increased. Among the genotypes, IE4795 showed superior response in terms of both disease resistance and better agronomic performance. A total of seven unambiguous QTLs were found to be associated with various agronomic traits including leaf blast resistance by association mapping analysis. The markers, UGEP101 and UGEP95, were strongly associated with blast resistance. UGEP98 was associated with tiller number and UGEP9 was associated with root length and seed yield. Cross species validation of markers revealed that 12 candidate genes were associated with 8 QTLs in the genomes of grass species such as rice, foxtail millet, maize, Brachypodium stacei, B. distachyon, Panicum hallii and switchgrass. Several candidate genes were found proximal to orthologous sequences of the identified QTLs such as 1,4-beta-glucanase for leaf blast resistance, cytokinin dehydrogenase (CKX) for tiller production, calmodulin (CaM) binding protein for seed yield and pectin methylesterase inhibitor (PMEI) for root growth and development. Most of these QTLs and their putatively associated candidate genes are reported for first time in finger millet. On validation, these novel QTLs may be utilized in future for marker assisted breeding for the development of fungal resistant and high yielding varieties of finger millet. | 4146 | NA | NA | NA | no | no |
| 4145 | 2017 | NA | Krams, IA; Krama, T; Trakimas, G; Kaasik, A; Rantala, MJ; Skute, A | 2017 | Reproduction is costly in an infected aquatic insect | Internal energy reserves of animals are limited, and the current investment in reproduction often decreases survival or future reproductive success. Some studies showed that copulatory activities impair the strength of immune function in insects, while the recent evidence is contradictory. In this study we tested whether copulatory activity affects the rate of encapsulation response in males of Calopteryx splendens damselfly in allopatric populations, and in sympatric populations where C. splendens stay together with their superior competitor C. virgo. We also counted the number of eugregarines, which are common parasites of damselflies. Copulation activity did not affect the immunity of C. splendens males in allopatric populations. In sympatric populations C. splendens males had more gut parasites, and we found a significant interaction between parasite number and copulatory activity on the rate of encapsulation. Our results suggest that the costs of reproduction are higher in infected males, which may affect reproductive investment and sexual selection. | 4147 | NA | NA | NA | no | no |
| 4146 | 2017 | NA | Wijayalath, W; Cheesman, S; Tanabe, K; Handunnetti, S; Carter, R; Pathirana, S | 2012 | Strain-Specific Protective Effect of the Immunity Induced by Live Malarial Sporozoites under Chloroquine Cover | The efficacy of a whole-sporozoite malaria vaccine would partly be determined by the strain-specificity of the protective responses against malarial sporozoites and liver-stage parasites. Evidence from previous reports were inconsistent, where some studies have shown that the protective immunity induced by irradiated or live sporozoites in rodents or humans were cross-protective and in others strain-specific. In the present work, we have studied the strain-specificity of live sporozoite-induced immunity using two genetically and immunologically different strains of Plasmodium cynomolgi, Pc746 and PcCeylon, in toque monkeys. Two groups of monkeys were immunized against live sporozoites of either the Pc746 (n = 5), or the PcCeylon (n = 4) strain, by the bites of 2-4 sporozoite-infected Anopheles tessellates mosquitoes per monkey under concurrent treatments with chloroquine and primaquine to abrogate detectable blood infections. Subsequently, a group of non-immunized monkeys (n = 4), and the two groups of immunized monkeys were challenged with a mixture of sporozoites of the two strains by the bites of 2-5 infective mosquitoes from each strain per monkey. In order to determine the strain-specificity of the protective immunity, the proportions of parasites of the two strains in the challenge infections were quantified using an allele quantification assay, Pyrosequencing (TM), based on a single nucleotide polymorphism (SNP) in the parasites’ circumsporozoite protein gene. The Pyrosequencing (TM) data showed that a significant reduction of parasites of the immunizing strain in each group of strain-specifically immunized monkeys had occurred, indicating a stronger killing effect on parasites of the immunizing strain. Thus, the protective immunity developed following a single, live sporozoite/chloroquine immunization, acted specifically against the immunizing strain and was, therefore, strain-specific. As our experiment does not allow us to determine the parasite stage at which the strain-specific protective immunity is directed, it is possible that the target of this immunity could be either the pre-erythrocytic stage, or the blood-stage, or both. | 4148 | NA | NA | NA | no | no |
| 4147 | 2017 | NA | Bender, HS; Graves, JM; Deakin, JE | 2014 | Pathogenesis and Molecular Biology of a Transmissible Tumor in the Tasmanian Devil | The emergence of a fatal transmissible cancer known as devil facial tumor disease (DFTD) is threatening the iconic Tasmanian devil with extinction in the wild within the next few decades. Since the first report of the disease in 1996, DFTD has spread to over 85% of the devils’ distribution and dramatically reduced devil numbers. Research into DFTD has focused on gaining a deeper understanding of the disease on multiple levels, including an accurate assessment of the tissue origin of the tumor, elucidation of how the tumor evades immune detection, and determination of how the tumor is transmitted between individuals and how it is evolving as it spreads through the population. Knowledge gained from these studies has important implications for DFTD management and devil conservation. | 4149 | NA | NA | NA | no | no |
| 4148 | 2017 | NA | Szmaragd, C; Balloux, F | 2007 | The population genomics of hepatitis B virus | Hepatitis B virus (HBV) infection is considered as the fifth leading cause of death due to infectious diseases and has a worldwide prevalence. The particular geographical distribution of the eight previously defined genotypes of HBV suggests that the viral population is highly structured. The presence of such population structure is likely to affect the geographical distribution of polymorphisms involved in disease progression. In this study, we determined the structure of the HBV population using a clustering approach based on the observed allele frequencies at the polymorphic loci. We used all full-genome sequences publicly available and obtained a significant clustering of the HBV population into four main clusters, strongly associated with the current classification into genotypes. One of these main clusters could itself be split into three well-supported subclusters, highlighting the hierarchical nature of the population differentiation between HBV strains. The extremely clear-cut subdivision of the HBV population further indicates that recombination in HBV is not as extensive as previously assumed. | 4150 | NA | NA | NA | no | no |
| 4149 | 2017 | NA | Valente, AXCN; Zischkau, J; Shin, JH; Gao, Y; Sarkar, A | 2012 | Genome-Wide Association Study Heterogeneous Cohort Homogenization via Subject Weight Knock-Down | Population structure can be a source of both false-positive and false-negative findings in a genome-wide association study. This article proposes an approach that helps to reduce the false-positives. It consists of homogenizing the diseased/healthy phenotype ratio across the cohort, by decreasing the statistical weight of selected individuals. After homogenization, the cohort is statistically handled as if originating from a single well-mixed population. The method was applied to homogenize a Parkinson’s disease genome-wide association study cohort. | 4151 | NA | NA | NA | no | no |
| 4150 | 2017 | NA | Poulsen, M; Bot, ANM; Currie, CR; Nielsen, MG; Boomsma, JJ | 2003 | Within-colony transmission and the cost of a mutualistic bacterium in the leaf-cutting ant Acromyrmex octospinosus | 1 Stable mutualistic interactions require the long‐term alignment of fitness interests of participating species. This condition is fulfilled when the benefits of the relationship exceed the costs for all partners.   2 One apparent stabilizing factor in mutualisms is the vertical (parent to offspring) transmission of symbionts, as this tends to reduce the expression of virulent traits and reproductive conflicts. This study examines the cost and mode of transmission of a mutualistic Streptomyces bacterium that grows on the cuticle of leaf‐cutting ants and produces antibiotics against a specialized fungal parasite of the ant fungus gardens.  3 It is shown that ant respiration rates are elevated by 10–20% when the bacterium is present on their cuticle. This increase is due to direct respiration of the bacterium and possible excess respiration by the ants. Although these two factors cannot be separated, it is clear that the total increase gives a reasonable quantification of the metabolic costs incurred by the Streptomyces symbiont.  4 Ants that actively maintain Streptomyces cultures on their cuticle tend to consume more of their mutualistic fungus garden than controls and this excess consumption increases with the amount of Streptomyces bacteria present.  5 Scanning electron microscopy showed that the mutualistic bacterium is not present on major workers immediately following eclosion, indicating that the bacterium is not transferred to callow workers until later. 6 The results of an experiment simulating within‐colony transmission to callow workers suggest that the bacterium is predominantly transmitted from older to newly eclosed major workers, but that transmission may also occur via the fungus garden. The presence of Streptomyces bacteria in the fungus garden implies that rare events of horizontal transmission of the fungal cultivar of attine ants may also imply horizontal transmission of strains of the mutualistic bacterium. | 4152 | NA | NA | NA | no | no |
| 4151 | 2017 | NA | Waugh, C; Khan, SA; Carver, S; Hanger, J; Loader, J; Polkinghorne, A; Beagley, K; Timms, P | 2016 | A Prototype Recombinant-Protein Based Chlamydia pecorum Vaccine Results in Reduced Chlamydial Burden and Less Clinical Disease in Free-Ranging Koalas (Phascolarctos cinereus) | Diseases associated with Chlamydia pecorum infection are a major cause of decline in koala populations in Australia. While koalas in care can generally be treated, a vaccine is considered the only option to effectively reduce the threat of infection and disease at the population level. In the current study, we vaccinated 30 free-ranging koalas with a prototype Chlamydia pecorum vaccine consisting of a recombinant chlamydial MOMP adjuvanted with an immune stimulating complex. An additional cohort of 30 animals did not receive any vaccine and acted as comparison controls. Animals accepted into this study were either uninfected (Chlamydia PCR negative) at time of initial vaccination, or infected (C. pecorum positive) at either urogenital (UGT) and/or ocular sites (Oc), but with no clinical signs of chlamydial disease. All koalas were vaccinated /sampled and then re-released into their natural habitat before re-capturing and re-sampling at 6 and 12 months. All vaccinated koalas produced a strong immune response to the vaccine, as indicated by high titres of specific plasma antibodies. The incidence of new infections in vaccinated koalas over the 12-month period post-vaccination was slightly less than koalas in the control group, however, this was not statistically significant. Importantly though, the vaccine was able to significantly reduce the infectious load in animals that were Chlamydia positive at the time of vaccination. This effect was evident at both the Oc and UGT sites and was stronger at 6 months than at 12 months post-vaccination. Finally, the vaccine was also able to reduce the number of animals that progressed to disease during the 12-month period. While the sample sizes were small (statistically speaking), results were nonetheless striking. This study highlights the potential for successful development of a Chlamydia vaccine for koalas in a wild setting. | 4153 | NA | NA | NA | no | no |
| 4152 | 2017 | NA | Keller, L; Liautard, C; Reuter, M; Brown, WD; Sundstrom, L; Chapuisat, M | 2001 | Sex ratio and Wolbachia infection in the ant Formica exsecta | Sex allocation data in social Hymenoptera provide some of the best tests of kin selection, parent-offspring conflict and sex ratio theories. However, these studies critically depend on controlling for confounding ecological factors and on identifying all parties that potentially manipulate colony sex ratio. It has been suggested that maternally inherited parasites may influence sex allocation in social Hymenoptera. If the parasites can influence sex allocation, infected colonies are predicted to invest more resources in females than non-infected colonies, because the parasites are transmitted through females but not males. Prime candidates for such sex ratio manipulation are Wolbachia, because these cytoplasmically transmitted bacteria have been shown to affect the sex ratio of host arthropods by cytoplasmic incompatibility, parthenogenesis, male-killing and feminization. In this study, we tested whether Wolbachia infection is associated with colony sex ratio in two populations of the ant Formica exsecta that have been the subject of extensive sex ratio studies. In these populations colonies specialize in the production of one sex or the other. We found that almost all F. exsecta colonies in both populations are infected with Wolbachia. However, in neither population did we find a significant association in the predicted direction between the prevalence of Wolbachia and colony sex ratio. In particular, colonies with a higher proportion of infected workers did not produce more females. Hence, we conclude that Wolbachia does not seem to alter the sex ratio of its hosts as a means to increase transmission rate in these two populations of ants. | 4154 | NA | NA | NA | no | no |
| 4153 | 2017 | NA | Velo-Suarez, L; Brosnahan, ML; Anderson, DM; McGillicuddy, DJ | 2013 | A Quantitative Assessment of the Role of the Parasite Amoebophrya in the Termination of Alexandrium fundyense Blooms within a Small Coastal Embayment | Parasitic dinoflagellates of the genus Amoebophrya infect free-living dinoflagellates, some of which can cause harmful algal blooms (HABs). High prevalence of Amoebophrya spp. has been linked to the decline of some HABs in marine systems. The objective of this study was to evaluate the impact of Amoebophrya spp. on the dynamics of dinoflagellate blooms in Salt Pond (MA, USA), particularly the harmful species Alexandrium fundyense. The abundance of Amoebophrya life stages was estimated 3-7 days per week through the full duration of an annual A. fundyense bloom using fluorescence in situ hybridization coupled with tyramide signal amplification (FISH-TSA). More than 20 potential hosts were recorded including Dinophysis spp., Protoperidinium spp. and Gonyaulax spp., but the only dinoflagellate cells infected by Amoebophrya spp. during the sampling period were A. fundyense. Maximum A. fundyense concentration co-occurred with an increase of infected hosts, followed by a massive release of Amoebophrya dinospores in the water column. On average, Amoebophrya spp. infected and killed similar to 30% of the A. fundyense population per day in the end phase of the bloom. The decline of the host A. fundyense population coincided with a dramatic life-cycle transition from vegetative division to sexual fusion. This transition occurred after maximum infected host concentrations and before peak infection percentages were observed, suggesting that most A. fundyense escaped parasite infection through sexual fusion. The results of this work highlight the importance of high frequency sampling of both parasite and host populations to accurately assess the impact of parasites on natural plankton assemblages. | 4155 | NA | NA | NA | no | no |
| 4154 | 2017 | NA | Karunaweera, ND; Ferreira, MU; Hartl, DL; Wirth, DF | 2007 | Fourteen polymorphic microsatellite DNA markers for the human malaria parasite Plasmodium vivax | We have optimized a set of 14 polymorphic microsatellite markers for the human malaria parasite Plasmodium vivax, all of them consisting of either tri- or tetranucleotide repeats. These markers, whose polymerase chain reaction amplification conditions are identical, were used to screen 25 parasite isolates from malaria-endemic areas in Sri Lanka. The total number of alleles per locus ranged between 6 and 13 (average, 7.8), and expected heterozygosity ranged from 0.627 to 0.913 (average, 0.790). These markers are now being used to characterize the population structure of P. vivax in other endemic areas. | 4156 | NA | NA | NA | no | no |
| 4155 | 2017 | NA | Takatsuka, J | 2016 | Lymantria mathura nucleopolyhedrovirus: Identification, occurrence and genetic diversity in Iwate Prefecture, Japan | A high prevalence of nuclear polyhedrosis has been observed in larval outbreaks of Lymantria mathura in Iwate Prefecture, Japan. However, the virus responsible has not been identified. Here the virus was designated Lymantria mathura nucleopolyhedrovirus (LymaNPV), based on partial sequence results of the lef-8, lef-9, and polh genes and transmission electron microscopic observations. Diagnosis by polymerase chain reaction targeting of the partial polh gene and a subsequent restriction fragment length polymorphism (RFLP) analysis indicated that LymaNPV was an exclusive causative agent for the nuclear polyhedrosis of the L. mathura larvae. LymaNPV was also detected from a very small fraction of L dispar larvae that co-occurred with L mathura larvae where the prevalence of LymaNPV-caused disease was high. A bioassay using LymaNPV against L. dispar larvae produced infection with the inoculated virus. This finding was consistent with the results of the field sample analysis. LymaNPV contained six genome types in the L. mathura populations sampled, as determined by RFLP of LymaNPV genomic DNA and southern blot hybridization analyses. None of the genome types was unique to any sampling site, indicating that some flow had occurred among sites. However, genome-type composition seemed to differ among sites. This study provides basic information about the interaction between L mathura and LymaNPV. (C) 2016 Elsevier Inc. All rights reserved. | 4157 | NA | NA | NA | no | no |
| 4156 | 2017 | NA | Kondgen, S; Schenk, S; Pauli, G; Boesch, C; Leendertz, FH | 2010 | Noninvasive Monitoring of Respiratory Viruses in Wild Chimpanzees | To diagnose respiratory disease among wild great apes, there is a need for noninvasive diagnostic methods. Therefore, we analyzed fecal samples from habituated chimpanzees from Ta < National Park, Cte d’Ivoire. Samples had been collected during four distinct outbreaks: two with known aetiology (March 2004 and February 2006) and two with unknown aetiology (October 2004 and August 2005). Fecal samples were screened by polymerase chain reaction (PCR) for the presence of human metapneumovirus (HMPV) and human respiratory syncytial virus (HRSV), two paramyxoviruses previously found in lung tissue of chimpanzees that died due to respiratory disease. In the March 2004 outbreak, 72% of the tested individuals were positive for HMPV, and during the 2006 epidemic, 25% tested HRSV-positive. In the outbreaks where no causative pathogen was previously known, fecal samples tested positive for either HRSV or HMPV, showing that reinfection occurred. Virus sequences were generated and compared with sequences previously found in tissue; nearly identical virus sequences in both tissue and fecal samples were found. These results demonstrate that fecal samples collected during outbreak times can be used for the diagnostic and phylogenetic analysis of HMPV and HRSV. Using such diagnostic tools, systematic noninvasive disease investigation of respiratory outbreaks in wild great apes becomes possible. The methods presented here may also be applied for the investigation of further acute diseases in great apes and other species. | 4158 | NA | NA | NA | no | no |
| 4157 | 2017 | NA | Mendoza, Y; Antunez, K; Branchiccela, B; Anido, M; Santos, E; Invernizzi, C | 2014 | Nosema ceranae and RNA viruses in European and Africanized honeybee colonies (Apis mellifera) in Uruguay | Nosema ceranae is one of the causative agents of Nosemosis, a severe disease that affects the honeybee Apis mellifera. The aim of the present work was to compare N. ceranae and RNA virus infections in Africanized bees (hybrid of Apis mellifera scutellata and A. m. mellifera) and European (Italian) bees (A. m. ligustica) under field conditions. Africanized and Italian healthy colonies were relocated to an Eucalyptus grandis plantation, a place where colonies inevitably acquire Nosemosis. Fifteen and 30 days after that, all colonies presented N. ceranae spores although Africanized bees were less infected than Italian bees. Sacbrood virus (SBV) and Black queen cell virus (BQCV) were detected in both races of bees, although Africanized bees presented a lower level of BQCV infection than Italian bees. At the end of the flowering period, Africanized colonies had a larger honeybee population and produced more honey than Italian colonies. These results suggest that Africanized bees may be able to limit N. ceranae and BQCV infections within the colony, and that this may allow them to be more productive. | 4159 | NA | NA | NA | no | no |
| 4158 | 2017 | NA | Garcia, SAL; Van der Lee, TAJ; Ferreira, CF; Hekkert, BTL; Zapater, MF; Goodwin, SB; Guzman, M; Kema, GHJ; Souza, MT | 2010 | Variable number of tandem repeat markers in the genome sequence of Mycosphaerella fijiensis, the causal agent of black leaf streak disease of banana (Musa spp) | We searched the genome of Mycosphaerella fijiensis for molecular markers that would allow population genetics analysis of this plant pathogen. M. fijiensis, the causal agent of banana leaf streak disease, also known as black Sigatoka, is the most devastating pathogen attacking bananas (Musa spp). Recently, the entire genome sequence of M. fijiensis became available. We screened this database for VNTR markers. Forty-two primer pairs were selected for validation, based on repeat type and length and the number of repeat units. Five VNTR markers showing multiple alleles were validated with a reference set of isolates from different parts of the world and a population from a banana plantation in Costa Rica. Polymorphism information content values varied from 0.6414 to 0.7544 for the reference set and from 0.0400 and 0.7373 for the population set. Eighty percent of the polymorphism information content values were above 0.60, indicating that the markers are highly informative. These markers allowed robust scoring of agarose gels and proved to be useful for variability and population genetics studies. In conclusion, the strategy we developed to identify and validate VNTR markers is an efficient means to incorporate markers that can be used for fungicide resistance management and to develop breeding strategies to control banana black leaf streak disease. This is the first report of VNTR-minisatellites from the M. fijiensis genome sequence. | 4160 | NA | NA | NA | no | no |
| 4159 | 2017 | NA | Meijerink, M; Ferrando, ML; Lammers, G; Taverne, N; Smith, HE; Wells, JM | 2012 | Immunomodulatory Effects of Streptococcus suis Capsule Type on Human Dendritic Cell Responses, Phagocytosis and Intracellular Survival | Streptococcus suis is a major porcine pathogen of significant commercial importance worldwide and an emerging zoonotic pathogen of humans. Given the important sentinel role of mucosal dendritic cells and their importance in induction of T cell responses we investigated the effect of different S. suis serotype strains and an isogenic capsule mutant of serotype 2 on the maturation, activation and expression of IL-10, IL-12p70 and TNF-alpha in human monocyte-derived dendritic cells. Additionally, we compared phagocytosis levels and bacterial survival after internalization. The capsule of serotype 2, the most common serotype associated with infection in humans and pigs, was highly anti-phagocytic and modulated the IL-10/IL-12 and IL-10/TNF-alpha cytokine production in favor of a more anti-inflammatory profile compared to other serotypes. This may have consequences for the induction of effective immunity to S. suis serotype 2 in humans. A shielding effect of the capsule on innate Toll-like receptor signaling was also demonstrated. Furthermore, we showed that 24 h after phagocytosis, significant numbers of viable intracellular S. suis were still present intracellularly. This may contribute to the dissemination of S. suis in the body. | 4161 | NA | NA | NA | no | no |
| 4160 | 2017 | NA | Hanson, DA; Britten, HB; Restani, M; Washburn, LR | 2007 | High prevalence of Yersinia pestis in black-tailed prairie dog colonies during an apparent enzootic phase of sylvatic plague | Sylvatic plague (Yersinia pestis) was introduced into North America over 100 years ago. The disease causes high mortality and extirpations in black-tailed prairie dogs (Cynomys ludovicianus), which is of conservation concern because prairie dogs provide habitat for the critically endangered black-footed ferret (Mustela nigripes). Our goal was to help elucidate the mechanism Y. pestis uses to persist in prairie ecosystems during enzootic and epizootic phases. We used a nested PCR protocol to assay for plague genomes in fleas collected from prairie dog burrows potentially exposed to plague in 1999 and 2000. No active plague epizootic was apparent in the 55 prairie dog colonies sampled in 2002 and 2003. However, 63% of the colonies contained plague-positive burrows in 2002, and 57% contained plague-positive burrows in 2003. Within plague-positive colonies, 23% of sampled burrows contained plague-positive fleas in 2002, and 26% contained plague-positive fleas in 2003. Of 15 intensively sampled colonies, there was no relationship between change in colony area and percentage of plague-positive burrows over the two years of the study. Some seasonality in plague prevalence was apparent because the highest percentages of plague-positive colonies were recorded in May and June. The surprisingly high prevalence of plague on study area colonies without any obvious epizootic suggested that the pathogen existed in an enzootic state in black-tailed prairie dogs. These findings have important implications for the management of prairie dogs and other species that are purported to be enzootic reservoir species. | 4162 | NA | NA | NA | no | no |
| 4161 | 2017 | NA | Sayler, KA; Loftis, AD; Beatty, SK; Boyce, CL; Garrison, E; Clemons, B; Cunningham, M; Alleman, AR; Barbet, AF | 2016 | Prevalence of Tick-Borne Pathogens in Host-Seeking Amblyomma americanum (Acari: Ixodidae) and Odocoileus virginianus (Artiodactyla: Cervidae) in Florida | Amblyomma americanum (L.), the lone star tick, is an aggressive tick that is expanding its geographic range within the United States. This tick is the vector for the human and veterinary pathogens Ehrlichia chaffeensis and Ehrlichia ewingii and is associated with other microbes of unspecified pathogenicity including Rickettsia amblyommii, Panola Mountain Ehrlichia, and Borrelia lonestari. In Florida, there has been sparse contemporary data on the prevalence of these organisms in host-seeking lone star ticks. To determine the prevalence of this tick and associated microbes in North Central Florida state parks, similar to 1,500 lone star tick specimens were collected between 2010 and 2012 analyzed by polymerase chain reaction (PCR) sequencing. Additionally, 393 white-tailed deer, Odocoileus virginianus (Zimmerman), samples were analyzed for pathogen prevalence using molecular methods and serology. In lone star ticks, 14.6, 15.6, and 57.1% were positive for E. chaffeensis, E. ewingii, and Rickettsia spp. DNA, respectively. Panola Mountain Ehrlichia or B. lonestari DNA were each detected in nearly 2% of tick specimens. In white-tailed deer, 7.3% were PCR positive for E. chaffeensis, 6.0% for E. ewingii, and 3.2% for rickettsial species. Approximately 45% of white-tailed deer specimens had antibodies to Ehrlichia spp., and <1% had antibodies to Borrelia burgdorferi. In summary, E. chaffeensis, E. ewingii, and spotted fever group rickettsia are highly prevalent in host-seeking lone star ticks and in white-tailed deer in Florida. The molecular and serological evidence of these microbes underscore their zoonotic potential in this region. | 4163 | NA | NA | NA | no | no |
| 4162 | 2017 | NA | Miao, M; Wang, ZL; Yang, ZQ; Yuan, LL; Parker, DM; Putaporntip, C; Jongwutiwes, S; Xangsayarath, P; Pongvongsa, T; Moji, H; Tuong, TD; Abe, T; Nakazawa, S; Kyaw, MP; Yan, GY; Sirichaisinthop, J; Sattabongkot, J; Mu, JB; Su, XZ; Kaneko, O; Cui, LW | 2013 | Genetic Diversity and Lack of Artemisinin Selection Signature on the Plasmodium falciparum ATP6 in the Greater Mekong Subregion | The recent detection of clinical Artemisinin (ART) resistance manifested as delayed parasite clearance in the Cambodia-Thailand border area raises a serious concern. The mechanism of ART resistance is not clear; but the P. falciparum sarco/endoplasmic reticulum Ca2+-ATPase (PfSERCA or PfATP6) has been speculated to be the target of ARTs and thus a potential marker for ART resistance. Here we amplified and sequenced pfatp6 gene (similar to 3.6 Kb) in 213 samples collected after 2005 from the Greater Mekong Subregion, where ART drugs have been used extensively in the past. A total of 24 single nucleotide polymorphisms (SNPs), including 8 newly found in this study and 13 nonsynonymous, were identified. However, these mutations were either uncommon or also present in other geographical regions with limited ART use. None of the mutations were suggestive of directional selection by ARTs. We further analyzed pfatp6 from a worldwide collection of 862 P. falciparum isolates in 19 populations from Asia, Africa, South America and Oceania, which include samples from regions prior to and after deployments ART drugs. A total of 71 SNPs were identified, resulting in 106 nucleotide haplotypes. Similarly, many of the mutations were continent-specific and present at frequencies below 5%. The most predominant and perhaps the ancestral haplotype occurred in 441 samples and was present in 16 populations from Asia, Africa, and Oceania. The 3D7 haplotype found in 54 samples was the second most common haplotype and present in nine populations from all four continents. Assessment of the selection strength on pfatp6 in the 19 parasite populations found that pfatp6 in most of these populations was under purifying selection with an average dN/dS ratio of 0.333. Molecular evolution analyses did not detect significant departures from neutrality in pfatp6 for most populations, challenging the suitability of this gene as a marker for monitoring ART resistance. | 4164 | NA | NA | NA | no | no |
| 4163 | 2017 | NA | Taylor, P | 2010 | Birth-death symmetry in the evolution of a social trait | Studies of the evolution of a social trait often make ecological assumptions (of population structure, life history), and thus a trait can be studied many different times with different assumptions. Here, I consider a Moran model of continuous reproduction and use an inclusive fitness analysis to investigate the relationships between fecundity or survival selection and birth-death (BD) or death-birth (DB) demography on the evolution of a social trait. A simple symmetry obtains: fecundity (respectively survival) effects under BD behave the same as survival (respectively fecundity) effects under DB. When these results are specialized to a homogeneous population, greatly simplified conditions for a positive inclusive fitness effect are obtained in both a finite and an infinite population. The results are established using the elegant formalism of mathematical group theory and are illustrated with an example of a finite population arranged in a cycle with asymmetric offspring dispersal. | 4165 | NA | NA | NA | no | no |
| 4164 | 2017 | NA | Hsieh, CY; Huang, CW; Pan, YC | 2016 | Crayfish plague Aphanomyces astaci detected in redclaw crayfish, Cherax quadricarinatus in Taiwan | Between December 2013 and January 2014, five outbreaks of an unknown disease with moderate to high cumulative mortality were observed among the freshwater redclaw crayfish (Cherax quadricarinatus) populations at four crayfish farms in Miaoli and Changhua counties (northern Taiwan) and at one crayfish farm in Pingtung County (southern Taiwan). Polymerase chain reaction (PCR) analysis allowed the detection of Aphanomyces astaci DNA in dead crayfish. Histopathological examination revealed an infection of host tissue by fungal hyphae that presented as typical non-septate hyphae within the soft abdominal cuticle from the first to second segment and in the tail fan. In PCR assays completed for the detection of crayfish plague, an expected 568-bp product, specific for the A. astaci ITS gene, was obtained from all sub-adults and adults examined. In a comparison of our strains with the known strains of A. astaci in Europe, nucleotide sequence identities were very similar, with 99.8-100% sequence similarity in that gene region. Positive reactions to in situ hybridization, using a digoxigenin (DIG)-labelled DNA probe, further confirmed A. astaci as the causative agent. This is the first report concerning natural infection of A. astaci in freshwater redclaw crayfish in Asia. (C) 2016 Elsevier Inc. All rights reserved. | 4166 | NA | NA | NA | no | no |
| 4165 | 2017 | NA | Hoppe, E; Pauly, M; Gillespie, TR; Akoua-Koffi, C; Hohmann, G; Fruth, B; Karhemere, S; Madinda, NF; Mugisha, L; Muyembe, JJ; Todd, A; Petrzelkova, KJ; Gray, M; Robbins, M; Bergl, RA; Wittig, RM; Zuberbuhler, K; Boesch, C; Schubert, G; Leendertz, FH; Ehlers, B; Calvignac-Spencer, S | 2015 | Multiple Cross-Species Transmission Events of Human Adenoviruses (HAdV) during Hominine Evolution | Human adenoviruses (HAdV; species HAdV-A to -G) are highly prevalent in the human population, and represent an important cause of morbidity and, to a lesser extent, mortality. Recent studies have identified close relatives of these viruses in African great apes, suggesting that some HAdV may be of zoonotic origin. We analyzed more than 800 fecal samples from wild African great apes and humans to further investigate the evolutionary history and zoonotic potential of hominine HAdV. HAdV-B and -E were frequently detected in wild gorillas (55%) and chimpanzees (25%), respectively. Bayesian ancestral host reconstruction under discrete diffusion models supported a gorilla and chimpanzee origin for these viral species. Host switches were relatively rare along HAdV evolution, with about ten events recorded in 4.5 My. Despite presumably rare direct contact between sympatric populations of the two species, transmission events from gorillas to chimpanzees were observed, suggesting that habitat and dietary overlap may lead to fecal-oral cross-hominine transmission of HAdV. Finally, we determined that two independent HAdV-B transmission events to humans occurred more than 100,000 years ago. We conclude that HAdV-B circulating in humans are of zoonotic origin and have probably affected global human health for most of our species lifetime. | 4167 | NA | NA | NA | no | no |
| 4166 | 2017 | NA | Andreassen, HP; Gundersen, G | 2006 | Male turnover reduces population growth: An enclosure experiment on voles | Turnover of individuals is assumed to cause disruptions of social organization, followed by reduced reproduction and survival. We tested how male turnover (removal of resident males and their replacement by unfamiliar males) affected population performance in experimental root vole (Microtus oeconomus) populations. The treatment simulated predation of adult males, with the subsequent replacement by immigrants, and provided insight into the interaction between extrinsic (i.e., predation) and intrinsic (i.e., social organization) factors. We showed that recruitment and female survival dramatically declined and that reproduction commenced slightly later in treatment populations compared with control populations. The treatment nearly halved the population growth rate. We suspect that recruitment failed due to infanticidal immigrating males. Reduced female survival was particularly apparent in treatment populations in which females exhibited a high degree of spatial overlap. Our experimental results show how males may significantly shape population dynamics and suggest how predation and social factors interact mechanistically. | 4168 | NA | NA | NA | no | no |
| 4167 | 2017 | NA | Roth, O; Keller, I; Landis, SH; Salzburger, W; Reusch, TBH | 2012 | HOSTS ARE AHEAD IN A MARINE HOST-PARASITE COEVOLUTIONARY ARMS RACE: INNATE IMMUNE SYSTEM ADAPTATION IN PIPEFISH SYNGNATHUS TYPHLE AGAINST VIBRIO PHYLOTYPES | Microparasites have a higher evolutionary potential than their hosts due to an increased mutation rate and a shorter generation time that usually results in parasites being locally adapted to their sympatric hosts. This pattern may not apply to generalist pathogens as adaptation to sympatric host genotypes is disadvantageous due to a narrowing of the host range, in particular under strong gene flow among host populations. Under this scenario, we predict that the immune defense of hosts reveals adaptation to locally common pathogen phylotypes. This was tested in four host populations of the pipefish Syngnathus typhle and associated bacteria of the genus Vibrio. We investigated the population divergence among host and bacteria populations and verified that gene flow is higher among host populations than among parasite populations. Next, we experimentally assessed the strength of innate immune defense of pipefish hosts using in vitro assays that measured antimicrobial activity of blood plasma against sympatric and allopatric Vibrio phylotypes. Pipefish plasma displays stronger antimicrobial activity against sympatric Vibrio phylotypes compared to allopatric ones. This suggests that host defense is genetically adapted against local bacteria with a broad and unspecialized host spectrum, a situation that is typical for marine systems with weak host population structure. | 4169 | NA | NA | NA | no | no |
| 4168 | 2017 | NA | Williams, CK; Lutz, RS; Applegate, RD | 2003 | Optimal group size and northern bobwhite coveys | Northern bobwhite, Colinus virginianus, form social units, called coveys, during the nonbreeding season (approximately September-April). Because the evolutionary advantage of this behaviour is generally unknown, we used controlled group size manipulations within an aviary to investigate whether group size influences (1) the time that the covey spends feeding, (2) the percentage of the covey that is vigilant, (3) the overall vigilance of the group and (4) the time to predator detection. We found that increasing group size increased the time that coveys spent in an exposed feeding area, reduced individual vigilance, improved group vigilance and decreased the time to detection of a potential predator. Additionally, we used experimental reductions of wild northern bobwhite coveys to test whether groups size influences (1) individual and covey survival, (2) daily movement in maintaining covey size and (3) mass change. We conducted field research on 12 independent 259-ha study areas (6 control plots and 6 treatments, where 60% of the population was removed) in east-central Kansas, U.S.A. between 9 November and 31 January, 1997-2000. We radio-marked 386 radiocollared individuals that comprised 137 groups on the study areas. Covey size did not differ between or within years or treatments ((X) over bar +/- SE: 10.98 +/- 0.22 individuals). Our results indicate that a stable group size existed between I and 22 individuals, with 11 being an optimal group size. Small coveys (1-7 individuals) had lower group persistence and individual survival, and used increased movement to create or join larger groups where survival was higher. Large groups (15-22) had lower individual survival, increased group movement and individual mass loss. Density-dependent feedbacks (e.g. lower survival and increased competition) may have lowered larger coveys to a stable size. Our results suggest the regulation of an optimal covey size of 11 was promoted by high group persistence, low group movement, improved feeding efficiency, improved individual predator detection and improved individual survival. (C) 2003 Published by Elsevier Ltd on behalf of The Association for the Study of Animal Behaviour. | 4170 | NA | NA | NA | no | no |
| 4169 | 2017 | NA | Wasitthankasem, R; Vongpunsawad, S; Siripon, N; Suya, C; Chulothok, P; Chaiear, K; Rujirojindakul, P; Kanjana, S; Theamboonlers, A; Tangkijvanich, P; Poovorawan, Y | 2015 | Genotypic Distribution of Hepatitis C Virus in Thailand and Southeast Asia | The majority of hepatitis C virus (HCV) infection results in chronic infection, which can lead to liver cirrhosis and hepatocellular carcinoma. Global burden of hepatitis C virus (HCV) is estimated at 150 million individuals, or 3% of the world’s population. The distribution of the seven major genotypes of HCV varies with geographical regions. Since Asia has a high incidence of HCV, we assessed the distribution of HCV genotypes in Thailand and Southeast Asia. From 588 HCV-positive samples obtained throughout Thailand, we characterized the HCV 5’ untranslated region, Core, and NS5B regions by nested PCR. Nucleotide sequences obtained from both the Core and NS5B of these isolates were subjected to phylogenetic analysis, and genotypes were assigned using published reference genotypes. Results were compared to the epidemiological data of HCV genotypes identified within Southeast Asian. Among the HCV subtypes characterized in the Thai samples, subtype 3a was the most predominant (36.4%), followed by 1a (19.9%), 1b (12.6%), 3b (9.7%) and 2a (0.5%). While genotype 1 was prevalent throughout Thailand (27-36%), genotype 3 was more common in the south. Genotype 6 (20.9%) constituted subtype 6f (7.8%), 6n (7.7%), 6i (3.4%), 6j and 6m (0.7% each), 6c (0.3%), 6v and 6xa (0.2% each) and its prevalence was significantly lower in southern Thailand compared to the north and northeast (p = 0.027 and p = 0.030, respectively). Within Southeast Asia, high prevalence of genotype 6 occurred in northern countries such as Myanmar, Laos, and Vietnam, while genotype 3 was prevalent in Thailand and Malaysia. Island nations of Singapore, Indonesia and Philippines demonstrated prevalence of genotype 1. This study further provides regional HCV genotype information that may be useful in fostering sound public health policy and tracking future patterns of HCV spread. | 4171 | NA | NA | NA | no | no |
| 4170 | 2017 | NA | Mu, JB; Awadalla, P; Duan, JH; McGee, KM; Keebler, J; Seydel, K; McVean, GAT; Su, XZ | 2007 | Genome-wide variation and identification of vaccine targets in the Plasmodium falciparum genome | One goal in sequencing the Plasmodium falciparum genome, the agent of the most lethal form of malaria, is to discover vaccine and drug targets(1). However, identifying those targets in a genome in which similar to 60% of genes have unknown functions is an enormous challenge. Because the majority of known malaria antigens and drug-resistant genes are highly polymorphic and under various selective pressures(2-6), genome-wide analysis for signatures of selection may lead to discovery of new vaccine and drug candidates. Here we surveyed 3,539 P. falciparum genes (similar to 65% of the predicted genes) for polymorphisms and identified various highly polymorphic loci and genes, some of which encode new antigens that we confirmed using human immune sera. Our collections of genome-wide SNPs (similar to 65% nonsynonymous) and polymorphic microsatellites and indels provide a high-resolution map (one marker per similar to 4 kb) for mapping parasite traits and studying parasite populations. In addition, we report new antigens, providing urgently needed vaccine candidates for disease control. | 4172 | NA | NA | NA | no | no |
| 4171 | 2017 | NA | Bekkevold, D; Boomsma, JJ | 2000 | Evolutionary transition to a semelparous life history in the socially parasitic ant Acromyrmex insinuator | The recently discovered social parasite Acromyrmex insinuator (Schultz, Bekkevold & Boomsma 1998) exploits colonies of the leafcutter ant A. echinatior. We document that A. insinuator represents a rare early stage in the evolution of social parasitism, because a worker caste is still partially present and mating phenology has remained at least partially similar to that of the host. A. insinuator is tolerant of host queens, and sexual offspring produced in parasitized colonies can be either exclusively A. insinuator or a mix of A. insinuator and A. echinatior. The remarkably high abundance of A. insinuator in nests of the investigated Panamanian host population and the fact that A. insinuator colonies readily reproduce under laboratory conditions allowed us to test evolutionary predictions on reproductive life history evolution that are not possible in most other socially parasitic ants. We show that (1) A. insinuator has a semelparous ‘big bang’ reproductive life history which exploits host colonies without leaving reserves for survival; (2) social parasite sexuals are significantly smaller than A. echinatior host sexuals, but still large compared to host workers, confirming an evolutionary scenario of gradual size reduction and loss of the worker caste after transition towards a socially parasitic life history; (3) major changes in the life history of ants can evolve relatively quickly compared to adaptations in morphology, caste differentiation and mating phenology. | 4173 | NA | NA | NA | no | no |
| 4172 | 2017 | NA | Mixao, VDP; Mendes, AM; Mauricio, IL; Calado, MM; Novo, MT; Belo, S; Almeida, APG | 2016 | Molecular detection of Wolbachia pipientis in natural populations of mosquito vectors of Dirofilaria immitis from continental Portugal: first detection in Culex theileri | Wolbachia pipientis (Rickettsiales: Rickettsiaceae) protects mosquitoes from infections with arboviruses and parasites. However, the effect of its co-infection on vector competence for Dirofilaria immitis (Spirurida: Onchocercidae) in the wild has not been investigated. This study aimed to screen vectors of D. immitis for wPip, to characterize these, and to investigate a possible association between the occurrence of W. pipientis and that of the nematode. The presence of W. pipientis was assessed in the five mosquito potential vectors of D. immitis in Portugal. Polymerase chain reaction (PCR) products were sequenced, and wPip haplotypes were determined by PCR-restricted fragment length polymorphism (RFLP). Results showed that wPip was detected in 61.5% of Culex pipiens (Diptera: Culicidae) pools and 6.3% of Culex theileri pools. wPip 16s rRNA sequences found in Cx. theileri exactly match those from Cx. pipiens, confirming a mosquito origin, rather than a nematode origin, as some specimens were infected with D. immitis. Only wPip haplotype I was found. No association was found between the presence of wPip and D. immitis in mosquitoes and hence a role for this endosymbiont in influencing vectorial competence is yet to be identified. This study contributes to understanding of wPip distribution in mosquito populations and, to the best of the authors’ knowledge, is the first report of natural infections by wPip in Cx. theileri. | 4174 | NA | NA | NA | no | no |
| 4173 | 2017 | NA | Bigio, G; Al Toufailia, H; Hughes, WOH; Ratnieks, FLW | 2014 | The effect of one generation of controlled mating on the expression of hygienic behaviour in honey bees | Honey bee mating cannot be directly controlled in the same way as in many agriculturally important animals. Instrumental insemination is, however, possible and can be used as an aid in selective breeding. Hygienic behaviour, in which worker bees detect and remove dead or diseased brood from capped cells, is a heritable trait that confers colony-level resistance against brood diseases. Using the freeze-killed brood (FKB) bioassay we compared the levels of hygiene in colonies headed by daughter queens reared from hygienic mother colonies that were either instrumentally inseminated with sperm from drones reared from hygienic colonies or allowed to mate naturally with naturally-occurring drones. Hygiene levels were significantly higher in the colonies of the instrumentally inseminated queens than in the colonies of the naturally-mated queens. However, the hygiene levels in the naturally-mated colonies were encouragingly high and indicate that supplying beekeepers with naturally-mated queens, or virgin queens to mate locally, can result in colonies with high levels of hygiene. | 4175 | NA | NA | NA | no | no |
| 4174 | 2017 | NA | Ricano, J; Guerri-Agullo, B; Serna-Sarrias, MJ; Rubio-Llorca, G; Asensio, L; Barranco, P; Lopez-Llorca, LV | 2013 | EVALUATION OF THE PATHOGENICITY OF MULTIPLE ISOLATES OF BEAUVERIA BASSIANA (HYPOCREALES: CLAVICIPITACEAE) ON RHYNCHOPHORUS FERRUGINEUS (COLEOPTERA: DRYOPHTHORIDAE) FOR THE ASSESSMENT OF A SOLID FORMULATION UNDER SIMULATED FIELD CONDITIONS | A solid state formulation of Beauveria bassiana (Balsamo) Vuillemin has been developed for biological control of the Red Palm Weevil (RPW), Rhynchophorus ferrugineus (Olivier, 1790). Two kinds of bioassays (dry conidia and dipping) using 10 isolates from several coleopterans in Mediterranean environments, identified 2 RPW derived isolates (193 and 203) as most pathogenic to RPW larvae and adults (zero survival within first 4-5 d for dry conidia, and 14 and 23 d for dipping bioassays). Isolate 203 (5.1 x 10(8) +/- 1.9 x 10(8) conidia g(-1)) was formulated with fragmented date seed into solid granules and tested in palms infested with RPW under semi-field conditions in Feb, Apr/May and Jun of both 2007 and 2008. Beauveria bassiana significantly reduced RPW adult survival with respect to controls in May 2007 and in the Apr/Jun 2008 experiments. Total RPW adult mortality was achieved within 30 days for all B. bassiana treatments, and was associated with increasing numbers of insects with signs of mycosis in 2008 experiments. Beauveria bassiana formulation reduced RPW multiplication in artificially infested palms compared to controls, and a positive correlation between numbers of larvae and time post-infestation was recorded. The suppression of RPW adult populations by B. bassiana persisted for at least 3 months under semi-field conditions. The Beauveria bassiana solid formulation, which induces great adult mortality and persistence in the field, could be applied as a preventive as well as a curative treatment for the integrated management of RPW. | 4176 | NA | NA | NA | no | no |
| 4175 | 2017 | NA | Marucco, F; Pletscher, DH; Boitani, L; Schwartz, MK; Pilgrim, KL; Lebreton, JD | 2009 | Wolf survival and population trend using non-invasive capture-recapture techniques in the Western Alps | 1. Reliable estimates of population parameters are often necessary for conservation management but these are hard to obtain for elusive, rare and wide‐ranging species such as wolves Canis lupus. This species has naturally recolonized parts of its former habitat in Western Europe; however, an accurate and cost‐effective method to assess population trend and survival has not been implemented yet. 2. We used open‐model capture–recapture (CR) sampling with non‐invasive individual identifications derived from faecal genotyping to estimate survival and trend in abundance for wolves in the Western Alps between 1999 and 2006. Our sampling strategy reduced individual heterogeneity in recaptures, thus minimizing bias and increasing the precision of the estimates. 3. Young wolves had lower apparent annual survival rates (0·24 ± 0·06) than adult wolves (0·82 ± 0·04); survival rates were lower in the summer than in the winter for both young and adults. The wolf population in the study area increased from 21 ± 9·6 wolves in 1999 to 47 ± 11·2 wolves in late winter 2005; the population growth rate (λ = 1·04 ± 0·27) was lower than that recorded for other recolonizing wolf populations. 4. We found a positive trend in wolf abundance, regardless of the method used. However, the abundance estimate based on snow‐tracking was on average 36·2% (SD = 13·6%) lower than that from CR modelling, because young dispersing wolves are likely to have lower sign detection rates in snow‐track surveys, a problem adequately addressed by CR sampling. 5. Synthesis and applications. We successfully implemented a new method to assess large carnivore population trend and survival at large spatial scales. These are the first such estimates for wolves in Italy and in the Alps and have important management implications. Our approach can be widely applied to broader spatial and temporal scales for other elusive and wide‐ranging species in Europe and elsewhere. | 4177 | NA | NA | NA | no | no |
| 4176 | 2017 | NA | Earl, JE; Gray, MJ | 2014 | Introduction of Ranavirus to Isolated Wood Frog Populations Could Cause Local Extinction | Amphibian declines and extinction have been attributed to many causes, including disease such as chytridiomycosis. Other pathogens may also contribute to declines, with ranavirus as the most likely candidate given reoccurring die-offs observed in the wild. We were interested in whether it is possible for ranavirus to cause extinction of a local, closed population of amphibians. We used susceptibility data from experimental challenges on different life stages combined with estimates of demographic parameters from a natural population to predict the likelihood of extinction using a stage-structured population model for wood frogs (Lithobates sylvaticus). Extinction was most likely when the larval or metamorph stage was exposed under frequent intervals in smaller populations. Extinction never occurred when only the egg stage was exposed to ranavirus. Under the worst-case scenario, extinction could occur in as quickly as 5 years with exposure every year and 25-44 years with exposure every 2 years. In natural wood frog populations, die-offs typically occur in the larval stage and can reoccur in subsequent years, indicating that our simulations represent possible scenarios. Additionally, wood frog populations are particularly sensitive to changes in survival during the pre-metamorphic stages when ranavirus tends to be most pathogenic. Our results suggest that ranavirus could contribute to amphibian species declines, especially for species that are very susceptible to ranavirus with closed populations. We recommend that ranavirus be considered in risk analyses for amphibian species. | 4178 | NA | NA | NA | no | no |
| 4177 | 2017 | NA | Nishiya, AS; de Almeida-Neto, C; Ferreira, SC; Alencar, CS; Di-Lorenzo-Oliveira, C; Levi, JE; Salles, NA; Mendrone, A; Sabino, EC | 2014 | HCV Genotypes, Characterization of Mutations Conferring Drug Resistance to Protease Inhibitors, and Risk Factors among Blood Donors in Sao Paulo, Brazil | Background: Hepatitis C virus (HCV) infection is a global health problem estimated to affect almost 200 million people worldwide. The aim of this study is to analyze the subtypes and existence of variants resistant to protease inhibitors and their association with potential HCV risk factors among blood donors in Brazil. Methods: Repeat anti-HCV reactive blood donors are systematically asked to return for retest, notification, and counseling in which they are interviewed for risk factors for transfusion-transmitted diseases. We analyzed 202 donors who returned for counseling from 2007 to 2010 and presented enzyme immunoassay-and immunoblot-reactive results. The HCV genotypes and resistance mutation analyses were determined by the direct sequencing of the NS5b and NS3 regions, respectively. The HCV viral load was determined using an in-house real-time PCR assay targeting the 5’-NCR. Results: HCV subtypes 1b, 1a, and 3a were found in 45.5%, 32.0%, and 18.0% of the donors, respectively. The mean viral load of genotype 1 was significantly higher than that of the genotype 3 isolates. Subtype 1a was more frequent among young donors and 3a was more frequent among older donors. Protease inhibitor-resistant variants were detected in 12.8% of the sequenced samples belonging to genotype 1, and a higher frequency was observed among subtype 1a (20%) in comparison to 1b (8%). There was no difference in the prevalence of HCV risk factors among the genotypes or drug-resistant variants. Conclusions: We found a predominance of subtype 1b, with an increase in the frequency of subtype 1a, in young subjects. Mutations conferring resistance to NS3 inhibitors were frequent in treatment-naive blood donors, particularly those infected with subtype 1a. These variants were detected in the major viral population of HCV quasispecies, have replicative capacities comparable to nonresistant strains, and could be important for predicting the response to antiviral triple therapy. | 4179 | NA | NA | NA | no | no |
| 4178 | 2017 | NA | Hartke, TR; Rosengaus, RB | 2013 | Costs of pleometrosis in a polygamous termite | Costs and benefits of pleometrosis, as understood from social Hymenoptera, have never been tested in the independently evolved termites. To understand the extent to which such co-founding may be advantageous for colony survival and growth, we tracked the survival and reproduction of 5000 laboratory-established incipient colonies of the facultatively polygamous neotropical termite Nasutitermes corniger. Significantly more pleometrotic groups than monogamous queen-king pairs failed within the first 90 days of establishment, and 99 per cent of pleometrotic groups lost at least one founding member. Oviposition commenced earlier in larger groups, but colony growth was slower and production of workers and soldiers was delayed compared with pairs. Thus, pleometrosis does not increase colony fitness and is in fact highly disadvantageous. | 4180 | NA | NA | NA | no | no |
| 4179 | 2017 | NA | Arruda, MP; Lipka, AE; Brown, PJ; Krill, AM; Thurber, C; Brown-Guedira, G; Dong, Y; Foresman, BJ; Kolb, FL | 2016 | Comparing genomic selection and marker-assisted selection for Fusarium head blight resistance in wheat (Triticum aestivum L.) | Genomic selection (GS) and marker-assisted selection (MAS) rely on marker-trait associations and are both routinely used for breeding purposes. Although similar, these two approaches differ in their applications and how markers are used to estimate breeding values. In this study, GS and MAS were compared in their ability to predict six traits associated with resistance to a destructive wheat disease, Fusarium head blight (FHB). A panel consisting in 273 soft red winter wheat lines from the US Midwestern and Eastern regions was used in this study. The statistical models for MAS were built using Fhb-1, the best-studied quantitative trait loci (QTL) for FHB resistance, and two sets of QTL: one independently identified by other groups and a newer set identified “in house”. In contrast, genomic selection models relied on 19,992 SNPs distributed throughout the genome. For the MAS and GS models, marker effects were estimated with ordinary least square and ridge regression best unbiased linear prediction, respectively. Intermediate to high values of prediction accuracy (0.4-0.9) were observed for most GS models, with lower values (<0.3) found for MAS models. Treating QTL as fixed effects in GS models resulted in higher prediction accuracy when compared with a GS model with only random effects, but overestimated accuracies were obtained with in house QTL. For the same selection intensity, GS resulted in higher selection differentials than MAS for all traits. Our results indicate that GS is a more appropriate strategy than MAS for FHB resistance. | 4181 | NA | NA | NA | no | no |
| 4180 | 2017 | NA | Flatz, R; Gerber, LR | 2010 | First Evidence for Adoption in California Sea Lions | Demographic parameters such as birth and death rates determine the persistence of populations. Understanding the mechanisms that influence these rates is essential to developing effective management strategies. Alloparental behavior, or the care of non-filial young, has been documented in many species and has been shown to influence offspring survival. However, the role of alloparental behavior in maintaining population viability has not been previously studied. Here, we provide the first evidence for adoption in California sea lions and show that adoption potentially works to maintain a high survival rate of young and may ultimately contribute to population persistence. Alloparental behavior should have a positive effect on the population growth rate when the sum of the effects on fitness for the alloparent and beneficiary is positive. | 4182 | NA | NA | NA | no | no |
| 4181 | 2017 | NA | Allen, LJS; McCormack, RK; Jonsson, CB | 2006 | Mathematical models for hantavirus infection in rodents | Hantavirus pulmonary syndrome is an emerging disease of humans that is carried by wild rodents. Humans arc usually exposed to the virus through geographically isolated outbreaks. The driving forces behind these outbreaks is poorly understood. Certainly. one key driver of the emergence of these viruses is the virus population dynamics within the rodent population. Two new mathematical models for hantavirus infection in rodents are formulated and studied. The new models include the dynamics of Susceptible, exposed, infective, and recovered male and female rodents. The first model is a system of ordinary differential equations while the second model is a system of stochastic differential equations. These new models capture some of the realistic dynamics of the male/female rodent hantavirus interaction: higher seroprevalence in males and variability in seroprevalence levels. | 4183 | NA | NA | NA | no | no |
| 4182 | 2017 | NA | Oldroyd, BP; Allsopp, MH; Lim, J; Beekman, M | 2011 | A THELYTOKOUS LINEAGE OF SOCIALLY PARASITIC HONEY BEES HAS RETAINED HETEROZYGOSITY DESPITE AT LEAST 10 YEARS OF INBREEDING | The honey bee population of South Africa is divided into two subspecies: a northern population in which queenless workers reproduce arrhenotokously and a southern one in which workers reproduce thelytokously. A hybrid zone separates the two, but on at least three occasions the northern population has become infested by reproductive workers derived from the southern population. These parasitic workers lay in host colonies parthenogenetically, resulting in yet more parasites. The current infestation is 20-year old-surprising because an asexual lineage is expected to show a decline in vigor over time due to increasing homozygosity. The decline is expected to be acute in honey bees, where homozygosity at the sex locus is lethal. We surveyed colonies from the zone of infestation and genotyped putative parasites at two sets of linked microsatellite loci. We confirm that there is a single clonal lineage of parasites that shows minor variations arising from recombination events. The lineage shows high levels of heterozygosity, which may be maintained by selection against homozygotes, or by a reduction in recombination frequency within the lineage. We suggest that the clonal lineage can endure the costs of asexual reproduction because of the fitness benefits of its parasitic life history. | 4184 | NA | NA | NA | no | no |
| 4183 | 2017 | NA | Dharia, NV; Plouffe, D; Bopp, SER; Gonzalez-Paez, GE; Lucas, C; Salas, C; Soberon, V; Bursulaya, B; Kochel, TJ; Bacon, DJ; Winzeler, EA | 2010 | Genome scanning of Amazonian Plasmodium falciparum shows subtelomeric instability and clindamycin-resistant parasites | Here, we fully characterize the genomes of 14 Plasmodium falciparum patient isolates taken recently from the Iquitos region using genome scanning, a microarray-based technique that delineates the majority of single-base changes, indels, and copy number variants distinguishing the coding regions of two clones. We show that the parasite population in the Peruvian Amazon bears a limited number of genotypes and low recombination frequencies. Despite the essentially clonal nature of some isolates, we see high frequencies of mutations in subtelomeric highly variable genes and internal var genes, indicating mutations arising during self-mating or mitotic replication. The data also reveal that one or two meioses separate different isolates, showing that P. falciparum clones isolated from different individuals in defined geographical regions could be useful in linkage analyses or quantitative trait locus studies. Through pairwise comparisons of different isolates we discovered point mutations in the apicoplast genome that are close to known mutations that confer clindamycin resistance in other species, but which were hitherto unknown in malaria parasites. Subsequent drug sensitivity testing revealed over 100-fold increase of clindamycin EC50 in strains harboring one of these mutations. This evidence of clindamycin-resistant parasites in the Amazon suggests that a shift should be made in health policy away from quinine + clindamycin therapy for malaria in pregnant women and infants, and that the development of new lincosamide antibiotics for malaria should be reconsidered. [Supplemental material is available online at http://www.genome.org. The microarray data from this study have been submitted to the NCBI Gene Expression Omnibus (http://www.ncbi.nlm.nih.gov/geo) under accession no. GSE22861 and are also at http://www.scripps.edu/cb/winzeler/resources/pf\_peru.] | 4185 | NA | NA | NA | no | no |
| 4184 | 2017 | NA | Zwick, ME; Mcafee, F; Cutler, DJ; Read, TD; Ravel, J; Bowman, GR; Galloway, DR; Mateczun, A | 2005 | Microarray-based resequencing of multiple Bacillus anthracis isolates | We used custom-designed resequencing arrays to generate 3.1 Mb of genomic sequence from a panel of 56 Bacillus anthracis strains. Sequence quality was shown to be very high by replication (discrepancy rate of 7.4 x 10(-7)) and by comparison to independently generated shotgun sequence (discrepancy rate < 2.5 x 10(-6)). Population genomics studies of microbial pathogens using rapid resequencing technologies such as resequencing arrays are critical for recognizing newly emerging or genetically engineered strains. | 4186 | NA | NA | NA | no | no |
| 4185 | 2017 | NA | Smith, JA; Schwarz, MP | 2006 | Strategic exploitation in a socially parasitic bee: a benefit in waiting? | Social parasitism has evolved at least ten times in the allodapine bees but studies that explore the parasite’s integration and exploitation of host colonies are lacking. Using colony content and dissection data, we examine how Inquilina schwarzi affects the social organisation of its host Exoneura robusta. Our samples include three critical periods in the host life cycle: initial formation of dominance hierarchies in late autumn, commencement of oviposition by host queens in late winter, and development of secondary reproductives in late spring. I. schwarzi preferentially parasitises larger host colonies in autumn, but during autumn and winter, the parasite appears to be socially invisible, living in the nest without disrupting the normal functioning of these colonies. Inquilines begin egg laying much later than their hosts, and by late spring, they have disrupted host reproductive hierarchies, leading to lower skew in ovarian sizes of their host nestmates. Living invisibly within the host nest for the first 6 months and waiting until well after host reproduction has begun before disrupting their social organisation appear to be unique among social insects. Such a change in strategy may be facilitated by the different social systems found in allodapine bees, with the social parasites possibly disrupting the reproductive hierarchies during spring to prevent or reduce the normal dispersal of some host females from their natal nests. | 4187 | NA | NA | NA | no | no |
| 4186 | 2017 | NA | Ujvari, B; Pearse, AM; Peck, S; Harmsen, C; Taylor, R; Pyecroft, S; Madsen, T; Papenfuss, AT; Belov, K | 2013 | Evolution of a contagious cancer: epigenetic variation in Devil Facial Tumour Disease | The emergence of Devil Facial Tumour Disease (DFTD), a highly contagious cancer, is driving Tasmanian devils (Sarcophilus harrisii) to extinction. The cancer is a genetically and chromosomally stable clonal cell line which is transmitted by biting during social interactions. In the present study, we explore the Devil Facial Tumour (DFT) epigenome and the genes involved in DNA methylation homeostasis. We show that tumour cells have similar levels of methylation to peripheral nerves, the tissue from which DFTD originated. We did not observe any strain or region-specific epimutations. However, we revealed a significant increase in hypomethylation in DFT samples over time (p<0.0001). We propose that loss of methylation is not because of a maintenance deficiency, as an upregulation of DNA methyltransferase 1 gene was observed in tumours compared with nerves (p<0.005). Instead, we believe that loss of methylation is owing to active demethylation, supported by the temporal increase in MBD2 and MBD4 (p<0.001). The implications of these changes on disease phenotypes need to be explored. Our work shows that DFTD should not be treated as a static entity, but rather as an evolving parasite with epigenetic plasticity. Understanding the role of epimutations in the evolution of this parasitic cancer will provide unique insights into the role of epigenetic plasticity in cancer evolution and progression in traditional cancers that arise and die with their hosts. | 4188 | NA | NA | NA | no | no |
| 4187 | 2017 | NA | Goldstein, O; Zangerl, B; Pearce-Kelling, S; Sidjanin, DJ; Kijas, JW; Felix, J; Acland, GM; Aguirre, GD | 2006 | Linkage disequilibrium mapping in domestic dog breeds narrows the progressive rod-cone degeneration interval and identifies ancestral disease-transmitting chromosome | Canine progressive rod-cone degeneration (prcd) is a retinal disease previously mapped to a broad, gene-rich centromeric region of canine chromosome 9. As allelic disorders are present in multiple breeds, we used linkage disequilibrium (LD) to narrow the similar to 6.4-Mb interval candidate region. Multiple dog breeds, each representing genetically isolated populations, were typed for SNPs and other polymorphisms identified from BACs. The candidate region was initially localized to a 1.5-Mb zero recombination interval between growth factor receptor-bound protein 2 (GRB2) and SEC 14-like 1 (SEC14L). A fine-scale haplotype of the region was developed, which reduced the LD interval to 106 kb and identified a conserved haplotype of 98 polymorphisms present in all prcd-affected chromosomes from 14 different dog breeds. The findings strongly suggest that a common ancestor transmitted the prcd disease allele to many of the modern dog breeds and demonstrate the power of the LD approach in the canine model. (c) 2006 Elsevier Inc. All rights reserved. | 4189 | NA | NA | NA | no | no |
| 4188 | 2017 | NA | van Heerwaarden, B; Lee, RFH; Overgaard, J; Sgro, CM | 2014 | No patterns in thermal plasticity along a latitudinal gradient in Drosophila simulans from eastern Australia | Phenotypic plasticity may be an important initial mechanism to counter environmental change, yet we know relatively little about the evolution of plasticity in nature. Species with widespread distributions are expected to have evolved higher levels of plasticity compared with those with more restricted, tropical distributions. At the intraspecific level, temperate populations are expected to have evolved higher levels of plasticity than their tropical counterparts. However, empirical support for these expectations is limited. In addition, no studies have comprehensively examined the evolution of thermal plasticity across life stages. Using populations of Drosophila simulans collected from a latitudinal cline spanning the entire east coast of Australia, we assessed thermal plasticity, measured as hardening capacity (the difference between basal and hardened thermal tolerance) for multiple measures of heat and cold tolerance across both adult and larval stages of development. This allowed us to explicitly ask whether the evolution of thermal plasticity is favoured in more variable, temperate environments. We found no relationship between thermal plasticity and latitude, providing little support for the hypothesis that temperate populations have evolved higher levels of thermal plasticity than their tropical counterparts. With the exception of adult heat survival, we also found no association between plasticity and ten climatic variables, indicating that the evolution of thermal plasticity is not easily predicted by the type of environment that a particular population occupies. We discuss these results in the context of the role of plasticity in a warming climate. | 4190 | NA | NA | NA | no | no |
| 4189 | 2017 | NA | Allsopp, MH; Beekman, M; Gloag, RS; Oldroyd, BP | 2010 | Maternity of replacement queens in the thelytokous Cape honey bee Apis mellifera capensis | Unlike workers of all other honey bee (Apis mellifera) subspecies, workers of the Cape honey bee of South Africa (A. mellifera capensis) reproduce thelytokously and are thus able to produce female offspring that are pseudoclones of themselves. This ability allows workers to compete with their queen over the maternity of daughter queens and, in one extreme case, has led to a clonal lineage of workers becoming a social parasite in commercially managed populations of A. mellifera scutellata. Previous work (Jordan et al., Proc R Soc Lond B Biol Sci 275:345, 2008) showed that, in A. mellifera capensis, 59% of queen cells produced during swarming events contained the offspring of workers and that, of these, 65% were the offspring of non-natal workers. Here, we confirm that a substantial proportion (38.5%) of offspring queens is worker-laid. We additionally show that: (1) Although queens produce most diploid female offspring sexually, we found some homozygous or hemizygous queen offspring, suggesting that queens also reproduce by thelytoky. These parthenogenetic individuals are probably nonviable beyond the larval stage. (2) Worker-laid offspring queens are viable and become the resident queen at the same frequency as do sexually produced queen-laid offspring queens. (3) In this study, all but one of the worker-derived queens were laid by natal workers rather than workers from another nest. This suggests that the very high rates of social parasitism observed in our previous study were enhanced by beekeeping manipulations, which increased movement of parasites between colonies. | 4191 | NA | NA | NA | no | no |
| 4190 | 2017 | NA | Palacin, C; Martin, B; Onrubia, A; Alonso, JC | 2016 | Assessing the extinction risk of the great bustard Otis tarda in Africa | We studied the dynamics and trend of the last extant population of great bustards Otis tarda in Africa. Moroccan great bustards are the southernmost population of this species, and thus show the characteristics of a peripheral population: small size, isolation and low gene flow. Available counts indicate a severe population decline (62% in the last 15 yr), as well as a contraction of the species’ distribution. We used a population viability analysis (PVA) to evaluate the quasi-extinction risk and to identify the most important threats. The estimated geometric growth rate of the more realistic of a set of possible scenarios was 0.87 (95% CI: 0.85, 0.89). This implies a 13% annual decline over 50 yr. However, projections derived from these results should be interpreted with caution, because models have a great deal of uncertainty and vital rates from Iberian populations may be different from those of the Moroccan population. PVA showed the negative consequence of human-induced mortality. According to the model that best fits our census data and if present threats remain in the coming years, this peripheral population could go extinct in ca. 20 yr. Agricultural intensification, infrastructure developments and new power lines in rural areas where the species occurs are causing habitat destruction and fragmentation and increasing artificial mortality. Urgent conservation measures, especially to reduce human-induced mortality, are needed to save African great bustards from extinction. We suggest that these findings can be generalized to other peripheral great bustard populations living in highly humanized landscapes. | 4192 | NA | NA | NA | no | no |
| 4191 | 2017 | NA | Chi, HS; Taik, P; Foley, EJ; Racicot, AC; Gray, HM; Guzzetta, KE; Lin, HY; Song, YL; Tung, CH; Zenke, K; Yoshinaga, T; Cheng, CY; Chang, WJ; Gong, H | 2017 | High genetic diversities between isolates of the fish parasite Cryptocaryon irritans (Ciliophora) suggest multiple cryptic species | The ciliate protozoan Cryptocaryon irritans parasitizes marine fish and causes lethal white spot disease. Sporadic infections as well as large-scale outbreaks have been reported globally and the parasite’s broad host range poses particular threat to the aquaculture and ornamental fish markets. In order to better understand C irritans’ population structure, we sequenced and compared mitochondrial cox-1, SSU rRNA, and ITS-1 sequences from 8 new isolates of C irritans collected in China, Japan, and Taiwan. We detected two SSU rRNA haplotypes, which differ at three positions, separating the isolates into two main groups (I and II). Cox-1 sequences also support the division into two groups, and the cox-1 divergence between these two groups is unexpectedly high (9.28% for 1582 nucleotide positions). The divergence is much greater than that detected in Ichthyophthirius multifiliis, the ciliate protozoan causing freshwater white spot disease in fish, where intraspecies divergence on cox-1 sequence is only 1.95%. ITS-1 sequences derived from these eight isolates and from all other C irritans isolates (deposited in the GenBank) not only support the two groups, but further suggest the presence of a third group with even greater sequence divergence. Finally, a small Ka/Ks ratio estimated from cox-1 sequences suggests that this gene in C. irritans remains under strong purifying selection. Taken together, the C irritans species may consists of many subspecies and/or syngens. Further work is needed to determine if there is reproductive isolation between the groups we have defined. (C) 2017 Elsevier Inc. All rights reserved. | 4193 | NA | NA | NA | no | no |
| 4192 | 2017 | NA | Al-Janabi, B; Kruse, I; Graiff, A; Winde, V; Lenz, M; Wahl, M | 2016 | Buffering and Amplifying Interactions among OAW (Ocean Acidification & Warming) and Nutrient Enrichment on Early Life-Stage Fucus vesiculosus L. (Phaeophyceae) and Their Carry Over Effects to Hypoxia Impact | Ocean acidification and warming (OAW) are occurring globally. Additionally, at a more local scale the spreading of hypoxic conditions is promoted by eutrophication and warming. In the semi-enclosed brackish Baltic Sea, occasional upwelling in late summer and autumn may expose even shallow-water communities including the macroalga Fucus vesiculosus to particularly acidified, nutrient-rich and oxygen-poor water bodies. During summer 2014 (July-September) sibling groups of early life-stage F. vesiculosus were exposed to OAW in the presence and absence of enhanced nutrient levels and, subsequently to a single upwelling event in a near-natural scenario which included all environmental fluctuations in the Kiel Fjord, southwestern Baltic Sea, Germany (54 degrees 27 ’N, 10 degrees 11 ’W). We strove to elucidate the single and combined impacts of these potential stressors, and how stress sensitivity varies among genetically different sibling groups. Enhanced by a circumstantial natural heat wave, warming and acidification increased mortalities and reduced growth in F. vesiculosus germlings. This impact, however, was mitigated by enhanced nutrient conditions. Survival under OAW conditions strongly varied among sibling groups hinting at a substantial adaptive potential of the natural Fucus populations in the Western Baltic. A three-day experimental upwelling caused severe mortality of Fucus germlings, which was substantially more severe in those sibling groups which previously had been exposed to OAW. Our results show that global (OAW), regional (nutrient enrichment) and local pressures (upwelling), both alone and co-occurring may have synergistic and antagonistic effects on survival and/or growth of Fucus germlings. This result emphasizes the need to consider combined stress effects. | 4194 | NA | NA | NA | no | no |
| 4193 | 2017 | NA | Wang, JH; Xi, DH; Liu, JJ; Chen, KL; Li, HW; Liu, X; Yuan, S; Ercisli, S; Lin, HH | 2012 | Genetic variability in Grapevine virus A from Vitis vinifera L. x Vitis labrusca L. in Sichuan, China | Grapevine virus A (GVA) has a quasispecies nature and is closely associated with the rugose-wood disease complex of Vitis vinifera L. In the different growing regions of table grape in Sichuan, southwest China, the incidence of GVA infection was found to be 16.9% by ELISA detection among 178 grape plants ( Vitis vinifera L. x Vitis labrusca L.). The results of restriction fragment length polymorphism (RFLP) studies on the polymerase chain reaction (PCR) products of a total of 139 plasmids, cloned from 15 GVA isolates, suggested that the GVA isolates contained highly divergent variants. The plasmids from each GVA isolate that yielded different PCR-RFLP profiles were preferentially chosen for sequencing and were designated as variants. Furthermore, a phylogenetic study based on the analysis of the GVA coat protein genes and RNA silencing suppressor genes showed that the 40 variants obtained from the 15 GVA isolates represented 4 clades, designated as molecular groups I, II, III, and IV, respectively. The variant-specific PCR detection results indicated that the parts of the tested grape plants were specifically infected by mild variants. | 4195 | NA | NA | NA | no | no |
| 4194 | 2017 | NA | Thornber, CS | 2006 | Functional properties of the isomorphic biphasic algal life cycle | Many species of marine algae have life cycles that involve multiple separate, free-living phases that frequently differ in ploidy levels. These complex life cycles have received increasing scientific attention over the past few decades, due to their usefulness for both ecological and evolutionary studies. I present a synthesis of our current knowledge of the ecological functioning and evolutionary implications of the isomorphic, biphasic life cycles commonly found in many species of marine algae. There are both costs and benefits to life cycles with 2 morphologically similar but separate, free-living phases that differ in ploidy levels (haploids and diploids). Evolutionary theory predicts that the existence of subtle yet important differences between the phases may be what allows these life cycles to be maintained. Different phases of the same species can vary in abundance, in demographic parameters such as mortality and fecundity, in their physiology, and in their resistance to herbivory. Some taxonomic groups within the red algae have received significant attention toward these issues, while our knowledge of these properties for brown and green algae remains limited. | 4196 | NA | NA | NA | no | no |
| 4195 | 2017 | NA | MOSS, R; PARR, R; LAMBIN, X | 1994 | EFFECTS OF TESTOSTERONE ON BREEDING DENSITY, BREEDING SUCCESS AND SURVIVAL OF RED GROUSE | Territorial intolerance can limit bird numbers by preventing subordinates from holding territories and breeding. We report the first controlled and replicated experiment in a natural population of territorial animals which shows that population density can be decreased by implants of testosterone inducing increased aggressiveness. Implanted territorial cocks expanded their territories at the expense of unimplanted neighbours, some of which left the study area. The probability of an unimplanted cock leaving the study area increased with his number of implanted neighbours. Implanted cocks had better breeding success but poorer subsequent survival than control cocks. | 4197 | NA | NA | NA | no | no |
| 4196 | 2017 | NA | Oh, KP; Badyaev, AV | 2009 | Isolation and characterization of 17 microsatellite loci for the house finch (Carpodacus mexicanus) | The house finch (Carpodacus mexicanus) has emerged recently as a model species in studies of sexual selection, reproductive physiology, population genetics, and epizootic disease ecology. Here we describe 17 highly polymorphic microsatellite loci for this species. In a sample of 36 individuals, we observed an average of 16 alleles per locus and heterozygosity ranged from 0.61 to 0.97. One locus showed significant deviation from Hardy-Weinberg proportions, but no significant gametic disequilibrium was observed among any of the loci. Amplification by polymerase chain reaction was optimized under similar parameters across loci, thereby facilitating multiplexing and rapid multilocus genotyping. | 4198 | NA | NA | NA | no | no |
| 4197 | 2017 | NA | Villain, L; Molina, A; Sierra, S; Decazy, B; Sarah, JL | 2000 | Effect of grafting and nematicide treatments on damage by root-lesion nematodes (Pratylenchus spp.) to Coffea arabica L. in Guatemala | Root-lesion nemattodes, Pratylenchus spp., are among the pests with the greatest negative impact on the economy of coffee production in Guatemala. A field experiment was undertaken in southwest Guatemala to assess damage due to a root-lesion nematode and to compare two methods of management: grafting onto Coffea canephora Pierre, 1897, and nematicide treatments (terbufos). The experiment was carried out for five years, and included the first three harvests. Root population densities of nematodes, coffee berry yield losses, and plant mortality rates were highest on ungrafted C. arabica, confirming its high degree of vulnerability to this root-lesion nematode population. Grafting onto C. canephora provided efficient control of populations of root-lesion nematodes and resulted in significantly greater yields compared to ungrafted plants. Nematicide treatments suppressed populations of this root-lesion nematodes only until the second year after planting. This was sufficient to significantly reduce rates of plant mortality in ungrafted plants. However, nematicide treatments did not result in significant Increase of yield regardless of the rootstocks used. This work also provided evidence that growing grafted coffee trees under shade may be a beneficial tactic for managing these root-lesion nematodes. | 4199 | NA | NA | NA | no | no |
| 4198 | 2017 | NA | Palomar, G; Voros, J; Bosch, J | 2017 | Tracking the introduction history of Ichthyosaura alpestris in a protected area of Central Spain | One of the main goals of protected areas, especially National Parks, is biodiversity conservation. Taking into account the multiple negative impacts that allochthonous animals can cause in an ecosystem, it is important to know and track the introductions in order to take efficient management measures to enhance biodiversity conservation. We study the origin of an Ichthyosaura alpestris population in Pealara Massif within the Guadarrama National Park, Central Spain. To accomplish this goal, we used nine microsatellite markers and sequences from 16S and ND4 genes. Both mitochondrial DNA and microsatellite analyses showed the close relationship between Pealara and Asturian populations, despite their geographical distance, confirming the allochthonous origin of this population. Interestingly, more than one introduction event seems to have occurred with individuals from Lago Ercina and Pozos de Lloroza populations participating in the founding of the Pealara population. Due to predation, competition and transmission of diseases enhanced by its ongoing expansion, I. alpestris population might be considered a threat to Pealara biodiversity. We advise an efficient population control to conserve the biodiversity within the Guadarrama National Park and an update of the position of this population in the Spanish indexes and list of species. | 4200 | NA | NA | NA | no | no |
| 4199 | 2017 | NA | Zhao, W; Zhang, WZ; Yang, ZY; Liu, AQ; Zhang, LX; Yang, FK; Wang, RJ; Ling, H | 2015 | Genotyping of Enterocytozoon bieneusi in Farmed Blue Foxes (Alopex lagopus) and Raccoon Dogs (Nyctereutes procyonoides) in China | Enterocytozoon bieneusi is the most common species of microsporidia found both in humans and animals. Farmed animals, particularly closely associated to humans, may play an important role of zoonotic reservoir in transmitting this disease to humans. The fur industry is a major economic component in some parts of China. To understand the prevalence, genotype variety and zoonotic risk of E. bieneusi in farmed foxes and raccoon dogs, two species of fur animals, fecal specimens of 110 blue foxes and 49 raccoon dogs from Heilongjiang and Jilin provinces in China were examined by internal transcribed spacer (ITS)-based PCR. E. bieneusi was detected in 16.4% (18/110) blue foxes and 4.1%(2/49) raccoon dogs. Altogether, four genotypes of E. bieneusi were identified, including two known genotypes D (n = 13) and EbpC (n = 5), and two novel genotypes named as CHN-F1 (n = 1) in a fox and CHN-R1 (n = 1) in a raccoon dog. Phylogenetic analysis revealed that all the four genotypes were the members of zoonotic group 1. Genotypes D and EbpC were found in humans previously. The findings of zoonotic genotypes of E. bieneusi in the foxes and raccoon dogs suggest these animals infected with E. bieneusi may pose a threat to human health. | 4201 | NA | NA | NA | no | no |
| 4200 | 2017 | NA | Markey, KL; Abdo, DA; Evans, SN; Bosserelle, C | 2016 | Keeping It Local: Dispersal Limitations of Coral Larvae to the High Latitude Coral Reefs of the Houtman Abrolhos Islands | In 2011 the first recorded bleaching event for the high latitude Houtman Abrolhos Islands (HAI) coral communities was documented. This bleaching event highlighted the question of whether a supply of `heat tolerant’ coral recruits from the tropical north would be sufficient to provide a level of resistance for these reefs to future warming events. Using Lagrangian modelling we showed that due to its regional isolation, large-scale larval input from potential tropical northern source populations to the HAI is unlikely, despite the southward flowing Leeuwin current. Successful recruitment to artificial substrates was recorded following the bleaching event. However, this was negligible (0.4 +/- 0.1 recruits per tile) compared to 2013 post impact recruitment (128.8 +/- 15.8 recruits per tile). Our data therefore provides preliminary evidence suggesting that the connectivity of the HAI with coral communities in the north is limited, and population maintenance and recovery is likely driven primarily by self-recruitment. Given the low thermal tolerance of the HAI coral communities, the dominance of Acropora, and the apparent reliance on self-recruitment, an increased frequency of thermally anomalous conditions at the HAI (such as experienced in 2011) has the potential to reduce the long-term stability of the HAI coral populations and species that depend upon them. | 4202 | NA | NA | NA | no | no |
| 4201 | 2017 | NA | Hardus, ME; Lameira, AR; Menken, SBJ; Wich, SA | 2012 | Effects of logging on orangutan behavior | The human footprint is increasing across the world’s natural habitats, causing large negative impacts on the survival of many species. In order to successfully mitigate the negative effects on species’ survival, it is crucial to understand their responses to human-induced changes. This paper examines the effect of one such disturbance, logging, on Sumatran orangutans - a critically endangered great ape. Orangutan population densities may decrease or remain stable after logging, but data on the effects of logging on the behavior of individuals is scant. Here, we provide individual-level behavioral data based on direct observations in 2003-2008 at the Ketambe (Sumatra, Indonesia) research area (partly subject to intense selective logging) in order to assess responses of Sumatran orangutans to logging. Logging significantly negatively affected forest structure and orangutan food resources, specifically important fallback and liana-derived foods. Individual orangutans behaved differently between logged and pristine forest; they moved more and rested less in logged forest. With the exception of figs, diet composition remained overall similar. Altogether, life after logging seems energetically more expensive for orangutans. Based on the results of this study, we provide recommendations for conservation research and guidelines for reduced-impact logging. (C) 2011 Elsevier Ltd. All rights reserved. | 4203 | NA | NA | NA | no | no |
| 4202 | 2017 | NA | Li, W; Sun, HY; Deng, YY; Zhang, AX; Chen, HG | 2014 | The heterogeneity of the rDNA-ITS sequence and its phylogeny in Rhizoctonia cerealis, the cause of sharp eyespot in wheat | The sequence heterogeneity of the ribosomal internal transcribed spacer (ITS) region was investigated for Rhizoctonia cerealis isolates from the anastomosis group AG-DI. Although sequence variability of the ITS has been reported in a few multinucleate R. solani isolates, it has very rarely been reported in binucleate Rhizoctonia spp. isolates and has never been described in R. cerealis, the pathogen of wheat sharp eyespot. In this study, the ITS regions of 15 R. cerealis isolates were cloned and sequenced. The results revealed more than one different ITS sequence within each isolate. This is the first evidence of ITS sequence heterogeneity in R. cerealis. Based on these ITS sequences, different sequences of one isolate did not cluster in one clade, but all of the sequences of the 15 isolates were clustered in the anastomosis subgroup AG-DI, suggesting that the heterogeneity of the ITS did not affect the molecular identification of their anastomosis group. Haplotype analyses indicated that there might be three evolutionary origins of R. cerealis, or a recombination event could be the cause of different ITS sequences in one genome. This study demonstrates the variability and the evolution of Rhizoctonia, especially binucleate R. cerealis. These findings will help design disease control strategies. | 4204 | NA | NA | NA | no | no |
| 4203 | 2017 | NA | Dutta, J; Thakur, D | 2017 | Evaluation of multifarious plant growth promoting traits, antagonistic potential and phylogenetic affiliation of rhizobacteria associated with commercial tea plants grown in Darjeeling, India | Plant growth promoting rhizobacteria (PGPR) are studied in different agricultural crops but the interaction of PGPR of tea crop is not yet studied well. In the present study, the indigenous tea rhizobacteria were isolated from seven tea estates of Darjeeling located in West Bengal, India. A total of 150 rhizobacterial isolates were screened for antagonistic activity against six different fungal pathogens i.e. Nigrospora sphaerica (KJ767520), Pestalotiopsis theae (ITCC 6599), Curvularia eragostidis (ITCC 6429), Glomerella cingulata (MTCC 2033), Rhizoctonia Solani (MTCC 4633) and Fusarium oxysporum (MTCC 284), out of which 48 isolates were antagonist to at least one fungal pathogen used. These 48 isolates exhibited multifarious antifungal properties like the production of siderophore, chitinase, protease and cellulase and also plant growth promoting (PGP) traits like IAA production, phosphate solubilization, ammonia and ACC deaminase production. Amplified ribosomal DNA restriction analysis (ARDRA) and BOX-PCR analysis based genotyping clustered the isolates into different groups. Finally, four isolates were selected for plant growth promotion study in two tea commercial cultivars TV-1 and Teenali-17 in nursery conditions. The plant growth promotion study showed that the inoculation of consortia of these four PGPR isolates significantly increased the growth of tea plant in nursery conditions. Thus this study underlines the commercial potential of these selected PGPR isolates for sustainable tea cultivation. | 4205 | NA | NA | NA | no | no |
| 4204 | 2017 | NA | Witek, M; Skorka, P; Sliwinska, EB; Nowicki, P; Moron, D; Settele, J; Woyciechowski, M | 2011 | Development of parasitic Maculinea teleius (Lepidoptera, Lycaenidae) larvae in laboratory nests of four Myrmica ant host species | Maculinea butterflies are social parasites of Myrmica ants. Methods to study the strength of host ant specificity in the Maculinea-Myrmica association include research on chemical and acoustic mimicry as well as experiments on ant adoption and rearing behaviour of Maculinea larvae. Here we present results of laboratory experiments on adoption, survival, development and integration of M. teleius larvae within the nests of different Myrmica host species, with the objective of quantifying the degree of specialization of this Maculinea species. In the laboratory, a total of 94 nests of four Myrmica species: M. scabrinodis, M. rubra, M. ruginodis and M. rugulosa were used. Nests of M. rubra and M. rugulosa adopted M. teleius larvae more readily and quickly than M. ruginodis colonies. No significant differences were found in the survival rates of M. teleius larvae reared by different ant species. Early larval growth of M. teleius larvae differed slightly among nests of four Myrmica host species. Larvae reared by colonies of M. rugulosa which were the heaviest at the beginning of larval development had the lowest mean larval body mass after 18 weeks compared to those reared by other Myrmica species. None of the M. teleius larvae was carried by M. scabrinodis or M. rubra workers after ant nests were destroyed, which suggests a lack of integration with host colonies. Results indicate that Myrmica species coming from the same site differ in their ability to adopt and rear M. teleius larvae but there was no obvious adaptation of this butterfly species to one of the host ant species. This may explain why, under natural conditions, all four ants can be used as hosts of this butterfly species. Slight advantages of particular Myrmica species as hosts at certain points in butterfly larval development can be explained by the ant species biology and colony structure rather than by specialization of M. teleius. | 4206 | NA | NA | NA | no | no |
| 4205 | 2017 | NA | Armitage, SAO; Broch, JF; Marin, HF; Nash, DR; Boomsma, JJ | 2011 | IMMUNE DEFENSE IN LEAF-CUTTING ANTS: A CROSS-FOSTERING APPROACH | To ameliorate the impact of disease, social insects combine individual innate immune defenses with collective social defenses. This implies that there are different levels of selection acting on investment in immunity, each with their own trade-offs. We present the results of a cross-fostering experiment designed to address the influences of genotype and social rearing environment upon individual and social immune defenses. We used a multiply mating leaf-cutting ant, enabling us to test for patriline effects within a colony, as well as cross-colony matriline effects. The worker’s father influenced both individual innate immunity (constitutive antibacterial activity) and the size of the metapleural gland, which secretes antimicrobial compounds and functions in individual and social defense, indicating multiple mating could have important consequences for both defense types. However, the primarily social defense, a Pseudonocardia bacteria that helps to control pathogens in the ants’ fungus garden, showed a significant colony of origin by rearing environment interaction, whereby ants that acquired the bacteria of a foster colony obtained a less abundant cover of bacteria: one explanation for this pattern would be co-adaptation between host colonies and their vertically transmitted mutualist. These results illustrate the complexity of the selection pressures that affect the expression of multilevel immune defenses. | 4207 | NA | NA | NA | no | no |
| 4206 | 2017 | NA | Andersone, U; Druva-Lusite, I; Ievina, B; Karlsons, A; Necajeva, J; Samsone, I; Ievinsh, G | 2011 | The use of nondestructive methods to assess a physiological status and conservation perspectives of Eryngium maritimum L. | Eryngium maritimum is a perennial species growing exclusively in a coastal littoral zone both on sand dunes and shingle beach and indicated as declining in Northern Europe. The objective of the present study was to prove the use of nondestructive physiological measurement methods to access physiological status of endangered plants, using E. maritimum as a model species. Plants from two Latvian sites were studied in comparison with other populations in Northern Europe (Estonia, Lithuania, Poland, United Kingdom) to find out if local differences in environmental factors affect vitality of E. maritimum individuals. Noninvasive chlorophyll analysis and chlorophyll a fluorescence measurements were used as indicators of plant physiological status through characterization of various aspects of photochemistry of photosystem II activity. Dynamics and morphology of individuals were investigated for evaluation of clonal growth potential of E. maritimum in natural conditions. Highly fluctuating trend of dynamics of individuals (within 40 groups at two Latvian sites) was established for E. maritimum in natural conditions over a five-year period. Disturbance of individuals lead to formation of new shoots from nodal root meristems. An exponential regression between fluorescence parameters F(V)/F(M) and Performance Index and summary monthly precipitation was found indicating that E. maritimum plants had significant tolerance to water shortage together with susceptibility to increased precipitation. Negative correlation between leaf chlorophyll content and more northern localization of individuals was found (r = 0.95 and r = 0.94, for generative and vegetative shoots, respectively). Northern populations of E. maritimum are endangered by high precipitation in conditions of low air temperature, leading to decrease of photosynthetic productivity and overall physiological status. High developmental plasticity at the root level leads to clonal growth and an efficient survival and relatively long life span of E. maritimum individuals. Chlorophyll a fluorescence is a useful method to search for the effect of suboptimal conditions on physiological status of endangered plant species without elimination and disturbance of individuals. | 4208 | NA | NA | NA | no | no |
| 4207 | 2017 | NA | Maura, M; Salvi, D; Bologna, MA; Nascetti, G; Canestrelli, D | 2014 | Northern richness and cryptic refugia: phylogeography of the Italian smooth newt Lissotriton vulgaris meridionalis | Recent phylogeographical studies have re-evaluated the role of refugia in central and northern Europe for glacial persistence and postglacial assembly of temperate biota. Yet, on a regional scale within Mediterranean peninsulas, putative northern’ refugia’s contribution to the current structure of biodiversity still needs to be fully appreciated. To this end, we investigated the phylogeographical structure and the evolutionary history of the Italian smooth newt, Lissotriton vulgaris meridionalis, through phylogeographical, molecular dating and historical demographic analyses. We found ten differentiated mitochondrial lineages with a clear geographical association, mainly distributed in northern Italy. The most ancient divergence among these lineages was estimated at the Early Pleistocene and was followed by a series of splits throughout the Middle Pleistocene. No haplogroup turned out to be derived from another one, each one occupying terminal positions within the phylogenetic network topologies. These results suggest an unprecedented scenario involving long-term survival of distinct evolutionary lineages in multiple northern Mediterranean refugia. This scenario mirrors on a smaller geographical scale what has been previously observed in the literature concerning northern European environments; it also sheds more light on how northern Italy has contributed to temperate species’ long-term survival and to the assembly of regional biota.(c) 2014 The Linnean Society of London, Biological Journal of the Linnean Society, 2014, 113, 590-603. | 4209 | NA | NA | NA | no | no |
| 4208 | 2017 | NA | Yu, MN; Yu, JJ; Gu, CH; Nie, YF; Chen, ZY; Yin, XL; Liu, YF | 2014 | De novo sequencing and transcriptome analysis of Ustilaginoidea virens by using Illumina paired-end sequencing and development of simple sequence repeat markers | Ustilaginoidea virens is the causal agent of rice false smut which is a rice disease of increasing importance worldwide that has caused with the quantitative and qualitative rice losses. However, research on the pathogenic mechanism of U. virens is limited. In this study, we reported a de novo assembling, annotation, and characterization of the transcriptome and developed simple sequence repeat (SSR) markers of U. virens. U. virens transcripts of the mycelia and conidia mixture were sequenced using Illumina RNA-seq technology. A total of 52,554,142 clean reads were assembled into 36,496 transcripts representing 18,534 unigenes. Assembled unigenes were annotated through sequence comparison with known protein databases, and 48.48% of the unigenes were without hits in any of these databases. Clusters of orthologous groups for eukaryotic complete genome analysis identified the largest set of genes associated with posttranslational modification, protein turnover and chaperones. Kyoto Encyclopedia of Genes and Genomes pathway analyses identified the number of genes associated with mitogen-activated protein kinase and calcium-calcineurin pathways. The study also identified several putative pathogenicity determinants and candidate effectors in U. virens by using the pathogen-host interaction database. In addition, bioinformatics analysis revealed the presence of 12,298 SSR markers. This study provides a better understanding of the biology of U. virens and is an excellent resource for candidate genes required for pathogenesis discovery. (C) 2014 Elsevier B.V. All rights reserved. | 4210 | NA | NA | NA | no | no |
| 4209 | 2017 | NA | Burton, OJ; Travis, JMJ | 2008 | Landscape structure and boundary effects determine the fate of mutations occurring during range expansions | The interplay between the spatial dynamics of range expansion and evolutionary processes is receiving considerable attention. Recent theory has demonstrated that mutations occurring towards the front of a spatially expanding population can sometimes ‘surf’ to high frequency and spatial extent. Here, we extend this work to consider how the fate of a novel mutation is influenced by where and when it occurs. Specifically, we are interested in establishing how the origin of a mutation relative to a habitat edge influences its dynamics, and in understanding how this is mediated by the behaviour of individuals at those boundaries. Using a coupled-map lattice model, we demonstrate that the survival probability, abundance and spatial extent of surviving mutants can depend on their origin. An edge effect is often observed and can be quite different both qualitatively and quantitatively depending on the behavioural rules assumed. Mutations, especially those that are deleterious, that arise at a habitat edge with reflective boundary conditions can be many more times likely to survive for substantial periods of time than those that arise away from the edge. Conversely, with absorbing boundary conditions, their survival is greater when they arise well away from the edge. Our results clearly illustrate that landscape structure, habitat edges and boundary conditions have a considerable influence on the likely fate of mutations that occur during a period of range expansion. | 4211 | NA | NA | NA | no | no |
| 4210 | 2017 | NA | MOSES, RA; MILLAR, JS | 1994 | PHILOPATRY AND MOTHER-DAUGHTER ASSOCIATIONS IN BUSHY-TAILED WOODRATS - SPACE USE AND REPRODUCTIVE SUCCESS | Social groups in mammals are often based on overlapping generations of philopatric females, yet few researchers have examined fitness consequences of associations between females and their daughters. We examined survival and reproductive consequences of sharing a natal site with one’s mother or daughter in wild bushy-tailed woodrats (Neotoma cinerea). Bushy-tailed woodrats reside on discrete rocky outcrops, and females tend to spend their entire reproductive life on their natal outcrop. Mothers and daughters that co-existed on the natal outcrop were closely associated in space. Juvenile females were more likely to survive their first winter to become breeding yearlings at their natal site if their mother was also present at the natal site during the winter. Juveniles survived equally well with or without their mother where densities of adult females were experimentally reduced, suggesting that females enhance their daughter’s survival by facilitating their access to limited resources. Yearling females exhibited no reproductive costs from association with their mothers at the natal site during the breeding season; in fact, yearlings that bred at the natal site simultaneously with their adult mothers tended (P = 0.07) to experience fewer reproductive failures than did those that bred in the absence of their mothers. Mothers did not experience any detectable costs when sharing a natal site with a daughter. We conclude that bushy-tailed woodrats exhibit stable, fitness-enhancing associations among mothers and their philopatric daughters. | 4212 | NA | NA | NA | no | no |
| 4211 | 2017 | NA | Schmitt, T; Habel, JC; Besold, J; Becker, T; Johnen, L; Knolle, M; Rzepecki, A; Schultze, J; Zapp, A | 2006 | The Chalk-hill Blue Polyommatus coridon (Lycaenidae, Lepidoptera) in a highly fragmented landscape: How sedentary is a sedentary butterfly? | The habitats of many species are fragmented. Therefore, the survival in a metapopulation depends on the stability of the single, populations and the amount of movements between patches. We chose the calcareous grassland specialist butterfly species Polyommatus coridon as a model. As study area,,we selected a mosaic-like landscape in Rhineland-Palatinate (western Germany) with several well preserved calcareous grassland fragments. We marked a total of 2,211 individuals during July and August 2003. The overall recapture ratio was 7.1%. The estimated mean butterfly densities over the whole flight season ranged from 52 to 487 individuals per hectare. The within-patch movements were relatively low (13.3%) compared with the between-patch movements (3.2%). Therefore, the metapopulation structure appears to be intact in our study area. | 4213 | NA | NA | NA | no | no |
| 4212 | 2017 | NA | Jones, CL; Anderson, TW; Edwards, MS | 2013 | Evaluating eelgrass site quality by the settlement, performance, and survival of a marine fish | The quantity of habitat can be defined by the distribution and abundance of organisms, but it is the quality of the habitat that may have more direct demographic consequences. Habitat quality is best assessed by determining demographic rates and the performance of the focal species residing within a habitat. Using this framework to develop a method for evaluating habitat quality among sites, we used two demographic rates (settlement and survival) and two performance measures (growth and condition) of the giant kelpfish (Heterostichus rostratus Girard) to determine whether eelgrass (Zostera marina L) habitat quality varies spatially (i.e., site quality) within San Diego Bay, California, USA. Seven sites were selected that encompass the northern and central areas of the bay. Settlement was estimated bi-weekly from May to July 2009 with artificial seagrass units (ASUs) and standard monitoring units for the recruitment of fishes (SMURFs). Eelgrass habitat complexity (shoot height and density) was measured at each site, and three complexity treatments were replicated with ASUs in large mesocosms to estimate survival of giant kelpfish after exposure to kelp bass (Paralabrax clathratus Girard), a common predator. Growth and condition were derived from juvenile giant kelpfish collected from sites two months after the settlement period using otolith daily growth increments and total lipid content, respectively. Settlement, growth, and condition were evaluated using a multivariate analysis to provide an assessment of habitat quality among sites and a framework for which site quality can be evaluated in different marine habitats. Combined with habitat-dependent survival from predators in laboratory trials that reflected site-specific habitat attributes, sites were ranked along a gradient of habitat quality. The multivariate analysis of site quality based on demographic and performance variables was then overlaid with a similar analysis of environmental variables. Environmental variables that would serve as proxies for habitat quality were not concordant with the suite of demographic and performance variables used to differentiate site quality, suggesting that a pluralistic approach using demographic rates and individual performance is a potentially more appropriate method for evaluating habitat quality, whether among different habitats or among sites of the same habitat. (C) 2013 Elsevier B.V. All rights reserved. | 4214 | NA | NA | NA | no | no |
| 4213 | 2017 | NA | Winget, DM; Williamson, KE; Helton, RR; Wommack, KE | 2005 | Tangential flow diafiltration: an improved technique for estimation of virioplankton production | Accurate estimates of viral production in natural environments are critical for assessing the impacts of viral lysis on bacterial mortality and dissolved organic matter release. Here, viral production was estimated using a tangential flow diafiltration (TFD) dilution method, which reduced viral abundance to about 25% of ambient while maintaining near ambient levels of bacterial abundance. In subsequent incubations, the rate of virus-like particle increase was measured and used to calculate viral production. TFD viral production estimates were compared to those from simultaneous incubations using a vacuum diafiltration procedure. At 4 stations in the Chesapeake and Delaware Bays, viral production averaged 4.8 +/- 1.7 x 10(10) and 5.9 +/- 4.4 x 10(10) viruses l(-1) d(-1) as assessed by the TFD and vacuum methods, respectively. The TFD procedure improved upon the vacuum-based method by recovering significantly more of the bacterial community and requiring less sample processing time. Optimization tests of the TFD procedure found that a 0.22 mu m pore size filter with a flushing rate of 40 ml min(-1), and a flushing volume 4-fold the initial sample volume gave the best combination of bacterial recovery, viral dilution, and processing time. Based on TFD viral production estimates, viral lysis was responsible for the loss of 14 to 93% of the bacterial standing stock and the release of 22 to 47 mu g C l(-1) d(-1) in the Chesapeake and Delaware Bays. These results indicate that viral lysis is a significant factor for microbial mortality and dissolved organic matter cycling within these estuaries. | 4215 | NA | NA | NA | no | no |
| 4214 | 2017 | NA | Segarra-Moragues, JG; Palop-Esteban, M; Gonzalez-Candelas, F; Catalan, P | 2007 | Nunatak survival vs. tabula rasa in the Central Pyrenees: a study on the endemic plant species Borderea pyrenaica (Dioscoreaceae) | Aim Borderea pyrenaica (Dioscoreaceae) is a Tertiary relict plant endemic to the Central Pyrenees. Because of its narrow distribution in a small geographical area and the fact that it is restricted to high alpine habitats, it constitutes an ideal model species for inferring the historical dynamics of population survival and migration during and after Quaternary glaciations in the Pyrenees. Location Central Pyrenees and pre-Pyrenees, Spain-France. Methods Eleven primer pairs were used to amplify 18 microsatellite loci in this allotetraploid species in a sample of 804 individuals from 15 populations, revealing a total of 77 alleles. Genotypic data of individuals and populations were analysed using clustering and Bayesian methods of analysis of population structure. Results A higher number of private alleles and a significantly higher allelic richness (A*) were found in the southern area (21, A* = 2.295) than in the northern area (5, A\* = 1.791). Furthermore, the allelic composition of the northern area represented a subset of that from the southern area. Main conclusion The hypothesis of in situ survival in northern Pyrenean nunataks was rejected, while peripheral refugia were considered to be restricted to the southern Pyrenees and pre-Pyrenees, where historical geographical fragmentation probably caused the divergence among southern Pyrenean populations. Molecular evidence indicates that these refugial populations probably colonized the northern area after sheet-ice retreat. Borderea pyrenaica lineages followed two migratory pathways in their northward colonization, suggesting several founder events for the populations that eventually reached the territory of the Gavarnie cirque. | 4216 | NA | NA | NA | no | no |
| 4215 | 2017 | NA | Goulet, TL | 2006 | Most corals may not change their symbionts | Many corals (stony corals and octocorals) rely on their symbiotic algae (zooxanthellae) for survival. Under stress, zooxanthellae are expelled, resulting in coral bleaching. The hypothesis that corals may survive climate change by exchanging algal types is shown here to be potentially applicable only to a minority of corals. Data on 442 coral species from 43 studies reveal that only a few coral species may be able to change their symbionts. The ability to change symbionts seems to be linked to whether a coral species can host multiple zooxanthella clades, either at different depths on the same reef, on different reefs or at different geographic locations, or concurrently within the same colony. The combined data set shows that only 23% of coral species host multiple zooxanthella clades. Most coral species (77%) exhibit fidelity to a narrow subset of a single zooxanthella clade, some even to specific algal genotypes within a clade, These specific algal genotypes in coral species hosting a single algal clade do not change over time. Furthermore, no algal change occurs when a coral colony is either transplanted to different environments, or subjected to stressors such as disease or increased temperatures. For the majority of corals, therefore, algal switching does not appear to occur. These coral species will survive only if the existing host-symbiont combination withstands the changing conditions. If climate warming continues, coral reefs may undergo a change in biodiversity such that only a subset of symbiotic corals may persist. | 4217 | NA | NA | NA | no | no |
| 4216 | 2017 | NA | Stucki, D; Brites, D; Jeljeli, L; Coscolla, M; Liu, QY; Trauner, A; Fenner, L; Rutaihwa, L; Borrell, S; Luo, T; Gao, Q; Kato-Maeda, M; Ballif, M; Egger, M; Macedo, R; Mardassi, H; Moreno, M; Vilanova, GT; Fyfe, J; Globan, M; Thomas, J; Jamieson, F; Guthrie, JL; Asante-Poku, A; Yeboah-Manu, D; Wampande, E; Ssengooba, W; Joloba, M; Boom, WH; Basu, I; Bower, J; Saraiva, M; Vasconcellos, SEG; Suffys, P; Koch, A; Wilkinson, R; Gail-Bekker, L; Malla, B; Ley, SD; Beck, HP; de Jong, BC; Toit, K; Sanchez-Padilla, E; Bonnet, M; Gil-Brusola, A; Frank, M; Beng, VNP; Eisenach, K; Alani, I; Ndung’u, PW; Revathi, G; Gehre, F; Akter, S; Ntoumi, F; Stewart-Isherwood, L; Ntinginya, NE; Rachow, A; Hoelscher, M; Cirillo, DM; Skenders, G; Hoffner, S; Bakonyte, D; Stakenas, P; Diel, R; Crudu, V; Moldovan, O; Al-Hajoj, S; Otero, L; Barletta, F; Carter, EJ; Diero, L; Supply, P; Comas, I; Niemann, S; Gagneux, S | 2016 | Mycobacterium tuberculosis lineage 4 comprises globally distributed and geographically restricted sublineages | Generalist and specialist species differ in the breadth of their ecological niches. Little is known about the niche width of obligate human pathogens. Here we analyzed a global collection of Mycobacterium tuberculosis lineage 4 clinical isolates, the most geographically widespread cause of human tuberculosis. We show that lineage 4 comprises globally distributed and geographically restricted sublineages, suggesting a distinction between generalists and specialists. Population genomic analyses showed that, whereas the majority of human T cell epitopes were conserved in all sublineages, the proportion of variable epitopes was higher in generalists. Our data further support a European origin for the most common generalist sublineage. Hence, the global success of lineage 4 reflects distinct strategies adopted by different sublineages and the influence of human migration. | 4218 | NA | NA | NA | no | no |
| 4217 | 2017 | NA | Calabuig, G; Ortego, J; Cordero, PJ; Aparicio, JM | 2010 | Colony foundation in the lesser kestrel: patterns and consequences of the occupation of empty habitat patches | Understanding the process of colonization of new habitat patches is critical to clarify the proximate mechanisms involved in the distribution of a species and particularly in the formation of breeding aggregations. We studied the process of colony foundation in a long-term monitored population of lesser kestrels, Falco naumanni. For this purpose, we first analysed which habitat/demographic features influence the occupation of empty habitat patches experimentally supplied with nestboxes. Second, we studied the individual characteristics of founders and the reproductive consequences of occupation of new breeding patches in comparison with individuals settled in already established colonies. We found that the probability of occupation of experimental breeding patches increased with the relative cover of cereal crops. Regardless of sex, founders and individuals that settled in pre-existing colonies did not differ in body condition or age. However, there was a higher proportion of unringed kestrels in new than in pre-existing colonies, suggesting that founders are mostly immigrants from distant populations. Founders and nonfounders had similar breeding success, but the former had a lower parasitic burden of feather lice, indicating that occupying new breeding patches could reduce parasite pressure and/or transmission. Our results suggest habitat characteristics influence settlement decisions in the absence of pre-existing social cues, but do not support the idea that founders are suboptimal individuals unable to gain access to previously established colonies. (C) 2010 The Association for the Study of Animal Behaviour. Published by Elsevier Ltd. All rights reserved. | 4219 | NA | NA | NA | no | no |
| 4218 | 2017 | NA | Pye, RJ; Pemberton, D; Tovar, C; Tubio, JMC; Dun, KA; Fox, S; Darby, J; Hayes, D; Knowles, GW; Kreiss, A; Siddle, HVT; Swift, K; Lyons, AB; Murchison, EP; Woods, GM | 2016 | A second transmissible cancer in Tasmanian devils | Clonally transmissible cancers are somatic cell lineages that are spread between individuals via the transfer of living cancer cells. There are only three known naturally occurring transmissible cancers, and these affect dogs, soft-shell clams, and Tasmanian devils, respectively. The Tasmanian devil transmissible facial cancer was first observed in 1996, and is threatening its host species with extinction. Until now, this disease has been consistently associated with a single aneuploid cancer cell lineage that we refer to as DFT1. Here we describe a second transmissible cancer, DFT2, in five devils located in southern Tasmania in 2014 and 2015. DFT2 causes facial tumors that are grossly indistinguishable but histologically distinct from those caused by DFT1. DFT2 bears no detectable cytogenetic similarity to DFT1 and carries a Y chromosome, which contrasts with the female origin of DFT1. DFT2 shows different alleles to both its hosts and DFT1 at microsatellite, structural variant, and major histocompatibility complex (MHC) loci, confirming that it is a second cancer that can be transmitted between devils as an allogeneic, MHC-discordant graft. These findings indicate that Tasmanian devils have spawned at least two distinct transmissible cancer lineages and suggest that transmissible cancers may arise more frequently in nature than previously considered. The discovery of DFT2 presents important challenges for the conservation of Tasmanian devils and raises the possibility that this species is particularly prone to the emergence of transmissible cancers. More generally, our findings highlight the potential for cancer cells to depart from their hosts and become dangerous transmissible pathogens. | 4220 | NA | NA | NA | no | no |
| 4219 | 2017 | NA | Schroder, R; Prasse, R | 2013 | From nursery into nature: A study on performance of cultivated varieties of native plants used in re-vegetation, their wild relatives and evolving wild x cultivar hybrids | Cultivated varieties may have life-history traits which are very different to those of their wild proge-nitors. Changes in life-history traits due to cultivation may not be desirable for plant material used for re-vegetation, as these plants may be adapted to constant human care and lack the finely tuned trade-off that allows wild plants to cope with the often harsh and heterogeneous environmental conditions outside of nurseries. Research is needed to identify differences in life-history traits between cultivated varieties used in re-vegetation and restoration and their wild relatives. If such traits are changed by cultivation, it is necessary to examine if they will persist also at the re-vegetation site and may be transferred to wild relatives by hybridization as that may reduce their survival or lead to undesirable changes on local ecosystems. In this study we examined whether cultivated varieties of Plantago lanceolata and Lotus corniculatus, two species frequently used in re-vegetation and restoration projects, differ from their wild relatives in morphology or flower phenology and whether differences could be transferred into wild relatives by hybridization. We tested if cultivated varieties are bred for high vegetative and generative yield in greenhouse studies. In common garden experiments we studied if the detected enhanced biomass production is persistent under the climatic conditions of the wild plant’s natural habitat in Central Europe. Additionally, we tested if hybrids show effects of outbreeding depression or heterosis. The cultivated varieties of both species grew very vigorously and showed a more erect growth pattern than their wild relatives. The cultivated varieties seem to be selected toward high vegetative and generative yield. However, if exposed to local climatic conditions, cultivated varieties suffered a fitness loss with a complete mortality for the L. corniculatus cultivar and a reduced vegetative biomass production for P. lanceolata cultivars. On the other hand, we detected heterosis effects in hybrids for survival and generative biomass production in L. corniculatus and number of leaves in P. lanceolata. As it remains possible that the use of cultivated varieties may result in strongly competitive genotypes or reduced local adaptation of wild plant populations (via hybridization), large-scale use of cultivated varieties of native plants should be avoided in re-vegetation and restoration. (C) 2013 Elsevier B.V. All rights reserved. | 4221 | NA | NA | NA | no | no |
| 4220 | 2017 | NA | Silva, AP; Parra, PP; Campos, VP; Costa, SS; Vicente, CSL; Ferreira, LG; Souza, RM; Mota, M | 2016 | Genetic diversity of Bursaphelenchus cocophilus in South America | Molecular characterisation of Bursaphelenchus cocophilus, the causal agent of ‘red ring disease’, is imperative for efficient identification procedures in Brazil and Colombia, because quarantine species such as B. xylophilus and B. mucronatus are already listed in both countries. ITS-1/2 region and D2-D3 segment of LSU rDNA were used to characterise isolates of B. cocophilus obtained from coconut plantations in Brazil and Colombia. Results from ITS-1/2 and LSU rDNA regions showed that all isolates of B. cocophilus from Brazil and Colombia formed a monophyletic group. The LSU rDNA region indicated that all isolates formed a single monophyletic group with high Bayesian posterior probability (100%). This is the first study on ITS-1/2 for the characterisation of B. cocophilus populations. A species-specific primer was designed for identification of B. cocophilus. | 4222 | NA | NA | NA | no | no |
| 4221 | 2017 | NA | Teo, YY; Fry, AE; Sanjoaquin, MA; Pederson, B; Small, KS; Rockett, KA; Kwiatkowski, DP; Clark, TG | 2009 | Assessing Genuine Parents-Offspring Trios for Genetic Association Studies | Objectives: Family-based association tests such as the transmission disequilibrium test (TDT) are dependent on the successful ascertainment of true nuclear family trios. Relationship misspecification inevitably occurs in a proportion of trios collected for genotyping which undetected can lead to a loss of power and increased Type I error due to biases in over-transmission of common alleles. Here, we introduce a method for evaluating the authenticity of nuclear family trios. Methods: Operating in a Bayesian framework, our approach assesses the extent of pedigree inconsistent genotype configurations in the presence of genotyping errors. Unlike other approaches, our method: (i) utilizes information from three individuals collectively (the whole trio) rather than consider two independent pairwise relationships; (ii) down-weighs SNPs with poor performance; (iii) does not require the user to pre-define a rate of genotyping error, which is often unknown to the user and seldom fixed across the different SNPs considered which available methods unrealistically assumed. Results: Simulation studies and comparisons with a real set of data showed that our approach is more likely to correctly identify the presence of true and misspecified trios compared to available software, accurately infers the extent of relationship misspecification in a trio and accurately estimates the genotyping error rates. Conclusions: Assessing relationship misspecification depends on the fidelity of the genotype data used. Available algorithms are not optimised for genotyping technology with varying rates of errors across the markers. Through our comparison studies, our approach is shown to outperform available methods for assessing relationship misspecifications. Copyright (C) 2008 S. Karger AG, Basel | 4223 | NA | NA | NA | no | no |
| 4222 | 2017 | NA | Aron, S; Passera, L | 1999 | Mode of colony foundation influences the primary sex ratio in ants | In ants, young queens can found new colonies independently (without the help of workers) or dependently (with the help of workers). It has been suggested that differences in the mode of colony founding strongly influence queen survival and colony development. This is because independent queens are constrained to produce a worker force rapidly, before they deplete their body reserves and to resist the intense intercolony competition during the founding stage. By contrast, queens that found colonies dependently remain with the workers, which probably results in a lower mortality rate and earlier production of reproductive offspring. Consequently, in species that found independently, queens of incipient colonies are expected to produce mostly worker brood by laying a lower fraction of haploid (male) eggs than queens in mature colonies; such a difference would not occur in species founding dependently. We compared the primary sex ratio (proportion of male-determined eggs) laid by queens in incipient and mature colonies of two ant species, Lasius niger and Linepithema humile, showing independent and dependent modes of colony founding, respectively. As predicted, L. niger queens of incipient colonies laid a lower proportion of haploid eggs than queens from mature colonies. By contrast, queens of L. humile laid a similar proportion of haploid eggs in both incipient and mature colonies. These results provide the first evidence that (1) the primary sex ratio varies according to the mode of colony foundation, and (2) queens can adjust the primary sex ratio according to the life history stage of the colony in ants. (C) 1999 The Association for the Study of Animal Behaviour. | 4224 | NA | NA | NA | no | no |
| 4223 | 2017 | NA | Lehmann, L; Rousset, F | 2012 | The evolution of social discounting in hierarchically clustered populations | The expression of a social behaviour may affect the fitness of actors and recipients living in the present and in the future of the population. When there is a risk that a future reward will not be experienced in such a context, the value of that reward should be discounted; but by how much? Here, we evaluate social discount rates for delayed fitness rewards to group of recipients living at different positions in both space and time than the actor in a hierarchically clustered population. This is a population where individuals are grouped into families, families into villages, villages into clans, and so on, possibly ad infinitum. The group-wide fitness effects are assumed to either increase or decrease the fecundity or the survival of recipients and can be arbitrarily extended in space and time. We find that actions changing the survival of individuals living in the future are generally more strongly discounted than fecundity-changing actions for all future times and that the value of future rewards increases as individuals live longer. We also find that delayed fitness effects may not only be discounted by a constant factor per unit delay (exponential discounting), but that, as soon as there is localized dispersal in a population, discounting per unit delay is likely to fall rapidly for small delays and then slowly for longer delays (hyperbolic discounting). As dispersal tends to be localized in natural populations, our results suggest that evolution is likely to favour individuals that express present-biased behaviours and that may be time-inconsistent with respect to their group-wide effects. | 4225 | NA | NA | NA | no | no |
| 4224 | 2017 | NA | MALLINSON, JJC | 1995 | CONSERVATION BREEDING PROGRAMS - AN IMPORTANT INGREDIENT FOR SPECIES SURVIVAL | Captive populations for the primary purpose of securing the survival of species through stable self-sustaining populations need to be founded and managed according to sound scientific principles. Given the current rate of habitat fragmentation and its effect on animal populations, species conservation over the long term increasingly will require management to reduce risk of extinction. This may include ex situ populations which can support and interact demographically and genetically with wild populations. This paper presents a review of what can be achieved through a multidisciplinary approach, involving science, interactive management, politics, environmental education, habitat preservation and habitat restoration. Examples of what conservation-oriented breeding programmes have contributed to date, what represents a viable population and what is meant by effective conservation are provided, and various scenarios for the future are discussed. | 4226 | NA | NA | NA | no | no |
| 4225 | 2017 | NA | Marigo, J; Cunha, HA; Bertozzi, CP; Souza, SP; Rosas, FCW; Cremer, MJ; Barreto, AS; de Oliveira, LR; Cappozzo, HL; Valente, ALS; Santos, CP; Vicente, ACP | 2015 | Genetic diversity and population structure of Synthesium pontoporiae (Digenea, Brachycladiidae) linked to its definitive host stocks, the endangered Franciscana dolphin, Pontoporia blainvillei (Pontoporiidae) off the coast of Brazil and Argentina | Pontoporia blainvillei (Gervais and d’Orbigny, 1844) is an endangered small cetacean endemic to South America with four Franciscana Management Areas (FMA) recognized as different population stocks. The role of the intestinal parasite Synthesium pontoporiae (Digenea: Brachycladiidae) as a possible biological marker to differentiate P. blainvillei stocks was evaluated using nuclear and mitochondrial DNA markers. Internal transcribed sequence 1 and 2 (ITS1 and ITS2) regions of S. pontoporiae did not show intraspecific variability. The mitochondrial NADH dehydrogenase subunit 3 (ND3) and cytochrome oxidase subunit I (COI) gene sequences suggested lack of population structure in S. pontoporiae and population expansion. The apparent panmixia of S. pontoporiae may be due to the high mobility of one or more of its intermediary hosts. Alternatively, it may be due to the small sample size. This result is incongruent with the previously proposed FMA. | 4227 | NA | NA | NA | no | no |
| 4226 | 2017 | NA | Lima, FED; Cibulski, SP; dos Santos, HF; Teixeira, TF; Varela, APM; Roehe, PM; Delwart, E; Franco, AC | 2015 | Genomic Characterization of Novel Circular ssDNA Viruses from Insectivorous Bats in Southern Brazil | Circoviruses are highly prevalent porcine and avian pathogens. In recent years, novel circular ssDNA genomes have recently been detected in a variety of fecal and environmental samples using deep sequencing approaches. In this study the identification of genomes of novel circoviruses and cycloviruses in feces of insectivorous bats is reported. Pan-reactive primers were used targeting the conserved rep region of circoviruses and cycloviruses to screen DNA bat fecal samples. Using this approach, partial rep sequences were detected which formed five phylogenetic groups distributed among the Circovirusand the recently proposed Cyclovirus genera of the Circoviridae. Further analysis using inverse PCR and Sanger sequencing led to the characterization of four new putative members of the family Circoviridae with genome size ranging from 1,608 to 1,790 nt, two inversely arranged ORFs, and canonical nonamer sequences atop a stem loop. | 4228 | NA | NA | NA | no | no |
| 4227 | 2017 | NA | Frezal, L; Felix, MA | 2015 | C. elegans outside the Petri dish | The roundworm Caenorhabditis elegans has risen to the status of a top model organism for biological research in the last fifty years. Among laboratory animals, this tiny nematode is one of the simplest and easiest organisms to handle. And its life outside the laboratory is beginning to be unveiled. Like other model organisms, C. elegans has a boom-and-bust lifestyle. It feasts on ephemeral bacterial blooms in decomposing fruits and stems. After resource depletion, its young larvae enter a migratory diapause stage, called the dauer. Organisms known to be associated with C. elegans include migration vectors (such as snails, slugs and isopods) and pathogens (such as microsporidia, fungi, bacteria and viruses). By deepening our understanding of the natural history of C. elegans, we establish a broader context and improved tools for studying its biology. | 4229 | NA | NA | NA | no | no |
| 4228 | 2017 | NA | Berglund, EC; Ellegaard, K; Granberg, F; Xie, ZP; Maruyama, S; Kosoy, MY; Birtles, RJ; Andersson, SGE | 2010 | Rapid diversification by recombination in Bartonella grahamii from wild rodents in Asia contrasts with low levels of genomic divergence in Northern Europe and America | Bartonella is a genus of vector-borne bacteria that infect the red blood cells of mammals, and includes several human-specific and zoonotic pathogens. Bartonella grahamii has a wide host range and is one of the most prevalent Bartonella species in wild rodents. We studied the population structure, genome content and genome plasticity of a collection of 26 B. grahamii isolates from 11 species of wild rodents in seven countries. We found strong geographic patterns, high recombination frequencies and large variations in genome size in B. grahamii compared with previously analysed cat- and human-associated Bartonella species. The extent of sequence divergence in B. grahamii populations was markedly lower in Europe and North America than in Asia, and several recombination events were predicted between the Asian strains. We discuss environmental and demographic factors that may underlie the observed differences. | 4230 | NA | NA | NA | no | no |
| 4229 | 2017 | NA | Lahlou, O; Millet, J; Chaoui, I; Sabouni, R; Filali-Maltouf, A; Akrim, M; El Mzibri, M; Rastogi, N; El Aouad, R | 2012 | The Genotypic Population Structure of Mycobacterium tuberculosis Complex from Moroccan Patients Reveals a Predominance of Euro-American Lineages | Background: Tuberculosis (TB) remains a major health problem in Morocco. Characterization of circulating Mycobacterium tuberculosis genotypic lineages, important to understand the dynamic of the disease, was hereby addressed for the first time at a national level. Methodology/Principal Findings: Spoligotyping was performed on a panel of 592 M. tuberculosis complex strains covering a 2-year period (2004-2006). It identified 129 patterns: 105 (n = 568 strains) corresponded to a SIT number in the SITVIT2 database, while 24 patterns were labeled as orphan. A total of 523 (88.3%) strains were clustered vs. 69 or 11.7% unclustered. Classification of strains within 3 large phylogenetical groups was as follows: group 1-ancestral/TbD1+/PGG1 (EAI, Bovis, Africanum), group 2-modern/TbD1-/PGG1 group (Beijing, CAS), group 3-evolutionary recent/TbD1-/PGG2/3 (Haarlem, X, S, T, LAM; alternatively designated as the Euro-American lineage). As opposed to group 3 strains (namely LAM, Haarlem, and T) that predominated (86.5% of all isolates), 6 strains belonged to group 2 (Beijing n = 5, CAS n = 1), and 3 strains (BOV\_1 n = 2, BOV\_4-CAPRAE) belonged to ancestral group 1 (EAI and AFRI lineage strains were absent). 12-loci MIRU-VNTR typing of the Casablanca subgroup (n = 114 strains) identified 71 patterns: 48 MITs and 23 orphan patterns; it allowed to reduce the clustering rate from 72.8% to 29.8% and the recent transmission rate from 64% to 20.2%. Conclusion: The M. tuberculosis population structure in Morocco is highly homogeneous, and is characterized by the predominance of the Euro-American lineages, namely LAM, Haarlem, and T, which belong to the "evolutionary recent’’ TbD1-/PGG2/3 phylogenetic group. The combination of spoligotyping and MIRUs decreased the clustering rate significantly, and should now be systematically applied in larger studies. The methods used in this study appear well suited to monitor the M. tuberculosis population structure for an enhanced TB management program in Morocco. | 4231 | NA | NA | NA | no | no |
| 4230 | 2017 | NA | Als, TD; Nash, DR; Boomsma, JJ | 2001 | Adoption of parasitic Maculinea alcon caterpillars (Lepidoptera : Lycaenidae) by three Myrmica ant species | Maculinea butterflies are parasites of Myrmica ant nests. The Alcon blue, Maculinea alcon, is unusual in that it parasitizes the nests of several Myrmica species, using M. rubra, M. ruginodis and M. scabrinodis as hosts in different parts of Europe. In Denmark it uses M. rubra and M. ruginodis, but never M. scabrinodis. Some populations use one of these species exclusively, despite the presence of the alternative host, while others use both hosts simultaneously. To examine the basis of this specificity, and local coadaptation between host and parasite, we offered freshly emerged caterpillars of M. alcon from three populations differing in their host use to laboratory nests of all three recorded host ant species collected from each of the M. alcon populations. We measured the attractiveness of the caterpillars to their host ants as the time taken for them to be adopted by each ant colony. Caterpillars from all populations took longer to be adopted to M. scabrinodis nests than to nests of the other two ant species. Adoption times to M. rubra and M. ruginodis colonies differed: caterpillars from each of the two populations that used a single host species were adopted most quickly by that species when local ant colonies were used. When ant colonies collected from the other two sites were used, this pattern broke down, and there was either no difference in adoption time, or M. rubra adopted caterpillars more quickly. Adoption of caterpillars from the population that used both M. rubra and M. ruginodis as hosts took an order of magnitude longer than caterpillars from populations using a single host species. (C) 2001 The Association for the Study of Animal Behaviour. | 4232 | NA | NA | NA | no | no |
| 4231 | 2017 | NA | Lester, PJ; Gruber, MAM | 2016 | Booms, busts and population collapses in invasive ants | The abundance of many invasive species can vary substantially over time, with dramatic population declines and local extinctions frequently observed in a wide range of taxa. We highlight population crashes of invasive ants, which are some of the most widespread and damaging invasive animals. Population collapse or substantial declines have been observed in nearly all of the major invasive ant species including the yellow crazy ant (Anoplolepis gracilipes), Argentine ants (Linepithema humile), big-headed or coastal brown ant (Pheidole megacephala), the tropical fire ant (Solenopsis geminata), red imported fire ants (Solenopsis invicta), and the little fire ant or electric ant (Wasmannia auropunctata). These declines frequently attract little attention, especially compared with their initial invasion phase. Suggested mechanisms for population collapse include pathogens or parasites, changes in the food availability, or even long-term effects of the reproductive biology of invasive ants. A critical component of the collapses may be a reduction in the densities of the invasive ant species, which are often competitively weak in low abundance. We propose that mechanisms causing a reduction in invasive ant abundance may initiate a local extinction vortex. Declines in abundance likely reduce the invasive ant’s competitive ability, resource acquisition and defense capability. These reductions could further reduce the abundance of an invasive ant species, and so on. Management of invasive ants through the use of pesticides is expensive, potentially ecologically harmful, and can be ineffective. We argue that pesticide use may even have the potential to forestall natural population declines and collapses. We propose that in order to better manage these invasive ants, we need to understand and capitalize on features of their population dynamics that promote population collapse. | 4233 | NA | NA | NA | no | no |
| 4232 | 2017 | NA | Paxton, RJ; Fries, I; Pieniazek, NJ; Tengo, J | 1997 | High incidence of infection of an undescribed microsporidium (Microspora) in the communal bee Andrena scotica (Hymenoptera, Andrenidae) | We document the abundance and distribution of the spores of an undescribed species of microsporidium within its host, the communal bee Andrena scotica, and relationships between this parasite and its host, Only the host’s adipose tissue (fat bodies) appeared infected, with up to 118 x 10(6) spores per bee. All hosts at one field site were infected. High spore load within hosts appeared to curtail female reproductive activity. However, we were unable to discern the microsporidium’s effects on male fecundity in that some males with a high spore load did undertake mate searching activity. Longevities of naturally infected A scotica males and females in a flight cage were apparently unaffected by the microsporidium. Spores were found in A scotica from other field sites but not in other species of bees, suggesting the microsporidium has a high degree of host specificity. | 4234 | NA | NA | NA | no | no |
| 4233 | 2017 | NA | Offenberg, J; Peng, RK; Nielsen, MG; Birkmose, D | 2012 | The Effect of Queen and Worker Adoption on Weaver Ant (Oecophylla smaragdina F.) Queen Fecundity | Incipient ant colonies are often under fierce competition, making fast growth crucial for survival. To increase production, colonies can adopt multiple queens (pleometrosis), fuse with other colonies or rob brood from neighboring colonies. However, different adoption strategies might have different impacts such as future queen fecundity or future colony size. O. smaragdina queen production was measured in incipient colonies with 2, 3 or 4 founding queens, following the transplantation of 0, 30 or 60 pupae from a donor colony. Pupae developed into mature workers, resulting in increased worker/queen ratios in pupae transplanted treatments and leading to increases in the per capita queen production. Conversely, more queens did not induce increased per capita fecundity. Thus, brood robbing added individuals to the worker force and increased future production of resident queens, whereas queen adoption increased the colony’s future production, but not the production of individual queens. | 4235 | NA | NA | NA | no | no |
| 4234 | 2017 | NA | Andayani, N; Morales, JC; Forstner, MRJ; Supriatna, J; Melnick, DJ | 2001 | Genetic variability in mtDNA of the silvery gibbon: Implications for the conservation of a critically endangered species | The silvery gibbon (Hylobates moloch), endemic to the island of Java, relies on closed-canopy lowland evergreen forest for its survival. Because Java has lost over 91% of its original forest, silvery gibbons currently occupy small, isolated forest fragments and are threatened with extinction. To contribute to a comprehensive conservation strategy for this species, we analyzed the mtDNA control region of 31 silvery gibbons representing most remaining populations. Our results suggest the presence of at least two genetically differentiated lineages: a “western” lineage, represented by the largest remaining natural population in Gunung Halimun National Park and a “central” lineage, consisting of smaller, more isolated populations in and around the Gunung Masigit/Simpang/Tilu complex, Gunung Gede/Pangrango, and Gunung Slamet. These two lineages, at a minimum, represent different management units that should, except in the most dire circumstances, be managed separately. | 4236 | NA | NA | NA | no | no |
| 4235 | 2017 | NA | Day, JL; Jacobs, JL; Rasmussen, J | 2017 | Considerations for the Propagation and Conservation of Endangered Lake Suckers of the Western United States | Decades of persistent natural and anthropogenic threats coupled with competing water needs have compromised numerous species of freshwater fishes, many of which are now artificially propagated in hatcheries. Low survival upon release is common, particularly in systems with substantial nonnative predator populations. Extensive sampling for Shortnose (Chasmistes brevirostris) and Lost River Suckers (Deltistes luxatus) in the Klamath River Basin on the California-Oregon border have failed to detect any new adult recruitment for at least two decades, prompting an investigation into artificial propagation as an extinction prevention measure. A comprehensive assessment of strategies and successes associated with propagation for conservation restocking has not been performed for any Catostomid. Here, we review available literature for all western lake sucker species to inform propagation and recovery efforts for Klamath suckers and summarize the relevance of these considerations to other endangered fishes. | 4237 | NA | NA | NA | no | no |
| 4236 | 2017 | NA | Avila, P; Fromhage, L | 2015 | No Synergy Needed: Ecological Constraints Favor the Evolution of Eusociality | In eusocial species, some individuals sacrifice their own reproduction for the benefit of others. It has been argued that the evolution of sterile helpers in eusocial insects requires synergistic efficiency gains through cooperation that are uncommon in cooperatively breeding vertebrates and that this precludes a universal ecological explanation of social systems with alloparental care. In contrast, using a model that incorporates realistic ecological mechanisms of population regulation, we show here that constraints on independent breeding (through nest-site limitation and dispersal mortality) eliminate any need for synergistic efficiency gains: sterile helpers may evolve even if they are relatively inefficient at rearing siblings, reducing their colony’s per-capita productivity. Our approach connects research fields by using hypotheses developed for cooperative breeding to explain the evolution of eusociality. The results suggest that these hypotheses may apply more generally than previously thought. | 4238 | NA | NA | NA | no | no |
| 4237 | 2017 | NA | Candido, M; Batinga, MCA; Alencar, ALF; de Almeida-Queiroz, SR; Buzinaro, MD; Livonesi, MC; Fernandes, AM; de Sousa, RLM | 2017 | Molecular characterization and genetic diversity of bovine Kobuvirus, Brazil | It is suggested that Bovine kobuvirus (BKV) is involved in the etiology of gastroenteric diseases especially among calves; however, this association remains unknown. This study evaluated 216 fecal samples from cattle with and without diarrhea symptoms obtained from different regions of Brazil. A 216 bp fragment of the BKV 3D gene was amplified by RT-PCR in 14.4 % (31/216) of the studied samples, and 17 samples were subjected to nucleotide sequencing. All positive samples were obtained from animals aged less than 5 months, and most of animals presented diarrhea (p < 0.05). Phylogenetic analyses showed that the obtained sequences were grouped within the genogroup 2 of BKV forming subclades specific for each Brazilian municipality sampled. In addition, the alignment of the sequences revealed differences of nucleotides between sequences from different locations. Our results indicate for the first time that there is a regional genotypic differentiation of BKV in Brazil. | 4239 | NA | NA | NA | no | no |
| 4238 | 2017 | NA | Pen, I | 2000 | Reproductive effort in viscous populations | Here I study a kin selection model of reproductive effort, the allocation of resources to fecundity versus survival, in a patch-structured population. Breeding females remain in the same patch for life. Offspring have costly, partial long-distance dispersal and compete for breeding sites, which become vacant upon the death of previous occupants. The main result is that the evolutionarily stable reproductive effort decreases as offspring dispersal rate increases. The result can be understood as follows: In a well-mixed population with global competition, neither adults nor juveniles compete with relatives, but in a patch-structured population with dispersal restricted to the juvenile phase, juveniles experience relatively less competition with relatives than adults, thus making juveniles relatively more valuable. Because this asymmetry between adults and juveniles decreases with the dispersal rate, so does the evolutionarily stable level of allocation to fecundity. | 4240 | NA | NA | NA | no | no |
| 4239 | 2017 | NA | Loeb, MLG; Zink, AG | 2006 | Fitness conflicts and the costs of sociality in communal egg layers: a theoretical model and empirical tests | Individuals within complex social groups often experience reduced reproduction owing to coercive or suppressive actions of other group members. However, the nature of social and ecological environments that favour individual acceptance of such costs of sociality is not well understood. Taxa with short periods of direct social interaction, such as some communal egg layers, are interesting models for study of the cost of social interaction because opportunities to control reproduction of others are limited to brief periods of reproduction. To understand the conditions under which communal egg layers are in fitness conflict and thus likely to influence each other’s reproduction, we develop an optimality model involving a brood guarding ‘host’ and a nonguarding disperser, or ‘egg dumper’. The model shows that when, where intermediate-sized broods have highest survival, lifetime inclusive fitnesses of hosts and dumpers are often optimized with different numbers of dumped eggs. We hypothesize that resolution of this conflict may involve attempts by one party to manipulate the other’s reproduction. To test model predictions we used a lace bug (Heteroptera: Tingidae) that shows both hosts and egg dumpers as well as increased offspring survival in response to communal egg laying. We found that egg-dumping lace bugs oviposit a number of eggs that very closely matches predicted fitness optimum for hosts rather than predicted optimum of dumpers. This result suggests that dumpers pay a social cost for communal egg laying, a cost that may occur through host suppression of dumper reproduction. Although dumper allocation of eggs is thus sub-optimal for dumpers, previous models show that the decision to egg dump is nevertheless evolutionarily stable, possibly because hosts permit just enough dumper oviposition to encourage commitment to the behaviour. | 4241 | NA | NA | NA | no | no |
| 4240 | 2017 | NA | Kaya, K; Gazel, M; Serce, CU; Elci, E; Cengiz, FC; Cambra, M; Caglayan, K | 2014 | Potential vectors of Plum pox virus in the Eastern Mediterranean Region of Turkey | Although Plum pox virus (PPV) was first detected in Turkey 44 years ago, the virus is present in a rather limited number of trees. Our recent studies on PPV incidence showed that PPV was introduced rapidly in PPV-free regions and that there are no data available about the role of aphid species and Prunus rootstocks on these new infections. In this study the epidemiological aspect of PPV was studied in Antakya-Hatay, located in the Eastern Mediterranean region of Turkey where PPV was first detected in 2011. The susceptibility of different Prunus rootstocks to PPV was evaluated in an established experimental plot next to a PPV-infected nectarine orchard. Aphid populations were monitored in 2011 and 2012 from the last week of April to the middle of June by the sticky-plant method in both the experimental plot (EP) and the surrounding infected nectarine orchard (SNO). Regularly collected plant samples and aphids were individually tested by DASI-ELISA and squash real-time RT-PCR, respectively. The highest aphid population densities were observed at the end of May in both years. The most abundant aphid species were Aphis gossypii and A. spiraecola both in EP and SNO in both years. The percentage of PPV-viruliferous Myzus persicae, A. fabae, A. gosypii, A. spiraecola, Hyalopterus pruni, Macrosiphon euphorbiae and A. craccivora as estimated by squash real-time RT-PCR were 39.47%, 25.00%, 24.56%, 22.60%, 22.22%, 20.00% and 8.00%, respectively. The percentages of viruliferous aphids collected from SNO were 12.5% in A. spiraecola, 12.42% in A. gossypii and 11.11% in H. pruni. At the end of 2012, three Myrobolan 29C and two Adesoto 101 rootstocks were found infected by PPV. Molecular characterization studies showed that PPV-M was the strain present in both the originally infected nectarine plot and the Myrobolan 29C rootstocks. | 4242 | NA | NA | NA | no | no |
| 4241 | 2017 | NA | Scheu, S; Drossel, B | 2007 | Sexual reproduction prevails in a world of structured resources in short supply | We present a model for the maintenance of sexual reproduction based on the availability of resources, which is the strongest factor determining the growth of populations. The model compares completely asexual species to species that switch between asexual and sexual reproduction (sexual species). Key features of the model are that sexual reproduction sets in when resources become scarce, and that at a given place only a few genotypes can be present at the same time. We show that under a wide range of conditions the sexual species outcompete the asexual ones. The asexual species win only when survival conditions are harsh and death rates are high, or when resources are so little structured or consumer genotypes are so manifold that all resources are exploited to the same extent. These conditions, largely represent the conditions in which sexuals predominate over asexuals in the field. | 4243 | NA | NA | NA | no | no |
| 4242 | 2017 | NA | dos Santos, AMPV; Matsumura, ATS; Van Der Sand, ST | 2002 | Intraspecific genetic diversity of Drechslera tritici-repentis as detected by random amplified polymorphic DNA analysis | The phytopathogenic fungus Drechslera tritici-repentis causes tan spot, an important disease of wheat in the southern Brazilian state of Rio Grande do Sul. Twelve D. tritici-repentis isolates were obtained from wheat seeds from different locations in the state. Their colony morphology on potato dextrose agar and polymorphisms in genomic DNA by the random amplified polymorphic DNA (RAPD) method were investigated. For the RAPD method, 23 primers were tested of which nine were selected for use in the study of D. tritici-repentis polymorphisms. The degree of similarity between isolates was calculated using a simple matching coefficient and dendrograms constructed by the unweighted pair-group method with arithmetical averages (UPGMA). The morphological and RAPD analyses showed intraspecific polymorphisms within the isolates, but it was not possible to establish a relationship between these polymorphisms and the geographical regions from where the host seeds were collected. | 4244 | NA | NA | NA | no | no |
| 4243 | 2017 | NA | Hovestadt, T; Thomas, JA; Mitesser, O; Elmes, GW; Schonrogge, K | 2012 | Unexpected Benefit of a Social Parasite for a Key Fitness Component of Its Ant Host | Numerous invertebrates inhabit social insect colonies, including the hoverfly genus Microdon, whose larvae typically live as brood predators. Formica lemani ant colonies apparently endure Microdon mutabilis infections over several years, despite losing a considerable fraction of young, and may even produce more gynes. We present a model for resource allocation within polygynous ant colonies, which assumes that whether an ant larva switches development into a worker or a gyne depends on the quantity of food received randomly from workers. Accordingly, Microdon predation promotes gyne development by increasing resource availability for surviving broods. Several model predictions are supported by empirical data. (i) Uninfected colonies seldom produce gynes. (ii) Infected colonies experience a short-lived peak in gyne production leading to a bimodal distribution in gyne production. (iii) Low brood : worker ratio is the critical mechanism controlling gyne production. (iv) Brood : worker ratio reduction must be substantial for increased gyne production to become noticeable. | 4245 | NA | NA | NA | no | no |
| 4244 | 2017 | NA | TABERLET, P; BOUVET, J | 1994 | MITOCHONDRIAL-DNA POLYMORPHISM, PHYLOGEOGRAPHY, AND CONSERVATION GENETICS OF THE BROWN BEAR URSUS-ARCTOS IN EUROPE | Some small European populations of the brown bear (Ursus arctos) are threatened by of extinction in the near future. The reinforcement of these populations with bears from other regions might provide a solution to their future survival. However, before any population transfer, the different conservation units must be identified. The phylogeographic approach has been advocated for this purpose. The different European populations were assayed for mitochondrial (mt) DNA polymorphism. A remarkable degree of concordance was found between the geographic distribution and the mtDNA haplotypes. Two clearly distinct lineages differing by more than 7 % in mtDNA control region sequences were found and, furthermore, the western lineage appears to be organized into two clades which correspond to two different ancestral refugia. The potential conservation units can be deduced from these results, and a management policy can consequently be inferred. This study clearly demonstrates the relevance of the molecular phylogeographic approach to the identification of conservation units. | 4246 | NA | NA | NA | no | no |
| 4245 | 2017 | NA | Hass, CC | 2002 | Home-range dynamics of white-nosed coatis in southeastern Arizona | I studied a population of white-nosed coatis (Nasua narica) in southeastern Arizona from 1996 to 2000 to quantify home-range size and overlap among matriarchal groups (troops) and solitary males. Home ranges of coatis averaged 13.57 km(2) +/- 1.72 SE for troops (n 9) and 6.11 +/- 1.42 km(2) for solitary males (n = 7; kernel-density estimator). Sizes of home ranges and core areas did not change during the study, even though density of coatis declined by greater than or equal to60%. Home ranges of males overlapped less than or equal to61%, home ranges of troops overlapped less than or equal to80%, and home ranges of males overlapped those of troops less than or equal to67%. Locations of annual home ranges and core areas shifted slightly from year to year, indicating minor range drift but no nomadism by radiocollared coatis in this population. Mortality due to predation was significantly higher in the periphery of the home range than that predicted based on the probability of occurrence. | 4247 | NA | NA | NA | no | no |
| 4246 | 2017 | NA | Chave, J; Leigh, EG | 2002 | A spatially explicit neutral model of beta-diversity in tropical forests | To represent species turnover in tropical rain forest, we use a neutral model where a tree’s fate is not affected by what species it belongs to, seeds disperse a limited distance from their parents, and speciation is in equilibrium with random extinction. We calculate the similarity function, the probability F(r) that two trees separated by a distance r belong to the same species, assuming that the dispersal kernel P(r), the distribution of seeds about their parents and the prospects of mortality and reproduction, are the same for all trees regardless of their species. If P(r) is radially symmetric Gaussian with mean-square dispersal distance a, F(r) can be expressed in closed form. If P(r) is a radially symmetric Cauchy distribution, then, in two-dimensional space, F(r) is proportional to 1/r for large r. Analytical results are compared with individual-based simulations, and the relevance to field observations is discussed. (C) 2002 Elsevier Science (USA). | 4248 | NA | NA | NA | no | no |
| 4247 | 2017 | NA | Sossai, S; Peconick, AP; Sales-Junior, A; Marcelino, FC; Vargas, MI; Neves, ES; Patarroyo, JH | 2005 | Polymorphism of the bm86 gene in South American strains of the cattle tick Boophilus microplus | Thirty Boophilus microplus strains from various geographic regions of Brazil, Argentina, Uruguay, Venezuela and Colombia were analyzed for the bm86 and bm95 gene. A fragment of cDNA of 794 base pairs of the parasite larvae, included between nucleotides 278-1071s, was amplified and cloned on the pGEM-T vector. Two random clones were sequenced for each population and the nucleotides 278-1071 and predicted amino acid sequences compared with the bm86 and bm95 genes. Variations from 1.76 to 3.65% were detected in the nucleotides sequence when compared with the homologous sequence of the bm86 gene and a 3.4-6.08% in the homologous amino acid sequence of the Bm86 protein. When the sequences obtained were compared with the bm95 gene, variations from 0.50 to 3.15% were detected. Variations from 1.14 to 4.56% were detected for the Bm95 protein homologous sequences in the deduced amino acid sequence. Only five of the 30 strains analyzed presented two different types of alleles expressed and the two alleles of the Alegre population and allele 1 of the Betim population were the most divergent of all those analyzed. | 4249 | NA | NA | NA | no | no |
| 4248 | 2017 | NA | Neumann, P; Blacquiere, T | 2017 | The Darwin cure for apiculture? Natural selection and managed honeybee health | Recent major losses of managed honeybee, Apis mellifera, colonies at a global scale have resulted in a multitude of research efforts to identify the underlying mechanisms. Numerous factors acting singly and/or in combination have been identified, ranging from pathogens, over nutrition to pesticides. However, the role of apiculture in limiting natural selection has largely been ignored. This is unfortunate, because honeybees are more exposed to environmental stressors compared to other livestock and management can severely compromise bee health. Here, we briefly review apicultural factors that influence bee health and focus on those most likely interfering with natural selection, which offers a broad range of evolutionary applications for field practice. Despite intense breeding over centuries, natural selection appears to be much more relevant for the health of managed A. mellifera colonies than previously thought. We conclude that sustainable solutions for the apicultural sector can only be achieved by taking advantage of natural selection and not by attempting to limit it. | 4250 | NA | NA | NA | no | no |
| 4249 | 2017 | NA | Knaepkens, G; Maerten, E; Eens, M | 2007 | Performance of a pool-and-weir fish pass for small bottom-dwelling freshwater fish species in a regulated lowland river | Although small benthic freshwater fish species are an important biological component of fish assemblages and free instrearn movement is indispensable for their survival, they are often neglected in fish pass performance studies. In this study, a capture-mark-recapture approach was used to assess whether small bottom-dwelling species, including gudgeon (Gobio gobio), stone loach (Barbatula barbatula), spined loach (Cobitis taenia) and bullhead (Cottus gobio), were able to cross a pool-and-weir fish pass in a regulated lowland river. Some tagged individuals of stone loach (18%), gudgeon (7%) and spined loach (2%) managed to successfully ascend the fish pass under study, despite the fact that water velocity levels in the different overflows of the facility (between 0.55-1.22 m/s) exceeded the critical swimming speed of all three species. Although this suggests that a pool-and-weir fish pass is a able to facilitate upstream movement of some small benthic species in a regulated river, more detailed research incorporating advanced tagging and retrieving techniques is necessary. | 4251 | NA | NA | NA | no | no |
| 4250 | 2017 | NA | Berlocher, SH; Dixon, PL | 2004 | Occurrence of Rhagoletis species in Newfoundland | The presence of Rhagoletis fruit flies in Newfoundland, Canada, was investigated by rearing large numbers of the fruits of known and potential host plant species. Three Rhagoletis species, R. tabellaria (Fitch), R. basiola (Osten Sacken), and the cherry pest species R. fausta (Osten Sacken), were found to occur naturally in Newfoundland, representing a range extension of some 500 km for R. tabellaria and R. basiola. Rhagoletis fausta had previously been reported from sticky traps used to monitor R. mendax Curran, which is not known from the province. To investigate the route by which the two new species may have colonized Newfoundland, R. tabellaria adults were sequenced for a mitochondrial genome fragment (COI-tRNA-Leu-COII-tRNA-Lys). This sample was genetically identical or similar to samples from other widely distributed sites in North America, suggesting that the presence of R. tabellaria in Newfoundland is the result of postglacial dispersal rather than survival in a glacial refugium. The implications of these findings for Rhagoletis management in Canada are discussed. | 4252 | NA | NA | NA | no | no |
| 4251 | 2017 | NA | Celdran, D; Marin, A | 2013 | Seed photosynthesis enhances Posidonia oceanica seedling growth | Posidonia oceanica seeds demonstrate photosynthetic activity during germination as well as throughout seedling development, a fact which suggests that seed photosynthesis can influence seedling growth. This study examines the contribution of seed photosynthesis to changes in leaf and root biomass, the concentration of seed carbohydrate reserves (free sugars and starch) and elemental C, N and P content in seedlings exposed to different light/dark treatments. Green seeds attached to seedlings remained photosynthetically active for three months and photosynthetic activity in the seeds enhanced seedling growth in leaves and roots. It was estimated that ca. 29.26% of leaf biomass and ca. 42.31% of root biomass production was due to seed photosynthetic activity. Photosynthesis occurred at a higher rate in seeds than in leaves during early seedling growth, and extreme light limitation reduced the mobilization of carbon reserves stored in the seeds. These adaptations control leaf self-shading and plant competition in well-established P. oceanica beds while also promoting increased seedling survival rates in open, uncolonised areas where clonal growth is limited. | 4253 | NA | NA | NA | no | no |
| 4252 | 2017 | NA | Wang, QS; Tian, F; Pan, YC; Buckler, ES; Zhang, ZW | 2014 | A SUPER Powerful Method for Genome Wide Association Study | Genome-Wide Association Studies shed light on the identification of genes underlying human diseases and agriculturally important traits. This potential has been shadowed by false positive findings. The Mixed Linear Model (MLM) method is flexible enough to simultaneously incorporate population structure and cryptic relationships to reduce false positives. However, its intensive computational burden is prohibitive in practice, especially for large samples. The newly developed algorithm, FaST-LMM, solved the computational problem, but requires that the number of SNPs be less than the number of individuals to derive a rank-reduced relationship. This restriction potentially leads to less statistical power when compared to using all SNPs. We developed a method to extract a small subset of SNPs and use them in FaST-LMM. This method not only retains the computational advantage of FaST-LMM, but also remarkably increases statistical power even when compared to using the entire set of SNPs. We named the method SUPER (Settlement of MLM Under Progressively Exclusive Relationship) and made it available within an implementation of the GAPIT software package. | 4254 | NA | NA | NA | no | no |
| 4253 | 2017 | NA | Monjeau, JA; Rotela, CH; Lamfri, M; Marquez, J; Scavuzzo, CM; Stanulescu, M; Nabte, MJ; Rial, EG | 2011 | Estimating habitat suitability for potential hantavirus reservoirs in north-western Patagonia using satellite imagery: Searching for the best predictive tools | We modelled potential distribution maps for four species of rodents known to be reservoirs of the hantavirus disease. These models provide general distribution hypotheses based on a 14-year period and 274 field capture sites. Satellite remote sensing were used to extrapolate climatic and environmental features of potentially suitable habitats for these rodents in Nahuel Huapi National Park and surrounding areas. We tested the accuracy of different satellite sensors for the geographic distribution of Oligoryzomys longicaudatus, Abrothrix longipilis, Abrothrix olivaceus and Loxodontomys micropus. When looking for the best method to predict the presence of potential hantavirus reservoir species assemblages, the best prediction levels were obtained using supervised parallelepiped classifications of the SAC-C Normalized Difference Vegetation Index (NDVI) combined with the Digital Elevation Model (DEM) (91.71% for A. longipilis, 95.35% for A. olivaceus, 91.92% for L micropus and 90.82% for O. longicaudatus). These results show that upper components of the landscape structure (e.g. climate and topography) determine the spatial distribution of hantavirus reservoirs independently of the floristic composition of the vegetation patches. (C) 2011 Deutsche Gesellschaft fur Saugetierkunde. Published by Elsevier GmbH. All rights reserved. | 4255 | NA | NA | NA | no | no |
| 4254 | 2017 | NA | Bar-Yam, Y | 2016 | From big data to important information | Advances in science are being sought in newly available opportunities to collect massive quantities of data about complex systems. While key advances are being made in detailed mapping of systems, how to relate these data to solving many of the challenges facing humanity is unclear. The questions we often wish to address require identifying the impact of interventions on the system and that impact is not apparent in the detailed data that is available. Here, we review key concepts and motivate a general framework for building larger scale views of complex systems and for characterizing the importance of information in physical, biological, and social systems. We provide examples of its application to evolutionary biology with relevance to ecology, biodiversity, pandemics, and human lifespan, and in the context of social systems with relevance to ethnic violence, global food prices, and stock market panic. Framing scientific inquiry as an effort to determine what is important and unimportant is a means for advancing our understanding and addressing many practical concerns, such as economic development or treating disease. (c) 2016 Wiley Periodicals, Inc. Complexity 21: 73-98, 2016 | 4256 | NA | NA | NA | no | no |
| 4255 | 2017 | NA | Islam, W; Zhang, J; Adnan, M; Noman, A; Zaynab, M; Wu, Z | 2017 | PLANT VIRUS ECOLOGY: A GLIMPSE OF RECENT ACCOMPLISHMENTS | Plant virus ecology mainly focuses upon populations and their interactions with host plants within environment. The subject includes interesting insights as many factors which affect the virus behavior, population, virus-vector interactions, biodiversity and host plants genotypes are involved in it. Moreover the achievements in the field of molecular ecology by application of recent molecular biology techniques are included which enhance the strength in understanding the economically important virus populations, growth and their world wide spread. Virus infection results direct and indirect effect on insect vectors by evolution of changes in their life cycles, health and interacting behaviors that support their spread. Similarly, the description of the recent information about how plant viruses disseminate towards the important agro-ecological zones in naturally managed vegetations and which factors play important role in ecological aspects are also included in this review. The modern era of science and technology requires a better understanding about movement of viruses in both directions which has become a highly important issue to levitate such kinds of aspects thus making plant virus ecology an exciting research discipline in future. | 4257 | NA | NA | NA | no | no |
| 4256 | 2017 | NA | Morris, DC; Schwarz, MP; Crespi, BJ; Cooper, SJB | 2001 | Phylogenetics of gall-inducing thrips on Australian Acacia | Analysis of DNA sequence data from four genes (Elongation Factor-1 alpha, wingless, 16S rDNA and cytochrome oxidase I) yielded a well-resolved, well-supported phylogeny for all 21 species of gall-inducing thrips found on Australian Acacia. This phylogeny was then used to investigate the evolution of various behavioural and life history traits, and to examine the level of agreement with the taxonomy of the group. Our results suggest that there may have been a single origin of soldier castes in gall-inducing thrips. Examination of the distribution of the three primary life history strategies employed by these thrips (pupating in the gall, pupating in soil with soldier castes and pupating in soil without soldier castes) indicates that two of the strategies may have evolved as a result of factors associated with host plant affiliations or through parasite pressure. Our phylogeny does not support the existing generic classification of the group in that the genera are not monophyletic, nor does it lend itself to a clear solution to improve the classification in accordance with the phylogeny. (C) 2001 The Linnean Society of London. | 4258 | NA | NA | NA | no | no |
| 4257 | 2017 | NA | Ishibashi, Y; Saitoh, T; Abe, S; Yoshida, MC | 1998 | Kin-related social organization in a winter population of the vole Clethrionomys rufocanus | Kinship among Clethrionomys rufocanus was investigated during the winter of 1992/93 in a 3-ha enclosure using both molecular and catch-mark-release techniques. Forty-six adult voles (22 males and 24 females) having high heterozygosities, which were collected from several natural populations, were released into the enclosure on 29 September 1992. Most fall-born individuals of both sexes stayed in their natal site during the non-breeding period (December-March), although reproductively active females dispersed during the fall breeding season (October-November). These philopatric individuals aggregated and formed an maternal family in the winter. Several females which failed to reproduce were solitary during this season. Some individuals which were derived from several families also aggregated into a mixed lineage group. Survival rate of fall-born voles from earlier litters was higher than that from later ones. Maternal families broke up soon after the onset of spring reproduction. Most females established a territory near the wintering site and made a kin-cluster, in which close relatives neighbored each other. Maternal families in winter bring about female kin-clusters in spring, which may influence reproductive output in the breeding season. | 4259 | NA | NA | NA | no | no |
| 4258 | 2017 | NA | Percy, DM | 2017 | A new endemic psyllid genus, Stevekenia gen. nov (Hemiptera: Psylloidea, Triozidae), from the Hawaiian Islands with two new and rare species on threatened host plants in the endemic genus Nothocestrum (Solanaceae) | A new endemic psyllid genus from the Hawaiian Islands, Stevekenia gen. nov., is described. Two new species, both single island endemics, feed on host plants in the endemic genus Nothocestrum (Solanaceae). Stevekenia nothocestri sp. nov. is found on Nothocestrum longifolium on the island of Oahu and Stevekenia aiea sp. nov. is found on N. peltatum on the island of Kauai. The host plants are montane rainforest species and both are on the IUCN Red List of Threatened Species. In particular, N. peltatum, which is endemic to the island of Kauai, is considered critically endangered, and therefore a similarly endangered status for these host-specific insects is likely. Only a few specimens of each Stevekenia species have been found, suggesting occurrence at low abundance. The host plants survive either as isolated individuals or small fragmented populations, which may already constitute less than the required critical host plant density for the long term survival of this psyllid genus. Unique morphological characters distinguish Stevekenia from other Hawaiian genera, and the placement of this genus within Triozidae is discussed. Mitochondrial DNA barcodes for the new species are provided. | 4260 | NA | NA | NA | no | no |
| 4259 | 2017 | NA | Van Zant, JL; Wooten, MC | 2003 | Translocation of Choctawhatchee beach mice (Peromyscus polionotus allophrys): hard lessons learned | Translocation of species for conservation purposes is a key element of many recovery programs. While the benefits of translocations seem obvious, potential negative effects have been described. For endangered beach mice (Peromyscus polionotus ssp.), repopulation of native, unoccupied habitat using translocated individuals has been extremely successful. Once populations are established, concerns over founder effects and isolation have led to proposals for continued secondary translocations. Unfortunately, little information is available to help formulate protocols for these actions. To test the effectiveness of translocation, the fates of 18 translocated Choctawhatchee beach mice (Peromyscus polionotus allophrys) were followed as they attempted to integrate into an established population. We found that translocated groups, repeated for two seasons, tended to use larger homeranges and significantly more burrows than did resident mice. The monthly (31 day) survival rate of resident mice was 3:4 times greater than for translocated individuals. We hypothesize that differential predation pressure was directly linked to the failure of mice to integrate successfully into the existing population. Given our results, we must advise caution and that additional knowledge be obtained before translocations are used to supplement existing populations. (C) 2003 Elsevier Science Ltd. All rights reserved. | 4261 | NA | NA | NA | no | no |
| 4260 | 2017 | NA | Silvestre, DAMM; Fontanari, JF | 2008 | Package models and the information crisis of prebiotic evolution | The coexistence between different types of templates has been the choice solution to the information crisis of prebiotic evolution, triggered by the finding that a single RNA-like template cannot carry enough information to code for any useful replicase. In principle, confining d distinct templates of length L in a package or protocell, whose Survival depends on the coexistence of the templates it holds in, could resolve this crisis provided that d is made sufficiently large. Here we review the prototypical package model of Niesert et al. [1981. Origin of life between Scylla and Charybdis. J. Mol. Evol. 17, 348-353] which guarantees the greatest possible region of viability of the protocell population, and show that this model, and hence the entire package approach, does not resolve the information crisis. In particular, we show that the total information stored in a viable protocell (Ld) tends to a constant value that depends only on the spontaneous error rate per nucleotide of the template replication mechanism. As a result, an increase of d must be followed by a decrease of L, so that the net information gain is null. (C) 2008 Elsevier Ltd. All rights reserved. | 4262 | NA | NA | NA | no | no |
| 4261 | 2017 | NA | Schroder, R; Prasse, R | 2013 | Cultivation and Hybridization Alter the Germination Behavior of Native Plants Used in Revegetation and Restoration | Native plants are increasingly used for revegetation and restoration. These plants are cultivated for several generations at plant nurseries and often they are of unknown provenance. Therefore, cultivated plants often differ from their wild progenitors in life-history traits. Using germination behavior as example, we tested the assumption that cultivated plants have different life-history traits than their uncultivated progenitors. Cultivated as well as wild individuals of Plantago lanceolata and Lotus corniculatus, two species frequently used in revegetation, were tested in a common garden experiment as well as in incubators for their germination behavior. We observed significantly faster and more abundant germination in cultivated varieties. Using artificial crossings, we found that also hybrids of cultivated varieties and wild relatives germinate faster and more abundant than the wilds. As wild plants acquire their life-history traits by natural selection, we have to assume that they represent the optimal adaptation to the environmental conditions. If these traits are changed by cultivation or by hybridization between cultivated varieties and local populations, the long-term survival probabilities of local populations may be altered. Therefore, the use of cultivated varieties of native plants should be avoided in revegetation. | 4263 | NA | NA | NA | no | no |
| 4262 | 2017 | NA | Hurtrez-Bousses, S; Pendino, A; Barnabe, C; Durand, P; Rondelaud, D; Durand, C; Meunier, C; Hurtrez, JE; Renaud, F | 2005 | Comparison between shell morphology and genetic diversity in two sympatric lymnaeid snails, vectors of fasciolosis | Species determination of freshwater snails is particularly important in the case of disease vectors. In central France, for example, it has been shown that Galba truncatula (Muller, 1774) and Omphiscola glabra (Muller, 1774), which can be found in sympatry, are intermediate hosts for fasciolosis. Although these two species are distinguishable based on their shell morphology, some snails present in sympatric zones possess shell characteristics that are common to both species, suggesting possible gene flow. To test this hypothesis, we carried out multilocus enzyme electrophoreses on individuals sampled in one zone of sympatry. Our results clearly show that all snails with shell characteristics common to both species are O. glabra. We also note an absence of hybridization between G. truncatula and O. glabra in the study area. Remarkably, we observe a total monomorphism at the six studied loci among individuals of G. truncatula. Similarly, we find a total lack of heterozygotes in the O. glabra sample (with classic or intermediate shell characteristics). These results suggest a predominance of selfing in the reproductive mode of both species. Our results suggest that conchological characteristics can provide information about the taxonomic position of lymnaeids. However, they are insufficient to confidently differentiate species. | 4264 | NA | NA | NA | no | no |
| 4263 | 2017 | NA | Xu, J; Lin, KK; Liu, SS | 2011 | Performance on different host plants of an alien and an indigenous Bemisia tabaci from China | The capacity of the Middle East-Asia Minor 1 putative species of the whitefly Bemisia tabaci (Gennadius) species complex, commonly referred to as the B biotype, to invade has often been linked to its presumed wider host range than the indigenous competitors. To determine whether this alien putative species and the indigenous Asia II 1 whitefly putative species, commonly referred to as the ZHJ2 biotype, differ in their ability to use different host plants, we compared their development, survival and reproduction on eight crop species/cultivars that are commonly cultivated in Zhejiang, China. Of the eight host plants tested, B performed substantially better than ZHJ2 on squash, tomato and tobacco, B and ZHJ2 preformed equally well on cotton and sweet potato, while ZHJ2 performed better than B on kidney bean and pepper. These results indicate that while B generally has a wider host range than many indigenous B. tabaci, an indigenous B. tabaci can perform as well as or better on some host plants. These results combined with the cropping patterns in Zhejiang suggested that the differential capacity to use various host plants between whitefly species is important in mediating the process of invasion by an alien whitefly species. | 4265 | NA | NA | NA | no | no |
| 4264 | 2017 | NA | Taylor, P; Lillicrap, T; Cownden, D | 2011 | INCLUSIVE FITNESS ANALYSIS ON MATHEMATICAL GROUPS | Recent work on the evolution of behaviour is set in a structured population, providing a systematic way to describe gene flow and behavioural interactions. To obtain analytical results one needs a structure with considerable regularity. Our results apply to such “homogeneous” structures (e.g., lattices, cycles, and island models). This regularity has been formally described by a “node-transitivity” condition but in mathematics, such internal symmetry is powerfully described by the theory of mathematical groups. Here, this theory provides elegant direct arguments for a more general version of a number of existing results. Our main result is that in large “group-structured” populations, primary fitness effects on others play no role in the evolution of the behaviour. The competitive effects of such a trait cancel the primary effects, and the inclusive fitness effect is given by the direct effect of the actor on its own fitness. This result is conditional on a number of assumptions such as (1) whether generations overlap, (2) whether offspring dispersal is symmetric, (3) whether the trait affects fecundity or survival, and (4) whether the underlying group is abelian. We formulate a number of results of this type in finite and infinite populations for both Moran and Wright-Fisher demographies. | 4266 | NA | NA | NA | no | no |
| 4265 | 2017 | NA | Larsen, A; Bryant, S | 1998 | Growth rate and toxicity of Prymnesium parvum and Prymnesium patelliferum (Haptophyta) in response to changes in salinity, light and temperature | Toxic blooms consisting of Prymnesium parvum CARTER and Prymnesium patelliferum GREEN, HIBBERD & PIENAAR have caused variable degrees of mortality of fanned fish in the Sandsfjord system, southwestern Norway annually between 1989 and 1996. Cultures of P. parvum and P. patelliferum were established from water samples during the bloom in the Sandsfjord system in 1993. In order to investigate possible differences in reproduction rates and autecology between the two species, growth as a function of different salinity, photon fluence rates (PFR) and temperatures of the two Norwegian strains was compared with the corresponding growth of strains of P. parvum from England and Denmark, and strains of P. patelliferum from Australia and England. The toxicities of the same strains grown under varying salinity, light and temperature conditions, were also compared. Maximum growth rates were generally higher in P. patelliferum than in P. parvum, but strain differences within the species varied significantly Both optimal growth conditions and toxicity differed greatly between the strains, and seem to be more linked to the individual strains than to species. Furthermore, there was no apparent relationship between toxicity and salinity, temperature or PFR. Thus, there were no marked differences in autecological and toxic properties between P. parvum and P. patelliferum apparent from this investigation. | 4267 | NA | NA | NA | no | no |
| 4266 | 2017 | NA | Lehmann, L; Feldman, MW; Rousset, F | 2009 | ON THE EVOLUTION OF HARMING AND RECOGNITION IN FINITE PANMICTIC AND INFINITE STRUCTURED POPULATIONS | Natural selection may favor two very different types of social behaviors that have costs in vital rates (fecundity and/or survival) to the actor: helping behaviors, which increase the vital rates of recipients, and harming behaviors, which reduce the vital rates of recipients. Although social evolutionary theory has mainly dealt with helping behaviors, competition for limited resources creates ecological conditions in which an actor may benefit from expressing behaviors that reduce the vital rates of neighbors. This may occur if the reduction in vital rates decreases the intensity of competition experienced by the actor or that experienced by its offspring. Here, we explore the joint evolution of neutral recognition markers and marker-based costly conditional harming whereby actors express harming, conditional on actor and recipient bearing different conspicuous markers. We do so for two complementary demographic scenarios: finite panmictic and infinite structured populations. We find that marker-based conditional harming can evolve under a large range of recombination rates and group sizes under both finite panmictic and infinite structured populations. A direct comparison with results for the evolution of marker-based conditional helping reveals that, if everything else is equal, marker-based conditional harming is often more likely to evolve than marker-based conditional helping. | 4268 | NA | NA | NA | no | no |
| 4267 | 2017 | NA | Oldroyd, BP; Allsopp, MH; Gloag, RS; Lim, J; Jordan, LA; Beekman, M | 2008 | Thelytokous parthenogenesis in unmated queen honeybees (Apis mellifera capensis): Central fusion and high recombination rates | The subspecies of honeybee indigenous to the Cape region of South Africa, Apis mellifera capensis, is unique because a high proportion of unmated workers can lay eggs that develop females via thelytokous parthenogenesis involving central fusion of melotic products. This ability allows pseudoclonal lineages of workers to establish, which are presently widespread as reproductive parasites within the honeybee populations Of South Africa. Successful long-term propagation of a parthenogen requires the maintenance of heterozygosity at the sex locus, Which in honeybees must be heterozygous for the expression of female traits. Thus, in successful lineages of parasitic workers, recombination events are reduced by an order of magnitude relative to meiosis in queens of other honeybee subspecies. Here we show that in unmated A.m. capensis queens treated to induce oviposition, no such reduction in recombination occurs, indicating that thelytoky and reduce recombination are not controlled by the same gene. Our virgin queens were able to lay both arrhenotokous male-producing haploid eggs and thelytokous female-producing diploid eggs at the same time, with evidence that they have some voluntary control over which king of egg was laid. If so, they are able to influence the kind of second-division meiosis that occurs in their eggs post partum. | 4269 | NA | NA | NA | no | no |
| 4268 | 2017 | NA | Charbonnel, N; Bryja, J; Galan, M; Deter, J; Tollenaere, C; Chaval, Y; Morand, S; Cosson, JF | 2010 | Negative relationships between cellular immune response, Mhc class II heterozygosity and secondary sexual trait in the montane water vole | Heterogeneities in immune responsiveness may affect key epidemiological parameters and the dynamics of pathogens. The roles of immunogenetics in these variations remain poorly explored. We analysed the influence of Major histocompatibility complex (Mhc) genes and epigamic traits on the response to phytohaemagglutinin in males from cyclic populations of the montane water vole (Arvicola scherman). Besides, we tested the relevance of lateral scent glands as honest signals of male quality. Our results did not corroborate neither the hypotheses of genome-wide heterozygosity-fitness correlation nor the Mhc heterozygote advantage. We found a negative relationship between Mhc hetetozygosity and response to phytohaemagglutinin, mediated by a specific Mhc homozygous genotype. Our results therefore support the hypothesis of the Arte-Dqa-05 homozygous genotype being a ‘good’ Mhc variant in terms of immunogenetic quality. The development of the scent glands seems to be an honest signal for mate choice as it is negatively correlated with helminth load. The ‘good gene’ hypothesis was not validated as Arte-Dqa-05 homozygous males did not exhibit larger glands. Besides, the negative relationship observed between the size of these glands and the response to phytohaemagglutinin, mainly for Mhc homozygotes, corroborates the immunocompetence handicap hypothesis. The Mhc variants associated with larger glands remain yet to be determined. | 4270 | NA | NA | NA | no | no |
| 4269 | 2017 | NA | Grubbs, KC; Small, RL; Schilling, EE | 2009 | Evidence for multiple, autoploid origins of agamospermous populations in Eupatorium sessilifolium (Asteraceae) | Comparative analyses were made of agamospermous populations of Eupatorium sessilifolium, which have previously been documented to be polyploid, to determine whether they are alloploid or autoploid in origin and to assess the possibility that they have arisen more than once. There was no variability in ITS sequences among seven agamospermous and eight sexual diploid populations of E. sessilifolium, which is consistent with morphological observations in suggesting that the agamospermous populations were autoploids. The ITS sequence characteristic of E. sessilifolium differs from all other North American species by a minimum of 15 changes, and heterogeneity or polymorphism would be expected if the agamospermous populations were alloploids. Analysis of the chloroplast-based trnC-psbM spacer region showed variability among both sexual diploid and agamospermous populations of E. sessilifolium, which suggested that the agamospermous populations stem from multiple origins. Analysis of ISSR data revealed considerable intraspecific variability within E. sessilifolium, and the distribution of variability, with agamospermous populations showing variability from one another, added further evidence for multiple origins of agamospermous populations. The results in conjunction with distributional evidence that the sexual diploid populations of E. sessilifolium are geographically restricted and uncommon suggest that monitoring of populations might be warranted to evaluate whether measures are needed to enhance their continued survival. | 4271 | NA | NA | NA | no | no |
| 4270 | 2017 | NA | Araki, S; Kadono, Y | 2003 | Restricted seed contribution and clonal dominance in a free-floating aquatic plant Utricularia australis R. Br. in southwestern Japan | Utricularia australis R. Br. is an aquatic angiosperm species common in natural and irrigation ponds in temperate regions. This species reproduces both sexually and vegetatively, but in southwestern Japan the occurrence of male-sterile populations, in which plants produce no pollen and propagate only vegetatively, has been recorded. We studied the reproductive contribution of seeds in normal pollen-producing populations using isozyme analyses, a pollination experiment under culture and field observations. Although seedlings obtained from controlled mating indicated segregation of isozyme, polymorphism of the isozyme genotype was detected mainly among populations, but rarely within each pond population. This suggested clonal dominance and rarity of seed or seedling survival in natural populations. In the pollination experiment, the mean seed set ratio in cross-pollination between plants of the same isozyme genotype (7.6%) did not differ significantly from self-pollination (7.6%), but was lower than cross-pollination between plants of different genotypes (45.7%). The low ratio in crossing between the same genotype plants was ascribed to the clonality of the parents. In general, these results corresponded with the low ratios in seed setting observed in natural populations (7.9-13.7%). All the male-sterile populations we surveyed showed the same genotype, thus male sterility in the study area was considered to have the same origin. | 4272 | NA | NA | NA | no | no |
| 4271 | 2017 | NA | BEE, CA; CLOSE, RL | 1993 | MITOCHONDRIAL-DNA ANALYSIS OF INTROGRESSION BETWEEN ADJACENT TAXA OF ROCK-WALLABIES, PETROGALE SPECIES (MARSUPIALIA, MACROPODIDAE) | Simple, inexpensive techniques were used to analyse the mtDNA of nine chromosomally distinct populations of Petrogale. Eight of these populations occur in sequence along the Great Dividing Range of eastern Australia; six have been described as species. Diagnostic mtDNA morphs were found throughout the latitudinal ranges of four of the described species. A fifth morph spanned the ranges of two described species and three additional taxa which have been designated chromosome races. These five mtDNA morphs, and others with local distributions, were used to assess interactions between the taxa. Limited introgression was indicated across the chromosomal boundaries of P. penicillata/P. herberti and P. inornata/P. assimilis; atypical mtDNA morphs were found within the P. herberti and P. inornata chromosomal distributions. No introgression was detected between P. herberti and P. inornata, whose distributions are separated by the Fitzroy River. Nor was there evidence of recent contact between P. assimilis, P. herberti and P. purpureicollis, despite the late occupancy of parts of the intervening area by unidentified Petrogale. These data, considered in the light of information obtained from previous studies on chromosomes, allozymes and parasites, have contributed to the decision to consider all the eastern representatives of the lateralis-penicillata group of Petrogale as being specifically distinct from each other. | 4273 | NA | NA | NA | no | no |
| 4272 | 2017 | NA | Dalton, CL | 2000 | Effects of female kin groups on reproduction and demography in the gray-tailed vole, Microtus canicaudus | I conducted an experiment with pray-tailed voles, Microtus canicaudus, to test the hypothesis proposed by Charnov and Finerty that populations of voles comprised of female kin groups would grow more rapidly and reach higher densities more quickly than populations in which female kin groups were disrupted. The experiment was conducted in 0.2-ha semi-natural enclosures planted with mixed grasses. In Four enclosures, females were unmanipulated (control) and in four enclosures all newly caught females were removed from their natal enclosures and replaced with females of comparable age From another enclosure, such that juvenile females did not settle near their siblings or parents (treatment). I found no significant differences in survival, reproduction, juvenile recruitment, population growth rates, or population size between control and treatment populations. The only difference was the time to sexual maturation for young females, which was 3.1 weeks for control enclosures compared with 4.2 weeks for treatment enclosures. I could not measure reproductive success for individual females, but my results did not support the hypothesis that the presence or absence of kin groups resulted in any biologically meaningful population-level effects. Female voles that have nesting territories adjacent to relatives may accrue some individual benefits, but these benefits are unlikely to contribute to population regulation in gray-tailed voles. | 4274 | NA | NA | NA | no | no |
| 4273 | 2017 | NA | Austin, CJ; Moehring, AJ | 2013 | Optimal temperature range of a plastic species, Drosophila simulans | When a species experiences a new climate, it can adapt in two main ways: become genetically adapted to the new temperature, or adopt a plastic approach that allows it to survive at a range of temperatures. The constraint on fitness for genetically adapted populations that are exposed to a new temperature has been well studied, but the range of optimal temperatures and their effect on fitness has never been examined across the worldwide distribution of a plastic species. Here, we determined the optimum temperature range of 11 populations of the phenotypically plastic species Drosophila simulans. We measured the influence of temperature on eggs, larvae and adults at six temperatures that span the natural range the flies experience during their primary breeding season. We found no correlation between optimum temperature and native temperature, an effect that is not likely due to laboratory maintenance, suggesting that the species has not locally adapted to temperature. We also found that this species had equal survival and reproductive success at most of the temperatures and life stages that we tested, regardless of the native temperature where the flies originated. Thus, this genetically plastic species has an optimum fitness at a surprisingly wide range of temperatures, and is the first example of a cosmopolitan species exhibiting this large amount of plasticity across its sampling distribution. | 4275 | NA | NA | NA | no | no |
| 4274 | 2017 | NA | Tanner, CE; Parham, T | 2010 | Growing Zostera marina (eelgrass) from Seeds in Land-Based Culture Systems for Use in Restoration Projects | The use of aquaculture systems to grow the seagrass Zostera marina (eelgrass) from seeds for restoration projects was evaluated through laboratory and mesocosm studies. Along the mid-Atlantic coast of North America Z. marina seeds are shed from late spring through early summer, but seeds typically do not begin to germinate until the late fall. Fall is the optimal season to plant both seeds and shoots in this region. We conducted studies to determine if Z. marina seeds can be induced to germinate in the summer and seedlings grown in mesocosms to a size sufficiently large enough for out-planting in the fall. Seeds in soil-less culture germinated in the summer when held at 14 degrees C, with percent germination increasing with lower salinities. Cold storage (4 degrees C) of seeds prior to planting in sediments enhanced germination and seedling survival. Growth rates of seedlings were significantly higher in nutrient enriched estuarine sediments. Results from preliminary studies were used in designing a large-scale culture project in which 15,000 shoots were grown and out-planted into the Potomac River estuary in the Chesapeake Bay and compared with an equal number of transplanted shoots. These studies demonstrate that growing Z. marina from seeds is an alternative approach to harvesting plants from donor beds when vegetative shoots are required for restoration projects. | 4276 | NA | NA | NA | no | no |
| 4275 | 2017 | NA | Lobato, E; Doutrelant, C; Melo, M; Reis, S; Covas, R | 2017 | Insularity effects on bird immune parameters: A comparison between island and mainland populations in West Africa | Oceanic islands share several environmental characteristics that have been shown to drive convergent evolutionary changes in island organisms. One change that is often assumed but has seldom been examined is the evolution of weaker immune systems in island species. The reduction in species richness on islands is expected to lead to a reduced parasite pressure and, given that immune function is costly, island animals should show a reduced immune response. However, alternative hypotheses exist; for example, the slower pace of life on islands could favor the reorganization of the immune system components (innate vs. acquired immunity) on islands. Thus far, few island species have been studied and no general patterns have emerged. Here, we compared two immune parameters of birds from SAo Tome and Principe islands to those of their close relatives at similar latitudes on the mainland (Gabon, West Africa). On islands, the acquired humoral component (total immunoglobulins) was lower for most species, whereas no clear pattern was detected for the innate component (haptoglobin levels). These different responses did not seem to arise from a reorganization of the two immune components, as both total immunoglobulins and haptoglobin levels were positively associated. This work adds to the few empirical studies conducted so far which suggest that changes in immune parameters in response to insularity are not as straightforward as initially thought. | 4277 | NA | NA | NA | no | no |
| 4276 | 2017 | NA | Salzburger, W; Brandstatter, A; Gilles, A; Parson, W; Hempel, M; Sturmbauer, C; Meyer, A | 2003 | Phylogeography of the vairone (Leuciscus souffia, Risso 1826) in Central Europe | The vairone Leuciscus souffia is a cyprinid fish that inhabits river systems in and around the Alps. The complete mitochondrial DNA control region (945 bp) was sequenced in 295 vairone from 22 populations in Central Europe. A total of 51 haplotypes were identified with a maximum pairwise distance between haplotypes of 5.6%. Phylogenetic analyses revealed two major clades in L. souffia, an ‘Italian’ clade, and an ‘Alpine’ clade. Two hybrid zones exist, in the Mediterranean Alps and in the Soca basin. The position of the sister species of L. souffia, L. turskyi, to the ‘Alpine’ and the ‘Italian’ clade could not be resolved unambiguously. However, a linearized tree analysis indicated that L. turskyi represents a third lineage, that originated at the same time as the ‘Alpine’ and the ‘Italian’ clades of L. souffia. In the ‘Alpine’ clade two groups were resolved, a subclade with haplotypes from the Rhone and Var basins and a cluster with haplotypes from the Danube and Rhine systems. Our data suggest a long history of the vairone in Central Europe, predating Pleistocene glacial cycles. Two main refugia during glaciations must have existed, one in Italy and another one most probably in the Danube system. However, age estimates based on molecular clock calibrations suggest the survival of ‘Alpine’ haplotypes in several drainages during the last glaciation cycles. The Rhine system was only recently colonized. | 4278 | NA | NA | NA | no | no |
| 4277 | 2017 | NA | Yagi, N; Hasegawa, E | 2011 | Social-Organization Shift in the Sweat Bee, Lasioglossum baleicum (Hymenoptera, Halictidae), Corresponds to Changes in Foraging Activity of the Predatory Ant Tetramorium tsushimae (Hymenoptera, Formicidae) | Ecological factors, such as predation pressure or survival rate, affect social structure (e.g., gyny or founding modes) in social insects. Multiple females may cooperatively found a nest under severe ecological conditions. In bivoltine sweat-bees, most nests in the first reproductive period include a single female, but nest organization changes to cooperative in the second period. This fact predicts low predation pressures during the first period. However, few studies have examined corresponding changes between nest organization and predation pressures in social insects. Here, we compare the predation pressure between the two reproductive periods by using a sweat bee, Lasioglossum baleicum, that shifts the social organization from solitary to cooperative between the two reproductive periods. We recorded foraging activities of the predatory ant, Tetramorium tsushimae, during the whole reproductive season of L. baleicum and compared those between the first and second periods. The foraging activities of T tsushimae were low in the first season but rapidly increased with the start of the second season. The foraging activities vary among bee populations depending on temperature conditions. The foraging activities were high during the cooperative period, suggesting that cooperation is a counter strategy to a high risk of predation. Further investigations on relationships between nest organization and strength of predation will bring us deeper insights into the effects of predators on the evolution of cooperation. | 4279 | NA | NA | NA | no | no |
| 4278 | 2017 | NA | Copete, MA; Herranz, JM; Ferrandis, P | 2008 | Reproductive biology of the critically endangered endemic Mediterranean plant Coincya rupestris subsp rupestris (Spain): the effects of competition and summer drought on seedling establishment | Flower. fruit, seed production, and flowering phenology (duration, intensity, moment and synchrony) were studied in the two main Populations (south east Spain) of the critically endangered endemic Mediterranean plant Coincya rupestris subsp. rupestris (Cruciferae). Production of flowers and fruits (mean +/- SD) ranged from 483 (+/- 688) to 748 (+/- 636) and from 317 (+/- 518) to 553 (+/- 500), respectively, between populations. In addition, the average seed production per plant was 1,607-2.798, thus concluding that fertility was not responsible for the rarity of this taxon. The fruit/flower ratio ranged from 0.60 to 0.75, showing significant inter-population differences. Flowering extended from February-March to the end of spring, with high synchrony (approximate to 85 %). This parameter was negatively correlated with duration of the flowering. period. The role of pollinator insects on reproductive Success, and the effect of watering treatments and the elimination of competitors on seedling recruitment were analysed in the classical locality. The exclusion of pollinators dramatically affected fructification, reducing reproductive Success from moderate values in plants exposed to insects (approximate to 0.5) to null values in those where insects were experimentally excluded. Seedling emergence wits autumnal and no influence of the factors analysed (i.e., water availability and inter-specific competition) was detected oil seedling establishment. A high interannual variability in the size and survival of cohorts originated each autumn was observed. It should be emphasized that the rarity of the taxon is not due to fecundity restrictions. | 4280 | NA | NA | NA | no | no |
| 4279 | 2017 | NA | Jiang, SC; Fu, W | 2001 | Seasonal abundance and distribution of Vibrio cholerae in coastal waters quantified by a 16S-23S intergenic spacer probe | Vibrio cholerae is the causative agent of the severe diarrheal disease cholera and is indigenous to brackish waters. To advance our understanding of the ecology of this bacterium, we have developed a molecular probing method for detection of V. cholerae in coastal waters. Water samples from 7 locations in the Newport Bay watershed, California were sampled monthly for a whole year. V. cholerae concentrations were determined by membrane filtration-colony hybridization using an oligonucleotide probe targeting the 16S-23S intergenic spacer (ITS) region. In addition to V. cholerae concentrations, environmental parameters, including temperature, salinity, total bacterial direct counts, total viable counts, and chlorophyll a concentrations, were determined for each site. V. cholerae was detected year-round throughout the watershed. Regression analysis indicated that the concentration of V. cholerae inversely correlated with salinity (p < 0.001). The sampling sites located nearest to the Pacific Ocean had lower concentrations, whereas sites located along the brackish San Diego Creek (salinity 0-12<parts per thousand>) routinely had higher concentrations. V. cholerae concentrations also correlated with temperature (p < 0.01) in the watershed, with concentrations ranging from less than 1 CFU mL(-1) to 2,930 CFU mL(-1) of water. The results of this study indicate that the dynamics of V. cholerae is mainly influenced, out of the parameters measured, by the temperature and salinity of the environment. This information is valuable for understanding the ecology of V. cholerae. | 4281 | NA | NA | NA | no | no |
| 4280 | 2017 | NA | Oualkacha, K; Dastani, Z; Li, R; Cingolani, PE; Spector, TD; Hammond, CJ; Richards, JB; Ciampi, A; Greenwood, CMT | 2013 | Adjusted Sequence Kernel Association Test for Rare Variants Controlling for Cryptic and Family Relatedness | Recent progress in sequencing technologies makes it possible to identify rare and unique variants that may be associated with complex traits. However, the results of such efforts depend crucially on the use of efficient statistical methods and study designs. Although family-based designs might enrich a data set for familial rare disease variants, most existing rare variant association approaches assume independence of all individuals. We introduce here a framework for association testing of rare variants in family-based designs. This framework is an adaptation of the sequence kernel association test (SKAT) which allows us to control for family structure. Our adjusted SKAT (ASKAT) combines the SKAT approach and the factored spectrally transformed linear mixed models (FaST-LMMs) algorithm to capture family effects based on a LMM incorporating the realized proportion of the genome that is identical by descent between pairs of individuals, and using restricted maximum likelihood methods for estimation. In simulation studies, we evaluated type I error and power of this proposed method and we showed that regardless of the level of the trait heritability, our approach has good control of type I error and good power. Since our approach uses FaST-LMM to calculate variance components for the proposed mixed model, ASKAT is reasonably fast and can analyze hundreds of thousands of markers. Data from the UK twins consortium are presented to illustrate the ASKAT methodology. | 4282 | NA | NA | NA | no | no |
| 4281 | 2017 | NA | Yu, JM; Ao, YY; Liu, N; Li, LL; Duan, ZJ | 2015 | Salivirus in Children and Its Association with Childhood Acute Gastroenteritis: A Paired Case-Control Study | Salivirus was recently discovered in children with gastroenteritis and in sewage. Though a causative role for salivirus in childhood gastroenteritis was suggested in the previous study, the relationship between salivirus and acute gastroenteritis has not yet been clearly clarified. The sewage strain reported by Ng, although represented by incomplete genome sequencing data, was distinct from previously reported saliviruses, and had not previously been detected in humans. A case-control study examining 461 paired stool samples from children with diarrhea and healthy controls (1:1) was conducted in this study. Also, common diarrheal viruses were detected and complete genome of a salivirus was determined. Results showed that salivirus was detected in 16 (3.5%) and 13 (2.8%) of the case and control samples, respectively; no differences in detection rates (p=0.571) or mean values of viral loads (p=0.400) were observed between the groups. Multivariate Cox regression revealed no association between salivirus and gastroenteritis (p=0.774). The data also demonstrated that salivirus infection did not exacerbate clinical symptoms of gastroenteritis in children. Furthermore, complete genome sequence of a salivirus recovered from the feces of a child with diarrhea (i.e., SaliV-FHB) shared a 99% nucleotide identity with the sewage strain. In conclusion, a paired case-control study did not support a causative role for salivirus strains detected in this study with pediatric gastroenteritis. This study also demonstrated that all known saliviruses can be detected in the feces of children with or without gastroenteritis. | 4283 | NA | NA | NA | no | no |
| 4282 | 2017 | NA | Moradi, Z; Mehrvar, M; Nazifi, E; Zakiaghl, M | 2016 | The complete genome sequences of two naturally occurring recombinant isolates of Sugarcane mosaic virus from Iran | Sugarcane mosaic virus (SCMV) is the most prevalent virus causing sugarcane mosaic and maize dwarf mosaic diseases. Here, we presented the first two complete genomic sequences of Iranian SCMV isolates, NRA and ZRA from sugarcane and maize. The complete genome sequences of NRA and ZRA were, respectively, 9571 and 9572 nucleotides (nt) in length, excluding the 3’-terminal poly(A) tail. Both isolates contained a 5’-untranslated region (UTR) of 149 nt, an open reading frame of 9192 nt encoding a polyprotein of 3063 amino acids (aa), and 3’-UTR of 230 nt for NRA and 231 nt for ZRA. SCMV-NRA and -ZRA genome nucleotide sequences were 97.3 % identical and shared nt identities of 79.1-92 % with those of other 21 SCMV isolates available in the GenBank, highest with the isolate Bris-A (AJ278405) (92 and 91.7 %) from Australia. When compared for separate genes, most of their genes shared the highest identities with Australian and Argentinean isolates. Phylogenetic analysis of the complete genomic sequences reveals that SCMV can be clustered to three groups. Both NRA and ZRA were clustered with sugarcane isolates from Australia and Argentina in group III but formed a separate sublineage. Recombination analysis showed that both isolates were intraspecific recombinants, and represented two novel recombination patterns of SCMV (in the P1 coding region). NRA had six recombination sites within the P1, HC-Pro, CI, NIa-Vpg, and NIa-pro coding regions, while ZRA had four within the P1, HC-Pro, NIa-Pro, and NIb coding regions. | 4284 | NA | NA | NA | no | no |
| 4283 | 2017 | NA | Garcia-Ortiz, MV; Marsin, S; Arana, ME; Gasparutto, D; Guerois, R; Kunkel, TA; Radicella, JP | 2011 | Unexpected Role for Helicobacter pylori DNA Polymerase I As a Source of Genetic Variability | Helicobacter pylori, a human pathogen infecting about half of the world population, is characterised by its large intraspecies variability. Its genome plasticity has been invoked as the basis for its high adaptation capacity. Consistent with its small genome, H. pylori possesses only two bona fide DNA polymerases, Pol I and the replicative Pol III, lacking homologues of translesion synthesis DNA polymerases. Bacterial DNA polymerases I are implicated both in normal DNA replication and in DNA repair. We report that H. pylori DNA Pol I 5’-3’ exonuclease domain is essential for viability, probably through its involvement in DNA replication. We show here that, despite the fact that it also plays crucial roles in DNA repair, Pol I contributes to genomic instability. Indeed, strains defective in the DNA polymerase activity of the protein, although sensitive to genotoxic agents, display reduced mutation frequencies. Conversely, overexpression of Pol I leads to a hypermutator phenotype. Although the purified protein displays an intrinsic fidelity during replication of undamaged DNA, it lacks a proofreading activity, allowing it to efficiently elongate mismatched primers and perform mutagenic translesion synthesis. In agreement with this finding, we show that the spontaneous mutator phenotype of a strain deficient in the removal of oxidised pyrimidines from the genome is in part dependent on the presence of an active DNA Pol I. This study provides evidence for an unexpected role of DNA polymerase I in generating genomic plasticity. | 4285 | NA | NA | NA | no | no |
| 4284 | 2017 | NA | de Silva, S; Webber, CE; Weerathunga, US; Pushpakumara, TV; Weerakoon, DK; Wittemyer, G | 2013 | Demographic Variables for Wild Asian Elephants Using Longitudinal Observations | Detailed demographic data on wild Asian elephants have been difficult to collect due to habitat characteristics of much of the species’ remaining range. Such data, however, are critical for understanding and modeling population processes in this endangered species. We present data from six years of an ongoing study of Asian elephants (Elephas maximus) in Uda Walawe National Park, Sri Lanka. This relatively undisturbed population numbering over one thousand elephants is individually monitored, providing cohort-based information on mortality and reproduction. Reproduction was seasonal, such that most births occurred during the long inter-monsoon dry season and peaked in May. During the study, the average age at first reproduction was 13.4 years and the 50th percentile inter-birth interval was approximately 6 years. Birth sex ratios did not deviate significantly from parity. Fecundity was relatively stable throughout the observed reproductive life of an individual (ages 11-60), averaging between 0.13-0.17 female offspring per individual per year. Mortalities and injuries based on carcasses and disappearances showed that males were significantly more likely than females to be killed or injured through anthropogenic activity. Overall, however, most observed injuries did not appear to be fatal. This population exhibits higher fecundity and density relative to published estimates on other Asian elephant populations, possibly enhanced by present range constriction. Understanding the factors responsible for these demographic dynamics can shed insight on the future needs of this elephant population, with probable parallels to other populations in similar settings. | 4286 | NA | NA | NA | no | no |
| 4285 | 2017 | NA | Edwards, S; Tolley, KA; Measey, GJ | 2017 | Habitat characteristics influence the breeding of Rose’s dwarf mountain toadlet Capensibufo rosei (Anura: Bufonidae) | Direct anthropogenic factors (e.g., habitat loss, fragmentation, and degradation) threaten many amphibian populations, however some declines have occurred in supposedly pristine environments with no obvious causes. These enigmatic declines may be due to shifts in environmental factors influencing development and ultimately adult survival. Rose’s mountain toadlet Capensibufo rosei has undergone such an enigmatic decline, with several populations presumed to be locally extinct at historic breeding sites. The two remaining breeding sites (Silvermine (SILV) and Cape of Good Hope (CGH)) on the Cape Peninsula of South Africa were monitored for three years (2012-2014) for life history traits and ecological requirements. Males congregate at ephemeral pools during the middle of the austral winter, with females arriving to lay eggs and then immediately leaving. Breeding only occurs in a few of the available pools. We hypothesised that larval development in colder, deeper pools would result in smaller-bodied tadpoles, and ultimately in relatively smaller adults. Pools at SILV were significantly deeper and colder compared to CGH, with breeding occurring in pools that were 27.05 +/- 10.21 mm and 21.55 +/- 6.95 mm deep at SILV and CGH, respectively. Contrary to expectations, breeding adults and developing tadpoles at SILV were larger than CGH individuals. The percentage of non-developing eggs at CGH was high compared to SILV and other anuran species. Development within this threatened species may be influenced by pool characteristics, which could provide clues as to the factors that influenced local extinctions in historical populations. | 4287 | NA | NA | NA | no | no |
| 4286 | 2017 | NA | Samara, RY; Monje, JC; Zebitz, CPW | 2008 | Comparison of different European strains of Trichogramma aurosum (Hymenoptera : Trichogrammatidae) using fertility life tables | Life table parameters were assessed for seven strains of Trichogramma aurosum Sugonjaev and Sorokina (Hymenoptera: Trichogrammatidae) collected in different European countries, in order to compare their performance when reared on eggs of Ephestia kuehniella Zeller (Lepidoptera: Pyralidae) as a potential factitious host for mass-rearing. The average number of progeny per female, cumulative fertility and emergence rate did not differ significantly, whereas female longevity and sex ratio significantly differed between the seven parasitoid strains. The Danish strain survived the longest (6.05 days) and the Dutch strain survived the shortest (2.75 days). Progeny was always female-biased with varying proportions (57.7-96.7%). Survival rates started to decrease after 3 days for some of the strains studied. The mean cohort generation duration (T-c) was 11.40, 10.15, 10.62, 10.63, 9.28, 9.70 and 11.30 days for the Austrian, Luxemburgian, Belgian, French, Dutch, Danish and German strains, respectively. Population doubling time (D-t) was 4.50, 7.96, 3.56, 5.30, 5.23, 7.36 and 3.30 days, respectively. Daily intrinsic rate of increase (r(m)) and finite rate of increase (exp. r(m)) ranged between 0.087 and 0.210 and 1.091-1.233, respectively. The German strain might be a potential candidate for mass rearing and releases against the codling moth, Cydia pomonella (L.) (Lepidoptera: Tortricidae), due to its high net reproduction rate (R-0=10.65 female), a high intrinsic rate of natural increase (r(m)=0.210), a high finite rate of increase (exp. r(m)=1.23), and a short population doubling time (D-t=3.3 days). The relevance of intra- and interstrain variability as well as the usefulness of fertility life tables for pre-introductory research is discussed. | 4288 | NA | NA | NA | no | no |
| 4287 | 2017 | NA | Gomez, I; Chet, I; HerreraEstrella, A | 1997 | Genetic diversity and vegetative compatibility among Trichoderma harzianum isolates | Trichoderma harzianum is the collective name of a set of asexual fungal strains which exhibit heterogeneity in genome structure, DNA sequence and behavior. Contour-clamped homogeneous field (CHEF) electrophoresis of the chromosomes of ten isolates of T. harzianum revealed six clearly distinct electrophoretic karyotypes. Of the ten isolates analyzed, four (GH12, G109, Y and YF) could be classified in a single group with identical karyotypes, while the strains T35 and 315 formed a second group. The genome size characteristic of the different isolates fell into a broad range varying from 29.6 to 56.1 Mb. Gene assignments to the resolved chromosomes showed that all genes analyzed were localized on equivalent chromosomes in the isolates belonging to the same group. Analysis of randomly amplified polymorphic DNAs from the ten isolates confirmed the classification into groups and allowed us to distinguish between isolates T35 and 315, as well as between isolates GH12, G109, Y and YF. Direct confrontation assays using isolates of the same group showed compatible interactions, whereas the same experiment carried out with isolates of different groups showed an incompatible interaction characterized by an area of cell damage. Microscopic observation of the compatible interactions showed hyphal fusions between the isolates, similar to those described for vegetative compatible groups in other fungi. The molecular karyotypes correlated well with the compatibility of the isolates. In addition, we have evaluated both electrophoretic karyotype and randomly amplified polymorphic DNAs analysis as criteria for grouping isolates within the genus according to their capacity for biocontrol of plant pathogens. | 4289 | NA | NA | NA | no | no |
| 4288 | 2017 | NA | Zhao, HD; Liu, SL; Dong, SK; Su, XK; Liu, Q; Deng, L | 2014 | Characterizing the importance of habitat patches in maintaining landscape connectivity for Tibetan antelope in the Altun Mountain National Nature Reserve, China | Connectivity of habitat patches is crucial for wildlife dispersal and survival, and identifying patches with high importance for maintaining connectivity can aid effective wildlife management. Knowledge of the habitat distribution of the Tibetan antelope in the Altun Mountain National Nature Reserve, which is essential for connectivity analysis, remains limited. We analyzed potential habitat distribution and priority patches using GIS-based habitat suitability modeling with three weighting factors and evaluated the connectivity of habitat patches under four dispersal distance scenarios. Patches with high habitat suitability covered 25.39 % of the total area, and these patches were selected for connectivity analysis as resource patches. Connectivity analysis indicated that, although the overall probability of connectivity (PC) showed an upward trend with increasing dispersal distance, the importance of each patch varied considerably under different dispersal distance scenarios. Transfer analysis of patch numbers between different importance levels revealed that the number of patches becoming less important was higher than the number of patches becoming more important when dispersal distance increased. In addition, nine patches covering 38.49 % of the suitable habitat area were identified as priority patches, in particular the patches near the Kardun and the Karchuka inspection stations. We also found that the habitat distribution of the Tibetan antelope obtained from the suitability model matched the population distribution determined by a field survey. Correlation analysis between patch area and the percentage of PC index value loss (dPC) revealed that the larger patches in this region were more likely to be important for maintaining connectivity. | 4290 | NA | NA | NA | no | no |
| 4289 | 2017 | NA | Udden, SMN; Zahid, MSH; Biswas, K; Ahmad, QS; Cravioto, A; Nair, GB; Mekalanos, JJ; Faruque, SM | 2008 | Acquisition of classical CTX prophage from Vibrio cholerae O141 by El Tor strains aided by lytic phages and chitin-induced competence | The El Tor biotype of Vibrio cholerae O1, causing the current seventh pandemic of cholera, has replaced the classical biotype, which caused the sixth pandemic. The CTX prophages encoding cholera toxin in the two biotypes have distinct repressor (rstR) genes. Recently, new variants of El Tor strains that carry the classical type (CTXclass) prophage have emerged. These “hybrid” strains apparently originate through lateral gene transfer and recombination events. To explore possible donors of the CTXclass prophage and its mode of transfer, we tested environmental V. cholerae isolates for the presence of CTXclass prophage and mobility of the phage genome. Of the 272 environmental V. cholerae isolates tested, 6 were found to carry the CTXclass prophage; all of these belonged to the O141 serogroup. These O141 strains were unable to produce infectious CTXclass phage or to transmit the prophage to recipient strains in the mouse model of infection; however, the CTXclass prophage was acquired by El Tor strains when cultured with the O141 strains in microcosms composed of filtered environmental water, a chitin substrate, and a V. cholerae O141-specific bacteriophage. The CTXclass, prophage either coexisted with or replaced the resident CTXET prophage, resulting in El Tor strains with CTX genotypes similar to those of the naturally occurring hybrid strains. Our results support a model involving phages and natural chitin substrate in the emergence of new variants of pathogenic V. cholerae. Furthermore, the O141 strains apparently represent an alternative reservoir of the CTXclass phage genome, because the classical V. cholerae O1 strains are possibly extinct. | 4291 | NA | NA | NA | no | no |
| 4290 | 2017 | NA | Laws, RJ; Kesler, DC | 2012 | A Bayesian network approach for selecting translocation sites for endangered island birds | Translocation has become increasingly important for conserving island species. Limited tools are available for guiding the selection of translocation sites, however, particularly when establishing rescue populations outside of historic ranges. We developed a Bayesian network model framework for translocation site selection for island birds. The model consisted of four primary components including ecological requirements for survival, anthropogenic threats at the population establishment site, effects the translocated species may have on native species, and operational support associated with the translocation process and ongoing management. We then used the model to identify potential sites for the establishment of a wild population of Guam Micronesian kingfishers (Todiramphus cinnamominus cinnamominus) on an island outside the bird’s historic range. Conditional probabilities that guided model evaluations were allocated using information from the literature, expert opinions, and a training set of islands outside the region under consideration for releases. The model was used to evaluate 239 islands where a translocation population of Micronesian kingfishers could be established. Five islands, all in the Federated States of Micronesia, were identified as being suitable for assisted colonization, including Kosrae, Yap, Faichuk, Weno and Fefan. Sensitivity analysis showed a correspondence between model variables and island characteristics indicated by the literature as being the most important for successful translocation. We found the Bayesian network model to be a useful tool for translocation site selection despite limited information on the natural history of the Guam Micronesian kingfisher and the factors that impact the success of translocations. (C) 2012 Elsevier Ltd. All rights reserved. | 4292 | NA | NA | NA | no | no |
| 4291 | 2017 | NA | Lopez-Mata, L | 2013 | The impact of seed extraction on the population dynamics of Pinus maximartinezii | Pinus maximartinezii is a rare, endemic, threatened species known from a single small population in the state of Zacatecas, Mexico. Among the pine species that produce edible nuts, it produces one of the largest and most nutritious seeds. The seeds of P. maximartinezii have historically been used for human consumption. The cones are harvested directly from the trees, and the seeds are sold illegally in local, national and international markets. However, the effects of seed extraction must be thoroughly evaluated to determine the potential impacts on population stability. To assess the impact of different rates of seed harvesting on the demography of this species, a 2-yr study of population dynamics was conducted in three 0.1-ha plots. A 9 x 9 size-structured matrix model was used to simulate changes in population growth over time in conjunction with increasing stepwise reductions in fecundity. The population growth rate (A) of P. maximartinezii was 1.1175, with a 95% confidence interval (Cl) from 1.1008 to 1.1321, and it was relatively insensitive to changes in fecundity and growth. Under a seed extraction intensity of 99%, lambda decreased to 1.0241, with a Cl from 1.0177 to 1.0361. Elasticity analysis was then performed to identify the combined effects of proportional changes in fecundities and the largest stasis elements on lambda. The results suggest that a sound conservation strategy should focus on improving the survival of juveniles and adults during their first reproductive events and on the largest adults, as well as on protecting the habitat of this threatened endemic species. (C) 2013 Elsevier Masson SAS. All rights reserved. | 4293 | NA | NA | NA | no | no |
| 4292 | 2017 | NA | Carvalho, RCT; Vasconcellos, SEG; Issa, MD; Soares, PM; Mota, PMPC; de Araujo, FR; Carvalho, ACD; Gomes, HM; Suffys, PN; Figueiredo, EED; Paschoalin, VMF | 2016 | Molecular Typing of Mycobacterium bovis from Cattle Reared in Midwest Brazil | Mycobacterium bovis is the causative agent of bovine tuberculosis (BTB), the pathogen responsible for serious economic impact on the livestock sector. In order to obtain data on isolated M. bovis strains and assist in the control and eradication program for BTB, a cross sectional descriptive molecular epidemiology study in the Brazilian Midwest was conducted. Through spoligotyping and 24-loci MIRU-VNTR methods, 37 clinical isolates of M. bovis circulating in the region were analyzed, 10 isolated from the state of Mato Grosso, 12 from the state of Mato Grosso do Sul and 15 from the state of Goias. The spoligotyping analysis identified 10 distinct M. bovis profiles (SB0121 n = 14, SB0295 n = 6, SB0140 n = 6, SB0881 n = 3, SB1144 n = 2, SB1145 n = 2, SB0134 n = 1, SB1050 n = 1, SB1055 n = 1, SB1136 n = 1) grouped in six clusters and four orphan patterns. The MIRU-VNTR 24-loci grouped the same isolates in six clusters and 22 unique orphan patterns, showing higher discriminatory power than spoligotyping. When associating the results of both techniques, the isolates were grouped in five clusters and 24 unique M. bovis profiles. Among the 24-loci MIRUVNTR evaluated, two, ETR-A and QUB 11b loci, showed high discriminatory ability (h = >= 0.50), while MIRU 16, MIRU 27, ETR-B, ETR-C, Mtub21 and QUB 26 loci showed moderate ability (h = 0.33 or h = 0.49) and were the most effective in evaluating the genotypic similarities among the clinical M. bovis isolate samples. Herein, the 29 patterns found amongst the 37 isolates of M. bovis circulating in the Brazilian Midwest can be due to the animal movement between regions, municipalities and farms, thus causing the spread of various M. bovis strains in herds from Midwest Brazil. | 4294 | NA | NA | NA | no | no |
| 4293 | 2017 | NA | Berry, RJ; Scriven, PN | 2005 | The house mouse: a model and motor for evolutionary understanding | Commensal house mice have spread from their probable origin in north India, differentiating into a number of forms described variously as species, semispecies or subspecies. The different taxa can breed together and exchange genes but they retain their distinctiveness (although Mus (musculus) molossinus of Japan seems to be the result of a complete fusion between M. (m.) musculus and M. (m.) castaneus). The most widespread form is M. (M.) domesticus, which has successfully colonized every continent as a commensal, albeit with varying contributions from other Mus genomes. It has also been domesticated as the laboratory mouse. This means that the same genome is exposed to a wide variety of environments and gives tremendous opportunities for exploring the operation of different evolutionary mechanisms. Mice have accompanied evolutionary understanding from early Darwinian days - confirming Mendelian ratios and showing they applied in mammals, providing data on rates of evolution, and representing examples of dominance modification, differential survival, competition and other indicators of a struggle for existence. However, they have an unfulfilled potential to drive as well as to illuminate evolutionary theory - by revealing more about, for example, the interactions between gene flow and social determinants, constraints on introgression and physiological adjustments. This potential can be explored through our ever-deepening knowledge of the genome and molecular mechanisms, and by the application of new techniques, but its most effective agent will always be the cross-disciplinary synergy of visionary scientists like Julian Huxley, Charles Elton and Louis Thaler. (c) 2005 The Linnean Society of London. | 4295 | NA | NA | NA | no | no |
| 4294 | 2017 | NA | Koen, EL; Tosa, MI; Nielsen, CK; Schauber, EM | 2017 | Does landscape connectivity shape local and global social network structure in white-tailed deer? | Intraspecific social behavior can be influenced by both intrinsic and extrinsic factors. While much research has focused on how characteristics of individuals influence their roles in social networks, we were interested in the role that landscape structure plays in animal sociality at both individual (local) and population (global) levels. We used female white-tailed deer (Odocoileus virginianus) in Illinois, USA, to investigate the potential effect of landscape on social network structure by weighting the edges of seasonal social networks with association rate (based on proximity inferred from GPS collar data). At the local level, we found that sociality among female deer in neighboring social groups (n = 36) was mainly explained by their home range overlap, with two exceptions: 1) during fawning in an area of mixed forest and grassland, deer whose home ranges had low forest connectivity were more social than expected; and 2) during the rut in an area of intensive agriculture, deer inhabiting home ranges with high amount and connectedness of agriculture were more social than expected. At the global scale, we found that deer populations (n = 7) in areas with highly connected forest-agriculture edge, a high proportion of agriculture, and a low proportion of forest tended to have higher weighted network closeness, although low sample size precluded statistical significance. This result implies that infectious disease could spread faster in deer populations inhabiting such landscapes. Our work advances the general understanding of animal social networks, demonstrating how landscape features can underlie differences in social behavior both within and among wildlife social networks. | 4296 | NA | NA | NA | no | no |
| 4295 | 2017 | NA | Barsoum, N | 2001 | Relative contributions of sexual and asexual regeneration strategies in Populus nigra and Salix alba during the first years of establishment on a braided gravel bed river | Populus nigra L. var betulifolia and Salix alba L. var alba are early successional riparian tree species threatened throughout Continental Europe by significant changes to the natural physical processes governing their natural habitat - geomorphologically active floodplains. River management activities have dramatically altered natural patterns of river flow and rates of sediment delivery along rivers, with possible consequences for the balance between sexual and asexual regeneration strategies in these species. Conservation strategies will benefit from a greater understanding of the ways in which dynamic physical processes on the floodplain influence sexual and asexual recruitment. This paper describes a field survey investigating the relative abundance and spatial distributions of P. nigra and S. alba sexual and asexual recruits during the first years of establishment along a braided gravel bed river. Sexual and asexual recruits were identified by excavation along transects in a wet and a dry field season and distributional differences were described in terms of elevation on the floodplain, local sediment type and exposure to floodwaters. Regeneration was overwhelmingly from seed in the first 2-3 years following recruitment, but poor survival rates among sexual recruits saw a shift in the relative abundance of regeneration strategies over time. In relating hydrological data to recruitment, unseasonal flood disturbances had a negative effect on recruitment from seed and a positive effect on vegetative regeneration. Seedlings were associated with fine sediment deposits and were restricted primarily to low elevations on the flood plain, while asexual recruits had a wider spatial distribution. Certain microsite types were unique to either regeneration strategy. | 4297 | NA | NA | NA | no | no |
| 4296 | 2017 | NA | Jezic, M; Krstin, L; Poljak, I; Liber, Z; Idzojtic, M; Jelic, M; Mestrovic, J; Zebec, M; Curkovic-Perica, M | 2014 | Castanea sativa: genotype-dependent recovery from chestnut blight | In Lovran (coastal Croatia), a unique forest/orchard of evenly mixed grafted marrons and naturally growing nongrafted sweet chestnut trees exists. This old chestnut population has been devastated by chestnut blight, caused by an aggressive introduced pathogenic fungus, Cryphonectria parasitica. However, initial observations indicated recovery of naturally growing chestnut trees in that area, mediated by Cryphonectria-associated hypovirus (Cryphonectria hypovirus 1 (CHV-1)). Such recovery was not observed on grafted trees. Genotyping both, we confirmed the clonal origin of the grafted ones-marrons. No significant difference was observed between fungal strains isolated from naturally growing trees and the ones from marrons regarding fungal vegetative compatibility types or the prevalence of CHV-1. A strong correlation was observed between the types of canker: active/deep-expanding versus healing callus or superficial necrosis and the absence or presence of CHV-1 in the fungal isolates, sampled from naturally growing trees (Spearman rho 0.686, p value 7.81 x 10(-5), Kendall tau 0.686, p value 5.18 x 10(-7)). Such correlation was not observed on marrons (Spearman rho 0.236, p value 0.235, Kendall tau 0.236, p value 0.084), because, unexpectedly, active/deep-expanding cankers were often associated with hypovirulent fungal isolates. These data indicate that the lack or unequal distribution of naturally occurring hypovirulence were not the cause of substantial marron decay in Lovran. Ecological and age-dependant differences were ruled out because all sampled trees are growing in close proximity and are of similar age. The results imply that the marron genotype is especially vulnerable and its ability to recover is limited even when the hypovirulent strain of the fungus is present in the canker. | 4298 | NA | NA | NA | no | no |
| 4297 | 2017 | NA | Evans, BJ; Pin, L; Melnick, DJ; Wright, SI | 2010 | Sex-Linked Inheritance in Macaque Monkeys: Implications for Effective Population Size and Dispersal to Sulawesi | Sex-specific differences in dispersal, survival, reproductive success, and natural selection differentially affect the effective population size (Ne) of genomic regions with different modes of inheritance such as sex chromosomes and mitochondrial DNA. In papionin monkeys (macaques, baboons, geladas, mandrills, drills, and mangabeys), for example, these factors are expected to reduce Ne of paternally inherited portions of the genome compared to maternally inherited portions. To explore this further, we quantified relative Ne of autosomal DNA, X and Y chromosomes, and mitochondrial DNA using molecular polymorphism and divergence information from pigtail macaque monkeys (Macaca nemestrina). Consistent with demographic expectations, we found that Ne of the Y is lower than expected from a Wright-Fisher idealized population with an equal proportion of males and females, whereas Ne of mitochondrial DNA is higher. However, Ne of 11 loci on the X chromosome was lower than expected, a finding that could be explained by pervasive hitchhiking effects on this chromosome. We evaluated the fit of these data to various models involving natural selection or sex-biased demography. Significant support was recovered for natural selection acting on the Y chromosome. A demographic model with a skewed sex ratio was more likely than one with sex-biased migration and explained the data about as well as an ideal model without sex-biased demography. We then incorporated these results into an evaluation of macaque divergence and migration on Borneo and Sulawesi islands. One X-linked locus was not monophyletic on Sulawesi, but multilocus data analyzed in a coalescent framework failed to reject a model without migration between these islands after both were colonized. | 4299 | NA | NA | NA | no | no |
| 4298 | 2017 | NA | Mougeot, F; Piertney, SB; Leckie, F; Evans, S; Moss, R; Redpath, SM; Hudson, PJ | 2005 | Experimentally increased aggressiveness reduces population kin structure and subsequent recruitment in red grouse Lagopus lagopus scoticus | 1. According to the ‘territorial behaviour’ hypothesis, red grouse population cycles are caused by delayed density-dependent changes in male aggressiveness influencing recruitment. These lagged changes in aggressiveness might be caused by changes in the kin structuring of male populations and differential aggressive behaviour between kin and non-kin (‘kinship’ hypothesis). 2. A population-level manipulation of male agressiveness in autumn affected the kin structure of male populations, their subsequent aggressiveness, and recruitment of both sexes. On two moors, we implanted the old territorial cocks in autumn with testosterone on an experimental area (T-areas) and with sham implants on a control area (C-areas). 3. Increased aggressiveness in autumn t reduced recruitment in autumns t and t+1, and breeding density of both sexes in springs t+1 and t+2, confirming previous studies elsewhere. A new observation was that cocks on T-areas had bigger combs (an ornament whose size is testosterone-dependent) than those on C-areas for at least 1.5 years after treatment, evidence that they remained more aggressive. 4. Increased aggressiveness reduced not only subsequent density but also kin structuring among territorial cocks. This is consistent with the ‘kinship’ hypothesis that changes in the kin structure of male populations mediate year-to-year changes in male aggressiveness. 5. Increased aggressiveness did not increase intensity of infection by the dominant intestinal nematode Trichostrongylus tenuis. which might have affected recruitment through reduced breeding success. Moreover, breeding success after treatment was no lower on the T- than on the C-areas. 6. The results show for the first time that increased aggressiveness affects both kin structure and subsequent recruitment, supporting a key assumption of the kinship hypothesis for red grouse population cycles. | 4300 | NA | NA | NA | no | no |
| 4299 | 2017 | NA | Sellers, GS; Griffin, LR; Hanfling, B; Gomez, A | 2017 | A new molecular diagnostic tool for surveying and monitoring Triops cancriformis populations | The tadpole shrimp, Triops cancriformis, is a freshwater crustacean listed as endangered in the UK and Europe living in ephemeral pools. Populations are threatened by habitat destruction due to land development for agriculture and increased urbanisation. Despite this, there is a lack of efficient methods for discovering and monitoring populations. Established macroinvertebrate monitoring methods, such as net sampling, are unsuitable given the organism’s life history, that include long lived diapausing eggs, benthic habits and ephemerally active populations. Conventional hatching methods, such as sediment incubation, are both time consuming and potentially confounded by bet-hedging hatching strategies of diapausing eggs. Here we develop a new molecular diagnostic method to detect viable egg banks of T. cancriformis, and compare its performance to two conventional monitoring methods involving diapausing egg hatching. We apply this method to a collection of pond sediments from the Wildfowl & Wetlands Trust Caerlaverock National Nature Reserve, which holds one of the two remaining British populations of T. cancriformis. DNA barcoding of isolated eggs, using newly designed species-specific primers for a large region of mtDNA, was used to estimate egg viability. These estimates were compared to those obtained by the conventional methods of sediment and isolation hatching. Our method outperformed the conventional methods, revealing six ponds holding viable T. cancriformis diapausing egg banks in Caerlaverock. Additionally, designed species-specific primers for a short region of mtDNA identified degraded, inviable eggs and were used to ascertain the levels of recent mortality within an egg bank. Together with efficient sugar flotation techniques to extract eggs from sediment samples, our molecular method proved to be a faster and more powerful alternative for assessing the viability and condition of T. cancriformis diapausing egg banks. | 4301 | NA | NA | NA | no | no |
| 4300 | 2017 | NA | Wang, CJ; Lin, YS; Lin, YH; Chung, WH | 2013 | Modified Primers for the Identification of Nonpathogenic Fusarium oxysporum Isolates That Have Biological Control Potential against Fusarium Wilt of Cucumber in Taiwan | Previous investigations demonstrated that Fusarium oxysporum (Fo), which is not pathogenic to cucumbers, could serve as a biological control agent for managing Fusarium wilt of cucumber caused by Fo f. sp. cucumerinum (Foc) in Taiwan. However, thus far it has not been possible to separate the populations of pathogenic Fo from the nonpathogenic isolates that have biological control potential through their morphological characteristics. Although these two populations can be distinguished from one another using a bioassay, the work is laborious and time-consuming. In this study, a fragment of the intergenic spacer (IGS) region of ribosomal DNA from an Fo biological control agent, Fo366, was PCR-amplified with published general primers, FIGS11/FIGS12 and sequenced. A new primer, NPIGS-R, which was designed based on the IGS sequence, was paired with the FIGS11 primer. These primers were then evaluated for their specificity to amplify DNA from nonpathogenic Fo isolates that have biological control potential. The results showed that the modified primer pair, FIGS11/ NPIGS-R, amplified a 500-bp DNA fragment from five of seven nonpathogenic Fo isolates. These five Fo isolates delayed symptom development of cucumber Fusarium wilt in greenhouse bioassay tests. Seventy-seven Fo isolates were obtained from the soil and plant tissues and then subjected to amplification using the modified primer pair; six samples showed positive amplification. These six isolates did not cause symptoms on cucumber seedlings when grown in peat moss infested with the isolates and delayed disease development when the same plants were subsequently inoculated with a virulent isolate of Foc. Therefore, the modified primer pair may prove useful for the identification of Fo isolates that are nonpathogenic to cucumber which can potentially act as biocontrol agents for Fusarium wilt of cucumber. | 4302 | NA | NA | NA | no | no |
| 4301 | 2017 | NA | Zang, LS; Chen, WQ; Liu, SS | 2006 | Comparison of performance on different host plants between the B biotype and a non-B biotype of Bemisia tabaci from Zhejiang, China | The capacity of the B biotype of the whitefly, Bemisia tabaci (Gennadius) (Homoptera: Aleyrodidae), to invade has often been linked to its presumably wider host range than the non-B indigenous biotypes. However, there are few experimental studies of the relative performance of the B biotype and non-B biotypes on different host-plant species. Here, we compared the performance of the B biotype and an indigenous non-B biotype (China-ZHJ-1) of B. tabaci from Zhejiang, China on five commonly cultivated host plants, each from a different family: cotton, tobacco, cabbage, squash, and kidney bean. We also examined the effect of rearing host plants on the performance of the B biotype. Overall, the performance of the B biotype on the five species of plants was much better than that of the indigenous non-B population. On tobacco, cabbage, and kidney bean, no individuals of ZHJ-1 completed development to adulthood, whereas the B biotype developed successfully from egg to adult on all three plants. On squash, the B biotype survived better, developed to adulthood earlier and had a higher fecundity than ZHJ-1. The two biotypes performed more equally on cotton, but even on this plant the B biotype female adults lived nearly twice as long as that of ZHJ-1 and may have realized a higher life-time fecundity. The B biotype also showed a substantial capacity to acclimatize to alternative host plants for improved survival and reproduction, on both highly suitable and marginally suitable host plants. We conclude that the host range of the B biotype of B. tabaci may be much wider than those of some indigenous biotypes, and this advantage of the B biotype over the non-B biotypes may assist in its invasion and displacement of some indigenous biotypes in the field. | 4303 | NA | NA | NA | no | no |
| 4302 | 2017 | NA | Cieslak, M; Mikkelsen, SS; Skall, HF; Baud, M; Diserens, N; Engelsma, MY; Haenen, OLM; Mousakhani, S; Panzarin, V; Wahli, T; Olesen, NJ; Schuetze, H | 2016 | Phylogeny of the Viral Hemorrhagic Septicemia Virus in European Aquaculture | One of the most valuable aquaculture fish in Europe is the rainbow trout, Oncorhynchus mykiss, but the profitability of trout production is threatened by a highly lethal infectious disease, viral hemorrhagic septicemia (VHS), caused by the VHS virus (VHSV). For the past few decades, the subgenogroup la of VHSV has been the main cause of VHS outbreaks in European freshwater-farmed rainbow trout. Little is currently known, however, about the phylogenetic radiation of this la lineage into subordinate la clades and their subsequent geographical spread routes. We investigated this topic using the largest la-isolate dataset ever compiled, comprising 651 complete G gene sequences: 209 GenBank la isolates and 442 la isolates from this study. The sequences come from 11 European countries and cover the period 1971-2015. Based on this dataset, we documented the extensive spread of the la population and the strong mixing of la isolates, assumed to be the result of the Europe-wide trout trade. For example, the la lineage underwent a radiation into nine la clades, most of which are difficult to allocate to a specific geographic distribution. Furthermore, we found indications for two rapid, large-scale population growth events, and identified three polytomies among the la clades, both of which possibly indicate a rapid radiation. However, only about 4% of la haplotypes (out of 398) occur in more than one European country. This apparently conflicting finding regarding the Europe-wide spread and mixing of la isolates can be explained by the high mutation rate of VHSV. Accordingly, the mean period of occurrence of a single la haplotype was less than a full year, and we found a substitution rate of up to 7.813 x 10(-4) nucleotides per site per year. Finally, we documented significant differences between Germany and Denmark regarding their VHS epidemiology, apparently due to those countries’ individual handling of VHS. | 4304 | NA | NA | NA | no | no |
| 4303 | 2017 | NA | Thorne, BL; Breisch, NL; Haverty, MI | 2002 | Longevity of kings and queens and first time of production of fertile progeny in dampwood termite (Isoptera; Termopsidae; Zootermopsis) colonies with different reproductive structures | 1 We report age‐specific survivorship of founding kings and queens and colony age of first production of fertile progeny (alates) of two cohorts, containing 52 and 42 complete colonies, respectively, in the primitive dampwood termite Zootermopsis nevadensis ssp. nevadensis.   2 Longevity of kings and queens was determined and compared in various experimental manipulations of reproductive structure of incipient colonies (removal of king or queen or both vs. unmanipulated pairs as controls) performed one year after pairing. Life span of reproductives was also evaluated in circumstances of interactions between young colonies, a treatment inspired by field observations of natural nest spacing and intraspecific competition between young dampwood termite colonies.  3 In our first experimental series, founding pairs survived 2–7 years (x̄ = 4·5 ± 0·3 SE) after pairing. Kings lived significantly longer (x̄ = 4·7 ± 0·3 years after pairing; range 2·2–7·3 years) in treatments initiated as pairs than when left as the remaining founder in the queen removal treatment (x̄ = 3·2 ± 0·6 years after pairing; range 1·5–5·3 years), even though one or more female replacement reproductives differentiated within several months after queen loss. Queen life span in the absence of kings averaged 4·9 ± 0·4 years (range 3·6–6·3 years) after pairing, not significantly different from the life span of queens living with kings. There was no significant difference between king and queen life spans in non‐interaction treatments in our first experiment. Results from the second experimental series (35 colonies) are presented to date.  4 Survival of pairs until death of one mate was significantly shorter in interaction treatments compared with control colonies, suggesting that intraspecific competition between young colonies may create opportunities for replacement reproductives.  5 The first alates were produced between 1·8 and 6·5 years after pairing in our first experimental series, and between 2·4 and 5 years, to date, in the second experiment. 6 Ecological and evolutionary correlates of long life span in social insect reproductives and other animals are discussed. | 4305 | NA | NA | NA | no | no |
| 4304 | 2017 | NA | Felix, MA; Duveau, F | 2012 | Population dynamics and habitat sharing of natural populations of Caenorhabditis elegans and C. briggsae | Background: The nematode Caenorhabditis elegans is a major model organism in laboratory biology. Very little is known, however, about its ecology, including where it proliferates. In the past, C. elegans was mainly isolated from human-made compost heaps, where it was overwhelmingly found in the non-feeding dauer diapause stage. Results: C. elegans and C. briggsae were found in large, proliferating populations in rotting plant material (fruits and stems) in several locations in mainland France. Both species were found to co-occur in samples isolated from a given plant species. Population counts spanned a range from one to more than 10,000 Caenorhabditis individuals on a single fruit or stem. Some populations with an intermediate census size (10 to 1,000) contained no dauer larvae at all, whereas larger populations always included some larvae in the pre-dauer or dauer stages. We report on associated micro-organisms, including pathogens. We systematically sampled a spatio-temporally structured set of rotting apples in an apple orchard in Orsay over four years. C. elegans and C. briggsae were abundantly found every year, but their temporal distributions did not coincide. C. briggsae was found alone in summer, whereas both species co-occurred in early fall and C. elegans was found alone in late fall. Competition experiments in the laboratory at different temperatures show that C. briggsae out-competes C. elegans at high temperatures, whereas C. elegans out-competes C. briggsae at lower temperatures. Conclusions: C. elegans and C. briggsae proliferate in the same rotting vegetal substrates. In contrast to previous surveys of populations in compost heaps, we found fully proliferating populations with no dauer larvae. The temporal sharing of the habitat by the two species coincides with their temperature preference in the laboratory, with C. briggsae populations growing faster than C. elegans at higher temperatures, and vice at lower temperatures. | 4306 | NA | NA | NA | no | no |
| 4305 | 2017 | NA | Chu, CJ; Adler, PB | 2015 | Large niche differences emerge at the recruitment stage to stabilize grassland coexistence | Niche differences and average fitness differences jointly determine coexistence. However, little empirical information about the magnitude of these two mechanisms is available. Using multispecies population models fit to long-term demographic data for common, co-occurring species in five grassland and shrubland plant communities in western North America, we estimated the strength of stabilizing niche differences and average fitness differences. In all five communities, both pairwise and full community comparisons showed evidence for strong stabilizing mechanisms and relatively small average fitness differences. For a total of 17 species pairs, a measure of niche differences based on simulations of invasion growth rates ranged from 0.59 to 0.93 with a mean of 0.81, where 0 indicates complete niche overlap and 1 indicates zero niche overlap. A corresponding measure of average fitness differences ranged from 1.02 to 2.54 with a mean of 1.53, where 1 indicates identical fitness and a value of 2 indicates a fourfold difference in sensitivity to competition. Comparisons of full communities displayed similar patterns: niche differences ranged from 0.58 to 0.69 with a mean of 0.64, and the average fitness differences ranged from 1.42 to 1.63 with a mean of 1.47. In almost every case, the stabilizing mechanisms were much stronger than minimally necessary to prevent competitive exclusion. Considering that all but one of the species we studied are perennial grasses, which are often grouped in the same functional type, the magnitude of these niche differences is surprising. In all five communities, differences between intra-and interspecific effects at the recruitment stage contributed far more to stabilization than interactions affecting growth and survival. Our results indicate that for these abundant, co-occurring species (1) dynamics are far from neutral, with strong niche differences and weak fitness differences combining to stabilize coexistence, and (2) processes operating at early life stages account for a large proportion of the stabilizing effect. Given the limitations of our inductive approach, both these findings represent hypotheses in need of experimental testing. | 4307 | NA | NA | NA | no | no |
| 4306 | 2017 | NA | Viswanathan, R; Karuppaiah, R; Balamuralikrishnan, M | 2009 | Identification of new variants of SCMV causing sugarcane mosaic in India and assessing their genetic diversity in relation to SCMV type strains | Sugarcane mosaic virus (SCMV) is one of the two causative viruses of mosaic in sugarcane, a sugar crop widely grown under tropical and subtropical conditions worldwide. Although molecular characterization of SCMV strains was reported from many countries, strains occurring in India, a major sugarcane producer have not been reported so far. Twenty-six sugarcane samples represented by seven major sugarcane growing states in India and USA were subjected to reverse-transcription polymerase chain reaction (RT-PCR) using a pair of newly designed coat protein specific primers. Among them 17 were found positive to the SCMV infection. The lengths of the sequences derived in this study using the new set of primers varied between 812 and 866 nt. The amino acid sequence comparison of 30 Indian SCMV isolates showed wide range of sequence similarities in core region (88.80-100%) and hyper variable region (51.3-100%). In the N-terminal region of the five Indian isolates, a deletion of 12 aa residues between aa 11 and 30 was observed, whereas the deletion was between aa 45 and 50 in SCMV-B and -D and between aa 61 and 70 in SCMV-A. The phylogenetic analyses performed with 46 SCMV CP sequences for both hyper variable region and core region separated the isolates mostly according to their geographical origin. The 30 Indian SCMV isolates were included exclusively in four groups besides SCMV-IND, which was grouped with SCMV-SC, a type of strain from Australia. Nearly 97.0% of the Indian isolates have no signs for close relationships with previously characterized SCMV type strains (SCMV-A, -B, -D, -E, and -SC) reported from other countries. Our studies revealed that the sugarcane mosaic in India are caused by at least nine new SCMV variants (IND-CC1, -CC2, -CC3, -CC4, -CO1, -CO2, -CP, -CS, and -J) and a type strain SCMV-SC represented by SCMV-IND. This is the first report on the variability and occurrence of new SCMV population in India. | 4308 | NA | NA | NA | no | no |
| 4307 | 2017 | NA | Wedekind, C; Furi, S | 1997 | Body odour preferences in men and women: do they aim for specific MHC combinations or simply heterozygosity? | The major histocompatibility complex (MHC) is an immunologically important group of genes that appears to be under natural as well as sexual selection. Several hypotheses suggest that certain MHC-allele combinations (usually heterozygous ones) are superior under selective pressure by pathogens. This could influence mate choice in a way that preferences function to create MHC-heterozygous offspring, or that they function to create specific allele combinations that are beneficial under the current environmental conditions through their complementary or epistatic effects. To test these hypotheses, we asked 121 men and women to score the odours of six T-shirts, worn by two women and four men. Their scorings of pleasantness correlated negatively with the degree of MHC similarity between smeller and T-shirt-wearer in men and women who were not using the contraceptive pill (but not in Pill-users). Depending on the T-shirt-wearer the amount of variance in the scorings of odour pleasantness that was explained by the degree of MHC similarity (r(2)) varied between nearly 0 and 23%. There was no apparent effect of gender in this correlation: the highest r(2) was actually reached with one of the male odours sniffed by male smellers. Men and women who were reminded of their own mate/ex-mate when sniffing a T-shirt had significantly fewer MHC-alleles in common with this T-shirt-wearer than expected by chance. This suggests that the MHC or linked genes influence human mate choice. We found no significant effect when we tested for an influence of the MHC on odour preferences after the degree of similarity between T-shirt-wearer and smeller was statistically controlled for. This suggests that in our study populations the MHC influences body odour preferences mainly, if not exclusively, by the degree of similarity or dissimilarity. The observed preferences would increase heterozygosity in the progeny. They do not seem to aim for more specific MHC combinations. | 4309 | NA | NA | NA | no | no |
| 4308 | 2017 | NA | Zavorka, L; Naslund, J; Aldven, D; Hojesjo, J; Johnsson, JI | 2015 | Effects of Familiarity and Population Density on Competitive Interactions and Growth: An Experimental Study on a Territorial Salmonid Fish | The deleterious effect of competition for space and food in animals increases with increasing population density. In contrast, familiarity towards conspecifics can relax the intensity of interference competition. Here, we hypothesized that familiarity towards conspecifics mitigates the effect of density-dependent growth and dispersal behaviour in territorial animals. To test this, wild-captured juvenile brown trout were subjected to two consecutive laboratory experiments. First, growth and fin erosion were measured for 40 d in a 2 x 2 factorial design manipulating density and familiarity. The density was manipulated via size of experimental tanks, while per capita food abundance and fish number was constant. All fish were subsequently exposed to an emergence test, giving them the option to leave their group and disperse to a novel unoccupied environment. The results show that familiarity increases growth and decreases the level of fin erosion (i.e. proxy of intensity of aggressive interactions). We found no significant effect of population density on growth rate. However, there was a tendency towards higher fin erosion in fish kept under high density. The growth of individuals was also affected by their size rank within the group, with the largest individuals in each group growing disproportionally faster than the rest of the group, probably due to their high social rank. However, the second and third fish in the size rank did not grow significantly faster and tended to suffer higher mortality than the rest of the group. During the emergence test, the largest individuals in the familiar groups left the shelter either as the first (six of 12 groups) or last (five of 12 groups) individual in the group, while no such pattern was observed in unfamiliar groups. Our results suggest that individuals in familiar groups receive less aggression and stress (i.e. fin damage) and grow faster than fish in unfamiliar groups. The mechanisms indicated in this laboratory study may be especially important in highly fecund organisms like fish which undergo density-dependent bottlenecks during early life. | 4310 | NA | NA | NA | no | no |
| 4309 | 2017 | NA | Sseruwagi, P; Maruthi, MN; Colvin, J; Rey, MEC; Brown, JK; Legg, JP | 2006 | Colonization of non-cassava plant species by cassava whiteflies (Bemisia tabaci) in Uganda | Bemisia tabaci (Genn.) (Homoptera: Aleyrodidae) is the vector of cassava mosaic geminiviruses (CMGs), which are the main production constraint to cassava [Manihot esculenta Crantz (Euphorbiaceae)], both in Uganda and elsewhere in Africa. Two B. tabaci genotype clusters, Ug1 and Ug2, differentiated at 8% nucleotide (nt) divergence within the mitochondrial cytochrome oxidase I (mtCOI) gene, have been shown to occur on cassava in Uganda. However, the role of alternative hosts in the ecology of cassava B. tabaci genotypes and their possible involvement in the epidemiology of cassava mosaic disease (CMD) in Uganda remain unknown. In this study, we investigated the restriction of cassava B. tabaci genotypes to cassava and the colonization of alternative host species in select cassava-growing areas of the country in 2003 and 2004. Bemisia tabaci adults and 4th instar nymphs were collected from cassava and 11 other cultivated and uncultivated species occurring adjacent to the sampled cassava fields. Phylogenetic analysis of mtCOI sequences revealed that only a single genotype cluster, Ug1, was present on both cassava and non-cassava plant species sampled in this study. The Ug1 genotypes (n = 49) shared 97-99% nt identity with the previously described cassava-associated B. tabaci populations in southern Africa, and were similar to 8% and similar to 13% divergent from Ug2 and the ‘Ivory Coast cassava’ genotypes in Uganda and Ivory Coast, respectively. The Ug1 genotypes occurred (as adults) on all 12 source-plant species sampled. However, based on the presence of B. tabaci 4th instar nymphs, the Ug1 genotypes (n = 13) colonized cassava and five other non-cassava plant species: Manihot glaziovii, Jatropha gossypifolia, Euphorbia heterophylla, Aspilia africana, and Abelmoschus esculentus, suggesting that cassava B. tabaci (Ug1 genotypes) are not restricted to cassava in Uganda. No Ug2-like genotypes were detected on any of the plant species sampled, including cassava, in this study. The identification of additional hosts for at least one genotype cluster, Ug1, known also to colonize cassava, and which was hitherto thought to be ‘cassava-restricted’ may have important epidemiological significance for the spread of CMGs in Uganda. | 4311 | NA | NA | NA | no | no |
| 4310 | 2017 | NA | Laufenberg, JS; Clark, JD; Hooker, MJ; Lowe, CL; O’Connell-Goode, KC; Troxler, JC; Davidson, MM; Chamberlain, MJ; Chandler, RB | 2016 | Demographic Rates and Population Viability of Black Bears in Louisiana | The Louisiana black bear (Ursus americanus luteolus) was reduced to a few small, fragmented, and isolated subpopulations in the Lower Mississippi Alluvial Valley by the mid-twentieth century resulting from loss and fragmentation of habitat. In 1992, the United States Fish and Wildlife Service (USFWS) granted the Louisiana black bear threatened status under the United States Endangered Species Act of 1973. Since that time, a recovery plan was developed, a reintroduced population was established, and habitat recovery has occurred. The Recovery Plan states that a minimum of 2 populations must be viable (i.e., persistence probabilities over 100 years >0.95), 1 in the Tensas River Basin and 1 in the Atchafalaya River Basin. Consequently, our objectives were to 1) estimate demographic rates of Louisiana black bear subpopulations, 2) develop data-driven stochastic population projection models, and 3) determine how different projection model assumptions affect population trajectories and predictions about long-term persistence. Our overall goal was to assess long-term persistence of the bear subpopulations in Louisiana, individually and as a whole. We collected data using varying combinations of non-invasive DNA sampling, live capture, winter den visits, and radio monitoring from 2002 to 2012 in the 4 areas currently supporting breeding subpopulations in Louisiana: Tensas River Basin (TRB), Upper Atchafalaya River Basin (UARB), Lower Atchafalaya River Basin (LARB), and a recently reintroduced population at the Three Rivers Complex (TRC). From 2002 to 2012, we radio monitored fates of 86 adult females within the TRB and 43 in the TRC. Mean estimates of annual adult survival for the TRB and TRC were 0.997 and 0.990, respectively, when unknown fates were assumed alive and 0.970 and 0.926 when unknown fates were assumed dead. From 2003 to 2013, we observed 130 cub litters from 74 females in the TRB, and 74 cub litters from 45 females in the TRC. During the same period, we observed 43 yearling litters for 33 females in the TRB and 21 yearling litters for 19 females in the TRC. The estimated number of cubs and number of yearlings produced per breeding adult female was 0.47 and 0.20, respectively, in the TRB and 0.32 and 0.18 in the TRC. On the basis of matrix projection models, asymptotic growth rates ranged from 1.053 to 1.078 for the TRB and from 1.005 to 1.062 for the TRC, depending on how we treated unresolved fates of adult females. Persistence probabilities estimated from stochastic population models based on telemetry data ranged from 0.997 to 0.998 for the TRC subpopulation depending on model assumptions and were > 0.999 for the TRB regardless of model assumptions. We extracted DNA from hair collected at baited, barbed-wire enclosures in the TRB, UARB, and LARB to determine individual identities for capture-mark-recapture (CMR) analysis. We used those detection histories to estimate apparent survival (phi), per-capita recruitment (f), abundance (N), realized growth rate (lambda), and long-term viability, based on Bayesian hierarchical modeling methods that allowed estimation of temporal process variance and parameter uncertainty. Based on 23,312 hair samples, annual N for females in the TRB ranged from 133 to 164 during 2006-2012, depending on year and how detection heterogeneity was modeled. Geometric mean of lambda ranged from 0.996 to 1.002. In the UARB, we collected 11,643 hair samples from 2007 to 2012, from which estimates of N for females ranged from 23 to 43 during the study period, depending on detection heterogeneity model. The geometric mean of lambda ranged from 1.038 to 1.059. Estimated N for females in LARB ranged from 69 to 96, and annual lambda ranged from 0.80 to 1.11 based on 3,698 hair samples collected during 2010-2012, also depending on year and heterogeneity model. Probabilities of persistence over 100 years for the TRC and TRB based on stochastic matrix projection models that used vital rate estimates from telemetry data were >0.95 for all scenarios. Probability of persistence at the TRB and the UARB based on projection models that used vital rate estimates from CMR analyses ranged from 0.928 to 0.954 and from 0.906 to 0.959, respectively, depending on model assumptions. Data from the LARB were insufficient for a viability assessment. Thus, individual persistence probabilities for TRB and UARB did not meet the strict definition of viability (i.e., >0.95) under some model assumptions. However, the joint probability of bears persisting either in the TRB or UARB was >0.993 assuming individual population dynamics were independent and was >0.958 assuming dynamics were perfectly correlated. Furthermore, including the TRC increased the joint probability of bears persisting somewhere in the TRB, UARB, or TRC to > 0.999 based on the most pessimistic individual persistence estimates from those subpopulations. Therefore, if the intent of specifying that 2 subpopulations should be viable was to ensure the persistence of Louisiana black bears somewhere within its historical range, then the viability threshold was met. (C) 2016 The Wildlife Society. | 4312 | NA | NA | NA | no | no |
| 4311 | 2017 | NA | Volz, EM | 2012 | Complex Population Dynamics and the Coalescent Under Neutrality | Estimates of the coalescent effective population size N-e can be poorly correlated with the true population size. The relationship between N-e and the population size is sensitive to the way in which birth and death rates vary over time. The problem of inference is exacerbated when the mechanisms underlying population dynamics are complex and depend on many parameters. In instances where nonparametric estimators of N-e such as the skyline struggle to reproduce the correct demographic history, model-based estimators that can draw on prior information about population size and growth rates may be more efficient. A coalescent model is developed for a large class of populations such that the demographic history is described by a deterministic nonlinear dynamical system of arbitrary dimension. This class of demographic model differs from those typically used in population genetics. Birth and death rates are not fixed, and no assumptions are made regarding the fraction of the population sampled. Furthermore, the population may be structured in such a way that gene copies reproduce both within and across demes. For this large class of models, it is shown how to derive the rate of coalescence, as well as the likelihood of a gene genealogy with heterochronous sampling and labeled taxa, and how to simulate a coalescent tree conditional on a complex demographic history. This theoretical framework encapsulates many of the models used by ecologists and epidemiologists and should facilitate the integration of population genetics with the study of mathematical population dynamics. | 4313 | NA | NA | NA | no | no |
| 4312 | 2017 | NA | Carrier, JA; Beebee, TJC | 2003 | Recent, substantial, and unexplained declines of the common toad Bufo bufo in lowland England | A decline in numbers of a large common toad (Bufo bufo) population in south-east England during the 1990s, together with anecdotal reports of similar trends in other toad populations, prompted a nation-wide survey of this species. The survey also included the common frog (Rana temporaria) as a control for which there was no comparable evidence of recent decline. A questionnaire requesting information on the fate of toad and frog populations in the last 15 years of the 20th century was distributed to professional and amateur herpetologists during autumn 2001. Ninety-five respondents provided data on a total of 277 sites, including 232 frog and 202 toad populations in England, Scotland and Wales. More than 80% of the reported sites were rural for both species. Rural frog populations were generally doing well in most parts of Britain, with almost as many increasing as decreasing populations and a high proportion of stable populations. Rural toads also showed no overall trend across the country as a whole. However, regional analysis indicated that although toads were faring at least as well as frogs in the north and west of Britain, they were declining seriously in central and eastern/south-eastern areas. In these regions 50% or more of toad populations have experienced recent declines, whereas frogs have fared as well as they have elsewhere. Toads breeding alone have fared significantly worse than toads breeding at sites where frogs were also present. Reasons for apparently toad-specific declines in lowland England remain unknown. (C) 2003 Elsevier Science Ltd. All rights reserved. | 4314 | NA | NA | NA | no | no |
| 4313 | 2017 | NA | Thorogood, R; Armstrong, DP; Low, M; Brekke, P; Ewen, JG | 2013 | The value of long-term ecological research: integrating knowledge for conservation of hihi on Tiritiri Matangi Island | In 1995 and 1996, release of 51 hihi (stitchbird, Notiomystis cincta) onto Tiritiri Matangi Island (wild caught on Hauturu, Little Barrier Island) marked the start of a research and ecological restoration success story. Although establishment of populations of hihi elsewhere in New Zealand has proven to be difficult, the population on Tiritiri Matangi Island has grown to c. 150 individuals and has become one of New Zealand’s few detailed case-study species. Here we review the major contributions from over 15 years of ongoing research on this dynamic species at this spectacular study location to demonstrate how behavioural ecology, molecular ecology, parasitology, and applied population ecology can inform conservation management. | 4315 | NA | NA | NA | no | no |
| 4314 | 2017 | NA | Di Fonzo, MMI; Pelletier, F; Clutton-Brock, TH; Pemberton, JM; Coulson, T | 2011 | The Population Growth Consequences of Variation in Individual Heterozygosity | Heterozygosity has been associated with components of fitness in numerous studies across a wide range of taxa. Because heterozygosity is associated with individual performance it is also expected to be associated with population dynamics. However, investigations into the association between heterozygosity and population dynamics have been rare because of difficulties in linking evolutionary and ecological processes. The choice of heterozygosity measure is a further issue confounding such studies as it can be biased by individual differences in the frequencies of the alleles studied, the number of alleles at each locus as well as the total number of loci typed. In this study, we first examine the differences between the principal metrics used to calculate heterozygosity using long-term data from a marked population of Soay sheep (Ovis aries). Next, by means of statistical transformation of the homozygosity weighted by loci index, we determine how heterozygosity contributes to population growth in Soay sheep by modelling individual contributions to population growth (p(t(i))) as a function of several covariates, including sex, weight and faecal egg count - a surrogate of parasitic nematode burden in the gut. We demonstrate that although heterozygosity is associated with some components of fitness, most notably adult male reproductive success, in general it is only weakly associated with population growth. | 4316 | NA | NA | NA | no | no |
| 4315 | 2017 | NA | Tentelier, C; Guillemaud, T; Ferry, S; Fauvergue, X | 2008 | Microsatellite-based parentage analysis reveals non-ideal free distribution in a parasitoid population | Habitat selection by dispersers is the focus of much theoretical models, most of which are based on the assumption of negative density dependence. The archetype of these models is the ideal free distribution, characterized by an evolutionary stable state where more competitors aggregate in better habitats, so that the fitness benefit of resource abundance is equally offset by the cost of competition in all habitats. In this study, we used parentage analysis on microsatellite genotypes to test the ideal free distribution in a natural population of aphid parasitoids. Parentage analysis was conducted on parasitoids emerging from aphid colonies. We inferred the number of foundress females which had reproduced in each colony, as well as the number of offspring for each foundress. As predicted by the ideal free distribution, the number of offspring per foundress per colony did not depend on the number of hosts per colony. However, contrary to ideal free distribution predictions, it was affected by the number of foundresses per colony. In surprising contrast with the basic assumption of negative density dependence, individual fitness increased with the number of foundresses. Moreover, parentage analysis revealed a very low number of offspring per foundress per colony (mean = 1.8). This observed distribution questions the validity of classical models of habitat choice based on competition. Indeed, our results provide a new illustration reinforcing a growing body of theory and data on positive density dependence. Our results also suggest that the avoidance of hyperparasitism and predation, although generally neglected, may shape the distribution of parasitoids in the field. | 4317 | NA | NA | NA | no | no |
| 4316 | 2017 | NA | Matthiopoulos, J; Moss, R; Lambin, X | 1998 | Models of red grouse cycles. A family affair? | The lack of parameterised models showing how intrinsic factors might affect population dynamics has contributed to the belief that they are unlikely to explain population cycles. In this paper we use mathematical models to show how a social process, differential territorial behaviour between kin and non-kin, might drive red grouse (Lagopus lagopus scoticus) population cycles. We develop a simple age-structured population model which has space as a limiting resource and examine two versions of it. The first contains no kin effect and is incapable of cyclic behaviour. The second, containing a modification to reflect the effect of kinship on recruitment, shows oscillatory behaviour over most of the parameter space and produces realistic output (period, amplitude, age structure, recruitment), while its quasi-cyclic behaviour is shown to be robust under random parameter variations. Further parameterisation with field data from two study sites in north-east Scotland yields output which resembles the course of observed population cycles. The model predicts that a young cock present in autumn will have a greater probability of recruitment into the territorial population during the increase than during the decline phase of the cycle. This is consistent with field data. The model further predicts that such fluctuations in the probability of recruitment will be synchronous with fluctuations in the size of kin-clusters. | 4318 | NA | NA | NA | no | no |
| 4317 | 2017 | NA | Lane, RS; Peavey, CA; Padgett, KA; Hendson, M | 1999 | Life history of Ixodes (Ixodes) jellisoni (Acari : Ixodidae) and its vector competence for Borrelia burgdorferi sensu lato | bodes (Ixodes) jellisoni Cooley & Kohls, a nonhuman biting and little known tick, is one of 4 members of the I. ricinus complex in the United States. A localized population of I. jellisoni inhabiting a grassland biotope in Mendocino County, CA, was studied from 1993 to 1997. Rodent trapping in all seasons revealed that the only host of both immature and adult I. jellisoni was the heteromyid rodent Dipodomys californicus Merriam. Field investigations suggested that I. jellisoni is nidicolous in habit, and laboratory findings demonstrated that it reproduces parthenogenetically. Known parthenogenetic females (n = 4) produced an average of 530 eggs of which 74% hatched, which was comparable to the fecundity and fertility of wild-caught females (n = 8). After the transstadial molt, 57 F-1 or F-2 nymphs derived from 2 wild-caught or 4 laboratory-reared, unmated females produced only females. Ixodes jellisoni males were not found on 112 wild-caught D. californicus individuals that were captured an average of 2 times. Collectively, these findings suggest that I. jellisoni may be obligatorily parthenogenetic. Borrelial isolates were obtained from 85% of 58 D. californicus and 33% of 21 I, jellisoni females removed from this rodent. None of the 7 infected female ticks passed borreliae ovarially to its F-1 larval progeny. Eight D. californicus and 5 I. jellisoni-derived isolates that were genetically characterized belonged to 2 restriction pattern groups of Borrelia burgdorferi s.l. Neither restriction pattern group has been assigned to a particular genospecies yet. After placement on naturally infected D. californicus, noninfected larval ticks acquired and transstadially passed spirochetes as efficiently as (group 1 borreliae) or 6 times more efficiently (group 2 borreliae) than bodes pacificus Cooley & Kohls. As few as 1-4 infected I. jellisoni nymphs were capable of transmitting group 1 or group 2 borreliae to naive D, californicus. We conclude that I. jellisoni is a competent vector of both restriction fragment groups when D. californicus is used as the animal model. | 4319 | NA | NA | NA | no | no |
| 4318 | 2017 | NA | Wirth, HP; Yang, MQ; Sanabria-Valentin, E; Berg, DE; Dubois, A; Blaser, MJ | 2006 | Host Lewis phenotype-dependent Helicobacter pylori Lewis antigen expression in rhesus monkeys | Both human and H. pylori populations are polymorphic for the expression of Lewis antigens. Using an experimental H. pylori challenge of rhesus monkeys of differing Lewis phenotypes, we aimed to determine whether H. pylori populations adapt their Lewis phenotypes to those of their hosts. After inoculation of four monkeys with a mixture of seven strains identified by RAPD-polymerase chain reaction, H. pylori Lewis expression was followed in 86 isolates obtained over 40 wk. Host Lewis(a/b) secretion status was characterized by immunological assays. Fingerprints of the predominating strain (J166) were identical in all four animals after 40 wk, but its Lewis phenotype had substantial variability in individual hosts. At 40 wk, J166 populations from two Lewis(a-b+) animals predominantly expressed Lewis(y). In contrast, J166 populations had switched to a Lewis(x) dominant phenotype in the two Lewis(a+b-) animals; a frame shift in futC, regulating conversion of Lewis(x) to Lewis(y), accounted for the phenotypic switch. The results indicate that individual cells in H. pylori populations can change Lewis phenotypes during long-term colonization of natural hosts to resemble those of their hosts, providing evidence for host selection for bacterial phenotypes. | 4320 | NA | NA | NA | no | no |
| 4319 | 2017 | NA | Polley, SD; Chokejindachai, W; Conway, DJ | 2003 | Allele frequency-based analyses robustly map sequence sites under balancing selection in a malaria vaccine candidate antigen | The Plasmodium falciparum apical membrane antigen I (AMA1) is a leading candidate for a malaria vaccine. Here, within-population analyses of alleles from 50 Thai P. falciparum isolates Yield significant evidence for balancing selection on polymorphisms within the disulfide-bonded domains I and III of tire surface accessible ectodomain of AMA1, a result very similar to that seen previously in a Nigerian population. Studying the frequency of nucleotide polymorphisms in both populations shows that the between-population component of variance (F-ST) is significantly lower in domains I and III compared to the intervening domain II and compared to 11 unlinked microsatellite loci. A nucleotide site-by-site analysis shows that sites with exceptionally high or low F-ST, values cluster significantly into serial runs, with four runs of low values in domain I and one in domain III. These runs may map the sequences that are consistently under the strongest balancing selection from naturally acquired immune responses. | 4321 | NA | NA | NA | no | no |
| 4320 | 2017 | NA | Vitale, K; Trbojevic-Cepe, M; Smolej-Narancic, N | 2002 | Apolipoprotein H genetic variability in the population of Krk Island, Croatia | Apolipoprotein polymorphisms are emerging as suitable markers for the study of the formation of human populations. In contrast to the data available for apolipoprotein E, the data regarding apolipoprotein H (protein, apoH; gene, APOH) variations are only beginning to accumulate. By blood plasma isoelectric focusing and immunoblotting, we analyzed the distribution of apoH phenotypes in 397 individuals (192 males; 205 females) from seven villages of an autochthonous population of the eastern Adriatic island of Krk. APOH allele frequencies were: APOH*2 = 0.877, APOH*3 = 0.098, APOH\*1 = 0.025, with the majority of the sample being homozygous. No significant differences between villages were observed. When these data were compared to those of other populations studied so far, a significant association between APOH allele frequencies and latitude was observed. We hypothesize that this association reflects differences in diet composition across different climatic zones. | 4322 | NA | NA | NA | no | no |
| 4321 | 2017 | NA | Hedenas, L | 2010 | Phylogeography and origin of European Sanionia uncinata (Amblystegiaceae, Bryophyta) | Based on information from ITS (112 specimens) and the chloroplast markers trnL-trnF and rpl16 (116), this study reconstructs the phylogeography of the moss Sanionia uncinata, with a focus on Europe. In a global context, especially ITS data, suggest a scenario with a major split, between one southern hemisphere and one eastern Asiatic lineage. Descendants of the first dispersed back to the northern hemisphere where they are presently widespread, and those of the second lineage likewise spread over much of the north. Within the latter, a separate group evolved which is the only one present in central Europe and has not reached arctic Europe. Members of this group also colonized tropical Africa. In addition, members of the two main lineages most likely survived the last glaciation in the north or north-east, whereas representatives of the last evolved group could have survived the glacial period in southern Europe. The results underline potential problems with too small data sets in phylogeographic studies. European ITS data suggest two cryptic species, but the global sampling includes representatives of several of the haplotypes that are ‘missing’ in Europe, and the chloroplast data suggest partly different groups than ITS. | 4323 | NA | NA | NA | no | no |
| 4322 | 2017 | NA | Margalida, A; Carrete, M; Hegglin, D; Serrano, D; Arenas, R; Donazar, JA | 2013 | Uneven Large-Scale Movement Patterns in Wild and Reintroduced Pre-Adult Bearded Vultures: Conservation Implications | After the quasi-extinction of much of the European vertebrate megafauna during the last few centuries, many reintroduction projects seek to restore decimated populations. However, the future of numerous species depends on the management scenarios of metapopulations where the flow of individuals can be critical to ensure their viability. This is the case of the bearded vulture Gypaetus barbatus, an Old World, large body-sized and long-lived scavenger living in mountain ranges. Although persecution in Western Europe restrained it to the Pyrenees, the species is nowadays present in other mountains thanks to reintroduction projects. We examined the movement patterns of pre-adult non-breeding individuals born in the wild population of the Pyrenees (n = 9) and in the reintroduced populations of the Alps (n = 24) and Andalusia (n = 13). Most birds were equipped with GPS-GSM radio transmitters, which allowed accurate determination of individual dispersal patterns. Two estimators were considered: i) step length (i. e., the distance travelled per day by each individual, calculated considering only successive days); and ii)total dispersal distance (i. e., the distance travelled between each mean daily location and the point of release). Both dispersal estimators showed a positive relationship with age but were also highly dependent on the source population, birds in Andalusia and Alps moving farther than in Pyrenees. Future research should confirm if differences in dispersal distances are the rule, in which case the dynamics of future populations would be strongly influenced. In summary, our findings highlight that inter-population differences can affect the flow of individuals among patches (a key aspect to ensure the viability of the European metapopulation of the endangered bearded vulture), and thus should be taken into account when planning reintroduction programs. This result also raises questions about whether similar scenarios may occur in other restoration projects of European megafauna. | 4324 | NA | NA | NA | no | no |
| 4323 | 2017 | NA | Jemielity, S; Keller, L | 2003 | Queen control over reproductive decisions - no sexual deception in the ant Lasius niger | Queen-worker conflicts in social insect societies have received much attention in the past decade. In many species workers modify the colony sex ratio to their own advantage or produce their own male offspring. In some other species, however, queens seem to be able to prevent workers from making selfish reproductive decisions. So far, little effort has been made to find out how queens may keep control over sex ratio and male parentage. In this study we use a Lasius niger population under apparent queen control to show that sexual deception cannot explain queen dominance in this population. The sexual deception hypothesis postulates that queens should prevent workers from discriminating against males by disguising male brood as females. Contrary to the predictions of this hypothesis, we found that workers are able to distinguish male and female larvae early in their development: in early spring workers generally placed only either female or male larvae in the uppermost chambers of the nest, although both types of larvae must have been present. At this time males were only at 11% of their final dry weight, a developmental stage at which (according to two models) workers would still have benefited from replacing queen-produced males by females or worker-produced males. This study thus demonstrates that sexual deception cannot account for the apparent queen control over colony sex ratio and male parentage in L. niger . | 4325 | NA | NA | NA | no | no |
| 4324 | 2017 | NA | Boulanger, J; Kendall, KC; Stetz, JB; Roon, DA; Waits, LP; Paetkau, D | 2008 | Multiple data sources improve DNA-based mark-recapture population estimates of grizzly bears | A fundamental challenge to estimating population size with mark-recapture methods is heterogeneous capture probabilities and subsequent bias of population estimates. Confronting this problem usually requires substantial sampling effort that can be difficult to achieve for some species, such as carnivores. We developed a methodology that uses two data sources to deal with heterogeneity and applied this to DNA mark-recapture data from grizzly bears ( Ursus arctos). We improved population estimates by incorporating additional DNA "captures’’ of grizzly bears obtained by collecting hair from unbaited bear rub trees concurrently with baited, grid-based, hair snag sampling. We consider a Lincoln-Petersen estimator with hair snag captures as the initial session and rub tree captures as the recapture session and develop an estimator in program MARK that treats hair snag and rub tree samples as successive sessions. Using empirical data from a large-scale project in the greater Glacier National Park, Montana, USA, area and simulation modeling we evaluate these methods and compare the results to hair-snag-only estimates. Empirical results indicate that, compared with hair-snag-only data, the joint hair-snag-rub-tree methods produce similar but more precise estimates if capture and recapture rates are reasonably high for both methods. Simulation results suggest that estimators are potentially affected by correlation of capture probabilities between sample types in the presence of heterogeneity. Overall, closed population Huggins-Pledger estimators showed the highest precision and were most robust to sparse data, heterogeneity, and capture probability correlation among sampling types. Results also indicate that these estimators can be used when a segment of the population has zero capture probability for one of the methods. We propose that this general methodology may be useful for other species in which mark-recapture data are available from multiple sources. | 4326 | NA | NA | NA | no | no |
| 4325 | 2017 | NA | Franzo, G; Tucciarone, CM; Cecchinato, M; Drigo, M | 2016 | Porcine circovirus type 2 (PCV2) evolution before and after the vaccination introduction: A large scale epidemiological study | Since their commercialization, vaccines against Porcine circovirus type 2 (PCV2) have been the cornerstone control strategy. Nevertheless, the periodic emergence of new genotype waves and the recent reports of vaccine failure outbreaks have raised the question if widespread vaccination strategies could have driven viral evolution and affected different genotype fitness. To investigate this issue an in-deep analysis, based on a bioinformatics and biostatistics approach, has been implemented. ORF2 sequences from vaccinated and non-vaccinated populations (i.e. domestic pigs before and after vaccine introduction and wild boars) were considered. The action of selective forces on PCV2 strains has been analyzed and compared among groups. Remarkable differences were found in the selective forces acting on viral populations circulating in different “immune environments”. Particularly for PCV2a, a directional selection promoting a change in the viral capsid away from the vaccine specific antigenic determinants has been detected after vaccine introduction. Involved amino acids were previously reported to be part of viral epitopes whose variability is responsible of immune escape. Our findings support a change in PCV2 evolutionary pattern after widespread vaccination introduction and stress once more the compulsoriness of a continuous monitoring of PCV2 epidemiology to promptly act in response to the emergence of possible vaccine-escaping mutants. | 4327 | NA | NA | NA | no | no |
| 4326 | 2017 | NA | Silva-Oliveira, GC; Silva, ABC; Blanchard, F; Nunes, Z; Torres, RA; Sampaio, I; Vallinoto, M | 2014 | Primers for the amplification of the MHC II beta chain exon 2 in the Atlantic goliath grouper (Epinephelus itajara) | In the present study we designed a pair of primers to amplify the exon 2 of the MHC II beta chain of the Atlantic goliath grouper, which is responsible for the recognition of pathogenic molecules and the regulation of the immune system. Future analyses of this region may provide an important database to understand the evolutionary processes affecting the populations of the goliath grouper, and to predict the conservation perspectives in the species. | 4328 | NA | NA | NA | no | no |
| 4327 | 2017 | NA | Sinclair, JS; Arnott, SE | 2017 | Relative importance of colonist quantity, quality, and arrival frequency to the extinction of two zooplankton species | Colonist quantity, quality, and arrival frequency can all individually drive the dynamics and extinction of new populations. However, we do not understand which has the strongest influence, nor the circumstances under which their relative importance may change. We conducted a field mesocosm experiment that manipulated colonist quantity, quality, and arrival frequency in two zooplankton species (Daphnia pulicaria and Skistodiaptomus oregonensis). Individuals of each species were cultured under either high or low food concentrations to produce, respectively, ‘good’ and ‘poor’ quality colonists. Each species was then introduced at either small (2 individuals) or large introduction quantities (8 individuals) divided over single or multiple introduction events. We found that the extinction of Daphnia pulicaria was not particularly affected by any of our treatments. Introductions of just two individuals performed as well as larger or more frequent introductions, regardless of quality. Conversely, Skistodiaptomus oregonensis extinction was strongly driven by arrival frequency. Populations that arrived in a single event exhibited high rates of extinction (75-83%), with this probability declining dramatically when colonists were introduced over multiple events (33% extinction). Our results show that other less studied aspects of the colonist pool, such as arrival frequency, could be as important to population persistence as the initial quantity of arriving colonists. Additionally, there are potentially numerous species that are well suited to succeeding with a small number of founders, and whose success is therefore not necessarily dependent upon colonist quantity, quality, or arrival frequency. | 4329 | NA | NA | NA | no | no |
| 4328 | 2017 | NA | Torang, P; Ehrlen, J; Agren, J | 2008 | Mutualists and antagonists mediate frequency-dependent selection on floral display | Theory predicts that, with conflicting selection pressures mediated by mutualists and antagonists, alternative reproductive strategies can be maintained by negative frequency-dependent selection if it results in rare-morph advantage. We combined field experiments and surveys of natural populations to determine whether selection on. oral display is frequency dependent in the self-incompatible herb Primula farinosa, which is polymorphic for inflorescence height and occurs in a short-scaped and a long-scaped morph. Among short-scaped plants, both pollination success, quantified as initiation of fruits and seeds, and seed predation were positively correlated with the relative frequency of the long-scaped morph. The relative strength of these effects and the direction of the resulting frequency-dependent selection on scape morph varied among years and populations. The results suggest that both mutualists and antagonists may mediate frequency-dependent selection and that frequency dependence may vary from positive to negative with rare-morph advantage, depending on the relative strength of these interactions. | 4330 | NA | NA | NA | no | no |
| 4329 | 2017 | NA | Vujovic, A; Ikovic, V; Golubovic, A; Dordevic, S; Pesic, V; Tomovic, L | 2015 | Effects of Fires and Roadkills on the Isolated Population of Testudo hermanni Gmelin, 1789 (Reptilia: Testudinidae) in Central Montenegro | In general, fires and traffic impose serious threats to wildlife. Chelonians are also gravely affected by these two perils, both directly (many die in fires and on the roads) and indirectly (due to habitat fragmentation and degradation). In the Hermann’s tortoise population subjected to capture-mark-recapture (CMR) study we analysed the impacts of fires and road kills. We found that the most vulnerable to both perils were the immatures and adult females, with most fatalities in summer. During the course of our research, along with the reconstruction and widening of a motorway, a rockslide-protective wire mesh was fixed along one section of the road under study. It reduced the number of road kills, but it also acted as a deadly trap for some of the tortoises trying to escape the fast-spreading fire. Here we presented the results of a short survey: in future more time and effort need to be devoted to the given population and its conservation needs. | 4331 | NA | NA | NA | no | no |
| 4330 | 2017 | NA | Kaufman, L; Vargas, AF; Coimbra, CEA; Santos, RV; Salzano, FM; Hutz, MH | 1999 | Apolipoprotein B genetic variability in Brazilian Indians | Three apolipoprotein B restriction fragment length polymorphisms (RFLPs) (XbaI, MspI, and EcoRI) and the signal peptide insertion-deletion polymorphism were investigated using the polymerase chain reaction in 140 individuals from 5 Brazilian Indian tribes, All studied markers were polymorphic in this ethnic group. The insertion allele 5’beta SP*29 at the signal peptide previously observed in Mexican Americans was detected in about 8% of the chromosomes of 2 tribes (Gaviao and Zoro), therefore confirming the Amerindian origin of this allele. Negative linkage disequilibrium was observed between alleles at the signal peptide and the EcoRI polymorphism in all tribes. In 3 populations (Gaviao, Surui, and Zoro) a negative disequilibrium was also detected between the insertion-deletion signal peptide markers and the XbaI polymorphism In the whole sample of Amerindians 14 of the 24 (58%) possible 4-marker extended haplotypes were identified, but only haplotype 2 (5’beta SP*24/*X+/*M+/*E+) and haplotype 5 (5’beta SP*27/*X-/*M+/\*E+) were shared by all tribes. No associations between plasma lipid levels or body mass index and these polymorphisms were observed in this sample. | 4332 | NA | NA | NA | no | no |
| 4331 | 2017 | NA | Geary, B; Green, MC; Ballard, BM | 2015 | Movements and survival of juvenile reddish egrets Egretta rufescens on the Gulf of Mexico coast | Understanding natal dispersal patterns of animals is critical to development of effective species conservation plans, as it ensures that population management takes place at appropriate scales. The reddish egret Egretta rufescens is a threatened waterbird species lacking documentation on many aspects of its ecology, including movement behaviors at all life stages. We attached satellite transmitters to 25 juvenile reddish egrets on their natal colonies and observed their dispersal patterns and subsequent movements over a period of 115 wk (May 2010-August 2012). Birds exhibited the greatest movement rates in the remainder of the first breeding season (through July 2010, similar to 10 to 13 wk of age, 11.07 km d(-1)) and steadily decreased in the post-breeding period (4.87 km d(-1)) and winter (1.96 km d(-1)) of their first year. Movements of 1 to 2.5 km d(-1) characterized the remainder of the tracking period, suggesting that surviving birds were able to establish local territories. Of the 25 tagged birds, 8 (32%) survived throughout the observation period, based on transmitter failure rates, with losses increasing each winter. The majority of birds remained on the Texas/Mexico coast of the Gulf of Mexico, indicating that the population is largely resident and therefore vulnerable to coastal habitat alterations in the region. Due to a combination of infrequent long-distance migration, specialized behaviors, and apparent limited gene flow, habitat maintenance should be of primary importance for management of this rare species. This is among the first published studies of heron movement ecology using telemetry, and should be followed by further tracking with developing technologies to characterize high-resolution movements and habitat associations. | 4333 | NA | NA | NA | no | no |
| 4332 | 2017 | NA | Sanz, L; Montero, M; Grondona, I; Vizcaino, J; Llobell, A; Hermosa, R; Monte, E | 2004 | Cell wall-degrading isoenzyme profiles of Trichoderma biocontrol strains show correlation with rDNA taxonomic species | Trichoderma is known for being the most frequently used biocontrol agent in agriculture. A fundamental part of the Trichoderma antifungal system relies on a series of genes coding for a variety of extracellular lytic enzymes. Characterization of the polymorphism between five putative isoenzymatic activities [beta-1,3-glucanase (EC 3.2.1.39, EC 3.2.1.58), beta-1,6- glucanase ( EC 3.2.1.75), cellulase ( EC 3.2.1.4; EC 3.2.1.21, EC 3.2.1.91), chitinase ( EC 3.2.1.30, EC 3.2.1.52), protease ( EC 3.4.11; EC 3.4.13 - 19; EC 3.4.21 - 24, EC 3.4.99)] was carried out using 18 strains from three sections of Trichoderma. Of these, seven strains were from T. sect. Pachybasium, nine from T. sect. Trichoderma and two from T. sect. Longibrachiatum. Thirty-seven different alleles in total were identified: 13 for beta-1,3-glucanase, four for beta-1,6- glucanase, three for cellulase, eight for chitinase and nine for protease activity. A dendrogram ( constructed by the unweighted pair group method with arithmetic averages) based on isoenzymatic data separated the 18 strains into three main enzymatic groups: T. harzianum, T. atroviride/T. viride/T. koningii and T. asperellum/ T. hamatum/T. longibrachiatum. Isoenzymatic groupings obtained from biocontrol strains are discussed in relation to their phylogenetic location, based on their sequence of internal transcribed spacer 1 in ribosomal DNA and their antifungal activities. | 4334 | NA | NA | NA | no | no |
| 4333 | 2017 | NA | Baxter-Gilbert, JH; Riley, JL; Lesbarreres, D; Litzgus, JD | 2015 | Mitigating Reptile Road Mortality: Fence Failures Compromise Ecopassage Effectiveness | Roadways pose serious threats to animal populations. The installation of roadway mitigation measures is becoming increasingly common, yet studies that rigorously evaluate the effectiveness of these conservation tools remain rare. A highway expansion project in Ontario, Canada included exclusion fencing and ecopassages as mitigation measures designed to offset detrimental effects to one of the most imperial groups of vertebrates, reptiles. Taking a multispecies approach, we used a Before-After-Control-Impact study design to compare reptile abundance on the highway before and after mitigation at an Impact site and a Control site from 1 May to 31 August in 2012 and 2013. During this time, radio telemetry, wildlife cameras, and an automated PIT-tag reading system were used to monitor reptile movements and use of ecopassages. Additionally, a willingness to utilize experiment was conducted to quantify turtle behavioral responses to ecopassages. We found no difference in abundance of turtles on the road between the un-mitigated and mitigated highways, and an increase in the percentage of both snakes and turtles detected dead on the road post-mitigation, suggesting that the fencing was not effective. Although ecopassages were used by reptiles, the number of crossings through ecopassages was lower than road-surface crossings. Furthermore, turtle willingness to use ecopassages was lower than that reported in previous arena studies, suggesting that effectiveness of ecopassages may be compromised when alternative crossing options are available (e.g., through holes in exclusion structures). Our rigorous evaluation of reptile roadway mitigation demonstrated that when exclusion structures fail, the effectiveness of population connectivity structures is compromised. Our project emphasizes the need to design mitigation measures with the biology and behavior of the target species in mind, to implement mitigation designs in a rigorous fashion, and quantitatively evaluate road mitigation to ensure allow for adaptive management and optimization of these increasingly important conservation tools. | 4335 | NA | NA | NA | no | no |
| 4334 | 2017 | NA | Mouyna, I; Renard, JL; Brygoo, Y | 1996 | DNA polymorphism among Fusarium oxysporum f sp elaeidis populations from oil palm, using a repeated and dispersed sequence ‘’palm’’ | A worldwide collection, of 76 F. oxysporum f.sp. elaeidis isolates (Foe), and of 21 F. oxysporum isolates from the soil of several palm grove was analysed by RFLP. As a probe; we used a random DNA fragment (probe 46) from a genomic library of a Foe isolate. This probe contains two different types of sequence, one being repeated and dispersed in the genome ‘’Palm’‘, the other being a single-copy sequence. All F. oxysporum isolates from the palm-grove soils were non-pathogenic to oil palm. They all had a simple restriction pattern with one band homologous to the single-copy sequence of probe 46. All Foe isolates were pathogenic to oil palm and they all had complex patterns due to hybridization with’‘Palm’’. This repetitive sequence reveals that Foe isolates are distinct from the other F. oxysporum palm-grove soils isolates. The sequence can reliably discriminate pathogenic from nonpathogenic oil palm isolates, Based on DNA fingerprint similarities, Foe populations were divided into ten groups consisting of isolates with the same geographic origin. Isolates from Brazil and Ecuador were an exception To that. rule as they had the same restriction pattern as a few isolates from the ivory Coast, suggesting they may originated from Africa. | 4336 | NA | NA | NA | no | no |
| 4335 | 2017 | NA | Tanovic, B; Delibasic, G; Milivojevic, J; Nikolic, M | 2009 | CHARACTERIZATION OF BOTRYTIS CINEREA ISOLATES FROM SMALL FRUITS AND GRAPEVINE IN SERBIA | Twenty-six single-spore isolates of Botrytis cinerea from blackberry, raspberry, strawberry, and grapevine were investigated using transposable elements, morphological characterization, and sensitivity to fungicides. Both transposable elements, Flipper and Boty, were detected among isolates from all the hosts. Six vacuma (without transposable elements) and seven transposa (containing both elements) isolates were found to be present in sympatry in Serbia. Isolates containing only the Boty element were detected. Eight morphological types of colonies on PDA and MA media were observed, confirming the great phenotypic variability of B. cinerea. Sensitivity to fungicides was various, depending on both the fungicide and the isolate. | 4337 | NA | NA | NA | no | no |
| 4336 | 2017 | NA | Satow, S; Saitow, Y; Yamaki, S; Hirota, T | 2013 | Japanese Queenless Ants, Pristomyrmex punctatus, Prefer the Traces of Both Nestmates and Strangers in Nest Selection | Conspecific avoidance may influence the spatial distribution of colonies in some ants. House-hunting ants (Temnothorax albipennis) avoid nesting in areas where non-nestmates have nested previously. However, no reports are available on conspecific avoidance during nest selection in other ants. In the present study, we investigated nest selection in another nomadic species, the Japanese queenless ant, Pristomyrmex punctatus. Two-choice tests revealed that, similar to house-hunting ants, P. punctatus preferred nests soiled by nestmates to clean nests. However, unlike house-hunting ants, P. punctatus also preferred nests soiled by non-nestmates to a clean nest. Given the choice between a nest soiled by nestmates and one soiled by strangers, P. punctatus, unlike house-haunting ants, showed no significant preference. Thus, conspecific avoidance in nest selection was not observed in P. punctatus. Interspecific differences in ecological factors may drive the evolution of different nest selection strategies. | 4338 | NA | NA | NA | no | no |
| 4337 | 2017 | NA | Takatsuka, J | 2007 | Specific PCR assays for the detection of DNA from Beauveria bassiana F-263, a highly virulent strain affecting the Japanese pine sawyer, Monochamus alternatus (Coleoptera : Cerambycidae), by a sequence-characterized amplified region (SCAR) marker | To monitor the Beauveria bassiana F-263 strain, which is under commercial development for controlling the Japanese pine sawyer Monochanuts alternatus, I developed molecular markers based on a sequence-characterized amplified region (SCAR). A diagnostic fragment generated from random amplified polymorphic DNA analysis (RAPD) was converted into two SCAR markers designated as C8(1068) and C8(352) using sequence information from the fragment. The two primer pairs for the SCAR markers were sensitive, but the primer pair for C8352 had superior sensitivity, capable of detecting as little as 10 pg B. bassiana F-263 strain DNA, regardless of whether the sample contained pure F-263 DNA or was contaminated with DNA from another strain. The markers were used in combination with dilution plating on selective medium, which allowed for both detection of B. bassiana F-263 presence and indirect density estimation of the strain. In addition, I developed a simple DNA extraction method by boiling fungi directly from colonies on medium. This DNA extraction method avoids laborious DNA extractions and expedites SCAR assays for monitoring the B. bassiana F-263 strain in the environment. | 4339 | NA | NA | NA | no | no |
| 4338 | 2017 | NA | Omar, AF | 2016 | Association of ‘Candidatus Phytoplasma cynodontis’ with Bermuda grass white leaf disease and its new hosts in Qassim province, Saudi Arabia | Typical symptoms of phytoplasma such as whitening of the leaves, shortening of the stolons on Bermuda grass, variegated leaves, yellows, stunting, little leaves and yellows on Giant reed, Cooba and sand olive shrub were observed in Qassim province, Saudi Arabia, during the autumn season of 2015. When tested for phytoplasma by universal primers P1/P7 followed by R16mF2/R16mR2, products of approximately 1400 bp (as expected) were amplified from 16 plants with symptoms but not from symptomless plants. Based on sequencing, phylogenetic analysis and virtual restriction fragment length polymorphism patterns of the 16S rDNA F2nR2 fragments of seven Qassim phytoplasma isolates, bermuda grass isolates 170, 175 and 177, giant reed isolate 180, sand olive isolates 181 and 182 and cooba isolate 185, the associated phytoplasma was identified as a member of ‘Candidatus Phytoplasma cynodontis’ which belong to the 16SrXIV-A subgroup. The 16S rDNA gene sequences of seven Qassim phytoplasma isolates exhibited over 99.2% identity with members of ‘Ca. Phytoplasma cynodontis’ group of phytoplasmas. This is the first report of characterization of ‘Ca. phytoplasma cynodonties’ (16SrXIV) associated with Cynodon dactylon in Saudi Arabia and its new hosts, Dodonaea angustifolia, Arundo donax and Acacia salicia. | 4340 | NA | NA | NA | no | no |
| 4339 | 2017 | NA | Rehan, SM; Rotella, A; Onuferko, TM; Richards, MH | 2013 | Colony disturbance and solitary nest initiation by workers in the obligately eusocial sweat bee, Halictus ligatus | Sweat bees are one of the most socially polymorphic lineages on the planet. In obligately eusocial species, newly enclosed females may become either queens or workers, depending on the environmental and social circumstances of the nest into which they emerge. In socially polymorphic species, females also have the option of nesting solitarily, founding a nest and raising future reproductives alone, without the help of other adult females. Halictus ligatus is a widespread Nearctic, ground-nesting sweat bee. It has been particularly well studied in Ontario, where detailed studies have described it as obligately eusocial. Here we report evidence that the flexibility of female H. ligatus actually extends to expressing behaviour more typical of socially polymorphic species, those in which some individuals reproduce solitarily. In a population in southern Ontario, black wasps (Astata sp.) emerged from the soil beneath the nesting aggregation and proceeded to excavate their own nesting tunnels, dislocating many H. ligatus nest entrances. Young workers whose natal nests were destroyed by the wasp activity constructed new nests, so under very specific circumstances, it is possible for potential altruists to nest solitarily. | 4341 | NA | NA | NA | no | no |
| 4340 | 2017 | NA | Pilotti, M; Brunetti, A; Gallelli, A; Loreti, S | 2008 | NPR1-like genes from cDNA of rosaceous trees: cloning strategy and genetic variation | In this work, we report the isolation and characterization of NPR1-like gene fragments from nine species belonging to the rosaceous subfamilies Pomoideae and Amygdaloideae. Seventy-three NPR1-like gene fragments with uninterrupted open reading frame (ORF) were isolated from the expressed mRNA through a reverse transcriptase PCR-based strategy, most of them ranging from 5 11 to 827 bp. The deduced amino acid sequences of these gene fragments shared structural features with known NPR1 (-like) proteins: ankyrin repeat and the broad complex tramtrack and bric-a-brac/pox virus and zinc finger domain. The highest homology values were found with the NPR1-like gene from Lycopersicon esculentum and genes within the NPR4 phylogenetic group from Arabidopsis thaliana, suggesting that they are the NPR4-like orthologs. Phylogenetic analysis confirmed this conclusion and demonstrated the complexity of the gene families. The ankyrin domain was analyzed in detail and an ankyrin consensus was determined. A. comparison of this consensus with a statistically derived ankyrin repeat, as reported by other authors, enabled the prediction of the ankyrin repeat secondary structural elements. In addition, a comparison with some important structural and functional features of NPR1 was performed. | 4342 | NA | NA | NA | no | no |
| 4341 | 2017 | NA | Xu, DL; Zhou, GH; Xie, YJ; Mock, R; Li, R | 2010 | Complete nucleotide sequence and taxonomy of Sugarcane streak mosaic virus, member of a novel genus in the family Potyviridae | The complete genomic sequence of a Pakistani isolate of Sugarcane streak mosaic virus (SCSMV-PAK) is determined to be 9782 nucleotides in length, excluding the 3’ poly(A) tail, and it comprises a large open reading frame encoding a polyprotein of 3130 amino acid residues. The deduced polyprotein is likely to be cleaved at nine putative protease sites by three viral proteases to ten mature proteins. Conserved motifs of orthologous proteins of other potyviruses are identified in corresponding positions of SCSMV-PAK. The genomic organization is virtually identical to the genera Ipomovirus, Potyvirus, Rymovirus, and Tritimovirus in the family Potyviridae. Sequence analyses indicate that the SCSMV-PAK genomic sequence is different from those of Sugarcane mosaic virus and Sorghum mosaic virus, two viruses with very similar symptoms and host range to SCSMV-PAK. SCSMV-PAK shares 52.7% identity with Triticum mosaic virus (TriMV) and 26.4-31.5% identities with species of the existing genera and unassigned viruses in the Potyviridae at the polyprotein sequence level. Phylogenetic analyses of the polyprotein and deduced mature protein amino acid sequences reveal that SCSMV, together with TriMV, forms a distinct group in the family at the genus level. Therefore, SCSMV should represent a new genus, Susmovirus, in the Potyviridae. | 4343 | NA | NA | NA | no | no |
| 4342 | 2017 | NA | Sarker, A; Kumar, S; Maalouf, F; Goya, A; Kemal, SA | 2014 | Genetic enhancement of food legumes for nutritional security and sustainable cereal-based cropping systems | Food legume crops are cultivated since the earliest days of agriculture for human food and animal feed. Today these crops are grown globally, providing food and nutritional security. In particular, they are the main source of protein, macro and micronutrients in the diets of medium to low-income people in most developing countries. Besides their nutritional value, food legumes are important nitrogen fixers, contributing to soil health improvement and thus providing sustainable cropping system. However, global food legume production of about 67 million tons per year is insufficient to meet demand from ever-increasing populations, particularly in Africa and Asia. Food legumes are grown mainly by small and marginal farmers under rainfed conditions in marginal areas, leading to low and unstable yields. Moreover, they have long been ‘orphan crops’, receiving very little attention from researchers and policy makers. To attain a sustainable increased production in food legumes, several international research centers are working closely with national institutions to address these issues. The International Center for Agricultural Research in the Dry Areas (ICARDA), located in the center of origin of many food legume species, is engaged in research to develop and deliver improved food legume technologies to farmers. The research covers multiple crops (lentil, faba bean, Kabuli chickpea, grasspea) and involves a multidisciplinary team comprising breeders, biotechnologists, pathologists, entomologists, and seed specialists, To date, a total of 368 improved varieties of these crops have been released for cultivation in various countries, jointly developed by IGARDA and national partners. | 4344 | NA | NA | NA | no | no |
| 4343 | 2017 | NA | Mokrousov, I; Vyazovaya, A; Iwamoto, T; Skiba, Y; Pole, I; Zhdanova, S; Arikawa, K; Sinkov, V; Umpeleva, T; Valcheva, V; Figueroa, MA; Ranka, R; Jansone, I; Ogarkov, O; Zhuravlev, V; Narvskaya, O | 2016 | Latin-American-Mediterranean lineage of Mycobacterium tuberculosis: Human traces across pathogen’s phylogeography | Currently, Mycobacterium tuberculosis isolates of Latin-American Mediterranean (LAM) family may be detected far beyond the geographic areas that coined its name 15 years ago. Here, we established the framework phylogeny of this geographically intriguing and pathobiologically important mycobacterial lineage and hypothesized how human demographics and migration influenced its phylogeography. Phylogenetic analysis of LAM isolates from all continents based on 24 variable number of tandem repeats (VNTR) loci and other markers identified three global sublineages with certain geographic affinities and defined by large deletions RD115, RD174, and by spoligotype SIT33. One minor sublineage (spoligotype SIT388) appears endemic in Japan. One-locus VNTR signatures were established for sublineages and served for their search in published literature and geographic mapping. We suggest that the LAM family originated in the Western Mediterranean region. The most widespread RD115 sublineage seems the most ancient and encompasses genetically and geographically distant branches, including extremely drug resistant KZN in South Africa and LAM-RUS recently widespread across Northern Eurasia. The RD174 sub lineage likely started its active spread in Brazil; its earlier branch is relatively dominated by isolates from South America and the derived one is dominated by Portuguese and South/Southeastern African isolates. The relatively most recent SIT33-sublineage is marked with enigmatic gaps and peaks across the Americas and includes South African Glade F11/RD761, which likely emerged within the SIT33 sub-population after its arrival to Africa. In addition to SIT388-sublineage, other deeply rooted, endemic LAM sub lineages may exist that remain to be discovered. As a general conclusion, human mass migration appears to be the major factor that shaped the M. tuberculosis phylogeography over large time-spans. (C) 2016 Elsevier Inc. All rights reserved. | 4345 | NA | NA | NA | no | no |
| 4344 | 2017 | NA | Rodriguez-Castro, K; Ciocheti, G; Ribeiro, JW; Ribeiro, MC; Galetti, PM | 2017 | Using DNA barcode to relate landscape attributes to small vertebrate roadkill | Large vertebrates are the main focus of roadkill studies because their greater size facilitates taxonomic identification and the collection of statistical data. However, these studies fail to effectively include and identify small vertebrates and correlate roadkill events with the surrounding landscape. Here we showed the effectiveness of molecular data to identify small vertebrate roadkill, and we correlated landscape structure attributes with the location of roadkill for functional groups of varying mobility. The extraction of DNA from roadkilled individuals was followed by the amplification of two mitochondrial genes. We compared each DNA sequence to a database and used the highest similarity values for species identification. The species were classified according to their taxa and degree of mobility: birds, reptilia and amphibia with low and intermediate movement capability. After calculating the landscape attributes for each roadkill point, we used a competing model approach based on Akaike Information Criteria to determine which landscape variable best explained the occurrence of roadkills. Combining molecular and morphological characteristics, we identified 82.93% of the roadkilled animals. DNA barcoding allowed the identification of 310% more specimens than by morphological characteristics alone. Roadkilled birds with intermediate movement capability were strongly influenced by dominated areas by agriculture and sugar cane monocultures. Roadkilled reptiles with low movement capability were positively correlated with the presence of forest remnants, while those with intermediate movement capability seemed to be more frequent in heavily anthropized landscapes. We showed that molecular data is a powerful tool for precisely identifying small-sized roadkilled animals. Our results also highlight that different landscape structure attributes enable the prediction of roadkill occurrence along roads, which in turn allows us to identify roadkill hotspots and plan appropriate mitigation actions. | 4346 | NA | NA | NA | no | no |
| 4345 | 2017 | NA | Portis, E; Portis, F; Valente, L; Moglia, A; Barchi, L; Lanteri, S; Acquadro, A | 2016 | A Genome-Wide Survey of the Microsatellite Content of the Globe Artichoke Genome and the Development of a Web-Based Database | The recently acquired genome sequence of globe artichoke (Cynara cardunculus var. scolymus) has been used to catalog the genome’s content of simple sequence repeat (SSR) markers. More than 177,000 perfect SSRs were revealed, equivalent to an overall density across the genome of 244.5 SSRs/Mbp, but some 224,000 imperfect SSRs were also identified. About 21% of these SSRs were complex (two stretches of repeats separated by <100 nt). Some 73% of the SSRs were composed of dinucleotide motifs. The SSRs were categorized for the numbers of repeats present, their overall length and were allocated to their linkage group. A total of 4,761 perfect and 6,583 imperfect SSRs were present in 3,781 genes (14.11% of the total), corresponding to an overall density across the gene space of 32,5 and 44,9 SSRs/Mbp for perfect and imperfect motifs, respectively. A putative function has been assigned, using the gene ontology approach, to the set of genes harboring at least one SSR. The same search parameters were applied to reveal the SSR content of 14 other plant species for which genome sequence is available. Certain species-specific SSR motifs were identified, along with a hexa-nucleotide motif shared only with the other two Compositae species (sunflower (Helianthus annuus) and horseweed (Conyza canadensis)) included in the study. Finally, a database, called “Cynara cardunculus MicroSatellite DataBase” (CyMSatDB) was developed to provide a searchable interface to the SSR data. CyMSatDB facilitates the retrieval of SSR markers, as well as suggested forward and reverse primers, on the basis of genomic location, genomic vs genic context, perfect vs imperfect repeat, motif type, motif sequence and repeat number. The SSR markers were validated via an in silico based PCR analysis adopting two available assembled transcriptomes, derived from contrasting globe artichoke accessions, as templates. | 4347 | NA | NA | NA | no | no |
| 4346 | 2017 | NA | Hogg, CJ; Grueber, CE; Pemberton, D; Fox, S; Lee, AV; Ivy, JA; Belov, K | 2017 | Devil Tools & Tech: A Synergy of Conservation Research and Management Practice | Biodiversity conservation continually presents new challenges, yet conservation resources are limited, and funding for applied conservation research projects more so. Recently, many have reported on the “research-implementation gap,” whereby conservation research findings are infrequently translated into conservation actions. In this perspective, we describe our experiences working in a large multi-institutional, multi-disciplinary team as we attempt to bridge the research-implementation gap by developing conservation tools needed to address the conservation challenges faced by Tasmanian devils (Sarcophilus harrisii). We discuss our project’s history, lessons learnt, outcomes, and future plans to provide insights that may help others develop multi-institutional projects, designed to target rapid and direct implementation of conservation research into management action. Key to our success is the needs-based prioritization of research measured against the management team’s questions, recognition of the different needs of academia, industry and government, a collegiate approach, and willingness to embrace adaptive management. Challenges include developing a project which meets all strategic targets of different institutions, in addition to sourcing funds. Overall, our goal has been to establish an enduring research-management framework, to facilitate improved integration of scientific research into the management needs of Tasmanian devil conservation, and serve as a template for other species management projects. | 4348 | NA | NA | NA | no | no |
| 4347 | 2017 | NA | Wu, BL; Guan, WH; Pankow, JS | 2016 | On Efficient and Accurate Calculation of Significance P-Values for Sequence Kernel Association Testing of Variant Set | The objective of this paper is to discuss and develop alternative computational methods to accurately and efficiently calculate significance P-values for the commonly used sequence kernel association test (SKAT) and adaptive sum of SKAT and burden test (SKAT-O) for variant set association. We show that the existing software can lead to either conservative or inflated type I errors. We develop alternative and efficient computational algorithms that quickly compute the SKAT P-value and have well-controlled type I errors. In addition, we derive an alternative and simplified formula for calculating the significance P-value of SKAT-O, which sheds light on the development of efficient and accurate numerical algorithms. We implement the proposed methods in the publicly available R package that can be readily used or adapted to large-scale sequencing studies. Given that more and more large-scale exome and whole genome sequencing or re-sequencing studies are being conducted, the proposed methods are practically very important. We conduct extensive numerical studies to investigate the performance of the proposed methods. We further illustrate their usefulness with application to associations between rare exonic variants and fasting glucose levels in the Atherosclerosis Risk in Communities (ARIC) study. | 4349 | NA | NA | NA | no | no |
| 4348 | 2017 | NA | Lively, CM; de Roode, JC; Duffy, MA; Graham, AL; Koskella, B | 2014 | Interesting Open Questions in Disease Ecology and Evolution | No abstract, scoring performed on title | 4350 | NA | NA | NA | no | no |
| 4349 | 2017 | NA | Zeng, B; Zhang, Y; Huang, LK; Jiang, XM; Luo, D; Yin, GH | 2014 | Genetic diversity of orchardgrass (Dactylis glomerata L.) germplasms with resistance to rust diseases revealed by Start Codon Targeted (SCoT) markers | No abstract, scoring performed on title | 4351 | NA | NA | NA | no | no |
| 4350 | 2017 | NA | Okamoto, N; Horak, A; Keeling, PJ | 2012 | Description of Two Species of Early Branching Dinoflagellates, Psammosa pacifica n. g., n. sp and P. atlantica n. sp | In alveolate evolution, dinoflagellates have developed many unique features, including the cell that has epicone and hypocone, the undulating transverse flagellum. However, it remains unclear how these features evolved. The early branching dinoflagellates so far investigated such as Hematodinium, Amoebophrya and Oxyrrhis marina differ in many ways from of core dinoflagellates, or dinokaryotes. Except those handful of well studied taxa, the vast majority of early branching dinoflagellates are known only by environmental sequences, and remain enigmatic. In this study we describe two new species of the early branching dinoflagellates, Psammosa pacifica n. g., n. sp. and P. atlantica n. sp. from marine intertidal sandy beach. Molecular phylogeny of the small subunit (SSU) ribosomal RNA and Hsp90 gene places Psammosa spp. as an early branch among the dinoflagellates. Morphologically (1) they lack the typical dinoflagellate epicone-hypocone structure, and (2) undulation in either flagella. Instead they display a mosaic c of dinokaryotes traits, i.e. (3) presence of bipartite trychocysts; Oxyrrhis marina-like traits, i.e. (4) presence of flagellar hairs, (5) presence of two-dimensional cobweb scales ornamenting both flagella (6) transversal cell division; a trait shared with some syndineansand Parvilucifera spp. i.e. (7) a nucleus with a conspicuous nucleolus and condensed chromatin distributed beneath the nuclear envelope; as well as Perkinsus marinus -like features i.e. (8) separate ventral grooves where flagella emerge and (9) lacking dinoflagellate-type undulating flagellum. Notably Psammosa retains an apical complex structure, which is shared between perkinsids, colpodellids, chromerids and apicomplexans, but is not found in dinokaryotic dinoflagellates. | 4352 | NA | NA | NA | no | no |
| 4351 | 2017 | NA | Weir, BS | 2010 | Statistical genetic issues for genome-wide association studies | Genotyping technology now allows the rapid and affordable generation of million-SNP profiles for humans, leading to considerable activity in association mapping. Similar activity is anticipated for many plant species, including Brassica. These plant association mapping activities will require the same care in quality control and quality assurance as for humans. The subsequent analyses may draw upon the same body of theory that is described here in the language of quantitative genetics. | 4353 | NA | NA | NA | no | no |
| 4352 | 2017 | NA | Imwong, M; Sudimack, D; Pukrittayakamee, S; Osorio, L; Carlton, JM; Day, NPJ; White, NJ; Anderson, TJC | 2006 | Microsatellite variation, repeat array length, and population history of plasmodium vivax | No abstract, scoring performed on title | 4354 | NA | NA | NA | no | no |
| 4353 | 2017 | NA | Walling, GA; Wilson, AD; Mcteir, BL; Bishop, SC | 2004 | Increased heterozygosity and allele variants are seen in Texel compared to Suffolk sheep | In this study, the Suffolk and Texel sheep breeds were compared for microsatellite marker heterozygosity throughout seven chromosomal regions in the sheep genome. A total of 623 Texel animals and 489 Suffolk animals in five and three half-sib families, respectively, were genotyped for microsatellite markers across the seven different chromosomes. Using the observed allele frequencies, the expected levels of heterozygosity were calculated for each family. The expected levels of heterozygosity did not significantly differ between the breeds across all regions studied. However, levels of expected heterozygosity were 32% higher in Texel animals on chromosome 4 due to a region of increased heterozygosity between BMS648 and BM3212. The number of allelic variants significantly differed between the breeds, solely due to a region of increased number of alleles on chromosome 20. This region of higher numbers of allele variants in the Texel breed extended from the MHC to c. 15cM distal to the MHC region incorporating markers OMHC1, CSRD226, TGLA387 and BM1818, which had 3.30, 7.02, 3.09 and 6.75 more alleles in Texel than in Suffolk animals, respectively. No difference was observed in the variance of allele frequency between the two breeds. It is proposed that previous selective sweeps may have reduced numbers of alleles and levels of heterozygosity in the Suffolk breed. | 4355 | NA | NA | NA | no | no |
| 4354 | 2017 | NA | Evdokimova, VN; Nazarenko, SA | 2001 | Genetic variability of the number of CAG repeats in the X-linked androgen receptor gene and human embryonic mortality | Distribution of the X-linked androgen receptor gene (AR) alleles with different number of CAG repeat units in the first exon was analyzed in spontaneous abortuses having the 46,XX karyotype. The increased frequency of alleles with low number of trinucleotide repeat units in these specimens compared to adult healthy women was revealed. For more detailed analysis, all AR alleles were grouped into three classes: A, with a low CAG repeat number (21 to 23); B, with a moderate repeat number (24 to 28); and C, with a high maximum repeat number (29 to 32). Statistically significant among-sample differences in the frequencies of AA genotypes, which prevailed in spontaneous abortuses, were revealed. The samples tested were also different by the mean squared distance between the AR allele lengths, which constituted 81.8 +/-0.4 and 53.2 +/-0.5 in healthy women and spontaneous abortuses, respectively. Based on the data on inverse correlation between the length of the CAG repeat sequence and transcriptional activity of the androgen receptor gene, a hypothesis on selection against female individuals with the small number of CAG repeats in the AR gene and high transcriptional activity of this locus in prenatal period of human ontogeny is advanced. | 4356 | NA | NA | NA | no | no |
| 4355 | 2017 | NA | Fong, MY; Rashdi, SAA; Yusof, R; Lau, YL | 2016 | Genetic Diversity, Natural Selection and Haplotype Grouping of Plasmodium knowlesi Gamma Protein Region II (Pk gamma RII): Comparison with the Duffy Binding Protein (PkDBP alpha RII) | Background Plasmodium knowlesi is a simian malaria parasite that has been reported to cause malaria in humans in Southeast Asia. This parasite invades the erythrocytes of humans and of its natural host, the macaque Macaca fascicularis, via interaction between the Duffy binding protein region II (PkDBP alpha RII) and the Duffy antigen receptor on the host erythrocytes. In contrast, the P. knowlesi gamma protein region II (Pk gamma RII) is not involved in the invasion of P. knowlesi into humans. Pk gamma RII, however, mediates the invasion of P. knowlesi into the erythrocytes of M. mulata, a non-natural host of P. knowlesi via a hitherto unknown receptor. The haplotypes of PkDBP alpha RII in P. knowlesi isolates from Peninsular Malaysia and North Borneo have been shown to be genetically distinct and geographically clustered. Also, the PkDBP alpha RII was observed to be undergoing purifying (negative) selection. The present study aimed to determine whether similar phenomena occur in Pk gamma RII. Methods Blood samples from 78 knowlesi malaria patients were used. Forty-eight of the samples were from Peninsular Malaysia, and 30 were from Malaysia Borneo. The genomic DNA of the samples was extracted and used as template for the PCR amplification of the Pk gamma RII. The PCR product was cloned and sequenced. The sequences obtained were analysed for genetic diversity and natural selection using MEGA6 and DnaSP (version 5.10.00) programmes. Genetic differentiation between the Pk gamma RII of Peninsular Malaysia and North Borneo isolates was estimated using the Wright’s F-ST fixation index in DnaSP (version 5.10.00). Haplotype analysis was carried out using the Median-Joining approach in NETWORK (version 4.6.1.3). Results A total of 78 Pk gamma RII sequences was obtained. Comparative analysis showed that the Pk gamma RII have similar range of haplotype (Hd) and nucleotide diversity (pi) with that of PkDBP alpha RII. Other similarities between Pk gamma RII and PkDBP alpha RII include undergoing purifying (negative) selection, geographical clustering of haplotypes, and high inter-population genetic differentiation (F-ST index). The main differences between Pk gamma RII and PkDBP alpha RII include length polymorphism and no departure from neutrality (as measured by Tajima’s D statistics) in the Pk gamma RII. Conclusion Despite the biological difference between Pk gamma RII and PkDBP alpha RII, both generally have similar genetic diversity level, natural selection, geographical haplotype clustering and interpopulation genetic differentiation index. | 4357 | NA | NA | NA | no | no |
| 4356 | 2017 | NA | Fong, MY; Ahmed, MA; Wong, SS; Lau, YL; Sitam, F | 2015 | Genetic Diversity and Natural Selection of the Plasmodium knowlesi Circumsporozoite Protein Nonrepeat Regions | Background Plasmodium knowlesi is a simian malaria parasite that has been identified to cause malaria in humans. To date, several thousand cases of human knowlesi malaria have been reported around Southeast Asia. Thus far, there is no detailed study on genetic diversity and natural selection of P. knowlesi circumsporozoite protein (CSP), a prominent surface antigen on the sporozoite of the parasite. In the present study, the genetic diversity and natural selection acting on the nonrepeat regions of the gene encoding P. knowlesi CSP were investigated, focusing on the T-cell epitope regions at the C-terminal of the protein. Methods Blood samples from 32 knowlesi malaria patients and 2 wild monkeys (Macaca fascicularis) were used. The CSP of the P. knowlesi isolates was amplified by PCR, cloned into Escherichia coil, and sequenced. The nonrepeat regions of the CSP gene were analysed for genetic diversity, natural selection and haplotypic grouping using MEGA5 and DnaSP version 5.10.00 programmes. A haplotype network was constructed based on the C-terminal (Th2R/Th3R) T-cell epitope regions using the Median-Joining method in the NETVVORK version 4.6.1.2 programme. Previously published sequences from other regions (Malaysia Borneo, Singapore) were also included in the analysis. Results A total of 123 P. knowlesi CSP sequences were analysed. Multiple sequence alignment revealed 58 amino acid changes, and 42 novel amino acid haplotypes were identified. Polymorphism was higher in the C-terminal Th2R/Th3R epitope (ii = 0.0293, n = 123) region compared to the overall combined nonrepeat regions (ii = 0.0120, n = 123). Negative natural selection was observed within the nonrepeat regions of the CSP gene. Within the C-terminal Th2R/Th3R epitope regions, there was evidence of slight positive selection. Based on haplotype network analysis of the Th2R/Th3R regions, five abundant haplotypes were identified. Sharing of haplotypes between humans and macaques were observed. Conclusion This study contributes to the understanding of the type and distribution of naturally occurring polymorphism in the P. knowlesi CSP gene. This study also provides a measurement of the genetic diversity of P. knowlesi and identifies the predominant haplotypes within Malaysia based on the C-terminal Th2R/Th3R regions. | 4358 | NA | NA | NA | no | no |
| 4357 | 2017 | NA | Pecon-Slattery, J; McCracken, CL; Troyer, JL; VandeWoude, S; Roelke, M; Sondgeroth, K; Winterbach, C; Winterbach, H; O’Brien, SJ | 2008 | Genomic organization, sequence divergence, and recombination of feline immunodeficiency virus from lions in the wild | Background: Feline immunodeficiency virus (FIV) naturally infects multiple species of cat and is related to human immunodeficiency virus in humans. FIV infection causes AIDS-like disease and mortality in the domestic cat ( Felis catus) and serves as a natural model for HIV infection in humans. In African lions ( Panthera leo) and other exotic felid species, disease etiology introduced by FIV infection are less clear, but recent studies indicate that FIV causes moderate to severe CD4 depletion. Results: In this study, comparative genomic methods are used to evaluate the full proviral genome of two geographically distinct FIV subtypes isolated from free-ranging lions. Genome organization of FIV(Ple) subtype B ( 9891 bp) from lions in the Serengeti National Park in Tanzania and FIVPle subtype E ( 9899 bp) isolated from lions in the Okavango Delta in Botswana, both resemble FIV genome sequence from puma, Pallas cat and domestic cat across 5’ LTR, gag, pol, vif, orfA, env, rev and 3’ LTR regions. Comparative analyses of available full-length FIV consisting of subtypes A, B and C from FIV(Fca), Pallas cat FIV(Oma) and two puma FIV(Pco) subtypes A and B recapitulate the species- specific monophyly of FIV marked by high levels of genetic diversity both within and between species. Across all FIVPle gene regions except env, lion subtypes B and E are monophyletic, and marginally more similar to Pallas cat FIVOma than to other FIV. Sequence analyses indicate the SU and TM regions of env vary substantially between subtypes, with FIVPle subtype E more related to domestic cat FIVFca than to FIVPle subtype B and FIVOma likely reflecting recombination between strains in the wild. Conclusion: This study demonstrates the necessity of whole-genome analysis to complement population/ gene-based studies, which are of limited utility in uncovering complex events such as recombination that may lead to functional differences in virulence and pathogenicity. These full-length lion lentiviruses are integral to the advancement of comparative genomics of human pathogens, as well as emerging disease in wild populations of endangered species. | 4359 | NA | NA | NA | no | no |
| 4358 | 2017 | NA | Xie, J; Yi, SJ; Zhu, JG; Li, P; Liang, BB; Li, H; Yang, XX; Wang, LG; Hao, RZ; Jia, LL; Wu, ZH; Qiu, SF; Song, HB | 2015 | Antimicrobial Resistance and Molecular Investigation of H2S-Negative Salmonella enterica subsp enterica serovar Choleraesuis Isolates in China | Salmonella enterica subsp. enterica serovar Choleraesuis is a highly invasive pathogen of swine that frequently causes serious outbreaks, in particular in Asia, and can also cause severe invasive disease in humans. In this study, 21 S. Choleraesuis isolates, detected from 21 patients with diarrhea in China between 2010 and 2011, were found to include 19 H2S-negative S. Choleraesuis isolates and two H2S-positive isolates. This is the first report of H2S-negative S. Choleraesuis isolated from humans. The majority of H2S-negative isolates exhibited high resistance to ampicillin, chloramphenicol, gentamicin, tetracycline, ticarcillin, and trimethoprim-sulfamethoxazole, but only six isolates were resistant to norfloxacin. In contrast, all of the isolates were sensitive to cephalosporins. Fifteen isolates were found to be multidrug resistant. In norfloxacin-resistant isolates, we detected mutations in the gyrA and parC genes and identified two new mutations in the parC gene. Pulsed-field gel electrophoresis (PFGE), multilocus sequence typing (MLST), and clustered regularly interspaced short palindromic repeat (CRISPR) analysis were employed to investigate the genetic relatedness of H2S-negative and H2S-positive S. Choleraesuis isolates. PFGE revealed two groups, with all 19 H2S-negative S. Choleraesuis isolates belonging to Group I and H2S-positive isolates belonging to Group II. By MLST analysis, the H2S-negative isolates were all found to belong to ST68 and H2S-positive isolates belong to ST145. By CRISPR analysis, no significant differences in CRISPR 1 were detected; however, one H2S-negative isolate was found to contain three new spacers in CRISPR 2. All 19 H2S-negative isolates also possessed a frame-shift mutation at position 760 of phsA gene compared with H2S-positive isolates, which may be responsible for the H2S-negative phenotype. Moreover, the 19 H2S-negative isolates have similar PFGE patterns and same mutation site in the phsA gene, these results indicated that these H2S-negative isolates may have been prevalent in China. These findings suggested that surveillance should be increased of H(2)Snegative S. Choleraesuis in China. | 4360 | NA | NA | NA | no | no |
| 4359 | 2017 | NA | Kauwe, JSK; Ridge, PG; Foster, NL; Cannon-Albright, LA | 2013 | Strong Evidence for a Genetic Contribution to Late-Onset Alzheimer’s Disease Mortality: A Population-Based Study | Background: Alzheimer’s disease (AD) is an international health concern that has a devastating effect on patients and families. While several genetic risk factors for AD have been identified much of the genetic variance in AD remains unexplained. There are limited published assessments of the familiality of Alzheimer’s disease. Here we present the largest genealogy-based analysis of AD to date. Methods: We assessed the familiality of AD in The Utah Population Database (UPDB), a population-based resource linking electronic health data repositories for the state with the computerized genealogy of the Utah settlers and their descendants. We searched UPDB for significant familial clustering of AD to evaluate the genetic contribution to disease. We compared the Genealogical Index of Familiality (GIF) between AD individuals and randomly selected controls and estimated the Relative Risk (RR) for a range of family relationships. Finally, we identified pedigrees with a significant excess of AD deaths. Results: The GIF analysis showed that pairs of individuals dying from AD were significantly more related than expected. This excess of relatedness was observed for both close and distant relationships. RRs for death from AD among relatives of individuals dying from AD were significantly increased for both close and more distant relatives. Multiple pedigrees had a significant excess of AD deaths. Conclusions: These data strongly support a genetic contribution to the observed clustering of individuals dying from AD. This report is the first large population-based assessment of the familiality of AD mortality and provides the only reported estimates of relative risk of AD mortality in extended relatives to date. The high-risk pedigrees identified show a true excess of AD mortality (not just multiple cases) and are greater in depth and width than published AD pedigrees. The presence of these high-risk pedigrees strongly supports the possibility of rare predisposition variants not yet identified. | 4361 | NA | NA | NA | no | no |
| 4360 | 2017 | NA | Salama, MS; Abu Shady, HM; El-Gameal, MMB; El Anany, MG; Abd-El-Rehem, GM | 2012 | Molecular Study on Relatedness between Clinical and Tap Water Isolates of Pseudomonas aeruginosa in Two Burn Units | Background: The role of tap water as an environmental source of disease causing P. aeruginosa strains was established. Objective: to study the relatedness between clinical and tap water P. aeruginosa isolates in the burn units of two different hospitals. Materials and methods: One hundred and fifty specimens were collected from two burn units of Cairo University Hospital and El Helmeya Military Hospital. These specimens included: 100 pus samples from infected wounds of burned patients, 25 water samples and 25 swabs were taken from hands of medical staff. The samples were cultured and P. aeruginosa isolates were identified as according to standards. The selected colonies were subjected to molecular identification as P. aeruginosa by PCR tesing using specific PAL1 andPAL2 primers, and tested for relatedness by plasmid profiling and protein electrophoresis. Results: All cultures from the hands of the medical personnel were negative for P. aeruginosa. The clinical and water samples yielded 52 P. aeruginosa isolates and only 19 isolates were confirmed by PCR. These isolates were from 16% of total water samples from 15% of total clinical swabs. Half of these isolates harbored plasmids. The phenotypic characteristics of isolates showed that 30% of isolates (from clinical and water specimens) were related. However, molecular studies did not prove any molecular evidence of relatedness between different clinical isolates or between clinical and environmental isolates. Conclusions: P. aeruginosa is the cause of 15% of infections in Egyptian burn units, and contaminates 16% of water samples of these units. The simple use of bacterial protein electrophoresis and plasmid profiling ruled out the relatedness between the clinical and the contaminated water samples. [Mohammed S. Salama, Hala M. Abu Shady, Mohammed M. B. El-Gameal, Mervat G. El Anany and Ghada M. Abd-El-Rehem. Molecular Study on Relatedness between Clinical and Tap Water Isolates of Pseudomonas aeruginosa in Two Burn Units. Life Science Journal. 2012;9(1):662-666] (ISSN: 1097-8135). http://www.lifesciencesite.com. 96 | 4362 | NA | NA | NA | no | no |
| 4361 | 2017 | NA | Gharbi, M; Flegg, JA; Pradines, B; Berenger, A; Ndiaye, M; Djimde, AA; Roper, C; Hubert, V; Kendjo, E; Venkatesan, M; Brasseur, P; Gaye, O; Offianan, AT; Penali, L; Le Bras, J; Guerin, PJ | 2013 | Surveillance of Travellers: An Additional Tool for Tracking Antimalarial Drug Resistance in Endemic Countries | Introduction: There are growing concerns about the emergence of resistance to artemisinin-based combination therapies (ACTs). Since the widespread adoption of ACTs, there has been a decrease in the systematic surveillance of antimalarial drug resistance in many malaria-endemic countries. The aim of this work was to test whether data on travellers returning from Africa with malaria could serve as an additional surveillance system of local information sources for the emergence of drug resistance in endemic-countries. Methodology: Data were collected from travellers with symptomatic Plasmodium falciparum malaria returning from Senegal (n = 1,993), Mali (n = 2,372), Cote d’Ivoire (n = 4,778) or Cameroon (n = 3,272) and recorded in the French Malaria Reference Centre during the period 1996-2011. Temporal trends of the proportion of parasite isolates that carried the mutant genotype, pfcrt 76T, a marker of resistance to chloroquine (CQ) and pfdhfr 108N, a marker of resistance to pyrimethamine, were compared for travellers and within-country surveys that were identified through a literature review in PubMed. The in vitro response to CQ was also compared between these two groups for parasites from Senegal. Results: The trends in the proportion of parasites that carried pfcrt 76T, and pfdhfr 108N, were compared for parasites from travellers and patients within-country using the slopes of the curves over time; no significant differences in the trends were found for any of the 4 countries. These results were supported by in vitro analysis of parasites from the field in Senegal and travellers returning to France, where the trends were also not significantly different. Conclusion: The results have not shown different trends in resistance between parasites derived from travellers or from parasites within-country. This work highlights the value of an international database of drug responses in travellers as an additional tool to assess the emergence of drug resistance in endemic areas where information is limited. | 4363 | NA | NA | NA | no | no |
| 4362 | 2017 | NA | Springfeld, C; Bugert, JJ; Schnitzler, P; Tobiasch, E; Kehm, R; Darai, G | 2000 | TT virus as a human pathogen: Significance and problems | In 1997 TTV was detected using representational difference analysis (RDA) in serum of a patient with posttransfusion hepatitis unrelated to known hepatitis viruses. The genome of TTV is a circular single-stranded DNA molecule of 3852 nt with negative polarity. TTV possibly can be grouped either into the existing family Circoviridae or into a recently established virus family “Circinoviridae”. Analysis of the complete DNA nucleotide sequence of TTV identified three partially overlapping open reading frames (ORFs). Neither DNA nucleotide nor corresponding amino acid sequences of TTV do show significant homologies to known sequences. TTV DNA nucleotide sequences amplified by PCR from sera of different patients show considerable sequence variations. Although the natural route of transmission of TTV is still unknown, there is clear evidence for a transmission of TTV through blood and blood products. TTV DNA can be detected in the feces of infected individuals suggesting that it may be possible to attract TTV infection from environmental sources. Since the discovery of TTV, numerous studies have investigated the prevalence of TTV infections in different human population groups all over the world. All these studies are based on PCR detection systems, but the technical aspects of the PCR systems vary significantly between the different investigators. The results of the epidemiological studies do not show a clear picture. The discovery of TTV as a viral agent and particularly the identification of a high percentage of infected carriers in the healthy human population raises the following questions: Firstly, what is the origin and molecular relatedness of TT virus. Secondly, what is the significance of TTV as a human pathogen. And thirdly, what are the exact molecular mechanisms of viral replication. To answer these questions it will be necessary to determine the primary structure and the coding capacity of several TTV patient isolates. | 4364 | NA | NA | NA | no | no |
| 4363 | 2017 | NA | Aguilar-Rodea, P; Zuniga, G; Rodriguez-Espino, BA; Cervantes, ALO; Arroyo, AEG; Moreno-Espinosa, S; Zamboni, DD; Martinez, BL; Castellanos-Cruz, MD; Parra-Ortega, I; Rojas, VLJ; Galindo, JCV; Velazquez-Guadarrama, N | 2017 | Identification of extensive drug resistant Pseudomonas aeruginosa strains: New clone ST1725 and high-risk clone ST233 | Several microorganisms produce nosocomial infections (NIs), among which Pseudomonas aeruginosa stands out as an opportunist pathogen with the capacity to develop multiresistance to first-choice antibiotics. From 2007 to 2013, forty-six NIs produced by P. aeruginosa were detected at a pediatric tertiary care hospital in Mexico with a significant mortality rate (17.39%). All isolates (n = 58/46 patients) were characterized by evaluating their response to several antibiotics as panresistant (PDR), extensively resistant (XDR), multiresistant (MDR) or sensitive (S). In addition, all isolates were typified through multilocus sequencing of seven genes: acsA, aroE, guaA, mutL, nuoD, ppsA and trpE. Furthermore, to establish the genetic relationships among these isolates, we carried out a phylogenetic inference analysis using maximum likelihood to construct a phylogenetic network. To assess evolutionary parameters, recombination was evaluated using the PHI test, and the ratio of nonsynonymous to synonymous substitutions was determined. Two of the strains were PDR (ST1725); 42 were XDR; four were MDR; and ten were S. Twenty-one new sequence types were detected. Thirty-three strains exhibited novel sequence type ST1725. The ratio of nonsynonym to synonym substitutions was 1: 1 considering all genes. Phylogenetic analysis showed that the genetic relationship of the PDR, XDR and MDR strains was mainly clonal; however, the PHI test and the phylogenetic network suggest that recombination events occurred to produce a non-clonal population. This study aimed not only to determine the genetic diversity of clinical P. aeruginosa but also to provide a warning regarding the identification and spreading of clone ST1725, its ability to cause outbreaks with high mortality rates, and to remain in the hospital environment for over seven years. These characteristics highlight the need to identify clonal outbreaks, especially where high resistance to most antibiotics is observed, and control measures are needed. This study also represents the first report of the PDR ST1725. | 4365 | NA | NA | NA | no | no |
| 4364 | 2017 | NA | Byrnes, EJ; Li, W; Lewit, Y; Perfect, JR; Carter, DA; Cox, GM; Heitman, J | 2009 | First Reported Case of Cryptococcus gattii in the Southeastern USA: Implications for Travel-Associated Acquisition of an Emerging Pathogen | In 2007, the first confirmed case of Cryptococcus gattii was reported in the state of North Carolina, USA. An otherwise healthy HIV negative male patient presented with a large upper thigh cryptococcoma in February, which was surgically removed and the patient was started on long-term high-dose fluconazole treatment. In May of 2007, the patient presented to the Duke University hospital emergency room with seizures. Magnetic resonance imaging revealed two large CNS lesions found to be cryptococcomas based on brain biopsy. Prior chest CT imaging had revealed small lung nodules indicating that C. gattii spores or desiccated yeast were likely inhaled into the lungs and dissemination occurred to both the leg and CNS. The patient’s travel history included a visit throughout the San Francisco, CA region in September through October of 2006, consistent with acquisition during this time period. Cultures from both the leg and brain biopsies were subjected to analysis. Based on phenotypic and molecular methods, both isolates were C. gattii, VGI molecular type, and distinct from the Vancouver Island outbreak isolates. Based on multilocus sequence typing of coding and noncoding regions and virulence in a heterologous host model, the leg and brain isolates are identical, but the two differed in mating fertility. Two clinical isolates, one from a transplant recipient in San Francisco and the other from Australia, were identical to the North Carolina clinical isolate at all markers tested. Closely related isolates that differ at only one or a few noncoding markers are present in the Australian environment. Taken together, these findings support a model in which C. gattii VGI was transferred from Australia to California, possibly though an association with its common host plant E. camaldulensis, and the patient was exposed in San Francisco and returned to present with disease in North Carolina. | 4366 | NA | NA | NA | no | no |
| 4365 | 2017 | NA | Kosuwin, R; Putaporntip, C; Tachibana, H; Jongwutiwes, S | 2014 | Spatial Variation in Genetic Diversity and Natural Selection on the Thrombospondin-Related Adhesive Protein Locus of Plasmodium vivax (PvTRAP) | Thrombospondin-related adhesive protein (TRAP) of malaria parasites is essential for sporozoite motility and invasions into mosquito’s salivary gland and vertebrate’s hepatocyte; thereby, it is a promising target for pre-erythrocytic vaccine. TRAP of Plasmodium vivax (PvTRAP) exhibits sequence heterogeneity among isolates, an issue relevant to vaccine development. To gain insights into variation in the complete PvTRAP sequences of parasites in Thailand, 114 vivax malaria patients were recruited in 2006-2007 from 4 major endemic provinces bordering Myanmar (Tak in the northwest, n = 30 and Prachuap Khirikhan in the southwest, n = 25), Cambodia (Chanthaburi in the east, n = 29) and Malaysia (Yala and Narathiwat in the south, n = 30). In total, 26 amino acid substitutions were detected and 9 of which were novel, resulting in 44 distinct haplotypes. Haplotype and nucleotide diversities were lowest in southern P. vivax population while higher levels of diversities were observed in other populations. Evidences of positive selection on PvTRAP were demonstrated in domains II and IV and purifying selection in domains I, II and VI. Genetic differentiation was significant between each population except that between populations bordering Myanmar where transmigration was common. Regression analysis of pairwise linearized Fst and geographic distance suggests that P. vivax populations in Thailand have been isolated by distance. Sequence diversity of PvTRAP seems to be temporally stable over one decade in Tak province based on comparison of isolates collected in 1996 (n = 36) and 2006-2007. Besides natural selection, evidences of intragenic recombination have been supported in this study that could maintain and further generate diversity in this locus. It remains to be investigated whether amino acid substitutions in PvTRAP could influence host immune responses although several predicted variant T cell epitopes drastically altered the epitope scores. Knowledge on geographic diversity in PvTRAP constitutes an important basis for vaccine design provided that vaccination largely confers variant-specific immunity. | 4367 | NA | NA | NA | no | no |
| 4366 | 2017 | NA | Ferianc, P; Harichova, J; Proksova, M; Kreposova, K; Chovanova, K; Toth, D | 2002 | The surface river water and clinical Escherichia coli isolates: characteristics, diversity and epidemiological significance | Using selective media for coliform bacteria, 487 isolates in total were obtained from five different sites of Danube and Morava river waters in and near to Bratislava metropolitan area from February to October during one year. The fifty-six randomly chosen representatives from 396 of these strains identified as Escherichia coli, on the basis of biochemical tests, and forty clinical E. coli strains isolated from patients with urinary disease, were further analysed. Most of isolates were resistant to one or more drugs belonging to different classes of antibiotics. Electrophoresis of outer membrane proteins diversified fifty-six river water isolates into 33 patterns, while corresponding figure for clinical strains was 19 patterns. Single protein membrane patterns were assigned to single bacterial clones. The results revealed the relationships between origin of bacterial clones and sample sites and/or sample times. The isolates also differed with regard to plasmid contents so that natural samples were represented by 28, and clinical isolates by 13 patterns. Both outer membrane protein patterns and plasmid patterns showed higher genetic variability inside the river water and inside the clinical strains. The presence of conjugative plasmid(s) in E. coli cells indicated horizontal gene transfer inside the E. coli populations within bacterial assemblages. Confirmation of the presence of stx2A gene carried on plasmid(s) inside clinical strains suggested the occurrence of Shiga toxin-producing E. coli (STEC) strains. The presence of stx2A gene in surface water of Danube river suggested the occurrence of some of Stx-producing bacterial strains, perhaps some representatives of enterohemorrhagic E. coli (EHEC). The epidemiological significance of Danube river water, and diagnostic samples were also discussed with regard to the occurrence of pathogenic E. coli strains in these samples, where they may pose a risk to human health. | 4368 | NA | NA | NA | no | no |
| 4367 | 2017 | NA | Restrepo, CM; De La Guardia, C; Sousa, OE; Calzada, JE; Fernandez, PL; Lleonart, R | 2013 | AFLP Polymorphisms Allow High Resolution Genetic Analysis of American Tegumentary Leishmaniasis Agents Circulating in Panama and Other Members of the Leishmania Genus | American Tegumentary Leishmaniasis is caused by parasites of the genus Leishmania, and causes significant health problems throughout the Americas. In Panama, Leishmania parasites are endemic, causing thousands of new cases every year, mostly of the cutaneous form. In the last years, the burden of the disease has increased, coincident with increasing disturbances in its natural sylvatic environments. The study of genetic variation in parasites is important for a better understanding of the biology, population genetics, and ultimately the evolution and epidemiology of these organisms. Very few attempts have been made to characterize genetic polymorphisms of parasites isolated from Panamanian patients of cutaneous leishmaniasis. Here we present data on the genetic variability of local isolates of Leishmania, as well as specimens from several other species, by means of Amplified Fragment Length Polymorphisms (AFLP), a technique seldom used to study genetic makeup of parasites. We demonstrate that this technique allows detection of very high levels of genetic variability in local isolates of Leishmania panamensis in a highly reproducible manner. The analysis of AFLP fingerprints generated by unique selective primer combinations in L. panamensis suggests a predominant clonal mode of reproduction. Using fluorescently labeled primers, many taxon-specific fragments were identified which may show potential as species diagnostic fragments. The AFLP permitted a high resolution genetic analysis of the Leishmania genus, clearly separating certain groups among L. panamensis specimens and highly related species such as L. panamensis and L. guyanensis. The phylogenetic networks reconstructed from our AFLP data are congruent with established taxonomy for the genus Leishmania, even when using single selective primer combinations. Results of this study demonstrate that AFLP polymorphisms can be informative for genetic characterization in Leishmania parasites, at both intra and inter-specific levels. | 4369 | NA | NA | NA | no | no |
| 4368 | 2017 | NA | EL Hadad, S; Al-Hamdan, H; Linjawi, S | 2017 | Partial sequencing analysis of the NS5B region confirmed the predominance of hepatitis C virus genotype 1 infection in Jeddah, Saudi Arabia | Chronic hepatitis C virus (HCV) infection and its progression are major health problems that many countries including Saudi Arabia are facing. Determination of HCV genotypes and subgenotypes is critical for epidemiological and clinical analysis and aids in the determination of the ideal treatment strategy that needs to be followed and the expected therapy response. Although HCV infection has been identified as the second most predominant type of hepatitis in Saudi Arabia, little is known about the molecular epidemiology and genetic variability of HCV circulating in the Jeddah province of Saudi Arabia. The aim of this study was to determine the dominance of various HCV genotypes and subgenotypes circulating in Jeddah using partial sequencing of the NS5B region. To the best of our knowledge, this is the first study of its kind in Saudi Arabia. To characterize HCV genotypes and subgenotypes, serum samples from 56 patients with chronic HCV infection were collected and subjected to partial NS5B gene amplification and sequence analysis. Phylogenetic analysis of the NS5B partial sequences revealed that HCV/1 was the predominant genotype (73%), followed by HCV/4 (24.49%) and HCV/3 (2.04%). Moreover, pairwise analysis also confirmed these results based on the average specific nucleotide distance identity: +/- 0.112, +/- 0.112, and +/- 0.179 for HCV/1, HCV/4, and HCV/3, respectively, without any interference between genotypes. Notably, the phylogenetic tree of the HCV/1 subgenotypes revealed that all the isolates (100%) from the present study belonged to the HCV/1a subgenotype. Our findings also revealed similarities in the nucleotide sequences between HCV circulating in Saudi Arabia and those circulating in countries such as Morocco, Egypt, Canada, India, Pakistan, and France. These results indicated that determination of HCV genotypes and subgenotypes based on partial sequence analysis of the NS5B region is accurate and reliable for HCV subtype determination. | 4370 | NA | NA | NA | no | no |
| 4369 | 2017 | NA | Al-abd, NM; Mahdy, MAK; Al-Mekhlafi, AMQ; Snounou, G; Abdul-Majid, NB; Al-Mekhlafi, HM; Fong, MY | 2013 | The Suitability of P. falciparum Merozoite Surface Proteins 1 and 2 as Genetic Markers for In Vivo Drug Trials in Yemen | Background: The accuracy of the conclusions from in vivo efficacy anti-malarial drug trials depends on distinguishing between recrudescences and re-infections which is accomplished by genotyping genes coding P. falciparum merozoite surface 1 (MSP1) and MSP2. However, the reliability of the PCR analysis depends on the genetic markers’ allelic diversity and variant frequency. In this study the genetic diversity of the genes coding for MSP1 and MSP2 was obtained for P. falciparum parasites circulating in Yemen. Methods: Blood samples were collected from 511 patients with fever and screened for malaria parasites using Giemsa-stained blood films. A total 74 samples were infected with P. falciparum, and the genetic diversity was assessed by nested PCR targeting Pfmsp1 (Block2) and Pfmsp2 (block 3). Results: Overall, 58%, 28% and 54% of the isolates harboured parasites of the Pfmsp1 K1, MAD20 and RO33 allelic families, and 55% and 89% harboured those of the Pfmsp2 FC27 and 3D7 allelic families, respectively. For both genetic makers, the multiplicity of the infection (MOI) was significantly higher in the isolates from the foothills/coastland areas as compared to those from the highland (P < 0.05). Pfmsp2 had higher number of distinct allelic variants than Pfmsp1 (20 vs 11). The expected heterozygosity (HE) for Pfmsp1 and Pfmsp2 were 0.82 and 0.94, respectively. Nonetheless, a bias in the frequency distribution of the Pfmsp1 allelic variants was noted from all areas, and of those of Pfmsp2 in the samples collected from the highland areas. Conclusions: Significant differences in the complexity and allelic diversity of Pfmsp1 and Pfmsp2 genes between areas probably reflect differences in the intensity of malaria transmission. The biased distribution of allelic variants suggests that in Yemen Pfmsp1 should not be used for PCR correction of in vivo clinical trials outcomes, and that caution should be exercised when employing Pfmsp2. | 4371 | NA | NA | NA | no | no |
| 4370 | 2017 | NA | Lieden, A; Winge, MCG; Saaf, A; Kockum, I; Ekelund, E; Rodriguez, E; Folster-Holst, R; Franke, A; Illig, T; Tengvall-Linder, M; Baurecht, H; Weidinger, S; Wahlgren, CF; Nordenskjold, M; Bradley, M | 2012 | Genetic Variation in the Epidermal Transglutaminase Genes Is Not Associated with Atopic Dermatitis | Background: Atopic dermatitis (AD) is a common chronic inflammatory skin disorder where epidermal barrier dysfunction is a major factor in the pathogenesis. The identification of AD susceptibility genes related to barrier dysfunction is therefore of importance. The epidermal transglutaminases (TGM1, TGM3 and TGM5) encodes essential cross-linking enzymes in the epidermis. Objective: To determine whether genetic variability in the epidermal transglutaminases contributes to AD susceptibility. Methods: Forty-seven single nucleotide polymorphisms (SNPs) in the TGM1, TGM3 and TGM5 gene region were tested for genetic association with AD, independently and in relation to FLG genotype, using a pedigree disequilibrium test (PDT) in a Swedish material consisting of 1753 individuals from 539 families. In addition, a German case-control material, consisting of 533 AD cases and 1996 controls, was used for in silico analysis of the epidermal TGM regions. Gene expression of the TGM1, TGM3 and TGM5 gene was investigated by relative quantification with Real Time PCR (qRT-PCR). Immunohistochemical (IHC) analysis was performed to detect TG1, TG3 and TG5 protein expression in the skin of patients and healthy controls. Results: PDT analysis identified a significant association between the TGM1 SNP rs941505 and AD with allergen-specific IgE in the Swedish AD family material. However, the association was not replicated in the German case-control material. No significant association was detected for analyzed SNPs in relation to FLG genotype. TG1, TG3 and TG5 protein expression was detected in AD skin and a significantly increased TGM3 mRNA expression was observed in lesional skin by qRT-PCR. Conclusion: Although TGM1 and TGM3 may be differentially expressed in AD skin, the results from the genetic analysis suggest that genetic variation in the epidermal transglutaminases is not an important factor in AD susceptibility. | 4372 | NA | NA | NA | no | no |
| 4371 | 2017 | NA | Lore, NI; Iraqi, FA; Bragonzi, A | 2015 | Host genetic diversity influences the severity of Pseudomonas aeruginosa pneumonia in the Collaborative Cross mice | Background: Pseudomonas aeruginosa is one of the top three causes of opportunistic infections in humans. Patients with a compromised immune system, due to immunosuppressive therapies or underlying diseases such as cancer, AIDS or the hereditary disease cystic fibrosis, are at risk of developing P. aeruginosa infection. However, clinical evidence indicates extremely variable outcomes of P. aeruginosa infections in individuals at risk, suggesting that host multi-complex genetic traits may influence the severity of this opportunistic infection. Here, we have used an innovative experimental model to dissect whether host genetic background, such as those found in the outbred population, could influence the risk of morbidity and mortality to P. aeruginosa pneumonia. Results: A highly genetically-diverse mouse resource population, Collaborative Cross (CC) mice, was infected with a clinical strain of P. aeruginosa and subsequently monitored for mortality, mean survival time, and morbidity, change in body weight for seven days post infection. Disease phenotypes ranged from complete resistance and recovery of body weight to lethal disease. Initial variables, including body weight, age and gender, have limited influence on P. aeruginosa outcome, emphasizing the role of host genetic background in defining the risk of morbidity and mortality. When broad-sense heritability of phenotypic traits was evaluated, it confirmed the influence of genetic profile rather than environmental factors among the CC lines during P. aeruginosa infection. Conclusion: This innovative model system can potentially reproduce the variables responses of disease severity observed in humans during P. aeruginosa pneumonia. Our results demonstrated that a widely-marked differential response to P. aeruginosa airway infection in term of morbidity and mortality, is mainly affected by host genetic factors, as multiple genetic loci or polymorphic variations. | 4373 | NA | NA | NA | no | no |
| 4372 | 2017 | NA | Navarro, KG; Agyingi, SE; Nwabuobi, CK; Thomas, BN | 2016 | Polymorphism of the endothelin-1 gene (rs5370) is a potential contributor to sickle cell disease pathophysiology | Sickle cell disease has been shown to demonstrate extensive variability in disease severity among and between individuals, the variability highlighted by differing genetic haplotypes. Despite the abundance of reports of functional significance due to polymorphisms of endothelial nitric oxide synthase (eNOS) and endothelin-1 (ET-1) genes, the role of these polymorphisms in mediating sickle cell disease pathophysiology among African Americans is presently unclear. To deconvolute their potential significance among African Americans with sickle cell disease, we examined the genetic diversity and haplotype frequency of eNOS and ET-1 polymorphisms in disease (n = 331) and control (n = 379) groups, with a polymerase-chain reaction restriction fragment length polymorphism assay. We report that genotypic and allelic frequencies of eNOS variants are not significantly different between groups. eNOS homozygote mutants, which had been shown to have clinical significance elsewhere, showed no statistical significance in our study. On the other hand, and contrary to previous report among Africans with sickle cell disease, the endothelin-1 homozygous mutant variant showed significant difference in genotypic (p = 2.84E-12) and allelic frequencies (p = 2.20E-16) between groups. The most common haplotype is the combination of T786C homozygote wildtype variant with homozygote mutant variants of G5665T (ET-1) and Glu298Asp (eNOS). These results show that endothelin-1 (rs5370) polymorphism, rather than endothelial nitric oxide synthase polymorphism might play a significant role in disease severity or individual clinical outcomes among African Americans with sickle cell disease. This would have profound implications for designing and/or advancing personalized care for sickle cell patients and relieving disease complications. Copyright (C) 2016, Chongqing Medical University. Production and hosting by Elsevier B.V. This is an open access article under the CC BY-NC-ND license (http://creativecommons.org/licenses/by-nc-nd/4.0/). | 4374 | NA | NA | NA | no | no |
| 4373 | 2017 | NA | Grosso-Becerra, MV; Santos-Medellin, C; Gonzalez-Valdez, A; Mendez, JL; Delgado, G; Morales-Espinosa, R; Servin-Gonzalez, L; Alcaraz, LD; Soberon-Chavez, G | 2014 | Pseudomonas aeruginosa clinical and environmental isolates constitute a single population with high phenotypic diversity | Background: Pseudomonas aeruginosa is an opportunistic pathogen with a high incidence of hospital infections that represents a threat to immune compromised patients. Genomic studies have shown that, in contrast to other pathogenic bacteria, clinical and environmental isolates do not show particular genomic differences. In addition, genetic variability of all the P. aeruginosa strains whose genomes have been sequenced is extremely low. This low genomic variability might be explained if clinical strains constitute a subpopulation of this bacterial species present in environments that are close to human populations, which preferentially produce virulence associated traits. Results: In this work, we sequenced the genomes and performed phenotypic descriptions for four non-human P. aeruginosa isolates collected from a plant, the ocean, a water-spring, and from dolphin stomach. We show that the four strains are phenotypically diverse and that this is not reflected in genomic variability, since their genomes are almost identical. Furthermore, we performed a detailed comparative genomic analysis of the four strains studied in this work with the thirteen previously reported P. aeruginosa genomes by means of describing their core and pan-genomes. Conclusions: Contrary to what has been described for other bacteria we have found that the P. aeruginosa core genome is constituted by a high proportion of genes and that its pan-genome is thus relatively small. Considering the high degree of genomic conservation between isolates of P. aeruginosa from diverse environments, including human tissues, some implications for the treatment of infections are discussed. This work also represents a methodological contribution for the genomic study of P. aeruginosa, since we provide a database of the comparison of all the proteins encoded by the seventeen strains analyzed. | 4375 | NA | NA | NA | no | no |
| 4374 | 2017 | NA | Linsuwanon, P; Puenpa, J; Suwannakarn, K; Auksornkitti, V; Vichiwattana, P; Korkong, S; Theamboonlers, A; Poovorawan, Y | 2012 | Molecular Epidemiology and Evolution of Human Enterovirus Serotype 68 in Thailand, 2006-2011 | Background: Publications worldwide have reported on the re-occurrence of human enterovirus 68 (EV68), a rarely detected pathogen usually causing respiratory illness. However, epidemiological data regarding this virus in particular on the Asian continent has so far been limited. Methodology/Findings: We investigated the epidemiology and genetic variability of EV68 infection among Thai children with respiratory illnesses from 2006-2011 (n = 1810). Semi-nested PCR using primer sets for amplification of the 5’-untranslated region through VP2 was performed for rhino-enterovirus detection. Altogether, 25 cases were confirmed as EV68 infection indicating a prevalence of 1.4% in the entire study population. Interestingly, the majority of samples were children aged >5 years (64%). Also, co-infection with other viruses was found in 28%, while pandemic H1N1 influenza/2009 virus was the most common co-infection. Of EV68-positive patients, 36% required hospitalizations with the common clinical presentations of fever, cough, dyspnea, and wheezing. The present study has shown that EV68 was extremely rare until 2009 (0.9%). An increasing annual prevalence was found in 2010 (1.6%) with the highest detection frequency in 2011 (4.3%). Based on analysis of the VP1 gene, the evolutionary rate of EV68 was estimated at 4.93x10(-3) substitutions/site/year. Major bifurcation of the currently circulating EV68 strains occurred 66 years ago (1945.31 with (1925.95-1960.46)95% HPD). Among the current lineages, 3 clusters of EV68 were categorized based on the different molecular signatures in the BC and DE loops of VP1 combined with high posterior probability values. Each cluster has branched off from their common ancestor at least 36 years ago (1975.78 with (1946.13-1984.97) 95% HPD). Conclusion: Differences in epidemiological characteristic and seasonal profile of EV68 have been found in this study. Results from Bayesian phylogenetic investigations also revealed that EV68 should be recognized as a genetically diverse virus with a substitution rate identical to that of enterovirus 71 genotype B (4.2x10(-3) s/s/y). | 4376 | NA | NA | NA | no | no |
| 4375 | 2017 | NA | Boldrin, E; Rumiato, E; Fassan, M; Cappellesso, R; Rugge, M; Chiarion-Sileni, V; Ruol, A; Alfieri, R; Cagol, M; Castoro, C; Amadori, A; Saggioro, D | 2015 | Genetic Features of Metachronous Esophageal Cancer Developed in Hodgkin’s Lymphoma or Breast Cancer Long-Term Survivors: An Exploratory Study | Background Development of novel therapeutic drugs and regimens for cancer treatment has led to improvements in patient long-term survival. This success has, however, been accompanied by the increased occurrence of second primary cancers. Indeed, patients who received regional radiotherapy for Hodgkin’s Lymphoma (HL) or breast cancer may develop, many years later, a solid metachronous tumor in the irradiated field. Despite extensive epidemiological studies, little information is available on the genetic changes involved in the pathogenesis of these solid therapy-related neoplasms. Methods Using microsatellite markers located in 7 chromosomal regions frequently deleted in sporadic esophageal cancer, we investigated loss of heterozygosity (LOH) and microsatellite instability (MSI) in 46 paired (normal and tumor) samples. Twenty samples were of esophageal carcinoma developed in HL or breast cancer long-term survivors: 14 squamous cell carcinomas (ESCC) and 6 adenocarcinomas (EADC), while 26 samples, used as control, were of sporadic esophageal cancer (15 ESCC and 11 EADC). Results We found that, though the overall LOH frequency at the studied chromosomal regions was similar among metachronous and sporadic tumors, the latter exhibited a statistically different higher LOH frequency at 17q21.31 (p = 0.018). By stratifying for tumor histotype we observed that LOH at 3p24.1, 5q11.2 and 9p21.3 were more frequent in ESCC than in EADC suggesting a different role of the genetic determinants located nearby these regions in the development of the two esophageal cancer histotypes. Conclusions Altogether, our results strengthen the genetic diversity among ESCC and EADC whether they occurred spontaneously or after therapeutic treatments. The presence of histotype-specific alterations in esophageal carcinoma arisen in HL or breast cancer long-term survivors suggests that their transformation process, though the putative different etiological origin, may retrace sporadic ESCC and EADC carcinogenesis. | 4377 | NA | NA | NA | no | no |
| 4376 | 2017 | NA | Carter, TE; Malloy, H; Existe, A; Memnon, G; St Victor, Y; Okech, BA; Mulligan, CJ | 2015 | Genetic Diversity of Plasmodium falciparum in Haiti: Insights from Microsatellite Markers | Hispaniola, comprising Haiti and the Dominican Republic, has been identified as a candidate for malaria elimination. However, incomplete surveillance data in Haiti hamper efforts to assess the impact of ongoing malaria control interventions. Characteristics of the genetic diversity of Plasmodium falciparum populations can be used to assess parasite transmission, which is information vital to evaluating malaria elimination efforts. Here we characterize the genetic diversity of P. falciparum samples collected from patients at seven sites in Haiti using 12 microsatellite markers previously employed in population genetic analyses of global P. falciparum populations. We measured multiplicity of infections, level of genetic diversity, degree of population geographic substructure, and linkage disequilibrium (defined as non-random association of alleles from different loci). For low transmission populations like Haiti, we expect to see few multiple infections, low levels of genetic diversity, high degree of population structure, and high linkage disequilibrium. In Haiti, we found low levels of multiple infections (12.9%), moderate to high levels of genetic diversity (mean number of alleles per locus = 4.9, heterozygosity = 0.61), low levels of population structure (highest pairwise F-st = 0.09 and no clustering in principal components analysis), and moderate linkage disequilibrium (ISA = 0.05, P<0.0001). In addition, population bottleneck analysis revealed no evidence for a reduction in the P. falciparum population size in Haiti. We conclude that the high level of genetic diversity and lack of evidence for a population bottleneck may suggest that Haiti’s P. falciparum population has been stable and discuss the implications of our results for understanding the impact of malaria control interventions. We also discuss the relevance of parasite population history and other host and vector factors when assessing transmission intensity from genetic diversity data. | 4378 | NA | NA | NA | no | no |
| 4377 | 2017 | NA | Marques, H; Freitas, J; Medeiros, R; Longatto, A | 2016 | Methodology for single nucleotide polymorphism selection in promoter regions for clinical use. An example of its applicability | Genetic variability in humans can explain many differences in disease risk factors. Polymorphism-related studies focus mainly on the single nucleotide polymorphisms (SNPs) of coding regions of the genes. SNPs on DNA binding motifs of the promoter region have been less explored. On a recent study of SNPs in patients with non-Hodgkin lymphomas we faced the problem of SNP selection from promoter regions and developed a practical methodology for clinical studies. The process consists in identifying SNPs in the coding and promoter regions of the antigen-processing system using the ‘dbSNP’ database. With the ‘HapMap’ program, we select SNPs with frequencies >20% in Caucasian populations. For coding regions, we sought biologically and clinically relevant SNPs described in the literature. For the promoter regions, we determined their chromosomal location on ‘QiagenSABioscience’ site database. The nucleotide sequence of ancestral and variant alleles is available in the ‘dbSNP’. These sequences were used in `Promoter TESS' to determine binding differences of transcription factors. Each sequence may have affinity to different TFs. Thus, SNP selection on the promoter regions was based in the differences on TF binding pattern between the old and the new allele. The potential clinical relevance of the new TFs was also evaluated before the final selection. With this approach, we found that almost half of the relevant SNP fall within the promoter region. In conclusion, we were able to develop a methodology of oriented selection of promoter regions of human genes, comparing the TF with affinity to the ancestral allele with the TF to a variant allele. We selected those SNPs that change the TF's affinity to a pattern with functional significance. </td> <td style="text-align:left;"> 4379 </td> <td style="text-align:left;"> NA </td> <td style="text-align:left;"> NA </td> <td style="text-align:left;"> NA </td> <td style="text-align:left;"> no </td> <td style="text-align:left;"> no </td> </tr> <tr> <td style="text-align:left;"> 4378 </td> <td style="text-align:left;"> 2017 </td> <td style="text-align:left;"> NA </td> <td style="text-align:left;"> Al-Mahruqi, SH; Zadjali, F; Koh, CY; Balkhair, A; Said, EA; Al-Balushi, MS; Hasson, SS; Al-Jabri, AA </td> <td style="text-align:left;"> 2014 </td> <td style="text-align:left;"> New genetic variants in the CCR5 gene and the distribution of known polymorphisms in Omani population </td> <td style="text-align:left;"> C-C motif chemokine receptor-5 (CCR5) is a pro-inflammatory receptor that binds to chemokines and facilitates the entry of the R5 strain of HIV-1. A number of polymorphisms were identified within the promoter and coding regions of the CCR5 gene, some of which have been found to affect the protein expression and thus receptor function. Although several CCR5 polymorphisms were shown to vary widely in their distribution among different ethnic populations, there has been no study addressing the potential variants of the CCR5 gene in the Omani population. The aim of this study was to identify the polymorphic sites that exist within the CCR5 gene in Omanis. Blood samples were collected from 89 Omani adult individuals, and genomic DNA was amplified by polymerase chain reaction and sequenced to identify the polymorphic sites. The distribution of the detected variants was examined and compared with the previously published data. Four new indels were detected of 32 variable positions, -2973A/-, -2894A/-, -2827TA/- and -2769T/-, and all were located in the 5UTR. Furthermore, two new mutations, -2248G/A and +658A/G, were observed for the first time; the -2248G/A was detected in the intron 1 region in one subject and +658A/G in the coding region of the CCR5 in another subject. In silico analysis showed that the novel variations in the 5UTR may have effects on the transcription factor binding sites. Therefore, this study demonstrates the presence of two new SNPs and four novel indels in the CCR5 gene in the Omani population. Our findings support the wide spectrum of genetic diversity reported within the CCR5 gene region among different ethnic groups. </td> <td style="text-align:left;"> 4380 </td> <td style="text-align:left;"> NA </td> <td style="text-align:left;"> NA </td> <td style="text-align:left;"> NA </td> <td style="text-align:left;"> no </td> <td style="text-align:left;"> no </td> </tr> <tr> <td style="text-align:left;"> 4379 </td> <td style="text-align:left;"> 2017 </td> <td style="text-align:left;"> NA </td> <td style="text-align:left;"> Amaral, EP; Kipnis, TL; de Carvalho, ECQ; da Silva, WD; Leao, SC; Lasunskaia, EB </td> <td style="text-align:left;"> 2011 </td> <td style="text-align:left;"> Difference in Virulence of Mycobacterium avium Isolates Sharing Indistinguishable DNA Fingerprint Determined in Murine Model of Lung Infection </td> <td style="text-align:left;"> Background: Opportunistic Mycobacterium avium typically causes disease in immunocompromised patients and in some groups of apparently healthy individuals. The high virulence of some bacterial lineages increases the disease risk. High-resolution molecular genotyping studies of M. avium clinical isolates demonstrated that some genotype patterns were more prevalent than others, suggesting that close genetic relatedness of these successful isolates sharing a similar genotype could determine similar biological properties associated with high virulence. Methods and Findings: In this study, we aimed to compare the virulence and pathogenic properties of two epidemiologically unrelated M. avium isolates sharing an indistinguishable DNA fingerprint in a well-characterized model of pulmonary infection in mice, resistant or susceptible to mycobacteria. The mice, C57BL/6 wild-type or IFN-gamma gene disrupted (GKO), respectively, were intratracheally infected with two isolates, H27 (human blood isolate) and P104 (pig lymph node isolate), and the lungs were examined for bacterial loads, histopathology and cytokine gene expression. The obtained data demonstrated significant differences in the virulence properties of these strains. Although the H27 strain grew significantly faster than P104 in the early stage of infection, this bacterium induced protective immunity that started to reduce bacterial numbers in the wild-type mice, whereas the P104 strain established a chronic infection. In the GKO mice, both strains were capable of causing a chronic infection, associated with higher bacterial burdens and severe lung pathology, in a similar manner. Conclusions/Significance: The results demonstrated that the studied isolates differed in the pathogenic properties although were indistinguishable by actually widely used genotyping techniques demonstrating that the genotype similarity does not predict similarity in virulence of M. avium isolates. </td> <td style="text-align:left;"> 4381 </td> <td style="text-align:left;"> NA </td> <td style="text-align:left;"> NA </td> <td style="text-align:left;"> NA </td> <td style="text-align:left;"> no </td> <td style="text-align:left;"> no </td> </tr> <tr> <td style="text-align:left;"> 4380 </td> <td style="text-align:left;"> 2017 </td> <td style="text-align:left;"> NA </td> <td style="text-align:left;"> Realpe, T; Correa, N; Rozo, JC; Ferro, BE; Gomez, V; Zapata, E; Ribon, W; Puerto, G; Castro, C; Nieto, LM; Diaz, ML; Rivera, O; Couvin, D; Rastogi, N; Arbelaez, MP; Robledo, J </td> <td style="text-align:left;"> 2014 </td> <td style="text-align:left;"> Population Structure among Mycobacterium tuberculosis Isolates from Pulmonary Tuberculosis Patients in Colombia </td> <td style="text-align:left;"> Background: Phylogeographic composition of M. tuberculosis populations reveals associations between lineages and human populations that might have implications for the development of strategies to control the disease. In Latin America, lineage 4 or the Euro-American, is predominant with considerable variations among and within countries. In Colombia, although few studies from specific localities have revealed differences in M. tuberculosis populations, there are still areas of the country where this information is lacking, as is a comparison of Colombian isolates with those from the rest of the world. Principal Findings: A total of 414 M. tuberculosis isolates from adult pulmonary tuberculosis cases from three Colombian states were studied. Isolates were genotyped using IS6110-restriction fragment length polymorphism (RFLP), spoligotyping, and 24-locus Mycobacterial interspersed repetitive units variable number tandem repeats (MIRU-VNTRs). SIT42 (LAM9) and SIT62 (H1) represented 53.3% of isolates, followed by 8.21% SIT50 (H3), 5.07% SIT53 (T1), and 3.14% SIT727 (H1). Composite spoligotyping and 24-locus MIRU-VNTR minimum spanning tree analysis suggest a recent expansion of SIT42 and SIT62 evolved originally from SIT53 (T1). The proportion of Haarlem sublineage (44.3%) was significantly higher than that in neighboring countries. Associations were found between M. tuberculosis MDR and SIT45 (H1), as well as HIV-positive serology with SIT727 (H1) and SIT53 (T1). Conclusions: This study showed the population structure of M. tuberculosis in several regions from Colombia with a dominance of the LAM and Haarlem sublineages, particularly in two major urban settings (Medellin and Cali). Dominant spoligotypes were LAM((SIT 42) and Haarlem (SIT62). The proportion of the Haarlem sublineage was higher in Colombia compared to that in neighboring countries, suggesting particular conditions of co-evolution with the corresponding human population that favor the success of this sublineage. </td> <td style="text-align:left;"> 4382 </td> <td style="text-align:left;"> NA </td> <td style="text-align:left;"> NA </td> <td style="text-align:left;"> NA </td> <td style="text-align:left;"> no </td> <td style="text-align:left;"> no </td> </tr> <tr> <td style="text-align:left;"> 4381 </td> <td style="text-align:left;"> 2017 </td> <td style="text-align:left;"> NA </td> <td style="text-align:left;"> Anderson, TJC; Williams, JT; Nair, S; Sudimack, D; Barends, M; Jaidee, A; Price, RN; Nosten, F </td> <td style="text-align:left;"> 2010 </td> <td style="text-align:left;"> Inferred relatedness and heritability in malaria parasites </td> <td style="text-align:left;"> Malaria parasites vary in phenotypic traits of biomedical or biological interest such as growth rate, virulence, sex ratio and drug resistance, and there is considerable interest in identifying the genes that underlie this variation. An important first step is to determine trait heritability (H-2). We evaluate two approaches to measuring H-2 in natural parasite populations using relatedness inferred from genetic marker data. We collected single-clone Plasmodium falciparum infections from 185 patients from the Thailand-Burma border, monitored parasite clearance following treatment with artemisinin combination therapy (ACT), measured resistance to six antimalarial drugs and genotyped parasites using 335 micro-satellites. We found strong relatedness structure. There were 27 groups of two to eight clonally identical (CI) parasites, and 74 per cent of parasites showed significant relatedness to one or more other parasites. Initially, we used matrices of allele sharing and variance components (VC) methods to estimate H-2. Inhibitory concentrations (IC50) for six drugs showed significant H-2 (0.24 to 0.79, p = 0.06 to 2.85 x 10(-9)), demonstrating that this study design has adequate power. However, a phenotype of current interest-parasite clearance following ACT-showed no detectable heritability (H-2 = 0-0.09, ns) in this population. The existence of CI parasites allows the use of a simple ANOVA approach for quantifying H-2, analogous to that used in human twin studies. This gave similar results to the VC method and requires considerably less genotyping information. We conclude (i) that H-2 can be effectively measured in malaria parasite populations using minimal genotype data, allowing rational design of genome-wide association studies; and (ii) while drug response (IC50) shows significant H-2, parasite clearance following ACT was not heritable in the population studied. </td> <td style="text-align:left;"> 4383 </td> <td style="text-align:left;"> NA </td> <td style="text-align:left;"> NA </td> <td style="text-align:left;"> NA </td> <td style="text-align:left;"> no </td> <td style="text-align:left;"> no </td> </tr> <tr> <td style="text-align:left;"> 4382 </td> <td style="text-align:left;"> 2017 </td> <td style="text-align:left;"> NA </td> <td style="text-align:left;"> Yu, CW; Yang, YA; Zou, L; Hu, ZX; Li, J; Liu, YQ; Ma, YX; Ma, MY; Su, D; Zhang, SZ </td> <td style="text-align:left;"> 2011 </td> <td style="text-align:left;"> Identification of novel mutations in Chinese Hans with autosomal dominant polycystic kidney disease </td> <td style="text-align:left;"> Background: Autosomal dominant polycystic kidney disease (ADPKD) is the most common inherited renal disease with an incidence of 1 in 400 to 1000. The disease is genetically heterogeneous, with two genes identified: PKD1 (16p13.3) and PKD2 (4q21). Molecular diagnosis of the disease in at-risk individuals is complicated due to the structural complexity of PKD1 gene and the high diversity of the mutations. This study is the first systematic ADPKD mutation analysis of both PKD1 and PKD2 genes in Chinese patients using denaturing high-performance liquid chromatography (DHPLC). Methods: Both PKD1 and PKD2 genes were mutation screened in each proband from 65 families using DHPLC followed by DNA sequencing. Novel variations found in the probands were checked in their family members available and 100 unrelated normal controls. Then the pathogenic potential of the variations of unknown significance was examined by evolutionary comparison, effects of amino acid substitutions on protein structure, and effects of splice site alterations using online mutation prediction resources. Results: A total of 92 variations were identified, including 27 reported previously. Definitely pathogenic mutations (ten frameshift, ten nonsense, two splicing defects and one duplication) were identified in 28 families, and probably pathogenic mutations were found in an additional six families, giving a total detection level of 52.3% (34/65). About 69% (20/29) of the mutations are first reported with a recurrent mutation rate of 31%. Conclusions: Mutation study of PKD1 and PKD2 genes in Chinese Hans with ADPKD may contribute to a better understanding of the genetic diversity between different ethnic groups and enrich the mutation database. Besides, evaluating the pathogenic potential of novel variations should also facilitate the clinical diagnosis and genetic counseling of the disease. </td> <td style="text-align:left;"> 4384 </td> <td style="text-align:left;"> NA </td> <td style="text-align:left;"> NA </td> <td style="text-align:left;"> NA </td> <td style="text-align:left;"> no </td> <td style="text-align:left;"> no </td> </tr> <tr> <td style="text-align:left;"> 4383 </td> <td style="text-align:left;"> 2017 </td> <td style="text-align:left;"> NA </td> <td style="text-align:left;"> Reiche, J; Jacobsen, S; Neubauer, K; Hafemann, S; Nitsche, A; Milde, J; Wolff, T; Schweiger, B </td> <td style="text-align:left;"> 2014 </td> <td style="text-align:left;"> Human Metapneumovirus: Insights from a Ten-Year Molecular and Epidemiological Analysis in Germany </td> <td style="text-align:left;"> Human metapneumovirus (HMPV) is a cause of respiratory tract illness at all ages. In this study the epidemiological and molecular diversity among patients of different ages was investigated. Between 2000-2001 and 2009-2010, HMPV was detected in 3% (138/4,549) of samples from outpatients with influenza-like illness with a new, sensitive real-time RT-PCR assay. Several hundred (797) clinical specimens from hospitalized children below the age of 4 years with acute respiratory illness were investigated and HMPV was detected in 11.9% of them. Investigation of outpatients revealed that HMPV infections occurred in individuals of all ages but were most prevalent in children (0-4 years) and the elderly (>60 years). The most present clinical features of HMPV infections were cough, bronchitis, fever/shivers and pneumonia. About two thirds of HMPV-positive samples were detected in February and March throughout the study period. Molecular characterization of HMPV revealed a complex cyclic pattern of group dominance where HMPV subgroup A and B viruses predominated in general for three consecutive seasons. German HMPV represented all genetic lineages including A1, A2, B1, B2, sub-clusters A2a and A2b. For Germany, not only time-dependent circulation of lineages and sub-clusters was observed but also co-circulation of two or three predominant lineages. Two newly emerging amino acid substitutions (positions 223 and 280) of lineage B2 were detected in seven German HMPV sequences. Our study gives new insights into the molecular epidemiology of HMPV in in-and outpatients over a time period of 10 years for the first time. It is one of only few long-term surveillance studies in Europe, and allows comparative molecular analyses of HMPV circulating worldwide. </td> <td style="text-align:left;"> 4385 </td> <td style="text-align:left;"> NA </td> <td style="text-align:left;"> NA </td> <td style="text-align:left;"> NA </td> <td style="text-align:left;"> no </td> <td style="text-align:left;"> no </td> </tr> <tr> <td style="text-align:left;"> 4384 </td> <td style="text-align:left;"> 2017 </td> <td style="text-align:left;"> NA </td> <td style="text-align:left;"> Zuercher, E; Butticaz, C; Wyniger, J; Martinez, R; Battegay, M; El Amari, EB; Dang, T; Egger, JF; Fehr, J; Mueller-Garamvogyi, E; Parini, A; Schaefer, SC; Schoeni-Affolter, F; Thurnheer, C; Tinguely, M; Telenti, A; Rothenberger, S </td> <td style="text-align:left;"> 2012 </td> <td style="text-align:left;"> Genetic Diversity of EBV-Encoded LMP1 in the Swiss HIV Cohort Study and Implication for NF-Kb Activation </td> <td style="text-align:left;"> Epstein-Barr virus (EBV) is associated with several types of cancers including Hodgkin's lymphoma (HL) and nasopharyngeal carcinoma (NPC). EBV-encoded latent membrane protein 1 (LMP1), a multifunctional oncoprotein, is a powerful activator of the transcription factor NF-kappa B, a property that is essential for EBV-transformed lymphoblastoid cell survival. Previous studies reported LMP1 sequence variations and induction of higher NF-kappa B activation levels compared to the prototype B95-8 LMP1 by some variants. Here we used biopsies of EBV-associated cancers and blood of individuals included in the Swiss HIV Cohort Study (SHCS) to analyze LMP1 genetic diversity and impact of sequence variations on LMP1-mediated NF-kappa B activation potential. We found that a number of variants mediate higher NF-kappa B activation levels when compared to B95-8 LMP1 and mapped three single polymorphisms responsible for this phenotype: F106Y, I124V and F144I. F106Y was present in all LMP1 isolated in this study and its effect was variant dependent, suggesting that it was modulated by other polymorphisms. The two polymorphisms I124V and F144I were present in distinct phylogenetic groups and were linked with other specific polymorphisms nearby, I152L and D150A/L151I, respectively. The two sets of polymorphisms, I124V/I152L and F144I/D150A/L151I, which were markers of increased NF-kappa B activation in vitro, were not associated with EBV-associated HL in the SHCS. Taken together these results highlighted the importance of single polymorphisms for the modulation of LMP1 signaling activity and demonstrated that several groups of LMP1 variants, through distinct mutational paths, mediated enhanced NF-kappa B activation levels compared to B95-8 LMP1. </td> <td style="text-align:left;"> 4386 </td> <td style="text-align:left;"> NA </td> <td style="text-align:left;"> NA </td> <td style="text-align:left;"> NA </td> <td style="text-align:left;"> no </td> <td style="text-align:left;"> no </td> </tr> <tr> <td style="text-align:left;"> 4385 </td> <td style="text-align:left;"> 2017 </td> <td style="text-align:left;"> NA </td> <td style="text-align:left;"> Gray, RR; Tanaka, Y; Takebe, Y; Magiorkinis, G; Buskell, Z; Seeff, L; Alter, HJ; Pybus, OG </td> <td style="text-align:left;"> 2013 </td> <td style="text-align:left;"> Evolutionary analysis of hepatitis C virus gene sequences from 1953 </td> <td style="text-align:left;"> Reconstructing the transmission history of infectious diseases in the absence of medical or epidemiological records often relies on the evolutionary analysis of pathogen genetic sequences. The precision of evolutionary estimates of epidemic history can be increased by the inclusion of sequences derived from 'archived' samples that are genetically distinct from contemporary strains. Historical sequences are especially valuable for viral pathogens that circulated for many years before being formally identified, including HIV and the hepatitis C virus (HCV). However, surprisingly few HCV isolates sampled before discovery of the virus in 1989 are currently available. Here, we report and analyse two HCV subgenomic sequences obtained from infected individuals in 1953, which represent the oldest genetic evidence of HCV infection. The pair-wise genetic diversity between the two sequences indicates a substantial period of HCV transmission prior to the 1950s, and their inclusion in evolutionary analyses provides new estimates of the common ancestor of HCV in the USA. To explore and validate the evolutionary information provided by these sequences, we used a new phylogenetic molecular clock method to estimate the date of sampling of the archived strains, plus the dates of four more contemporary reference genomes. Despite the short fragments available, we conclude that the archived sequences are consistent with a proposed sampling date of 1953, although statistical uncertainty is large. Our cross-validation analyses suggest that the bias and low statistical power observed here likely arise from a combination of high evolutionary rate heterogeneity and an unstructured, star-like phylogeny. We expect that attempts to date other historical viruses under similar circumstances will meet similar problems. </td> <td style="text-align:left;"> 4387 </td> <td style="text-align:left;"> NA </td> <td style="text-align:left;"> NA </td> <td style="text-align:left;"> NA </td> <td style="text-align:left;"> no </td> <td style="text-align:left;"> no </td> </tr> <tr> <td style="text-align:left;"> 4386 </td> <td style="text-align:left;"> 2017 </td> <td style="text-align:left;"> NA </td> <td style="text-align:left;"> Weinmaier, T; Hoser, J; Eck, S; Kaufhold, I; Shima, K; Strom, TM; Rattei, T; Rupp, J </td> <td style="text-align:left;"> 2015 </td> <td style="text-align:left;"> Genomic factors related to tissue tropism in Chlamydia pneumoniae infection </td> <td style="text-align:left;"> Background: Chlamydia pneumoniae (Cpn) are obligate intracellular bacteria that cause acute infections of the upper and lower respiratory tract and have been implicated in chronic inflammatory diseases. Although of significant clinical relevance, complete genome sequences of only four clinical Cpn strains have been obtained. All of them were isolated from the respiratory tract and shared more than 99% sequence identity. Here we investigate genetic differences on the whole-genome level that are related to Cpn tissue tropism and pathogenicity. Results: We have sequenced the genomes of 18 clinical isolates from different anatomical sites (e.g. lung, blood, coronary arteries) of diseased patients, and one animal isolate. In total 1,363 SNP loci and 184 InDels have been identified in the genomes of all clinical Cpn isolates. These are distributed throughout the whole chlamydial genome and enriched in highly variable regions. The genomes show clear evidence of recombination in at least one potential region but no phage insertions. The tyrP gene was always encoded as single copy in all vascular isolates. Phylogenetic reconstruction revealed distinct evolutionary lineages containing primarily non-respiratory Cpn isolates. In one of these, clinical isolates from coronary arteries and blood monocytes were closely grouped together. They could be distinguished from all other isolates by characteristic nsSNPs in genes involved in RB to EB transition, inclusion membrane formation, bacterial stress response and metabolism. Conclusions: This study substantially expands the genomic data of Cpn and elucidates its evolutionary history. The translation of the observed Cpn genetic differences into biological functions and the prediction of novel pathogen-oriented diagnostic strategies have to be further explored. </td> <td style="text-align:left;"> 4388 </td> <td style="text-align:left;"> NA </td> <td style="text-align:left;"> NA </td> <td style="text-align:left;"> NA </td> <td style="text-align:left;"> no </td> <td style="text-align:left;"> no </td> </tr> <tr> <td style="text-align:left;"> 4387 </td> <td style="text-align:left;"> 2017 </td> <td style="text-align:left;"> NA </td> <td style="text-align:left;"> Kim, JY; Goo, YK; Zo, YG; Ji, SY; Trimarsanto, H; To, S; Clark, TG; Price, RN; Auburn, S </td> <td style="text-align:left;"> 2016 </td> <td style="text-align:left;"> Further Evidence of Increasing Diversity of Plasmodium vivax in the Republic of Korea in Recent Years </td> <td style="text-align:left;"> Background Vivax malaria was successfully eliminated from the Republic of Korea (ROK) in the late 1970s but re-emerged in 1993. Two decades later as the ROK enters the final stages of malaria elimination, dedicated surveillance of the local P. vivax population is critical. We apply a population genetic approach to gauge P. vivax transmission dynamics in the ROK between 2010 and 2012. Methodology/Principal Findings P. vivax positive blood samples from 98 autochthonous cases were collected from patients attending health centers in the ROK in 2010 (n = 27), 2011 (n = 48) and 2012 (n = 23). Parasite genotyping was undertaken at 9 tandem repeat markers. Although not reaching significance, a trend of increasing population diversity was observed from 2010 (H-E = 0.50 +/- 0.11) to 2011 (H-E = 0.56 +/- 0.08) and 2012 (H-E = 0.60 +/- 0.06). Conversely, linkage disequilibrium declined during the same period: I-AS = 0.15 in 2010 (P = 0.010), 0.09 in 2011 (P = 0.010) and 0.05 in 2012 (P = 0.010). In combination with data from other ROK studies undertaken between 1994 and 2007, our results are consistent with increasing parasite divergence since re-emergence. Polyclonal infections were rare (3% infections) suggesting that local out-crossing alone was unlikely to explain the increased divergence. Cases introduced from an external reservoir may therefore have contributed to the increased diversity. Aside from one isolate, all infections carried a short MS20 allele (142 or 149 bp), not observed in other studies in tropical endemic countries despite high diversity, inferring that these regions are unlikely reservoirs. Conclusions Whilst a number of factors may explain the observed population genetic trends, the available evidence suggests that an external geographic reservoir with moderate diversity sustains the majority of P. vivax infection in the ROK, with important implications for malaria elimination. </td> <td style="text-align:left;"> 4389 </td> <td style="text-align:left;"> NA </td> <td style="text-align:left;"> NA </td> <td style="text-align:left;"> NA </td> <td style="text-align:left;"> no </td> <td style="text-align:left;"> no </td> </tr> <tr> <td style="text-align:left;"> 4388 </td> <td style="text-align:left;"> 2017 </td> <td style="text-align:left;"> NA </td> <td style="text-align:left;"> Bassaganyas, L; Riveira-Munoz, E; Garcia-Aragones, M; Gonzalez, JR; Caceres, M; Armengol, L; Estivill, X </td> <td style="text-align:left;"> 2013 </td> <td style="text-align:left;"> Worldwide population distribution of the common LCE3C-LCE3B deletion associated with psoriasis and other autoimmune disorders </td> <td style="text-align:left;"> Background: There is increasing evidence of the importance of copy number variants (CNV) in genetic diversity among individuals and populations, as well as in some common genetic diseases. We previously characterized a common 32-kb insertion/deletion variant of the PSORS4 locus at chromosome 1q21 that harbours the LCE3C and LCE3B genes. This variant allele (LCE3C_LCE3B-del) is common in patients with psoriasis and other autoimmune disorders from certain ethnic groups. Results: Using array-CGH (Agilent 244 K) in samples from the HapMap and Human Genome Diversity Panel (HGDP) collections, we identified 54 regions showing population differences in comparison to Africans. We provided here a comprehensive population-genetic analysis of one of these regions, which involves the 32-kb deletion of the PSORS4 locus. By a PCR-based genotyping assay we characterised the profiles of the LCE3C_LCE3B-del and the linkage disequilibrium (LD) pattern between the variant allele and the tag SNP rs4112788. Our results show that most populations tend to have a higher frequency of the deleted allele than Sub-Saharan Africans. Furthermore, we found strong LD between rs4112788G and LCE3C_LCE3B-del in most non-African populations (r2>0.8), in contrast to the low concordance between loci (r2<0.3) in the African populations. Conclusions: These results are another example of population variability in terms of biomedical interesting CNV. The frequency distribution of the LCE3C_LCE3B-del allele and the LD pattern across populations suggest that the differences between ethnic groups might not be due to natural selection, but the consequence of genetic drift caused by the strong bottleneck that occurred during "out of Africa" expansion. </td> <td style="text-align:left;"> 4390 </td> <td style="text-align:left;"> NA </td> <td style="text-align:left;"> NA </td> <td style="text-align:left;"> NA </td> <td style="text-align:left;"> no </td> <td style="text-align:left;"> no </td> </tr> <tr> <td style="text-align:left;"> 4389 </td> <td style="text-align:left;"> 2017 </td> <td style="text-align:left;"> NA </td> <td style="text-align:left;"> Cheeseman, IH; McDew-White, M; Phyo, AP; Sriprawat, K; Nosten, F; Anderson, TJC </td> <td style="text-align:left;"> 2015 </td> <td style="text-align:left;"> Pooled Sequencing and Rare Variant Association Tests for Identifying the Determinants of Emerging Drug Resistance in Malaria Parasites </td> <td style="text-align:left;"> We explored the potential of pooled sequencing to swiftly and economically identify selective sweeps due to emerging artemisinin (ART) resistance in a South-East Asian malaria parasite population. ART resistance is defined by slow parasite clearance from the blood of ART-treated patients and mutations in the kelch gene (chr. 13) have been strongly implicated to play a role. We constructed triplicate pools of 70 slow-clearing (resistant) and 70 fast-clearing (sensitive) infections collected from the Thai-Myanmar border and sequenced these to high (similar to 150-fold) read depth. Allele frequency estimates from pools showed almost perfect correlation (Lin's concordance = 0.98) with allele frequencies at 93 single nucleotide polymorphisms measured directly from individual infections, giving us confidence in the accuracy of this approach. By mapping genome-wide divergence (F-ST) between pools of drug-resistant and drug-sensitive parasites, we identified two large (> 150 kb) regions (on chrs. 13 and 14) and 17 smaller candidate genome regions. To identify individual genes within these genome regions, we resequenced an additional 38 parasite genomes (16 slow and 22 fast-clearing) and performed rare variant association tests. These confirmed kelch as a major molecular marker for ART resistance (P = 6.03 x 10(-6)). This two-tier approach is powerful because pooled sequencing rapidly narrows down genome regions of interest, while targeted rare variant association testing within these regions can pinpoint the genetic basis of resistance. We show that our approach is robust to recurrent mutation and the generation of soft selective sweeps, which are predicted to be common in pathogen populations with large effective population sizes, and may confound more traditional gene mapping approaches. </td> <td style="text-align:left;"> 4391 </td> <td style="text-align:left;"> NA </td> <td style="text-align:left;"> NA </td> <td style="text-align:left;"> NA </td> <td style="text-align:left;"> no </td> <td style="text-align:left;"> no </td> </tr> <tr> <td style="text-align:left;"> 4390 </td> <td style="text-align:left;"> 2017 </td> <td style="text-align:left;"> NA </td> <td style="text-align:left;"> Hargreaves, KR; Otieno, JR; Thanki, A; Blades, MJ; Millard, AD; Browne, HP; Lawley, TD; Clokie, MRJ </td> <td style="text-align:left;"> 2015 </td> <td style="text-align:left;"> As Clear as Mud? Determining the Diversity and Prevalence of Prophages in the Draft Genomes of Estuarine Isolates of Clostridium difficile </td> <td style="text-align:left;"> The bacterium Clostridium difficile is a significant cause of nosocomial infections worldwide. The pathogenic success of this organism can be attributed to its flexible genome which is characterized by the exchange of mobile genetic elements, and by ongoing genome evolution. Despite its pathogenic status, C. difficile can also be carried asymptomatically, and has been isolated from natural environments such as water and sediments where multiple strain types (ribotypes) are found in close proximity. These include ribotypes which are associated with disease, as well as those that are less commonly isolated from patients. Little is known about the genomic content of strains in such reservoirs in the natural environment. In this study, draft genomes have been generated for 13 C. difficile isolates from estuarine sediments including clinically relevant and environmental associated types. To identify the genetic diversity within this strain collection, whole-genome comparisons were performed using the assemblies. The strains are highly genetically diverse with regards to the C. difficile "mobilome," which includes transposons and prophage elements. We identified a novel transposon-like element in two R078 isolates. Multiple, related and unrelated, prophages were detected in isolates across ribotype groups, including two novel prophage elements and those related to the transducing phage phi C2. The susceptibility of these isolates to lytic phage infection was tested using a panel of characterized phages found from the same locality. In conclusion, estuarine sediments are a source of genetically diverse C. difficile strains with a complex network of prophages, which could contribute to the emergence of new strains in clinics. </td> <td style="text-align:left;"> 4392 </td> <td style="text-align:left;"> NA </td> <td style="text-align:left;"> NA </td> <td style="text-align:left;"> NA </td> <td style="text-align:left;"> no </td> <td style="text-align:left;"> no </td> </tr> <tr> <td style="text-align:left;"> 4391 </td> <td style="text-align:left;"> 2017 </td> <td style="text-align:left;"> NA </td> <td style="text-align:left;"> Cheeseman, IH; Miller, B; Tan, JC; Tan, A; Nair, S; Nkhoma, SC; De Donato, M; Rodulfo, H; Dondorp, A; Branch, OH; Mesia, LR; Newton, P; Mayxay, M; Amambua-Ngwa, A; Conway, DJ; Nosten, F; Ferdig, MT; Anderson, TJC </td> <td style="text-align:left;"> 2016 </td> <td style="text-align:left;"> Population Structure Shapes Copy Number Variation in Malaria Parasites </td> <td style="text-align:left;"> If copy number variants (CNVs) are predominantly deleterious, we would expect them to be more efficiently purged from populations with a large effective population size (Ne) than from populations with a small Ne. Malaria parasites (Plasmodium falciparum) provide an excellent organism to examine this prediction, because this protozoan shows a broad spectrum of population structures within a single species, with large, stable, outbred populations in Africa, small unstable inbred populations in South America and with intermediate population characteristics in South East Asia. We characterized 122 single-clone parasites, without prior laboratory culture, from malaria-infected patients in seven countries in Africa, South East Asia and South America using a high-density single-nucleotide polymorphism/CNV microarray. We scored 134 high-confidence CNVs across the parasite exome, including 33 deletions and 102 amplifications, which ranged in size from <500 bp to 59 kb, as well as 10,107 flanking, biallelic single-nucleotide polymorphisms. Overall, CNVs were rare, small, and skewed toward low frequency variants, consistent with the deleterious model. Relative to African and South East Asian populations, CNVs were significantly more common in South America, showed significantly less skew in allele frequencies, and were significantly larger. On this background of low frequency CNV, we also identified several high-frequency CNVs under putative positive selection using an FST outlier analysis. These included known adaptive CNVs containing rh2b and pfmdr1, and several other CNVs (e.g., DNA helicase and three conserved proteins) that require further investigation. Our data are consistent with a significant impact of genetic structure on CNV burden in an important human pathogen. </td> <td style="text-align:left;"> 4393 </td> <td style="text-align:left;"> NA </td> <td style="text-align:left;"> NA </td> <td style="text-align:left;"> NA </td> <td style="text-align:left;"> no </td> <td style="text-align:left;"> no </td> </tr> <tr> <td style="text-align:left;"> 4392 </td> <td style="text-align:left;"> 2017 </td> <td style="text-align:left;"> NA </td> <td style="text-align:left;"> Perricone, C; Ciccacci, C; Ceccarelli, F; Di Fusco, D; Spinelli, FR; Cipriano, E; Novelli, G; Valesini, G; Conti, F; Borgiani, P </td> <td style="text-align:left;"> 2013 </td> <td style="text-align:left;"> TRAF3IP2 gene and systemic lupus erythematosus: association with disease susceptibility and pericarditis development </td> <td style="text-align:left;"> Systemic lupus erythematosus (SLE) is a multifactorial autoimmune disease. Although genetic factors confer susceptibility to the disease, only 15 % of the genetic contribution has been identified. TRAF3IP2 gene, associated with susceptibility to psoriatic arthritis and psoriasis, encodes for Act1, a negative regulator of adaptive immunity and a positive signaling adaptor in IL-17-mediated immune responses. The aim of this study was to assess the role of TRAF3IP2 gene variability in SLE susceptibility and disease phenotype in an Italian population. Two hundred thirty-nine consecutive SLE patients were enrolled. Study protocol included complete physical examination; the clinical and laboratory data were collected. Two hundred seventy-eight age- and ethnicity-matched healthy subjects served as controls. TRAF3IP2 polymorphisms (rs33980500, rs13190932, and rs13193677) were analyzed in both cases and controls. Genotype analysis was performed by allelic discrimination assays. A case-control association study and a genotype-phenotype correlation were performed. The rs33980500 and rs13193677 resulted significantly associated with SLE susceptibility (P = 0.021, odds ratio (OR) = 1.71, and P = 0.046, OR = 1.73, respectively). All three TRAF3IP2 single nucleotide polymorphisms resulted associated with the development of pericarditis; in particular, rs33980500 showed the strongest association (P = 0.002, OR 2.59). This association was further highlighted by binary logistic regression analysis. In conclusion, our data show for the first time the contribution of TRAF3IP2 genetic variability in SLE susceptibility, providing further suggestions that common variation in genes that function in the adaptive and innate arms of the immune system are important in establishing SLE risk. Our study also shows that this gene may affect disease phenotype and, particularly, the occurrence of pericarditis. </td> <td style="text-align:left;"> 4394 </td> <td style="text-align:left;"> NA </td> <td style="text-align:left;"> NA </td> <td style="text-align:left;"> NA </td> <td style="text-align:left;"> no </td> <td style="text-align:left;"> no </td> </tr> <tr> <td style="text-align:left;"> 4393 </td> <td style="text-align:left;"> 2017 </td> <td style="text-align:left;"> NA </td> <td style="text-align:left;"> Bolnick, DI; Snowberg, LK; Caporaso, JG; Lauber, C; Knight, R; Stutz, WE </td> <td style="text-align:left;"> 2014 </td> <td style="text-align:left;"> Major Histocompatibility Complex class IIb polymorphism influences gut microbiota composition and diversity </td> <td style="text-align:left;"> Animals harbour diverse communities of symbiotic bacteria, which differ dramatically among host individuals. This heterogeneity poses an immunological challenge: distinguishing between mutualistic and pathogenic members of diverse and host-specific microbial communities. We propose that Major Histocompatibility class II (MHC) genotypes contribute to recognition and regulation of gut microbes, and thus, MHC polymorphism contributes to microbial variation among hosts. Here, we show that MHC IIb polymorphism is associated with among-individual variation in gut microbiota within a single wild vertebrate population of a small fish, the threespine stickleback. We sampled stickleback from Cedar Lake, on Vancouver Island, and used next-generation sequencing to genotype the sticklebacks' gut microbiota (16S sequencing) and their MHC class IIb exon 2 sequences. The presence of certain MHC motifs was associated with altered relative abundance (increase or decrease) of some microbial Families. The effect sizes are modest and entail a minority of microbial taxa, but these results represent the first indication that MHC genotype may affect gut microbiota composition in natural populations (MHC-microbe associations have also been found in a few studies of lab mice). Surprisingly, these MHC effects were frequently sex-dependent. Finally, hosts with more diverse MHC motifs had less diverse gut microbiota. One implication is that MHC might influence the efficacy of therapeutic strategies to treat dysbiosis-associated disease, including the outcome of microbial transplants between healthy and diseased patients. We also speculate that macroparasite-driven selection on MHC has the potential to indirectly alter the host gut microbiota, and vice versa. </td> <td style="text-align:left;"> 4395 </td> <td style="text-align:left;"> NA </td> <td style="text-align:left;"> NA </td> <td style="text-align:left;"> NA </td> <td style="text-align:left;"> no </td> <td style="text-align:left;"> no </td> </tr> <tr> <td style="text-align:left;"> 4394 </td> <td style="text-align:left;"> 2017 </td> <td style="text-align:left;"> NA </td> <td style="text-align:left;"> Runcharoen, C; Moradigaravand, D; Blane, B; Paksanont, S; Thammachote, J; Anun, S; Parkhill, J; Chantratita, N; Peacock, SJ </td> <td style="text-align:left;"> 2017 </td> <td style="text-align:left;"> Whole genome sequencing reveals high-resolution epidemiological links between clinical and environmental Klebsiella pneumoniae </td> <td style="text-align:left;"> Background: Klebsiella pneumoniae is a gram-negative bacterial species capable of occupying a broad range of environmental and clinical habitats. Known as an opportunistic pathogen, it has recently become a major causative agent of clinical infections worldwide. Despite growing knowledge about the highly diverse population of K. pneumoniae, the evolution and clinical significance of environmental K. pneumoniae, as well as the relationship between clinical and environmental K. pneumoniae, are poorly defined. Methods: We isolated and sequenced K. pneumoniae from in-patients in a single hospital in Thailand, as well as hospital sewage, and surrounding canals and farms within a 20-km radius. Results: Phylogenetic analysis of 77 K. pneumoniae (48 clinical and 29 non-clinical isolates) demonstrated that the two groups were intermixed throughout the tree and in some cases resided in the same clade, suggesting recent divergence from a common ancestor. Phylogenetic comparison of the 77 Thai genomes with 286 K. pneumoniae from a global collection showed that Thai isolates were closely related to the clinical sub-population of the global collection, indicating that Thai clinical isolates belonged to globally circulating lineages. Dating of four Thai K. pneumoniae clades indicated that they emerged between 50 and 150 years ago. Despite their phylogenetic relatedness, virulence factors and beta-lactamase resistance genes were more numerous in clinical than in environmental isolates. Our results indicate that clinical and environmental K. pneumoniae are closely related, but that hospitals may select for isolates with a more resistant and virulent genotype. Conclusions: These findings highlight the clinical relevance of environmental K. pneumoniae isolates. </td> <td style="text-align:left;"> 4396 </td> <td style="text-align:left;"> NA </td> <td style="text-align:left;"> NA </td> <td style="text-align:left;"> NA </td> <td style="text-align:left;"> no </td> <td style="text-align:left;"> no </td> </tr> <tr> <td style="text-align:left;"> 4395 </td> <td style="text-align:left;"> 2017 </td> <td style="text-align:left;"> NA </td> <td style="text-align:left;"> Trauner, A; Liu, QY; Via, LE; Liu, X; Ruan, XL; Liang, LL; Shi, HM; Chen, Y; Wang, ZL; Liang, RX; Zhang, W; Wei, W; Gao, JC; Sun, G; Brites, D; England, K; Zhang, GL; Gagneux, S; Barry, CE; Gao, Q </td> <td style="text-align:left;"> 2017 </td> <td style="text-align:left;"> The within-host population dynamics of Mycobacterium tuberculosis vary with treatment efficacy </td> <td style="text-align:left;"> Background: Combination therapy is one of the most effective tools for limiting the emergence of drug resistance in pathogens. Despite the widespread adoption of combination therapy across diseases, drug resistance rates continue to rise, leading to failing treatment regimens. The mechanisms underlying treatment failure are well studied, but the processes governing successful combination therapy are poorly understood. We address this question by studying the population dynamics of Mycobacterium tuberculosis within tuberculosis patients undergoing treatment with different combinations of antibiotics. Results: By combining very deep whole genome sequencing (similar to 1000-fold genome-wide coverage) with sequential sputum sampling, we were able to detect transient genetic diversity driven by the apparently continuous turnover of minor alleles, which could serve as the source of drug-resistant bacteria. However, we report that treatment efficacy has a clear impact on the population dynamics: sufficient drug pressure bears a clear signature of purifying selection leading to apparent genetic stability. In contrast, M. tuberculosis populations subject to less drug pressure show markedly different dynamics, including cases of acquisition of additional drug resistance. Conclusions: Our findings show that for a pathogen like M. tuberculosis, which is well adapted to the human host, purifying selection constrains the evolutionary trajectory to resistance in effectively treated individuals. Nonetheless, we also report a continuous turnover of minor variants, which could give rise to the emergence of drug resistance in cases of drug pressure weakening. Monitoring bacterial population dynamics could therefore provide an informative metric for assessing the efficacy of novel drug combinations. </td> <td style="text-align:left;"> 4397 </td> <td style="text-align:left;"> NA </td> <td style="text-align:left;"> NA </td> <td style="text-align:left;"> NA </td> <td style="text-align:left;"> no </td> <td style="text-align:left;"> no </td> </tr> <tr> <td style="text-align:left;"> 4396 </td> <td style="text-align:left;"> 2017 </td> <td style="text-align:left;"> NA </td> <td style="text-align:left;"> Wilson, DJ; Gabriel, E; Leatherbarrow, AJH; Cheesbrough, J; Gee, S; Bolton, E; Fox, A; Fearnhead, P; Hart, CA; Diggle, PJ </td> <td style="text-align:left;"> 2008 </td> <td style="text-align:left;"> Tracing the Source of Campylobacteriosis </td> <td style="text-align:left;"> Campylobacter jejuni is the leading cause of bacterial gastro-enteritis in the developed world. It is thought to infect 2 3 million people a year in the US alone, at a cost to the economy in excess of US $ 4 billion. C. jejuni is a widespread zoonotic pathogen that is carried by animals farmed for meat and poultry. A connection with contaminated food is recognized, but C. jejuni is also commonly found in wild animals and water sources. Phylogenetic studies have suggested that genotypes pathogenic to humans bear greatest resemblance to non-livestock isolates. Moreover, seasonal variation in campylobacteriosis bears the hallmarks of water-borne disease, and certain outbreaks have been attributed to contamination of drinking water. As a result, the relative importance of these reservoirs to human disease is controversial. We use multilocus sequence typing to genotype 1,231 cases of C. jejuni isolated from patients in Lancashire, England. By modeling the DNA sequence evolution and zoonotic transmission of C. jejuni between host species and the environment, we assign human cases probabilistically to source populations. Our novel population genetics approach reveals that the vast majority (97%) of sporadic disease can be attributed to animals farmed for meat and poultry. Chicken and cattle are the principal sources of C. jejuni pathogenic to humans, whereas wild animal and environmental sources are responsible for just 3% of disease. Our results imply that the primary transmission route is through the food chain, and suggest that incidence could be dramatically reduced by enhanced on-farm biosecurity or preventing food-borne transmission. </td> <td style="text-align:left;"> 4398 </td> <td style="text-align:left;"> NA </td> <td style="text-align:left;"> NA </td> <td style="text-align:left;"> NA </td> <td style="text-align:left;"> no </td> <td style="text-align:left;"> no </td> </tr> <tr> <td style="text-align:left;"> 4397 </td> <td style="text-align:left;"> 2017 </td> <td style="text-align:left;"> NA </td> <td style="text-align:left;"> Torres, AU; Chu, A; Read, R; MacDonald, J; Gregson, D; Louie, T; Delongchamp, J; Ward, L; McClure, J; Zhang, KY; Conly, J </td> <td style="text-align:left;"> 2017 </td> <td style="text-align:left;"> The epidemiology of Staphylococcus aureus carriage in patients attending inner city sexually transmitted infections and community clinics in Calgary, Canada </td> <td style="text-align:left;"> Background Although the nares represent the most common carriage site for traditional hospital-associated strains of Staphylococcus aureus (SA), the predominant site of carriage of SA in the community is less certain. Methods We conducted a cross-sectional study in 285 patients attending sexually transmitted diseases and inner-city clinics to evaluate the prevalence, body site colonisation and risk factors associated with carriage of methicillin susceptible SA (MSSA). All isolates were characterized by pulsed field gel electrophoresis, staphylococcal cassette chromosome mec, staphylococcal protein A and multilocus sequence typing. Results The prevalence of colonisation with SA was 57.5% (164/285); 162 (56.8%) participants were colonized with MSSA, and 4 (1.4%) with methicillin-resistant SA (MRSA), 2 of them were co-colonised with both MRSA and MSSA. The most common sites of colonisation were the throat (73.1%), nares (65.2%) and interdigital web spaces of the hand (21.3%). Three out of 4 MRSA isolates were USA300-MRSA strains. Twelve MSSA isolates were closely related to the USA300 CA-MRSA. We identified sexual behaviours such as having more than 6 heterosexual sexual partners in the last 6 months and trimming pubic hair to be independently associated with MSSA colonisation, and more specifically practicing oral sex as a risk factor for throat colonisation. Conclusion There is a high prevalence of MSSA carriage in this population, with a low prevalence of MRSA. The throat was the most common site of carriage and sexual behaviours were found to be risk factors for MSSA colonisation. Close strain relatedness of MSSA and USA300-MRSA isolates suggests either gain or loss of the SCCmec element, respectively. </td> <td style="text-align:left;"> 4399 </td> <td style="text-align:left;"> NA </td> <td style="text-align:left;"> NA </td> <td style="text-align:left;"> NA </td> <td style="text-align:left;"> no </td> <td style="text-align:left;"> no </td> </tr> <tr> <td style="text-align:left;"> 4398 </td> <td style="text-align:left;"> 2017 </td> <td style="text-align:left;"> NA </td> <td style="text-align:left;"> Getachew, S; To, S; Trimarsanto, H; Thriemer, K; Clark, TG; Petros, B; Aseffa, A; Price, RN; Auburn, S </td> <td style="text-align:left;"> 2015 </td> <td style="text-align:left;"> Variation in Complexity of Infection and Transmission Stability between Neighbouring Populations of Plasmodium vivax in Southern Ethiopia </td> <td style="text-align:left;"> Background P. vivax is an important public health burden in Ethiopia, accounting for almost half of all malaria cases. Owing to heterogeneous transmission across the country, a stronger evidence base on local transmission dynamics is needed to optimise allocation of resources and improve malaria interventions. Methodology and Principal Findings In a pilot evaluation of local level P. vivax molecular surveillance in southern Ethiopia, the diversity and population structure of isolates collected between May and November 2013 were investigated. Blood samples were collected from microscopy positive P. vivax patients recruited to clinical and cross-sectional surveys from four sites: Arbaminch, Halaba, Badawacho and Hawassa. Parasite genotyping was undertaken at nine tandem repeat markers. Eight loci were successfully genotyped in 197 samples (between 36 and 59 per site). Heterogeneity was observed in parasite diversity and structure amongst the sites. Badawacho displayed evidence of unstable transmission, with clusters of identical clonal infections. Linkage disequilibrium in Badawacho was higher (I-AS = 0.32, P = 0.010) than in the other populations (I-AS range = 0.01-0.02) and declined markedly after adjusting for identical infections (I-AS = 0.06, P = 0.010). Other than Badawacho (H-E = 0.70), population diversity was equivalently high across the sites (H-E = 0.83). Polyclonal infections were more frequent in Hawassa (67%) than the other populations (range: 8-44%). Despite the variable diversity, differentiation between the sites was low (FST range: 5 x 10(-3)-0.03). Conclusions Marked variation in parasite population structure likely reflects differing local transmission dynamics. Parasite genotyping in these heterogeneous settings has potential to provide important complementary information with which to optimise malaria control interventions. </td> <td style="text-align:left;"> 4400 </td> <td style="text-align:left;"> NA </td> <td style="text-align:left;"> NA </td> <td style="text-align:left;"> NA </td> <td style="text-align:left;"> no </td> <td style="text-align:left;"> no </td> </tr> <tr> <td style="text-align:left;"> 4399 </td> <td style="text-align:left;"> 2017 </td> <td style="text-align:left;"> NA </td> <td style="text-align:left;"> Santos, CL; Nebenzahl-Guimaraes, H; Mendes, MV; van Soolingen, D; Correia-Neves, M </td> <td style="text-align:left;"> 2015 </td> <td style="text-align:left;"> To Be or Not to Be a Pseudogene: A Molecular Epidemiological Approach to the mclx Genes and Its Impact in Tuberculosis </td> <td style="text-align:left;"> Tuberculosis presents a myriad of symptoms, progression routes and propagation patterns not yet fully understood. Whereas for a long time research has focused solely on the patient immunity and overall susceptibility, it is nowadays widely accepted that the genetic diversity of its causative agent, Mycobacterium tuberculosis, plays a key role in this dynamic. This study focuses on a particular family of genes, the mclxs (Mycobacterium cyclase/LuxR-like genes), which codify for a particular and nearly mycobacterial-exclusive combination of protein domains. mclxs genes were found to be pseudogenized by frameshift-causing insertion (s)/deletion(s) in a considerable number of M. tuberculosis complex strains and clinical isolates. To discern the functional implications of the pseudogenization, we have analysed the pattern of frameshift-causing mutations in a group of M. tuberculosis isolates while taking into account their microbial-, patient- and disease-related traits. Our logistic regression-based analyses have revealed disparate effects associated with the transcriptional inactivation of two mclx genes. In fact, mclx2 (Rv1358) pseudogenization appears to be primarily driven by the microbial phylogenetic background, being mainly related to the Euro-American (EAm) lineage; on the other hand, mclx3 (Rv2488c) presents a higher tendency for pseudogenization among isolates from patients born on the Western Pacific area, and from isolates causing extra-pulmonary infections. These results contribute to the overall knowledge on the biology of M. tuberculosis infection, whereas at the same time launch the necessary basis for the functional assessment of these so far overlooked genes. </td> <td style="text-align:left;"> 4401 </td> <td style="text-align:left;"> NA </td> <td style="text-align:left;"> NA </td> <td style="text-align:left;"> NA </td> <td style="text-align:left;"> no </td> <td style="text-align:left;"> no </td> </tr> <tr> <td style="text-align:left;"> 4400 </td> <td style="text-align:left;"> 2017 </td> <td style="text-align:left;"> NA </td> <td style="text-align:left;"> Eppinger, M; Mammel, MK; Leclerc, JE; Ravel, J; Cebula, TA </td> <td style="text-align:left;"> 2011 </td> <td style="text-align:left;"> Genomic anatomy of Escherichia coli O157:H7 outbreaks </td> <td style="text-align:left;"> The rapid emergence of Escherichia coli O157:H7 from an unknown strain in 1982 to the dominant hemorrhagic E. coli serotype in the United States and the cause of widespread outbreaks of human food-borne illness highlights a need to evaluate critically the extent to which genomic plasticity of this important enteric pathogen contributes to its pathogenic potential and its evolution as well as its adaptation in different ecological niches. Aimed at a better understanding of the evolution of the E. coli O157:H7 pathogenome, the present study presents the high-quality sequencing and comparative phylogenomic analysis of a comprehensive panel of 25 E. coli O157:H7 strains associated with three nearly simultaneous food-borne outbreaks of human disease in the United States. Here we present a population genetic analysis of more than 200 related strains recovered from patients, contaminated produce, and zoonotic sources. High-resolution phylogenomic approaches allow the dynamics of pathogenome evolution to be followed at a high level of phylogenetic accuracy and resolution. SNP discovery and study of genome architecture and prophage content identified numerous biomarkers to assess the extent of genetic diversity within a set of clinical and environmental strains. A total of 1,225 SNPs were identified in the present study and are now available for typing of the E. coli O157:H7 lineage. These data should prove useful for the development of a refined phylogenomic framework for forensic, diagnostic, and epidemiological studies to define better risk in response to novel and emerging E. coli O157:H7 resistance and virulence phenotypes. </td> <td style="text-align:left;"> 4402 </td> <td style="text-align:left;"> NA </td> <td style="text-align:left;"> NA </td> <td style="text-align:left;"> NA </td> <td style="text-align:left;"> no </td> <td style="text-align:left;"> no </td> </tr> <tr> <td style="text-align:left;"> 4401 </td> <td style="text-align:left;"> 2017 </td> <td style="text-align:left;"> NA </td> <td style="text-align:left;"> Narayanan, H; Sankar, S; Simoes, E; Nandagopal, B; Sridharan, G </td> <td style="text-align:left;"> 2013 </td> <td style="text-align:left;"> Analysis of Sequence Diversity of Human Metapneumovirus Collected from Young Children with Acute Respiratory Tract Infections in South India </td> <td style="text-align:left;"> Human metapneumovirus (hMPV), which has a global distribution, is an important cause of acute respiratory tract infections, especially in children and immunocompromised patients. We investigated the genetic variability of partial nucleoprotein (N) gene sequences of hMPV strains identified among young children in South India. The sequences of the N gene were compared with previously reported sequences available in the GenBank repository. The results showed that strains are localized in a geographically circumscribed area (topotype). The results also demonstrates that viruses from the same genetic lineage can circulate concurrently within a given location during a given season. The close clustering of the majority of our hMPV isolates indicates that the N gene sequences in the virus population are relatively homogeneous, and suggests temporal rather than geographic variations in the evolutionary pattern. In our study, the majority of the strains belonged to genetic lineage B2 (71.1 %), followed by A2b (18.4 %), A2a (7.9 %), and B1 (2.6 %), demonstrating the presence of 4 of the 5 known genotypes of hMPV. Global alignment of the nucleotide sequences showed that the strains are closely related to sequences from Canada, The Netherlands, and Australasia. Differences at the nucleotide level and the amino acid level were identified. The results provide evidence for the diversity of the N gene of hMPV in samples collected from South India compared with global strains. When investigated for selective pressure, the sequences showed 1 positively selected site and 19 negatively selected sites. These data should prove useful in further investigations of the evolutionary dynamics of hMPV infection. </td> <td style="text-align:left;"> 4403 </td> <td style="text-align:left;"> NA </td> <td style="text-align:left;"> NA </td> <td style="text-align:left;"> NA </td> <td style="text-align:left;"> no </td> <td style="text-align:left;"> no </td> </tr> <tr> <td style="text-align:left;"> 4402 </td> <td style="text-align:left;"> 2017 </td> <td style="text-align:left;"> NA </td> <td style="text-align:left;"> Diskin, SJ; Hou, CP; Glessner, JT; Attiyeh, EF; Laudenslager, M; Bosse, K; Cole, K; Mosse, YP; Wood, A; Lynch, JE; Pecor, K; Diamond, M; Winter, C; Wang, K; Kim, C; Geiger, EA; McGrady, PW; Blakemore, AIF; London, WB; Shaikh, TH; Bradfield, J; Grant, SFA; Li, HZ; Devoto, M; Rappaport, ER; Hakonarson, H; Maris, JM </td> <td style="text-align:left;"> 2009 </td> <td style="text-align:left;"> Copy number variation at 1q21.1 associated with neuroblastoma </td> <td style="text-align:left;"> Common copy number variations (CNVs) represent a significant source of genetic diversity, yet their influence on phenotypic variability, including disease susceptibility, remains poorly understood. To address this problem in human cancer, we performed a genome-wide association study of CNVs in the childhood cancer neuroblastoma, a disease in which single nucleotide polymorphism variations are known to influence susceptibility(1,2). We first genotyped 846 Caucasian neuroblastoma patients and 803 healthy Caucasian controls at similar to 550,000 single nucleotide polymorphisms, and performed a CNV-based test for association. We then replicated significant observations in two independent sample sets comprised of a total of 595 cases and 3,357 controls. Here we describe the identification of a common CNV at chromosome 1q21.1 associated with neuroblastoma in the discovery set, which was confirmed in both replication sets. This CNV was validated by quantitative polymerase chain reaction, fluorescent in situ hybridization and analysis of matched tumour specimens, and was shown to be heritable in an independent set of 713 cancer-free parent offspring trios. We identified a previously unknown transcript within the CNV that showed high sequence similarity to several neuroblastoma breakpoint family (NBPF) genes(3,4) and represents a new member of this gene family (NBPF23). This transcript was preferentially expressed in fetal brain and fetal sympathetic nervous tissues, and the expression level was strictly correlated with CNV state in neuroblastoma cells. These data demonstrate that inherited copy number variation at 1q21.1 is associated with neuroblastoma and implicate a previously unknown neuroblastoma breakpoint family gene in early tumorigenesis of this childhood cancer. </td> <td style="text-align:left;"> 4404 </td> <td style="text-align:left;"> NA </td> <td style="text-align:left;"> NA </td> <td style="text-align:left;"> NA </td> <td style="text-align:left;"> no </td> <td style="text-align:left;"> no </td> </tr> <tr> <td style="text-align:left;"> 4403 </td> <td style="text-align:left;"> 2017 </td> <td style="text-align:left;"> NA </td> <td style="text-align:left;"> Kalnina, I; Kapa, I; Pirags, V; Ignatovica, V; Schioth, HB; Klovins, J </td> <td style="text-align:left;"> 2009 </td> <td style="text-align:left;"> Association between a rare SNP in the second intron of human Agouti related protein gene and increased BMI </td> <td style="text-align:left;"> Background: The agouti related protein (AGRP) is an endogenous antagonist of the melanocortin 4 receptor and is one of the most potent orexigenic factors. The aim of the present study was to assess the genetic variability of AGRP gene and investigate whether the previously reported SNP rs5030980 and the rs11575892, a SNP that so far has not been studied with respect to obesity is associated with increased body mass index (BMI). Methods: We determined the complete sequence of the AGRP gene and upstream promoter region in 95 patients with severe obesity (BMI > 35 kg/m(2)). Three polymorphisms were identified: silent mutation c. 123G> A (rs34123523) in the second exon, non-synonymous mutation c. 199G> A (rs5030980) and c. 131-42C> T (rs11575892) located in the second intron. We further screened rs11575892 in a selected group of 1135 and rs5030980 in group of 789 participants from the Genome Database of Latvian Population and Latvian State Research Program Database. Results: The CT heterozygotes of rs11575892 had significantly higher mean BMI value (p = 0.027). After adjustment for age, gender and other significant non-genetic factors (presence of diseases), the BMI levels remained significantly higher in carriers of the rs11575892 T allele (p = 0.001). The adjusted mean BMI value of CC genotype was 27.92 +/- 1.01 kg/m(2) (mean, SE) as compared to 30.97 +/- 1.03 kg/m(2) for the CT genotype. No association was found between rs5030980 and BMI. Conclusion: This study presents an association of rare allele of AGRP polymorphism in heterozygous state with increased BMI. The possible functional effects of this polymorphism are unclear but may relate to splicing defects. </td> <td style="text-align:left;"> 4405 </td> <td style="text-align:left;"> NA </td> <td style="text-align:left;"> NA </td> <td style="text-align:left;"> NA </td> <td style="text-align:left;"> no </td> <td style="text-align:left;"> no </td> </tr> <tr> <td style="text-align:left;"> 4404 </td> <td style="text-align:left;"> 2017 </td> <td style="text-align:left;"> NA </td> <td style="text-align:left;"> Griffing, SM; Mixson-Hayden, T; Sridaran, S; Alam, MT; McCollum, AM; Cabezas, C; Quezada, WM; Barnwell, JW; De Oliveira, AM; Lucas, C; Arrospide, N; Escalante, AA; Bacon, DJ; Udhayakumar, V </td> <td style="text-align:left;"> 2011 </td> <td style="text-align:left;"> South American Plasmodium falciparum after the Malaria Eradication Era: Clonal Population Expansion and Survival of the Fittest Hybrids </td> <td style="text-align:left;"> Malaria has reemerged in many regions where once it was nearly eliminated. Yet the source of these parasites, the process of repopulation, their population structure, and dynamics are ill defined. Peru was one of malaria eradication's successes, where Plasmodium falciparum was nearly eliminated for two decades. It reemerged in the 1990s. In the new era of malaria elimination, Peruvian P. falciparum is a model of malaria reinvasion. We investigated its population structure and drug resistance profiles. We hypothesized that only populations adapted to local ecological niches could expand and repopulate and originated as vestigial populations or recent introductions. We investigated the genetic structure (using microsatellites) and drug resistant genotypes of 220 parasites collected from patients immediately after peak epidemic expansion (19992000) from seven sites across the country. The majority of parasites could be grouped into five clonal lineages by networks and AMOVA. The distribution of clonal lineages and their drug sensitivity profiles suggested geographic structure. In 2001, artesunate combination therapy was introduced in Peru. We tested 62 parasites collected in 2006-2007 for changes in genetic structure. Clonal lineages had recombined under selection for the fittest parasites. Our findings illustrate that local adaptations in the post-eradication era have contributed to clonal lineage expansion. Within the shifting confluence of drug policy and malaria incidence, populations continue to evolve through genetic outcrossing influenced by antimalarial selection pressure. Understanding the population substructure of P. falciparum has implications for vaccine, drug, and epidemiologic studies, including monitoring malaria during and after the elimination phase. </td> <td style="text-align:left;"> 4406 </td> <td style="text-align:left;"> NA </td> <td style="text-align:left;"> NA </td> <td style="text-align:left;"> NA </td> <td style="text-align:left;"> no </td> <td style="text-align:left;"> no </td> </tr> <tr> <td style="text-align:left;"> 4405 </td> <td style="text-align:left;"> 2017 </td> <td style="text-align:left;"> NA </td> <td style="text-align:left;"> Kaul, S; Kour, H; Pandita, D; Kour, S; Dhar, MK </td> <td style="text-align:left;"> 2013 </td> <td style="text-align:left;"> Polymerase Chain Reaction: Restriction Fragment Length Polymorphism Differentiates the Environmental and Clinically Important Fungal Isolates </td> <td style="text-align:left;"> Soil rich in keratinous material represents the main reservoir of keratinophilic fungi. Majority of soil saprophytes are known to cause fungal disease (mycoses) in human beings and animals. Considering the importance of soil saprophytes as potential human pathogens, this study aimed at assessing their presence in various soil samples collected from different locations of Jammu, India. Soil samples were screened for the isolation of keratinophilic fungi by using hair bait technique. A total of 16 fungal isolates were identified on the basis of macro and microscopic characteristics. Out of these, 12 fungal isolates were obtained from soil and four were clinical isolates. PCR-RFLP approach was used for detecting genetic relatedness between the environmental and clinical isolates. ITS (ITS1-5.8S-ITS2) region of 16 fungal isolate was amplified with primer pair ITS1 and ITS4. Gel electrophoresis after PCR of ITS region showed that DNA from all the fungal isolates yielded single fragment ranging from 550 to 650 bp. This was followed by Seminested PCR to detect variations in ITS2 region using primer pair ITS86 and ITS4. The PCR product was subjected to restriction digestion with ten restriction enzymes out of which four enzymes MboI, MseI, MvaI, HpaII showed best results. Restriction fragments obtained were scored for the presence and absence of bands. Dendrogram was generated which divided the fungal isolates into four main groups. PCR-RFLP made possible the detection of interspecies and intrastrainal variations in keratinophilic fungi isolated from soil and clinically relevant isolates from patients with opportunistic infections. </td> <td style="text-align:left;"> 4407 </td> <td style="text-align:left;"> NA </td> <td style="text-align:left;"> NA </td> <td style="text-align:left;"> NA </td> <td style="text-align:left;"> no </td> <td style="text-align:left;"> no </td> </tr> <tr> <td style="text-align:left;"> 4406 </td> <td style="text-align:left;"> 2017 </td> <td style="text-align:left;"> NA </td> <td style="text-align:left;"> Wetmore, JB; Johansen, KL; Sen, S; Hung, AM; Lovett, DH </td> <td style="text-align:left;"> 2006 </td> <td style="text-align:left;"> An angiotensin converting enzyme haplotype predicts survival in patients with end stage renal disease </td> <td style="text-align:left;"> The renin-angiotensin system is implicated in the development of a variety of human diseases. Many studies have sought to characterize the clinical implications of polymorphisms in the angiotensin converting enzyme (ACE) gene. Given the high mortality rate of individuals on chronic hemodialysis (HD), we sought to investigate whether genetic diversity in the ACE gene correlates with mortality in this population. We assembled a racially diverse cohort of prevalent individuals on chronic outpatient HD, and followed it prospectively for a mean of 2.1 years. Subjects were genotyped for seven single nucleotide polymorphisms (SNPs) in the ACE gene. Haplotype probabilities were calculated using an expectation-maximization algorithm. Cox proportional hazards regression was used to determine associations between haplotype and time to mortality from initiation of HD. There was strong linkage disequilibrium (LD) across the ACE gene, with three tagging SNPs found to account for all seven-SNP haplotypes that had a frequency of greater than 4%. After adjustment for age, race, gender, and diabetes status, a three-locus haplotype was associated with a 72% risk reduction in mortality (P = 0.004). The majority of this association was captured by the TT genotype of A-239T promoter polymorphism. The TGG (non-wild-type) haplotype, consisting of three tagging SNPs in the ACE gene, is associated with significantly decreased risk of all-cause mortality in HD patients independent of age, race, gender, and diabetic status. This "protective" haplotype may encompass loci with functional significance in the ACE gene. </td> <td style="text-align:left;"> 4408 </td> <td style="text-align:left;"> NA </td> <td style="text-align:left;"> NA </td> <td style="text-align:left;"> NA </td> <td style="text-align:left;"> no </td> <td style="text-align:left;"> no </td> </tr> <tr> <td style="text-align:left;"> 4407 </td> <td style="text-align:left;"> 2017 </td> <td style="text-align:left;"> NA </td> <td style="text-align:left;"> Farci, P; Quinti, I; Farci, S; Alter, HJ; Strazzera, R; Palomba, E; Coiana, A; Cao, D; Casadei, AM; Ledda, R; Iorio, R; Vegnente, A; Diaz, G; Tovo, PA </td> <td style="text-align:left;"> 2006 </td> <td style="text-align:left;"> Evolution of hepatitis C viral quasispecies and hepatic injury in perinatally infected children followed prospectively </td> <td style="text-align:left;"> Perinatal infection with hepatitis C virus (HCV) is characterized by a wide range of alanine aminotransferase (ALT) levels. The mechanisms responsible for this variability are unknown. We examined whether the evolution of the HCV quasispecies was associated with different ALT profiles in perinatally infected children. Sequences within HCV envelope 1 and 2 genes, inclusive of the hypervariable region 1, the viral load, and the nascent humoral immunity were analyzed in serial serum samples from 12 perinatally infected children prospectively followed for a median of 53 months. These patients were selected to represent two different ALT patterns during the first year of life: 6 had high levels (maximum values ranging from 4.2 to 30 times the normal upper limit), and 6 had normal or slightly elevated levels (< 2 times the normal upper limit). Two patterns of viral evolution were identified according to the ALT profiles. Biochemical evidence of hepatic injury was invariably associated with a mono- or oligoclonal viral population, whereas mild or no liver damage correlated with the early emergence of a heterogeneous viral quasispecies. Consistent with selective immune pressure, amino acid changes occurred almost exclusively within the hypervariable region 1 and were temporally associated with antibody seroconversion; at this time, the difference in genetic diversity between the two groups was highly significant (P = 0.002). The two patterns of viral evolution persisted over time and did not correlate with viral load or genotype. Our study demonstrates that, in perinatally infected children, the evolution of HCV quasispecies correlates with hepatic injury. </td> <td style="text-align:left;"> 4409 </td> <td style="text-align:left;"> NA </td> <td style="text-align:left;"> NA </td> <td style="text-align:left;"> NA </td> <td style="text-align:left;"> no </td> <td style="text-align:left;"> no </td> </tr> <tr> <td style="text-align:left;"> 4408 </td> <td style="text-align:left;"> 2017 </td> <td style="text-align:left;"> NA </td> <td style="text-align:left;"> Batista, PP; Santos, JF; Oliveira, NT; Pires, APD; Motta, CMS; Lima, EALA </td> <td style="text-align:left;"> 2008 </td> <td style="text-align:left;"> Genetic characterization of Brazilian strains of Aspergillus flavus using DNA markers </td> <td style="text-align:left;"> The Aspergillus genus belongs to a filamentous fungal group characterized by wide dispersion in the environment. Some species are associated with diseases, especially in immunocompromised patients, while others are of economical importance due to aflatoxin production or biotechnological applications. Its species identification is nowadays performed by traditional techniques combined with molecular markers, resulting in a higher efficiency of isolate characterization. In the present study, internal transcribed spacer, inter-simple sequence repeats (ISSR), and random amplified polymorphic DNA ( RAPD) molecular markers were used, with the aim of genetically characterizing strains of Aspergillus flavus and strains of other species of the A. flavus group. High genetic diversity was revealed by RAPD and by ISSR, in which the use of the (GACA) 4 primer yielded a higher diversity than with the (GTG) 5 primer, although the latter showed a characteristic banding profile for each species. These data were used to create a similarity matrix for the construction of dendrograms by means of the UPGMA method. The ISSR and RAPD profiles showed that among the strains previously identificated as A. flavus, one should be A. oryzae, one A. parasiticus and two A. tamarii. On the other hand, a strain previously identified as A. parasiticus should be A. flavus. All these strains were retested by traditional methods and their new species identification was confirmed. These results strongly support the need for using molecular markers as an auxiliary tool in differentiating fungal species and strains. </td> <td style="text-align:left;"> 4410 </td> <td style="text-align:left;"> NA </td> <td style="text-align:left;"> NA </td> <td style="text-align:left;"> NA </td> <td style="text-align:left;"> no </td> <td style="text-align:left;"> no </td> </tr> <tr> <td style="text-align:left;"> 4409 </td> <td style="text-align:left;"> 2017 </td> <td style="text-align:left;"> NA </td> <td style="text-align:left;"> Wolf, DM; Vazirani, VV; Arkin, AP </td> <td style="text-align:left;"> 2005 </td> <td style="text-align:left;"> Diversity in times of adversity: probabilistic strategies in microbial survival games </td> <td style="text-align:left;"> Population diversification strategies are ubiquitous among microbes, encompassing random phase-variation (RP V) of pathogenic bacteria, viral latency as observed in some bacteriophage and HIV, and the non-genetic diversity of bacterial stress responses. Precise conditions under which these diversification strategies confer an advantage have not been well defined. We develop a model of population growth conditioned on dynamical environmental and cellular states. Transitions among cellular states, in turn, may be biased by possibly noisy readings of the environment from cellular sensors. For various types of environmental dynamics and cellular sensor capability, we apply game-theoretic analysis to derive the evolutionarily stable strategy (ESS) for an organism and determine when that strategy is diversification. We find that: (1) RPV, effecting a sort of Parrondo paradox wherein random alternations between losing strategies produce a winning strategy, is selected when transitions between different selective environments cannot be sensed, (2) optimal RP V cell switching rates are a function of environmental lifecycle asymmetries and environmental autocorrelation, (3) probabilistic diversification upon entering a new environment is selected when sensors can detect environmental transitions but have poor precision in identifying new environments, and (4) in the presence of excess additive noise, low-pass filtering is required for evolutionary stability. We show that even when RP V is not the ESS, it may minimize growth rate variance and the risk of extinction due to 'unlucky' environmental dynamics. (c) 2005 Elsevier Ltd. All rights reserved. </td> <td style="text-align:left;"> 4411 </td> <td style="text-align:left;"> NA </td> <td style="text-align:left;"> NA </td> <td style="text-align:left;"> NA </td> <td style="text-align:left;"> no </td> <td style="text-align:left;"> no </td> </tr> <tr> <td style="text-align:left;"> 4410 </td> <td style="text-align:left;"> 2017 </td> <td style="text-align:left;"> NA </td> <td style="text-align:left;"> D'Amato, M; Zucchelli, M; Seddighzadeh, M; Anedda, F; Lindblad, S; Kere, J; Alfredsson, L; Klareskog, L; Padyukov, L </td> <td style="text-align:left;"> 2010 </td> <td style="text-align:left;"> Analysis of Neuropeptide S Receptor Gene (NPSR1) Polymorphism in Rheumatoid Arthritis </td> <td style="text-align:left;"> Background: Polymorphism in the neuropeptide S receptor gene NPSR1 is associated with asthma and inflammatory bowel disease. NPSR1 is expressed in the brain, where it modulates anxiety and responses to stress, but also in other tissues and cell types including lymphocytes, the lungs, and the intestine, where it appears to be up-regulated in inflammation. We sought to determine whether genetic variability at the NPSR1 locus influences the susceptibility and clinical manifestation of rheumatoid arthritis (RA). Methodology/Principal Findings: From the Epidemiological Investigation of Rheumatoid Arthritis (EIRA) case-control study, 1,888 rheumatoid arthritis patients and 888 controls were genotyped for 19 single-nucleotide polymorphisms (SNPs) spanning the entire NPSR1 gene and 220 KB of DNA on chromosome 7p14. The association between individual genetic markers and their haplotypic combinations, respectively, and diagnosis of RA, presence of autoantibodies to citrullinated proteins (ACPA), and disease activity score based on 28 joints (DAS28) was tested. There was no association between diagnosis of RA and NPSR1 variants. However, several associations of nominal significance were detected concerning susceptibility to ACPA-negative RA and disease activity measures (DAS28). Among these, the association of SNP rs324987 with ACPA-negative RA [(p = 0.004, OR = 0.674 (95% CI 0.512-0.888)] and that of SNP rs10263447 with DAS28 [p = 0.0002, OR = 0.380 (95% CI 0.227-0.635)] remained significant after correction for multiple comparisons. Conclusions/Significance: NPSR1 polymorphism may be relevant to RA susceptibility and its clinical manifestation. Specific alleles at the NPSR1 locus may represent common risk factors for chronic inflammatory diseases, including RA. </td> <td style="text-align:left;"> 4412 </td> <td style="text-align:left;"> NA </td> <td style="text-align:left;"> NA </td> <td style="text-align:left;"> NA </td> <td style="text-align:left;"> no </td> <td style="text-align:left;"> no </td> </tr> <tr> <td style="text-align:left;"> 4411 </td> <td style="text-align:left;"> 2017 </td> <td style="text-align:left;"> NA </td> <td style="text-align:left;"> Fode, P; Larsen, AR; Feenstra, B; Jespersgaard, C; Skov, RL; Stegger, M; Fowler, VG; Andersen, PS </td> <td style="text-align:left;"> 2012 </td> <td style="text-align:left;"> Genetic Variability in Beta-Defensins Is Not Associated with Susceptibility to Staphylococcus aureus Bacteremia </td> <td style="text-align:left;"> Introduction: Human beta-defensins are key components of human innate immunity to a variety of pathogens, including Staphylococcus aureus. The aim of the present study was to investigate a potential association between gene variations in DEFB1 and DEFB103/DEFB4 and the development of S. aureus bacteremia (SAB) employing a case-control design. Methods: Cases were unique patients with documented SAB, identified with the National S. aureus Bacteremia Register, a comprehensive dataset of all episodes of community associated-SABs (CA-SAB) occurring in children (<= 20 yrs) in Denmark from 1990 to 2006. Controls were age-matched healthy individuals with no history of SAB. DNA obtained from cases and controls using the Danish Newborn Screening Biobank were genotyped for functional polymorphisms of DEFB1 by Sanger sequencing and copy number variation of the DEFB103 and DEFB4 genes using Pyrosequencing-based Paralogue Ratio Test (P-PRT). Results: 193 ethnic Danish SAB cases with 382 age-matched controls were used for this study. S. aureus isolates represented a variety of bacterial (i.e., different spa types) types similar to SAB isolates in general. DEFB1 minor allele frequencies of rs11362 (cases vs. controls 0.47/0.44), rs1800972 (0.21/0.24), and rs1799946 (0.32/0.33) were not significantly different in cases compared with controls. Also, DEFB4/DEFB103 gene copy numbers (means 4.83/4.92) were not significantly different in cases compared with controls. Conclusions: Using a large, unique cohort of pediatric CA-SAB, we found no significant association between DEFB1 genetic variation or DEFB4/DEFB103 gene copy number and susceptibility for SAB. </td> <td style="text-align:left;"> 4413 </td> <td style="text-align:left;"> NA </td> <td style="text-align:left;"> NA </td> <td style="text-align:left;"> NA </td> <td style="text-align:left;"> no </td> <td style="text-align:left;"> no </td> </tr> <tr> <td style="text-align:left;"> 4412 </td> <td style="text-align:left;"> 2017 </td> <td style="text-align:left;"> NA </td> <td style="text-align:left;"> Akparova, A; Abdrakhmanova, B; Nilanjana, BB; Bersimbaev, R </td> <td style="text-align:left;"> 2017 </td> <td style="text-align:left;"> EPHX1 Y113H polymorphism is associated with increased risk of chronic obstructive pulmonary disease in Kazakhstan population </td> <td style="text-align:left;"> Chronic obstructive pulmonary disease (COPD) is a type of obstructive lung disease characterized by long term poor airflow which worsens over time. It is considered to be one of the top five chronic diseases of the world in terms of morbidity and mortality. Genetic variability has been found to contribute to the development of COPD. Although association between gene polymorphisms in EPHX1 and TNF-alpha genes and chronic obstructive pulmonary disease (COPD) have been found but till date no genetic association studies have been done in the COPD affected Kazakhstan population. The aim of the present work was to investigate the association between the Y113H polymorphism (rs1051740) in EPHX1 gene and -308G/A polymorphism (rsl 800629) in TNF-alpha gene and COPD in Kazakhstan population. A case-control study was conducted in Astana and Akmola regions of Kazakhstan, involving 55 cases with COPD and 52 healthy individuals who served as the controls. The polymorphisms were determined using conventional PCR and Sanger sequencing method. Results show that for the EPHX1 gene Y113H polymorphism, the presence of an "C" allele (TC/CC genotype) was significantly overrepresented in the COPD patients compared to the controls. For the TNF-alpha gene -308G/A polymorphism, no significant difference was found between the two groups. Thus we found that, Y113H polymorphism in EPHX1 gene contributed to increased susceptibility to COPD in the Kazakhstan population. (C) 2017 Elsevier B.V. All rights reserved. </td> <td style="text-align:left;"> 4414 </td> <td style="text-align:left;"> NA </td> <td style="text-align:left;"> NA </td> <td style="text-align:left;"> NA </td> <td style="text-align:left;"> no </td> <td style="text-align:left;"> no </td> </tr> <tr> <td style="text-align:left;"> 4413 </td> <td style="text-align:left;"> 2017 </td> <td style="text-align:left;"> NA </td> <td style="text-align:left;"> Vasconcellos, SEG; Acosta, CC; Gomes, LL; Conceicao, EC; Lima, KV; de Araujo, MI; Leite, MD; Tannure, F; Caldas, PCD; Gomes, HM; Santos, AR; Gomgnimbou, MK; Sola, C; Couvin, D; Rastogi, N; Boechat, N; Suffys, PN </td> <td style="text-align:left;"> 2014 </td> <td style="text-align:left;"> Strain Classification of Mycobacterium tuberculosis Isolates in Brazil Based on Genotypes Obtained by Spoligotyping, Mycobacterial Interspersed Repetitive Unit Typing and the Presence of Large Sequence and Single Nucleotide Polymorphism </td> <td style="text-align:left;"> Rio de Janeiro is endemic for tuberculosis (TB) and presents the second largest prevalence of the disease in Brazil. Here, we present the bacterial population structure of 218 isolates of Mycobacterium tuberculosis, derived from 186 patients that were diagnosed between January 2008 and December 2009. Genotypes were generated by means of spoligotyping, 24 MIRU-VNTR typing and presence of fbpC(103), RDRio and RD174. The results confirmed earlier data that predominant genotypes in Rio de Janeiro are those of the Euro American Lineages (99%). However, we observed differences between the classification by spoligotyping when comparing to that of 24 MIRU-VNTR typing, being respectively 43.6% vs. 62.4% of LAM, 34.9% vs. 9.6% of T and 18.3% vs. 21.5% of Haarlem. Among isolates classified as LAM by MIRU typing, 28.0% did not present the characteristic spoligotype profile with absence of spacers 21 to 24 and 32 to 36 and we designated these conveniently as "LAM-like", 79.3% of these presenting the LAM-specific SNP fbpC(103). The frequency of RDRio and RD174 in the LAM strains, as defined both by spoligotyping and 24 MIRU-VNTR loci, were respectively 11% and 15.4%, demonstrating that RD174 is not always a marker for LAM/RDRio strains. We conclude that, although spoligotyping alone is a tool for classification of strains of the Euro-American lineage, when combined with MIRU-VNTRs, SNPs and RD typing, it leads to a much better understanding of the bacterial population structure and phylogenetic relationships among strains of M. tuberculosis in regions with high incidence of TB. </td> <td style="text-align:left;"> 4415 </td> <td style="text-align:left;"> NA </td> <td style="text-align:left;"> NA </td> <td style="text-align:left;"> NA </td> <td style="text-align:left;"> no </td> <td style="text-align:left;"> no </td> </tr> <tr> <td style="text-align:left;"> 4414 </td> <td style="text-align:left;"> 2017 </td> <td style="text-align:left;"> NA </td> <td style="text-align:left;"> Caramalho, R; Gusmao, L; Lackner, M; Amorim, A; Araujo, R </td> <td style="text-align:left;"> 2013 </td> <td style="text-align:left;"> SNaPAfu: A Novel Single Nucleotide Polymorphism Multiplex Assay for Aspergillus fumigatus Direct Detection, Identification and Genotyping in Clinical Specimens </td> <td style="text-align:left;"> Objective: Early diagnosis of invasive aspergillosis is essential for positive patient outcome. Likewise genotyping of fungal isolates is desirable for outbreak control in clinical setting. We designed a molecular assay that combines detection, identification, and genotyping of Aspergillus fumigatus in a single reaction. Methods: To this aim we combined 20 markers in a multiplex reaction and the results were seen following mini-sequencing readings. Pure culture extracts were firstly tested. Thereafter, Aspergillus-DNA samples obtained from clinical specimens of patients with possible, probable, or proven aspergillosis according to European Organization for the Research and Treatment of Cancer/Mycoses Study Group (EORTC/MSG) criteria. Results: A new set of designed primers allowed multilocus sequence typing (MLST) gene amplification in a single multiplex reaction. The newly proposed SNaPAfu assay had a specificity of 100%, a sensitivity of 89% and detection limit of 1 ITS copy/mL (similar to 0.5 fg genomic Aspergillus-DNA/mL). The marker A49_F was detected in 89% of clinical samples. The SNaPAfu assay was accurately performed on clinical specimens using only 1% of DNA extract (total volume 50 mu L) from 1 mL of used bronchoalveolar lavage. Conclusions: The first highly sensitive and specific, time-and cost-economic multiplex assay was implemented that allows detection, identification, and genotyping of A. fumigatus strains in a single amplification followed by mini-sequencing reaction. The new test is suitable to clinical routine and will improve patient management. </td> <td style="text-align:left;"> 4416 </td> <td style="text-align:left;"> NA </td> <td style="text-align:left;"> NA </td> <td style="text-align:left;"> NA </td> <td style="text-align:left;"> no </td> <td style="text-align:left;"> no </td> </tr> <tr> <td style="text-align:left;"> 4415 </td> <td style="text-align:left;"> 2017 </td> <td style="text-align:left;"> NA </td> <td style="text-align:left;"> Sironi, M; Guerini, FR; Agliardi, C; Biasin, M; Cagliani, R; Fumagalli, M; Caputo, D; Cassinotti, A; Ardizzone, S; Zanzottera, M; Bolognesi, E; Riva, S; Kanari, Y; Miyazawa, M; Clerici, M </td> <td style="text-align:left;"> 2011 </td> <td style="text-align:left;"> An Evolutionary Analysis of RAC2 Identifies Haplotypes Associated with Human Autoimmune Diseases </td> <td style="text-align:left;"> The human RAC2 gene encodes a small GTP-binding protein with a pivotal role in immune activation and in the induction of peripheral immune tolerance through restimulation-induced cell death (RICD). Different human pathogens target the protein product of RAC2, suggesting that the gene may be subject to natural selection, and that variants in RAC2 may affect immunological phenotypes in humans. We scanned the genomic region encompassing the entire transcription unit for the presence of putative noncoding regulatory elements conserved across mammals. This information was used to select two RAC2 gene regions and analyze their intraspecific genetic diversity. Results suggest that a region covering the 3' untranslated region has been a target of multiallelic balancing selection (or diversifying selection), and three major RAC2 haplogroups occur in human populations. Haplotypes belonging to one of these clades are associated with increased susceptibility to multiple sclerosis (P = 0.022) and earlier onset of disease symptoms (P = 0.025). This same haplogroup is significantly more common in patients with Crohn's disease compared with healthy controls (P = 0.048). These data reinforce recent evidences that susceptibility alleles/haplotypes are shared among multiple autoimmune disorders and support a causal "role for RAC2" variants in the pathogenesis of autoimmune diseases. Other genes with a role in RICD have previously been associated with autoimmunity in humans, suggesting that this pathway and RAC2 may represent novel therapeutic targets in autoimmune disorders. </td> <td style="text-align:left;"> 4417 </td> <td style="text-align:left;"> NA </td> <td style="text-align:left;"> NA </td> <td style="text-align:left;"> NA </td> <td style="text-align:left;"> no </td> <td style="text-align:left;"> no </td> </tr> <tr> <td style="text-align:left;"> 4416 </td> <td style="text-align:left;"> 2017 </td> <td style="text-align:left;"> NA </td> <td style="text-align:left;"> Crawford, MH; Banerjee, R; Demarchi, DA; Zlojutro, M; McComb, J; Livshits, J; Henneberg, M; Mosher, MJ; Schanfield, MS; Knowles, JA </td> <td style="text-align:left;"> 2005 </td> <td style="text-align:left;"> Applications of pooled DNA samples to the assessment of population affinities: Short tandem repeats </td> <td style="text-align:left;"> Pooled DNA samples have been used in association studies of Mendelian disease genes. This method involves combining equal quantities of DNA from patients and control subjects into separate pools and comparing the pools for distributions of genetic markers. In this study identical quantities of DNA from 300 individuals representing 6 populations were pooled and amplified for 296 loci using the touchdown polymerase chain reaction (PCR) method. The purpose of this study is to test the efficacy of pooled DNA markers in the reconstruction of the genetic structure of human populations. The populations sampled included Chuvash, Buryats, Kizhi, Native Americans, South Africans, and New York City whites. To test the accuracy of the allele-frequency distributions, we genotyped the Buryats and New York samples individually for six microsatellite markers and compared their frequencies to the allele frequencies derived from the electropherogram peak heights for the pooled DNA, producing a correlation of 0.9811 with a variance of less than 0.04. Two-dimensional scaling of genetic distances among the six populations produced clusters that reflected known historical relationships. A distance matrix was created using all 296 loci, and matrices based on individual chromosomes were correlated against the total matrix. As expected, the largest chromosomes had the highest correlations with the total matrix, whereas one of the smallest chromosomes, chromosome 22, had the lowest correlation and differed most from the combined STR distance matrix. </td> <td style="text-align:left;"> 4418 </td> <td style="text-align:left;"> NA </td> <td style="text-align:left;"> NA </td> <td style="text-align:left;"> NA </td> <td style="text-align:left;"> no </td> <td style="text-align:left;"> no </td> </tr> <tr> <td style="text-align:left;"> 4417 </td> <td style="text-align:left;"> 2017 </td> <td style="text-align:left;"> NA </td> <td style="text-align:left;"> Gloria-Bottini, F; Antonacci, E; Bottini, N; Ogana, A; Borgiani, P; De Santis, G; Lucarini, N </td> <td style="text-align:left;"> 2000 </td> <td style="text-align:left;"> RH blood groups and diabetic disorders: Is there an effect on glycosylated hemoglobin level? </td> <td style="text-align:left;"> Recent cloning of RH genes has elucidated their structure, suggesting that RH proteins are part of an oligomeric complex with transport function in the erythrocyte. This observation prompted us to investigate a possible relationship between the RH system and the glycosylated hemoglobin level (Hb A(lc)) in diabetes. This compound is considered an important indicator of glycemic control in diabetic disorders. We studied 278 subjects with non-insulin-dependent diabetes mellitus (NIDDM) from the population of Penne, Italy. Glycemic and glycosylated hemoglobin (Hb A(lc)) levels are associated with RH phenotype. Glucose and Hb A,, levels are increased in DCcEe subjects and decreased in ddccee subjects as compared to the mean values for other genotypes. Sex, age at onset of disease, duration of disease, and age of patients were also considered. Correlation analysis suggests that these variables influence glycemia directly and Hb A,, indirectly. The RH system, on the other hand, seems to influence the Hb A(lc) level directly. Preliminary data on 53 children with insulin-dependent diabetes mellitus (IDDM) from Sardinia seem to confirm the relationship between RH and Hb A(lc) observed in NIDDM. Since glycosylated hemoglobin is found inside red blood cells, the relationship between RH genetic variability and Hb A(lc) level suggests that RH proteins may influence glucose transport through red cell membrane and/or hemoglobin glycation. </td> <td style="text-align:left;"> 4419 </td> <td style="text-align:left;"> NA </td> <td style="text-align:left;"> NA </td> <td style="text-align:left;"> NA </td> <td style="text-align:left;"> no </td> <td style="text-align:left;"> no </td> </tr> <tr> <td style="text-align:left;"> 4418 </td> <td style="text-align:left;"> 2017 </td> <td style="text-align:left;"> NA </td> <td style="text-align:left;"> Papiol, S; Begemann, M; Rosenberger, A; Friedrichs, H; Ribbe, K; Grube, S; Schwab, MH; Jahn, H; Gunkel, S; Benseler, F; Nave, KA; Ehrenreich, H </td> <td style="text-align:left;"> 2011 </td> <td style="text-align:left;"> A Phenotype-Based Genetic Association Study Reveals the Contribution of Neuregulin1 Gene Variants to Age of Onset and Positive Symptom Severity in Schizophrenia </td> <td style="text-align:left;"> By pure endpoint diagnosis of the disease, the risk of developing schizophrenia has been repeatedly associated with specific variants of the neuregulin1 (NRG1) gene. However, the role of NRG1 in the etiology of schizophrenia has remained unclear. Since Nrg1 serves vital functions in early brain development of mice, we hypothesized that human NRG1 alleles codetermine developmentally influenced readouts of the disease: age of onset and positive symptom severity. We analyzed 1,071 comprehensively phenotyped schizophrenic/schizoaffective patients, diagnosed according to DSM-IV-TR, from the GRAS (Gottingen Research Association for Schizophrenia) Data Collection for genetic variability in the Icelandic region of risk in the NRG1 gene. For the case-control analysis part of the study, we included 1,056 healthy individuals with comparable ethnicity. The phenotype-based genetic association study (PGAS) was performed on the GRAS sample. Instead of a risk constellation, we detected that several haplotypic variants of NRG1 were, unexpectedly, less frequent in the schizophrenic than in the control sample (mean OR = 0.78, range between 0.68 and 0.85). In the PGAS we found that these "protective" NRG1 variants are specifically underrepresented in subgroups of schizophrenic subjects with early age of onset and high positive symptom load. The GRAS Data Collection as a prerequisite for PGAS has enabled us to associate protective NRG1 genotypes with later onset and milder course of schizophrenia. (C) 2011 Wiley-Liss, Inc. </td> <td style="text-align:left;"> 4420 </td> <td style="text-align:left;"> NA </td> <td style="text-align:left;"> NA </td> <td style="text-align:left;"> NA </td> <td style="text-align:left;"> no </td> <td style="text-align:left;"> no </td> </tr> <tr> <td style="text-align:left;"> 4419 </td> <td style="text-align:left;"> 2017 </td> <td style="text-align:left;"> NA </td> <td style="text-align:left;"> Udo, EE; Al-Sweih, N </td> <td style="text-align:left;"> 2017 </td> <td style="text-align:left;"> Dominance of community-associated methicillin-resistant Staphylococcus aureus clones in a maternity hospital </td> <td style="text-align:left;"> Background Methicillin-resistant Staphylococcus aureus (MRSA) is a major pathogen causing healthcare-and community-acquired infections. The purpose of this study was to characterize MRSA isolated at the Maternity Hospital between 2006 and 2011 for their genetic relatedness. Materials and methods The MRSA isolates were investigated using a combination of antibiogram, Staphylococcal chromosome cassette mec (SCCmec) and spa typing to determine their relatedness to MRSA isolated in other Kuwait hospitals. The isolates were also investigated for the carriage of genes for Pantone valentine Leukocidin (PVL). Results A total of 103 MRSA obtained from 64 neonates, 17 adult patients and 12 healthcare workers. The isolates were resistant to Kanamycin (46.6%), gentamicin (40.8%), trimethoprim (32%), ciprofloxacin (22.3%), fusidic acid (16.5%), tetracycline (19.4%), erythromycin (15.5%), clindamycin (15.5%), streptomycin (11.6%) high-level mupirocin (2.9%) and chloramphenicol (0.9%). Twenty (19.4%) of the isolates were multiresistant. Thirty-one (30.0%) isolates were positive for PVL. Molecular typing revealed the presence of 11 clonal complexes and 23 clones with ST5-V-t002, (N = 22), ST22-IV-t223 (N = 18), ST22-IV-t852 (N = 10), ST80-IV-t044 (N = 7), ST5-V-t688 (N = 5), ST772-V-t657 (N = 5) and ST239-III-t860 (N = 4) constituting 66.9% of the isolates. Other clones were isolated sporadically. The number of MRSA isolates increased from two in 2006 to 22 in 2011 with a peak of 43 in 2008. Conclusion The study revealed a high prevalence of community-associated MRSA Maternity hospital. The MRSA population consisted of known strains, such as ST239-III-t680, ST22-IV-t223/t852 and ST80-IV-t044, that were reported previously in Kuwait and novel strains such as </td> <td style="text-align:left;"> 4421 </td> <td style="text-align:left;"> NA </td> <td style="text-align:left;"> NA </td> <td style="text-align:left;"> NA </td> <td style="text-align:left;"> no </td> <td style="text-align:left;"> no </td> </tr> <tr> <td style="text-align:left;"> 4420 </td> <td style="text-align:left;"> 2017 </td> <td style="text-align:left;"> NA </td> <td style="text-align:left;"> Olival, KJ; Hosseini, PR; Zambrana-Torrelio, C; Ross, N; Bogich, TL; Daszak, P </td> <td style="text-align:left;"> 2017 </td> <td style="text-align:left;"> Host and viral traits predict zoonotic spillover from mammals </td> <td style="text-align:left;"> The majority of human emerging infectious diseases are zoonotic, with viruses that originate in wild mammals of particular concern (for example, HIV, Ebola and SARS)(1-3). Understanding patterns of viral diversity in wildlife and determinants of successful cross-species transmission, or spillover, are therefore key goals for pandemic surveillance programs(4). However, few analytical tools exist to identify which host species are likely to harbour the next human virus, or which viruses can cross species boundaries(5-7). Here we conduct a comprehensive analysis of mammalian host-virus relationships and show that both the total number of viruses that infect a given species and the proportion likely to be zoonotic are predictable. After controlling for research effort, the proportion of zoonotic viruses per species is predicted by phylogenetic relatedness to humans, host taxonomy and human population within a species range-which may reflect human-wildlife contact. We demonstrate that bats harbour a significantly higher proportion of zoonotic viruses than all other mammalian orders. We also identify the taxa and geographic regions with the largest estimated number of 'missing viruses' and 'missing zoonoses' and therefore of highest value for future surveillance. We then show that phylogenetic host breadth and other viral traits are significant predictors of zoonotic potential, providing a novel framework to assess if a newly discovered mammalian virus could infect people. </td> <td style="text-align:left;"> 4422 </td> <td style="text-align:left;"> NA </td> <td style="text-align:left;"> NA </td> <td style="text-align:left;"> NA </td> <td style="text-align:left;"> no </td> <td style="text-align:left;"> no </td> </tr> <tr> <td style="text-align:left;"> 4421 </td> <td style="text-align:left;"> 2017 </td> <td style="text-align:left;"> NA </td> <td style="text-align:left;"> Koehler, RN; Walsh, AM; Sanders-Buell, EE; Eller, LA; Eller, M; Currier, JR; Bautista, CT; Wabwire-Mangen, F; Hoelscher, M; Maboko, L; Kim, J; Michael, NL; Robb, ML; McCutchan, FE; Kijak, GH </td> <td style="text-align:left;"> 2010 </td> <td style="text-align:left;"> High-Throughput High- Resolution Class I HLA Genotyping in East Africa </td> <td style="text-align:left;"> HLA, the most genetically diverse loci in the human genome, play a crucial role in host-pathogen interaction by mediating innate and adaptive cellular immune responses. A vast number of infectious diseases affect East Africa, including HIV/AIDS, malaria, and tuberculosis, but the HLA genetic diversity in this region remains incompletely described. This is a major obstacle for the design and evaluation of preventive vaccines. Available HLA typing techniques, that provide the 4-digit level resolution needed to interpret immune responses, lack sufficient throughput for large immunoepidemiological studies. Here we present a novel HLA typing assay bridging the gap between high resolution and high throughput. The assay is based on real-time PCR using sequence-specific primers (SSP) and can genotype carriers of the 49 most common East African class I HLA-A, -B, and -C alleles, at the 4-digit level. Using a validation panel of 175 samples from Kampala, Uganda, previously defined by sequence-based typing, the new assay performed with 100% sensitivity and specificity. The assay was also implemented to define the HLA genetic complexity of a previously uncharacterized Tanzanian population, demonstrating its inclusion in the major East African genetic cluster. The availability of genotyping tools with this capacity will be extremely useful in the identification of correlates of immune protection and the evaluation of candidate vaccine efficacy. </td> <td style="text-align:left;"> 4423 </td> <td style="text-align:left;"> NA </td> <td style="text-align:left;"> NA </td> <td style="text-align:left;"> NA </td> <td style="text-align:left;"> no </td> <td style="text-align:left;"> no </td> </tr> <tr> <td style="text-align:left;"> 4422 </td> <td style="text-align:left;"> 2017 </td> <td style="text-align:left;"> NA </td> <td style="text-align:left;"> Firouzabadi, N; Ghazanfari, N; Shoushtari, AA; Erfani, N; Fathi, F; Bazrafkan, M; Bahramali, E </td> <td style="text-align:left;"> 2016 </td> <td style="text-align:left;"> Genetic Variants of Angiotensin-Converting Enzyme Are Linked to Autism: A Case-Control Study </td> <td style="text-align:left;"> Background Autism is a disease of complex nature with a significant genetic component. The importance of renin-angiotensin system (RAS) elements in cognition and behavior besides the interaction of angiotensin II (Ang II), the main product of angiotensin-converting enzyme (ACE), with neurotransmitters in CNS, especially dopamine, proposes the involvement of RAS in autism. Since the genetic architecture of autism has remained elusive, here we postulated that genetic variations in RAS are associated with autism. Methods Considering the relation between the three polymorphisms of ACE (I/D, rs4343 and rs4291) with the level of ACE activity, we have investigated this association with autism, in a case-control study. Genotype and allele frequencies of polymorphisms were determined in DNAs extracted from venous blood of 120 autistic patients and their age and sex-matched healthy controls, using polymerase chain reaction (PCR) and PCR-restriction fragment length polymorphism (PCR-RFLP) methods. Results There were strong associations between both DD genotype of ACE I/D and the D allele, with autism (P = 0.006, OR = 2.9, 95% CI = 1.64-5.13 and P = 0.006, OR = 2.18, 95% CI = 1.37-3.48 respectively). Furthermore, a significant association between the G allele of rs4343 and autism was observed (P = 0.006, OR = 1.84, 95% CI = 1.26-2.67). Moreover, haplotype analysis revealed an association between DTG haplotype and autism (P = 0.008). Conclusion Our data suggests the involvement of RAS genetic diversity in increasing the risk of autism. </td> <td style="text-align:left;"> 4424 </td> <td style="text-align:left;"> NA </td> <td style="text-align:left;"> NA </td> <td style="text-align:left;"> NA </td> <td style="text-align:left;"> no </td> <td style="text-align:left;"> no </td> </tr> <tr> <td style="text-align:left;"> 4423 </td> <td style="text-align:left;"> 2017 </td> <td style="text-align:left;"> NA </td> <td style="text-align:left;"> Coker, OO; Chaiprasert, A; Ngamphiw, C; Tongsima, S; Regmi, SM; Clark, TG; Ong, RTH; Teo, YY; Prammananan, T; Palittapongarnpim, P </td> <td style="text-align:left;"> 2016 </td> <td style="text-align:left;"> Genetic signatures of Mycobacterium tuberculosis Nonthaburi genotype revealed by whole genome analysis of isolates from tuberculous meningitis patients in Thailand </td> <td style="text-align:left;"> Genome sequencing plays a key role in understanding the genetic diversity of Mycobacterium tuberculosis (M.tb). The genotype-specific character of M. tb contributes to tuberculosis severity and emergence of drug resistance. Strains of M. tb complex can be classified into seven lineages. The Nonthaburi (NB) genotype, belonging to the Indo-Oceanic lineage (lineage 1), has a unique spoligotype and IS6110-RFLP pattern but has not previously undergone a detailed whole genome analysis. In addition, there is not much information available on the whole genome analysis of M. tb isolates from tuberculous meningitis (TBM) patients in public databases. Isolates CSF3053, 46-5069 and 43-13838 of NB genotype were obtained from the cerebrospinal fluids of TBM Thai patients in Siriraj Hospital, Bangkok. The whole genomes were subjected to high throughput sequencing. The sequence data of each isolate were assembled into draft genome. The sequences were also aligned to reference genome, to determine genornic variations. Single nucleotide polymorphisrns (SNPs) were obtained and grouped according to the functions of the genes containing them. They were compared with SNPs from 1,601 genomes, representing the seven lineages of M. tb complex, to determine the uniqueness of NB genotype. Susceptibility to first-line, second-line and other antituberculosis drugs were determined and related to the SNPs previously reported in drug-resistant related genes. The assembled genomes have an average size of 4,364,461 bp, 4,154 genes, 48 RNAs and 64 pseudogenes. A 500 base pairs deletion, which includes ppe50, was found in all isolates. RD239, specific for members of Indo Oceanic lineage, and RD147c were identified. A total of 2,202 SNPs were common to the isolates and used to classiyy the NB strains as members of sublineage 1.2.1. Compared with 1,601 genomes from the seven lineages of M. tb complex, mutation G2342203C was found novel to the isolates in this study. Three mutations (T28910C, C1180580T and C152178T) were found only in Thai NB isolates, including isolates from previous study. Although drug susceptibility tests indicated pan-susceptibility, non-synonymous SNPs previously reported to be associated with resistance to anti-tuberculous drugs; isoniazid, ethambutol, and ethionamide were identified in all the isolates. Non-synonymous SNPs were found in virulence genes such as the genes playing roles in apoptosis inhibition and phagosome arrest. We also report polymorphisms in essential genes, efflux pumps associated genes and genes with known epitopes. The analysis of the TBM isolates and the availability of the variations obtained will provide additional resources for global comparison of isolates from Pulmonary tuberculosis and TBM. It will also contribute to the richness of genomic databases towards the prediction of antibiotic resistance, level of virulence and of origin of infection. </td> <td style="text-align:left;"> 4425 </td> <td style="text-align:left;"> NA </td> <td style="text-align:left;"> NA </td> <td style="text-align:left;"> NA </td> <td style="text-align:left;"> no </td> <td style="text-align:left;"> no </td> </tr> <tr> <td style="text-align:left;"> 4424 </td> <td style="text-align:left;"> 2017 </td> <td style="text-align:left;"> NA </td> <td style="text-align:left;"> Bozek, K; Lengauer, T </td> <td style="text-align:left;"> 2010 </td> <td style="text-align:left;"> Positive selection of HIV host factors and the evolution of lentivirus genes </td> <td style="text-align:left;"> Background: Positive selection of host proteins that interact with pathogens can indicate factors relevant for infection and potentially be a measure of pathogen driven evolution. Results: Our analysis of 1439 primate genes and 175 lentivirus genomes points to specific host factors of high genetic variability that could account for differences in susceptibility to disease and indicate specific mechanisms of host defense and pathogen adaptation. We find that the largest amount of genetic change occurs in genes coding for cellular membrane proteins of the host as well as in the viral envelope genes suggesting cell entry and immune evasion as the primary evolutionary interface between host and pathogen. We additionally detect the innate immune response as a gene functional group harboring large differences among primates that could potentially account for the different levels of immune activation in the HIV/SIV primate infection. We find a significant correlation between the evolutionary rates of interacting host and viral proteins pointing to processes of the host-pathogen biology that are relatively conserved among species and to those undergoing accelerated genetic evolution. Conclusions: These results indicate specific host factors and their functional groups experiencing pathogen driven evolutionary selection pressures. Individual host factors pointed to by our analysis might merit further study as potential targets of antiretroviral therapies. </td> <td style="text-align:left;"> 4426 </td> <td style="text-align:left;"> NA </td> <td style="text-align:left;"> NA </td> <td style="text-align:left;"> NA </td> <td style="text-align:left;"> no </td> <td style="text-align:left;"> no </td> </tr> <tr> <td style="text-align:left;"> 4425 </td> <td style="text-align:left;"> 2017 </td> <td style="text-align:left;"> NA </td> <td style="text-align:left;"> Wootton, PTE; Arora, NL; Drenos, F; Thompson, SR; Cooper, JA; Stephens, JW; Hurel, SJ; Hurt-Camejo, E; Wiklund, O; Humphries, SE; Talmud, PJ </td> <td style="text-align:left;"> 2007 </td> <td style="text-align:left;"> Tagging SNP haplotype analysis of the secretory PLA2-V gene, PLA2G5, shows strong association with LDL and oxLDL levels, suggesting functional distinction from sPLA2-IIA: results from the UDACS study </td> <td style="text-align:left;"> Animal and human studies suggest that both secretory PLA2 (sPLA2)-V and sPLA2-IIA (encoded, respectively, by the neighbouring PLA2G5 and PLA2G2A genes) contribute to atherogenesis. Elevated plasma sPLA2-IIA predicts coronary heart disease (CHD) risk, but no mass assay for sPLA2-V is available. We previously reported that tagging single nucleotide polymorphism (tSNP) haplotypes of PLA2G2A are strongly associated with sPLA2-IIA mass, but not lipid levels. Here, we use tSNPs of the sPLA2-V gene to investigate the association of PLA2G5 with CHD risk markers. Seven PLA2G5 tSNPs genotypes, explaining > 92% of the locus genetic variability, were determined in 519 patients with Type 11 diabetes (in whom PLA2G2A tSNP data was available), and defined seven common haplotypes (frequencies > 5%). PLA2G5 and PLA2G2A tSNPs showed linkage disequilibrium (LD). Compared to the common PLA2G5 haplotype, H1 (frequency 34.9%), haplotypes H2-7 were associated with overall higher plasma LDL (P < 0.00004) and total cholesterol (P < 0.00003) levels yet lower oxLD/LDL (P = 0.006) and sPLA2-IIA mass (P = 0.04), probably reflecting LD with PLA2G2A. Intronic tSNP (rs11573248), unlikely itself to be functional, distinguished H1 from LDL-raising haplotypes and may mark a functional site. In conclusion, PLA2G5tSNP haplotypes demonstrate an association with total and LDL cholesterol and oxLDL/LDL, not seen with PLA2G2A, thus confirming distinct functional roles for these two sPLA2s. </td> <td style="text-align:left;"> 4427 </td> <td style="text-align:left;"> NA </td> <td style="text-align:left;"> NA </td> <td style="text-align:left;"> NA </td> <td style="text-align:left;"> no </td> <td style="text-align:left;"> no </td> </tr> <tr> <td style="text-align:left;"> 4426 </td> <td style="text-align:left;"> 2017 </td> <td style="text-align:left;"> NA </td> <td style="text-align:left;"> Ergoren, MC; Pirzada, RH; Arici, M; Serakinci, N </td> <td style="text-align:left;"> 2015 </td> <td style="text-align:left;"> Near East University Genetic Mutation Database (NEU-GD): The first mutation database of Northern Cyprus </td> <td style="text-align:left;"> The health care system is negatively affected by the genetic disorders that lead to an increasing rate of morbidity and neonatal deaths and affect adults as well. These create a substantial government's psychosocial and economic burden on clinicians, patients and their families with the advancement in the field of genetics. There has been a tremendous increase in the rate in which diseases associated with variant DNA sequences are being sought and identified. The goal behind the creation of Near East University Genetic Mutation Database (NEU-GD) is to map and apprehend the patterns of common genetic diversity in the human genetic makeup in order to accelerate the search for the genetic causes of human disease. NEU-GD will allow scientists to generate extraordinarily useful information such as allelic variations among population, and description of the genetic blueprint of mutations occurring in human beings. In this communication we report the construction of the first genetic mutation database for the people belonging to different ethnic groups living in North Cyprus (http://genetics-db.neu.edu.tr/). Therefore NEU-GD can serve as an important tool available online for molecular genetic testing of inherited disorder and persuade for further investigation of novel genetic disorders in North Cyprus population. (C) 2015 Elsevier B.V. All rights reserved. </td> <td style="text-align:left;"> 4428 </td> <td style="text-align:left;"> NA </td> <td style="text-align:left;"> NA </td> <td style="text-align:left;"> NA </td> <td style="text-align:left;"> no </td> <td style="text-align:left;"> no </td> </tr> <tr> <td style="text-align:left;"> 4427 </td> <td style="text-align:left;"> 2017 </td> <td style="text-align:left;"> NA </td> <td style="text-align:left;"> O'Brien, SJ; Troyer, JL; Roelke, M; Marker, L; Pecon-Slattery, J </td> <td style="text-align:left;"> 2006 </td> <td style="text-align:left;"> Plagues and adaptation: Lessons from the Felidae models for SARS and AIDS </td> <td style="text-align:left;"> Research studies of infectious disease outbreaks in wild species of the cat family Felidae have revealed unusual details regarding forces that shape population survival and genetic resistance in these species. A highly virulent feline coronavirus epidemic in African cheetahs, a disease model for human SARS, illustrates the critical role of ancestral population genetic variation. Widespread prevalence of species specific feline immunodeficiency virus (FM, a relative of HIV-AIDS, occurs with little pathogenesis in felid species, except in domestic cats, suggesting immunological adaptation in species where FIV is endemic. Resolving the interaction of host and pathogen genomes can shed new light on the process of disease outbreak in wildlife and in humankind. The role of disease in endangered populations and species is difficult to access as opportunities to monitor outbreaks in natural populations are limited. Conservation management may benefit greatly from advances in molecular genetic tools developed for human biomedical research to assay the biodiversity of both host species and emerging pathogen. As these examples illustrate, strong parallels exist between disease in human and endangered wildlife and argue for an integration of the research fields of comparative genomics, infectious disease, epidemiology, molecular genetics and population biology for an effective proactive conservation approach. (c) 2006 Elsevier Ltd. All rights reserved. </td> <td style="text-align:left;"> 4429 </td> <td style="text-align:left;"> NA </td> <td style="text-align:left;"> NA </td> <td style="text-align:left;"> NA </td> <td style="text-align:left;"> no </td> <td style="text-align:left;"> no </td> </tr> <tr> <td style="text-align:left;"> 4428 </td> <td style="text-align:left;"> 2017 </td> <td style="text-align:left;"> NA </td> <td style="text-align:left;"> Yu, GX; Greninger, AL; Isa, P; Phan, TG; Martinez, MA; Sanchez, MD; Contreras, JF; Santos-Preciado, JI; Parsonnet, J; Miller, S; DeRisi, JL; Delwart, E; Arias, CF; Chiu, CY </td> <td style="text-align:left;"> 2012 </td> <td style="text-align:left;"> Discovery of a Novel Polyomavirus in Acute Diarrheal Samples from Children </td> <td style="text-align:left;"> Polyomaviruses are small circular DNA viruses associated with chronic infections and tumors in both human and animal hosts. Using an unbiased deep sequencing approach, we identified a novel, highly divergent polyomavirus, provisionally named MX polyomavirus (MXPyV), in stool samples from children. The similar to 5.0 kB viral genome exhibits little overall homology (<46% amino acid identity) to known polyomaviruses, and, due to phylogenetic variation among its individual proteins, cannot be placed in any existing taxonomic group. PCR-based screening detected MXPyV in 28 of 834 (3.4%) fecal samples collected from California, Mexico, and Chile, and 1 of 136 (0.74%) of respiratory samples from Mexico, but not in blood or urine samples from immunocompromised patients. By quantitative PCR, the measured titers of MXPyV in human stool at 10% (weight/volume) were as high as 15,075 copies. No association was found between the presence of MXPyV and diarrhea, although girls were more likely to shed MXPyV in the stool than boys (p = 0.012). In one child, viral shedding was observed in two stools obtained 91 days apart, raising the possibility of chronic infection by MXPyV. A multiple sequence alignment revealed that MXPyV is a closely related variant of the recently reported MWPyV and HPyV10 polyomaviruses. Further studies will be important to determine the association, if any, of MXPyV with disease in humans. </td> <td style="text-align:left;"> 4430 </td> <td style="text-align:left;"> NA </td> <td style="text-align:left;"> NA </td> <td style="text-align:left;"> NA </td> <td style="text-align:left;"> no </td> <td style="text-align:left;"> no </td> </tr> <tr> <td style="text-align:left;"> 4429 </td> <td style="text-align:left;"> 2017 </td> <td style="text-align:left;"> NA </td> <td style="text-align:left;"> de Lucio, A; Martinez-Ruiz, R; Merino, FJ; Bailo, B; Aguilera, M; Fuentes, I; Carmena, D </td> <td style="text-align:left;"> 2015 </td> <td style="text-align:left;"> Molecular Genotyping of Giardia duodenalis Isolates from Symptomatic Individuals Attending Two Major Public Hospitals in Madrid, Spain </td> <td style="text-align:left;"> Background The flagellate protozoan Giardia duodenalis is an enteric parasite causing human giardiasis, a major gastrointestinal disease of global distribution affecting both developing and industrialised countries. In Spain, sporadic cases of giardiasis have been regularly identified, particularly in pediatric and immigrant populations. However, there is limited information on the genetic variability of circulating G. duodenalis isolates in the country. Methods In this longitudinal molecular epidemiological study we report the diversity and frequency of the G. duodenalis assemblages and sub-assemblages identified in 199 stool samples collected from 184 individual with symptoms compatible with giardiasis presenting to two major public hospitals in Madrid for the period December 2013-January 2015. G. duodenalis cysts were initially detected by conventional microscopy and/or immunochomatography on stool samples. Confirmation of the infection was performed by direct immunofluorescence and real-time PCR methods. G. duodenalis assemblages and sub-assemblages were determined by multi-locus genotyping of the glutamate dehydrogenase (GDH) and beta-giardin (BG) genes of the parasite. Sociodemographic and clinical features of patients infected with G. duodenalis were also analysed. Principal findings Of 188 confirmed positive samples from 178 giardiasis cases a total of 124 G. duodenalis isolates were successfully typed at the GDH and/or the BG loci, revealing the presence of sub-assemblages BIV (62.1%), AII (15.3%), BIII (4.0%), AI (0.8%), and AIII (0.8%). Additionally, 6.5% of the isolates were only characterised at the assemblage level, being all of them assigned to assemblage B. Discordant genotype results AII/AIII or BIII/BIV were also observed in 10.5% of DNA isolates. A large number of multi-locus genotypes were identified in G. duodenalis assemblage B, but not assemblage A, isolates at both the GDH and BG loci, confirming the high degree of genetic variability observed in other molecular surveys. BIV was the most prevalent genetic variant of G. duodenalis found in individuals with symptomatic giardiasis in the population under study. Conclusions Human giardiasis is an ongoing public health problem in Spain affecting primarily young children under four years of age but also individuals of all age groups. Our typing and sub-typing results demonstrate that assemblage B is the most prevalent G. duodenalis assemblage circulating in patients with clinical giardiasis in Central Spain. Our analyses also revealed a large genetic variability in assemblage B (but not assemblage A) isolates of the parasite, corroborating the information obtained in similar studies in other geographical regions. We believe that molecular data presented here provide epidemiological evidence at the population level in support of the existence of genetic exchange within assemblages of G. duodenalis. </td> <td style="text-align:left;"> 4431 </td> <td style="text-align:left;"> NA </td> <td style="text-align:left;"> NA </td> <td style="text-align:left;"> NA </td> <td style="text-align:left;"> no </td> <td style="text-align:left;"> no </td> </tr> <tr> <td style="text-align:left;"> 4430 </td> <td style="text-align:left;"> 2017 </td> <td style="text-align:left;"> NA </td> <td style="text-align:left;"> Malheiros, D; Petzl-Erler, ML </td> <td style="text-align:left;"> 2009 </td> <td style="text-align:left;"> Individual and epistatic effects of genetic polymorphisms of B-cell co-stimulatory molecules on susceptibility to pemphigus foliaceus </td> <td style="text-align:left;"> Following the candidate gene approach we analyzed the CD40L, CD40, BLYS and CD19 genes that participate of B-cell co-stimulation, for association with pemphigus foliaceus (PF), an organ-specific autoimmune disease, characterized by the detachment of epidermal cells from each other ( acantholysis) and presence of autoantibodies specific for desmoglein 1 (dsg1), an epidermal cell-adhesion molecule. The disease is endemic in certain regions of Brazil and also is known as fogo selvagem. Complex interactions among environmental and genetic susceptibility factors contribute to the manifestation of this multifactorial disease. The sample included 179 patients and 317 controls. Strong significant association was found with CD40L-726T>C ( odds ratio, OR=5.54 and 0.30 for T+ and C+ genotypes, respectively). In addition, there were significant negative associations with CD40 - 1T (OR=0.61) and BLYS-871T (OR=0.62) due to the decrease of the frequency of both homo- and heterozygotes in the patient group. No associations were found with variants of CD19 gene. Gene-gene interactions were observed between CD40 and BLYS, and between CD40L and BLYS. So, the dominant protective effects of CD40L-726C and of CD40 - 1T only manifest in BLYS-871T+ individuals, and vice versa. We conclude that genetic variability of CD40L, CD40 and BLYS is an important factor for PF pathogenesis. Genes and Immunity (2009) 10, 547-558; doi:10.1038/gene.2009.36; published online 7 May 2009 </td> <td style="text-align:left;"> 4432 </td> <td style="text-align:left;"> NA </td> <td style="text-align:left;"> NA </td> <td style="text-align:left;"> NA </td> <td style="text-align:left;"> no </td> <td style="text-align:left;"> no </td> </tr> <tr> <td style="text-align:left;"> 4431 </td> <td style="text-align:left;"> 2017 </td> <td style="text-align:left;"> NA </td> <td style="text-align:left;"> Grandjean, L; Iwamoto, T; Lithgow, A; Gilman, RH; Arikawa, K; Nakanishi, N; Martin, L; Castillo, E; Alarcon, V; Coronel, J; Solano, W; Aminian, M; Guezala, C; Rastogi, N; Couvin, D; Sheen, P; Zimic, M; Moore, DAJ </td> <td style="text-align:left;"> 2015 </td> <td style="text-align:left;"> The Association between Mycobacterium Tuberculosis Genotype and Drug Resistance in Peru </td> <td style="text-align:left;"> Background The comparison of Mycobacterium tuberculosis bacterial genotypes with phenotypic, demographic, geospatial and clinical data improves our understanding of how strain lineage influences the development of drug-resistance and the spread of tuberculosis. Methods To investigate the association of Mycobacterium tuberculosis bacterial genotype with drugresistance. Drug susceptibility testing together with genotyping using both 15-loci MIRU-typing and spoligotyping, was performed on 2,139 culture positive isolates, each from a different patient in Lima, Peru. Demographic, geospatial and socio-economic data were collected using questionnaires, global positioning equipment and the latest national census. Results The Latin American Mediterranean (LAM) clade (OR 2.4, p<0.001) was significantly associated with drug-resistance and alone accounted for more than half of all drug resistance in the region. Previously treated patients, prisoners and genetically clustered cases were also significantly associated with drug-resistance (OR's 2.5, 2.4 and 1.8, p<0.001, p<0.05, p<0.001 respectively). Conclusions Tuberculosis disease caused by the LAM clade was more likely to be drug resistant independent of important clinical, genetic and socio-economic confounding factors. Explanations for this include; the preferential co-evolution of LAM strains in a Latin American population, a LAM strain bacterial genetic background that favors drug-resistance or the "founder effect" from pre-existing LAM strains disproportionately exposed to drugs. </td> <td style="text-align:left;"> 4433 </td> <td style="text-align:left;"> NA </td> <td style="text-align:left;"> NA </td> <td style="text-align:left;"> NA </td> <td style="text-align:left;"> no </td> <td style="text-align:left;"> no </td> </tr> <tr> <td style="text-align:left;"> 4432 </td> <td style="text-align:left;"> 2017 </td> <td style="text-align:left;"> NA </td> <td style="text-align:left;"> Awan, AR; Babar, ME; Ali, A; Awan, Z; Saleem, Z; Zahoor, Y </td> <td style="text-align:left;"> 2011 </td> <td style="text-align:left;"> Phylogenetics and Evolutionary Association of Hepatitis B Virus Isolated from Pakistan </td> <td style="text-align:left;"> Genetic variations of Hepatitis B Virus (HBV) are closely associated with viral pathogenesis The generation of variants of Hepatitis B virus (HBV) with altered functional properties and increased pathogenesis are the result of mutational events that can affect the disease patterns and response to antiviral treatments Due to high rate of mutational changes in viral genomic structure the resulting variables become resistant to antiviral drugs therefore making it imperative to develop therapeutic alternatives by identifying rapidly occurring variants This study describes genetic variability of Pakistani HBV isolates based upon DNA sequences of cloned PreS1/PreS2/S region or PreS/S Open Reading Frame (ORF) HBV DNA was isolated from blood samples of 10 unrelated patients and PreS/S ORF of HBV was amplified through PCR from these isolates The DNA fragments were cloned and sequenced using standard methodologies The surface genes from HBV isolates showed 96-99% homology with reported HBV genotype D but Pakistani isolates demonstrated sequence novelties never reported elsewhere Phylogenetic analysis of the sequences revealed that these isolates clustered within the genotype D group reported in different regions of the world This study describes the phylogenetic and evolutionary characterization of HBV isolated from Pakistan based on DNA sequence of complete PreS/S ORF </td> <td style="text-align:left;"> 4434 </td> <td style="text-align:left;"> NA </td> <td style="text-align:left;"> NA </td> <td style="text-align:left;"> NA </td> <td style="text-align:left;"> no </td> <td style="text-align:left;"> no </td> </tr> <tr> <td style="text-align:left;"> 4433 </td> <td style="text-align:left;"> 2017 </td> <td style="text-align:left;"> NA </td> <td style="text-align:left;"> Mohammed, H; Kassa, M; Assefa, A; Tadesse, M; Kebede, A </td> <td style="text-align:left;"> 2017 </td> <td style="text-align:left;"> Genetic polymorphism of Merozoite Surface Protein-2 (MSP-2) in Plasmodium falciparum isolates from Pawe District, North West Ethiopia </td> <td style="text-align:left;"> Background In malaria endemic regions, Plasmodium falciparum infection is characterized by extensive genetic diversity. Describing this diversity provides important information about the local malaria situation. This study was conducted to evaluate the extent of genetic diversity of P. falciparum in Pawe district, North West Ethiopia, using the highly polymorphic merozoite surface protein 2 gene. Methods Atotal of 92 isolates from patients with uncomplicated P. falciparum attending Pawe Health Centre were collected from September to December 2013. Genomic DNA was extracted using the Chelex method and analyzed by length polymorphism following gel electrophoresis of DNA products from nested PCR of msp2 (block 3), targeting allelic families of FC27 and 3D7/IC. Results There were twenty-two different msp2 alleles, 11 corresponding to the 3D7/IC and 11 to the FC27 allelic family. The frequency of isolates of the msp2 3D7/IC allelic family was higher (51%) compared to FC27 (49%). The majority of the isolates (76%) contained multiple infections and the overall mean multiplicity of infection was 2.8 (CI 95% 2.55-3.03). The heterozygosity index was 0.66 for msp2. There was no statically significant difference in the multiplicity of infection by age or parasite density. Conclusions The results of this study show that P. falciparum polymorphismsare extensive in Northwest Ethiopia and most of the infections are composed of multiple clones. </td> <td style="text-align:left;"> 4435 </td> <td style="text-align:left;"> NA </td> <td style="text-align:left;"> NA </td> <td style="text-align:left;"> NA </td> <td style="text-align:left;"> no </td> <td style="text-align:left;"> no </td> </tr> <tr> <td style="text-align:left;"> 4434 </td> <td style="text-align:left;"> 2017 </td> <td style="text-align:left;"> NA </td> <td style="text-align:left;"> Jacquot, M; Abrial, D; Gasqui, P; Bord, S; Marsot, M; Masseglia, S; Pion, A; Poux, V; Zilliox, L; Chapuis, JL; Vourc'h, G; Bailly, X </td> <td style="text-align:left;"> 2016 </td> <td style="text-align:left;"> Multiple independent transmission cycles of a tick-borne pathogen within a local host community </td> <td style="text-align:left;"> Many pathogens are maintained by multiple host species and involve multiple strains with potentially different phenotypic characteristics. Disentangling transmission patterns in such systems is often challenging, yet investigating how different host species contribute to transmission is crucial to properly assess and manage disease risk. We aim to reveal transmission cycles of bacteria within the Borrelia burgdorferi species complex, which include Lyme disease agents. We characterized Borrelia genotypes found in 488 infected Ixodes ricinus nymphs collected in the Senart Forest located near Paris (France). These genotypes were compared to those observed in three sympatric species of small mammals and network analyses reveal four independent transmission cycles. Statistical modelling shows that two cycles involving chipmunks, an introduced species, and non-sampled host species such as birds, are responsible for the majority of tick infections. In contrast, the cycle involving native bank voles only accounts for a small proportion of infected ticks. Genotypes associated with the two primary transmission cycles were isolated from Lyme disease patients, confirming the epidemiological threat posed by these strains. Our work demonstrates that combining high-throughput sequence typing with networks tools and statistical modeling is a promising approach for characterizing transmission cycles of multi-host pathogens in complex ecological settings. </td> <td style="text-align:left;"> 4436 </td> <td style="text-align:left;"> NA </td> <td style="text-align:left;"> NA </td> <td style="text-align:left;"> NA </td> <td style="text-align:left;"> no </td> <td style="text-align:left;"> no </td> </tr> <tr> <td style="text-align:left;"> 4435 </td> <td style="text-align:left;"> 2017 </td> <td style="text-align:left;"> NA </td> <td style="text-align:left;"> Djoko, CF; Wolfe, ND; Aghokeng, AF; LeBreton, M; Liegeois, F; Tamoufe, U; Schneider, B; Ortiz, N; Mbacham, WF; Carr, JK; Rimoin, AW; Fair, JN; Pike, BL; Mpoudi-Ngole, E; Delaporte, E; Burke, DS; Peeters, M </td> <td style="text-align:left;"> 2012 </td> <td style="text-align:left;"> Failure to Detect Simian Immunodeficiency Virus Infection in a Large Cameroonian Cohort with High Non-human Primate Exposure </td> <td style="text-align:left;"> Hunting and butchering of wildlife in Central Africa are known risk factors for a variety of human diseases, including HIV/AIDS. Due to the high incidence of human exposure to body fluids of non-human primates, the significant prevalence of simian immunodeficiency virus (SIV) in non-human primates, and hunting/butchering associated cross-species transmission of other retroviruses in Central Africa, it is possible that SIV is actively transmitted to humans from primate species other than mangabeys, chimpanzees, and/or gorillas. We evaluated SIV transmission to humans by screening 2,436 individuals that hunt and butcher non-human primates, a population in which simian foamy virus and simian T-lymphotropic virus were previously detected. We identified 23 individuals with high seroreactivity to SIV. Nucleic acid sequences of SIV genes could not be detected, suggesting that SIV infection in humans could occur at a lower frequency than infections with other retroviruses, including simian foamy virus and simian T-lymphotropic virus. Additional studies on human populations at risk for non-human primate zoonosis are necessary to determine whether these results are due to viral/host characteristics or are indicative of low SIV prevalence in primate species consumed as bushmeat as compared to other retroviruses in Cameroon. </td> <td style="text-align:left;"> 4437 </td> <td style="text-align:left;"> NA </td> <td style="text-align:left;"> NA </td> <td style="text-align:left;"> NA </td> <td style="text-align:left;"> no </td> <td style="text-align:left;"> no </td> </tr> <tr> <td style="text-align:left;"> 4436 </td> <td style="text-align:left;"> 2017 </td> <td style="text-align:left;"> NA </td> <td style="text-align:left;"> Wong, W; Griggs, AD; Daniels, RF; Schaffner, SF; Ndiaye, D; Bei, AK; Deme, AB; MacInnis, B; Volkman, SK; Hartl, DL; Neafsey, DE; Wirth, DF </td> <td style="text-align:left;"> 2017 </td> <td style="text-align:left;"> Genetic relatedness analysis reveals the cotransmission of genetically related Plasmodium falciparum parasites in Thies, Senegal </td> <td style="text-align:left;"> Background: As public health interventions drive parasite populations to elimination, genetic epidemiology models that incorporate population genomics can be powerful tools for evaluating the effectiveness of continued intervention. However, current genetic epidemiology models may not accurately simulate the population genetic profile of parasite populations, particularly with regard to polygenomic (multi-strain) infections. Current epidemiology models simulate polygenomic infections via superinfection (multiple mosquito bites), despite growing evidence that cotransmission (a single mosquito bite) may contribute to polygenomic infections. Methods: Here, we quantified the relatedness of strains within 31 polygenomic infections collected from patients in Thies, Senegal using a hidden Markov model to measure the proportion of the genome that is inferred to be identical by descent. Results: We found that polygenomic infections can be composed of highly related parasites and that superinfection models drastically underestimate the relatedness of strains within polygenomic infections. Conclusions: Our findings suggest that cotransmission is a major contributor to polygenomic infections in Thies, Senegal. The incorporation of cotransmission into existing genetic epidemiology models may enhance our ability to characterize and predict changes in population structure associated with reduced transmission intensities and the emergence of important phenotypes like drug resistance that threaten to undermine malaria elimination activities. </td> <td style="text-align:left;"> 4438 </td> <td style="text-align:left;"> NA </td> <td style="text-align:left;"> NA </td> <td style="text-align:left;"> NA </td> <td style="text-align:left;"> no </td> <td style="text-align:left;"> no </td> </tr> <tr> <td style="text-align:left;"> 4437 </td> <td style="text-align:left;"> 2017 </td> <td style="text-align:left;"> NA </td> <td style="text-align:left;"> Reddy, R; Fahiminiya, S; El Zir, E; Mansour, A; Megarbane, A; Majewski, J; Slim, R </td> <td style="text-align:left;"> 2014 </td> <td style="text-align:left;"> Molecular Genetics of the Usher Syndrome in Lebanon: Identification of 11 Novel Protein Truncating Mutations by Whole Exome Sequencing </td> <td style="text-align:left;"> Background: Usher syndrome (USH) is a genetically heterogeneous condition with ten disease-causing genes. The spectrum of genes and mutations causing USH in the Lebanese and Middle Eastern populations has not been described. Consequently, diagnostic approaches designed to screen for previously reported mutations were unlikely to identify the mutations in 11 unrelated families, eight of Lebanese and three of Middle Eastern origins. In addition, six of the ten USH genes consist of more than 20 exons, each, which made mutational analysis by Sanger sequencing of PCR-amplified exons from genomic DNA tedious and costly. The study was aimed at the identification of USH causing genes and mutations in 11 unrelated families with USH type I or II. Methods: Whole exome sequencing followed by expanded familial validation by Sanger sequencing. Results: We identified disease-causing mutations in all the analyzed patients in four USH genes, MYO7A, USH2A, GPR98 and CDH23. Eleven of the mutations were novel and protein truncating, including a complex rearrangement in GPR98. Conclusion: Our data highlight the genetic diversity of Usher syndrome in the Lebanese population and the time and cost-effectiveness of whole exome sequencing approach for mutation analysis of genetically heterogeneous conditions caused by large genes. </td> <td style="text-align:left;"> 4439 </td> <td style="text-align:left;"> NA </td> <td style="text-align:left;"> NA </td> <td style="text-align:left;"> NA </td> <td style="text-align:left;"> no </td> <td style="text-align:left;"> no </td> </tr> <tr> <td style="text-align:left;"> 4438 </td> <td style="text-align:left;"> 2017 </td> <td style="text-align:left;"> NA </td> <td style="text-align:left;"> Cagliani, R; Guerini, FR; Rubio-Acero, R; Baglio, F; Forni, D; Agliardi, C; Griffanti, L; Fumagalli, M; Pozzoli, U; Riva, S; Calabrese, E; Sikora, M; Casals, F; Comi, GP; Bresolin, N; Caceres, M; Clerici, M; Sironi, M </td> <td style="text-align:left;"> 2013 </td> <td style="text-align:left;"> Long-Standing Balancing Selection in the THBS4 Gene: Influence on Sex-Specific Brain Expression and Gray Matter Volumes in Alzheimer Disease </td> <td style="text-align:left;"> The THBS4 gene encodes a glycoprotein involved in inflammatory responses and synaptogenesis. THBS4 is expressed at higher levels in the brain of humans compared with nonhuman primates, and the protein accumulates in -amyloid plaques. We analyzed THBS4 genetic variability in humans and show that two haplotypes (hap1 and hap2) are maintained by balancing selection and modulate THBS4 expression in lymphocytes. Indeed, the balancing selection region covers a predicted transcriptional enhancer. In humans, but not in macaques and chimpanzees, THBS4 brain expression increases with age, and variants in the balancing selection region interact with sex in influencing THBS4 expression (pinteraction = 0.038), with hap1 homozygous females showing lowest expression. In Alzheimer disease (AD) patients, significant interactions between sex and THBS4 genotype were detected for peripheral gray matter (pinteraction = 0.014) and total gray matter (pinteraction = 0.012) volumes. Similarly to the gene expression results, the interaction is mainly mediated by hap1 homozygous AD females, who show reduced volumes. Thus, the balancing selection target in THBS4 is likely represented by one or more variants that regulate tissue-specific and sex-specific gene expression. The selection signature associated with THBS4 might not be related to AD pathogenesis, but rather to inflammatory responses. </td> <td style="text-align:left;"> 4440 </td> <td style="text-align:left;"> NA </td> <td style="text-align:left;"> NA </td> <td style="text-align:left;"> NA </td> <td style="text-align:left;"> no </td> <td style="text-align:left;"> no </td> </tr> <tr> <td style="text-align:left;"> 4439 </td> <td style="text-align:left;"> 2017 </td> <td style="text-align:left;"> NA </td> <td style="text-align:left;"> Curtis, J; Fraga, LA; de Souza, CP; Correa-Oliveira, R; Minchella, DJ </td> <td style="text-align:left;"> 2001 </td> <td style="text-align:left;"> Widespread heteroplasmy in schistosomes makes an mtVNTR marker "nearsighted" </td> <td style="text-align:left;"> Mitochondrial markers are often hailed as the preferred DNA elements for analyses of population subdivision. To this end we have employed a mitochondrial repeat element to examine the population structure in Schistosoma mansoni (human blood flukes). Schistosome isolates were collected from each of 21 different patients representing seven different areas of a Brazilian village. These parasite isolates demonstrate substantial genetic polymorphism, with an average of 10 genotypes infecting each patient, which is more readily detected because of high levels of heteroplasmy (i.e., 72.5% of the individual worms exhibit multiple versions of this repeat region with different numbers of repeats). Due to the high number of common haplotypes in the population, this repeat element from S. mansoni has a large proportion (47%) of its genetic variation described by differences among mitochondrial genomes within individual worms. However, when only rare haplotypes are considered, population structure can be defected. It seems that heteroplasmy in the schistosome population of Melquiades is both the source of plentiful genetic variation and a confounding factor in the analysis of that variation. Thus the schistosome population in Melquiades may actually be more strongly subdivided than we are able to detect using this mitochondrial marker. </td> <td style="text-align:left;"> 4441 </td> <td style="text-align:left;"> NA </td> <td style="text-align:left;"> NA </td> <td style="text-align:left;"> NA </td> <td style="text-align:left;"> no </td> <td style="text-align:left;"> no </td> </tr> <tr> <td style="text-align:left;"> 4440 </td> <td style="text-align:left;"> 2017 </td> <td style="text-align:left;"> NA </td> <td style="text-align:left;"> Alonso, S; Izagirre, N; Lopez, S; Smith-Zubiaga, I; Hervella, M; Boyano, MD; Arroyo-Berdugo, Y; Gardeazabal, J; Diaz-Ramon, JL; Diez, AS; Careaga, JM; de la Rua, C </td> <td style="text-align:left;"> 2010 </td> <td style="text-align:left;"> The Diversity Profile of TP53 Is Influenced by Positive Selection on the Immediately Upstream Locus WDR79 </td> <td style="text-align:left;"> Background/Aim: TP53 is an efficient central node in a signal transduction network that responds to minimize cancer. However, over 50% of tumors show some mutation in TP53. Thus, one might argue that this single central node network lacks robustness. Therefore, we wanted to investigate if natural selection has played a role in shaping the genomic region containing TP53. Methods: We have analyzed the HapMap data for evidence of selection using FST pairwise comparisons and the extended haplotype homozygosity test on a 200-kb region encompassing TP53. We have also resequenced 4 kb upstream TP53 in Europeans (including melanoma patients), Asians, Australian Aborigines and Africans. Results: Genetic hitchhiking by a linked, positively selected allele at the nearby gene WDR79 may be partly responsible for the sequence diversity profile of TP53. It can help explain why the TP53 Arg72 allele is the major allele in Europeans even when the alternative allele, 72Pro, has been reported to offer an increased longevity after disease. Conclusions: Despite the important role of TP53, a complex interplay with other evolutionary forces, which are extrinsic to TP53 function, may have driven the genetic diversity pattern of this locus, and, as a consequence, its structure and function. Copyright (C) 2009 S. Karger AG, Basel </td> <td style="text-align:left;"> 4442 </td> <td style="text-align:left;"> NA </td> <td style="text-align:left;"> NA </td> <td style="text-align:left;"> NA </td> <td style="text-align:left;"> no </td> <td style="text-align:left;"> no </td> </tr> <tr> <td style="text-align:left;"> 4441 </td> <td style="text-align:left;"> 2017 </td> <td style="text-align:left;"> NA </td> <td style="text-align:left;"> Tumapa, S; Holden, MTG; Vesaratchavest, M; Wuthiekanun, V; Limmathurotsakul, D; Chierakul, W; Feil, EJ; Currie, BJ; Day, NPJ; Nierman, WC; Peacock, SJ </td> <td style="text-align:left;"> 2008 </td> <td style="text-align:left;"> Burkholderia pseudomallei genome plasticity associated with genomic island variation </td> <td style="text-align:left;"> Background: Burkholderia pseudomallei is a soil-dwelling saprophyte and the cause of melioidosis. Horizontal gene transfer contributes to the genetic diversity of this pathogen and may be an important determinant of virulence potential. The genome contains genomic island (GI) regions that encode a broad array of functions. Although there is some evidence for the variable distribution of genomic islands in B. pseudomallei isolates, little is known about the extent of variation between related strains or their association with disease or environmental survival. Results: Five islands from B. pseudomallei strain K96243 were chosen as representatives of different types of genomic islands present in this strain, and their presence investigated in other B. pseudomallei. In silico analysis of 10 B. pseudomallei genome sequences provided evidence for the variable presence of these regions, together with micro-evolutionary changes that generate GI diversity. The diversity of GIs in 186 isolates from NE Thailand (83 environmental and 103 clinical isolates) was investigated using multiplex PCR screening. The proportion of all isolates positive by PCR ranged from 12% for a prophage-like island (GI 9), to 76% for a metabolic island (GI 16). The presence of each of the five GIs did not differ between environmental and disease-associated isolates (p > 0.05 for all five islands). The cumulative number of GIs per isolate for the 186 isolates ranged from 0 to 5 (median 2, IQR 1 to 3). The distribution of cumulative GI number did not differ between environmental and disease-associated isolates (p = 0.27). The presence of GIs was defined for the three largest clones in this collection (each defined as a single sequence type, ST, by multilocus sequence typing); these were ST 70 (n = 15 isolates), ST 54 (n = 11), and ST 167 (n = 9). The rapid loss and/or acquisition of gene islands was observed within individual clones. Comparisons were drawn between isolates obtained from the environment and from patients with melioidosis in order to examine the role of genomic islands in virulence and clinical associations. There was no reproducible association between the individual or cumulative presence of five GIs and a range of clinical features in 103 patients with melioidosis. Conclusion: Horizontal gene transfer of mobile genetic elements can rapidly alter the gene repertoire of B. pseudomallei. This study confirms the utility of a range of approaches in defining the presence and significance of genomic variation in natural populations of B. pseudomallei. </td> <td style="text-align:left;"> 4443 </td> <td style="text-align:left;"> NA </td> <td style="text-align:left;"> NA </td> <td style="text-align:left;"> NA </td> <td style="text-align:left;"> no </td> <td style="text-align:left;"> no </td> </tr> <tr> <td style="text-align:left;"> 4442 </td> <td style="text-align:left;"> 2017 </td> <td style="text-align:left;"> NA </td> <td style="text-align:left;"> Santi-Rocca, J; Fernandez-Cortes, F; Chillon-Marinas, C; Gonzalez-Rubio, ML; Martin, D; Girones, N; Fresno, M </td> <td style="text-align:left;"> 2017 </td> <td style="text-align:left;"> A multi-parametric analysis of Trypanosoma cruzi infection: common pathophysiologic patterns beyond extreme heterogeneity of host responses </td> <td style="text-align:left;"> The extreme genetic diversity of the protozoan Trypanosoma cruzi has been proposed to be associated with the clinical outcomes of the disease it provokes: Chagas disease (CD). To address this question, we analysed the similarities and differences in the CD pathophysiogenesis caused by different parasite strains. Using syngeneic mice infected acutely or chronically with 6 distant parasite strains, we integrated simultaneously 66 parameters: parasite tropism (7 parameters), organ and immune responses (local and systemic; 57 parameters), and clinical presentations of CD (2 parameters). While the parasite genetic background consistently impacts most of these parameters, they remain highly variable, as observed in patients, impeding reliable one-dimensional association with phases, strains, and damage. However, multi-dimensional statistics overcame this extreme intra-group variability for each individual parameter and revealed some pathophysiological patterns that accurately allow defining (i) the infection phase, (ii) the infecting parasite strains, and (iii) organ damage type and intensity. Our results demonstrated a greater variability of clinical outcomes and host responses to T. cruzi infection than previously thought, while our multi-parametric analysis defined common pathophysiological patterns linked to clinical outcome of CD, conserved among the genetically diverse infecting strains. </td> <td style="text-align:left;"> 4444 </td> <td style="text-align:left;"> NA </td> <td style="text-align:left;"> NA </td> <td style="text-align:left;"> NA </td> <td style="text-align:left;"> no </td> <td style="text-align:left;"> no </td> </tr> <tr> <td style="text-align:left;"> 4443 </td> <td style="text-align:left;"> 2017 </td> <td style="text-align:left;"> NA </td> <td style="text-align:left;"> Al-Mahruqi, SH; Zadjali, F; Beja-Pereira, A; Koh, CY; Balkhair, A; Al-Jabri, AA </td> <td style="text-align:left;"> 2014 </td> <td style="text-align:left;"> Genetic diversity and prevalence of CCR2-CCR5 gene polymorphisms in the Omani population </td> <td style="text-align:left;"> Polymorphisms in the regulatory region of the CCR5 gene affect protein expression and modulate the progress of HIV-1 disease. Because of this prominent role, variations in this gene have been under differential pressure and their frequencies vary among human populations. The CCR2V64I mutation is tightly linked to certain polymorphisms in the CCR5 gene. The current Omani population is genetically diverse, a reflection of their history as traders who ruled extensive regions around the Indian Ocean. In this study, we examined the CCR2-CCR5 haplotypes in Omanis and compared the patterns of genetic diversity with those of other populations. Blood samples were collected from 115 Omani adults and genomic DNA was screened to identify the polymorphic sites in theCCR5 gene and the CCR2V64I mutation. Four minor alleles were common: CCR5-2554T and CCR5-2086G showed frequencies of 49% and 46%, respectively, whereas CCR5-2459A and CCR5-2135C both had a frequency of 36%. These alleles showed moderate levels of heterozygosity, indicating that they were under balancing selection. However, the well-known allele CCR5 Delta 32 was relatively rare. Eleven haplotypes were identified, four of which were common: HHC (46%), HHE (20%), HHA (14%) and HHF*2 (12%). </td> <td style="text-align:left;"> 4445 </td> <td style="text-align:left;"> NA </td> <td style="text-align:left;"> NA </td> <td style="text-align:left;"> NA </td> <td style="text-align:left;"> no </td> <td style="text-align:left;"> no </td> </tr> <tr> <td style="text-align:left;"> 4444 </td> <td style="text-align:left;"> 2017 </td> <td style="text-align:left;"> NA </td> <td style="text-align:left;"> Mak, CM; Lam, CW; Tam, S; Lai, CL; Chan, LY; Fan, ST; Lau, YL; Lai, JY; Yuen, P; Hui, J; Fu, CC; Wong, KS; Mak, WL; Tze, K; Tong, SF; Lau, A; Leung, N; Hui, A; Cheung, KM; Ko, CH; Chan, YK; Ma, O; Chau, TN; Chiu, A; Chan, YW </td> <td style="text-align:left;"> 2008 </td> <td style="text-align:left;"> Mutational analysis of 65 Wilson disease patients in Hong Kong Chinese: Identification of 17 novel mutations and its genetic heterogeneity </td> <td style="text-align:left;"> Wilson disease (WD), an autosomal recessive disorder of copper transport, is the most common inherited liver disorder in Hong Kong Chinese. This was the first local study to elucidate the molecular basis and establish an effective DNA-based diagnostic protocol. The ATP7B genes of 65 patients were amplified by polymerase chain reaction (PCR) and sequenced. Haplotype analysis was performed using D13S301, D13S314, and D13S316. The p.L770L/p.R778L status in 660 subjects was determined to estimate WD prevalence. Allele age of p.R778L was determined by the smallest homozygosity region between D13S301 and D13S270. We identified 42 different mutations with 17 being novel. p.R778L (17.3%) was the most prevalent. Exons 2, 8, 12, 13, and 16 harbored 70% mutations. Thirty-two haplotypes were associated with WD chromosomes. The estimated prevalence rate was 1 in 5,400. Three out of 660 normal subjects had p.L770L/p.R778L. In the remaining 657 individuals, neither p.L770L nor p.R778L was found. We characterized a Hong Kong Chinese-specific ATP7B mutation spectrum with great genetic diversity. Exons 2, 8, 12, 13, and 16 should be screened first. The perfect linkage disequilibrium suggested that p.R778L and its private polymorphism p.L770L originated from a single ancestor. This East-Asian-specific mutation p.R778L/p.L770L is aged at least 5,500 years. </td> <td style="text-align:left;"> 4446 </td> <td style="text-align:left;"> NA </td> <td style="text-align:left;"> NA </td> <td style="text-align:left;"> NA </td> <td style="text-align:left;"> no </td> <td style="text-align:left;"> no </td> </tr> <tr> <td style="text-align:left;"> 4445 </td> <td style="text-align:left;"> 2017 </td> <td style="text-align:left;"> NA </td> <td style="text-align:left;"> Liu, C; He, T; Rong, YX; Du, FJ; Ma, DX; Wei, YJ; Mei, ZQ; Wang, YL; Wang, HB; Zhu, YH; Zhang, ZD; Zheng, L; Wu, XQ; Liu, HL; Ding, WJ </td> <td style="text-align:left;"> 2016 </td> <td style="text-align:left;"> Association of Mannose-binding Lectin Polymorphisms with Tuberculosis Susceptibility among Chinese </td> <td style="text-align:left;"> Tuberculosis (TB) is caused by infection of Mycobacterium tuberculosis. Host genetic variability is an important determinant of the risk of developing TB in humans. Although the association between MBL2 polymorphisms and TB has been studied in various populations, the results are controversial. In this study four functional single-nucleotide polymorphisms (SNPs, H/L, X/Y, P/Q and A/B) across the MBL2 gene were genotyped by direct DNA sequencing of PCR products in a case-control population of Chinese Han origin, consisting of 1,020 patients with pulmonary TB and 1,020 controls. We found that individuals carrying variant allele at A/B (namely BB or AB genotypes) was associated with increased susceptibility to TB (odds ratios [OR] = 1.57, 95% confidence interval [CI] 1.30-1.91, P = 1.3 x 10(-6)). Additionally, LYPB haplotype showed a significant association with increased risk of TB (OR = 1.54, 95% CI 1.27-1.87, P = 4.2 x 10(-6); global haplotype association P = 3.5 x 10(-5)). Furthermore, individuals bearing low-or medium-MBL expression haplotype pairs had an increased risk of TB (OR = 1.56, 95% CI 1.29-1.90, P = 1.4 x 10(-6)). Thus, the reduced expression of functional MBL secondary to having MBL2 variants may partially mediate the increased susceptibility to TB risk. </td> <td style="text-align:left;"> 4447 </td> <td style="text-align:left;"> NA </td> <td style="text-align:left;"> NA </td> <td style="text-align:left;"> NA </td> <td style="text-align:left;"> no </td> <td style="text-align:left;"> no </td> </tr> <tr> <td style="text-align:left;"> 4446 </td> <td style="text-align:left;"> 2017 </td> <td style="text-align:left;"> NA </td> <td style="text-align:left;"> Vale, FF; Nunes, A; Oleastro, M; Gomes, JP; Sampaio, DA; Rocha, R; Vitor, JMB; Engstrand, L; Pascoe, B; Berthenet, E; Sheppard, SK; Hitchings, MD; Megraud, F; Vadivelu, J; Lehours, P </td> <td style="text-align:left;"> 2017 </td> <td style="text-align:left;"> Genomic structure and insertion sites of Helicobacter pylori prophages from various geographical origins </td> <td style="text-align:left;"> Helicobacter pylori genetic diversity is known to be influenced by mobile genomic elements. Here we focused on prophages, the least characterized mobile elements of H. pylori. We present the full genomic sequences, insertion sites and phylogenetic analysis of 28 prophages found in H. pylori isolates from patients of distinct disease types, ranging from gastritis to gastric cancer, and geographic origins, covering most continents. The genome sizes of these prophages range from 22.6-33.0 Kbp, consisting of 27-39 open reading frames. A 36.6% GC was found in prophages in contrast to 39% in H. pylori genome. Remarkably a conserved integration site was found in over 50% of the cases. Nearly 40% of the prophages harbored insertion sequences (IS) previously described in H. pylori. Tandem repeats were frequently found in the intergenic region between the prophage at the 3' end and the bacterial gene. Furthermore, prophage genomes present a robust phylogeographic pattern, revealing four distinct clusters: one African, one Asian and two European prophage populations. Evidence of recombination was detected within the genome of some prophages, resulting in genome mosaics composed by different populations, which may yield additional H. pylori phenotypes. </td> <td style="text-align:left;"> 4448 </td> <td style="text-align:left;"> NA </td> <td style="text-align:left;"> NA </td> <td style="text-align:left;"> NA </td> <td style="text-align:left;"> no </td> <td style="text-align:left;"> no </td> </tr> <tr> <td style="text-align:left;"> 4447 </td> <td style="text-align:left;"> 2017 </td> <td style="text-align:left;"> NA </td> <td style="text-align:left;"> Merino, AM; Zhang, K; Kaslow, RA; Aissani, B </td> <td style="text-align:left;"> 2013 </td> <td style="text-align:left;"> Structure of tumor necrosis factor-alpha haploblocks in European populations </td> <td style="text-align:left;"> DNA variants in the tumor necrosis factor-alpha (TNF) and linked lymphotoxin-alpha genes, and specific alleles of the highly polymorphic human leukocyte antigen B (HLA-B) gene have been implicated in a plethora of immune and infectious diseases. However, the tight linkage disequilibrium characterizing the central region of the human major histocompatibility complex (MHC) containing these gene loci has made difficult the unequivocal interpretation of genetic association data. To alleviate these difficulties and facilitate the design of more focused follow-up studies, we investigated the structure and distribution of HLA-B-specific MHC haplotypes reconstructed in a European population from unphased genotypes at a set of 25 single nucleotide polymorphism sites spanning a 66-kilobase long region across TNF. Consistent with the published data, we found limited genetic diversity across the so-called TNF block, with the emergence of seven common MHC haplotypes, termed TNF block super-haplotypes. We also found that the ancestral haplotype 8.1 shares a TNF block haplotype with HLA-B*4402. HLA-B*5701, a known protective allele in HIV-1 pathogenesis, occurred in a unique TNF block haplotype. </td> <td style="text-align:left;"> 4449 </td> <td style="text-align:left;"> NA </td> <td style="text-align:left;"> NA </td> <td style="text-align:left;"> NA </td> <td style="text-align:left;"> no </td> <td style="text-align:left;"> no </td> </tr> <tr> <td style="text-align:left;"> 4448 </td> <td style="text-align:left;"> 2017 </td> <td style="text-align:left;"> NA </td> <td style="text-align:left;"> Gautam, V; Patil, PP; Kumar, S; Midha, S; Kaur, M; Kaur, S; Singh, M; Mali, S; Shastri, J; Arora, A; Ray, P; Patil, PB </td> <td style="text-align:left;"> 2016 </td> <td style="text-align:left;"> Multilocus sequence analysis reveals high genetic diversity in clinical isolates of Burkholderia cepacia complex from India </td> <td style="text-align:left;"> Burkholderia cepacia complex (Bcc) is a complex group of bacteria causing opportunistic infections in immunocompromised and cystic fibrosis (CF) patients. Herein, we report multilocus sequence typing and analysis of the 57 clinical isolates of Bcc collected over the period of seven years (2005-2012) from several hospitals across India. A total of 21 sequence types (ST) including two STs from cystic fibrosis patient's isolates and twelve novel STs were identified in the population reflecting the extent of genetic diversity. Multilocus sequence analysis revealed two lineages in population, a major lineage belonging to B. cenocepacia and a minor lineage belonging to B. cepacia. Split-decomposition analysis suggests absence of interspecies recombination and intraspecies recombination contributed in generating genotypic diversity amongst isolates. Further linkage disequilibrium analysis indicates that recombination takes place at a low frequency, which is not sufficient to break down the clonal relationship. This knowledge of the genetic structure of Bcc population from a rapidly developing country will be invaluable in the epidemiology, surveillance and understanding global diversity of this group of a pathogen. </td> <td style="text-align:left;"> 4450 </td> <td style="text-align:left;"> NA </td> <td style="text-align:left;"> NA </td> <td style="text-align:left;"> NA </td> <td style="text-align:left;"> no </td> <td style="text-align:left;"> no </td> </tr> <tr> <td style="text-align:left;"> 4449 </td> <td style="text-align:left;"> 2017 </td> <td style="text-align:left;"> NA </td> <td style="text-align:left;"> Biunno, I; Aceto, G; Awadelkarim, KD; Morgano, A; Elhaj, A; Eltayeb, EA; Abuidris, DO; Elwali, NE; Spinelli, C; De Blasio, P; Rovida, E; Mariani-Costantini, R </td> <td style="text-align:left;"> 2014 </td> <td style="text-align:left;"> BRCA1 point mutations in premenopausal breast cancer patients from Central Sudan </td> <td style="text-align:left;"> Premenopausal breast cancer (BC) is one of the most common cancers of women in rural Africa and part of the disease load may be related to hereditary predisposition, including mutations in the BRCA1 gene. However, the BRCA1 mutations associated with BC in Africa are scarcely characterized. We report here 33 BRCA1 point mutations, among which 2 novel missense variants, found in 59 Central Sudanese premenopausal BC patients. The high fractions of mutations with intercontinental and uniquely African distribution (17/33, 51.5 % and 14/33, 42.4 %, respectively) are in agreement with the high genetic diversity expected in an African population. Overall 24/33 variants (72.7 %) resulted neutral; 8/33 of unknown significance (24.3 %, including the 2 novel missense mutations); 1 (3.0 %) overtly deleterious. Notably, in silico studies predict that the novel C-terminal missense variant c.5090G > A (p.Cys1697Tyr) affects phosphopeptide recognition by the BRCA1 BRCT1 domain and may have a pathogenic impact. Genetic variation and frequency of unique or rare mutations of uncertain clinical relevance pose significant challenges to BRCA1 testing in Sudan, as it might happen in other low-resource rural African contexts. </td> <td style="text-align:left;"> 4451 </td> <td style="text-align:left;"> NA </td> <td style="text-align:left;"> NA </td> <td style="text-align:left;"> NA </td> <td style="text-align:left;"> no </td> <td style="text-align:left;"> no </td> </tr> <tr> <td style="text-align:left;"> 4450 </td> <td style="text-align:left;"> 2017 </td> <td style="text-align:left;"> NA </td> <td style="text-align:left;"> Newport, MJ; Finan, C </td> <td style="text-align:left;"> 2011 </td> <td style="text-align:left;"> Genome-wide association studies and susceptibility to infectious diseases </td> <td style="text-align:left;"> Progress in genomics and the associated technological, statistical and bioinformatics advances have facilitated the successful implementation of genome-wide association studies (GWAS) towards understanding the genetic basis of common diseases. Infectious diseases contribute significantly to the global burden of disease and there is robust epidemiological evidence that host genetic factors are important determinants of the outcome of interactions between host and pathogen. Indeed, infectious diseases have exerted profound selective pressure on human evolution. However, the application of GWAS to infectious diseases has been relatively limited compared with non-communicable diseases. Here we review GWAS findings for important infectious diseases, including malaria, tuberculosis and HIV. We highlight some of the pitfalls recognized more generally for GWAS, as well as issues specific to infection, including the role of the pathogen which also has a genome. We also discuss the challenges encountered when studying African populations which are genetically more ancient and more diverse that other populations and disproportionately bear the main global burden of serious infectious diseases. </td> <td style="text-align:left;"> 4452 </td> <td style="text-align:left;"> NA </td> <td style="text-align:left;"> NA </td> <td style="text-align:left;"> NA </td> <td style="text-align:left;"> no </td> <td style="text-align:left;"> no </td> </tr> <tr> <td style="text-align:left;"> 4451 </td> <td style="text-align:left;"> 2017 </td> <td style="text-align:left;"> NA </td> <td style="text-align:left;"> Gomes, KB; Pardini, VC; Ferreira, ACS; Fonseca, CG; Fernandes, AP </td> <td style="text-align:left;"> 2007 </td> <td style="text-align:left;"> Founder effect of the 669insA mutation in BSCL2 gene causing Berardinelli-Seip congenital lipodystrophy in a cluster from Brazil </td> <td style="text-align:left;"> Congenital generalized lipodystrophy (CGL) or Berardinelli-Seip Syndrome (BSCL) is a rare autosomal recessive disease characterized by a nearly-complete absence of adipose tissue from birth and severe metabolic alterations. The 669insA mutation in exon 4 of the BSCL2 gene was identified as the major genetic alteration leading to BSCL in a group of 22 patients from the northeastern Brazilian state of Rio Grande do Norte. Aiming to investigate the causes of the high frequency of BSCL in this region, a molecular genetic study was conducted using eight microsatelite markers located in chromosome 11. Additional investigations concerning the proportion of expected homozygous and heterozygous individuals, genetic diversity, fixation index and coefficient of endogamy were undertaken, and indicated significant differences by comparing the allelic and haplotypic frequencies observed for the BSCL affected families and the control group. It was concluded that a founder effect, genetic drift and consanguineous marriages have significantly affected the structure of this population, resulting in the highest frequency of BSCL in Brazil. </td> <td style="text-align:left;"> 4453 </td> <td style="text-align:left;"> NA </td> <td style="text-align:left;"> NA </td> <td style="text-align:left;"> NA </td> <td style="text-align:left;"> no </td> <td style="text-align:left;"> no </td> </tr> <tr> <td style="text-align:left;"> 4452 </td> <td style="text-align:left;"> 2017 </td> <td style="text-align:left;"> NA </td> <td style="text-align:left;"> Trifonova, EA; Spiridonova, MG; Gabidulina, TV; Urnov, FD; Puzyrev, VP; Stepanov, VA </td> <td style="text-align:left;"> 2012 </td> <td style="text-align:left;"> Analysis of the MTHFR gene linkage disequilibrium structure and association of polymorphic gene variants with coronary atherosclerosis </td> <td style="text-align:left;"> Analysis of the genome-specific linkage disequilibrium patterns in certain populations is a highly promising approach to the identification of functional variants that underlie susceptibility to complex diseases. In the present study, the linkage disequilibrium patterns of the methylenetetrahydrofolate reductase gene (MTHFR) were examined in a group of patients with coronary atherosclerosis (coronary artery disease, CAD) and in a control sample from the Russian population. It was demonstrated that in the samples from one population, which were differentiated by the presence or absence of CAD, the MTHFR linkage disequilibrium patterns had similar features. Association of the MTHFR rs7533315 and rs2066462 polymorphisms with CAD was demonstrated. In addition, the evolution of the haplotypes and their role in the formation of CAD in the Russian population was reconstructed. The data on the association between genetic variability in the MTHFR locus and pathogenetically important indices of lipid metabolism were obtained. The high informativeness of the haplotype approach in case-control tests for associations with CAD was demonstrated. </td> <td style="text-align:left;"> 4454 </td> <td style="text-align:left;"> NA </td> <td style="text-align:left;"> NA </td> <td style="text-align:left;"> NA </td> <td style="text-align:left;"> no </td> <td style="text-align:left;"> no </td> </tr> <tr> <td style="text-align:left;"> 4453 </td> <td style="text-align:left;"> 2017 </td> <td style="text-align:left;"> NA </td> <td style="text-align:left;"> Papeo, L; Cecchetto, C; Mazzon, G; Granello, G; Cattaruzza, T; Verriello, L; Eleopra, R; Rumiati, RI </td> <td style="text-align:left;"> 2015 </td> <td style="text-align:left;"> The processing of actions and action-words in amyotrophic lateral sclerosis patients </td> <td style="text-align:left;"> Amyotrophic lateral sclerosis (ALS) is a neurodegenerative disease with prime consequences on the motor function and concomitant cognitive changes, most frequently in the domain of executive functions. Moreover, poorer performance with action-verbs versus object-nouns has been reported in ALS patients, raising the hypothesis that the motor dysfunction deteriorates the semantic representation of actions. Using action-verbs and manipulable-object nouns sharing semantic relationship with the same motor representations, the verb-noun difference was assessed in a group of 21 ALS-patients with severely impaired motor behavior, and compared with a normal sample's performance. ALS-group performed better on nouns than verbs, both in production (action and object naming) and comprehension (word-picture matching). This observation implies that the interpretation of the verb-noun difference in ALS cannot be accounted by the relatedness of verbs to motor representations, but has to consider the role of other semantic and/or morphophonological dimensions that distinctively define the two grammatical classes. Moreover, this difference in the ALS-group was not greater than the noun-verb difference in the normal sample. The mental representation of actions also involves an executive-control component to organize, in logical/temporal order, the individual motor events (or subgoals) that form a purposeful action. We assessed this ability with action sequencing tasks, requiring participants to re-construct a purposeful action from the scrambled presentation of its constitutive motor events, shown in the form of photographs or short sentences. In those tasks, ALS-group's performance was significantly poorer than controls'. Thus, the executive dysfunction manifested in the sequencing deficit but not the selective verb deficit appears as a consistent feature of the cognitive profile associated with ALS. We suggest that ALS can offer a valuable model to study the relationship between (frontal) motor centers and the executive-control machinery housed in the frontal brain, and the implications of executive dysfunctions in tasks such as action processing. (C) 2014 Elsevier Ltd. All rights reserved. </td> <td style="text-align:left;"> 4455 </td> <td style="text-align:left;"> NA </td> <td style="text-align:left;"> NA </td> <td style="text-align:left;"> NA </td> <td style="text-align:left;"> no </td> <td style="text-align:left;"> no </td> </tr> <tr> <td style="text-align:left;"> 4454 </td> <td style="text-align:left;"> 2017 </td> <td style="text-align:left;"> NA </td> <td style="text-align:left;"> Perez-Losada, M; Alamri, L; Crandall, KA; Freishtat, RJ </td> <td style="text-align:left;"> 2017 </td> <td style="text-align:left;"> Nasopharyngeal Microbiome Diversity Changes over Time in Children with Asthma </td> <td style="text-align:left;"> Background The nasopharynx is a reservoir for pathogens associated with respiratory illnesses such as asthma. Next-generation sequencing (NGS) has been used to characterize the nasopharyngeal microbiome of infants and adults during health and disease; less is known, however, about the composition and temporal dynamics (i. e., longitudinal variation) of microbiotas from children and adolescents. Here we use NGS technology to characterize the nasopharyngeal microbiomes of asthmatic children and adolescents (6 to 18 years) and determine their stability over time. Methods Two nasopharyngeal washes collected 5.5 to 6.5 months apart were taken from 40 children and adolescents with asthma living in the Washington D. C. area. Sequence data from the 16S-V4 rRNA gene region (similar to 250 bp) were collected from the samples using the MiSeq platform. Raw data were processed in mothur (SILVA123 reference database) and Operational Taxonomic Units (OTU)-based alpha-and beta-diversity metrics were estimated. Relatedness among samples was assessed using PCoA ordination and Procrustes analyses. Differences in microbial diversity and taxon mean relative proportions were assessed using linear mixed effects models. Core microbiome analyses were also performed to identify stable and consistent microbes of the nasopharynx. Results and Discussion A total of 2,096,584 clean 16S sequences corresponding to an average of 167 OTUs per sample were generated. Representatives of Moraxella*, Staphylococcus*, Dolosigranulum, Corynebacterium, Prevotella, Streptococcus*, Haemophilus*, Fusobacterium* and a Neisseriaceae genus accounted for 86% of the total reads. These nine genera have been previously found in the nasopharynxes of both infants and adults, but in different proportions. OTUs from the five genera highlighted (*) above defined the nasopharyngeal core microbiome at the 95% level. No significant differences in alpha-and beta-diversity were observed between seasons, but bacterial mean relative proportions of Haemophilus, Moraxella, Staphylococcus and Corynebacterium varied significantly between summer-fall and age groups (inter-patient variation). Additionally, OTUs varied significantly within patients between time points in 35 of the 40 patients analyzed. Future cross-sectional studies should be mindful of the temporal dynamics of the nasopharyngeal microbiota. </td> <td style="text-align:left;"> 4456 </td> <td style="text-align:left;"> NA </td> <td style="text-align:left;"> NA </td> <td style="text-align:left;"> NA </td> <td style="text-align:left;"> no </td> <td style="text-align:left;"> no </td> </tr> <tr> <td style="text-align:left;"> 4455 </td> <td style="text-align:left;"> 2017 </td> <td style="text-align:left;"> NA </td> <td style="text-align:left;"> Shahzad, N; Aslam, B; Hussain, I; Ijaz, M; Rasool, MH; Tasneem, F; Hamid, T; Tayyeb, A; Hussain, T </td> <td style="text-align:left;"> 2016 </td> <td style="text-align:left;"> Distribution and Phylogenetic Analysis of Bacterial Isolates from Urinary Tract Infection Patients of Pakistan </td> <td style="text-align:left;"> Urinary Tract Infections (UTIs) are the second most common infections that infect millions of people worldwide. UTI can occur anywhere along the urinary tract and may cause symptomatic infection to complete renal failure. Most UTIs are caused by Escherichia coli (E. coli) which exhibit a high degree of both phenotypic and genetic diversity and disseminated in various geographical regions of the world. The aim of the present study was to determine the foremost pathogen responsible for UTIs and to investigate its genetic lineage in Pakistan. The clean clutch midstream urine samples of 174 (78% females and 22% males) patients with clinical symptoms of UTI from Gujranwala and Faisalabad districts of Pakistan, were cultured for the isolation of bacterial agents. The bacterial species were identified by standard biochemical and molecular tests. Out of total 174 samples, 84 (48%) were found urine culture positive with CFU >104. Among them, E. colt was the most frequent bacterium (n=51, 60.7%) isolated from patients followed by Pseudomonas sp. (n=13,15.4 %), Klebsiella sp. (n=9, 10.7%), Proteus sp. (n=7, 8.3%), and Staphylococcus aureus (n=4, 4.7%). To perform phylogenetic analysis of E. coli isolates, partial 16S rRNA was amplified and sequenced. Sequences were aligned and edited using CodonCode Aligner. Phylogenetic tree constructed on the basis of 613 bp fragment of 16S rRNA, using MEGA6.1 through Neighbor Joining method revealed that majority of E. coli isolates (n=16) had close relatedness with established uropathogenic strain ST-131. Eight isolates resembled closely and grouped with each of ST-101 and ST-14 strains while seven isolates showed high similarity with ST-648 strain followed by ST-73 strain indicating genetic closeness with four local isolates. It is concluded that genetically diverse uropathogenic E. coli are prevalent in the clinical setting of Pakistan in the studied area. The data of these isolated E. coli strains would improve our understanding about the epidemiology of this organism and may serve as baseline information for future researchers in the days ahead. </td> <td style="text-align:left;"> 4457 </td> <td style="text-align:left;"> NA </td> <td style="text-align:left;"> NA </td> <td style="text-align:left;"> NA </td> <td style="text-align:left;"> no </td> <td style="text-align:left;"> no </td> </tr> <tr> <td style="text-align:left;"> 4456 </td> <td style="text-align:left;"> 2017 </td> <td style="text-align:left;"> NA </td> <td style="text-align:left;"> Manske, M; Miotto, O; Campino, S; Auburn, S; Almagro-Garcia, J; Maslen, G; O'Brien, J; Djimde, A; Doumbo, O; Zongo, I; Ouedraogo, JB; Michon, P; Mueller, I; Siba, P; Nzila, A; Borrmann, S; Kiara, SM; Marsh, K; Jiang, H; Su, XZ; Amaratunga, C; Fairhurst, R; Socheat, D; Nosten, F; Imwong, M; White, NJ; Sanders, M; Anastasi, E; Alcock, D; Drury, E; Oyola, S; Quail, MA; Turner, DJ; Ruano-Rubio, V; Jyothi, D; Amenga-Etego, L; Hubbart, C; Jeffreys, A; Rowlands, K; Sutherland, C; Roper, C; Mangano, V; Modiano, D; Tan, JC; Ferdig, MT; Amambua-Ngwa, A; Conway, DJ; Takala-Harrison, S; Plowe, CV; Rayner, JC; Rockett, KA; Clark, TG; Newbold, CI; Berriman, M; MacInnis, B; Kwiatkowski, DP </td> <td style="text-align:left;"> 2012 </td> <td style="text-align:left;"> Analysis of Plasmodium falciparum diversity in natural infections by deep sequencing </td> <td style="text-align:left;"> Malaria elimination strategies require surveillance of the parasite population for genetic changes that demand a public health response, such as new forms of drug resistance(1,2). Here we describe methods for the large-scale analysis of genetic variation in Plasmodium falciparum by deep sequencing of parasite DNA obtained from the blood of patients with malaria, either directly or after short-term culture. Analysis of 86,158 exonic single nucleotide polymorphisms that passed genotyping quality control in 227 samples from Africa, Asia and Oceania provides genome-wide estimates of allele frequency distribution, population structure and linkage disequilibrium. By comparing the genetic diversity of individual infections with that of the local parasite population, we derive a metric of within-host diversity that is related to the level of inbreeding in the population. An open-access web application has been established for the exploration of regional differences in allele frequency and of highly differentiated loci in the P. falciparum genome. </td> <td style="text-align:left;"> 4458 </td> <td style="text-align:left;"> NA </td> <td style="text-align:left;"> NA </td> <td style="text-align:left;"> NA </td> <td style="text-align:left;"> no </td> <td style="text-align:left;"> no </td> </tr> <tr> <td style="text-align:left;"> 4457 </td> <td style="text-align:left;"> 2017 </td> <td style="text-align:left;"> NA </td> <td style="text-align:left;"> Buckee, CO; Bull, PC; Gupta, S </td> <td style="text-align:left;"> 2009 </td> <td style="text-align:left;"> Inferring malaria parasite population structure from serological networks </td> <td style="text-align:left;"> The malaria parasite Plasmodium falciparum is characterized by high levels of genetic diversity at antigenic loci involved in virulence and immune evasion. Knowledge of the population structure and dynamics of these genes is important for designing control programmes and understanding the acquisition of immunity to malaria; however, high rates of homologous and non-homologous recombination as well as complex patterns of expression within hosts have hindered attempts to elucidate these structures experimentally. Here, we analyse serological data from Kenya using a novel network technique to deconstruct the relationships between patients' immune responses to different parasite isolates. We show that particular population structures and expression patterns produce distinctive signatures within serological networks of parasite recognition, which can be used to discriminate between competing hypotheses regarding the organization of these genes. Our analysis suggests that different levels of immune selection occur within different groups of the same multigene family leading to mixed population structures. </td> <td style="text-align:left;"> 4459 </td> <td style="text-align:left;"> NA </td> <td style="text-align:left;"> NA </td> <td style="text-align:left;"> NA </td> <td style="text-align:left;"> no </td> <td style="text-align:left;"> no </td> </tr> <tr> <td style="text-align:left;"> 4458 </td> <td style="text-align:left;"> 2017 </td> <td style="text-align:left;"> NA </td> <td style="text-align:left;"> Deshpande, NP; Kaakoush, NO; Wilkins, MR; Mitchell, HM </td> <td style="text-align:left;"> 2013 </td> <td style="text-align:left;"> Comparative genomics of Campylobacter concisus isolates reveals genetic diversity and provides insights into disease association </td> <td style="text-align:left;"> Background: In spite of its association with gastroenteritis and inflammatory bowel diseases, the isolation of Campylobacter concisus from both diseased and healthy individuals has led to controversy regarding its role as an intestinal pathogen. One proposed reason for this is the presence of high genetic diversity among the genomes of C. concisus strains. Results: In this study the genomes of six C. concisus strains were sequenced, assembled and annotated including two strains isolated from Crohn's disease patients (UNSW2 and UNSW3), three from gastroenteritis patients (UNSW1, UNSWCS and ATCC 51562) and one from a healthy individual (ATCC 51561). The genomes of C. concisus BAA-1457 and UNSWCD, available from NCBI, were included in subsequent comparative genomic analyses. The Pan and Core genomes for the sequenced C. concisus strains consisted of 3254 and 1556 protein coding genes, respectively. Conclusion: Genes were identified with specific conservation in C. concisus strains grouped by phenotypes such as invasiveness, adherence, motility and diseased states. Phylogenetic trees based on ribosomal RNA sequences and concatenated host-related pathways for the eight C. concisus strains were generated using the neighbor-joining method, of which the 16S rRNA gene and peptidoglycan biosynthesis grouped the C. concisus strains according to their pathogenic phenotypes. Furthermore, 25 non-synonymous amino acid changes with 14 affecting functional domains, were identified within proteins of conserved host-related pathways, which had possible associations with the pathogenic potential of C. concisus strains. Finally, the genomes of the eight C. concisus strains were compared to the nine available genomes of the well-established pathogen Campylobacter jejuni, which identified several important differences in the respiration pathways of these two species. Our findings indicate that C. concisus strains are genetically diverse, and suggest the genomes of this bacterium contain respiration pathways and modifications in the peptidoglycan layer that may play an important role in its virulence. </td> <td style="text-align:left;"> 4460 </td> <td style="text-align:left;"> NA </td> <td style="text-align:left;"> NA </td> <td style="text-align:left;"> NA </td> <td style="text-align:left;"> no </td> <td style="text-align:left;"> no </td> </tr> <tr> <td style="text-align:left;"> 4459 </td> <td style="text-align:left;"> 2017 </td> <td style="text-align:left;"> NA </td> <td style="text-align:left;"> Ghaloul-Gonzalez, L; Goldstein, A; Vockley, CW; Dobrowolski, SF; Biery, A; Irani, A; Ibarra, J; Morton, DH; Mohsen, AW; Vockley, J </td> <td style="text-align:left;"> 2016 </td> <td style="text-align:left;"> Mitochondrial respiratory chain disorders in the Old Order Amish population </td> <td style="text-align:left;"> The Old Order Amish populations in the US are one of the Plain People groups and are descendants of the Swiss Anabaptist immigrants who came to North America in the early eighteenth century. They live in numerous small endogamous demes that have resulted in reduced genetic diversity along with a high prevalence of specific genetic disorders, many of them autosomal recessive. Mitochondrial respiratory chain deficiencies arising from mitochondrial or nuclear DNA mutations have not previously been reported in the Plain populations. Here we present four different Amish families with mitochondrial respiratory chain disorders. Mutations in two mitochondrial encoded genes leading to mitochondrial respiratory chain disorder were identified in two patients. In the first case, MELAS syndrome caused by a mitochondrial DNA (mtDNA) mutation (m.3243A>G) was identified in an extended Amish pedigree following a presentation of metabolic strokes in the proband. Characterization of the extended family of the proband by a high resolution melting assay identified the same mutation in many previously undiagnosed family members with a wide range of clinical symptoms. A MELAS/Leigh syndrome phenotype caused by a mtDNA mutation [m.13513G>A; p.Asp393Asn] in the ND5 gene encoding the ND5 subunit of respiratory chain complex I was identified in a patient in a second family. Mutations in two nuclear encoded genes leading to mitochondrial respiratory chain disorder were also identified in two patients. One patient presented with Leigh syndrome and had a homozygous deletion in the NDUFAF2 gene, while the second patient had a homozygous mutation in the POLG gene, [c.1399G>A; p.Ala467Thr]. Our findings identify mitochondrial respiratory chain deficiency as a cause of disease in the Old Order Amish that must be considered in the context of otherwise unexplained systemic disease, especially if neuromuscular symptoms are present. (C) 2016 Elsevier Inc. All rights reserved. </td> <td style="text-align:left;"> 4461 </td> <td style="text-align:left;"> NA </td> <td style="text-align:left;"> NA </td> <td style="text-align:left;"> NA </td> <td style="text-align:left;"> no </td> <td style="text-align:left;"> no </td> </tr> <tr> <td style="text-align:left;"> 4460 </td> <td style="text-align:left;"> 2017 </td> <td style="text-align:left;"> NA </td> <td style="text-align:left;"> Rueffert, H; Olthoff, D; Deutrich, C; Froster, UG </td> <td style="text-align:left;"> 2001 </td> <td style="text-align:left;"> Determination of a positive malignant hyperthermia (MH) disposition without the in vitro contracture test in families carrying the RYR1 Arg614Cys mutation </td> <td style="text-align:left;"> Molecular genetic methods are used with caution for determining positive malignant hyperthermia (MH) disposition in clinical MH diagnosis because of the genetic variability of this disease. But under defined conditions, genotyping can have an advantage over the standardized in vitro contracture test (IVCT) in respect of invasive approach, specificity, patient compliance, and the work and expense involved in the method. We aim to demonstrate this using 10 families with the Arg614Cys mutation in the ryanodine receptor as an example. Fifty-one index patients who had been classified as MH-susceptible (MHS) in the IVCT (European MH protocol) after a clinical MH incident or suspected MH were screened for the Arg614Cys (C1840 --> T) mutation in the RYR1 gene because this mutation is more common in German MH families (9%). The family members or those index patients, in whom a Arg614Cys mutation was detectable, were also screened for the presence of this mutation (n = 136), and the results were subjected to a more detailed analysis including existing IVCT findings (n = 71). The Arg614Cys mutation was identified in a total of 64 members of the 10 independent families. In 35 individuals in this group, there was a definite concordance between the MHS diagnosis in. the IVCT and the presence of the Arg614Cys mutation. No individual phenotyped as MH-negative carried the mutation. On the basis of the guidelines of the EMHG for molecular genetic detection of MH susceptibility, 29 individuals who bore the Arg614Cys mutation were classified as MHS without the IVCT. If a causal mutation is detected in an MH family, the MHS diagnosis can be deduced without the invasive IVCT in all other mutation carriers. Despite inclusion of only one (Arg614Cys) of all known MH mutations, the study emphasizes the practical use of a genetic approach for determination of a positive MH diagnosis. </td> <td style="text-align:left;"> 4462 </td> <td style="text-align:left;"> NA </td> <td style="text-align:left;"> NA </td> <td style="text-align:left;"> NA </td> <td style="text-align:left;"> no </td> <td style="text-align:left;"> no </td> </tr> <tr> <td style="text-align:left;"> 4461 </td> <td style="text-align:left;"> 2017 </td> <td style="text-align:left;"> NA </td> <td style="text-align:left;"> Darini, ALC; Magalhaes, VD; Levy, CL; Barth, AL; Coscina, AL </td> <td style="text-align:left;"> 1999 </td> <td style="text-align:left;"> Phenotyping and genotyping methods applied to investigate the relatedness of Brazilian isolates of Enterobacter cloacae </td> <td style="text-align:left;"> In order to evaluate the resolving power of several typing methods to identify relatedness among Brazilian strains of Enterobacter cloacae, we selected twenty isolates from different patients on three wards of a University Hospital (Orthopedics, Nephrology, and Hematology). Traditional phenotyping methods applied to isolates included biotyping, antibiotic sensitivity, phage-typing, and O-serotyping. Plasmid profile analysis, ribotyping, and macrorestriction analysis by pulsed-field gel electrophoresis (PFGE) were used as genotyping methods. Sero- and phage-typing were not useful since the majority of isolates could not be subtyped by these methods. Biotyping, antibiogram and plasmid profile permitted us to classify the samples into different groups depending on the method used, and consequently were not reliable. Ribotyping and PFGE were significantly correlated with the clinical epidemiological analysis. PFGE did not type strains containing nonspecific DNase. Ribotyping was the most discriminative method for typing Brazilian isolates of E. cloacae. </td> <td style="text-align:left;"> 4463 </td> <td style="text-align:left;"> NA </td> <td style="text-align:left;"> NA </td> <td style="text-align:left;"> NA </td> <td style="text-align:left;"> no </td> <td style="text-align:left;"> no </td> </tr> <tr> <td style="text-align:left;"> 4462 </td> <td style="text-align:left;"> 2017 </td> <td style="text-align:left;"> NA </td> <td style="text-align:left;"> Ikonen, N; Haanpaa, M; Ronkko, E; Lyytikainen, O; Kuusi, M; Ruutu, P; Kallio-Kokko, H; Mannonen, L; Lappalainen, M; Ziegler, T; Julkunen, I </td> <td style="text-align:left;"> 2010 </td> <td style="text-align:left;"> Genetic Diversity of the 2009 Pandemic Influenza A(H1N1) Viruses in Finland </td> <td style="text-align:left;"> Background: In Finland, the first infections caused by the 2009 pandemic influenza A(H1N1) virus were identified on May 10. During the next three months almost all infections were found from patients who had recently traveled abroad. In September 2009 the pandemic virus started to spread in the general population, leading to localized outbreaks and peak epidemic activity was reached during weeks 43-48. Methods/Results: The nucleotide sequences of the hemagglutinin (HA) and neuraminidase (NA) genes from viruses collected from 138 patients were determined. The analyzed viruses represented mild and severe infections and different geographic regions and time periods. Based on HA and NA gene sequences, the Finnish pandemic viruses clustered in four groups. Finnish epidemic viruses and A/California/07/2009 vaccine virus strain varied from 2-8 and 0-5 amino acids in HA and NA molecules, respectively, giving a respective maximal evolution speed of 1.4% and 1.1%. Most amino acid changes in HA and NA molecules accumulated on the surface of the molecule and were partly located in antigenic sites. Three severe infections were detected with a mutation at HA residue 222, in two viruses with a change D222G, and in one virus D222Y. Also viruses with change D222E were identified. All Finnish pandemic viruses were sensitive to oseltamivir having the amino acid histidine at residue 275 of the neuraminidase molecule. Conclusions: The Finnish pandemic viruses were quite closely related to A/California/07/2009 vaccine virus. Neither in the HA nor in the NA were changes identified that may lead to the selection of a virus with increased epidemic potential or exceptionally high virulence. Continued laboratory-based surveillance of the 2009 pandemic influenza A(H1N1) is important in order to rapidly identify drug resistant viruses and/or virus variants with potential ability to cause severe forms of infection and an ability to circumvent vaccine-induced immunity. </td> <td style="text-align:left;"> 4464 </td> <td style="text-align:left;"> NA </td> <td style="text-align:left;"> NA </td> <td style="text-align:left;"> NA </td> <td style="text-align:left;"> no </td> <td style="text-align:left;"> no </td> </tr> <tr> <td style="text-align:left;"> 4463 </td> <td style="text-align:left;"> 2017 </td> <td style="text-align:left;"> NA </td> <td style="text-align:left;"> Pakhomov, SVS; Hemmy, LS; Lim, KO </td> <td style="text-align:left;"> 2012 </td> <td style="text-align:left;"> Automated semantic indices related to cognitive function and rate of cognitive decline </td> <td style="text-align:left;"> The objective of our study is to introduce a fully automated, computational linguistic technique to quantify semantic relations between words generated on a standard semantic verbal fluency test and to determine its cognitive and clinical correlates. Cognitive differences between patients with Alzheimer's disease and mild cognitive impairment are evident in their performance on the semantic verbal fluency test. In addition to the semantic verbal fluency test score, several other performance characteristics sensitive to disease status and predictive of future cognitive decline have been defined in terms of words generated from semantically related categories (clustering) and shifting between categories (switching). However, the traditional assessment of clustering and switching has been performed manually in a qualitative fashion resulting in subjective scoring with limited reproducibility and scalability. Our approach uses word definitions and hierarchical relations between the words in WordNet (R), a large electronic lexical database, to quantify the degree of semantic similarity and relatedness between words. We investigated the novel semantic fluency indices of mean cumulative similarity and relatedness between all pairs of words regardless of their order, and mean sequential similarity and relatedness between pairs of adjacent words in a sample of patients with clinically diagnosed probable (n=55) or possible (n=27) Alzheimer's disease or mild cognitive impairment (n=31). The semantic fluency indices differed significantly between the diagnostic groups, and were strongly associated with neuropsychological tests of executive function, as well as the rate of global cognitive decline. Our results suggest that word meanings and relations between words shared across individuals and computationally modeled via WordNet and large text corpora provide the necessary context to account for the variability in language-based behavior and relate it to cognitive dysfunction observed in mild cognitive impairment and Alzheimer's disease. (C) 2012 Elsevier Ltd. All rights reserved. </td> <td style="text-align:left;"> 4465 </td> <td style="text-align:left;"> NA </td> <td style="text-align:left;"> NA </td> <td style="text-align:left;"> NA </td> <td style="text-align:left;"> no </td> <td style="text-align:left;"> no </td> </tr> <tr> <td style="text-align:left;"> 4464 </td> <td style="text-align:left;"> 2017 </td> <td style="text-align:left;"> NA </td> <td style="text-align:left;"> SAYADA, C; VRETOU, E; ORFILA, J; ELION, J; DENAMUR, E </td> <td style="text-align:left;"> 1995 </td> <td style="text-align:left;"> HETEROGENEITY WITHIN THE FIRST CONSTANT SEGMENT OF THE MAJOR OUTER-MEMBRANE PROTEIN GENE IN CHLAMYDIA-TRACHOMATIS SEROVAR D/DA DISTINGUISHES 2 LINEAGES </td> <td style="text-align:left;"> Restriction fragment length polymorphism of the the major outer membrane protein gene (omp 1) was studied in 73 epidemiologically unrelated Chlamydia trachomaris serovar D (n = 64) and Da (n = 9) strains isolated between 1983 and 1991 in various european countries from patients suffering from sexually transmitted diseases, as well as 3 reference strains D/IC-Cal-8 (USA), D/UW3 (UK) and Da/TW-448 (Taiwan). Among these strains, 5 different genotypic groups were distinguished: 3 for the serovar D strains and 2 for the serovar Da strains. Comparisons of the complete omp1 nucleotide sequence of 1 member of each group revealed 2 distinct lineages, independently of the D/Da classification, presumably as the result of an intragenic DNA recombination event within the first constant segment. The observed omp1 genetic diversity of serovar D/Da strains in regions involved in T- and B-cell responses might add another level of complexity to the design of a subunit vaccine. </td> <td style="text-align:left;"> 4466 </td> <td style="text-align:left;"> NA </td> <td style="text-align:left;"> NA </td> <td style="text-align:left;"> NA </td> <td style="text-align:left;"> no </td> <td style="text-align:left;"> no </td> </tr> <tr> <td style="text-align:left;"> 4465 </td> <td style="text-align:left;"> 2017 </td> <td style="text-align:left;"> NA </td> <td style="text-align:left;"> Bennabi, M; Delorme, R; Oliveira, J; Fortier, C; Lajnef, M; Boukouaci, W; Feugeas, JP; Marzais, F; Gaman, A; Charron, D; Ghaleh, B; Krishnamoorthy, R; Leboyer, M; Tamouza, R </td> <td style="text-align:left;"> 2015 </td> <td style="text-align:left;"> Dectin-1 Polymorphism: A Genetic Disease Specifier in Autism Spectrum Disorders? </td> <td style="text-align:left;"> Introduction In autism spectrum disorders (ASD), complex gene-environment interactions contribute to disease onset and progress. Given that gastro-intestinal dysfunctions are common in ASD, we postulated involvement of microbial dysbiosis in ASD and investigated, under a case-control design, the influence of DNA polymorphisms in the CLEC7A gene that encodes a pivotal fungal sensor, Dectin-1. Material and methods DNAs from 478 ASD patients and 351 healthy controls (HC) were analyzed for the CLEC7A rs16910631G/A and rs2078178 A/G single nucleotide polymorphisms (SNPs). Differences in the distribution of allele, genotype and haplotype by Chi-square testing and nonparametric analysis by Kruskal-Wallis/Mann-Whitney tests, where appropriate, were performed. The free statistical package R. 2.13 software was used for the statistical analysis. Results We found that the CLEC7A rs2078178 G allele and GG genotype were more prevalent in HC as compared to ASD but failed to reach statistical significance for the latter (pc = 0.01, 0.06 respectively). However, after phenotype-based stratification, the CLEC7A rs2078178 G allele and GG genotype were found to be significantly more frequent in the Asperger group as compared to other ASD subsets (pc = 0.02, 0.01), a finding reinforced by haplotype analysis (rs2078178/rs16910631 G-G/G-G) (pc = 0.002). Further, intellectual quotient (IQ)-based stratification of ASD patients revealed that IQ values increase linearly along the CLEC7A rs2078178 AA, AG and GG genotypes (p = 0.05) and in a recessive manner (GG vs. AA+AG p = 0.02), further confirmed by haplotype distribution (CLEC7A rs2078178-16910631; A-G/A-G, A-G/G-G and G-G/G-G, p = 0.02, G-G/G-G vs. others, p = 0.01). Conclusion Our data suggest that the genetic diversity of CLEC7A gene influences the ASD phenotype by behaving as a disease specifier and imply that the genetic control of innate immune response could determine the ASD phenotype. </td> <td style="text-align:left;"> 4467 </td> <td style="text-align:left;"> NA </td> <td style="text-align:left;"> NA </td> <td style="text-align:left;"> NA </td> <td style="text-align:left;"> no </td> <td style="text-align:left;"> no </td> </tr> <tr> <td style="text-align:left;"> 4466 </td> <td style="text-align:left;"> 2017 </td> <td style="text-align:left;"> NA </td> <td style="text-align:left;"> Tsai, CT; Hsieh, CS; Chang, SN; Chuang, EY; Juang, JMJ; Lin, LY; Lai, LP; Hwang, JJ; Chiang, FT; Lin, JL </td> <td style="text-align:left;"> 2015 </td> <td style="text-align:left;"> Next-generation sequencing of nine atrial fibrillation candidate genes identified novel de novo mutations in patients with extreme trait of atrial fibrillation </td> <td style="text-align:left;"> Background Atrial fibrillation (AF) is the most common sustained cardiac arrhythmia. Genome-wide association studies (GWAS) have identified common variants in nine genomic regions associated with AF (KCNN3, PRRX1, PITX2, WNT8A, CAV1, C9orf3, SYNE2, HCN4 and ZFHX3 genes); however, the genetic variability of these risk variants does not explain the entire genetic susceptibility to AF. Rare variants missed by GWAS may also contribute to genetic risk of AF. Methods We used an extreme trait design to sequence carefully selected probands with extreme phenotypes and their unaffected parents to identify rare de novo variants or mutations. Based on the hypothesis that common and rare variants may colocate in the same disease susceptibility gene, we used next-generation sequencing to sequence these nine published AF susceptibility genes identified by GWAS (a total of 179 exons) in 20 trios, 200 unrelated patients with AF and 200 non-AF controls. Results We identified a novel mutation in the 50 untranslated region of the PITX2 gene, which localised in the transcriptionally active enhancer region. We also identified one missense exon mutation in KCNN3, two in ZFHX3 and one in SYNE2. None of these mutations were present in other unrelated patients with AF, healthy controls, unaffected parents and are thus novel and de novo (p<10(-4)). Functional study showed that the mutation in the 50 untranslated region of the PITX2 gene significantly downregulated PITX2 expression in atrial myocytes, either in basal condition or during rapid pacing. In silico analysis showed that the missense mutation in ZFHX3 results in damage of the ZFHX3 protein structure. Conclusions The genetic architecture of subjects with extreme phenotypes of AF is similar to that of rare or Mendelian diseases, and mutations may be the underlying cause. </td> <td style="text-align:left;"> 4468 </td> <td style="text-align:left;"> NA </td> <td style="text-align:left;"> NA </td> <td style="text-align:left;"> NA </td> <td style="text-align:left;"> no </td> <td style="text-align:left;"> no </td> </tr> <tr> <td style="text-align:left;"> 4467 </td> <td style="text-align:left;"> 2017 </td> <td style="text-align:left;"> NA </td> <td style="text-align:left;"> De Araujo, ME; Erhart, G; Buck, K; Muller-Holzner, E; Hubalek, M; Fiegl, H; Campa, D; Canzian, F; Eilber, U; Chang-Claude, J; Coassin, S; Haun, M; Kedenko, L; Paulweber, B; Reitsamer, R; Himmel, I; Flesch-Janys, D; Lamina, C; Kronenberg, F; Huber, LA; Kloss-Brandstatter, A </td> <td style="text-align:left;"> 2013 </td> <td style="text-align:left;"> Polymorphisms in the Gene Regions of the Adaptor Complex LAMTOR2/LAMTOR3 and Their Association with Breast Cancer Risk </td> <td style="text-align:left;"> Background: The late endosomal LAMTOR complex serves as a convergence point for both the RAF/MEK/ERK and the PI3K/AKT/mTOR pathways. Interestingly, both of these signalling cascades play a significant role in the aetiology of breast cancer. Our aim was to address the possible role of genetic polymorphisms in LAMTOR2 and LAMTOR3 as genetic risk factors for breast cancer. Methodology/Results: We sequenced the exons and exon-intron boundaries of LAMTOR2 (p14) and LAMTOR3 (MP1) in 50 prospectively collected pairs of cancerous tissue and blood samples from breast cancer patients and compared their genetic variability. We found one single nucleotide polymorphism (SNP) in LAMTOR2 (rs7541) and two SNPs in LAMTOR3 (rs2298735 and rs148972953) in both tumour and blood samples, but no somatic mutations in cancerous tissues. In addition, we genotyped all three SNPs in 296 samples from the Risk Prediction of Breast Cancer Metastasis Study and found evidence of a genetic association between rs148972953 and oestrogen (ER) and progesterone receptor negative status (PR) (ER: OR = 3.60 (1.15-11.28); PR: OR = 4.27 (1.43-12.72)). However, when we additionally genotyped rs148972953 in the MARIE study including 2,715 breast cancer cases and 5,216 controls, we observed neither a difference in genotype frequencies between patients and controls nor was the SNP associated with ER or PR. Finally, all three SNPs were equally frequent in breast cancer samples and female participants (n = 640) of the population-based SAPHIR Study. Conclusions: The identified polymorphisms in LAMTOR2 and LAMTOR3 do not seem to play a relevant role in breast cancer. Our work does not exclude a role of other not yet identified SNPs or that the here annotated polymorphism may in fact play a relevant role in other diseases. Our results underscore the importance of replication in association studies. </td> <td style="text-align:left;"> 4469 </td> <td style="text-align:left;"> NA </td> <td style="text-align:left;"> NA </td> <td style="text-align:left;"> NA </td> <td style="text-align:left;"> no </td> <td style="text-align:left;"> no </td> </tr> <tr> <td style="text-align:left;"> 4468 </td> <td style="text-align:left;"> 2017 </td> <td style="text-align:left;"> NA </td> <td style="text-align:left;"> Robinson, T; Campino, SG; Auburn, S; Assefa, SA; Polley, SD; Manske, M; MacInnis, B; Rockett, KA; Maslen, GL; Sanders, M; Quail, MA; Chiodini, PL; Kwiatkowski, DP; Clark, TG; Sutherland, CJ </td> <td style="text-align:left;"> 2011 </td> <td style="text-align:left;"> Drug-Resistant Genotypes and Multi-Clonality in Plasmodium falciparum Analysed by Direct Genome Sequencing from Peripheral Blood of Malaria Patients </td> <td style="text-align:left;"> Naturally acquired blood-stage infections of the malaria parasite Plasmodium falciparum typically harbour multiple haploid clones. The apparent number of clones observed in any single infection depends on the diversity of the polymorphic markers used for the analysis, and the relative abundance of rare clones, which frequently fail to be detected among PCR products derived from numerically dominant clones. However, minority clones are of clinical interest as they may harbour genes conferring drug resistance, leading to enhanced survival after treatment and the possibility of subsequent therapeutic failure. We deployed new generation sequencing to derive genome data for five non-propagated parasite isolates taken directly from 4 different patients treated for clinical malaria in a UK hospital. Analysis of depth of coverage and length of sequence intervals between paired reads identified both previously described and novel gene deletions and amplifications. Full-length sequence data was extracted for 6 loci considered to be under selection by antimalarial drugs, and both known and previously unknown amino acid substitutions were identified. Full mitochondrial genomes were extracted from the sequencing data for each isolate, and these are compared against a panel of polymorphic sites derived from published or unpublished but publicly available data. Finally, genome-wide analysis of clone multiplicity was performed, and the number of infecting parasite clones estimated for each isolate. Each patient harboured at least 3 clones of P. falciparum by this analysis, consistent with results obtained with conventional PCR analysis of polymorphic merozoite antigen loci. We conclude that genome sequencing of peripheral blood P. falciparum taken directly from malaria patients provides high quality data useful for drug resistance studies, genomic structural analyses and population genetics, and also robustly represents clonal multiplicity. </td> <td style="text-align:left;"> 4470 </td> <td style="text-align:left;"> NA </td> <td style="text-align:left;"> NA </td> <td style="text-align:left;"> NA </td> <td style="text-align:left;"> no </td> <td style="text-align:left;"> no </td> </tr> <tr> <td style="text-align:left;"> 4469 </td> <td style="text-align:left;"> 2017 </td> <td style="text-align:left;"> NA </td> <td style="text-align:left;"> Fichera, M; Spalletta, A; Fiorenza, F; Lombardo, T; Schiliro, G; Tamouza, R; Lapoumeroulie, C; Labie, D; Ragusa, A </td> <td style="text-align:left;"> 1997 </td> <td style="text-align:left;"> Molecular basis of alpha-thalassemia in Sicily </td> <td style="text-align:left;"> To evaluate the allelic frequency and genetic diversity of alpha-thalassemia defects in Sicily, both epidemiological and patient-oriented studies were carried out, For the epidemiological study, phenotypic data were collected on more than 1000 Sicilian individuals. Among them, 427 were explored at the molecular level for nine a-thalassemic variants known to be common in the Mediterranean region. Our data reveal an allele frequency of 4.1% for alpha(+)-thalassemia matching that of beta-thalassemia in this region. The presence of alpha degrees-thalassemia (--(MEDI) and --(CAL)) was observed only in the group of referred patients. Newly acquired nucleotide sequence data on the deletional breakpoint of --(CAL) allowed us to design a simple PCR-based procedure for exploring this allele, The data also provide additional information concerning the genetic mechanisms involved in such large deletions. </td> <td style="text-align:left;"> 4471 </td> <td style="text-align:left;"> NA </td> <td style="text-align:left;"> NA </td> <td style="text-align:left;"> NA </td> <td style="text-align:left;"> no </td> <td style="text-align:left;"> no </td> </tr> <tr> <td style="text-align:left;"> 4470 </td> <td style="text-align:left;"> 2017 </td> <td style="text-align:left;"> NA </td> <td style="text-align:left;"> Koppens, PFJ; Hoogenboezem, T; Degenhart, HJ </td> <td style="text-align:left;"> 2000 </td> <td style="text-align:left;"> CYP21 and CYP21P variability in steroid 21-hydroxylase deficiency patients and in the general population in the Netherlands </td> <td style="text-align:left;"> Steroid 21-hydroxylase deficiency is caused by defectiveness of the CYP21 gene. Such defects have presumably originated from interactions with the nearby CYP21P pseudogene during evolution. We studied these mechanisms by comparing the genetic variability of CYP21, CYP21P, and CYP21P/CYP21 hybrids (resulting from large-scale rearrangements) at eight mutation sites in a group of Dutch steroid 21-hydroxylase deficiency patients, their family members, and controls. The most common CYP21 defect in patients with salt-losing steroid 21-hydroxylase deficiency was a splice junction mutation in intron 2. The most common defect in the simple virilising form of the disease was ile72-->asn. CYP21P showed considerable sequence variation in its central and 3' sections; the 5' section was constant. A single nucleotide (T) insert in exon 7 was found in all CYP21P genes. During the course of evolution, this was probably the third defect introduced into CYP21P after the splice junction mutation in intron 2 and the 8 bp deletion in exon 3. Gene conversions introducing CYP21-like sequences contribute to CYP21P variability. Such an event has occurred de novo in one family. A comparison of CYP21 and CYP21P mutations on the same chromosome shows that at least some of the small-scale gene conversions that supposedly transfer defects to CYP21 involve interaction between homologous chromosomes. The majority of the putative CYP21P-CYP21 transitions in hybrid genes appears to occur in a distinct zone that lies 5' of nucleotide 2108, which is further downstream than previously hypothesised. The other transitions lie upstream of nucleotide 999. Apparent 'large-scale' CYP21-CYP21P gene conversions lead to hybrid genes that are very similar to those found in CYP21 deletions, so these haplotypes have probably resulted from a meiotic double unequal crossover. </td> <td style="text-align:left;"> 4472 </td> <td style="text-align:left;"> NA </td> <td style="text-align:left;"> NA </td> <td style="text-align:left;"> NA </td> <td style="text-align:left;"> no </td> <td style="text-align:left;"> no </td> </tr> <tr> <td style="text-align:left;"> 4471 </td> <td style="text-align:left;"> 2017 </td> <td style="text-align:left;"> NA </td> <td style="text-align:left;"> Viswanathan, J; Makinen, P; Helisalmni, S; Haapasalo, A; Soininen, H; Hiltunen, M </td> <td style="text-align:left;"> 2009 </td> <td style="text-align:left;"> An Association Study Between Granulin Gene Polymorphisms and Alzheimer's Disease in Finnish Population </td> <td style="text-align:left;"> Granulin protein plays an important role in neurite outgrowth and neuronal survival. Recently, it was shown that mutations in granulin (GRN) gene cause tau-negative frontotemporal dementia supporting the idea that granulin is involved in neurodegeneration. Here we have investigated whether genetic variability in the GRN gene influences also the risk of developing Alzheimer's disease (AD). Genotyping of six single nucleotide polymorphisms (SNPs) in the GRN gene among 512 AD patients and 649 control subjects originating from Finland did not show significant association with AD. However, stratification according to gender revealed a significant male-specific allele, genotype and haplotype association between AD and GRN SNPs rs4792939, rs850713, and rs5848. These data suggest that genetic variability in the GRN gene may also increase the risk for developing AD in a gender-specific manner. (C) 2008 Wiley-Liss, Inc. </td> <td style="text-align:left;"> 4473 </td> <td style="text-align:left;"> NA </td> <td style="text-align:left;"> NA </td> <td style="text-align:left;"> NA </td> <td style="text-align:left;"> no </td> <td style="text-align:left;"> no </td> </tr> <tr> <td style="text-align:left;"> 4472 </td> <td style="text-align:left;"> 2017 </td> <td style="text-align:left;"> NA </td> <td style="text-align:left;"> Alam, MM; Khurshid, A; Shaukat, S; Rana, MS; Sharif, S; Angez, M; Nisar, N; Naeem, M; Zaidi, SSZ </td> <td style="text-align:left;"> 2013 </td> <td style="text-align:left;"> Human Parechovirus Genotypes-10,-13 and-15 in Pakistani Children with Acute Dehydrating Gastroenteritis </td> <td style="text-align:left;"> Human parechoviruses are known to cause asymptomatic to severe clinical illness predominantly respiratory and gastroenetric infections. Despite their global prevalence, epidemiological studies have not been performed in Pakistan. In this study, we retrospectively analyzed 110 fecal specimen and found 26 (24%) positive for viral RNA with HPeV-10 (n = 3, 23%), HPeV-13 (n = 4, 31%) and HPeV-15 (n = 6, 46%) genotypes. Clinical features of patients with different HPeV genotypes were compared. All HPeV positive children were aged <= 4 years (mean 13.92 months). The male-to-female ratio was 1:1.17 (46.2 vs 53.8%) with significant association (p=.031) to HPeV infectivity. HPeV-10 and -13 were found during summer while HPeV-15 was only detected during late winter season. Disease symptoms were more severe in children infected with HPeV-10 and -13 as compared to HPeV-15. Fever and vomiting were observed in 100% cases of HPeV-10 and -13 while only 17% patients of HPeV-15 had these complaints. Phylogenetic analyses showed that HPeV-10, -13 and -15 strains found in this study have 9-13%, 16.8% and 21.8% nucleotide divergence respectively from the prototype strains and were clustered to distinct genetic lineages. This is the first report of HPeV-15 infection in humans although first identified in rhesus macaques. The arginine-glycine-aspartic acid (RGD) motif present at the C-terminal of VP1 responsible for the viral attachment to cellular integrins was not found in all of these strains. In conclusion, these findings enhance our knowledge related to the epidemiology and genetic diversity of the HPeV in Pakistan and support the need for continued laboratory based surveillance programs especially in infants and neonatal clinical settings. Further, the parechovirus pathogenesis, cross-species transmission and disease reservoirs must be ascertained to adopt better prevention measures. </td> <td style="text-align:left;"> 4474 </td> <td style="text-align:left;"> NA </td> <td style="text-align:left;"> NA </td> <td style="text-align:left;"> NA </td> <td style="text-align:left;"> no </td> <td style="text-align:left;"> no </td> </tr> <tr> <td style="text-align:left;"> 4473 </td> <td style="text-align:left;"> 2017 </td> <td style="text-align:left;"> NA </td> <td style="text-align:left;"> Feng, ZL; Curtis, J; Minchella, DJ </td> <td style="text-align:left;"> 2001 </td> <td style="text-align:left;"> The influence of drug treatment on the maintenance of schistosome genetic diversity </td> <td style="text-align:left;"> Drug treatment of patients with schistosomiasis may select for drug-resistant parasites. In this article, we formulate a deterministic model with multiple strains of schistosomes (helminth parasites with a two-host life cycles) in order to explore the role of drug treatment in the maintenance of a polymorphism of parasite strains that differ in their resistance levels. The basic reproductive numbers for all strains are computed, and are shown to determine the stabilities of equilibria of the model and consequently the distribution of parasite phenotypes with different levels of drug tolerance. Analysis of our model shows that the likelihood that resistant strains will increase in frequency depends on the interplay between their relative fitness, the cost of resistance, and the degree of selection pressure exerted by the drug treatments. </td> <td style="text-align:left;"> 4475 </td> <td style="text-align:left;"> NA </td> <td style="text-align:left;"> NA </td> <td style="text-align:left;"> NA </td> <td style="text-align:left;"> no </td> <td style="text-align:left;"> no </td> </tr> <tr> <td style="text-align:left;"> 4474 </td> <td style="text-align:left;"> 2017 </td> <td style="text-align:left;"> NA </td> <td style="text-align:left;"> Hornberger, M; Bell, B; Graham, KS; Rogers, TT </td> <td style="text-align:left;"> 2009 </td> <td style="text-align:left;"> Are judgments of semantic relatedness systematically impaired in Alzheimer's disease? </td> <td style="text-align:left;"> We employed a triadic comparison task in patients with Alzheimer's disease (AD) and healthy controls to contrast (a) multidimensional scaling (MDS) and accuracy-based assessments of semantic memory, and (b) degraded-store versus degraded-access accounts of semantic impairment in Alzheimer's disease (AD). Similar to other studies using triadic comparison tasks, participants were asked to indicate which two out of three words (animal names) were most similar in meaning. Novel to this investigation, we contrasted performance on two semantic dimensions of strong and equal saliency to controls, but varying in their specificity (land/water versus bird/non-bird). Degraded-store accounts predict that the more specific bird/non-bird dimension should be more consistently impaired in AD, whereas degraded-access accounts predict that both dimensions, because they are equally salient, should be equivalently impaired in the disorder. The MDS results suggested that both patient and control group responses were not discriminable from random responding, consistent with previous studies. By contrast an accuracy-based analysis on the same data showed that controls showed good knowledge of both salient dimensions, and were evenly split in their individual preference for one dimension over another. In contrast, patients showed higher accuracy and sensitivity to the broader land/water dimension than to the more specific bird/non-bird dimension, consistent with a storage-based account of the semantic impairment in AD. Our results further suggest that MDS methods can fail to reveal important and systematic behaviour in semantic tasks, in both patient and control groups. (C) 2009 Elsevier Ltd. All rights reserved. </td> <td style="text-align:left;"> 4476 </td> <td style="text-align:left;"> NA </td> <td style="text-align:left;"> NA </td> <td style="text-align:left;"> NA </td> <td style="text-align:left;"> no </td> <td style="text-align:left;"> no </td> </tr> <tr> <td style="text-align:left;"> 4475 </td> <td style="text-align:left;"> 2017 </td> <td style="text-align:left;"> NA </td> <td style="text-align:left;"> Dubois, B; Bertin, P; Mingeot, D </td> <td style="text-align:left;"> 2016 </td> <td style="text-align:left;"> Molecular diversity of alpha-gliadin expressed genes in genetically contrasted spelt (Triticum aestivum ssp spelta) accessions and comparison with bread wheat (T. aestivum ssp aestivum) and related diploid Triticum and Aegilops species </td> <td style="text-align:left;"> The gluten proteins of cereals such as bread wheat (Triticum aestivum ssp. aestivum) and spelt (T. aestivum ssp. spelta) are responsible for celiac disease (CD). The alpha-gliadins constitute the most immunogenic class of gluten proteins as they include four main T-cell stimulatory epitopes that affect CD patients. Spelt has been less studied than bread wheat and could constitute a source of valuable diversity. The objective of this work was to study the genetic diversity of spelta-gliadin transcripts and to compare it with those of bread wheat. Genotyping data from 85 spelt accessions obtained with 19 simple sequence repeat (SSR) markers were used to select 11 contrasted accessions, from which 446 full open reading frame alpha-gliadin genes were cloned and sequenced, which revealed a high allelic diversity. High variations among the accessions were highlighted, in terms of the proportion of alpha-gliadin sequences from each of the three genomes (A, B and D), and their composition in the four T-cell stimulatory epitopes. An accession from Tajikistan stood out, having a particularly high proportion of alpha-gliadins from the B genome and a low immunogenic content. Even if no clear separation between spelt and bread wheat sequences was shown, spelt alpha-gliadins displayed specific features concerning e.g. the frequencies of some amino acid substitutions. Given this observation and the variations in toxicity revealed in the spelt accessions in this study, the high genetic diversity held in spelt germplasm collections could be a valuable resource in the development of safer varieties for CD patients. </td> <td style="text-align:left;"> 4477 </td> <td style="text-align:left;"> NA </td> <td style="text-align:left;"> NA </td> <td style="text-align:left;"> NA </td> <td style="text-align:left;"> no </td> <td style="text-align:left;"> no </td> </tr> <tr> <td style="text-align:left;"> 4476 </td> <td style="text-align:left;"> 2017 </td> <td style="text-align:left;"> NA </td> <td style="text-align:left;"> Warby, SC; Montpetit, A; Hayden, AR; Carroll, JB; Butland, SL; Visscher, F; Collins, JA; Semaka, A; Hudson, TJ; Hayden, MR </td> <td style="text-align:left;"> 2009 </td> <td style="text-align:left;"> CAG Expansion in the Huntington Disease Gene Is Associated with a Specific and Targetable Predisposing Haplogroup </td> <td style="text-align:left;"> Huntington disease (HD) is an autosomal-dominant disorder that results from >36 CAG repeats in the HD gene (HTT). Approximately 10% of patients inherit a chromosome that underwent CAG expansion from an unaffected parent with <36 CAG repeats. This study is a comprehensive analysis of genetic diversity in HTTand reveals that HD patients of European origin (n = 65) have a significant enrichment (95%) of a specific set of 22 tagging single nucleotide polymorphisms (SNPs) that constitute a single haplogroup. The disease association of many SNPs is much stronger than any previously reported polymorphism and was confirmed in a replication cohort (n = 203). Importantly, the same haplogroup is also significantly enriched (83%) in individuals with 27-35 CAG repeats (intermediate alleles, n = 66), who are unaffected by the disease, but have increased CAG tract sizes relative to the general Population (n = 116). These data Support a stepwise model for CAG expansion into the affected range (>36 CAG) and identifies specific haplogroup variants in the general population associated with this instability. The specific variants at risk for CAG expansion are not present in the general population in China, Japan, and Nigeria where the prevalence of I ID is much lower. The current data argue that cis-elements have a major predisposing influence on CAG Instability in HTT. The strong association between specific SNP alleles and CAG expansion also provides an opportunity of personalized therapeutics in HD where the clinical development of only a small number of allele-specific targets may be sufficient to treat up to 88% of the HD patient population. </td> <td style="text-align:left;"> 4478 </td> <td style="text-align:left;"> NA </td> <td style="text-align:left;"> NA </td> <td style="text-align:left;"> NA </td> <td style="text-align:left;"> no </td> <td style="text-align:left;"> no </td> </tr> <tr> <td style="text-align:left;"> 4477 </td> <td style="text-align:left;"> 2017 </td> <td style="text-align:left;"> NA </td> <td style="text-align:left;"> Khamrin, P; Chaimongkol, N; Malasao, R; Suantai, B; Saikhruang, W; Kongsricharoern, T; Ukarapol, N; Okitsu, S; Shimizu, H; Hayakawa, S; Ushijima, H; Maneekarn, N </td> <td style="text-align:left;"> 2012 </td> <td style="text-align:left;"> Detection and molecular characterization of cosavirus in adults with diarrhea, Thailand </td> <td style="text-align:left;"> Human cosavirus (HCoSV) is a newly discovered virus in Picornaviridae family. At present it is not clear whether HCoSV is associated with diseases, including gastroenteritis in humans, as epidemiological data is limited. Epidemiological surveillance of HCoSV was conducted on 150 fecal specimens collected from children and 150 samples from adults with diarrhea in Thailand by RT-PCR screening. HCoSV was found in a single adult specimen and not in any of the fecal specimens from children. This represents the first report of HCoSV infection in patients with diarrhea in Thailand. Extensive epidemiological surveillance of novel viruses associated with diarrhea in other populations may provide a better understanding of the distribution, genetic diversity, and association of the viral agents associated with acute gastroenteritis in humans. </td> <td style="text-align:left;"> 4479 </td> <td style="text-align:left;"> NA </td> <td style="text-align:left;"> NA </td> <td style="text-align:left;"> NA </td> <td style="text-align:left;"> no </td> <td style="text-align:left;"> no </td> </tr> <tr> <td style="text-align:left;"> 4478 </td> <td style="text-align:left;"> 2017 </td> <td style="text-align:left;"> NA </td> <td style="text-align:left;"> Alomar, SY; Alkhuriji, A; Trayhyrn, P; Alhetheel, A; Al-jurayyan, A; Mansour, L </td> <td style="text-align:left;"> 2017 </td> <td style="text-align:left;"> Association of the genetic diversity of killer cell immunoglobulin-like receptor genes and HLA-C ligand in Saudi women with breast cancer </td> <td style="text-align:left;"> Breast cancer (BC) progression and metastases have been linked to antitumor immunity inefficiency and particularly to natural killer (NK) cells. Killer cell immunoglobulin-like receptors (KIRs) are the most polymorphic receptors of NK cells. Through their interactions with human leukocyte antigen (HLA)-C ligands, they modulate NK and T cell actions against target cells. Therefore, we studied the combinatorial effect of KIR genes and their HLA-C ligands on the susceptibility to development of BC in Saudi women. The presence of KIR genes and HLA-C1 and HLA-C2 groups was typed in 50 Saudi patients living in Riyadh and 65 healthy controls using polymerase chain reaction with sequence-specific primers. Our results indicated a protective effect by the KIR2DS2, 2DS3, and 2DL5A genes against BC (OR = 0.25, 0.21, and 0.27, respectively, and p < 0.01). The synergistic action of the three genes was observed when they occurred together, and the absence of the three genes increased BC occurrence by 6.5-fold. Distribution of the HLA-C1/C2 ligand between patients and controls showed an increase in the risk of BC occurrence for the heterozygote C1/C2 (OR = 2.33; 95 % CI = 1.08-5.02; p = 0.037) and a protective effect of the homozygote C2C2 (OR = 0.03; 95 % CI = 0.009-0.098; p < 0.001). Combinatory analyses of KIR genes and their HLA-C ligands showed protective effects of KIR2DL2 and 2DL3 in the absence of their HLA-C1 ligand. These results suggested that KIR-gene content combined with their ligand could influence the risk of BC development in women in Saudi Arabia. </td> <td style="text-align:left;"> 4480 </td> <td style="text-align:left;"> NA </td> <td style="text-align:left;"> NA </td> <td style="text-align:left;"> NA </td> <td style="text-align:left;"> no </td> <td style="text-align:left;"> no </td> </tr> <tr> <td style="text-align:left;"> 4479 </td> <td style="text-align:left;"> 2017 </td> <td style="text-align:left;"> NA </td> <td style="text-align:left;"> Bob, NS; Diop, BM; Renaud, F; Marrama, L; Durand, P; Tall, A; Ka, B; Ekala, MT; Bouchier, C; Mercereau-Puijalon, O; Jambou, R </td> <td style="text-align:left;"> 2010 </td> <td style="text-align:left;"> Parasite Polymorphism and Severe Malaria in Dakar (Senegal): A West African Urban Area </td> <td style="text-align:left;"> Background: Transmission of malaria in West African urban areas is low and healthcare facilities are well organized. However, malaria mortality remains high. We conducted a survey in Dakar with the general objective to establish who died from severe malaria (SM) in urban areas (particularly looking at the age-groups) and to compare parasite isolates associated with mild or severe malaria. Methodology/Principal Findings: The current study included mild-(MM) and severe malaria (SM) cases, treated in dispensaries (n = 2977) and hospitals (n = 104), We analysed Pfdhfr/Pfcrt-exon2 and nine microsatellite loci in 102 matched cases of SM and MM. Half of the malaria cases recorded at the dispensaries and 87% of SM cases referred to hospitals, occurred in adults, although adults only accounted for 26% of all dispensary consultations. This suggests that, in urban settings, whatever the reason for this adult over-representation, health-workers are forced to take care of increasing numbers of malaria cases among adults. Inappropriate self treatment and mutations in genes associated with drug resistance were found associated with SM in adults. SM was also associated with a specific pool of isolates highly polymorphic and different from those associated with MM. Conclusion: In this urban setting, adults currently represent one of the major groups of patients attending dispensaries for malaria treatment. For these patients, despite the low level of transmission, SM was associated with a specific and highly polymorphic pool of parasites which may have been selected by inappropriate treatment. </td> <td style="text-align:left;"> 4481 </td> <td style="text-align:left;"> NA </td> <td style="text-align:left;"> NA </td> <td style="text-align:left;"> NA </td> <td style="text-align:left;"> no </td> <td style="text-align:left;"> no </td> </tr> <tr> <td style="text-align:left;"> 4480 </td> <td style="text-align:left;"> 2017 </td> <td style="text-align:left;"> NA </td> <td style="text-align:left;"> Kim, JM; Park, SK; Yang, JJ; Shin, ES; Lee, JY; Yun, JY; Kim, JS; Park, SS; Jeon, BS </td> <td style="text-align:left;"> 2011 </td> <td style="text-align:left;"> SNPs in axon guidance pathway genes and susceptibility for Parkinson's disease in the Korean population </td> <td style="text-align:left;"> Single-nucleotide polymorphisms (SNPs) in genes of the axon guidance pathway have been reported to be a possible susceptibility factor for Parkinson's disease (PD). This study investigated whether the genetic variability in the axon guidance pathway is a susceptibility factor in PD patients in the Korean population. A total of 373 patients and 384 healthy subjects were included. A set of 22 SNPs was analyzed, and the risk of PD was evaluated using odds ratios in an unconditional and conditional logistic regression models of age- and gender-matched subsets. A multidimensionality reduction (MDR) analysis was performed to explore potential gene-gene interactions. SNPs in the DCC, CHP, RRAS2 and EPHB1 genes of the axon guidance pathway showed significant associations with PD. The DCC rs17468382 and EPHB1 rs2030737 SNPs may be associated with increased PD risk, and the CHP rs6492998 and RRAS2 rs2970332 SNPs may be associated with reduced PD risk. However, no significant interactions for PD risk were found in the MDR analysis and logistic regression analysis using SNP interaction terms. This study supports that only four of the selected 22 SNPs are regulating factors associated with PD in the Korean population. However, no interactions were found among the SNPs, suggesting that the effect for the pathway as a whole is not greater than that for single genes in the Korean population. Further investigations involving populations of various ethnicities and other genetic markers and models are warranted. Journal of Human Genetics (2011) 56, 125-129; doi: 10.1038/jhg.2010.130; published online 18 November 2010 </td> <td style="text-align:left;"> 4482 </td> <td style="text-align:left;"> NA </td> <td style="text-align:left;"> NA </td> <td style="text-align:left;"> NA </td> <td style="text-align:left;"> no </td> <td style="text-align:left;"> no </td> </tr> <tr> <td style="text-align:left;"> 4481 </td> <td style="text-align:left;"> 2017 </td> <td style="text-align:left;"> NA </td> <td style="text-align:left;"> Eutsey, RA; Hiller, NL; Earl, JP; Janto, BA; Dahlgren, ME; Ahmed, A; Powell, E; Schultz, MP; Gilsdorf, JR; Zhang, L; Smith, A; Murphy, TF; Sethi, S; Shen, K; Post, JC; Hu, FZ; Ehrlich, GD </td> <td style="text-align:left;"> 2013 </td> <td style="text-align:left;"> Design and validation of a supragenome array for determination of the genomic content of Haemophilus influenzae isolates </td> <td style="text-align:left;"> Background: Haemophilus influenzae colonizes the human nasopharynx as a commensal, and is etiologically associated with numerous opportunistic infections of the airway; it is also less commonly associated with invasive disease. Clinical isolates of H. influenzae display extensive genomic diversity and plasticity. The development of strategies to successfully prevent, diagnose and treat H. influenzae infections depends on tools to ascertain the gene content of individual isolates. Results: We describe and validate a Haemophilus influenzae supragenome hybridization (SGH) array that can be used to characterize the full genic complement of any strain within the species, as well as strains from several highly related species. The array contains 31,307 probes that collectively cover essentially all alleles of the 2890 gene clusters identified from the whole genome sequencing of 24 clinical H. influenzae strains. The finite supragenome model predicts that these data include greater than 85% of all non-rare genes (where rare genes are defined as those present in less than 10% of sequenced strains). The veracity of the array was tested by comparing the whole genome sequences of eight strains with their hybridization data obtained using the supragenome array. The array predictions were correct and reproducible for similar to 98% of the gene content of all of the sequenced strains. This technology was then applied to an investigation of the gene content of 193 geographically and clinically diverse H. influenzae clinical strains. These strains came from multiple locations from five different continents and Papua New Guinea and include isolates from: the middle ears of persons with otitis media and otorrhea; lung aspirates and sputum samples from pneumonia and COPD patients, blood specimens from patients with sepsis; cerebrospinal fluid from patients with meningitis, as well as from pharyngeal specimens from healthy persons. Conclusions: These analyses provided the most comprehensive and detailed genomic/phylogenetic look at this species to date, and identified a subset of highly divergent strains that form a separate lineage within the species. This array provides a cost-effective and high-throughput tool to determine the gene content of any H. influenzae isolate or lineage. Furthermore, the method for probe selection can be applied to any species, given a group of available whole genome sequences. </td> <td style="text-align:left;"> 4483 </td> <td style="text-align:left;"> NA </td> <td style="text-align:left;"> NA </td> <td style="text-align:left;"> NA </td> <td style="text-align:left;"> no </td> <td style="text-align:left;"> no </td> </tr> <tr> <td style="text-align:left;"> 4482 </td> <td style="text-align:left;"> 2017 </td> <td style="text-align:left;"> NA </td> <td style="text-align:left;"> Reid, SD; Green, NM; Buss, JK; Lei, B; Musser, JM </td> <td style="text-align:left;"> 2001 </td> <td style="text-align:left;"> Multilocus analysis of extracellular putative virulence proteins made by group A Streptococcus: Population genetics, human serologic response, and gene transcription </td> <td style="text-align:left;"> Species of pathogenic microbes are composed of an array of evolutionarily distinct chromosomal genotypes characterized by diversity in gene content and sequence (allelic variation). The occurrence of substantial genetic diversity has hindered progress in developing a comprehensive understanding of the molecular basis of virulence and new therapeutics such as vaccines. To provide new information that bears on these issues, 11 genes encoding extracellular proteins in the human bacterial pathogen group A Streptococcus identified by analysis of four genomes were studied. Eight of the 11 genes encode proteins with a LPXTC(L) motif that covalently links Cram-positive virulence factors to the bacterial cell surface. Sequence analysis of the 11 genes in 37 geographically and phylogenetically diverse group A Streptococcos strains cultured from patients with different infection types found that recent horizontal gene transfer has contributed substantially to chromosomal diversity. Regions of the inferred proteins likely to interact with the host were identified by molecular population genetic analysis, and Western immunoblot analysis with sera from infected patients confirmed that they were antigenic. Real-time reverse transcriptase-PCR (TaqMan) assays found that transcription of six of the 11 genes was substantially up-regulated in the stationary phase. In addition, transcription of many genes was influenced by the covR and mga trans-acting gene regulatory loci. Multilocus investigation of putative virulence genes by the integrated approach described herein provides an important strategy to aid microbial pathogenesis research and rapidly identify new targets for therapeutics research. </td> <td style="text-align:left;"> 4484 </td> <td style="text-align:left;"> NA </td> <td style="text-align:left;"> NA </td> <td style="text-align:left;"> NA </td> <td style="text-align:left;"> no </td> <td style="text-align:left;"> no </td> </tr> <tr> <td style="text-align:left;"> 4483 </td> <td style="text-align:left;"> 2017 </td> <td style="text-align:left;"> NA </td> <td style="text-align:left;"> Bouchami, O; Ben Hassen, A; de Lencastre, H; Miragaia, M </td> <td style="text-align:left;"> 2011 </td> <td style="text-align:left;"> Molecular Epidemiology of Methicillin-Resistant Staphylococcus hominis (MRSHo): Low Clonality and Reservoirs of SCCmec Structural Elements </td> <td style="text-align:left;"> Background: Methicillin resistant Staphylococcus hominis (MRSHo) are important human pathogens in immunocompromised patients. However, little is known regarding its population structure and staphylococcal chromosomal cassette mec (SCCmec) content. Methodology/Principal Findings: To assess the population structure and the SCCmec content of S. hominis, 34 MRSHo and 11 methicillin-susceptible S. hominis (MSSHo) from neutropenic patients collected over a 3-year period were studied. The genetic backgrounds of S. hominis isolates were analyzed by pulsed-field gel electrophoresis (PFGE) and SCCmec types were determined by PCR. Cassette chromosome recombinases (ccr) were characterized by PCR and ccrB sequencing. The 34 S. hominis isolates were classified into as many as 28 types and 32 subtypes (SID = 99.82%); clonal dissemination was occasionally observed. The main SCCmec structures identified were SCCmec type VI (4B) (20%), SCCmec VIII (4A) (15%), and a new SCCmec composed of mec complex A in association with ccrAB1 (38%); 27% of the isolates harbored non-typeable SCCmec. Overall, a high prevalence of mec complex A (73.5%), ccrAB1 (50%) and ccrAB4 (44%) were found. Importantly, ccrB1 and ccrB4 from both MRSHo and MSSHo showed a high nucleotide sequence homology with those found in S. aureus SCCmec I, VI and VIII respectively (>95%). Conclusions/Significance: The S. hominis population showed a limited clonality and a low genetic diversity in the allotypes of ccr and classes of mec complex. Moreover, our data suggest that S. hominis might have been a privileged source of mec complex A, ccrB1 and ccrB4, for the assembly of primordial SCCmec types. </td> <td style="text-align:left;"> 4485 </td> <td style="text-align:left;"> NA </td> <td style="text-align:left;"> NA </td> <td style="text-align:left;"> NA </td> <td style="text-align:left;"> no </td> <td style="text-align:left;"> no </td> </tr> <tr> <td style="text-align:left;"> 4484 </td> <td style="text-align:left;"> 2017 </td> <td style="text-align:left;"> NA </td> <td style="text-align:left;"> Gosadi, IM; Goyder, EC; Teare, MD </td> <td style="text-align:left;"> 2014 </td> <td style="text-align:left;"> Investigating the Potential Effect of Consanguinity on Type 2 Diabetes Susceptibility in a Saudi Population </td> <td style="text-align:left;"> Objective: To examine mechanisms by which consanguinity might increase the risk of type 2 diabetes (T2D) in a Saudi population. Methodology: 362 adult male participants were recruited, 179 were T2D patients and 183 healthy siblings. T2D severity was assessed in patients by recording age at diagnosis. In healthy subjects, diabetes risk was studied by measuring the body mass index, fasting blood glucose (FBG) level, and waist circumference. Extended pedigrees were constructed to calculate inbreeding coefficients. To account for tribal relatedness degrees reported in the constructed pedigrees, assumed inbreeding coefficients for tribal relatedness were added to the calculated inbreeding coefficients. A total of 23 SNPs associated with a higher risk of T2D were genotyped. Results : A significant inverse association was detected between inbreeding coefficients and age at diagnosis (Spearman's coefficient: -0.186, p = 0.013). In 42 families, we were able to recruit 2 healthy siblings. Pearson's correlation coefficient of FBG between siblings was 0.317 (p = 0.04). The correlation between the siblings' FBG increased with an increasing degree of consanguinity. The effect of consanguinity on the FBG level was further assessed by regression line analysis and by controlling for differences in age, caloric intake, and level of physical activity (beta: -0.118, p = 0.024). No significant association between the number of T2D risk alleles and the traits was found. Conclusion: Our findings suggest that consanguinity might increase the risk of T2D by an earlier onset of the disease and by strengthening possible genetic effects on FBG. (C) 2014 S. Karger AG, Basel </td> <td style="text-align:left;"> 4486 </td> <td style="text-align:left;"> NA </td> <td style="text-align:left;"> NA </td> <td style="text-align:left;"> NA </td> <td style="text-align:left;"> no </td> <td style="text-align:left;"> no </td> </tr> <tr> <td style="text-align:left;"> 4485 </td> <td style="text-align:left;"> 2017 </td> <td style="text-align:left;"> NA </td> <td style="text-align:left;"> Yu, WH; Wang, YX; Guo, JQ; Wang, YL; Zheng, JS; Zhu, KX </td> <td style="text-align:left;"> 2015 </td> <td style="text-align:left;"> Genetic variability of ERCC1 and ERCC2 influences treatment outcomes in gastric cancer </td> <td style="text-align:left;"> We performed a study to investigate the role of ERCC1 (rs11615, rs2298881, and rs3212986) and ERCC2 (rs13181, rs238406, and rs1799793) polymorphisms in the prognosis of gastric cancer. A total of 346 patients with gastric cancer were recruited between May 2009 and May 2012. Single nucleotide polymorphism genotyping was performed using the Sequenom MassARRAY platform. The GA+AA genotype of ERCC2 rs1799793 showed significant and favorable response to chemotherapy than the wide-type GG genotype in multivariate analysis (OR = 1.78, 95% CI = 1.13-2.81). In a Cox proportional hazard model, carriers of ERCC2 rs1799793 GA+AA genotype exhibited longer duration of survival than did those with the GG genotype (hazards ratio = 0.57, 95% CI = 0.35-0.92). In conclusion, our study suggests that ERCC2 rs1799793 polymorphic variation could be used as a predictor for the prognosis of gastric cancer. </td> <td style="text-align:left;"> 4487 </td> <td style="text-align:left;"> NA </td> <td style="text-align:left;"> NA </td> <td style="text-align:left;"> NA </td> <td style="text-align:left;"> no </td> <td style="text-align:left;"> no </td> </tr> <tr> <td style="text-align:left;"> 4486 </td> <td style="text-align:left;"> 2017 </td> <td style="text-align:left;"> NA </td> <td style="text-align:left;"> Reinauer, C; Rosenbauer, J; Bachle, C; Herder, C; Roden, M; Ellard, S; De Franco, E; Karges, B; Holl, RW; Enczmann, J; Meissner, T </td> <td style="text-align:left;"> 2017 </td> <td style="text-align:left;"> The Clinical Course of Patients with Preschool Manifestation of Type 1 Diabetes Is Independent of the HLA DR-DQ Genotype </td> <td style="text-align:left;"> Introduction: Major histocompatibility complex class II genes are considered major genetic risk factors for autoimmune diabetes. We analysed Human Leukocyte Antigen (HLA) DR and DQ haplotypes in a cohort with early-onset (age < 5 years), long term type 1 diabetes (T1D) and explored their influence on clinical and laboratory parameters. Methods: Intermediate resolution HLA-DRB1, DQA1 and DQB1 typing was performed in 233 samples from the German Paediatric Diabetes Biobank and compared with a local control cohort of 19,544 cases. Clinical follow-up data of 195 patients (diabetes duration 14.2 +/- 2.9 years) and residual C-peptide levels were compared between three HLA risk groups using multiple linear regression analysis. Results: Genetic variability was low, 44.6% (104/233) of early-onset T1D patients carried the highest-risk genotype HLA-DRB1*03: 01-DQA1*05: 01-DQB1*02: 01/DRB1*04-DQA1* 03: 01-DQB1*03: 02 (HLA-DRB1*04 denoting 04:01/02/04/05), and 231 of 233 individuals carried at least one of six risk haplotypes. Comparing clinical data between the highest (n = 83), moderate (n = 106) and low risk (n = 6) genotypes, we found no difference in age at diagnosis (mean age 2.8 +/- 1.1 vs. 2.8 +/- 1.2 vs. 3.2 +/- 1.5 years), metabolic control, or frequency of associated autoimmune diseases between HLA risk groups (each p > 0.05). Residual C-peptide was detectable in 23.5% and C-peptide levels in the highest-risk group were comparable to levels in moderate to high risk genotypes. Conclusion: In this study, we saw no evidence for a different clinical course of early-onset T1D based on the HLA genotype within the first ten years after manifestation. </td> <td style="text-align:left;"> 4488 </td> <td style="text-align:left;"> NA </td> <td style="text-align:left;"> NA </td> <td style="text-align:left;"> NA </td> <td style="text-align:left;"> no </td> <td style="text-align:left;"> no </td> </tr> <tr> <td style="text-align:left;"> 4487 </td> <td style="text-align:left;"> 2017 </td> <td style="text-align:left;"> NA </td> <td style="text-align:left;"> Chia, JH; Lai, HC; Su, LH; Kuo, AJ; Wu, TL </td> <td style="text-align:left;"> 2013 </td> <td style="text-align:left;"> Molecular Epidemiology of Clostridium difficile at a Medical Center in Taiwan: Persistence of Genetically Clustering of A(-)B(+) Isolates and Increase of A(+)B(+) Isolates </td> <td style="text-align:left;"> Introduction: We investigated the changing trend of various toxigenic Clostridium difficile isolates at a 3 500-bed hospital in Taiwan. Genetic relatedness and antimicrobial susceptibility of toxigenic C. difficile isolates were also examined. Methods: A total of 110 non-repeat toxigenic C. difficile isolates from different patients were collected between 2002 and 2007. Characterization of the 110 toxigenic isolates was performed using agar dilution method, multilocus variable-number tandem-repeat analysis (MLVA) genotyping, tcdC genotyping, and toxinotyping. Results: Among the 110 toxigenic isolates studied, 70 isolates harbored tcdA and tcdB (A(+)B(+)) and 40 isolates harbored tcdB only (A(-)B(+)). The annual number of A(+)B(+) isolates considerably increased over the 6-year study (P = 0.055). A total of 109 different MLVA genotypes were identified, in which A(+)B(+) isolates and A(-)B(+) isolates were differentiated into two genetic clusters with similarity of 17.6%. Twenty-four (60%) of the 40 A(-)B(+) isolates formed a major cluster, MLVA-group 1, with a similarity of 85%. Seven (6.4%) resistant isolates were identified, including two metronidazole-resistant and five vancomycin-resistant isolates. Conclusions: This study indicated a persistence of a MLVA group 1 A(-)B(+) isolates and an increase of A(+)B(+) isolates with diverse MLVA types. Moreover, C. difficile isolates with antimicrobial resistance to metronidazole or vancomycin were found to have emerged. Continuous surveillance is warranted to understand the recent situation and control the further spread of the toxigenic C. difficile isolates, especially among hospitalized patients. </td> <td style="text-align:left;"> 4489 </td> <td style="text-align:left;"> NA </td> <td style="text-align:left;"> NA </td> <td style="text-align:left;"> NA </td> <td style="text-align:left;"> no </td> <td style="text-align:left;"> no </td> </tr> <tr> <td style="text-align:left;"> 4488 </td> <td style="text-align:left;"> 2017 </td> <td style="text-align:left;"> NA </td> <td style="text-align:left;"> Fontes, FL; de Araujo, LF; Coutinho, LG; Leib, SL; Agnez-Lima, LF </td> <td style="text-align:left;"> 2015 </td> <td style="text-align:left;"> Genetic polymorphisms associated with the inflammatory response in bacterial meningitis7 </td> <td style="text-align:left;"> Background: Bacterial meningitis (BM) is an infectious disease that results in high mortality and morbidity. Despite efficacious antibiotic therapy, neurological sequelae are often observed in patients after disease. Currently, the main challenge in BM treatment is to develop adjuvant therapies that reduce the occurrence of sequelae. In recent papers published by our group, we described the associations between the single nucleotide polymorphisms (SNPs) AADAT +401C > T, APEX1 Asn148Glu, OGG1 Ser326Cys and PARP1 Val762Ala and BM. In this study, we analyzed the associations between the SNPs TNF -308G > A, TNF -857C > T, IL-8 -251A > T and BM and investigated gene-gene interactions, including the SNPs that we published previously. Methods: The study was conducted with 54 BM patients and 110 healthy volunteers (as the control group). The genotypes were investigated via primer-introduced restriction analysis-polymerase chain reaction (PIRA-PCR) or polymerase chain reaction-based restriction fragment length polymorphism (PCR-RFLP) analysis. Allelic and genotypic frequencies were also associated with cytokine and chemokine levels, as measured with the x-MAP method, and cell counts. We analyzed gene-gene interactions among SNPs using the generalized multifactor dimensionality reduction (GMDR) method. Results: We did not find significant association between the SNPs TNF -857C > T and IL-8 -251A > T and the disease. However, a higher frequency of the variant allele TNF -308A was observed in the control group, associated with changes in cytokine levels compared to individuals with wild type genotypes, suggesting a possible protective role. In addition, combined inter-gene interaction analysis indicated a significant association between certain genotypes and BM, mainly involving the alleles APEX1 148Glu, IL-8 -251 T and AADAT +401 T. These genotypic combinations were shown to affect cyto/chemokine levels and cell counts in CSF samples from BM patients. Conclusions: In conclusion, this study revealed a significant association between genetic variability and altered inflammatory responses, involving important pathways that are activated during BM. This knowledge may be useful for a better understanding of BM pathogenesis and the development of new therapeutic approaches. </td> <td style="text-align:left;"> 4490 </td> <td style="text-align:left;"> NA </td> <td style="text-align:left;"> NA </td> <td style="text-align:left;"> NA </td> <td style="text-align:left;"> no </td> <td style="text-align:left;"> no </td> </tr> <tr> <td style="text-align:left;"> 4489 </td> <td style="text-align:left;"> 2017 </td> <td style="text-align:left;"> NA </td> <td style="text-align:left;"> Perez-Losada, M; Viscidi, RP; Demma, JC; Zenilman, J; Crandall, KA </td> <td style="text-align:left;"> 2005 </td> <td style="text-align:left;"> Population genetics of Neisseria gonorrhoeae in a high-prevalence community using a hypervariable outer membrane porB and 13 slowly evolving housekeeping genes </td> <td style="text-align:left;"> Baltimore, Md., is an urban community with a high prevalence of Neisseria gonorrhoeae. Due to partially protective immune responses, introduction of new strains from other host populations, and exposure of N. gonorrhoeae to antibiotics, the phenotypic and genotypic characteristics of the circulating strains can fluctuate over time. Understanding the overall genetic diversity and population structure of N. gonorrhoeae is essential for informing public health interventions to eliminate this pathogen. We studied gonococci population genetics in Baltimore by analyzing a hypervariable and strongly selected outer membrane porB gene and 13 slowly evolving and presumably neutral housekeeping genes (abcZ, adk, aroE, fumC, gdh, glnA, gnd, pdhC, pgm, pilA, ppk, pyrD, and serC) in 204 isolates collected in 1991, 1996, and 2001 from male and female patients of two public sexually transmitted diseases clinics. Genetic diversity (0), recombination (C), growth (g), population structure, and adaptive selection under codon-substitution and amino acid property models were estimated and compared between these two gene classes. Estimates of the F(ST) fixation index and the X 2 test of sequence absolute frequencies revealed significant temporal substructuring for both gene types. Baltimore's N. gonorrhoeae populations have increased since 1991 as indicated by consistent positive values of g. Female patients showed similar or lower levels of 0 and C than male patients. Within the MLST housekeeping genes, levels of 0 and C ranged from 0.001-0.013 and 0.000-0.018, respectively. Overall recombination seems to be the dominant force driving evolution in these populations. All loci showed amino acid sites and physicochemical properties under adaptive (or positive-destabilizing) selection, rejecting the generally assumed hypothesis of stabilizing selection for these MLST genes. Within the porB gene, protein I B showed higher 0 and C values than protein I A. Directional positive selection possibly mediated by the immune system operates to a significant extent in the protein I sequences, as indicated by the distribution of the positively selected sites in the surface-exposed loops. Thirteen amino acid physicochemical properties seem to drive protein evolution of the PI porins in N. gonorrhoeae. </td> <td style="text-align:left;"> 4491 </td> <td style="text-align:left;"> NA </td> <td style="text-align:left;"> NA </td> <td style="text-align:left;"> NA </td> <td style="text-align:left;"> no </td> <td style="text-align:left;"> no </td> </tr> <tr> <td style="text-align:left;"> 4490 </td> <td style="text-align:left;"> 2017 </td> <td style="text-align:left;"> NA </td> <td style="text-align:left;"> Moreira, MAM; Bonvicino, CR; Soares, MA; Seuanez, HN </td> <td style="text-align:left;"> 2010 </td> <td style="text-align:left;"> Genetic Diversity of Neotropical Primates: Phylogeny, Population Genetics, and Animal Models for Infectious Diseases </td> <td style="text-align:left;"> The classification of neotropical primates has been controversial, and different arrangements have been proposed based on disparate taxonomic criteria and on the traits selected for elucidating phylogenetic reconstructions, like morphologic characters, nuclear DNA and mitochondrial DNA. Population studies of some neotropical primates have been useful for assessing their extant genetic variability and for understanding their social structure and dynamics. Finally, neotropical primates have become valuable models for some human infectious deseases, especially for HIV studies related to viral resistance. In this review, we comment on these aspects that make neotropical primates a group of highly valuable species for basic and applied research. Copyright (C) 2010 S. Karger AG, Basel </td> <td style="text-align:left;"> 4492 </td> <td style="text-align:left;"> NA </td> <td style="text-align:left;"> NA </td> <td style="text-align:left;"> NA </td> <td style="text-align:left;"> no </td> <td style="text-align:left;"> no </td> </tr> <tr> <td style="text-align:left;"> 4491 </td> <td style="text-align:left;"> 2017 </td> <td style="text-align:left;"> NA </td> <td style="text-align:left;"> Heirali, A; McKeon, S; Purighalla, S; Storey, DG; Rossi, L; Costilhes, G; Drews, SJ; Rabin, HR; Surette, MG; Parkins, MD </td> <td style="text-align:left;"> 2016 </td> <td style="text-align:left;"> Assessment of the Microbial Constituents of the Home Environment of Individuals with Cystic Fibrosis (CF) and Their Association with Lower Airways Infections </td> <td style="text-align:left;"> Introduction Cystic fibrosis (CF) airways are colonized by a polymicrobial community of organisms, termed the CF microbiota. We sought to define the microbial constituents of the home environment of individuals with CF and determine if it may serve as a latent reservoir for infection. Methods Six patients with newly identified CF pathogens were included. An investigator collected repeat sputum and multiple environmental samples from their homes. Bacteria were cultured under both aerobic and anaerobic conditions. Morphologically distinct colonies were selected, purified and identified to the genus and species level through 16S rRNA gene sequencing. When concordant organisms were identified in sputum and environment, pulsed-field gel electrophoresis (PFGE) was performed to determine relatedness. Culture-independent bacterial profiling of each sample was carried out by Illumina sequencing of the V3 region of the 16s RNA gene. Results New respiratory pathogens prompting investigation included: Mycobacterium abscessus (2), Stenotrophomonas maltophilia(3), Pseudomonas aeruginosa(3), Pseudomonas fluorescens (1), Nocardia spp.(1), and Achromobacter xylosoxidans(1). A median 25 organisms/patient were cultured from sputum. A median 125 organisms/home were cultured from environmental sites. Several organisms commonly found in the CF lung microbiome were identified within the home environments of these patients. Concordant species included members of the following genera: Brevibacterium(1), Microbacterium(1), Staphylococcus (3), Stenotrophomonas(2), Streptococcus(2), Sphingomonas(1), and Pseudomonas(4). PFGE confirmed related strains (one episode each of Sphinogomonas and P. aeruginosa) from the environment and airways were identified in two patients. Culture-independent assessment confirmed that many organisms were not identified using culture-dependent techniques. Conclusions Members of the CF microbiota can be found as constituents of the home environment in individuals with CF. While the majority of isolates from the home environment were not genetically related to those isolated from the lower airways of individuals with CF suggesting alternate sources of infection were more common, a few genetically related isolates were indeed identified. As such, the home environment may rarely serve as either the source of infection or a persistent reservoir for re-infection after clearance. </td> <td style="text-align:left;"> 4493 </td> <td style="text-align:left;"> NA </td> <td style="text-align:left;"> NA </td> <td style="text-align:left;"> NA </td> <td style="text-align:left;"> no </td> <td style="text-align:left;"> no </td> </tr> <tr> <td style="text-align:left;"> 4492 </td> <td style="text-align:left;"> 2017 </td> <td style="text-align:left;"> NA </td> <td style="text-align:left;"> Georgi, E; Walter, MC; Pfalzgraf, MT; Northoff, BH; Holdt, LM; Scholz, HC; Zoeller, L; Zange, S; Antwerpen, MH </td> <td style="text-align:left;"> 2017 </td> <td style="text-align:left;"> Whole genome sequencing of Brucella melitensis isolated from 57 patients in Germany reveals high diversity in strains from Middle East </td> <td style="text-align:left;"> Brucellosis, a worldwide common bacterial zoonotic disease, has become quite rare in Northern and Western Europe. However, since 2014 a significant increase of imported infections caused by Brucella (B.) melitensis has been noticed in Germany. Patients predominantly originated from Middle East including Turkey and Syria. These circumstances afforded an opportunity to gain insights into the population structure of Brucella strains. Brucella-isolates from 57 patients were recovered between January 2014 and June 2016 with culture confirmed brucellosis by the National Consultant Laboratory for Brucella. Their whole genome sequences were generated using the Illumina MiSeq platform. A whole genome-based SNP typing assay was developed in order to resolve geographically attributed genetic clusters. Results were compared to MLVA typing results, the current gold-standard of Brucella typing. In addition, sequences were examined for possible genetic variation within target regions of molecular diagnostic assays. Phylogenetic analyses revealed spatial clustering and distinguished strains from different patients in either case, whereas multiple isolates from a single patient or technical replicates showed identical SNP and MLVA profiles. By including WGS data from the NCBI database, five major genotypes were identified. Notably, strains originating from Turkey showed a high diversity and grouped into seven subclusters of genotype II. MLVA analysis congruently clustered all isolates and predominantly matched the East Mediterranean genetic clade. This study confirms whole-genome based SNP-analysis as a powerful tool for accurate typing of B. melitensis. Furthermore it allows special allocation and therefore provides useful information on the geographic origin for trace-back analysis. However, the lack of reliable metadata in public databases often prevents a resolution below geographic regions or country levels and corresponding precise trace-back analysis. Once this obstacle is resolved, WGS-derived bacterial typing adds an important method to complement epidemiological surveys during outbreak investigations. This is the first report of a detailed genetic investigation of an extensive collection of B. melitensis strains isolated from human cases in Germany. </td> <td style="text-align:left;"> 4494 </td> <td style="text-align:left;"> NA </td> <td style="text-align:left;"> NA </td> <td style="text-align:left;"> NA </td> <td style="text-align:left;"> no </td> <td style="text-align:left;"> no </td> </tr> <tr> <td style="text-align:left;"> 4493 </td> <td style="text-align:left;"> 2017 </td> <td style="text-align:left;"> NA </td> <td style="text-align:left;"> Waldron-Lynch, F; Adams, C; Amos, C; Zhu, DK; McDermott, MF; Shanahan, F; Molloy, MG; O'Gara, F </td> <td style="text-align:left;"> 2001 </td> <td style="text-align:left;"> Tumour necrosis factor 5 ' promoter single nucleotide polymorphisms influence susceptibility to rheumatoid arthritis (RA) in immunogenetically defined multiplex RA families </td> <td style="text-align:left;"> Tumour necrosis factor (TNF) plays a pivotal role in the pathogenesis of rheumatoid arthritis (RA) and it has been shown that the TNF-lymphotoxin (TNF-LT) region influences susceptibility to RA. To investigate the role of the TNF-LT locus further, inheritance of TNF 5' promoter alleles was determined in multiplex RA families. Six previously defined TNF promoter single nucleotide polymorphisms (SNPs) (-233 -308, -376, -857 -863 -1031) were observed in these families and in addition. a heretofore undocumented adenine (A) to cytosine (C) substitution at position -572 relative to the transcription start site was defined. TNF 5' promoter SNPs were found to cc-segregate with specific TNF microsatellite haplotypes. In particular, the SNP -308A allele was found to be inherited with the TNF a2, b3, c1, d1, e3 (H2) microsatellite haplotype (P < 0.001) which had previously been found to be associated with RA in individuals heterozygous for the HLA-DR 'shared epitope' (SE). When the data were stratified by the presence of the SE with further stratification according to SE DR subtypes and analysed by transmission disequilibrium test (TDT) for which offspring were assumed independent, the -303A and -857T alleles were found to be associated with RA in patients carrying the SE (P = 0.0076 and 0.0063 respectively). The data were further stratified to analyse for association in individuals homozygous or heterozygous for SE alleles. Results showed that the -308A allele was significantly associated with RA susceptibility in individuals heterozygous for the SE (P < 0.001) with the significance only occurring in patients carrying HLA-DR4 (P < 0.001), while the -857T allele was significant in individuals homozygous for the SE (P = 0.0039). Further analysis using the pedigree disequilibrium test (PDT) which conservatively adjusts for all sources of familial correlation except that conferred by linkage disequilibrium still indicated a significant role for the -308A and -857T alleles. These data provide evidence that TNF promoter SNPs may play an independent role in RA susceptibility in specific immunogenetically-defined groups of RA patients. </td> <td style="text-align:left;"> 4495 </td> <td style="text-align:left;"> NA </td> <td style="text-align:left;"> NA </td> <td style="text-align:left;"> NA </td> <td style="text-align:left;"> no </td> <td style="text-align:left;"> no </td> </tr> <tr> <td style="text-align:left;"> 4494 </td> <td style="text-align:left;"> 2017 </td> <td style="text-align:left;"> NA </td> <td style="text-align:left;"> Komarova, TY; Korneva, VA; Kuznetsova, TY; Golovina, AS; Vasilyev, VB; Mandelshtam, MY </td> <td style="text-align:left;"> 2013 </td> <td style="text-align:left;"> Familial hypercholesterolemia mutations in Petrozavodsk: no similarity to St. Petersburg mutation spectrum </td> <td style="text-align:left;"> Background: Familial hypercholesterolemia (FH) is a human monogenic disease induced by a variety of mutations with striking genetic diversity. Despite this variability recurrent mutations occur in each population studied, which allows both elucidating prevalent mutations and developing DNA diagnostic tools for the disease. Recent research of FH in St. Petersburg, Moscow and Novosibirsk (major cities in Russia) demonstrates that each megapolis has its own FH mutation spectrum sharing only small part of mutations with other populations in Russia and Europe. In order to optimize molecular-genetic diagnostic protocols for FH in Russia we studied mutation spectrum in other regions including Petrozavodsk, a smaller town in relatively close proximity to St. Petersburg. Methods: The principal method was automated detection of single-strand conformation polymorphism followed by direct PCR amplified DNA sequencing. Results: Twelve different mutations of the low density lipoprotein (LDL) receptor gene were detected in the Petrozavodsk sample (80 patients). Out of these twelve mutations, seven have never been described before (c. 192_201delinsGGACTTCA, c. 195_196insT, c. 618 T > G, c. 1340C > G, c. 1686_1693delinsT, c. 1936C > A, c. 2191delG). Other five mutations (c. 58G > A, c. 925_931del, c. 1194C > T, c. 1532 T > C, c. 1920C > T) were previously characterized elsewhere. All new mutations are considered to be a probable cause of the FH in their carriers. Direct evidence of the neutral character of c. 58G > A or p. (Gly20Arg) is provided for the first time. Each pathogenic mutation was a trait of its own unique pedigree and so far has not been found in other patients. Conclusions: Strikingly, out of twelve mutations characterized in the Petrozavodsk sample only one mutation, c. 925_931del, has previously been found in patients from St. Petersburg and Finland (most closely located studied populations), suggesting some common roots in origin of these populations in the past or limited gene exchange between them nowadays. No recurrent mutations were detected. </td> <td style="text-align:left;"> 4496 </td> <td style="text-align:left;"> NA </td> <td style="text-align:left;"> NA </td> <td style="text-align:left;"> NA </td> <td style="text-align:left;"> no </td> <td style="text-align:left;"> no </td> </tr> <tr> <td style="text-align:left;"> 4495 </td> <td style="text-align:left;"> 2017 </td> <td style="text-align:left;"> NA </td> <td style="text-align:left;"> Wroblewski, EE; Norman, PJ; Guethlein, LA; Rudicell, RS; Ramirez, MA; Li, YY; Hahn, BH; Pusey, AE; Parham, P </td> <td style="text-align:left;"> 2015 </td> <td style="text-align:left;"> Signature Patterns of MHC Diversity in Three Gombe Communities of Wild Chimpanzees Reflect Fitness in Reproduction and Immune Defense against SIVcpz </td> <td style="text-align:left;"> Major histocompatibility complex (MHC) class I molecules determine immune responses to viral infections. These polymorphic cell-surface glycoproteins bind peptide antigens, forming ligands for cytotoxic T and natural killer cell receptors. Under pressure from rapidly evolving viruses, hominoid MHC class I molecules also evolve rapidly, becoming diverse and species-specific. Little is known of the impact of infectious disease epidemics on MHC class I variant distributions in human populations, a context in which the chimpanzee is the superior animal model. Population dynamics of the chimpanzees inhabiting Gombe National Park, Tanzania have been studied for over 50 years. This population is infected with SIVcpz, the precursor of human HIV-1. Because HLA-B is the most polymorphic human MHC class I molecule and correlates strongly with HIV-1 progression, we determined sequences for its ortholog, Patr-B, in 125 Gombe chimpanzees. Eleven Patr-B variants were defined, as were their frequencies in Gombe's three communities, changes in frequency with time, and effect of SIVcpz infection. The growing populations of the northern and central communities, where SIVcpz is less prevalent, have stable distributions comprising a majority of low-frequency Patr-B variants and a few high-frequency variants. Driving the latter to high frequency has been the fecundity of immigrants to the northern community, whereas in the central community, it has been the fecundity of socially dominant individuals. In the declining population of the southern community, where greater SIVcpz prevalence is associated with mortality and emigration, Patr-B variant distributions have been changing. Enriched in this community are Patr-B variants that engage with natural killer cell receptors. Elevated among SIVcpz-infected chimpanzees, the Patr-B*06:03 variant has striking structural and functional similarities to HLA-B*57, the human allotype most strongly associated with delayed HIV-1 progression. Like HLA-B*57, Patr-B*06:03 correlates with reduced viral load, as assessed by detection of SIVcpz RNA in feces. </td> <td style="text-align:left;"> 4497 </td> <td style="text-align:left;"> NA </td> <td style="text-align:left;"> NA </td> <td style="text-align:left;"> NA </td> <td style="text-align:left;"> no </td> <td style="text-align:left;"> no </td> </tr> <tr> <td style="text-align:left;"> 4496 </td> <td style="text-align:left;"> 2017 </td> <td style="text-align:left;"> NA </td> <td style="text-align:left;"> Wu, KF; Luo, T; Li, L; Zhang, QQ; Zhu, JH; Gao, Q; Chen, M; Zhu, M </td> <td style="text-align:left;"> 2015 </td> <td style="text-align:left;"> Multilocus Sequence Typing of Pathogenic Candida albicans Isolates Collected from a Teaching Hospital in Shanghai, China: A Molecular Epidemiology Study </td> <td style="text-align:left;"> Molecular typing of Candida albicansis important for studying the population structure and epidemiology of this opportunistic yeast, such as population dynamics, nosocomial infections, multiple infections and microevolution. The genetic diversity of C. albicanshas been rarely studied in China. In the present study, multilocus sequence typing (MLST) was used to characterize the genetic diversity and population structure of 62 C. albicansisolates collected from 40 patients from Huashan Hospital in Shanghai, China. A total of 50 diploid sequence types (DSTs) were identified in the 62 C. albicansisolates, with 41 newly identified DSTs. Based on cluster analysis, the 62 isolates were classified into nine existing clades and two new clades (namely clades New 1 and New 2). The majority of the isolates were clustered into three clades, clade 6 (37.5%), clade 1 (15.0%) and clade 17 (15.0%). Isolates of clade New 2 were specifically identified in East Asia. We identified three cases of potential nosocomial transmission based on association analysis between patients' clinical data and the genotypes of corresponding isolates. Finally, by analyzing the genotypes of serial isolates we further demonstrated that the microevolution of C. albicans was due to loss of heterozygosity. Our study represents the first molecular typing of C. albicansin eastern China, and we confirmed that MLST is a useful tool for studying the epidemiology and evolution of C. albicans. </td> <td style="text-align:left;"> 4498 </td> <td style="text-align:left;"> NA </td> <td style="text-align:left;"> NA </td> <td style="text-align:left;"> NA </td> <td style="text-align:left;"> no </td> <td style="text-align:left;"> no </td> </tr> <tr> <td style="text-align:left;"> 4497 </td> <td style="text-align:left;"> 2017 </td> <td style="text-align:left;"> NA </td> <td style="text-align:left;"> Sazzini, M; Zuntini, R; Farjadian, S; Quinti, I; Ricci, G; Romeo, G; Ferrari, S; Calafell, F; Luiselli, D </td> <td style="text-align:left;"> 2009 </td> <td style="text-align:left;"> An evolutionary approach to the medical implications of the tumor necrosis factor receptor superfamily member 13B (TNFRSF13B) gene </td> <td style="text-align:left;"> Coding variants in tumor necrosis factor receptor superfamily member 13B (TNFRSF13B) have been implicated in common variable immunodeficiency (CVID), but the functional effects of such mutations in relation to the development of the disease have not been entirely established. To examine the potential contribution of TNFRSF13B variants to CVID, we have applied an evolutionary approach by sequencing its coding region in 451 individuals belonging to 26 worldwide populations, in addition to controls, patients with CVID and selective IgA deficiency (IgAD) from Italy. The low level of geographical structure for the observed genetic diversity and the several neutrality tests performed confirm the absence of recent population-specific selective pressures, suggesting that TNFRSF13B may be involved also in innate immune functions, rather than in adaptive immunity only. A slight excess of rare derived alleles was found in patients with CVID, and thus some of these variants may contribute to the disease, implying that CVID probably fits the rare variants rather than the common disease/common variant paradigm. This also confirms the previous suggestion that TNFRSF13B defects alone do not cause CVID and that such an extremely heterogeneous immunodeficiency might be more likely related to additional, still unknown environmental and genetic factors. Genes and Immunity (2009) 10, 566-578; doi:10.1038/gene.2009.43; published online 4 June 2009 </td> <td style="text-align:left;"> 4499 </td> <td style="text-align:left;"> NA </td> <td style="text-align:left;"> NA </td> <td style="text-align:left;"> NA </td> <td style="text-align:left;"> no </td> <td style="text-align:left;"> no </td> </tr> <tr> <td style="text-align:left;"> 4498 </td> <td style="text-align:left;"> 2017 </td> <td style="text-align:left;"> NA </td> <td style="text-align:left;"> Mbenda, HGN; Das, A </td> <td style="text-align:left;"> 2014 </td> <td style="text-align:left;"> Molecular Evidence of Plasmodium vivax Mono and Mixed Malaria Parasite Infections in Duffy-Negative Native Cameroonians </td> <td style="text-align:left;"> The malaria parasite Plasmodium vivax is known to be majorly endemic to Asian and Latin American countries with no or very few reports of Africans infected with this parasite. Since the human Duffy antigens act as receptors for P. vivax to invade human RBCs and Africans are generally Duffy-negative, non-endemicity of P. vivax in Africa has been attributed to this fact. However, recent reports describing P. vivax infections in Duffy-negative Africans from West and Central parts of Africa have been surfaced including a recent report on P. vivax infection in native Cameroonians. In order to know if Cameroonians living in the southern regions are also susceptible to P. vivax infection, we collected finger-prick blood samples from 485 malarial symptomatic patients in five locations and followed PCR diagnostic assays with DNA sequencing of the 18S ribosomal RNA gene. Out of the 201 malaria positive cases detected, 193 were pure P. falciparum, six pure P. vivax and two mixed parasite infections (P. falciparum + P. vivax). The eight P. vivax infected samples (six single + two mixed) were further subjected to DNA sequencing of the P. vivax multidrug resistance 1 (pvmdr1) and the P. vivax circumsporozoite (pvcsp) genes. Alignment of the eight Cameroonian pvmdr1 sequences with the reference sequence showed high sequence similarities, reconfirming P. vivax infection in all the eight patients. DNA sequencing of the pvcsp gene indicated all the eight P. vivax to be of VK247 type. Interestingly, DNA sequencing of a part of the human Duffy gene covering the promoter region in the eight P. vivax-infected Cameroonians to identify the T-33C mutation revealed all these patients as Duffy-negative. The results provide evidence of single P. vivax as well as mixed malaria parasite infection in native Cameroonians and add knowledge to the growing evidences of P. vivax infection in Duffy-negative Africans. </td> <td style="text-align:left;"> 4500 </td> <td style="text-align:left;"> NA </td> <td style="text-align:left;"> NA </td> <td style="text-align:left;"> NA </td> <td style="text-align:left;"> no </td> <td style="text-align:left;"> no </td> </tr> <tr> <td style="text-align:left;"> 4499 </td> <td style="text-align:left;"> 2017 </td> <td style="text-align:left;"> NA </td> <td style="text-align:left;"> Pennings, PS; Kryazhimskiy, S; Wakeley, J </td> <td style="text-align:left;"> 2014 </td> <td style="text-align:left;"> Loss and Recovery of Genetic Diversity in Adapting Populations of HIV </td> <td style="text-align:left;"> The evolution of drug resistance in HIV occurs by the fixation of specific, well-known, drug-resistance mutations, but the underlying population genetic processes are not well understood. By analyzing within-patient longitudinal sequence data, we make four observations that shed a light on the underlying processes and allow us to infer the short-term effective population size of the viral population in a patient. Our first observation is that the evolution of drug resistance usually occurs by the fixation of one drug-resistance mutation at a time, as opposed to several changes simultaneously. Second, we find that these fixation events are accompanied by a reduction in genetic diversity in the region surrounding the fixed drug-resistance mutation, due to the hitchhiking effect. Third, we observe that the fixation of drug-resistance mutations involves both hard and soft selective sweeps. In a hard sweep, a resistance mutation arises in a single viral particle and drives all linked mutations with it when it spreads in the viral population, which dramatically reduces genetic diversity. On the other hand, in a soft sweep, a resistance mutation occurs multiple times on different genetic backgrounds, and the reduction of diversity is weak. Using the frequency of occurrence of hard and soft sweeps we estimate the effective population size of HIV to be 1.5 x 10(5) (95% confidence interval [0: 8 x 10(5),4.8 x 10(5)]). This number is much lower than the actual number of infected cells, but much larger than previous population size estimates based on synonymous diversity. We propose several explanations for the observed discrepancies. Finally, our fourth observation is that genetic diversity at non-synonymous sites recovers to its pre-fixation value within 18 months, whereas diversity at synonymous sites remains depressed after this time period. These results improve our understanding of HIV evolution and have potential implications for treatment strategies. </td> <td style="text-align:left;"> 4501 </td> <td style="text-align:left;"> NA </td> <td style="text-align:left;"> NA </td> <td style="text-align:left;"> NA </td> <td style="text-align:left;"> no </td> <td style="text-align:left;"> no </td> </tr> <tr> <td style="text-align:left;"> 4500 </td> <td style="text-align:left;"> 2017 </td> <td style="text-align:left;"> NA </td> <td style="text-align:left;"> Nkhoma, SC; Nair, S; Cheeseman, IH; Rohr-Allegrini, C; Singlam, S; Nosten, F; Anderson, TJC </td> <td style="text-align:left;"> 2012 </td> <td style="text-align:left;"> Close kinship within multiple-genotype malaria parasite infections </td> <td style="text-align:left;"> Malaria infections containing multiple parasite genotypes are ubiquitous in nature, and play a central role in models of recombination, intra-host dynamics, virulence, sex ratio, immunity and drug resistance evolution in Plasmodium. While these multiple infections (MIs) are often assumed to result from superinfection (bites from multiple infected mosquitoes), we know remarkably little about their composition or generation. We isolated 336 parasite clones from eight patients from Malawi (high transmission) and six from Thailand (low transmission) by dilution cloning. These were genotyped using 384 single-nucleotide polymorphisms, revealing 22 independent haplotypes in Malawi (2-6 per MI) and 15 in Thailand (2-5 per MI). Surprisingly, all six patients from Thailand and six of eight from Malawi contained related haplotypes, and haplotypes were more similar within-than between-infections. These results argue against a simple superinfection model. Instead, the observed kinship patterns may be explained by inoculation of multiple related haploid sporozoites from single mosquito bites, by immune suppression of parasite subpopulations within infections, and serial transmission of related parasites between people. That relatedness is maintained in endemic areas in the face of repeated bites from infected mosquitoes has profound implications for understanding malaria transmission, immunity and intra-host dynamics of co-infecting parasite genotypes. </td> <td style="text-align:left;"> 4502 </td> <td style="text-align:left;"> NA </td> <td style="text-align:left;"> NA </td> <td style="text-align:left;"> NA </td> <td style="text-align:left;"> no </td> <td style="text-align:left;"> no </td> </tr> <tr> <td style="text-align:left;"> 4501 </td> <td style="text-align:left;"> 2017 </td> <td style="text-align:left;"> NA </td> <td style="text-align:left;"> Horne, BD; Carlquist, JF; Cannon-Albright, LA; Muhlestein, JB; McKinney, JT; Kolek, MJ; Clarke, JL; Anderson, JL; Camp, NJ </td> <td style="text-align:left;"> 2006 </td> <td style="text-align:left;"> High-resolution characterization of linkage disequilibrium structure and selection of tagging single nucleotide polymorphisms: Application to the cholesteryl ester transfer protein gene </td> <td style="text-align:left;"> Full characterization of intragenic variation may improve candidate gene associations. This study selected tagging (t) single nucleotide polymorphisms (SNPs) to comprehensively represent genetic variability in the cholesteryl ester transfer protein (CETP) gene. Nineteen SNPs were identified in 50 unrelated individuals in the SNP discovery phase, and 13 intronic SNPs were added from the literature. These 32 SNPs were genotyped in 339 apparently healthy individuals and 190 coronary artery disease (CAD) patients. Using phased haplotypes, linkage disequilibrium (LD) structure was characterized and tSNPs selected using a principal component analysis (PCA) method. In healthy individuals, seven LD groups were identified that accounted for 93.4% of the observed genetic variation. These LD groups highlighted a complex LD structure for CETP, including both recombination and mutation, and eleven tSNPs were selected. Among CAD patients the results were essentially the same. Results from PCA using diploid genotype data were reasonably comparable. Finally, the selected tSNPs successfully represented the association evidence discovered for all of the other SNPs studied. This study provides an optimal set of tSNPs for association analyses of CETP. The observed complexity of LD structure highlights the importance of using methods, such as PCA, that allow for multiple dynamics in intragenic LD structure. </td> <td style="text-align:left;"> 4503 </td> <td style="text-align:left;"> NA </td> <td style="text-align:left;"> NA </td> <td style="text-align:left;"> NA </td> <td style="text-align:left;"> no </td> <td style="text-align:left;"> no </td> </tr> <tr> <td style="text-align:left;"> 4502 </td> <td style="text-align:left;"> 2017 </td> <td style="text-align:left;"> NA </td> <td style="text-align:left;"> Vargas-Sanchez, GB; Romero-Valdovinos, M; Ramirez-Guerrero, C; Vargas-Hernandez, I; Ramirez-Miranda, ME; Martinez-Ocana, J; Valadez, A; Ximenez, C; Lopez-Escamilla, E; Hernandez-Campos, ME; Villalobos, G; Martinez-Hernandez, F; Maravilla, P </td> <td style="text-align:left;"> 2015 </td> <td style="text-align:left;"> Blastocystis Isolates from Patients with Irritable Bowel Syndrome and from Asymptomatic Carriers Exhibit Similar Parasitological Loads, but Significantly Different Generation Times and Genetic Variability across Multiple Subtypes </td> <td style="text-align:left;"> Blastocystis spp is a common intestinal parasite of humans and animals that has been associated to the etiology of irritable bowel syndrome (IBS); however, some studies have not found this association. Furthermore, many biological features of Blastocystis are little known. The objective of present study was to assess the generation times of Blastocystis cultures, from IBS patients and from asymptomatic carriers. A total of 100 isolates were obtained from 50 IBS patients and from 50 asymptomatic carriers. Up to 50 mg of feces from each participant were cultured in Barret's and in Pavlova's media during 48 h. Initial and final parasitological load were measured by microscopy and by quantitative PCR. Amplicons were purified, sequenced and submitted to GenBank; sequences were analysed for genetic diversity and a Bayesian inference allowed identifying genetic subtypes (ST). Generation times for Blastocystis isolates in both media, based on microscopic measures and molecular assays, were calculated. The clinical symptoms of IBS patients and distribution of Blastocystis ST 1, 2 and 3 in both groups was comparable to previous reports. Interestingly, the group of cases showed scarce mean nucleotide diversity (pi) as compared to the control group (0.011 +/- 0.016 and 0.118 +/- 0.177, respectively), whilst high gene flow and small genetic differentiation indexes between different ST were found. Besides, Tajima's D test showed negative values for ST1-ST3. No statistical differences regarding parasitological load between cases and controls in both media, as searched by microscopy and by qPCR, were detected except that parasites grew faster in Barret's than in Pavlova's medium. Interestingly, slow growth of isolates recovered from cases in comparison to those of controls was observed (p<0.05). We propose that generation times of Blastocystis might be easily affected by intestinal environmental changes due to IBS probably because virulent strains with slow growth may be selected, reducing their genetic variability. </td> <td style="text-align:left;"> 4504 </td> <td style="text-align:left;"> NA </td> <td style="text-align:left;"> NA </td> <td style="text-align:left;"> NA </td> <td style="text-align:left;"> no </td> <td style="text-align:left;"> no </td> </tr> <tr> <td style="text-align:left;"> 4503 </td> <td style="text-align:left;"> 2017 </td> <td style="text-align:left;"> NA </td> <td style="text-align:left;"> Moyo, SJ; Blomberg, B; Hanevik, K; Kommedal, O; Vainio, K; Maselle, SY; Langeland, N </td> <td style="text-align:left;"> 2014 </td> <td style="text-align:left;"> Genetic Diversity of Circulating Rotavirus Strains in Tanzania Prior to the Introduction of Vaccination </td> <td style="text-align:left;"> Background: Tanzania currently rolls out vaccination against rotavirus-diarrhea, a major cause of child illness and death. As the vaccine covers a limited number of rotavirus variants, this study describes the molecular epidemiology of rotavirus among children under two years in Dar es Salaam, Tanzania, prior to implementation of vaccination. Methods: Stool specimens, demographic and clinical information, were collected from 690 children admitted to hospital due to diarrhea (cases) and 545 children without diarrhea (controls) during one year. Controls were inpatient or children attending child health clinics. Rotavirus antigen was detected using ELISA and positive samples were typed by multiplex semi-nested PCR and sequencing. Results: The prevalence of rotavirus was higher in cases (32.5%) than in controls (7.7%, P<0.001). The most common G genotypes were G1 followed by G8, G12, and G4 in cases and G1, G12 and G8 in controls. The Tanzanian G1 variants displayed 94% similarity with the Rotarix vaccine G1 variant. The commonest P genotypes were P[8], P[4] and P[6], and the commonest G/P combination G1 P[8] (n = 123), G8 P[4] and G12 P[6]. Overall, rotavirus prevalence was higher in cool (23.9%) than hot months (17.1%) of the year (P = 0.012). We also observed significant seasonal variation of G genotypes. Rotavirus was most frequently found in the age group of four to six months. The prevalence of rotavirus in cases was lower in stunted children (28.9%) than in non-stunted children (40.1%, P = 0.003) and lower in HIV-infected (15.4%, 4/26) than in HIV-uninfected children (55.3%, 42/76, P<0.001). Conclusion: This pre-vaccination study shows predominance of genotype G1 in Tanzania, which is phylogenetically distantly related to the vaccine strains. We confirm the emergence of genotype G8 and G12. Rotavirus infection and circulating genotypes showed seasonal variation. This study also suggests that rotavirus may not be an opportunistic pathogen in children infected with HIV. </td> <td style="text-align:left;"> 4505 </td> <td style="text-align:left;"> NA </td> <td style="text-align:left;"> NA </td> <td style="text-align:left;"> NA </td> <td style="text-align:left;"> no </td> <td style="text-align:left;"> no </td> </tr> <tr> <td style="text-align:left;"> 4504 </td> <td style="text-align:left;"> 2017 </td> <td style="text-align:left;"> NA </td> <td style="text-align:left;"> Takakura, S; Kohno, T; Shimizu, K; Ohwada, S; Okamoto, A; Yokota, J </td> <td style="text-align:left;"> 2000 </td> <td style="text-align:left;"> Somatic mutations and genetic polymorphisms of the PPP1R3 gene in patients with several types of cancers </td> <td style="text-align:left;"> Recently, we found nonsense and missense mutations of the PPP1R3 (protein phosphatase 1, regulatory submit 3) gene in diverse human cancer cell lines and primary lung carcinomas, indicating that PPP1R3 functions as a tumor suppressor in human carcinogenesis. In this study, to assess the prevalence of PPP1R3 mutations in human primary cancers and the genetic diversity of the PPP1R3 gene in the human population, somatic mutations and genetic polymorphisms in the PPP1R3 gene were examined in 137 pairs of cancerous and non-cancerous tissues of patients with cancers of colon, ovary, and liver. Five somatic mutations including two missense mutations were detected in three cancerous tissues consisting of two colorectal carcinomas and one ovarian carcinoma, Five novel single nucleotide polymorphisms (SNPs) associated with the substitution of amino acids were also identified in cancer patients, in addition to five known nonsynonymous SNPs, including three previously reported ones as having an impact on the susceptibility to insulin resistant disorders. Differences in the activities and properties of multiple PPP1R3 proteins, which are produced in human cells due to variable somatic mutations and genetic polymorphisms in the PPP1R3 gene, can be involved in human carcinogenesis and susceptibility to diseases. </td> <td style="text-align:left;"> 4506 </td> <td style="text-align:left;"> NA </td> <td style="text-align:left;"> NA </td> <td style="text-align:left;"> NA </td> <td style="text-align:left;"> no </td> <td style="text-align:left;"> no </td> </tr> <tr> <td style="text-align:left;"> 4505 </td> <td style="text-align:left;"> 2017 </td> <td style="text-align:left;"> NA </td> <td style="text-align:left;"> Tapparel, C; Cordey, S; Junier, T; Farinelli, L; Van Belle, S; Soccal, PM; Aubert, JD; Zdobnov, E; Kaiser, L </td> <td style="text-align:left;"> 2011 </td> <td style="text-align:left;"> Rhinovirus Genome Variation during Chronic Upper and Lower Respiratory Tract Infections </td> <td style="text-align:left;"> Routine screening of lung transplant recipients and hospital patients for respiratory virus infections allowed to identify human rhinovirus (HRV) in the upper and lower respiratory tracts, including immunocompromised hosts chronically infected with the same strain over weeks or months. Phylogenetic analysis of 144 HRV-positive samples showed no apparent correlation between a given viral genotype or species and their ability to invade the lower respiratory tract or lead to protracted infection. By contrast, protracted infections were found almost exclusively in immunocompromised patients, thus suggesting that host factors rather than the virus genotype modulate disease outcome, in particular the immune response. Complete genome sequencing of five chronic cases to study rhinovirus genome adaptation showed that the calculated mutation frequency was in the range observed during acute human infections. Analysis of mutation hot spot regions between specimens collected at different times or in different body sites revealed that non-synonymous changes were mostly concentrated in the viral capsid genes VP1, VP2 and VP3, independent of the HRV type. In an immunosuppressed lung transplant recipient infected with the same HRV strain for more than two years, both classical and ultra-deep sequencing of samples collected at different time points in the upper and lower respiratory tracts showed that these virus populations were phylogenetically indistinguishable over the course of infection, except for the last month. Specific signatures were found in the last two lower respiratory tract populations, including changes in the 5'UTR polypyrimidine tract and the VP2 immunogenic site 2. These results highlight for the first time the ability of a given rhinovirus to evolve in the course of a natural infection in immunocompromised patients and complement data obtained from previous experimental inoculation studies in immunocompetent volunteers. </td> <td style="text-align:left;"> 4507 </td> <td style="text-align:left;"> NA </td> <td style="text-align:left;"> NA </td> <td style="text-align:left;"> NA </td> <td style="text-align:left;"> no </td> <td style="text-align:left;"> no </td> </tr> <tr> <td style="text-align:left;"> 4506 </td> <td style="text-align:left;"> 2017 </td> <td style="text-align:left;"> NA </td> <td style="text-align:left;"> Molenaar-de Backer, MWA; Lukashov, VV; van Binnendijk, RS; Boot, HJ; Zaaijer, HL </td> <td style="text-align:left;"> 2012 </td> <td style="text-align:left;"> Global Co-Existence of Two Evolutionary Lineages of Parvovirus B19 1a, Different in Genome-Wide Synonymous Positions </td> <td style="text-align:left;"> Parvovirus B19 (B19V) can cause infection in humans. To date, three genotypes of B19V, with subtypes, are known, of which genotype 1a is the most prevalent genotype in the Western world. We sequenced the genome of B19V strains of 65 asymptomatic, recently infected Dutch blood donors, to investigate the spatio-temporal distribution of B19V strains, in the years 2003-2009. The sequences were compared to B19V sequences from Dutch patients with fifth disease, and to global B19V sequences as available from GenBank. All Dutch B19V strains belonged to genotype 1a. Phylogenetic analysis of the strains from Dutch blood donors showed that two groups of genotype 1a co-exist. A clear-cut division into the two groups was also found among the B19V strains from Dutch patients, and among the B19V sequences in GenBank. The two groups of genotype 1a co-exist around the world and do not appear to differ in their ability to cause disease. Strikingly, the two groups of B19V predominantly differ in synonymous mutations, distributed throughout the entire genome of B19V. We propose to call the two groups of B19V genotype 1a respectively subtype 1a1 and 1a2. </td> <td style="text-align:left;"> 4508 </td> <td style="text-align:left;"> NA </td> <td style="text-align:left;"> NA </td> <td style="text-align:left;"> NA </td> <td style="text-align:left;"> no </td> <td style="text-align:left;"> no </td> </tr> <tr> <td style="text-align:left;"> 4507 </td> <td style="text-align:left;"> 2017 </td> <td style="text-align:left;"> NA </td> <td style="text-align:left;"> Zhao, DM; Li, NN; Zhang, JH; Chang, XL; Mao, XY; Liao, Q; Tan, EK; Peng, R </td> <td style="text-align:left;"> 2012 </td> <td style="text-align:left;"> GSK3 beta Reduces Risk of Sporadic Parkinson's Disease in Ethnic Chinese </td> <td style="text-align:left;"> Genetic variability of glycogen synthase kinase-3 beta (GSK3 beta) may be linked to Parkinson's disease (PD). Its role in ethnic Chinese population is still unclear. We examined the association between GSK3 beta variation and PD in a Han Chinese population from mainland China. Using a case-control methodology, we genotyped the single nucleotide polymorphism (SNP) in GSK3 beta (rs334558) to investigate the association with risk of PD. A total of 1,280 ethnic Han Chinese study subjects comprising 761 sporadic PD patients and 519 controls were recruited. The T allele of a promoter SNP rs334558 was found to reduce the risk of PD (OR = 0.82, 95% CI: 0.696-0.960, P = 0.014). Patients with CT + TT genotypes have a reduced risk of PD compared to those with CC genotype (OR = 0.61, 95% CI: 0.477-0.776, P = 6.09E-5). In addition, we demonstrated that CT + TT subjects cannot be distinguished from CC subjects based on their clinical features. Our data suggest that rs334558 variant in GSK3 beta reduces the risk of PD in a Han Chinese population from mainland China. Further studies of large series of subjects are necessary to fully elucidate the true role of GSK3 beta in PD. (C) 2012 Wiley Periodicals, Inc. </td> <td style="text-align:left;"> 4509 </td> <td style="text-align:left;"> NA </td> <td style="text-align:left;"> NA </td> <td style="text-align:left;"> NA </td> <td style="text-align:left;"> no </td> <td style="text-align:left;"> no </td> </tr> <tr> <td style="text-align:left;"> 4508 </td> <td style="text-align:left;"> 2017 </td> <td style="text-align:left;"> NA </td> <td style="text-align:left;"> MUSSER, JM; HAUSER, AR; KIM, MH; SCHLIEVERT, PM; NELSON, K; SELANDER, RK </td> <td style="text-align:left;"> 1991 </td> <td style="text-align:left;"> STREPTOCOCCUS-PYOGENES CAUSING TOXIC-SHOCK-LIKE SYNDROME AND OTHER INVASIVE DISEASES - CLONAL DIVERSITY AND PYROGENIC EXOTOXIN EXPRESSION </td> <td style="text-align:left;"> Genetic diversity and relationships among 108 isolates of the bacterium Streptococcus pyogenes recently recovered from patients in the United States with toxic-shock-like syndrome or other invasive diseases were estimated by multilocus enzyme electrophoresis. Thirty-three electrophoretic types (ETs), representing distinctive multilocus clonal genotypes, were identified, but nearly half the disease episodes, including more than two-thirds of the cases of toxic-shock-like syndrome, were caused by strains of two related clones (ET 1 and ET 2). These two clones were also represented by recent pathogenic European isolates. A previous report of a relatively high frequency of expression of exotoxin A among isolates recovered from toxic-shock-like syndrome patients in the United States was confirmed; and the demonstration of this association both within clones and among distantly related clones supports the hypothesis that exotoxin A is a causal factor in pathogenesis of this disease. Near identity of the nucleotide sequences of the exotoxin A structural gene of six isolates of five ETs in diverse phylogenetic lineages was interpreted as evidence that the gene has been horizontally distributed among clones, presumably by bacteriophage-mediated transfer. </td> <td style="text-align:left;"> 4510 </td> <td style="text-align:left;"> NA </td> <td style="text-align:left;"> NA </td> <td style="text-align:left;"> NA </td> <td style="text-align:left;"> no </td> <td style="text-align:left;"> no </td> </tr> <tr> <td style="text-align:left;"> 4509 </td> <td style="text-align:left;"> 2017 </td> <td style="text-align:left;"> NA </td> <td style="text-align:left;"> Bittar, C; Jardim, ACG; Yamasaki, LHT; Carareto, CMA; Pinho, JRR; Lemey, P; de Carvalho-Mello, IMVG; Rahal, P </td> <td style="text-align:left;"> 2013 </td> <td style="text-align:left;"> On Hepatitis C Virus Evolution: The Interaction between Virus and Host towards Treatment Outcome </td> <td style="text-align:left;"> Background: Hepatitis C is a disease spread throughout the world. Hepatitis C virus (HCV), the etiological agent of this disease, is a single-stranded positive RNA virus. Its genome encodes a single precursor protein that yields ten proteins after processing. NS5A, one of the non-structural viral proteins, is most associated with interferon-based therapy response, the approved treatment for hepatitis C in Brazil. HCV has a high mutation rate and therefore high variability, which may be important for evading the immune system and response to therapy. The aim of this study was to analyze the evolution of NS5A quasispecies before, during, and after treatment in patients infected with HCV genotype 3a who presented different therapy responses. Methods: Viral RNA was extracted, cDNA was synthesized, the NS5A region was amplified and cloned, and 15 clones from each time-point were sequenced. The sequences were analyzed for evolutionary history, genetic diversity and selection. Results: This analysis shows that the viral population that persists after treatment for most non-responder patients is present in before-treatment samples, suggesting it is adapted to evade treatment. In contrast, the population found in before treatment samples from most end-of-treatment responder patients either are selected out or appears in low frequency after relapse, therefore changing the population structure. The exceptions illustrate the uniqueness of the evolutionary process, and therefore the treatment resistance process, in each patient. Conclusion: Although evolutionary behavior throughout treatment showed that each patient presented different population dynamics unrelated to therapy outcome, it seems that the viral population from non-responders that resists the treatment already had strains that could evade therapy before it started. </td> <td style="text-align:left;"> 4511 </td> <td style="text-align:left;"> NA </td> <td style="text-align:left;"> NA </td> <td style="text-align:left;"> NA </td> <td style="text-align:left;"> no </td> <td style="text-align:left;"> no </td> </tr> <tr> <td style="text-align:left;"> 4510 </td> <td style="text-align:left;"> 2017 </td> <td style="text-align:left;"> NA </td> <td style="text-align:left;"> Tuteja, A; Siddiqui, AB; Madan, K; Goyal, R; Shalimar; Sreenivas, V; Kaur, N; Panda, SK; Narayanasamy, K; Subodh, S; Acharya, SK </td> <td style="text-align:left;"> 2014 </td> <td style="text-align:left;"> Mutation Profiling of the Hepatitis B Virus Strains Circulating in North Indian Population </td> <td style="text-align:left;"> Aims: The aim of this study was to investigate the genomic mutations in the circulating Hepatitis B virus strains causing infection in the Indian population. Further, we wanted to analyze the biological significance of these mutations in HBV mediated disease. Methods: 222 HBsAg positive patients were enrolled in the study. The genotype and mutation profile was determined for the infecting HBV isolate by sequencing overlapping fragments. These sequences were analyzed by using different tools and compared with previously available HBV sequence information. Mutation Frequency Index (MFI) for the Genes and Diagnosis group was also calculated. Results: HBV Genotype D was found in 55% (n = 121) of the patient group and genotype A was found in 30% (n = 66) of samples. The majority (52%) of the HBV-infected individuals in the present study were HBeAg-negative in all the age groups studied. Spontaneous drug associated mutations implicated in resistance to antiviral therapy were also identified in about quarter of our patients, which is of therapeutic concern. The MFI approach used in the study indicated that Core peptide was the most conserved region in both genotypes and Surface peptide had highest mutation frequency. Few mutations in X gene (T36A and G50R) showed high frequency of association with HCC. A rare recombinant strain of HBV genotype A and D was also identified in the patient group. Conclusions: HBV genotype D was found out to be most prevalent. More than half of the patients studied had HBeAg negative disease. Core region was found to be most conserved. Drug Associated mutations were detected in 22% of the patient group and T36A and G50R mutations in X gene were found to be associated with HCC. </td> <td style="text-align:left;"> 4512 </td> <td style="text-align:left;"> NA </td> <td style="text-align:left;"> NA </td> <td style="text-align:left;"> NA </td> <td style="text-align:left;"> no </td> <td style="text-align:left;"> no </td> </tr> <tr> <td style="text-align:left;"> 4511 </td> <td style="text-align:left;"> 2017 </td> <td style="text-align:left;"> NA </td> <td style="text-align:left;"> Mhandire, D; Lacerda, M; Castel, S; Mhandire, K; Zhou, DA; Swart, M; Shamu, T; Smith, P; Musingwini, T; Wiesner, L; Stray-Pedersen, B; Dandara, C </td> <td style="text-align:left;"> 2015 </td> <td style="text-align:left;"> Effects of CYP2B6 and CYP1A2 Genetic Variation on Nevirapine Plasma Concentration and Pharmacodynamics as Measured by CD4 Cell Count in Zimbabwean HIV-Infected Patients </td> <td style="text-align:left;"> The extremely high prevalence of HIV/AIDS in sub-Saharan Africa and limitations of current antiretroviral medicines demand new tools to optimize therapy such as pharmacogenomics for person-to-person variations. African populations exhibit greater genetic diversity than other world populations, thus making it difficult to extrapolate findings from one population to another. Nevirapine, an antiretroviral medicine, displays large plasma concentration variability which adversely impacts therapeutic virological response. This study, therefore, aimed to identify sources of variability in nevirapine pharmacokinetics and pharmacodynamics, focusing on genetic variation in CYP2B6 and CYP1A2. Using a cross-sectional study design, 118 HIV-infected adult Zimbabwean patients on nevirapine-containing highly active antiretroviral therapy (HAART) were characterized for three key functional single nucleotide polymorphisms (SNPs), CYP2B6 c.516G>T (rs3745274), CYP2B6 c.983T>C (rs28399499), and CYP1A2 g.-163C>A (rs762551). We investigated whether genotypes at these loci were associated with nevirapine plasma concentration, a therapeutic biomarker, and CD4 cell count, a biomarker of disease progression. CYP2B6 and CYP1A2 were chosen as the candidate genes based on reports in literature, as well as their prominence in the metabolism of efavirenz, a drug in the same class with nevirapine. Nevirapine plasma concentration was determined using LC-MS/MS. The mean nevirapine concentration for CYP2B6 c.516T/T genotype differed significantly from that of 516G/G (p < 0.001) and 516G/T (p < 0.01) genotypes, respectively. There were also significant differences in mean nevirapine concentration between CYP2B6 c.983T > C genotypes (p = 0.04). Importantly, the CYP1A2 g.-163C>A SNP was significantly associated with the pharmacodynamics endpoint, the CD4 cell count (p = 0.012). Variant allele frequencies for the three SNPs observed in this Zimbabwean group were similar to other African population groups but different to observations among Caucasian and Asian populations. We conclude that CYP2B6 c.516G>T and CYP2B6 c.983T>C could be important sources of nevirapine pharmacokinetic variability that could be considered for dosage optimization, while CYP1A2 g.-163C>A seems to be associated with HIV disease progression. These inter- and intra-population pharmacokinetic and pharmacodynamics differences suggest that a single prescribed dosage may not be appropriate for the treatment of disease. Further research into a personalized nevirapine regimen is required. </td> <td style="text-align:left;"> 4513 </td> <td style="text-align:left;"> NA </td> <td style="text-align:left;"> NA </td> <td style="text-align:left;"> NA </td> <td style="text-align:left;"> no </td> <td style="text-align:left;"> no </td> </tr> <tr> <td style="text-align:left;"> 4512 </td> <td style="text-align:left;"> 2017 </td> <td style="text-align:left;"> NA </td> <td style="text-align:left;"> Fisher, MC; De Hoog, S; Vanittanakom, N </td> <td style="text-align:left;"> 2004 </td> <td style="text-align:left;"> A highly discriminatory multilocus microsatellite typing (MLMT) system for Penicillium marneffei </td> <td style="text-align:left;"> For eukaryotic pathogens that are depauperate in genetic variation, multilocus microsatellite typing (MLMT) offers an accurate and reproducible method of characterizing genetic diversity; herein we describe the development of an MLMT system for the emerging pathogenic fungus Penicillium marneffei based on 23 microsatellite loci. Screening isolates held within the Centraalbureau voor Schimmelcultures culture collection demonstrate high levels of genetic diversity and 100% reproducibility. This MLMT system provides a powerful epidemiological tool to analyse the underlying parameters that are responsible for the emergence of P. marneffei in human HIV-positive populations. </td> <td style="text-align:left;"> 4514 </td> <td style="text-align:left;"> NA </td> <td style="text-align:left;"> NA </td> <td style="text-align:left;"> NA </td> <td style="text-align:left;"> no </td> <td style="text-align:left;"> no </td> </tr> <tr> <td style="text-align:left;"> 4513 </td> <td style="text-align:left;"> 2017 </td> <td style="text-align:left;"> NA </td> <td style="text-align:left;"> Rios, DLS; Vargas, AF; Torres, AMR; Zago, AJ; Callegari-Jacques, SM; Hutz, MH </td> <td style="text-align:left;"> 2003 </td> <td style="text-align:left;"> Interaction between SREBP-1a and APOB polymorphisms influences total and low-density lipoprotein cholesterol levels in patients with coronary artery disease </td> <td style="text-align:left;"> In this study, we examined the insertion/deletion (Ins/Del) and XbaI polymorphisms of the apolipoprotein B (APOB) gene and the -36delG polymorphism in the sterol regulatory element binding protein-1a (SREBP-1a) gene in 298 patients with non-diabetic angiographically assessed coronary artery disease (CAD), and 188 healthy controls, from a Brazilian population of European descent. Del/X+ haplotype carriers had higher levels of total cholesterol (TC) and low-density lipoprotein cholesterol (LDL-C) in patients (TC, p = 0.05; LDL-C, p = 0.049) and controls (TC, p = 0.004; LDL-C, p = 0.013). No association was detected between the SREBP-1a -36delG polymorphism and lipid levels, but a significant interaction effect between APOB and SREBP-la polymorphisms was observed in the patient sample on TC (p = 0.005) and on LDL-C (p = 0.019) levels. Carriers of the APOB Del/X+ haplotype and SREBP-la G-G- genotype showed the highest levels of these lipid parameters. This effect of interaction was not observed in the control sample. Despite the associations with lipids, these polymorphisms were not associated with CAD risk or severity in this sample. </td> <td style="text-align:left;"> 4515 </td> <td style="text-align:left;"> NA </td> <td style="text-align:left;"> NA </td> <td style="text-align:left;"> NA </td> <td style="text-align:left;"> no </td> <td style="text-align:left;"> no </td> </tr> <tr> <td style="text-align:left;"> 4514 </td> <td style="text-align:left;"> 2017 </td> <td style="text-align:left;"> NA </td> <td style="text-align:left;"> Li, LN; Chen, WF; Bouvier, M </td> <td style="text-align:left;"> 2005 </td> <td style="text-align:left;"> A biochemical and structural analysis of genetic diversity within the HLA-A*11 subtype </td> <td style="text-align:left;"> The HLA-A*11 subtype includes 17 naturally occurring variants (-A*1101 to -A*1117) distributed among different ethnic groups worldwide. At present, only HLA-A*1101 has been characterized at the molecular, structural, and immunological level. Developing similar knowledge on other HLA-A*11 alleles is highly important for bone marrow and graft transplantation. This is also important to better understand disease linkages within the HLA-A*11 subtype given that HLA-A*11 molecules are associated with resistance to acquisition of HIV-1 infection and various autoimmune diseases. To broaden our understanding of HLA-A*11 molecules, we have determined the impact of natural polymorphism on the peptide-binding properties of several HLA-A*11 molecules: -A*1103, -A*1106, -A*1108, -A*1110, -A*1111, and -A*1114. We used an approach that combines data from thermal stability studies of recombinant, soluble forms of these molecules in complex with HIV-1 peptides, together with a detailed structural analysis of the resulting HLA-A*11 molecule/peptide complexes based on crystal and molecular model structures. Our analysis shows that natural polymorphism within the HLA-A*11 subtype is distributed along the alpha 1 and alpha 2 helices of the peptide- binding groove, in marked contrast to the pattern of polymorphism in HLA-A*2 and HLA-B*27 subtypes. Natural polymorphism greatly altered the abilities of individual -A*11 molecules to form stable complexes with HIV-1 peptides. In comparison to -A*1101, natural polymorphism altered the peptide-presenting properties of -A*1103, -A*1108, and -A*1114 and has the potential to affect the peptide-selecting properties of -A*1106, -A*1110, and -A*1111 as well. Overall, our findings suggest that HLA-A*11 molecules may stimulate alloreactive CD8+ cytotoxic T-cell responses. </td> <td style="text-align:left;"> 4516 </td> <td style="text-align:left;"> NA </td> <td style="text-align:left;"> NA </td> <td style="text-align:left;"> NA </td> <td style="text-align:left;"> no </td> <td style="text-align:left;"> no </td> </tr> <tr> <td style="text-align:left;"> 4515 </td> <td style="text-align:left;"> 2017 </td> <td style="text-align:left;"> NA </td> <td style="text-align:left;"> Chen, Y; Litvintseva, AP; Frazzitta, AE; Haverkamp, MR; Wang, LY; Fang, C; Muthoga, C; Mitchell, TG; Perfect, JR </td> <td style="text-align:left;"> 2015 </td> <td style="text-align:left;"> Comparative analyses of clinical and environmental populations of Cryptococcus neoformans in Botswana </td> <td style="text-align:left;"> Cryptococcus neoformans var. grubii (Cng) is the most common cause of fungal meningitis, and its prevalence is highest in sub-Saharan Africa. Patients become infected by inhaling airborne spores or desiccated yeast cells from the environment, where the fungus thrives in avian droppings, trees and soil. To investigate the prevalence and population structure of Cng in southern Africa, we analysed isolates from 77 environmental samples and 64 patients. We detected significant genetic diversity among isolates and strong evidence of geographic structure at the local level. High proportions of isolates with the rare MATa allele were observed in both clinical and environmental isolates; however, the mating-type alleles were unevenly distributed among different subpopulations. Nearly equal proportions of the MATa and MAT mating types were observed among all clinical isolates and in one environmental subpopulation from the eastern part of Botswana. As previously reported, there was evidence of both clonality and recombination in different geographic areas. These results provide a foundation for subsequent genomewide association studies to identify genes and genotypes linked to pathogenicity in humans. </td> <td style="text-align:left;"> 4517 </td> <td style="text-align:left;"> NA </td> <td style="text-align:left;"> NA </td> <td style="text-align:left;"> NA </td> <td style="text-align:left;"> no </td> <td style="text-align:left;"> no </td> </tr> <tr> <td style="text-align:left;"> 4516 </td> <td style="text-align:left;"> 2017 </td> <td style="text-align:left;"> NA </td> <td style="text-align:left;"> Boltina, IV </td> <td style="text-align:left;"> 2007 </td> <td style="text-align:left;"> The usage of the index "Frequency of chromosomal aberrations" for the risk group formation in respect to oncologic diseases </td> <td style="text-align:left;"> It has been well established that chromosome instability involves the effect of environmental mutagenic factors. In addition, chromosomal aberrations can be markers of gene changes such as in oncology. So far, there is no unequivocal explanation of age relatedness for the frequency of chromosomal aberrations in the examined individuals or the increased level of this index in oncologic patients. Therefore, the value of the index "frequency of chromosomal aberrations" can be used to form the risk groups, including oncologic, for more profound studies using other methods. </td> <td style="text-align:left;"> 4518 </td> <td style="text-align:left;"> NA </td> <td style="text-align:left;"> NA </td> <td style="text-align:left;"> NA </td> <td style="text-align:left;"> no </td> <td style="text-align:left;"> no </td> </tr> <tr> <td style="text-align:left;"> 4517 </td> <td style="text-align:left;"> 2017 </td> <td style="text-align:left;"> NA </td> <td style="text-align:left;"> Aranda-Romo, S; Garcia-Sepulveda, CA; Comas-Garcia, A; Lovato-Salas, F; Salgado-Bustamante, M; Gomez-Gomez, A; Noyola, DE </td> <td style="text-align:left;"> 2012 </td> <td style="text-align:left;"> Killer-cell immunoglobulin-like receptors (KIR) in severe A (H1N1) 2009 influenza infections </td> <td style="text-align:left;"> Introduction of a novel influenza virus into the human population leads to the occurrence of pandemic events, such as the one caused by pandemic influenza A (H1N1) 2009 virus. The severity of infections caused by this virus in young adults was greater than that observed in patients with seasonal influenza. Fatal cases have been associated with an abnormal innate, proinflammatory immune response. A critical role for natural killer cells during the initial responses to influenza infections has been suggested. In this study, we assessed the association of killer-cell immunoglobulin-like receptors (KIRs) with disease severity by comparing KIR gene content in patients with mild and severe pandemic influenza virus infections to a control group. We found that activator (KIR3DS1 and KIR2DS5) and inhibitory (KIR2DL5) genes, encoded in group B haplotypes containing the cB01, cB03 and tB01 motifs, are associated with severe pandemic influenza A (H1N1) 2009 infections. Better understanding of how genetic variability contributes to influenza virus pathogenesis may help to the development of immune intervention strategies aiming at controlling the severity of disease. </td> <td style="text-align:left;"> 4519 </td> <td style="text-align:left;"> NA </td> <td style="text-align:left;"> NA </td> <td style="text-align:left;"> NA </td> <td style="text-align:left;"> no </td> <td style="text-align:left;"> no </td> </tr> <tr> <td style="text-align:left;"> 4518 </td> <td style="text-align:left;"> 2017 </td> <td style="text-align:left;"> NA </td> <td style="text-align:left;"> Williamson, S; Perry, SM; Bustamante, CD; Orive, ME; Stearns, MN; Kelly, JK </td> <td style="text-align:left;"> 2005 </td> <td style="text-align:left;"> A statistical characterization of consistent patterns of human immunodeficiency virus evolution within infected patients </td> <td style="text-align:left;"> Within-patient HIV populations evolve rapidly because of a high mutation rate, short generation time, and strong positive selection pressures. Previous studies have identified "consistent patterns" of viral sequence evolution. Just before HIV infection progresses to AIDS, evolution seems to slow markedly, and the genetic diversity of the viral population drops. This evolutionary slowdown could be caused either by a reduction in the average viral replication rate or because selection pressures weaken with the collapse of the immune system. The former hypothesis (which we denote "cellular exhaustion") predicts a simultaneous reduction in both synonymous and nonsynonymous evolution, whereas the latter hypothesis (denoted "immune relaxation") predicts that only nonsynonymous evolution will slow. In this paper, we present a set of statistical procedures for distinguishing between these alternative hypotheses using DNA sequences sampled over the course of infection. The first component is a new method for estimating evolutionary rates that takes advantage of the temporal information in longitudinal DNA sequence samples. Second, we develop a set of probability models for the analysis of evolutionary rates in HIV populations in vivo. Application of these models to both synonymous and nonsynonymous evolution affords a comparison of the cellular-exhaustion and immune-relaxation hypotheses. We apply the procedures to longitudinal data sets in which sequences of the em, gene were sampled over the entire course of infection. Our analyses (1) statistically confirm that an evolutionary slowdown occurs late in infection, (2) strongly support the immune-relaxation hypothesis, and (3) indicate that the cessation of nonsynonymous evolution is associated with disease progression. </td> <td style="text-align:left;"> 4520 </td> <td style="text-align:left;"> NA </td> <td style="text-align:left;"> NA </td> <td style="text-align:left;"> NA </td> <td style="text-align:left;"> no </td> <td style="text-align:left;"> no </td> </tr> <tr> <td style="text-align:left;"> 4519 </td> <td style="text-align:left;"> 2017 </td> <td style="text-align:left;"> NA </td> <td style="text-align:left;"> TULINIUS, H; OLAFSDOTTIR, GH; SIGVALDASON, H; TRYGGVADOTTIR, L; BJARNADOTTIR, K </td> <td style="text-align:left;"> 1994 </td> <td style="text-align:left;"> NEOPLASTIC DISEASES IN FAMILIES SF BREAST-CANCER PATIENTS </td> <td style="text-align:left;"> Objective - To investigate whether the risk of cancer at all sites, and at individual sites other than breast, prostate, ovaries, and endometrium, is increased among relatives of breast cancer patients compared with the general population. Design - A cohort of family members of breast cancer patients was established. The probands were chosen by year of birth or time of diagnosis. Any influence of knowledge of the cancer experience of the relatives has been avoided. The risk estimates are based on expected numbers computed from age and time specific incidence rates for the Icelandic population. Setting - Iceland. Subjects - The population of Iceland. Main outcome measures - Relative risks by degree of relatedness and age of proband. Results - The relative risk of cancer at all sites is raised for males and females. This is more than expected based on the known familial risk of breast cancer, prostate, and ovarian cancer. The excess risk of breast, prostate, and ovarian cancer is confirmed, but not that of cancer of the endometrium. The risk of cancer of the pancreas in both sexes and the stomach and kidneys in females is significantly raised. No evidence was found for decreased risk for any cancer type. Conclusions - The risk of cancer at all sites in relatives of breast cancer patients is increased. In addition to the risk of breast, prostate, and ovarian cancer, the risk of pancreas cancer and cancer of the stomach and kidneys in females is raised, but the last mentioned observations need further confirmation. </td> <td style="text-align:left;"> 4521 </td> <td style="text-align:left;"> NA </td> <td style="text-align:left;"> NA </td> <td style="text-align:left;"> NA </td> <td style="text-align:left;"> no </td> <td style="text-align:left;"> no </td> </tr> <tr> <td style="text-align:left;"> 4520 </td> <td style="text-align:left;"> 2017 </td> <td style="text-align:left;"> NA </td> <td style="text-align:left;"> Mejri, A; Siala, H; Ouali, F; Bibi, A; Messaoud, T </td> <td style="text-align:left;"> 2012 </td> <td style="text-align:left;"> Identification of candidate genes involved in clinical variability among Tunisian patients with beta-thalassemia </td> <td style="text-align:left;"> The short tandem repeats (STR) are undoubtedly the most used molecular markers in genetic diversity studies. beta-thalassemia is a hereditary hemolytic anemia which is a common health problem all over the world. The disease has two main clinical forms: the severe form or beta-thalassemia major (TM) and the moderate form or beta-thalassemia intermedia (TI). To help understand this inconsistency, five STR have been studied in a group of 27 beta-thalassemic patients divided into 15 (TM) and 12 (TI). For each STR, the distribution of alleles, has been compared among patients with beta-thalassemia major (TM) and those with beta-thalassemia intermedia (TI). The comparison has shown, for the STR D14S72, one specific allele to (TM) and for the SIR D14S990 and D14S68, two specific alleles to TI patients. The combination of these alleles with severe beta-thalassemia genotypes leads to the phenotype of beta-thalassemia intermedia even though there are no any attenuating factors such as XmnI Gg polymorphism at position - 158 of the HBG2 promotor or the alpha-globin defects. This suggests that these alleles are associated with factors reducing beta-thalassemia clinical effects. One of this STR is located in the 5'UTR of the CEBP epsilon gene which incodes a transcription factor expressed in the myeloid lineage. Thus, the involvement of the transcription factor CEBP epsilon in the myeloid cells suggests that CEBP epsilon is a candidate gene involved in the modulation of the beta-thalassemia expression through immune system reactions. (C) 2012 Elsevier B.V. All rights reserved. </td> <td style="text-align:left;"> 4522 </td> <td style="text-align:left;"> NA </td> <td style="text-align:left;"> NA </td> <td style="text-align:left;"> NA </td> <td style="text-align:left;"> no </td> <td style="text-align:left;"> no </td> </tr> <tr> <td style="text-align:left;"> 4521 </td> <td style="text-align:left;"> 2017 </td> <td style="text-align:left;"> NA </td> <td style="text-align:left;"> Fesel, C; Barreto, M; Ferreira, RC; Costa, N; Venda, LL; Pereira, C; Carvalho, C; Moraes-Fontes, MF; Ferreira, CM; Vasconcelos, C; Viana, JF; Santos, E; Martins, B; Demengeot, J; Vicente, AM </td> <td style="text-align:left;"> 2012 </td> <td style="text-align:left;"> Compensatory T-Cell Regulation in Unaffected Relatives of SLE Patients, and Opposite IL-2/CD25-Mediated Effects Suggested by Coreferentiality Modeling </td> <td style="text-align:left;"> In human systemic lupus erythematosus (SLE), diverse autoantibodies accumulate over years before disease manifestation. Unaffected relatives of SLE patients frequently share a sustained production of autoantibodies with indiscriminable specificity, usually without ever acquiring the disease. We studied relations of IgG autoantibody profiles and peripheral blood activated regulatory T-cells (aTregs), represented by CD4(+)CD25(bright) T-cells that were regularly 70-90% Foxp3(+). We found consistent positive correlations of broad-range as well as specific SLE-associated IgG with aTreg frequencies within unaffected relatives, but not patients or unrelated controls. Our interpretation: unaffected relatives with shared genetic factors compensated pathogenic effects by aTregs engaged in parallel with the individual autoantibody production. To study this further, we applied a novel analytic approach named coreferentiality that tests the indirect relatedness of parameters in respect to multivariate phenotype data. Results show that independently of their direct correlation, aTreg frequencies and specific SLE-associated IgG were likely functionally related in unaffected relatives: they significantly parallelled each other in their relations to broad-range immunoblot autoantibody profiles. In unaffected relatives, we also found coreferential effects of genetic variation in the loci encoding IL-2 and CD25. A model of CD25 functional genetic effects constructed by coreferentiality maximization suggests that IL-2-CD25 interaction, likely stimulating aTregs in unaffected relatives, had an opposed effect in SLE patients, presumably triggering primarily T-effector cells in this group. Coreferentiality modeling as we do it here could also be useful in other contexts, particularly to explore combined functional genetic effects. </td> <td style="text-align:left;"> 4523 </td> <td style="text-align:left;"> NA </td> <td style="text-align:left;"> NA </td> <td style="text-align:left;"> NA </td> <td style="text-align:left;"> no </td> <td style="text-align:left;"> no </td> </tr> <tr> <td style="text-align:left;"> 4522 </td> <td style="text-align:left;"> 2017 </td> <td style="text-align:left;"> NA </td> <td style="text-align:left;"> Smith, JA; Knight, RG </td> <td style="text-align:left;"> 2002 </td> <td style="text-align:left;"> Memory processing in Alzheimer's disease </td> <td style="text-align:left;"> The aim of the present research was to examine automatic and controlled influences on memory processing in patients with Alzheimer's disease using the process-dissociation procedure. In Experiment 1, a source recognition procedure was used, and the patients were found to have significantly reduced estimates of automatic processing and capacity to recognise words seen during the study phase of the procedure. In Experiment 2, a detection of repetition procedure was used to determine whether automatic influences on memory decline as a dementia progressed. The patients showed the expected inability to detect repetition in their responding. but there was no evidence that estimates of automatic processing were predicted by mental status scores or by ratings of the severity of dementia. In the third study, a novel method for estimating parameters in the process-dissociation model, developed from the task used in Experiment 2 was tested in a student sample. In this procedure. participants first produce semantic associates with either high or low relatedness to a list of cue words. These responses are subsequently used in a paired associate learning paradigm to determine independent estimates of recollection and automatic processing. Evidence for the validity of this procedure was found in Experiment 3 and the procedure used to examine memory processing in a sample of persons with dementia (Experiment 4) The patient group was found to have a substantial deficit in controlled recollection and a reduced capacity for automatic memory processing. (C) 2002 Elsevier Science Ltd. All rights reserved. </td> <td style="text-align:left;"> 4524 </td> <td style="text-align:left;"> NA </td> <td style="text-align:left;"> NA </td> <td style="text-align:left;"> NA </td> <td style="text-align:left;"> no </td> <td style="text-align:left;"> no </td> </tr> <tr> <td style="text-align:left;"> 4523 </td> <td style="text-align:left;"> 2017 </td> <td style="text-align:left;"> NA </td> <td style="text-align:left;"> Cvjeticanin, S; Marinkovic, D </td> <td style="text-align:left;"> 2005 </td> <td style="text-align:left;"> Genetic variability in the group of patients with congenital hip dislocation </td> <td style="text-align:left;"> Our study of genetic homozygosity degree includes an analysis of the presence, distribution, and individual combination of 20 selected genetically controlled morphophysiological traits in the group of patients (N = 93) with congenital hip dislocation (CDH) and in control sample consisting of schoolchildren from Belgrade (N = 200). Assuming that CDH is a genetically controlled disease, we made a hypothesis that an increased homozygosity level, as well as the changed variability among the patients, could be a population-genetic parameter for the prediction of the illness. Taking into consideration our experience, as well as the experience of numerous scientists who studied the nature of the inheritance of mono- and oligogenically controlled qualitative traits, we applied a methodology to estimate the proportion of such homozygously recessive characters (HRC-TEST). This population-genetic study did not only show statistically significant difference of the middle values of genetic homozygosity (CDH: 7.1 +/- 0.2; control: 5.2 +/- 0.1), but of the differences in the type of distribution too, as well as the differences in the presence of certain individual combinations of such traits. The described methodology can be used in further analyses, with hope that it can be applied as an early prognosis for decreased resistance to different diseases. The frequencies of ABO blood types in the sample of CDH patients were similar to the average value of the Serbian population, while the percentage of blood group A is slightly increased. Comparing frequencies of Rh blood groups, there is no difference between tested samples. </td> <td style="text-align:left;"> 4525 </td> <td style="text-align:left;"> NA </td> <td style="text-align:left;"> NA </td> <td style="text-align:left;"> NA </td> <td style="text-align:left;"> no </td> <td style="text-align:left;"> no </td> </tr> <tr> <td style="text-align:left;"> 4524 </td> <td style="text-align:left;"> 2017 </td> <td style="text-align:left;"> NA </td> <td style="text-align:left;"> Faisel, F; Romppanen, EL; Hiltunen, M; Helisalmi, S; Laasanen, J; Punnonen, K; Salonen, JT; Heinonen, S </td> <td style="text-align:left;"> 2004 </td> <td style="text-align:left;"> Susceptibility to pre-eclampsia in Finnish women is associated with R485K polymorphism in the factor V gene, not with Leiden mutation </td> <td style="text-align:left;"> This study determines whether genetic variability in the gene-encoding factor V contributes to differences in pre-eclampsia susceptibility. Allele and genotype frequencies of three single-nucleotide polymorphisms ( SNPs) in the factor V gene leading to nonsynonymous changes (M385T in exon 8, and R485K and R506Q ( Leiden mutation) in exon 10) were studied in 133 Caucasian women with pre-eclampsia and 112 healthy controls. Single-point analysis was expanded to haplotype analysis, and haplotype frequencies were estimated using an expectation-maximization algorithm. Comparison of single-point allele and genotype distributions of SNPs in exons 8 and 10 of the factor V gene revealed statistically significant differences in R485K allele ( P = 0.003) and genotype ( P = 0.03) frequencies between the patients and the control subjects. The A allele of SNP R485K was over-represented among the patients (12%) vs the control subjects (4%), at an odds ratio ( OR) of 2.8 (95% confidence interval (CI) 1.2 - 6.2) for combined A genotypes (GA+AA vs GG). Allele and genotype differences between the patients and control subjects as regards M385T and Leiden mutation were not significant. In haplotype estimation analysis, there was a significantly elevated frequency of haplotype T-A-G encoding the M385-K485-R506 variant in the preeclamptic group vs the control group ( P = 0.01), at an OR of 2.6 ( 95% CI 1.2 - 5.5). We conclude that the T-A-G haplotype was more frequent among the patient group than in the control group, and genetic variations in the factor V gene other than the Leiden mutation may play a role in disease susceptibility. </td> <td style="text-align:left;"> 4526 </td> <td style="text-align:left;"> NA </td> <td style="text-align:left;"> NA </td> <td style="text-align:left;"> NA </td> <td style="text-align:left;"> no </td> <td style="text-align:left;"> no </td> </tr> <tr> <td style="text-align:left;"> 4525 </td> <td style="text-align:left;"> 2017 </td> <td style="text-align:left;"> NA </td> <td style="text-align:left;"> Zahnleiter, D; Uebe, S; Ekici, AB; Hoyer, J; Wiesener, A; Wieczorek, D; Kunstmann, E; Reis, A; Doerr, HG; Rauch, A; Thiel, CT </td> <td style="text-align:left;"> 2013 </td> <td style="text-align:left;"> Rare Copy Number Variants Are a Common Cause of Short Stature </td> <td style="text-align:left;"> Human growth has an estimated heritability of about 80%-90%. Nevertheless, the underlying cause of shortness of stature remains unknown in the majority of individuals. Genome-wide association studies (GWAS) showed that both common single nucleotide polymorphisms and copy number variants (CNVs) contribute to height variation under a polygenic model, although explaining only a small fraction of overall genetic variability in the general population. Under the hypothesis that severe forms of growth retardation might also be caused by major gene effects, we searched for rare CNVs in 200 families, 92 sporadic and 108 familial, with idiopathic short stature compared to 820 control individuals. Although similar in number, patients had overall significantly larger CNVs (p-value <1 x 10(-7)). In a gene-based analysis of all non-polymorphic CNVs >50 kb for gene function, tissue expression, and murine knock-out phenotypes, we identified 10 duplications and 10 deletions ranging in size from 109 kb to 14 Mb, of which 7 were de novo (p < 0.03) and 13 inherited from the likewise affected parent but absent in controls. Patients with these likely disease causing 20 CNVs were smaller than the remaining group (p < 0.01). Eleven (55%) of these CNVs either overlapped with known microaberration syndromes associated with short stature or contained GWAS loci for height. Haploinsufficiency (HI) score and further expression profiling suggested dosage sensitivity of major growth-related genes at these loci. Overall 10% of patients carried a disease-causing CNV indicating that, like in neurodevelopmental disorders, rare CNVs are a frequent cause of severe growth retardation. </td> <td style="text-align:left;"> 4527 </td> <td style="text-align:left;"> NA </td> <td style="text-align:left;"> NA </td> <td style="text-align:left;"> NA </td> <td style="text-align:left;"> no </td> <td style="text-align:left;"> no </td> </tr> <tr> <td style="text-align:left;"> 4526 </td> <td style="text-align:left;"> 2017 </td> <td style="text-align:left;"> NA </td> <td style="text-align:left;"> Cvjeticanin, S; Marinkovic, D </td> <td style="text-align:left;"> 2005 </td> <td style="text-align:left;"> Genetic variability and frequencies of ABO blood types among different samples of patients from Serbia </td> <td style="text-align:left;"> Our study of genetic homozygosity degree includes an analysis of the presence, distribution and individual combination of 20 to 30 selected genetically controlled morpho-physiological traits in the four samples of patients from Belgrade affected with different diseases (CHD-congenital hip dislocation N = 93, DM-diabetes mellitus N = 95, TBC-tuberculosis N = 79, BC-bronchogen carcinoma N = 109). Taking into consideration our experience, as well as the experience of numerous scientists studying the nature of the inheritance of mono- and oligo-genetically controlled qualitative traits, we applied this methodology to estimate the proportion of such homozygously recessive characteristics (HRC-test). This population-genetic study did not only show statistically significant difference in the middle value of genetic homozygosity (CHD - 7.1 +/- 0.1, control - 5.2 +/- 0.1; DM - 6.3 +/- 0.3, control - 4.4 +/- 0.2; TBC - 6.1 +/- 0.3, BC - 5.9 +/- 0.3, control - 4.9 +/- 0.3), but also in the differences in the distribution type, as well as the differences in the presence of certain individual combinations of such traits. Further applications of HRC-testing may have practical use in genetic counselling, since homozygosity seems to be correlated with an individual's susceptibility to different diseases. The frequency of ABO blood types in the samples of patients with CHD, DM and BC were quite similar to the percentage of blood groups in the Serbian population. In the sample of TBC-affected patients, 0 blood group is almost significantly on the decrease, while the other blood groups are slightly on the increase, according to the average value of those blood groups in Serbian popoulation. </td> <td style="text-align:left;"> 4528 </td> <td style="text-align:left;"> NA </td> <td style="text-align:left;"> NA </td> <td style="text-align:left;"> NA </td> <td style="text-align:left;"> no </td> <td style="text-align:left;"> no </td> </tr> <tr> <td style="text-align:left;"> 4527 </td> <td style="text-align:left;"> 2017 </td> <td style="text-align:left;"> NA </td> <td style="text-align:left;"> Boukhris, SA; Amarti, A; El Rhazi, K; El Khadir, M; Benajah, DA; Ibrahimi, SA; Nejjari, C; Mahmoud, M; Souleimani, A; Bennani, B </td> <td style="text-align:left;"> 2013 </td> <td style="text-align:left;"> Helicobacter pylori Genotypes Associated with Gastric Histo-Pathological Damages in a Moroccan Population </td> <td style="text-align:left;"> H. pylori persistent infection induces chronic gastritis and is associated with peptic ulcer disease and gastric carcinoma development. The severity of these diseases is related to human's genetic diversity, H. pylori genetic variability and environmental factors. To identify the prevalence of histo-pathological damages caused by H. pylori infection in Moroccan population, and to determine their association to H. pylori genotypes, a prospective study has been conducted during 3 years on patients attending the gastroenterology department of Hassan II University Hospital (CHU) of Fez, Morocco. A total of 801 Moroccan adults' patients were recruited; H. pylori was diagnosed and genotyped by PCR in biopsy specimens and histological exam was performed. We found a high rate of glandular atrophy. Chronic inflammation, neutrophil activity and glandular atrophy showed statistically significant association with H. pylori infection. However, intestinal metaplasia was inversely associated to this infection and no association was observed with gastric cancer cases. A statistically significant association was found between intestinal metaplasia and vacAs1 and vac Am1 genotypes in patients aged 50 years and more but not in younger. This last genotype is also associated to gastric cancer. In this study, gastric cancer showed no significant association with H. pylori. Further studies are warranted to determine the role of other etiological agents such as Epstein-Barr virus, human papillomavirus and possibly environmental and dietetic factors in the occurrence of this pathology. </td> <td style="text-align:left;"> 4529 </td> <td style="text-align:left;"> NA </td> <td style="text-align:left;"> NA </td> <td style="text-align:left;"> NA </td> <td style="text-align:left;"> no </td> <td style="text-align:left;"> no </td> </tr> <tr> <td style="text-align:left;"> 4528 </td> <td style="text-align:left;"> 2017 </td> <td style="text-align:left;"> NA </td> <td style="text-align:left;"> Feng, Y; Zhao, WH; Feng, YM; Dai, JJ; Li, Z; Zhang, XY; Liu, L; Bai, J; Zhang, HT; Lu, L; Xia, XS </td> <td style="text-align:left;"> 2011 </td> <td style="text-align:left;"> A Novel Genotype of GB Virus C: Its Identification and Predominance among Injecting Drug Users in Yunnan, China </td> <td style="text-align:left;"> GB virus C (GBV-C) is prevalent globally and particularly among individuals at risk of parental exposures. Based on genetic diversity, this virus is now classified into six genotypes and many subtypes with distinct geographical distribution. In this study, 120 Injecting Drug Users (IDUs) were recruited from Yunnan province, China. Among them, 43 (35.8%) were positive for GBV-C RNA, 70 (58.3%) and 103 (85.8%) sero-positive for HIV-1 and HCV respectively. This revealed 18.3% of IDUs having GBV-C/HIV/HCV triple infection, which is significantly higher than 7.5% of GBV-C/HIV-1 and 10% of GBV-C/HCV dual infection rates (P<0.05). Based on 5'UTR sequences, the identified 43 viral isolates can be classified into three phylogenetic groups: one (2.3%) and two (4.7%) belonged to genotype 3 and 4, respectively, and the remaining 40 (93%) formed a new group with 97% of bootstrap support. This new GBV-C group was further confirmed by characterizing the E2 region and full-length genome sequences. Analysis of 187 nt 5'UTR sequence showed three previous reported isolates from Southeast Asia were re-classified into this new group. It implies they have the same origin with strains from Yunnan. Although we provisionally assigned this new group as GBV-C genotype 7, a simpler five groups of GBV-C nomenclature is recommended. Genotype 4, 6 and the newly designated genotype 7 could be reclassified as one group, which may represent a single GBV-C genotype. The classification of the other four groups was corresponding to that of previous reported genotype 1, 2, 3 and 5. Furthermore, the diversity of amino acid sequence in the E2 region was analyzed. The inhibitory effect of GBV-C genotype 7 on HIV-1 cell entry could be deduced. Since GBV-C may have a beneficial effect on AIDS disease progression and interact with HCV during co-infection, this finding may raise interests in future studies on this virus that was previously thought to be a "non-pathogenic virus''. </td> <td style="text-align:left;"> 4530 </td> <td style="text-align:left;"> NA </td> <td style="text-align:left;"> NA </td> <td style="text-align:left;"> NA </td> <td style="text-align:left;"> no </td> <td style="text-align:left;"> no </td> </tr> <tr> <td style="text-align:left;"> 4529 </td> <td style="text-align:left;"> 2017 </td> <td style="text-align:left;"> NA </td> <td style="text-align:left;"> Patra, R; Chattopadhyay, S; De, R; Ghosh, P; Ganguly, M; Chowdhury, A; Ramamurthy, T; Nair, GB; Mukhopadhyay, AK </td> <td style="text-align:left;"> 2012 </td> <td style="text-align:left;"> Multiple Infection and Microdiversity among Helicobacter pylori Isolates in a Single Host in India </td> <td style="text-align:left;"> Helicobacter pylori is one of the most diverse bacterial species that chronically infects more than 70% of Indian population. Interestingly, data showing microdiversity of the H. pylori strains within a particular gastric niche remained scarce. To understand the extent of genetic diversity among H. pylori strains within a given host, 30 patients with gastro-duodenal problems were subjected to endoscopy and from each patient 10 single colonies were isolated. Characterization of each of these 10 single colonies by DNA fingerprinting as well as genotyping of several important genetic markers viz. cagA, vacA, iceA, vapD, cag PAI empty site, IS605, RFLP and two other genetic segments within cag PAI revealed that all of the 30 patients were infected with more than one strain and sometimes strains with 5 to 6 types of genetic variants. Analyses of certain genetic loci showed the microdiversity among the colonies from single patient, which may be due to the recombination events during long-term carriage of the pathogen. These results suggest that most of the patients have acquired H. pylori due to repeated exposure to this pathogen with different genetic make-up, which may increase the possibility of super infections. Genetic exchanges between these unrelated H. pylori strains may support certain H. pylori variant to grow better in a given host than the parental strain and thereby increasing the possibility for the severity of the infection. </td> <td style="text-align:left;"> 4531 </td> <td style="text-align:left;"> NA </td> <td style="text-align:left;"> NA </td> <td style="text-align:left;"> NA </td> <td style="text-align:left;"> no </td> <td style="text-align:left;"> no </td> </tr> <tr> <td style="text-align:left;"> 4530 </td> <td style="text-align:left;"> 2017 </td> <td style="text-align:left;"> NA </td> <td style="text-align:left;"> Weiss, SL; Yu, M; Jennings, L; Haymond, S; Zhang, G; Wainwright, MS </td> <td style="text-align:left;"> 2012 </td> <td style="text-align:left;"> Pilot Study of the Association of the DDAH2-449G Polymorphism with Asymmetric Dimethylarginine and Hemodynamic Shock in Pediatric Sepsis </td> <td style="text-align:left;"> Background: Genetic variability in the regulation of the nitric oxide (NO) pathway may influence hemodynamic changes in pediatric sepsis. We sought to determine whether functional polymorphisms in DDAH2, which metabolizes the NO synthase inhibitor asymmetric dimethylarginine (ADMA), are associated with susceptibility to sepsis, plasma ADMA, distinct hemodynamic states, and vasopressor requirements in pediatric septic shock. Methodology/Principal Findings: In a prospective study, blood and buccal swabs were obtained from 82 patients <= 18 years (29 with severe sepsis/septic shock plus 27 febrile and 26 healthy controls). Plasma ADMA was measured using tandem mass spectrometry. DDAH2 gene was partially sequenced to determine the -871 6g/7g insertion/deletion and -449G/C single nucleotide polymorphisms. Shock type ("warm'' versus "cold'') was characterized by clinical assessment. The -871 7g allele was more common in septic (17%) then febrile (4%) and healthy (8%) patients, though this was not significant after controlling for sex and race (p = 0.96). ADMA did not differ between -871 6g/7g genotypes. While genotype frequencies also did not vary between groups for the -449G/C SNP (p = 0.75), septic patients with at least one -449G allele had lower ADMA (median, IQR 0.36, 0.30-0.41 mu mol/L) than patients with the -449CC genotype (0.55, 0.49-0.64 mu mol/L, p = 0.008) and exhibited a higher incidence of "cold'' shock (45% versus 0%, p = 0.01). However, after controlling for race, the association with shock type became non-significant (p = 0.32). Neither polymorphism was associated with inotrope score or vasoactive infusion duration. Conclusions/Significance: The -449G polymorphism in the DDAH2 gene was associated with both low plasma ADMA and an increased likelihood of presenting with "cold'' shock in pediatric sepsis, but not with vasopressor requirement. Race, however, was an important confounder. These results support and justify the need for larger studies in racially homogenous populations to further examine whether genotypic differences in NO metabolism contribute to phenotypic variability in sepsis pathophysiology. </td> <td style="text-align:left;"> 4532 </td> <td style="text-align:left;"> NA </td> <td style="text-align:left;"> NA </td> <td style="text-align:left;"> NA </td> <td style="text-align:left;"> no </td> <td style="text-align:left;"> no </td> </tr> <tr> <td style="text-align:left;"> 4531 </td> <td style="text-align:left;"> 2017 </td> <td style="text-align:left;"> NA </td> <td style="text-align:left;"> Wang, Q; Li, B; Tsang, AKL; Yi, Y; Woo, PCY; Liu, CH </td> <td style="text-align:left;"> 2013 </td> <td style="text-align:left;"> Genotypic Analysis of Klebsiella pneumoniae Isolates in a Beijing Hospital Reveals High Genetic Diversity and Clonal Population Structure of Drug-Resistant Isolates </td> <td style="text-align:left;"> Background: The genetic diversity and the clinical relevance of the drug-resistant Klebsiella pneumoniae isolates from hospital settings are largely unknown. We thus conducted this prospective study to analyze the molecular epidemiology of K. pneumoniae isolates from patients being treated in the 306 Hospital in Beijing, China for the period of November 1, 2010-October 31, 2011. Methodology/Principal Findings: Antibiotic susceptibility testing, PCR amplification and sequencing of the drug resistance-associated genes, and multilocus sequence typing (MLST) were conducted. A total of 163 isolates were analyzed. The percentage of MDR, XDR and PDR isolates were 63.8% (104), 20.9 (34), and 1.8% (3), respectively. MLST results showed that 60 sequence types (STs) were identified, which were further separated by eBURST into 13 clonal complexes and 18 singletons. The most dominant ST was ST15 (10.4%). Seven new alleles and 24 new STs were first identified in this study. Multiple logistic regression analysis revealed that certain clinical characteristics were associated with those prevalent STs such as: from ICU, from medical ward, from community acquired infection, from patients without heart disease, from patients with treatment success, susceptible to extended spectrum cephalosporin, susceptible to cephamycins, susceptible to fluoroquinolones, and with MDR. Conclusions/Significance: Our data indicate that certain drug-resistant K. pneumoniae clones are highly prevalent and are associated with certain clinical characteristics in hospital settings. Our study provides evidence demonstrating that intensive nosocomial infection control measures are urgently needed. </td> <td style="text-align:left;"> 4533 </td> <td style="text-align:left;"> NA </td> <td style="text-align:left;"> NA </td> <td style="text-align:left;"> NA </td> <td style="text-align:left;"> no </td> <td style="text-align:left;"> no </td> </tr> <tr> <td style="text-align:left;"> 4532 </td> <td style="text-align:left;"> 2017 </td> <td style="text-align:left;"> NA </td> <td style="text-align:left;"> Gotts, SJ; Plaut, DC </td> <td style="text-align:left;"> 2002 </td> <td style="text-align:left;"> The impact of synaptic depression following brain damage: A connectionist account of "access/refractory" and "degraded-store" semantic impairments </td> <td style="text-align:left;"> Neuropsychological studies of patients with acquired semantic impairments have yielded two distinct and contrasting patterns of performance in a spoken-word/picture-matching task (Warrington & Cipolotti, 1996). Patients labeled access/refractory are strongly influenced by presentation rate, semantic relatedness of distractors, and repetition, yet they seem relatively unaffected by lexical frequency. Degraded-store patients, on the other hand, are strongly affected by lexical frequency but are less affected by presentation rate, semantic relatedness, or repetition. Our account of these patterns of performance is based on the distinction between two different types of neurological damage: (1) damage to neuromodulatory systems that function to amplify neural signals while suppressing normal refractory-like effects and (2) damage to connections between groups of neurons that encode semantic information and are sensitive to frequency/familiarity. We present a connectionist model that learns to map spoken-word input to semantic representations and that incorporates a particular form of neural refractoriness referred to as synaptic depression, as well as a simple form of neuromodulation. We show that the model is capable of accounting for the contrasting patterns of semantic impairment under these two different forms of damage and, furthermore, demonstrate how it is capable of handling several documented cases that are exceptions to the basic patterns of impairment. Several predictions and limitations of the present model are discussed. </td> <td style="text-align:left;"> 4534 </td> <td style="text-align:left;"> NA </td> <td style="text-align:left;"> NA </td> <td style="text-align:left;"> NA </td> <td style="text-align:left;"> no </td> <td style="text-align:left;"> no </td> </tr> <tr> <td style="text-align:left;"> 4533 </td> <td style="text-align:left;"> 2017 </td> <td style="text-align:left;"> NA </td> <td style="text-align:left;"> Santos, M; Carvalho, S; Lima, L; Nogueira, A; Assis, J; Mota-Pereira, J; Pimentel, P; Maia, D; Correia, D; Gomes, S; Cruz, A; Medeiros, R </td> <td style="text-align:left;"> 2014 </td> <td style="text-align:left;"> Common Genetic Polymorphisms in the ABCB1 Gene Are Associated with Risk of Major Depressive Disorder in Male Portuguese Individuals </td> <td style="text-align:left;"> Major depressive disorder (MDD) is a highly prevalent disorder, which has been associated with an abnormal response of the hypothalamus-pituitary-adrenal (HPA) axis. Reports have argued that an abnormal HPA axis response can be due to an altered P-Glycoprotein (P-GP) function. This argument suggests that genetic polymorphisms in ABCB1 may have an effect on the HPA axis activity; however, it is still not clear if this influences the risk of MDD. Our study aims to evaluate the effect of ABCB1 C1236T, G2677TA and C3435T genetic polymorphisms on MDD risk in a subset of Portuguese patients. DNA samples from 80 MDD patients and 160 control subjects were genotyped using TaqMan((R)) SNP Genotyping assays. A significant protection for MDD males carrying the T allele was observed (C1236T: odds ratio (OR)=0.360, 95% confidence interval [CI]: [0.140-0.950], p=0.022; C3435T: OR=0.306, 95% CI: [0.096-0.980], p=0.042; and G2677TA: OR=0.300, 95% CI: [0.100-0.870], p=0.013). Male Portuguese individuals carrying the 1236T/2677T/3435T haplotype had nearly 70% less risk of developing MDD (OR=0.313, 95% CI: [0.118-0.832], p=0.016, FDR p=0.032). No significant differences were observed regarding the overall subjects. Our results suggest that genetic variability of the ABCB1 is associated with MDD development in male Portuguese patients. To the best of our knowledge, this is the first report in Caucasian samples to analyze the effect of these ABCB1 genetic polymorphisms on MDD risk. </td> <td style="text-align:left;"> 4535 </td> <td style="text-align:left;"> NA </td> <td style="text-align:left;"> NA </td> <td style="text-align:left;"> NA </td> <td style="text-align:left;"> no </td> <td style="text-align:left;"> no </td> </tr> <tr> <td style="text-align:left;"> 4534 </td> <td style="text-align:left;"> 2017 </td> <td style="text-align:left;"> NA </td> <td style="text-align:left;"> Starkova, DA; Otten, TF; Mokrousov, IV; Vyazovaya, AA; Vishnevsky, BI; Narvskaya, OV </td> <td style="text-align:left;"> 2013 </td> <td style="text-align:left;"> Genotypic characteristics of Mycobacterium avium subsp hominissuis strains </td> <td style="text-align:left;"> Mycobacterium avium are typical environmental, non-tuberculosis microorganisms that occasionally cause mycobacteriosis, an infectious disease in wild and domestic animals, birds, and humans. Here, we report the results of the first study on the genetic diversity of the Russian population of M. avium. A total of 85 M. avium subsp. hominissuis (MAH) clinical strains were isolated from patients (including 30 HIV-positive individuals) with mycobacteriosis in St. Petersburg, 2008-2011. The identification of the microorganisms was carried out using biochemical tests and the PCR detection of the mobile elements IS901 and IS900, as well as of the polymorphism of restriction fragments of the hsp65 gene. The genetic diversity of the isolates was evaluated by VNTR typing based on eight variable-number tandem repeats (VNTRs) (292, X3, 25, 47, 3, 7, 10, and 32 [Thibault et al., 2007]). The MAH population studied was characterized by 15 VNTR types, including nine unique patterns and six clusters of isolates with identical eight-digit profiles. The largest clusters (22221128 and 24221128) included 45 (59.2%) and 15 (19.7%) isolates, respectively; the others contained 2-7 strains. The strains of the cluster 2533112'8 possessed a truncated TR10 locus (allele 2'). Taking into account the absence of the epidemiological links between the patients and the fact that the infection was presumably delivered from the environment, the high rate of clustering of MAH isolates can be explained by the low discriminatory power of the eight-locus VNTR-typing scheme (HGDI 0-0.61). </td> <td style="text-align:left;"> 4536 </td> <td style="text-align:left;"> NA </td> <td style="text-align:left;"> NA </td> <td style="text-align:left;"> NA </td> <td style="text-align:left;"> no </td> <td style="text-align:left;"> no </td> </tr> <tr> <td style="text-align:left;"> 4535 </td> <td style="text-align:left;"> 2017 </td> <td style="text-align:left;"> NA </td> <td style="text-align:left;"> Hernandez, DG; Nalls, MA; Ylikotila, P; Keller, M; Hardy, JA; Majamaa, K; Singleton, AB </td> <td style="text-align:left;"> 2012 </td> <td style="text-align:left;"> Genome Wide Assessment of Young Onset Parkinson's Disease from Finland </td> <td style="text-align:left;"> In the current study we undertook a series of experiments to test the hypothesis that a monogenic cause of disease may be detectable within a cohort of Finnish young onset Parkinson's disease patients. In the first instance we performed standard genome wide association analyses, and subsequent risk profile analysis. In addition we performed a series of analyses that involved testing measures of global relatedness within the cases compared to controls, searching for excess homozygosity in the cases, and examining the cases for signs of excess local genomic relatedness using a sliding window approach. This work suggested that the previously identified common, low risk alleles, and the risk models associated with these alleles, were generalizable to the Finnish Parkinson's disease population. However, we found no evidence that would suggest a single common high penetrance mutation exists in this cohort of young onset patients. </td> <td style="text-align:left;"> 4537 </td> <td style="text-align:left;"> NA </td> <td style="text-align:left;"> NA </td> <td style="text-align:left;"> NA </td> <td style="text-align:left;"> no </td> <td style="text-align:left;"> no </td> </tr> <tr> <td style="text-align:left;"> 4536 </td> <td style="text-align:left;"> 2017 </td> <td style="text-align:left;"> NA </td> <td style="text-align:left;"> Beres, SB; Sylva, GL; Sturdevant, DE; Granville, CN; Liu, MY; Ricklefs, SM; Whitney, AR; Parkins, LD; Hoe, NP; Adams, GJ; Low, DE; DeLeo, FR; McGeer, A; Musser, JM </td> <td style="text-align:left;"> 2004 </td> <td style="text-align:left;"> Genome-wide molecular dissection of serotype M3 group A Streptococcus strains causing two epidemics of invasive infections </td> <td style="text-align:left;"> Molecular factors that contribute to the emergence of new virulent bacterial subdones and epidemics are poorly understood. We hypothesized that analysis of a population-based strain sample of serotype M3 group A Streptococcus (GAS) recovered from patients with invasive infection by using genome-wide investigative methods would provide new insight into this fundamental infectious disease problem. Serotype M3 GAS strains (n = 255) cultured from patients in Ontario, Canada, over 11 years and representing two distinct infection peaks were studied. Genetic diversity was indexed by pulsed-field gel electrophoresis, DNA-DNA microarray, whole-genome PCR scanning, prophage genotyping, targeted gene sequencing, and single-nucleotide polymorphism genotyping. All variation in gene content was attributable to acquisition or loss of prophages, a molecular process that generated unique combinations of proven or putative virulence genes. Distinct serotype M3 genotypes experienced rapid population expansion and caused infections that differed significantly in character and severity. Molecular genetic analysis, combined with immunologic studies, implicated a 4-aa duplication in the extreme N terminus of M protein as a factor contributing to an epidemic wave of serotype M3 invasive infections. This finding has implications for GAS vaccine research. Genome-wide analysis of population-based strain samples cultured from clinically well defined patients is crucial for understanding the molecular events underlying bacterial epidemics. </td> <td style="text-align:left;"> 4538 </td> <td style="text-align:left;"> NA </td> <td style="text-align:left;"> NA </td> <td style="text-align:left;"> NA </td> <td style="text-align:left;"> no </td> <td style="text-align:left;"> no </td> </tr> <tr> <td style="text-align:left;"> 4537 </td> <td style="text-align:left;"> 2017 </td> <td style="text-align:left;"> NA </td> <td style="text-align:left;"> Kidd, TJ; Ritchie, SR; Ramsay, KA; Grimwood, K; Bell, SC; Rainey, PB </td> <td style="text-align:left;"> 2012 </td> <td style="text-align:left;"> Pseudomonas aeruginosa Exhibits Frequent Recombination, but Only a Limited Association between Genotype and Ecological Setting </td> <td style="text-align:left;"> Pseudomonas aeruginosa is an opportunistic pathogen and an important cause of infection, particularly amongst cystic fibrosis (CF) patients. While specific strains capable of patient-to-patient transmission are known, many infections appear to be caused by unique and unrelated strains. There is a need to understand the relationship between strains capable of colonising the CF lung and the broader set of P. aeruginosa isolates found in natural environments. Here we report the results of a multilocus sequence typing (MLST)-based study designed to understand the genetic diversity and population structure of an extensive regional sample of P. aeruginosa isolates from South East Queensland, Australia. The analysis is based on 501 P. aeruginosa isolates obtained from environmental, animal and human (CF and non-CF) sources with particular emphasis on isolates from the Lower Brisbane River and isolates from CF patients obtained from the same geographical region. Overall, MLST identified 274 different sequence types, of which 53 were shared between one or more ecological settings. Our analysis revealed a limited association between genotype and environment and evidence of frequent recombination. We also found that genetic diversity of P. aeruginosa in Queensland, Australia was indistinguishable from that of the global P. aeruginosa population. Several CF strains were encountered frequently in multiple ecological settings; however, the most frequently encountered CF strains were confined to CF patients. Overall, our data confirm a non-clonal epidemic structure and indicate that most CF strains are a random sample of the broader P. aeruginosa population. The increased abundance of some CF strains in different geographical regions is a likely product of chance colonisation events followed by adaptation to the CF lung and horizontal transmission among patients. </td> <td style="text-align:left;"> 4539 </td> <td style="text-align:left;"> NA </td> <td style="text-align:left;"> NA </td> <td style="text-align:left;"> NA </td> <td style="text-align:left;"> no </td> <td style="text-align:left;"> no </td> </tr> <tr> <td style="text-align:left;"> 4538 </td> <td style="text-align:left;"> 2017 </td> <td style="text-align:left;"> NA </td> <td style="text-align:left;"> Dunford, L; Carr, MJ; Dean, J; Waters, A; Nguyen, LT; Thu, HTT; Lan, ABT; Do, HD; Thu, TDT; Nguyen, HT; Trinh, TDD; Luu, QP; Connell, J; Coughlan, S; Nguyen, HT; Hall, WW; Lan, ANT </td> <td style="text-align:left;"> 2012 </td> <td style="text-align:left;"> Hepatitis C Virus in Vietnam: High Prevalence of Infection in Dialysis and Multi-Transfused Patients Involving Diverse and Novel Virus Variants </td> <td style="text-align:left;"> Hepatitis C virus (HCV) is a genetically diverse pathogen infecting approximately 2-3% of the world's population. Herein, we describe results of a large, multicentre serological and molecular epidemiological study cataloguing the prevalence and genetic diversity of HCV in five regions of Vietnam; Ha Noi, Hai Phong, Da Nang, Khanh Hoa and Can Tho. Individuals (n = 8654) with varying risk factors for infection were analysed for the presence of HCV Ab/Ag and, in a subset of positive specimens, for HCV RNA levels (n = 475) and genotype (n = 282). In lower risk individuals, including voluntary blood donors, military recruits and pregnant women, the prevalence of infection was 0.5% (n = 26/5250). Prevalence rates were significantly higher (p < 0.001) in intravenous drug users (IDUs; 55.6%, n = 556/1000), dialysis patients (26.6%, n = 153/575) commercial sex workers (CSWs; 8.7%, n = 87/1000), and recipients of multiple blood transfusions (6.0%, n = 32/529). The prevalence of HCV in dialysis patients varied but remained high in all regions (11-43%) and was associated with the receipt of blood transfusions [OR: 2.08 (1.85-2.34), p = 0.001], time from first transfusion [OR: 1.07 (1.01-1.13), p = 0.023], duration of dialysis [OR: 1.31 (1.19-1.43), p < 0.001] and male gender [OR: 1.60 (1.06-2.41), p = 0.026]. Phylogenetic analysis revealed high genetic diversity, particularly amongst dialysis and multi-transfused patients, identifying subtypes 1a (33%), 1b (27%), 2a (0.4%), 3a (0.7%), 3b (1.1%), 6a (18.8%), 6e (6.0%), 6h (4.6%), 6l (6.4%) and 2 clusters of novel genotype 6 variants (2.1%). HCV genotype 1 predominated in Vietnam (60%, n = 169/282) but the proportion of infections attributable to genotype 1 varied between regions and risk groups and, in the Southern part of Vietnam, genotype 6 viruses dominated in dialysis and multi-transfused patients (73.9%). This study confirms a high prevalence of HCV infection in Vietnamese IDUs and, notably, reveals high levels of HCV infection associated with dialysis and blood transfusion. </td> <td style="text-align:left;"> 4540 </td> <td style="text-align:left;"> NA </td> <td style="text-align:left;"> NA </td> <td style="text-align:left;"> NA </td> <td style="text-align:left;"> no </td> <td style="text-align:left;"> no </td> </tr> <tr> <td style="text-align:left;"> 4539 </td> <td style="text-align:left;"> 2017 </td> <td style="text-align:left;"> NA </td> <td style="text-align:left;"> Fladvad, T; Klepstad, P; Langaas, M; Dale, O; Kaasa, S; Caraceni, A; Skorpen, F </td> <td style="text-align:left;"> 2013 </td> <td style="text-align:left;"> Variability in UDP-glucuronosyltransferase genes and morphine metabolism: observations from a cross-sectional multicenter study in advanced cancer patients with pain </td> <td style="text-align:left;"> Objective The objective of the present study was to determine whether genetic variability in UDP-glucuronosyltransferase (UGT) genes, together with clinical factors, contribute to variability in morphine glucuronide (M6G and M3G) to morphine serum concentration ratios in patients with advanced cancer receiving chronic morphine therapy. Materials and methods A total of 41 polymorphisms and predicted haplotypes in the UGT2B7, UGT1A1, and UGT1A8 genes were analyzed in 759 patients who were recruited from the European Pharmacogenetic Opioid Study and received chronic morphine therapy by the oral route (n = 635) or parenterally (n = 124). The administration groups were analyzed separately by multiple linear regression analyses. Results Two haplotypes in UGT1A1/UGT1A8 were weak predictors of reduced M6G/morphine and M3G/morphine serum ratios after oral administration (false discovery rate-corrected P-values < 0.1). No effect of genotype was seen in the parenteral group. Of the clinical variables (age, sex, BMI, renal function, Karnofsky performance status, and presence of liver metastases), renal function was the major contributor to variation in serum concentration ratios. Concomitant administration of paracetamol predicted significantly higher morphine metabolic ratios after oral administration of morphine (false discovery rate-corrected values < 2.1E - 12). The regression models explained about 35% of the total variability in the data. Conclusion Genetic variation in the UGT genes together with clinical factors influence morphine metabolic ratios in patients with advanced cancer disease and who are scheduled with oral morphine. This information may be included in future research that develop and test new classification systems for opioid treatment in patients with advanced cancer. Pharmacogenetics and Genomics 23:117-126 (c) 2013 Wolters Kluwer Health vertical bar Lippincott Williams & Wilkins. </td> <td style="text-align:left;"> 4541 </td> <td style="text-align:left;"> NA </td> <td style="text-align:left;"> NA </td> <td style="text-align:left;"> NA </td> <td style="text-align:left;"> no </td> <td style="text-align:left;"> no </td> </tr> <tr> <td style="text-align:left;"> 4540 </td> <td style="text-align:left;"> 2017 </td> <td style="text-align:left;"> NA </td> <td style="text-align:left;"> Brisson, D; Baxamusa, N; Schwartz, I; Wormser, GP </td> <td style="text-align:left;"> 2011 </td> <td style="text-align:left;"> Biodiversity of Borrelia burgdorferi Strains in Tissues of Lyme Disease Patients </td> <td style="text-align:left;"> Plant and animal biodiversity are essential to ecosystem health and can provide benefits to humans ranging from aesthetics to maintaining air quality. Although the importance of biodiversity to ecology and conservation biology is obvious, such measures have not been applied to strains of an invasive bacterium found in human tissues during infection. In this study, we compared the strain biodiversity of Borrelia burgdorferi found in tick populations with that found in skin, blood, synovial fluid or cerebrospinal fluid of Lyme disease patients. The biodiversity of B. burgdorferi strains is significantly greater in tick populations than in the skin of patients with erythema migrans. In turn, strains from skin are significantly more diverse than strains at any of the disseminated sites. The cerebrospinal fluid of patients with neurologic Lyme disease harbored the least pathogen biodiversity. These results suggest that human tissues act as niches that can allow entry to or maintain only a subset of the total pathogen population. These data help to explain prior clinical observations on the natural history of B. burgdorferi infection and raise several questions that may help to direct future research to better understand the pathogenesis of this infection. </td> <td style="text-align:left;"> 4542 </td> <td style="text-align:left;"> NA </td> <td style="text-align:left;"> NA </td> <td style="text-align:left;"> NA </td> <td style="text-align:left;"> no </td> <td style="text-align:left;"> no </td> </tr> <tr> <td style="text-align:left;"> 4541 </td> <td style="text-align:left;"> 2017 </td> <td style="text-align:left;"> NA </td> <td style="text-align:left;"> Elattar, N; Swelam, EE; Hamed, E; Elnahal, A; Mostafa, E </td> <td style="text-align:left;"> 2012 </td> <td style="text-align:left;"> PARAOXONASE 1 Gene Polymorphism Relationship with Type 2 Diabetes Mellitus </td> <td style="text-align:left;"> Background: Human serum paraoxonase1 (PON1) an antioxidant enzyme closely associated with high density lipoprotein (HDL) has been implicated in the prevention of low density lipoprotein (LDL) oxidation and these may provide HDL-associated protection against atherosclerosis. The aim of this study is to investigate PON1 activity and its gene polymorphism in type 2 diabetes mellitus and its potential significance in the occurrence of diabetic complications. Patients and Methods: This study includes 60 subjects divided into: Twenty healthy subjects as a control (group I), Twenty diabetic patients without vascular complications (group II), and Twenty diabetic patients with vascular complications (group III). Laboratory investigations included: estimation of PON1 enzymatic activity by hydrolysis of paraoxon and PON1gene polymorphism by polymerase chain reaction (PCR) followed by polymorphism specific restriction enzyme digestion, other investigations included fasting and post prandial plasma glucose (FPG and PPPG), glycosylated hemoglobin (HbA1c) and lipid profile. Results : It revealed that PON1 activity was significantly lower in diabetics than control (p<0.001) and is lower in group III (183.6 +/- 52.01) than group II (230.05 +/- 59.75). PON1 was negatively correlated to HbA1c(r = -0.540, P<0.001). Gene and allele frequencies was significantly different in diabetics than control at 192 polymorphism (X-2 = 7.645, P<0.05) but not at 55 polymorphism with QQ higher in diabetics (77.5%) and RR higher in controls (25%). In both control and diabetics QQ and MM genotypes have the lowest activity of PON1 and RR and LL genotypes have the highest activity and QR and LM genotypes have intermediate activity, HbA1c was highest in QQ and MM genotypes, intermediate in QR and LM genotypes and lowest in RR and LL genotypes. The allelic frequency of Q and M genotypes were higher in group III than in group II and R and L genotypes were lower in group III than in group II. Conclusion: Paraoxonase activity is affected by PON1 genetic variability in type2 diabetic patients. The PON1 QQ and MM genotypes are associated with lower PON1 activity than RR and LL genotypes. In type 2 diabetic patients the QQ and MM genotypes are more common and associated with poor glucose control mainly in diabetic patients with vascular complication which suggest their essential roles in occurrence of diabetic vascular complications. [Elatar N., Swelam E.E1, Hamed E; Elnahal S, and Mostafa E. PARAOXONASE 1 Gene Polymorphism Relationship with Type 2 Diabetes Mellitus. Life Sci J 2012;9(3):1742-1751] (ISSN: 1097-8135). http://www.lifesciencesite.com.253 </td> <td style="text-align:left;"> 4543 </td> <td style="text-align:left;"> NA </td> <td style="text-align:left;"> NA </td> <td style="text-align:left;"> NA </td> <td style="text-align:left;"> no </td> <td style="text-align:left;"> no </td> </tr> <tr> <td style="text-align:left;"> 4542 </td> <td style="text-align:left;"> 2017 </td> <td style="text-align:left;"> NA </td> <td style="text-align:left;"> Dialdestoro, K; Sibbesen, JA; Maretty, L; Raghwani, J; Gall, A; Kellam, P; Pybus, OG; Hein, J; Jenkins, PA </td> <td style="text-align:left;"> 2016 </td> <td style="text-align:left;"> Coalescent Inference Using Serially Sampled, High-Throughput Sequencing Data from Intrahost HIV Infection </td> <td style="text-align:left;"> Human immunodeficiency virus (HIV) is a rapidly evolving pathogen that causes chronic infections, so genetic diversity within a single infection can be very high. High-throughput deep sequencing can now measure this diversity in unprecedented detail, particularly since it can be performed at different time points during an infection, and this offers a potentially powerful way to infer the evolutionary dynamics of the intrahost viral population. However, population genomic inference from HIV sequence data is challenging because of high rates of mutation and recombination, rapid demographic changes, and ongoing selective pressures. In this article we develop a new method for inference using HIV deep sequencing data, using an approach based on importance sampling of ancestral recombination graphs under a multilocus coalescent model. The approach further extends recent progress in the approximation of so-called conditional sampling distributions, a quantity of key interest when approximating coalescent likelihoods. The chief novelties of our method are that it is able to infer rates of recombination and mutation, as well as the effective population size, while handling sampling over different time points and missing data without extra computational difficulty. We apply our method to a data set of HIV-1, in which several hundred sequences were obtained from an infected individual at seven time points over 2 years. We find mutation rate and effective population size estimates to be comparable to those produced by the software BEAST. Additionally, our method is able to produce local recombination rate estimates. The software underlying our method, Coalescenator, is freely available. </td> <td style="text-align:left;"> 4544 </td> <td style="text-align:left;"> NA </td> <td style="text-align:left;"> NA </td> <td style="text-align:left;"> NA </td> <td style="text-align:left;"> no </td> <td style="text-align:left;"> no </td> </tr> <tr> <td style="text-align:left;"> 4543 </td> <td style="text-align:left;"> 2017 </td> <td style="text-align:left;"> NA </td> <td style="text-align:left;"> Stepanov, VA; Puzyrev, VP; Karpov, RS; Kutmin, AI </td> <td style="text-align:left;"> 1998 </td> <td style="text-align:left;"> Genetic markers in coronary artery disease in a Russian population </td> <td style="text-align:left;"> The association of genetic markers at the apolipoprotein B gene (APOB) (XbaI, MspI, and EcoRI polymorphisms) and the lipoprotein lipase gene (LPL) (PvuII polymorphism) with coronary artery disease (CAD) and with variation in plasma lipid levels (total cholesterol, high-density lipoprotein cholesterol, and total triglycerides) was studied in 94 male patients with CAD diagnosed by angiography and in 122 unrelated men of Russian descent free of clinical signs of CAD. The frequent allele (M+) of the APOB MspI RFLP was seen more frequently in CAD patients than in control subjects (0.963 vs. 0.898, p < 0.025). No significant differences in allele frequencies were observed for the APOB XbaI and EcoRI polymorphisms or for the LPL PVuII polymorphism, In patients, carriers of the rare allele (E-) of the EcoRI RFLP had higher mean triglyceride levels than homozygotes for E+ (p < 0.05). No significant differences in lipid variables were determined for the other three polymorphisms studied. Analysis of intragenotype variances and the multivariate measure of mean values of lipid concentrations showed that genetic variability within the APOB locus may contribute to a certain extent to the level and variability of serum Lipid levels in this Russian population. </td> <td style="text-align:left;"> 4545 </td> <td style="text-align:left;"> NA </td> <td style="text-align:left;"> NA </td> <td style="text-align:left;"> NA </td> <td style="text-align:left;"> no </td> <td style="text-align:left;"> no </td> </tr> <tr> <td style="text-align:left;"> 4544 </td> <td style="text-align:left;"> 2017 </td> <td style="text-align:left;"> NA </td> <td style="text-align:left;"> Edwards, CTT; Holmes, EC; Pybus, OG; Wilson, DJ; Viscidi, RP; Abrams, EJ; Phillips, RE; Drummond, AJ </td> <td style="text-align:left;"> 2006 </td> <td style="text-align:left;"> Evolution of the human immunodeficiency virus envelope gene is dominated by purifying selection </td> <td style="text-align:left;"> The evolution of the human immunodeficiency virus (HIV-1) during chronic infection involves the rapid, continuous turnover of genetic diversity. However, the role of natural selection, relative to random genetic drift, in governing this process is unclear. We tested a stochastic model of genetic drift using partial envelope sequences sampled longitudinally in 28 infected children. In each case the Bayesian posterior (empirical) distribution of coalescent genealogies was estimated using Markov chain Monte Carlo methods. Posterior predictive simulation was then used to generate a null distribution of genealogies assuming neutrality, with the null and empirical distributions compared using four genealogy-based summary statistics sensitive to nonneutral evolution. Because both null and empirical distributions were generated within a coalescent framework, we were able to explicitly account for the confounding influence of demography. From the distribution of corrected P-values across patients, we conclude that empirical genealogies are more asymmetric than expected if evolution is driven by mutation and genetic drift only, with an excess of low-frequency polymorphisms in the population. This indicates that although drift may still play an important role, natural selection has a strong influence on the evolution of HIV-1 envelope. A negative relationship between effective population size and substitution rate indicates that as the efficacy of selection increases, a smaller proportion of mutations approach fixation in the population. This suggests the presence of deleterious mutations. We therefore conclude that intrahost HIV-1 evolution in envelope is dominated by purifying selection against low-frequency deleterious mutations that do not reach fixation. </td> <td style="text-align:left;"> 4546 </td> <td style="text-align:left;"> NA </td> <td style="text-align:left;"> NA </td> <td style="text-align:left;"> NA </td> <td style="text-align:left;"> no </td> <td style="text-align:left;"> no </td> </tr> <tr> <td style="text-align:left;"> 4545 </td> <td style="text-align:left;"> 2017 </td> <td style="text-align:left;"> NA </td> <td style="text-align:left;"> Perri, R; Zannino, GD; Caltagirone, C; Carlesimo, GA </td> <td style="text-align:left;"> 2011 </td> <td style="text-align:left;"> Semantic priming for coordinate distant concepts in Alzheimer's disease patients </td> <td style="text-align:left;"> Semantic priming paradigms have been used to investigate semantic knowledge in patients with Alzheimer's disease (AD). While priming effects produced by prime-target pairs with associative relatedness reflect processes at both lexical and semantic levels, priming effects produced by words that are semantically related but not associated should reflect only semantic activation processes. This study was aimed at further investigating automatic semantic priming effects in AD patients when semantically related concepts with little to no lexical association are used. Twenty patients with mild to moderate AD and 20 matched controls (NCs) performed a lexical decision task on 30 concept pairs (15 in the living and 15 in the non-living domain) in an automatic semantic priming paradigm. In order to investigate the relationship between priming alteration and semantic damage, we chose concepts from a database. This allowed us to quantify semantic indexes relative to the structural representation at the feature level. No priming was found in NCs or mild AD patients, probably because feature similarity was insufficient in the concept pairs used. Similar to the hyperpriming observed in previous studies, the appearance of priming in the moderate AD group suggests early semantic damage in which attribute knowledge is partially affected. Furthermore, the finding that priming was predicted by the level of sharing (in the semantic system) of features common to the two concepts in the pairs indicates that the level of redundancy of attribute information is the main factor responsible for resiliency to neurological damage in AD. (C) 2011 Elsevier Ltd. All rights reserved. </td> <td style="text-align:left;"> 4547 </td> <td style="text-align:left;"> NA </td> <td style="text-align:left;"> NA </td> <td style="text-align:left;"> NA </td> <td style="text-align:left;"> no </td> <td style="text-align:left;"> no </td> </tr> <tr> <td style="text-align:left;"> 4546 </td> <td style="text-align:left;"> 2017 </td> <td style="text-align:left;"> NA </td> <td style="text-align:left;"> Aiamkitsumrit, B; Dampier, W; Martin-Garcia, J; Nonnemacher, MR; Pirrone, V; Ivanova, T; Zhong, W; Kilareski, E; Aldigun, H; Frantz, B; Rimbey, M; Wojno, A; Passic, S; Williams, JW; Shah, S; Blakey, B; Parikh, N; Jacobson, JM; Moldover, B; Wigdahl, B </td> <td style="text-align:left;"> 2014 </td> <td style="text-align:left;"> Defining Differential Genetic Signatures in CXCR4-and the CCR5-Utilizing HIV-1 Co-Linear Sequences </td> <td style="text-align:left;"> The adaptation of human immunodeficiency virus type-1 (HIV-1) to an array of physiologic niches is advantaged by the plasticity of the viral genome, encoded proteins, and promoter. CXCR4-utilizing (X4) viruses preferentially, but not universally, infect CD4(+) T cells, generating high levels of virus within activated HIV-1-infected T cells that can be detected in regional lymph nodes and peripheral blood. By comparison, the CCR5-utilizing (R5) viruses have a greater preference for cells of the monocyte-macrophage lineage; however, while R5 viruses also display a propensity to enter and replicate in T cells, they infect a smaller percentage of CD4(+) T cells in comparison to X4 viruses. Additionally, R5 viruses have been associated with viral transmission and CNS disease and are also more prevalent during HIV-1 disease. Specific adaptive changes associated with X4 and R5 viruses were identified in co-linear viral sequences beyond the Env-V3. The in silico position-specific scoring matrix (PSSM) algorithm was used to define distinct groups of X4 and R5 sequences based solely on sequences in Env-V3. Bioinformatic tools were used to identify genetic signatures involving specific protein domains or long terminal repeat (LTR) transcription factor sites within co-linear viral protein R (Vpr), trans-activator of transcription (Tat), or LTR sequences that were preferentially associated with X4 or R5 Env-V3 sequences. A number of differential amino acid and nucleotide changes were identified across the co-linear Vpr, Tat, and LTR sequences, suggesting the presence of specific genetic signatures that preferentially associate with X4 or R5 viruses. Investigation of the genetic relatedness between X4 and R5 viruses utilizing phylogenetic analyses of complete sequences could not be used to definitively and uniquely identify groups of R5 or X4 sequences; in contrast, differences in the genetic diversities between X4 and R5 were readily identified within these co-linear sequences in HIV-1-infected patients. </td> <td style="text-align:left;"> 4548 </td> <td style="text-align:left;"> NA </td> <td style="text-align:left;"> NA </td> <td style="text-align:left;"> NA </td> <td style="text-align:left;"> no </td> <td style="text-align:left;"> no </td> </tr> <tr> <td style="text-align:left;"> 4547 </td> <td style="text-align:left;"> 2017 </td> <td style="text-align:left;"> NA </td> <td style="text-align:left;"> Swarovskaya, MG; Stepanova, SK; Marussin, AV; Sukhomyasova, AL; Maximova, NR; Stepanov, VA </td> <td style="text-align:left;"> 2015 </td> <td style="text-align:left;"> Genetic variability and structure of SNP haplotypes in the DMPK gene in Yakuts and other ethnic groups of northern Eurasia in relation to myotonic dystrophy </td> <td style="text-align:left;"> The genetic variability of the DMPK locus has been studied in relation to six SNP markers (rs2070736, rs572634, rs1799894, rs527221, rs915915, and rs10415988) in Yakuts with myotonic dystrophy (MD) in the Yakut population and in populations of northern Eurasia. Significant differences were observed in the allele frequencies between patients and a population sample of Yakuts for three SNP loci (rs915915, rs1799894, and rs0415988) associated with a high chance of disease manifestation. The odds ratios (OR) of MD development in representatives of the Yakut population for these three loci were 2.59 (95% CI, p = 0,004), 4.99 (95% CI, p = 0.000), and 3.15 (95% CI, p = 0.01), respectively. Haplotype TTTCTC, which is associated with MD, and haplotype GTCCTT, which was observed only in Yakut MD patients (never in MD patients of non-Yakut origin), were revealed. A low level of variability in the locus of DMRK gene in Yakuts (H (e) = 0.283) compared with other examined populations was noted. An analysis of pairwise genetic relationships between populations revealed their significant differentiation for all the examined loci. In addition, a low level of differentiation in territorial groups of Yakut populations (F (ST) = 0.79%), which was related to the high subdivision of the northern Eurasian population (F (ST) = 11.83%), was observed. </td> <td style="text-align:left;"> 4549 </td> <td style="text-align:left;"> NA </td> <td style="text-align:left;"> NA </td> <td style="text-align:left;"> NA </td> <td style="text-align:left;"> no </td> <td style="text-align:left;"> no </td> </tr> <tr> <td style="text-align:left;"> 4548 </td> <td style="text-align:left;"> 2017 </td> <td style="text-align:left;"> NA </td> <td style="text-align:left;"> Wang, MJ; Zhu, Y; Guo, XJ; Tian, ZZ </td> <td style="text-align:left;"> 2015 </td> <td style="text-align:left;"> Genetic variability of genes involved in DNA repair influence treatment outcome in osteosarcoma </td> <td style="text-align:left;"> We conducted a perspective study to investigate the role of ERCC1 (rs11615), ERCC2 (rs13181 and rs1799793), ERCC4 (rs1800067), and ERCC5 (rs17655) in NER pathway in the prognosis of osteosarcoma patients. In total, 146 osteosarcoma patients were recruited between 2008 and 2013. ERCC1 rs11615, ERCC2 rs13181 and rs1799793, ERCC4 rs1800067, and ERCC5 rs17655 gene polymorphisms were assessed by polymerase chain reaction-restriction fragment length polymorphism assay. By multivariate Cox proportional hazards models, we found that carriers of ERCC1 rs11615 TT genotype showed significantly favorable survival compared to wide-type CC genotype, and the adjusted OR (95%CI) was 0.24 (0.08-0.96). Moreover, we found that subjects with ERCC2 rs1799793 AA genotype were associated with decreased hazards of death in multivariate analysis (HR = 0.22, 95%CI = 0.12-0.93). In conclusion, our results suggest that ERCC1 rs11615 and ERCC2 rs1799793 may be useful genetic prognostic markers for osteosarcoma in a Chinese population. </td> <td style="text-align:left;"> 4550 </td> <td style="text-align:left;"> NA </td> <td style="text-align:left;"> NA </td> <td style="text-align:left;"> NA </td> <td style="text-align:left;"> no </td> <td style="text-align:left;"> no </td> </tr> <tr> <td style="text-align:left;"> 4549 </td> <td style="text-align:left;"> 2017 </td> <td style="text-align:left;"> NA </td> <td style="text-align:left;"> Wollmer, MA; Papassotiropoulos, A; Streffer, JR; Grimaldi, LME; Kapaki, E; Salani, G; Paraskevas, GP; Maddelena, A; de Quervain, D; Bieber, C; Umbricht, D; Lemke, U; Bosshardt, S; Degonda, N; Henke, K; Hegi, T; Jung, HH; Pasch, T; Hock, C; Nitsch, RM </td> <td style="text-align:left;"> 2002 </td> <td style="text-align:left;"> Genetic polymorphisms and cerebrospinal fluid levels of tissue inhibitor of metalloproteinases 1 in sporadic Alzheimer's disease </td> <td style="text-align:left;"> Tissue inhibitor of metalloproteinases I (TIMP-1) inhibits several proteinases including a disintegrin and metalloproteinase 10 (ADAM10), a major alpha-secretase that cleaves the beta-amyloid precursor protein within its amyloidogenic Abeta domain. The gene encoding TIMP-1 (TIMP1) maps to the short arm of the X chromosome, in a region previously suggested as conferring genetic susceptibility for Alzheimer's disease (AD). To determine whether genetic variability of TIMP1 contributes to the pathogenesis of AD, we analysed one single nucleotide polymorphism within TIMP1 and one single nucleotide polymorphism in the 5'-untranslated region of TIMP1 in patients with AD and control subjects from two independent and ethnically different populations. We did not observe any association between TIMP1 genotypes and the diagnosis of AD in men or women. We also measured TIMP-1 protein levels in the cerebrospinal fluid of patients with AD, healthy control subjects, and patients with other neurological disorders. TIMP-I levels were similar in all groups. In addition, no significant differences were observed after stratification for TIMP1 genotypes. Our data show that neither genetic variability nor protein levels of TIMP-1 are associated with AD. (C) 2002 Lippincott Williams Wilkins. </td> <td style="text-align:left;"> 4551 </td> <td style="text-align:left;"> NA </td> <td style="text-align:left;"> NA </td> <td style="text-align:left;"> NA </td> <td style="text-align:left;"> no </td> <td style="text-align:left;"> no </td> </tr> <tr> <td style="text-align:left;"> 4550 </td> <td style="text-align:left;"> 2017 </td> <td style="text-align:left;"> NA </td> <td style="text-align:left;"> Law, J; Jovel, J; Patterson, J; Ford, G; O'keefe, S; Wang, WW; Meng, B; Song, DY; Zhang, Y; Tian, ZJ; Wasilenko, ST; Rahbari, M; Mitchell, T; Jordan, T; Carpenter, E; Mason, AL; Wong, GKS </td> <td style="text-align:left;"> 2013 </td> <td style="text-align:left;"> Identification of Hepatotropic Viruses from Plasma Using Deep Sequencing: A Next Generation Diagnostic Tool </td> <td style="text-align:left;"> We conducted an unbiased metagenomics survey using plasma from patients with chronic hepatitis B, chronic hepatitis C, autoimmune hepatitis (AIH), non-alcoholic steatohepatitis (NASH), and patients without liver disease (control). RNA and DNA libraries were sequenced from plasma filtrates enriched in viral particles to catalog virus populations. Hepatitis viruses were readily detected at high coverage in patients with chronic viral hepatitis B and C, but only a limited number of sequences resembling other viruses were found. The exception was a library from a patient diagnosed with hepatitis C virus (HCV) infection that contained multiple sequences matching GB virus C (GBV-C). Abundant GBV-C reads were also found in plasma from patients with AIH, whereas Torque teno virus (TTV) was found at high frequency in samples from patients with AIH and NASH. After taxonomic classification of sequences by BLASTn, a substantial fraction in each library, ranging from 35% to 76%, remained unclassified. These unknown sequences were assembled into scaffolds along with virus, phage and endogenous retrovirus sequences and then analyzed by BLASTx against the non-redundant protein database. Nearly the full genome of a heretofore-unknown circovirus was assembled and many scaffolds that encoded proteins with similarity to plant, insect and mammalian viruses. The presence of this novel circovirus was confirmed by PCR. BLASTx also identified many polypeptides resembling nucleo-cytoplasmic large DNA viruses (NCLDV) proteins. We re-evaluated these alignments with a profile hidden Markov method, HHblits, and observed inconsistencies in the target proteins reported by the different algorithms. This suggests that sequence alignments are insufficient to identify NCLDV proteins, especially when these alignments are only to small portions of the target protein. Nevertheless, we have now established a reliable protocol for the identification of viruses in plasma that can also be adapted to other patient samples such as urine, bile, saliva and other body fluids. </td> <td style="text-align:left;"> 4552 </td> <td style="text-align:left;"> NA </td> <td style="text-align:left;"> NA </td> <td style="text-align:left;"> NA </td> <td style="text-align:left;"> no </td> <td style="text-align:left;"> no </td> </tr> <tr> <td style="text-align:left;"> 4551 </td> <td style="text-align:left;"> 2017 </td> <td style="text-align:left;"> NA </td> <td style="text-align:left;"> Gentle, NL; Paximadis, M; Puren, A; Tiemessen, CT </td> <td style="text-align:left;"> 2013 </td> <td style="text-align:left;"> Genetic Variability in Markers of HLA-C Expression in Two Diverse South African Populations </td> <td style="text-align:left;"> An insertion-deletion (indel) polymorphism within the 39 untranslated region (UTR) of HLA-C has been shown to be involved in the regulation of HLA-C expression. Individuals who carry a deletion at this position exhibit increased HLA-C expression, which associates with lower viral set point in HIV-1 infected individuals. This 263 indel (rs67384697) is reported to be in strong linkage disequilibrium (LD) with a single nucleotide polymorphism (SNP) 35 kilobases upstream of HLA-C (-35T/C; rs9264942) in Caucasian individuals, making this SNP a potential marker for both HLA-C expression and HIV-1 disease progression. We therefore examined genetic variation within the HLA-C 3' UTR of 265 Black and Caucasian South Africans by direct sequencing and identified haplotypes encompassing the 263 indel and another indel at position 230 in both populations. Concomitant evaluation of variability at the -35 SNP revealed this polymorphism to be an inappropriate marker for the 263 indel in these populations. These findings provide important insights into genetic variability within the regulatory regions of HLA-C that have potential implications for our understanding of the regulation of HLA-C expression and its impact on HIV-1 disease progression. </td> <td style="text-align:left;"> 4553 </td> <td style="text-align:left;"> NA </td> <td style="text-align:left;"> NA </td> <td style="text-align:left;"> NA </td> <td style="text-align:left;"> no </td> <td style="text-align:left;"> no </td> </tr> <tr> <td style="text-align:left;"> 4552 </td> <td style="text-align:left;"> 2017 </td> <td style="text-align:left;"> NA </td> <td style="text-align:left;"> Makhoul, I; Todorova, VK; Siegel, ER; Erickson, SW; Dhakal, I; Raj, VR; Lee, JY; Orloff, MS; Griffin, RJ; Henry-Tillman, RS; Klimberg, S; Hutchins, LF; Kadlubar, SA </td> <td style="text-align:left;"> 2017 </td> <td style="text-align:left;"> Germline Genetic Variants in TEK, ANGPT1, ANGPT2, MMP9, FGF2 and VEGFA Are Associated with Pathologic Complete Response to Bevacizumab in Breast Cancer Patients </td> <td style="text-align:left;"> Background We previously reported improved pathologic complete response (pCR) in a prospective phase II study using neoadjuvant bevacizumab in combination with chemotherapy compared to chemotherapy alone in breast cancer patients (41% vs. 25%, p = 0.0291). In this study, we queried germline single-nucleotide polymorphisms (SNPs) in angiogenesis-related genes for their impact on pCR and overall survival (OS). Methods DNA for genotyping was available from 34 subjects who received bevacizumab in addition to chemotherapy and 29 subjects who did not. Using Illumina (R) technology, we queried 504 SNPs with a minor allele frequency (MAF) of at least 5%, located in 10 angiogenesis-related genes, for their effect on pCR via logistic regression with an additive-inheritance model while adjusting for race and bevacizumab treatment. SNPs that showed significant associations with pCR were selected for additional characterization. Results After adjusting for race and tumor type, patients who had bevacizumab added to their neoadjuvant therapy were found to experience a significantly improved rate of pCR compared to patients who did not (adjusted OR 8.40, 95% CI 1.90-37.1). When patients were analyzed for SNP effects via logistic regression with race and bevacizumab treatment included as covariates, two SNPs in angiopoietin 1 (ANGPT1), six in ANGPT2, three in fibroblast growth factor 2 (FGF2), four in matrix metalloproteinase 9 (MMP9), three in tyrosine kinase, endothelial (TEK) and two in vascular endothelial growth factor A (VEGFA) were associated with pCR (P<0.05). However, when overall survival was considered, there was no difference between treatment groups or between genotypes. Conclusion Genetic variability in TEK, ANGPT1, ANGPT2, FGF2, MMP9 and VEGFA is associated with pCR in bevacizumab-treated patients. Consistent with other studies, adding bevacizumab to standard chemotherapy did not impact OS, likely due to other factors and thus, while SNPs in TEK, ANGPT1, ANGPT2, FGF2, MMP9 and VEGFA were associated with pCR, they were not predictive of OS in this patient population. </td> <td style="text-align:left;"> 4554 </td> <td style="text-align:left;"> NA </td> <td style="text-align:left;"> NA </td> <td style="text-align:left;"> NA </td> <td style="text-align:left;"> no </td> <td style="text-align:left;"> no </td> </tr> <tr> <td style="text-align:left;"> 4553 </td> <td style="text-align:left;"> 2017 </td> <td style="text-align:left;"> NA </td> <td style="text-align:left;"> Pichat, C; Couvin, D; Carret, G; Fredenucci, I; Jacomo, V; Carricajo, A; Boisset, S; Dumitrescu, O; Flandrois, JP; Lina, G; Rastogi, N </td> <td style="text-align:left;"> 2016 </td> <td style="text-align:left;"> Combined Genotypic, Phylogenetic, and Epidemiologic Analyses of Mycobacterium tuberculosis Genetic Diversity in the Rhone Alpes Region, France </td> <td style="text-align:left;"> Background The present work relates to identification and a deep molecular characterization of circulating Mycobacterium tuberculosis complex (MTBC) strains in the Rhone-Alpes region, France from 2000 to 2010. It aimed to provide with a first snapshot of MTBC genetic diversity in conjunction with bacterial drug resistance, type of disease and available demographic and epidemiologic characteristics over an eleven-year period, in the south-east of France. Methods Mycobacterium tuberculosis complex (MTBC) strains isolated in the Rhone-Alpes region, France (n = 2257, 1 isolate per patient) between 2000 and 2010 were analyzed by spoligotyping. MIRU-VNTR typing was applied on n = 1698 strains (with full results available for 974 strains). The data obtained were compared with the SITVIT2 database, followed by detailed genotyping, phylogenetic, and epidemiologic analyses in correlation with anonymized data on available demographic, and epidemiologic characteristics, and location of disease (pulmonary or extrapulmonary TB). Results The most predominant spoligotyping clusters were SIT53/T1 (n = 346, 15.3%) > SIT50/H3 (n = 166, 7.35%) > SIT42/LAM9 (n = 125, 5.5%) > SIT1/Beijing (n = 72, 3.2%) > SIT47/H1 (n = 71, 3.1%). Evolutionary-recent strains belonging to the Principal Genetic Group (PGG) 2/3, or Euro-American lineages (T, LAM, Haarlem, X, S) were predominant and represented 1768 or 78.33% of all isolates. For strains having drug resistance information (n = 1119), any drug resistance accounted for 14.83% cases vs. 1.52% for multidrug resistance (MDR); and was significantly more associated with age group 21-40 years (pvalue<0.001). Extra-pulmonary TB was more common among female patients while pulmonary TB predominated among men (p-value<0.001; OR = 2.16 95% CI [1.69; 2.77]). Also, BOV and CAS lineages were significantly well represented in patients affected by extra-pulmonary TB (p-value<0.001). The origin was known for 927/2257 patients: 376 (40.6%) being French-born vs. 551 (59.4%) Foreign-born. French patients were significantly older (mean age: 58.42 yrs 95% CI [56.04; 60.80]) than Foreign-born patients (mean age: 42.38 yrs. 95% CI [40.75; 44.0]). Conclusion The study underlined the importance of imported TB cases on the genetic diversity and epidemiologic characteristics of circulating MTBC strains in Rhone-Alpes region, France over a large time-period. It helps better understand intricate relationships between certain lineages and geographic origin of the patients, and pinpoints genotypic and phylogenetic specificities of prevailing MTBC strains. Lastly, it also demonstrated a slow decline in isolation of M. africanum lineage in this region between 2000 and 2010. </td> <td style="text-align:left;"> 4555 </td> <td style="text-align:left;"> NA </td> <td style="text-align:left;"> NA </td> <td style="text-align:left;"> NA </td> <td style="text-align:left;"> no </td> <td style="text-align:left;"> no </td> </tr> <tr> <td style="text-align:left;"> 4554 </td> <td style="text-align:left;"> 2017 </td> <td style="text-align:left;"> NA </td> <td style="text-align:left;"> Marusin, AV; Kurtanov, HA; Maksimova, NR; Swarovsakaja, MG; Stepanov, VA </td> <td style="text-align:left;"> 2016 </td> <td style="text-align:left;"> Haplotype analysis of oculopharyngeal muscular dystrophy (OPMD) locus in Yakutia </td> <td style="text-align:left;"> Oculopharyngeal muscular dystrophy (OPMD) is a hereditary neuromuscular disease with autosomal dominant and rarely with autosomal recessive inheritance types. This study included 50 patients with a clinical diagnosis of OPMD, 23 asymptomatic carriers of the mutation from 45 unrelated families, and 56 healthy relatives, as well as population samples of four ethnic groups of Yakutia: Yakuts, Evens, Evenks, Yukaghirs. It was found that the cause of OPMD development in all investigated families is the same increase in GCN repeats to 14 copies in the PABPN1 gene. The molecular structure of the (GCN)(14) mutant allele is (GCG)(10)(GCA)(3)GCG. The genetic variability of ten SNPs at the OPMD locus was studied in patient families and population samples. The haplotypes of OPMD were determined by a segregation analysis technique and using the EM algorithm in the groups of patients, mutation carriers, and population samples. Only one haplotype of four SNPs (ATCG) linked with the (GCN)(14) mutant allele was found in Yakuts and Russian patients and OPMD mutation carriers. Probably, this indicates the accumulation of mutations as a result of the founder effect. </td> <td style="text-align:left;"> 4556 </td> <td style="text-align:left;"> NA </td> <td style="text-align:left;"> NA </td> <td style="text-align:left;"> NA </td> <td style="text-align:left;"> no </td> <td style="text-align:left;"> no </td> </tr> <tr> <td style="text-align:left;"> 4555 </td> <td style="text-align:left;"> 2017 </td> <td style="text-align:left;"> NA </td> <td style="text-align:left;"> Beh-Pajooh, A; Fasihi-Ramandi, M; Tavallaie, M </td> <td style="text-align:left;"> 2017 </td> <td style="text-align:left;"> Association analysis of IL-4 VNTR polymorphism with rheumatoid arthritis in Iranian patients </td> <td style="text-align:left;"> Rheumatoid arthritis (RA) is an autoimmune disease characterized by movement disability and pain in the joints. The affected individuals are susceptible to other subsequent diseases, exacerbating the condition. To find out the genetic variability of this disease at the genomic level for the first time in the Iranian population, we carried out an investigation on the VNTR of IL-4 gene within its third intron. For this goal we isolated the genomic DNA from blood samples of 576 rheumatoid arthritis patients and 546 healthy controls and investigated the presence or absence of specific amplicons via polymerase chain reaction (PCR). The size of each amplicon on a 1.5% agarose gel corresponded to a certain number of tandem repeats which indicated a specific allele. Statistical test of chi(2) Fisher's exact test and odds ratio (OR) was used to analyze the data. The results showed that RA1/RA1 genotype was the dominant genotype in both healthy controls and patients and the heterozygote genotype of RA1/RA2 was observed more in the healthy controls than patients (108 vs. 66) with significant difference with P value < 0.005 and odds ratio of 0.214. However two genotypes of RA2/RA2 and RA2/RA3 were exclusively observed in the patients' samples with P value = 0.023 and odds ratio of 0.988. We concluded that IL-4 VNTR polymorphism has a strong association with rheumatoid arthritis and might be a high risk factor for development of rheumatoid arthritis in the investigated Iranian population. </td> <td style="text-align:left;"> 4557 </td> <td style="text-align:left;"> NA </td> <td style="text-align:left;"> NA </td> <td style="text-align:left;"> NA </td> <td style="text-align:left;"> no </td> <td style="text-align:left;"> no </td> </tr> <tr> <td style="text-align:left;"> 4556 </td> <td style="text-align:left;"> 2017 </td> <td style="text-align:left;"> NA </td> <td style="text-align:left;"> Ronsard, L; Raja, R; Panwar, V; Saini, S; Mohankumar, K; Sridharan, S; Padmapriya, R; Chaudhuri, S; Ramachandran, VG; Banerjea, AC </td> <td style="text-align:left;"> 2015 </td> <td style="text-align:left;"> Genetic and functional characterization of HIV-1 Vif on APOBEC3G degradation: First report of emergence of B/C recombinants from North India </td> <td style="text-align:left;"> HIV-1 is characterized by high genetic heterogeneity which is a challenge for developing therapeutics. Therefore, it is necessary to understand the extent of genetic variations that HIV is undergoing in North India. The objective of this study was to determine the role of genetic and functional role of Vif on APOBEC3G degradation. Vif is an accessory protein involved in counteracting APOBEC3/F proteins. Genetic analysis of Vif variants revealed that Vif C variants were closely related to South African Vif C whereas Vif B variants and Vif B/C showed distinct geographic locations. This is the first report to show the emergence of Vif B/C in our population. The functional domains, motifs and phosphorylation sites were well conserved. Vif C variants differed in APOBEC3G degradation from Vif B variants. Vif B/C revealed similar levels of APOBEC3G degradation to Vif C confirming the presence of genetic determinants in C-terminal region. High genetic diversity was observed in Vif variants which may cause the emergence of more complex and divergent strains. These results reveal the genetic determinants of Vif in mediating APOBEC3G degradation and highlight the genetic information for the development of anti-viral drugs against HIV. Importance: Vif is an accessory HIV-1 protein which plays significant role in the degradation of human DNA-editing factor APOBEC3G, thereby impeding the antiretroviral activity of APOBEC3G. It is known that certain natural polymorphisms in Vif could degrade APOBEC3G relatively higher rate, suggesting its role in HIV-1 pathogenesis. This is the first report from North India showcasing genetic variations and novel polymorphisms in Vif gene. Subtype C is prevalent in India, but for the first time we observed putative B/C recombinants with a little high ability to degrade APOBEC3G indicating adaptation and evolving nature of virus in our population. Indian Vif C variants were able to degrade APOBEC3G well in comparison to Vif B variants. These genetic changes were most likely selected during adaptation of HIV to our population. These results elucidate that the genetic determinants of Vif and highlights the potential targets for therapeutics. </td> <td style="text-align:left;"> 4558 </td> <td style="text-align:left;"> NA </td> <td style="text-align:left;"> NA </td> <td style="text-align:left;"> NA </td> <td style="text-align:left;"> no </td> <td style="text-align:left;"> no </td> </tr> <tr> <td style="text-align:left;"> 4557 </td> <td style="text-align:left;"> 2017 </td> <td style="text-align:left;"> NA </td> <td style="text-align:left;"> Bettens, K; Brouwers, N; Engelborghs, S; Van Miegroet, H; De Deyn, PP; Theuns, J; Sleegers, K; Van Broeckhoven, C </td> <td style="text-align:left;"> 2009 </td> <td style="text-align:left;"> APP and BACE1 miRNA Genetic Variability Has No Major Role in Risk for Alzheimer Disease </td> <td style="text-align:left;"> Expression levels of the amyloid precursor protein (APP) and beta-site amyloid (A beta) cleaving enzyme 1 (BACE1) have been implicated in Alzheimer disease (AD) progression. In a well-characterized Belgian group of 358 AD patients and 462 controls, we examined whether genetic variability in microRNA (miPNA) binding sites of APP and BACE1 or in associated miRNAs influenced risk for AD. Direct sequencing identified six variant, in the 3' untranslated region (UTR) of APP and 29 variants in the 3' UTR of BACE1, of which few variants were restricted to patients: in APP, 4 variants in 6 patients (similar to 2%) and in BACE1; 7 variants in 11 patients (similar to 3.5%). Further genetic screening of the miR-29 cluster encoding the miR-29a/b-1 genes showed 10 variants in close proximity of this cluster. Association studies using all common variants detected in the 3' UTR of BACE1 and the miR-29 gene cluster did not identify an association,with AD risk. However, we did observe statistical interaction between rs535860 (BACE1 3' UTR) and rs34772568 (near miR29a; odds ratio [OR](interaction), 0.4; 95% confidence interval [CI] 0.17-0.96; P = 0.033). While the exact role of the patient, specific miRNA variants within the 3' UTR region of APP and BACE1 demands further analyses, this study does not support a major contribution of miRNA genetic variability to AD pathogenesis. Hum Mutat 30, 1207-1213, 2009. (C) 2009 Wiley-Liss, Inc. </td> <td style="text-align:left;"> 4559 </td> <td style="text-align:left;"> NA </td> <td style="text-align:left;"> NA </td> <td style="text-align:left;"> NA </td> <td style="text-align:left;"> no </td> <td style="text-align:left;"> no </td> </tr> <tr> <td style="text-align:left;"> 4558 </td> <td style="text-align:left;"> 2017 </td> <td style="text-align:left;"> NA </td> <td style="text-align:left;"> Poveda, C; Fresno, M; Girones, N; Martins, OA; Ramirez, JD; Santi-Rocca, J; Marin-Neto, JA; Morillo, CA; Rosas, F; Guhl, F </td> <td style="text-align:left;"> 2014 </td> <td style="text-align:left;"> Cytokine Profiling in Chagas Disease: Towards Understanding the Association with Infecting Trypanosoma cruzi Discrete Typing Units (A BENEFIT TRIAL Sub-Study) </td> <td style="text-align:left;"> Background: Chagas disease caused by the protozoan Trypanosoma cruzi is an important public health problem in Latin America. The immunological mechanisms involved in Chagas disease pathogenesis remain incompletely elucidated. The aim of this study was to explore cytokine profiles and their possible association to the infecting DTU and the pathogenesis of Chagas disease. Methods: 109 sero-positive T. cruzi patients and 21 negative controls from Bolivia and Colombia, were included. Flow cytometry assays for 13 cytokines were conducted on human sera. Patients were divided into two groups: in one we compared the quantification of cytokines between patients with and without chronic cardiomyopathy; in second group we compared the levels of cytokines and the genetic variability of T. cruzi. Results: Significant difference in anti-inflammatory and pro-inflammatory cytokines profiles was observed between the two groups cardiac and non-cardiac. Moreover, serum levels of IFN-gamma, IL-12, IL-22 and IL-10 presented an association with the genetic variability of T. cruzi, with significant differences in TcI and mixed infections TcI/TcII. Conclusion: Expression of anti-inflammatory and pro-inflammatory cytokines may play a relevant role in determining the clinical presentation of chronic patients with Chagas disease and suggests the occurrence of specific immune responses, probably associated to different T. cruzi DTUs. </td> <td style="text-align:left;"> 4560 </td> <td style="text-align:left;"> NA </td> <td style="text-align:left;"> NA </td> <td style="text-align:left;"> NA </td> <td style="text-align:left;"> no </td> <td style="text-align:left;"> no </td> </tr> <tr> <td style="text-align:left;"> 4559 </td> <td style="text-align:left;"> 2017 </td> <td style="text-align:left;"> NA </td> <td style="text-align:left;"> MacFie, S; Nerrienet, E; de Groot, NG; Bontrop, RE; Mundy, NI </td> <td style="text-align:left;"> 2009 </td> <td style="text-align:left;"> Patterns of Diversity in HIV-Related Loci among Subspecies of Chimpanzee: Concordance at CCR5 and Differences at CXCR4 and CX(3)CR1 </td> <td style="text-align:left;"> Human immunodeficiency virus type 1 (HIV-1) arose in humans via zoonotic transmissions of simian immunodeficiency viruses (SIVcpz) from common chimpanzees, Pan troglodytes. Despite the close relatedness of the two viruses and their hosts, we do not yet understand what causes SIVcpz to be nonpathogenic in chimpanzees, and HIV/AIDS to be one of the most devastating infectious diseases to have emerged in humans. There have been a number of genes identified in humans that confer disease resistance/susceptibility toward HIV-1, but little is known about the evolution and diversity of most of these chemokine receptor genes in chimpanzees. Here we show that genetic variation in chimpanzees differs across the various loci related to HIV-1, and that the pattern of variation differs among the chimpanzee subspecies. For all three subspecies, low diversity at CCR5 is confined to a small area of chromosome 3, suggesting that a selective sweep at this locus may have predated subspeciation. In contrast, diversity and neutrality tests suggest differing evolutionary forces among subspecies at CXCR4 and CX(3)CR1, with directional selection (in Pan troglodytes vellerosus) and demographic expansion (Pan troglodytes troglodytes) offering the most likely scenarios. These are some of the first data demonstrating differentiation in functional loci among chimpanzee subspecies. </td> <td style="text-align:left;"> 4561 </td> <td style="text-align:left;"> NA </td> <td style="text-align:left;"> NA </td> <td style="text-align:left;"> NA </td> <td style="text-align:left;"> no </td> <td style="text-align:left;"> no </td> </tr> <tr> <td style="text-align:left;"> 4560 </td> <td style="text-align:left;"> 2017 </td> <td style="text-align:left;"> NA </td> <td style="text-align:left;"> Nebavi, F; Ayala, FJ; Renaud, F; Bertout, S; Eholie, S; Moussa, K; Mallie, M; de Meeus, T </td> <td style="text-align:left;"> 2006 </td> <td style="text-align:left;"> Clonal population structure and genetic diversity of Candida albicans in AIDS patients from Abidjan (Cote d'Ivoire) </td> <td style="text-align:left;"> We have investigated the genotype at 14 enzyme-encoding loci in 275 isolates of the pathogenic yeast Candida albicans sampled from 42 HIV-positive patients (all but one with AIDS) from Abidjan (Cote d'Ivoire). We separately analyzed the following variables: patient, residence, age, gender, T cell count, hospitalization (yes or no), drug treatment, date of sampling, multilocus genotype, and serotype. The most important factors contributing to the genetic variability of C albicans are individual patient and gender. Our data manifest that the population size of the parasite is relatively small within each patient, although larger in women than in men, and that, at least for the patients involved in the study, the transmission rate of C albicans between human adults is very low. Most important is the inference that the prevailing mode of reproduction of C albicans in natural populations is clonal, so that sexual reproduction is extremely rare, if it occurs at all. </td> <td style="text-align:left;"> 4562 </td> <td style="text-align:left;"> NA </td> <td style="text-align:left;"> NA </td> <td style="text-align:left;"> NA </td> <td style="text-align:left;"> no </td> <td style="text-align:left;"> no </td> </tr> <tr> <td style="text-align:left;"> 4561 </td> <td style="text-align:left;"> 2017 </td> <td style="text-align:left;"> NA </td> <td style="text-align:left;"> Christine, S; Narcisse, E; Philippe, J; Tania, V; Mathieu, N </td> <td style="text-align:left;"> 2016 </td> <td style="text-align:left;"> Genetic Modulators of Sickle Cell Disease in French Guiana: Markers of the Slave Trade </td> <td style="text-align:left;"> Objectives: Sickle cell disease (SCD) is the leading genetic disease in French Guiana, reflecting the predominantly African ancestry of the Guianese population. Our purpose was to characterize the genetic modulators of SCD in order to retrace the origin of the population in light of the slave trade. Methods: We have studied the sickle cell genotype, the beta S haplotypes, the alpha and beta thalassemia and the UGT1A1 promoter polymorphisms in 224 Guianese patients with SCD. Results: The genotypes of SCD were HbSS 65.6%, HbSC 24.5%, and HbS-beta thalassemia 9.4%. The most frequent beta S haplotypes were the Benin haplotype (65.9% of the chromosomes) and the Bantu (20.5%). Alpha thalassemic deletions were present in 37% of the patients and homozygosity for the (TA) 7 allele of the UGT1A1 promoter in 21.4%. When the patients' origins were considered, 3 groups, Noir Marron, Haitians and Creoles, displayed distinctive characteristics. The HbSC genotype, the Benin haplotype, and the homozygous UGT1A1 genotype TA7/TA7 were significantly more frequent in Noir Marron. The Haitian patients were characterized by the occurrence of alpha-thalassemia and beta-thalassemia and by a higher prevalence of the Bantu haplotype. In the group of Creole patients, the genotype HbSS was predominant but the other modulators of SCD were associated with intermediate risk. Conclusions: The results highlight the genetic diversity of the Guianese population and are concordant with historical data on the slave trade showing a West African origin for Noir Marron and a Central African origin for Haitians, while Guianese Creoles are highly admixed. (C) 2016 Wiley Periodicals, Inc. </td> <td style="text-align:left;"> 4563 </td> <td style="text-align:left;"> NA </td> <td style="text-align:left;"> NA </td> <td style="text-align:left;"> NA </td> <td style="text-align:left;"> no </td> <td style="text-align:left;"> no </td> </tr> <tr> <td style="text-align:left;"> 4562 </td> <td style="text-align:left;"> 2017 </td> <td style="text-align:left;"> NA </td> <td style="text-align:left;"> Xu, YM; Bai, XN; Zhao, AL; Zhang, W; Ba, PB; Liu, K; Jin, YJ; Wang, H; Guo, QS; Sun, H; Xu, JG; Xiong, YW </td> <td style="text-align:left;"> 2016 </td> <td style="text-align:left;"> Genetic Diversity of Intimin Gene of Atypical Enteropathogenic Escherichia coli Isolated from Human, Animals and Raw Meats in China </td> <td style="text-align:left;"> Atypical enteropathogenic Escherichia coli (aEPEC) is considered to be an emerging enteropathogen that is more prevalent than typical EPEC in developing and developed countries. The major adherence factor, intimin, an outer membrane protein encoded by eae, plays a pivotal role in the pathogenesis of aEPEC. This study investigated the distribution and polymorphisms of intimin subtypes of 143 aEPEC strains from diarrheal patients, healthy carriers, animals, and raw meats in China. These aEPEC strains belonged to more than 71 different serotypes, which comprised 52 O serogroups and 24 H types. Sixty-eight different eae genotypes and 19 intimin subtypes were detected. Eighteen, eight, seven, and five intimin subtypes were identified from 86 diarrheal patients, 14 healthy carriers, 19 animals, and 24 raw meats strains, respectively. Intimin beta 1 was the most prevalent subtype in strains from diarrheal patients (34.88%) and animals (47.37%). There was a statistically significant difference in the distribution of eae-beta 1 between diarrheal patients and healthy carriers (P = 0.004). Intimin-theta was more predominant among raw meat strains (50%) than among diarrheal patients strains (12.79%, P = 0.0003), healthy carrier strains (7.14%, P = 0.007), or animal strains (15.79%, P = 0.020). The two predominant subtypes (eae-beta 1 and eae-theta) had considerable polymorphisms with no significant differences among the four sources. PFGE analysis revealed 119 distinct patterns and the strains were clustered into 11 groups with similarity indices ranging from 63% to 100%. These results suggest that in China, aEPEC strains from different sources are highly heterogeneous. Animals and raw meats are important sources of genetically diverse intimin-harboring aEPEC, which might serve as important transmission vehicles of these bacteria. </td> <td style="text-align:left;"> 4564 </td> <td style="text-align:left;"> NA </td> <td style="text-align:left;"> NA </td> <td style="text-align:left;"> NA </td> <td style="text-align:left;"> no </td> <td style="text-align:left;"> no </td> </tr> <tr> <td style="text-align:left;"> 4563 </td> <td style="text-align:left;"> 2017 </td> <td style="text-align:left;"> NA </td> <td style="text-align:left;"> Dalmau, J; Codoner, FM; Erkizia, I; Pino, M; Pou, C; Paredes, R; Clotet, B; Martinez-Picado, J; Prado, JG </td> <td style="text-align:left;"> 2012 </td> <td style="text-align:left;"> In-Depth Characterization of Viral Isolates from Plasma and Cells Compared with Plasma Circulating Quasispecies in Early HIV-1 Infection </td> <td style="text-align:left;"> Background: The use of in vitro models to unravel the phenotypic characteristics of circulating viral variants is key to understanding HIV-1 pathogenesis but limited by the availability of primary viral isolates from biological samples. However, overall in vivo genetic variability of HIV-1 within a subject may not be reflected in the viable viral population obtained after isolation. Although several studies have tried to determine whether viral populations expanded in vitro are representative of in vivo findings, the answer remains unclear due to the reduced number of clonal sequences analyzed or samples compared. In order to overcome previous experimental limitations, here we applied Deep Pyrosequencing (DPS) technology in combination with phenotypic experiments to analyze and compare with unprecedented detail the composition of viral isolates and in vivo quasispecies. Methodology/Principal Findings: We amplified by DPS HIV-1 genomic regions covering gag, protease, integrase and env-V3 to characterize paired isolates from plasma and peripheral blood mononuclear cells and compare them with total plasma viral RNA in four recently HIV-1 infected subjects. Our study demonstrated the presence of unique haplotypes scattered between sample types with conservation of major variants. In addition, no differences in intra- and inter-population encoded protein variability were found between the different types of isolates or when these were compared to plasma viral RNA within subjects. Additionally, in vitro experiments demonstrated phenotypic similarities in terms of replicative capacity and co-receptor usage between viral isolates and plasma viral RNA. Conclusion: This study is the first in-depth comparison and characterization of viral isolates from different sources and plasma circulating quasispecies using DPS in recently HIV-1 infected subjects. Our data supports the use of primary isolates regardless of their plasma or cellular origin to define genetic variability and biological traits of circulating HIV-1 quasispecies. </td> <td style="text-align:left;"> 4565 </td> <td style="text-align:left;"> NA </td> <td style="text-align:left;"> NA </td> <td style="text-align:left;"> NA </td> <td style="text-align:left;"> no </td> <td style="text-align:left;"> no </td> </tr> <tr> <td style="text-align:left;"> 4564 </td> <td style="text-align:left;"> 2017 </td> <td style="text-align:left;"> NA </td> <td style="text-align:left;"> Nobuhara, Y; Usuku, K; Saito, M; Izumo, S; Arimura, K; Bangham, CRM; Osame, M </td> <td style="text-align:left;"> 2006 </td> <td style="text-align:left;"> Genetic variability in the extracellular matrix protein as a determinant of risk for developing HTLV-I-associated neurological disease </td> <td style="text-align:left;"> Aggrecan, which is a well-known proteoglycan in joint cartilage, also exists in the spinal cord and plays an important role in maintaining water content in the extracellular matrix structure. In this study, we first examined the variable number of tandem repeat ( VNTR) polymorphism of the aggrecan gene in 227 HTLV-I associated myelopathy/ tropical spastic paraparesis (HAM/TSP) patients, in 217 HTLV-I-infected healthy carriers (HCs), and in 85 normal controls. The VNTR allele 28 ( 1,630 bp) was more frequently observed in HAM/TSP patients than in HCs (chi(2)=12.02, p=0.0005, odds ratio 1.79, 95% C.I. 1.29-2.50) and in controls (chi(2)=13.43, p=0.0002, odds ratio 2.54, 95% C.I. 1.52-4.25), although this allele was not related to disease progression or to HTLV-I provirus load. We also found that the aggrecan concentration in cerebrospinal fluid (CSF) from rapidly progressive HAM/TSP patients was significantly higher than in slowly progressive patients (corrected p=0.0145) but not in infected non-inflammatory neurological other disease controls (OND) (corrected p=0.078). We then analyzed this aggrecan VNTR polymorphism in the different set of patients with HAM/TSP (n=58) and healthy carriers (n=70). This analysis, again, revealed that allele 28 was detected more frequently in HAM/TSP group than in HCs (chi(2)=11.03, p=0.0009, odd ratio 3.04, 95% C.I. 1.55-5.97). The reproducibility of our study was regarded as a second- or third-class association by comparing combined p values and the Better Associations for Disease and GEnes ( BADGE) system. Our results suggest that aggrecan polymorphism can be a novel genetic risk factor for developing HAM/TSP. </td> <td style="text-align:left;"> 4566 </td> <td style="text-align:left;"> NA </td> <td style="text-align:left;"> NA </td> <td style="text-align:left;"> NA </td> <td style="text-align:left;"> no </td> <td style="text-align:left;"> no </td> </tr> <tr> <td style="text-align:left;"> 4565 </td> <td style="text-align:left;"> 2017 </td> <td style="text-align:left;"> NA </td> <td style="text-align:left;"> Solano, AR; Aceto, GM; Delettieres, D; Veschi, S; Neuman, MI; Alonso, E; Chialina, S; Chacon, RD; Renato, MC; Podesta, EJ </td> <td style="text-align:left;"> 2012 </td> <td style="text-align:left;"> BRCA1 And BRCA2 analysis of Argentinean breast/ovarian cancer patients selected for age and family history highlights a role for novel mutations of putative south-American origin </td> <td style="text-align:left;"> Background: The spectrum of BRCA1/2 genetic variation in breast-ovarian cancer patients has been scarcely investigated outside Europe and North America, with few reports for South America, where Amerindian founder effects and recent multiracial immigration are predicted to result in high genetic diversity. We describe here the results of BRCA1/BRCA2 germline analysis in an Argentinean series of breast/ovarian cancer patients selected for young age at diagnosis or breast/ovarian cancer family history. Methods: The study series (134 patients) included 37 cases diagnosed within 40 years of age and no family history (any ethnicity, fully-sequenced), and 97 cases with at least 2 affected relatives (any age), of which 57 were non-Ashkenazi (fully-sequenced) and 40 Ashkenazi (tested only for the founder mutations c.66_67delAG and c.5263insC in BRCA1 and c.5946delT in BRCA2). Discussion: We found 24 deleterious mutations (BRCA1: 16; BRCA2: 8) in 38/134 (28.3%) patients, of which 6/37 (16.2%) within the young age group, 15/57 (26.3%) within the non-Ahkenazi positive for family history; and 17/40 (42.5%) within the Ashkenazi. Seven pathogenetic mutations were novel, five in BRCA1: c.1502_1505delAATT, c.2626_2627delAA c.2686delA, c.2728 C > T, c.3758_3759delCT, two in BRCA2: c.7105insA, c.793 + 1delG. We also detected 72 variants of which 54 previously reported and 17 novel, 33 detected in an individual patient. Four missense variants of unknown clinical significance, identified in 5 patients, are predicted to affect protein function. While global and European variants contributed near 45% of the detected BRCA1/2 variation, the significant fraction of new variants (25/96, 26%) suggests the presence of a South American genetic component. This study, the first conducted in Argentinean patients, highlights a significant impact of novel BRCA1/2 mutations and genetic variants, which may be regarded as putatively South American, and confirms the important role of founder BRCA1 and BRCA2 mutations in Argentinean Ashkenazi Jews. </td> <td style="text-align:left;"> 4567 </td> <td style="text-align:left;"> NA </td> <td style="text-align:left;"> NA </td> <td style="text-align:left;"> NA </td> <td style="text-align:left;"> no </td> <td style="text-align:left;"> no </td> </tr> <tr> <td style="text-align:left;"> 4566 </td> <td style="text-align:left;"> 2017 </td> <td style="text-align:left;"> NA </td> <td style="text-align:left;"> Caiola, E; Porcu, L; Fruscio, R; Giuliani, D; Milani, R; Torri, V; Broggini, M; Marabese, M </td> <td style="text-align:left;"> 2013 </td> <td style="text-align:left;"> DNA-damage response gene polymorphisms and therapeutic outcomes in ovarian cancer </td> <td style="text-align:left;"> Epithelial ovarian cancer has a poor prognosis owing to late diagnosis and frequent relapse after first-line therapy. Analysis of individual genetic variability could aid in the identification of markers, which could help in stratifying patients with the aim of optimizing individual therapy. In this study we assessed polymorphisms in three genes important in drugs' response in 97 early and 235 late-stage ovarian cancer patients. The Asp1104His polymorphism in xpg, a gene important for removal of platinum adducts, was associated with progression-free survival in early-and late-stage ovarian cancer. Our data indicate that a simple diagnostic analysis such as xpg genotyping can help in predicting response, and extension to other possibly relevant genotypes could be useful in selecting patients with epithelial ovarian cancer for optimal therapy and hence increase the chance of response. The Pharmacogenomics Journal (2013) 13, 159-172; doi:10.1038/tpj.2011.50; published online 13 December 2011 </td> <td style="text-align:left;"> 4568 </td> <td style="text-align:left;"> NA </td> <td style="text-align:left;"> NA </td> <td style="text-align:left;"> NA </td> <td style="text-align:left;"> no </td> <td style="text-align:left;"> no </td> </tr> <tr> <td style="text-align:left;"> 4567 </td> <td style="text-align:left;"> 2017 </td> <td style="text-align:left;"> NA </td> <td style="text-align:left;"> Jie, L; Ma, ZH; Lou, MZ; Shao, YM </td> <td style="text-align:left;"> 2004 </td> <td style="text-align:left;"> HIV-1 population dynamics in vivo: Implications for pathogenesis, effect of cytokine and therapy strategy </td> <td style="text-align:left;"> In this paper, we present a dynamical model to study the spread of HIV-1 in vivo. Our goal is to better understand the interaction between HIV-1 and the human immune system. Making use of the Hill function, we describe two kinds of processions occurring in the immune response: the activation interactions or inhibitory interactions occurring between different components in the immune response, and the autocatalytic maintenance of the CD4+ T cells and CD8+ T cells populations. We also consider the impact of the cytokine interleukin-2 (IL-2) and the CD8 antiviral factor (CAF) on HIV-1 infection. Through numerical simulations we get several results. First, we find that the effects of IL-2 and CAF in the treatment for the infected are limiting. Namely, the curative effect will not always increase along with the dose of IL-2 or CAF or both. The increasing trend will stagnate at a certain dose that we used. Second, we find some possible reasons for the collapse of the lymph system in HIV-1 infection - the loss of these restraining functions, and/or the genetic variability of the virus due to immune escape that enhances the virulence, which then bring the collapse of the immune system. In some conditions the system will produce a Hopf bifurcation. We also simulate the theoretical warrant of the feasibility of the combined chemotherapy strategies for the HIV-1 infected patient. </td> <td style="text-align:left;"> 4569 </td> <td style="text-align:left;"> NA </td> <td style="text-align:left;"> NA </td> <td style="text-align:left;"> NA </td> <td style="text-align:left;"> no </td> <td style="text-align:left;"> no </td> </tr> <tr> <td style="text-align:left;"> 4568 </td> <td style="text-align:left;"> 2017 </td> <td style="text-align:left;"> NA </td> <td style="text-align:left;"> Bielaszewska, M; Kock, R; Friedrich, AW; von Eiff, C; Zimmerhackl, LB; Karch, H; Mellmann, A </td> <td style="text-align:left;"> 2007 </td> <td style="text-align:left;"> Shiga Toxin-Mediated Hemolytic Uremic Syndrome: Time to Change the Diagnostic Paradigm? </td> <td style="text-align:left;"> Background. Hemolytic uremic syndrome (HUS) is caused by enterohemorrhagic Escherichia coli (EHEC) which possess genes encoding Shiga toxin (stx), the major virulence factor, and adhesin intimin (eae). However, the frequency of stx-negative/eae-positive E. coli in stools of HUS patients and the clinical significance of such strains are unknown. Methodology/Principal Findings. Between 1996 and 2006, we sought stx-negative/eae-positive E. coli in stools of HUS patients using colony blot hybridization with the eae probe and compared the isolates to EHEC causing HUS. stx-negative/eae-positive E. coli were isolated as the only pathogens from stools of 43 (5.5%) of 787 HUS patients; additional 440 (55.9%) patients excreted EHEC. The majority (90.7%) of the stx-negative/eae-positive isolates belonged to serotypes O26: H11/NM (nonmotile), O103:H2/NM, O145:H28/NM, and O157:H7/NM, which were also the most frequent serotypes identified among EHEC. The stx-negative isolates shared non-stx virulence and fitness genes with EHEC of the corresponding serotypes and clustered with them into the same clonal complexes in multilocus sequence typing, demonstrating their close relatedness to EHEC. Conclusions/Significance. At the time of microbiological analysis, similar to 5% of HUS patients shed no longer the causative EHEC, but do excrete stx-negative derivatives of EHEC that lost stx during infection. In such patients, the EHEC etiology of HUS is missed using current methods detecting solely stx or Shiga toxin; this can hamper epidemiological investigations and lead to inappropriate clinical management. While maintaining the paradigm that HUS is triggered by Shiga toxin, our data demonstrate the necessity of considering genetic changes of the pathogen during infection to adapt appropriately diagnostic strategies. </td> <td style="text-align:left;"> 4570 </td> <td style="text-align:left;"> NA </td> <td style="text-align:left;"> NA </td> <td style="text-align:left;"> NA </td> <td style="text-align:left;"> no </td> <td style="text-align:left;"> no </td> </tr> <tr> <td style="text-align:left;"> 4569 </td> <td style="text-align:left;"> 2017 </td> <td style="text-align:left;"> NA </td> <td style="text-align:left;"> De Marco, EV; Tarantino, P; Rocca, FE; Provenzano, G; Civitelli, D; De Luca, V; Annesi, F; Carrideo, S; Candiano, ICC; Romeo, N; Nicoletti, G; Marconi, R; Novellino, F; Morelli, M; Quattrone, A; Annesi, G </td> <td style="text-align:left;"> 2008 </td> <td style="text-align:left;"> Alpha-synuclein promoter haplotypes and dementia in Parkinson's disease </td> <td style="text-align:left;"> Dementia is a common complication of Parkinson's disease (PD). It correlates significantly with the presence of cortical, limbic or nigral Lewy bodies, mainly constituted of alpha-synuclein. Mutations of the alpha-synuclein gene (SNCA) have been linked to rare familial forms of PD, while association studies on the promoter polymorphisms have given conflicting results in sporadic patients. We have performed a case control study to investigate whether genetic variability in the promoter of the alpha-synuclein gene could predispose to dementia in PD. A total of 114 demented patients and 114 non-demented patients with sporadic PD were included in the study. Six polymorphic loci (including the Rep1 microsatellite) in the promoter of the SNCA gene were examined. Each marker, taken individually, did not show association to dementia and no significant differences were observed in the inferred haplotype frequencies of demented and non-demented patients. Our data suggest the lack of involvement of the SNCA promoter in the pathogenesis of dementia in PD. Further studies in other populations are needed to confirm these results. (C) 2007 Wiley-Liss, Inc. </td> <td style="text-align:left;"> 4571 </td> <td style="text-align:left;"> NA </td> <td style="text-align:left;"> NA </td> <td style="text-align:left;"> NA </td> <td style="text-align:left;"> no </td> <td style="text-align:left;"> no </td> </tr> <tr> <td style="text-align:left;"> 4570 </td> <td style="text-align:left;"> 2017 </td> <td style="text-align:left;"> NA </td> <td style="text-align:left;"> Gampenrieder, SP; Hufnagl, C; Brechelmacher, S; Huemer, F; Hackl, H; Rinnerthaler, G; Romeder, F; Fuentes, CM; Morre, P; Hauser-Kronberger, C; Mlineritsch, B; Greil, R </td> <td style="text-align:left;"> 2017 </td> <td style="text-align:left;"> Endothelin-1 genetic polymorphism as predictive marker for bevacizumab in metastatic breast cancer </td> <td style="text-align:left;"> Biomarkers for bevacizumab efficacy in metastatic breast cancer (MBC) are of urgent need. The genetic variability of genes involved in angiogenesis could explain the interpatient variability of drug effects. For this biomarker study DNA was extracted from tumor blocks or blood samples of patients with human epidermal growth factor receptor 2 (HER2)-negative MBC treated with bevacizumab in combination with chemotherapy (bevacizumab cohort, 163 patients) or chemotherapy only (control cohort, 105 patients). We assessed the correlation of 10 single-nucleotide polymorphisms (SNPs) in genes modulating angiogenesis (vascular endothelial growth factor-A (VEGF-A), VEGF receptor 1 (VEGFR-1), serine threonine kinase 39 (STK39)) or hypertension (endothelin-1 and uromodulin) with outcome and toxicity. In the bevacizumab cohort, the SNP rs5370-TT in endothelin-1 (EDN1) showed a significantly shorter median overall survival (OS, 6.3 vs 21.3 months; hazard ratio (HR) 2.89, 95% confidence interval (CI) 1.34 - 6.26; log-rank P = 0.0069) and a trend toward worse median progression-free survival (3.5 vs 7.9 months; HR 2.05, 95% CI 0.96 - 4.39; log-rank P = 0.065) compared with the alternate genotypes combined. Similarly, patients harboring the VEGF-936 (rs3025039) TT alleles showed a significantly shorter median OS than patients with VEGF-936 CC or CT (14.9 vs 21.3 months; HR 2.37, 95% CI 1.09 - 5.13; P = 0.0286). In multivariate analysis including important clinical parameters like disease-free survival (DFS), adjuvant chemotherapy, ECOG (Eastern Cooperative Oncology Group) performance score, histologic subtype, grade, hormone receptor status, visceral metastases and treatment line, only the association of rs5370 (EDN1) with OS was still statistically significant (P = 0.012). In the control cohort, no association of the EDN1 genotype with outcome was seen, suggesting a predictive value for bevacizumab. In conclusion, the SNP rs5370 in endothelin-1 could help identifying patients who unlikely gain any benefit from bevacizumab. </td> <td style="text-align:left;"> 4572 </td> <td style="text-align:left;"> NA </td> <td style="text-align:left;"> NA </td> <td style="text-align:left;"> NA </td> <td style="text-align:left;"> no </td> <td style="text-align:left;"> no </td> </tr> <tr> <td style="text-align:left;"> 4571 </td> <td style="text-align:left;"> 2017 </td> <td style="text-align:left;"> NA </td> <td style="text-align:left;"> Volz, EM; Romero-Severson, E; Leitner, T </td> <td style="text-align:left;"> 2017 </td> <td style="text-align:left;"> Phylodynamic Inference across Epidemic Scales </td> <td style="text-align:left;"> Within-host genetic diversity and large transmission bottlenecks confound phylodynamic inference of epidemiological dynamics. Conventional phylodynamic approaches assume that nodes in a time-scaled pathogen phylogeny correspond closely to the time of transmission between hosts that are ancestral to the sample. However, when hosts harbor diverse pathogen populations, node times can substantially pre-date infection times. Imperfect bottlenecks can cause lineages sampled in different individuals to coalesce in unexpected patterns. To address realistic violations of standard phylodynamic assumptions we developed a new inference approach based on a multi-scale coalescent model, accounting for nonlinear epidemiological dynamics, heterogeneous sampling through time, non-negligible genetic diversity of pathogens within hosts, and imperfect transmission bottlenecks. We apply this method to HIV-1 and Ebola virus (EBOV) outbreak sequence data, illustrating how and when conventional phylodynamic inference may give misleading results. Within-host diversity of HIV-1 causes substantial upwards bias in the number of infected hosts using conventional coalescent models, but estimates using the multi-scale model have greater consistency with reported number of diagnoses through time. In contrast, we find that within-host diversity of EBOV has little influence on estimated numbers of infected hosts or reproduction numbers, and estimates are highly consistent with the reported number of diagnoses through time. The multi-scale coalescent also enables estimation of within-host effective population size using single sequences from a random sample of patients. We find within-host population genetic diversity of HIV-1 p17 to be 2N mu = 0.012 (95% CI 0.0066-0.023), which is lower than estimates based on HIV envelope serial sequencing of individual patients. </td> <td style="text-align:left;"> 4573 </td> <td style="text-align:left;"> NA </td> <td style="text-align:left;"> NA </td> <td style="text-align:left;"> NA </td> <td style="text-align:left;"> no </td> <td style="text-align:left;"> no </td> </tr> <tr> <td style="text-align:left;"> 4572 </td> <td style="text-align:left;"> 2017 </td> <td style="text-align:left;"> NA </td> <td style="text-align:left;"> Arenas, M; Lorenzo-Redondo, R; Lopez-Galindez, C </td> <td style="text-align:left;"> 2016 </td> <td style="text-align:left;"> Influence of mutation and recombination on HIV-1 in vitro fitness recovery </td> <td style="text-align:left;"> The understanding of the evolutionary processes underlying HIV-1 fitness recovery is fundamental for HIV-1 pathogenesis, antiretroviral treatment and vaccine design. It is known that HIV-1 can present very high mutation and recombination rates, however the specific contribution of these evolutionary forces in the "in vitro" viral fitness recovery has not been simultaneously quantified. To this aim, we analyzed substitution, recombination and molecular adaptation rates in a variety of HIV-1 biological clones derived from a viral isolate after severe population bottlenecks and a number of large population cell culture passages. These clones presented an overall but uneven fitness gain, mean of 3-fold, respect to the initial passage values. We found a significant relationship between the fitness increase and the appearance and fixation of mutations. In addition, these fixed mutations presented molecular signatures of positive selection through the accumulation of non-synonymous substitutions. Interestingly, viral recombination correlated with fitness recovery in most of studied viral quasispecies. The genetic diversity generated by these evolutionary processes was positively correlated with the viral fitness. We conclude that HIV-1 fitness recovery can be derived from the genetic heterogeneity generated through both mutation and recombination, and under diversifying molecular adaptation. The findings also suggest nonrandom evolutionary pathways for in vitro fitness recovery. (C) 2015 Elsevier Inc. All rights reserved. </td> <td style="text-align:left;"> 4574 </td> <td style="text-align:left;"> NA </td> <td style="text-align:left;"> NA </td> <td style="text-align:left;"> NA </td> <td style="text-align:left;"> no </td> <td style="text-align:left;"> no </td> </tr> <tr> <td style="text-align:left;"> 4573 </td> <td style="text-align:left;"> 2017 </td> <td style="text-align:left;"> NA </td> <td style="text-align:left;"> Schliemann, C; Gerss, J; Wiebe, S; Mikesch, JH; Knoblauch, N; Sauer, T; Angenendt, L; Kewitz, T; Urban, M; Butterfass-Bahloul, T; Edemir, S; Vehring, K; Muller-Tidow, C; Berdel, WE; Krug, U </td> <td style="text-align:left;"> 2016 </td> <td style="text-align:left;"> A Phase I Dose Escalation Study of the Triple Angiokinase Inhibitor Nintedanib Combined with Low-Dose Cytarabine in Elderly Patients with Acute Myeloid Leukemia </td> <td style="text-align:left;"> Nintedanib (BIBF 1120), a potent multikinase inhibitor of VEGFR-1/-2/-3, FGFR-1/-2/-3 and PDGFR-alpha/-beta, exerts growth inhibitory and pro-apoptotic effects in myeloid leukemic cells, especially when used in combination with cytarabine. This phase I study evaluated nintedanib in combination with low-dose cytarabine (LDAC) in elderly patients with untreated or relapsed/refractory acute myeloid leukemia (AML) ineligible for intensive chemotherapy in a 3+ 3 design. Nintedanib (dose levels 100, 150, and 200 mg orally twice daily) and LDAC (20 mg subcutaneous injection twice daily for 10 days) were administered in 28-day cycles. Dose-limiting toxicity (DLT) was defined as non-hematological severe adverse reaction CTC grade >= 4 with possible or definite relationship to nintedanib. Between April 2012 and October 2013, 13 patients (median age 73 [range: 62-86] years) were enrolled. One patient did not receive study medication and was replaced. Nine (69%) patients had relapsed or refractory disease and 6 (46%) patients had unfavorable cytogenetics. The most frequently reported treatment-related adverse events (AE) were gastrointestinal events. Twelve SAEs irrespective of relatedness were reported. Two SUSARs were observed, one fatal hypercalcemia and one fatal gastrointestinal infection. Two patients (17%) with relapsed AML achieved a complete remission (one CR, one CRi) and bone marrow blast reductions without fulfilling PR criteria were observed in 3 patients (25%). One-year overall survival was 33%. Nintedanib combined with LDAC shows an adequate safety profile and survival data are promising in a difficult-to-treat patient population. Continuation of this trial with a phase II recommended dose of 2 x 200 mg nintedanib in a randomized, placebo-controlled phase II study is planned. The trial is registered to EudraCT as 2011-001086-41. </td> <td style="text-align:left;"> 4575 </td> <td style="text-align:left;"> NA </td> <td style="text-align:left;"> NA </td> <td style="text-align:left;"> NA </td> <td style="text-align:left;"> no </td> <td style="text-align:left;"> no </td> </tr> <tr> <td style="text-align:left;"> 4574 </td> <td style="text-align:left;"> 2017 </td> <td style="text-align:left;"> NA </td> <td style="text-align:left;"> Amiri, A; Sabooteh, T; Shahsavar, F; Anbari, K; Pouremadi, F </td> <td style="text-align:left;"> 2017 </td> <td style="text-align:left;"> Mannose-Binding Lectin (MBL) gene polymorphisms in susceptibility to pulmonary tuberculosis among the Lur population of Lorestan Province of Iran </td> <td style="text-align:left;"> Objective: Tuberculosis (TB) is caused by infection of Mycobacterium tuberculosis. Host genetic variability is an important determinant of the risk of developing TB in humans. Although the association between MBL polymorphisms and TB has been studied in various populations, the results are controversial. The aim of this study was to investigate mannose-binding lectin (MBL) gene polymorphisms with susceptibility to pulmonary tuberculosis (PTB) in a Lur population of Iran. Methods: In this case-control study, four functional MBL gene polymorphisms (HL, XY, PQ and AB) were genotyped by using PCR Single Strand Conformation Polymorphism (SSCP) technique in a Lur population living in Lorestan Province, consisting of 100 patients with pulmonary tuberculosis (PTB) age and sex matched 100 healthy controls (HCs). Association analyses were performed with the SPSS 21 statistical software. Results: We found that MBL (HH) genotype polymorphism significantly was associated with increased susceptibility to TB (35% in patients vs. 22% in controls, P = 0.0417, OR = 1.909, % 95 CI = 1.020-3.573). Additionally, H allele showed a significant association with increased risk of TB (56.5% in patients vs. 46% in controls, P = 0.0357, OR = 1.525, % 95 CI = 1.028-2.262). Also, the distribution of L allele in patients was significantly lower frequency in TB patients compared to controls (43.5% vs. 54%, P = 0.0357, OR = 0.656, % 95 CI = 0.442-0.973). However, the allelic and genotypic frequencies of AB, XY and PQ polymorphisms were not significantly different between the patients and the controls. We couldn't detect any significant differences between haplotypes among TB patients and healthy controls. Conclusions: Our findings demonstrated that HH genotype and H allele may increase the susceptibility to pulmonary TB in the Lur population of Iran, although L allele may decrease the susceptibility to pulmonary TB in this population. We suggest that it is necessary to further more studies with larger sample size and other ethnic population. </td> <td style="text-align:left;"> 4576 </td> <td style="text-align:left;"> NA </td> <td style="text-align:left;"> NA </td> <td style="text-align:left;"> NA </td> <td style="text-align:left;"> no </td> <td style="text-align:left;"> no </td> </tr> <tr> <td style="text-align:left;"> 4575 </td> <td style="text-align:left;"> 2017 </td> <td style="text-align:left;"> NA </td> <td style="text-align:left;"> Goodreau, SM </td> <td style="text-align:left;"> 2006 </td> <td style="text-align:left;"> Assessing the effects of human mixing patterns on human immunodeficiency virus-1 interhost phylogenetics through social network simulation </td> <td style="text-align:left;"> Geneticists seeking to understand HIV-1 evolution among human hosts generally assume that hosts represent a panmictic population. Social science research demonstrates that the network patterns over which HIV-1 spreads are highly nonrandom, but the effect of these patterns Oil the genetic diversity of HIV-1 and other sexually transmitted pathogens has yet to be thoroughly examined. In addition, interhost phylogenetic models rarely account explicitly for genetic diversity arising from intrahost dynamics. This study outlines a graph-theoretic framework (exponential random graph modeling, ERGN4) for the estimation, inference, and simulation of dynamic partnership networks. This approach is used to simulate HIV-1 transmission and evolution under eight mixing patterns resembling those observed in empirical human populations, while simultaneously incorporating intrahost viral diversity. Models of parametric growth fit panmictic Populations well, yielding estimates of total viral effective population oil the order of the product of infected host size an intrahost effective viral population size. Populations exhibiting patterns of nonrandom mixing differ more widely in estimates of effective population size they yield, however, and reconstructions of population dynamics call exhibit severe errors if panmixis is assumed. I discuss implications for HIV-1 phylogenetics and the potential for ERGM to provide a general framework for addressing these issues. </td> <td style="text-align:left;"> 4577 </td> <td style="text-align:left;"> NA </td> <td style="text-align:left;"> NA </td> <td style="text-align:left;"> NA </td> <td style="text-align:left;"> no </td> <td style="text-align:left;"> no </td> </tr> <tr> <td style="text-align:left;"> 4576 </td> <td style="text-align:left;"> 2017 </td> <td style="text-align:left;"> NA </td> <td style="text-align:left;"> Sargsyan, O </td> <td style="text-align:left;"> 2014 </td> <td style="text-align:left;"> A Framework Including Recombination for Analyzing the Dynamics of Within-Host HIV Genetic Diversity </td> <td style="text-align:left;"> This paper presents a novel population genetic model and a computationally and statistically tractable framework for analyzing within-host HIV diversity based on serial samples of HIV DNA sequences. This model considers within-host HIV evolution during the chronic phase of infection and assumes that the HIV population is homogeneous at the beginning, corresponding to the time of seroconversion, and evolves according to the Wright-Fisher reproduction model with recombination and variable mutation rate across nucleotide sites. In addition, the population size and generation time vary over time as piecewise constant functions of time. Under this model I approximate the genealogical and mutational processes for serial samples of DNA sequences by a continuous coalescent-recombination process and an inhomogeneous Poisson process, respectively. Based on these derivations, an efficient algorithm is described for generating polymorphisms in serial samples of DNA sequences under the model including various substitution models. Extensions of the algorithm are also described for other demographic scenarios that can be more suitable for analyzing the dynamics of genetic diversity of other pathogens in vitro and in vivo. For the case of the infinite-sites model, I derive analytical formulas for the expected number of polymorphic sites in sample of DNA sequences, and apply the developed simulation and analytical methods to explore the fit of the model to HIV genetic diversity based on serial samples of HIV DNA sequences from 9 HIV-infected individuals. The results particularly show that the estimates of the ratio of recombination rate over mutation rate can vary over time between very high and low values, which can be considered as a consequence of the impact of selection forces. </td> <td style="text-align:left;"> 4578 </td> <td style="text-align:left;"> NA </td> <td style="text-align:left;"> NA </td> <td style="text-align:left;"> NA </td> <td style="text-align:left;"> no </td> <td style="text-align:left;"> no </td> </tr> <tr> <td style="text-align:left;"> 4577 </td> <td style="text-align:left;"> 2017 </td> <td style="text-align:left;"> NA </td> <td style="text-align:left;"> Luk, KC; Berg, MG; Naccache, SN; Kabre, B; Federman, S; Mbanya, D; Kaptue, L; Chiu, CY; Brennan, CA; Hackett, J </td> <td style="text-align:left;"> 2015 </td> <td style="text-align:left;"> Utility of Metagenomic Next-Generation Sequencing for Characterization of HIV and Human Pegivirus Diversity </td> <td style="text-align:left;"> Given the dynamic changes in HIV-1 complexity and diversity, next-generation sequencing (NGS) has the potential to revolutionize strategies for effective HIV global surveillance. In this study, we explore the utility of metagenomic NGS to characterize divergent strains of HIV-1 and to simultaneously screen for other co-infecting viruses. Thirty-five HIV-1-infected Cameroonian blood donor specimens with viral loads of >4.4 log(10) copies/ml were selected to include a diverse representation of group M strains. Random-primed NGS libraries, prepared from plasma specimens, resulted in greater than 90% genome coverage for 88% of specimens. Correct subtype designations based on NGS were concordant with sub-region PCR data in 31 of 35 (89%) cases. Complete genomes were assembled for 25 strains, including circulating recombinant forms with relatively limited data available (7 CRF11_cpx, 2 CRF13_cpx, 1 CRF18_cpx, and 1 CRF37_cpx), as well as 9 unique recombinant forms. HPgV (formerly designated GBV-C) co-infection was detected in 9 of 35 (25%) specimens, of which eight specimens yielded complete genomes. The recovered HPgV genomes formed a diverse cluster with genotype 1 sequences previously reported from Ghana, Uganda, and Japan. The extensive genome coverage obtained by NGS improved accuracy and confidence in phylogenetic classification of the HIV-1 strains present in the study population relative to conventional sub-region PCR. In addition, these data demonstrate the potential for metagenomic analysis to be used for routine characterization of HIV-1 and identification of other viral co-infections. </td> <td style="text-align:left;"> 4579 </td> <td style="text-align:left;"> NA </td> <td style="text-align:left;"> NA </td> <td style="text-align:left;"> NA </td> <td style="text-align:left;"> no </td> <td style="text-align:left;"> no </td> </tr> <tr> <td style="text-align:left;"> 4578 </td> <td style="text-align:left;"> 2017 </td> <td style="text-align:left;"> NA </td> <td style="text-align:left;"> Clark, TG; Mallard, K; Coll, F; Preston, M; Assefa, S; Harris, D; Ogwang, S; Mumbowa, F; Kirenga, B; O'Sullivan, DM; Okwera, A; Eisenach, KD; Joloba, M; Bentley, SD; Ellner, JJ; Parkhill, J; Jones-Lopez, EC; McNerney, R </td> <td style="text-align:left;"> 2013 </td> <td style="text-align:left;"> Elucidating Emergence and Transmission of Multidrug-Resistant Tuberculosis in Treatment Experienced Patients by Whole Genome Sequencing </td> <td style="text-align:left;"> Background: Understanding the emergence and spread of multidrug-resistant tuberculosis (MDR-TB) is crucial for its control. MDR-TB in previously treated patients is generally attributed to the selection of drug resistant mutants during inadequate therapy rather than transmission of a resistant strain. Traditional genotyping methods are not sufficient to distinguish strains in populations with a high burden of tuberculosis and it has previously been difficult to assess the degree of transmission in these settings. We have used whole genome analysis to investigate M. tuberculosis strains isolated from treatment experienced patients with MDR-TB in Uganda over a period of four years. Methods and Findings: We used high throughput genome sequencing technology to investigate small polymorphisms and large deletions in 51 Mycobacterium tuberculosis samples from 41 treatment-experienced TB patients attending a TB referral and treatment clinic in Kampala. This was a convenience sample representing 69% of MDR-TB cases identified over the four year period. Low polymorphism was observed in longitudinal samples from individual patients (2-15 SNPs). Clusters of samples with less than 50 SNPs variation were examined. Three clusters comprising a total of 8 patients were found with almost identical genetic profiles, including mutations predictive for resistance to rifampicin and isoniazid, suggesting transmission of MDR-TB. Two patients with previous drug susceptible disease were found to have acquired MDR strains, one of which shared its genotype with an isolate from another patient in the cohort. Conclusions: Whole genome sequence analysis identified MDR-TB strains that were shared by more than one patient. The transmission of multidrug-resistant disease in this cohort of retreatment patients emphasises the importance of early detection and need for infection control. Consideration should be given to rapid testing for drug resistance in patients undergoing treatment to monitor the emergence of resistance and permit early intervention to avoid onward transmission. </td> <td style="text-align:left;"> 4580 </td> <td style="text-align:left;"> NA </td> <td style="text-align:left;"> NA </td> <td style="text-align:left;"> NA </td> <td style="text-align:left;"> no </td> <td style="text-align:left;"> no </td> </tr> <tr> <td style="text-align:left;"> 4579 </td> <td style="text-align:left;"> 2017 </td> <td style="text-align:left;"> NA </td> <td style="text-align:left;"> Balding, J; Healy, CM; Livingstone, WJ; White, B; Mynett-Johnson, L; Cafferkey, M; Smith, OP </td> <td style="text-align:left;"> 2003 </td> <td style="text-align:left;"> Genomic polymorphic profiles in an Irish population with meningococcaemia: is it possible to predict severity and outcome of disease? </td> <td style="text-align:left;"> Patients with meningococcal disease have increased plasma levels of proinflammatory cytokines IL-6, IL-1beta, and TNF-alpha, with higher levels associated with fatal outcome. This study investigated whether polymorphisms in genes encoding these cytokines, and in those encoding anti-inflammatory IL-10 and IL-1Ra, are associated with the outcome in patients with meningococcal disease. Seven polymorphisms were genotyped in 183 meningococcal disease patients and 389 controls. The IL-6 - 174 G/G and IL-10 - 1082 A/A genotypes were more frequent in nonsurvivors compared with survivors (P = 0.023 IL-6, 0.25 IL-10), and in patients with severe disease compared to those with mild disease (P = 0.037 IL-6, 0.0078 IL-10). An association was also found between meningococcal disease and the IL-1RN VNTR polymorphism, but no association was observed with the LTA + 252, TNF - 308, IL-10 - 592, or IL-1B + 3953 polymorphisms. We conclude that genetic variability in the IL-6, IL-10, and IL-1RN genes is associated with a poor outcome in meningococcal disease. </td> <td style="text-align:left;"> 4581 </td> <td style="text-align:left;"> NA </td> <td style="text-align:left;"> NA </td> <td style="text-align:left;"> NA </td> <td style="text-align:left;"> no </td> <td style="text-align:left;"> no </td> </tr> <tr> <td style="text-align:left;"> 4580 </td> <td style="text-align:left;"> 2017 </td> <td style="text-align:left;"> NA </td> <td style="text-align:left;"> Hunt, GM; Johnson, D; Tiemessen, CT </td> <td style="text-align:left;"> 2001 </td> <td style="text-align:left;"> Characterisation of the long terminal repeat regions of south african human immunodeficiency virus type 1 isolates </td> <td style="text-align:left;"> The 5' long terminal repeat (LTR) region of the integrated proviral human immunodeficiency virus type 1 (HIV-1) template encodes cis-acting sequences for cellular proteins that are responsible for initiating viral transcription. The objective of this study was to analyse the LTR regions of isolates from a broad spectrum of South African HIV-1 infected individuals to (i) determine if sequence diversity was sufficient to allow for subtyping on the basis of this region, and (ii) to note any specific or unusual alterations in promoter binding motifs that may be common to this group of isolates or specific HIV-1 subtypes within this group. A total of 60 isolates were subtyped by heteroduplex mobility assay (HMA) and by phylogenetic analysis, using both the env and gag regions. Phylogenetic relatedness within the LTR region demonstrated the suitability of this region for use in HIV-1 subtype designation. The presence of additional NF-kappaB binding elements as well as altered USF binding sites were features common to subtype C HIV-1 isolates. Although the biological relevance of these alterations within the HIV-1 LTR with respect to viral replicative capacity and patient disease progression is unknown, there is strong support to suggest that in the presence of these features, there is increased gene transcription in subtype C isolates, and that this would be further increased in the presence of secondary infection. </td> <td style="text-align:left;"> 4582 </td> <td style="text-align:left;"> NA </td> <td style="text-align:left;"> NA </td> <td style="text-align:left;"> NA </td> <td style="text-align:left;"> no </td> <td style="text-align:left;"> no </td> </tr> <tr> <td style="text-align:left;"> 4581 </td> <td style="text-align:left;"> 2017 </td> <td style="text-align:left;"> NA </td> <td style="text-align:left;"> Pan, WH; Khayhan, K; Hagen, F; Wahyuningsih, R; Chakrabarti, A; Chowdhary, A; Ikeda, R; Taj-Aldeen, SJ; Khan, Z; Imran, D; Sjam, R; Sriburee, P; Liao, WQ; Chaicumpar, K; Ingviya, N; Mouton, JW; Curfs-Breuker, I; Boekhout, T; Meis, JF; Klaassen, CHW </td> <td style="text-align:left;"> 2012 </td> <td style="text-align:left;"> Resistance of Asian Cryptococcus neoformans Serotype A Is Confined to Few Microsatellite Genotypes </td> <td style="text-align:left;"> Background: Cryptococcus neoformans is a pathogenic yeast that causes cryptococcosis, a life threatening disease. The prevalence of cryptococcosis in Asia has been rising after the onset of the AIDS epidemic and estimates indicate more than 120 cases per 1,000 HIV-infected individuals per year. Almost all cryptococcal disease cases in both immunocompromised and immunocompetent patients in Asia are caused by C. neoformans var. grubii. Epidemiological studies on C. neoformans in pan-Asia have not been reported. The present work studies the genetic diversity of the fungus by microsatellite typing and susceptibility analysis of approximately 500 isolates from seven Asian countries. Methodology/Principal Findings: Genetic diversity of Asian isolates of C. neoformans was determined using microsatellite analysis with nine microsatellite markers. The analysis revealed eight microsatellite complexes (MCs) which showed different distributions among geographically defined populations. A correlation between MCs and HIV-status was observed. Microsatellite complex 2 was mainly associated with isolates from HIV-negative patients, whereas MC8 was associated with those from HIV-positive patients. Most isolates were susceptible to amphotericin B, itraconazole, voriconazole, posaconazole, and isavuconazole, but 17 (3.4%) and 10 (2%) were found to be resistant to 5-flucytosine and fluconazole, respectively. Importantly, five Indonesian isolates (approximately 12.5% from all Indonesian isolates investigated and 1% from the total studied isolates) were resistant to both antifungals. The majority of 5-flucytosine resistant isolates belonged to MC17. Conclusions: The findings showed a different distribution of genotypes of C. neoformans var. grubii isolates from various countries in Asia, as well as a correlation of the microsatellite genotypes with the original source of the strains and resistance to 5-flucytosine. </td> <td style="text-align:left;"> 4583 </td> <td style="text-align:left;"> NA </td> <td style="text-align:left;"> NA </td> <td style="text-align:left;"> NA </td> <td style="text-align:left;"> no </td> <td style="text-align:left;"> no </td> </tr> <tr> <td style="text-align:left;"> 4582 </td> <td style="text-align:left;"> 2017 </td> <td style="text-align:left;"> NA </td> <td style="text-align:left;"> Garcia-Martin, E; Ayuso, P; Luengo, A; Martinez, C; Agundez, JAG </td> <td style="text-align:left;"> 2008 </td> <td style="text-align:left;"> Genetic variability of histamine receptors in patients with Parkinson's disease </td> <td style="text-align:left;"> Background: Changes in the density and expression of histamine receptors (HRH) have been detected in Parkinson's disease (PD) patients, and HRH antagonists bring about improvements in motor and other symptoms, thus suggesting that HRH play a role in the clinical response of PD patients. This study is aimed to analyse polymorphic variations of HRH in patients with PD. Methods: Leukocytary DNA from 195 PD patients and a control group of 231 unrelated healthy individuals was studied for the nonsynonymous HRH1Leu449Ser and the promoter HRH2G1018A polymorphisms by using amplification-restriction analyses. Results: The HRH1Leu449Ser amino acid substitution was identified in two women with late-onset PD whereas it was not observed among healthy subjects. The HRH2G1018A polymorphism was observed with allele frequencies = 3.59 (95% CI = 1.74-5.44) and 5.0 (95% CI = 3.00-6.96) for patients with PD and healthy controls, respectively. These frequencies were independent of gender and age of onset of the disease. Multiple comparison analyses revealed that differences were not statistically significant. Conclusion: These results indicate that the polymorphisms analyzed are not a major risk factor for PD, although the HRH1Leu449Ser amino acid substitution might be related to PD. </td> <td style="text-align:left;"> 4584 </td> <td style="text-align:left;"> NA </td> <td style="text-align:left;"> NA </td> <td style="text-align:left;"> NA </td> <td style="text-align:left;"> no </td> <td style="text-align:left;"> no </td> </tr> <tr> <td style="text-align:left;"> 4583 </td> <td style="text-align:left;"> 2017 </td> <td style="text-align:left;"> NA </td> <td style="text-align:left;"> Roberts, RL; Gearry, RB; Barclay, ML; Kennedy, MA </td> <td style="text-align:left;"> 2007 </td> <td style="text-align:left;"> IMPDH1 promoter mutations in a patient exhibiting azathioprine resistance </td> <td style="text-align:left;"> Around 9% of inflammatory bowel disease (IBD) patients are resistant to azathioprine. We hypothesized that these patients may carry mutations within inosine-5 '-monophosphate dehydrogenase (IMPDH). To test this hypothesis, we screened 20 azathioprine-resistant patients for variations in the two IMPDH genes (IMPDH1 and IMPDH2) using dHPLC and DNA sequencing. A 9 bp insertion within the IMPDH1 P3 promoter was found in a patient exhibiting severe azathioprine resistance. The insertion is predicted to abolish a cAMP-response element (CRE) and was found to significantly reduce IMPDH1 P3 promoter activity in a luciferase reporter gene assay (P-value < 0.001). This in vitro assay suggests the variant promoter has altered function in vivo and consequently may have contributed to the thiopurine resistance observed in this patient. The absence of functional variants within the other patients indicates that if IMPDH genetic variability contributes to azathioprine resistance it does so infrequently. </td> <td style="text-align:left;"> 4585 </td> <td style="text-align:left;"> NA </td> <td style="text-align:left;"> NA </td> <td style="text-align:left;"> NA </td> <td style="text-align:left;"> no </td> <td style="text-align:left;"> no </td> </tr> <tr> <td style="text-align:left;"> 4584 </td> <td style="text-align:left;"> 2017 </td> <td style="text-align:left;"> NA </td> <td style="text-align:left;"> Yang, J; Xia, XY; He, X; Yang, SL; Ruan, YH; Zhao, QB; Wang, ZX; Shao, YM; Pan, XM </td> <td style="text-align:left;"> 2012 </td> <td style="text-align:left;"> A new pattern-based method for identifying recent HIV-1 infections from the viral sequence </td> <td style="text-align:left;"> The long asymptomatic stage of HIV infection poses a great challenge in identifying recent HIV infections. This is a bottleneck for monitoring HIV epidemic trends and evaluating the effectiveness of national AIDS control programs. Several serological methods were used to address this issue with some success. Because of high false-positive rates in patients with advanced infection or in ART treatment, UNAIDS still hesitates to recommend their use in routine surveillance. We developed a new pattern-based method for measuring intra-patient viral genetic diversity for determination of recent infections and estimation of population incidence. This method is verified by using several datasets (424 subtype B and 77 CRF07_BC samples) with clearly identified HIV-1 infection times. Pattern-based diversities of recent infections are significantly lower than that of chronic ones. With larger window periods varying from 200 to 350 days, a higher accuracy (90%-95%) not affected by advanced disease nor ART treatment could be obtained. The pattern-based genetic method is supplementary to the existing serology-based assays, both of which could be suitable for use in low and high epidemic regions, respectively. </td> <td style="text-align:left;"> 4586 </td> <td style="text-align:left;"> NA </td> <td style="text-align:left;"> NA </td> <td style="text-align:left;"> NA </td> <td style="text-align:left;"> no </td> <td style="text-align:left;"> no </td> </tr> <tr> <td style="text-align:left;"> 4585 </td> <td style="text-align:left;"> 2017 </td> <td style="text-align:left;"> NA </td> <td style="text-align:left;"> Bartona, JP; Kardar, M; Chakraborty, AK </td> <td style="text-align:left;"> 2015 </td> <td style="text-align:left;"> Scaling laws describe memories of host-pathogen riposte in the HIV population </td> <td style="text-align:left;"> The enormous genetic diversity and mutability of HIV has prevented effective control of this virus by natural immune responses or vaccination. Evolution of the circulating HIV population has thus occurred in response to diverse, ultimately ineffective, immune selection pressures that randomly change from host to host. We show that the interplay between the diversity of human immune responses and the ways that HIV mutates to evade them results in distinct sets of sequences defined by similar collectively coupled mutations. Scaling laws that relate these sets of sequences resemble those observed in linguistics and other branches of inquiry, and dynamics reminiscent of neural networks are observed. Like neural networks that store memories of past stimulation, the circulating HIV population stores memories of host-pathogen combat won by the virus. We describe an exactly solvable model that captures the main qualitative features of the sets of sequences and a simple mechanistic model for the origin of the observed scaling laws. Our results define collective mutational pathways used by HIV to evade human immune responses, which could guide vaccine design. </td> <td style="text-align:left;"> 4587 </td> <td style="text-align:left;"> NA </td> <td style="text-align:left;"> NA </td> <td style="text-align:left;"> NA </td> <td style="text-align:left;"> no </td> <td style="text-align:left;"> no </td> </tr> <tr> <td style="text-align:left;"> 4586 </td> <td style="text-align:left;"> 2017 </td> <td style="text-align:left;"> NA </td> <td style="text-align:left;"> Pandit, A; Sinha, S </td> <td style="text-align:left;"> 2011 </td> <td style="text-align:left;"> Differential Trends in the Codon Usage Patterns in HIV-1 Genes </td> <td style="text-align:left;"> Host-pathogen interactions underlie one of the most complex evolutionary phenomena resulting in continual adaptive genetic changes, where pathogens exploit the host's molecular resources for growth and survival, while hosts try to eliminate the pathogen. Deciphering the molecular basis of host-pathogen interactions is useful in understanding the factors governing pathogen evolution and disease propagation. In host-pathogen context, a balance between mutation, selection, and genetic drift is known to maintain codon bias in both organisms. Studies revealing determinants of the bias and its dynamics are central to the understanding of host-pathogen evolution. We considered the Human Immunodeficiency Virus (HIV) type 1 and its human host to search for evolutionary signatures in the viral genome. Positive selection is known to dominate intra-host evolution of HIV-1, whereas high genetic variability underlies the belief that neutral processes drive inter-host differences. In this study, we analyze the codon usage patterns of HIV-1 genomes across all subtypes and clades sequenced over a period of 23 years. We show presence of unique temporal correlations in the codon bias of three HIV-1 genes illustrating differential adaptation of the HIV-1 genes towards the host preferred codons. Our results point towards gene-specific translational selection to be an important force driving the evolution of HIV-1 at the population level. </td> <td style="text-align:left;"> 4588 </td> <td style="text-align:left;"> NA </td> <td style="text-align:left;"> NA </td> <td style="text-align:left;"> NA </td> <td style="text-align:left;"> no </td> <td style="text-align:left;"> no </td> </tr> <tr> <td style="text-align:left;"> 4587 </td> <td style="text-align:left;"> 2017 </td> <td style="text-align:left;"> NA </td> <td style="text-align:left;"> Tsipi, M; Tzetis, M; Kosma, K; Moschos, M; Braoudaki, M; Poulou, M; Kanavakis, E; Kitsiou-Tzeli, S </td> <td style="text-align:left;"> 2016 </td> <td style="text-align:left;"> Genomic screening of ABCA4 and array CGH analysis underline the genetic variability of Greek patients with inherited retinal diseases </td> <td style="text-align:left;"> Background: Retinal dystrophies are a clinically and genetically heterogeneous group of disorders which affect more than two million people worldwide. The present study focused on the role of the ABCA4 gene in the pathogenesis of hereditary retinal dystrophies (autosomal recessive Stargardt disease, autosomal recessive cone-rod dystrophy, and autosomal recessive retinitis pigmentosa) in patients of Greek origin. Materials and methods: Our cohort included 26 unrelated patients and their first degree healthy relatives. The ABCA4 mutation screening involved Sanger sequencing of all exons and flanking regions. Evaluation of novel variants included sequencing of control samples, family segregation analysis and characterization by in silico prediction tools. Twenty five patients were also screened for copy number variations by array-comparative genomic hybridization. Results: Excluding known disease-causing mutations and polymorphisms, two novel variants were identified in coding and non-coding regions of ABCA4. Array-CGH analysis revealed two partial deletions of USH2A and MYO3A in two patients with nonsyndromic autosomal recessive retinitis pigmentosa. Conclusions: The ABCA4 mutation spectrumin Greek patients differs from other populations. Bioinformatic tools, segregation analysis along with clinical data from the patients seemed to be crucial for the evaluation of genetic variants and particularly for the discrimination between causative and non-causative variants. (C) 2016 The Authors. Published by Elsevier B.V. </td> <td style="text-align:left;"> 4589 </td> <td style="text-align:left;"> NA </td> <td style="text-align:left;"> NA </td> <td style="text-align:left;"> NA </td> <td style="text-align:left;"> no </td> <td style="text-align:left;"> no </td> </tr> <tr> <td style="text-align:left;"> 4588 </td> <td style="text-align:left;"> 2017 </td> <td style="text-align:left;"> NA </td> <td style="text-align:left;"> Divella, R; Mazzocca, A; Gadaleta, C; Simone, G; Paradiso, A; Quaranta, M; Daniele, A </td> <td style="text-align:left;"> 2012 </td> <td style="text-align:left;"> Influence of Plasminogen Activator Inhibitor-1 (SERPINE1) 4G/5G Polymorphism on Circulating SERPINE-1 Antigen Expression in HCC Associated with Viral Infection </td> <td style="text-align:left;"> Hepatocarcinogenesis is heavily influenced by chronic hepatitis B (HBV) and C (HCV) infection. Elevated levels of plasminogen activator inhibitor-1 (SERPINE1/PAI-1) have been reported in patients with hepatocellular carcinoma (HCC) associated with viral infection. The gene encoding SERPINE1 is highly polymorphic and the frequently associated 4/5 guanosine (4G/5G) polymorphism in the gene promoter may influence its expression. Here, we investigated the distribution of genotypes and the frequency of alleles of the 4G/5G polymorphism in patients with HCC, the influence of the 4G/5G polymorphism on plasma SERPINE1 levels and its association with viral infection. A total of 75 patients with HCC were enrolled: 32 (42.6%) were HBV+/HCV+, 11 (14.6%) were only HCV+, and 32 (42.6%) were negative for both viruses. A control group of healthy donors was also enrolled (n=50). SERPINE1 plasma concentrations were determined by ELISA and the detection of the promoter 4G/5G polymorphism was performed by an allele-specific PCR analysis. We found that the frequency of both the 4G/4G genotype (p=0.02) and the 4G allele (p=0.006) were significantly higher in patients with HCC compared to the control group, and particularly higher in patients with HCC co-infected with HBV+/HCV+ than in those with no viral infection. We also found that patients with the 4G/4G genotype had significantly higher plasma SERPINE1 protein levels when compared with patients with the 4G/5G or 5G/5G genotype (p<0.001). Differences in frequency of 4G allele and genetic variability of 4G/5G SERPINE1 polymorphism with a higher level of SERPINE1 protein in patients with HCC with HBV+/HCV+ than those without infection, suggest the presence of two distinct pathogenic mechanisms in hepatocarcinogenesis, depending on the etiology. </td> <td style="text-align:left;"> 4590 </td> <td style="text-align:left;"> NA </td> <td style="text-align:left;"> NA </td> <td style="text-align:left;"> NA </td> <td style="text-align:left;"> no </td> <td style="text-align:left;"> no </td> </tr> <tr> <td style="text-align:left;"> 4589 </td> <td style="text-align:left;"> 2017 </td> <td style="text-align:left;"> NA </td> <td style="text-align:left;"> Jefferies, E; Baker, SS; Doran, M; Ralph, MAL </td> <td style="text-align:left;"> 2007 </td> <td style="text-align:left;"> Refractory effects in stroke aphasia: A consequence of poor semantic control </td> <td style="text-align:left;"> This study examined the full range of effects associated with "semantic access impairment" - namely, refractory variables (semantic relatedness, speed of presentation and item repetition), inconsistency, the absence of frequency effects and facilitation by cues - in a series of stroke patients with multimodal semantically impairment. By investigating all of these factors in a group of patients who were not specifically selected to show access" effects, we were able to establish (1) whether this pattern is a common consequence of infarcts that produce semantic impairment and (2) if these symptoms co-occur. All of the patients showed effects of cueing and an absence of frequency effects in comprehension. Patients whose brain damage included the left inferior prefrontal cortex (LIPC) also showed marked effects of refractory variables; in contrast, two patients with temporal-parietal but not frontal lesions were less sensitive to these variables. Parallel results were obtained for cyclical naming and word-picture matching tasks suggesting that the LIPC plays a role in semantic selection as well as lexical retrieval. Rapid presentation and item repetition is likely to have increased the selection demands in both of these tasks in a similar fashion. Unlike patients with classical "semantic access impairment", our semantically impaired stroke patients showed significant test-retest consistency, indicating that their difficulties did not result from an unpredictable failure of semantic access-instead, their deficits were interpreted as arising from failures of semantic control. (c) 2006 Elsevier Ltd. All rights reserved. </td> <td style="text-align:left;"> 4591 </td> <td style="text-align:left;"> NA </td> <td style="text-align:left;"> NA </td> <td style="text-align:left;"> NA </td> <td style="text-align:left;"> no </td> <td style="text-align:left;"> no </td> </tr> <tr> <td style="text-align:left;"> 4590 </td> <td style="text-align:left;"> 2017 </td> <td style="text-align:left;"> NA </td> <td style="text-align:left;"> Brumme, ZL; John, M; Carlson, JM; Brumme, CJ; Chan, D; Brockman, MA; Swenson, LC; Tao, I; Szeto, S; Rosato, P; Sela, J; Kadie, CM; Frahm, N; Brander, C; Haas, DW; Riddler, SA; Haubrich, R; Walker, BD; Harrigan, PR; Heckerman, D; Mallal, S </td> <td style="text-align:left;"> 2009 </td> <td style="text-align:left;"> HLA-Associated Immune Escape Pathways in HIV-1 Subtype B Gag, Pol and Nef Proteins </td> <td style="text-align:left;"> Background: Despite the extensive genetic diversity of HIV-1, viral evolution in response to immune selective pressures follows broadly predictable mutational patterns. Sites and pathways of Human Leukocyte-Antigen (HLA)-associated polymorphisms in HIV-1 have been identified through the analysis of population-level data, but the full extent of immune escape pathways remains incompletely characterized. Here, in the largest analysis of HIV-1 subtype B sequences undertaken to date, we identify HLA-associated polymorphisms in the three HIV-1 proteins most commonly considered in cellular-based vaccine strategies. Results are organized into protein-wide escape maps illustrating the sites and pathways of HLA-driven viral evolution. Methodology/Principal Findings: HLA-associated polymorphisms were identified in HIV-1 Gag, Pol and Nef in a multicenter cohort of >1500 chronically subtype-B infected, treatment-naive individuals from established cohorts in Canada, the USA and Western Australia. At q <= 0.05, 282 codons commonly mutating under HLA-associated immune pressures were identified in these three proteins. The greatest density of associations was observed in Nef (where close to 40% of codons exhibited a significant HLA association), followed by Gag then Pol (where similar to 15-20% of codons exhibited HLA associations), confirming the extensive impact of immune selection on HIV evolution and diversity. Analysis of HIV codon covariation patterns identified over 2000 codon-codon interactions at q <= 0.05, illustrating the dense and complex networks of linked escape and secondary/compensatory mutations. Conclusions/Significance: The immune escape maps and associated data are intended to serve as a user-friendly guide to the locations of common escape mutations and covarying codons in HIV-1 subtype B, and as a resource facilitating the systematic identification and classification of immune escape mutations. These resources should facilitate research in HIV epitope discovery and host-pathogen co-evolution, and are relevant to the continued search for an effective CTL-based AIDS vaccine. </td> <td style="text-align:left;"> 4592 </td> <td style="text-align:left;"> NA </td> <td style="text-align:left;"> NA </td> <td style="text-align:left;"> NA </td> <td style="text-align:left;"> no </td> <td style="text-align:left;"> no </td> </tr> <tr> <td style="text-align:left;"> 4591 </td> <td style="text-align:left;"> 2017 </td> <td style="text-align:left;"> NA </td> <td style="text-align:left;"> Khayhan, K; Hagen, F; Pan, WH; Simwami, S; Fisher, MC; Wahyuningsih, R; Chakrabarti, A; Chowdhary, A; Ikeda, R; Taj-Aldeen, SJ; Khan, Z; Ip, M; Imran, D; Sjam, R; Sriburee, P; Liao, WQ; Chaicumpar, K; Vuddhakul, V; Meyer, W; Trilles, L; van Iersel, LJJ; Meis, JF; Klaassen, CHW; Boekhout, T </td> <td style="text-align:left;"> 2013 </td> <td style="text-align:left;"> Geographically Structured Populations of Cryptococcus neoformans Variety grubii in Asia Correlate with HIV Status and Show a Clonal Population Structure </td> <td style="text-align:left;"> Cryptococcosis is an important fungal disease in Asia with an estimated 140,000 new infections annually the majority of which occurs in patients suffering from HIV/AIDS. Cryptococcus neoformans variety grubii (serotype A) is the major causative agent of this disease. In the present study, multilocus sequence typing (MLST) using the ISHAM MLST consensus scheme for the C. neoformans/C. gattii species complex was used to analyse nucleotide polymorphisms among 476 isolates of this pathogen obtained from 8 Asian countries. Population genetic analysis showed that the Asian C. neoformans var. grubii population shows limited genetic diversity and demonstrates a largely clonal mode of reproduction when compared with the global MLST dataset. HIV-status, sequence types and geography were found to be confounded. However, a correlation between sequence types and isolates from HIV-negative patients was observed among the Asian isolates. Observations of high gene flow between the Middle Eastern and the Southeastern Asian populations suggest that immigrant workers in the Middle East were originally infected in Southeastern Asia. </td> <td style="text-align:left;"> 4593 </td> <td style="text-align:left;"> NA </td> <td style="text-align:left;"> NA </td> <td style="text-align:left;"> NA </td> <td style="text-align:left;"> no </td> <td style="text-align:left;"> no </td> </tr> <tr> <td style="text-align:left;"> 4592 </td> <td style="text-align:left;"> 2017 </td> <td style="text-align:left;"> NA </td> <td style="text-align:left;"> Gervas-Arruga, J; Cebolla, JJ; de Blas, I; Roca, M; Pocovi, M; Giraldo, P </td> <td style="text-align:left;"> 2015 </td> <td style="text-align:left;"> The Influence of Genetic Variability and Proinflammatory Status on the Development of Bone Disease in Patients with Gaucher Disease </td> <td style="text-align:left;"> Gaucher disease, the most common lysosomal storage disorder, is caused by alpha-glucocerebrosidase deficiency. Bone complications are the major cause of morbidity in patients with type 1 Gaucher disease (GD1). Genetic components strongly influence bone remodelling. In addition, chronic inflammation produced by Gaucher cells induces the production of several cytokines, which leads to direct changes in the bone remodelling process and can also affect the process indirectly through other immune cells. In this study, we analysed the association between bone mineral density (BMD), bone marrow burden score, and relevant genetic polymorphisms related to bone metabolism, as well as profiles of proinflammatory cytokines in a GD1 cohort. This study included 83 patients distributed according to bone status. BMD was measured with DXA and broadband ultrasound attenuation; bone marrow involvement was evaluated using MRI. We also analysed 26 SNPs located in 14 genes related to bone metabolism. To assess proinflammatory status, we analysed IL-4, IL-6, IL-7, IL-10, IL-13, MIP-1 alpha, MIP-1 alpha, and TNF alpha in plasma samples from 71 control participants and GD1 patients. SNP genotype proportions and BMD differed significantly between ESRI c.453-397T>C and VDR c. 1024+283G>A variants. We also observed significant associations between GD1 genotypes and bone affectation. When patients were stratified by spleen status, we observed significant correlations between non-/splenectomized groups and Spanish MRI (S-MRI) score. Across genotype proportions of non-/splenectomized patients and S-MRI, we observed significant differences in ESRI c. 453-397T>C, VDR c.-8325988G>A, and TNFRSF11B c.9C>G polymorphisms. We observed different significant proinflammatory profiles between control participants, treatment-naive patients, and patients on enzyme replacement therapy (ERT); between non-/splenectomized patients (between untreated and ERT-treated patients) and among those with differing GBA genotypes. The data suggest that patients with GD1 have increased susceptibility to developing bone disease owing to the coexistence of genetic variants, and that genetic background in GD1 is fundamental to regulate the impact of proinflammatory status on the development of bone disease. </td> <td style="text-align:left;"> 4594 </td> <td style="text-align:left;"> NA </td> <td style="text-align:left;"> NA </td> <td style="text-align:left;"> NA </td> <td style="text-align:left;"> no </td> <td style="text-align:left;"> no </td> </tr> <tr> <td style="text-align:left;"> 4593 </td> <td style="text-align:left;"> 2017 </td> <td style="text-align:left;"> NA </td> <td style="text-align:left;"> Zeh, C; Inzaule, SC; Ondoa, P; Nafisa, LG; Kasembeli, A; Otieno, F; Vandenhoudt, H; Amornkul, PN; Mills, LA; Nkengasong, JN </td> <td style="text-align:left;"> 2016 </td> <td style="text-align:left;"> Molecular Epidemiology and Transmission Dynamics of Recent and Long-Term HIV-1 Infections in Rural Western Kenya </td> <td style="text-align:left;"> Objective To identify unique characteristics of recent versus established HIV infections and describe sexual transmission networks, we characterized circulating HIV-1 strains from two randomly selected populations of ART-naive participants in rural western Kenya. Methods Recent HIV infections were identified by the HIV-1 subtype B, E and D, immunoglobulin G capture immunoassay (IgG BED-CEIA) and BioRad avidity assays. Genotypic and phylogenetic analyses were performed on the pol gene to identify transmitted drug resistance (TDR) mutations, characterize HIV subtypes and potential transmission clusters. Factors associated with recent infection and clustering were assessed by logistic regression. Results Of the 320 specimens, 40 (12.5%) were concordantly identified by the two assays as recent infections. Factors independently associated with being recently infected were age <= 19 years (P = 0.001) and history of sexually transmitted infections (STIs) in the past six months (P = 0.004). HIV subtype distribution differed in recently versus chronically infected participants, with subtype A observed among 53% recent vs. 68% chronic infections (p = 0.04) and subtype D among 26% recent vs. 12% chronic infections (p = 0.012). Overall, the prevalence of primary drug resistance was 1.16%. Of the 258 sequences, 11.2% were in monophyletic clusters of between 2-4 individuals. In multivariate analysis factors associated with clustering included having recent HIV infection P = 0.043 and being from Gem region P = 0.002. Conclusions Recent HIV-1 infection was more frequent among 13-19 year olds compared with older age groups, underscoring the ongoing risk and susceptibility of younger persons for acquiring HIV infection. Our findings also provide evidence of sexual networks. The association of recent infections with clustering suggests that early infections may be contributing significant proportions of onward transmission highlighting the need for early diagnosis and treatment as prevention for ongoing prevention. Larger studies are needed to better understand the structure of these networks and subsequently implement and evaluate targeted interventions. </td> <td style="text-align:left;"> 4595 </td> <td style="text-align:left;"> NA </td> <td style="text-align:left;"> NA </td> <td style="text-align:left;"> NA </td> <td style="text-align:left;"> no </td> <td style="text-align:left;"> no </td> </tr> <tr> <td style="text-align:left;"> 4594 </td> <td style="text-align:left;"> 2017 </td> <td style="text-align:left;"> NA </td> <td style="text-align:left;"> Rangel, HR; Maes, M; Villalba, J; Sulbaran, Y; de Waard, JH; Bello, G; Pujol, FH </td> <td style="text-align:left;"> 2012 </td> <td style="text-align:left;"> Evidence of at Least Two Introductions of HIV-1 in the Amerindian Warao Population from Venezuela </td> <td style="text-align:left;"> Background: The Venezuelan Amerindians were, until recently, free of human immunodeficiency virus (HIV) infection. However, in 2007, HIV-1 infection was detected for the first time in the Warao Amerindian population living in the Eastern part of Venezuela, in the delta of the Orinoco river. The aim of this study was to analyze the genetic diversity of the HIV-1 circulating in this population. Methodology/Principal Findings: The pol genomic region was sequenced for 16 HIV-1 isolates and for some of them, sequences from env, vif and nef genomic regions were obtained. All HIV-1 isolates were classified as subtype B, with exception of one that was classified as subtype C. The 15 subtype B isolates exhibited a high degree of genetic similarity and formed a highly supported monophyletic cluster in each genomic region analyzed. Evolutionary analyses of the pol genomic region indicated that the date of the most recent common ancestor of the Waraos subtype B clade dates back to the late 1990s. Conclusions/Significance: At least two independent introductions of HIV-1 have occurred in the Warao Amerindians from Venezuela. The HIV-1 subtype B was successfully established and got disseminated in the community, while no evidence of local dissemination of the HIV-1 subtype C was detected in this study. These results warrant further surveys to evaluate the burden of this disease, which can be particularly devastating in this Amerindian population, with a high prevalence of tuberculosis, hepatitis B, among other infectious diseases, and with limited access to primary health care. </td> <td style="text-align:left;"> 4596 </td> <td style="text-align:left;"> NA </td> <td style="text-align:left;"> NA </td> <td style="text-align:left;"> NA </td> <td style="text-align:left;"> no </td> <td style="text-align:left;"> no </td> </tr> <tr> <td style="text-align:left;"> 4595 </td> <td style="text-align:left;"> 2017 </td> <td style="text-align:left;"> NA </td> <td style="text-align:left;"> Yan, M; Zhao, K; Du, J; Li, LZ; Wu, DL; Xu, SM; Zeng, XC; Wang, GJ; Yu, XF </td> <td style="text-align:left;"> 2014 </td> <td style="text-align:left;"> HIV-1 Diversity and Drug-Resistant Mutations in Infected Individuals in Changchun, China </td> <td style="text-align:left;"> Objectives: Human immunodeficiency virus type 1 (HIV-1) infection has been detected in all provinces of China. Although epidemiological and phylogenetic studies have been conducted in many regions, such analyses are lacking from Jilin province in northeastern China. Method: Epidemiological and phylogenetic analyses, as well as detection of drug-resistant mutations, were conducted on 57 HIV-1 infected patients from Changchun city identified and confirmed through annual surveillance by local Centers for Disease Control in Jilin province of northeastern China in 2012. Results: Sexual contact was determined to be the major pathway for HIV-1 transmission in Jilin, where hetero- and homosexual activities contributed almost equally. Phylogenetic analyses detected multiple subtypes of HIV-1 including subtype G circulating in Jilin, with multiple origins for each of them. Both subtype B and CRF01_AE were dominant, and evidence of subtype B transmitting between different high-risk groups was observed. Mutations in the viral protease at position 71 indicated the presence of a selective pressure. Several drug-resistant mutations were detected, although they were predicted with low-level resistance to antiviral treatments. Conclusions: Information from this study fills the gap in knowledge of HIV-1 transmission in Changchun city, Jilin province, China. By revealing the origin and evolutionary status of local HIV-1 strains, this work contributes to ongoing efforts in the control and prevention of AIDS. </td> <td style="text-align:left;"> 4597 </td> <td style="text-align:left;"> NA </td> <td style="text-align:left;"> NA </td> <td style="text-align:left;"> NA </td> <td style="text-align:left;"> no </td> <td style="text-align:left;"> no </td> </tr> <tr> <td style="text-align:left;"> 4596 </td> <td style="text-align:left;"> 2017 </td> <td style="text-align:left;"> NA </td> <td style="text-align:left;"> Li, XY; Xue, YL; Cheng, H; Lin, Y; Zhou, LM; Ning, Z; Wang, XQ; Yu, XL; Zhang, W; Shen, FW; Zheng, XH; Gai, J; Li, XS; Kang, LY; Nyambi, P; Wang, Y; Zhuang, MH; Pan, QC; Zhuang, X; Zhong, P </td> <td style="text-align:left;"> 2015 </td> <td style="text-align:left;"> HIV-1 Genetic Diversity and Its Impact on Baseline CD4+T Cells and Viral Loads among Recently Infected Men Who Have Sex with Men in Shanghai, China </td> <td style="text-align:left;"> The HIV-1 epidemic among men who have sex with men (MSM) has been spreading throughout China. Shanghai, a central gathering place for MSM, is facing a continuously increasing incidence of HIV-1 infection. In order to better understand the dynamics of HIV-1 diversity and its influence on patient's immune status at baseline on diagnosis, 1265 newly HIV-1-infected MSM collected from January 2009 to December 2013 in Shanghai were retrospectively analyzed for genetic subtyping, CD4+T cell counts, and viral loads. HIV-1 phylogenetic analysis revealed a broad viral diversity including CRF01_AE (62.13%), CRF07_BC (24.51%), subtype B (8.06%), CRF55_01B (3.24%), CER67_01B (0.95%), CRF68_01B (0.4%), CRF08_BC (0.08%) and CRF59_01B (0.08%). Twenty-four unique recombination forms (URFs) (1.98%) were identified as well. Bayesian inference analysis indicated that the introduction of CRF01_AE strain (1997) was earlier than CRF07_BC strain (2001) into MSM population in Shanghai based on the time of the most recent common ancestor (tMRCA). Three epidemic clusters and five sub-clusters were found in CRF01_AE. Significantly lower CD4+T cell count was found in individuals infected with CRF01_AE than in those infected with CRF07_BC infection (P<0.01), whereas viral load was significantly higher those infected with CRF01_AE than with CRF07_BC (P<0.01). In addition, the patients with >45 years of age were found to have lower CD4+T cell counts and higher viral loads than the patients with <25 years of age (P<0.05). This study reveals the presence of HIV-1 subtype diversity in Shanghai and its remarkable influence on clinical outcome. A real-time surveillance of HIV-1 viral diversity and phylodynamics of epidemic cluster, patient's baseline CD4+T cell count and viral load would be of great value to monitoring of disease progression, intervention for transmission, improvement of antiretroviral therapy strategy and design of vaccines. </td> <td style="text-align:left;"> 4598 </td> <td style="text-align:left;"> NA </td> <td style="text-align:left;"> NA </td> <td style="text-align:left;"> NA </td> <td style="text-align:left;"> no </td> <td style="text-align:left;"> no </td> </tr> <tr> <td style="text-align:left;"> 4597 </td> <td style="text-align:left;"> 2017 </td> <td style="text-align:left;"> NA </td> <td style="text-align:left;"> Koch, AS; Brites, D; Stucki, D; Evans, JC; Seldon, R; Heekes, A; Mulder, N; Nicol, M; Oni, T; Mizrahi, V; Warner, DF; Parkhill, J; Gagneux, S; Martin, DP; Wilkinson, RJ </td> <td style="text-align:left;"> 2017 </td> <td style="text-align:left;"> The Influence of HIV on the Evolution of Mycobacterium tuberculosis </td> <td style="text-align:left;"> HIV significantly affects the immunological environment during tuberculosis coinfection, and therefore may influence the selective landscape upon which M. tuberculosis evolves. To test this hypothesis whole genome sequences were determined for 169 South African M. tuberculosis strains from HIV-1 coinfected and uninfected individuals and analyzed using two Bayesian codon-model based selection analysis approaches: FUBAR which was used to detect persistent positive and negative selection (selection respectively favoring and disfavoring nonsynonymous substitutions); and MEDS which was used to detect episodic directional selection specifically favoring nonsynonymous substitutions within HIV-1 infected individuals. Among the 25,251 polymorphic codon sites analyzed, FUBAR revealed that 189-fold more were detectably evolving under persistent negative selection than were evolving under persistent positive selection. Three specific codon sites within the genes celA2b, katG, and cyp138 were identified by MEDS as displaying significant evidence of evolving under directional selection influenced by HIV-1 coinfection. All three genes encode proteins that may indirectly interact with human proteins that, in turn, interact functionally with HIV proteins. Unexpectedly, epitope encoding regions were enriched for sites displaying weak evidence of directional selection influenced by HIV-1. Although the low degree of genetic diversity observed in our M. tuberculosis data set means that these results should be interpreted carefully, the effects of HIV-1 on epitope evolution in M. tuberculosis may have implications for the design of M. tuberculosis vaccines that are intended for use in populations with high HIV-1 infection rates. </td> <td style="text-align:left;"> 4599 </td> <td style="text-align:left;"> NA </td> <td style="text-align:left;"> NA </td> <td style="text-align:left;"> NA </td> <td style="text-align:left;"> no </td> <td style="text-align:left;"> no </td> </tr> <tr> <td style="text-align:left;"> 4598 </td> <td style="text-align:left;"> 2017 </td> <td style="text-align:left;"> NA </td> <td style="text-align:left;"> Zanini, F; Brodin, J; Thebo, L; Lanz, C; Bratt, G; Albert, J; Neher, RA </td> <td style="text-align:left;"> 2015 </td> <td style="text-align:left;"> Population genomics of intrapatient HIV-1 evolution </td> <td style="text-align:left;"> Many microbial populations rapidly adapt to changing environments with multiple variants competing for survival. To quantify such complex evolutionary dynamics in vivo, time resolved and genome wide data including rare variants are essential. We performed whole-genome deep sequencing of HIV-1 populations in 9 untreated patients, with 6-12 longitudinal samples per patient spanning 5-8 years of infection. The data can be accessed and explored via an interactive web application. We show that patterns of minor diversity are reproducible between patients and mirror global HIV-1 diversity, suggesting a universal landscape of fitness costs that control diversity. Reversions towards the ancestral HIV-1 sequence are observed throughout infection and account for almost one third of all sequence changes. Reversion rates depend strongly on conservation. Frequent recombination limits linkage disequilibrium to about 100bp in most of the genome, but strong hitch-hiking due to short range linkage limits diversity. </td> <td style="text-align:left;"> 4600 </td> <td style="text-align:left;"> NA </td> <td style="text-align:left;"> NA </td> <td style="text-align:left;"> NA </td> <td style="text-align:left;"> no </td> <td style="text-align:left;"> no </td> </tr> <tr> <td style="text-align:left;"> 4599 </td> <td style="text-align:left;"> 2017 </td> <td style="text-align:left;"> NA </td> <td style="text-align:left;"> Perez-Losada, M; Jobes, DV; Sinangil, F; Crandall, KA; Arenas, M; Posada, D; Berman, PW </td> <td style="text-align:left;"> 2011 </td> <td style="text-align:left;"> Phylodynamics of HIV-1 from a Phase III AIDS Vaccine Trial in Bangkok, Thailand </td> <td style="text-align:left;"> Background: In 2003, a phase III placebo-controlled trial (VAX003) was completed in Bangkok, Thailand. Of the 2,546 individuals enrolled in the trial based on high risk for infection through injection drug use (IDU), we obtained clinical samples and HIV-1 sequence data (envelope glycoprotein gene gp120) from 215 individuals who became infected during the trial. Here, we used these data in combination with other publicly available gp120 sequences to perform a molecular surveillance and phylodynamic analysis of HIV-1 in Thailand. Methodology and Findings: Phylogenetic and population genetic estimators were used to assess HIV-1 gp120 diversity as a function of vaccination treatment, viral load (VL) and CD4(+) counts, to indentify transmission clusters and to investigate the timescale and demographics of HIV-1 in Thailand. Three HIV-1 subtypes were identified: CRF01_AE (85% of the infections), subtype B (13%) and CRF15_AE (2%). The Bangkok IDU cohort showed more gp120 diversity than other Asian IDU cohorts and similar diversity to that observed in sexually infected individuals. Moreover, significant differences (P < 0.02) in genetic diversity were observed in CRF01_AE IDU with different VL and CD4(+) counts. No phylogenetic structure was detected regarding any of the epidemiological and clinical factors tested, although high proportions (35% to 50%) of early infections fell into clusters, which suggests that transmission chains associated with acute infection play a key role on HIV-1 spread among IDU. CRF01_AE was estimated to have emerged in Thailand in 1984.5 (1983-1986), 3-6 years before the first recognition of symptomatic patients (1989). The relative genetic diversity of the HIV-1 population has remained high despite decreasing prevalence rates since the mid 1990s. Conclusions: Our study and recent epidemiological reports indicate that HIV-1 is still a major threat in Thailand and suggest that HIV awareness and prevention needs to be strengthened to avoid AIDS resurgence. </td> <td style="text-align:left;"> 4601 </td> <td style="text-align:left;"> NA </td> <td style="text-align:left;"> NA </td> <td style="text-align:left;"> NA </td> <td style="text-align:left;"> no </td> <td style="text-align:left;"> no </td> </tr> <tr> <td style="text-align:left;"> 4600 </td> <td style="text-align:left;"> 2017 </td> <td style="text-align:left;"> NA </td> <td style="text-align:left;"> Cembranelli, SBS; Souto, FO; Ferreira-Paim, K; Richinho, TT; Nunes, PL; Nascentes, GAN; Ferreira, TB; Correia, D; Lages-Silva, E </td> <td style="text-align:left;"> 2013 </td> <td style="text-align:left;"> First Evidence of Genetic Intraspecific Variability and Occurrence of Entamoeba gingivalis in HIV(+)/AIDS </td> <td style="text-align:left;"> Entamoeba gingivalis is considered an oral commensal but demonstrates a pathogenic potential associated with periodontal disease in immunocompromised individuals. Therefore, this study evaluated the occurrence, opportunistic conditions, and intraspecific genetic variability of E. gingivalis in HIV(+)/AIDS patients. Entamoeba gingivalis was studied using fresh examination (FE), culture, and PCR from bacterial plaque samples collected from 82 HIV(+)/AIDS patients. Genetic characterization of the lower ribosomal subunit of region 18S (18S-SSU rRNA) was conducted in 9 positive samples using low-stringency single specific primer PCR (LSSP-PCR) and sequencing analysis. Entamoeba gingivalis was detected in 63.4% (52/82) of the samples. No association was detected between the presence of E. gingivalis and the CD4(+) lymphocyte count (<= 200 cells/mm(3) (p = 0.912) or viral load (p = 0.429). The LSSP-PCR results helped group E. gingivalis populations into 2 polymorphic groups (68.3% similarity): group I, associated with 63.6% (7/11) of the samples, and group II, associated with 36.4% (4/11) of the samples, which shared 74% and 83.7% similarity and association with C and E isolates from HIV(-) individuals, respectively. Sequencing of 4 samples demonstrated 99% identity with the reference strain ATCC 30927 and also showed 2 divergent clusters, similar to those detected by LSSP-PCR. Opportunistic behavior of E. gingivalis was not detected, which may be related to the use of highly active antiretroviral therapy by all HIV(+)/AIDS patients. The high occurrence of E. gingivalis in these patients can be influenced by multifactorial components not directly related to the CD4(+) lymphocyte counts, such as cholesterol and the oral microbiota host, which could mask the potential opportunistic ability of E. gingivalis. The identification of the 18S SSU-rRNA polymorphism by LSSP-PCR and sequencing analysis provides the first evidence of genetic variability in E. gingivalis isolated from HIV patients. </td> <td style="text-align:left;"> 4602 </td> <td style="text-align:left;"> NA </td> <td style="text-align:left;"> NA </td> <td style="text-align:left;"> NA </td> <td style="text-align:left;"> no </td> <td style="text-align:left;"> no </td> </tr> <tr> <td style="text-align:left;"> 4601 </td> <td style="text-align:left;"> 2017 </td> <td style="text-align:left;"> NA </td> <td style="text-align:left;"> Switzer, WM; Tang, SH; Zheng, HQ; Shankar, A; Sprinkle, PS; Sullivan, V; Granade, TC; Heneine, W </td> <td style="text-align:left;"> 2016 </td> <td style="text-align:left;"> Dual Simian Foamy Virus/Human Immunodeficiency Virus Type 1 Infections in Persons from Cote d'Ivoire </td> <td style="text-align:left;"> Zoonotic transmission of simian retroviruses in West-Central Africa occurring in primate hunters has resulted in pandemic spread of human immunodeficiency viruses (HIVs) and human T-lymphotropic viruses (HTLVs). While simian foamy virus (SFV) and simian T-lymphotropic virus (STLV)-like infection were reported in healthy persons exposed to nonhuman primates (NHPs) in West-Central Africa, less is known about the distribution of these viruses in Western Africa and in hospitalized populations. We serologically screened for SFV and STLV infection using 1,529 specimens collected between 1985 and 1997 from Cote d'Ivoire patients with high HIV prevalence. PCR amplification and analysis of SFV, STLV, and HIV/SIV sequences from PBMCs was used to investigate possible simian origin of infection. We confirmed SFV antibodies in three persons (0.2%), two of whom were HIV1- infected. SFV polymerase (pol) and LTR sequences were detected in PBMC DNA available for one HIV-infected person. Phylogenetic comparisons with new SFV sequences from African guenons showed infection likely originated from a Chlorocebus sabaeus monkey endemic to Cote d'Ivoire. 4.6% of persons were HTLV seropositive and PCR testing of PBMCs from 15 HTLV seroreactive persons identified nine with HTLV-1 and one with HTLV-2 LTR sequences. Phylogenetic analysis showed that two persons had STLV-1-like infections, seven were HTLV-1, and one was an HTLV-2 infection. 310/858 (53%), 8/858 (0.93%), and 18/858 (2.1%) were HIV-1, HIV-2, and HIV-positive but undifferentiated by serology, respectively. No SIV sequences were found in persons with HIV-2 antibodies (n = 1) or with undifferentiated HIV results (n = 7). We document SFV, STLV-1-like, and dual SFV/HIV infection in Cote d'Ivoire expanding the geographic range for zoonotic simian retrovirus transmission to West Africa. These findings highlight the need to define the public health consequences of these infections. Studying dual HIV-1/SFV infections in immunocompromised populations may provide a new opportunity to better understand SFV pathogenicity and transmissibility in humans. </td> <td style="text-align:left;"> 4603 </td> <td style="text-align:left;"> NA </td> <td style="text-align:left;"> NA </td> <td style="text-align:left;"> NA </td> <td style="text-align:left;"> no </td> <td style="text-align:left;"> no </td> </tr> <tr> <td style="text-align:left;"> 4602 </td> <td style="text-align:left;"> 2017 </td> <td style="text-align:left;"> NA </td> <td style="text-align:left;"> Sousa, JD; Temudo, MP; Hewlett, BS; Camacho, RJ; Muller, V; Vandamme, AM </td> <td style="text-align:left;"> 2016 </td> <td style="text-align:left;"> Male Circumcision and the Epidemic Emergence of HIV-2 in West Africa </td> <td style="text-align:left;"> Background Epidemic HIV-2 (groups A and B) emerged in humans circa 1930-40. Its closest ancestors are SIVsmm infecting sooty mangabeys from southwestern Cote d'Ivoire. The earliest large-scale serological surveys of HIV-2 in West Africa (1985-91) show a patchy spread. Cote d'Ivoire and Guinea-Bissau had the highest prevalence rates by then, and phylogeographical analysis suggests they were the earliest epicenters. Wars and parenteral transmission have been hypothesized to have promoted HIV-2 spread. Male circumcision (MC) is known to correlate negatively with HIV-1 prevalence in Africa, but studies examining this issue for HIV-2 are lacking. Methods We reviewed published HIV-2 serosurveys for 30 cities of all West African countries and obtained credible estimates of real prevalence through Bayesian estimation. We estimated past MC rates of 218 West African ethnic groups, based on ethnographic literature and fieldwork. We collected demographic tables specifying the ethnic partition in cities. Uncertainty was incorporated by defining plausible ranges of parameters (e.g. timing of introduction, proportion circumcised). We generated 1,000 sets of past MC rates per city using Latin Hypercube Sampling with different parameter combinations, and explored the correlation between HIV-2 prevalence and estimated MC rate (both logit-transformed) in the 1,000 replicates. Results and Conclusions Our survey reveals that, in the early 20th century, MC was far less common and geographically more variable than nowadays. HIV-2 prevalence in 1985-91 and MC rates in 1950 were negatively correlated (Spearman rho = -0.546, IQR: -0.553--0.546, p <= 0.0021). Guinea-Bissau and Cote d'Ivoire cities had markedly lower MC rates. In addition, MC was uncommon in rural southwestern Cote d'Ivoire in 1930. The differential HIV-2 spread in West Africa correlates with different historical MC rates. We suggest HIV-2 only formed early substantial foci in cities with substantial uncircumcised populations. Lack of MC in rural areas exposed to bushmeat may have had a role in successful HIV-2 emergence. </td> <td style="text-align:left;"> 4604 </td> <td style="text-align:left;"> NA </td> <td style="text-align:left;"> NA </td> <td style="text-align:left;"> NA </td> <td style="text-align:left;"> no </td> <td style="text-align:left;"> no </td> </tr> <tr> <td style="text-align:left;"> 4603 </td> <td style="text-align:left;"> 2017 </td> <td style="text-align:left;"> NA </td> <td style="text-align:left;"> de Arellano, ER; Martin, C; Soriano, V; Alcami, J; Holguin, A </td> <td style="text-align:left;"> 2007 </td> <td style="text-align:left;"> Genetic analysis of the long terminal repeat (LTR) promoter region in HIV-1-infected individuals with different rates of disease progression </td> <td style="text-align:left;"> The long terminal repeat (LTR) region of HIV-1 promotes and modulates proviral transcription. LTR genetic variability might influence viral replication and disease progression. Proviral LTR sequences from 32 HIV-1-infected individuals showing different rates of disease progression were examined. Non-progressors (NP, n = 11) were individuals with high and stable CD4 counts and persistently low or undetectable plasma HIV-RNA. Slow progressors (SP, n = 6) were subjects with minimal CD4 decays over time and low plasma HIV-RNA. Typical progressors (TP, n = 15) were individuals with chronic infection showing CD4 counts repeatedly below 500 cells/mu l. The mutation frequency within distinct LTR functional regions involved in HIV-1 transcription were compared in these three groups of patients. No significant differences were observed in the mutation frequency in most LTR regulatory sites when comparing the three groups. However, changes in USF regulatory binding sites were more frequent in TP than in SP/NP, while changes in Sp1 binding sites were less common in the former. This is the first study examining the genetic variability of the HIV-1 LTR region in long-term non-progressors showing further divergent outcomes. </td> <td style="text-align:left;"> 4605 </td> <td style="text-align:left;"> NA </td> <td style="text-align:left;"> NA </td> <td style="text-align:left;"> NA </td> <td style="text-align:left;"> no </td> <td style="text-align:left;"> no </td> </tr> <tr> <td style="text-align:left;"> 4604 </td> <td style="text-align:left;"> 2017 </td> <td style="text-align:left;"> NA </td> <td style="text-align:left;"> Almeida, RR; Rosa, DS; Ribeiro, SP; Santana, VC; Kallas, EG; Sidney, J; Sette, A; Kalil, J; Cunha-Neto, E </td> <td style="text-align:left;"> 2012 </td> <td style="text-align:left;"> Broad and Cross-Clade CD4(+) T-Cell Responses Elicited by a DNA Vaccine Encoding Highly Conserved and Promiscuous HIV-1 M-Group Consensus Peptides </td> <td style="text-align:left;"> T-cell based vaccine approaches have emerged to counteract HIV-1/AIDS. Broad, polyfunctional and cytotoxic CD4(+) T-cell responses have been associated with control of HIV-1 replication, which supports the inclusion of CD4(+) T-cell epitopes in vaccines. A successful HIV-1 vaccine should also be designed to overcome viral genetic diversity and be able to confer immunity in a high proportion of immunized individuals from a diverse HLA-bearing population. In this study, we rationally designed a multiepitopic DNA vaccine in order to elicit broad and cross-clade CD4(+) T-cell responses against highly conserved and promiscuous peptides from the HIV-1 M-group consensus sequence. We identified 27 conserved, multiple HLA-DR-binding peptides in the HIV-1 M-group consensus sequences of Gag, Pol, Nef, Vif, Vpr, Rev and Vpu using the TEPITOPE algorithm. The peptides bound in vitro to an average of 12 out of the 17 tested HLA-DR molecules and also to several molecules such as HLA-DP, -DQ and murine IA(b) and IA(d). Sixteen out of the 27 peptides were recognized by PBMC from patients infected with different HIV-1 variants and 72% of such patients recognized at least 1 peptide. Immunization with a DNA vaccine (HIVBr27) encoding the identified peptides elicited IFN-gamma secretion against 11 out of the 27 peptides in BALB/c mice; CD4(+) and CD8(+) T-cell proliferation was observed against 8 and 6 peptides, respectively. HIVBr27 immunization elicited cross-clade T-cell responses against several HIV-1 peptide variants. Polyfunctional CD4(+) and CD8(+) T cells, able to simultaneously proliferate and produce IFN-gamma and TNF-alpha, were also observed. This vaccine concept may cope with HIV-1 genetic diversity as well as provide increased population coverage, which are desirable features for an efficacious strategy against HIV-1/AIDS. </td> <td style="text-align:left;"> 4606 </td> <td style="text-align:left;"> NA </td> <td style="text-align:left;"> NA </td> <td style="text-align:left;"> NA </td> <td style="text-align:left;"> no </td> <td style="text-align:left;"> no </td> </tr> <tr> <td style="text-align:left;"> 4605 </td> <td style="text-align:left;"> 2017 </td> <td style="text-align:left;"> NA </td> <td style="text-align:left;"> Laddha, NC; Dwivedi, M; Mansuri, MS; Singh, M; Patel, HH; Agarwal, N; Shah, AM; Begum, R </td> <td style="text-align:left;"> 2014 </td> <td style="text-align:left;"> Association of Neuropeptide Y (NPY), Interleukin-1B (IL1B) Genetic Variants and Correlation of IL1B Transcript Levels with Vitiligo Susceptibility </td> <td style="text-align:left;"> Background: Vitiligo is a depigmenting disorder resulting from loss of functional melanocytes in the skin. NPY plays an important role in induction of immune response by acting on a variety of immune cells. NPY synthesis and release is governed by IL1B. Moreover, genetic variability in IL1B is reported to be associated with elevated NPY levels. Objectives: Aim of the present study was to explore NPY promoter 2399T/C (rs16147) and exon2 +1128T/C (rs16139) polymorphisms as well as IL1B promoter 2511C/T (rs16944) polymorphism and to correlate IL1B transcript levels with vitiligo. Methods: PCR-RFLP method was used to genotype NPY -399T/C SNP in 454 patients and 1226 controls; +1128T/C SNP in 575 patients and 1279 controls and IL1B -511C/T SNP in 448 patients and 785 controls from Gujarat. IL1B transcript levels in blood were also assessed in 105 controls and 95 patients using real-time PCR. Results: Genotype and allele frequencies for NPY -399T/C, +1128T/C and IL1B -511C/T SNPs differed significantly (p<0.0001, p<0.0001; p = 0.0161, p = 0.0035 and p<0.0001, p<0.0001) between patients and controls. 'TC' haplotype containing minor alleles of NPY polymorphisms was significantly higher in patients and increased the risk of vitiligo by 2.3 fold (p<0.0001). Transcript levels of IL1B were significantly higher, in patients compared to controls (p = 0.0029), in patients with active than stable vitiligo (p = 0.015), also in female patients than male patients (p = 0.026). Genotype-phenotype correlation showed moderate association of IL1B -511C/T polymorphism with higher IL1B transcript levels. Trend analysis revealed significant difference between patients and controls for IL1B transcript levels with respect to different genotypes. Conclusion: Our results suggest that NPY -399T/C, +1128T/C and IL1B -511C/T polymorphisms are associated with vitiligo and IL1B -511C/T SNP influences its transcript levels leading to increased risk for vitiligo in Gujarat population. Upregulation of IL1B transcript in patients advocates its possible role in autoimmune pathogenesis of vitiligo. </td> <td style="text-align:left;"> 4607 </td> <td style="text-align:left;"> NA </td> <td style="text-align:left;"> NA </td> <td style="text-align:left;"> NA </td> <td style="text-align:left;"> no </td> <td style="text-align:left;"> no </td> </tr> <tr> <td style="text-align:left;"> 4606 </td> <td style="text-align:left;"> 2017 </td> <td style="text-align:left;"> NA </td> <td style="text-align:left;"> Orru, S; Manolakos, E; Orru, N; Kokotas, H; Mascia, V; Carcassi, C; Petersen, MB </td> <td style="text-align:left;"> 2012 </td> <td style="text-align:left;"> High frequency of the TARDBP p.Ala382Thr mutation in Sardinian patients with amyotrophic lateral sclerosis </td> <td style="text-align:left;"> Recently, rare mutations in the TARDBP gene have been identified in familial and sporadic amyotrophic lateral sclerosis (ALS) patients. The purpose of this study was to characterize the genetic variability of the TARDBP gene in a cohort of Sardinian ALS patients. The coding region of the gene was analyzed in 97 unrelated patients previously tested negative for superoxide dismutase (SOD1) mutations. The p.Ala382Thr (c.1144G>A) mutation was found in 30 patients (30.9%). The mutation was predominant in familial ALS patients (FALS) as it was represented in 24 of 30 FALS cases (80%) (p < 0.0003). Six cases were apparently sporadic (9% of sporadic ALS patients). No further mutation of TARDBP was found in our cohort of ALS patients. Patients carrying the mutation showed spinal site of onset in 24 cases (80%), an average age at onset of 54.7 +/- 11.1 years, not significantly different from patients not harboring TARDBP mutations (56.7 +/- 9.6) and a female: male gender ratio of 1:1.1. The haplotype analysis carried out using eight microsatellite markers flanking the gene showed a founder effect for this mutation. Finally, we estimated the age-specific penetrance of the TARDBP p.Ala382Thr mutation in an additional sample of 47 carriers (20 affected and 27 unaffected). The average penetrance to 70 years was 60% (95% confidence interval 41-79%). A trend toward a higher penetrance in males was observed. Even in the presence of a causal mutation, most of the ALS clinical heterogeneity, however, draws upon from a multifactorial context. </td> <td style="text-align:left;"> 4608 </td> <td style="text-align:left;"> NA </td> <td style="text-align:left;"> NA </td> <td style="text-align:left;"> NA </td> <td style="text-align:left;"> no </td> <td style="text-align:left;"> no </td> </tr> <tr> <td style="text-align:left;"> 4607 </td> <td style="text-align:left;"> 2017 </td> <td style="text-align:left;"> NA </td> <td style="text-align:left;"> Glasner, C; van Timmeren, MM; Stobernack, T; Omansen, TF; Raangs, EC; Rossen, JW; de Goffau, MC; Arends, JP; Kampinga, GA; Koedijk, DGAM; Neef, J; Buist, G; Tavakol, M; van Wamel, WJB; Rutgers, A; Stegeman, CA; Kallenberg, CGM; Heeringa, P; van Dijl, JM </td> <td style="text-align:left;"> 2015 </td> <td style="text-align:left;"> Low anti-staphylococcal IgG responses in granulomatosis with polyangiitis patients despite long-term Staphylococcus aureus exposure </td> <td style="text-align:left;"> Chronic nasal carriage of the bacterium Staphylococcus aureus in patients with the autoimmune disease granulomatosis with polyangiitis (GPA) is a risk factor for disease relapse. To date, it was neither known whether GPA patients show similar humoral immune responses to S. aureus as healthy carriers, nor whether specific S. aureus types are associated with GPA. Therefore, this study was aimed at assessing humoral immune responses of GPA patients against S. aureus antigens in relation to the genetic diversity of their nasal S. aureus isolates. A retrospective cohort study was conducted, including 85 GPA patients and 18 healthy controls (HC). Humoral immune responses against S. aureus were investigated by determining serum IgG levels against 59 S. aureus antigens. Unexpectedly, patient sera contained lower anti-staphylococcal IgG levels than sera from HC, regardless of the patients' treatment, while total IgG levels were similar or higher. Furthermore, 210 S. aureus isolates obtained from GPA patients were characterized by different typing approaches. This showed that the S. aureus population of GPA patients is highly diverse and mirrors the general S. aureus population. Our combined findings imply that GPA patients are less capable of mounting a potentially protective antibody response to S. aureus than healthy individuals. </td> <td style="text-align:left;"> 4609 </td> <td style="text-align:left;"> NA </td> <td style="text-align:left;"> NA </td> <td style="text-align:left;"> NA </td> <td style="text-align:left;"> no </td> <td style="text-align:left;"> no </td> </tr> <tr> <td style="text-align:left;"> 4608 </td> <td style="text-align:left;"> 2017 </td> <td style="text-align:left;"> NA </td> <td style="text-align:left;"> Castley, A; Sawleshwarkar, S; Varma, R; Herring, B; Thapa, K; Dwyer, D; Chibo, D; Nguyen, N; Hawke, K; Ratcliff, R; Garsia, R; Kelleher, A; Nolan, D </td> <td style="text-align:left;"> 2017 </td> <td style="text-align:left;"> A national study of the molecular epidemiology of HIV-1 in Australia 2005-2012 </td> <td style="text-align:left;"> Introduction Rates of new HIV-1 diagnoses are increasing in Australia, with evidence of an increasing proportion of non-B HIV-1 subtypes reflecting a growing impact of migration and travel. The present study aims to define HIV-1 subtype diversity patterns and investigate possible HIV-1 transmission networks within Australia. Methods The Australian Molecular Epidemiology Network (AMEN) HIV collaborating sites in Western Australia, South Australia, Victoria, Queensland and western Sydney (New South Wales), provided baseline HIV-1 partial pol sequence, age and gender information for 4,873 patients who had genotypes performed during 2005-2012. HIV-1 phylogenetic analyses utilised MEGA V6, with a stringent classification of transmission pairs or clusters (bootstrap >= 98%, genetic distance <= 1.5% from at least one other sequence in the cluster). Results HIV-1 subtype B represented 74.5% of the 4,873 sequences (WA 59%, SA 68.4%, w-Syd 73.8%, Vic 75.6%, Qld 82.1%), with similar proportion of transmission pairs and clusters found in the B and non-B cohorts (23% vs 24.5% of sequences, p = 0.3). Significantly more subtype B clusters were comprised of =3 sequences compared with non-B clusters (45.0% vs 24.0%, p = 0.021) and significantly more subtype B pairs and clusters were male-only (88% compared to 53% CRF01_AE and 17% subtype C clusters). Factors associated with being in a cluster of any size included; being sequenced in a more recent time period (p<0.001), being younger (p<0.001), being male (p = 0.023) and having a B subtype (p = 0.02). Being in a larger cluster (>3) was associated with being sequenced in a more recent time period (p = 0.05) and being male (p = 0.008). Conclusion This nationwide HIV-1 study of 4,873 patient sequences highlights the increased diversity of HIV-1 subtypes within the Australian epidemic, as well as differences in transmission networks associated with these HIV-1 subtypes. These findings provide epidemiological insights not readily available using standard surveillance methods and can inform the development of effective public health strategies in the current paradigm of HIV prevention in Australia. </td> <td style="text-align:left;"> 4610 </td> <td style="text-align:left;"> NA </td> <td style="text-align:left;"> NA </td> <td style="text-align:left;"> NA </td> <td style="text-align:left;"> no </td> <td style="text-align:left;"> no </td> </tr> <tr> <td style="text-align:left;"> 4609 </td> <td style="text-align:left;"> 2017 </td> <td style="text-align:left;"> NA </td> <td style="text-align:left;"> Casseb, J; Hong, MA; Gonsalez, C; Brigido, LF; Duarte, AJS; Michel-Hendry, R </td> <td style="text-align:left;"> 1998 </td> <td style="text-align:left;"> Two variants of HIV-1 B serotype are transmitted heterosexually in Sao Paulo, Brazil </td> <td style="text-align:left;"> HIV-1 variability may have an important impact on transmission and pathogenicity. Better characterization of the HIV epidemic in Brazil is necessary for the development of vaccine trials in this country. We analyzed sera from 108 HIV-1-infected volunteers from Sao Paulo City to determine serotype and reactivity for V3 motifs of HIV in this population, and the relationship to transmission mode. We concluded that the HIV-1 B serotype is frequent among heterosexually infected women, even in the absence of anal sex, and that two major V3 motifs, GPGR and GWGR, had similar prevalence among women (48% and 52%, respectively) and men (56% and 44%, respectively). We also observed an equal distribution of these strains regardless of their CD4+ T cell counts, clinical status, and mode of transmission. Even though V3 serology for HIV-1 subtyping is an inexpensive tool for use in developing countries, additional methods, such as heteroduplex mobility assay and direct DNA sequencing, should be included to determine HIV-1 genetic diversity. </td> <td style="text-align:left;"> 4611 </td> <td style="text-align:left;"> NA </td> <td style="text-align:left;"> NA </td> <td style="text-align:left;"> NA </td> <td style="text-align:left;"> no </td> <td style="text-align:left;"> no </td> </tr> <tr> <td style="text-align:left;"> 4610 </td> <td style="text-align:left;"> 2017 </td> <td style="text-align:left;"> NA </td> <td style="text-align:left;"> Pagan, I; Rojas, P; Ramos, JT; Holguin, A </td> <td style="text-align:left;"> 2016 </td> <td style="text-align:left;"> Clinical Determinants of HIV-1B Between-Host Evolution and their Association with Drug Resistance in Pediatric Patients </td> <td style="text-align:left;"> Understanding the factors that modulate the evolution of virus populations is essential to design efficient control strategies. Mathematical models predict that factors affecting viral within-host evolution may also determine that at the between-host level. Although HIV-1 within-host evolution has been associated with clinical factors used to monitor AIDS progression, such as patient age, CD4 cells count, viral load, and antiretroviral experience, little is known about the role of these clinical factors in determining between-host HIV-1 evolution. Moreover, whether the relative importance of such factors in HIV-1 evolution vary in adult and children patients, in which the course of infection is different, has seldom been analysed. To address these questions, HIV-1 subtype B (HIV-1B) pol sequences of 163 infected children and 450 adults of Madrid, Spain, were used to estimate genetic diversity, rates of synonymous and non-synonymous mutations, selection pressures and frequency of drug-resistance mutations (DRMs). The role and relative importance of patient age, % CD4, CD4/mm(3), viral load, and antiretroviral experience in HIV-1B evolution was analysed. In the pediatric HIV-1B population, three clinical factors were primary predictors of virus evolution: Higher HIV-1B genetic diversity was observed with increasing children age, decreasing CD4/mm(3) and upon antiretroviral experience. This was mostly due to higher rates of non-synonymous mutations, which were associated with higher frequency of DRMs. Using this data, we have also constructed a simple multivariate model explaining between 55% and 66% of the variance in HIV-1B evolutionary parameters in pediatric populations. On the other hand, the analysed clinical factors had little effect in adult-infecting HIV-1B evolution. These findings highlight the different evolutionary dynamics of HIV-1B in children and adults, and contribute to understand the factors shaping HIV-1B evolution and the appearance of drug-resistance mutation in pediatric patients. </td> <td style="text-align:left;"> 4612 </td> <td style="text-align:left;"> NA </td> <td style="text-align:left;"> NA </td> <td style="text-align:left;"> NA </td> <td style="text-align:left;"> no </td> <td style="text-align:left;"> no </td> </tr> <tr> <td style="text-align:left;"> 4611 </td> <td style="text-align:left;"> 2017 </td> <td style="text-align:left;"> NA </td> <td style="text-align:left;"> Lima, K; Leal, ED; Cavalcanti, AMS; Salustiano, DM; de Medeiros, LB; da Silva, SP; Lacerda, HR </td> <td style="text-align:left;"> 2016 </td> <td style="text-align:left;"> Epidemiological, Clinical and Antiretroviral Susceptibility Characterization of Human Immunodeficiency Virus Subtypes B and Non-B in Pernambuco, Northeast Brazil </td> <td style="text-align:left;"> Background HIV-1 diversity causes important differences in the virus' biological properties and their interactions with hosts, such as cell tropism, responses to antiretroviral therapy, drug-resistance, and disease progression. Objectives We evaluated the interrelationship of phylogenetic inference with epidemiological and laboratory data for HIV-1 isolates circulating in Pernambuco, Northeast Region-Brazil. Study design A total of 168 HIV-1 pol sequences were analysed, 64 were obtained from 2002-2003, and 104, from 2007-2009. Socio-demographic, clinical, and behavioural data were obtained from medical records. Laboratory testing enabled the determination of recent HIV-1 infections and co-infections with HBV, HCV, HTLV, or syphilis. Surveillance drug-resistance mutation analysis and antiretroviral susceptibility profiling were performed using HIV Drug-Resistance Database. Results HIV-1 non-B was associated with female, lower education, lower viral loads, and higher T cell counts mean. Frequencies of co-infection HIV-HBV, HIV-HCV, and HIV-syphilis were 27.8% (95% CI: 19.8-37.7), 1.04% (95% CI: 0.05-5.00) and 14.7% (95% CI: 8.6-23.0), respectively. Drug-resistant mutations rate was 2.98%(95% CI: 1.10-6.47). HIV-HBV subtype B co-infection was associated with men who have sex with men (MSM), higher education, higher viral loads and males. HIV-syphilis subtype non-B co-infection was associated with MSM status, lower T cell counts and males. Conclusions Data showed the importance of molecular characterisations of the HIV-1 epidemic and its relation with epidemiological and clinical characteristics of the population, as well as its association with other infectious diseases, so they can effort to improve preventive measures for health services and more information about the progress and effects of the epidemic in Northeastern-Brazil. </td> <td style="text-align:left;"> 4613 </td> <td style="text-align:left;"> NA </td> <td style="text-align:left;"> NA </td> <td style="text-align:left;"> NA </td> <td style="text-align:left;"> no </td> <td style="text-align:left;"> no </td> </tr> <tr> <td style="text-align:left;"> 4612 </td> <td style="text-align:left;"> 2017 </td> <td style="text-align:left;"> NA </td> <td style="text-align:left;"> Rubio, AE; Abraha, A; Carpenter, CA; Troyer, RM; Reyes-Rodriguez, AL; Salomon, H; Arts, EJ; Tebit, DM </td> <td style="text-align:left;"> 2014 </td> <td style="text-align:left;"> Similar Replicative Fitness Is Shared by the Subtype B and Unique BF Recombinant HIV-1 Isolates that Dominate the Epidemic in Argentina </td> <td style="text-align:left;"> The HIV-1 epidemic in South America is dominated by pure subtypes (mostly B and C) and more than 7 BF and BC recombinant forms. In Argentina, circulating recombinant forms (CRFs) comprised of subtypes B and F make up more than 50% of HIV infections. For this study, 28 HIV-1 primary isolates were obtained from patients in Buenos Aires, Argentina and initially classified into subtype B (n = 9, 32.1%), C (n = 1, 3.6%), and CRFs (n = 18, 64.3%) using partial pol and vpu-env sequences, which proved to be inconsistent and inaccurate for these phylogenetic analyses. Near full length genome sequences of these primary HIV-1 isolates revealed that nearly all intersubtype BF recombination sites were unique and countered previous "CRF'' B/F classifications. The majority of these Argentinean HIV-1 isolates were CCR5-using but 4 had a dual/mixed tropism as predicted by both phenotypic and genotypic assays. Comparison of the replicative fitness of these BF primary HIV-1 isolates to circulating B, F, and C HIV-1 using pairwise competitions in peripheral blood mononuclear cells (PBMCs) indicated a similarity in fitness of these BF recombinants to subtypes B and F HIV-1 (of the same co-receptor usage) whereas subtype C HIV-1 was significantly less fit than all as previously reported. These results suggest that the multitude of BF HIV-1 strains present within the Argentinean population do not appear to have gained replicative fitness following recent B and F recombination events. </td> <td style="text-align:left;"> 4614 </td> <td style="text-align:left;"> NA </td> <td style="text-align:left;"> NA </td> <td style="text-align:left;"> NA </td> <td style="text-align:left;"> no </td> <td style="text-align:left;"> no </td> </tr> <tr> <td style="text-align:left;"> 4613 </td> <td style="text-align:left;"> 2017 </td> <td style="text-align:left;"> NA </td> <td style="text-align:left;"> Bukowska-Osko, I; Pawelczyk, A; Perlejewski, K; Kubisa, N; Cortes, KC; Rosinska, M; Ploski, R; Fic, M; Kazmierczak, J; Popiel, M; Zabek, P; Horban, A; Radkowski, M; Laskus, T </td> <td style="text-align:left;"> 2015 </td> <td style="text-align:left;"> Genetic Variability of Hepatitis C Virus (HCV) 5' Untranslated Region in HIV/HCV Coinfected Patients Treated with Pegylated Interferon and Ribavirin </td> <td style="text-align:left;"> Association between hepatitis C virus (HCV) quasispecies and treatment outcome among patients with chronic hepatitis C has been the subject of many studies. However, these studies focused mainly on viral variable regions (E1 and E2) and usually did not include human immunodeficiency virus (HIV)-positive patients. The aim of the present study was to analyze heterogeneity of the 5'untranslated region (5'UTR) in HCV/HIV coinfected patients treated with interferon and ribavirin. The HCV 5'UTR was amplified from serum and peripheral blood mononuclear cells (PBMC) samples in 37 HCV/HIV coinfected patients treated for chronic hepatitis C. Samples were collected right before treatment, and at 2, 4, 6, 8, 12, 20, 24, 36, 44, 48, 60, and 72 weeks. Heterogeneity of the 5'UTR was analyzed by single strand conformational polymorphism (SSCP), cloning and sequencing. Sustained virological response (SVR) was achieved in 46% of analyzed HCV/HIV co-infected patients. Stable SSCP band pattern was observed in 22 patients (62.9%) and SVR rate among these patients was 23%. Decline in the number of bands and/or shift in band positions were found in 6 patients (17.1%), 5 (83%) of whom achieved SVR (p=0.009). A novel viral genotype was identified in all but one of these patients. In 5 of these 6 patients a new genotype was dominant. 5'UTR heterogeneity may correlate with interferon and ribavirin treatment outcome. In the analyzed group of HCV/HIV coinfected patients, viral quasispecies stability during treatment favored viral persistence, whereas decrease in the number of variants and/or emergence of new variants was associated with SVR. Among injection drug users (IDU) patients, a new genotype may become dominant during treatment, probably due to the presence of mixed infections with various strains, which have different susceptibility to treatment. </td> <td style="text-align:left;"> 4615 </td> <td style="text-align:left;"> NA </td> <td style="text-align:left;"> NA </td> <td style="text-align:left;"> NA </td> <td style="text-align:left;"> no </td> <td style="text-align:left;"> no </td> </tr> <tr> <td style="text-align:left;"> 4614 </td> <td style="text-align:left;"> 2017 </td> <td style="text-align:left;"> NA </td> <td style="text-align:left;"> de Pina-Araujo, IIM; Guimaraes, ML; Bello, G; Vicente, ACP; Morgado, MG </td> <td style="text-align:left;"> 2014 </td> <td style="text-align:left;"> Profile of the HIV Epidemic in Cape Verde: Molecular Epidemiology and Drug Resistance Mutations among HIV-1 and HIV-2 Infected Patients from Distinct Islands of the Archipelago </td> <td style="text-align:left;"> HIV-1 and HIV-2 have been detected in Cape Verde since 1987, but little is known regarding the genetic diversity of these viruses in this archipelago, located near the West African coast. In this study, we characterized the molecular epidemiology of HIV-1 and HIV-2 and described the occurrence of drug resistance mutations (DRM) among antiretroviral therapy naive (ARTn) patients and patients under treatment (ARTexp) from different Cape Verde islands. Blood samples, socio-demographic and clinical-laboratory data were obtained from 221 HIV-positive individuals during 2010-2011. Phylogenetic and bootscan analyses of the pol region (1300 bp) were performed for viral subtyping. HIV-1 and HIV-2 DRM were evaluated for ARTn and ARTexp patients using the Stanford HIV Database and HIV-GRADE e.V. Algorithm Homepage, respectively. Among the 221 patients (169 [76.5%] HIV-1, 43 [19.5%] HIV-2 and 9 [4.1%] HIV-1/HIV-2 co-infections), 67% were female. The median ages were 34 (IQR = 1-75) and 47 (IQR = 12-84) for HIV-1 and HIV-2, respectively. HIV-1 infections were due to subtypes G (36.6%), CRF02_AG (30.6%), F1 (9.7%), URFs (10.4%), B (5.2%), CRF05_DF (3.0%), C (2.2%), CRF06_cpx (0.7%), CRF25_cpx (0.7%) and CRF49_cpx (0.7%), whereas all HIV-2 infections belonged to group A. Transmitted DRM (TDRM) was observed in 3.4% (2/58) of ARTn HIV-1-infected patients (1.7% NRTI, 1.7% NNRTI), but not among those with HIV-2. Among ARTexp patients, DRM was observed in 47.8% (33/69) of HIV-1 (37.7% NRTI, 37.7% NNRTI, 7.4% PI, 33.3% for two classes) and 17.6% (3/17) of HIV-2-infections (17.6% NRTI, 11.8% PI, 11.8% both). This study indicates that Cape Verde has a complex and unique HIV-1 molecular epidemiological scenario dominated by HIV-1 subtypes G, CRF02_AG and F1 and HIV-2 subtype A. The occurrence of TDRM and the relatively high level of DRM among treated patients are of concern. Continuous monitoring of patients on ART, including genotyping, are public policies to be implemented. </td> <td style="text-align:left;"> 4616 </td> <td style="text-align:left;"> NA </td> <td style="text-align:left;"> NA </td> <td style="text-align:left;"> NA </td> <td style="text-align:left;"> no </td> <td style="text-align:left;"> no </td> </tr> <tr> <td style="text-align:left;"> 4615 </td> <td style="text-align:left;"> references </td> <td style="text-align:left;"> NA </td> <td style="text-align:left;"> Meyer-Lucht, Yvonne; Otten, Celine; Puettker, Thomas; et al. </td> <td style="text-align:left;"> 2010 </td> <td style="text-align:left;"> Variety matters: Adaptive genetic diversity and parasite load in two mouse opossums from the Brazilian Atlantic forest </td> <td style="text-align:left;"> The adaptive potential of a species to a changing environment and in disease defence is primarily based on genetic variation. Immune genes, such as genes of the major histocompatibility complex (MHC), may thereby be of particular importance. In marsupials, however, there is very little knowledge about natural levels and functional importance of MHC polymorphism, despite their key role in the mammalian evolution. In a previous study, we discovered remarkable differences in the MHC class II diversity between two species of mouse opossums (Gracilinanus microtarsus, Marmosops incanus) from the Brazilian Atlantic forest, which is one of the most endangered hotspots for biodiversity conservation. Since the main forces in generating MHC diversity are assumed to be pathogens, we investigated in this study gastrointestinal parasite burden and functional associations between the individual MHC constitution and parasite load. We tested two contrasting scenarios, which might explain differences in MHC diversity between species. We predicted that a species with low MHC diversity would either be under relaxed selection pressure by low parasite diversity (‘Evolutionary equilibrium’ scenario), or there was a recent loss in MHC diversity leading to a lack of resistance alleles and increased parasite burden (‘Unbalanced situation’ scenario). In both species it became apparent that the MHC class II is functionally important in defence against gastrointestinal helminths, which was shown here for the first time in marsupials. On the population level, parasite diversity did not markedly differ between the two host species. However, we did observe considerable differences in the individual parasite load (parasite prevalence and infection intensity): while M. incanus revealed low MHC DAB diversity and high parasite load, G. microtarsus showed a tenfold higher population wide MHC DAB diversity and lower parasite burden. These results support the second scenario of an unbalanced situation. </td> <td style="text-align:left;"> NA </td> <td style="text-align:left;"> no </td> <td style="text-align:left;"> reference check list </td> <td style="text-align:left;"> NA </td> <td style="text-align:left;"> yes </td> <td style="text-align:left;"> no </td> </tr> <tr> <td style="text-align:left;"> 4616 </td> <td style="text-align:left;"> 2020 </td> <td style="text-align:left;"> 44113 </td> <td style="text-align:left;"> Desvars-Larrive, A; Pascal, M; Gasqui, P; Cosson, JF; Benoit, E; Lattard, V; Crespin, L; Lorvelec, O; Pisanu, B; Teynie, A; Vayssier-Taussat, M; Bonnet, S; Marianneau, P; Lacote, S; Bourhy, P; Berny, P; Pavio, N; Le Poder, S; Gilot-Fromont, E; Jourdain, E; Hammed, A; Fourel, I; Chikh, F; Vourc'h, G </td> <td style="text-align:left;"> 2017 </td> <td style="text-align:left;"> Population genetics, community of parasites, and resistance to rodenticides in an urban brown rat (Rattus norvegicus) population </td> <td style="text-align:left;"> Brown rats are one of the most widespread urban species worldwide. Despite the nuisances they induce and their potential role as a zoonotic reservoir, knowledge on urban rat populations remains scarce. The main purpose of this study was to characterize an urban brown rat population from Chanteraines park (Hauts-de-Seine, France), with regards to haematology, population genetics, immunogenic diversity, resistance to anticoagulant rodenticides, and community of parasites. Haematological parameters were measured. Population genetics was investigated using 13 unlinked microsatellite loci. Immunogenic diversity was assessed for Mhc-Drb. Frequency of the Y139F mutation (conferring resistance to rodenticides) and two linked microsatellites were studied, concurrently with the presence of anticoagulant residues in the liver. Combination of microscopy and molecular methods were used to investigate the occurrence of 25 parasites. Statistical approaches were used to explore multiple parasite relationships and model parasite occurrence. Eighty-six rats were caught. The first haematological data for a wild urban R. norvegicus population was reported. Genetic results suggested high genetic diversity and connectivity between Chanteraines rats and surrounding population(s). We found a high prevalence (55.8%) of the mutation Y139F and presence of rodenticide residues in 47.7% of the sampled individuals. The parasite species richness was high (16). Seven potential zoonotic pathogens were identified, together with a surprisingly high diversity of Leptospira species (4). Chanteraines rat population is not closed, allowing gene flow and making eradication programs challenging, particularly because rodenticide resistance is highly prevalent. Parasitological results showed that co-infection is more a rule than an exception. Furthermore, the presence of several potential zoonotic pathogens, of which four Leptospira species, in this urban rat population raised its role in the maintenance and spread of these pathogens. Our findings should stimulate future discussions about the development of a long-term rat-control management program in Chanteraines urban park. </td> <td style="text-align:left;"> NA </td> <td style="text-align:left;"> maybe </td> <td style="text-align:left;"> ok gd and fitness measure group level? </td> <td style="text-align:left;"> No match between gd of multiple groups and parasites </td> <td style="text-align:left;"> no </td> <td style="text-align:left;"> no </td> </tr> <tr> <td style="text-align:left;"> 4617 </td> <td style="text-align:left;"> 2020 </td> <td style="text-align:left;"> 44039 </td> <td style="text-align:left;"> Heni, AC; Schmid, J; Rasche, A; Corman, VM; Drosten, C; Sommer, S </td> <td style="text-align:left;"> 2020 </td> <td style="text-align:left;"> Pathogen-associated selection on innate immunity genes (TLR4, TLR7) in a neotropical rodent in landscapes differing in anthropogenic disturbance </td> <td style="text-align:left;"> Toll-like receptors (TLRs) form part of the innate immune system and can recognize structurally conserved pathogen-associated molecular pattern (PAMP) molecules. Their functional importance in the resistance to pathogens has been documented in laboratory experimental settings and in humans. TLR diversity, however, has been rarely investigated in wildlife species. How the genetic diversity of TLRs is associated with various pathogens and how it is shaped by habitat disturbance are understudied. Therefore, we investigated the role of genetic diversity in the functionally important parts of TLR4 and TLR7 genes in resistance towards gastrointestinal nematodes andHepacivirusinfection. We chose a generalist study species, the rodentProechimys semispinosus, because it is highly abundant in three Panamanian landscapes that differ in their degree of anthropogenic modification. We detected only two TLR7 haplotypes that differed by one synonymous single-nucleotide polymorphism (SNP) position. The TLR4 variability was higher, and we detected four TLR4 haplotypes that differed at one synonymous SNP and at three amino acid positions within the leucine-rich repeat region. Only TLR4 haplotypes had different frequencies in each landscape. Using generalized linear models, we found evidence that nematode loads and virus prevalence were influenced by both specific TLR4 haplotypes and landscape. Here, the variable landscape served as a surrogate for the important influential ecological factors distinguishing landscapes in our study, i.e. species diversity and host population density. Individuals carrying the common TLR4_Ht1 haplotype were less intensely infected by the most abundant strongyle nematode. Individuals carrying the rare TLR4_Ht3 haplotype were allHepacivirus-positive, where those carrying the rare haplotype TLR4_Ht4 were less often infected byHepacivirusthan individuals with other haplotypes. Our study highlights the role of TLR diversity in pathogen resistance and the importance of considering immune genetic as well as ecological factors in order to understand the effects of anthropogenic changes on wildlife health. </td> <td style="text-align:left;"> NA </td> <td style="text-align:left;"> maybe </td> <td style="text-align:left;"> any good GD measure of pop? Or all ind level? </td> <td style="text-align:left;"> Diversity in TLR measured, not relatedness </td> <td style="text-align:left;"> no </td> <td style="text-align:left;"> no </td> </tr> <tr> <td style="text-align:left;"> 4618 </td> <td style="text-align:left;"> 2020 </td> <td style="text-align:left;"> 44042 </td> <td style="text-align:left;"> Paczesniak, D; Klappert, K; Kopp, K; Neiman, M; Seppala, K; Lively, CM; Jokela, J </td> <td style="text-align:left;"> 2019 </td> <td style="text-align:left;"> Parasite resistance predicts fitness better than fecundity in a natural population of the freshwater snail Potamopyrgus antipodarum </td> <td style="text-align:left;"> The cost of males should give asexual females an advantage when in competition with sexual females. In addition, high-fecundity asexual genotypes should have an advantage over low-fecundity clones, leading to reduction in clonal diversity over time. To evaluate fitness components in a natural population, we measured the annual reproductive rate of individual sexual and asexual female Potamopyrgus antipodarum, a New Zealand freshwater snail, in field enclosures that excluded competitors and predators. We used allozyme genotyping to assign the asexual females to particular clonal genotypes. We found that the most fecund asexual clones had similar or higher fecundity as the top 10% of sexual families, suggesting that fecundity selection, even without the cost of males, would lead to replacement of the sexual population by clones. Consequently, we expected that the clones with the highest fecundity would dominate the natural population. Counter to this prediction, we found that high annual reproductive rates did not correlate with the frequency of clones in the natural population. When we exposed the same clones to parasites in the laboratory, we found that resistance to infection was positively correlated with the frequency of clones in the population. The correlation between fecundity and parasite resistance was negative, suggesting a trade-off between these two traits. Our results thus suggest that parasite resistance is an important short-term predictor of the success of asexual P. antipodarum in this population. </td> <td style="text-align:left;"> NA </td> <td style="text-align:left;"> maybe </td> <td style="text-align:left;"> probably no - how to measure GD and fitness of a group here? </td> <td style="text-align:left;"> Don't look at genetic diversity of groups </td> <td style="text-align:left;"> no </td> <td style="text-align:left;"> no </td> </tr> <tr> <td style="text-align:left;"> 4619 </td> <td style="text-align:left;"> 2020 </td> <td style="text-align:left;"> 44112 </td> <td style="text-align:left;"> Miguez-Soto, B; Fernandez-Cruz, J; Fernandez-Lopez, J </td> <td style="text-align:left;"> 2019 </td> <td style="text-align:left;"> Mediterranean and Northern Iberian gene pools of wild Castanea sativa Mill. are two differentiated ecotypes originated under natural divergent selection </td> <td style="text-align:left;"> Nine wild Iberian provenances of Castanea sativa Mill. grouped in two gene pools, North Iberian Peninsula and Mediterranean, were evaluated for several adaptive traits in two provenance-progeny trials with the aim of evaluating the role of natural selection in shaping adaptive variation and increasing our understanding of the genetic structure of this species, as well as reporting complete information on the genetic variation among and within the studied populations. An annual growth rhythm experiment was evaluated during the first 3 years after establishment for phenology, growth, stem form and survival, and a periodic drought-stress experiment was evaluated for dry weight, growth, survival and other related drought traits in both well-watered and drought-stress treatments. The high genetic variability reported in both trials is largely due to the genetic variation among populations. The significant differences reported between quantitative genetic and neutral marker differentiation indicated the local adaptation of these populations through directional selection, mainly for phenology, growth and biomass allocation. A clinal variation among populations was determined through correlations of phenology with latitude and xerothermic index of the provenances, showing that central and southern Mediterranean populations had earlier phenology than northern populations and that drought played a relevant role in this differentiation. The significant correlation between phenological traits and the ancestry values in the Mediterranean gene pool supported the different pattern of behavior between both gene pools and also indicated the existence of two ecotypes: xeric and mesophytic ecotypes, corresponding to Mediterranean and North Iberian gene pools, respectively. The results obtained in the drought-stress experiment confirmed that, in general terms, xeric populations showed a greater adaptability to drought, with more developed root systems and higher survival than northern populations. Moreover, the genetic variability observed within populations indicated the potential response capacity of Iberian C. sativa populations to undergo fast adaptive evolution. </td> <td style="text-align:left;"> NA </td> <td style="text-align:left;"> maybe </td> <td style="text-align:left;"> probably the gd does not work? Any group level fitness? </td> <td style="text-align:left;"> Estimates of genetic variation ar from quant gen analyses. Molecular genetic work doesn't presented gd estimates </td> <td style="text-align:left;"> no </td> <td style="text-align:left;"> no </td> </tr> <tr> <td style="text-align:left;"> 4620 </td> <td style="text-align:left;"> 2020 </td> <td style="text-align:left;"> 44111 </td> <td style="text-align:left;"> Orsted, M; Hoffmann, AA; Sverrisdottir, E; Nielsen, KL; Kristensen, TN </td> <td style="text-align:left;"> 2019 </td> <td style="text-align:left;"> Genomic variation predicts adaptive evolutionary responses better than population bottleneck history </td> <td style="text-align:left;"> The relationship between population size, inbreeding, loss of genetic variation and evolutionary potential of fitness traits is still unresolved, and large-scale empirical studies testing theoretical expectations are surprisingly scarce. Here we present a highly replicated experimental evolution setup with 120 lines of Drosophila melanogaster having experienced inbreeding caused by low population size for a variable number of generations. Genetic variation in inbred lines and in outbred control lines was assessed by genotyping-by-sequencing (GBS) of pooled samples consisting of 15 males per line. All lines were reared on a novel stressful medium for 10 generations during which body mass, productivity, and extinctions were scored in each generation. In addition, we investigated egg-to-adult viability in the benign and the stressful environments before and after rearing at the stressful conditions for 10 generations. We found strong positive correlations between levels of genetic variation and evolutionary response in all investigated traits, and showed that genomic variation was more informative in predicting evolutionary responses than population history reflected by expected inbreeding levels. We also found that lines with lower genetic diversity were at greater risk of extinction. For viability, the results suggested a trade-off in the costs of adapting to the stressful environments when tested in a benign environment. This work presents convincing support for long-standing evolutionary theory, and it provides novel insights into the association between genetic variation and evolutionary capacity in a gradient of diversity rather than dichotomous inbred/outbred groups. Author summary Genetic variation is a prerequisite for evolution to occur. Quantifying, utilizing and understanding the role of this variation for the survival and persistence of populations is central to several research disciplines including evolutionary biology, animal and plant breeding and conservation genetics. Environments are changing at a rate unpreceded for millennia and rates of local and global extinction of populations and species are alarming. The ability to cope with environmental change through genetic changes is therefore key in order to adapt and survive. Here we provide insights into the association between genetic variation and evolutionary and demographic response to environmental stress. We do this based on results from a long-term experimental evolution study on 130 genome-wide sequenced inbred and outbred lines of vinegar flies (Drosophila melanogaster). We found 1) strong positive correlations between levels of genetic variation and evolutionary response, 2) that genomic variation is predicting evolutionary responses better than expected inbreeding levels, and 3) that lines with lower genetic diversity are at greater risk of extinction. This work presents convincing support for long-standing evolutionary theory, reinforces the importance on maintaining genetic variation in wild and domestic populations and pinpoints applied benefits of high-throughput sequencing. </td> <td style="text-align:left;"> NA </td> <td style="text-align:left;"> maybe </td> <td style="text-align:left;"> too inbred lines? </td> <td style="text-align:left;"> Examines inbred lines </td> <td style="text-align:left;"> no </td> <td style="text-align:left;"> no </td> </tr> <tr> <td style="text-align:left;"> 4621 </td> <td style="text-align:left;"> 2020 </td> <td style="text-align:left;"> 44042 </td> <td style="text-align:left;"> Berg, EC; Lind, MI; Monahan, S; Bricout, S; Maklakov, AA </td> <td style="text-align:left;"> 2019 </td> <td style="text-align:left;"> Kin but less than kind: within-group male relatedness does not increase female fitness in seed beetles </td> <td style="text-align:left;"> Theory maintains within-group male relatedness can mediate sexual conflict by reducing male-male. competition and collateral harm to females. We tested whether male relatedness can lessen female harm in the seed beetle Callosobruchus maculatus. Male relatedness did not influence female lifetime reproductive success or individual fitness across two different ecologically relevant scenarios of mating competition. However, male relatedness marginally improved female survival. Because male relatedness improved female-survival in late life when C. maculatus females are no longer producing offspring, our results do not provide support for the role of within-group male relatedness in mediating sexual conflict. The fact that male relatedness improves the post-reproductive part of the female life cycle strongly suggests that the effect is non-adaptive. We discuss adaptive and non-adaptive mechanisms that could result in reduced female harm in this and previous studies, and suggest that cognitive error is a likely explanation. </td> <td style="text-align:left;"> NA </td> <td style="text-align:left;"> maybe </td> <td style="text-align:left;"> could we use this gd and fitness somehow? </td> <td style="text-align:left;"> Male relatedness manipulated but only female survival measured </td> <td style="text-align:left;"> no </td> <td style="text-align:left;"> no </td> </tr> <tr> <td style="text-align:left;"> 4622 </td> <td style="text-align:left;"> 2020 </td> <td style="text-align:left;"> 44113 </td> <td style="text-align:left;"> Evans, SM; Sinclair, EA; Poore, AGB; Bain, KF; Verges, A </td> <td style="text-align:left;"> 2018 </td> <td style="text-align:left;"> Assessing the effect of genetic diversity on the early establishment of the threatened seagrass Posidonia australis using a reciprocal-transplant experiment </td> <td style="text-align:left;"> Two common goals for restoration are rapid plant establishment and long-term plant persistence. The success of transplanted populations may be jeopardized if the donor transplants are not genetically diverse, and/or poorly matched to their new environment. Here, we test the effects of local adaptation and plot-level genetic diversity on the early establishment phase of a threatened seagrass species, Posidonia australis, by performing a reciprocal transplant experiment across two genetically and geographically distinct populations in southeastern Australia. Posidonia australis is a long-lived, slow-growing species that has no seed bank, and the successful transplantation of live shoots and seedlings is the only available restoration method. Our results show a strong effect of local adaptation and genetic diversity on P. australis survivorship and performance over the first 6months following transplantation. High-genetic diversity plots displayed higher survival rates and exhibited reduced productivity and increased carbohydrate reserves within the rhizome. This suggests that high-diversity plots included shoots that were conserving energy stores by actively reducing growth rates during the early stages of transplantation. The lowest diversity plots exhibited high leaf and root productivity and corresponding low carbohydrate reserves. This may be a sign of stress in the low-diversity transplants, potentially explaining the very low survival rate. We suggest that future restoration efforts source donor transplants from multiple local sources to ensure both local adaptation and sufficient genetic diversity to increase the likelihood of early establishment success. </td> <td style="text-align:left;"> NA </td> <td style="text-align:left;"> maybe </td> <td style="text-align:left;"> "is the gd of ""groups"" ok?" </td> <td style="text-align:left;"> N=2, high vs low comparison genetic diversity comparison </td> <td style="text-align:left;"> no </td> <td style="text-align:left;"> no </td> </tr> <tr> <td style="text-align:left;"> 4623 </td> <td style="text-align:left;"> 2020 </td> <td style="text-align:left;"> 44111 </td> <td style="text-align:left;"> Kosch, TA; Silva, CNS; Brannelly, LA; Roberts, AA; Lau, Q; Marantelli, G; Berger, L; Skerratt, LF </td> <td style="text-align:left;"> 2019 </td> <td style="text-align:left;"> Genetic potential for disease resistance in critically endangered amphibians decimated by chytridiomycosis </td> <td style="text-align:left;"> Hundreds of amphibian species have declined worldwide after the emergence of the amphibian fungal pathogen Batrachochytrium dendrobatidis (Bd). Despite captive breeding efforts, it is unlikely that wild populations for many species will be reestablished unless Bd resistance increases. We performed a Bd-challenge study in the functionally extinct southern corroboree frog Pseudophryne corroboree to investigate differences in Bd susceptibility among individuals and populations, identify genetic [major histocompatibility complex (MHC) class I] and genome-wide variants associated with Bd resistance, and measure genetic diversity and population genetic structure. We found three MHC variants and one MHC supertype associated with Bd infection load and survival along with a suggestively associated single nucleotide polymorphism. We also showed that genome-wide heterozygosity is associated with increased survival. Additionally, we found evidence of significant population structure among the four P. corroboree populations studied and high MHC genetic diversity. Our results indicate that there are immunogenetic differences among captive southern corroboree frogs; such differences could be manipulated to increase disease resistance and mitigate the significant threat of chytridiomycosis. These results demonstrate a potential long-term solution to chytridiomycosis that could include breeding more resistant individuals and returning them to the wild. </td> <td style="text-align:left;"> NA </td> <td style="text-align:left;"> maybe </td> <td style="text-align:left;"> ok gd measure? </td> <td style="text-align:left;"> No clear measure of between individual genetic diversity / relatedness </td> <td style="text-align:left;"> no </td> <td style="text-align:left;"> no </td> </tr> <tr> <td style="text-align:left;"> 4624 </td> <td style="text-align:left;"> 2020 </td> <td style="text-align:left;"> 44043 </td> <td style="text-align:left;"> Vanderplanck, M; Roger, N; Moerman, R; Ghisbain, G; Gerard, M; Popowski, D; Granica, S; Fournier, D; Meeus, I; Piot, N; Smagghe, G; Terrana, L; Michez, D </td> <td style="text-align:left;"> 2019 </td> <td style="text-align:left;"> Bumble bee parasite prevalence but not genetic diversity impacted by the invasive plant Impatiens glandulifera </td> <td style="text-align:left;"> While many bee species are experiencing population declines, some host plant generalist bees remain common in Europe, partly because they seem able to shift to new resources. However, foraging on a new alternative plant, such as an invasive species, can modify diet quality and have a potentially detrimental effect on bee health. Herein, we investigated whether the spread of the invasive plant Impatiens glandulifera affects Bombus pascuorum population regarding parasite prevalence, genetic structure, and nest density in Belgium. While no difference in bumble bee genetic structure was detected between invaded and uninvaded sites, we show that I. glandulifera occurrence was significantly correlated with a decrease in the prevalence of Apicystis bombi but not the prevalence of three other parasite species (i.e., Crithidia bombi, Nosema bombi, Nosema ceranae, and Nosema sp.). Regarding our investigations, this effect was likely not due to variation in local bumble bee population fitness before I. glandulifera flowering, nor to the relative abundance of other pollinators such as Apis mellifera, but the unique chemical composition (i.e., polyphenol rich) of the pollen of I. glandulifera remained as an interesting hypothesis. Whereas B. pascuorum queens probably colonize all the potential nesting sites in an area, invaded by I. glandulifera or not, the abundance of polyphenol ampelopsin in pollen from I. glandulifera pollen might reduce local parasite prevalence. Our field study confirms that bumble bee parasite prevalence is potentially related to the particular chemical composition of collected pollen. Plant traits such as secondary metabolite occurrence could play a key role in the health and conservation of bumble bees. </td> <td style="text-align:left;"> NA </td> <td style="text-align:left;"> maybe </td> <td style="text-align:left;"> ok fitness and gd? </td> <td style="text-align:left;"> No colony level data and sibship details not given </td> <td style="text-align:left;"> no </td> <td style="text-align:left;"> no </td> </tr> <tr> <td style="text-align:left;"> 4625 </td> <td style="text-align:left;"> 2020 </td> <td style="text-align:left;"> 44039 </td> <td style="text-align:left;"> Rothstein, AP; Knapp, RA; Bradburd, GS; Boiano, DM; Briggs, CJ; Rosenblum, EB </td> <td style="text-align:left;"> 2020 </td> <td style="text-align:left;"> Stepping into the past to conserve the future: Archived skin swabs from extant and extirpated populations inform genetic management of an endangered amphibian </td> <td style="text-align:left;"> Moving animals on a landscape through translocations and reintroductions is an important management tool used in the recovery of endangered species, particularly for the maintenance of population genetic diversity and structure. Management of imperiled amphibian species rely heavily on translocations and reintroductions, especially for species that have been brought to the brink of extinction by habitat loss, introduced species, and disease. One striking example of amphibian declines and associated management efforts is in California's Sequoia and Kings Canyon National Parks with the mountain yellow-legged frog species complex (Rana sierrae/muscosa). Mountain yellow-legged frogs have been extirpated from more than 93% of their historic range, and limited knowledge of their population genetics has made long-term conservation planning difficult. To address this, we used 598 archived skin swabs from both extant and extirpated populations across 48 lake basins to generate a robust Illumina-based nuclear amplicon data set. We found that samples grouped into three main genetic clusters, concordant with watershed boundaries. We also found evidence for historical gene flow across watershed boundaries with a north-to-south axis of migration. Finally, our results indicate that genetic diversity is not significantly different between populations with different disease histories. Our study offers specific management recommendations for imperiled mountain yellow-legged frogs and, more broadly, provides a population genetic framework for leveraging minimally invasive samples for the conservation of threatened species. </td> <td style="text-align:left;"> NA </td> <td style="text-align:left;"> maybe </td> <td style="text-align:left;"> any good GD and fitness measure of pops? </td> <td style="text-align:left;"> No good fitness measure of groups </td> <td style="text-align:left;"> no </td> <td style="text-align:left;"> no </td> </tr> <tr> <td style="text-align:left;"> 4626 </td> <td style="text-align:left;"> 2020 </td> <td style="text-align:left;"> 44042 </td> <td style="text-align:left;"> Lynch, EC; Lummaa, V; Htut, W; Landenpera, M </td> <td style="text-align:left;"> 2019 </td> <td style="text-align:left;"> Evolutionary significance of maternal kinship in a long-lived mammal </td> <td style="text-align:left;"> Preferential treatment of kin is widespread across social species and is considered a central prerequisite to the evolution of cooperation through kin selection. Though it is well known that, among most social mammals, females will remain within their natal group and often bias social behaviour towards female maternal kin, less is known about the fitness consequences of these relationships. We test the fitness benefits of living with maternal sisters, measured by age-specific female reproduction, using an unusually large database of a semi-captive Asian elephant (Elephas maximus) population. This study system is particularly valuable to an exploration of reproductive trends in a long-lived mammal, because it includes life-history data that span multiple generations, enabling a study of the effects of kinship across a female's lifespan. We find that living near a sister significantly increased the likelihood of annual reproduction among young female elephants, and this effect was strongest when living near a sister 0-5 years younger. Our results show that fitness benefits gained from relationships with kin are age-specific, establish the basis necessary for the formation and maintenance of close social relationships with female kin, and highlight the adaptive importance of matriliny in a long-lived mammal. This article is part of the theme issue 'The evolution of female-biased kinship in humans and other mammals'. </td> <td style="text-align:left;"> NA </td> <td style="text-align:left;"> maybe </td> <td style="text-align:left;"> probably no - how to measure GD and fitness of a group here? </td> <td style="text-align:left;"> no good gd and fitness groups </td> <td style="text-align:left;"> no </td> <td style="text-align:left;"> no </td> </tr> <tr> <td style="text-align:left;"> 4627 </td> <td style="text-align:left;"> 2020 </td> <td style="text-align:left;"> 44040 </td> <td style="text-align:left;"> Fang, CC; Chang, FH; Duong, P; Kurian, J; Mueller, UG </td> <td style="text-align:left;"> 2020 </td> <td style="text-align:left;"> Colony fitness and garden growth in the asexual fungus-growing ant Mycocepurus smithii (Attini, Formicidae) </td> <td style="text-align:left;"> Nest-founding in the fungus-growing ant Mycocepurus smithii is typically by single queens (monogyny) and colonies transition to multiple queens (polygyny) as they grow larger. Here, we study the transition from monogyny to polygyny of M. smithii under lab conditions. We hypothesize that the worker-to-queen ratio affects colony growth, fungus-garden growth, and colony survival. Monogyne colonies with small gardens (0.1 g) had greater garden growth than polygyne colonies, suggesting that monogyny may be the superior strategy for small colonies after initial nest establishment. In monogyne colonies with small gardens and either 30, 60, or 90 workers, colonies with 60 workers produced the largest gardens, suggesting that an intermediate worker-to-queen ratio is optimal for monogyne colonies with small gardens. For monogyne colonies with larger gardens (0.45 g) and with either 6, 18, 60, or 90 workers, colonies with 60 or 90 workers had significantly greater garden growth than those with 6 or 18 workers. New daughter queens were produced only by colonies with a worker-to-queen ratio of 60 or 90, suggesting that only colonies with sufficient worker numbers and garden growth are stimulated to produce new queens. A single queen lays only 1.12 +/- 0.06 (SE) eggs within 24 h, therefore, limiting growth of monogyne colonies. The transition from monogyny to polygyny through the addition of supernumary queens, as well as a worker-to-queen ratio of around 60, are, therefore, critical for colony growth and reproduction in M. smithii. </td> <td style="text-align:left;"> NA </td> <td style="text-align:left;"> maybe </td> <td style="text-align:left;"> probably not a good gd measure </td> <td style="text-align:left;"> no measure of mortality </td> <td style="text-align:left;"> no </td> <td style="text-align:left;"> no </td> </tr> <tr> <td style="text-align:left;"> 4628 </td> <td style="text-align:left;"> 2020 </td> <td style="text-align:left;"> 44040 </td> <td style="text-align:left;"> Pacioglu, O; Theissinger, K; Alexa, A; Samoila, C; S?rbu, OI; Schrimpf, A; Zubrod, JP; Schulz, R; P?rvu, M; Lele, SF; Jones, JI; P?rvulescu, L </td> <td style="text-align:left;"> 2020 </td> <td style="text-align:left;"> Multifaceted implications of the competition between native and invasive crayfish: a glimmer of hope for the native's long-term survival </td> <td style="text-align:left;"> Biological invasions represent a complex phenomenon driven by multiple factors. In this study, a real-time invasion process between a native (Pontastacus leptodactylus) and an invasive (Faxonius limosus) crayfish species was investigated in the Lower Danube (South-East Europe) through an interdisciplinary approach, by measuring various ecological, genetic, physiological and biometric endpoints. The results revealed that the prolonged competition in old invaded sites of the river (at least a decade) either drove the native species to extinction, or, unexpectedly, allowed its survival as highly fragmented populations. However, for the latter situation, several biological and ecological traits differed in the remnant populations: increased trophic position and elemental imbalance for two major macronutrients (C:N molar ratio), low growth, as strongly contracted trophic niche widths and low overlap degree with the invasive crayfish. The data suggest that the prolonged competition induced potential resource partitioning between species, potentially driving their coexistence, as the development of larger and heavier claws within the native males' population. On the contrary, in more recently invaded sectors of the Lower Danube (3 years), the trophic niche of the native species was significantly larger compared to old invaded sites and characterised by high level of niche overlap, indicating almost identical diet with the invasive crayfish, but characterised by the lowest trophic position compared to other invasion sectors. The genetic diversity of the native crayfish populations was strongly reduced in the invaded sectors of the river, but without signs of genetic bottleneck, which may be explained by a drift-mutational equilibrium reached as a consequence of diminishing population size. Our findings suggest strong coexistence potential in the future for both species in the Lower Danube. </td> <td style="text-align:left;"> NA </td> <td style="text-align:left;"> maybe </td> <td style="text-align:left;"> probably not a good fitness measure </td> <td style="text-align:left;"> no measure of mortality or parasitism </td> <td style="text-align:left;"> no </td> <td style="text-align:left;"> no </td> </tr> <tr> <td style="text-align:left;"> 4629 </td> <td style="text-align:left;"> 2020 </td> <td style="text-align:left;"> 44111 </td> <td style="text-align:left;"> Ferreira, D; Pinho, C; Brito, JC; Santos, X </td> <td style="text-align:left;"> 2019 </td> <td style="text-align:left;"> Increase of genetic diversity indicates ecological opportunities in recurrent-fire landscapes for wall lizards </td> <td style="text-align:left;"> Socioeconomic and climatic factors are modifying fire regimes with an increase of fire frequency and extension. Unfortunately, the effects of recurrent fires on biological processes that ultimately affect the genetic diversity of animal populations are mostly unknown. We examined genetic patterns of diversity in the wall lizard Podarcis guadarramae in northern Portugal, one of the European regions with the highest percentage of burnt land. This species is a small saxicolous lizard as it inhabits natural outcrops and artificial stone walls, likely in recurrent-fire landscapes. We genotyped nine microsatellites from ten populations selected according to a gradient in fire recurrence, and compared genetic diversity indexes and demographic patterns among them. At the population level, we hypothesize that a high level of mortality and population bottlenecks are expected to reduce genetic heterozygosity in sampled localities affected by recurrent fires. Alternatively, genetic signatures are expected to be absent whether fire did not cause high mortality. Regardless of levels of mortality, we expect a gain in genetic diversity whether recurrent fires facilitate lizard dispersal and migration due to the increased quality of the habitat for wall lizards. At the regional level, we examine whether a recurrent fire regime may disrupt the spatial structure of populations. Our results showed an increase in genetic diversity in recurrently burnt populations, and a decline in longer-unburnt populations. We did not detect bottleneck effects in repeatedly-burnt populations. High genetic diversity in recurrent fire populations suggests a high dispersion rate between adjacent metapopulations and perhaps immigration from outside the fire boundary. At the regional level, lizard populations show low differentiation and weak genetic structure, suggesting no effects of fire. This study confirms field-based censuses showing that recurrent-fire regimes give ecological opportunities to wall lizards that benefit from habitat openness. </td> <td style="text-align:left;"> NA </td> <td style="text-align:left;"> maybe </td> <td style="text-align:left;"> ok fitness&GD, on group level? </td> <td style="text-align:left;"> No measure of survival or pathogens + no clear measurement of group/population genetic diversity </td> <td style="text-align:left;"> no </td> <td style="text-align:left;"> no </td> </tr> <tr> <td style="text-align:left;"> 4630 </td> <td style="text-align:left;"> 2020 </td> <td style="text-align:left;"> 44039 </td> <td style="text-align:left;"> Rangel, J; Traver, B; Stoner, M; Hatter, A; Trevelline, B; Garza, C; Shepherd, T; Seeley, TD; Wenzel, J </td> <td style="text-align:left;"> 2020 </td> <td style="text-align:left;"> Genetic diversity of wild and managed honey bees (Apis mellifera) in Southwestern Pennsylvania, and prevalence of the microsporidian gut pathogens Nosema ceranae and N. apis </td> <td style="text-align:left;"> The populations of wild honey bee (Apis mellifera) colonies in the USA were decimated after the arrival of a parasitic mite Varroa destructor in the 1980s. However, in some places, wild honey bee colonies survived. In this 3-year study, we analyzed 32 wild and 11 managed colonies in Southwestern Pennsylvania for their maternal genetic ancestries and their levels of Nosema spp. infection. We detected nine mtDNA haplotypes in the 32 wild colonies sampled: six belonged to the Eastern European lineage (C) and three belonged to the Western European lineage (M). We found only three mtDNA haplotypes in the eleven managed colonies sampled, all belonging to the C lineage. Infection levels of N. ceranae were relatively high and fluctuated over time while those of N. apis remained relatively low and constant. There were no differences in N. ceranae or N. apis levels between wild and managed colonies. This study shows that wild honey bee colonies can represent old lineages despite being susceptible to Nosema. </td> <td style="text-align:left;"> NA </td> <td style="text-align:left;"> maybe </td> <td style="text-align:left;"> GD individual or colony level? </td> <td style="text-align:left;"> Only mtDNA haplotypes measured and not related to parasites </td> <td style="text-align:left;"> no </td> <td style="text-align:left;"> no </td> </tr> <tr> <td style="text-align:left;"> 4631 </td> <td style="text-align:left;"> 2020 </td> <td style="text-align:left;"> 44043 </td> <td style="text-align:left;"> Berbel, WM; de Leaniz, CG; Morn, P; Cable, J; Lima, SMQ; Consuegra, S </td> <td style="text-align:left;"> 2019 </td> <td style="text-align:left;"> Local parasite pressures and host genotype modulate epigenetic diversity in a mixed-mating fish </td> <td style="text-align:left;"> Parasite-mediated selection is one of the main drivers of genetic variation in natural populations. The persistence of long-term self-fertilization, however, challenges the notion that low genetic variation and inbreeding compromise the host's ability to respond to pathogens. DNA methylation represents a potential mechanism for generating additional adaptive variation under low genetic diversity. We compared genetic diversity (microsatellites and AFLPs), variation in DNA methylation (MS-AFLPs), and parasite loads in three populations of Kryptolebias hermaphroditus, a predomintanly self-fertilizing fish, to analyze the potential adaptive value of DNA methylation in relation to genetic diversity and parasite loads. We found strong genetic population structuring, as well as differences in parasite loads and methylation levels among sampling sites and selfing lineages. Globally, the interaction between parasites and inbreeding with selfing lineages influenced DNA methylation, but parasites seemed more important in determining methylation levels at the local scale. </td> <td style="text-align:left;"> NA </td> <td style="text-align:left;"> maybe </td> <td style="text-align:left;"> gd of group ok? </td> <td style="text-align:left;"> Within individual genetic diversity not group </td> <td style="text-align:left;"> no </td> <td style="text-align:left;"> no </td> </tr> <tr> <td style="text-align:left;"> 4632 </td> <td style="text-align:left;"> 2020 </td> <td style="text-align:left;"> 44113 </td> <td style="text-align:left;"> Dagan, Y; Kosman, E; Ben-Ami, F </td> <td style="text-align:left;"> 2017 </td> <td style="text-align:left;"> Cost of resistance to trematodes in freshwater snail populations with low clonal diversity </td> <td style="text-align:left;"> Background: The persistence of high genetic variability in natural populations garners considerable interest among ecologists and evolutionary biologists. One proposed hypothesis for the maintenance of high levels of genetic diversity relies on frequency-dependent selection imposed by parasites on host populations (Red Queen hypothesis). A complementary hypothesis suggests that a trade-off between fitness costs associated with tolerance to stress factors and fitness costs associated with resistance to parasites is responsible for the maintenance of host genetic diversity. Results: The present study investigated whether host resistance to parasites is traded off with tolerance to environmental stress factors (high/low temperatures, high salinity), by comparing populations of the freshwater snail Melanoides tuberculata with low vs. high clonal diversity. Since polyclonal populations were found to be more parasitized than populations with low clonal diversity, we expected them to be tolerant to environmental stress factors. We found that clonal diversity explained most of the variation in snail survival under high temperature, thereby suggesting that tolerance to high temperatures of clonally diverse populations is higher than that of populations with low clonal diversity. Conclusions: Our results suggest that resistance to parasites may come at a cost of reduced tolerance to certain environmental stress factors. </td> <td style="text-align:left;"> NA </td> <td style="text-align:left;"> maybe </td> <td style="text-align:left;"> ok fitness measure? </td> <td style="text-align:left;"> NA </td> <td style="text-align:left;"> no </td> <td style="text-align:left;"> yes </td> </tr> <tr> <td style="text-align:left;"> 4633 </td> <td style="text-align:left;"> 2020 </td> <td style="text-align:left;"> 44112 </td> <td style="text-align:left;"> Mott, CL; Dzaferbegovic, H; Timm, SR; Whiteman, HH </td> <td style="text-align:left;"> 2019 </td> <td style="text-align:left;"> Influences of facultative paedomorphosis on kin selection in a larval salamander, Ambystoma talpoideum </td> <td style="text-align:left;"> Kin selection in larval amphibians is hypothesized to increase survival to metamorphosis. While kin selection may benefit amphibians with obligate metamorphosis, increased survival within sibships may exert fitness costs on facultatively paedomorphic species, such as increased competition among kin. Consequently, it is unclear whether such species should engage in kin selection. We investigated kin selection in a facultatively paedomorphic salamander, Ambystoma talpoideum, using laboratory behavioural trials and microcosm experiments. Individuals were most aggressive towards familiar siblings, and full-sibship groups incurred more injuries than mixed-sibship groups; however, familiar siblings ultimately exhibited higher survival. Thus, while short-term responses appeared to reflect the hypothesized costs of kin recognition, long-term patterns of survival did not support this hypothesis. The inconsistencies between results suggest that short-term studies may not capture ontogenetic variation in kin selection, and that long-term studies are needed to better test the hypothesized effects of kin selection on survival and metamorphosis. </td> <td style="text-align:left;"> NA </td> <td style="text-align:left;"> maybe </td> <td style="text-align:left;"> any ok fitness and how are the sibling groups formed? </td> <td style="text-align:left;"> NA </td> <td style="text-align:left;"> no </td> <td style="text-align:left;"> yes </td> </tr> <tr> <td style="text-align:left;"> 4634 </td> <td style="text-align:left;"> 2020 </td> <td style="text-align:left;"> 44111 </td> <td style="text-align:left;"> Fayad, N; Awad, MK; Mahillon, J </td> <td style="text-align:left;"> 2019 </td> <td style="text-align:left;"> Diversity of Bacillus cereus sensu lato mobilome </td> <td style="text-align:left;"> BackgroundBacillus cereus sensu lato s.l.) is a group of bacteria displaying close phylogenetic relationships but a high ecological diversity. The three most studied species are Bacillus anthracis, Bacillus cereus sensu stricto and Bacillus thuringiensis. While some species are pathogenic to mammals or associated with food poisoning, Bacillus thuringiensis is a well-known entomopathogenic bacterium used as biopesticide worldwide. B. cereus s.l. also contains a large variety of mobile genetic elements (MGEs).ResultsIn this study, we detail the occurrence and plasmid vs. chromosome distribution of several MGEs in 102 complete and annotated genomes of B. cereus s.l. These MGEs include 16 Insertion Sequence (IS) families, the Tn3 family, 18 different Bacillus cereus repeats (BCRs) and 30 known group II introns.ConclusionsOur analysis not only shows the diversity of these MGEs among strains of the same species and between different species within the B. cereus s.l. group, but also highlights the potential impact of these elements on the plasticity of the plasmid pool, and the TEs (Transposable Elements) - species relationship within B. cereus s.l. </td> <td style="text-align:left;"> NA </td> <td style="text-align:left;"> no </td> <td style="text-align:left;"> No good fitness measure </td> <td style="text-align:left;"> NA </td> <td style="text-align:left;"> no </td> <td style="text-align:left;"> no </td> </tr> <tr> <td style="text-align:left;"> 4635 </td> <td style="text-align:left;"> 2020 </td> <td style="text-align:left;"> 44111 </td> <td style="text-align:left;"> Jaffe, R; Veiga, JC; Pope, NS; Lanes, ECM; Carvalho, CS; Alves, R; Andrade, SCS; Arias, MC; Bonatti, V; Carvalho, AT; de Castro, MS; Contrera, FAL; Francoy, TM; Freitas, BM; Giannini, TC; Hrncir, M; Martins, CF; Oliveira, G; Saraiva, AM; Souza, BA; Imperatriz-Fonseca, VL </td> <td style="text-align:left;"> 2019 </td> <td style="text-align:left;"> Landscape genomics to the rescue of a tropical bee threatened by habitat loss and climate change </td> <td style="text-align:left;"> Habitat degradation and climate change are currently threatening wild pollinators, compromising their ability to provide pollination services to wild and cultivated plants. Landscape genomics offers powerful tools to assess the influence of landscape modifications on genetic diversity and functional connectivity, and to identify adaptations to local environmental conditions that could facilitate future bee survival. Here, we assessed range-wide patterns of genetic structure, genetic diversity, gene flow, and local adaptation in the stingless bee Melipona subnitida, a tropical pollinator of key biological and economic importance inhabiting one of the driest and hottest regions of South America. Our results reveal four genetic clusters across the species' full distribution range. All populations were found to be under a mutation-drift equilibrium, and genetic diversity was not influenced by the amount of reminiscent natural habitats. However, genetic relatedness was spatially autocorrelated and isolation by landscape resistance explained range-wide relatedness patterns better than isolation by geographic distance, contradicting earlier findings for stingless bees. Specifically, gene flow was enhanced by increased thermal stability, higher forest cover, lower elevations, and less corrugated terrains. Finally, we detected genomic signatures of adaptation to temperature, precipitation, and forest cover, spatially distributed in latitudinal and altitudinal patterns. Taken together, our findings shed important light on the life history of M. subnitida and highlight the role of regions with large thermal fluctuations, deforested areas, and mountain ranges as dispersal barriers. Conservation actions such as restricting long-distance colony transportation, preserving local adaptations, and improving the connectivity between highlands and lowlands are likely to assure future pollination services. </td> <td style="text-align:left;"> NA </td> <td style="text-align:left;"> no </td> <td style="text-align:left;"> No good fitness measure and measure host GD </td> <td style="text-align:left;"> NA </td> <td style="text-align:left;"> no </td> <td style="text-align:left;"> no </td> </tr> <tr> <td style="text-align:left;"> 4636 </td> <td style="text-align:left;"> 2020 </td> <td style="text-align:left;"> 44042 </td> <td style="text-align:left;"> Reyda, FB; Wells, SM; Ermolenko, AV; Zietara, MS; Lumme, JI </td> <td style="text-align:left;"> 2020 </td> <td style="text-align:left;"> Global parasite trafficking: Asian Gyrodactylus (Monogenea) arrived to the USA via invasive fish Misgurnus anguillicaudatus as a threat to amphibians </td> <td style="text-align:left;"> A monogenean flatworm Gyrodactylus jennyae Paetow, Cone, Huyse, McLaughlin & Marcogliese, 2009 was previously described as a pathogen on bullfrog Lithobates catesbeianus Shaw, 1802, in a Canadian captive population originating in Missouri, U.S.A. The ITS barcoding of G. jennyae showed relatedness to Asian Gyrodactylus macracanthus Hukuda 1940, a parasite of the Asian loach Misgurnus anguillicaudatus Cantor, 1842. The resulting suggestion that the globally invasive pet-trade of fish may be a mechanism for arrival of Gyrodactylus species to North America provided the framework for the current study. The present study was undertaken following the discovery of two other species of Gyrodactylus in a population of illegally introduced M. anguillicaudatus in New York State. Here the invasion hypothesis was tested via DNA sequencing of the ITS of the two Gyrodactylus species obtained from M. anguillicaudatus from New York, termed Gyrodactylus sp. A and Gyrodactylus sp. B. Both Gyrodactylus sp. A and Gyrodactylus sp. B were closely related to G. jennyae and G. macracanthus, and all belong to a molecularly well-supported monophyletic Asian freshwater group. In conclusion, this invasive fish has trafficked at least three parasite species to the U.S.A., one of them also found on frog. This route from the Asian wetlands to other continents is similar to that of amphibian chytrid fungi of genus Batrachochytrium Longcore, Pessier & Nichols, 1999. </td> <td style="text-align:left;"> NA </td> <td style="text-align:left;"> no </td> <td style="text-align:left;"> No good fitness measure and measure host GD </td> <td style="text-align:left;"> NA </td> <td style="text-align:left;"> no </td> <td style="text-align:left;"> no </td> </tr> <tr> <td style="text-align:left;"> 4637 </td> <td style="text-align:left;"> 2020 </td> <td style="text-align:left;"> 44112 </td> <td style="text-align:left;"> Wang, S; Lin, ZG; Dietemann, V; Neumann, P; Wu, YQ; Hu, FL; Zheng, HQ </td> <td style="text-align:left;"> 2019 </td> <td style="text-align:left;"> Ectoparasitic Mites Varroa underwoodi (Acarina: Varroidae) in Eastern Honeybees, but not in Western Honeybees </td> <td style="text-align:left;"> The ectoparasitic mite Varroa destructor Anderson and Trueman (Acarina: Varroidae) shifted host from Eastern honeybees Apis cerana Fabricius (Hymenoptera: Apidae) to Western honeybees Apis mellifera Linnaeus (Hymenoptera: Apidae) with disastrous consequences globally. The high genetic diversity of V. destructor and abundant opportunities for cross-species transmission probably promoted this host shift. Whether Varroa underwoodi (Acarina: Varroidae) also exhibits these traits is unknown. Here, we conduct a large-scale survey across China on the occurrence, morphology, reproduction, and genetics of V. underwoodi in A. cerana and A. mellifera colonies to fill gaps in our knowledge of this mite and to determine whether host shifts occurred. Despite the large number of colonies screened, V. underwoodi was exclusively found in A. cerana, where it occurred at low infestation rates. Three genetic clades were detected in the V. underwoodi population, which differed neither in morphology nor in reproductive ability. Nevertheless, the genetic diversity of V. underwoodi is likely to increase chances for host shifts, even though opportunities for cross-species transmission seem low. More studies of the neglected Varroa species seem appropriate to enable a better understanding of host shifts in the Apis spp./Varroa spp. system and evaluate the potential risk they pose to apiculture with A. mellifera. </td> <td style="text-align:left;"> NA </td> <td style="text-align:left;"> no </td> <td style="text-align:left;"> no good gd measure host pop </td> <td style="text-align:left;"> NA </td> <td style="text-align:left;"> no </td> <td style="text-align:left;"> no </td> </tr> <tr> <td style="text-align:left;"> 4638 </td> <td style="text-align:left;"> 2020 </td> <td style="text-align:left;"> 44040 </td> <td style="text-align:left;"> Abdelraheem, A; Elassbli, H; Zhu, Y; Kuraparthy, V; Hinze, L; Stelly, D; Wedegaertner, T; Zhang, JF </td> <td style="text-align:left;"> 2020 </td> <td style="text-align:left;"> A genome-wide association study uncovers consistent quantitative trait loci for resistance to Verticillium wilt and Fusarium wilt race 4 in the US Upland cotton </td> <td style="text-align:left;"> Key message A high-resolution GWAS detected consistent QTL for resistance to Verticillium wilt and Fusarium wilt race 4 in 376 U.S. Upland cotton accessions based on six independent replicated greenhouse tests. Verticillium wilt (VW, caused by Verticillium dahliae Kleb.) and Fusarium wilt (FOV, caused by Fusarium oxysporum f.sp. vasinfectum Atk. Sny & Hans) are the most important soil-borne fungal diseases in cotton. To augment and refine resistance quantitative trait loci (QTL), we conducted a genome-wide association study (GWAS) using high-density genotyping with the CottonSNP63K array. Resistance of 376 US Upland cotton accessions to a defoliating VW and virulent FOV4 was evaluated in four and two independent replicated greenhouse tests, respectively. A total of 15 and 13 QTL for VW and FOV4 resistances were anchored by 30 (on five chromosomes) and 56 (on six chromosomes) significant single nucleotide polymorphic (SNPs) markers, respectively. QTL on c8, c10, c16, and c21 were consistent in two or more tests for VW resistance, while two QTL on c8 and c14 were consistent for FOV4 resistance in two tests. Two QTL clusters on c16 and c19 were observed for both VW and FOV4 resistance, suggesting that these genomic regions may harbor genes in response to both diseases. Using BLAST search against the sequenced TM-1 genome, 30 and 35 candidate genes were identified on four QTL for VW resistance and on three QTL for FOV4 resistance, respectively. These genomic regions were rich in NBS-LRR genes presented in clusters. The results create opportunities for further studies to determine the correlations of field resistance with these QTL, molecular examinations of VW and FOV4 resistances, marker-assisted selection (MAS) and eventual cloning of QTL for disease resistance in cotton. </td> <td style="text-align:left;"> NA </td> <td style="text-align:left;"> no </td> <td style="text-align:left;"> dometicated host </td> <td style="text-align:left;"> NA </td> <td style="text-align:left;"> no </td> <td style="text-align:left;"> no </td> </tr> <tr> <td style="text-align:left;"> 4639 </td> <td style="text-align:left;"> 2020 </td> <td style="text-align:left;"> 44113 </td> <td style="text-align:left;"> Abdelraheem, MH; Bansal, D; Idris, MA; Mukhtar, MM; Hamid, MMA; Imam, ZS; Getachew, S; Sehgal, R; Kaur, H; Gadalla, AH; Al-Hamidhi, S; Al-Hashami, Z; Al-Jabri, A; Sultan, AA; Babiker, HA </td> <td style="text-align:left;"> 2018 </td> <td style="text-align:left;"> Genetic diversity and transmissibility of imported Plasmodium vivax in Qatar and three countries of origin </td> <td style="text-align:left;"> Malaria control program in the Arabian Peninsula, backed by adequate logistical support, has interrupted transmission with exception of limited sites in Saudi Arabia and sporadic outbreaks in Oman. However, sustained influx of imported malaria represents a direct threat to the above success. Here we examined the extent of genetic diversity among imported P. vivax in Qatar, and its ability to produce gametocytes, compared to parasites in main sites of imported cases, the Indian subcontinent (india) and East Africa (Sudan and Ethiopia). High diversity was seen among imported P. vivax in Qatar, comparable to parasites in the Indian subcontinent and East Africa. Limited genetic differentiation was seen among imported P. vivax, which overlapped with parasites in India, but differentiated from that in Sudan and Ethiopia. Parasite density among imported cases, ranged widely between 26.25-7985934.1 Pv18S rRNA copies/mu l blood, with a high prevalence of infections carried gametocytes detectable by qRT-PCR. Parasitaemia was a stronger predictor for P. vivax gametocytes density (r = 0.211, P = 0.04). The extensive diversity of imported P. vivax and its ability to produce gametocytes represent a major threat for re-introduction of malaria in Qatar. The genetic relatedness between P. vivax reported in Qatar and those in India suggest that elimination strategy should target flow and dispersal of imported malaria into the region. </td> <td style="text-align:left;"> NA </td> <td style="text-align:left;"> no </td> <td style="text-align:left;"> gd of pathogen </td> <td style="text-align:left;"> NA </td> <td style="text-align:left;"> no </td> <td style="text-align:left;"> no </td> </tr> <tr> <td style="text-align:left;"> 4640 </td> <td style="text-align:left;"> 2020 </td> <td style="text-align:left;"> 44040 </td> <td style="text-align:left;"> Abdi, AI; Nichols, PGH; Kaur, P; Wintle, BJ; Erskine, W </td> <td style="text-align:left;"> 2020 </td> <td style="text-align:left;"> Morphological diversity within a core collection of subterranean clover (Trifolium subterraneum L.): Lessons in pasture adaptation from the wild </td> <td style="text-align:left;"> Subterranean clover (Trifolium subterraneum L.) is a diploid self-pollinated annual pasture legume native to the Mediterranean region and widely sown in southern Australia and other countries with Mediterranean-type climates. This study utilised a core collection of 97 lines, representing around 80% of the genetic diversity of the species, to examine morphological diversity within subterranean clover. A total of 23 quantitative agro-morphological and 13 semi-quantitative morphological marker traits were assayed on the core collection and 28 diverse Australian cultivars as spaced plants in a replicated common garden experiment. Relationships between these traits and 24 climatic and edaphic parameters at their sites of origin were also examined within the core collection. Significant diversity was present for all traits. The Australian cultivars had similar levels of diversity to the core collection for several traits. Among the agro-morphological traits, time to flowering, leaf size and petiole diameter in mid-winter, plant area in late winter, maximum stem length, content of the oestogenic isoflavone biochanin A and total isoflavone content, were correlated with seven or more environmental variables. These can be considered highly adaptive, being the result of strong environmental selection pressure over time. For the first time in a clover species, morphological markers, including leaf mark, anthocyanin pigmentation and pubescence traits, have been associated with rainfall and soil parameters. This suggests they either have an adaptive role or the genes controlling them may be linked to other genes controlling adaptive traits. This study demonstrated the value of core collections to examine diversity within much larger global collections. It also identified adaptive traits from wild plants that can be utilised to develop more productive and persistent subterranean clover cultivars. The high heritability of these traits indicates that selection gains can be readily made. </td> <td style="text-align:left;"> NA </td> <td style="text-align:left;"> no </td> <td style="text-align:left;"> no good gd measure host pop </td> <td style="text-align:left;"> NA </td> <td style="text-align:left;"> no </td> <td style="text-align:left;"> no </td> </tr> <tr> <td style="text-align:left;"> 4641 </td> <td style="text-align:left;"> 2020 </td> <td style="text-align:left;"> 44113 </td> <td style="text-align:left;"> Abduriyim, S; Nishita, Y; Kosintsev, PA; Raichev, E; Vainola, R; Kryukov, AP; Abramov, AV; Kaneko, Y; Masuda, R </td> <td style="text-align:left;"> 2017 </td> <td style="text-align:left;"> Diversity and evolution of MHC class II DRB gene in the Eurasian badger genus Meles (Mammalia: Mustelidae) </td> <td style="text-align:left;"> Major histocompatibility complex (MHC) genes encode proteins that play a key role in the adaptive immune system of vertebrates and are generally highly polymorphic for defence against various pathogens. To understand the diversity and evolution of MHC variation in badgers in the genus Meles (Carnivora, Mustelidae), we analysed sequence variation of the MHC class II DRB gene exon 2 in the Japanese (Meles anakuma), Asian (Meles leucurus), European (Meles meles) and Southwest Asian (Meles canescens) badgers. Variation was higher in the Meles species than in other species in Mustelidae, and altogether 60 alleles were isolated from 28 individuals. The variable number of three to eight putative alleles per individual was observed, indicating the presence of two to four DRB loci per haploid genome. Non-synonymous substitutions exceeded synonymous substitutions at putative antigen-binding sites. Selection analyses of PAML models, fixed-effect likelihood and mixed-effect model evolution, together with the single breakpoint recombination, indicated that recombination and selection could be responsible for driving and maintaining the diversity of Meles DRBs. In a phylogenetic analysis, the DRB sequences from Meles were distributed in several clusters, which were dispersed among sequences of other mustelid family, even five alleles comprised a monophyletic group of Meles DRBs within a canid clade. The data demonstrate trans-species polymorphisms at different taxonomic and temporal scales, transgressing family-, genus- and species-level splits. Some allele sequences were shared by two to four of the Meles species, in line with a close phylogenetic relationship among these species. </td> <td style="text-align:left;"> NA </td> <td style="text-align:left;"> no </td> <td style="text-align:left;"> mhc diversity </td> <td style="text-align:left;"> NA </td> <td style="text-align:left;"> no </td> <td style="text-align:left;"> no </td> </tr> <tr> <td style="text-align:left;"> 4642 </td> <td style="text-align:left;"> 2020 </td> <td style="text-align:left;"> 44111 </td> <td style="text-align:left;"> Abeid, SN; Motrane, M; Farhane, H; Harich, N </td> <td style="text-align:left;"> 2019 </td> <td style="text-align:left;"> Alu elements within the human major histocompatibility class I region in the Comoros Islands: genetic variation and population relationships </td> <td style="text-align:left;"> Background: Alu elements are attractive markers for population genetics, disease, forensics and paternity analyses, due to their particular characteristics. Five polymorphic Alu insertions within the MHC class I region have been little examined in human populations. Aim: The analysis of the genetic diversity of autochthonous Comorians from the three major islands of the archipelago by these polymorphic MHC Alus and to assess their relationships together and with other populations. Subjects and methods: Two hundred and fifty-seven unrelated participants from the Comoros archipelago, Grande Comore (86), Anjouan (93) and Moheli (78), were examined for five MHC Alu insertions. The data were analysed for intra- and inter-population genetic variation. Results: All MHC Alu were polymorphic in the three samples and only one significant differentiation was observed between Anjouan and Moheli. According to the MDS and AMOVA results, the populations included in the inter-population analyses were grouped in three major clusters according to their genetic ancestry. The haplotype diversity showed by the Comorians is higher than in previously studied African populations and occupies an intermediate position between African and Asian clusters. Conclusion: MHC Alu insertions are useful markers to study micro-geographical genetic variations. Using these polymorphisms, new insights have been obtained about the biological history and evolution of the Comoros. </td> <td style="text-align:left;"> NA </td> <td style="text-align:left;"> no </td> <td style="text-align:left;"> human </td> <td style="text-align:left;"> NA </td> <td style="text-align:left;"> no </td> <td style="text-align:left;"> no </td> </tr> <tr> <td style="text-align:left;"> 4643 </td> <td style="text-align:left;"> 2020 </td> <td style="text-align:left;"> 44042 </td> <td style="text-align:left;"> Abeli, T; Dalrymple, S; Godefroid, S; Mondoni, A; Mller, JV; Rossi, G; Orsenigo, S </td> <td style="text-align:left;"> 2020 </td> <td style="text-align:left;"> Ex situ collections and their potential for the restoration of extinct plants </td> <td style="text-align:left;"> The alarming current and predicted species extinction rates have galvanized conservationists in their efforts to avoid future biodiversity losses, but for species extinct in the wild, few options exist. We posed the questions, can these species be restored, and, if so, what role can ex situ plant collections (i.e., botanic gardens, germplasm banks, herbaria) play in the recovery of plant genetic diversity? We reviewed the relevant literature to assess the feasibility of recovering lost plant genetic diversity with using ex situ material and the probability of survival of subsequent translocations. Thirteen attempts to recover species extinct in the wild were found, most of which used material preserved in botanic gardens (12) and seed banks (2). One case of a locally extirpated population was recovered from herbarium material. Eight (60%) of these cases were successful or partially successful translocations of the focal species or population; the other 5 failed or it was too early to determine the outcome. Limiting factors of the use of ex situ source material for the restoration of plant genetic diversity in the wild include the scarcity of source material, low viability and reduced longevity of the material, low genetic variation, lack of evolution (especially for material stored in germplasm banks and herbaria), and socioeconomic factors. However, modern collecting practices present opportunities for plant conservation, such as improved collecting protocols and improved cultivation and storage conditions. Our findings suggest that all types of ex situ collections may contribute effectively to plant species conservation if their use is informed by a thorough understanding of the aforementioned problems. We conclude that the recovery of plant species currently classified as extinct in the wild is not 100% successful, and the possibility of successful reintroduction should not be used to justify insufficient in situ conservation. </td> <td style="text-align:left;"> NA </td> <td style="text-align:left;"> no </td> <td style="text-align:left;"> No good fitness measure and measure host GD </td> <td style="text-align:left;"> NA </td> <td style="text-align:left;"> no </td> <td style="text-align:left;"> no </td> </tr> <tr> <td style="text-align:left;"> 4644 </td> <td style="text-align:left;"> 2020 </td> <td style="text-align:left;"> 44111 </td> <td style="text-align:left;"> Abukari, Z; Okonu, R; Nyarko, SB; Lo, AC; Dieng, CC; Salifu, SP; Gyan, B; Lo, E; Amoah, LE </td> <td style="text-align:left;"> 2019 </td> <td style="text-align:left;"> The Diversity, Multiplicity of Infection and Population Structure of P. falciparum Parasites Circulating in Asymptomatic Carriers Living in High and Low Malaria Transmission Settings of Ghana </td> <td style="text-align:left;"> Background: Diversity in Plasmodium falciparum poses a major threat to malaria control and elimination interventions. This study utilized 12 polymorphic microsatellite (MS) markers and the Msp2 marker to examine diversity, multiplicity of infection (MOI) as well as the population structure of parasites circulating in two sites separated by about 92 km and with varying malaria transmission intensities within the Greater Accra Region of Ghana. Methods: The diversity and MOI of P. falciparum parasites in 160 non-symptomatic volunteers living in Obom (high malaria transmission intensity) and Asutsuare (low malaria transmission intensity) aged between 8 and 60 years was determined using Msp2 genotyping and microsatellite analysis. Results: The prevalence of asymptomatic P. falciparum carriers as well as the parasite density of infections was significantly higher in Obom than in Asutsuare. Samples from Asutsuare and Obom were 100% and 65% clonal, respectively, by Msp2 genotyping but decreased to 50% and 5%, respectively, when determined by MS analysis. The genetic composition of parasites from Obom and Asutsuare were highly distinct, with parasites from Obom being more diverse than those from Asutsuare. Conclusion: Plasmodium falciparum parasites circulating in Obom are genetically more diverse and distinct from those circulating in Asutsuare. The MOI in samples from both Obom and Asutsuare increased when assessed by MS analysis relative to MSP2 genotyping. The TA40 and TA87 loci are useful markers for estimating MOI in high and low parasite prevalence settings. </td> <td style="text-align:left;"> NA </td> <td style="text-align:left;"> no </td> <td style="text-align:left;"> No good fitness measure and measure host GD </td> <td style="text-align:left;"> NA </td> <td style="text-align:left;"> no </td> <td style="text-align:left;"> no </td> </tr> <tr> <td style="text-align:left;"> 4645 </td> <td style="text-align:left;"> 2020 </td> <td style="text-align:left;"> 44112 </td> <td style="text-align:left;"> Acevedo-Whitehouse, K </td> <td style="text-align:left;"> 2019 </td> <td style="text-align:left;"> Physiological Thresholds in the Context of Marine Mammal Conservation </td> <td style="text-align:left;"> There is growing evidence of the adverse effects of global environmental change on marine mammals, particularly in terms of changes in abundance, distribution, habitat use, migratory phenology, feeding habits, risk of infectious diseases, bioaccumulation of contaminants, declines in reproductive success, and reductions in genetic diversity. These anthropogenic stressors have led to an evident conservation crisis: a quarter of the extant marine mammal species that have been assessed and now considered at risk of extinction. However, we still know very little about the subtle, subclinical effects of environmental stressors on various aspects of physiology that could impacts their survival and long-term fertility, or that could impact the performance of future generations. This chapter argues for the need of considering physiological thresholds when examining how drivers of global environmental change can impact marine mammal populations. It also poses questions regarding our understanding of individual phenotypic plasticity and resilience of species in the face of environmental stressors, particularly under abrupt and unpredictable environmental changes. Specifically, I present evidence of how drivers of environmental change can exert effects at different levels of biological organization to influence the viability of individuals and populations, and discuss which aspects of environmental change could be the most likely to shift the physiological thresholds of different marine mammal species. Finally, the chapter identifies areas of further research in marine mammal conservation physiology in the current context of global environmental change. </td> <td style="text-align:left;"> NA </td> <td style="text-align:left;"> no </td> <td style="text-align:left;"> No good fitness measure and measure host GD </td> <td style="text-align:left;"> NA </td> <td style="text-align:left;"> no </td> <td style="text-align:left;"> no </td> </tr> <tr> <td style="text-align:left;"> 4646 </td> <td style="text-align:left;"> 2020 </td> <td style="text-align:left;"> 44040 </td> <td style="text-align:left;"> Achari, SR; Kaur, J; Dinh, Q; Mann, R; Sawbridge, T; Summerell, BA; Edwards, J </td> <td style="text-align:left;"> 2020 </td> <td style="text-align:left;"> Phylogenetic relationship between Australian Fusarium oxysporum isolates and resolving the species complex using the multispecies coalescent model </td> <td style="text-align:left;"> BackgroundThe Fusarium oxysporum species complex (FOSC) is a ubiquitous group of fungal species readily isolated from agroecosystem and natural ecosystem soils which includes important plant and human pathogens. Genetic relatedness within the complex has been studied by sequencing either the genes or the barcoding gene regions within those genes. Phylogenetic analyses have demonstrated a great deal of diversity which is reflected in the differing number of clades identified: three, five and eight. Genetic limitation within the species in the complex has been studied through Genealogical Concordance Phylogenetic Species Recognition (GCPSR) analyses with varying number of phylogenetic 'species' identified ranging from two to 21. Such differing views have continued to confuse users of these taxonomies.ResultsThe phylogenetic relationships between Australian F. oxysporum isolates from both natural and agricultural ecosystems were determined using three datasets: whole genome, nuclear genes, and mitochondrial genome sequences. The phylogenies were concordant except for three isolates. There were three concordant clades from all the phylogenies suggesting similar evolutionary history for mitochondrial genome and nuclear genes for the isolates in these three clades. Applying a multispecies coalescent (MSC) model on the eight single copy nuclear protein coding genes from the nuclear gene dataset concluded that the three concordant clades correspond to three phylogenetic species within the FOSC. There was 100% posterior probability support for the formation of three species within the FOSC. This is the first report of using the MSC model to estimate species within the F. oxysporum species complex. The findings from this study were compared with previously published phylogenetics and species delimitation studies.ConclusionPhylogenetic analyses using three different gene datasets from Australian F. oxysporum isolates have all supported the formation of three major clades which delineated into three species. Species 2 (Clade 3) may be called F. oxysporum as it contains the neotype for F. oxysporum. </td> <td style="text-align:left;"> NA </td> <td style="text-align:left;"> no </td> <td style="text-align:left;"> No good fitness measure and measure host GD </td> <td style="text-align:left;"> NA </td> <td style="text-align:left;"> no </td> <td style="text-align:left;"> no </td> </tr> <tr> <td style="text-align:left;"> 4647 </td> <td style="text-align:left;"> 2020 </td> <td style="text-align:left;"> 44040 </td> <td style="text-align:left;"> Adams, LG; Gordon, MS; Buth, DG; Hutchings, EM </td> <td style="text-align:left;"> 2020 </td> <td style="text-align:left;"> A Comparison of Isogenic Homozygous Clone and Wildtype Zebrafish (Danio rerio): Survival and Developmental Responses to Low pH Conditions </td> <td style="text-align:left;"> The value of bioassays as analytical methods for assessing the potency of particular stressors on live animal models depends on the precision of their results, which are greatly influenced by the choice of test subjects. The genetic makeup of experimental subjects varies, and, as such, so will their responses to the test environment. Genetic diversity of test populations may contribute to statistical variability; therefore, the use of genetically similar subjects may enhance the utility of bioassays. This study addresses the efficacy of using isogenic homozygous zebrafish (Danio rerio) as subjects for bioassays. Stress responses (acidic conditions) were compared during early development for gynogenetically produced isogenic homozygous line of zebrafish (C32) and wildtype (WT) zebrafish. Experiments evaluated early life stage milestones after exposure to low pH in water of a different electrolyte composition. Because the isogenic homozygous clonal (IHC) fish possessed far less genetic variability than the WT fish tested, it was predicted that the IHC fish would exhibit less variability in their response to stress. Although we found no significant differences in the variability between the responses of the IHC and WT fish, pH and water hardness level had a differential effect on the two groups. Simple strain differences may be the probable cause of the response differences to environmental stress. Factors that may affect stress response, such as heterogeneity, co-adapted gene complexes, and domestication, are discussed. Our findings and review of recent zebrafish literature stress the need for researchers to carefully consider breeding histories and trait characteristics for each potential test subject to maximize the sensitivity of the assay. </td> <td style="text-align:left;"> NA </td> <td style="text-align:left;"> no </td> <td style="text-align:left;"> No group level </td> <td style="text-align:left;"> NA </td> <td style="text-align:left;"> no </td> <td style="text-align:left;"> no </td> </tr> <tr> <td style="text-align:left;"> 4648 </td> <td style="text-align:left;"> 2020 </td> <td style="text-align:left;"> 44113 </td> <td style="text-align:left;"> Adamson, K; Mullett, MS; Solheim, H; Barnes, I; Muller, MM; Hantula, J; Vuorinen, M; Kacergius, A; Markovskaja, S; Musolin, DL; Davydenko, K; Keca, N; Ligi, K; Priedite, RD; Millberg, H; Drenkhan, R </td> <td style="text-align:left;"> 2018 </td> <td style="text-align:left;"> Looking for relationships between the populations of Dothistroma septosporum in northern Europe and Asia </td> <td style="text-align:left;"> Dothistroma septosporum, a notorious pine needle pathogen with an unknown historical geographic origin and poorly known distribution pathways, is nowadays found almost in all areas inhabited by pines (Pinus spp.). The main aim of this study was to determine the relationship between North European and East Asian populations. In total, 238 Eurasian D. septosporum isolates from 11 countries, including 211 isolates from northern Europe, 16 isolates from Russian Far East and 11 isolates from Bhutan were analysed using 11 species-specific microsatellite and mating type markers. The most diverse populations were found in northern Europe, including the Baltic countries, Finland and European Russia. Notably, D. septosporum has not caused heavy damage to P. sylvestris in northern Europe, which may suggest a long co-existence of the host and the pathogen. No indication was obtained that the Russian Far East or Bhutan could be the indigenous area of D. septosporum, as the genetic diversity of the fungus there was low and evidence suggests gene flow from northern Europe to Russian Far East. On the western coast of Norway, a unique genetic pattern was observed, which differed from haplotypes dominating other Fennoscandian populations. As an agent of dothistroma needle blight, only D. septosporum was documented in northern Europe and Asia, while D. pini was found in Ukraine and Serbia. </td> <td style="text-align:left;"> NA </td> <td style="text-align:left;"> no </td> <td style="text-align:left;"> No good fitness measure and measure host GD </td> <td style="text-align:left;"> NA </td> <td style="text-align:left;"> no </td> <td style="text-align:left;"> no </td> </tr> <tr> <td style="text-align:left;"> 4649 </td> <td style="text-align:left;"> 2020 </td> <td style="text-align:left;"> 44112 </td> <td style="text-align:left;"> Afandi, A; Murayama, E; Yin-Ling; Hieno, A; Suga, H; Kageyama, K </td> <td style="text-align:left;"> 2018 </td> <td style="text-align:left;"> Population structures of the water-borne plant pathogen Phytopythium helicoides reveal its possible origins and transmission modes in Japan </td> <td style="text-align:left;"> The purpose of this study was to clarify the genetic diversity of Phytopythium helicoides and to understand the transmission mode of the pathogen in Japan. In total, 232 P. helicoides isolates were collected from various host plants and geographic origins, including farms and natural environments. We developed 6 novel microsatellite markers for use in the study and found 90 alleles among the 6 markers in the 232 isolates. The analysis of molecular variance suggested that P. helicoides has high variance within individuals and low fixation indices between populations. A phylogenetic analysis revealed that isolates collected from the same hosts and/or geographic origins were often grouped together. For example, several isolates from natural environments were grouped with isolates from nearby agricultural areas. On the other hand, 2 geographically distant populations collected from the same host plant had similar genotypes. Our results suggested that migration of the pathogen could be facilitated naturally via drainage systems or by human activity in the transport of agricultural materials. </td> <td style="text-align:left;"> NA </td> <td style="text-align:left;"> no </td> <td style="text-align:left;"> gd of pathogen </td> <td style="text-align:left;"> NA </td> <td style="text-align:left;"> no </td> <td style="text-align:left;"> no </td> </tr> <tr> <td style="text-align:left;"> 4650 </td> <td style="text-align:left;"> 2020 </td> <td style="text-align:left;"> 44111 </td> <td style="text-align:left;"> Agren, A; Vainikka, A; Janhunen, M; Hyvarinen, P; Piironen, J; Kortet, R </td> <td style="text-align:left;"> 2019 </td> <td style="text-align:left;"> Experimental crossbreeding reveals strain-specific variation in mortality, growth and personality in the brown trout (Salmo trutta) </td> <td style="text-align:left;"> Although hybridization between populations with low genetic diversity may induce heterosis, it can also lead to reduced fitness of hybrid offspring through outbreeding depression and loss of local adaptations. Using a half-sib mating design, we studied on brown trout (Salmo trutta) how hybridization of migratory hatchery-strain females with males from various strains would affect early mortality, growth and personality in F-1 offspring. No differences in mortality or alevin body length were found between the crossing groups by the end of the yolk-sac stage. At later developmental stages, higher mortality and slower growth in one of the geographically distant hybrid groups indicated potential outbreeding depression. The personality component indicating boldness and exploration tendency showed fairly low genetic variation and no phenotypic differences among the crossing groups while the personality component related to freezing behavior indicated stronger freezing responses in the purebred and local cross strain when compared to the two other strains. However, the purebred hatchery strain possessed stronger additive genetic tendency for boldness and explorative behavior, and weaker genetic tendency for freezing behavior, when compared to the wild x hatchery hybrid group. Our results add to the cumulating evidence of risks related to the stocking of fish strains from non-native origins. </td> <td style="text-align:left;"> NA </td> <td style="text-align:left;"> no </td> <td style="text-align:left;"> no good gd measure host pop </td> <td style="text-align:left;"> NA </td> <td style="text-align:left;"> no </td> <td style="text-align:left;"> no </td> </tr> <tr> <td style="text-align:left;"> 4651 </td> <td style="text-align:left;"> 2020 </td> <td style="text-align:left;"> 44111 </td> <td style="text-align:left;"> Aguilar, R; Cristobal-Perez, EJ; Balvino-Olvera, FJ; Aguilar-Aguilar, MD; Aguirre-Acosta, N; Ashworth, L; Lobo, JA; Marten-Rodriguez, S; Fuchs, EJ; Sanchez-Montoya, G; Bernardello, G; Quesada, M </td> <td style="text-align:left;"> 2019 </td> <td style="text-align:left;"> Habitat fragmentation reduces plant progeny quality: a global synthesis </td> <td style="text-align:left;"> Most of the world's land surface is currently under human use and natural habitats remain as fragmented samples of the original landscapes. Measuring the quality of plant progeny sired in these pervasive environments represents a fundamental endeavour for predicting the evolutionary potential of plant populations remaining in fragmented habitats and thus their ability to adapt to changing environments. By means of hierarchical and phylogenetically independent meta-analyses we reviewed habitat fragmentation effects on the genetic and biological characteristics of progenies across 179 plant species. Progeny sired in fragmented habitats showed overall genetic erosion in contrast with progeny sired in continuous habitats, with the exception of plants pollinated by vertebrates. Similarly, plant progeny in fragmented habitats showed reduced germination, survival and growth. Habitat fragmentation had stronger negative effects on the progeny vigour of outcrossing- than mixed-mating plant species, except for vertebrate-pollinated species. Finally, we observed that increased inbreeding coefficients due to fragmentation correlated negatively with progeny vigour. Our findings reveal a gloomy future for angiosperms remaining in fragmented habitats as fewer sired progeny of lower quality may decrease recruitment of plant populations, thereby increasing their probability of extinction. </td> <td style="text-align:left;"> NA </td> <td style="text-align:left;"> no </td> <td style="text-align:left;"> No good fitness measure and measure host GD </td> <td style="text-align:left;"> NA </td> <td style="text-align:left;"> no </td> <td style="text-align:left;"> no </td> </tr> <tr> <td style="text-align:left;"> 4652 </td> <td style="text-align:left;"> 2020 </td> <td style="text-align:left;"> 44043 </td> <td style="text-align:left;"> Agyenim-Boateng, KG; Lu, JN; Shi, YZ; Zhang, D; Yin, XG </td> <td style="text-align:left;"> 2019 </td> <td style="text-align:left;"> SRAP analysis of the genetic diversity of wild castor (Ricinus communis L.) in South China </td> <td style="text-align:left;"> Castor bean is an important seed oil crop. Castor oil is a highly demanded oil for several industrial uses. Currently, castor bean varieties suffer from low productivity and high risk of insect pests and diseases. It is in urgent need to mine elite genes from wild materials for castor breeding. 29 pairs of polymorphic SRAP primers out of 361 pairs were used to analyse the genetic diversity of 473 wild castor materials from South China. 203 bands were amplified by the 29 pairs of primers, of which 169 bands were polymorphic, with a polymorphic percentage of 83.25%. With an average number of alleles per locus (A(p)) of 1.801, average number of effective alleles per locus (A(e)) of 1.713 and average percentage of polymorphic loci (P) of 90.04%, these primers were proven to be useful and effective. Nei' genetic distance between the materials ranged from 1.04 to 25.02, with an average of 13.03. At the genetic distance of 25.02, the materials clustered into two major groups, consistent with the result of population structure analysis. However, more subgroups existed between 5.21 and 13.32. Although not all the materials from the same region were clustered in the same group, an obvious trend existed where the groups were related to regions to a great extent. Based on multiple indices, the genetic diversity of materials from Hainan was the lowest. However, there was not much difference between West Guangdong and Guangxi, although the former was slightly higher. Moderate genetic differentiation was observed in wild materials in South China. The genetic differentiation mainly occurred within population, with maximum differentiation in Guangxi, followed by West Guangdong and the minimum in Hainan. Nonetheless, there was an extensive geneflow between populations. The above results provided a direction for the conservation and breeding application of these materials. </td> <td style="text-align:left;"> NA </td> <td style="text-align:left;"> no </td> <td style="text-align:left;"> dometicated host </td> <td style="text-align:left;"> NA </td> <td style="text-align:left;"> no </td> <td style="text-align:left;"> no </td> </tr> <tr> <td style="text-align:left;"> 4653 </td> <td style="text-align:left;"> 2020 </td> <td style="text-align:left;"> 44112 </td> <td style="text-align:left;"> Ahirwar, RN; Mishra, VK; Chand, R; Budhlakoti, N; Mishra, DC; Kumar, S; Singh, S; Joshi, AK </td> <td style="text-align:left;"> 2018 </td> <td style="text-align:left;"> Genome-wide association mapping of spot blotch resistance in wheat association mapping initiative (WAMI) panel of spring wheat (Triticum aestivum L.) </td> <td style="text-align:left;"> Spot blotch (SB) caused by Bipolaris sorokiniana, is one of the most important diseases of wheat in the eastern part of south Asia causing considerable yield loss to the wheat crop. There is an urgent need to identify genetic loci closely associated with resistance to this pathogen for developing resistant cultivars. Hence, genomic regions responsible for SB resistance were searched using a wheat association mapping initiative (WAMI) panel involving 287 spring wheat genotypes of different origin. Genome-wide association mapping (GWAM) was performed using single nucleotide polymorphism (SNP) markers from a custom 90 K wheat SNP array. A mixed linear model (MLM) was used for assessing the association of SNP markers with spot blotch resistance in three consecutive years. Three traits were measured: incubation period, lesion number and area under the disease progress curve (AUDPC). Significant SNP markers were found linked to five, six and four quantitative trait loci (QTLs) for incubation period, lesion number and AUDPC respectively. They were detected on 11 different chromosomes: 1A, 1B, 1D, 4A, 5A, 5B, 6A, 6B, 6D, 7A, 7B with marker R-2 range of 0.083 to 0.11. The greatest number of significant SNP-markers was found for lesion number and AUDPC on chromosome 6B and 5B, respectively, representing a better coverage of B-genome by SNPs. On the other hand, the most significant and largest SNP markers for incubation period were detected on 6A and 4A chromosomes indicating that this trait is associated with the A-genome of wheat. Although, QTLs for spot blotch resistance have been reported in wheat on these same chromosomes, the association of incubation period and lesion number with SB resistance has not been reported in previous studies. The panel exhibits considerable variation for SB resistance and also provides a good scope of marker-assisted selection using the identified SNP markers linked to resistant QTLs. </td> <td style="text-align:left;"> NA </td> <td style="text-align:left;"> no </td> <td style="text-align:left;"> gd of pathogen </td> <td style="text-align:left;"> NA </td> <td style="text-align:left;"> no </td> <td style="text-align:left;"> no </td> </tr> <tr> <td style="text-align:left;"> 4654 </td> <td style="text-align:left;"> 2020 </td> <td style="text-align:left;"> 44112 </td> <td style="text-align:left;"> Ahmed, MA; Chu, KB; Quan, FS </td> <td style="text-align:left;"> 2018 </td> <td style="text-align:left;"> The Plasmodium knowlesi Pk41 surface protein diversity, natural selection, sub population and geographical clustering: a 6-cysteine protein family member </td> <td style="text-align:left;"> Introduction: The zoonotic malaria parasite Plasmodium knowlesi has currently become the most dominant form of infection in humans in Malaysia and is an emerging infectious disease in most Southeast Asian countries. The P41 is a merozoite surface protein belonging to the 6-cysteine family and is a well-characterized vaccine candidate in P. vivax and P. falciparum; however, no study has been done in the orthologous gene of P. knowlesi. This study investigates the level of polymorphism, haplotypes and natural selection of pk41 genes in clinical isolates from Malaysia. Method: Thirty-five full-length pk41 sequences from clinical isolates of Malaysia along with four laboratory lines (along with H-strain) were downloaded from public databases. For comparative analysis between species, orthologous P41 genes from P. falciparum, P. vivax, P. coatneyi and P. cynomolgi were also downloaded. Genetic diversity, polymorphism, haplotype and natural selection were determined using DnaSP 5.10 software. Phylogenetic relationships between Pk41 genes were determined using MEGA 5.0 software. Results: Analysis of 39 full-length pk41 sequences along with the H-strain identified 36 SNPs (20 non-synonymous and 16 synonymous substitutions) resulting in 31 haplotypes. Nucleotide diversity across the full-length gene was low and was similar to its ortholog in P. vivax; pv41. Domain-wise amino acid analysis of the two s48/45 domains indicated low level of polymorphisms for both the domains, and the glutamic acid rich region had extensive size variations. In the central domain, upstream to the glutamate rich region, a unique two to six (K-E)(n) repeat region was identified within the clinical isolates. Overall, the pk41 genes were indicative of negative/purifying selection due to functional constraints. Domain-wise analysis of the s48/45 domains also indicated purifying selection. However, analysis of Tajima's D across the genes identified non-synonymous SNPs in the s48/45 domain II with high positive values indicating possible epitope binding regions. All the 6-cysteine residues within the s48/45 domains were conserved within the clinical isolates indicating functional conservation of these regions. Phylogenetic analysis of full-length pk41 genes indicated geographical clustering and identified three subpopulations of P. knowlesi; one originating in the laboratory lines and two originating from Sarawak, Malaysian Borneo. Conclusion: This is the first study to report on the polymorphism and natural selection of pk41 genes from clinical isolates of Malaysia. The results reveal that there is low level of polymorphism in both s48/45 domains, indicating that this antigen could be a potential vaccine target. However, genetic and molecular immunology studies involving higher number of samples from various parts of Malaysia would be necessary to validate this antigen's candidacy as a vaccine target for P. knowlesi. </td> <td style="text-align:left;"> NA </td> <td style="text-align:left;"> no </td> <td style="text-align:left;"> gd of pathogen </td> <td style="text-align:left;"> NA </td> <td style="text-align:left;"> no </td> <td style="text-align:left;"> no </td> </tr> <tr> <td style="text-align:left;"> 4655 </td> <td style="text-align:left;"> 2020 </td> <td style="text-align:left;"> 44111 </td> <td style="text-align:left;"> Ailloud, F; Didelot, X; Woltemate, S; Pfaffinger, G; Overmann, J; Bader, RC; Schulz, C; Malfertheiner, P; Suerbaum, S </td> <td style="text-align:left;"> 2019 </td> <td style="text-align:left;"> Within-host evolution of Helicobacter pylori shaped by niche-specific adaptation, intragastric migrations and selective sweeps </td> <td style="text-align:left;"> The human pathogen Helicobacter pylori displays extensive genetic diversity. While H. pylori is known to evolve during infection, population dynamics inside the gastric environment have not been extensively investigated. Here we obtained gastric biopsies from multiple stomach regions of 16 H. pylori-infected adults, and analyze the genomes of 10 H. pylori isolates from each biopsy. Phylogenetic analyses suggest location-specific evolution and bacterial migration between gastric regions. Migration is significantly more frequent between the corpus and the fundus than with the antrum, suggesting that physiological differences between antral and oxyntic mucosa contribute to spatial partitioning of H. pylori populations. Associations between H. pylori gene polymorphisms and stomach niches suggest that chemotaxis, regulatory functions and outer membrane proteins contribute to specific adaptation to the antral and oxyntic mucosa. Moreover, we show that antibiotics can induce severe population bottlenecks and likely play a role in shaping the population structure of H. pylori. </td> <td style="text-align:left;"> NA </td> <td style="text-align:left;"> no </td> <td style="text-align:left;"> gd of pathogen </td> <td style="text-align:left;"> NA </td> <td style="text-align:left;"> no </td> <td style="text-align:left;"> no </td> </tr> <tr> <td style="text-align:left;"> 4656 </td> <td style="text-align:left;"> 2020 </td> <td style="text-align:left;"> 44112 </td> <td style="text-align:left;"> Akbar, A; Hussain, S; Ullah, K; Fahim, M; Ali, GS </td> <td style="text-align:left;"> 2018 </td> <td style="text-align:left;"> Detection, virulence and genetic diversity of Fusarium species infecting tomato in Northern Pakistan </td> <td style="text-align:left;"> In addition to the well-known Fusarium oxysporum f.sp. lycopersici, several other Fusarium species are known to cause extensive worldwide crop losses in tomatoes. Prevalence and identities of Fusarium species infecting tomatoes in Northwest Pakistan is currently not known. In this study, we surveyed and characterized Fusarium species associated with symptomatic tomatoes in Northwest Pakistan using morphological and molecular analyses. Pathogenicity tests revealed varying degrees of virulence with some Fusarium sp. causing severe disease symptoms whereas others displaying mild symptoms. Molecular identification based on Internal Transcribed Spacer (ITS) region and TEF-1 alpha gene sequencing classified all isolates into four major species with a majority (68.9%) belonging to Fusarium incarnatum-equiseti species complex (FIESC), followed by F. graminearum (20.7%), F. acuminatum (6.8%), and F. solani (6.8%). ISSR analyses revealed substantial genetic variability among all the Fusarium population infecting tomatoes. Genetic distance between populations from the central region and the type strain F.o.f.sp. lycopersici from Florida was the highest (0.3662), whereas between the south and central region was the lowest (0.0298), which showed that genetic exchange is negatively effected by distance. High genetic variability suggests that these Fusarium species have the potential to become a major production constraint for tomato growers. Findings in this report would greatly facilitate identification of Fusarium species in developing countries and would provide groundwork for devising and implementing disease management measures for minimizing losses caused by Fusarium species in tomatoes. </td> <td style="text-align:left;"> NA </td> <td style="text-align:left;"> no </td> <td style="text-align:left;"> gd of pathogen </td> <td style="text-align:left;"> NA </td> <td style="text-align:left;"> no </td> <td style="text-align:left;"> no </td> </tr> <tr> <td style="text-align:left;"> 4657 </td> <td style="text-align:left;"> 2020 </td> <td style="text-align:left;"> 44112 </td> <td style="text-align:left;"> Akindolire, MA; Ateba, CN </td> <td style="text-align:left;"> 2018 </td> <td style="text-align:left;"> Use of pulsed field gel electrophoresis genetic typing for tracing contamination with virulent Escherichia coli O157:H7 in beef-cattle producing farms </td> <td style="text-align:left;"> E. coli. O157:H7 is an important causative agent of a variety of foodborne infections including hemorrhagic colitis (HC), hemolytic uremic syndrome (HUS) and thrombotic thrombocytopenic purpura (TTP) that may lead to renal failure in humans. The main source of infection is contaminated food of animal origin and a number of animal species especially cattle have been associated with this pathogen. Contamination usually occurs when faeces of animals get into contact with carcass during slaughtering. The aim of the present study was to determine the virulence profiles and genetic relationships of E. coli. O157:H7 strains isolated from cattle some commercial farms in the North West Province, South Africa. A total of 260 faecal samples were collected from eight farms over a six-month period and assessed for the presence of E. coli. O157:H7 through PCR analysis. Bacteria 16S rRNA gene fragments were amplified as an internal control while the rfb(O157) and flic(H7), gene PCR were used to confirm identities of isolates. STEC virulence genes stxl, stx2, eaeA and the hlyA were detected using PCR and the genetic relatedness of the isolates was determined using Pulse Field Gel electrophoresis (PFGE). A total of 69 (26.5%) E. coli O157:H7 isolates were detected in samples obtained from all the eight farms. A large proportions of the E. coli isolates possessed the stx(1) (66.7%) and stx(2) (97.1%) genes respectively. In addition, other accessory virulence genes eaeA and hIyA were detected in 30 (43.5%) and 43 (62.3%) of the E. coli. isolates respectively. Pulsed-Filed Gel Electrophoresis (PFGE) analysis grouped these isolates into 6 clusters and the largest cluster contained 17 isolates originating from different farms. These results provide valid evidence that cattle harbour virulent and genetically similar E. coll. strains particularly those belonging to the serotype O157 and therefore these animals may serve as possible source for zoonotic transfer of these pathogens to humans. The implication is that these isolates may have severe public health consequences on consumers. </td> <td style="text-align:left;"> NA </td> <td style="text-align:left;"> no </td> <td style="text-align:left;"> dometicated host </td> <td style="text-align:left;"> NA </td> <td style="text-align:left;"> no </td> <td style="text-align:left;"> no </td> </tr> <tr> <td style="text-align:left;"> 4658 </td> <td style="text-align:left;"> 2020 </td> <td style="text-align:left;"> 44112 </td> <td style="text-align:left;"> Akindolire, MA; Kumar, A; Ateba, CN </td> <td style="text-align:left;"> 2018 </td> <td style="text-align:left;"> Genetic characterization of antibiotic-resistant Staphylococcus aureus from milk in the North-West Province, South Africa </td> <td style="text-align:left;"> Food borne diseases are a major public health concern worldwide. Staphylococcus aureus is one of the potential food borne pathogens which causes nosocomial and community acquired infections. In the present study, 74 representative strains of S. aureus isolated and characterized in previous study from different milk samples were subjected to random amplified polymorphic DNA (RAPD) polymerase chain reaction (PCR) and enterobacterial repetitive intergenic consensus (ERIC)-PCR to generate fingerprints to determine the genetic relationships of the isolated strains. A total of 20 RAPD patterns were generated and the number of amplified fragments obtained ranged from 0 to 8 with molecular weight ranging from 250 to 2000 bp. A dendrogram based on fingerprinting pattern grouped isolates into twelve major clusters (I-XII). In the case of ERIC-PCR 9 banding patterns were obtained with amplicons ranging from 1 to 8 and band sizes ranging from 250 to 2000 bp. A total of four major clusters (I-IV) were observed in the dendrogram based on ERIC fingerprints. The discrete banding patterns obtained both from ERIC-PCR and RAPD-PCR showed remarkably the genetic diversity of S. aureus. The findings of this study indicate that raw, bulk and pasteurized milk in the North-West Province was contaminated with toxigenic and multi-drug resistant S. aureus strains. This emphasizes the need to implement appropriate control measures to reduce contamination as well as the spread of virulent S. aureus strains to reduce the burden of disease in humans. (C) 2015 The Authors. Production and hosting by Elsevier B.V. on behalf of King Saud University. This is an open access article under the CCBY-NC-ND license (http://creativecommons.org/licenses/by-nc-nd/4.0/). </td> <td style="text-align:left;"> NA </td> <td style="text-align:left;"> no </td> <td style="text-align:left;"> No good fitness measure and measure host GD </td> <td style="text-align:left;"> NA </td> <td style="text-align:left;"> no </td> <td style="text-align:left;"> no </td> </tr> <tr> <td style="text-align:left;"> 4659 </td> <td style="text-align:left;"> 2020 </td> <td style="text-align:left;"> 44039 </td> <td style="text-align:left;"> Al-Dakhil, AA </td> <td style="text-align:left;"> 2020 </td> <td style="text-align:left;"> Genetic Variability of the Populations of Phlebotomus papatasi, the Main Vector of Leishmania major, in Al-Madinah Al-Munawarah, Saudi Arabia </td> <td style="text-align:left;"> Sand flies are vectors of several human pathogens. Phlebotomus papatasi has been confirmed as the vector of Leishmania major, the causative agent of cutaneous leishmaniasis (CL) in Saudi Arabia. This species is the predominant in Al-Madinah which is in agreement with the expansion of CL. In the present study, the random amplified polymorphic DNA-polymerase chain reaction (RAPD-PCR) technique was applied with six primers to evaluate the relationships among the geographic populations of P. papatasi collected from four villages in Al-Madinah: Almondasa, Almaliliah, Abyar Al-Mashy and Agool. The results showed the mean proportion of the polymorphic loci within the populations was (84.89%), while between them it was (94.51%). The average total genetic diversity across the all studied populations was slightly higher (0.35) than the genetic diversity within the populations (0.31). The average of the gene flow between the four populations was rated to have a worth of 4.363. This higher level of gene flow corresponds to less differentiation between the populations, which could result from the limited distances between the sampling areas. The phylogenetic and genetic distance analyses generated from the RAPD-PCR profiles showed close relationships between Almondasa and Almaliliah populations, while the largest genetic distance was detected between the populations of Almondasa and Agool. In conclusion, the genetic variability among and within P. papatasi populations was identified based on the efficient RAPD-PCR technique. The identification of the vector of cutaneous leishmaniasis in Al-Madinah and the differentiation of its populations and individuals is vital to the field of medical entomology. </td> <td style="text-align:left;"> NA </td> <td style="text-align:left;"> no </td> <td style="text-align:left;"> No good fitness measure </td> <td style="text-align:left;"> NA </td> <td style="text-align:left;"> no </td> <td style="text-align:left;"> no </td> </tr> <tr> <td style="text-align:left;"> 4660 </td> <td style="text-align:left;"> 2020 </td> <td style="text-align:left;"> 44112 </td> <td style="text-align:left;"> Albinsky, D; Wham, D; Shinzato, N; Reimer, JD </td> <td style="text-align:left;"> 2018 </td> <td style="text-align:left;"> Population Connectivity in the Common Reef Zoantharian Zoanthus sansibaricus (Anthozoa: Hexacorallia) in Southern Japan </td> <td style="text-align:left;"> Tropical and subtropical shallow benthic marine communities are highly diverse and balanced systems that constitute an important natural resource. Knowledge of the genetic diversity, connectivity and reproduction mode of each population is critical to understanding the fate of whole assemblages in times of disturbances. Importantly, the capability of populations to adapt to environmental challenges will be crucial to determining their survival. Here, we report on the population structure of the common reef zoantharian Zoanthus sansibaricus in the northwestern Pacific, by examining populations at three different locations in southern Japan using five highly variable microsatellite markers. Analyses of a population at the species' northern distribution limit combined with analyses of two subtropical populations suggest that habitat characteristics and ocean currents influence the connectivity and genetic diversity of this species. Our findings emphasize the adaptive ability of Z. sansibaricus to different environmental conditions and may help explain the wide distribution and generalist nature of this species. </td> <td style="text-align:left;"> NA </td> <td style="text-align:left;"> no </td> <td style="text-align:left;"> No good fitness measure </td> <td style="text-align:left;"> NA </td> <td style="text-align:left;"> no </td> <td style="text-align:left;"> no </td> </tr> <tr> <td style="text-align:left;"> 4661 </td> <td style="text-align:left;"> 2020 </td> <td style="text-align:left;"> 44040 </td> <td style="text-align:left;"> Alemu, K; Adugna, G; Lemessa, F; Muleta, D </td> <td style="text-align:left;"> 2020 </td> <td style="text-align:left;"> Variation among colletotrichum isolates associated with coffee berry disease in Ethiopia </td> <td style="text-align:left;"> Variations among the populations of Colletotrichum species from diseased coffee berries were studied. A total of 35 Colletotrichum isolates representing 24 districts from major coffee-producing regions of Ethiopia was studied on the basis of cultural, morphological, and pathological characteristics. The isolates differed significantly on their colony color, density, sector, and acervuli. Mycelial growth rate varied significantly (p < 0.05) among the isolates and ranged between 3.97 and 8.69 mm/day. Sporulation capacity, conidial length (12.3-17.7 mu m) and conidia width (3.6-5.1 mu m) also varied significantly (p < 0.05) among the isolates. The dominant forms of conidia were cylindrical and round at both ends followed by cylindrical acute at one and round at the other ends. Cluster analysis based on cultural, morphological, and pathological characteristics showed that isolates of Colletotrichum species associated diseased coffee berries fell into three distinct groups and were identified as C. kahawae, C. gloeosporioides, and C. acutatum. Among these fungal isolates, only C. kahawae was pathogenic to both detached coffee berries and coffee hypocotyls tested. The other Colletotrichum species exist as saprophytic or sequential colonizer of dead tissues. In conclusion, our study indicates the existence of variation in cultural, morphological characteristics and pathogenicity among the C. kahawae and the other related Colletotrichum isolates. However, molecular methods and other detail biochemical tests would provide the genetic diversity of the CBD pathogen populations in the country. </td> <td style="text-align:left;"> NA </td> <td style="text-align:left;"> no </td> <td style="text-align:left;"> dometicated host </td> <td style="text-align:left;"> NA </td> <td style="text-align:left;"> no </td> <td style="text-align:left;"> no </td> </tr> <tr> <td style="text-align:left;"> 4662 </td> <td style="text-align:left;"> 2020 </td> <td style="text-align:left;"> 44113 </td> <td style="text-align:left;"> Ali, Z; Zulfiqar, S; Klar, J; Wikstrom, J; Ullah, F; Khan, A; Abdullah, U; Baig, S; Dahl, N </td> <td style="text-align:left;"> 2017 </td> <td style="text-align:left;"> Homozygous GRID2 missense mutation predicts a shift in the D-serine binding domain of GluD2 in a case with generalized brain atrophy and unusual clinical features </td> <td style="text-align:left;"> Background: Spinocerebellar ataxias comprise a large and heterogeneous group of disorders that may present with isolated ataxia, or ataxia in combination with other neurologic or non-neurologic symptoms. Monoallelic or biallelic GRID2 mutations were recently reported in rare cases with cerebellar syndrome and variable degree of ataxia, ocular symptoms, hypotonia and developmental delay. Case presentation: We report on a consanguineous family with autosomal recessive childhood onset of slowly progressive cerebellar ataxia and delayed psychomotor development in three siblings. MRI of an adult and affected family member revealed slightly widened cerebral and cerebellar sulci, suggesting generalized brain atrophy, and mild cerebellar atrophy. Using whole exome sequencing we identified a novel homozygous missense variant [c. 2128C > T, p. (Arg710Trp)] in GRID2 that segregates with the disease. The missense variant is located in a conserved region encoding the extracellular serine-binding domain of the GluD2 protein and predicts a change in conformation of the protein. Conclusion: The widespread supratentorial brain abnormalities, absence of oculomotor symptoms, increased peripheral muscle tone and the novel missense mutation add to the clinical and genetic variability in GRID2 associated cerebellar syndrome. The neuroradiological findings in our family indicate a generalized neurodegenerative process to be taken into account in other families segregating complex clinical features and GRID2 mutations. </td> <td style="text-align:left;"> NA </td> <td style="text-align:left;"> no </td> <td style="text-align:left;"> No good fitness measure and measure host GD </td> <td style="text-align:left;"> NA </td> <td style="text-align:left;"> no </td> <td style="text-align:left;"> no </td> </tr> <tr> <td style="text-align:left;"> 4663 </td> <td style="text-align:left;"> 2020 </td> <td style="text-align:left;"> 44039 </td> <td style="text-align:left;"> Alkafajy, A; Al-Karagoly, H; Brujeni, GN </td> <td style="text-align:left;"> 2020 </td> <td style="text-align:left;"> Comparison of cattle BoLA-DRB3 typing by PCR-RFLP, direct sequencing, and high-resolution DNA melting curve analysis </td> <td style="text-align:left;"> Major histocompatibility complex (MHC) represents an important genetic marker for manipulation to improve the health and productivity of cattle. It is closely associated with numerous disease susceptibilities and immune responses. Bovine MHC, also called bovine leukocyte antigen (BoLA), is considered as a suitable marker for genetic diversity studies. In cattle, most of the polymorphisms are located in exon 2 of BoLA-DRB3, which encodes the peptide-binding cleft. In this study, the polymorphism of the BoLA-DRB3.2 gene in Holstein's calves was studied using high resolution melting curve analysis (HRM). Observed HRM results were compared to PCR-RFLP and direct sequencing techniques. Eight different HRM and seven different RFLP profiles were identified among the population studied. By comparing to sequencing data, HRM could completely discriminate all genotypes (eight profiles), while the RFLP failed to distinguish between the genotypes *1101/*1001 and *1104/*1501. According to the results, the HRM analysis method gave more accurate results than RFLP by differentiating between the BoLA-DRB3.2 genotypes. Due to the Co-dominant nature of the MHC alleles, HRM technique could be used for investigating the polymorphisms of genotypes and their associations with immune responses. (C) 2020 Urmia University. All rights reserved. </td> <td style="text-align:left;"> NA </td> <td style="text-align:left;"> no </td> <td style="text-align:left;"> no group level </td> <td style="text-align:left;"> NA </td> <td style="text-align:left;"> no </td> <td style="text-align:left;"> no </td> </tr> <tr> <td style="text-align:left;"> 4664 </td> <td style="text-align:left;"> 2020 </td> <td style="text-align:left;"> 44042 </td> <td style="text-align:left;"> Ally, HM; El Hamss, H; Simiand, C; Maruthi, MN; Colvin, J; Omongo, CA; Delatte, H </td> <td style="text-align:left;"> 2019 </td> <td style="text-align:left;"> What has changed in the outbreaking populations of the severe crop pest whitefly species in cassava in two decades? </td> <td style="text-align:left;"> High populations of African cassava whitefly (Bemisia tabaci) have been associated with epidemics of two viral diseases in Eastern Africa. We investigated population dynamics and genetic patterns by comparing whiteflies collected on cassava in 1997, during the first whitefly upsurges in Uganda, with collections made in 2017 from the same locations. Nuclear markers and mtCOI barcoding sequences were used on 662 samples. The composition of the SSA1 population changed significantly over the 20-year period with the SSA1-SG2 percentage increasing from 0.9 to 48.6%. SSA1-SG1 and SSA1-SG2 clearly interbreed, confirming that they are a single biological species called SSA1. The whitefly species composition changed: in 1997, SSA1, SSA2 and B. afer were present; in 2017, no SSA2 was found. These data and those of other publications do not support the 'invader' hypothesis. Our evidence shows that no new species or new population were found in 20 years, instead, the distribution of already present genetic clusters composing SSA1 species have changed over time and that this may be in response to several factors including the introduction of new cassava varieties or climate changes. The practical implications are that cassava genotypes possessing both whitefly and disease resistances are needed urgently. </td> <td style="text-align:left;"> NA </td> <td style="text-align:left;"> no </td> <td style="text-align:left;"> No good fitness measure and measure host GD </td> <td style="text-align:left;"> NA </td> <td style="text-align:left;"> no </td> <td style="text-align:left;"> no </td> </tr> <tr> <td style="text-align:left;"> 4665 </td> <td style="text-align:left;"> 2020 </td> <td style="text-align:left;"> 44111 </td> <td style="text-align:left;"> Almiron, MG; Carretero, EEM; Kaluza, MDN; Rothis, LM </td> <td style="text-align:left;"> 2019 </td> <td style="text-align:left;"> Linking hydrological, geomorphological and biological properties in hyper-arid conditions: Distribution processes of a dominant clonal cactus by run-off </td> <td style="text-align:left;"> The objective was to analyse the asexual spread of Tephrocactus aoracanthus (Cactaceae) related to run-off triggered by torrential summer rains in a hyper-arid desert. Through a manipulative essay, 30 joints (asexual dispersing stems) were placed in the mainstream of an alluvial fan and 30 joints out of the alluvial fan, without run-off influence (control). All joints were labelled, and their weight and diameter were measured. After each rain event with run-off, joints were surveyed identifying their spatial coordinates and environmental context. For this, two factors with two levels each one was selected: position respect to both microrelief (channels with concentrated run-off, or inter-channels as flat areas between channels, without run-off) and vegetation (entangled or not entangled in shrubs). Through a geographic information system, changes in slope angle and rugosity of terrain were analysed. Linear regressions were performed between the partial displacement of joints and their weight and diameter, grouped according to environmental context before each rainfall. None of the joints of the control treatment were moved, but all joints located in the mainstream were relocated. Three rainfall events triggered a mean displacement of 480.4 +/- 210.0m (SD); 103.0 +/- 138.9m (SD) and 33.2 +/- 52.3m (SD), respectively. The mean accumulated displacement of all joints during the experience was 577.12 +/- 220.0m (SD). As they moved, the joints passed through several different environmental contexts, increasing the proportion of joints entangled in vegetation and in inter-channel position. Until rooted, joints could be displaced by run-off in an anisotropic process, with an evident effect of slope angle and rugosity of terrain. On the other hand, no direct relationship was found between the displacement and the magnitude of the event, or both the size and the weight of joints. We assess the strategies related to the dispersal mechanism of clonal plants, related to conditions and enabling its agamic dispersal over long distances. </td> <td style="text-align:left;"> NA </td> <td style="text-align:left;"> no </td> <td style="text-align:left;"> No good fitness measure and measure host GD </td> <td style="text-align:left;"> NA </td> <td style="text-align:left;"> no </td> <td style="text-align:left;"> no </td> </tr> <tr> <td style="text-align:left;"> 4666 </td> <td style="text-align:left;"> 2020 </td> <td style="text-align:left;"> 44041 </td> <td style="text-align:left;"> Alotaibi, NM; Kenyon, EJ; Cook, KJ; Borger, L; Bull, JC </td> <td style="text-align:left;"> 2019 </td> <td style="text-align:left;"> Low genotypic diversity and long-term ecological decline in a spatially structured seagrass population </td> <td style="text-align:left;"> In isolated or declining populations, viability may be compromised further by loss of genetic diversity. Therefore, it is important to understand the relationship between long-term ecological trajectories and population genetic structure. However, opportunities to combine these types of data are rare, especially in natural systems. Using an existing panel of 15 microsatellites, we estimated allelic diversity in seagrass, Zostera marina, at five sites around the Isles of Scilly Special Area of Conservation, UK, in 2010 and compared this to 23 years of annual ecological monitoring (1996-2018). We found low diversity and long-term declines in abundance in this relatively pristine but isolated location. Inclusion of the snapshot of genotypic, but less-so genetic, diversity improved prediction of abundance trajectories; however, this was spatial scale-dependent. Selection of the appropriate level of genetic organization and spatial scale for monitoring is, therefore, important to identify drivers of eco-evolutionary dynamics. This has implications for the use of population genetic information in conservation, management, and spatial planning. </td> <td style="text-align:left;"> NA </td> <td style="text-align:left;"> no </td> <td style="text-align:left;"> No good fitness measure </td> <td style="text-align:left;"> NA </td> <td style="text-align:left;"> no </td> <td style="text-align:left;"> no </td> </tr> <tr> <td style="text-align:left;"> 4667 </td> <td style="text-align:left;"> 2020 </td> <td style="text-align:left;"> 44112 </td> <td style="text-align:left;"> Alves, CL; Repoles, BM; da Silva, MS; Mendes, IC; Marin, PA; Aguiar, PHN; Santos, SD; Franco, GR; Macedo, AM; Pena, SDJ; Andrade, LD; Guarneri, AA; Tahara, EB; Elias, MC; Machado, CR </td> <td style="text-align:left;"> 2018 </td> <td style="text-align:left;"> The recombinase Rad51 plays a key role in events of genetic exchange in Trypanosoma cruzi </td> <td style="text-align:left;"> Detection of genetic exchange has been a limiting factor to deepen the knowledge on the mechanisms by which Trypanosoma cruzi is able to generate progeny and genetic diversity. Here we show that incorporation of halogenated thymidine analogues, followed by immunostaining, is a reliable method not only to detect T. cruzifused-cell hybrids, but also to quantify their percentage in populations of this parasite. Through this approach, we were able to detect and quantify fused-cell hybrids of T. cruzi clones CL Brener andY. Given the increased detection of fused-cell hybrids in naturally-occurring hybrid CL Brener strain, which displays increased levels of RAD51 and BRCA2 transcripts, we further investigated the role of Rad51 - a recombinase involved in homologous recombination - in the process of genetic exchange. We also verified that the detection of fused-cell hybrids in T. cruzi overexpressing RAD51 is increased when compared to wild-type cells, suggesting a key role for Rad51 either in the formation or in the stabilization of fused-cell hybrids in this organism. </td> <td style="text-align:left;"> NA </td> <td style="text-align:left;"> no </td> <td style="text-align:left;"> No good fitness measure and measure host GD </td> <td style="text-align:left;"> NA </td> <td style="text-align:left;"> no </td> <td style="text-align:left;"> no </td> </tr> <tr> <td style="text-align:left;"> 4668 </td> <td style="text-align:left;"> 2020 </td> <td style="text-align:left;"> 44112 </td> <td style="text-align:left;"> Amari, T; Zaabar, W; Semmar, N; Souty-Grosset, C; Achouri, MS </td> <td style="text-align:left;"> 2019 </td> <td style="text-align:left;"> PORCELLIO LAEVIS (CRUSTACEA, ISOPODA) FROM SOIL DETRITIVORE TO VEGETABLE CROP PEST: CASE STUDY OF THE MELON (CUCUMIS MELO L., CUCURBITACEAE) </td> <td style="text-align:left;"> Terrestrial isopods play a particularly important ecosystem function in mineralizing organic matter but, according to the species and the cultivated agroecosystem, their status of pest is yet to be confirmed. In this context, Porcellio laevis Latreille, 1804 is considered as a serious pest for some varieties of melon (Cucumis melo L.) in Tunisia. Monthly samples (March 2013 to December 2015) showed that the important variation in density of P. laevis depended both on season and melon variety: afamia, canary, cantaloupe and tziri. Furthermore, this density was positively correlated with maturity stage of melon (immature, ripe and overripe). In melon, analysis of total phenolic contents and hydrophilic antioxidant activity exhibited a significant difference between varieties and maturity stages (P < 0.01). Our results showed that the most damaged varieties afamia and cantaloupe, characterized by (i) thinner cortex, (ii) higher total phenolic contents, (iii) high concentration of internal ethylene and (iv) liberation of aromatic odor during mature and post mature stages, offered favorable conditions for fast growth, longer survival and higher fertility of P. laevis leading to an increase in population and turning it into a crop pest. </td> <td style="text-align:left;"> NA </td> <td style="text-align:left;"> no </td> <td style="text-align:left;"> dometicated host </td> <td style="text-align:left;"> NA </td> <td style="text-align:left;"> no </td> <td style="text-align:left;"> no </td> </tr> <tr> <td style="text-align:left;"> 4669 </td> <td style="text-align:left;"> 2020 </td> <td style="text-align:left;"> 44111 </td> <td style="text-align:left;"> Ambrosio, L; Neri, A; Fazio, C; Rossolini, GM; Vacca, P; Riccobono, E; Voller, F; Miglietta, A; Stefanelli, P </td> <td style="text-align:left;"> 2019 </td> <td style="text-align:left;"> Genomic analysis of Neisseria meningitidis carriage isolates during an outbreak of serogroup C clonal complex 11, Tuscany, Italy </td> <td style="text-align:left;"> Background In 2015-2016, a cross-sectional carriage survey was performed in Tuscany Region, Italy, during an outbreak of invasive meningococcal disease due to Neisseria meningitidis serogroup C clonal complex 11 (MenC:cc11). This study aims to evaluate the genomic profile of meningococcal carriage isolates collected during the survey. Methods Whole-genome sequencing (WGS) was performed using Illumina MiSeq on 85 cultivated meningococcal carriage isolates received at the Dept. of Infectious Disease, National Institute of Health (Istituto Superiore di Sanita, ISS), as National Reference Laboratory (NRL) for Invasive Meningococcal Disease (IMD). De novo assembled genomes were scanned by the BIGSdb platform to assign: the genotypic profiles, the cgMLST, the vaccine antigen variants and allele types of antimicrobial resistance associated genes, together with denitrification pathway loci. Results Capsule null and non-groupable meningococci accounted for 52.9% and 10.6%, respectively. Among the remaining carriage isolates, serogroup B was the predominant (71.0%). Serogroup C meningococci were culture negative and unavailable for WGS. Overall, 64 genotypic profiles were identified and, based on cgMLST, isolates clustered according to clonal complexes. Eight isolates (9.4%) harbored at least one gene encoding a 4CMenB vaccine antigen. Mutated penA alleles were found in more than 82%. Finally, complete aniA and norB coding sequences were detected among 71.8% of carriage isolates. Conclusions Meningococcal carriage isolates collected during the MenC:cc11 outbreak were characterized by an extensive genetic diversity. The lack of outbreak-related isolates among carriage might be attributable to the high transmissibility with low duration of colonization of MenC: cc11 meningococci. </td> <td style="text-align:left;"> NA </td> <td style="text-align:left;"> no </td> <td style="text-align:left;"> No good fitness measure and measure host GD </td> <td style="text-align:left;"> NA </td> <td style="text-align:left;"> no </td> <td style="text-align:left;"> no </td> </tr> <tr> <td style="text-align:left;"> 4670 </td> <td style="text-align:left;"> 2020 </td> <td style="text-align:left;"> 44113 </td> <td style="text-align:left;"> Amherd, M; Velicer, GJ; Rendueles, O </td> <td style="text-align:left;"> 2018 </td> <td style="text-align:left;"> Spontaneous nongenetic variation of group size creates cheater-free groups of social microbes </td> <td style="text-align:left;"> Social microbes are exposed to cheaters that exploit the advantages of cooperative actions without paying their fair share. Upon starvation, myxobacterial cells cooperatively aggregate into discrete fruiting bodies containing stress-resistant spores. Fruiting bodies formed from a genetically identical population can vary greatly in the number of spores that they carry. We show that such variation allows the exclusion of cheaters from some fruiting bodies and can be a mechanism to maintain cooperation.In social organisms, cheaters that gain a fitness advantage by defecting from the costs of cooperation reduce the average level of cooperation in a population. Such cheating load can be severe enough to cause local extinction events when cooperation is necessary for survival, but can also mediate group-level selection against cheaters across spatially structured groups that vary in cheater frequency. In cheater-laden populations, such variation could be generated by the formation of new homogeneous groups by small numbers of identical cells. Here, we use the model social bacterium Myxococcus xanthus to test whether population bottlenecks inherent to the starvation-induced formation of multicellular fruiting bodies can generate cheater-free groups within an initially cheater-laden population. We first show that genetically identical fruiting bodies vary greatly in their numbers of stress-resistant spores. We further show mathematically and experimentally that this variation can include small cheater-free groups. Such nongenetic variation in group size was found to occur in a variety of M. xanthus isolates and Myxococcus species. Our results suggest that stress-induced reductions in group size may serve as a general process that repeatedly purges genetic diversity from a minority of social groups, thus recurrently generating high-relatedness social environments unburdened by cheating load. </td> <td style="text-align:left;"> NA </td> <td style="text-align:left;"> no </td> <td style="text-align:left;"> No good fitness measure and measure host GD </td> <td style="text-align:left;"> NA </td> <td style="text-align:left;"> no </td> <td style="text-align:left;"> no </td> </tr> <tr> <td style="text-align:left;"> 4671 </td> <td style="text-align:left;"> 2020 </td> <td style="text-align:left;"> 44113 </td> <td style="text-align:left;"> Ammar, E; Hall, DG; Hosseinzadeh, S; Heck, M </td> <td style="text-align:left;"> 2018 </td> <td style="text-align:left;"> The quest for a non-vector psyllid: Natural variation in acquisition and transmission of the huanglongbing pathogen 'Candidatus Liberibacter asiaticus' by Asian citrus psyllid isofemale lines </td> <td style="text-align:left;"> Genetic variability in insect vectors is valuable to study vector competence determinants and to select non-vector populations that may help reduce the spread of vector-borne pathogens. We collected and tested vector competency of 15 isofemale lines of Asian citrus psyllid, Diaphorina citri, vector of`Candidatus Liberibacter asiaticus’ (CLas). CLas is associated with huanglongbing (citrus greening), the most serious citrus disease worldwide. D. citri adults were collected from orange jasmine (Murraya paniculata) hedges in Florida, and individual pairs (females and males) were caged on healthy Murraya plants for egg laying. The progeny from each pair that tested CLas-negative by qPCR were maintained on Murraya plants and considered an isofemale line. Six acquisition tests on D. citri adults that were reared as nymphs on CLas-infected citrus, from various generations of each line, were conducted to assess their acquisition rates (percentage of qPCR-positive adults). Three lines with mean acquisition rates of 28 to 32%, were classified as ‘good’ acquirers and three other lines were classified as ‘poor’ acquirers, with only 5 to 8% acquisition rates. All lines were further tested for their ability to inoculate CLas by confining CLas-exposed psyllids for one week onto healthy citrus leaves (6 +/- 10 adults/leaf/week), and testing the leaves for CLas by qPCR. Mean inoculation rates were 19 to 28% for the three good acquirer lines and 0 to 3% for the three poor acquirer lines. Statistical analyses indicated positive correlations between CLas acquisition and inoculation rates, as well as between CLas titer in the psyllids and CLas acquisition or inoculation rates. Phenotypic and molecular characterization of one of the good and one of the poor acquirer lines revealed differences between them in color morphs and hemocyanin expression, but not the composition of bacterial endosymbionts. Understanding the genetic architecture of CLas transmission will enable the development of new tools for combating this devastating citrus disease. | NA | no | No good fitness measure and measure host GD | NA | no | no |
| 4672 | 2020 | 44111 | Anacleto, O; Cabaleiro, S; Villanueva, B; Saura, M; Houston, RD; Woolliams, JA; Doeschl-Wilson, AB | 2019 | Genetic differences in host infectivity affect disease spread and survival in epidemics | Survival during an epidemic is partly determined by host genetics. While quantitative genetic studies typically consider survival as an indicator for disease resistance (an individual’s propensity to avoid becoming infected or diseased), mortality rates of populations undergoing an epidemic are also affected by endurance (the propensity of diseased individual to survive the infection) and infectivity (i.e. the propensity of an infected individual to transmit disease). Few studies have demonstrated genetic variation in disease endurance, and no study has demonstrated genetic variation in host infectivity, despite strong evidence for considerable phenotypic variation in this trait. Here we propose an experimental design and statistical models for estimating genetic diversity in all three host traits. Using an infection model in fish we provide, for the first time, direct evidence for genetic variation in host infectivity, in addition to variation in resistance and endurance. We also demonstrate how genetic differences in these three traits contribute to survival. Our results imply that animals can evolve different disease response types affecting epidemic survival rates, with important implications for understanding and controlling epidemics. | NA | no | n | NA | no | no |
| 4673 | 2020 | 44042 | Andersen, JC; Camp, CP; Davis, MJ; Havill, NP; Elkinton, JS | 2019 | Development of microsatellite markers for an outbreaking species of oak gall wasp, Zapatella davisae (Hymenoptera: Cynipidae), in the northeastern United States | Populations of the recently described black oak gall wasp, Zapatella davisae Buffington (Hymenoptera: Cynipidae), have been identified as the cause of extensive tree damage and mortality to black oaks, Quercus velutina Lamarck (Fagales: Fagaceae), in the northeastern United States. Relatively little is known, however, about the distribution, phylogenetic placement, and lifecycle of this important tree pest. Therefore, we conducted next-generation sequencing using the Ion Torrent (TM) PGM (ThermoFisher Scientific, Inc.) platform to develop genomic resources for the study of Z. davisae and for other closely related species of oak gall wasps. Individual sequence reads were aligned, assembled into unique contigs, and the contigs were then utilized for the in silico isolation and development of microsatellite markers. In total, we screened 36 candidate microsatellite loci, of which 23 amplified consistently (five polymorphic and 18 monomorphic). We then examined whether the polymorphic loci could be used to infer whether populations of Z. davisae from Cape Cod and Nantucket are sexual or asexual by calculating several metrics of genetic diversity that might indicate the mode of reproduction. These included testing for statistical deviations from Hardy-Weinberg equilibrium (HWE) and for linkage disequilibrium (LD), observations for the presence of the Meselson effect, and by calculating the probability that clonal individuals are more prevalent than would be expected in a randomly mating population. While we found significant deviations from HWE and more clonal individuals than expected, our estimates of the Meselson effect were inconclusive due to limited sampling, and we found no evidence of LD. Therefore, the sexual/asexual status of Z. davisae populations remains uncertain. | NA | no | No good fitness measure and measure host GD | NA | no | no |
| 4674 | 2020 | 44112 | Andersson, M; Ahlund, M; Waldeck, P | 2019 | Brood parasitism, relatedness and sociality: a kinship role in female reproductive tactics | Conspecific brood parasitism (CBP) is a reproductive tactic in which parasitic females lay eggs in nests of other females of the same species that then raise the joint brood. Parasites benefit by increased reproduction, without costs of parental care for the parasitic eggs. CBP occurs in many egg-laying animals, among birds most often in species with large clutches and self-feeding young: two major factors facilitating successful parasitism. CBP is particularly common in waterfowl (Anatidae), a group with female-biased natal philopatry and locally related females. Theory suggests that relatedness between host and parasite can lead to inclusive fitness benefits for both, but if host costs are high, parasites should instead target unrelated females. Pairwise relatedness (r) in host-parasite (h-p) pairs of females has been estimated using molecular genetic methods in seven waterfowl (10 studies). In many h-p pairs, the two females were unrelated (with low r, near the local population mean). However, close relatives (r = 0.5) were over-represented in h-p pairs, which in all 10 studies had higher mean relatedness than other females. In one species where this was studied, h-p relatedness was higher than between nesting close neighbours, and hosts parasitized by non-relatives aggressively rejected other females. In another species, birth nest-mates (mother-daughters, sisters) associated in the breeding area as adults, and became h-p pairs more often than expected by chance. These and other results point to recognition of birth nest-mates and perhaps other close relatives. For small to medium host clutch sizes, addition of a few parasitic eggs need not reduce host offspring success. Estimates in two species suggest that hosts can then gain inclusive fitness if parasitized by relatives. Other evidence of female cooperation is incubation by old eider Somateria mollissima females of clutches laid by their relatives, and merging and joint care of broods of young. Merging females tended to be more closely related. Eiders associate with kin in many situations, and in some geese and swans, related females may associate over many years. Recent genetic evidence shows that also New World quails (Odontophoridae) have female-biased natal philopatry, CBP and brood merging, inviting further study and comparison with waterfowl. Kin-related parasitism also occurs in some insects, with revealing parallels and differences compared to birds. In hemipteran bugs, receiving extra eggs is beneficial for hosts by diluting offspring predation. In eggplant lace bugs Gargaphia solani, host and parasite are closely related, and kin selection favours egg donation to related females. Further studies of kinship in CBP, brood merging and other contexts can test if some of these species are socially more advanced than presently known. | NA | no | No good fitness measure and measure host GD | NA | no | no |
| 4675 | 2020 | 44113 | Andras, JP; Fields, PD; Ebert, D | 2018 | Spatial population genetic structure of a bacterial parasite in close coevolution with its host | Knowledge of a species’ population genetic structure can provide insight into fundamental ecological and evolutionary processes including gene flow, genetic drift and adaptive evolution. Such inference is of particular importance for parasites, as an understanding of their population structure can illuminate epidemiological and coevolutionary dynamics. Here, we describe the population genetic structure of the bacterium Pasteuria ramosa, a parasite that infects planktonic crustaceans of the genus Daphnia. This system has become a model for investigations of host-parasite interactions and represents an example of coevolution via negative frequency-dependent selection (aka Red Queen dynamics). To sample P. ramosa, we experimentally infected a panel of Daphnia hosts with natural spore banks from the sediments of 25 ponds throughout much of the species range in Europe and western Asia. Using 12 polymorphic variable number tandem repeat loci (VNTR loci), we identified substantial genetic diversity, both within and among localities, that was structured geographically among ponds. Genetic diversity was also structured among host genotypes within ponds, although this pattern varied by locality, with P. ramosa at some localities partitioned into distinct host-specific lineages, and other localities where recombination had shuffled genetic variation among different infection phenotypes. Across the sample range, there was a pattern of isolation by distance, and principal components analysis coupled with Procrustes rotation identified congruence between patterns of genetic variation and geography. Our findings support the hypothesis that Pasteuria is an endemic parasite coevolving closely with its host. These results provide important context for previous studies of this model system and inform hypotheses for future research. | NA | no | No good fitness measure and measure host GD | NA | no | no |
| 4676 | 2020 | 44113 | Andre, A; Millien, V; Galan, M; Ribas, A; Michaux, JR | 2017 | Effects of parasite and historic driven selection on the diversity and structure of a MHC-II gene in a small mammal species (Peromyscus leucopus) undergoing range expansion | Genetic diversity may decrease from the centre to the margin of a species distribution range due to neutral stochastic processes. Selection may also alter genetic diversity in non-neutral markers, such as genes associated with the immune system. Both neutral processes and selection on the immune system are thus expected to affect the spatial distribution of such markers, but the relative strength of each has been scarcely studied. Here, we compared the diversity of a neutral marker (mitochondrial cytochrome b) and a selected marker (DRB gene from the MHC-II), in eastern-North American populations of white-footed mice (Peromyscus leucopus), a species known for its role of main reservoir of the Lyme disease. We observed distinct phylogeographic patterns with these two markers, which may be the result of selection pressure acting upon the DRB gene. As predicted by the central marginal hypothesis, we observed a loss of neutral genetic diversity toward the margin of the species distribution. A decrease in diversity was also observed for the DRB gene, likely due to genetic drift and positive selection operated by helminth parasites. Such a loss in genetic diversity at the range margin may slow down the ongoing expansion of P. leucopus, by counterbalancing the effect of global warming on the mouse survival at higher latitude. | NA | no | mhc diversity | NA | no | no |
| 4677 | 2020 | 44111 | Angles, F; Hutt, DM; Balch, WE | 2019 | HDAC inhibitors rescue multiple disease-causing CFTR variants | Understanding the role of the epigenome in protein-misfolding diseases remains a challenge in light of genetic diversity found in the world-wide population revealed by human genome sequencing efforts and the highly variable response of the disease population to therapeutics. An ever-growing body of evidence has shown that histone deacetylase (HDAC) inhibitors (HDACi) can have significant benefit in correcting protein-misfolding diseases that occur in response to both familial and somatic mutation. Cystic fibrosis (CF) is a familial autosomal recessive disease, caused by genetic diversity in the CF transmembrane conductance regulator (CFTR) gene, a cyclic Adenosine MonoPhosphate (cAMP)-dependent chloride channel expressed at the apical plasma membrane of epithelial cells in multiple tissues. The potential utility of HDACi in correcting the phenylalanine 508 deletion (F508del) CFTR variant as well as the over 2000 CF-associated variants remains controversial. To address this concern, we examined the impact of US Food and Drug Administration-approved HDACi on the trafficking and function of a panel of CFTR variants. Our data reveal that panobinostat (LBH-589) and romidepsin (FK-228) provide functional correction of Class II and III CFTR variants, restoring cell surface chloride channel activity in primary human bronchial epithelial cells. We further demonstrate a synergistic effect of these HDACi with Vx809, which can significantly restore channel activity for multiple CFTR variants. These data suggest that HDACi can serve to level the cellular playing field for correcting CF-causing mutations, a leveling effect that might also extend to other protein-misfolding diseases. | NA | no | No good fitness measure and measure host GD | NA | no | no |
| 4678 | 2020 | 44111 | Antonides, J; Mathur, S; Sundaram, M; Ricklefs, R; DeWoody, JA | 2019 | Immunogenetic response of the bananaquit in the face of malarial parasites | Background: In the arms race between hosts and parasites, genes involved in the immune response are targets for natural selection. Toll-Like Receptor (TLR) genes play a role in parasite detection as part of the innate immune system whereas Major Histocompatibility Complex (MHC) genes encode proteins that display antigens as part of the vertebrate adaptive immune system. Thus, both gene families are under selection pressure from pathogens. The bananaquit (Coereba flaveola) is a passerine bird that is a common host of avian malarial parasites (Plasmodium sp. and Haemoproteus sp.). We assessed molecular variation of TLR and MHC genes in a wild population of bananaquits and identified allelic associations with resistance/susceptibility to parasitic infection to address hypotheses of avian immune response to haemosporidian parasites. Results: We found that allele frequencies are associated with infection status at the immune loci studied. A consistent general trend showed the infected groups possessed more alleles at lower frequencies, and exhibited unique alleles, compared to the uninfected group. Conclusions: Our results support the theory of natural selection favoring particular alleles for resistance while maintaining overall genetic diversity in the population, a mechanism which has been demonstrated in some systems in MHC previously but understudied in TLRs. | NA | no | no good gd measure host pop | NA | no | no |
| 4679 | 2020 | 44112 | Antunes, B; Lourenco, A; Caeiro-Dias, G; Dinis, M; Goncalves, H; Martinez-Solano, I; Tarroso, P; Velo-Anton, G | 2018 | Combining phylogeography and landscape genetics to infer the evolutionary history of a short-range Mediterranean relict, Salamandra salamandra longirostris | Examining historical and contemporary processes underlying current patterns of genetic variation is key to reconstruct the evolutionary history of species and implement conservation measures promoting their long-term persistence. Combining phylogeographic and landscape genetic approaches can provide valuable insights, especially in regions harboring high levels of biodiversity that are currently threatened by climate and land cover changes, like southern Iberia. We used genetic (mtDNA and microsatellites) and spatial data (climate and land cover) to infer the evolutionary history and contemporary genetic connectivity in a short-range endemic salamander subspecies, Salamandra salamandra longirostris, using a combination of ecological niche modelling, phylogeographic, and landscape genetic analyses. Ecological-based analyses support a role of the Guadalquivir River Basin as a major vicariant agent in this taxon. The lower genetic diversity and greater differentiation of peripheral populations, together with analyses of climatically stable areas throughout time, suggest the persistence of a population in the central part of the current range since the Last Inter Glacial [LIG; 120,000-140,000years BP], and a micro refugium in the eastern end of the range. Habitat heterogeneity plays a major role in shaping patterns of genetic differentiation in S. s. longirostris, with forests representing key areas for its long-term persistence under scenarios of environmental change. Our study stresses the importance of maintaining population genetic connectivity in low-dispersal organisms under rapidly changing environments, and will inform management plans for the long-term survival of this evolutionarily distinct Mediterranean endemic. | NA | no | No good fitness measure | NA | no | no |
| 4680 | 2020 | 44113 | Ao, YY; Li, XY; Li, LL; Xie, XL; Jin, D; Yu, JM; Lu, S; Duan, ZJ | 2017 | Two novel bocaparvovirus species identified in wild Himalayan marmots | Bocaparvovirus (BOV) is a genetically diverse group of DNA viruses and a possible cause of respiratory, enteric, and neurological diseases in humans and animals. Here, two highly divergent BOVs (tentatively named as Himalayan marmot BOV, HMBOV1 and HMBOV2) were identified in the livers and feces of wild Himalayan marmots in China, by viral metagenomic analysis. Five of 300 liver samples from Himalayan marmots were positive for HMBOV1 and five of 99 fecal samples from these animals for HMBOV2. Their nearly complete genome sequences are 4,672 and 4,887 nucleotides long, respectively, with a standard genomic organization and containing protein-coding motifs typical for BOVs. Based on their NS1, NP1, and VP1, HMBOV1 and HMBOV2 are most closely related to porcine BOV SX/1-2 (approximately 77.0%/50.0%, 50.0%/53.0%, and 79.0%/54.0% amino acid identity, respectively). Phylogenetic analysis of these three proteins showed that HMBOV1 and HMBOV2 formed two distinctly independent branches in BOVs. According to these results, HMBOV1 and HMBOV2 are two different novel species in the Bocaparvovirus genus. Their identification expands our knowledge of the genetic diversity and evolution of BOVs. Further studies are needed to investigate their potential pathogenicity and their impact on Himalayan marmots and humans. | NA | no | No good fitness measure and measure host GD | NA | no | no |
| 4681 | 2020 | 44113 | Appelgren, ASC; Saladin, V; Richner, H; Doligez, B; McCoy, KD | 2018 | Gene flow and adaptive potential in a generalist ectoparasite | Background: In host-parasite systems, relative dispersal rates condition genetic novelty within populations and thus their adaptive potential. Knowledge of host and parasite dispersal rates can therefore help us to understand current interaction patterns in wild populations and why these patterns shift over time and space. For generalist parasites however, estimates of dispersal rates depend on both host range and the considered spatial scale. Here, we assess the relative contribution of these factors by studying the population genetic structure of a common avian ectoparasite, the hen flea Ceratophyllus gallinae, exploiting two hosts that are sympatric in our study population, the great tit Paws major and the collared flycatcher Ficedula albicollis. Previous experimental studies have indicated that the hen flea is both locally maladapted to great tit populations and composed of subpopulations specialized on the two host species, suggesting limited parasite dispersal in space and among hosts, and a potential interaction between these two structuring factors. Results: C gallinae fleas were sampled from old nests of the two passerine species in three replicate wood patches and were genotyped at microsatellite markers to assess population genetic structure at different scales (among individuals within a nest among nests and between host species within a patch and among patches). As expected, significant structure was found at all spatial scales and between host species, supporting the hypothesis of limited dispersal in this parasite. Clustering analyses and estimates of relatedness further suggested that inbreeding regularly occurs within nests. Patterns of isolation by distance within wood patches indicated that flea dispersal likely occurs in a stepwise manner among neighboring nests. From these data, we estimated that gene flow in the hen flea is approximately half that previously described for its great tit hosts. Conclusion: Our results fall in line with predictions based on observed patterns of adaptation in this host-parasite system, suggesting that parasite dispersal is limited and impacts its adaptive potential with respect to its hosts. More generally, this study sheds light on the complex interaction between parasite gene flow, local adaptation and host specialization within a single host-parasite system. | NA | no | no good gd measure host pop | NA | no | no |
| 4682 | 2020 | 44112 | Arabaghian, H; Salloum, T; Alousi, S; Panossian, B; Araj, GF; Tokajian, S | 2019 | Molecular Characterization of Carbapenem Resistant Klebsiella pneumoniae and Klebsiella quasipneumoniae Isolated from Lebanon | Klebsiella pneumoniae is a Gram-negative organism and a major public health threat. In this study, we used whole-genome sequences to characterize 32 carbapenem-resistant K. pneumoniae (CRKP) and two carbapenem-resistant K. quasipneumoniae (CRKQ). Antimicrobial resistance was assessed using disk diffusion and E-test, while virulence was assessed in silico. The capsule type was determined by sequencing the wzi gene. The plasmid diversity was assessed by PCR-based replicon typing to detect the plasmid incompatibility (Inc) groups. The genetic relatedness was determined by multilocus sequence typing, pan-genome, and recombination analysis. All of the isolates were resistant to ertapenem together with imipenem and/or meropenem. Phenotypic resistance was due to blaOXA-48, blaNDM-1, blaNDM-7, or the coupling of ESBLs and outer membrane porin modifications. This is the first comprehensive study reporting on the WGS of CRKP and the first detection of CRKQ in the region. The presence and dissemination of CRKP and CRKQ, with some additionally having characteristics of hypervirulent clones such as the hypermucoviscous phenotype and the capsular type K2, are particularly concerning. Additionally, mining the completely sequenced K. pneumoniae genomes revealed the key roles of mobile genetic elements in the spread of antibiotic resistance and in understanding the epidemiology of these clinically significant pathogens. | NA | no | gd of pathogen | NA | no | no |
| 4683 | 2020 | 44040 | Arafa, RA; Kamel, SM; Rakha, MT; Soliman, NEK; Moussa, OM; Shirasawa, K | 2020 | Analysis of the lineage of Phytophthora infestans isolates using mating type assay, traditional markers, and next generation sequencing technologies | Phytophthora infestans (Mont.) de Bary, a hemibiotrophic oomycete, has caused severe epidemics of late blight in tomato and potato crops around the world since the Irish Potato Famine in the 1840s. Breeding of late blight resistant cultivars is one of the most effective strategies to overcome this disruptive disease. However, P. infestans is able to break down host resistance and acquire resistance to various fungicides, possibly because of the existence of high genetic variability among P. infestans isolates via sexual and asexual reproduction. Therefore, to manage this disease, it is important to understand the genetic divergence of P. infestans isolates. In this study, we analyzed the genomes of P. infestans isolates collected from Egypt and Japan using various molecular approaches including the mating type assay and genotyping simple sequence repeats, mitochondria DNA, and effector genes. We also analyzed genome-wide single nucleotide polymorphisms using doubledigest restriction-site associated DNA sequencing and whole genome resequencing (WGRS). The isolates were classified adequately using high-resolution genome-wide approaches. Moreover, these analyses revealed new clusters of P. infestans isolates in the Egyptian population. Monitoring the genetic divergence of P. infestans isolates as well as breeding of resistant cultivars would facilitate the elimination of the late blight disease. | NA | no | gd of pathogen | NA | no | no |
| 4684 | 2020 | 44042 | Armstrong, C; Davies, RG; Gonzalez-Quevedo, C; Dunne, M; Spurgin, LG; Richardson, DS | 2019 | Adaptive landscape genetics and malaria across divergent island bird populations | Environmental conditions play a major role in shaping the spatial distributions of pathogens, which in turn can drive local adaptation and divergence in host genetic diversity. Haemosporidians, such as Plasmodium (malaria), are a strong selective force, impacting survival and fitness of hosts, with geographic distributions largely determined by habitat suitability for their insect vectors. Here, we have tested whether patterns of fine-scale local adaptation to malaria are replicated across discrete, ecologically differing island populations of Berthelot’s pipits Anthus berthelotii. We sequenced TLR4, an innate immunity gene that is potentially under positive selection in Berthelot’s pipits, and two SNPs previously identified as being associated with malaria infection in a genome-wide association study (GWAS) in Berthelot’s pipits in the Canary Islands. We determined the environmental predictors of malaria infection, using these to estimate variation in malaria risk on Porto Santo, and found some congruence with previously identified environmental risk factors on Tenerife. We also found a negative association between malaria infection and a TLR4 variant in Tenerife. In contrast, one of the GWAS SNPs showed an association with malaria risk in Porto Santo, but in the opposite direction to that found in the Canary Islands GWAS. Together, these findings suggest that disease-driven local adaptation may be an important factor in shaping variation among island populations. | NA | no | n | NA | no | no |
| 4685 | 2020 | 44114 | Armstrong, C; Richardson, DS; Hipperson, H; Horsburgh, GJ; Kupper, C; Percival-Alwyn, L; Clark, M; Burke, T; Spurgin, LG | 2018 | Genomic associations with bill length and disease reveal drift and selection across island bird populations | Island species provide excellent models for investigating how selection and drift operate in wild populations, and for determining how these processes act to influence local adaptation and speciation. Here, we examine the role of selection and drift in shaping genomic and phenotypic variation across recently separated populations of Berthelot’s pipit (Anthus berthelotii), a passerine bird endemic to three archipelagos in the Atlantic. We first characterized genetic diversity and population structuring that supported previous inferences of a history of recent colonizations and bottlenecks. We then tested for regions of the genome associated with the ecologically important traits of bill length and malaria infection, both of which vary substantially across populations in this species. We identified a SNP associated with variation in bill length among individuals, islands, and archipelagos; patterns of variation at this SNP suggest that both phenotypic and genotypic variation in bill length is largely shaped by founder effects. Malaria was associated with SNPs near/within genes involved in the immune response, but this relationship was not consistent among archipelagos, supporting the view that disease resistance is complex and rapidly evolving. Although we found little evidence for divergent selection at candidate loci for bill length and malaria resistance, genome scan analyses pointed to several genes related to immunity and metabolism as having important roles in divergence and adaptation. Our findings highlight the utility and challenges involved with combining association mapping and population genetic analysis in nonequilibrium populations, to disentangle the effects of drift and selection on shaping genotypes and phenotypes. | NA | no | No good fitness measure and measure host GD | NA | no | no |
| 4686 | 2020 | 44040 | Armstrong, EE; Taylor, RW; Miller, DE; Kaelin, CB; Barsh, GS; Hadly, EA; Petrov, D | 2020 | Long live the king: chromosome-level assembly of the lion (Panthera leo) using linked-read, Hi-C, and long-read data | Background The lion (Panthera leo) is one of the most popular and iconic feline species on the planet, yet in spite of its popularity, the last century has seen massive declines for lion populations worldwide. Genomic resources for endangered species represent an important way forward for the field of conservation, enabling high-resolution studies of demography, disease, and population dynamics. Here, we present a chromosome-level assembly from a captive African lion from the Exotic Feline Rescue Center (Center Point, IN) as a resource for current and subsequent genetic work of the sole social species of the Panthera clade. Results Our assembly is composed of 10x Genomics Chromium data, Dovetail Hi-C, and Oxford Nanopore long-read data. Synteny is highly conserved between the lion, other Panthera genomes, and the domestic cat. We find variability in the length of runs of homozygosity across lion genomes, indicating contrasting histories of recent and possibly intense inbreeding and bottleneck events. Demographic analyses reveal similar ancient histories across all individuals during the Pleistocene except the Asiatic lion, which shows a more rapid decline in population size. We show a substantial influence on the reference genome choice in the inference of demographic history and heterozygosity. Conclusions We demonstrate that the choice of reference genome is important when comparing heterozygosity estimates across species and those inferred from different references should not be compared to each other. In addition, estimates of heterozygosity or the amount or length of runs of homozygosity should not be taken as reflective of a species, as these can differ substantially among individuals. This high-quality genome will greatly aid in the continuing research and conservation efforts for the lion, which is rapidly moving towards becoming a species in danger of extinction. | NA | no | n | NA | no | no |
| 4687 | 2020 | 44113 | Arnold, C; Bachmann, O; Schnitzler, A | 2017 | Insights into the Vitis complex in the Danube floodplain (Austria) | European grapevine populations quickly disappeared from most of their range, massively killed by the spread of North American grapevine pests and diseases. Nowadays taxonomic pollution represents a new threat. A large Vitis complex involves escaped cultivars, rootstocks, and wild grapevines. The study aimed to provide insight into the Vitis complex in the Danube region through field and genetic analyses. Among the five other major rivers in Europe which still host wild grapevine populations, the Danube floodplain is the only one benefiting from an extensive protected forest area (93 km(2)) and an relatively active dynamic flood pulse. The Donau-Auen National Park also re-groups the largest wild grapevine population in Europe. Ninety-two percent of the individuals collected in the park were true wild grapevines, and 8% were hybrids and introgressed individuals of rootstocks, wild grapevines, and cultivars. These three groups are interfertile acting either as pollen donor or receiver. Hybrids were established within and outside the dykes, mostly in anthropized forest edges. The best-developed individuals imply rootstock genes. They establish in the most erosive parts of the floodplain. 42% of the true wild grapevines lived at the edges of forest/meadow, 33.3% at the edges forest/channels, and 23.9% in forest gaps. DBH (Diameter Breast Height) varied significantly with the occurrence of flooding. Clones were found in both true wild and hybrids/introgressed grapevines. The process of cloning seemed to be prevented in places where flooding dynamics is reduced. The current global distribution of true wild grapevines shows a strong tendency toward clustering, in sites where forestry practices were the most extensive. However, the reduced flooding activity is a danger for long-term sustainability of the natural wild grapevine population. | NA | no | No good fitness measure and measure host GD | NA | no | no |
| 4688 | 2020 | 44112 | Arsenault-Labrecque, G; Sonah, H; Lebreton, A; Labbe, C; Marchand, G; Xue, A; Belzile, F; Knaus, BJ; Grunwald, NJ; Belanger, RR | 2018 | Stable predictive markers for Phytophthora sojae avirulence genes that impair infection of soybean uncovered by whole genome sequencing of 31 isolates | Background: The interaction between oomycete plant pathogen Phytophthora sojae and soybean is characterized by the presence of avirulence (Avr) genes in P. sojae, which encode for effectors that trigger immune responses and resistance in soybean via corresponding resistance genes (Rps). A recent survey highlighted a rapid diversification of P. sojae Avr genes in soybean fields and the need to deploy new Rps genes. However, the full genetic diversity of P. sojae isolates remains complex and dynamic and is mostly characterized on the basis of phenotypic associations with differential soybean lines. Results: We sequenced the genomes of 31 isolates of P. sojae, representing a large spectrum of the pathotypes found in soybean fields, and compared all the genetic variations associated with seven Avr genes (1a, 1b, 1c, 1d, 1k, 3a, 6) and how the derived haplotypes matched reported phenotypes in 217 interactions. We discovered new variants, copy number variations and some discrepancies with the virulence of previously described isolates with Avr genes, notably with Avr1b and Avr1c. In addition, genomic signatures revealed 11.5% potentially erroneous phenotypes. When these interactions were re-phenotyped, and the Avr genes re-sequenced over time and analyzed for expression, our results showed that genomic signatures alone accurately predicted 99.5% of the interactions. Conclusions: This comprehensive genomic analysis of seven Avr genes of p. sojae in a population of 31 isolates highlights that genomic signatures can be used as accurate predictors of phenotypes for compatibility with Rps genes in soybean. Our findings also show that spontaneous mutations, often speculated as a source of aberrant phenotypes, did not occur within the confines of our experiments and further suggest that epigenesis or gene silencing do not account alone for previous discordance between genotypes and phenotypes. Furthermore, on the basis of newly identified virulence patterns within Avr1c, our results offer an explanation why Rps1c has failed more rapidly in the field than the reported information on virulence pathotypes. | NA | no | dometicated host | NA | no | no |
| 4689 | 2020 | 44041 | Aruge, A; Batool, H; Khan, FM; Fakhar-i-Abbas; Janjua, S | 2019 | A pilot study-genetic diversity and population structure of snow leopards of Gilgit-Baltistan, Pakistan, using molecular techniques | Background: The Hindu Kush and Karakoram mountain ranges in Pakistan’s northern areas are a natural habitat of the snow leopard (Panthera uncia syn. Uncia uncia) but the ecological studies on this animal are scarce since it is human shy by nature and lives in difficult mountainous tracts. The pilot study is conducted to exploit the genetic diversity and population structure of the snow leopard in this selected natural habitat of the member of the wildcat family in Pakistan. Method: About 50 putative scat samples of snow leopard from five localities of Gilgit-Baltistan (Pakistan) along with a control sample of zoo maintained male snow leopard were collected for comparison. Significant quality and quantity of genomic DNA was extracted from scat samples using combined Zhang-phenol-chloroform method and successful amplification of cytochrome c oxidase I gene (190 bp) using mini-barcode primers, seven simple sequence repeats (SSR) markers and Y-linked AMELY gene (200 bp) was done. Results: Cytochrome c oxidase I gene sequencing suggested that 33/50 (66%) scat samples were of snow leopard. AMELY primer suggested that out of 33 amplified samples, 21 (63.63%) scats were from male and 12 (36.36%) from female leopards. Through successful amplification of DNA of 25 out of 33 (75.75%) scat samples using SSR markers, a total of 68 alleles on seven SSR loci were identified, showing low heterozygosity, while high gene flow between population. Discussion: The low gene flow rate among the population results in low genetic diversity causing decreased diversification. This affects the adaptability to climatic changes, thus ultimately resulting in decreased population size of the species. | NA | no | No good fitness measure and measure host GD | NA | no | no |
| 4690 | 2020 | 44042 | Asadzadeh, M; Alanazi, AF; Ahmad, S; Al-Sweih, N; Khan, Z | 2019 | Lack of detection of Candida nivariensis and Candida bracarensis among 440 clinical Candida glabrata sensu lato isolates in Kuwait | Occurrence of Candida nivariensis and Candida bracarensis, two species phenotypically similar to Candida glabrata sensu stricto, in human clinical samples from different geographical settings remains unknown. This study developed a low-cost multiplex PCR (mPCR) and three species-specific singleplex PCR assays. Reference strains of common Candida species were used during development and the performance of mPCR and singleplex PCR assays was evaluated with 440 clinical C. glabrata sensu lato isolates. The internal transcribed spacer (ITS) region of rDNA was also sequenced from 85 selected isolates and rDNA sequence variations were used for determining genetic relatedness among the isolates by using MEGA X software. Species-specific amplicons for C. glabrata (similar to 360 bp), C. nivariensis (similar to 250 bp) and C. bracarensis (similar to 180 bp) were obtained in mPCR while no amplicon was obtained from other Candida species. The three singleplex PCR assays also yielded expected results with reference strains of Candida species. The mPCR amplified similar to 360 bp amplicon from all 440 C. glabrata sensu lato isolates thus identifying all clinical isolates in Kuwait as C. glabrata sensu stricto. The results of mPCR were confirmed for all 440 isolates as they yielded an amplicon only in C. glabrata sensu stricto-specific singleplex PCR assay. The rDNA sequence data identified 28 ITS haplotypes among 85 isolates with 18 isolates belonging to unique haplotypes and 67 isolates belonging to 10 cluster haplotypes. In conclusion, we have developed a simple, low-cost mPCR assay for rapid differentiation of C. glabrata sensu stricto from C. nivariensis and C. bracarensis. Our data obtained from a large collection of clinical C. glabrata sensu lato isolates show that C. nivariensis and C. bracarensis are rare pathogens in Kuwait. Considerable genetic diversity among C. glabrata sensu stricto isolates was also indicated by rDNA sequence analyses. | NA | no | human | NA | no | no |
| 4691 | 2020 | 44113 | Aslam, M; Haq, MA; Bandesha, AA; Haidar, S | 2018 | NIAB-777: AN EARLY MATURING, HIGH YIELDING AND BETTER QUALITY COTTON MUTANT DEVELOPED THROUGH POLLEN IRRADIATION TECHNIQUE-SUITABLE FOR HIGH DENSITY PLANTING | This paper describes development of mutant NIAB-777 with improved yield and fibre characters through pollen irradiation method. A cross between NIAB-78 and REBA-288 using gamma irradiated pollen at the rate of 10 Gray (Gy) of gamma rays before cross pollination was attempted. The objective was to create new genetic variability and select the desirable new cotton mutants. After irradiation followed by hybridization subsequent generations were raised to investigate the effect of irradiation treatment. Significant variations from control/parents were observed. From M1 generation, the M-2 population was grown and different desirable mutants having higher yield, early maturity, resistance/tolerance to diseases were selected. These were evaluated for yield potential and desirable other economic traits in different generations till uniformity was achieved. Of these, M-7/02, later named as cv. NIAB-777 was finally selected. It produced 23.4% & 18.5% higher seed cotton yield from standards at local trials. It produced higher seed cotton yield in regional adaptability trials (17.4%), provincial coordinated cotton trials (16.6 %) and in national coordinated varietal trials (8.8 %) compared to standard cv. CIM-496. It has desirable fibre quality traits i. e. ginning out turn (GOT) of 37.6%, fibre length of 28.81 mm, fibre fineness of 4.67 mu g/inch, uniformity index of 85.0%, fibre maturity 81.2% and fibre strength 91.12 thousand pounds per square inch (TPPSI). NIAB-777 is suitable for high density planting. It has better tolerance to cotton leaf curl virus (CLCuV) disease and insect pests. It is concluded that 10 Gray gamma irradiation in cotton has effectively stimulate/increase the agronomical characters and tolerance to disease. | NA | no | dometicated host | NA | no | no |
| 4692 | 2020 | 44113 | Astuti, G; Roma-Marzio, F; D’Antraccoli, M; Bedini, G; Carta, A; Sebastiani, F; Bruschi, P; Peruzzi, L | 2017 | Conservation biology of the last Italian population of Cistus laurifolius (Cistaceae): demographic structure, reproductive success and population genetics | Isolated populations are usually subject to low fitness and reduced genetic diversity, both of which may negatively affect their survival and adaptive potential. Hence, these issues cannot be neglected when planning conservation actions for isolated populations. The Italian population of Cistus laurifolius subsp. laurifolius is extremely isolated. Furthermore, it is affected by fragmentation, being constituted by a single larger subpopulation, surrounded by three much smaller subpopulations, a few hundred metres to a few kilometres apart. In order to fill gaps in demographic and genetic knowledge concerning the Italian population, its area of occupancy, size, age-stage structure and phenology were investigated and its reproductive fitness, pollination strategies and genetic variability were assessed. The population was inferred as fully xenogamous and showed good reproductive performance. Despite this, its genetic variability was low and it showed relatively high levels of inbreeding depression (FIS), seemingly not affected by sub-population size. These results suggest that the Italian population recently suffered fragmentation and reduction in size. The low genetic diversity observed could be explained by the high percentage of mature individuals found in the population, possibly established before fragmentation. For these reasons, the Italian population of C. laurifolius subsp. laurifolius should be monitored and concrete actions aimed at its conservation planned. | NA | no | No good fitness measure and measure host GD | NA | no | no |
| 4693 | 2020 | 44112 | Athrey, G; Faust, N; Hieke, ASC; Brisbin, IL | 2018 | Effective population sizes and adaptive genetic variation in a captive bird population | Captive populations are considered a key component of ex situ conservation programs. Research on multiple taxa has shown the differential success of maintaining demographic versus genetic stability and viability in captive populations. In typical captive populations, usually founded by few or related individuals, genetic diversity can be lost and inbreeding can accumulate rapidly, calling into question their ultimate utility for release into the wild. Furthermore, domestication selection for survival in captive conditions is another concern. Therefore, it is crucial to understand the dynamics of population sizes, particularly the effective population size, and genetic diversity at nonneutral and adaptive loci in captive populations. In this study, we assessed effective population sizes and genetic variation at both neutral microsatellite markers, as well as SNP variants from the MHC-B locus of a captive Red Junglefowl population. This population represents a rare instance of a population with a well-documented history in captivity, following a realistic scenario of chain-of-custody, unlike many captive lab populations. Our analyses, which included 27 individuals comprising the entirety of one captive population show very low neutral and adaptive genetic variation, as well as low effective sizes, which correspond with the known demographic history. Finally, our study also shows the divergent impacts of small effective size and inbreeding in captive populations on microsatellite versus adaptive genetic variation in the MHC-B locus. Our study provides insights into the difficulties of maintaining adaptive genetic variation in small captive populations. | NA | no | No good fitness measure and measure host GD | NA | no | no |
| 4694 | 2020 | 44113 | Atkinson, KE; Kitchener, AC; Tobe, SS; O’Donoghue, P | 2018 | An assessment of the genetic diversity of the founders of the European captive population of Asian lion (Panthera leo leo), using microsatellite markers and studbook analysis | A European Endangered Species Programme (EEP) was established in the early 1990s, in order to manage a captive population of Asian lions (Panthera leo leo) within European zoos. The founders of this population comprised of nine individuals that originated from a captive population in India. During 2007-2009, 57 lions were born in the European captive population. Of these births, 35 individuals died within 20 days, three died within two months and one individual was euthanased at four months old. Indeed, over 50% of the total historical captive population died within 30 days of birth. The ‘European Studbook for the Asian Lion’ shows that the EEP founder population contains individuals from matings of full and half siblings, including all female founders. It is probable that high levels of inbreeding within this captive population are causing high levels of stillbirths and infant mortality. Previous research has shown that there is limited genetic variation in the captive population in India. This study uses the same microsatellite markers to establish the level of genetic variation that was present when the EEP population was established in comparison with that observed in the Indian zoo population, from which it was derived. Only three of the 12 microsatellite markers, showing variation in the Indian captive population, showed bi-allelic heterozygosity in the EEP founders, indicating that most variation was not present during the establishment of the EEP population. Therefore, the future of the Asian lion EEP is compromised by lack of genetic variation and high levels of inbreeding, which can only be alleviated by importing further individuals with different genotypes from India. (c) 2017 Deutsche Gesellschaft fur Saugetierkunde. Published by Elsevier GmbH. All rights reserved. | NA | no | No good fitness measure and measure host GD | NA | no | no |
| 4695 | 2020 | 44113 | Auer, SK; Anderson, GJ; McKelvey, S; Bassar, RD; McLennan, D; Armstrong, JD; Nislow, KH; Downie, HK; McKelvey, L; Morgan, TAJ; Salin, K; Orrell, DL; Gauthey, A; Reid, TC; Metcalfe, NB | 2018 | Nutrients from salmon parents alter selection pressures on their offspring | Organisms can modify their surrounding environment, but whether these changes are large enough to feed back and alter their evolutionary trajectories is not well understood, particularly in wild populations. Here we show that nutrient pulses from decomposing Atlantic salmon (Salmo salar) parents alter selection pressures on their offspring with important consequences for their phenotypic and genetic diversity. We found a strong survival advantage to larger eggs and faster juvenile metabolic rates in streams lacking carcasses but not in streams containing this parental nutrient input. Differences in selection intensities led to significant phenotypic divergence in these two traits among stream types. Stronger selection in streams with low parental nutrient input also decreased the number of surviving families compared to streams with high parental nutrient levels. Observed effects of parent-derived nutrients on selection pressures provide experimental evidence for key components of eco-evolutionary feedbacks in wild populations. | NA | no | No good fitness measure and measure host GD | NA | no | no |
| 4696 | 2020 | 44113 | Auld, SKJR; Brand, J | 2017 | Simulated climate change, epidemic size, and host evolution across host-parasite populations | Climate change is causing warmer and more variable temperatures as well as physical flux in natural populations, which will affect the ecology and evolution of infectious disease epidemics. Using replicate seminatural populations of a coevolving freshwater invertebrate-parasite system (host: Daphnia magna, parasite: Pasteuria ramosa), we quantified the effects of ambient temperature and population mixing (physical flux within populations) on epidemic size and population health. Each population was seeded with an identical suite of host genotypes and dose of parasite transmission spores. Biologically reasonable increases in environmental temperature caused larger epidemics, and population mixing reduced overall epidemic size. Mixing also had a detrimental effect on host populations independent of disease. Epidemics drove parasite-mediated selection, leading to a loss of host genetic diversity, and mixed populations experienced greater evolution due to genetic drift over the season. These findings further our understanding of how diversity loss will reduce the host populations’ capacity to respond to changes in selection, therefore stymying adaptation to further environmental change. | NA | no | No good fitness measure and measure host GD | NA | no | no |
| 4697 | 2020 | 44112 | Awadi, A; Ben Slimen, H; Smith, S; Kahlen, J; Makni, M; Suchentrunk, F | 2018 | Genetic diversity of the toll-like receptor 2 (TLR2) in hare (Lepus capensis) populations from Tunisia | Toll-like receptors (TLRs) are a major group of proteins that recognize molecular components of infectious agents, known as pathogen associated molecular patterns (PAMPs). The structure of these genes is similar and characterized by the presence of an ectodomain, a signal transmembrane segment and a highly conserved cytoplasmic domain. The latter domain is homologous to the human interleukin-1 receptor (IL1R) and human IL-18 receptor (IL-18R) and designated TIR domain. The latter domain of the TLR genes was suggested to be very conservative and its evolution is driven by purifying selection. Variability and evolution of the TIR sequences of TLR2 gene were studied in three hare populations from Tunisia with different ecological characteristics (NT-North Tunisia with Mediterranean, CT-Central Tunisia with semi-arid, and ST-South Tunisia with arid climate). Sequencing of a 372 bp fragment of TIR2 revealed 25 alleles among 110 hares. Twenty variable nucleotide positions were detected, of which 7 were non-synonymous. The highest variability was observed in CT, with 16 polymorphic positions. In ST, only 4 polymorphic nucleotide positions were detected with all diversity values lower than those recorded for the other two populations. By using several approaches, no positive selection was detected. However, evidence of purifying selection was found at two positions. The logistic models of the most common TIR2 protein variant that we run to examine whether itsn occurrence was affected by climatic variationin dependent of the geographic sample location suggested only a longitudinal effect. Finally, the mapping of the non-synonymous mutations to the inferred tertiary protein structure showed that they were all localized in the different loop regions. Among all non-synonymous substitutions, three were suggested to be deleterious as evidenced by PROVEAN analysis. The observed patterns of variability characterized by low genetic diversity in ST might suggest that the TIR region was more affected, than other markers, by genetic drift or/and that these patterns were shaped by different selective pressures under different ecological conditions. Notably, this low diversity was not detected by other (putatively neutral) microsatellite markers analysed in the course of other studies. But low diversity was also found for two MHC class II adaptive immune genes. As expected from functionally important regions, the evolution of the TIR2 domain is mainly driven by purifying selection. However, the occurrence of deleterious non-synonymous substitutions might highlight the flexible evolution of the TIR genes and/or their interactions with other proteins. (C) 2018 Academie des sciences. Published by Elsevier Masson SAS. All rights reserved. | NA | no | No good fitness measure and measure host GD | NA | no | no |
| 4698 | 2020 | 44042 | Aziz-ul Rahman; Munir, M; Shabbir, MZ | 2019 | A comparative genomic and evolutionary analysis of circulating strains of Avian avulavirus 1 in Pakistan | Newcastle disease, caused by Avian avulavirus 1 (AAvV 1), is endemic to many developing countries around the globe including Pakistan. Frequent epidemics are not uncommon even in vaccinated populations and are largely attributed to the genetic divergence of prevailing isolates and their transmission in the environment. With the strengthening of laboratory capabilities in Pakistan, a number of genetically diverse AAvV 1 strains have recently been isolated and individually characterized in comparison with isolates reported elsewhere in the world. However, there lacks sufficient comparative genomic and phylogenomic analyses of field circulating strains that can elucidate the evolutionary dynamics over a period of time. Herein, we enriched the whole genome sequences of AAvV reported so far (n = 35) from Pakistan and performed comparative genomic, phylogenetic and evolutionary analyses. Based on these analyses, we found only isolates belonging to genotypes VI, VII and XIII of AAvV 1 in a wide range of avian and human hosts. Comparative phylogeny revealed the concurrent circulation of avulaviruses representing different sub-genotypes such as VIg, VIm, VIIa, VIIb, VIIe, VIIf, VIIi, XIIIb and XIIId. We found that the isolates of genotype VII were more closely associated with viruses of genotype XIII than genotype VI. An inter-genotype comparative residue analysis revealed a few substitutions in structurally and functionally important motifs. Putative recombination events were reported for only one of the captive-wild bird (pheasant)-origin isolates. The viruses of genotype VII had a high genetic diversity as compared to isolates from genotypes VI and XIII and, therefore, have more potential to evolve over a period of time. Taken together, the current study provides an insight into the genetic diversity and evolutionary dynamics of AAvV 1 strains circulating in Pakistan. Such findings are expected to facilitate better intervention strategies for the prevention and control of ND in disease-endemic countries across the globe particularly Pakistan. | NA | no | gd of pathogen | NA | no | no |
| 4699 | 2020 | 44113 | Aziz, A; Sarovich, DS; Harris, TM; Kaestli, M; McRobb, E; Mayo, M; Currie, BJ; Price, EP | 2017 | Suspected cases of intracontinental Burkholderia pseudomallei sequence type homoplasy resolved using whole-genome sequencing | Burkholderia pseudomallei is a Gram-negative environmental bacterium that causes melioidosis, a disease of high mortality in humans and animals. Multilocus sequence typing (MLST) is a popular and portable genotyping method that has been used extensively to characterise the genetic diversity of B. pseudomallei populations. MLST has been central to our understanding of the underlying phylogeographical signal present in the B. pseudomallei genome, revealing distinct populations on both the intra- and the inter-continental level. However, due to its high recombination rate, it is possible for B. pseudomallei isolates to share the same multilocus sequence type (ST) despite being genetically and geographically distinct, with two cases of ‘ST homoplasy’ recently reported between Cambodian and Australian B. pseudomallei isolates. This phenomenon can dramatically confound conclusions about melioidosis transmission patterns and source attribution, a critical issue for bacteria such as B. pseudomallei that are of concern due to their potential for use as bioweapons. In this study, we used whole-genome sequencing to identify the first reported instances of intracontinental ST homoplasy, which involved ST-722 and ST-804 B. pseudomallei isolates separated by large geographical distances. In contrast, a third suspected homoplasy case was shown to be a true long-range (460 km) dispersal event between a remote Australian island and the Australian mainland. Our results show that, whilst a highly useful and portable method, MLST can occasionally lead to erroneous conclusions about isolate origin and disease attribution. In cases where a shared ST is identified between geographically distant locales, whole-genome sequencing should be used to resolve strain origin. | NA | no | No good fitness measure and measure host GD | NA | no | no |
| 4700 | 2020 | 44112 | Babushkina, NP; Kucher, AN; Bragina, EY; Garaeva, AF; Goncharova, IA; Tcitrikov, DY; Gomboeva, DE; Rudko, AA; Freidin, MB | 2018 | Ethnic and Geographical Aspects of the Prevalence of the Polymorphic Variants of Genes Associated with Tuberculosis | Specificity of the structure of gene pools of different ethno-territorial groups of the human population can underlie the epidemiological features of the spread of tuberculosis (TB) and the structure of the genetic component of the susceptibility to the disease. The variability of 62 genetic variants potentially associated with the risk of the development of TB in the Russian population of the city of Tomsk has been studied and the differentiation of various ethno-territorial groups of the world by these markers has been assessed. The studied sample comprised 445 Russian residents of the city of Tomsk without bronchopulmonary pathology. For comparison, the data on the variability of the genetic markers of interest in 26 populations from the 1000 Genomes Project was involved. In the Tomsk population, only the ancestral allele was found for seven of the 58 SNPs studied; the allele frequencies for 36 markers were within the limits of the values seen in other European populations; for 12 SNPs, the observed frequencies were closer to populations with a significant Mongoloid component. By the total of the SNPs, the Tomsk population, despite the geographical distance from the rest of the European populations, did not differ from them (in genetic distances and G st statistics), although it had some features of the gene pool. Intergroup differentiation of the world populations by these SNPs reflects mainly interracial differences. The greatest differences in the genetic structure between the studied populations were seen for the markers localized in intergenic regions. Statistically significant differences were found when comparing the levels of the average expected heterozygosity between groups of L4 carrier populations of mycobacteria and non-L4 populations, which indicates the impact of the prevalence of different pathogenic lineages of M. tuberculosis on the formation of population specificity of the allelic frequencies. | NA | no | No good fitness measure and measure host GD | NA | no | no |
| 4701 | 2020 | 44041 | Baden, AL; Mancini, AN; Federman, S; Holmes, SM; Johnson, SE; Kamilar, J; Louis, EE; Bradley, BJ | 2019 | Anthropogenic pressures drive population genetic structuring across a Critically Endangered lemur species range | In recent decades Madagascar has experienced significant habitat loss and modification, with minimal understanding of how human land use practices have impacted the evolution of its flora and fauna. In light of ongoing and intensifying anthropogenic pressures, we seek new insight into mechanisms driving genetic variability on this island, using a Critically Endangered lemur species, the black-and-white ruffed lemur (Varecia variegata), as a test case. Here, we examine the relative influence of natural and anthropogenic landscape features that we predict will impose barriers to dispersal and promote genetic structuring across the species range. Using circuit theory, we model functional connectivity among 18 sampling localities using population-based genetic distance (FST). We optimized resistance surfaces using genetic algorithms and assessed their performance using maximum-likelihood population-effects mixed models. The best supported resistance model was a composite surface that included two anthropogenic features, habitat cover and distance to villages, suggesting that rapid land cover modification by humans has driven change in the genetic structure of wild lemurs. Primary conservation priority should be placed on mitigating further forest loss and connecting regions identified as having low dispersal potential to prevent further loss of genetic diversity and promote the survival of other moist forest specialists. | NA | no | No good fitness measure and measure host GD | NA | no | no |
| 4702 | 2020 | 44113 | Baena-Diaz, F; Ramirez-Barahona, S; Ornelas, JF | 2018 | Hybridization and differential introgression associated with environmental shifts in a mistletoe species complex | Host specialization after host shifting is traditionally viewed as the pathway to speciation in parasitic plants. However, geographical and environmental changes can also influence parasite speciation, through hybridization processes. Here we investigated the impact of past climatic fluctuations, environment, and host shifts on the genetic structure and patterns of hybridization and gene flow between Psittacanthus calyculatus and P. schiedeanus, a Mesoamerican species complex. Using microsatellites (408 individuals), we document moderate genetic diversity but high genetic differentiation between widespread parental clusters, calyculatus in dry pine-oak forests and schiedeanus in cloud forests. Bayesian analyses identified a third cluster, with admixture between parental clusters in areas of xeric and tropical dry forests and high levels of migration rates following secondary contact. Coincidently host associations in these areas differ from those in areas of parental species, suggesting that past hybridization played a role in environmental and host shifts. Overall, the observed genetic and geographic patterns suggest that these Psittacanthus populations could have entered a distinct evolutionary pathway. The results provide evidence for highlights on the importance of the Pleistocene climate changes, habitat differences, and potential host shifts in the evolutionary history of Neotropical mistletoes. | NA | no | No good fitness measure | NA | no | no |
| 4703 | 2020 | 44113 | Bagheri, A; Fathipour, Y; Askari-Seyahooei, M; Zeinalabedini, M | 2018 | Ommatissus lybicus (Hemiptera: Tropiduchidae), an economically important pest of date palm (Arecaceae) with highly divergent populations | Ommatissus lybicus de Bergevin (Hemiptera: Tropiduchidae) is a key pest of date palm (Phoenix dactylifera Linnaeus; Arecaceae) with worldwide distribution and various management strategies. To study genetic diversity of date palm hopper, a series of experiments was conducted on genetic structure and genetic diversity of 15 geographic populations of O. lybicus (Abu Musa, Bam, Bushehr, Behbahan, Tezerj, Fin, Jiroft, Shahdad, Jahrom, Ghire Karzin, Ghasre Shirin, Iran; Pakistan; Oman; Egypt; and Tunisia) by amplified fragment length polymorphism, cytochrome c oxidase subunit I (COI), and 28S rRNA markers. Analysis of molecular variance analysis of amplified fragment length polymorphism data and COI sequences revealed a significant variation among O. lybicus populations (94.12% and 65.08% similarities for amplified fragment length polymorphism and COI, respectively). The 28S rDNA sequences from different populations were identical. Phylogenetic network inferred from amplified fragment length polymorphism data and COI sequences grouped two geographically close populations (Tezerj and Bam) in the two distinct clades while far apart geographical populations bunched in the same or close clades. These two populations experience repeated exposure to heavy pesticide applications annually. In conclusion, study of the genetic structure revealed a considerable variation between O. lybicus populations under intensive chemical strategies. | NA | no | gd of pathogen | NA | no | no |
| 4704 | 2020 | 44113 | Bakht, J; Iqbal, M; Shafi, M | 2017 | Genetic diversity and phylogenetic relationship in different genotypes of cotton for future breeding | Background: To make the plants well adapted and more resistant to diseases and other environmental stresses there is always a need to improve the quality of plant’s genome i.e. to increase its genetic diversity. & para;& para;Methods: In the present study six variety and six lines of cotton were investigated for their genetic diversity and phylogenetic relationship. For this purpose 35 different RAPD primers obtained from the Gene Link Technologies, USA were used. & para;& para;Results: Among 35 RAPD primers, 13 primers produced reproducible PCR bands while the rest failed to show any amplification product. Our results indicated that the total count of the reproducible bands was 670 and polymorphic loci were counted to be 442 which constitute 66% of total loci. Phylogenetic analysis revealed two major groups each consists of 7 and 5 genotypes respectively. Genotypes Lp1 and Tp4 were placed at maximum genetic distance and in separate groups and could be utilized for future cotton breeding. & para;& para;Conclusions: RAPD analysis is a cheaper and time saving technique for the determination of genetic diversity of different cotton genotypes. Cotton genotype Lp1 and Tp4 could be the best candidates for future breeding programs as both genotypes are genetically distant from each other. | NA | no | No good fitness measure and measure host GD | NA | no | no |
| 4705 | 2020 | 44112 | Balakrishnan, P; Vaidya, D; Voruganti, VS; Haack, K; Kent, AW; North, KE; Laston, S; Howard, BV; Umans, JG; Lee, ET; Best, LG; MacCluer, JW; Cole, SA; Navas-Acien, A; Franceschini, N | 2018 | Genetic Variants Related to Cardiometabolic Traits Are Associated to B Cell Function, Insulin Resistance, and Diabetes Among AmeriCan Indians: The Strong Heart Family Study | Background: Genetic research may inform underlying mechanisms for disparities in the burden of type 2 diabetes mellitus among American Indians. Our objective was to assess the association of genetic variants in cardiometabolic candidate genes with B cell dysfunction via HOMA-B, insulin resistance via HOMA-IR, and type 2 diabetes mellitus in the Strong Heart Family Study (SHFS). Methods and Results: We examined the association of variants, previously associated with cardiometabolic traits (similar to 200,000 from Illumina Cardio MetaboChip), using mixed models of HOMA-B residuals corrected for HOMA-IR (cHOMA-B), log transformed HOMA-IR, and incident diabetes, adjusted for age, sex, population stratification, and familial relatedness. Center-specific estimates were combined using fixed effect meta-analyses. We used Bonferroni correction to account for multiple testing (P < 4.13 x 10(-7)). We also assessed the association between variants in candidate diabetes genes with these metabolic traits. We explored the top SNPs in an independent, replication sample from Southwestern Arizona. We identified significant associations with cHOMA-B for common variants at 26 loci of which 8 were novel (PRSS7, FCRL5, PEL1, LRP12, IGLL1, ARHGEF10, PARVA, FLJ16686). The most significant variant association with cHOMA-B was observed on chromosome 5 for an intergenic variant near PARP8 (rs2961831, P = 6.39 x 10(-9)). In the replication study, we found a signal at rs4607517 near GCK/YKT6 (P = 0.01). Variants near candidate diabetes genes (especially GCK and KCNQ1) were also nominally associated with HOMA-IR and cHOMA-B. Conclusion: We identified variants at novel loci and confirmed those at known candidate diabetes loci associations for cHOMA-B. This study also provided evidence for association of variants at KCNQ2, CTNAA2, and KCNQ1with cHOMA-B among American Indians. Further studies are needed to account for the high heritability of diabetes among the American Indian participants of the SHFS cohort. | NA | no | human | NA | no | no |
| 4706 | 2020 | 44043 | Balanza, V; Mendoza, JE; Bielza, P | 2019 | Variation in susceptibility and selection for resistance to imidacloprid and thiamethoxam in Mediterranean populations of Orius laevigatus | Imidacloprid and thiamethoxam are neonicotinoids that have been tested in several Orius species, including Orius laevigatus (Fieber) (Hemiptera: Anthocoridae), but not the variability in their effect among Orius populations of a single species. In this study, the variation in susceptibility to imidacloprid and thiamethoxam in 30 Mediterranean wild populations and four commercial populations of O. laevigatus was investigated in the laboratory using a standard dip bioassay method. Lethal concentration values (LC50) and the mortality of adults at the maximum field rate (MFR) were calculated. The range of LC50 of thiamethoxam was from 0.7 to 5.9 mg l(-1), an 8.4-fold variability, obtaining mortality at MFR (100 mg l(-1)) of >89.1% in all populations. The baseline obtained a value of 2.1 mg l(-1), which is very low compared to the MFR. For imidacloprid, the LC50 varied from 7.7 to 94.7 mg l(-1) (12.3-fold variability). Mortalities at the MFR (150 mg l(-1)) were 57.7-99.2%, that is, more variable than for thiamethoxam. The LC50 value of the baseline was 48.7 mg l(-1), also low compared to the MFR. This variation was exploited to select two populations resistant to thiamethoxam and imidacloprid, respectively. Artificial selection for on average 40 cycles significantly increased the resistance to thiamethoxam (LC50 = 149.1 mg l(-1)) and imidacloprid (LC50 = 309.9 mg l(-1)). Mortalities at the MFR in the thiamethoxam- and imidacloprid-resistant populations were 44.5 and 36.9%, respectively. These results demonstrate that resistance can be enhanced in biocontrol agents by artificial selection under laboratory conditions, starting with populations showing no or very low tolerance. Our neonicotinoid-resistant populations might enhance the wider adoption of biological control by allowing punctual or hotspot applications of neonicotinoids to control several main and secondary pests. | NA | no | no good gd measure host | NA | no | no |
| 4707 | 2020 | 44113 | Balasubramaniam, K; Beisner, B; Guan, JH; Vandeleest, J; Fushing, H; Atwill, E; McCowan, B | 2018 | Social network community structure and the contact-mediated sharing of commensal E. coli among captive rhesus macaques (Macaca mulatta) | In group-living animals, heterogeneity in individuals’ social connections may mediate the sharing of microbial infectious agents. In this regard, the genetic relatedness of individuals’ commensal gut bacterium Escherichia coli may be ideal to assess the potential for pathogen transmission through animal social networks. Here we use microbial phylogenetics and population genetics approaches, as well as host social network reconstruction, to assess evidence for the contact-mediated sharing of E. coli among three groups of captively housed rhesus macaques (Macaca mulatta), at multiple organizational scales. For each group, behavioral data on grooming, huddling, and aggressive interactions collected for a six-week period were used to reconstruct social network communities via the Data Cloud Geometry (DCG) clustering algorithm. Further, an E. coli isolate was biochemically confirmed and genotypically fingerprinted from fecal swabs collected from each macaque. Population genetics approaches revealed that Group Membership, in comparison to intrinsic attributes like age, sex, and/or matriline membership of individuals, accounted for the highest proportion of variance in E. coli genotypic similarity. Social network approaches revealed that such sharing was evident at the community-level rather than the dyadic level. Specifically, although we found no links between dyadic E. coil similarity, and social contact frequencies, similarity was significantly greater among macaques within the same social network communities compared to those across different communities. Moreover, tests for one of our study-groups confirmed that E. coli isolated from macaque rectal swabs were more genotypically similar to each other than they were to isolates from environmentally deposited feces. In summary, our results suggest that among frequently interacting, spatially constrained macaques with complex social relationships, microbial sharing via fecal-oral, social contact-mediated routes may depend on both individuals’ direct connections and on secondary network pathways that define community structure. They lend support to the hypothesis that social network communities may act as bottlenecks to contain the spread of infectious agents, thereby encouraging disease control strategies to focus on multiple organizational scales. Future directions include increasing microbial sampling effort per individual to better-detect dyadic transmission events, and assessments of the co-evolutionary links between sociality, infectious agent risk, and host immune function. | NA | no | No good fitness measure | NA | no | no |
| 4708 | 2020 | 44113 | Balik-Meisner, M; Truong, L; Scholl, EH; Tanguay, RL; Reif, DM | 2018 | Population genetic diversity in zebrafish lines | Toxicological and pharmacological researchers have seized upon the many benefits of zebrafish, including the short generation time, well-characterized development, and early maturation as clear embryos. A major difference from many model organisms is that standard husbandry practices in zebrafish are designed to maintain population diversity. While this diversity is attractive for translational applications in human and ecological health, it raises critical questions on how interindividual genetic variation might contribute to chemical exposure or disease susceptibility differences. Findings from pooled samples of zebrafish support this supposition of diversity yet cannot directly measure allele frequencies for reference versus alternate alleles. Using the Tanguay lab Tropical 5D zebrafish line (T5D), we performed whole genome sequencing on a large group (n = 276) of individual zebrafish embryos. Paired-end reads were collected on an Illumina 3000HT, then aligned to the most recent zebrafish reference genome (GRCz10). These data were used to compare observed population genetic variation across species (humans, mice, zebrafish), then across lines within zebrafish. We found more single nucleotide polymorphisms (SNPs) in T5D than have been reported in SNP databases for any of the WIK, TU, TL, or AB lines. We theorize that some subset of the novel SNPs may be shared with other zebrafish lines but have not been identified in other studies due to the limitations of capturing population diversity in pooled sequencing strategies. We establish T5D as a model that is representative of diversity levels within laboratory zebrafish lines and demonstrate that experimental design and analysis can exert major effects when characterizing genetic diversity in heterogeneous populations. | NA | no | No good fitness measure | NA | no | no |
| 4709 | 2020 | 44113 | Banks, SC; Davies, ID; Cary, GJ | 2017 | When can refuges mediate the genetic effects of fire regimes? A simulation study of the effects of topography and weather on neutral and adaptive genetic diversity in fire-prone landscapes | Understanding how landscape heterogeneity mediates the effects of fire on biodiversity is increasingly important under global changes in fire regimes. We used a simulation experiment to investigate how fire regimes interact with topography and weather to shape neutral and selection-driven genetic diversity under alternative dispersal scenarios, and to explore the conditions under which microrefuges can maintain genetic diversity of populations exposed to recurrent fire. Spatial heterogeneity in simulated fire frequency occurred in topographically complex landscapes, with fire refuges and fire-prone hotspots apparent. Interannual weather variability reduced the effect of topography on fire patterns, with refuges less apparent under high weather variability. Neutral genetic diversity was correlated with long-term fire frequency under spatially heterogeneous fire regimes, being higher in fire refuges than fire-prone areas, except under high dispersal or low fire severity (low mortality). This generated different spatial genetic structures in fire-prone and fire-refuge components of the landscape, despite similar dispersal. In contrast, genetic diversity was only associated with time since the most recent fire in flat landscapes without predictable refuges and hotspots. Genetic effects of selection driven by fire-related conditions depended on selection pressure, migration distance and spatial heterogeneity in fire regimes. Allele frequencies at a locus conferring higher fitness under successional environmental conditions followed a pattern of temporal adaptation to contemporary conditions under strong selection pressure and high migration. However, selected allele frequencies were correlated with spatial variation in long-term mean fire frequency (relating to environmental predictability) under weak dispersal, low selection pressure and strong spatial heterogeneity in fire regimes. | NA | no | No good fitness measure and measure host GD | NA | no | no |
| 4710 | 2020 | 44113 | Banks, SC; McBurney, L; Blair, D; Davies, ID; Lindenmayer, DB | 2017 | Where do animals come from during post-fire population recovery? Implications for ecological and genetic patterns in post-fire landscapes | Identifying where animals come from during population recovery can help to understand the impacts of disturbance events and regimes on species distributions and genetic diversity. Alternative recovery processes for animal populations affected by fire include external recolonization, nucleated recovery from refuges, or in situ survival and population growth. We used simulations to develop hypotheses about ecological and genetic patterns corresponding to these alternative models. We tested these hypotheses in a study of the recovery of two small mammals, the Australian bush rat and the agile antechinus, after a large (> 50 000 ha), severe wildfire. The abundance of both species was severely reduced by fire and recovered to near or above pre-fire levels within two generations, yet we rejected a hypothesis of recovery by external recolonization. While the agile antechinus showed genetic evidence for far greater dispersal capacity than the bush rat, neither species showed gradients in abundance or genetic diversity with distance from unburnt forest during population recovery. Population recovery was driven by local-scale processes. However, the mechanisms differed between species, resulting from the spatial impacts of fire on habitat suitability. Agile antechinus populations recovered through population growth from in situ survivors. The bush rat followed a model of nucleated recovery, involving local recolonization from micro-refuges in topographic drainage lines. Nucleated recovery by the bush rat was associated with changes in dispersal, and fine-scale patterns of genetic admixture. We identified increased dispersal by females during recovery, contrasting with male-biased dispersal in unburnt forest. Such flexibility in dispersal can potentially increase recovery rates compared to expectations based on dispersal behavior within undisturbed populations. Our study shows how the initial distribution of survivors, determined by fire effects on resource distribution, determines the subsequent scaling of population recovery patterns, and the sensitivity of population distribution and genetic diversity to changing disturbance regimes. | NA | no | No good fitness measure and measure host GD | NA | no | no |
| 4711 | 2020 | 44111 | Barbanti, A; Martin, C; Blumenthal, JM; Boyle, J; Broderick, AC; Collyer, L; Ebanks-Petrie, G; Godley, BJ; Mustin, W; Ordonez, V; Pascual, M; Carreras, C | 2019 | How many came home? Evaluating ex situ conservation of green turtles in the Cayman Islands | Ex situ management is an important conservation tool that allows the preservation of biological diversity outside natural habitats while supporting survival in the wild. Captive breeding followed by re-introduction is a possible approach for endangered species conservation and preservation of genetic variability. The Cayman Turtle Centre Ltd was established in 1968 to market green turtle (Chelonia mydas) meat and other products and replenish wild populations, thought to be locally extirpated, through captive breeding. We evaluated the effects of this re-introduction programmme using molecular markers (13 microsatellites, 800-bp D-loop and simple tandem repeat mitochondrial DNA sequences) from captive breeders (N = 257) and wild nesting females (N = 57) (sampling period: 2013-2015). We divided the captive breeders into three groups: founders (from the original stock), and then two subdivisions of F1 individuals corresponding to two different management strategies, cohort 1995 (C1995) and multicohort F1 (MCF1). Loss of genetic variability and increased relatedness was observed in the captive stock over time. We found no significant differences in diversity among captive and wild groups, and similar or higher levels of haplotype variability when compared to other natural populations. Using parentage and sibship assignment, we determined that 90% of the wild individuals were related to the captive stock. Our results suggest a strong impact of the re-introduction programmme on the present recovery of the wild green turtle population nesting in the Cayman Islands. Moreover, genetic relatedness analyses of captive populations are necessary to improve future management actions to maintain genetic diversity in the long term and avoid inbreeding depression. | NA | no | No good fitness measure | NA | no | no |
| 4712 | 2020 | 44112 | Barbosa, S; Mestre, F; White, TA; Pauperio, J; Alves, PC; Searle, JB | 2018 | Integrative approaches to guide conservation decisions: Using genomics to define conservation units and functional corridors | Climate change and increasing habitat loss greatly impact species survival, requiring range shifts, phenotypic plasticity and/or evolutionary change for long-term persistence, which may not readily occur unaided in threatened species. Therefore, defining conservation actions requires a detailed assessment of evolutionary factors. Existing genetic diversity needs to be thoroughly evaluated and spatially mapped to define conservation units (CUs) in an evolutionary context, and we address that here. We also propose a multidisciplinary approach to determine corridors and functional connectivity between CUs by including genetic diversity in the modelling while controlling for isolation by distance and phylogeographic history. We evaluate our approach on a Near Threatened Iberian endemic rodent by analysing genotyping-by-sequencing (GBS) genomic data from 107 Cabrera voles (Microtus cabrerae), screening the entire species distribution to define categories of CUs and their connectivity: We defined six management units (MUs) which can be grouped into four evolutionarily significant units (ESUs) and three (putatively) adaptive units (AUs). We demonstrate that the three different categories of CU can be objectively defined using genomic data, and their characteristics and connectivity can inform conservation decision-making. In particular, we show that connectivity of the Cabrera vole is very limited in eastern Iberia and that the pre-Pyrenean and part of the Betic geographic nuclei contribute the most to the species genetic diversity. We argue that a multidisciplinary framework for CU definition is essential and that this framework needs a strong evolutionary basis. | NA | no | No good fitness measure and measure host GD | NA | no | no |
| 4713 | 2020 | 44111 | Barboza, N; Hernandez, E; Inoue-Nagata, AK; Moriones, E; Hilje, L | 2019 | Achievements in the epidemiology of begomoviruses and their vector Bemisia tabaci in Costa Rica | Since the early 1990s, and almost simultaneously, unknown diseases started to be observed in many crops, especially in tropical and subtropical regions. These diseases were predominantly caused by begomoviruses, which were poorly known at that time. Their vector, the whitefly Bemisia tabaci (Hemiptera: Aleyrodidae), often reached unprecedented huge populations in agricultural areas. This elicited a serious production crisis worldwide, that caused losses of millions of dollars for farmers in many countries, including the Mesoamerican region. Fortunately, in Costa Rica, some local research centers, with the collaboration of foreign specialists, have been able to study the causes of this phenomenon, in the search for solutions based on solid epidemiological information. In addition to the previously reported native Bemisia tabaci species, New World (NW), two exotic species, - Mediterranean (MED) and Middle East-Asia Minor 1 (MEAM1) - were found. Moreover, native and exotic bipartite begomoviruses have been detected, especially in common bean, cucurbits, tomato and sweet pepper, as well as the worldwide spread monopartite begomovirus Tomato yellow leaf curl virus (TYLCV). Based upon biological and ecological knowledge accumulated to date, this review offers a comprehensive overview of the very dynamic ways in which the interactions of the different whiteflies and begomovirus species have expressed in Costa Rica, with emphasis on vegetable pathosystems. Hopefully, the information provided in this paper may allow farmers, extension agents, and researchers involved in vegetable production to develop sound practical responses to current and unforeseen problems regarding whiteflies and their associated viruses. | NA | no | No good fitness measure and measure host GD | NA | no | no |
| 4714 | 2020 | 44113 | Barony, GM; Tavares, GC; Pereira, FL; Carvalho, AF; Dorella, FA; Leal, CAG; Figueiredo, HCP | 2017 | Large-scale genomic analyses reveal the population structure and evolutionary trends of Streptococcus agalactiae strains in Brazilian fish farms | Streptococcus agalactiae is a major pathogen and a hindrance on tilapia farming worldwide. The aims of this work were to analyze the genomic evolution of Brazilian strains of S. agalactiae and to establish spatial and temporal relations between strains isolated from different outbreaks of streptococcosis. A total of 39 strains were obtained from outbreaks and their whole genomes were sequenced and annotated for comparative analysis of multilocus sequence typing, genomic similarity and whole genome multilocus sequence typing (wgMLST). The Brazilian strains presented two sequence types, including a newly described ST, and a non-typeable lineage. The use of wgMLST could differentiate each strain in a single clone and was used to establish temporal and geographical correlations among strains. Bayesian phylogenomic analysis suggests that the studied Brazilian population was co-introduced in the country with their host, approximately 60 years ago. Brazilian strains of S. agalactiae were shown to be heterogeneous in their genome sequences and were distributed in different regions of the country according to their genotype, which allowed the use of wgMLST analysis to track each outbreak event individually. | NA | no | gd of pathogen | NA | no | no |
| 4715 | 2020 | 44042 | Barrera-Reyes, PK; Tejero, ME | 2019 | Genetic variation influencing hemoglobin levels and risk for anemia across populations | Hemoglobin (Hb) concentration is the outcome of the interaction between genetic variation and environmental factors, including nutritional status, sex, age, and altitude. Genetic diversity influencing this protein is complex and varies widely across populations. Variants related to abnormal Hb or altered characteristics of the erythrocytes increase the risk for anemia. The most prevalent are related to the inherited globin abnormalities affecting Hb production and structure. Malaria-endemic regions harbor the highest frequencies of variants associated with the most frequent monogenic diseases and the risk for nonnutritional anemia and are considered as public health problems. Variation in genes encoding for enzymes and membrane proteins in red blood cells also influence erythrocyte life span and risk for anemia. Most of these variants are rare. Interindividual variability of hematological parameters is also influenced by common genetic variation across the whole genome. Some of the identified variants are associated with Hb production, erythropoiesis, and iron metabolism. Specialized databases have been developed to organize and update the large body of available information on genetic variation related to Hb variation, their frequency, geographical distribution, and clinical significance. Our present review analyzed the underlying genetic factors that affect Hb concentrations, their clinical relevance, and geographical distribution across populations. | NA | no | No good fitness measure and measure host GD | NA | no | no |
| 4716 | 2020 | 44041 | Barroso, GV; Puzovic, N; Dutheil, JY | 2019 | Inference of recombination maps from a single pair of genomes and its application to ancient samples | Author summary In sexually-reproducing species, meiotic recombination causes the genome of each individual to be a mosaic of DNA sequences that existed in its ancestral population. As a result, genealogical ancestry changes along the genome and shapes genetic diversity. The importance of recombination in genome evolution has motivated a surge in the development of statistical tools to infer genome-wide variation in the recombination rate using polymorphism data. For the most part, however, these methods rely on relatively large sample sizes. Here, we introduce iSMC-a new tool that infers recombination maps while simultaneously modelling the demographic history. A critical improvement over existing methods is that iSMC has high accuracy using as little as a single diploid genome. Using experimentally derived recombination maps from fruit-flies and a fungal pathogen, we demonstrate that iSMC compares well to state-of-the-art methods that require larger sample sizes. We further analyse data from ancient hominins, showcasing that our method can extract information in intrinsically limited datasets. These results suggest that iSMC is a valuable tool that can foster studies in non-model organisms. Moreover, its joint-inference approach of demography and the recombination landscape represents a step towards more realistic models in population genomics. Understanding the causes and consequences of recombination landscape evolution is a fundamental goal in genetics that requires recombination maps from across the tree of life. Such maps can be obtained from population genomic datasets, but require large sample sizes. Alternative methods are therefore necessary to research organisms where such datasets cannot be generated easily, such as non-model or ancient species. Here we extend the sequentially Markovian coalescent model to jointly infer demography and the spatial variation in recombination rate. Using extensive simulations and sequence data from humans, fruit-flies and a fungal pathogen, we demonstrate that iSMC accurately infers recombination maps under a wide range of scenarios-remarkably, even from a single pair of unphased genomes. We exploit this possibility and reconstruct the recombination maps of ancient hominins. We report that the ancient and modern maps are correlated in a manner that reflects the established phylogeny of Neanderthals, Denisovans, and modern human populations. | NA | no | No good fitness measure and measure host GD | NA | no | no |
| 4717 | 2020 | 44112 | Bartakova, V; Bryja, J; Reichard, M | 2018 | Fine-scale genetic structure of the European bitterling at the intersection of three major European watersheds | Background: Anthropogenic factors can have a major impact on the contemporary distribution of intraspecific genetic diversity. Many freshwater fishes have finely structured and locally adapted populations, but their natural genetic structure can be affected by river engineering schemes across river basins, fish transfers in aquaculture industry and conservation management. The European bitterling (Rhodeus amarus) is a small fish that is a brood parasite of freshwater mussels and is widespread across continental Europe. Its range recently expanded, following sharp declines in the 1970s and 1980s. We investigated its genetic variability and spatial structure at the centre of its distribution at the boundary of three watersheds, testing the role of natural and anthropogenic factors in its genetic structure. Results: Sequences of mitochondrial cytochrome B (CYTB) revealed that bitterling colonised central Europe from two Ponto-Caspian refugia, which partly defines its contemporary genetic structure. Twelve polymorphic microsatellite loci revealed pronounced interpopulation differentiation, with significant small-scale differentiation within the same river basins. At a large scale, populations from the Baltic Sea watershed (middle Oder and Vistula basins) were distinct from those from the Black Sea watershed (Danube basin), while populations from rivers of the North Sea watershed (Rhine, Elbe) originated from the admixture of both original sources. Notable exceptions demonstrated the potential role of human translocations across watersheds, with the upper River Oder (Baltic watershed) inhabited by fish from the Danube basin (Black Sea watershed) and a population in the southern part of the River Elbe (North Sea watershed) basin possessing a signal of admixture from the Danube basin. Conclusions: Hydrography and physical barriers to dispersal are only partly reflected in the genetic structure of the European bitterling at the intersection of three major watersheds in central Europe. Drainage boundaries have been obscured by human-mediated translocations, likely related to common carp, Cyprinus carpio, cultivation and game-fish management. Despite these translocations, populations of bitterling are significantly structured by genetic drift, possibly reinforced by its low dispersal ability. Overall, the impact of anthropogenic factors on the genetic structure of the bitterling populations in central Europe is limited. | NA | no | No good fitness measure | NA | no | no |
| 4718 | 2020 | 44040 | Bartocillo, AMF; Nishita, Y; Abramov, AV; Masuda, R | 2020 | Molecular evolution of MHC class II DRB exon 2 in Japanese and Russian raccoon dogs, Nyctereutes procyonoides (Carnivora: Canidae) | Raccoon dogs, Nyctereutes procyonoides, are native to East Asia, but have been introduced into western Russia and eastern Europe. To determine allelic diversity and elucidate the evolution of major histocompatibility complex (MHC) class II genes in the raccoon dog, we analysed a 237-bp region of DRB exon 2 from 36 individuals of native and introduced populations from Japan and Russia. We detected 23 DRB alleles (Nypr-DRBs), 22 of which were novel. Some alleles were found across the species’ range, while others were geographically restricted. For both native and introduced populations, the ratio of non-synonymous to synonymous substitution rates for codons at predicted antigen-binding sites was significantly greater than 2, indicating that Nypr-DRBs have evolved under positive selection. Mixed effect model evolution analysis and an algorithm to detect recombination showed five positively selected codons and one recombination breakpoint, respectively. Overall, our results suggest that the diversity of MHC class II DRB in N. procyonoides was influenced and maintained by recombination, pathogen-driven positive selection, geographical barriers and the founder effect. A Bayesian phylogenetic tree revealed no evidence of trans-species polymorphism (TSP), but instead showed monophyly for the Nypr-DRB alleles within a larger clade of canid sequences. The lack of TSP may have been due to long-term divergence of raccoon dogs from other canids, or to their having encountered different sets of pathogens due to occupying a different ecological niche. | NA | no | No group level | NA | no | no |
| 4719 | 2020 | 44040 | Bashevkin, SM; Dibble, CD; Dunn, RP; Hollarsmith, JA; Ng, G; Satterthwaite, EV; Morgan, SC | 2020 | Larval dispersal in a changing ocean with an emphasis on upwelling regions | Dispersal of benthic species in the sea is mediated primarily through small, vulnerable larvae that must survive minutes to months as members of the plankton community while being transported by strong, dynamic currents. As climate change alters ocean conditions, the dispersal of these larvae will be affected, with pervasive ecological and evolutionary consequences. We review the impacts of oceanic changes on larval transport, physiology, and behavior. We then discuss the implications for population connectivity and recruitment and evaluate life history strategies that will affect susceptibility to the effects of climate change on their dispersal patterns, with implications for understanding selective regimes in a future ocean. We find that physical oceanographic changes will impact dispersal by transporting larvae in different directions or inhibiting their movements while changing environmental factors, such as temperature, pH, salinity, oxygen, ultraviolet radiation, and turbidity, will affect the survival of larvae and alter their behavior. Reduced dispersal distance may make local adaptation more likely in well-connected populations with high genetic variation while reduced dispersal success will lower recruitment with implications for fishery stocks. Increased dispersal may spur adaptation by increasing genetic diversity among previously disconnected populations as well as increasing the likelihood of range expansions. We hypothesize that species with planktotrophic (feeding), calcifying, or weakly swimming larvae with specialized adult habitats will be most affected by climate change. We also propose that the adaptive value of retentive larval behaviors may decrease where transport trajectories follow changing climate envelopes and increase where transport trajectories drive larvae toward increasingly unsuitable conditions. Our holistic framework, combined with knowledge of regional ocean conditions and larval traits, can be used to produce powerful predictions of expected impacts on larval dispersal as well as the consequences for connectivity, range expansion, or recruitment. Based on our findings, we recommend that future studies take a holistic view of dispersal incorporating biological and oceanographic impacts of climate change rather than solely focusing on oceanography or physiology. Genetic and paleontological techniques can be used to examine evolutionary impacts of altered dispersal in a future ocean, while museum collections and expedition records can inform modern-day range shifts. | NA | no | n | NA | no | no |
| 4720 | 2020 | 44040 | Bashyal, BM; Aggarwal, R; Rawat, K; Sharma, S; Gupta, AK; Choudhary, R; Bhat, J; Krishnan, SG; Singh, AK | 2020 | Genetic diversity and population structure of Fusarium fujikuroi causing Bakanae, an emerging disease of rice in India | Bakanae caused by Fusarium fujikuroi (Nirenberg), is emerging as a serious threat for rice (Oryza sativa. L.) cultivation in India. In this study, 63 isolates of Fusarium fujikuroi isolated from symptomatic diseased plants were characterized for their morphology, pathogenicity and molecular variability using universal rice primers (URP). Of the 12 URPs used in the study, 6 primers could produce polymorphic fragments in all the isolates. The URP 17R primer was highly polymorphic (100%), whereas, the URP 1F primer produced 75% polymorphic fragments. A dendrogram obtained from the combined analysis of 6 URP primers categorized the isolates into four clusters, where most of the isolates from Punjab and Haryana were clustered separately. Mating type of the population was identified based on MAT-1 and MAT-2 region universal primers for Gibberella fujikuroi. Among the 63 isolates, 18 (28.57%) were identified as MAT-1 and 45 (71.42%) as MAT-2. The effective population number for mating type was 89% of the total population. Since the distribution frequencies of both mating types were not equal in the Indian population of E fujikuroi, it could conclude that majority of the multiplication of isolates under field conditions was through asexual reproduction. However, the presence of both mating types in F. fujikuroi indicates that the population is also capable of sexual reproduction. Therefore, it is important to develop cultivars with inbuilt resistance to bakanae disease, taking into consideration the factors such as environmental conditions and variability of the pathogen in the area of intended cultivation. | NA | no | gd of pathogen | NA | no | no |
| 4721 | 2020 | 44112 | Basim, H; Basim, E; Ersoy, A | 2019 | PHENOTYPIC AND GENOTYPIC CHARACTERIZATION OF PSEUDOMONAS SAVASTANOI PV. SAVASTANOI CAUSING OLIVE KNOT DISEASE IN TURKEY | Olive knot disease caused by Pseudomonas savastanoi pv. savastanoi (Psv) is one of the major diseases influencing olive (Oleae europaea L.) production in Turkey. The disease incidence rate was found to range between 4 and 80% according to the 2015 and 2018 surveys. A total of 67 isolates were recovered from 7 symptomatic Turkish olive cultivars on a semi -selective medium, PVF-1, and then identified as Psv by biochemical and molecular tests. The isolates produced characteristic gall symptoms on olive plants and were consistently re -isolated. Fatty acid methyl ester (FAME) analysis indicated that the major fatty acid components were oleic acid (18:1), palmitoleic acid (16:1), and palmitic acid (16:0), and also clustered the olive strains into 2 groups. The repetitive element palindromic PCR (rep-PCR) primer, the BOXA1R primer, produced the discriminatory profile, with amplicon sizes ranging from similar to 200 bp to 2 kb, and categorized the olive strains into 2 separate groups. Pulsed-Field Gel Electrophoresis (PFGE) differentiated the olive Psv isolates into 3 discrete haplotype groups after the genomic DNA was digested with Spa This is the first study using PFGE to determine the genetic diversity of the Psv olive population. | NA | no | gd of pathogen | NA | no | no |
| 4722 | 2020 | 44112 | Basnet, S; Kamble, ST | 2019 | Advances in Molecular Research on Bed Bugs (Hemiptera: Cimicidae) | With the resurgence and increase in infestations of the common bed bug, Cimex lectularius L. (Hemiptera: Cimicidae), across the world, there has been renewed interest in molecular research on this pest. In this paper, we present current information on the biology, medical importance, management practices, behavior and physiology, and molecular research conducted on bed bugs. The majority of molecular studies are focused towards understanding the molecular mechanism of insecticide resistance. Bed bugs are hematophagous insects with no prior record of vectoring any disease organisms. An improved understanding of how bed bugs lack vector competency may provide information to prevent disease transmission in other hematophagous insects. The genome of bed bugs has been sequenced, and genomic studies may provide a better understanding of bed bug behavior that might be utilized in developing effective management strategies. Recently, with the advancement of RNA interference (RNAi) as a tool to suppress insects, a few RNAi studies have been conducted in bed bugs. RNAi in bed bugs shows potential to suppress populations in laboratory conditions. However, delivery of double-stranded RNA (dsRNA) into bed bugs under field condition requires extensive research. | NA | no | No good fitness measure and measure host GD | NA | no | no |
| 4723 | 2020 | 44113 | Batista, MRD; Rocha, FB; Klaczko, LB | 2018 | Altitudinal distribution of two sibling species of the Drosophila tripunctata group in a preserved tropical forest and their male sterility thermal thresholds | Variation of ecophysiological traits may help to explain geographic distribution patterns of Drosophila sibling species. Many traits in ectotherms have optimal performance within specific temperature ranges. Altitudinal gradients are potentially informative for characterizing differences of sibling species distributions. We collected two sibling species of the tripunctata group - Drosophila mediopunctata (MPT) and D. unipunctata (UNI) - at eight altitudes (ranging from 593 to 1185 m above sea level) located at a continuous Atlantic Rainforest reserve in consecutive years (2009-2011), with two collections at the hot-rainy season and two at the cold-dry season. Mean altitude was significantly different between species and seasons. Their distributions showed a consistent pattern with MPT always occurring at higher altitudes than UNI. A significant correlation was found between altitude and species relative abundance. We characterized the thermal range of fertility, an important fitness component, for each species and found evidence for differential thermal adaptation. Our results suggest that the two species altitudinal distributions and seasonal relative abundances are consistent with their differential thermal adaptations: MPT seems to be adapted to lower temperatures, occupies higher altitudes and occurs at higher relative abundances in the cold-dry season; while UNI tolerates higher temperatures and occurs at lower altitudes and higher relative abundances in the hot-rainy season. However, their thermal ranges overlap at most temperatures, suggesting that additional variables (e.g. habitat choice, competition, differential survival etc.) may also play a role to determine their distribution in the field. | NA | no | No good fitness measure and measure host GD | NA | no | no |
| 4724 | 2020 | 44040 | Bauer, ML; Ferry, B; Holman, H; Kovach, AI | 2020 | Monitoring a New England Cottontail Reintroduction with Noninvasive Genetic Sampling | Careful monitoring of reintroduced threatened species is essential for informing conservation strategies and evaluating reintroduction efforts in an adaptive management context. We used noninvasive genetic sampling to monitor a reintroduction of a threatened shrubland specialist, the New England cottontail (Sylvilagus transitionalis), in southeastern New Hampshire, USA. We monitored the apparent survival and breeding success of founder individuals and tracked changes in population size and genetic diversity for 5 years following an initial reintroduction in 2013. We released 42 rabbits, documented 29 unique offspring in years following releases through noninvasive surveys, and identified 6 founder individuals and 9 recruited offspring that bred. Apparent survival of founders was variable and greatest in the first year of the reintroduction. Predation was the primary cause of mortality and greatest in the first month after release and after heavy snowfall. Population size remained small but relatively stable until a stochastic decline in the fourth year following reintroduction, followed by a slight rebound after population augmentation and offspring production by wild-born rabbits. Genetic diversity increased after the initial founders with diverse genetic backgrounds were released and then they and their subsequent offspring bred. We documented successful dispersal 700 m from the release site to a high-quality patch of habitat, which remained occupied throughout the study. For New England cottontail reintroductions to be successful in the long term, releases will be needed at multiple patches within dispersal distance, and habitat corridors need to be restored among patches to create a functioning metapopulation. For small or isolated reintroduced populations, continued intensive monitoring is needed to detect stochastic declines in population size or changes in sex ratios and guide subsequent management reactions via additional reintroductions or population augmentations. Noninvasive genetic sampling is a valuable tool to monitor reintroductions of the New England cottontail and other threatened species to provide managers with detailed information to inform decision-making in an adaptive management framework. (c) 2020 The Wildlife Society. | NA | no | No good fitness measure and measure host GD | NA | no | no |
| 4725 | 2020 | 44041 | Baums, IB; Baker, AC; Davies, SW; Grottoli, AG; Kenkel, CD; Kitchen, SA; Kuffner, IB; LaJeunesse, TC; Matz, MV; Miller, MW; Parkinson, JE; Shantz, AA | 2019 | Considerations for maximizing the adaptive potential of restored coral populations in the western Atlantic | Active coral restoration typically involves two interventions: crossing gametes to facilitate sexual larval propagation; and fragmenting, growing, and outplanting adult colonies to enhance asexual propagation. From an evolutionary perspective, the goal of these efforts is to establish self-sustaining, sexually reproducing coral populations that have sufficient genetic and phenotypic variation to adapt to changing environments. Here, we provide concrete guidelines to help restoration practitioners meet this goal for most Caribbean species of interest. To enable the persistence of coral populations exposed to severe selection pressure from many stressors, a mixed provenance strategy is suggested: genetically unique colonies (genets) should be sourced both locally as well as from more distant, environmentally distinct sites. Sourcing three to four genets per reef along environmental gradients should be sufficient to capture a majority of intraspecies genetic diversity. It is best for practitioners to propagate genets with one or more phenotypic traits that are predicted to be valuable in the future, such as low partial mortality, high wound healing rate, high skeletal growth rate, bleaching resilience, infectious disease resilience, and high sexual reproductive output. Some effort should also be reserved for underperforming genets because colonies that grow poorly in nurseries sometimes thrive once returned to the reef and may harbor genetic variants with as yet unrecognized value. Outplants should be clustered in groups of four to six genets to enable successful fertilization upon maturation. Current evidence indicates that translocating genets among distant reefs is unlikely to be problematic from a population genetic perspective but will likely provide substantial adaptive benefits. Similarly, inbreeding depression is not a concern given that current practices only raise first-generation offspring. Thus, proceeding with the proposed management strategies even in the absence of a detailed population genetic analysis of the focal species at sites targeted for restoration is the best course of action. These basic guidelines should help maximize the adaptive potential of reef-building corals facing a rapidly changing environment. | NA | no | No good fitness measure and measure host GD | NA | no | no |
| 4726 | 2020 | 44113 | Bayas-Rea, RD; Felix, F; Montufar, R | 2018 | Genetic divergence and fine scale population structure of the common bottlenose dolphin (Tursiops truncatus, Montagu) found in the Gulf of Guayaquil, Ecuador | The common bottlenose dolphin, Tursiops truncatus, is widely distributed along the western coast of South America. In Ecuador, a resident population of bottlenose dolphins inhabits the inner estuarine area of the Gulf of Guayaquil located in the southwestern part of the country and is under threat from different human activities in the area. Only one genetic study on South American common bottlenose dolphins has been carried out to date, and understanding genetic variation of wildlife populations, especially species that are identified as threatened, is crucial for defining conservation units and developing appropriate conservation strategies. In order to evaluate the evolutionary link of this population, we assessed the phylogenetic relationships, phylogeographic patterns, and population structure using mitochondrial DNA (mtDNA). The sampling comprised: (i) 31 skin samples collected from free-ranging dolphins at three locations in the Gulf of Guayaquil inner estuary, (ii) 38 samples from stranded dolphins available at the collection of the Museo de Ballenas de Salinas, (iii) 549 mtDNA control region (mtDNA CR) sequences from GenBank, and (iv) 66 concatenated sequences from 7-mtDNA regions (12S rRNA, 16S rRNA, NADH dehydrogenase subunit I-II, cytochrome oxidase I and II, cytochrome b, and CR) obtained from mitogenomes available in GenBank. Our analyses indicated population structure between both inner and outer estuary dolphin populations as well as with distinct populations of T. truncatus using mtDNA CR. Moreover, the inner estuary bottlenose dolphin (estuarine bottlenose dolphin) population exhibited lower levels of genetic diversity than the outer estuary dolphin population according to the mtDNA CR. Finally, the estuarine bottlenose dolphin population was genetically distinct from other T. truncatus populations based on mtDNA CR and 7-mtDNA regions. From these results, we suggest that the estuarine bottlenose dolphin population should be considered a distinct lineage. This dolphin population faces a variety of anthropogenic threats in this area; thus, we highlight its fragility and urge authorities to issue prompt management and conservation measures. | NA | no | No good fitness measure | NA | no | no |
| 4727 | 2020 | 44043 | Bayjanov, JR; Baan, J; Rogers, MRC; Troelstra, A; Willems, RJL; van Schaik, W | 2019 | Enterococcus faecium genome dynamics during long-term asymptomatic patient gut colonization | Enterococcus faecium is a gut commensal of humans and animals. In addition, it has recently emerged as an important nosocomial pathogen through the acquisition of genetic elements that confer resistance to antibiotics and virulence. We performed a whole-genome sequencing-based study on 96 multidrug-resistant E. faecium strains that asymptomatically colonized five patients with the aim of describing the genome dynamics of this species. The patients were hospitalized on multiple occasions and isolates were collected over periods ranging from 15 months to 6.5 years. Ninety-five of the sequenced isolates belonged to E. faecium Glade Al, which was previously determined to be responsible for the vast majority of clinical infections. The Glade Al strains clustered into six clonal groups of highly similar isolates, three of which consisted entirely of isolates from a single patient. We also found evidence of concurrent colonization of patients by multiple distinct lineages and transfer of strains between patients during hospitalization. We estimated the evolutionary rate of two clonal groups that each colonized single patients at 12.6 and 25.2 single-nucleotide polymorphisms (SNPs)/genome/year. A detailed analysis of the accessory genome of one of the clonal groups revealed considerable variation due to gene gain and loss events, including the chromosomal acquisition of a 37 kbp prophage and the loss of an element containing carbohydrate metabolism-related genes. We determined the presence and location of 12 different insertion sequence (IS) elements, with ISEfa5 showing a unique pattern of location in 24 of the 25 isolates. suggesting widespread ISEfa5 excision and insertion into the genome during gut colonization. Our findings show that the E. faecium genome is highly dynamic during asymptomatic colonization of the human gut. We observed considerable genomic flexibility due to frequent horizontal gene transfer and recombination. which can contribute to the generation of genetic diversity within the species and, ultimately, can contribute to its success as a nosocomial pathogen. | NA | no | human | NA | no | no |
| 4728 | 2020 | 44112 | Beaurepaire, AL; Ellis, JD; Krieger, KJ; Moritz, RFA | 2019 | Association of Varroa destructor females in multiply infested cells of the honeybee Apis mellifera | The genetic diversity of Varroa destructor (Anderson & Trueman) is limited outside its natural range due to population bottlenecks and its propensity to inbreed. In light of the arms race between V. destructor and its honeybee (Apis mellifera L.) host, any mechanism enhancing population admixture of the mite may be favored. One way that admixture can occur is when two genetically dissimilar mites coinvade a brood cell, with the progeny of the foundresses admixing. We determined the relatedness of 393 pairs of V. destructor foundresses, each pair collected from a single bee brood cell (n = five colonies). We used six microsatellites to identify the genotypes of mites coinvading a cell and calculated the frequency of pairs with different or the same genotypes. We found no deviation from random coinvasion, but the frequency of cells infested by mites with different genotypes was high. This rate of recombination, coupled with a high transmission rate of mites, homogenized the allelic pool of mites within the apiary. | NA | no | No good fitness measure and measure host GD | NA | no | no |
| 4729 | 2020 | 44040 | Beck, EA; Currey, MC; Small, CM; Cresko, WA | 2020 | QTL Mapping of Intestinal Neutrophil Variation in Threespine Stickleback Reveals Possible Gene Targets Connecting Intestinal Inflammation and Systemic Health | Selection, via host immunity, is often required to foster beneficial microbial symbionts and suppress deleterious pathogens. In animals, the host immune system is at the center of this relationship. Failed host immune system-microbial interactions can result in a persistent inflammatory response in which the immune system indiscriminately attacks resident microbes, and at times the host cells themselves, leading to diseases such as Ulcerative Colitis, Crohn’s Disease, and Psoriasis. Host genetic variation has been linked to both microbiome diversity and to severity of such inflammatory disease states in humans. However, the microbiome and inflammatory states manifest as quantitative traits, which encompass many genes interacting with one another and the environment. The mechanistic relationships among all of these interacting components are still not clear. Developing natural genetic models of host-microbe interactions is therefore fundamental to understanding the complex genetics of these and other diseases. Threespine stickleback (Gasterosteus aculeatus) fish are a tractable model for attacking this problem because of abundant population-level genetic and phenotypic variation in the gut inflammatory response. Previous work in our laboratory identified genetically divergent stickleback populations exhibiting differences in intestinal neutrophil activity. We took advantage of this diversity to genetically map variation in an emblematic element of gut inflammation - intestinal neutrophil recruitment - using an F2-intercross mapping framework. We identified two regions of the genome associated with increased intestinal inflammation containing several promising candidate genes. Within these regions we found candidates in the Coagulation/Complement System, NFkB and MAPK pathways along with several genes associated with intestinal diseases and neurological diseases commonly accompanying intestinal inflammation as a secondary symptom. These findings highlight the utility of using naturally genetically diverse ‘evolutionary mutant models’ such as threespine stickleback to better understand interactions among host genetic diversity and microbiome variation in health and disease states. | NA | no | No good fitness measure and measure host GD | NA | no | no |
| 4730 | 2020 | 44041 | Becker, DJ; Downs, CJ; Martin, LB | 2019 | Multi-Scale Drivers of Immunological Variation and Consequences for Infectious Disease Dynamics | Synopsis The immune system is the primary barrier to parasite infection, replication, and transmission following exposure, and variation in immunity can accordingly manifest in heterogeneity in traits that govern population-level infectious disease dynamics. While much work in ecoimmunology has focused on individual-level determinants of host immune defense (e.g., reproductive status and body condition), an ongoing challenge remains to understand the broader evolutionary and ecological contexts of this variation (e.g., phylogenetic relatedness and landscape heterogeneity) and to connect these differences into epidemiological frameworks. Ultimately, such efforts could illuminate general principles about the drivers of host defense and improve predictions and control of infectious disease. Here, we highlight recent work that synthesizes the complex drivers of immunological variation across biological scales of organization and scales these within-host differences to population-level infection outcomes. Such studies note the limitations involved in making species-level comparisons of immune phenotypes, stress the importance of spatial scale for immunology research, showcase several statistical tools for translating within-host data into epidemiological parameters, and provide theoretical frameworks for linking within- and between-host scales of infection processes. Building from these studies, we highlight several promising avenues for continued work, including the application of machine learning tools and phylogenetically controlled meta-analyses to immunology data and quantifying the joint spatial and temporal dependencies in immune defense using range expansions as model systems. We also emphasize the use of organismal traits (e.g., host tolerance, competence, and resistance) as a way to interlink various scales of analysis. Such continued collaboration and disciplinary cross-talk among ecoimmunology, disease ecology, and mathematical modeling will facilitate an improved understanding of the multi-scale drivers and consequences of variation in host defense. | NA | no | No good fitness measure and measure host GD | NA | no | no |
| 4731 | 2020 | 44040 | Becker, DJ; Speer, KA; Brown, AM; Fenton, MB; Washburne, AD; Altizer, S; Streicker, DG; Plowright, RK; Chizhikov, VE; Simmons, NB; Volokhov, DV | 2020 | Ecological and evolutionary drivers of haemoplasma infection and bacterial genotype sharing in a Neotropical bat community | Most emerging pathogens can infect multiple species, underlining the importance of understanding the ecological and evolutionary factors that allow some hosts to harbour greater infection prevalence and share pathogens with other species. However, our understanding of pathogen jumps is based primarily around viruses, despite bacteria accounting for the greatest proportion of zoonoses. Because bacterial pathogens in bats (order Chiroptera) can have conservation and human health consequences, studies that examine the ecological and evolutionary drivers of bacterial prevalence and barriers to pathogen sharing are crucially needed. Here were studied haemotropic Mycoplasma spp. (i.e., haemoplasmas) across a species-rich bat community in Belize over two years. Across 469 bats spanning 33 species, half of individuals and two-thirds of species were haemoplasma positive. Infection prevalence was higher for males and for species with larger body mass and colony sizes. Haemoplasmas displayed high genetic diversity (21 novel genotypes) and strong host specificity. Evolutionary patterns supported codivergence of bats and bacterial genotypes alongside phylogenetically constrained host shifts. Bat species centrality to the network of shared haemoplasma genotypes was phylogenetically clustered and unrelated to prevalence, further suggesting rare-but detectable-bacterial sharing between species. Our study highlights the importance of using fine phylogenetic scales when assessing host specificity and suggests phylogenetic similarity may play a key role in host shifts not only for viruses but also for bacteria. Such work more broadly contributes to increasing efforts to understand cross-species transmission and the epidemiological consequences of bacterial pathogens. | NA | no | No good fitness measure and measure host GD | NA | no | no |
| 4732 | 2020 | 44113 | Beghini, F; Pasolli, E; Truong, TD; Putignani, L; Caccio, SM; Segata, N | 2017 | Large-scale comparative metagenomics of Blastocystis, a common member of the human gut microbiome | The influence of unicellular eukaryotic microorganisms on human gut health and disease is still largely unexplored. Blastocystis spp. commonly colonize the gut, but its clinical significance and ecological role are currently unsettled. We have developed a high-sensitivity bioinformatic pipeline to detect Blastocystis subtypes (STs) from shotgun metagenomics, and applied it to 12 large data sets, comprising 1689 subjects of different geographic origin, disease status and lifestyle. We confirmed and extended previous observations on the high prevalence the microrganism in the population (14.9%), its non-random and ST-specific distribution, and its ability to cause persistent (asymptomatic) colonization. These findings, along with the higher prevalence observed in non-westernized individuals, the lack of positive association with any of the disease considered, and decreased presence in individuals with dysbiosis associated with colorectal cancer and Crohn’s disease, strongly suggest that Blastocystis is a component of the healthy gut microbiome. Further, we found an inverse association between body mass index and Blastocystis, and strong co-occurrence with archaeal organisms (Methanobrevibacter smithii) and several bacterial species. The association of specific microbial community structures with Blastocystis was confirmed by the high predictability (up to 0.91 area under the curve) of the microorganism colonization based on the species-level composition of the microbiome. Finally, we reconstructed and functionally profiled 43 new draft Blastocystis genomes and discovered a higher intra subtype variability of ST1 and ST2 compared with ST3 and ST4. Altogether, we provide an in-depth epidemiologic, ecological, and genomic analysis of Blastocystis, and show how metagenomics can be crucial to advance population genomics of human parasites. | NA | no | No good fitness measure and measure host GD | NA | no | no |
| 4733 | 2020 | 44112 | Belasen, AM; Bletz, MC; Leite, DD; Toledo, LF; James, TY | 2019 | Long-Term Habitat Fragmentation Is Associated With Reduced MHC IIB Diversity and Increased Infections in Amphibian Hosts | Habitat fragmentation and wildlife disease are two widespread drivers of biodiversity loss, yet few empirical studies have explored their interactions. In this study, we utilized a naturally fragmented island system to examine the impacts of fragmentation on genetic diversity and amphibian infection dynamics. We determined the impacts of fragmentation on genetic diversity at the immunity locus MHC IIB, a hypothesized predictor of disease susceptibility. Contrary to the expectation that MHC diversity would remain high due to balancing selection, island populations lost genetic diversity at this locus while simultaneously experiencing positive selection at MHC IIB. We then used Next-Generation Sequencing to identify a variety of potential eukaryotic parasites from amphibian skin swabs. Island populations exhibited higher potential parasite richness (proportion of eukaryotic microbe operational taxonomic units or OTUs from parasitic taxa) relative to mainland populations. MHC homozygotes hosted a lower diversity of potential parasites, and population-level MHC diversity was negatively associated with parasite richness. Our results show that genetic erosion can occur at the MHC IIB locus following fragmentation, which may contribute to increased susceptibility to parasites. | NA | no | no good gd measure host pop | NA | no | no |
| 4734 | 2020 | 44113 | Belbin, GM; Odgis, J; Sorokin, EP; Yee, MC; Kohli, S; Glicksberg, BS; Gignoux, CR; Wojcik, GL; Van Vleck, T; Jeff, JM; Linderman, M; Schurmann, C; Ruderfer, D; Cai, XQ; Merkelson, A; Justice, AE; Young, KL; Graff, M; North, KE; Peters, U; James, R; Hindorff, L; Kornreich, R; Edelman, L; Gottesman, O; Stahl, EEA; Cho, JH; Loos, RJF; Bottinger, EP; Nadkarni, GN; Abul-Husn, NS; Kenny, EE | 2017 | Genetic identification of a common collagen disease in puerto ricans via identity-by-descent mapping in a health system | Achieving confidence in the causality of a disease locus is a complex task that often requires supporting data from both statistical genetics and clinical genomics. Here we describe a combined approach to identify and characterize a genetic disorder that leverages distantly related patients in a health system and population-scale mapping. We utilize genomic data to uncover components of distant pedigrees, in the absence of recorded pedigree information, in the multi-ethnic BioMe biobank in New York City. By linking to medical records, we discover a locus associated with both elevated genetic relatedness and extreme short stature. We link the gene, COL27A1, with a little-known genetic disease, previously thought to be rare and recessive. We demonstrate that disease manifests in both heterozygotes and homozygotes, indicating a common collagen disorder impacting up to 2% of individuals of Puerto Rican ancestry, leading to a better understanding of the continuum of complex and Mendelian disease. | NA | no | No good fitness measure and measure host GD | NA | no | no |
| 4735 | 2020 | 44040 | Bellis, ES; Kelly, EA; Lorts, CM; Gao, HR; Deleo, VL; Rouhan, G; Budden, A; Bhaskara, GB; Hu, ZB; Muscarella, R; Timko, MP; Nebie, B; Runo, SM; Chilcoat, ND; Juenger, TE; Morris, GP; dePamphilis, CW; Lasky, JR | 2020 | Genomics of sorghum local adaptation to a parasitic plant | Host-parasite coevolution can maintain high levels of genetic diversity in traits involved in species interactions. In many systems, host traits exploited by parasites are constrained by use in other functions, leading to complex selective pressures across space and time. Here, we study genome-wide variation in the staple crop Sorghum bicolor (L.) Moench and its association with the parasitic weed Striga hermonthica (Delile) Benth., a major constraint to food security in Africa. We hypothesize that geographic selection mosaics across gradients of parasite occurrence maintain genetic diversity in sorghum landrace resistance. Suggesting a role in local adaptation to parasite pressure, multiple independent loss-of-function alleles at sorghum LOW GERMINATION STIMULANT 1 (LGS1) are broadly distributed among African landraces and geographically associated with S. hermonthica occurrence. However, low frequency of these alleles within S. hermonthica-prone regions and their absence elsewhere implicate potential trade-offs restricting their fixation. LGS1 is thought to cause resistance by changing stereochemistry of strigolactones, hormones that control plant architecture and below-ground signaling to mycorrhizae and are required to stimulate parasite germination. Consistent with trade-offs, we find signatures of balancing selection surrounding LGS1 and other candidates from analysis of genome-wide associations with parasite distribution. Experiments with CRISPR-Cas9-edited sorghum further indicate that the benefit of LGS1-mediated resistance strongly depends on parasite genotype and abiotic environment and comes at the cost of reduced photosystem gene expression. Our study demonstrates long-term maintenance of diversity in host resistance genes across smallholder agroecosystems, providing a valuable comparison to both industrial farming systems and natural communities. | NA | no | dometicated host | NA | no | no |
| 4736 | 2020 | 44112 | Benavente, ED; Oresegun, DR; de Sessions, PF; Walker, EM; Roper, C; Dombrowski, JG; de Souza, RM; Marinho, CRF; Sutherland, CJ; Hibberd, ML; Mohareb, F; Baker, DA; Clark, TG; Campino, S | 2018 | Global genetic diversity of var2csa in Plasmodium falciparum with implications for malaria in pregnancy and vaccine development | Malaria infection during pregnancy, caused by the sequestering of Plasmodium falciparum parasites in the placenta, leads to high infant mortality and maternal morbidity. The parasite-placenta adherence mechanism is mediated by the VAR2CSA protein, a target for natural occurring immunity. Currently, vaccine development is based on its ID1-DBL2Xb domain however little is known about the global genetic diversity of the encoding var2csa gene, which could influence vaccine efficacy. In a comprehensive analysis of the var2csa gene in >2,000 P. falciparum field isolates across 23 countries, we found that var2csa is duplicated in high prevalence (>25%), African and Oceanian populations harbour a much higher diversity than other regions, and that insertions/deletions are abundant leading to an underestimation of the diversity of the locus. Further, ID1-DBL2Xb haplotypes associated with adverse birth outcomes are present globally, and African-specific haplotypes exist, which should be incorporated into vaccine design. | NA | no | gd of pathogen | NA | no | no |
| 4737 | 2020 | 44112 | Bensasson, D; Dicks, J; Ludwig, JM; Bond, CJ; Elliston, A; Roberts, IN; James, SA | 2019 | Diverse Lineages of Candida albicans Live on Old Oaks | The human pathogen Candida albicans is considered an obligate commensal of animals, yet it is occasionally isolated from trees, shrubs, and grass. We generated genome sequence data for three strains of C. albicans that we isolated from oak trees in an ancient wood pasture, and compared these to the genomes of over 200 clinical strains. C. albicans strains from oak are similar to clinical C. albicans in that they are predominantly diploid and can become homozygous at the mating locus through whole-chromosome loss of heterozygosity. Oak strains differed from clinical strains in showing slightly higher levels of heterozygosity genome-wide. Using phylogenomic analyses and in silico chromosome painting, we show that each oak strain is more closely related to strains from humans and other animals than to strains from other oaks. The high genetic diversity of C. albicans from old oaks shows that they can live in this environment for extended periods of time. | NA | no | gd of pathogen | NA | no | no |
| 4738 | 2020 | 44111 | Benson, JF; Mahoney, PJ; Vickers, TW; Sikich, JA; Beier, P; Riley, SPD; Ernest, HB; Boyce, WM | 2019 | Extinction vortex dynamics of top predators isolated by urbanization | Extinction risk is elevated in small, isolated populations due to demographic and genetic interactions. Therefore, it is critical to model these processes realistically in population viability analyses (PVA) to inform local management and contribute to a greater understanding of mechanisms within the extinction vortex. We conducted PVA’s for two small mountain lion populations isolated by urbanization in southern California to predict population growth, extinction probability, and loss of genetic diversity with empirical data. Specifically, we (1) provide the first PVA for isolated mountain lions in the Santa Ana Mountains (SAM) that considers both demographic and genetic risk factors and (2) test the hypothesis that variation in abundance and mortality between the SAM and Santa Monica Mountains (SMM) result in differences in population growth, loss of heterozygosity, and extinction probability. Our models predicted 16-21% probability of local extinction in the SAM due purely to demographic processes over 50 yr with current low levels or no immigration. Our models also predicted that genetic diversity will further erode in the SAM such that concern regarding inbreeding depression is warranted unless gene flow is increased, and that if inbreeding depression occurs, rapid local extinction will be highly likely. Dynamics of the two populations were broadly similar, but they also exhibited differences driven by larger population size and higher mortality in the SAM. Density-independent scenarios predicted a rapidly increasing population in the SMM, whereas growth potential did not differ from a stable trend in the SAM. Demographic extinction probability and loss of heterozygosity were greater in the SMM for density-dependent scenarios without immigration. However, higher levels of immigration had stronger, positive influences on both demographic viability and retention of genetic diversity in the SMM driven by lower abundance and higher adult survival. Our results elucidate demographic and genetic threats to small populations within the extinction vortex, and how these vary relative to demographic structure. importantly, simulating seemingly attainable increases in connectivity was sufficient to greatly reduce extinction probability. Our work highlights that conservation of large carnivores is achievable within urbanized landscapes, but requires land protection, connectivity, and strategies to promote coexistence with humans. | NA | no | only two pop | NA | no | no |
| 4739 | 2020 | 44040 | Bentley, AR; Callier, SL; Rotimi, CN | 2020 | Evaluating the promise of inclusion of African ancestry populations in genomics | The lack of representation of diverse ancestral backgrounds in genomic research is well-known, and the resultant scientific and ethical limitations are becoming increasingly appreciated. The paucity of data on individuals with African ancestry is especially noteworthy as Africa is the birthplace of modern humans and harbors the greatest genetic diversity. It is expected that greater representation of those with African ancestry in genomic research will bring novel insights into human biology, and lead to improvements in clinical care and improved understanding of health disparities. Now that major efforts have been undertaken to address this failing, is there evidence of these anticipated advances? Here, we evaluate the promise of including diverse individuals in genomic research in the context of recent literature on individuals of African ancestry. In addition, we discuss progress and achievements on related technological challenges and diversity among scientists conducting genomic research. | NA | no | human | NA | no | no |
| 4740 | 2020 | 44112 | Benton, CH; Delahay, RJ; Smith, FAP; Robertson, A; McDonald, RA; Young, AJ; Burke, TA; Hodgson, D | 2018 | Inbreeding intensifies sex- and age-dependent disease in a wild mammal | The mutation accumulation theory of senescence predicts that age-related deterioration of fitness can be exaggerated when inbreeding causes homozygosity for deleterious alleles. A vital component of fitness, in natural populations, is the incidence and progression of disease. Evidence is growing for natural links between inbreeding and ageing; between inbreeding and disease; between sex and ageing; and between sex and disease. However, there is scant evidence, to date, for links among age, disease, inbreeding and sex in a single natural population. Using ecological and epidemiological data from a long-term longitudinal field study, we show that in wild European badgers (Meles meles) exposed naturally to bovine tuberculosis (bTB), inbreeding (measured as multilocus homozygosity) intensifies a positive correlation between age and evidence of progressed infection (measured as an antibody response to bTB), but only among females. Male badgers suffer a steeper relationship between age and progressed infection than females, with no influence of inbred status. We found no link between inbreeding and the incidence of progressed infection during early life in either sex. Our findings highlight an age-related increase in the impact of inbreeding on a fitness-relevant trait (disease state) among females. This relationship is consistent with the predictions of the mutation accumulation theory of senescence, but other mechanisms could also play a role. For example, late-life declines in condition, arising through mechanisms other than mutation accumulation might have increased the magnitude of inbreeding depression in late life. Whichever mechanism causes the observed patterns, we have shown that inbreeding can influence age-dependent patterns of disease and, by extension, is likely to affect the magnitude and timing of the late-life declines in components of fitness that characterise senescence. Better understanding of sex-specific links between inbreeding, disease and ageing provides insights into population-level pathogen dynamics and could influence management strategies for wildlife reservoirs of zoonotic disease. | NA | no | inbreeding | NA | no | no |
| 4741 | 2020 | 44113 | Berchi, GM; Copilas-Ciocianu, D; Kment, P; Buzzetti, FM; Petrusek, A; Rakosy, L; Cianferoni, F; Damgaard, J | 2018 | Molecular phylogeny and biogeography of the West-Palaearctic Velia (Heteroptera: Gerromorpha: Veliidae) | We investigated the systematics and biogeography of the West-Palaearctic water cricket genus Velia Latreille based on a phylogenetic analysis of five molecular markers obtained from 79% of all known taxa of the subgenera Velia (s.s.) and Velia (Plesiovelia) Tamanini. The results revealed a sister-group relationship between Velia (Plesiovelia) and the monotypic subgenus Velia (s.s.), and showed that the former is divided into three major clades. All taxa of Velia (Plesiovelia) were recovered as monophyletic, except for V.(P.) serbica Tamanini, which was paraphyletic with respect to V.(P.) mancinii mancinii Tamanini. Our results also indicate the existence of several unrecognized species. Molecular dating based on fossil data and COI rates indicates that the split between Velia (s.s.) and Velia (Plesiovelia) occurred between 40 and 22Ma. An ancestral area reconstruction suggests that the latter originated in southeastern Europe, from where it radiated to the west and east, along the Neogene archipelagos of Europe and Asia Minor. Northwestern Africa served as the second most important diversification centre of the subgenus. The low genetic variability in the widespread V.(P.) caprai caprai Tamanini and V.(P.) saulii Tamanini implies a rapid postglacial colonization of Europe, whereas high diversity within the lineages of V.(P.) serbica indicates survival of Pleistocene glaciations in microrefugia throughout southeastern Europe. These results serve as a useful framework for future studies ranging from the systematics of the group to historical biogeography, ecology and biodiversity conservation. | NA | no | No good fitness measure and measure host GD | NA | no | no |
| 4742 | 2020 | 44112 | Berdjeb, L; Parada, A; Needham, DM; Fuhrman, JA | 2018 | Short-term dynamics and interactions of marine protist communities during the spring-summer transition | We examined the short-term variability, by daily to weekly sampling, of protist assemblages from March to July in surface water of the San Pedro Ocean Time-series station (eastern North Pacific), by V4 Illumina sequencing of the 18S rRNA gene. The sampling period encompassed a spring bloom followed by progression to summer conditions. Several protistan taxa displayed sharp increases and declines, with whole community Bray-Curtis dissimilarities of adjacent days being 66% in March and 40% in May. High initial abundance of parasitic Cercozoa Cryothecomonas longipes and Protaspis grandis coincided with a precipitous decline of blooming Pseudo-nitzschia diatoms, possibly suggesting their massive infection by these parasites; these cercozoans were hardly detectable afterwards. Canonical correspondence analysis indicated a limited predictability of community variability from environmental factors. This indicates that other factors are relevant in explaining changes in protist community composition at short temporal scales, such as interspecific relationships, stochastic processes, mixing with adjacent water, or advection of patches with different protist communities. Association network analysis revealed that interactions between the many parasitic OTUs and other taxa were overwhelmingly positive and suggest that although sometimes parasites may cause a crash of host populations, they may often follow their hosts and do not regularly cause enough mortality to potentially create negative correlations at the daily to weekly time scales we studied. | NA | no | No good fitness measure and measure host GD | NA | no | no |
| 4743 | 2020 | 44043 | Bererhi, B; Wapstra, E; Schwartz, TS; Olsson, M | 2019 | Inconsistent inbreeding effects during lizard ontogeny | Reported effects of inbreeding vary among taxa and may depend on a number of factors, including what trait is measured, temporal variability, parental effects, or life history stage. To understand the effects of inbreeding during early life history stages, we estimated the effects of individual-level heterozygosity on hatching success and first year survival in a Swedish population of sand lizards (Lacerta agilis) over a period of almost a decade, using over 4000 eggs, 400 clutches, and over 3000 juveniles. Heterozygosity had a positive effect on hatching success, in standardized laboratory conditions, but no effect on first year survival. Also, both of these measures of viability varied across the years of the study, demonstrating the importance of temporal heterogeneity in pre and post-hatching conditions. Finally, we identified both paternal and maternal identity effects on hatching success. Thus, we show that selection on heterozygosity was not consistent across developmental life stages, emphasizing the need of considering a number of ontogenic stages, as well as potential parental and environmental effects, when studying the effects of heterozygosity on viability in natural populations. | NA | no | inbreeding | NA | no | no |
| 4744 | 2020 | 44042 | Bergner, LM; Orton, RJ; Benavides, JA; Becker, DJ; Tello, C; Biek, R; Streicker, DG | 2020 | Demographic and environmental drivers of metagenomic viral diversity in vampire bats | Viruses infect all forms of life and play critical roles as agents of disease, drivers of biochemical cycles and sources of genetic diversity for their hosts. Our understanding of viral diversity derives primarily from comparisons among host species, precluding insight into how intraspecific variation in host ecology affects viral communities or how predictable viral communities are across populations. Here we test spatial, demographic and environmental hypotheses explaining viral richness and community composition across populations of common vampire bats, which occur in diverse habitats of North, Central and South America. We demonstrate marked variation in viral communities that was not consistently predicted by a null model of declining community similarity with increasing spatial or genetic distances separating populations. We also find no evidence that larger bat colonies host greater viral diversity. Instead, viral diversity follows an elevational gradient, is enriched by juvenile-biased age structure, and declines with local anthropogenic food resources as measured by livestock density. Our results establish the value of linking the modern influx of metagenomic sequence data with comparative ecology, reveal that snapshot views of viral diversity are unlikely to be representative at the species level, and affirm existing ecological theories that link host ecology not only to single pathogen dynamics but also to viral communities. | NA | no | no good gd measure host | NA | no | no |
| 4745 | 2020 | 44112 | Bestehorn, M; Weigold, S; Kern, WV; Chitimia-Dobler, L; Mackenstedt, U; Dobler, G; Borde, JP | 2018 | Phylogenetics of tick-borne encephalitis virus in endemic foci in the upper Rhine region in France and Germany | Objective Tick-borne encephalitis (TBE) caused by the tick-borne encephalitis virus (TBEV) is the most important tick-borne arboviral disease in Europe and Asia. The Upper Rhine Valley is thought to be the very western border of TBEV distribution in Europe. The aim of our study was to identify natural foci and isolate TBEV from ticks, to determine the prevalence of TBEV in local tick populations and to study the phylogenetic relatedness of circulating TBEV strains in this region. Material and methods Ticks were collected between 2016, 2017 and 2018 by flagging. TBEV was isolated from collected ticks and phylogenetic analyses were performed. Minimal infection rates (MIR) of the collected ticks were calculated. Results At 12 sampling sites, a total of 4,064 Ixodes ticks were collected in 2016 and 2017 -(and one single collection 2018). 953 male, 856 female adult ticks and 2,255 nymphs were identified. The MIR rates were 0,17% (1/595) for Schiltach (Germany) and 0,11% (1/944) for Foret de la Robertsau (France), respectively. Overall, the three newly described TBEV strains, isolated in the years 2016 and 2017 from the Upper Rhine Valley have no close phylogenetic relation and show a genetic relationship with strains from eastern Europe. The 2018 TBEV strain from Aubachstrasse (Germany), however, is closely related to the TBEV found in Schiltach (Germany). Conclusion In conclusion, we demonstrate, to our knowledge for the first time, the phylogenetic relations of the newly isolated TBEV strains on both sides of the upper Rhine river. | NA | no | No good fitness measure and measure host GD | NA | no | no |
| 4746 | 2020 | 44112 | Bichet, C; Vedder, O; Sauer-Gurth, H; Becker, PH; Wink, M; Bouwhuis, S | 2019 | Contrasting heterozygosity-fitness correlations across life in a long-lived seabird | Selection is a central force underlying evolutionary change and can vary in strength and direction, for example across time and space. The fitness consequences of individual genetic diversity have often been investigated by testing for multilocus heterozygosity-fitness correlations (HFCs), but few studies have been able to assess HFCs across life stages and in both sexes. Here, we test for HFCs using a 26-year longitudinal individual-based data set from a large population of a long-lived seabird (the common tern, Sterna hirundo), where 7,974 chicks and breeders of known age were genotyped at 15 microsatellite loci and sampled for life-history traits over the complete life cycle. Heterozygosity was not correlated with fledging or post-fledging prospecting probabilities, but was positively correlated with recruitment probability. For breeders, annual survival was not correlated with heterozygosity, but annual fledgling production was negatively correlated with heterozygosity in males and highest in intermediately heterozygous females. The contrasting HFCs among life stages and sexes indicate differential selective processes and emphasize the importance of assessing fitness consequences of traits over complete life histories. | NA | no | no good gd measure host pop | NA | no | no |
| 4747 | 2020 | 44042 | Bieber, C; Rauchenschwandtner, E; Michel, V; Suchentrunk, F; Smith, S; Vetter, SG | 2019 | Forming a group in the absence of adult females? Social Networks in yearling wild boars | Wild boar populations are strongly increasing across Europe. Although details of population dynamics and reproductive potential are known in this species, our knowledge about the social structure and a possible impact of hunting on this structure is poorly understood. Here we investigated the grouping behaviour of yearling wild boar females under enclosure housed conditions. All females were at the same age and older females (i.e., potential group leaders in a family group) were not present. The wild boar females were purchased at the age of six months from four different breeding sites. After a habituation period of (similar to)8 weeks the females were released randomly into two large enclosures, but ensuring that animals of different origin and body mass were evenly distributed. We analysed data from 111 female wild boars kept in two enclosures (enclosure east (EE): 72 females on (similar to)33 ha and enclosure west (EW): 39 females on (similar to)22 ha). Observations were carried out between March and August the year after birth (at the age of yearlings). The yearling females did not reproduce in the observation year. Data were analysed using social network analysis (SNA). We observed that the females formed stable groups during the study period. Group size did not differ significantly between the enclosures (group sizes between 16 and 20 individuals in the EE and between 11 and 17 individuals in the EW). Interestingly, the higher number of individuals lead to more instead of larger groups in the EE. Further, the group compositions were not based on the relatedness between the animals, as females from different breeding sites formed stable groups together. Rather, common habituation (familiarity) had a stronger effect on subsequent group formation. Within the groups we did not observe a linear hierarchy, although the groups were stable over time. Two females showed high betweenness values (i.e., an individual was often part of the shortest pathways in a network) compared with the average, indicating that females show differences in their connectivity within the social network of a population. Individuals with a high betweenness are very likely to rapidly spread diseases in a population and might play a key role in health management strategies. | NA | no | No good fitness measure and measure host GD | NA | no | no |
| 4748 | 2020 | 44040 | Biedrzycka, A; Konopinski, M; Hoffman, E; Trujillo, A; Zalewski, A | 2020 | Comparing raccoon major histocompatibility complex diversity in native and introduced ranges: Evidence for the importance of functional immune diversity for adaptation and survival in novel environments | The adaptive potential of invasive species is related to the genetic diversity of the invader, which is influenced by genetic drift and natural selection. Typically, the genetic diversity of invaders is studied with neutral genetic markers; however, the expectation of reduced diversity has not been consistently supported by empirical studies. Here, we describe and interpret genetic diversity at both neutral microsatellite loci and the immune-related MHC-DRB locus of native and invasive populations of raccoon to better understand of how drift and selection impact patterns of genetic diversity during the invasion process. We found that despite the loss of many MHC (major histocompatibility complex) alleles in comparison with native populations, functional MHC supertypes are preserved in the invasive region. In the native raccoon population, the number of supertypes within individuals was higher than expected under a neutral model. The high level of individual functional divergence may facilitate the adaptation to local conditions in the invasive range. In the invasive populations, we also detected increased population structure at microsatellites compared to the MHC locus, further suggesting that balancing selection is acting on adaptively important regions of the raccoon genome. Finally, we found that alleles known to exhibit resistance to rabies in the native range, Prlo-DRB*4, Prlo-DRB*16 and Prlo-DRB\*102, were the most common alleles in the European populations, suggesting directional selection is acting on this locus. Our research shows empirical support for the importance of functional immune diversity for adaptation and survival in novel environments. | NA | no | No good fitness measure | NA | no | no |
| 4749 | 2020 | 44040 | Biedrzycka, A; Popiolek, M; Zalewski, A | 2020 | Host-parasite interactions in non-native invasive species are dependent on the levels of standing genetic variation at the immune locus | Background Parasites may mediate the success of biological invasions through their effect on host fitness and thus, on host population growth and stability. However, a release from the pressure of parasites is strongly related to the genetic differentiation of the host. In invasive host populations, the number of available genetic variants, allowing them to ‘fight’ the infection, are likely to be influenced by founder events and genetic drift. The level standing genetic variation of invasive populations may be crucial in successfully adapting to new environments and resisting diseases. We studied invasive populations of raccoon that experienced a random reduction in genetic diversity during the establishment and evaluated the relationship between host immune genetic diversity and intestinal parasites infection. Results We distinguished two different genetic clusters that are characterized by different sets of functionally relevant MHC-DRB alleles. Both clusters were characterized by considerably different allele-parasite associations and different levels of parasite infection. The specific resistance MHC-DRB alleles explained the lower prevalence of Digenea parasites. An increased infection intensity was related to the presence of two MHC-DRB alleles. One of these alleles significantly decreased in frequency over time, causing a decrease of Digenea abundance in raccoons in consecutive years. Conclusions Our findings suggest that intestinal parasites can exert selective pressure on an invasive host with lowered levels of immune genetic diversity and contribute to promoting local adaptation over time. The random genetic drift that created the two different genetic clusters in the invasive raccoon range imposed completely different MHC-parasite associations, strongly associated with the infection status of populations. Our findings underline the role of standing genetic variation in shaping host-parasite relationships and provide empirical support that functional genetic variation may be, at least partly, responsible for differences in the success of invasive populations. | NA | no | No good fitness measure and measure host GD | NA | no | no |
| 4750 | 2020 | 44113 | Biersma, EM; Jackson, JA; Bracegirdle, TJ; Griffiths, H; Linse, K; Convey, P | 2018 | Low genetic variation between South American and Antarctic populations of the bank-forming moss Chorisodontium aciphyllum (Dicranaceae) | The Antarctic-South American bank-forming moss Chorisodontium aciphyllum is known for having the oldest sub-fossils of any extant plant in Antarctica as well as extreme survival abilities, making it a candidate species for possible long-term survival in Antarctica. Applying phylogeographic and population genetic methods using the plastid markers trnL-F and rps4 and the nuclear internal transcribed spacer, we investigated the genetic diversity within C. aciphyllum throughout its range. Low genetic variation was found in all loci, both between and within Antarctic and southern South American populations, suggesting a relatively recent (likely within the last million years) colonization of this moss to the Antarctic, as well as a likely severe bottleneck during Pleistocene glaciations in southern South America. We also performed a simple atmospheric transfer modeling approach to study potential colonization rates of small (microscopic/microbial) or spore-dispersed organisms (such as many mosses and lichens). These suggested that the northern Antarctic Peninsula shows potentially regular connectivity from southern South America, with air masses transferring, particularly southbound, between the two regions. We found elevated genetic variation of C. aciphyllum in Elephant Island, also the location of the oldest known moss banks (> 5500 years), suggesting this location to be a genetic hotspot for this species in the Antarctic. | NA | no | No good fitness measure | NA | no | no |
| 4751 | 2020 | 44113 | Biersma, EM; Jackson, JA; Stech, M; Griffiths, H; Linse, K; Convey, P | 2018 | Molecular Data Suggest Long-Term in Situ Antarctic Persistence Within Antarctica’s Most Speciose Plant Genus, Schistidium | From glacial reconstructions it is clear that Antarctic terrestrial life must have been extremely limited throughout Quaternary glacial periods. In contrast, recent biological studies provide clear evidence for long-term in situ persistence throughout glacial times within most extant Antarctic faunal and several microbial groups. However, even now, the evolutionary history of the Antarctic flora-despite playing major role in Antarctic ecosystems- remains poorly studied. We assessed the diversity, richness and relative age divergences within Schistidium (Grimmiaceae, Bryophyta), the most species-rich plant genus in the Antarctic, as well as the plant genus containing most Antarctic endemic species. We applied phylogenetic and molecular dating methods based on nuclear ribosomal Internal Transcribed Spacer sequences, including all known Antarctic Schistidium species with available sample material. We additionally investigated the continent-wide genetic diversity within the most common Antarctic representative of the genus-the endemic species Schistidium antarctici-and performed preliminary phylogeographic analyses of the bipolar species Schistidium rivulare. Most previously described Antarctic Schistidium species were genetically distinct, confirming their specific status. Interspecific divergences of all species took place at least similar to 1 Mya, suggesting a likely in situ persistence in Antarctica for (at least) all endemic Schistidium species. The widespread endemic species, Schistidium antarctici, diverged from other Antarctic congeners in the late Miocene, thereby revealing the oldest extant plant species currently known in Antarctica, and providing increasing support for the hypothesis of vegetation survival through multiple glacial periods. Within S. antarctic! we identified several distinct clades dividing the eastern Antarctic Peninsula and Scotia Arc islands from the western Antarctic Peninsula and all continental locations. This suggests that the mountainous spine on the Antarctic Peninsula forms a strong barrier to gene flow in this species, while increased genetic diversity in the northern Maritime Antarctic indicates likely glacial refugia in this area. This study provides an important first step toward assessing the diversity and evolutionary history of the most speciose moss genus in the Antarctic. The multi-million year presence of several endemic species contributes to studies on their adaptive potential to survive climate change over both historical and contemporary timescales. | NA | no | No good fitness measure and measure host GD | NA | no | no |
| 4752 | 2020 | 44113 | Bitencourth, K; Amorim, M; De Oliveira, SV; Caetano, RL; Voloch, CM; Gazeta, GS | 2017 | Amblyomma sculptum: genetic diversity and rickettsias in the Brazilian Cerrado biome | Amblyomma sculptum (Ixodida: Ixodidae) Berlese, 1888 is the most important tick vector in Brazil, transmitting the bioagent of the most severe form of spotted fever (SF) in part of the Cerrado (in the states of Minas Gerais and SAo Paulo). In another part of the Cerrado (Central-West region of Brazil), a milder form of SF has been recorded. However, neither the rickettsia nor the vector involved have been characterized. The aim of the current study was to analyse genetic variation and the presence of rickettsia in A. sculptum in Cerrado, from silent areas and with the milder form of SF. Samples were subjected to DNA extraction, amplification and sequencing of 12S rDNA, cytochrome oxidase subunit II and D-loop mitochondrial genes (for tick population analyses), and gltA, htrA, ompA and geneD (sca4) genes for rickettsia researches. Exclusive haplotypes with low frequencies, high haplotype diversity and low nucleotide diversity, star-shaped networks and significant results in neutrality tests indicate A. sculptum population expansions in some areas. Rickettsia amblyommatis, CandidatusRickettsia andeanae and Rickettsia felis were detected. The A. sculptum diversity is not geographically, or biome delimited, pointing to a different potential in vector capacity, possibly associated with differing tick genetic profiles. | NA | no | No good fitness measure and measure host GD | NA | no | no |
| 4753 | 2020 | 44111 | Bitencourth, K; Amorim, M; de Oliveira, SV; Voloch, CM; Gazeta, GS | 2019 | Genetic diversity, population structure and rickettsias in Amblyomma ovale in areas of epidemiological interest for spotted fever in Brazil | Amblyomma ovale (Ixodida: Ixodidae) Koch, 1844 is widely-reported in the neotropical region and is the main vector in the epidemic cycle of Rickettsia parkeri strain Atlantic rainforest, a bioagent of a milder variety of spotted fever (SF). Because species with wide geographical distributions are known to exhibit variations that influence their vectorial capacity, the present study aimed to analyze genetic diversity and rickettsia infection of A. ovale collected during the investigation and surveillance of SF cases in the Cerrado and Atlantic rainforest (ARF) Brazilian biomes. Samples had their DNA extracted, amplified and sequenced for 16S rDNA, 12S rDNA, cytochrome oxidase subunit II and D-loop markers for tick analyses, as well as the gltA, htrA, ompA and ompB genes for rickettsia detection. Between 11 and 33 A. ovale haplotypes were identified, all of them exclusive to areas within individual analyzed biome areas. The A. ovale populations appeared to be structured, with Cluster I restricted to Cerrado + ARF isolated in Caatinga and Cluster II to ARF continuous area. Rickettsia bellii, R. parkeri strain Atlantic rainforest (first report for Goias state, Cerrado), Rickettsia asemboensis (first record in A. ovale for Brazil) and Rickettsia felis (first detection in this ixodid) were identified. A. ovale clusters were not associated with rickettsia types. | NA | no | No good fitness measure and measure host GD | NA | no | no |
| 4754 | 2020 | 44040 | Bittar, C; Machado, RRG; Comelis, MT; Bueno, LM; Beguelini, MR; Morielle-Versute, E; Nogueira, ML; Rahal, P | 2020 | Alphacoronavirus Detection in Lungs, Liver, and Intestines of Bats from Brazil | Bats are flying mammals distributed worldwide known to host several types of Coronavirus (CoV). Since they were reported as the probable source of spillover of highly pathogenic CoV into the human population, investigating the circulation of this virus in bats around the world became of great importance. We analyzed samples from 103 bats from two distinct regions in Brazil. Coronavirus from the Alphacoronavirus genus was detected in 12 animals, 11 from Sao Jose do Rio Preto-SP region and 1 from Barreiras-BA region, resulting in a prevalence of 17.18% and 2.56% respectively. The virus was detected not only in intestines but also in lungs and liver. Phylogenetic analysis based on nsP12 genomic region suggests that the sequences group according to host family and sampling location. Studies on the circulation of these viruses in bats remain important to understand the ecology and evolutionary relationship of these pathogens. | NA | no | No group level | NA | no | no |
| 4755 | 2020 | 44040 | Bittebiere, AK; Benot, ML; Mony, C | 2020 | Clonality as a key but overlooked driver of biotic interactions in plants | Most plants are clonal i.e. able to laterally propagate by producing new genetically identical ramets connected by specialized clonal organs. Clonality determines ramet aggregation patterns, the presence of physical connections between ramets, the sharing of resources, hormones, and signaling molecules within the clonal fragment. We thus argue that clonal traits drive not only the individual plant fitness, but also the whole plant community assembly and ecosystem functioning through their involvement in plant biotic interactions with other micro- and macro-organisms. In an extensive literature review, we investigated how clonality influences a wide range of processes in space and over time, and subsequently affects biotic interactions. These processes occur both at the plant and the population levels, including spatial patterns of below- and aboveground organs, micro-environmental heterogeneity and genetic diversity. We highlight the responses of clonal plant traits to biotic interactions, and reciprocally, the effects of clonality on plant-plant, plant-animal (with herbivores, pollinators), and plant-microorganism interactions (with pathogens, mutualists). Based on this knowledge, we suggest future prospects are promising, in particular if clonal traits are integrated in studies of multitrophic level interactions, thereby enabling a new understanding of plant community assembly rules and the ability to predict changes in biodiversity due to modifications in biotic interactions. | NA | no | No good fitness measure and measure host GD | NA | no | no |
| 4756 | 2020 | 44043 | Blacquiere, T; Boot, W; Calis, J; Moro, A; Neumann, P; Panziera, D | 2019 | Darwinian black box selection for resistance to settled invasive Varroa destructor parasites in honey bees | Established invasive species can pose a continuous threat to biodiversity and food security, thereby calling for sustainable mitigation. There is a consensus that the ubiquitous ecto-parasitic mite Varroa destructor, an invasive species from Asia, is the main biological threat to global apiculture with Apis mellifera. V. destructor has almost completely wiped out wild European honey bee (Apis mellifera) populations. The only remedy for apiculture, to date, is frequent control measures against the mite throughout the season, which prevents possible adaptations. While targeted breeding efforts have, so far, not achieved the selection of tolerant or resistant bees, natural selection approaches have succeeded at least seven times. Here, we propose to take advantage of natural selection for honey bee resistance by stopping mite treatment in managed colonies. The main principles are within population mating of the colonies’ own virgin queens and drones and selection based on survival and proliferous development of colonies. Being used for 10years, it has shown to result in grosso modo normal’ colonies with a high level of resistance to V. destructor. Here, we call for local groups of beekeepers and scientists to join a novel natural selection program that has started so far on three locations. This will eventually lead to several locally adapted V. destructor resistant honey bee populations around the world, and help global apiculture becoming more sustainable. | NA | no | No good fitness measure and measure host GD | NA | no | no |
| 4757 | 2020 | 44042 | Blakeslee, AMH; Haram, LE; Altman, I; Kennedy, K; Ruiz, GM; Miller, AW | 2020 | Founder effects and species introductions: A host versus parasite perspective | Species colonizations (both natural and anthropogenic) can be associated with genetic founder effects, where founding populations demonstrate significant genetic bottlenecks compared to native populations. Yet, many successfully established free-living species exhibit little reduction in genetic diversity-possibly due to multiple founding events and/or high propagule pressure during introductions. Less clear, however, is whether parasites may show differential signatures to their free-living hosts. Parasites with indirect life cycles may particularly be more prone to founder effects (i.e., more genetically depauperate) because of inherently smaller founding populations and complex life cycles. We investigated this question in native (east coast) and introduced (west coast) North American populations of a host snail Tritia obsoleta (formerly Ilyanassa obsoleta, the eastern mudsnail) and four trematode parasite species that obligately infect it. We examined genetic diversity, gene flow, and population structure using two molecular markers (mitochondrial and nuclear) for the host and the parasites. In the host snail, we found little to no evidence of genetic founder effects, while the trematode parasites showed significantly lower genetic diversity in the introduced versus native ranges. Moreover, the parasite’s final host influenced infection prevalence and genetic diversity: Trematode species that utilized fish as final hosts demonstrated lower parasite diversity and heightened founder effects in the introduced range than those trematodes using birds as final hosts. In addition, inter-regional gene flow was strongest for comparisons that included the putative historical source region (mid-Atlantic populations of the US east coast). Overall, our results broaden understanding of the role that colonization events (including recent anthropogenic introductions) have on genetic diversity in non-native organisms by also evaluating less studied groups like parasites. | NA | no | No good fitness measure and measure host GD | NA | no | no |
| 4758 | 2020 | 44040 | Blakeslee, AMH; Manousaki, T; Vasileiadou, K; Tepolt, CK | 2020 | An evolutionary perspective on marine invasions | Species distributions are rapidly changing as human globalization increasingly moves organisms to novel environments. In marine systems, species introductions are the result of a number of anthropogenic mechanisms, notably shipping, aquaculture/mariculture, the pet and bait trades, and the creation of canals. Marine invasions are a global threat to human and non-human populations alike and are often listed as one of the top conservation concerns worldwide, having ecological, evolutionary, and social ramifications. Evolutionary investigations of marine invasions can provide crucial insight into an introduced species’ potential impacts in its new range, including: physiological adaptation and behavioral changes to exploit new environments; changes in resident populations, community interactions, and ecosystems; and severe reductions in genetic diversity that may limit evolutionary potential in the introduced range. This special issue focuses on current research advances in the evolutionary biology of marine invasions and can be broadly classified into a few major avenues of research: the evolutionary history of invasive populations, post-invasion reproductive changes, and the role of evolution in parasite introductions. Together, they demonstrate the value of investigating marine invasions from an evolutionary perspective, with benefits to both fundamental and applied evolutionary biology at local and broad scales. | NA | no | n | NA | no | no |
| 4759 | 2020 | 44111 | Blanchard, KR; Kalyanasundaram, A; Henry, C; Brym, MZ; Surles, JG; Kendall, RJ | 2019 | Predicting seasonal infection of eyeworm (Oxyspirura petrowi) and caecal worm (Aulonocephalus pennula) in northern bobwhite quail (Colinus virginianus) of the Rolling Plains Ecoregion of Texas, USA | The northern bobwhite quail (Colinus virginianus) is a popular gamebird in the Rolling Plains Ecoregion of West Texas. However, there has been a population decline in this area over recent decades. Consistent reports indicate a high prevalence of the eyeworm (Oxyspirura petrowi) and caecal worm (Aulonocephalus pennula), which may be of major influence on the bobwhite population. While research has suggested pathological consequences and genetic relatedness to other pathologically significant parasites, little is known about the influence of climate on these parasites. In this study, we examined whether seasonal temperature and precipitation influences the intensity of these parasites in bobwhite. We also analyzed quantitative PCR results for bobwhite feces and cloacal swabs against temperature and precipitation to identify climatic impacts on parasite reproduction in this region. Multiple linear regression analyses were used for parasite intensity investigation while binary logistic regression analyses were used for parasite reproduction studies. Our analyses suggest that caecal worm intensity, caecal worm reproduction, and eyeworm reproduction are influenced by temperature and precipitation. Temperature data was collected 15, 30, and 60 days prior to the date of collection of individual bobwhite and compared to qPCR results to generate a temperature range that may influence future eyeworm reproduction. This is the first preliminary study investigating climatic influences with predictive statistics on eyeworm and caecal worm infection of northern bobwhite in the Rolling Plains. | NA | no | No good fitness measure and measure host GD | NA | no | no |
| 4760 | 2020 | 44113 | Blanco-Meneses, M; Carbone, I; Ristaino, JB | 2018 | Population structure and migration of the Tobacco Blue Mold Pathogen, Peronospora tabacina, into North America and Europe | Tobacco blue mold, caused by Peronospora tabacina, is an oomycete plant pathogen that causes yearly epidemics in tobacco (Nicotiana tabacum) in the United States and Europe. The genetic structure of P. tabacina was examined to understand genetic diversity, population structure and patterns of migration. Two nuclear loci, Igs2 and Ypt1, and one mitochondrial locus, cox2, were amplified, cloned and sequenced from fifty-four isolates of P. tabacina from the United States, Central America-Caribbean-Mexico (CCAM), Europe and the Middle East (EULE). Cloned sequences from the three genes showed high genetic variability across all populations. Nucleotide diversity and the population mean mutation parameter per site (Watterson’s theta) were higher in EULE and CCAM and lower in U.S. populations. Neutrality tests were significant and the equilibrium model of neutral evolution was rejected, indicating an excess of recent mutations or rare alleles. Hudson’s S-nn tests were performed to examine population subdivision and gene flow among populations. An isolation-with-migration analysis (IM) supported the hypothesis of long-distance migration of P. tabacina from the Caribbean region, Florida and Texas into other states in the United States. Within the European populations, the model documented migration from North Central Europe into western Europe and Lebanon, and migration from western Europe into Lebanon. The migration patterns observed support historical observations about the first disease introductions and movement in Europe. The models developed are applicable to other aerial dispersed emerging pathogens and document that high-evolutionary-risk plant pathogens can move over long distances to cause disease due to their large effective population size, population expansion and dispersal. | NA | no | gd of pathogen | NA | no | no |
| 4761 | 2020 | 44113 | Blanco, RY; Loureiro, CL; Villalba, JA; Sulbaran, YF; Maes, M; de Waard, JH; Rangel, HR; Jaspe, RC; Pujol, FH | 2018 | Decreasing prevalence of Hepatitis B and absence of Hepatitis C Virus infection in the Warao indigenous population of Venezuela | Prevalence and molecular epidemiology studies for hepatitis B (HBV) and C (HCV) virus are scarce in Warao Amerindians from Venezuela, where an epidemic of human immunodeficiency virus type 1 (HIV-1) has recently been documented. To carry out a molecular epidemiology analysis of hepatitis B (HBV) and C (HCV) virus in Warao individuals from the Delta Amacuro State of Venezuela. A total of 548 sera were tested for serological and molecular markers for HBV and HCV. The prevalence of active infection (presence of HBV surface antigen, HBsAg), exposure to HBV (presence of Antibody to HBV core antigen, anti-HBc) and anti-HCV, was 1.8%, 13% and 0% respectively. HBV exposure was significantly lower in men below 18 years old and also lower than rates previously reported in other Amerindian communities from Venezuela. Thirty one percent (31%, 25/80) of individuals without evidence of HBV infection exhibited anti-HBs titer >= 10U.I/ml, being significantly more frequent in individuals younger than 20 years. A higher HBV exposure was observed among HIV-1 positive individuals (33% vs 11%, p<0.005). A high prevalence of occult HBV infection was also observed (5.6%, 11/195). Phylogenetic analysis of S gene and complete HBV genomes showed that F3 is the only circulating subgenotype, different from the F2 subgenotype found in 1991 in this population. These results suggest a recent introduction of subgenotype F3, with a low divergence among the isolates. These results highlight the importance of molecular epidemiology studies for viral control, and support the effectiveness of vaccination in reducing transmission of HBV. | NA | no | human | NA | no | no |
| 4762 | 2020 | 44040 | Blumstein, M; Richardson, A; Weston, D; Zhang, J; Muchero, W; Hopkins, R | 2020 | A New Perspective on Ecological Prediction Reveals Limits to Climate Adaptation in a Temperate Tree Species | Forests absorb a large fraction of anthropogenic CO2 emission, but their ability to continue to act as a sink under climate change depends in part on plant species undergoing rapid adaptation. Yet models of forest response to climate change currently ignore local adaptation as a response mechanism. Thus, considering the evolution of intraspecific trait variation is necessary for reliable, long-term species and climate projections. Here, we combine ecophysiology and predictive climate modeling with analyses of genomic variation to determine whether sugar and starch storage, energy reserves for trees under extreme conditions, have the heritable variation and genetic diversity necessary to evolve in response to climate change within populations of black cottonwood (Populus trichocarpa). Despite current patterns of local adaptation and extensive range-wide heritable variation in storage, we demonstrate that adaptive evolution in response to climate change will be limited by a lack of heritable variation within northern populations and by a need for extreme genetic changes in southern populations. Our method can help design more targeted species management interventions and highlights the power of using genomic tools in ecological prediction to scale from molecular to regional processes to determine the ability of a species to respond to future climates. | NA | no | No good fitness measure and measure host GD | NA | no | no |
| 4763 | 2020 | 44112 | Bocanegra-Gonzalez, KT; Thomas, E; Guillemin, ML; de Carvalho, D; Gutierrez, JP; Caicedo, CA; Higuita, LGM; Becerra, LA; Gonzalez, MA | 2018 | Genetic diversity of Ceiba pentandra in Colombian seasonally dry tropical forest: Implications for conservation and management | Seasonally dry tropical forests (SDTFs) are one of the most degraded vegetation types worldwide and in Colombia < 10% of the original cover remains. This calls for urgent conservation measures and restoration efforts. Understanding the genetic diversity and structure of tree species is crucial to inform not only conservation measures, but also sourcing of planting materials to ensure the long-term success of tree planting efforts, particularly in light of climate change. We assessed the genetic diversity distribution and structure of Ceiba pentandra from twelve representative locations of SDTF in Colombia, and how they may have been shaped by past climatic changes and human influence. We found three different genetic groups which may be the result of differentiation due to isolation of the Caribbean region, the Upper Cauca River Valley and the Pada River Valley in pre-glacial times. Range expansion of SDTF during the last glacial period, followed by more recent range contraction during the Holocene can explain the current distribution and mixture of genetic groups across contemporary STDF fragments. Most of the sampled localities showed heterozygosity scores close to Hardy-Weinberg expectations. Only two sites, among which the Pada River valley, an area with high conservation value, displayed significantly positive values of inbreeding coefficient, potentially affecting their survival and use as seed sources. While the effects of climate change might threaten C. pentandra populations across their current distribution ranges, opportunities remain for the in situ persistence of the most genetically diverse and unique ones. Based on our findings we identify priority areas for the in situ conservation of C. pentandra in Colombian SDTF and propose a pragmatic approach to guide the selection of appropriate planting material for use in restoration. | NA | no | No good fitness measure, maybe also inbreeding effects | NA | no | no |
| 4764 | 2020 | 44040 | Bohorquez, MD; Ordonez, D; Suarez, CF; Vicente, B; Vieira, C; Lopez-Aban, J; Muro, A; Ordonez, I; Patarroyo, MA | 2020 | Major Histocompatibility Complex Class II (DRB3) Genetic Diversity in Spanish Morucha and Colombian Normande Cattle Compared to Taurine and Zebu Populations | Bovine leukocyte antigens (BoLA) have been used as disease markers and immunological traits in cattle due to their primary role in pathogen recognition by the immune system. A higher MHC allele diversity in a population will allow presenting a broader peptide repertoire. However, loss of overall diversity due to domestication process can decrease a population’s peptide repertoire. Within the context of zebu and taurine cattle populations, BoLA-DRB3 genetic diversity in Spanish Morucha and Colombian Normande cattle was analyzed and an approach to estimate functional diversity was performed. Sequence-based typing was used for identifying 29, 23, 27, and 28 alleles in Spanish Morucha, Narino-, Boyaca-, and Cundinamarca-Normande cattle, respectively. These breeds had remarkably low heterozygosity levels and the Hardy-Weinberg principle revealed significant heterozygote deficiency. F-ST and D-A genetic distance showed that Colombian Normande populations had greater variability than other phenotypically homogeneous breeds, such as Holstein. It was also found that Spanish Morucha cattle were strongly differentiated from other cattle breeds. Spanish Morucha had greater divergence in the peptide-binding region regarding other cattle breeds. However, peptide-binding region covariation indicated that the potential peptide repertoire seemed equivalent among cattle breeds. Despite the genetic divergence observed, the extent of the potential peptide repertoire in the cattle populations studied appears to be similar and thus their pathogen recognition potential should be equivalent, suggesting that functional diversity might persist in the face of bottlenecks imposed by domestication and breeding. | NA | no | dometicated host | NA | no | no |
| 4765 | 2020 | 44112 | Bonato, L; Corbetta, A; Giovine, G; Romanazzi, E; Sunje, E; Vernesi, C; Crestanello, B | 2018 | Diversity among peripheral populations: genetic and evolutionary differentiation of Salamandra atra at the southern edge of the Alps | Separate populations at the edge of a species range are receiving great attention and have been shown to be often different from populations in the core area. However, it has rarely been tested whether neighboring peripheral populations are genetically and evolutionarily similar to each other, as expected for their geographical proximity and similar ecological conditions, or differ due to historical contingency. We investigated isolation and differentiation, within-population genetic diversity and evolutionary relationships among multiple peripheral populations of a cold-adapted terrestrial salamander, Salamandra atra, at the southern edge of the species core range. We carried out population genetic, phylogeographic, and phylogenetic analyses on various molecular markers (10 autosomal microsatellite loci, three mitochondrial loci with total length -2,100 bp, two protein-coding nuclear genes) sampled from more than 100 individuals from 13 sites along the southern Prealps. We found at least seven isolated peripheral populations, all highly differentiated from the remaining populations and differentiated from each other at various levels. The within-population genetic diversity was variable in the peripheral populations, but consistently lower than in the remaining populations. All peripheral populations along the southern Prealps belong to an ancient lineage that is also found in the Dinarides but did not contribute to the postglacial recolonization of the inner and northern Alps. All fully melanistic populations from the Orobian mountains to the southern Dinarides represent a single clade, to the exclusion of the two yellow-patched populations inhabiting the Pasubio massif and the Sette Comuni plateau, which are distinguished as S. atra pasubiensis and S. atra aurorae, respectively. In conclusion, multiple populations of S. atra at the southern edge of the species core area have different levels of differentiation, different amount of within-population genetic diversity, and different evolutionary origin. Therefore, they should be regarded as complementary conservation targets to preserve the overall genetic and evolutionary diversity of the species. | NA | no | No good fitness measure | NA | no | no |
| 4766 | 2020 | 44111 | Bope, CD; Chimusa, ER; Nembaware, V; Mazandu, GK; de Vries, J; Wonkam, A | 2019 | Dissecting in silico Mutation Prediction of Variants in African Genomes: Challenges and Perspectives | Genomic medicine is set to drastically improve clinical care globally due to high throughput technologies which enable speedy in silico detection and analysis of clinically relevant mutations. However, the variability in the in silico prediction methods and categorization of functionally relevant genetic variants can pose specific challenges in some populations. In silico mutation prediction tools could lead to high rates of false positive/negative results, particularly in African genomes that harbor the highest genetic diversity and that are disproportionately underrepresented in public databases and reference panels. These issues are particularly relevant with the recent increase in initiatives, such as the Human Heredity and Health (H3Africa), that are generating huge amounts of genomic sequence data in the absence of policies to guide genomic researchers to return results of variants in so-called actionable genes to research participants. This report (i) provides an inventory of publicly available Whole Exome/Genome data from Africa which could help improve reference panels and explore the frequency of pathogenic variants in actionable genes and related challenges, (ii) reviews available in silico prediction mutation tools and the criteria for categorization of pathogenicity of novel variants, and (iii) proposes recommendations for analyzing pathogenic variants in African genomes for their use in research and clinical practice. In conclusion, this work proposes criteria to define mutation pathogenicity and actionability in human genetic research and clinical practice in Africa and recommends setting up an African expert panel to oversee the proposed criteria. | NA | no | No good fitness measure and measure host GD | NA | no | no |
| 4767 | 2020 | 44113 | Borowiec, BG; O’Connor, CM; Goodick, K; Scott, GR; Balshine, S | 2018 | The Preference for Social Affiliation Renders Fish Willing to Accept Lower O-2 Levels | Animals are bombarded with information about their environment and must select and interpret the relevant cues to make behavioral adjustments critical to survival. How animals integrate and balance the many signals they receive about their environment is rarely assessed. We investigated how signals from the social and physical environment interact to influence environmental preferences in the endemic Tanganyikan cichlid Neolamprologus pulcher. Specifically, we explored how fish respond to the physiological challenge of declining O-2 levels in light of embedded social preferences using a modified shuttle box apparatus to test O-2 preferences. In the presence of a conspecific, the average (preferred) partial pressure of oxygen (Po-2) and minimum Po-2 experienced were significantly lower (14.90 +/- 2.13 and 12.35 +/- 3.15 kPa, respectively) than in trials without a conspecific (17.18 +/- 2.55 and 15.62 +/- 3.09 kPa, respectively). Fish with conspecifics also spent more time in the low Po-2 zone of the shuttle box and moved between the high and low Po-2 zones less frequently. Hence, O-2 preferences were modified, and fish willingly remained in an area of continuously declining O-2 availability to associate with a conspecific. The O-2 preferences of an individual during social trials correlated with its excess postexercise O-2 consumption following an exhaustive chase but not with its aerobic scope, routine O-2 consumption rate, or body mass. These results suggest that some aspects of respiratory and metabolic physiology (such as the propensity to use anaerobic metabolism) but not others (such as O-2 transport capacity) underpin some variation in social behavior under environmental stress. | NA | no | No good fitness measure and measure host GD | NA | no | no |
| 4768 | 2020 | 44042 | Botelho, ABRZ; Alves-Pereira, A; Prado, RC; Zucchi, MI; Delalibera, I | 2019 | Metarhizium species in soil from Brazilian biomes: a study of diversity, distribution, and association with natural and agricultural environments | Metarhizium is a diverse genus of fungi adapted for different ecologies, including soil saprotrophs, entomopathogens, and endophytes. We characterized the genetic diversity and distribution of Metarhizium species in soils found in native and agricultural landscapes within Brazilian biomes (Amazon, Cerrado, Atlantic Forest, Caatinga. and Pampa), Current species limits were determined with 5’-TEF, and the genetic diversity discerned using MzIGS3 sequences. Metarhizium robertsii, Metarhizium anisopliae, Metarhizium pingshaense, and three other lineages that lie beyond currently recognized species were found. Only soils from the Amazon contained all the species. The diversity of Metarhizium species associated with native vegetation was greater than that identified in annual and perennial crops. M. robertsii was the most abundant species (65%), followed by Metarhizium sp. indet. 1, which exhibited the highest haplotype and nucleotide diversities. Metarhizium sp. indet. 3 was found predominantly in the Caatinga biome. This information increases the knowledge about diversity and belowground species composition of Metarhizium in Brazil. (C) 2019 Elsevier Ltd and British Mycological Society. All rights reserved. | NA | no | No good fitness measure and measure host GD | NA | no | no |
| 4769 | 2020 | 44112 | Boyce, RM; Hathaway, N; Fulton, T; Reyes, R; Matte, M; Ntaro, M; Mulogo, E; Waltmann, A; Bailey, JA; Siedner, MJ; Juliano, JJ | 2018 | Reuse of malaria rapid diagnostic tests for amplicon deep sequencing to estimate Plasmodium falciparum transmission intensity in western Uganda | Molecular techniques are not routinely employed for malaria surveillance, while cross-sectional, community-based parasite surveys require significant resources. Here, we describe a novel use of malaria rapid diagnostic tests (RDTs) collected at a single facility as source material for sequencing to esimtate malaria transmission intensity across a relatively large catchment area. We extracted Plasmodium falciparum DNA from RDTs, then amplified and sequenced a region of the apical membrane antigen 1 (pfama1) using targeted amplicon deep sequencing. We determined the multiplicity of infection (MOI) for each sample and examined associations with demographic, clinical, and spatial factors. We successfully genotyped 223 of 287 (77.7%) of the samples. We demonstrated an inverse relationship between the MOI and elevation with individuals presenting from the highest elevation villages harboring infections approximately half as complex as those from the lowest (MOI 1.85 vs. 3.51, AOR 0.25, 95% CI 0.09-0.65, p = 0.004). This study demonstrates the feasibility and validity of using routinely-collected RDTs for molecular surveillance of malaria and has real-world utility, especially as the cost of high-throughpout sequencing continues to decline. | NA | no | gd of pathogen | NA | no | no |
| 4770 | 2020 | 44112 | Bradke, DR; Hileman, ET; Bartman, JF; Faust, LJ; King, RB; Kudla, N; Moore, JA | 2018 | Implications of Small Population Size in a Threatened Pitviper Species | Destruction and fragmentation of wildlife habitat often results in small, isolated populations that are highly susceptible to extirpation. In many cases, however, estimates of population size are lacking, precluding accurate assessments of population viability and sound conservation management recommendations. The Eastern Massasauga (Sistrurus catenatus) is a federally threatened pitviper species that has been extirpated throughout much of its historic range attributable to agricultural conversion of wetland habitat and other synergistic threats. Population size is generally unknown among extant massasauga populations, making site-specific management difficult. In this study, we estimated genetic effective population size (N-e) and census population size (N-c) for Eastern Massasaugas at two sites in southwest Michigan. For each population, we used mark-recapture models to estimate N-e, and the linkage disequilibrium method to estimate N-e. Our results revealed small N-c with approximately 108 (95% CI = 87-165) and 148 (95% CI = 102-295) adults estimated at our study sites in Cass County and Barry County, respectively. Estimates of N-e were even smaller: approximately 29.5 (95% CI = 21.2-43.1) for Cass County and 44.2 (95% CI = 30.8-69.3) for Barry County. Additionally, N-e/N-c, ratios were similar across study sites, suggesting some stability in this ratio for Eastern Massasaugas, at least for populations in close proximity. Although we did not detect high levels of inbreeding or relatedness in either population, we caution that these small populations could become increasingly vulnerable to extirpation from unpredictable threats such as disease and climate change. | NA | no | No good fitness measure and measure host GD | NA | no | no |
| 4771 | 2020 | 44113 | Braidwood, L; Quito-Avila, DF; Cabanas, D; Bressan, A; Wangai, A; Baulcombe, DC | 2018 | Maize chlorotic mottle virus exhibits low divergence between differentiated regional sub-populations | Maize chlorotic mottle virus has been rapidly spreading around the globe over the past decade. The interactions of maize chlorotic mottle virus with Potyviridae viruses causes an aggressive synergistic viral condition - maize lethal necrosis, which can cause total yield loss. Maize production in sub-Saharan Africa, where it is the most important cereal, is threatened by the arrival of maize lethal necrosis. We obtained maize chlorotic mottle virus genome sequences from across East Africa and for the first time from Ecuador and Hawaii, and constructed a phylogeny which highlights the similarity of Chinese to African isolates, and Ecuadorian to Hawaiian isolates. We used a measure of clustering, the adjusted Rand index, to extract region-specific SNPs and coding variation that can be used for diagnostics. The population genetics analysis we performed shows that the majority of sequence diversity is partitioned between populations, with diversity extremely low within China and East Africa. | NA | no | gd of pathogen | NA | no | no |
| 4772 | 2020 | 44113 | Brambilla, A; Keller, L; Bassano, B; Grossen, C | 2018 | Heterozygosity-fitness correlation at the major histocompatibility complex despite low variation in Alpine ibex (Capra ibex) | Crucial for the long-term survival of wild populations is their ability to fight diseases. Disease outbreaks can lead to severe population size reductions, which makes endangered and reintroduced species especially vulnerable. In vertebrates, the major histocompatibility complex (MHC) plays an important role in determining the immune response. Species that went through severe bottlenecks often show very low levels of genetic diversity at the MHC. Due to the known link between the MHC and immune response, such species are expected to be at particular risk in case of disease outbreaks. However, so far, only few studies have shown that low MHC diversity is correlated with increased disease susceptibility in species after severe bottlenecks. We investigated genetic variation at the MHC and its correlations with disease resistance and other fitness-related traits in Alpine ibex (Capra ibex), a wild goat species that underwent a strong bottleneck in the last century and that is known to have extremely low genetic variability, both genome-wide and at the MHC. We studied MHC variation in male ibex of Gran Paradiso National Park, the population used as a source for all postbottleneck reintroductions. We found that individual MHC heterozygosity (based on six microsatellites) was not correlated with genome-wide neutral heterozygosity. MHC heterozygosity, but not genome-wide heterozygosity, was positively correlated with resistance to infectious keratoconjunctivitis and with body mass. Our results show that genetic variation at the MHC plays an important role in disease resistance and, hence, should be taken into account for successfully managing species conservation. | NA | no | mhc diversity | NA | no | no |
| 4773 | 2020 | 44041 | Brandies, P; Peel, E; Hogg, CJ; Belov, K | 2019 | The Value of Reference Genomes in the Conservation of Threatened Species | Conservation initiatives are now more crucial than ever-over a million plant and animal species are at risk of extinction over the coming decades. The genetic management of threatened species held in insurance programs is recommended; however, few are taking advantage of the full range of genomic technologies available today. Less than 1% of the 13505 species currently listed as threated by the International Union for Conservation of Nature (IUCN) have a published genome. While there has been much discussion in the literature about the importance of genomics for conservation, there are limited examples of how having a reference genome has changed conservation management practice. The Tasmanian devil (Sarcophilus harrisii), is an endangered Australian marsupial, threatened by an infectious clonal cancer devil facial tumor disease (DFTD). Populations have declined by 80% since the disease was first recorded in 1996. A reference genome for this species was published in 2012 and has been crucial for understanding DFTD and the management of the species in the wild. Here we use the Tasmanian devil as an example of how a reference genome has influenced management actions in the conservation of a species. | NA | no | No good fitness measure and measure host GD | NA | no | no |
| 4774 | 2020 | 44111 | Branham, SE; Levi, A; Katawczik, ML; Wechter, WP | 2019 | QTL mapping of resistance to bacterial fruit blotch in Citrullus amarus | Key messageSix QTLs were associated with affected leaf area in response to inoculation with Acidovorax citrulli in a recombinant inbred line population of Citrullus amarus.AbstractAcidovorax citrulli, the causal agent of bacterial fruit blotch (BFB) of cucurbits, has the potential to devastate production of watermelon and other cucurbits. Despite decades of research on host-plant resistance to A. citrulli, no germplasm has been found with immunity and only a few sources with various levels of BFB resistance have been identified, but the genetic basis of resistance in these watermelon sources are not known. Most sources of resistance are plant introductions of Citrullus amarus (citron melon), a closely related species that crosses readily with cultivated watermelon (Citrullus lanatus L.). In this study, we evaluated a recombinant inbred line population (N=200), derived from a cross between BFB-resistant (USVL246-FR2) and BFB-susceptible (USVL114) C. amarus lines, for foliar resistance to A. citrulli in three replicated greenhouse trials. We found the genetics of BFB resistance to be complicated by strong environmental influence, low heritability and significant genotype-by-environment interactions. QTL mapping of affected leaf area identified six QTL that each explained between 5 and 15% of the variation in BFB resistance in the population. This study represents the first identification of QTL associated with resistance to A. citrulli in any cucurbit. | NA | no | inbreeding | NA | no | no |
| 4775 | 2020 | 44040 | Branham, SE; Wechter, WP; Ling, KS; Chanda, B; Massey, L; Zhao, G; Guner, N; Bello, M; Kabelka, E; Fei, Z; Levi, A | 2020 | QTL mapping of resistance to Fusarium oxysporum f. sp. niveum race 2 and Papaya ringspot virus in Citrullus amarus | Key message A Citrullus amarus mapping population segregating for resistance to Fusarium oxysporum f. sp. niveum race 2 and Papaya ringspot virus was used to identify novel QTL, important for the improvement in watermelon disease resistance. Multiple disease screens of the USDA Citrullus spp. germplasm collection have highlighted the value of Citrullus amarus (citron melon or wild watermelon) as a resource for enhancing modern watermelon cultivars (Citrullus lanatus) with resistance to a broad range of fungal, bacterial and viral diseases of watermelon. We have generated a genetic population of C. amarus segregating for resistance to two important watermelon diseases: Fusarium wilt (caused by the fungus Fusarium oxysporum f. sp. niveum; Fon race 2) and Papaya ringspot virus-watermelon strain (PRSV-W). QTL mapping of Fon race 2 resistance identified seven significant QTLs, with the major QTL representing a novel genetic source of resistance and an opportunity for gene pyramiding. A single QTL was associated with resistance to PRSV-W, which adhered to expectations of a prior study indicating a single-gene recessive inheritance in watermelon. The resistance loci identified here provide valuable genetic resources for introgression into cultivated watermelon for the improvement in disease resistance. | NA | no | No good fitness measure and measure host GD | NA | no | no |
| 4776 | 2020 | 44042 | Brante, A; Guzman-Rendon, G; Barria, EM; Guillemin, ML; Vera-Escalona, I; Hernandez, CE | 2019 | Post-Disturbance Genetic Changes: The Impact of the 2010 Mega-Earthquake and Tsunami on Chilean Sandy Beach Fauna | Earthquake/tsunamis can have profound impacts on species and their genetic patterns. It is expected that the magnitude of this impact might depend on the species and the time since the disturbance occurs, nevertheless these assumptions remain mostly unexplored. Here we studied the genetic responses of the crustacean species Emerita analoga, Excirolana hirsuticauda, and Orchestoidea tuberculata to the 27F mega-earthquake/tsunami that occurred in Chile in February 2010. mtDNA sequence analyses revealed a lower haplotype diversity for E. analoga and E. hirsuticauda in impacted areas one month after the 27F, and the opposite for O. tuberculata. Three years after the 27F we observed a recovery in the genetic diversity of E. analoga and E. hirsuticauda and decrease in the genetic diversity in O. tuberculata in 2/3 of sampled areas. Emerita analoga displayed decrease of genetic differentiation and increase in gene flow explained by long-range population expansion. The other two species revealed slight increase in the number of genetic groups, little change in gene flow and no signal of population expansion associated to adult survival, rapid colonization, and capacity to burrow in the sand. Our results reveal that species response to a same disturbance event could be extremely diverse and depending on life-history traits and the magnitude of the effect. | NA | no | No good fitness measure and measure host GD | NA | no | no |
| 4777 | 2020 | 44113 | Brasington, LF; Wikberg, EC; Kawamura, S; Fedigan, LM; Jack, KM | 2017 | Infant mortality in white-faced capuchins: The impact of alpha male replacements | Infanticide is common in the context of alpha male replacements (AMR), particularly in groups where alpha males experience high reproductive skew and the infants are unlikely to be related to a new alpha male. We examined the relationship between the rate of infant mortality, infant age, and the occurrence and type ofAMR in white-faced capuchin monkeys (Cebus capucinus imitator) of the Santa Rosa population in Sector Santa Rosa, Area de Conservacion Guanacaste. Specifically, we investigated how the source of the new alpha male (coresident or extragroup) and relative aggression level during AMRs influenced infant mortality in this species. Between 1986 and 2015, we recorded 221 births in five study groups. Infants present at the time of an AMR, or born within 5.5 months following an AMR (i.e., conceived prior to AMR), experienced significantly higher mortality than those born during periods of group stability. Infant age was a significant predictor of infant survival, with the probability of surviving increasing by 0.4% for each additional day older an infant was at the time of the AMR. Infant mortality rates did not differ between AMRs by coresident males and extragroup males, possibly because the degree of relatedness between infants and new alphas did not significantly differ between coresident and extragroup AMRs. Infant mortality rates did not differ significantly between aggressive AMRs and more peaceful AMRs. Our results are consistent with predictions derived from the sexual selection hypothesis (SSH) of infanticide and suggest that future studies examine the role of testosterone as an underlying proximate mechanism for the aggression leading to this behavior. We argue that the sexual selection and generalized aggression hypotheses (GAH) of infanticide are best considered as different levels of analysis rather than competing hypotheses. | NA | no | No good fitness measure and measure host GD | NA | no | no |
| 4778 | 2020 | 44113 | Brehme, CS; Hathaway, SA; Fisher, RN | 2018 | An objective road risk assessment method for multiple species: ranking 166 reptiles and amphibians in California | Transportation and wildlife agencies may consider the need for barrier structures and safe wildlife road-crossings to maintain the long-term viability of wildlife populations. In order to prioritize these efforts, it is important to identify species that are most at risk of extirpation from road-related impacts. Our goal was to identify reptiles and amphibians in California most susceptible to road mortality and fragmentation. With over 160 species and a lack of species-specific research data, we developed an objective risk assessment method based upon road ecology science. Risk scoring was based upon a suite of life history and space-use characteristics associated with negative road effects applied in a hierarchical manner from individuals to species. We evaluated risk to both aquatic and terrestrial connectivity and calculated buffer distances to encompass 95% of population-level movements. We ranked species into five relative categories of road-related risk (very-high to very-low) based upon 20% increments of all species scores. All chelonids, 72% of snakes, 50% of anurans, 18% of lizards and 17% of salamander species in California were ranked at high or very-high risk from negative road impacts. Results were largely consistent with local and global scientific literature in identifying high risk species and groups. This comparative risk assessment method provides a science-based framework to identify species most susceptible to negative road impacts. The results can inform regional-scale road mitigation planning and prioritization efforts and threat assessments for special-status species. We believe this approach is applicable to numerous landscapes and taxonomic groups. | NA | no | No good fitness measure and measure host GD | NA | no | no |
| 4779 | 2020 | 44112 | Brenton-Rule, EC; Dobelmann, J; Baty, JW; Brown, RL; Dvorak, L; Grangier, J; Masciocchi, M; McGrannachan, C; Shortall, CR; Schmack, J; van Zyl, C; Veldtman, R; Lester, PJ | 2018 | The origins of global invasions of the German wasp (Vespula germanica) and its infection with four honey bee viruses | A successful control or eradication programme using biological control or genetically-mediated methods requires knowledge of the origin and the extent of wasp genetic diversity. Mitochondrial DNA variation in the native and invaded range of the social wasp Vespula germanica was used to examine intra-specific genetic variation and invasive source populations. We also examined wasps for the presence of four viruses found in honey bees: Acute bee paralysis virus, Deformed wing virus, Israeli acute paralysis virus and Kashmir bee virus. German wasps showed reduced genetic diversity in the invaded range compared to that of their native range. Populations in the introduced range are likely to have arrived from different source populations. All four viral honey bee pathogens were found in V. germanica, although they varied in their distribution and strain. Multiple introductions of German wasps have occurred for most invaded regions, though some populations are genetically homogenous. The differing locations of origin will guide researchers searching for biocontrol agents and the reduced genetic diversity may make these wasps a potentially viable target for control via gene drives. | NA | no | no good gd measure host pop | NA | no | no |
| 4780 | 2020 | 44111 | Breton, G; Thibault, S; Werts, M; Baudry, E | 2019 | Captive ring-tailed lemur breeding in semi-free ranging conditions and genetic parentage analysis | The ring-tailed lemur (Lemur catta), one of the most iconic and widely recognized primates in the world, is threatened in its native range and classified as endangered by the International Union for Conservation of Nature. The global conservation strategy for the species includes ex situ conservation efforts led by zoological institutions in the framework of regional captive breeding programs. To maximize the conservation of genetic diversity and optimize breeding programs, an accurate pedigree must be established in captive populations. Our study documents the formation and subsequent monitoring of a large ring-tailed lemur group in a French zoological institution. Based on staff’s behavioral observations and DNA analyses using 11 microsatellite markers, we were able to document the survival and reproduction rates of the lemurs in this situation and reconstruct the pedigree of infants born in the institution. In total, 22 of the 28 imported animals as well as 4 locally born individuals gave birth to 58 infants of which 28 survived. The consistent genotypes obtained from the 53 sampled animals allowed us to identify 25 of the sires and 27 of the dams for the 28 surviving infants. A total of 12 different females and 14 different males produced the 28 surviving infants, suggesting that no individual dominates reproduction. This pioneering study has practical applications for the captive management of the species, which can be raised to the individual level instead of the group level. | NA | no | no good gd measure host pop | NA | no | no |
| 4781 | 2020 | 44113 | Brinkmeyer-Langford, C; Chu, C; Balog-Alvarez, C; Yu, X; Cai, JJ; Nabity, M; Kornegay, JN | 2018 | Expression profiling of disease progression in canine model of Duchenne muscular dystrophy | Duchenne muscular dystrophy (DMD) causes progressive disability in 1 of every 5,000 boys due to the lack of functional dystrophin protein. Despite much advancement in knowledge about DMD disease presentation and progression-attributable in part to studies using mouse and canine models of the disease-current DMD treatments are not equally effective in all patients. There remains, therefore, a need for translational animal models in which novel treatment targets can be identified and evaluated. Golden Retriever muscular dystrophy (GRMD) is a phenotypically and genetically homologous animal model of DMD. As with DMD, speed of disease progression in GRMD varies substantially. However, unlike DMD, all GRMD dogs possess the same causal mutation; therefore genetic modifiers of phenotypic variation are relatively easier to identify. Furthermore, the GRMD dogs used in this study reside within the same colony, reducing the confounding effects of environment on phenotypic variation. To detect modifiers of disease progression, we developed gene expression profiles using RNA sequencing for 9 dogs: 6 GRMD dogs (3 with faster-progressing and 3 with slower-progressing disease, based on quantitative, objective biomarkers) and 3 control dogs from the same colony. All dogs were evaluated at 2 time points: early disease onset (3 months of age) and the point at which GRMD stabilizes (6 months of age) using quantitative, objective biomarkers identified as robust against the effects of relatedness/inbreeding. Across all comparisons, the most differentially expressed genes fell into 3 categories: myogenesis/muscle regeneration, metabolism, and inflammation. Our findings are largely in concordance with DMD and mouse model studies, reinforcing the utility of GRMD as a translational model. Novel findings include the strong up-regulation of chitinase 3-like 1 (CHI3L1) in faster-progressing GRMD dogs, suggesting previously unexplored mechanisms underlie progression speed in GRMD and DMD. In summary, our findings support the utility of RNA sequencing for evaluating potential biomarkers of GRMD progression speed, and are valuable for identifying new avenues of exploration in DMD research. | NA | no | dog | NA | no | no |
| 4782 | 2020 | 44043 | Broekman, MJE; Muller-Landau, HC; Visser, MD; Jongejans, E; Wright, SJ; de Kroon, H | 2019 | Signs of stabilisation and stable coexistence | Many empirical studies motivated by an interest in stable coexistence have quantified negative density dependence, negative frequency dependence, or negative plant-soil feedback, but the links between these empirical results and ecological theory are not straightforward. Here, we relate these analyses to theoretical conditions for stabilisation and stable coexistence in classical competition models. By stabilisation, we mean an excess of intraspecific competition relative to interspecific competition that inherently slows or even prevents competitive exclusion. We show that most, though not all, tests demonstrating negative density dependence, negative frequency dependence, and negative plant-soil feedback constitute sufficient conditions for stabilisation of two-species interactions if applied to data for per capita population growth rates of pairs of species, but none are necessary or sufficient conditions for stable coexistence of two species. Potential inferences are even more limited when communities involve more than two species, and when performance is measured at a single life stage or vital rate. We then discuss two approaches that enable stronger tests for stable coexistence-invasibility experiments and model parameterisation. The model parameterisation approach can be applied to typical density-dependence, frequency-dependence, and plant-soil feedback data sets, and generally enables better links with mechanisms and greater insights, as demonstrated by recent studies. | NA | no | No good fitness measure and measure host GD | NA | no | no |
| 4783 | 2020 | 44112 | Bromham, L; Hua, X; Cardillo, M; Schneemann, H; Greenhill, SJ | 2018 | Parasites and politics: why cross-cultural studies must control for relatedness, proximity and covariation | A growing number of studies seek to identify predictors of broad-scale patterns in human cultural diversity, but three sources of non-independence in human cultural variables can bias the results of cross-cultural studies. First, related cultures tend to have many traits in common, regardless of whether those traits are functionally linked. Second, societies in geographical proximity will share many aspects of culture, environment and demography. Third, many cultural traits covary, leading to spurious relationships between traits. Here, we demonstrate tractable methods for dealing with all three sources of bias. We use cross-cultural analyses of proposed associations between human cultural traits and parasite load to illustrate the potential problems of failing to correct for these three forms of statistical non-independence. Associations between parasite stress and sociosexuality, authoritarianism, democracy and language diversity are weak or absent once relatedness and proximity are taken into account, and parasite load has no more power to explain variation in traditionalism, religiosity and collectivism than other measures of biodiversity, climate or population size do. Without correction for statistical non-independence and covariation in cross-cultural analyses, we risk misinterpreting associations between culture and environment. | NA | no | No good fitness measure and measure host GD | NA | no | no |
| 4784 | 2020 | 44113 | Bronowski, C; Mustafa, K; Goodhead, I; James, CE; Nelson, C; Lucaci, A; Wigley, P; Humphrey, TJ; Williams, NJ; Winstanley, C | 2017 | Campylobacter jejuni transcriptome changes during loss of culturability in water | Background Water serves as a potential reservoir for Campylobacter, the leading cause of bacterial gastroenteritis in humans. However, little is understood about the mechanisms underlying variations in survival characteristics between different strains of C. jejuni in natural environments, including water. Results We identified three Campylobacter jejuni strains that exhibited variability in their ability to retain culturability after suspension in tap water at two different temperatures (4 degrees C and 25 degrees C). Of the three, strains C. jejuni M1 exhibited the most rapid loss of culturability whilst retaining viability. Using RNAseq transcriptomics, we characterised C. jejuni M1 gene expression in response to suspension in water by analyzing bacterial suspensions recovered immediately after introduction into water (Time 0), and from two sampling time/temperature combinations where considerable loss of culturability was evident, namely (i) after 24 h at 25 degrees C, and (ii) after 72 h at 4 degrees C. Transcript data were compared with a culture-grown control. Some gene expression characteristics were shared amongst the three populations recovered from water, with more genes being up-regulated than down. Many of the up-regulated genes were identified in the Time 0 sample, whereas the majority of down-regulated genes occurred in the 25 degrees C (24 h) sample. Conclusions Variations in expression were found amongst genes associated with oxygen tolerance, starvation and osmotic stress. However, we also found upregulation of flagellar assembly genes, accompanied by down-regulation of genes involved in chemotaxis. Our data also suggested a switch from secretion via the sec system to via the tat system, and that the quorum sensing gene luxS may be implicated in the survival of strain M1 in water. Variations in gene expression also occurred in accessory genome regions. Our data suggest that despite the loss of culturability, C. jejuni M1 remains viable and adapts via specific changes in gene expression. | NA | no | No good fitness measure and measure host GD | NA | no | no |
| 4785 | 2020 | 44113 | Brown, GP; Madsen, T; Dubey, S; Shine, R | 2017 | The causes and ecological correlates of head scale asymmetry and fragmentation in a tropical snake | The challenge of identifying the proximate causes and ecological consequences of phenotypic variation can be facilitated by studying traits that are usually but not always bilaterally symmetrical; deviations from symmetry likely reflect disrupted embryogenesis. Based on a 19-year mark-recapture study of >1300 slatey-grey snakes (Stegonotus cucullatus) in tropical Australia, and incubation of >700 eggs, we document developmental and ecological correlates of two morphological traits: asymmetry and fragmentation of head scales. Asymmetry was directional (more scales on the left side) and was higher in individuals with lower heterozygosity, but was not heritable. In contrast, fragmentation was heritable and was higher in females than males. Both scale asymmetry and fragmentation were increased by rapid embryogenesis but were not affected by hydric conditions during incubation. Snakes with asymmetry and fragmentation exhibited slightly lower survival and increased (sex-specific) movements, and females with more scale fragmentation produced smaller eggs. Counterintuitively, snakes with more asymmetry had higher growth rates (possibly reflecting trade-offs with other traits), and snakes with more fragmentation had fewer parasites (possibly due to lower feeding rates). Our data paint an unusually detailed picture of the complex genetic and environmental factors that, by disrupting early embryonic development, generate variations in morphology that have detectable correlations with ecological performance. | NA | no | no good gd measure host pop | NA | no | no |
| 4786 | 2020 | 44112 | Bruggemann, H; Migliorini, LB; de Sales, RO; Koga, PCM; de Souza, AV; Jensen, A; Poehlein, A; Brzuszkiewicz, E; Doi, AM; Pasternak, J; Martino, MD; Severino, P | 2018 | Comparative Genomics of Nonoutbreak Pseudomonas aeruginosa Strains Underlines Genome Plasticity and Geographic Relatedness of the Global Clone ST235 | Pseudomonas aeruginosa is an important opportunistic pathogen in hospitals, responsible for various infections that are difficult to treat due to intrinsic and acquired antibiotic resistance. Here, 20 epidemiologically unrelated strains isolated from patients in a general hospital over a time period of two decades were analyzed using whole genome sequencing. The genomes were compared in order to assess the presence of a predominant clone or sequence type (ST). No clonal structure was identified, but core genome-based single nucleotide polymorphism (SNP) analysis distinguished two major, previously identified phylogenetic groups. Interestingly, most of the older strains isolated between 1994 and 1998 harbored exoU, encoding a cytotoxic phospholipase. In contrast, most strains isolated between 2011 and 2016 were exoU-negative and phylogenetically very distinct from the older strains, suggesting a population shift of nosocomial P. aeruginosa over time. Three out of 20 strains were ST235 strains, a global high-risk clonal lineage; these carried several additional resistance determinants including aac(6’)Ib-cr encoding an aminoglycoside N-acetyltransferase that confers resistance to fluoroquinolones. Core genome comparison with ST235 strains from other parts of the world showed that the three strains clustered together with other Brazilian/Argentinean isolates. Despite this regional relatedness, the individuality of each of the three ST235 strains was revealed by core genome-based SNPs and the presence of genomic islands in the accessory genome. Similarly, strain-specific characteristics were detected for the remaining strains, indicative of individual evolutionary histories and elevated genome plasticity. | NA | no | gd of pathogen | NA | no | no |
| 4787 | 2020 | 44113 | Bruniche-Olsen, A; Jones, ME; Burridge, CP; Murchison, EP; Holland, BR; Austin, JJ | 2018 | Ancient DNA tracks the mainland extinction and island survival of the Tasmanian devil | Aim: The Tasmanian devil (Sarcophilus harrisii), currently restricted to the island of Tasmania, was found over most of the Australian mainland prior to its extinction similar to 3,000years ago. Recent debate has focused on the roles of humans, climate change and dingoes as drivers of the mainland extinction. Determining past genetic diversity and population dynamics of both populations is a fundamental component to understand why the species went extinct on mainland Australia, but survived in Tasmania. Here, we investigate the phylogeography and demographic history of the Tasmanian devil across southern Australia over the last similar to 30k years. Location: Australia. Taxon: Tasmanian devil (Sarcophilus harrisii). Methods: We used complete and partial mitochondrial DNA (mtDNA) genomes from 202 devils representing the extinct mainland (n = 17) and the extant Tasmanian (n = 185) populations to investigate the population dynamics of southern mainland and Tasmanian devils. The samples were sub-fossil bones, historical museum specimens and modern tissue samples, dating from the present to 17k years before present. Using summary statistics, frequentist inference and Bayesian phylogenetic analysis we explored whether levels of genetic diversity were similar, and if the southern mainland experienced a gradual rather than an abrupt decline prior to its extinction. Results: MtDNA genomes from mainland devils suggest that this population was larger and had more genetic diversity than the Tasmanian population. Directly dated samples indicates that the southern mainland population expanded after the last glacial maximum and remained stable until its extinction. The Tasmanian population has much lower diversity and descends from a single mtDNA lineage similar to 3,000years ago. The recent origin for all Tasmanian mtDNA diversity is concordant with a previously documented late-Holocene population bottleneck and is broadly contemporaneous with the extinction of the southern mainland population. Main conclusions: This pattern shows striking similarity to the demographic history of thylacines, suggesting that a shared factor initiated population declines in both species on the southern mainland and in Tasmania. El Nino Southern Oscillation (ENSO)-related climate change is the only factor common to both mainland Australia and Tasmania. Additional, direct or indirect, pressures from humans and/or dingoes on the mainland may have ultimately resulted in their extinction. | NA | no | No good fitness measure and measure host GD | NA | no | no |
| 4788 | 2020 | 44113 | Brunt, J; Carter, AT; Pye, HV; Peck, MW | 2018 | The orphan germinant receptor protein GerXAO (but not GerX3b) is essential for L-alanine induced germination in Clostridium botulinum Group II | Clostridium botulinum is an anaerobic spore forming bacterium that produces the potent botulinum neurotoxin that causes a severe and fatal neuro-paralytic disease of humans and animals (botulism). C. botulinum Group II is a psychrotrophic saccharolytic bacterium that forms spores of moderate heat resistance and is a particular hazard in minimally heated chilled foods. Spore germination is a fundamental process that allows the spore to transition to a vegetative cell and typically involves a germinant receptor (GR) that responds to environmental signals. Analysis of C. botulinum Group II genomes shows they contain a single GR cluster (gerX3b), and an additional single gerA subunit (gerXAO). Spores of C. botulinum Group II strain Eklund 17B germinated in response to the addition of L-alanine, but did not germinate following the addition of exogenous Ca2+-DPA. Insertional inactivation experiments in this strain unexpectedly revealed that the orphan GR GerXAO is essential for L-alanine stimulated germination. GerX3bA and GerX3bC affected the germination rate but were unable to induce germination in the absence of GerXAO. No role could be identified for GerX3bB. This is the first study to identify the functional germination receptor of C. botulinum Group II. | NA | no | No good fitness measure and measure host GD | NA | no | no |
| 4789 | 2020 | 44113 | Brutsch, T; Avril, A; Chapuisat, M | 2017 | No evidence for social immunity in co-founding queen associations | Ant queens often associate to found new colonies, yet the benefits of this behaviour remain unclear. A major hypothesis is that queens founding in groups are protected by social immunity and can better resist disease than solitary queens, due to mutual grooming, sharing of antimicrobials, or higher genetic diversity among their workers. We tested this hypothesis by manipulating the number of queens in incipient colonies of Lasius niger and measuring their resistance to the fungal entomopathogen Metarhizium brunneum. We found no evidence for social immunity in associations of founding queens. First, co-founding queens engaged in self-grooming, but performed very little allo-grooming or trophallaxis. Second, co-founding queens did not exhibit higher pathogen resistance than solitary queens, and their respective workers did not differ in disease resistance. Finally, queens founding in groups increased their investment in a component of individual immunity, as expected if they do not benefit from social immunity but respond to a higher risk of disease. Overall, our results provide no evidence that joint colony founding by L. niger queens increases their ability to resist fungal pathogens. | NA | no | No good fitness measure and measure host GD | NA | no | no |
| 4790 | 2020 | 44113 | Bryan, GJ; McLean, K; Waugh, R; Spooner, DM | 2017 | Levels of Intra-specific AFLP Diversity in Tuber-Bearing Potato Species with Different Breeding Systems and Ploidy Levels | DNA-based marker analysis of plant genebank material has become a useful tool in the evaluation of levels of genetic diversity and for the informed use and maintenance of germplasm. In this study, we quantify levels of amplified fragment length polymorphism (AFLP) in representative accessions of wild and cultivated potato species of differing geographic origin, ploidy, and breeding system. We generated 449 polymorphic AFLP fragments in 619 plants, representing multiple plants (16-23) from 17 accessions of 14 potato taxa as well as single plants sampled from available accessions (from 3 to 56) of the same 14 taxa. Intra-accession diversities were compared to those of a synthetic ‘taxon-wide’ population comprising a single individual from a variable number of available accessions of each sampled taxon. Results confirm the expected considerably lower levels of polymorphism within accessions of self-compatible as compared to self-incompatible taxa. We observed broadly similar levels of ‘taxon-wide’ polymorphism among self-compatible and self-incompatible species, with self-compatible taxa showing only slightly lower rates of polymorphism. The most diverse accessions were the two cultivated potato accessions examined, the least diverse being the Mexican allohexaploids Solanum demissum and S. iopetalum. Generally allopolyploid self-compatible accessions exhibited lower levels of diversity. Some purported self-incompatible accessions showed relatively low levels of marker diversity, similar to the more diverse self-compatible material surveyed. Our data indicate that for self-compatible species a single plant is highly representative of a genebank accession. The situation for self-incompatible taxa is less clear, and sampling strategies used will depend on the type of investigation. These results have important implications for those seeking novel trait variation (e.g., disease resistance) in gene banks as well as for the selection of individuals for genomics studies. We also show that AFLPs, despite having been largely replaced by other marker types, is highly suitable for the evaluation of within and between accession diversity in genebanks. | NA | no | No good fitness measure and measure host GD | NA | no | no |
| 4791 | 2020 | 44040 | Bubier, JA; Philip, VM; Quince, C; Campbell, J; Zhou, YJ; Vishnivetskaya, T; Duvvuru, S; Blair, RH; Ndukum, J; Donohue, KD; Foster, CM; Mellert, DJ; Weinstock, G; Culiat, CT; O’Hara, BF; Palumbo, AV; Podar, M; Chesler, EJ | 2020 | A Microbe Associated with Sleep Revealed by a Novel Systems Genetic Analysis of the Microbiome in Collaborative Cross Mice | Host genetic diversity provides a variable selection environment and physiological context for microbiota and their interaction with host physiology. Using a highly diverse mouse population, Bubier et al. identified that Odoribacter abundance influences sleep archi-tecture in a manner… The microbiome influences health and disease through complex networks of host genetics, genomics, microbes, and environment. Identifying the mechanisms of these interactions has remained challenging. Systems genetics in laboratory mice (Mus musculus) enables data-driven discovery of biological network components and mechanisms of host-microbial interactions underlying disease phenotypes. To examine the interplay among the whole host genome, transcriptome, and microbiome, we mapped QTL and correlated the abundance of cecal messenger RNA, luminal microflora, physiology, and behavior in a highly diverse Collaborative Cross breeding population. One such relationship, regulated by a variant on chromosome 7, was the association of Odoribacter (Bacteroidales) abundance and sleep phenotypes. In a test of this association in the BKS.Cg-Dock7(m) +/+ Lepr(db)/J mouse model of obesity and diabetes, known to have abnormal sleep and colonization by Odoribacter, treatment with antibiotics altered sleep in a genotype-dependent fashion. The many other relationships extracted from this study can be used to interrogate other diseases, microbes, and mechanisms. | NA | no | No good fitness measure and measure host GD | NA | no | no |
| 4792 | 2020 | 44041 | Buckley, J; Daly, R; Cobbold, CA; Burgess, K; Mable, BK | 2019 | Changing environments and genetic variation: natural variation in inbreeding does not compromise short-term physiological responses | Selfing plant lineages are surprisingly widespread and successful in a broad range of environments, despite showing reduced genetic diversity, which is predicted to reduce their long-term evolutionary potential. However, appropriate short-term plastic responses to new environmental conditions might not require high levels of standing genetic variation. In this study, we tested whether mating system variation among populations, and associated changes in genetic variability, affected short-term responses to environmental challenges. We compared relative fitness and metabolome profiles of naturally outbreeding (genetically diverse) and inbreeding (genetically depauperate) populations of a perennial plant, Arabidopsis lyrata, under constant growth chamber conditions and an outdoor common garden environment outside its native range. We found no effect of inbreeding on survival, flowering phenology or short-term physiological responses. Specifically, naturally occurring inbreeding had no significant effects on the plasticity of metabolome profiles, using either multivariate approaches or analysis of variation in individual metabolites, with inbreeding populations showing similar physiological responses to outbreeding populations over time in both growing environments. We conclude that low genetic diversity in naturally inbred populations may not always compromise fitness or short-term physiological capacity to respond to environmental change, which could help to explain the global success of selfing mating strategies. | NA | no | inbreeding | NA | no | no |
| 4793 | 2020 | 44112 | Buckley, SJ; Timms, P; Davies, MR; McMillan, DJ | 2018 | In silico characterisation of the two-component system regulators of Streptococcus pyogenes | Bacteria respond to environmental changes through the co-ordinated regulation of gene expression, often mediated by two-component regulatory systems (TCS). Group A Streptococcus (GAS), a bacterium which infects multiple human body sites and causes multiple diseases, possesses up to 14 TCS. In this study we examined genetic variation in the coding sequences and non-coding DNA upstream of these TCS as a method for evaluating relationships between different GAS emm-types, and potential associations with GAS disease. Twelve of the 14 TCS were present in 90% of the genomes examined. The length of the inter genic regions (IGRs) upstream of TCS coding regions varied from 39 to 345 nucleotides, with an average nucleotide diversity of 0.0064. Overall, IGR allelic variation was generally conserved with an emm-type. Subsequent phylogenetic analysis of concatenated sequences based on all TCS IGR sequences grouped genomes of the same emm-type together. However grouping with emm-pattern and emm-cluster-types was much weaker, suggesting epidemiological and functional properties associated with the latter are not due to evolutionary relatedness of emm-types. All emm5, emm6 and most of the emm18 genomes, all historically considered rheumatogenic emm-types clustered together, suggesting a shared evolutionary history. However emm1, emm3 and several emm18 genomes did not cluster within this group. These latter emm18 isolates were epidemiologically distinct from other emm18 genomes in study, providing evidence for local variation. emm-types associated with invasive disease or nephritogenicity also did not cluster together. Considering the TCS coding sequences (cds), correlation with emm-type was weaker than for the IGRs, and no strong correlation with disease was observed. Deletion of the malate transporter, maeP, was identified that serves as a putative marker for the emm89.0 subtype, which has been implicated in invasive outbreaks. A recombination-related, subclade-forming DNA motif was identified in the putative receiver domain of the Spy1556 response regulator that correlated with throat associated emm-pattern-type A-C strains. | NA | no | gd of pathogen | NA | no | no |
| 4794 | 2020 | 44112 | Budd, K; Berkman, LK; Anderson, M; Koppelman, J; Eggert, LS | 2018 | Genetic Structure and Recovery of White-Tailed Deer in Missouri | The white-tailed deer (Odocoileus virginianus) of Missouri have undergone changes in the past 100 years. From a historical estimate of 700,000 individuals, deer were nearly extirpated from the state following overexploitation in the late 1800s. Despite this, deer have since undergone a rapid expansion to the current population of 1.4 million. Because of this complex recent history, we sought to understand the levels and distribution of genetic diversity in deer across Missouri. We genotyped 751 deer at 10 microsatellite loci and used genetic distance metrics and Bayesian clustering analyses to identify distinct genetic groups. We also sequenced the mitochondrial control region from a subset (n=248) of deer to identify historical lineages. We found high levels of nuclear marker diversity (H-E = 0.818) and low, but statistically significant, levels of differentiation among regions. The high level of mitochondrial diversity (48 haplotypes; haplotype diversity = 0.621-0.964) is reflected in a haplotype network that suggests the persistent signature of translocated lineages. Overall, the white-tailed deer of Missouri now represent a highly diverse population with no indication that there are barriers to gene flow. This will be an important consideration for the management of disease resistance and spread throughout the state. (C) 2018 The Wildlife Society. | NA | no | No good fitness measure | NA | no | no |
| 4795 | 2020 | 44112 | Bujan, N; Balboa, S; Romalde, JL; Toranzo, AE; Magarinos, B | 2018 | Population genetic and evolution analysis of controversial genus Edwardsiella by multilocus sequence typing | At present, the genus Edwardsiella compiles five species: E. tarda, E. hoshinae, E. ictaluri, E. piscicida and E. anguillarum. Some species of this genus such us E. ictaluri and E. piscicida are important pathogens of numerous fish species. With the description of the two latter species, the phylogeny of Edwardsiella became more complicated. With the aim to clarify the relationships among all species in the genus, a multilocus sequence typing (MLST) approach was developed and applied to characterize 56 isolates and 6 reference strains belonging to the five Edwardsiella species. Moreover, several analyses based on the MLST scheme were performed to investigate the evolution within the genus, as well as the influence of recombination and mutation in the speciation. Edwardsiella isolates presented a high genetic variability reflected in the fourteen sequence types (ST) represented by a single isolates out of eighteen total ST. Mutation events were considerably more frequent than recombination, although both approximately equal influenced the genetic diversification. However, the speciation among species occurred mostly by recombination. Edwardsiella genus displays a non-clonal population structure with some degree of geographical isolation followed by a population expansion of E. piscicida. A database from this study was created and hosted on pubmlst.org (http://pubmlst.org/edwardsiella/). | NA | no | No good fitness measure and measure host GD | NA | no | no |
| 4796 | 2020 | 44112 | Buk, KG; van der Merwe, VC; Marnewick, K; Funston, PJ | 2018 | Conservation of severely fragmented populations: lessons from the transformation of uncoordinated reintroductions of cheetahs (Acinonyx jubatus) into a managed metapopulation with self-sustained growth | We document and evaluate the use of metapopulation management to conserve a declining population of 217 cheetahs in 40 subpopulations. Metapopulation management resembles a natural metapopulation, but dispersal success, demographic rescue effects and genetic viability are enhanced by moving suitable individuals to selected habitat fragments. Unfortunately, history and results of metapopulation management are rarely published. Cheetahs, extirpated from 85% of South Africa, were reintroduced from Namibian and South African ranches into fenced reserves. During 1965-2009 343 cheetahs were reintroduced, yet reserves held only 289 in 2009. Then translocations of free-roaming cheetahs were halted, and numbers dropped to 217 on 40 reserves by 2012. A metapopulation project was launched, and key conservation problems indentified from interviews and records. Thirty-five percent of reserves had no breeding cheetahs, 13% were inbreeding, fence quality was erratic, 3% of cheetahs were sold into captivity annually, and 28% of cheetah mortalities were anthropogenic. Lions accounted for 31% of mortality, perhaps elevated by lion-inexperienced cheetahs and high lion densities. These problems were addressed, and cheetahs were translocated between reserves. Although the median reserve size was only 125km(2) holding four cheetahs, and 80% of reserves were privately owned, in 6years the metapopulation grew by 51% to 328 cheetahs on 51 reserves, while genetic diversity was managed and monitored. Thus, using metapopulation management, low density species and associated key processes, including carnivory or mega-herbivory, can be conserved in relatively small reserves in regions withdensehuman populations precluding natural gene flow. | NA | no | No good fitness measure and measure host GD | NA | no | no |
| 4797 | 2020 | 44042 | Burghardt, LT; Epstein, B; Tiffin, P | 2019 | Legacy of prior host and soil selection on rhizobial fitness in planta | Measuring selection acting on microbial populations in natural or even seminatural environments is challenging because many microbial populations experience variable selection. The majority of rhizobial bacteria are found in the soil. However, they also live symbiotically inside nodules of legume hosts and each nodule can release thousands of daughter cells back into the soil. We tested how past selection (i.e., legacies) by two plant genotypes and by the soil alone affected selection and genetic diversity within a population of 101 strains of Ensifer meliloti. We also identified allelic variants most strongly associated with soil- and host-dependent fitness. In addition to imposing direct selection on rhizobia populations, soil and host environments had lasting effects across host generations. Host presence and genotype during the legacy period explained 22% and 12% of the variance in the strain composition of nodule communities in the second cohort, respectively. Although strains with high host fitness in the legacy cohort tended to be enriched in the second cohort, the diversity of the strain community was greater when the second cohort was preceded by host rather than soil legacies. Our results indicate the potential importance of soil selection driving the evolution of these plant-associated microbes. | NA | no | No good fitness measure and measure host GD | NA | no | no |
| 4798 | 2020 | 44112 | Bushakra, JM; Dossett, M; Carter, KA; Vining, KJ; Lee, JC; Bryant, DW; VanBuren, R; Lee, J; Mockler, TC; Finn, CE; Bassil, NV | 2018 | Characterization of aphid resistance loci in black raspberry (Rubus occidentalis L.) | Viruses vectored by the aphid Amphorophora agathonica cause decline in black raspberry plant health resulting in a shortened life and poor fruit quality of the infected plantings. New aphid resistant cultivars could increase the longevity of plantings providing growers and processors with consistent fruit production. Recent exploration of the native range of black raspberry identified three sources of aphid resistance: Ag (4) from Ontario (ON), Canada, Ag (5) from Maine (ME), and a third source from Michigan (MI) with no formal designation. The objectives of this study were to assess segregation of these three sources of aphid resistance in populations with single and combined sources and develop markers that can identify each source of resistance. A genetic linkage map constructed for ORUS 4305 placed the ON aphid resistance locus on Rubus linkage group (RLG) 6. Segregation ratios in populations with single and combined sources, and linkage mapping in two populations (ORUS 4304 and ORUS 4812) segregating for the Ag (5) and MI sources, respectively, indicated that these three sources of resistance are each conferred by single dominant genes/alleles that are linked on RLG6. Confirmation of marker association in 16 validation populations identified four markers that could be used to predict resistance; however, none could distinguish between the ON and MI sources. These four markers may be useful for screening populations to enrich the field-planted progeny for aphid resistance. Fine mapping of the resistance loci is needed to develop functional markers at each of the resistance loci to enable pyramiding and durable aphid resistance. | NA | no | No good fitness measure and measure host GD | NA | no | no |
| 4799 | 2020 | 44112 | Bycroft, C; Freeman, C; Petkova, D; Band, G; Elliott, LT; Sharp, K; Motyer, A; Vukcevic, D; Delaneau, O; O’Connell, J; Cortes, A; Welsh, S; Young, A; Effingham, M; McVean, G; Leslie, S; Allen, N; Donnelly, P; Marchini, J | 2018 | The UK Biobank resource with deep phenotyping and genomic data | The UK Biobank project is a prospective cohort study with deep genetic and phenotypic data collected on approximately 500,000 individuals from across the United Kingdom, aged between 40 and 69 at recruitment. The open resource is unique in its size and scope. A rich variety of phenotypic and health-related information is available on each participant, including biological measurements, lifestyle indicators, biomarkers in blood and urine, and imaging of the body and brain. Follow-up information is provided by linking health and medical records. Genome-wide genotype data have been collected on all participants, providing many opportunities for the discovery of new genetic associations and the genetic bases of complex traits. Here we describe the centralized analysis of the genetic data, including genotype quality, properties of population structure and relatedness of the genetic data, and efficient phasing and genotype imputation that increases the number of testable variants to around 96 million. Classical allelic variation at 11 human leukocyte antigen genes was imputed, resulting in the recovery of signals with known associations between human leukocyte antigen alleles and many diseases. | NA | no | No good fitness measure and measure host GD | NA | no | no |
| 4800 | 2020 | 44111 | Byers, JE; Schmidt, JP; Pappalardo, P; Haas, SE; Stephens, PR | 2019 | What factors explain the geographical range of mammalian parasites? | Free-living species vary substantially in the extent of their spatial distributions. However, distributions of parasitic species have not been comprehensively compared in this context. We investigated which factors most influence the geographical extent of mammal parasites. Using the Global Mammal Parasite Database we analysed 17 818 individual geospatial records on 1806 parasite species (encompassing viruses, bacteria, protozoa, arthropods and helminths) that infect 396 carnivore, ungulate and primate host species. As a measure of the geographical extent of each parasite species we quantified the number and area of world ecoregions occupied by each. To evaluate the importance of variables influencing the summed area of ecoregions occupied by a parasite species, we used Bayesian network analysis of a subset (n = 866) of the parasites in our database that had at least two host species and complete information on parasite traits. We found that parasites that covered more geographical area had a greater number of host species, higher average phylogenetic relatedness between host species and more sampling effort. Host and parasite taxonomic groups had weak and indirect effects on parasite ecoregion area; parasite transmission mode had virtually no effect. Mechanistically, a greater number of host species probably increases both the collective abundance and habitat breadth of hosts, providing more opportunities for a parasite to have an expansive range. Furthermore, even though mammals are one of the best-studied animal classes, the ecoregion area occupied by their parasites is strongly sensitive to sampling effort, implying mammal parasites are undersampled. Overall, our results support that parasite geographical extent is largely controlled by host characteristics, many of which are subsumed within host taxonomic identity. | NA | no | No good fitness measure and measure host GD | NA | no | no |
| 4801 | 2020 | 44113 | Byrne, AQ; Rothstein, AP; Poorten, TJ; Erens, J; Settles, ML; Rosenblum, EB | 2017 | Unlocking the story in the swab: A new genotyping assay for the amphibian chytrid fungus Batrachochytrium dendrobatidis | One of the most devastating emerging pathogens of wildlife is the chytrid fungus, Batrachochytrium dendrobatidis (Bd), which affects hundreds of amphibian species around the world. Genomic data from pure Bd cultures have advanced our understanding of Bd phylogenetics, genomic architecture and mechanisms of virulence. However, pure cultures are laborious to obtain and whole-genome sequencing is comparatively expensive, so relatively few isolates have been genetically characterized. Thus, we still know little about the genetic diversity of Bd in natural systems. The most common noninvasive method of sampling Bd from natural populations is to swab amphibian skin. Hundreds of thousands of swabs have been collected from amphibians around the world, but Bd DNA collected via swabs is often low in quality and/or quantity. In this study, we developed a custom Bd genotyping assay using the Fluidigm Access Array platform to amplify 192 carefully selected regions of the Bd genome. We obtained robust sequence data for pure Bd cultures and field-collected skin swabs. This new assay has the power to accurately discriminate among the major Bd clades, recovering the basic tree topology previously revealed using whole-genome data. Additionally, we established a critical value for initial Bd load for swab samples (150 Bd genomic equivalents) above which our assay performs well. By leveraging advances in microfluidic multiplex PCR technology and the globally distributed resource of amphibian swab samples, noninvasive skin swabs can now be used to address critical spatial and temporal questions about Bd and its effects on declining amphibian populations. | NA | no | gd of pathogen | NA | no | no |
| 4802 | 2020 | 44111 | Cabezas, MP; Ros, M; dos Santos, AM; Martinez-Laiz, G; Xavier, R; Montelli, L; Hoffman, R; Fersi, A; Dauvin, JC; Guerra-Garcia, JM | 2019 | Unravelling the origin and introduction pattern of the tropical species Paracaprella pusilla Mayer, 1890 (Crustacea, Amphipoda, Caprellidae) in temperate European waters: first molecular insights from a spatial and temporal perspective | Paracaprella pusilla Mayer, 1890 is a tropical caprellid species recently introduced to the Eastern Atlantic coast of the Iberian Peninsula and the Mediterranean Sea. In this study, we used direct sequencing of mitochondrial (COI and 16S) and nuclear (28S and ITS) genes to compare genetic differences in presumed native and introduced populations in order to infer its introduction pattern and to shed light on the native range of this species. The temporal pattern of genetic diversity at the westernmost limit of the geographic range of P. pusilla in Europe (the Atlantic coast of southern Spain) over an eight-year period was also investigated. Our results confirm P. pusilla as a neocosmopolitan species and suggest that the species is native to the Atlantic coast of Central and South America. Paracaprella pusilla seems to have been introduced into European waters from multiple introduction pathways and source populations, which are likely to include populations from coastal waters of Brazil. Multiple introduction pathways may have been involved, with the most important being commercial shipping through the Strait of Gibraltar. While this tropical species appears to be expanding in the Mediterranean, populations from the westernmost limit of its geographic range in Europe showed a temporal instability. This study constitutes the first molecular approach focused on this species, but it is also the first study of temporal change in genetic diversity of any introduced marine amphipod. Additional intensive sampling of this species, including both native and non-native populations, and detailed temporal studies are still necessary to properly understand how genetic diversity influences the introduction and survival of P. pusilla in invaded areas. | NA | no | No good fitness measure | NA | no | no |
| 4803 | 2020 | 44111 | Cabrera-Mora, JA; Guzman-Franco, AW; Santillan-Galicia, MT; Tamayo-Mejia, E | 2019 | Niche separation of species of entomopathogenic fungi within the genera Metarhizium and Beauveria in different cropping systems in Mexico | Entomopathogenic fungi from the genera Beauveria and Metarhizium, were isolated from soil using the Galleria mellonella baiting method, and from infected white grub larvae from a diversity of cropping systems in Puebla and Guanajuato, Mexico. Isolates were identified to species level using Bloc and Elongation Factor 1-alpha sequence information. Although widespread, Beauveria bassiana (41 isolates) was only isolated from soil and not from infected white grubs. In contrast, Beauveria pseudobassiana (six isolates) was predominantly isolated from white grub larvae (only one isolate from soil). Haplotype analysis of B. bassiana Bloc sequences identified 25 haplotypes indicating substantial genetic diversity; neither geographical origin nor crop type explained this genetic variation. Metarhizium brunneum (three isolates) and Metarhizium robertsii (17 isolates) were also only isolated from soil, while Metarhizium anisopliae (six isolates) and Metarhizium pingshaense (four isolates) were only isolated from white grub larvae. M. anisopliae was only found infecting Paranomala species while M. pingshaense was only found infecting Phyllophaga species. Species diversity in Metarhizium was influenced by crop type. Our results showed that entomopathogenic fungi species could co-exist in the same soil ecosystem but in separate niches. The potential ecological roles of these species are discussed. (C) 2019 Elsevier Ltd and British Mycological Society. All rights reserved. | NA | no | No good fitness measure and measure host GD | NA | no | no |
| 4804 | 2020 | 44040 | Cai, SS; Gao, TX; Yan, BL; Zhu, AY; Zhang, XM | 2020 | Preliminary Assessment of Stock Enhancement in Swimming Crab (Portunus trituberculatus) Based on Molecular Markers | Assessment of the stock enhancement programs is crucial for fishery resources recovery, yet lack of proper methods hinders the precision and accuracy of such assessment. The swimming crab, Portunus trituberculatus is a commercially important species in Chinese fishery industry. However the natural resources of swimming crabs are declining and enhancement programs are being conducted for resources restoration for decades. In this study, 524 female broodstock from 10 hatcheries and 547 recaptured crabs from 6 investigations were used to assess the proportion of released individuals and evaluate the effect of the program in Shandong Province in 2014. Parentage determination between broodstock and recaptured individuals was implemented by using mitochondrial control region fragments and three microsatellite markers. When using mtDNA loci, 242 individuals (44.24%) were excluded for no shared haplotype with broodstock. Further 81 (14.81%) crabs were identified as hatchery-reared individuals based on microsatellite loci from the remaining 305 crabs. Our results also showed high genetic diversity and a certain degree of heterozygote deficiency of natural swimming crab populations. The advancing technology and its unique advantages will make molecular markers as novel and highly-efficient approaches for assessment of stock enhancement. | NA | no | No good fitness measure and measure host GD | NA | no | no |
| 4805 | 2020 | 44042 | Caicedo-Garzon, V; Salgado-Roa, FC; Sanchez-Herrera, M; Hernandez, C; Arias-Giraldo, LM; Garcia, L; Vallejo, G; Cantillo, O; Tovar, C; da Rosa, JA; Carrasco, HJ; Segovia, M; Salazar, C; Ramirez, JD | 2019 | Genetic diversification of Panstrongylus geniculatus (Reduviidae: Triatominae) in northern South America | Triatomines are the vectors of Trypanosoma cruzi, the etiological agent of Chagas disease. Although Triatoma and Rhodnius are the most-studied vector genera, other triatomines, such as Panstrongylus, also transmit T. cruzi, creating new epidemiological scenarios. Panstrongylus has at least 13 reported species but there is limited information about its intraspecific genetic variation and patterns of diversification. Here, we begin to fill this gap by studying populations of P. geniculatus from Colombia and Venezuela and including other epidemiologically important species from the region. We examined the pattern of diversification of P. geniculatus in Colombia using mitochondrial and nuclear ribosomal data. Genetic diversity and differentiation were calculated within and among populations of P. geniculatus. Moreover, we constructed maximum likelihood and Bayesian inference phylogenies and haplotype networks using P. geniculatus and other species from the genus (P. megistus, P. lignarius, P. lutzi, P. tupynambai, P. chinai, P. rufotuberculatus and P. howardi). Using a coalescence framework, we also dated the P. geniculatus lineages. The total evidence tree showed that P. geniculatus is a monophyletic species, with four clades that are concordant with its geographic distribution and are partly explained by the Andes orogeny. However, other factors, including anthropogenic and eco-epidemiological effects must be investigated to explain the existence of recent geographic P. geniculatus lineages. The epidemiological dynamics in structured vector populations, such as those found here, warrant further investigation. Extending our knowledge of P. geniculatus is necessary for the accurate development of effective strategies for the control of Chagas disease vectors. | NA | no | No good fitness measure and measure host GD | NA | no | no |
| 4806 | 2020 | 44112 | Calarco, L; Barratt, J; Ellis, J | 2018 | Genome Wide Identification of Mutational Hotspots in the Apicomplexan Parasite Neospora caninum and the Implications for Virulence | Neospora caninum is an apicomplexan parasite responsible for neosporosis, a disease causing hind limb paralysis in dogs and abortion in cattle, resulting in substantial economic losses to beef and dairy industries. Marked differences in pathogenicity exist between N. caninum strains suggesting that intrinsic genetic differences exist between them. These differences likely exist in genes expressed during the tachyzoite lifecycle stage which is responsible for the pathogenesis of neosporosis. An improved understanding of these genetic differences is essential to understanding N. caninum virulence, though such knowledge is scarce. Using a variant detection workflow we compared the tachyzoite transcriptomes of two N. caninum strains with different virulence properties: NC-Liverpool (virulent) and NC-Nowra (avirulent). This workflow identified 3130 SNPs and 6123 indels between the strains, and nine markers capturing 30 variants were Sanger sequenced for both strains. Sequencing of these loci was extended to an additional eight strains and subsequent phylogenetic analysis supported a genetic population structure comprised of two major clades with no geographical segregation. Sequence polymorphisms within coding regions of tachyzoite-associated genes were concentrated on chromosomes XI and XII, with 19 distinct tachyzoite-associated SNP hotspot regions identified within coding regions of the N. caninum nuclear genome. The variants were predominantly located in loci associated with protein binding, protein-protein interactions, transcription, and translation. Furthermore, 468 nonsynonymous SNPs identified within protein-coding genes were associated with protein kinase activity, protein binding, protein phosphorylation, and proteolysis. This work may implicate these processes and the specific proteins involved as novel effectors of N. caninum tachyzoite virulence. | NA | no | gd of pathogen | NA | no | no |
| 4807 | 2020 | 44113 | Callet, T; Medale, F; Larroquet, L; Surget, A; Aguirre, P; Kerneis, T; Labbe, L; Quillet, E; Geurden, I; Skiba-Cassy, S; Dupont-Nivet, M | 2017 | Successful selection of rainbow trout (Oncorhynchus mykiss) on their ability to grow with a diet completely devoid of fishmeal and fish oil, and correlated changes in nutritional traits | In the context of limited marine resources, the exponential growth of aquaculture requires the substitution of fish oil and fishmeal, the traditional components of fish feeds by terrestrial plant ingredients. High levels of such substitution are known to negatively impact fish performance such as growth and survival in rainbow trout (Oncorhynchus mykiss) as in other salmonids. In this respect, genetic selection is a key enabler for improving those performances and hence for the further sustainable development of aquaculture. We selected a rainbow trout line over three generations for its ability to survive and grow on a 100% plant-based diet devoid of both fish oil and fishmeal (V diet) from the very first meal. In the present study, we compared the control line and the selected line after 3 generations of selection, both fed either the V diet or a marine resources-based diet (M diet). The objective of the study was to assess the efficiency of selection and the consequences on various correlated nutritional traits: feed intake, feed efficiency, digestibility, composition of whole fish, nutrient retention and fatty acid (FA) profile. We demonstrated that the genetic variability present in our rainbow trout population can be selected to improve survival and growth. The major result of the study is that after only three generations of selection, selected fish fed the V diet grew at the same rate as the control line fed the M diet, whilst the relative reduction of body weight was 36.8% before the selection. This enhanced performance on the V diet seems to be mostly linked to a higher feed intake for the selected fish. | NA | no | no good gd measure host pop | NA | no | no |
| 4808 | 2020 | 44112 | Camargo-Ayala, PA; Garzon-Ospina, D; Moreno-Perez, DA; Ricaurte-Contreras, LA; Noya, O; Patarroyo, MA | 2018 | On the Evolution and Function of Plasmodium vivax Reticulocyte Binding Surface Antigen (pvrbsa) | The RBSA protein is encoded by a gene described in Plasmodium species having tropism for reticulocytes. Since this protein is antigenic in natural infections and can bind to target cells, it has been proposed as a potential candidate for an anti-Plasmodium vivax vaccine. However, genetic diversity (a challenge which must be overcome for ensuring fully effective vaccine design) has not been described at this locus. Likewise, the minimum regions mediating specific parasite-host interaction have not been determined. This is why the rbsa gene’s evolutionary history is being here described, as well as the P. vivax rbsa (pvrbsa) genetic diversity and the specific regions mediating parasite adhesion to reticulocytes. Unlike what has previously been reported, rbsa was also present in several parasite species belonging to the monkey-malaria clade; paralogs were also found in Plasmodium parasites invading reticulocytes. The pvrbsa locus had less diversity than other merozoite surface proteins where natural selection and recombination were the main evolutionary forces involved in causing the observed polymorphism. The N-terminal end (PvRBSA-A) was conserved and under functional constraint; consequently, it was expressed as recombinant protein for binding assays. This protein fragment bound to reticulocytes whilst the C-terminus, included in recombinant PvRBSA-B (which was not under functional constraint), did not. Interestingly, two PvRBSA-A-derived peptides were able to inhibit protein binding to reticulocytes. Specific conserved and functionally important peptides within PvRBSA-A could thus be considered when designing a fully-effective vaccine against P. vivax. | NA | no | No good fitness measure | NA | no | no |
| 4809 | 2020 | 44113 | Cambridge, JM; Blinkova, AL; Rocha, EIS; Hernandez, AB; Moreno, M; Gines-Candelaria, E; Goetz, BM; Hunicke-Smith, S; Satterwhite, E; Tucker, HO; Walker, JR | 2018 | Genomics of &ITClostridium taeniosporum&IT, an organism which forms endospores with ribbon-like appendages | Clostridium taeniosporum, a non-pathogenic anaerobe closely related to the C. botulinum Group II members, was isolated from Crimean lake silt about 60 years ago. Its endospores are surrounded by an encasement layer which forms a trunk at one spore pole to which about 12-14 large, ribbon-like appendages are attached. The genome consists of one 3,264,813 bp, circular chromosome (with 26.6% GC) and three plasmids. The chromosome contains 2,892 potential protein coding sequences: 2,124 have specific functions, 147 have general functions, 228 are conserved but without known function and 393 are hypothetical based on the fact that no statistically significant orthologs were found. The chromosome also contains 101 genes for stable RNAs, including 7 rRNA clusters. Over 84% of the protein coding sequences and 96% of the stable RNA coding regions are oriented in the same direction as replication. The three known appendage genes are located within a single cluster with five other genes, the protein products of which are closely related, in terms of sequence, to the known appendage proteins. The relatedness of the deduced protein products suggests that all or some of the closely related genes might code for minor appendage proteins or assembly factors. The appendage genes might be unique among the known clostridia; no statistically significant orthologs were found within other clostridial genomes for which sequence data are available. The C. taeniosporum chromosome contains two functional prophages, one Siphoviridae and one Myoviridae, and one defective prophage. Three plasmids of 5.9, 69.7 and 163.1 Kbp are present. These data are expected to contribute to future studies of developmental, structural and evolutionary biology and to potential industrial applications of this organism. | NA | no | No good fitness measure and measure host GD | NA | no | no |
| 4810 | 2020 | 44113 | Cambronero, MC; Zeis, B; Orsini, L | 2018 | Haemoglobin-mediated response to hyper-thermal stress in the keystone species Daphnia magna | Anthropogenic global warming has become a major geological and environmental force driving drastic changes in natural ecosystems. Due to the high thermal conductivity of water and the effects of temperature on metabolic processes, freshwater ecosystems are among the most impacted by these changes. The ability to tolerate changes in temperature may determine species long-term survival and fitness. Therefore, it is critical to identify coping mechanisms to thermal and hyper-thermal stress in aquatic organisms. A central regulatory element compensating for changes in oxygen supply and ambient temperature is the respiratory protein haemoglobin (Hb). Here, we quantify Hb plastic and evolutionary response in Daphnia magna subpopulations resurrected from the sedimentary archive of a lake with known history of increase in average temperature and recurrence of heat waves. By measuring constitutive changes in crude Hb protein content among subpopulations, we assessed evolution of the Hb gene family in response to temperature increase. To quantify the contribution of plasticity in the response of this gene family to hyper-thermal stress, we quantified changes in Hb content in all subpopulations under hyper-thermal stress as compared to nonstressful temperature. Further, we tested competitive abilities of genotypes as a function of their Hb content, constitutive and induced. We found that Hb-rich genotypes have superior competitive abilities as compared to Hb-poor genotypes under hyper-thermal stress after a period of acclimation. These findings suggest that whereas long-term adjustment to higher occurrence of heat waves may require a combination of plasticity and genetic adaptation, plasticity is most likely the coping mechanism to hyper-thermal stress in the short term. Our study suggests that with higher occurrence of heat waves, Hb-rich genotypes may be favoured with potential long-term impact on population genetic diversity. | NA | no | No good fitness measure and measure host GD | NA | no | no |
| 4811 | 2020 | 44043 | Can, H; Kal, U; Ozyigit, II; Paksoy, M; Turkmen, O | 2019 | Construction, characteristics and high throughput molecular screening methodologies in some special breeding populations: a horticultural perspective | Advanced marker technologies are widely used for evaluation of genetic diversity in cultivated crops, wild ancestors, landraces or any special plant genotypes. Developing agricultural cultivars requires the following steps: (i) determining desired characteristics to be improved, (ii) screening genetic resources to help find a superior cultivar, (iii) intercrossing selected individuals, (iv) generating genetically hybrid populations and screening them for agro-morphological or molecular traits, (v) evaluating the superior cultivar candidates, (vi) testing field performance at different locations, and (vii) certifying. In the cultivar development process valuable genes can be identified by creating special biparental or multiparental populations and analysing their association using suitable markers in given populations. These special populations and advanced marker technologies give us a deeper knowledge about the inherited agronomic characteristics. Unaffected by the changing environmental conditions, these provide a higher understanding of genome dynamics in plants. The last decade witnessed new applications for advanced molecular techniques in the area of breeding, with low costs per sample. These, especially, include next-generation sequencing technologies like reduced representation genome sequencing (genotyping by sequencing, restriction site-associated DNA). These enabled researchers to develop new markers, such as simple sequence repeat and single- nucleotide polymorphism, for expanding the qualitative and quantitative information on population dynamics. Thus, the knowledge acquired from novel technologies is a valuable asset for the breeding process and to better understand the population dynamics, their properties, and analysis methods. | NA | no | No good fitness measure and measure host GD | NA | no | no |
| 4812 | 2020 | 44112 | Caniglia, R; Fabbri, E; Hulva, P; Bolfikova, BC; Jindrichova, M; Stronen, AV; Dykyy, I; Camatta, A; Carnier, P; Randi, E; Galaverni, M | 2018 | Wolf outside, dog inside? The genomic make-up of the Czechoslovakian Wolfdog | Background: Genomic methods can provide extraordinary tools to explore the genetic background of wild species and domestic breeds, optimize breeding practices, monitor and limit the spread of recessive diseases, and discourage illegal crossings. in this study we analysed a panel of 170k Single Nucleotide Polymorphisms with a combination of multivariate, Bayesian and outlier gene approaches to examine the genome-wide diversity and inbreeding levels in a recent wolf x dog cross-breed, the Czechoslovakian Wolfdog, which is becoming increasingly popular across Europe. Results: Pairwise F-ST values, multivariate and assignment procedures indicated that the Czechoslovakian Wolfdog was significantly differentiated from all the other analysed breeds and also well-distinguished from both parental populations (Carpathian wolves and German Shepherds). Coherently with the low number of founders involved in the breed selection, the individual inbreeding levels calculated from homozygosity legions were relatively high and comparable with those derived from the pedigree data. In contrast, the coefficient of relatedness between individuals estimated from the pedigrees often underestimated the identity-by-descent scores determined using genetic profiles. The timing of the admixture and the effective population size trends estimated from the LD patterns reflected the documented history/ of the breed. Ancestry reconstruction methods identified more than 300 genes with excess of wolf ancestry compared to random expectations, mainly related to key morphological features, and more than 2000 genes with excess of dog ancestry, playing important roles in lipid metabolism, in the regulation of circadian rhythms, in learning and memory processes, and in sociability, such as the COMT gene, which has been described as a candidate gene for the latter trait in dogs. Conclusions: In this study we successfully applied genome wide procedures to reconstruct the history of the Czechoslovakian Wolfdog, assess individual wolf ancestry proportions and, thanks to the availability of a well- annotated reference genome, identify possible candidate genes for wolf-like and dog-like phenotypic traits typical of this bleed, including commonly inherited disorders. Moreover, through the identification of ancestry-informative markers, these genomic approaches could provide tools for forensic applications to unmask illegal crossings with wolves and uncontrolled trades of recent and undeclared wolfdog hybrids. | NA | no | No good fitness measure and measure host GD | NA | no | no |
| 4813 | 2020 | 44112 | Cannon-Albright, LA; Dintelman, S; Maness, T; Cerny, J; Thomas, A; Backus, S; Farnham, JM; Teerlink, CC; Contreras, J; Kauwe, JSK; Meyer, LJ | 2018 | Population genealogy resource shows evidence of familial clustering for Alzheimer disease | Objective To show the potential of a resource consisting of a genealogy of the US record linked to National Veterans Health Administration (VHA) patient data for investigation of the genetic contribution to health-related phenotypes, we present an analysis of familial clustering of VHA patients diagnosed with Alzheimer disease (AD). Methods Patients with AD were identified by the International Classification of Diseases code. The Genealogical Index of Familiality method was used to compare the average relatedness of VHA patients with AD with expected relatedness. Relative risks for AD were estimated in first-to fifth-degree relatives of patients with AD using population rates for AD. Results Evidence for significant excess relatedness and significantly elevated risks for AD in relatives was observed; multiple pedigrees with a significant excess of VHA patients with AD were identified. Conclusions This analysis of AD shows the nascent power of the US Veterans Genealogy Resource, in early stages, to provide evidence for familial clustering of multiple phenotypes, and shows the utility of this VHA genealogic resource for future genetic studies. | NA | no | human | NA | no | no |
| 4814 | 2020 | 44113 | Cantanhede, LM; Fernandes, FG; Ferreira, GEM; Porrozzi, R; Ferreira, RDGM; Cupolillo, E | 2018 | New insights into the genetic diversity of Leishmania RNA Virus 1 and its species-specific relationship with Leishmania parasites | Cutaneous leishmaniasis is a neglected parasitic disease that manifests in infected individuals under different phenotypes, with a range of factors contributing to its broad clinical spectrum. One factor, Leishmania RNA Virus 1 (LRV1), has been described as an endosymbiont present in different species of Leishmania. LRV1 significantly worsens the lesion, exacerbating the immune response in both experimentally infected animals and infected individuals. Little is known about the composition and genetic diversity of these viruses. Here, we investigated the relationship between the genetic composition of LRV1 detected in strains of Leishmania (Viannia) braziliensis and L. (V.) guyanensis and the interaction between the endosymbiont and the parasitic species, analyzing an approximately 850 base pair region of the viral genome. We also included one LRV1 sequence detected in L. (V.) shawi, representing the first report of LRV1 in a species other than L. braziliensis and L. guyanensis. The results illustrate the genetic diversity of the LRV1 strains analyzed here, with smaller divergences detected among viral sequences from the same parasite species. Phylogenetic analyses showed that the LRV1 sequences are grouped according to the parasite species and possibly according to the population of the parasite in which the virus was detected, corroborating the hypothesis of joint evolution of the viruses with the speciation of Leishmania parasites. | NA | no | gd of pathogen | NA | no | no |
| 4815 | 2020 | 44042 | Cappucci, U; Noro, F; Casale, AM; Fanti, L; Berloco, M; Alagia, AA; Grassi, L; Le Pera, L; Piacentini, L; Pimpinelli, S | 2019 | The Hsp70 chaperone is a major player in stress-induced transposable element activation | Previous studies have shown that heat shock stress may activate transposable elements (TEs) in Drosophila and other organisms. Such an effect depends on the disruption of a chaperone complex that is normally involved in biogenesis of Piwi-interacting RNAs (piRNAs), the largest class of germline-enriched small noncoding RNAs implicated in the epigenetic silencing of TEs. However, a satisfying picture of how chaperones could be involved in repressing TEs in germ cells is still unknown. Here we show that, in Drosophila, heat shock stress increases the expression of TEs at a posttranscriptional level by affecting piRNA biogenesis through the action of the inducible chaperone Hsp70. We found that stress-induced TE activation is triggered by an interaction of Hsp70 with the Hsc70-Hsp90 complex and other factors all involved in piRNA biogenesis in both ovaries and testes. Such interaction induces a displacement of all such factors to the lysosomes, resulting in a functional collapse of piRNA biogenesis. This mechanism has clear evolutionary implications. In the presence of drastic environmental changes, Hsp70 plays a key dual role in increasing both the survival probability of individuals and the genetic variability in their germ cells. The consequent increase of genetic variation in a population potentiates evolutionary plasticity and evolvability. | NA | no | No good fitness measure and measure host GD | NA | no | no |
| 4816 | 2020 | 44040 | Carrillo-Galvan, G; Bye, R; Eguiarte, LE; Cristians, S; Perez-Lopez, P; Vergara-Silva, F; Luna-Cavazos, M | 2020 | Domestication of aromatic medicinal plants in Mexico: Agastache (Lamiaceae)-an ethnobotanical, morpho-physiological, and phytochemical analysis | Background Most reports of domesticated plants that involve a domestication gradient or inter-specific hybridization in Mexico have focused on those used as food. This study provides knowledge about these processes in two aromatic medicinal plants, Agastache mexicana (Lamiaceae) and A. m. subsp. xolocotziana, widely used in Mexican traditional medicine for the treatment of gastrointestinal ailments and for their sedative effect. Different populations of A. mexicana along a gradient of domestication are found in the foothills of the Popocatepetl volcano of central Mexico, while in this same area the subsp. xolocotziana grows only in the cultivation, possibly a product of hybridization between A. mexicana and Agastache palmeri. This study links ethnobotanical, morpho-physiological, and phytochemical evidence to document the domestication of both taxa as well as elucidates the possible hybrid origin of the subsp. xolocotziana. Method We analyze three groups of data derived from (1) 80 semi-structured interviews aimed at documenting the selection criteria related to the use and management of A. mexicana; (2) a cultivation experiment under homogeneous conditions, evaluating 21 floral, vegetative, and seed characters (that were important according to ethnobotanical information) in 97 plants corresponding to 13 populations of the taxa under study; and (3) the chemical profiles of the essential oils of these plants by means of a thin-layer chromatography. Results By linking the three types of evidence, two evolutionary processes are distinguished: (1) A. mexicana occurs in the encouraged-cultivated phases of the domestication gradient and (2) A. m. subsp. xolocotziana may have originated through inbreeding depression or hybridization. These two cultivated plants show a domestication syndrome based upon organoleptic differentiation due to their dissimilar phytochemical composition and gigantism in flowers, seeds, and rhizomes (the last enhancing their asexual reproductive capacity). In addition to this, A. mexicana exhibits more intense floral pigmentation and foliar gigantism while subsp. xolocotziana presents floral albinism and partial seed sterility. Conclusion Two divergent evolutionary processes are reported for the domestication of A. mexicana as a result of the intensification of its use and management. The selection processes of these plants have resulted in alternation of the organoleptic properties based upon the divergence of the phytochemical composition. Also, gigantism has been selected in culturally preferred plant parts and in correlated structures. The preceding characteristics reinforce the joint use of these plants in infusion in Mexican traditionalmedicine for the treatment of gastrointestinal diseases and for their sedative effects. | NA | no | dometicated host | NA | no | no |
| 4817 | 2020 | 44113 | Carvalho, N; Canela, FM; Leite, PHS; Ferreira, MA; Oliveira, VR; Santos, MF; Souza, NOS; Buso, GSC | 2017 | Analysis of genetic variability of commercial melon cultivars using SSR molecular markers | Taking into account the scenario of melon production (Cucumis melo L.) in Brazil, it is notable the importance of the culture in the fruit market. The preference of the national market is concentrated in the fruits of the yellow type. Therefore, it is possible that the genetic base of the commercial cultivars is relatively narrow due to the loss of important genes in the breeding process, and it is possible to introgress external genes for increasing recombination possibilities that generate sources of resistance to the main diseases and superior genotypes. This study evaluated the genetic variability of 87 melon accessions composed of 72 commercial cultivars belonging to the Inodorus and Cantaloupensis groups and 15 plant introductions (PIs). Forty-four polymorphic SSR primers were used to amplify a total of 202 alleles. The expected heterozygosity presented an average of 0.519 and the PIC index of 0.458, being moderately informative. The dendrogram generated for the 72 cultivars grouped the genotypes into 2 main groups, and there was no association with the classification of groups of the genotypes in the grouping. The number of SSR markers was sufficient to predict wide genetic variability among the cultivars studied, with the similarity between 0.35 and 0.98. The dendrogram presented for the 72 cultivars and the 15 PI genotypes was not associated with the classification of the genotypes in the grouping, and the 15 PIs were well dispersed with similarity indexes that resemble the two groups studied. A set of 25 primers was identified that were useful in distinguishing the 72 cultivars. These primers can be used in later research with the cultivars, as well as in crop protection situations, being an important tool in the useful and rapid distinction of genotypes, and in commercial disputes regarding the certification of the main melon cultivars used in the country. | NA | no | No good fitness measure | NA | no | no |
| 4818 | 2020 | 44040 | Casado, C; Galvez, C; Pernas, M; Tarancon-Diez, L; Rodriguez, C; Sanchez-Merino, V; Vera, M; Olivares, I; De Pablo-Bernal, R; Merino-Mansilla, A; Del Romero, J; Lorenzo-Redondo, R; Ruiz-Mateos, E; Salgado, M; Martinez-Picado, J; Lopez-Galindez, C | 2020 | Permanent control of HIV-1 pathogenesis in exceptional elite controllers: a model of spontaneous cure | Elite controllers (EC) represent a small subset of HIV-1-infected people that spontaneously control viral replication. However, natural virological suppression and absence of immune dysfunction are not always long-term sustained. We define exceptional EC (EEC) as HIV-1 subjects who maintain the EC characteristics without disease progression for more than 25 years. We analyzed three EEC, diagnosed between 1988 and 1992, who never showed signs of clinical disease progression in absence of any antiretroviral treatment. A comprehensive clinical, virological, and immunological study was performed. The individuals simultaneously exhibited >= 3 described host protective alleles, low levels of total HIV-1 DNA (<20 copies/10(6) CD4(+) T-cells) without evidence of replication-competent viruses (<0.025 IUPM), consistent with high levels of defective genomes, strong cellular HIV-1-specific immune response, and a high poly-functionality index (>0.50). Inflammation levels of EEC were similar to HIV-1 negative donors. Remarkably, they showed an exceptional lack of viral evolution and 8-fold lower genetic diversity (<0.01s/n) in env gene than other EC. We postulate that these EEC represent cases of spontaneous functional HIV-1 cure. A non-functional and non-genetically evolving viral reservoir along with an HIV-1-specific immune response seems to be key for the spontaneous functional cure. | NA | no | hiv | NA | no | no |
| 4819 | 2020 | 44112 | Casey, CS; Orozco-terWengel, P; Yaya, K; Kadwell, M; Fernandez, M; Marin, JC; Rosadio, R; Maturrano, L; Hoces, D; Hu, Y; Wheeler, JC; Bruford, MW | 2018 | Comparing genetic diversity and demographic history in co-distributed wild South American camelids | Vicunas and guanacos are two species of wild South American camelids that are key ruminants in the ecosystems where they occur. Although closely related, these species feature differing ecologies and life history characters, which are expected to influence both their genetic diversity and population differentiation at different spatial scales. Here, using mitochondrial and microsatellite genetic markers, we show that vicuna display lower genetic diversity within populations than guanaco but exhibit more structure across their Peruvian range, which may reflect a combination of natural genetic differentiation linked to geographic isolation and recent anthropogenic population declines. Coalescent-based demographic analyses indicate that both species have passed through a strong bottleneck, reducing their effective population sizes from over 20,000 to less than 1000 individuals. For vicuna, this bottleneck is inferred to have taken place similar to 3300 years ago, but to have occurred more recently for guanaco at similar to 2000 years ago. These inferred dates are considerably later than the onset of domestication (when the alpaca was domesticated from the vicuna while the llama was domesticated from the guanaco), coinciding instead with a major human population expansion following the mid-Holocene cold period. As importantly, they imply earlier declines than the well-documented Spanish conquest, where major mass mortality events were recorded for Andean human and camelid populations. We argue that underlying species’ differences and recent demographic perturbations have influenced genetic diversity in modern vicuna and guanaco populations, and these processes should be carefully evaluated in the development and implementation of management strategies for these important genetic resources. | NA | no | No good fitness measure and measure host GD | NA | no | no |
| 4820 | 2020 | 44039 | Castillo, AI; Chacon-Diaz, C; Rodriguez-Murillo, N; Coletta, HD; Almeida, RPP | 2020 | Impacts of local population history and ecology on the evolution of a globally dispersed pathogen | BackgroundPathogens with a global distribution face diverse biotic and abiotic conditions across populations. Moreover, the ecological and evolutionary history of each population is unique. Xylella fastidiosa is a xylem-dwelling bacterium infecting multiple plant hosts, often with detrimental effects. As a group, X. fastidiosa is divided into distinct subspecies with allopatric historical distributions and patterns of multiple introductions from numerous source populations. The capacity of X. fastidiosa to successfully colonize and cause disease in naive plant hosts varies among subspecies, and potentially, among populations. Within Central America (i.e. Costa Rica) two X. fastidiosa subspecies coexist: the native subsp. fastidiosa and the introduced subsp. pauca. Using whole genome sequences, the patterns of gene gain/loss, genomic introgression, and genetic diversity were characterized within Costa Rica and contrasted to other X. fastidiosa populations.ResultsWithin Costa Rica, accessory and core genome analyses showed a highly malleable genome with numerous intra- and inter-subspecific gain/loss events. Likewise, variable levels of inter-subspecific introgression were found within and between both coexisting subspecies; nonetheless, the direction of donor/recipient subspecies to the recombinant segments varied. Some strains appeared to recombine more frequently than others; however, no group of genes or gene functions were overrepresented within recombinant segments. Finally, the patterns of genetic diversity of subsp. fastidiosa in Costa Rica were consistent with those of other native populations (i.e. subsp. pauca in Brazil).ConclusionsOverall, this study shows the importance of characterizing local evolutionary and ecological history in the context of world-wide pathogen distribution. | NA | no | No good fitness measure and measure host GD | NA | no | no |
| 4821 | 2020 | 44111 | Castillo, JA; Agathos, SN | 2019 | A genome-wide scan for genes under balancing selection in the plant pathogen Ralstonia solanacearum | BackgroundPlant pathogens are under significant selective pressure by the plant host. Consequently, they are expected to have adapted to this condition or contribute to evading plant defenses. In order to acquire long-term fitness, plant bacterial pathogens are usually forced to maintain advantageous genetic diversity in populations. This strategy ensures that different alleles in the pathogen’s gene pool are maintained in a population at frequencies larger than expected under neutral evolution. This selective process, known as balancing selection, is the subject of this work in the context of a common bacterial phytopathogen. We performed a genome-wide scan of Ralstonia solanacearum species complex, an aggressive plant bacterial pathogen that shows broad host range and causes a devastating disease called bacterial wilt’.ResultsUsing a sliding window approach, we analyzed 57 genomes from three phylotypes of the R. solanacearum species complex to detect signatures of balancing selection. A total of 161 windows showed extreme values in three summary statistics of population genetics: Tajima’s D, (w) and Fu & Li’s D\*. We discarded any confounding effects due to demographic events by means of coalescent simulations of genetic data. The prospective windows correspond to 78 genes with known function that map in any of the two main replicons (1.7% of total number of genes). The candidate genes under balancing selection are related to primary metabolism and other basal activities (51.3%) or directly associated to virulence (48.7%), the latter being involved in key functions targeted to dismantle plant defenses or to participate in critical stages in the pathogenic process.ConclusionsWe identified various genes under balancing selection that play a significant role in basic metabolism as well as in virulence of the R. solanacearum species complex. These genes are useful to understand and monitor the evolution of bacterial pathogen populations and emerge as potential candidates for future treatments to induce specific plant immune responses. | NA | no | gd of pathogen | NA | no | no |
| 4822 | 2020 | 44113 | Castro-Cerritos, KV; Villegas-Negrete, N; Ramirez-Ramirez, N; Robleto, EA; Pedraza-Reyes, M | 2017 | Stationary-phase Mutagenesis Soft-agar Overlay Assays in Bacillus subtilis | Elucidating how a population of non-growing bacteria generates mutations improves our understanding of phenomena like antibiotic resistance, bacterial pathogenesis, genetic diversity and evolution. To evaluate mutations that occur in nutritionally stressed non-growing bacteria, we have employed the strain B. subtilis YB955, which measures the reversions rates to the chromosomal auxotrophies hisC952, metB5 and leuC427 (Sung and Yasbin, 2002). This gain-of-function system has successfully allowed establishing the role played by repair systems and transcriptional factors in stress-associated mutagenesis (SPM) (Barajas-Ornelas et al., 2014; Gomez-Marroquin et al., 2016). In a recent study (Castro-Cerritos et al., 2017), it was found that Ribonucleotide Reductase (RNR) was necessary for SPM; this enzyme is essential in this bacterium. We engineered a conditional mutant of strain B. subtilis YB955 in which expression of the nrdEF operon was modulated by isopropyl-p-D-thiogalactopyranoside (IPTG) (Castro-Cerritos et al., 2017). The conditions to determine mutation frequencies conferring amino acid prototrophy in three genes (hisC952, metB5, leuC427) under nutritional stress in this conditional mutant are detailed here. This technique could be used to evaluate the participation of essential genes in the mutagenic processes occurring in stressed B. subtilis cells. | NA | no | No good fitness measure and measure host GD | NA | no | no |
| 4823 | 2020 | 44113 | Cattelan, S; Di Nisio, A; Pilastro, A | 2018 | Stabilizing selection on sperm number revealed by artificial selection and experimental evolution | Sperm competition is taxonomically widespread in animals and is usually associated with large sperm production, being the number of sperm in the competing pool the prime predictor of fertilization success. Despite the strong postcopulatory selection acting directionally on sperm production, its genetic variance is often very high. This can be explained by trade-offs between sperm production and traits associated with mate acquisition or survival, that may contribute to generate an overall stabilizing selection. To investigate this hypothesis, we first artificially selected male guppies (Poecilia reticulata) for high and low sperm production for three generations, while simultaneously removing sexual selection. Then, we interrupted artificial selection and restored sexual selection. Sperm production responded to divergent selection in one generation, and when we restored sexual selection, both high and low lines converged back to the mean sperm production of the original population within two generations, indicating that sperm number is subject to strong stabilizing total sexual selection (i.e., selection acting simultaneously on all traits associated with reproductive success). We discuss the possible mechanisms responsible for the maintenance of high genetic variability in sperm production despite strong selection acting on it. | NA | no | No good fitness measure and measure host GD | NA | no | no |
| 4824 | 2020 | 44113 | Ceballos, FC; Hazelhurst, S; Ramsay, M | 2018 | Assessing runs of Homozygosity: a comparison of SNP Array and whole genome sequence low coverage data | Background: Runs of Homozygosity (ROH) are genomic regions where identical haplotypes are inherited from each parent. Since their first detection due to technological advances in the late 1990s, ROHs have been shedding light on human population history and deciphering the genetic basis of monogenic and complex traits and diseases. ROH studies have predominantly exploited SNP array data, but are gradually moving to whole genome sequence (WGS) data as it becomes available. WGS data, covering more genetic variability, can add value to ROH studies, but require additional considerations during analysis. Results: Using SNP array and low coverage WGS data from 1885 individuals from 20 world populations, our aims were to compare ROH from the two datasets and to establish software conditions to get comparable results, thus providing guidelines for combining disparate datasets in joint ROH analyses. By allowing heterozygous SNPs per window, using the PLINK homozygosity function and non-parametric analysis, we were able to obtain non-significant differences in number ROH, mean ROH size and total sum of ROH between data sets using the different technologies for almost all populations. Conclusions: By allowing 3 heterozygous SNPs per ROH when dealing with WGS low coverage data, it is possible to establish meaningful comparisons between data using SNP array and WGS low coverage technologies. | NA | no | No good fitness measure and measure host GD | NA | no | no |
| 4825 | 2020 | 44113 | Cecchi, F; Carlini, G; Giuliotti, L; Russo, C | 2018 | Inbreeding may affect phenotypic traits in an Italian population of Basset Hound dogs | The influence of inbreeding depression on phenotypic traits of a Basset Hound dog Italian population was evaluated. Moreover, this paper reports the first results of a survey on morphological traits and analyse the genetic variability of the population using genealogical information. The following traits were studied: height at withers, height of chest, depth of chest, body length, length at rump, ischiatic width of rump, circumference of chest, circumference of cannon, length of ear, and length of nose. The inbreeding coefficient (F) of each dog was computed by genealogical data. Traits were taken from 75 adult (mean age 2.17 +/- 1.545 years) dogs (36 males and 39 females) belonging to 19 different Italian farms. A linear regression of each trait on inbreeding coefficient was used to estimate inbreeding depression. ANOVA was used to test the differences in morphological traits between the sexes, among breeders and among animals with different F level. Despite the good level of genetic variability and the low coefficients of inbreeding observed in the population, a significant weakly effect of inbreeding on the depth of chest was observed. In addition, the present study revealed no significant differences in morphological traits between sexes and among farms. | NA | no | dog | NA | no | no |
| 4826 | 2020 | 44039 | Cecchi, F; Vannucchi, I; Carlini, G; Macchioni, F | 2020 | Inbreeding, phenotypic traits, coat colours and prevalence of health problems in a population of English Cocker Spaniels: the first survey in Italy | This paper presents a survey on inbreeding (F), phenotypic traits, coat colours and prevalence of health problems in a population of English Cocker Spaniels. The aims were to analyse the health and genetic variability of the population and to check whether the population falls within the standards for the breed from a morphological point of view. In addition, we studied the influence of F on phenotypic traits and on the occurrence of various genetic diseases (progressive retinal atrophy, familiar nephropathy, hip dysplasia, patellar luxation and the juvenile renal dysplasia). All data were collected in relation to 83 adult (mean age 4.42 +/- 2.528 years) dogs (20 males and 63 females) born in the period 2011-2018 and belonging to one Italian breeder. The F of each dog and other genealogical parameters were calculated. The prevalence of each highlighted disease within the population was performed. ANOVA was used to test the differences in F values among the animals subdivided into different groups (healthy and un-healthy dogs, coat colour, birth year and sex). Differences between sexes in morphological traits and proportions were also analysed showing sexual dimorphism. The predominant colours were Blue-Roan (30.12%) and Red (26.51%). We found an excellent health status and a good genetic variability of the analysed population. The only disorder that caused concern was juvenile renal dysplasia (26.51%, 95% CI 14.27-38.74%). A relationship was suggested between inbreeding and the disorder as F was significantly higher (P < 0.01) in sick (F = 0.068) than in healthy animals (F = 0.014). | NA | no | Inbreeding | NA | no | no |
| 4827 | 2020 | 44112 | Chabas, H; Lion, S; Nicot, A; Meaden, S; van Houte, S; Moineau, S; Wahl, LM; Westra, ER; Gandon, S | 2018 | Evolutionary emergence of infectious diseases in heterogeneous host populations | The emergence and re-emergence of pathogens remains a major public health concern. Unfortunately, when and where pathogens will (re-)emerge is notoriously difficult to predict, as the erratic nature of those events is reinforced by the stochastic nature of pathogen evolution during the early phase of an epidemic. For instance, mutations allowing pathogens to escape host resistance may boost pathogen spread and promote emergence. Yet, the ecological factors that govern such evolutionary emergence remain elusive because of the lack of ecological realism of current theoretical frameworks and the difficulty of experimentally testing their predictions. Here, we develop a theoretical model to explore the effects of the heterogeneity of the host population on the probability of pathogen emergence, with or without pathogen evolution. We show that evolutionary emergence and the spread of escape mutations in the pathogen population is more likely to occur when the host population contains an intermediate proportion of resistant hosts. We also show that the probability of pathogen emergence rapidly declines with the diversity of resistance in the host population. Experimental tests using lytic bacteriophages infecting their bacterial hosts containing Clustered Regularly Interspaced Short Palindromic Repeat and CRISPR-associated (CRISPR-Cas) immune defenses confirm these theoretical predictions. These results suggest effective strategies for cross-species spillover and for the management of emerging infectious diseases. | NA | no | No good fitness measure and measure host GD | NA | no | no |
| 4828 | 2020 | 44041 | Chaintoutis, SC; Papa, A; Pervanidou, D; Dovas, CI | 2019 | Evolutionary dynamics of lineage 2 West Nile virus in Europe, 2004-2018: Phylogeny, selection pressure and phylogeography | West Nile virus (WNV) is an arbovirus causing neuroinvasive disease to humans and equines. Since 2004, lineage 2 WNV strains have been identified in Europe and have been implicated in severe outbreaks, with that of 2018 exceeding the total number from the previous seven years. The aim of this study was to explore the evolutionary process that shapes the genetic diversity of lineage 2 WNV strains (belonging to the Central European/Hungarian subclade) and reconstruct the origin and transmission routes in Europe, and especially in the Balkans. For this purpose, a high number of whole genome sequences (WGSs) were analyzed, along with newly characterized sequences, including strains from the 2018 WNV transmission season in Greece. Maximum likelihood and Bayesian inference methods were used to perform the phylogenetic and phylodynamic analyses and phylogeographic reconstruction. The majority of the Central European/Hungarian lineage 2 strains are grouped in 2 phylogenetic subgroups (Central/South-West European and Balkan) with bush-like topology. Purifying selection shapes their evolution, however, strong evidence of positive selection was revealed in 7 non-structural protein codons of NS1, NS4B and NS5. Thirty-two amino-acid substitutions were fixed in different phylogenetic subgroups, indicating that random genetic drift is responsible for the majority of evolutionary changes. Virus migration, followed by subsequent local evolution is responsible for continuously evolving strains throughout Europe. In total, 10 virus transitions between discrete geographical locations, involving virus spread from Central Europe to other regions, were highly supported. Three novel, independent introductions from Hungary and Bulgaria were responsible for the 2018 re-emergence of WNV in Northern Greece, indicating that Hungary remains an important ecological niche for the virus and has a central role for the dissemination of novel strains in the Balkans. In Northern Greece, tMRCA estimations indicated that a 1-to 2-year period of silent enzootic transmission precedes spread to dead-end hosts. Reconstruction of WNV population dynamics, from WGS data, revealed epidemic patterns characterized by 3- to 5-year oscillations in Europe. Future studies are necessary to determine the possible driving factors for these fluctuations i.e. avian herd immunity and climatic conditions affecting mosquito and bird populations. Maintaining adequate epidemiological surveillance with emphasis on obtaining WGS data, in areas at risk, is crucial for understanding the epidemiology and transmission patterns of WNV. It can further support integrated programs for risk assessment of virus circulation dynamics, aiming to targeted prevention and response measures for veterinary and public health in Europe. | NA | no | gd of pathogen | NA | no | no |
| 4829 | 2020 | 44040 | Chaiphongpachara, T | 2020 | Comparison of genetic variation in the blow fly, Chrysomya megacephala (Diptera: Calliphoridae), thirteen countries | Chrysomya megacephala (Fabricius, 1794), also called the oriental latrine fly, is a member of the family Calliphoridae and is a medically important blow fly species. C. megacephala has a wide geographical distribution, including Asia, Australasia, and the Palaearctic, Afrotropical, Nearctic, and Neotropical realms. The study of genetic variation in organisms to understand organismal biodiversity is very important to know the genetic structure of populations, one of the basic data required for disease control. In this research, we conducted a study of worldwide genetic variation in the blow fly C. megacephala by comparing mitochondrial DNA sequences (cytochrome c oxidase subunit I [COI]) obtained from the National Center for Biotechnology Information (NCBI) GenBank and the Barcode of Life Database (BOLD). Fifty COI sequences of C. megacephala from 13 countries comprising Australia, Brazil, China, Costa Rica, Ecuador, Egypt, India, Malaysia, Peru, Philippines, Saudi Arabia, Thailand, and the USA were obtained. The results of this analysis revealed that 62.65% of the variation occurred within the populations with statistically significant difference (p< 0.05). Only eight haplotypes of C. megacephala populations comprising four singular and four shared species were detected in this study, indicating that C. megacephala has low genetic diversity. A Pairwise FST analysis showed significant genetic differences between the populations from Malaysia and Philippines, Malaysia and Thailand, the USA and Philippines, and the USA and Thailand. | NA | no | n | NA | no | no |
| 4830 | 2020 | 44111 | Chaisi, ME; Osinubi, ST; Dalton, DL; Suleman, E | 2019 | Occurrence and diversity of avian haemosporidia in Afrotropical landbirds | Avian haemosporidian infections are widespread and can result in the decline of wild bird populations or in some cases contribute to extinction of species. We determined the prevalence and genetic diversity of avian haemosporidia in 93 samples from 22 landbird species from South Africa (N = 76) and West Africa (N = 17), of which six are intra-African migrants and one is a Palearctic migrant. The samples were analysed for the presence of avian haemosporidian DNA using real-time quantitative PCR (qPCR) and nested PCR assays targeting specific mitochondrial genes of these parasites. The cytochrome b (cytb) gene was sequenced for all samples that tested positive and phylogenetic analysis was conducted in order to determine the relationship of the new sequences with previously published sequences from the MalAvi database. The overall prevalence of avian haemosporidiosis was 68.82% (95% CI: 56.4%-78.87%) and 82.80% (95% CI: 65.68%-86.11%) as determined by qPCR and nested PCR respectively. Eighteen (19.36%; 95% CI; 10.78%-29.97%) samples had mixed infections. Infection prevalence of all haemosporidian spp. were significantly higher (p < 0.05) in samples from West Africa. Forty-six mitochondrial sequences obtained from 14 avian species grouped into three distinct clusters of Haemoproteus (36), Leucocytozoon (8) and Plasmodium (2). These represent eight published and nine new cytb lineages. The most common lineage was Haemoproteus sp. (VIMWE1) which was identified in two bird species from West Africa and seven bird species from South Africa. This study adds to our knowledge of host-parasite relationships of avian haemosporidia of Afrotropical birds. | NA | no | no good gd measure host pop | NA | no | no |
| 4831 | 2020 | 44113 | Chakrabarti, P; Sarkar, S; Basu, P | 2018 | Field Populations of Wild Apis cerana Honey Bees Exhibit Increased Genetic Diversity Under Pesticide Stress Along an Agricultural Intensification Gradient in Eastern India | Pesticides have been reported to be one of the major drivers in the global pollinator losses. The large-scale decline in honey bees, an important pollinator group, has resulted in comprehensive studies on honey bee colonies. Lack of information on native wild pollinators has paved the way for this study, which highlights the underlying evolutionary changes occurring in the wild honey bee populations exposed to pesticides along an agricultural intensification landscape. The study reports an increased genetic diversity in native Apis cerana Fabricius (Hymenoptera: Apidae) populations continually exposed to pesticide stress. An increased heterozygosity, evidenced by a higher electrophoretic banding pattern, was observed in the pesticide-exposed populations for two isozymes involved with xenobiotic metabolism-esterase and glucose-6-phosphate dehydrogenase. Differential banding patterns also revealed a higher percentage of polymorphic loci, number of polymorphic bands, Nei’s genetic distance, etc. observed in these populations in the Randomly Amplified Polymorphic DNA-Polymerase Chain Reaction (RAPD-PCR) experiments using three random decamer primers. Higher heterozygosity, being indicative of a more resistant population, implies population survival within the threshold pesticide stress. This study reports such changes for the first time in native wild Indian honey bee populations exposed to pesticides and has far-reaching implications on the population adaptability under pesticide stress. | NA | no | No good fitness measure | NA | no | no |
| 4832 | 2020 | 44111 | Chan, JM; Isik, F | 2019 | Genetic variation in resistance to Uromycladium acaciae fungus, growth, gummosis, and stem form in Acacia mearnsii populations | A new epidemic disease caused by the rust fungus Uromycladium acaciae emerged in 2013/2014 causing defoliation, branch, and stem deformations in plantations of Acacia mearnsii (black wattle) in South Africa. This study investigated the level of genetic resistance to the rust fungus Uromycladium acaciae in six progeny tests from two different trial series. A total of 269 open pollinated families of different levels of improvement, ranging from wild selections to fourth cycle generations were included in the study. Families originated from land race subpopulations of South Africa and from Australian provenances. Rust severity was assessed across all the trials at 6months of age. In the rust screening trials, different measures of rust incidence were assessed at 6, 12, and 17months, as well as tree height and mirid damage at 12months. In the progeny trial series, tree height, stem defects, and gummosis were measured at 36months. Results suggested that rust incidence is under moderate genetic control at the family level. The coefficient of additive genetic variation was large (59.4%) for the disease incidence. The family mean heritability for rust disease was hfm2=0.48 +/- 0.16 in the progeny trial series, whereas it was hfm2=0.33 +/- 0.06 in the rust screening trials. Among the rust assessment methods, 6-month rust incidence (yes/no) assessment produced higher heritability estimates. With some exceptions, Australian provenances from low altitude sites along the eastern coast of Victoria and the south east coast of New South Wales showed high tolerance to rust, better growth, low gummosis incidence but poor stem form. All the South African sources showed high rust susceptibility and had average or below average growth, gummosis incidence, and stem form. For rust disease improvement, the South African material should be avoided and the best existing Australian families should be used for further breeding. In addition, new seed sources from Australia should be infused to the breeding program in order to increase the genetic diversity while increasing genetic gain for the rust, gummosis, and growth. | NA | no | No good fitness measure and measure host GD | NA | no | no |
| 4833 | 2020 | 44112 | Chapman, NC; Cocenza, RD; Blanchard, B; Nguyen, LM; Lim, J; Buchmann, G; Oldroyd, BP | 2019 | Genetic Diversity in the Progeny of Commercial Australian Queen Honey Bees (Hymenoptera: Apidae) Produced in Autumn and Early Spring | Honey bee [Apis mellifera L. (Hymenoptera: Apidae)] queens are polyandrous, mating with an average 12 males (drones). Polyandry has been shown to confer benefits to queens and the colonies they head, including avoidance of inviable brood that can arise via sex locus homozygosity, increased resilience to pests and pathogens, and increased survival and productivity, leading to improved colony-level fitness. Queens with an effective mating frequency (k(e)) greater than 7 are considered adequately mated, whereas queens that fall below this threshold head colonies that have increased likelihood of failure and may be less productive for beekeepers. We determined k(e) in queens produced in early Spring and Autumn by five Australian commercial queen producers to determine whether the queens they produced were suitably mated. Drone populations are low at these times of year, and therefore, there is an increased risk that queens would fall below the k(e) > 7 threshold. We found that 33.8% of Autumn-produced queens did not meet the threshold, whereas 93.8% of Spring queens were adequately mated. The number of colonies contributing drones to the mating pool was similarly high in both seasons, suggesting that although many colonies have drones, their numbers may be decreased in Autumn and management strategies may be required to boost drone numbers at this time. Finally, queens had similar levels of homozygosity to workers, and inbreeding coefficients were very low, suggesting that inbreeding is not a problem. | NA | no | No good fitness measure | NA | no | no |
| 4834 | 2020 | 44113 | Chapuis, E; Lamy, T; Pointier, JP; Juillet, N; Segard, A; Jarne, P; David, P | 2017 | Bioinvasion Triggers Rapid Evolution of Life Histories in Freshwater Snails | Biological invasions offer interesting situations for observing how novel interactions between closely related, formerly allopatric species may trigger phenotypic evolution in situ. Assuming that successful invaders are usually filtered to be competitively dominant, invasive and native species may follow different trajectories. Natives may evolve traits that minimize the negative impact of competition, while trait shifts in invasives should mostly reflect expansion dynamics, through selection for colonization ability and transiently enhanced mutation load at the colonization front. These ideas were tested through a large-scale common-garden experiment measuring life-history traits in two closely related snail species, one invasive and one native, co-occurring in a network of freshwater ponds in Guadeloupe. We looked for evidence of recent evolution by comparing uninvaded or recently invaded sites with long-invaded ones. The native species adopted a life history favoring rapid population growth (i.e., increased fecundity, earlier reproduction, and increased juvenile survival) that may increase its prospects of coexistence with the more competitive invader. We discuss why these effects are more likely to result from genetic change than from maternal effects. The invader exhibited slightly decreased overall performances in recently colonized sites, consistent with a moderate expansion load resulting from local founder effects. Our study highlights a rare example of rapid life-history evolution following invasion. | NA | no | No good fitness measure and measure host GD | NA | no | no |
| 4835 | 2020 | 44111 | Chasse, P; Pelosi, C; Lata, JC; Barot, S | 2019 | Impact of crop genetic diversity on a litter consumer | Belowground communities, and especially earthworms, provide numerous services in agroecosystems. In the current context of crop genetic erosion, this study aims at assessing the effect of wheat cultivar diversity on earthworm growth and survival. Our laboratory experiment was divided into two periods: (1) the feeding period (4 months), simulating conditions in autumn with cool temperatures and high food availability, and (2) the non-feeding period (1 month) simulating conditions in winter with cold temperatures and low food availability because litter had already been consumed without replacement. The aerial biomass of mixtures of wheat cultivars was used as food for earthworms and their growth was measured twice during the feeding period and once during the non-feeding period. We found two major effects on earthworm growth. Increasing the cultivar number (i) increased juvenile earthworm growth and earthworm survival and (ii) decreased adult earthworm growth. A consistent positive effect of nitrogen concentration of the biomass was found on growth and this effect was independent of biodiversity effects. This study suggests that the loss of litter intraspecific genetic diversity has an impact on earthworm populations, probably through mechanisms linked to the variance in edibility or the balanced diet hypotheses. While they remain to be tested in the field, these results suggest that the current losses of crop genetic diversity could potentially impact processes at the community or ecosystem level through a decrease in food quality. (C) 2019 Gesellschaft fur Okologie. Published by Elsevier GmbH. All rights reserved. | NA | no | no good gd measure host pop | NA | no | no |
| 4836 | 2020 | 44113 | Chavarria-Pizarro, L; Silva, M; Noll, FB | 2018 | Neotropical Swarm-Founding Wasps (Vespidae: Polistinae: Epiponini) Accept Expelled Queens in Case of Queen Loss | In the Epiponini, queen number declines through colony cycle, because some queens are expelled from colonies. Here we demonstrate that Epiponini wasps may accept expelled queens in situations of queenlessness. One colony of Protopolybia exigua was observed at the University of Sao Paulo in Brazil; and another of Metapolybia docilis was observed at La Selva Biological Station in Costa Rica. Queen removal tests were performed to study workers’ acceptance of expelled queens and queens from other colonies. In P. exigua, the experimental queen elimination caused a change in the workers’ behavior, ranging from aggressive expulsion of non-selected queens to re-acceptance. In M. docilis workers were willing to accept queens from other colonies after queen elimination. Our results indicate that because of a decrease in workers aggressiveness during the colony cycle, workers may accept expelled queens (even foreign ones, in experimental situations) in order to ensure colony survival. | NA | no | No good fitness measure and measure host GD | NA | no | no |
| 4837 | 2020 | 44112 | Che-Castaldo, J; Che-Castaldo, C; Neel, MC | 2018 | Predictability of demographic rates based on phylogeny and biological similarity | Lack of demographic data for most of the world’s threatened species is a widespread problem that precludes viability-based status assessments for species conservation. A commonly suggested solution is to use data from species that are closely related or biologically similar to the focal species. This approach assumes similar species and populations of the same species have similar demographic rates, an assumption that has yet to be thoroughly tested. We constructed a Bayesian hierarchical model with data on 425 plant species to predict demographic rates (intrinsic rate of population growth, recruit survival, juvenile survival, adult survival, and fecundity) based on biological traits and phylogenetic relatedness. Generally, we found small effects of species-level traits (except woody polycarpic species tended to have high adult survival rates that increased with plant height) and a weak phylogenetic signal for 4 of the 5 demographic parameters examined. Patterns were stronger in adult survival and fecundity than other demographic rates; however, the unexplained variances at both the species and population levels were high for all demographic rates. For species lacking demographic data, our model produced large, often inaccurate, prediction intervals that may not be useful in a management context. Our findings do not support the assumption that biologically similar or closely related species have similar demographic rates and provide further evidence that direct monitoring of focal species and populations is necessary for informing conservation status assessments. | NA | no | No good fitness measure and measure host GD | NA | no | no |
| 4838 | 2020 | 44113 | Cheddadi, R; Henrot, AJ; Francois, L; Boyer, F; Bush, M; Carre, M; Coissac, E; De Oliveira, PE; Ficetola, F; Hambuckers, A; Huang, KY; Lezine, AM; Nourelbait, M; Rhoujjati, A; Taberlet, P; Sarmiento, F; Abel-Schaad, D; Alba-Sanchez, F; Zheng, Z | 2017 | Microrefugia, Climate Change, and Conservation of Cedrus atlantica in the Rif Mountains, Morocco | This study reconstructs and interprets the changing range of Atlas cedar in northern Morocco over the last 9,000 years. A synthesis of fossil pollen records indicated that Atlas cedars occupied a wider range at lower elevations during the mid-Holocene than today. The mid-Holocene geographical expansion reflected low winter temperatures and higher water availability over the whole range of the Rif Mountains relative to modern conditions. A trend of increasing aridity observed after 6,000 years BP progressively reduced the range of Atlas cedar and prompted its migration toward elevations above 1,400 masl. To assess the impact of climate change on cedar populations over the last decades, we performed a transient model simulation for the period between 1960 and 2010. Our simulation showed that the range of Atlas cedar decreased by about 75% over the last 50 years and that the eastern populations of the range in the Rif Mountains were even more threatened by the overall lack of water availability than the western ones. Today, Atlas cedar populations in the Rif Mountains are persisting in restricted and isolated areas (Jbel Kelti, Talassemtane, Jbel Tiziren, Oursane, Tidighine) that we consider to bemodern microrefugia. Conservation of these isolated populations is essential for the future survival of the species, preserving polymorphisms and the potential for population recovery under different climatic conditions. | NA | no | No good fitness measure and measure host GD | NA | no | no |
| 4839 | 2020 | 44112 | Chen, H; Huffman, JE; Brody, JA; Wang, CL; Lee, S; Li, ZL; Gogarten, SM; Sofer, T; Bielak, LF; Bis, JC; Blangero, J; Bowler, RP; Cade, BE; Cho, MH; Correa, A; Curran, JE; de Vries, PS; Glahn, DC; Guo, XQ; Johnson, AD; Kardia, S; Kooperberg, C; Lewis, JP; Liu, XM; Mathias, RA; Mitchell, BD; O’Connell, JR; Peyser, PA; Post, WS; Reiner, AP; Rich, SS; Rotter, JI; Silverman, EK; Smith, JA; Vasan, RS; Wilson, JG; Yanek, LR; Redline, S; Smith, NL; Boerwinkle, E; Borecki, IB; Cupples, LA; Laurie, CC; Morrison, AC; Rice, KM; Lin, XH | 2019 | Efficient Variant Set Mixed Model Association Tests for Continuous and Binary Traits in Large-Scale Whole-Genome Sequencing Studies | With advances in whole-genome sequencing (WGS) technology, more advanced statistical methods for testing genetic association with rare variants are being developed. Methods in which variants are grouped for analysis are also known as variant-set, gene-based, and aggregate unit tests. The burden test and sequence kernel association test (SKAT) are two widely used variant-set tests, which were originally developed for samples of unrelated individuals and later have been extended to family data with known pedigree structures. However, computationally efficient and powerful variant-set tests are needed to make analyses tractable in large-scale WGS studies with complex study samples. In this paper, we propose the variant-set mixed model association tests (SMMAT) for continuous and binary traits using the generalized linear mixed model framework. These tests can be applied to large-scale WGS studies involving samples with population structure and relatedness, such as in the National Heart, Lung, and Blood Institute’s Trans-Omics for Precision Medicine (TOPMed) program. SMMATs share the same null model for different variant sets, and a virtue of this null model, which includes covariates only, is that it needs to be fit only once for all tests in each genome-wide analysis. Simulation studies show that all the proposed SMMATs correctly control type I error rates for both continuous and binary traits in the presence of population structure and relatedness. We also illustrate our tests in a real data example of analysis of plasma fibrinogen levels in the TOPMed program (n = 23,763), using the Analysis Commons, a cloud-based computing platform. | NA | no | No good fitness measure and measure host GD | NA | no | no |
| 4840 | 2020 | 44112 | Chen, HZ; Yang, HF; Yao, KB; Shu, ZL; Zhuang, YQ | 2019 | Molecular Identification of the Gibberella fujikuroi Species Complex Associated with Rice Bakanae and its Management in Jiangsu Province, China | Rice bakanae disease (RBD) caused by Gibberella fujikuroi species complex (GFSC) is a rice (Oryza sativa L.) seed-borne disease worldwide. In this report, 122 strains of Fusarium spp. isolated from RBD-infected rice plants were identified. Phylogenetic analysis based on translation elongation factor 1-alpha (TEF1-alpha) sequences, as well as two-year field trials against RBD were conducted using eight fungicide products-representative products registered by the Institute for the Control of Agrochemicals, Ministry of Agriculture of China (ICAMA). Among the 122 strains, four species of GFSC were identified: F. fujikuroi (representing 74.59%), F. verticillioides (18.03%), F. proliferatum (5.74%) and F. andiyazi (1.64%). Phylogenetic analysis showed broad genetic diversity among the strains, which were clearly grouped into four different clusters. In field trials in 2016 and 2017, the control effect of recommended doses of the eight fungicides were in the following order: penflufen > phenamacril > mixture of fludioxonil and metalaxyl-M > mixture of thiophanate-methyl, metalaxyl, and azoxystrobin > prochloraz > fludioxonil > mixture of carbendazim (MBC) and thiram > hymexazol. Penflufen and phenamacril provided a > 97% control effect. Prochloraz, fludioxonil, and mixture of MBC and thiram, which are representative of about 86% of the relevant products registered by ICAMA, provided a weak control effect. This was the first report of the population structure of GFSC associated with RBD in Jiangsu Province, as well as the second record of F. andiyazi associated with RBD in China. (C) 2019 Friends Science Publishers | NA | no | gd of pathogen | NA | no | no |
| 4841 | 2020 | 44040 | Chen, LX; Xu, ST; Ding, WH; Li, JM; Alpert, P | 2020 | Genetic diversity and offspring fitness in the red and white fruit color morphs of the wild strawberry Fragaria pentaphylla | Aims Fruit color polymorphisms are widespread in plants, but what maintains them is largely unclear. One hypothesis is that some morphs are preferred by dispersers while others have higher pre- or postdispersal fitness. This leads to the prediction that fruit color morphs will differ in pre- or postdispersal fitness. Methods We compared genetic and clonal diversity, mating system, morphological traits that might be associated with resistance to freezing, and germination, survival and seed production of progeny of the red and white fruit morphs in a population of a diploid, wild strawberry, Fragaria pentaphylla, from south-central China. Important Findings The red morph was much more abundant than the white but did not show higher genetic diversity as measured by observed and effective numbers of alleles, Shannon information index, or expected or observed heterozygosities. AMOVA showed that most of the genetic variation in the population was within rather than between morphs. Morphs did not differ in mating system parameters, and no significant biparental inbreeding was found in either morph. Gene flow between two morphs was high (N m = 6.89). Seeds of the red morph germinated about 2 days earlier and had a 40% higher rate of germination than those of the white morph, but survival of seedlings and seed production by surviving offspring did not differ between morphs. The whole postdispersal fitness of the red morph was about two times higher than that of the white morph. Red morphs had hairier petioles but not more surface wax on leaves. Overall, results showed partial evidence for difference in pre- arid postdispersal fitness between fruit color morphs in F. pentaphylla. Differences in fitness independent of dispersal may thus partially account for fruit color polymorphism in all cases. | NA | no | n | NA | no | no |
| 4842 | 2020 | 44112 | Chen, S; Chai, ML; Tian, C; Li, YZ; Deng, T; Wu, H; Liu, XL | 2018 | Genetic variants of fatty acid elongase 6 in Chinese Holstein cow | In the dairy industry, genetic variants have contributed to the improvement of milk production traits. Fatty acid elongase 6 (ELOVL6), which elongates saturated fatty acids (SFAs) and monounsaturated fatty acids (MUFAs), plays a distinct role in the balance of long-chain fatty acids composition in animals. ELOVL6 catalyzes the elongation of palmitic acids (C16:0) which is the most common saturated fatty acid found in animals and also an essential precursor to synthesize other long-chain fatty acids. However, the genetic variant research of bovine ELOVL6 on milk quality traits is still unknown. Therefore, our study aimed to detect the single nucleotide polymorphism (SNPs) of bovine ELOVL6 and explored the relationship between SNPs and milk quality traits including milk yield, fat content, protein content and somatic cell score. In this study, three SNPs, including SNP1 (g16379651A > G), SNP2 (g16458976A > G) and SNP3 (g16511290A > G), have been identified in intron 3 and 3’UTR regions of ELOVL6 in Chinese Holstein (CH) cows. Besides, the results of genetic diversity analysis, linkage disequilibrium and haplotype analysis indicated that these SNPs presented moderate polymorphisms which reflected relatively high genetic diversity. No strong linkage among these SNPs were detected in sampled population of cows. Moreover, the results of correlation analyses demonstrated that these SNPs of bovine ELOVL6 were significantly related to milk yield (P < 0.05). The SNP1 was also correlated with somatic cell score, whereas the SNP3 was associated with fat content. The 21 combined genotypes (diplotypes) were highly significantly correlated (P < 0.01) with milk yield. These results revealed that the genetic variants of bovine ELOVL6 influenced the milk production of CH cows. Hence, the three SNPs could be regarded as molecular markers in marker-assisted selection (MAS) of the dairy cow breeding. | NA | no | No good fitness measure and measure host GD | NA | no | no |
| 4843 | 2020 | 44111 | Chen, WB; Wosula, EN; Hasegawa, DK; Casinga, C; Shirima, RR; Fiaboe, KKM; Hanna, R; Fosto, A; Goergen, G; Tamo, M; Mahuku, G; Murithi, HM; Tripathi, L; Mware, B; Kumar, LP; Ntawuruhunga, P; Moyo, C; Yomeni, M; Boahen, S; Edet, M; Awoyale, W; Wintemantel, WM; Ling, KS; Legg, JP; Fei, ZJ | 2019 | Genome of the African cassava whitefly Bemisia tabaci and distribution and genetic diversity of cassava-colonizing whiteflies in Africa | The whitefy Bemisia tabaci, a species complex consisting of many morphologically indistinguishable species divided into distinct clades, is one of the most globally important agricultural pests and plant virus vectors. Cassava-colonizing B. tabaci transmits viruses that cause cassava mosaic disease (CMD) and cassava brown streak disease (CBSD). Half of all cassava plants in Africa are affected by these viral diseases, resulting in annual production losses of more than US$ 1 billion. Here we report the draft genome of the cassava whitefly B. tabaci Sub-Saharan Africa - East and Central Africa (SSA-ECA), the super-abundant population that has been associated with the rapid spread of viruses causing the pandemics of CMD and CBSD. The SSA-ECA genome assembled from Illumina short reads has a total size of 513.7 Mb and a scaffold N50 length of 497 kb, and contains 15,084 predicted protein-coding genes. Phylogenetic analysis suggests that SSA-ECA diverged from MEAM1 around 5.26 million years ago. A comprehensive genetic analysis of cassava-colonizing B. tabaci in Africa was also conducted, in which a total of 243 whitefly specimens were collected from 18 countries representing all major cassava growing regions in the continent and genotyped using NextRAD sequencing. Population genomic analyses confirmed the existence of six major populations linked by gene flow and inferred the distribution patterns of these populations across the African continent. The genome of SSA-ECA and the genetic findings provide valuable resources and guidance to facilitate whitefly research and the development of strategies to control cassava viral diseases spread by whiteflies. | NA | no | no good gd measure host | NA | no | no |
| 4844 | 2020 | 44040 | Chen, Y; Chen, YH; Pouillot, R; Dennis, S; Xian, ZH; Luchansky, JB; Porto-Fett, ACS; Lindsay, JA; Hammack, TS; Allard, M; Van Doren, JM; Brown, EW | 2020 | Genetic diversity and profiles of genes associated with virulence and stress resistance among isolates from the 2010-2013 interagency Listeria monocytogenes market basket survey | Whole genome sequencing (WGS) was performed on 201 Listeria monocytogenes isolates recovered from 102 of 27,389 refrigerated ready-to-eat (RTE) food samples purchased at retail in U.S. FoodNet sites as part of the 2010-2013 interagency L. monocytogenes Market Basket Survey (Lm MBS). Core genome multi-locus sequence typing (cgMLST) and in-silico analyses were conducted, and these data were analyzed with metadata for isolates from five food groups: produce, seafood, dairy, meat, and combination foods. Six of 201 isolates, from 3 samples, were subsequently confirmed as L. welshimeri. Three samples contained one isolate per sample; mmong the 96 samples that contained two isolates per sample, 3 samples each contained two different strains and 93 samples each contained duplicate isolates. After 93 duplicate isolates were removed, the remaining 102 isolates were delineated into 29 clonal complexes (CCs) or singletons based on their sequence type. The five most prevalent CCs were CC155, CC1, CC5, CC87, and CC321. The Shannon’s diversity index for clones per food group ranged from 1.49 for dairy to 2.32 for produce isolates, which were not significantly different in pairwise comparisons. The most common molecular serogroup as determined by in-silico analysis was IIa (45.6%), followed by IIb (27.2%), IVb (20.4%), and IIc (4.9%). The proportions of isolates within lineages I, II, and III were 48.0%, 50.0% and 2.0%, respectively. Full-length inlA was present in 89.3% of isolates. Listeria pathogenicity island 3 (LIPI-3) and LIPI-4 were found in 51% and 30.6% of lineage I isolates, respectively. Stress survival islet 1 (SSI-1) was present in 34.7% of lineage I isolates, 80.4% of lineage II isolates and the 2 lineage III isolates; SSI-2 was present only in the CC121 isolate. Plasmids were found in 48% of isolates, including 24.5% of lineage I isolates and 72.5% of lineage II isolates. Among the plasmid-carrying isolates, 100% contained at least one cadmium resistance cassette and 89.8% contained bcrABC, involved in quaternary ammonium compound tolerance. Multiple clusters of isolates from different food samples were identified by cgMLST which, along with available metadata, could aid in the investigation of possible cross-contamination and persistence events. | NA | no | No good fitness measure and measure host GD | NA | no | no |
| 4845 | 2020 | 44113 | Cheng, CW; Putaporntip, C; Jongwutiwes, S | 2018 | Polymorphism in merozoite surface protein-7E of Plasmodium vivax in Thailand: Natural selection related to protein secondary structure | Merozoite surface protein 7 (MSP-7) is a multigene family expressed during malaria blood-stage infection. MSP-7 forms complex with MSP-1 prior to merozoite egress from erythrocytes, and could affect merozoite invasion of erythrocytes. To characterize sequence variation in the orthologue in P. vivax (PvMSP-7), a gene member encoding PvMSP-7E was analyzed among 92 Thai isolates collected from 3 major endemic areas of Thailand (Northwest: Tak, North-east: Ubon Ratchathani, and South: Yala and Narathiwat provinces). In total, 52 distinct haplotypes were found to circulate in these areas. Although population structure based on this locus was observed between each endemic area, no genetic differentiation occurred between populations collected from different periods in the same endemic area, suggesting spatial but not temporal genetic variation. Sequence microhetero-geneity in both N- and C-terminal regions was predicted to display 4 and 6 alpha-helical domains, respectively. Signals of purifying selection were observed in alpha-helices II-X, suggesting structural or functional constraint in these domains. By contrast, alpha-helix-I spanning the putative signal peptide was under positive selection, in which amino acid substitutions could alter predicted CD4+ T helper cell epitopes. The central region of PvMSP-7E comprised the 5’-trimorphic and the 3’-dimorphic subregions. Positive selection was identified in the 3’ dimorphic subregion of the central domain. A consensus of intrinsically unstructured or disordered protein was predicted to encompass the entire central domain that contained a number of putative B cell epitopes and putative protein binding regions. Evidences of intra-genic recombination were more common in the central region than the remainders of the gene. These results suggest that the extent of sequence variation, recombination events and selective pressures in the PvMSP-7E locus seem to be differentially affected by protein secondary structure. | NA | no | No good fitness measure and measure host GD | NA | no | no |
| 4846 | 2020 | 44113 | Cheng, YY; Polkinghorne, A; Gillett, A; Jones, EA; O’Meally, D; Timms, P; Belov, K | 2018 | Characterisation of MHC class I genes in the koala | Koala (Phascolarctos cinereus) populations are on the decline across the majority of Australia’s mainland. Two major diseases threatening the long-term survival of affected koala populations are caused by obligate intracellular pathogens: Chlamydia and koala retrovirus (KoRV). To improve our understanding of the koala immune system, we characterised their major histocompatibility complex (MHC) class I genes, which are centrally involved in presenting foreign peptides derived from intracellular pathogens to cytotoxic T cells. A total of 11 class I genes were identified in the koala genome. Three genes, Phci-UA, UB and UC, showed relatively high genetic variability and were expressed in all 12 examined tissues, whereas the other eight genes had tissue-specific expression and limited polymorphism. Evidence of diversifying selection was detected in Phci-UA and UC, while gene conversion may have played a role in creating new alleles at Phci-UB. We propose that Phci-UA, UB and UC are likely classical MHC genes of koalas, and further research is needed to understand their role in koala chlamydial and KoRV infections. | NA | no | mhc diversity | NA | no | no |
| 4847 | 2020 | 44112 | Chien, CC; Chou, MY; Chen, CY; Shih, MC | 2019 | Analysis of genetic diversity of Xanthomonas oryzae pv. oryzae populations in Taiwan | Rice bacterial blight caused by Xanthomonas oryzae pv. oryzae (Xoo) is a major rice disease. In Taiwan, the tropical indica type of Oryza sativa originally grown in this area is mix-cultivated with the temperate japonica type of O. sativa, and this might have led to adaptive changes of both rice host and Xoo isolates. In order to better understand how Xoo adapts to this unique environment, we collected and analyzed fifty-one Xoo isolates in Taiwan. Three different genetic marker systems consistently identified five groups. Among these groups, two of them had unique sequences in the last acquired ten spacers in the clustered regularly interspaced short palindromic repeats (CRISPR) region, and the other two had sequences that were similar to the Japanese isolate MAFF311018 and the Philippines isolate PXO563, respectively. The genomes of two Taiwanese isolates with unique CRISPR sequence features, XF89b and XM9, were further completely sequenced. Comparison of the genome sequences suggested that XF89b is phylogenetically close to MAFF311018, and XM9 is close to PXO563. Here, documentation of the diversity of groups of Xoo in Taiwan provides evidence of the populations from different sources and hitherto missing information regarding distribution of Xoo populations in East Asia. | NA | no | gd of pathogen | NA | no | no |
| 4848 | 2020 | 44112 | Chinn, SM; Monson, DH; Tinker, MT; Staedler, MM; Crocker, DE | 2018 | Lactation and resource limitation affect stress responses, thyroid hormones, immune function, and antioxidant capacity of sea otters (Enhydra lutris) | Lactation is the most energetically demanding stage of reproduction in female mammals. Increased energetic allocation toward current reproduction may result in fitness costs, although the mechanisms underlying these trade-offs are not well understood. Trade-offs during lactation may include reduced energetic allocation to cellular maintenance, immune response, and survival and may be influenced by resource limitation. As the smallest marine mammal, sea otters (Enhydra lutris) have the highest mass-specific metabolic rate necessitating substantial energetic requirements for survival. To provide the increased energy needed for lactation, female sea otters significantly increase foraging effort, especially during late-lactation. Caloric insufficiency during lactation is reflected in the high numbers of maternal deaths due to End-Lactation Syndrome in the California subpopulation. We investigated the effects of lactation and resource limitation on maternal stress responses, metabolic regulation, immune function, and antioxidant capacity in two subspecies of wild sea otters (northern: E.l.nereis and southern: E.l.kenyoni) within the California, Washington, and Alaska subpopulations. Lactation and resource limitation were associated with reduced glucocorticoid responses to acute capture stress. Corticosterone release was lower in lactating otters. Cortisol release was lower under resource limitation and suppression during lactation was only evident under resource limitation. Lactation and resource limitation were associated with alterations in thyroid hormones. Immune responses and total antioxidant capacity were not reduced by lactation or resource limitation. Southern sea otters exhibited higher concentrations of antioxidants, immunoglobulins, and thyroid hormones than northern sea otters. These data provide evidence for allocation trade-offs during reproduction and in response to nutrient limitation but suggest self-maintenance of immune function and antioxidant defenses despite energetic constraints. Income-breeding strategists may be especially vulnerable to the consequences of stress and modulation of thyroid function when food resources are insufficient to support successful reproduction and may come at a cost to survival, and thereby influence population trends. | NA | no | No good fitness measure and measure host GD | NA | no | no |
| 4849 | 2020 | 44111 | Chislock, MF; Kaul, RB; Durham, KA; Sarnelle, O; Wilson, AE | 2019 | Eutrophication mediates rapid clonal evolution in Daphnia pulicaria | Laboratory studies have revealed that Daphnia species can evolve to tolerate toxic cyanobacteria in the diet. Specifically, Daphnia from eutrophic lakes where cyanobacteria are common tend to have higher growth rates and survival when fed toxic cyanobacteria than populations from oligotrophic environments with low abundance of cyanobacteria. We conducted an in-lake mesocosm (i.e. limnocorral) experiment during the autumn of 2009 to assess the effects of nutrient enrichment on clonal evolution in Daphnia pulicaria. As nutrient enrichment often favours grazing-resistant cyanobacteria, we hypothesised that fertilisation would influence the genotypic composition of D. pulicaria that vary in tolerance to cyanobacteria. Mesocosms were fertilised to manipulate phytoplankton and cyanobacterial abundance and concentrations of a cyanobacterial toxin (microcystin). Thus, half of the mesocosms were high-nutrient and half were low-nutrient. We then stocked half of the mesocosms with a mixture of six genetically-distinct D. pulicaria genotypes (three genotypes from oligotrophic lakes and three from eutrophic lakes) leaving half of the mesocosms Daphnia-free to assess grazing effects, using a fully factorial design. When compared to the low nutrient treatment, high nutrient mesocosms had nearly five-fold higher chlorophyll a concentrations, eight-fold higher cyanobacterial dry biomass, and three-fold higher microcystin levels at the start of the experiment. In contrast, low nutrient mesocosms had phytoplankton concentrations typical of mesotrophic lakes. Fertilisation strongly affected Daphnia genetic diversity in the mesocosms. Final Daphnia genotype diversity in the mesocosms with low-cyanobacteria (richness = 5.83, Shannon-Weiner index = 1.55, evenness = 0.88) was similar to the initial stocked diversity (richness = 5.50, Shannon-Weiner index = 1.48, evenness = 0.87). In contrast, final diversity in fertilised mesocosms with high cyanobacteria was greatly reduced (richness = 2, Shannon-Weiner index = 0.17), with one Daphnia genotype that originated from the most-eutrophic lake being highly dominant (evenness = 0.25). Thus, eutrophication mediated strong clonal selection of a cyanobacteria-tolerant Daphnia genotype over just 10 weeks. By the end of the experiment, Daphnia significantly reduced phytoplankton biomass in the high-nutrient, but not in the low-nutrient treatment. This difference in effect size was largely driven by the five-fold higher initial phytoplankton biomass in the high-nutrient treatment. Thus, the ability of Daphnia to reduce phytoplankton biomass in eutrophic lakes may be driven more so by the abundance of planktivorous fishes, as opposed to the prevalence of cyanobacteria and their associated toxins. | NA | no | No good fitness measure | NA | no | no |
| 4850 | 2020 | 44040 | Choi, YJ; Fontenla, S; Fischer, PU; Le, TH; Costabile, A; Blair, D; Brindley, PJ; Tort, JF; Cabada, MM; Mitreva, M | 2020 | Adaptive Radiation of the Flukes of the Family Fasciolidae Inferred from Genome-Wide Comparisons of Key Species | Liver and intestinal flukes of the family Fasciolidae cause zoonotic food-borne infections that impact both agriculture and human health throughout the world. Their evolutionary history and the genetic basis underlying their phenotypic and ecological diversity are not well understood. To close that knowledge gap, we compared the whole genomes of Fasciola hepatica, Fasciola gigantica, and Fasciolopsis buski and determined that the split between Fasciolopsis and Fasciola took place similar to 90 Ma in the late Cretaceous period, and that between 65 and 50 Ma an intermediate host switch and a shift from intestinal to hepatic habitats occurred in the Fasciola lineage. The rapid climatic and ecological changes occurring during this period may have contributed to the adaptive radiation of these flukes. Expansion of cathepsins, fatty-acid-binding proteins, protein disulfide-isomerases, and molecular chaperones in the genus Fasciola highlights the significance of excretory-secretory proteins in these liver-dwelling flukes. Fasciola hepatica and Fasciola gigantica diverged similar to 5 Ma near the Miocene-Pliocene boundary that coincides with reduced faunal exchange between Africa and Eurasia. Severe decrease in the effective population size similar to 10ka in Fasciola is consistent with a founder effect associated with its recent global spread through ruminant domestication. G-protein-coupled receptors may have key roles in adaptation of physiology and behavior to new ecological niches. This study has provided novel insights about the genome evolution of these important pathogens, has generated genomic resources to enable development of improved interventions and diagnosis, and has laid a solid foundation for genomic epidemiology to trace drug resistance and to aid surveillance. | NA | no | No good fitness measure and measure host GD | NA | no | no |
| 4851 | 2020 | 44113 | Chong, JP; Roe, KJ | 2018 | A comparison of genetic diversity and population structure of the endangered scaleshell mussel (Leptodea leptodon), the fragile papershell (Leptodea fragilis) and their host-fish the freshwater drum (Aplodinotus grunniens) | The larvae of freshwater mussels in the order Unionoida are obligate parasites on fishes. Because adult mussels are infaunal and largely sessile, it is generally assumed that the majority of gene flow among mussel populations relies on the dispersal of larvae by their hosts. The objective of this study was to compare the genetic diversity and the degree of congruence between the population structures of two related freshwater mussels Leptodea leptodon and Leptodea fragilis and their fish host, Aplodinotus grunniens. Host specificity in parasites has been shown to result in greater congruence between the population structures of the two interacting species, and assessing the congruence of genetic structure of the endangered L. leptodon with its sister species L. fragilis and their sole host is an important step in understanding the impact of host dispersal on population structure. Analysis of microsatellite data indicated that despite its imperiled status, L. leptodon displayed greater genetic diversity than the more common L. fragilis. However, the population structures of all three species were incongruent even in the presence of substantial gene flow. Other factors such as habitat specificity may play a role in generating the differences in population structure observed. This study indicates that barriers to gene flow or lack of available host fish are not the cause of decline of the federally endangered L. leptodon, and suggests that alternative explanations should be considered. | NA | no | No good fitness measure | NA | no | no |
| 4852 | 2020 | 44113 | Choudhury, A; Ramsay, M; Hazelhurst, S; Aron, S; Bardien, S; Botha, G; Chimusa, ER; Christoffels, A; Gamieldien, J; Sefid-Dashti, MJ; Joubert, F; Meintjes, A; Mulder, N; Ramesar, R; Rees, J; Scholtz, K; Sengupta, D; Soodyall, H; Venter, P; Warnich, L; Pepper, MS | 2017 | Whole-genome sequencing for an enhanced understanding of genetic variation among South Africans | The Southern African Human Genome Programme is a national initiative that aspires to unlock the unique genetic character of southern African populations for a better understanding of human genetic diversity. In this pilot study the Southern African Human Genome Programme characterizes the genomes of 24 individuals (8 Coloured and 16 black southeastern Bantu-speakers) using deep whole-genome sequencing. A total of similar to 16 million unique variants are identified. Despite the shallow time depth since divergence between the two main southeastern Bantu-speaking groups (Nguni and Sotho-Tswana), principal component analysis and structure analysis reveal significant (p < 10(-6)) differentiation, and FST analysis identifies regions with high divergence. The Coloured individuals show evidence of varying proportions of admixture with Khoesan, Bantu-speakers, Europeans, and populations from the Indian sub-continent. Whole-genome sequencing data reveal extensive genomic diversity, increasing our understanding of the complex and region-specific history of African populations and highlighting its potential impact on biomedical research and genetic susceptibility to disease. | NA | no | human | NA | no | no |
| 4853 | 2020 | 44113 | Christie, MR; Searle, CL | 2018 | Evolutionary rescue in a host-pathogen system results in coexistence not clearance | The evolutionary rescue of host populations may prevent extinction from novel pathogens. However, the conditions that facilitate rapid evolution of hosts, in particular the population variation in host susceptibility, and the effects of host evolution in response to pathogens on population outcomes remain largely unknown. We constructed an individual-based model to determine the relationships between genetic variation in host susceptibility and population persistence in an amphibian-fungal pathogen (Batrachochytrium dendrobatidis) system. We found that host populations can rapidly evolve reduced susceptibility to a novel pathogen and that this rapid evolution led to a 71-fold increase in the likelihood of host-pathogen coexistence. However, the increased rates of coexistence came at a cost to host populations; fewer populations cleared infection, population sizes were depressed, and neutral genetic diversity was lost. Larger adult host population sizes and greater adaptive genetic variation prior to the onset of pathogen introduction led to substantially reduced rates of extinction, suggesting that populations with these characteristics should be prioritized for conservation when species are threatened by novel infectious diseases. | NA | no | No good fitness measure and measure host GD | NA | no | no |
| 4854 | 2020 | 44043 | Chu, CC; Hoffmann, M; Braswell, WE; Pelz-Stelinski, KS | 2019 | Genetic variation and potential coinfection of Wolbachia among widespread Asian citrus psyllid (Diaphorina citri Kuwayama) populations | Wolbachia can profoundly influence the survival, reproduction, and defenses of insect hosts. These interactions could potentially be harnessed for managing pests or insect-transmitted diseases. Diaphorina citri Kuwayama is a phloem-feeding pest capable of transmitting the putative causal agent of citrus greening, Candidatus Liberibacter asiaticus (CLas). Like many insects, D. citri is also infected with Wolbachia (wDi). Recent studies indicate that the relative abundance of wDi could be associated with the abundance of CLas, and that wDi may contribute to regulating expression of phage lytic cycle genes in CLas, suggesting the need for better understanding of wDi biology in general. This study investigated the genetic diversity of wDi among D. citri in populations spanning eleven countries and two U.S. territories. Six Wolbachia genes, wsp, coxA, fbpA, ftsZ, gatB, and hcpA, were sequenced and compared across samples. Two prevalent wDi strains were identified across the samples, and screening of clone libraries revealed possible coinfection of wDi strains in specific populations. D. citri mitochondrial cytochrome oxidase subunit I gene (mtCOI) were more divergent between D. citri populations that were infected with different wDi strains or had different infection statuses (single infection vs. coinfection). While we could not eliminate the possibility that maternal transmission may contribute to such patterns, it is also possible that wDi may induce cytoplasmic incompatibility in their host. These findings should contribute to the understanding of wDi population ecology, which may facilitate manipulation of this endosymbiont for management of citrus greening disease worldwide. | NA | no | No good fitness measure | NA | no | no |
| 4855 | 2020 | 44113 | Chung, CL; Lee, TJ; Akiba, M; Lee, HH; Kuo, TH; Liu, D; Ke, HM; Yokoi, T; Roa, MB; Lu, MYJ; Chang, YY; Ann, PJ; Tsai, JN; Chen, CY; Tzean, SS; Ota, Y; Hattori, T; Sahashi, N; Liou, RF; Kikuchi, T; Tsai, IJ | 2017 | Comparative and population genomic landscape of Phellinus noxius: A hypervariable fungus causing root rot in trees | The order Hymenochaetales of white rot fungi contain some of the most aggressive wood decayers causing tree deaths around the world. Despite their ecological importance and the impact of diseases they cause, little is known about the evolution and transmission patterns of these pathogens. Here, we sequenced and undertook comparative genomic analyses of Hymenochaetales genomes using brown root rot fungus Phellinus noxius, wood-decomposing fungus Phellinus lamaensis, laminated root rot fungus Phellinus sulphurascens and trunk pathogen Porodaedalea pini. Many gene families of lignin-degrading enzymes were identified from these fungi, reflecting their ability as white rot fungi. Comparing against distant fungi highlighted the expansion of 1,3-beta-glucan synthases in P.noxius, which may account for its fast-growing attribute. We identified 13 linkage groups conserved within Agaricomycetes, suggesting the evolution of stable karyotypes. We determined that P.noxius has a bipolar heterothallic mating system, with unusual highly expanded similar to 60kb A locus as a result of accumulating gene transposition. We investigated the population genomics of 60 P.noxius isolates across multiple islands of the Asia Pacific region. Whole-genome sequencing showed this multinucleate species contains abundant poly-allelic single nucleotide polymorphisms with atypical allele frequencies. Different patterns of intra-isolate polymorphism reflect mono-/heterokaryotic states which are both prevalent in nature. We have shown two genetically separated lineages with one spanning across many islands despite the geographical barriers. Both populations possess extraordinary genetic diversity and show contrasting evolutionary scenarios. These results provide a framework to further investigate the genetic basis underlying the fitness and virulence of white rot fungi. | NA | no | No good fitness measure and measure host GD | NA | no | no |
| 4856 | 2020 | 44113 | Ciancaleoni, S; Raggi, L; Negri, V | 2018 | Assessment of spatial-temporal variation in natural populations of Brassica incana in south Italy: implications for conservation | Brassica incana is a secondary-gene pool wild relative of Brassica oleracea. Twenty-two B. incana populations are recorded in Italy, where the species has recently been pointed out as in priority need of conservation. While data on the spatial and temporal variation of B. incana Italian populations are completely lacking, this information is useful in conservation planning for this species. Three populations from the Sorrento peninsula and from the islands of Ischia and Capri, collected in 1984 and 2012, were characterised for 12 morpho-phenological and 21 genetic traits to assess their spatial-temporal variation. The populations were quite different for morpho-phenological and genetic traits. Spatial differentiation was high and easily explained by the isolation. Temporal differentiation between the Sorrento and Ischia populations was high and explained by a reduction in the population census across time, while it was not significant between the two Capri accessions as such pointing to a major effect of genetic drift. Numerical dimension is extremely relevant in evaluating conservation priorities since it has a major impact on population dynamics over time. The Sorrento and Ischia populations are under threat and urgently need conservation actions, suggesting an alarming scenario for the survival of other crop wild relative populations which are similar in census. Our data also show that, in an allogamous and self-incompatible species like B. incana, populations of 100-200 individuals maintain high allelic diversity. According to obtained results, natural populations of species with similar reproductive system and census can be considered at low risk of genetic erosion. | NA | no | No good fitness measure | NA | no | no |
| 4857 | 2020 | 44113 | Cicala, F; Moore, JD; Caceres-Martinez, J; Del Rio-Portilla, MA; Hernandez-Rodriguez, M; Vasquez-Yeomans, R; Rocha-Olivares, A | 2018 | Monomorphic pathogens: The case of Candidatus Xenohaliotis californiensis from abalone in California, USA and Baja California, Mexico | Withering syndrome (WS) is a chronic wasting disease affecting abalone species attributed to the pathogen Candidatus Xenohaliotis califomiensis (CXc). Wild populations of blue (Haliotis fulgens) and yellow (H. corrugata) abalone have experienced unusual mortality rates since 2009 off the peninsula of Baja California and WS has been hypothesized as a possible cause. Currently, little information is available about the genetic diversity of CXc and particularly the possible existence of strains differing in pathogenicity. In a recent phylogenetic analysis, we characterized five coding genes from this rickettsial pathogen. Here, we analyze those genes and two additional intergenic non-coding regions following multi-locus sequence typing (MLST) and multi-spacer typing (MST) approaches to assess the genetic variability of CXc and its relationship with blue, yellow and red (H. rufescens) abalone. Moreover, we used 16S rRNA pyrosequencing reads from gut microbiomes of blue and yellow abalone to complete the genetic characterization of this prokaryote. The presence of CXc was investigated in more than 150 abalone of the three species; furthermore, a total of 385 DNA sequences and 7117 16S rRNA reads from Candidatus Xenohaliotis califomiensis were used to evaluate its population genetic structure. Our findings suggest the absence of polymorphism in the DNA sequences of analyzed loci and the presence of a single lineage of CXc infecting abalone from California (USA) and Baja California (Mexico). We posit that the absence of genetic variably in this marine rickettsia may be the result of evolutionary and ecological processes. | NA | no | No good fitness measure | NA | no | no |
| 4858 | 2020 | 44042 | Cirak, C; Radusiene, J | 2019 | Factors affecting the variation of bioactive compounds in Hypericum species | The genus Hypericum (Hypericaceae) consists of 484 species from 36 sections with worldwide distribution in different areas. Turkey is considered as hot spot for diversity of Hypericum genus. Despite numerous publications, Hypericum species still attracted considerable scientific interest due to pharmaceutically relevant secondary metabolites: naphthodianthrones, acylphloroglucinol derivatives, phenolic acids, flavonoid glycosides, biflavonoids, and some other valuable constituents. Phytochemical investigations carried out on different Hypericum species provided highly heterogeneous results. The content of bioactive compounds varies significantly due to many internal and external factors, including plant organs, phenological stage, genetic profile, environmental abiotic and biotic factors, such as growing site, light, temperature, radiation, soil drought and salinity, pathogens, and herbivores attack. The variations in content of bioactive compounds in plants are regarded as the main problem in the standardization of Hypericum-derived pharmaceuticals and dietary supplements. The review discusses the main factors contributing to the variations of bioactive compounds and what kind of modulations can increase quality of Hypericum raw material. | NA | no | No good fitness measure and measure host GD | NA | no | no |
| 4859 | 2020 | 44113 | Clark, CN; Nicholas, JM; Agustus, JL; Hardy, CJD; Russell, LL; Brotherhood, EV; Dick, KM; Marshall, CR; Mummery, CJ; Rohrer, JD; Warren, JD | 2017 | Auditory conflict and congruence in frontotemporal dementia | Impaired analysis of signal conflict and congruence may contribute to diverse socio-emotional symptoms in frontotemporal dementias, however the underlying mechanisms have not been defined. Here we addressed this issue in patients with behavioural variant frontotemporal dementia (bvFTD; n = 19) and semantic dementia (SD; n = 10) relative to healthy older individuals (n = 20). We created auditory scenes in which semantic and emotional congruity of constituent sounds were independently probed; associated tasks controlled for auditory perceptual similarity, scene parsing and semantic competence. Neuroanatomical correlates of auditory congruity processing were assessed using voxel-based morphometry. Relative to healthy controls, both the bvFTD and SD groups had impaired semantic and emotional congruity processing (after taking auditory control task performance into account) and reduced affective integration of sounds into scenes. Grey matter correlates of auditory semantic congruity processing were identified in distributed regions encompassing prefrontal, parieto-temporal and insular areas and correlates of auditory emotional congruity in partly overlapping temporal, insular and striatal regions. Our findings suggest that decoding of auditory signal relatedness may probe a generic cognitive mechanism and neural architecture underpinning frontotemporal dementia syndromes. | NA | no | No good fitness measure and measure host GD | NA | no | no |
| 4860 | 2020 | 44112 | Cleary, D; Szalanski, AL; Trammel, C; Williams, MK; Tripodi, A; Downey, D | 2018 | MITOCHONDRIAL DNA VARIATION OF FERAL HONEY BEES (APIS MELLIFERA L.) FROM UTAH (USA) | A study was conducted on the mitochondria! DNA genetic diversity of feral colonies and swarms of Apis mellifera from ten counties in Utah by sequencing the intergenic region of the cytochrome oxidase (COI-COII) gene region. A total of 20 haplotypes were found from 174 honey bee colony samples collected from 2008 to 2017. Samples belonged to the A (African) (48%); C (Eastern Europe) (43%); M (Western Europe) (4%); and 0 (Oriental) lineages (5%). Ten African A lineage haplotypes were observed with two unique to Utah among A lineage haplotypes recorded in the US. Haplotypes belonging to the A lineage were observed from six Utah counties located in the southern portion of the State, from elevations as high as 1357 m. All five C lineage haplotypes that were found have been observed from queen breeders in the US. Three haplotypes of the M lineage (n=7) and two of the 0 lineage (n=9) were also observed. This study provides evidence that honey bees of African descent are both common and diverse in wild populations of honey bees in southern Utah. The high levels of genetic diversity of A lineage honey bee colonies in Utah provide evidence that the lineage may have been established in Utah before the introduction of A lineage honey bees from Brazil to Texas in 1990. | NA | no | No good fitness measure | NA | no | no |
| 4861 | 2020 | 44113 | Clifton, EH; Jaronski, ST; Coates, BS; Hodgson, EW; Gassmann, AJ | 2018 | Effects of endophytic entomopathogenic fungi on soybean aphid and identification of Metarhizium isolates from agricultural fields | Terrestrial plants can harbor endophytic fungi that may induce changes in plant physiology that in turn affect interactions with herbivorous insects. We evaluated whether the application of entomopathogenic fungi Beauveria bassiana and Metarhizium brunneum to soybean seeds could become endophytic and affect interactions with soybean aphid (Aphis glycines Matsumura). It was found that A. glycines population sizes increased on plants with M. brunneum (strain F52) seed inoculum, but no significant effects were shown with analogous treatments with B. bassiana (strain GHA). Fungi recovered from soybean plant tissues indicate that endophytism was established, and that B. bassiana was more prevalent. Metarhizium brunneum was only recovered from stems, but B. bassiana was recovered from stems and leaves. This work confirms that some entomopathogenic fungi can be endophytic in soybean, however, some of these fungi may have a negative effect on the plants by increasing susceptibility of soybean to A. glycines. We also used DNA sequence data to identify species of Metarhizium obtained from agricultural fields in Iowa. Phylogenetic analyses, based on DNA sequence data, found that all isolates were Metarhizium robertsii, which is consistent with past studies indicating a cosmopolitan distribution and wide host range for this species. These results are important for understanding the dynamics of implementing environmentally sustainable measures for the control of pest insects. | NA | no | dometicated host | NA | no | no |
| 4862 | 2020 | 44112 | Clulow, J; Upton, R; Trudeau, VL; Clulow, S | 2019 | Amphibian Assisted Reproductive Technologies: Moving from Technology to Application | Amphibians have experienced a catastrophic decline since the 1980s driven by disease, habitat loss, and impacts of invasive species and face ongoing threats from climate change. About 40% of extant amphibians are under threat of extinction and about 200 species have disappeared completely. Reproductive technologies and biobanking of cryopreserved materials offer technologies that could increase the efficiency and effectiveness of conservation programs involving management of captive breeding and wild populations through reduced costs, better genetic management and reduced risk of species extinctions. However, there are relatively few examples of applications of these technologies in practice in on-the-ground conservation programs, and no example that we know of where genetic diversity has been restored to a threatened amphibian species in captive breeding or in wild populations using cryopreserved genetic material. This gap in the application of technology to conservation programs needs to be addressed if assisted reproductive technologies (ARTs) and biobanking are to realise their potential in amphibian conservation. We review successful technologies including non-invasive gamete collection, IVF and sperm cryopreservation that work well enough to be applied to many current conservation programs. We consider new advances in technology (vitrification and laser warming) of cryopreservation of aquatic embryos of fish and some marine invertebrates that may help us to overcome factors limiting amphibian oocyte and embryo cryopreservation. Finally, we address two case studies that illustrate the urgent need and the opportunity to implement immediately ARTs, cryopreservation and biobanking to amphibian conservation. These are (1) managing the biosecurity (disease risk) of the frogs of New Guinea which are currently free of chytridiomycosis, but are at high risk (2) the Sehuencas water frog of Bolivia, which until recently had only one known surviving male. | NA | no | No good fitness measure and measure host GD | NA | no | no |
| 4863 | 2020 | 44112 | Cobben, MMP; van Noordwijk, AJ | 2017 | Consequences of the genetic threshold model for observing partial migration under climate change scenarios | Migration is a widespread phenomenon across the animal kingdom as a response to seasonality in environmental conditions. Partially migratory populations are populations that consist of both migratory and residential individuals. Such populations are very common, yet their stability has long been debated. The inheritance of migratory activity is currently best described by the threshold model of quantitative genetics. The inclusion of such a genetic threshold model for migratory behavior leads to a stable zone in time and space of partially migratory populations under a wide range of demographic parameter values, when assuming stable environmental conditions and unlimited genetic diversity. Migratory species are expected to be particularly sensitive to global warming, as arrival at the breeding grounds might be increasingly mistimed as a result of the uncoupling of long-used cues and actual environmental conditions, with decreasing reproduction as a consequence. Here, we investigate the consequences for migratory behavior and the stability of partially migratory populations under five climate change scenarios and the assumption of a genetic threshold value for migratory behavior in an individual-based model. The results show a spatially and temporally stable zone of partially migratory populations after different lengths of time in all scenarios. In the scenarios in which the species expands its range from a particular set of starting populations, the genetic diversity and location at initialization determine the species’ colonization speed across the zone of partial migration and therefore across the entire landscape. Abruptly changing environmental conditions after model initialization never caused a qualitative change in phenotype distributions, or complete extinction. This suggests that climate change-induced shifts in species’ ranges as well as changes in survival probabilities and reproductive success can be met with flexibility in migratory behavior at the species level, which will reduce the risk of extinction. | NA | no | No good fitness measure and measure host GD | NA | no | no |
| 4864 | 2020 | 44111 | Cognato, AI; Smith, SM; Li, Y; Pham, TH; Hulcr, J | 2019 | Genetic Variability Among Xyleborus glabratus Populations Native to Southeast Asia (Coleoptera: Curculionidae: Scolytinae: Xyleborini) and the Description of Two Related Species | The redbay ambrosia beetle, Xyleborus glabratus Eichhoff, is native to Southeast Asia, where it specializes on Lauraceae trees. It forms a symbiosis with the ambrosia fungus Raffaelea lauricola T.C. Harr., Fraedrich & Aghayeva, which can act as a pathogen in living host trees. The beetle and fungus were recently introduced into the United States, where they have killed millions of native Lauraceae trees and threaten the avocado industry. These introduced populations have limited genetic variation. In the native range, the fungi are genetically variable, but the native genetic variability of the beetles is unknown. It is important to assess the beetle’s native genetic variation because different lineages may vary in the capacity to vector this fungus, which may affect disease etiology. Here, we analyzed genetic variation in several Chinese, Taiwanese, and Vietnamese populations of X. glabratus using mitochondrial (COI) and nuclear DNA (CAD) markers. Phylogenetic analysis revealed nine COI haplotypes and four CAD genotypes. Uncorrected ‘p’ distance for intrapopulation comparisons ranged from 0 to 0.1 and 0 to 0.013 and interpopulation comparisons ranged from 0.137 to 0.168 and 0.015 to 0.032 for COI and CAD, respectively. Two populations exceeded the range of intraspecific nucleotide differences for both genes. Given that individuals from these populations also exhibited consistent morphological differences, they are described as two new species: Xyleborus insidiosus Cognato & Smith, n. sp. and Xyleborus mysticulus Cognato & Smith, n. sp. Xyleborus glabratus was redescribed and a lectotype was designated to facilitate its recognition in light of these new species. These results indicate that X. glabratus is genetically variable and is related to two morphologically similar species. Whether these new species and X. glabratus lineages associate with different fungal strains is unknown. Given that the biology and host colonization of these new species are unknown, preventing their introduction to other regions is prudent. | NA | no | No good fitness measure | NA | no | no |
| 4865 | 2020 | 44113 | Coker, SM; Hernandez, SM; Kistler, WM; Curry, SE; Welch, CN; Barron, HW; Harsch, S; Murray, MH; Yabsley, MJ | 2017 | Diversity and prevalence of hemoparasites of wading birds in southern Florida, USA | Relatively few studies on hemoparasites have been conducted on wading birds in the families Ardeidae and Threskiornithidae (order Pelecaniformes), especially in the United States. In this study, we obtained baseline data on the prevalence and genetic diversity of haemosporidian parasites in wading birds opportunistically sampled from southern Florida, USA. We detected blood parasites in White Ibis (Eudocimus albus), Glossy Ibis (Plegadis falcinellus), Green Heron (Butorides virescens), and Roseate Spoonbill (Platalea ajaja) with several novel host-parasite relationships. Infected birds had low parasitemias (average 0.77%, range 0-4%) suggesting that infections were chronic. Despite the low sample sizes for several of our sampled species, these data highlight the diversity of parasites in this understudied group of birds and suggest that additional studies are needed to investigate the potential impacts of these parasites on their health, especially since southern Florida is becoming increasingly urbanized which can alter parasite transmission or host susceptibility. (C) 2017 The Authors. Published by Elsevier Ltd on behalf of Australian Society for Parasitology. | NA | no | no good gd measure host pop | NA | no | no |
| 4866 | 2020 | 44113 | Coller, KE; Butler, EK; Luk, KC; Rodgers, MA; Cassidy, M; Gersch, J; McNamara, AL; Kuhns, MC; Dawson, GJ; Kaptue, L; Bremer, B; Wedemeyer, H; Cloherty, GA | 2018 | Development and performance of prototype serologic and molecular tests for hepatitis delta infection | Worldwide, an estimated 5% of hepatitis B virus (HBV) infected people are coinfected with hepatitis delta virus (HDV). HDV infection leads to increased mortality over HBV mono-infection, yet HDV diagnostics are not widely available. Prototype molecular (RNA) and serologic (IgG) assays were developed for high-throughput testing on the Abbott m2000 and ARCHITECT systems, respectively. RNA detection was achieved through amplification of a ribozyme region target, with a limit of detection of 5 IU/ml. The prototype serology assay (IgG) was developed using peptides derived from HDV large antigen (HDAg), and linear epitopes were further identified by peptide scan. Specificity of an HBV negative population was 100% for both assays. A panel of 145 HBsAg positive samples from Cameroon with unknown HDV status was tested using both assays: 16 (11.0%) had detectable HDV RNA, and 23 (15.7%) were sero-positive including the 16 HDV RNA positive samples. Additionally, an archival serial bleed panel from an HDV superinfected chimpanzee was tested with both prototypes; data was consistent with historic testing data using a commercial total anti-Delta test. Overall, the two prototype assays provide sensitive and specific methods for HDV detection using high throughput automated platforms, allowing opportunity for improved diagnosis of HDV infected patients. | NA | no | No good fitness measure and measure host GD | NA | no | no |
| 4867 | 2020 | 44039 | Collis, RM; Biggs, PJ; Midwinter, AC; Browne, AS; Wilkinson, DA; Irshad, H; French, NP; Brightwell, G; Cookson, AL | 2020 | Genomic epidemiology and carbon metabolism ofEscherichia coliserogroup O145 reflect contrasting phylogenies | Shiga toxin-producingEscherichia coli(STEC) are a leading cause of foodborne outbreaks of human disease, but they reside harmlessly as an asymptomatic commensal in the ruminant gut. STEC serogroup O145 are difficult to isolate as routine diagnostic methods are unable to distinguish non-O157 serogroups due to their heterogeneous metabolic characteristics, resulting in under-reporting which is likely to conceal their true prevalence. In light of these deficiencies, the purpose of this study was a twofold approach to investigate enhanced STEC O145 diagnostic culture-based methods: firstly, to use a genomic epidemiology approach to understand the genetic diversity and population structure of serogroup O145 at both a local (New Zealand) (n = 47) and global scale (n = 75) and, secondly, to identify metabolic characteristics that will help the development of a differential media for this serogroup. Analysis of a subset ofE.coliserogroup O145 strains demonstrated considerable diversity in carbon utilisation, which varied in association witheaesubtype and sequence type. Several carbon substrates, such as D-serine and D-malic acid, were utilised by the majority of serogroup O145 strains, which, when coupled with current molecular and culture-based methods, could aid in the identification of presumptiveE.coliserogroup O145 isolates. These carbon substrates warrant subsequent testing with additional serogroup O145 strains and non-O145 strains. Serogroup O145 strains displayed extensive genetic heterogeneity that was correlated with sequence type andeaesubtype, suggesting these genetic markers are good indicators for distinctE.coliphylogenetic lineages. Pangenome analysis identified a core of 3,036 genes and an open pangenome of >14,000 genes, which is consistent with the identification of distinct phylogenetic lineages. Overall, this study highlighted the phenotypic and genotypic heterogeneity withinE.coliserogroup O145, suggesting that the development of a differential media targeting this serogroup will be challenging. | NA | no | No good fitness measure and measure host GD | NA | no | no |
| 4868 | 2020 | 44113 | Conlon, BH; Frey, E; Rosenkranz, P; Locke, B; Moritz, RFA; Routtu, J | 2018 | The role of epistatic interactions underpinning resistance to parasitic Varroa mites in haploid honey bee (Apis mellifera) drones | The Red Queen hypothesis predicts that host-parasite coevolutionary dynamics can select for host resistance through increased genetic diversity, recombination and evolutionary rates. However, in haplodiploid organisms such as the honeybee (Apis mellifera), models suggest the selective pressure is weaker than in diploids. Haplodiploid sex determination, found in A.mellifera, can allow deleterious recessive alleles to persist in the population through the diploid sex with negative effects predominantly expressed in the haploid sex. To overcome these negative effects in haploid genomes, epistatic interactions have been hypothesized to play an important role. Here, we use the interaction between A.mellifera and the parasitic mite Varroa destructor to test epistasis in the expression of resistance, through the inhibition of parasite reproduction, in haploid drones. We find novel loci on three chromosomes which explain over 45% of the resistance phenotype. Two of these loci interact only additively, suggesting their expression is independent of each other, but both loci interact epistatically with the third locus. With drone offspring inheriting only one copy of the queen’s chromosomes, the drones will only possess one of two queen alleles throughout the years-long lifetime of the honeybee colony. Varroa, in comparison, completes its highly inbred reproductive cycle in a matter of weeks, allowing it to rapidly evolve resistance. Faced with the rapidly evolving Varroa, a diversity of pathways and epistatic interactions for the inhibition of Varroa reproduction could therefore provide a selective advantage to the high levels of recombination seen in A.mellifera. This allows for the remixing of phenotypes despite a fixed queen genotype. | NA | no | No good fitness measure and measure host GD | NA | no | no |
| 4869 | 2020 | 44112 | Connallon, T; Chenoweth, SF | 2019 | Dominance reversals and the maintenance of genetic variation for fitness | Antagonistic selection between different fitness components (e.g., survival versus fertility) or different types of individuals in a population (e.g., females versus males) can potentially maintain genetic diversity and thereby account for the high levels of fitness variation observed in natural populations. However, the degree to which antagonistic selection can maintain genetic variation critically depends on the dominance relations between antagonistically selected alleles in diploid individuals. Conditions for stable polymorphism of antagonistically selected alleles are narrow, particularly when selection is weak, unless the alleles exhibit dominance reversals-in which each allele is partially or completely dominant in selective contexts in which it is favored and recessive in contexts in which it is harmful. Although theory predicts that dominance reversals should emerge under biologically plausible conditions, evidence for dominance reversals is sparse. In this primer, we review theoretical arguments and data supporting a role for dominance reversals in the maintenance of genetic variation. We then highlight an illuminating new study by Grieshop and Arnqvist, which reports a genome-wide signal of dominance reversals between male and female fitness in seed beetles. | NA | no | No good fitness measure and measure host GD | NA | no | no |
| 4870 | 2020 | 44111 | Corbi-Botto, CM; Morales-Durand, H; Zappa, ME; Sadaba, SA; Peral-Garcia, P; Giovambattista, G; Diaz, S | 2019 | Genomic structural diversity in Criollo Argentino horses: Analysis of copy number variations | Copy number variation (CNV) has been proved to be widespread in human, animal and plant genomes. Together with single nucleotide polymorphisms (SNPs), CVNs play a key role in genetic diversity. In this study, genome-wide detection of CNVs was performed based on SNP data from 24 Criollo Argentino horses genotyped with the GGP Equine70k array. Overall, 165 CNVs meeting stringent quality control criteria were identified and then aggregated into 87 CNV regions (CNVRs), representing a horse genome coverage of 13.69 Mb. Functional analysis of CNVRs allowed the identification of 337 genes implicated in a wide range of biological functions such as signal transducer activity (olfactory receptors), receptor activities and binding. Furthermore, enrichment analysis showed that the most represented protein classes (over 25%) were immunoglobulin receptor subfamily, immunoglobulins and major histocompatibility complex antigen (beta-2-microglobulin). To the best of our knowledge, this is the first report of CNV in Criollo Argentino horses. | NA | no | No good fitness measure and measure host GD | NA | no | no |
| 4871 | 2020 | 44041 | Cordero, AIH; Gonzales, NM; Parker, CC; Sokolof, G; Vandenbergh, DJ; Cheng, RY; Abney, M; Sko, A; Douglas, A; Palmer, AA; Gregory, JS; Lionikas, A | 2019 | Genome-wide Associations Reveal Human-Mouse Genetic Convergence and Modifiers of Myogenesis, CPNE1 and STC2 | Muscle bulk in adult healthy humans is highly variable even after height, age, and sex are accounted for. Low muscle mass, due to fewer and/or smaller constituent muscle fibers, would exacerbate the impact of muscle loss occurring in aging or disease. Genetic variability substantially influences muscle mass differences, but causative genes remain largely unknown. In a genome-wide association study (GWAS) on appendicular lean mass (ALM) in a population of 85,750 middle-aged (aged 38-49 years) individuals from the UK Biobank (UKB), we found 182 loci associated with ALM (p < 5 x 10(-8)). We replicated associations for 78% of these loci (p < 5 x 10(-8)) with ALM in a population of 181,862 elderly (aged 60-74 years) individuals from UKB. We also conducted a GWAS on hindlimb skeletal muscle mass of 1,867 mice from an advanced intercross between two inbred strains (LG/J and SM/J); this GWAS identified 23 quantitative trait loci. Thirty-eight positional candidates distributed across five loci overlapped between the two species. In vitro studies of positional candidates confirmed CPNE1 and STC2 as modifiers of myogenesis. Collectively, these findings shed light on the genetics of muscle mass variability in humans and identify targets for the development of interventions for treatment of muscle loss. The overlapping results between humans and the mouse model GWAS point to shared genetic mechanisms across species. | NA | no | human | NA | no | no |
| 4872 | 2020 | 44040 | Cornuault, JK; Moncaut, E; Loux, V; Mathieu, A; Sokol, H; Petit, MA; De Paepe, M | 2020 | The enemy from within: a prophage of Roseburia intestinalis systematically turns lytic in the mouse gut, driving bacterial adaptation by CRISPR spacer acquisition | Despite an overall temporal stability in time of the human gut microbiota at the phylum level, strong variations in species abundance have been observed. We are far from a clear understanding of what promotes or disrupts the stability of microbiome communities. Environmental factors, like food or antibiotic use, modify the gut microbiota composition, but their overall impacts remain relatively low. Phages, the viruses that infect bacteria, might constitute important factors explaining temporal variations in species abundance. Gut bacteria harbour numerous prophages, or dormant viruses, which can evolve to become ultravirulent phage mutants, potentially leading to important bacterial death. Whether such phenomenon occurs in the mammal’s microbiota has been largely unexplored. Here we studied temperate phage-bacteria coevolution in gnotoxenic mice colonised with Roseburia intestinalis, a dominant symbiont of the human gut microbiota, and Escherichia coli, a sub-dominant member of the same microbiota. We show that R. intestinalis L1-82 harbours two active prophages, Jekyll and Shimadzu. We observed the systematic evolution in mice of ultravirulent Shimadzu phage mutants, which led to a collapse of R. intestinalis population. In a second step, phage infection drove the fast counter-evolution of host phage resistance mainly through phage-derived spacer acquisition in a clustered regularly interspaced short palindromic repeats array. Alternatively, phage resistance was conferred by a prophage originating from an ultravirulent phage with a restored ability to lysogenize. Our results demonstrate that prophages are a potential source of ultravirulent phages that can successfully infect most of the susceptible bacteria. This suggests that prophages can play important roles in the short-term temporal variations observed in the composition of the gut microbiota. | NA | no | No good fitness measure and measure host GD | NA | no | no |
| 4873 | 2020 | 44113 | Cornwall, DH; Kubinak, JL; Zachary, E; Stark, DL; Seipel, D; Potts, WK | 2018 | Experimental manipulation of population-level MHC diversity controls pathogen virulence evolution in Mus musculus | The virulence levels attained by serial passage of pathogens through similar host genotypes are much higher than observed in natural systems; however, it is unknown what keeps natural virulence levels below these empirically demonstrated maximum levels. One hypothesis suggests that host diversity impedes pathogen virulence, because adaptation to one host genotype carries trade-offs in the ability to replicate and cause disease in other host genotypes. To test this hypothesis, with the simplest level of population diversity within the loci of the major histocompatibility complex (MHC), we serially passaged Friend virus complex (FVC) through two rounds, in hosts with either the same MHC genotypes (pure passage) or hosts with different MHC genotypes (alternated passage). Alternated passages showed a significant overall reduction in viral titre (31%) and virulence (54%) when compared to pure passages. Furthermore, a resistant host genotype initially dominated any effects due to MHC diversity; however, when FVC was allowed to adapt to the resistant host genotype, predicted MHC effects emerged; that is, alternated lines show reduced virulence. These data indicate serial exposure to diverse MHC genotypes is an impediment to pathogen adaptation, suggesting genetic variation at MHC loci is important for limiting virulence in a rapidly evolving pathogen and supports negative frequency-dependent selection as a force maintaining MHC diversity in host populations. | NA | no | mhc diversity | NA | no | no |
| 4874 | 2020 | 44113 | Corona, E; Wang, LY; Ko, D; Patel, CJ | 2018 | Systematic detection of positive selection in the human-pathogen interactome and lasting effects on infectious disease susceptibility | Infectious disease has shaped the natural genetic diversity of humans throughout the world. A new approach to capture positive selection driven by pathogens would provide information regarding pathogen exposure in distinct human populations and the constantly evolving arms race between host and disease-causing agents. We created a human pathogen interaction database and used the integrated haplotype score (iHS) to detect recent positive selection in genes that interact with proteins from 26 different pathogens. We used the Human Genome Diversity Panel to identify specific populations harboring pathogen-interacting genes that have undergone positive selection. We found that human genes that interact with 9 pathogen species show evidence of recent positive selection. These pathogens are Yersenia pestis, human immunodeficiency virus (HIV) 1, Zaire ebolavirus, Francisella tularensis, dengue virus, human respiratory syncytial virus, measles virus, Rubella virus, and Bacillus anthracis. For HIV-1, GWAS demonstrate that some naturally selected variants in the host-pathogen protein interaction networks continue to have functional consequences for susceptibility to these pathogens. We show that selected human genes were enriched for HIV susceptibility variants (identified through GWAS), providing further support for the hypothesis that ancient humans were exposed to lentivirus pandemics. Human genes in the Italian, Miao, and Biaka Pygmy populations that interact with Y. pestis show significant signs of selection. These results reveal some of the genetic footprints created by pathogens in the human genome that may have left lasting marks on susceptibility to infectious disease. | NA | no | hiv | NA | no | no |
| 4875 | 2020 | 44042 | Correa-Macedo, W; Cambri, G; Schurr, E | 2019 | The Interplay of Human and Mycobacterium Tuberculosis Genomic Variability | Tuberculosis (TB), caused by the human pathogens Mycobacterium tuberculosis (Mtb) and Mycobacterium africanum, has plagued humanity for millennia and remains the deadliest infectious disease in the modern world. Mycobacterium tuberculosis and M. africanum can be subdivided phylogenetically into seven lineages exhibiting a low but significant degree of genomic diversity and preferential geographic distributions. Human genetic variability impacts all stages of TB pathogenesis ranging from susceptibility to infection with Mtb, progression of infection to disease, and the development of distinct clinical subtypes. The genetic study of severe childhood TB identified strong inborn single-gene errors revealing crucial pathways of vulnerability to TB. However, the identification of major TB-susceptibility genes on the population level has remained elusive. In particular, the replication of findings from candidate and genome-wide association studies across distinct human populations has proven difficult, thus hampering the characterization of reliable host molecular markers of susceptibility. Among the possible confounding factors of genetic association studies is Mtb genomic variability, which generally was not taken into account by human genetic studies. In support of this possibility, Mtb lineage was found to be a contributing factor to clinical presentation of TB and epidemiological spread of Mtb in exposed populations. The confluence of pathogen and human host genetic variability to TB pathogenesis led to the consideration of a possible coadaptation of Mtb strains and their human hosts, which should reveal itself in significant interaction effects between Mtb strain and TB-susceptibility/resistance alleles. Here, we present some of the most consistent findings of genetic susceptibility factors in human TB and review studies that point to genome-to-genome interaction between humans and Mtb lineages. The limited results available so far suggest that analyses considering joint human-Mtb genomic variability may provide improved power for the discovery of pathogenic drivers of the ongoing TB epidemic. | NA | no | human | NA | no | no |
| 4876 | 2020 | 44113 | Correa, F; Stoore, C; Horlacher, P; Jimenez, M; Hidalgo, C; Alvarez Rojas, CA; Barros, GF; Ferreira, HB; Hernandez, M; Cabrera, G; Paredes, R | 2018 | First description of Echinococcus ortleppi and cystic echinococcosis infection status in Chile | Cystic echinococcosis (CE), a parasitic disease caused by the cestode Echinococcus granulosus sensu lato (s.l.), is a worldwide zoonotic infection. Although endemic in Chile, information on the molecular characteristics of CE in livestock remains scarce. Therefore we aimed to describe the status of infection with E. granulosus s.l. in cattle from central Chile and also to contribute to the study of the molecular epidemiology of this parasite. According to our results, the prevalence of CE is 18.84% in cattle, similar to previous reports from Chile, suggesting that the prevalence in Santiago Metropolitan area has not changed in the last 30 years. Most of the cysts were found only in lungs (51%), followed by concurrent infection in liver and lungs (30%), and only liver (19%). Molecular characterization of the genetic diversity and population structure of E. granulosus s.l. from cattle in central Chile was performed using a section of the cytochrome c oxidase subunit 1 (cox1) mitochondrial gene. E. granulosus sensu stricto (s.s.) (G1-G3 genotypes) was confirmed by RFLP-PCR to be the dominant species affecting cattle (284 samples/290 samples); we also report for the first time in Chile the presence of E. ortleppi (G5 genotype) (2 samples/61 samples). The Chilean E. granulosus s.s. parsimony network displayed 1 main haplotype. Additional studies using isolates from many locations across Chile and different intermediate hosts will provide more data on the molecular structure of E. granulosus s.s. within this region. Likewise, investigations of the importance of E. ortleppi in human infection in Chile deserve future attention. | NA | no | No good fitness measure and measure host GD | NA | no | no |
| 4877 | 2020 | 44112 | Correia, M; Campoy, A; Madeira, C; Andrade, JP | 2018 | Is filament clipping an effective tool for tissue sampling in Hippocampus guttulatus? | Examining genetic diversity, population structure, and geographic distribution has become an important part in the conservation strategies of endangered populations. However, these studies rely on tissue samples collection for DNA analysis which may be problematic for species with a sensitive conservation status. Partial fin-clipping has been employed for tissue collection, due to the increased popularity of DNA-based analysis for ecological and fisheries studies, however, fin clipping can potentially affect behavior, swimming performance, predator avoidance and the ability to find and capture prey. This study aimed to test the effects of filament clipping using captive breed seahorses (Hippocampus guttulatus) as an alternative tool for tissue sample. Clipped filaments regrew on average 0.05 +/- 0.02 mm/day for 3 months and no mortality or disease was observed during the experiment. Filaments provided enough tissue for DNA analysis. This study provided valuable information regarding a new sampling technique that does not impair the seahorse locomotion. This methodology may be used in population’s genetic studies of other species that have skin filaments as a morphological trait. | NA | no | No good fitness measure and measure host GD | NA | no | no |
| 4878 | 2020 | 44112 | Cortes, EDJ; Sieck, MAC; Perea, AJA; Medrano, RMM; Jaime, AB; Martinez, PE; Zuniga, J; Alonzo, VA; Granados, J; Barquera, R | 2018 | Genetic Differentiation in a Sample from Northern Mexico City Detected by HLA System Analysis: Impact in the Study of Population Immunogenetics | The major histocompatibility complex is directly involved in the immune response, and thus the genes coding for its proteins are useful markers for the study of genetic diversity, susceptibility to disease (autoimmunity and infections), transplant medicine, and pharmacogenetics, among others. The polymorphism of the system also allows researchers to use it as a proxy for population genetics analysis, such as genetic admixture and genetic structure. In order to determine the immunogenetic characteristics of a sample from the northern part of Mexico City and to use them to analyze the genetic diffferentiation from other admixed populations, including those from previous studies of Mexico City population, we analyzed molecular typing results of donors and patients from the Histocompatibility Laboratory of the Central Blood Bank of the Centro Medico Nacional La Raza selected according to their geographic origin. HLA-A, -B, -DRB1, and -DQB1 alleles were typed by polymerase chain reaction with sequence-specific primers. Allelic and haplotype frequencies, as well as population genetics parameters, were obtained by maximum likelihood methods. The most frequent haplotypes found were HLA-A*02/-B*39/-DRB1*04/-DQB1*03:02P, HLA-A*02/-B*35/-DRB1*04/-DQB1*03:02P, HLA-A*68/-B*39/-DRB1*04/-DQB1*03:02P, and HLA-A*02/-B*35/-DRB1*08/-DQB1*04. Importantly, the second most frequent haplotype found in our sample (HLA-A*02/-B*35/-DRB1*04/-DQB1*03:02P) has not been previously reported in any mixed-ancestry populations from Mexico but is commonly encountered in Native American human groups, which can reflect the impact of migration dynamics in the genetic conformation of the northern part of Mexico City, and the limitations of previous studies with regard to the genetic diversity of the analyzed groups. Diffferences found in haplotype frequencies demonstrated that large urban conglomerates cannot be analyzed as one homogeneous entity but, rather, should be understood as a set of structures in which social, political, and economical factors influence their genesis and dynamics. | NA | no | mhc diversity | NA | no | no |
| 4879 | 2020 | 44113 | Costa, EN; Nogueira, L; de Souza, BHS; Ribeiro, ZA; Louvandini, H; Zukoff, SN; Boica, AL | 2018 | Characterization of Antibiosis to Diabrotica speciosa (Coleoptera: Chrysomelidae) in Brazilian Maize Landraces | Resistance to insect pests can be found in several native, landrace plants and can be an important alternative to conventional control methods. Diabrotica speciosa (Germar) (Coleoptera: Chrysomelidae) larvae are important maize (Zea mays L.) (Cyperales: Poaceae) root pests and finding native resistance in landraces would greatly contribute to maize-breeding programs aimed at controlling this pest. This study investigated whether the growth, survival, oviposition rhythm, fecundity, and fertility of D. speciosa are negatively influenced by specific maize landraces, and the existence of any morphological barriers in the roots that may correlate with plant resistance to the larval attack. Nineteen genotypes (17 landraces and 2 cultivars) were screened for antibiosis in assays that were conducted in the laboratory using seedling maize plants where the development time, longevity, weight, total survival, and sex ratio of adults were evaluated. Out of nineteen genotypes, eight were selected according to their resistance levels for an additional rearing study evaluating oviposition and fecundity. Landrace Perola and cultivar SCS 154-Fortuna were classified as resistant because they increased the maturation period from larva to adult and decreased survivorship; and the landrace Palha Roxa was also classified as resistant for showing a lower fertility rate than other landraces. Resistant landraces that were infested by D. speciosa larvae showed greater amounts of some morphological barriers comparing with uninfested plants. The landraces classified as resistant may be considered in future plant-breeding programs, aiming to develop resistant maize cultivars to D. speciosa larval attack. | NA | no | No good fitness measure and measure host GD | NA | no | no |
| 4880 | 2020 | 44112 | Costa, SS; Sobkowiak, B; Parreira, R; Edgeworth, JD; Viveiros, M; Clark, TG; Couto, I | 2019 | Genetic Diversity of norA, Coding for a Main Efflux Pump of Staphylococcus aureus | NorA is the best studied efflux system of Staphylococcus aureus and therefore frequently used as a model for investigating efflux-mediated resistance in this pathogen. NorA activity is associated with resistance to fluoroquinolones, several antiseptics and disinfectants and several reports have pointed out the role of efflux systems, including NorA, as a first-line response to antimicrobials in S. aureus. Genetic diversity studies of the gene norA have described three alleles; norAI, norAII and norAIII. However, the epidemiology of these alleles and their impact on NorA activity remains unclear. Additionally, increasing studies do not account for norA variability when establishing relations between resistance phenotypes and norA presence or reported absence, which actually corresponds, as we now demonstrate, to different norA alleles. In the present study we assessed the variability of the norA gene present in the genome of over 1,000 S. aureus isolates, corresponding to 112 S. aureus strains with whole genome sequences publicly available; 917 MRSA strains sourced from a London-based study and nine MRSA isolates collected in a major Hospital in Lisbon, Portugal. Our analyses show that norA is part of the core genome of S. aureus. It also suggests that occurrence of norA variants reflects the population structure of this major pathogen. Overall, this work highlights the ubiquitous nature of norA in S. aureus which must be taken into account when studying the role played by this important determinant on S. aureus resistance to antimicrobials. | NA | no | No good fitness measure and measure host GD | NA | no | no |
| 4881 | 2020 | 44113 | Costantini, F; Rugiu, L; Cerrano, C; Abbiati, M | 2018 | Living upside down: patterns of red coral settlement in a cave | Background, Larval settlement and intra-specific interactions during the recruitment phase are crucial in determining the distribution and density of sessile marine populations Marine caves are confined and stable habitats. As such, they provide a natural laboratory to study the settlement and recruitment processes in sessile invertebrates, including the valuable Mediterranean red coral Corallium rubrum, In the present study, the spatial and temporal variability of red coral settlers in an underwater cave was investigated by demographic and genetic approaches. Methods. Sixteen PVC tiles were positioned on the walls and ceiling of the Colombara Cave, Ligurian Sea, and recovered after twenty months. A total of 372 individuals of red coral belonging to two different reproductive events were recorded. Basal diameter, height, and number of polyps were measured, land seven microsatellites loci were used to evaluate the genetic relationships among individuals and the genetic structure. Results. Significant differences in the colonization rate were observed both between the two temporal cohorts and between ceiling and walls. No genetic structuring was observed between cohorts. Overall, high levels of relatedness among individuals were found. Conclusion. The results show that C. rubrum individuals on tiles are highly related at very small spatial scales, suggesting that nearby recruits are likely to be sibs. Self-recruitment and the synchronous settlement of clouds of larvae could be possible explanations for the observed pattern. | NA | no | No good fitness measure and measure host GD | NA | no | no |
| 4882 | 2020 | 44040 | Covo, S | 2020 | Genomic Instability in Fungal Plant Pathogens | Fungi and fungal-like organisms (oomycetes) that cause diseases in plants have impacted human communities for centuries and probably from the dawn of agriculture. In modern agriculture, there is a constant race between new strategies to manage fungal plant pathogens and their ability to adapt. An important component in this race is fungal genetic diversity. Mechanisms such as sexual and parasexual recombination that contribute to the creation of novel allele combinations in fungal plant pathogens are briefly discussed in the first part of this review. Advances in genomics have enabled the investigation of chromosomal aberrations of agriculturally important fungal isolates at the nucleotide level. Some of these cases are summarized in the second part of this review; it is claimed that the effect of chromosomal aberrations on pathogenicity should be studied mechanistically. More data on the effect of gene copy number variations on phenotypes that are relevant to agriculture are especially needed. Genome rearrangements through translocations have shaped the genome of fungal plant pathogens by creating lineage-specific chromosome territories encoding for genes participating in plant diseases. Pathogenicity chromosomes are unique cases of such lineage-specific genetic elements, interestingly these chromosomes can be transferred horizontally and thus transforming a non-pathogenic strain to a pathogenic one. The third part of this review describes our attempts to reveal mutators in fungal plant pathogens by identifying fungi that lack important DNA repair genes or respond to DNA damage in an unconventional way. We found that a group of fungal plant pathogens lack conserved genes that are needed for an important Holliday junction resolution pathway. In addition, in Fusarium oxysporum, the rate-limiting step in dNTP production is not induced under DNA replication stress. This is very different from organisms from bacteria to humans. It remains to be seen if these mechanisms promote genetic instability in fungal plant pathogens. | NA | no | No good fitness measure and measure host GD | NA | no | no |
| 4883 | 2020 | 44112 | Cowell, AN; Valdivia, HO; Bishop, DK; Winzeler, EA | 2018 | Exploration of Plasmodium vivax transmission dynamics and recurrent infections in the Peruvian Amazon using whole genome sequencing | Background: Plasmodium vivax poses a significant challenge to malaria elimination due to its ability to cause relapsed infections from reactivation of dormant liver parasites called hypnozoites. We analyzed 69 P. vivax whole genome sequences obtained from subjects residing in three different villages along the Peruvian Amazon. This included 23 paired P. vivax samples from subjects who experienced recurrent P. vivax parasitemia following observed treatment with chloroquine and primaquine. Methods: Genomic DNA was extracted from whole blood samples collected from subjects. P. vivax DNA was enriched using selective whole genome amplification and whole genome sequencing. We used single nucleotide polymorphisms (SNPs) from the core P. vivax genome to determine characteristics of the parasite population using discriminant analysis of principal components, maximum likelihood estimation of individual ancestries, and phylogenetic analysis. We estimated the relatedness of the paired samples by calculating the number of segregating sites and using a hidden Markov model approach to estimate identity by descent. Results: We present a comprehensive dataset of population genetics of Plasmodium vivax in the Peruvian Amazonian. We define the parasite population structure in this region and demonstrate a novel method for distinguishing homologous relapses from reinfections or heterologous relapses with improved accuracy. The parasite population in this area was quite diverse with an estimated five subpopulations and evidence of a highly heterogeneous ancestry of some of the isolates, similar to previous analyses of P. vivax in this region. Pairwise comparison of recurrent infections determined that there were 12 homologous relapses and 3 likely heterologous relapses with highly related parasites. To the best of our knowledge, this is the first large-scale study to evaluate recurrent P. vivax infections using whole genome sequencing. Conclusions: Whole genome sequencing is a high-resolution tool that can identify P. vivax homologous relapses with increased sensitivity, while also providing data about drug resistance and parasite population genetics. This information is important for evaluating the efficacy of known and novel antirelapse medications in endemic areas and thus advancing the campaign to eliminatemalaria. | NA | no | gd of pathogen | NA | no | no |
| 4884 | 2020 | 44040 | Crawford, JW; Schrader, M; Hall, SR; Caceres, CE | 2020 | Intraspecific variation in resource use is not explained by population persistence or seasonality | Populations of generalist grazers often contain genotypes with powerful and efficient strategies. Powerful genotypes grow rapidly on rich-quality resources, but slowly on poorer-quality ones, while efficient genotypes grow relatively better on poorer resources but cannot exploit richer resources as well. Via a power-efficiency trade-off, variation in resource quality could maintain genetic diversity. To evaluate this mechanism, we sampled six populations of the freshwater cladoceran Daphnia pulicaria. In persisting (year-round) populations, Daphnia consume resources that vary in quality, whereas in non-persisting (spring-only) populations, Daphnia primarily encounter rich-quality resources. We hypothesized that non-persisting populations harbor no efficient clones (hence should show lower growth on poor-quality resources). Although individuals from non-persisting populations remained smaller than individuals from persisting populations, no evidence arose for a trade-off between powerful and efficient strategies. In fact, growth rates on the two diets were positively correlated (instead of negatively, as predicted). Furthermore, in the persisting populations, we predicted that clonal selection from spring to summer should shift the distribution of genotypes from powerful (specialists on richer spring resources) to efficient (poorer, summer resources). Genetic composition of populations shifted from spring to summer, but not toward more efficient genotypes. Therefore, in these lakes, maintenance of variation among genotypes must stem from more complicated factors than population persistence patterns or seasonal shifts in resource quality alone. | NA | no | No good fitness measure and measure host GD | NA | no | no |
| 4885 | 2020 | 44040 | Creedy, TJ; Norman, H; Tang, CQ; Chin, KQ; Andujar, C; Arribas, P; O’Connor, RS; Carvell, C; Notton, DG; Vogler, AP | 2020 | A validated workflow for rapid taxonomic assignment and monitoring of a national fauna of bees (Apiformes) using high throughput DNA barcoding | Improved taxonomic methods are needed to quantify declining populations of insect pollinators. This study devises a high-throughput DNA barcoding protocol for a regional fauna (United Kingdom) of bees (Apiformes), consisting of reference library construction, a proof-of-concept monitoring scheme, and the deep barcoding of individuals to assess potential artefacts and organismal associations. A reference database of cytochrome oxidase c subunit 1 (cox1) sequences including 92.4% of 278 bee species known from the UK showed high congruence with morphological taxon concepts, but molecular species delimitations resulted in numerous split and (fewer) lumped entities within the Linnaean species. Double tagging permitted deep Illumina sequencing of 762 separate individuals of bees from a UK-wide survey. Extracting the target barcode from the amplicon mix required a new protocol employing read abundance and phylogenetic position, which revealed 180 molecular entities of Apiformes identifiable to species. An additional 72 entities were ascribed to nuclear pseudogenes based on patterns of read abundance and phylogenetic relatedness to the reference set. Clustering of reads revealed a range of secondary operational taxonomic units (OTUs) in almost all samples, resulting from traces of insect species caught in the same traps, organisms associated with the insects including a known mite parasite of bees, and the common detection of human DNA, besides evidence for low-level cross-contamination in pan traps and laboratory procedures. Custom scripts were generated to conduct critical steps of the bioinformatics protocol. The resources built here will greatly aid DNA-based monitoring to inform management and conservation policies for the protection of pollinators. | NA | no | n | NA | no | no |
| 4886 | 2020 | 44040 | Criscione, CD; van Paridon, BJ; Gilleard, JS; Goater, CP | 2020 | Clonemate cotransmission supports a role for kin selection in a puppeteer parasite | Host manipulation by parasites is a fascinating evolutionary outcome, but adaptive scenarios that often accompany even iconic examples in this popular field of study are speculative. Kin selection has been invoked as a means of explaining the evolution of an altruistic-based, host-manipulating behavior caused by larvae of the lancet fluke Dicrocoelium dendriticum in ants. Specifically, cotransmission of larval clonemates from a snail first host to an ant second host is presumed to lead to a puppeteer parasite in the ant’s brain that has clonemates in the ant abdomen. Clonal relatedness between the actor (brain fluke) and recipients (abdomen flukes) enables kin selection of the parasite’s host-manipulating trait, which facilitates transmission of the recipients to the final host. However, the hypothesis that asexual reproduction in the snail leads to a high abundance of clonemates in the same ant is untested. Clonal relationships between the manipulator in the brain and the nonmanipulators in the abdomen are also untested. We provide empirical data on the lancet fluke’s clonal diversity within its ant host. In stark contrast to other trematodes, which do not exhibit the same host-manipulating behavioral trait, the lancet fluke has a high abundance of clonemates. Moreover, our data support existing theory that indicates that the altruistic behavior can evolve even in the presence of multiple clones within the same ant host. Importantly, our analyses conclusively show clonemate cotransmission into ants, and, as such, we find support for kin selection to drive the evolution and maintenance of this iconic host manipulation. | NA | no | No good fitness measure and measure host GD | NA | no | no |
| 4887 | 2020 | 44042 | Crispim, MAE; Reis, MNG; Abrahim, C; Kiesslich, D; Fraiji, N; Bello, G; Stefani, MMA | 2019 | Homogenous HIV-1 subtype B from the Brazilian Amazon with infrequent diverse BF1 recombinants, subtypes F1 and C among blood donors | In the last decade a growing HIV/AIDS epidemic with increased incidence and AIDS-related mortality has been reported in Northern Brazil from which molecular data are scarce. Also, apparently healthy, adult blood donors, recently diagnosed with HIV-1 represent important sentinel populations for molecular studies. This cross-sectional study describes HIV-1 sub-types in blood donors from three reference public blood centers located in three States in Northern Brazil. HIV-1 pol sequencing (protease/PR, reverse transcriptase/RT) was performed on plasma samples of HIV-1 positive donors from HEMOAM, Manaus, Amazonas (n = 198), HEMERON, Porto Velho, Rondonia (n = 20) and HEMORAIMA, Boa Vista, Roraima (n = 9) collected from 2011-2017. HIV-1 subtypes were identified by REGA, phylogenetic inference; recombinant viruses were characterized by SIMPLOT. Young, single, males predominated, around half was first-time donors. Syphilis co-infection was detected in 17% (39 out of 227), 8% (18 out of 227) was anti-HBc positive. Subtype B represented >= 90% in Amazonas, Rondonia and Roraima, subtype C (3.1%) was found in Amazonas and Rondonia; subtype F1 (0.9%) and BF1 recombinants (5.3%) were only detected in Amazonas. Subtype B sequences from Amazonas (n = 179), Rondonia (n = 18) and Roraima (n = 9) were combined with viral strains representative of the B-PANDEMIC (n = 300) and B-CARIBBEAN/B-CAR (n = 200) lineages. The B-PANDEMIC lineage predominated (78%) although B-CAR lineages were frequent in Roraima (56%) and Amazonas (22%). Subtype C and subtype F1 sequences identified here clustered within Brazilian C-BR and F1(BR) lineages, respectively. Twelve BF1 mosaics showed 11 different recombination profiles: six were singleton unique-recombinant-forms/URFs, one displays a CRF28/29\_BF-like recombinant pattern and the remaining four BF1 isolates branched with other Brazilian BF1 viruses previously described and may represent putative new CRF\_BF1 from Northern Brazil. Our study shows a highly homogeneous molecular pattern with prevalent subtype B, followed by BF1, and sporadic subtype C and F1 in blood donors from the Northern region. Surveillance studies are important to monitor HIV-1 diversity which can reveal patterns of viral dissemination, especially in a highly endemic, remote and geographically isolated region as Northern Brazil. | NA | no | hiv | NA | no | no |
| 4888 | 2020 | 44111 | Crocco, P; Montesanto, A; Dato, S; Geracitano, S; Iannone, F; Passarino, G; Rose, G | 2019 | Inter-Individual Variability in Xenobiotic-Metabolizing Enzymes: Implications for Human Aging and Longevity | Xenobiotic-metabolizing enzymes (XME) mediate the body’s response to potentially harmful compounds of exogenous/endogenous origin to which individuals are exposed during their lifetime. Aging adversely affects such responses, making the elderly more susceptible to toxics. Of note, XME genetic variability was found to impact the ability to cope with xenobiotics and, consequently, disease predisposition. We hypothesized that the variability of these genes influencing the interaction with the exposome could affect the individual chance of becoming long-lived. We tested this hypothesis by screening a cohort of 1112 individuals aged 20-108 years for 35 variants in 23 XME genes. Four variants in different genes (CYP2B6/rs3745274-G/T, CYP3A5/rs776746-G/A, COMT/rs4680-G/A and ABCC2/rs2273697-G/A) differently impacted the longevity phenotype. In particular, the highest impact was observed in the age group 65-89 years, known to have the highest incidence of age-related diseases. In fact, genetic variability of these genes we found to account for 7.7% of the chance to survive beyond the age of 89 years. Results presented herein confirm that XME genes, by mediating the dynamic and the complex gene-environment interactions, can affect the possibility to reach advanced ages, pointing to them as novel genes for future studies on genetic determinants for age-related traits. | NA | no | No good fitness measure and measure host GD | NA | no | no |
| 4889 | 2020 | 44043 | Cui, ZJ; Zhang, YL; Zhang, X; Luo, ZH; Zhang, P; Golec, J; Poland, TM; Zalucki, MP; Han, P; Lu, ZZ | 2019 | Life history and mortality factors of Agrilus mali Matsumura (Coleoptera: Buprestidae) in wild apples in Northwestern China | Wild apple Malus sieversii (Ledeb.) M. Roem. (Rosales: Rosaceae), the ancestor of cultivated apples, is widely distributed in Central Asia and is recognized as an important germplasm bank. Recently, the invasive pest Agrilus mali Matsumura (Coleoptera: Buprestidae), originally distributed in eastern Asia, has damaged endemic apple forests in the Yili River valley, Xinjiang, China, and has spread rapidly, infesting more than 80% of wild apple trees in this region. We investigated the life-history traits and native natural enemies in the recently invaded range during 2016 and 2017. Agrilus mali has a univoltine life cycle and overwinters as young larvae in galleries in the cambium. Adults emerged from early June to mid-August and their density peaked in mid-July. Several native natural enemies were identified from Agrilus mali larvae, including Atanycolus denigrator (L.) (Hymenoptera: Braconidae), the mite Pyemotes moseri Yu et Liang (Acari: Pyemotidae) and fungal entomopathogens. Combined, these natural enemies were responsible for mortality rates ranging from 20% to 80% during the summer and autumn. The most abundant and important natural enemy was A. denigrator, which was responsible for up to 15% mortality of A. mali. The results of the present study suggest that augmentation and conservation of A. denigrator and P. moseri should be considered with respect to biological control against this devastating pest. | NA | no | No good fitness measure and measure host GD | NA | no | no |
| 4890 | 2020 | 44111 | da Cunha, LM; Uppal, A; Seddon, E; Nusbaumer, D; Vermeirssen, ELM; Wedekind, C | 2019 | No additive genetic variance for tolerance to ethynylestradiol exposure in natural populations of brown trout (Salmo trutta) | One of the most common and potent pollutants of freshwater habitats is 17-alpha-ethynylestradiol (EE2), a synthetic component of oral contraceptives that is not completely eliminated during sewage treatment and that threatens natural populations of fish. Previous studies found additive genetic variance for the tolerance against EE2 in different salmonid fishes and concluded that rapid evolution to this type of pollution seems possible. However, these previous studies were done with fishes that are lake-dwelling and hence typically less exposed to EE2 than river-dwelling species. Here, we test whether there is additive genetic variance for the tolerance against EE2 also in river-dwelling salmonid populations that have been exposed to various concentrations of EE2 over the last decades. We sampled 287 adult brown trout (Salmo trutta) from seven populations that show much genetic diversity within populations, are genetically differentiated, and that vary in their exposure to sewage-treated effluent. In order to estimate their potential to evolve tolerance to EE2, we collected their gametes to produce 730 experimental families in blockwise full-factorial in vitro fertilizations. We then raised 7,302 embryos singly in 2-ml containers each and either exposed them to 1 ng/L EE2 (an ecologically relevant concentration, i.e., 2 pg per embryo added in a single spike to the water) or sham-treated them. Exposure to EE2 increased embryo mortality, delayed hatching time, and decreased hatchling length. We found no population differences and no additive genetic variance for tolerance to EE2. We conclude that EE2 has detrimental effects that may adversely affect population even at a very low concentration, but that our study populations lack the potential for rapid genetic adaptation to this type of pollution. One possible explanation for the latter is that continuous selection over the last decades has depleted genetic variance for tolerance to this synthetic stressor. | NA | no | no good gd measure host pop | NA | no | no |
| 4891 | 2020 | 44113 | Da Re, F; Graczyk, M; Cwiertnia, P; Cassandro, M; Szwaczkowski, T; Sell-Kubiak, E | 2018 | Explorative statistical analysis to identify factors affecting the survival of pygmy hippopotamus (Choeropsis liberiensis) kept in zoological gardens | This study aimed to evaluate the genetic and environmental factors affecting the survival of the pygmy hippopotamus (Choeropsis liberiensis) population kept in zoological gardens, including the estimation of the inbreeding level. Survival was examined at three time points: birth (0-1 days), 1 month (30 days) and 5 years of age. The data were obtained from the Zoological Information Management System, and covered observations on 1196 pygmy hippo, collected between 1873 and April 2016 and grouped into five geographical regions (Africa, Australasia, Europe, USA and Canada, and South America). The analyses were performed using LOGIT and PROBIT transformations of the binominal survival data. No statistically significant differences (P-value > 0.05) were observed between the results obtained from the two transformations. The survival of individuals was found to decrease with increased level of inbreeding, the most significant effect being on survival at birth. At all three survival time points the highest survival was observed among females. Additionally, the Australasia region showed the highest overall survival. We hypothesize that the environment, climate and similarity of local food to the native diet of pygmy hippo allows animals kept in zoological gardens in that region to perform better than in other regions. The results of this study indicated that it is necessary to exercise vigilance in reducing inbreeding as it negatively impacts survival. This can be achieved by continued efforts to maintain and improve genetic diversity through management of breeding and associated transfer of animals between zoological facilities to reduce relatedness in breeding pairs. | NA | no | No good fitness measure and measure host GD | NA | no | no |
| 4892 | 2020 | 44112 | Da Silva, JZ; Dos Reis, MS | 2019 | Consumption of Euterpe edulis fruit by wildlife: implications for conservation and management of the Southern Brazilian Atlantic Forest | This study aimed to measure the wildlife consumption of Euterpe edulis fruit and use this data to discuss management possibilities. To estimate infructescence fruit volume consumed, collectors were installed in fruit-bearing palms. To characterize consumption from the ground, samples were placed next to fruiting palms. To identify wildlife and their activities, camera traps were installed in infructescences and on the ground. The results suggested that there was a small fruit surplus (1.8 %), and this finding indicated the possibility of a harvest to reduce food for the wildlife. However, recurrent variations in the annual fruit production (21.4 %) were also noted, and suggested that wildlife could tolerate some fruit harvesting. Thus, a harvest could be restricted to fruit volume that exceeds the annual average (94 kg/ha/year). Turdus flavipes, a migratory’ bird, was the most active species in the dispersal of seeds; this finding indicates the need for broader conservation strategies. Wildlife composition also changed along with the fruiting, and this alteration suggests that dependence on the fruit is variable among different species. Seed germination and seedling mortality were high, results that indicate that local conditions may have a predominant effect on seed volume in natural regeneration density. | NA | no | No good fitness measure and measure host GD | NA | no | no |
| 4893 | 2020 | 44112 | Daas, MS; Rosana, ARR; Acedo, JZ; Douzane, M; Nateche, F; Kebbouche-Gana, S; Vederas, JC | 2018 | Insights into the draft genome sequence of bioactives-producing Bacillus thuringiensis DNG9 isolated from Algerian soil-oil slough | Bacillus thuringiensis is widely used as a bioinsecticide due to its ability to form parasporal crystals containing proteinaceous toxins. It is a member of the Bacillus cereus sensu lato, a group with low genetic diversity but produces several promising antimicrobial compounds. B. thuringiensis DNG9, isolated from an oil-contaminated slough in Algeria, has strong antibacterial, antifungal and biosurfactant properties. Here, we report the 6.06 Mbp draft genome sequence of B. thuringiensis DNG9. The genome encodes several gene inventories for the biosynthesis of bioactive compounds such as zwittermycin A, petrobactin, insecticidal toxins, polyhydroxyalkanoates and multiple bacteriocins. We expect the genome information of strain DNG9 will provide another model system to study pathogenicity against insect pests, plant diseases, and antimicrobial compound mining and comparative phylogenesis among the Bacillus cereus sensu lato group. | NA | no | No good fitness measure and measure host GD | NA | no | no |
| 4894 | 2020 | 44040 | Dai, CZL; Vazifeh, MM; Yeang, CH; Tachet, R; Wells, RS; Vilar, MG; Daly, MJ; Ratti, C; Martin, AR | 2020 | Population Histories of the United States Revealed through Fine-Scale Migration and Haplotype Analysis | The population of the United States is shaped by centuries of migration, isolation, growth, and admixture between ancestors of global origins. Here, we assemble a comprehensive view of recent population history by studying the ancestry and population structure of more than 32,000 individuals in the US using genetic, ancestral birth origin, and geographic data from the National Geographic Genographic Project. We identify migration routes and barriers that reflect historical demographic events. We also uncover the spatial patterns of relatedness in subpopulations through the combination of haplotype clustering, ancestral birth origin analysis, and local ancestry inference. Examples of these patterns include substantial substructure and heterogeneity in Hispanics/Latinos, isolationby-distance in African Americans, elevated levels of relatedness and homozygosity in Asian immigrants, and fine-scale structure in European descents. Taken together, our results provide detailed insights into the genetic structure and demographic history of the diverse US population. | NA | no | human | NA | no | no |
| 4895 | 2020 | 44039 | Damchuay, K; Longya, A; Sriwongchai, T; Songkumarn, P; Parinthawong, N; Darwell, K; Talumphai, S; Tasanasuwan, P; Jantasuriyarat, C | 2020 | High nucleotide sequence variation of avirulent gene,AVR-Pita1, in Thai rice blast fungus population | Rice blast disease, caused byMagnaporthe oryzae, is one of the most importance diseases of rice production worldwide. The key role of defense mechanism to combat this fungus in rice follows the gene-for-gene concept, which a plant resistant (R) gene product recognizes a fungal avirulent (AVR) effector and triggers the hypersensitive response. However, theAVRgenes have been shown to be rapidly evolving resulting in high level of genetic diversity. The aims of this study were to examine the nucleotide sequence variation ofAVR-Pita1gene in Thai rice blast isolates and to identify the severity of blast disease using isogenic line ofPitagene. Seventy-six rice blast isolates collected from different parts of Thailand were used. Gene specific primers forAVR-Pita1gene coding sequence were designed and used for identifying the genetic diversity ofAVR-Pita1gene by PCR amplification and sequencing. The obtained sequences were analysed for genetic variation and genetic relationship. Our results revealed the association between the sequence variations ofAVR-Pita1and selective forces fromPitagene. This phenomenon demonstrated the coevolution between rice blast resistant gene in rice and avirulent gene in blast fungus. The information about variation and evolutionary mechanisms ofAVRgene obtained from this study can be used in rice blast resistant breeding programme. | NA | no | GD of pathogen | NA | no | no |
| 4896 | 2020 | 44113 | Dapp, MJ; Kober, KM; Chen, LN; Westfall, DH; Wong, K; Zhao, H; Hall, BM; Deng, WJ; Sibley, T; Ghorai, S; Kim, K; Chen, N; McHugh, S; Au, L; Cohen, M; Anastos, K; Mullins, JI | 2017 | Patterns and rates of viral evolution in HIV-1 subtype B infected females and males | Biological sex differences affect the course of HIV infection, with untreated women having lower viral loads compared to their male counterparts but, for a given viral load, women have a higher rate of progression to AIDS. However, the vast majority of data on viral evolution, a process that is clearly impacted by host immunity and could be impacted by sex differences, has been derived from men. We conducted an intensive analysis of HIV-1 gag and env-gp120 evolution taken over the first 6-11 years of infection from 8 Women’s Interagency HIV Study (WIHS) participants who had not received combination antiretroviral therapy (ART). This was compared to similar data previously collected from men, with both groups infected with HIV-1 subtype B. Early virus populations in men and women were generally homogenous with no differences in diversity between sexes. No differences in ensuing nucleotide substitution rates were found between the female and male cohorts studied herein. As previously reported for men, time to peak diversity in env-gp120 in women was positively associated with time to CD4+ cell count below 200 (P = 0.017), and the number of predicted N- linked glycosylation sites generally increased over time, followed by a plateau or decline, with the majority of changes localized to the V1-V2 region. These findings strongly suggest that the sex differences in HIV-1 disease progression attributed to immune system composition and sensitivities are not revealed by, nor do they impact, global patterns of viral evolution, the latter of which proceeds similarly in women and men. | NA | no | hiv | NA | no | no |
| 4897 | 2020 | 44112 | Davidson-Phillips, S; Davidson-Phillips, P; Canning, G; Schroder, B; Swart, J; Burger, A | 2019 | Canine distemper virus management in lions (Panthera leo) on Welgevonden Game Reserve | Large charismatic predators have been re-introduced to several fenced reserves (<1000 km(2)); consequently, their populations can be at risk to declining genetic diversity and catastrophic events such as disease. Welgevonden Game Reserve (WGR) experienced an outbreak of canine distemper virus (CDV) in its lion (Panthera leo) population between October 2015 and February 2016. The decline was attributed to both direct CDV mortalities and indirect management interventions with a population decrease of 93%. In addition, the first documented cases of CDV were recorded in a brown hyaena (Hyaena brunnea) population. This article shares the management of a disease outbreak in WGR’s lion population. It discusses the population history, the making of clinical observations leading to diagnoses and recommends strategies that can be implemented to prevent extirpation. The sourcing of new lions from established meta-populations. and the use of vaccinations to proactively prevent future outbreaks in managed wild lions is discussed. In less than two years the population has recovered. This paper provides a case report for wildlife practitioners of small populations of managed wild lions. | NA | no | No good fitness measure and measure host GD | NA | no | no |
| 4898 | 2020 | 44112 | de Castro, APG; Costa, AP; Peixoto, JR; Faleiro, FG; Vilela, MD; Vendrame, W | 2018 | MOLECULAR CHARACTERIZATION OF YELLOW PASSION FRUIT GENOTYPES WITH DIFFERENT YIELD AND DISEASE RESISTANCE LEVELS | Brazil is the largest passion fruit producer in the world. However, the yield is still considered low, and the cultivation of unsuitable varieties is one of the factors directly influencing this trait. As a consequence, breeding studies have been developed with the purpose of obtaining genetic materials with high yield, high fruit quality, and disease resistance. The objective of this study was to characterize and quantify the genetic variability in 18 genotypes of yellow passion fruit (Passiflora edulis Sims) with different levels of yield and disease resistance, using RAPD markers. The RAPD markers were obtained from 10 decamer primers and converted into a matrix of binary data. Estimations of the genetic dissimilarities between different genotypes and cluster analysis were performed. A total of 58 markers were generated, 63.80% of which were polymorphic. The genetic distances among genotypes varied from 0.040 to 0.354 and genotypes were subdivided into at least 5 groups of similarity. The dispersion graphs showed a low clustering tendency for yield and resistance to different diseases (septoriosis, anthracnose, scab, bacterial spot, and passion fruit woodiness disease). These results demonstrate a high genetic variability among the evaluated genotypes, which is valuable information when selecting promising materials to be used perse or as parents in genetic breeding programs. | NA | no | dometicated host | NA | no | no |
| 4899 | 2020 | 44040 | De Moor, D; Roos, C; Ostner, J; Schulke, O | 2020 | Female Assamese macaques bias their affiliation to paternal and maternal kin | Forming strong social bonds can lead to higher reproductive success, increased longevity, and/or increased infant survival in several mammal species. Given these adaptive benefits, understanding what determines partner preferences in social bonding is important. Maternal relatedness strongly predicts partner preference across many mammalian taxa. The role of paternal relatedness, however, has received relatively little attention, even though paternal and maternal kin share the same number of genes, and theoretically similar preferences would therefore be expected for paternal kin. Here, we investigate the role of maternal and paternal relatedness in female affiliation in Assamese macaques (Macaca assamensis), a species characterized by a relatively low male reproductive skew. We studied a wild population under natural conditions using extensive behavioral data and relatedness analyses based on pedigree reconstruction. We found stronger affiliative relationships and more time spent grooming between maternal kin and paternal half-sisters compared with nonkin, with no preference of maternal over paternal kin. Paternally related and nonrelated dyads did not form stronger relationships when they had less close maternal kin available, but we would need a bigger sample size to confirm this. As expected given the low reproductive skew, affiliative relationships between paternal half-sisters closer in age were not stronger than between paternal half-sisters with larger age differences, suggesting that the kin bias toward paternal kin was not mediated by age similarity. An alternative way through which paternal kin could get familiarized is mother- and/or father-mediated familiarity. Lay Summary: Family first! Wild female Assamese macaques form strong relationships with their paternal and maternal kin. Although maternal kin can be discriminated because mothers take care of their offspring, discriminating paternal kin is more complicated: Females often mate with multiple males, making it unclear who the father is. Even so, we show that female Assamese macaques are capable of discriminating their paternal kin from unrelated group members, replicating results from an increasing number of species. | NA | no | No good fitness measure and measure host GD | NA | no | no |
| 4900 | 2020 | 44040 | de Oliveira, CM; de Souza, IT; Almeida, NO; de Freitas, MA; da Rocha, MR; Petrofeza, S | 2020 | DEVELOPMENT OF QUANTITATIVE DETECTION METHOD FOR Meloidogyne incognita BY qPCR | The root-knot nematode (Meloidogyne spp.) is the most important plant-parasitic nematode genus, they are the most common and destructive pathogens in this group. They produce some of the most drastic symptoms in plants and can significantly reduce the yield of crops. In order to achieve deploy an efficient method of plant-parasitic nematode management, is necessary an identification and quantification accurate and reliable of plant-parasitic nematodes. The aim of this study was to analyze samples in qPCR to detect and quantify M. incognita, in the field samples, comparing different methods of extraction of DNA and its efficacy in establishing the number of individuals. For this purpose the effectiveness of different DNA methods of extraction was compared through the values of CT intervals. For standard curve and method comparisons, we used nematodes multiplied in a greenhouse and carefully separated in the specific quantities of the experiments. For the number of individuals experiment field samples previously counted under an optical microscope were used. The DNA extraction was made from 100 nematodes by the methods: CTAB, Phenol: Chloroform and commercial kit (PureLink (R) Genomic DNA Kit, Invitrogen). In the comparative analysis using the three methods of DNA extracting from 100 nematodes, it was observed that commercial kit and CTAB methods obtained CT values similar. The CTAB method of extraction, showed less variation in the repeats and greater linearity of standard curve in comparison with other methods tested. So, it was possible to quantify the samples through the CT value intervals, established from different numbers of individuals (1, 10, 25, 100, 250, 500 and 750), in field samples. This study demonstrated that qPCR technique is an alternative sensitive and reliable for the quantification of M. incognita to support laboratories of diagnose and field survey. | NA | no | No good fitness measure and measure host GD | NA | no | no |
| 4901 | 2020 | 44111 | de Oliveira, LR; Fraga, LD; Ott, PH; Siciliano, S; Lopes, F; Almeida, R; Wickert, JC; Milmann, L; Danilewicz, D; Emin-Lima, NR; Meirelles, AC; Luz, V; do Nascimento, LF; de Thoisy, B; Tavares, M; Zerbini, AN; Baumgarten, M; Valiati, VH; Bonatto, SL | 2019 | Population structure, phylogeography, and genetic diversity of the common bottlenose dolphin in the tropical and subtropical southwestern Atlantic Ocean | We assessed the level of genetic variability and population structure of the common bottlenose dolphin (Tursiops truncatus) in the tropical and subtropical portions of the southwestern Atlantic Ocean and compared the results with previous morphological findings. We analyzed 109 samples of common bottlenose dolphins that were sequenced for control region mtDNA and genotyped for seven polymorphic microsatellite loci. The results suggested that the species in this region can be separated in two major biological units, northern and southern, with an area of parapatry at southern Brazil. The northern unit seems to occur in a wide range of depths, including offshore waters, is consistent with the canonical morphology of T. truncatus, and can be divided into three management units: 1) Saint Paul’s Rocks, 2) north and northeast of Brazil, and 3) Campos and Santos Basins (that extend at least to southernmost Brazil). The southern unit is coastal, occurring exclusively in very shallow waters (< 10 m) and estuaries, and is consistent with the previously described (putative) Tursiops gephyreus. Nevertheless, a formal decision on the taxonomic status of T. gephyreus should wait for a more geographically comprehensive and data-integrative study. | NA | no | No good fitness measure | NA | no | no |
| 4902 | 2020 | 44113 | De Paiva, BAR; Wendland, A; Borba, TC; Araujo, LG | 2018 | Molecular characterization and differential interaction of Xanthomonas axonopodis pv. phaseoli and Xanthomonas fuscans subsp fuscans with common beans (Phaseolus vulgaris) | The common bacterial blight, caused by Xanthomonas axonopodis pv. phaseoli (Xap) and X. fuscans subsp. fuscans (Xff), is one of the most important dry bean diseases of bacterial etiology. The present work aimed to study the genetic structure among and within populations, and also identify the pathogenic variability of Xap and Xff isolates evaluating the horizontal and vertical resistance of the host. A total of 42 Xap and Xff isolates were obtained were submitted to molecular characterization by rep-PCR markers, and inoculated in the genotypes CNFP 10120, BRS Notavel, and Rosinha G-2, for differential interaction studies. In the analysis of genetic structure, the coefficient of genetic differentiation (GST=0.5154) revealed diversity both within and among the pathogenic populations, confirming the result obtained by AMOVA (FST=0.5198). Of a total of 51 loci, 98% were polymorphic, among Xap 59% and within Xff only 21% were polymorphic. In studies of pathogenic variability, diallelic analyses showed significant effects of the general combination ability (GCA), of the general aggression ability (GAA), and of the specific interaction ability (SIA). Therefore, seven races were classified through the differential behavior of all isolates in the three genotypes of bean. | NA | no | gd of pathogen | NA | no | no |
| 4903 | 2020 | 44113 | de Pina-Araujo, IIM; Spitz, N; Soares, CC; Niel, C; Lago, BV; Gomes, SA | 2018 | Hepatitis B virus genotypes A1, A2 and E in Cape Verde: Unequal distribution through the islands and association with human flows | Hepatitis B virus (HBV) diversity has not been previously studied in Cape Verde. The archipelago was discovered in 1460 by Portuguese explorers, who brought African slaves to colonise the islands. In this study, we investigated the HBV characteristics from 183 HBsAg-positive Cape Verdean individuals. Phylogenetic analysis of the pre-S/S region and the full-length genomes revealed 54 isolates with HBV/A1 (57%), 21 with HBV/A2 (22%), 19 with HBV/E (20%), and one with HBV/D (1%). HBV genotypes and subgenotypes were unequally distributed through the islands. In Sao Vicente, the main northern island, most isolates (84%) belonged to the African-originated HBV/A1, with the remaining isolates belonging to HBV/A2, which is prevalent in Europe. Interestingly, the HBV/A1 isolates from Sao Vicente were closely related to Brazilian sequences into the Asian-American clade, which suggests the dissemination of common African ancestors through slave trade. In contrast, in Santiago and nearby southern islands, where a recent influx from different populations circulates, a higher diversity of HBV was observed: HBV/A1 (40%); HBV/E (32%); HBV/A2 (28%); and HBV/D (1%). HBV/E is a recent genotype disseminated in Africa that was absent in the era of the slave trade. African and European human flows at different times of the history may explain the HBV diversity in Cape Verde. The possible origin and specifics of each HBV genotype circulating in Cape Verde are discussed. | NA | no | gd of pathogen | NA | no | no |
| 4904 | 2020 | 44041 | De Silvestri, A; Capittini, C; Mallucci, G; Bergamaschi, R; Rebuffi, C; Pasi, A; Martinetti, M; Tinelli, C | 2019 | The Involvement of HLA Class II Alleles in Multiple Sclerosis: A Systematic Review with Meta-analysis | Multiple Sclerosis (MS) displays a heterogeneous clinical onset and progression, which are mostly unpredictable, but demyelination of the central nervous system (CNS) leads to substantial deficits of sensory, motor, autonomic, and neurocognitive functions. Considering all genetic studies on MS, including the advanced genome-wide association studies, the risk linked to HLA alleles remains the highest among other susceptibility genetic variants. However, given the genetic variability of HLA alleles in different ethnic groups, we conducted a systematic review of reviews and meta-analyses aiming at summarizing all the results on the association between MS and HLA class II genes. We systematically searched meta-analyses and systematic reviews dealing with MS and HLA in all ethnicities. From 154 records, we included 5 articles collecting HLA data from 15,232 MS patients and 24,194 ethnically matched controls. DRB115 (OR ranging from 1.39 in Chinese Han to 2.59 in Caucasians) and DQB106:02 (OR ranging from 1.91 in Caucasians to 2.49 in Colombian) alleles confer an increased risk for MS transethnically (Caucasians, Chinese, South Americans, Carribeans, Middle Easterners, Japanese, and North Africans). DRB101, DRB109, DRB111, DRB112, and DRB116 alleles were protective, in agreement with the type of amino-acidic (aa) residues (ranging from position 9 to 90) included in pockets 1, 4, 6, 7, and 9, which are most involved in peptide presentation. Changes in aa residues affect the capability of HLA molecules in binding myelin peptides. DQB106:02 risk allele seems to be the most interesting target as humanized mice expressing only DQB106:02 develop MS-like disease mediated by autoimmune reactions against myelin oligodendrocytic basic protein that stabilizes the myelin. Our summary of results from a high number of patients and controls suggests that allelic variants from both DQB1 and DRB1 genes are equally involved in MS susceptibility/protection transethnically. | NA | no | No good fitness measure and measure host GD | NA | no | no |
| 4905 | 2020 | 44111 | de Villemereuil, P; Rutschmann, A; Ewen, JG; Santure, AW; Brekke, P | 2019 | Can threatened species adapt in a restored habitat? No expected evolutionary response in lay date for the New Zealand hihi | Many bird species have been observed shifting their laying date to earlier in the year in response to climate change. However, the vast majority of these studies were performed on non-threatened species, less impacted by reduced genetic diversity (which is expected to limit evolutionary response) as a consequence of genetic bottlenecks, drift and population isolation. Here, we study the relationship between lay date and fitness, as well as its genetic basis, to understand the evolutionary constraints on phenology faced by threatened species using a recently reintroduced population of the endangered New Zealand passerine, the hihi (Notiomystis cincta). A large discrepancy between the optimal laying date and the mode of laying date creates a strong selection differential of -11.24. The impact of this discrepancy on fitness is principally mediated through survival of offspring from hatchling to fledgling. This discrepancy does not seem to arise from a difference in female quality or a trade-off with lifetime breeding success. We find that start of breeding season depends on female age and average temperature prior to the breeding season. Laying date is not found to be significantly heritable. Overall, our research suggests that this discrepancy is a burden on hihi fitness, which will not be resolved through evolution or phenotypic plasticity. More generally, these results show that threatened species introduced to restored habitats might lack adaptive potential and plasticity to adjust their phenology to their new environment. This constraint is also likely to limit their ability to face future challenges, including climate change. | NA | no | No good fitness measure | NA | no | no |
| 4906 | 2020 | 44113 | de Vos, MGJ; Zagorski, M; McNally, A; Bollenbach, T | 2017 | Interaction networks, ecological stability, and collective antibiotic tolerance in polymicrobial infections | Polymicrobial infections constitute small ecosystems that accommodate several bacterial species. Commonly, these bacteria are investigated in isolation. However, it is unknown to what extent the isolates interact and whether their interactions alter bacterial growth and ecosystem resilience in the presence and absence of antibiotics. We quantified the complete ecological interaction network for 72 bacterial isolates collected from 23 individuals diagnosed with polymicrobial urinary tract infections and found that most interactions cluster based on evolutionary relatedness. Statistical network analysis revealed that competitive and cooperative reciprocal interactions are enriched in the global network, while cooperative interactions are depleted in the individual host community networks. A population dynamics model parameterized by our measurements suggests that interactions restrict community stability, explaining the observed species diversity of these communities. We further show that the clinical isolates frequently protect each other from clinically relevant antibiotics. Together, these results highlight that ecological interactions are crucial for the growth and survival of bacteria in polymicrobial infection communities and affect their assembly and resilience. | NA | no | No good fitness measure and measure host GD | NA | no | no |
| 4907 | 2020 | 44112 | de Winter, II; Qurkhuli, T; de Groot, N; de Vos-Rouweler, AJM; van Hooft, P; Heitkonig, IMA; Prins, HHT; Bontrop, RE; Doxiadis, GGM | 2019 | Determining Mhc-DRB profiles in wild populations of three congeneric true lemur species by noninvasive methods | The major histocompatibility complex (MHC) is a highly polymorphic and polygenic genomic region that plays a crucial role in immune-related diseases. Given the need for comparative studies on the variability of immunologically important genes among wild populations and species, we investigated the allelic variation of MHC class II DRB among three congeneric true lemur species: the red-fronted lemur (Eulemur rufifrons), red-bellied lemur (Eulemur rubriventer), and black lemur (Eulemur macaco). We noninvasively collected hair and faecal samples from these species across different regions in Madagascar. We assessed DRB exon 2 polymorphism with a newly developed primer set, amplifying nearly all non-synonymous codons of the antigen-binding sites. We defined 26 DRB alleles from 45 individuals (17 alleles from E. rufifrons (N=18); 5 from E. rubriventer (N=7); and 4 from E. macaco (N=20). All detected alleles are novel and show high levels of nucleotide (26.8%) and non-synonymous codon polymorphism (39.4%). In these lemur species, we found neither evidence of a duplication of DRB genes nor a sharing of alleles among sympatric groups or allopatric populations of the same species. The non-sharing of alleles may be the result of a geographical separation over a long time span and/or different pathogen selection pressures. We found dN/dS rates >1 in the functionally important antigen recognition sites, providing evidence for balancing selection. Especially for small and isolated populations, quantifying and monitoring DRB variation are recommended to establish successful conservation plans that mitigate the possible loss of immunogenetic diversity in lemurs. | NA | no | No good fitness measure and measure host GD | NA | no | no |
| 4908 | 2020 | 44112 | DeCandia, AL; Brzeski, KE; Heppenheimer, E; Caro, CV; Camenisch, G; Wandeler, P; Driscoll, C; vonHoldt, BM | 2019 | Urban colonization through multiple genetic lenses: The city-fox phenomenon revisited | Urbanization is driving environmental change on a global scale, creating novel environments for wildlife to colonize. Through a combination of stochastic and selective processes, urbanization is also driving evolutionary change. For instance, difficulty in traversing human-modified landscapes may isolate newly established populations from rural sources, while novel selective pressures, such as altered disease risk, toxicant exposure, and light pollution, may further diverge populations through local adaptation. Assessing the evolutionary consequences of urban colonization and the processes underlying them is a principle aim of urban evolutionary ecology. In the present study, we revisited the genetic effects of urbanization on red foxes (Vulpes vulpes) that colonized Zurich, Switzerland. Through use of genome-wide single nucleotide polymorphisms and microsatellite markers linked to the major histocompatibility complex (MHC), we expanded upon a previous neutral microsatellite study to assess population structure, characterize patterns of genetic diversity, and detect outliers associated with urbanization. Our results indicated the presence of one large evolutionary cluster, with substructure evident between geographic sampling areas. In urban foxes, we observed patterns of neutral and functional diversity consistent with founder events and reported increased differentiation between populations separated by natural and anthropogenic barriers. We additionally reported evidence of selection acting on MHC-linked markers and identified outlier loci with putative gene functions related to energy metabolism, behavior, and immunity. We concluded that demographic processes primarily drove patterns of diversity, with outlier tests providing preliminary evidence of possible urban adaptation. This study contributes to our overall understanding of urban colonization ecology and emphasizes the value of combining datasets when examining evolutionary change in an increasingly urban world. | NA | no | No good fitness measure and measure host GD | NA | no | no |
| 4909 | 2020 | 44112 | DeCandia, AL; Dobson, AP; vonHoldt, BM | 2018 | Toward an integrative molecular approach to wildlife disease | Pathogens pose serious threats to human health, agricultural investment, and biodiversity conservation through the emergence of zoonoses, spillover to domestic livestock, and epizootic outbreaks. As such, wildlife managers are often tasked with mitigating the negative effects of disease. Yet, parasites form a major component of biodiversity that often persist. This is due to logistical challenges of implementing management strategies and to insufficient understanding of host-parasite dynamics. We advocate for an inclusive understanding of molecular diversity in driving parasite infection and variable host disease states in wildlife systems. More specifically, we examine the roles of genetic, epigenetic, and commensal microbial variation in disease pathogenesis. These include mechanisms underlying parasite virulence and host resistance and tolerance, and the development, regulation, and parasite subversion of immune pathways, among other processes. Case studies of devil facial tumor disease in Tasmanian devils (Sarcophilus harrisii) and chytridiomycosis in globally distributed amphibians exemplify the broad range of questions that can be addressed by examining different facets of molecular diversity. For particularly complex systems, integrative molecular analyses present a promising frontier that can provide critical insights necessary to elucidate disease dynamics operating across scales. These insights enable more accurate risk assessment, reconstruction of transmission pathways, discernment of optimal intervention strategies, and development of more effective and ecologically sound treatments that minimize damage to the host population and environment. Such measures are crucial when mitigating threats posed by wildlife disease to humans, domestic animals, and species of conservation concern. | NA | no | No good fitness measure and measure host GD | NA | no | no |
| 4910 | 2020 | 44039 | Dekelaita, DJ; Epps, CW; Stewart, KM; Sedinger, JS; Powers, JG; Gonzales, B; Abella-Vu, RK; Darby, NW; Hughson, DL | 2020 | Survival of Adult Female Bighorn Sheep Following a Pneumonia Epizootic | Beginning in the early 1900s, poly-factorial, poly-microbial pneumonia was identified as a disease affecting bighorn sheep (Ovis canadensis) and it continues to threaten bighorn populations, posing an ongoing management challenge. In May and June 2013, a pneumonia outbreak linked to the pathogenMycoplasma ovipneumoniaeled to an all-age die-off of desert bighorn sheep (O. c. nelsoni) at Old Dad Peak in the Kelso Mountains of the Mojave Desert in California, USA. Subsequently, we observed clinical signs of respiratory disease among bighorn sheep in multiple neighboring ranges. Our objective was to investigate post-outbreak survival of adult female bighorn across 9 populations from 2014 to 2017 in the Mojave Desert and evaluate the relationship betweenM. ovipneumoniaeinfection and survival, while testing effects of range factors that could potentially influence differences in adult female survival (i.e., forage quality, winter precipitation, population abundance). We fitted adult females with radio-collars following the outbreak and collected serum and nasal swab samples for competitive enzyme-linked immunosorbent assay (cELISA) and polymerase chain reaction (PCR) testing to determine exposure and infection status at time of capture. We tracked survival of 115 adult females with radio-collars and used the known-fate model in Program MARK to evaluate effects and estimate survival from November 2013 to March 2017. Annual survival was negatively correlated with positive infection status at capture but varied across populations with respect to differences in range conditions. Summer and autumn forage quality, as represented by mean normalized difference vegetation index (NDVI) values for summer and autumn, was positively correlated with overwinter survival, whereas winter precipitation (a proxy for winter severity) was negatively correlated with overwinter survival. Population abundance was negatively correlated with annual survival, suggesting a potential density-dependent effect. Model-averaged annual survival estimates ranged from 0.700 +/- 0.07 (SE) to 0.945 +/- 0.026 for infected individuals and 0.896 +/- 0.03 to 0.983 +/- 0.011 for uninfected individuals. We conclude that summer and autumn forage quality, indexed by NDVI, may partially offset the negative effect associated withM. ovipneumoniaeinfection on host survival. Our survival modeling results suggest that chronic infection may have afflicted adult females that were PCR-positive (i.e., infected withM. ovipneumoniae) at time of capture. We propose programmatic re-testing of infected individuals to assess pathogen persistence at the individual level and evaluate whether selective culling might potentially help to reduce prevalence and transmission within populations. (c) 2020 The Wildlife Society. | NA | no | No good measureGD of host | NA | no | no |
| 4911 | 2020 | 44043 | del Valle-Mendoza, J; Orellana-Peralta, F; del Valle, LJ; Verne, E; Ugarte, C; Weilg, C; Silva-Caso, W; Valverde-Ezeta, J; Carrillo-Ng, H; Pena-Tuesta, I; Palomares-Reyes, C; Cornejo-Tapia, A; Aguilar-Luis, MA | 2019 | Detection of human Metapneumovirus infection in children under 18 years old hospitalized in Lima-Peru | Background. Human Metapneumovirus (hMPV) is a negative single-stranded RNA virus. Infection by hMPV mainly affects the pediatric population and can cause upper or lower respiratory tract pathologies which can develop life threating complications. This study was carried out between 2009 and 2010 in a high complexity national hospital in Lima, Peru. The time frame corresponds to the pandemic of influenza A H1N1. Methods. A prospective study was performed between September 2009 and September 2010. Patients with a clinical diagnosis suggestive of an acute respiratory infection were included. RT-PCR was utilized to attain the amplification and identification of the hMPV. Results. A total of 539 samples were analyzed from patients with a clinical context suggestive of an acute respiratory tract infection. Of these samples 73, (13.54%) were positive for hMPV. Out of the positive cases, 63% were under one year old, and increased to nearly 80% when considering children younger than two years old. Cough was the most frequent symptom presented by our population with a number of 62 cases (84.93%). Viral seasonality was also established, noting its predominance during the months of summer in the southern hemisphere. The infection by hMPV has an important prevalence in Peru. It mainly affects children under one year old and should be considered an important differential diagnosis in a patient with an acute respiratory infection. | NA | no | human | NA | no | no |
| 4912 | 2020 | 44112 | Delgado-Suarez, EJ; Selem-Mojica, N; Ortiz-Lopez, R; Gebreyes, WA; Allard, MW; Barona-Gomez, F; Rubio-Lozano, MS | 2018 | Whole genome sequencing reveals widespread distribution of typhoidal toxin genes and VirB/D4 plasmids in bovine-associated nontyphoidal Salmonella | Nontyphoidal Salmonella (NTS) is a common pathogen in food-producing animals and a public health concern worldwide. Various NTS serovars may be present in apparently healthy animals. This could result in carcass contamination during the slaughter process leading to human exposure. While most genomic research has focused on Salmonella pathogenesis, little is known on the factors associated with subclinical infections and environmental persistence. We report here the widespread distribution of typhoidal toxin genes (i.e. the cdtB islet, hlyE, taiA), among NTS strains from a beef slaughter operation (n = 39) and from epidemiologically unconnected ground beef (n = 20). These genes were present in 76% of the strains, regardless of serovar, isolation source or geographical location. Moreover, strains that predominated in the slaughterhouse carry plasmid-borne type IV secretion systems (T4SS), which have been linked to persistent infections in numerous pathogens. Population genomics supports clonal dissemination of NTS along the food production chain, highlighting its role as reservoir of genetic variability in the environment. Overall, the study provides a thorough characterization of serovar diversity and genomic features of beef-associated NTS in Mexico. Furthermore, it reveals how common genetic factors could partially explain the emergence and persistence of certain NTS serovars in the beef industry. | NA | no | gd of pathogen | NA | no | no |
| 4913 | 2020 | 44042 | Delices, G; Ovalle, ORL; Mota-Vargas, C; Pastrana, RN; Pastrana, RG; Meza, PA; Serna-Lagunes, R | 2019 | Biogeography of tomato Solanum lycopersicum var. cerasiform (Solanaceae) in its center of origin (South America) and domestication (Mexico) | y The tomato (Solanum lycopersicum) is one of the main horticultural crops in the world because of the important economic benefits that its commercialization generates. Even though the species is widely cultivated in the world, it is susceptible to pests, diseases, and environmental stresses due to the loss of its genetic diversity. There is a wild form called S. l. var ceraciforme that is distributed from South America (its center of origin) to Mexico (its area of domestication), specifically on the slope of the Gulf of Mexico in Veracruz. Due to the large genetic diversity of these wild tomatoes, it is important to improve its crop. The objective of this investigation was to identify patterns of environmental diversification of the tomato, infer the processes of adaptation of the populations in the area of domestication with respect to their center of origin and propose measures of conservation and variation of S. l. var. cerasiforme. We generated two ecological niche models (MNE) with Maxent version 3.4.1 (evaluated with AUC and partial-ROC) to determine the suitability of environmental conditions including their respective projections from the domestication center to the area of origin and vice versa. We used eight variables of temperature and precipitation. Additionally, we included tomato records from bibliographical sources and fieldwork. We also used the niche comparison test (equivalency and similarity) proposed by Broennimann et al. (2012) to evaluate the similarity of environmental conditions in both regions. Subsequently, we carried out an analysis of variance followed by a mean comparison test (Tukey, P = 0.05) with all environmental variables measured. The values of AUC (0.93 and 0.80) and partial-ROC (1.86 and 1.71, P = 0.0001) of the MNE showed that they were good predictive models. We observed that, in the center of Mexico, there are environmental conditions similar to those of the center of origin, unlike the slope of the Gulf of Mexico where they are different. The equivalency test showed that the environmental comparison between both regions is less similar than expected by chance (P = 0.003). The similarity test indicated that the existing similarity between both regions can also be obtained by chance (P = 0.683). We also found significant differences in three temperature and precipitation variables. In conclusion, we determined that the center of origin and domestication of S. l. var. cerasiforme has similar environmental characteristics despite the geographic distance; nevertheless, there are geographical zones (the Gulf of Mexico in Veracruz) in the area of domestication with different environmental conditions. Those places have the potential to contain valuable germplasms. | NA | no | No good fitness measure and measure host GD | NA | no | no |
| 4914 | 2020 | 44042 | Della Rocca, G; Danti, R; Williams, N; Eyre, C; Garbelotto, M | 2019 | Molecular analyses indicate that both native and exotic pathogen populations serve as sources of novel outbreaks of Cypress Canker Disease | Invasive pathogens may follow stepwise spread pathways in which novel disease outbreaks become themselves sources of genotypes initiating novel infestations. Due to its worldwide distribution, Cypress Canker Disease (CCD) provides an opportunity to understand patterns of global spread of a forest pathogen. A comparative genetic analysis was performed on 110 isolates of the causal agent of CCD, the fungus Seiridium cardinale, from Europe, North America, Africa and Oceania using beta-tubulin gene sequences and repeat numbers of seven nuclear SSRs. SSR data were used to calculate genetic indices for each geographic population, including those calculating allele and private allele diversity; clonal genotype, haplotype, and gene diversity; expected heterozygosity; and presence of linkage disequilibrium. Additionally, SSR data were used to calculate migration rates between regions, and to draw a minimum spanning network to visualize genetic relatedness among genotypes. Results identified the California population as a sexually reproducing, highly diverse, native population, and the Mediterranean population as a large, clonally reproducing, and exotic population directly derived from the California population. The New Zealand population appeared to be non-native, with intermediate values of genetic diversity and an asexual reproductive mode. Unexpectedly, two sources were identified for the New Zealand population: an older source was identified in California, while a more recent one was identified in the Mediterranean. Results allowed to infer migration intensity between continents, and are among the first to show that an exotic outbreak of a forest disease can become a source of pathogen genotypes for novel outbreaks in disjunct geographic regions. | NA | no | No good fitness measure and measure host GD | NA | no | no |
| 4915 | 2020 | 44041 | DeLong, JA; Stewart, JE; Valencia-Botin, A; Pedley, KF; Buck, JW; Brewer, MT | 2019 | Invasions of gladiolus rust in North America are caused by a widely-distributed clone of Uromyces transversalis | Uromyces transversalis, the causal agent of Gladiolus rust, is an invasive plant pathogen in the United States and is regulated as a quarantine pathogen in Europe. The aim of this research was to: (i) determine the origin of introductions of U. transversalis to the United States, (ii) track the movement of genotypes, and (iii) understand the worldwide genetic diversity of the species. To develop molecular markers for genotyping, whole genome sequencing was performed on three isolates collected in the United States. Genomes were assembled de novo and searched for microsatellite regions. Primers were developed and tested on ten isolates from the United States resulting in the identification of 24 polymorphic markers. Among 92 isolates collected from Costa Rica, Mexico, New Zealand, Australia, and the United States there were polymorphisms within isolates with no genotypic diversity detected among isolates; however, missing data among the New Zealand and Australia isolates due to either poor amplification of degraded DNA or null alleles as a result of genetic differences made it difficult to generate conclusions about these populations. The microsatellite loci and flanking regions showed high diversity and two divergent genomes within dikaryotic individuals, yet no diversity among individuals, suggesting that the invasive U. transversalis populations from North America are strictly clonal. | NA | no | gd of pathogen | NA | no | no |
| 4916 | 2020 | 44112 | Delser, PM; Ravnik-Glavac, M; Gasparini, P; Glavac, D; Mezzavilla, M | 2018 | Genetic Landscape of Slovenians: Past Admixture and Natural Selection Pattern | The Slovenian territory played a crucial role in the past serving as gateway for several human migrations. Previous studies used Slovenians as a source population to interpret different demographic events happened in Europe but not much is known about the genetic background and the demographic history of this population. Here, we analyzed genome-wide data from 96 individuals to shed light on the genetic role and history of the Slovenian population. Y chromosome diversity splits into two major haplogroups Rib and R1a with the latter suggesting a genetic contribution from the steppe. Slovenian individuals are more closely related to Northern and Eastern European populations than Southern European populations even though they are geographically closer. This pattern is confirmed by an admixture and clustering analysis. We also identified a single stream of admixture events between the Slovenians with Sardinians and Russians around similar to 2630 BCE (2149-3112). Using ancient samples, we found a significant admixture in Slovenians using Yamnaya and the early Neolithic Hungarians as sources, dated around similar to 1762 BCE (1099-2426) suggesting a strong contribution from the steppe to the foundation of the observed modern genetic diversity. Finally, we looked for signals of selection in candidate variants and we found significant hits in HERC2 and FADS responsible for blue eye color and synthesis of long-chain unsaturated fatty acids, respectively, when Slovenians were compared to Southern Europeans. While the comparison was done with Eastern Europeans, we identified significant signals in PKD2L1 and 1L6R which are genes associated with taste and coronary artery disease, respectively. | NA | no | human | NA | no | no |
| 4917 | 2020 | 44112 | Demastes, JW; Hafner, DJ; Hafner, MS; Light, JE; Spradling, TA | 2019 | Loss of genetic diversity, recovery and allele surfing in a colonizing parasite, Geomydoecus aurei | Understanding the genetic consequences of changes in species distributions has wide-ranging implications for predicting future outcomes of climate change, for protecting threatened or endangered populations and for understanding the history that has led to current genetic patterns within species. Herein, we examine the genetic consequences of range expansion over a 25-year period in a parasite (Geomydoecus aurei) that is in the process of expanding its geographic range via invasion of a novel host. By sampling the genetics of 1,935 G. aurei lice taken from 64 host individuals collected over this time period using 12 microsatellite markers, we test hypotheses concerning linear spatial expansion, genetic recovery time and allele surfing. We find evidence of decreasing allelic richness (AR) with increasing distance from the source population, supporting a linear, stepping stone model of spatial expansion that emphasizes the effects of repeated bottleneck events during colonization. We provide evidence of post-bottleneck genetic recovery, with average AR of infrapopulations increasing about 30% over the 225-generation span of time observed directly in this study. Our estimates of recovery rate suggest, however, that recovery has plateaued and that this population may not reach genetic diversity levels of the source population without further immigration from the source population. Finally, we employ a grid-based sampling scheme in the region of ongoing population expansion and provide empirical evidence for the power of allele surfing to impart genetic structure on a population, even under conditions of selective neutrality and in a place that lacks strong barriers to gene flow. | NA | no | gd of pathogen | NA | no | no |
| 4918 | 2020 | 44042 | Deng, K; Liu, W; Wang, DH | 2019 | Relatedness and spatial distance modulate intergroup interactions: experimental evidence from a social rodent | Kin selection theory predicts that individuals should generally behave less aggressively or more amicably towards relatives than nonkin. However, how individuals treat conspecifics depends on genetic relatedness but also on the ecological context, which influences the benefits and costs of their interactions. In this study, we used microsatellite DNA markers and behavioral tests to examine the influence of kinship and proximity on the social behavior of Mongolian gerbils Meriones unguiculatus living in different social groups, and whether these effects varied with sex and season. We recorded the duration of 4 behavioral categories (investigative, neutral, amicable, and agonistic) during a 10-min pairwise test. We found that genetic relatedness had significant effects on the duration of investigative, neutral, and amicable behavior, but not on agonistic behavior. We also found significant interaction effects of relatedness and distance between burrow systems (i.e., spatial distance) on investigative, neutral, and amicable behavior, which suggests that the effects of kinship on social behavior were restricted by spatial proximity. The interaction effect between sex and relatedness on amicable behavior showed that male gerbils became more intimate with individuals of the same sex that had higher pairwise relatedness than females. Furthermore, both male and female gerbils enhanced their aggression during the food-hoarding season, but the intensity of these changes was significantly higher in females. Overall, our results suggest that the effects of kinship and spatial proximity on social behavior exhibit sexual or seasonal patterns, thereby implying ecological context-dependent responses to out-group individuals in Mongolian gerbils. | NA | no | No good fitness measure | NA | no | no |
| 4919 | 2020 | 44112 | Dervishi, A; Jakse, J; Ismaili, H; Javornik, B; Stajner, N | 2018 | Comparative assessment of genetic diversity in Albanian olive (Olea europaea L.) using SSRs from anonymous and transcribed genomic regions | A total of 560,578 sequencing reads obtained from the de novo NGS transcriptome sequencing of Olea europaea were searched for the presence of tri- and tetra-nucleotide repeat motifs, which resulted in 375 tri-nucleotide microsatellites and 252 tetra-nucleotides. Twelve expressed sequence tags (EST)-derived SSRs that gave a reproducible pattern, enabling easy scoring and allele size determination, were further tested for polymorphism on 165 cultivars O. europaea subsp. sativa and on 18 oleasters (O. europaea subsp. sylvestris). The main diversity parameters of EST-SSRs were compared to the genomic SSRs that have been most frequently used so far in olive genotyping and mapping studies. Diversity parameters were also compared for different lengths of repeat motifs (di-nucleotide, tri-nucleotide, and tetra-nucleotide microsatellites). The occurrence of null alleles was only observed in anonymous SSRs, while their frequencies were negative for all EST-SSR loci. Eight out of 12 EST-SSRs were classified as highly informative, indicating their applicability in assessing molecular diversity and genetic relatedness in olive. The 12 novel EST-SSR markers selected as a subset of all identified loci containing SSR motifs showed their suitability for evaluation of genetic diversity and population structure and for genetic mapping. They were also linked to genes and, as shown by annotation analysis, four of them have potential for interpretation of allelic variability related to disease resistance and can be evaluated as a potential diagnostic marker for disease resistance. The development of new EST-SSRs provides a valuable tool in molecular breeding programs for both cultivated and wild olives. | NA | no | No good fitness measure and measure host GD | NA | no | no |
| 4920 | 2020 | 44113 | Dettori, CA; Serreli, L; Lombrana, AC; Fois, M; Tamburini, E; Porceddu, M; Fenu, G; Cogoni, D; Bacchetta, G | 2018 | The genetic structure and diversity of Gentiana lutea subsp lutea (Gentianaceae) in Sardinia: further insights for its conservation planning | Knowledge of the levels of genetic diversity and of the spatial genetic structure of plant species is important to ensure their effective management and conservation, especially in the case of endangered species. Gentiana lutea L. subsp. lutea is a long-lived plant which occurs in central and southern European mountains. It has a long-standing history of human exploitation, mainly in the liqueur and in the pharmaceutical industries and it is currently listed in the EU Habitats Directive 92/43/EEC Annex V. Mainly due to a prolonged root harvesting, its current distribution range in Sardinia consists of only a few groups of individuals limited to small areas of the Gennargentu massif (Central-Eastern area of the island). In this study, we investigated the levels of genetic diversity and the genetic structure of the species in Sardinia. We used AFLP (amplified fragment length polymorphism) markers to investigate the genetic variability of 182 samples from 13 subpopulations. A total of 433 fragments were detected, of which 75.5% were polymorphic. The levels of genetic diversity were generally high, but they tended to decrease in smaller subpopulations. Of the genetic variability 88% was found within subpopulations, while the genetic structure among them was fairly weak. In order to ensure the survival of these subpopulations, especially the smaller ones, ex situ and in situ management actions should be planned, such as the long term conservation of its seeds in germplasm repositories and their population reinforcements and monitoring. | NA | no | No good fitness measure and measure host GD | NA | no | no |
| 4921 | 2020 | 44042 | Dev, SA; Balakrishnan, S; Kurian, A; Sreekumar, VB | 2019 | Narrow gene pool can threaten the survival of Calamus nagbettai R. R. Fernald & Dey: a highly, endemic dioecious rattan species in the Western Ghats of India | Rattans, the spiny climbing palms of Arecaceae (Palmae) family exhibit high endemism to the biodiversity hot spots in India. Of the five rattan genera, Calamus is the only genus found in peninsular India with 15 of 21 species, endemic to the Western Ghats. The extensive utilization of rattans owing to their strength, durability and huge demand has resulted in depletion of their natural resources. Of the 15 endemic species, C. nagbettai is the most affected species on account of endemism, low population size and restricted distribution with fragmented populations. The present study revealed high amount of genetic diversity in the surviving scattered populations of the species using microsatellite markers. High gene flow (N-m=1.498) observed across the populations resulted in low genetic differentiation (14%). A clear genetic admixture could be seen in Kerala as well as one of the Karnataka’s populations while the remaining two populations were genetically distinct. UPGMA, PCoA and STRUCTURE analyses showed significantly different genetic composition in Kerala population compared to other populations. Kerala and Karnataka populations of C. nagbettai were also unique in their genetic structure and allelic composition. Therefore, effective management and conservation strategies have to be implemented to preserve the rare alleles with adaptive potential to protect this economically valuable Calamus species from endangerment. Overexploitation, low seed set and poor regeneration, as well as habitat fragmentation can further threaten the survival of this endemic, narrowly distributed dioecious rattan species in the Western Ghats region. | NA | no | No good fitness measure and measure host GD | NA | no | no |
| 4922 | 2020 | 44041 | Di Giannatale, E; Calistri, P; Di Donato, G; Decastelli, L; Goffredo, E; Adriano, D; Mancini, ME; Galleggiante, A; Neri, D; Antoci, S; Marfoglia, C; Marotta, F; Nuvoloni, R; Migliorati, G | 2019 | Thermotolerant Campylobacter spp. in chicken and bovine meat in Italy: Prevalence, level of contamination and molecular characterization of isolates | Campylobacter species are common foodborne pathogens associated with cases of human gastroenteritis worldwide. A detailed understanding of the prevalence, contamination levels and molecular characteristics of Campylobacter spp. in cattle and chicken, which are likely the most important sources of human contamination, is imperative. A collection of 1243 poultry meat samples (665 chicken breasts and 578 chicken thighs) and 1203 bovine meat samples (689 hamburgers and 514 knife-cut meat preparations) were collected at retail outlets, in randomly selected supermarkets located in different Italian regions during one year. Of these samples, 17.38% of the poultry meat and 0.58% of the bovine meat samples tested positive for Campylobacter, of which 131 were Campylobacter jejuni (57.96%) and 95 were Campylobacter coli (42.03%). Campylobacter isolates were genotyped with the aim of assessing the genetic diversity, population structure, source distribution and Campylobacter transmission route to humans. All isolates were molecularly characterized by pulse field gel electrophoresis (PFGE), and further genotyped using multilocus sequence typing (MLST) and fla-SVR sequencing to gain better insight into the population structure. Antibiotic resistance was also investigate. The highest levels of resistance among chicken strains were observed for ciprofloxacin (88.25%), nalidixic acid (81.45%) and tetracycline (75.6%). PFGE analysis revealed 73 pulsotypes for C. jejuni and 54 pulsotypes for C. coli, demonstrating the existance of different and specific clones circulating in Italy. MLST of C.jejuni isolates mainly clustered in the CC353, CC354, CC21, CC206 and CC443; while C.coli isolates clustered only in CC828. The most common flaA alleles were 287 for C. jejuni and 66 for C. coli. Our study confirms that poultry meat is the main source of Campylobacteriosis, whereas red meat had a low level of contamination suggesting a minor role in transmission. The high presence of Campylobacter in retail chicken meat, paired with its increased resistance to antimicrobials with several multidrug resistance profiles detected, is alarming and represents a persistent threat to public health. | NA | no | No good fitness measure and measure host GD | NA | no | no |
| 4923 | 2020 | 44042 | Diao, QY; Yang, DH; Zhao, HX; Deng, S; Wang, XL; Hou, CS; Wilfert, L | 2019 | Prevalence and population genetics of the emerging honey bee pathogen DWV in Chinese apiculture | Honey bees are agriculturally important, both as pollinators and by providing products such as honey. The sustainability of beekeeping is at risk through factors of global change such as habitat loss, as well as through the spread of infectious diseases. In China and other parts of Asia, beekeepers rely both on native Apis cerana and non-native Apis mellifera, putting bee populations at particular risk of disease emergence from multi-host pathogens. Indeed, two important honey bee parasites have emerged from East Asian honey bees, the mite Varroa destructor and the microsporidian Nosema ceranae. As V. destructor vectors viral bee diseases, we investigated whether another key bee pathogen, Deformed Wing Virus (DWV), may also have originated in East Asian honey bee populations. We use a large-scale survey of apiaries across China to investigate the prevalence and seasonality of DWV in managed A. mellifera and A. cerana colonies, showing that DWV-A prevalence was higher in A. mellifera, with a seasonal spike in prevalence in autumn and winter. Using phylogenetic and population genetic approaches, we show that while China and East Asian DWV isolates show comparatively high levels of genetic diversity, these bee populations are not a source for the current global DWV epidemic. | NA | no | gd of pathogen | NA | no | no |
| 4924 | 2020 | 44043 | Dibble, CJ; Rudolf, VHW | 2019 | Phenotype-Environment Matching Predicts Both Positive and Negative Effects of Intraspecific Variation | Natural populations can vary considerably in their genotypic and/or phenotypic diversity. Differences in this intraspecific diversity can have important consequences for contemporary ecological dynamics, but the direction and magnitude of these effects appear inconsistent across studies and systems. Here we proposed and tested the hypothesis that context-dependent ecological effects of altering phenotypic variance are predictable and arise from the relationship between a population’s mean phenotype and the local environmental optimum. By factorially manipulating the mean and variance of a key host trait in environments with and without a lethal parasite, we demonstrate that increasing phenotypic variance can have beneficial effects for host populations (e.g., smaller disease epidemics) but only when the population’s initial phenotype was poorly matched to the local environment. When phenotypes were initially well suited to environmental conditions, in contrast, greater phenotypic variance led to larger disease epidemics. Significant reductions in individual susceptibility occurred in both contexts over time, but the mechanisms leading to those reductions differed; strong selection was caused by either a suboptimal trait mean and insufficient trait variance or a near-optimal trait mean and too much trait variance. Increasing intraspecific variation is clearly not always beneficial for populations, instead producing predictable ecological and evolutionary effects that depend on environmental context and biological interactions. | NA | no | No good fitness measure and measure host GD | NA | no | no |
| 4925 | 2020 | 44112 | Dickey, AM; Schuller, G; Loy, JD; Clawson, ML | 2018 | Whole genome sequencing of Moraxella bovoculi reveals high genetic diversity and evidence for interspecies recombination at multiple loci | Moraxella bovoculi is frequently cultured from the ocular secretions and conjunctiva of cattle with Infectious Bovine Keratoconjunctivitis (IBK). Previous work has shown that single nucleotide polymorphism (SNP) diversity in this species is quite high with 81,284 SNPs identified in eight genomes representing two distinct genotypes isolated from IBK affected eyes (genotype 1) and the nasopharynx of cattle without clinical IBK signs (genotype 2), respectively. The goals of this study were to identify SNPs from a collection of geographically diverse and epidemiologically unlinked M. bovoculi strains from the eyes of IBK positive cattle (n = 183) and another from the eyes of cattle (most from a single population at a single time-point) without signs of IBK (n = 63) and to characterize the genetic diversity. Strains of both genotypes were identified from the eyes of cattle without IBK signs. Only genotype 1 strains were identified from IBK affected eyes, however, these strains were isolated before the discovery of genotype 2, and the protocol for their isolation would have preferentially selected genotype 1 M. bovoculi. The core genome comprised similar to 74% of the whole and contained >127,000 filtered SNPs. More than 80% of these characterize diversity within genotype 1 while 23,611 SNPs (similar to 18%) delimit the two major genotypes. Genotype 2 strains lacked a repeats-in-toxin (RTX) putative pathogenesis factor and any of ten putative antibiotic resistance genes carried within a genomic island. Within genotype 1, prevalence of these elements was 0.85 and 0.12 respectively in strains from eyes that were IBK positive. Recombination appears to be an important source of genetic diversity for genotype 1 and undermines the utility of ribosomal-locus-based species identification. The extremely high genetic diversity in genotype 1 presents a challenge to the development of an efficacious vaccine directed against them, however, several low-diversity pilin-like genes were identified. Finally, the genotype-defining SNPs described in this study are a resource that can facilitate the development of more accurate M. bovoculi diagnostic tests. | NA | no | No good fitness measure and measure host GD | NA | no | no |
| 4926 | 2020 | 44112 | Dillon, MM; Thakur, S; Almeida, RND; Wang, PW; Weir, BS; Guttman, DS | 2019 | Recombination of ecologically and evolutionarily significant loci maintains genetic cohesion in the Pseudomonas syringae species complex | BackgroundPseudomonas syringae is a highly diverse bacterial species complex capable of causing a wide range of serious diseases on numerous agronomically important crops. We examine the evolutionary relationships of 391 agricultural and environmental strains using whole-genome sequencing and evolutionary genomic analyses.ResultsWe describe the phylogenetic distribution of all 77,728 orthologous gene families in the pan-genome, reconstruct the core genome phylogeny using the 2410 core genes, hierarchically cluster the accessory genome, identify the diversity and distribution of type III secretion systems and their effectors, predict ecologically and evolutionary relevant loci, and establish the molecular evolutionary processes operating on gene families. Phylogenetic and recombination analyses reveals that the species complex is subdivided into primary and secondary phylogroups, with the former primarily comprised of agricultural isolates, including all of the well-studied P. syringae strains. In contrast, the secondary phylogroups include numerous environmental isolates. These phylogroups also have levels of genetic diversity typically found among distinct species. An analysis of rates of recombination within and between phylogroups revealed a higher rate of recombination within primary phylogroups than between primary and secondary phylogroups. We also find that ecologically significant virulence-associated loci and evolutionarily significant loci under positive selection are over-represented among loci that undergo inter-phylogroup genetic exchange.ConclusionsWhile inter-phylogroup recombination occurs relatively rarely, it is an important force maintaining the genetic cohesion of the species complex, particularly among primary phylogroup strains. This level of genetic cohesion, and the shared plant-associated niche, argues for considering the primary phylogroups as a single biological species. | NA | no | gd of pathogen | NA | no | no |
| 4927 | 2020 | 44043 | Dimitromanolakis, A; Paterson, AD; Sun, L | 2019 | Fast and Accurate Shared Segment Detection and Relatedness Estimation in Un-phased Genetic Data via TRUFFLE | Relationship estimation and segment detection between individuals is an important aspect of disease gene mapping. Existing methods are either tailored for computational efficiency or require phasing to improve accuracy. We developed TRUFFLE, a method that integrates computational techniques and statistical principles for the identification and visualization of identity-by-descent (IBD) segments using un-phased data. By skipping the haplotype phasing step and, instead, relying on a simpler region-based approach, our method is computationally efficient while maintaining inferential accuracy. In addition, an error model corrects for segment break-ups that occur as a consequence of genotyping errors. TRUFFLE can estimate relatedness for 3.1 million pairs from the 1000 Genomes Project data in a few minutes on a typical laptop computer. Consistent with expectation, we identified only three second cousin or closer pairs across different populations, while commonly used methods identified a large number of such pairs. Similarly, within populations, we identified many fewer related pairs. Compared to methods relying on phased data, TRUFFLE has comparable accuracy but is drastically faster and has fewer broken segments. We also identified specific local genomic regions that are commonly shared within populations, suggesting selection. When applied to pedigree data, we observed 99.6% accuracy in detecting 1st to 5th degree relationships. As genomic datasets become much larger, TRUFFLE can enable disease gene mapping through implicit shared haplotypes by accurate IBD segment detection. | NA | no | human | NA | no | no |
| 4928 | 2020 | 44112 | Dimova, M; Madyarova, E; Gurkov, A; Drozdova, P; Lubyaga, Y; Kondrateva, E; Adelshin, R; Timofeyev, M | 2018 | Genetic diversity of Microsporidia in the circulatory system of endemic amphipods from different locations and depths of ancient Lake Baikal | Endemic amphipods (Amphipoda, Crustacea) of the most ancient and large freshwater Lake Baikal (Siberia, Russia) are a highly diverse group comprising > 15% of all known species of continental amphipods. The extensive endemic biodiversity of Baikal amphipods provides the unique opportunity to study interactions and possible coevolution of this group and their parasites, such as Microsporidia. In this study, we investigated microsporidian diversity in the circulatory system of 22 endemic species of amphipods inhabiting littoral, sublittoral and deep-water zones in all three basins of Lake Baikal. Using molecular genetic techniques, we found microsporidian DNA in two littoral (Eulinmogammarus verrucosus, Eulimnogammarus cyaneus), two littorksublittoral (Pallasea cancellus, Eulimnogammarus marituji) and two sublittoralideepwater (Acanthogarnmarus lappaceus longispinus, Acanthogarnmarus victorii maculosus) endemic species. Twenty sequences of the small subunit ribosomal (SSU) rDNA were obtained from the haemolymph of the six endemic amphipod species sampled from 0-60 m depths at the Southern Lake Baikal’s basin (only the Western shore) and at the Central Baikal. They form clusters with similarity to Enterocytospora Cucumispora Dictyocoela, and several unassigned Microsporidia sequences, respectively. Our sequence data show similarity to previously identified microsporidian DNA from inhabitants of both Lake Baikal and other water reservoirs. The results of our study suggest that the genetic diversity of Microsporidia in haemolymph of endemic amphipods from Lake Baikal does not correlate with host species, geographic location or depth factors but is homogeneously diverse. | NA | no | No good fitness measure and measure host GD | NA | no | no |
| 4929 | 2020 | 44113 | Ding, G; Xu, H; Oldroyd, BP; Gloag, RS | 2017 | Extreme polyandry aids the establishment of invasive populations of a social insect | Although monandry is believed to have facilitated the evolution of eusociality, many highly eusocial insects have since evolved extreme polyandry. The transition to extreme polyandry was likely driven by the benefits of within-colony genetic variance to task specialization and/or disease resistance, but the extent to which it confers secondary benefits, once evolved, is unclear. Here we investigate the consequences of extreme polyandry on the invasive potential of the Asian honey bee, Apis cerana. In honey bees and other Hymenoptera, small newly founded invasive populations must overcome the genetic constraint of their sex determination system that requires heterozygosity at a sex-determining locus to produce viable females. We find A. cerana queens in an invasive population mate with an average of 27 males (range 16-42) that would result in the founding queen/s carrying 75% of their source population’s sex alleles in stored sperm. This mating frequency is similar to native-range Chinese A. cerana (mean 29 males, range 19-46). Simulations reveal that extreme polyandry reduces the risk, relative to monandry or moderate polyandry, that colonies produce a high incidence of inviable brood in populations that have experienced a founder event, that is, when sex allele diversity is low and/or allele frequencies are unequal. Thus, extreme polyandry aids the invasiveness of A. cerana in two ways: (1) by increasing the sex locus allelic richness carried to new populations with each founder, thereby increasing sex locus heterozygosity; and (2) by reducing the population variance in colony fitness following a founder event. | NA | no | No good fitness measure | NA | no | no |
| 4930 | 2020 | 44113 | Djaout, A; Chiappini, B; Gaouar, SBS; Afri-Bouzebda, F; Conte, M; Chekkal, F; El-Bouyahiaoui, R; Boukhari, R; Agrimi, U; Vaccari, G | 2018 | Biodiversity and selection for scrapie resistance in sheep: genetic polymorphism in eight breeds of Algeria | Scrapie is a prion disease that affects the sheep and goats. It belongs to the group of transmissible spongiform encephalopathies (TSE). TSEs are characterized by the accumulation of the pathological form (PrPSc) of the cellular prion protein (PrPC). The susceptibility of sheep to scrapie is influenced by polymorphisms in the PrP gene (PRNP). The aim of this study was to identify the genetic variability of sheep PRNP in Algerian sheep. Two-hundred and thirteen Algerian sheep from eight breeds (Ouled Djellal, Rembi, Hamra, Berbere, Barbarine, Sidaou, Taadmit and Tazegzawt) with no clinical manifestation of scrapie were analysed. Sequencing of the entire coding sequence of PRNP showed four main alleles (ARQ, ARR, AHQ and ARH) based on codons 136, 154 and 171 with different frequencies among the investigated breeds. Moreover, 14 additional nonsynonymous polymorphisms (Q101R, N103K, M112T, A116P, M137I, L141F, I142M, H143R, N146S, R151G, Y172D, N176K, H180Y and S240P) as well as two synonymous polymorphisms at codons 231 and 237 were found in the PRNP gene. Interestingly, the N103K, M137I and I142M polymorphisms were not described in sheep. The ARQ, ARR and ARH haplotypes were present in all breeds with a highest frequency of ARQ in Barbarine. The ARH was absent in Barbarine breed and the VRQ haplotype was absent in all Algerian breeds studied. The ARQ and ARR alleles were the most common with frequencies ranging from 30 to 65% and from 8 to 26%, respectively, in different breeds. These results represent the first study on PRNP variability in Algerian sheep and may serve as a basis for the development of breeding programmes to render national sheep breeds resistant to scrapie. | NA | no | dometicated host | NA | no | no |
| 4931 | 2020 | 44112 | do Prado, FD; Vera, M; Hermida, M; Bouza, C; Pardo, BG; Vilas, R; Blanco, A; Fernandez, C; Maroso, F; Maes, GE; Turan, C; Volckaert, FAM; Taggart, JB; Carr, A; Ogden, R; Nielsen, EE; Martinez, P | 2018 | Parallel evolution and adaptation to environmental factors in a marine flatfish: Implications for fisheries and aquaculture management of the turbot (Scophthalmus maximus) | Unraveling adaptive genetic variation represents, in addition to the estimate of population demographic parameters, a cornerstone for the management of aquatic natural living resources, which, in turn, represent the raw material for breeding programs. The turbot (Scophthalmus maximus) is a marine flatfish of high commercial value living on the European continental shelf. While wild populations are declining, aquaculture is flourishing in southern Europe. We evaluated the genetic structure of turbot throughout its natural distribution range (672 individuals; 20 populations) by analyzing allele frequency data from 755 single nucleotide polymorphism discovered and genotyped by double-digest RAD sequencing. The species was structured into four main regions: Baltic Sea, Atlantic Ocean, Adriatic Sea, and Black Sea, with subtle differentiation apparent at the distribution margins of the Atlantic region. Genetic diversity and effective population size estimates were highest in the Atlantic populations, the area of greatest occurrence, while turbot from other regions showed lower levels, reflecting geographical isolation and reduced abundance. Divergent selection was detected within and between the Atlantic Ocean and Baltic Sea regions, and also when comparing these two regions with the Black Sea. Evidence of parallel evolution was detected between the two low salinity regions, the Baltic and Black seas. Correlation between genetic and environmental variation indicated that temperature and salinity were probably the main environmental drivers of selection. Mining around the four genomic regions consistently inferred to be under selection identified candidate genes related to osmoregulation, growth, and resistance to diseases. The new insights are useful for the management of turbot fisheries and aquaculture by providing the baseline for evaluating the consequences of turbot releases from restocking and farming. | NA | no | No good fitness measure and measure host GD | NA | no | no |
| 4932 | 2020 | 44113 | Dogan, MV; Grumbach, IM; Michaelson, JJ; Philibert, RA | 2018 | Integrated genetic and epigenetic prediction of coronary heart disease in the Framingham Heart Study | An improved method for detecting coronary heart disease (CHD) could have substantial clinical impact. Building on the idea that systemic effects of CHD risk factors are a conglomeration of genetic and environmental factors, we use machine learning techniques and integrate genetic, epigenetic and phenotype data from the Framingham Heart Study to build and test a Random Forest classification model for symptomatic CHD. Our classifier was trained on n = 1,545 individuals and consisted of four DNA methylation sites, two SNPs, age and gender. The methylation sites and SNPs were selected during the training phase. The final trained model was then tested on n = 142 individuals. The test data comprised of individuals removed based on relatedness to those in the training dataset. This integrated classifier was capable of classifying symptomatic CHD status of those in the test set with an accuracy, sensitivity and specificity of 78%, 0.75 and 0.80, respectively. In contrast, a model using only conventional CHD risk factors as predictors had an accuracy and sensitivity of only 65% and 0.42, respectively, but with a specificity of 0.89 in the test set. Regression analyses of the methylation signatures illustrate our ability to map these signatures to known risk factors in CHD pathogenesis. These results demonstrate the capability of an integrated approach to effectively model symptomatic CHD status. These results also suggest that future studies of biomaterial collected from longitudinally informative cohorts that are specifically characterized for cardiac disease at follow-up could lead to the introduction of sensitive, readily employable integrated genetic-epigenetic algorithms for predicting onset of future symptomatic CHD. | NA | no | No good fitness measure and measure host GD | NA | no | no |
| 4933 | 2020 | 44111 | Doke, MA; McGrady, CM; Otieno, M; Grozinger, CM; Frazier, M | 2019 | Colony Size, Rather Than Geographic Origin of Stocks, Predicts Overwintering Success in Honey Bees (Hymenoptera: Apidae) in the Northeastern United States | Honey bees (Apis mellifera L.) are key pollinators of agricultural crops. However, approximately 30% of managed colonies die each winter in the United States. There has been great interest in breeding for locally adapted stocks which survive winter conditions in a particular region. Here, we evaluate the impact of geographic origin of stock on colony weight, population size, and overwintering survival. Comparing four different U.S. honey bee stocks (two bred in southern and two bred in northern regions) under standard beekeeping practices in three different apiary locations in central Pennsylvania, we examined possible adaptation of these stocks to temperate conditions. We confirmed the genotypic difference among the stocks from different geographic origins via microsatellite analysis. We found that stock or region of origin was not correlated with weight, population size, or overwintering success. However, overwintering success was influenced by the weight and population size the colonies reached prior to winter where higher colony weight is a strong predictor of overwintering survival. Although the number of locations used in this study was limited, the difference in average colony sizes from different locations may be attributable to the abundance and diversity of floral resources near the honey bee colonies. Our results suggest that 1) honey bees may use similar strategies to cope with environmental conditions in both southern and northern regions, 2) colonies must reach a population size threshold to survive adverse conditions (an example of the Allee effect), and 3) landscape nutrition is a key component to colony survival. | NA | no | No good fitness measure | NA | no | no |
| 4934 | 2020 | 44113 | Dolka, B; Chrobak-Chmiel, D; Czopowicz, M; Szeleszczuk, P | 2017 | Characterization of pathogenic Enterococcus cecorum from different poultry groups: Broiler chickens, layers, turkeys, and waterfowl | Enterococcus cecorum (EC) is known as a commensal in the intestines of mammals and birds. However, it has been described as an emerging pathogen in poultry industry worldwide. The aim of this study was to analyze and compare EC isolated from clinical material collected from poultry groups with different production purposes. The genetic diversity among pathogenic EC in relation to each specific poultry type was examined. In total, 148 isolates from independent infection outbreaks (2011 - 2016) were used: 76 broiler chickens (CB), 37 broiler breeders (BB), 23 layers (CL), 7 waterfowl (W) and 5 turkey (T) flocks (1 iso-late/1 flock). We provided age ranges at diagnosis of EC-infection for 5 poultry groups. Isolates obtained from CB were significantly more frequently retrieved from bone marrow, joints, spine, and contrary to BB, CL less frequently retrieved from respiratory system. The study showed differences between EC of various poultry types in relation to 10/32 (31.3%) biochemical parameters. EC isolates from CB were significantly more often positive for beta GAL, beta NAG, MLZ, and less often positive for PAL and beta MAN than isolates from other poultry types. However, BB and W isolates showed higher ability to metabolise mannitol than CB, CL, and T. CB isolates showed lower ability to survive at 60 degrees C. Only chicken EC-isolates harbored virulence genes: CB (8.1%) > BB (3.4%) > CL (2%). No specific pulsotype of EC was associated with a specific poultry. One or several various (up to 6) genetic types of EC may be involved in outbreaks in CB flocks within one year in one region. Outbreaks reported in following years in the same region were usually caused by a distinct set of EC-genetic types. PFGE results indicated at the genetic heterogeneity among pathogenic isolates involved in outbreaks in relation to each poultry type. To our best knowledge, this is the first study which provides a comparison between clinical EC from 5 poultry groups. The study provides a new insight into EC as pathogen of different bird species. The obtained data may be useful in further studies on EC-infections more focused on a specific type of poultry. | NA | no | gd of pathogen | NA | no | no |
| 4935 | 2020 | 44113 | Domman, D; Quilici, ML; Dorman, MJ; Njamkepo, E; Mutreja, A; Mather, AE; Delgado, G; Morales-Espinosa, R; Grimont, PAD; Lizarraga-Partida, ML; Bouchier, C; Aanensen, DM; Kuri-Morales, P; Tarr, CL; Dougan, G; Parkhill, J; Campos, J; Cravioto, A; Weill, FX; Thomson, NR | 2017 | Integrated view of Vibrio cholerae in the Americas | Latin America has experienced two of the largest cholera epidemics in modern history; one in 1991 and the other in 2010. However, confusion still surrounds the relationships between globally circulating pandemic Vibrio cholerae clones and local bacterial populations. We used whole-genome sequencing to characterize cholera across the Americas over a 40-year time span. We found that both epidemics were the result of intercontinental introductions of seventh pandemic El Tor V. cholerae and that at least seven lineages local to the Americas are associated with disease that differs epidemiologically from epidemic cholera. Our results consolidate historical accounts of pandemic cholera with data to show the importance of local lineages, presenting an integrated view of cholera that is important to the design of future disease control strategies. | NA | no | No good fitness measure and measure host GD | NA | no | no |
| 4936 | 2020 | 44042 | Dong, CJ; Jiang, P; Zhang, JF; Li, XJ; Li, SJ; Bai, JJ; Fan, JJ; Xu, P | 2019 | High-Density Linkage Map and Mapping for Sex and Growth-Related Traits of Largemouth Bass (Micropterus salmoides) | The largemouth bass is an important species, and its culture has risen sharply with the surge in fish aquaculture in China. Due to the lack of selective breeding technology for the largemouth bass, the growth rate and disease resistance are low, its sexual maturation is slow, and other serious problems are contributing to a sharp decline in the safety and quality of largemouth bass products in recent decades. Therefore, comprehensive breeding programs to improve the economic performance and promote the modern industrial development of largemouth bass must be considered a priority. Here, a total of 152 adult largemouth bass, including two parents and 150 progenies, were selected to produce the genetic mapping family. Then, a high-density linkage map was constructed based on restriction site-associated DNA sequencing using 6,917 single-nucleotide polymorphisms (SNPs) located in 24 linkage groups (LGs). The total genetic length of the linkage map was 1,261.96 cM, and the length of each LG varied from 24.72 cM for LG02 to 117.53 cM for LG16, with an average length of 52.58 cM and an average SNP number of 286. Thirteen significant quantitative trait loci (QTLs) for sex determination were located on LG04, LG05, LG08, LG12, LG15, LG21, and LG23. An informative QTL cluster that included six QTLs was detected on LG12. However, one notable QTL, which accounted for 71.48% of the total phenotypic variation, was located in the region of 1.85 cM on LG05. In addition, 32 identified QTLs were related to growth, including body weight, body length, body height, and head length. The QTLs for these growth-related traits are located in 13 LG regions and have little effect on phenotypic variation. This high-density genetic linkage map will enable the fine-mapping of economic traits and support the future genome assembly of the largemouth bass. Additionally, our study will be useful for future selective culture of largemouth bass and could potentially be used in molecularassisted breeding of largemouth bass for aquaculture. | NA | no | No good fitness measure and measure host GD | NA | no | no |
| 4937 | 2020 | 44111 | Dong, ZK; Men, XY; Liu, S; Zhang, ZY | 2019 | Food web structure of parasitoids in greenhouses is affected by surrounding landscape at different spatial scales | Landscape management affects species interactions and can have notable effects on food web structure. Local parasitoid populations in greenhouses usually migrate from outside crops; biological control of greenhouse aphids may be thus highly dependent on the composition of surrounding landscape. However, it is less clear how surrounding landscape composition affects primary-hyperparasitoid food webs and pest control services in greenhouses. We investigated the food web of parasitoids on melon-cotton aphid (Aphis gossypii Glover) in watermelon greenhouses in two suburban Beijing counties over two years. We used the quantitative food web metrics (generality, vulnerability, link density, and interaction evenness) to assess the effects of landscape composition on primary-hyperparasitoid food web structure. We found that landscape with more cropland within 1-3 km tended to have more primary parasitoids per hyperparasitoid species (generality). Higher proportions of woodland at the 0.5 km scale were negatively correlated with the mean numbers of hyperparasitoid per primary parasitoid species (vulnerability), as well as with hyperparasitism rate and hyperparasitoid richness. Link density, interaction evenness and aphid mortality caused by parasitoids (parasitism rate) were not affected by landscape factors. However, active primary parasitism (biocontrol potential) increased with the proportion of woodland. This suggested that the bottom-up effect induced by primary parasitoids might benefit hyperparasitoids, thus exerting little influence of primary parasitoids on pest control. The top-down effect of hyperparasitoids may reduce with increasing woodland proportion. To enhance the effects of primary parasitoids, landscape management programs should also target, and thus limit the impact of hyperparasitoids. | NA | no | no good gd measure host | NA | no | no |
| 4938 | 2020 | 44112 | Donkersley, P; Blanford, JM; Queiroz, RB; Silva, FWS; Carvalho, CM; Al-Sadi, AM; Elliot, SL | 2018 | Witch ’ s Broom Disease of Lime (Candidatus Phytoplasma aurantifolia): Identifying High-Risk Areas by Climatic Mapping | Biological invasions of vectorborne diseases can be devastating. Bioclimatic modeling provides an opportunity to assess and predict areas at risk from complex multitrophic interactions of pathogens, highlighting areas in need of increased monitoring effort. Here, we model the distribution of an economically critical vectorborne plant pathogen ‘Candidatus Phytoplasma aurantifolia’, the etiological agent of Witches’ Broom Disease of Lime. This disease is a significant limiting factor on acid lime production (Citrus aurantifolia, Swingle) in the Middle East and threatens its production globally. We found that temperature, humidity, and the vector populations significantly determine disease distribution. Following this, we used bioclimatic modeling to predict potential novel sites of infections. The model outputs identified potential novel sites of infection in the citrus producing regions of Brazil and China. We also used our model to explore sites in Oman where the pathogen may not be infectious, and suggest nurseries be established there. Recent major turbulence in the citrus agricultural economy has highlighted the importance of this work and the need for appropriate and targeted monitoring programs to safeguard lime production. | NA | no | No good fitness measure and measure host GD | NA | no | no |
| 4939 | 2020 | 44112 | dos Santos, LF; Polveiro, RC; Moreira, TS; Vidigal, PMP; Chang, YF; Moreira, MAS | 2018 | Polymorphism analysis of the apxIA gene of Actinobacillus pleuropneumoniae serovar 5 isolated in swine herds from Brazil | The bacterium Actinobacillus pleuropneumoniae is the etiological agent of Contagious Porcine Pleuropneumonia, a disease responsible for economic losses in the swine industry worldwide. A. pleuropneumoniae is capable of producing proteinaceous exotoxins responsible for inducing hemorrhagic lesions, one of which is ApxI. Few studies have conducted an in-depth evaluation of polymorphisms of the nucleotides that make up the ApxI toxin gene. Here we analyze the polymorphisms of the apxIA gene region of A. pleuropneumoniae serovar 5 isolated from swine in different regions in Brazil and report the results of molecular sequencing and phylogenetic analysis. Analysis of the apxIA gene in 60 isolates revealed the presence of genetic diversity and variability. The polymorphisms in the nucleotide sequences determined the grouping of the Brazilian sequences and five more sequences from the GenBank database into 14 different haplotypes, which formed three main groups and revealed the presence of mutations in the nucleotide sequences. The estimation of selection pressures suggests the occurrence of genetic variations by positive selective pressure on A. pleuropneumoniae in large groups of animals in relatively small spaces. These conditions presumably favor the horizontal dissemination of apxIA gene mutations within bacterial populations with host reservoirs. As a result, the same serovar can demonstrate different antigenic capacities due to mutations in the apxIA gene. These alterations in sequences of the apxIA gene could occur in other areas of countries with intense swine production, which could lead to differences in the pathogenicity and immunogenicity of each serovar and have implications for the clinical status or diagnosis of A. pleuropneumoniae. | NA | no | No good fitness measure and measure host GD | NA | no | no |
| 4940 | 2020 | 44111 | dos Santos, VO; Viana, AP; Preisigke, SC; Santos, EA | 2019 | Characterization of a segregating population of passion fruit with resistance to Cowpea aphid-borne mosaic virus through morpho-agronomic descriptors | In passion-fruit breeding programs, characterizing genotypes developed through morpho-agronomic descriptors helps quantify genetic diversity and identify individuals with desirable qualities. We examined the discriminatory ability of passion-fruit descriptors and determined their relative importance in the characterization of 91 genotypes from a breeding program for resistance to Cowpea aphid-borne mosaic virus. Twenty-four quantitative and 14 multi-category qualitative descriptors related to plant, leaf, flower, and fruit characteristics were used. The quantitative descriptors were subjected to correlation and principal-component analyses and selected based on direct selection and the Singh method. The traits were used to obtain a distance matrix, based on Gower’s algorithm, and a comparative clustering between the dendrograms for the morpho-agronomic variables was obtained using the unweighted pair group method with arithmetic mean procedure. Based on the principal components method, the traits that most contributed to genetic variability were number of seeds (23.241%), petiole length (19.438%), and petal width (10.440%). In its turn by the Singh method, the traits androgynophore length (6.68%), followed by flower length (5.47%), area under the disease progress curve (5.27%), and peduncle length (5.17%) were those which most contributed to the differentiation of genotypes. Although seven descriptors (leaf length, bract width, corona long filament length, sepal length, fruit width, fruit mass, and mass of fruit pulp) showed little contribution to the characterization of genotypes, their discard is not suggested, as they significantly contribute to the discrimination of genetic divergence in the population. Comparative analysis between the dendrogram containing all descriptors and the dendrogram containing only flower, leaf, or fruit descriptors evidenced the need for using a large number of descriptors in the characterization of genetic diversity in Passiflora. The use of all 38 descriptors increased the efficiency in the discrimination of groups. | NA | no | No good fitness measure and measure host GD | NA | no | no |
| 4941 | 2020 | 44042 | Doyle, JM; Willoughby, JR; Bell, DA; Bloom, PH; Bragin, EA; Fernandez, NB; Katzner, TE; Leonard, K; DeWoody, JA | 2019 | Elevated Heterozygosity in Adults Relative to Juveniles Provides Evidence of Viability Selection on Eagles and Falcons | Viability selection yields adult populations that are more genetically variable than those of juveniles, producing a positive correlation between heterozygosity and survival. Viability selection could be the result of decreased heterozygosity across many loci in inbred individuals and a subsequent decrease in survivorship resulting from the expression of the deleterious alleles. Alternatively, locus-specific differences in genetic variability between adults and juveniles may be driven by forms of balancing selection, including heterozygote advantage, frequency-dependent selection, or selection across temporal and spatial scales. We use a pooled-sequencing approach to compare genome-wide and locus-specific genetic variability between 74 golden eagle (Aquila chrysaetos), 62 imperial eagle (Aquila heliaca), and 69 prairie falcon (Falco mexicanus) juveniles and adults. Although genome-wide genetic variability is comparable between juvenile and adult golden eagles and prairie falcons, imperial eagle adults are significantly more heterozygous than juveniles. This evidence of viability selection may stem from a relatively smaller imperial eagle effective population size and potentially greater genetic load. We additionally identify similar to 2000 single-nucleotide polymorphisms across the 3 species with extreme differences in heterozygosity between juveniles and adults. Many of these markers are associated with genes implicated in immune function or olfaction. These loci represent potential targets for studies of how heterozygote advantage, frequency-dependent selection, and selection over spatial and temporal scales influence survivorship in avian species. Overall, our genome-wide data extend previous studies that used allozyme or microsatellite markers and indicate that viability selection may be a more common evolutionary phenomenon than often appreciated. | NA | no | No good fitness measure and measure host GD | NA | no | no |
| 4942 | 2020 | 44111 | Doyle, SR; Illingworth, CJR; Laing, R; Bartley, DJ; Redman, E; Martinelli, A; Holroyd, N; Morrison, AA; Rezansoff, A; Tracey, A; Devaney, E; Berriman, M; Sargison, N; Cotton, JA; Gilleard, JS | 2019 | Population genomic and evolutionary modelling analyses reveal a single major QTL for ivermectin drug resistance in the pathogenic nematode, Haemonchus contortus | BackgroundInfections with helminths cause an enormous disease burden in billions of animals and plants worldwide. Large scale use of anthelmintics has driven the evolution of resistance in a number of species that infect livestock and companion animals, and there are growing concerns regarding the reduced efficacy in some human-infective helminths. Understanding the mechanisms by which resistance evolves is the focus of increasing interest; robust genetic analysis of helminths is challenging, and although many candidate genes have been proposed, the genetic basis of resistance remains poorly resolved.ResultsHere, we present a genome-wide analysis of two genetic crosses between ivermectin resistant and sensitive isolates of the parasitic nematode Haemonchus contortus, an economically important gastrointestinal parasite of small ruminants and a model for anthelmintic research. Whole genome sequencing of parental populations, and key stages throughout the crosses, identified extensive genomic diversity that differentiates populations, but after backcrossing and selection, a single genomic quantitative trait locus (QTL) localised on chromosome V was revealed to be associated with ivermectin resistance. This QTL was common between the two geographically and genetically divergent resistant populations and did not include any leading candidate genes, suggestive of a previously uncharacterised mechanism and/or driver of resistance. Despite limited resolution due to low recombination in this region, population genetic analyses and novel evolutionary models supported strong selection at this QTL, driven by at least partial dominance of the resistant allele, and that large resistance-associated haplotype blocks were enriched in response to selection.ConclusionsWe have described the genetic architecture and mode of ivermectin selection, revealing a major genomic locus associated with ivermectin resistance, the most conclusive evidence to date in any parasitic nematode. This study highlights a novel genome-wide approach to the analysis of a genetic cross in non-model organisms with extreme genetic diversity, and the importance of a high-quality reference genome in interpreting the signals of selection so identified. | NA | no | No good fitness measure and measure host GD | NA | no | no |
| 4943 | 2020 | 44039 | Drury, C; Portela, RP; Serrano, XM; Oleksiak, M; Baker, AC | 2020 | Fine-scale structure among mesophotic populations of the great star coral Montastraea cavernosa revealed by SNP genotyping | Mesophotic reefs (30-150 m) have been proposed as potential refugia that facilitate the recovery of degraded shallow reefs following acute disturbances such as coral bleaching and disease. However, because of the technical difficulty of collecting samples, the connectivity of adjacent mesophotic reefs is relatively unknown compared with shallower counterparts. We used genotyping by sequencing to assess fine-scale genetic structure of Montastraea cavernosa at two sites at Pulley Ridge, a mesophotic coral reef ecosystem in the Gulf of Mexico, and downstream sites along the Florida Reef Tract. We found differentiation between reefs at Pulley Ridge (similar to 68 m) and corals at downstream upper mesophotic depths in the Dry Tortugas (28-36 m) and shallow reefs in the northern Florida Keys (Key Biscayne, similar to 5 m). The spatial endpoints of our study were distinct, with the Dry Tortugas as a genetic intermediate. Most striking were differences in population structure among northern and southern sites at Pulley Ridge that were separated by just 12km. Unique patterns of clonality and outlier loci allele frequency support these sites as different populations and suggest that the long-distance horizontal connectivity typical of shallow-water corals may not be typical for mesophotic systems in Florida and the Gulf of Mexico. We hypothesize that this may be due to the spawning of buoyant gametes, which commits propagules to the surface, resulting in greater dispersal and lower connectivity than typically found between nearby shallow sites. Differences in population structure over small spatial scales suggest that demographic constraints and/or environmental disturbances may be more variable in space and time on mesophotic reefs compared with their shallow-water counterparts. | NA | no | No good fitness measure and measure host GD | NA | no | no |
| 4944 | 2020 | 44111 | Du, YH; Ma, JJ; Yin, ZQ; Liu, K; Yao, G; Xu, WF; Fan, LC; Du, BH; Ding, YQ; Wang, CQ | 2019 | Comparative genomic analysis of Bacillus paralicheniformis MDJK30 with its closely related species reveals an evolutionary relationship between B. paralicheniformis and B. licheniformis | Background: Members of the genus Bacillus are important plant growth-promoting rhizobacteria that serve as biocontrol agents. Bacillus paralicheniformis MDJK30 is a PGPR isolated from the peony rhizosphere and can suppress plant-pathogenic bacteria and fungi. To further uncover the genetic mechanism of the plant growth-promoting traits of MDJK30 and its closely related strains, we used comparative genomics to provide insights into the genetic diversity and evolutionary relationship between B. paralicheniformis and B. licheniformis. Results: A comparative genomics analysis based on B. paralicheniformis MDJK30 and 55 other previously reported Bacillus strains was performed. The evolutionary position of MDJK30 and the evolutionary relationship between B. paralicheniformis and B. licheniformis were evaluated by studying the phylogeny of the core genomes, a population structure analysis and ANI results. Comparative genomic analysis revealed various features of B. paralicheniformis that contribute to its commensal lifestyle in the rhizosphere, including an opening pan genome, a diversity of transport and the metabolism of the carbohydrates and amino acids. There are notable differences in the numbers and locations of the insertion sequences, prophages, genomic islands and secondary metabolic synthase operons between B. paralicheniformis and B. licheniformis. In particular, we found most gene clusters of Fengycin, Bacitracin and Lantipeptide were only present in B. paralicheniformis and were obtained by horizontal gene transfer (HGT), and these clusters may be used as genetic markers for distinguishing B. paralicheniformis and B. licheniformis. Conclusions: This study reveals that MDJK30 and the other strains of lineage paralicheniformis present plant growth-promoting traits at the genetic level and can be developed and commercially formulated in agriculture as PGPR. Core genome phylogenies and population structure analysis has proven to be a powerful tool for differentiating B. paralicheniformis and B. licheniformis. Comparative genomic analyses illustrate the genetic differences between the paralicheniformis-licheniformis group with respect to rhizosphere adaptation. | NA | no | No good fitness measure and measure host GD | NA | no | no |
| 4945 | 2020 | 44111 | Du, ZL; Ma, L; Qu, HZ; Chen, W; Zhang, B; Lu, X; Zhai, WB; Sheng, X; Sun, YQ; Li, WJ; Lei, M; Qi, QH; Yuan, N; Shi, S; Zeng, JY; Wang, JY; Yang, YD; Liu, Q; Hong, YQ; Dong, LL; Zhang, ZW; Zou, D; Wang, YQ; Song, SH; Liu, F; Fang, XD; Chen, H; Liu, X; Xiao, JF; Zeng, CQ | 2019 | Whole Genome Analyses of Chinese Population and De Novo Assembly of A Northern Han Genome | To unravel the genetic mechanisms of disease and physiological traits, it requires comprehensive sequencing analysis of large sample size in Chinese populations. Here, we report the primary results of the Chinese Academy of Sciences Precision Medicine Initiative (CASPMI) project launched by the Chinese Academy of Sciences, including the de novo assembly of a northern Han reference genome (NH1.0) and whole genome analyses of 597 healthy people coming from most areas in China. Given the two existing reference genomes for Han Chinese (YH and HX1) were both from the south, we constructed NH1.0, a new reference genome from a northern individual, by combining the sequencing strategies of PacBio, 10x Genomics, and Bionano mapping. Using this integrated approach, we obtained an N50 scaffold size of 46.63 Mb for the NH1.0 genome and performed a comparative genome analysis of NH1.0 with YH and HX1. In order to generate a genomic variation map of Chinese populations, we performed the whole-genome sequencing of 597 participants and identified 24.85 million (M) single nucleotide variants (SNVs), 3.85 M small indels, and 106,382 structural variations. In the association analysis with collected phenotypes, we found that the T allele of rs1549293 in KAT8 significantly correlated with the waist circumference in northern Han males. Moreover, significant genetic diversity in MTHFR, TCN2, FADS1, and FADS2, which associate with circulating folate, vitamin B12, or lipid metabolism, was observed between northerners and southerners. Especially, for the homocysteine-increasing allele of rs1801133 (MTHFR 677T), we hypothesize that there exists a comfort zone for a high frequency of 677T between latitudes of 35-45 degree North. Taken together, our results provide a high-quality northern Han reference genome and novel population-specific data sets of genetic variants for use in the personalized and precision medicine. | NA | no | human | NA | no | no |
| 4946 | 2020 | 44112 | Dubs, F; Vergnes, A; Mirlicourtois, E; Le Viol, I; Kerbiriou, C; Goulnik, J; Belghali, S; Bentze, L; Barot, S; Porcher, E | 2018 | Positive effects of wheat variety mixtures on aboveground arthropods are weak and variable | Although modern agriculture generally relies on homogeneous varieties that are usually grown in pure stands, crop variety mixtures have been used for a long time, notably to improve resistance to fungal diseases. A growing number of studies suggest that intraspecific plant diversity may also enhance the abundance and diversity of wild species and thereby some ecosystem services such as biological control by natural predators. However, positive effects of the genetic diversity of plant species on the diversity of associated communities have mostly been documented in natural systems, with only a handful of studies targeting crop species in agroecosystems. Here, we investigated the ecological effects of the number of winter wheat varieties (Triticum aestivum) on aboveground arthropods and particularly predatory species. We manipulated the number of wheat varieties (1, 2,4 or 8) in 120 plots (80 m(2) each) to examine how wheat diversity and stand characteristics impact communities of three dominant aboveground arthropod groups that include many predatory species: ground beetles, rove beetles and spiders. The number of wheat varieties had a weak, but positive effect on predator abundance, notably spider abundance. In contrast, wheat functional diversity, as assessed by the number of wheat functional groups, was only negatively related to the diversity of spiders. Among wheat stand characteristics, the variance in plant height, wheat biomass and the Green Area Index were weakly correlated with ground beetle, rove beetle and predatory diversity, respectively. The Green Area Index was also weakly correlated with ground beetle abundance. Our study suggests that wheat variety mixtures have variable and limited effects on aboveground arthropods and probably low effectiveness to enhance biological control, but these results should be further tested under low-input agriculture in real fields. (C) 2018 Gesellschaft fur Okologie. Published by Elsevier GmbH. All rights reserved. | NA | no | dometicated host | NA | no | no |
| 4947 | 2020 | 44111 | Dumont, BL | 2019 | Significant Strain Variation in the Mutation Spectra of Inbred Laboratory Mice | Mutation provides the ultimate source of all new alleles in populations, including variants that cause disease and fuel adaptation. Recent whole genome sequencing studies have uncovered variation in the mutation rate among individuals and differences in the relative frequency of specific nucleotide changes (the mutation spectrum) between populations. Although parental age is a major driver of differences in overall mutation rate among individuals, the causes of variation in the mutation spectrum remain less well understood. Here, I use high-quality whole genome sequences from 29 inbred laboratory mouse strains to explore the root causes of strain variation in the mutation spectrum. My analysis leverages the unique, mosaic patterns of genetic relatedness among inbredmouse strains to identify strain private variants residing on haplotypes shared between multiple strains due to their recent descent from a common ancestor. I show that these strain-private alleles are strongly enriched for recent de novo mutations and lack signals of widespread purifying selection, suggesting their faithful recapitulation of the spontaneous mutation landscape in single strains. The spectrum of strain-private variants varies significantly among inbred mouse strains reared under standardized laboratory conditions. This variation is not solely explained by strain differences in age at reproduction, raising the possibility that segregating genetic differences affect the constellation of new mutations that arise in a given strain. Collectively, these findings imply the action of remarkably precise nucleotide-specific genetic mechanisms for tuning the de novo mutation landscape in mammals and underscore the genetic complexity of mutation rate control. | NA | no | inbreeding | NA | no | no |
| 4948 | 2020 | 44113 | Durand, S; Loiseau, V; Prigot, C; Braquart-Varnier, C; Beltran-Bech, S | 2018 | Producing offspring in Armadillidium vulgare: Effects of genetic diversity and inbreeding | Genetic diversity is known to be correlated to fitness traits, and inbred individuals often display lower values for life history traits. In this study, we attempt to quantify how inbreeding affects such traits in the terrestrial isopod Armadillidium vulgare by performing inbred and non-inbred crosses under laboratory conditions. We estimated genetic characteristics of parents and offspring, and related them to fecundity and fertility measures, as well as offspring growth and survival. Our study shows that a decrease in offspring number might result from mortality around birth, but not to changes in fecundity, fertilization rate, or developmental failure between inbred and non-inbred crosses. More heterozygous females tended to be bigger and had a higher fecundity, which could have implications in mate choice. No effect of inbreeding was detected on offspring growth and survival. These results can be related to previously observed effects of genetic characteristics on mating strategies in A. vulgare, and could shed light on mechanisms of inbreeding avoidance in this species. | NA | no | inbreeding | NA | no | no |
| 4949 | 2020 | 44113 | Dussex, N; Robertson, BC | 2018 | Contemporary effective population size and predicted maintenance of genetic diversity in the endangered kea (Nestor notabilis) | Population size and the potential for maintenance of genetic diversity are critical information for the monitoring of species of conservation concern. However, direct estimates of population size are not always feasible, making indirect genetic approaches a valuable alternative. We estimated contemporary effective population size (Ne) in the endangered kea (Nestor notabilis) using three different methods. We then inferred the census size (NC) using published Ne/NC ratios and modelled the future maintenance of genetic diversity assuming a number of demographic parameters. Short-term Ne was small with a rangewide Ne < 250-700, while NC was within the range of the current estimate (c. 1000-5000). Forward simulations showed low probability of retaining 90% of rare alleles without immigration. However, the probability of maintaining genetic diversity was high with immigration, juvenile survival of >= 30%, and an initial sex ratio of c. 0.5-0.6. Despite the low Ne in kea, predator control and/ or artificial immigration might be sufficient to maintain the present genetic diversity. | NA | no | no good gd measure host pop | NA | no | no |
| 4950 | 2020 | 44039 | E, GX; Duan, XH; Yang, BG; Na, RS; Han, YG; Zeng, Y | 2020 | Genetic Diversity Pattern of the MHC-LEI0258 Locus across Asian Populations of Chickens | The major histocompatibility complex (MHC) is surprisingly well associated with epidemiological and pathogenic environments, and the MHC-related microsatellite LEI0258 is a useful biomarker for indicating MHC haplotypes. In the present study, we conducted a comprehensive analysis of LEI0258 diversity in 492 Asian chickens and White Leghorns using multiple strategies to gain insight into the effectiveness of this adaptive locus for assessing the diversity and genetics of populations and the potential forces that affect MHC evolution. Comparisons between five Asian local breeds with wide geographic distributions and one commercial breed (White Leghorn chickens) revealed a total of 82 different alleles. The overall observed heterozygosity was 85.55% in Asian chickens and 30.98% in White Leghorn chickens, indicating higher LEI0258 diversity in Asian chickens than in the commercial breed. In addition, a population structure analysis of nine wild jungle fowl populations and several geographically varied Asian chicken populations revealed consistent clustering. Moreover, the results of AMOVA among jungle fowl populations suggested that most of the diversity was found among individuals within populations and within individuals. High levels of polymorphism and divergence were observed in the MHC region within and among populations, whereas limited polymorphisms and divergence were present among geographic groups. Hence, we concluded that the MHC in chickens has low intraspecific and interbreed variation and cannot be adapted as a tool to estimate diversity and migration based on drift and neutral events. | NA | no | No good fitness measure | NA | no | no |
| 4951 | 2020 | 44113 | Eamsobhana, P; Yong, HS; Song, SL; Prasartvit, A; Boonyong, S; Tungtrongchitr, A | 2018 | Cytochrome c oxidase subunit I haplotype reveals high genetic diversity of Angiostrongylus malaysiensis (Nematoda: Angiostrongylidae) | The rat lungworm Angiostrongylus malaysiensis is a metastrongyloid nematode parasite. It has been reported in Malaysia, Thailand, Laos, Myanmar, Indonesia and Japan. In this study, A. malaysiensis adult worms recovered from the lungs of wild rats in different geographical regions/provinces in Thailand were used to determine their haplotype by means of the mitochondrial partial cytochrome c oxidase subunit I (COI) gene sequence. The results revealed high COI haplotype diversity of A. malaysiensis from Thailand. The geographical isolates of A. malaysiensis from Thailand and other countries formed a monophyletic clade distinct from the closely related A. cantonensis. In the present study, five new haplotypes were identified in addition to the four haplotypes reported in the literature. Phylogenetic analysis revealed that four of these five new haplotypes - one from Mae Hong Song (northern region), two from Tak (western region) and one from Phang Nga (southern region) - formed a distinct clade with those from Phatthalung (southern region) and Malaysia. The haplotype from Malaysia was identical to that of Phatthalung (haplotype AM1). In general, the COI sequences did not differentiate unambiguously the various geographical isolates of A. malaysiensis. This study has confirmed the presence of high COI genetic diversity in various geographical isolates of A. malaysiensis. The COI gene sequence will be suitable for studying genetic diversity, population structure and phylogeography. | NA | no | gd of pathogen | NA | no | no |
| 4952 | 2020 | 44113 | Early, AM; Lievens, M; MacInnis, BL; Ockenhouse, CF; Volkman, SK; Adjei, S; Agbenyega, T; Ansong, D; Gondi, S; Greenwood, B; Hamel, M; Odero, C; Otieno, K; Otieno, W; Owusu-Agyei, S; Asante, KP; Sorgho, H; Tina, L; Tinto, H; Valea, I; Wirth, DF; Neafsey, DE | 2018 | Host-mediated selection impacts the diversity of Plasmodium falciparum antigens within infections | Host immunity exerts strong selective pressure on pathogens. Population-level genetic analysis can identify signatures of this selection, but these signatures reflect the net selective effect of all hosts and vectors in a population. In contrast, analysis of pathogen diversity within hosts provides information on individual, host-specific selection pressures. Here, we combine these complementary approaches in an analysis of the malaria parasite Plasmodium falciparum using haplotype sequences from thousands of natural infections in sub-Saharan Africa. We find that parasite genotypes show preferential clustering within multi-strain infections in young children, and identify individual amino acid positions that may contribute to strain-specific immunity. Our results demonstrate that natural host defenses to P. falciparum act in an allele-specific manner to block specific parasite haplotypes from establishing blood-stage infections. This selection partially explains the extreme amino acid diversity of many parasite antigens and suggests that vaccines targeting such proteins should account for allele-specific immunity. | NA | no | human | NA | no | no |
| 4953 | 2020 | 44113 | Eastwood, JR; Ribot, RFH; Rollins, LA; Buchanan, KL; Walder, K; Bennett, ATD; Berg, ML | 2017 | Host heterozygosity and genotype rarity affect viral dynamics in an avian subspecies complex | Genetic diversity at community, population and individual levels is thought to influence the spread of infectious disease. At the individual level, inbreeding and heterozygosity are associated with increased risk of infection and disease severity. Host genotype rarity may also reduce infection risk if pathogens are co-adapted to common or local hosts, but to date, no studies have investigated the relative importance of genotype rarity and heterozygosity for infection in a wild, sexually reproducing vertebrate. With beak and feather disease virus (BFDV) infection in a wild parrot (Platycercus elegans), we show that both heterozygosity and genotype rarity of individual hosts predicted infection, but in contrasting ways. Heterozygosity was negatively associated with probability of infection, but not with infection load. In contrast, increased host genotype rarity was associated with lower viral load in infected individuals, but did not predict infection probability. These effects were largely consistent across subspecies, but were not evident at the population level. Subspecies and age were also strongly associated with infection. Our study provides novel insights into infection dynamics by quantifying rarity and diversity simultaneously. We elucidate roles that host genetic diversity can play in infection dynamics, with implications for understanding population divergence, intraspecific diversity and conservation. | NA | no | no good gd measure host pop | NA | no | no |
| 4954 | 2020 | 44112 | Ebbs, ET; Loker, ES; Brant, SV | 2018 | Phylogeography and genetics of the globally invasive snail physa acuta Draparnaud 1805, and its potential to serve as an intermediate host to larval digenetic trematodes | Background: Physa acuta is a globally invasive freshwater snail native to North America. Prior studies have led to conflicting views of how P. acuta populations are connected and genetic diversity is partitioned globally. This study aims to characterize phylogeographic and population genetic structure within the native range of P. acuta, elucidate its invasion history and assess global patterns of genetic diversity. Further, using meta-analytic methods, we test the ‘Enemy Release hypothesis’ within the P. acuta - digenetic trematode system. The ‘Enemy Release hypothesis’ refers to the loss of native parasites following establishment of their host within an invasive range. Population genetic data is combined with surveys of trematode infections to map range-wide trematode species richness associated with P. acuta, and to identify relevant host-population parameters important in modeling host parasite invasion. Results: Phylogenetic analyses using mtDNA uncovered two major clades (A & B). Clade A occurs globally while clade B was only recovered from the Western USA. All invasive populations sampled grouped within Clade A, where multiple independent source populations were identified from across North America. Significant population genetic structure was found within the native range of P. acuta, with some evidence for contemporary geographic barriers between western and eastern populations. Mito-nuclear discordance was found suggesting historical isolation with secondary contact between the two mitochondrial clades. Trematode species richness was found to differ significantly between native and invasive populations, in concordance with the ‘Enemy Release hypothesis’. Further, our data suggests a positive relationship between nucleotide diversity of invasive populations and trematode prevalence and richness. Conclusions: This study includes a wider geographic sampling of P. acuta within its native range that provides insight into phylogeographic and population genetic structure, range-wide genetic diversity and estimation of the invasion history. Meta-analysis of P. acuta trematode surveys globally is consistent with the ‘Enemy-Release hypothesis’. Additionally, results from this study suggest that host demographic parameters, namely genetic diversity as a proxy for population size, may play an essential role in how parasite communities assemble within invasive host populations. This knowledge can be used to begin to construct a framework to model host-parasite invasion dynamics over time. | NA | no | No good fitness measure and measure host GD | NA | no | no |
| 4955 | 2020 | 44111 | Eck, JL; Stump, SM; Delavaux, CS; Mangan, SA; Comita, LS | 2019 | Evidence of within-species specialization by soil microbes and the implications for plant community diversity | Microbes are thought to maintain diversity in plant communities by specializing on particular species, but it is not known whether microbes that specialize within species (i.e., on genotypes) affect diversity or dynamics in plant communities. Here we show that soil microbes can specialize at the within-population level in a wild plant species, and that such specialization could promote species diversity and seed dispersal in plant communities. In a shadehouse experiment in Panama, we found that seedlings of the native tree species, Virola surinamensis (Myristicaceae), had reduced performance in the soil microbial community of their maternal tree compared with in the soil microbial community of a nonmaternal tree from the same population. Performance differences were unrelated to soil nutrients or to colonization by my-corrhizal fungi, suggesting that highly specialized pathogens were the mechanism reducing seedling performance in maternal soils. We then constructed a simulation model to explore the ecological and evolutionary consequences of genotype-specific pathogens in multispecies plant communities. Model results indicated that genotype-specific pathogens promote plant species coexistence-albeit less strongly than species-specific pathogens-and are most effective at maintaining species richness when genetic diversity is relatively low. Simulations also revealed that genotype-specific pathogens select for increased seed dispersal relative to species-specific pathogens, potentially helping to create seed dispersal landscapes that allow pathogens to more effectively promote diversity. Combined, our results reveal that soil microbes can specialize within wild plant populations, affecting seedling performance near conspecific adults and influencing plant community dynamics on ecological and evolutionary time scales. | NA | no | No good fitness measure and measure host GD | NA | no | no |
| 4956 | 2020 | 44111 | Eckardt, W; Stoinski, TS; Rosenbaum, S; Santymire, R | 2019 | Social and ecological factors alter stress physiology of Virunga mountain gorillas (Gorilla beringei beringei) | Living in a rapidly changing environment can alter stress physiology at the population level, with negative impacts on health, reproductive rates, and mortality that may ultimately result in species decline. Small, isolated animal populations where genetic diversity is low are at particular risks, such as endangered Virunga mountain gorillas (Gorilla beringei beringei). Along with climate change-associated environmental shifts that are affecting the entire population, subpopulations of the Virunga gorillas have recently experienced extreme changes in their social environment. As the growing population moves closer to the forest’s carrying capacity, the gorillas are coping with rising population density, increased frequencies of interactions between social units, and changing habitat use (e.g., more overlapping home ranges and routine ranging at higher elevations). Using noninvasive monitoring of fecal glucocorticoid metabolites (FGM) on 115 habituated Virunga gorillas, we investigated how social and ecological variation are related to baseline FGM levels, to better understand the adaptive capacity of mountain gorillas and monitor potential physiological indicators of population decline risks. Generalized linear mixed models revealed elevated mean monthly baseline FGM levels in months with higher rainfall and higher mean maximum and minimum temperature, suggesting that Virunga gorillas might be sensitive to predicted warming and rainfall trends involving longer, warmer dry seasons and more concentrated and extreme rainfall occurrences. Exclusive use of smaller home range areas was linked to elevated baseline FGM levels, which may reflect reduced feeding efficiency and increased travel efforts to actively avoid neighboring groups. The potential for additive effects of stress-inducing factors could have short- and long-term impacts on the reproduction, health, and ultimately survival of the Virunga gorilla population. The ongoing effects of environmental changes and population dynamics must be closely monitored and used to develop effective long-term conservation strategies that can help address these risk factors. | NA | no | no good gd measure host pop | NA | no | no |
| 4957 | 2020 | 44112 | Edworthy, AB; Langmore, NE; Heinsohn, R | 2019 | Native fly parasites are the principal cause of nestling mortality in endangered Tasmanian pardalotes | Established host-parasite interactions at an evolutionary equilibrium are not predicted to result in host population decline. However, parasites may become a major threat to host species weakened by other factors such as habitat degradation or loss of genetic diversity in small populations. We investigate an unusually virulent Tasmanian ectoparasite, Passeromyia longicornis, in its long-term hosts, the endangered forty-spotted pardalote (Pardalotus quadragintus) and striated pardalote (Pardalotus striatus) in southeastern Tasmania, Australia. We conducted a parasite elimination experiment to determine the net effect of parasites on forty-spotted pardalote nestling mortality, and monitored nestling parasite load and mortality in forty-spotted and striated pardalote nestlings during two breeding seasons (Aug-Jan, 2013-2015). Passeromyia longicornis larvae killed 81% of all forty-spotted pardalotes nestlings. Across 2 years, forty-spotted pardalotes fledged fewer nestlings (18%) than sympatric striated pardalotes (26%), and this difference was generated by a combination of higher parasite load and virulence in forty-spotted pardalote nests. These results show that native ectoparasites can cause substantial and potentially unsustainable mortality in their hosts. | NA | no | no good gd measure host pop | NA | no | no |
| 4958 | 2020 | 44042 | Ekroth, AKE; Rafaluk-Mohr, C; King, KC | 2019 | Host genetic diversity limits parasite success beyond agricultural systems: a meta-analysis | There is evidence that human activities are reducing the population genetic diversity of species worldwide. Given the prediction that parasites better exploit genetically homogeneous host populations, many species could be vulnerable to disease outbreaks. While agricultural studies have shown the devastating effects of infectious disease in crop monocultures, the wide-spread nature of this diversity-disease relationship remains unclear in natural systems. Here, we provide broad support that high population genetic diversity can protect against infectious disease by conducting a meta-analysis of 23 studies, with a total of 67 effect sizes. We found that parasite functional group (micro- or macroparasite) affects the presence of the effect and study setting (field or laboratory-based environment) influences the magnitude. Our study also suggests that host genetic diversity is overall a robust defence against infection regardless of host reproduction, parasite host range, parasite diversity, virulence and the method by which parasite success was recorded. Combined, these results highlight the importance of monitoring declines of host population genetic diversity as shifts in parasite distributions could have devastating effects on at-risk populations in nature. | NA | no | meta -analysis, screened reference list. Found one new study (Strauss 2017) | NA | yes | no |
| 4959 | 2020 | 44113 | El Hadad, S; Alakilli, S; Rabah, S; Sabir, J | 2018 | Sequence analysis of sub-genotype D hepatitis B surface antigens isolated from Jeddah, Saudi Arabia | Little is known about the prevalence of HBV genotypes/sub-genotypes in Jeddah province, although the hepatitis B virus (HBV) was identified as the most predominant type of hepatitis in Saudi Arabia. To characterize HBV genotypes/sub-genotypes, serum samples from 15 patients with chronic HBV were collected and subjected to HBsAg gene amplification and sequence analysis. Phylogenetic analysis of the HBsAg gene sequences revealed that 11 (48%) isolates belonged to HBV/D while 4 (18%) were associated with HBV/C. Notably, a HBV/D sub-genotype phylogenetic tree identified that eight current isolates (72%) belonged to HBV/D1, whereas three isolates (28%) appeared to be more closely related to HBV/D5, although they formed a novel cluster supported by a branch with 99% bootstrap value. Isolates belonging to D1 were grouped in one branch and seemed to be more closely related to various strains isolated from different countries. For further determination of whether the three current isolates belonged to HBV/D5 or represented a novel sub-genotype, HBV/DA, whole HBV genome sequences would be required. In the present study, we verified that HBV/D1 is the most prevalent HBV sub-genotype in Jeddah, and identified novel variant mutations suggesting that an additional sub-genotype designated HBV/DA should be proposed. Overall, the results of the present HBsAg sequence analyses provide us with insights regarding the nucleotide differences between the present HBsAg/D isolates identified in the populace of Jeddah, Saudi Arabia and those previously isolated worldwide. Additional studies with large numbers of subjects in other areas might lead to the discovery of the specific HBV strain genotypes or even additional new sub-genotypes that are circulating in Saudi Arabia. (C) 2016 The Authors. Production and hosting by Elsevier B.V. on behalf of King Saud University. | NA | no | gd of pathogen | NA | no | no |
| 4960 | 2020 | 44042 | Eliades, NGH; Papageorgiou, AC; Fady, B; Gailing, O; Leinemann, L; Finkeldey, R | 2019 | An approach to genetic resources conservation of peripheral isolated plant populations: the case of an island narrow endemic species | This study reinforces the argument that forest genetic resources for peripheral isolated plant populations (PIPP) need to come under rationalistic assessment, for developing and proposing conservation strategies of forest genetic resources. Cedrus brevifolia is a narrow endemic island tree occurring in a sole population. Using biparentally and paternally inherited microsatellites, the genetic contribution and genetic variation patterns within sites (subpopulations) and within plantations of this species were assessed. The C. brevifolia population recorded uneven partition of genetic diversity at the site and at local scales; mirroring the demography and genetic evolutionary factors that occurred in long-term at the intra-species level. Results from the plantations imply that screening and rejection of unsuitable seedlings before plantation establishment did not influence the genetic divergence of planted trees from the general genetic pool of natural population. Based on these outcomes, numerous conservation measures are proposed, towards maintaining the species’ genetic resources. These measures need to ensure the dynamic processes within stands. Thus, this study argues that for PIPP which recorded high genetic diversity and non-uniform distribution of genetic viability (complex genetic patterns), the several small conservation units seem to be the most rationalistic conservation units. In such case, forest genetic micro-reserves (GMR) can be enacted, where on-the-point dynamic in situ conservation activities can be adopted, while the GMR could be used for ex situ conservation purposes. In conclusion, in PIPP the maintenance of genetic diversity should be regarded as one of the main prerequisites for sustainable management and long-term survival of the species. | NA | no | No good fitness measure and measure host GD | NA | no | no |
| 4961 | 2020 | 44039 | Ennos, RA; Sjokvist, EI; Piotrowska, MJ; Riddell, C; Hoebe, PN | 2020 | Using genome resequencing to investigate racial structure, genetic diversity, sexual reproduction and hybridisation in the pine pathogen Dothistroma septosporum | Whole genome resequencing of 25 isolates of the ascomycete pine pathogen Dothistroma septosporum revealed the presence of three genetically distinct races in Scotland, confirming previous results based on microsatellite markers. The genetically uniform indigenous race from Scotland (NPR), is closely related to an introduced and genetically diverse southern race (SR) from England and France. Race LPR, confined to lodge-pole pine, is genetically divergent from these, but has close affinity with Canadian (CAN) isolates. Genome comparisons between individuals within CAN and SR races revealed short and scattered regions of near sequence identity, indicating significant sexual reproduction. Sequence identity patterns within LPR suggest that it derives from introduction of a single sexual fruit body from North America. Finally a first generation interracial hybrid between indigenous NPR and introduced SR was identified, signalling an increase in the evolutionary potential of D. septosporum in Scotland, and an enhanced risk to native Scots pine (Pinus sylvestris). (C) 2020 Elsevier Ltd and British Mycological Society. All rights reserved. | NA | no | Gd of pathogen | NA | no | no |
| 4962 | 2020 | 44113 | Epps, CW; Crowhurst, RS; Nickerson, BS | 2018 | Assessing changes in functional connectivity in a desert bighorn sheep metapopulation after two generations | Determining how species move across complex and fragmented landscapes and interact with human-made barriers is a major research focus in conservation. Studies estimating functional connectivity from movement, dispersal or gene flow usually rely on a single study period and rarely consider variation over time. We contrasted genetic structure and gene flow across barriers for a metapopulation of desert bighorn sheep (Ovis canadensis nelsoni) using genotypes collected 2000-2003 and 2013-2015. Based on the recently observed but unexpected spread of a respiratory pathogen across an interstate highway previously identified as a barrier to gene flow, we hypothesized that bighorn sheep changed how they interacted with that barrier, and that shifts in metapopulation structure influenced gene flow, genetic diversity and connectivity. Population assignment tests, genetic structure and genetic recapture demonstrated that bighorn sheep crossed the interstate highway in at least one location in 2013-2015, sharply reducing genetic structure between two populations, but supported conclusions of an earlier study that such crossings were very infrequent or unknown in 2000-2003. A recently expanded population established new links and caused decreases in genetic structure among multiple populations. Genetic diversity showed only slight increases in populations linked by new connections. Genetic structure and assignments revealed other previously undetected changes in movements and distribution, but much was consistent. Thus, we observed changes in both structural and functional connectivity over just two generations, but only in specific locations. Movement patterns of species should be revisited periodically to enable informed management, particularly in dynamic and fragmented systems. | NA | no | No good fitness measure and measure host GD | NA | no | no |
| 4963 | 2020 | 44112 | Erdem, G; Singh, AK; Brusnahan, AJ; Moore, AN; Barson, WJ; Leber, A; Vidal, JE; Atici, S; King, SJ | 2018 | Pneumococcal colonization among tracheostomy tube dependent children | Streptococcus pneumoniae colonization is a precursor to pneumococcal disease. Although children with a tracheostomy have an increased risk of pneumococcal pneumonia, the pneumococci colonizing their lower airways remain largely uncharacterized. We sought to compare lower respiratory tract isolates colonizing tracheostomy patients and a convenience sample of isolates from individuals intubated for acute conditions. We collected pneumococcal isolates from the lower respiratory tract of 27 patients with a tracheostomy and 42 patients intubated for acute conditions. We compared the penicillin susceptibility, rates of co-colonization, genetic background, and serotype of isolates colonizing these patient populations. Isolates from both groups showed high genetic diversity. Forty multi-locus sequence types and 20 serotypes were identified. There was no significant difference in serotype distribution, co-colonization rates, vaccine coverage, or non-susceptibility to penicillin among pneumococcal isolates from the two groups. Colonization of the lower airways with non-vaccine serotypes 15B/C, 23B and 35B was noted for the first time in patients with tracheostomies and supports recently observed increases in nasopharyngeal colonization and disease due to these serotypes. | NA | no | human | NA | no | no |
| 4964 | 2020 | 44111 | Eriksson, TH; Holldobler, B; Taylor, JE; Gadau, J | 2019 | Intraspecific variation in colony founding behavior and social organization in the honey ant Myrmecocystus mendax | Persistent cooperation between unrelated reproductives occurs rarely in mature eusocial insect societies, and when present, is frequently geographically constrained. Here we present genetic and behavioral evidence showing that primary polygyny occurs in some, but not all populations of the honey ant Myrmecocystus mendax. Specifically, we found that all mature colonies sampled in a population in the Sierra Ancha Mountains of central Arizona (USA) were polygynous with a relatively high number of queens (average=6.27), while the majority of mature colonies sampled in the Chiricahua Mountains of southeastern Arizona were monogynous. Field and laboratory observations showed that Chiricahua foundresses are primarily haplometrotic, whereas Sierra Ancha foundresses can be either haplometrotic or facultatively pleometrotic. Nestmate relatedness of mature Sierra Ancha field colonies suggests that the reproductive individuals within these colonies are unrelated, consistent with primary polygyny. In the laboratory, Sierra Ancha foundresses cooperatively established incipient colonies without queen reduction, and colonies with multiple queens produced more minims andworkers that may serve the role ofrepletes (honeypots) than haplometrotic colonies. This was in stark contrast to foundresses from the Chiricahua population, which showed strong aggression when forced to found colonies together in the laboratory. When brood raiding was experimentally induced between laboratory Sierra Ancha colonies, queens from colonies with more workers had a higher survival probability, although in some cases the competing colonies fused and queens from both colonies continued to reproduce. Fusion between incipient ant colonies is a rare phenomenon, but could contribute to the high frequency of polygyny and high queen number in mature colonies in the Sierra Ancha population. | NA | no | No good fitness measure | NA | no | no |
| 4965 | 2020 | 44042 | Erin, NI; Benesh, DP; Henrich, T; Samonte, IE; Jakobsen, PJ; Kalbe, M | 2019 | Examining the role of parasites in limiting unidirectional gene flow between lake and river sticklebacks | 1. Parasites are important selective agents with the potential to limit gene flow between host populations by shaping local host immunocompetence. 2. We report on a contact zone between lake and river three-spined sticklebacks (Gasterosteus aculeatus) that offers the ideal biogeographic setting to explore the role of parasite-mediated selection on reproductive isolation. A waterfall acts as a natural barrier and enforces unidirectional migration from the upstream river stickleback population to the downstream river and lake populations. 3. We assessed population genetic structure and parasite communities over four years. In a set of controlled experimental infections, we compared parasite susceptibility of upstream and downstream fish by exposing laboratory-bred upstream river and lake fish, as well as hybrids, to two common lake parasite species: a generalist trematode parasite, Diplostomum pseudospathaceum, and a host-specific cestode, Schistocephalus solidus. 4. We found consistent genetic differentiation between upstream and downstream populations across four sampling years, even though the downstream river consisted of similar to 10% first-generation migrants from the upstream population as detected by parentage analysis. Fish in the upstream population had lower genetic diversity and were strikingly devoid of macroparasites. Through experimental infections, we demonstrated that upstream fish and their hybrids had higher susceptibility to parasite infections than downstream fish. Despite this, naturally sampled upstream migrants were less infected than downstream residents. Thus, migrants coming from a parasite-free environment may enjoy an initial fitness advantage, but their descendants seem likely to suffer from higher parasite loads. 5. Our results suggest that adaptation to distinct parasite communities can influence stickleback invasion success and may represent a barrier to gene flow, even between close and connected populations. | NA | no | no good gd measure host | NA | no | no |
| 4966 | 2020 | 44111 | Erlandson, MA; Mori, BA; Coutu, C; Holowachuk, J; Olfert, OO; Gariepy, TD; Hegedus, DD | 2019 | Examining population structure of a bertha armyworm, Mamestra configurata (Lepidoptera: Noctuidae), outbreak in western North America: Implications for gene flow and dispersal | The bertha armyworm (BAW), Mamestra configurata, is a significant pest of canola (Brassica napus L. and B. rapa L.) in western North America that undergoes cyclical outbreaks every 6-8 years. During peak outbreaks millions of dollars are spent on insecticidal control and, even with control efforts, subsequent damage can result in losses worth millions of dollars. Despite the importance of this pest insect, information is lacking on the dispersal ability of BAW and the genetic variation of populations from across its geographic range which may underlie potential differences in their susceptibility to insecticides or pathogens. Here, we examined the genetic diversity of BAW populations during an outbreak across its geographic range in western North America. First, mitochondrial cytochrome oxidase 1 (CO1) barcode sequences were used to confirm species identification of insects captured in a network of pheromone traps across the range, followed by haplotype analyses. We then sequenced the BAW genome and used double-digest restriction site associated DNA sequencing, mapped to the genome, to identify 1000s of single nucleotide polymorphisms (SNP) markers. CO1 haplotype analysis identified 9 haplotypes distributed across 28 sample locations and three laboratory-reared colonies. Analysis of genotypic data from both the CO1 and SNP markers revealed little population structure across BAW’s vast range. The CO1 haplotype pattern showed a star-like phylogeny which is often associated with species whose population abundance and range has recently expanded and combined with pheromone trap data, indicates the outbreak may have originated from a single focal point in central Saskatchewan. The relatively recent introduction of canola and rapid expansion of the canola growing region across western North America, combined with the cyclical outbreaks of BAW caused by precipitous population crashes, has likely selected for a genetically homogenous BAW population adapted to this crop. | NA | no | No good fitness measure | NA | no | no |
| 4967 | 2020 | 44039 | Erofeeva, MN; Vasilieva, NA; Naidenko, SV | 2020 | Effect of inbreeding on kittens’ body mass in Eurasian lynx (Lynx lynx) | The development of industry and agriculture and the continuous growth of human population on Earth have dramatically increased anthropogenic pressure on natural ecosystems and wildlife. As a result, fragmentation of the natural habitats and the decrease in genetic diversity in these populations increase the probability of reproduction with genetically closely related individuals (inbreeding). However, the effect of inbreeding in felids has been studied insufficiently and data are scarce. We decided to study the effect of inbreeding on kittens’ body mass in captivity in the Eurasian lynx (Lynx lynx) colony which we keep near Moscow to study behavior and reproductive physiology of this species. This study analyzed the data on lynx kittens’ body mass in inbred and non-inbred litters. Newborn kittens’ body mass correlated negatively with the parents’ inbreeding coefficient. Inbred parents had smaller kittens at birth in comparison with non-inbred ones. These differences disappeared when kittens reached 2 months of age. It is probable that this correlates with the maternal effect (higher investment in smaller kittens) and changes in the diet of kittens. | NA | no | Inbreeding | NA | no | no |
| 4968 | 2020 | 44111 | Ershov, NI; Mordvinov, VA; Prokhortchouk, EB; Pakharukova, MY; Gunbin, KV; Ustyantsev, K; Genaev, MA; Blinov, AG; Mazur, A; Boulygina, E; Tsygankova, S; Khrameeva, E; Chekanov, N; Fan, GY; Xiao, A; Zhang, H; Xu, X; Yang, HM; Solovyev, V; Lee, SMY; Liu, X; Afonnikov, DA; Skryabin, KG | 2019 | New insights from Opisthorchis felineus genome: update on genomics of the epidemiologically important liver flukes | BackgroundThe three epidemiologically important Opisthorchiidae liver flukes Opisthorchis felineus, O. viverrini, and Clonorchis sinensis, are believed to harbour similar potencies to provoke hepatobiliary diseases in their definitive hosts, although their populations have substantially different ecogeographical aspects including habitat, preferred hosts, population structure. Lack of O. felineus genomic data is an obstacle to the development of comparative molecular biological approaches necessary to obtain new knowledge about the biology of Opisthorchiidae trematodes, to identify essential pathways linked to parasite-host interaction, to predict genes that contribute to liver fluke pathogenesis and for the effective prevention and control of the disease.ResultsHere we present the first draft genome assembly of O. felineus and its gene repertoire accompanied by a comparative analysis with that of O. viverrini and Clonorchis sinensis. We observed both noticeably high heterozygosity of the sequenced individual and substantial genetic diversity in a pooled sample. This indicates that potency of O. felineus population for rapid adaptive response to control and preventive measures of opisthorchiasis is higher than in O. viverrini and C. sinensis. We also have found that all three species are characterized by more intensive involvement of trans-splicing in RNA processing compared to other trematodes.ConclusionAll revealed peculiarities of structural organization of genomes are of extreme importance for a proper description of genes and their products in these parasitic species. This should be taken into account both in academic and applied research of epidemiologically important liver flukes. Further comparative genomics studies of liver flukes and non-carcinogenic flatworms allow for generation of well-grounded hypotheses on the mechanisms underlying development of cholangiocarcinoma associated with opisthorchiasis and clonorchiasis as well as species-specific mechanisms of these diseases. | NA | no | No good fitness measure and measure host GD | NA | no | no |
| 4969 | 2020 | 44113 | Eskildsen, GA; Rovira, JR; Smith, O; Miller, MJ; Bennett, KL; McMillan, WO; Loaiza, J | 2018 | Maternal invasion history of Aedes aegypti and Aedes albopictus into the Isthmus of Panama: Implications for the control of emergent viral disease agents | Despite an increase in dengue outbreaks and the arrival of chikungunya and Zika disease in Panama, studies on the demographic history of the invasive Aedes mosquitoes that are the principle vectors of these diseases are still lacking in this region. Here, we assess the genetic diversity of these mosquitoes in order to decipher their invasion histories into the Isthmus of Panama. DNA sequences from the mitochondrial cytochrome C oxidase I gene obtained from 30 localities in 10 provinces confirmed the presence of more than one mitochondrial haplogroup (i.e., maternal lineage) in each species. The invasion of Aedes albopictus was likely from temperate European countries, as the most frequent and widespread haplogroup in Panama harbored variants that are uncommon elsewhere in the Americas. Two infrequent and geographically restricted Ae. albopictus haplotypes appear to have subsequently invaded Panama from neighboring Costa Rica and the USA, respectively. In addition, we recovered two deeply divergent mitochondrial clades in Panamanian Aedes aegypti. The geographic origins of these clades is unknown, given that divergence in the mitochondrial genome is probably due to ancient population processes within the native range of Ae. aegypti, rather than due to its global expansion out of Africa. However, Panamanian Ae. aegypti mitochondrial sequences within the first Glade were closely related to others from Colombia, Bolivia, Brazil, Mexico and the USA, suggesting two separate invasions from Western Hemisphere source populations. The pattern of increased genetic diversity in Aedes mosquitoes in Panama is likely facilitated by the numerous land and water inter-connections across the country, which allows them to enter via sea- and land-transportation from Europe, North, Central and South America. Our results here should be considered in disease mitigation programs if emergent arboviruses are to be effectively diminished in Panama through vector suppression. | NA | no | No good fitness measure and measure host GD | NA | no | no |
| 4970 | 2020 | 44040 | Esmaeilnezhad, K; Mehrjerdi, MZ; Neghab, MG | 2020 | Genetic diversity and structure of Buxus hyrcana (Pojark) populations in the Caspian forests of northern Iran revealed by ISSR markers | The Caspian boxwood (Buxus hyrcana) is an endangered, evergreen species in the Hyrcanian forests. The prevalence of pests and boxwood blight disease are bound to reduce the genetic diversity of this species in the near future. Accordingly, the current study considered habitat conservation and restoration by evaluating the genetic diversity of different Caspian boxwood populations. Ninety-seven accessions from seven geographic regions of Hyrcanian forests in Iran were investigated to determine genetic diversity and differentiation among and between populations using 10 ISSR primers. Results showed that the inter-population genetic diversity in B. hyrcana was estimated to be higher than intra-population genetic diversity. Principal coordinates analysis was performed on the populations, along with cluster (based on the Nei distance) and Bayesian structure analysis which classified the populations based on their distance from the sea and their altitude above sea level. Our findings suggest that geographic and environmental factors have an essential role in shaping regional gene flow and influence the genetic structure of B. hyrcana populations in the Hyrcanian forests. | NA | no | n | NA | no | no |
| 4971 | 2020 | 44113 | Espinosa, J; Lopez-Olvera, JR; Cano-Manuel, FJ; Fandos, P; Perez, JM; Lopez-Graells, C; Raez-Bravo, A; Mentaberre, G; Romero, D; Soriguer, RC; Granados, JE | 2017 | Guidelines for managing captive Iberian ibex herds for conservation purposes | Overexploitation, pollution, habitat loss or emerging diseases have led to a large number of species to extinction. This has made zoos and wildlife enclosures expand their goals beyond entertainment and fun; their participation in conservation and research programs is important for the recovery of multiple species. To ensure success, staff need to know the specific requirements of each species. In case of the Iberian ibex (Capra pyreanica), a wild ungulate endemic to the Iberian Peninsula, different sarcoptic mange outbreaks caused dramatic declines of some ibex populations, which led managers and researchers to explore strategies aimed at preventing and controlling this disease and to reduce its impact on ibex populations. Such management plans included the creation of stock reservoirs as an in-situ conservation measure. The objective of the stock reservoir El Toril, as a key part of a general management plan, is to keep in captivity (in range) a sex and age structured representation of the free-ranging population, with most of its genetic variability, destined for conservation programs. However, under captivity conditions with potential for high concentration of animals, direct contact and stress occur and the appearance, transmission, and severity of diseases could be favored. Therefore, it is necessary to establish health protocols in order to guarantee animal welfare. Spanish Animal Health laws establish specific requirements and preventive measures for controlling diseases in captive populations: sarcoptic mange, tuberculosis, brucellosis and bluetongue are notifiable diseases, and the staff of the reservoir must apply specific diagnostic methods to detect them. The management recommendations presented here may be very useful for other managers involved in the conservation of wild ruminants. | NA | no | No good fitness measure and measure host GD | NA | no | no |
| 4972 | 2020 | 44111 | Estrada-Pena, A; Cabezas-Cruz, A | 2019 | Phyloproteomic and functional analyses do not support a split in the genus Borrelia (phylum Spirochaetes) | Background: The evolutionary history of a species is frequently derived from molecular sequences, and the resulting phylogenetic trees do not include explicit functional information. Here, we aimed to assess the functional relationships among bacteria in the Spirochaetes phylum, based on the biological processes of 42,489 proteins in reference proteomes of 34 Spirochaetes species. We tested the hypothesis that the species in the genus Borrelia might be sufficiently different to warrant splitting them into two separate genera. Results: A detrended canonical analysis demonstrated that the presence/absence of biological processes among selected bacteria contained a strong phylogenetic signal, which did not separate species of Borrelia. We examined the ten biological processes in which most proteins were involved consistently. This analysis demonstrated that species in Borrelia were more similar to each other than to free-life species (Sediminispirochaeta, Spirochaeta, Sphaerochaeta) or to pathogenic species without vectors (Leptospira, Treponema, Brachyspira), which are highly divergent. A dendrogram based on the presence/absence of proteins in the reference proteomes demonstrated that distances between species of the same genus among free-life or pathogenic non-vector species were higher than the distances between the 19 species (27 strains) of Borrelia. A phyloproteomic network supported the close functional association between species of Borrelia. In the proteome of 27 strains of Borrelia, only a few proteins had evolved separately, in the relapsing fever and Lyme borreliosis groups. The most prominent Borrelia proteins and processes were a subset of those also found in free-living and non-vectored pathogenic species. In addition, the functional innovation (i.e., unique biological processes or proteins) of Borrelia was very low, compared to other genera of Spirochaetes. Conclusions: We found only marginal functional differences among Borrelia species. Phyloproteomic networks that included all pairwise combinations between species, proteins, and processes were more effective than other methods for evaluating the evolutionary relationships among taxa. With the limitations of data availability, our results did not support a split of the arthropod-transmitted spirochaetes into the proposed genera, Borrelia and Borreliella. | NA | no | no good gd measure host pop | NA | no | no |
| 4973 | 2020 | 44042 | Estrada-Reyes, ZM; Tsukahara, Y; Amadeu, RR; Goetsch, AL; Gipson, TA; Sahlu, T; Puchala, R; Wang, Z; Hart, SP; Mateescu, RG | 2019 | Signatures of selection for resistance to Haemonchus contortus in sheep and goats | Background Gastrointestinal nematode infection (GNI) is the most important disease affecting the small ruminant industry in U.S. The environmental conditions in the southern United States are ideal for the survival of the most pathogenic gastrointestinal nematode, Haemonchus contortus. Host genetic variation for resistance to H. contortus allows selective breeding for increased resistance of animals. This selection process increases the prevalence of particular alleles in sheep and goats and creates unique genetic patterns in the genome of these species. The aim of this study was to identify loci with divergent allelic frequencies in a candidate gene panel of 100 genes using two different approaches (frequentist and Bayesian) to estimate Fst outliers in three different breeds of sheep and goats exposed to H. contortus. Results Our results for sheep populations showed SNPs under selection in C3AR1, CSF3, SOCS2, NOS2, STAT5B, TGFB2 and IL2RA genes using frequentist and Bayesian approaches. For goats, SNPs in CD1D, ITGA9, IL12A, IL13RA1, CD86 and TGFB2 genes were under selection. Common signatures of selection in both species were observed in NOS2, TGFB2 and TLR4 genes. Directional selection was present in all SNPs evaluated in the present study. Conclusions A total of 13 SNPs within 7 genes of our candidate gene panel related to H. contortus exposure were identified under selection in sheep populations. For goats, 11 SNPs within 7 genes were identified under selection. Results from this study support the hypothesis that resistance to H. contortus is likely to be controlled by many loci. Shared signatures of selection related to mechanisms of immune protection against H. contortus infection in sheep and goats could be useful targets in breeding programs aimed to produce resistant animals with low FEC. | NA | no | No good fitness measure and measure host GD | NA | no | no |
| 4974 | 2020 | 44113 | Everaert, H; Rottiers, H; Pham, PHD; Ha, LTV; Nguyen, TPD; Tran, PD; De Wever, J; Maebe, K; Smagghe, G; Dewettinck, K; Messens, K | 2017 | Molecular characterization of Vietnamese cocoa genotypes (Theobroma cacao L.) using microsatellite markers | Vietnam has the appropriate climate, soil, and humidity for cocoa cultivation and is growing as a cocoaproducing country. To supply the international cocoa market, trees have been planted in Southern Vietnam. Cocoa quality depends on various factors, such as the genotype/cultivar, environment, and post-harvest processing. Until now, little research has been done on the genetic background of Vietnamese cocoa. Therefore, this study focused on the genetic relationships of 75 cocoa cultivars, sampled in Vietnam. Fourteen microsatellite markers were used to assay the genetic diversity and to genotype and differentiate the accessions. Descriptive statistics showed that most of the used microsatellite markers were sufficient to be used in further analysis, of which mTcCIR 15, 33, and 37 were the most polymorphic. The principal coordinate analysis (PCoA) and Bayesian clustering approach divided the samples in two groups, which were linked to the Trinitario and Forastero varieties. The Vietnamese Can Tho (CT) cultivars showed little variation, while the Thu Duc (TD) cultivars showed a continuous variation between the different reference cultivars. This indicates that the CT cultivars were Trinitario and that the TD cultivars were hybrids of Forastero or Forastero and Trinitario. The molecular characterization is an important step towards developing a strong genetic basis for the Vietnamese cocoa industry. It can be used to conserve valuable genetic material and to select promising cocoa cultivars which are disease resistant, high yielding, and fine flavored. In this way, the high-quality Vietnamese cocoa production can be improved and maintained. | NA | no | no good gd measure host pop | NA | no | no |
| 4975 | 2020 | 44112 | Eyer, PA; McDowell, B; Johnson, LNL; Calcaterra, LA; Fernandez, MB; Shoemaker, D; Puckett, RT; Vargo, EL | 2018 | Supercolonial structure of invasive populations of the tawny crazy ant Nylanderia fulva in the US | BackgroundSocial insects are among the most serious invasive pests in the world, particularly successful at monopolizing environmental resources to outcompete native species and achieve ecological dominance. The invasive success of some social insects is enhanced by their unicolonial structure, under which the presence of numerous queens and the lack of aggression against non-nestmates allow high worker densities, colony growth, and survival while eliminating intra-specific competition. In this study, we investigated the population genetics, colony structure and levels of aggression in the tawny crazy ant, Nylanderia fulva, which was recently introduced into the United States from South America.ResultsWe found that this species experienced a genetic bottleneck during its invasion lowering its genetic diversity by 60%. Our results show that the introduction of N. fulva is associated with a shift in colony structure. This species exhibits a multicolonial organization in its native range, with colonies clearly separated from one another, whereas it displays a unicolonial system with no clear boundaries among nests in its invasive range. We uncovered an absence of genetic differentiation among populations across the entire invasive range, and a lack of aggressive behaviors towards conspecifics from different nests, even ones separated by several hundreds of kilometers.ConclusionsOverall, these results suggest that across its entire invasive range in the U.S.A., this species forms a single supercolony spreading more than 2000km. In each invasive nest, we found several, up to hundreds, of reproductive queens, each being mated with a single male. The many reproductive queens per nests, together with the free movement of individuals between nests, leads to a relatedness coefficient among nestmate workers close to zero in introduced populations, calling into question the stability of this unicolonial system in which indirect fitness benefits to workers is apparently absent. | NA | no | No good fitness measure | NA | no | no |
| 4976 | 2020 | 44113 | Faburay, B; McGill, J; Jongejan, F | 2017 | A glycosylated recombinant subunit candidate vaccine consisting of Ehrlichia ruminantium major antigenic protein1 induces specific humoral and Th1 type cell responses in sheep | Heartwater, or cowdriosis, is a tick-borne disease of domestic and wild ruminants that is endemic in the Caribbean and sub-Saharan Africa. The disease is caused by an intracellular pathogen, Ehrlichia ruminantium and may be fatal within days of the onset of clinical signs with mortality rates of up to 90% in susceptible hosts. Due to the presence of competent tick vectors in North America, there is substantial risk of introduction of heartwater with potentially devastating consequences to the domestic livestock industry. There is currently no reliable or safe vaccine for use globally. To develop a protective DIVA (differentiate infected from vaccinated animals) subunit vaccine for heartwater, we targeted the E. ruminantium immunodominant major antigenic protein1 (MAP1) with the hypothesis that MAP1 is a glycosylated protein and glycans contained in the antigenic protein are important epitope determinants. Using a eukaryotic recombinant baculovirus expression system, we expressed and characterized, for the first time, a glycoform profile of MAP1 of two Caribbean E. ruminantium isolates, Antigua and Gardel. We have shown that the 37-38 kDa protein corresponded to a glycosylated form of the MAP1 protein, whereas the 31-32 kDa molecular weight band represented the non-glycosylated form of the protein frequently reported in scientific literature. Three groups of sheep (n = 3-6) were vaccinated with increasing doses of a bivalent (Antigua and Gardel MAP1) rMAP1 vaccine cocktail formulation with montanide ISA25 as an adjuvant. The glycosylated recombinant subunit vaccine induced E. ruminantium-specific humoral and Th1 type T cell responses, which are critical for controlling intracellular pathogens, including E. ruminantium, in infected hosts. These results provide an important basis for development of a subunit vaccine as a novel strategy to protect susceptible livestock against heartwater in non-endemic and endemic areas. | NA | no | No good fitness measure and measure host GD | NA | no | no |
| 4977 | 2020 | 44040 | Fackovcova, Z; Slovak, M; Vd’acny, P; Melicharkova, A; Zozomova-Lihova, J; Guttova, A | 2020 | Spatio-temporal formation of the genetic diversity in the Mediterranean dwelling lichen during the Neogene and Quaternary epochs | Genetic patterns of lichenized fungi often display a mosaic-like and difficult to interpret structure blurring their evolutionary history. The genetic diversity and phylogeographic pattern of a mycobiont of the predominantly Mediterranean dwelling lichen Solenopsora candicans were investigated on the base of extensive sampling (361 individuals, 77 populations) across its entire distribution range. We tested whether the genetic pattern of S. candicans mirrors paleoclimatic and paleogeological events in the Mediterranean and adjacent regions. The divergence time estimates indicated a Tertiary origin for S. candicans, with formation of intraspecific diversity initiated in the Late Miocene. The distribution of the most divergent haplotypes, mostly of a pre-Pleistocene origin, was restricted to the eastern or western extremities of the Mediterranean exhibiting Kiermack disjunction. The population genetic diversity analyses indicated multiple diversity centres and refugia for S. candicans across the entire Mediterranean Basin. While the south Mediterranean regions harboured both the Tertiary and Quaternary born diversity, conforming to the ‘cumulative refugia’ paradigm, the Apennine and Balkan Peninsulas in the north hosted mostly younger Pleistocene haplotypes and lineages. The recent population expansion of S. candicans might have occurred in the middle Pleistocene with a population burst in the Apennine and Balkan peninsulas. The presence of unique haplotypes in Central Europe indicates the existence of extra-Mediterranean microrefugia. This study presents the first comprehensive lichen phylogeography from the Mediterranean region and simultaneously reports for the first time the glacial survival of a warm-adapted lichen in the temperate zone. | NA | no | No good fitness measure and measure host GD | NA | no | no |
| 4978 | 2020 | 44040 | Falque, M; Jebreen, K; Paux, E; Knaak, C; Mezmouk, S; Martin, OC | 2020 | CNVmap: A Method and Software To Detect and Map Copy Number Variants from Segregation Data | Single nucleotide polymorphisms (SNPs) are used widely for detecting quantitative trait loci, or for searching for causal variants of diseases. Nevertheless, structural variations such as copy-number variants (CNVs) represent a large part of natural genetic diversity, and contribute significantly to trait variation. Numerous methods and softwares based on different technologies (amplicons, CGH, tiling, or SNP arrays, or sequencing) have already been developed to detect CNVs, but they bypass a wealth of information such as genotyping data from segregating populations, produced, e.g., for QTL mapping. Here, we propose an original method to both detect and genetically map CNVs using mapping panels. Specifically, we exploit the apparent heterozygous state of duplicated loci: peaks in appropriately defined genome-wide allelic profiles provide highly specific signatures that identify the nature and position of the CNVs. Our original method and software can detect and map automatically up to 33 different predefined types of CNVs based on segregation data only. We validate this approach on simulated and experimental biparental mapping panels in two maize populations and one wheat population. Most of the events found correspond to having just one extra copy in one of the parental lines, but the corresponding allelic value can be that of either parent. We also find cases with two or more additional copies, especially in wheat, where these copies locate to homeologues. More generally, our computational tool can be used to give additional value, at no cost, to many datasets produced over the past decade from genetic mapping panels. Copy-number variants (CNVs) represent a large part of natural genetic diversity and contribute significantly to trait variation. As a complement to sequence-based approaches, Falque et al. propose an original method to both detect and map… | NA | no | No good fitness measure and measure host GD | NA | no | no |
| 4979 | 2020 | 44112 | Fan, WL; Xu, LY; Cheng, H; Li, M; Liu, HH; Jiang, Y; Guo, YM; Zhou, ZK; Hou, SS | 2018 | Characterization of Duck (Anas platyrhynchos) Short Tandem Repeat Variation by Population-Scale Genome Resequencing | Short tandem repeats (STRs) are usually associated with genetic diseases and gene regulatory functions, and are also important genetic markers for analysis of evolutionary, genetic diversity and forensic. However, for the majority of STRs in the duck genome, their population genetic properties and functional impacts remain poorly defined. Recent advent of next generation sequencing (NGS) has offered an opportunity for profiling large numbers of polymorphic STRs. Here, we reported a population-scale analysis of STR variation using genome resequencing in mallard and Pekin duck. Our analysis provided the first genome-wide duck STR reference including 198,022 STR loci with motif size of 2-6 base pairs. We observed a relatively uneven distribution of STRs in different genomic regions, which indicates that the occurrence of STRs in duck genome is not random, but undergoes a directional selection pressure. Using genome resequencing data of 23 mallard and 26 Pekin ducks, we successfully identified 89,891 polymorphic STR loci. Intensive analysis of this dataset suggested that shorter repeat motif, longer reference tract length, higher purity, and residing outside of a coding region are all associated with an increase in STR variability. STR genotypes were utilized for population genetic analysis, and the results showed that population structure and divergence patterns among population groups can be efficiently captured. In addition, comparison between Pekin duck and mallard identified 3,122 STRs with extremely divergent allele frequency, which overlapped with a set of genes related to nervous system, energy metabolism and behavior. The evolutionary analysis revealed that the genes containing divergent STRs may play important roles in phenotypic changes during duck domestication. The variation analysis of STRs in population scale provides valuable resource for future study of genetic diversity and genome evolution in duck. | NA | no | No good fitness measure | NA | no | no |
| 4980 | 2020 | 44113 | Fan, ZX; Li, WJ; Jin, JZ; Cui, K; Yan, CC; Peng, CJ; Jian, ZY; Bu, P; Price, M; Zhang, XY; Shen, YM; Li, J; Wenhua, Q; Yue, BS | 2018 | The draft genome sequence of forest musk deer (Moschus berezovskii) | Background: The forest musk deer, Moschus berezovskii, is one of seven musk deer (Moschus spp.) and is distributed in Southwest China. Akin to other musk deer, the forest musk deer has been traditionally and is currently hunted for its musk (i.e., global perfume industry). Considerable hunting pressure and habitat loss have caused significant population declines. Consequently, the Chinese government commenced captive breeding programs for musk harvesting in the 1950s. However, the prevalence of fatal diseases is considerably restricting population increases. Disease severity and extent are exacerbated by inbreeding and genetic diversity declines in captive musk deer populations. It is essential that knowledge of captive and wild forest musk deer populations’ immune system and genome be gained in order to improve their physical and genetic health. We have thus sequenced the whole genome of the forest musk deer, completed the genomic assembly and annotation, and performed preliminary bioinformatic analyses. Findings: A total of 407 Gb raw reads from whole-genome sequencing were generated using the Illumina HiSeq 4000 platform. The final genome assembly is around 2.72 Gb, with a contig N50 length of 22.6 kb and a scaffold N50 length of 2.85 Mb. We identified 24,352 genes and found that 42.05% of the genome is composed of repetitive elements. We also detected 1,236 olfactory receptor genes. The genome- wide phylogenetic tree indicated that the forest musk deer was within the order Artiodactyla, and it appeared as the sister clade of four members of Bovidae. In total, 576 genes were under positive selection in the forest musk deer lineage. Conclusions: We provide the first genome sequence and gene annotation for the forest musk deer. The availability of these resources will be very useful for the conservation and captive breeding of this endangered and economically important species and for reconstructing the evolutionary history of the order Artiodactyla. | NA | no | No good fitness measure and measure host GD | NA | no | no |
| 4981 | 2020 | 44039 | Fang, HT; Liu, HY; Ma, RS; Liu, YX; Li, JN; Yu, XY; Zhang, HY; Yang, YL; Zhang, GD | 2020 | Genome-wide assessment of population structure and genetic diversity of Chinese Lou onion using specific length amplified fragment (SLAF) sequencing | Lou onion (Allium fistulosum L. var. viviparum) is an abundant source of flavonols which provides additional health benefits to diseases. Genome-wide specific length amplified fragment (SLAF) sequencing method is a rapidly developed deep sequencing technologies used for selection and identification of genetic loci or markers. This study aimed to elucidate the genetic diversity of 122 onion accessions in China using the SLAF-seq method. A set of 122 onion accessions including 107 A.fistulosum L. var. viviparum Makino, 3 A.fistulosum L. var. gigantum Makino, 3 A.mongolicum Regel and 9 A.cepa L. accessions (3 whites, 3 reds and 3 yellows) from different regions in China were enrolled. Genomic DNA was isolated from young leaves and prepared for the SLAF-seq, which generated a total of 1,387.55 M reads and 162,321 high quality SNPs (integrity > 0.5 and MAF > 0.05). These SNPs were used for the construction of neighbor-joining phylogenetic tree, in which 10 A.fistulosum L. var. viviparum Makino accessions from Yinchuan (Ningxia province) and Datong (Qinghai province) had close genetic relationship. The 3 A.cepa L. clusters (red, white and yellow) had close genetic relationship especially with the 97 A.fistulosum L. var. viviparum Makino accessions. Population structure analysis suggested entire population could be clustered into 3 groups, while principal component analysis (PCA) showed there were 4 genetic groups. We confirmed the SLAF-seq approach was effective in genetic diversity analysis in red onion accessions. The key findings would provide a reference to the Lou onion germplasm in China. | NA | no | No good fitness measure and measure host GD | NA | no | no |
| 4982 | 2020 | 44042 | Fang, HY; Hui, Q; Lynch, J; Honerlaw, J; Assimes, TL; Huang, J; Vujkovic, M; Damrauer, SM; Pyarajan, S; Gaziano, JM; DuVall, SL; O’Donnell, CJ; Cho, K; Chang, KM; Wilson, PWF; Tsao, PS; Sun, YV; Tang, H | 2019 | Harmonizing Genetic Ancestry and Self-identified Race/Ethnicity in Genome-wide Association Studies | Large-scale multi-ethnic cohorts offer unprecedented opportunities to elucidate the genetic factors influencing complex traits related to health and disease among minority populations. At the same time, the genetic diversity in these cohorts presents new challenges for analysis and interpretation. We consider the utility of race and/or ethnicity categories in genome-wide association studies (GWASs) of multi-ethnic cohorts. We demonstrate that race/ethnicity information enhances the ability to understand population-specific genetic architecture. To address the practical issue that self-identified racial/ethnic information may be incomplete, we propose a machine learning algorithm that produces a surrogate variable, termed HARE. We use height as a model trait to demonstrate the utility of HARE and ethnicity-specific GWASs. | NA | no | human | NA | no | no |
| 4983 | 2020 | 44043 | Faria, CMA; Shaw, P; Emerson, BC | 2019 | Evidence for the Pleistocene persistence of Collembola in Great Britain | Aim Using two genera of springtail, Lepidocyrtus and Entomobrya (Collembola), we test for genetic signatures of Pleistocene persistence of soil arthropods in Great Britain. Location Great Britain. Methods A region of the mitochondrial cytochrome oxidase I (COI) gene was sequenced for 1,150 Collembola specimens from the genera Lepidocyrtus and Entomobrya across Great Britain. Individuals were clustered into Operational Taxonomic Units (OTUs), and both OTU richness and geographical patterns of genetic variation within OTUs were compared between glaciated and unglaciated areas to identify signatures of OTU persistence through Pleistocene glacial events. Results Our analyses identified 12 Entomobrya and 18 Lepidocyrtus OTUs in Great Britain. Lepidocyrtus OTU richness was significantly lower in glaciated than unglaciated areas, whereas there was no difference for Entomobrya OTU richness. However, both genera presented clear patterns of geographically disjunct genetic variation and geographically localized diversification of OTUs. Estimated dates for the onset of in situ diversification events indicate population persistence that pre-dates the Last Glacial Maximum. Main conclusions Patterns of genetic diversity within Collembola OTUs in Great Britain add to a growing body of evidence that elements of the invertebrate fauna have persisted in situ through Pleistocene glacial cycles. Genetic signatures of population persistence in more northern glaciated areas of Great Britain support a hypothesis of geothermal glacial refugia that call for further investigation with other soil mesofaunal taxa. | NA | no | No good fitness measure and measure host GD | NA | no | no |
| 4984 | 2020 | 44112 | Farjamfar, M; Saboori, A; Gonzalez-Cabrera, J; Rodriguez, CSH | 2018 | Genetic variability and pyrethroid susceptibility of the parasitic honey bee mite Varroa destructor (Acari: Varroidae) in Iran | The ectoparasitic honey bee mite Varroa destructor Anderson & Trueman (Acari: Varroidae) is one of the major concerns for worldwide beekeeping. The use of synthetic pyrethroids for controlling the mite was among the most popular treatments until resistance evolved in the mid 1990’s. In Iran, beekeepers are dealing with the parasite and they also used pyrethroids for controlling the mite for a long time. After the evolution of resistance to pyrethroids, they based mite control mostly on treatments with amitraz, organic acids and several management practices. Here we conducted a comprehensive characterization of V. destructor populations parasitizing Apis mellifera in Iran. We determined the genetic variability of mites collected from 28 localities distributed throughout the country. The haplotype of V. destructor was determined by PCR-RFLP, analyzing a fragment of the mitochondrial cox1 gene. It was found that only the Korean haplotype was present in samples from all localities. DNA fragments from cox1, atp6, cox3 and cytb mitochondrial genes were sequenced and the results showed that all samples were identical to the K1-1 or the K1-2 V. destructor haplotypes. Moreover, as it has been reported that resistance to pyrethroids in V. destructor is associated with mutations at position 925 of the voltage-gated sodium channel, a TaqMan (R)-based allelic discrimination assay was conducted to genotype the mites collected. The results showed that all the mites tested were homozygous for the wild-type allele and, therefore, susceptible to treatment with pyrethroids. | NA | no | gd of pathogen | NA | no | no |
| 4985 | 2020 | 44113 | Farquharson, KA; Hogg, CJ; Grueber, CE | 2018 | A meta-analysis of birth-origin effects on reproduction in diverse captive environments | Successfully establishing captive breeding programs is a priority across diverse industries to address food security, demand for ethical laboratory research animals, and prevent extinction. Differences in reproductive success due to birth origin may threaten the long-term sustainability of captive breeding. Our meta-analysis examining 115 effect sizes from 44 species of invertebrates, fish, birds, and mammals shows that, overall, captive-born animals have a 42% decreased odds of reproductive success in captivity compared to their wild-born counterparts. The largest effects are seen in commercial aquaculture, relative to conservation or laboratory settings, and offspring survival and offspring quality were the most sensitive traits. Although a somewhat weaker trend, reproductive success in conservation and laboratory research breeding programs is also in a negative direction for captive-born animals. Our study provides the foundation for future investigation of non-genetic and genetic drivers of change in captivity, and reveals areas for the urgent improvement of captive breeding. | NA | no | No good fitness measure and measure host GD | NA | no | no |
| 4986 | 2020 | 44043 | Farrington, HL; Lawson, LP; Petren, K | 2019 | Predicting population extinctions in Darwin’s finches | Genetic data are increasingly used for fast, efficient, and cost-effective monitoring of natural populations and assessment of extinction risk in species management. A single modern molecular snapshot is typically used to infer population size and vulnerability, yet for species with unknown and potentially complex genetic metapopulation structure, this technique may not effectively predict vulnerability. Darwin’s finches, which are well-represented in museum collections, offer a unique opportunity to test the effectiveness of predicting extinction vulnerability in species with complex structure, such as naturally fragmented populations. In this study, we compared ancient DNA from similar to 100year old extinct and extant Darwin’s finch populations in the Galapagos Islands to determine whether single time point genetic assessments in the past accurately predicted extinction risk, or if other factors such as metapopulation dynamics could mask population declines. Of eight extinct populations, only one had significantly reduced genetic variation compared to an extant population of similar characteristics. Contrary to our prediction that populations would have decreased genetic diversity prior to extinction when compared to persisting populations, at least one measure of genetic diversity was significantly higher in six of the eight extinct populations when compared to extant populations. Simulations lend support to the hypothesis that unaccounted for metapopulation structure may explain the observed pattern in many species. Therefore, models of genetic diversity reflecting population extinction potential may be inadequate for highly-mobile species with metapopulation dynamics such as the Galapagos finches. | NA | no | No good fitness measure and measure host GD | NA | no | no |
| 4987 | 2020 | 44042 | Feis, ME; Goedknegt, MA; Arzul, I; Chenuil, A; den Boon, O; Gottschalck, L; Kondo, Y; Ohtsuka, S; Shama, LNS; Thieltges, DW; Wegner, KM; Luttikhuizen, PC | 2019 | Global invasion genetics of two parasitic copepods infecting marine bivalves | Invasive species, and especially invasive parasites, represent excellent models to study ecological and evolutionary mechanisms in the wild. To understand these processes, it is crucial to obtain more knowledge on the native range, invasion routes and invasion history of invasive parasites. We investigated the consecutive invasions of two parasitic copepods (Mytilicola intestinalis and Mytilicola orientalis) by combining an extensive literature survey covering the reported putative native regions and the present-day invaded regions with a global phylogeography of both species. The population genetic analyses based on partial COI sequences revealed significant population differentiation for M. orientalis within the native region in Japan, while introduced populations in North America and Europe could not be distinguished from the native ones. Thus, M. orientalis’ invasion history resembles the genetic structure and recent spread of its principal host, the Pacific oyster, Crassostrea gigas, while M. intestinalis lacks population genetic structure and has an overall low genetic diversity. Therefore, the native origin of M. intestinalis remains unclear. With this study, we demonstrate that even highly related and biologically similar invasive species can differ in their invasion genetics. From this, we conclude that extrapolating invasion genetics dynamics from related invasive taxa may not always be possible. | NA | no | gd of pathogen | NA | no | no |
| 4988 | 2020 | 44041 | Ferguson, ME; Shah, T; Kulakow, P; Ceballos, H | 2019 | A global overview of cassava genetic diversity | Although numerous studies of diversity have been conducted in cassava, there is no comprehensive assessment of global genetic diversity. Here we draw on previous studies and breeders’ knowledge to select diversity sets from the International Institute of Tropical Agriculture (IITA) and the International Center for Tropical Agriculture (CIAT) genebanks and breeders’ germplasm, as well as elite germplasm and landraces from eastern, southern and central (ESC) Africa to make a global assessment of diversity in cassava, using a SNP based GoldenGate (Illumina Inc.) assay. A synthesis of results from genetic distance and ADMIXTURE analysis essentially revealed four populations (i) South American germplasm characterised by relatively higher genetic diversity with hypothetical ancestral founder genotypes from Brazil, (ii) a smaller group of African introduction germplasm which is more distantly related to all other germplasm, (iii) West Africa germplasm dominated by IITA breeding lines, containing sources of cassava mosaic disease resistance, and IITA gene bank accessions from West Africa, both characterised by slightly lower diversity, and (iv) a less cohesive group of African germplasm, termed ‘Other’, with moderate levels of diversity and a majority of germplasm from ESC Africa. This study highlights opportunities for heterosis breeding, purging of duplicates in genebanks and the need for conservation of ESC Africa landraces. | NA | no | dometicated host | NA | no | no |
| 4989 | 2020 | 44041 | Feurtey, A; Stevens, DM; Stephan, W; Stukenbrock, EH | 2019 | Interspecific Gene Exchange Introduces High Genetic Variability in Crop Pathogen | Genome analyses have revealed a profound role of hybridization and introgression in the evolution of many eukaryote lineages, including fungi. The impact of recurrent introgression on fungal evolution however remains elusive. Here, we analyzed signatures of introgression along the genome of the fungal wheat pathogen Zymoseptoria tritici. We applied a comparative population genomics approach, including genome data from five Zymoseptoria species, to characterize the distribution and composition of introgressed regions representing segments with an exceptional haplotypepattern. These regions are found throughout the genome, comprising 5% of the total genome and overlapping with > 1,000 predicted genes. We performed window-based phylogenetic analyses along the genome to distinguish regions which have a monophyletic or nonmonophyletic origin with Z. tritici sequences. A majority of nonmonophyletic windows overlap with the highly variable regions suggesting that these originate from introgression. We verified that incongruent gene genealogies do not result from incomplete lineage sorting by comparing the observed and expected length distribution of haplotype blocks resulting from incomplete lineage sorting. Although protein-coding genes are not enriched in these regions, we identify 18 that encode putative virulence determinants. Moreover, we find an enrichment of transposable elements in these regions implying that hybridization may contribute to the horizontal spread of transposable elements. We detected a similar pattern in the closely related species Zymoseptoria ardabiliae, suggesting that hybridization is widespread among these closely related grass pathogens. Overall, our results demonstrate a significant impact of recurrent hybridization on overall genome evolution of this important wheat pathogen. | NA | no | gd of pathogen | NA | no | no |
| 4990 | 2020 | 44041 | Figueiredo, L; Krauss, J; Steffan-Dewenter, I; Cabral, JS | 2019 | Understanding extinction debts: spatio-temporal scales, mechanisms and a roadmap for future research | Extinction debt refers to delayed species extinctions expected as a consequence of ecosystem perturbation. Quantifying such extinctions and investigating long-term consequences of perturbations has proven challenging, because perturbations are not isolated and occur across various spatial and temporal scales, from local habitat losses to global warming. Additionally, the relative importance of eco-evolutionary processes varies across scales, because levels of ecological organization, i.e. individuals, (meta)populations and (meta)communities, respond hierarchically to perturbations. To summarize our current knowledge of the scales and mechanisms influencing extinction debts, we reviewed recent empirical, theoretical and methodological studies addressing either the spatio-temporal scales of extinction debts or the eco-evolutionary mechanisms delaying extinctions. Extinction debts were detected across a range of ecosystems and taxonomic groups, with estimates ranging from 9 to 90% of current species richness. The duration over which debts have been sustained varies from 5 to 570 yr, and projections of the total period required to settle a debt can extend to 1000 yr. Reported causes of delayed extinctions are 1) life-history traits that prolong individual survival, and 2) population and metapopulation dynamics that maintain populations under deteriorated conditions. Other potential factors that may extend survival time such as microevolutionary dynamics, or delayed extinctions of interaction partners, have rarely been analyzed. Therefore, we propose a roadmap for future research with three key avenues: 1) the microevolutionary dynamics of extinction processes, 2) the disjunctive loss of interacting species and 3) the impact of multiple regimes of perturbation on the payment of debts. For their ability to integrate processes occurring at different levels of ecological organization, we highlight mechanistic simulation models as tools to address these knowledge gaps and to deepen our understanding of extinction dynamics. | NA | no | No good fitness measure and measure host GD | NA | no | no |
| 4991 | 2020 | 44112 | Fischer, D; Ziegler, L; Hail, K; Heckmann, J; Reiners, TE; Bauer, C; Lierz, M; Lang, J | 2018 | Clinical examination methods and investigation into the occurrence of endoparasites, Borna disease virus and genetic variability in the garden dormouse, Eliomys quercinus | Facing the ongoing decline in garden dormice (Eliomys quercinus) in large parts of Europe, data about genetic diversity, weight, size, and parasite burden were collected from live individuals (n = 156) from Hesse and Rhineland-Palatinate, Germany. Blood was collected under Isoflurane-oxygen-anaesthesia from the anterior vena cava or jugular vein, respectively. Sex, weight, tibiotarsal and tail length of all animals were recorded. Genetic analysis (n = 64) using hair samples, viral examinations of oral, ocular and anal swabs (n = 156) and parasitological examination of faecal samples (n = 57) were performed. Genetic analysis of mitochondrial DNA revealed three haplotypes in the study area (WE4, WE4-3, WE5). Microsatellite analysis demonstrated a comparably high genetic diversity with 66 % overlap of alleles, a low genetic distance (D-Jost 5.6 %) and a low index of differentiation (F-ST = 0.02) between individuals from Hesse and Rhineland-Palatinate. All swab samples were negative for Borna disease virus. In 47 % of the faecal samples Entamoeba sp. cysts, Eimeria sp. oocysts, and eggs of trematodes, cestodes (Hymenolepis spp.) and nematodes (Capillaria spp., Strongylida) were detected. To our knowledge, this is the first report of inhalation anaesthesia, clinical examination, blood collection and assessment of Borna disease virus in living garden dormice. | NA | no | no good gd measure host pop | NA | no | no |
| 4992 | 2020 | 44113 | Fitzsimmons, WJ; Woods, RJ; McCrone, JT; Woodman, A; Arnold, JJ; Yennawar, M; Evans, R; Cameron, CE; Lauring, AS | 2018 | A speed-fidelity trade-off determines the mutation rate and virulence of an RNA virus | Mutation rates can evolve through genetic drift, indirect selection due to genetic hitchhiking, or direct selection on the physicochemical cost of high fidelity. However, for many systems, it has been difficult to disentangle the relative impact of these forces empirically. In RNA viruses, an observed correlation between mutation rate and virulence has led many to argue that their extremely high mutation rates are advantageous because they may allow for increased adaptability. This argument has profound implications because it suggests that pathogenesis in many viral infections depends on rare or de novo mutations. Here, we present data for an alternative model whereby RNA viruses evolve high mutation rates as a byproduct of selection for increased replicative speed. We find that a poliovirus antimutator, 3D(G64S), has a significant replication defect and that wild-type (WT) and 3D(G64S) populations have similar adaptability in 2 distinct cellular environments. Experimental evolution of 3D(G64S) under selection for replicative speed led to reversion and compensation of the fidelity phenotype. Mice infected with 3D(G64S) exhibited delayed morbidity at doses well above the lethal level, consistent with attenuation by slower growth as opposed to reduced mutational supply. Furthermore, compensation of the 3D(G64S) growth defect restored virulence, while compensation of the fidelity phenotype did not. Our data are consistent with the kinetic proofreading model for biosynthetic reactions and suggest that speed is more important than accuracy. In contrast with what has been suggested for many RNA viruses, we find that within-host spread is associated with viral replicative speed and not standing genetic diversity. | NA | no | gd of pathogen | NA | no | no |
| 4993 | 2020 | 44042 | Flores-Trevino, S; Rodriguez-Noriega, E; Garza-Gonzalez, E; Gonzalez-Diaz, E; Esparza-Ahumada, S; Escobedo-Sanchez, R; Perez-Gomez, HR; Leon-Garnica, G; Morfin-Otero, R | 2019 | Clinical predictors of drug-resistant tuberculosis in Mexico | Drug-resistant tuberculosis (DR-TB) remains a major global health problem. Early treatment of TB is critical; in the absence of rapid-susceptibility testing, the empiric selection of drugs should be guided by clinical data. This study aimed to determine the clinical predictors of DR-TB. From September 2010 to August 2017, sociodemographic and clinical characteristics were collected from 144 patients with tuberculosis at the Hospital Civil de Guadalajara, Mexico. Isolates were subjected to drug-susceptibility testing. Clinical predictors of DR-TB were determined using univariate and multivariate analysis. Any drug, isoniazid, and rifampin resistance rates were 47.7, 23.0, and 11.6%, respectively. The visualization of cavities and nodules through either chest radiography or computed tomography were independent predictors of DR-TB. In conclusion, early detection of DR-TB in this population could be based on multiple cavities being observed using chest imaging. This study’s results can be applied to future patients with TB in our community to optimize the DR-TB diagnostic process. | NA | no | human | NA | no | no |
| 4994 | 2020 | 44111 | Flori, L; Moazami-Goudarzi, K; Alary, V; Araba, A; Boujenane, I; Boushaba, N; Casabianca, F; Casu, S; Ciampolini, R; D’Acier, AC; Coquelle, C; Delgado, JV; El-Beltagi, A; Hadjipavlou, G; Jousselin, E; Landi, V; Lauyie, A; Lecomte, P; Ligda, C; Marinthe, C; Martinez, A; Mastrangelo, S; Menni, D; Moulin, CH; Osman, MA; Pineau, O; Portolano, B; Rodellar, C; Saidi-Mehtar, N; Sechi, T; Sempere, G; Thevenon, S; Tsiokos, D; Laloe, D; Gautier, M | 2019 | A genomic map of climate adaptation in Mediterranean cattle breeds | Domestic species such as cattle (Bos taurus taurus and B. t. indicus) represent attractive biological models to characterize the genetic basis of short-term evolutionary response to climate pressure induced by their post-domestication history. Here, using newly generated dense SNP genotyping data, we assessed the structuring of genetic diversity of 21 autochtonous cattle breeds from the whole Mediterranean basin and performed genome-wide association analyses with covariables discriminating the different Mediterranean climate subtypes. This provided insights into both the demographic and adaptive histories of Mediterranean cattle. In particular, a detailed functional annotation of genes surrounding variants associated with climate variations highlighted several biological functions involved in Mediterranean climate adaptation such as thermotolerance, UV protection, pathogen resistance or metabolism with strong candidate genes identified (e.g., NDUFB3, FBN1, METTL3, LEF1, ANTXR2 and TCF7). Accordingly, our results suggest that main selective pressures affecting cattle in Mediterranean area may have been related to variation in heat and UV exposure, in food resources availability and in exposure to pathogens, such as anthrax bacteria (Bacillus anthracis). Furthermore, the observed contribution of the three main bovine ancestries (indicine, European and African taurine) in these different populations suggested that adaptation to local climate conditions may have either relied on standing genomic variation of taurine origin, or adaptive introgression from indicine origin, depending on the local breed origins. Taken together, our results highlight the genetic uniqueness of local Mediterranean cattle breeds and strongly support conservation of these populations. | NA | no | dometicated host | NA | no | no |
| 4995 | 2020 | 44111 | Fogell, DJ; Groombridge, JJ; Tollington, S; Canessa, S; Henshaw, S; Zuel, N; Jones, CG; Greenwood, A; Ewen, JG | 2019 | Hygiene and biosecurity protocols reduce infection prevalence but do not improve fledging success in an endangered parrot | Emerging Infectious Diseases (EIDs) are recognised as global extinction drivers of threatened species. Unfortunately, biodiversity managers have few tested solutions to manage them when often the desperate need for solutions necessitates a response. Here we test in situ biosecurity protocols to assess the efficacy of managing Psittacine beak and feather disease (PBFD), one of the most common and emergent viral diseases in wild parrots (Psittaciformes) that is currently affecting numerous threatened species globally. In response to an outbreak of PBFD in Mauritius echo parakeets (Psittacula eques), managers implemented a set of biosecurity protocols to limit transmission and impact of Beak and feather disease virus (BFDV). Here we used a reciprocal design experiment on the wild population to test whether BFDV management reduced viral prevalence and viral load, and improved nestling body condition and fledge success. Whilst management reduced the probability of nestling infection by approximately 11% there was no observed impact on BFDV load and nestling body condition. In contrast to expectations there was lower fledge success in nests with added BFDV biosecurity (83% in untreated vs. 79% in treated nests). Our results clearly illustrate that management for wildlife conservation should be critically evaluated through targeted monitoring and experimental manipulation, and this evaluation should always focus on the fundamental objective of conservation. | NA | no | no good gd measure host pop | NA | no | no |
| 4996 | 2020 | 44112 | Fogell, DJ; Martin, RO; Bunbury, N; Lawson, B; Sells, J; McKeand, AM; Tatayah, V; Trung, CT; Groombridge, JJ | 2018 | Trade and conservation implications of new beak and feather disease virus detection in native and introduced parrots | Psittacine beak and feather disease (PBFD), caused by Beak and feather disease virus (BFDV), has spread rapidly around the world, raising concerns for threatened species conservation and biosecurity associated with the global pet bird trade. The virus has been reported in several wild parrot populations, but data are lacking for many taxa and geographical areas with high parrot endemism. We aimed to advance understanding of BFDV distribution in many data-deficient areas and determine phylogenetic and biogeographic associations of the virus in 5 parrot species across Africa, the Indian Ocean islands, Asia, and Europe and focused specifically on the highly traded and invasive Psittacula krameri. Blood, feather, and tissue samples were screened for BFDV through standard polymerase chain reaction. Isolates obtained from positive individuals were then analyzed in a maximum likelihood phylogeny along with all other publically available global BFDV sequences. We detected BFDV in 8 countries where it was not known to occur previously, indicating the virus is more widely distributed than currently recognized. We documented for the first time the presence of BFDV in wild populations of P. krameri within its native range in Asia and Africa. We detected BFDV among introduced P. krameri in Mauritius and the Seychelles, raising concerns for island endemic species in the region. Phylogenetic relationships between viral sequences showed likely pathways of transmission between populations in southern Asia and western Africa. A high degree of phylogenetic relatedness between viral variants from geographically distant populations suggests recent introductions, likely driven by global trade. These findings highlight the need for effective regulation of international trade in live parrots, particularly in regions with high parrot endemism or vulnerable taxa where P. krameri could act as a reservoir host. | NA | no | No good fitness measure and measure host GD | NA | no | no |
| 4997 | 2020 | 44111 | Fong, MY; Lau, YL; Jelip, J; Ooi, CH; Cheong, FW | 2019 | Genetic characterisation of the erythrocyte-binding protein (PkII) of <bold>Plasmodium knowlesi</bold> isolates from Malaysia | Plasmodium knowlesi contributes to the majority of human malaria incidences in Malaysia. Its uncontrollable passage among the natural monkey hosts can potentially lead to zoonotic outbreaks. The merozoite of this parasite invades host erythrocytes through interaction between its erythrocyte-binding proteins (EBPs) and their respective receptor on the erythrocytes. The region II of P. knowlesi EBP, P. knowlesi beta (PkII) protein is found to be mediating merozoite invasion into monkey erythrocytes by interacting with sialic acid receptors. Hence, the objective of this study was to investigate the genetic diversity, natural selection and haplotype grouping of PkII of P. knowlesi isolates in Malaysia. Polymerase chain reaction amplifications of PkII were performed on archived blood samples from Malaysia and 64 PkII sequences were obtained. Sequence analysis revealed length polymorphism, and its amino acids at critical residues indicate the ability of PkII to mediate P. knowlesi invasion into monkey erythrocytes. Low genetic diversity (=0.007) was observed in the PkII of Malaysia Borneo compared to Peninsular Malaysia (=0.015). The PkII was found to be under strong purifying selection to retain infectivity in monkeys and it plays a limited role in the zoonotic potential of P. knowlesi. Its haplotypes could be clustered into Peninsular Malaysia and Malaysia Borneo groups, indicating the existence of two distinct P. knowlesi parasites in Malaysia as reported in an earlier study. | NA | no | No good fitness measure | NA | no | no |
| 4998 | 2020 | 44041 | Foote, A; Simma, D; Khatkar, M; Raadsma, H; Guppy, J; Coman, G; Giardina, E; Jerry, D; Zenger, K; Wade, N | 2019 | Considerations for Maintaining Family Diversity in Commercially Mass-Spawned Penaeid Shrimp: A Case Study on Penaeus monodon | Skewed family distributions are common in aquaculture species that are highly fecund, communally (mass) spawned, and/or communally reared. The magnitude of skews pose challenges for maintaining family-specific genetic diversity, as increased resources are required to detect individuals from underrepresented families, or reliably determine relative survival as a measure of family performance. There is limited understanding of family skews or changes in family proportion of communally reared shrimp under commercial rearing conditions and particularly how this may affect genotyping strategies to recover family performance data in breeding programs. In this study, three separate batches of shrimp, Penaeus monodon, were communally spawned and reared, and then sampled as larvae when ponds were stocked at 30 days of culture (DOC) and as juveniles from commercial ponds during harvest at 150 DOC. A total of 199 broodstock contributed to the 5,734 progeny that were genotyped with a custom multiplex single nucleotide polymorphism (SNP) panel, and family assignments were cross-referenced using two parentage assignment methods, CERVUS and COLONY. A total of 121 families were detected, with some families contributing up to 11% of progeny at 30 DOC and up to 18% of progeny at harvest. Significant changes were detected for 20% of families from 30 to 150 DOC, with up to a 9% change in relative contribution. Family skew data was applied in several models to determine the optimal sample size to detect families, along with the ability to detect changes in relative family contribution over time. Results showed that an order of magnitude increase in sampling was required to capture the lowest represented 25% of families, as well as significantly improve the accuracy to determine changes in family proportion from 30 to 150 DOC. Practical measures may be implemented at the hatchery to reduce family skews; a cost-effective measure may be to address the initial magnitude differences in viable progeny produced among families, by pooling equal quantities of hatched larvae from each family. This study demonstrates the relationships between skews in families under commercial conditions, the ability to accurately detect families, and the balance of sampling effort and genotyping cost in highly fecund species such as shrimp. | NA | no | No good fitness measure and measure host GD | NA | no | no |
| 4999 | 2020 | 44042 | Ford, BM; Weir, RD; Lewis, JC; Larsen, KW; Russell, MA | 2019 | Fine-scale genetic structure and conservation status of American badgers at their northwestern range periphery | Peripheral populations are often characterized by small population size and low genetic diversity, with many at risk of extirpation. These characteristics may be even more pronounced in human-modified landscapes that further reduce the resiliency of populations to environmental change. Situated at the northwestern edge of the species’ range, the western American badger (Taxidea taxus jeffersonii) is an endangered mammal in Canada, where it inhabits the interior grassland and open forest ecosystems of British Columbia (BC) and continues to be threatened by severe vehicle-induced mortality rates and other anthropogenic factors. Here, we collected mitochondrial DNA haplotypic and microsatellite genotypic data to investigate the extent and distribution of American badger genetic variation within and among sites in British Columbia, and relative to adjacent populations in the USA, including in Washington state. From these data, we reconstructed population structure and connectivity, and examined current designatable unit status. Patterns of genetic variation for American badgers in British Columbia were as expected for peripheral populations, including reduced genetic diversity, increased population differentiation, and evidence of demographic contraction. Furthermore, we found limited connectivity between regional populations in our study area and identified significant substructure isolating the most northwestern sampling unit (Cariboo), findings that starkly contrast with the high levels of gene flow observed between populations across the species’ range core. These results have important implications for current designatable unit status for western American badgers in British Columbia and emphasize the need for further population monitoring and mitigation of potential barriers to gene flow. | NA | no | No good fitness measure | NA | no | no |
| 5000 | 2020 | 44112 | Ford, MJ; Parsons, KM; Ward, EJ; Hempelmann, JA; Emmons, CK; Hanson, MB; Balcomb, KC; Park, LK | 2018 | Inbreeding in an endangered killer whale population | There are genetic risks associated with small population sizes, including loss of genetic diversity and inbreeding depression. The southern resident killer whale Orcinus orca population is a group of similar to 80 whales listed as ‘endangered’ under the U.S. Endangered Species Act. Recovery efforts are focused on increasing prey and reducing impacts from environmental disturbance, but the population’s small size and insularity suggest that inbreeding depression could also be important. We analyzed genotypes at 68-94 nuclear loci from 105 individuals to refine a population pedigree to evaluate inbreeding and the relationship between multi-locus heterozygosity and fitness. Our results expand upon an earlier study and shed new light on both inbreeding within this population and the mating patterns of killer whales. We found that only two adult males sired 52% of the sampled progeny born since 1990. Confirming earlier results, we found male reproductive success increased with age. Based on the pedigree, four sampled offspring were the result of inbred mating - two between a parent and offspring, one between paternal half-siblings, and one between uncle and half-niece. There is no evidence to date that the survival or fecundity of these individuals is lower than normal. There was some evidence for inbreeding depression in the form of a weakly supported relationship between multi-locus heterozygosity and annual survival probability, but the power of our data to quantify this effect was low. We found no evidence of inbreeding avoidance in the population, but a late age of breeding success for males may indirectly limit the frequency of parent/offspring mating. The effective number of breeders in the population is currently similar to 26, and was estimated to have ranged from 12-53 over the past 40 years. The population that produced the oldest (pre-1970) sampled individuals was estimated to have 24 effective breeders. Overall, our results indicate that inbreeding is likely common in the population, but the fitness effects continue to be uncertain. | NA | no | inbreeding | NA | no | no |
| 5001 | 2020 | 44112 | Fortin, M; Vitet, C; Souty-Grosset, C; Richard, FJ | 2018 | How do familiarity and relatedness influence mate choice in Armadillidium vulgare? | Mate choice is an important process in sexual selection and usually prevents inbreeding depression in populations. In the terrestrial isopod Armadillidium vulgare, the close physical proximity between individuals may increase the risk of reproducing with siblings. Moreover, individuals of this species can be infected with the feminizing bacteria of Wolbachia, which influence male mate choice. However, little is known about the kinship or familiarity assessment of the selected partner that occurs when a male can choose between females with or without Wolbachia. To investigate the potential mechanisms leading to mate choice and the potential impact of the parasite, we performed behavioral choice tests on males where they could choose between sibling vs. nonsibling females, familiar vs. unfamiliar females, and sibling familiar vs. unfamiliar nonsibling females. To investigate the costs of inbreeding, we compared the reproductive success of both sibling and nonsibling mates. Our results revealed that male copulation attempts were higher for familiar females and for nonsibling females when both females were Wolbachia-infected, but the duration was longer when both females were Wolbachia-free. When males mated with a sibling female, their fecundity was severely decreased, consistent with inbreeding depression. Overall, we observed copulations with all types of females and demonstrated discrimination capacities and potential preferences. We highlight the complexity of the tradeoff between kinship, familiarity and parasite transmission assessment for mate choice. | NA | no | No good fitness measure and measure host GD | NA | no | no |
| 5002 | 2020 | 44112 | Fortuna, TM; Namias, A; Snirc, A; Branca, A; Hood, ME; Raquin, C; Shykoff, JA; Giraud, T | 2018 | Multiple infections, relatedness and virulence in the anther-smut fungus castrating Saponaria plants | Multiple infections (co-occurrence of multiple pathogen genotypes within an individual host) can have important impacts on diseases. Relatedness among pathogens can affect the likelihood of multiple infections and their consequences through kin selection. Previous studies on the castrating anther-smut fungus Microbotryum lychnidis-dioicae have shown that multiple infections occur in its host plant Silene latifolia. Relatedness was high among fungal genotypes within plants, which could result from competitive exclusion between unrelated fungal genotypes, from population structure or from interactions between plant and fungal genotypes for infection ability. Here, we aimed at disentangling these hypotheses using M. saponariae and its host Saponaria officinalis, both experimentally tractable for these questions. By analysing populations using microsatellite markers, we also found frequent occurrence of multiple infections and high relatedness among strains within host plants. Infections resulting from experimental inoculations in the greenhouse also revealed high relatedness among strains co-infecting host plants, even in clonally replicated plant genotypes, indicating that high relatedness within plants did not result merely from plant x fungus interactions or population structure. Furthermore, hyphal growth in vitro was affected by the presence of a competitor growing nearby and by its genetic similarity, although this latter effect was strain-dependent. Altogether, our results support the hypothesis that relatedness-dependent competitive exclusion occurs in Microbotryum fungi within plants. These microorganisms can thus respond to competitors and to their level of relatedness. | NA | no | gd of pathogen | NA | no | no |
| 5003 | 2020 | 44113 | Fountain, ED; Kang, JK; Tempel, DJ; Palsboll, PJ; Pauli, JN; Peery, MZ | 2018 | Genomics meets applied ecology: Characterizing habitat quality for sloths in a tropical agroecosystem | Understanding how habitat quality in heterogeneous landscapes governs the distribution and fitness of individuals is a fundamental aspect of ecology. While mean individual fitness is generally considered a key to assessing habitat quality, a comprehensive understanding of habitat quality in heterogeneous landscapes requires estimates of dispersal rates among habitat types. The increasing accessibility of genomic approaches, combined with field-based demographic methods, provides novel opportunities for incorporating dispersal estimation into assessments of habitat quality. In this study, we integrated genomic kinship approaches with field-based estimates of fitness components and approximate Bayesian computation (ABC) procedures to estimate habitat-specific dispersal rates and characterize habitat quality in two-toed sloths (Choloepus hoffmanni) occurring in a Costa Rican agricultural ecosystem. Field-based observations indicated that birth and survival rates were similar in a sparsely shaded cacao farm and adjacent cattle pasture-forest mosaic. Sloth density was threefold higher in pasture compared with cacao, whereas home range size and overlap were greater in cacao compared with pasture. Dispersal rates were similar between the two habitats, as estimated using ABC procedures applied to the spatial distribution of pairs of related individuals identified using 3,431 single nucleotide polymorphism and 11 microsatellite locus genotypes. Our results indicate that crops produced under a sparse overstorey can, in some cases, constitute lower-quality habitat than pasture-forest mosaics for sloths, perhaps because of differences in food resources or predator communities. Finally, our study demonstrates that integrating field-based demographic approaches with genomic methods can provide a powerful means for characterizing habitat quality for animal populations occurring in heterogeneous landscapes. | NA | no | No good fitness measure and measure host GD | NA | no | no |
| 5004 | 2020 | 44039 | Fraik, AK; Margres, MJ; Epstein, B; Barbosa, S; Jones, M; Hendricks, S; Schonfeld, B; Stahlke, AR; Veillet, A; Hamede, R; McCallum, H; Lopez-Contreras, E; Kallinen, SJ; Hohenlohe, PA; Kelley, JL; Storfer, A | 2020 | Disease swamps molecular signatures of genetic-environmental associations to abiotic factors in Tasmanian devil (Sarcophilus harrisii) populations | Landscape genomics studies focus on identifying candidate genes under selection via spatial variation in abiotic environmental variables, but rarely by biotic factors (i.e., disease). The Tasmanian devil (Sarcophilus harrisii) is found only on the environmentally heterogeneous island of Tasmania and is threatened with extinction by a transmissible cancer, devil facial tumor disease (DFTD). Devils persist in regions of long-term infection despite epidemiological model predictions of species’ extinction, suggesting possible adaptation to DFTD. Here, we test the extent to which spatial variation and genetic diversity are associated with the abiotic environment (i.e., climatic variables, elevation, vegetation cover) and/or DFTD. We employ genetic-environment association analyses using 6886 SNPs from 3287 individuals sampled pre- and post-disease arrival across the devil’s geographic range. Pre-disease, we find significant correlations of allele frequencies with environmental variables, including 365 unique loci linked to 71 genes, suggesting local adaptation to abiotic environment. The majority of candidate loci detected pre-DFTD are not detected post-DFTD arrival. Several post-DFTD candidate loci are associated with disease prevalence and were in linkage disequilibrium with genes involved in tumor suppression and immune response. Loss of apparent signal of abiotic local adaptation post-disease suggests swamping by strong selection resulting from the rapid onset of DFTD. | NA | no | No good fitness measure | NA | no | no |
| 5005 | 2020 | 44113 | Frank, SC; Leclerc, M; Pelletier, F; Rosell, F; Swenson, JE; Bischof, R; Kindberg, J; Eiken, HG; Hagen, SB; Zedrosser, A | 2018 | Sociodemographic factors modulate the spatial response of brown bears to vacancies created by hunting | 1. There is a growing recognition of the importance of indirect effects from hunting on wildlife populations, e.g. social and behavioural changes due to harvest, which occur after the initial offtake. Nonetheless, little is known about how the removal of members of a population influences the spatial configuration of the survivors. 2. We studied how surviving brown bears (Ursus arctos) used former home ranges that had belonged to casualties of the annual bear hunting season in southcentral Sweden (2007-2015). We used resource selection functions to explore the effects of the casualty’s and survivor’s sex, age and their pairwise genetic relatedness, population density and hunting intensity on survivors’ spatial responses to vacated home ranges. 3. We tested the competitive release hypothesis, whereby survivors that increase their use of a killed bear’s home range are presumed to have been released from intraspecific competition. We found strong support for this hypothesis, as survivors of the same sex as the casualty consistently increased their use of its vacant home range. Patterns were less pronounced or absent when the survivor and casualty were of opposite sex. 4. Genetic relatedness between the survivor and the casualty emerged as the most important factor explaining increased use of vacated male home ranges by males, with a stronger response from survivors of lower relatedness. Relatedness was also important for females, but it did not influence use following removal; female survivors used home ranges of higher related female casualties more, both before and after death. Spatial responses by survivors were further influenced by bear age, population density and hunting intensity. 5. We have shown that survivors exhibit a spatial response to vacated home ranges caused by hunting casualties, even in nonterritorial species such as the brown bear. This spatial reorganization can have unintended consequences for population dynamics and interfere with management goals. Altogether, our results underscore the need to better understand the short- and long-term indirect effects of hunting on animal social structure and their resulting distribution in space. | NA | no | No good fitness measure and measure host GD | NA | no | no |
| 5006 | 2020 | 44040 | Franssen, SU; Durrant, C; Stark, O; Moser, B; Downing, T; Imamura, H; Dujardin, JC; Sanders, MJ; Mauricio, I; Miles, MA; Schnur, LF; Jaffe, CL; Nasereddin, A; Schallig, H; Yeo, M; Bhattacharyya, T; Alam, MZ; Berriman, M; Wirth, T; Schonian, G; Cotton, JA | 2020 | Global genome diversity of the Leishmania donovani complex | Protozoan parasites of the Leishmania donovani complex - L. donovani and L. infantum - cause the fatal disease visceral leishmaniasis. We present the first comprehensive genome-wide global study, with 151 cultured field isolates representing most of the geographical distribution. L. donovani isolates separated into five groups that largely coincide with geographical origin but vary greatly in diversity. In contrast, the majority of L. infantum samples fell into one globally-distributed group with little diversity. This picture is complicated by several hybrid lineages. Identified genetic groups vary in heterozygosity and levels of linkage, suggesting different recombination histories. We characterise chromosome-specific patterns of aneuploidy and identified extensive structural variation, including known and suspected drug resistance loci. This study reveals greater genetic diversity than suggested by geographically-focused studies, provides a resource of genomic variation for future work and sets the scene for a new understanding of the evolution and genetics of the Leishmania donovani complex. | NA | no | No good fitness measure | NA | no | no |
| 5007 | 2020 | 44112 | Franzo, G; Cecchinato, M; Tosi, G; Fiorentini, L; Faccin, F; Tucciarone, CM; Trogu, T; Barbieri, I; Massi, P; Moreno, A | 2018 | GI-16 lineage (624/I or Q1), there and back again: The history of one of the major threats for poultry farming of our era | The genetic variability of Infectious bronchitis virus (IBV) is one of the main challenges for its control, hindering not only the development of effective vaccination strategies but also its classification and, consequently, epidemiology understanding. The 624/I and Q1 genotypes, now recognized to be part of the GI-16 lineage, represent an excellent example of the practical consequences of IBV molecular epidemiology limited knowledge. In fact, being their common origin unrecognized for a long time, independent epidemiological pictures were drawn for the two genotypes. To fix this misinterpretation, the present study reconstructs the history, population dynamics and spreading patterns of GI-16 lineage as a whole using a phylodynamic approach. A collection of worldwide available hypervariable region 1 and 2 (HVR12) and 3 (HVR3) sequences of the S1 protein was analysed together with 258 HVR3 sequences obtained from samples collected in Italy (the country where this genotype was initially identified) since 1963. The results demonstrate that after its emergence at the beginning of the XX century, GI-16 was able to persist until present days in Italy. Approximately in the late 1980s, it migrated to Asia, which became the main nucleus for further spreading to Middle East, Europe and especially South America, likely through multiple introduction events. A remarkable among-country diffusion was also demonstrated in Asia and South America. Interestingly, although most of the recent Italian GI-16 strains originated from ancestral viruses detected in the same country, a couple were closely related to Chinese ones, supporting a backward viral flow from China to Italy. Besides to the specific case-study results, this work highlights the misconceptions that originate from the lack of a unified nomenclature and poor molecular epidemiology data generation and sharing. This shortcoming appears particularly relevant since the described scenario could likely be shared by many other IBV genotypes and pathogens in general. | NA | no | gd of pathogen | NA | no | no |
| 5008 | 2020 | 44111 | Fregel, R; Ordonez, AC; Santana-Cabrera, J; Cabrera, VM; Velasco-Vazquez, J; Alberto, V; Moreno-Benitez, MA; Delgado-Darias, T; Rodriguez-Rodriguez, A; Hernandez, JC; Pais, J; Gonzalez-Montelongo, R; Lorenzo-Salazar, JM; Flores, C; Cruz-de-Mercadal, MC; Alvarez-Rodriguez, N; Shapiro, B; Arnay, M; Bustamante, CD | 2019 | Mitogenomes illuminate the origin and migration patterns of the indigenous people of the Canary Islands | The Canary Islands’ indigenous people have been the subject of substantial archaeological, anthropological, linguistic and genetic research pointing to a most probable North African Berber source. However, neither agreement about the exact point of origin nor a model for the indigenous colonization of the islands has been established. To shed light on these questions, we analyzed 48 ancient mitogenomes from 25 archaeological sites from the seven main islands. Most lineages observed in the ancient samples have a Mediterranean distribution, and belong to lineages associated with the Neolithic expansion in the Near East and Europe (T2c, J2a, X3a…). This phylogeographic analysis of Canarian ancient mitogenomes, the first of its kind, shows that some lineages are restricted to Central North Africa (H1cf, J2a2d and T2c1d3), while others have a wider distribution, including both West and Central North Africa, and, in some cases, Europe and the Near East (U6a1a1, U6a7a1, U6b, X3a, U6c1). In addition, we identify four new Canarian-specific lineages (H1e1a9, H4a1e, J2a2d1a and L3b1a12) whose coalescence dates correlate with the estimated time for the colonization of the islands (1st millennia CE). Additionally, we observe an asymmetrical distribution of mtDNA haplogroups in the ancient population, with certain haplogroups appearing more frequently in the islands closer to the continent. This reinforces results based on modern mtDNA and Y-chromosome data, and archaeological evidence suggesting the existence of two distinct migrations. Comparisons between insular populations show that some populations had high genetic diversity, while others were probably affected by genetic drift and/or bottlenecks. In spite of observing interinsular differences in the survival of indigenous lineages, modern populations, with the sole exception of La Gomera, are homogenous across the islands, supporting the theory of extensive human mobility after the European conquest. | NA | no | human | NA | no | no |
| 5009 | 2020 | 44113 | Fresnedo-Ramirez, J; Yang, SS; Sun, Q; Cote, LM; Schweitzer, PA; Reisch, BI; Ledbetter, CA; Luby, JJ; Clark, MD; Londo, JP; Gadoury, DM; Kozma, P; Cadle-Davidson, L | 2017 | An integrative AmpSeq platform for highly multiplexed marker-assisted pyramiding of grapevine powdery mildew resistance loci | Resistance breeding often requires the introgression and tracking of resistance loci from wild species into domesticated backgrounds, typically with the goal of pyramiding multiple resistance genes, to provide durable disease resistance to breeding selections and ultimately cultivars. While molecular markers are commonly used to facilitate these efforts, high genetic diversity and divergent marker technologies can complicate marker-assisted breeding strategies. Here, amplicon sequencing (AmpSeq) was used to integrate SNP markers with dominant presence/absence markers derived from genotyping-by-sequencing and other genotyping technologies, for the simultaneous tracking of five loci for resistance to grapevine powdery mildew. SNP haploblocks defined the loci for REN1, REN2 and REN3, which confer quantitative resistance phenotypes that are challenging to measure via field ratings of natural infections. Presence/absence markers for RUN1 and REN4 were validated to predict qualitative resistance phenotypes and corresponded with previous presence/absence fluorescent electrophoretic assays. Thus, 37 AmpSeq-derived markers were identified for the five loci, and markers for REN1, REN2, REN4 and RUN1 were used for multiplexed screening and selection within diverse breeding germplasm. Poor transferability of SNP markers indicated imperfect marker-trait association in some families. Together, AmpSeq SNP haploblocks and presence/absence markers provide a high-throughput, cost-effective tool to integrate divergent technologies for marker-assisted selection and genetic analysis of introgressed disease resistance loci in grapevine. | NA | no | No good fitness measure and measure host GD | NA | no | no |
| 5010 | 2020 | 44111 | Frias, L; Stark, DJ; Lynn, MS; Nathan, S; Goossens, B; Okamoto, M; MacIntosh, AJJ | 2019 | Molecular characterization of nodule worm in a community of Bornean primates | Strongyles are commonly reported parasites in studies of primate parasite biodiversity. Among them, nodule worm species are often overlooked as a serious concern despite having been observed to cause serious disease in nonhuman primates and humans. In this study, we investigated whether strongyles found in Bornean primates are the nodule worm Oesophagostomum spp., and to what extent these parasites are shared among members of the community. To test this, we propose two hypotheses that use the parasite genetic structure to infer transmission processes within the community. In the first scenario, the absence of parasite genetic substructuring would reflect high levels of parasite transmission among primate hosts, as primates’ home ranges overlap in the study area. In the second scenario, the presence of parasite substructuring would suggest cryptic diversity within the parasite genus and the existence of phylogenetic barriers to cross-species transmission. By using molecular markers, we identify strongyles infecting this primate community as O. aculeatum, the only species of nodule worm currently known to infect Asian nonhuman primates. Furthermore, the little to no genetic substructuring supports a scenario with no phylogenetic barriers to transmission and where host movements across the landscape would enable gene flow between host populations. This work shows that the parasite’s high adaptability could act as a buffer against local parasite extinctions. Surveys targeting human populations living in close proximity to nonhuman primates could help clarify whether this species of nodule worm presents the zoonotic potential found in the other two species infecting African nonhuman primates. | NA | no | gd of pathogen | NA | no | no |
| 5011 | 2020 | 44112 | Frias, L; Stark, DJ; Lynn, MS; Nathan, SKSS; Goossens, B; Okamoto, M; MacIntosh, AJJ | 2018 | Lurking in the dark: Cryptic Strongyloides in a Bornean slow loris | Within host communities, related species are more likely to share common parasitic agents, and as a result, morphological similarities have led researchers to conclude that parasites infecting closely related hosts within a community represent a single species. However, genetic diversity within parasite genera and host range remain poorly investigated in most systems. Strongyloides is a genus of soil-transmitted nematode that has been reported from several primate species in Africa and Asia, and has been estimated to infect hundreds of millions of people worldwide, although no precise estimates are available. Here we describe a case of infection with a cryptic species of Strongyloides in a Bornean (Philippine) slow loris (Nycticebus menagensis) living within a diverse community of several primate species in the Lower Kinabatangan Wildlife Sanctuary, Malaysian Borneo. Fresh fecal samples were collected from five primate species and nematode larvae cultured from these samples were selected for phylogenetic analyses. Sequences obtained for most larvae were identified as S. fuelleborni, grouping into three different clusters and showing no aggregation within specific hosts or geographic location. In contrast, a set of parasite sequences obtained from a slow loris clustered closely with S. stercoralis into a different group, being genetically distinct to sequences reported from other primate hosts, humans included. Our results suggest that although S. fuelleborni infects all haplorrhines sampled in this primate community, a different species might be infecting the slow loris, the only strepsirrhine in Borneo and one of the least studied primates in the region. Although more data are needed to support this conclusion, we propose that Strongyloides species in primates might be more diverse than previously thought, with potential implications for ecological and evolutionary host-parasite associations, as well as epidemiological dynamics. | NA | no | No good fitness measure and measure host GD | NA | no | no |
| 5012 | 2020 | 44112 | Fronhofer, EA; Liebig, J; Mitesser, O; Poethke, HJ | 2018 | Eusociality outcompetes egalitarian and solitary strategies when resources are limited and reproduction is costly | Explaining the evolution and maintenance of animal groups remains a challenge. Surprisingly, fundamental ecological factors, such as resource variance and competition for limited resources, tend to be ignored in models of cooperation. We use a mathematical model previously developed to quantify the influence of different group sizes on resource use efficiency in egalitarian groups and extend its scope to groups with severe reproductive skew (eusocial groups). Accounting for resource limitation, the model allows calculation of optimal group sizes (highest resource use efficiency) and equilibrium population sizes in egalitarian as well as eusocial groups for a broad spectrum of environmental conditions (variance of resource supply). We show that, in contrast to egalitarian groups, eusocial groups may not only reduce variance in resource supply for survival, thus reducing the risk of starvation, they may also increase variance in resource supply for reproduction. The latter effect allows reproduction even in situations when resources are scarce. These two facets of eusocial groups, resource sharing for survival and resource pooling for reproduction, constitute two beneficial mechanisms of group formation. In a majority of environmental situations, these two benefits of eusociality increase resource use efficiency and lead to supersaturation-a strong increase in carrying capacity. The increase in resource use efficiency provides indirect benefits to group members even for low intra-group relatedness and may represent one potential explanation for the evolution and especially the maintenance of eusociality and cooperative breeding. | NA | no | No good fitness measure and measure host GD | NA | no | no |
| 5013 | 2020 | 44042 | Fu, CZ; Guang, XM; Wan, QH; Fang, SG | 2019 | Genome Resequencing Reveals Congenital Causes of Embryo and Nestling Death in Crested Ibis (Nipponia nippon) | The crested ibis (Nipponia nippon) is endangered worldwide. Although a series of conservation measures have markedly increased the population size and distribution area of these birds, the high mortality of embryos and nestlings considerably decreases the survival potential of this bird species. High-throughput sequencing technology was utilized to compare whole genomes between ten samples from dead crested ibises (including six dead embryos and four dead nestlings aged 0-45 days) and 32 samples from living birds. The results indicated that the dead samples all shared the genetic background of a specific ancestral subpopulation. Furthermore, the dead individuals were less genetically diverse and suffered higher degrees of inbreeding compared with these measures in live birds. Several candidate genes (KLHL3, SETDB2, TNNT2, PKP1, AK1, and EXOSC3) associated with detrimental diseases were identified in the genomic regions that differed between the alive and dead samples, which are likely responsible for the death of embryos and nestlings. In addition, in these regions, we also found several genes involved in the protein catabolic process (UBE4A and LONP1), lipid metabolism (ACOT1), glycan biosynthesis and metabolism (HYAL1 and HYAL4), and the immune system (JAM2) that are likely to promote the normal development of embryos and nestlings. The aberrant conditions of these genes and biological processes may contribute to the death of embryos and nestlings. Our data identify congenital factors underlying the death of embryos and nestlings at the whole genome level, which may be useful toward informing more effective conservation efforts for this bird species. | NA | no | No good fitness measure and measure host GD | NA | no | no |
| 5014 | 2020 | 44113 | Fu, SS; Bai, XN; Fan, RY; Sun, H; Xu, YM; Xiong, YW | 2018 | Genetic diversity of the enterohaemolysin gene (ehxA) in non-O157 Shiga toxin-producing Escherichia coli strains in China | Non-O157 Shiga toxin-producing Escherichia coli (STEC) is increasingly recognized as an important enteric foodborne pathogen. The hallmark of the disease is the production of Shiga toxins; however, there are other virulence factors that contribute to the pathogenesis of STEC. This study aimed to investigate the prevalence and genetic diversity of the enterohaemolysin gene, ehxA, among non-O157 STEC strains from human, animal, and food sources. The ehxA gene was amplified from 138 (31.8%) of 434 non-O157 STEC strains, among which 36 unique ehxA sequences were identified. Based on ehxA sequence analysis, three phylogenetic ehxA groups (I II, and III) were determined. Correlations between ehxA groups and sources, serotypes, and virulent gene profiles were observed. The ehxA group II strains were mostly diarrhoeal patient-derived and may demonstrate higher pathogenic potential compared with the ehxA group I and group III strains. Five types of replicons (I1-Ig, FIB, K, F, and B/O) were identified in the 138 ehxA-positive strains, and 3.6%, 5.8%, and 52.2% of the strains harboured toxB, katP and espP genes, respectively, implying marked genetic diversity of ehxA containing plasmids in non-O157 STEC strains. Sequence-based ehxA genotyping might be important in modern strain typing and in epidemiological surveillance of non-O157 STEC infections. | NA | no | No good fitness measure and measure host GD | NA | no | no |
| 5015 | 2020 | 44112 | Fuentes, A; Braswell, WE; Ruiz-Arce, R; Racelis, A | 2018 | Genetic variation and population structure of Diaphorina citri using cytochrome oxidase I sequencing | Citrus greening disease, or huanglongbing (HLB), is currently one of the most devastating diseases of citrus. The bacteria thought to be responsible for the disease, Candidatus Liberi-bacter asiaticus impact the majority of commercial citrus species worldwide. These bacteria are transmitted by the Asian citrus psyllid (ACP), Diaphorina citri Kuwayama, which is now found in most citrus growing regions. With no known cure, ACP-vectored HLB is responsible for significant economic losses to the global citrus industry. A better understanding of the global genetic diversity of D. citri would improve current and future pest management and mitigation programs. To assess the genetic diversity of D. citri in worldwide collections, a total of 1,108 sequences belonging to ACP gathered from 27 countries in the Americas, the Caribbean, Southeast and Southwest Asia were examined for the study. 883 D. citri came from 98 locations in 18 different countries, and were sequenced using a 678bp fragment of the mitochondrial cytochrome oxidase I (COI) gene. Additionally, 225 previously-reported D. citri COI sequences, were also included in our analysis. Analyses revealed 28 haplotypes and a low genetic diversity. This is in accordance with previous reports on the little diversity of D. citri in worldwide populations. Our analyses reveal population structure with 21 haplotypes showing geographic association, increasing the resolution for the source estimation of ACP. This study reveals the distribution of haplotypes observed in different geographic regions and likely geographic sources for D. citri introductions. | NA | no | gd of pathogen | NA | no | no |
| 5016 | 2020 | 44040 | Fukumoto, J; Yamano, A; Matsuzaki, M; Kyan, H; Masatani, T; Matsuo, T; Matsui, T; Murakami, M; Takashima, Y; Matsubara, R; Tahara, M; Sakura, T; Takeuchi, F; Nagamune, K | 2020 | Molecular and biological analysis revealed genetic diversity and high virulence strain of Toxoplasma gondii in Japan | Toxoplasma gondii is classified into 16 haplogroups based on a worldwide genotyping study of the parasite. However, only a few isolates from Japan were included in this analysis. To conduct more precise genotyping of T. gondii, we examined the genotypes of Japanese isolates in this study. DNA sequences of 6 loci were determined in 17 Japanese isolates and compared with those of strains of 16 haplogroups. As a result, Japanese isolates were classified into four groups. We investigated the virulence of some Japanese isolates and found a highly virulent strain in mice, comparable to that of RH strain, although this Japanese isolate was sister to strains of haplogroup 2, which show moderate virulence in mice. We further investigated whether this high virulence isolate had different virulence mechanism and strategy to adapt to Japanese host from other strains by comparing the virulence-related genes, ROP5, 18 and the immunomodulatory gene, ROP16 of the isolate with those of archetypical strains (GT1, ME49 and VEG). This analysis indicated the high virulence of the isolate in mice was partly explained by gene sequences of ROP5 and ROP16. These findings lead to the elucidation of biodiversity of T. gondii and have potential to optimize the diagnostic protocol. | NA | no | gd of pathogen | NA | no | no |
| 5017 | 2020 | 44039 | Fung, C; Gonzalez-Moreno, P; Pratt, C; Oliver, TH; Bourchier, RS; Gonzalez-Suarez, M | 2020 | Effect of humidity and temperature on the performance of three strains of Aphalara itadori, a biocontrol agent for Japanese Knotweed | Japanese knotweed (Fallopia japonica) is a highly damaging invasive species affecting UK infrastructure and biodiversity. Under laboratory conditions, the psyllid Aphalara itadori has demonstrated its potential to be a successful biocontrol agent for F. japonica. However, this potential has not materialised in the field where long-term establishment of A. itadori has been unsuccessful and faces the added challenge of climate change. Intraspecific variation (variation among individuals of a species) has been shown to support establishment in alien species and improve resilience to changing environmental conditions. Here we propose it could improve the performance of biocontrols. To test this possibility we compared the performance and impact on F. japonica of three strains of A. itadori with different genetic backgrounds, including a newly created hybrid. We hypothesize that genetic variability would be increased in hybrids resulting in greater biocontrol effectiveness (greater impact on plant growth). We also explored the potential influence of changing climate on performance, testing all strains under two humidity conditions (with the same temperature). Contrary to our expectation, the hybrid strain had the worst performance (slowest development rate and lower survival from egg to adult emergence) under both environmental conditions. Exposure to different strains of A. itadori did not result in consistent differences in plant growth, suggesting similar biocontrol effectiveness among strains. Under the drier, more stressful, conditions plants exposed to A. itadori had fewer leaves and accumulated less above-ground biomass. Overall, our results suggest that genetic variability may not be the key to improve A. itadori biocontrol effectiveness, but that predicted climate change, which anticipates drier and hotter summers in the UK, could reduce the growth potential of F. japonica when exposed to A. itadori. | NA | no | No good fitness measure and measure host GD | NA | no | no |
| 5018 | 2020 | 44040 | Furlan, EM; Gruber, B; Attard, CRM; Wager, RNE; Kerezsy, A; Faulks, LK; Beheregaray, LB; Unmack, PJ | 2020 | Assessing the benefits and risks of translocations in depauperate species: A theoretical framework with an empirical validation | Conservation translocations are becoming more common to assist in the management of threatened native species. While many translocation programs focus on maximizing survival in newly established populations, consideration is also required for the persistence of source populations. Here, we present and test a theoretical framework that assesses the translocation trade-off between increasing a species probability of survival and decreasing a species’ overall genetic diversity. We anticipate that (a) the genetic diversity of translocated populations will be reduced compared to the source due to a failure to capture and retain genetic diversity and (b) the genetic diversity of the source population will decline due to the removal of founder individuals. We test this framework with an empirical study of redfin blue eye Scaturiginichthys vermeilipinnis, a critically endangered fish species which has undergone several replicate translocations, established with founders sourced from a single remnant population. Several generations after reintroduction, we show that the predicted survival of the species has improved as a result of these translocations. While the species’ genetic diversity has been retained across all populations combined (translocated and source), we show that genetic diversity in each individual population (including the source) is reduced compared to the source population prior to translocation. Synthesis and applications. Conservation translocations can provide great benefits to species survival, enabling extinction risk to be spread across multiple populations. Translocated populations, however, often harbour reduced genetic diversity compared to source populations and initiating translocated populations can decrease the genetic diversity of source populations, placing them at an increased risk of extinction. The framework presented here enables the trade-off between extinction risk and retention of genetic diversity to be established. This will enable the optimal conservation strategy to be employed to increase the long-term persistence and evolutionary potential of a species. | NA | no | No good fitness measure and measure host GD | NA | no | no |
| 5019 | 2020 | 44039 | Fusco, NA; Pehek, E; Munshi-South, J | 2020 | Urbanization reduces gene flow but not genetic diversity of stream salamander populations in the New York City metropolitan area | Natural landscape heterogeneity and barriers resulting from urbanization can reduce genetic connectivity between populations. The evolutionary, demographic, and ecological effects of reduced connectivity may lead to population isolation and ultimately extinction. Alteration to the terrestrial and aquatic environment caused by urban influence can affect gene flow, specifically for stream salamanders who depend on both landscapes for survival and reproduction. To examine how urbanization affects a relatively common stream salamander species, we compared genetic connectivity ofEurycea bislineata(northern two-lined salamander) populations within and between streams in an urban, suburban, and rural habitat around the New York City (NYC) metropolitan area. We report reduced genetic connectivity between streams within the urban landscape found to correspond with potential barriers to gene flow, that is, areas with more dense urbanization (roadways, industrial buildings, and residential housing). The suburban populations also exhibited areas of reduced connectivity correlated with areas of greater human land use and greater connectivity within a preserve protected from development. Connectivity was relatively high among neighboring rural streams, but a major roadway corresponded with genetic breaks even though the habitat contained more connected green space overall. Despite greater human disturbance across the landscape, urban and suburban salamander populations maintained comparable levels of genetic diversity to their rural counterparts. Yet small effective population size in the urban habitats yielded a high probability of loss of heterozygosity due to genetic drift in the future. In conclusion, urbanization impacted connectivity among stream salamander populations where its continual influence may eventually hinder population persistence for this native species in urban habitats. | NA | no | No good fitness measure and measure host GD | NA | no | no |
| 5020 | 2020 | 44042 | Gagnon, M; Yannic, G; Perrier, C; Cote, SD | 2019 | No evidence of inbreeding depression in fast declining herds of migratory caribou | Identifying inbreeding depression early in small and declining populations is essential for management and conservation decisions. Correlations between heterozygosity and fitness (HFCs) provide a way to identify inbreeding depression without prior knowledge of kinship among individuals. In Northern Quebec and Labrador, the size of two herds of migratory caribou (Riviere-George, RG and Riviere-aux-Feuilles, RAF) has declined by one to two orders of magnitude in the last three decades. This raises the question of a possible increase in inbreeding depression originating from, and possibly contributing to, the demographic decline in those populations. Here, we tested for the association of genomic inbreeding indices (estimated with 22,073 SNPs) with body mass and survival in 400 caribou sampled in RG and RAF herds between 1996 and 2016. We found no association of individual heterozygosity or inbreeding coefficient with body mass or annual survival. Furthermore, those genomic inbreeding indices remained stable over the period monitored. These results suggest that the rapid and intense demographic decline of the herds did not cause inbreeding depression in those populations. Although we found no evidence for HFCs, if demographic decline continues, it is possible that such inbreeding depression would be triggered. | NA | no | inbreeding | NA | no | no |
| 5021 | 2020 | 44113 | Gaigher, A; Roulin, A; Gharib, WH; Taberlet, P; Burri, R; Fumagalli, L | 2018 | Lack of evidence for selection favouring MHC haplotypes that combine high functional diversity | High rates of gene duplication and the highest levels of functional allelic diversity in vertebrate genomes are the main hallmarks of the major histocompatibility complex (MHC), a multigene family with a primordial role in pathogen recognition. The usual tight linkage among MHC gene duplicates may provide an opportunity for the evolution of haplotypes that associate functionally divergent alleles and thus grant the transmission of optimal levels of diversity to coming generations. Even though such associations may be a crucial component of disease resistance, this hypothesis has been given little attention in wild populations. Here, we leveraged pedigree data from a barn owl (Tyto alba) population to characterize MHC haplotype structure across two MHC class I (MHC-I) and two MHC class IIB (MHC-IIB) duplicates, in order to test the hypothesis that haplotypes’ genetic diversity is higher than expected from randomly associated alleles. After showing that MHC loci are tightly linked within classes, we found limited evidence for shifts towards MHC haplotypes combining high diversity. Neither amino acid nor functional within-haplotype diversity were significantly higher than in random sets of haplotypes, regardless of MHC class. Our results therefore provide no evidence for selection towards high-diversity MHC haplotypes in barn owls. Rather, high rates of concerted evolution may constrain the evolution of high-diversity haplotypes at MHC-I, while, in contrast, for MHC-IIB, fixed differences among loci may provide barn owls with already optimized functional diversity. This suggests that at the MHC-I and MHC-IIB respectively, different evolutionary dynamics may govern the evolution of within-haplotype diversity. | NA | no | mhc diversity | NA | no | no |
| 5022 | 2020 | 44043 | Gajic, B; Munoz, I; De la Rua, P; Stevanovic, J; Lakic, N; Kulisic, Z; Stanimirovic, Z | 2019 | Coexistence of genetically different Varroa destructor in Apis mellifera colonies | The aim of this study was to investigate the genetic diversity of Varroa destructor parasitizing Apis mellifera colonies and to test for possible host-parasite association at the mitochondrial DNA (mtDNA) level. Six A. mellifera haplotypes (including a novel C2aa) and five haplotypes of V. destructor were detected in 29 analyzed colonies from eight sampling sites in Serbia. We revealed the presence of the K and S1 haplotypes as well as KS1 and KP1 heteroplasmic mite individuals in all localities, while the P1 haplotype was only found in four sampling sites. Significant differences in V. destructor genetic diversity were found at both apiary and colony levels, with mite haplotypes coexisting in almost all tested colonies. In addition, a significant correlation between the number of analyzed mites per colony and the number of identified V. destructor haplotypes was observed. However, no significant host-parasite relationship was found, suggesting that mites bearing different haplotypes as well as those heteroplasmic individuals are well adapted to the host, A. mellifera, independently of the identified haplotype present in each colony. Our results will contribute to future population and biogeographic studies concerning V. destructor infesting A. mellifera, as well as to better understanding their host-parasite relationship. | NA | no | gd of pathogen | NA | no | no |
| 5023 | 2020 | 44113 | Galvan, I; Moller, AP | 2018 | Dispersal capacity explains the evolution of lifespan variability | The evolutionary explanation for lifespan variation is still based on the antagonistic pleiotropy hypothesis, which has been challenged by several studies. Alternative models assume the existence of genes that favor aging and group benefits at the expense of reductions in individual lifespans. Here we propose a new model without making such assumptions. It considers that limited dispersal can generate, through reduced gene flow, spatial segregation of individual organisms according to lifespan. Individuals from subpopulations with shorter lifespan could thus resist collapse in a growing population better than individuals from subpopulations with longer lifespan, hence reducing lifespan variability within species. As species that disperse less may form more homogeneous subpopulations regarding lifespan, this may lead to a greater capacity to maximize lifespan that generates viable subpopulations, therefore creating negative associations between dispersal capacity and lifespan across species. We tested our model with individual-based simulations and a comparative study using empirical data of maximum lifespan and natal dispersal distance in 26 species of birds, controlling for the effects of genetic variability, body size, and phylogeny. Simulations resulted in maximum lifespans arising from lowest dispersal probabilities, and comparative analyses resulted in a negative association between lifespan and natal dispersal distance, thus consistent with our model. Our findings therefore suggest that the evolution of lifespan variability is the result of the ecological process of dispersal. | NA | no | No good fitness measure and measure host GD | NA | no | no |
| 5024 | 2020 | 44113 | Gamez, G; Castro, A; Gomez-Mejia, A; Gallego, M; Bedoya, A; Camargo, M; Hammerschmidt, S | 2018 | The variome of pneumococcal virulence factors and regulators | Background: In recent years, the idea of a highly immunogenic protein-based vaccine to combat Streptococcus pneumoniae and its severe invasive infectious diseases has gained considerable interest. However, the target proteins to be included in a vaccine formulation have to accomplish several genetic and immunological characteristics, (such as conservation, distribution, immunogenicity and protective effect), in order to ensure its suitability and effectiveness. This study aimed to get comprehensive insights into the genomic organization, population distribution and genetic conservation of all pneumococcal surface-exposed proteins, genetic regulators and other virulence factors, whose important function and role in pathogenesis has been demonstrated or hypothesized. Results: After retrieving the complete set of DNA and protein sequences reported in the databases GenBank, KEGG, VFDB, P2CS and Uniprot for pneumococcal strains whose genomes have been fully sequenced and annotated, a comprehensive bioinformatic analysis and systematic comparison has been performed for each virulence factor, standalone regulator and two-component regulatory system (TCS) encoded in the pan-genome of S. pneumoniae. A total of 25 S. pneumoniae strains, representing different pneumococcal phylogenetic lineages and serotypes, were considered. A set of 92 different genes and proteins were identified, classified and studied to construct a pan-genomic variability map (variome) for S. pneumoniae. Both, pneumococcal virulence factors and regulatory genes, were well-distributed in the pneumococcal genome and exhibited a conserved feature of genome organization, where replication and transcription are co-oriented. The analysis of the population distribution for each gene and protein showed that 49 of them are part of the core genome in pneumococci, while 43 belong to the accessory-genome. Estimating the genetic variability revealed that pneumolysin, enolase and Usp45 (SP\_2216 in S. p. TIGR4) are the pneumococcal virulence factors with the highest conservation, while TCS08, TCS05, and TCS02 represent the most conserved pneumococcal genetic regulators. Conclusions: The results identified well-distributed and highly conserved pneumococcal virulence factors as well as regulators, representing promising candidates for a new generation of serotype-independent protein-based vaccine(s) to combat pneumococcal infections. | NA | no | No good fitness measure and measure host GD | NA | no | no |
| 5025 | 2020 | 44040 | Gandhi, R; Yadav, KK; Patil, PB; Bihani, P; Char, B; Dasgupta, SK; Zehr, UB; Barwale, SR | 2020 | Molecular analysis of mitochondrial cytochrome oxidase I gene of Aedes aegypti L. mosquitoes | Aedes aegypti is the most important arboviral vector worldwide. Recent studies reported that genetic variations and gene flow among same mosquito species is responsible for different disease transmission rate. Hence, to understand the relationship between genetic diversity and disease transmission potential, study on genetic variations among mosquito populations is essential. The aim of present study was to investigate the genetic variations of Ae. aegypti targeting COI gene from nine villages of Jalna District, Maharashtra and three laboratory strains originated from Aurangabad, Delhi and transgenic OX513A strain imported from OXITEC, UK. OX513A strain consists of a self-limiting dominant lethal gene construct intended for its use in suppression of Ae. aegypti population by sustained male adult releases in the environment. Mosquito eggs from field and laboratory strains were reared to adults and identified on the basis of morphological characteristics followed by COI gene sequence. Result of MSA and haplotype analysis revealed low genetic variations among field samples and Aurangabad strain, belonged to two haplotypes (H1 and H2) except Ramkheda village represented by separate haplotype H3. Other laboratory DEL strain and transgenic OX513A have great genetic variability to all isolates and have a separate haplotypes H4 and H5. Similar results were observed in phylogenetic analysis. Our observation of phylogenies revealed close relationship among the DEL and transgenic strain OX513A with few Indian and worldwide isolates. The information on genetic variability of mosquito population could help to understand and design the strategies for risk mitigation and effective implementation of new vector control tools like genetically modified mosquitoes. | NA | no | No good fitness measure | NA | no | no |
| 5026 | 2020 | 44041 | Gandhi, SR; Korolev, KS; Gore, J | 2019 | Cooperation mitigates diversity loss in a spatially expanding microbial population | The evolution and potentially even the survival of a spatially expanding population depends on its genetic diversity, which can decrease rapidly due to a serial founder effect. The strength of the founder effect is predicted to depend strongly on the details of the growth dynamics. Here, we probe this dependence experimentally using a single microbial species, Saccharomyces cerevisiae, expanding inmultiple environments that induce varying levels of cooperativity during growth. We observe a drastic reduction in diversity during expansions when yeast grows noncooperatively on simple sugars, but almost no loss of diversity when cooperation is required to digest complex metabolites. These results are consistent with theoretical expectations: When cells grow independently from each other, the expansion proceeds as a pulled wave driven by growth at the low-density tip of the expansion front. Such populations lose diversity rapidly because of the strong genetic drift at the expansion edge. In contrast, diversity loss is substantially reduced in pushed waves that arise due to cooperative growth. In such expansions, the low-density tip of the front grows much more slowly and is often reseeded from the genetically diverse population core. Additionally, in both pulled and pushed expansions, we observe a few instances of abrupt changes in allele fractions due to rare fluctuations of the expansion front and show how to distinguish such rapid genetic drift from selective sweeps. | NA | no | No good fitness measure and measure host GD | NA | no | no |
| 5027 | 2020 | 44113 | Gao, MC; Liu, Y; Guo, YL; Wang, YJ; Dai, HY; Song, ZF; Wang, JW; Han, WY | 2018 | Identification and characterization of a rabbit novel IFN-alpha unlocated in genome | The multigene family of rabbit IFN-alpha (RbIFN-alpha) is located on chromosome 1, which shows seven functional genes in type I IFN locus. A novel RbIFN-alpha that remains unlocated in the rabbit genome was amplified and designated as the first novel rabbit IFN-alpha (RblFN-alpha Nov1), which possesses the typical molecular characteristics of type I IFNs and could be induced in RK-13 cells and peripheral blood mononuclear cells. After the mature peptide of RblFN-alpha Nov1 was expressed, its antiviral activity, physicochemical characteristics, and cytotoxicity were determined in vitro. Results indicated that RblFN-alpha Nov1 exerted a high specific antiviral activity against VSV and a low cytotoxic effect on RK-13 cells. RblFN-alpha Nov1 showed high sensitivity to trypsin and remained relatively stable after acid, alkali, or heat treatment. RblFN-alpha Nov1 could induce Mx1 expression on RK-13 ceils and activate the NF-kappa B, ISRE and BoIFN-beta promoter activities on bovine testicular cells. Overall, our research on RblFN-alpha Nov1 not only enriches the knowledge about rabbit IFNs but also makes RblFN-alpha Nov1 have the potential to be used as an effective therapeutic agent for rabbit viral diseases. (C) 2017 Elsevier Ltd. All rights reserved. | NA | no | No good fitness measure and measure host GD | NA | no | no |
| 5028 | 2020 | 44112 | Garant, D; Bourret, A; Schmitt, C; Turcotte, A; Pelletier, F; Belisle, M | 2018 | Effects of blood parasite infection and innate immune genetic diversity on mating patterns in a passerine bird breeding in contrasted habitats | Genetic diversity at immune genes and levels of parasitism are known to affect patterns of (dis)assortative mating in several species. Heterozygote advantage and/or good genes should shape mate choice originating from pathogen/parasite-driven selection at immune genes. However, the stability of these associations, and whether they vary with environmental conditions, are still rarely documented. In this study, we describe mating patterns in a wild population of tree swallows (Tachycineta bicolor) over 4 years and assess the effects of haemosporidian parasite infection and immune genetic diversity at beta-defensin genes on those patterns within two habitats of contrasting environmental quality, in southern Quebec, Canada. We first show that mating patterns were only very weakly related to individual status of infection by haemosporidian parasites. However, we found a difference between habitats in mating patterns related to infection status, which was likely due to a non-random distribution of individuals, as non-infected mating pairs were more frequent in lower quality habitats. Mating patterns also differed depending on beta-defensin heterozygosity at AvBD2, but only for genetic partners outside of the social couple, with heterozygous individuals pairing together. Our study underlines the importance of considering habitat heterogeneity in studies of sexual selection. | NA | no | no good gd measure host pop | NA | no | no |
| 5029 | 2020 | 44043 | Gaspari, S; Marsili, L; Natali, C; Airoldi, S; Lanfredi, C; Deeming, C; Moura, AE | 2019 | Spatio-temporal patterns of genetic diversity in the Mediterranean striped dolphin (Stenella coeruleoalba) | Comparing the genetic composition of wild animals between geographic regions with distinct environments is common in evolutionary studies. However, genetic composition can also change through time in response to environmental changes but studies examining this are carried out less often. In this study, we characterize striped dolphin genetic composition in the Mediterranean Sea across both geography and time. We provide genotype data for 15 microsatellite loci and 919 bp of mtDNA control region, collected over 21 years across all main Mediterranean Sea basins. We investigated spatial genetic structure using both classical and Bayesian population structure methods, and compared it with temporal patterns of genetic change using time-series statistics. We integrated the temporal datasets with known environmental pressures and data on social structure, to infer potential drivers of observed changes. Geographic analyses suggest weak differentiation for striped dolphin in the Mediterranean Sea, with evidence for a recent expansion. Temporal analyses show significant cyclical fluctuations in genetic composition over 21 years, which correspond well with recurrent morbillivirus epizootics. Similarly, social group composition shows changes in the relative number of juveniles and adults per group, and an overall increase in the number of adults per group relative to juveniles over the time period. We suggest that the observed changes in genetic and group composition could relate to specific dynamics of morbillivirus resistance. Overall, our study highlights the importance of tracking long-term genetic variation and the potential for this species as a model in studying genetic adaptation to environmental stress. | NA | no | n | NA | no | no |
| 5030 | 2020 | 44112 | Gaudeul, M; Delahaye, T; Muller, S | 2019 | AFLP markers show low levels of clonal propagation and high genotypic diversity in the rare, southernmost populations of Linnaea borealis L. (Caprifoliaceae) in the Western Alps | In plants, clonal propagation is a common reproductive strategy in parallel to sexual reproduction. It has both advantages and drawbacks, and the potential complete loss of sexual reproduction causes serious conservation concerns, especially because population maintenance then only relies on adult survival and low genetic diversity leads to decreased adaptive potential. We investigated the rare, southernmost populations of the mostly circumboreal twinflower Linnaea borealis, located in the Western Alps. Based on 105 AFLP markers and 118 leaf samples, including replicates, we estimated the genetic similarity threshold above which samples belong to a single clone. Although the species is known for extensive clonal propagation, we observed high genotypic diversity within the seven studied populations and almost all samples were genetically distinct. Nevertheless, some clonal samples were detected in two populations, separated by up to 180m. We found a strong genetic differentiation among populations (overall F-st=0.38), which was congruent with the previously documented high plastid diversity in the region. We therefore hypothesize that Alpine populations are relicts of the Quaternary glacial periods, when the species probably survived at these lower latitudes before colonizing Northern Europe. Regarding conservation, our results suggest that most extant plants result from sexual reproduction and that populations are not highly threatened. Nevertheless, since clones can be very long-lived and almost no seedlings were observed in recent years, events of sexual reproduction may be ancient. The current reproductive dynamics should therefore be studied to estimate e.g. pollinators activity, proportions of flowering plants, and seed set. | NA | no | No good fitness measure | NA | no | no |
| 5031 | 2020 | 44111 | Gaughan, S; Steffensen, K; Lu, GQ | 2019 | Habitat use and population structure of the shoal chub (Macrhybopsis hyostoma) in the upper Mississippi River basin | Anthropogenic alterations of river systems may have a profound effect on native fish community and habitat use; however, it’s difficult to understand the extent of these impacts without establishing well-defined habitat preferences. We investigated the Shoal chub, Macrhybopsis hyostoma, a native obligate river species from nine sampling locations in the upper Mississippi River Basin (UMRB). Field surveys demonstrated that overall Shoal chubs preferred tributaries, yet this was statistically significant only for gravid females. Diet analysis and comparative morphology suggested that the Shoal chub is insectivorous and prefer benthic habitats. Our analysis of habitat use suggested that juvenile Shoal chubs preferred sand substrate and adults preferred medium to large gravel. Shoal chubs developed more melanophores as they aged, which is a likely an adaptation to their habitat shifts. The field survey identified possible sites where spawning was occurring and may be important for future conservation efforts for the Shoal chub. In addition, we conducted population genomic analysis of Shoal chub samples collected from the streams in three Midwest states (Illinois, Missouri, and Nebraska) and found low genetic diversity among the chubs that raises a concern in conservation. This preliminary study provides insights into further investigation of the impact caused by stream habitat alteration on native species and into the conservation of Shoal chubs in the UMRB. | NA | no | No good fitness measure | NA | no | no |
| 5032 | 2020 | 44042 | Ge, DY; Lu, L; Abramov, AV; Wen, ZX; Cheng, JL; Xia, L; Vogler, AP; Yang, QS | 2019 | Coalescence Models Reveal the Rise of the White-Bellied Rat (Niviventer confucianus) Following the Loss of Asian Megafauna | Rodents are the major remaining mammals in many terrestrial ecosystems after the historical loss of megafauna and large-bodied taxa. Niviventer confucianus is a dominant habitat generalist in natural forests in most of China. It is also recorded as an important vector of diverse zoonotic diseases. Here, three mitochondrial and one nuclear DNA fragments were sequenced from samples covering most of the species range to study intraspecific genetic diversification and demographic history. Molecular voucher specimens of N. confucianus revealed that its assumed distribution range has been overestimated because of the hitherto unrecognized separation from parapatric species. Phylogenetic inferences recognized three geographically delimited intraspecific lineages that diverged at approximately 1.28 and 0.68 Mya. Hengduan Mountains, the east margin of Qinghai Tibetan Plateau, and the mountains surrounding Sichuan Basin were recognized as the major geographical barriers. Demographic analysis revealed dramatic population growth in southwest, central, and northern China in the late Pleistocene, but only slight growth in Yunnan/Tibet. The population boom apparently coincided with the reduction of predation and competition from the loss of megafauna in the late Pleistocene. Distributional ranges were inferred to be fairly stable through the late Quaternary glacial-interglacial climatic oscillations, possibly enabled by the species’ seed hoarding behavior and wide climatic tolerance. The demographic history of N. confucianus suggests that these rodents directly profited from the loss of megafauna, while their most recent increases potentially led to the proliferation of zoonotic disease by this species. | NA | no | No good fitness measure and measure host GD | NA | no | no |
| 5033 | 2020 | 44043 | Gelmi-Candusso, TA; Bialozyt, R; Slana, D; Gomez, RZ; Heymann, EW; Heer, K | 2019 | Estimating seed dispersal distance: A comparison of methods using animal movement and plant genetic data on two primate-dispersed Neotropical plant species | Seed dispersal distance (SDD) critically influences the survival of seedlings, spatial patterns of genetic diversity within plant populations, and gene flow among plant populations. In animal-dispersed species, foraging behavior and movement patterns determine SDD. Direct observations of seed dispersal events by animals in natural plant populations are mostly constrained by the high mobility and low visibility of seed dispersers. Therefore, diverse alternative methods are used to estimate seed dispersal distance, but direct comparisons of these approaches within the same seed dispersal system are mostly missing. We investigated two plant species with different life history traits, Leonia cymosa and Parkia panurensis, exclusively dispersed by two tamarin species, Saguinus mystax and Leontocebus nigrifrons. We compared SDD estimates obtained from direct observations, genetic identification of mother plants from seed coats, parentage analysis of seedlings/saplings, and phenomenological and mechanistic modeling approaches. SDD derived from the different methods ranged between 158 and 201 m for P. panurensis and between 178 and 318 m for L. cymosa. In P. panurensis, the modeling approaches resulted in moderately higher estimates than observations and genotyping of seed coats. In L. cymosa, parentage analysis resulted in a lower estimate than all other methods. Overall, SDD estimates for P. panurensis (179 +/- 16 m; mean +/- SD) were significantly lower than for L. cymosa (266 +/- 59 m; mean +/- SD). Differences among methods were related to processes of the seed dispersal loop integrated by the respective methods (e.g., seed deposition or seedling distribution). We discuss the merits and limitations of each method and highlight the aspects to be considered when comparing SDD derived from different methodologies. Differences among plant species were related to differences in reproductive traits influencing gut passage time and feeding behavior, highlighting the importance of plant traits on animal-mediated seed dispersal distance. | NA | no | No good fitness measure and measure host GD | NA | no | no |
| 5034 | 2020 | 44042 | Gentiluomo, M; Peduzzi, G; Lu, Y; Campa, D; Canzian, F | 2019 | Genetic polymorphisms in inflammatory genes and pancreatic cancer risk: a two-phase study on more than 14 000 individuals | There is overwhelming evidence that inflammation plays a key role in the pathogenesis of cancer and its progression. Inflammation is regulated through a complex network of genes and polymorphic variants in these genes have been found to be associated to risk of various human cancers, alone or in combination with environmental variables. Despite this, not much is known on the genetic variability of genes that regulate inflammation and risk of pancreatic ductal adenocarcinoma (PDAC). We performed a two-phase association study considering the genetic variability of 76 genes that are key players in inflammatory response. We analysed tagging single nucleotide polymorphisms (SNPs) and regulatory SNPs on 7207 PDAC cases and 7063 controls and observed several associations with PDAC risk. The most significant association was between the carriers of the A allele of the CCL4-rs1719217 polymorphism, which was reported to be also associated with the expression level of the CCL4 gene, and increased risk of developing PDAC (odds ratio = 1.12, 95% confidence interval = 1.06-1.18, P = 3.34 x 10(-5)). This association was significant also after correction for multiple testing, highlighting the importance of using potentially functional SNPs in order to discover more genetic variants associated with PDAC risk. | NA | no | human | NA | no | no |
| 5035 | 2020 | 44113 | Ghosh, S; Bouvaine, S; Richardson, SCW; Ghanim, M; Maruthi, MN | 2018 | Fitness costs associated with infections of secondary endosymbionts in the cassava whitefly species Bemisia tabaci | We investigated the dual effects of bacterial infections and diseased cassava plants on the fitness and biology of the Bemisia tabaci infesting cassava in Africa. Isofemale B. tabaci colonies of sub-Saharan Africa 1-subgroup 3 (SSA1-SG3), infected with two secondary endosymbiotic bacteria Arsenophonus and Rickettsia (AR+) and those free of AR infections (AR-), were compared for fitness parameters on healthy and East African cassava mosaic virus-Uganda variant (EACMV-UG)-infected cassava plants. The whitefly fecundity and nymph development was not affected by bacterial infections or the infection of cassava by the virus. However, emergence of adults from nymphs was 50 and 17% higher by AR- on healthy and virus-infected plants, respectively, than AR+ flies. Development time of adults also was 10 days longer in AR+ than AR-. The whiteflies were further compared for acquisition and retention of EACMV-UG. Higher proportion of AR- acquired (91.8%) and retained (87.6%) the virus than AR+ (71.8, 61.2%, respectively). Similarly, the AR- flies retained higher quantities of virus (ninefold more) than AR+. These results indicated that bacteria-free whiteflies were superior and better transmitters of EACMV-UG, as they had higher adult emergence, quicker life cycle and better virus retention abilities than those infected with bacteria. | NA | no | No good fitness measure and measure host GD | NA | no | no |
| 5036 | 2020 | 44039 | Gibson, AK; Baffoe-Bonnie, H; Penley, MJ; Lin, JL; Owens, R; Khalid, A; Morran, LT | 2020 | The evolution of parasite host range in heterogeneous host populations | Theory on the evolution of niche width argues that resource heterogeneity selects for niche breadth. For parasites, this theory predicts that parasite populations will evolve, or maintain, broader host ranges when selected in genetically diverse host populations relative to homogeneous host populations. To test this prediction, we selected the bacterial parasite Serratia marcescens to kill Caenorhabditis elegans in populations that were genetically heterogeneous (50% mix of two experimental genotypes) or homogeneous (100% of either genotype). After 20 rounds of selection, we compared the host range of selected parasites by measuring parasite fitness (i.e. virulence, the selected fitness trait) on the two focal host genotypes and on a novel host genotype. As predicted, heterogeneous host populations selected for parasites with a broader host range: these parasite populations gained or maintained virulence on all host genotypes. This result contrasted with selection in homogeneous populations of one host genotype. Here, host range contracted, with parasite populations gaining virulence on the focal host genotype and losing virulence on the novel host genotype. This pattern was not, however, repeated with selection in homogeneous populations of the second host genotype: these parasite populations did not gain virulence on the focal host genotype, nor did they lose virulence on the novel host genotype. Our results indicate that host heterogeneity can maintain broader host ranges in parasite populations. Individual host genotypes, however, vary in the degree to which they select for specialization in parasite populations. | NA | no | fitness and diversity of parasite | NA | no | no |
| 5037 | 2020 | 44113 | Gim, J; Kim, W; Kwak, SH; Choi, H; Park, C; Park, KS; Kwon, S; Park, T; Won, S | 2017 | Improving Disease Prediction by Incorporating Family Disease History in Risk Prediction Models with Large-Scale Genetic Data | Despite the many successes of genome-wide association studies (GWAS), the known susceptibility variants identified by GWAS have modest effect sizes, leading to notable skepticism about the effectiveness of building a risk prediction model from large-scale genetic data. However, in contrast to genetic variants, the family history of diseases has been largely accepted as an important risk factor in clinical diagnosis and risk prediction. Nevertheless, the complicated structures of the family history of diseases have limited their application in clinical practice. Here, we developed a new method that enables incorporation of the general family history of diseases with a liability threshold model, and propose a new analysis strategy for risk prediction with penalized regression analysis that incorporates both large numbers of genetic variants and clinical risk factors. Application of our model to type 2 diabetes in the Korean population (1846 cases and 1846 controls) demonstrated that single-nucleotide polymorphisms accounted for 32.5% of the variation explained by the predicted risk scores in the test data set, and incorporation of family history led to an additional 6.3% improvement in prediction. Our results illustrate that family medical history provides valuable information on the variation of complex diseases and improves prediction performance. | NA | no | No good fitness measure and measure host GD | NA | no | no |
| 5038 | 2020 | 44112 | Giometto, A; Nelson, DR; Murray, AW | 2018 | Physical interactions reduce the power of natural selection in growing yeast colonies | Microbial populations often assemble in dense populations in which proliferating individuals exert mechanical forces on the nearby cells. Here, we use yeast strains whose doubling times depend differently on temperature to show that physical interactions among cells affect the competition between different genotypes in growing yeast colonies. Our experiments demonstrate that these physical interactions have two related effects: they cause the prolonged survival of slower-growing strains at the actively-growing frontier of the colony and cause faster-growing strains to increase their frequency more slowly than expected in the absence of physical interactions. These effects also promote the survival of slower-growing strains and the maintenance of genetic diversity in colonies grown in time-varying environments. A continuum model inspired by overdamped hydrodynamics reproduces the experiments and predicts that the strength of natural selection depends on the width of the actively growing layer at the colony frontier. We verify these predictions experimentally. The reduced power of natural selection observed here may favor the maintenance of drug-resistant cells in microbial populations and could explain the apparent neutrality of interclone competition within tumors. | NA | no | No good fitness measure and measure host GD | NA | no | no |
| 5039 | 2020 | 44039 | Gioria, M; Pysek, P; Baskin, CC; Carta, A | 2020 | Phylogenetic relatedness mediates persistence and density of soil seed banks | Soil seed banks can strongly affect survival and expansion of plant populations by spreading mortality risks and distributing genetic diversity through time. Knowledge of the main factors regulating the ability of seeds to persist in the soil beyond the first germination season is however limited. While morphological and physiological seed traits, and the degree of environmental uncertainty are considered important in shaping the seed banking strategies of plants, global assessments that explicitly account for phylogenetic relatedness are lacking. Using a global seed bank database comprising data for 2,350 angiosperms, we examined the extent to which two seed bank properties, i.e. seed bank type (transient vs. persistent) and density of viable seed banks, are determined by phylogenetic relatedness. We then tested phylogenetic correlations between these properties with seed mass and seed dormancy (dormant vs. non-dormant), and the contribution of phylogenetic relatedness relative to that of climatic and habitat-related variables in shaping seed bank properties. We found significant phylogenetic signal in seed bank type and density, providing evidence that the ability to form persistent seed banks is not randomly distributed across the phylogeny. While the ability to persist in the soil was phylogenetically correlated to the production of dormant and smaller seeds, seed mass and seed dormancy per se were poor predictors of seed persistence. Interestingly, habitat-related variables (mainly disturbance and canopy openness) but not climate significantly affect the ability of seed plants to form persistent seed banks. Synthesis. Our study is the first to show that phylogenetic relatedness plays an important role in explaining seed bank properties in angiosperms and how these properties relate to early life-history traits, climate and habitat-related variables. These findings represent a starting point to assess the generality of persistent seed banks as a bet-hedging strategy in unpredictable environments and provide important insights into how seed plants might respond to global environmental changes. | NA | no | No good fitness measure and measure host GD | NA | no | no |
| 5040 | 2020 | 44041 | Giudicelli, GC; Turchetto, C; Silva-Arias, GA; Freitas, LB | 2019 | Influence of climate changes on the potential distribution of a widespread grassland species in South America | The consequences of Quaternary climatic fluctuations on plant evolutionary history have been well documented in North America and Europe but have been far less studied in the Neotropical region. Diversification patterns in South American grasslands have been neglected, especially in the Pampas. In this study, we applied an ensemble-modeling framework and a bioregionalization method to test the hypothesis that Petunia axillaris subspecies responded differently to past climate changes in the South American grasslands. Scenarios for the future were projected to better understand the consequences of climatic shifts on the species’ survival. Our results revealed that each subspecies is currently distributed in different bioregions and displays substantial geographic separation as a consequence of past climate oscillations. The projected future scenarios suggest that all subspecies would be affected by habitat fragmentation. This could influence the genetic structuring among P. axillaris populations, as the diversity of grasslands species depends on the genetic characteristics of the remaining populations over the fragmented regions. These results reinforce the importance of considering intraspecific diversity when planning conservation efforts in grasslands in southern South America. | NA | no | No good fitness measure and measure host GD | NA | no | no |
| 5041 | 2020 | 44113 | Gleichsner, AM; Reinhart, K; Minchella, DJ | 2018 | The influence of related and unrelated co-infections on parasite dynamics and virulence | Many parasitic infections increase the morbidity and mortality of host populations. Interactions between co-infecting parasites can influence virulence, the damage done to a host. Previous studies investigating the impacts of parasite co-infection on hosts have been limited by their inability to control parasite dosage, use consistent virulence metrics, or verify co-infection status. This study used molecular tools, known infection dosage, and multiple assessments over time to test whether parasite relatedness can predict virulence in co-infections, as well as whether competitive interactions between different parasite strains within a host are predictable over time. In addition, we examined the impacts of other parasite traits, such as infectivity, as alternative predictors of virulence and competition outcomes. Hosts with single-strain (related) parasite infections were found to have lower virulence in terms of host and parasite reproduction, supporting kin selection predictions. However, these infections also resulted in higher host mortality. We argue that mortality should not be used as a measurement of virulence in parasite systems that castrate hosts. Hosts were more susceptible to mixed strain (unrelated) parasite infections, indicating that co-infections may make resistance more costly to hosts. Co-infections were dynamic, with changes in parasite dominance over the course of the infection. The more infective parasite strain appeared to suppress the less infective strain, ultimately increasing host longevity. Our findings suggest that unrelated, or more diverse, parasite infections are associated with higher virulence, but that studies must consider their methodology and possible alternative explanations beyond kin selection to understand virulence outcomes. | NA | no | No good fitness measure and measure host GD | NA | no | no |
| 5042 | 2020 | 44111 | Gloag, RS; Christie, JR; Ding, GL; Stephens, RE; Buchmann, G; Oldroyd, BP | 2019 | Workers’ sons rescue genetic diversity at the sex locus in an invasive honey bee population | The hallmark of eusociality is the division of labour between reproductive (queen) and nonreproductive (worker) females. Yet in many eusocial insects, workers retain the ability to produce haploid male offspring from unfertilized eggs. The reproductive potential of workers has well-documented consequences for the structure and function of insect colonies, but its implications at the population level are less often considered. We show that worker reproduction in honey bees can have an important role in maintaining genetic diversity at the sex locus in invasive populations. The honey bee sex locus is homozygous-lethal, and, all else being equal, a higher allele number in the population lead to higher mean brood survival. In an invasive population of the honey bee Apis cerana in Australia, workers contribute significantly to male production: 38% of male-producing colonies are queenless, and these contribute one-third of all males at mating congregations. Using a model, we show that such male production by queenless workers will increase the number of sex alleles retained in nascent invasive populations following founder events, relative to a scenario in which only queens reproduce. We conclude that by rescuing sex locus diversity that would otherwise be lost, workers’ sons help honey bee populations to minimize the negative effects of inbreeding after founder events and so contribute to their success as invaders. | NA | no | No good fitness measure and measure host GD | NA | no | no |
| 5043 | 2020 | 44113 | Gloria-Soria, A; Armstrong, PM; Powell, JR; Turner, PE | 2017 | Infection rate of Aedes aegypti mosquitoes with dengue virus depends on the interaction between temperature and mosquito genotype | Dengue fever is the most prevalent arthropod-transmitted viral disease worldwide, with endemic transmission restricted to tropical and subtropical regions of different temperature profiles. Temperature is epidemiologically relevant because it affects dengue infection rates in Aedes aegypti mosquitoes, the major vector of the dengue virus (DENV). Aedes aegypti populations are also known to vary in competence for different DENV genotypes. We assessed the effects of mosquito and virus genotype on DENV infection in the context of temperature by challenging Ae. aegypti from two locations in Vietnam, which differ in temperature regimes, with two isolates of DENV-2 collected from the same two localities, followed by incubation at 25, 27 or 32 degrees C for 10 days. Genotyping of the mosquito populations and virus isolates confirmed that each group was genetically distinct. Extrinsic incubation temperature (EIT) and DENV-2 genotype had a direct effect on the infection rate, consistent with previous studies. However, our results show that the EIT impacts the infection rate differently in each mosquito population, indicating a genotype by environment interaction. These results suggest that the magnitude of DENV epidemics may not only depend on the virus and mosquito genotypes present, but also on how they interact with local temperature. This information should be considered when estimating vector competence of local and introduced mosquito populations during disease risk evaluation. | NA | no | No good fitness measure and measure host GD | NA | no | no |
| 5044 | 2020 | 44042 | Godbout, J; Gros-Louis, MC; Lamothe, M; Isabel, N | 2020 | Going with the flow: Intraspecific variation may act as a natural ally to counterbalance the impacts of global change for the riparian species Populus deltoides | The speed and magnitude of global change will have major impacts on riparian ecosystems, thereby leading to greater forest vulnerability. Assessing species’ adaptive capacities to provide relevant information for vulnerability assessments remains challenging, especially for nonmodel species like the North American Populus deltoides W. Bartram ex Marshall. The objective of this study was to understand how genomic diversity of this foundation species was shaped by its environment (climate, soil, and biotic interactions) to gauge its adaptive capacity. We used two complementary approaches to get a full portrait of P. deltoides genetic diversity at both the species and whole-genome ranges. First, we used a set of 93 nuclear and three chloroplastic SNP markers in 946 individuals covering most of the species’ natural distribution. Then, to measure the degree of intraspecific divergence at the whole-genome level and to support the outlier and genomic-environment association analyses, we used a sequence capture approach on DNA pools. Three distinct lineages for P. deltoides were detected, and their current distribution was associated with abiotic and biotic variations. The comparison between both cpDNA and ncDNA patterns showed that gene flow between the lineages is unbalanced. The southern and northeastern populations may benefit from the input, through river flow, of novel alleles located upstream to their local gene pools. These alleles could migrate from populations that are already adapted to conditions that fit the predicted climates in the receiving local populations, hotter at the northeastern limit and drier in the Central United States. These preadapted incoming alleles may help to cope with maladaptation in populations facing changing conditions. | NA | no | No good fitness measure and measure host GD | NA | no | no |
| 5045 | 2020 | 44113 | Goethert, HK; Molloy, P; Berardi, V; Weeks, K; Telford, SR | 2018 | Zoonotic Babesia microti in the northeastern US: Evidence for the expansion of a specific parasite lineage | The recent range expansion of human babesiosis in the northeastern United States, once found only in restricted coastal sites, is not well understood. This study sought to utilize a large number of samples to examine the population structure of the parasites on a fine scale to provide insights into the mode of emergence across the region. 228 B. microti samples collected in endemic northeastern U.S. sites were genotyped using published Variable number tandem repeat (VNTR) markers. The genetic diversity and population structure were analysed on a geographic scale using Phyloviz and TESS, programs that utilize two different methods to identify population membership without predefined population data. Three distinct populations were detected in northeastern US, each dominated by a single ancestral type. In contrast to the limited range of the Nantucket and Cape Cod populations, the mainland population dominated from New Jersey eastward to Boston. Ancestral populations of B. microti were sufficiently isolated to differentiate into distinct populations. Despite this, a single population was detected across a large geographic area of the northeast that historically had at least 3 distinct foci of transmission, central New Jersey, Long Island and southeastern Connecticut. We conclude that a single B. microti genotype has expanded across the northeastern U.S. The biological attributes associated with this parasite genotype that have contributed to such a selective sweep remain to be identified. | NA | no | gd of pathogen | NA | no | no |
| 5046 | 2020 | 44111 | Goldenberg, SZ; Owen, MA; Brown, JL; Wittemyer, G; Oo, ZM; Leimgruber, P | 2019 | Increasing conservation translocation success by building social functionality in released populations | The importance of animal behavior to successful wildlife translocations has been acknowledged in recent decades, and it has been increasingly considered and more frequently incorporated into translocation management and research. However, explicit consideration of social behavior is often overlooked in this context. Social relationships take a variety of forms (e.g., cooperative partners, members of a dominance hierarchy, territorial neighbors) and play important roles in survival, reproduction, and resource exploitation. We review the ways in which concepts from studies of social behavior in wild populations may be leveraged to increase translocation success. Social structure and cohesion, social roles, social learning, and social competency may all be important to consider in building populations that are resilient and likely to persist. We argue that relevant data collected at all stages of translocation, including candidate selection, and during pre-release, release, and post-release monitoring, may inform the establishment of functional social structure post-release in species dependent on social processes. Integrating knowledge of social behavior into management decisions may be particularly useful when comparing the success of alternative release protocols or release candidate behavioral traits. Complementary datasets on a range of fitness-related metrics post-release will further leverage our understanding of social establishment in translocated populations. We illustrate the potential of these ideas using Asian and African elephants as a model. Both species are particularly challenging to manage but are translocated frequently; thus, evidence-based protocols for conservation translocations of elephants are urgently needed. (C) 2019 The Authors. Published by Elsevier B.V. | NA | no | No good fitness measure and measure host GD | NA | no | no |
| 5047 | 2020 | 44113 | Goldenberg, SZ; Wittemyer, G | 2017 | Orphaned female elephant social bonds reflect lack of access to mature adults | Compensatory social behavior in nonhuman animals following maternal loss has been documented, but understanding of how orphans allocate bonding to reconstruct their social networks is limited. Successful social integration may be critical to survival and reproduction for highly social species and, therefore, may be tied to population persistence. We examined the social partners involved in affiliative interactions of female orphans and non-orphans in an elephant population in Samburu, northern Kenya that experienced heightened adult mortality driven by drought and intense ivory poaching. We contrasted partners across different competitive contexts to gain insight to the influence of resource availability on social interactions. Though the number of partners did not differ between orphans and non-orphans, their types of social partners did. Orphans interacted with sisters and matriarchs less while feeding than did non-orphans, but otherwise their affiliates were similar. While resting under spatially concentrated shade, orphans had markedly less access to mature adults but affiliated instead with sisters, bulls, and age mates. Orphan propensity to strengthen bonds with non- dominant animals appears to offer routes to social integration following maternal loss, but lack of interaction with adult females suggests orphans may experience decreased resource access and associated fitness costs in this matriarchal society. | NA | no | No good fitness measure and measure host GD | NA | no | no |
| 5048 | 2020 | 44042 | Gomez-Ruiz, EP; Lacher, TE | 2019 | Climate change, range shifts, and the disruption of a pollinator-plant complex | Climate change has significant impacts on the distribution of species and alters ecological processes that result from species interactions. There is concern that such distribution shifts will affect animal-plant pollination networks. We modelled the potential future (2050 and 2070) distribution of an endangered migratory bat species (Leptonycteris nivalis) and the plants they pollinate (Agave spp) during their annual migration from central Mexico to the southern United States. Our models show that the overlap between the Agave and the endangered pollinating bat will be reduced by at least 75%. The reduction of suitable areas for Agave species will restrict the foraging resources available for the endangered bat, threatening the survival of its populations and the maintenance of their pollination service. The potential extinction of the bat L. nivalis will likely have negative effects on the sexual reproduction and genetic variability of Agave plants increasing their vulnerability to future environmental changes. | NA | no | n | NA | no | no |
| 5049 | 2020 | 44113 | Gong, SP; Vamberger, M; Auer, M; Praschag, P; Fritz, U | 2018 | Millennium-old farm breeding of Chinese softshell turtles (Pelodiscus spp.) results in massive erosion of biodiversity | Chinese softshell turtles (Pelodiscus spp.) are widely distributed, ranging from the Amur and Ussuri Rivers in the Russian Far East through the Korean Peninsula, Japan, and eastern, central, and southern China to southern Vietnam. In East and Southeast Asia, Chinese softshell turtles are traditionally exploited for food and have been farm-bred in China since the Spring and Autumn Period, more than 2400 years ago. Currently, the annual production of Pelodiscus amounts to 340,000 t in China alone. Using mitochondrial DNA (2428 bp) and five nuclear loci (3704 bp), we examined broad sampling of wild and farm-bred Pelodiscus to infer genetic and taxonomic differentiation. We discovered four previously unknown mitochondrial lineages, all from China. One lineage from Jiangxi is deeply divergent and sister to the mitochondrial lineage of Pelodiscus axenaria. The nuclear loci supported species status for P. axenaria and the new lineage from Jiangxi. Pelodiscus maackii and P. parviformis, both harboring distinct mitochondrial lineages, were not differentiated from P. sinensis in the studied nuclear markers. The same is true for two new mitochondrial lineages from Zhejiang, China, represented by only one individual each, and another new lineage from Anhui, Guangdong, Jiangxi and Zhejiang, China. However, Vietnamese turtles yielding a mitochondrial lineage clustering within P. sinensis were distinct in nuclear markers, suggesting that these populations could represent another unknown species with introgressed mitochondria. Its species status is also supported by the syntopic occurrence with P. sinensis in northern Vietnam and by morphology. In addition, we confirmed sympatry of P. axenaria and P. parviformis in Guangxi, China, and found evidence for sympatry of P. sinensis and the new putative species from Jiangxi, China. We also discovered evidence for hybridization in turtle farms and for the occurrence of alien lineages in the wild (Zhejiang, China), highlighting the risk of genetic pollution of native stock. In the face of the large-scale breeding of Pelodiscus, we claim that the long-term survival of distinct genetic lineages and species can only be assured when an upscale market segment for pure-bred softshell turtles is established, making the breeding of pure lineages lucrative for turtle farms. Our findings underline that the diversity of Pelodiscus is currently underestimated and threatened by anthropogenic admixture. We recommend mass screening of genetic and morphological variation of Chinese softshell turtles as a first step to understand and preserve their diversity. | NA | no | dometicated host | NA | no | no |
| 5050 | 2020 | 44041 | Gonzalez, AV; Gomez-Silva, V; Ramirez, MJ; Fonturbel, FE | 2020 | Meta-analysis of the differential effects of habitat fragmentation and degradation on plant genetic diversity | Genetic diversity is a key factor for population survival and evolution. However, anthropogenic habitat disturbance can erode it, making populations more prone to extinction. Aiming to assess the global effects of habitat disturbance on plant genetic variation, we conducted a meta-analysis based on 92 case studies obtained from published literature. We compared the effects of habitat fragmentation and degradation on plant allelic richness and gene diversity (equivalent to expected heterozygosity) and tested whether such changes are sensitive to different life-forms, life spans, mating systems, and commonness. Anthropogenic disturbance had a negative effect on allelic richness, but not on gene diversity. Habitat fragmentation had a negative effect on genetic variation, whereas habitat degradation had no effect. When we examined the individual effects in fragmented habitats, allelic richness and gene diversity decreased, but this decrease was strongly dependent on certain plant traits. Specifically, common long-lived trees and self-incompatible species were more susceptible to allelic richness loss. Conversely, gene diversity decreased in common short-lived species (herbs) with self-compatible reproduction. In a wider geographical context, tropical plant communities were more sensitive to allelic richness loss, whereas temperate plant communities were more sensitive to gene diversity loss. Our synthesis showed complex responses to habitat disturbance among plant species. In many cases, the absence of effects could be the result of the time elapsed since the disturbance event or reproductive systems favoring self-pollination, but attention must be paid to those plant species that are more susceptible to losing genetic diversity, and appropriate conservation should be actions taken. | NA | no | No good fitness measure and measure host GD | NA | no | no |
| 5051 | 2020 | 44112 | Gonzalez, HM; Tarraf, W; Jian, XQ; Vasquez, PM; Kaplan, R; Thyagarajan, B; Daviglus, M; Lamar, M; Gallo, LC; Zeng, DL; Fornage, M | 2018 | Apolipoprotein E genotypes among diverse middle-aged and older Latinos: Study of Latinos-Investigation of Neurocognitive Aging results (HCHS/SOL) | The apoE4 isoform is associated with increased cholesterol, cardiovascular risk, and Alzheimer’s Disease risk, however, its distribution is not well-understood among US Latinos. Latinos living in the US are highly Amerindian, European and African admixed, which varies by region and country of origin. However, Latino genetic diversity is understudied and consequently poorly understood, which has significant implications for understanding disease risk in nearly one-fifth of the US population. In this report we describe apoE distributions in a large and representative sample of diverse, genetically determined US Latinos. | NA | no | human | NA | no | no |
| 5052 | 2020 | 44111 | Goodman, J; Copplestone, D; Laptev, GV; Gashchak, S; Auld, SKJR | 2019 | Variation in chronic radiation exposure does not drive life history divergence among Daphnia populations across the Chernobyl Exclusion Zone | Ionizing radiation is a mutagen with known negative impacts on individual fitness. However, much less is known about how these individual fitness effects translate into population-level variation in natural environments that have experienced varying levels of radiation exposure. In this study, we sampled genotypes of the freshwater crustacean, Daphnia pulex, from the eight inhabited lakes across the Chernobyl Exclusion Zone (CEZ). Each lake has experienced very different levels of chronic radiation exposure since a nuclear power reactor exploded there over thirty years ago. The sampled Daphnia genotypes represent genetic snapshots of current populations and allowed us to examine fitness-related traits under controlled laboratory conditions at UK background dose rates. We found that whilst there was variation in survival and schedules of reproduction among populations, there was no compelling evidence that this was driven by variation in exposure to radiation. Previous studies have shown that controlled exposure to radiation at dose rates included in the range measured in the current study reduce survival, or fecundity, or both. One limitation of this study is the lack of available sites at high dose rates, and future work could test life history variation in various organisms at other high radiation areas. Our results are nevertheless consistent with the idea that other ecological factors, for example competition, predation or parasitism, are likely to play a much bigger role in driving variation among populations than exposure to the high radiation dose rates found in the CEZ. These findings clearly demonstrate that it is important to examine the potential negative effects of radiation across wild populations that are subject to many and varied selection pressures as a result of complex ecological interactions. | NA | no | No good fitness measure and measure host GD | NA | no | no |
| 5053 | 2020 | 44039 | Gooley, RM; Hogg, CJ; Fox, S; Pemberton, D; Belov, K; Grueber, CE | 2020 | Inbreeding depression in one of the last DFTD-free wild populations of Tasmanian devils | Background. Vulnerable species experiencing inbreeding depression are prone to localised extinctions because of their reduced fitness. For Tasmanian devils, the rapid spread of devil facial tumour disease (DFTD) has led to population declines and fragmentation across the species’ range. Here we show that one of the few remaining DFTD-free populations of Tasmanian devils is experiencing inbreeding depression. Moreover, this population has experienced a significant reduction in reproductive success over recent years. Methods. We used 32 microsatellite loci to examine changes in genetic diversity and inbreeding in the wild population at Woolnorth, alongside field data on breeding success from females to test for inbreeding depression. Results. We found that maternal internal relatedness has a negative impact on litter sizes. The results of this study imply that this population may be entering an extinction vortex and that to protect the population genetic rescue should be considered. This study provides conservation managers with useful information for managing wild devils and provides support for the Wild Devil Recovery Program’’, which is currently augmenting small, isolated populations. | NA | no | Inbreeding | NA | no | no |
| 5054 | 2020 | 44113 | Gordeeva, NV; Salmenkova, EA | 2017 | Genetic Markers of Adaptive Processes in the Far Eastern Pink Salmon &ITOncorhynchus gorbuscha&IT: Allelic Diversity at the Locus of Major Histocompatibility Complex MHC I-&ITA1&IT | To clarify allelic diversity at the locus of major histocompatibility complex MHC class l-A1 in the Far Eastern pink salmon Oncorhynchus gorbuscha, sequencing of the electrophoretic alleles isolated from the gel (DGGE alleles) was performed. In 47 individuals, the genotypes of which consisted of ten DGGE alleles, 18 MHC l-A1 nucleotide sequences were revealed, and thus, eight cryptic alleles not detected by electrophoresis were identified. Eleven of these alleles were identified earlier in pink salmon from Hokkaido, Alaska, and British Columbia, and seven, possibly, were unique to the populations from some Far Eastern regions. Six of the previously determined DGGE alleles corresponded to more than one nucleotide sequence. However, the sequences attributed to the same DGGE allele differed on average by less than 1 nucleotide. These findings point to sufficient sensitivity of the DGGE method, although the genetic diversity and differentiation estimates obtained with it will obviously be somewhat underestimated. Considerable predominance of nonsynonymous substitutions over the synonymous ones in the codons of the MHC l-A1 antigen-binding site confirms the presence of positive selection aimed at providing the population resistance to local spectrum of pathogens. Refinement of the allelic composition of the adaptively important MHC genetic marker will contribute to more complete understanding of the adaptive genetic structure of pink salmon as an important element of the overall population structure of the species. | NA | no | mhc diversity | NA | no | no |
| 5055 | 2020 | 44040 | Gould, L; Cowen, LLE | 2020 | Lemur catta in small forest fragments: Which variables best predict population viability? | Habitat fragmentation is an increasingly serious issue affecting primates in most regions where they are found today. Populations of Lemur catta (ring-tailed lemur) in Madagascar’s south-central region are increasingly restricted to small, isolated forest fragments, surrounded by grasslands or small-scale agriculture. Our aim was to evaluate the potential for population viability of L. catta in nine forest fragments of varying sizes (2-46 ha, population range: 6-210 animals) in south-central Madagascar, using a set of comparative, quantitative ecological measures. We used Poisson regression models with a log link function to examine the effects of fragment size, within-fragment food availability, and abundance of matrix resources (food and water sources) on L. catta population sizes and juvenile recruitment. We found a strong association between overall population size and (a) fragment size and (b) abundance of key food resources Melia azedarach and Ficus spp. (per 100 m along transect lines). Juvenile recruitment was also associated with fragment size and abundance of the two above-mentioned food resources. When the largest population, an outlier, was removed from the analysis, again, the model containing fragment size and abundance of M. azedarach and Ficus spp. was the best fitting, but the model that best predicted juvenile recruitment contained only fragment size. While our results are useful for predicting population presence and possible persistence in these fragments, both the potential for male dispersal and the extent of human disturbance within most fragments play crucial roles regarding the likelihood of long-term L. catta survival. While seven of the nine fragments were reasonably protected from human disturbance, only three offered the strong potential for male dispersal, thus the long-term viability of many of these populations is highly uncertain. | NA | no | No good measureGD of host | NA | no | no |
| 5056 | 2020 | 44040 | Govindan, BN; Hutchison, WD | 2020 | Influence of Temperature on Age-Stage, Two-Sex Life Tables for a Minnesota-Acclimated Population of the Brown Marmorated Stink Bug (Halyomorpha halys) | Temperature is a critical single factor influencing insect population dynamics, and is foundational for improving our understanding of the phenology of invasive species adapting to new agroecosystems or in the process of range expansion. An age-stage, two-sex life table was therefore developed to analyze fundamental demographic features such as development, survival, and reproduction of a Minnesota-acclimated population of the invasive brown marmorated stink bug (Halyomorpha halys), in the north central USA. All salient life history parameters were estimated to better understand the population growth potential of H. halys at the current limit of its northern range in North America. We examined the effect of selected constant temperatures on immature development and survival (15-39 degrees C), adult reproduction and longevity (17-36 degrees C) of H. halys in the laboratory. The Minnesota population developed faster and survived at higher rates relative to a population that had previously established in Pennsylvania, USA. Mean generation time for the Minnesota population was minimized at 30 degrees C, while survival and fecundity were maximized at 27 and 23 degrees C, respectively. Given these findings, we assessed the effect of temperature on the intrinsic rate of increase (rm), the life table parameter that integrates the effects of temperature on development, survival, and reproduction. A Ratkowsky model predicted rm was maximized (0.0899) at 27.5 degrees C. We discuss the implications of our findings for understanding population growth rates for H. halys in the context of a warming climate, and potential to emerge as a serious crop pest in the Midwest U.S. region. | NA | no | n | NA | no | no |
| 5057 | 2020 | 44113 | Gowane, GR; Akram, N; Misra, SS; Prakash, V; Kumar, A | 2018 | Genetic diversity of Cahi DRB and DQB genes of caprine MHC class II in Sirohi goat | The objective of this study was to assess the genetic diversity of the Sirohi goat for DRB and DQB1 loci, and to study their association with antibody response induced by the Peste des petits ruminants (PPR) vaccine. A total of 360 Sirohi kids were studied using single stranded confirmation polymorphism (SSCP) followed by polymerase chain reaction sequence-based typing (PCR-SBT) for DRB and DQB1 diversities. The competitive enzyme-linked immuno-sorbent assay (C-ELISA) was used to evaluate immune response post-PPR vaccination. Study revealed rich diversity of major histocompatibility complex (MHC) region in goat. A total of 18 DRB and 15 DQB1 alleles were obtained which were new. Alleles DRB*0104 and DQB1*0101 were the most common. The approach of SSCP combined with PCR-SBT reflects cost-effective and most powerful approach to decipher the genetic diversity in complex MHC region. Study revealed variation in DRB and DQB1 genes in Sirohi flock along with high Wu Kabat index. A total of 16 of the 89 amino acid residue sites in DRB and 19 of 86 residue sites in DQB1 had more than three amino acid substitutions. Positive evolutionary selection was evident in Sirohi for MHC region. Nonsignificant association of DRB and DQB1 genotypes with PPR virus (PPRV) vaccine response revealed complexity of the phenotype and importance of other factors for vaccine response. Rich diversity of DRB and DQB1 genes reflects the fitness of the population and importance of this locus for future selection programmes. | NA | no | mhc diversity | NA | no | no |
| 5058 | 2020 | 44113 | Gracia, ES; de Bekker, C; Hanks, EM; Hughes, DP | 2018 | Within the fortress: A specialized parasite is not discriminated against in a social insect society | Social insect colonies function cohesively due, in part, to altruistic behaviors performed towards related individuals. These colonies can be affected by parasites in two distinct ways, either at the level of the individual or the entire colony. As such, colonies of social insects can experience conflict with infected individuals reducing the cohesiveness that typifies them. Parasites of social insects therefore offer us a framework to study conflicts within social insect colonies in addition to the traditionally viewed conflicts afforded by groups of low genetic relatedness due to multiple mating for example. In our study, we use the behavior manipulating fungal pathogen, Ophiocordyceps kimflemingiae (= unilateralis) and its host, Camponotus castaneus, to ask if colony members are able to detect infected individuals. Such detection would be optimal for the colony since infected workers die near foraging trails where the fungus develops its external structures and releases spores that infect other colony members. To determine if C. castaneus workers can detect these future threats, we used continuous -time point observations coupled with longer continuous observations to discern any discrimination towards infected individuals. After observing 1,240 hours of video footage we found that infected individuals are not removed from the colony and continuously received food during the course of fungal infection. We also calculated the distances between workers and the nest entrance in a total of 35,691 data points to find infected workers spent more time near the entrance of the nest. Taken together, these results suggest healthy individuals do not detect the parasite inside their nestmates. The colony’s inability to detect infected individuals allows O. kimflemingiae to develop within the colony, while receiving food and protection from natural enemies, which could damage or kill its ant host before the parasite has completed its development. | NA | no | No good fitness measure and measure host GD | NA | no | no |
| 5059 | 2020 | 44111 | Graitson, E; Barbraud, C; Bonnet, X | 2019 | Catastrophic impact of wild boars: insufficient hunting pressure pushes snakes to the brink | Climatic changes, habitat loss and invasive species are important threats for many animal populations. Here, we report rapid declines and extinctions of adder populations monitored during 12 years in Belgium where none of the above causes was involved. This study provides the first large-scale data showing that wild boars represent a major risk for snakes, pushing populations to extinction. Drastic population declines were observed in the sites impacted by wild boars (N = 14), while in other sites spared by wild boars populations remained stable (N = 9). Wild boars are highly fertile and their main predators have been extirpated from Western Europe; yet, recreational hunting kept populations in check during decades. Hunting pressure is currently insufficient to control the rapid expansion of wild boars, demographic outbreaks are increasingly frequent. Wild boars are omnivorous; they can destroy snakes directly and indirectly through the depletion of snake’s preys and via the destruction of key microhabitats. Snakes exhibit limited dispersal capacities; they cannot escape local perturbations and thus are highly vulnerable. Because wild boars have a very eclectic diet, are prolific breeders and are able to devastate a range of habitats their negative impact under relaxed hunting pressure applies to a huge variety of organisms, including reptiles. Policies to limit wild boar populations are urgently needed. Recommendations to target reproductive females and piglets should be generalized and applied. | NA | no | No good fitness measure and measure host GD | NA | no | no |
| 5060 | 2020 | 44111 | Grandaubert, J; Duthei, JY; Stukenbrock, EH | 2019 | The genomic determinants of adaptive evolution in a fungal pathogen | Unravelling the strength, frequency, and distribution of selective variants along the genome as well as the underlying factors shaping this distribution are fundamental goals of evolutionary biology. Antagonistic host-pathogen coevolution is thought to be a major driver of genome evolution between interacting species. While rapid evolution of pathogens has been documented in several model organisms, the genetic mechanisms of their adaptation are still poorly understood and debated, particularly the role of sexual reproduction. Here, we apply a population genomic approach to infer genome-wide patterns of selection among 13 isolates of Zymoseptoria tritici, a fungal pathogen characterized by extremely high genetic diversity, gene density, and recombination rates. We report that the genome of Z. tritici undergoes a high rate of adaptive substitutions, with 44% of nonsynonymous substitutions being adaptive on average. This fraction reaches 68% in so-called effector genes encoding determinants of pathogenicity, and the distribution of fitness effects differs in this class of genes as they undergo adaptive mutations with stronger positive fitness effects, but also more slightly deleterious mutations. Besides the globally high rate of adaptive substitutions, we report a negative relationship between pN/pS and the fine-scale recombination rate and a strong positive correlation between the rate of adaptive nonsynonymous substitutions (omega(a)) and recombination rate. This result suggests a pervasive role of both background selection and Hill-Robertson interference even in a species with an exceptionally high recombination rate (60 cM/Mb on average). While transposable elements (TEs) have been suggested to contribute to adaptation by creating compartments of fast-evolving genomic regions, we do not find a significant effect of TEs on the rate of adaptive mutations. Overall our study suggests that sexual recombination is a significant driver of genome evolution, even in rapidly evolving organisms subject to recurrent mutations with large positive effects. | NA | no | gd of pathogen | NA | no | no |
| 5061 | 2020 | 44112 | Grassl, J; Holt, S; Cremen, N; Peso, M; Hahne, D; Baer, B | 2018 | Synergistic effects of pathogen and pesticide exposure on honey bee (Apis mellifera) survival and immunity | Declines in native insect pollinator populations and substantial losses in managed honey bees have been reported on a global scale and become a widespread concern because of the importance of these insects for human food production and ecosystem stability. Several potential factors have been studied as possible causes of declining pollinator health, such as parasites and pathogens, exposure to agricultural pesticides, habitat loss and/or climate change. More recently, a combination of these factors rather than a single cause have been blamed for observed pollinator losses, but field studies of such interactions are challenging, especially in the presence of confounding environmental stressors. We therefore examined the impact of single and combined stressors on the honey bee (Apis mellifera) in a generally healthy Australian population. We exposed workers during their larval development and drones until they reached sexual maturity to the neonicotinoid pesticide Thiamethoxam, at concentrations more than 20 times lower than we initially measured in the field, the microsporidian gut pathogen Nosema apis or both stressors at the same time. We found that simultaneous exposure significantly reduced bee health. We observed a substantial increase in mortality and a reduction of immunocompetence in workers exposed to both the pathogen and the pesticide. We conclude that the exposure of generally healthy bees to multiple environmental stressors results in synergistic effects where the effects are expected to negatively impact performance and could be sufficient to trigger colony collapse. We found that the vast majority of males did not survive to sexual maturity after exposure to very low levels of Thiamethoxam. This would not only reduce the reproductive success of individual colonies, but can also impact gene flow and genetic diversity at the population level, which are both known as key components of honey bee health. | NA | no | no good gd measure host pop | NA | no | no |
| 5062 | 2020 | 44043 | Graziano, F; Biino, G; Bonati, MT; Neale, BM; Do, R; Concas, MP; Vaccargiu, S; Pirastu, M; Terradura-Vagnarelli, O; Cirillo, M; Laurenzi, M; Mancini, M; Zanchetti, A; Grassi, M | 2019 | Estimation of metabolic syndrome heritability in three large populations including full pedigree and genomic information | Metabolic syndrome is a complex human disorder characterized by a cluster of conditions (increased blood pressure, hyperglycemia, excessive body fat around the waist, and abnormal cholesterol or triglyceride levels). Any of these conditions increases the risk of serious disorders such as diabetes or cardiovascular disease. Currently, the degree of genetic regulation of this syndrome is under debate and partially unknown. The principal aim of this study was to estimate the genetic component and the common environmental effects in different populations using full pedigree and genomic information. We used three large populations (Gubbio, ARIC, and Ogliastra cohorts) to estimate the heritability of metabolic syndrome. Due to both pedigree and genotyped data, different approaches were applied to summarize relatedness conditions. Linear mixed models (LLM) using average information restricted maximum likelihood (AIREML) algorithm were applied to partition the variances and estimate heritability (h(2)) and common sib-household effect (c(2)). Globally, results obtained from pedigree information showed a significant heritability (h(2): 0.286 and 0.271 in Gubbio and Ogliastra, respectively), whereas a lower, but still significant heritability was found using SNPs data (hSNP2: 0.167and0.254 in ARIC and Ogliastra). The remaining heritability between h(2) and hSNP2 ranged between 0.031 and 0.237. Finally, the common environmental c(2) in Gubbio and Ogliastra were also significant accounting for about 11% of the phenotypic variance. Availability of different kinds of populations and data helped us to better understand what happened when heritability of metabolic syndrome is estimated and account for different possible confounding. Furthermore, the opportunity of comparing different results provided more precise and less biased estimation of heritability. | NA | no | human | NA | no | no |
| 5063 | 2020 | 44041 | Grieves, LA; Gloor, GB; Bernards, MA; MacDougall-Shackleton, EA | 2019 | Songbirds show odour-based discrimination of similarity and diversity at the major histocompatibility complex | The major histocompatibility complex (MHC) is the most polymorphic region of the vertebrate genome. Individuals with more MHC alleles can respond to a broader suite of pathogens, suggesting that selection should favour the ability to assess the MHC genotype of potential mates. Indeed, MHC-based mate choice, particularly preferences for MHC-dissimilar or MHC-diverse partners, appears widespread among vertebrates. Mammals, fish and seabirds assess MHC through odour cues. However, despite the prominence of songbirds in mate choice studies, the mechanisms by which this group might assess MHC remain speculative. Motivated by the discovery that chemical similarity in the preen oil of song sparrows, Melospiza melodia, predicts similarity at MHC class II, we examined preen oil odour preferences for MHC dissimilarity and diversity. We presented breeding-condition song sparrows with preen oil from opposite-sex conspecifics using a two-choice design. We compared time spent with odour from MHC-dissimilar versus MHC-similar birds, and MHC-diverse versus less MHC-diverse birds. Both sexes spent more time with odour from MHC-dissimilar compared to MHC-similar birds and with odour from more MHC-diverse than less MHC-diverse birds. We conclude that song sparrows, and presumably other passerines, can use preen oil odour to discriminate MHC similarity and diversity of potential mates. (C) 2019 The Association for the Study of Animal Behaviour. Published by Elsevier Ltd. All rights reserved. | NA | no | n | NA | no | no |
| 5064 | 2020 | 44112 | Griffiths, SM; Harrison, XA; Weldon, C; Wood, MD; Pretorius, A; Hopkins, K; Fox, G; Preziosi, RF; Antwis, RE | 2018 | Genetic variability and ontogeny predict microbiome structure in a disease-challenged montane amphibian | Amphibian populations worldwide are at risk of extinction from infectious diseases, including chytridiomycosis caused by the fungal pathogen Batrachochytrium dendrobatidis (Bd). Amphibian cutaneous microbiomes interact with Bd and can confer protective benefits to the host. The composition of the microbiome itself is influenced by many environment- and host-related factors. However, little is known about the interacting effects of host population structure, genetic variation and developmental stage on microbiome composition and Bd prevalence across multiple sites. Here we explore these questions in Amietia hymenopus, a disease-affected frog in southern Africa. We use microsatellite genotyping and 16S amplicon sequencing to show that the microbiome associated with tadpole mouthparts is structured spatially, and is influenced by host genotype and developmental stage. We observed strong genetic structure in host populations based on rivers and geographic distances, but this did not correspond to spatial patterns in microbiome composition. These results indicate that demographic and host genetic factors affect microbiome composition within sites, but different factors are responsible for host population structure and microbiome structure at the between-site level. Our results help to elucidate complex within- and among-population drivers of microbiome structure in amphibian populations. That there is a genetic basis to microbiome composition in amphibians could help to inform amphibian conservation efforts against infectious diseases. | NA | no | No good fitness measure and measure host GD | NA | no | no |
| 5065 | 2020 | 44040 | Griffiths, SM; Taylor-Cox, ED; Behringer, DC; Butler, MJ; Preziosi, RF | 2020 | Using genetics to inform restoration and predict resilience in declining populations of a keystone marine sponge | Genetic tools can have a key role in informing conservation management of declining populations. Genetic diversity is an important determinant of population fitness and resilience, and can require careful management to ensure sufficient variation is present. In addition, population genetics data reveal patterns of connectivity and gene flow between locations, enabling mangers to predict recovery and resilience, identify areas of local adaptation, and generate restoration plans. Here, we demonstrate a conservation genetics approach to inform restoration and management of the loggerhead sponge (Spheciospongia vesparium) in the Florida Keys, USA. This species is a dominant, habitat-forming component of marine ecosystems in the Caribbean region, but in Florida has suffered numerous mass mortality events. We developed microsatellite markers and used them to genotype sponges from 14 locations in Florida and a site each in The Bahamas, Belize and Barbuda. We found that genetic diversity levels were similar across all sites, but inbreeding and bottleneck signatures were present in Florida. Populations are highly structured at the regional scale, whilst within Florida connectivity is present in a weak isolation by distance pattern, coupled with chaotic genetic patchiness. Evidence of a weak barrier to gene flow was found in Florida among sites situated on opposite sides of the islands in the Middle Keys. Loggerhead sponge populations in Florida are vulnerable in the face of mass mortalities due to low connectivity with other areas in the region, as well as distance-limited and unpredictable local connectivity patterns. However, our discovery of Florida’s high genetic diversity increases hope for resilience to future perturbations. These results provide valuable insight for sponge restoration practice in Florida. | NA | no | inbreeding | NA | no | no |
| 5066 | 2020 | 44112 | Grinde, KE; Qi, QB; Thornton, TA; Liu, SM; Shadyab, AH; Chan, KHK; Reiner, AP; Sofer, T | 2019 | Generalizing polygenic risk scores from Europeans to Hispanics/Latinos | Polygenic risk scores (PRSs) are weighted sums of risk allele counts of single-nucleotide polymorphisms (SNPs) associated with a disease or trait. PRSs are typically constructed based on published results from Genome-Wide Association Studies (GWASs), and the majority of which has been performed in large populations of European ancestry (EA) individuals. Although many genotype-trait associations have generalized across populations, the optimal choice of SNPs and weights for PRSs may differ between populations due to different linkage disequilibrium (LD) and allele frequency patterns. We compare various approaches for PRS construction, using GWAS results from both large EA studies and a smaller study in Hispanics/Latinos: The Hispanic Community Health Study/Study of Latinos (HCHS/SOL, n=12,803). We consider multiple approaches for selecting SNPs and for computing SNP weights. We study the performance of the resulting PRSs in an independent study of Hispanics/Latinos from the Women’s Health Initiative (WHI, n=3,582). We support our investigation with simulation studies of potential genetic architectures in a single locus. We observed that selecting variants based on EA GWASs generally performs well, except for blood pressure trait. However, the use of EA GWASs for weight estimation was suboptimal. Using non-EA GWAS results to estimate weights improved results. | NA | no | No good fitness measure and measure host GD | NA | no | no |
| 5067 | 2020 | 44112 | Gritsenko, D; Pozharsky, A; Deryabina, N; Kassenova, A; Galiakparov, N | 2019 | GENETIC ANALYSIS OF HEMAGGLUTININ PROTEINS OF H3 AND H1 SUBTYPES IN KAZAKHSTAN | The influenza is one of the most dangerous and widespread infectious diseases on the planet. A natural reservoir of the influenza A virus is wild waterfowl, which subsequently contribute to the spread of viral infection among domestic animals Currently, different influenza A subtypes were isolated from various animal species and their genetic diversity were investigated with the subsequent possibility to predict the places of outbreaks and the transfer between species. An investigation of genetic diversity of influenza A virus is also important for a timely response by developing suitable vaccines to the emergence of new strains. In the work, we investigated two subtypes of hemagglutinin (H3, H1) from wild waterfowl in the Republic of Kazakhstan (RK). This work was aimed to determine the homology between these subtypes and currently known isolates from the NCBI database. H3N8 isolates from Kazakhstan were located in a monophyletic group together with isolates from Mongolia and Altai according to phylogenetic analysis of the hemagglutinin sequences . The hemagglutinin homology of H3N8 isolates from Kazakhstan and A/eq/Richmond/1/2007 vaccine strain was 86.07% and with A/eq/Ohio/2003 vaccine strain was 86, 24%. In the case of H1N1 isolates from Kazakhstan, the highest hemagglutinin homology was with isolates from Europe. The homology with the A/California/07/2009 (H1N1) vaccine strain was 81.27%. Important amino acids of cleavage and receptor binding sites were not variable in both H3 and H1 subtypes. The investigation of antigenic sites showed presence of variations in all five sites for H3 subtype and in 4 sites for H1. | NA | no | gd of pathogen | NA | no | no |
| 5068 | 2020 | 44112 | Grobler, P; van Wyk, AM; Dalton, DL; van Vuuren, BJ; Kotze, A | 2018 | Assessing introgressive hybridization between blue wildebeest (Connochaetes taurinus) and black wildebeest (Connochaetes gnou) from South Africa | Introgressive hybridization poses a threat to the genetic integrity of black wildebeest (Connochaetes gnou) and blue wildebeest (Connochaetes taurinus) populations in South Africa. Black wildebeest is endemic to South Africa and was driven to near extinction in the early 1900s due to habitat destruction, hunting pressure and disease outbreaks. Blue wildebeest on the other hand are widely distributed in southern and east Africa. In South Africa the natural distribution ranges of both species overlap, however, extensive translocation of black wildebeest outside of its normal distribution range in South Africa have led to potential hybridization between the two species. The molecular identification of pure and admixed populations is necessary to design viable and sustainable conservation strategies, since phenotypic evidence of hybridization is inconclusive after successive generations of backcrossing. The aim of this study was to assess levels of hybridization in wildebeest using both species-specific and cross-species microsatellite markers. Black wildebeest (157) and blue wildebeest (122) from provincial and national parks and private localities were included as reference material, with 180 putative hybrid animals also screened. A molecular marker panel consisting of 13 cross-species and 11 species-specific microsatellite markers was developed. We used a Bayesian clustering model to confirm the uniqueness of blue- and black wildebeest reference groups, assign individuals to each of the two clusters, and determine levels of admixture. Results indicated a clear partition between black wildebeest and blue wildebeest (the average proportions of membership to black wildebeest and blue wildebeest clusters were QI = 0.994 and QI = 0.955 respectively). From the putative hybrid samples, only five hybrid individuals were confirmed. However, high levels of linkage disequilibrium were observed in the putative hybrid populations which may indicate historical hybridization. Measures of genetic diversity in the black wildebeest populations were found to be lower than that of the blue wildebeest. The observed lower level of genetic diversity was expected due to the demographic history of the specie. This study will make a significant contribution to inform a national conservation strategy to conserve the genetic integrity of both species. | NA | no | No good fitness measure and measure host GD | NA | no | no |
| 5069 | 2020 | 44112 | Groenewoud, F; Kingma, SA; Hammers, M; Dugdale, HL; Burke, T; Richardson, DS; Komdeur, J | 2018 | Subordinate females in the cooperatively breeding Seychelles warbler obtain direct benefits by joining unrelated groups | In many cooperatively breeding animals, a combination of ecological constraints and benefits of philopatry favours offspring taking a subordinate position on the natal territory instead of dispersing to breed independently. However, in many species individuals disperse to a subordinate position in a non-natal group (subordinate between-group dispersal), despite losing the kin-selected and nepotistic benefits of remaining in the natal group. It is unclear which social, genetic and ecological factors drive between-group dispersal. We aim to elucidate the adaptive significance of subordinate between-group dispersal by examining which factors promote such dispersal, whether subordinates gain improved ecological and social conditions by joining a non-natal group, and whether between-group dispersal results in increased lifetime reproductive success and survival. Using a long-term dataset on the cooperatively breeding Seychelles warbler (Acrocephalus sechellensis), we investigated how a suite of proximate factors (food availability, group composition, age and sex of focal individuals, population density) promote subordinate between-group dispersal by comparing such dispersers with subordinates that dispersed to a dominant position or became floaters. We then analysed whether subordinates that moved to a dominant or non-natal subordinate position, or became floaters, gained improved conditions relative to the natal territory and compared fitness components between the three dispersal strategies. We show that individuals that joined another group as non-natal subordinates were mainly female and that, similar to floating, between-group dispersal was associated with social and demographic factors that constrained dispersal to an independent breeding position. Between-group dispersal was not driven by improved ecological or social conditions in the new territory and did not result in higher survival. Instead, between-group dispersing females often became cobreeders, obtaining maternity in the new territory, and were likely to inherit the territory in the future, leading to higher lifetime reproductive success compared to females that floated. Males never reproduced as subordinates, which may be one explanation why subordinate between-group dispersal by males is rare. Our results suggest that subordinate between-group dispersal is used by females to obtain reproductive benefits when options to disperse to an independent breeding position are limited. This provides important insight into the additional strategies that individuals can use to obtain reproductive benefits. | NA | no | No good fitness measure and measure host GD | NA | no | no |
| 5070 | 2020 | 44113 | Grogan, KE; Sauther, ML; Cuozzo, FP; Drea, CM | 2017 | Genetic wealth, population health: Major histocompatibility complex variation in captive and wild ring-tailed lemurs (Lemur catta) | Across species, diversity at the major histocompatibility complex (MHC) is critical to individual disease resistance and, hence, to population health; however, MHC diversity can be reduced in small, fragmented, or isolated populations. Given the need for comparative studies of functional genetic diversity, we investigated whether MHC diversity differs between populations which are open, that is experiencing gene flow, versus populations which are closed, that is isolated from other populations. Using the endangered ring-tailed lemur (Lemur catta) as a model, we compared two populations under long-term study: a relatively open, wild population (n=180) derived from Beza Mahafaly Special Reserve, Madagascar (2003-2013) and a closed, captive population (n=121) derived from the Duke Lemur Center (DLC, 1980-2013) and from the Indianapolis and Cincinnati Zoos (2012). For all animals, we assessed MHC-DRB diversity and, across populations, we compared the number of unique MHC-DRB alleles and their distributions. Wild individuals possessed more MHC-DRB alleles than did captive individuals, and overall, the wild population had more unique MHC-DRB alleles that were more evenly distributed than did the captive population. Despite management efforts to maintain or increase genetic diversity in the DLC population, MHC diversity remained static from 1980 to 2010. Since 2010, however, captive-breeding efforts resulted in the MHC diversity of offspring increasing to a level commensurate with that found in wild individuals. Therefore, loss of genetic diversity in lemurs, owing to small founder populations or reduced gene flow, can be mitigated by managed breeding efforts. Quantifying MHC diversity within individuals and between populations is the necessary first step to identifying potential improvements to captive management and conservation plans. | NA | no | mhc diversity | NA | no | no |
| 5071 | 2020 | 44113 | Grossen, C; Biebach, I; Angelone-Alasaad, S; Keller, LF; Croll, D | 2018 | Population genomics analyses of European ibex species show lower diversity and higher inbreeding in reintroduced populations | Restoration of lost species ranges to their native distribution is key for the survival of endangered species. However, reintroductions often fail and long-term genetic consequences are poorly understood. Alpine ibex (Capra ibex) are wild goats that recovered from <100 individuals to similar to 50,000 within a century by population reintroductions. We analyzed the population genomic consequences of the Alpine ibex reintroduction strategy. We genotyped 101,822 genomewide single nucleotide polymorphism loci in 173 Alpine ibex, the closely related Iberian ibex (Capra pyrenaica) and domestic goat (Capra hircus). The source population of all Alpine ibex maintained genetic diversity comparable to Iberian ibex, which experienced less severe bottlenecks. All reintroduced Alpine ibex populations had individually and combined lower levels of genetic diversity than the source population. The reintroduction strategy consisted of primary reintroductions from captive breeding and secondary reintroductions from established populations. This stepwise reintroduction strategy left a strong genomic footprint of population differentiation, which increased with subsequent rounds of reintroductions. Furthermore, analyses of genomewide runs of homozygosity showed recent inbreeding primarily in individuals of reintroduced populations. We showed that despite the rapid census recovery, Alpine ibex carry a persistent genomic signature of their reintroduction history. We discuss how genomic monitoring can serve as an early indicator of inbreeding. | NA | no | inbreeding | NA | no | no |
| 5072 | 2020 | 44111 | Grossman, JJ; Cavender-Bares, J | 2019 | Consequences of biodiversity shift across phylogenetic scales for aspen and willow growth, survival, and herbivory | Question It has been established that community biodiversity has consequences for ecosystem function. Yet research assessing these biodiversity-ecosystem function (BEF) relationships usually occurs at only one phylogenetic scale; as such, the dependence of BEF relationships on phylogenetic scale has not been characterized. We present a novel framework for considering the consequences of biodiversity across phylogenetic scales, allowing us to ask: Do the consequences of intraspecific and interspecific diversity affect the growth, survival, and leaf herbivory of three temperate tree species? Study site Salicaceous tree plantation, Minnesota, northern USA. Methods We established an experimental plantation consisting of trees of three species within the willow (Salicaceae) family. Two aspen (Populus tremuloides, P. alba) and one willow (Salix nigra) species were represented by three unique genotypes such that tree neighborhoods varied both in genotype richness (intraspecific diversity) and species richness (interspecific diversity). We assessed the consequences of tree identity and diversity across these two phylogenetic scales for all trees’ aboveground productivity and survival, and for herbivore damage (on P. tremuloides) at the end of the second full growing season of the experiment. Results Diversity at any phylogenetic scale had no effect on the growth and survival of P. alba or S. nigra. However, intraspecific diversity increased the likelihood of P. tremuloides survival while interspecific diversity reduced P. tremuloides survival. Intraspecific diversity also reduced leaf removal and galling herbivory on P. tremuloides, while interspecific diversity had no effect on leaf removal and increased galling herbivory. Neither scale of diversity affected leaf mining. Conclusions Tree diversity within and among populations and species affected plant performance and ecosystem properties differentially, demonstrating that BEF relationships shift across phylogenetic scales in a taxon-specific manner. We call for further experiments that explicitly span these scales by measuring ecosystem and physiological responses to the manipulation of diversity within and among species. | NA | no | no good gd measure host pop | NA | no | no |
| 5073 | 2020 | 44111 | Grueber, CE; Peel, E; Wright, B; Hogg, CJ; Belov, K | 2019 | A Tasmanian devil breeding program to support wild recovery | Tasmanian devils are threatened in the wild by devil facial tumour disease: a transmissible cancer with a high fatality rate. In response, the Save the Tasmanian Devil Program (STDP) established an insurance population’ to enable the preservation of genetic diversity and natural behaviours of devils. This breeding program includes a range of institutions and facilities, from zoo-based intensive enclosures to larger, more natural environments, and a strategic approach has been required to capture and maintain genetic diversity, natural behaviours and to ensure reproductive success. Laboratory-based research, particularly genetics, in tandem with adaptive management has helped the STDP reach its goals, and has directly contributed to the conservation of the species in the wild. Here we review this work and show that the Tasmanian devil breeding program is a powerful example of how genetic research can be used to understand and improve reproductive success in a threatened species. | NA | no | No good fitness measure and measure host GD | NA | no | no |
| 5074 | 2020 | 44111 | Grueter, CC; Hale, J; Jin, RB; Judge, D; Stoinski, T | 2019 | Infant handling by female mountain gorillas: Establishing its frequency, function, and (ir)relevance for life history evolution | Objectives Infant handling describes cases in which youngsters are temporarily removed from the care of their mothers and taken care of (held, carried, etc.) by other conspecifics. Handlers may gain indirect fitness benefits from these actions and can practice mothering skills, thereby improving the odds of survival of their own infants. Great apes are notable for displaying little infant handling. Apart from anecdotal observations, no published data exist on infant handling in wild mountain gorillas. We tested two of the most pertinent explanations (kin selection and learning to mother) in a wild population of mountain gorillas in Rwanda. We predicted that (a) nulliparous females would exhibit infant handling (i.e., carrying) more than parous females and (b) maternal kin would exhibit more infant handling than nonkin. Methods We collated 8 years of data on infant carrying behavior collected in 13 groups monitored by the Dian Fossey Gorilla Fund’s Karisoke Research Center. Results Infant handling is an infrequent behavior (1,783 instances over 25,600 observation hours). A strong positive effect of relatedness and handler parity on the frequency of infant handling emerged. Conclusions While the nature of handler-infant interactions (affiliative, abusive, etc.) remains unstudied, they could constitute alloparental care and could therefore attenuate maternal energetic burden and ultimately allow increased birth rates. However, the rarity of this behavior makes it an unlikely contributor to mountain gorillas’ relatively short interbirth intervals. | NA | no | No good fitness measure and measure host GD | NA | no | no |
| 5075 | 2020 | 44113 | Gui, Y; Lei, X; Huang, S | 2018 | Collective effects of common single nucleotide polymorphisms and genetic risk prediction in type 1 diabetes | Type 1 diabetes (T1D) is a common autoimmune disease and may be related to multiple genetic and environmental risk factors. Previous genetic studies have focused on looking for individual polymorphic risk variants. Here, we studied the overall levels of genetic diversity in T1D patients by making use of a previously published study including 1865 cases and 2828 reference samples with genotyping data for 500 K common single nucleotide polymorphisms (SNPs). We determined the minor allele (MA) status of each SNP in the reference samples and calculated the total number of MAs or minor allele contents (MAC) of each individual. We found the average MAC of cases to be greater than that of the reference samples. By focusing on MAs with strong linkage to cases, we further identified a set of 112 SNPs that could predict 19.19% of cases. These results suggest that overall genetic variation over a threshold level may be a risk factor in T1D and provide a new genetic method for predicting the disorder. | NA | no | human | NA | no | no |
| 5076 | 2020 | 44042 | Guichard, M; Neuditschko, M; Fried, P; Soland, G; Dainat, B | 2019 | A future resistance breeding strategy against Varroa destructor in a small population of the dark honey bee | In scientific literature, host resistance of Apis mellifera against its parasite Varroa destructor is often presented as a potential solution to enable a sustainable relationship between both species and secure pollination and beekeeping services. Surprisingly, there are only few studies investigating the interest of beekeepers for this topic. In this paper, we propose a method to assess the desirability of resistance as part of a breeding goal, with a particular focus on small closed populations. The Swiss population of Apis mellifera mellifera is taken as case study. The general importance of this selection objective as well as the variability of the acceptance of beekeepers are studied. Thereafter, the willingness to pay for resistance is assessed to highlight possible compromises beekeepers could accept between different qualities they expect from their honey bees. Finally, the main characteristics of the breeding program desired by the beekeepers are presented. In our example, beekeepers are generally in favour of resistant honey bees even if differences in the expected breeding goals were identified. The majority is interested in a breeding strategy to select for resistant stock even though honey bees would produce less honey, swarm more often or be less gentle, showing a clear desirability for resistant traits. Furthermore, we noticed that beekeepers prefer locally selected honey bees displaying a broad genetic diversity. The presented method can be easily applied to evaluate the acceptance of new breeding objectives and to assess the potential of future strategies focusing on resistance against V. destructor in breeding programs involving beekeepers and research institutes. | NA | no | No good fitness measure and measure host GD | NA | no | no |
| 5077 | 2020 | 44039 | Gultyaeva, EI; Kokhmetova, AM; Shreyder, ER; Shaydayuk, EL; Atishova, MN; Madenova, A; Malysheva, A; Galymbek, K | 2020 | GENETIC VARIABILITY OF PERSPECTIVE BTREEDING MATERIAL OF SPRING BREAD WHEAT FOR RESISTANCE TO LEAF RUST IN RUSSIA AND KAZAKHSTAN | Leaf rust, caused by Puccinia triticina Erikss., is one of the major diseases of wheat in Russia and Kazakhstan. The resistance and genetic diversity of 61 spring wheat genotypes from Russia and 50 ones from Kazakhstan were studied. Field evaluation of Kazakhstani wheat material allowed to select 27 lines as resistant and 8 lines as moderate resistant to leaf rust. Molecular screening revealed 29 wheat lines characterized by the presence of Lr genes. As a result of phytopathological and molecular screening of Kazakhstani material, carriers of 2, 3, and 4 Lr genes were selected. The 92 % of Chelyabinsk’ lines were characterized by high level of resistance to leaf rust both at the adult and seedling stages. Using molecular markers Lr24, Lr9, Lr19, LrSp, Lr1, Lr3, Lr10, Lr26, Lr21 and Lr34 genes separately or in different combinations has been revealed in Russian wheat lines. In the field conditions of the Southern Urals, isogenic lines with genes Lr24, Lr25, Lr26, Lr28, Lr45, Lr47, Lr49, Lr51 and Lr57 showed high resistance, and in lines with the genes Lr17, Lr23, Lr29 and Lr64 moderate resistance was observed. At the seedling stage all single pustule isolates were avirulent to Tc-lines with Lr16, Lr19, Lr24, Lr28 and Lr29 genes. In Kazakhstan virulence of P. triticina population collected in South-East and North-Kazakh regions was studied. Against the Almaty population Lr9, Lr19, Lr24, Lr25 and Lr28 genes with high efficacy, and gene Lr45 with moderate efficacy were identified. All of the lines with the rest Lr genes were susceptible to the pathogen. The isogenic lines with genes Lr19, Lr24, Lr25, Lr28, Lr36 and Lr45 were characterized by high efficacy to the Kostanai P. triticina population. Virulence studies of P. triticina showed a similarity of pathogen structure on the Southern Urals of Russia and Northern Kazakhstan. | NA | no | No good fitness measure and measure host GD | NA | no | no |
| 5078 | 2020 | 44112 | Gundorova, P; Zinchenko, RA; Kuznetsova, IA; Bliznetz, EA; Stepanova, AA; Polyakov, AV | 2018 | Molecular-genetic causes for the high frequency of phenylketonuria in the population from the North Caucasus | Phenylketonuria is an inherited disease caused by mutations in the phenylalanine hydroxylase gene PAH. Different PAH pathogenic variants occur in different ethnic groups with various frequencies and the incidence of the disease itself varies from country to country. In the Caucasus region of Russia, some ethnoses are geographically and culturally isolated from each other. The tradition of monoethnic marriages may cause decreased genetic variability in those populations. In the Karachay-Cherkess Republic (Russia), the highest incidence of phenylketonuria in the world has been detected (1:850 newborns) in the region and 1:332 among the titular nation Karachays. Here, we showed that this phenomenon is due to the widespread prevalence of the p.Arg261\* variant. Its allele frequency among Karachay patients with PKU was 68.4% and the carrier frequency in Karachays was 1:16 healthy individuals. PAH haplotype analysis showed a unique common origin. The founder haplotype and mutation age were estimated by analyzing the linkage disequilibrium between p.Arg261\* and extragenic short tandem repeat loci. The p.Arg261\* variant occurred in the Karachays population 10.2 +/- 2.7 generations ago (275 +/- 73 years) and its spread occurred in parallel with the growth of the population. | NA | no | gd of pathogen | NA | no | no |
| 5079 | 2020 | 44042 | Gupta, MK; Vadde, R | 2019 | Genetic Basis of Adaptation and Maladaptation via Balancing Selection | Since human left Africa about 100 thousand years ago, they experienced numerous environmental as well as social transitions. During these transitions, their genome too experiences various forms of selective pressure and retained favorable advantageous alleles in their genome by either positive selection or balancing selection, while removing deleterious alleles through purifying selection, However, when an individual with certain advantageous genetic diversity is migrated to new contrasting environment or lifestyle, the advantageous genetic diversity becomes disadvantageous and finally causing maladaptation. Thus, understanding the role of evolution in adaptation and also in the regulation of population dynamics, is highly important for identifying naturally occurring advantageous or disease risk allele in contemporary populations. Recent advancements in high-throughput sequence technologies have made it easier for understanding the impact of evolutionary forces on the genetic make-up of human in different environmental and social conditions in a far better way. Statistical tests described in this review will enable reader to identify various signatures of balancing selection in different time scales in a more comprehensive way. Additionally, these tests will also help in identifying naturally occurring advantageous or disease risk alleles with applications in animal breeding, nature conservation and human medicine. | NA | no | No good fitness measure and measure host GD | NA | no | no |
| 5080 | 2020 | 44112 | Gurgul, A; Jasielczuk, I; Semik-Gurgul, E; Pawlina-Tyszko, K; Stefaniuk-Szmukier, M; Szmatola, T; Polak, G; Tomczyk-Wrona, I; Bugno-Poniewierska, M | 2019 | A genome-wide scan for diversifying selection signatures in selected horse breeds | The genetic differentiation of the current horse population was evolutionarily created by natural or artificial selection which shaped the genomes of individual breeds in several unique ways. The availability of high throughput genotyping methods created the opportunity to study this genetic variation on a genome-wide level allowing detection of genome regions divergently selected between separate breeds as well as among different horse types sharing similar phenotypic features. In this study, we used the population differentiation index (F-ST) that is generally used for measuring locus-specific allele frequencies variation between populations, to detect selection signatures among six horse breeds maintained in Poland. These breeds can be classified into three major categories, including light, draft and primitive horses, selected mainly in terms of type (utility), exterior, performance, size, coat color and appearance. The analysis of the most pronounced selection signals found in this study allowed us to detect several genomic regions and genes connected with processes potentially important for breed phenotypic differentiation and associated with energy homeostasis during physical effort, heart functioning, fertility, disease resistance and motor coordination. Our results also confirmed previously described association of loci on ECA3 (spanning LCORL and NCAPG genes) and ECA11 (spanning LASP1 gene) with the regulation of body size in our draft and primitive (small size) horses. The efficiency of the applied F-ST-based approach was also confirmed by the identification of a robust selection signal in the blue dun colored Polish Konik horses at the locus of TBX3 gene, which was previously shown to be responsible for dun coat color dilution in other horse breeds. F-ST-based method showed to be efficient in detection of diversifying selection signatures in the analyzed horse breeds. Especially pronounced signals were observed at the loci responsible for fixed breed-specific features. Several candidate genes under selection were proposed in this study for traits selected in separate breeds and horse types, however, further functional and comparative studies are needed to confirm and explain their effect on the observed genetic diversity of the horse breeds. | NA | no | No good fitness measure and measure host GD | NA | no | no |
| 5081 | 2020 | 44113 | Gutierrez-Ruacho, O; Coronado, ML; Sanchez-Teyer, F; Sanchez, A; Gutierrez, A; Esqueda, M | 2018 | Abundance of rhizospheric bacteria and fungi associated with Fouquieria columnaris at Punta Cirio, Sonora, Mexico | A unique relictual population of Fouquieria columnaris is located in Sonora, along a 45-km strip of land encompassing Punta Cirio in the Sonoran Desert. The interaction of plants with microorganisms is an important adaptation to survival in desertic areas. The present study reports the abundance of bacteria, actinohacteria, and filamentous and arbuscular mycorrhizal fungi (AMF) in the rhizosphere of F. columnaris at Punta Cirio. Also, the genetic variability of F. columnaris was analyzed using amplied fragment length polymorphism markers. Bacteria and fungi in 27 soil samples from the rhizosphere of F. columnaris at 3 hills with low, medium, and high elevations were analyzed. The total bacterial, actinobacterial, and filamentous fungal counts did not differ among sites or elevations; no interaction between site and elevation was found. Nitrogen-fixing bacteria varied according to site but not elevation, whereas phosphorus-solubilizing bacteria were undetected. Filamentous fungi were dominated by Aspergillus spp. (48%) and Penicillium spp. (28%), which together represented 76% of the total average colony-forming units. The spore density of AMF ranged from 109 to 245 per 100 g of soil, and 65 to 80% of roots were colonized by hyphae, vesicles, or arbuscles of AMF. The DNA analysis generated an average similarity coefficient of 0.89, which could indicate high genetic homogeneity within the evaluated F. columnaris population. | NA | no | No good fitness measure and measure host GD | NA | no | no |
| 5082 | 2020 | 44040 | Haag, KL; Pombert, JF; Sun, YK; de Albuquerque, NRM; Batliner, B; Fields, P; Lopes, TF; Ebert, D | 2020 | Microsporidia with Vertical Transmission Were Likely Shaped by Nonadaptive Processes | Microsporidia have the leanest genomes among eukaryotes, and their physiological and genomic simplicity has been attributed to their intracellular, obligate parasitic life-style. However, not all microsporidia genomes are small or lean, with the largest dwarfing the smallest ones by at least an order of magnitude. To better understand the evolutionary mechanisms behind this genomic diversification, we explore here two clades of microsporidia with distinct life histories, Ordospora and Hamiltosporidium, parasitizing the same host species, Daphnia magna. Based on seven newly assembled genomes, we show that mixed-mode transmission (the combination of horizontal and vertical transmission), which occurs in Hamiltosporidium, is found to be associated with larger and AT-biased genomes, more genes, and longer intergenic regions, as compared with the exclusively horizontally transmitted Ordospora. Furthermore, the Hamiltosporidium genome assemblies contain a variety of repetitive elements and long segmental duplications. We show that there is an excess of nonsynonymous substitutions in the microsporidia with mixed-mode transmission, which cannot be solely attributed to the lack of recombination, suggesting that bursts of genome size in these microsporidia result primarily from genetic drift. Overall, these findings suggest that the switch from a horizontal-only to a mixed mode of transmission likely produces population bottlenecks in Hamiltosporidium species, therefore reducing the effectiveness of natural selection, and allowing their genomic features to be largely shaped by nonadaptive processes. | NA | no | No good fitness measure and measure host GD | NA | no | no |
| 5083 | 2020 | 44112 | Hacking, JD; Stuart-Fox, D; Godfrey, SS; Gardner, MG | 2018 | Specific MHC class I supertype associated with parasite infection and color morph in a wild lizard population | The major histocompatibility complex (MHC) is a large gene family that plays a central role in the immune system of all jawed vertebrates. Nonavian reptiles are underrepresented within the MHC literature and little is understood regarding the mechanisms maintaining MHC diversity in this vertebrate group. Here, we examined the relative roles of parasite-mediated selection and sexual selection in maintaining MHC class I diversity of a color polymorphic lizard. We discovered evidence for parasite-mediated selection acting via rare-allele advantage or fluctuating selection as ectoparasite load was significantly lower in the presence of a specific MHC supertype (functional clustering of alleles): supertype four. Based on comparisons between ectoparasite prevalence and load, and assessment of the impact of ectoparasite load on host fitness, we suggest that supertype four confers quantitative resistance to ticks or an intracellular tickborne parasite. We found no evidence for MHC-associated mating in terms of pair genetic distance, number of alleles, or specific supertypes. An association was uncovered between supertype four and male throat color morph. However, it is unlikely that male throat coloration acts as a signal of MHC genotype to conspecifics because we found no evidence to suggest that male throat coloration predicts male mating status. Overall, our results suggest that parasite-mediated selection plays a role in maintaining MHC diversity in this population via rare-allele advantage and/or fluctuating selection. Further work is required to determine whether sexual selection also plays a role in maintaining MHC diversity in agamid lizards. | NA | no | no good gd measure host pop | NA | no | no |
| 5084 | 2020 | 44111 | Hadifar, S; Shamkhali, L; Kamakoli, MK; Mostafaei, S; Khanipour, S; Mansoori, N; Fateh, A; Siadat, SD; Vaziri, F | 2019 | Genetic diversity of Mycobacterium tuberculosis isolates causing pulmonary and extrapulmonary tuberculosis in the capital of Iran | Objectives: Evaluation of the genetic diversity of Mycobacterium tuberculosis (M.tb) and determining if the association between a specific genotype and the site of infection is crucial. Accordingly, the current study aimed at comparing predominant M.tb genotypes in pulmonary (PTB) and extrapulmonary tuberculosis (EPTB) isolates circulating in the capital of Iran. Methods: The genetic diversity of culture-confirmed PTB and EPTB isolates were evaluated by Spoligotyping and MIRU-VNTR (mycobacterial interspersed repetitive-unit-variable-number tandem-repeat) typing methods. Genotyping data were analyzed with SITVIT, MIRU-VNTRplus, and TBminer databases. To assess adjusted associations, chi-square/the Fisher exact test and multiple logistic regression model were applied. Results: URAL2 (NEW-1) (28/88; 31.8%) and CAS1-DELHI (25/84; 29.8%) genotypes were predominant in EPTB and PTB strains, respectively. Based on MIRU-VNTR typing, 158 different MIRU-VNTR patterns were identified. Clustering rate and minimum estimate of the proportion of TB caused by recent transmission was 4.1% and 8.1%, respectively. Conclusions: The current study provided new insight into circulating genotypes of M.tb in PTB and EPTB patients in Tehran, Iran. This low percentage of TB transmission rate, demonstrated that mode of TB transmission was mainly associated with reactivation of latent TB rather than recently transmitted infection in this region. There was no significant difference in the association between the genotypes of M.tb strains and the site of the disease. | NA | no | gd of pathogen | NA | no | no |
| 5085 | 2020 | 44112 | Hagedorn, M; Spindler, R; Daly, J | 2019 | Cryopreservation as a Tool for Reef Restoration: 2019 | Throughout the world coral reefs are being degraded at unprecedented rates. Locally, reefs are damaged by pollution, nutrient overload and sedimentation from out-dated land-use, fishing and mining practices. Globally, increased greenhouse gases are warming and acidifying oceans, making corals more susceptible to stress, bleaching and newly emerging diseases. The coupling of climate change impacts and local anthropogenic stressors has caused a widespread and well-recognized reef crisis. While the establishment and enforcement of marine protected areas and preventing the acceleration of climate change are essential to management of these stressors, the inexorable impacts of climate change will continue to cause declines in genetic diversity and population viability. Gamete cryopreservation has already acted as an effective insurance policy to maintain the genetic diversity of many wildlife species, and has now begun to be explored and applied to coral conservation. Cryopreservation can act to preserve reef biodiversity and genetic diversity. To date, we have had a great deal of success with cryopreserving sperm from similar to 30 coral species of coral species. Moreover, we are creating the basic science to freeze and thaw coral larvae that can soon be used to help secure and restore reefs. Building on these successes, we have established genetic banks using frozen samples and use those samples to help mitigate threats to the Great Barrier Reef and other areas. | NA | no | No good fitness measure and measure host GD | NA | no | no |
| 5086 | 2020 | 44113 | Hailemariam, Z; Krucken, J; Baumann, M; Ahmed, JS; Clausen, PH; Nijhof, AM | 2017 | Molecular detection of tick-borne pathogens in cattle from Southwestern Ethiopia | Tick-borne diseases (TBDs) cause significant losses among livestock and impact the livelihoods of resource-poor farming communities worldwide. In Ethiopia, detailed studies on the epidemiology of tick-borne pathogens (TBPs) in cattle using sensitive molecular detection methods are scarce. The objective of this study was to determine the prevalence and species composition of bovine TBPs of veterinary significance in local cattle populations. A comprehensive cross-sectional epidemiological study was conducted in cattle populations of Illubabor zone in Southwestern Ethiopia from June to August 2013. For this purpose, blood samples were collected from 392 cattle. A combination of polymerase chain reaction (PCR) and a Reverse Line Blot (RLB) hybridization assay was employed for the detection of TBPs in these samples. The PCR/RLB results of the 392 blood samples indicated a high overall prevalence of 96.9% for TBPs, including Theileria mutans (66.1%), Theileria orientalis (51.8%), Anaplasma sp. Omatjenne (25.5%), Anaplasma marginale (14.5%), Babesia bigemina (14.0%) and Theileria velifera (13.0%) and minor occurrences of Ehrlichia ruminantium (0.5%) and Ehrlichia minasensis (0.26%). Moreover, three novel Anaplasma genotypes were detected in bovine blood samples. A phylogenetic analysis revealed that they most likely represent three, but at least two, new species. The prevalence of the three novel Anaplasma species, preliminary designated as Anaplasma sp. Hadesa, Anaplasma sp. Saso and Anaplasma sp. Dedessa, was 12.5%, 14.3% and 5.6%, respectively. Overall, a total of 227 cattle (57.9%) were found to be co-infected with two or more TBPs simultaneously and 86 different species combinations were observed. The findings show a very high burden of infection of cattle with TBPs in Ethiopia. The high frequency of co-infections suggests that clinical manifestations might be complex. Further research is required to determine the pathogenicity, host cell types and vector of the three novel Anaplasma species identified in this study. | NA | no | gd of pathogen | NA | no | no |
| 5087 | 2020 | 44112 | Hajduk, GK; Cockburn, A; Margraf, N; Osmond, HL; Walling, CA; Kruuk, LEB | 2018 | Inbreeding, inbreeding depression, and infidelity in a cooperatively breeding bird | Inbreeding depression plays a major role in shaping mating systems: in particular, inbreeding avoidance is often proposed as a mechanism explaining extra-pair reproduction in socially monogamous species. This suggestion relies on assumptions that are rarely comprehensively tested: that inbreeding depression is present, that higher kinship between social partners increases infidelity, and that infidelity reduces the frequency of inbreeding. Here, we test these assumptions using 26 years of data for a cooperatively breeding, socially monogamous bird with high female infidelity, the superb fairy-wren (Malurus cyaneus). Although inbred individuals were rare (similar to 6% of offspring), we found evidence of inbreeding depression in nestling mass (but not in fledgling survival). Mother-son social pairings resulted in 100% infidelity, but kinship between a social pair did not otherwise predict female infidelity. Nevertheless, extra-pair offspring were less likely to be inbred than within-pair offspring. Finally, the social environment (the number of helpers in a group) did not affect offspring inbreeding coefficients or inbreeding depression levels. In conclusion, despite some agreement with the assumptions that are necessary for inbreeding avoidance to drive infidelity, the apparent scarcity of inbreeding events and the observed levels of inbreeding depression seem insufficient to explain the ubiquitous infidelity in this system, beyond the mother-son mating avoidance. | NA | no | inbreeding | NA | no | no |
| 5088 | 2020 | 44040 | Ham-Duenas, JG; Canales-del-Castillo, R; Voelker, G; Ruvalcaba-Ortega, I; Aguirre-Calderon, CE; Gonzalez-Rojas, JI | 2020 | Adaptive genetic diversity and evidence of population genetic structure in the endangered Sierra Madre Sparrow (Xenospiza baileyi) | The magnitude and distribution of genetic diversity through space and time can provide useful information relating to evolutionary potential and conservation status in threatened species. In assessing genetic diversity in species that are of conservation concern, several studies have focused on the use of Toll-like receptors (TLRs). TLRs are innate immune genes related to pathogen resistance, and polymorphisms may reflect not only levels of functional diversity, but may also be used to assess genetic diversity within and among populations. Here, we combined four potentially adaptive markers (TLRs) with one mitochondrial (COI) marker to evaluate genetic variation in the endangered Sierra Madre Sparrow (Xenospiza baileyi). This species offers an ideal model to investigate population and evolutionary genetic processes that may be occurring in a habitat restricted endangered species with disjunct populations (Mexico City and Durango), the census sizes of which differ by an order of magnitude. TLRs diversity in the Sierra Madre Sparrow was relatively high, which was not expected given its two small, geographically isolated populations. Genetic diversity was different (but not significantly so) between the two populations, with less diversity seen in the smaller Durango population. Population genetic structure between populations was due to isolation and different selective forces acting on different TLRs; population structure was also evident in COI. Reduction of genetic diversity in COI was observed over 20 years in the Durango population, a result likely caused by habitat loss, a factor which may be the main cause of diversity decline generally. Our results provide information related to the ways in which adaptive variation can be altered by demographic changes due to human-mediated habitat alterations. Furthermore, our findings may help to guide conservation schemes for both populations and their restricted habitat. | NA | no | only two pop | NA | no | no |
| 5089 | 2020 | 44040 | Hamdi, Y; Jerbi, M; Romdhane, L; Ben Rekaya, M; El Benna, H; Chouchane, L; Boubaker, MS; Abdelhak, S; Yacoub-Youssef, H | 2020 | Genetic diversity and functional effect of common polymorphisms in genes involved in the first heterodimeric complex of the Nucleotide Excision Repair pathway | Nucleotide excision repair is a multistep process that recognizes and eliminates a spectrum of DNA damages. Five proteins, namely XPC, RAD23, Centrin 2, DDB1 and DDB2 act as a heterodimeric complex at the early steps of the NER pathway and play a crucial role in the removal of DNA lesions. Several exonic mutations on genes coding for these proteins have been identified as associated with Xeroderma-pigmentosum (XP), a rare monogenic disorder. However, the role of regulatory polymorphisms in disease development and inter-ethnic diversity is still not well documented. Due to the high incidence rate of XP in Tunisia, we performed a genotyping analysis of 140 SNPs found on these 5 genes in a set of 135-subjects representing the general Tunisian-population. An inter-ethnic comparison based on the genotype frequency of these SNPs have been also conducted. For the most relevant variants, we performed a comprehensive assessment of their functional effects. Linkage disequilibrium and principal component analysis showed that the Tunisian-population is an admixed and intermediate population between Sub-Saharan Africans and Europeans. Using variable factor maps, we identified a list of 20 polymorphisms that contribute considerably to the inter-ethnic diversity of the NER complex. In-silica functional analysis showed that SNPs on XPC, DDB1 and DDB2 are associated with eQTLs mainly DDB2-rs10838681 that seems to decrease significantly the expression level of ACP2 (p = 6.1 x 10(-26)). Statistical analysis showed that the allelic frequency of DDB2-rs10838681 in Tunisia is significantly different from all other populations. Using rVarBase, we identified 5 variants on XPC, DDB1 and DDB2 that seem to alter the binding sites of several transcription factors considered as key players in DNA-repair pathways. Results presented in this study provide the first report on regulatory polymorphisms of the NER-complex genes in Tunisia. These results may also help to establish a baseline database for future association and functional studies. | NA | no | human | NA | no | no |
| 5090 | 2020 | 44042 | Hamelin, RC; Roe, AD | 2020 | Genomic biosurveillance of forest invasive alien enemies: A story written in code | The world’s forests face unprecedented threats from invasive insects and pathogens that can cause large irreversible damage to the ecosystems. This threatens the world’s capacity to provide long-term fiber supply and ecosystem services that range from carbon storage, nutrient cycling, and water and air purification, to soil preservation and maintenance of wildlife habitat. Reducing the threat of forest invasive alien species requires vigilant biosurveillance, the process of gathering, integrating, interpreting, and communicating essential information about pest and pathogen threats to achieve early detection and warning and to enable better decision-making. This process is challenging due to the diversity of invasive pests and pathogens that need to be identified, the diverse pathways of introduction, and the difficulty in assessing the risk of establishment. Genomics can provide powerful new solutions to biosurveillance. The process of invasion is a story written in four chapters: transport, introduction, establishment, and spread. The series of processes that lead to a successful invasion can leave behind a DNA signature that tells the story of an invasion. This signature can help us understand the dynamic, multistep process of invasion and inform management of current and future introductions. This review describes current and future application of genomic tools and pipelines that will provide accurate identification of pests and pathogens, assign outbreak or survey samples to putative sources to identify pathways of spread, and assess risk based on traits that impact the outbreak outcome. | NA | no | No good fitness measure | NA | no | no |
| 5091 | 2020 | 44113 | Hammarsjo, A; Wang, Z; Vaz, R; Taylan, F; Sedghi, M; Girisha, KM; Chitayat, D; Neethukrishna, K; Shannon, P; Godoy, R; Gowrishankar, K; Lindstrand, A; Nasiri, J; Baktashian, M; Newton, PT; Guo, L; Hofmeister, W; Pettersson, M; Chagin, AS; Nishimura, G; Yan, L; Matsumoto, N; Nordgren, A; Miyake, N; Grigelioniene, G; Ikegawa, S | 2017 | Novel KIAA0753 mutations extend the phenotype of skeletal ciliopathies | The skeletal ciliopathies are a heterogeneous group of disorders with a significant clinical and genetic variability and the main clinical features are thoracic hypoplasia and short tubular bones. To date, 25 genes have been identified in association with skeletal ciliopathies. Mutations in the KIAA0753 gene have recently been associated with Joubert syndrome (JBTS) and orofaciodigital (OFD) syndrome. We report biallelic pathogenic variants in KIAA0753 in four patients with short-rib type skeletal dysplasia. The manifestations in our patients are variable and ranging from fetal lethal to viable and moderate skeletal dysplasia with narrow thorax and abnormal metaphyses. We demonstrate that KIAA0753 is expressed in normal fetal human growth plate and show that the affected fetus, with a compound heterozygous frameshift and a nonsense mutation in KIAA0753, has an abnormal proliferative zone and a broad hypertrophic zone. The importance of KIAA0753 for normal skeletal development is further confirmed by our findings that zebrafish embryos homozygous for a nonsense mutation in kiaa0753 display altered cartilage patterning. | NA | no | No good fitness measure and measure host GD | NA | no | no |
| 5092 | 2020 | 44112 | Harrington, AM; Tudor, MS; Reese, HR; Bouchard, DA; Hamlin, HJ | 2019 | Effects of temperature on larval American lobster (Homarus americanus): Is there a trade-off between growth rate and developmental stability? | The American lobster supports the most economically valuable fishery in the Gulf of Maine and Atlantic Canada. Across much of its range, ocean temperatures have increased at rates faster than almost anywhere in the world. Studies of warming effects on larvae have largely focused on survival and development, but rarely have examined sub-lethal effects that could influence settlement and subsequent recruitment to the fishery. We explored how warming influences rate of development, survival, stress, and developmental stability of larval lobsters reared under four nominal temperatures: 14, 16, 18, and 22 degrees C. Our study is the first to evaluate the use of fluctuating asymmetry as a biomarker for developmental instability in larval American lobster. We also recorded total hemocyte counts in postlarvae as an indicator of stress, a novel technique for work in larval lobster. Development proceeded significantly faster as temperature increased, and cumulative survival was significantly positively correlated with temperature. However, postlarvae reared under temperature extremes exhibited elevated hemocyte counts and had significantly lower levels of variance in midline asymmetry compared to intermediate temperature groups. Together, this suggests that warmer temperatures may facilitate faster growth at the expense of increased physiological stress and a loss of genetic diversity, potentially affecting the species’ ability to adapt to changing environmental conditions. Fluctuating asymmetry could prove to be a useful bioindicator for population resilience. | NA | no | no good gd measure host pop | NA | no | no |
| 5093 | 2020 | 44112 | Harris, AR; Mullett, MS; Webber, JF | 2018 | Changes in the population structure and sporulation behaviour of Phytophthora ramorum associated with the epidemic on Larix (larch) in Britain | During a decade of invasion, the exotic pathogen Phytophthora ramorum has undergone an unexpected change in behaviour during spread into the woodlands and forests of Great Britain. From 2002 to 2008 most outbreaks centred on nurseries and managed gardens with affected hosts almost exclusively broadleaf shrubs and trees. However 2009 saw a major shift as larch tree plantations (Larix) were affected by widespread infection and mortality incited by P. ramorum. To understand the processes underlying the host jump to larch, isolates of the EU1 lineage of P. ramorum collected from 2002 to 2012 were investigated using seven polymorphic microsatellite markers. Analysis of 347 isolates resolved 51 multilocus genotypes (MLGs) which partitioned into two distinct clusters. One comprised MLGs unique to Britain and unknown elsewhere in Europe, the other cluster was primarily of MLGs already known in other European countries but dominated by one genotype, EU1MG1. Pre-2009 isolates were predominantly of the unique British cluster with only a few typical of the European cluster. This reversed after 2009 with European MLGs, especially EU1MG1, becoming increasingly common as the larch epidemic expanded. We hypothesise that the growing dominance of EU1MLG1 has been an important driver in the emergence of the epidemic on larch, aided by its ability to sporulate more abundantly compared with the dominant unique British MLG. European MLGs appear closely associated with the distribution of larch along the west coast of Britain whereas unique British MLGs tend to be concentrated in south west England. The two population clusters suggest at least two separate introductions of the EU1 lineage into Britain with subsequent diversification. | NA | no | No good fitness measure and measure host GD | NA | no | no |
| 5094 | 2020 | 44113 | Hartmann, AC; Petras, D; Quinn, RA; Protsyuk, I; Archer, FI; Ransome, E; Williams, GJ; Bailey, BA; Vermeij, MJA; Alexandrov, T; Dorrestein, PC; Rohwer, FL | 2017 | Meta-mass shift chemical profiling of metabolomes from coral reefs | Untargeted metabolomics of environmental samples routinely detects thousands of small molecules, the vast majority of which cannot be identified. Meta-mass shift chemical (MeMSChem) profiling was developed to identify mass differences between related molecules using molecular networks. This approach illuminates metabolome- wide relationships between molecules and the putative chemical groups that differentiate them (e.g., H-2, CH2, COCH2). MeMSChem profiling was used to analyze a publicly available metabolomic dataset of coral, algal, and fungal mat holobionts (i.e., the host and its associated microbes and viruses) sampled from some of Earth’s most remote and pristine coral reefs. Each type of holo-biont had distinct mass shift profiles, even when the analysis was restricted to molecules found in all samples. This result suggests that holobionts modify the same molecules in different ways and offers insights into the generation of molecular diversity. Three genera of stony corals had distinct patterns of molecular relatedness despite their high degree of taxonomic relatedness. MeMSChem profiles also partially differentiated between individuals, suggesting that every coral reef holobiont is a potential source of novel chemical diversity. | NA | no | No good fitness measure and measure host GD | NA | no | no |
| 5095 | 2020 | 44113 | Hartmann, M; Stefanek, M; Zdvorak, P; Herman, P; Chrtek, J; Mraz, P | 2017 | The Red Queen hypothesis and geographical parthenogenesis in the alpine hawkweed Hieracium alpinum (Asteraceae) | The Red Queen hypothesis (RQH) suggests that sexuals have an evolutionary advantage in habitats with many biotic interactions (typically in southern latitudes) while asexuals are more frequent in habitats with harsher climate and consequently lowered biotic pressure (typically in northern latitudes/higher altitudes). The biotic interactions are thus considered to be a possible driver of differential geographical distributions often observed between closely related sexuals and asexuals: a pattern referred to as geographical parthenogenesis (GP). We tested the RQH in Hieracium alpinum L., a vascular plant species exhibiting a striking pattern of GP, by assessing the frequency and intensity with which seed predator specialists occur and the level of interspecific plant-plant competition expressed through both the surrounding vegetation density and the species richness across its distributional range. Furthermore, we tested whether there were negative associations between intra-population genetic diversity, as assessed by amplified fragment length polymorphism markers, and incidences of seed predation. Our results indicate no significant differences in frequency and intensity of seed predation nor in the level of interspecific plant-plant interactions between diploid sexual and triploid asexual populations. Furthermore, there were no consistent latitudinal and/or altitudinal patterns for the assessed parameters. Contrary to our expectations, there were either positive or non-significant associations between genetic diversity and the level of seed predation. To conclude, the RQH cannot thoroughly explain the GP in H. alpinum, and other alternative scenarios should be considered and tested. | NA | no | No good fitness measure and measure host GD | NA | no | no |
| 5096 | 2020 | 44113 | Hashmi, TR; Dey, D; Prasad, R | 2018 | Distribution frequency of endosymbionts and genetic characterisation of Bemisia tabaci (Hemiptera: Aleyrodidae) on Fabaceous host plants in India | A study was carried out to determine the genetic diversity and distribution of known endosymbionts in the populations of Bemisia tabaci from three host plants (soya bean, mungbean and subabool). The presence of five endosymbiont (Portiera, Rickettsia, Wolbachia, Cardinium and Arsenophonus) were checked in B. tabaci populations. Phylogenetic analyses based on the mitochondrial cytochrome I gene showed the presence of Asia II-1 and Asia II-7 genetic groups for B. tabaci. Individual were perused for symbiotic bacterial infection using specific primers targeting 16S rRNA and 23S rRNA gene. Portiera was present in all the B. tabaci samples. However, differences were observed in the circulation frequencies of secondary endosymbionts among the B. tabaci populations. A noteworthy difference was observed in the distribution frequency of Cardinium in between soyabean and subabool along with mungbean and subabool with the p-values as 0.037 and 0.001, respectively. The irregular presence of secondary endosymbionts supports the supposition that each endosymbiotic bacterium not only has a role in the survival but may also have a role in the polyphagous nature of B. tabaci. It also delivers an elementary evidence for progressive studies by targeting the endosymbionts for control measures of this notorious insect pest. | NA | no | No good fitness measure and measure host GD | NA | no | no |
| 5097 | 2020 | 44112 | Haueisen, J; Moller, M; Eschenbrenner, CJ; Grandaubert, J; Seybold, H; Adamiak, H; Stukenbrock, EH | 2019 | Highly flexible infection programs in a specialized wheat pathogen | Many filamentous plant pathogens exhibit high levels of genomic variability, yet the impact of this variation on host-pathogen interactions is largely unknown. We have addressed host specialization in the wheat pathogen Zymoseptoria tritici. Our study builds on comparative analyses of infection and gene expression phenotypes of three isolates and reveals the extent to which genomic variation translates into phenotypic variation. The isolates exhibit genetic and genomic variation but are similarly virulent. By combining confocal microscopy, disease monitoring, staining of ROS, and comparative transcriptome analyses, we conducted a detailed comparison of the infection processes of these isolates in a susceptible wheat cultivar. We characterized four core infection stages: establishment, biotrophic growth, lifestyle transition, and necrotrophic growth and asexual reproduction that are shared by the three isolates. However, we demonstrate differentiated temporal and spatial infection development and significant differences in the expression profiles of the three isolates during the infection stages. More than 20% of the genes were differentially expressed and these genes were located significantly closer to transposable elements, suggesting an impact of epigenetic regulation. Further, differentially expressed genes were enriched in effector candidates suggesting that isolate-specific strategies for manipulating host defenses are present in Z. tritici. We demonstrate that individuals of a host-specialized pathogen have highly differentiated infection programs characterized by flexible infection development and functional redundancy. This illustrates how high genetic diversity in pathogen populations results in highly differentiated infection phenotypes, which fact needs to be acknowledged to understand host-pathogen interactions and pathogen evolution. | NA | no | gd of pathogen | NA | no | no |
| 5098 | 2020 | 44040 | Haugen, H; Linlokken, A; Ostbye, K; Heggenes, J | 2020 | Landscape genetics of northern crested newt Triturus cristatus populations in a contrasting natural and human-impacted boreal forest | Among vertebrates, amphibians currently have the highest proportion of threatened species worldwide, mainly through loss of habitat, leading to increased population isolation. Smaller amphibian populations may lose more genetic diversity, and become more dependent on immigration for survival. Investigations of landscape factors and patterns mediating migration and population genetic differentiation are fundamental for knowledge-based conservation. The pond-breeding northern crested newt (Triturus cristatus) populations are decreasing throughout Europe, and are a conservation concern. Using microsatellites, we studied the genetic structure of the northern crested newt in a boreal forest ecosystem containing two contrasting landscapes, one subject to recent change and habitat loss by clear-cutting and roadbuilding, and one with little anthropogenic disturbance. Newts from 12 breeding ponds were analyzed for 13 microsatellites and 7 landscape and spatial variables. With a Maximum-likelihood population-effects model we investigated important landscape factors potentially explaining genetic patterns. Results indicate that intervening landscape factors between breeding ponds, explain the genetic differentiation in addition to an isolation-by-distance effect. Geographic distance, gravel roads, and south/south-west facing slopes reduced landscape permeability and increased genetic differentiation for these newts. The effect was opposite for streams, presumably being more favorable for newt dispersal. Populations within or bordering on old growth forest had a higher allelic richness than populations in managed forest outside these areas. Old growth forest areas may be important source habitats in the conservation of northern crested newt populations. | NA | no | No good fitness measure | NA | no | no |
| 5099 | 2020 | 44040 | Hayati, R; Basyuni, M; Chalil, D | 2020 | Genetic Diversity, Sequence and Bioinformatic Analysis of Ganoderma boninense Isolates | Ganoderma boninense is the most severe pathogen of Elaeis guineensis in Sumatra and Sulawesi, Indonesia, owing to the fact that it causes upper or basal stem rot disease. This study aimed to determine the genetic diversity, sequence and bioinformatics of E. guineensis, Borassus flabellifer, Hevea brasiliensis and Cocos nucifera. As many as 49 individuals of G. boninense from six populations were analyzed using the four SSR marker loci. The genetic structure was carried out by the GenAlEx ver 6.502 software. The sequences compared database with the BLASTX program. Physico-chemical, possible transfer peptide, and subcellular localization relationships were analyzed using the bioinformatics methods. The clustering UPGMA and phylogenetic were generated using MVSP ver. 3.22 and CLUSTAL W ver. 1.83 software. Results of the analysis showed that the observed heterozygosity (Ho) was 0.32. The two loci that revealed polymorphism with a polymorphic information content (PIC) value was 0.61 (KT124397 and KT12440). Further, the distribution of the BLASTX analyses indicated that the isolates were more similar to the Transcription factor (G. sinense strain ZZ0214-1) homologs in some other fungi. The potential peptides prediction in Ganoderma was similarly localized to the chloroplasts and mitochondria (0.1). The two subcellular principal predicted in the cytoplasm and mitochondria localization were stable (2.0-8.5). They were UPGMA clustered into two distinct geographical distributions. The isolates from the North Sulawesi Province have a genetic correlation close to the isolates from the North Sumatra Province. However, the phylogenetic clearly separated the G. boninense and G. sinense. (C) 2020 Friends Science Publishers | NA | no | gd of pathogen | NA | no | no |
| 5100 | 2020 | 44112 | Hayes, BJ; Daetwyler, HD | 2019 | 1000 Bull Genomes Project to Map Simple and Complex Genetic Traits in Cattle: Applications and Outcomes | The 1000 Bull Genomes Project is a collection of whole-genome sequences from 2,703 individuals capturing a significant proportion of the world’s cattle diversity. So far, 84 million single-nucleotide polymorphisms (SNPs) and 2.5 million small insertion deletions have been identified in the collection, a very high level of genetic diversity. The project has greatly accelerated the identification of deleterious mutations for a range of genetic diseases, as well as for embryonic lethals. The rate of identification of causal mutations for complex traits has been slower, reflecting the typically small effect size of these mutations and the fact that many are likely in as-yet-unannotated regulatory regions. Both the deleterious mutations that have been identified and the mutations associated with complex trait variation have been included in low-cost SNP array designs, and these arrays are being genotyped in tens of thousands of dairy and beef cattle, enabling management of deleterious mutations in these populations as well as genomic selection. | NA | no | No good fitness measure and measure host GD | NA | no | no |
| 5101 | 2020 | 44112 | Hayes, LD; Correa, LA; Abades, S; Gao, CL; Ebensperger, LA | 2019 | Male group members are costly to plurally breeding Octodon degus females | We report the results of a 6-year study of social (number of adult males/females, relatedness of females, communal litter size) and ecological (mean/CV of food abundance, soil hardness, burrow openings) factors influencing the direct fitness of plurally breeding degu (Octodon degus) females. The best fit models for per capita offspring weaned and standardized variance in direct fitness (within-group variation) included the number of adult males per group. Per capita number of offspring weaned decreased and standardized variance in direct fitness increased with increasing number of adult males per group. Thus, females experience a cost associated with males that is not shared equally. Standardize variance in direct fitness decreased with increasing communal litter size. All other factors were not significant predictors of direct fitness variation. Our study suggests that plural breeding may not be as egalitarian as previously thought. Consequences of plural breeding may be influenced by intra-and inter-sexual conflict. | NA | no | No good fitness measure and measure host GD | NA | no | no |
| 5102 | 2020 | 44039 | He, CH; Du, JJ; Zhu, D; Zhang, L | 2020 | Population viability analysis of small population: a case study for Asian elephant in China | Small populations are at risk of extinction from deterministic and stochastic factors. Less than 250 Asian elephants (Elephas maximus) remain in China, and are distributed in a few isolated areas; yet, population viability analyses of this endangered population have not been conducted. Here, the current genetic status of the Pu’Er-Mengyang Asian elephant populations in China was analyzed, and the risk of extinction was predicted over the next 500 years. Factors affecting the viability of this population were determined through simulations. The genetic diversity of the population was very low (mean allele number: 3.1; expected heterozygosity: 0.463), even though a recent population bottleneck was not detected. The effective population size was approximately 24.1 adult elephants. Enough adult breeding individuals exist to maintain population viability. VORTEX simulation model showed that this population would not go extinct in the next 500 years. However, illegal poaching and harvesting could negatively affect population size. A sensitivity analysis showed that the mean stochastic growth rate of the study population is sensitive to sex ratio, number of breeding females, mortality of females of different age classes, carrying capacity, and lethal equivalents. Based on our results, we suggest that action should be taken to alleviate inbreeding and any further loss of genetic diversity, by connecting fragmented elephant habitat or by translocating individual elephants. In addition, human-elephant conflict should be mitigated using various modern approaches, including crop guarding techniques, and by encouraging farmers to switch to crops and income sources not vulnerable to elephant raids. | NA | no | only one pop, no good fitness measure, maybe inbred? | NA | no | no |
| 5103 | 2020 | 44040 | He, L; Kulminski, AM | 2020 | Fast Algorithms for Conducting Large-Scale GWAS of Age-at-Onset Traits Using Cox Mixed-Effects Models | Age-at-onset traits are of paramount importance in studies of age-related diseases and behavioral genetics. Cox mixed-effects models (CMEMs) are widely used when sample dependence is taken into account. However, the application of CMEMs in genome-wide association… Age-at-onset is one of the critical traits in cohort studies of age-related diseases. Large-scale genome-wide association studies (GWAS) of age-at-onset traits can provide more insights into genetic effects on disease progression and transitions between stages. Moreover, proportional hazards (or Cox) regression models can achieve higher statistical power in a cohort study than a case-control trait using logistic regression. Although mixed-effects models are widely used in GWAS to correct for sample dependence, application of Cox mixed-effects models (CMEMs) to large-scale GWAS is so far hindered by intractable computational cost. In this work, we propose COXMEG, an efficient R package for conducting GWAS of age-at-onset traits using CMEMs. COXMEG introduces fast estimation algorithms for general sparse relatedness matrices including, but not limited to, block-diagonal pedigree-based matrices. COXMEG also introduces a fast and powerful score test for dense relatedness matrices, accounting for both population stratification and family structure. In addition, COXMEG generalizes existing algorithms to support positive semidefinite relatedness matrices, which are common in twin and family studies. Our simulation studies suggest that COXMEG, depending on the structure of the relatedness matrix, is orders of magnitude computationally more efficient than coxme and coxph with frailty for GWAS. We found that using sparse approximation of relatedness matrices yielded highly comparable results in controlling false-positive rate and retaining statistical power for an ethnically homogeneous family-based sample. By applying COXMEG to a study of Alzheimer’s disease (AD) with a Late-Onset Alzheimer’s Disease Family Study from the National Institute on Aging sample comprising 3456 non-Hispanic whites and 287 African Americans, we identified the APOE epsilon 4 variant with strong statistical power (P = 1e-101), far more significant than that reported in a previous study using a transformed variable and a marginal Cox model. Furthermore, we identified novel SNP rs36051450 (P = 2e-9) near GRAMD1B, the minor allele of which significantly reduced the hazards of AD in both genders. These results demonstrated that COXMEG greatly facilitates the application of CMEMs in GWAS of age-at-onset traits. | NA | no | No good fitness measure and measure host GD | NA | no | no |
| 5104 | 2020 | 44113 | He, Q; Pilosof, S; Tiedje, KE; Ruybal-Pesantez, S; Artzy-Randrup, Y; Baskerville, EB; Day, KP; Pascual, M | 2018 | Networks of genetic similarity reveal non-neutral processes shape strain structure in Plasmodium falciparum | Pathogens compete for hosts through patterns of cross-protection conferred by immune responses to antigens. In Plasmodium falciparum malaria, the var multigene family encoding for the major blood-stage antigen PfEMP1 has evolved enormous genetic diversity through ectopic recombination and mutation. With 50-60 var genes per genome, it is unclear whether immune selection can act as a dominant force in structuring var repertoires of local populations. The combinatorial complexity of the var system remains beyond the reach of existing strain theory and previous evidence for non-random structure cannot demonstrate immune selection without comparison with neutral models. We develop two neutral models that encompass malaria epidemiology but exclude competitive interactions between parasites. These models, combined with networks of genetic similarity, reveal non-neutral strain structure in both simulated systems and an extensively sampled population in Ghana. The unique population structure we identify underlies the large transmission reservoir characteristic of highly endemic regions in Africa. | NA | no | No good fitness measure and measure host GD | NA | no | no |
| 5105 | 2020 | 44043 | Healy, K; Ezard, THG; Jones, OR; Salguero-Gomez, R; Buckley, YM | 2019 | Animal life history is shaped by the pace of life and the distribution of age-specific mortality and reproduction | Animals exhibit an extraordinary diversity of life history strategies. These realized combinations of survival, development and reproduction are predicted to be constrained by physiological limitations and by trade-offs in resource allocation. However, our understanding of these patterns is restricted to a few taxonomic groups. Using demographic data from 121 species, ranging from humans to sponges, we test whether such trade-offs universally shape animal life history strategies. We show that, after accounting for body mass and phylogenetic relatedness, 71% of the variation in animal life history strategies can be explained by life history traits associated with the fast-slow continuum (pace of life) and with a second axis defined by the distribution of age-specific mortality hazards and the spread of reproduction. While we found that life history strategies are associated with metabolic rate and ecological modes of life, surprisingly similar life history strategies can be found across the phylogenetic and physiological diversity of animals. | NA | no | No good fitness measure and measure host GD | NA | no | no |
| 5106 | 2020 | 44112 | Heckwolf, MJ; Meyer, BS; Doring, T; Eizaguirre, C; Reusch, TBH | 2018 | Transgenerational plasticity and selection shape the adaptive potential of sticklebacks to salinity change | In marine climate change research, salinity shifts have been widely overlooked. While widespread desalination effects are expected in higher latitudes, salinity is predicted to increase closer to the equator. We took advantage of the steep salinity gradient of the Baltic Sea as a space-for-time design to address effects of salinity change on populations. Additionally, genetic diversity, a prerequisite for adaptive responses, is reduced in Baltic compared to Atlantic populations. On the one hand, adaptive transgenerational plasticity (TGP) might buffer the effects of environmental change, which may be of particular importance under reduced genetic variation. On the other hand, physiological trade-offs due to environmental stress may hamper parental provisioning to offspring thereby intensifying the impact of climate change across generations (nonadaptive TGP). Here, we studied both hypothesis of adaptive and nonadaptive TGP in the three-spined stickleback (Gasterosteus aculeatus) fish model along the strong salinity gradient of the Baltic Sea in a space-for-time experiment. Each population tolerated desalination well, which was not altered by parental exposure to low salinity. Despite a common marine ancestor, populations locally adapted to low salinity lost their ability to cope with fully marine conditions, resulting in lower survival and reduced relative fitness. Negative transgenerational effects were evident in early life stages, but disappeared after selection via mortality occurred during the first 12-30 days posthatch. Modeling various strengths of selection, we showed that nonadaptive transgenerational plasticity accelerated evolution by increasing directional selection within the offspring generation. Qualitatively, when genetic diversity is large, we predict that such effects will facilitate rapid adaptation and population persistence, while below a certain threshold populations suffer a higher risk of local extinction. Overall, our results suggest that transgenerational plasticity and selection are not independent of each other and thereby highlight a current gap in TGP studies. | NA | no | No good fitness measure and measure host GD | NA | no | no |
| 5107 | 2020 | 44042 | Hector, TE; Sgr?, CM; Hall, MD | 2019 | Pathogen exposure disrupts an organism’s ability to cope with thermal stress | As a result of global climate change, species are experiencing an escalation in the severity and regularity of extreme thermal events. With patterns of disease distribution and transmission predicted to undergo considerable shifts in the coming years, the interplay between temperature and pathogen exposure will likely determine the capacity of a population to persist under the dual threat of global change and infectious disease. In this study, we investigated how exposure to a pathogen affects an individual’s ability to cope with extreme temperatures. Using experimental infections of Daphnia magna with its obligate bacterial pathogen Pasteuria ramosa, we measured upper thermal limits of multiple host and pathogen genotype combinations across the dynamic process of infection and under various forms (static and ramping) of thermal stress. We find that pathogens substantially limit the thermal tolerance of their host, with the reduction in upper thermal limits on par with the breadth of variation seen across similar species entire geographical ranges. The precise magnitude of any reduction, however, was specific to the host and pathogen genotype combination. In addition, as thermal ramping rate slowed, upper thermal limits of both healthy and infected individuals were reduced. Our results suggest that the capacity of a population to evolve new thermal limits, when also faced with the threat of infection, will depend not only on a host’s genetic variability in warmer environments, but also on the frequency of host and pathogen genotypes. We suggest that pathogen-induced alterations of host thermal performance should be taken into account when assessing the resilience of any population and its potential for adaptation to global change. | NA | no | No good fitness measure and measure host GD | NA | no | no |
| 5108 | 2020 | 44113 | Helmkampf, M; Wolfgruber, TK; Bellinger, MR; Paudel, R; Kantar, MB; Miyasaka, SC; Kimball, HL; Brown, A; Veillet, A; Read, A; Shintaku, M | 2018 | Phylogenetic Relationships, Breeding Implications, and Cultivation History of Hawaiian Taro (Colocasia Esculenta) Through Genome-Wide SNP Genotyping | Taro, Colocasia esculenta, is one of the world’s oldest root crops and is of particular economic and cultural significance in Hawai’i, where historically more than 150 different landraces were grown. We developed a genome-wide set of more than 2400 high-quality single nucleotide polymorphism (SNP) markers from 70 taro accessions of Hawaiian, South Pacific, Palauan, and mainland Asian origins, with several objectives: 1) uncover the phylogenetic relationships between Hawaiian and other Pacific landraces, 2) shed light on the history of taro cultivation in Hawai’i, and 3) develop a tool to discriminate among Hawaiian and other taros. We found that almost all existing Hawaiian landraces fall into 5 monophyletic groups that are largely consistent with the traditional Hawaiian classification based on morphological characters, for example, leaf shape and petiole color. Genetic diversity was low within these clades but considerably higher between them. Population structure analyses further indicated that the diversification of taro in Hawai’i most likely occurred by a combination of frequent somatic mutation and occasional hybridization. Unexpectedly, the South Pacific accessions were found nested within the clades mainly composed of Hawaiian accessions, rather than paraphyletic to them. This suggests that the origin of clades identified here preceded the colonization of Hawai’i and that early Polynesian settlers brought taro landraces from different clades with them. In the absence of a sequenced genome, this marker set provides a valuable resource towards obtaining a genetic linkage map and to study the genetic basis of phenotypic traits of interest to taro breeding such as disease resistance. | NA | no | No good fitness measure and measure host GD | NA | no | no |
| 5109 | 2020 | 44111 | Hemsley, CM; O’Neill, PA; Essex-Lopresti, A; Norville, IH; Atkins, TP; Titball, RW | 2019 | Extensive genome analysis of Coxiella burnetii reveals limited evolution within genomic groups | BackgroundCoxiella burnetii is a zoonotic pathogen that resides in wild and domesticated animals across the globe and causes a febrile illness, Q fever, in humans. An improved understanding of the genetic diversity of C. burnetii is essential for the development of diagnostics, vaccines and therapeutics, but genotyping data is lacking from many parts of the world. Sporadic outbreaks of Q fever have occurred in the United Kingdom, but the local genetic make-up of C. burnetii has not been studied in detail.ResultsHere, we report whole genome data for nine C. burnetii sequences obtained in the UK. All four genomes of C. burnetii from cattle, as well as one sheep sample, belonged to Multi-spacer sequence type (MST) 20, whereas the goat samples were MST33 (three genomes) and MST32 (one genome), two genotypes that have not been described to be present in the UK to date. We established the phylogenetic relationship between the UK genomes and 67 publically available genomes based on single nucleotide polymorphisms (SNPs) in the core genome, which confirmed tight clustering of strains within genomic groups, but also indicated that sub-groups exist within those groups. Variation is mainly achieved through SNPs, many of which are non-synonymous, thereby confirming that evolution of C. burnetii is based on modification of existing genes. Finally, we discovered genomic-group specific genome content, which supports a model of clonal expansion of previously established genotypes, with large scale dissemination of some of these genotypes across continents being observed.ConclusionsThe genetic make-up of C. burnetii in the UK is similar to the one in neighboring European countries. As a species, C. burnetii has been considered a clonal pathogen with low genetic diversity at the nucleotide level. Here, we present evidence for significant variation at the protein level between isolates of different genomic groups, which mainly affects secreted and membrane-associated proteins. Our results thereby increase our understanding of the global genetic diversity of C. burnetii and provide new insights into the evolution of this emerging zoonotic pathogen. | NA | no | no good gd and fitness | NA | no | no |
| 5110 | 2020 | 44113 | Henden, L; Lee, S; Mueller, I; Barry, A; Bahlo, M | 2018 | Identity-by-descent analyses for measuring population dynamics and selection in recombining pathogens | Identification of genomic regions that are identical by descent (IBD) has proven useful for human genetic studies where analyses have led to the discovery of familial relatedness and fine-mapping of disease critical regions. Unfortunately however, IBD analyses have been underutilized in analysis of other organisms, including human pathogens. This is in part due to the lack of statistical methodologies for non-diploid genomes in addition to the added complexity of multiclonal infections. As such, we have developed an IBD methodology, called isoRelate, for analysis of haploid recombining microorganisms in the presence of multiclonal infections. Using the inferred IBD status at genomic locations, we have also developed a novel statistic for identifying loci under positive selection and propose relatedness networks as a means of exploring shared haplotypes within populations. We evaluate the performance of our methodologies for detecting IBD and selection, including comparisons with existing tools, then perform an exploratory analysis of whole genome sequencing data from a global Plasmodium falciparum dataset of more than 2500 genomes. This analysis identifies Southeast Asia as having many highly related isolates, possibly as a result of both reduced transmission from intensified control efforts and population bottlenecks following the emergence of antimalarial drug resistance. Many signals of selection are also identified, most of which overlap genes that are known to be associated with drug resistance, in addition to two novel signals observed in multiple countries that have yet to be explored in detail. Additionally, we investigate relatedness networks over the selected loci and determine that one of these sweeps has spread between continents while the other has arisen independently in different countries. IBD analysis of microorganisms using isoRelate can be used for exploring population structure, positive selection and haplotype distributions, and will be a valuable tool for monitoring disease control and elimination efforts of many diseases. | NA | no | No good fitness measure and measure host GD | NA | no | no |
| 5111 | 2020 | 44113 | Hendry, LM; Sahibdeen, V; Choudhury, A; Norris, SA; Ramsay, M; Lombard, Z | 2018 | Insights into the genetics of blood pressure in black South African individuals: the Birth to Twenty cohort | Background: Cardiovascular diseases (CVDs) are the leading cause of non-communicable disease deaths globally, with hypertension being a major risk factor contributing to CVDs. Blood pressure is a heritable trait, with relatively few genetic studies having been performed in Africans. This study aimed to identify genetic variants associated with variance in systolic (SBP) and diastolic (DBP) blood pressure in black South Africans. Methods: Genotyping was performed using the Metabochip in a subset of participants (mixed sex; median age 17.9) and their adult female caregivers (median age 41.0) from the Birth to Twenty cohort (n = 1947). Data were analysed as a merged dataset (all participants and caregivers together) in GEMMA (v0.94.1) using univariate linear mixed models, incorporating a centered relatedness matrix to account for the relatedness between individuals and with adjustments for age, sex, BMI and principal components of the genotype information. Results: Association analysis identified regions of interest in the NOS1AP (DBP: rs112468105 - p = 7.18 x 10(-5) and SBP: rs4657181 - p = 4.04 x 10(-5)), MYRF (SBP: rs11230796 -p = 2.16 x 10(-7), rs400075 - p = 2.88 x 10(-7)) and POC1B (SBP: rs770373 - p = 7.05 x 10(-5), rs770374 -p = 9.05 x 10(-5)) genes and some intergenic regions (DACH1\| LOC440145 (DBP: rs17240498 - p = 4.91 x 10(-6) and SBP: rs17240498 -p = 2.10 x 10(-5)) and INTS10\| LPL (SBP: rs55830938 -p = 1.30 x 10(-5), rs73599609 - p = 5.78 x 10(-5), rs73667448 -p = 6.86x10(-5))). Conclusions: The study provided further insight into the contribution of genetic variants to blood pressure in black South Africans. Future functional and replication studies in larger samples are required to confirm the role of the identified loci in blood pressure regulation and whether or not these variants are African-specific. | NA | no | human | NA | no | no |
| 5112 | 2020 | 44041 | Henkel, J; Saif, R; Jagannathan, V; Schmocker, C; Zeindler, F; Bangerter, E; Herren, U; Posantzis, D; Bulut, Z; Ammann, P; Drogemuller, C; Flury, C; Leeb, T | 2019 | Selection signatures in goats reveal copy number variants underlying breed-defining coat color phenotypes | Domestication and human selection have formed diverse goat breeds with characteristic phenotypes. This process correlated with the fixation of causative genetic variants controlling breed-specific traits within regions of reduced genetic diversity, so called selection signatures or selective sweeps. Using whole genome sequencing of DNA pools (pool-seq) from 20 genetically diverse modern goat breeds and bezoars, we identified 2,239 putative selection signatures. In two Pakistani goat breeds, Pak Angora and Barbari, we found selection signatures in a region harboring KIT, a gene involved in melanoblast development, migration, and survival. The search for candidate causative variants responsible for these selective sweeps revealed two different copy number variants (CNVs) downstream of KIT that were exclusively present in white Pak Angora and white-spotted Barbari goats. Several Swiss goat breeds selected for specific coat colors showed selection signatures at the ASIP locus encoding the agouti signaling protein. Analysis of these selective sweeps revealed four different CNVs associated with the white or tan (A(Wt)), Swiss markings (A(sm)), badger-face (A(b)), and the newly proposed peacock (A(pc)) allele. RNA-seq analyses on skin samples from goats with the different CNV alleles suggest that the identified structural variants lead to an altered expression of ASIP between eumelanistic and pheomelanistic body areas. Our study yields novel insights into the genetic control of pigmentation by identifying six functionally relevant CNVs. It illustrates how structural changes of the genome have contributed to phenotypic evolution in domestic goats. | NA | no | dometicated host | NA | no | no |
| 5113 | 2020 | 44112 | Hermes, R; Hildebrandt, TB; Goritz, F | 2018 | Cryopreservation in rhinoceros-Setting a new benchmark for sperm cryosurvival | At times when rhinoceros are fiercely poached, when some rhinoceros species are closer than ever to extinction, and when the scientific community is in debate over the use of advanced cell technologies as a remaining resort it is time to simplify and improve existing assisted reproduction techniques to enhance breeding and genetic diversity in the living populations under our care. Semen cryopreservation has been performed in all captive rhinoceros species with limited degree of success. Here we tested three freezing extenders, containing different cryoprotectants and various freezing rates for the cryopreservation of rhinoceros sperm from 14 bulls. In experiment I, semen from 9 bulls was used to determine the most suitable diluent, cryoprotectant and freezing rate for the successful cryopreservation of rhinoceros sperm. In experiment II, semen from 5 bulls was used to assess whether the removal of seminal plasma could further improve post thaw sperm quality following cryopreservation with conditions identified in Experiment I. Semen was diluted with Berliner Cryomedia, ButoCrio (R) or INRA Freeze (R), packaged in 0.5 mL straws and frozen 3, 4, and 5 cm over liquid nitrogen (LN) vapour or directly in a dryshipper. It was found that semen extended with ButoCrio (R) (containing glycerol and methylformamide) and frozen 3cm over LN vapour provided the best protection to rhinoceros spermatozoa during cryopreservation. When pooled over treatments, total and progressive post thaw motility was 75.3 +/- 4.2% and 68.5 +/- 5.7%, respectively marking a new benchmark for the cryopreservation of rhinoceros sperm. Post thaw total and progressive motility, viability and acrosome integrity of semen diluted in ButoCrio (R) was significantly higher than semen extended in Berliner Cryomedia or INRA Freeze (R). The removal of seminal plasma did not improve post thaw sperm survival (p > 0.05). In conclusion, the cryosurvival of rhinoceros spermatozoa was significantly improved when using a mixture of glycerol and methylformamide in combination with a fast freezing rate at 3cm. These results describe a new protocol for the improved cryosurvival of rhinoceros spermatozoa and will enable a more successful preservation of genetic diversity between males, especially in donors whose spermatozoa may already be compromised prior to or during collection. The successful reduction of glycerol concentration in favour of methylformamide as a cryoprotectant could be a novel suggestion for the improvement of cryopreservation techniques in other wildlife species. | NA | no | No good fitness measure and measure host GD | NA | no | no |
| 5114 | 2020 | 44111 | Hernandez-Triana, LM; Brugman, VA; Nikolova, NI; Ruiz-Arrondo, I; Barrero, E; Thorne, L; de Marco, MF; Kruger, A; Lumley, S; Johnson, N; Fooks, AR | 2019 | DNA barcoding of British mosquitoes (Diptera, Culicidae) to support species identification, discovery of cryptic genetic diversity and monitoring invasive species | Correct mosquito species identification is essential for mosquito and disease control programs. However, this is complicated by the difficulties in morphologically identifying some mosquito species. In this study, variation of a partial sequence of the cytochrome c oxidase unit I (COI) gene was used for the molecular identification of British mosquito species and to facilitate the discovery of cryptic diversity, and monitoring invasive species. Three DNA extraction methods were compared to obtain DNA barcodes from adult specimens. In total, we analyzed 42 species belonging to the genera Aedes Meigen, 1818 (21 species), Anopheles Meigen, 1818 (7 species), Coquillettidia Theobald, 1904 (1 species), Culex Linnacus, 1758 (6 species), Culiseta Felt, 1904 (7 species), and Orthopodomyia Theobald, 1904 (1 species). Intraspecific genetic divergence ranged from 0% to 5.4%, while higher interspecific divergences were identified between Aedes geminus Peus, 1971/Culiseta litorea (Shute, 1928) (24.6%) and Ae. geminusl An. plumbeus Stephens, 1828 (22.5%). Taxonomic discrepancy was shown between An. daciae Linton, Nicolescu & Harbach, 2004 and An. messeae Falleroni, 1828 indicating the poor resolution of the COIDNA barcoding region in separating these taxa. Other species such as Ae. cancans (Meigen, 1818)/Ae. annulipes (Meigen, 1830) showed similar discrepancies indicating some limitation of this genetic marker to identify certain mosquito species. The combination of morphology and DNA barcoding is an effective approach for the identification of British mosquitoes, for invasive mosquitoes posing a threat to the UK, and for the detection of hidden diversity within species groups. | NA | no | No good fitness measure and measure host GD | NA | no | no |
| 5115 | 2020 | 44113 | Herrera, A; Smith-Herron, A; Traub, N; Yount, K; Chapman, BR | 2017 | PREVALENCE OF HONEY BEE (APIS MELLIFERA) PARASITES ACROSS TEXAS | We conducted a statewide study, in June and July 2016, of feral colonies and domesticated hives to understand the current and potential impacts of invasive parasites and pests on honey bee (Apis mellifera) populations in Texas. We identify the subspecies for each colony sampled in addition to reporting the presence of parasites. The most common subspecies found were Apis mellifera scutellata, Apis mellifera carnica, and Apis mellifera ligustica. We confirmed the presence of Apis mellifera syriaca and Apis mellifera macedonica in Texas. Varroa destructor and Nosema ceranae were the most common parasites, found in eight and nine ecoregions, respectively. Aethina tumida was found in only 4 of the 10 ecoregions. | NA | no | no good gd measure host pop | NA | no | no |
| 5116 | 2020 | 44042 | Hervey, SD; Barnas, AF; Stechmann, TJ; Rockwell, RF; Ellis-Felege, SN; Darby, BJ | 2019 | Kin grouping is insufficient to explain the inclusive fitness gains of conspecific brood parasitism in the common eider | Conspecific brood parasitism allows females to exploit other females’ nests and enhance their reproductive output. Here, we test a recent theoretical model of how host females gain inclusive fitness from brood parasitism. High levels of relatedness between host and parasitizer can be maintained either by: (a) kin recognizing and parasitizing each other as a form of cooperative breeding or (b) natal philopatry and nest site fidelity facilitating the formation of kin groups, thereby increasing the probability of parasitism between relatives nesting in close proximity. To address these two hypotheses we genotyped feathers and hatch membranes of common eiders (Somateria mollissima) from western Hudson Bay, Canada, using a noninvasive sampling methodology. We found that most instances of brood parasitism do result in inclusive fitness gains. Furthermore, females with failed nests moved an average of 492 m from their previous year’s nest site, while successful females only moved an average of 13 m. Therefore, we observed host-parasite relatedness can occur at levels higher than would be expected by chance even in the absence of kin grouping, suggesting that closely related females nesting near one another is not essential to maintain high host-parasitizer relatedness. In addition, kin grouping is only a transient phenomenon that cannot occur every year due to the propensity for females of failed nests to nest farther away from their nest site in subsequent years than females with successful nests, which provides support for kin recognition as a more likely mechanism to maintain high host-parasitizer relatedness over time. | NA | no | brood parasitism | NA | no | no |
| 5117 | 2020 | 44113 | Hibino, A; Saito, R; Taniguchi, K; Zaraket, H; Shobugawa, Y; Matsui, T; Suzuki, H | 2018 | Molecular epidemiology of human respiratory syncytial virus among children in Japan during three seasons and hospitalization risk of genotype ON1 | We investigated the genetic diversity, the circulation patterns, and risk for hospital admission of human respiratory syncytial virus (HRSV) strains in Japan between 2012 through 2015. During the study period, 744 HRSV-positive cases were identified by rapid diagnostic test. Of these, 572 samples were positive by real-time PCR; 400 (69.9%) were HRSV-A, and 172 (30.1%) were HRSV-B. HRSV-A and -B alternated as the dominant strain in the subsequent seasons. Phylogenetic tree analysis of the second hyper-variable region of the G protein classified the HRSV-A specimens into NA1 (n = 242) and ON1 (n = 114) genotypes and the HRSV-B specimens into BA9 (n = 60), and BA10 (n = 27). The ON1 genotype, containing a 72-nucleotide duplication in the G protein’s second hyper-variable region, was first detected in the 2012-2013 season but it predominated and replaced the older NA1 HRSV-A in the 2014-2015 season, which also coincided with a record number of HRSV cases reported to the National Infectious Disease Surveillance in Japan. The risk of hospitalization was 6.9 times higher for the ON1 genotype compared to NA1. In conclusion, our data showed that the emergence and predominance of the relatively new ON1 genotype in Japan was associated with a record high number of cases and increased risk for hospitalization. | NA | no | gd of pathogen | NA | no | no |
| 5118 | 2020 | 44042 | Hill, E; Linacre, A; Toop, S; Murphy, N; Strugnell, J | 2019 | Widespread hybridization in the introduced hog deer population of Victoria, Australia, and its implications for conservation | In Australia, many species have been introduced that have since undergone drastic declines in their native range. One species of note is the hog deer (Axis porcinus) which was introduced in the 1860s to Victoria, Australia, and has since become endangered in its native range throughout South-East Asia. There is increased interest in using non-native populations as a source for genetic rescue; however, considerations need to be made of the genetic suitability of the non-native population. Three mitochondrial markers and two nuclear markers were sequenced to assess the genetic variation of the Victorian population of hog deer, which identified that the Victorian population has hybrid origins with the closely related chital (Axis axis), a species that is no longer present in the wild in Victoria. In addition, the mitochondrial D-loop region within the Victorian hog deer is monomorphic, demonstrating that mitochondrial genetic diversity is very low within this population. This study is the first to report of long-term persistence of hog deer and chital hybrids in a wild setting, and the continual survival of this population suggests that hybrids of these two species are fertile. Despite the newly discovered hybrid status in Victorian hog deer, this population may still be beneficial for future translocations within the native range. However, more in-depth analysis of genetic diversity within the Victorian hog deer population and investigation of hybridization rates within the native range are necessary before translocations are attempted. | NA | no | No good fitness measure and measure host GD | NA | no | no |
| 5119 | 2020 | 44113 | Hillary, RM; Bravington, MV; Patterson, TA; Grewe, P; Bradford, R; Feutry, P; Gunasekera, R; Peddemors, V; Werry, J; Francis, MP; Duffy, CAJ; Bruce, BD | 2018 | Genetic relatedness reveals total population size of white sharks in eastern Australia and New Zealand | Conservation concerns exist for many sharks but robust estimates of abundance are often lacking. Improving population status is a performance measure for species under conservation or recovery plans, yet the lack of data permitting estimation of population size means the efficacy of management actions can be difficult to assess, and achieving the goal of removing species from conservation listing challenging. For potentially dangerous species, like the white shark, balancing conservation and public safety demands is politically and socially complex, often leading to vigorous debate about their population status. This increases the need for robust information to inform policy decisions. We developed a novel method for estimating the total abundance of white sharks in eastern Australia and New Zealand using the genetic-relatedness of juveniles and applying a close-kin mark-recapture framework and demographic model. Estimated numbers of adults are small (ca. 280-650), as is total population size (ca. 2,500-6,750). However, estimates of survival probability are high for adults (over 90%), and fairly high for juveniles (around 73%). This represents the first direct estimate of total white shark abundance and survival calculated from data across both the spatial and temporal life-history of the animal and provides a pathway to estimate population trend. | NA | no | No good fitness measure | NA | no | no |
| 5120 | 2020 | 44043 | Hoadley, KD; Lewis, AM; Wham, DC; Pettay, DT; Grasso, C; Smith, R; Kemp, DW; LaJeunesse, TC; Warner, ME | 2019 | Host-symbiont combinations dictate the photo-physiological response of reef-building corals to thermal stress | High sea surface temperatures often lead to coral bleaching wherein reef-building corals lose significant numbers of their endosymbiotic dinoflagellates (Symbiodiniaceae). These increasingly frequent bleaching events often result in large scale coral mortality, thereby devasting reef systems throughout the world. The reef habitats surrounding Palau are ideal for investigating coral responses to climate perturbation, where many inshore bays are subject to higher water temperature as compared with offshore barrier reefs. We examined fourteen physiological traits in response to high temperature across various symbiotic dinoflagellates in four common Pacific coral species, Acropora muricata, Coelastrea aspera, Cyphastrea chalcidicum and Pachyseris rugosa found in both offshore and inshore habitats. Inshore corals were dominated by a single homogenous population of the stress tolerant symbiont Durusdinium trenchii, yet symbiont thermal response and physiology differed significantly across coral species. In contrast, offshore corals harbored specific species of Cladocopium spp. (ITS2 rDNA type-C) yet all experienced similar patterns of photoinactivation and symbiont loss when heated. Additionally, cell volume and light absorption properties increased in heated Cladocopium spp., leading to a greater loss in photo-regulation. While inshore coral temperature response was consistently muted relative to their offshore counterparts, high physiological variability in D. trenchii across inshore corals suggests that bleaching resilience among even the most stress tolerant symbionts is still heavily influenced by their host environment. | NA | no | No good fitness measure and measure host GD | NA | no | no |
| 5121 | 2020 | 44112 | Hohenlohe, PA; McCallum, HI; Jones, ME; Lawrance, MF; Hamede, RK; Storfer, A | 2019 | Conserving adaptive potential: lessons from Tasmanian devils and their transmissible cancer | Maintenance of adaptive genetic variation has long been a goal of management of natural populations, but only recently have genomic tools allowed identification of specific loci associated with fitness-related traits in species of conservation concern. This raises the possibility of managing for genetic variation directly relevant to specific threats, such as those due to climate change or emerging infectious disease. Tasmanian devils (Sarcophilus harrisii) face the threat of a transmissible cancer, devil facial tumor disease (DFTD), that has decimated wild populations and led to intensive management efforts. Recent discoveries from genomic and modeling studies reveal how natural devil populations are responding to DFTD, and can inform management of both captive and wild devil populations. Notably, recent studies have documented genetic variation for disease-related traits and rapid evolution in response to DFTD, as well as potential mechanisms for disease resistance such as immune response and tumor regression in wild devils. Recent models predict dynamic persistence of devils with or without DFTD under a variety of modeling scenarios, although at much lower population densities than before DFTD emerged, contrary to previous predictions of extinction. As a result, current management that focuses on captive breeding and release for maintaining genome-wide genetic diversity or demographic supplementation of populations could have negative consequences. Translocations of captive devils into wild populations evolving with DFTD can cause outbreeding depression and/or increases in the force of infection and thereby the severity of the epidemic, and we argue that these risks outweigh any benefits of demographic supplementation in wild populations. We also argue that genetic variation at loci associated with DFTD should be monitored in both captive and wild populations, and that as our understanding of DFTD-related genetic variation improves, considering genetic management approaches to target this variation is warranted in developing conservation strategies for Tasmanian devils. | NA | no | No good fitness measure and measure host GD | NA | no | no |
| 5122 | 2020 | 44042 | Hokken, MWJ; Zwaan, BJ; Melchers, WJG; Verweij, PE | 2019 | Facilitators of adaptation and antifungal resistance mechanisms in clinically relevant fungi | Opportunistic fungal pathogens can cause a diverse range of diseases in humans. The increasing rate of fungal infections caused by strains that are resistant to commonly used antifungals results in difficulty to treat diseases, with accompanying high mortality rates. Existing and newly emerging molecular resistance mechanisms rapidly spread in fungal populations and need to be monitored. Fungi exhibit a diversity of mechanisms to maintain physiological resilience and create genetic variation; processes which eventually lead to the selection and spread of resistant fungal pathogens. To prevent and anticipate this dispersion, the role of evolutionary factors that drive fungal adaptation should be investigated. In this review, we provide an overview of resistance mechanisms against commonly used antifungal compounds in the clinic and for which fungal resistance has been reported. Furthermore, we aim to summarize and elucidate potent generators of genetic variability across the fungal kingdom that aid adaptation to stressful environments. This knowledge can lead to recognizing potential niches that facilitate fast resistance development and can provide leads for new management strategies to battle the emerging resistant populations in the clinic and the environment. | NA | no | No good fitness measure and measure host GD | NA | no | no |
| 5123 | 2020 | 44113 | Holtz, MD; Hwang, SF; Strelkov, SE | 2018 | Genotyping of Plasmodiophora brassicae reveals the presence of distinct populations | Background: Plasmodiophora brassicae is a soilborne pathogen of the family Brassicaceae and the causal agent of clubroot disease. In Canada, P. brassicae is now one of the most important constraints to canola (Brassica napus) production, and is managed mainly by the deployment of resistant cultivars. In recent years, however, new strains of the pathogen have emerged that are capable of overcoming host resistance, posing new challenges for disease management. Despite its economic significance, molecular studies of P. brassicae are rare, mainly because this microorganism cannot be cultured outside of its host. Results: Restriction site-associated DNA sequencing (RADseq) was used to examine the genetic diversity within P. brassicae single-spore and field isolates collected from across Canada. The isolates included individuals that were either capable or incapable of causing disease on clubroot resistant canola cultivars. Over 8750 variants were identified through RADseq. Population analysis indicated that most isolates belonged to one of two distinct populations, corresponding with the ability of isolates to cause disease on resistant cultivars. Within each population, there were low levels of genetic diversity. One thousand and fifty of the genetic variants that distinguished the two populations were nonsynonymous, altering the coding sequences of genes. Conclusion: The application of RADseq revealed two distinct populations of P. brassicae in Canada, suggesting multiple introductions of the pathogen into the country. The genetic variation found here will be important for future research and monitoring of the pathogen. | NA | no | No good fitness measure | NA | no | no |
| 5124 | 2020 | 44113 | Holz, PH; Lumsden, LF; Druce, J; Legione, AR; Vaz, P; Devlin, JM; Hufschmid, J | 2018 | Virus survey in populations of two subspecies of bent-winged bats (Miniopterus orianae bassanii and oceanensis) in south-eastern Australia reveals a high prevalence of diverse herpesviruses | While bats are often viewed as carriers of infectious disease agents, little research has been conducted on the effects these potential pathogens may have on the bat populations themselves. The southern bent-winged bat (Miniopterus orianae bassanii) is a critically endangered subspecies endemic to south-eastern Australia. Population numbers of this bat have been declining for the past 50 years, but the reasons for this are unclear. As part of a larger study to determine if disease could be a contributing factor to this decline, 351 southern bent-winged bats from four locations were captured, and oral swabs were collected and tested for the presence of potentially pathogenic viruses. Results were compared with those obtained from 116 eastern bent-winged bats (Miniopterus orianae oceanensis) from three different locations. The eastern bent-winged bat is a related but more common and widespread subspecies whose geographical range overlaps partly with southern bent-winged bats. Herpesviruses were detected in bent-winged bats from all seven locations. At least six novel herpesviruses (five betaherpesviruses and one gammaherpesvirus) were identified. The prevalence of herpesvirus infection was higher in eastern bent-winged bats (44%, 51/116), compared to southern bent-winged bats (27%, 95/351), although this varied across the locations and sampling periods. Adenoviruses and a range of different RNA viruses (lyssaviruses, filoviruses, coronaviruses and henipaviruses) were also tested for but not detected. The detected herpesviruses did not appear to be associated with obvious ill health, and may thus not be playing a role in the population decline of the southern bent-winged bat. The detection of multiple novel herpesviruses at a high prevalence of infection is consistent with our understanding of bats as hosts to a rich diversity of viruses. | NA | no | No good fitness measure and measure host GD | NA | no | no |
| 5125 | 2020 | 44113 | Hong, Y; Kim, KS; Kimura, J; Kauhala, K; Voloshina, I; Goncharuk, MS; Yu, L; Zhang, YP; Sashika, M; Lee, H; Min, MS | 2018 | Genetic Diversity and Population Structure of East Asian Raccoon Dog (Nyctereutes procyonoides): Genetic Features in Central and Marginal Populations | The raccoon dog (Nyctereutes procyonoides) is endemic to East Asia but has been introduced in Europe. Its high adaptability enabled its rapid colonization of European countries, where population growth has been raising concerns regarding ecosystem disturbance and the spread of zoonotic diseases. The genetic diversity and structure of endemic, source, and introduced populations from seven locations across South Korea, China, Russian Far East, Finland (spread to Finland after introduction to European part of Russia from Russian Far East), Vietnam, and Japan (Honshu and Hokkaido) were examined based on 16 microsatellite loci. Two major and significantly different (F-ST = 0.236) genetic clusters were found: continental (South Korean, Chinese, Russian, Finnish, and Vietnamese) and island (Japanese) populations. The continental raccoon dog population comprises three subpopulations (Chinese\_Russian\_Finnish, South Korean, and Vietnamese) and the Japanese population consists of Honshu and Hokkaido subpopulations. The genetic diversity and geographic structure of raccoon dogs in East Asia has been influenced by natural barriers to gene flow and reveals a typical central-marginal trend in genetic diversity (continental vs. island, and central vs. marginal or source vs. introduced within continental populations). The detected differences between continental and island populations agree with those reported in previous studies that considered these populations as different species. | NA | no | No good fitness measure | NA | no | no |
| 5126 | 2020 | 44111 | Hooker, MJ; Bond, BT; Chamberlain, MJ | 2019 | Population genetics of American black bears in Georgia, USA | There are 3 American black bear (Ursus americanus) populations in the state of Georgia, USA. We used multi-locus microsatellite genotypes derived from bear hair and tissue samples collected across these populations to assess levels of genetic diversity within and between populations. We used population assignment clustering to evaluate whether there has been recent immigration into the smallest of the 3 populations, the Central Georgia Bear Population. Compared with other bear populations in the United States, the North Georgia and South Georgia Bear Populations have relatively high rates of genetic diversity (H-0 = 0.72 +/- 0.02, A = 6.68 +/- 0.32, and H-0 = 0.72 +/- 0.02, A = 6.82 +/- 0.35, respectively). In contrast, the Central Georgia Bear Population has relatively low rates (H-0 = 0.46 +/- 0.03, and A = 3.96 +/- 0.20). Fixation indices for pairings between Georgia bear populations indicated that the North Georgia Bear Population was more similar to the South Georgia Bear Population than either was to the Central Georgia Bear Population. Our findings suggest that the Central Georgia Bear population has experienced long-term genetic isolation and genetic drift. Of a sample of 365 bears from Central Georgia, we only detected 1 immigrant and no evidence of gene flow into the population. We recommend development and implementation of plans to encourage gene flow toward the Central Georgia Bear Population. | NA | no | No good fitness measure | NA | no | no |
| 5127 | 2020 | 44112 | Horns, F; Vollmers, C; Dekker, CL; Quake, SR | 2019 | Signatures of selection in the human antibody repertoire: Selective sweeps, competing subclones, and neutral drift | Antibodies are created and refined by somatic evolution in B cell populations, which endows the human immune system with the ability to recognize and eliminate diverse pathogens. However, the evolutionary processes that sculpt antibody repertoires remain poorly understood. Here, using an unbiased repertoire-scale approach, we show that the population genetic signatures of evolution are evident in human B cell lineages and reveal how antibodies evolve somatically. We measured the dynamics and genetic diversity of B cell responses in five adults longitudinally before and after influenza vaccination using high-throughput antibody repertoire sequencing. We identified vaccine-responsive B cell lineages that carry signatures of selective sweeps driven by positive selection, and discovered that they often display evidence for selective sweeps favoring multiple subclones. We also found persistent B cell lineages that exhibit stable population dynamics and carry signatures of neutral drift. By exploiting the relationship between B cell fitness and antibody binding affinity, we demonstrate the potential for using phylogenetic approaches to identify antibodies with high binding affinity. This quantitative characterization reveals that antibody repertoires are shaped by an unexpectedly broad spectrum of evolutionary processes and shows how signatures of evolutionary history can be harnessed for antibody discovery and engineering. | NA | no | human | NA | no | no |
| 5128 | 2020 | 44039 | Hosotani, S; Nishita, Y; Masuda, R | 2020 | Genetic diversity and evolution of the MHC class II DRB gene in the Japanese marten, Martes melampus (Carnivora: Mustelidae) | High polymorphism in major histocompatibility complex (MHC) genes plays an essential role in the adaptive immune system of vertebrates for defense against various pathogens. To further understand the evolution of MHC in wildlife, partial sequences of exon 2 in MHC class II DRB genes were determined and analyzed for the Japanese marten (Martes melampus), which is endemic to Japan. From 26 individuals, 17 DRB alleles (Mame-DRBs) and four presumed pseudogenes were identified, some of them geographically widespread and others of limited distribution. Mixed-effect model evolution (MEME) and single breakpoint recombination (SBP) analyses indicated six amino acid positions under positive selection and one recombination site, respectively, that are implicated in maintaining the diversity of Mame-DRBs. In a Bayesian phylogenetic tree, Mame-DRBs did not group monophyletically, but were scattered among seven sub-clades, each containing DRBs from one or more other mustelid species. This trans-species polymorphism of Mame-DRBs has resulted from long-term balancing selection. | NA | no | no group level or good fitness | NA | no | no |
| 5129 | 2020 | 44042 | Hovestadt, T; Thomas, JA; Mitesser, O; Schonrogge, K | 2019 | Multiple host use and the dynamics of host switching in host-parasite systems | The link between multi-host use and host switching in host-parasite interactions is a continuing area of debate. Lycaenid butterflies in the genus Maculinea, for example, exploit societies of different Myrmica ant species across their ranges, but there is only rare evidence that they simultaneously utilise multiple hosts at a local site, even where alternative hosts are present. We present a simple population-genetic model accounting for the proportion of two alternative hosts and the fitness of parasite genotypes on each host. In agreement with standard models, we conclude that simultaneous host use is possible whenever fitness of heterozygotes on alternative hosts is not too low. We specifically focus on host-shifting dynamics when the frequency of hosts changes. We find that (i) host shifting may proceed so rapidly that multiple host use is unlikely to be observed, (ii) back and forth transition in host use can exhibit a hysteresis loop, (iii) the parasites’ host use may not be proportional to local host frequencies and be restricted to the rarer host under some conditions, and (iv) that a substantial decline in parasite abundance may typically precede a shift in host use. We conclude that focusing not just on possible equilibrium conditions but also considering the dynamics of host shifting in non-equilibrium situations may provide added insights into host-parasite systems. | NA | no | No good fitness measure and measure host GD | NA | no | no |
| 5130 | 2020 | 44112 | Howell, HJ; Seigel, RA | 2019 | The Effects of Road Mortality on Small, Isolated Turtle Populations | Roads impact wildlife in a variety of direct and indirect ways. Roads may act as barriers to dispersal, lead to decreasing population size and genetic diversity, change animal behavior, result in direct mortality, and increase habitat disturbance. Road mortality is especially detrimental to long-lived species, such as freshwater turtles, whose population persistence relies on high adult and subadult survivorship to counter high egg and hatchling mortality. The Spotted Turtle (Clemmys guttata) is a small-bodied, freshwater turtle species that is listed as endangered in Canada and proposed for federal listing in the United States. We used a population viability analysis to attempt to quantify the impact that road mortality has on two distinct populations of Spotted Turtles. The baseline model for the North Wetland Complex (NWC) population predicted a probability of quasi-extinction within 150 yr of 20%. The baseline model for the South Wetland Complex (SWC) predicted a probability of quasi-extinction within 150 yr of 24%. Including an estimate of road mortality (modeled as a reduction in adult survival through annual catastrophic events) into the models, the probability of quasiextinction within 150 yr changed to 93% for the NWC and 94% for the SWC Our results highlight the critical importance that anthropogenic additive adult mortality has on small populations of turtles and the necessity of detailed demographic studies to detect potential declines in populations of long-lived species. | NA | no | no good gd measure host pop | NA | no | no |
| 5131 | 2020 | 44040 | Hu, JY; Hao, ZQ; Frantz, L; Wu, SF; Chen, W; Jiang, YF; Wu, H; Kuang, WM; Li, HP; Zhang, YP; Yu, L | 2020 | Genomic consequences of population decline in critically endangered pangolins and their demographic histories | Pangolins are among the most critically endangered animals due to heavy poaching and worldwide trafficking. However, their demographic histories and the genomic consequences of their recent population declines remain unknown. We generated high-quality de novo reference genomes for critically endangered Malayan (Manis javanica, MJ) and Chinese (M. pentadactyla, MP) pangolins and re-sequencing population genomic data from 74 MJs and 23 MPs. We recovered the population identities of illegally traded pangolins and previously unrecognized genetic populations that should be protected as evolutionarily distinct conservation units. Demographic reconstruction suggested environmental changes have resulted in a population size fluctuation of pangolins. Additionally, recent population size declines due to human activities have resulted in an increase in inbreeding and genetic load. Deleterious mutations were enriched in genes related to cancer/diseases and cholesterol homeostasis, which may have increased their susceptibility to diseases and decreased their survival potential to adapt to environmental changes and high-cholesterol diets. This comprehensive study provides not only high-quality pangolin reference genomes, but also valuable information concerning the driving factors of long-term population size fluctuations and the genomic impact of recent population size declines due to human activities, which is essential for pangolin conservation management and global action planning. | NA | no | No good fitness measure and measure host GD | NA | no | no |
| 5132 | 2020 | 44041 | Huang, XL; Zhou, ZY; Liu, HF; Deng, L; Bi, B; Chai, YJ; Zhong, ZJ; Hu, YC; Fu, HL; Peng, GN | 2019 | New genotypes and molecular characterization of Enterocytozoon bieneusi in captive black bears in China | Enterocytozoon bieneusi, a common eukaryotic obligate intracellular parasite, can infect a wide range of hosts, including humans and domestic animals. There have been some reports of this organism in captive wildlife animals worldwide, but few studies have reported its detection in the captive black bears in Sichuan province of southwestern China. The present study was performed to determine the prevalence, genetic diversity, and zoonotic potential of E. bieneusi in captive Asiatic black bears from three farms in Sichuan province. Fecal specimens from Asiatic black bears in three farms were collected and analyzed for the prevalence of E. bieneusi. The overall prevalence of E. bieneusi was 18.7% (57/305) as determined by nested PCR amplification of the Internal Transcribed Spacer (ITS) gene on the rRNA of E. bieneusi, with the highest prevalence in the farm being 47.8% (44/92). Altogether, five genotypes of E. bieneusi were identified among the 57 E. bieneusi-positive samples, comprising three known genotypes (SC02, MJ2, and MJ5) and two novel genotypes named SCBB1 and SCBB2. Phylogenetic analysis showed that the genotypes SC02 and MJ2 were clustered into group 1 of zoonotic potential and that the genotypes MJ5, SCBB1, and SCBB2 were clustered into group 10. In conclusion, two known genotypes, SC02 and MJ2, were found to belong to the zoonotic potential group 1 and this evidence points to the fact that the E. bieneusi from these black bears could infect humans. | NA | no | No good fitness measure and measure host GD | NA | no | no |
| 5133 | 2020 | 44042 | Hughes, AR; Hanley, TC; Byers, JE; Grabowski, JH; McCrudden, T; Piehler, MF; Kimbro, DL | 2019 | Genetic diversity and phenotypic variation within hatchery-produced oyster cohorts predict size and success in the field | The rapid growth of the aquaculture industry to meet global seafood demand offers both risks and opportunities for resource management and conservation. In particular, hatcheries hold promise for stock enhancement and restoration, yet cultivation practices may lead to enhanced variation between populations at the expense of variation within populations, with uncertain implications for performance and resilience. To date, few studies have assessed how production techniques impact genetic diversity and population structure, as well as resultant trait variation in and performance of cultivated offspring. We collaborated with a commercial hatchery to produce multiple cohorts of the eastern oyster (Crassostrea virginica) from field-collected broodstock using standard practices. We recorded key characteristics of the broodstock (male : female ratio, effective population size), quantified the genetic diversity of the resulting cohorts, and tested their trait variation and performance across multiple field sites and experimental conditions. Oyster cohorts produced under the same conditions in a single hatchery varied almost twofold in genetic diversity. In addition, cohort genetic diversity was a significant positive predictor of oyster performance traits, including initial size and survival in the field. Oyster cohorts produced in the hatchery had lower within-cohort genetic variation and higher among-cohort genetic structure than adults surveyed from the same source sites. These findings are consistent with sweepstakes reproduction in oysters, even when manually spawned. A readily measured characteristic of broodstock, the ratio of males to females, was positively correlated with within-cohort genetic diversity of the resulting offspring. Thus, this metric may offer a tractable way both to meet short-term production goals for seafood demand and to ensure the capacity of hatchery-produced stock to achieve conservation objectives, such as the recovery of self-sustaining wild populations. | NA | no | No good fitness measure and measure host GD | NA | no | no |
| 5134 | 2020 | 44113 | Huguin, M; Arechiga-Ceballos, N; Delaval, M; Guidez, A; de Castro, IJ; Lacoste, V; Salmier, A; Setien, AA; Silva, CR; Lavergne, A; de Thoisy, B | 2018 | How Social Structure Drives the Population Dynamics of the Common Vampire Bat (Desmodus rotundus, Phyllostomidae) | Social systems are major drivers of population structure and gene flow, with important effects on dynamics and dispersal of associated populations of parasites. Among bats, the common vampire bat (Desmodus rotundus) has likely one of the most complex social structures. Using autosomal and mitochondrial markers on vampires from Mexico, French Guiana, and North Brazil, from both roosting and foraging areas, we observed an isolation by distance at the wider scale and lower but significant differentiation between closer populations (< 50 km). All populations had a low level of relatedness and showed deviations from Hardy-Weinberg equilibrium and a low but significant inbreeding coefficient. The associated heterozygote deficiency was likely related to a Wahlund effect and to cryptic structures, reflecting social groups living in syntopy, both in roosting and foraging areas, with only limited admixture. Discrepancy between mitochondrial and nuclear markers suggests female philopatry and higher dispersal rates in males, associated with peripheral positions in the groups. Vampires are also the main neotropical reservoir for rabies virus, one of the main lethal pathogens for humans. Female social behaviors and trophallaxis may favor a rapid spread of virus to related and unrelated offspring and females. The high dispersal capacity of males may explain the wider circulation of viruses and the inefficacy of bat population controls. In such opportunistic species, gene connectivity should be considered for management decision making. Strategies such as culling could induce immigration of bats from neighboring colonies to fill vacant roosts and feeding areas, associated with the dispersal of viral strains. | NA | no | No good fitness measure and measure host GD | NA | no | no |
| 5135 | 2020 | 44043 | Humphries, MB; Stacy, MT; Ricklefs, RE | 2019 | Population structure of avian malaria parasites | The geographic distribution of genetic diversity in malaria parasite populations (Apicomplexa: Haemosporida) presumably influences local patterns of virulence and the evolution of host-resistance, but little is known about population genetic structure in these parasites. We assess the distribution of genetic diversity in the partial Domain I of apical membrane antigen 1 (AMA1) in three mtDNA-defined lineages of avian Plasmodium to determine spatial population structure and host-parasite genetic relationships. We find that one parasite lineage is genetically differentiated in association with a single host genus and among some locations, but not with respect to other hosts. Two other parasite lineages are undifferentiated with respect to host species but exhibit geographic differentiation that is inconsistent with shared geographic barriers or with isolation-by-distance. Additional differentiation within two other lineages is unassociated with host species or location; in one case, we tentatively interpret this differentiation as the result of mitochondrial introgression from one of the lineages into a second lineage. More sampling of nuclear genetic diversity within populations of avian Plasmodium is needed to rule out coinfection as a possible confounding factor. If coinfections are not responsible for these findings, further assessment is needed to determine the frequency of mitonuclear discordance and its implications for defining parasite lineages based on mitochondrial genetic variation. | NA | no | gd of pathogen | NA | no | no |
| 5136 | 2020 | 44112 | Hurt, C; Ellis, N; Harman, A; Savage, C | 2019 | Genetic Structure at the Major Histocompatibility Complex in the Endangered Barrens Topminnow (Fundulus julisia) | Genetic diversity at the Major Histocompatibility Complex (MHC) is particularly important for species viability as it allows populations to respond to emerging pathogens and infectious disease. Patterns of variation at this gene complex serve as a useful complement to information obtained from neutral loci for planning management and conservation strategies that seek to ensure the adaptive potential of at-risk species. In this study, we investigated patterns of genetic variation at exon 2 of the MHC class II gene in the critically endangered Fundulus julisia (Barrens Topminnow). This species has undergone dramatic declines over the last 30 years, leading to its recent proposal for federal protection under the Endangered Species Act. Patterns of nucleotide substitution and phylogenetic analyses revealing trans-species polymorphisms suggest that this locus has been of adaptive importance in the history of this species. Despite recent population declines and documented population bottlenecks, measures of genetic diversity were high in comparison to patterns observed at putatively neutral microsatellite and mitochondrial markers. Results from this study are discussed in the context of recovery plans for the Barrens Topminnow and lend support to the previous designation of evolutionary significant units and management units based on neutral markers. | NA | no | no good gd measure host pop | NA | no | no |
| 5137 | 2020 | 44113 | Hurt, C; Kuhajda, B; Harman, A; Ellis, N; Nalan, M | 2017 | Genetic diversity and population structure in the Barrens Topminnow (Fundulus julisia): implications for conservation and management of a critically endangered species | The Barrens Topminnow (Fundulus julisia) has undergone a rapid and dramatic decline. In the 1980s, at least twenty localities with Barrens Topminnows were known to exist in the Barrens Plateau region of middle Tennessee; currently only three areas with natural (not stocked) populations remain. The long-term survival of the Barrens Topminnow will depend entirely on effective management and conservation efforts. Captive propagation and stocking of captive-reared juveniles to suitable habitats have successfully established a handful of self-sustaining populations. However, very little is known about the genetic composition of source and introduced populations including levels of genetic diversity and structuring of genetic variation. Here we use both mitochondrial sequence data and genotypes from 14 microsatellite loci to examine patterns of genetic variation among ten sites, including all sites with natural populations and a subset of sites with introduced (stocked) populations of this species. Mitochondrial sequence analysis reveals extremely low levels of variation within populations and fixed differences between drainages. Microsatellite genotype data shows higher levels of genetic variability and a molecular signature consistent with a recent history of population bottlenecks. Measures of genetic diversity at microsatellite loci including allelic richness are similar within source and introduced populations. Bayesian assignment tests and analysis of molecular variation (AMOVA) support two distinct populations, consistent with drainage boundaries. Results from AMOVA analysis also suggest low levels of genetic connectivity between isolated populations within the same drainage. Here we propose two distinct evolutionary significant units (ESUs) and two management units that reflect this population substructure and warrant consideration in future management efforts. | NA | no | No good fitness measure | NA | no | no |
| 5138 | 2020 | 44112 | Hurtado, R; Carhuaricra, D; Soare, S; Viana, MVC; Azevedo, V; Maturrano, L; Aburjaile, F | 2018 | Pan-genomic approach shows insight of genetic divergence and pathogenic-adaptation of Pasteurella multocida | Pasteurella multocida is a gram-negative, non-motile bacterial pathogen, which is associated with chronic and acute infections as snuffles, pneumonia, atrophic rhinitis, fowl cholera and hemorrhagic septicemia. These diseases affect a wide range of domestic animals, leading to significant morbidity and mortality and causing significant economic losses worldwide. Due to the interest in deciphering the genetic diversity and process adaptive between P. multocida strains, this work aimed was to perform a pan-genome analysis to evidence horizontal gene transfer and positive selection among 23 P. multocida strains isolated from distinct diseases and hosts. The results revealed an open pan-genome containing 3585 genes and an accessory genome presenting 1200 genes. The phylogenomic analysis based on the presence/absence of genes and islands exhibit high levels of plasticity, which reflects a high intraspecific diversity and a possible adaptive mechanism responsible for the specific disease manifestation between the established groups (pneumonia, fowl cholera, hemorrhagic septicemia and snuffles). Additionally, we identified differences in accessory genes among groups, which are involved in sugar metabolism and transport systems, virulence-related genes and a high concentration of hypothetical proteins. However, there was no specific indispensable functional mechanism to decisively correlate the presence of genes and their adaptation to a specific host/disease. Also, positive selection was found only for two genes from sub-group hemorrhagic septicemia, serotype B. This comprehensive comparative genome analysis will provide new insights of horizontal gene transfers that play an essential role in the diversification and adaptation mechanism into P. multocida species to a specific disease. | NA | no | gd of pathogen | NA | no | no |
| 5139 | 2020 | 44113 | Hydeman, ME; Longo, AV; Velo-Anton, G; Rodriguez, D; Zamudio, KR; Bell, RC | 2017 | Prevalence and genetic diversity of Batrachochytrium dendrobatidis in Central African island and continental amphibian communities | The fungal pathogen Batrachochytrium dendrobatidis (Bd) infects hundreds of amphibian species and is implicated in global amphibian declines. Bd is comprised of several lineages that differ in pathogenicity, thus, identifying which Bd strains are present in a given amphibian community is essential for understanding host-pathogen dynamics. The presence of Bd has been confirmed in Central Africa, yet vast expanses of this region have not yet been surveyed for Bd prevalence, and the genetic diversity of Bd is largely unknown in this part of the world. Using retrospective surveys of museum specimens and contemporary field surveys, we estimated the prevalence of Bd in Central African island and continental amphibian assemblages, and genotyped strains of Bd present in each community. Our sampling of museum specimens included just a few individuals collected in the Gulf of Guinea archipelago prior to 1998, yet one of these individuals was Bd-positive indicating that the pathogen has been on Bioko Island since 1966. We detected Bd across all subsequent sample years in our study and found modest support for a relationship between host life history and Bd prevalence, a positive relationship between prevalence and host community species richness, and no significant relationship between elevation and prevalence. The Global Panzootic Lineage (BdGPL) was present in all the island and continental amphibian communities we surveyed. Our results are consistent with a long-term and widespread distribution of Bd in amphibian communities of Gabon and the Gulf of Guinea archipelago. | NA | no | gd of pathogen | NA | no | no |
| 5140 | 2020 | 44113 | Hyvarinen, K; Ritari, J; Koskela, S; Niittyvuopio, R; Nihtinen, A; Volin, L; Gallardo, D; Partanen, J | 2017 | Genetic polymorphism related to monocyte-macrophage function is associated with graft-versus-host disease | Despite detailed human leukocyte antigen (HLA) matching and modern immunosuppressive therapy, severe graft-versus-host disease (GvHD) remains a major hurdle for successful allogeneic hematopoietic stem cell transplantation (HSCT). As the genetic diversity in GvHD complicates the systematic discovery of associated variants across populations, we studied 122 GvHD-associated single nucleotide polymorphisms (SNPs) in 492 HLA-matched sibling HSCT donor-recipient pairs from Finland and Spain. The association between these candidate SNPs and grade III-IV acute GvHD and extensive chronic GvHD was assessed. The functional effects of the variants were determined using expression and cytokine quantitative trait loci (QTL) database analyses. Clear heterogeneity was observed in the associated markers between the two populations. Interestingly, the majority of markers, such as those annotated to IL1, IL23R, TLR9, TNF, and NOD2 genes, are related to the immunological response by monocytes-macrophages to microbes, a step that precedes GvHD as a result of intestinal lesions. Furthermore, cytokine QTL analysis showed that the GvHD-associated markers regulate IL1 beta, IFN gamma, and IL6 responses. These results support a crucial role for the anti-microbial response in GvHD risk. Furthermore, despite apparent heterogeneity in the genetic markers associated with GvHD, it was possible to identify a biological pathway shared by most markers in both populations. | NA | no | No good fitness measure and measure host GD | NA | no | no |
| 5141 | 2020 | 44113 | Iannucci, A; Natali, C; Ciofi, C; Sposimo, P; Fratini, S | 2018 | Population genetic analysis of invasive black rats: Defining eradication units in the Tuscan Archipelago National Park | Invasive species are one of the main causes of biodiversity loss, and rodents in particular are regarded as a real threat worldwide, especially to island ecosystems. The Tuscan Archipelago National Park is the largest in the Mediterranean basin, it harbours a large number of autochthonous endemic species, mostly reptiles and insects, and hosts many migratory birds during their seasonal movements. Although a number of sites in the Archipelago are under strict protection regimes, the invasive black rat Rattus rattus has significantly affected survival of local wildlife. As part of an eradication campaign conducted in 2012 and 2017, we assessed genetic diversity and population differentiation of black rats from a total of six locations on the largest Elba Island, a possible source of invasion, and the southern, small islands of Pianosa and Montecristo using six nuclear DNA microsatellite loci. We recorded a strong population structure and identified the islands of Elba, Pianosa and Montecristo as three distinct eradication units. Despite some degree of admixture was recorded on Elba, the largest island of the archipelago was unlikely the main source of invasive rats to Pianosa and Montecristo. We also recorded evidence of past reduction in population size, particularly in Montecristo, probably due to repeated past founding events. Biodiversity management plans should consider monitoring vessels arriving to the Tuscan Archipelago from the mainland and the major Tyrrhenian islands in order to limit alien invasion. Moreover, as reinvasion can occur a few years after eradication, regular monitoring should be conducted thus to rapidly intercept the arrival of new invaders. | NA | no | No good fitness measure | NA | no | no |
| 5142 | 2020 | 44040 | Ijaz, S; Ul Haq, I; Nasir, B | 2020 | In silico identification of expressed sequence tags based simple sequence repeats (EST-SSRs) markers in Trifolium species | The EST-SSRs (genic SSRs) are the microsatellites in expressed sequence tags (ESTs). Advancement in functional genomics has contributed mainly for the identification of ESTs in different species and made them available in databases. They are of transcribe regions, so are more conserved and considered with more cross transferability across the taxonomic borders. In Trifolium alexandrinum (Berseem), no expressed sequence tags (ESTs) are available in NCBI database yet. Therefore, in this study by considering the cross transferability potential of EST-SSRs, expressed sequence tags of Trifolium pratense and Trifolium repens were retrieved from NCBI database and their EST-SSRs were in silico identified. We retrieved 1014 ESTs for SSR identification from both Trifolium species and identified 198 EST-SSRs from them. In identified EST-SSRs, trinucleotide SSRs repeats were found to be more frequent. Of these 198 in silico identified EST-SSRs, 44 showed cross species amplification in T. alexandrinum and was proved for their transferability potential. The identified EST-SSRs could be a great source for genetic diversity and population genetics studies of Trifolium species. These EST-SSRs could be further screened to investigate their linkage to disease resistance in Trifolium species. | NA | no | No good fitness measure and measure host GD | NA | no | no |
| 5143 | 2020 | 44112 | Ijaz, S; Ul Haq, I; Razzaq, HA; Nasir, B; Babar, M | 2019 | ISSR-BASED POPULATION GENETICS STUDY FOR TAGGING A DIVERSE POPULATION OF SHISHAM (DALBERGIA SISSOO) IN PAKISTAN | Population genetics is a subject of modelling the variations in gene and allele frequencies resulting genetic diversity between and within the population. The molecular marker assisted approaches in juxtaposition to computational biology have become powerful tools to explore the genetic pattern and diversity of a particular population. Therefore, population genetics of shisham population in terms of their diversity and genetic pattern was investigated. Twenty-one ISSR markers were used to investigate 30 shisham genotypes collected from Sindh, KPK and Balochistan. Genetic diversity analysis showed 337 loci were amplified with percent polymorphic loci, 99.41. Maximum diversity was found between genotypes SKP3 and KPK P3, however, genotypes TTP1 & TTP2 was found to be more similar. Principal Coordinate analysis depicted, first II coordinates accounted for 40.69% total genetic variation in 2-D plotting. AMOVA predicted high level of percent genetic variance within population (99%). Population genetic structure prediction using admixture model of Bayesian inference showed selected shisham population shared three gene pools (K = 3), with diverse genetic pattern that were grouped as distinct or even sharing maximum genetics. These findings could either be helpful for tagging and shaping scattered shisham population of Pakistan or a practical step toward identifying dieback disease resistant shisham germplasm. | NA | no | No good fitness measure and measure host GD | NA | no | no |
| 5144 | 2020 | 44112 | Ilvonen, JJ; Kaunisto, KM; Suhonen, J | 2018 | Odonates, gregarines and water mites: why are the same host species infected by both parasites? | 1. Damselflies and dragonflies are widely parasitised insects and numerous studies have tried to understand this host-parasite relationship. However, most of these studies have concentrated on a single host species, neglecting the larger pattern within the Odonata order. 2. The aim of this paper was to examine different damselfly and dragonfly species for common endo- and ectoparasites and whether a general infection pattern can be found. Additionally, the goal was to investigate whether the phylogeny of the host species could explain these possible infection patterns. To this end, a dataset from the existing literature was compiled and the prevalence of endoparasitic gregarines and ectoparasitic water mites was analysed for 46 different odonate species. 3. Three distinct patterns were found: (i) most of the odonate host species had both gregarines and water mites, rather than only either one or neither; (ii) there appears to be a positive association between gregarine and water mite prevalences across host species; (iii) a weak phylogenetic signal was detected in gregarine prevalence and a strong one in water mite prevalence. 4. It is hypothesised that, due to the infection and transmission mechanisms by which water mites and gregarines infect different odonate host species, parasitism is aggregated to common, high-density species. However, much research is needed in order to fully understand this relationship between odonates and their parasites, especially within the same host populations and host species assemblages. | NA | no | No good fitness measure and measure host GD | NA | no | no |
| 5145 | 2020 | 44111 | Imura, Y; Hatakeyama, Y; Takahashi, M; Ohbayashi, T; Mizobe, S; Iwano, H | 2019 | A novel approach using microsporidia to estimate the flight route of the common cutworm, Spodoptera litura (Lepidoptera: Noctuidae) | Microsporidia are unique fungi that exist as obligate intracellular parasites. Approximately 40% of the known microsporidian strains infect various insects. Due to their high host specificity, microsporidia have potential use as powerful biological pesticides. Here, we analyzed microsporidian strains isolated from the common cutworm, Spodoptera litura Fabricius (Lepidoptera: Noctuidae) of Japan and Taiwan, which is considered a pest insect. Two experiments were performed: a comparison of spore size and a phylogenetic analysis using small subunit ribosomal RNA gene sequencing. We then estimated the flight routes of S. litura by using the phylogenetic relationships of the isolated microsporidian strains as a marker. An analysis of spore size indicated that the test strains comprised three groups: Pleistophora, Nosema or Vairimorpha, and Trachipleistophora or Vavraia. The results of the phylogenetic analysis suggested a classification into five genera, including Vavraia. The genus Vavraia was first detected from lepidopteran insects in Japan. We estimated the sources and flight routes of S. litura using phylogenetic data for the genera Nosema and Trachipleistophora. In this study, we used microsporidia as a novel marker to estimate sources and flight routes of S. litura from Ogasawara and Southern China to mainland Japan, demonstrating the usefulness of this approach. | NA | no | gd of pathogen | NA | no | no |
| 5146 | 2020 | 44113 | Iqbal, A; Shah, S; Nisar, M; Ghafoor, A | 2017 | Morphological Characterization and Selection for High Yielding and Powdery Mildew Resistant Pea (Pisum sativum) Lines | Twenty lines of Pisum sativum particularly developed for high yield and resistant to powdery mildew were evaluated along with two parents (Fallon(er) and 11760-3(ER)) and two checks (Climex and a local cultivar) with the objectives to determine morphological characterization, yield potential and resistance to powdery mildew. On the basis of one way cluster, the 24 lines were mainly grouped into four clusters, especially on the vegetative and yield contributing traits. It was observed that the tall and high yielding lines were grouped in cluster-III while the dwarf and high yielding lines were grouped in cluster-IV. Analysis of variance (ANOVA) showed significant difference (p<0.05) in the yield of 24 pea lines. The average grain yield of the 24 pea lines ranged from 22.87 to 102.54 g. The highest grain yield was produced by PL-4 (102.54 g plant(-1)) followed by PL-5 (82.14 g plant-1). Of the 24 pea lines, two lines (PL-4 and PL-5) were highly resistant to powdery mildew disease. Therefore, the newly developed PL-4 and PL-5 lines were high yielding and highly resistant. Among the 19 morphological traits, six (Eigenvalue > 1.0) contributed more than 80% variability among the materials. | NA | no | dometicated host | NA | no | no |
| 5147 | 2020 | 44112 | Iranmehr, A; Stobdan, T; Zhou, D; Poulsen, O; Strohl, KP; Aldashev, A; Telenti, A; Wong, EHM; Kirkness, EF; Venter, JC; Bafna, V; Haddad, GG | 2019 | Novel insight into the genetic basis of high-altitude pulmonary hypertension in Kyrgyz highlanders | The Central Asian Kyrgyz highland population provides a unique opportunity to address genetic diversity and understand the genetic mechanisms underlying high-altitude pulmonary hypertension (HAPH). Although a significant fraction of the population is unaffected, there are susceptible individuals who display HAPH in the absence of any lung, cardiac or hematologic disease. We report herein the analysis of the whole-genome sequencing of healthy individuals compared with HAPH patients and other controls (total n = 33). Genome scans reveal selection signals in various regions, encompassing multiple genes from the first whole-genome sequences focusing on HAPH. We show here evidence of three candidate genes MTMR4, TMOD3 and VCAM1 that are functionally associated with well-known molecular and pathophysiological processes and which likely lead to HAPH in this population. These processes are (a) dysfunctional BMP signaling, (b) disrupted tissue repair processes and (c) abnormal endothelial cell function. Whole-genome sequence of well-characterized patients and controls and using multiple statistical tools uncovered novel candidate genes that belong to pathways central to the pathogenesis of HAPH. These studies on high-altitude human populations are pertinent to the understanding of sea level diseases involving hypoxia as a main element of their pathophysiology. | NA | no | human | NA | no | no |
| 5148 | 2020 | 44040 | Isabel, N; Holliday, JA; Aitken, SN | 2020 | Forest genomics: Advancing climate adaptation, forest health, productivity, and conservation | Forest ecosystems provide important ecological services and resources, from habitat for biodiversity to the production of environmentally friendly products, and play a key role in the global carbon cycle. Humanity is counting on forests to sequester and store a substantial portion of the anthropogenic carbon dioxide produced globally. However, the unprecedented rate of climate change, deforestation, and accidental importation of invasive insects and diseases are threatening the health and productivity of forests, and their capacity to provide these services. Knowledge of genetic diversity, local adaptation, and genetic control of key traits is required to predict the adaptive capacity of tree populations, inform forest management and conservation decisions, and improve breeding for productive trees that will withstand the challenges of the 21st century. Genomic approaches have well accelerated the generation of knowledge of the genetic and evolutionary underpinnings of nonmodel tree species, and advanced their applications to address these challenges. This special issue of Evolutionary Applications features 14 papers that demonstrate the value of a wide range of genomic approaches that can be used to better understand the biology of forest trees, including species that are widespread and managed for timber production, and others that are threatened or endangered, or serve important ecological roles. We highlight some of the major advances, ranging from understanding the evolution of genomes since the period when gymnosperms separated from angiosperms 300 million years ago to using genomic selection to accelerate breeding for tree health and productivity. We also discuss some of the challenges and future directions for applying genomic tools to address long-standing questions about forest trees. | NA | no | No good fitness measure and measure host GD | NA | no | no |
| 5149 | 2020 | 44040 | Iwu, CD; Okoh, AI | 2020 | Characterization of antibiogram fingerprints in Listeria monocytogenes recovered from irrigation water and agricultural soil samples | Listeria monocytogenes (L. monocytogenes) is a foodborne pathogen and the etiologic agent of listeriosis, which can be disseminated within the agricultural environment particularly soil and irrigation water, contaminate farm produce and cause high mortality and morbidity among vulnerable individuals. This study assessed the incidence and antibiogram of L. monocytogenes recovered from irrigation water and agricultural soil samples collected from Chris Hani and Amathole District Municipalities (DMs) in Eastern Cape Province, South Africa. The distribution of presumptive L. monocytogenes in irrigation water and agricultural soil samples was done using the standard plate count method, while polymerase chain reaction (PCR) was used to identify the isolates. The confirmed isolates were screened for 9 key virulence markers using PCR after which they were subjected to antibiotic susceptibility testing against 18 antibiotics used for the alleviation of listeriosis using the disk diffusion method. Relevant putative antibiotic resistance genes in the resistant variants were screened for using PCR. The distribution of L. monocytogenes in irrigation water samples was statistically significant (P < 0.05) and ranged from log(10) 1.00 CFU/100ml to log(10) 3.75 CFU/100 ml. In agricultural soil samples, the distribution ranged significantly (P <= 0.05) from log(10) 2.10 CFU/g to log(10) 3.51 CFU/g. Of the 117 presumptive L. monocytogenes recovered from irrigation water samples and 183 presumptive L. monocytogenes isolated from agricultural soil samples, 8 (6.8%) and 12 (6.6%) isolates were confirmed respectively. Nine virulence genes including in/A, in/B, in/C, inIJ, actA, hlyA, plcA, plcB, and lap were detected in all the isolates. The proportion of the isolates exhibiting phenotypic resistance against the test antimicrobials followed the order: tetracycline (90%), doxycycline (85%), cefotaxime (80%), penicillin (80%), chloramphenicol (70%), linezolid (65%), erythromycin (60%) and trimethoprim/sulfamethoxazole (55%). The isolates exhibited multiple antibiotic resistance against 3 or more antibiotics and the MAR indices of all the multidrug isolates were >= 0.2. The isolates harboured antibiotic resistance genes including tetA, tetB, tetC, sun, sulll, aadA, aac(3)-IIa and ESBLs including bla(TEM), bla(CTX-M) group 9, blav(EB) as well as AmpC. None of the isolates harboured the carbapenemases. We conclude that irrigation water and agricultural soil collected from Chris Hani and Amathole District Municipalities (DMs) in Eastern Cape Province of South Africa are reservoirs and potential transmission routes of multidrug-resistant L. monocytogenes to the food web and consequently threat to public health. | NA | no | n | NA | no | no |
| 5150 | 2020 | 44113 | Izzo, VM; Chen, YH; Schoville, SD; Wang, C; Hawthorne, DJ | 2018 | Origin of Pest Lineages of the Colorado Potato Beetle (Coleoptera: Chrysomelidae) | Colorado potato beetle (Leptinotarsa decemlineata Say [Coleoptera: Chrysomelidae]) is a pest of potato throughout the Northern Hemisphere, but little is known about the beetle’s origins as a pest. We sampled the beetle from uncultivated Solanum host plants in Mexico, and from pest and non-pest populations in the United States and used mitochondrial DNA and nuclear loci to examine three hypotheses on the origin of the pest lineages: 1) the pest beetles originated from Mexican populations, 2) they descended from hybridization between previously divergent populations, or 3) they descended from populations that are native to the Plains states in the United States. Mitochondrial haplotypes of non-pest populations from Mexico and Arizona differed substantially from beetles collected from the southern plains and potato fields in the United States, indicating that beetles from Mexico and Arizona did not contribute to founding the pest lineages. Similar results were observed for AFLP and microsatellite data. In contrast, non-pest populations from the states of Colorado, Kansas, Nebraska, New Mexico, and Texas were genetically similar to U.S. pest populations, indicating that they contributed to the founding of the pest lineages. Most of the pest populations do not show a significant reduction in genetic diversity compared to the plains populations in the United States. We conclude that genetically heterogeneous beetle populations expanded onto potato from native Solanum hosts. This mode of host range expansion may have contributed to the abundant genetic diversity of contemporary populations, perhaps contributing to the rapid evolution of climate tolerance, host range, and insecticide resistance. | NA | no | No good fitness measure and measure host GD | NA | no | no |
| 5151 | 2020 | 44042 | Jacobs, CE; Ausband, DE | 2019 | Wolves in space: locations of individuals and their effect on pup survival in groups of a cooperatively breeding canid | In some cooperatively breeding species, breeding and nonbreeding individuals within a group care for young through provisioning and guarding, commonly referred to as alloparental care. The question of why nonbreeders contribute to the care of young is at the centre of research on the evolution of cooperative societies, and the mechanisms that drive the behaviour are a topic of debate. We employed a novel approach to examine grey wolf, Canis lupus, space use at pup-rearing sites by using genotyped scats to measure the distance between wolves and evaluate the influence of genetic relatedness and other group characteristics on this space use. Specifically, we evaluated how group composition and individual characteristics influenced space use and whether the distance between adults and pups influenced pup survival. We genotyped 3093 wolf scats collected at pup-rearing sites during June -August 2008-2016 and calculated the distance between scats. We found that space use at puprearing sites did not influence pup survival. We also found that nonbreeders, especially yearlings, remained closer to pups than did breeders, and that individuals more genetically related to the pups were found closer to them, suggesting kin-directed attendance behaviour. Finally, we found evidence suggesting that direct fitness benefits, such as gaining access to food, may influence space use as well and help to explain why yearling nonbreeders were found the closest to the pups. This is the first study to provide indirect evidence for kin-directed attendance behaviour in wolves and it contributes novel methodology for investigating space use and helping behaviour in cooperative breeders at reproductive sites. Published by Elsevier Ltd on behalf of The Association for the Study of Animal Behaviour. | NA | no | no good gd measure host | NA | no | no |
| 5152 | 2020 | 44112 | Jafari, M; Akram, W; Pang, YJ; Ahmad, A; Ahmed, S; Yasin, NA; Anjum, T; Ali, B; Hu, XD; Li, XH; Dong, S; Cai, Q; Ciprian, M; Bielec, M; Hu, S; Sefidkon, F; Hu, XB | 2018 | Genetic diversity and biogeography of T. officinale inferred from multi locus sequence typing approach | Taraxacum officinale (Asteraceae) is widely distributed weedy plant used as a traditional medicinal herb. The population genetics and historical biogeography of this plant have remained relatively unexplored. This study explores phylogeny, population genetics and ancestral reconstructions adopting multi locus sequence typing (MLST) approach. MLST sequences dataset was generated from genomics and chloroplast DNA sequences obtained from 31 T. officinale haplotypes located in 16 different countries. Phylogenetic analysis distributed these haplotypes in well differentiated geographic clades. The study suggested a close relationship between Europe and adjacent Asian countries. Populations of these regions predominantly formed common haplogroups, showed considerable level of gene flow and evidence for recombination events across European and Asian population. Biogeographical inferences obtained by applying statistical dispersal-vicariance analysis (S-DIVA) and Bayesian binary MCMC (BBM) analysis showed that T. officinale was putatively originated in Europe. Molecular clock analysis based on ITS dataset suggested that the divergence between Europe and East Asian populations can be dated to 1.07 Mya with sub-sequent dispersal and vicariance events. Among different spatial process long distance seed dispersal mediated by wind had potentially assisted the population expansion of T. officinale. | NA | no | No good fitness measure and measure host GD | NA | no | no |
| 5153 | 2020 | 44112 | Jafari, R; Sanei, B; Baradaran, A; Spotin, A; Bagherpour, B; Darani, HY | 2018 | Genetic characterization of Echinococcus granulosus strains isolated from humans based on nad1 and cox1 gene analysis in Isfahan, central Iran | Cystic echinococcosis (CE) is a medically important parasite-caused human disease. Humans may acquire the infection accidentally by ingestion of E. granulosus eggs. The parasite has a broad range of hosts and genotypes, which may affect its aetiological and biological characteristics. The present study aimed to determine the genetic characteristics of human isolates of E. granulosus in Isfahan, Iran. In this cross-sectional study, 50 surgically removed hydatid cysts were collected from hospitalized patients in Al-Zahra Hospital, Isfahan, Iran, over a period of 2 years (2015-2017). DNA was extracted from cyst material, and polymerase chain reactions (PCR) were performed targeting cox1 and nad1 genes. Amplicons were sequenced directly and the resulting sequences were aligned and analysed. Phylogenetic and genetic diversity analyses were also performed. Among the isolates, 43 (86%), 3 (6%) and 4 (8%) out of 50 were E. granulosus (G1), E. granulosus (G3) and E. intermedius (G6), respectively. In total, nine and eight haplotypes were identified by nad1 and cox1 gene analysis, respectively. The haplotype diversity index was higher by cox1 gene analysis (0.547) in G1 strains compared with nad1 (0.433). The G1 genotype was the most predominant isolate from human cases of CE, and the presence of G6 is indicative of an important role of camels in the development of human CE in Isfahan. This is the first report of the G3 genotype causing human CE in Isfahan. Moreover, cox1 gene analysis enables a higher resolution of the genetic diversity of the E. granulosus population compared with nad1 gene analysis. | NA | no | human | NA | no | no |
| 5154 | 2020 | 44041 | Jaffal, L; Joumaa, WH; Assi, A; Helou, C; Cherfan, G; Zibara, K; Audo, I; Zeitz, C; El Shamieh, S | 2019 | Next Generation Sequencing Identifies Five Novel Mutations in Lebanese Patients with Bardet-Biedl and Usher Syndromes | Aim: To identify disease-causing mutations in four Lebanese families: three families with Bardet-Biedl and one family with Usher syndrome (BBS and USH respectively), using next generation sequencing (NGS). Methods: We applied targeted NGS in two families and whole exome sequencing (WES) in two other families. Pathogenicity of candidate mutations was evaluated according to frequency, conservation, in silico prediction tools, segregation with disease, and compatibility with inheritance pattern. The presence of pathogenic variants was confirmed via Sanger sequencing followed by segregation analysis. Results: Most likely disease-causing mutations were identified in all included patients. In BBS patients, we found (M1): c.2258A > T, p. (G1u753Val) in BBS9, (M2): c.68T > C; p. (Leu23Pro) in ARL6, (M3): c.265 266delTT; p. (Leu89Valfs\*11) and (M4): c.880T > G; p. (Tyr294Asp) in BBS12. A previously known variant (M5): c.551A > G; p. (Asp184Ser) was also detected in BBS5. In the USH patient, we found (M6): c.188A > C, p. (Tyr63Ser) in CLRN1. M2, M3, M4, and M6 were novel. All of the candidate mutations were shown to be likely disease-causing through our bioinformatic analysis. They also segregated with the corresponding phenotype in available family members. Conclusion: This study expanded the mutational spectrum and showed the genetic diversity of BBS and USH. It also spotlighted the efficiency of NGS techniques in revealing mutations underlying clinically and genetically heterogeneous disorders. | NA | no | human | NA | no | no |
| 5155 | 2020 | 44112 | Jagadeesh, D; Kumar, MKP; Devaki, NS | 2018 | Population analysis of Magnaporthe oryzae by using endogenous repetitive DNA sequences and mating-type alleles in different districts of Karnataka, India | Rice is the staple food crop of more than 60% of the population of the world. This crop suffers from blast disease caused by Magnaporthe oryzae. Information on the mating-type allele distribution and diversity of the pathogen population for the state of Karnataka, India is scanty. With this background, a total of 72 isolates of M. oryzae from rice in different districts of Karnataka were examined for identifying sexual mating alleles MAT1, MAT2 and understanding the genetic diversity based on DNA fingerprint of pot2, an inverted repeat transposon. Among 72 isolates, 44 isolates belonged to MAT1 type (male fertile) and 28 isolates were of MAT2 (female fertile) and there were no hermaphrodite isolates. In a given geographical location, only one mating type was identified. Results revealed that the isolates obtained from these regions are not sexually fertile showing predominant asexual reproduction. Hence, genetic variation observed in the pathogen may be mainly because of high copy number of transposons. A high copy number transposon, namely Pot2, was selected in our study to detect genetic diversity of the pathogen. Pot2 rep-PCR DNA fingerprinting profile showed 27 polymorphic bands with bands ranging in size from 0.65 to 4.0 kb and an average of 10 to 14 bands per isolate. Five distinct clusters were formed with two major, two minor, and one outlier. Clusters 4 and 5 are further subdivided into three sub-clusters. Some of the isolates belonging to clusters 3, 4, and 5 are interlinked as these locations are close to one another sharing common geographical parameters and boundaries. This knowledge on the sexual behavior and genetic diversity of M. oryzae is important with respect to breeding for disease resistance. | NA | no | gd of pathogen | NA | no | no |
| 5156 | 2020 | 44112 | Jahnke, M; D’Esposito, D; Orru, L; Lamontanara, A; Dattolo, E; Badalamenti, F; Mazzuca, S; Procaccini, G; Orsini, L | 2019 | Adaptive responses along a depth and a latitudinal gradient in the endemic seagrass Posidonia oceanica | Seagrass meadows provide important ecosystem services and are critical for the survival of the associated invertebrate community. However, they are threatened worldwide by human-driven environmental change. Understanding the seagrasses’ potential for adaptation is critical to assess not only their ability to persist under future global change scenarios, but also to assess the persistence of the associated communities. Here we screened a wild population of Posidonia oceanica, an endemic long-lived seagrass in the Mediterranean Sea, for genes that may be target of environmental selection, using an outlier and a genome-wide transcriptome analysis. We identified loci where polymorphism or differential expression was associated with either a latitudinal or a bathymetric gradient, as well as with both gradients in an effort to identify loci associated with temperature and light. We found the candidate genes underlying growth and immunity to be divergent between populations adapted to different latitudes and/or depths, providing evidence for local adaptation. Furthermore, we found evidence of reduced gene flow among populations including adjacent populations. Reduced gene flow, combined with low sexual recombination, small effective population size, and long generation time of P. oceanica raises concerns for the long-term persistence of this species, especially in the face of rapid environmental change driven by human activities. | NA | no | No good fitness measure and measure host GD | NA | no | no |
| 5157 | 2020 | 44040 | Jamaluddin, J; Khair, NKM; Vinodamaney, SD; Othman, Z; Abubakar, S | 2020 | Copy number variation of CCL3L1 among three major ethnic groups in Malaysia | Background C-C motif Chemokine Ligand 3 Like 1 (CCL3L1) is a multiallelic copy number variable, which plays a crucial role in immunoregulatory and hosts defense through the production of macrophage inflammatory protein (MIP)-1 alpha. Variable range of the CCL3L1 copies from 0 to 14 copies have been documented in several different populations. However, there is still lack of report on the range of CCL3L1 copy number exclusively among Malaysians who are a multi-ethnic population. Thus, this study aims to extensively examine the distribution of CCL3L1 copy number in the three major populations from Malaysia namely Malay, Chinese and Indian. A diploid copy number of CCL3L1 for 393 Malaysians (Malay = 178, Indian = 90, and Chinese = 125) was quantified using Paralogue Ratio Tests (PRTs) and then validated with microsatellites analysis. Results To our knowledge, this is the first report on the CCL3L1 copy number that has been attempted among Malaysians and the Chinese ethnic group exhibits a diverse pattern of CCL3L1 distribution copy number from the Malay and Indian (p < 0.0001). The CCL3L1 ranged from 0 to 8 copies for both the Malay and Indian ethnic groups while 0 to 10 copies for the Chinese ethnic. Consequently, the CCL3L1 copy number among major ethnic groups in the Malaysian population is found to be significantly varied when compared to the European population (p < 0.0001). The mean/median reported for the Malay, Chinese, Indian, and European are 2.759/2.869, 3.453/3.290, 2.437/1.970 and 2.001/1.940 respectively. Conclusion This study reveals the existence of genetic variation of CCL3L1 in the Malaysian population, and suggests by examining genetic diversity on the ethnicity, and specific geographical region could help in reconstructing human evolutionary history and for the prediction of disease risk related to the CCL3L1 copy number. | NA | no | human | NA | no | no |
| 5158 | 2020 | 44042 | Jarman, R; Mattioni, C; Russell, K; Chambers, FM; Bartlett, D; Martin, MA; Cherubini, M; Villani, F; Webb, J | 2019 | DNA analysis of Castanea sativa (sweet chestnut) in Britain and Ireland: Elucidating European origins and genepool diversity | Castanea sativa is classified as non-indigenous in Britain and Ireland. It was long held that it was first introduced into Britain by the Romans, until a recent study found no corroborative evidence of its growing here before c. AD 650. This paper presents new data on the genetic diversity of C. sativa in Britain and Ireland and potential ancestral sources in continental Europe. Microsatellite markers and analytical methods tested in previous European studies were used to genotype over 600 C. sativa trees and coppice stools, sampled from ancient semi-natural woodlands, secondary woodlands and historic cultural sites across Britain and Ireland. A single overall genepool with a diverse admixture of genotypes was found, containing two sub groups differentiating Wales from Ireland, with discrete geographical and typological clusters. C. sativa genotypes in Britain and Ireland were found to relate predominantly to some sites in Portugal, Spain, France, Italy and Romania, but not to Greece, Turkey or eastern parts of Europe. C. sativa has come to Britain and Ireland from these western European areas, which had acted as refugia in the Last Glacial Maximum; we compare its introduction with the colonization/translocation of oak, ash, beech and hazel into Britain and Ireland. Clones of C. sativa were identified in Britain, defining for the first time the antiquity of some ancient trees and coppice stools, evincing both natural regeneration and anthropogenic propagation over many centuries and informing the chronology of the species’ arrival in Britain. This new evidence on the origins and antiquity of British and Irish C. sativa trees enhances their conservation and economic significance, important in the context of increasing threats from environmental change, pests and pathogens. | NA | no | n | NA | no | no |
| 5159 | 2020 | 44040 | Jarosova, J; Antolova, D; Snabel, V; Guimaraes, N; Stofik, J; Urban, P; Cavallero, S; Miterpakova, M | 2020 | The fox tapeworm,Echinococcus multilocularis, in grey wolves and dogs in Slovakia: epidemiology and genetic analysis | Echinococcus multilocularis, the causative agent of human alveolar echinococcosis, is an important emerging parasite in the northern hemisphere. In epidemiological studies, the highest attention is being paid to foxes as the main reservoir hosts responsible for geographic expansion from multiple focal populations and the invasion of urban habitats, but little information is available on the parasite distribution in other carnivores. Hence, the study was designed to obtain updated information about the occurrence and genetic diversity ofE. multilocularisin grey wolves and dogs in Slovakia. Faecal samples of wolves were collected from three locations under a certain level of environmental protection in the central and eastern parts of the country, and the presence of the parasite DNA was detected in 35.7% of 112 samples, with the highest rate (51.2%) recorded in the Poloniny National Park in north-eastern Slovakia. Among 110 faecal dog samples,E. multiloculariswas detected in three faeces from segregated Roma settlements in the eastern part of the country, which accounted for an overall positivity of 2.7%. Sequence analysis of two mitochondrial genes, 12S rRNA and NADH dehydrogenase subunit 1, revealed four haplotypes in 13 isolates from wolves and dogs originating from four sites in eastern and central Slovakia, with all samples bearing a European-type pattern ofE. multilocularis. The more than one-third positivity rate ofE. multilocularisin wolf faecal samples dispersed over a large part of the country has corroborated the extensive circulation of the parasite in wildlife and confirmed the need to improve intervention control strategies. | NA | no | No good fitness measure and measure host GD | NA | no | no |
| 5160 | 2020 | 44040 | Jaworska, J; Ropka-Molik, K; Woclawek-Potocka, I; Siemieniuch, M | 2020 | Inter- and intrabreed diversity of the major histocompatibility complex (MHC) in primitive and draft horse breeds | Background Polymorphism of major histocompatibility complex (MHC) genes ensures effective immune responses against a wide array of pathogens. However, artificial selection, as performed in the case of domestic animals, may influence MHC diversity. Here, we investigate and compare the MHC diversity of three populations of horses, for which different breeding policies were applied, to evaluate the impact of artificial selection and the environment on MHC polymorphism. Methods Samples of DNA were taken from 100 Polish draft horses, 38 stabled Konik Polski horses and 32 semiferal Konik Polski horses. MHC alleles and haplotype diversity within and between these populations of horses was estimated from 11 MHC microsatellite loci. Results MHC diversity measured based on allelic richness, observed heterozygosity, expected heterozygosity and polymorphism content was similar across the MHC microsatellite loci in all three populations. The highest expected heterozygosity was detected in semiferal primitive horses (He = 0.74), while the lowest was calculated for draft horses (He = 0.65). In total, 203 haplotypes were determined (111 in Polish draft horses, 43 in semiferal Konik Polski horses and 49 in stabled Konik Polski horses), and four haplotypes were shared between the two populations of Koniks. None of these haplotypes were present in any of the previously investigated horse breeds. Intra-MHC recombination events were detected in all three populations. However, the population of semiferal Konik horses showed the highest recombination frequency among the three horse populations. In addition, three recombination events were detected. Conclusions These results showed that despite the different breeding policies, the MHC allele and haplotype diversity was similarly high in all three horse populations. Nevertheless, the proportion of new haplotypes in the offspring was the highest in semiferal Konik Polski horses, which indicates the influence of the environment on MHC diversity in horses. Thus, we speculate that the genetic makeup of the domestic horse MHC might be more strongly influenced by the environment than by artificial selection. Moreover, intra-MHC conversion, insertion, and deletion and intra-MHC recombination may be proposed as mechanisms underlying the generation of new MHC haplotypes in horses. | NA | no | dometicated host | NA | no | no |
| 5161 | 2020 | 44113 | Jelocnik, M; Islam, MM; Madden, D; Jenkins, C; Branley, J; Carve, S; Polkinghorne, A | 2017 | Development and evaluation of rapid novel isothermal amplification assays for important veterinary pathogens: Chlamydia psittaci and Chlamydia pecorum | Background. Chlamydia psittaci and Chlamydia pecorum are important veterinary pathogens, with the former also being responsible for zoonoses, and the latter adversely affecting koala populations in Australia and livestock globally. The rapid detection of these organisms is still challenging, particularly at the point-of-care (POC). In the present study, we developed and evaluated rapid, sensitive and robust C. psittaci-specific and C. pecorum-specific Loop Mediated Isothermal Amplification (LAMP) assays for detection of these pathogens. Methods and Materials. The LAMP assays, performed in a Genie III real-time fluorometer, targeted a 263 bp region of the C. psittaci-specific Cps\_0607 gene or a 209 bp region of a C. pecorum-specific conserved gene CpecG\_0573, and were evaluated using a range of samples previously screened using species-specific quantitative PCRs (qPCRs). Species-specificity for C. psittaci and C. pecorum LAMP targets was tested against DNA samples from related chlamydial species and a range of other bacteria. In order to evaluate pathogen detection in clinical samples, C. psittaci LAMP was evaluated using a total of 26 DNA extracts from clinical samples from equine and avian hosts, while for C. pecorum LAMP, we tested a total of 63 DNA extracts from clinical samples from koala, sheep and cattle hosts. A subset of 36 C. pecorum samples was also tested in a thermal cycler (instead of a real-time fluorometer) using newly developed LAMP and results were determined as an end point detection. We also evaluated rapid swab processing (without DNA extraction) to assess the robustness of these assays. Results. Both LAMP assays were demonstrated to species-specific, highly reproducible and to be able to detect as little as 10 genome copy number/reaction, with a mean amplification time of 14 and 24 min for C. psittaci and C. pecorum , respectively. When testing clinical samples, the overall congruence between the newly developed LAMP assays and qPCR was 92.3% for C. psittaci (91.7% sensitivity and 92.9% specificity); and 84.1% for C. pecorum (90.6% sensitivity and 77.4% specificity). For a subset of 36 C. pecorum samples tested in a thermal cycler using newly developed LAMP, we observed 34/36 (94.4%) samples result being congruent between LAMP performed in fluorometer and in thermal cycler. Rapid swab processing method evaluated in this study also allows for chlamydial DNA detection using LAMP. Discussion. In this study, we describe the development of novel, rapid and robust C. psittaci-specific and C. pecorum-specific LAMP assays that are able to detect these bacteria in clinical samples in either the laboratory or POC settings. With further development and a focus on the preparation of these assays at the POC, it is anticipated that both tests may fill an important niche in the repertoire of ancillary diagnostic tools available to clinicians | NA | no | No good fitness measure and measure host GD | NA | no | no |
| 5162 | 2020 | 44111 | Jenkins, SN; Okello, E; Rossitto, PV; Lehenbauer, TW; Champagne, J; Penedo, MCT; Arruda, AG; Godden, S; Rapnicki, P; Gorden, PJ; Timms, LL; Aly, SS | 2019 | Molecular epidemiology of coagulase-negative Staphylococcus species isolated at different lactation stages from dairy cattle in the United States | Background. Coagulase negative Staphylococcus (CNS) species are currently the most prevalent intra-mammary pathogens causing subclinical mastitis and occasional clinical mastitis or persistent infection in lactating dairy cattle. More than 10 CNS species have been identified, but they are generally managed as one group on most dairies in the United States. However, improved management decisions and treatment outcomes may be achieved with better understanding of the prevalent species, pathogenicity and strain diversity within and across dairies. Methodology. A total of 604 CNS isolates were cultured from milk samples collected during a dry-cow treatment clinical trial conducted on 6 dairy herds in 4 states in the US. All the study cows were randomized to receive 1 of the 3 different infra-mammary antimicrobial infusions (Quatermaster, Spectramast DC or ToMorrow Dry Cow) at dry-off. Milk samples were collected at dry-off, calving (0-6 days in milk, DIM), post-calving (7-13 DIM) and at mastitis events within the first 100 DIM. The CNS isolates were identified to species level by partial sequencing of the rpo beta gene, and genetic relatedness within species was investigated by phylogenetic analysis of the pulse-field gel electrophoresis profiles of the isolates. Results. The major CNS species identified were S. chromogenes (48.3%), S. haemolyticus (17.9%), S. simulans and S. epidermidis (each at 6.5%). Other CNS species identified at lower frequencies included S. hominis, S. auricularis, S. sciuri, S. spp KS-SP, S. capitis, S. cohnii, S. warneri, S. pasteuri, S. xylosus, S. hyicus, S. equorum, S. microti, S. rostri, S. gallinarum, S. saprophyticus and S. succinus. Phylogenetic analyses of the major species types demonstrated an association between genetic relatedness and epidemiological distributions of S. chromogenes, S. simulans, S. haemolyticus and S. auricularis. Additionally, identical strains of S. chromogenes and S. simulans were isolated from the same udder quarter of several cows at consecutive sample stages. The rest of the minor species had no deducible genetic-epidemiological link. Discussion. The observed association between genetic and epidemiological distributions indicated animal-adapted nature of four CNS species, suggesting possible host-adapted and environmental transmission of these species. Multi-stage isolation of the same udder quarter strain was evidence for chronic intra-mammary infection. Conclusion. The different CNS species and strains circulating on US dairy herds were genetically diverse. Four species identified were likely udder-adapted pathogens, 2 of which caused persistent infection. Our findings are important in guiding the design of effective mastitis control strategies. | NA | no | No good fitness measure and measure host GD | NA | no | no |
| 5163 | 2020 | 44113 | Jensen, LF; Ejbye-Ernst, R; Michaelsen, TY; Jensen, A; Hansen, DM; Nielsen, ME; Pertoldi, C | 2017 | Assessing the genetic effects of rehabilitating harbor seals (Phoca vitulina) in the Wadden Sea using stochastic simulations | Rehabilitation of marine mammals with the intent of releasing them back into nature is carried out for several species. Rehabilitation can help supporting critically endangered species, increase public awareness, and serve scientific purposes, but rehabilitation also causes concern due to risk of introducing diseases into the wild populations and genetic changes from disruption of natural selection and host-pathogen co-evolution. In this study, we investigate another potential risk from rehabilitation that has not previously been considered, i.e., anthropogenic increase in population inbreeding level. For this purpose, we performed stochastic population viability analyses of the Wadden Sea harbor seal population using VORTEX. In the Wadden Sea, rehabilitation takes place in several rehabilitation centers, and many of the seals suffer from parasitic bronchopneumonia or are orphaned. Several studies have found a correlation between helminth infection and inbreeding level. Moreover, a relation between fitness traits such as pup survival and inbreeding has been demonstrated. On this basis, we assess the effects from rehabilitating relatively inbred seals on population size and genetic diversity. We find that releasing seals significantly affect population inbreeding level and to a lesser extent population size. The effect depends on the level of inbreeding in rehabilitated seals as well as the actual number of inbred seals released. | NA | no | inbreeding | NA | no | no |
| 5164 | 2020 | 44041 | Jeong, K; Munoz-Bodnar, A; Rojas, NA; Poulin, L; Rodriguez-R, LM; Gagnevin, L; Verniere, C; Pruvost, O; Koebnik, R | 2019 | CRISPR elements provide a new framework for the genealogy of the citrus canker pathogen Xanthomonas citri pv. citri | Background Xanthomonads are an important clade of Gram-negative bacteria infecting a plethora of economically important host plants, including citrus. Knowledge about the pathogen’s diversity and population structure are prerequisite for epidemiological surveillance and efficient disease management. Rapidly evolving genetic loci, such as Clustered Regularly Interspaced Short Palindromic Repeats (CRISPR), are of special interest to develop new molecular typing tools. Results We analyzed CRISPR loci of 56 Xanthomonas citri pv. citri strains of world-wide origin, a regulated pathogen causing Asiatic citrus canker in several regions of the world. With one exception, 23 unique sequences built up the repertoire of spacers, suggesting that this set of strains originated from a common ancestor that already harbored these 23 spacers. One isolate originating from Pakistan contained a string of 14 additional, probably more recently acquired spacers indicating that this genetic lineage has or had until recently the capacity to acquire new spacers. Comparison of CRISPR arrays with previously obtained molecular typing data, such as amplified fragment length polymorphisms (AFLP), variable-number of tandem-repeats (VNTR) and genome-wide single-nucleotide polymorphisms (SNP), demonstrated that these methods reveal similar evolutionary trajectories. Notably, genome analyses allowed to generate a model for CRISPR array evolution in X. citri pv. citri, which provides a new framework for the genealogy of the citrus canker pathogen. Conclusions CRISPR-based typing will further improve the accuracy of the genetic identification of X. citri pv. citri outbreak strains in molecular epidemiology analyses, especially when used concomitantly with another genotyping method. | NA | no | gd of pathogen | NA | no | no |
| 5165 | 2020 | 44042 | Jhala, YV; Banerjee, K; Chakrabarti, S; Basu, P; Singh, K; Dave, C; Gogoi, K | 2019 | Asiatic Lion: Ecology, Economics, and Politics of Conservation | Asiatic lions typify most challenges faced by large carnivores: single population, historical bottlenecks, habitat loss, poaching, and conflict with humans. Their recovery from <50 in a few hundred km(2) to >500 occupying 13,000 km(2) of agro-pastoral Saurashtra landscape, Gujarat, India is an enigma. We review and evaluate the multidisciplinary aspects of lion conservation-strategy that covers ecology, conflict, community perceptions, economics, management, and politics. The history of modern lions in India dates back to similar to 4-6,000 BP, but evidence suggests presence as early as 10-15,000 BP. Asiatic lions can be distinguished from African lions by their belly-folds; adult males and females weighing 160 (SE 4.7) and 116 (SE 3.7) kg, respectively. Lion density ranged from 2 to 15/100 km(2) in the Saurashtra landscape. Demographic parameters of Asiatic lions were comparable to African lions. Prides were related females and cubs; males lived separately in hierarchical coalitions having overlapping ranges with multiple prides. Lionesses mated with multiple coalitions to reduce infanticide and enhance genetic diversity of their progeny. Few hectares of scrub sufficed as daytime refuges, while >4 km(2) patches were required for breeding. Sink populations outside Gir Protected Area (PA) were maintained by immigrants. Lions within PA fed primarily on wild-prey, while scavenging and predation on livestock was the mainstay outside. Monetary compensation for livestock-depredation, legal-protection, lion-related profits, combined with religious and cultural sentiments were major drivers of population recovery. The lion has become a socio-political instrument in Gujarat, which despite a Supreme Court directive, has not parted with founders to establish another population. Threats from epidemics loom large and currently a canine distemper virus outbreak is prevalent. Attacks on humans were rare, however, with increasing lion density the intensity of conflict is increasing. This, coupled with lowered tolerance of communities due to erosion of traditional values sets the stage for retaliation. Future of lions outside PA is uncertain as breeding refuges and their connecting corridors are vanishing rapidly. A human-free National Park of similar to 1,000 km(2) is essential for ensuring a viable population that retains its ecological role and evolutionary potential. Legalizing lion based ecotourism by forming village consortia holds promise to prevent land conversion and promoting lion-human coexistence. | NA | no | No good fitness measure and measure host GD | NA | no | no |
| 5166 | 2020 | 44043 | Ji, DM; Xing, WH; Li, F; Huang, Z; Zheng, WK; Hu, BY; Niu, FL; Zhu, Y; Yang, XJ | 2019 | Correlation of EYS polymorphisms with lumbar disc herniation risk among Han Chinese population | Background Lumbar disc herniation (LDH) is a common spinal disease in clinical practice. Once lumbar disc herniation occurs, it seriously reduces patient’s quality of life. The EYS (eyes shut homolog) was discovered in recent years and it may be related to lumbar disc herniation. So we conducted a case-control study to explore the relationship between EYS polymorphism and lumbar disc herniation risk. Methods We selected 5 single-nucleotide polymorphisms (SNPs) of EYS gene in a case-control study with 508 cases and 508 healthy controls to evaluate the relatedness by using genetic model, haplotype, and stratification analysis. Results We found that the minor alleles of rs62413038 (OR = 1.21, 95%CI: 1.01-1.43, p = .036) and rs9450607 (OR = 1.26, 95% CI: 1.05-1.53, p = .016) were associated with an increased risk of lumbar disc herniation in the allelic model analysis. In the genotypic model analysis, rs62413038 displayed a significantly increased risk of lumbar disc herniation in log-additive models (OR = 1.20, 95% CI: 1.01-1.43, p = .039). While the rs9450607 was also obviously associated with an increased lumbar disc herniation risk in recessive (OR = 1.98, 95% CI: 1.24-3.13, p = .004) and log-additive models (OR = 1.27, 95% CI: 1.05-1.55, p = .014). In addition, in the haplotype analyses of the SNPs, we found that the CGGA haplotype of rs1482456, rs9342097, rs9450607, and rs7757884 was associated with lumbar disc herniation. (OR = 0.52, 95% CI: 0.30-0.89, p = .017). Conclusion These results suggest that EYS polymorphism may be associated with lumbar disc herniation among Han Chinese population. It also opens up a new exploration direction for the etiology of lumbar disc herniation. | NA | no | human | NA | no | no |
| 5167 | 2020 | 44113 | Jiang, CM; Shen, QXJ; Wang, B; He, B; Xiao, SQ; Chen, L; Yu, TQ; Ke, X; Zhong, QF; Fu, J; Chen, Y; Wang, LX; Yin, FY; Zhang, DY; Ghidan, W; Huang, XQ; Cheng, ZQ | 2017 | Transcriptome analysis of WRKY gene family in Oryza officinalis Wall ex Watt and WRKY genes involved in responses to Xanthomonas oryzae pv. oryzae stress | Oryza officinalis Wall ex Watt, a very important and special wild rice species, shows abundant genetic diversity and disease resistance features, especially high resistance to bacterial blight. The molecular mechanisms of bacterial blight resistance in O. officinalis have not yet been elucidated. The WRKY transcription factor family is one of the largest gene families involved in plant growth, development and stress response. However, little is known about the numbers, structure, molecular phylogenetics, and expression of the WRKY genes under Xanthomonas oryzae pv. oryzae (Xoo) stress in O. officinalis due to lacking of O. officinalis genome. Therefore, based on the RNA-sequencing data of O. officinalis, we performed a comprehensive study of WRKY genes in O. officinalis and identified 89 OoWRKY genes. Then 89 OoWRKY genes were classified into three groups based on the WRKY domains and zinc finger motifs. Phylogenetic analysis strongly supported that the evolution of OoWRKY genes were consistent with previous studies of WRKYs, and subgroup IIc OoWRKY genes were the original ancestors of some group II and group III OoWRKYs. Among the 89 OoWRKY genes, eight OoWRKYs displayed significantly different expression (>2-fold, p<0.01) in the O. officinalis transcriptome under Xoo strains PXO99 and C5 stress 48 h, suggesting these genes might play important role in PXO99 and C5 stress responses in O. officinalis. QRT-PCR analysis and confirmation of eight OoWRKYs expression patterns revealed that they responded strongly to PXO99 and C5 stress 24 h, 48 h, and 72 h, and the trends of these genes displaying marked changes were consistent with the 48 h RNA-sequencing data, demonstrated these genes played important roles in response to biotic stress and might even involved in the bacterial blight resistance. Tissue expression profiles of eight OoWRKY genes revealed that they were highly expressed in root, stem, leaf, and flower, especially in leaf (except OoWRKY71), suggesting these genes might be also important for plant growth and organ development. In this study, we analyzed the WRKY family of transcription factors in O. officinalis. Insight was gained into the classification, evolution, and function of the OoWRKY genes, revealing the putative roles of eight significantly different expression OoWRKYs in Xoo strains PXO99 and C5 stress responses in O. officinalis. This study provided a better understanding of the evolution and functions of O. officinalis WRKY genes, and suggested that manipulating eight significantly different expression OoWRKYs would enhance resistance to bacterial blight. | NA | no | No good fitness measure and measure host GD | NA | no | no |
| 5168 | 2020 | 44042 | Jiang, S; Luo, MX; Gao, RH; Zhang, W; Yang, YZ; Li, YJ; Liao, PC | 2019 | Isolation-by-environment as a driver of genetic differentiation among populations of the only broad-leaved evergreen shrub Ammopiptanthus mongolicus in Asian temperate deserts | Whether the effect of migration-selection-drift equilibrium on population structure is governed by spatial or environmental differences is usually elucidated by isolation-by-distance (IBD), isolation-by-environment (IBE), and isolation-by-resistance (IBR) tests. The population structure of Ammopiptanthus mongolicus, a broad-leaved evergreen psammophyte in eastern Central Asia, was previously thought to follow an isolation by distance pattern. However, recent studies have emphasized the effects of environmental factors on its growth and distribution, suggesting an important influence of local adaptation on the genetic structure of the species. Using inter-simple sequence repeat (ISSR) markers, we verified the previously inferred low intra-population variation and high inter-population differentiation. However, in contrast to previous studies, the results of partial Mantel tests and a maximum likelihood population effects mixed model (MLPE) suggested that local climate differences, rather than geographic distances or resistance distances, are the main factor affecting population differentiation. Further analysis with removal of multicollinear climatic variables and univariate MLPE found that summer and winter precipitation were crucial for shaping the current population genetic structure. Since local precipitation is related to the regeneration, colonization, and overwintering survival of A. mongolicus, its influence on demographic change may explain its effect on the population genetic structure. In addition, precipitation is related to terrain despite westward decreases, which explains the independence of genetic difference and geographic distance. The identified role of IBE suggests that collecting germplasm resources from genetically differentiated populations could be a more effective strategy to preserve the overall genetic diversity of the species than the establishment of corridors to enhance gene flow among populations. | NA | no | No good fitness measure and measure host GD | NA | no | no |
| 5169 | 2020 | 44113 | Jighly, A; Joukhadar, R; Singh, S; Ogbonnaya, FC | 2018 | Decomposing Additive Genetic Variance Revealed Novel Insights into Trait Evolution in Synthetic Hexaploid Wheat | Whole genome duplication (WGD) is an evolutionary phenomenon, which causes significant changes to genomic structure and trait architecture. In recent years, a number of studies decomposed the additive genetic variance explained by different sets of variants. However, they investigated diploid populations only and none of the studies examined any polyploid organism. In this research, we extended the application of this approach to polyploids, to differentiate the additive variance explained by the three subgenomes and seven sets of homoeologous chromosomes in synthetic allohexaploid wheat (SHW) to gain a better understanding of trait evolution after WGD. Our SHW population was generated by crossing improved durum parents (Triticum turgidum; 2n = 4x = 28, AABB subgenomes) with the progenitor species Aegilops tauschii (syn Ae. squarrosa, T. tauschii; 2n = 2x = 14, DD subgenome). The population was phenotyped for 10 fungal/nematode resistance traits as well as two abiotic stresses. We showed that the wild D subgenome dominated the additive effect and this dominance affected the A more than the B subgenome. We provide evidence that this dominance was not inflated by population structure, relatedness among individuals or by longer linkage disequilibrium blocks observed in the D subgenome within the population used for this study. The cumulative size of the three homoeologs of the seven chromosomal groups showed a weak but significant positive correlation with their cumulative explained additive variance. Furthermore, an average of 69% for each chromosomal group’s cumulative additive variance came from one homoeolog that had the highest explained variance within the group across all 12 traits. We hypothesize that structural and functional changes during diploidization may explain chromosomal group relations as allopolyploids keep balanced dosage for many genes. Our results contribute to a better understanding of trait evolution mechanisms in polyploidy, which will facilitate the effective utilization of wheat wild relatives in breeding. | NA | no | No good fitness measure and measure host GD | NA | no | no |
